# Comprehensive and bias-aware analysis of small RNA NGS data for biomarker detection with caRNAge

**DOI:** 10.20517/evcna.2026.78

**Published:** 2026-09-24

**Authors:** Benedikt Kirchner, Christian Grätz, Johannes Kersting, Martina Schuster, Marlene Reithmair, Markus List, Michael W. Pfaffl

**Affiliations:** ^1^Department of Animal Physiology and Immunology, School of Life Sciences, Technical University of Munich, Freising 85354, Germany.; ^2^Data Science in Systems Biology, School of Life Sciences, Technical University of Munich, Freising 85354, Germany.; ^3^Institute of Human Genetics, University Hospital, Ludwig-Maximilians-University Munich, Munich 80336, Germany.; ^4^Munich Data Science Institute (MDSI), Technical University of Munich, Garching 85748, Germany.; ^#^These authors contributed equally to this work.

**Keywords:** Small RNA-Seq, next-generation sequencing, biomarker discovery, extracellular vesicles, miRNA, data analysis

## Abstract

**Aim:** Small RNA sequencing (small RNA-Seq) is a widely used and important method for biomarker discovery in extracellular vesicles (EVs), which carry small RNAs among their cargo and protect them from degradation in liquid biopsies. However, existing computational tools for small RNA-Seq data are not tailored to EV-RNA.

**Methods:** We present comprehensive and bias-aware analysis of small RNA gene expression (caRNAge), a biomarker discovery pipeline for small RNA-Seq data intended for users without extensive bioinformatic expertise. Ready to use as a Snakemake pipeline, caRNAge assists users in bias-aware normalization, differential gene expression analysis, and pathway over-representation analysis.

**Results:** Possible confounding factors can be identified and corrected to maximize data comparability between experiments and biomarker robustness. caRNAge is not limited to miRNAs; it also supports the detection of miRNA isomer (isomiRs), tRNA-derived fragments (tRFs), transfer RNA (tRNAs), and other small RNA species, thereby enhancing the specificity of biomarker signatures. Specific tissues can be selected for target prediction and pathway analysis to eliminate false-positive results. All results are provided in accessible and interactive HTML reports. We provide example data for each step of the pipeline to demonstrate caRNAge’s capabilities and advantages across three EV-miRNA biomarker discovery use cases. The pipeline is not limited to EV-miRNA data but can be used for data evaluation in any small RNA-Seq experiment.

**Conclusion:** By assisting users at every step of the discovery workflow, caRNAge fills an important gap in the field of EV-associated transcriptomic biomarker development. The pipeline is freely available at https://gitlab.lrz.de/kirchner/carnage.

## INTRODUCTION

Ever since it was demonstrated in 2007 that extracellular vesicles (EVs) carry RNA, including microRNA (miRNA), among their cargo^[1]^, the number of studies on EV-associated small RNA has skyrocketed. This can be demonstrated by a literature search in the PubMed^[2]^ database, using the query “((extracellular vesicles[Title/Abstract]) OR (exosome[Title/Abstract])) AND ((miRNA[Title/Abstract]) OR (small RNA[Title/Abstract]))”, which identified an increase in annual publications from 3 in 2009 to 1,036 in 2025. A major reason for this increased interest in EV-miRNAs is that RNAs associated with EVs - in the EV lumen or as part of the EV corona - are protected from degradation^[3-5]^. Due to their short length, miRNAs are found in EVs unfragmented (in contrast to EV-mRNA) and can therefore actively influence the translation in recipient cells, representing a form of intercellular communication. Other small RNA species commonly found associated with EVs include transfer RNA (tRNA)-derived fragments (tRFs) and small interfering RNAs (siRNAs)^[6]^. Besides the extensively studied miRNAs, especially the highly abundant and long-overlooked tRFs have recently sparked interest as potential EV-associated disease biomarkers^[7-10]^. Sequence variants (isomers) of canonical miRNAs, termed isomiRs, have been known to exert distinct roles different from the corresponding canonical miRNA^[11-14]^. There is evidence that specific isomiRs and tRFs are enriched in EVs compared to their cellular levels^[15,16]^, making these two small RNA biotypes additional interesting biomarker candidates, besides the extensively studied miRNAs.

Among several profiling methods for (EV-associated) miRNA biomarker studies, small RNA sequencing (small RNA-Seq) has proven to be the most accurate, sensitive, and specific; however, it is less reproducible than other methods for miRNA analysis^[17]^. In fact, a lack of reproducibility in small RNA-Seq experiments has been reported previously^[18-20]^. A major reason for these reproducibility issues is the incomplete reporting of the analysis pipeline used for data evaluation^[21]^. Detailed reporting is crucial because different analysis pipelines can yield very different results for the same data set, e.g., due to differences introduced by the algorithms used for normalization and bias correction^[22-24]^. However, those are not the only choices in data analysis that can affect the results. It has been demonstrated, for instance, that the choice of reference genome used for transcript mapping can have a dramatic effect on RNA-Seq data analysis^[25]^. In addition to the bias introduced *in silico*, the underlying issues of biological variation and experimental bias remain, as in every biological experiment^[26,27]^. While it is possible to correct for batch effects^[28]^ and other biases introduced during sampling or pre-analytics in the wet lab using bioinformatic tools, these tools are only of help when the researcher is aware of the bias and its cause. Therefore, it is important that data visualization is one of the first steps in the analysis pipeline, helping the researcher to easily identify potential bias in the data by visual inspection and choose the analysis parameters accordingly.

Additional issues might arise later during the complex, multi-step data analysis. Since small RNA-Seq library preparation protocols include a size-selection step to capture mostly libraries with short inserts in the range of miRNA length, the libraries are comparably short [usually < 200 base pairs (bp)]. These short insert sizes result in adapter sequences being covered in almost all reads, making adapter trimming a prerequisite for data analysis^[29]^. Further, small RNA-Seq reads generally fail several checkpoints during analysis in tools developed for total RNA-Seq data, due to their short length and the limited pool of small RNA sequences compared to mRNAs, for example. Sequence bias in miRNAs, such as the first 5’ base and the seed region^[30]^, also contributes to this. Recently, concerns have been raised that miRNA target determination yields many false positives. Researchers have shown that the most commonly used test for functional enrichment yielded significant *P*-values for targets of randomly selected miRNAs^[31,32]^. A major problem is that miRNA binding sites are very short (down to 7 nucleotides), which makes *in silico* target prediction approaches prone to producing many false positives^[33]^. It is also well known that each miRNA regulates a multitude of target mRNAs, further complicating and often inflating target prediction^[34,35]^. Restricting the possible targets to those expected to be expressed in the tissue of interest can therefore reduce the number of false-positive results and improve both the efficiency and the meaningfulness of the analysis.

Here, we present a novel bioinformatic pipeline for comprehensive and bias-aware analysis of small RNA gene expression (caRNAge) in small RNA next-generation sequencing (NGS) data. caRNAge utilizes and combines several publicly available tools and databases in a modular fashion to facilitate small RNA-Seq data analysis. After the initial setup, the pipeline is very accessible for life science researchers and does not require extensive bioinformatic expertise. While caRNAge was developed for studies of EV-associated small RNA, it can be applied to any small RNA-Seq dataset. Demanding only raw sequencing data and experimental metadata as input, caRNAge supports users in identifying and correcting for potential batch effects, selecting the appropriate normalization strategy, and detecting differentially expressed small RNA genes and degraded mRNA fragments. With caRNAge, users can further identify predicted and experimentally validated targets of differentially expressed miRNAs and evaluate their biological relevance through pathway analysis, while minimizing false positives through tissue selection. The results of caRNAge’s differential gene expression (DGE) capabilities are also an optimal basis for identifying stable reference transcripts, e.g., using the miREV^[36]^ tool. Small RNA biomarker identification is enhanced by employing unsupervised clustering methods alongside discriminant analysis, as well as by in-depth characterization of EV small RNA cargo. Furthermore, the pipeline enables researchers to fully leverage the dataset, incorporating isomiR analysis and tRF detection to improve biomarker specificity. Additionally, caRNAge allows users to reduce false-positive results in miRNA target prediction and pathway over-representation analysis (ORA) by setting a minimal expression threshold for the analyzed tissues or tissues expected to take up the analyzed EVs. To facilitate sharing and browsing of the results and visualizations, all reports are generated as explorative HTML files. The novelty of caRNAge does not primarily lie in the individual preprocessing or statistical tools, many of which are established methods, but in their integration into a bias-aware and iterative decision-support framework. In particular, caRNAge places systematic assessment of normalization effects and potential confounding factors before differential testing and allows their consequences to be evaluated across multiple small-RNA feature spaces. This enables users to adapt the statistical analysis to the observed structure of their dataset rather than applying a fixed analysis strategy. Table 1 summarizes key capabilities, modular components, and optional bias-correction steps of caRNAge alongside those of other pipelines, highlighting the unique functionalities offered by our workflow.

**Table 1:** Comparison of the features included in caRNAge with five established small RNA-Seq data analysis pipelines

**Feature**	**caRNAge**	**miRge3.0^[37]^**	**sRNAnalyzer^[38]^**	**sRNAflow^[39]^**	**SPAR^[40]^**	**nf-core/smrnaseq^[41]^**
**RNA species support**						
Canonical small RNAs and their functional variants (miRNAs, tRNAs, tRFs, isomiRs)	✓	✓	✓	✓	✓^*^	✓
Common long RNA fragments (rRNA, mRNA)	✓	✓	✓	✓	✓	✓
LncRNAs	✓	✗	✓	✓	✗	✓
Other, more specialized RNA species (piRNA, Y RNA, siRNA, snoRNA)	✓	✓^*^	✓	✓	✓	✓
Exogenous RNAs	✗	✗	✓	✓	✗	✓
**Pre-processing**						
Adapter trimming (cutadapt)^[42]^	✓	✓	✓	✓	✗	✓
UMI handling	✗	✓	✓	✓	✗	✓
**Core analysis**						
Multiple, sequential alignment (bowtie)^[43]^	✓	✓	✓	✓	✗	✓
Specialized, comprehensive tRF mapping (MINTmap)^[44]^	✓	✗	✗	✗	✗	✗
Custom normalization	✓	✗	✗	✗	✗	✗
Integrated detection and correction of batch effects	✓	✗	✗	✗	✗	✗
DGE analysis with DESeq2	✓	✓	✗	✓	✗	✓
**Functional and downstream analysis for biomarker applications**						
sPLS-DA and AUROC	✓	✗	✗	✗	✗	✗
miRNA target prediction	✓	✗	✗	✗	✗	✗
Tissue-specific miRNA DGE and target prediction	✓	✗	✗	✗	✗	✗
Pathway analysis	✓	✗	✗	✗	✗	✗
**Further modules**						
Sequencing error correction with miREC^[45]^	✗	✓	✗	✗	✗	✗
Prediction of novel miRNAs	✗	✓	✗	✗	✓	✗
RNA editing detection	✓^*^	✓	✓	✗	✗	✗
**Technical features**						
GUI	✗	✓	✓^*^	✓	✓	✗
Interactive output files	✓	✓	✓^*^	✓^*^	✓	✓
Runs locally	✓	✓	✓	✓	✓	✓
Web-based	✗	✓	✓	✓	✓	✓

^*^Partial or limited support. Check marks and cross marks indicate features included in or missing from the pipelines, respectively. AUROC: Area under the receiver operating characteristic curve; DGE: differential gene expression; GUI: graphical user interface; isomiR: miRNA isomer; lncRNA: long non-coding RNA; miRNA: microRNA; mRNA: messenger RNA; piRNA: piwi-interacting RNA; rRNA: ribosomal RNA; siRNA: small interfering RNA; snoRNA: small nucleolar RNA; sPLS-DA: sparse partial least squares discriminant analysis; tRF: tRNA-derived fragment; tRNA: transfer RNA; UMI: unique molecular identifier; DESeq2: differential expression sequence analysis 2; MINTmap: mitochondrial and nuclear tRF mapping; miREC: miRNA error rectification; miRge: a multiplexed method of processing small RNA-Seq data to determine microRNA entropy; nf-core/smrnaseq: Nextflow core / small RNA sequencing; SPAR: small RNA-seq portal for analysis of sequencing experiments; sRNA: small RNA.

Throughout this manuscript, we will provide more details on the workflow and benefits of caRNAge, and highlight specific use cases where the pipeline has improved the results of small RNA-Seq data analysis. For visualization of the caRNAge output, a small RNA-Seq dataset obtained from a previously published study by Schuster *et al.* was used^[46]^. This study compared the miRNA cargo of EVs shed by primary glioblastoma cells cultured as 2D cultures and 3D organoid models.

## METHODS

### Raw data processing and alignment

All raw small RNA-Seq data were processed using the reproducible and modular caRNAge workflow implemented in Snakemake (v7.14.1)^[47,48]^ - a tool to create reproducible and scalable data analyses, ensuring rule-based dependency tracking, transparent logging, and version control of all intermediate steps. The workflow was executed in a Conda-managed environment (a software environment management system that handles dependencies and package versions) to guarantee computational reproducibility across platforms. The pipeline is freely available at https://gitlab.lrz.de/kirchner/carnage. caRNAge generally expects single-end sequencing data but can alternatively run on single reads from paired-end data as well. Throughout the pipeline, users have the option to adjust important parameters for each step as needed in the YAML configuration file. The file already provides parameter values, but users are highly encouraged to adapt them to their specific needs, as different experiments may profit from different adapter settings.

Adapter trimming was performed using Cutadapt (v1.17)^[42]^ with parameters as specified in the YAML configuration file (adapters: “-a AGATCGGAAGAGCAC”, extra: “-e 0.1 -m 16”). Adapter sequences were removed from single-end reads, allowing configurable mismatch tolerances and length constraints. Reads failing trimming criteria were discarded. Quality control of trimmed FASTQ files was conducted using FastQC (v0.12.1)^[49]^ to evaluate per-base quality scores, nucleotide composition, GC distribution, sequence length distribution, and adapter contamination. To enable comprehensive annotation of EV-associated and cellular small RNAs while minimizing ambiguous assignments, a hierarchical alignment strategy was applied using the sequence aligner Bowtie (v1.3.1)^[43]^. Bowtie was selected due to its suitability for short-read alignment and its deterministic mismatch handling for small RNA sequences. Alignment was performed with the --best and --norc options, suppressing reverse-complement alignments to preserve strand specificity. Mismatch allowance (-v) was configurable per reference index.

Trimmed reads were first aligned to a human tRNA reference derived from the genomic resources for *Homo sapiens* (GRCh38 build). This step captures mature and precursor tRNA-derived sequences and reduces cross-mapping to other RNA classes. This ordering represents a conservative annotation strategy, as several tRF subclasses overlap in length and sequence space with mature miRNAs and may therefore be misassigned during miRNA/isomiR mapping. By removing reads with strong sequence support for a tRNA origin before the more permissive isomiR mapping step, caRNAge reduces potential false-positive miRNA/isomiR assignments, although ambiguous reads cannot always be unequivocally assigned to their transcript of origin. tRFs were subsequently identified using MINTmap (v1.0)^[44]^, which enables exact and high-resolution mapping of tRFs using a precompiled lookup table and curated tRNA reference space. Both exclusive and ambiguous tRF assignments were retained and annotated. MINTmap reference files (LookupTable.tRFs.MINTmap_v1, tRNAspace sequences, and annotation files) were obtained from the official release package. If no tRNA alignments were detected for a sample, the pipeline generated structurally valid empty output files to ensure workflow continuity. Reads not aligning to tRNA references were stratified by length (16-34 nt) and aligned against length-specific isomiR reference indices derived from miRBase^[50,51]^ annotations for *Homo sapiens* that include isomiR sequences with up to 3 nt additions or up to 6 nt on both ends. The isomiR reference is available together with the Snakemake workflow and users can change the alignment parameters if needed through the YAML file. Duplicate sequences were collapsed to obtain per-sequence read counts. Reads remaining unmapped after tRNA and isomiR alignment were aligned to a combined RefSeq/piRNA (piwi-interacting RNA) transcriptome reference (NCBI RefSeq select human release; piRNAdb)^[52,53]^ to capture additional small RNA species, including annotated piRNAs and small coding/non-coding transcripts. Sequence-level counts were generated by collapsing identical alignments. Residual unmapped reads were aggregated and length-characterized to enable downstream inspection of potentially novel small RNA species or sequencing artifacts.

### Quality control, normalization, differential expression, and functional downstream analyses

All post-alignment analyses were executed in R (v4.5.2)^[54]^ using an isolated, project-local package environment managed with renv^[55]^, an R dependency management system that records and restores exact package versions via a lockfile (lockfile-based dependency pinning), to ensure computational reproducibility across systems and time. The analysis stage consumes the per-sample count/sequence summaries generated by the Snakemake workflow (mapping outputs for tRNA, tRFs, isomiR/miRNA, transcriptome classes, and unmapped reads), along with the user-provided experimental metadata table (design.tsv) and the pipeline configuration (config.yaml). Per-sample FastQC^[49]^ outputs generated during raw data processing were aggregated in R using fastqcr^[56]^, producing a sample-by-module quality control (QC) summary and enabling selective visualization of diagnostic plots for flagged modules. Input metadata and count tables were imported using data.table^[57]^ for high-throughput I/O and aggregation. Sequence-level outputs were consolidated into feature-level matrices as follows:

1. tRF quantification: MINTmap-derived outputs were read per sample and merged by MINTbase_Unique_ID and tRF_type. Missing/empty files were handled defensively by generating valid zero-filled placeholders to preserve sample-wise matrix integrity.

2. tRNA quantification: Per-sample tRNA sequence tables were merged by gene/sequence/length and then collapsed to gene-level counts; gene identifiers were normalized by removing isoacceptor copy suffixes where present.

3. isomiR parsing and canonical miRNA definition: IsomiR identifiers were decomposed into canonical miRNA name and sequence-variation descriptors (5’/3’ end modifications and mismatches). Canonical miRNA counts were derived by excluding isomiRs exceeding user-defined thresholds for 5’ shifts, 3’ shifts, or polymorphic substitutions (as specified in the editable config.yaml).

4. Transcriptome (RefSeq + piRNA) quantification: Transcriptome-mapped reads were aggregated by gene and length, then joined to annotation mapping tables (refseq_piRNAdb_info*.txt) to assign gene symbol, RNA class, and description. Lowly expressed transcriptome features were removed using a user-defined mean-count threshold to reduce noise and stabilize downstream normalization.

5. Library composition summaries: Length distributions and RNA-biotype distributions were computed for pre-trimming, mapped fractions, and unmapped reads; where requested, counts were additionally aggregated to group-wise means over metadata categories (e.g., cell line, treatment, isolation method).

For each analysis scope (total small-RNA set and user-specified subsets including miRNA-only, miRNA + tRNA, isomiR-only, tRF-only, tRF + isomiR, and a user-defined “custom” RNA class set), a DESeq2 (v1.50.2)^[58]^ dataset was constructed from the corresponding count matrix using the experimental design formula assembled from three configuration-driven components: (i) variables to correct as potential batch effects; (ii) dependency/paired factors (if supplied); and (iii) the primary grouping variable for downstream contrasts. DESeq2 fits generalized linear models with a negative binomial distribution to RNA-seq count data to identify genes with statistically significant expression changes between conditions. Size factors were estimated using the default DESeq2 approach; when the default method failed due to pervasive zeros, the pipeline automatically re-ran DESeq2 using sfType = “poscounts” for robust size-factor estimation in sparse count regimes. A variance-stabilizing transformation (VST) was applied to obtain approximately homoscedastic expression values for clustering and principal component analysis (PCA). To support bias-aware visualization, optional batch correction for plotting and downstream analysis was performed on VST-transformed expression values using limma’s (v3.66.0)^[59]^ removeBatchEffect() while preserving the primary biological design term. Limma fits linear models to gene expression data, applies empirical Bayes moderation to improve variance estimates, and allows correction for batch effects via model design or direct adjustment of expression values. Unsupervised sample structure was assessed by (i) hierarchical clustering on transformed expression matrices and (ii) PCA. To evaluate normalization sensitivity, these multivariate summaries were computed for each normalization scope and reported side-by-side in the overview report.

Differential expression was computed with DESeq2^[58]^ under the negative binomial generalized linear model framework. For the user-specified primary grouping factor, all pairwise contrasts between factor levels were generated and evaluated for each normalization scope. For each contrast, DESeq2 result tables (including log2 fold changes, Wald test *P*-values, and multiple-testing adjusted *P*-values) were merged with group-mean normalized counts to support downstream interpretability and report display. Full, unfiltered result tables were written to disk for reproducible reuse. Interactive reporting of DGE results was produced using EnhancedVolcano (v1.28.2)^[60]^ for volcano plots and DT^[61]^ for sortable/exportable tables, embedded in the caRNAge_DGE.html report. Where multiple contrasts were available, overlap of significant feature sets was summarized via UpSet visualizations (ComplexUpset^[62,63]^). To complement univariate DGE, supervised sparse partial least squares discriminant analysis (sPLS-DA) was performed using mixOmics (v6.34.0)^[64]^ on VST-transformed expression matrices after expression filtering (baseMean threshold) and optional variance-based feature restriction to the top-N most variable features. Model sparsity was optimized using repeated cross-validation according to the parameters specified in config.yaml. Classification performance of each fitted model was additionally assessed by internal cross-validation and summarized using the overall classification error and balanced error rate (BER). The selected sparsity parameters, validation scheme, number of folds and repeats, and distance metric are reported in the interactive DGE report. Area under the receiver operating characteristic curve (AUROC) values are additionally provided as descriptive summaries of the fitted training data and are not interpreted as unbiased estimates of performance in an independent cohort.

For each DGE contrast, significantly regulated canonical miRNAs were submitted to ORA using microRNA Enrichment Analysis and Annotation (miEAA) 2.0^[65]^, a microRNA enrichment analysis tool, via the rbioapi (v0.8.3)^[66]^ client, which provides programmatic access to bioinformatics web services via RESTful application programming interfaces (APIs) using standardized web requests. ORA results were stored per contrast and rendered in the caRNAge_ORA.html report with interactive tables and database-specific annotation summaries. Predicted miRNA-mRNA interactions were retrieved programmatically with multiMiR (v1.32.0)^[67]^, an R package that aggregates validated and predicted miRNA-target interactions from multiple databases, together with TargetScan^[68]^ predictions, which identify conserved miRNA binding sites in mRNAs. To add tissue context and reduce false-positive interpretability, predicted target genes were annotated with consensus tissue expression values [normalized transcripts per million (nTPM)] derived from Human Protein Atlas^[69]^ RNA tissue consensus data, which integrates evidence from the Genotype-Tissue Expression Portal (GTEx)^[70]^. Target lists were filtered by a configurable nTPM threshold (set to 50 for the example data) and summarized (i) as tissue-by-target heatmaps and (ii) as tissue-stratified interaction tables. For each contrast, ORA was additionally performed on (i) all predicted targets and (ii) tissue-filtered expressed targets using the gene set enrichment analysis tool Enrichr^[71]^ via rbioapi (v0.8.3)^[66]^. ORA was executed across the databases Gene Ontology (GO), Kyoto Encyclopedia of Genes and Genomes (KEGG), Reactome, WikiPathways, and disease/phenotype association libraries, and results were written to structured output directories and summarized in caRNAge_ORA.html. All three interactive HTML reports were generated using R Markdown/knitr^[72,73]^ and embedding interactive graphics and tables (plotly^[74]^, htmlwidgets^[75]^, ggplot2^[76]^, DT^[61]^). The reports are intended as exploratory, parameter-guiding documents that expose configuration choices, software versions, QC summaries, and intermediate results to support transparent, bias-aware iterative analysis.

Figure 1 provides an overview of the caRNAge workflow in a subway map style.

**Figure 1:**
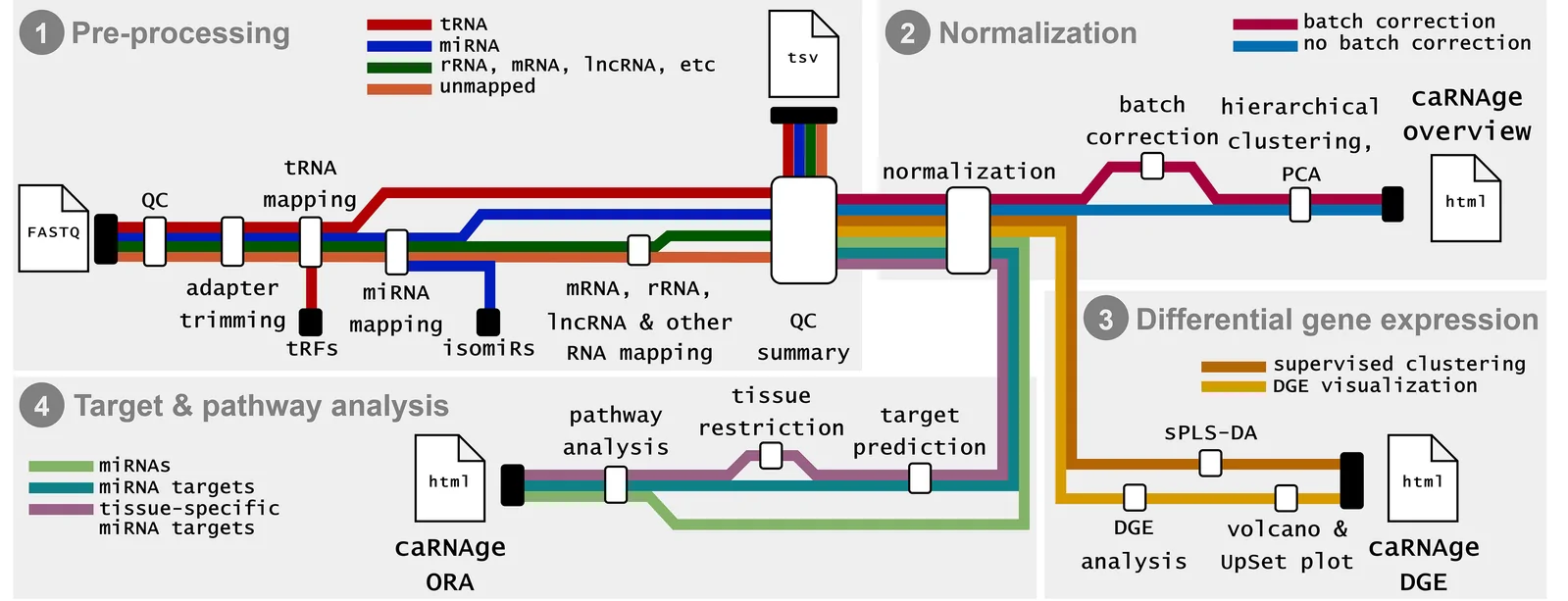
Overview map of the caRNAge workflow. DGE: Differential gene expression; lncRNA: long non-coding RNA; miRNA: microRNA; mRNA: messenger RNA; ORA: over-representation analysis; PCA: principal component analysis; QC: quality control; rRNA: ribosomal RNA; sPLS-DA: sparse partial least squares discriminant analysis; tRNA: transfer RNA; tRF: tRNA-derived fragment; miRNA: isomer; rRNA: ribosomal RNA.

### Example and independent validation datasets

The example dataset used in this article was originally generated for a study of Schuster *et al*. (2024)^[46]^. Methodological details are reported in the original article. Briefly, six patient-derived, primary glioblastoma cell lines were cultured as 2D and 3D models in cell culture flasks and as spheroids in Petri dishes, respectively. EVs were isolated from pre-cleared cell culture supernatants using two independent approaches: precipitation and immunoaffinity. After EV characterization, EV-RNA was isolated using the miRNeasy mini kit (Qiagen, Hilden, Germany) and small RNA libraries were prepared using the NEBNext Multiplex SmallRNA Library Prep Set for Illumina (New England Biolabs, Ipswich, MA, USA) as described in Buschmann *et al*. (2018)^[77]^. Library size selection was performed on a 4% agarose gel, cutting the area between the 140 and 150 bp marker bands. The final libraries were sequenced on a NovaSeq 6000 platform (Illumina, San Diego, CA, USA) after successful quality assessment by capillary electrophoresis.

For independent validation, caRNAge was additionally applied to the publicly available GSE181216 small RNA-Seq dataset generated by Céspedes *et al*.^[78]^. The study compared small-RNA cargo from primary human CD4^+^ and CD8^+^ T cells, constitutively released EVs, trans-synaptic vesicles (tSVs), and bead-supported lipid bilayer background controls. The complete dataset comprised 185 sequencing libraries and was initially evaluated using the caRNAge overview module to assess library composition and potential technical or biological sources of variation. Because sequencing platform and intracellular sample origin were partially confounded in the complete dataset, inferential analyses were subsequently restricted to a donor-matched subset of 120 libraries from eight donors generated within the same sequencing-platform subset. For downstream functional interpretation, predicted miRNA targets were additionally restricted to genes expressed in lymph node or spleen at nTPM ≥ 10.

## RESULTS

caRNAge produces multiple result folders, summarized in three successively generated HTML reports, to facilitate adjusting parameters for subsequent steps based on the current output. Each report file includes a detailed description, but it will also be explained briefly below.

### First report: data overview

The first HTML report generated by caRNAge, saved as *carnage_overview.html*, provides a comprehensive overview of the dataset and is designed to assist in QC, visualization of mapping distributions, and, most importantly, detection of potential biases [Supplementary File 1]. Figure 2 shows an excerpt from the caRNAge overview report, including read mapping distributions, hierarchical clustering results, and PCA results before and after batch correction. The *Data processing* section in the report summarizes the tools and software packages used throughout the pipeline. Metadata and processing information are reported to ensure reproducibility.

**Figure 2:**
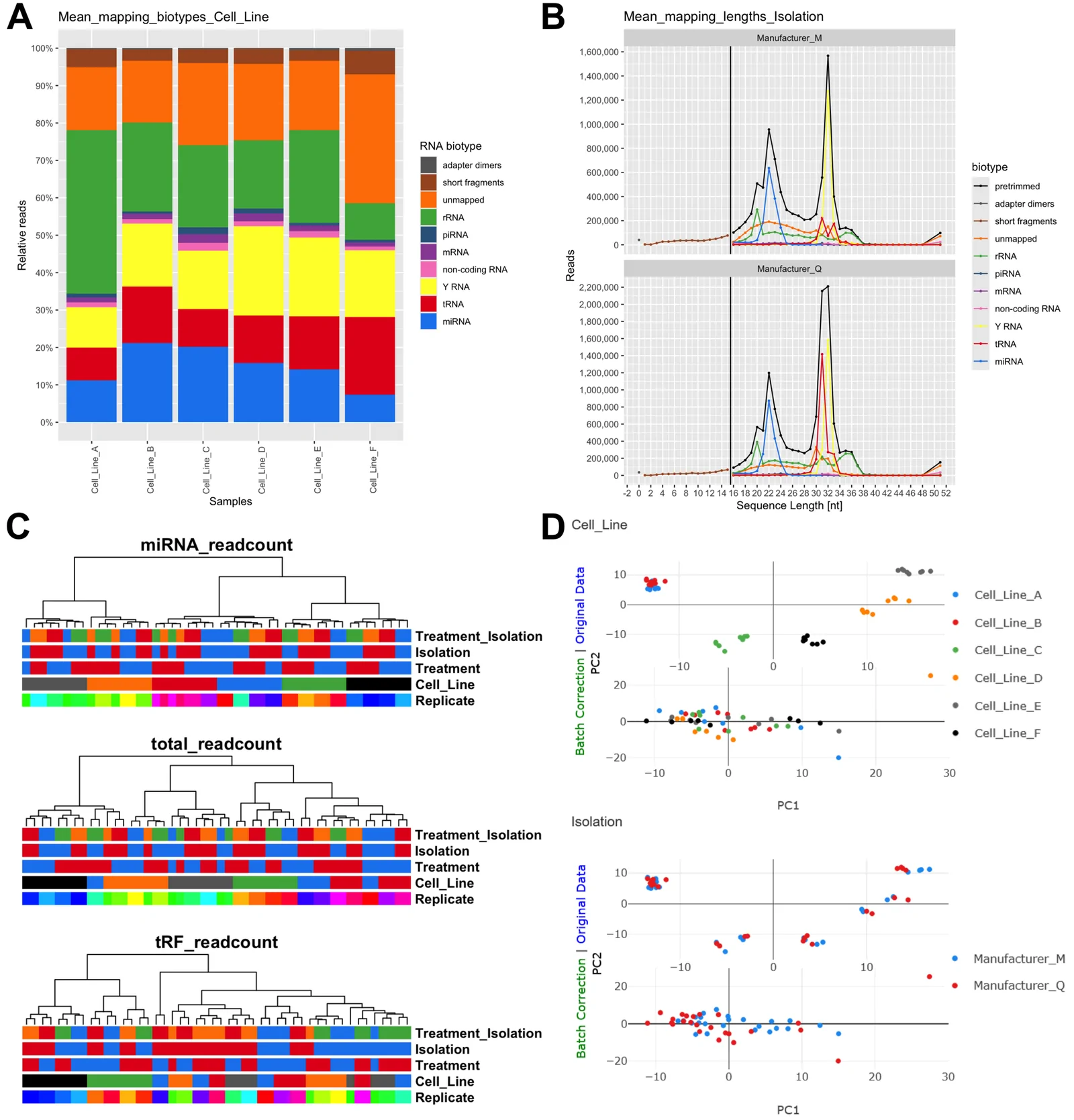
Overview of example output generated for the caRNAge overview report. (A) Relative read mapping distributions aggregated by cell line and stratified by RNA biotype; (B) Read mapping length distribution plots after adapter trimming, aggregated by EV isolation method and stratified by RNA biotype; (C) Excerpts of hierarchical clustering results showing dendrograms with the five associated metadata categories. Clusterings after normalization to miRNA read count (top), total read count (middle), and tRF read count (bottom) are shown; (D) Two-dimensional PCA (PC1 vs. PC2) illustrating sample separation before and after batch correction. The panels show PCAs computed from the 500 most variable features after miRNA read count normalization, with samples colored by cell line (top) and EV isolation method (bottom). EV: Extracellular vesicle; miRNA: microRNA; mRNA: messenger RNA; PC: principal component; PCA: principal component analysis; piRNA: piwi-interacting RNA; rRNA: ribosomal RNA; tRF: tRNA-derived fragment; tRNA: transfer RNA.

The QC module contains a summary of multiple quality checkpoints for all samples, based on FastQC^[49]^. However, users should be aware that FastQC was originally designed for whole-genome and total RNA-Seq data. Due to their short length and strong nucleotide composition biases, such as the frequent presence of uracil as the first 5’ base and conserved sequences in the seed region critical for target binding, miRNAs typically fail several FastQC thresholds^[30]^. Further, a very small group of miRNAs is generally much more abundant than others, something that typically only occurs in total RNA sequencing to a much lesser extent. Therefore, some quality checkpoints are typically not met in small RNA-Seq experiments, and these experiments are flagged by FastQC. These warnings are expected to appear and do not necessarily indicate library issues. Generally, the *per base N content*, *per base sequence quality*, and *per sequence quality scores* metrics provide more informative assessments for small RNA-Seq experiments than some of the other checkpoints. Detailed plots for these checkpoints are shown if one or more samples fail the respective quality criteria.

Mapping statistics reveal the distribution of reads across small RNA biotypes and their corresponding length profiles [Figure 2A and B]. These figures enable users to assess miRNA enrichment in their sequencing results and detect atypical size or biotype profiles that may indicate suboptimal library preparation or experimental bias.

caRNAge further helps compare the effects of normalization strategies applied by DESeq2 based on different RNA biotype read counts specified in the *config.yaml* file. A tabular overview of s*ize factor* distributions and a visual heatmap generated by *hierarchical clustering* [Figure 2C] for each strategy allows users to select the normalization approach best suited to their experimental design and biological expectations. The final section of the first caRNAge report enables interactive exploration of potential confounding variables. Experimental factors suspected of introducing potential systematic bias (e.g., RNA isolation method, cell line) can be defined in the configuration file (*config.yaml*) used for batch-effect modeling and correction during modeling of DGE. For each normalization strategy, two-dimensional PCA plots [Figure 2D] before (top) and after (bottom) batch correction allow for immediate visual assessment of the correction effectiveness. A complementary 3D PCA representation is also provided in the report. Data points are colored and grouped by metadata and a slider widget underneath each set of plots allows restricting the PCA to the top *n* small RNAs with the highest variance (all, 1,000, 500, 100, 50, or 10) to reduce noise from low-abundance sequences.

### Second report: differential gene expression analysis

The second caRNAge report (*caRNAge_DGE.html*) contains the differentially expressed genes identified by DESeq2^[58]^ after the chosen normalization strategy, including confounder correction of identified bias [Supplementary File 2]. Volcano plots visualize the genes with an adjusted *P*-value (padj) < 0.05 and a log2 fold change (log2FC) ≥ 1 or ≤ -1, stratified by RNA biotype to highlight differences between small RNA classes [Figure 3A]. A separate volcano plot is provided for each chosen analysis setting (total, miRNA, tRNA + miRNA, isomiR, tRF, and tRF + isomiR read counts, as well as the individually defined set of RNA species termed custom analysis - miRNA, tRNA, and Y RNA read counts in this example dataset). Additionally, an expanded summary table reports the total number of genes with nonzero counts, the number and percentage of significantly regulated genes (padj < 0.1), and the proportion of outliers and genes filtered by DESeq2 due to low mean counts, enabling users to quickly assess data quality and model behavior^[58,79]^. Below the volcano plots, the DESeq2 result tables list the up- and downregulated genes according to padj (≤ 0.05) and log2FC (> 1 or < -1). The full, unfiltered result tables are stored in the automatically generated *DGE_results* folder for downstream use.

**Figure 3:**
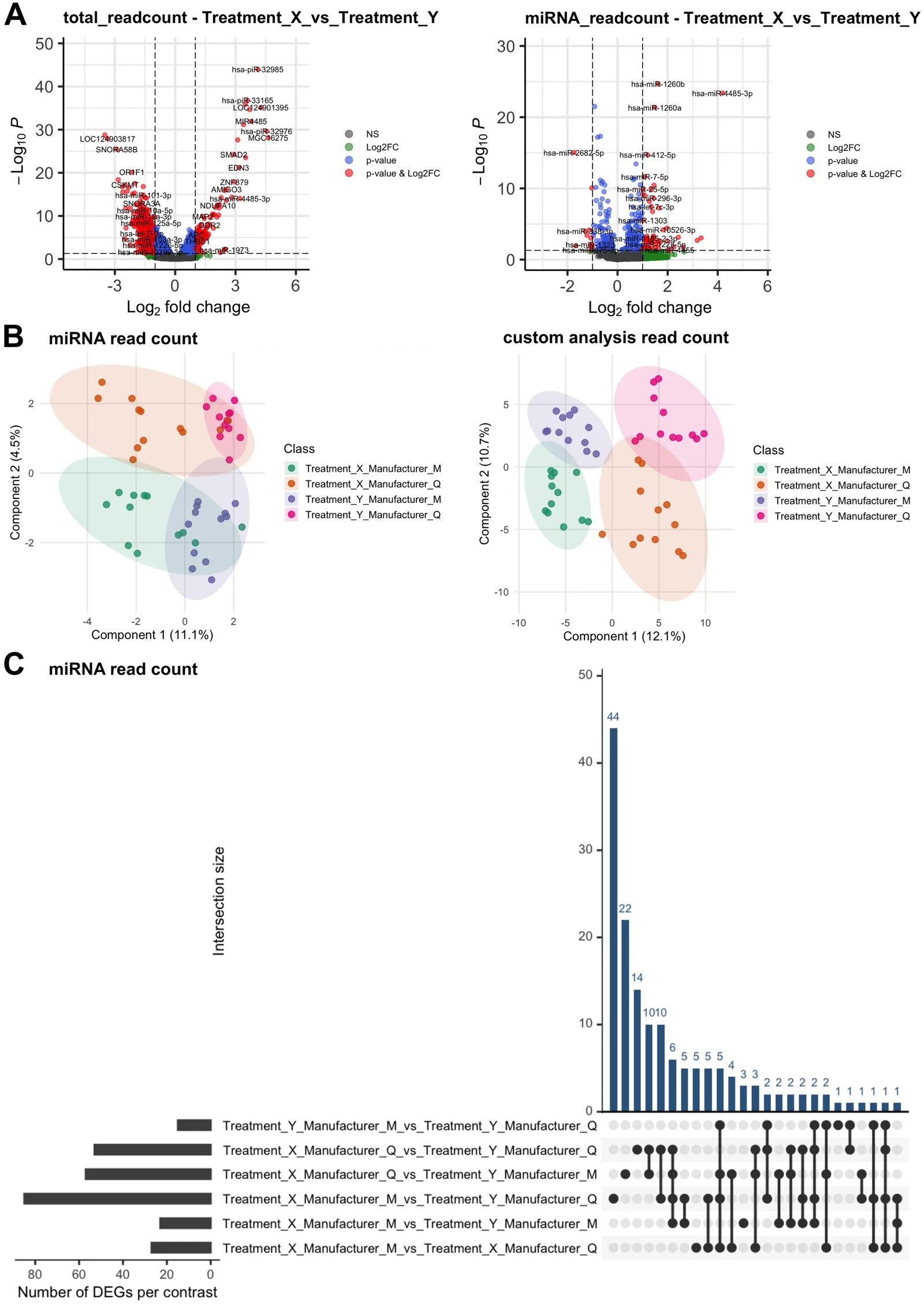
Differential expression and multivariate analysis results generated by caRNAge for the second report. (A) Volcano plots from the caRNAge DGE report for total read count analysis (left) and miRNA read count analysis (right). Features significant by padj only (≤ 0.05, -1 < log2FC < 1) are shown in blue, those meeting FC criteria only (|log2FC| ≥ 1, padj > 0.05) in green, and high-confidence DEGs meeting both criteria (padj ≤ 0.05, |log2FC| ≥ 1) in red; (B) Two-dimensional sPLS-DA plots from the caRNAge DGE report showing sample separation by treatment and EV isolation method, based on miRNA read count (left) and tRF read count analysis (right); (C) UpSet plot showing overlap and uniqueness of DEGs identified based on miRNA read count between the different treatment and EV isolation method combinations. DEGs: Differentially expressed genes; DGE: differential gene expression; FC: fold change; isomiR: miRNA isomer; miRNA: microRNA; NS: non-significant; padj: adjusted P-value; sPLS-DA: sparse partial least squares discriminant analysis; tRF: tRNA-derived fragment; tRNA: transfer RNA; EV: extracellular vesicle; NS: non-significant.

For users interested in finding novel biomarker candidates from the dataset, caRNAge performs sPLS-DA for each comparison. The two-dimensional sample separation plot [Figure 3B] is accompanied by bar charts that visualize the loadings of the genes in each component, representing the degree to which individual features contribute to sample discrimination. In addition, caRNAge reports the parameters selected during sPLS-DA tuning and internally cross-validated classification performance as overall classification error and BER. Receiver operating characteristic (ROC)/AUROC analyses are additionally provided as descriptive summaries of the fitted training data but are explicitly distinguished from cross-validated performance estimates. If more than one comparison is selected for DGE analysis, the dropdown menu above the DESeq2 results table in the report allows users to switch from the volcano plot to an UpSet plot that visualizes the intersection between analyses [Figure 3C]. The UpSet plot is automatically omitted when only one comparison is provided or when only a single analysis yields differentially expressed genes, as observed in the example dataset.

### Third report: miRNA over-representation analysis and target prediction

The final, third report created by caRNAge (*carnage_ORA.html*) summarizes information about miRNA targets and enriched pathways, with options for tissue-specific analyses [Supplementary File 3]. It contains the results of several miRNA ORAs performed using the miEAA platform^[80]^ in various databases for biological functions, pathways, cellular processes, and disease associations. The different analysis results can be selected through dropdown menus. Table 2 provides an overview of the miRNA ORAs performed by caRNAge and the databases used. For each individual analysis, an interactive results table is provided, that includes the ORA category, subcategory, enrichment (over- or under-represented), *P*-value, padj, *q*-value, and the expected and observed counts.

**Table 2:** Overview of the types of miRNA ORAs performed by caRNAge and the databases used for each analysis

**Information**	**Databases/Packages**
Pathway	miRPathDB^[81]^ KEGG^[82-84]^ Reactome^[85]^ WikiPathways^[86]^ miRTarBase^[87-89]^ miRWalk^[90]^
Gene sets & interactions	GO^[91,92]^ biological process & molecular function miRTarBase miRWalk^[90]^ miRandola^[93]^ miRBase^[50,94]^ NPInter^[95,96]^
Localization	GO Cellular component miRTarBase miRWalk^[90]^ RNALocate^[97]^
Tissue/cell type specificity	MiRGeneDB^[98-100]^ miRNA TissueAtlas^[101]^ isomiRdb^[102]^
Structural information	miRandola^[93]^ miRBase^[50,94]^
Disease association & drug interaction	miEAA^[65,80]^ MNDR^[103]^ SM2miR^[104]^

GO: Gene Ontology; isomiR: miRNA isomer; isomiRdb: isomiR database; KEGG: Kyoto Encyclopedia of Genes and Genomes; miEAA: microRNA Enrichment Analysis and Annotation; miRNA: microRNA; MiRGeneDB: microRNA Gene Database; miRPathDB: miRNA Pathway Dictionary Database; miRTarBase: microRNA-target interactions database; MNDR: Mammalian ncRNA-Disease Repository; ncRNA: non-coding RNA; NPInter: The noncoding RNAs and protein related biomacromolecules interaction database; ORA: over-representation analysis; RNALocate: RNA Subcellular Localization Repository; SM2miR: Small Molecules to microRNAs.

A heatmap of log10-transformed consensus tissue expression values across target genes predicted by *TargetScan*^[105]^ for each of the chosen analyses is also included in the third caRNAge report [Figure 4]. While this heatmap provides a general overview at a glance, users can navigate through the different tissue types in detail, each yielding a table with detailed information. However, some miRNAs can bind to the same mRNA target at different sites, leading to two identical interactions occurring in the table with different scores. The cutoff nTPM value for the heatmap and tissue-specific result tables can be adjusted in the *config.yaml* file (default = 50).

**Figure 4:**
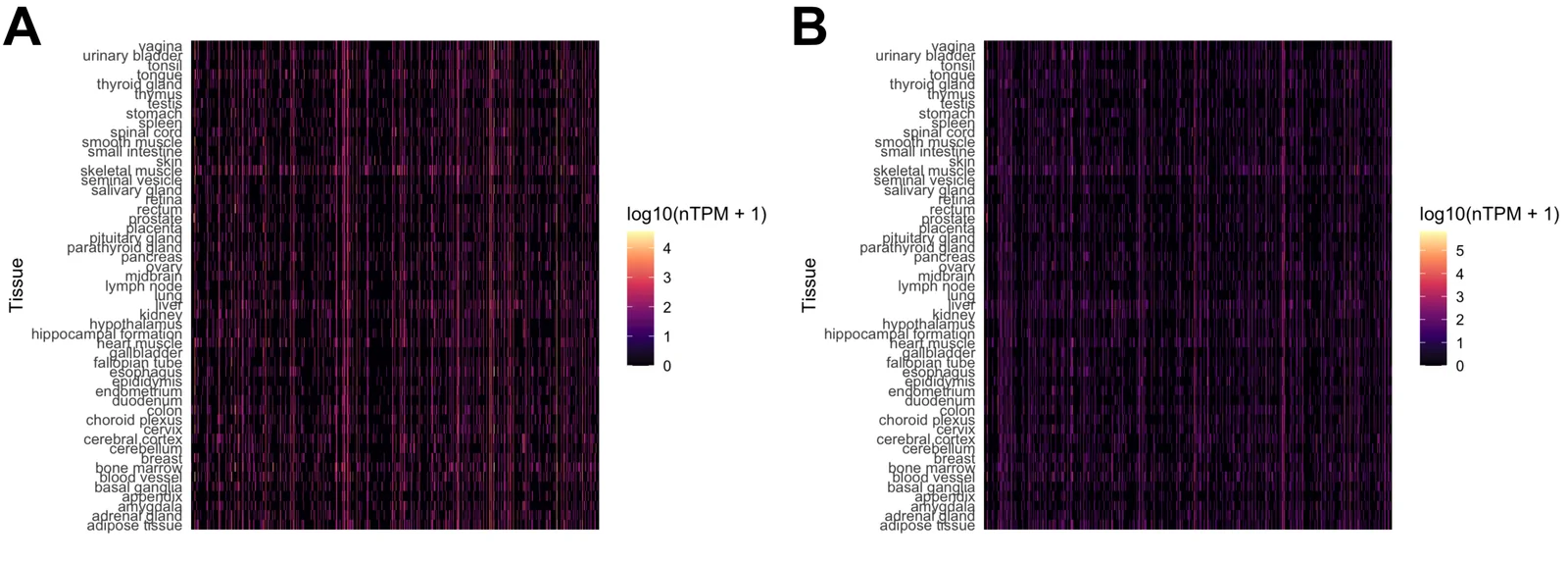
Tissue expression heatmaps of miRNA targets generated from the example dataset by caRNAge for the third report, using log10-transformed nTPM values, based on the DEGs for the two different treatments. (A) Isolation with manufacturer M; (B) Isolation with manufacturer Q. nTPM: Normalized transcripts per million.

To reduce the number of false positive pathway predictions, caRNAge performs tissue-specific pathway ORAs for all analyses, based on all miRNA target genes and filtered by tissues specified in the *config.yaml* file. Each result table shows the pathway name, overlap ratio, *P*-value, padj, odds ratio as a measure for enrichment, Enrichr^[71,106,107]^ combined score, and the list of gene names involved in the pathway. The different databases used for ORA can be navigated through the dropdown menu. An overview of the databases used for tissue-specific ORA is provided in Table 3.

**Table 3:** Overview of the databases used in caRNAge for tissue-specific pathway ORA of miRNA targets

**Information**	**Databases**
Pathway	KEGG^[82-84]^ Reactome^[85]^ WikiPathways^[86]^
Gene sets	GO^[91,92]^ biological process & molecular function
Localization	GO Cellular component
Disease association	DisGeNET^[108]^ OMIM^[109]^ HPO^[110]^

DisGeNET: Disease Gene Network; GO: Gene Ontology; HPO: Human Phenotype Ontology; KEGG: Kyoto Encyclopedia of Genes and Genomes; OMIM: Online Mendelian Inheritance in Man.

## DISCUSSION

### Detection of outliers and bias as a prerequisite for robust normalization and downstream inference

A central objective of caRNAge is not merely to provide multiple normalization options, but to enable systematic detection of outliers and dominant sources of variation prior to statistical testing. In small EV-RNA sequencing experiments, where library composition can differ substantially across samples and RNA classes, undetected outliers or confounding factors can profoundly distort normalization, dispersion estimation, and ultimately differential expression results^[23,111,112]^. Careful exploratory assessment of the data structure is therefore a prerequisite for valid inference.

Normalization in count-based RNA-seq analysis aims to correct for differences in sequencing depth and global composition while preserving biologically meaningful variation^[58]^. However, in small RNA-seq datasets, the relative abundance of RNA classes (e.g., miRNAs, tRFs, other RNA fragments) can vary markedly between samples due to biological or technical factors. When normalization is restricted to a subset of RNA species, such as miRNAs alone, the estimated size factors reflect only that subset’s composition. Samples in which this subset is unusually enriched or depleted may therefore exhibit extreme scaling factors relative to the remainder of the cohort. In caRNAge, inspection of size-factor distributions across alternative feature scopes enables detection of such composition-driven outliers, a feature notably absent in most other pipelines where such distributions are typically not exposed or visualized. A sample with consistently extreme size factors within a given scope may represent (i) a true biological deviation; (ii) technical bias such as extraction efficiency or amplification skew; or (iii) library imbalance caused by overrepresentation of a limited number of sequences. In the example dataset [Supplementary File 1], we observed substantial variation in size factors for multiple samples based on the normalization strategy used. For instance, samples 12, 18, and 23 displayed pronounced discrepancies in size factors when normalized to the total read count or tRF read count compared to the other normalization strategies. Across the dataset, size factors ranged from 0.1 (sample 3, *miRNA_readcount* and *isomiR_readcount*) to 6.82 (sample 36, *miRNA_readcount*). Importantly, large size factors are not intrinsically indicative of incorrect normalization; rather, they signal that the underlying library composition deviates substantially from the majority of samples. Identifying such samples prior to modeling is critical, as disproportionate scaling can influence dispersion estimation and shrinkage behavior in negative binomial frameworks^[58,113]^. In contrast to widely used small RNA-seq pipelines such as miRge3.0, sRNAnalyzer, sRNAflow, SPAR, and nf-core/smrnaseq, which typically provide a single predefined normalization strategy with limited transparency, caRNAge explicitly exposes normalization behavior across multiple feature scopes and enables direct comparison of their effects on scaling [Table 1].

Beyond individual outliers, the global data structure must be evaluated to determine which variables drive inter-sample similarity. Unsupervised methods such as hierarchical clustering and PCA are widely recommended for detecting latent structure and potential confounding in high-dimensional transcriptomic data^[111,114]^. If samples cluster primarily according to a technical covariate - such as isolation method, sequencing batch, or replicate^[115]^ - rather than the primary biological factor of interest, this indicates that the technical variable explains a substantial fraction of the variance. In the example dataset, hierarchical clustering revealed that cell line identity constituted the dominant axis of variation prior to correction [Figure 2C]. This pattern is not unexpected, as distinct cell lines often exhibit extensive transcriptional differences. However, when the scientific objective is to compare treatment effects across cell lines, strong cell-line-driven clustering highlights a potential confounding structure: treatment-associated variation may be masked by between-line differences. Such scenarios have been shown to compromise reproducibility and to inflate false discoveries if not appropriately modeled or corrected^[111,114]^. caRNAge facilitates explicit assessment of this issue by providing clustering and PCA visualizations under alternative normalization scopes. If the dominant clustering pattern persists across scopes, the variable likely represents a fundamental biological axis of variation. If clustering patterns shift substantially depending on which RNA classes are used for normalization, this suggests that compositional differences between RNA biotypes are influencing the global similarity structure. Whereas most existing pipelines focus primarily on generating count matrices and differential expression outputs, caRNAge integrates exploratory diagnostics as a central component of the workflow, enabling users to iteratively assess how normalization choices influence global data structure prior to statistical modeling.

Once a potential confounder has been identified, it is essential to determine whether accounting for this factor alters the apparent data structure. Batch-effect correction methods, such as those implemented in limma^[59]^, have been shown to reduce systematic technical variation when appropriately applied^[114,116]^. In caRNAge, PCA plots before and after removal of specified covariates enable visualization of how strongly the identified bias shapes the expression space. In the example dataset, PCA prior to correction separated samples predominantly by cell line [Figure 2D, top]. After adjusting for this variable for visualization purposes, treatment-associated separation became more apparent [Figure 2D, bottom]. This indicates that the treatment signal was present but partially obscured by a stronger cell-line effect. Such findings underscore the importance of incorporating relevant covariates into the statistical design rather than relying solely on uncorrected clustering results. Failure to model dominant confounders has been repeatedly identified as a major source of irreproducibility in high-throughput studies^[111,114]^. It is critical to emphasize that visualization-based correction does not replace formal modeling. Rather, it serves as a diagnostic tool to assess whether the intended biological contrast can be isolated once major confounders are accounted for. Differential expression testing must subsequently include appropriate design terms to control for these variables within the generalized linear modeling framework^[58]^. This explicit coupling of normalization, visualization, and covariate-aware interpretation is not systematically implemented in current small RNA-seq pipelines, which generally require users to export data into external tools for such assessments.

#### Conceptual implications for normalization strategy selection

The choice of which RNA species to include as input for normalization should be guided primarily by the biological question under investigation. A study focused on EV-miRNA regulation necessarily requires inclusion of miRNA counts, whereas broader exploratory analyses may justify inclusion of additional small RNA classes. However, exploratory diagnostics demonstrate that the selected feature scope can influence both scaling behavior and the apparent structure of the dataset.

Therefore, the normalization strategy should not be selected solely based on numerical dispersion or minimal variance. Instead, it should be evaluated in the context of:

1. Whether extreme scaling identifies biologically implausible outliers; 2. Whether clustering reveals dominance of unwanted technical variables; 3. Whether removal or modeling of identified biases reveals the biological contrast of interest.

By integrating size-factor summaries, clustering heatmaps, and PCA before and after covariate adjustment, caRNAge operationalizes established recommendations for bias detection in high-dimensional transcriptomic studies^[58,111,114]^. This structured exploratory phase reduces the risk of proceeding to differential testing under an inappropriate normalization scope or with unmodeled confounding, thereby strengthening the validity and interpretability of downstream results. By embedding these diagnostic layers directly into the analysis workflow, caRNAge extends beyond conventional pipelines that treat normalization as a fixed preprocessing step, instead framing it as a hypothesis-dependent and data-driven decision process.

### Biotype-resolved analysis enhances biomarker specificity but requires biological and statistical restraint

Small RNA-Seq captures a heterogeneous population of molecules that extends beyond canonical mature miRNAs, including isomiRs, tRFs, piRNAs, and additional small non-coding RNAs (ncRNAs) species present in cells or associated with EVs^[117,118]^. While many analytical workflows collapse this complexity and rely solely on gene-level miRNA counts, increasing evidence suggests that biotype- and isoform-resolved analyses can uncover biologically meaningful signals that are otherwise obscured^[11,12]^. caRNAge enables differential expression and supervised modeling at multiple levels of granularity, allowing users to evaluate the contribution of canonical miRNAs, isomiRs, and tRFs separately or in combination.

IsomiRs - sequence variants of canonical miRNAs differing in 5’ or 3’ termini or internal edits - are not merely technical artifacts but can exhibit distinct targeting properties and functional roles^[11,12]^. In particular, 5’-end variations alter the seed sequence and thereby redirect target recognition, potentially modifying regulatory networks in a context-dependent manner^[11]^. Moreover, non-templated nucleotide additions have been shown to influence intracellular localization and exosomal enrichment, suggesting that specific isomiRs may carry biologically distinct export or retention signals^[16]^. Similarly, tRFs represent an evolutionarily conserved class of regulatory RNAs with documented functional roles, including association with Argonaute proteins and modulation of translation^[119-121]^. Distinct tRF sub-classes differ in their origin (5’-derived, 3’-derived, or internal fragments) and biological activity. In EV studies, selective enrichment of specific tRF lengths and subclasses has been reported, supporting their potential utility as biomarkers^[15]^. Disease-associated alterations in RNA processing pathways may further shift the abundance of specific isomiRs and tRFs, rendering them more informative than their canonical counterparts in certain contexts^[122,123]^. Taken together, these studies provide a mechanistic rationale for extending biomarker discovery beyond canonical miRNA counts and considering biotype-resolved feature spaces.

Length distribution plots constitute a critical first step in determining which RNA classes are robustly represented in a given dataset. Distinct small RNA species exhibit characteristic length peaks - miRNAs typically around 21-23 nucleotides, certain tRF subclasses around 30-34 nucleotides, and for example Y RNA fragments at defined intermediate lengths. Inspection of mean mapping-length distributions allows assessment of whether these peaks are consistently present across biological groups and replicates [Figure 2B]. If a specific length class shows consistent and treatment-associated shifts in abundance, this supports its inclusion in downstream modeling. Conversely, irregular or highly sample-specific peaks may indicate technical artifacts (e.g., adapter contamination, degradation fragments) rather than biologically meaningful species. Thus, length profiling serves as an empirical guide to determine whether isomiR- or tRF-focused analyses are biologically justified in a given dataset, as expanding the feature space from canonical miRNAs to isomiRs and tRFs increases both dimensionality and biological resolution.

In the EV-RNA example dataset, sPLS-DA demonstrated that treatment separation could be achieved using different feature scopes [Supplementary File 2]. However, the identity of selected discriminative features differed substantially depending on whether canonical miRNAs, isomiRs, tRFs, or combined classes were analyzed [Supplementary Table 1]. For instance, total read count analysis selected six miRNAs, three piRNAs, two uncharacterized ncRNAs, a long non-coding RNA (lncRNA), and three protein-coding genes. Interestingly, only three of these six miRNAs were also selected by the model based on miRNA-focused analyses: The mature miRNAs miR-4485-3p, miR-181a-5p, and miR-196a-5p. When tRNA counts were included in the analysis alongside miRNAs, the resulting sPLS model selected 11 tRNAs as discriminating factors. Approximately half of the miRNAs were shared between that model and the one based on miRNA read count alone, while the remaining were unique to each analysis, further indicating the method’s substantial sensitivity to the composition of the available feature space. Further, isomiR-based analysis identified 14 isomiRs corresponding to six distinct miRNAs that were detected in the miRNA-based analyses, as well as 61 isomiRs from 41 additional miRNAs. When adding tRF read count information to the isomiR analysis, the resulting sPLS model primarily selected isomiRs already found in the isomiR-only model, along with four unique isomiRs and two tRFs. The same two tRFs were also selected by the tRF-only model, which otherwise showed no overlap with any of the other analyses, underscoring the distinct biological signal that tRFs can provide. Such side-by-side evaluation of feature-space-dependent model behavior is generally not supported in existing workflows, where analyses are typically restricted to a single annotation level per run.

These examples underscore an important principle: biomarker signatures are conditional on the feature space from which they are derived. Total read-count analyses may prioritize highly abundant or broadly variable transcripts, potentially overlooking isoform-specific signals. Conversely, isomiR- or tRF-specific analyses may identify variants that more precisely capture treatment-associated biology. Such subclass-specific features may enhance biomarker specificity, particularly if disease- or context-dependent RNA processing mechanisms selectively alter isoform abundance^[122,123]^. In contrast to many established pipelines that predominantly summarize reads at the canonical miRNA level, caRNAge natively supports parallel, biotype-resolved analyses, allowing systematic comparison of miRNAs, isomiRs, tRFs, and combined feature spaces within a unified framework.

While expanded feature resolution can increase sensitivity, it also introduces statistical challenges. As the example data shows [Supplementary File 2 and Supplementary Table 1], isoform-level analyses substantially inflate the number of candidate features, increasing the risk of overfitting in supervised models, particularly in datasets with limited sample size. High-dimensional feature spaces can produce apparently strong class separation that does not generalize to independent cohorts unless appropriate cross-validation and regularization are applied. Overfitting is a well-recognized risk in biomarker development, especially in omics-based classification studies^[124,125]^. Furthermore, the biological interpretation of isoform-level biomarkers requires caution. Some sequence variants may arise from stochastic processing variation, sequencing bias, or mapping ambiguity rather than functional diversification. Collapsing biologically distinct isomiRs into canonical miRNA counts can obscure relevant biology^[12,126]^, but, conversely, over-interpreting poorly validated variants may inflate false discovery rates. By integrating feature selection, visualization, and validation-aware workflows, caRNAge enables more controlled exploration of high-dimensional feature spaces compared to pipelines that primarily emphasize preprocessing and annotation [Table 1].

Therefore, high-resolution analyses should be accompanied by^[19,20,127-130]^:

1. Stringent cross-validation and, where possible, external validation cohorts; 2. Independent experimental confirmation of candidate biomarkers, e.g., using reverse transcription quantitative polymerase chain reaction (RT-qPCR) or reverse transcription digital polymerase chain reaction (RT-dPCR); 3. Biological plausibility assessment based on known processing mechanisms and functional evidence, e.g., by using caRNAges pathway ORA capabilities.

#### Conceptual implications for biomarker discovery strategies

The example analyses illustrate that canonical miRNAs represent merely a subset of the regulatory information embedded within small RNA-seq data. In certain contexts, specific isomiRs or tRF subclasses may provide improved discrimination between biological states. However, the choice to include additional RNA species should be guided by empirical evidence from length distributions, expression stability, and model robustness rather than by default expansion of the feature space. By enabling parallel analyses across multiple RNA classes and visualizing their impact on sample separation, caRNAge supports informed decision-making regarding feature inclusion. This structured comparison encourages researchers to evaluate whether subclass-specific signals enhance classification performance while maintaining statistical rigor and biological credibility. Ultimately, integrating isomiR and tRF analyses can increase biomarker specificity and mechanistic insight, provided that feature selection is constrained by robust validation and awareness of the risk of overfitting. Such an approach aligns with current recommendations for reproducible biomarker development in high-dimensional transcriptomic studies^[124,125]^.

### Tissue-informed target restriction enhances biological interpretability of miRNA pathway analysis

Computational miRNA target prediction remains intrinsically prone to inflation of candidate interactions. Because miRNA seed sequences are short and partially degenerate, *in silico* prediction algorithms frequently generate large numbers of putative targets per miRNA^[68,131,132]^. When these extensive target lists are used as input for ORA, enrichment results can be dominated by combinatorial target multiplicity rather than by biologically plausible regulatory events. Consequently, unrestricted miRNA target ORA often yields a high number of statistically significant pathways, many of which may not be functionally relevant in the biological context under investigation.

A fundamental biological constraint, however, is frequently underutilized in such analyses: miRNAs can exert regulatory effects only on mRNAs expressed in the same cellular or tissue environment. Predicted interactions involving genes that are not expressed in the relevant tissue are unlikely to have functional consequences. Incorporating tissue-level expression information into target filtering therefore represents a principled strategy to reduce false-positive functional inference and to increase contextual relevance^[105,133]^. caRNAge operationalizes this concept by allowing users to restrict predicted miRNA-mRNA interactions to genes that exceed a configurable expression threshold in selected tissues, based on consensus expression resources that integrate large-scale transcriptomic datasets^[69,70]^. This approach does not alter the underlying prediction algorithm; rather, it constrains downstream interpretation to interactions that are biologically accessible in the tissue(s) of interest.

In the example dataset, heatmap visualization of log-transformed tissue expression values for predicted EV-miRNA target genes demonstrates that a subset of targets is broadly expressed across tissues, whereas other genes show tissue-specific or -enriched expression patterns [Figure 4]. When restricting interactions to genes with nTPM values above the predefined threshold, the number and identity of retained miRNA-mRNA pairs varied substantially between tissues. A more detailed examination of the data [Supplementary File 3] reveals that tissues with globally high expression of target genes in the heatmap (such as liver, skeletal muscle, and blood vessels) had approximately 1,800-2,400 predicted miRNA-mRNA combinations with an mRNA nTPM value over the threshold of 50. In contrast, substantially fewer (1,200-1,400) miRNA-mRNA combinations were predicted in immune-related tissues (spleen, lymph nodes). This quantitative reduction is not a loss of information per se; rather, it reflects increased biological specificity. By excluding targets not expressed in the selected tissue, the regulatory network becomes more focused and potentially more interpretable. Importantly, tissue restriction can also alter the ranking of predicted interactions. When interactions were sorted by TargetScan context++ score, certain high-scoring interactions were retained across multiple broadly expressing tissues, whereas others emerged as tissue-specific top-ranked candidates. For example, interactions involving genes such as XPO1 - an exportin frequently dysregulated in cancer^[134]^ - were retained only in specific tissues, highlighting how tissue context can reveal distinct regulatory hypotheses that would be diluted in an unrestricted analysis.

The influence of tissue restriction becomes particularly evident at the pathway level. When ORA was performed using all predicted target genes, a large number of significantly enriched KEGG pathways were identified (73 and 85 for isolates from EV isolation kit manufacturers M and Q, respectively). Restricting the analysis to genes expressed in the cerebral cortex - a biologically relevant microenvironment for glioblastoma - substantially reduced the number of significant pathways to 37 and 27, respectively. Approximately half of the previously enriched pathways were no longer detected after tissue restriction. This reduction should not be interpreted as a loss of analytical sensitivity; rather, it likely reflects removal of pathways driven by targets not expressed in the tumor’s anatomical context. Notably, cortex-restricted analyses exhibited improved enrichment of neural and brain-associated pathways, enhancing tissue-level interpretability. While existing pipelines often provide miRNA target prediction as a downstream add-on [Table 1], caRNAge uniquely incorporates tissue-informed filtering directly into the analytical workflow, thereby linking target prediction with biological context in a systematic manner.

In the specific context of EV research, tissue selection requires additional consideration. EV-associated miRNAs may act not only in the tissue of origin but also in distant recipient tissues^[1]^. Consequently, caRNAge allows flexible selection of multiple tissues reflecting hypothesized EV uptake sites. When restricting analysis to lymph node-expressed targets, immune-related pathways - such as B cell receptor signaling and antigen processing - emerged more prominently, consistent with potential immunomodulatory roles of tumor-derived EVs. Thus, tissue-informed filtering can both reduce spurious enrichments and uncover biologically plausible, context-specific regulatory effects.

#### Conceptual implications for miRNA functional inference

These results underscore a broader conceptual principle: miRNA target prediction is necessary but insufficient for biological interpretation. Without contextual constraints, enrichment analyses risk conflating computational possibility with physiological relevance. Integrating tissue expression data introduces a biologically grounded filter that aligns computational predictions with the spatial constraints of gene regulation. From a methodological perspective, tissue restriction functions as a structured reduction of the hypothesis space. By limiting analysis to expressed targets, it reduces the effective multiple-testing burden and enhances the interpretability of pathway enrichment results. At the same time, it preserves flexibility by allowing users to tailor tissue selection to the biological system under study, including scenarios involving inter-tissue communication such as EV-mediated signaling.

However, tissue-informed restriction should not be applied indiscriminately. Expression atlases represent averaged measurements across individuals and cell types and may not fully capture intra-tissue heterogeneity or disease-specific transcriptional rewiring. Therefore, tissue filtering should be regarded as a biologically informed approximation rather than a definitive exclusion criterion. Whenever possible, tissue expression assumptions should be corroborated with experimental data from the specific model system. In summary, tissue-specific target restriction enhances the biological credibility of miRNA pathway analysis by aligning predicted regulatory interactions with spatial expression constraints. In the EV-RNA example dataset, this approach refined pathway interpretation and revealed tissue-dependent regulatory hypotheses that were obscured in global analyses. By embedding tissue selection directly into the analytical workflow, caRNAge supports context-aware biomarker discovery and mitigates overinterpretation of computationally inflated target networks.

### Independent validation using a complex T-cell small RNA-Seq dataset

To assess the generalizability of caRNAge beyond the glioblastoma EV example dataset, we independently re-analyzed GSE181216 from Céspedes *et al*.^[78]^ [Supplementary Files 4-7]. The complete dataset comprised 185 sequencing libraries, including intracellular CD4^+^ and CD8^+^ T-cell RNA as well as constitutive EVs, tSVs, and background controls. Exploratory analysis with caRNAge revealed partial confounding between sequencing platform and biological source, because the NextSeq subset consisted of intracellular samples. Sequencing platform could therefore not be interpreted as an independently correctable technical effect in analyses directly comparing cellular and extracellular material. Formal differential-expression analysis was consequently performed on the donor-matched 120-library subset, with donor explicitly incorporated into the statistical model.

Direct comparison of constitutive EV and tSV miRNA profiles identified 59 and 67 differentially abundant miRNAs in CD4^+^ and CD8^+^ samples, respectively (padj ≤ 0.05, |log2FC| ≥ 1), confirming pronounced differences between both vesicle populations. Comparison with the vesicle-specific miRNAs reported in Supplementary Files 4-6 from data by Céspedes *et al*.^[78]^ identified concordant candidates including miR-21-3p and miR-221-3p in CD4^+^ EVs, miR-184 in CD4^+^ tSVs, miR-486-5p and miR-744-5p in CD8^+^ EVs, and miR-1248 and miR-142-3p in CD8^+^ tSVs. Exact feature-level overlap was partial, consistent with the different analytical definitions: the original study classified miRNAs according to enrichment and conservation across vesicle, cellular, donor and sequencing-run contexts, whereas caRNAge performed direct donor-adjusted EV-tSV differential-expression testing.

Despite this difference at the individual-miRNA level, functional concordance was pronounced. Of the 18 specific signaling and functional categories highlighted in the original study, 15 were independently recovered as significant KEGG pathways in the unfiltered caRNAge EV-tSV target analyses, including Ras, MAPK, AMPK, PI3K-Akt, IL-17, TNF, JAK-STAT, FoxO and PD-L1/PD-1 checkpoint signaling. When tissue-informed target analyses were additionally considered, B-cell receptor signaling was also recovered, resulting in concordance for 16 of 18 original functional categories. These results support the original conclusion that distinct EV- and tSV-associated miRNA populations converge on substantially overlapping immune-regulatory functions. Tissue-informed analysis additionally altered the interpretation of specific immune pathways. For example, in the CD8^+^ tSV-associated target set, T-cell receptor signaling changed from padj = 0.092 using unrestricted predicted targets to padj = 0.0042 after restriction to lymph-node-expressed genes. Similarly, PD-L1/PD-1 checkpoint signaling changed from padj = 0.195 to padj = 0.0042. Thus, the independent dataset illustrates both the ability of caRNAge to recover previously reported biological patterns and the additional value of integrating experimental-design diagnostics and tissue context into downstream interpretation.

The independent GSE181216 analysis further illustrates that reproducibility across analysis workflows should not be assessed solely by exact agreement of individual miRNA lists. The original study and caRNAge use distinct definitions of vesicle-associated miRNAs and different statistical frameworks, resulting in only partial feature-level overlap. Nevertheless, the extensive concordance of downstream immune-regulatory pathways indicates that the principal biological conclusions are robust to these analytical differences. At the same time, caRNAge provided additional information by exposing confounding between sequencing platform and biological source and by allowing tissue-informed restriction of predicted targets. These results exemplify the intended role of caRNAge as a decision-support framework in which exploratory assessment of experimental structure and biological context informs subsequent statistical and functional analysis.

### Limitations and future perspectives

Despite the comprehensive analytical framework provided by caRNAge, several limitations should be considered when interpreting its current scope and future applicability. First, caRNAge was developed for small RNA-seq data generated using Illumina sequencing platforms and currently relies on Illumina-compatible input formats and sequencing characteristics. Although the general analytical concepts are not restricted to this single sequencing technology, differences in read length, error profiles, and library preparation strategies between platforms may influence adapter trimming, alignment, and small RNA annotation^[111]^. Therefore, expansion towards a platform-independent framework would require systematic evaluation and optimization using datasets generated from alternative sequencing technologies. Nevertheless, Illumina-based sequencing remains the most widely used approach for small RNA-Seq experiments, and the current implementation was designed to address the analytical challenges most commonly encountered in this context.

A further limitation is that caRNAge currently focuses on the analysis of small RNA abundance rather than sequence-level epitranscriptomic modifications. RNA editing and other nucleotide modifications represent an emerging layer of small RNA regulation that may influence RNA stability, target recognition, and biological function^[135]^. However, reliable detection of such modifications requires dedicated computational approaches and appropriate sequencing strategies to distinguish biological variation from technical sequencing errors^[136]^. Future extensions of caRNAge could incorporate analysis of small RNA editing and related sequence variations to further increase the biological resolution of biomarker discovery.

The interpretation of downstream functional analyses is also dependent on the availability and completeness of external reference resources. For example, tissue-informed miRNA target restriction relies on expression atlases that represent averaged transcriptomic profiles and may not fully capture disease-specific alterations, cellular heterogeneity, or cell-type-specific expression patterns^[137,138]^. Therefore, tissue filtering should be regarded as a biologically informed approximation rather than a definitive exclusion criterion. Integration of disease-specific and single-cell expression resources may further improve the contextual interpretation of predicted miRNA targets in future versions.

A formal benchmark of runtime, peak memory consumption, and computational scalability was not performed in the present study. The computationally intensive preprocessing steps of caRNAge rely predominantly on established tools such as Cutadapt, Bowtie, FastQC, and MINTmap, while the workflow provides their integration with downstream R-based analyses. Absolute resource requirements are therefore strongly dependent on sequencing depth, sample number, enabled annotation and downstream modules, parallelization, storage performance, and the underlying hardware. The successful analysis of the substantially larger independent validation dataset demonstrates practical applicability to larger studies but should not be interpreted as a systematic computational scalability benchmark.

Finally, although caRNAge provides visualization and diagnostic tools to identify potential sources of variation, these analyses do not replace appropriate statistical modeling. Batch-effect visualization and correction support the interpretation of data structure but should be complemented by inclusion of relevant covariates in differential expression models^[139]^. Similarly, biomarker candidates identified from high-dimensional small RNA feature spaces require independent validation and careful biological interpretation to ensure robustness and reproducibility^[127]^. Likewise, the internally cross-validated performance metrics reported for sPLS-DA provide an assessment of model stability within the analyzed cohort but cannot substitute for evaluation of predictive performance in an independent validation cohort.

### Conclusion

caRNAge represents a comprehensive pipeline for small RNA biomarker discovery that encompasses the entire analytical workflow, from preprocessing to downstream functional interpretation. To the best of our knowledge, no other existing pipeline combines all those features together. Importantly, our pipeline incorporates several specialized features not commonly integrated into existing small RNA-seq pipelines, including dedicated tRF mapping using MINTmap, flexible and customizable normalization strategies, and integrated detection and correction of batch effects. Furthermore, it provides advanced support for biomarker discovery studies through supervised multivariate approaches such as sPLS-DA, combined application of miRNA target prediction, pathway ORA, and the ability to restrict analyses to tissue-specific contexts. Its user-friendly design enables researchers without advanced bioinformatics expertise to apply state-of-the-art analytical strategies within their experimental studies. The EV-miRNA use cases presented in this article illustrate several key strengths of caRNAge, including bias detection and correction, the identification of non-miRNA small RNA biomarkers, and the integration of tissue-specific analyses. Together, these features may enhance robustness, biological specificity, and interpretability of the resulting EV-associated biomarker signatures. We demonstrated how caRNAge facilitates the selection of an appropriate normalization strategy and supports the identification and correction of the cell line-specific biases. In addition, we highlighted its capabilities to detect diverse classes of small RNA biomarker candidates, such as miRNAs, tRNAs and their variations (tRFs and isomiRs), through DGE analysis and showed that different analyses can yield partially distinct biomarker sets. Finally, we emphasized the pipeline’s functionality for tissue-specific miRNA target prediction and over-representation analysis, which resulted in fewer but more biologically contextualized targets and pathways when tissue expression data were incorporated. Overall, caRNAge is a flexible and customizable framework for the discovery of miRNA and other small RNA biomarkers from any small RNA-Seq dataset, including EV-RNA studies. By generating accessible reports and requiring only limited expertise in bioinformatics and statistics, it is specifically designed to support researchers with predominantly experimental and biological backgrounds. In this way, caRNAge may help to potentially promote more reproducible, transparent, and biologically meaningful small RNA biomarker studies across diverse research contexts.

## DECLARATIONS

### Availability of data and materials

The pipeline is freely available at https://gitlab.lrz.de/kirchner/carnage. The dataset used as an example for this article was first published in the original article by Schuster *et al*.^[46]^ and is available in the ENA repository under accession number PRJEB112197.

### Copyright

© The Author(s) 2026.
