# Supplementary material for: Comprehensive and bias-aware analysis of small RNA NGS data for biomarker detection with caRNAge
Source: Extracell Vesicles Circ Nucl Acids. 2026 Sep 24;7(3):1554–81. doi: 10.20517/evcna.2026.78 (PMC13678173; doi:10.20517/evcna.2026.78)
Supplement: Supplementary file 1 [file evcna-7-3-1554-SupplementaryMaterials.zip › evcna7078-SupplementaryMaterials/Supplementary File 2.html]

caRNAge - comprehensive analysis of small RNA gene expression


# caRNAge - comprehensive analysis of small RNA gene expression

### Differential gene expression

#### 13 February, 2026

## Purpose of this overview (Disclaimer)

This report is meant to give you a complete overview of
differentially expressed genes for the group comparison you have chosen
with their respective sources of bias you have identified in the data
overview. Please refer to the guidelines for best practice of caRNAge
and the respective manuals and vignettes of the applied tools. You are
strongly advised to thoroughly check all available information in
addition to this data overview. caRNAge is built upon the work of many
excellent scientists who developed the necessary tools to process small
RNA NGS data, so please make sure to cite the corresponding original
authors in addition to caRNAge when publishing your work.

If you encounter any technical problems, feel free to contact the
corresponding author at bkirchner@tum.de.

## Data processing

Raw sequencing reads were processed with Cutadapt to trim
adapter sequences from 3’ ends if present and to remove degraded reads
(shorter than 16nt). Trimmed reads were analysed with FastQC
to evaluate phred scores and read lengths before aligning them against
the human tRNA (GtRNAdb), miRNA
(miRBase) including isomiRs (isomiRROR), piRNAs (piRNAdb) and refseq transcriptome
sequences (refseq)
with bowtie.
Aligned reads were then analyzed to generate length and mapping
distributions as well as reads counts. Finally, gene read counts were
processed with R and mainly DESeq2
to normalize and transform data and calculate expression changes.
Differential gene expression is visualized as volcano plots with the
help of the EnhancedVolcano
package

Fig 1: Simplified workflow for NGS data analysis

|  | Tools | Version | Parameters |
| --- | --- | --- | --- |
| Trimming | Cutadapt | 5.1 | -a AGATCGGAAGAGCAC, -e 0.1 -m 16 |
| Quality Control | FastQC | 0.12.1 |  |
| Alignment | bowtie | 1.3.1 | –quantMode TranscriptomeSAM –outSAMtype BAM SortedByCoordinate; mismatches tRNA: 1 , isomiR: 3 , transcriptome: 1 |
| Normalisation | DESeq2 | R version 4.5.2 (2025-10-31), DESeq2 1.50.2 | ~Cell\_Line + Treatment\_Isolation |
| DGE calling | DESeq2 | R version 4.5.2 (2025-10-31), DESeq2 1.50.2 |  |
| Visualization | EnhancedVolcano | R version 4.5.2 (2025-10-31), EnhancedVolcano 1.28.2 |  |
| Supervised Clustering | mixOmics | R version 4.5.2 (2025-10-31), mixOmics 6.34.0 | Genes per component: comp1=5, comp2=10, comp3=5 , Distance metric: centroids.dist , Minimum mean expression: 10 , Maximum number of genes sorted by Variance: 1000 Crossfold validation: Mfold , Validation repeats: 100 |

## Sample overview

The starting point for all analyses, is a well designed study design.
In addition to the classification of the main research question, it
should also incorporate all available information that could in any way
affect the outcome. This includes potential confounding factors such as
batch effects as well as statistical relevant information such as
patient cohorts or cell lines. Please make sure that the classifications
given in your *design.tsv* are correct and that all relevant
information is present.

### Study design

The row names and “Sample” column should be matching and indicate
that samples in design were correctly assigned in the analysis. A column
named “replaceable” maybe present if groups are large enough (>7) to
replace single outlier values.

|  | Sample | Replicate | Cell\_Line | Treatment | Isolation | Treatment\_Isolation |
| --- | --- | --- | --- | --- | --- | --- |
| Sample\_1 | Sample\_1 | R1 | Cell\_Line\_A | Treatment\_X | Manufacturer\_M | Treatment\_X\_Manufacturer\_M |
| Sample\_10 | Sample\_10 | R10 | Cell\_Line\_C | Treatment\_Y | Manufacturer\_M | Treatment\_Y\_Manufacturer\_M |
| Sample\_11 | Sample\_11 | R11 | Cell\_Line\_C | Treatment\_X | Manufacturer\_Q | Treatment\_X\_Manufacturer\_Q |
| Sample\_12 | Sample\_12 | R12 | Cell\_Line\_C | Treatment\_Y | Manufacturer\_Q | Treatment\_Y\_Manufacturer\_Q |
| Sample\_13 | Sample\_13 | R13 | Cell\_Line\_D | Treatment\_X | Manufacturer\_M | Treatment\_X\_Manufacturer\_M |
| Sample\_14 | Sample\_14 | R14 | Cell\_Line\_D | Treatment\_Y | Manufacturer\_M | Treatment\_Y\_Manufacturer\_M |
| Sample\_15 | Sample\_15 | R15 | Cell\_Line\_D | Treatment\_X | Manufacturer\_Q | Treatment\_X\_Manufacturer\_Q |
| Sample\_16 | Sample\_16 | R16 | Cell\_Line\_D | Treatment\_Y | Manufacturer\_Q | Treatment\_Y\_Manufacturer\_Q |
| Sample\_17 | Sample\_17 | R17 | Cell\_Line\_E | Treatment\_X | Manufacturer\_M | Treatment\_X\_Manufacturer\_M |
| Sample\_18 | Sample\_18 | R18 | Cell\_Line\_E | Treatment\_Y | Manufacturer\_M | Treatment\_Y\_Manufacturer\_M |
| Sample\_19 | Sample\_19 | R19 | Cell\_Line\_E | Treatment\_X | Manufacturer\_Q | Treatment\_X\_Manufacturer\_Q |
| Sample\_2 | Sample\_2 | R2 | Cell\_Line\_A | Treatment\_Y | Manufacturer\_M | Treatment\_Y\_Manufacturer\_M |
| Sample\_20 | Sample\_20 | R20 | Cell\_Line\_E | Treatment\_Y | Manufacturer\_Q | Treatment\_Y\_Manufacturer\_Q |
| Sample\_21 | Sample\_21 | R21 | Cell\_Line\_F | Treatment\_X | Manufacturer\_M | Treatment\_X\_Manufacturer\_M |
| Sample\_22 | Sample\_22 | R22 | Cell\_Line\_F | Treatment\_Y | Manufacturer\_M | Treatment\_Y\_Manufacturer\_M |
| Sample\_23 | Sample\_23 | R23 | Cell\_Line\_F | Treatment\_X | Manufacturer\_Q | Treatment\_X\_Manufacturer\_Q |
| Sample\_24 | Sample\_24 | R24 | Cell\_Line\_F | Treatment\_Y | Manufacturer\_Q | Treatment\_Y\_Manufacturer\_Q |
| Sample\_25 | Sample\_25 | R1 | Cell\_Line\_A | Treatment\_X | Manufacturer\_M | Treatment\_X\_Manufacturer\_M |
| Sample\_26 | Sample\_26 | R2 | Cell\_Line\_A | Treatment\_Y | Manufacturer\_M | Treatment\_Y\_Manufacturer\_M |
| Sample\_27 | Sample\_27 | R3 | Cell\_Line\_A | Treatment\_X | Manufacturer\_Q | Treatment\_X\_Manufacturer\_Q |
| Sample\_28 | Sample\_28 | R4 | Cell\_Line\_A | Treatment\_Y | Manufacturer\_Q | Treatment\_Y\_Manufacturer\_Q |
| Sample\_29 | Sample\_29 | R5 | Cell\_Line\_B | Treatment\_X | Manufacturer\_M | Treatment\_X\_Manufacturer\_M |
| Sample\_3 | Sample\_3 | R3 | Cell\_Line\_A | Treatment\_X | Manufacturer\_Q | Treatment\_X\_Manufacturer\_Q |
| Sample\_30 | Sample\_30 | R6 | Cell\_Line\_B | Treatment\_Y | Manufacturer\_M | Treatment\_Y\_Manufacturer\_M |
| Sample\_31 | Sample\_31 | R7 | Cell\_Line\_B | Treatment\_X | Manufacturer\_Q | Treatment\_X\_Manufacturer\_Q |
| Sample\_32 | Sample\_32 | R8 | Cell\_Line\_B | Treatment\_Y | Manufacturer\_Q | Treatment\_Y\_Manufacturer\_Q |
| Sample\_33 | Sample\_33 | R9 | Cell\_Line\_C | Treatment\_X | Manufacturer\_M | Treatment\_X\_Manufacturer\_M |
| Sample\_34 | Sample\_34 | R10 | Cell\_Line\_C | Treatment\_Y | Manufacturer\_M | Treatment\_Y\_Manufacturer\_M |
| Sample\_35 | Sample\_35 | R11 | Cell\_Line\_C | Treatment\_X | Manufacturer\_Q | Treatment\_X\_Manufacturer\_Q |
| Sample\_36 | Sample\_36 | R12 | Cell\_Line\_C | Treatment\_Y | Manufacturer\_Q | Treatment\_Y\_Manufacturer\_Q |
| Sample\_37 | Sample\_37 | R13 | Cell\_Line\_D | Treatment\_X | Manufacturer\_M | Treatment\_X\_Manufacturer\_M |
| Sample\_38 | Sample\_38 | R14 | Cell\_Line\_D | Treatment\_Y | Manufacturer\_M | Treatment\_Y\_Manufacturer\_M |
| Sample\_39 | Sample\_39 | R15 | Cell\_Line\_D | Treatment\_X | Manufacturer\_Q | Treatment\_X\_Manufacturer\_Q |
| Sample\_4 | Sample\_4 | R4 | Cell\_Line\_A | Treatment\_Y | Manufacturer\_Q | Treatment\_Y\_Manufacturer\_Q |
| Sample\_40 | Sample\_40 | R16 | Cell\_Line\_D | Treatment\_Y | Manufacturer\_Q | Treatment\_Y\_Manufacturer\_Q |
| Sample\_41 | Sample\_41 | R17 | Cell\_Line\_E | Treatment\_X | Manufacturer\_M | Treatment\_X\_Manufacturer\_M |
| Sample\_42 | Sample\_42 | R18 | Cell\_Line\_E | Treatment\_Y | Manufacturer\_M | Treatment\_Y\_Manufacturer\_M |
| Sample\_43 | Sample\_43 | R19 | Cell\_Line\_E | Treatment\_X | Manufacturer\_Q | Treatment\_X\_Manufacturer\_Q |
| Sample\_44 | Sample\_44 | R20 | Cell\_Line\_E | Treatment\_Y | Manufacturer\_Q | Treatment\_Y\_Manufacturer\_Q |
| Sample\_45 | Sample\_45 | R21 | Cell\_Line\_F | Treatment\_X | Manufacturer\_M | Treatment\_X\_Manufacturer\_M |
| Sample\_46 | Sample\_46 | R22 | Cell\_Line\_F | Treatment\_Y | Manufacturer\_M | Treatment\_Y\_Manufacturer\_M |
| Sample\_47 | Sample\_47 | R23 | Cell\_Line\_F | Treatment\_X | Manufacturer\_Q | Treatment\_X\_Manufacturer\_Q |
| Sample\_48 | Sample\_48 | R24 | Cell\_Line\_F | Treatment\_Y | Manufacturer\_Q | Treatment\_Y\_Manufacturer\_Q |
| Sample\_5 | Sample\_5 | R5 | Cell\_Line\_B | Treatment\_X | Manufacturer\_M | Treatment\_X\_Manufacturer\_M |
| Sample\_6 | Sample\_6 | R6 | Cell\_Line\_B | Treatment\_Y | Manufacturer\_M | Treatment\_Y\_Manufacturer\_M |
| Sample\_7 | Sample\_7 | R7 | Cell\_Line\_B | Treatment\_X | Manufacturer\_Q | Treatment\_X\_Manufacturer\_Q |
| Sample\_8 | Sample\_8 | R8 | Cell\_Line\_B | Treatment\_Y | Manufacturer\_Q | Treatment\_Y\_Manufacturer\_Q |
| Sample\_9 | Sample\_9 | R9 | Cell\_Line\_C | Treatment\_X | Manufacturer\_M | Treatment\_X\_Manufacturer\_M |

### Consolidated sample groups

```
$Replicate

 R1 R10 R11 R12 R13 R14 R15 R16 R17 R18 R19  R2 R20 R21 R22 R23 R24  R3  R4  R5 
  2   2   2   2   2   2   2   2   2   2   2   2   2   2   2   2   2   2   2   2 
 R6  R7  R8  R9 
  2   2   2   2 

$Cell_Line

Cell_Line_A Cell_Line_B Cell_Line_C Cell_Line_D Cell_Line_E Cell_Line_F 
          8           8           8           8           8           8 

$Treatment

Treatment_X Treatment_Y 
         24          24 

$Isolation

Manufacturer_M Manufacturer_Q 
            24             24 

$Treatment_Isolation

Treatment_X_Manufacturer_M Treatment_X_Manufacturer_Q 
                        12                         12 
Treatment_Y_Manufacturer_M Treatment_Y_Manufacturer_Q 
                        12                         12
```

## Differential Gene Expression

DESeq2 performs an internal normalization where geometric mean is
calculated for each gene across all samples. The counts for a gene in
each sample is then divided by this mean. The median of these ratios in
a sample is the size factor for that sample. This procedure corrects for
library size and RNA composition bias, which can arise for example when
only a small number of genes are very highly expressed in one experiment
condition but not in the other.

As small numbers of replicates make it impossible to estimate
within-group variance reliably, DESeq2 uses shrinkage estimation for
dispersions and fold changes. A dispersion value is estimated for each
gene through a model fit procedure. You need to have biological
replicates of each experiment condition in order to estimate dispersion
properly. If there are no replicates, DESeq will estimate dispersion
using the samples from the different conditions as replicates. DESeq2
fits negative binomial generalized linear models for each gene and uses
the Wald test for significance testing. In addition to the group
information, you can give an additional experimental factor like pairing
to the analysis. DESeq2 detects automatically count outliers using
Cooks’s distance and removes these genes from analysis. It also
automatically removes genes whose mean of normalized counts is below a
threshold determined by an optimization procedure. Removing these genes
with low counts improves the detection power by making the multiple
testing adjustment of the p-values less severe.

The chosen RNA classes for your custom analysis were:
**miRNA,tRNA,Y RNA** and the threshold for defining
canonical miRNAs from isomiRs were **0 change(s) on the 5’end, 2
change(s) on the 3’end and 1 substitution(s) within the
sequence** (see config.yaml). The study design parameter chosen
for DGE testing was **Treatment\_Isolation** and the
parameter(s) chosen for correction were **Cell\_Line**

### total\_readcount

#### Treatment\_X\_Manufacturer\_M\_vs\_Treatment\_X\_Manufacturer\_Q

**Summary of DESeq2 results:**

```
out of 5631 with nonzero total read count
adjusted p-value < 0.1
LFC > 0 (up)       : 319, 5.7%
LFC < 0 (down)     : 380, 6.7%
outliers [1]       : 0, 0%
low counts [2]     : 874, 16%
(mean count < 0)
```

**Volcano plot:**

**Upregulated genes (padj ≤ 0.05, log2FC > 1):**

**Downregulated genes (padj ≤ 0.05, log2FC <
-1):**

#### Treatment\_X\_Manufacturer\_M\_vs\_Treatment\_Y\_Manufacturer\_M

**Summary of DESeq2 results:**

```
out of 5631 with nonzero total read count
adjusted p-value < 0.1
LFC > 0 (up)       : 199, 3.5%
LFC < 0 (down)     : 312, 5.5%
outliers [1]       : 0, 0%
low counts [2]     : 655, 12%
(mean count < 0)
```

**Volcano plot:**

**Upregulated genes (padj ≤ 0.05, log2FC > 1):**

**Downregulated genes (padj ≤ 0.05, log2FC <
-1):**

#### Treatment\_X\_Manufacturer\_M\_vs\_Treatment\_Y\_Manufacturer\_Q

**Summary of DESeq2 results:**

```
out of 5631 with nonzero total read count
adjusted p-value < 0.1
LFC > 0 (up)       : 1322, 23%
LFC < 0 (down)     : 1283, 23%
outliers [1]       : 0, 0%
low counts [2]     : 655, 12%
(mean count < 0)
```

**Volcano plot:**

**Upregulated genes (padj ≤ 0.05, log2FC > 1):**

**Downregulated genes (padj ≤ 0.05, log2FC <
-1):**

#### Treatment\_X\_Manufacturer\_Q\_vs\_Treatment\_Y\_Manufacturer\_M

**Summary of DESeq2 results:**

```
out of 5631 with nonzero total read count
adjusted p-value < 0.1
LFC > 0 (up)       : 508, 9%
LFC < 0 (down)     : 492, 8.7%
outliers [1]       : 0, 0%
low counts [2]     : 765, 14%
(mean count < 0)
```

**Volcano plot:**

**Upregulated genes (padj ≤ 0.05, log2FC > 1):**

**Downregulated genes (padj ≤ 0.05, log2FC <
-1):**

#### Treatment\_X\_Manufacturer\_Q\_vs\_Treatment\_Y\_Manufacturer\_Q

**Summary of DESeq2 results:**

```
out of 5631 with nonzero total read count
adjusted p-value < 0.1
LFC > 0 (up)       : 760, 13%
LFC < 0 (down)     : 918, 16%
outliers [1]       : 0, 0%
low counts [2]     : 655, 12%
(mean count < 0)
```

**Volcano plot:**

**Upregulated genes (padj ≤ 0.05, log2FC > 1):**

**Downregulated genes (padj ≤ 0.05, log2FC <
-1):**

#### Treatment\_Y\_Manufacturer\_M\_vs\_Treatment\_Y\_Manufacturer\_Q

**Summary of DESeq2 results:**

```
out of 5631 with nonzero total read count
adjusted p-value < 0.1
LFC > 0 (up)       : 957, 17%
LFC < 0 (down)     : 877, 16%
outliers [1]       : 0, 0%
low counts [2]     : 765, 14%
(mean count < 0)
```

**Volcano plot:**

**Upregulated genes (padj ≤ 0.05, log2FC > 1):**

**Downregulated genes (padj ≤ 0.05, log2FC <
-1):**

#### Similarities in of pairwise comparisons (UpSet Plot)

### miRNA\_readcount

#### Treatment\_X\_Manufacturer\_M\_vs\_Treatment\_X\_Manufacturer\_Q

**Summary of DESeq2 results:**

```
out of 1762 with nonzero total read count
adjusted p-value < 0.1
LFC > 0 (up)       : 46, 2.6%
LFC < 0 (down)     : 68, 3.9%
outliers [1]       : 0, 0%
low counts [2]     : 1127, 64%
(mean count < 1)
```

**Volcano plot:**

**Upregulated genes (padj ≤ 0.05, log2FC > 1):**

**Downregulated genes (padj ≤ 0.05, log2FC <
-1):**

#### Treatment\_X\_Manufacturer\_M\_vs\_Treatment\_Y\_Manufacturer\_M

**Summary of DESeq2 results:**

```
out of 1762 with nonzero total read count
adjusted p-value < 0.1
LFC > 0 (up)       : 56, 3.2%
LFC < 0 (down)     : 52, 3%
outliers [1]       : 0, 0%
low counts [2]     : 1230, 70%
(mean count < 3)
```

**Volcano plot:**

**Upregulated genes (padj ≤ 0.05, log2FC > 1):**

**Downregulated genes (padj ≤ 0.05, log2FC <
-1):**

#### Treatment\_X\_Manufacturer\_M\_vs\_Treatment\_Y\_Manufacturer\_Q

**Summary of DESeq2 results:**

```
out of 1762 with nonzero total read count
adjusted p-value < 0.1
LFC > 0 (up)       : 131, 7.4%
LFC < 0 (down)     : 122, 6.9%
outliers [1]       : 0, 0%
low counts [2]     : 991, 56%
(mean count < 1)
```

**Volcano plot:**

**Upregulated genes (padj ≤ 0.05, log2FC > 1):**

**Downregulated genes (padj ≤ 0.05, log2FC <
-1):**

#### Treatment\_X\_Manufacturer\_Q\_vs\_Treatment\_Y\_Manufacturer\_M

**Summary of DESeq2 results:**

```
out of 1762 with nonzero total read count
adjusted p-value < 0.1
LFC > 0 (up)       : 121, 6.9%
LFC < 0 (down)     : 75, 4.3%
outliers [1]       : 0, 0%
low counts [2]     : 956, 54%
(mean count < 1)
```

**Volcano plot:**

**Upregulated genes (padj ≤ 0.05, log2FC > 1):**

**Downregulated genes (padj ≤ 0.05, log2FC <
-1):**

#### Treatment\_X\_Manufacturer\_Q\_vs\_Treatment\_Y\_Manufacturer\_Q

**Summary of DESeq2 results:**

```
out of 1762 with nonzero total read count
adjusted p-value < 0.1
LFC > 0 (up)       : 102, 5.8%
LFC < 0 (down)     : 65, 3.7%
outliers [1]       : 0, 0%
low counts [2]     : 991, 56%
(mean count < 1)
```

**Volcano plot:**

**Upregulated genes (padj ≤ 0.05, log2FC > 1):**

**Downregulated genes (padj ≤ 0.05, log2FC <
-1):**

#### Treatment\_Y\_Manufacturer\_M\_vs\_Treatment\_Y\_Manufacturer\_Q

**Summary of DESeq2 results:**

```
out of 1762 with nonzero total read count
adjusted p-value < 0.1
LFC > 0 (up)       : 57, 3.2%
LFC < 0 (down)     : 52, 3%
outliers [1]       : 0, 0%
low counts [2]     : 1161, 66%
(mean count < 2)
```

**Volcano plot:**

**Upregulated genes (padj ≤ 0.05, log2FC > 1):**

**Downregulated genes (padj ≤ 0.05, log2FC <
-1):**

#### Similarities in of pairwise comparisons (UpSet Plot)

### tRNA\_miRNA\_readcount

#### Treatment\_X\_Manufacturer\_M\_vs\_Treatment\_X\_Manufacturer\_Q

**Summary of DESeq2 results:**

```
out of 1811 with nonzero total read count
adjusted p-value < 0.1
LFC > 0 (up)       : 47, 2.6%
LFC < 0 (down)     : 61, 3.4%
outliers [1]       : 0, 0%
low counts [2]     : 1088, 60%
(mean count < 1)
```

**Volcano plot:**

**Upregulated genes (padj ≤ 0.05, log2FC > 1):**

**Downregulated genes (padj ≤ 0.05, log2FC <
-1):**

#### Treatment\_X\_Manufacturer\_M\_vs\_Treatment\_Y\_Manufacturer\_M

**Summary of DESeq2 results:**

```
out of 1811 with nonzero total read count
adjusted p-value < 0.1
LFC > 0 (up)       : 49, 2.7%
LFC < 0 (down)     : 62, 3.4%
outliers [1]       : 0, 0%
low counts [2]     : 1088, 60%
(mean count < 1)
```

**Volcano plot:**

**Upregulated genes (padj ≤ 0.05, log2FC > 1):**

**Downregulated genes (padj ≤ 0.05, log2FC <
-1):**

#### Treatment\_X\_Manufacturer\_M\_vs\_Treatment\_Y\_Manufacturer\_Q

**Summary of DESeq2 results:**

```
out of 1811 with nonzero total read count
adjusted p-value < 0.1
LFC > 0 (up)       : 123, 6.8%
LFC < 0 (down)     : 156, 8.6%
outliers [1]       : 0, 0%
low counts [2]     : 1018, 56%
(mean count < 1)
```

**Volcano plot:**

**Upregulated genes (padj ≤ 0.05, log2FC > 1):**

**Downregulated genes (padj ≤ 0.05, log2FC <
-1):**

#### Treatment\_X\_Manufacturer\_Q\_vs\_Treatment\_Y\_Manufacturer\_M

**Summary of DESeq2 results:**

```
out of 1811 with nonzero total read count
adjusted p-value < 0.1
LFC > 0 (up)       : 112, 6.2%
LFC < 0 (down)     : 95, 5.2%
outliers [1]       : 0, 0%
low counts [2]     : 1088, 60%
(mean count < 1)
```

**Volcano plot:**

**Upregulated genes (padj ≤ 0.05, log2FC > 1):**

**Downregulated genes (padj ≤ 0.05, log2FC <
-1):**

#### Treatment\_X\_Manufacturer\_Q\_vs\_Treatment\_Y\_Manufacturer\_Q

**Summary of DESeq2 results:**

```
out of 1811 with nonzero total read count
adjusted p-value < 0.1
LFC > 0 (up)       : 100, 5.5%
LFC < 0 (down)     : 93, 5.1%
outliers [1]       : 0, 0%
low counts [2]     : 948, 52%
(mean count < 1)
```

**Volcano plot:**

**Upregulated genes (padj ≤ 0.05, log2FC > 1):**

**Downregulated genes (padj ≤ 0.05, log2FC <
-1):**

#### Treatment\_Y\_Manufacturer\_M\_vs\_Treatment\_Y\_Manufacturer\_Q

**Summary of DESeq2 results:**

```
out of 1811 with nonzero total read count
adjusted p-value < 0.1
LFC > 0 (up)       : 56, 3.1%
LFC < 0 (down)     : 58, 3.2%
outliers [1]       : 0, 0%
low counts [2]     : 1159, 64%
(mean count < 2)
```

**Volcano plot:**

**Upregulated genes (padj ≤ 0.05, log2FC > 1):**

**Downregulated genes (padj ≤ 0.05, log2FC <
-1):**

#### Similarities in of pairwise comparisons (UpSet Plot)

### isomiR\_readcount

#### Treatment\_X\_Manufacturer\_M\_vs\_Treatment\_X\_Manufacturer\_Q

**Summary of DESeq2 results:**

```
out of 5364 with nonzero total read count
adjusted p-value < 0.1
LFC > 0 (up)       : 597, 11%
LFC < 0 (down)     : 757, 14%
outliers [1]       : 0, 0%
low counts [2]     : 0, 0%
(mean count < 4)
```

**Volcano plot:**

**Upregulated genes (padj ≤ 0.05, log2FC > 1):**

**Downregulated genes (padj ≤ 0.05, log2FC <
-1):**

#### Treatment\_X\_Manufacturer\_M\_vs\_Treatment\_Y\_Manufacturer\_M

**Summary of DESeq2 results:**

```
out of 5364 with nonzero total read count
adjusted p-value < 0.1
LFC > 0 (up)       : 474, 8.8%
LFC < 0 (down)     : 644, 12%
outliers [1]       : 0, 0%
low counts [2]     : 0, 0%
(mean count < 4)
```

**Volcano plot:**

**Upregulated genes (padj ≤ 0.05, log2FC > 1):**

**Downregulated genes (padj ≤ 0.05, log2FC <
-1):**

#### Treatment\_X\_Manufacturer\_M\_vs\_Treatment\_Y\_Manufacturer\_Q

**Summary of DESeq2 results:**

```
out of 5364 with nonzero total read count
adjusted p-value < 0.1
LFC > 0 (up)       : 1169, 22%
LFC < 0 (down)     : 1737, 32%
outliers [1]       : 0, 0%
low counts [2]     : 0, 0%
(mean count < 4)
```

**Volcano plot:**

**Upregulated genes (padj ≤ 0.05, log2FC > 1):**

**Downregulated genes (padj ≤ 0.05, log2FC <
-1):**

#### Treatment\_X\_Manufacturer\_Q\_vs\_Treatment\_Y\_Manufacturer\_M

**Summary of DESeq2 results:**

```
out of 5364 with nonzero total read count
adjusted p-value < 0.1
LFC > 0 (up)       : 990, 18%
LFC < 0 (down)     : 984, 18%
outliers [1]       : 0, 0%
low counts [2]     : 0, 0%
(mean count < 4)
```

**Volcano plot:**

**Upregulated genes (padj ≤ 0.05, log2FC > 1):**

**Downregulated genes (padj ≤ 0.05, log2FC <
-1):**

#### Treatment\_X\_Manufacturer\_Q\_vs\_Treatment\_Y\_Manufacturer\_Q

**Summary of DESeq2 results:**

```
out of 5364 with nonzero total read count
adjusted p-value < 0.1
LFC > 0 (up)       : 946, 18%
LFC < 0 (down)     : 1085, 20%
outliers [1]       : 0, 0%
low counts [2]     : 0, 0%
(mean count < 4)
```

**Volcano plot:**

**Upregulated genes (padj ≤ 0.05, log2FC > 1):**

**Downregulated genes (padj ≤ 0.05, log2FC <
-1):**

#### Treatment\_Y\_Manufacturer\_M\_vs\_Treatment\_Y\_Manufacturer\_Q

**Summary of DESeq2 results:**

```
out of 5364 with nonzero total read count
adjusted p-value < 0.1
LFC > 0 (up)       : 808, 15%
LFC < 0 (down)     : 847, 16%
outliers [1]       : 0, 0%
low counts [2]     : 0, 0%
(mean count < 4)
```

**Volcano plot:**

**Upregulated genes (padj ≤ 0.05, log2FC > 1):**

**Downregulated genes (padj ≤ 0.05, log2FC <
-1):**

#### Similarities in of pairwise comparisons (UpSet Plot)

### tRF\_readcount

#### Treatment\_X\_Manufacturer\_M\_vs\_Treatment\_X\_Manufacturer\_Q

**Summary of DESeq2 results:**

```
out of 736 with nonzero total read count
adjusted p-value < 0.1
LFC > 0 (up)       : 120, 16%
LFC < 0 (down)     : 171, 23%
outliers [1]       : 0, 0%
low counts [2]     : 0, 0%
(mean count < 6)
```

**Volcano plot:**

**Upregulated genes (padj ≤ 0.05, log2FC > 1):**

**Downregulated genes (padj ≤ 0.05, log2FC <
-1):**

#### Treatment\_X\_Manufacturer\_M\_vs\_Treatment\_Y\_Manufacturer\_M

**Summary of DESeq2 results:**

```
out of 736 with nonzero total read count
adjusted p-value < 0.1
LFC > 0 (up)       : 50, 6.8%
LFC < 0 (down)     : 61, 8.3%
outliers [1]       : 0, 0%
low counts [2]     : 0, 0%
(mean count < 6)
```

**Volcano plot:**

**Upregulated genes (padj ≤ 0.05, log2FC > 1):**

**Downregulated genes (padj ≤ 0.05, log2FC <
-1):**

#### Treatment\_X\_Manufacturer\_M\_vs\_Treatment\_Y\_Manufacturer\_Q

**Summary of DESeq2 results:**

```
out of 736 with nonzero total read count
adjusted p-value < 0.1
LFC > 0 (up)       : 207, 28%
LFC < 0 (down)     : 238, 32%
outliers [1]       : 0, 0%
low counts [2]     : 0, 0%
(mean count < 6)
```

**Volcano plot:**

**Upregulated genes (padj ≤ 0.05, log2FC > 1):**

**Downregulated genes (padj ≤ 0.05, log2FC <
-1):**

#### Treatment\_X\_Manufacturer\_Q\_vs\_Treatment\_Y\_Manufacturer\_M

**Summary of DESeq2 results:**

```
out of 736 with nonzero total read count
adjusted p-value < 0.1
LFC > 0 (up)       : 207, 28%
LFC < 0 (down)     : 164, 22%
outliers [1]       : 0, 0%
low counts [2]     : 0, 0%
(mean count < 6)
```

**Volcano plot:**

**Upregulated genes (padj ≤ 0.05, log2FC > 1):**

**Downregulated genes (padj ≤ 0.05, log2FC <
-1):**

#### Treatment\_X\_Manufacturer\_Q\_vs\_Treatment\_Y\_Manufacturer\_Q

**Summary of DESeq2 results:**

```
out of 736 with nonzero total read count
adjusted p-value < 0.1
LFC > 0 (up)       : 169, 23%
LFC < 0 (down)     : 164, 22%
outliers [1]       : 0, 0%
low counts [2]     : 0, 0%
(mean count < 6)
```

**Volcano plot:**

**Upregulated genes (padj ≤ 0.05, log2FC > 1):**

**Downregulated genes (padj ≤ 0.05, log2FC <
-1):**

#### Treatment\_Y\_Manufacturer\_M\_vs\_Treatment\_Y\_Manufacturer\_Q

**Summary of DESeq2 results:**

```
out of 736 with nonzero total read count
adjusted p-value < 0.1
LFC > 0 (up)       : 144, 20%
LFC < 0 (down)     : 190, 26%
outliers [1]       : 0, 0%
low counts [2]     : 0, 0%
(mean count < 6)
```

**Volcano plot:**

**Upregulated genes (padj ≤ 0.05, log2FC > 1):**

**Downregulated genes (padj ≤ 0.05, log2FC <
-1):**

#### Similarities in of pairwise comparisons (UpSet Plot)

### tRF\_isomiR\_readcount

#### Treatment\_X\_Manufacturer\_M\_vs\_Treatment\_X\_Manufacturer\_Q

**Summary of DESeq2 results:**

```
out of 6100 with nonzero total read count
adjusted p-value < 0.1
LFC > 0 (up)       : 688, 11%
LFC < 0 (down)     : 893, 15%
outliers [1]       : 0, 0%
low counts [2]     : 0, 0%
(mean count < 5)
```

**Volcano plot:**

**Upregulated genes (padj ≤ 0.05, log2FC > 1):**

**Downregulated genes (padj ≤ 0.05, log2FC <
-1):**

#### Treatment\_X\_Manufacturer\_M\_vs\_Treatment\_Y\_Manufacturer\_M

**Summary of DESeq2 results:**

```
out of 6100 with nonzero total read count
adjusted p-value < 0.1
LFC > 0 (up)       : 465, 7.6%
LFC < 0 (down)     : 667, 11%
outliers [1]       : 0, 0%
low counts [2]     : 0, 0%
(mean count < 5)
```

**Volcano plot:**

**Upregulated genes (padj ≤ 0.05, log2FC > 1):**

**Downregulated genes (padj ≤ 0.05, log2FC <
-1):**

#### Treatment\_X\_Manufacturer\_M\_vs\_Treatment\_Y\_Manufacturer\_Q

**Summary of DESeq2 results:**

```
out of 6100 with nonzero total read count
adjusted p-value < 0.1
LFC > 0 (up)       : 1285, 21%
LFC < 0 (down)     : 2035, 33%
outliers [1]       : 0, 0%
low counts [2]     : 0, 0%
(mean count < 5)
```

**Volcano plot:**

**Upregulated genes (padj ≤ 0.05, log2FC > 1):**

**Downregulated genes (padj ≤ 0.05, log2FC <
-1):**

#### Treatment\_X\_Manufacturer\_Q\_vs\_Treatment\_Y\_Manufacturer\_M

**Summary of DESeq2 results:**

```
out of 6100 with nonzero total read count
adjusted p-value < 0.1
LFC > 0 (up)       : 1178, 19%
LFC < 0 (down)     : 1143, 19%
outliers [1]       : 0, 0%
low counts [2]     : 0, 0%
(mean count < 5)
```

**Volcano plot:**

**Upregulated genes (padj ≤ 0.05, log2FC > 1):**

**Downregulated genes (padj ≤ 0.05, log2FC <
-1):**

#### Treatment\_X\_Manufacturer\_Q\_vs\_Treatment\_Y\_Manufacturer\_Q

**Summary of DESeq2 results:**

```
out of 6100 with nonzero total read count
adjusted p-value < 0.1
LFC > 0 (up)       : 1099, 18%
LFC < 0 (down)     : 1367, 22%
outliers [1]       : 0, 0%
low counts [2]     : 0, 0%
(mean count < 5)
```

**Volcano plot:**

**Upregulated genes (padj ≤ 0.05, log2FC > 1):**

**Downregulated genes (padj ≤ 0.05, log2FC <
-1):**

#### Treatment\_Y\_Manufacturer\_M\_vs\_Treatment\_Y\_Manufacturer\_Q

**Summary of DESeq2 results:**

```
out of 6100 with nonzero total read count
adjusted p-value < 0.1
LFC > 0 (up)       : 885, 15%
LFC < 0 (down)     : 1124, 18%
outliers [1]       : 0, 0%
low counts [2]     : 0, 0%
(mean count < 5)
```

**Volcano plot:**

**Upregulated genes (padj ≤ 0.05, log2FC > 1):**

**Downregulated genes (padj ≤ 0.05, log2FC <
-1):**

#### Similarities in of pairwise comparisons (UpSet Plot)

### custom\_analysis\_readcount

#### Treatment\_X\_Manufacturer\_M\_vs\_Treatment\_X\_Manufacturer\_Q

**Summary of DESeq2 results:**

```
out of 1815 with nonzero total read count
adjusted p-value < 0.1
LFC > 0 (up)       : 46, 2.5%
LFC < 0 (down)     : 65, 3.6%
outliers [1]       : 0, 0%
low counts [2]     : 1056, 58%
(mean count < 1)
```

**Volcano plot:**

**Upregulated genes (padj ≤ 0.05, log2FC > 1):**

**Downregulated genes (padj ≤ 0.05, log2FC <
-1):**

#### Treatment\_X\_Manufacturer\_M\_vs\_Treatment\_Y\_Manufacturer\_M

**Summary of DESeq2 results:**

```
out of 1815 with nonzero total read count
adjusted p-value < 0.1
LFC > 0 (up)       : 50, 2.8%
LFC < 0 (down)     : 59, 3.3%
outliers [1]       : 0, 0%
low counts [2]     : 1056, 58%
(mean count < 1)
```

**Volcano plot:**

**Upregulated genes (padj ≤ 0.05, log2FC > 1):**

**Downregulated genes (padj ≤ 0.05, log2FC <
-1):**

#### Treatment\_X\_Manufacturer\_M\_vs\_Treatment\_Y\_Manufacturer\_Q

**Summary of DESeq2 results:**

```
out of 1815 with nonzero total read count
adjusted p-value < 0.1
LFC > 0 (up)       : 129, 7.1%
LFC < 0 (down)     : 161, 8.9%
outliers [1]       : 0, 0%
low counts [2]     : 1091, 60%
(mean count < 1)
```

**Volcano plot:**

**Upregulated genes (padj ≤ 0.05, log2FC > 1):**

**Downregulated genes (padj ≤ 0.05, log2FC <
-1):**

#### Treatment\_X\_Manufacturer\_Q\_vs\_Treatment\_Y\_Manufacturer\_M

**Summary of DESeq2 results:**

```
out of 1815 with nonzero total read count
adjusted p-value < 0.1
LFC > 0 (up)       : 118, 6.5%
LFC < 0 (down)     : 93, 5.1%
outliers [1]       : 0, 0%
low counts [2]     : 1091, 60%
(mean count < 1)
```

**Volcano plot:**

**Upregulated genes (padj ≤ 0.05, log2FC > 1):**

**Downregulated genes (padj ≤ 0.05, log2FC <
-1):**

#### Treatment\_X\_Manufacturer\_Q\_vs\_Treatment\_Y\_Manufacturer\_Q

**Summary of DESeq2 results:**

```
out of 1815 with nonzero total read count
adjusted p-value < 0.1
LFC > 0 (up)       : 103, 5.7%
LFC < 0 (down)     : 94, 5.2%
outliers [1]       : 0, 0%
low counts [2]     : 950, 52%
(mean count < 1)
```

**Volcano plot:**

**Upregulated genes (padj ≤ 0.05, log2FC > 1):**

**Downregulated genes (padj ≤ 0.05, log2FC <
-1):**

#### Treatment\_Y\_Manufacturer\_M\_vs\_Treatment\_Y\_Manufacturer\_Q

**Summary of DESeq2 results:**

```
out of 1815 with nonzero total read count
adjusted p-value < 0.1
LFC > 0 (up)       : 57, 3.1%
LFC < 0 (down)     : 61, 3.4%
outliers [1]       : 0, 0%
low counts [2]     : 1161, 64%
(mean count < 2)
```

**Volcano plot:**

**Upregulated genes (padj ≤ 0.05, log2FC > 1):**

**Downregulated genes (padj ≤ 0.05, log2FC <
-1):**

#### Similarities in of pairwise comparisons (UpSet Plot)

## Supervised clustering (sPLS-DA)

Sparse Partial Least Squares Discriminant Analysis (sPLS-DA) is a
supervised multivariate classification method designed to identify the
minimal set of variables (here: genes) that best discriminate between
predefined experimental groups. Unlike unsupervised techniques such as
PCA, which only capture the major axes of variance in the data, sPLS-DA
explicitly incorporates sample class labels during model training. This
allows the method to focus on variation that is predictive of group
membership, which is particularly advantageous in high-dimensional
RNA-seq datasets where the number of genes greatly exceeds the number of
samples.

Important note on overfitting:   
 Because sPLS-DA is a supervised
method, it is highly susceptible to overfitting, especially when the
number of samples is small, the number of variables (genes) is very
large, the same dataset is used for both training and evaluation and
tuning parameters are optimized on the same data used to assess
performance. In this analysis, the ROC and AUC values are computed from
the training data only, not from an independent validation dataset. The
AUC values represent the model’s performance on the data it was trained
on and should not be interpreted as unbiased estimates of predictive
ability. They often overestimate true performance. To reduce this risk,
cross-validation was performed, so results are more reliable but still
subject to optimistic bias unless a completely independent test set is
used.

### total\_readcount

#### Treatment\_X\_Manufacturer\_M\_vs\_Treatment\_X\_Manufacturer\_Q

##### Sample separation plot

##### Loadings per component

###### Component 1

###### Component 2

###### Component 3

##### ROC / AUC curves

#### Treatment\_X\_Manufacturer\_M\_vs\_Treatment\_Y\_Manufacturer\_M

##### Sample separation plot

##### Loadings per component

###### Component 1

###### Component 2

###### Component 3

##### ROC / AUC curves

#### Treatment\_X\_Manufacturer\_M\_vs\_Treatment\_Y\_Manufacturer\_Q

##### Sample separation plot

##### Loadings per component

###### Component 1

###### Component 2

###### Component 3

##### ROC / AUC curves

#### Treatment\_X\_Manufacturer\_Q\_vs\_Treatment\_Y\_Manufacturer\_M

##### Sample separation plot

##### Loadings per component

###### Component 1

###### Component 2

###### Component 3

##### ROC / AUC curves

#### Treatment\_X\_Manufacturer\_Q\_vs\_Treatment\_Y\_Manufacturer\_Q

##### Sample separation plot

##### Loadings per component

###### Component 1

###### Component 2

###### Component 3

##### ROC / AUC curves

#### Treatment\_Y\_Manufacturer\_M\_vs\_Treatment\_Y\_Manufacturer\_Q

##### Sample separation plot

##### Loadings per component

###### Component 1

###### Component 2

###### Component 3

##### ROC / AUC curves

### miRNA\_readcount

#### Treatment\_X\_Manufacturer\_M\_vs\_Treatment\_X\_Manufacturer\_Q

##### Sample separation plot

##### Loadings per component

###### Component 1

###### Component 2

###### Component 3

##### ROC / AUC curves

#### Treatment\_X\_Manufacturer\_M\_vs\_Treatment\_Y\_Manufacturer\_M

##### Sample separation plot

##### Loadings per component

###### Component 1

###### Component 2

###### Component 3

##### ROC / AUC curves

#### Treatment\_X\_Manufacturer\_M\_vs\_Treatment\_Y\_Manufacturer\_Q

##### Sample separation plot

##### Loadings per component

###### Component 1

###### Component 2

###### Component 3

##### ROC / AUC curves

#### Treatment\_X\_Manufacturer\_Q\_vs\_Treatment\_Y\_Manufacturer\_M

##### Sample separation plot

##### Loadings per component

###### Component 1

###### Component 2

###### Component 3

##### ROC / AUC curves

#### Treatment\_X\_Manufacturer\_Q\_vs\_Treatment\_Y\_Manufacturer\_Q

##### Sample separation plot

##### Loadings per component

###### Component 1

###### Component 2

###### Component 3

##### ROC / AUC curves

#### Treatment\_Y\_Manufacturer\_M\_vs\_Treatment\_Y\_Manufacturer\_Q

##### Sample separation plot

##### Loadings per component

###### Component 1

###### Component 2

###### Component 3

##### ROC / AUC curves

### tRNA\_miRNA\_readcount

#### Treatment\_X\_Manufacturer\_M\_vs\_Treatment\_X\_Manufacturer\_Q

##### Sample separation plot

##### Loadings per component

###### Component 1

###### Component 2

###### Component 3

##### ROC / AUC curves

#### Treatment\_X\_Manufacturer\_M\_vs\_Treatment\_Y\_Manufacturer\_M

##### Sample separation plot

##### Loadings per component

###### Component 1

###### Component 2

###### Component 3

##### ROC / AUC curves

#### Treatment\_X\_Manufacturer\_M\_vs\_Treatment\_Y\_Manufacturer\_Q

##### Sample separation plot

##### Loadings per component

###### Component 1

###### Component 2

###### Component 3

##### ROC / AUC curves

#### Treatment\_X\_Manufacturer\_Q\_vs\_Treatment\_Y\_Manufacturer\_M

##### Sample separation plot

##### Loadings per component

###### Component 1

###### Component 2

###### Component 3

##### ROC / AUC curves

#### Treatment\_X\_Manufacturer\_Q\_vs\_Treatment\_Y\_Manufacturer\_Q

##### Sample separation plot

##### Loadings per component

###### Component 1

###### Component 2

###### Component 3

##### ROC / AUC curves

#### Treatment\_Y\_Manufacturer\_M\_vs\_Treatment\_Y\_Manufacturer\_Q

##### Sample separation plot

##### Loadings per component

###### Component 1

###### Component 2

###### Component 3

##### ROC / AUC curves

### isomiR\_readcount

#### Treatment\_X\_Manufacturer\_M\_vs\_Treatment\_X\_Manufacturer\_Q

##### Sample separation plot

##### Loadings per component

###### Component 1

###### Component 2

###### Component 3

##### ROC / AUC curves

#### Treatment\_X\_Manufacturer\_M\_vs\_Treatment\_Y\_Manufacturer\_M

##### Sample separation plot

##### Loadings per component

###### Component 1

###### Component 2

###### Component 3

##### ROC / AUC curves

#### Treatment\_X\_Manufacturer\_M\_vs\_Treatment\_Y\_Manufacturer\_Q

##### Sample separation plot

##### Loadings per component

###### Component 1

###### Component 2

###### Component 3

##### ROC / AUC curves

#### Treatment\_X\_Manufacturer\_Q\_vs\_Treatment\_Y\_Manufacturer\_M

##### Sample separation plot

##### Loadings per component

###### Component 1

###### Component 2

###### Component 3

##### ROC / AUC curves

#### Treatment\_X\_Manufacturer\_Q\_vs\_Treatment\_Y\_Manufacturer\_Q

##### Sample separation plot

##### Loadings per component

###### Component 1

###### Component 2

###### Component 3

##### ROC / AUC curves

#### Treatment\_Y\_Manufacturer\_M\_vs\_Treatment\_Y\_Manufacturer\_Q

##### Sample separation plot

##### Loadings per component

###### Component 1

###### Component 2

###### Component 3

##### ROC / AUC curves

### tRF\_readcount

#### Treatment\_X\_Manufacturer\_M\_vs\_Treatment\_X\_Manufacturer\_Q

##### Sample separation plot

##### Loadings per component

###### Component 1

###### Component 2

###### Component 3

##### ROC / AUC curves

#### Treatment\_X\_Manufacturer\_M\_vs\_Treatment\_Y\_Manufacturer\_M

##### Sample separation plot

##### Loadings per component

###### Component 1

###### Component 2

###### Component 3

##### ROC / AUC curves

#### Treatment\_X\_Manufacturer\_M\_vs\_Treatment\_Y\_Manufacturer\_Q

##### Sample separation plot

##### Loadings per component

###### Component 1

###### Component 2

###### Component 3

##### ROC / AUC curves

#### Treatment\_X\_Manufacturer\_Q\_vs\_Treatment\_Y\_Manufacturer\_M

##### Sample separation plot

##### Loadings per component

###### Component 1

###### Component 2

###### Component 3

##### ROC / AUC curves

#### Treatment\_X\_Manufacturer\_Q\_vs\_Treatment\_Y\_Manufacturer\_Q

##### Sample separation plot

##### Loadings per component

###### Component 1

###### Component 2

###### Component 3

##### ROC / AUC curves

#### Treatment\_Y\_Manufacturer\_M\_vs\_Treatment\_Y\_Manufacturer\_Q

##### Sample separation plot

##### Loadings per component

###### Component 1

###### Component 2

###### Component 3

##### ROC / AUC curves

### tRF\_isomiR\_readcount

#### Treatment\_X\_Manufacturer\_M\_vs\_Treatment\_X\_Manufacturer\_Q

##### Sample separation plot

##### Loadings per component

###### Component 1

###### Component 2

###### Component 3

##### ROC / AUC curves

#### Treatment\_X\_Manufacturer\_M\_vs\_Treatment\_Y\_Manufacturer\_M

##### Sample separation plot

##### Loadings per component

###### Component 1

###### Component 2

###### Component 3

##### ROC / AUC curves

#### Treatment\_X\_Manufacturer\_M\_vs\_Treatment\_Y\_Manufacturer\_Q

##### Sample separation plot

##### Loadings per component

###### Component 1

###### Component 2

###### Component 3

##### ROC / AUC curves

#### Treatment\_X\_Manufacturer\_Q\_vs\_Treatment\_Y\_Manufacturer\_M

##### Sample separation plot

##### Loadings per component

###### Component 1

###### Component 2

###### Component 3

##### ROC / AUC curves

#### Treatment\_X\_Manufacturer\_Q\_vs\_Treatment\_Y\_Manufacturer\_Q

##### Sample separation plot

##### Loadings per component

###### Component 1

###### Component 2

###### Component 3

##### ROC / AUC curves

#### Treatment\_Y\_Manufacturer\_M\_vs\_Treatment\_Y\_Manufacturer\_Q

##### Sample separation plot

##### Loadings per component

###### Component 1

###### Component 2

###### Component 3

##### ROC / AUC curves

### custom\_analysis\_readcount

#### Treatment\_X\_Manufacturer\_M\_vs\_Treatment\_X\_Manufacturer\_Q

##### Sample separation plot

##### Loadings per component

###### Component 1

###### Component 2

###### Component 3

##### ROC / AUC curves

#### Treatment\_X\_Manufacturer\_M\_vs\_Treatment\_Y\_Manufacturer\_M

##### Sample separation plot

##### Loadings per component

###### Component 1

###### Component 2

###### Component 3

##### ROC / AUC curves

#### Treatment\_X\_Manufacturer\_M\_vs\_Treatment\_Y\_Manufacturer\_Q

##### Sample separation plot

##### Loadings per component

###### Component 1

###### Component 2

###### Component 3

##### ROC / AUC curves

#### Treatment\_X\_Manufacturer\_Q\_vs\_Treatment\_Y\_Manufacturer\_M

##### Sample separation plot

##### Loadings per component

###### Component 1

###### Component 2

###### Component 3

##### ROC / AUC curves

#### Treatment\_X\_Manufacturer\_Q\_vs\_Treatment\_Y\_Manufacturer\_Q

##### Sample separation plot

##### Loadings per component

###### Component 1

###### Component 2

###### Component 3

##### ROC / AUC curves

#### Treatment\_Y\_Manufacturer\_M\_vs\_Treatment\_Y\_Manufacturer\_Q

##### Sample separation plot

##### Loadings per component

###### Component 1

###### Component 2

###### Component 3

##### ROC / AUC curves
