# Supplementary material for: Comprehensive and bias-aware analysis of small RNA NGS data for biomarker detection with caRNAge
Source: Extracell Vesicles Circ Nucl Acids. 2026 Sep 24;7(3):1554–81. doi: 10.20517/evcna.2026.78 (PMC13678173; doi:10.20517/evcna.2026.78)
Supplement: Supplementary file 1 [file evcna-7-3-1554-SupplementaryMaterials.zip › evcna7078-SupplementaryMaterials/Supplementary File 3-2.html]

caRNAge - comprehensive analysis of small RNA gene expression


# caRNAge - comprehensive analysis of small RNA gene expression

### Over representation analysis of miRNAs and their mRNA targets

#### 16 February, 2026

## Purpose of this overview (Disclaimer)

This report is meant to give you a complete overview of
differentially expressed genes for the group comparison you have chosen
with their respective sources of bias you have identified in the data
overview. Please refer to the guidelines for best practice of caRNAge
and the respective manuals and vignettes of the applied tools. You are
strongly advised to thoroughly check all available information in
addition to this data overview. caRNAge is built upon the work of many
excellent scientists who developed the necessary tools to process small
RNA NGS data, so please make sure to cite the corresponding original
authors in addition to caRNAge when publishing your work.

If you encounter any technical problems, feel free to contact the
corresponding author at bkirchner@tum.de.

## Data processing

Raw sequencing reads were processed with Cutadapt to trim
adapter sequences from 3’ ends if present and to remove degraded reads
(shorter than 16nt). Trimmed reads were analysed with FastQC
to evaluate phred scores and read lengths before aligning them against
the human tRNA (GtRNAdb), miRNA
(miRBase) including isomiRs (isomiRROR), piRNAs (piRNAdb) and refseq transcriptome
sequences (refseq)
with bowtie.
Finally, gene read counts were processed with R and mainly DESeq2
to normalize and transform data and calculate expression changes.

Fig 1: Simplified workflow for NGS data analysis

|  | Tools | Version | Parameters |
| --- | --- | --- | --- |
| Trimming | Cutadapt | 5.1 | -a AGATCGGAAGAGCAC, -e 0.1 -m 16 |
| Quality Control | FastQC | 0.12.1 |  |
| Alignment | bowtie | 1.3.1 | –quantMode TranscriptomeSAM –outSAMtype BAM SortedByCoordinate; mismatches tRNA: 1 , isomiR: 3 , transcriptome: 1 |
| Normalisation | DESeq2 | R version 4.5.2 (2025-10-31), DESeq2 1.50.2 | ~Cell\_Line + Treatment\_Isolation |
| DGE calling | DESeq2 | R version 4.5.2 (2025-10-31), DESeq2 1.50.2 |  |
| miRNA ORA | rbioapi, miEAA | R version 4.5.2 (2025-10-31), rbioapi 0.8.3, miEEA |  |
| miRNA target prediction | multiMiR Targetscan | R version 4.5.2 (2025-10-31), multiMiR 1.32.0, TargetScan |  |
| miRNA target ORA | rbioapi, enrichr | R version 4.5.2 (2025-10-31), rbioapi 0.8.3, enrichr |  |

## Sample overview

The starting point for all analyses, is a well designed study design.
In addition to the classification of the main research question, it
should also incorporate all available information that could in any way
affect the outcome. This includes potential confounding factors such as
batch effects as well as statistical relevant information such as
patient cohorts or cell lines. Please make sure that the classifications
given in your *design.tsv* are correct and that all relevant
information is present.

### Study design

The row names and “Sample” column should be matching and indicate
that samples in design were correctly assigned in the analysis. A column
named “replaceable” maybe present if groups are large enough (>7) to
replace single outlier values.

|  | Sample | Replicate | Cell\_Line | Treatment | Isolation | Treatment\_Isolation |
| --- | --- | --- | --- | --- | --- | --- |
| Sample\_1 | Sample\_1 | R1 | Cell\_Line\_A | Treatment\_X | Manufacturer\_M | Treatment\_X\_Manufacturer\_M |
| Sample\_10 | Sample\_10 | R10 | Cell\_Line\_C | Treatment\_Y | Manufacturer\_M | Treatment\_Y\_Manufacturer\_M |
| Sample\_11 | Sample\_11 | R11 | Cell\_Line\_C | Treatment\_X | Manufacturer\_Q | Treatment\_X\_Manufacturer\_Q |
| Sample\_12 | Sample\_12 | R12 | Cell\_Line\_C | Treatment\_Y | Manufacturer\_Q | Treatment\_Y\_Manufacturer\_Q |
| Sample\_13 | Sample\_13 | R13 | Cell\_Line\_D | Treatment\_X | Manufacturer\_M | Treatment\_X\_Manufacturer\_M |
| Sample\_14 | Sample\_14 | R14 | Cell\_Line\_D | Treatment\_Y | Manufacturer\_M | Treatment\_Y\_Manufacturer\_M |
| Sample\_15 | Sample\_15 | R15 | Cell\_Line\_D | Treatment\_X | Manufacturer\_Q | Treatment\_X\_Manufacturer\_Q |
| Sample\_16 | Sample\_16 | R16 | Cell\_Line\_D | Treatment\_Y | Manufacturer\_Q | Treatment\_Y\_Manufacturer\_Q |
| Sample\_17 | Sample\_17 | R17 | Cell\_Line\_E | Treatment\_X | Manufacturer\_M | Treatment\_X\_Manufacturer\_M |
| Sample\_18 | Sample\_18 | R18 | Cell\_Line\_E | Treatment\_Y | Manufacturer\_M | Treatment\_Y\_Manufacturer\_M |
| Sample\_19 | Sample\_19 | R19 | Cell\_Line\_E | Treatment\_X | Manufacturer\_Q | Treatment\_X\_Manufacturer\_Q |
| Sample\_2 | Sample\_2 | R2 | Cell\_Line\_A | Treatment\_Y | Manufacturer\_M | Treatment\_Y\_Manufacturer\_M |
| Sample\_20 | Sample\_20 | R20 | Cell\_Line\_E | Treatment\_Y | Manufacturer\_Q | Treatment\_Y\_Manufacturer\_Q |
| Sample\_21 | Sample\_21 | R21 | Cell\_Line\_F | Treatment\_X | Manufacturer\_M | Treatment\_X\_Manufacturer\_M |
| Sample\_22 | Sample\_22 | R22 | Cell\_Line\_F | Treatment\_Y | Manufacturer\_M | Treatment\_Y\_Manufacturer\_M |
| Sample\_23 | Sample\_23 | R23 | Cell\_Line\_F | Treatment\_X | Manufacturer\_Q | Treatment\_X\_Manufacturer\_Q |
| Sample\_24 | Sample\_24 | R24 | Cell\_Line\_F | Treatment\_Y | Manufacturer\_Q | Treatment\_Y\_Manufacturer\_Q |
| Sample\_25 | Sample\_25 | R1 | Cell\_Line\_A | Treatment\_X | Manufacturer\_M | Treatment\_X\_Manufacturer\_M |
| Sample\_26 | Sample\_26 | R2 | Cell\_Line\_A | Treatment\_Y | Manufacturer\_M | Treatment\_Y\_Manufacturer\_M |
| Sample\_27 | Sample\_27 | R3 | Cell\_Line\_A | Treatment\_X | Manufacturer\_Q | Treatment\_X\_Manufacturer\_Q |
| Sample\_28 | Sample\_28 | R4 | Cell\_Line\_A | Treatment\_Y | Manufacturer\_Q | Treatment\_Y\_Manufacturer\_Q |
| Sample\_29 | Sample\_29 | R5 | Cell\_Line\_B | Treatment\_X | Manufacturer\_M | Treatment\_X\_Manufacturer\_M |
| Sample\_3 | Sample\_3 | R3 | Cell\_Line\_A | Treatment\_X | Manufacturer\_Q | Treatment\_X\_Manufacturer\_Q |
| Sample\_30 | Sample\_30 | R6 | Cell\_Line\_B | Treatment\_Y | Manufacturer\_M | Treatment\_Y\_Manufacturer\_M |
| Sample\_31 | Sample\_31 | R7 | Cell\_Line\_B | Treatment\_X | Manufacturer\_Q | Treatment\_X\_Manufacturer\_Q |
| Sample\_32 | Sample\_32 | R8 | Cell\_Line\_B | Treatment\_Y | Manufacturer\_Q | Treatment\_Y\_Manufacturer\_Q |
| Sample\_33 | Sample\_33 | R9 | Cell\_Line\_C | Treatment\_X | Manufacturer\_M | Treatment\_X\_Manufacturer\_M |
| Sample\_34 | Sample\_34 | R10 | Cell\_Line\_C | Treatment\_Y | Manufacturer\_M | Treatment\_Y\_Manufacturer\_M |
| Sample\_35 | Sample\_35 | R11 | Cell\_Line\_C | Treatment\_X | Manufacturer\_Q | Treatment\_X\_Manufacturer\_Q |
| Sample\_36 | Sample\_36 | R12 | Cell\_Line\_C | Treatment\_Y | Manufacturer\_Q | Treatment\_Y\_Manufacturer\_Q |
| Sample\_37 | Sample\_37 | R13 | Cell\_Line\_D | Treatment\_X | Manufacturer\_M | Treatment\_X\_Manufacturer\_M |
| Sample\_38 | Sample\_38 | R14 | Cell\_Line\_D | Treatment\_Y | Manufacturer\_M | Treatment\_Y\_Manufacturer\_M |
| Sample\_39 | Sample\_39 | R15 | Cell\_Line\_D | Treatment\_X | Manufacturer\_Q | Treatment\_X\_Manufacturer\_Q |
| Sample\_4 | Sample\_4 | R4 | Cell\_Line\_A | Treatment\_Y | Manufacturer\_Q | Treatment\_Y\_Manufacturer\_Q |
| Sample\_40 | Sample\_40 | R16 | Cell\_Line\_D | Treatment\_Y | Manufacturer\_Q | Treatment\_Y\_Manufacturer\_Q |
| Sample\_41 | Sample\_41 | R17 | Cell\_Line\_E | Treatment\_X | Manufacturer\_M | Treatment\_X\_Manufacturer\_M |
| Sample\_42 | Sample\_42 | R18 | Cell\_Line\_E | Treatment\_Y | Manufacturer\_M | Treatment\_Y\_Manufacturer\_M |
| Sample\_43 | Sample\_43 | R19 | Cell\_Line\_E | Treatment\_X | Manufacturer\_Q | Treatment\_X\_Manufacturer\_Q |
| Sample\_44 | Sample\_44 | R20 | Cell\_Line\_E | Treatment\_Y | Manufacturer\_Q | Treatment\_Y\_Manufacturer\_Q |
| Sample\_45 | Sample\_45 | R21 | Cell\_Line\_F | Treatment\_X | Manufacturer\_M | Treatment\_X\_Manufacturer\_M |
| Sample\_46 | Sample\_46 | R22 | Cell\_Line\_F | Treatment\_Y | Manufacturer\_M | Treatment\_Y\_Manufacturer\_M |
| Sample\_47 | Sample\_47 | R23 | Cell\_Line\_F | Treatment\_X | Manufacturer\_Q | Treatment\_X\_Manufacturer\_Q |
| Sample\_48 | Sample\_48 | R24 | Cell\_Line\_F | Treatment\_Y | Manufacturer\_Q | Treatment\_Y\_Manufacturer\_Q |
| Sample\_5 | Sample\_5 | R5 | Cell\_Line\_B | Treatment\_X | Manufacturer\_M | Treatment\_X\_Manufacturer\_M |
| Sample\_6 | Sample\_6 | R6 | Cell\_Line\_B | Treatment\_Y | Manufacturer\_M | Treatment\_Y\_Manufacturer\_M |
| Sample\_7 | Sample\_7 | R7 | Cell\_Line\_B | Treatment\_X | Manufacturer\_Q | Treatment\_X\_Manufacturer\_Q |
| Sample\_8 | Sample\_8 | R8 | Cell\_Line\_B | Treatment\_Y | Manufacturer\_Q | Treatment\_Y\_Manufacturer\_Q |
| Sample\_9 | Sample\_9 | R9 | Cell\_Line\_C | Treatment\_X | Manufacturer\_M | Treatment\_X\_Manufacturer\_M |

### Consolidated sample groups

```
$Replicate

 R1 R10 R11 R12 R13 R14 R15 R16 R17 R18 R19  R2 R20 R21 R22 R23 R24  R3  R4  R5 
  2   2   2   2   2   2   2   2   2   2   2   2   2   2   2   2   2   2   2   2 
 R6  R7  R8  R9 
  2   2   2   2 

$Cell_Line

Cell_Line_A Cell_Line_B Cell_Line_C Cell_Line_D Cell_Line_E Cell_Line_F 
          8           8           8           8           8           8 

$Treatment

Treatment_X Treatment_Y 
         24          24 

$Isolation

Manufacturer_M Manufacturer_Q 
            24             24 

$Treatment_Isolation

Treatment_X_Manufacturer_M Treatment_X_Manufacturer_Q 
                        12                         12 
Treatment_Y_Manufacturer_M Treatment_Y_Manufacturer_Q 
                        12                         12
```

## miRNA ORA Results

Over-representation analysis (ORA) is a statistical framework used to
determine whether a predefined set of genes or miRNAs is enriched for
specific biological functions, pathways, cellular processes, or disease
associations more than would be expected by chance. Instead of
evaluating individual miRNAs in isolation, ORA quantifies whether entire
categories occur disproportionately within the input list. For this
analysis, ORA was performed using miEAA (microRNA Enrichment Analysis
and Annotation), a specialized platform designed for miRNA-centric
functional enrichment. miEAA integrates heterogeneous annotation sources
including miRBase, miRPathDB, miRWalk, miRTarBase, miRandola, RNALocate,
isomiRdb, MNDR, and others.

The study design parameter chosen for DGE testing was
**Treatment\_Isolation** and the parameter(s) chosen for
correction were **Cell\_Line**.

### Treatment\_X\_Manufacturer\_M\_vs\_Treatment\_X\_Manufacturer\_Q

#### WikiPathways (miRPathDB)

This category represents pathway annotations curated within the
WikiPathways consortium, integrated into miRPathDB for miRNA-centric
enrichment. It includes signaling cascades, metabolic routes,
developmental pathways, and disease-specific modules. WikiPathways often
captures emerging or highly specialized pathways not present in more
canonical databases. Enrichment here highlights functional modules
potentially coordinated by sets of dysregulated miRNAs.

#### Reactome (miRPathDB)

This category maps miRNAs onto high-quality Reactome pathway
annotations, which include precise descriptions of molecular processes
such as signaling, DNA repair, apoptosis, and cell-cycle regulation.
Reactome’s hierarchical structure provides fine-resolution insights into
biologically meaningful subprocesses. Enrichment indicates regulatory
programs influenced by miRNA-driven target repression. These results are
mechanistically interpretable and highly reliable due to Reactome’s
expert curation.

#### GO Biological process (miRPathDB)

GO Biological Process terms represent coordinated biological
functions such as proliferation, apoptosis, migration, and
differentiation. miRPathDB maps miRNAs to these processes using
validated or computationally inferred target pathways. Enrichment
indicates higher-order functional programs influenced by the miRNA set.
These results provide biologically interpretable insight into the
systemic effects of miRNA dysregulation.

#### GO Cellular component (miRPathDB)

This category captures the subcellular structures or compartments
where gene products act, such as mitochondria, membranes, vesicles, or
the cytoskeleton. miRPathDB associates miRNAs with GO CC terms through
target gene annotation. Enrichment highlights specific cellular
locations under miRNA-driven regulatory pressure. This is important when
studying processes like secretion, organelle stress, or vesicular
biology.

#### GO Molecular function (miRPathDB)

GO Molecular Function terms describe biochemical activities such as
enzyme binding, kinase activity, or receptor interactions. miRPathDB
connects miRNAs to these functions through experimentally supported or
computationally inferred target genes. Enrichment reveals which
molecular activities are disproportionately targeted by deregulated
miRNAs. This helps identify mechanistic consequences of miRNA
changes.

#### Pathways (KEGG)

This category uses miEAA’s internal mapping of miRNAs to KEGG
pathways, independent of miRPathDB integration. These mappings often
produce complementary enrichment because they rely on internal curated
interactions. Enrichment suggests miRNA-driven modulation of classical
biological pathways. This category is particularly robust for widely
studied signaling and metabolic cascades.

#### Annotation (Gene Ontology)

This category contains direct GO annotations applied to miRNAs by
miEAA without relying on inferred target genes. It captures
experimentally observed functional roles attributed directly to specific
miRNAs. Enrichment indicates established biological functions associated
with these miRNAs beyond target-based inference. This may reveal
phenotypic or regulatory roles assigned in the literature.

#### Annotations derived over miRTarBase (Gene Ontology)

This category derives GO terms indirectly by mapping miRNAs through
experimentally validated miRNA–mRNA targets in miRTarBase. Because these
interactions are validated, this enrichment layer provides
high-confidence functional inference. Enrichment indicates biological
processes regulated by direct miRNA–target interactions rather than
prediction alone. This category often yields mechanistically
interpretable results.

#### Gene Ontology (miRWalk)

GO terms in this category are supplied through the miRWalk platform
using comprehensive predicted and validated miRNA–target data.
Enrichment highlights biological processes, molecular functions, or
cellular components regulated by the miRNA set. Because miRWalk
integrates numerous prediction models, it offers broad functional
coverage. This helps cross-validate findings from other GO
categories.

#### Pathways (miRWalk)

This category maps miRNAs to pathway annotations defined in miRWalk
through target-gene associations. It includes metabolic, signaling, and
disease pathways. Enrichment reveals biological processes
disproportionately targeted by the miRNA set. These results are often
broader or more exploratory compared to canonical pathway databases.

#### exRNA forms (miRandola)

This category captures extracellular RNA (exRNA) carriers such as
exosomes, microvesicles, AGO2 complexes, and HDL particles. Enrichment
indicates whether dysregulated miRNAs are preferentially associated with
specific extracellular transport mechanisms. These patterns may reflect
intercellular communication or secretion dynamics. This category is
highly relevant for studies involving circulating miRNAs or liquid
biopsies.

#### Chromosomal location (miRBase)

This category maps miRNAs to their precise chromosomal loci,
including clusters and host gene configurations. Enrichment highlights
genomic regions that may contribute disproportionately to observed
dysregulation. Chromosomal clustering may indicate coordinated
transcription or shared epigenetic regulation. These insights can reveal
genomic hotspots of miRNA regulation.

#### Seed family (miRBase)

This category organizes miRNAs based on shared seed sequences, which
define their target specificity. Enrichment indicates whether particular
seed families dominate the differential expression pattern, suggesting
coordinated targeting of the same gene networks. Seed families are
crucial determinants of regulatory logic. This category provides
mechanistic insight into shared targeting programs.

#### Target genes (miRTarBase)

This category uses experimentally validated interactions from
miRTarBase to infer enrichment of biological processes based on target
genes. Enrichment highlights functions regulated through direct
miRNA–mRNA interactions with the strongest empirical support. These
results often represent the highest-confidence mechanistic insights.
This category is valuable for identifying actionable biological
pathways.

#### Diseases (MNDR)

This category contains curated disease–miRNA associations from MNDR,
integrating literature, experiments, and computational analyses.
Enrichment indicates potential disease relevance of the miRNA set. MNDR
captures a wide range of pathological conditions, including cancer,
neurological disorders, and immune diseases. These findings are highly
useful for translational and clinical interpretation.

#### Interactions (NPInter)

This category catalogs miRNA interactions with noncoding RNAs and
other biomolecules from NPInter, including lncRNAs, protein complexes,
and RNA–RNA interactions. Enrichment suggests participation of the miRNA
set in complex regulatory networks such as ceRNA competition. These
interactions underlie broad post-transcriptional regulatory
architecture. This category provides insights into multilayered RNA
crosstalk.

#### Published Diseases

This category captures disease-associated miRNAs curated from
peer-reviewed literature. Enrichment suggests that the miRNA set is
involved in known disease-associated pathways or phenotypes. These
associations often overlap with major pathological processes such as
inflammation, tumor progression, or metabolic dysregulation. It is
particularly useful for translational interpretation.

#### Immune cells

This category includes immune cell–specific miRNA signatures from
published datasets. Enrichment indicates the involvement of the miRNA
set in immune regulation, inflammation, or immune cell differentiation.
Many of these miRNAs regulate cytokine signaling, antigen presentation,
or immune activation. This category is highly relevant in studies of
cancer, infection, or immunotherapy.

#### Cell-type specific (Cellular microRNAome)

This category is based on high-resolution miRNA expression profiles
across numerous cell types. Enrichment identifies cell-type-specific
regulatory patterns at finer granularity than other databases. These
associations can reveal lineage relationships, differentiation
trajectories, or microenvironmental contributions. This category is
especially valuable for interpreting complex tissue samples.

#### Localization (RNALocate)

This category annotates miRNAs based on subcellular localization
within structures such as the nucleus, cytoplasm, mitochondria,
vesicles, and stress granules. Enrichment indicates that dysregulated
miRNAs preferentially function within specific cellular compartments.
Subcellular localization influences miRNA mechanisms, including nuclear
feedback regulation or mitochondrial signaling. This category clarifies
compartment-specific regulatory dynamics.

#### Drugs (SM2miR)

This category catalogs drug–miRNA interactions based on experimental
evidence of drug-induced miRNA expression changes. Enrichment suggests
that the miRNA set may modulate or respond to specific therapeutic
agents. These associations support drug mechanism investigation and
potential repositioning strategies. This category is especially
informative for pharmacological and toxicological studies.

#### Expressed in tissue (Tissue Atlas)

This category integrates consensus miRNA expression across tissues
using HPA, GTEx, and FANTOM datasets. Enrichment indicates
tissue-of-origin signals or tissues most influenced by the dysregulated
miRNA set. These patterns support interpretation in heterogeneous tissue
samples or organ-specific diseases. This is highly relevant for
biomarker discovery and tissue classification.

#### Tissue specific (isomiRdb)

This category includes tissue-enriched isomiR variants from isomiRdb,
reflecting tissue-specific differences in miRNA processing and isoform
expression. Enrichment suggests regulatory variation specific to certain
tissues. IsomiRs can alter target specificity and regulatory potency.
This category captures subtle regulatory layers beyond canonical
miRNAs.

#### isomiRs (isomiRdb)

This category represents general isomiR annotations including
trimming, extension, or nucleotide substitution variants. Enrichment
indicates broader variation in miRNA processing mechanisms. IsomiRs
often modulate targeting precision and functional impact. This category
helps characterize post-transcriptional diversity within the miRNA
population.

### Treatment\_X\_Manufacturer\_M\_vs\_Treatment\_Y\_Manufacturer\_M

#### WikiPathways (miRPathDB)

This category represents pathway annotations curated within the
WikiPathways consortium, integrated into miRPathDB for miRNA-centric
enrichment. It includes signaling cascades, metabolic routes,
developmental pathways, and disease-specific modules. WikiPathways often
captures emerging or highly specialized pathways not present in more
canonical databases. Enrichment here highlights functional modules
potentially coordinated by sets of dysregulated miRNAs.

#### Reactome (miRPathDB)

This category maps miRNAs onto high-quality Reactome pathway
annotations, which include precise descriptions of molecular processes
such as signaling, DNA repair, apoptosis, and cell-cycle regulation.
Reactome’s hierarchical structure provides fine-resolution insights into
biologically meaningful subprocesses. Enrichment indicates regulatory
programs influenced by miRNA-driven target repression. These results are
mechanistically interpretable and highly reliable due to Reactome’s
expert curation.

#### KEGG (miRPathDB)

This category links miRNAs to KEGG pathways using transcript-level
mappings from miRPathDB. KEGG includes canonical pathways covering
metabolism, cell communication, genetic information processing, and
human diseases. Enrichment suggests convergence of miRNAs on
well-characterized biological systems. This is particularly useful for
highlighting miRNA-driven perturbation of established signaling
networks.

#### GO Biological process (miRPathDB)

GO Biological Process terms represent coordinated biological
functions such as proliferation, apoptosis, migration, and
differentiation. miRPathDB maps miRNAs to these processes using
validated or computationally inferred target pathways. Enrichment
indicates higher-order functional programs influenced by the miRNA set.
These results provide biologically interpretable insight into the
systemic effects of miRNA dysregulation.

#### GO Cellular component (miRPathDB)

This category captures the subcellular structures or compartments
where gene products act, such as mitochondria, membranes, vesicles, or
the cytoskeleton. miRPathDB associates miRNAs with GO CC terms through
target gene annotation. Enrichment highlights specific cellular
locations under miRNA-driven regulatory pressure. This is important when
studying processes like secretion, organelle stress, or vesicular
biology.

#### GO Molecular function (miRPathDB)

GO Molecular Function terms describe biochemical activities such as
enzyme binding, kinase activity, or receptor interactions. miRPathDB
connects miRNAs to these functions through experimentally supported or
computationally inferred target genes. Enrichment reveals which
molecular activities are disproportionately targeted by deregulated
miRNAs. This helps identify mechanistic consequences of miRNA
changes.

#### Pathways (KEGG)

This category uses miEAA’s internal mapping of miRNAs to KEGG
pathways, independent of miRPathDB integration. These mappings often
produce complementary enrichment because they rely on internal curated
interactions. Enrichment suggests miRNA-driven modulation of classical
biological pathways. This category is particularly robust for widely
studied signaling and metabolic cascades.

#### Annotation (Gene Ontology)

This category contains direct GO annotations applied to miRNAs by
miEAA without relying on inferred target genes. It captures
experimentally observed functional roles attributed directly to specific
miRNAs. Enrichment indicates established biological functions associated
with these miRNAs beyond target-based inference. This may reveal
phenotypic or regulatory roles assigned in the literature.

#### Annotations derived over miRTarBase (Gene Ontology)

This category derives GO terms indirectly by mapping miRNAs through
experimentally validated miRNA–mRNA targets in miRTarBase. Because these
interactions are validated, this enrichment layer provides
high-confidence functional inference. Enrichment indicates biological
processes regulated by direct miRNA–target interactions rather than
prediction alone. This category often yields mechanistically
interpretable results.

#### Gene Ontology (miRWalk)

GO terms in this category are supplied through the miRWalk platform
using comprehensive predicted and validated miRNA–target data.
Enrichment highlights biological processes, molecular functions, or
cellular components regulated by the miRNA set. Because miRWalk
integrates numerous prediction models, it offers broad functional
coverage. This helps cross-validate findings from other GO
categories.

#### Pathways (miRWalk)

This category maps miRNAs to pathway annotations defined in miRWalk
through target-gene associations. It includes metabolic, signaling, and
disease pathways. Enrichment reveals biological processes
disproportionately targeted by the miRNA set. These results are often
broader or more exploratory compared to canonical pathway databases.

#### exRNA forms (miRandola)

This category captures extracellular RNA (exRNA) carriers such as
exosomes, microvesicles, AGO2 complexes, and HDL particles. Enrichment
indicates whether dysregulated miRNAs are preferentially associated with
specific extracellular transport mechanisms. These patterns may reflect
intercellular communication or secretion dynamics. This category is
highly relevant for studies involving circulating miRNAs or liquid
biopsies.

#### Chromosomal location (miRBase)

This category maps miRNAs to their precise chromosomal loci,
including clusters and host gene configurations. Enrichment highlights
genomic regions that may contribute disproportionately to observed
dysregulation. Chromosomal clustering may indicate coordinated
transcription or shared epigenetic regulation. These insights can reveal
genomic hotspots of miRNA regulation.

#### Seed family (miRBase)

This category organizes miRNAs based on shared seed sequences, which
define their target specificity. Enrichment indicates whether particular
seed families dominate the differential expression pattern, suggesting
coordinated targeting of the same gene networks. Seed families are
crucial determinants of regulatory logic. This category provides
mechanistic insight into shared targeting programs.

#### Target genes (miRTarBase)

This category uses experimentally validated interactions from
miRTarBase to infer enrichment of biological processes based on target
genes. Enrichment highlights functions regulated through direct
miRNA–mRNA interactions with the strongest empirical support. These
results often represent the highest-confidence mechanistic insights.
This category is valuable for identifying actionable biological
pathways.

#### Diseases (MNDR)

This category contains curated disease–miRNA associations from MNDR,
integrating literature, experiments, and computational analyses.
Enrichment indicates potential disease relevance of the miRNA set. MNDR
captures a wide range of pathological conditions, including cancer,
neurological disorders, and immune diseases. These findings are highly
useful for translational and clinical interpretation.

#### Interactions (NPInter)

This category catalogs miRNA interactions with noncoding RNAs and
other biomolecules from NPInter, including lncRNAs, protein complexes,
and RNA–RNA interactions. Enrichment suggests participation of the miRNA
set in complex regulatory networks such as ceRNA competition. These
interactions underlie broad post-transcriptional regulatory
architecture. This category provides insights into multilayered RNA
crosstalk.

#### Published Diseases

This category captures disease-associated miRNAs curated from
peer-reviewed literature. Enrichment suggests that the miRNA set is
involved in known disease-associated pathways or phenotypes. These
associations often overlap with major pathological processes such as
inflammation, tumor progression, or metabolic dysregulation. It is
particularly useful for translational interpretation.

#### Immune cells

This category includes immune cell–specific miRNA signatures from
published datasets. Enrichment indicates the involvement of the miRNA
set in immune regulation, inflammation, or immune cell differentiation.
Many of these miRNAs regulate cytokine signaling, antigen presentation,
or immune activation. This category is highly relevant in studies of
cancer, infection, or immunotherapy.

#### Cell-type specific (Cellular microRNAome)

This category is based on high-resolution miRNA expression profiles
across numerous cell types. Enrichment identifies cell-type-specific
regulatory patterns at finer granularity than other databases. These
associations can reveal lineage relationships, differentiation
trajectories, or microenvironmental contributions. This category is
especially valuable for interpreting complex tissue samples.

#### Localization (RNALocate)

This category annotates miRNAs based on subcellular localization
within structures such as the nucleus, cytoplasm, mitochondria,
vesicles, and stress granules. Enrichment indicates that dysregulated
miRNAs preferentially function within specific cellular compartments.
Subcellular localization influences miRNA mechanisms, including nuclear
feedback regulation or mitochondrial signaling. This category clarifies
compartment-specific regulatory dynamics.

#### Drugs (SM2miR)

This category catalogs drug–miRNA interactions based on experimental
evidence of drug-induced miRNA expression changes. Enrichment suggests
that the miRNA set may modulate or respond to specific therapeutic
agents. These associations support drug mechanism investigation and
potential repositioning strategies. This category is especially
informative for pharmacological and toxicological studies.

#### Expressed in tissue (Tissue Atlas)

This category integrates consensus miRNA expression across tissues
using HPA, GTEx, and FANTOM datasets. Enrichment indicates
tissue-of-origin signals or tissues most influenced by the dysregulated
miRNA set. These patterns support interpretation in heterogeneous tissue
samples or organ-specific diseases. This is highly relevant for
biomarker discovery and tissue classification.

#### Tissue specific (isomiRdb)

This category includes tissue-enriched isomiR variants from isomiRdb,
reflecting tissue-specific differences in miRNA processing and isoform
expression. Enrichment suggests regulatory variation specific to certain
tissues. IsomiRs can alter target specificity and regulatory potency.
This category captures subtle regulatory layers beyond canonical
miRNAs.

#### isomiRs (isomiRdb)

This category represents general isomiR annotations including
trimming, extension, or nucleotide substitution variants. Enrichment
indicates broader variation in miRNA processing mechanisms. IsomiRs
often modulate targeting precision and functional impact. This category
helps characterize post-transcriptional diversity within the miRNA
population.

### Treatment\_X\_Manufacturer\_M\_vs\_Treatment\_Y\_Manufacturer\_Q

#### WikiPathways (miRPathDB)

This category represents pathway annotations curated within the
WikiPathways consortium, integrated into miRPathDB for miRNA-centric
enrichment. It includes signaling cascades, metabolic routes,
developmental pathways, and disease-specific modules. WikiPathways often
captures emerging or highly specialized pathways not present in more
canonical databases. Enrichment here highlights functional modules
potentially coordinated by sets of dysregulated miRNAs.

#### Reactome (miRPathDB)

This category maps miRNAs onto high-quality Reactome pathway
annotations, which include precise descriptions of molecular processes
such as signaling, DNA repair, apoptosis, and cell-cycle regulation.
Reactome’s hierarchical structure provides fine-resolution insights into
biologically meaningful subprocesses. Enrichment indicates regulatory
programs influenced by miRNA-driven target repression. These results are
mechanistically interpretable and highly reliable due to Reactome’s
expert curation.

#### KEGG (miRPathDB)

This category links miRNAs to KEGG pathways using transcript-level
mappings from miRPathDB. KEGG includes canonical pathways covering
metabolism, cell communication, genetic information processing, and
human diseases. Enrichment suggests convergence of miRNAs on
well-characterized biological systems. This is particularly useful for
highlighting miRNA-driven perturbation of established signaling
networks.

#### GO Biological process (miRPathDB)

GO Biological Process terms represent coordinated biological
functions such as proliferation, apoptosis, migration, and
differentiation. miRPathDB maps miRNAs to these processes using
validated or computationally inferred target pathways. Enrichment
indicates higher-order functional programs influenced by the miRNA set.
These results provide biologically interpretable insight into the
systemic effects of miRNA dysregulation.

#### GO Cellular component (miRPathDB)

This category captures the subcellular structures or compartments
where gene products act, such as mitochondria, membranes, vesicles, or
the cytoskeleton. miRPathDB associates miRNAs with GO CC terms through
target gene annotation. Enrichment highlights specific cellular
locations under miRNA-driven regulatory pressure. This is important when
studying processes like secretion, organelle stress, or vesicular
biology.

#### GO Molecular function (miRPathDB)

GO Molecular Function terms describe biochemical activities such as
enzyme binding, kinase activity, or receptor interactions. miRPathDB
connects miRNAs to these functions through experimentally supported or
computationally inferred target genes. Enrichment reveals which
molecular activities are disproportionately targeted by deregulated
miRNAs. This helps identify mechanistic consequences of miRNA
changes.

#### Pathways (KEGG)

This category uses miEAA’s internal mapping of miRNAs to KEGG
pathways, independent of miRPathDB integration. These mappings often
produce complementary enrichment because they rely on internal curated
interactions. Enrichment suggests miRNA-driven modulation of classical
biological pathways. This category is particularly robust for widely
studied signaling and metabolic cascades.

#### Annotation (Gene Ontology)

This category contains direct GO annotations applied to miRNAs by
miEAA without relying on inferred target genes. It captures
experimentally observed functional roles attributed directly to specific
miRNAs. Enrichment indicates established biological functions associated
with these miRNAs beyond target-based inference. This may reveal
phenotypic or regulatory roles assigned in the literature.

#### Annotations derived over miRTarBase (Gene Ontology)

This category derives GO terms indirectly by mapping miRNAs through
experimentally validated miRNA–mRNA targets in miRTarBase. Because these
interactions are validated, this enrichment layer provides
high-confidence functional inference. Enrichment indicates biological
processes regulated by direct miRNA–target interactions rather than
prediction alone. This category often yields mechanistically
interpretable results.

#### Gene Ontology (miRWalk)

GO terms in this category are supplied through the miRWalk platform
using comprehensive predicted and validated miRNA–target data.
Enrichment highlights biological processes, molecular functions, or
cellular components regulated by the miRNA set. Because miRWalk
integrates numerous prediction models, it offers broad functional
coverage. This helps cross-validate findings from other GO
categories.

#### Pathways (miRWalk)

This category maps miRNAs to pathway annotations defined in miRWalk
through target-gene associations. It includes metabolic, signaling, and
disease pathways. Enrichment reveals biological processes
disproportionately targeted by the miRNA set. These results are often
broader or more exploratory compared to canonical pathway databases.

#### exRNA forms (miRandola)

This category captures extracellular RNA (exRNA) carriers such as
exosomes, microvesicles, AGO2 complexes, and HDL particles. Enrichment
indicates whether dysregulated miRNAs are preferentially associated with
specific extracellular transport mechanisms. These patterns may reflect
intercellular communication or secretion dynamics. This category is
highly relevant for studies involving circulating miRNAs or liquid
biopsies.

#### Chromosomal location (miRBase)

This category maps miRNAs to their precise chromosomal loci,
including clusters and host gene configurations. Enrichment highlights
genomic regions that may contribute disproportionately to observed
dysregulation. Chromosomal clustering may indicate coordinated
transcription or shared epigenetic regulation. These insights can reveal
genomic hotspots of miRNA regulation.

#### Seed family (miRBase)

This category organizes miRNAs based on shared seed sequences, which
define their target specificity. Enrichment indicates whether particular
seed families dominate the differential expression pattern, suggesting
coordinated targeting of the same gene networks. Seed families are
crucial determinants of regulatory logic. This category provides
mechanistic insight into shared targeting programs.

#### Target genes (miRTarBase)

This category uses experimentally validated interactions from
miRTarBase to infer enrichment of biological processes based on target
genes. Enrichment highlights functions regulated through direct
miRNA–mRNA interactions with the strongest empirical support. These
results often represent the highest-confidence mechanistic insights.
This category is valuable for identifying actionable biological
pathways.

#### Diseases (MNDR)

This category contains curated disease–miRNA associations from MNDR,
integrating literature, experiments, and computational analyses.
Enrichment indicates potential disease relevance of the miRNA set. MNDR
captures a wide range of pathological conditions, including cancer,
neurological disorders, and immune diseases. These findings are highly
useful for translational and clinical interpretation.

#### Interactions (NPInter)

This category catalogs miRNA interactions with noncoding RNAs and
other biomolecules from NPInter, including lncRNAs, protein complexes,
and RNA–RNA interactions. Enrichment suggests participation of the miRNA
set in complex regulatory networks such as ceRNA competition. These
interactions underlie broad post-transcriptional regulatory
architecture. This category provides insights into multilayered RNA
crosstalk.

#### Published Diseases

This category captures disease-associated miRNAs curated from
peer-reviewed literature. Enrichment suggests that the miRNA set is
involved in known disease-associated pathways or phenotypes. These
associations often overlap with major pathological processes such as
inflammation, tumor progression, or metabolic dysregulation. It is
particularly useful for translational interpretation.

#### Cell-type specific (Cellular microRNAome)

This category is based on high-resolution miRNA expression profiles
across numerous cell types. Enrichment identifies cell-type-specific
regulatory patterns at finer granularity than other databases. These
associations can reveal lineage relationships, differentiation
trajectories, or microenvironmental contributions. This category is
especially valuable for interpreting complex tissue samples.

#### Localization (RNALocate)

This category annotates miRNAs based on subcellular localization
within structures such as the nucleus, cytoplasm, mitochondria,
vesicles, and stress granules. Enrichment indicates that dysregulated
miRNAs preferentially function within specific cellular compartments.
Subcellular localization influences miRNA mechanisms, including nuclear
feedback regulation or mitochondrial signaling. This category clarifies
compartment-specific regulatory dynamics.

#### Drugs (SM2miR)

This category catalogs drug–miRNA interactions based on experimental
evidence of drug-induced miRNA expression changes. Enrichment suggests
that the miRNA set may modulate or respond to specific therapeutic
agents. These associations support drug mechanism investigation and
potential repositioning strategies. This category is especially
informative for pharmacological and toxicological studies.

#### Expressed in tissue (Tissue Atlas)

This category integrates consensus miRNA expression across tissues
using HPA, GTEx, and FANTOM datasets. Enrichment indicates
tissue-of-origin signals or tissues most influenced by the dysregulated
miRNA set. These patterns support interpretation in heterogeneous tissue
samples or organ-specific diseases. This is highly relevant for
biomarker discovery and tissue classification.

#### Tissue specific (isomiRdb)

This category includes tissue-enriched isomiR variants from isomiRdb,
reflecting tissue-specific differences in miRNA processing and isoform
expression. Enrichment suggests regulatory variation specific to certain
tissues. IsomiRs can alter target specificity and regulatory potency.
This category captures subtle regulatory layers beyond canonical
miRNAs.

#### isomiRs (isomiRdb)

This category represents general isomiR annotations including
trimming, extension, or nucleotide substitution variants. Enrichment
indicates broader variation in miRNA processing mechanisms. IsomiRs
often modulate targeting precision and functional impact. This category
helps characterize post-transcriptional diversity within the miRNA
population.

### Treatment\_X\_Manufacturer\_Q\_vs\_Treatment\_Y\_Manufacturer\_M

#### WikiPathways (miRPathDB)

This category represents pathway annotations curated within the
WikiPathways consortium, integrated into miRPathDB for miRNA-centric
enrichment. It includes signaling cascades, metabolic routes,
developmental pathways, and disease-specific modules. WikiPathways often
captures emerging or highly specialized pathways not present in more
canonical databases. Enrichment here highlights functional modules
potentially coordinated by sets of dysregulated miRNAs.

#### Reactome (miRPathDB)

This category maps miRNAs onto high-quality Reactome pathway
annotations, which include precise descriptions of molecular processes
such as signaling, DNA repair, apoptosis, and cell-cycle regulation.
Reactome’s hierarchical structure provides fine-resolution insights into
biologically meaningful subprocesses. Enrichment indicates regulatory
programs influenced by miRNA-driven target repression. These results are
mechanistically interpretable and highly reliable due to Reactome’s
expert curation.

#### KEGG (miRPathDB)

This category links miRNAs to KEGG pathways using transcript-level
mappings from miRPathDB. KEGG includes canonical pathways covering
metabolism, cell communication, genetic information processing, and
human diseases. Enrichment suggests convergence of miRNAs on
well-characterized biological systems. This is particularly useful for
highlighting miRNA-driven perturbation of established signaling
networks.

#### GO Biological process (miRPathDB)

GO Biological Process terms represent coordinated biological
functions such as proliferation, apoptosis, migration, and
differentiation. miRPathDB maps miRNAs to these processes using
validated or computationally inferred target pathways. Enrichment
indicates higher-order functional programs influenced by the miRNA set.
These results provide biologically interpretable insight into the
systemic effects of miRNA dysregulation.

#### GO Cellular component (miRPathDB)

This category captures the subcellular structures or compartments
where gene products act, such as mitochondria, membranes, vesicles, or
the cytoskeleton. miRPathDB associates miRNAs with GO CC terms through
target gene annotation. Enrichment highlights specific cellular
locations under miRNA-driven regulatory pressure. This is important when
studying processes like secretion, organelle stress, or vesicular
biology.

#### GO Molecular function (miRPathDB)

GO Molecular Function terms describe biochemical activities such as
enzyme binding, kinase activity, or receptor interactions. miRPathDB
connects miRNAs to these functions through experimentally supported or
computationally inferred target genes. Enrichment reveals which
molecular activities are disproportionately targeted by deregulated
miRNAs. This helps identify mechanistic consequences of miRNA
changes.

#### Pathways (KEGG)

This category uses miEAA’s internal mapping of miRNAs to KEGG
pathways, independent of miRPathDB integration. These mappings often
produce complementary enrichment because they rely on internal curated
interactions. Enrichment suggests miRNA-driven modulation of classical
biological pathways. This category is particularly robust for widely
studied signaling and metabolic cascades.

#### Annotation (Gene Ontology)

This category contains direct GO annotations applied to miRNAs by
miEAA without relying on inferred target genes. It captures
experimentally observed functional roles attributed directly to specific
miRNAs. Enrichment indicates established biological functions associated
with these miRNAs beyond target-based inference. This may reveal
phenotypic or regulatory roles assigned in the literature.

#### Annotations derived over miRTarBase (Gene Ontology)

This category derives GO terms indirectly by mapping miRNAs through
experimentally validated miRNA–mRNA targets in miRTarBase. Because these
interactions are validated, this enrichment layer provides
high-confidence functional inference. Enrichment indicates biological
processes regulated by direct miRNA–target interactions rather than
prediction alone. This category often yields mechanistically
interpretable results.

#### Diseases (miRWalk)

This category includes disease-associated gene sets from miRWalk
mapped through predicted or validated miRNA–target interactions.
Enrichment reflects potential involvement of the miRNA set in specific
diseases or pathological processes. miRWalk’s broad disease coverage
makes this category particularly informative for exploring disease
relevance. Results often reveal connections to cancer, immune
dysfunction, or metabolic conditions.

#### Gene Ontology (miRWalk)

GO terms in this category are supplied through the miRWalk platform
using comprehensive predicted and validated miRNA–target data.
Enrichment highlights biological processes, molecular functions, or
cellular components regulated by the miRNA set. Because miRWalk
integrates numerous prediction models, it offers broad functional
coverage. This helps cross-validate findings from other GO
categories.

#### Pathways (miRWalk)

This category maps miRNAs to pathway annotations defined in miRWalk
through target-gene associations. It includes metabolic, signaling, and
disease pathways. Enrichment reveals biological processes
disproportionately targeted by the miRNA set. These results are often
broader or more exploratory compared to canonical pathway databases.

#### exRNA forms (miRandola)

This category captures extracellular RNA (exRNA) carriers such as
exosomes, microvesicles, AGO2 complexes, and HDL particles. Enrichment
indicates whether dysregulated miRNAs are preferentially associated with
specific extracellular transport mechanisms. These patterns may reflect
intercellular communication or secretion dynamics. This category is
highly relevant for studies involving circulating miRNAs or liquid
biopsies.

#### Chromosomal location (miRBase)

This category maps miRNAs to their precise chromosomal loci,
including clusters and host gene configurations. Enrichment highlights
genomic regions that may contribute disproportionately to observed
dysregulation. Chromosomal clustering may indicate coordinated
transcription or shared epigenetic regulation. These insights can reveal
genomic hotspots of miRNA regulation.

#### Seed family (miRBase)

This category organizes miRNAs based on shared seed sequences, which
define their target specificity. Enrichment indicates whether particular
seed families dominate the differential expression pattern, suggesting
coordinated targeting of the same gene networks. Seed families are
crucial determinants of regulatory logic. This category provides
mechanistic insight into shared targeting programs.

#### Target genes (miRTarBase)

This category uses experimentally validated interactions from
miRTarBase to infer enrichment of biological processes based on target
genes. Enrichment highlights functions regulated through direct
miRNA–mRNA interactions with the strongest empirical support. These
results often represent the highest-confidence mechanistic insights.
This category is valuable for identifying actionable biological
pathways.

#### Diseases (MNDR)

This category contains curated disease–miRNA associations from MNDR,
integrating literature, experiments, and computational analyses.
Enrichment indicates potential disease relevance of the miRNA set. MNDR
captures a wide range of pathological conditions, including cancer,
neurological disorders, and immune diseases. These findings are highly
useful for translational and clinical interpretation.

#### Interactions (NPInter)

This category catalogs miRNA interactions with noncoding RNAs and
other biomolecules from NPInter, including lncRNAs, protein complexes,
and RNA–RNA interactions. Enrichment suggests participation of the miRNA
set in complex regulatory networks such as ceRNA competition. These
interactions underlie broad post-transcriptional regulatory
architecture. This category provides insights into multilayered RNA
crosstalk.

#### Published Diseases

This category captures disease-associated miRNAs curated from
peer-reviewed literature. Enrichment suggests that the miRNA set is
involved in known disease-associated pathways or phenotypes. These
associations often overlap with major pathological processes such as
inflammation, tumor progression, or metabolic dysregulation. It is
particularly useful for translational interpretation.

#### Immune cells

This category includes immune cell–specific miRNA signatures from
published datasets. Enrichment indicates the involvement of the miRNA
set in immune regulation, inflammation, or immune cell differentiation.
Many of these miRNAs regulate cytokine signaling, antigen presentation,
or immune activation. This category is highly relevant in studies of
cancer, infection, or immunotherapy.

#### Cell-type specific (Cellular microRNAome)

This category is based on high-resolution miRNA expression profiles
across numerous cell types. Enrichment identifies cell-type-specific
regulatory patterns at finer granularity than other databases. These
associations can reveal lineage relationships, differentiation
trajectories, or microenvironmental contributions. This category is
especially valuable for interpreting complex tissue samples.

#### Localization (RNALocate)

This category annotates miRNAs based on subcellular localization
within structures such as the nucleus, cytoplasm, mitochondria,
vesicles, and stress granules. Enrichment indicates that dysregulated
miRNAs preferentially function within specific cellular compartments.
Subcellular localization influences miRNA mechanisms, including nuclear
feedback regulation or mitochondrial signaling. This category clarifies
compartment-specific regulatory dynamics.

#### Drugs (SM2miR)

This category catalogs drug–miRNA interactions based on experimental
evidence of drug-induced miRNA expression changes. Enrichment suggests
that the miRNA set may modulate or respond to specific therapeutic
agents. These associations support drug mechanism investigation and
potential repositioning strategies. This category is especially
informative for pharmacological and toxicological studies.

#### Expressed in tissue (Tissue Atlas)

This category integrates consensus miRNA expression across tissues
using HPA, GTEx, and FANTOM datasets. Enrichment indicates
tissue-of-origin signals or tissues most influenced by the dysregulated
miRNA set. These patterns support interpretation in heterogeneous tissue
samples or organ-specific diseases. This is highly relevant for
biomarker discovery and tissue classification.

#### Tissue specific (isomiRdb)

This category includes tissue-enriched isomiR variants from isomiRdb,
reflecting tissue-specific differences in miRNA processing and isoform
expression. Enrichment suggests regulatory variation specific to certain
tissues. IsomiRs can alter target specificity and regulatory potency.
This category captures subtle regulatory layers beyond canonical
miRNAs.

#### isomiRs (isomiRdb)

This category represents general isomiR annotations including
trimming, extension, or nucleotide substitution variants. Enrichment
indicates broader variation in miRNA processing mechanisms. IsomiRs
often modulate targeting precision and functional impact. This category
helps characterize post-transcriptional diversity within the miRNA
population.

### Treatment\_X\_Manufacturer\_Q\_vs\_Treatment\_Y\_Manufacturer\_Q

#### WikiPathways (miRPathDB)

This category represents pathway annotations curated within the
WikiPathways consortium, integrated into miRPathDB for miRNA-centric
enrichment. It includes signaling cascades, metabolic routes,
developmental pathways, and disease-specific modules. WikiPathways often
captures emerging or highly specialized pathways not present in more
canonical databases. Enrichment here highlights functional modules
potentially coordinated by sets of dysregulated miRNAs.

#### Reactome (miRPathDB)

This category maps miRNAs onto high-quality Reactome pathway
annotations, which include precise descriptions of molecular processes
such as signaling, DNA repair, apoptosis, and cell-cycle regulation.
Reactome’s hierarchical structure provides fine-resolution insights into
biologically meaningful subprocesses. Enrichment indicates regulatory
programs influenced by miRNA-driven target repression. These results are
mechanistically interpretable and highly reliable due to Reactome’s
expert curation.

#### KEGG (miRPathDB)

This category links miRNAs to KEGG pathways using transcript-level
mappings from miRPathDB. KEGG includes canonical pathways covering
metabolism, cell communication, genetic information processing, and
human diseases. Enrichment suggests convergence of miRNAs on
well-characterized biological systems. This is particularly useful for
highlighting miRNA-driven perturbation of established signaling
networks.

#### GO Biological process (miRPathDB)

GO Biological Process terms represent coordinated biological
functions such as proliferation, apoptosis, migration, and
differentiation. miRPathDB maps miRNAs to these processes using
validated or computationally inferred target pathways. Enrichment
indicates higher-order functional programs influenced by the miRNA set.
These results provide biologically interpretable insight into the
systemic effects of miRNA dysregulation.

#### GO Cellular component (miRPathDB)

This category captures the subcellular structures or compartments
where gene products act, such as mitochondria, membranes, vesicles, or
the cytoskeleton. miRPathDB associates miRNAs with GO CC terms through
target gene annotation. Enrichment highlights specific cellular
locations under miRNA-driven regulatory pressure. This is important when
studying processes like secretion, organelle stress, or vesicular
biology.

#### GO Molecular function (miRPathDB)

GO Molecular Function terms describe biochemical activities such as
enzyme binding, kinase activity, or receptor interactions. miRPathDB
connects miRNAs to these functions through experimentally supported or
computationally inferred target genes. Enrichment reveals which
molecular activities are disproportionately targeted by deregulated
miRNAs. This helps identify mechanistic consequences of miRNA
changes.

#### Pathways (KEGG)

This category uses miEAA’s internal mapping of miRNAs to KEGG
pathways, independent of miRPathDB integration. These mappings often
produce complementary enrichment because they rely on internal curated
interactions. Enrichment suggests miRNA-driven modulation of classical
biological pathways. This category is particularly robust for widely
studied signaling and metabolic cascades.

#### Annotation (Gene Ontology)

This category contains direct GO annotations applied to miRNAs by
miEAA without relying on inferred target genes. It captures
experimentally observed functional roles attributed directly to specific
miRNAs. Enrichment indicates established biological functions associated
with these miRNAs beyond target-based inference. This may reveal
phenotypic or regulatory roles assigned in the literature.

#### Annotations derived over miRTarBase (Gene Ontology)

This category derives GO terms indirectly by mapping miRNAs through
experimentally validated miRNA–mRNA targets in miRTarBase. Because these
interactions are validated, this enrichment layer provides
high-confidence functional inference. Enrichment indicates biological
processes regulated by direct miRNA–target interactions rather than
prediction alone. This category often yields mechanistically
interpretable results.

#### Gene Ontology (miRWalk)

GO terms in this category are supplied through the miRWalk platform
using comprehensive predicted and validated miRNA–target data.
Enrichment highlights biological processes, molecular functions, or
cellular components regulated by the miRNA set. Because miRWalk
integrates numerous prediction models, it offers broad functional
coverage. This helps cross-validate findings from other GO
categories.

#### Pathways (miRWalk)

This category maps miRNAs to pathway annotations defined in miRWalk
through target-gene associations. It includes metabolic, signaling, and
disease pathways. Enrichment reveals biological processes
disproportionately targeted by the miRNA set. These results are often
broader or more exploratory compared to canonical pathway databases.

#### exRNA forms (miRandola)

This category captures extracellular RNA (exRNA) carriers such as
exosomes, microvesicles, AGO2 complexes, and HDL particles. Enrichment
indicates whether dysregulated miRNAs are preferentially associated with
specific extracellular transport mechanisms. These patterns may reflect
intercellular communication or secretion dynamics. This category is
highly relevant for studies involving circulating miRNAs or liquid
biopsies.

#### Chromosomal location (miRBase)

This category maps miRNAs to their precise chromosomal loci,
including clusters and host gene configurations. Enrichment highlights
genomic regions that may contribute disproportionately to observed
dysregulation. Chromosomal clustering may indicate coordinated
transcription or shared epigenetic regulation. These insights can reveal
genomic hotspots of miRNA regulation.

#### Seed family (miRBase)

This category organizes miRNAs based on shared seed sequences, which
define their target specificity. Enrichment indicates whether particular
seed families dominate the differential expression pattern, suggesting
coordinated targeting of the same gene networks. Seed families are
crucial determinants of regulatory logic. This category provides
mechanistic insight into shared targeting programs.

#### Target genes (miRTarBase)

This category uses experimentally validated interactions from
miRTarBase to infer enrichment of biological processes based on target
genes. Enrichment highlights functions regulated through direct
miRNA–mRNA interactions with the strongest empirical support. These
results often represent the highest-confidence mechanistic insights.
This category is valuable for identifying actionable biological
pathways.

#### Diseases (MNDR)

This category contains curated disease–miRNA associations from MNDR,
integrating literature, experiments, and computational analyses.
Enrichment indicates potential disease relevance of the miRNA set. MNDR
captures a wide range of pathological conditions, including cancer,
neurological disorders, and immune diseases. These findings are highly
useful for translational and clinical interpretation.

#### Interactions (NPInter)

This category catalogs miRNA interactions with noncoding RNAs and
other biomolecules from NPInter, including lncRNAs, protein complexes,
and RNA–RNA interactions. Enrichment suggests participation of the miRNA
set in complex regulatory networks such as ceRNA competition. These
interactions underlie broad post-transcriptional regulatory
architecture. This category provides insights into multilayered RNA
crosstalk.

#### Published Diseases

This category captures disease-associated miRNAs curated from
peer-reviewed literature. Enrichment suggests that the miRNA set is
involved in known disease-associated pathways or phenotypes. These
associations often overlap with major pathological processes such as
inflammation, tumor progression, or metabolic dysregulation. It is
particularly useful for translational interpretation.

#### Immune cells

This category includes immune cell–specific miRNA signatures from
published datasets. Enrichment indicates the involvement of the miRNA
set in immune regulation, inflammation, or immune cell differentiation.
Many of these miRNAs regulate cytokine signaling, antigen presentation,
or immune activation. This category is highly relevant in studies of
cancer, infection, or immunotherapy.

#### Cell-type specific (Cellular microRNAome)

This category is based on high-resolution miRNA expression profiles
across numerous cell types. Enrichment identifies cell-type-specific
regulatory patterns at finer granularity than other databases. These
associations can reveal lineage relationships, differentiation
trajectories, or microenvironmental contributions. This category is
especially valuable for interpreting complex tissue samples.

#### Localization (RNALocate)

This category annotates miRNAs based on subcellular localization
within structures such as the nucleus, cytoplasm, mitochondria,
vesicles, and stress granules. Enrichment indicates that dysregulated
miRNAs preferentially function within specific cellular compartments.
Subcellular localization influences miRNA mechanisms, including nuclear
feedback regulation or mitochondrial signaling. This category clarifies
compartment-specific regulatory dynamics.

#### Drugs (SM2miR)

This category catalogs drug–miRNA interactions based on experimental
evidence of drug-induced miRNA expression changes. Enrichment suggests
that the miRNA set may modulate or respond to specific therapeutic
agents. These associations support drug mechanism investigation and
potential repositioning strategies. This category is especially
informative for pharmacological and toxicological studies.

#### Expressed in tissue (Tissue Atlas)

This category integrates consensus miRNA expression across tissues
using HPA, GTEx, and FANTOM datasets. Enrichment indicates
tissue-of-origin signals or tissues most influenced by the dysregulated
miRNA set. These patterns support interpretation in heterogeneous tissue
samples or organ-specific diseases. This is highly relevant for
biomarker discovery and tissue classification.

#### Tissue specific (isomiRdb)

This category includes tissue-enriched isomiR variants from isomiRdb,
reflecting tissue-specific differences in miRNA processing and isoform
expression. Enrichment suggests regulatory variation specific to certain
tissues. IsomiRs can alter target specificity and regulatory potency.
This category captures subtle regulatory layers beyond canonical
miRNAs.

#### isomiRs (isomiRdb)

This category represents general isomiR annotations including
trimming, extension, or nucleotide substitution variants. Enrichment
indicates broader variation in miRNA processing mechanisms. IsomiRs
often modulate targeting precision and functional impact. This category
helps characterize post-transcriptional diversity within the miRNA
population.

### Treatment\_Y\_Manufacturer\_M\_vs\_Treatment\_Y\_Manufacturer\_Q

#### WikiPathways (miRPathDB)

This category represents pathway annotations curated within the
WikiPathways consortium, integrated into miRPathDB for miRNA-centric
enrichment. It includes signaling cascades, metabolic routes,
developmental pathways, and disease-specific modules. WikiPathways often
captures emerging or highly specialized pathways not present in more
canonical databases. Enrichment here highlights functional modules
potentially coordinated by sets of dysregulated miRNAs.

#### Reactome (miRPathDB)

This category maps miRNAs onto high-quality Reactome pathway
annotations, which include precise descriptions of molecular processes
such as signaling, DNA repair, apoptosis, and cell-cycle regulation.
Reactome’s hierarchical structure provides fine-resolution insights into
biologically meaningful subprocesses. Enrichment indicates regulatory
programs influenced by miRNA-driven target repression. These results are
mechanistically interpretable and highly reliable due to Reactome’s
expert curation.

#### KEGG (miRPathDB)

This category links miRNAs to KEGG pathways using transcript-level
mappings from miRPathDB. KEGG includes canonical pathways covering
metabolism, cell communication, genetic information processing, and
human diseases. Enrichment suggests convergence of miRNAs on
well-characterized biological systems. This is particularly useful for
highlighting miRNA-driven perturbation of established signaling
networks.

#### GO Biological process (miRPathDB)

GO Biological Process terms represent coordinated biological
functions such as proliferation, apoptosis, migration, and
differentiation. miRPathDB maps miRNAs to these processes using
validated or computationally inferred target pathways. Enrichment
indicates higher-order functional programs influenced by the miRNA set.
These results provide biologically interpretable insight into the
systemic effects of miRNA dysregulation.

#### GO Cellular component (miRPathDB)

This category captures the subcellular structures or compartments
where gene products act, such as mitochondria, membranes, vesicles, or
the cytoskeleton. miRPathDB associates miRNAs with GO CC terms through
target gene annotation. Enrichment highlights specific cellular
locations under miRNA-driven regulatory pressure. This is important when
studying processes like secretion, organelle stress, or vesicular
biology.

#### GO Molecular function (miRPathDB)

GO Molecular Function terms describe biochemical activities such as
enzyme binding, kinase activity, or receptor interactions. miRPathDB
connects miRNAs to these functions through experimentally supported or
computationally inferred target genes. Enrichment reveals which
molecular activities are disproportionately targeted by deregulated
miRNAs. This helps identify mechanistic consequences of miRNA
changes.

#### Pathways (KEGG)

This category uses miEAA’s internal mapping of miRNAs to KEGG
pathways, independent of miRPathDB integration. These mappings often
produce complementary enrichment because they rely on internal curated
interactions. Enrichment suggests miRNA-driven modulation of classical
biological pathways. This category is particularly robust for widely
studied signaling and metabolic cascades.

#### Annotation (Gene Ontology)

This category contains direct GO annotations applied to miRNAs by
miEAA without relying on inferred target genes. It captures
experimentally observed functional roles attributed directly to specific
miRNAs. Enrichment indicates established biological functions associated
with these miRNAs beyond target-based inference. This may reveal
phenotypic or regulatory roles assigned in the literature.

#### Annotations derived over miRTarBase (Gene Ontology)

This category derives GO terms indirectly by mapping miRNAs through
experimentally validated miRNA–mRNA targets in miRTarBase. Because these
interactions are validated, this enrichment layer provides
high-confidence functional inference. Enrichment indicates biological
processes regulated by direct miRNA–target interactions rather than
prediction alone. This category often yields mechanistically
interpretable results.

#### Gene Ontology (miRWalk)

GO terms in this category are supplied through the miRWalk platform
using comprehensive predicted and validated miRNA–target data.
Enrichment highlights biological processes, molecular functions, or
cellular components regulated by the miRNA set. Because miRWalk
integrates numerous prediction models, it offers broad functional
coverage. This helps cross-validate findings from other GO
categories.

#### Pathways (miRWalk)

This category maps miRNAs to pathway annotations defined in miRWalk
through target-gene associations. It includes metabolic, signaling, and
disease pathways. Enrichment reveals biological processes
disproportionately targeted by the miRNA set. These results are often
broader or more exploratory compared to canonical pathway databases.

#### exRNA forms (miRandola)

This category captures extracellular RNA (exRNA) carriers such as
exosomes, microvesicles, AGO2 complexes, and HDL particles. Enrichment
indicates whether dysregulated miRNAs are preferentially associated with
specific extracellular transport mechanisms. These patterns may reflect
intercellular communication or secretion dynamics. This category is
highly relevant for studies involving circulating miRNAs or liquid
biopsies.

#### Seed family (miRBase)

This category organizes miRNAs based on shared seed sequences, which
define their target specificity. Enrichment indicates whether particular
seed families dominate the differential expression pattern, suggesting
coordinated targeting of the same gene networks. Seed families are
crucial determinants of regulatory logic. This category provides
mechanistic insight into shared targeting programs.

#### Target genes (miRTarBase)

This category uses experimentally validated interactions from
miRTarBase to infer enrichment of biological processes based on target
genes. Enrichment highlights functions regulated through direct
miRNA–mRNA interactions with the strongest empirical support. These
results often represent the highest-confidence mechanistic insights.
This category is valuable for identifying actionable biological
pathways.

#### Diseases (MNDR)

This category contains curated disease–miRNA associations from MNDR,
integrating literature, experiments, and computational analyses.
Enrichment indicates potential disease relevance of the miRNA set. MNDR
captures a wide range of pathological conditions, including cancer,
neurological disorders, and immune diseases. These findings are highly
useful for translational and clinical interpretation.

#### Interactions (NPInter)

This category catalogs miRNA interactions with noncoding RNAs and
other biomolecules from NPInter, including lncRNAs, protein complexes,
and RNA–RNA interactions. Enrichment suggests participation of the miRNA
set in complex regulatory networks such as ceRNA competition. These
interactions underlie broad post-transcriptional regulatory
architecture. This category provides insights into multilayered RNA
crosstalk.

#### Published Diseases

This category captures disease-associated miRNAs curated from
peer-reviewed literature. Enrichment suggests that the miRNA set is
involved in known disease-associated pathways or phenotypes. These
associations often overlap with major pathological processes such as
inflammation, tumor progression, or metabolic dysregulation. It is
particularly useful for translational interpretation.

#### Immune cells

This category includes immune cell–specific miRNA signatures from
published datasets. Enrichment indicates the involvement of the miRNA
set in immune regulation, inflammation, or immune cell differentiation.
Many of these miRNAs regulate cytokine signaling, antigen presentation,
or immune activation. This category is highly relevant in studies of
cancer, infection, or immunotherapy.

#### Cell-type specific (Cellular microRNAome)

This category is based on high-resolution miRNA expression profiles
across numerous cell types. Enrichment identifies cell-type-specific
regulatory patterns at finer granularity than other databases. These
associations can reveal lineage relationships, differentiation
trajectories, or microenvironmental contributions. This category is
especially valuable for interpreting complex tissue samples.

#### Localization (RNALocate)

This category annotates miRNAs based on subcellular localization
within structures such as the nucleus, cytoplasm, mitochondria,
vesicles, and stress granules. Enrichment indicates that dysregulated
miRNAs preferentially function within specific cellular compartments.
Subcellular localization influences miRNA mechanisms, including nuclear
feedback regulation or mitochondrial signaling. This category clarifies
compartment-specific regulatory dynamics.

#### Drugs (SM2miR)

This category catalogs drug–miRNA interactions based on experimental
evidence of drug-induced miRNA expression changes. Enrichment suggests
that the miRNA set may modulate or respond to specific therapeutic
agents. These associations support drug mechanism investigation and
potential repositioning strategies. This category is especially
informative for pharmacological and toxicological studies.

#### Expressed in tissue (Tissue Atlas)

This category integrates consensus miRNA expression across tissues
using HPA, GTEx, and FANTOM datasets. Enrichment indicates
tissue-of-origin signals or tissues most influenced by the dysregulated
miRNA set. These patterns support interpretation in heterogeneous tissue
samples or organ-specific diseases. This is highly relevant for
biomarker discovery and tissue classification.

#### Tissue specific (isomiRdb)

This category includes tissue-enriched isomiR variants from isomiRdb,
reflecting tissue-specific differences in miRNA processing and isoform
expression. Enrichment suggests regulatory variation specific to certain
tissues. IsomiRs can alter target specificity and regulatory potency.
This category captures subtle regulatory layers beyond canonical
miRNAs.

#### isomiRs (isomiRdb)

This category represents general isomiR annotations including
trimming, extension, or nucleotide substitution variants. Enrichment
indicates broader variation in miRNA processing mechanisms. IsomiRs
often modulate targeting precision and functional impact. This category
helps characterize post-transcriptional diversity within the miRNA
population.

## miRNA Target Prediction Results

This section summarizes predicted miRNA–mRNA regulatory interactions
identified using TargetScan, a widely used algorithm that infers miRNA
targeting based on seed sequence complementarity, evolutionary
conservation, and contextual features of 3′UTR binding sites. The
resulting interaction set represents putative post-transcriptional
regulatory relationships through which differentially expressed miRNAs
may influence downstream gene expression programs.

To contextualize these predicted interactions at the tissue level,
target genes were annotated with consensus tissue expression values
(nTPM) derived from the Human Protein Atlas (HPA) and the
Genotype-Tissue Expression (GTEx) project. These resources integrate
large-scale transcriptomic measurements across a broad spectrum of human
tissues, providing a robust estimate of physiological gene expression
patterns. Predicted miRNA target genes are presented grouped by tissue,
with each table containing targets that show detectable expression in
the respective tissue context.

The threshold chosen for tissue specificity was nTPM =
**50** and tissue specific ORA was analyzed for
**lymph node, spleen**.

### Treatment\_X\_Manufacturer\_M\_vs\_Treatment\_X\_Manufacturer\_Q

#### Tissue expression heatmap (log10-transformed)

#### liver

#### basal ganglia

#### cerebellum

#### cerebral cortex

#### choroid plexus

#### hypothalamus

#### midbrain

#### pancreas

#### parathyroid gland

#### retina

#### salivary gland

#### skeletal muscle

#### spinal cord

#### thyroid gland

#### colon

#### rectum

#### small intestine

#### blood vessel

#### cervix

#### endometrium

#### fallopian tube

#### ovary

#### urinary bladder

#### stomach

#### adrenal gland

#### amygdala

#### bone marrow

#### breast

#### duodenum

#### epididymis

#### heart muscle

#### hippocampal formation

#### kidney

#### lymph node

#### pituitary gland

#### placenta

#### thymus

#### tongue

#### tonsil

#### prostate

#### adipose tissue

#### appendix

#### esophagus

#### gallbladder

#### lung

#### seminal vesicle

#### skin

#### smooth muscle

#### spleen

#### testis

#### vagina

### Treatment\_X\_Manufacturer\_M\_vs\_Treatment\_Y\_Manufacturer\_M

#### Tissue expression heatmap (log10-transformed)

#### liver

#### bone marrow

#### kidney

#### colon

#### rectum

#### blood vessel

#### cervix

#### endometrium

#### fallopian tube

#### ovary

#### urinary bladder

#### amygdala

#### basal ganglia

#### cerebral cortex

#### hippocampal formation

#### stomach

#### cerebellum

#### hypothalamus

#### skeletal muscle

#### prostate

#### testis

#### breast

#### parathyroid gland

#### adipose tissue

#### adrenal gland

#### appendix

#### choroid plexus

#### duodenum

#### epididymis

#### esophagus

#### gallbladder

#### heart muscle

#### lung

#### lymph node

#### midbrain

#### pancreas

#### pituitary gland

#### placenta

#### salivary gland

#### seminal vesicle

#### skin

#### small intestine

#### smooth muscle

#### spleen

#### thymus

#### thyroid gland

#### tongue

#### vagina

#### spinal cord

#### retina

#### tonsil

### Treatment\_X\_Manufacturer\_M\_vs\_Treatment\_Y\_Manufacturer\_Q

#### Tissue expression heatmap (log10-transformed)

#### liver

#### adipose tissue

#### adrenal gland

#### amygdala

#### basal ganglia

#### blood vessel

#### breast

#### cerebellum

#### cerebral cortex

#### cervix

#### choroid plexus

#### colon

#### duodenum

#### endometrium

#### esophagus

#### fallopian tube

#### gallbladder

#### heart muscle

#### hippocampal formation

#### hypothalamus

#### kidney

#### midbrain

#### ovary

#### pancreas

#### parathyroid gland

#### pituitary gland

#### placenta

#### prostate

#### rectum

#### salivary gland

#### seminal vesicle

#### skeletal muscle

#### skin

#### small intestine

#### spinal cord

#### spleen

#### stomach

#### testis

#### thymus

#### thyroid gland

#### tongue

#### urinary bladder

#### vagina

#### retina

#### bone marrow

#### epididymis

#### lymph node

#### tonsil

#### appendix

#### lung

#### smooth muscle

### Treatment\_X\_Manufacturer\_Q\_vs\_Treatment\_Y\_Manufacturer\_M

#### Tissue expression heatmap (log10-transformed)

#### adipose tissue

#### adrenal gland

#### amygdala

#### basal ganglia

#### blood vessel

#### breast

#### cerebellum

#### cerebral cortex

#### cervix

#### choroid plexus

#### colon

#### duodenum

#### endometrium

#### esophagus

#### fallopian tube

#### gallbladder

#### heart muscle

#### hippocampal formation

#### hypothalamus

#### kidney

#### liver

#### midbrain

#### ovary

#### pancreas

#### parathyroid gland

#### pituitary gland

#### placenta

#### prostate

#### rectum

#### salivary gland

#### seminal vesicle

#### skeletal muscle

#### skin

#### small intestine

#### spinal cord

#### spleen

#### stomach

#### testis

#### thymus

#### thyroid gland

#### tongue

#### urinary bladder

#### vagina

#### retina

#### appendix

#### bone marrow

#### epididymis

#### lymph node

#### tonsil

#### lung

#### smooth muscle

### Treatment\_X\_Manufacturer\_Q\_vs\_Treatment\_Y\_Manufacturer\_Q

#### Tissue expression heatmap (log10-transformed)

#### liver

#### bone marrow

#### cerebellum

#### choroid plexus

#### retina

#### skeletal muscle

#### stomach

#### kidney

#### colon

#### rectum

#### epididymis

#### pancreas

#### small intestine

#### amygdala

#### basal ganglia

#### cerebral cortex

#### hippocampal formation

#### spleen

#### hypothalamus

#### prostate

#### testis

#### urinary bladder

#### adipose tissue

#### adrenal gland

#### appendix

#### blood vessel

#### breast

#### cervix

#### duodenum

#### endometrium

#### esophagus

#### fallopian tube

#### gallbladder

#### heart muscle

#### lung

#### lymph node

#### midbrain

#### ovary

#### parathyroid gland

#### pituitary gland

#### placenta

#### salivary gland

#### seminal vesicle

#### skin

#### smooth muscle

#### thymus

#### thyroid gland

#### tongue

#### vagina

#### spinal cord

#### tonsil

### Treatment\_Y\_Manufacturer\_M\_vs\_Treatment\_Y\_Manufacturer\_Q

#### Tissue expression heatmap (log10-transformed)

#### liver

#### adipose tissue

#### adrenal gland

#### amygdala

#### basal ganglia

#### blood vessel

#### breast

#### cerebellum

#### cerebral cortex

#### cervix

#### choroid plexus

#### colon

#### duodenum

#### endometrium

#### esophagus

#### fallopian tube

#### gallbladder

#### heart muscle

#### hippocampal formation

#### hypothalamus

#### kidney

#### midbrain

#### ovary

#### pancreas

#### parathyroid gland

#### pituitary gland

#### placenta

#### prostate

#### rectum

#### salivary gland

#### seminal vesicle

#### skeletal muscle

#### skin

#### small intestine

#### spinal cord

#### spleen

#### stomach

#### testis

#### thymus

#### thyroid gland

#### tongue

#### urinary bladder

#### vagina

#### retina

#### bone marrow

#### epididymis

#### lymph node

#### tonsil

#### appendix

#### lung

#### smooth muscle

## Treatment\_X\_Manufacturer\_M\_vs\_Treatment\_X\_Manufacturer\_Q - Tissue specific ORA

### all\_genes

#### GO\_Biological\_Process\_2025

This category comprises Gene Ontology (GO) Biological Process terms
describing coordinated and temporally ordered biological programs such
as cell cycle progression, immune activation, apoptosis,
differentiation, and metabolic regulation. GO Biological Process
annotations are curated by the Gene Ontology Consortium and integrate
experimental evidence, computational inference, and expert review.
Enrichment in this category indicates that the differentially expressed
target genes participate disproportionately in shared biological
programs rather than isolated molecular events. These results provide
high-level functional insight into systemic biological changes and are
particularly useful for interpreting phenotypic consequences of gene
expression alterations.

#### GO\_Cellular\_Component\_2025

This category captures Gene Ontology Cellular Component annotations
describing the subcellular localization of gene products, including
organelles, macromolecular complexes, membranes, and specialized
cellular structures. GO Cellular Component terms are curated through
experimental localization studies and integrative annotation pipelines.
Enrichment reveals whether target genes preferentially localize to
specific cellular compartments, suggesting spatial organization of
regulatory effects. These insights are valuable for understanding
compartment-specific processes such as mitochondrial regulation, vesicle
trafficking, nuclear control mechanisms, or membrane-associated
signaling.

#### GO\_Molecular\_Function\_2025

GO Molecular Function terms describe the elemental biochemical
activities of gene products, including binding affinities, catalytic
activities, transporter functions, and receptor interactions. These
annotations are curated by the Gene Ontology Consortium based on
experimental data and validated inference. Enrichment in this category
highlights specific molecular activities that are over-represented among
the target genes, offering mechanistic insight into how regulatory
changes may alter protein function or signaling capacity. This category
is especially informative for identifying enzymatic classes, signaling
mediators, or binding modules affected by miRNA regulation.

#### KEGG\_2021\_Human

This category is based on pathway annotations from the Kyoto
Encyclopedia of Genes and Genomes (KEGG), a manually curated resource
integrating molecular interaction networks, metabolic pathways, and
disease-associated signaling cascades. KEGG pathways represent canonical
biological systems including metabolism, signal transduction, cell
communication, and genetic information processing. Enrichment in KEGG
pathways suggests coordinated perturbation of well-characterized
biological networks. This category is particularly useful for
identifying conserved signaling routes and metabolic programs affected
by gene expression changes.

#### Reactome\_Pathways\_2024

Reactome is an expert-curated, peer-reviewed pathway database that
describes biological processes as detailed molecular reaction networks.
It emphasizes mechanistic precision, causal relationships, and
hierarchical pathway structure. Enrichment in Reactome pathways
indicates that target genes participate in specific molecular events or
subprocesses such as signal transduction modules, DNA repair mechanisms,
immune cascades, or cell-cycle transitions. Due to its high curation
standards, Reactome enrichment results are considered highly reliable
and mechanistically interpretable.

#### WikiPathways\_2024\_Human

WikiPathways is a community-curated pathway resource that complements
canonical databases by incorporating emerging biological knowledge and
specialized pathways. Pathways are contributed and maintained by domain
experts and are frequently updated to reflect current research.
Enrichment in WikiPathways highlights involvement of target genes in
less standardized or rapidly evolving biological processes, disease
mechanisms, or tissue-specific pathways. This category is particularly
useful for capturing novel or non-canonical regulatory programs not yet
represented in traditional pathway databases.

#### DisGeNET

DisGeNET integrates gene–disease associations derived from curated
databases, genome-wide association studies (GWAS), animal models, and
text-mined literature. Associations span a wide range of human diseases
and phenotypes. Enrichment in DisGeNET indicates that the target genes
are disproportionately associated with specific pathological conditions,
suggesting disease relevance of the observed gene expression changes.
This category is valuable for translational interpretation and for
linking molecular findings to clinical phenotypes.

#### OMIM\_Disease

This category is based on disease annotations from Online Mendelian
Inheritance in Man (OMIM), a comprehensive, manually curated database
focusing on inherited human disorders and gene–phenotype relationships.
OMIM emphasizes high-confidence links supported by genetic and clinical
evidence. Enrichment suggests that target genes are involved in
monogenic or well-characterized genetic diseases, providing insight into
potential hereditary components or fundamental biological mechanisms
underlying the observed expression patterns.

#### OMIM\_Expanded

OMIM Expanded extends the core OMIM disease annotations by
incorporating additional phenotype descriptions, allelic variants, and
gene–trait associations. This broader annotation set captures a wider
spectrum of genetic and phenotypic relationships beyond classical
Mendelian disorders. Enrichment in this category highlights genes with
established roles in human traits or disease susceptibility, offering a
more inclusive view of genetic contributions to biological and
pathological processes.

#### Human\_Phenotype\_Ontology

The Human Phenotype Ontology (HPO) provides a standardized vocabulary
of phenotypic abnormalities encountered in human disease, linking
clinical features to underlying genetic causes. HPO integrates clinical
observations, rare disease data, and curated gene–phenotype
associations. Enrichment in HPO terms indicates that target genes are
associated with specific clinical signs or symptom clusters. This
category is especially useful for connecting molecular findings to
observable phenotypes and for supporting clinical or diagnostic
interpretation.

### lymph node

#### GO\_Biological\_Process\_2025

This category comprises Gene Ontology (GO) Biological Process terms
describing coordinated and temporally ordered biological programs such
as cell cycle progression, immune activation, apoptosis,
differentiation, and metabolic regulation. GO Biological Process
annotations are curated by the Gene Ontology Consortium and integrate
experimental evidence, computational inference, and expert review.
Enrichment in this category indicates that the differentially expressed
target genes participate disproportionately in shared biological
programs rather than isolated molecular events. These results provide
high-level functional insight into systemic biological changes and are
particularly useful for interpreting phenotypic consequences of gene
expression alterations.

#### GO\_Cellular\_Component\_2025

This category captures Gene Ontology Cellular Component annotations
describing the subcellular localization of gene products, including
organelles, macromolecular complexes, membranes, and specialized
cellular structures. GO Cellular Component terms are curated through
experimental localization studies and integrative annotation pipelines.
Enrichment reveals whether target genes preferentially localize to
specific cellular compartments, suggesting spatial organization of
regulatory effects. These insights are valuable for understanding
compartment-specific processes such as mitochondrial regulation, vesicle
trafficking, nuclear control mechanisms, or membrane-associated
signaling.

#### GO\_Molecular\_Function\_2025

GO Molecular Function terms describe the elemental biochemical
activities of gene products, including binding affinities, catalytic
activities, transporter functions, and receptor interactions. These
annotations are curated by the Gene Ontology Consortium based on
experimental data and validated inference. Enrichment in this category
highlights specific molecular activities that are over-represented among
the target genes, offering mechanistic insight into how regulatory
changes may alter protein function or signaling capacity. This category
is especially informative for identifying enzymatic classes, signaling
mediators, or binding modules affected by miRNA regulation.

#### KEGG\_2021\_Human

This category is based on pathway annotations from the Kyoto
Encyclopedia of Genes and Genomes (KEGG), a manually curated resource
integrating molecular interaction networks, metabolic pathways, and
disease-associated signaling cascades. KEGG pathways represent canonical
biological systems including metabolism, signal transduction, cell
communication, and genetic information processing. Enrichment in KEGG
pathways suggests coordinated perturbation of well-characterized
biological networks. This category is particularly useful for
identifying conserved signaling routes and metabolic programs affected
by gene expression changes.

#### Reactome\_Pathways\_2024

Reactome is an expert-curated, peer-reviewed pathway database that
describes biological processes as detailed molecular reaction networks.
It emphasizes mechanistic precision, causal relationships, and
hierarchical pathway structure. Enrichment in Reactome pathways
indicates that target genes participate in specific molecular events or
subprocesses such as signal transduction modules, DNA repair mechanisms,
immune cascades, or cell-cycle transitions. Due to its high curation
standards, Reactome enrichment results are considered highly reliable
and mechanistically interpretable.

#### WikiPathways\_2024\_Human

WikiPathways is a community-curated pathway resource that complements
canonical databases by incorporating emerging biological knowledge and
specialized pathways. Pathways are contributed and maintained by domain
experts and are frequently updated to reflect current research.
Enrichment in WikiPathways highlights involvement of target genes in
less standardized or rapidly evolving biological processes, disease
mechanisms, or tissue-specific pathways. This category is particularly
useful for capturing novel or non-canonical regulatory programs not yet
represented in traditional pathway databases.

#### DisGeNET

DisGeNET integrates gene–disease associations derived from curated
databases, genome-wide association studies (GWAS), animal models, and
text-mined literature. Associations span a wide range of human diseases
and phenotypes. Enrichment in DisGeNET indicates that the target genes
are disproportionately associated with specific pathological conditions,
suggesting disease relevance of the observed gene expression changes.
This category is valuable for translational interpretation and for
linking molecular findings to clinical phenotypes.

#### OMIM\_Disease

This category is based on disease annotations from Online Mendelian
Inheritance in Man (OMIM), a comprehensive, manually curated database
focusing on inherited human disorders and gene–phenotype relationships.
OMIM emphasizes high-confidence links supported by genetic and clinical
evidence. Enrichment suggests that target genes are involved in
monogenic or well-characterized genetic diseases, providing insight into
potential hereditary components or fundamental biological mechanisms
underlying the observed expression patterns.

#### OMIM\_Expanded

OMIM Expanded extends the core OMIM disease annotations by
incorporating additional phenotype descriptions, allelic variants, and
gene–trait associations. This broader annotation set captures a wider
spectrum of genetic and phenotypic relationships beyond classical
Mendelian disorders. Enrichment in this category highlights genes with
established roles in human traits or disease susceptibility, offering a
more inclusive view of genetic contributions to biological and
pathological processes.

#### Human\_Phenotype\_Ontology

The Human Phenotype Ontology (HPO) provides a standardized vocabulary
of phenotypic abnormalities encountered in human disease, linking
clinical features to underlying genetic causes. HPO integrates clinical
observations, rare disease data, and curated gene–phenotype
associations. Enrichment in HPO terms indicates that target genes are
associated with specific clinical signs or symptom clusters. This
category is especially useful for connecting molecular findings to
observable phenotypes and for supporting clinical or diagnostic
interpretation.

### spleen

#### GO\_Biological\_Process\_2025

This category comprises Gene Ontology (GO) Biological Process terms
describing coordinated and temporally ordered biological programs such
as cell cycle progression, immune activation, apoptosis,
differentiation, and metabolic regulation. GO Biological Process
annotations are curated by the Gene Ontology Consortium and integrate
experimental evidence, computational inference, and expert review.
Enrichment in this category indicates that the differentially expressed
target genes participate disproportionately in shared biological
programs rather than isolated molecular events. These results provide
high-level functional insight into systemic biological changes and are
particularly useful for interpreting phenotypic consequences of gene
expression alterations.

#### GO\_Cellular\_Component\_2025

This category captures Gene Ontology Cellular Component annotations
describing the subcellular localization of gene products, including
organelles, macromolecular complexes, membranes, and specialized
cellular structures. GO Cellular Component terms are curated through
experimental localization studies and integrative annotation pipelines.
Enrichment reveals whether target genes preferentially localize to
specific cellular compartments, suggesting spatial organization of
regulatory effects. These insights are valuable for understanding
compartment-specific processes such as mitochondrial regulation, vesicle
trafficking, nuclear control mechanisms, or membrane-associated
signaling.

#### GO\_Molecular\_Function\_2025

GO Molecular Function terms describe the elemental biochemical
activities of gene products, including binding affinities, catalytic
activities, transporter functions, and receptor interactions. These
annotations are curated by the Gene Ontology Consortium based on
experimental data and validated inference. Enrichment in this category
highlights specific molecular activities that are over-represented among
the target genes, offering mechanistic insight into how regulatory
changes may alter protein function or signaling capacity. This category
is especially informative for identifying enzymatic classes, signaling
mediators, or binding modules affected by miRNA regulation.

#### KEGG\_2021\_Human

This category is based on pathway annotations from the Kyoto
Encyclopedia of Genes and Genomes (KEGG), a manually curated resource
integrating molecular interaction networks, metabolic pathways, and
disease-associated signaling cascades. KEGG pathways represent canonical
biological systems including metabolism, signal transduction, cell
communication, and genetic information processing. Enrichment in KEGG
pathways suggests coordinated perturbation of well-characterized
biological networks. This category is particularly useful for
identifying conserved signaling routes and metabolic programs affected
by gene expression changes.

#### Reactome\_Pathways\_2024

Reactome is an expert-curated, peer-reviewed pathway database that
describes biological processes as detailed molecular reaction networks.
It emphasizes mechanistic precision, causal relationships, and
hierarchical pathway structure. Enrichment in Reactome pathways
indicates that target genes participate in specific molecular events or
subprocesses such as signal transduction modules, DNA repair mechanisms,
immune cascades, or cell-cycle transitions. Due to its high curation
standards, Reactome enrichment results are considered highly reliable
and mechanistically interpretable.

#### WikiPathways\_2024\_Human

WikiPathways is a community-curated pathway resource that complements
canonical databases by incorporating emerging biological knowledge and
specialized pathways. Pathways are contributed and maintained by domain
experts and are frequently updated to reflect current research.
Enrichment in WikiPathways highlights involvement of target genes in
less standardized or rapidly evolving biological processes, disease
mechanisms, or tissue-specific pathways. This category is particularly
useful for capturing novel or non-canonical regulatory programs not yet
represented in traditional pathway databases.

#### DisGeNET

DisGeNET integrates gene–disease associations derived from curated
databases, genome-wide association studies (GWAS), animal models, and
text-mined literature. Associations span a wide range of human diseases
and phenotypes. Enrichment in DisGeNET indicates that the target genes
are disproportionately associated with specific pathological conditions,
suggesting disease relevance of the observed gene expression changes.
This category is valuable for translational interpretation and for
linking molecular findings to clinical phenotypes.

#### OMIM\_Disease

This category is based on disease annotations from Online Mendelian
Inheritance in Man (OMIM), a comprehensive, manually curated database
focusing on inherited human disorders and gene–phenotype relationships.
OMIM emphasizes high-confidence links supported by genetic and clinical
evidence. Enrichment suggests that target genes are involved in
monogenic or well-characterized genetic diseases, providing insight into
potential hereditary components or fundamental biological mechanisms
underlying the observed expression patterns.

#### OMIM\_Expanded

OMIM Expanded extends the core OMIM disease annotations by
incorporating additional phenotype descriptions, allelic variants, and
gene–trait associations. This broader annotation set captures a wider
spectrum of genetic and phenotypic relationships beyond classical
Mendelian disorders. Enrichment in this category highlights genes with
established roles in human traits or disease susceptibility, offering a
more inclusive view of genetic contributions to biological and
pathological processes.

#### Human\_Phenotype\_Ontology

The Human Phenotype Ontology (HPO) provides a standardized vocabulary
of phenotypic abnormalities encountered in human disease, linking
clinical features to underlying genetic causes. HPO integrates clinical
observations, rare disease data, and curated gene–phenotype
associations. Enrichment in HPO terms indicates that target genes are
associated with specific clinical signs or symptom clusters. This
category is especially useful for connecting molecular findings to
observable phenotypes and for supporting clinical or diagnostic
interpretation.

## Treatment\_X\_Manufacturer\_M\_vs\_Treatment\_Y\_Manufacturer\_M - Tissue specific ORA

### all\_genes

#### GO\_Biological\_Process\_2025

This category comprises Gene Ontology (GO) Biological Process terms
describing coordinated and temporally ordered biological programs such
as cell cycle progression, immune activation, apoptosis,
differentiation, and metabolic regulation. GO Biological Process
annotations are curated by the Gene Ontology Consortium and integrate
experimental evidence, computational inference, and expert review.
Enrichment in this category indicates that the differentially expressed
target genes participate disproportionately in shared biological
programs rather than isolated molecular events. These results provide
high-level functional insight into systemic biological changes and are
particularly useful for interpreting phenotypic consequences of gene
expression alterations.

#### GO\_Cellular\_Component\_2025

This category captures Gene Ontology Cellular Component annotations
describing the subcellular localization of gene products, including
organelles, macromolecular complexes, membranes, and specialized
cellular structures. GO Cellular Component terms are curated through
experimental localization studies and integrative annotation pipelines.
Enrichment reveals whether target genes preferentially localize to
specific cellular compartments, suggesting spatial organization of
regulatory effects. These insights are valuable for understanding
compartment-specific processes such as mitochondrial regulation, vesicle
trafficking, nuclear control mechanisms, or membrane-associated
signaling.

#### GO\_Molecular\_Function\_2025

GO Molecular Function terms describe the elemental biochemical
activities of gene products, including binding affinities, catalytic
activities, transporter functions, and receptor interactions. These
annotations are curated by the Gene Ontology Consortium based on
experimental data and validated inference. Enrichment in this category
highlights specific molecular activities that are over-represented among
the target genes, offering mechanistic insight into how regulatory
changes may alter protein function or signaling capacity. This category
is especially informative for identifying enzymatic classes, signaling
mediators, or binding modules affected by miRNA regulation.

#### KEGG\_2021\_Human

This category is based on pathway annotations from the Kyoto
Encyclopedia of Genes and Genomes (KEGG), a manually curated resource
integrating molecular interaction networks, metabolic pathways, and
disease-associated signaling cascades. KEGG pathways represent canonical
biological systems including metabolism, signal transduction, cell
communication, and genetic information processing. Enrichment in KEGG
pathways suggests coordinated perturbation of well-characterized
biological networks. This category is particularly useful for
identifying conserved signaling routes and metabolic programs affected
by gene expression changes.

#### Reactome\_Pathways\_2024

Reactome is an expert-curated, peer-reviewed pathway database that
describes biological processes as detailed molecular reaction networks.
It emphasizes mechanistic precision, causal relationships, and
hierarchical pathway structure. Enrichment in Reactome pathways
indicates that target genes participate in specific molecular events or
subprocesses such as signal transduction modules, DNA repair mechanisms,
immune cascades, or cell-cycle transitions. Due to its high curation
standards, Reactome enrichment results are considered highly reliable
and mechanistically interpretable.

#### WikiPathways\_2024\_Human

WikiPathways is a community-curated pathway resource that complements
canonical databases by incorporating emerging biological knowledge and
specialized pathways. Pathways are contributed and maintained by domain
experts and are frequently updated to reflect current research.
Enrichment in WikiPathways highlights involvement of target genes in
less standardized or rapidly evolving biological processes, disease
mechanisms, or tissue-specific pathways. This category is particularly
useful for capturing novel or non-canonical regulatory programs not yet
represented in traditional pathway databases.

#### DisGeNET

DisGeNET integrates gene–disease associations derived from curated
databases, genome-wide association studies (GWAS), animal models, and
text-mined literature. Associations span a wide range of human diseases
and phenotypes. Enrichment in DisGeNET indicates that the target genes
are disproportionately associated with specific pathological conditions,
suggesting disease relevance of the observed gene expression changes.
This category is valuable for translational interpretation and for
linking molecular findings to clinical phenotypes.

#### OMIM\_Disease

This category is based on disease annotations from Online Mendelian
Inheritance in Man (OMIM), a comprehensive, manually curated database
focusing on inherited human disorders and gene–phenotype relationships.
OMIM emphasizes high-confidence links supported by genetic and clinical
evidence. Enrichment suggests that target genes are involved in
monogenic or well-characterized genetic diseases, providing insight into
potential hereditary components or fundamental biological mechanisms
underlying the observed expression patterns.

#### OMIM\_Expanded

OMIM Expanded extends the core OMIM disease annotations by
incorporating additional phenotype descriptions, allelic variants, and
gene–trait associations. This broader annotation set captures a wider
spectrum of genetic and phenotypic relationships beyond classical
Mendelian disorders. Enrichment in this category highlights genes with
established roles in human traits or disease susceptibility, offering a
more inclusive view of genetic contributions to biological and
pathological processes.

#### Human\_Phenotype\_Ontology

The Human Phenotype Ontology (HPO) provides a standardized vocabulary
of phenotypic abnormalities encountered in human disease, linking
clinical features to underlying genetic causes. HPO integrates clinical
observations, rare disease data, and curated gene–phenotype
associations. Enrichment in HPO terms indicates that target genes are
associated with specific clinical signs or symptom clusters. This
category is especially useful for connecting molecular findings to
observable phenotypes and for supporting clinical or diagnostic
interpretation.

### lymph node

#### GO\_Biological\_Process\_2025

This category comprises Gene Ontology (GO) Biological Process terms
describing coordinated and temporally ordered biological programs such
as cell cycle progression, immune activation, apoptosis,
differentiation, and metabolic regulation. GO Biological Process
annotations are curated by the Gene Ontology Consortium and integrate
experimental evidence, computational inference, and expert review.
Enrichment in this category indicates that the differentially expressed
target genes participate disproportionately in shared biological
programs rather than isolated molecular events. These results provide
high-level functional insight into systemic biological changes and are
particularly useful for interpreting phenotypic consequences of gene
expression alterations.

#### GO\_Cellular\_Component\_2025

This category captures Gene Ontology Cellular Component annotations
describing the subcellular localization of gene products, including
organelles, macromolecular complexes, membranes, and specialized
cellular structures. GO Cellular Component terms are curated through
experimental localization studies and integrative annotation pipelines.
Enrichment reveals whether target genes preferentially localize to
specific cellular compartments, suggesting spatial organization of
regulatory effects. These insights are valuable for understanding
compartment-specific processes such as mitochondrial regulation, vesicle
trafficking, nuclear control mechanisms, or membrane-associated
signaling.

#### GO\_Molecular\_Function\_2025

GO Molecular Function terms describe the elemental biochemical
activities of gene products, including binding affinities, catalytic
activities, transporter functions, and receptor interactions. These
annotations are curated by the Gene Ontology Consortium based on
experimental data and validated inference. Enrichment in this category
highlights specific molecular activities that are over-represented among
the target genes, offering mechanistic insight into how regulatory
changes may alter protein function or signaling capacity. This category
is especially informative for identifying enzymatic classes, signaling
mediators, or binding modules affected by miRNA regulation.

#### KEGG\_2021\_Human

This category is based on pathway annotations from the Kyoto
Encyclopedia of Genes and Genomes (KEGG), a manually curated resource
integrating molecular interaction networks, metabolic pathways, and
disease-associated signaling cascades. KEGG pathways represent canonical
biological systems including metabolism, signal transduction, cell
communication, and genetic information processing. Enrichment in KEGG
pathways suggests coordinated perturbation of well-characterized
biological networks. This category is particularly useful for
identifying conserved signaling routes and metabolic programs affected
by gene expression changes.

#### Reactome\_Pathways\_2024

Reactome is an expert-curated, peer-reviewed pathway database that
describes biological processes as detailed molecular reaction networks.
It emphasizes mechanistic precision, causal relationships, and
hierarchical pathway structure. Enrichment in Reactome pathways
indicates that target genes participate in specific molecular events or
subprocesses such as signal transduction modules, DNA repair mechanisms,
immune cascades, or cell-cycle transitions. Due to its high curation
standards, Reactome enrichment results are considered highly reliable
and mechanistically interpretable.

#### WikiPathways\_2024\_Human

WikiPathways is a community-curated pathway resource that complements
canonical databases by incorporating emerging biological knowledge and
specialized pathways. Pathways are contributed and maintained by domain
experts and are frequently updated to reflect current research.
Enrichment in WikiPathways highlights involvement of target genes in
less standardized or rapidly evolving biological processes, disease
mechanisms, or tissue-specific pathways. This category is particularly
useful for capturing novel or non-canonical regulatory programs not yet
represented in traditional pathway databases.

#### DisGeNET

DisGeNET integrates gene–disease associations derived from curated
databases, genome-wide association studies (GWAS), animal models, and
text-mined literature. Associations span a wide range of human diseases
and phenotypes. Enrichment in DisGeNET indicates that the target genes
are disproportionately associated with specific pathological conditions,
suggesting disease relevance of the observed gene expression changes.
This category is valuable for translational interpretation and for
linking molecular findings to clinical phenotypes.

#### OMIM\_Disease

This category is based on disease annotations from Online Mendelian
Inheritance in Man (OMIM), a comprehensive, manually curated database
focusing on inherited human disorders and gene–phenotype relationships.
OMIM emphasizes high-confidence links supported by genetic and clinical
evidence. Enrichment suggests that target genes are involved in
monogenic or well-characterized genetic diseases, providing insight into
potential hereditary components or fundamental biological mechanisms
underlying the observed expression patterns.

#### OMIM\_Expanded

OMIM Expanded extends the core OMIM disease annotations by
incorporating additional phenotype descriptions, allelic variants, and
gene–trait associations. This broader annotation set captures a wider
spectrum of genetic and phenotypic relationships beyond classical
Mendelian disorders. Enrichment in this category highlights genes with
established roles in human traits or disease susceptibility, offering a
more inclusive view of genetic contributions to biological and
pathological processes.

#### Human\_Phenotype\_Ontology

The Human Phenotype Ontology (HPO) provides a standardized vocabulary
of phenotypic abnormalities encountered in human disease, linking
clinical features to underlying genetic causes. HPO integrates clinical
observations, rare disease data, and curated gene–phenotype
associations. Enrichment in HPO terms indicates that target genes are
associated with specific clinical signs or symptom clusters. This
category is especially useful for connecting molecular findings to
observable phenotypes and for supporting clinical or diagnostic
interpretation.

### spleen

#### GO\_Biological\_Process\_2025

This category comprises Gene Ontology (GO) Biological Process terms
describing coordinated and temporally ordered biological programs such
as cell cycle progression, immune activation, apoptosis,
differentiation, and metabolic regulation. GO Biological Process
annotations are curated by the Gene Ontology Consortium and integrate
experimental evidence, computational inference, and expert review.
Enrichment in this category indicates that the differentially expressed
target genes participate disproportionately in shared biological
programs rather than isolated molecular events. These results provide
high-level functional insight into systemic biological changes and are
particularly useful for interpreting phenotypic consequences of gene
expression alterations.

#### GO\_Cellular\_Component\_2025

This category captures Gene Ontology Cellular Component annotations
describing the subcellular localization of gene products, including
organelles, macromolecular complexes, membranes, and specialized
cellular structures. GO Cellular Component terms are curated through
experimental localization studies and integrative annotation pipelines.
Enrichment reveals whether target genes preferentially localize to
specific cellular compartments, suggesting spatial organization of
regulatory effects. These insights are valuable for understanding
compartment-specific processes such as mitochondrial regulation, vesicle
trafficking, nuclear control mechanisms, or membrane-associated
signaling.

#### GO\_Molecular\_Function\_2025

GO Molecular Function terms describe the elemental biochemical
activities of gene products, including binding affinities, catalytic
activities, transporter functions, and receptor interactions. These
annotations are curated by the Gene Ontology Consortium based on
experimental data and validated inference. Enrichment in this category
highlights specific molecular activities that are over-represented among
the target genes, offering mechanistic insight into how regulatory
changes may alter protein function or signaling capacity. This category
is especially informative for identifying enzymatic classes, signaling
mediators, or binding modules affected by miRNA regulation.

#### KEGG\_2021\_Human

This category is based on pathway annotations from the Kyoto
Encyclopedia of Genes and Genomes (KEGG), a manually curated resource
integrating molecular interaction networks, metabolic pathways, and
disease-associated signaling cascades. KEGG pathways represent canonical
biological systems including metabolism, signal transduction, cell
communication, and genetic information processing. Enrichment in KEGG
pathways suggests coordinated perturbation of well-characterized
biological networks. This category is particularly useful for
identifying conserved signaling routes and metabolic programs affected
by gene expression changes.

#### Reactome\_Pathways\_2024

Reactome is an expert-curated, peer-reviewed pathway database that
describes biological processes as detailed molecular reaction networks.
It emphasizes mechanistic precision, causal relationships, and
hierarchical pathway structure. Enrichment in Reactome pathways
indicates that target genes participate in specific molecular events or
subprocesses such as signal transduction modules, DNA repair mechanisms,
immune cascades, or cell-cycle transitions. Due to its high curation
standards, Reactome enrichment results are considered highly reliable
and mechanistically interpretable.

#### WikiPathways\_2024\_Human

WikiPathways is a community-curated pathway resource that complements
canonical databases by incorporating emerging biological knowledge and
specialized pathways. Pathways are contributed and maintained by domain
experts and are frequently updated to reflect current research.
Enrichment in WikiPathways highlights involvement of target genes in
less standardized or rapidly evolving biological processes, disease
mechanisms, or tissue-specific pathways. This category is particularly
useful for capturing novel or non-canonical regulatory programs not yet
represented in traditional pathway databases.

#### DisGeNET

DisGeNET integrates gene–disease associations derived from curated
databases, genome-wide association studies (GWAS), animal models, and
text-mined literature. Associations span a wide range of human diseases
and phenotypes. Enrichment in DisGeNET indicates that the target genes
are disproportionately associated with specific pathological conditions,
suggesting disease relevance of the observed gene expression changes.
This category is valuable for translational interpretation and for
linking molecular findings to clinical phenotypes.

#### OMIM\_Disease

This category is based on disease annotations from Online Mendelian
Inheritance in Man (OMIM), a comprehensive, manually curated database
focusing on inherited human disorders and gene–phenotype relationships.
OMIM emphasizes high-confidence links supported by genetic and clinical
evidence. Enrichment suggests that target genes are involved in
monogenic or well-characterized genetic diseases, providing insight into
potential hereditary components or fundamental biological mechanisms
underlying the observed expression patterns.

#### OMIM\_Expanded

OMIM Expanded extends the core OMIM disease annotations by
incorporating additional phenotype descriptions, allelic variants, and
gene–trait associations. This broader annotation set captures a wider
spectrum of genetic and phenotypic relationships beyond classical
Mendelian disorders. Enrichment in this category highlights genes with
established roles in human traits or disease susceptibility, offering a
more inclusive view of genetic contributions to biological and
pathological processes.

#### Human\_Phenotype\_Ontology

The Human Phenotype Ontology (HPO) provides a standardized vocabulary
of phenotypic abnormalities encountered in human disease, linking
clinical features to underlying genetic causes. HPO integrates clinical
observations, rare disease data, and curated gene–phenotype
associations. Enrichment in HPO terms indicates that target genes are
associated with specific clinical signs or symptom clusters. This
category is especially useful for connecting molecular findings to
observable phenotypes and for supporting clinical or diagnostic
interpretation.

## Treatment\_X\_Manufacturer\_M\_vs\_Treatment\_Y\_Manufacturer\_Q - Tissue specific ORA

### all\_genes

#### GO\_Biological\_Process\_2025

This category comprises Gene Ontology (GO) Biological Process terms
describing coordinated and temporally ordered biological programs such
as cell cycle progression, immune activation, apoptosis,
differentiation, and metabolic regulation. GO Biological Process
annotations are curated by the Gene Ontology Consortium and integrate
experimental evidence, computational inference, and expert review.
Enrichment in this category indicates that the differentially expressed
target genes participate disproportionately in shared biological
programs rather than isolated molecular events. These results provide
high-level functional insight into systemic biological changes and are
particularly useful for interpreting phenotypic consequences of gene
expression alterations.

#### GO\_Cellular\_Component\_2025

This category captures Gene Ontology Cellular Component annotations
describing the subcellular localization of gene products, including
organelles, macromolecular complexes, membranes, and specialized
cellular structures. GO Cellular Component terms are curated through
experimental localization studies and integrative annotation pipelines.
Enrichment reveals whether target genes preferentially localize to
specific cellular compartments, suggesting spatial organization of
regulatory effects. These insights are valuable for understanding
compartment-specific processes such as mitochondrial regulation, vesicle
trafficking, nuclear control mechanisms, or membrane-associated
signaling.

#### GO\_Molecular\_Function\_2025

GO Molecular Function terms describe the elemental biochemical
activities of gene products, including binding affinities, catalytic
activities, transporter functions, and receptor interactions. These
annotations are curated by the Gene Ontology Consortium based on
experimental data and validated inference. Enrichment in this category
highlights specific molecular activities that are over-represented among
the target genes, offering mechanistic insight into how regulatory
changes may alter protein function or signaling capacity. This category
is especially informative for identifying enzymatic classes, signaling
mediators, or binding modules affected by miRNA regulation.

#### KEGG\_2021\_Human

This category is based on pathway annotations from the Kyoto
Encyclopedia of Genes and Genomes (KEGG), a manually curated resource
integrating molecular interaction networks, metabolic pathways, and
disease-associated signaling cascades. KEGG pathways represent canonical
biological systems including metabolism, signal transduction, cell
communication, and genetic information processing. Enrichment in KEGG
pathways suggests coordinated perturbation of well-characterized
biological networks. This category is particularly useful for
identifying conserved signaling routes and metabolic programs affected
by gene expression changes.

#### Reactome\_Pathways\_2024

Reactome is an expert-curated, peer-reviewed pathway database that
describes biological processes as detailed molecular reaction networks.
It emphasizes mechanistic precision, causal relationships, and
hierarchical pathway structure. Enrichment in Reactome pathways
indicates that target genes participate in specific molecular events or
subprocesses such as signal transduction modules, DNA repair mechanisms,
immune cascades, or cell-cycle transitions. Due to its high curation
standards, Reactome enrichment results are considered highly reliable
and mechanistically interpretable.

#### WikiPathways\_2024\_Human

WikiPathways is a community-curated pathway resource that complements
canonical databases by incorporating emerging biological knowledge and
specialized pathways. Pathways are contributed and maintained by domain
experts and are frequently updated to reflect current research.
Enrichment in WikiPathways highlights involvement of target genes in
less standardized or rapidly evolving biological processes, disease
mechanisms, or tissue-specific pathways. This category is particularly
useful for capturing novel or non-canonical regulatory programs not yet
represented in traditional pathway databases.

#### DisGeNET

DisGeNET integrates gene–disease associations derived from curated
databases, genome-wide association studies (GWAS), animal models, and
text-mined literature. Associations span a wide range of human diseases
and phenotypes. Enrichment in DisGeNET indicates that the target genes
are disproportionately associated with specific pathological conditions,
suggesting disease relevance of the observed gene expression changes.
This category is valuable for translational interpretation and for
linking molecular findings to clinical phenotypes.

#### OMIM\_Disease

This category is based on disease annotations from Online Mendelian
Inheritance in Man (OMIM), a comprehensive, manually curated database
focusing on inherited human disorders and gene–phenotype relationships.
OMIM emphasizes high-confidence links supported by genetic and clinical
evidence. Enrichment suggests that target genes are involved in
monogenic or well-characterized genetic diseases, providing insight into
potential hereditary components or fundamental biological mechanisms
underlying the observed expression patterns.

#### OMIM\_Expanded

OMIM Expanded extends the core OMIM disease annotations by
incorporating additional phenotype descriptions, allelic variants, and
gene–trait associations. This broader annotation set captures a wider
spectrum of genetic and phenotypic relationships beyond classical
Mendelian disorders. Enrichment in this category highlights genes with
established roles in human traits or disease susceptibility, offering a
more inclusive view of genetic contributions to biological and
pathological processes.

#### Human\_Phenotype\_Ontology

The Human Phenotype Ontology (HPO) provides a standardized vocabulary
of phenotypic abnormalities encountered in human disease, linking
clinical features to underlying genetic causes. HPO integrates clinical
observations, rare disease data, and curated gene–phenotype
associations. Enrichment in HPO terms indicates that target genes are
associated with specific clinical signs or symptom clusters. This
category is especially useful for connecting molecular findings to
observable phenotypes and for supporting clinical or diagnostic
interpretation.

### lymph node

#### GO\_Biological\_Process\_2025

This category comprises Gene Ontology (GO) Biological Process terms
describing coordinated and temporally ordered biological programs such
as cell cycle progression, immune activation, apoptosis,
differentiation, and metabolic regulation. GO Biological Process
annotations are curated by the Gene Ontology Consortium and integrate
experimental evidence, computational inference, and expert review.
Enrichment in this category indicates that the differentially expressed
target genes participate disproportionately in shared biological
programs rather than isolated molecular events. These results provide
high-level functional insight into systemic biological changes and are
particularly useful for interpreting phenotypic consequences of gene
expression alterations.

#### GO\_Cellular\_Component\_2025

This category captures Gene Ontology Cellular Component annotations
describing the subcellular localization of gene products, including
organelles, macromolecular complexes, membranes, and specialized
cellular structures. GO Cellular Component terms are curated through
experimental localization studies and integrative annotation pipelines.
Enrichment reveals whether target genes preferentially localize to
specific cellular compartments, suggesting spatial organization of
regulatory effects. These insights are valuable for understanding
compartment-specific processes such as mitochondrial regulation, vesicle
trafficking, nuclear control mechanisms, or membrane-associated
signaling.

#### GO\_Molecular\_Function\_2025

GO Molecular Function terms describe the elemental biochemical
activities of gene products, including binding affinities, catalytic
activities, transporter functions, and receptor interactions. These
annotations are curated by the Gene Ontology Consortium based on
experimental data and validated inference. Enrichment in this category
highlights specific molecular activities that are over-represented among
the target genes, offering mechanistic insight into how regulatory
changes may alter protein function or signaling capacity. This category
is especially informative for identifying enzymatic classes, signaling
mediators, or binding modules affected by miRNA regulation.

#### KEGG\_2021\_Human

This category is based on pathway annotations from the Kyoto
Encyclopedia of Genes and Genomes (KEGG), a manually curated resource
integrating molecular interaction networks, metabolic pathways, and
disease-associated signaling cascades. KEGG pathways represent canonical
biological systems including metabolism, signal transduction, cell
communication, and genetic information processing. Enrichment in KEGG
pathways suggests coordinated perturbation of well-characterized
biological networks. This category is particularly useful for
identifying conserved signaling routes and metabolic programs affected
by gene expression changes.

#### Reactome\_Pathways\_2024

Reactome is an expert-curated, peer-reviewed pathway database that
describes biological processes as detailed molecular reaction networks.
It emphasizes mechanistic precision, causal relationships, and
hierarchical pathway structure. Enrichment in Reactome pathways
indicates that target genes participate in specific molecular events or
subprocesses such as signal transduction modules, DNA repair mechanisms,
immune cascades, or cell-cycle transitions. Due to its high curation
standards, Reactome enrichment results are considered highly reliable
and mechanistically interpretable.

#### WikiPathways\_2024\_Human

WikiPathways is a community-curated pathway resource that complements
canonical databases by incorporating emerging biological knowledge and
specialized pathways. Pathways are contributed and maintained by domain
experts and are frequently updated to reflect current research.
Enrichment in WikiPathways highlights involvement of target genes in
less standardized or rapidly evolving biological processes, disease
mechanisms, or tissue-specific pathways. This category is particularly
useful for capturing novel or non-canonical regulatory programs not yet
represented in traditional pathway databases.

#### DisGeNET

DisGeNET integrates gene–disease associations derived from curated
databases, genome-wide association studies (GWAS), animal models, and
text-mined literature. Associations span a wide range of human diseases
and phenotypes. Enrichment in DisGeNET indicates that the target genes
are disproportionately associated with specific pathological conditions,
suggesting disease relevance of the observed gene expression changes.
This category is valuable for translational interpretation and for
linking molecular findings to clinical phenotypes.

#### OMIM\_Disease

This category is based on disease annotations from Online Mendelian
Inheritance in Man (OMIM), a comprehensive, manually curated database
focusing on inherited human disorders and gene–phenotype relationships.
OMIM emphasizes high-confidence links supported by genetic and clinical
evidence. Enrichment suggests that target genes are involved in
monogenic or well-characterized genetic diseases, providing insight into
potential hereditary components or fundamental biological mechanisms
underlying the observed expression patterns.

#### OMIM\_Expanded

OMIM Expanded extends the core OMIM disease annotations by
incorporating additional phenotype descriptions, allelic variants, and
gene–trait associations. This broader annotation set captures a wider
spectrum of genetic and phenotypic relationships beyond classical
Mendelian disorders. Enrichment in this category highlights genes with
established roles in human traits or disease susceptibility, offering a
more inclusive view of genetic contributions to biological and
pathological processes.

#### Human\_Phenotype\_Ontology

The Human Phenotype Ontology (HPO) provides a standardized vocabulary
of phenotypic abnormalities encountered in human disease, linking
clinical features to underlying genetic causes. HPO integrates clinical
observations, rare disease data, and curated gene–phenotype
associations. Enrichment in HPO terms indicates that target genes are
associated with specific clinical signs or symptom clusters. This
category is especially useful for connecting molecular findings to
observable phenotypes and for supporting clinical or diagnostic
interpretation.

### spleen

#### GO\_Biological\_Process\_2025

This category comprises Gene Ontology (GO) Biological Process terms
describing coordinated and temporally ordered biological programs such
as cell cycle progression, immune activation, apoptosis,
differentiation, and metabolic regulation. GO Biological Process
annotations are curated by the Gene Ontology Consortium and integrate
experimental evidence, computational inference, and expert review.
Enrichment in this category indicates that the differentially expressed
target genes participate disproportionately in shared biological
programs rather than isolated molecular events. These results provide
high-level functional insight into systemic biological changes and are
particularly useful for interpreting phenotypic consequences of gene
expression alterations.

#### GO\_Cellular\_Component\_2025

This category captures Gene Ontology Cellular Component annotations
describing the subcellular localization of gene products, including
organelles, macromolecular complexes, membranes, and specialized
cellular structures. GO Cellular Component terms are curated through
experimental localization studies and integrative annotation pipelines.
Enrichment reveals whether target genes preferentially localize to
specific cellular compartments, suggesting spatial organization of
regulatory effects. These insights are valuable for understanding
compartment-specific processes such as mitochondrial regulation, vesicle
trafficking, nuclear control mechanisms, or membrane-associated
signaling.

#### GO\_Molecular\_Function\_2025

GO Molecular Function terms describe the elemental biochemical
activities of gene products, including binding affinities, catalytic
activities, transporter functions, and receptor interactions. These
annotations are curated by the Gene Ontology Consortium based on
experimental data and validated inference. Enrichment in this category
highlights specific molecular activities that are over-represented among
the target genes, offering mechanistic insight into how regulatory
changes may alter protein function or signaling capacity. This category
is especially informative for identifying enzymatic classes, signaling
mediators, or binding modules affected by miRNA regulation.

#### KEGG\_2021\_Human

This category is based on pathway annotations from the Kyoto
Encyclopedia of Genes and Genomes (KEGG), a manually curated resource
integrating molecular interaction networks, metabolic pathways, and
disease-associated signaling cascades. KEGG pathways represent canonical
biological systems including metabolism, signal transduction, cell
communication, and genetic information processing. Enrichment in KEGG
pathways suggests coordinated perturbation of well-characterized
biological networks. This category is particularly useful for
identifying conserved signaling routes and metabolic programs affected
by gene expression changes.

#### Reactome\_Pathways\_2024

Reactome is an expert-curated, peer-reviewed pathway database that
describes biological processes as detailed molecular reaction networks.
It emphasizes mechanistic precision, causal relationships, and
hierarchical pathway structure. Enrichment in Reactome pathways
indicates that target genes participate in specific molecular events or
subprocesses such as signal transduction modules, DNA repair mechanisms,
immune cascades, or cell-cycle transitions. Due to its high curation
standards, Reactome enrichment results are considered highly reliable
and mechanistically interpretable.

#### WikiPathways\_2024\_Human

WikiPathways is a community-curated pathway resource that complements
canonical databases by incorporating emerging biological knowledge and
specialized pathways. Pathways are contributed and maintained by domain
experts and are frequently updated to reflect current research.
Enrichment in WikiPathways highlights involvement of target genes in
less standardized or rapidly evolving biological processes, disease
mechanisms, or tissue-specific pathways. This category is particularly
useful for capturing novel or non-canonical regulatory programs not yet
represented in traditional pathway databases.

#### DisGeNET

DisGeNET integrates gene–disease associations derived from curated
databases, genome-wide association studies (GWAS), animal models, and
text-mined literature. Associations span a wide range of human diseases
and phenotypes. Enrichment in DisGeNET indicates that the target genes
are disproportionately associated with specific pathological conditions,
suggesting disease relevance of the observed gene expression changes.
This category is valuable for translational interpretation and for
linking molecular findings to clinical phenotypes.

#### OMIM\_Disease

This category is based on disease annotations from Online Mendelian
Inheritance in Man (OMIM), a comprehensive, manually curated database
focusing on inherited human disorders and gene–phenotype relationships.
OMIM emphasizes high-confidence links supported by genetic and clinical
evidence. Enrichment suggests that target genes are involved in
monogenic or well-characterized genetic diseases, providing insight into
potential hereditary components or fundamental biological mechanisms
underlying the observed expression patterns.

#### OMIM\_Expanded

OMIM Expanded extends the core OMIM disease annotations by
incorporating additional phenotype descriptions, allelic variants, and
gene–trait associations. This broader annotation set captures a wider
spectrum of genetic and phenotypic relationships beyond classical
Mendelian disorders. Enrichment in this category highlights genes with
established roles in human traits or disease susceptibility, offering a
more inclusive view of genetic contributions to biological and
pathological processes.

#### Human\_Phenotype\_Ontology

The Human Phenotype Ontology (HPO) provides a standardized vocabulary
of phenotypic abnormalities encountered in human disease, linking
clinical features to underlying genetic causes. HPO integrates clinical
observations, rare disease data, and curated gene–phenotype
associations. Enrichment in HPO terms indicates that target genes are
associated with specific clinical signs or symptom clusters. This
category is especially useful for connecting molecular findings to
observable phenotypes and for supporting clinical or diagnostic
interpretation.

## Treatment\_X\_Manufacturer\_Q\_vs\_Treatment\_Y\_Manufacturer\_M - Tissue specific ORA

### all\_genes

#### GO\_Biological\_Process\_2025

This category comprises Gene Ontology (GO) Biological Process terms
describing coordinated and temporally ordered biological programs such
as cell cycle progression, immune activation, apoptosis,
differentiation, and metabolic regulation. GO Biological Process
annotations are curated by the Gene Ontology Consortium and integrate
experimental evidence, computational inference, and expert review.
Enrichment in this category indicates that the differentially expressed
target genes participate disproportionately in shared biological
programs rather than isolated molecular events. These results provide
high-level functional insight into systemic biological changes and are
particularly useful for interpreting phenotypic consequences of gene
expression alterations.

#### GO\_Cellular\_Component\_2025

This category captures Gene Ontology Cellular Component annotations
describing the subcellular localization of gene products, including
organelles, macromolecular complexes, membranes, and specialized
cellular structures. GO Cellular Component terms are curated through
experimental localization studies and integrative annotation pipelines.
Enrichment reveals whether target genes preferentially localize to
specific cellular compartments, suggesting spatial organization of
regulatory effects. These insights are valuable for understanding
compartment-specific processes such as mitochondrial regulation, vesicle
trafficking, nuclear control mechanisms, or membrane-associated
signaling.

#### GO\_Molecular\_Function\_2025

GO Molecular Function terms describe the elemental biochemical
activities of gene products, including binding affinities, catalytic
activities, transporter functions, and receptor interactions. These
annotations are curated by the Gene Ontology Consortium based on
experimental data and validated inference. Enrichment in this category
highlights specific molecular activities that are over-represented among
the target genes, offering mechanistic insight into how regulatory
changes may alter protein function or signaling capacity. This category
is especially informative for identifying enzymatic classes, signaling
mediators, or binding modules affected by miRNA regulation.

#### KEGG\_2021\_Human

This category is based on pathway annotations from the Kyoto
Encyclopedia of Genes and Genomes (KEGG), a manually curated resource
integrating molecular interaction networks, metabolic pathways, and
disease-associated signaling cascades. KEGG pathways represent canonical
biological systems including metabolism, signal transduction, cell
communication, and genetic information processing. Enrichment in KEGG
pathways suggests coordinated perturbation of well-characterized
biological networks. This category is particularly useful for
identifying conserved signaling routes and metabolic programs affected
by gene expression changes.

#### Reactome\_Pathways\_2024

Reactome is an expert-curated, peer-reviewed pathway database that
describes biological processes as detailed molecular reaction networks.
It emphasizes mechanistic precision, causal relationships, and
hierarchical pathway structure. Enrichment in Reactome pathways
indicates that target genes participate in specific molecular events or
subprocesses such as signal transduction modules, DNA repair mechanisms,
immune cascades, or cell-cycle transitions. Due to its high curation
standards, Reactome enrichment results are considered highly reliable
and mechanistically interpretable.

#### WikiPathways\_2024\_Human

WikiPathways is a community-curated pathway resource that complements
canonical databases by incorporating emerging biological knowledge and
specialized pathways. Pathways are contributed and maintained by domain
experts and are frequently updated to reflect current research.
Enrichment in WikiPathways highlights involvement of target genes in
less standardized or rapidly evolving biological processes, disease
mechanisms, or tissue-specific pathways. This category is particularly
useful for capturing novel or non-canonical regulatory programs not yet
represented in traditional pathway databases.

#### DisGeNET

DisGeNET integrates gene–disease associations derived from curated
databases, genome-wide association studies (GWAS), animal models, and
text-mined literature. Associations span a wide range of human diseases
and phenotypes. Enrichment in DisGeNET indicates that the target genes
are disproportionately associated with specific pathological conditions,
suggesting disease relevance of the observed gene expression changes.
This category is valuable for translational interpretation and for
linking molecular findings to clinical phenotypes.

#### OMIM\_Disease

This category is based on disease annotations from Online Mendelian
Inheritance in Man (OMIM), a comprehensive, manually curated database
focusing on inherited human disorders and gene–phenotype relationships.
OMIM emphasizes high-confidence links supported by genetic and clinical
evidence. Enrichment suggests that target genes are involved in
monogenic or well-characterized genetic diseases, providing insight into
potential hereditary components or fundamental biological mechanisms
underlying the observed expression patterns.

#### OMIM\_Expanded

OMIM Expanded extends the core OMIM disease annotations by
incorporating additional phenotype descriptions, allelic variants, and
gene–trait associations. This broader annotation set captures a wider
spectrum of genetic and phenotypic relationships beyond classical
Mendelian disorders. Enrichment in this category highlights genes with
established roles in human traits or disease susceptibility, offering a
more inclusive view of genetic contributions to biological and
pathological processes.

#### Human\_Phenotype\_Ontology

The Human Phenotype Ontology (HPO) provides a standardized vocabulary
of phenotypic abnormalities encountered in human disease, linking
clinical features to underlying genetic causes. HPO integrates clinical
observations, rare disease data, and curated gene–phenotype
associations. Enrichment in HPO terms indicates that target genes are
associated with specific clinical signs or symptom clusters. This
category is especially useful for connecting molecular findings to
observable phenotypes and for supporting clinical or diagnostic
interpretation.

### lymph node

#### GO\_Biological\_Process\_2025

This category comprises Gene Ontology (GO) Biological Process terms
describing coordinated and temporally ordered biological programs such
as cell cycle progression, immune activation, apoptosis,
differentiation, and metabolic regulation. GO Biological Process
annotations are curated by the Gene Ontology Consortium and integrate
experimental evidence, computational inference, and expert review.
Enrichment in this category indicates that the differentially expressed
target genes participate disproportionately in shared biological
programs rather than isolated molecular events. These results provide
high-level functional insight into systemic biological changes and are
particularly useful for interpreting phenotypic consequences of gene
expression alterations.

#### GO\_Cellular\_Component\_2025

This category captures Gene Ontology Cellular Component annotations
describing the subcellular localization of gene products, including
organelles, macromolecular complexes, membranes, and specialized
cellular structures. GO Cellular Component terms are curated through
experimental localization studies and integrative annotation pipelines.
Enrichment reveals whether target genes preferentially localize to
specific cellular compartments, suggesting spatial organization of
regulatory effects. These insights are valuable for understanding
compartment-specific processes such as mitochondrial regulation, vesicle
trafficking, nuclear control mechanisms, or membrane-associated
signaling.

#### GO\_Molecular\_Function\_2025

GO Molecular Function terms describe the elemental biochemical
activities of gene products, including binding affinities, catalytic
activities, transporter functions, and receptor interactions. These
annotations are curated by the Gene Ontology Consortium based on
experimental data and validated inference. Enrichment in this category
highlights specific molecular activities that are over-represented among
the target genes, offering mechanistic insight into how regulatory
changes may alter protein function or signaling capacity. This category
is especially informative for identifying enzymatic classes, signaling
mediators, or binding modules affected by miRNA regulation.

#### KEGG\_2021\_Human

This category is based on pathway annotations from the Kyoto
Encyclopedia of Genes and Genomes (KEGG), a manually curated resource
integrating molecular interaction networks, metabolic pathways, and
disease-associated signaling cascades. KEGG pathways represent canonical
biological systems including metabolism, signal transduction, cell
communication, and genetic information processing. Enrichment in KEGG
pathways suggests coordinated perturbation of well-characterized
biological networks. This category is particularly useful for
identifying conserved signaling routes and metabolic programs affected
by gene expression changes.

#### Reactome\_Pathways\_2024

Reactome is an expert-curated, peer-reviewed pathway database that
describes biological processes as detailed molecular reaction networks.
It emphasizes mechanistic precision, causal relationships, and
hierarchical pathway structure. Enrichment in Reactome pathways
indicates that target genes participate in specific molecular events or
subprocesses such as signal transduction modules, DNA repair mechanisms,
immune cascades, or cell-cycle transitions. Due to its high curation
standards, Reactome enrichment results are considered highly reliable
and mechanistically interpretable.

#### WikiPathways\_2024\_Human

WikiPathways is a community-curated pathway resource that complements
canonical databases by incorporating emerging biological knowledge and
specialized pathways. Pathways are contributed and maintained by domain
experts and are frequently updated to reflect current research.
Enrichment in WikiPathways highlights involvement of target genes in
less standardized or rapidly evolving biological processes, disease
mechanisms, or tissue-specific pathways. This category is particularly
useful for capturing novel or non-canonical regulatory programs not yet
represented in traditional pathway databases.

#### DisGeNET

DisGeNET integrates gene–disease associations derived from curated
databases, genome-wide association studies (GWAS), animal models, and
text-mined literature. Associations span a wide range of human diseases
and phenotypes. Enrichment in DisGeNET indicates that the target genes
are disproportionately associated with specific pathological conditions,
suggesting disease relevance of the observed gene expression changes.
This category is valuable for translational interpretation and for
linking molecular findings to clinical phenotypes.

#### OMIM\_Disease

This category is based on disease annotations from Online Mendelian
Inheritance in Man (OMIM), a comprehensive, manually curated database
focusing on inherited human disorders and gene–phenotype relationships.
OMIM emphasizes high-confidence links supported by genetic and clinical
evidence. Enrichment suggests that target genes are involved in
monogenic or well-characterized genetic diseases, providing insight into
potential hereditary components or fundamental biological mechanisms
underlying the observed expression patterns.

#### OMIM\_Expanded

OMIM Expanded extends the core OMIM disease annotations by
incorporating additional phenotype descriptions, allelic variants, and
gene–trait associations. This broader annotation set captures a wider
spectrum of genetic and phenotypic relationships beyond classical
Mendelian disorders. Enrichment in this category highlights genes with
established roles in human traits or disease susceptibility, offering a
more inclusive view of genetic contributions to biological and
pathological processes.

#### Human\_Phenotype\_Ontology

The Human Phenotype Ontology (HPO) provides a standardized vocabulary
of phenotypic abnormalities encountered in human disease, linking
clinical features to underlying genetic causes. HPO integrates clinical
observations, rare disease data, and curated gene–phenotype
associations. Enrichment in HPO terms indicates that target genes are
associated with specific clinical signs or symptom clusters. This
category is especially useful for connecting molecular findings to
observable phenotypes and for supporting clinical or diagnostic
interpretation.

### spleen

#### GO\_Biological\_Process\_2025

This category comprises Gene Ontology (GO) Biological Process terms
describing coordinated and temporally ordered biological programs such
as cell cycle progression, immune activation, apoptosis,
differentiation, and metabolic regulation. GO Biological Process
annotations are curated by the Gene Ontology Consortium and integrate
experimental evidence, computational inference, and expert review.
Enrichment in this category indicates that the differentially expressed
target genes participate disproportionately in shared biological
programs rather than isolated molecular events. These results provide
high-level functional insight into systemic biological changes and are
particularly useful for interpreting phenotypic consequences of gene
expression alterations.

#### GO\_Cellular\_Component\_2025

This category captures Gene Ontology Cellular Component annotations
describing the subcellular localization of gene products, including
organelles, macromolecular complexes, membranes, and specialized
cellular structures. GO Cellular Component terms are curated through
experimental localization studies and integrative annotation pipelines.
Enrichment reveals whether target genes preferentially localize to
specific cellular compartments, suggesting spatial organization of
regulatory effects. These insights are valuable for understanding
compartment-specific processes such as mitochondrial regulation, vesicle
trafficking, nuclear control mechanisms, or membrane-associated
signaling.

#### GO\_Molecular\_Function\_2025

GO Molecular Function terms describe the elemental biochemical
activities of gene products, including binding affinities, catalytic
activities, transporter functions, and receptor interactions. These
annotations are curated by the Gene Ontology Consortium based on
experimental data and validated inference. Enrichment in this category
highlights specific molecular activities that are over-represented among
the target genes, offering mechanistic insight into how regulatory
changes may alter protein function or signaling capacity. This category
is especially informative for identifying enzymatic classes, signaling
mediators, or binding modules affected by miRNA regulation.

#### KEGG\_2021\_Human

This category is based on pathway annotations from the Kyoto
Encyclopedia of Genes and Genomes (KEGG), a manually curated resource
integrating molecular interaction networks, metabolic pathways, and
disease-associated signaling cascades. KEGG pathways represent canonical
biological systems including metabolism, signal transduction, cell
communication, and genetic information processing. Enrichment in KEGG
pathways suggests coordinated perturbation of well-characterized
biological networks. This category is particularly useful for
identifying conserved signaling routes and metabolic programs affected
by gene expression changes.

#### Reactome\_Pathways\_2024

Reactome is an expert-curated, peer-reviewed pathway database that
describes biological processes as detailed molecular reaction networks.
It emphasizes mechanistic precision, causal relationships, and
hierarchical pathway structure. Enrichment in Reactome pathways
indicates that target genes participate in specific molecular events or
subprocesses such as signal transduction modules, DNA repair mechanisms,
immune cascades, or cell-cycle transitions. Due to its high curation
standards, Reactome enrichment results are considered highly reliable
and mechanistically interpretable.

#### WikiPathways\_2024\_Human

WikiPathways is a community-curated pathway resource that complements
canonical databases by incorporating emerging biological knowledge and
specialized pathways. Pathways are contributed and maintained by domain
experts and are frequently updated to reflect current research.
Enrichment in WikiPathways highlights involvement of target genes in
less standardized or rapidly evolving biological processes, disease
mechanisms, or tissue-specific pathways. This category is particularly
useful for capturing novel or non-canonical regulatory programs not yet
represented in traditional pathway databases.

#### DisGeNET

DisGeNET integrates gene–disease associations derived from curated
databases, genome-wide association studies (GWAS), animal models, and
text-mined literature. Associations span a wide range of human diseases
and phenotypes. Enrichment in DisGeNET indicates that the target genes
are disproportionately associated with specific pathological conditions,
suggesting disease relevance of the observed gene expression changes.
This category is valuable for translational interpretation and for
linking molecular findings to clinical phenotypes.

#### OMIM\_Disease

This category is based on disease annotations from Online Mendelian
Inheritance in Man (OMIM), a comprehensive, manually curated database
focusing on inherited human disorders and gene–phenotype relationships.
OMIM emphasizes high-confidence links supported by genetic and clinical
evidence. Enrichment suggests that target genes are involved in
monogenic or well-characterized genetic diseases, providing insight into
potential hereditary components or fundamental biological mechanisms
underlying the observed expression patterns.

#### OMIM\_Expanded

OMIM Expanded extends the core OMIM disease annotations by
incorporating additional phenotype descriptions, allelic variants, and
gene–trait associations. This broader annotation set captures a wider
spectrum of genetic and phenotypic relationships beyond classical
Mendelian disorders. Enrichment in this category highlights genes with
established roles in human traits or disease susceptibility, offering a
more inclusive view of genetic contributions to biological and
pathological processes.

#### Human\_Phenotype\_Ontology

The Human Phenotype Ontology (HPO) provides a standardized vocabulary
of phenotypic abnormalities encountered in human disease, linking
clinical features to underlying genetic causes. HPO integrates clinical
observations, rare disease data, and curated gene–phenotype
associations. Enrichment in HPO terms indicates that target genes are
associated with specific clinical signs or symptom clusters. This
category is especially useful for connecting molecular findings to
observable phenotypes and for supporting clinical or diagnostic
interpretation.

## Treatment\_X\_Manufacturer\_Q\_vs\_Treatment\_Y\_Manufacturer\_Q - Tissue specific ORA

### all\_genes

#### GO\_Biological\_Process\_2025

This category comprises Gene Ontology (GO) Biological Process terms
describing coordinated and temporally ordered biological programs such
as cell cycle progression, immune activation, apoptosis,
differentiation, and metabolic regulation. GO Biological Process
annotations are curated by the Gene Ontology Consortium and integrate
experimental evidence, computational inference, and expert review.
Enrichment in this category indicates that the differentially expressed
target genes participate disproportionately in shared biological
programs rather than isolated molecular events. These results provide
high-level functional insight into systemic biological changes and are
particularly useful for interpreting phenotypic consequences of gene
expression alterations.

#### GO\_Cellular\_Component\_2025

This category captures Gene Ontology Cellular Component annotations
describing the subcellular localization of gene products, including
organelles, macromolecular complexes, membranes, and specialized
cellular structures. GO Cellular Component terms are curated through
experimental localization studies and integrative annotation pipelines.
Enrichment reveals whether target genes preferentially localize to
specific cellular compartments, suggesting spatial organization of
regulatory effects. These insights are valuable for understanding
compartment-specific processes such as mitochondrial regulation, vesicle
trafficking, nuclear control mechanisms, or membrane-associated
signaling.

#### GO\_Molecular\_Function\_2025

GO Molecular Function terms describe the elemental biochemical
activities of gene products, including binding affinities, catalytic
activities, transporter functions, and receptor interactions. These
annotations are curated by the Gene Ontology Consortium based on
experimental data and validated inference. Enrichment in this category
highlights specific molecular activities that are over-represented among
the target genes, offering mechanistic insight into how regulatory
changes may alter protein function or signaling capacity. This category
is especially informative for identifying enzymatic classes, signaling
mediators, or binding modules affected by miRNA regulation.

#### KEGG\_2021\_Human

This category is based on pathway annotations from the Kyoto
Encyclopedia of Genes and Genomes (KEGG), a manually curated resource
integrating molecular interaction networks, metabolic pathways, and
disease-associated signaling cascades. KEGG pathways represent canonical
biological systems including metabolism, signal transduction, cell
communication, and genetic information processing. Enrichment in KEGG
pathways suggests coordinated perturbation of well-characterized
biological networks. This category is particularly useful for
identifying conserved signaling routes and metabolic programs affected
by gene expression changes.

#### Reactome\_Pathways\_2024

Reactome is an expert-curated, peer-reviewed pathway database that
describes biological processes as detailed molecular reaction networks.
It emphasizes mechanistic precision, causal relationships, and
hierarchical pathway structure. Enrichment in Reactome pathways
indicates that target genes participate in specific molecular events or
subprocesses such as signal transduction modules, DNA repair mechanisms,
immune cascades, or cell-cycle transitions. Due to its high curation
standards, Reactome enrichment results are considered highly reliable
and mechanistically interpretable.

#### WikiPathways\_2024\_Human

WikiPathways is a community-curated pathway resource that complements
canonical databases by incorporating emerging biological knowledge and
specialized pathways. Pathways are contributed and maintained by domain
experts and are frequently updated to reflect current research.
Enrichment in WikiPathways highlights involvement of target genes in
less standardized or rapidly evolving biological processes, disease
mechanisms, or tissue-specific pathways. This category is particularly
useful for capturing novel or non-canonical regulatory programs not yet
represented in traditional pathway databases.

#### DisGeNET

DisGeNET integrates gene–disease associations derived from curated
databases, genome-wide association studies (GWAS), animal models, and
text-mined literature. Associations span a wide range of human diseases
and phenotypes. Enrichment in DisGeNET indicates that the target genes
are disproportionately associated with specific pathological conditions,
suggesting disease relevance of the observed gene expression changes.
This category is valuable for translational interpretation and for
linking molecular findings to clinical phenotypes.

#### OMIM\_Disease

This category is based on disease annotations from Online Mendelian
Inheritance in Man (OMIM), a comprehensive, manually curated database
focusing on inherited human disorders and gene–phenotype relationships.
OMIM emphasizes high-confidence links supported by genetic and clinical
evidence. Enrichment suggests that target genes are involved in
monogenic or well-characterized genetic diseases, providing insight into
potential hereditary components or fundamental biological mechanisms
underlying the observed expression patterns.

#### OMIM\_Expanded

OMIM Expanded extends the core OMIM disease annotations by
incorporating additional phenotype descriptions, allelic variants, and
gene–trait associations. This broader annotation set captures a wider
spectrum of genetic and phenotypic relationships beyond classical
Mendelian disorders. Enrichment in this category highlights genes with
established roles in human traits or disease susceptibility, offering a
more inclusive view of genetic contributions to biological and
pathological processes.

#### Human\_Phenotype\_Ontology

The Human Phenotype Ontology (HPO) provides a standardized vocabulary
of phenotypic abnormalities encountered in human disease, linking
clinical features to underlying genetic causes. HPO integrates clinical
observations, rare disease data, and curated gene–phenotype
associations. Enrichment in HPO terms indicates that target genes are
associated with specific clinical signs or symptom clusters. This
category is especially useful for connecting molecular findings to
observable phenotypes and for supporting clinical or diagnostic
interpretation.

### lymph node

#### GO\_Biological\_Process\_2025

This category comprises Gene Ontology (GO) Biological Process terms
describing coordinated and temporally ordered biological programs such
as cell cycle progression, immune activation, apoptosis,
differentiation, and metabolic regulation. GO Biological Process
annotations are curated by the Gene Ontology Consortium and integrate
experimental evidence, computational inference, and expert review.
Enrichment in this category indicates that the differentially expressed
target genes participate disproportionately in shared biological
programs rather than isolated molecular events. These results provide
high-level functional insight into systemic biological changes and are
particularly useful for interpreting phenotypic consequences of gene
expression alterations.

#### GO\_Cellular\_Component\_2025

This category captures Gene Ontology Cellular Component annotations
describing the subcellular localization of gene products, including
organelles, macromolecular complexes, membranes, and specialized
cellular structures. GO Cellular Component terms are curated through
experimental localization studies and integrative annotation pipelines.
Enrichment reveals whether target genes preferentially localize to
specific cellular compartments, suggesting spatial organization of
regulatory effects. These insights are valuable for understanding
compartment-specific processes such as mitochondrial regulation, vesicle
trafficking, nuclear control mechanisms, or membrane-associated
signaling.

#### GO\_Molecular\_Function\_2025

GO Molecular Function terms describe the elemental biochemical
activities of gene products, including binding affinities, catalytic
activities, transporter functions, and receptor interactions. These
annotations are curated by the Gene Ontology Consortium based on
experimental data and validated inference. Enrichment in this category
highlights specific molecular activities that are over-represented among
the target genes, offering mechanistic insight into how regulatory
changes may alter protein function or signaling capacity. This category
is especially informative for identifying enzymatic classes, signaling
mediators, or binding modules affected by miRNA regulation.

#### KEGG\_2021\_Human

This category is based on pathway annotations from the Kyoto
Encyclopedia of Genes and Genomes (KEGG), a manually curated resource
integrating molecular interaction networks, metabolic pathways, and
disease-associated signaling cascades. KEGG pathways represent canonical
biological systems including metabolism, signal transduction, cell
communication, and genetic information processing. Enrichment in KEGG
pathways suggests coordinated perturbation of well-characterized
biological networks. This category is particularly useful for
identifying conserved signaling routes and metabolic programs affected
by gene expression changes.

#### Reactome\_Pathways\_2024

Reactome is an expert-curated, peer-reviewed pathway database that
describes biological processes as detailed molecular reaction networks.
It emphasizes mechanistic precision, causal relationships, and
hierarchical pathway structure. Enrichment in Reactome pathways
indicates that target genes participate in specific molecular events or
subprocesses such as signal transduction modules, DNA repair mechanisms,
immune cascades, or cell-cycle transitions. Due to its high curation
standards, Reactome enrichment results are considered highly reliable
and mechanistically interpretable.

#### WikiPathways\_2024\_Human

WikiPathways is a community-curated pathway resource that complements
canonical databases by incorporating emerging biological knowledge and
specialized pathways. Pathways are contributed and maintained by domain
experts and are frequently updated to reflect current research.
Enrichment in WikiPathways highlights involvement of target genes in
less standardized or rapidly evolving biological processes, disease
mechanisms, or tissue-specific pathways. This category is particularly
useful for capturing novel or non-canonical regulatory programs not yet
represented in traditional pathway databases.

#### DisGeNET

DisGeNET integrates gene–disease associations derived from curated
databases, genome-wide association studies (GWAS), animal models, and
text-mined literature. Associations span a wide range of human diseases
and phenotypes. Enrichment in DisGeNET indicates that the target genes
are disproportionately associated with specific pathological conditions,
suggesting disease relevance of the observed gene expression changes.
This category is valuable for translational interpretation and for
linking molecular findings to clinical phenotypes.

#### OMIM\_Disease

This category is based on disease annotations from Online Mendelian
Inheritance in Man (OMIM), a comprehensive, manually curated database
focusing on inherited human disorders and gene–phenotype relationships.
OMIM emphasizes high-confidence links supported by genetic and clinical
evidence. Enrichment suggests that target genes are involved in
monogenic or well-characterized genetic diseases, providing insight into
potential hereditary components or fundamental biological mechanisms
underlying the observed expression patterns.

#### OMIM\_Expanded

OMIM Expanded extends the core OMIM disease annotations by
incorporating additional phenotype descriptions, allelic variants, and
gene–trait associations. This broader annotation set captures a wider
spectrum of genetic and phenotypic relationships beyond classical
Mendelian disorders. Enrichment in this category highlights genes with
established roles in human traits or disease susceptibility, offering a
more inclusive view of genetic contributions to biological and
pathological processes.

#### Human\_Phenotype\_Ontology

The Human Phenotype Ontology (HPO) provides a standardized vocabulary
of phenotypic abnormalities encountered in human disease, linking
clinical features to underlying genetic causes. HPO integrates clinical
observations, rare disease data, and curated gene–phenotype
associations. Enrichment in HPO terms indicates that target genes are
associated with specific clinical signs or symptom clusters. This
category is especially useful for connecting molecular findings to
observable phenotypes and for supporting clinical or diagnostic
interpretation.

### spleen

#### GO\_Biological\_Process\_2025

This category comprises Gene Ontology (GO) Biological Process terms
describing coordinated and temporally ordered biological programs such
as cell cycle progression, immune activation, apoptosis,
differentiation, and metabolic regulation. GO Biological Process
annotations are curated by the Gene Ontology Consortium and integrate
experimental evidence, computational inference, and expert review.
Enrichment in this category indicates that the differentially expressed
target genes participate disproportionately in shared biological
programs rather than isolated molecular events. These results provide
high-level functional insight into systemic biological changes and are
particularly useful for interpreting phenotypic consequences of gene
expression alterations.

#### GO\_Cellular\_Component\_2025

This category captures Gene Ontology Cellular Component annotations
describing the subcellular localization of gene products, including
organelles, macromolecular complexes, membranes, and specialized
cellular structures. GO Cellular Component terms are curated through
experimental localization studies and integrative annotation pipelines.
Enrichment reveals whether target genes preferentially localize to
specific cellular compartments, suggesting spatial organization of
regulatory effects. These insights are valuable for understanding
compartment-specific processes such as mitochondrial regulation, vesicle
trafficking, nuclear control mechanisms, or membrane-associated
signaling.

#### GO\_Molecular\_Function\_2025

GO Molecular Function terms describe the elemental biochemical
activities of gene products, including binding affinities, catalytic
activities, transporter functions, and receptor interactions. These
annotations are curated by the Gene Ontology Consortium based on
experimental data and validated inference. Enrichment in this category
highlights specific molecular activities that are over-represented among
the target genes, offering mechanistic insight into how regulatory
changes may alter protein function or signaling capacity. This category
is especially informative for identifying enzymatic classes, signaling
mediators, or binding modules affected by miRNA regulation.

#### KEGG\_2021\_Human

This category is based on pathway annotations from the Kyoto
Encyclopedia of Genes and Genomes (KEGG), a manually curated resource
integrating molecular interaction networks, metabolic pathways, and
disease-associated signaling cascades. KEGG pathways represent canonical
biological systems including metabolism, signal transduction, cell
communication, and genetic information processing. Enrichment in KEGG
pathways suggests coordinated perturbation of well-characterized
biological networks. This category is particularly useful for
identifying conserved signaling routes and metabolic programs affected
by gene expression changes.

#### Reactome\_Pathways\_2024

Reactome is an expert-curated, peer-reviewed pathway database that
describes biological processes as detailed molecular reaction networks.
It emphasizes mechanistic precision, causal relationships, and
hierarchical pathway structure. Enrichment in Reactome pathways
indicates that target genes participate in specific molecular events or
subprocesses such as signal transduction modules, DNA repair mechanisms,
immune cascades, or cell-cycle transitions. Due to its high curation
standards, Reactome enrichment results are considered highly reliable
and mechanistically interpretable.

#### WikiPathways\_2024\_Human

WikiPathways is a community-curated pathway resource that complements
canonical databases by incorporating emerging biological knowledge and
specialized pathways. Pathways are contributed and maintained by domain
experts and are frequently updated to reflect current research.
Enrichment in WikiPathways highlights involvement of target genes in
less standardized or rapidly evolving biological processes, disease
mechanisms, or tissue-specific pathways. This category is particularly
useful for capturing novel or non-canonical regulatory programs not yet
represented in traditional pathway databases.

#### DisGeNET

DisGeNET integrates gene–disease associations derived from curated
databases, genome-wide association studies (GWAS), animal models, and
text-mined literature. Associations span a wide range of human diseases
and phenotypes. Enrichment in DisGeNET indicates that the target genes
are disproportionately associated with specific pathological conditions,
suggesting disease relevance of the observed gene expression changes.
This category is valuable for translational interpretation and for
linking molecular findings to clinical phenotypes.

#### OMIM\_Disease

This category is based on disease annotations from Online Mendelian
Inheritance in Man (OMIM), a comprehensive, manually curated database
focusing on inherited human disorders and gene–phenotype relationships.
OMIM emphasizes high-confidence links supported by genetic and clinical
evidence. Enrichment suggests that target genes are involved in
monogenic or well-characterized genetic diseases, providing insight into
potential hereditary components or fundamental biological mechanisms
underlying the observed expression patterns.

#### OMIM\_Expanded

OMIM Expanded extends the core OMIM disease annotations by
incorporating additional phenotype descriptions, allelic variants, and
gene–trait associations. This broader annotation set captures a wider
spectrum of genetic and phenotypic relationships beyond classical
Mendelian disorders. Enrichment in this category highlights genes with
established roles in human traits or disease susceptibility, offering a
more inclusive view of genetic contributions to biological and
pathological processes.

#### Human\_Phenotype\_Ontology

The Human Phenotype Ontology (HPO) provides a standardized vocabulary
of phenotypic abnormalities encountered in human disease, linking
clinical features to underlying genetic causes. HPO integrates clinical
observations, rare disease data, and curated gene–phenotype
associations. Enrichment in HPO terms indicates that target genes are
associated with specific clinical signs or symptom clusters. This
category is especially useful for connecting molecular findings to
observable phenotypes and for supporting clinical or diagnostic
interpretation.

## Treatment\_Y\_Manufacturer\_M\_vs\_Treatment\_Y\_Manufacturer\_Q - Tissue specific ORA

### all\_genes

#### GO\_Biological\_Process\_2025

This category comprises Gene Ontology (GO) Biological Process terms
describing coordinated and temporally ordered biological programs such
as cell cycle progression, immune activation, apoptosis,
differentiation, and metabolic regulation. GO Biological Process
annotations are curated by the Gene Ontology Consortium and integrate
experimental evidence, computational inference, and expert review.
Enrichment in this category indicates that the differentially expressed
target genes participate disproportionately in shared biological
programs rather than isolated molecular events. These results provide
high-level functional insight into systemic biological changes and are
particularly useful for interpreting phenotypic consequences of gene
expression alterations.

#### GO\_Cellular\_Component\_2025

This category captures Gene Ontology Cellular Component annotations
describing the subcellular localization of gene products, including
organelles, macromolecular complexes, membranes, and specialized
cellular structures. GO Cellular Component terms are curated through
experimental localization studies and integrative annotation pipelines.
Enrichment reveals whether target genes preferentially localize to
specific cellular compartments, suggesting spatial organization of
regulatory effects. These insights are valuable for understanding
compartment-specific processes such as mitochondrial regulation, vesicle
trafficking, nuclear control mechanisms, or membrane-associated
signaling.

#### GO\_Molecular\_Function\_2025

GO Molecular Function terms describe the elemental biochemical
activities of gene products, including binding affinities, catalytic
activities, transporter functions, and receptor interactions. These
annotations are curated by the Gene Ontology Consortium based on
experimental data and validated inference. Enrichment in this category
highlights specific molecular activities that are over-represented among
the target genes, offering mechanistic insight into how regulatory
changes may alter protein function or signaling capacity. This category
is especially informative for identifying enzymatic classes, signaling
mediators, or binding modules affected by miRNA regulation.

#### KEGG\_2021\_Human

This category is based on pathway annotations from the Kyoto
Encyclopedia of Genes and Genomes (KEGG), a manually curated resource
integrating molecular interaction networks, metabolic pathways, and
disease-associated signaling cascades. KEGG pathways represent canonical
biological systems including metabolism, signal transduction, cell
communication, and genetic information processing. Enrichment in KEGG
pathways suggests coordinated perturbation of well-characterized
biological networks. This category is particularly useful for
identifying conserved signaling routes and metabolic programs affected
by gene expression changes.

#### Reactome\_Pathways\_2024

Reactome is an expert-curated, peer-reviewed pathway database that
describes biological processes as detailed molecular reaction networks.
It emphasizes mechanistic precision, causal relationships, and
hierarchical pathway structure. Enrichment in Reactome pathways
indicates that target genes participate in specific molecular events or
subprocesses such as signal transduction modules, DNA repair mechanisms,
immune cascades, or cell-cycle transitions. Due to its high curation
standards, Reactome enrichment results are considered highly reliable
and mechanistically interpretable.

#### WikiPathways\_2024\_Human

WikiPathways is a community-curated pathway resource that complements
canonical databases by incorporating emerging biological knowledge and
specialized pathways. Pathways are contributed and maintained by domain
experts and are frequently updated to reflect current research.
Enrichment in WikiPathways highlights involvement of target genes in
less standardized or rapidly evolving biological processes, disease
mechanisms, or tissue-specific pathways. This category is particularly
useful for capturing novel or non-canonical regulatory programs not yet
represented in traditional pathway databases.

#### DisGeNET

DisGeNET integrates gene–disease associations derived from curated
databases, genome-wide association studies (GWAS), animal models, and
text-mined literature. Associations span a wide range of human diseases
and phenotypes. Enrichment in DisGeNET indicates that the target genes
are disproportionately associated with specific pathological conditions,
suggesting disease relevance of the observed gene expression changes.
This category is valuable for translational interpretation and for
linking molecular findings to clinical phenotypes.

#### OMIM\_Disease

This category is based on disease annotations from Online Mendelian
Inheritance in Man (OMIM), a comprehensive, manually curated database
focusing on inherited human disorders and gene–phenotype relationships.
OMIM emphasizes high-confidence links supported by genetic and clinical
evidence. Enrichment suggests that target genes are involved in
monogenic or well-characterized genetic diseases, providing insight into
potential hereditary components or fundamental biological mechanisms
underlying the observed expression patterns.

#### OMIM\_Expanded

OMIM Expanded extends the core OMIM disease annotations by
incorporating additional phenotype descriptions, allelic variants, and
gene–trait associations. This broader annotation set captures a wider
spectrum of genetic and phenotypic relationships beyond classical
Mendelian disorders. Enrichment in this category highlights genes with
established roles in human traits or disease susceptibility, offering a
more inclusive view of genetic contributions to biological and
pathological processes.

#### Human\_Phenotype\_Ontology

The Human Phenotype Ontology (HPO) provides a standardized vocabulary
of phenotypic abnormalities encountered in human disease, linking
clinical features to underlying genetic causes. HPO integrates clinical
observations, rare disease data, and curated gene–phenotype
associations. Enrichment in HPO terms indicates that target genes are
associated with specific clinical signs or symptom clusters. This
category is especially useful for connecting molecular findings to
observable phenotypes and for supporting clinical or diagnostic
interpretation.

### lymph node

#### GO\_Biological\_Process\_2025

This category comprises Gene Ontology (GO) Biological Process terms
describing coordinated and temporally ordered biological programs such
as cell cycle progression, immune activation, apoptosis,
differentiation, and metabolic regulation. GO Biological Process
annotations are curated by the Gene Ontology Consortium and integrate
experimental evidence, computational inference, and expert review.
Enrichment in this category indicates that the differentially expressed
target genes participate disproportionately in shared biological
programs rather than isolated molecular events. These results provide
high-level functional insight into systemic biological changes and are
particularly useful for interpreting phenotypic consequences of gene
expression alterations.

#### GO\_Cellular\_Component\_2025

This category captures Gene Ontology Cellular Component annotations
describing the subcellular localization of gene products, including
organelles, macromolecular complexes, membranes, and specialized
cellular structures. GO Cellular Component terms are curated through
experimental localization studies and integrative annotation pipelines.
Enrichment reveals whether target genes preferentially localize to
specific cellular compartments, suggesting spatial organization of
regulatory effects. These insights are valuable for understanding
compartment-specific processes such as mitochondrial regulation, vesicle
trafficking, nuclear control mechanisms, or membrane-associated
signaling.

#### GO\_Molecular\_Function\_2025

GO Molecular Function terms describe the elemental biochemical
activities of gene products, including binding affinities, catalytic
activities, transporter functions, and receptor interactions. These
annotations are curated by the Gene Ontology Consortium based on
experimental data and validated inference. Enrichment in this category
highlights specific molecular activities that are over-represented among
the target genes, offering mechanistic insight into how regulatory
changes may alter protein function or signaling capacity. This category
is especially informative for identifying enzymatic classes, signaling
mediators, or binding modules affected by miRNA regulation.

#### KEGG\_2021\_Human

This category is based on pathway annotations from the Kyoto
Encyclopedia of Genes and Genomes (KEGG), a manually curated resource
integrating molecular interaction networks, metabolic pathways, and
disease-associated signaling cascades. KEGG pathways represent canonical
biological systems including metabolism, signal transduction, cell
communication, and genetic information processing. Enrichment in KEGG
pathways suggests coordinated perturbation of well-characterized
biological networks. This category is particularly useful for
identifying conserved signaling routes and metabolic programs affected
by gene expression changes.

#### Reactome\_Pathways\_2024

Reactome is an expert-curated, peer-reviewed pathway database that
describes biological processes as detailed molecular reaction networks.
It emphasizes mechanistic precision, causal relationships, and
hierarchical pathway structure. Enrichment in Reactome pathways
indicates that target genes participate in specific molecular events or
subprocesses such as signal transduction modules, DNA repair mechanisms,
immune cascades, or cell-cycle transitions. Due to its high curation
standards, Reactome enrichment results are considered highly reliable
and mechanistically interpretable.

#### WikiPathways\_2024\_Human

WikiPathways is a community-curated pathway resource that complements
canonical databases by incorporating emerging biological knowledge and
specialized pathways. Pathways are contributed and maintained by domain
experts and are frequently updated to reflect current research.
Enrichment in WikiPathways highlights involvement of target genes in
less standardized or rapidly evolving biological processes, disease
mechanisms, or tissue-specific pathways. This category is particularly
useful for capturing novel or non-canonical regulatory programs not yet
represented in traditional pathway databases.

#### DisGeNET

DisGeNET integrates gene–disease associations derived from curated
databases, genome-wide association studies (GWAS), animal models, and
text-mined literature. Associations span a wide range of human diseases
and phenotypes. Enrichment in DisGeNET indicates that the target genes
are disproportionately associated with specific pathological conditions,
suggesting disease relevance of the observed gene expression changes.
This category is valuable for translational interpretation and for
linking molecular findings to clinical phenotypes.

#### OMIM\_Disease

This category is based on disease annotations from Online Mendelian
Inheritance in Man (OMIM), a comprehensive, manually curated database
focusing on inherited human disorders and gene–phenotype relationships.
OMIM emphasizes high-confidence links supported by genetic and clinical
evidence. Enrichment suggests that target genes are involved in
monogenic or well-characterized genetic diseases, providing insight into
potential hereditary components or fundamental biological mechanisms
underlying the observed expression patterns.

#### OMIM\_Expanded

OMIM Expanded extends the core OMIM disease annotations by
incorporating additional phenotype descriptions, allelic variants, and
gene–trait associations. This broader annotation set captures a wider
spectrum of genetic and phenotypic relationships beyond classical
Mendelian disorders. Enrichment in this category highlights genes with
established roles in human traits or disease susceptibility, offering a
more inclusive view of genetic contributions to biological and
pathological processes.

#### Human\_Phenotype\_Ontology

The Human Phenotype Ontology (HPO) provides a standardized vocabulary
of phenotypic abnormalities encountered in human disease, linking
clinical features to underlying genetic causes. HPO integrates clinical
observations, rare disease data, and curated gene–phenotype
associations. Enrichment in HPO terms indicates that target genes are
associated with specific clinical signs or symptom clusters. This
category is especially useful for connecting molecular findings to
observable phenotypes and for supporting clinical or diagnostic
interpretation.

### spleen

#### GO\_Biological\_Process\_2025

This category comprises Gene Ontology (GO) Biological Process terms
describing coordinated and temporally ordered biological programs such
as cell cycle progression, immune activation, apoptosis,
differentiation, and metabolic regulation. GO Biological Process
annotations are curated by the Gene Ontology Consortium and integrate
experimental evidence, computational inference, and expert review.
Enrichment in this category indicates that the differentially expressed
target genes participate disproportionately in shared biological
programs rather than isolated molecular events. These results provide
high-level functional insight into systemic biological changes and are
particularly useful for interpreting phenotypic consequences of gene
expression alterations.

#### GO\_Cellular\_Component\_2025

This category captures Gene Ontology Cellular Component annotations
describing the subcellular localization of gene products, including
organelles, macromolecular complexes, membranes, and specialized
cellular structures. GO Cellular Component terms are curated through
experimental localization studies and integrative annotation pipelines.
Enrichment reveals whether target genes preferentially localize to
specific cellular compartments, suggesting spatial organization of
regulatory effects. These insights are valuable for understanding
compartment-specific processes such as mitochondrial regulation, vesicle
trafficking, nuclear control mechanisms, or membrane-associated
signaling.

#### GO\_Molecular\_Function\_2025

GO Molecular Function terms describe the elemental biochemical
activities of gene products, including binding affinities, catalytic
activities, transporter functions, and receptor interactions. These
annotations are curated by the Gene Ontology Consortium based on
experimental data and validated inference. Enrichment in this category
highlights specific molecular activities that are over-represented among
the target genes, offering mechanistic insight into how regulatory
changes may alter protein function or signaling capacity. This category
is especially informative for identifying enzymatic classes, signaling
mediators, or binding modules affected by miRNA regulation.

#### KEGG\_2021\_Human

This category is based on pathway annotations from the Kyoto
Encyclopedia of Genes and Genomes (KEGG), a manually curated resource
integrating molecular interaction networks, metabolic pathways, and
disease-associated signaling cascades. KEGG pathways represent canonical
biological systems including metabolism, signal transduction, cell
communication, and genetic information processing. Enrichment in KEGG
pathways suggests coordinated perturbation of well-characterized
biological networks. This category is particularly useful for
identifying conserved signaling routes and metabolic programs affected
by gene expression changes.

#### Reactome\_Pathways\_2024

Reactome is an expert-curated, peer-reviewed pathway database that
describes biological processes as detailed molecular reaction networks.
It emphasizes mechanistic precision, causal relationships, and
hierarchical pathway structure. Enrichment in Reactome pathways
indicates that target genes participate in specific molecular events or
subprocesses such as signal transduction modules, DNA repair mechanisms,
immune cascades, or cell-cycle transitions. Due to its high curation
standards, Reactome enrichment results are considered highly reliable
and mechanistically interpretable.

#### WikiPathways\_2024\_Human

WikiPathways is a community-curated pathway resource that complements
canonical databases by incorporating emerging biological knowledge and
specialized pathways. Pathways are contributed and maintained by domain
experts and are frequently updated to reflect current research.
Enrichment in WikiPathways highlights involvement of target genes in
less standardized or rapidly evolving biological processes, disease
mechanisms, or tissue-specific pathways. This category is particularly
useful for capturing novel or non-canonical regulatory programs not yet
represented in traditional pathway databases.

#### DisGeNET

DisGeNET integrates gene–disease associations derived from curated
databases, genome-wide association studies (GWAS), animal models, and
text-mined literature. Associations span a wide range of human diseases
and phenotypes. Enrichment in DisGeNET indicates that the target genes
are disproportionately associated with specific pathological conditions,
suggesting disease relevance of the observed gene expression changes.
This category is valuable for translational interpretation and for
linking molecular findings to clinical phenotypes.

#### OMIM\_Disease

This category is based on disease annotations from Online Mendelian
Inheritance in Man (OMIM), a comprehensive, manually curated database
focusing on inherited human disorders and gene–phenotype relationships.
OMIM emphasizes high-confidence links supported by genetic and clinical
evidence. Enrichment suggests that target genes are involved in
monogenic or well-characterized genetic diseases, providing insight into
potential hereditary components or fundamental biological mechanisms
underlying the observed expression patterns.

#### OMIM\_Expanded

OMIM Expanded extends the core OMIM disease annotations by
incorporating additional phenotype descriptions, allelic variants, and
gene–trait associations. This broader annotation set captures a wider
spectrum of genetic and phenotypic relationships beyond classical
Mendelian disorders. Enrichment in this category highlights genes with
established roles in human traits or disease susceptibility, offering a
more inclusive view of genetic contributions to biological and
pathological processes.

#### Human\_Phenotype\_Ontology

The Human Phenotype Ontology (HPO) provides a standardized vocabulary
of phenotypic abnormalities encountered in human disease, linking
clinical features to underlying genetic causes. HPO integrates clinical
observations, rare disease data, and curated gene–phenotype
associations. Enrichment in HPO terms indicates that target genes are
associated with specific clinical signs or symptom clusters. This
category is especially useful for connecting molecular findings to
observable phenotypes and for supporting clinical or diagnostic
interpretation.
