# Supplementary material for: Comprehensive and bias-aware analysis of small RNA NGS data for biomarker detection with caRNAge
Source: Extracell Vesicles Circ Nucl Acids. 2026 Sep 24;7(3):1554–81. doi: 10.20517/evcna.2026.78 (PMC13678173; doi:10.20517/evcna.2026.78)
Supplement: Supplementary file 1 [file evcna-7-3-1554-SupplementaryMaterials.zip › evcna7078-SupplementaryMaterials/Supplementary File 4.html]

caRNAge - comprehensive analysis of small RNA gene expression


# caRNAge - comprehensive analysis of small RNA gene expression

### Data overview - quality control, mapping distribution and bias detection

#### 22 August, 2026

## Purpose of this overview (Disclaimer)

This short overview is meant as a first step in data exploration for
your sequencing experiment. It tries to identify sources of bias like
batch effects or design flaws and supports you in finding correct
normalization strategies but it is neither fully comprehensive nor a
final analysis. Please refer to the guidelines for best practice of
caRNAge and the respective manuals and vignettes of the applied tools.
You are strongly advised to thoroughly check all available information
in addition to this data overview. caRNAge is built upon the work of
many excellent scientists who developed the necessary tools to process
small RNA NGS data, so please make sure to cite the corresponding
original authors in addition to caRNAge when publishing your work.

If you encounter any technical problems, feel free to contact the
corresponding author at bkirchner@tum.de.

## Data processing

Raw sequencing reads were processed with Cutadapt to trim
adapter sequences from 3’ ends if present and to remove degraded reads
(shorter than 16nt). Trimmed reads were analysed with FastQC
to evaluate phred scores and read lengths before aligning them against
the human tRNA (GtRNAdb), miRNA
(miRBase) including isomiRs (isomiRROR), piRNAs (piRNAdb) and refseq transcriptome
sequences (refseq)
with bowtie.
Aligned reads were then analyzed to generate length and mapping
distributions as well as reads counts. Finally, gene read counts were
processed with R and mainly DESeq2
to normalize and transform data and generate unsupervised clustering
plots (PCA). Potential batch effects were visualized by removing them
for plotting with limma.
For a full list of incorporated tools and R packages, please see
[References].

Fig 1: Simplified workflow for NGS data analysis

|  | Tools | Version | Parameters |
| --- | --- | --- | --- |
| Trimming | Cutadapt | 4.4 | -a AGATCGGAAGAGCAC, -e 0.1 -m 16 |
| Quality Control | FastQC | 0.12.1 |  |
| Alignment | bowtie | 1.3.1 | Bowtie -v mismatches: tRNA=1, isomiR=3, transcriptome=1; –best –norc |
| Normalisation | DESeq2 | R version 4.6.1 (2026-06-24), DESeq2 1.52.0 | ~compartment |
| Data Transformation | DESeq2 | R version 4.6.1 (2026-06-24), DESeq2 1.52.0 | varianceStabilizingTransformation() |
| Unsupervised Clustering | limma | R version 4.6.1 (2026-06-24), limma 3.68.5 | hclust(dist(method = ‘euclidean’), method=‘ward.D’), prcomp(scale. = TRUE), removeBatchEffect() |

## Sample overview

The starting point for all analyses, is a well designed study design.
In addition to the classification of the main research question, it
should also incorporate all available information that could in any way
affect the outcome. This includes potential confounding factors such as
batch effects as well as statistical relevant information such as
patient cohorts or cell lines. Please make sure that the classifications
given in your *design.tsv* are correct and that all relevant
information is present.

### Study design

The row names and “Sample” column should be matching and indicate
that samples in design were correctly assigned in the analysis. A column
named “replaceable” maybe present if groups are large enough (>7) to
replace single outlier values.

|  | Sample | source\_name | cell\_type | condition | compartment | experimental\_block | sequencing\_platform | sequencing\_cycles | replaceable |
| --- | --- | --- | --- | --- | --- | --- | --- | --- | --- |
| group7\_cd4cells\_rep1 | group7\_cd4cells\_rep1 | CD4 T cells | cd4\_t\_cell | cell | intracellular | group7\_cd4\_cells | illumina\_nextseq\_500 | 75 | TRUE |
| group7\_cd4cells\_rep10 | group7\_cd4cells\_rep10 | CD4 T cells | cd4\_t\_cell | cell | intracellular | group7\_cd4\_cells | illumina\_nextseq\_500 | 75 | TRUE |
| group7\_cd4cells\_rep11 | group7\_cd4cells\_rep11 | CD4 T cells | cd4\_t\_cell | cell | intracellular | group7\_cd4\_cells | illumina\_nextseq\_500 | 75 | TRUE |
| group7\_cd4cells\_rep12 | group7\_cd4cells\_rep12 | CD4 T cells | cd4\_t\_cell | cell | intracellular | group7\_cd4\_cells | illumina\_nextseq\_500 | 75 | TRUE |
| group7\_cd4cells\_rep13 | group7\_cd4cells\_rep13 | CD4 T cells | cd4\_t\_cell | cell | intracellular | group7\_cd4\_cells | illumina\_nextseq\_500 | 75 | TRUE |
| group7\_cd4cells\_rep14 | group7\_cd4cells\_rep14 | CD4 T cells | cd4\_t\_cell | cell | intracellular | group7\_cd4\_cells | illumina\_nextseq\_500 | 75 | TRUE |
| group7\_cd4cells\_rep15 | group7\_cd4cells\_rep15 | CD4 T cells | cd4\_t\_cell | cell | intracellular | group7\_cd4\_cells | illumina\_nextseq\_500 | 75 | TRUE |
| group7\_cd4cells\_rep16 | group7\_cd4cells\_rep16 | CD4 T cells | cd4\_t\_cell | cell | intracellular | group7\_cd4\_cells | illumina\_nextseq\_500 | 75 | TRUE |
| group7\_cd4cells\_rep17 | group7\_cd4cells\_rep17 | CD4 T cells | cd4\_t\_cell | cell | intracellular | group7\_cd4\_cells | illumina\_nextseq\_500 | 75 | TRUE |
| group7\_cd4cells\_rep18 | group7\_cd4cells\_rep18 | CD4 T cells | cd4\_t\_cell | cell | intracellular | group7\_cd4\_cells | illumina\_nextseq\_500 | 75 | TRUE |
| group7\_cd4cells\_rep19 | group7\_cd4cells\_rep19 | CD4 T cells | cd4\_t\_cell | cell | intracellular | group7\_cd4\_cells | illumina\_nextseq\_500 | 75 | TRUE |
| group7\_cd4cells\_rep2 | group7\_cd4cells\_rep2 | CD4 T cells | cd4\_t\_cell | cell | intracellular | group7\_cd4\_cells | illumina\_nextseq\_500 | 75 | TRUE |
| group7\_cd4cells\_rep20 | group7\_cd4cells\_rep20 | CD4 T cells | cd4\_t\_cell | cell | intracellular | group7\_cd4\_cells | illumina\_nextseq\_500 | 75 | TRUE |
| group7\_cd4cells\_rep21 | group7\_cd4cells\_rep21 | CD4 T cells | cd4\_t\_cell | cell | intracellular | group7\_cd4\_cells | illumina\_nextseq\_500 | 75 | TRUE |
| group7\_cd4cells\_rep22 | group7\_cd4cells\_rep22 | CD4 T cells | cd4\_t\_cell | cell | intracellular | group7\_cd4\_cells | illumina\_nextseq\_500 | 75 | TRUE |
| group7\_cd4cells\_rep23 | group7\_cd4cells\_rep23 | CD4 T cells | cd4\_t\_cell | cell | intracellular | group7\_cd4\_cells | illumina\_nextseq\_500 | 75 | TRUE |
| group7\_cd4cells\_rep24 | group7\_cd4cells\_rep24 | CD4 T cells | cd4\_t\_cell | cell | intracellular | group7\_cd4\_cells | illumina\_nextseq\_500 | 75 | TRUE |
| group7\_cd4cells\_rep3 | group7\_cd4cells\_rep3 | CD4 T cells | cd4\_t\_cell | cell | intracellular | group7\_cd4\_cells | illumina\_nextseq\_500 | 75 | TRUE |
| group7\_cd4cells\_rep4 | group7\_cd4cells\_rep4 | CD4 T cells | cd4\_t\_cell | cell | intracellular | group7\_cd4\_cells | illumina\_nextseq\_500 | 75 | TRUE |
| group7\_cd4cells\_rep5 | group7\_cd4cells\_rep5 | CD4 T cells | cd4\_t\_cell | cell | intracellular | group7\_cd4\_cells | illumina\_nextseq\_500 | 75 | TRUE |
| group7\_cd4cells\_rep6 | group7\_cd4cells\_rep6 | CD4 T cells | cd4\_t\_cell | cell | intracellular | group7\_cd4\_cells | illumina\_nextseq\_500 | 75 | TRUE |
| group7\_cd4cells\_rep7 | group7\_cd4cells\_rep7 | CD4 T cells | cd4\_t\_cell | cell | intracellular | group7\_cd4\_cells | illumina\_nextseq\_500 | 75 | TRUE |
| group7\_cd4cells\_rep8 | group7\_cd4cells\_rep8 | CD4 T cells | cd4\_t\_cell | cell | intracellular | group7\_cd4\_cells | illumina\_nextseq\_500 | 75 | TRUE |
| group7\_cd4cells\_rep9 | group7\_cd4cells\_rep9 | CD4 T cells | cd4\_t\_cell | cell | intracellular | group7\_cd4\_cells | illumina\_nextseq\_500 | 75 | TRUE |
| group7\_cd8cells\_rep1 | group7\_cd8cells\_rep1 | CD8 T cells | cd8\_t\_cell | cell | intracellular | group7\_cd8\_cells | illumina\_nextseq\_500 | 75 | TRUE |
| group7\_cd8cells\_rep10 | group7\_cd8cells\_rep10 | CD8 T cells | cd8\_t\_cell | cell | intracellular | group7\_cd8\_cells | illumina\_nextseq\_500 | 75 | TRUE |
| group7\_cd8cells\_rep11 | group7\_cd8cells\_rep11 | CD8 T cells | cd8\_t\_cell | cell | intracellular | group7\_cd8\_cells | illumina\_nextseq\_500 | 75 | TRUE |
| group7\_cd8cells\_rep12 | group7\_cd8cells\_rep12 | CD8 T cells | cd8\_t\_cell | cell | intracellular | group7\_cd8\_cells | illumina\_nextseq\_500 | 75 | TRUE |
| group7\_cd8cells\_rep13 | group7\_cd8cells\_rep13 | CD8 T cells | cd8\_t\_cell | cell | intracellular | group7\_cd8\_cells | illumina\_nextseq\_500 | 75 | TRUE |
| group7\_cd8cells\_rep14 | group7\_cd8cells\_rep14 | CD8 T cells | cd8\_t\_cell | cell | intracellular | group7\_cd8\_cells | illumina\_nextseq\_500 | 75 | TRUE |
| group7\_cd8cells\_rep15 | group7\_cd8cells\_rep15 | CD8 T cells | cd8\_t\_cell | cell | intracellular | group7\_cd8\_cells | illumina\_nextseq\_500 | 75 | TRUE |
| group7\_cd8cells\_rep16 | group7\_cd8cells\_rep16 | CD8 T cells | cd8\_t\_cell | cell | intracellular | group7\_cd8\_cells | illumina\_nextseq\_500 | 75 | TRUE |
| group7\_cd8cells\_rep17 | group7\_cd8cells\_rep17 | CD8 T cells | cd8\_t\_cell | cell | intracellular | group7\_cd8\_cells | illumina\_nextseq\_500 | 75 | TRUE |
| group7\_cd8cells\_rep18 | group7\_cd8cells\_rep18 | CD8 T cells | cd8\_t\_cell | cell | intracellular | group7\_cd8\_cells | illumina\_nextseq\_500 | 75 | TRUE |
| group7\_cd8cells\_rep19 | group7\_cd8cells\_rep19 | CD8 T cells | cd8\_t\_cell | cell | intracellular | group7\_cd8\_cells | illumina\_nextseq\_500 | 75 | TRUE |
| group7\_cd8cells\_rep2 | group7\_cd8cells\_rep2 | CD8 T cells | cd8\_t\_cell | cell | intracellular | group7\_cd8\_cells | illumina\_nextseq\_500 | 75 | TRUE |
| group7\_cd8cells\_rep20 | group7\_cd8cells\_rep20 | CD8 T cells | cd8\_t\_cell | cell | intracellular | group7\_cd8\_cells | illumina\_nextseq\_500 | 75 | TRUE |
| group7\_cd8cells\_rep21 | group7\_cd8cells\_rep21 | CD8 T cells | cd8\_t\_cell | cell | intracellular | group7\_cd8\_cells | illumina\_nextseq\_500 | 75 | TRUE |
| group7\_cd8cells\_rep22 | group7\_cd8cells\_rep22 | CD8 T cells | cd8\_t\_cell | cell | intracellular | group7\_cd8\_cells | illumina\_nextseq\_500 | 75 | TRUE |
| group7\_cd8cells\_rep23 | group7\_cd8cells\_rep23 | CD8 T cells | cd8\_t\_cell | cell | intracellular | group7\_cd8\_cells | illumina\_nextseq\_500 | 75 | TRUE |
| group7\_cd8cells\_rep24 | group7\_cd8cells\_rep24 | CD8 T cells | cd8\_t\_cell | cell | intracellular | group7\_cd8\_cells | illumina\_nextseq\_500 | 75 | TRUE |
| group7\_cd8cells\_rep3 | group7\_cd8cells\_rep3 | CD8 T cells | cd8\_t\_cell | cell | intracellular | group7\_cd8\_cells | illumina\_nextseq\_500 | 75 | TRUE |
| group7\_cd8cells\_rep4 | group7\_cd8cells\_rep4 | CD8 T cells | cd8\_t\_cell | cell | intracellular | group7\_cd8\_cells | illumina\_nextseq\_500 | 75 | TRUE |
| group7\_cd8cells\_rep5 | group7\_cd8cells\_rep5 | CD8 T cells | cd8\_t\_cell | cell | intracellular | group7\_cd8\_cells | illumina\_nextseq\_500 | 75 | TRUE |
| group7\_cd8cells\_rep6 | group7\_cd8cells\_rep6 | CD8 T cells | cd8\_t\_cell | cell | intracellular | group7\_cd8\_cells | illumina\_nextseq\_500 | 75 | TRUE |
| group7\_cd8cells\_rep7 | group7\_cd8cells\_rep7 | CD8 T cells | cd8\_t\_cell | cell | intracellular | group7\_cd8\_cells | illumina\_nextseq\_500 | 75 | TRUE |
| group7\_cd8cells\_rep8 | group7\_cd8cells\_rep8 | CD8 T cells | cd8\_t\_cell | cell | intracellular | group7\_cd8\_cells | illumina\_nextseq\_500 | 75 | TRUE |
| group7\_cd8cells\_rep9 | group7\_cd8cells\_rep9 | CD8 T cells | cd8\_t\_cell | cell | intracellular | group7\_cd8\_cells | illumina\_nextseq\_500 | 75 | TRUE |
| wtchg\_834291\_208\_1 | wtchg\_834291\_208\_1 | CD4 EV | cd4\_t\_cell | ev | extracellular\_vesicle | wtchg\_208 | illumina\_hiseq\_4000 | 75 | TRUE |
| wtchg\_834291\_209\_1 | wtchg\_834291\_209\_1 | CD8 EV | cd8\_t\_cell | ev | extracellular\_vesicle | wtchg\_209 | illumina\_hiseq\_4000 | 75 | TRUE |
| wtchg\_834291\_210\_1 | wtchg\_834291\_210\_1 | CD8 EV | cd8\_t\_cell | ev | extracellular\_vesicle | wtchg\_210 | illumina\_hiseq\_4000 | 75 | TRUE |
| wtchg\_834291\_211\_1 | wtchg\_834291\_211\_1 | CD4 EV | cd4\_t\_cell | ev | extracellular\_vesicle | wtchg\_211 | illumina\_hiseq\_4000 | 75 | TRUE |
| wtchg\_834291\_212\_1 | wtchg\_834291\_212\_1 | CD8 EV | cd8\_t\_cell | ev | extracellular\_vesicle | wtchg\_212 | illumina\_hiseq\_4000 | 75 | TRUE |
| wtchg\_834291\_213\_1 | wtchg\_834291\_213\_1 | CD4 EV | cd4\_t\_cell | ev | extracellular\_vesicle | wtchg\_213 | illumina\_hiseq\_4000 | 75 | TRUE |
| wtchg\_834291\_220\_1 | wtchg\_834291\_220\_1 | CD4 S0 | cd4\_t\_cell | s0 | bslb\_background\_control | wtchg\_220 | illumina\_hiseq\_4000 | 75 | TRUE |
| wtchg\_834291\_225\_1 | wtchg\_834291\_225\_1 | CD8 S1 | cd8\_t\_cell | s1 | trans\_synaptic\_vesicle | wtchg\_225 | illumina\_hiseq\_4000 | 75 | TRUE |
| wtchg\_834291\_226\_1 | wtchg\_834291\_226\_1 | CD4 S0 | cd4\_t\_cell | s0 | bslb\_background\_control | wtchg\_226 | illumina\_hiseq\_4000 | 75 | TRUE |
| wtchg\_834291\_227\_1 | wtchg\_834291\_227\_1 | CD4 S1 | cd4\_t\_cell | s1 | trans\_synaptic\_vesicle | wtchg\_227 | illumina\_hiseq\_4000 | 75 | TRUE |
| wtchg\_834291\_228\_1 | wtchg\_834291\_228\_1 | CD8 S1 | cd8\_t\_cell | s1 | trans\_synaptic\_vesicle | wtchg\_228 | illumina\_hiseq\_4000 | 75 | TRUE |
| wtchg\_834291\_233\_1 | wtchg\_834291\_233\_1 | CD4 S1 | cd4\_t\_cell | s1 | trans\_synaptic\_vesicle | wtchg\_233 | illumina\_hiseq\_4000 | 75 | TRUE |
| wtchg\_834291\_234\_1 | wtchg\_834291\_234\_1 | CD4 S1 | cd4\_t\_cell | s1 | trans\_synaptic\_vesicle | wtchg\_234 | illumina\_hiseq\_4000 | 75 | TRUE |
| wtchg\_834291\_237\_1 | wtchg\_834291\_237\_1 | CD8 S0 | cd8\_t\_cell | s0 | bslb\_background\_control | wtchg\_237 | illumina\_hiseq\_4000 | 75 | TRUE |
| wtchg\_834291\_238\_1 | wtchg\_834291\_238\_1 | CD8 S1 | cd8\_t\_cell | s1 | trans\_synaptic\_vesicle | wtchg\_238 | illumina\_hiseq\_4000 | 75 | TRUE |
| wtchg\_834292\_208\_1 | wtchg\_834292\_208\_1 | CD4 EV | cd4\_t\_cell | ev | extracellular\_vesicle | wtchg\_208 | illumina\_hiseq\_4000 | 75 | TRUE |
| wtchg\_834292\_209\_1 | wtchg\_834292\_209\_1 | CD8 EV | cd8\_t\_cell | ev | extracellular\_vesicle | wtchg\_209 | illumina\_hiseq\_4000 | 75 | TRUE |
| wtchg\_834292\_210\_1 | wtchg\_834292\_210\_1 | CD8 EV | cd8\_t\_cell | ev | extracellular\_vesicle | wtchg\_210 | illumina\_hiseq\_4000 | 75 | TRUE |
| wtchg\_834292\_211\_1 | wtchg\_834292\_211\_1 | CD4 EV | cd4\_t\_cell | ev | extracellular\_vesicle | wtchg\_211 | illumina\_hiseq\_4000 | 75 | TRUE |
| wtchg\_834292\_212\_1 | wtchg\_834292\_212\_1 | CD8 EV | cd8\_t\_cell | ev | extracellular\_vesicle | wtchg\_212 | illumina\_hiseq\_4000 | 75 | TRUE |
| wtchg\_834292\_213\_1 | wtchg\_834292\_213\_1 | CD4 EV | cd4\_t\_cell | ev | extracellular\_vesicle | wtchg\_213 | illumina\_hiseq\_4000 | 75 | TRUE |
| wtchg\_834292\_220\_1 | wtchg\_834292\_220\_1 | CD4 S0 | cd4\_t\_cell | s0 | bslb\_background\_control | wtchg\_220 | illumina\_hiseq\_4000 | 75 | TRUE |
| wtchg\_834292\_225\_1 | wtchg\_834292\_225\_1 | CD8 S1 | cd8\_t\_cell | s1 | trans\_synaptic\_vesicle | wtchg\_225 | illumina\_hiseq\_4000 | 75 | TRUE |
| wtchg\_834292\_226\_1 | wtchg\_834292\_226\_1 | CD4 S0 | cd4\_t\_cell | s0 | bslb\_background\_control | wtchg\_226 | illumina\_hiseq\_4000 | 75 | TRUE |
| wtchg\_834292\_227\_1 | wtchg\_834292\_227\_1 | CD4 S1 | cd4\_t\_cell | s1 | trans\_synaptic\_vesicle | wtchg\_227 | illumina\_hiseq\_4000 | 75 | TRUE |
| wtchg\_834292\_228\_1 | wtchg\_834292\_228\_1 | CD8 S1 | cd8\_t\_cell | s1 | trans\_synaptic\_vesicle | wtchg\_228 | illumina\_hiseq\_4000 | 75 | TRUE |
| wtchg\_834292\_233\_1 | wtchg\_834292\_233\_1 | CD4 S1 | cd4\_t\_cell | s1 | trans\_synaptic\_vesicle | wtchg\_233 | illumina\_hiseq\_4000 | 75 | TRUE |
| wtchg\_834292\_234\_1 | wtchg\_834292\_234\_1 | CD4 S1 | cd4\_t\_cell | s1 | trans\_synaptic\_vesicle | wtchg\_234 | illumina\_hiseq\_4000 | 75 | TRUE |
| wtchg\_834292\_237\_1 | wtchg\_834292\_237\_1 | CD8 S0 | cd8\_t\_cell | s0 | bslb\_background\_control | wtchg\_237 | illumina\_hiseq\_4000 | 75 | TRUE |
| wtchg\_834292\_238\_1 | wtchg\_834292\_238\_1 | CD8 S1 | cd8\_t\_cell | s1 | trans\_synaptic\_vesicle | wtchg\_238 | illumina\_hiseq\_4000 | 75 | TRUE |
| wtchg\_834293\_208\_1 | wtchg\_834293\_208\_1 | CD4 EV | cd4\_t\_cell | ev | extracellular\_vesicle | wtchg\_208 | illumina\_hiseq\_4000 | 75 | TRUE |
| wtchg\_834293\_209\_1 | wtchg\_834293\_209\_1 | CD8 EV | cd8\_t\_cell | ev | extracellular\_vesicle | wtchg\_209 | illumina\_hiseq\_4000 | 75 | TRUE |
| wtchg\_834293\_210\_1 | wtchg\_834293\_210\_1 | CD8 EV | cd8\_t\_cell | ev | extracellular\_vesicle | wtchg\_210 | illumina\_hiseq\_4000 | 75 | TRUE |
| wtchg\_834293\_211\_1 | wtchg\_834293\_211\_1 | CD4 EV | cd4\_t\_cell | ev | extracellular\_vesicle | wtchg\_211 | illumina\_hiseq\_4000 | 75 | TRUE |
| wtchg\_834293\_212\_1 | wtchg\_834293\_212\_1 | CD8 EV | cd8\_t\_cell | ev | extracellular\_vesicle | wtchg\_212 | illumina\_hiseq\_4000 | 75 | TRUE |
| wtchg\_834293\_213\_1 | wtchg\_834293\_213\_1 | CD4 EV | cd4\_t\_cell | ev | extracellular\_vesicle | wtchg\_213 | illumina\_hiseq\_4000 | 75 | TRUE |
| wtchg\_834293\_220\_1 | wtchg\_834293\_220\_1 | CD4 S0 | cd4\_t\_cell | s0 | bslb\_background\_control | wtchg\_220 | illumina\_hiseq\_4000 | 75 | TRUE |
| wtchg\_834293\_225\_1 | wtchg\_834293\_225\_1 | CD8 S1 | cd8\_t\_cell | s1 | trans\_synaptic\_vesicle | wtchg\_225 | illumina\_hiseq\_4000 | 75 | TRUE |
| wtchg\_834293\_226\_1 | wtchg\_834293\_226\_1 | CD4 S0 | cd4\_t\_cell | s0 | bslb\_background\_control | wtchg\_226 | illumina\_hiseq\_4000 | 75 | TRUE |
| wtchg\_834293\_227\_1 | wtchg\_834293\_227\_1 | CD4 S1 | cd4\_t\_cell | s1 | trans\_synaptic\_vesicle | wtchg\_227 | illumina\_hiseq\_4000 | 75 | TRUE |
| wtchg\_834293\_228\_1 | wtchg\_834293\_228\_1 | CD8 S1 | cd8\_t\_cell | s1 | trans\_synaptic\_vesicle | wtchg\_228 | illumina\_hiseq\_4000 | 75 | TRUE |
| wtchg\_834293\_233\_1 | wtchg\_834293\_233\_1 | CD4 S1 | cd4\_t\_cell | s1 | trans\_synaptic\_vesicle | wtchg\_233 | illumina\_hiseq\_4000 | 75 | TRUE |
| wtchg\_834293\_234\_1 | wtchg\_834293\_234\_1 | CD4 S1 | cd4\_t\_cell | s1 | trans\_synaptic\_vesicle | wtchg\_234 | illumina\_hiseq\_4000 | 75 | TRUE |
| wtchg\_834293\_237\_1 | wtchg\_834293\_237\_1 | CD8 S0 | cd8\_t\_cell | s0 | bslb\_background\_control | wtchg\_237 | illumina\_hiseq\_4000 | 75 | TRUE |
| wtchg\_834293\_238\_1 | wtchg\_834293\_238\_1 | CD8 S1 | cd8\_t\_cell | s1 | trans\_synaptic\_vesicle | wtchg\_238 | illumina\_hiseq\_4000 | 75 | TRUE |
| wtchg\_834294\_208\_1 | wtchg\_834294\_208\_1 | CD4 EV | cd4\_t\_cell | ev | extracellular\_vesicle | wtchg\_208 | illumina\_hiseq\_4000 | 75 | TRUE |
| wtchg\_834294\_209\_1 | wtchg\_834294\_209\_1 | CD8 EV | cd8\_t\_cell | ev | extracellular\_vesicle | wtchg\_209 | illumina\_hiseq\_4000 | 75 | TRUE |
| wtchg\_834294\_210\_1 | wtchg\_834294\_210\_1 | CD8 EV | cd8\_t\_cell | ev | extracellular\_vesicle | wtchg\_210 | illumina\_hiseq\_4000 | 75 | TRUE |
| wtchg\_834294\_211\_1 | wtchg\_834294\_211\_1 | CD4 EV | cd4\_t\_cell | ev | extracellular\_vesicle | wtchg\_211 | illumina\_hiseq\_4000 | 75 | TRUE |
| wtchg\_834294\_212\_1 | wtchg\_834294\_212\_1 | CD8 EV | cd8\_t\_cell | ev | extracellular\_vesicle | wtchg\_212 | illumina\_hiseq\_4000 | 75 | TRUE |
| wtchg\_834294\_213\_1 | wtchg\_834294\_213\_1 | CD4 EV | cd4\_t\_cell | ev | extracellular\_vesicle | wtchg\_213 | illumina\_hiseq\_4000 | 75 | TRUE |
| wtchg\_834294\_220\_1 | wtchg\_834294\_220\_1 | CD4 S0 | cd4\_t\_cell | s0 | bslb\_background\_control | wtchg\_220 | illumina\_hiseq\_4000 | 75 | TRUE |
| wtchg\_834294\_225\_1 | wtchg\_834294\_225\_1 | CD8 S1 | cd8\_t\_cell | s1 | trans\_synaptic\_vesicle | wtchg\_225 | illumina\_hiseq\_4000 | 75 | TRUE |
| wtchg\_834294\_226\_1 | wtchg\_834294\_226\_1 | CD4 S0 | cd4\_t\_cell | s0 | bslb\_background\_control | wtchg\_226 | illumina\_hiseq\_4000 | 75 | TRUE |
| wtchg\_834294\_227\_1 | wtchg\_834294\_227\_1 | CD4 S1 | cd4\_t\_cell | s1 | trans\_synaptic\_vesicle | wtchg\_227 | illumina\_hiseq\_4000 | 75 | TRUE |
| wtchg\_834294\_228\_1 | wtchg\_834294\_228\_1 | CD8 S1 | cd8\_t\_cell | s1 | trans\_synaptic\_vesicle | wtchg\_228 | illumina\_hiseq\_4000 | 75 | TRUE |
| wtchg\_834294\_233\_1 | wtchg\_834294\_233\_1 | CD4 S1 | cd4\_t\_cell | s1 | trans\_synaptic\_vesicle | wtchg\_233 | illumina\_hiseq\_4000 | 75 | TRUE |
| wtchg\_834294\_234\_1 | wtchg\_834294\_234\_1 | CD4 S1 | cd4\_t\_cell | s1 | trans\_synaptic\_vesicle | wtchg\_234 | illumina\_hiseq\_4000 | 75 | TRUE |
| wtchg\_834294\_237\_1 | wtchg\_834294\_237\_1 | CD8 S0 | cd8\_t\_cell | s0 | bslb\_background\_control | wtchg\_237 | illumina\_hiseq\_4000 | 75 | TRUE |
| wtchg\_834294\_238\_1 | wtchg\_834294\_238\_1 | CD8 S1 | cd8\_t\_cell | s1 | trans\_synaptic\_vesicle | wtchg\_238 | illumina\_hiseq\_4000 | 75 | TRUE |
| wtchg\_836235\_208\_1 | wtchg\_836235\_208\_1 | CD4 EV | cd4\_t\_cell | ev | extracellular\_vesicle | wtchg\_208 | illumina\_hiseq\_4000 | 75 | TRUE |
| wtchg\_836235\_209\_1 | wtchg\_836235\_209\_1 | CD8 EV | cd8\_t\_cell | ev | extracellular\_vesicle | wtchg\_209 | illumina\_hiseq\_4000 | 75 | TRUE |
| wtchg\_836235\_210\_1 | wtchg\_836235\_210\_1 | CD8 EV | cd8\_t\_cell | ev | extracellular\_vesicle | wtchg\_210 | illumina\_hiseq\_4000 | 75 | TRUE |
| wtchg\_836235\_211\_1 | wtchg\_836235\_211\_1 | CD4 EV | cd4\_t\_cell | ev | extracellular\_vesicle | wtchg\_211 | illumina\_hiseq\_4000 | 75 | TRUE |
| wtchg\_836235\_212\_1 | wtchg\_836235\_212\_1 | CD8 EV | cd8\_t\_cell | ev | extracellular\_vesicle | wtchg\_212 | illumina\_hiseq\_4000 | 75 | TRUE |
| wtchg\_836235\_213\_1 | wtchg\_836235\_213\_1 | CD4 EV | cd4\_t\_cell | ev | extracellular\_vesicle | wtchg\_213 | illumina\_hiseq\_4000 | 75 | TRUE |
| wtchg\_836235\_220\_1 | wtchg\_836235\_220\_1 | CD4 S0 | cd4\_t\_cell | s0 | bslb\_background\_control | wtchg\_220 | illumina\_hiseq\_4000 | 75 | TRUE |
| wtchg\_836235\_225\_1 | wtchg\_836235\_225\_1 | CD8 S1 | cd8\_t\_cell | s1 | trans\_synaptic\_vesicle | wtchg\_225 | illumina\_hiseq\_4000 | 75 | TRUE |
| wtchg\_836235\_226\_1 | wtchg\_836235\_226\_1 | CD4 S0 | cd4\_t\_cell | s0 | bslb\_background\_control | wtchg\_226 | illumina\_hiseq\_4000 | 75 | TRUE |
| wtchg\_836235\_227\_1 | wtchg\_836235\_227\_1 | CD4 S1 | cd4\_t\_cell | s1 | trans\_synaptic\_vesicle | wtchg\_227 | illumina\_hiseq\_4000 | 75 | TRUE |
| wtchg\_836235\_228\_1 | wtchg\_836235\_228\_1 | CD8 S1 | cd8\_t\_cell | s1 | trans\_synaptic\_vesicle | wtchg\_228 | illumina\_hiseq\_4000 | 75 | TRUE |
| wtchg\_836235\_233\_1 | wtchg\_836235\_233\_1 | CD4 S1 | cd4\_t\_cell | s1 | trans\_synaptic\_vesicle | wtchg\_233 | illumina\_hiseq\_4000 | 75 | TRUE |
| wtchg\_836235\_234\_1 | wtchg\_836235\_234\_1 | CD4 S1 | cd4\_t\_cell | s1 | trans\_synaptic\_vesicle | wtchg\_234 | illumina\_hiseq\_4000 | 75 | TRUE |
| wtchg\_836235\_237\_1 | wtchg\_836235\_237\_1 | CD8 S0 | cd8\_t\_cell | s0 | bslb\_background\_control | wtchg\_237 | illumina\_hiseq\_4000 | 75 | TRUE |
| wtchg\_836235\_238\_1 | wtchg\_836235\_238\_1 | CD8 S1 | cd8\_t\_cell | s1 | trans\_synaptic\_vesicle | wtchg\_238 | illumina\_hiseq\_4000 | 75 | TRUE |
| wtchg\_836236\_208\_1 | wtchg\_836236\_208\_1 | CD4 EV | cd4\_t\_cell | ev | extracellular\_vesicle | wtchg\_208 | illumina\_hiseq\_4000 | 75 | TRUE |
| wtchg\_836236\_209\_1 | wtchg\_836236\_209\_1 | CD8 EV | cd8\_t\_cell | ev | extracellular\_vesicle | wtchg\_209 | illumina\_hiseq\_4000 | 75 | TRUE |
| wtchg\_836236\_210\_1 | wtchg\_836236\_210\_1 | CD8 EV | cd8\_t\_cell | ev | extracellular\_vesicle | wtchg\_210 | illumina\_hiseq\_4000 | 75 | TRUE |
| wtchg\_836236\_211\_1 | wtchg\_836236\_211\_1 | CD4 EV | cd4\_t\_cell | ev | extracellular\_vesicle | wtchg\_211 | illumina\_hiseq\_4000 | 75 | TRUE |
| wtchg\_836236\_212\_1 | wtchg\_836236\_212\_1 | CD8 EV | cd8\_t\_cell | ev | extracellular\_vesicle | wtchg\_212 | illumina\_hiseq\_4000 | 75 | TRUE |
| wtchg\_836236\_213\_1 | wtchg\_836236\_213\_1 | CD4 EV | cd4\_t\_cell | ev | extracellular\_vesicle | wtchg\_213 | illumina\_hiseq\_4000 | 75 | TRUE |
| wtchg\_836236\_220\_1 | wtchg\_836236\_220\_1 | CD4 S0 | cd4\_t\_cell | s0 | bslb\_background\_control | wtchg\_220 | illumina\_hiseq\_4000 | 75 | TRUE |
| wtchg\_836236\_225\_1 | wtchg\_836236\_225\_1 | CD8 S1 | cd8\_t\_cell | s1 | trans\_synaptic\_vesicle | wtchg\_225 | illumina\_hiseq\_4000 | 75 | TRUE |
| wtchg\_836236\_226\_1 | wtchg\_836236\_226\_1 | CD4 S0 | cd4\_t\_cell | s0 | bslb\_background\_control | wtchg\_226 | illumina\_hiseq\_4000 | 75 | TRUE |
| wtchg\_836236\_227\_1 | wtchg\_836236\_227\_1 | CD4 S1 | cd4\_t\_cell | s1 | trans\_synaptic\_vesicle | wtchg\_227 | illumina\_hiseq\_4000 | 75 | TRUE |
| wtchg\_836236\_228\_1 | wtchg\_836236\_228\_1 | CD8 S1 | cd8\_t\_cell | s1 | trans\_synaptic\_vesicle | wtchg\_228 | illumina\_hiseq\_4000 | 75 | TRUE |
| wtchg\_836236\_233\_1 | wtchg\_836236\_233\_1 | CD4 S1 | cd4\_t\_cell | s1 | trans\_synaptic\_vesicle | wtchg\_233 | illumina\_hiseq\_4000 | 75 | TRUE |
| wtchg\_836236\_234\_1 | wtchg\_836236\_234\_1 | CD4 S1 | cd4\_t\_cell | s1 | trans\_synaptic\_vesicle | wtchg\_234 | illumina\_hiseq\_4000 | 75 | TRUE |
| wtchg\_836236\_237\_1 | wtchg\_836236\_237\_1 | CD8 S0 | cd8\_t\_cell | s0 | bslb\_background\_control | wtchg\_237 | illumina\_hiseq\_4000 | 75 | TRUE |
| wtchg\_836236\_238\_1 | wtchg\_836236\_238\_1 | CD8 S1 | cd8\_t\_cell | s1 | trans\_synaptic\_vesicle | wtchg\_238 | illumina\_hiseq\_4000 | 75 | TRUE |
| wtchg\_836237\_208\_1 | wtchg\_836237\_208\_1 | CD4 EV | cd4\_t\_cell | ev | extracellular\_vesicle | wtchg\_208 | illumina\_hiseq\_4000 | 75 | TRUE |
| wtchg\_836237\_209\_1 | wtchg\_836237\_209\_1 | CD8 EV | cd8\_t\_cell | ev | extracellular\_vesicle | wtchg\_209 | illumina\_hiseq\_4000 | 75 | TRUE |
| wtchg\_836237\_210\_1 | wtchg\_836237\_210\_1 | CD8 EV | cd8\_t\_cell | ev | extracellular\_vesicle | wtchg\_210 | illumina\_hiseq\_4000 | 75 | TRUE |
| wtchg\_836237\_211\_1 | wtchg\_836237\_211\_1 | CD4 EV | cd4\_t\_cell | ev | extracellular\_vesicle | wtchg\_211 | illumina\_hiseq\_4000 | 75 | TRUE |
| wtchg\_836237\_212\_1 | wtchg\_836237\_212\_1 | CD8 EV | cd8\_t\_cell | ev | extracellular\_vesicle | wtchg\_212 | illumina\_hiseq\_4000 | 75 | TRUE |
| wtchg\_836237\_213\_1 | wtchg\_836237\_213\_1 | CD4 EV | cd4\_t\_cell | ev | extracellular\_vesicle | wtchg\_213 | illumina\_hiseq\_4000 | 75 | TRUE |
| wtchg\_836237\_220\_1 | wtchg\_836237\_220\_1 | CD4 S0 | cd4\_t\_cell | s0 | bslb\_background\_control | wtchg\_220 | illumina\_hiseq\_4000 | 75 | TRUE |
| wtchg\_836237\_225\_1 | wtchg\_836237\_225\_1 | CD8 S1 | cd8\_t\_cell | s1 | trans\_synaptic\_vesicle | wtchg\_225 | illumina\_hiseq\_4000 | 75 | TRUE |
| wtchg\_836237\_226\_1 | wtchg\_836237\_226\_1 | CD4 S0 | cd4\_t\_cell | s0 | bslb\_background\_control | wtchg\_226 | illumina\_hiseq\_4000 | 75 | TRUE |
| wtchg\_836237\_227\_1 | wtchg\_836237\_227\_1 | CD4 S1 | cd4\_t\_cell | s1 | trans\_synaptic\_vesicle | wtchg\_227 | illumina\_hiseq\_4000 | 75 | TRUE |
| wtchg\_836237\_228\_1 | wtchg\_836237\_228\_1 | CD8 S1 | cd8\_t\_cell | s1 | trans\_synaptic\_vesicle | wtchg\_228 | illumina\_hiseq\_4000 | 75 | TRUE |
| wtchg\_836237\_233\_1 | wtchg\_836237\_233\_1 | CD4 S1 | cd4\_t\_cell | s1 | trans\_synaptic\_vesicle | wtchg\_233 | illumina\_hiseq\_4000 | 75 | TRUE |
| wtchg\_836237\_234\_1 | wtchg\_836237\_234\_1 | CD4 S1 | cd4\_t\_cell | s1 | trans\_synaptic\_vesicle | wtchg\_234 | illumina\_hiseq\_4000 | 75 | TRUE |
| wtchg\_836237\_237\_1 | wtchg\_836237\_237\_1 | CD8 S0 | cd8\_t\_cell | s0 | bslb\_background\_control | wtchg\_237 | illumina\_hiseq\_4000 | 75 | TRUE |
| wtchg\_836237\_238\_1 | wtchg\_836237\_238\_1 | CD8 S1 | cd8\_t\_cell | s1 | trans\_synaptic\_vesicle | wtchg\_238 | illumina\_hiseq\_4000 | 75 | TRUE |
| wtchg\_836238\_208\_1 | wtchg\_836238\_208\_1 | CD4 EV | cd4\_t\_cell | ev | extracellular\_vesicle | wtchg\_208 | illumina\_hiseq\_4000 | 75 | TRUE |
| wtchg\_836238\_209\_1 | wtchg\_836238\_209\_1 | CD8 EV | cd8\_t\_cell | ev | extracellular\_vesicle | wtchg\_209 | illumina\_hiseq\_4000 | 75 | TRUE |
| wtchg\_836238\_210\_1 | wtchg\_836238\_210\_1 | CD8 EV | cd8\_t\_cell | ev | extracellular\_vesicle | wtchg\_210 | illumina\_hiseq\_4000 | 75 | TRUE |
| wtchg\_836238\_211\_1 | wtchg\_836238\_211\_1 | CD4 EV | cd4\_t\_cell | ev | extracellular\_vesicle | wtchg\_211 | illumina\_hiseq\_4000 | 75 | TRUE |
| wtchg\_836238\_212\_1 | wtchg\_836238\_212\_1 | CD8 EV | cd8\_t\_cell | ev | extracellular\_vesicle | wtchg\_212 | illumina\_hiseq\_4000 | 75 | TRUE |
| wtchg\_836238\_213\_1 | wtchg\_836238\_213\_1 | CD4 EV | cd4\_t\_cell | ev | extracellular\_vesicle | wtchg\_213 | illumina\_hiseq\_4000 | 75 | TRUE |
| wtchg\_836238\_220\_1 | wtchg\_836238\_220\_1 | CD4 S0 | cd4\_t\_cell | s0 | bslb\_background\_control | wtchg\_220 | illumina\_hiseq\_4000 | 75 | TRUE |
| wtchg\_836238\_225\_1 | wtchg\_836238\_225\_1 | CD8 S1 | cd8\_t\_cell | s1 | trans\_synaptic\_vesicle | wtchg\_225 | illumina\_hiseq\_4000 | 75 | TRUE |
| wtchg\_836238\_226\_1 | wtchg\_836238\_226\_1 | CD4 S0 | cd4\_t\_cell | s0 | bslb\_background\_control | wtchg\_226 | illumina\_hiseq\_4000 | 75 | TRUE |
| wtchg\_836238\_227\_1 | wtchg\_836238\_227\_1 | CD4 S1 | cd4\_t\_cell | s1 | trans\_synaptic\_vesicle | wtchg\_227 | illumina\_hiseq\_4000 | 75 | TRUE |
| wtchg\_836238\_228\_1 | wtchg\_836238\_228\_1 | CD8 S1 | cd8\_t\_cell | s1 | trans\_synaptic\_vesicle | wtchg\_228 | illumina\_hiseq\_4000 | 75 | TRUE |
| wtchg\_836238\_233\_1 | wtchg\_836238\_233\_1 | CD4 S1 | cd4\_t\_cell | s1 | trans\_synaptic\_vesicle | wtchg\_233 | illumina\_hiseq\_4000 | 75 | TRUE |
| wtchg\_836238\_234\_1 | wtchg\_836238\_234\_1 | CD4 S1 | cd4\_t\_cell | s1 | trans\_synaptic\_vesicle | wtchg\_234 | illumina\_hiseq\_4000 | 75 | TRUE |
| wtchg\_836238\_237\_1 | wtchg\_836238\_237\_1 | CD8 S0 | cd8\_t\_cell | s0 | bslb\_background\_control | wtchg\_237 | illumina\_hiseq\_4000 | 75 | TRUE |
| wtchg\_836238\_238\_1 | wtchg\_836238\_238\_1 | CD8 S1 | cd8\_t\_cell | s1 | trans\_synaptic\_vesicle | wtchg\_238 | illumina\_hiseq\_4000 | 75 | TRUE |
| x11\_lolafdez\_rna\_seq\_small\_s11\_l001\_r1\_001 | x11\_lolafdez\_rna\_seq\_small\_s11\_l001\_r1\_001 | CD4 EV | cd4\_t\_cell | ev | extracellular\_vesicle | legacy\_lolafdez | illumina\_hiseq\_4000 | 61 | TRUE |
| x14\_lolafdez\_rna\_seq\_small\_s14\_l001\_r1\_001 | x14\_lolafdez\_rna\_seq\_small\_s14\_l001\_r1\_001 | CD8 T cells | cd8\_t\_cell | cell | intracellular | legacy\_lolafdez | illumina\_hiseq\_4000 | 61 | TRUE |
| x15\_lolafdez\_rna\_seq\_small\_s15\_l001\_r1\_001 | x15\_lolafdez\_rna\_seq\_small\_s15\_l001\_r1\_001 | CD8 T cells | cd8\_t\_cell | cell | intracellular | legacy\_lolafdez | illumina\_hiseq\_4000 | 61 | TRUE |
| x17\_lolafdez\_rna\_seq\_small\_s17\_l001\_r1\_001 | x17\_lolafdez\_rna\_seq\_small\_s17\_l001\_r1\_001 | CD4 T cells | cd4\_t\_cell | cell | intracellular | legacy\_lolafdez | illumina\_hiseq\_4000 | 61 | TRUE |
| x18\_lolafdez\_rna\_seq\_small\_s18\_l001\_r1\_001 | x18\_lolafdez\_rna\_seq\_small\_s18\_l001\_r1\_001 | CD8 T cells | cd8\_t\_cell | cell | intracellular | legacy\_lolafdez | illumina\_hiseq\_4000 | 61 | TRUE |
| x19\_lolafdez\_rna\_seq\_small\_s19\_l001\_r1\_001 | x19\_lolafdez\_rna\_seq\_small\_s19\_l001\_r1\_001 | CD4 T cells | cd4\_t\_cell | cell | intracellular | legacy\_lolafdez | illumina\_hiseq\_4000 | 61 | TRUE |
| x1\_lolafdez\_rna\_seq\_small\_s1\_l001\_r1\_001 | x1\_lolafdez\_rna\_seq\_small\_s1\_l001\_r1\_001 | CD8 S1 | cd8\_t\_cell | s1 | trans\_synaptic\_vesicle | legacy\_lolafdez | illumina\_hiseq\_4000 | 61 | TRUE |
| x20\_lolafdez\_rna\_seq\_small\_s20\_l001\_r1\_001 | x20\_lolafdez\_rna\_seq\_small\_s20\_l001\_r1\_001 | CD4 T cells | cd4\_t\_cell | cell | intracellular | legacy\_lolafdez | illumina\_hiseq\_4000 | 61 | TRUE |
| x21\_lolafdez\_rna\_seq\_small\_s21\_l001\_r1\_001 | x21\_lolafdez\_rna\_seq\_small\_s21\_l001\_r1\_001 | CD4 T cells | cd4\_t\_cell | cell | intracellular | legacy\_lolafdez | illumina\_hiseq\_4000 | 61 | TRUE |
| x23\_lolafdez\_rna\_seq\_small\_s23\_l001\_r1\_001 | x23\_lolafdez\_rna\_seq\_small\_s23\_l001\_r1\_001 | CD8 T cells | cd8\_t\_cell | cell | intracellular | legacy\_lolafdez | illumina\_hiseq\_4000 | 61 | TRUE |
| x2\_lolafdez\_rna\_seq\_small\_s2\_l001\_r1\_001 | x2\_lolafdez\_rna\_seq\_small\_s2\_l001\_r1\_001 | CD8 S1 | cd8\_t\_cell | s1 | trans\_synaptic\_vesicle | legacy\_lolafdez | illumina\_hiseq\_4000 | 61 | TRUE |
| x3\_lolafdez\_rna\_seq\_small\_s3\_l001\_r1\_001 | x3\_lolafdez\_rna\_seq\_small\_s3\_l001\_r1\_001 | CD8 S1 | cd8\_t\_cell | s1 | trans\_synaptic\_vesicle | legacy\_lolafdez | illumina\_hiseq\_4000 | 61 | TRUE |
| x5\_lolafdez\_rna\_seq\_small\_s5\_l001\_r1\_001 | x5\_lolafdez\_rna\_seq\_small\_s5\_l001\_r1\_001 | CD4 EV | cd4\_t\_cell | ev | extracellular\_vesicle | legacy\_lolafdez | illumina\_hiseq\_4000 | 61 | TRUE |
| x6\_lolafdez\_rna\_seq\_small\_s6\_l001\_r1\_001 | x6\_lolafdez\_rna\_seq\_small\_s6\_l001\_r1\_001 | CD8 S1 | cd8\_t\_cell | s1 | trans\_synaptic\_vesicle | legacy\_lolafdez | illumina\_hiseq\_4000 | 61 | TRUE |
| x7\_lolafdez\_rna\_seq\_small\_s7\_l001\_r1\_001 | x7\_lolafdez\_rna\_seq\_small\_s7\_l001\_r1\_001 | CD4 EV | cd4\_t\_cell | ev | extracellular\_vesicle | legacy\_lolafdez | illumina\_hiseq\_4000 | 61 | TRUE |
| x8\_lolafdez\_rna\_seq\_small\_s8\_l001\_r1\_001 | x8\_lolafdez\_rna\_seq\_small\_s8\_l001\_r1\_001 | CD4 EV | cd4\_t\_cell | ev | extracellular\_vesicle | legacy\_lolafdez | illumina\_hiseq\_4000 | 61 | TRUE |
| x9\_lolafdez\_rna\_seq\_small\_s9\_l001\_r1\_001 | x9\_lolafdez\_rna\_seq\_small\_s9\_l001\_r1\_001 | CD4 EV | cd4\_t\_cell | ev | extracellular\_vesicle | legacy\_lolafdez | illumina\_hiseq\_4000 | 61 | TRUE |

### Consolidated sample groups

```
$source_name

     CD4 EV      CD4 S0      CD4 S1 CD4 T cells      CD8 EV      CD8 S0 
         29          16          24          28          24           8 
     CD8 S1 CD8 T cells 
         28          28 

$cell_type

cd4_t_cell cd8_t_cell 
        97         88 

$condition

cell   ev   s0   s1 
  56   53   24   52 

$compartment

          intracellular   extracellular_vesicle bslb_background_control 
                     56                      53                      24 
 trans_synaptic_vesicle 
                     52 

$experimental_block

group7_cd4_cells group7_cd8_cells  legacy_lolafdez        wtchg_208 
              24               24               17                8 
       wtchg_209        wtchg_210        wtchg_211        wtchg_212 
               8                8                8                8 
       wtchg_213        wtchg_220        wtchg_225        wtchg_226 
               8                8                8                8 
       wtchg_227        wtchg_228        wtchg_233        wtchg_234 
               8                8                8                8 
       wtchg_237        wtchg_238 
               8                8 

$sequencing_platform

 illumina_hiseq_4000 illumina_nextseq_500 
                 137                   48 

$sequencing_cycles

 61  75 
 17 168
```

## Quality control

We can assess the technical quality of the sequencing run and to some
extent of the library preparation by looking at phred score
and length as well as mapping distributions. While the following
summaries can give you hints if some samples behave abnormally, it is
strongly recommended to closely inspect the FastQC report for each
sample separately. Individual FastQC reports can be found in the
*FastQC* folder located in your **results** data
directory. Explanations to each category can be found at the tools
homepage.

### Summary

GC content per sequence or base is directly related to the
duplication level of RNAs in the analyzed sample. Since small RNA NGS
experiments tend to be dominated by relatively few transcripts,
distributions of A/T/C/G are not expected to be uniform in most
cases.

| Module Name | Samples (Sum) | Failed (Sum) | Passed (Sum) | Warning (Sum) | Failed | Warning |
| --- | --- | --- | --- | --- | --- | --- |
| Adapter Content | 185 | 0 | 185 | 0 | NA | NA |
| Basic Statistics | 185 | 0 | 185 | 0 | NA | NA |
| Per base sequence content | 185 | 149 | 0 | 36 | group7\_cd4cells\_rep1, group7\_cd4cells\_rep10, group7\_cd4cells\_rep11, group7\_cd4cells\_rep12, group7\_cd4cells\_rep13, group7\_cd4cells\_rep14, group7\_cd4cells\_rep15, group7\_cd4cells\_rep16, group7\_cd4cells\_rep17, group7\_cd4cells\_rep18, group7\_cd4cells\_rep19, group7\_cd4cells\_rep2, group7\_cd4cells\_rep20, group7\_cd4cells\_rep21, group7\_cd4cells\_rep22, group7\_cd4cells\_rep23, group7\_cd4cells\_rep24, group7\_cd4cells\_rep3, group7\_cd4cells\_rep4, group7\_cd4cells\_rep5, group7\_cd4cells\_rep6, group7\_cd4cells\_rep7, group7\_cd4cells\_rep8, group7\_cd4cells\_rep9, group7\_cd8cells\_rep1, group7\_cd8cells\_rep10, group7\_cd8cells\_rep11, group7\_cd8cells\_rep12, group7\_cd8cells\_rep13, group7\_cd8cells\_rep14, group7\_cd8cells\_rep15, group7\_cd8cells\_rep16, group7\_cd8cells\_rep17, group7\_cd8cells\_rep18, group7\_cd8cells\_rep19, group7\_cd8cells\_rep2, group7\_cd8cells\_rep20, group7\_cd8cells\_rep21, group7\_cd8cells\_rep22, group7\_cd8cells\_rep23, group7\_cd8cells\_rep24, group7\_cd8cells\_rep3, group7\_cd8cells\_rep4, group7\_cd8cells\_rep5, group7\_cd8cells\_rep6, group7\_cd8cells\_rep7, group7\_cd8cells\_rep8, group7\_cd8cells\_rep9, wtchg\_834291\_208\_1, wtchg\_834291\_209\_1, wtchg\_834291\_210\_1, wtchg\_834291\_211\_1, wtchg\_834291\_212\_1, wtchg\_834291\_213\_1, wtchg\_834291\_220\_1, wtchg\_834291\_225\_1, wtchg\_834291\_226\_1, wtchg\_834291\_227\_1, wtchg\_834291\_237\_1, wtchg\_834292\_208\_1, wtchg\_834292\_209\_1, wtchg\_834292\_210\_1, wtchg\_834292\_211\_1, wtchg\_834292\_212\_1, wtchg\_834292\_220\_1, wtchg\_834292\_225\_1, wtchg\_834292\_226\_1, wtchg\_834292\_227\_1, wtchg\_834292\_237\_1, wtchg\_834293\_208\_1, wtchg\_834293\_209\_1, wtchg\_834293\_210\_1, wtchg\_834293\_211\_1, wtchg\_834293\_212\_1, wtchg\_834293\_213\_1, wtchg\_834293\_220\_1, wtchg\_834293\_225\_1, wtchg\_834293\_226\_1, wtchg\_834293\_227\_1, wtchg\_834293\_237\_1, wtchg\_834294\_208\_1, wtchg\_834294\_209\_1, wtchg\_834294\_210\_1, wtchg\_834294\_211\_1, wtchg\_834294\_212\_1, wtchg\_834294\_220\_1, wtchg\_834294\_225\_1, wtchg\_834294\_226\_1, wtchg\_834294\_227\_1, wtchg\_834294\_237\_1, wtchg\_836235\_208\_1, wtchg\_836235\_209\_1, wtchg\_836235\_210\_1, wtchg\_836235\_211\_1, wtchg\_836235\_212\_1, wtchg\_836235\_220\_1, wtchg\_836235\_225\_1, wtchg\_836235\_226\_1, wtchg\_836235\_227\_1, wtchg\_836235\_237\_1, wtchg\_836236\_208\_1, wtchg\_836236\_209\_1, wtchg\_836236\_210\_1, wtchg\_836236\_211\_1, wtchg\_836236\_212\_1, wtchg\_836236\_220\_1, wtchg\_836236\_225\_1, wtchg\_836236\_226\_1, wtchg\_836236\_227\_1, wtchg\_836236\_237\_1, wtchg\_836237\_208\_1, wtchg\_836237\_209\_1, wtchg\_836237\_210\_1, wtchg\_836237\_211\_1, wtchg\_836237\_212\_1, wtchg\_836237\_213\_1, wtchg\_836237\_220\_1, wtchg\_836237\_225\_1, wtchg\_836237\_226\_1, wtchg\_836237\_227\_1, wtchg\_836237\_234\_1, wtchg\_836237\_237\_1, wtchg\_836238\_208\_1, wtchg\_836238\_209\_1, wtchg\_836238\_210\_1, wtchg\_836238\_211\_1, wtchg\_836238\_212\_1, wtchg\_836238\_220\_1, wtchg\_836238\_225\_1, wtchg\_836238\_226\_1, wtchg\_836238\_227\_1, wtchg\_836238\_237\_1, x1\_lolafdez\_rna\_seq\_small\_s1\_l001\_r1\_001, x11\_lolafdez\_rna\_seq\_small\_s11\_l001\_r1\_001, x14\_lolafdez\_rna\_seq\_small\_s14\_l001\_r1\_001, x15\_lolafdez\_rna\_seq\_small\_s15\_l001\_r1\_001, x17\_lolafdez\_rna\_seq\_small\_s17\_l001\_r1\_001, x18\_lolafdez\_rna\_seq\_small\_s18\_l001\_r1\_001, x19\_lolafdez\_rna\_seq\_small\_s19\_l001\_r1\_001, x2\_lolafdez\_rna\_seq\_small\_s2\_l001\_r1\_001, x20\_lolafdez\_rna\_seq\_small\_s20\_l001\_r1\_001, x21\_lolafdez\_rna\_seq\_small\_s21\_l001\_r1\_001, x23\_lolafdez\_rna\_seq\_small\_s23\_l001\_r1\_001, x3\_lolafdez\_rna\_seq\_small\_s3\_l001\_r1\_001, x5\_lolafdez\_rna\_seq\_small\_s5\_l001\_r1\_001, x6\_lolafdez\_rna\_seq\_small\_s6\_l001\_r1\_001, x7\_lolafdez\_rna\_seq\_small\_s7\_l001\_r1\_001, x8\_lolafdez\_rna\_seq\_small\_s8\_l001\_r1\_001, x9\_lolafdez\_rna\_seq\_small\_s9\_l001\_r1\_001 | wtchg\_834291\_228\_1, wtchg\_834291\_233\_1, wtchg\_834291\_234\_1, wtchg\_834291\_238\_1, wtchg\_834292\_213\_1, wtchg\_834292\_228\_1, wtchg\_834292\_233\_1, wtchg\_834292\_234\_1, wtchg\_834292\_238\_1, wtchg\_834293\_228\_1, wtchg\_834293\_233\_1, wtchg\_834293\_234\_1, wtchg\_834293\_238\_1, wtchg\_834294\_213\_1, wtchg\_834294\_228\_1, wtchg\_834294\_233\_1, wtchg\_834294\_234\_1, wtchg\_834294\_238\_1, wtchg\_836235\_213\_1, wtchg\_836235\_228\_1, wtchg\_836235\_233\_1, wtchg\_836235\_234\_1, wtchg\_836235\_238\_1, wtchg\_836236\_213\_1, wtchg\_836236\_228\_1, wtchg\_836236\_233\_1, wtchg\_836236\_234\_1, wtchg\_836236\_238\_1, wtchg\_836237\_228\_1, wtchg\_836237\_233\_1, wtchg\_836237\_238\_1, wtchg\_836238\_213\_1, wtchg\_836238\_228\_1, wtchg\_836238\_233\_1, wtchg\_836238\_234\_1, wtchg\_836238\_238\_1 |
| Per sequence GC content | 185 | 121 | 19 | 45 | group7\_cd4cells\_rep1, group7\_cd4cells\_rep10, group7\_cd4cells\_rep11, group7\_cd4cells\_rep12, group7\_cd4cells\_rep13, group7\_cd4cells\_rep14, group7\_cd4cells\_rep15, group7\_cd4cells\_rep16, group7\_cd4cells\_rep17, group7\_cd4cells\_rep18, group7\_cd4cells\_rep19, group7\_cd4cells\_rep2, group7\_cd4cells\_rep20, group7\_cd4cells\_rep21, group7\_cd4cells\_rep22, group7\_cd4cells\_rep23, group7\_cd4cells\_rep24, group7\_cd4cells\_rep3, group7\_cd4cells\_rep4, group7\_cd4cells\_rep5, group7\_cd4cells\_rep6, group7\_cd4cells\_rep7, group7\_cd4cells\_rep8, group7\_cd4cells\_rep9, group7\_cd8cells\_rep1, group7\_cd8cells\_rep10, group7\_cd8cells\_rep11, group7\_cd8cells\_rep12, group7\_cd8cells\_rep13, group7\_cd8cells\_rep14, group7\_cd8cells\_rep15, group7\_cd8cells\_rep16, group7\_cd8cells\_rep17, group7\_cd8cells\_rep18, group7\_cd8cells\_rep19, group7\_cd8cells\_rep2, group7\_cd8cells\_rep20, group7\_cd8cells\_rep21, group7\_cd8cells\_rep22, group7\_cd8cells\_rep23, group7\_cd8cells\_rep24, group7\_cd8cells\_rep3, group7\_cd8cells\_rep4, group7\_cd8cells\_rep5, group7\_cd8cells\_rep6, group7\_cd8cells\_rep7, group7\_cd8cells\_rep8, group7\_cd8cells\_rep9, wtchg\_834291\_208\_1, wtchg\_834291\_209\_1, wtchg\_834291\_210\_1, wtchg\_834291\_211\_1, wtchg\_834291\_213\_1, wtchg\_834291\_220\_1, wtchg\_834291\_225\_1, wtchg\_834291\_226\_1, wtchg\_834292\_208\_1, wtchg\_834292\_209\_1, wtchg\_834292\_210\_1, wtchg\_834292\_211\_1, wtchg\_834292\_220\_1, wtchg\_834292\_225\_1, wtchg\_834292\_226\_1, wtchg\_834293\_208\_1, wtchg\_834293\_209\_1, wtchg\_834293\_210\_1, wtchg\_834293\_211\_1, wtchg\_834293\_213\_1, wtchg\_834293\_220\_1, wtchg\_834293\_225\_1, wtchg\_834293\_226\_1, wtchg\_834294\_208\_1, wtchg\_834294\_209\_1, wtchg\_834294\_210\_1, wtchg\_834294\_211\_1, wtchg\_834294\_220\_1, wtchg\_834294\_225\_1, wtchg\_834294\_226\_1, wtchg\_836235\_208\_1, wtchg\_836235\_209\_1, wtchg\_836235\_210\_1, wtchg\_836235\_211\_1, wtchg\_836235\_213\_1, wtchg\_836235\_220\_1, wtchg\_836235\_225\_1, wtchg\_836235\_226\_1, wtchg\_836236\_208\_1, wtchg\_836236\_209\_1, wtchg\_836236\_210\_1, wtchg\_836236\_211\_1, wtchg\_836236\_220\_1, wtchg\_836236\_225\_1, wtchg\_836236\_226\_1, wtchg\_836237\_208\_1, wtchg\_836237\_209\_1, wtchg\_836237\_210\_1, wtchg\_836237\_211\_1, wtchg\_836237\_213\_1, wtchg\_836237\_220\_1, wtchg\_836237\_225\_1, wtchg\_836237\_226\_1, wtchg\_836238\_208\_1, wtchg\_836238\_209\_1, wtchg\_836238\_210\_1, wtchg\_836238\_211\_1, wtchg\_836238\_220\_1, wtchg\_836238\_225\_1, wtchg\_836238\_226\_1, x11\_lolafdez\_rna\_seq\_small\_s11\_l001\_r1\_001, x14\_lolafdez\_rna\_seq\_small\_s14\_l001\_r1\_001, x15\_lolafdez\_rna\_seq\_small\_s15\_l001\_r1\_001, x17\_lolafdez\_rna\_seq\_small\_s17\_l001\_r1\_001, x18\_lolafdez\_rna\_seq\_small\_s18\_l001\_r1\_001, x19\_lolafdez\_rna\_seq\_small\_s19\_l001\_r1\_001, x20\_lolafdez\_rna\_seq\_small\_s20\_l001\_r1\_001, x21\_lolafdez\_rna\_seq\_small\_s21\_l001\_r1\_001, x23\_lolafdez\_rna\_seq\_small\_s23\_l001\_r1\_001, x5\_lolafdez\_rna\_seq\_small\_s5\_l001\_r1\_001, x7\_lolafdez\_rna\_seq\_small\_s7\_l001\_r1\_001, x8\_lolafdez\_rna\_seq\_small\_s8\_l001\_r1\_001, x9\_lolafdez\_rna\_seq\_small\_s9\_l001\_r1\_001 | wtchg\_834291\_212\_1, wtchg\_834291\_227\_1, wtchg\_834291\_234\_1, wtchg\_834291\_237\_1, wtchg\_834291\_238\_1, wtchg\_834292\_212\_1, wtchg\_834292\_213\_1, wtchg\_834292\_227\_1, wtchg\_834292\_234\_1, wtchg\_834292\_237\_1, wtchg\_834292\_238\_1, wtchg\_834293\_212\_1, wtchg\_834293\_227\_1, wtchg\_834293\_234\_1, wtchg\_834293\_237\_1, wtchg\_834293\_238\_1, wtchg\_834294\_212\_1, wtchg\_834294\_213\_1, wtchg\_834294\_227\_1, wtchg\_834294\_234\_1, wtchg\_834294\_237\_1, wtchg\_834294\_238\_1, wtchg\_836235\_212\_1, wtchg\_836235\_227\_1, wtchg\_836235\_234\_1, wtchg\_836235\_237\_1, wtchg\_836235\_238\_1, wtchg\_836236\_212\_1, wtchg\_836236\_213\_1, wtchg\_836236\_227\_1, wtchg\_836236\_234\_1, wtchg\_836236\_237\_1, wtchg\_836236\_238\_1, wtchg\_836237\_212\_1, wtchg\_836237\_227\_1, wtchg\_836237\_234\_1, wtchg\_836237\_237\_1, wtchg\_836237\_238\_1, wtchg\_836238\_212\_1, wtchg\_836238\_213\_1, wtchg\_836238\_227\_1, wtchg\_836238\_234\_1, wtchg\_836238\_237\_1, wtchg\_836238\_238\_1, x6\_lolafdez\_rna\_seq\_small\_s6\_l001\_r1\_001 |
| Per tile sequence quality | 185 | 0 | 0 | 2 | NA | x19\_lolafdez\_rna\_seq\_small\_s19\_l001\_r1\_001, x3\_lolafdez\_rna\_seq\_small\_s3\_l001\_r1\_001 |
| Sequence Duplication Levels | 185 | 185 | 0 | 0 | group7\_cd4cells\_rep1, group7\_cd4cells\_rep10, group7\_cd4cells\_rep11, group7\_cd4cells\_rep12, group7\_cd4cells\_rep13, group7\_cd4cells\_rep14, group7\_cd4cells\_rep15, group7\_cd4cells\_rep16, group7\_cd4cells\_rep17, group7\_cd4cells\_rep18, group7\_cd4cells\_rep19, group7\_cd4cells\_rep2, group7\_cd4cells\_rep20, group7\_cd4cells\_rep21, group7\_cd4cells\_rep22, group7\_cd4cells\_rep23, group7\_cd4cells\_rep24, group7\_cd4cells\_rep3, group7\_cd4cells\_rep4, group7\_cd4cells\_rep5, group7\_cd4cells\_rep6, group7\_cd4cells\_rep7, group7\_cd4cells\_rep8, group7\_cd4cells\_rep9, group7\_cd8cells\_rep1, group7\_cd8cells\_rep10, group7\_cd8cells\_rep11, group7\_cd8cells\_rep12, group7\_cd8cells\_rep13, group7\_cd8cells\_rep14, group7\_cd8cells\_rep15, group7\_cd8cells\_rep16, group7\_cd8cells\_rep17, group7\_cd8cells\_rep18, group7\_cd8cells\_rep19, group7\_cd8cells\_rep2, group7\_cd8cells\_rep20, group7\_cd8cells\_rep21, group7\_cd8cells\_rep22, group7\_cd8cells\_rep23, group7\_cd8cells\_rep24, group7\_cd8cells\_rep3, group7\_cd8cells\_rep4, group7\_cd8cells\_rep5, group7\_cd8cells\_rep6, group7\_cd8cells\_rep7, group7\_cd8cells\_rep8, group7\_cd8cells\_rep9, wtchg\_834291\_208\_1, wtchg\_834291\_209\_1, wtchg\_834291\_210\_1, wtchg\_834291\_211\_1, wtchg\_834291\_212\_1, wtchg\_834291\_213\_1, wtchg\_834291\_220\_1, wtchg\_834291\_225\_1, wtchg\_834291\_226\_1, wtchg\_834291\_227\_1, wtchg\_834291\_228\_1, wtchg\_834291\_233\_1, wtchg\_834291\_234\_1, wtchg\_834291\_237\_1, wtchg\_834291\_238\_1, wtchg\_834292\_208\_1, wtchg\_834292\_209\_1, wtchg\_834292\_210\_1, wtchg\_834292\_211\_1, wtchg\_834292\_212\_1, wtchg\_834292\_213\_1, wtchg\_834292\_220\_1, wtchg\_834292\_225\_1, wtchg\_834292\_226\_1, wtchg\_834292\_227\_1, wtchg\_834292\_228\_1, wtchg\_834292\_233\_1, wtchg\_834292\_234\_1, wtchg\_834292\_237\_1, wtchg\_834292\_238\_1, wtchg\_834293\_208\_1, wtchg\_834293\_209\_1, wtchg\_834293\_210\_1, wtchg\_834293\_211\_1, wtchg\_834293\_212\_1, wtchg\_834293\_213\_1, wtchg\_834293\_220\_1, wtchg\_834293\_225\_1, wtchg\_834293\_226\_1, wtchg\_834293\_227\_1, wtchg\_834293\_228\_1, wtchg\_834293\_233\_1, wtchg\_834293\_234\_1, wtchg\_834293\_237\_1, wtchg\_834293\_238\_1, wtchg\_834294\_208\_1, wtchg\_834294\_209\_1, wtchg\_834294\_210\_1, wtchg\_834294\_211\_1, wtchg\_834294\_212\_1, wtchg\_834294\_213\_1, wtchg\_834294\_220\_1, wtchg\_834294\_225\_1, wtchg\_834294\_226\_1, wtchg\_834294\_227\_1, wtchg\_834294\_228\_1, wtchg\_834294\_233\_1, wtchg\_834294\_234\_1, wtchg\_834294\_237\_1, wtchg\_834294\_238\_1, wtchg\_836235\_208\_1, wtchg\_836235\_209\_1, wtchg\_836235\_210\_1, wtchg\_836235\_211\_1, wtchg\_836235\_212\_1, wtchg\_836235\_213\_1, wtchg\_836235\_220\_1, wtchg\_836235\_225\_1, wtchg\_836235\_226\_1, wtchg\_836235\_227\_1, wtchg\_836235\_228\_1, wtchg\_836235\_233\_1, wtchg\_836235\_234\_1, wtchg\_836235\_237\_1, wtchg\_836235\_238\_1, wtchg\_836236\_208\_1, wtchg\_836236\_209\_1, wtchg\_836236\_210\_1, wtchg\_836236\_211\_1, wtchg\_836236\_212\_1, wtchg\_836236\_213\_1, wtchg\_836236\_220\_1, wtchg\_836236\_225\_1, wtchg\_836236\_226\_1, wtchg\_836236\_227\_1, wtchg\_836236\_228\_1, wtchg\_836236\_233\_1, wtchg\_836236\_234\_1, wtchg\_836236\_237\_1, wtchg\_836236\_238\_1, wtchg\_836237\_208\_1, wtchg\_836237\_209\_1, wtchg\_836237\_210\_1, wtchg\_836237\_211\_1, wtchg\_836237\_212\_1, wtchg\_836237\_213\_1, wtchg\_836237\_220\_1, wtchg\_836237\_225\_1, wtchg\_836237\_226\_1, wtchg\_836237\_227\_1, wtchg\_836237\_228\_1, wtchg\_836237\_233\_1, wtchg\_836237\_234\_1, wtchg\_836237\_237\_1, wtchg\_836237\_238\_1, wtchg\_836238\_208\_1, wtchg\_836238\_209\_1, wtchg\_836238\_210\_1, wtchg\_836238\_211\_1, wtchg\_836238\_212\_1, wtchg\_836238\_213\_1, wtchg\_836238\_220\_1, wtchg\_836238\_225\_1, wtchg\_836238\_226\_1, wtchg\_836238\_227\_1, wtchg\_836238\_228\_1, wtchg\_836238\_233\_1, wtchg\_836238\_234\_1, wtchg\_836238\_237\_1, wtchg\_836238\_238\_1, x1\_lolafdez\_rna\_seq\_small\_s1\_l001\_r1\_001, x11\_lolafdez\_rna\_seq\_small\_s11\_l001\_r1\_001, x14\_lolafdez\_rna\_seq\_small\_s14\_l001\_r1\_001, x15\_lolafdez\_rna\_seq\_small\_s15\_l001\_r1\_001, x17\_lolafdez\_rna\_seq\_small\_s17\_l001\_r1\_001, x18\_lolafdez\_rna\_seq\_small\_s18\_l001\_r1\_001, x19\_lolafdez\_rna\_seq\_small\_s19\_l001\_r1\_001, x2\_lolafdez\_rna\_seq\_small\_s2\_l001\_r1\_001, x20\_lolafdez\_rna\_seq\_small\_s20\_l001\_r1\_001, x21\_lolafdez\_rna\_seq\_small\_s21\_l001\_r1\_001, x23\_lolafdez\_rna\_seq\_small\_s23\_l001\_r1\_001, x3\_lolafdez\_rna\_seq\_small\_s3\_l001\_r1\_001, x5\_lolafdez\_rna\_seq\_small\_s5\_l001\_r1\_001, x6\_lolafdez\_rna\_seq\_small\_s6\_l001\_r1\_001, x7\_lolafdez\_rna\_seq\_small\_s7\_l001\_r1\_001, x8\_lolafdez\_rna\_seq\_small\_s8\_l001\_r1\_001, x9\_lolafdez\_rna\_seq\_small\_s9\_l001\_r1\_001 | NA |

### Per base N content

Reads should contain very low numbers of undefined nucleotides (N
instead of A,C,G,T). Ligation errors can sometimes lead to N’s at the
beginning of sequences and flowcells with very high cluster density or
extremely abundant transcripts can sometimes cause overlay of
neighboring signals. If no plots are displayed, all samples passed
quality criteria.

```
[1] "Base calling accuracy was sufficiently high across all reads"
```

### Per base sequence quality

Reads should have high Phred Scores (>20) over all separate
sequencing cycles especially for short reads (~ 50nt). Quality can drop
when sequencing longer reads (~150 nt) and necessitate additional
trimmings. If no plots are displayed, all samples passed quality
criteria.

```
[1] "All samples had sufficient mean Phred Scores per base"
```

### Per sequence quality scores

Reads should have high mean Phred Scores (>20) over their complete
sequence. Quality can drop when the flowcell gets clustered too densely
or if samples are dominated by overrepresented sequences and
necessitiate additional trimmings. If no plots are displayed, all
samples passed quality criteria.

```
[1] "All samples had sufficient mean Phred Scores per sequence"
```

## Mapping and length distribution

In addition to checking for technical errors, it is important to take
a closer look at the composition of sequencing libraries to identify
potential outliers and systemic or batch effects.

**Current compatibility note:** reads of length 35 nt or
longer that remain unmapped after the tRNA step are intentionally
excluded from the subsequent isomiR/transcriptome branch in this version
of the workflow. They therefore do not appear in transcriptome or
final-unmapped distributions.

### Raw read counts

#### Mean\_mapping\_lengths\_source\_name

#### Mean\_mapping\_lengths\_cell\_type

#### Mean\_mapping\_lengths\_condition

#### Mean\_mapping\_lengths\_compartment

#### Mean\_mapping\_lengths\_experimental\_block

#### Mean\_mapping\_lengths\_sequencing\_platform

### Relative read counts (%)

#### Mean\_mapping\_lengths\_source\_name

#### Mean\_mapping\_lengths\_cell\_type

#### Mean\_mapping\_lengths\_condition

#### Mean\_mapping\_lengths\_compartment

#### Mean\_mapping\_lengths\_experimental\_block

#### Mean\_mapping\_lengths\_sequencing\_platform

## Influence of normalization strategy

Normalization of raw read count data is crucial for accurately
detecting differential gene expression in small RNA NGS experiments. The
need for normalization arises due to inherent biases and technical
variations introduced during sampling, library preparation and
sequencing. These variations can obscure the true biological differences
between samples, potentially leading to erroneous conclusions in
downstream analyses. Systemic biases in your data can include
differences in library size and therefore sequencing depth, sequence
composition and batch effects. One major source of bias in small RNA NGS
is the size selection often performed during library preparation. While
enriching the library significantly in small RNA biotypes such as miRNA,
it can also cause a severe shift in relative abundances due to the
finite number of sequencing clusters that are available for each
sequencing run, especially in the presence of other small RNA classes or
degradational products.

### Size factors

Differences in normalization strategies are most apparent when
comparing size factors, which are calculated as the median ratio of the
sample over geometric mean of all samples for each gene. Individual read
counts are then divided by this size factor to generate normalized
reads.

|  | total\_readcount | miRNA\_readcount | tRNA\_miRNA\_readcount | isomiR\_readcount | tRF\_readcount | tRF\_isomiR\_readcount | custom\_analysis\_readcount |
| --- | --- | --- | --- | --- | --- | --- | --- |
| group7\_cd4cells\_rep1 | 0.87 | 4.74 | 1.95 | 4.34 | 2.15 | 3.29 | 1.83 |
| group7\_cd4cells\_rep10 | 0.86 | 4.66 | 1.89 | 4.55 | 2.26 | 3.37 | 1.62 |
| group7\_cd4cells\_rep11 | 0.42 | 3.77 | 1.46 | 3.09 | 0.58 | 1.19 | 1.28 |
| group7\_cd4cells\_rep12 | 0.61 | 23.58 | 3.74 | 14.24 | 1.70 | 5.46 | 3.49 |
| group7\_cd4cells\_rep13 | 0.85 | 4.64 | 1.92 | 4.35 | 2.07 | 3.47 | 1.89 |
| group7\_cd4cells\_rep14 | 0.43 | 3.39 | 1.36 | 2.93 | 0.63 | 1.32 | 1.10 |
| group7\_cd4cells\_rep15 | 0.62 | 23.27 | 3.93 | 14.47 | 1.62 | 5.55 | 3.55 |
| group7\_cd4cells\_rep16 | 0.91 | 4.62 | 1.97 | 4.34 | 2.13 | 3.38 | 1.59 |
| group7\_cd4cells\_rep17 | 0.41 | 3.52 | 1.37 | 3.12 | 0.65 | 1.17 | 0.95 |
| group7\_cd4cells\_rep18 | 0.57 | 23.12 | 3.83 | 14.11 | 1.49 | 5.43 | 3.76 |
| group7\_cd4cells\_rep19 | 0.85 | 4.46 | 1.99 | 3.91 | 2.21 | 3.35 | 1.81 |
| group7\_cd4cells\_rep2 | 0.42 | 3.67 | 1.64 | 2.92 | 0.62 | 1.25 | 1.14 |
| group7\_cd4cells\_rep20 | 0.44 | 3.46 | 1.50 | 3.05 | 0.55 | 1.41 | 1.02 |
| group7\_cd4cells\_rep21 | 0.59 | 22.61 | 3.85 | 13.71 | 1.45 | 5.14 | 3.53 |
| group7\_cd4cells\_rep22 | 0.90 | 4.62 | 1.99 | 4.33 | 2.14 | 3.21 | 1.72 |
| group7\_cd4cells\_rep23 | 0.42 | 3.60 | 1.54 | 3.11 | 0.68 | 1.33 | 1.06 |
| group7\_cd4cells\_rep24 | 0.59 | 23.20 | 3.85 | 14.48 | 1.66 | 5.93 | 3.46 |
| group7\_cd4cells\_rep3 | 0.59 | 23.21 | 3.73 | 14.24 | 1.57 | 6.01 | 3.63 |
| group7\_cd4cells\_rep4 | 0.86 | 4.64 | 2.13 | 4.32 | 2.36 | 3.12 | 1.78 |
| group7\_cd4cells\_rep5 | 0.45 | 3.51 | 1.39 | 3.05 | 0.51 | 1.23 | 1.07 |
| group7\_cd4cells\_rep6 | 0.58 | 22.97 | 3.79 | 14.47 | 1.50 | 5.58 | 3.35 |
| group7\_cd4cells\_rep7 | 0.90 | 4.70 | 1.85 | 4.51 | 2.30 | 3.45 | 1.68 |
| group7\_cd4cells\_rep8 | 0.43 | 3.73 | 1.46 | 3.13 | 0.52 | 1.15 | 1.14 |
| group7\_cd4cells\_rep9 | 0.60 | 23.03 | 3.74 | 14.03 | 1.48 | 6.01 | 3.60 |
| group7\_cd8cells\_rep1 | 0.90 | 4.16 | 1.60 | 3.27 | 0.99 | 1.87 | 1.52 |
| group7\_cd8cells\_rep10 | 0.88 | 4.08 | 1.68 | 3.52 | 0.88 | 1.77 | 1.62 |
| group7\_cd8cells\_rep11 | 0.45 | 2.00 | 1.07 | 1.51 | 0.52 | 0.76 | 0.99 |
| group7\_cd8cells\_rep12 | 0.91 | 12.84 | 2.75 | 9.72 | 2.18 | 3.88 | 2.51 |
| group7\_cd8cells\_rep13 | 0.87 | 4.08 | 1.57 | 3.20 | 0.99 | 1.77 | 1.56 |
| group7\_cd8cells\_rep14 | 0.41 | 2.03 | 1.04 | 1.58 | 0.52 | 0.77 | 0.99 |
| group7\_cd8cells\_rep15 | 0.90 | 13.02 | 2.83 | 9.43 | 2.01 | 4.12 | 2.57 |
| group7\_cd8cells\_rep16 | 0.86 | 3.96 | 1.42 | 3.29 | 0.93 | 1.67 | 1.38 |
| group7\_cd8cells\_rep17 | 0.43 | 1.95 | 1.08 | 1.44 | 0.51 | 0.79 | 0.96 |
| group7\_cd8cells\_rep18 | 0.89 | 12.75 | 2.64 | 9.76 | 2.32 | 4.15 | 2.57 |
| group7\_cd8cells\_rep19 | 0.91 | 4.09 | 1.84 | 3.31 | 0.86 | 1.78 | 1.73 |
| group7\_cd8cells\_rep2 | 0.44 | 2.02 | 1.11 | 1.53 | 0.53 | 0.80 | 0.96 |
| group7\_cd8cells\_rep20 | 0.42 | 1.86 | 0.98 | 1.45 | 0.55 | 0.77 | 0.93 |
| group7\_cd8cells\_rep21 | 0.91 | 12.35 | 2.74 | 9.54 | 2.21 | 3.98 | 2.59 |
| group7\_cd8cells\_rep22 | 0.87 | 3.83 | 1.72 | 3.17 | 0.92 | 1.83 | 1.71 |
| group7\_cd8cells\_rep23 | 0.45 | 2.04 | 1.06 | 1.58 | 0.50 | 0.80 | 0.93 |
| group7\_cd8cells\_rep24 | 0.94 | 12.71 | 2.68 | 9.51 | 2.12 | 4.12 | 2.53 |
| group7\_cd8cells\_rep3 | 0.91 | 12.66 | 2.58 | 9.60 | 2.23 | 4.05 | 2.44 |
| group7\_cd8cells\_rep4 | 0.88 | 4.06 | 1.65 | 3.20 | 0.91 | 1.73 | 1.62 |
| group7\_cd8cells\_rep5 | 0.47 | 2.00 | 1.11 | 1.32 | 0.50 | 0.73 | 0.93 |
| group7\_cd8cells\_rep6 | 0.94 | 12.96 | 2.65 | 9.66 | 2.07 | 3.82 | 2.55 |
| group7\_cd8cells\_rep7 | 0.85 | 4.13 | 1.68 | 3.33 | 0.89 | 1.89 | 1.62 |
| group7\_cd8cells\_rep8 | 0.41 | 2.01 | 1.08 | 1.47 | 0.45 | 0.79 | 0.98 |
| group7\_cd8cells\_rep9 | 0.93 | 12.73 | 2.86 | 9.95 | 2.11 | 4.09 | 2.61 |
| wtchg\_834291\_208\_1 | 1.81 | 1.93 | 2.15 | 1.79 | 5.53 | 2.00 | 2.37 |
| wtchg\_834291\_209\_1 | 1.77 | 2.01 | 2.11 | 1.65 | 5.10 | 2.19 | 2.26 |
| wtchg\_834291\_210\_1 | 1.60 | 1.87 | 1.96 | 1.41 | 5.74 | 1.81 | 2.07 |
| wtchg\_834291\_211\_1 | 2.62 | 1.21 | 3.63 | 1.31 | 7.71 | 2.05 | 4.66 |
| wtchg\_834291\_212\_1 | 0.80 | 0.12 | 0.33 | 0.15 | 0.86 | 0.42 | 0.37 |
| wtchg\_834291\_213\_1 | 1.76 | 0.38 | 0.70 | 0.42 | 1.26 | 0.69 | 0.80 |
| wtchg\_834291\_220\_1 | 0.91 | 0.40 | 0.55 | 0.56 | 0.39 | 0.51 | 0.56 |
| wtchg\_834291\_225\_1 | 0.73 | 0.46 | 0.60 | 0.57 | 0.46 | 0.47 | 0.60 |
| wtchg\_834291\_226\_1 | 0.65 | 0.20 | 0.31 | 0.33 | 0.25 | 0.27 | 0.34 |
| wtchg\_834291\_227\_1 | 0.73 | 0.44 | 0.42 | 0.46 | 0.17 | 0.40 | 0.45 |
| wtchg\_834291\_228\_1 | 0.54 | 0.17 | 0.29 | 0.23 | 0.20 | 0.23 | 0.26 |
| wtchg\_834291\_233\_1 | 0.39 | 0.07 | 0.10 | 0.09 | 0.07 | 0.08 | 0.10 |
| wtchg\_834291\_234\_1 | 0.62 | 0.09 | 0.12 | 0.11 | 0.14 | 0.11 | 0.11 |
| wtchg\_834291\_237\_1 | 1.45 | 0.55 | 0.74 | 0.70 | 1.41 | 0.86 | 0.74 |
| wtchg\_834291\_238\_1 | 0.67 | 0.15 | 0.23 | 0.13 | 0.30 | 0.21 | 0.22 |
| wtchg\_834292\_208\_1 | 1.78 | 1.91 | 2.28 | 1.80 | 5.67 | 2.04 | 2.58 |
| wtchg\_834292\_209\_1 | 1.74 | 1.97 | 2.15 | 1.75 | 5.34 | 2.09 | 2.39 |
| wtchg\_834292\_210\_1 | 1.62 | 1.71 | 2.08 | 1.46 | 5.75 | 1.82 | 2.20 |
| wtchg\_834292\_211\_1 | 2.82 | 1.31 | 3.41 | 1.42 | 7.56 | 2.16 | 4.43 |
| wtchg\_834292\_212\_1 | 0.83 | 0.11 | 0.33 | 0.12 | 0.90 | 0.40 | 0.37 |
| wtchg\_834292\_213\_1 | 1.78 | 0.39 | 0.69 | 0.54 | 1.31 | 0.78 | 0.78 |
| wtchg\_834292\_220\_1 | 0.94 | 0.42 | 0.50 | 0.56 | 0.51 | 0.52 | 0.50 |
| wtchg\_834292\_225\_1 | 0.71 | 0.46 | 0.60 | 0.59 | 0.44 | 0.48 | 0.60 |
| wtchg\_834292\_226\_1 | 0.63 | 0.19 | 0.30 | 0.28 | 0.19 | 0.23 | 0.35 |
| wtchg\_834292\_227\_1 | 0.73 | 0.41 | 0.39 | 0.47 | 0.17 | 0.40 | 0.40 |
| wtchg\_834292\_228\_1 | 0.57 | 0.21 | 0.30 | 0.20 | 0.19 | 0.20 | 0.26 |
| wtchg\_834292\_233\_1 | 0.41 | 0.08 | 0.10 | 0.10 | 0.08 | 0.10 | 0.10 |
| wtchg\_834292\_234\_1 | 0.64 | 0.09 | 0.12 | 0.11 | 0.11 | 0.11 | 0.11 |
| wtchg\_834292\_237\_1 | 1.41 | 0.51 | 0.75 | 0.66 | 1.30 | 0.89 | 0.75 |
| wtchg\_834292\_238\_1 | 0.61 | 0.16 | 0.24 | 0.17 | 0.35 | 0.24 | 0.23 |
| wtchg\_834293\_208\_1 | 1.79 | 1.99 | 2.28 | 1.90 | 5.79 | 2.08 | 2.50 |
| wtchg\_834293\_209\_1 | 1.73 | 2.08 | 2.21 | 1.77 | 5.47 | 2.05 | 2.26 |
| wtchg\_834293\_210\_1 | 1.61 | 1.73 | 2.12 | 1.36 | 6.06 | 1.90 | 2.15 |
| wtchg\_834293\_211\_1 | 2.65 | 1.26 | 3.72 | 1.40 | 8.10 | 2.16 | 5.07 |
| wtchg\_834293\_212\_1 | 0.81 | 0.11 | 0.39 | 0.13 | 0.95 | 0.41 | 0.41 |
| wtchg\_834293\_213\_1 | 1.85 | 0.39 | 0.70 | 0.54 | 1.10 | 0.70 | 0.74 |
| wtchg\_834293\_220\_1 | 0.96 | 0.41 | 0.53 | 0.47 | 0.48 | 0.48 | 0.54 |
| wtchg\_834293\_225\_1 | 0.74 | 0.45 | 0.61 | 0.60 | 0.39 | 0.48 | 0.61 |
| wtchg\_834293\_226\_1 | 0.62 | 0.17 | 0.29 | 0.27 | 0.23 | 0.25 | 0.32 |
| wtchg\_834293\_227\_1 | 0.75 | 0.41 | 0.42 | 0.50 | 0.22 | 0.40 | 0.42 |
| wtchg\_834293\_228\_1 | 0.56 | 0.19 | 0.28 | 0.21 | 0.22 | 0.21 | 0.25 |
| wtchg\_834293\_233\_1 | 0.41 | 0.07 | 0.09 | 0.10 | 0.11 | 0.10 | 0.09 |
| wtchg\_834293\_234\_1 | 0.65 | 0.08 | 0.14 | 0.10 | 0.14 | 0.10 | 0.13 |
| wtchg\_834293\_237\_1 | 1.47 | 0.55 | 0.84 | 0.65 | 1.44 | 0.76 | 0.80 |
| wtchg\_834293\_238\_1 | 0.70 | 0.16 | 0.25 | 0.17 | 0.32 | 0.21 | 0.24 |
| wtchg\_834294\_208\_1 | 1.83 | 2.06 | 2.23 | 1.93 | 5.80 | 2.15 | 2.40 |
| wtchg\_834294\_209\_1 | 1.77 | 2.08 | 2.23 | 1.76 | 5.37 | 2.22 | 2.28 |
| wtchg\_834294\_210\_1 | 1.61 | 1.80 | 2.06 | 1.45 | 6.06 | 2.01 | 2.11 |
| wtchg\_834294\_211\_1 | 2.69 | 1.36 | 3.83 | 1.51 | 8.03 | 2.20 | 5.16 |
| wtchg\_834294\_212\_1 | 0.90 | 0.13 | 0.35 | 0.10 | 0.94 | 0.43 | 0.40 |
| wtchg\_834294\_213\_1 | 1.84 | 0.37 | 0.66 | 0.48 | 1.23 | 0.75 | 0.77 |
| wtchg\_834294\_220\_1 | 0.93 | 0.41 | 0.56 | 0.58 | 0.50 | 0.52 | 0.56 |
| wtchg\_834294\_225\_1 | 0.76 | 0.48 | 0.53 | 0.62 | 0.48 | 0.54 | 0.53 |
| wtchg\_834294\_226\_1 | 0.61 | 0.21 | 0.40 | 0.30 | 0.25 | 0.27 | 0.40 |
| wtchg\_834294\_227\_1 | 0.77 | 0.47 | 0.43 | 0.49 | 0.21 | 0.43 | 0.45 |
| wtchg\_834294\_228\_1 | 0.57 | 0.20 | 0.28 | 0.21 | 0.17 | 0.21 | 0.27 |
| wtchg\_834294\_233\_1 | 0.39 | 0.08 | 0.09 | 0.10 | 0.09 | 0.09 | 0.09 |
| wtchg\_834294\_234\_1 | 0.62 | 0.09 | 0.13 | 0.12 | 0.15 | 0.13 | 0.12 |
| wtchg\_834294\_237\_1 | 1.58 | 0.57 | 0.74 | 0.68 | 1.45 | 0.83 | 0.74 |
| wtchg\_834294\_238\_1 | 0.66 | 0.16 | 0.25 | 0.17 | 0.34 | 0.25 | 0.24 |
| wtchg\_836235\_208\_1 | 1.79 | 1.91 | 2.13 | 1.83 | 5.56 | 2.00 | 2.24 |
| wtchg\_836235\_209\_1 | 1.71 | 2.01 | 2.31 | 1.72 | 5.32 | 2.08 | 2.42 |
| wtchg\_836235\_210\_1 | 1.64 | 1.75 | 2.07 | 1.46 | 5.66 | 1.92 | 2.17 |
| wtchg\_836235\_211\_1 | 2.67 | 1.28 | 3.89 | 1.42 | 7.94 | 2.07 | 4.09 |
| wtchg\_836235\_212\_1 | 0.93 | 0.11 | 0.36 | 0.14 | 1.08 | 0.37 | 0.40 |
| wtchg\_836235\_213\_1 | 1.78 | 0.35 | 0.65 | 0.47 | 1.16 | 0.82 | 0.73 |
| wtchg\_836235\_220\_1 | 0.91 | 0.42 | 0.53 | 0.52 | 0.44 | 0.52 | 0.54 |
| wtchg\_836235\_225\_1 | 0.78 | 0.43 | 0.60 | 0.61 | 0.46 | 0.50 | 0.60 |
| wtchg\_836235\_226\_1 | 0.61 | 0.21 | 0.36 | 0.29 | 0.22 | 0.25 | 0.36 |
| wtchg\_836235\_227\_1 | 0.73 | 0.43 | 0.45 | 0.49 | 0.21 | 0.40 | 0.46 |
| wtchg\_836235\_228\_1 | 0.56 | 0.17 | 0.27 | 0.20 | 0.20 | 0.20 | 0.23 |
| wtchg\_836235\_233\_1 | 0.38 | 0.08 | 0.10 | 0.10 | 0.07 | 0.10 | 0.09 |
| wtchg\_836235\_234\_1 | 0.60 | 0.08 | 0.11 | 0.10 | 0.10 | 0.10 | 0.11 |
| wtchg\_836235\_237\_1 | 1.50 | 0.55 | 0.76 | 0.59 | 1.36 | 0.78 | 0.74 |
| wtchg\_836235\_238\_1 | 0.66 | 0.16 | 0.23 | 0.16 | 0.33 | 0.23 | 0.23 |
| wtchg\_836236\_208\_1 | 1.79 | 2.03 | 2.24 | 1.93 | 5.77 | 2.07 | 2.38 |
| wtchg\_836236\_209\_1 | 1.66 | 2.04 | 2.19 | 1.65 | 5.33 | 2.04 | 2.33 |
| wtchg\_836236\_210\_1 | 1.58 | 1.69 | 1.83 | 1.39 | 5.76 | 1.77 | 2.17 |
| wtchg\_836236\_211\_1 | 2.59 | 1.31 | 3.66 | 1.40 | 7.90 | 2.30 | 4.84 |
| wtchg\_836236\_212\_1 | 0.92 | 0.13 | 0.37 | 0.16 | 0.99 | 0.41 | 0.38 |
| wtchg\_836236\_213\_1 | 1.71 | 0.36 | 0.69 | 0.47 | 1.22 | 0.69 | 0.74 |
| wtchg\_836236\_220\_1 | 0.93 | 0.44 | 0.52 | 0.56 | 0.47 | 0.53 | 0.52 |
| wtchg\_836236\_225\_1 | 0.74 | 0.47 | 0.55 | 0.58 | 0.47 | 0.52 | 0.55 |
| wtchg\_836236\_226\_1 | 0.61 | 0.21 | 0.31 | 0.31 | 0.21 | 0.24 | 0.31 |
| wtchg\_836236\_227\_1 | 0.71 | 0.40 | 0.40 | 0.49 | 0.22 | 0.36 | 0.40 |
| wtchg\_836236\_228\_1 | 0.55 | 0.18 | 0.28 | 0.21 | 0.21 | 0.21 | 0.25 |
| wtchg\_836236\_233\_1 | 0.37 | 0.09 | 0.09 | 0.11 | 0.08 | 0.10 | 0.09 |
| wtchg\_836236\_234\_1 | 0.61 | 0.09 | 0.11 | 0.09 | 0.15 | 0.10 | 0.10 |
| wtchg\_836236\_237\_1 | 1.44 | 0.53 | 0.70 | 0.55 | 1.35 | 0.82 | 0.70 |
| wtchg\_836236\_238\_1 | 0.64 | 0.17 | 0.26 | 0.16 | 0.39 | 0.24 | 0.24 |
| wtchg\_836237\_208\_1 | 1.74 | 1.91 | 2.25 | 1.78 | 5.76 | 2.10 | 2.60 |
| wtchg\_836237\_209\_1 | 1.71 | 2.25 | 2.27 | 1.67 | 5.37 | 2.28 | 2.46 |
| wtchg\_836237\_210\_1 | 1.58 | 1.85 | 2.11 | 1.43 | 5.80 | 1.90 | 2.27 |
| wtchg\_836237\_211\_1 | 2.56 | 1.31 | 3.49 | 1.45 | 7.43 | 2.05 | 5.09 |
| wtchg\_836237\_212\_1 | 0.82 | 0.10 | 0.38 | 0.11 | 0.92 | 0.38 | 0.43 |
| wtchg\_836237\_213\_1 | 1.71 | 0.36 | 0.62 | 0.52 | 1.22 | 0.71 | 0.69 |
| wtchg\_836237\_220\_1 | 0.89 | 0.40 | 0.51 | 0.51 | 0.47 | 0.50 | 0.51 |
| wtchg\_836237\_225\_1 | 0.73 | 0.47 | 0.50 | 0.59 | 0.41 | 0.48 | 0.50 |
| wtchg\_836237\_226\_1 | 0.60 | 0.20 | 0.28 | 0.30 | 0.21 | 0.26 | 0.30 |
| wtchg\_836237\_227\_1 | 0.76 | 0.38 | 0.41 | 0.49 | 0.20 | 0.36 | 0.41 |
| wtchg\_836237\_228\_1 | 0.58 | 0.18 | 0.25 | 0.21 | 0.17 | 0.21 | 0.25 |
| wtchg\_836237\_233\_1 | 0.37 | 0.07 | 0.09 | 0.09 | 0.08 | 0.09 | 0.09 |
| wtchg\_836237\_234\_1 | 0.64 | 0.09 | 0.11 | 0.09 | 0.11 | 0.09 | 0.10 |
| wtchg\_836237\_237\_1 | 1.48 | 0.56 | 0.73 | 0.66 | 1.38 | 0.90 | 0.73 |
| wtchg\_836237\_238\_1 | 0.66 | 0.17 | 0.25 | 0.15 | 0.37 | 0.27 | 0.24 |
| wtchg\_836238\_208\_1 | 1.83 | 1.96 | 2.23 | 1.81 | 5.72 | 2.07 | 2.29 |
| wtchg\_836238\_209\_1 | 1.67 | 2.08 | 2.16 | 1.80 | 5.38 | 2.12 | 2.29 |
| wtchg\_836238\_210\_1 | 1.56 | 1.79 | 1.91 | 1.41 | 5.83 | 1.98 | 2.30 |
| wtchg\_836238\_211\_1 | 2.74 | 1.31 | 4.29 | 1.39 | 7.47 | 2.15 | 4.55 |
| wtchg\_836238\_212\_1 | 0.88 | 0.13 | 0.35 | 0.15 | 1.14 | 0.36 | 0.40 |
| wtchg\_836238\_213\_1 | 1.80 | 0.37 | 0.65 | 0.46 | 1.21 | 0.73 | 0.73 |
| wtchg\_836238\_220\_1 | 0.97 | 0.43 | 0.58 | 0.59 | 0.53 | 0.54 | 0.57 |
| wtchg\_836238\_225\_1 | 0.71 | 0.45 | 0.58 | 0.67 | 0.45 | 0.51 | 0.58 |
| wtchg\_836238\_226\_1 | 0.59 | 0.20 | 0.33 | 0.30 | 0.24 | 0.24 | 0.34 |
| wtchg\_836238\_227\_1 | 0.73 | 0.39 | 0.38 | 0.50 | 0.18 | 0.38 | 0.39 |
| wtchg\_836238\_228\_1 | 0.59 | 0.19 | 0.28 | 0.21 | 0.18 | 0.21 | 0.23 |
| wtchg\_836238\_233\_1 | 0.39 | 0.07 | 0.10 | 0.09 | 0.09 | 0.09 | 0.09 |
| wtchg\_836238\_234\_1 | 0.59 | 0.09 | 0.11 | 0.09 | 0.13 | 0.10 | 0.11 |
| wtchg\_836238\_237\_1 | 1.44 | 0.57 | 0.75 | 0.68 | 1.39 | 0.84 | 0.75 |
| wtchg\_836238\_238\_1 | 0.68 | 0.17 | 0.24 | 0.18 | 0.30 | 0.26 | 0.23 |
| x11\_lolafdez\_rna\_seq\_small\_s11\_l001\_r1\_001 | 7.67 | 10.65 | 11.82 | 10.27 | 77.21 | 17.31 | 12.70 |
| x14\_lolafdez\_rna\_seq\_small\_s14\_l001\_r1\_001 | 2.10 | 10.10 | 4.66 | 12.92 | 3.28 | 6.78 | 3.96 |
| x15\_lolafdez\_rna\_seq\_small\_s15\_l001\_r1\_001 | 1.38 | 6.30 | 3.06 | 7.76 | 2.93 | 5.62 | 2.77 |
| x17\_lolafdez\_rna\_seq\_small\_s17\_l001\_r1\_001 | 2.13 | 22.98 | 10.33 | 30.59 | 4.21 | 10.38 | 10.28 |
| x18\_lolafdez\_rna\_seq\_small\_s18\_l001\_r1\_001 | 2.76 | 18.15 | 8.98 | 26.60 | 3.27 | 11.01 | 7.91 |
| x19\_lolafdez\_rna\_seq\_small\_s19\_l001\_r1\_001 | 2.80 | 9.60 | 3.93 | 12.02 | 3.52 | 7.53 | 3.67 |
| x1\_lolafdez\_rna\_seq\_small\_s1\_l001\_r1\_001 | 0.17 | 0.13 | 0.25 | 0.18 | 0.67 | 0.27 | 0.25 |
| x20\_lolafdez\_rna\_seq\_small\_s20\_l001\_r1\_001 | 3.38 | 20.95 | 16.10 | 23.39 | 22.45 | 23.26 | 15.20 |
| x21\_lolafdez\_rna\_seq\_small\_s21\_l001\_r1\_001 | 1.23 | 22.63 | 8.94 | 19.36 | 1.68 | 8.06 | 7.53 |
| x23\_lolafdez\_rna\_seq\_small\_s23\_l001\_r1\_001 | 2.39 | 30.28 | 11.18 | 35.02 | 2.61 | 11.19 | 9.75 |
| x2\_lolafdez\_rna\_seq\_small\_s2\_l001\_r1\_001 | 0.09 | 0.04 | 0.10 | 0.04 | 0.08 | 0.06 | 0.10 |
| x3\_lolafdez\_rna\_seq\_small\_s3\_l001\_r1\_001 | 1.35 | 0.80 | 1.37 | 1.37 | 2.06 | 1.59 | 1.27 |
| x5\_lolafdez\_rna\_seq\_small\_s5\_l001\_r1\_001 | 1.65 | 1.58 | 1.62 | 2.03 | 1.80 | 2.00 | 1.66 |
| x6\_lolafdez\_rna\_seq\_small\_s6\_l001\_r1\_001 | 2.72 | 2.36 | 2.33 | 3.53 | 2.09 | 2.65 | 2.33 |
| x7\_lolafdez\_rna\_seq\_small\_s7\_l001\_r1\_001 | 15.72 | 17.04 | 18.99 | 20.90 | 110.54 | 32.04 | 19.35 |
| x8\_lolafdez\_rna\_seq\_small\_s8\_l001\_r1\_001 | 1.84 | 2.65 | 2.86 | 2.43 | 15.60 | 5.16 | 3.95 |
| x9\_lolafdez\_rna\_seq\_small\_s9\_l001\_r1\_001 | 3.14 | 5.94 | 6.41 | 5.83 | 29.68 | 10.28 | 6.82 |

### Hierarchical Clustering - Heatmap

In agglomerative, hierarchical clustering, every sample starts in its
own cluster and a distance matrix is calculated between every cluster in
the multi-dimensional space created by the normalized, transformed read
count data. Similarity between samples is determined by the distance
between them and depends on the chosen distance metric (Euclidian) and
linkage criteria (Ward). The two most similar clusters are combined into
a new cluster and the procedure is repeated iteratively until only a
single cluster remains. In a related way, similarity of samples is
visualized by color coded Z-scores of the gene expression.

#### total\_readcount

#### miRNA\_readcount

#### tRNA\_miRNA\_readcount

#### isomiR\_readcount

#### tRF\_readcount

#### tRF\_isomiR\_readcount

#### custom\_analysis\_readcount

## Influence of batch effects

Batch effect detection is crucial in small RNA NGS sequencing
analysis because technical variation introduced during sample
preparation, library construction, or sequencing can obscure true
biological signals and lead to misleading results. Even when biological
replicates are consistent, differences between sequencing runs, reagent
lots, RNA extraction methods, or sample handling can introduce
systematic biases that cluster samples by technical factors rather than
biology. Common batch effects in small RNA datasets include differences
in adapter ligation efficiency, RNA degradation during extraction,
variations in sequencing depth or read quality across lanes, and
instrument-specific artifacts. Detecting and correcting for these
effects ensures that observed variation reflects genuine biological
differences, such as differential expression of miRNAs or other small
RNAs, rather than confounding technical noise. Batch effects can often
be identified by unwanted groupings of technical factors of the study
design in unsupervised clustering such as HCA (see above) or PCA (see
below).

### Principal component analysis (PCA)

PCA is an orthogonal linear transformation of your original read
count data to a new coordinate system defined by a decreasing explained
variance of each coordinates. Therefore the first coordinate, also
called first component, is a scalar projection of the read count data
that describes the greatest variance within the data set and subsequent
components are orthogonal to all preceding components. By restricting
plots to the first 2 or 3 components, predominant sources of variances
can be visualized. In comparison to hierarchical clustering that takes
the complete data set into account, this enables suppression of
background noise which will be restricted to later components.

#### Total readcount

##### source\_name

##### cell\_type

##### condition

##### compartment

##### experimental\_block

##### sequencing\_platform

##### sequencing\_cycles

#### miRNA readcount

##### source\_name

##### cell\_type

##### condition

##### compartment

##### experimental\_block

##### sequencing\_platform

##### sequencing\_cycles

#### tRNA + miRNA readcount

##### source\_name

##### cell\_type

##### condition

##### compartment

##### experimental\_block

##### sequencing\_platform

##### sequencing\_cycles

#### isomiR readcount

##### source\_name

##### cell\_type

##### condition

##### compartment

##### experimental\_block

##### sequencing\_platform

##### sequencing\_cycles

#### tRF readcount

##### source\_name

##### cell\_type

##### condition

##### compartment

##### experimental\_block

##### sequencing\_platform

##### sequencing\_cycles

#### tRF + isomiR readcount

##### source\_name

##### cell\_type

##### condition

##### compartment

##### experimental\_block

##### sequencing\_platform

##### sequencing\_cycles

#### Custom analysis readcount

##### source\_name

##### cell\_type

##### condition

##### compartment

##### experimental\_block

##### sequencing\_platform

##### sequencing\_cycles

#### Explained variance original data

##### Total readcount

| Number\_of\_Genes | PC1 | PC2 | PC3 | PC4 | PC5 | PC6 | PC7 | PC8 | PC9 | PC10 |
| --- | --- | --- | --- | --- | --- | --- | --- | --- | --- | --- |
| 1594 | 22.9 | 8.7 | 4.9 | 3.6 | 2.6 | 1.8 | 1.7 | 1.6 | 1.5 | 1.4 |
| 1000 | 34.7 | 13.6 | 7.0 | 5.2 | 3.3 | 2.0 | 1.8 | 1.5 | 1.1 | 1.0 |
| 500 | 45.5 | 21.5 | 7.6 | 5.7 | 3.6 | 1.9 | 1.7 | 1.2 | 1.0 | 0.7 |
| 100 | 63.8 | 21.8 | 3.6 | 2.5 | 1.8 | 1.1 | 0.9 | 0.5 | 0.4 | 0.3 |
| 50 | 64.3 | 26.0 | 2.7 | 2.1 | 1.4 | 0.6 | 0.6 | 0.3 | 0.2 | 0.2 |
| 10 | 97.6 | 1.6 | 0.1 | 0.1 | 0.1 | 0.1 | 0.1 | 0.1 | 0.1 | 0.1 |

##### miRNA readcount

| Number\_of\_Genes | PC1 | PC2 | PC3 | PC4 | PC5 | PC6 | PC7 | PC8 | PC9 | PC10 |
| --- | --- | --- | --- | --- | --- | --- | --- | --- | --- | --- |
| 1124 | 7.0 | 2.8 | 2.6 | 2.2 | 2.1 | 1.5 | 1.4 | 1.4 | 1.3 | 1.3 |
| 1000 | 7.5 | 3.0 | 2.8 | 2.3 | 2.2 | 1.4 | 1.3 | 1.2 | 1.1 | 1.1 |
| 500 | 11.2 | 5.6 | 4.1 | 3.6 | 2.9 | 2.1 | 1.8 | 1.7 | 1.6 | 1.5 |
| 100 | 23.6 | 13.3 | 9.8 | 6.1 | 4.7 | 4.0 | 3.4 | 2.7 | 2.1 | 1.9 |
| 50 | 32.7 | 16.4 | 12.2 | 5.9 | 5.0 | 4.4 | 2.7 | 2.0 | 1.8 | 1.6 |
| 10 | 62.0 | 15.6 | 7.7 | 6.8 | 3.9 | 1.6 | 1.0 | 0.6 | 0.6 | 0.3 |

##### tRNA + miRNA readcount

| Number\_of\_Genes | PC1 | PC2 | PC3 | PC4 | PC5 | PC6 | PC7 | PC8 | PC9 | PC10 |
| --- | --- | --- | --- | --- | --- | --- | --- | --- | --- | --- |
| 1173 | 15.2 | 2.8 | 2.6 | 2.2 | 1.8 | 1.8 | 1.7 | 1.5 | 1.4 | 1.4 |
| 1000 | 17.5 | 3.1 | 2.6 | 2.3 | 2.1 | 1.6 | 1.3 | 1.2 | 1.2 | 1.1 |
| 500 | 29.6 | 5.3 | 3.9 | 3.5 | 2.7 | 2.2 | 1.7 | 1.5 | 1.3 | 1.2 |
| 100 | 56.0 | 11.9 | 5.6 | 3.7 | 2.5 | 2.3 | 1.9 | 1.4 | 1.2 | 1.1 |
| 50 | 57.7 | 15.0 | 5.7 | 4.4 | 2.5 | 2.2 | 1.9 | 1.3 | 1.1 | 1.0 |
| 10 | 56.2 | 18.3 | 9.9 | 4.4 | 4.0 | 3.3 | 1.6 | 1.0 | 0.7 | 0.5 |

##### isomiR readcount

| Number\_of\_Genes | PC1 | PC2 | PC3 | PC4 | PC5 | PC6 | PC7 | PC8 | PC9 | PC10 |
| --- | --- | --- | --- | --- | --- | --- | --- | --- | --- | --- |
| 574 | 33.2 | 10.8 | 7.0 | 5.1 | 3.8 | 2.9 | 2.7 | 1.8 | 1.5 | 1.3 |
| 500 | 34.7 | 10.8 | 7.2 | 4.8 | 3.9 | 3.0 | 2.3 | 1.7 | 1.6 | 1.3 |
| 100 | 80.4 | 9.6 | 4.2 | 1.4 | 0.8 | 0.6 | 0.5 | 0.3 | 0.2 | 0.1 |
| 50 | 85.3 | 7.7 | 2.8 | 1.1 | 0.6 | 0.4 | 0.3 | 0.3 | 0.1 | 0.1 |
| 10 | 95.3 | 2.2 | 1.1 | 0.4 | 0.3 | 0.2 | 0.2 | 0.1 | 0.1 | 0.1 |

##### tRF readcount

| Number\_of\_Genes | PC1 | PC2 | PC3 | PC4 | PC5 | PC6 | PC7 | PC8 | PC9 | PC10 |
| --- | --- | --- | --- | --- | --- | --- | --- | --- | --- | --- |
| 132 | 31.7 | 12.6 | 11.5 | 6.5 | 3.7 | 3.1 | 2.8 | 1.9 | 1.5 | 1.4 |
| 100 | 34.1 | 13.9 | 11.5 | 6.2 | 3.8 | 2.9 | 2.5 | 1.6 | 1.3 | 1.3 |
| 50 | 41.8 | 18.9 | 7.1 | 5.1 | 4.4 | 2.8 | 1.8 | 1.6 | 1.3 | 1.2 |
| 10 | 61.8 | 12.0 | 11.8 | 4.1 | 3.2 | 1.8 | 1.6 | 1.4 | 1.3 | 1.0 |

##### tRF + isomiR readcount

| Number\_of\_Genes | PC1 | PC2 | PC3 | PC4 | PC5 | PC6 | PC7 | PC8 | PC9 | PC10 |
| --- | --- | --- | --- | --- | --- | --- | --- | --- | --- | --- |
| 706 | 38.6 | 15.7 | 6.4 | 4.8 | 3.3 | 2.5 | 1.9 | 1.8 | 1.2 | 1.2 |
| 500 | 40.8 | 19.0 | 6.5 | 4.4 | 3.5 | 2.1 | 1.7 | 1.3 | 1.2 | 1.0 |
| 100 | 79.3 | 8.5 | 4.3 | 2.6 | 1.0 | 0.7 | 0.6 | 0.5 | 0.2 | 0.2 |
| 50 | 85.4 | 6.0 | 2.8 | 1.9 | 1.0 | 0.7 | 0.4 | 0.3 | 0.1 | 0.1 |
| 10 | 96.5 | 1.5 | 0.9 | 0.3 | 0.2 | 0.2 | 0.1 | 0.1 | 0.1 | 0.1 |

##### Custom analysis readcount

| Number\_of\_Genes | PC1 | PC2 | PC3 | PC4 | PC5 | PC6 | PC7 | PC8 | PC9 | PC10 |
| --- | --- | --- | --- | --- | --- | --- | --- | --- | --- | --- |
| 1177 | 15.5 | 2.8 | 2.6 | 2.3 | 1.8 | 1.8 | 1.7 | 1.5 | 1.4 | 1.4 |
| 1000 | 17.9 | 3.1 | 2.7 | 2.4 | 2.0 | 1.6 | 1.4 | 1.3 | 1.2 | 1.2 |
| 500 | 30.6 | 5.6 | 3.8 | 3.5 | 2.7 | 2.2 | 1.7 | 1.5 | 1.3 | 1.2 |
| 100 | 56.5 | 13.3 | 5.2 | 3.4 | 2.5 | 2.1 | 1.9 | 1.3 | 1.1 | 1.0 |
| 50 | 59.0 | 15.0 | 5.7 | 4.0 | 2.3 | 2.0 | 1.8 | 1.3 | 1.0 | 1.0 |
| 10 | 57.5 | 17.9 | 8.3 | 6.2 | 4.2 | 2.4 | 1.7 | 0.9 | 0.7 | 0.4 |

#### Explained variance batch-corrected data

##### Total readcount

| Number\_of\_Genes | PC1 | PC2 | PC3 | PC4 | PC5 | PC6 | PC7 | PC8 | PC9 | PC10 |
| --- | --- | --- | --- | --- | --- | --- | --- | --- | --- | --- |
| 1594 | 22.9 | 8.7 | 4.9 | 3.6 | 2.6 | 1.8 | 1.7 | 1.6 | 1.5 | 1.4 |
| 1000 | 34.7 | 13.6 | 7.0 | 5.2 | 3.3 | 2.0 | 1.8 | 1.5 | 1.1 | 1.0 |
| 500 | 45.5 | 21.5 | 7.6 | 5.7 | 3.6 | 1.9 | 1.7 | 1.2 | 1.0 | 0.7 |
| 100 | 63.8 | 21.8 | 3.6 | 2.5 | 1.8 | 1.1 | 0.9 | 0.5 | 0.4 | 0.3 |
| 50 | 64.3 | 26.0 | 2.7 | 2.1 | 1.4 | 0.6 | 0.6 | 0.3 | 0.2 | 0.2 |
| 10 | 97.6 | 1.6 | 0.1 | 0.1 | 0.1 | 0.1 | 0.1 | 0.1 | 0.1 | 0.1 |

##### miRNA readcount

| Number\_of\_Genes | PC1 | PC2 | PC3 | PC4 | PC5 | PC6 | PC7 | PC8 | PC9 | PC10 |
| --- | --- | --- | --- | --- | --- | --- | --- | --- | --- | --- |
| 1124 | 7.0 | 2.8 | 2.6 | 2.2 | 2.1 | 1.5 | 1.4 | 1.4 | 1.3 | 1.3 |
| 1000 | 7.5 | 3.0 | 2.8 | 2.3 | 2.2 | 1.4 | 1.3 | 1.2 | 1.1 | 1.1 |
| 500 | 11.2 | 5.6 | 4.1 | 3.6 | 2.9 | 2.1 | 1.8 | 1.7 | 1.6 | 1.5 |
| 100 | 23.6 | 13.3 | 9.8 | 6.1 | 4.7 | 4.0 | 3.4 | 2.7 | 2.1 | 1.9 |
| 50 | 32.7 | 16.4 | 12.2 | 5.9 | 5.0 | 4.4 | 2.7 | 2.0 | 1.8 | 1.6 |
| 10 | 62.0 | 15.6 | 7.7 | 6.8 | 3.9 | 1.6 | 1.0 | 0.6 | 0.6 | 0.3 |

##### tRNA + miRNA readcount

| Number\_of\_Genes | PC1 | PC2 | PC3 | PC4 | PC5 | PC6 | PC7 | PC8 | PC9 | PC10 |
| --- | --- | --- | --- | --- | --- | --- | --- | --- | --- | --- |
| 1173 | 15.2 | 2.8 | 2.6 | 2.2 | 1.8 | 1.8 | 1.7 | 1.5 | 1.4 | 1.4 |
| 1000 | 17.5 | 3.1 | 2.6 | 2.3 | 2.1 | 1.6 | 1.3 | 1.2 | 1.2 | 1.1 |
| 500 | 29.6 | 5.3 | 3.9 | 3.5 | 2.7 | 2.2 | 1.7 | 1.5 | 1.3 | 1.2 |
| 100 | 56.0 | 11.9 | 5.6 | 3.7 | 2.5 | 2.3 | 1.9 | 1.4 | 1.2 | 1.1 |
| 50 | 57.7 | 15.0 | 5.7 | 4.4 | 2.5 | 2.2 | 1.9 | 1.3 | 1.1 | 1.0 |
| 10 | 56.2 | 18.3 | 9.9 | 4.4 | 4.0 | 3.3 | 1.6 | 1.0 | 0.7 | 0.5 |

##### isomiR readcount

| Number\_of\_Genes | PC1 | PC2 | PC3 | PC4 | PC5 | PC6 | PC7 | PC8 | PC9 | PC10 |
| --- | --- | --- | --- | --- | --- | --- | --- | --- | --- | --- |
| 574 | 33.2 | 10.8 | 7.0 | 5.1 | 3.8 | 2.9 | 2.7 | 1.8 | 1.5 | 1.3 |
| 500 | 34.7 | 10.8 | 7.2 | 4.8 | 3.9 | 3.0 | 2.3 | 1.7 | 1.6 | 1.3 |
| 100 | 80.4 | 9.6 | 4.2 | 1.4 | 0.8 | 0.6 | 0.5 | 0.3 | 0.2 | 0.1 |
| 50 | 85.3 | 7.7 | 2.8 | 1.1 | 0.6 | 0.4 | 0.3 | 0.3 | 0.1 | 0.1 |
| 10 | 95.3 | 2.2 | 1.1 | 0.4 | 0.3 | 0.2 | 0.2 | 0.1 | 0.1 | 0.1 |

##### tRF readcount

| Number\_of\_Genes | PC1 | PC2 | PC3 | PC4 | PC5 | PC6 | PC7 | PC8 | PC9 | PC10 |
| --- | --- | --- | --- | --- | --- | --- | --- | --- | --- | --- |
| 132 | 31.7 | 12.6 | 11.5 | 6.5 | 3.7 | 3.1 | 2.8 | 1.9 | 1.5 | 1.4 |
| 100 | 34.1 | 13.9 | 11.5 | 6.2 | 3.8 | 2.9 | 2.5 | 1.6 | 1.3 | 1.3 |
| 50 | 41.8 | 18.9 | 7.1 | 5.1 | 4.4 | 2.8 | 1.8 | 1.6 | 1.3 | 1.2 |
| 10 | 61.8 | 12.0 | 11.8 | 4.1 | 3.2 | 1.8 | 1.6 | 1.4 | 1.3 | 1.0 |

##### tRF + isomiR readcount

| Number\_of\_Genes | PC1 | PC2 | PC3 | PC4 | PC5 | PC6 | PC7 | PC8 | PC9 | PC10 |
| --- | --- | --- | --- | --- | --- | --- | --- | --- | --- | --- |
| 706 | 38.6 | 15.7 | 6.4 | 4.8 | 3.3 | 2.5 | 1.9 | 1.8 | 1.2 | 1.2 |
| 500 | 40.8 | 19.0 | 6.5 | 4.4 | 3.5 | 2.1 | 1.7 | 1.3 | 1.2 | 1.0 |
| 100 | 79.3 | 8.5 | 4.3 | 2.6 | 1.0 | 0.7 | 0.6 | 0.5 | 0.2 | 0.2 |
| 50 | 85.4 | 6.0 | 2.8 | 1.9 | 1.0 | 0.7 | 0.4 | 0.3 | 0.1 | 0.1 |
| 10 | 96.5 | 1.5 | 0.9 | 0.3 | 0.2 | 0.2 | 0.1 | 0.1 | 0.1 | 0.1 |

##### Custom analysis readcount

| Number\_of\_Genes | PC1 | PC2 | PC3 | PC4 | PC5 | PC6 | PC7 | PC8 | PC9 | PC10 |
| --- | --- | --- | --- | --- | --- | --- | --- | --- | --- | --- |
| 1177 | 15.5 | 2.8 | 2.6 | 2.3 | 1.8 | 1.8 | 1.7 | 1.5 | 1.4 | 1.4 |
| 1000 | 17.9 | 3.1 | 2.7 | 2.4 | 2.0 | 1.6 | 1.4 | 1.3 | 1.2 | 1.2 |
| 500 | 30.6 | 5.6 | 3.8 | 3.5 | 2.7 | 2.2 | 1.7 | 1.5 | 1.3 | 1.2 |
| 100 | 56.5 | 13.3 | 5.2 | 3.4 | 2.5 | 2.1 | 1.9 | 1.3 | 1.1 | 1.0 |
| 50 | 59.0 | 15.0 | 5.7 | 4.0 | 2.3 | 2.0 | 1.8 | 1.3 | 1.0 | 1.0 |
| 10 | 57.5 | 17.9 | 8.3 | 6.2 | 4.2 | 2.4 | 1.7 | 0.9 | 0.7 | 0.4 |
