# Supplementary material for: Comprehensive and bias-aware analysis of small RNA NGS data for biomarker detection with caRNAge
Source: Extracell Vesicles Circ Nucl Acids. 2026 Sep 24;7(3):1554–81. doi: 10.20517/evcna.2026.78 (PMC13678173; doi:10.20517/evcna.2026.78)
Supplement: Supplementary file 1 [file evcna-7-3-1554-SupplementaryMaterials.zip › evcna7078-SupplementaryMaterials/Supplementary File 5.html]

caRNAge - comprehensive analysis of small RNA gene expression


# caRNAge - comprehensive analysis of small RNA gene expression

### Differential gene expression

#### 22 August, 2026

## Purpose of this overview (Disclaimer)

This report is meant to give you a complete overview of
differentially expressed genes for the group comparison you have chosen
with their respective sources of bias you have identified in the data
overview. Please refer to the guidelines for best practice of caRNAge
and the respective manuals and vignettes of the applied tools. You are
strongly advised to thoroughly check all available information in
addition to this data overview. caRNAge is built upon the work of many
excellent scientists who developed the necessary tools to process small
RNA NGS data, so please make sure to cite the corresponding original
authors in addition to caRNAge when publishing your work.

If you encounter any technical problems, feel free to contact the
corresponding author at bkirchner@tum.de.

## Data processing

Raw sequencing reads were processed with Cutadapt to trim
adapter sequences from 3’ ends if present and to remove degraded reads
(shorter than 16nt). Trimmed reads were analysed with FastQC
to evaluate phred scores and read lengths before aligning them against
the human tRNA (GtRNAdb), miRNA
(miRBase) including isomiRs (isomiRROR), piRNAs (piRNAdb) and refseq transcriptome
sequences (refseq)
with bowtie.
Aligned reads were then analyzed to generate length and mapping
distributions as well as reads counts. Finally, gene read counts were
processed with R and mainly DESeq2
to normalize and transform data and calculate expression changes.
Differential gene expression is visualized as volcano plots with the
help of the EnhancedVolcano
package

Fig 1: Simplified workflow for NGS data analysis

|  | Tools | Version | Parameters |
| --- | --- | --- | --- |
| Trimming | Cutadapt | 4.4 | -a AGATCGGAAGAGCAC, -e 0.1 -m 16 |
| Quality Control | FastQC | 0.12.1 |  |
| Alignment | bowtie | 1.3.1 | Bowtie -v mismatches: tRNA=1, isomiR=3, transcriptome=1; –best –norc |
| Normalisation | DESeq2 | R version 4.6.1 (2026-06-24), DESeq2 1.52.0 | ~donor + analysis\_group |
| DGE calling | DESeq2 | R version 4.6.1 (2026-06-24), DESeq2 1.52.0 |  |
| Visualization | EnhancedVolcano | R version 4.6.1 (2026-06-24), EnhancedVolcano 1.30.0 |  |
| Supervised Clustering | mixOmics | R version 4.6.1 (2026-06-24), mixOmics 6.36.0 | Pairwise tuning stored in splsda\_tuning\_table; Distance metric: centroids.dist , Minimum mean expression: 10 , Maximum number of genes sorted by variance: 1000 , Cross-validation: Mfold , Validation repeats: 100 |

## Sample overview

The starting point for all analyses, is a well designed study design.
In addition to the classification of the main research question, it
should also incorporate all available information that could in any way
affect the outcome. This includes potential confounding factors such as
batch effects as well as statistical relevant information such as
patient cohorts or cell lines. Please make sure that the classifications
given in your *design.tsv* are correct and that all relevant
information is present.

### Study design

The row names and “Sample” column should be matching and indicate
that samples in design were correctly assigned in the analysis. A column
named “replaceable” maybe present if groups are large enough (>7) to
replace single outlier values.

|  | Sample | source\_name | cell\_type | condition | compartment | experimental\_block | sequencing\_platform | donor | analysis\_group | sequencing\_cycles |
| --- | --- | --- | --- | --- | --- | --- | --- | --- | --- | --- |
| wtchg\_834291\_208\_1 | wtchg\_834291\_208\_1 | CD4 EV | cd4\_t\_cell | ev | extracellular\_vesicle | wtchg\_208 | illumina\_hiseq\_4000 | donor\_834291 | cd4\_ev | 75 |
| wtchg\_834291\_209\_1 | wtchg\_834291\_209\_1 | CD8 EV | cd8\_t\_cell | ev | extracellular\_vesicle | wtchg\_209 | illumina\_hiseq\_4000 | donor\_834291 | cd8\_ev | 75 |
| wtchg\_834291\_210\_1 | wtchg\_834291\_210\_1 | CD8 EV | cd8\_t\_cell | ev | extracellular\_vesicle | wtchg\_210 | illumina\_hiseq\_4000 | donor\_834291 | cd8\_ev | 75 |
| wtchg\_834291\_211\_1 | wtchg\_834291\_211\_1 | CD4 EV | cd4\_t\_cell | ev | extracellular\_vesicle | wtchg\_211 | illumina\_hiseq\_4000 | donor\_834291 | cd4\_ev | 75 |
| wtchg\_834291\_212\_1 | wtchg\_834291\_212\_1 | CD8 EV | cd8\_t\_cell | ev | extracellular\_vesicle | wtchg\_212 | illumina\_hiseq\_4000 | donor\_834291 | cd8\_ev | 75 |
| wtchg\_834291\_213\_1 | wtchg\_834291\_213\_1 | CD4 EV | cd4\_t\_cell | ev | extracellular\_vesicle | wtchg\_213 | illumina\_hiseq\_4000 | donor\_834291 | cd4\_ev | 75 |
| wtchg\_834291\_220\_1 | wtchg\_834291\_220\_1 | CD4 S0 | cd4\_t\_cell | s0 | bslb\_background\_control | wtchg\_220 | illumina\_hiseq\_4000 | donor\_834291 | cd4\_s0 | 75 |
| wtchg\_834291\_225\_1 | wtchg\_834291\_225\_1 | CD8 S1 | cd8\_t\_cell | s1 | trans\_synaptic\_vesicle | wtchg\_225 | illumina\_hiseq\_4000 | donor\_834291 | cd8\_s1 | 75 |
| wtchg\_834291\_226\_1 | wtchg\_834291\_226\_1 | CD4 S0 | cd4\_t\_cell | s0 | bslb\_background\_control | wtchg\_226 | illumina\_hiseq\_4000 | donor\_834291 | cd4\_s0 | 75 |
| wtchg\_834291\_227\_1 | wtchg\_834291\_227\_1 | CD4 S1 | cd4\_t\_cell | s1 | trans\_synaptic\_vesicle | wtchg\_227 | illumina\_hiseq\_4000 | donor\_834291 | cd4\_s1 | 75 |
| wtchg\_834291\_228\_1 | wtchg\_834291\_228\_1 | CD8 S1 | cd8\_t\_cell | s1 | trans\_synaptic\_vesicle | wtchg\_228 | illumina\_hiseq\_4000 | donor\_834291 | cd8\_s1 | 75 |
| wtchg\_834291\_233\_1 | wtchg\_834291\_233\_1 | CD4 S1 | cd4\_t\_cell | s1 | trans\_synaptic\_vesicle | wtchg\_233 | illumina\_hiseq\_4000 | donor\_834291 | cd4\_s1 | 75 |
| wtchg\_834291\_234\_1 | wtchg\_834291\_234\_1 | CD4 S1 | cd4\_t\_cell | s1 | trans\_synaptic\_vesicle | wtchg\_234 | illumina\_hiseq\_4000 | donor\_834291 | cd4\_s1 | 75 |
| wtchg\_834291\_237\_1 | wtchg\_834291\_237\_1 | CD8 S0 | cd8\_t\_cell | s0 | bslb\_background\_control | wtchg\_237 | illumina\_hiseq\_4000 | donor\_834291 | cd8\_s0 | 75 |
| wtchg\_834291\_238\_1 | wtchg\_834291\_238\_1 | CD8 S1 | cd8\_t\_cell | s1 | trans\_synaptic\_vesicle | wtchg\_238 | illumina\_hiseq\_4000 | donor\_834291 | cd8\_s1 | 75 |
| wtchg\_834292\_208\_1 | wtchg\_834292\_208\_1 | CD4 EV | cd4\_t\_cell | ev | extracellular\_vesicle | wtchg\_208 | illumina\_hiseq\_4000 | donor\_834292 | cd4\_ev | 75 |
| wtchg\_834292\_209\_1 | wtchg\_834292\_209\_1 | CD8 EV | cd8\_t\_cell | ev | extracellular\_vesicle | wtchg\_209 | illumina\_hiseq\_4000 | donor\_834292 | cd8\_ev | 75 |
| wtchg\_834292\_210\_1 | wtchg\_834292\_210\_1 | CD8 EV | cd8\_t\_cell | ev | extracellular\_vesicle | wtchg\_210 | illumina\_hiseq\_4000 | donor\_834292 | cd8\_ev | 75 |
| wtchg\_834292\_211\_1 | wtchg\_834292\_211\_1 | CD4 EV | cd4\_t\_cell | ev | extracellular\_vesicle | wtchg\_211 | illumina\_hiseq\_4000 | donor\_834292 | cd4\_ev | 75 |
| wtchg\_834292\_212\_1 | wtchg\_834292\_212\_1 | CD8 EV | cd8\_t\_cell | ev | extracellular\_vesicle | wtchg\_212 | illumina\_hiseq\_4000 | donor\_834292 | cd8\_ev | 75 |
| wtchg\_834292\_213\_1 | wtchg\_834292\_213\_1 | CD4 EV | cd4\_t\_cell | ev | extracellular\_vesicle | wtchg\_213 | illumina\_hiseq\_4000 | donor\_834292 | cd4\_ev | 75 |
| wtchg\_834292\_220\_1 | wtchg\_834292\_220\_1 | CD4 S0 | cd4\_t\_cell | s0 | bslb\_background\_control | wtchg\_220 | illumina\_hiseq\_4000 | donor\_834292 | cd4\_s0 | 75 |
| wtchg\_834292\_225\_1 | wtchg\_834292\_225\_1 | CD8 S1 | cd8\_t\_cell | s1 | trans\_synaptic\_vesicle | wtchg\_225 | illumina\_hiseq\_4000 | donor\_834292 | cd8\_s1 | 75 |
| wtchg\_834292\_226\_1 | wtchg\_834292\_226\_1 | CD4 S0 | cd4\_t\_cell | s0 | bslb\_background\_control | wtchg\_226 | illumina\_hiseq\_4000 | donor\_834292 | cd4\_s0 | 75 |
| wtchg\_834292\_227\_1 | wtchg\_834292\_227\_1 | CD4 S1 | cd4\_t\_cell | s1 | trans\_synaptic\_vesicle | wtchg\_227 | illumina\_hiseq\_4000 | donor\_834292 | cd4\_s1 | 75 |
| wtchg\_834292\_228\_1 | wtchg\_834292\_228\_1 | CD8 S1 | cd8\_t\_cell | s1 | trans\_synaptic\_vesicle | wtchg\_228 | illumina\_hiseq\_4000 | donor\_834292 | cd8\_s1 | 75 |
| wtchg\_834292\_233\_1 | wtchg\_834292\_233\_1 | CD4 S1 | cd4\_t\_cell | s1 | trans\_synaptic\_vesicle | wtchg\_233 | illumina\_hiseq\_4000 | donor\_834292 | cd4\_s1 | 75 |
| wtchg\_834292\_234\_1 | wtchg\_834292\_234\_1 | CD4 S1 | cd4\_t\_cell | s1 | trans\_synaptic\_vesicle | wtchg\_234 | illumina\_hiseq\_4000 | donor\_834292 | cd4\_s1 | 75 |
| wtchg\_834292\_237\_1 | wtchg\_834292\_237\_1 | CD8 S0 | cd8\_t\_cell | s0 | bslb\_background\_control | wtchg\_237 | illumina\_hiseq\_4000 | donor\_834292 | cd8\_s0 | 75 |
| wtchg\_834292\_238\_1 | wtchg\_834292\_238\_1 | CD8 S1 | cd8\_t\_cell | s1 | trans\_synaptic\_vesicle | wtchg\_238 | illumina\_hiseq\_4000 | donor\_834292 | cd8\_s1 | 75 |
| wtchg\_834293\_208\_1 | wtchg\_834293\_208\_1 | CD4 EV | cd4\_t\_cell | ev | extracellular\_vesicle | wtchg\_208 | illumina\_hiseq\_4000 | donor\_834293 | cd4\_ev | 75 |
| wtchg\_834293\_209\_1 | wtchg\_834293\_209\_1 | CD8 EV | cd8\_t\_cell | ev | extracellular\_vesicle | wtchg\_209 | illumina\_hiseq\_4000 | donor\_834293 | cd8\_ev | 75 |
| wtchg\_834293\_210\_1 | wtchg\_834293\_210\_1 | CD8 EV | cd8\_t\_cell | ev | extracellular\_vesicle | wtchg\_210 | illumina\_hiseq\_4000 | donor\_834293 | cd8\_ev | 75 |
| wtchg\_834293\_211\_1 | wtchg\_834293\_211\_1 | CD4 EV | cd4\_t\_cell | ev | extracellular\_vesicle | wtchg\_211 | illumina\_hiseq\_4000 | donor\_834293 | cd4\_ev | 75 |
| wtchg\_834293\_212\_1 | wtchg\_834293\_212\_1 | CD8 EV | cd8\_t\_cell | ev | extracellular\_vesicle | wtchg\_212 | illumina\_hiseq\_4000 | donor\_834293 | cd8\_ev | 75 |
| wtchg\_834293\_213\_1 | wtchg\_834293\_213\_1 | CD4 EV | cd4\_t\_cell | ev | extracellular\_vesicle | wtchg\_213 | illumina\_hiseq\_4000 | donor\_834293 | cd4\_ev | 75 |
| wtchg\_834293\_220\_1 | wtchg\_834293\_220\_1 | CD4 S0 | cd4\_t\_cell | s0 | bslb\_background\_control | wtchg\_220 | illumina\_hiseq\_4000 | donor\_834293 | cd4\_s0 | 75 |
| wtchg\_834293\_225\_1 | wtchg\_834293\_225\_1 | CD8 S1 | cd8\_t\_cell | s1 | trans\_synaptic\_vesicle | wtchg\_225 | illumina\_hiseq\_4000 | donor\_834293 | cd8\_s1 | 75 |
| wtchg\_834293\_226\_1 | wtchg\_834293\_226\_1 | CD4 S0 | cd4\_t\_cell | s0 | bslb\_background\_control | wtchg\_226 | illumina\_hiseq\_4000 | donor\_834293 | cd4\_s0 | 75 |
| wtchg\_834293\_227\_1 | wtchg\_834293\_227\_1 | CD4 S1 | cd4\_t\_cell | s1 | trans\_synaptic\_vesicle | wtchg\_227 | illumina\_hiseq\_4000 | donor\_834293 | cd4\_s1 | 75 |
| wtchg\_834293\_228\_1 | wtchg\_834293\_228\_1 | CD8 S1 | cd8\_t\_cell | s1 | trans\_synaptic\_vesicle | wtchg\_228 | illumina\_hiseq\_4000 | donor\_834293 | cd8\_s1 | 75 |
| wtchg\_834293\_233\_1 | wtchg\_834293\_233\_1 | CD4 S1 | cd4\_t\_cell | s1 | trans\_synaptic\_vesicle | wtchg\_233 | illumina\_hiseq\_4000 | donor\_834293 | cd4\_s1 | 75 |
| wtchg\_834293\_234\_1 | wtchg\_834293\_234\_1 | CD4 S1 | cd4\_t\_cell | s1 | trans\_synaptic\_vesicle | wtchg\_234 | illumina\_hiseq\_4000 | donor\_834293 | cd4\_s1 | 75 |
| wtchg\_834293\_237\_1 | wtchg\_834293\_237\_1 | CD8 S0 | cd8\_t\_cell | s0 | bslb\_background\_control | wtchg\_237 | illumina\_hiseq\_4000 | donor\_834293 | cd8\_s0 | 75 |
| wtchg\_834293\_238\_1 | wtchg\_834293\_238\_1 | CD8 S1 | cd8\_t\_cell | s1 | trans\_synaptic\_vesicle | wtchg\_238 | illumina\_hiseq\_4000 | donor\_834293 | cd8\_s1 | 75 |
| wtchg\_834294\_208\_1 | wtchg\_834294\_208\_1 | CD4 EV | cd4\_t\_cell | ev | extracellular\_vesicle | wtchg\_208 | illumina\_hiseq\_4000 | donor\_834294 | cd4\_ev | 75 |
| wtchg\_834294\_209\_1 | wtchg\_834294\_209\_1 | CD8 EV | cd8\_t\_cell | ev | extracellular\_vesicle | wtchg\_209 | illumina\_hiseq\_4000 | donor\_834294 | cd8\_ev | 75 |
| wtchg\_834294\_210\_1 | wtchg\_834294\_210\_1 | CD8 EV | cd8\_t\_cell | ev | extracellular\_vesicle | wtchg\_210 | illumina\_hiseq\_4000 | donor\_834294 | cd8\_ev | 75 |
| wtchg\_834294\_211\_1 | wtchg\_834294\_211\_1 | CD4 EV | cd4\_t\_cell | ev | extracellular\_vesicle | wtchg\_211 | illumina\_hiseq\_4000 | donor\_834294 | cd4\_ev | 75 |
| wtchg\_834294\_212\_1 | wtchg\_834294\_212\_1 | CD8 EV | cd8\_t\_cell | ev | extracellular\_vesicle | wtchg\_212 | illumina\_hiseq\_4000 | donor\_834294 | cd8\_ev | 75 |
| wtchg\_834294\_213\_1 | wtchg\_834294\_213\_1 | CD4 EV | cd4\_t\_cell | ev | extracellular\_vesicle | wtchg\_213 | illumina\_hiseq\_4000 | donor\_834294 | cd4\_ev | 75 |
| wtchg\_834294\_220\_1 | wtchg\_834294\_220\_1 | CD4 S0 | cd4\_t\_cell | s0 | bslb\_background\_control | wtchg\_220 | illumina\_hiseq\_4000 | donor\_834294 | cd4\_s0 | 75 |
| wtchg\_834294\_225\_1 | wtchg\_834294\_225\_1 | CD8 S1 | cd8\_t\_cell | s1 | trans\_synaptic\_vesicle | wtchg\_225 | illumina\_hiseq\_4000 | donor\_834294 | cd8\_s1 | 75 |
| wtchg\_834294\_226\_1 | wtchg\_834294\_226\_1 | CD4 S0 | cd4\_t\_cell | s0 | bslb\_background\_control | wtchg\_226 | illumina\_hiseq\_4000 | donor\_834294 | cd4\_s0 | 75 |
| wtchg\_834294\_227\_1 | wtchg\_834294\_227\_1 | CD4 S1 | cd4\_t\_cell | s1 | trans\_synaptic\_vesicle | wtchg\_227 | illumina\_hiseq\_4000 | donor\_834294 | cd4\_s1 | 75 |
| wtchg\_834294\_228\_1 | wtchg\_834294\_228\_1 | CD8 S1 | cd8\_t\_cell | s1 | trans\_synaptic\_vesicle | wtchg\_228 | illumina\_hiseq\_4000 | donor\_834294 | cd8\_s1 | 75 |
| wtchg\_834294\_233\_1 | wtchg\_834294\_233\_1 | CD4 S1 | cd4\_t\_cell | s1 | trans\_synaptic\_vesicle | wtchg\_233 | illumina\_hiseq\_4000 | donor\_834294 | cd4\_s1 | 75 |
| wtchg\_834294\_234\_1 | wtchg\_834294\_234\_1 | CD4 S1 | cd4\_t\_cell | s1 | trans\_synaptic\_vesicle | wtchg\_234 | illumina\_hiseq\_4000 | donor\_834294 | cd4\_s1 | 75 |
| wtchg\_834294\_237\_1 | wtchg\_834294\_237\_1 | CD8 S0 | cd8\_t\_cell | s0 | bslb\_background\_control | wtchg\_237 | illumina\_hiseq\_4000 | donor\_834294 | cd8\_s0 | 75 |
| wtchg\_834294\_238\_1 | wtchg\_834294\_238\_1 | CD8 S1 | cd8\_t\_cell | s1 | trans\_synaptic\_vesicle | wtchg\_238 | illumina\_hiseq\_4000 | donor\_834294 | cd8\_s1 | 75 |
| wtchg\_836235\_208\_1 | wtchg\_836235\_208\_1 | CD4 EV | cd4\_t\_cell | ev | extracellular\_vesicle | wtchg\_208 | illumina\_hiseq\_4000 | donor\_836235 | cd4\_ev | 75 |
| wtchg\_836235\_209\_1 | wtchg\_836235\_209\_1 | CD8 EV | cd8\_t\_cell | ev | extracellular\_vesicle | wtchg\_209 | illumina\_hiseq\_4000 | donor\_836235 | cd8\_ev | 75 |
| wtchg\_836235\_210\_1 | wtchg\_836235\_210\_1 | CD8 EV | cd8\_t\_cell | ev | extracellular\_vesicle | wtchg\_210 | illumina\_hiseq\_4000 | donor\_836235 | cd8\_ev | 75 |
| wtchg\_836235\_211\_1 | wtchg\_836235\_211\_1 | CD4 EV | cd4\_t\_cell | ev | extracellular\_vesicle | wtchg\_211 | illumina\_hiseq\_4000 | donor\_836235 | cd4\_ev | 75 |
| wtchg\_836235\_212\_1 | wtchg\_836235\_212\_1 | CD8 EV | cd8\_t\_cell | ev | extracellular\_vesicle | wtchg\_212 | illumina\_hiseq\_4000 | donor\_836235 | cd8\_ev | 75 |
| wtchg\_836235\_213\_1 | wtchg\_836235\_213\_1 | CD4 EV | cd4\_t\_cell | ev | extracellular\_vesicle | wtchg\_213 | illumina\_hiseq\_4000 | donor\_836235 | cd4\_ev | 75 |
| wtchg\_836235\_220\_1 | wtchg\_836235\_220\_1 | CD4 S0 | cd4\_t\_cell | s0 | bslb\_background\_control | wtchg\_220 | illumina\_hiseq\_4000 | donor\_836235 | cd4\_s0 | 75 |
| wtchg\_836235\_225\_1 | wtchg\_836235\_225\_1 | CD8 S1 | cd8\_t\_cell | s1 | trans\_synaptic\_vesicle | wtchg\_225 | illumina\_hiseq\_4000 | donor\_836235 | cd8\_s1 | 75 |
| wtchg\_836235\_226\_1 | wtchg\_836235\_226\_1 | CD4 S0 | cd4\_t\_cell | s0 | bslb\_background\_control | wtchg\_226 | illumina\_hiseq\_4000 | donor\_836235 | cd4\_s0 | 75 |
| wtchg\_836235\_227\_1 | wtchg\_836235\_227\_1 | CD4 S1 | cd4\_t\_cell | s1 | trans\_synaptic\_vesicle | wtchg\_227 | illumina\_hiseq\_4000 | donor\_836235 | cd4\_s1 | 75 |
| wtchg\_836235\_228\_1 | wtchg\_836235\_228\_1 | CD8 S1 | cd8\_t\_cell | s1 | trans\_synaptic\_vesicle | wtchg\_228 | illumina\_hiseq\_4000 | donor\_836235 | cd8\_s1 | 75 |
| wtchg\_836235\_233\_1 | wtchg\_836235\_233\_1 | CD4 S1 | cd4\_t\_cell | s1 | trans\_synaptic\_vesicle | wtchg\_233 | illumina\_hiseq\_4000 | donor\_836235 | cd4\_s1 | 75 |
| wtchg\_836235\_234\_1 | wtchg\_836235\_234\_1 | CD4 S1 | cd4\_t\_cell | s1 | trans\_synaptic\_vesicle | wtchg\_234 | illumina\_hiseq\_4000 | donor\_836235 | cd4\_s1 | 75 |
| wtchg\_836235\_237\_1 | wtchg\_836235\_237\_1 | CD8 S0 | cd8\_t\_cell | s0 | bslb\_background\_control | wtchg\_237 | illumina\_hiseq\_4000 | donor\_836235 | cd8\_s0 | 75 |
| wtchg\_836235\_238\_1 | wtchg\_836235\_238\_1 | CD8 S1 | cd8\_t\_cell | s1 | trans\_synaptic\_vesicle | wtchg\_238 | illumina\_hiseq\_4000 | donor\_836235 | cd8\_s1 | 75 |
| wtchg\_836236\_208\_1 | wtchg\_836236\_208\_1 | CD4 EV | cd4\_t\_cell | ev | extracellular\_vesicle | wtchg\_208 | illumina\_hiseq\_4000 | donor\_836236 | cd4\_ev | 75 |
| wtchg\_836236\_209\_1 | wtchg\_836236\_209\_1 | CD8 EV | cd8\_t\_cell | ev | extracellular\_vesicle | wtchg\_209 | illumina\_hiseq\_4000 | donor\_836236 | cd8\_ev | 75 |
| wtchg\_836236\_210\_1 | wtchg\_836236\_210\_1 | CD8 EV | cd8\_t\_cell | ev | extracellular\_vesicle | wtchg\_210 | illumina\_hiseq\_4000 | donor\_836236 | cd8\_ev | 75 |
| wtchg\_836236\_211\_1 | wtchg\_836236\_211\_1 | CD4 EV | cd4\_t\_cell | ev | extracellular\_vesicle | wtchg\_211 | illumina\_hiseq\_4000 | donor\_836236 | cd4\_ev | 75 |
| wtchg\_836236\_212\_1 | wtchg\_836236\_212\_1 | CD8 EV | cd8\_t\_cell | ev | extracellular\_vesicle | wtchg\_212 | illumina\_hiseq\_4000 | donor\_836236 | cd8\_ev | 75 |
| wtchg\_836236\_213\_1 | wtchg\_836236\_213\_1 | CD4 EV | cd4\_t\_cell | ev | extracellular\_vesicle | wtchg\_213 | illumina\_hiseq\_4000 | donor\_836236 | cd4\_ev | 75 |
| wtchg\_836236\_220\_1 | wtchg\_836236\_220\_1 | CD4 S0 | cd4\_t\_cell | s0 | bslb\_background\_control | wtchg\_220 | illumina\_hiseq\_4000 | donor\_836236 | cd4\_s0 | 75 |
| wtchg\_836236\_225\_1 | wtchg\_836236\_225\_1 | CD8 S1 | cd8\_t\_cell | s1 | trans\_synaptic\_vesicle | wtchg\_225 | illumina\_hiseq\_4000 | donor\_836236 | cd8\_s1 | 75 |
| wtchg\_836236\_226\_1 | wtchg\_836236\_226\_1 | CD4 S0 | cd4\_t\_cell | s0 | bslb\_background\_control | wtchg\_226 | illumina\_hiseq\_4000 | donor\_836236 | cd4\_s0 | 75 |
| wtchg\_836236\_227\_1 | wtchg\_836236\_227\_1 | CD4 S1 | cd4\_t\_cell | s1 | trans\_synaptic\_vesicle | wtchg\_227 | illumina\_hiseq\_4000 | donor\_836236 | cd4\_s1 | 75 |
| wtchg\_836236\_228\_1 | wtchg\_836236\_228\_1 | CD8 S1 | cd8\_t\_cell | s1 | trans\_synaptic\_vesicle | wtchg\_228 | illumina\_hiseq\_4000 | donor\_836236 | cd8\_s1 | 75 |
| wtchg\_836236\_233\_1 | wtchg\_836236\_233\_1 | CD4 S1 | cd4\_t\_cell | s1 | trans\_synaptic\_vesicle | wtchg\_233 | illumina\_hiseq\_4000 | donor\_836236 | cd4\_s1 | 75 |
| wtchg\_836236\_234\_1 | wtchg\_836236\_234\_1 | CD4 S1 | cd4\_t\_cell | s1 | trans\_synaptic\_vesicle | wtchg\_234 | illumina\_hiseq\_4000 | donor\_836236 | cd4\_s1 | 75 |
| wtchg\_836236\_237\_1 | wtchg\_836236\_237\_1 | CD8 S0 | cd8\_t\_cell | s0 | bslb\_background\_control | wtchg\_237 | illumina\_hiseq\_4000 | donor\_836236 | cd8\_s0 | 75 |
| wtchg\_836236\_238\_1 | wtchg\_836236\_238\_1 | CD8 S1 | cd8\_t\_cell | s1 | trans\_synaptic\_vesicle | wtchg\_238 | illumina\_hiseq\_4000 | donor\_836236 | cd8\_s1 | 75 |
| wtchg\_836237\_208\_1 | wtchg\_836237\_208\_1 | CD4 EV | cd4\_t\_cell | ev | extracellular\_vesicle | wtchg\_208 | illumina\_hiseq\_4000 | donor\_836237 | cd4\_ev | 75 |
| wtchg\_836237\_209\_1 | wtchg\_836237\_209\_1 | CD8 EV | cd8\_t\_cell | ev | extracellular\_vesicle | wtchg\_209 | illumina\_hiseq\_4000 | donor\_836237 | cd8\_ev | 75 |
| wtchg\_836237\_210\_1 | wtchg\_836237\_210\_1 | CD8 EV | cd8\_t\_cell | ev | extracellular\_vesicle | wtchg\_210 | illumina\_hiseq\_4000 | donor\_836237 | cd8\_ev | 75 |
| wtchg\_836237\_211\_1 | wtchg\_836237\_211\_1 | CD4 EV | cd4\_t\_cell | ev | extracellular\_vesicle | wtchg\_211 | illumina\_hiseq\_4000 | donor\_836237 | cd4\_ev | 75 |
| wtchg\_836237\_212\_1 | wtchg\_836237\_212\_1 | CD8 EV | cd8\_t\_cell | ev | extracellular\_vesicle | wtchg\_212 | illumina\_hiseq\_4000 | donor\_836237 | cd8\_ev | 75 |
| wtchg\_836237\_213\_1 | wtchg\_836237\_213\_1 | CD4 EV | cd4\_t\_cell | ev | extracellular\_vesicle | wtchg\_213 | illumina\_hiseq\_4000 | donor\_836237 | cd4\_ev | 75 |
| wtchg\_836237\_220\_1 | wtchg\_836237\_220\_1 | CD4 S0 | cd4\_t\_cell | s0 | bslb\_background\_control | wtchg\_220 | illumina\_hiseq\_4000 | donor\_836237 | cd4\_s0 | 75 |
| wtchg\_836237\_225\_1 | wtchg\_836237\_225\_1 | CD8 S1 | cd8\_t\_cell | s1 | trans\_synaptic\_vesicle | wtchg\_225 | illumina\_hiseq\_4000 | donor\_836237 | cd8\_s1 | 75 |
| wtchg\_836237\_226\_1 | wtchg\_836237\_226\_1 | CD4 S0 | cd4\_t\_cell | s0 | bslb\_background\_control | wtchg\_226 | illumina\_hiseq\_4000 | donor\_836237 | cd4\_s0 | 75 |
| wtchg\_836237\_227\_1 | wtchg\_836237\_227\_1 | CD4 S1 | cd4\_t\_cell | s1 | trans\_synaptic\_vesicle | wtchg\_227 | illumina\_hiseq\_4000 | donor\_836237 | cd4\_s1 | 75 |
| wtchg\_836237\_228\_1 | wtchg\_836237\_228\_1 | CD8 S1 | cd8\_t\_cell | s1 | trans\_synaptic\_vesicle | wtchg\_228 | illumina\_hiseq\_4000 | donor\_836237 | cd8\_s1 | 75 |
| wtchg\_836237\_233\_1 | wtchg\_836237\_233\_1 | CD4 S1 | cd4\_t\_cell | s1 | trans\_synaptic\_vesicle | wtchg\_233 | illumina\_hiseq\_4000 | donor\_836237 | cd4\_s1 | 75 |
| wtchg\_836237\_234\_1 | wtchg\_836237\_234\_1 | CD4 S1 | cd4\_t\_cell | s1 | trans\_synaptic\_vesicle | wtchg\_234 | illumina\_hiseq\_4000 | donor\_836237 | cd4\_s1 | 75 |
| wtchg\_836237\_237\_1 | wtchg\_836237\_237\_1 | CD8 S0 | cd8\_t\_cell | s0 | bslb\_background\_control | wtchg\_237 | illumina\_hiseq\_4000 | donor\_836237 | cd8\_s0 | 75 |
| wtchg\_836237\_238\_1 | wtchg\_836237\_238\_1 | CD8 S1 | cd8\_t\_cell | s1 | trans\_synaptic\_vesicle | wtchg\_238 | illumina\_hiseq\_4000 | donor\_836237 | cd8\_s1 | 75 |
| wtchg\_836238\_208\_1 | wtchg\_836238\_208\_1 | CD4 EV | cd4\_t\_cell | ev | extracellular\_vesicle | wtchg\_208 | illumina\_hiseq\_4000 | donor\_836238 | cd4\_ev | 75 |
| wtchg\_836238\_209\_1 | wtchg\_836238\_209\_1 | CD8 EV | cd8\_t\_cell | ev | extracellular\_vesicle | wtchg\_209 | illumina\_hiseq\_4000 | donor\_836238 | cd8\_ev | 75 |
| wtchg\_836238\_210\_1 | wtchg\_836238\_210\_1 | CD8 EV | cd8\_t\_cell | ev | extracellular\_vesicle | wtchg\_210 | illumina\_hiseq\_4000 | donor\_836238 | cd8\_ev | 75 |
| wtchg\_836238\_211\_1 | wtchg\_836238\_211\_1 | CD4 EV | cd4\_t\_cell | ev | extracellular\_vesicle | wtchg\_211 | illumina\_hiseq\_4000 | donor\_836238 | cd4\_ev | 75 |
| wtchg\_836238\_212\_1 | wtchg\_836238\_212\_1 | CD8 EV | cd8\_t\_cell | ev | extracellular\_vesicle | wtchg\_212 | illumina\_hiseq\_4000 | donor\_836238 | cd8\_ev | 75 |
| wtchg\_836238\_213\_1 | wtchg\_836238\_213\_1 | CD4 EV | cd4\_t\_cell | ev | extracellular\_vesicle | wtchg\_213 | illumina\_hiseq\_4000 | donor\_836238 | cd4\_ev | 75 |
| wtchg\_836238\_220\_1 | wtchg\_836238\_220\_1 | CD4 S0 | cd4\_t\_cell | s0 | bslb\_background\_control | wtchg\_220 | illumina\_hiseq\_4000 | donor\_836238 | cd4\_s0 | 75 |
| wtchg\_836238\_225\_1 | wtchg\_836238\_225\_1 | CD8 S1 | cd8\_t\_cell | s1 | trans\_synaptic\_vesicle | wtchg\_225 | illumina\_hiseq\_4000 | donor\_836238 | cd8\_s1 | 75 |
| wtchg\_836238\_226\_1 | wtchg\_836238\_226\_1 | CD4 S0 | cd4\_t\_cell | s0 | bslb\_background\_control | wtchg\_226 | illumina\_hiseq\_4000 | donor\_836238 | cd4\_s0 | 75 |
| wtchg\_836238\_227\_1 | wtchg\_836238\_227\_1 | CD4 S1 | cd4\_t\_cell | s1 | trans\_synaptic\_vesicle | wtchg\_227 | illumina\_hiseq\_4000 | donor\_836238 | cd4\_s1 | 75 |
| wtchg\_836238\_228\_1 | wtchg\_836238\_228\_1 | CD8 S1 | cd8\_t\_cell | s1 | trans\_synaptic\_vesicle | wtchg\_228 | illumina\_hiseq\_4000 | donor\_836238 | cd8\_s1 | 75 |
| wtchg\_836238\_233\_1 | wtchg\_836238\_233\_1 | CD4 S1 | cd4\_t\_cell | s1 | trans\_synaptic\_vesicle | wtchg\_233 | illumina\_hiseq\_4000 | donor\_836238 | cd4\_s1 | 75 |
| wtchg\_836238\_234\_1 | wtchg\_836238\_234\_1 | CD4 S1 | cd4\_t\_cell | s1 | trans\_synaptic\_vesicle | wtchg\_234 | illumina\_hiseq\_4000 | donor\_836238 | cd4\_s1 | 75 |
| wtchg\_836238\_237\_1 | wtchg\_836238\_237\_1 | CD8 S0 | cd8\_t\_cell | s0 | bslb\_background\_control | wtchg\_237 | illumina\_hiseq\_4000 | donor\_836238 | cd8\_s0 | 75 |
| wtchg\_836238\_238\_1 | wtchg\_836238\_238\_1 | CD8 S1 | cd8\_t\_cell | s1 | trans\_synaptic\_vesicle | wtchg\_238 | illumina\_hiseq\_4000 | donor\_836238 | cd8\_s1 | 75 |

### Consolidated sample groups

```
$source_name

CD4 EV CD4 S0 CD4 S1 CD8 EV CD8 S0 CD8 S1 
    24     16     24     24      8     24 

$cell_type

cd4_t_cell cd8_t_cell 
        64         56 

$condition

ev s0 s1 
48 24 48 

$compartment

bslb_background_control   extracellular_vesicle  trans_synaptic_vesicle 
                     24                      48                      48 

$experimental_block

wtchg_208 wtchg_209 wtchg_210 wtchg_211 wtchg_212 wtchg_213 wtchg_220 wtchg_225 
        8         8         8         8         8         8         8         8 
wtchg_226 wtchg_227 wtchg_228 wtchg_233 wtchg_234 wtchg_237 wtchg_238 
        8         8         8         8         8         8         8 

$sequencing_platform

illumina_hiseq_4000 
                120 

$donor

donor_834291 donor_834292 donor_834293 donor_834294 donor_836235 donor_836236 
          15           15           15           15           15           15 
donor_836237 donor_836238 
          15           15 

$analysis_group

cd4_ev cd8_ev cd4_s0 cd8_s1 cd4_s1 cd8_s0 
    24     24     16     24     24      8 

$sequencing_cycles

 75 
120
```

## Differential Gene Expression

DESeq2 performs an internal normalization where geometric mean is
calculated for each gene across all samples. The counts for a gene in
each sample is then divided by this mean. The median of these ratios in
a sample is the size factor for that sample. This procedure corrects for
library size and RNA composition bias, which can arise for example when
only a small number of genes are very highly expressed in one experiment
condition but not in the other.

DESeq2 estimates gene-wise dispersions and shrinks those estimates
toward a fitted dispersion trend, which improves stability when
replicate numbers are limited. Biological replication is required for
differential-expression testing; designs without replication cannot
provide a valid within-group dispersion estimate. The log2 fold changes
shown in this report are the standard DESeq2 maximum-likelihood
estimates from `results()`; no additional
`lfcShrink()` step is applied. DESeq2 fits negative binomial
generalized linear models for each gene and uses the Wald test for
significance testing. In addition to the group information, you can give
an additional experimental factor like pairing to the analysis. DESeq2
uses Cook’s distance to flag count outliers; depending on replicate
number and settings, affected tests may receive an `NA`
p-value rather than being treated as ordinary significant results.
Independent filtering can also set adjusted p-values to `NA`
for low-information features, improving multiple-testing power without
altering the fitted count model.

The chosen RNA classes for your custom analysis were:
**miRNA** and the threshold for defining canonical miRNAs
from isomiRs were **0 change(s) on the 5’end, 2 change(s) on the
3’end and 1 substitution(s) within the sequence** (see
config.yaml). The study design parameter chosen for DGE testing was
**analysis\_group** and the parameter(s) chosen for
correction were \*\*\*\*

### total\_readcount

#### cd4\_ev\_vs\_cd8\_ev

**Summary of DESeq2 results:**

```
out of 1180 with nonzero total read count
adjusted p-value < 0.1
LFC > 0 (up)       : 91, 7.7%
LFC < 0 (down)     : 225, 19%
outliers [1]       : 0, 0%
low counts [2]     : 481, 41%
(mean count < 0)
```

**Volcano plot:**

**Upregulated genes (padj ≤ 0.05, log2FC ≥ 1):**

**Downregulated genes (padj ≤ 0.05, log2FC ≤ -1):**

#### cd4\_ev\_vs\_cd4\_s0

**Summary of DESeq2 results:**

```
out of 1180 with nonzero total read count
adjusted p-value < 0.1
LFC > 0 (up)       : 211, 18%
LFC < 0 (down)     : 224, 19%
outliers [1]       : 0, 0%
low counts [2]     : 503, 43%
(mean count < 0)
```

**Volcano plot:**

**Upregulated genes (padj ≤ 0.05, log2FC ≥ 1):**

**Downregulated genes (padj ≤ 0.05, log2FC ≤ -1):**

#### cd4\_ev\_vs\_cd8\_s1

**Summary of DESeq2 results:**

```
out of 1180 with nonzero total read count
adjusted p-value < 0.1
LFC > 0 (up)       : 206, 17%
LFC < 0 (down)     : 228, 19%
outliers [1]       : 0, 0%
low counts [2]     : 526, 45%
(mean count < 0)
```

**Volcano plot:**

**Upregulated genes (padj ≤ 0.05, log2FC ≥ 1):**

**Downregulated genes (padj ≤ 0.05, log2FC ≤ -1):**

#### cd4\_ev\_vs\_cd4\_s1

**Summary of DESeq2 results:**

```
out of 1180 with nonzero total read count
adjusted p-value < 0.1
LFC > 0 (up)       : 249, 21%
LFC < 0 (down)     : 226, 19%
outliers [1]       : 0, 0%
low counts [2]     : 458, 39%
(mean count < 0)
```

**Volcano plot:**

**Upregulated genes (padj ≤ 0.05, log2FC ≥ 1):**

**Downregulated genes (padj ≤ 0.05, log2FC ≤ -1):**

#### cd4\_ev\_vs\_cd8\_s0

**Summary of DESeq2 results:**

```
out of 1180 with nonzero total read count
adjusted p-value < 0.1
LFC > 0 (up)       : 242, 21%
LFC < 0 (down)     : 204, 17%
outliers [1]       : 0, 0%
low counts [2]     : 526, 45%
(mean count < 0)
```

**Volcano plot:**

**Upregulated genes (padj ≤ 0.05, log2FC ≥ 1):**

**Downregulated genes (padj ≤ 0.05, log2FC ≤ -1):**

#### cd8\_ev\_vs\_cd4\_s0

**Summary of DESeq2 results:**

```
out of 1180 with nonzero total read count
adjusted p-value < 0.1
LFC > 0 (up)       : 247, 21%
LFC < 0 (down)     : 184, 16%
outliers [1]       : 0, 0%
low counts [2]     : 458, 39%
(mean count < 0)
```

**Volcano plot:**

**Upregulated genes (padj ≤ 0.05, log2FC ≥ 1):**

**Downregulated genes (padj ≤ 0.05, log2FC ≤ -1):**

#### cd8\_ev\_vs\_cd8\_s1

**Summary of DESeq2 results:**

```
out of 1180 with nonzero total read count
adjusted p-value < 0.1
LFC > 0 (up)       : 284, 24%
LFC < 0 (down)     : 191, 16%
outliers [1]       : 0, 0%
low counts [2]     : 321, 27%
(mean count < 0)
```

**Volcano plot:**

**Upregulated genes (padj ≤ 0.05, log2FC ≥ 1):**

**Downregulated genes (padj ≤ 0.05, log2FC ≤ -1):**

#### cd8\_ev\_vs\_cd4\_s1

**Summary of DESeq2 results:**

```
out of 1180 with nonzero total read count
adjusted p-value < 0.1
LFC > 0 (up)       : 306, 26%
LFC < 0 (down)     : 195, 17%
outliers [1]       : 0, 0%
low counts [2]     : 481, 41%
(mean count < 0)
```

**Volcano plot:**

**Upregulated genes (padj ≤ 0.05, log2FC ≥ 1):**

**Downregulated genes (padj ≤ 0.05, log2FC ≤ -1):**

#### cd8\_ev\_vs\_cd8\_s0

**Summary of DESeq2 results:**

```
out of 1180 with nonzero total read count
adjusted p-value < 0.1
LFC > 0 (up)       : 292, 25%
LFC < 0 (down)     : 179, 15%
outliers [1]       : 0, 0%
low counts [2]     : 435, 37%
(mean count < 0)
```

**Volcano plot:**

**Upregulated genes (padj ≤ 0.05, log2FC ≥ 1):**

**Downregulated genes (padj ≤ 0.05, log2FC ≤ -1):**

#### cd4\_s0\_vs\_cd8\_s1

**Summary of DESeq2 results:**

```
out of 1180 with nonzero total read count
adjusted p-value < 0.1
LFC > 0 (up)       : 91, 7.7%
LFC < 0 (down)     : 113, 9.6%
outliers [1]       : 0, 0%
low counts [2]     : 572, 48%
(mean count < 0)
```

**Volcano plot:**

**Upregulated genes (padj ≤ 0.05, log2FC ≥ 1):**

**Downregulated genes (padj ≤ 0.05, log2FC ≤ -1):**

#### cd4\_s0\_vs\_cd4\_s1

**Summary of DESeq2 results:**

```
out of 1180 with nonzero total read count
adjusted p-value < 0.1
LFC > 0 (up)       : 200, 17%
LFC < 0 (down)     : 149, 13%
outliers [1]       : 0, 0%
low counts [2]     : 481, 41%
(mean count < 0)
```

**Volcano plot:**

**Upregulated genes (padj ≤ 0.05, log2FC ≥ 1):**

**Downregulated genes (padj ≤ 0.05, log2FC ≤ -1):**

#### cd4\_s0\_vs\_cd8\_s0

**Summary of DESeq2 results:**

```
out of 1180 with nonzero total read count
adjusted p-value < 0.1
LFC > 0 (up)       : 163, 14%
LFC < 0 (down)     : 95, 8.1%
outliers [1]       : 0, 0%
low counts [2]     : 481, 41%
(mean count < 0)
```

**Volcano plot:**

**Upregulated genes (padj ≤ 0.05, log2FC ≥ 1):**

**Downregulated genes (padj ≤ 0.05, log2FC ≤ -1):**

#### cd8\_s1\_vs\_cd4\_s1

**Summary of DESeq2 results:**

```
out of 1180 with nonzero total read count
adjusted p-value < 0.1
LFC > 0 (up)       : 126, 11%
LFC < 0 (down)     : 113, 9.6%
outliers [1]       : 0, 0%
low counts [2]     : 549, 47%
(mean count < 0)
```

**Volcano plot:**

**Upregulated genes (padj ≤ 0.05, log2FC ≥ 1):**

**Downregulated genes (padj ≤ 0.05, log2FC ≤ -1):**

#### cd8\_s1\_vs\_cd8\_s0

**Summary of DESeq2 results:**

```
out of 1180 with nonzero total read count
adjusted p-value < 0.1
LFC > 0 (up)       : 69, 5.8%
LFC < 0 (down)     : 29, 2.5%
outliers [1]       : 0, 0%
low counts [2]     : 572, 48%
(mean count < 0)
```

**Volcano plot:**

**Upregulated genes (padj ≤ 0.05, log2FC ≥ 1):**

**Downregulated genes (padj ≤ 0.05, log2FC ≤ -1):**

#### cd4\_s1\_vs\_cd8\_s0

**Summary of DESeq2 results:**

```
out of 1180 with nonzero total read count
adjusted p-value < 0.1
LFC > 0 (up)       : 69, 5.8%
LFC < 0 (down)     : 42, 3.6%
outliers [1]       : 0, 0%
low counts [2]     : 503, 43%
(mean count < 0)
```

**Volcano plot:**

**Upregulated genes (padj ≤ 0.05, log2FC ≥ 1):**

**Downregulated genes (padj ≤ 0.05, log2FC ≤ -1):**

#### Similarities in of pairwise comparisons (UpSet Plot)

### miRNA\_readcount

#### cd4\_ev\_vs\_cd8\_ev

**Summary of DESeq2 results:**

```
out of 766 with nonzero total read count
adjusted p-value < 0.1
LFC > 0 (up)       : 38, 5%
LFC < 0 (down)     : 59, 7.7%
outliers [1]       : 0, 0%
low counts [2]     : 549, 72%
(mean count < 0)
```

**Volcano plot:**

**Upregulated genes (padj ≤ 0.05, log2FC ≥ 1):**

**Downregulated genes (padj ≤ 0.05, log2FC ≤ -1):**

#### cd4\_ev\_vs\_cd4\_s0

**Summary of DESeq2 results:**

```
out of 766 with nonzero total read count
adjusted p-value < 0.1
LFC > 0 (up)       : 43, 5.6%
LFC < 0 (down)     : 57, 7.4%
outliers [1]       : 0, 0%
low counts [2]     : 475, 62%
(mean count < 0)
```

**Volcano plot:**

**Upregulated genes (padj ≤ 0.05, log2FC ≥ 1):**

**Downregulated genes (padj ≤ 0.05, log2FC ≤ -1):**

#### cd4\_ev\_vs\_cd8\_s1

**Summary of DESeq2 results:**

```
out of 766 with nonzero total read count
adjusted p-value < 0.1
LFC > 0 (up)       : 50, 6.5%
LFC < 0 (down)     : 51, 6.7%
outliers [1]       : 0, 0%
low counts [2]     : 564, 74%
(mean count < 0)
```

**Volcano plot:**

**Upregulated genes (padj ≤ 0.05, log2FC ≥ 1):**

**Downregulated genes (padj ≤ 0.05, log2FC ≤ -1):**

#### cd4\_ev\_vs\_cd4\_s1

**Summary of DESeq2 results:**

```
out of 766 with nonzero total read count
adjusted p-value < 0.1
LFC > 0 (up)       : 48, 6.3%
LFC < 0 (down)     : 47, 6.1%
outliers [1]       : 0, 0%
low counts [2]     : 520, 68%
(mean count < 0)
```

**Volcano plot:**

**Upregulated genes (padj ≤ 0.05, log2FC ≥ 1):**

**Downregulated genes (padj ≤ 0.05, log2FC ≤ -1):**

#### cd4\_ev\_vs\_cd8\_s0

**Summary of DESeq2 results:**

```
out of 766 with nonzero total read count
adjusted p-value < 0.1
LFC > 0 (up)       : 52, 6.8%
LFC < 0 (down)     : 37, 4.8%
outliers [1]       : 0, 0%
low counts [2]     : 534, 70%
(mean count < 0)
```

**Volcano plot:**

**Upregulated genes (padj ≤ 0.05, log2FC ≥ 1):**

**Downregulated genes (padj ≤ 0.05, log2FC ≤ -1):**

#### cd8\_ev\_vs\_cd4\_s0

**Summary of DESeq2 results:**

```
out of 766 with nonzero total read count
adjusted p-value < 0.1
LFC > 0 (up)       : 56, 7.3%
LFC < 0 (down)     : 59, 7.7%
outliers [1]       : 0, 0%
low counts [2]     : 520, 68%
(mean count < 0)
```

**Volcano plot:**

**Upregulated genes (padj ≤ 0.05, log2FC ≥ 1):**

**Downregulated genes (padj ≤ 0.05, log2FC ≤ -1):**

#### cd8\_ev\_vs\_cd8\_s1

**Summary of DESeq2 results:**

```
out of 766 with nonzero total read count
adjusted p-value < 0.1
LFC > 0 (up)       : 64, 8.4%
LFC < 0 (down)     : 41, 5.4%
outliers [1]       : 0, 0%
low counts [2]     : 549, 72%
(mean count < 0)
```

**Volcano plot:**

**Upregulated genes (padj ≤ 0.05, log2FC ≥ 1):**

**Downregulated genes (padj ≤ 0.05, log2FC ≤ -1):**

#### cd8\_ev\_vs\_cd4\_s1

**Summary of DESeq2 results:**

```
out of 766 with nonzero total read count
adjusted p-value < 0.1
LFC > 0 (up)       : 62, 8.1%
LFC < 0 (down)     : 54, 7%
outliers [1]       : 0, 0%
low counts [2]     : 534, 70%
(mean count < 0)
```

**Volcano plot:**

**Upregulated genes (padj ≤ 0.05, log2FC ≥ 1):**

**Downregulated genes (padj ≤ 0.05, log2FC ≤ -1):**

#### cd8\_ev\_vs\_cd8\_s0

**Summary of DESeq2 results:**

```
out of 766 with nonzero total read count
adjusted p-value < 0.1
LFC > 0 (up)       : 71, 9.3%
LFC < 0 (down)     : 34, 4.4%
outliers [1]       : 0, 0%
low counts [2]     : 490, 64%
(mean count < 0)
```

**Volcano plot:**

**Upregulated genes (padj ≤ 0.05, log2FC ≥ 1):**

**Downregulated genes (padj ≤ 0.05, log2FC ≤ -1):**

#### cd4\_s0\_vs\_cd8\_s1

**Summary of DESeq2 results:**

```
out of 766 with nonzero total read count
adjusted p-value < 0.1
LFC > 0 (up)       : 33, 4.3%
LFC < 0 (down)     : 19, 2.5%
outliers [1]       : 0, 0%
low counts [2]     : 520, 68%
(mean count < 0)
```

**Volcano plot:**

**Upregulated genes (padj ≤ 0.05, log2FC ≥ 1):**

**Downregulated genes (padj ≤ 0.05, log2FC ≤ -1):**

#### cd4\_s0\_vs\_cd4\_s1

**Summary of DESeq2 results:**

```
out of 766 with nonzero total read count
adjusted p-value < 0.1
LFC > 0 (up)       : 41, 5.4%
LFC < 0 (down)     : 35, 4.6%
outliers [1]       : 0, 0%
low counts [2]     : 564, 74%
(mean count < 0)
```

**Volcano plot:**

**Upregulated genes (padj ≤ 0.05, log2FC ≥ 1):**

**Downregulated genes (padj ≤ 0.05, log2FC ≤ -1):**

#### cd4\_s0\_vs\_cd8\_s0

**Summary of DESeq2 results:**

```
out of 766 with nonzero total read count
adjusted p-value < 0.1
LFC > 0 (up)       : 36, 4.7%
LFC < 0 (down)     : 18, 2.3%
outliers [1]       : 0, 0%
low counts [2]     : 505, 66%
(mean count < 0)
```

**Volcano plot:**

**Upregulated genes (padj ≤ 0.05, log2FC ≥ 1):**

**Downregulated genes (padj ≤ 0.05, log2FC ≤ -1):**

#### cd8\_s1\_vs\_cd4\_s1

**Summary of DESeq2 results:**

```
out of 766 with nonzero total read count
adjusted p-value < 0.1
LFC > 0 (up)       : 33, 4.3%
LFC < 0 (down)     : 32, 4.2%
outliers [1]       : 0, 0%
low counts [2]     : 623, 81%
(mean count < 1)
```

**Volcano plot:**

**Upregulated genes (padj ≤ 0.05, log2FC ≥ 1):**

**Downregulated genes (padj ≤ 0.05, log2FC ≤ -1):**

#### cd8\_s1\_vs\_cd8\_s0

**Summary of DESeq2 results:**

```
out of 766 with nonzero total read count
adjusted p-value < 0.1
LFC > 0 (up)       : 14, 1.8%
LFC < 0 (down)     : 10, 1.3%
outliers [1]       : 0, 0%
low counts [2]     : 520, 68%
(mean count < 0)
```

**Volcano plot:**

**Upregulated genes (padj ≤ 0.05, log2FC ≥ 1):**

**Downregulated genes (padj ≤ 0.05, log2FC ≤ -1):**

#### cd4\_s1\_vs\_cd8\_s0

**Summary of DESeq2 results:**

```
out of 766 with nonzero total read count
adjusted p-value < 0.1
LFC > 0 (up)       : 12, 1.6%
LFC < 0 (down)     : 9, 1.2%
outliers [1]       : 0, 0%
low counts [2]     : 549, 72%
(mean count < 0)
```

**Volcano plot:**

**Upregulated genes (padj ≤ 0.05, log2FC ≥ 1):**

**Downregulated genes (padj ≤ 0.05, log2FC ≤ -1):**

#### Similarities in of pairwise comparisons (UpSet Plot)

### tRNA\_miRNA\_readcount

#### cd4\_ev\_vs\_cd8\_ev

**Summary of DESeq2 results:**

```
out of 815 with nonzero total read count
adjusted p-value < 0.1
LFC > 0 (up)       : 35, 4.3%
LFC < 0 (down)     : 76, 9.3%
outliers [1]       : 0, 0%
low counts [2]     : 521, 64%
(mean count < 0)
```

**Volcano plot:**

**Upregulated genes (padj ≤ 0.05, log2FC ≥ 1):**

**Downregulated genes (padj ≤ 0.05, log2FC ≤ -1):**

#### cd4\_ev\_vs\_cd4\_s0

**Summary of DESeq2 results:**

```
out of 815 with nonzero total read count
adjusted p-value < 0.1
LFC > 0 (up)       : 45, 5.5%
LFC < 0 (down)     : 105, 13%
outliers [1]       : 0, 0%
low counts [2]     : 521, 64%
(mean count < 0)
```

**Volcano plot:**

**Upregulated genes (padj ≤ 0.05, log2FC ≥ 1):**

**Downregulated genes (padj ≤ 0.05, log2FC ≤ -1):**

#### cd4\_ev\_vs\_cd8\_s1

**Summary of DESeq2 results:**

```
out of 815 with nonzero total read count
adjusted p-value < 0.1
LFC > 0 (up)       : 58, 7.1%
LFC < 0 (down)     : 94, 12%
outliers [1]       : 0, 0%
low counts [2]     : 537, 66%
(mean count < 0)
```

**Volcano plot:**

**Upregulated genes (padj ≤ 0.05, log2FC ≥ 1):**

**Downregulated genes (padj ≤ 0.05, log2FC ≤ -1):**

#### cd4\_ev\_vs\_cd4\_s1

**Summary of DESeq2 results:**

```
out of 815 with nonzero total read count
adjusted p-value < 0.1
LFC > 0 (up)       : 44, 5.4%
LFC < 0 (down)     : 118, 14%
outliers [1]       : 0, 0%
low counts [2]     : 506, 62%
(mean count < 0)
```

**Volcano plot:**

**Upregulated genes (padj ≤ 0.05, log2FC ≥ 1):**

**Downregulated genes (padj ≤ 0.05, log2FC ≤ -1):**

#### cd4\_ev\_vs\_cd8\_s0

**Summary of DESeq2 results:**

```
out of 815 with nonzero total read count
adjusted p-value < 0.1
LFC > 0 (up)       : 43, 5.3%
LFC < 0 (down)     : 68, 8.3%
outliers [1]       : 0, 0%
low counts [2]     : 537, 66%
(mean count < 0)
```

**Volcano plot:**

**Upregulated genes (padj ≤ 0.05, log2FC ≥ 1):**

**Downregulated genes (padj ≤ 0.05, log2FC ≤ -1):**

#### cd8\_ev\_vs\_cd4\_s0

**Summary of DESeq2 results:**

```
out of 815 with nonzero total read count
adjusted p-value < 0.1
LFC > 0 (up)       : 60, 7.4%
LFC < 0 (down)     : 84, 10%
outliers [1]       : 0, 0%
low counts [2]     : 506, 62%
(mean count < 0)
```

**Volcano plot:**

**Upregulated genes (padj ≤ 0.05, log2FC ≥ 1):**

**Downregulated genes (padj ≤ 0.05, log2FC ≤ -1):**

#### cd8\_ev\_vs\_cd8\_s1

**Summary of DESeq2 results:**

```
out of 815 with nonzero total read count
adjusted p-value < 0.1
LFC > 0 (up)       : 65, 8%
LFC < 0 (down)     : 62, 7.6%
outliers [1]       : 0, 0%
low counts [2]     : 474, 58%
(mean count < 0)
```

**Volcano plot:**

**Upregulated genes (padj ≤ 0.05, log2FC ≥ 1):**

**Downregulated genes (padj ≤ 0.05, log2FC ≤ -1):**

#### cd8\_ev\_vs\_cd4\_s1

**Summary of DESeq2 results:**

```
out of 815 with nonzero total read count
adjusted p-value < 0.1
LFC > 0 (up)       : 71, 8.7%
LFC < 0 (down)     : 84, 10%
outliers [1]       : 0, 0%
low counts [2]     : 506, 62%
(mean count < 0)
```

**Volcano plot:**

**Upregulated genes (padj ≤ 0.05, log2FC ≥ 1):**

**Downregulated genes (padj ≤ 0.05, log2FC ≤ -1):**

#### cd8\_ev\_vs\_cd8\_s0

**Summary of DESeq2 results:**

```
out of 815 with nonzero total read count
adjusted p-value < 0.1
LFC > 0 (up)       : 73, 9%
LFC < 0 (down)     : 52, 6.4%
outliers [1]       : 0, 0%
low counts [2]     : 427, 52%
(mean count < 0)
```

**Volcano plot:**

**Upregulated genes (padj ≤ 0.05, log2FC ≥ 1):**

**Downregulated genes (padj ≤ 0.05, log2FC ≤ -1):**

#### cd4\_s0\_vs\_cd8\_s1

**Summary of DESeq2 results:**

```
out of 815 with nonzero total read count
adjusted p-value < 0.1
LFC > 0 (up)       : 31, 3.8%
LFC < 0 (down)     : 17, 2.1%
outliers [1]       : 0, 0%
low counts [2]     : 506, 62%
(mean count < 0)
```

**Volcano plot:**

**Upregulated genes (padj ≤ 0.05, log2FC ≥ 1):**

**Downregulated genes (padj ≤ 0.05, log2FC ≤ -1):**

#### cd4\_s0\_vs\_cd4\_s1

**Summary of DESeq2 results:**

```
out of 815 with nonzero total read count
adjusted p-value < 0.1
LFC > 0 (up)       : 52, 6.4%
LFC < 0 (down)     : 59, 7.2%
outliers [1]       : 0, 0%
low counts [2]     : 569, 70%
(mean count < 0)
```

**Volcano plot:**

**Upregulated genes (padj ≤ 0.05, log2FC ≥ 1):**

**Downregulated genes (padj ≤ 0.05, log2FC ≤ -1):**

#### cd4\_s0\_vs\_cd8\_s0

**Summary of DESeq2 results:**

```
out of 815 with nonzero total read count
adjusted p-value < 0.1
LFC > 0 (up)       : 38, 4.7%
LFC < 0 (down)     : 16, 2%
outliers [1]       : 0, 0%
low counts [2]     : 490, 60%
(mean count < 0)
```

**Volcano plot:**

**Upregulated genes (padj ≤ 0.05, log2FC ≥ 1):**

**Downregulated genes (padj ≤ 0.05, log2FC ≤ -1):**

#### cd8\_s1\_vs\_cd4\_s1

**Summary of DESeq2 results:**

```
out of 815 with nonzero total read count
adjusted p-value < 0.1
LFC > 0 (up)       : 36, 4.4%
LFC < 0 (down)     : 47, 5.8%
outliers [1]       : 0, 0%
low counts [2]     : 521, 64%
(mean count < 0)
```

**Volcano plot:**

**Upregulated genes (padj ≤ 0.05, log2FC ≥ 1):**

**Downregulated genes (padj ≤ 0.05, log2FC ≤ -1):**

#### cd8\_s1\_vs\_cd8\_s0

**Summary of DESeq2 results:**

```
out of 815 with nonzero total read count
adjusted p-value < 0.1
LFC > 0 (up)       : 13, 1.6%
LFC < 0 (down)     : 8, 0.98%
outliers [1]       : 0, 0%
low counts [2]     : 553, 68%
(mean count < 0)
```

**Volcano plot:**

**Upregulated genes (padj ≤ 0.05, log2FC ≥ 1):**

**Downregulated genes (padj ≤ 0.05, log2FC ≤ -1):**

#### cd4\_s1\_vs\_cd8\_s0

**Summary of DESeq2 results:**

```
out of 815 with nonzero total read count
adjusted p-value < 0.1
LFC > 0 (up)       : 30, 3.7%
LFC < 0 (down)     : 16, 2%
outliers [1]       : 0, 0%
low counts [2]     : 442, 54%
(mean count < 0)
```

**Volcano plot:**

**Upregulated genes (padj ≤ 0.05, log2FC ≥ 1):**

**Downregulated genes (padj ≤ 0.05, log2FC ≤ -1):**

#### Similarities in of pairwise comparisons (UpSet Plot)

### isomiR\_readcount

#### cd4\_ev\_vs\_cd8\_ev

**Summary of DESeq2 results:**

```
out of 278 with nonzero total read count
adjusted p-value < 0.1
LFC > 0 (up)       : 41, 15%
LFC < 0 (down)     : 98, 35%
outliers [1]       : 0, 0%
low counts [2]     : 0, 0%
(mean count < 4)
```

**Volcano plot:**

**Upregulated genes (padj ≤ 0.05, log2FC ≥ 1):**

**Downregulated genes (padj ≤ 0.05, log2FC ≤ -1):**

#### cd4\_ev\_vs\_cd4\_s0

**Summary of DESeq2 results:**

```
out of 278 with nonzero total read count
adjusted p-value < 0.1
LFC > 0 (up)       : 136, 49%
LFC < 0 (down)     : 86, 31%
outliers [1]       : 0, 0%
low counts [2]     : 0, 0%
(mean count < 4)
```

**Volcano plot:**

**Upregulated genes (padj ≤ 0.05, log2FC ≥ 1):**

**Downregulated genes (padj ≤ 0.05, log2FC ≤ -1):**

#### cd4\_ev\_vs\_cd8\_s1

**Summary of DESeq2 results:**

```
out of 278 with nonzero total read count
adjusted p-value < 0.1
LFC > 0 (up)       : 106, 38%
LFC < 0 (down)     : 129, 46%
outliers [1]       : 0, 0%
low counts [2]     : 0, 0%
(mean count < 4)
```

**Volcano plot:**

**Upregulated genes (padj ≤ 0.05, log2FC ≥ 1):**

**Downregulated genes (padj ≤ 0.05, log2FC ≤ -1):**

#### cd4\_ev\_vs\_cd4\_s1

**Summary of DESeq2 results:**

```
out of 278 with nonzero total read count
adjusted p-value < 0.1
LFC > 0 (up)       : 114, 41%
LFC < 0 (down)     : 126, 45%
outliers [1]       : 0, 0%
low counts [2]     : 0, 0%
(mean count < 4)
```

**Volcano plot:**

**Upregulated genes (padj ≤ 0.05, log2FC ≥ 1):**

**Downregulated genes (padj ≤ 0.05, log2FC ≤ -1):**

#### cd4\_ev\_vs\_cd8\_s0

**Summary of DESeq2 results:**

```
out of 278 with nonzero total read count
adjusted p-value < 0.1
LFC > 0 (up)       : 104, 37%
LFC < 0 (down)     : 121, 44%
outliers [1]       : 0, 0%
low counts [2]     : 0, 0%
(mean count < 4)
```

**Volcano plot:**

**Upregulated genes (padj ≤ 0.05, log2FC ≥ 1):**

**Downregulated genes (padj ≤ 0.05, log2FC ≤ -1):**

#### cd8\_ev\_vs\_cd4\_s0

**Summary of DESeq2 results:**

```
out of 278 with nonzero total read count
adjusted p-value < 0.1
LFC > 0 (up)       : 156, 56%
LFC < 0 (down)     : 75, 27%
outliers [1]       : 0, 0%
low counts [2]     : 0, 0%
(mean count < 4)
```

**Volcano plot:**

**Upregulated genes (padj ≤ 0.05, log2FC ≥ 1):**

**Downregulated genes (padj ≤ 0.05, log2FC ≤ -1):**

#### cd8\_ev\_vs\_cd8\_s1

**Summary of DESeq2 results:**

```
out of 278 with nonzero total read count
adjusted p-value < 0.1
LFC > 0 (up)       : 131, 47%
LFC < 0 (down)     : 112, 40%
outliers [1]       : 0, 0%
low counts [2]     : 0, 0%
(mean count < 4)
```

**Volcano plot:**

**Upregulated genes (padj ≤ 0.05, log2FC ≥ 1):**

**Downregulated genes (padj ≤ 0.05, log2FC ≤ -1):**

#### cd8\_ev\_vs\_cd4\_s1

**Summary of DESeq2 results:**

```
out of 278 with nonzero total read count
adjusted p-value < 0.1
LFC > 0 (up)       : 129, 46%
LFC < 0 (down)     : 119, 43%
outliers [1]       : 0, 0%
low counts [2]     : 0, 0%
(mean count < 4)
```

**Volcano plot:**

**Upregulated genes (padj ≤ 0.05, log2FC ≥ 1):**

**Downregulated genes (padj ≤ 0.05, log2FC ≤ -1):**

#### cd8\_ev\_vs\_cd8\_s0

**Summary of DESeq2 results:**

```
out of 278 with nonzero total read count
adjusted p-value < 0.1
LFC > 0 (up)       : 129, 46%
LFC < 0 (down)     : 114, 41%
outliers [1]       : 0, 0%
low counts [2]     : 0, 0%
(mean count < 4)
```

**Volcano plot:**

**Upregulated genes (padj ≤ 0.05, log2FC ≥ 1):**

**Downregulated genes (padj ≤ 0.05, log2FC ≤ -1):**

#### cd4\_s0\_vs\_cd8\_s1

**Summary of DESeq2 results:**

```
out of 278 with nonzero total read count
adjusted p-value < 0.1
LFC > 0 (up)       : 38, 14%
LFC < 0 (down)     : 148, 53%
outliers [1]       : 0, 0%
low counts [2]     : 0, 0%
(mean count < 4)
```

**Volcano plot:**

**Upregulated genes (padj ≤ 0.05, log2FC ≥ 1):**

**Downregulated genes (padj ≤ 0.05, log2FC ≤ -1):**

#### cd4\_s0\_vs\_cd4\_s1

**Summary of DESeq2 results:**

```
out of 278 with nonzero total read count
adjusted p-value < 0.1
LFC > 0 (up)       : 57, 21%
LFC < 0 (down)     : 176, 63%
outliers [1]       : 0, 0%
low counts [2]     : 0, 0%
(mean count < 4)
```

**Volcano plot:**

**Upregulated genes (padj ≤ 0.05, log2FC ≥ 1):**

**Downregulated genes (padj ≤ 0.05, log2FC ≤ -1):**

#### cd4\_s0\_vs\_cd8\_s0

**Summary of DESeq2 results:**

```
out of 278 with nonzero total read count
adjusted p-value < 0.1
LFC > 0 (up)       : 38, 14%
LFC < 0 (down)     : 146, 53%
outliers [1]       : 0, 0%
low counts [2]     : 0, 0%
(mean count < 4)
```

**Volcano plot:**

**Upregulated genes (padj ≤ 0.05, log2FC ≥ 1):**

**Downregulated genes (padj ≤ 0.05, log2FC ≤ -1):**

#### cd8\_s1\_vs\_cd4\_s1

**Summary of DESeq2 results:**

```
out of 278 with nonzero total read count
adjusted p-value < 0.1
LFC > 0 (up)       : 44, 16%
LFC < 0 (down)     : 115, 41%
outliers [1]       : 0, 0%
low counts [2]     : 0, 0%
(mean count < 4)
```

**Volcano plot:**

**Upregulated genes (padj ≤ 0.05, log2FC ≥ 1):**

**Downregulated genes (padj ≤ 0.05, log2FC ≤ -1):**

#### cd8\_s1\_vs\_cd8\_s0

**Summary of DESeq2 results:**

```
out of 278 with nonzero total read count
adjusted p-value < 0.1
LFC > 0 (up)       : 15, 5.4%
LFC < 0 (down)     : 7, 2.5%
outliers [1]       : 0, 0%
low counts [2]     : 0, 0%
(mean count < 4)
```

**Volcano plot:**

**Upregulated genes (padj ≤ 0.05, log2FC ≥ 1):**

**Downregulated genes (padj ≤ 0.05, log2FC ≤ -1):**

#### cd4\_s1\_vs\_cd8\_s0

**Summary of DESeq2 results:**

```
out of 278 with nonzero total read count
adjusted p-value < 0.1
LFC > 0 (up)       : 37, 13%
LFC < 0 (down)     : 20, 7.2%
outliers [1]       : 0, 0%
low counts [2]     : 6, 2.2%
(mean count < 6)
```

**Volcano plot:**

**Upregulated genes (padj ≤ 0.05, log2FC ≥ 1):**

**Downregulated genes (padj ≤ 0.05, log2FC ≤ -1):**

#### Similarities in of pairwise comparisons (UpSet Plot)

### tRF\_readcount

#### cd4\_ev\_vs\_cd8\_ev

**Summary of DESeq2 results:**

```
out of 115 with nonzero total read count
adjusted p-value < 0.1
LFC > 0 (up)       : 40, 35%
LFC < 0 (down)     : 12, 10%
outliers [1]       : 0, 0%
low counts [2]     : 0, 0%
(mean count < 2)
```

**Volcano plot:**

**Upregulated genes (padj ≤ 0.05, log2FC ≥ 1):**

**Downregulated genes (padj ≤ 0.05, log2FC ≤ -1):**

#### cd4\_ev\_vs\_cd4\_s0

**Summary of DESeq2 results:**

```
out of 115 with nonzero total read count
adjusted p-value < 0.1
LFC > 0 (up)       : 61, 53%
LFC < 0 (down)     : 34, 30%
outliers [1]       : 0, 0%
low counts [2]     : 0, 0%
(mean count < 2)
```

**Volcano plot:**

**Upregulated genes (padj ≤ 0.05, log2FC ≥ 1):**

**Downregulated genes (padj ≤ 0.05, log2FC ≤ -1):**

#### cd4\_ev\_vs\_cd8\_s1

**Summary of DESeq2 results:**

```
out of 115 with nonzero total read count
adjusted p-value < 0.1
LFC > 0 (up)       : 70, 61%
LFC < 0 (down)     : 28, 24%
outliers [1]       : 0, 0%
low counts [2]     : 0, 0%
(mean count < 2)
```

**Volcano plot:**

**Upregulated genes (padj ≤ 0.05, log2FC ≥ 1):**

**Downregulated genes (padj ≤ 0.05, log2FC ≤ -1):**

#### cd4\_ev\_vs\_cd4\_s1

**Summary of DESeq2 results:**

```
out of 115 with nonzero total read count
adjusted p-value < 0.1
LFC > 0 (up)       : 77, 67%
LFC < 0 (down)     : 26, 23%
outliers [1]       : 0, 0%
low counts [2]     : 0, 0%
(mean count < 2)
```

**Volcano plot:**

**Upregulated genes (padj ≤ 0.05, log2FC ≥ 1):**

**Downregulated genes (padj ≤ 0.05, log2FC ≤ -1):**

#### cd4\_ev\_vs\_cd8\_s0

**Summary of DESeq2 results:**

```
out of 115 with nonzero total read count
adjusted p-value < 0.1
LFC > 0 (up)       : 77, 67%
LFC < 0 (down)     : 18, 16%
outliers [1]       : 0, 0%
low counts [2]     : 0, 0%
(mean count < 2)
```

**Volcano plot:**

**Upregulated genes (padj ≤ 0.05, log2FC ≥ 1):**

**Downregulated genes (padj ≤ 0.05, log2FC ≤ -1):**

#### cd8\_ev\_vs\_cd4\_s0

**Summary of DESeq2 results:**

```
out of 115 with nonzero total read count
adjusted p-value < 0.1
LFC > 0 (up)       : 61, 53%
LFC < 0 (down)     : 38, 33%
outliers [1]       : 0, 0%
low counts [2]     : 0, 0%
(mean count < 2)
```

**Volcano plot:**

**Upregulated genes (padj ≤ 0.05, log2FC ≥ 1):**

**Downregulated genes (padj ≤ 0.05, log2FC ≤ -1):**

#### cd8\_ev\_vs\_cd8\_s1

**Summary of DESeq2 results:**

```
out of 115 with nonzero total read count
adjusted p-value < 0.1
LFC > 0 (up)       : 68, 59%
LFC < 0 (down)     : 33, 29%
outliers [1]       : 0, 0%
low counts [2]     : 0, 0%
(mean count < 2)
```

**Volcano plot:**

**Upregulated genes (padj ≤ 0.05, log2FC ≥ 1):**

**Downregulated genes (padj ≤ 0.05, log2FC ≤ -1):**

#### cd8\_ev\_vs\_cd4\_s1

**Summary of DESeq2 results:**

```
out of 115 with nonzero total read count
adjusted p-value < 0.1
LFC > 0 (up)       : 70, 61%
LFC < 0 (down)     : 30, 26%
outliers [1]       : 0, 0%
low counts [2]     : 0, 0%
(mean count < 2)
```

**Volcano plot:**

**Upregulated genes (padj ≤ 0.05, log2FC ≥ 1):**

**Downregulated genes (padj ≤ 0.05, log2FC ≤ -1):**

#### cd8\_ev\_vs\_cd8\_s0

**Summary of DESeq2 results:**

```
out of 115 with nonzero total read count
adjusted p-value < 0.1
LFC > 0 (up)       : 74, 64%
LFC < 0 (down)     : 23, 20%
outliers [1]       : 0, 0%
low counts [2]     : 0, 0%
(mean count < 2)
```

**Volcano plot:**

**Upregulated genes (padj ≤ 0.05, log2FC ≥ 1):**

**Downregulated genes (padj ≤ 0.05, log2FC ≤ -1):**

#### cd4\_s0\_vs\_cd8\_s1

**Summary of DESeq2 results:**

```
out of 115 with nonzero total read count
adjusted p-value < 0.1
LFC > 0 (up)       : 44, 38%
LFC < 0 (down)     : 10, 8.7%
outliers [1]       : 0, 0%
low counts [2]     : 3, 2.6%
(mean count < 2)
```

**Volcano plot:**

**Upregulated genes (padj ≤ 0.05, log2FC ≥ 1):**

**Downregulated genes (padj ≤ 0.05, log2FC ≤ -1):**

#### cd4\_s0\_vs\_cd4\_s1

**Summary of DESeq2 results:**

```
out of 115 with nonzero total read count
adjusted p-value < 0.1
LFC > 0 (up)       : 58, 50%
LFC < 0 (down)     : 14, 12%
outliers [1]       : 0, 0%
low counts [2]     : 0, 0%
(mean count < 2)
```

**Volcano plot:**

**Upregulated genes (padj ≤ 0.05, log2FC ≥ 1):**

**Downregulated genes (padj ≤ 0.05, log2FC ≤ -1):**

#### cd4\_s0\_vs\_cd8\_s0

**Summary of DESeq2 results:**

```
out of 115 with nonzero total read count
adjusted p-value < 0.1
LFC > 0 (up)       : 63, 55%
LFC < 0 (down)     : 8, 7%
outliers [1]       : 0, 0%
low counts [2]     : 0, 0%
(mean count < 2)
```

**Volcano plot:**

**Upregulated genes (padj ≤ 0.05, log2FC ≥ 1):**

**Downregulated genes (padj ≤ 0.05, log2FC ≤ -1):**

#### cd8\_s1\_vs\_cd4\_s1

**Summary of DESeq2 results:**

```
out of 115 with nonzero total read count
adjusted p-value < 0.1
LFC > 0 (up)       : 23, 20%
LFC < 0 (down)     : 6, 5.2%
outliers [1]       : 0, 0%
low counts [2]     : 0, 0%
(mean count < 2)
```

**Volcano plot:**

**Upregulated genes (padj ≤ 0.05, log2FC ≥ 1):**

**Downregulated genes (padj ≤ 0.05, log2FC ≤ -1):**

#### cd8\_s1\_vs\_cd8\_s0

**Summary of DESeq2 results:**

```
out of 115 with nonzero total read count
adjusted p-value < 0.1
LFC > 0 (up)       : 43, 37%
LFC < 0 (down)     : 1, 0.87%
outliers [1]       : 0, 0%
low counts [2]     : 0, 0%
(mean count < 2)
```

**Volcano plot:**

**Upregulated genes (padj ≤ 0.05, log2FC ≥ 1):**

**Downregulated genes (padj ≤ 0.05, log2FC ≤ -1):**

#### cd4\_s1\_vs\_cd8\_s0

**Summary of DESeq2 results:**

```
out of 115 with nonzero total read count
adjusted p-value < 0.1
LFC > 0 (up)       : 27, 23%
LFC < 0 (down)     : 8, 7%
outliers [1]       : 0, 0%
low counts [2]     : 0, 0%
(mean count < 2)
```

**Volcano plot:**

**Upregulated genes (padj ≤ 0.05, log2FC ≥ 1):**

**Downregulated genes (padj ≤ 0.05, log2FC ≤ -1):**

#### Similarities in of pairwise comparisons (UpSet Plot)

### tRF\_isomiR\_readcount

#### cd4\_ev\_vs\_cd8\_ev

**Summary of DESeq2 results:**

```
out of 393 with nonzero total read count
adjusted p-value < 0.1
LFC > 0 (up)       : 84, 21%
LFC < 0 (down)     : 99, 25%
outliers [1]       : 0, 0%
low counts [2]     : 0, 0%
(mean count < 3)
```

**Volcano plot:**

**Upregulated genes (padj ≤ 0.05, log2FC ≥ 1):**

**Downregulated genes (padj ≤ 0.05, log2FC ≤ -1):**

#### cd4\_ev\_vs\_cd4\_s0

**Summary of DESeq2 results:**

```
out of 393 with nonzero total read count
adjusted p-value < 0.1
LFC > 0 (up)       : 199, 51%
LFC < 0 (down)     : 127, 32%
outliers [1]       : 0, 0%
low counts [2]     : 0, 0%
(mean count < 3)
```

**Volcano plot:**

**Upregulated genes (padj ≤ 0.05, log2FC ≥ 1):**

**Downregulated genes (padj ≤ 0.05, log2FC ≤ -1):**

#### cd4\_ev\_vs\_cd8\_s1

**Summary of DESeq2 results:**

```
out of 393 with nonzero total read count
adjusted p-value < 0.1
LFC > 0 (up)       : 164, 42%
LFC < 0 (down)     : 167, 42%
outliers [1]       : 0, 0%
low counts [2]     : 0, 0%
(mean count < 3)
```

**Volcano plot:**

**Upregulated genes (padj ≤ 0.05, log2FC ≥ 1):**

**Downregulated genes (padj ≤ 0.05, log2FC ≤ -1):**

#### cd4\_ev\_vs\_cd4\_s1

**Summary of DESeq2 results:**

```
out of 393 with nonzero total read count
adjusted p-value < 0.1
LFC > 0 (up)       : 177, 45%
LFC < 0 (down)     : 150, 38%
outliers [1]       : 0, 0%
low counts [2]     : 0, 0%
(mean count < 3)
```

**Volcano plot:**

**Upregulated genes (padj ≤ 0.05, log2FC ≥ 1):**

**Downregulated genes (padj ≤ 0.05, log2FC ≤ -1):**

#### cd4\_ev\_vs\_cd8\_s0

**Summary of DESeq2 results:**

```
out of 393 with nonzero total read count
adjusted p-value < 0.1
LFC > 0 (up)       : 180, 46%
LFC < 0 (down)     : 143, 36%
outliers [1]       : 0, 0%
low counts [2]     : 0, 0%
(mean count < 3)
```

**Volcano plot:**

**Upregulated genes (padj ≤ 0.05, log2FC ≥ 1):**

**Downregulated genes (padj ≤ 0.05, log2FC ≤ -1):**

#### cd8\_ev\_vs\_cd4\_s0

**Summary of DESeq2 results:**

```
out of 393 with nonzero total read count
adjusted p-value < 0.1
LFC > 0 (up)       : 207, 53%
LFC < 0 (down)     : 122, 31%
outliers [1]       : 0, 0%
low counts [2]     : 0, 0%
(mean count < 3)
```

**Volcano plot:**

**Upregulated genes (padj ≤ 0.05, log2FC ≥ 1):**

**Downregulated genes (padj ≤ 0.05, log2FC ≤ -1):**

#### cd8\_ev\_vs\_cd8\_s1

**Summary of DESeq2 results:**

```
out of 393 with nonzero total read count
adjusted p-value < 0.1
LFC > 0 (up)       : 169, 43%
LFC < 0 (down)     : 148, 38%
outliers [1]       : 0, 0%
low counts [2]     : 0, 0%
(mean count < 3)
```

**Volcano plot:**

**Upregulated genes (padj ≤ 0.05, log2FC ≥ 1):**

**Downregulated genes (padj ≤ 0.05, log2FC ≤ -1):**

#### cd8\_ev\_vs\_cd4\_s1

**Summary of DESeq2 results:**

```
out of 393 with nonzero total read count
adjusted p-value < 0.1
LFC > 0 (up)       : 202, 51%
LFC < 0 (down)     : 148, 38%
outliers [1]       : 0, 0%
low counts [2]     : 0, 0%
(mean count < 3)
```

**Volcano plot:**

**Upregulated genes (padj ≤ 0.05, log2FC ≥ 1):**

**Downregulated genes (padj ≤ 0.05, log2FC ≤ -1):**

#### cd8\_ev\_vs\_cd8\_s0

**Summary of DESeq2 results:**

```
out of 393 with nonzero total read count
adjusted p-value < 0.1
LFC > 0 (up)       : 190, 48%
LFC < 0 (down)     : 129, 33%
outliers [1]       : 0, 0%
low counts [2]     : 0, 0%
(mean count < 3)
```

**Volcano plot:**

**Upregulated genes (padj ≤ 0.05, log2FC ≥ 1):**

**Downregulated genes (padj ≤ 0.05, log2FC ≤ -1):**

#### cd4\_s0\_vs\_cd8\_s1

**Summary of DESeq2 results:**

```
out of 393 with nonzero total read count
adjusted p-value < 0.1
LFC > 0 (up)       : 74, 19%
LFC < 0 (down)     : 155, 39%
outliers [1]       : 0, 0%
low counts [2]     : 0, 0%
(mean count < 3)
```

**Volcano plot:**

**Upregulated genes (padj ≤ 0.05, log2FC ≥ 1):**

**Downregulated genes (padj ≤ 0.05, log2FC ≤ -1):**

#### cd4\_s0\_vs\_cd4\_s1

**Summary of DESeq2 results:**

```
out of 393 with nonzero total read count
adjusted p-value < 0.1
LFC > 0 (up)       : 125, 32%
LFC < 0 (down)     : 183, 47%
outliers [1]       : 0, 0%
low counts [2]     : 0, 0%
(mean count < 3)
```

**Volcano plot:**

**Upregulated genes (padj ≤ 0.05, log2FC ≥ 1):**

**Downregulated genes (padj ≤ 0.05, log2FC ≤ -1):**

#### cd4\_s0\_vs\_cd8\_s0

**Summary of DESeq2 results:**

```
out of 393 with nonzero total read count
adjusted p-value < 0.1
LFC > 0 (up)       : 102, 26%
LFC < 0 (down)     : 147, 37%
outliers [1]       : 0, 0%
low counts [2]     : 16, 4.1%
(mean count < 5)
```

**Volcano plot:**

**Upregulated genes (padj ≤ 0.05, log2FC ≥ 1):**

**Downregulated genes (padj ≤ 0.05, log2FC ≤ -1):**

#### cd8\_s1\_vs\_cd4\_s1

**Summary of DESeq2 results:**

```
out of 393 with nonzero total read count
adjusted p-value < 0.1
LFC > 0 (up)       : 97, 25%
LFC < 0 (down)     : 125, 32%
outliers [1]       : 0, 0%
low counts [2]     : 0, 0%
(mean count < 3)
```

**Volcano plot:**

**Upregulated genes (padj ≤ 0.05, log2FC ≥ 1):**

**Downregulated genes (padj ≤ 0.05, log2FC ≤ -1):**

#### cd8\_s1\_vs\_cd8\_s0

**Summary of DESeq2 results:**

```
out of 393 with nonzero total read count
adjusted p-value < 0.1
LFC > 0 (up)       : 49, 12%
LFC < 0 (down)     : 8, 2%
outliers [1]       : 0, 0%
low counts [2]     : 0, 0%
(mean count < 3)
```

**Volcano plot:**

**Upregulated genes (padj ≤ 0.05, log2FC ≥ 1):**

**Downregulated genes (padj ≤ 0.05, log2FC ≤ -1):**

#### cd4\_s1\_vs\_cd8\_s0

**Summary of DESeq2 results:**

```
out of 393 with nonzero total read count
adjusted p-value < 0.1
LFC > 0 (up)       : 82, 21%
LFC < 0 (down)     : 37, 9.4%
outliers [1]       : 0, 0%
low counts [2]     : 0, 0%
(mean count < 3)
```

**Volcano plot:**

**Upregulated genes (padj ≤ 0.05, log2FC ≥ 1):**

**Downregulated genes (padj ≤ 0.05, log2FC ≤ -1):**

#### Similarities in of pairwise comparisons (UpSet Plot)

### custom\_analysis\_readcount

#### cd4\_ev\_vs\_cd8\_ev

**Summary of DESeq2 results:**

```
out of 766 with nonzero total read count
adjusted p-value < 0.1
LFC > 0 (up)       : 38, 5%
LFC < 0 (down)     : 59, 7.7%
outliers [1]       : 0, 0%
low counts [2]     : 549, 72%
(mean count < 0)
```

**Volcano plot:**

**Upregulated genes (padj ≤ 0.05, log2FC ≥ 1):**

**Downregulated genes (padj ≤ 0.05, log2FC ≤ -1):**

#### cd4\_ev\_vs\_cd4\_s0

**Summary of DESeq2 results:**

```
out of 766 with nonzero total read count
adjusted p-value < 0.1
LFC > 0 (up)       : 43, 5.6%
LFC < 0 (down)     : 57, 7.4%
outliers [1]       : 0, 0%
low counts [2]     : 475, 62%
(mean count < 0)
```

**Volcano plot:**

**Upregulated genes (padj ≤ 0.05, log2FC ≥ 1):**

**Downregulated genes (padj ≤ 0.05, log2FC ≤ -1):**

#### cd4\_ev\_vs\_cd8\_s1

**Summary of DESeq2 results:**

```
out of 766 with nonzero total read count
adjusted p-value < 0.1
LFC > 0 (up)       : 50, 6.5%
LFC < 0 (down)     : 51, 6.7%
outliers [1]       : 0, 0%
low counts [2]     : 564, 74%
(mean count < 0)
```

**Volcano plot:**

**Upregulated genes (padj ≤ 0.05, log2FC ≥ 1):**

**Downregulated genes (padj ≤ 0.05, log2FC ≤ -1):**

#### cd4\_ev\_vs\_cd4\_s1

**Summary of DESeq2 results:**

```
out of 766 with nonzero total read count
adjusted p-value < 0.1
LFC > 0 (up)       : 48, 6.3%
LFC < 0 (down)     : 47, 6.1%
outliers [1]       : 0, 0%
low counts [2]     : 520, 68%
(mean count < 0)
```

**Volcano plot:**

**Upregulated genes (padj ≤ 0.05, log2FC ≥ 1):**

**Downregulated genes (padj ≤ 0.05, log2FC ≤ -1):**

#### cd4\_ev\_vs\_cd8\_s0

**Summary of DESeq2 results:**

```
out of 766 with nonzero total read count
adjusted p-value < 0.1
LFC > 0 (up)       : 52, 6.8%
LFC < 0 (down)     : 37, 4.8%
outliers [1]       : 0, 0%
low counts [2]     : 534, 70%
(mean count < 0)
```

**Volcano plot:**

**Upregulated genes (padj ≤ 0.05, log2FC ≥ 1):**

**Downregulated genes (padj ≤ 0.05, log2FC ≤ -1):**

#### cd8\_ev\_vs\_cd4\_s0

**Summary of DESeq2 results:**

```
out of 766 with nonzero total read count
adjusted p-value < 0.1
LFC > 0 (up)       : 56, 7.3%
LFC < 0 (down)     : 59, 7.7%
outliers [1]       : 0, 0%
low counts [2]     : 520, 68%
(mean count < 0)
```

**Volcano plot:**

**Upregulated genes (padj ≤ 0.05, log2FC ≥ 1):**

**Downregulated genes (padj ≤ 0.05, log2FC ≤ -1):**

#### cd8\_ev\_vs\_cd8\_s1

**Summary of DESeq2 results:**

```
out of 766 with nonzero total read count
adjusted p-value < 0.1
LFC > 0 (up)       : 64, 8.4%
LFC < 0 (down)     : 41, 5.4%
outliers [1]       : 0, 0%
low counts [2]     : 549, 72%
(mean count < 0)
```

**Volcano plot:**

**Upregulated genes (padj ≤ 0.05, log2FC ≥ 1):**

**Downregulated genes (padj ≤ 0.05, log2FC ≤ -1):**

#### cd8\_ev\_vs\_cd4\_s1

**Summary of DESeq2 results:**

```
out of 766 with nonzero total read count
adjusted p-value < 0.1
LFC > 0 (up)       : 62, 8.1%
LFC < 0 (down)     : 54, 7%
outliers [1]       : 0, 0%
low counts [2]     : 534, 70%
(mean count < 0)
```

**Volcano plot:**

**Upregulated genes (padj ≤ 0.05, log2FC ≥ 1):**

**Downregulated genes (padj ≤ 0.05, log2FC ≤ -1):**

#### cd8\_ev\_vs\_cd8\_s0

**Summary of DESeq2 results:**

```
out of 766 with nonzero total read count
adjusted p-value < 0.1
LFC > 0 (up)       : 71, 9.3%
LFC < 0 (down)     : 34, 4.4%
outliers [1]       : 0, 0%
low counts [2]     : 490, 64%
(mean count < 0)
```

**Volcano plot:**

**Upregulated genes (padj ≤ 0.05, log2FC ≥ 1):**

**Downregulated genes (padj ≤ 0.05, log2FC ≤ -1):**

#### cd4\_s0\_vs\_cd8\_s1

**Summary of DESeq2 results:**

```
out of 766 with nonzero total read count
adjusted p-value < 0.1
LFC > 0 (up)       : 33, 4.3%
LFC < 0 (down)     : 19, 2.5%
outliers [1]       : 0, 0%
low counts [2]     : 520, 68%
(mean count < 0)
```

**Volcano plot:**

**Upregulated genes (padj ≤ 0.05, log2FC ≥ 1):**

**Downregulated genes (padj ≤ 0.05, log2FC ≤ -1):**

#### cd4\_s0\_vs\_cd4\_s1

**Summary of DESeq2 results:**

```
out of 766 with nonzero total read count
adjusted p-value < 0.1
LFC > 0 (up)       : 41, 5.4%
LFC < 0 (down)     : 35, 4.6%
outliers [1]       : 0, 0%
low counts [2]     : 564, 74%
(mean count < 0)
```

**Volcano plot:**

**Upregulated genes (padj ≤ 0.05, log2FC ≥ 1):**

**Downregulated genes (padj ≤ 0.05, log2FC ≤ -1):**

#### cd4\_s0\_vs\_cd8\_s0

**Summary of DESeq2 results:**

```
out of 766 with nonzero total read count
adjusted p-value < 0.1
LFC > 0 (up)       : 36, 4.7%
LFC < 0 (down)     : 18, 2.3%
outliers [1]       : 0, 0%
low counts [2]     : 505, 66%
(mean count < 0)
```

**Volcano plot:**

**Upregulated genes (padj ≤ 0.05, log2FC ≥ 1):**

**Downregulated genes (padj ≤ 0.05, log2FC ≤ -1):**

#### cd8\_s1\_vs\_cd4\_s1

**Summary of DESeq2 results:**

```
out of 766 with nonzero total read count
adjusted p-value < 0.1
LFC > 0 (up)       : 33, 4.3%
LFC < 0 (down)     : 32, 4.2%
outliers [1]       : 0, 0%
low counts [2]     : 623, 81%
(mean count < 1)
```

**Volcano plot:**

**Upregulated genes (padj ≤ 0.05, log2FC ≥ 1):**

**Downregulated genes (padj ≤ 0.05, log2FC ≤ -1):**

#### cd8\_s1\_vs\_cd8\_s0

**Summary of DESeq2 results:**

```
out of 766 with nonzero total read count
adjusted p-value < 0.1
LFC > 0 (up)       : 14, 1.8%
LFC < 0 (down)     : 10, 1.3%
outliers [1]       : 0, 0%
low counts [2]     : 520, 68%
(mean count < 0)
```

**Volcano plot:**

**Upregulated genes (padj ≤ 0.05, log2FC ≥ 1):**

**Downregulated genes (padj ≤ 0.05, log2FC ≤ -1):**

#### cd4\_s1\_vs\_cd8\_s0

**Summary of DESeq2 results:**

```
out of 766 with nonzero total read count
adjusted p-value < 0.1
LFC > 0 (up)       : 12, 1.6%
LFC < 0 (down)     : 9, 1.2%
outliers [1]       : 0, 0%
low counts [2]     : 549, 72%
(mean count < 0)
```

**Volcano plot:**

**Upregulated genes (padj ≤ 0.05, log2FC ≥ 1):**

**Downregulated genes (padj ≤ 0.05, log2FC ≤ -1):**

#### Similarities in of pairwise comparisons (UpSet Plot)

## Supervised clustering (sPLS-DA)

Sparse Partial Least Squares Discriminant Analysis (sPLS-DA) is a
supervised multivariate classification method designed to identify the
minimal set of variables (here: genes) that best discriminate between
predefined experimental groups. Unlike unsupervised techniques such as
PCA, which only capture the major axes of variance in the data, sPLS-DA
explicitly incorporates sample class labels during model training. This
allows the method to focus on variation that is predictive of group
membership, which is particularly advantageous in high-dimensional
RNA-seq datasets where the number of genes greatly exceeds the number of
samples.

Important note on overfitting:   
 Because sPLS-DA is a supervised
method, it is highly susceptible to overfitting, especially when the
number of samples is small, the number of variables (genes) is very
large, the same dataset is used for both training and evaluation and
tuning parameters are optimized on the same data used to assess
performance. In this analysis, the ROC and AUC values are computed from
the training data only, not from an independent validation dataset. The
AUC values represent the model’s performance on the data it was trained
on and should not be interpreted as unbiased estimates of predictive
ability. They often overestimate true performance. To reduce this risk,
cross-validation was performed, so results are more reliable but still
subject to optimistic bias unless a completely independent test set is
used.

### sPLS-DA tuning choices

| analysis | contrast | component | keepX | validation | folds | nrepeat | distance\_metric |
| --- | --- | --- | --- | --- | --- | --- | --- |
| total\_readcount | cd4\_ev\_vs\_cd8\_ev | 1 | 10 | Mfold | 5 | 100 | centroids.dist |
| total\_readcount | cd4\_ev\_vs\_cd8\_ev | 2 | 20 | Mfold | 5 | 100 | centroids.dist |
| total\_readcount | cd4\_ev\_vs\_cd8\_ev | 3 | 10 | Mfold | 5 | 100 | centroids.dist |
| total\_readcount | cd4\_ev\_vs\_cd4\_s0 | 1 | 5 | Mfold | 5 | 100 | centroids.dist |
| total\_readcount | cd4\_ev\_vs\_cd4\_s0 | 2 | 5 | Mfold | 5 | 100 | centroids.dist |
| total\_readcount | cd4\_ev\_vs\_cd4\_s0 | 3 | 5 | Mfold | 5 | 100 | centroids.dist |
| total\_readcount | cd4\_ev\_vs\_cd8\_s1 | 1 | 5 | Mfold | 5 | 100 | centroids.dist |
| total\_readcount | cd4\_ev\_vs\_cd8\_s1 | 2 | 5 | Mfold | 5 | 100 | centroids.dist |
| total\_readcount | cd4\_ev\_vs\_cd8\_s1 | 3 | 5 | Mfold | 5 | 100 | centroids.dist |
| total\_readcount | cd4\_ev\_vs\_cd4\_s1 | 1 | 5 | Mfold | 5 | 100 | centroids.dist |
| total\_readcount | cd4\_ev\_vs\_cd4\_s1 | 2 | 5 | Mfold | 5 | 100 | centroids.dist |
| total\_readcount | cd4\_ev\_vs\_cd4\_s1 | 3 | 5 | Mfold | 5 | 100 | centroids.dist |
| total\_readcount | cd4\_ev\_vs\_cd8\_s0 | 1 | 5 | Mfold | 5 | 100 | centroids.dist |
| total\_readcount | cd4\_ev\_vs\_cd8\_s0 | 2 | 5 | Mfold | 5 | 100 | centroids.dist |
| total\_readcount | cd4\_ev\_vs\_cd8\_s0 | 3 | 5 | Mfold | 5 | 100 | centroids.dist |
| total\_readcount | cd8\_ev\_vs\_cd4\_s0 | 1 | 5 | Mfold | 5 | 100 | centroids.dist |
| total\_readcount | cd8\_ev\_vs\_cd4\_s0 | 2 | 5 | Mfold | 5 | 100 | centroids.dist |
| total\_readcount | cd8\_ev\_vs\_cd4\_s0 | 3 | 5 | Mfold | 5 | 100 | centroids.dist |
| total\_readcount | cd8\_ev\_vs\_cd8\_s1 | 1 | 5 | Mfold | 5 | 100 | centroids.dist |
| total\_readcount | cd8\_ev\_vs\_cd8\_s1 | 2 | 5 | Mfold | 5 | 100 | centroids.dist |
| total\_readcount | cd8\_ev\_vs\_cd8\_s1 | 3 | 5 | Mfold | 5 | 100 | centroids.dist |
| total\_readcount | cd8\_ev\_vs\_cd4\_s1 | 1 | 5 | Mfold | 5 | 100 | centroids.dist |
| total\_readcount | cd8\_ev\_vs\_cd4\_s1 | 2 | 5 | Mfold | 5 | 100 | centroids.dist |
| total\_readcount | cd8\_ev\_vs\_cd4\_s1 | 3 | 5 | Mfold | 5 | 100 | centroids.dist |
| total\_readcount | cd8\_ev\_vs\_cd8\_s0 | 1 | 5 | Mfold | 5 | 100 | centroids.dist |
| total\_readcount | cd8\_ev\_vs\_cd8\_s0 | 2 | 5 | Mfold | 5 | 100 | centroids.dist |
| total\_readcount | cd8\_ev\_vs\_cd8\_s0 | 3 | 5 | Mfold | 5 | 100 | centroids.dist |
| total\_readcount | cd4\_s0\_vs\_cd8\_s1 | 1 | 5 | Mfold | 5 | 100 | centroids.dist |
| total\_readcount | cd4\_s0\_vs\_cd8\_s1 | 2 | 5 | Mfold | 5 | 100 | centroids.dist |
| total\_readcount | cd4\_s0\_vs\_cd8\_s1 | 3 | 5 | Mfold | 5 | 100 | centroids.dist |
| total\_readcount | cd4\_s0\_vs\_cd4\_s1 | 1 | 5 | Mfold | 5 | 100 | centroids.dist |
| total\_readcount | cd4\_s0\_vs\_cd4\_s1 | 2 | 5 | Mfold | 5 | 100 | centroids.dist |
| total\_readcount | cd4\_s0\_vs\_cd4\_s1 | 3 | 5 | Mfold | 5 | 100 | centroids.dist |
| total\_readcount | cd4\_s0\_vs\_cd8\_s0 | 1 | 5 | Mfold | 5 | 100 | centroids.dist |
| total\_readcount | cd4\_s0\_vs\_cd8\_s0 | 2 | 5 | Mfold | 5 | 100 | centroids.dist |
| total\_readcount | cd4\_s0\_vs\_cd8\_s0 | 3 | 5 | Mfold | 5 | 100 | centroids.dist |
| total\_readcount | cd8\_s1\_vs\_cd4\_s1 | 1 | 5 | Mfold | 5 | 100 | centroids.dist |
| total\_readcount | cd8\_s1\_vs\_cd4\_s1 | 2 | 5 | Mfold | 5 | 100 | centroids.dist |
| total\_readcount | cd8\_s1\_vs\_cd4\_s1 | 3 | 5 | Mfold | 5 | 100 | centroids.dist |
| total\_readcount | cd8\_s1\_vs\_cd8\_s0 | 1 | 50 | Mfold | 5 | 100 | centroids.dist |
| total\_readcount | cd8\_s1\_vs\_cd8\_s0 | 2 | 5 | Mfold | 5 | 100 | centroids.dist |
| total\_readcount | cd8\_s1\_vs\_cd8\_s0 | 3 | 5 | Mfold | 5 | 100 | centroids.dist |
| total\_readcount | cd4\_s1\_vs\_cd8\_s0 | 1 | 5 | Mfold | 5 | 100 | centroids.dist |
| total\_readcount | cd4\_s1\_vs\_cd8\_s0 | 2 | 5 | Mfold | 5 | 100 | centroids.dist |
| total\_readcount | cd4\_s1\_vs\_cd8\_s0 | 3 | 5 | Mfold | 5 | 100 | centroids.dist |
| miRNA\_readcount | cd4\_ev\_vs\_cd8\_ev | 1 | 5 | Mfold | 5 | 100 | centroids.dist |
| miRNA\_readcount | cd4\_ev\_vs\_cd8\_ev | 2 | 10 | Mfold | 5 | 100 | centroids.dist |
| miRNA\_readcount | cd4\_ev\_vs\_cd8\_ev | 3 | 5 | Mfold | 5 | 100 | centroids.dist |
| miRNA\_readcount | cd4\_ev\_vs\_cd4\_s0 | 1 | 5 | Mfold | 5 | 100 | centroids.dist |
| miRNA\_readcount | cd4\_ev\_vs\_cd4\_s0 | 2 | 5 | Mfold | 5 | 100 | centroids.dist |
| miRNA\_readcount | cd4\_ev\_vs\_cd4\_s0 | 3 | 5 | Mfold | 5 | 100 | centroids.dist |
| miRNA\_readcount | cd4\_ev\_vs\_cd8\_s1 | 1 | 5 | Mfold | 5 | 100 | centroids.dist |
| miRNA\_readcount | cd4\_ev\_vs\_cd8\_s1 | 2 | 5 | Mfold | 5 | 100 | centroids.dist |
| miRNA\_readcount | cd4\_ev\_vs\_cd8\_s1 | 3 | 5 | Mfold | 5 | 100 | centroids.dist |
| miRNA\_readcount | cd4\_ev\_vs\_cd4\_s1 | 1 | 50 | Mfold | 5 | 100 | centroids.dist |
| miRNA\_readcount | cd4\_ev\_vs\_cd4\_s1 | 2 | 5 | Mfold | 5 | 100 | centroids.dist |
| miRNA\_readcount | cd4\_ev\_vs\_cd4\_s1 | 3 | 5 | Mfold | 5 | 100 | centroids.dist |
| miRNA\_readcount | cd4\_ev\_vs\_cd8\_s0 | 1 | 5 | Mfold | 5 | 100 | centroids.dist |
| miRNA\_readcount | cd4\_ev\_vs\_cd8\_s0 | 2 | 5 | Mfold | 5 | 100 | centroids.dist |
| miRNA\_readcount | cd4\_ev\_vs\_cd8\_s0 | 3 | 5 | Mfold | 5 | 100 | centroids.dist |
| miRNA\_readcount | cd8\_ev\_vs\_cd4\_s0 | 1 | 5 | Mfold | 5 | 100 | centroids.dist |
| miRNA\_readcount | cd8\_ev\_vs\_cd4\_s0 | 2 | 5 | Mfold | 5 | 100 | centroids.dist |
| miRNA\_readcount | cd8\_ev\_vs\_cd4\_s0 | 3 | 5 | Mfold | 5 | 100 | centroids.dist |
| miRNA\_readcount | cd8\_ev\_vs\_cd8\_s1 | 1 | 10 | Mfold | 5 | 100 | centroids.dist |
| miRNA\_readcount | cd8\_ev\_vs\_cd8\_s1 | 2 | 5 | Mfold | 5 | 100 | centroids.dist |
| miRNA\_readcount | cd8\_ev\_vs\_cd8\_s1 | 3 | 5 | Mfold | 5 | 100 | centroids.dist |
| miRNA\_readcount | cd8\_ev\_vs\_cd4\_s1 | 1 | 5 | Mfold | 5 | 100 | centroids.dist |
| miRNA\_readcount | cd8\_ev\_vs\_cd4\_s1 | 2 | 5 | Mfold | 5 | 100 | centroids.dist |
| miRNA\_readcount | cd8\_ev\_vs\_cd4\_s1 | 3 | 5 | Mfold | 5 | 100 | centroids.dist |
| miRNA\_readcount | cd8\_ev\_vs\_cd8\_s0 | 1 | 5 | Mfold | 5 | 100 | centroids.dist |
| miRNA\_readcount | cd8\_ev\_vs\_cd8\_s0 | 2 | 5 | Mfold | 5 | 100 | centroids.dist |
| miRNA\_readcount | cd8\_ev\_vs\_cd8\_s0 | 3 | 5 | Mfold | 5 | 100 | centroids.dist |
| miRNA\_readcount | cd4\_s0\_vs\_cd8\_s1 | 1 | 20 | Mfold | 5 | 100 | centroids.dist |
| miRNA\_readcount | cd4\_s0\_vs\_cd8\_s1 | 2 | 50 | Mfold | 5 | 100 | centroids.dist |
| miRNA\_readcount | cd4\_s0\_vs\_cd8\_s1 | 3 | 20 | Mfold | 5 | 100 | centroids.dist |
| miRNA\_readcount | cd4\_s0\_vs\_cd4\_s1 | 1 | 5 | Mfold | 5 | 100 | centroids.dist |
| miRNA\_readcount | cd4\_s0\_vs\_cd4\_s1 | 2 | 5 | Mfold | 5 | 100 | centroids.dist |
| miRNA\_readcount | cd4\_s0\_vs\_cd4\_s1 | 3 | 5 | Mfold | 5 | 100 | centroids.dist |
| miRNA\_readcount | cd4\_s0\_vs\_cd8\_s0 | 1 | 5 | Mfold | 5 | 100 | centroids.dist |
| miRNA\_readcount | cd4\_s0\_vs\_cd8\_s0 | 2 | 5 | Mfold | 5 | 100 | centroids.dist |
| miRNA\_readcount | cd4\_s0\_vs\_cd8\_s0 | 3 | 5 | Mfold | 5 | 100 | centroids.dist |
| miRNA\_readcount | cd8\_s1\_vs\_cd4\_s1 | 1 | 50 | Mfold | 5 | 100 | centroids.dist |
| miRNA\_readcount | cd8\_s1\_vs\_cd4\_s1 | 2 | 50 | Mfold | 5 | 100 | centroids.dist |
| miRNA\_readcount | cd8\_s1\_vs\_cd4\_s1 | 3 | 50 | Mfold | 5 | 100 | centroids.dist |
| miRNA\_readcount | cd8\_s1\_vs\_cd8\_s0 | 1 | 10 | Mfold | 5 | 100 | centroids.dist |
| miRNA\_readcount | cd8\_s1\_vs\_cd8\_s0 | 2 | 5 | Mfold | 5 | 100 | centroids.dist |
| miRNA\_readcount | cd8\_s1\_vs\_cd8\_s0 | 3 | 5 | Mfold | 5 | 100 | centroids.dist |
| miRNA\_readcount | cd4\_s1\_vs\_cd8\_s0 | 1 | 10 | Mfold | 5 | 100 | centroids.dist |
| miRNA\_readcount | cd4\_s1\_vs\_cd8\_s0 | 2 | 5 | Mfold | 5 | 100 | centroids.dist |
| miRNA\_readcount | cd4\_s1\_vs\_cd8\_s0 | 3 | 5 | Mfold | 5 | 100 | centroids.dist |
| tRNA\_miRNA\_readcount | cd4\_ev\_vs\_cd8\_ev | 1 | 5 | Mfold | 5 | 100 | centroids.dist |
| tRNA\_miRNA\_readcount | cd4\_ev\_vs\_cd8\_ev | 2 | 5 | Mfold | 5 | 100 | centroids.dist |
| tRNA\_miRNA\_readcount | cd4\_ev\_vs\_cd8\_ev | 3 | 5 | Mfold | 5 | 100 | centroids.dist |
| tRNA\_miRNA\_readcount | cd4\_ev\_vs\_cd4\_s0 | 1 | 5 | Mfold | 5 | 100 | centroids.dist |
| tRNA\_miRNA\_readcount | cd4\_ev\_vs\_cd4\_s0 | 2 | 5 | Mfold | 5 | 100 | centroids.dist |
| tRNA\_miRNA\_readcount | cd4\_ev\_vs\_cd4\_s0 | 3 | 5 | Mfold | 5 | 100 | centroids.dist |
| tRNA\_miRNA\_readcount | cd4\_ev\_vs\_cd8\_s1 | 1 | 5 | Mfold | 5 | 100 | centroids.dist |
| tRNA\_miRNA\_readcount | cd4\_ev\_vs\_cd8\_s1 | 2 | 5 | Mfold | 5 | 100 | centroids.dist |
| tRNA\_miRNA\_readcount | cd4\_ev\_vs\_cd8\_s1 | 3 | 5 | Mfold | 5 | 100 | centroids.dist |
| tRNA\_miRNA\_readcount | cd4\_ev\_vs\_cd4\_s1 | 1 | 10 | Mfold | 5 | 100 | centroids.dist |
| tRNA\_miRNA\_readcount | cd4\_ev\_vs\_cd4\_s1 | 2 | 5 | Mfold | 5 | 100 | centroids.dist |
| tRNA\_miRNA\_readcount | cd4\_ev\_vs\_cd4\_s1 | 3 | 5 | Mfold | 5 | 100 | centroids.dist |
| tRNA\_miRNA\_readcount | cd4\_ev\_vs\_cd8\_s0 | 1 | 5 | Mfold | 5 | 100 | centroids.dist |
| tRNA\_miRNA\_readcount | cd4\_ev\_vs\_cd8\_s0 | 2 | 5 | Mfold | 5 | 100 | centroids.dist |
| tRNA\_miRNA\_readcount | cd4\_ev\_vs\_cd8\_s0 | 3 | 5 | Mfold | 5 | 100 | centroids.dist |
| tRNA\_miRNA\_readcount | cd8\_ev\_vs\_cd4\_s0 | 1 | 5 | Mfold | 5 | 100 | centroids.dist |
| tRNA\_miRNA\_readcount | cd8\_ev\_vs\_cd4\_s0 | 2 | 5 | Mfold | 5 | 100 | centroids.dist |
| tRNA\_miRNA\_readcount | cd8\_ev\_vs\_cd4\_s0 | 3 | 5 | Mfold | 5 | 100 | centroids.dist |
| tRNA\_miRNA\_readcount | cd8\_ev\_vs\_cd8\_s1 | 1 | 5 | Mfold | 5 | 100 | centroids.dist |
| tRNA\_miRNA\_readcount | cd8\_ev\_vs\_cd8\_s1 | 2 | 5 | Mfold | 5 | 100 | centroids.dist |
| tRNA\_miRNA\_readcount | cd8\_ev\_vs\_cd8\_s1 | 3 | 5 | Mfold | 5 | 100 | centroids.dist |
| tRNA\_miRNA\_readcount | cd8\_ev\_vs\_cd4\_s1 | 1 | 5 | Mfold | 5 | 100 | centroids.dist |
| tRNA\_miRNA\_readcount | cd8\_ev\_vs\_cd4\_s1 | 2 | 5 | Mfold | 5 | 100 | centroids.dist |
| tRNA\_miRNA\_readcount | cd8\_ev\_vs\_cd4\_s1 | 3 | 5 | Mfold | 5 | 100 | centroids.dist |
| tRNA\_miRNA\_readcount | cd8\_ev\_vs\_cd8\_s0 | 1 | 5 | Mfold | 5 | 100 | centroids.dist |
| tRNA\_miRNA\_readcount | cd8\_ev\_vs\_cd8\_s0 | 2 | 5 | Mfold | 5 | 100 | centroids.dist |
| tRNA\_miRNA\_readcount | cd8\_ev\_vs\_cd8\_s0 | 3 | 5 | Mfold | 5 | 100 | centroids.dist |
| tRNA\_miRNA\_readcount | cd4\_s0\_vs\_cd8\_s1 | 1 | 10 | Mfold | 5 | 100 | centroids.dist |
| tRNA\_miRNA\_readcount | cd4\_s0\_vs\_cd8\_s1 | 2 | 20 | Mfold | 5 | 100 | centroids.dist |
| tRNA\_miRNA\_readcount | cd4\_s0\_vs\_cd8\_s1 | 3 | 20 | Mfold | 5 | 100 | centroids.dist |
| tRNA\_miRNA\_readcount | cd4\_s0\_vs\_cd4\_s1 | 1 | 5 | Mfold | 5 | 100 | centroids.dist |
| tRNA\_miRNA\_readcount | cd4\_s0\_vs\_cd4\_s1 | 2 | 5 | Mfold | 5 | 100 | centroids.dist |
| tRNA\_miRNA\_readcount | cd4\_s0\_vs\_cd4\_s1 | 3 | 5 | Mfold | 5 | 100 | centroids.dist |
| tRNA\_miRNA\_readcount | cd4\_s0\_vs\_cd8\_s0 | 1 | 5 | Mfold | 5 | 100 | centroids.dist |
| tRNA\_miRNA\_readcount | cd4\_s0\_vs\_cd8\_s0 | 2 | 5 | Mfold | 5 | 100 | centroids.dist |
| tRNA\_miRNA\_readcount | cd4\_s0\_vs\_cd8\_s0 | 3 | 5 | Mfold | 5 | 100 | centroids.dist |
| tRNA\_miRNA\_readcount | cd8\_s1\_vs\_cd4\_s1 | 1 | 20 | Mfold | 5 | 100 | centroids.dist |
| tRNA\_miRNA\_readcount | cd8\_s1\_vs\_cd4\_s1 | 2 | 20 | Mfold | 5 | 100 | centroids.dist |
| tRNA\_miRNA\_readcount | cd8\_s1\_vs\_cd4\_s1 | 3 | 5 | Mfold | 5 | 100 | centroids.dist |
| tRNA\_miRNA\_readcount | cd8\_s1\_vs\_cd8\_s0 | 1 | 20 | Mfold | 5 | 100 | centroids.dist |
| tRNA\_miRNA\_readcount | cd8\_s1\_vs\_cd8\_s0 | 2 | 10 | Mfold | 5 | 100 | centroids.dist |
| tRNA\_miRNA\_readcount | cd8\_s1\_vs\_cd8\_s0 | 3 | 5 | Mfold | 5 | 100 | centroids.dist |
| tRNA\_miRNA\_readcount | cd4\_s1\_vs\_cd8\_s0 | 1 | 10 | Mfold | 5 | 100 | centroids.dist |
| tRNA\_miRNA\_readcount | cd4\_s1\_vs\_cd8\_s0 | 2 | 5 | Mfold | 5 | 100 | centroids.dist |
| tRNA\_miRNA\_readcount | cd4\_s1\_vs\_cd8\_s0 | 3 | 5 | Mfold | 5 | 100 | centroids.dist |
| isomiR\_readcount | cd4\_ev\_vs\_cd8\_ev | 1 | 5 | Mfold | 5 | 100 | centroids.dist |
| isomiR\_readcount | cd4\_ev\_vs\_cd8\_ev | 2 | 5 | Mfold | 5 | 100 | centroids.dist |
| isomiR\_readcount | cd4\_ev\_vs\_cd8\_ev | 3 | 5 | Mfold | 5 | 100 | centroids.dist |
| isomiR\_readcount | cd4\_ev\_vs\_cd4\_s0 | 1 | 5 | Mfold | 5 | 100 | centroids.dist |
| isomiR\_readcount | cd4\_ev\_vs\_cd4\_s0 | 2 | 5 | Mfold | 5 | 100 | centroids.dist |
| isomiR\_readcount | cd4\_ev\_vs\_cd4\_s0 | 3 | 5 | Mfold | 5 | 100 | centroids.dist |
| isomiR\_readcount | cd4\_ev\_vs\_cd8\_s1 | 1 | 5 | Mfold | 5 | 100 | centroids.dist |
| isomiR\_readcount | cd4\_ev\_vs\_cd8\_s1 | 2 | 5 | Mfold | 5 | 100 | centroids.dist |
| isomiR\_readcount | cd4\_ev\_vs\_cd8\_s1 | 3 | 5 | Mfold | 5 | 100 | centroids.dist |
| isomiR\_readcount | cd4\_ev\_vs\_cd4\_s1 | 1 | 5 | Mfold | 5 | 100 | centroids.dist |
| isomiR\_readcount | cd4\_ev\_vs\_cd4\_s1 | 2 | 5 | Mfold | 5 | 100 | centroids.dist |
| isomiR\_readcount | cd4\_ev\_vs\_cd4\_s1 | 3 | 5 | Mfold | 5 | 100 | centroids.dist |
| isomiR\_readcount | cd4\_ev\_vs\_cd8\_s0 | 1 | 5 | Mfold | 5 | 100 | centroids.dist |
| isomiR\_readcount | cd4\_ev\_vs\_cd8\_s0 | 2 | 5 | Mfold | 5 | 100 | centroids.dist |
| isomiR\_readcount | cd4\_ev\_vs\_cd8\_s0 | 3 | 5 | Mfold | 5 | 100 | centroids.dist |
| isomiR\_readcount | cd8\_ev\_vs\_cd4\_s0 | 1 | 5 | Mfold | 5 | 100 | centroids.dist |
| isomiR\_readcount | cd8\_ev\_vs\_cd4\_s0 | 2 | 5 | Mfold | 5 | 100 | centroids.dist |
| isomiR\_readcount | cd8\_ev\_vs\_cd4\_s0 | 3 | 5 | Mfold | 5 | 100 | centroids.dist |
| isomiR\_readcount | cd8\_ev\_vs\_cd8\_s1 | 1 | 5 | Mfold | 5 | 100 | centroids.dist |
| isomiR\_readcount | cd8\_ev\_vs\_cd8\_s1 | 2 | 5 | Mfold | 5 | 100 | centroids.dist |
| isomiR\_readcount | cd8\_ev\_vs\_cd8\_s1 | 3 | 20 | Mfold | 5 | 100 | centroids.dist |
| isomiR\_readcount | cd8\_ev\_vs\_cd4\_s1 | 1 | 5 | Mfold | 5 | 100 | centroids.dist |
| isomiR\_readcount | cd8\_ev\_vs\_cd4\_s1 | 2 | 5 | Mfold | 5 | 100 | centroids.dist |
| isomiR\_readcount | cd8\_ev\_vs\_cd4\_s1 | 3 | 5 | Mfold | 5 | 100 | centroids.dist |
| isomiR\_readcount | cd8\_ev\_vs\_cd8\_s0 | 1 | 5 | Mfold | 5 | 100 | centroids.dist |
| isomiR\_readcount | cd8\_ev\_vs\_cd8\_s0 | 2 | 5 | Mfold | 5 | 100 | centroids.dist |
| isomiR\_readcount | cd8\_ev\_vs\_cd8\_s0 | 3 | 5 | Mfold | 5 | 100 | centroids.dist |
| isomiR\_readcount | cd4\_s0\_vs\_cd8\_s1 | 1 | 10 | Mfold | 5 | 100 | centroids.dist |
| isomiR\_readcount | cd4\_s0\_vs\_cd8\_s1 | 2 | 20 | Mfold | 5 | 100 | centroids.dist |
| isomiR\_readcount | cd4\_s0\_vs\_cd8\_s1 | 3 | 10 | Mfold | 5 | 100 | centroids.dist |
| isomiR\_readcount | cd4\_s0\_vs\_cd4\_s1 | 1 | 5 | Mfold | 5 | 100 | centroids.dist |
| isomiR\_readcount | cd4\_s0\_vs\_cd4\_s1 | 2 | 5 | Mfold | 5 | 100 | centroids.dist |
| isomiR\_readcount | cd4\_s0\_vs\_cd4\_s1 | 3 | 5 | Mfold | 5 | 100 | centroids.dist |
| isomiR\_readcount | cd4\_s0\_vs\_cd8\_s0 | 1 | 5 | Mfold | 5 | 100 | centroids.dist |
| isomiR\_readcount | cd4\_s0\_vs\_cd8\_s0 | 2 | 5 | Mfold | 5 | 100 | centroids.dist |
| isomiR\_readcount | cd4\_s0\_vs\_cd8\_s0 | 3 | 5 | Mfold | 5 | 100 | centroids.dist |
| isomiR\_readcount | cd8\_s1\_vs\_cd4\_s1 | 1 | 5 | Mfold | 5 | 100 | centroids.dist |
| isomiR\_readcount | cd8\_s1\_vs\_cd4\_s1 | 2 | 5 | Mfold | 5 | 100 | centroids.dist |
| isomiR\_readcount | cd8\_s1\_vs\_cd4\_s1 | 3 | 10 | Mfold | 5 | 100 | centroids.dist |
| isomiR\_readcount | cd8\_s1\_vs\_cd8\_s0 | 1 | 5 | Mfold | 5 | 100 | centroids.dist |
| isomiR\_readcount | cd8\_s1\_vs\_cd8\_s0 | 2 | 10 | Mfold | 5 | 100 | centroids.dist |
| isomiR\_readcount | cd8\_s1\_vs\_cd8\_s0 | 3 | 5 | Mfold | 5 | 100 | centroids.dist |
| isomiR\_readcount | cd4\_s1\_vs\_cd8\_s0 | 1 | 20 | Mfold | 5 | 100 | centroids.dist |
| isomiR\_readcount | cd4\_s1\_vs\_cd8\_s0 | 2 | 10 | Mfold | 5 | 100 | centroids.dist |
| isomiR\_readcount | cd4\_s1\_vs\_cd8\_s0 | 3 | 5 | Mfold | 5 | 100 | centroids.dist |
| tRF\_readcount | cd4\_ev\_vs\_cd8\_ev | 1 | 5 | Mfold | 5 | 100 | centroids.dist |
| tRF\_readcount | cd4\_ev\_vs\_cd8\_ev | 2 | 10 | Mfold | 5 | 100 | centroids.dist |
| tRF\_readcount | cd4\_ev\_vs\_cd8\_ev | 3 | 5 | Mfold | 5 | 100 | centroids.dist |
| tRF\_readcount | cd4\_ev\_vs\_cd4\_s0 | 1 | 5 | Mfold | 5 | 100 | centroids.dist |
| tRF\_readcount | cd4\_ev\_vs\_cd4\_s0 | 2 | 5 | Mfold | 5 | 100 | centroids.dist |
| tRF\_readcount | cd4\_ev\_vs\_cd4\_s0 | 3 | 5 | Mfold | 5 | 100 | centroids.dist |
| tRF\_readcount | cd4\_ev\_vs\_cd8\_s1 | 1 | 5 | Mfold | 5 | 100 | centroids.dist |
| tRF\_readcount | cd4\_ev\_vs\_cd8\_s1 | 2 | 5 | Mfold | 5 | 100 | centroids.dist |
| tRF\_readcount | cd4\_ev\_vs\_cd8\_s1 | 3 | 5 | Mfold | 5 | 100 | centroids.dist |
| tRF\_readcount | cd4\_ev\_vs\_cd4\_s1 | 1 | 5 | Mfold | 5 | 100 | centroids.dist |
| tRF\_readcount | cd4\_ev\_vs\_cd4\_s1 | 2 | 5 | Mfold | 5 | 100 | centroids.dist |
| tRF\_readcount | cd4\_ev\_vs\_cd4\_s1 | 3 | 5 | Mfold | 5 | 100 | centroids.dist |
| tRF\_readcount | cd4\_ev\_vs\_cd8\_s0 | 1 | 5 | Mfold | 5 | 100 | centroids.dist |
| tRF\_readcount | cd4\_ev\_vs\_cd8\_s0 | 2 | 5 | Mfold | 5 | 100 | centroids.dist |
| tRF\_readcount | cd4\_ev\_vs\_cd8\_s0 | 3 | 5 | Mfold | 5 | 100 | centroids.dist |
| tRF\_readcount | cd8\_ev\_vs\_cd4\_s0 | 1 | 5 | Mfold | 5 | 100 | centroids.dist |
| tRF\_readcount | cd8\_ev\_vs\_cd4\_s0 | 2 | 5 | Mfold | 5 | 100 | centroids.dist |
| tRF\_readcount | cd8\_ev\_vs\_cd4\_s0 | 3 | 5 | Mfold | 5 | 100 | centroids.dist |
| tRF\_readcount | cd8\_ev\_vs\_cd8\_s1 | 1 | 10 | Mfold | 5 | 100 | centroids.dist |
| tRF\_readcount | cd8\_ev\_vs\_cd8\_s1 | 2 | 5 | Mfold | 5 | 100 | centroids.dist |
| tRF\_readcount | cd8\_ev\_vs\_cd8\_s1 | 3 | 5 | Mfold | 5 | 100 | centroids.dist |
| tRF\_readcount | cd8\_ev\_vs\_cd4\_s1 | 1 | 5 | Mfold | 5 | 100 | centroids.dist |
| tRF\_readcount | cd8\_ev\_vs\_cd4\_s1 | 2 | 5 | Mfold | 5 | 100 | centroids.dist |
| tRF\_readcount | cd8\_ev\_vs\_cd4\_s1 | 3 | 5 | Mfold | 5 | 100 | centroids.dist |
| tRF\_readcount | cd8\_ev\_vs\_cd8\_s0 | 1 | 5 | Mfold | 5 | 100 | centroids.dist |
| tRF\_readcount | cd8\_ev\_vs\_cd8\_s0 | 2 | 5 | Mfold | 5 | 100 | centroids.dist |
| tRF\_readcount | cd8\_ev\_vs\_cd8\_s0 | 3 | 5 | Mfold | 5 | 100 | centroids.dist |
| tRF\_readcount | cd4\_s0\_vs\_cd8\_s1 | 1 | 50 | Mfold | 5 | 100 | centroids.dist |
| tRF\_readcount | cd4\_s0\_vs\_cd8\_s1 | 2 | 50 | Mfold | 5 | 100 | centroids.dist |
| tRF\_readcount | cd4\_s0\_vs\_cd8\_s1 | 3 | 50 | Mfold | 5 | 100 | centroids.dist |
| tRF\_readcount | cd4\_s0\_vs\_cd4\_s1 | 1 | 50 | Mfold | 5 | 100 | centroids.dist |
| tRF\_readcount | cd4\_s0\_vs\_cd4\_s1 | 2 | 5 | Mfold | 5 | 100 | centroids.dist |
| tRF\_readcount | cd4\_s0\_vs\_cd4\_s1 | 3 | 5 | Mfold | 5 | 100 | centroids.dist |
| tRF\_readcount | cd4\_s0\_vs\_cd8\_s0 | 1 | 5 | Mfold | 5 | 100 | centroids.dist |
| tRF\_readcount | cd4\_s0\_vs\_cd8\_s0 | 2 | 5 | Mfold | 5 | 100 | centroids.dist |
| tRF\_readcount | cd4\_s0\_vs\_cd8\_s0 | 3 | 5 | Mfold | 5 | 100 | centroids.dist |
| tRF\_readcount | cd8\_s1\_vs\_cd4\_s1 | 1 | 5 | Mfold | 5 | 100 | centroids.dist |
| tRF\_readcount | cd8\_s1\_vs\_cd4\_s1 | 2 | 10 | Mfold | 5 | 100 | centroids.dist |
| tRF\_readcount | cd8\_s1\_vs\_cd4\_s1 | 3 | 5 | Mfold | 5 | 100 | centroids.dist |
| tRF\_readcount | cd8\_s1\_vs\_cd8\_s0 | 1 | 5 | Mfold | 5 | 100 | centroids.dist |
| tRF\_readcount | cd8\_s1\_vs\_cd8\_s0 | 2 | 10 | Mfold | 5 | 100 | centroids.dist |
| tRF\_readcount | cd8\_s1\_vs\_cd8\_s0 | 3 | 5 | Mfold | 5 | 100 | centroids.dist |
| tRF\_readcount | cd4\_s1\_vs\_cd8\_s0 | 1 | 10 | Mfold | 5 | 100 | centroids.dist |
| tRF\_readcount | cd4\_s1\_vs\_cd8\_s0 | 2 | 20 | Mfold | 5 | 100 | centroids.dist |
| tRF\_readcount | cd4\_s1\_vs\_cd8\_s0 | 3 | 20 | Mfold | 5 | 100 | centroids.dist |
| tRF\_isomiR\_readcount | cd4\_ev\_vs\_cd8\_ev | 1 | 5 | Mfold | 5 | 100 | centroids.dist |
| tRF\_isomiR\_readcount | cd4\_ev\_vs\_cd8\_ev | 2 | 5 | Mfold | 5 | 100 | centroids.dist |
| tRF\_isomiR\_readcount | cd4\_ev\_vs\_cd8\_ev | 3 | 5 | Mfold | 5 | 100 | centroids.dist |
| tRF\_isomiR\_readcount | cd4\_ev\_vs\_cd4\_s0 | 1 | 5 | Mfold | 5 | 100 | centroids.dist |
| tRF\_isomiR\_readcount | cd4\_ev\_vs\_cd4\_s0 | 2 | 5 | Mfold | 5 | 100 | centroids.dist |
| tRF\_isomiR\_readcount | cd4\_ev\_vs\_cd4\_s0 | 3 | 5 | Mfold | 5 | 100 | centroids.dist |
| tRF\_isomiR\_readcount | cd4\_ev\_vs\_cd8\_s1 | 1 | 5 | Mfold | 5 | 100 | centroids.dist |
| tRF\_isomiR\_readcount | cd4\_ev\_vs\_cd8\_s1 | 2 | 5 | Mfold | 5 | 100 | centroids.dist |
| tRF\_isomiR\_readcount | cd4\_ev\_vs\_cd8\_s1 | 3 | 5 | Mfold | 5 | 100 | centroids.dist |
| tRF\_isomiR\_readcount | cd4\_ev\_vs\_cd4\_s1 | 1 | 5 | Mfold | 5 | 100 | centroids.dist |
| tRF\_isomiR\_readcount | cd4\_ev\_vs\_cd4\_s1 | 2 | 5 | Mfold | 5 | 100 | centroids.dist |
| tRF\_isomiR\_readcount | cd4\_ev\_vs\_cd4\_s1 | 3 | 5 | Mfold | 5 | 100 | centroids.dist |
| tRF\_isomiR\_readcount | cd4\_ev\_vs\_cd8\_s0 | 1 | 5 | Mfold | 5 | 100 | centroids.dist |
| tRF\_isomiR\_readcount | cd4\_ev\_vs\_cd8\_s0 | 2 | 5 | Mfold | 5 | 100 | centroids.dist |
| tRF\_isomiR\_readcount | cd4\_ev\_vs\_cd8\_s0 | 3 | 5 | Mfold | 5 | 100 | centroids.dist |
| tRF\_isomiR\_readcount | cd8\_ev\_vs\_cd4\_s0 | 1 | 5 | Mfold | 5 | 100 | centroids.dist |
| tRF\_isomiR\_readcount | cd8\_ev\_vs\_cd4\_s0 | 2 | 5 | Mfold | 5 | 100 | centroids.dist |
| tRF\_isomiR\_readcount | cd8\_ev\_vs\_cd4\_s0 | 3 | 5 | Mfold | 5 | 100 | centroids.dist |
| tRF\_isomiR\_readcount | cd8\_ev\_vs\_cd8\_s1 | 1 | 5 | Mfold | 5 | 100 | centroids.dist |
| tRF\_isomiR\_readcount | cd8\_ev\_vs\_cd8\_s1 | 2 | 5 | Mfold | 5 | 100 | centroids.dist |
| tRF\_isomiR\_readcount | cd8\_ev\_vs\_cd8\_s1 | 3 | 5 | Mfold | 5 | 100 | centroids.dist |
| tRF\_isomiR\_readcount | cd8\_ev\_vs\_cd4\_s1 | 1 | 5 | Mfold | 5 | 100 | centroids.dist |
| tRF\_isomiR\_readcount | cd8\_ev\_vs\_cd4\_s1 | 2 | 5 | Mfold | 5 | 100 | centroids.dist |
| tRF\_isomiR\_readcount | cd8\_ev\_vs\_cd4\_s1 | 3 | 5 | Mfold | 5 | 100 | centroids.dist |
| tRF\_isomiR\_readcount | cd8\_ev\_vs\_cd8\_s0 | 1 | 5 | Mfold | 5 | 100 | centroids.dist |
| tRF\_isomiR\_readcount | cd8\_ev\_vs\_cd8\_s0 | 2 | 5 | Mfold | 5 | 100 | centroids.dist |
| tRF\_isomiR\_readcount | cd8\_ev\_vs\_cd8\_s0 | 3 | 5 | Mfold | 5 | 100 | centroids.dist |
| tRF\_isomiR\_readcount | cd4\_s0\_vs\_cd8\_s1 | 1 | 10 | Mfold | 5 | 100 | centroids.dist |
| tRF\_isomiR\_readcount | cd4\_s0\_vs\_cd8\_s1 | 2 | 20 | Mfold | 5 | 100 | centroids.dist |
| tRF\_isomiR\_readcount | cd4\_s0\_vs\_cd8\_s1 | 3 | 10 | Mfold | 5 | 100 | centroids.dist |
| tRF\_isomiR\_readcount | cd4\_s0\_vs\_cd4\_s1 | 1 | 5 | Mfold | 5 | 100 | centroids.dist |
| tRF\_isomiR\_readcount | cd4\_s0\_vs\_cd4\_s1 | 2 | 5 | Mfold | 5 | 100 | centroids.dist |
| tRF\_isomiR\_readcount | cd4\_s0\_vs\_cd4\_s1 | 3 | 5 | Mfold | 5 | 100 | centroids.dist |
| tRF\_isomiR\_readcount | cd4\_s0\_vs\_cd8\_s0 | 1 | 5 | Mfold | 5 | 100 | centroids.dist |
| tRF\_isomiR\_readcount | cd4\_s0\_vs\_cd8\_s0 | 2 | 5 | Mfold | 5 | 100 | centroids.dist |
| tRF\_isomiR\_readcount | cd4\_s0\_vs\_cd8\_s0 | 3 | 5 | Mfold | 5 | 100 | centroids.dist |
| tRF\_isomiR\_readcount | cd8\_s1\_vs\_cd4\_s1 | 1 | 5 | Mfold | 5 | 100 | centroids.dist |
| tRF\_isomiR\_readcount | cd8\_s1\_vs\_cd4\_s1 | 2 | 10 | Mfold | 5 | 100 | centroids.dist |
| tRF\_isomiR\_readcount | cd8\_s1\_vs\_cd4\_s1 | 3 | 20 | Mfold | 5 | 100 | centroids.dist |
| tRF\_isomiR\_readcount | cd8\_s1\_vs\_cd8\_s0 | 1 | 10 | Mfold | 5 | 100 | centroids.dist |
| tRF\_isomiR\_readcount | cd8\_s1\_vs\_cd8\_s0 | 2 | 5 | Mfold | 5 | 100 | centroids.dist |
| tRF\_isomiR\_readcount | cd8\_s1\_vs\_cd8\_s0 | 3 | 5 | Mfold | 5 | 100 | centroids.dist |
| tRF\_isomiR\_readcount | cd4\_s1\_vs\_cd8\_s0 | 1 | 5 | Mfold | 5 | 100 | centroids.dist |
| tRF\_isomiR\_readcount | cd4\_s1\_vs\_cd8\_s0 | 2 | 5 | Mfold | 5 | 100 | centroids.dist |
| tRF\_isomiR\_readcount | cd4\_s1\_vs\_cd8\_s0 | 3 | 5 | Mfold | 5 | 100 | centroids.dist |
| custom\_analysis\_readcount | cd4\_ev\_vs\_cd8\_ev | 1 | 5 | Mfold | 5 | 100 | centroids.dist |
| custom\_analysis\_readcount | cd4\_ev\_vs\_cd8\_ev | 2 | 10 | Mfold | 5 | 100 | centroids.dist |
| custom\_analysis\_readcount | cd4\_ev\_vs\_cd8\_ev | 3 | 5 | Mfold | 5 | 100 | centroids.dist |
| custom\_analysis\_readcount | cd4\_ev\_vs\_cd4\_s0 | 1 | 5 | Mfold | 5 | 100 | centroids.dist |
| custom\_analysis\_readcount | cd4\_ev\_vs\_cd4\_s0 | 2 | 5 | Mfold | 5 | 100 | centroids.dist |
| custom\_analysis\_readcount | cd4\_ev\_vs\_cd4\_s0 | 3 | 5 | Mfold | 5 | 100 | centroids.dist |
| custom\_analysis\_readcount | cd4\_ev\_vs\_cd8\_s1 | 1 | 5 | Mfold | 5 | 100 | centroids.dist |
| custom\_analysis\_readcount | cd4\_ev\_vs\_cd8\_s1 | 2 | 5 | Mfold | 5 | 100 | centroids.dist |
| custom\_analysis\_readcount | cd4\_ev\_vs\_cd8\_s1 | 3 | 5 | Mfold | 5 | 100 | centroids.dist |
| custom\_analysis\_readcount | cd4\_ev\_vs\_cd4\_s1 | 1 | 50 | Mfold | 5 | 100 | centroids.dist |
| custom\_analysis\_readcount | cd4\_ev\_vs\_cd4\_s1 | 2 | 5 | Mfold | 5 | 100 | centroids.dist |
| custom\_analysis\_readcount | cd4\_ev\_vs\_cd4\_s1 | 3 | 5 | Mfold | 5 | 100 | centroids.dist |
| custom\_analysis\_readcount | cd4\_ev\_vs\_cd8\_s0 | 1 | 5 | Mfold | 5 | 100 | centroids.dist |
| custom\_analysis\_readcount | cd4\_ev\_vs\_cd8\_s0 | 2 | 5 | Mfold | 5 | 100 | centroids.dist |
| custom\_analysis\_readcount | cd4\_ev\_vs\_cd8\_s0 | 3 | 5 | Mfold | 5 | 100 | centroids.dist |
| custom\_analysis\_readcount | cd8\_ev\_vs\_cd4\_s0 | 1 | 5 | Mfold | 5 | 100 | centroids.dist |
| custom\_analysis\_readcount | cd8\_ev\_vs\_cd4\_s0 | 2 | 5 | Mfold | 5 | 100 | centroids.dist |
| custom\_analysis\_readcount | cd8\_ev\_vs\_cd4\_s0 | 3 | 5 | Mfold | 5 | 100 | centroids.dist |
| custom\_analysis\_readcount | cd8\_ev\_vs\_cd8\_s1 | 1 | 20 | Mfold | 5 | 100 | centroids.dist |
| custom\_analysis\_readcount | cd8\_ev\_vs\_cd8\_s1 | 2 | 5 | Mfold | 5 | 100 | centroids.dist |
| custom\_analysis\_readcount | cd8\_ev\_vs\_cd8\_s1 | 3 | 5 | Mfold | 5 | 100 | centroids.dist |
| custom\_analysis\_readcount | cd8\_ev\_vs\_cd4\_s1 | 1 | 5 | Mfold | 5 | 100 | centroids.dist |
| custom\_analysis\_readcount | cd8\_ev\_vs\_cd4\_s1 | 2 | 5 | Mfold | 5 | 100 | centroids.dist |
| custom\_analysis\_readcount | cd8\_ev\_vs\_cd4\_s1 | 3 | 5 | Mfold | 5 | 100 | centroids.dist |
| custom\_analysis\_readcount | cd8\_ev\_vs\_cd8\_s0 | 1 | 5 | Mfold | 5 | 100 | centroids.dist |
| custom\_analysis\_readcount | cd8\_ev\_vs\_cd8\_s0 | 2 | 5 | Mfold | 5 | 100 | centroids.dist |
| custom\_analysis\_readcount | cd8\_ev\_vs\_cd8\_s0 | 3 | 5 | Mfold | 5 | 100 | centroids.dist |
| custom\_analysis\_readcount | cd4\_s0\_vs\_cd8\_s1 | 1 | 10 | Mfold | 5 | 100 | centroids.dist |
| custom\_analysis\_readcount | cd4\_s0\_vs\_cd8\_s1 | 2 | 50 | Mfold | 5 | 100 | centroids.dist |
| custom\_analysis\_readcount | cd4\_s0\_vs\_cd8\_s1 | 3 | 20 | Mfold | 5 | 100 | centroids.dist |
| custom\_analysis\_readcount | cd4\_s0\_vs\_cd4\_s1 | 1 | 5 | Mfold | 5 | 100 | centroids.dist |
| custom\_analysis\_readcount | cd4\_s0\_vs\_cd4\_s1 | 2 | 5 | Mfold | 5 | 100 | centroids.dist |
| custom\_analysis\_readcount | cd4\_s0\_vs\_cd4\_s1 | 3 | 5 | Mfold | 5 | 100 | centroids.dist |
| custom\_analysis\_readcount | cd4\_s0\_vs\_cd8\_s0 | 1 | 5 | Mfold | 5 | 100 | centroids.dist |
| custom\_analysis\_readcount | cd4\_s0\_vs\_cd8\_s0 | 2 | 5 | Mfold | 5 | 100 | centroids.dist |
| custom\_analysis\_readcount | cd4\_s0\_vs\_cd8\_s0 | 3 | 5 | Mfold | 5 | 100 | centroids.dist |
| custom\_analysis\_readcount | cd8\_s1\_vs\_cd4\_s1 | 1 | 50 | Mfold | 5 | 100 | centroids.dist |
| custom\_analysis\_readcount | cd8\_s1\_vs\_cd4\_s1 | 2 | 50 | Mfold | 5 | 100 | centroids.dist |
| custom\_analysis\_readcount | cd8\_s1\_vs\_cd4\_s1 | 3 | 50 | Mfold | 5 | 100 | centroids.dist |
| custom\_analysis\_readcount | cd8\_s1\_vs\_cd8\_s0 | 1 | 10 | Mfold | 5 | 100 | centroids.dist |
| custom\_analysis\_readcount | cd8\_s1\_vs\_cd8\_s0 | 2 | 5 | Mfold | 5 | 100 | centroids.dist |
| custom\_analysis\_readcount | cd8\_s1\_vs\_cd8\_s0 | 3 | 5 | Mfold | 5 | 100 | centroids.dist |
| custom\_analysis\_readcount | cd4\_s1\_vs\_cd8\_s0 | 1 | 10 | Mfold | 5 | 100 | centroids.dist |
| custom\_analysis\_readcount | cd4\_s1\_vs\_cd8\_s0 | 2 | 5 | Mfold | 5 | 100 | centroids.dist |
| custom\_analysis\_readcount | cd4\_s1\_vs\_cd8\_s0 | 3 | 10 | Mfold | 5 | 100 | centroids.dist |

### total\_readcount

#### cd4\_ev\_vs\_cd8\_ev

##### Sample separation plot

##### Loadings per component

###### Component 1

###### Component 2

###### Component 3

##### ROC / AUC curves

#### cd4\_ev\_vs\_cd4\_s0

##### Sample separation plot

##### Loadings per component

###### Component 1

###### Component 2

###### Component 3

##### ROC / AUC curves

#### cd4\_ev\_vs\_cd8\_s1

##### Sample separation plot

##### Loadings per component

###### Component 1

###### Component 2

###### Component 3

##### ROC / AUC curves

#### cd4\_ev\_vs\_cd4\_s1

##### Sample separation plot

##### Loadings per component

###### Component 1

###### Component 2

###### Component 3

##### ROC / AUC curves

#### cd4\_ev\_vs\_cd8\_s0

##### Sample separation plot

##### Loadings per component

###### Component 1

###### Component 2

###### Component 3

##### ROC / AUC curves

#### cd8\_ev\_vs\_cd4\_s0

##### Sample separation plot

##### Loadings per component

###### Component 1

###### Component 2

###### Component 3

##### ROC / AUC curves

#### cd8\_ev\_vs\_cd8\_s1

##### Sample separation plot

##### Loadings per component

###### Component 1

###### Component 2

###### Component 3

##### ROC / AUC curves

#### cd8\_ev\_vs\_cd4\_s1

##### Sample separation plot

##### Loadings per component

###### Component 1

###### Component 2

###### Component 3

##### ROC / AUC curves

#### cd8\_ev\_vs\_cd8\_s0

##### Sample separation plot

##### Loadings per component

###### Component 1

###### Component 2

###### Component 3

##### ROC / AUC curves

#### cd4\_s0\_vs\_cd8\_s1

##### Sample separation plot

##### Loadings per component

###### Component 1

###### Component 2

###### Component 3

##### ROC / AUC curves

#### cd4\_s0\_vs\_cd4\_s1

##### Sample separation plot

##### Loadings per component

###### Component 1

###### Component 2

###### Component 3

##### ROC / AUC curves

#### cd4\_s0\_vs\_cd8\_s0

##### Sample separation plot

##### Loadings per component

###### Component 1

###### Component 2

###### Component 3

##### ROC / AUC curves

#### cd8\_s1\_vs\_cd4\_s1

##### Sample separation plot

##### Loadings per component

###### Component 1

###### Component 2

###### Component 3

##### ROC / AUC curves

#### cd8\_s1\_vs\_cd8\_s0

##### Sample separation plot

##### Loadings per component

###### Component 1

###### Component 2

###### Component 3

##### ROC / AUC curves

#### cd4\_s1\_vs\_cd8\_s0

##### Sample separation plot

##### Loadings per component

###### Component 1

###### Component 2

###### Component 3

##### ROC / AUC curves

### miRNA\_readcount

#### cd4\_ev\_vs\_cd8\_ev

##### Sample separation plot

##### Loadings per component

###### Component 1

###### Component 2

###### Component 3

##### ROC / AUC curves

#### cd4\_ev\_vs\_cd4\_s0

##### Sample separation plot

##### Loadings per component

###### Component 1

###### Component 2

###### Component 3

##### ROC / AUC curves

#### cd4\_ev\_vs\_cd8\_s1

##### Sample separation plot

##### Loadings per component

###### Component 1

###### Component 2

###### Component 3

##### ROC / AUC curves

#### cd4\_ev\_vs\_cd4\_s1

##### Sample separation plot

##### Loadings per component

###### Component 1

###### Component 2

###### Component 3

##### ROC / AUC curves

#### cd4\_ev\_vs\_cd8\_s0

##### Sample separation plot

##### Loadings per component

###### Component 1

###### Component 2

###### Component 3

##### ROC / AUC curves

#### cd8\_ev\_vs\_cd4\_s0

##### Sample separation plot

##### Loadings per component

###### Component 1

###### Component 2

###### Component 3

##### ROC / AUC curves

#### cd8\_ev\_vs\_cd8\_s1

##### Sample separation plot

##### Loadings per component

###### Component 1

###### Component 2

###### Component 3

##### ROC / AUC curves

#### cd8\_ev\_vs\_cd4\_s1

##### Sample separation plot

##### Loadings per component

###### Component 1

###### Component 2

###### Component 3

##### ROC / AUC curves

#### cd8\_ev\_vs\_cd8\_s0

##### Sample separation plot

##### Loadings per component

###### Component 1

###### Component 2

###### Component 3

##### ROC / AUC curves

#### cd4\_s0\_vs\_cd8\_s1

##### Sample separation plot

##### Loadings per component

###### Component 1

###### Component 2

###### Component 3

##### ROC / AUC curves

#### cd4\_s0\_vs\_cd4\_s1

##### Sample separation plot

##### Loadings per component

###### Component 1

###### Component 2

###### Component 3

##### ROC / AUC curves

#### cd4\_s0\_vs\_cd8\_s0

##### Sample separation plot

##### Loadings per component

###### Component 1

###### Component 2

###### Component 3

##### ROC / AUC curves

#### cd8\_s1\_vs\_cd4\_s1

##### Sample separation plot

##### Loadings per component

###### Component 1

###### Component 2

###### Component 3

##### ROC / AUC curves

#### cd8\_s1\_vs\_cd8\_s0

##### Sample separation plot

##### Loadings per component

###### Component 1

###### Component 2

###### Component 3

##### ROC / AUC curves

#### cd4\_s1\_vs\_cd8\_s0

##### Sample separation plot

##### Loadings per component

###### Component 1

###### Component 2

###### Component 3

##### ROC / AUC curves

### tRNA\_miRNA\_readcount

#### cd4\_ev\_vs\_cd8\_ev

##### Sample separation plot

##### Loadings per component

###### Component 1

###### Component 2

###### Component 3

##### ROC / AUC curves

#### cd4\_ev\_vs\_cd4\_s0

##### Sample separation plot

##### Loadings per component

###### Component 1

###### Component 2

###### Component 3

##### ROC / AUC curves

#### cd4\_ev\_vs\_cd8\_s1

##### Sample separation plot

##### Loadings per component

###### Component 1

###### Component 2

###### Component 3

##### ROC / AUC curves

#### cd4\_ev\_vs\_cd4\_s1

##### Sample separation plot

##### Loadings per component

###### Component 1

###### Component 2

###### Component 3

##### ROC / AUC curves

#### cd4\_ev\_vs\_cd8\_s0

##### Sample separation plot

##### Loadings per component

###### Component 1

###### Component 2

###### Component 3

##### ROC / AUC curves

#### cd8\_ev\_vs\_cd4\_s0

##### Sample separation plot

##### Loadings per component

###### Component 1

###### Component 2

###### Component 3

##### ROC / AUC curves

#### cd8\_ev\_vs\_cd8\_s1

##### Sample separation plot

##### Loadings per component

###### Component 1

###### Component 2

###### Component 3

##### ROC / AUC curves

#### cd8\_ev\_vs\_cd4\_s1

##### Sample separation plot

##### Loadings per component

###### Component 1

###### Component 2

###### Component 3

##### ROC / AUC curves

#### cd8\_ev\_vs\_cd8\_s0

##### Sample separation plot

##### Loadings per component

###### Component 1

###### Component 2

###### Component 3

##### ROC / AUC curves

#### cd4\_s0\_vs\_cd8\_s1

##### Sample separation plot

##### Loadings per component

###### Component 1

###### Component 2

###### Component 3

##### ROC / AUC curves

#### cd4\_s0\_vs\_cd4\_s1

##### Sample separation plot

##### Loadings per component

###### Component 1

###### Component 2

###### Component 3

##### ROC / AUC curves

#### cd4\_s0\_vs\_cd8\_s0

##### Sample separation plot

##### Loadings per component

###### Component 1

###### Component 2

###### Component 3

##### ROC / AUC curves

#### cd8\_s1\_vs\_cd4\_s1

##### Sample separation plot

##### Loadings per component

###### Component 1

###### Component 2

###### Component 3

##### ROC / AUC curves

#### cd8\_s1\_vs\_cd8\_s0

##### Sample separation plot

##### Loadings per component

###### Component 1

###### Component 2

###### Component 3

##### ROC / AUC curves

#### cd4\_s1\_vs\_cd8\_s0

##### Sample separation plot

##### Loadings per component

###### Component 1

###### Component 2

###### Component 3

##### ROC / AUC curves

### isomiR\_readcount

#### cd4\_ev\_vs\_cd8\_ev

##### Sample separation plot

##### Loadings per component

###### Component 1

###### Component 2

###### Component 3

##### ROC / AUC curves

#### cd4\_ev\_vs\_cd4\_s0

##### Sample separation plot

##### Loadings per component

###### Component 1

###### Component 2

###### Component 3

##### ROC / AUC curves

#### cd4\_ev\_vs\_cd8\_s1

##### Sample separation plot

##### Loadings per component

###### Component 1

###### Component 2

###### Component 3

##### ROC / AUC curves

#### cd4\_ev\_vs\_cd4\_s1

##### Sample separation plot

##### Loadings per component

###### Component 1

###### Component 2

###### Component 3

##### ROC / AUC curves

#### cd4\_ev\_vs\_cd8\_s0

##### Sample separation plot

##### Loadings per component

###### Component 1

###### Component 2

###### Component 3

##### ROC / AUC curves

#### cd8\_ev\_vs\_cd4\_s0

##### Sample separation plot

##### Loadings per component

###### Component 1

###### Component 2

###### Component 3

##### ROC / AUC curves

#### cd8\_ev\_vs\_cd8\_s1

##### Sample separation plot

##### Loadings per component

###### Component 1

###### Component 2

###### Component 3

##### ROC / AUC curves

#### cd8\_ev\_vs\_cd4\_s1

##### Sample separation plot

##### Loadings per component

###### Component 1

###### Component 2

###### Component 3

##### ROC / AUC curves

#### cd8\_ev\_vs\_cd8\_s0

##### Sample separation plot

##### Loadings per component

###### Component 1

###### Component 2

###### Component 3

##### ROC / AUC curves

#### cd4\_s0\_vs\_cd8\_s1

##### Sample separation plot

##### Loadings per component

###### Component 1

###### Component 2

###### Component 3

##### ROC / AUC curves

#### cd4\_s0\_vs\_cd4\_s1

##### Sample separation plot

##### Loadings per component

###### Component 1

###### Component 2

###### Component 3

##### ROC / AUC curves

#### cd4\_s0\_vs\_cd8\_s0

##### Sample separation plot

##### Loadings per component

###### Component 1

###### Component 2

###### Component 3

##### ROC / AUC curves

#### cd8\_s1\_vs\_cd4\_s1

##### Sample separation plot

##### Loadings per component

###### Component 1

###### Component 2

###### Component 3

##### ROC / AUC curves

#### cd8\_s1\_vs\_cd8\_s0

##### Sample separation plot

##### Loadings per component

###### Component 1

###### Component 2

###### Component 3

##### ROC / AUC curves

#### cd4\_s1\_vs\_cd8\_s0

##### Sample separation plot

##### Loadings per component

###### Component 1

###### Component 2

###### Component 3

##### ROC / AUC curves

### tRF\_readcount

#### cd4\_ev\_vs\_cd8\_ev

##### Sample separation plot

##### Loadings per component

###### Component 1

###### Component 2

###### Component 3

##### ROC / AUC curves

#### cd4\_ev\_vs\_cd4\_s0

##### Sample separation plot

##### Loadings per component

###### Component 1

###### Component 2

###### Component 3

##### ROC / AUC curves

#### cd4\_ev\_vs\_cd8\_s1

##### Sample separation plot

##### Loadings per component

###### Component 1

###### Component 2

###### Component 3

##### ROC / AUC curves

#### cd4\_ev\_vs\_cd4\_s1

##### Sample separation plot

##### Loadings per component

###### Component 1

###### Component 2

###### Component 3

##### ROC / AUC curves

#### cd4\_ev\_vs\_cd8\_s0

##### Sample separation plot

##### Loadings per component

###### Component 1

###### Component 2

###### Component 3

##### ROC / AUC curves

#### cd8\_ev\_vs\_cd4\_s0

##### Sample separation plot

##### Loadings per component

###### Component 1

###### Component 2

###### Component 3

##### ROC / AUC curves

#### cd8\_ev\_vs\_cd8\_s1

##### Sample separation plot

##### Loadings per component

###### Component 1

###### Component 2

###### Component 3

##### ROC / AUC curves

#### cd8\_ev\_vs\_cd4\_s1

##### Sample separation plot

##### Loadings per component

###### Component 1

###### Component 2

###### Component 3

##### ROC / AUC curves

#### cd8\_ev\_vs\_cd8\_s0

##### Sample separation plot

##### Loadings per component

###### Component 1

###### Component 2

###### Component 3

##### ROC / AUC curves

#### cd4\_s0\_vs\_cd8\_s1

##### Sample separation plot

##### Loadings per component

###### Component 1

###### Component 2

###### Component 3

##### ROC / AUC curves

#### cd4\_s0\_vs\_cd4\_s1

##### Sample separation plot

##### Loadings per component

###### Component 1

###### Component 2

###### Component 3

##### ROC / AUC curves

#### cd4\_s0\_vs\_cd8\_s0

##### Sample separation plot

##### Loadings per component

###### Component 1

###### Component 2

###### Component 3

##### ROC / AUC curves

#### cd8\_s1\_vs\_cd4\_s1

##### Sample separation plot

##### Loadings per component

###### Component 1

###### Component 2

###### Component 3

##### ROC / AUC curves

#### cd8\_s1\_vs\_cd8\_s0

##### Sample separation plot

##### Loadings per component

###### Component 1

###### Component 2

###### Component 3

##### ROC / AUC curves

#### cd4\_s1\_vs\_cd8\_s0

##### Sample separation plot

##### Loadings per component

###### Component 1

###### Component 2

###### Component 3

##### ROC / AUC curves

### tRF\_isomiR\_readcount

#### cd4\_ev\_vs\_cd8\_ev

##### Sample separation plot

##### Loadings per component

###### Component 1

###### Component 2

###### Component 3

##### ROC / AUC curves

#### cd4\_ev\_vs\_cd4\_s0

##### Sample separation plot

##### Loadings per component

###### Component 1

###### Component 2

###### Component 3

##### ROC / AUC curves

#### cd4\_ev\_vs\_cd8\_s1

##### Sample separation plot

##### Loadings per component

###### Component 1

###### Component 2

###### Component 3

##### ROC / AUC curves

#### cd4\_ev\_vs\_cd4\_s1

##### Sample separation plot

##### Loadings per component

###### Component 1

###### Component 2

###### Component 3

##### ROC / AUC curves

#### cd4\_ev\_vs\_cd8\_s0

##### Sample separation plot

##### Loadings per component

###### Component 1

###### Component 2

###### Component 3

##### ROC / AUC curves

#### cd8\_ev\_vs\_cd4\_s0

##### Sample separation plot

##### Loadings per component

###### Component 1

###### Component 2

###### Component 3

##### ROC / AUC curves

#### cd8\_ev\_vs\_cd8\_s1

##### Sample separation plot

##### Loadings per component

###### Component 1

###### Component 2

###### Component 3

##### ROC / AUC curves

#### cd8\_ev\_vs\_cd4\_s1

##### Sample separation plot

##### Loadings per component

###### Component 1

###### Component 2

###### Component 3

##### ROC / AUC curves

#### cd8\_ev\_vs\_cd8\_s0

##### Sample separation plot

##### Loadings per component

###### Component 1

###### Component 2

###### Component 3

##### ROC / AUC curves

#### cd4\_s0\_vs\_cd8\_s1

##### Sample separation plot

##### Loadings per component

###### Component 1

###### Component 2

###### Component 3

##### ROC / AUC curves

#### cd4\_s0\_vs\_cd4\_s1

##### Sample separation plot

##### Loadings per component

###### Component 1

###### Component 2

###### Component 3

##### ROC / AUC curves

#### cd4\_s0\_vs\_cd8\_s0

##### Sample separation plot

##### Loadings per component

###### Component 1

###### Component 2

###### Component 3

##### ROC / AUC curves

#### cd8\_s1\_vs\_cd4\_s1

##### Sample separation plot

##### Loadings per component

###### Component 1

###### Component 2

###### Component 3

##### ROC / AUC curves

#### cd8\_s1\_vs\_cd8\_s0

##### Sample separation plot

##### Loadings per component

###### Component 1

###### Component 2

###### Component 3

##### ROC / AUC curves

#### cd4\_s1\_vs\_cd8\_s0

##### Sample separation plot

##### Loadings per component

###### Component 1

###### Component 2

###### Component 3

##### ROC / AUC curves

### custom\_analysis\_readcount

#### cd4\_ev\_vs\_cd8\_ev

##### Sample separation plot

##### Loadings per component

###### Component 1

###### Component 2

###### Component 3

##### ROC / AUC curves

#### cd4\_ev\_vs\_cd4\_s0

##### Sample separation plot

##### Loadings per component

###### Component 1

###### Component 2

###### Component 3

##### ROC / AUC curves

#### cd4\_ev\_vs\_cd8\_s1

##### Sample separation plot

##### Loadings per component

###### Component 1

###### Component 2

###### Component 3

##### ROC / AUC curves

#### cd4\_ev\_vs\_cd4\_s1

##### Sample separation plot

##### Loadings per component

###### Component 1

###### Component 2

###### Component 3

##### ROC / AUC curves

#### cd4\_ev\_vs\_cd8\_s0

##### Sample separation plot

##### Loadings per component

###### Component 1

###### Component 2

###### Component 3

##### ROC / AUC curves

#### cd8\_ev\_vs\_cd4\_s0

##### Sample separation plot

##### Loadings per component

###### Component 1

###### Component 2

###### Component 3

##### ROC / AUC curves

#### cd8\_ev\_vs\_cd8\_s1

##### Sample separation plot

##### Loadings per component

###### Component 1

###### Component 2

###### Component 3

##### ROC / AUC curves

#### cd8\_ev\_vs\_cd4\_s1

##### Sample separation plot

##### Loadings per component

###### Component 1

###### Component 2

###### Component 3

##### ROC / AUC curves

#### cd8\_ev\_vs\_cd8\_s0

##### Sample separation plot

##### Loadings per component

###### Component 1

###### Component 2

###### Component 3

##### ROC / AUC curves

#### cd4\_s0\_vs\_cd8\_s1

##### Sample separation plot

##### Loadings per component

###### Component 1

###### Component 2

###### Component 3

##### ROC / AUC curves

#### cd4\_s0\_vs\_cd4\_s1

##### Sample separation plot

##### Loadings per component

###### Component 1

###### Component 2

###### Component 3

##### ROC / AUC curves

#### cd4\_s0\_vs\_cd8\_s0

##### Sample separation plot

##### Loadings per component

###### Component 1

###### Component 2

###### Component 3

##### ROC / AUC curves

#### cd8\_s1\_vs\_cd4\_s1

##### Sample separation plot

##### Loadings per component

###### Component 1

###### Component 2

###### Component 3

##### ROC / AUC curves

#### cd8\_s1\_vs\_cd8\_s0

##### Sample separation plot

##### Loadings per component

###### Component 1

###### Component 2

###### Component 3

##### ROC / AUC curves

#### cd4\_s1\_vs\_cd8\_s0

##### Sample separation plot

##### Loadings per component

###### Component 1

###### Component 2

###### Component 3

##### ROC / AUC curves
