# Supplementary material for: Comprehensive and bias-aware analysis of small RNA NGS data for biomarker detection with caRNAge
Source: Extracell Vesicles Circ Nucl Acids. 2026 Sep 24;7(3):1554–81. doi: 10.20517/evcna.2026.78 (PMC13678173; doi:10.20517/evcna.2026.78)
Supplement: Supplementary file 1 [file evcna-7-3-1554-SupplementaryMaterials.zip › evcna7078-SupplementaryMaterials/Supplementary File 6.html]

 

 

 

 
 
 


 caRNAge - comprehensive analysis of small RNA gene expression 

 
 
 
 
 
 
 
 
 
 
 
 
 
 
 
 
 
 

 

 
 


 


 

 

 


 


 

 


 


 
 
 
 
 
 

 


 


 caRNAge - comprehensive analysis of small
RNA gene expression 
 Over representation analysis of miRNAs and their
mRNA targets 
 22 August, 2026 

 


 
 
 
 Purpose of this overview (Disclaimer) 
 This report is meant to give you a complete overview of
differentially expressed genes for the group comparison you have chosen
with their respective sources of bias you have identified in the data
overview. Please refer to the guidelines for best practice of caRNAge
and the respective manuals and vignettes of the applied tools. You are
strongly advised to thoroughly check all available information in
addition to this data overview. caRNAge is built upon the work of many
excellent scientists who developed the necessary tools to process small
RNA NGS data, so please make sure to cite the corresponding original
authors in addition to caRNAge when publishing your work. 
 If you encounter any technical problems, feel free to contact the
corresponding author at  bkirchner@tum.de . 
 
 
 Data processing 
 Raw sequencing reads were processed with  Cutadapt  to trim
adapter sequences from 3’ ends if present and to remove degraded reads
(shorter than 16nt). Trimmed reads were analysed with  FastQC 
to evaluate phred scores and read lengths before aligning them against
the human tRNA ( GtRNAdb ), miRNA
( miRBase ) including isomiRs ( isomiRROR ), piRNAs ( piRNAdb ) and refseq transcriptome
sequences ( refseq )
with  bowtie .
Finally, gene read counts were processed with  R  and mainly  DESeq2 
to normalize and transform data and calculate expression changes. 
 
  
 
 
Fig 1: Simplified workflow for NGS data analysis
 
 
   
 
 
 
 
 
 
 
 
 
  
 Tools 
 Version 
 Parameters 
 
 
 
 
 Trimming 
 Cutadapt 
 4.4 
 -a AGATCGGAAGAGCAC, -e 0.1 -m 16 
 
 
 Quality Control 
 FastQC 
 0.12.1 
  
 
 
 Alignment 
 bowtie 
 1.3.1 
 Bowtie -v mismatches: tRNA=1, isomiR=3,
transcriptome=1; –best –norc 
 
 
 Normalisation 
 DESeq2 
 R version 4.6.1 (2026-06-24), DESeq2 1.52.0 
 ~donor + analysis_group 
 
 
 DGE calling 
 DESeq2 
 R version 4.6.1 (2026-06-24), DESeq2 1.52.0 
  
 
 
 miRNA ORA 
 rbioapi, miEAA 
 R version 4.6.1 (2026-06-24), rbioapi 0.8.3, miEEA 
  
 
 
 miRNA target prediction 
 multiMiR Targetscan 
 R version 4.6.1 (2026-06-24), multiMiR 1.34.0,
TargetScan 
  
 
 
 miRNA target ORA 
 rbioapi, enrichr 
 R version 4.6.1 (2026-06-24), rbioapi 0.8.3,
enrichr 
  
 
 
 
     
 
 
 Sample overview 
 The starting point for all analyses, is a well designed study design.
In addition to the classification of the main research question, it
should also incorporate all available information that could in any way
affect the outcome. This includes potential confounding factors such as
batch effects as well as statistical relevant information such as
patient cohorts or cell lines. Please make sure that the classifications
given in your  design.tsv  are correct and that all relevant
information is present. 
   
 
 Study design 
 The row names and “Sample” column should be matching and indicate
that samples in design were correctly assigned in the analysis. A column
named “replaceable” maybe present if groups are large enough (>7) to
replace single outlier values. 
 
 
 
 
 
 
 
 
 
 
 
 
 
 
 
 
  
 Sample 
 source_name 
 cell_type 
 condition 
 compartment 
 experimental_block 
 sequencing_platform 
 donor 
 analysis_group 
 sequencing_cycles 
 
 
 
 
 wtchg_834291_208_1 
 wtchg_834291_208_1 
 CD4 EV 
 cd4_t_cell 
 ev 
 extracellular_vesicle 
 wtchg_208 
 illumina_hiseq_4000 
 donor_834291 
 cd4_ev 
 75 
 
 
 wtchg_834291_211_1 
 wtchg_834291_211_1 
 CD4 EV 
 cd4_t_cell 
 ev 
 extracellular_vesicle 
 wtchg_211 
 illumina_hiseq_4000 
 donor_834291 
 cd4_ev 
 75 
 
 
 wtchg_834291_213_1 
 wtchg_834291_213_1 
 CD4 EV 
 cd4_t_cell 
 ev 
 extracellular_vesicle 
 wtchg_213 
 illumina_hiseq_4000 
 donor_834291 
 cd4_ev 
 75 
 
 
 wtchg_834291_220_1 
 wtchg_834291_220_1 
 CD4 S0 
 cd4_t_cell 
 s0 
 bslb_background_control 
 wtchg_220 
 illumina_hiseq_4000 
 donor_834291 
 cd4_s0 
 75 
 
 
 wtchg_834291_226_1 
 wtchg_834291_226_1 
 CD4 S0 
 cd4_t_cell 
 s0 
 bslb_background_control 
 wtchg_226 
 illumina_hiseq_4000 
 donor_834291 
 cd4_s0 
 75 
 
 
 wtchg_834291_227_1 
 wtchg_834291_227_1 
 CD4 S1 
 cd4_t_cell 
 s1 
 trans_synaptic_vesicle 
 wtchg_227 
 illumina_hiseq_4000 
 donor_834291 
 cd4_s1 
 75 
 
 
 wtchg_834291_233_1 
 wtchg_834291_233_1 
 CD4 S1 
 cd4_t_cell 
 s1 
 trans_synaptic_vesicle 
 wtchg_233 
 illumina_hiseq_4000 
 donor_834291 
 cd4_s1 
 75 
 
 
 wtchg_834291_234_1 
 wtchg_834291_234_1 
 CD4 S1 
 cd4_t_cell 
 s1 
 trans_synaptic_vesicle 
 wtchg_234 
 illumina_hiseq_4000 
 donor_834291 
 cd4_s1 
 75 
 
 
 wtchg_834292_208_1 
 wtchg_834292_208_1 
 CD4 EV 
 cd4_t_cell 
 ev 
 extracellular_vesicle 
 wtchg_208 
 illumina_hiseq_4000 
 donor_834292 
 cd4_ev 
 75 
 
 
 wtchg_834292_211_1 
 wtchg_834292_211_1 
 CD4 EV 
 cd4_t_cell 
 ev 
 extracellular_vesicle 
 wtchg_211 
 illumina_hiseq_4000 
 donor_834292 
 cd4_ev 
 75 
 
 
 wtchg_834292_213_1 
 wtchg_834292_213_1 
 CD4 EV 
 cd4_t_cell 
 ev 
 extracellular_vesicle 
 wtchg_213 
 illumina_hiseq_4000 
 donor_834292 
 cd4_ev 
 75 
 
 
 wtchg_834292_220_1 
 wtchg_834292_220_1 
 CD4 S0 
 cd4_t_cell 
 s0 
 bslb_background_control 
 wtchg_220 
 illumina_hiseq_4000 
 donor_834292 
 cd4_s0 
 75 
 
 
 wtchg_834292_226_1 
 wtchg_834292_226_1 
 CD4 S0 
 cd4_t_cell 
 s0 
 bslb_background_control 
 wtchg_226 
 illumina_hiseq_4000 
 donor_834292 
 cd4_s0 
 75 
 
 
 wtchg_834292_227_1 
 wtchg_834292_227_1 
 CD4 S1 
 cd4_t_cell 
 s1 
 trans_synaptic_vesicle 
 wtchg_227 
 illumina_hiseq_4000 
 donor_834292 
 cd4_s1 
 75 
 
 
 wtchg_834292_233_1 
 wtchg_834292_233_1 
 CD4 S1 
 cd4_t_cell 
 s1 
 trans_synaptic_vesicle 
 wtchg_233 
 illumina_hiseq_4000 
 donor_834292 
 cd4_s1 
 75 
 
 
 wtchg_834292_234_1 
 wtchg_834292_234_1 
 CD4 S1 
 cd4_t_cell 
 s1 
 trans_synaptic_vesicle 
 wtchg_234 
 illumina_hiseq_4000 
 donor_834292 
 cd4_s1 
 75 
 
 
 wtchg_834293_208_1 
 wtchg_834293_208_1 
 CD4 EV 
 cd4_t_cell 
 ev 
 extracellular_vesicle 
 wtchg_208 
 illumina_hiseq_4000 
 donor_834293 
 cd4_ev 
 75 
 
 
 wtchg_834293_211_1 
 wtchg_834293_211_1 
 CD4 EV 
 cd4_t_cell 
 ev 
 extracellular_vesicle 
 wtchg_211 
 illumina_hiseq_4000 
 donor_834293 
 cd4_ev 
 75 
 
 
 wtchg_834293_213_1 
 wtchg_834293_213_1 
 CD4 EV 
 cd4_t_cell 
 ev 
 extracellular_vesicle 
 wtchg_213 
 illumina_hiseq_4000 
 donor_834293 
 cd4_ev 
 75 
 
 
 wtchg_834293_220_1 
 wtchg_834293_220_1 
 CD4 S0 
 cd4_t_cell 
 s0 
 bslb_background_control 
 wtchg_220 
 illumina_hiseq_4000 
 donor_834293 
 cd4_s0 
 75 
 
 
 wtchg_834293_226_1 
 wtchg_834293_226_1 
 CD4 S0 
 cd4_t_cell 
 s0 
 bslb_background_control 
 wtchg_226 
 illumina_hiseq_4000 
 donor_834293 
 cd4_s0 
 75 
 
 
 wtchg_834293_227_1 
 wtchg_834293_227_1 
 CD4 S1 
 cd4_t_cell 
 s1 
 trans_synaptic_vesicle 
 wtchg_227 
 illumina_hiseq_4000 
 donor_834293 
 cd4_s1 
 75 
 
 
 wtchg_834293_233_1 
 wtchg_834293_233_1 
 CD4 S1 
 cd4_t_cell 
 s1 
 trans_synaptic_vesicle 
 wtchg_233 
 illumina_hiseq_4000 
 donor_834293 
 cd4_s1 
 75 
 
 
 wtchg_834293_234_1 
 wtchg_834293_234_1 
 CD4 S1 
 cd4_t_cell 
 s1 
 trans_synaptic_vesicle 
 wtchg_234 
 illumina_hiseq_4000 
 donor_834293 
 cd4_s1 
 75 
 
 
 wtchg_834294_208_1 
 wtchg_834294_208_1 
 CD4 EV 
 cd4_t_cell 
 ev 
 extracellular_vesicle 
 wtchg_208 
 illumina_hiseq_4000 
 donor_834294 
 cd4_ev 
 75 
 
 
 wtchg_834294_211_1 
 wtchg_834294_211_1 
 CD4 EV 
 cd4_t_cell 
 ev 
 extracellular_vesicle 
 wtchg_211 
 illumina_hiseq_4000 
 donor_834294 
 cd4_ev 
 75 
 
 
 wtchg_834294_213_1 
 wtchg_834294_213_1 
 CD4 EV 
 cd4_t_cell 
 ev 
 extracellular_vesicle 
 wtchg_213 
 illumina_hiseq_4000 
 donor_834294 
 cd4_ev 
 75 
 
 
 wtchg_834294_220_1 
 wtchg_834294_220_1 
 CD4 S0 
 cd4_t_cell 
 s0 
 bslb_background_control 
 wtchg_220 
 illumina_hiseq_4000 
 donor_834294 
 cd4_s0 
 75 
 
 
 wtchg_834294_226_1 
 wtchg_834294_226_1 
 CD4 S0 
 cd4_t_cell 
 s0 
 bslb_background_control 
 wtchg_226 
 illumina_hiseq_4000 
 donor_834294 
 cd4_s0 
 75 
 
 
 wtchg_834294_227_1 
 wtchg_834294_227_1 
 CD4 S1 
 cd4_t_cell 
 s1 
 trans_synaptic_vesicle 
 wtchg_227 
 illumina_hiseq_4000 
 donor_834294 
 cd4_s1 
 75 
 
 
 wtchg_834294_233_1 
 wtchg_834294_233_1 
 CD4 S1 
 cd4_t_cell 
 s1 
 trans_synaptic_vesicle 
 wtchg_233 
 illumina_hiseq_4000 
 donor_834294 
 cd4_s1 
 75 
 
 
 wtchg_834294_234_1 
 wtchg_834294_234_1 
 CD4 S1 
 cd4_t_cell 
 s1 
 trans_synaptic_vesicle 
 wtchg_234 
 illumina_hiseq_4000 
 donor_834294 
 cd4_s1 
 75 
 
 
 wtchg_836235_208_1 
 wtchg_836235_208_1 
 CD4 EV 
 cd4_t_cell 
 ev 
 extracellular_vesicle 
 wtchg_208 
 illumina_hiseq_4000 
 donor_836235 
 cd4_ev 
 75 
 
 
 wtchg_836235_211_1 
 wtchg_836235_211_1 
 CD4 EV 
 cd4_t_cell 
 ev 
 extracellular_vesicle 
 wtchg_211 
 illumina_hiseq_4000 
 donor_836235 
 cd4_ev 
 75 
 
 
 wtchg_836235_213_1 
 wtchg_836235_213_1 
 CD4 EV 
 cd4_t_cell 
 ev 
 extracellular_vesicle 
 wtchg_213 
 illumina_hiseq_4000 
 donor_836235 
 cd4_ev 
 75 
 
 
 wtchg_836235_220_1 
 wtchg_836235_220_1 
 CD4 S0 
 cd4_t_cell 
 s0 
 bslb_background_control 
 wtchg_220 
 illumina_hiseq_4000 
 donor_836235 
 cd4_s0 
 75 
 
 
 wtchg_836235_226_1 
 wtchg_836235_226_1 
 CD4 S0 
 cd4_t_cell 
 s0 
 bslb_background_control 
 wtchg_226 
 illumina_hiseq_4000 
 donor_836235 
 cd4_s0 
 75 
 
 
 wtchg_836235_227_1 
 wtchg_836235_227_1 
 CD4 S1 
 cd4_t_cell 
 s1 
 trans_synaptic_vesicle 
 wtchg_227 
 illumina_hiseq_4000 
 donor_836235 
 cd4_s1 
 75 
 
 
 wtchg_836235_233_1 
 wtchg_836235_233_1 
 CD4 S1 
 cd4_t_cell 
 s1 
 trans_synaptic_vesicle 
 wtchg_233 
 illumina_hiseq_4000 
 donor_836235 
 cd4_s1 
 75 
 
 
 wtchg_836235_234_1 
 wtchg_836235_234_1 
 CD4 S1 
 cd4_t_cell 
 s1 
 trans_synaptic_vesicle 
 wtchg_234 
 illumina_hiseq_4000 
 donor_836235 
 cd4_s1 
 75 
 
 
 wtchg_836236_208_1 
 wtchg_836236_208_1 
 CD4 EV 
 cd4_t_cell 
 ev 
 extracellular_vesicle 
 wtchg_208 
 illumina_hiseq_4000 
 donor_836236 
 cd4_ev 
 75 
 
 
 wtchg_836236_211_1 
 wtchg_836236_211_1 
 CD4 EV 
 cd4_t_cell 
 ev 
 extracellular_vesicle 
 wtchg_211 
 illumina_hiseq_4000 
 donor_836236 
 cd4_ev 
 75 
 
 
 wtchg_836236_213_1 
 wtchg_836236_213_1 
 CD4 EV 
 cd4_t_cell 
 ev 
 extracellular_vesicle 
 wtchg_213 
 illumina_hiseq_4000 
 donor_836236 
 cd4_ev 
 75 
 
 
 wtchg_836236_220_1 
 wtchg_836236_220_1 
 CD4 S0 
 cd4_t_cell 
 s0 
 bslb_background_control 
 wtchg_220 
 illumina_hiseq_4000 
 donor_836236 
 cd4_s0 
 75 
 
 
 wtchg_836236_226_1 
 wtchg_836236_226_1 
 CD4 S0 
 cd4_t_cell 
 s0 
 bslb_background_control 
 wtchg_226 
 illumina_hiseq_4000 
 donor_836236 
 cd4_s0 
 75 
 
 
 wtchg_836236_227_1 
 wtchg_836236_227_1 
 CD4 S1 
 cd4_t_cell 
 s1 
 trans_synaptic_vesicle 
 wtchg_227 
 illumina_hiseq_4000 
 donor_836236 
 cd4_s1 
 75 
 
 
 wtchg_836236_233_1 
 wtchg_836236_233_1 
 CD4 S1 
 cd4_t_cell 
 s1 
 trans_synaptic_vesicle 
 wtchg_233 
 illumina_hiseq_4000 
 donor_836236 
 cd4_s1 
 75 
 
 
 wtchg_836236_234_1 
 wtchg_836236_234_1 
 CD4 S1 
 cd4_t_cell 
 s1 
 trans_synaptic_vesicle 
 wtchg_234 
 illumina_hiseq_4000 
 donor_836236 
 cd4_s1 
 75 
 
 
 wtchg_836237_208_1 
 wtchg_836237_208_1 
 CD4 EV 
 cd4_t_cell 
 ev 
 extracellular_vesicle 
 wtchg_208 
 illumina_hiseq_4000 
 donor_836237 
 cd4_ev 
 75 
 
 
 wtchg_836237_211_1 
 wtchg_836237_211_1 
 CD4 EV 
 cd4_t_cell 
 ev 
 extracellular_vesicle 
 wtchg_211 
 illumina_hiseq_4000 
 donor_836237 
 cd4_ev 
 75 
 
 
 wtchg_836237_213_1 
 wtchg_836237_213_1 
 CD4 EV 
 cd4_t_cell 
 ev 
 extracellular_vesicle 
 wtchg_213 
 illumina_hiseq_4000 
 donor_836237 
 cd4_ev 
 75 
 
 
 wtchg_836237_220_1 
 wtchg_836237_220_1 
 CD4 S0 
 cd4_t_cell 
 s0 
 bslb_background_control 
 wtchg_220 
 illumina_hiseq_4000 
 donor_836237 
 cd4_s0 
 75 
 
 
 wtchg_836237_226_1 
 wtchg_836237_226_1 
 CD4 S0 
 cd4_t_cell 
 s0 
 bslb_background_control 
 wtchg_226 
 illumina_hiseq_4000 
 donor_836237 
 cd4_s0 
 75 
 
 
 wtchg_836237_227_1 
 wtchg_836237_227_1 
 CD4 S1 
 cd4_t_cell 
 s1 
 trans_synaptic_vesicle 
 wtchg_227 
 illumina_hiseq_4000 
 donor_836237 
 cd4_s1 
 75 
 
 
 wtchg_836237_233_1 
 wtchg_836237_233_1 
 CD4 S1 
 cd4_t_cell 
 s1 
 trans_synaptic_vesicle 
 wtchg_233 
 illumina_hiseq_4000 
 donor_836237 
 cd4_s1 
 75 
 
 
 wtchg_836237_234_1 
 wtchg_836237_234_1 
 CD4 S1 
 cd4_t_cell 
 s1 
 trans_synaptic_vesicle 
 wtchg_234 
 illumina_hiseq_4000 
 donor_836237 
 cd4_s1 
 75 
 
 
 wtchg_836238_208_1 
 wtchg_836238_208_1 
 CD4 EV 
 cd4_t_cell 
 ev 
 extracellular_vesicle 
 wtchg_208 
 illumina_hiseq_4000 
 donor_836238 
 cd4_ev 
 75 
 
 
 wtchg_836238_211_1 
 wtchg_836238_211_1 
 CD4 EV 
 cd4_t_cell 
 ev 
 extracellular_vesicle 
 wtchg_211 
 illumina_hiseq_4000 
 donor_836238 
 cd4_ev 
 75 
 
 
 wtchg_836238_213_1 
 wtchg_836238_213_1 
 CD4 EV 
 cd4_t_cell 
 ev 
 extracellular_vesicle 
 wtchg_213 
 illumina_hiseq_4000 
 donor_836238 
 cd4_ev 
 75 
 
 
 wtchg_836238_220_1 
 wtchg_836238_220_1 
 CD4 S0 
 cd4_t_cell 
 s0 
 bslb_background_control 
 wtchg_220 
 illumina_hiseq_4000 
 donor_836238 
 cd4_s0 
 75 
 
 
 wtchg_836238_226_1 
 wtchg_836238_226_1 
 CD4 S0 
 cd4_t_cell 
 s0 
 bslb_background_control 
 wtchg_226 
 illumina_hiseq_4000 
 donor_836238 
 cd4_s0 
 75 
 
 
 wtchg_836238_227_1 
 wtchg_836238_227_1 
 CD4 S1 
 cd4_t_cell 
 s1 
 trans_synaptic_vesicle 
 wtchg_227 
 illumina_hiseq_4000 
 donor_836238 
 cd4_s1 
 75 
 
 
 wtchg_836238_233_1 
 wtchg_836238_233_1 
 CD4 S1 
 cd4_t_cell 
 s1 
 trans_synaptic_vesicle 
 wtchg_233 
 illumina_hiseq_4000 
 donor_836238 
 cd4_s1 
 75 
 
 
 wtchg_836238_234_1 
 wtchg_836238_234_1 
 CD4 S1 
 cd4_t_cell 
 s1 
 trans_synaptic_vesicle 
 wtchg_234 
 illumina_hiseq_4000 
 donor_836238 
 cd4_s1 
 75 
 
 
 
   
 
 
 Consolidated sample groups 
  $source_name

CD4 EV CD4 S0 CD4 S1 
    24     16     24 

$cell_type

cd4_t_cell 
        64 

$condition

ev s0 s1 
24 16 24 

$compartment

bslb_background_control   extracellular_vesicle  trans_synaptic_vesicle 
                     16                      24                      24 

$experimental_block

wtchg_208 wtchg_211 wtchg_213 wtchg_220 wtchg_226 wtchg_227 wtchg_233 wtchg_234 
        8         8         8         8         8         8         8         8 

$sequencing_platform

illumina_hiseq_4000 
                 64 

$donor

donor_834291 donor_834292 donor_834293 donor_834294 donor_836235 donor_836236 
           8            8            8            8            8            8 
donor_836237 donor_836238 
           8            8 

$analysis_group

cd4_ev cd8_ev cd4_s0 cd8_s1 cd4_s1 cd8_s0 
    24      0     16      0     24      0 

$sequencing_cycles

75 
64   
     
 
 
 
 miRNA ORA Results 
 Over-representation analysis (ORA) is a statistical framework used to
determine whether a predefined set of genes or miRNAs is enriched for
specific biological functions, pathways, cellular processes, or disease
associations more than would be expected by chance. Instead of
evaluating individual miRNAs in isolation, ORA quantifies whether entire
categories occur disproportionately within the input list. For this
analysis, ORA was performed using miEAA (microRNA Enrichment Analysis
and Annotation), a specialized platform designed for miRNA-centric
functional enrichment. miEAA integrates heterogeneous annotation sources
including miRBase, miRPathDB, miRWalk, miRTarBase, miRandola, RNALocate,
isomiRdb, MNDR, and others. 
 The study design parameter chosen for DGE testing was
 analysis_group  and the parameter(s) chosen for
correction were ****. 
 For downstream ORA and target prediction, miRNAs are separated by
direction and must satisfy  adjusted p-value ≤ 0.05 and |log2FC|
≥ 1 . Upregulated and downregulated miRNAs are analyzed
independently. 
 
 cd4_ev_vs_cd4_s0_upregulated 
 
 
 cd4_ev_vs_cd4_s0_downregulated 
 
 
 cd4_ev_vs_cd4_s1_upregulated 
 
 
 cd4_ev_vs_cd4_s1_downregulated 
 
 
 cd4_s0_vs_cd4_s1_upregulated 
 
 
 cd4_s0_vs_cd4_s1_downregulated 
 
 
 
 miRNA Target Prediction Results 
 This section summarizes predicted miRNA–mRNA regulatory interactions
identified using TargetScan, a widely used algorithm that infers miRNA
targeting based on seed sequence complementarity, evolutionary
conservation, and contextual features of 3′UTR binding sites. The
resulting interaction set represents putative post-transcriptional
regulatory relationships through which differentially expressed miRNAs
may influence downstream gene expression programs. 
 To contextualize these predicted interactions at the tissue level,
target genes were annotated with consensus tissue expression values
(nTPM) derived from the Human Protein Atlas (HPA) and the
Genotype-Tissue Expression (GTEx) project. These resources integrate
large-scale transcriptomic measurements across a broad spectrum of human
tissues, providing a robust estimate of physiological gene expression
patterns. Predicted miRNA target genes are presented grouped by tissue,
with each table containing targets that show detectable expression in
the respective tissue context. 
 Target tables are shown as paginated interactive tables.
Enrichment-result tables are capped at the top 200 rows after sorting by
adjusted p-value. 
 The threshold chosen for tissue specificity was nTPM =
 10  and tissue specific ORA was analyzed for
 lymph node, spleen . 
 
 cd4_ev_vs_cd4_s0_upregulated 
 
 Tissue expression heatmap (log10-transformed) 
   
 
 
 liver 
 
 
 
 
target_symbol
 
 
mature_mirna_id
 
 
score
 
 
nTPM
 
 
 
 
 
 
A1BG
 
 
hsa-miR-23a-3p
 
 
-0.434
 
 
565.1
 
 
 
 
A1BG
 
 
hsa-miR-23b-3p
 
 
-0.434
 
 
565.1
 
 
 
 
AAGAB
 
 
hsa-miR-34a-5p
 
 
-0.319
 
 
16.9
 
 
 
 
AAR2
 
 
hsa-miR-29a-3p
 
 
-0.327
 
 
22.0
 
 
 
 
ABCB10
 
 
hsa-miR-152-3p
 
 
-0.291
 
 
17.7
 
 
 
 
ABCB10
 
 
hsa-miR-148b-3p
 
 
-0.291
 
 
17.7
 
 
 
 
ABCB6
 
 
hsa-miR-29a-3p
 
 
-0.48
 
 
12.7
 
 
 
 
ABCB7
 
 
hsa-miR-148b-3p
 
 
-0.515
 
 
14.1
 
 
 
 
ABCB7
 
 
hsa-miR-152-3p
 
 
-0.515
 
 
14.1
 
 
 
 
ABCD3
 
 
hsa-miR-148b-3p
 
 
-0.388
 
 
118.4
 
 
 
 
ABCD3
 
 
hsa-miR-152-3p
 
 
-0.388
 
 
118.4
 
 
 
 
ABCE1
 
 
hsa-miR-29a-3p
 
 
-0.404
 
 
37.1
 
 
 
 
ABHD3
 
 
hsa-miR-125a-5p
 
 
-0.453
 
 
36.7
 
 
 
 
ABHD4
 
 
hsa-miR-29a-3p
 
 
-0.312
 
 
24.2
 
 
 
 
ABHD5
 
 
hsa-miR-132-3p
 
 
-0.323
 
 
20.3
 
 
 
 
ABHD5
 
 
hsa-miR-27a-3p
 
 
-0.407
 
 
20.3
 
 
 
 
ABHD6
 
 
hsa-miR-125a-5p
 
 
-0.501
 
 
60.7
 
 
 
 
ABRAXAS2
 
 
hsa-miR-34a-5p
 
 
-0.538
 
 
12.8
 
 
 
 
ABTB1
 
 
hsa-miR-125a-5p
 
 
-0.519
 
 
18.5
 
 
 
 
ACBD3
 
 
hsa-miR-34a-5p
 
 
-0.415
 
 
22.7
 
 
 
 
ACSL1
 
 
hsa-miR-34a-5p
 
 
-0.306
 
 
1068.9
 
 
 
 
ACSL4
 
 
hsa-miR-34a-5p
 
 
-0.372
 
 
12.3
 
 
 
 
ACTA2
 
 
hsa-miR-27a-3p
 
 
-0.577
 
 
154.5
 
 
 
 
ACVR1
 
 
hsa-miR-152-3p
 
 
-0.397
 
 
15.3
 
 
 
 
ACVR1
 
 
hsa-miR-148b-3p
 
 
-0.397
 
 
15.3
 
 
 
 
ADAM10
 
 
hsa-miR-152-3p
 
 
-0.348
 
 
15.0
 
 
 
 
ADAM10
 
 
hsa-miR-148b-3p
 
 
-0.356
 
 
15.0
 
 
 
 
ADGRA3
 
 
hsa-miR-27a-3p
 
 
-0.296
 
 
54.5
 
 
 
 
ADGRF5
 
 
hsa-miR-148b-3p
 
 
-0.354
 
 
12.3
 
 
 
 
ADGRF5
 
 
hsa-miR-152-3p
 
 
-0.354
 
 
12.3
 
 
 
 
ADIPOR2
 
 
hsa-miR-150-5p
 
 
-0.287
 
 
77.7
 
 
 
 
ADIPOR2
 
 
hsa-miR-34a-5p
 
 
-0.33
 
 
77.7
 
 
 
 
ADK
 
 
hsa-miR-34a-5p
 
 
-0.374
 
 
141.2
 
 
 
 
AFG3L2
 
 
hsa-miR-181b-5p
 
 
-0.491
 
 
25.9
 
 
 
 
AFG3L2
 
 
hsa-miR-181a-5p
 
 
-0.491
 
 
25.9
 
 
 
 
AGFG1
 
 
hsa-miR-148b-3p
 
 
-0.332
 
 
33.8
 
 
 
 
AGFG1
 
 
hsa-miR-152-3p
 
 
-0.332
 
 
33.8
 
 
 
 
AGGF1
 
 
hsa-miR-125a-5p
 
 
-0.331
 
 
12.9
 
 
 
 
AGMAT
 
 
hsa-miR-22-3p
 
 
-0.294
 
 
58.0
 
 
 
 
AGPAT3
 
 
hsa-miR-27a-3p
 
 
-0.29
 
 
50.8
 
 
 
 
AGTRAP
 
 
hsa-miR-34a-5p
 
 
-0.328
 
 
27.6
 
 
 
 
AHR
 
 
hsa-miR-29a-3p
 
 
-0.316
 
 
42.0
 
 
 
 
AHSG
 
 
hsa-miR-27a-3p
 
 
-0.29
 
 
5439.8
 
 
 
 
AIDA
 
 
hsa-miR-22-3p
 
 
-0.316
 
 
45.8
 
 
 
 
AIFM1
 
 
hsa-miR-125a-5p
 
 
-0.3
 
 
77.0
 
 
 
 
AK1
 
 
hsa-miR-29a-3p
 
 
-0.333
 
 
35.8
 
 
 
 
AKAP1
 
 
hsa-miR-148b-3p
 
 
-0.381
 
 
48.4
 
 
 
 
AKAP1
 
 
hsa-miR-152-3p
 
 
-0.381
 
 
48.4
 
 
 
 
AKIRIN1
 
 
hsa-miR-27a-3p
 
 
-0.368
 
 
49.7
 
 
 
 
ALCAM
 
 
hsa-miR-34a-5p
 
 
-0.303
 
 
44.8
 
 
 
 
ALCAM
 
 
hsa-miR-148b-3p
 
 
-0.339
 
 
44.8
 
 
 
 
ALCAM
 
 
hsa-miR-152-3p
 
 
-0.347
 
 
44.8
 
 
 
 
ALDOA
 
 
hsa-miR-34a-5p
 
 
-0.655
 
 
253.5
 
 
 
 
ALPL
 
 
hsa-miR-10a-5p
 
 
-0.32
 
 
52.2
 
 
 
 
ANAPC16
 
 
hsa-miR-125a-5p
 
 
-0.578
 
 
25.2
 
 
 
 
ANK3
 
 
hsa-miR-34a-5p
 
 
-0.29
 
 
15.4
 
 
 
 
ANK3
 
 
hsa-miR-34a-5p
 
 
-0.347
 
 
15.4
 
 
 
 
ANKHD1
 
 
hsa-miR-23a-3p
 
 
-0.368
 
 
51.5
 
 
 
 
ANKHD1
 
 
hsa-miR-23b-3p
 
 
-0.368
 
 
51.5
 
 
 
 
ANKRD13A
 
 
hsa-miR-22-3p
 
 
-0.478
 
 
14.3
 
 
 
 
ANKRD40
 
 
hsa-miR-27a-3p
 
 
-0.35
 
 
16.6
 
 
 
 
ANP32A
 
 
hsa-miR-132-3p
 
 
-0.374
 
 
71.3
 
 
 
 
ANP32B
 
 
hsa-miR-34a-5p
 
 
-0.29
 
 
96.8
 
 
 
 
ANPEP
 
 
hsa-miR-125a-5p
 
 
-0.372
 
 
256.4
 
 
 
 
ANTXR2
 
 
hsa-miR-29a-3p
 
 
-0.347
 
 
32.2
 
 
 
 
AP3S2
 
 
hsa-miR-34a-5p
 
 
-0.413
 
 
37.7
 
 
 
 
APH1A
 
 
hsa-miR-34a-5p
 
 
-0.304
 
 
127.5
 
 
 
 
APOC4
 
 
hsa-miR-125a-5p
 
 
-0.515
 
 
585.9
 
 
 
 
APOO
 
 
hsa-miR-181b-5p
 
 
-0.5
 
 
17.0
 
 
 
 
APOO
 
 
hsa-miR-181a-5p
 
 
-0.5
 
 
17.0
 
 
 
 
APPBP2
 
 
hsa-miR-27a-3p
 
 
-0.378
 
 
10.3
 
 
 
 
AQP11
 
 
hsa-miR-27a-3p
 
 
-0.666
 
 
15.1
 
 
 
 
ARF4
 
 
hsa-miR-152-3p
 
 
-0.487
 
 
161.3
 
 
 
 
ARF4
 
 
hsa-miR-148b-3p
 
 
-0.487
 
 
161.3
 
 
 
 
ARF5
 
 
hsa-miR-29a-3p
 
 
-0.491
 
 
153.7
 
 
 
 
ARF6
 
 
hsa-miR-181b-5p
 
 
-0.33
 
 
45.4
 
 
 
 
ARF6
 
 
hsa-miR-181a-5p
 
 
-0.33
 
 
45.4
 
 
 
 
ARFGEF1
 
 
hsa-miR-148b-3p
 
 
-0.306
 
 
14.2
 
 
 
 
ARFGEF1
 
 
hsa-miR-152-3p
 
 
-0.306
 
 
14.2
 
 
 
 
ARFIP1
 
 
hsa-miR-152-3p
 
 
-0.474
 
 
14.4
 
 
 
 
ARFIP1
 
 
hsa-miR-148b-3p
 
 
-0.474
 
 
14.4
 
 
 
 
ARHGAP1
 
 
hsa-miR-34a-5p
 
 
-0.369
 
 
44.2
 
 
 
 
ARHGAP21
 
 
hsa-miR-152-3p
 
 
-0.287
 
 
11.9
 
 
 
 
ARHGAP21
 
 
hsa-miR-148b-3p
 
 
-0.287
 
 
11.9
 
 
 
 
ARHGEF26
 
 
hsa-miR-22-3p
 
 
-0.374
 
 
14.5
 
 
 
 
ARL4A
 
 
hsa-miR-29a-3p
 
 
-0.386
 
 
14.6
 
 
 
 
ARL5B
 
 
hsa-miR-29a-3p
 
 
-0.304
 
 
27.9
 
 
 
 
ARL6IP1
 
 
hsa-miR-148b-3p
 
 
-0.579
 
 
336.2
 
 
 
 
ARL6IP1
 
 
hsa-miR-152-3p
 
 
-0.579
 
 
336.2
 
 
 
 
ARL8B
 
 
hsa-miR-152-3p
 
 
-0.337
 
 
41.1
 
 
 
 
ARL8B
 
 
hsa-miR-148b-3p
 
 
-0.337
 
 
41.1
 
 
 
 
ARMCX3
 
 
hsa-miR-425-5p
 
 
-0.325
 
 
22.5
 
 
 
 
ARPC5
 
 
hsa-miR-22-3p
 
 
-0.679
 
 
60.1
 
 
 
 
ARPIN-AP3S2
 
 
hsa-miR-34a-5p
 
 
-0.348
 
 
21.4
 
 
 
 
ARPP19
 
 
hsa-miR-29a-3p
 
 
-0.371
 
 
39.5
 
 
 
 
ARPP19
 
 
hsa-miR-34a-5p
 
 
-0.497
 
 
39.5
 
 
 
 
ARRDC3
 
 
hsa-miR-152-3p
 
 
-0.305
 
 
71.0
 
 
 
 
ARRDC3
 
 
hsa-miR-148b-3p
 
 
-0.305
 
 
71.0
 
 
 
 
ARRDC3
 
 
hsa-miR-29a-3p
 
 
-0.402
 
 
71.0
 
 
 
 
ASAH1
 
 
hsa-miR-27a-3p
 
 
-0.398
 
 
56.9
 
 
 
 
 
 
 stomach 
 
 
 
 
target_symbol
 
 
mature_mirna_id
 
 
score
 
 
nTPM
 
 
 
 
 
 
A4GNT
 
 
hsa-miR-22-3p
 
 
-0.601
 
 
22.3
 
 
 
 
AAGAB
 
 
hsa-miR-34a-5p
 
 
-0.319
 
 
22.5
 
 
 
 
AAR2
 
 
hsa-miR-29a-3p
 
 
-0.327
 
 
21.7
 
 
 
 
ABCB7
 
 
hsa-miR-148b-3p
 
 
-0.515
 
 
11.6
 
 
 
 
ABCB7
 
 
hsa-miR-152-3p
 
 
-0.515
 
 
11.6
 
 
 
 
ABCC5
 
 
hsa-miR-125a-5p
 
 
-0.288
 
 
77.7
 
 
 
 
ABCD3
 
 
hsa-miR-148b-3p
 
 
-0.388
 
 
41.1
 
 
 
 
ABCD3
 
 
hsa-miR-152-3p
 
 
-0.388
 
 
41.1
 
 
 
 
ABCE1
 
 
hsa-miR-29a-3p
 
 
-0.404
 
 
19.0
 
 
 
 
ABHD17C
 
 
hsa-miR-27a-3p
 
 
-0.435
 
 
34.0
 
 
 
 
ABHD3
 
 
hsa-miR-125a-5p
 
 
-0.453
 
 
33.6
 
 
 
 
ABHD4
 
 
hsa-miR-29a-3p
 
 
-0.312
 
 
26.1
 
 
 
 
ABHD6
 
 
hsa-miR-125a-5p
 
 
-0.501
 
 
12.2
 
 
 
 
ABTB1
 
 
hsa-miR-125a-5p
 
 
-0.519
 
 
28.0
 
 
 
 
ACBD3
 
 
hsa-miR-34a-5p
 
 
-0.415
 
 
29.9
 
 
 
 
ACER2
 
 
hsa-miR-125a-5p
 
 
-0.338
 
 
58.3
 
 
 
 
ACSL1
 
 
hsa-miR-34a-5p
 
 
-0.306
 
 
50.6
 
 
 
 
ACSL4
 
 
hsa-miR-34a-5p
 
 
-0.372
 
 
17.6
 
 
 
 
ACTA2
 
 
hsa-miR-27a-3p
 
 
-0.577
 
 
1353.4
 
 
 
 
ACVR1
 
 
hsa-miR-152-3p
 
 
-0.397
 
 
11.0
 
 
 
 
ACVR1
 
 
hsa-miR-148b-3p
 
 
-0.397
 
 
11.0
 
 
 
 
ADA
 
 
hsa-miR-29a-3p
 
 
-0.376
 
 
44.4
 
 
 
 
ADAM10
 
 
hsa-miR-152-3p
 
 
-0.348
 
 
19.0
 
 
 
 
ADAM10
 
 
hsa-miR-148b-3p
 
 
-0.356
 
 
19.0
 
 
 
 
ADAP1
 
 
hsa-miR-27a-3p
 
 
-0.302
 
 
18.4
 
 
 
 
ADCY3
 
 
hsa-miR-132-3p
 
 
-0.303
 
 
23.8
 
 
 
 
ADIPOR2
 
 
hsa-miR-150-5p
 
 
-0.287
 
 
42.5
 
 
 
 
ADIPOR2
 
 
hsa-miR-34a-5p
 
 
-0.33
 
 
42.5
 
 
 
 
ADK
 
 
hsa-miR-34a-5p
 
 
-0.374
 
 
11.3
 
 
 
 
AFG3L2
 
 
hsa-miR-181b-5p
 
 
-0.491
 
 
26.3
 
 
 
 
AFG3L2
 
 
hsa-miR-181a-5p
 
 
-0.491
 
 
26.3
 
 
 
 
AGFG1
 
 
hsa-miR-148b-3p
 
 
-0.332
 
 
31.3
 
 
 
 
AGFG1
 
 
hsa-miR-152-3p
 
 
-0.332
 
 
31.3
 
 
 
 
AGPAT3
 
 
hsa-miR-27a-3p
 
 
-0.29
 
 
16.9
 
 
 
 
AGTRAP
 
 
hsa-miR-34a-5p
 
 
-0.328
 
 
31.9
 
 
 
 
AHCYL2
 
 
hsa-miR-34a-5p
 
 
-0.359
 
 
39.6
 
 
 
 
AHDC1
 
 
hsa-miR-148b-3p
 
 
-0.413
 
 
10.8
 
 
 
 
AHDC1
 
 
hsa-miR-152-3p
 
 
-0.413
 
 
10.8
 
 
 
 
AHR
 
 
hsa-miR-29a-3p
 
 
-0.316
 
 
23.8
 
 
 
 
AIDA
 
 
hsa-miR-22-3p
 
 
-0.316
 
 
26.6
 
 
 
 
AIFM1
 
 
hsa-miR-125a-5p
 
 
-0.3
 
 
29.8
 
 
 
 
AK1
 
 
hsa-miR-29a-3p
 
 
-0.333
 
 
28.6
 
 
 
 
AKAP1
 
 
hsa-miR-148b-3p
 
 
-0.381
 
 
53.0
 
 
 
 
AKAP1
 
 
hsa-miR-152-3p
 
 
-0.381
 
 
53.0
 
 
 
 
AKIRIN1
 
 
hsa-miR-27a-3p
 
 
-0.368
 
 
31.7
 
 
 
 
ALCAM
 
 
hsa-miR-34a-5p
 
 
-0.303
 
 
31.6
 
 
 
 
ALCAM
 
 
hsa-miR-148b-3p
 
 
-0.339
 
 
31.6
 
 
 
 
ALCAM
 
 
hsa-miR-152-3p
 
 
-0.347
 
 
31.6
 
 
 
 
ALDOA
 
 
hsa-miR-34a-5p
 
 
-0.655
 
 
700.2
 
 
 
 
ANAPC16
 
 
hsa-miR-125a-5p
 
 
-0.578
 
 
24.4
 
 
 
 
ANK3
 
 
hsa-miR-34a-5p
 
 
-0.29
 
 
14.2
 
 
 
 
ANK3
 
 
hsa-miR-34a-5p
 
 
-0.347
 
 
14.2
 
 
 
 
ANKHD1
 
 
hsa-miR-23a-3p
 
 
-0.368
 
 
39.4
 
 
 
 
ANKHD1
 
 
hsa-miR-23b-3p
 
 
-0.368
 
 
39.4
 
 
 
 
ANKHD1-EIF4EBP3
 
 
hsa-miR-22-3p
 
 
-0.479
 
 
11.7
 
 
 
 
ANKRD13A
 
 
hsa-miR-22-3p
 
 
-0.478
 
 
16.1
 
 
 
 
ANKRD29
 
 
hsa-miR-132-3p
 
 
-0.47
 
 
14.3
 
 
 
 
ANKRD40
 
 
hsa-miR-27a-3p
 
 
-0.35
 
 
17.6
 
 
 
 
ANP32A
 
 
hsa-miR-132-3p
 
 
-0.374
 
 
74.4
 
 
 
 
ANP32B
 
 
hsa-miR-34a-5p
 
 
-0.29
 
 
125.6
 
 
 
 
ANTXR2
 
 
hsa-miR-29a-3p
 
 
-0.347
 
 
46.6
 
 
 
 
APH1A
 
 
hsa-miR-34a-5p
 
 
-0.304
 
 
90.3
 
 
 
 
APOO
 
 
hsa-miR-181b-5p
 
 
-0.5
 
 
17.0
 
 
 
 
APOO
 
 
hsa-miR-181a-5p
 
 
-0.5
 
 
17.0
 
 
 
 
APPBP2
 
 
hsa-miR-27a-3p
 
 
-0.378
 
 
10.1
 
 
 
 
AREG
 
 
hsa-miR-34a-5p
 
 
-0.62
 
 
19.1
 
 
 
 
ARF4
 
 
hsa-miR-152-3p
 
 
-0.487
 
 
123.5
 
 
 
 
ARF4
 
 
hsa-miR-148b-3p
 
 
-0.487
 
 
123.5
 
 
 
 
ARF5
 
 
hsa-miR-29a-3p
 
 
-0.491
 
 
126.0
 
 
 
 
ARF6
 
 
hsa-miR-181b-5p
 
 
-0.33
 
 
44.4
 
 
 
 
ARF6
 
 
hsa-miR-181a-5p
 
 
-0.33
 
 
44.4
 
 
 
 
ARFGEF1
 
 
hsa-miR-148b-3p
 
 
-0.306
 
 
18.5
 
 
 
 
ARFGEF1
 
 
hsa-miR-152-3p
 
 
-0.306
 
 
18.5
 
 
 
 
ARFIP1
 
 
hsa-miR-152-3p
 
 
-0.474
 
 
21.5
 
 
 
 
ARFIP1
 
 
hsa-miR-148b-3p
 
 
-0.474
 
 
21.5
 
 
 
 
ARHGAP1
 
 
hsa-miR-34a-5p
 
 
-0.369
 
 
46.3
 
 
 
 
ARHGAP21
 
 
hsa-miR-152-3p
 
 
-0.287
 
 
25.5
 
 
 
 
ARHGAP21
 
 
hsa-miR-148b-3p
 
 
-0.287
 
 
25.5
 
 
 
 
ARHGAP26
 
 
hsa-miR-34a-5p
 
 
-0.384
 
 
14.4
 
 
 
 
ARID1B
 
 
hsa-miR-29a-3p
 
 
-0.342
 
 
11.1
 
 
 
 
ARL4A
 
 
hsa-miR-29a-3p
 
 
-0.386
 
 
24.0
 
 
 
 
ARL6IP1
 
 
hsa-miR-148b-3p
 
 
-0.579
 
 
118.7
 
 
 
 
ARL6IP1
 
 
hsa-miR-152-3p
 
 
-0.579
 
 
118.7
 
 
 
 
ARL8B
 
 
hsa-miR-152-3p
 
 
-0.337
 
 
32.2
 
 
 
 
ARL8B
 
 
hsa-miR-148b-3p
 
 
-0.337
 
 
32.2
 
 
 
 
ARMCX3
 
 
hsa-miR-425-5p
 
 
-0.325
 
 
26.2
 
 
 
 
ARPC5
 
 
hsa-miR-22-3p
 
 
-0.679
 
 
83.5
 
 
 
 
ARPP19
 
 
hsa-miR-29a-3p
 
 
-0.371
 
 
36.4
 
 
 
 
ARPP19
 
 
hsa-miR-34a-5p
 
 
-0.497
 
 
36.4
 
 
 
 
ARRB1
 
 
hsa-miR-22-3p
 
 
-0.616
 
 
31.7
 
 
 
 
ARRDC3
 
 
hsa-miR-152-3p
 
 
-0.305
 
 
24.3
 
 
 
 
ARRDC3
 
 
hsa-miR-148b-3p
 
 
-0.305
 
 
24.3
 
 
 
 
ARRDC3
 
 
hsa-miR-29a-3p
 
 
-0.402
 
 
24.3
 
 
 
 
ASAH1
 
 
hsa-miR-27a-3p
 
 
-0.398
 
 
57.7
 
 
 
 
ASAP2
 
 
hsa-miR-29a-3p
 
 
-0.348
 
 
22.9
 
 
 
 
ASF1A
 
 
hsa-miR-23a-3p
 
 
-0.506
 
 
10.3
 
 
 
 
ASF1A
 
 
hsa-miR-23b-3p
 
 
-0.506
 
 
10.3
 
 
 
 
ASPH
 
 
hsa-miR-152-3p
 
 
-0.391
 
 
96.9
 
 
 
 
ASPH
 
 
hsa-miR-148b-3p
 
 
-0.391
 
 
96.9
 
 
 
 
ATG4B
 
 
hsa-miR-34a-5p
 
 
-0.58
 
 
18.1
 
 
 
 
 
 
 adipose tissue 
 
 
 
 
target_symbol
 
 
mature_mirna_id
 
 
score
 
 
nTPM
 
 
 
 
 
 
AAGAB
 
 
hsa-miR-34a-5p
 
 
-0.319
 
 
14.1
 
 
 
 
AAR2
 
 
hsa-miR-29a-3p
 
 
-0.327
 
 
21.1
 
 
 
 
ABCB7
 
 
hsa-miR-148b-3p
 
 
-0.515
 
 
12.4
 
 
 
 
ABCB7
 
 
hsa-miR-152-3p
 
 
-0.515
 
 
12.4
 
 
 
 
ABCC5
 
 
hsa-miR-125a-5p
 
 
-0.288
 
 
15.3
 
 
 
 
ABCD3
 
 
hsa-miR-148b-3p
 
 
-0.388
 
 
15.7
 
 
 
 
ABCD3
 
 
hsa-miR-152-3p
 
 
-0.388
 
 
15.7
 
 
 
 
ABCE1
 
 
hsa-miR-29a-3p
 
 
-0.404
 
 
28.1
 
 
 
 
ABHD3
 
 
hsa-miR-125a-5p
 
 
-0.453
 
 
16.0
 
 
 
 
ABHD4
 
 
hsa-miR-29a-3p
 
 
-0.312
 
 
24.3
 
 
 
 
ABHD5
 
 
hsa-miR-132-3p
 
 
-0.323
 
 
27.9
 
 
 
 
ABHD5
 
 
hsa-miR-27a-3p
 
 
-0.407
 
 
27.9
 
 
 
 
ABHD6
 
 
hsa-miR-125a-5p
 
 
-0.501
 
 
11.4
 
 
 
 
ABTB1
 
 
hsa-miR-125a-5p
 
 
-0.519
 
 
38.9
 
 
 
 
ACBD3
 
 
hsa-miR-34a-5p
 
 
-0.415
 
 
20.3
 
 
 
 
ACER3
 
 
hsa-miR-22-3p
 
 
-0.453
 
 
10.2
 
 
 
 
ACSL1
 
 
hsa-miR-34a-5p
 
 
-0.306
 
 
680.0
 
 
 
 
ACSL4
 
 
hsa-miR-34a-5p
 
 
-0.372
 
 
38.3
 
 
 
 
ACTA2
 
 
hsa-miR-27a-3p
 
 
-0.577
 
 
979.1
 
 
 
 
ACVR1
 
 
hsa-miR-152-3p
 
 
-0.397
 
 
12.6
 
 
 
 
ACVR1
 
 
hsa-miR-148b-3p
 
 
-0.397
 
 
12.6
 
 
 
 
ADA
 
 
hsa-miR-29a-3p
 
 
-0.376
 
 
16.5
 
 
 
 
ADAM10
 
 
hsa-miR-152-3p
 
 
-0.348
 
 
14.0
 
 
 
 
ADAM10
 
 
hsa-miR-148b-3p
 
 
-0.356
 
 
14.0
 
 
 
 
ADAMTS10
 
 
hsa-miR-27a-3p
 
 
-0.354
 
 
13.8
 
 
 
 
ADAMTS10
 
 
hsa-miR-29a-3p
 
 
-0.516
 
 
13.8
 
 
 
 
ADAMTS9
 
 
hsa-miR-29a-3p
 
 
-0.305
 
 
39.1
 
 
 
 
ADAMTS9
 
 
hsa-miR-29a-3p
 
 
-0.485
 
 
39.1
 
 
 
 
ADCY3
 
 
hsa-miR-132-3p
 
 
-0.303
 
 
90.0
 
 
 
 
ADGRF5
 
 
hsa-miR-148b-3p
 
 
-0.354
 
 
66.6
 
 
 
 
ADGRF5
 
 
hsa-miR-152-3p
 
 
-0.354
 
 
66.6
 
 
 
 
ADIPOR2
 
 
hsa-miR-150-5p
 
 
-0.287
 
 
53.2
 
 
 
 
ADIPOR2
 
 
hsa-miR-34a-5p
 
 
-0.33
 
 
53.2
 
 
 
 
ADK
 
 
hsa-miR-34a-5p
 
 
-0.374
 
 
22.4
 
 
 
 
ADRA2A
 
 
hsa-miR-23a-3p
 
 
-0.371
 
 
46.9
 
 
 
 
ADRA2A
 
 
hsa-miR-23b-3p
 
 
-0.371
 
 
46.9
 
 
 
 
AFG3L2
 
 
hsa-miR-181b-5p
 
 
-0.491
 
 
17.1
 
 
 
 
AFG3L2
 
 
hsa-miR-181a-5p
 
 
-0.491
 
 
17.1
 
 
 
 
AGFG1
 
 
hsa-miR-148b-3p
 
 
-0.332
 
 
64.8
 
 
 
 
AGFG1
 
 
hsa-miR-152-3p
 
 
-0.332
 
 
64.8
 
 
 
 
AGPAT3
 
 
hsa-miR-27a-3p
 
 
-0.29
 
 
19.6
 
 
 
 
AGTRAP
 
 
hsa-miR-34a-5p
 
 
-0.328
 
 
41.4
 
 
 
 
AHDC1
 
 
hsa-miR-148b-3p
 
 
-0.413
 
 
11.7
 
 
 
 
AHDC1
 
 
hsa-miR-152-3p
 
 
-0.413
 
 
11.7
 
 
 
 
AHR
 
 
hsa-miR-29a-3p
 
 
-0.316
 
 
21.0
 
 
 
 
AIDA
 
 
hsa-miR-22-3p
 
 
-0.316
 
 
67.8
 
 
 
 
AIFM1
 
 
hsa-miR-125a-5p
 
 
-0.3
 
 
17.4
 
 
 
 
AK1
 
 
hsa-miR-29a-3p
 
 
-0.333
 
 
30.1
 
 
 
 
AKAP1
 
 
hsa-miR-148b-3p
 
 
-0.381
 
 
38.1
 
 
 
 
AKAP1
 
 
hsa-miR-152-3p
 
 
-0.381
 
 
38.1
 
 
 
 
AKIRIN1
 
 
hsa-miR-27a-3p
 
 
-0.368
 
 
43.1
 
 
 
 
AKT3
 
 
hsa-miR-29a-3p
 
 
-0.287
 
 
16.9
 
 
 
 
AKT3
 
 
hsa-miR-22-3p
 
 
-0.451
 
 
16.9
 
 
 
 
ALDH1A2
 
 
hsa-miR-23b-3p
 
 
-0.346
 
 
20.6
 
 
 
 
ALDH1A2
 
 
hsa-miR-23a-3p
 
 
-0.354
 
 
20.6
 
 
 
 
ALDOA
 
 
hsa-miR-34a-5p
 
 
-0.655
 
 
657.0
 
 
 
 
ALPL
 
 
hsa-miR-10a-5p
 
 
-0.32
 
 
23.7
 
 
 
 
ANAPC16
 
 
hsa-miR-125a-5p
 
 
-0.578
 
 
18.9
 
 
 
 
ANK2
 
 
hsa-miR-34a-5p
 
 
-0.317
 
 
23.6
 
 
 
 
ANKHD1
 
 
hsa-miR-23a-3p
 
 
-0.368
 
 
37.3
 
 
 
 
ANKHD1
 
 
hsa-miR-23b-3p
 
 
-0.368
 
 
37.3
 
 
 
 
ANKRD13A
 
 
hsa-miR-22-3p
 
 
-0.478
 
 
12.3
 
 
 
 
ANKRD29
 
 
hsa-miR-132-3p
 
 
-0.47
 
 
17.0
 
 
 
 
ANKRD40
 
 
hsa-miR-27a-3p
 
 
-0.35
 
 
40.1
 
 
 
 
ANP32A
 
 
hsa-miR-132-3p
 
 
-0.374
 
 
47.7
 
 
 
 
ANP32B
 
 
hsa-miR-34a-5p
 
 
-0.29
 
 
136.8
 
 
 
 
ANPEP
 
 
hsa-miR-125a-5p
 
 
-0.372
 
 
50.1
 
 
 
 
ANTXR2
 
 
hsa-miR-29a-3p
 
 
-0.347
 
 
57.1
 
 
 
 
AP3S2
 
 
hsa-miR-34a-5p
 
 
-0.413
 
 
22.7
 
 
 
 
APH1A
 
 
hsa-miR-34a-5p
 
 
-0.304
 
 
69.8
 
 
 
 
APPBP2
 
 
hsa-miR-27a-3p
 
 
-0.378
 
 
13.9
 
 
 
 
AREG
 
 
hsa-miR-34a-5p
 
 
-0.62
 
 
45.9
 
 
 
 
ARF4
 
 
hsa-miR-152-3p
 
 
-0.487
 
 
118.7
 
 
 
 
ARF4
 
 
hsa-miR-148b-3p
 
 
-0.487
 
 
118.7
 
 
 
 
ARF5
 
 
hsa-miR-29a-3p
 
 
-0.491
 
 
109.3
 
 
 
 
ARF6
 
 
hsa-miR-181b-5p
 
 
-0.33
 
 
30.0
 
 
 
 
ARF6
 
 
hsa-miR-181a-5p
 
 
-0.33
 
 
30.0
 
 
 
 
ARFGEF1
 
 
hsa-miR-148b-3p
 
 
-0.306
 
 
10.7
 
 
 
 
ARFGEF1
 
 
hsa-miR-152-3p
 
 
-0.306
 
 
10.7
 
 
 
 
ARFIP1
 
 
hsa-miR-152-3p
 
 
-0.474
 
 
15.8
 
 
 
 
ARFIP1
 
 
hsa-miR-148b-3p
 
 
-0.474
 
 
15.8
 
 
 
 
ARHGAP1
 
 
hsa-miR-34a-5p
 
 
-0.369
 
 
79.6
 
 
 
 
ARHGAP21
 
 
hsa-miR-152-3p
 
 
-0.287
 
 
26.1
 
 
 
 
ARHGAP21
 
 
hsa-miR-148b-3p
 
 
-0.287
 
 
26.1
 
 
 
 
ARHGAP26
 
 
hsa-miR-34a-5p
 
 
-0.384
 
 
11.2
 
 
 
 
ARID1B
 
 
hsa-miR-29a-3p
 
 
-0.342
 
 
14.1
 
 
 
 
ARL4A
 
 
hsa-miR-29a-3p
 
 
-0.386
 
 
30.8
 
 
 
 
ARL5B
 
 
hsa-miR-29a-3p
 
 
-0.304
 
 
10.5
 
 
 
 
ARL6IP1
 
 
hsa-miR-148b-3p
 
 
-0.579
 
 
133.2
 
 
 
 
ARL6IP1
 
 
hsa-miR-152-3p
 
 
-0.579
 
 
133.2
 
 
 
 
ARL8B
 
 
hsa-miR-152-3p
 
 
-0.337
 
 
39.1
 
 
 
 
ARL8B
 
 
hsa-miR-148b-3p
 
 
-0.337
 
 
39.1
 
 
 
 
ARMCX3
 
 
hsa-miR-425-5p
 
 
-0.325
 
 
32.7
 
 
 
 
ARPC5
 
 
hsa-miR-22-3p
 
 
-0.679
 
 
80.7
 
 
 
 
ARPIN-AP3S2
 
 
hsa-miR-34a-5p
 
 
-0.348
 
 
11.1
 
 
 
 
ARPP19
 
 
hsa-miR-29a-3p
 
 
-0.371
 
 
57.4
 
 
 
 
ARPP19
 
 
hsa-miR-34a-5p
 
 
-0.497
 
 
57.4
 
 
 
 
ARRB1
 
 
hsa-miR-22-3p
 
 
-0.616
 
 
28.8
 
 
 
 
ARRDC3
 
 
hsa-miR-152-3p
 
 
-0.305
 
 
34.5
 
 
 
 
ARRDC3
 
 
hsa-miR-148b-3p
 
 
-0.305
 
 
34.5
 
 
 
 
 
 
 adrenal gland 
 
 
 
 
target_symbol
 
 
mature_mirna_id
 
 
score
 
 
nTPM
 
 
 
 
 
 
AAGAB
 
 
hsa-miR-34a-5p
 
 
-0.319
 
 
17.7
 
 
 
 
AAR2
 
 
hsa-miR-29a-3p
 
 
-0.327
 
 
24.5
 
 
 
 
ABCB7
 
 
hsa-miR-148b-3p
 
 
-0.515
 
 
12.5
 
 
 
 
ABCB7
 
 
hsa-miR-152-3p
 
 
-0.515
 
 
12.5
 
 
 
 
ABCC5
 
 
hsa-miR-125a-5p
 
 
-0.288
 
 
23.1
 
 
 
 
ABCD3
 
 
hsa-miR-148b-3p
 
 
-0.388
 
 
17.6
 
 
 
 
ABCD3
 
 
hsa-miR-152-3p
 
 
-0.388
 
 
17.6
 
 
 
 
ABCE1
 
 
hsa-miR-29a-3p
 
 
-0.404
 
 
20.3
 
 
 
 
ABHD4
 
 
hsa-miR-29a-3p
 
 
-0.312
 
 
29.6
 
 
 
 
ABHD6
 
 
hsa-miR-125a-5p
 
 
-0.501
 
 
13.2
 
 
 
 
ABTB1
 
 
hsa-miR-125a-5p
 
 
-0.519
 
 
19.3
 
 
 
 
ACBD3
 
 
hsa-miR-34a-5p
 
 
-0.415
 
 
16.0
 
 
 
 
ACSL1
 
 
hsa-miR-34a-5p
 
 
-0.306
 
 
41.7
 
 
 
 
ACSL4
 
 
hsa-miR-34a-5p
 
 
-0.372
 
 
34.9
 
 
 
 
ACTA2
 
 
hsa-miR-27a-3p
 
 
-0.577
 
 
227.6
 
 
 
 
ADCY3
 
 
hsa-miR-132-3p
 
 
-0.303
 
 
16.9
 
 
 
 
ADGRF5
 
 
hsa-miR-148b-3p
 
 
-0.354
 
 
22.0
 
 
 
 
ADGRF5
 
 
hsa-miR-152-3p
 
 
-0.354
 
 
22.0
 
 
 
 
ADIPOR2
 
 
hsa-miR-150-5p
 
 
-0.287
 
 
69.8
 
 
 
 
ADIPOR2
 
 
hsa-miR-34a-5p
 
 
-0.33
 
 
69.8
 
 
 
 
ADK
 
 
hsa-miR-34a-5p
 
 
-0.374
 
 
14.6
 
 
 
 
AFG3L2
 
 
hsa-miR-181b-5p
 
 
-0.491
 
 
12.9
 
 
 
 
AFG3L2
 
 
hsa-miR-181a-5p
 
 
-0.491
 
 
12.9
 
 
 
 
AGFG1
 
 
hsa-miR-148b-3p
 
 
-0.332
 
 
37.2
 
 
 
 
AGFG1
 
 
hsa-miR-152-3p
 
 
-0.332
 
 
37.2
 
 
 
 
AGGF1
 
 
hsa-miR-125a-5p
 
 
-0.331
 
 
10.0
 
 
 
 
AGPAT3
 
 
hsa-miR-27a-3p
 
 
-0.29
 
 
19.9
 
 
 
 
AGTRAP
 
 
hsa-miR-34a-5p
 
 
-0.328
 
 
46.3
 
 
 
 
AHDC1
 
 
hsa-miR-148b-3p
 
 
-0.413
 
 
23.0
 
 
 
 
AHDC1
 
 
hsa-miR-152-3p
 
 
-0.413
 
 
23.0
 
 
 
 
AHR
 
 
hsa-miR-29a-3p
 
 
-0.316
 
 
14.2
 
 
 
 
AIDA
 
 
hsa-miR-22-3p
 
 
-0.316
 
 
34.1
 
 
 
 
AIFM1
 
 
hsa-miR-125a-5p
 
 
-0.3
 
 
50.1
 
 
 
 
AK1
 
 
hsa-miR-29a-3p
 
 
-0.333
 
 
40.4
 
 
 
 
AKAP1
 
 
hsa-miR-148b-3p
 
 
-0.381
 
 
14.9
 
 
 
 
AKAP1
 
 
hsa-miR-152-3p
 
 
-0.381
 
 
14.9
 
 
 
 
AKIRIN1
 
 
hsa-miR-27a-3p
 
 
-0.368
 
 
28.2
 
 
 
 
ALCAM
 
 
hsa-miR-34a-5p
 
 
-0.303
 
 
30.9
 
 
 
 
ALCAM
 
 
hsa-miR-148b-3p
 
 
-0.339
 
 
30.9
 
 
 
 
ALCAM
 
 
hsa-miR-152-3p
 
 
-0.347
 
 
30.9
 
 
 
 
ALDOA
 
 
hsa-miR-34a-5p
 
 
-0.655
 
 
799.7
 
 
 
 
ALPL
 
 
hsa-miR-10a-5p
 
 
-0.32
 
 
57.7
 
 
 
 
ANAPC16
 
 
hsa-miR-125a-5p
 
 
-0.578
 
 
24.7
 
 
 
 
ANK2
 
 
hsa-miR-34a-5p
 
 
-0.317
 
 
10.7
 
 
 
 
ANKHD1
 
 
hsa-miR-23a-3p
 
 
-0.368
 
 
41.3
 
 
 
 
ANKHD1
 
 
hsa-miR-23b-3p
 
 
-0.368
 
 
41.3
 
 
 
 
ANKHD1-EIF4EBP3
 
 
hsa-miR-22-3p
 
 
-0.479
 
 
14.6
 
 
 
 
ANKRD40
 
 
hsa-miR-27a-3p
 
 
-0.35
 
 
18.5
 
 
 
 
ANP32A
 
 
hsa-miR-132-3p
 
 
-0.374
 
 
32.7
 
 
 
 
ANP32B
 
 
hsa-miR-34a-5p
 
 
-0.29
 
 
72.7
 
 
 
 
ANTXR2
 
 
hsa-miR-29a-3p
 
 
-0.347
 
 
28.3
 
 
 
 
AP3S2
 
 
hsa-miR-34a-5p
 
 
-0.413
 
 
19.2
 
 
 
 
APH1A
 
 
hsa-miR-34a-5p
 
 
-0.304
 
 
90.1
 
 
 
 
APOO
 
 
hsa-miR-181b-5p
 
 
-0.5
 
 
22.6
 
 
 
 
APOO
 
 
hsa-miR-181a-5p
 
 
-0.5
 
 
22.6
 
 
 
 
APPBP2
 
 
hsa-miR-27a-3p
 
 
-0.378
 
 
10.6
 
 
 
 
AQP11
 
 
hsa-miR-27a-3p
 
 
-0.666
 
 
14.1
 
 
 
 
ARF4
 
 
hsa-miR-152-3p
 
 
-0.487
 
 
151.0
 
 
 
 
ARF4
 
 
hsa-miR-148b-3p
 
 
-0.487
 
 
151.0
 
 
 
 
ARF5
 
 
hsa-miR-29a-3p
 
 
-0.491
 
 
102.3
 
 
 
 
ARF6
 
 
hsa-miR-181b-5p
 
 
-0.33
 
 
24.5
 
 
 
 
ARF6
 
 
hsa-miR-181a-5p
 
 
-0.33
 
 
24.5
 
 
 
 
ARFIP1
 
 
hsa-miR-152-3p
 
 
-0.474
 
 
13.9
 
 
 
 
ARFIP1
 
 
hsa-miR-148b-3p
 
 
-0.474
 
 
13.9
 
 
 
 
ARHGAP1
 
 
hsa-miR-34a-5p
 
 
-0.369
 
 
46.3
 
 
 
 
ARHGAP21
 
 
hsa-miR-152-3p
 
 
-0.287
 
 
16.9
 
 
 
 
ARHGAP21
 
 
hsa-miR-148b-3p
 
 
-0.287
 
 
16.9
 
 
 
 
ARHGAP36
 
 
hsa-miR-34a-5p
 
 
-0.367
 
 
99.5
 
 
 
 
ARID1B
 
 
hsa-miR-29a-3p
 
 
-0.342
 
 
10.7
 
 
 
 
ARL5B
 
 
hsa-miR-29a-3p
 
 
-0.304
 
 
19.6
 
 
 
 
ARL6IP1
 
 
hsa-miR-148b-3p
 
 
-0.579
 
 
161.7
 
 
 
 
ARL6IP1
 
 
hsa-miR-152-3p
 
 
-0.579
 
 
161.7
 
 
 
 
ARL8B
 
 
hsa-miR-152-3p
 
 
-0.337
 
 
48.3
 
 
 
 
ARL8B
 
 
hsa-miR-148b-3p
 
 
-0.337
 
 
48.3
 
 
 
 
ARMCX3
 
 
hsa-miR-425-5p
 
 
-0.325
 
 
55.6
 
 
 
 
ARPC5
 
 
hsa-miR-22-3p
 
 
-0.679
 
 
46.4
 
 
 
 
ARPIN-AP3S2
 
 
hsa-miR-34a-5p
 
 
-0.348
 
 
11.3
 
 
 
 
ARPP19
 
 
hsa-miR-29a-3p
 
 
-0.371
 
 
48.8
 
 
 
 
ARPP19
 
 
hsa-miR-34a-5p
 
 
-0.497
 
 
48.8
 
 
 
 
ARRDC3
 
 
hsa-miR-152-3p
 
 
-0.305
 
 
75.7
 
 
 
 
ARRDC3
 
 
hsa-miR-148b-3p
 
 
-0.305
 
 
75.7
 
 
 
 
ARRDC3
 
 
hsa-miR-29a-3p
 
 
-0.402
 
 
75.7
 
 
 
 
ASAH1
 
 
hsa-miR-27a-3p
 
 
-0.398
 
 
67.8
 
 
 
 
ASPH
 
 
hsa-miR-152-3p
 
 
-0.391
 
 
150.8
 
 
 
 
ASPH
 
 
hsa-miR-148b-3p
 
 
-0.391
 
 
150.8
 
 
 
 
ATG4B
 
 
hsa-miR-34a-5p
 
 
-0.58
 
 
19.2
 
 
 
 
ATMIN
 
 
hsa-miR-34a-5p
 
 
-0.409
 
 
12.0
 
 
 
 
ATP2B4
 
 
hsa-miR-152-3p
 
 
-0.337
 
 
23.7
 
 
 
 
ATP2B4
 
 
hsa-miR-148b-3p
 
 
-0.337
 
 
23.7
 
 
 
 
ATP5MC3
 
 
hsa-miR-425-5p
 
 
-0.328
 
 
190.4
 
 
 
 
ATP5MC3
 
 
hsa-miR-29a-3p
 
 
-0.33
 
 
190.4
 
 
 
 
ATP5MC3
 
 
hsa-miR-27a-3p
 
 
-0.416
 
 
190.4
 
 
 
 
ATP6AP2
 
 
hsa-miR-148b-3p
 
 
-0.592
 
 
67.5
 
 
 
 
ATP6AP2
 
 
hsa-miR-152-3p
 
 
-0.592
 
 
67.5
 
 
 
 
ATP6AP2
 
 
hsa-miR-148b-3p
 
 
-0.668
 
 
67.5
 
 
 
 
ATP6AP2
 
 
hsa-miR-152-3p
 
 
-0.668
 
 
67.5
 
 
 
 
ATP6V1E1
 
 
hsa-miR-23a-3p
 
 
-0.294
 
 
163.9
 
 
 
 
ATP6V1E1
 
 
hsa-miR-23b-3p
 
 
-0.294
 
 
163.9
 
 
 
 
ATXN10
 
 
hsa-miR-27a-3p
 
 
-0.295
 
 
56.3
 
 
 
 
ATXN2
 
 
hsa-miR-10a-5p
 
 
-0.362
 
 
24.4
 
 
 
 
 
 
 amygdala 
 
 
 
 
target_symbol
 
 
mature_mirna_id
 
 
score
 
 
nTPM
 
 
 
 
 
 
AAGAB
 
 
hsa-miR-34a-5p
 
 
-0.319
 
 
10.9
 
 
 
 
AAR2
 
 
hsa-miR-29a-3p
 
 
-0.327
 
 
10.0
 
 
 
 
ABCC5
 
 
hsa-miR-125a-5p
 
 
-0.288
 
 
24.0
 
 
 
 
ABCD3
 
 
hsa-miR-148b-3p
 
 
-0.388
 
 
16.0
 
 
 
 
ABCD3
 
 
hsa-miR-152-3p
 
 
-0.388
 
 
16.0
 
 
 
 
ABCE1
 
 
hsa-miR-29a-3p
 
 
-0.404
 
 
11.9
 
 
 
 
ABHD17C
 
 
hsa-miR-27a-3p
 
 
-0.435
 
 
17.9
 
 
 
 
ABHD3
 
 
hsa-miR-125a-5p
 
 
-0.453
 
 
10.3
 
 
 
 
ABHD4
 
 
hsa-miR-29a-3p
 
 
-0.312
 
 
16.0
 
 
 
 
ABHD6
 
 
hsa-miR-125a-5p
 
 
-0.501
 
 
15.3
 
 
 
 
ABTB1
 
 
hsa-miR-125a-5p
 
 
-0.519
 
 
23.8
 
 
 
 
ACHE
 
 
hsa-miR-125a-5p
 
 
-0.308
 
 
14.7
 
 
 
 
ACHE
 
 
hsa-miR-125a-5p
 
 
-0.361
 
 
14.7
 
 
 
 
ACSL1
 
 
hsa-miR-34a-5p
 
 
-0.306
 
 
24.5
 
 
 
 
ACSL6
 
 
hsa-miR-125a-5p
 
 
-0.307
 
 
20.3
 
 
 
 
ACTA2
 
 
hsa-miR-27a-3p
 
 
-0.577
 
 
36.7
 
 
 
 
ADAP1
 
 
hsa-miR-27a-3p
 
 
-0.302
 
 
109.3
 
 
 
 
ADCY2
 
 
hsa-miR-152-3p
 
 
-0.299
 
 
28.0
 
 
 
 
ADCY2
 
 
hsa-miR-148b-3p
 
 
-0.299
 
 
28.0
 
 
 
 
ADCY3
 
 
hsa-miR-132-3p
 
 
-0.303
 
 
27.0
 
 
 
 
ADD2
 
 
hsa-miR-34a-5p
 
 
-0.305
 
 
12.1
 
 
 
 
ADGRA3
 
 
hsa-miR-27a-3p
 
 
-0.296
 
 
19.9
 
 
 
 
ADGRB3
 
 
hsa-miR-152-3p
 
 
-0.516
 
 
12.8
 
 
 
 
ADGRB3
 
 
hsa-miR-148b-3p
 
 
-0.524
 
 
12.8
 
 
 
 
ADIPOR2
 
 
hsa-miR-150-5p
 
 
-0.287
 
 
43.7
 
 
 
 
ADIPOR2
 
 
hsa-miR-34a-5p
 
 
-0.33
 
 
43.7
 
 
 
 
ADK
 
 
hsa-miR-34a-5p
 
 
-0.374
 
 
16.1
 
 
 
 
AGFG1
 
 
hsa-miR-148b-3p
 
 
-0.332
 
 
25.1
 
 
 
 
AGFG1
 
 
hsa-miR-152-3p
 
 
-0.332
 
 
25.1
 
 
 
 
AGGF1
 
 
hsa-miR-125a-5p
 
 
-0.331
 
 
10.3
 
 
 
 
AGPAT3
 
 
hsa-miR-27a-3p
 
 
-0.29
 
 
41.1
 
 
 
 
AGPAT4
 
 
hsa-miR-29a-3p
 
 
-0.376
 
 
10.0
 
 
 
 
AGTRAP
 
 
hsa-miR-34a-5p
 
 
-0.328
 
 
23.7
 
 
 
 
AHCYL2
 
 
hsa-miR-34a-5p
 
 
-0.359
 
 
17.8
 
 
 
 
AHDC1
 
 
hsa-miR-148b-3p
 
 
-0.413
 
 
10.1
 
 
 
 
AHDC1
 
 
hsa-miR-152-3p
 
 
-0.413
 
 
10.1
 
 
 
 
AIDA
 
 
hsa-miR-22-3p
 
 
-0.316
 
 
28.0
 
 
 
 
AIFM1
 
 
hsa-miR-125a-5p
 
 
-0.3
 
 
10.3
 
 
 
 
AK1
 
 
hsa-miR-29a-3p
 
 
-0.333
 
 
104.5
 
 
 
 
AKAP1
 
 
hsa-miR-148b-3p
 
 
-0.381
 
 
22.8
 
 
 
 
AKAP1
 
 
hsa-miR-152-3p
 
 
-0.381
 
 
22.8
 
 
 
 
AKIRIN1
 
 
hsa-miR-27a-3p
 
 
-0.368
 
 
32.7
 
 
 
 
AKT3
 
 
hsa-miR-29a-3p
 
 
-0.287
 
 
17.4
 
 
 
 
AKT3
 
 
hsa-miR-22-3p
 
 
-0.451
 
 
17.4
 
 
 
 
ALCAM
 
 
hsa-miR-34a-5p
 
 
-0.303
 
 
31.7
 
 
 
 
ALCAM
 
 
hsa-miR-148b-3p
 
 
-0.339
 
 
31.7
 
 
 
 
ALCAM
 
 
hsa-miR-152-3p
 
 
-0.347
 
 
31.7
 
 
 
 
ALDOA
 
 
hsa-miR-34a-5p
 
 
-0.655
 
 
853.4
 
 
 
 
ALPL
 
 
hsa-miR-10a-5p
 
 
-0.32
 
 
10.6
 
 
 
 
AMER2
 
 
hsa-miR-34a-5p
 
 
-0.48
 
 
25.2
 
 
 
 
AMOT
 
 
hsa-miR-29a-3p
 
 
-0.304
 
 
18.6
 
 
 
 
ANGEL2
 
 
hsa-miR-132-3p
 
 
-0.357
 
 
10.6
 
 
 
 
ANGEL2
 
 
hsa-miR-22-3p
 
 
-0.601
 
 
10.6
 
 
 
 
ANK2
 
 
hsa-miR-34a-5p
 
 
-0.317
 
 
17.3
 
 
 
 
ANK3
 
 
hsa-miR-34a-5p
 
 
-0.29
 
 
37.4
 
 
 
 
ANK3
 
 
hsa-miR-34a-5p
 
 
-0.347
 
 
37.4
 
 
 
 
ANKHD1
 
 
hsa-miR-23a-3p
 
 
-0.368
 
 
18.9
 
 
 
 
ANKHD1
 
 
hsa-miR-23b-3p
 
 
-0.368
 
 
18.9
 
 
 
 
ANKRD40
 
 
hsa-miR-27a-3p
 
 
-0.35
 
 
96.8
 
 
 
 
ANP32A
 
 
hsa-miR-132-3p
 
 
-0.374
 
 
49.8
 
 
 
 
ANP32B
 
 
hsa-miR-34a-5p
 
 
-0.29
 
 
123.1
 
 
 
 
AP3S2
 
 
hsa-miR-34a-5p
 
 
-0.413
 
 
14.5
 
 
 
 
APC
 
 
hsa-miR-29a-3p
 
 
-0.413
 
 
38.7
 
 
 
 
APH1A
 
 
hsa-miR-34a-5p
 
 
-0.304
 
 
50.4
 
 
 
 
APOO
 
 
hsa-miR-181b-5p
 
 
-0.5
 
 
45.1
 
 
 
 
APOO
 
 
hsa-miR-181a-5p
 
 
-0.5
 
 
45.1
 
 
 
 
ARF4
 
 
hsa-miR-152-3p
 
 
-0.487
 
 
60.1
 
 
 
 
ARF4
 
 
hsa-miR-148b-3p
 
 
-0.487
 
 
60.1
 
 
 
 
ARF5
 
 
hsa-miR-29a-3p
 
 
-0.491
 
 
176.5
 
 
 
 
ARHGAP1
 
 
hsa-miR-34a-5p
 
 
-0.369
 
 
37.0
 
 
 
 
ARHGAP21
 
 
hsa-miR-152-3p
 
 
-0.287
 
 
40.6
 
 
 
 
ARHGAP21
 
 
hsa-miR-148b-3p
 
 
-0.287
 
 
40.6
 
 
 
 
ARL4A
 
 
hsa-miR-29a-3p
 
 
-0.386
 
 
14.1
 
 
 
 
ARL6IP1
 
 
hsa-miR-148b-3p
 
 
-0.579
 
 
241.3
 
 
 
 
ARL6IP1
 
 
hsa-miR-152-3p
 
 
-0.579
 
 
241.3
 
 
 
 
ARL8B
 
 
hsa-miR-152-3p
 
 
-0.337
 
 
53.8
 
 
 
 
ARL8B
 
 
hsa-miR-148b-3p
 
 
-0.337
 
 
53.8
 
 
 
 
ARMCX3
 
 
hsa-miR-425-5p
 
 
-0.325
 
 
32.9
 
 
 
 
ARPC5
 
 
hsa-miR-22-3p
 
 
-0.679
 
 
19.6
 
 
 
 
ARPIN-AP3S2
 
 
hsa-miR-34a-5p
 
 
-0.348
 
 
10.5
 
 
 
 
ARPP19
 
 
hsa-miR-29a-3p
 
 
-0.371
 
 
178.5
 
 
 
 
ARPP19
 
 
hsa-miR-34a-5p
 
 
-0.497
 
 
178.5
 
 
 
 
ARRB1
 
 
hsa-miR-22-3p
 
 
-0.616
 
 
32.5
 
 
 
 
ASAH1
 
 
hsa-miR-27a-3p
 
 
-0.398
 
 
50.6
 
 
 
 
ASB1
 
 
hsa-miR-34a-5p
 
 
-0.406
 
 
16.3
 
 
 
 
ASF1A
 
 
hsa-miR-23a-3p
 
 
-0.506
 
 
14.0
 
 
 
 
ASF1A
 
 
hsa-miR-23b-3p
 
 
-0.506
 
 
14.0
 
 
 
 
ASPH
 
 
hsa-miR-152-3p
 
 
-0.391
 
 
62.6
 
 
 
 
ASPH
 
 
hsa-miR-148b-3p
 
 
-0.391
 
 
62.6
 
 
 
 
ATCAY
 
 
hsa-miR-10a-5p
 
 
-0.322
 
 
44.9
 
 
 
 
ATG4B
 
 
hsa-miR-34a-5p
 
 
-0.58
 
 
21.3
 
 
 
 
ATMIN
 
 
hsa-miR-34a-5p
 
 
-0.409
 
 
19.5
 
 
 
 
ATP1A2
 
 
hsa-miR-34a-5p
 
 
-0.306
 
 
434.8
 
 
 
 
ATP5MC3
 
 
hsa-miR-425-5p
 
 
-0.328
 
 
183.1
 
 
 
 
ATP5MC3
 
 
hsa-miR-29a-3p
 
 
-0.33
 
 
183.1
 
 
 
 
ATP5MC3
 
 
hsa-miR-27a-3p
 
 
-0.416
 
 
183.1
 
 
 
 
ATP6AP2
 
 
hsa-miR-148b-3p
 
 
-0.592
 
 
69.8
 
 
 
 
ATP6AP2
 
 
hsa-miR-152-3p
 
 
-0.592
 
 
69.8
 
 
 
 
ATP6AP2
 
 
hsa-miR-148b-3p
 
 
-0.668
 
 
69.8
 
 
 
 
ATP6AP2
 
 
hsa-miR-152-3p
 
 
-0.668
 
 
69.8
 
 
 
 
 
 
 appendix 
 
 
 
 
target_symbol
 
 
mature_mirna_id
 
 
score
 
 
nTPM
 
 
 
 
 
 
AAGAB
 
 
hsa-miR-34a-5p
 
 
-0.319
 
 
18.2
 
 
 
 
AAR2
 
 
hsa-miR-29a-3p
 
 
-0.327
 
 
13.4
 
 
 
 
ABCC5
 
 
hsa-miR-125a-5p
 
 
-0.288
 
 
15.4
 
 
 
 
ABCD3
 
 
hsa-miR-148b-3p
 
 
-0.388
 
 
15.3
 
 
 
 
ABCD3
 
 
hsa-miR-152-3p
 
 
-0.388
 
 
15.3
 
 
 
 
ABCE1
 
 
hsa-miR-29a-3p
 
 
-0.404
 
 
31.7
 
 
 
 
ABHD3
 
 
hsa-miR-125a-5p
 
 
-0.453
 
 
23.7
 
 
 
 
ABTB1
 
 
hsa-miR-125a-5p
 
 
-0.519
 
 
17.8
 
 
 
 
ACBD3
 
 
hsa-miR-34a-5p
 
 
-0.415
 
 
19.2
 
 
 
 
ACSL1
 
 
hsa-miR-34a-5p
 
 
-0.306
 
 
84.1
 
 
 
 
ACSL4
 
 
hsa-miR-34a-5p
 
 
-0.372
 
 
34.5
 
 
 
 
ACTA2
 
 
hsa-miR-27a-3p
 
 
-0.577
 
 
687.0
 
 
 
 
ADA
 
 
hsa-miR-29a-3p
 
 
-0.376
 
 
15.1
 
 
 
 
ADAM10
 
 
hsa-miR-152-3p
 
 
-0.348
 
 
17.9
 
 
 
 
ADAM10
 
 
hsa-miR-148b-3p
 
 
-0.356
 
 
17.9
 
 
 
 
ADAMTS9
 
 
hsa-miR-29a-3p
 
 
-0.305
 
 
11.5
 
 
 
 
ADAMTS9
 
 
hsa-miR-29a-3p
 
 
-0.485
 
 
11.5
 
 
 
 
ADAP1
 
 
hsa-miR-27a-3p
 
 
-0.302
 
 
10.6
 
 
 
 
ADCY3
 
 
hsa-miR-132-3p
 
 
-0.303
 
 
16.3
 
 
 
 
ADIPOR2
 
 
hsa-miR-150-5p
 
 
-0.287
 
 
10.0
 
 
 
 
ADIPOR2
 
 
hsa-miR-34a-5p
 
 
-0.33
 
 
10.0
 
 
 
 
ADK
 
 
hsa-miR-34a-5p
 
 
-0.374
 
 
15.9
 
 
 
 
AFG3L2
 
 
hsa-miR-181b-5p
 
 
-0.491
 
 
12.5
 
 
 
 
AFG3L2
 
 
hsa-miR-181a-5p
 
 
-0.491
 
 
12.5
 
 
 
 
AGFG1
 
 
hsa-miR-148b-3p
 
 
-0.332
 
 
45.7
 
 
 
 
AGFG1
 
 
hsa-miR-152-3p
 
 
-0.332
 
 
45.7
 
 
 
 
AGGF1
 
 
hsa-miR-125a-5p
 
 
-0.331
 
 
10.9
 
 
 
 
AGPAT3
 
 
hsa-miR-27a-3p
 
 
-0.29
 
 
18.1
 
 
 
 
AGTRAP
 
 
hsa-miR-34a-5p
 
 
-0.328
 
 
28.5
 
 
 
 
AHCYL2
 
 
hsa-miR-34a-5p
 
 
-0.359
 
 
14.7
 
 
 
 
AHR
 
 
hsa-miR-29a-3p
 
 
-0.316
 
 
24.3
 
 
 
 
AIDA
 
 
hsa-miR-22-3p
 
 
-0.316
 
 
36.9
 
 
 
 
AIFM1
 
 
hsa-miR-125a-5p
 
 
-0.3
 
 
16.6
 
 
 
 
AK1
 
 
hsa-miR-29a-3p
 
 
-0.333
 
 
10.8
 
 
 
 
AKAP1
 
 
hsa-miR-148b-3p
 
 
-0.381
 
 
16.8
 
 
 
 
AKAP1
 
 
hsa-miR-152-3p
 
 
-0.381
 
 
16.8
 
 
 
 
AKIRIN1
 
 
hsa-miR-27a-3p
 
 
-0.368
 
 
31.6
 
 
 
 
ALCAM
 
 
hsa-miR-34a-5p
 
 
-0.303
 
 
11.5
 
 
 
 
ALCAM
 
 
hsa-miR-148b-3p
 
 
-0.339
 
 
11.5
 
 
 
 
ALCAM
 
 
hsa-miR-152-3p
 
 
-0.347
 
 
11.5
 
 
 
 
ALDOA
 
 
hsa-miR-34a-5p
 
 
-0.655
 
 
417.5
 
 
 
 
ALPL
 
 
hsa-miR-10a-5p
 
 
-0.32
 
 
26.3
 
 
 
 
ANAPC16
 
 
hsa-miR-125a-5p
 
 
-0.578
 
 
14.0
 
 
 
 
ANKHD1
 
 
hsa-miR-23a-3p
 
 
-0.368
 
 
34.9
 
 
 
 
ANKHD1
 
 
hsa-miR-23b-3p
 
 
-0.368
 
 
34.9
 
 
 
 
ANKRD13A
 
 
hsa-miR-22-3p
 
 
-0.478
 
 
34.4
 
 
 
 
ANKRD40
 
 
hsa-miR-27a-3p
 
 
-0.35
 
 
14.6
 
 
 
 
ANP32A
 
 
hsa-miR-132-3p
 
 
-0.374
 
 
82.3
 
 
 
 
ANP32B
 
 
hsa-miR-34a-5p
 
 
-0.29
 
 
117.0
 
 
 
 
ANPEP
 
 
hsa-miR-125a-5p
 
 
-0.372
 
 
34.6
 
 
 
 
ANTXR2
 
 
hsa-miR-29a-3p
 
 
-0.347
 
 
33.4
 
 
 
 
AP3S2
 
 
hsa-miR-34a-5p
 
 
-0.413
 
 
10.5
 
 
 
 
APH1A
 
 
hsa-miR-34a-5p
 
 
-0.304
 
 
57.1
 
 
 
 
AREG
 
 
hsa-miR-34a-5p
 
 
-0.62
 
 
34.6
 
 
 
 
ARF4
 
 
hsa-miR-152-3p
 
 
-0.487
 
 
101.3
 
 
 
 
ARF4
 
 
hsa-miR-148b-3p
 
 
-0.487
 
 
101.3
 
 
 
 
ARF5
 
 
hsa-miR-29a-3p
 
 
-0.491
 
 
68.5
 
 
 
 
ARF6
 
 
hsa-miR-181b-5p
 
 
-0.33
 
 
41.0
 
 
 
 
ARF6
 
 
hsa-miR-181a-5p
 
 
-0.33
 
 
41.0
 
 
 
 
ARFGEF1
 
 
hsa-miR-148b-3p
 
 
-0.306
 
 
13.8
 
 
 
 
ARFGEF1
 
 
hsa-miR-152-3p
 
 
-0.306
 
 
13.8
 
 
 
 
ARFIP1
 
 
hsa-miR-152-3p
 
 
-0.474
 
 
14.9
 
 
 
 
ARFIP1
 
 
hsa-miR-148b-3p
 
 
-0.474
 
 
14.9
 
 
 
 
ARHGAP1
 
 
hsa-miR-34a-5p
 
 
-0.369
 
 
35.8
 
 
 
 
ARHGAP26
 
 
hsa-miR-34a-5p
 
 
-0.384
 
 
16.7
 
 
 
 
ARID1B
 
 
hsa-miR-29a-3p
 
 
-0.342
 
 
13.6
 
 
 
 
ARL4A
 
 
hsa-miR-29a-3p
 
 
-0.386
 
 
15.8
 
 
 
 
ARL6IP1
 
 
hsa-miR-148b-3p
 
 
-0.579
 
 
73.3
 
 
 
 
ARL6IP1
 
 
hsa-miR-152-3p
 
 
-0.579
 
 
73.3
 
 
 
 
ARL8B
 
 
hsa-miR-152-3p
 
 
-0.337
 
 
34.1
 
 
 
 
ARL8B
 
 
hsa-miR-148b-3p
 
 
-0.337
 
 
34.1
 
 
 
 
ARMCX3
 
 
hsa-miR-425-5p
 
 
-0.325
 
 
23.0
 
 
 
 
ARPC5
 
 
hsa-miR-22-3p
 
 
-0.679
 
 
139.8
 
 
 
 
ARPP19
 
 
hsa-miR-29a-3p
 
 
-0.371
 
 
36.4
 
 
 
 
ARPP19
 
 
hsa-miR-34a-5p
 
 
-0.497
 
 
36.4
 
 
 
 
ARRB1
 
 
hsa-miR-22-3p
 
 
-0.616
 
 
20.7
 
 
 
 
ARRDC3
 
 
hsa-miR-152-3p
 
 
-0.305
 
 
25.3
 
 
 
 
ARRDC3
 
 
hsa-miR-148b-3p
 
 
-0.305
 
 
25.3
 
 
 
 
ARRDC3
 
 
hsa-miR-29a-3p
 
 
-0.402
 
 
25.3
 
 
 
 
ASAH1
 
 
hsa-miR-27a-3p
 
 
-0.398
 
 
48.7
 
 
 
 
ASPH
 
 
hsa-miR-152-3p
 
 
-0.391
 
 
47.6
 
 
 
 
ASPH
 
 
hsa-miR-148b-3p
 
 
-0.391
 
 
47.6
 
 
 
 
ATMIN
 
 
hsa-miR-34a-5p
 
 
-0.409
 
 
10.8
 
 
 
 
ATP2B4
 
 
hsa-miR-152-3p
 
 
-0.337
 
 
24.2
 
 
 
 
ATP2B4
 
 
hsa-miR-148b-3p
 
 
-0.337
 
 
24.2
 
 
 
 
ATP5MC3
 
 
hsa-miR-425-5p
 
 
-0.328
 
 
123.3
 
 
 
 
ATP5MC3
 
 
hsa-miR-29a-3p
 
 
-0.33
 
 
123.3
 
 
 
 
ATP5MC3
 
 
hsa-miR-27a-3p
 
 
-0.416
 
 
123.3
 
 
 
 
ATP6AP2
 
 
hsa-miR-148b-3p
 
 
-0.592
 
 
57.3
 
 
 
 
ATP6AP2
 
 
hsa-miR-152-3p
 
 
-0.592
 
 
57.3
 
 
 
 
ATP6AP2
 
 
hsa-miR-148b-3p
 
 
-0.668
 
 
57.3
 
 
 
 
ATP6AP2
 
 
hsa-miR-152-3p
 
 
-0.668
 
 
57.3
 
 
 
 
ATP6V1E1
 
 
hsa-miR-23a-3p
 
 
-0.294
 
 
64.3
 
 
 
 
ATP6V1E1
 
 
hsa-miR-23b-3p
 
 
-0.294
 
 
64.3
 
 
 
 
ATXN10
 
 
hsa-miR-27a-3p
 
 
-0.295
 
 
32.7
 
 
 
 
ATXN2
 
 
hsa-miR-10a-5p
 
 
-0.362
 
 
10.6
 
 
 
 
AXL
 
 
hsa-miR-34a-5p
 
 
-0.512
 
 
14.6
 
 
 
 
AZIN1
 
 
hsa-miR-132-3p
 
 
-0.29
 
 
44.7
 
 
 
 
AZIN1
 
 
hsa-miR-132-3p
 
 
-0.299
 
 
44.7
 
 
 
 
B4GALT2
 
 
hsa-miR-34a-5p
 
 
-0.314
 
 
12.3
 
 
 
 
 
 
 basal ganglia 
 
 
 
 
target_symbol
 
 
mature_mirna_id
 
 
score
 
 
nTPM
 
 
 
 
 
 
AAGAB
 
 
hsa-miR-34a-5p
 
 
-0.319
 
 
12.7
 
 
 
 
AAR2
 
 
hsa-miR-29a-3p
 
 
-0.327
 
 
11.5
 
 
 
 
ABCC5
 
 
hsa-miR-125a-5p
 
 
-0.288
 
 
35.1
 
 
 
 
ABCD3
 
 
hsa-miR-148b-3p
 
 
-0.388
 
 
17.4
 
 
 
 
ABCD3
 
 
hsa-miR-152-3p
 
 
-0.388
 
 
17.4
 
 
 
 
ABCE1
 
 
hsa-miR-29a-3p
 
 
-0.404
 
 
11.5
 
 
 
 
ABHD17C
 
 
hsa-miR-27a-3p
 
 
-0.435
 
 
20.2
 
 
 
 
ABHD3
 
 
hsa-miR-125a-5p
 
 
-0.453
 
 
11.4
 
 
 
 
ABHD4
 
 
hsa-miR-29a-3p
 
 
-0.312
 
 
15.4
 
 
 
 
ABHD6
 
 
hsa-miR-125a-5p
 
 
-0.501
 
 
18.4
 
 
 
 
ABTB1
 
 
hsa-miR-125a-5p
 
 
-0.519
 
 
41.3
 
 
 
 
ACHE
 
 
hsa-miR-125a-5p
 
 
-0.308
 
 
38.3
 
 
 
 
ACHE
 
 
hsa-miR-125a-5p
 
 
-0.361
 
 
38.3
 
 
 
 
ACSL1
 
 
hsa-miR-34a-5p
 
 
-0.306
 
 
28.3
 
 
 
 
ACSL6
 
 
hsa-miR-125a-5p
 
 
-0.307
 
 
18.7
 
 
 
 
ACTA2
 
 
hsa-miR-27a-3p
 
 
-0.577
 
 
45.5
 
 
 
 
ACTN2
 
 
hsa-miR-23a-3p
 
 
-0.294
 
 
36.8
 
 
 
 
ACTN2
 
 
hsa-miR-29a-3p
 
 
-0.311
 
 
36.8
 
 
 
 
ADAP1
 
 
hsa-miR-27a-3p
 
 
-0.302
 
 
77.3
 
 
 
 
ADCY2
 
 
hsa-miR-152-3p
 
 
-0.299
 
 
23.7
 
 
 
 
ADCY2
 
 
hsa-miR-148b-3p
 
 
-0.299
 
 
23.7
 
 
 
 
ADCY3
 
 
hsa-miR-132-3p
 
 
-0.303
 
 
57.0
 
 
 
 
ADD2
 
 
hsa-miR-34a-5p
 
 
-0.305
 
 
16.7
 
 
 
 
ADGRA3
 
 
hsa-miR-27a-3p
 
 
-0.296
 
 
18.8
 
 
 
 
ADGRB3
 
 
hsa-miR-152-3p
 
 
-0.516
 
 
21.1
 
 
 
 
ADGRB3
 
 
hsa-miR-148b-3p
 
 
-0.524
 
 
21.1
 
 
 
 
ADGRF5
 
 
hsa-miR-148b-3p
 
 
-0.354
 
 
13.3
 
 
 
 
ADGRF5
 
 
hsa-miR-152-3p
 
 
-0.354
 
 
13.3
 
 
 
 
ADIPOR2
 
 
hsa-miR-150-5p
 
 
-0.287
 
 
50.5
 
 
 
 
ADIPOR2
 
 
hsa-miR-34a-5p
 
 
-0.33
 
 
50.5
 
 
 
 
ADK
 
 
hsa-miR-34a-5p
 
 
-0.374
 
 
22.3
 
 
 
 
ADO
 
 
hsa-miR-34a-5p
 
 
-0.377
 
 
11.0
 
 
 
 
AGFG1
 
 
hsa-miR-148b-3p
 
 
-0.332
 
 
27.0
 
 
 
 
AGFG1
 
 
hsa-miR-152-3p
 
 
-0.332
 
 
27.0
 
 
 
 
AGPAT3
 
 
hsa-miR-27a-3p
 
 
-0.29
 
 
44.7
 
 
 
 
AGPAT4
 
 
hsa-miR-29a-3p
 
 
-0.376
 
 
10.7
 
 
 
 
AGTRAP
 
 
hsa-miR-34a-5p
 
 
-0.328
 
 
21.3
 
 
 
 
AHCYL2
 
 
hsa-miR-34a-5p
 
 
-0.359
 
 
31.1
 
 
 
 
AHDC1
 
 
hsa-miR-148b-3p
 
 
-0.413
 
 
12.3
 
 
 
 
AHDC1
 
 
hsa-miR-152-3p
 
 
-0.413
 
 
12.3
 
 
 
 
AIDA
 
 
hsa-miR-22-3p
 
 
-0.316
 
 
28.2
 
 
 
 
AIFM1
 
 
hsa-miR-125a-5p
 
 
-0.3
 
 
12.5
 
 
 
 
AK1
 
 
hsa-miR-29a-3p
 
 
-0.333
 
 
100.7
 
 
 
 
AKAP1
 
 
hsa-miR-148b-3p
 
 
-0.381
 
 
26.7
 
 
 
 
AKAP1
 
 
hsa-miR-152-3p
 
 
-0.381
 
 
26.7
 
 
 
 
AKAP5
 
 
hsa-miR-29a-3p
 
 
-0.298
 
 
17.6
 
 
 
 
AKIRIN1
 
 
hsa-miR-27a-3p
 
 
-0.368
 
 
33.9
 
 
 
 
AKT3
 
 
hsa-miR-29a-3p
 
 
-0.287
 
 
15.0
 
 
 
 
AKT3
 
 
hsa-miR-22-3p
 
 
-0.451
 
 
15.0
 
 
 
 
ALCAM
 
 
hsa-miR-34a-5p
 
 
-0.303
 
 
37.8
 
 
 
 
ALCAM
 
 
hsa-miR-148b-3p
 
 
-0.339
 
 
37.8
 
 
 
 
ALCAM
 
 
hsa-miR-152-3p
 
 
-0.347
 
 
37.8
 
 
 
 
ALDOA
 
 
hsa-miR-34a-5p
 
 
-0.655
 
 
972.8
 
 
 
 
ALPL
 
 
hsa-miR-10a-5p
 
 
-0.32
 
 
12.3
 
 
 
 
AMER2
 
 
hsa-miR-34a-5p
 
 
-0.48
 
 
31.7
 
 
 
 
AMOT
 
 
hsa-miR-29a-3p
 
 
-0.304
 
 
18.8
 
 
 
 
ANAPC16
 
 
hsa-miR-125a-5p
 
 
-0.578
 
 
11.6
 
 
 
 
ANGEL2
 
 
hsa-miR-132-3p
 
 
-0.357
 
 
14.7
 
 
 
 
ANGEL2
 
 
hsa-miR-22-3p
 
 
-0.601
 
 
14.7
 
 
 
 
ANK2
 
 
hsa-miR-34a-5p
 
 
-0.317
 
 
20.3
 
 
 
 
ANK3
 
 
hsa-miR-34a-5p
 
 
-0.29
 
 
40.8
 
 
 
 
ANK3
 
 
hsa-miR-34a-5p
 
 
-0.347
 
 
40.8
 
 
 
 
ANKHD1
 
 
hsa-miR-23a-3p
 
 
-0.368
 
 
20.4
 
 
 
 
ANKHD1
 
 
hsa-miR-23b-3p
 
 
-0.368
 
 
20.4
 
 
 
 
ANKRD13B
 
 
hsa-miR-29a-3p
 
 
-0.421
 
 
12.3
 
 
 
 
ANKRD40
 
 
hsa-miR-27a-3p
 
 
-0.35
 
 
100.5
 
 
 
 
ANP32A
 
 
hsa-miR-132-3p
 
 
-0.374
 
 
53.9
 
 
 
 
ANP32B
 
 
hsa-miR-34a-5p
 
 
-0.29
 
 
112.9
 
 
 
 
AP3S2
 
 
hsa-miR-34a-5p
 
 
-0.413
 
 
15.3
 
 
 
 
APC
 
 
hsa-miR-29a-3p
 
 
-0.413
 
 
15.7
 
 
 
 
APH1A
 
 
hsa-miR-34a-5p
 
 
-0.304
 
 
58.7
 
 
 
 
APOO
 
 
hsa-miR-181b-5p
 
 
-0.5
 
 
38.6
 
 
 
 
APOO
 
 
hsa-miR-181a-5p
 
 
-0.5
 
 
38.6
 
 
 
 
ARF4
 
 
hsa-miR-152-3p
 
 
-0.487
 
 
71.0
 
 
 
 
ARF4
 
 
hsa-miR-148b-3p
 
 
-0.487
 
 
71.0
 
 
 
 
ARF5
 
 
hsa-miR-29a-3p
 
 
-0.491
 
 
195.0
 
 
 
 
ARF6
 
 
hsa-miR-181b-5p
 
 
-0.33
 
 
10.2
 
 
 
 
ARF6
 
 
hsa-miR-181a-5p
 
 
-0.33
 
 
10.2
 
 
 
 
ARHGAP1
 
 
hsa-miR-34a-5p
 
 
-0.369
 
 
41.0
 
 
 
 
ARHGAP21
 
 
hsa-miR-152-3p
 
 
-0.287
 
 
37.1
 
 
 
 
ARHGAP21
 
 
hsa-miR-148b-3p
 
 
-0.287
 
 
37.1
 
 
 
 
ARHGAP26
 
 
hsa-miR-34a-5p
 
 
-0.384
 
 
10.6
 
 
 
 
ARL4A
 
 
hsa-miR-29a-3p
 
 
-0.386
 
 
10.5
 
 
 
 
ARL6IP1
 
 
hsa-miR-148b-3p
 
 
-0.579
 
 
196.4
 
 
 
 
ARL6IP1
 
 
hsa-miR-152-3p
 
 
-0.579
 
 
196.4
 
 
 
 
ARL8B
 
 
hsa-miR-152-3p
 
 
-0.337
 
 
53.5
 
 
 
 
ARL8B
 
 
hsa-miR-148b-3p
 
 
-0.337
 
 
53.5
 
 
 
 
ARMCX3
 
 
hsa-miR-425-5p
 
 
-0.325
 
 
33.9
 
 
 
 
ARPC5
 
 
hsa-miR-22-3p
 
 
-0.679
 
 
21.4
 
 
 
 
ARPIN-AP3S2
 
 
hsa-miR-34a-5p
 
 
-0.348
 
 
11.8
 
 
 
 
ARPP19
 
 
hsa-miR-29a-3p
 
 
-0.371
 
 
481.4
 
 
 
 
ARPP19
 
 
hsa-miR-34a-5p
 
 
-0.497
 
 
481.4
 
 
 
 
ARRB1
 
 
hsa-miR-22-3p
 
 
-0.616
 
 
40.7
 
 
 
 
ARRDC3
 
 
hsa-miR-152-3p
 
 
-0.305
 
 
10.6
 
 
 
 
ARRDC3
 
 
hsa-miR-148b-3p
 
 
-0.305
 
 
10.6
 
 
 
 
ARRDC3
 
 
hsa-miR-29a-3p
 
 
-0.402
 
 
10.6
 
 
 
 
ASAH1
 
 
hsa-miR-27a-3p
 
 
-0.398
 
 
51.7
 
 
 
 
ASB1
 
 
hsa-miR-34a-5p
 
 
-0.406
 
 
16.2
 
 
 
 
ASF1A
 
 
hsa-miR-23a-3p
 
 
-0.506
 
 
15.3
 
 
 
 
ASF1A
 
 
hsa-miR-23b-3p
 
 
-0.506
 
 
15.3
 
 
 
 
 
 
 blood vessel 
 
 
 
 
target_symbol
 
 
mature_mirna_id
 
 
score
 
 
nTPM
 
 
 
 
 
 
AAGAB
 
 
hsa-miR-34a-5p
 
 
-0.319
 
 
11.8
 
 
 
 
AAR2
 
 
hsa-miR-29a-3p
 
 
-0.327
 
 
28.2
 
 
 
 
ABCB7
 
 
hsa-miR-148b-3p
 
 
-0.515
 
 
12.3
 
 
 
 
ABCB7
 
 
hsa-miR-152-3p
 
 
-0.515
 
 
12.3
 
 
 
 
ABCC5
 
 
hsa-miR-125a-5p
 
 
-0.288
 
 
17.4
 
 
 
 
ABCD3
 
 
hsa-miR-148b-3p
 
 
-0.388
 
 
14.4
 
 
 
 
ABCD3
 
 
hsa-miR-152-3p
 
 
-0.388
 
 
14.4
 
 
 
 
ABCE1
 
 
hsa-miR-29a-3p
 
 
-0.404
 
 
15.1
 
 
 
 
ABHD3
 
 
hsa-miR-125a-5p
 
 
-0.453
 
 
10.2
 
 
 
 
ABHD4
 
 
hsa-miR-29a-3p
 
 
-0.312
 
 
38.3
 
 
 
 
ABTB1
 
 
hsa-miR-125a-5p
 
 
-0.519
 
 
47.2
 
 
 
 
ACBD3
 
 
hsa-miR-34a-5p
 
 
-0.415
 
 
19.2
 
 
 
 
ACSL1
 
 
hsa-miR-34a-5p
 
 
-0.306
 
 
119.9
 
 
 
 
ACSL4
 
 
hsa-miR-34a-5p
 
 
-0.372
 
 
15.9
 
 
 
 
ACTA2
 
 
hsa-miR-27a-3p
 
 
-0.577
 
 
10509.9
 
 
 
 
ACTN2
 
 
hsa-miR-23a-3p
 
 
-0.294
 
 
12.2
 
 
 
 
ACTN2
 
 
hsa-miR-29a-3p
 
 
-0.311
 
 
12.2
 
 
 
 
ADAMTS10
 
 
hsa-miR-27a-3p
 
 
-0.354
 
 
20.0
 
 
 
 
ADAMTS10
 
 
hsa-miR-29a-3p
 
 
-0.516
 
 
20.0
 
 
 
 
ADAMTS7
 
 
hsa-miR-29a-3p
 
 
-0.427
 
 
14.2
 
 
 
 
ADAMTS9
 
 
hsa-miR-29a-3p
 
 
-0.305
 
 
18.0
 
 
 
 
ADAMTS9
 
 
hsa-miR-29a-3p
 
 
-0.485
 
 
18.0
 
 
 
 
ADCY3
 
 
hsa-miR-132-3p
 
 
-0.303
 
 
43.2
 
 
 
 
ADGRF5
 
 
hsa-miR-148b-3p
 
 
-0.354
 
 
12.6
 
 
 
 
ADGRF5
 
 
hsa-miR-152-3p
 
 
-0.354
 
 
12.6
 
 
 
 
ADIPOR2
 
 
hsa-miR-150-5p
 
 
-0.287
 
 
23.0
 
 
 
 
ADIPOR2
 
 
hsa-miR-34a-5p
 
 
-0.33
 
 
23.0
 
 
 
 
ADK
 
 
hsa-miR-34a-5p
 
 
-0.374
 
 
15.2
 
 
 
 
AGFG1
 
 
hsa-miR-148b-3p
 
 
-0.332
 
 
37.3
 
 
 
 
AGFG1
 
 
hsa-miR-152-3p
 
 
-0.332
 
 
37.3
 
 
 
 
AGPAT3
 
 
hsa-miR-27a-3p
 
 
-0.29
 
 
11.3
 
 
 
 
AGTRAP
 
 
hsa-miR-34a-5p
 
 
-0.328
 
 
38.4
 
 
 
 
AHDC1
 
 
hsa-miR-148b-3p
 
 
-0.413
 
 
27.7
 
 
 
 
AHDC1
 
 
hsa-miR-152-3p
 
 
-0.413
 
 
27.7
 
 
 
 
AHR
 
 
hsa-miR-29a-3p
 
 
-0.316
 
 
15.2
 
 
 
 
AIDA
 
 
hsa-miR-22-3p
 
 
-0.316
 
 
49.9
 
 
 
 
AK1
 
 
hsa-miR-29a-3p
 
 
-0.333
 
 
58.4
 
 
 
 
AKAP1
 
 
hsa-miR-148b-3p
 
 
-0.381
 
 
84.5
 
 
 
 
AKAP1
 
 
hsa-miR-152-3p
 
 
-0.381
 
 
84.5
 
 
 
 
AKIRIN1
 
 
hsa-miR-27a-3p
 
 
-0.368
 
 
32.7
 
 
 
 
AKT3
 
 
hsa-miR-29a-3p
 
 
-0.287
 
 
34.5
 
 
 
 
AKT3
 
 
hsa-miR-22-3p
 
 
-0.451
 
 
34.5
 
 
 
 
ALCAM
 
 
hsa-miR-34a-5p
 
 
-0.303
 
 
85.5
 
 
 
 
ALCAM
 
 
hsa-miR-148b-3p
 
 
-0.339
 
 
85.5
 
 
 
 
ALCAM
 
 
hsa-miR-152-3p
 
 
-0.347
 
 
85.5
 
 
 
 
ALDOA
 
 
hsa-miR-34a-5p
 
 
-0.655
 
 
986.4
 
 
 
 
ALPL
 
 
hsa-miR-10a-5p
 
 
-0.32
 
 
12.2
 
 
 
 
ANAPC16
 
 
hsa-miR-125a-5p
 
 
-0.578
 
 
31.1
 
 
 
 
ANK3
 
 
hsa-miR-34a-5p
 
 
-0.29
 
 
22.9
 
 
 
 
ANK3
 
 
hsa-miR-34a-5p
 
 
-0.347
 
 
22.9
 
 
 
 
ANKHD1
 
 
hsa-miR-23a-3p
 
 
-0.368
 
 
40.7
 
 
 
 
ANKHD1
 
 
hsa-miR-23b-3p
 
 
-0.368
 
 
40.7
 
 
 
 
ANKRD13A
 
 
hsa-miR-22-3p
 
 
-0.478
 
 
16.9
 
 
 
 
ANKRD40
 
 
hsa-miR-27a-3p
 
 
-0.35
 
 
29.5
 
 
 
 
ANP32A
 
 
hsa-miR-132-3p
 
 
-0.374
 
 
51.8
 
 
 
 
ANP32B
 
 
hsa-miR-34a-5p
 
 
-0.29
 
 
163.8
 
 
 
 
ANPEP
 
 
hsa-miR-125a-5p
 
 
-0.372
 
 
14.7
 
 
 
 
ANTXR2
 
 
hsa-miR-29a-3p
 
 
-0.347
 
 
50.3
 
 
 
 
AP3S2
 
 
hsa-miR-34a-5p
 
 
-0.413
 
 
10.1
 
 
 
 
APH1A
 
 
hsa-miR-34a-5p
 
 
-0.304
 
 
45.6
 
 
 
 
AREG
 
 
hsa-miR-34a-5p
 
 
-0.62
 
 
10.0
 
 
 
 
ARF4
 
 
hsa-miR-152-3p
 
 
-0.487
 
 
151.4
 
 
 
 
ARF4
 
 
hsa-miR-148b-3p
 
 
-0.487
 
 
151.4
 
 
 
 
ARF5
 
 
hsa-miR-29a-3p
 
 
-0.491
 
 
115.2
 
 
 
 
ARF6
 
 
hsa-miR-181b-5p
 
 
-0.33
 
 
22.6
 
 
 
 
ARF6
 
 
hsa-miR-181a-5p
 
 
-0.33
 
 
22.6
 
 
 
 
ARFIP1
 
 
hsa-miR-152-3p
 
 
-0.474
 
 
14.2
 
 
 
 
ARFIP1
 
 
hsa-miR-148b-3p
 
 
-0.474
 
 
14.2
 
 
 
 
ARHGAP1
 
 
hsa-miR-34a-5p
 
 
-0.369
 
 
119.0
 
 
 
 
ARHGAP21
 
 
hsa-miR-152-3p
 
 
-0.287
 
 
14.0
 
 
 
 
ARHGAP21
 
 
hsa-miR-148b-3p
 
 
-0.287
 
 
14.0
 
 
 
 
ARHGEF26
 
 
hsa-miR-22-3p
 
 
-0.374
 
 
10.5
 
 
 
 
ARID1B
 
 
hsa-miR-29a-3p
 
 
-0.342
 
 
13.6
 
 
 
 
ARL4A
 
 
hsa-miR-29a-3p
 
 
-0.386
 
 
19.2
 
 
 
 
ARL6IP1
 
 
hsa-miR-148b-3p
 
 
-0.579
 
 
52.2
 
 
 
 
ARL6IP1
 
 
hsa-miR-152-3p
 
 
-0.579
 
 
52.2
 
 
 
 
ARL8B
 
 
hsa-miR-152-3p
 
 
-0.337
 
 
49.5
 
 
 
 
ARL8B
 
 
hsa-miR-148b-3p
 
 
-0.337
 
 
49.5
 
 
 
 
ARMCX3
 
 
hsa-miR-425-5p
 
 
-0.325
 
 
32.6
 
 
 
 
ARPC5
 
 
hsa-miR-22-3p
 
 
-0.679
 
 
68.6
 
 
 
 
ARPP19
 
 
hsa-miR-29a-3p
 
 
-0.371
 
 
47.3
 
 
 
 
ARPP19
 
 
hsa-miR-34a-5p
 
 
-0.497
 
 
47.3
 
 
 
 
ARRB1
 
 
hsa-miR-22-3p
 
 
-0.616
 
 
10.5
 
 
 
 
ARRDC3
 
 
hsa-miR-152-3p
 
 
-0.305
 
 
25.4
 
 
 
 
ARRDC3
 
 
hsa-miR-148b-3p
 
 
-0.305
 
 
25.4
 
 
 
 
ARRDC3
 
 
hsa-miR-29a-3p
 
 
-0.402
 
 
25.4
 
 
 
 
ASAH1
 
 
hsa-miR-27a-3p
 
 
-0.398
 
 
83.2
 
 
 
 
ASAP2
 
 
hsa-miR-29a-3p
 
 
-0.348
 
 
16.3
 
 
 
 
ASB1
 
 
hsa-miR-34a-5p
 
 
-0.406
 
 
11.1
 
 
 
 
ASPH
 
 
hsa-miR-152-3p
 
 
-0.391
 
 
72.5
 
 
 
 
ASPH
 
 
hsa-miR-148b-3p
 
 
-0.391
 
 
72.5
 
 
 
 
ATG4B
 
 
hsa-miR-34a-5p
 
 
-0.58
 
 
16.6
 
 
 
 
ATMIN
 
 
hsa-miR-34a-5p
 
 
-0.409
 
 
11.8
 
 
 
 
ATP10D
 
 
hsa-miR-125a-5p
 
 
-0.375
 
 
11.2
 
 
 
 
ATP1A2
 
 
hsa-miR-34a-5p
 
 
-0.306
 
 
72.3
 
 
 
 
ATP2B4
 
 
hsa-miR-152-3p
 
 
-0.337
 
 
24.9
 
 
 
 
ATP2B4
 
 
hsa-miR-148b-3p
 
 
-0.337
 
 
24.9
 
 
 
 
ATP5MC3
 
 
hsa-miR-425-5p
 
 
-0.328
 
 
129.6
 
 
 
 
ATP5MC3
 
 
hsa-miR-29a-3p
 
 
-0.33
 
 
129.6
 
 
 
 
ATP5MC3
 
 
hsa-miR-27a-3p
 
 
-0.416
 
 
129.6
 
 
 
 
 
 
 bone marrow 
 
 
 
 
target_symbol
 
 
mature_mirna_id
 
 
score
 
 
nTPM
 
 
 
 
 
 
AAGAB
 
 
hsa-miR-34a-5p
 
 
-0.319
 
 
18.7
 
 
 
 
AAR2
 
 
hsa-miR-29a-3p
 
 
-0.327
 
 
21.0
 
 
 
 
ABCB10
 
 
hsa-miR-152-3p
 
 
-0.291
 
 
56.7
 
 
 
 
ABCB10
 
 
hsa-miR-148b-3p
 
 
-0.291
 
 
56.7
 
 
 
 
ABCB7
 
 
hsa-miR-148b-3p
 
 
-0.515
 
 
10.5
 
 
 
 
ABCB7
 
 
hsa-miR-152-3p
 
 
-0.515
 
 
10.5
 
 
 
 
ABCC5
 
 
hsa-miR-125a-5p
 
 
-0.288
 
 
21.2
 
 
 
 
ABCD3
 
 
hsa-miR-148b-3p
 
 
-0.388
 
 
23.6
 
 
 
 
ABCD3
 
 
hsa-miR-152-3p
 
 
-0.388
 
 
23.6
 
 
 
 
ABCE1
 
 
hsa-miR-29a-3p
 
 
-0.404
 
 
25.7
 
 
 
 
ABHD3
 
 
hsa-miR-125a-5p
 
 
-0.453
 
 
16.6
 
 
 
 
ABHD4
 
 
hsa-miR-29a-3p
 
 
-0.312
 
 
12.9
 
 
 
 
ABHD5
 
 
hsa-miR-132-3p
 
 
-0.323
 
 
37.5
 
 
 
 
ABHD5
 
 
hsa-miR-27a-3p
 
 
-0.407
 
 
37.5
 
 
 
 
ABRAXAS2
 
 
hsa-miR-34a-5p
 
 
-0.538
 
 
11.6
 
 
 
 
ABTB1
 
 
hsa-miR-125a-5p
 
 
-0.519
 
 
48.3
 
 
 
 
ACBD3
 
 
hsa-miR-34a-5p
 
 
-0.415
 
 
24.4
 
 
 
 
ACSL1
 
 
hsa-miR-34a-5p
 
 
-0.306
 
 
237.5
 
 
 
 
ACSL4
 
 
hsa-miR-34a-5p
 
 
-0.372
 
 
11.1
 
 
 
 
ACSL6
 
 
hsa-miR-125a-5p
 
 
-0.307
 
 
21.4
 
 
 
 
ADA
 
 
hsa-miR-29a-3p
 
 
-0.376
 
 
14.3
 
 
 
 
ADAM10
 
 
hsa-miR-152-3p
 
 
-0.348
 
 
17.3
 
 
 
 
ADAM10
 
 
hsa-miR-148b-3p
 
 
-0.356
 
 
17.3
 
 
 
 
ADCY3
 
 
hsa-miR-132-3p
 
 
-0.303
 
 
16.7
 
 
 
 
ADD2
 
 
hsa-miR-34a-5p
 
 
-0.305
 
 
62.7
 
 
 
 
ADK
 
 
hsa-miR-34a-5p
 
 
-0.374
 
 
27.0
 
 
 
 
AFG3L2
 
 
hsa-miR-181b-5p
 
 
-0.491
 
 
23.4
 
 
 
 
AFG3L2
 
 
hsa-miR-181a-5p
 
 
-0.491
 
 
23.4
 
 
 
 
AGFG1
 
 
hsa-miR-148b-3p
 
 
-0.332
 
 
146.9
 
 
 
 
AGFG1
 
 
hsa-miR-152-3p
 
 
-0.332
 
 
146.9
 
 
 
 
AGO2
 
 
hsa-miR-132-3p
 
 
-0.406
 
 
10.7
 
 
 
 
AGO2
 
 
hsa-miR-27a-3p
 
 
-0.486
 
 
10.7
 
 
 
 
AGO2
 
 
hsa-miR-23a-3p
 
 
-0.576
 
 
10.7
 
 
 
 
AGO2
 
 
hsa-miR-23b-3p
 
 
-0.587
 
 
10.7
 
 
 
 
AGO2
 
 
hsa-miR-27a-3p
 
 
-0.646
 
 
10.7
 
 
 
 
AGO2
 
 
hsa-miR-125a-5p
 
 
-0.811
 
 
10.7
 
 
 
 
AGO4
 
 
hsa-miR-34a-5p
 
 
-0.306
 
 
19.2
 
 
 
 
AGPAT3
 
 
hsa-miR-27a-3p
 
 
-0.29
 
 
10.4
 
 
 
 
AGTRAP
 
 
hsa-miR-34a-5p
 
 
-0.328
 
 
125.0
 
 
 
 
AHR
 
 
hsa-miR-29a-3p
 
 
-0.316
 
 
41.5
 
 
 
 
AIDA
 
 
hsa-miR-22-3p
 
 
-0.316
 
 
20.7
 
 
 
 
AIFM1
 
 
hsa-miR-125a-5p
 
 
-0.3
 
 
11.2
 
 
 
 
AK1
 
 
hsa-miR-29a-3p
 
 
-0.333
 
 
27.0
 
 
 
 
AKIRIN1
 
 
hsa-miR-27a-3p
 
 
-0.368
 
 
72.8
 
 
 
 
ALDOA
 
 
hsa-miR-34a-5p
 
 
-0.655
 
 
691.0
 
 
 
 
ANAPC16
 
 
hsa-miR-125a-5p
 
 
-0.578
 
 
13.0
 
 
 
 
ANKHD1
 
 
hsa-miR-23a-3p
 
 
-0.368
 
 
69.9
 
 
 
 
ANKHD1
 
 
hsa-miR-23b-3p
 
 
-0.368
 
 
69.9
 
 
 
 
ANKRD13A
 
 
hsa-miR-22-3p
 
 
-0.478
 
 
34.0
 
 
 
 
ANP32A
 
 
hsa-miR-132-3p
 
 
-0.374
 
 
129.5
 
 
 
 
ANP32B
 
 
hsa-miR-34a-5p
 
 
-0.29
 
 
344.9
 
 
 
 
ANPEP
 
 
hsa-miR-125a-5p
 
 
-0.372
 
 
97.7
 
 
 
 
ANTXR2
 
 
hsa-miR-29a-3p
 
 
-0.347
 
 
17.7
 
 
 
 
APH1A
 
 
hsa-miR-34a-5p
 
 
-0.304
 
 
74.2
 
 
 
 
AREG
 
 
hsa-miR-34a-5p
 
 
-0.62
 
 
122.6
 
 
 
 
ARF4
 
 
hsa-miR-152-3p
 
 
-0.487
 
 
68.5
 
 
 
 
ARF4
 
 
hsa-miR-148b-3p
 
 
-0.487
 
 
68.5
 
 
 
 
ARF5
 
 
hsa-miR-29a-3p
 
 
-0.491
 
 
135.0
 
 
 
 
ARF6
 
 
hsa-miR-181b-5p
 
 
-0.33
 
 
78.0
 
 
 
 
ARF6
 
 
hsa-miR-181a-5p
 
 
-0.33
 
 
78.0
 
 
 
 
ARFGEF1
 
 
hsa-miR-148b-3p
 
 
-0.306
 
 
26.0
 
 
 
 
ARFGEF1
 
 
hsa-miR-152-3p
 
 
-0.306
 
 
26.0
 
 
 
 
ARHGAP1
 
 
hsa-miR-34a-5p
 
 
-0.369
 
 
31.5
 
 
 
 
ARHGAP26
 
 
hsa-miR-34a-5p
 
 
-0.384
 
 
34.3
 
 
 
 
ARID1B
 
 
hsa-miR-29a-3p
 
 
-0.342
 
 
27.3
 
 
 
 
ARID2
 
 
hsa-miR-132-3p
 
 
-0.304
 
 
11.7
 
 
 
 
ARL4A
 
 
hsa-miR-29a-3p
 
 
-0.386
 
 
92.7
 
 
 
 
ARL5B
 
 
hsa-miR-29a-3p
 
 
-0.304
 
 
18.5
 
 
 
 
ARL6IP1
 
 
hsa-miR-148b-3p
 
 
-0.579
 
 
197.7
 
 
 
 
ARL6IP1
 
 
hsa-miR-152-3p
 
 
-0.579
 
 
197.7
 
 
 
 
ARL8B
 
 
hsa-miR-152-3p
 
 
-0.337
 
 
56.4
 
 
 
 
ARL8B
 
 
hsa-miR-148b-3p
 
 
-0.337
 
 
56.4
 
 
 
 
ARMCX3
 
 
hsa-miR-425-5p
 
 
-0.325
 
 
13.9
 
 
 
 
ARPC5
 
 
hsa-miR-22-3p
 
 
-0.679
 
 
65.3
 
 
 
 
ARPP19
 
 
hsa-miR-29a-3p
 
 
-0.371
 
 
97.5
 
 
 
 
ARPP19
 
 
hsa-miR-34a-5p
 
 
-0.497
 
 
97.5
 
 
 
 
ARRB1
 
 
hsa-miR-22-3p
 
 
-0.616
 
 
14.8
 
 
 
 
ARRDC3
 
 
hsa-miR-152-3p
 
 
-0.305
 
 
158.3
 
 
 
 
ARRDC3
 
 
hsa-miR-148b-3p
 
 
-0.305
 
 
158.3
 
 
 
 
ARRDC3
 
 
hsa-miR-29a-3p
 
 
-0.402
 
 
158.3
 
 
 
 
ASAH1
 
 
hsa-miR-27a-3p
 
 
-0.398
 
 
101.8
 
 
 
 
ASPH
 
 
hsa-miR-152-3p
 
 
-0.391
 
 
15.8
 
 
 
 
ASPH
 
 
hsa-miR-148b-3p
 
 
-0.391
 
 
15.8
 
 
 
 
ATG4B
 
 
hsa-miR-34a-5p
 
 
-0.58
 
 
13.9
 
 
 
 
ATP2B4
 
 
hsa-miR-152-3p
 
 
-0.337
 
 
14.9
 
 
 
 
ATP2B4
 
 
hsa-miR-148b-3p
 
 
-0.337
 
 
14.9
 
 
 
 
ATP5MC3
 
 
hsa-miR-425-5p
 
 
-0.328
 
 
133.0
 
 
 
 
ATP5MC3
 
 
hsa-miR-29a-3p
 
 
-0.33
 
 
133.0
 
 
 
 
ATP5MC3
 
 
hsa-miR-27a-3p
 
 
-0.416
 
 
133.0
 
 
 
 
ATP6AP2
 
 
hsa-miR-148b-3p
 
 
-0.592
 
 
117.4
 
 
 
 
ATP6AP2
 
 
hsa-miR-152-3p
 
 
-0.592
 
 
117.4
 
 
 
 
ATP6AP2
 
 
hsa-miR-148b-3p
 
 
-0.668
 
 
117.4
 
 
 
 
ATP6AP2
 
 
hsa-miR-152-3p
 
 
-0.668
 
 
117.4
 
 
 
 
ATP6V1E1
 
 
hsa-miR-23a-3p
 
 
-0.294
 
 
85.4
 
 
 
 
ATP6V1E1
 
 
hsa-miR-23b-3p
 
 
-0.294
 
 
85.4
 
 
 
 
ATP8A1
 
 
hsa-miR-22-3p
 
 
-0.468
 
 
22.1
 
 
 
 
ATXN10
 
 
hsa-miR-27a-3p
 
 
-0.295
 
 
39.6
 
 
 
 
AZIN1
 
 
hsa-miR-132-3p
 
 
-0.29
 
 
123.6
 
 
 
 
AZIN1
 
 
hsa-miR-132-3p
 
 
-0.299
 
 
123.6
 
 
 
 
B3GNT7
 
 
hsa-miR-27a-3p
 
 
-0.355
 
 
13.4
 
 
 
 
 
 
 breast 
 
 
 
 
target_symbol
 
 
mature_mirna_id
 
 
score
 
 
nTPM
 
 
 
 
 
 
AAGAB
 
 
hsa-miR-34a-5p
 
 
-0.319
 
 
21.1
 
 
 
 
AAR2
 
 
hsa-miR-29a-3p
 
 
-0.327
 
 
20.3
 
 
 
 
ABCB7
 
 
hsa-miR-148b-3p
 
 
-0.515
 
 
11.6
 
 
 
 
ABCB7
 
 
hsa-miR-152-3p
 
 
-0.515
 
 
11.6
 
 
 
 
ABCC5
 
 
hsa-miR-125a-5p
 
 
-0.288
 
 
12.8
 
 
 
 
ABCD3
 
 
hsa-miR-148b-3p
 
 
-0.388
 
 
30.8
 
 
 
 
ABCD3
 
 
hsa-miR-152-3p
 
 
-0.388
 
 
30.8
 
 
 
 
ABCE1
 
 
hsa-miR-29a-3p
 
 
-0.404
 
 
30.9
 
 
 
 
ABHD17C
 
 
hsa-miR-27a-3p
 
 
-0.435
 
 
10.9
 
 
 
 
ABHD3
 
 
hsa-miR-125a-5p
 
 
-0.453
 
 
14.7
 
 
 
 
ABHD4
 
 
hsa-miR-29a-3p
 
 
-0.312
 
 
18.7
 
 
 
 
ABHD5
 
 
hsa-miR-132-3p
 
 
-0.323
 
 
18.8
 
 
 
 
ABHD5
 
 
hsa-miR-27a-3p
 
 
-0.407
 
 
18.8
 
 
 
 
ABRAXAS2
 
 
hsa-miR-34a-5p
 
 
-0.538
 
 
11.4
 
 
 
 
ABTB1
 
 
hsa-miR-125a-5p
 
 
-0.519
 
 
33.9
 
 
 
 
ACBD3
 
 
hsa-miR-34a-5p
 
 
-0.415
 
 
22.8
 
 
 
 
ACSL1
 
 
hsa-miR-34a-5p
 
 
-0.306
 
 
321.1
 
 
 
 
ACSL4
 
 
hsa-miR-34a-5p
 
 
-0.372
 
 
16.3
 
 
 
 
ACTA2
 
 
hsa-miR-27a-3p
 
 
-0.577
 
 
802.4
 
 
 
 
ACVR1
 
 
hsa-miR-152-3p
 
 
-0.397
 
 
11.1
 
 
 
 
ACVR1
 
 
hsa-miR-148b-3p
 
 
-0.397
 
 
11.1
 
 
 
 
ADA
 
 
hsa-miR-29a-3p
 
 
-0.376
 
 
11.4
 
 
 
 
ADAM10
 
 
hsa-miR-152-3p
 
 
-0.348
 
 
14.2
 
 
 
 
ADAM10
 
 
hsa-miR-148b-3p
 
 
-0.356
 
 
14.2
 
 
 
 
ADAMTS9
 
 
hsa-miR-29a-3p
 
 
-0.305
 
 
19.0
 
 
 
 
ADAMTS9
 
 
hsa-miR-29a-3p
 
 
-0.485
 
 
19.0
 
 
 
 
ADCY3
 
 
hsa-miR-132-3p
 
 
-0.303
 
 
72.4
 
 
 
 
ADGRA3
 
 
hsa-miR-27a-3p
 
 
-0.296
 
 
10.2
 
 
 
 
ADGRF5
 
 
hsa-miR-148b-3p
 
 
-0.354
 
 
44.5
 
 
 
 
ADGRF5
 
 
hsa-miR-152-3p
 
 
-0.354
 
 
44.5
 
 
 
 
ADIPOR2
 
 
hsa-miR-150-5p
 
 
-0.287
 
 
44.0
 
 
 
 
ADIPOR2
 
 
hsa-miR-34a-5p
 
 
-0.33
 
 
44.0
 
 
 
 
ADK
 
 
hsa-miR-34a-5p
 
 
-0.374
 
 
25.7
 
 
 
 
ADRA2A
 
 
hsa-miR-23a-3p
 
 
-0.371
 
 
31.1
 
 
 
 
ADRA2A
 
 
hsa-miR-23b-3p
 
 
-0.371
 
 
31.1
 
 
 
 
AFG3L2
 
 
hsa-miR-181b-5p
 
 
-0.491
 
 
15.5
 
 
 
 
AFG3L2
 
 
hsa-miR-181a-5p
 
 
-0.491
 
 
15.5
 
 
 
 
AGFG1
 
 
hsa-miR-148b-3p
 
 
-0.332
 
 
48.8
 
 
 
 
AGFG1
 
 
hsa-miR-152-3p
 
 
-0.332
 
 
48.8
 
 
 
 
AGPAT3
 
 
hsa-miR-27a-3p
 
 
-0.29
 
 
14.5
 
 
 
 
AGTRAP
 
 
hsa-miR-34a-5p
 
 
-0.328
 
 
41.1
 
 
 
 
AHDC1
 
 
hsa-miR-148b-3p
 
 
-0.413
 
 
11.9
 
 
 
 
AHDC1
 
 
hsa-miR-152-3p
 
 
-0.413
 
 
11.9
 
 
 
 
AHR
 
 
hsa-miR-29a-3p
 
 
-0.316
 
 
18.4
 
 
 
 
AIDA
 
 
hsa-miR-22-3p
 
 
-0.316
 
 
39.1
 
 
 
 
AIFM1
 
 
hsa-miR-125a-5p
 
 
-0.3
 
 
11.7
 
 
 
 
AK1
 
 
hsa-miR-29a-3p
 
 
-0.333
 
 
20.7
 
 
 
 
AKAP1
 
 
hsa-miR-148b-3p
 
 
-0.381
 
 
34.0
 
 
 
 
AKAP1
 
 
hsa-miR-152-3p
 
 
-0.381
 
 
34.0
 
 
 
 
AKIRIN1
 
 
hsa-miR-27a-3p
 
 
-0.368
 
 
21.6
 
 
 
 
AKT3
 
 
hsa-miR-29a-3p
 
 
-0.287
 
 
13.8
 
 
 
 
AKT3
 
 
hsa-miR-22-3p
 
 
-0.451
 
 
13.8
 
 
 
 
ALCAM
 
 
hsa-miR-34a-5p
 
 
-0.303
 
 
76.0
 
 
 
 
ALCAM
 
 
hsa-miR-148b-3p
 
 
-0.339
 
 
76.0
 
 
 
 
ALCAM
 
 
hsa-miR-152-3p
 
 
-0.347
 
 
76.0
 
 
 
 
ALDOA
 
 
hsa-miR-34a-5p
 
 
-0.655
 
 
590.2
 
 
 
 
ALPL
 
 
hsa-miR-10a-5p
 
 
-0.32
 
 
21.4
 
 
 
 
ANAPC16
 
 
hsa-miR-125a-5p
 
 
-0.578
 
 
20.2
 
 
 
 
ANK2
 
 
hsa-miR-34a-5p
 
 
-0.317
 
 
14.7
 
 
 
 
ANK3
 
 
hsa-miR-34a-5p
 
 
-0.29
 
 
19.6
 
 
 
 
ANK3
 
 
hsa-miR-34a-5p
 
 
-0.347
 
 
19.6
 
 
 
 
ANKHD1
 
 
hsa-miR-23a-3p
 
 
-0.368
 
 
37.8
 
 
 
 
ANKHD1
 
 
hsa-miR-23b-3p
 
 
-0.368
 
 
37.8
 
 
 
 
ANKRD13A
 
 
hsa-miR-22-3p
 
 
-0.478
 
 
15.4
 
 
 
 
ANKRD29
 
 
hsa-miR-132-3p
 
 
-0.47
 
 
15.0
 
 
 
 
ANKRD40
 
 
hsa-miR-27a-3p
 
 
-0.35
 
 
24.7
 
 
 
 
ANP32A
 
 
hsa-miR-132-3p
 
 
-0.374
 
 
66.9
 
 
 
 
ANP32B
 
 
hsa-miR-34a-5p
 
 
-0.29
 
 
132.4
 
 
 
 
ANPEP
 
 
hsa-miR-125a-5p
 
 
-0.372
 
 
49.7
 
 
 
 
ANTXR2
 
 
hsa-miR-29a-3p
 
 
-0.347
 
 
38.4
 
 
 
 
AP3S2
 
 
hsa-miR-34a-5p
 
 
-0.413
 
 
18.1
 
 
 
 
APH1A
 
 
hsa-miR-34a-5p
 
 
-0.304
 
 
91.6
 
 
 
 
APOO
 
 
hsa-miR-181b-5p
 
 
-0.5
 
 
12.5
 
 
 
 
APOO
 
 
hsa-miR-181a-5p
 
 
-0.5
 
 
12.5
 
 
 
 
APPBP2
 
 
hsa-miR-27a-3p
 
 
-0.378
 
 
10.6
 
 
 
 
AREG
 
 
hsa-miR-34a-5p
 
 
-0.62
 
 
57.8
 
 
 
 
ARF4
 
 
hsa-miR-152-3p
 
 
-0.487
 
 
129.6
 
 
 
 
ARF4
 
 
hsa-miR-148b-3p
 
 
-0.487
 
 
129.6
 
 
 
 
ARF5
 
 
hsa-miR-29a-3p
 
 
-0.491
 
 
108.8
 
 
 
 
ARF6
 
 
hsa-miR-181b-5p
 
 
-0.33
 
 
31.6
 
 
 
 
ARF6
 
 
hsa-miR-181a-5p
 
 
-0.33
 
 
31.6
 
 
 
 
ARFGEF1
 
 
hsa-miR-148b-3p
 
 
-0.306
 
 
12.7
 
 
 
 
ARFGEF1
 
 
hsa-miR-152-3p
 
 
-0.306
 
 
12.7
 
 
 
 
ARFIP1
 
 
hsa-miR-152-3p
 
 
-0.474
 
 
22.0
 
 
 
 
ARFIP1
 
 
hsa-miR-148b-3p
 
 
-0.474
 
 
22.0
 
 
 
 
ARHGAP1
 
 
hsa-miR-34a-5p
 
 
-0.369
 
 
59.0
 
 
 
 
ARHGAP21
 
 
hsa-miR-152-3p
 
 
-0.287
 
 
15.1
 
 
 
 
ARHGAP21
 
 
hsa-miR-148b-3p
 
 
-0.287
 
 
15.1
 
 
 
 
ARHGAP26
 
 
hsa-miR-34a-5p
 
 
-0.384
 
 
11.5
 
 
 
 
ARID1B
 
 
hsa-miR-29a-3p
 
 
-0.342
 
 
11.7
 
 
 
 
ARL4A
 
 
hsa-miR-29a-3p
 
 
-0.386
 
 
51.6
 
 
 
 
ARL6IP1
 
 
hsa-miR-148b-3p
 
 
-0.579
 
 
105.7
 
 
 
 
ARL6IP1
 
 
hsa-miR-152-3p
 
 
-0.579
 
 
105.7
 
 
 
 
ARL8B
 
 
hsa-miR-152-3p
 
 
-0.337
 
 
39.7
 
 
 
 
ARL8B
 
 
hsa-miR-148b-3p
 
 
-0.337
 
 
39.7
 
 
 
 
ARMCX3
 
 
hsa-miR-425-5p
 
 
-0.325
 
 
30.1
 
 
 
 
ARPC5
 
 
hsa-miR-22-3p
 
 
-0.679
 
 
45.7
 
 
 
 
ARPIN-AP3S2
 
 
hsa-miR-34a-5p
 
 
-0.348
 
 
10.4
 
 
 
 
ARPP19
 
 
hsa-miR-29a-3p
 
 
-0.371
 
 
43.2
 
 
 
 
ARPP19
 
 
hsa-miR-34a-5p
 
 
-0.497
 
 
43.2
 
 
 
 
 
 
 cerebral cortex 
 
 
 
 
target_symbol
 
 
mature_mirna_id
 
 
score
 
 
nTPM
 
 
 
 
 
 
AAGAB
 
 
hsa-miR-34a-5p
 
 
-0.319
 
 
20.5
 
 
 
 
AAR2
 
 
hsa-miR-29a-3p
 
 
-0.327
 
 
10.6
 
 
 
 
ABCC5
 
 
hsa-miR-125a-5p
 
 
-0.288
 
 
41.2
 
 
 
 
ABCD3
 
 
hsa-miR-148b-3p
 
 
-0.388
 
 
17.1
 
 
 
 
ABCD3
 
 
hsa-miR-152-3p
 
 
-0.388
 
 
17.1
 
 
 
 
ABCE1
 
 
hsa-miR-29a-3p
 
 
-0.404
 
 
21.8
 
 
 
 
ABHD17C
 
 
hsa-miR-27a-3p
 
 
-0.435
 
 
16.3
 
 
 
 
ABHD4
 
 
hsa-miR-29a-3p
 
 
-0.312
 
 
14.6
 
 
 
 
ABHD6
 
 
hsa-miR-125a-5p
 
 
-0.501
 
 
21.5
 
 
 
 
ABTB1
 
 
hsa-miR-125a-5p
 
 
-0.519
 
 
51.2
 
 
 
 
ACBD3
 
 
hsa-miR-34a-5p
 
 
-0.415
 
 
15.8
 
 
 
 
ACHE
 
 
hsa-miR-125a-5p
 
 
-0.308
 
 
23.4
 
 
 
 
ACHE
 
 
hsa-miR-125a-5p
 
 
-0.361
 
 
23.4
 
 
 
 
ACSL1
 
 
hsa-miR-34a-5p
 
 
-0.306
 
 
19.9
 
 
 
 
ACSL4
 
 
hsa-miR-34a-5p
 
 
-0.372
 
 
27.8
 
 
 
 
ACSL6
 
 
hsa-miR-125a-5p
 
 
-0.307
 
 
29.5
 
 
 
 
ACTA2
 
 
hsa-miR-27a-3p
 
 
-0.577
 
 
56.2
 
 
 
 
ACTN2
 
 
hsa-miR-23a-3p
 
 
-0.294
 
 
14.9
 
 
 
 
ACTN2
 
 
hsa-miR-29a-3p
 
 
-0.311
 
 
14.9
 
 
 
 
ADAM10
 
 
hsa-miR-152-3p
 
 
-0.348
 
 
16.0
 
 
 
 
ADAM10
 
 
hsa-miR-148b-3p
 
 
-0.356
 
 
16.0
 
 
 
 
ADAMTS10
 
 
hsa-miR-27a-3p
 
 
-0.354
 
 
10.8
 
 
 
 
ADAMTS10
 
 
hsa-miR-29a-3p
 
 
-0.516
 
 
10.8
 
 
 
 
ADAP1
 
 
hsa-miR-27a-3p
 
 
-0.302
 
 
126.8
 
 
 
 
ADCY2
 
 
hsa-miR-152-3p
 
 
-0.299
 
 
35.4
 
 
 
 
ADCY2
 
 
hsa-miR-148b-3p
 
 
-0.299
 
 
35.4
 
 
 
 
ADCY3
 
 
hsa-miR-132-3p
 
 
-0.303
 
 
37.4
 
 
 
 
ADD2
 
 
hsa-miR-34a-5p
 
 
-0.305
 
 
25.2
 
 
 
 
ADGRA3
 
 
hsa-miR-27a-3p
 
 
-0.296
 
 
17.8
 
 
 
 
ADGRB3
 
 
hsa-miR-152-3p
 
 
-0.516
 
 
28.2
 
 
 
 
ADGRB3
 
 
hsa-miR-148b-3p
 
 
-0.524
 
 
28.2
 
 
 
 
ADIPOR2
 
 
hsa-miR-150-5p
 
 
-0.287
 
 
29.8
 
 
 
 
ADIPOR2
 
 
hsa-miR-34a-5p
 
 
-0.33
 
 
29.8
 
 
 
 
ADK
 
 
hsa-miR-34a-5p
 
 
-0.374
 
 
16.5
 
 
 
 
ADO
 
 
hsa-miR-34a-5p
 
 
-0.377
 
 
15.9
 
 
 
 
ADORA2B
 
 
hsa-miR-27a-3p
 
 
-0.638
 
 
12.5
 
 
 
 
AFG3L2
 
 
hsa-miR-181b-5p
 
 
-0.491
 
 
17.1
 
 
 
 
AFG3L2
 
 
hsa-miR-181a-5p
 
 
-0.491
 
 
17.1
 
 
 
 
AGFG1
 
 
hsa-miR-148b-3p
 
 
-0.332
 
 
33.6
 
 
 
 
AGFG1
 
 
hsa-miR-152-3p
 
 
-0.332
 
 
33.6
 
 
 
 
AGGF1
 
 
hsa-miR-125a-5p
 
 
-0.331
 
 
12.2
 
 
 
 
AGPAT3
 
 
hsa-miR-27a-3p
 
 
-0.29
 
 
38.5
 
 
 
 
AGPAT4
 
 
hsa-miR-29a-3p
 
 
-0.376
 
 
13.7
 
 
 
 
AGTRAP
 
 
hsa-miR-34a-5p
 
 
-0.328
 
 
16.4
 
 
 
 
AHCYL2
 
 
hsa-miR-34a-5p
 
 
-0.359
 
 
30.6
 
 
 
 
AHDC1
 
 
hsa-miR-148b-3p
 
 
-0.413
 
 
15.5
 
 
 
 
AHDC1
 
 
hsa-miR-152-3p
 
 
-0.413
 
 
15.5
 
 
 
 
AIDA
 
 
hsa-miR-22-3p
 
 
-0.316
 
 
30.3
 
 
 
 
AIFM1
 
 
hsa-miR-125a-5p
 
 
-0.3
 
 
10.3
 
 
 
 
AK1
 
 
hsa-miR-29a-3p
 
 
-0.333
 
 
81.2
 
 
 
 
AKAP1
 
 
hsa-miR-148b-3p
 
 
-0.381
 
 
24.9
 
 
 
 
AKAP1
 
 
hsa-miR-152-3p
 
 
-0.381
 
 
24.9
 
 
 
 
AKAP5
 
 
hsa-miR-29a-3p
 
 
-0.298
 
 
12.9
 
 
 
 
AKIRIN1
 
 
hsa-miR-27a-3p
 
 
-0.368
 
 
38.1
 
 
 
 
AKT3
 
 
hsa-miR-29a-3p
 
 
-0.287
 
 
23.7
 
 
 
 
AKT3
 
 
hsa-miR-22-3p
 
 
-0.451
 
 
23.7
 
 
 
 
ALCAM
 
 
hsa-miR-34a-5p
 
 
-0.303
 
 
54.7
 
 
 
 
ALCAM
 
 
hsa-miR-148b-3p
 
 
-0.339
 
 
54.7
 
 
 
 
ALCAM
 
 
hsa-miR-152-3p
 
 
-0.347
 
 
54.7
 
 
 
 
ALDOA
 
 
hsa-miR-34a-5p
 
 
-0.655
 
 
1334.6
 
 
 
 
ALPL
 
 
hsa-miR-10a-5p
 
 
-0.32
 
 
13.5
 
 
 
 
AMER2
 
 
hsa-miR-34a-5p
 
 
-0.48
 
 
35.0
 
 
 
 
AMOT
 
 
hsa-miR-29a-3p
 
 
-0.304
 
 
17.9
 
 
 
 
ANK2
 
 
hsa-miR-34a-5p
 
 
-0.317
 
 
79.8
 
 
 
 
ANK3
 
 
hsa-miR-34a-5p
 
 
-0.29
 
 
51.5
 
 
 
 
ANK3
 
 
hsa-miR-34a-5p
 
 
-0.347
 
 
51.5
 
 
 
 
ANKHD1
 
 
hsa-miR-23a-3p
 
 
-0.368
 
 
22.1
 
 
 
 
ANKHD1
 
 
hsa-miR-23b-3p
 
 
-0.368
 
 
22.1
 
 
 
 
ANKRD13B
 
 
hsa-miR-29a-3p
 
 
-0.421
 
 
17.2
 
 
 
 
ANKRD13C
 
 
hsa-miR-29a-3p
 
 
-0.411
 
 
10.2
 
 
 
 
ANKRD40
 
 
hsa-miR-27a-3p
 
 
-0.35
 
 
54.5
 
 
 
 
ANP32A
 
 
hsa-miR-132-3p
 
 
-0.374
 
 
64.2
 
 
 
 
ANP32B
 
 
hsa-miR-34a-5p
 
 
-0.29
 
 
70.2
 
 
 
 
AP3S2
 
 
hsa-miR-34a-5p
 
 
-0.413
 
 
25.3
 
 
 
 
APC
 
 
hsa-miR-29a-3p
 
 
-0.413
 
 
34.5
 
 
 
 
APH1A
 
 
hsa-miR-34a-5p
 
 
-0.304
 
 
45.1
 
 
 
 
APOO
 
 
hsa-miR-181b-5p
 
 
-0.5
 
 
56.2
 
 
 
 
APOO
 
 
hsa-miR-181a-5p
 
 
-0.5
 
 
56.2
 
 
 
 
APPBP2
 
 
hsa-miR-27a-3p
 
 
-0.378
 
 
17.6
 
 
 
 
ARF4
 
 
hsa-miR-152-3p
 
 
-0.487
 
 
61.5
 
 
 
 
ARF4
 
 
hsa-miR-148b-3p
 
 
-0.487
 
 
61.5
 
 
 
 
ARF5
 
 
hsa-miR-29a-3p
 
 
-0.491
 
 
222.4
 
 
 
 
ARF6
 
 
hsa-miR-181b-5p
 
 
-0.33
 
 
13.0
 
 
 
 
ARF6
 
 
hsa-miR-181a-5p
 
 
-0.33
 
 
13.0
 
 
 
 
ARHGAP1
 
 
hsa-miR-34a-5p
 
 
-0.369
 
 
40.8
 
 
 
 
ARHGAP21
 
 
hsa-miR-152-3p
 
 
-0.287
 
 
47.3
 
 
 
 
ARHGAP21
 
 
hsa-miR-148b-3p
 
 
-0.287
 
 
47.3
 
 
 
 
ARHGAP26
 
 
hsa-miR-34a-5p
 
 
-0.384
 
 
16.8
 
 
 
 
ARID1B
 
 
hsa-miR-29a-3p
 
 
-0.342
 
 
12.2
 
 
 
 
ARL4A
 
 
hsa-miR-29a-3p
 
 
-0.386
 
 
23.3
 
 
 
 
ARL6IP1
 
 
hsa-miR-148b-3p
 
 
-0.579
 
 
314.1
 
 
 
 
ARL6IP1
 
 
hsa-miR-152-3p
 
 
-0.579
 
 
314.1
 
 
 
 
ARL8B
 
 
hsa-miR-152-3p
 
 
-0.337
 
 
75.1
 
 
 
 
ARL8B
 
 
hsa-miR-148b-3p
 
 
-0.337
 
 
75.1
 
 
 
 
ARMCX3
 
 
hsa-miR-425-5p
 
 
-0.325
 
 
42.1
 
 
 
 
ARPC5
 
 
hsa-miR-22-3p
 
 
-0.679
 
 
53.1
 
 
 
 
ARPIN-AP3S2
 
 
hsa-miR-34a-5p
 
 
-0.348
 
 
13.1
 
 
 
 
ARPP19
 
 
hsa-miR-29a-3p
 
 
-0.371
 
 
292.4
 
 
 
 
ARPP19
 
 
hsa-miR-34a-5p
 
 
-0.497
 
 
292.4
 
 
 
 
ARRB1
 
 
hsa-miR-22-3p
 
 
-0.616
 
 
40.1
 
 
 
 
 
 
 cervix 
 
 
 
 
target_symbol
 
 
mature_mirna_id
 
 
score
 
 
nTPM
 
 
 
 
 
 
AAGAB
 
 
hsa-miR-34a-5p
 
 
-0.319
 
 
16.7
 
 
 
 
AAR2
 
 
hsa-miR-29a-3p
 
 
-0.327
 
 
19.6
 
 
 
 
ABCB7
 
 
hsa-miR-148b-3p
 
 
-0.515
 
 
10.8
 
 
 
 
ABCB7
 
 
hsa-miR-152-3p
 
 
-0.515
 
 
10.8
 
 
 
 
ABCC5
 
 
hsa-miR-125a-5p
 
 
-0.288
 
 
31.4
 
 
 
 
ABCD3
 
 
hsa-miR-148b-3p
 
 
-0.388
 
 
21.0
 
 
 
 
ABCD3
 
 
hsa-miR-152-3p
 
 
-0.388
 
 
21.0
 
 
 
 
ABCE1
 
 
hsa-miR-29a-3p
 
 
-0.404
 
 
19.5
 
 
 
 
ABHD17C
 
 
hsa-miR-27a-3p
 
 
-0.435
 
 
18.1
 
 
 
 
ABHD3
 
 
hsa-miR-125a-5p
 
 
-0.453
 
 
10.5
 
 
 
 
ABHD4
 
 
hsa-miR-29a-3p
 
 
-0.312
 
 
27.3
 
 
 
 
ABTB1
 
 
hsa-miR-125a-5p
 
 
-0.519
 
 
42.5
 
 
 
 
ACBD3
 
 
hsa-miR-34a-5p
 
 
-0.415
 
 
25.9
 
 
 
 
ACSL1
 
 
hsa-miR-34a-5p
 
 
-0.306
 
 
55.5
 
 
 
 
ACSL4
 
 
hsa-miR-34a-5p
 
 
-0.372
 
 
43.6
 
 
 
 
ACTA2
 
 
hsa-miR-27a-3p
 
 
-0.577
 
 
2602.4
 
 
 
 
ACVR1
 
 
hsa-miR-152-3p
 
 
-0.397
 
 
20.6
 
 
 
 
ACVR1
 
 
hsa-miR-148b-3p
 
 
-0.397
 
 
20.6
 
 
 
 
ADAM10
 
 
hsa-miR-152-3p
 
 
-0.348
 
 
15.1
 
 
 
 
ADAM10
 
 
hsa-miR-148b-3p
 
 
-0.356
 
 
15.1
 
 
 
 
ADAMTS10
 
 
hsa-miR-27a-3p
 
 
-0.354
 
 
11.2
 
 
 
 
ADAMTS10
 
 
hsa-miR-29a-3p
 
 
-0.516
 
 
11.2
 
 
 
 
ADAMTS9
 
 
hsa-miR-29a-3p
 
 
-0.305
 
 
11.0
 
 
 
 
ADAMTS9
 
 
hsa-miR-29a-3p
 
 
-0.485
 
 
11.0
 
 
 
 
ADCY3
 
 
hsa-miR-132-3p
 
 
-0.303
 
 
60.4
 
 
 
 
ADGRA3
 
 
hsa-miR-27a-3p
 
 
-0.296
 
 
10.2
 
 
 
 
ADIPOR2
 
 
hsa-miR-150-5p
 
 
-0.287
 
 
25.7
 
 
 
 
ADIPOR2
 
 
hsa-miR-34a-5p
 
 
-0.33
 
 
25.7
 
 
 
 
ADK
 
 
hsa-miR-34a-5p
 
 
-0.374
 
 
20.2
 
 
 
 
ADRA2A
 
 
hsa-miR-23a-3p
 
 
-0.371
 
 
35.0
 
 
 
 
ADRA2A
 
 
hsa-miR-23b-3p
 
 
-0.371
 
 
35.0
 
 
 
 
AFG3L2
 
 
hsa-miR-181b-5p
 
 
-0.491
 
 
14.0
 
 
 
 
AFG3L2
 
 
hsa-miR-181a-5p
 
 
-0.491
 
 
14.0
 
 
 
 
AGFG1
 
 
hsa-miR-148b-3p
 
 
-0.332
 
 
27.5
 
 
 
 
AGFG1
 
 
hsa-miR-152-3p
 
 
-0.332
 
 
27.5
 
 
 
 
AGPAT3
 
 
hsa-miR-27a-3p
 
 
-0.29
 
 
12.1
 
 
 
 
AGTRAP
 
 
hsa-miR-34a-5p
 
 
-0.328
 
 
35.6
 
 
 
 
AHDC1
 
 
hsa-miR-148b-3p
 
 
-0.413
 
 
23.6
 
 
 
 
AHDC1
 
 
hsa-miR-152-3p
 
 
-0.413
 
 
23.6
 
 
 
 
AHR
 
 
hsa-miR-29a-3p
 
 
-0.316
 
 
13.6
 
 
 
 
AIDA
 
 
hsa-miR-22-3p
 
 
-0.316
 
 
44.9
 
 
 
 
AIFM1
 
 
hsa-miR-125a-5p
 
 
-0.3
 
 
14.8
 
 
 
 
AK1
 
 
hsa-miR-29a-3p
 
 
-0.333
 
 
35.3
 
 
 
 
AKAP1
 
 
hsa-miR-148b-3p
 
 
-0.381
 
 
22.6
 
 
 
 
AKAP1
 
 
hsa-miR-152-3p
 
 
-0.381
 
 
22.6
 
 
 
 
AKIRIN1
 
 
hsa-miR-27a-3p
 
 
-0.368
 
 
20.5
 
 
 
 
AKT3
 
 
hsa-miR-29a-3p
 
 
-0.287
 
 
15.6
 
 
 
 
AKT3
 
 
hsa-miR-22-3p
 
 
-0.451
 
 
15.6
 
 
 
 
ALCAM
 
 
hsa-miR-34a-5p
 
 
-0.303
 
 
26.3
 
 
 
 
ALCAM
 
 
hsa-miR-148b-3p
 
 
-0.339
 
 
26.3
 
 
 
 
ALCAM
 
 
hsa-miR-152-3p
 
 
-0.347
 
 
26.3
 
 
 
 
ALDH1A2
 
 
hsa-miR-23b-3p
 
 
-0.346
 
 
49.1
 
 
 
 
ALDH1A2
 
 
hsa-miR-23a-3p
 
 
-0.354
 
 
49.1
 
 
 
 
ALDOA
 
 
hsa-miR-34a-5p
 
 
-0.655
 
 
694.9
 
 
 
 
ANAPC16
 
 
hsa-miR-125a-5p
 
 
-0.578
 
 
18.3
 
 
 
 
ANK2
 
 
hsa-miR-34a-5p
 
 
-0.317
 
 
25.2
 
 
 
 
ANK3
 
 
hsa-miR-34a-5p
 
 
-0.29
 
 
11.0
 
 
 
 
ANK3
 
 
hsa-miR-34a-5p
 
 
-0.347
 
 
11.0
 
 
 
 
ANKHD1
 
 
hsa-miR-23a-3p
 
 
-0.368
 
 
41.2
 
 
 
 
ANKHD1
 
 
hsa-miR-23b-3p
 
 
-0.368
 
 
41.2
 
 
 
 
ANKRD13A
 
 
hsa-miR-22-3p
 
 
-0.478
 
 
13.2
 
 
 
 
ANKRD29
 
 
hsa-miR-132-3p
 
 
-0.47
 
 
12.2
 
 
 
 
ANKRD40
 
 
hsa-miR-27a-3p
 
 
-0.35
 
 
18.9
 
 
 
 
ANP32A
 
 
hsa-miR-132-3p
 
 
-0.374
 
 
50.7
 
 
 
 
ANP32B
 
 
hsa-miR-34a-5p
 
 
-0.29
 
 
143.2
 
 
 
 
ANPEP
 
 
hsa-miR-125a-5p
 
 
-0.372
 
 
38.9
 
 
 
 
ANTXR2
 
 
hsa-miR-29a-3p
 
 
-0.347
 
 
49.9
 
 
 
 
AP3S2
 
 
hsa-miR-34a-5p
 
 
-0.413
 
 
16.2
 
 
 
 
APH1A
 
 
hsa-miR-34a-5p
 
 
-0.304
 
 
80.9
 
 
 
 
APOO
 
 
hsa-miR-181b-5p
 
 
-0.5
 
 
12.3
 
 
 
 
APOO
 
 
hsa-miR-181a-5p
 
 
-0.5
 
 
12.3
 
 
 
 
APPBP2
 
 
hsa-miR-27a-3p
 
 
-0.378
 
 
11.0
 
 
 
 
AREG
 
 
hsa-miR-34a-5p
 
 
-0.62
 
 
18.0
 
 
 
 
ARF4
 
 
hsa-miR-152-3p
 
 
-0.487
 
 
196.1
 
 
 
 
ARF4
 
 
hsa-miR-148b-3p
 
 
-0.487
 
 
196.1
 
 
 
 
ARF5
 
 
hsa-miR-29a-3p
 
 
-0.491
 
 
129.6
 
 
 
 
ARF6
 
 
hsa-miR-181b-5p
 
 
-0.33
 
 
31.8
 
 
 
 
ARF6
 
 
hsa-miR-181a-5p
 
 
-0.33
 
 
31.8
 
 
 
 
ARFGEF1
 
 
hsa-miR-148b-3p
 
 
-0.306
 
 
13.3
 
 
 
 
ARFGEF1
 
 
hsa-miR-152-3p
 
 
-0.306
 
 
13.3
 
 
 
 
ARFIP1
 
 
hsa-miR-152-3p
 
 
-0.474
 
 
21.1
 
 
 
 
ARFIP1
 
 
hsa-miR-148b-3p
 
 
-0.474
 
 
21.1
 
 
 
 
ARHGAP1
 
 
hsa-miR-34a-5p
 
 
-0.369
 
 
62.9
 
 
 
 
ARHGAP21
 
 
hsa-miR-152-3p
 
 
-0.287
 
 
18.5
 
 
 
 
ARHGAP21
 
 
hsa-miR-148b-3p
 
 
-0.287
 
 
18.5
 
 
 
 
ARHGAP26
 
 
hsa-miR-34a-5p
 
 
-0.384
 
 
11.2
 
 
 
 
ARID1B
 
 
hsa-miR-29a-3p
 
 
-0.342
 
 
15.1
 
 
 
 
ARL4A
 
 
hsa-miR-29a-3p
 
 
-0.386
 
 
46.9
 
 
 
 
ARL6IP1
 
 
hsa-miR-148b-3p
 
 
-0.579
 
 
74.5
 
 
 
 
ARL6IP1
 
 
hsa-miR-152-3p
 
 
-0.579
 
 
74.5
 
 
 
 
ARL8B
 
 
hsa-miR-152-3p
 
 
-0.337
 
 
42.9
 
 
 
 
ARL8B
 
 
hsa-miR-148b-3p
 
 
-0.337
 
 
42.9
 
 
 
 
ARMCX3
 
 
hsa-miR-425-5p
 
 
-0.325
 
 
42.3
 
 
 
 
ARPC5
 
 
hsa-miR-22-3p
 
 
-0.679
 
 
45.8
 
 
 
 
ARPP19
 
 
hsa-miR-29a-3p
 
 
-0.371
 
 
35.8
 
 
 
 
ARPP19
 
 
hsa-miR-34a-5p
 
 
-0.497
 
 
35.8
 
 
 
 
ARRB1
 
 
hsa-miR-22-3p
 
 
-0.616
 
 
10.1
 
 
 
 
ARRDC3
 
 
hsa-miR-152-3p
 
 
-0.305
 
 
42.0
 
 
 
 
ARRDC3
 
 
hsa-miR-148b-3p
 
 
-0.305
 
 
42.0
 
 
 
 
ARRDC3
 
 
hsa-miR-29a-3p
 
 
-0.402
 
 
42.0
 
 
 
 
 
 
 choroid plexus 
 
 
 
 
target_symbol
 
 
mature_mirna_id
 
 
score
 
 
nTPM
 
 
 
 
 
 
AAGAB
 
 
hsa-miR-34a-5p
 
 
-0.319
 
 
27.4
 
 
 
 
AAR2
 
 
hsa-miR-29a-3p
 
 
-0.327
 
 
27.3
 
 
 
 
ABCB7
 
 
hsa-miR-148b-3p
 
 
-0.515
 
 
15.2
 
 
 
 
ABCB7
 
 
hsa-miR-152-3p
 
 
-0.515
 
 
15.2
 
 
 
 
ABCC5
 
 
hsa-miR-125a-5p
 
 
-0.288
 
 
137.7
 
 
 
 
ABCD3
 
 
hsa-miR-148b-3p
 
 
-0.388
 
 
13.3
 
 
 
 
ABCD3
 
 
hsa-miR-152-3p
 
 
-0.388
 
 
13.3
 
 
 
 
ABHD3
 
 
hsa-miR-125a-5p
 
 
-0.453
 
 
24.1
 
 
 
 
ABHD4
 
 
hsa-miR-29a-3p
 
 
-0.312
 
 
31.8
 
 
 
 
ABHD6
 
 
hsa-miR-125a-5p
 
 
-0.501
 
 
15.1
 
 
 
 
ABTB1
 
 
hsa-miR-125a-5p
 
 
-0.519
 
 
29.8
 
 
 
 
ACBD3
 
 
hsa-miR-34a-5p
 
 
-0.415
 
 
12.2
 
 
 
 
ACSL1
 
 
hsa-miR-34a-5p
 
 
-0.306
 
 
57.8
 
 
 
 
ACTA2
 
 
hsa-miR-27a-3p
 
 
-0.577
 
 
76.7
 
 
 
 
ACTN2
 
 
hsa-miR-23a-3p
 
 
-0.294
 
 
16.8
 
 
 
 
ACTN2
 
 
hsa-miR-29a-3p
 
 
-0.311
 
 
16.8
 
 
 
 
ADCY3
 
 
hsa-miR-132-3p
 
 
-0.303
 
 
17.0
 
 
 
 
ADGRA3
 
 
hsa-miR-27a-3p
 
 
-0.296
 
 
23.6
 
 
 
 
ADIPOR2
 
 
hsa-miR-150-5p
 
 
-0.287
 
 
17.0
 
 
 
 
ADIPOR2
 
 
hsa-miR-34a-5p
 
 
-0.33
 
 
17.0
 
 
 
 
ADK
 
 
hsa-miR-34a-5p
 
 
-0.374
 
 
13.6
 
 
 
 
ADORA2B
 
 
hsa-miR-27a-3p
 
 
-0.638
 
 
11.1
 
 
 
 
AGFG1
 
 
hsa-miR-148b-3p
 
 
-0.332
 
 
23.3
 
 
 
 
AGFG1
 
 
hsa-miR-152-3p
 
 
-0.332
 
 
23.3
 
 
 
 
AGPAT3
 
 
hsa-miR-27a-3p
 
 
-0.29
 
 
12.0
 
 
 
 
AGTRAP
 
 
hsa-miR-34a-5p
 
 
-0.328
 
 
114.6
 
 
 
 
AHCYL2
 
 
hsa-miR-34a-5p
 
 
-0.359
 
 
258.9
 
 
 
 
AIDA
 
 
hsa-miR-22-3p
 
 
-0.316
 
 
24.6
 
 
 
 
AIFM1
 
 
hsa-miR-125a-5p
 
 
-0.3
 
 
37.9
 
 
 
 
AK1
 
 
hsa-miR-29a-3p
 
 
-0.333
 
 
522.7
 
 
 
 
AKIRIN1
 
 
hsa-miR-27a-3p
 
 
-0.368
 
 
30.6
 
 
 
 
ALDOA
 
 
hsa-miR-34a-5p
 
 
-0.655
 
 
1143.6
 
 
 
 
ALPL
 
 
hsa-miR-10a-5p
 
 
-0.32
 
 
17.0
 
 
 
 
ANAPC16
 
 
hsa-miR-125a-5p
 
 
-0.578
 
 
16.8
 
 
 
 
ANKHD1
 
 
hsa-miR-23a-3p
 
 
-0.368
 
 
42.2
 
 
 
 
ANKHD1
 
 
hsa-miR-23b-3p
 
 
-0.368
 
 
42.2
 
 
 
 
ANKHD1-EIF4EBP3
 
 
hsa-miR-22-3p
 
 
-0.479
 
 
11.6
 
 
 
 
ANKRD40
 
 
hsa-miR-27a-3p
 
 
-0.35
 
 
17.4
 
 
 
 
ANP32A
 
 
hsa-miR-132-3p
 
 
-0.374
 
 
28.9
 
 
 
 
ANP32B
 
 
hsa-miR-34a-5p
 
 
-0.29
 
 
148.4
 
 
 
 
AP3S2
 
 
hsa-miR-34a-5p
 
 
-0.413
 
 
15.9
 
 
 
 
APH1A
 
 
hsa-miR-34a-5p
 
 
-0.304
 
 
54.0
 
 
 
 
APOO
 
 
hsa-miR-181b-5p
 
 
-0.5
 
 
102.5
 
 
 
 
APOO
 
 
hsa-miR-181a-5p
 
 
-0.5
 
 
102.5
 
 
 
 
ARF4
 
 
hsa-miR-152-3p
 
 
-0.487
 
 
115.4
 
 
 
 
ARF4
 
 
hsa-miR-148b-3p
 
 
-0.487
 
 
115.4
 
 
 
 
ARF5
 
 
hsa-miR-29a-3p
 
 
-0.491
 
 
140.5
 
 
 
 
ARF6
 
 
hsa-miR-181b-5p
 
 
-0.33
 
 
20.9
 
 
 
 
ARF6
 
 
hsa-miR-181a-5p
 
 
-0.33
 
 
20.9
 
 
 
 
ARHGAP1
 
 
hsa-miR-34a-5p
 
 
-0.369
 
 
26.4
 
 
 
 
ARHGAP26
 
 
hsa-miR-34a-5p
 
 
-0.384
 
 
14.6
 
 
 
 
ARL4A
 
 
hsa-miR-29a-3p
 
 
-0.386
 
 
29.1
 
 
 
 
ARL6IP1
 
 
hsa-miR-148b-3p
 
 
-0.579
 
 
185.0
 
 
 
 
ARL6IP1
 
 
hsa-miR-152-3p
 
 
-0.579
 
 
185.0
 
 
 
 
ARL8B
 
 
hsa-miR-152-3p
 
 
-0.337
 
 
19.0
 
 
 
 
ARL8B
 
 
hsa-miR-148b-3p
 
 
-0.337
 
 
19.0
 
 
 
 
ARMCX3
 
 
hsa-miR-425-5p
 
 
-0.325
 
 
25.1
 
 
 
 
ARPC5
 
 
hsa-miR-22-3p
 
 
-0.679
 
 
17.2
 
 
 
 
ARPIN-AP3S2
 
 
hsa-miR-34a-5p
 
 
-0.348
 
 
12.4
 
 
 
 
ARPP19
 
 
hsa-miR-29a-3p
 
 
-0.371
 
 
32.4
 
 
 
 
ARPP19
 
 
hsa-miR-34a-5p
 
 
-0.497
 
 
32.4
 
 
 
 
ARRDC3
 
 
hsa-miR-152-3p
 
 
-0.305
 
 
24.1
 
 
 
 
ARRDC3
 
 
hsa-miR-148b-3p
 
 
-0.305
 
 
24.1
 
 
 
 
ARRDC3
 
 
hsa-miR-29a-3p
 
 
-0.402
 
 
24.1
 
 
 
 
ASAH1
 
 
hsa-miR-27a-3p
 
 
-0.398
 
 
113.7
 
 
 
 
ASPH
 
 
hsa-miR-152-3p
 
 
-0.391
 
 
79.9
 
 
 
 
ASPH
 
 
hsa-miR-148b-3p
 
 
-0.391
 
 
79.9
 
 
 
 
ATG4B
 
 
hsa-miR-34a-5p
 
 
-0.58
 
 
17.1
 
 
 
 
ATP1A2
 
 
hsa-miR-34a-5p
 
 
-0.306
 
 
41.0
 
 
 
 
ATP5MC3
 
 
hsa-miR-425-5p
 
 
-0.328
 
 
264.7
 
 
 
 
ATP5MC3
 
 
hsa-miR-29a-3p
 
 
-0.33
 
 
264.7
 
 
 
 
ATP5MC3
 
 
hsa-miR-27a-3p
 
 
-0.416
 
 
264.7
 
 
 
 
ATP6AP2
 
 
hsa-miR-148b-3p
 
 
-0.592
 
 
85.2
 
 
 
 
ATP6AP2
 
 
hsa-miR-152-3p
 
 
-0.592
 
 
85.2
 
 
 
 
ATP6AP2
 
 
hsa-miR-148b-3p
 
 
-0.668
 
 
85.2
 
 
 
 
ATP6AP2
 
 
hsa-miR-152-3p
 
 
-0.668
 
 
85.2
 
 
 
 
ATP6V1E1
 
 
hsa-miR-23a-3p
 
 
-0.294
 
 
285.9
 
 
 
 
ATP6V1E1
 
 
hsa-miR-23b-3p
 
 
-0.294
 
 
285.9
 
 
 
 
ATXN10
 
 
hsa-miR-27a-3p
 
 
-0.295
 
 
46.3
 
 
 
 
ATXN2
 
 
hsa-miR-10a-5p
 
 
-0.362
 
 
17.3
 
 
 
 
AUH
 
 
hsa-miR-23a-3p
 
 
-0.495
 
 
34.6
 
 
 
 
AUH
 
 
hsa-miR-23b-3p
 
 
-0.503
 
 
34.6
 
 
 
 
AUH
 
 
hsa-miR-23a-3p
 
 
-0.512
 
 
34.6
 
 
 
 
AUH
 
 
hsa-miR-23b-3p
 
 
-0.512
 
 
34.6
 
 
 
 
AZIN1
 
 
hsa-miR-132-3p
 
 
-0.29
 
 
28.1
 
 
 
 
AZIN1
 
 
hsa-miR-132-3p
 
 
-0.299
 
 
28.1
 
 
 
 
B4GALT2
 
 
hsa-miR-34a-5p
 
 
-0.314
 
 
21.4
 
 
 
 
B4GALT3
 
 
hsa-miR-27a-3p
 
 
-0.41
 
 
19.1
 
 
 
 
B4GAT1
 
 
hsa-miR-23a-3p
 
 
-0.361
 
 
93.8
 
 
 
 
B4GAT1
 
 
hsa-miR-23b-3p
 
 
-0.361
 
 
93.8
 
 
 
 
BAG5
 
 
hsa-miR-22-3p
 
 
-0.318
 
 
18.0
 
 
 
 
BAK1
 
 
hsa-miR-125a-5p
 
 
-0.402
 
 
16.1
 
 
 
 
BAK1
 
 
hsa-miR-29a-3p
 
 
-0.409
 
 
16.1
 
 
 
 
BATF3
 
 
hsa-miR-22-3p
 
 
-0.786
 
 
24.8
 
 
 
 
BAZ1B
 
 
hsa-miR-10a-5p
 
 
-0.309
 
 
12.0
 
 
 
 
BCL2L2
 
 
hsa-miR-29a-3p
 
 
-0.312
 
 
22.4
 
 
 
 
BCL3
 
 
hsa-miR-27a-3p
 
 
-0.413
 
 
12.1
 
 
 
 
BDH1
 
 
hsa-miR-125a-5p
 
 
-0.389
 
 
39.2
 
 
 
 
BET1
 
 
hsa-miR-23a-3p
 
 
-0.402
 
 
13.1
 
 
 
 
BET1
 
 
hsa-miR-23b-3p
 
 
-0.418
 
 
13.1
 
 
 
 
 
 
 colon 
 
 
 
 
target_symbol
 
 
mature_mirna_id
 
 
score
 
 
nTPM
 
 
 
 
 
 
AAGAB
 
 
hsa-miR-34a-5p
 
 
-0.319
 
 
26.1
 
 
 
 
AAR2
 
 
hsa-miR-29a-3p
 
 
-0.327
 
 
25.8
 
 
 
 
ABCB10
 
 
hsa-miR-152-3p
 
 
-0.291
 
 
10.4
 
 
 
 
ABCB10
 
 
hsa-miR-148b-3p
 
 
-0.291
 
 
10.4
 
 
 
 
ABCB7
 
 
hsa-miR-148b-3p
 
 
-0.515
 
 
13.4
 
 
 
 
ABCB7
 
 
hsa-miR-152-3p
 
 
-0.515
 
 
13.4
 
 
 
 
ABCC5
 
 
hsa-miR-125a-5p
 
 
-0.288
 
 
24.4
 
 
 
 
ABCD3
 
 
hsa-miR-148b-3p
 
 
-0.388
 
 
41.8
 
 
 
 
ABCD3
 
 
hsa-miR-152-3p
 
 
-0.388
 
 
41.8
 
 
 
 
ABCE1
 
 
hsa-miR-29a-3p
 
 
-0.404
 
 
21.3
 
 
 
 
ABHD17C
 
 
hsa-miR-27a-3p
 
 
-0.435
 
 
61.2
 
 
 
 
ABHD3
 
 
hsa-miR-125a-5p
 
 
-0.453
 
 
59.5
 
 
 
 
ABHD4
 
 
hsa-miR-29a-3p
 
 
-0.312
 
 
20.3
 
 
 
 
ABHD5
 
 
hsa-miR-132-3p
 
 
-0.323
 
 
11.4
 
 
 
 
ABHD5
 
 
hsa-miR-27a-3p
 
 
-0.407
 
 
11.4
 
 
 
 
ABHD6
 
 
hsa-miR-125a-5p
 
 
-0.501
 
 
13.2
 
 
 
 
ABTB1
 
 
hsa-miR-125a-5p
 
 
-0.519
 
 
39.8
 
 
 
 
ACBD3
 
 
hsa-miR-34a-5p
 
 
-0.415
 
 
21.8
 
 
 
 
ACSL1
 
 
hsa-miR-34a-5p
 
 
-0.306
 
 
33.2
 
 
 
 
ACSL4
 
 
hsa-miR-34a-5p
 
 
-0.372
 
 
11.3
 
 
 
 
ACTA2
 
 
hsa-miR-27a-3p
 
 
-0.577
 
 
2601.4
 
 
 
 
ADAM10
 
 
hsa-miR-152-3p
 
 
-0.348
 
 
20.8
 
 
 
 
ADAM10
 
 
hsa-miR-148b-3p
 
 
-0.356
 
 
20.8
 
 
 
 
ADAP1
 
 
hsa-miR-27a-3p
 
 
-0.302
 
 
41.4
 
 
 
 
ADCY3
 
 
hsa-miR-132-3p
 
 
-0.303
 
 
26.7
 
 
 
 
ADGRA3
 
 
hsa-miR-27a-3p
 
 
-0.296
 
 
16.0
 
 
 
 
ADIPOR2
 
 
hsa-miR-150-5p
 
 
-0.287
 
 
31.0
 
 
 
 
ADIPOR2
 
 
hsa-miR-34a-5p
 
 
-0.33
 
 
31.0
 
 
 
 
ADK
 
 
hsa-miR-34a-5p
 
 
-0.374
 
 
16.7
 
 
 
 
AFG3L2
 
 
hsa-miR-181b-5p
 
 
-0.491
 
 
38.7
 
 
 
 
AFG3L2
 
 
hsa-miR-181a-5p
 
 
-0.491
 
 
38.7
 
 
 
 
AGFG1
 
 
hsa-miR-148b-3p
 
 
-0.332
 
 
30.2
 
 
 
 
AGFG1
 
 
hsa-miR-152-3p
 
 
-0.332
 
 
30.2
 
 
 
 
AGPAT3
 
 
hsa-miR-27a-3p
 
 
-0.29
 
 
20.6
 
 
 
 
AGTRAP
 
 
hsa-miR-34a-5p
 
 
-0.328
 
 
27.8
 
 
 
 
AHCYL2
 
 
hsa-miR-34a-5p
 
 
-0.359
 
 
147.3
 
 
 
 
AHDC1
 
 
hsa-miR-148b-3p
 
 
-0.413
 
 
15.6
 
 
 
 
AHDC1
 
 
hsa-miR-152-3p
 
 
-0.413
 
 
15.6
 
 
 
 
AHR
 
 
hsa-miR-29a-3p
 
 
-0.316
 
 
12.9
 
 
 
 
AIDA
 
 
hsa-miR-22-3p
 
 
-0.316
 
 
47.7
 
 
 
 
AIFM1
 
 
hsa-miR-125a-5p
 
 
-0.3
 
 
23.9
 
 
 
 
AK1
 
 
hsa-miR-29a-3p
 
 
-0.333
 
 
41.6
 
 
 
 
AKAP1
 
 
hsa-miR-148b-3p
 
 
-0.381
 
 
72.8
 
 
 
 
AKAP1
 
 
hsa-miR-152-3p
 
 
-0.381
 
 
72.8
 
 
 
 
AKIRIN1
 
 
hsa-miR-27a-3p
 
 
-0.368
 
 
33.0
 
 
 
 
AKT3
 
 
hsa-miR-29a-3p
 
 
-0.287
 
 
20.3
 
 
 
 
AKT3
 
 
hsa-miR-22-3p
 
 
-0.451
 
 
20.3
 
 
 
 
ALCAM
 
 
hsa-miR-34a-5p
 
 
-0.303
 
 
15.7
 
 
 
 
ALCAM
 
 
hsa-miR-148b-3p
 
 
-0.339
 
 
15.7
 
 
 
 
ALCAM
 
 
hsa-miR-152-3p
 
 
-0.347
 
 
15.7
 
 
 
 
ALDOA
 
 
hsa-miR-34a-5p
 
 
-0.655
 
 
907.5
 
 
 
 
ANAPC16
 
 
hsa-miR-125a-5p
 
 
-0.578
 
 
22.7
 
 
 
 
ANK2
 
 
hsa-miR-34a-5p
 
 
-0.317
 
 
18.0
 
 
 
 
ANK3
 
 
hsa-miR-34a-5p
 
 
-0.29
 
 
24.1
 
 
 
 
ANK3
 
 
hsa-miR-34a-5p
 
 
-0.347
 
 
24.1
 
 
 
 
ANKHD1
 
 
hsa-miR-23a-3p
 
 
-0.368
 
 
38.3
 
 
 
 
ANKHD1
 
 
hsa-miR-23b-3p
 
 
-0.368
 
 
38.3
 
 
 
 
ANKHD1-EIF4EBP3
 
 
hsa-miR-22-3p
 
 
-0.479
 
 
11.6
 
 
 
 
ANKRD13A
 
 
hsa-miR-22-3p
 
 
-0.478
 
 
20.4
 
 
 
 
ANKRD40
 
 
hsa-miR-27a-3p
 
 
-0.35
 
 
29.8
 
 
 
 
ANP32A
 
 
hsa-miR-132-3p
 
 
-0.374
 
 
75.8
 
 
 
 
ANP32B
 
 
hsa-miR-34a-5p
 
 
-0.29
 
 
136.6
 
 
 
 
ANPEP
 
 
hsa-miR-125a-5p
 
 
-0.372
 
 
250.5
 
 
 
 
ANTXR2
 
 
hsa-miR-29a-3p
 
 
-0.347
 
 
83.9
 
 
 
 
AP3S2
 
 
hsa-miR-34a-5p
 
 
-0.413
 
 
18.1
 
 
 
 
APH1A
 
 
hsa-miR-34a-5p
 
 
-0.304
 
 
94.2
 
 
 
 
APOO
 
 
hsa-miR-181b-5p
 
 
-0.5
 
 
16.2
 
 
 
 
APOO
 
 
hsa-miR-181a-5p
 
 
-0.5
 
 
16.2
 
 
 
 
AREG
 
 
hsa-miR-34a-5p
 
 
-0.62
 
 
17.5
 
 
 
 
ARF4
 
 
hsa-miR-152-3p
 
 
-0.487
 
 
148.9
 
 
 
 
ARF4
 
 
hsa-miR-148b-3p
 
 
-0.487
 
 
148.9
 
 
 
 
ARF5
 
 
hsa-miR-29a-3p
 
 
-0.491
 
 
119.1
 
 
 
 
ARF6
 
 
hsa-miR-181b-5p
 
 
-0.33
 
 
50.4
 
 
 
 
ARF6
 
 
hsa-miR-181a-5p
 
 
-0.33
 
 
50.4
 
 
 
 
ARFGEF1
 
 
hsa-miR-148b-3p
 
 
-0.306
 
 
18.0
 
 
 
 
ARFGEF1
 
 
hsa-miR-152-3p
 
 
-0.306
 
 
18.0
 
 
 
 
ARFIP1
 
 
hsa-miR-152-3p
 
 
-0.474
 
 
26.4
 
 
 
 
ARFIP1
 
 
hsa-miR-148b-3p
 
 
-0.474
 
 
26.4
 
 
 
 
ARHGAP1
 
 
hsa-miR-34a-5p
 
 
-0.369
 
 
59.8
 
 
 
 
ARHGAP21
 
 
hsa-miR-152-3p
 
 
-0.287
 
 
15.5
 
 
 
 
ARHGAP21
 
 
hsa-miR-148b-3p
 
 
-0.287
 
 
15.5
 
 
 
 
ARHGAP26
 
 
hsa-miR-34a-5p
 
 
-0.384
 
 
12.1
 
 
 
 
ARHGEF26
 
 
hsa-miR-22-3p
 
 
-0.374
 
 
16.6
 
 
 
 
ARID1B
 
 
hsa-miR-29a-3p
 
 
-0.342
 
 
11.7
 
 
 
 
ARL4A
 
 
hsa-miR-29a-3p
 
 
-0.386
 
 
38.1
 
 
 
 
ARL6IP1
 
 
hsa-miR-148b-3p
 
 
-0.579
 
 
132.0
 
 
 
 
ARL6IP1
 
 
hsa-miR-152-3p
 
 
-0.579
 
 
132.0
 
 
 
 
ARL8B
 
 
hsa-miR-152-3p
 
 
-0.337
 
 
34.2
 
 
 
 
ARL8B
 
 
hsa-miR-148b-3p
 
 
-0.337
 
 
34.2
 
 
 
 
ARMCX3
 
 
hsa-miR-425-5p
 
 
-0.325
 
 
27.7
 
 
 
 
ARPC5
 
 
hsa-miR-22-3p
 
 
-0.679
 
 
98.1
 
 
 
 
ARPIN-AP3S2
 
 
hsa-miR-34a-5p
 
 
-0.348
 
 
13.7
 
 
 
 
ARPP19
 
 
hsa-miR-29a-3p
 
 
-0.371
 
 
35.7
 
 
 
 
ARPP19
 
 
hsa-miR-34a-5p
 
 
-0.497
 
 
35.7
 
 
 
 
ARRB1
 
 
hsa-miR-22-3p
 
 
-0.616
 
 
24.1
 
 
 
 
ARRDC3
 
 
hsa-miR-152-3p
 
 
-0.305
 
 
18.9
 
 
 
 
ARRDC3
 
 
hsa-miR-148b-3p
 
 
-0.305
 
 
18.9
 
 
 
 
ARRDC3
 
 
hsa-miR-29a-3p
 
 
-0.402
 
 
18.9
 
 
 
 
ASAH1
 
 
hsa-miR-27a-3p
 
 
-0.398
 
 
79.0
 
 
 
 
ASAP2
 
 
hsa-miR-29a-3p
 
 
-0.348
 
 
16.6
 
 
 
 
 
 
 duodenum 
 
 
 
 
target_symbol
 
 
mature_mirna_id
 
 
score
 
 
nTPM
 
 
 
 
 
 
AAGAB
 
 
hsa-miR-34a-5p
 
 
-0.319
 
 
14.8
 
 
 
 
AAR2
 
 
hsa-miR-29a-3p
 
 
-0.327
 
 
14.2
 
 
 
 
ABCB10
 
 
hsa-miR-152-3p
 
 
-0.291
 
 
16.1
 
 
 
 
ABCB10
 
 
hsa-miR-148b-3p
 
 
-0.291
 
 
16.1
 
 
 
 
ABCB7
 
 
hsa-miR-148b-3p
 
 
-0.515
 
 
15.1
 
 
 
 
ABCB7
 
 
hsa-miR-152-3p
 
 
-0.515
 
 
15.1
 
 
 
 
ABCC5
 
 
hsa-miR-125a-5p
 
 
-0.288
 
 
10.1
 
 
 
 
ABCD3
 
 
hsa-miR-148b-3p
 
 
-0.388
 
 
42.1
 
 
 
 
ABCD3
 
 
hsa-miR-152-3p
 
 
-0.388
 
 
42.1
 
 
 
 
ABCE1
 
 
hsa-miR-29a-3p
 
 
-0.404
 
 
19.0
 
 
 
 
ABHD17C
 
 
hsa-miR-27a-3p
 
 
-0.435
 
 
21.9
 
 
 
 
ABHD3
 
 
hsa-miR-125a-5p
 
 
-0.453
 
 
41.5
 
 
 
 
ABHD4
 
 
hsa-miR-29a-3p
 
 
-0.312
 
 
15.6
 
 
 
 
ABHD6
 
 
hsa-miR-125a-5p
 
 
-0.501
 
 
37.1
 
 
 
 
ABTB1
 
 
hsa-miR-125a-5p
 
 
-0.519
 
 
11.6
 
 
 
 
ACBD3
 
 
hsa-miR-34a-5p
 
 
-0.415
 
 
16.5
 
 
 
 
ACSL1
 
 
hsa-miR-34a-5p
 
 
-0.306
 
 
28.3
 
 
 
 
ACSL4
 
 
hsa-miR-34a-5p
 
 
-0.372
 
 
13.8
 
 
 
 
ACTA2
 
 
hsa-miR-27a-3p
 
 
-0.577
 
 
234.0
 
 
 
 
ADA
 
 
hsa-miR-29a-3p
 
 
-0.376
 
 
454.0
 
 
 
 
ADAM10
 
 
hsa-miR-152-3p
 
 
-0.348
 
 
14.7
 
 
 
 
ADAM10
 
 
hsa-miR-148b-3p
 
 
-0.356
 
 
14.7
 
 
 
 
ADAP1
 
 
hsa-miR-27a-3p
 
 
-0.302
 
 
18.5
 
 
 
 
ADCY3
 
 
hsa-miR-132-3p
 
 
-0.303
 
 
10.3
 
 
 
 
ADIPOR2
 
 
hsa-miR-150-5p
 
 
-0.287
 
 
20.9
 
 
 
 
ADIPOR2
 
 
hsa-miR-34a-5p
 
 
-0.33
 
 
20.9
 
 
 
 
ADK
 
 
hsa-miR-34a-5p
 
 
-0.374
 
 
15.9
 
 
 
 
AFG3L2
 
 
hsa-miR-181b-5p
 
 
-0.491
 
 
48.3
 
 
 
 
AFG3L2
 
 
hsa-miR-181a-5p
 
 
-0.491
 
 
48.3
 
 
 
 
AGFG1
 
 
hsa-miR-148b-3p
 
 
-0.332
 
 
40.8
 
 
 
 
AGFG1
 
 
hsa-miR-152-3p
 
 
-0.332
 
 
40.8
 
 
 
 
AGGF1
 
 
hsa-miR-125a-5p
 
 
-0.331
 
 
13.2
 
 
 
 
AGMAT
 
 
hsa-miR-22-3p
 
 
-0.294
 
 
16.8
 
 
 
 
AGPAT3
 
 
hsa-miR-27a-3p
 
 
-0.29
 
 
42.0
 
 
 
 
AGTRAP
 
 
hsa-miR-34a-5p
 
 
-0.328
 
 
10.5
 
 
 
 
AHCYL2
 
 
hsa-miR-34a-5p
 
 
-0.359
 
 
54.3
 
 
 
 
AHR
 
 
hsa-miR-29a-3p
 
 
-0.316
 
 
15.4
 
 
 
 
AIDA
 
 
hsa-miR-22-3p
 
 
-0.316
 
 
23.9
 
 
 
 
AIFM1
 
 
hsa-miR-125a-5p
 
 
-0.3
 
 
49.8
 
 
 
 
AK1
 
 
hsa-miR-29a-3p
 
 
-0.333
 
 
16.7
 
 
 
 
AKAP1
 
 
hsa-miR-148b-3p
 
 
-0.381
 
 
51.5
 
 
 
 
AKAP1
 
 
hsa-miR-152-3p
 
 
-0.381
 
 
51.5
 
 
 
 
AKIRIN1
 
 
hsa-miR-27a-3p
 
 
-0.368
 
 
22.8
 
 
 
 
ALCAM
 
 
hsa-miR-34a-5p
 
 
-0.303
 
 
16.0
 
 
 
 
ALCAM
 
 
hsa-miR-148b-3p
 
 
-0.339
 
 
16.0
 
 
 
 
ALCAM
 
 
hsa-miR-152-3p
 
 
-0.347
 
 
16.0
 
 
 
 
ALDOA
 
 
hsa-miR-34a-5p
 
 
-0.655
 
 
486.2
 
 
 
 
ANAPC16
 
 
hsa-miR-125a-5p
 
 
-0.578
 
 
14.5
 
 
 
 
ANK3
 
 
hsa-miR-34a-5p
 
 
-0.29
 
 
36.7
 
 
 
 
ANK3
 
 
hsa-miR-34a-5p
 
 
-0.347
 
 
36.7
 
 
 
 
ANKHD1
 
 
hsa-miR-23a-3p
 
 
-0.368
 
 
29.8
 
 
 
 
ANKHD1
 
 
hsa-miR-23b-3p
 
 
-0.368
 
 
29.8
 
 
 
 
ANKRD13A
 
 
hsa-miR-22-3p
 
 
-0.478
 
 
13.4
 
 
 
 
ANKRD40
 
 
hsa-miR-27a-3p
 
 
-0.35
 
 
11.1
 
 
 
 
ANP32A
 
 
hsa-miR-132-3p
 
 
-0.374
 
 
66.5
 
 
 
 
ANP32B
 
 
hsa-miR-34a-5p
 
 
-0.29
 
 
89.5
 
 
 
 
ANPEP
 
 
hsa-miR-125a-5p
 
 
-0.372
 
 
2245.0
 
 
 
 
ANTXR2
 
 
hsa-miR-29a-3p
 
 
-0.347
 
 
21.6
 
 
 
 
AP3S2
 
 
hsa-miR-34a-5p
 
 
-0.413
 
 
10.3
 
 
 
 
APH1A
 
 
hsa-miR-34a-5p
 
 
-0.304
 
 
77.4
 
 
 
 
APOO
 
 
hsa-miR-181b-5p
 
 
-0.5
 
 
13.6
 
 
 
 
APOO
 
 
hsa-miR-181a-5p
 
 
-0.5
 
 
13.6
 
 
 
 
AQP11
 
 
hsa-miR-27a-3p
 
 
-0.666
 
 
27.0
 
 
 
 
ARF4
 
 
hsa-miR-152-3p
 
 
-0.487
 
 
116.2
 
 
 
 
ARF4
 
 
hsa-miR-148b-3p
 
 
-0.487
 
 
116.2
 
 
 
 
ARF5
 
 
hsa-miR-29a-3p
 
 
-0.491
 
 
86.9
 
 
 
 
ARF6
 
 
hsa-miR-181b-5p
 
 
-0.33
 
 
43.6
 
 
 
 
ARF6
 
 
hsa-miR-181a-5p
 
 
-0.33
 
 
43.6
 
 
 
 
ARFGEF1
 
 
hsa-miR-148b-3p
 
 
-0.306
 
 
15.9
 
 
 
 
ARFGEF1
 
 
hsa-miR-152-3p
 
 
-0.306
 
 
15.9
 
 
 
 
ARFIP1
 
 
hsa-miR-152-3p
 
 
-0.474
 
 
21.8
 
 
 
 
ARFIP1
 
 
hsa-miR-148b-3p
 
 
-0.474
 
 
21.8
 
 
 
 
ARHGAP1
 
 
hsa-miR-34a-5p
 
 
-0.369
 
 
24.2
 
 
 
 
ARHGAP21
 
 
hsa-miR-152-3p
 
 
-0.287
 
 
12.1
 
 
 
 
ARHGAP21
 
 
hsa-miR-148b-3p
 
 
-0.287
 
 
12.1
 
 
 
 
ARHGAP26
 
 
hsa-miR-34a-5p
 
 
-0.384
 
 
11.3
 
 
 
 
ARID1B
 
 
hsa-miR-29a-3p
 
 
-0.342
 
 
13.4
 
 
 
 
ARL4A
 
 
hsa-miR-29a-3p
 
 
-0.386
 
 
28.1
 
 
 
 
ARL6IP1
 
 
hsa-miR-148b-3p
 
 
-0.579
 
 
130.6
 
 
 
 
ARL6IP1
 
 
hsa-miR-152-3p
 
 
-0.579
 
 
130.6
 
 
 
 
ARL8B
 
 
hsa-miR-152-3p
 
 
-0.337
 
 
28.9
 
 
 
 
ARL8B
 
 
hsa-miR-148b-3p
 
 
-0.337
 
 
28.9
 
 
 
 
ARMCX3
 
 
hsa-miR-425-5p
 
 
-0.325
 
 
19.0
 
 
 
 
ARPC5
 
 
hsa-miR-22-3p
 
 
-0.679
 
 
93.6
 
 
 
 
ARPP19
 
 
hsa-miR-29a-3p
 
 
-0.371
 
 
27.2
 
 
 
 
ARPP19
 
 
hsa-miR-34a-5p
 
 
-0.497
 
 
27.2
 
 
 
 
ARRB1
 
 
hsa-miR-22-3p
 
 
-0.616
 
 
20.6
 
 
 
 
ASAH1
 
 
hsa-miR-27a-3p
 
 
-0.398
 
 
51.6
 
 
 
 
ASAP2
 
 
hsa-miR-29a-3p
 
 
-0.348
 
 
13.0
 
 
 
 
ASPH
 
 
hsa-miR-152-3p
 
 
-0.391
 
 
42.9
 
 
 
 
ASPH
 
 
hsa-miR-148b-3p
 
 
-0.391
 
 
42.9
 
 
 
 
ATG4B
 
 
hsa-miR-34a-5p
 
 
-0.58
 
 
10.7
 
 
 
 
ATMIN
 
 
hsa-miR-34a-5p
 
 
-0.409
 
 
12.1
 
 
 
 
ATOH1
 
 
hsa-miR-22-3p
 
 
-0.566
 
 
10.4
 
 
 
 
ATP2B4
 
 
hsa-miR-152-3p
 
 
-0.337
 
 
11.8
 
 
 
 
ATP2B4
 
 
hsa-miR-148b-3p
 
 
-0.337
 
 
11.8
 
 
 
 
ATP5MC3
 
 
hsa-miR-425-5p
 
 
-0.328
 
 
355.5
 
 
 
 
ATP5MC3
 
 
hsa-miR-29a-3p
 
 
-0.33
 
 
355.5
 
 
 
 
ATP5MC3
 
 
hsa-miR-27a-3p
 
 
-0.416
 
 
355.5
 
 
 
 
ATP6AP2
 
 
hsa-miR-148b-3p
 
 
-0.592
 
 
30.3
 
 
 
 
 
 
 endometrium 
 
 
 
 
target_symbol
 
 
mature_mirna_id
 
 
score
 
 
nTPM
 
 
 
 
 
 
AAGAB
 
 
hsa-miR-34a-5p
 
 
-0.319
 
 
13.5
 
 
 
 
AAR2
 
 
hsa-miR-29a-3p
 
 
-0.327
 
 
23.5
 
 
 
 
ABCB7
 
 
hsa-miR-148b-3p
 
 
-0.515
 
 
10.5
 
 
 
 
ABCB7
 
 
hsa-miR-152-3p
 
 
-0.515
 
 
10.5
 
 
 
 
ABCC5
 
 
hsa-miR-125a-5p
 
 
-0.288
 
 
22.5
 
 
 
 
ABCD3
 
 
hsa-miR-148b-3p
 
 
-0.388
 
 
13.8
 
 
 
 
ABCD3
 
 
hsa-miR-152-3p
 
 
-0.388
 
 
13.8
 
 
 
 
ABCE1
 
 
hsa-miR-29a-3p
 
 
-0.404
 
 
22.8
 
 
 
 
ABHD4
 
 
hsa-miR-29a-3p
 
 
-0.312
 
 
26.6
 
 
 
 
ABTB1
 
 
hsa-miR-125a-5p
 
 
-0.519
 
 
33.5
 
 
 
 
ACBD3
 
 
hsa-miR-34a-5p
 
 
-0.415
 
 
21.4
 
 
 
 
ACSL1
 
 
hsa-miR-34a-5p
 
 
-0.306
 
 
36.5
 
 
 
 
ACSL4
 
 
hsa-miR-34a-5p
 
 
-0.372
 
 
17.4
 
 
 
 
ACTA2
 
 
hsa-miR-27a-3p
 
 
-0.577
 
 
5917.5
 
 
 
 
ACVR1
 
 
hsa-miR-152-3p
 
 
-0.397
 
 
15.9
 
 
 
 
ACVR1
 
 
hsa-miR-148b-3p
 
 
-0.397
 
 
15.9
 
 
 
 
ADAM10
 
 
hsa-miR-152-3p
 
 
-0.348
 
 
15.2
 
 
 
 
ADAM10
 
 
hsa-miR-148b-3p
 
 
-0.356
 
 
15.2
 
 
 
 
ADAMTS10
 
 
hsa-miR-27a-3p
 
 
-0.354
 
 
10.1
 
 
 
 
ADAMTS10
 
 
hsa-miR-29a-3p
 
 
-0.516
 
 
10.1
 
 
 
 
ADAMTS9
 
 
hsa-miR-29a-3p
 
 
-0.305
 
 
12.4
 
 
 
 
ADAMTS9
 
 
hsa-miR-29a-3p
 
 
-0.485
 
 
12.4
 
 
 
 
ADCY3
 
 
hsa-miR-132-3p
 
 
-0.303
 
 
52.0
 
 
 
 
ADGRA3
 
 
hsa-miR-27a-3p
 
 
-0.296
 
 
14.1
 
 
 
 
ADGRF5
 
 
hsa-miR-148b-3p
 
 
-0.354
 
 
16.0
 
 
 
 
ADGRF5
 
 
hsa-miR-152-3p
 
 
-0.354
 
 
16.0
 
 
 
 
ADIPOR2
 
 
hsa-miR-150-5p
 
 
-0.287
 
 
24.5
 
 
 
 
ADIPOR2
 
 
hsa-miR-34a-5p
 
 
-0.33
 
 
24.5
 
 
 
 
ADK
 
 
hsa-miR-34a-5p
 
 
-0.374
 
 
10.1
 
 
 
 
AFG3L2
 
 
hsa-miR-181b-5p
 
 
-0.491
 
 
12.4
 
 
 
 
AFG3L2
 
 
hsa-miR-181a-5p
 
 
-0.491
 
 
12.4
 
 
 
 
AGFG1
 
 
hsa-miR-148b-3p
 
 
-0.332
 
 
23.1
 
 
 
 
AGFG1
 
 
hsa-miR-152-3p
 
 
-0.332
 
 
23.1
 
 
 
 
AGTRAP
 
 
hsa-miR-34a-5p
 
 
-0.328
 
 
23.7
 
 
 
 
AHDC1
 
 
hsa-miR-148b-3p
 
 
-0.413
 
 
26.0
 
 
 
 
AHDC1
 
 
hsa-miR-152-3p
 
 
-0.413
 
 
26.0
 
 
 
 
AIDA
 
 
hsa-miR-22-3p
 
 
-0.316
 
 
33.8
 
 
 
 
AIFM1
 
 
hsa-miR-125a-5p
 
 
-0.3
 
 
10.6
 
 
 
 
AK1
 
 
hsa-miR-29a-3p
 
 
-0.333
 
 
16.9
 
 
 
 
AKAP1
 
 
hsa-miR-148b-3p
 
 
-0.381
 
 
24.8
 
 
 
 
AKAP1
 
 
hsa-miR-152-3p
 
 
-0.381
 
 
24.8
 
 
 
 
AKIRIN1
 
 
hsa-miR-27a-3p
 
 
-0.368
 
 
26.8
 
 
 
 
AKT3
 
 
hsa-miR-29a-3p
 
 
-0.287
 
 
19.5
 
 
 
 
AKT3
 
 
hsa-miR-22-3p
 
 
-0.451
 
 
19.5
 
 
 
 
ALCAM
 
 
hsa-miR-34a-5p
 
 
-0.303
 
 
14.4
 
 
 
 
ALCAM
 
 
hsa-miR-148b-3p
 
 
-0.339
 
 
14.4
 
 
 
 
ALCAM
 
 
hsa-miR-152-3p
 
 
-0.347
 
 
14.4
 
 
 
 
ALDH1A2
 
 
hsa-miR-23b-3p
 
 
-0.346
 
 
76.9
 
 
 
 
ALDH1A2
 
 
hsa-miR-23a-3p
 
 
-0.354
 
 
76.9
 
 
 
 
ALDOA
 
 
hsa-miR-34a-5p
 
 
-0.655
 
 
580.3
 
 
 
 
ANAPC16
 
 
hsa-miR-125a-5p
 
 
-0.578
 
 
18.6
 
 
 
 
ANK2
 
 
hsa-miR-34a-5p
 
 
-0.317
 
 
13.0
 
 
 
 
ANK3
 
 
hsa-miR-34a-5p
 
 
-0.29
 
 
21.4
 
 
 
 
ANK3
 
 
hsa-miR-34a-5p
 
 
-0.347
 
 
21.4
 
 
 
 
ANKHD1
 
 
hsa-miR-23a-3p
 
 
-0.368
 
 
40.5
 
 
 
 
ANKHD1
 
 
hsa-miR-23b-3p
 
 
-0.368
 
 
40.5
 
 
 
 
ANKRD13A
 
 
hsa-miR-22-3p
 
 
-0.478
 
 
11.2
 
 
 
 
ANKRD29
 
 
hsa-miR-132-3p
 
 
-0.47
 
 
14.7
 
 
 
 
ANKRD40
 
 
hsa-miR-27a-3p
 
 
-0.35
 
 
22.4
 
 
 
 
ANP32A
 
 
hsa-miR-132-3p
 
 
-0.374
 
 
55.3
 
 
 
 
ANP32B
 
 
hsa-miR-34a-5p
 
 
-0.29
 
 
111.3
 
 
 
 
ANTXR2
 
 
hsa-miR-29a-3p
 
 
-0.347
 
 
89.2
 
 
 
 
AP3S2
 
 
hsa-miR-34a-5p
 
 
-0.413
 
 
12.2
 
 
 
 
APH1A
 
 
hsa-miR-34a-5p
 
 
-0.304
 
 
57.8
 
 
 
 
APPBP2
 
 
hsa-miR-27a-3p
 
 
-0.378
 
 
12.0
 
 
 
 
ARF4
 
 
hsa-miR-152-3p
 
 
-0.487
 
 
113.9
 
 
 
 
ARF4
 
 
hsa-miR-148b-3p
 
 
-0.487
 
 
113.9
 
 
 
 
ARF5
 
 
hsa-miR-29a-3p
 
 
-0.491
 
 
111.9
 
 
 
 
ARF6
 
 
hsa-miR-181b-5p
 
 
-0.33
 
 
19.5
 
 
 
 
ARF6
 
 
hsa-miR-181a-5p
 
 
-0.33
 
 
19.5
 
 
 
 
ARFIP1
 
 
hsa-miR-152-3p
 
 
-0.474
 
 
20.3
 
 
 
 
ARFIP1
 
 
hsa-miR-148b-3p
 
 
-0.474
 
 
20.3
 
 
 
 
ARHGAP1
 
 
hsa-miR-34a-5p
 
 
-0.369
 
 
81.8
 
 
 
 
ARHGAP21
 
 
hsa-miR-152-3p
 
 
-0.287
 
 
18.5
 
 
 
 
ARHGAP21
 
 
hsa-miR-148b-3p
 
 
-0.287
 
 
18.5
 
 
 
 
ARID1B
 
 
hsa-miR-29a-3p
 
 
-0.342
 
 
15.0
 
 
 
 
ARL4A
 
 
hsa-miR-29a-3p
 
 
-0.386
 
 
17.7
 
 
 
 
ARL6IP1
 
 
hsa-miR-148b-3p
 
 
-0.579
 
 
66.4
 
 
 
 
ARL6IP1
 
 
hsa-miR-152-3p
 
 
-0.579
 
 
66.4
 
 
 
 
ARL8B
 
 
hsa-miR-152-3p
 
 
-0.337
 
 
42.3
 
 
 
 
ARL8B
 
 
hsa-miR-148b-3p
 
 
-0.337
 
 
42.3
 
 
 
 
ARMCX3
 
 
hsa-miR-425-5p
 
 
-0.325
 
 
38.3
 
 
 
 
ARPC5
 
 
hsa-miR-22-3p
 
 
-0.679
 
 
63.7
 
 
 
 
ARPP19
 
 
hsa-miR-29a-3p
 
 
-0.371
 
 
44.7
 
 
 
 
ARPP19
 
 
hsa-miR-34a-5p
 
 
-0.497
 
 
44.7
 
 
 
 
ARRDC3
 
 
hsa-miR-152-3p
 
 
-0.305
 
 
38.0
 
 
 
 
ARRDC3
 
 
hsa-miR-148b-3p
 
 
-0.305
 
 
38.0
 
 
 
 
ARRDC3
 
 
hsa-miR-29a-3p
 
 
-0.402
 
 
38.0
 
 
 
 
ASAH1
 
 
hsa-miR-27a-3p
 
 
-0.398
 
 
43.1
 
 
 
 
ASAP2
 
 
hsa-miR-29a-3p
 
 
-0.348
 
 
12.0
 
 
 
 
ASB1
 
 
hsa-miR-34a-5p
 
 
-0.406
 
 
10.1
 
 
 
 
ASPH
 
 
hsa-miR-152-3p
 
 
-0.391
 
 
45.6
 
 
 
 
ASPH
 
 
hsa-miR-148b-3p
 
 
-0.391
 
 
45.6
 
 
 
 
ATG4B
 
 
hsa-miR-34a-5p
 
 
-0.58
 
 
19.1
 
 
 
 
ATMIN
 
 
hsa-miR-34a-5p
 
 
-0.409
 
 
12.9
 
 
 
 
ATP2B4
 
 
hsa-miR-152-3p
 
 
-0.337
 
 
98.2
 
 
 
 
ATP2B4
 
 
hsa-miR-148b-3p
 
 
-0.337
 
 
98.2
 
 
 
 
ATP5MC3
 
 
hsa-miR-425-5p
 
 
-0.328
 
 
118.3
 
 
 
 
ATP5MC3
 
 
hsa-miR-29a-3p
 
 
-0.33
 
 
118.3
 
 
 
 
ATP5MC3
 
 
hsa-miR-27a-3p
 
 
-0.416
 
 
118.3
 
 
 
 
 
 
 epididymis 
 
 
 
 
target_symbol
 
 
mature_mirna_id
 
 
score
 
 
nTPM
 
 
 
 
 
 
AAGAB
 
 
hsa-miR-34a-5p
 
 
-0.319
 
 
24.4
 
 
 
 
AAR2
 
 
hsa-miR-29a-3p
 
 
-0.327
 
 
23.5
 
 
 
 
ABCD3
 
 
hsa-miR-148b-3p
 
 
-0.388
 
 
20.0
 
 
 
 
ABCD3
 
 
hsa-miR-152-3p
 
 
-0.388
 
 
20.0
 
 
 
 
ABCE1
 
 
hsa-miR-29a-3p
 
 
-0.404
 
 
15.1
 
 
 
 
ABHD4
 
 
hsa-miR-29a-3p
 
 
-0.312
 
 
28.3
 
 
 
 
ABHD6
 
 
hsa-miR-125a-5p
 
 
-0.501
 
 
13.9
 
 
 
 
ACBD3
 
 
hsa-miR-34a-5p
 
 
-0.415
 
 
19.4
 
 
 
 
ACSL1
 
 
hsa-miR-34a-5p
 
 
-0.306
 
 
50.9
 
 
 
 
ACSL4
 
 
hsa-miR-34a-5p
 
 
-0.372
 
 
10.2
 
 
 
 
ACTA2
 
 
hsa-miR-27a-3p
 
 
-0.577
 
 
1284.3
 
 
 
 
ACVR1
 
 
hsa-miR-152-3p
 
 
-0.397
 
 
10.8
 
 
 
 
ACVR1
 
 
hsa-miR-148b-3p
 
 
-0.397
 
 
10.8
 
 
 
 
ADAM17
 
 
hsa-miR-125a-5p
 
 
-0.292
 
 
10.4
 
 
 
 
ADAM17
 
 
hsa-miR-152-3p
 
 
-0.301
 
 
10.4
 
 
 
 
ADAM17
 
 
hsa-miR-148b-3p
 
 
-0.301
 
 
10.4
 
 
 
 
ADCY3
 
 
hsa-miR-132-3p
 
 
-0.303
 
 
19.3
 
 
 
 
ADK
 
 
hsa-miR-34a-5p
 
 
-0.374
 
 
15.6
 
 
 
 
ADO
 
 
hsa-miR-34a-5p
 
 
-0.377
 
 
12.4
 
 
 
 
AGFG1
 
 
hsa-miR-148b-3p
 
 
-0.332
 
 
18.8
 
 
 
 
AGFG1
 
 
hsa-miR-152-3p
 
 
-0.332
 
 
18.8
 
 
 
 
AGGF1
 
 
hsa-miR-125a-5p
 
 
-0.331
 
 
20.2
 
 
 
 
AGPAT3
 
 
hsa-miR-27a-3p
 
 
-0.29
 
 
11.9
 
 
 
 
AGTRAP
 
 
hsa-miR-34a-5p
 
 
-0.328
 
 
45.1
 
 
 
 
AIDA
 
 
hsa-miR-22-3p
 
 
-0.316
 
 
52.9
 
 
 
 
AK1
 
 
hsa-miR-29a-3p
 
 
-0.333
 
 
27.0
 
 
 
 
AKAP1
 
 
hsa-miR-148b-3p
 
 
-0.381
 
 
18.6
 
 
 
 
AKAP1
 
 
hsa-miR-152-3p
 
 
-0.381
 
 
18.6
 
 
 
 
AKIRIN1
 
 
hsa-miR-27a-3p
 
 
-0.368
 
 
39.9
 
 
 
 
ALCAM
 
 
hsa-miR-34a-5p
 
 
-0.303
 
 
33.4
 
 
 
 
ALCAM
 
 
hsa-miR-148b-3p
 
 
-0.339
 
 
33.4
 
 
 
 
ALCAM
 
 
hsa-miR-152-3p
 
 
-0.347
 
 
33.4
 
 
 
 
ALDOA
 
 
hsa-miR-34a-5p
 
 
-0.655
 
 
189.5
 
 
 
 
AMOT
 
 
hsa-miR-29a-3p
 
 
-0.304
 
 
137.7
 
 
 
 
ANAPC16
 
 
hsa-miR-125a-5p
 
 
-0.578
 
 
20.9
 
 
 
 
ANK3
 
 
hsa-miR-34a-5p
 
 
-0.29
 
 
116.7
 
 
 
 
ANK3
 
 
hsa-miR-34a-5p
 
 
-0.347
 
 
116.7
 
 
 
 
ANKHD1
 
 
hsa-miR-23a-3p
 
 
-0.368
 
 
45.6
 
 
 
 
ANKHD1
 
 
hsa-miR-23b-3p
 
 
-0.368
 
 
45.6
 
 
 
 
ANKHD1-EIF4EBP3
 
 
hsa-miR-22-3p
 
 
-0.479
 
 
18.5
 
 
 
 
ANKRD40
 
 
hsa-miR-27a-3p
 
 
-0.35
 
 
13.0
 
 
 
 
ANP32A
 
 
hsa-miR-132-3p
 
 
-0.374
 
 
47.9
 
 
 
 
ANP32B
 
 
hsa-miR-34a-5p
 
 
-0.29
 
 
98.9
 
 
 
 
ANTXR2
 
 
hsa-miR-29a-3p
 
 
-0.347
 
 
20.7
 
 
 
 
AP1S3
 
 
hsa-miR-181b-5p
 
 
-0.383
 
 
45.7
 
 
 
 
AP1S3
 
 
hsa-miR-181a-5p
 
 
-0.383
 
 
45.7
 
 
 
 
AP3S2
 
 
hsa-miR-34a-5p
 
 
-0.413
 
 
15.3
 
 
 
 
APH1A
 
 
hsa-miR-34a-5p
 
 
-0.304
 
 
79.3
 
 
 
 
APOO
 
 
hsa-miR-181b-5p
 
 
-0.5
 
 
18.5
 
 
 
 
APOO
 
 
hsa-miR-181a-5p
 
 
-0.5
 
 
18.5
 
 
 
 
ARF4
 
 
hsa-miR-152-3p
 
 
-0.487
 
 
214.1
 
 
 
 
ARF4
 
 
hsa-miR-148b-3p
 
 
-0.487
 
 
214.1
 
 
 
 
ARF5
 
 
hsa-miR-29a-3p
 
 
-0.491
 
 
107.7
 
 
 
 
ARF6
 
 
hsa-miR-181b-5p
 
 
-0.33
 
 
27.6
 
 
 
 
ARF6
 
 
hsa-miR-181a-5p
 
 
-0.33
 
 
27.6
 
 
 
 
ARFGEF1
 
 
hsa-miR-148b-3p
 
 
-0.306
 
 
10.4
 
 
 
 
ARFGEF1
 
 
hsa-miR-152-3p
 
 
-0.306
 
 
10.4
 
 
 
 
ARFIP1
 
 
hsa-miR-152-3p
 
 
-0.474
 
 
19.3
 
 
 
 
ARFIP1
 
 
hsa-miR-148b-3p
 
 
-0.474
 
 
19.3
 
 
 
 
ARHGAP1
 
 
hsa-miR-34a-5p
 
 
-0.369
 
 
20.3
 
 
 
 
ARID1B
 
 
hsa-miR-29a-3p
 
 
-0.342
 
 
10.3
 
 
 
 
ARL4A
 
 
hsa-miR-29a-3p
 
 
-0.386
 
 
13.0
 
 
 
 
ARL6IP1
 
 
hsa-miR-148b-3p
 
 
-0.579
 
 
116.0
 
 
 
 
ARL6IP1
 
 
hsa-miR-152-3p
 
 
-0.579
 
 
116.0
 
 
 
 
ARL8B
 
 
hsa-miR-152-3p
 
 
-0.337
 
 
37.5
 
 
 
 
ARL8B
 
 
hsa-miR-148b-3p
 
 
-0.337
 
 
37.5
 
 
 
 
ARMCX3
 
 
hsa-miR-425-5p
 
 
-0.325
 
 
99.4
 
 
 
 
ARPC5
 
 
hsa-miR-22-3p
 
 
-0.679
 
 
14.8
 
 
 
 
ARPIN-AP3S2
 
 
hsa-miR-34a-5p
 
 
-0.348
 
 
10.7
 
 
 
 
ARPP19
 
 
hsa-miR-29a-3p
 
 
-0.371
 
 
34.2
 
 
 
 
ARPP19
 
 
hsa-miR-34a-5p
 
 
-0.497
 
 
34.2
 
 
 
 
ARRDC3
 
 
hsa-miR-152-3p
 
 
-0.305
 
 
25.6
 
 
 
 
ARRDC3
 
 
hsa-miR-148b-3p
 
 
-0.305
 
 
25.6
 
 
 
 
ARRDC3
 
 
hsa-miR-29a-3p
 
 
-0.402
 
 
25.6
 
 
 
 
ASAH1
 
 
hsa-miR-27a-3p
 
 
-0.398
 
 
124.2
 
 
 
 
ASF1A
 
 
hsa-miR-23a-3p
 
 
-0.506
 
 
12.3
 
 
 
 
ASF1A
 
 
hsa-miR-23b-3p
 
 
-0.506
 
 
12.3
 
 
 
 
ASPH
 
 
hsa-miR-152-3p
 
 
-0.391
 
 
42.3
 
 
 
 
ASPH
 
 
hsa-miR-148b-3p
 
 
-0.391
 
 
42.3
 
 
 
 
ATMIN
 
 
hsa-miR-34a-5p
 
 
-0.409
 
 
16.9
 
 
 
 
ATP1A2
 
 
hsa-miR-34a-5p
 
 
-0.306
 
 
11.8
 
 
 
 
ATP2B4
 
 
hsa-miR-152-3p
 
 
-0.337
 
 
18.9
 
 
 
 
ATP2B4
 
 
hsa-miR-148b-3p
 
 
-0.337
 
 
18.9
 
 
 
 
ATP5MC3
 
 
hsa-miR-425-5p
 
 
-0.328
 
 
119.2
 
 
 
 
ATP5MC3
 
 
hsa-miR-29a-3p
 
 
-0.33
 
 
119.2
 
 
 
 
ATP5MC3
 
 
hsa-miR-27a-3p
 
 
-0.416
 
 
119.2
 
 
 
 
ATP6AP2
 
 
hsa-miR-148b-3p
 
 
-0.592
 
 
86.1
 
 
 
 
ATP6AP2
 
 
hsa-miR-152-3p
 
 
-0.592
 
 
86.1
 
 
 
 
ATP6AP2
 
 
hsa-miR-148b-3p
 
 
-0.668
 
 
86.1
 
 
 
 
ATP6AP2
 
 
hsa-miR-152-3p
 
 
-0.668
 
 
86.1
 
 
 
 
ATP6V1E1
 
 
hsa-miR-23a-3p
 
 
-0.294
 
 
128.2
 
 
 
 
ATP6V1E1
 
 
hsa-miR-23b-3p
 
 
-0.294
 
 
128.2
 
 
 
 
ATP8A1
 
 
hsa-miR-22-3p
 
 
-0.468
 
 
11.5
 
 
 
 
ATXN10
 
 
hsa-miR-27a-3p
 
 
-0.295
 
 
36.8
 
 
 
 
ATXN2
 
 
hsa-miR-10a-5p
 
 
-0.362
 
 
13.1
 
 
 
 
AUH
 
 
hsa-miR-23a-3p
 
 
-0.495
 
 
13.4
 
 
 
 
AUH
 
 
hsa-miR-23b-3p
 
 
-0.503
 
 
13.4
 
 
 
 
AUH
 
 
hsa-miR-23a-3p
 
 
-0.512
 
 
13.4
 
 
 
 
AUH
 
 
hsa-miR-23b-3p
 
 
-0.512
 
 
13.4
 
 
 
 
AZIN1
 
 
hsa-miR-132-3p
 
 
-0.29
 
 
28.4
 
 
 
 
 
 
 esophagus 
 
 
 
 
target_symbol
 
 
mature_mirna_id
 
 
score
 
 
nTPM
 
 
 
 
 
 
AAGAB
 
 
hsa-miR-34a-5p
 
 
-0.319
 
 
19.3
 
 
 
 
AAR2
 
 
hsa-miR-29a-3p
 
 
-0.327
 
 
16.7
 
 
 
 
ABCB7
 
 
hsa-miR-148b-3p
 
 
-0.515
 
 
11.1
 
 
 
 
ABCB7
 
 
hsa-miR-152-3p
 
 
-0.515
 
 
11.1
 
 
 
 
ABCC5
 
 
hsa-miR-125a-5p
 
 
-0.288
 
 
44.1
 
 
 
 
ABCD3
 
 
hsa-miR-148b-3p
 
 
-0.388
 
 
25.0
 
 
 
 
ABCD3
 
 
hsa-miR-152-3p
 
 
-0.388
 
 
25.0
 
 
 
 
ABCE1
 
 
hsa-miR-29a-3p
 
 
-0.404
 
 
33.0
 
 
 
 
ABHD17C
 
 
hsa-miR-27a-3p
 
 
-0.435
 
 
45.1
 
 
 
 
ABHD3
 
 
hsa-miR-125a-5p
 
 
-0.453
 
 
17.3
 
 
 
 
ABHD4
 
 
hsa-miR-29a-3p
 
 
-0.312
 
 
40.8
 
 
 
 
ABHD5
 
 
hsa-miR-132-3p
 
 
-0.323
 
 
18.4
 
 
 
 
ABHD5
 
 
hsa-miR-27a-3p
 
 
-0.407
 
 
18.4
 
 
 
 
ABTB1
 
 
hsa-miR-125a-5p
 
 
-0.519
 
 
13.8
 
 
 
 
ACBD3
 
 
hsa-miR-34a-5p
 
 
-0.415
 
 
29.0
 
 
 
 
ACSL1
 
 
hsa-miR-34a-5p
 
 
-0.306
 
 
63.8
 
 
 
 
ACSL4
 
 
hsa-miR-34a-5p
 
 
-0.372
 
 
14.6
 
 
 
 
ACTA2
 
 
hsa-miR-27a-3p
 
 
-0.577
 
 
1416.9
 
 
 
 
ACTN2
 
 
hsa-miR-23a-3p
 
 
-0.294
 
 
19.6
 
 
 
 
ACTN2
 
 
hsa-miR-29a-3p
 
 
-0.311
 
 
19.6
 
 
 
 
ADAM10
 
 
hsa-miR-152-3p
 
 
-0.348
 
 
14.2
 
 
 
 
ADAM10
 
 
hsa-miR-148b-3p
 
 
-0.356
 
 
14.2
 
 
 
 
ADCY3
 
 
hsa-miR-132-3p
 
 
-0.303
 
 
29.3
 
 
 
 
ADIPOR2
 
 
hsa-miR-150-5p
 
 
-0.287
 
 
44.0
 
 
 
 
ADIPOR2
 
 
hsa-miR-34a-5p
 
 
-0.33
 
 
44.0
 
 
 
 
ADK
 
 
hsa-miR-34a-5p
 
 
-0.374
 
 
46.4
 
 
 
 
ADORA2B
 
 
hsa-miR-27a-3p
 
 
-0.638
 
 
10.4
 
 
 
 
AFG3L2
 
 
hsa-miR-181b-5p
 
 
-0.491
 
 
25.7
 
 
 
 
AFG3L2
 
 
hsa-miR-181a-5p
 
 
-0.491
 
 
25.7
 
 
 
 
AGFG1
 
 
hsa-miR-148b-3p
 
 
-0.332
 
 
44.3
 
 
 
 
AGFG1
 
 
hsa-miR-152-3p
 
 
-0.332
 
 
44.3
 
 
 
 
AGPAT3
 
 
hsa-miR-27a-3p
 
 
-0.29
 
 
24.6
 
 
 
 
AGTRAP
 
 
hsa-miR-34a-5p
 
 
-0.328
 
 
51.2
 
 
 
 
AHDC1
 
 
hsa-miR-148b-3p
 
 
-0.413
 
 
34.3
 
 
 
 
AHDC1
 
 
hsa-miR-152-3p
 
 
-0.413
 
 
34.3
 
 
 
 
AHR
 
 
hsa-miR-29a-3p
 
 
-0.316
 
 
16.2
 
 
 
 
AIDA
 
 
hsa-miR-22-3p
 
 
-0.316
 
 
42.4
 
 
 
 
AIFM1
 
 
hsa-miR-125a-5p
 
 
-0.3
 
 
15.9
 
 
 
 
AK1
 
 
hsa-miR-29a-3p
 
 
-0.333
 
 
30.1
 
 
 
 
AKAP1
 
 
hsa-miR-148b-3p
 
 
-0.381
 
 
34.2
 
 
 
 
AKAP1
 
 
hsa-miR-152-3p
 
 
-0.381
 
 
34.2
 
 
 
 
AKIRIN1
 
 
hsa-miR-27a-3p
 
 
-0.368
 
 
27.4
 
 
 
 
ALDOA
 
 
hsa-miR-34a-5p
 
 
-0.655
 
 
1511.5
 
 
 
 
ANAPC16
 
 
hsa-miR-125a-5p
 
 
-0.578
 
 
13.6
 
 
 
 
ANK3
 
 
hsa-miR-34a-5p
 
 
-0.29
 
 
15.5
 
 
 
 
ANK3
 
 
hsa-miR-34a-5p
 
 
-0.347
 
 
15.5
 
 
 
 
ANKHD1
 
 
hsa-miR-23a-3p
 
 
-0.368
 
 
42.1
 
 
 
 
ANKHD1
 
 
hsa-miR-23b-3p
 
 
-0.368
 
 
42.1
 
 
 
 
ANKHD1-EIF4EBP3
 
 
hsa-miR-22-3p
 
 
-0.479
 
 
14.8
 
 
 
 
ANKRD13A
 
 
hsa-miR-22-3p
 
 
-0.478
 
 
29.8
 
 
 
 
ANKRD40
 
 
hsa-miR-27a-3p
 
 
-0.35
 
 
17.3
 
 
 
 
ANP32A
 
 
hsa-miR-132-3p
 
 
-0.374
 
 
61.9
 
 
 
 
ANP32B
 
 
hsa-miR-34a-5p
 
 
-0.29
 
 
133.7
 
 
 
 
ANTXR2
 
 
hsa-miR-29a-3p
 
 
-0.347
 
 
23.0
 
 
 
 
AP3S2
 
 
hsa-miR-34a-5p
 
 
-0.413
 
 
19.4
 
 
 
 
APH1A
 
 
hsa-miR-34a-5p
 
 
-0.304
 
 
113.5
 
 
 
 
APOO
 
 
hsa-miR-181b-5p
 
 
-0.5
 
 
12.1
 
 
 
 
APOO
 
 
hsa-miR-181a-5p
 
 
-0.5
 
 
12.1
 
 
 
 
AREG
 
 
hsa-miR-34a-5p
 
 
-0.62
 
 
42.1
 
 
 
 
ARF4
 
 
hsa-miR-152-3p
 
 
-0.487
 
 
102.8
 
 
 
 
ARF4
 
 
hsa-miR-148b-3p
 
 
-0.487
 
 
102.8
 
 
 
 
ARF5
 
 
hsa-miR-29a-3p
 
 
-0.491
 
 
248.7
 
 
 
 
ARF6
 
 
hsa-miR-181b-5p
 
 
-0.33
 
 
81.9
 
 
 
 
ARF6
 
 
hsa-miR-181a-5p
 
 
-0.33
 
 
81.9
 
 
 
 
ARFGEF1
 
 
hsa-miR-148b-3p
 
 
-0.306
 
 
14.2
 
 
 
 
ARFGEF1
 
 
hsa-miR-152-3p
 
 
-0.306
 
 
14.2
 
 
 
 
ARFIP1
 
 
hsa-miR-152-3p
 
 
-0.474
 
 
19.6
 
 
 
 
ARFIP1
 
 
hsa-miR-148b-3p
 
 
-0.474
 
 
19.6
 
 
 
 
ARHGAP1
 
 
hsa-miR-34a-5p
 
 
-0.369
 
 
71.8
 
 
 
 
ARHGAP21
 
 
hsa-miR-152-3p
 
 
-0.287
 
 
10.2
 
 
 
 
ARHGAP21
 
 
hsa-miR-148b-3p
 
 
-0.287
 
 
10.2
 
 
 
 
ARID1B
 
 
hsa-miR-29a-3p
 
 
-0.342
 
 
13.7
 
 
 
 
ARL4A
 
 
hsa-miR-29a-3p
 
 
-0.386
 
 
56.3
 
 
 
 
ARL6IP1
 
 
hsa-miR-148b-3p
 
 
-0.579
 
 
65.8
 
 
 
 
ARL6IP1
 
 
hsa-miR-152-3p
 
 
-0.579
 
 
65.8
 
 
 
 
ARL8B
 
 
hsa-miR-152-3p
 
 
-0.337
 
 
71.1
 
 
 
 
ARL8B
 
 
hsa-miR-148b-3p
 
 
-0.337
 
 
71.1
 
 
 
 
ARMCX3
 
 
hsa-miR-425-5p
 
 
-0.325
 
 
17.0
 
 
 
 
ARPC5
 
 
hsa-miR-22-3p
 
 
-0.679
 
 
73.2
 
 
 
 
ARPIN-AP3S2
 
 
hsa-miR-34a-5p
 
 
-0.348
 
 
11.9
 
 
 
 
ARPP19
 
 
hsa-miR-29a-3p
 
 
-0.371
 
 
49.0
 
 
 
 
ARPP19
 
 
hsa-miR-34a-5p
 
 
-0.497
 
 
49.0
 
 
 
 
ARRB1
 
 
hsa-miR-22-3p
 
 
-0.616
 
 
17.9
 
 
 
 
ARRDC3
 
 
hsa-miR-152-3p
 
 
-0.305
 
 
36.4
 
 
 
 
ARRDC3
 
 
hsa-miR-148b-3p
 
 
-0.305
 
 
36.4
 
 
 
 
ARRDC3
 
 
hsa-miR-29a-3p
 
 
-0.402
 
 
36.4
 
 
 
 
ASAH1
 
 
hsa-miR-27a-3p
 
 
-0.398
 
 
68.8
 
 
 
 
ASPH
 
 
hsa-miR-152-3p
 
 
-0.391
 
 
66.3
 
 
 
 
ASPH
 
 
hsa-miR-148b-3p
 
 
-0.391
 
 
66.3
 
 
 
 
ATG4B
 
 
hsa-miR-34a-5p
 
 
-0.58
 
 
18.3
 
 
 
 
ATMIN
 
 
hsa-miR-34a-5p
 
 
-0.409
 
 
17.8
 
 
 
 
ATP1A2
 
 
hsa-miR-34a-5p
 
 
-0.306
 
 
31.5
 
 
 
 
ATP2B4
 
 
hsa-miR-152-3p
 
 
-0.337
 
 
30.6
 
 
 
 
ATP2B4
 
 
hsa-miR-148b-3p
 
 
-0.337
 
 
30.6
 
 
 
 
ATP5MC3
 
 
hsa-miR-425-5p
 
 
-0.328
 
 
293.6
 
 
 
 
ATP5MC3
 
 
hsa-miR-29a-3p
 
 
-0.33
 
 
293.6
 
 
 
 
ATP5MC3
 
 
hsa-miR-27a-3p
 
 
-0.416
 
 
293.6
 
 
 
 
ATP6AP2
 
 
hsa-miR-148b-3p
 
 
-0.592
 
 
65.8
 
 
 
 
ATP6AP2
 
 
hsa-miR-152-3p
 
 
-0.592
 
 
65.8
 
 
 
 
ATP6AP2
 
 
hsa-miR-148b-3p
 
 
-0.668
 
 
65.8
 
 
 
 
 
 
 fallopian tube 
 
 
 
 
target_symbol
 
 
mature_mirna_id
 
 
score
 
 
nTPM
 
 
 
 
 
 
AAGAB
 
 
hsa-miR-34a-5p
 
 
-0.319
 
 
15.5
 
 
 
 
AAR2
 
 
hsa-miR-29a-3p
 
 
-0.327
 
 
21.8
 
 
 
 
ABCB7
 
 
hsa-miR-148b-3p
 
 
-0.515
 
 
11.3
 
 
 
 
ABCB7
 
 
hsa-miR-152-3p
 
 
-0.515
 
 
11.3
 
 
 
 
ABCC5
 
 
hsa-miR-125a-5p
 
 
-0.288
 
 
16.2
 
 
 
 
ABCD3
 
 
hsa-miR-148b-3p
 
 
-0.388
 
 
15.8
 
 
 
 
ABCD3
 
 
hsa-miR-152-3p
 
 
-0.388
 
 
15.8
 
 
 
 
ABCE1
 
 
hsa-miR-29a-3p
 
 
-0.404
 
 
21.1
 
 
 
 
ABHD17C
 
 
hsa-miR-27a-3p
 
 
-0.435
 
 
10.3
 
 
 
 
ABHD4
 
 
hsa-miR-29a-3p
 
 
-0.312
 
 
18.8
 
 
 
 
ABTB1
 
 
hsa-miR-125a-5p
 
 
-0.519
 
 
39.7
 
 
 
 
ACBD3
 
 
hsa-miR-34a-5p
 
 
-0.415
 
 
16.8
 
 
 
 
ACSL1
 
 
hsa-miR-34a-5p
 
 
-0.306
 
 
34.0
 
 
 
 
ACSL4
 
 
hsa-miR-34a-5p
 
 
-0.372
 
 
12.4
 
 
 
 
ACTA2
 
 
hsa-miR-27a-3p
 
 
-0.577
 
 
3776.0
 
 
 
 
ACVR1
 
 
hsa-miR-152-3p
 
 
-0.397
 
 
13.2
 
 
 
 
ACVR1
 
 
hsa-miR-148b-3p
 
 
-0.397
 
 
13.2
 
 
 
 
ADAM10
 
 
hsa-miR-152-3p
 
 
-0.348
 
 
13.1
 
 
 
 
ADAM10
 
 
hsa-miR-148b-3p
 
 
-0.356
 
 
13.1
 
 
 
 
ADAMTS9
 
 
hsa-miR-29a-3p
 
 
-0.305
 
 
10.3
 
 
 
 
ADAMTS9
 
 
hsa-miR-29a-3p
 
 
-0.485
 
 
10.3
 
 
 
 
ADCY3
 
 
hsa-miR-132-3p
 
 
-0.303
 
 
52.6
 
 
 
 
ADGRA3
 
 
hsa-miR-27a-3p
 
 
-0.296
 
 
20.3
 
 
 
 
ADGRF5
 
 
hsa-miR-148b-3p
 
 
-0.354
 
 
13.6
 
 
 
 
ADGRF5
 
 
hsa-miR-152-3p
 
 
-0.354
 
 
13.6
 
 
 
 
ADIPOR2
 
 
hsa-miR-150-5p
 
 
-0.287
 
 
23.2
 
 
 
 
ADIPOR2
 
 
hsa-miR-34a-5p
 
 
-0.33
 
 
23.2
 
 
 
 
ADK
 
 
hsa-miR-34a-5p
 
 
-0.374
 
 
15.5
 
 
 
 
AFG3L2
 
 
hsa-miR-181b-5p
 
 
-0.491
 
 
12.9
 
 
 
 
AFG3L2
 
 
hsa-miR-181a-5p
 
 
-0.491
 
 
12.9
 
 
 
 
AGFG1
 
 
hsa-miR-148b-3p
 
 
-0.332
 
 
23.3
 
 
 
 
AGFG1
 
 
hsa-miR-152-3p
 
 
-0.332
 
 
23.3
 
 
 
 
AGPAT3
 
 
hsa-miR-27a-3p
 
 
-0.29
 
 
11.6
 
 
 
 
AGTRAP
 
 
hsa-miR-34a-5p
 
 
-0.328
 
 
45.4
 
 
 
 
AHDC1
 
 
hsa-miR-148b-3p
 
 
-0.413
 
 
19.8
 
 
 
 
AHDC1
 
 
hsa-miR-152-3p
 
 
-0.413
 
 
19.8
 
 
 
 
AIDA
 
 
hsa-miR-22-3p
 
 
-0.316
 
 
29.5
 
 
 
 
AIFM1
 
 
hsa-miR-125a-5p
 
 
-0.3
 
 
20.8
 
 
 
 
AK1
 
 
hsa-miR-29a-3p
 
 
-0.333
 
 
321.8
 
 
 
 
AKAP1
 
 
hsa-miR-148b-3p
 
 
-0.381
 
 
33.9
 
 
 
 
AKAP1
 
 
hsa-miR-152-3p
 
 
-0.381
 
 
33.9
 
 
 
 
AKIRIN1
 
 
hsa-miR-27a-3p
 
 
-0.368
 
 
23.3
 
 
 
 
AKT3
 
 
hsa-miR-29a-3p
 
 
-0.287
 
 
15.1
 
 
 
 
AKT3
 
 
hsa-miR-22-3p
 
 
-0.451
 
 
15.1
 
 
 
 
ALCAM
 
 
hsa-miR-34a-5p
 
 
-0.303
 
 
46.2
 
 
 
 
ALCAM
 
 
hsa-miR-148b-3p
 
 
-0.339
 
 
46.2
 
 
 
 
ALCAM
 
 
hsa-miR-152-3p
 
 
-0.347
 
 
46.2
 
 
 
 
ALDH1A2
 
 
hsa-miR-23b-3p
 
 
-0.346
 
 
70.1
 
 
 
 
ALDH1A2
 
 
hsa-miR-23a-3p
 
 
-0.354
 
 
70.1
 
 
 
 
ALDOA
 
 
hsa-miR-34a-5p
 
 
-0.655
 
 
522.8
 
 
 
 
AMOT
 
 
hsa-miR-29a-3p
 
 
-0.304
 
 
11.3
 
 
 
 
ANAPC16
 
 
hsa-miR-125a-5p
 
 
-0.578
 
 
21.7
 
 
 
 
ANK3
 
 
hsa-miR-34a-5p
 
 
-0.29
 
 
17.4
 
 
 
 
ANK3
 
 
hsa-miR-34a-5p
 
 
-0.347
 
 
17.4
 
 
 
 
ANKHD1
 
 
hsa-miR-23a-3p
 
 
-0.368
 
 
37.7
 
 
 
 
ANKHD1
 
 
hsa-miR-23b-3p
 
 
-0.368
 
 
37.7
 
 
 
 
ANKRD13A
 
 
hsa-miR-22-3p
 
 
-0.478
 
 
11.6
 
 
 
 
ANKRD29
 
 
hsa-miR-132-3p
 
 
-0.47
 
 
14.4
 
 
 
 
ANKRD40
 
 
hsa-miR-27a-3p
 
 
-0.35
 
 
18.4
 
 
 
 
ANP32A
 
 
hsa-miR-132-3p
 
 
-0.374
 
 
70.6
 
 
 
 
ANP32B
 
 
hsa-miR-34a-5p
 
 
-0.29
 
 
117.2
 
 
 
 
ANTXR2
 
 
hsa-miR-29a-3p
 
 
-0.347
 
 
42.2
 
 
 
 
AP3S2
 
 
hsa-miR-34a-5p
 
 
-0.413
 
 
16.2
 
 
 
 
APH1A
 
 
hsa-miR-34a-5p
 
 
-0.304
 
 
78.3
 
 
 
 
APOO
 
 
hsa-miR-181b-5p
 
 
-0.5
 
 
19.5
 
 
 
 
APOO
 
 
hsa-miR-181a-5p
 
 
-0.5
 
 
19.5
 
 
 
 
ARF4
 
 
hsa-miR-152-3p
 
 
-0.487
 
 
79.6
 
 
 
 
ARF4
 
 
hsa-miR-148b-3p
 
 
-0.487
 
 
79.6
 
 
 
 
ARF5
 
 
hsa-miR-29a-3p
 
 
-0.491
 
 
100.8
 
 
 
 
ARF6
 
 
hsa-miR-181b-5p
 
 
-0.33
 
 
26.6
 
 
 
 
ARF6
 
 
hsa-miR-181a-5p
 
 
-0.33
 
 
26.6
 
 
 
 
ARFGEF1
 
 
hsa-miR-148b-3p
 
 
-0.306
 
 
11.1
 
 
 
 
ARFGEF1
 
 
hsa-miR-152-3p
 
 
-0.306
 
 
11.1
 
 
 
 
ARFIP1
 
 
hsa-miR-152-3p
 
 
-0.474
 
 
17.4
 
 
 
 
ARFIP1
 
 
hsa-miR-148b-3p
 
 
-0.474
 
 
17.4
 
 
 
 
ARHGAP1
 
 
hsa-miR-34a-5p
 
 
-0.369
 
 
71.5
 
 
 
 
ARHGAP21
 
 
hsa-miR-152-3p
 
 
-0.287
 
 
16.4
 
 
 
 
ARHGAP21
 
 
hsa-miR-148b-3p
 
 
-0.287
 
 
16.4
 
 
 
 
ARHGAP26
 
 
hsa-miR-34a-5p
 
 
-0.384
 
 
14.6
 
 
 
 
ARID1B
 
 
hsa-miR-29a-3p
 
 
-0.342
 
 
16.6
 
 
 
 
ARL4A
 
 
hsa-miR-29a-3p
 
 
-0.386
 
 
26.0
 
 
 
 
ARL6IP1
 
 
hsa-miR-148b-3p
 
 
-0.579
 
 
81.6
 
 
 
 
ARL6IP1
 
 
hsa-miR-152-3p
 
 
-0.579
 
 
81.6
 
 
 
 
ARL8B
 
 
hsa-miR-152-3p
 
 
-0.337
 
 
29.3
 
 
 
 
ARL8B
 
 
hsa-miR-148b-3p
 
 
-0.337
 
 
29.3
 
 
 
 
ARMCX3
 
 
hsa-miR-425-5p
 
 
-0.325
 
 
25.7
 
 
 
 
ARPC5
 
 
hsa-miR-22-3p
 
 
-0.679
 
 
50.4
 
 
 
 
ARPP19
 
 
hsa-miR-29a-3p
 
 
-0.371
 
 
31.4
 
 
 
 
ARPP19
 
 
hsa-miR-34a-5p
 
 
-0.497
 
 
31.4
 
 
 
 
ARRB1
 
 
hsa-miR-22-3p
 
 
-0.616
 
 
15.0
 
 
 
 
ARRDC3
 
 
hsa-miR-152-3p
 
 
-0.305
 
 
23.4
 
 
 
 
ARRDC3
 
 
hsa-miR-148b-3p
 
 
-0.305
 
 
23.4
 
 
 
 
ARRDC3
 
 
hsa-miR-29a-3p
 
 
-0.402
 
 
23.4
 
 
 
 
ASAH1
 
 
hsa-miR-27a-3p
 
 
-0.398
 
 
66.2
 
 
 
 
ASAP2
 
 
hsa-miR-29a-3p
 
 
-0.348
 
 
10.1
 
 
 
 
ASPH
 
 
hsa-miR-152-3p
 
 
-0.391
 
 
45.0
 
 
 
 
ASPH
 
 
hsa-miR-148b-3p
 
 
-0.391
 
 
45.0
 
 
 
 
ATG4B
 
 
hsa-miR-34a-5p
 
 
-0.58
 
 
19.3
 
 
 
 
ATMIN
 
 
hsa-miR-34a-5p
 
 
-0.409
 
 
10.8
 
 
 
 
ATP1A2
 
 
hsa-miR-34a-5p
 
 
-0.306
 
 
15.4
 
 
 
 
 
 
 gallbladder 
 
 
 
 
target_symbol
 
 
mature_mirna_id
 
 
score
 
 
nTPM
 
 
 
 
 
 
AAGAB
 
 
hsa-miR-34a-5p
 
 
-0.319
 
 
17.3
 
 
 
 
AAR2
 
 
hsa-miR-29a-3p
 
 
-0.327
 
 
15.5
 
 
 
 
ABCC5
 
 
hsa-miR-125a-5p
 
 
-0.288
 
 
16.8
 
 
 
 
ABCD3
 
 
hsa-miR-148b-3p
 
 
-0.388
 
 
25.2
 
 
 
 
ABCD3
 
 
hsa-miR-152-3p
 
 
-0.388
 
 
25.2
 
 
 
 
ABCE1
 
 
hsa-miR-29a-3p
 
 
-0.404
 
 
21.0
 
 
 
 
ABHD3
 
 
hsa-miR-125a-5p
 
 
-0.453
 
 
19.5
 
 
 
 
ABHD4
 
 
hsa-miR-29a-3p
 
 
-0.312
 
 
25.4
 
 
 
 
ABHD5
 
 
hsa-miR-132-3p
 
 
-0.323
 
 
10.2
 
 
 
 
ABHD5
 
 
hsa-miR-27a-3p
 
 
-0.407
 
 
10.2
 
 
 
 
ABTB1
 
 
hsa-miR-125a-5p
 
 
-0.519
 
 
17.0
 
 
 
 
ACBD3
 
 
hsa-miR-34a-5p
 
 
-0.415
 
 
22.0
 
 
 
 
ACSL1
 
 
hsa-miR-34a-5p
 
 
-0.306
 
 
32.1
 
 
 
 
ACSL4
 
 
hsa-miR-34a-5p
 
 
-0.372
 
 
25.8
 
 
 
 
ACTA2
 
 
hsa-miR-27a-3p
 
 
-0.577
 
 
849.5
 
 
 
 
ACVR1
 
 
hsa-miR-152-3p
 
 
-0.397
 
 
14.1
 
 
 
 
ACVR1
 
 
hsa-miR-148b-3p
 
 
-0.397
 
 
14.1
 
 
 
 
ADAM10
 
 
hsa-miR-152-3p
 
 
-0.348
 
 
17.7
 
 
 
 
ADAM10
 
 
hsa-miR-148b-3p
 
 
-0.356
 
 
17.7
 
 
 
 
ADCY3
 
 
hsa-miR-132-3p
 
 
-0.303
 
 
21.6
 
 
 
 
ADGRF5
 
 
hsa-miR-148b-3p
 
 
-0.354
 
 
16.1
 
 
 
 
ADGRF5
 
 
hsa-miR-152-3p
 
 
-0.354
 
 
16.1
 
 
 
 
ADK
 
 
hsa-miR-34a-5p
 
 
-0.374
 
 
10.7
 
 
 
 
ADRA2A
 
 
hsa-miR-23a-3p
 
 
-0.371
 
 
18.7
 
 
 
 
ADRA2A
 
 
hsa-miR-23b-3p
 
 
-0.371
 
 
18.7
 
 
 
 
AFG3L2
 
 
hsa-miR-181b-5p
 
 
-0.491
 
 
10.6
 
 
 
 
AFG3L2
 
 
hsa-miR-181a-5p
 
 
-0.491
 
 
10.6
 
 
 
 
AGFG1
 
 
hsa-miR-148b-3p
 
 
-0.332
 
 
36.5
 
 
 
 
AGFG1
 
 
hsa-miR-152-3p
 
 
-0.332
 
 
36.5
 
 
 
 
AGPAT3
 
 
hsa-miR-27a-3p
 
 
-0.29
 
 
12.9
 
 
 
 
AGTRAP
 
 
hsa-miR-34a-5p
 
 
-0.328
 
 
23.6
 
 
 
 
AHCYL2
 
 
hsa-miR-34a-5p
 
 
-0.359
 
 
52.3
 
 
 
 
AHR
 
 
hsa-miR-29a-3p
 
 
-0.316
 
 
31.5
 
 
 
 
AIDA
 
 
hsa-miR-22-3p
 
 
-0.316
 
 
38.3
 
 
 
 
AK1
 
 
hsa-miR-29a-3p
 
 
-0.333
 
 
21.1
 
 
 
 
AKAP1
 
 
hsa-miR-148b-3p
 
 
-0.381
 
 
16.8
 
 
 
 
AKAP1
 
 
hsa-miR-152-3p
 
 
-0.381
 
 
16.8
 
 
 
 
AKIRIN1
 
 
hsa-miR-27a-3p
 
 
-0.368
 
 
39.1
 
 
 
 
ALCAM
 
 
hsa-miR-34a-5p
 
 
-0.303
 
 
21.9
 
 
 
 
ALCAM
 
 
hsa-miR-148b-3p
 
 
-0.339
 
 
21.9
 
 
 
 
ALCAM
 
 
hsa-miR-152-3p
 
 
-0.347
 
 
21.9
 
 
 
 
ALDH1A2
 
 
hsa-miR-23b-3p
 
 
-0.346
 
 
22.5
 
 
 
 
ALDH1A2
 
 
hsa-miR-23a-3p
 
 
-0.354
 
 
22.5
 
 
 
 
ALDOA
 
 
hsa-miR-34a-5p
 
 
-0.655
 
 
394.6
 
 
 
 
ANAPC16
 
 
hsa-miR-125a-5p
 
 
-0.578
 
 
11.6
 
 
 
 
ANK2
 
 
hsa-miR-34a-5p
 
 
-0.317
 
 
18.1
 
 
 
 
ANK3
 
 
hsa-miR-34a-5p
 
 
-0.29
 
 
19.8
 
 
 
 
ANK3
 
 
hsa-miR-34a-5p
 
 
-0.347
 
 
19.8
 
 
 
 
ANKHD1
 
 
hsa-miR-23a-3p
 
 
-0.368
 
 
23.4
 
 
 
 
ANKHD1
 
 
hsa-miR-23b-3p
 
 
-0.368
 
 
23.4
 
 
 
 
ANKRD13A
 
 
hsa-miR-22-3p
 
 
-0.478
 
 
21.4
 
 
 
 
ANKRD40
 
 
hsa-miR-27a-3p
 
 
-0.35
 
 
15.1
 
 
 
 
ANP32A
 
 
hsa-miR-132-3p
 
 
-0.374
 
 
56.0
 
 
 
 
ANP32B
 
 
hsa-miR-34a-5p
 
 
-0.29
 
 
56.6
 
 
 
 
ANPEP
 
 
hsa-miR-125a-5p
 
 
-0.372
 
 
113.0
 
 
 
 
ANTXR2
 
 
hsa-miR-29a-3p
 
 
-0.347
 
 
51.4
 
 
 
 
AP3S2
 
 
hsa-miR-34a-5p
 
 
-0.413
 
 
16.4
 
 
 
 
APH1A
 
 
hsa-miR-34a-5p
 
 
-0.304
 
 
64.7
 
 
 
 
APPBP2
 
 
hsa-miR-27a-3p
 
 
-0.378
 
 
10.9
 
 
 
 
AREG
 
 
hsa-miR-34a-5p
 
 
-0.62
 
 
69.2
 
 
 
 
ARF4
 
 
hsa-miR-152-3p
 
 
-0.487
 
 
145.1
 
 
 
 
ARF4
 
 
hsa-miR-148b-3p
 
 
-0.487
 
 
145.1
 
 
 
 
ARF5
 
 
hsa-miR-29a-3p
 
 
-0.491
 
 
76.6
 
 
 
 
ARF6
 
 
hsa-miR-181b-5p
 
 
-0.33
 
 
30.6
 
 
 
 
ARF6
 
 
hsa-miR-181a-5p
 
 
-0.33
 
 
30.6
 
 
 
 
ARFGEF1
 
 
hsa-miR-148b-3p
 
 
-0.306
 
 
11.7
 
 
 
 
ARFGEF1
 
 
hsa-miR-152-3p
 
 
-0.306
 
 
11.7
 
 
 
 
ARFIP1
 
 
hsa-miR-152-3p
 
 
-0.474
 
 
18.7
 
 
 
 
ARFIP1
 
 
hsa-miR-148b-3p
 
 
-0.474
 
 
18.7
 
 
 
 
ARHGAP1
 
 
hsa-miR-34a-5p
 
 
-0.369
 
 
47.5
 
 
 
 
ARHGAP21
 
 
hsa-miR-152-3p
 
 
-0.287
 
 
16.7
 
 
 
 
ARHGAP21
 
 
hsa-miR-148b-3p
 
 
-0.287
 
 
16.7
 
 
 
 
ARHGAP26
 
 
hsa-miR-34a-5p
 
 
-0.384
 
 
13.3
 
 
 
 
ARID1B
 
 
hsa-miR-29a-3p
 
 
-0.342
 
 
12.6
 
 
 
 
ARL4A
 
 
hsa-miR-29a-3p
 
 
-0.386
 
 
18.0
 
 
 
 
ARL5B
 
 
hsa-miR-29a-3p
 
 
-0.304
 
 
13.2
 
 
 
 
ARL6IP1
 
 
hsa-miR-148b-3p
 
 
-0.579
 
 
123.8
 
 
 
 
ARL6IP1
 
 
hsa-miR-152-3p
 
 
-0.579
 
 
123.8
 
 
 
 
ARL8B
 
 
hsa-miR-152-3p
 
 
-0.337
 
 
36.3
 
 
 
 
ARL8B
 
 
hsa-miR-148b-3p
 
 
-0.337
 
 
36.3
 
 
 
 
ARMCX3
 
 
hsa-miR-425-5p
 
 
-0.325
 
 
31.9
 
 
 
 
ARPC5
 
 
hsa-miR-22-3p
 
 
-0.679
 
 
49.6
 
 
 
 
ARPP19
 
 
hsa-miR-29a-3p
 
 
-0.371
 
 
36.7
 
 
 
 
ARPP19
 
 
hsa-miR-34a-5p
 
 
-0.497
 
 
36.7
 
 
 
 
ARRB1
 
 
hsa-miR-22-3p
 
 
-0.616
 
 
12.1
 
 
 
 
ARRDC3
 
 
hsa-miR-152-3p
 
 
-0.305
 
 
36.6
 
 
 
 
ARRDC3
 
 
hsa-miR-148b-3p
 
 
-0.305
 
 
36.6
 
 
 
 
ARRDC3
 
 
hsa-miR-29a-3p
 
 
-0.402
 
 
36.6
 
 
 
 
ASAH1
 
 
hsa-miR-27a-3p
 
 
-0.398
 
 
92.8
 
 
 
 
ASPH
 
 
hsa-miR-152-3p
 
 
-0.391
 
 
56.5
 
 
 
 
ASPH
 
 
hsa-miR-148b-3p
 
 
-0.391
 
 
56.5
 
 
 
 
ATMIN
 
 
hsa-miR-34a-5p
 
 
-0.409
 
 
10.8
 
 
 
 
ATP2B4
 
 
hsa-miR-152-3p
 
 
-0.337
 
 
23.7
 
 
 
 
ATP2B4
 
 
hsa-miR-148b-3p
 
 
-0.337
 
 
23.7
 
 
 
 
ATP5MC3
 
 
hsa-miR-425-5p
 
 
-0.328
 
 
70.9
 
 
 
 
ATP5MC3
 
 
hsa-miR-29a-3p
 
 
-0.33
 
 
70.9
 
 
 
 
ATP5MC3
 
 
hsa-miR-27a-3p
 
 
-0.416
 
 
70.9
 
 
 
 
ATP6AP2
 
 
hsa-miR-148b-3p
 
 
-0.592
 
 
74.7
 
 
 
 
ATP6AP2
 
 
hsa-miR-152-3p
 
 
-0.592
 
 
74.7
 
 
 
 
ATP6AP2
 
 
hsa-miR-148b-3p
 
 
-0.668
 
 
74.7
 
 
 
 
 
 
 heart muscle 
 
 
 
 
target_symbol
 
 
mature_mirna_id
 
 
score
 
 
nTPM
 
 
 
 
 
 
AAGAB
 
 
hsa-miR-34a-5p
 
 
-0.319
 
 
14.8
 
 
 
 
AAR2
 
 
hsa-miR-29a-3p
 
 
-0.327
 
 
17.9
 
 
 
 
ABCB7
 
 
hsa-miR-148b-3p
 
 
-0.515
 
 
20.0
 
 
 
 
ABCB7
 
 
hsa-miR-152-3p
 
 
-0.515
 
 
20.0
 
 
 
 
ABCC5
 
 
hsa-miR-125a-5p
 
 
-0.288
 
 
47.0
 
 
 
 
ABCD3
 
 
hsa-miR-148b-3p
 
 
-0.388
 
 
24.0
 
 
 
 
ABCD3
 
 
hsa-miR-152-3p
 
 
-0.388
 
 
24.0
 
 
 
 
ABCE1
 
 
hsa-miR-29a-3p
 
 
-0.404
 
 
23.3
 
 
 
 
ABHD18
 
 
hsa-miR-29a-3p
 
 
-0.321
 
 
12.6
 
 
 
 
ABHD18
 
 
hsa-miR-29a-3p
 
 
-0.34
 
 
12.6
 
 
 
 
ABHD4
 
 
hsa-miR-29a-3p
 
 
-0.312
 
 
21.4
 
 
 
 
ABHD5
 
 
hsa-miR-132-3p
 
 
-0.323
 
 
10.6
 
 
 
 
ABHD5
 
 
hsa-miR-27a-3p
 
 
-0.407
 
 
10.6
 
 
 
 
ABTB1
 
 
hsa-miR-125a-5p
 
 
-0.519
 
 
42.4
 
 
 
 
ACBD3
 
 
hsa-miR-34a-5p
 
 
-0.415
 
 
20.1
 
 
 
 
ACHE
 
 
hsa-miR-125a-5p
 
 
-0.308
 
 
11.4
 
 
 
 
ACHE
 
 
hsa-miR-125a-5p
 
 
-0.361
 
 
11.4
 
 
 
 
ACSL1
 
 
hsa-miR-34a-5p
 
 
-0.306
 
 
161.9
 
 
 
 
ACSL4
 
 
hsa-miR-34a-5p
 
 
-0.372
 
 
26.9
 
 
 
 
ACTA2
 
 
hsa-miR-27a-3p
 
 
-0.577
 
 
1015.4
 
 
 
 
ACTN2
 
 
hsa-miR-23a-3p
 
 
-0.294
 
 
695.9
 
 
 
 
ACTN2
 
 
hsa-miR-29a-3p
 
 
-0.311
 
 
695.9
 
 
 
 
ACVR1
 
 
hsa-miR-152-3p
 
 
-0.397
 
 
14.3
 
 
 
 
ACVR1
 
 
hsa-miR-148b-3p
 
 
-0.397
 
 
14.3
 
 
 
 
ADA
 
 
hsa-miR-29a-3p
 
 
-0.376
 
 
18.4
 
 
 
 
ADAM10
 
 
hsa-miR-152-3p
 
 
-0.348
 
 
11.3
 
 
 
 
ADAM10
 
 
hsa-miR-148b-3p
 
 
-0.356
 
 
11.3
 
 
 
 
ADAMTS7
 
 
hsa-miR-29a-3p
 
 
-0.427
 
 
28.7
 
 
 
 
ADAMTS9
 
 
hsa-miR-29a-3p
 
 
-0.305
 
 
14.2
 
 
 
 
ADAMTS9
 
 
hsa-miR-29a-3p
 
 
-0.485
 
 
14.2
 
 
 
 
ADCY3
 
 
hsa-miR-132-3p
 
 
-0.303
 
 
35.0
 
 
 
 
ADGRA3
 
 
hsa-miR-27a-3p
 
 
-0.296
 
 
10.1
 
 
 
 
ADGRF5
 
 
hsa-miR-148b-3p
 
 
-0.354
 
 
50.1
 
 
 
 
ADGRF5
 
 
hsa-miR-152-3p
 
 
-0.354
 
 
50.1
 
 
 
 
ADIPOR2
 
 
hsa-miR-150-5p
 
 
-0.287
 
 
31.9
 
 
 
 
ADIPOR2
 
 
hsa-miR-34a-5p
 
 
-0.33
 
 
31.9
 
 
 
 
ADK
 
 
hsa-miR-34a-5p
 
 
-0.374
 
 
25.4
 
 
 
 
ADO
 
 
hsa-miR-34a-5p
 
 
-0.377
 
 
13.5
 
 
 
 
AFG3L2
 
 
hsa-miR-181b-5p
 
 
-0.491
 
 
48.7
 
 
 
 
AFG3L2
 
 
hsa-miR-181a-5p
 
 
-0.491
 
 
48.7
 
 
 
 
AGFG1
 
 
hsa-miR-148b-3p
 
 
-0.332
 
 
33.8
 
 
 
 
AGFG1
 
 
hsa-miR-152-3p
 
 
-0.332
 
 
33.8
 
 
 
 
AGPAT3
 
 
hsa-miR-27a-3p
 
 
-0.29
 
 
25.4
 
 
 
 
AGTRAP
 
 
hsa-miR-34a-5p
 
 
-0.328
 
 
34.3
 
 
 
 
AHDC1
 
 
hsa-miR-148b-3p
 
 
-0.413
 
 
17.4
 
 
 
 
AHDC1
 
 
hsa-miR-152-3p
 
 
-0.413
 
 
17.4
 
 
 
 
AIDA
 
 
hsa-miR-22-3p
 
 
-0.316
 
 
42.8
 
 
 
 
AIFM1
 
 
hsa-miR-125a-5p
 
 
-0.3
 
 
74.9
 
 
 
 
AK1
 
 
hsa-miR-29a-3p
 
 
-0.333
 
 
313.1
 
 
 
 
AKAP1
 
 
hsa-miR-148b-3p
 
 
-0.381
 
 
78.5
 
 
 
 
AKAP1
 
 
hsa-miR-152-3p
 
 
-0.381
 
 
78.5
 
 
 
 
AKIRIN1
 
 
hsa-miR-27a-3p
 
 
-0.368
 
 
36.6
 
 
 
 
AKT3
 
 
hsa-miR-29a-3p
 
 
-0.287
 
 
10.6
 
 
 
 
AKT3
 
 
hsa-miR-22-3p
 
 
-0.451
 
 
10.6
 
 
 
 
ALDH1A2
 
 
hsa-miR-23b-3p
 
 
-0.346
 
 
27.5
 
 
 
 
ALDH1A2
 
 
hsa-miR-23a-3p
 
 
-0.354
 
 
27.5
 
 
 
 
ALDOA
 
 
hsa-miR-34a-5p
 
 
-0.655
 
 
2567.3
 
 
 
 
ALPL
 
 
hsa-miR-10a-5p
 
 
-0.32
 
 
15.8
 
 
 
 
ANAPC16
 
 
hsa-miR-125a-5p
 
 
-0.578
 
 
42.0
 
 
 
 
ANK2
 
 
hsa-miR-34a-5p
 
 
-0.317
 
 
73.9
 
 
 
 
ANK3
 
 
hsa-miR-34a-5p
 
 
-0.29
 
 
77.4
 
 
 
 
ANK3
 
 
hsa-miR-34a-5p
 
 
-0.347
 
 
77.4
 
 
 
 
ANKHD1
 
 
hsa-miR-23a-3p
 
 
-0.368
 
 
34.0
 
 
 
 
ANKHD1
 
 
hsa-miR-23b-3p
 
 
-0.368
 
 
34.0
 
 
 
 
ANKRD13A
 
 
hsa-miR-22-3p
 
 
-0.478
 
 
10.0
 
 
 
 
ANKRD40
 
 
hsa-miR-27a-3p
 
 
-0.35
 
 
25.8
 
 
 
 
ANP32A
 
 
hsa-miR-132-3p
 
 
-0.374
 
 
119.7
 
 
 
 
ANP32B
 
 
hsa-miR-34a-5p
 
 
-0.29
 
 
87.4
 
 
 
 
ANTXR2
 
 
hsa-miR-29a-3p
 
 
-0.347
 
 
26.5
 
 
 
 
AP3S2
 
 
hsa-miR-34a-5p
 
 
-0.413
 
 
28.7
 
 
 
 
APH1A
 
 
hsa-miR-34a-5p
 
 
-0.304
 
 
68.4
 
 
 
 
APOO
 
 
hsa-miR-181b-5p
 
 
-0.5
 
 
61.9
 
 
 
 
APOO
 
 
hsa-miR-181a-5p
 
 
-0.5
 
 
61.9
 
 
 
 
AREG
 
 
hsa-miR-34a-5p
 
 
-0.62
 
 
20.3
 
 
 
 
ARF4
 
 
hsa-miR-152-3p
 
 
-0.487
 
 
106.8
 
 
 
 
ARF4
 
 
hsa-miR-148b-3p
 
 
-0.487
 
 
106.8
 
 
 
 
ARF5
 
 
hsa-miR-29a-3p
 
 
-0.491
 
 
127.9
 
 
 
 
ARF6
 
 
hsa-miR-181b-5p
 
 
-0.33
 
 
15.7
 
 
 
 
ARF6
 
 
hsa-miR-181a-5p
 
 
-0.33
 
 
15.7
 
 
 
 
ARHGAP1
 
 
hsa-miR-34a-5p
 
 
-0.369
 
 
105.1
 
 
 
 
ARHGAP21
 
 
hsa-miR-152-3p
 
 
-0.287
 
 
19.7
 
 
 
 
ARHGAP21
 
 
hsa-miR-148b-3p
 
 
-0.287
 
 
19.7
 
 
 
 
ARHGAP26
 
 
hsa-miR-34a-5p
 
 
-0.384
 
 
14.8
 
 
 
 
ARID1B
 
 
hsa-miR-29a-3p
 
 
-0.342
 
 
12.9
 
 
 
 
ARL4A
 
 
hsa-miR-29a-3p
 
 
-0.386
 
 
14.4
 
 
 
 
ARL6IP1
 
 
hsa-miR-148b-3p
 
 
-0.579
 
 
30.1
 
 
 
 
ARL6IP1
 
 
hsa-miR-152-3p
 
 
-0.579
 
 
30.1
 
 
 
 
ARL8B
 
 
hsa-miR-152-3p
 
 
-0.337
 
 
51.7
 
 
 
 
ARL8B
 
 
hsa-miR-148b-3p
 
 
-0.337
 
 
51.7
 
 
 
 
ARMCX3
 
 
hsa-miR-425-5p
 
 
-0.325
 
 
47.8
 
 
 
 
ARPC5
 
 
hsa-miR-22-3p
 
 
-0.679
 
 
33.5
 
 
 
 
ARPIN-AP3S2
 
 
hsa-miR-34a-5p
 
 
-0.348
 
 
17.1
 
 
 
 
ARPP19
 
 
hsa-miR-29a-3p
 
 
-0.371
 
 
76.2
 
 
 
 
ARPP19
 
 
hsa-miR-34a-5p
 
 
-0.497
 
 
76.2
 
 
 
 
ARRB1
 
 
hsa-miR-22-3p
 
 
-0.616
 
 
16.5
 
 
 
 
ARRDC3
 
 
hsa-miR-152-3p
 
 
-0.305
 
 
23.2
 
 
 
 
ARRDC3
 
 
hsa-miR-148b-3p
 
 
-0.305
 
 
23.2
 
 
 
 
ARRDC3
 
 
hsa-miR-29a-3p
 
 
-0.402
 
 
23.2
 
 
 
 
ASAH1
 
 
hsa-miR-27a-3p
 
 
-0.398
 
 
537.2
 
 
 
 
ASB1
 
 
hsa-miR-34a-5p
 
 
-0.406
 
 
15.2
 
 
 
 
 
 
 hippocampal formation 
 
 
 
 
target_symbol
 
 
mature_mirna_id
 
 
score
 
 
nTPM
 
 
 
 
 
 
AAGAB
 
 
hsa-miR-34a-5p
 
 
-0.319
 
 
10.0
 
 
 
 
AAR2
 
 
hsa-miR-29a-3p
 
 
-0.327
 
 
10.6
 
 
 
 
ABCC5
 
 
hsa-miR-125a-5p
 
 
-0.288
 
 
30.8
 
 
 
 
ABCD3
 
 
hsa-miR-148b-3p
 
 
-0.388
 
 
15.1
 
 
 
 
ABCD3
 
 
hsa-miR-152-3p
 
 
-0.388
 
 
15.1
 
 
 
 
ABCE1
 
 
hsa-miR-29a-3p
 
 
-0.404
 
 
13.9
 
 
 
 
ABHD17C
 
 
hsa-miR-27a-3p
 
 
-0.435
 
 
14.4
 
 
 
 
ABHD4
 
 
hsa-miR-29a-3p
 
 
-0.312
 
 
14.2
 
 
 
 
ABHD6
 
 
hsa-miR-125a-5p
 
 
-0.501
 
 
25.8
 
 
 
 
ABTB1
 
 
hsa-miR-125a-5p
 
 
-0.519
 
 
29.3
 
 
 
 
ACHE
 
 
hsa-miR-125a-5p
 
 
-0.308
 
 
14.5
 
 
 
 
ACHE
 
 
hsa-miR-125a-5p
 
 
-0.361
 
 
14.5
 
 
 
 
ACSL1
 
 
hsa-miR-34a-5p
 
 
-0.306
 
 
39.9
 
 
 
 
ACSL4
 
 
hsa-miR-34a-5p
 
 
-0.372
 
 
12.3
 
 
 
 
ACSL6
 
 
hsa-miR-125a-5p
 
 
-0.307
 
 
15.3
 
 
 
 
ACTA2
 
 
hsa-miR-27a-3p
 
 
-0.577
 
 
32.3
 
 
 
 
ACTN2
 
 
hsa-miR-23a-3p
 
 
-0.294
 
 
10.1
 
 
 
 
ACTN2
 
 
hsa-miR-29a-3p
 
 
-0.311
 
 
10.1
 
 
 
 
ADAP1
 
 
hsa-miR-27a-3p
 
 
-0.302
 
 
123.8
 
 
 
 
ADCY2
 
 
hsa-miR-152-3p
 
 
-0.299
 
 
18.5
 
 
 
 
ADCY2
 
 
hsa-miR-148b-3p
 
 
-0.299
 
 
18.5
 
 
 
 
ADCY3
 
 
hsa-miR-132-3p
 
 
-0.303
 
 
30.1
 
 
 
 
ADD2
 
 
hsa-miR-34a-5p
 
 
-0.305
 
 
12.8
 
 
 
 
ADGRA3
 
 
hsa-miR-27a-3p
 
 
-0.296
 
 
12.8
 
 
 
 
ADGRB3
 
 
hsa-miR-152-3p
 
 
-0.516
 
 
12.8
 
 
 
 
ADGRB3
 
 
hsa-miR-148b-3p
 
 
-0.524
 
 
12.8
 
 
 
 
ADGRF5
 
 
hsa-miR-148b-3p
 
 
-0.354
 
 
10.4
 
 
 
 
ADGRF5
 
 
hsa-miR-152-3p
 
 
-0.354
 
 
10.4
 
 
 
 
ADIPOR2
 
 
hsa-miR-150-5p
 
 
-0.287
 
 
57.6
 
 
 
 
ADIPOR2
 
 
hsa-miR-34a-5p
 
 
-0.33
 
 
57.6
 
 
 
 
ADK
 
 
hsa-miR-34a-5p
 
 
-0.374
 
 
13.3
 
 
 
 
ADO
 
 
hsa-miR-34a-5p
 
 
-0.377
 
 
11.3
 
 
 
 
AGFG1
 
 
hsa-miR-148b-3p
 
 
-0.332
 
 
29.8
 
 
 
 
AGFG1
 
 
hsa-miR-152-3p
 
 
-0.332
 
 
29.8
 
 
 
 
AGPAT3
 
 
hsa-miR-27a-3p
 
 
-0.29
 
 
37.1
 
 
 
 
AGPAT4
 
 
hsa-miR-29a-3p
 
 
-0.376
 
 
16.8
 
 
 
 
AGTRAP
 
 
hsa-miR-34a-5p
 
 
-0.328
 
 
20.9
 
 
 
 
AHCYL2
 
 
hsa-miR-34a-5p
 
 
-0.359
 
 
15.5
 
 
 
 
AIDA
 
 
hsa-miR-22-3p
 
 
-0.316
 
 
22.9
 
 
 
 
AIFM1
 
 
hsa-miR-125a-5p
 
 
-0.3
 
 
10.5
 
 
 
 
AK1
 
 
hsa-miR-29a-3p
 
 
-0.333
 
 
93.4
 
 
 
 
AKAP1
 
 
hsa-miR-148b-3p
 
 
-0.381
 
 
17.0
 
 
 
 
AKAP1
 
 
hsa-miR-152-3p
 
 
-0.381
 
 
17.0
 
 
 
 
AKIRIN1
 
 
hsa-miR-27a-3p
 
 
-0.368
 
 
35.2
 
 
 
 
AKT3
 
 
hsa-miR-29a-3p
 
 
-0.287
 
 
17.7
 
 
 
 
AKT3
 
 
hsa-miR-22-3p
 
 
-0.451
 
 
17.7
 
 
 
 
ALCAM
 
 
hsa-miR-34a-5p
 
 
-0.303
 
 
33.8
 
 
 
 
ALCAM
 
 
hsa-miR-148b-3p
 
 
-0.339
 
 
33.8
 
 
 
 
ALCAM
 
 
hsa-miR-152-3p
 
 
-0.347
 
 
33.8
 
 
 
 
ALDOA
 
 
hsa-miR-34a-5p
 
 
-0.655
 
 
777.3
 
 
 
 
ALPL
 
 
hsa-miR-10a-5p
 
 
-0.32
 
 
13.7
 
 
 
 
AMER2
 
 
hsa-miR-34a-5p
 
 
-0.48
 
 
32.9
 
 
 
 
AMOT
 
 
hsa-miR-29a-3p
 
 
-0.304
 
 
12.0
 
 
 
 
ANGEL2
 
 
hsa-miR-132-3p
 
 
-0.357
 
 
11.0
 
 
 
 
ANGEL2
 
 
hsa-miR-22-3p
 
 
-0.601
 
 
11.0
 
 
 
 
ANK2
 
 
hsa-miR-34a-5p
 
 
-0.317
 
 
18.2
 
 
 
 
ANK3
 
 
hsa-miR-34a-5p
 
 
-0.29
 
 
51.4
 
 
 
 
ANK3
 
 
hsa-miR-34a-5p
 
 
-0.347
 
 
51.4
 
 
 
 
ANKHD1
 
 
hsa-miR-23a-3p
 
 
-0.368
 
 
18.9
 
 
 
 
ANKHD1
 
 
hsa-miR-23b-3p
 
 
-0.368
 
 
18.9
 
 
 
 
ANKRD13B
 
 
hsa-miR-29a-3p
 
 
-0.421
 
 
10.6
 
 
 
 
ANKRD40
 
 
hsa-miR-27a-3p
 
 
-0.35
 
 
105.0
 
 
 
 
ANP32A
 
 
hsa-miR-132-3p
 
 
-0.374
 
 
49.8
 
 
 
 
ANP32B
 
 
hsa-miR-34a-5p
 
 
-0.29
 
 
139.9
 
 
 
 
AP3S2
 
 
hsa-miR-34a-5p
 
 
-0.413
 
 
12.8
 
 
 
 
APC
 
 
hsa-miR-29a-3p
 
 
-0.413
 
 
34.3
 
 
 
 
APH1A
 
 
hsa-miR-34a-5p
 
 
-0.304
 
 
46.6
 
 
 
 
APOO
 
 
hsa-miR-181b-5p
 
 
-0.5
 
 
45.8
 
 
 
 
APOO
 
 
hsa-miR-181a-5p
 
 
-0.5
 
 
45.8
 
 
 
 
ARF4
 
 
hsa-miR-152-3p
 
 
-0.487
 
 
61.3
 
 
 
 
ARF4
 
 
hsa-miR-148b-3p
 
 
-0.487
 
 
61.3
 
 
 
 
ARF5
 
 
hsa-miR-29a-3p
 
 
-0.491
 
 
168.1
 
 
 
 
ARF6
 
 
hsa-miR-181b-5p
 
 
-0.33
 
 
10.0
 
 
 
 
ARF6
 
 
hsa-miR-181a-5p
 
 
-0.33
 
 
10.0
 
 
 
 
ARHGAP1
 
 
hsa-miR-34a-5p
 
 
-0.369
 
 
48.9
 
 
 
 
ARHGAP21
 
 
hsa-miR-152-3p
 
 
-0.287
 
 
50.4
 
 
 
 
ARHGAP21
 
 
hsa-miR-148b-3p
 
 
-0.287
 
 
50.4
 
 
 
 
ARL4A
 
 
hsa-miR-29a-3p
 
 
-0.386
 
 
12.2
 
 
 
 
ARL6IP1
 
 
hsa-miR-148b-3p
 
 
-0.579
 
 
196.8
 
 
 
 
ARL6IP1
 
 
hsa-miR-152-3p
 
 
-0.579
 
 
196.8
 
 
 
 
ARL8B
 
 
hsa-miR-152-3p
 
 
-0.337
 
 
53.8
 
 
 
 
ARL8B
 
 
hsa-miR-148b-3p
 
 
-0.337
 
 
53.8
 
 
 
 
ARMCX3
 
 
hsa-miR-425-5p
 
 
-0.325
 
 
33.7
 
 
 
 
ARPC5
 
 
hsa-miR-22-3p
 
 
-0.679
 
 
23.8
 
 
 
 
ARPIN-AP3S2
 
 
hsa-miR-34a-5p
 
 
-0.348
 
 
10.6
 
 
 
 
ARPP19
 
 
hsa-miR-29a-3p
 
 
-0.371
 
 
120.9
 
 
 
 
ARPP19
 
 
hsa-miR-34a-5p
 
 
-0.497
 
 
120.9
 
 
 
 
ARRB1
 
 
hsa-miR-22-3p
 
 
-0.616
 
 
25.3
 
 
 
 
ARRDC3
 
 
hsa-miR-152-3p
 
 
-0.305
 
 
11.5
 
 
 
 
ARRDC3
 
 
hsa-miR-148b-3p
 
 
-0.305
 
 
11.5
 
 
 
 
ARRDC3
 
 
hsa-miR-29a-3p
 
 
-0.402
 
 
11.5
 
 
 
 
ASAH1
 
 
hsa-miR-27a-3p
 
 
-0.398
 
 
51.2
 
 
 
 
ASB1
 
 
hsa-miR-34a-5p
 
 
-0.406
 
 
15.5
 
 
 
 
ASF1A
 
 
hsa-miR-23a-3p
 
 
-0.506
 
 
11.7
 
 
 
 
ASF1A
 
 
hsa-miR-23b-3p
 
 
-0.506
 
 
11.7
 
 
 
 
ASPH
 
 
hsa-miR-152-3p
 
 
-0.391
 
 
45.5
 
 
 
 
ASPH
 
 
hsa-miR-148b-3p
 
 
-0.391
 
 
45.5
 
 
 
 
ATCAY
 
 
hsa-miR-10a-5p
 
 
-0.322
 
 
38.9
 
 
 
 
ATG4B
 
 
hsa-miR-34a-5p
 
 
-0.58
 
 
19.9
 
 
 
 
ATMIN
 
 
hsa-miR-34a-5p
 
 
-0.409
 
 
18.7
 
 
 
 
 
 
 hypothalamus 
 
 
 
 
target_symbol
 
 
mature_mirna_id
 
 
score
 
 
nTPM
 
 
 
 
 
 
AAGAB
 
 
hsa-miR-34a-5p
 
 
-0.319
 
 
14.4
 
 
 
 
ABCC5
 
 
hsa-miR-125a-5p
 
 
-0.288
 
 
33.4
 
 
 
 
ABCD3
 
 
hsa-miR-148b-3p
 
 
-0.388
 
 
13.5
 
 
 
 
ABCD3
 
 
hsa-miR-152-3p
 
 
-0.388
 
 
13.5
 
 
 
 
ABCE1
 
 
hsa-miR-29a-3p
 
 
-0.404
 
 
12.9
 
 
 
 
ABHD17C
 
 
hsa-miR-27a-3p
 
 
-0.435
 
 
11.1
 
 
 
 
ABHD4
 
 
hsa-miR-29a-3p
 
 
-0.312
 
 
13.5
 
 
 
 
ABHD6
 
 
hsa-miR-125a-5p
 
 
-0.501
 
 
12.9
 
 
 
 
ABTB1
 
 
hsa-miR-125a-5p
 
 
-0.519
 
 
20.4
 
 
 
 
ACHE
 
 
hsa-miR-125a-5p
 
 
-0.308
 
 
103.9
 
 
 
 
ACHE
 
 
hsa-miR-125a-5p
 
 
-0.361
 
 
103.9
 
 
 
 
ACSL1
 
 
hsa-miR-34a-5p
 
 
-0.306
 
 
28.9
 
 
 
 
ACSL4
 
 
hsa-miR-34a-5p
 
 
-0.372
 
 
13.7
 
 
 
 
ACSL6
 
 
hsa-miR-125a-5p
 
 
-0.307
 
 
11.9
 
 
 
 
ACTA2
 
 
hsa-miR-27a-3p
 
 
-0.577
 
 
34.8
 
 
 
 
ADAP1
 
 
hsa-miR-27a-3p
 
 
-0.302
 
 
58.0
 
 
 
 
ADCY2
 
 
hsa-miR-152-3p
 
 
-0.299
 
 
14.0
 
 
 
 
ADCY2
 
 
hsa-miR-148b-3p
 
 
-0.299
 
 
14.0
 
 
 
 
ADCY3
 
 
hsa-miR-132-3p
 
 
-0.303
 
 
32.1
 
 
 
 
ADD2
 
 
hsa-miR-34a-5p
 
 
-0.305
 
 
12.7
 
 
 
 
ADGRA3
 
 
hsa-miR-27a-3p
 
 
-0.296
 
 
10.8
 
 
 
 
ADGRB3
 
 
hsa-miR-152-3p
 
 
-0.516
 
 
16.0
 
 
 
 
ADGRB3
 
 
hsa-miR-148b-3p
 
 
-0.524
 
 
16.0
 
 
 
 
ADIPOR2
 
 
hsa-miR-150-5p
 
 
-0.287
 
 
40.5
 
 
 
 
ADIPOR2
 
 
hsa-miR-34a-5p
 
 
-0.33
 
 
40.5
 
 
 
 
ADK
 
 
hsa-miR-34a-5p
 
 
-0.374
 
 
15.8
 
 
 
 
AGFG1
 
 
hsa-miR-148b-3p
 
 
-0.332
 
 
26.0
 
 
 
 
AGFG1
 
 
hsa-miR-152-3p
 
 
-0.332
 
 
26.0
 
 
 
 
AGPAT3
 
 
hsa-miR-27a-3p
 
 
-0.29
 
 
28.2
 
 
 
 
AGPAT4
 
 
hsa-miR-29a-3p
 
 
-0.376
 
 
14.0
 
 
 
 
AGTRAP
 
 
hsa-miR-34a-5p
 
 
-0.328
 
 
18.3
 
 
 
 
AHCYL2
 
 
hsa-miR-34a-5p
 
 
-0.359
 
 
16.7
 
 
 
 
AHDC1
 
 
hsa-miR-148b-3p
 
 
-0.413
 
 
10.9
 
 
 
 
AHDC1
 
 
hsa-miR-152-3p
 
 
-0.413
 
 
10.9
 
 
 
 
AIDA
 
 
hsa-miR-22-3p
 
 
-0.316
 
 
30.0
 
 
 
 
AK1
 
 
hsa-miR-29a-3p
 
 
-0.333
 
 
100.1
 
 
 
 
AKAP1
 
 
hsa-miR-148b-3p
 
 
-0.381
 
 
15.1
 
 
 
 
AKAP1
 
 
hsa-miR-152-3p
 
 
-0.381
 
 
15.1
 
 
 
 
AKIRIN1
 
 
hsa-miR-27a-3p
 
 
-0.368
 
 
39.9
 
 
 
 
AKT3
 
 
hsa-miR-29a-3p
 
 
-0.287
 
 
14.5
 
 
 
 
AKT3
 
 
hsa-miR-22-3p
 
 
-0.451
 
 
14.5
 
 
 
 
ALCAM
 
 
hsa-miR-34a-5p
 
 
-0.303
 
 
36.1
 
 
 
 
ALCAM
 
 
hsa-miR-148b-3p
 
 
-0.339
 
 
36.1
 
 
 
 
ALCAM
 
 
hsa-miR-152-3p
 
 
-0.347
 
 
36.1
 
 
 
 
ALDOA
 
 
hsa-miR-34a-5p
 
 
-0.655
 
 
780.8
 
 
 
 
AMER2
 
 
hsa-miR-34a-5p
 
 
-0.48
 
 
16.5
 
 
 
 
AMOT
 
 
hsa-miR-29a-3p
 
 
-0.304
 
 
11.9
 
 
 
 
ANK2
 
 
hsa-miR-34a-5p
 
 
-0.317
 
 
16.1
 
 
 
 
ANK3
 
 
hsa-miR-34a-5p
 
 
-0.29
 
 
37.6
 
 
 
 
ANK3
 
 
hsa-miR-34a-5p
 
 
-0.347
 
 
37.6
 
 
 
 
ANKHD1
 
 
hsa-miR-23a-3p
 
 
-0.368
 
 
17.8
 
 
 
 
ANKHD1
 
 
hsa-miR-23b-3p
 
 
-0.368
 
 
17.8
 
 
 
 
ANKRD40
 
 
hsa-miR-27a-3p
 
 
-0.35
 
 
80.8
 
 
 
 
ANP32A
 
 
hsa-miR-132-3p
 
 
-0.374
 
 
37.9
 
 
 
 
ANP32B
 
 
hsa-miR-34a-5p
 
 
-0.29
 
 
120.3
 
 
 
 
AP3S2
 
 
hsa-miR-34a-5p
 
 
-0.413
 
 
14.6
 
 
 
 
APC
 
 
hsa-miR-29a-3p
 
 
-0.413
 
 
11.2
 
 
 
 
APH1A
 
 
hsa-miR-34a-5p
 
 
-0.304
 
 
40.7
 
 
 
 
APOO
 
 
hsa-miR-181b-5p
 
 
-0.5
 
 
43.8
 
 
 
 
APOO
 
 
hsa-miR-181a-5p
 
 
-0.5
 
 
43.8
 
 
 
 
ARF4
 
 
hsa-miR-152-3p
 
 
-0.487
 
 
75.3
 
 
 
 
ARF4
 
 
hsa-miR-148b-3p
 
 
-0.487
 
 
75.3
 
 
 
 
ARF5
 
 
hsa-miR-29a-3p
 
 
-0.491
 
 
134.0
 
 
 
 
ARF6
 
 
hsa-miR-181b-5p
 
 
-0.33
 
 
10.2
 
 
 
 
ARF6
 
 
hsa-miR-181a-5p
 
 
-0.33
 
 
10.2
 
 
 
 
ARHGAP1
 
 
hsa-miR-34a-5p
 
 
-0.369
 
 
31.0
 
 
 
 
ARHGAP21
 
 
hsa-miR-152-3p
 
 
-0.287
 
 
30.5
 
 
 
 
ARHGAP21
 
 
hsa-miR-148b-3p
 
 
-0.287
 
 
30.5
 
 
 
 
ARHGAP36
 
 
hsa-miR-34a-5p
 
 
-0.367
 
 
77.0
 
 
 
 
ARL6IP1
 
 
hsa-miR-148b-3p
 
 
-0.579
 
 
212.8
 
 
 
 
ARL6IP1
 
 
hsa-miR-152-3p
 
 
-0.579
 
 
212.8
 
 
 
 
ARL8B
 
 
hsa-miR-152-3p
 
 
-0.337
 
 
54.9
 
 
 
 
ARL8B
 
 
hsa-miR-148b-3p
 
 
-0.337
 
 
54.9
 
 
 
 
ARMCX3
 
 
hsa-miR-425-5p
 
 
-0.325
 
 
32.9
 
 
 
 
ARPC5
 
 
hsa-miR-22-3p
 
 
-0.679
 
 
21.6
 
 
 
 
ARPIN-AP3S2
 
 
hsa-miR-34a-5p
 
 
-0.348
 
 
13.7
 
 
 
 
ARPP19
 
 
hsa-miR-29a-3p
 
 
-0.371
 
 
103.9
 
 
 
 
ARPP19
 
 
hsa-miR-34a-5p
 
 
-0.497
 
 
103.9
 
 
 
 
ARRB1
 
 
hsa-miR-22-3p
 
 
-0.616
 
 
21.9
 
 
 
 
ARRDC3
 
 
hsa-miR-152-3p
 
 
-0.305
 
 
10.3
 
 
 
 
ARRDC3
 
 
hsa-miR-148b-3p
 
 
-0.305
 
 
10.3
 
 
 
 
ARRDC3
 
 
hsa-miR-29a-3p
 
 
-0.402
 
 
10.3
 
 
 
 
ASAH1
 
 
hsa-miR-27a-3p
 
 
-0.398
 
 
58.0
 
 
 
 
ASB1
 
 
hsa-miR-34a-5p
 
 
-0.406
 
 
13.8
 
 
 
 
ASF1A
 
 
hsa-miR-23a-3p
 
 
-0.506
 
 
13.0
 
 
 
 
ASF1A
 
 
hsa-miR-23b-3p
 
 
-0.506
 
 
13.0
 
 
 
 
ASIC2
 
 
hsa-miR-34a-5p
 
 
-0.496
 
 
10.2
 
 
 
 
ASPH
 
 
hsa-miR-152-3p
 
 
-0.391
 
 
51.0
 
 
 
 
ASPH
 
 
hsa-miR-148b-3p
 
 
-0.391
 
 
51.0
 
 
 
 
ATCAY
 
 
hsa-miR-10a-5p
 
 
-0.322
 
 
56.2
 
 
 
 
ATG4B
 
 
hsa-miR-34a-5p
 
 
-0.58
 
 
22.5
 
 
 
 
ATMIN
 
 
hsa-miR-34a-5p
 
 
-0.409
 
 
20.4
 
 
 
 
ATP1A2
 
 
hsa-miR-34a-5p
 
 
-0.306
 
 
193.2
 
 
 
 
ATP5MC3
 
 
hsa-miR-425-5p
 
 
-0.328
 
 
193.1
 
 
 
 
ATP5MC3
 
 
hsa-miR-29a-3p
 
 
-0.33
 
 
193.1
 
 
 
 
ATP5MC3
 
 
hsa-miR-27a-3p
 
 
-0.416
 
 
193.1
 
 
 
 
ATP6AP2
 
 
hsa-miR-148b-3p
 
 
-0.592
 
 
69.0
 
 
 
 
ATP6AP2
 
 
hsa-miR-152-3p
 
 
-0.592
 
 
69.0
 
 
 
 
ATP6AP2
 
 
hsa-miR-148b-3p
 
 
-0.668
 
 
69.0
 
 
 
 
ATP6AP2
 
 
hsa-miR-152-3p
 
 
-0.668
 
 
69.0
 
 
 
 
 
 
 kidney 
 
 
 
 
target_symbol
 
 
mature_mirna_id
 
 
score
 
 
nTPM
 
 
 
 
 
 
AAGAB
 
 
hsa-miR-34a-5p
 
 
-0.319
 
 
25.5
 
 
 
 
AAR2
 
 
hsa-miR-29a-3p
 
 
-0.327
 
 
19.2
 
 
 
 
ABCC5
 
 
hsa-miR-125a-5p
 
 
-0.288
 
 
48.3
 
 
 
 
ABCD3
 
 
hsa-miR-148b-3p
 
 
-0.388
 
 
55.5
 
 
 
 
ABCD3
 
 
hsa-miR-152-3p
 
 
-0.388
 
 
55.5
 
 
 
 
ABCE1
 
 
hsa-miR-29a-3p
 
 
-0.404
 
 
22.2
 
 
 
 
ABHD17C
 
 
hsa-miR-27a-3p
 
 
-0.435
 
 
13.6
 
 
 
 
ABHD3
 
 
hsa-miR-125a-5p
 
 
-0.453
 
 
13.5
 
 
 
 
ABHD4
 
 
hsa-miR-29a-3p
 
 
-0.312
 
 
26.4
 
 
 
 
ABHD5
 
 
hsa-miR-132-3p
 
 
-0.323
 
 
10.4
 
 
 
 
ABHD5
 
 
hsa-miR-27a-3p
 
 
-0.407
 
 
10.4
 
 
 
 
ABHD6
 
 
hsa-miR-125a-5p
 
 
-0.501
 
 
33.2
 
 
 
 
ABRAXAS2
 
 
hsa-miR-34a-5p
 
 
-0.538
 
 
11.5
 
 
 
 
ABTB1
 
 
hsa-miR-125a-5p
 
 
-0.519
 
 
26.5
 
 
 
 
ACBD3
 
 
hsa-miR-34a-5p
 
 
-0.415
 
 
18.8
 
 
 
 
ACSL1
 
 
hsa-miR-34a-5p
 
 
-0.306
 
 
173.2
 
 
 
 
ACSL4
 
 
hsa-miR-34a-5p
 
 
-0.372
 
 
42.0
 
 
 
 
ACTA2
 
 
hsa-miR-27a-3p
 
 
-0.577
 
 
281.9
 
 
 
 
ACVR1
 
 
hsa-miR-152-3p
 
 
-0.397
 
 
15.0
 
 
 
 
ACVR1
 
 
hsa-miR-148b-3p
 
 
-0.397
 
 
15.0
 
 
 
 
ADAM10
 
 
hsa-miR-152-3p
 
 
-0.348
 
 
16.8
 
 
 
 
ADAM10
 
 
hsa-miR-148b-3p
 
 
-0.356
 
 
16.8
 
 
 
 
ADAP1
 
 
hsa-miR-27a-3p
 
 
-0.302
 
 
21.6
 
 
 
 
ADCY3
 
 
hsa-miR-132-3p
 
 
-0.303
 
 
27.1
 
 
 
 
ADGRF5
 
 
hsa-miR-148b-3p
 
 
-0.354
 
 
55.8
 
 
 
 
ADGRF5
 
 
hsa-miR-152-3p
 
 
-0.354
 
 
55.8
 
 
 
 
ADIPOR2
 
 
hsa-miR-150-5p
 
 
-0.287
 
 
20.9
 
 
 
 
ADIPOR2
 
 
hsa-miR-34a-5p
 
 
-0.33
 
 
20.9
 
 
 
 
ADK
 
 
hsa-miR-34a-5p
 
 
-0.374
 
 
33.4
 
 
 
 
ADO
 
 
hsa-miR-34a-5p
 
 
-0.377
 
 
10.5
 
 
 
 
AFG3L2
 
 
hsa-miR-181b-5p
 
 
-0.491
 
 
36.4
 
 
 
 
AFG3L2
 
 
hsa-miR-181a-5p
 
 
-0.491
 
 
36.4
 
 
 
 
AGFG1
 
 
hsa-miR-148b-3p
 
 
-0.332
 
 
30.6
 
 
 
 
AGFG1
 
 
hsa-miR-152-3p
 
 
-0.332
 
 
30.6
 
 
 
 
AGGF1
 
 
hsa-miR-125a-5p
 
 
-0.331
 
 
12.1
 
 
 
 
AGMAT
 
 
hsa-miR-22-3p
 
 
-0.294
 
 
125.0
 
 
 
 
AGPAT3
 
 
hsa-miR-27a-3p
 
 
-0.29
 
 
49.9
 
 
 
 
AGTRAP
 
 
hsa-miR-34a-5p
 
 
-0.328
 
 
61.8
 
 
 
 
AHCYL2
 
 
hsa-miR-34a-5p
 
 
-0.359
 
 
26.6
 
 
 
 
AHDC1
 
 
hsa-miR-148b-3p
 
 
-0.413
 
 
12.2
 
 
 
 
AHDC1
 
 
hsa-miR-152-3p
 
 
-0.413
 
 
12.2
 
 
 
 
AIDA
 
 
hsa-miR-22-3p
 
 
-0.316
 
 
36.7
 
 
 
 
AIFM1
 
 
hsa-miR-125a-5p
 
 
-0.3
 
 
69.0
 
 
 
 
AK1
 
 
hsa-miR-29a-3p
 
 
-0.333
 
 
37.0
 
 
 
 
AKAP1
 
 
hsa-miR-148b-3p
 
 
-0.381
 
 
24.5
 
 
 
 
AKAP1
 
 
hsa-miR-152-3p
 
 
-0.381
 
 
24.5
 
 
 
 
AKIRIN1
 
 
hsa-miR-27a-3p
 
 
-0.368
 
 
40.0
 
 
 
 
ALCAM
 
 
hsa-miR-34a-5p
 
 
-0.303
 
 
20.8
 
 
 
 
ALCAM
 
 
hsa-miR-148b-3p
 
 
-0.339
 
 
20.8
 
 
 
 
ALCAM
 
 
hsa-miR-152-3p
 
 
-0.347
 
 
20.8
 
 
 
 
ALDH1A2
 
 
hsa-miR-23b-3p
 
 
-0.346
 
 
19.1
 
 
 
 
ALDH1A2
 
 
hsa-miR-23a-3p
 
 
-0.354
 
 
19.1
 
 
 
 
ALDOA
 
 
hsa-miR-34a-5p
 
 
-0.655
 
 
878.7
 
 
 
 
ALPL
 
 
hsa-miR-10a-5p
 
 
-0.32
 
 
42.7
 
 
 
 
ANAPC16
 
 
hsa-miR-125a-5p
 
 
-0.578
 
 
23.1
 
 
 
 
ANK2
 
 
hsa-miR-34a-5p
 
 
-0.317
 
 
76.8
 
 
 
 
ANK3
 
 
hsa-miR-34a-5p
 
 
-0.29
 
 
87.6
 
 
 
 
ANK3
 
 
hsa-miR-34a-5p
 
 
-0.347
 
 
87.6
 
 
 
 
ANKHD1
 
 
hsa-miR-23a-3p
 
 
-0.368
 
 
34.2
 
 
 
 
ANKHD1
 
 
hsa-miR-23b-3p
 
 
-0.368
 
 
34.2
 
 
 
 
ANKHD1-EIF4EBP3
 
 
hsa-miR-22-3p
 
 
-0.479
 
 
11.4
 
 
 
 
ANKRD13A
 
 
hsa-miR-22-3p
 
 
-0.478
 
 
16.4
 
 
 
 
ANKRD40
 
 
hsa-miR-27a-3p
 
 
-0.35
 
 
16.6
 
 
 
 
ANP32A
 
 
hsa-miR-132-3p
 
 
-0.374
 
 
70.0
 
 
 
 
ANP32B
 
 
hsa-miR-34a-5p
 
 
-0.29
 
 
90.4
 
 
 
 
ANPEP
 
 
hsa-miR-125a-5p
 
 
-0.372
 
 
235.6
 
 
 
 
ANTXR2
 
 
hsa-miR-29a-3p
 
 
-0.347
 
 
16.0
 
 
 
 
AP3S2
 
 
hsa-miR-34a-5p
 
 
-0.413
 
 
33.9
 
 
 
 
APH1A
 
 
hsa-miR-34a-5p
 
 
-0.304
 
 
75.0
 
 
 
 
APOO
 
 
hsa-miR-181b-5p
 
 
-0.5
 
 
43.3
 
 
 
 
APOO
 
 
hsa-miR-181a-5p
 
 
-0.5
 
 
43.3
 
 
 
 
APPBP2
 
 
hsa-miR-27a-3p
 
 
-0.378
 
 
12.2
 
 
 
 
ARF4
 
 
hsa-miR-152-3p
 
 
-0.487
 
 
106.7
 
 
 
 
ARF4
 
 
hsa-miR-148b-3p
 
 
-0.487
 
 
106.7
 
 
 
 
ARF5
 
 
hsa-miR-29a-3p
 
 
-0.491
 
 
140.7
 
 
 
 
ARF6
 
 
hsa-miR-181b-5p
 
 
-0.33
 
 
29.7
 
 
 
 
ARF6
 
 
hsa-miR-181a-5p
 
 
-0.33
 
 
29.7
 
 
 
 
ARFGEF1
 
 
hsa-miR-148b-3p
 
 
-0.306
 
 
21.3
 
 
 
 
ARFGEF1
 
 
hsa-miR-152-3p
 
 
-0.306
 
 
21.3
 
 
 
 
ARFIP1
 
 
hsa-miR-152-3p
 
 
-0.474
 
 
18.5
 
 
 
 
ARFIP1
 
 
hsa-miR-148b-3p
 
 
-0.474
 
 
18.5
 
 
 
 
ARHGAP1
 
 
hsa-miR-34a-5p
 
 
-0.369
 
 
38.9
 
 
 
 
ARHGAP21
 
 
hsa-miR-152-3p
 
 
-0.287
 
 
10.2
 
 
 
 
ARHGAP21
 
 
hsa-miR-148b-3p
 
 
-0.287
 
 
10.2
 
 
 
 
ARID1B
 
 
hsa-miR-29a-3p
 
 
-0.342
 
 
10.7
 
 
 
 
ARL6IP1
 
 
hsa-miR-148b-3p
 
 
-0.579
 
 
140.2
 
 
 
 
ARL6IP1
 
 
hsa-miR-152-3p
 
 
-0.579
 
 
140.2
 
 
 
 
ARL8B
 
 
hsa-miR-152-3p
 
 
-0.337
 
 
50.2
 
 
 
 
ARL8B
 
 
hsa-miR-148b-3p
 
 
-0.337
 
 
50.2
 
 
 
 
ARMCX3
 
 
hsa-miR-425-5p
 
 
-0.325
 
 
34.8
 
 
 
 
ARPC5
 
 
hsa-miR-22-3p
 
 
-0.679
 
 
36.4
 
 
 
 
ARPIN-AP3S2
 
 
hsa-miR-34a-5p
 
 
-0.348
 
 
14.2
 
 
 
 
ARPP19
 
 
hsa-miR-29a-3p
 
 
-0.371
 
 
52.6
 
 
 
 
ARPP19
 
 
hsa-miR-34a-5p
 
 
-0.497
 
 
52.6
 
 
 
 
ARRB1
 
 
hsa-miR-22-3p
 
 
-0.616
 
 
16.5
 
 
 
 
ARRDC3
 
 
hsa-miR-152-3p
 
 
-0.305
 
 
49.3
 
 
 
 
ARRDC3
 
 
hsa-miR-148b-3p
 
 
-0.305
 
 
49.3
 
 
 
 
ARRDC3
 
 
hsa-miR-29a-3p
 
 
-0.402
 
 
49.3
 
 
 
 
ARSB
 
 
hsa-miR-29a-3p
 
 
-0.36
 
 
19.9
 
 
 
 
ASAH1
 
 
hsa-miR-27a-3p
 
 
-0.398
 
 
190.4
 
 
 
 
 
 
 lung 
 
 
 
 
target_symbol
 
 
mature_mirna_id
 
 
score
 
 
nTPM
 
 
 
 
 
 
AAGAB
 
 
hsa-miR-34a-5p
 
 
-0.319
 
 
14.7
 
 
 
 
AAR2
 
 
hsa-miR-29a-3p
 
 
-0.327
 
 
18.1
 
 
 
 
ABCC5
 
 
hsa-miR-125a-5p
 
 
-0.288
 
 
15.3
 
 
 
 
ABCD3
 
 
hsa-miR-148b-3p
 
 
-0.388
 
 
22.0
 
 
 
 
ABCD3
 
 
hsa-miR-152-3p
 
 
-0.388
 
 
22.0
 
 
 
 
ABCE1
 
 
hsa-miR-29a-3p
 
 
-0.404
 
 
17.4
 
 
 
 
ABHD17C
 
 
hsa-miR-27a-3p
 
 
-0.435
 
 
18.6
 
 
 
 
ABHD3
 
 
hsa-miR-125a-5p
 
 
-0.453
 
 
14.8
 
 
 
 
ABHD4
 
 
hsa-miR-29a-3p
 
 
-0.312
 
 
18.5
 
 
 
 
ABHD5
 
 
hsa-miR-132-3p
 
 
-0.323
 
 
11.2
 
 
 
 
ABHD5
 
 
hsa-miR-27a-3p
 
 
-0.407
 
 
11.2
 
 
 
 
ABTB1
 
 
hsa-miR-125a-5p
 
 
-0.519
 
 
33.0
 
 
 
 
ACBD3
 
 
hsa-miR-34a-5p
 
 
-0.415
 
 
21.1
 
 
 
 
ACSL1
 
 
hsa-miR-34a-5p
 
 
-0.306
 
 
169.5
 
 
 
 
ACSL4
 
 
hsa-miR-34a-5p
 
 
-0.372
 
 
35.4
 
 
 
 
ACTA2
 
 
hsa-miR-27a-3p
 
 
-0.577
 
 
687.9
 
 
 
 
ACVR1
 
 
hsa-miR-152-3p
 
 
-0.397
 
 
12.2
 
 
 
 
ACVR1
 
 
hsa-miR-148b-3p
 
 
-0.397
 
 
12.2
 
 
 
 
ADAM10
 
 
hsa-miR-152-3p
 
 
-0.348
 
 
19.7
 
 
 
 
ADAM10
 
 
hsa-miR-148b-3p
 
 
-0.356
 
 
19.7
 
 
 
 
ADAM17
 
 
hsa-miR-125a-5p
 
 
-0.292
 
 
12.5
 
 
 
 
ADAM17
 
 
hsa-miR-152-3p
 
 
-0.301
 
 
12.5
 
 
 
 
ADAM17
 
 
hsa-miR-148b-3p
 
 
-0.301
 
 
12.5
 
 
 
 
ADAMTS9
 
 
hsa-miR-29a-3p
 
 
-0.305
 
 
13.3
 
 
 
 
ADAMTS9
 
 
hsa-miR-29a-3p
 
 
-0.485
 
 
13.3
 
 
 
 
ADAP1
 
 
hsa-miR-27a-3p
 
 
-0.302
 
 
10.1
 
 
 
 
ADCY3
 
 
hsa-miR-132-3p
 
 
-0.303
 
 
39.8
 
 
 
 
ADGRF5
 
 
hsa-miR-148b-3p
 
 
-0.354
 
 
99.9
 
 
 
 
ADGRF5
 
 
hsa-miR-152-3p
 
 
-0.354
 
 
99.9
 
 
 
 
ADIPOR2
 
 
hsa-miR-150-5p
 
 
-0.287
 
 
42.5
 
 
 
 
ADIPOR2
 
 
hsa-miR-34a-5p
 
 
-0.33
 
 
42.5
 
 
 
 
ADK
 
 
hsa-miR-34a-5p
 
 
-0.374
 
 
23.0
 
 
 
 
AFG3L2
 
 
hsa-miR-181b-5p
 
 
-0.491
 
 
12.4
 
 
 
 
AFG3L2
 
 
hsa-miR-181a-5p
 
 
-0.491
 
 
12.4
 
 
 
 
AGFG1
 
 
hsa-miR-148b-3p
 
 
-0.332
 
 
51.7
 
 
 
 
AGFG1
 
 
hsa-miR-152-3p
 
 
-0.332
 
 
51.7
 
 
 
 
AGO4
 
 
hsa-miR-34a-5p
 
 
-0.306
 
 
10.5
 
 
 
 
AGPAT3
 
 
hsa-miR-27a-3p
 
 
-0.29
 
 
18.0
 
 
 
 
AGTRAP
 
 
hsa-miR-34a-5p
 
 
-0.328
 
 
58.9
 
 
 
 
AHCYL2
 
 
hsa-miR-34a-5p
 
 
-0.359
 
 
62.0
 
 
 
 
AHDC1
 
 
hsa-miR-148b-3p
 
 
-0.413
 
 
11.4
 
 
 
 
AHDC1
 
 
hsa-miR-152-3p
 
 
-0.413
 
 
11.4
 
 
 
 
AHR
 
 
hsa-miR-29a-3p
 
 
-0.316
 
 
35.5
 
 
 
 
AIDA
 
 
hsa-miR-22-3p
 
 
-0.316
 
 
43.6
 
 
 
 
AK1
 
 
hsa-miR-29a-3p
 
 
-0.333
 
 
70.4
 
 
 
 
AKAP1
 
 
hsa-miR-148b-3p
 
 
-0.381
 
 
18.4
 
 
 
 
AKAP1
 
 
hsa-miR-152-3p
 
 
-0.381
 
 
18.4
 
 
 
 
AKIRIN1
 
 
hsa-miR-27a-3p
 
 
-0.368
 
 
28.3
 
 
 
 
AKT3
 
 
hsa-miR-29a-3p
 
 
-0.287
 
 
13.9
 
 
 
 
AKT3
 
 
hsa-miR-22-3p
 
 
-0.451
 
 
13.9
 
 
 
 
ALCAM
 
 
hsa-miR-34a-5p
 
 
-0.303
 
 
69.9
 
 
 
 
ALCAM
 
 
hsa-miR-148b-3p
 
 
-0.339
 
 
69.9
 
 
 
 
ALCAM
 
 
hsa-miR-152-3p
 
 
-0.347
 
 
69.9
 
 
 
 
ALDOA
 
 
hsa-miR-34a-5p
 
 
-0.655
 
 
498.0
 
 
 
 
ALPL
 
 
hsa-miR-10a-5p
 
 
-0.32
 
 
53.5
 
 
 
 
ANAPC16
 
 
hsa-miR-125a-5p
 
 
-0.578
 
 
17.6
 
 
 
 
ANK3
 
 
hsa-miR-34a-5p
 
 
-0.29
 
 
22.3
 
 
 
 
ANK3
 
 
hsa-miR-34a-5p
 
 
-0.347
 
 
22.3
 
 
 
 
ANKHD1
 
 
hsa-miR-23a-3p
 
 
-0.368
 
 
38.2
 
 
 
 
ANKHD1
 
 
hsa-miR-23b-3p
 
 
-0.368
 
 
38.2
 
 
 
 
ANKHD1-EIF4EBP3
 
 
hsa-miR-22-3p
 
 
-0.479
 
 
10.9
 
 
 
 
ANKRD13A
 
 
hsa-miR-22-3p
 
 
-0.478
 
 
25.4
 
 
 
 
ANKRD29
 
 
hsa-miR-132-3p
 
 
-0.47
 
 
29.5
 
 
 
 
ANKRD40
 
 
hsa-miR-27a-3p
 
 
-0.35
 
 
21.2
 
 
 
 
ANP32A
 
 
hsa-miR-132-3p
 
 
-0.374
 
 
66.9
 
 
 
 
ANP32B
 
 
hsa-miR-34a-5p
 
 
-0.29
 
 
116.3
 
 
 
 
ANPEP
 
 
hsa-miR-125a-5p
 
 
-0.372
 
 
31.9
 
 
 
 
ANTXR2
 
 
hsa-miR-29a-3p
 
 
-0.347
 
 
23.3
 
 
 
 
AP3S2
 
 
hsa-miR-34a-5p
 
 
-0.413
 
 
15.3
 
 
 
 
APH1A
 
 
hsa-miR-34a-5p
 
 
-0.304
 
 
78.9
 
 
 
 
AREG
 
 
hsa-miR-34a-5p
 
 
-0.62
 
 
35.6
 
 
 
 
ARF4
 
 
hsa-miR-152-3p
 
 
-0.487
 
 
116.3
 
 
 
 
ARF4
 
 
hsa-miR-148b-3p
 
 
-0.487
 
 
116.3
 
 
 
 
ARF5
 
 
hsa-miR-29a-3p
 
 
-0.491
 
 
100.1
 
 
 
 
ARF6
 
 
hsa-miR-181b-5p
 
 
-0.33
 
 
29.7
 
 
 
 
ARF6
 
 
hsa-miR-181a-5p
 
 
-0.33
 
 
29.7
 
 
 
 
ARFGEF1
 
 
hsa-miR-148b-3p
 
 
-0.306
 
 
13.0
 
 
 
 
ARFGEF1
 
 
hsa-miR-152-3p
 
 
-0.306
 
 
13.0
 
 
 
 
ARFIP1
 
 
hsa-miR-152-3p
 
 
-0.474
 
 
15.8
 
 
 
 
ARFIP1
 
 
hsa-miR-148b-3p
 
 
-0.474
 
 
15.8
 
 
 
 
ARHGAP1
 
 
hsa-miR-34a-5p
 
 
-0.369
 
 
36.0
 
 
 
 
ARHGAP21
 
 
hsa-miR-152-3p
 
 
-0.287
 
 
20.4
 
 
 
 
ARHGAP21
 
 
hsa-miR-148b-3p
 
 
-0.287
 
 
20.4
 
 
 
 
ARHGAP26
 
 
hsa-miR-34a-5p
 
 
-0.384
 
 
19.9
 
 
 
 
ARHGEF26
 
 
hsa-miR-22-3p
 
 
-0.374
 
 
15.4
 
 
 
 
ARID1B
 
 
hsa-miR-29a-3p
 
 
-0.342
 
 
13.4
 
 
 
 
ARL4A
 
 
hsa-miR-29a-3p
 
 
-0.386
 
 
18.9
 
 
 
 
ARL6IP1
 
 
hsa-miR-148b-3p
 
 
-0.579
 
 
75.1
 
 
 
 
ARL6IP1
 
 
hsa-miR-152-3p
 
 
-0.579
 
 
75.1
 
 
 
 
ARL8B
 
 
hsa-miR-152-3p
 
 
-0.337
 
 
41.1
 
 
 
 
ARL8B
 
 
hsa-miR-148b-3p
 
 
-0.337
 
 
41.1
 
 
 
 
ARMCX3
 
 
hsa-miR-425-5p
 
 
-0.325
 
 
26.1
 
 
 
 
ARPC5
 
 
hsa-miR-22-3p
 
 
-0.679
 
 
97.1
 
 
 
 
ARPP19
 
 
hsa-miR-29a-3p
 
 
-0.371
 
 
42.5
 
 
 
 
ARPP19
 
 
hsa-miR-34a-5p
 
 
-0.497
 
 
42.5
 
 
 
 
ARRB1
 
 
hsa-miR-22-3p
 
 
-0.616
 
 
50.0
 
 
 
 
ARRDC3
 
 
hsa-miR-152-3p
 
 
-0.305
 
 
42.0
 
 
 
 
ARRDC3
 
 
hsa-miR-148b-3p
 
 
-0.305
 
 
42.0
 
 
 
 
ARRDC3
 
 
hsa-miR-29a-3p
 
 
-0.402
 
 
42.0
 
 
 
 
ASAH1
 
 
hsa-miR-27a-3p
 
 
-0.398
 
 
179.4
 
 
 
 
 
 
 lymph node 
 
 
 
 
target_symbol
 
 
mature_mirna_id
 
 
score
 
 
nTPM
 
 
 
 
 
 
AAGAB
 
 
hsa-miR-34a-5p
 
 
-0.319
 
 
18.6
 
 
 
 
AAR2
 
 
hsa-miR-29a-3p
 
 
-0.327
 
 
16.4
 
 
 
 
ABCB10
 
 
hsa-miR-152-3p
 
 
-0.291
 
 
10.3
 
 
 
 
ABCB10
 
 
hsa-miR-148b-3p
 
 
-0.291
 
 
10.3
 
 
 
 
ABCB7
 
 
hsa-miR-148b-3p
 
 
-0.515
 
 
11.2
 
 
 
 
ABCB7
 
 
hsa-miR-152-3p
 
 
-0.515
 
 
11.2
 
 
 
 
ABCC5
 
 
hsa-miR-125a-5p
 
 
-0.288
 
 
12.3
 
 
 
 
ABCD3
 
 
hsa-miR-148b-3p
 
 
-0.388
 
 
12.4
 
 
 
 
ABCD3
 
 
hsa-miR-152-3p
 
 
-0.388
 
 
12.4
 
 
 
 
ABCE1
 
 
hsa-miR-29a-3p
 
 
-0.404
 
 
37.8
 
 
 
 
ABHD3
 
 
hsa-miR-125a-5p
 
 
-0.453
 
 
27.9
 
 
 
 
ABRAXAS2
 
 
hsa-miR-34a-5p
 
 
-0.538
 
 
11.7
 
 
 
 
ABTB1
 
 
hsa-miR-125a-5p
 
 
-0.519
 
 
20.7
 
 
 
 
ACBD3
 
 
hsa-miR-34a-5p
 
 
-0.415
 
 
19.9
 
 
 
 
ACSL1
 
 
hsa-miR-34a-5p
 
 
-0.306
 
 
17.6
 
 
 
 
ACSL4
 
 
hsa-miR-34a-5p
 
 
-0.372
 
 
20.7
 
 
 
 
ACTA2
 
 
hsa-miR-27a-3p
 
 
-0.577
 
 
162.6
 
 
 
 
ADA
 
 
hsa-miR-29a-3p
 
 
-0.376
 
 
27.8
 
 
 
 
ADAM10
 
 
hsa-miR-152-3p
 
 
-0.348
 
 
20.8
 
 
 
 
ADAM10
 
 
hsa-miR-148b-3p
 
 
-0.356
 
 
20.8
 
 
 
 
ADCY3
 
 
hsa-miR-132-3p
 
 
-0.303
 
 
17.7
 
 
 
 
ADK
 
 
hsa-miR-34a-5p
 
 
-0.374
 
 
21.3
 
 
 
 
ADRA2A
 
 
hsa-miR-23a-3p
 
 
-0.371
 
 
10.5
 
 
 
 
ADRA2A
 
 
hsa-miR-23b-3p
 
 
-0.371
 
 
10.5
 
 
 
 
AFG3L2
 
 
hsa-miR-181b-5p
 
 
-0.491
 
 
12.0
 
 
 
 
AFG3L2
 
 
hsa-miR-181a-5p
 
 
-0.491
 
 
12.0
 
 
 
 
AGFG1
 
 
hsa-miR-148b-3p
 
 
-0.332
 
 
44.0
 
 
 
 
AGFG1
 
 
hsa-miR-152-3p
 
 
-0.332
 
 
44.0
 
 
 
 
AGGF1
 
 
hsa-miR-125a-5p
 
 
-0.331
 
 
12.7
 
 
 
 
AGPAT3
 
 
hsa-miR-27a-3p
 
 
-0.29
 
 
15.3
 
 
 
 
AGTRAP
 
 
hsa-miR-34a-5p
 
 
-0.328
 
 
21.9
 
 
 
 
AHCYL2
 
 
hsa-miR-34a-5p
 
 
-0.359
 
 
10.0
 
 
 
 
AHR
 
 
hsa-miR-29a-3p
 
 
-0.316
 
 
14.4
 
 
 
 
AIDA
 
 
hsa-miR-22-3p
 
 
-0.316
 
 
47.9
 
 
 
 
AIFM1
 
 
hsa-miR-125a-5p
 
 
-0.3
 
 
15.1
 
 
 
 
AKAP1
 
 
hsa-miR-148b-3p
 
 
-0.381
 
 
10.0
 
 
 
 
AKAP1
 
 
hsa-miR-152-3p
 
 
-0.381
 
 
10.0
 
 
 
 
AKIRIN1
 
 
hsa-miR-27a-3p
 
 
-0.368
 
 
39.5
 
 
 
 
ALDOA
 
 
hsa-miR-34a-5p
 
 
-0.655
 
 
335.0
 
 
 
 
ANAPC16
 
 
hsa-miR-125a-5p
 
 
-0.578
 
 
23.5
 
 
 
 
ANKHD1
 
 
hsa-miR-23a-3p
 
 
-0.368
 
 
38.6
 
 
 
 
ANKHD1
 
 
hsa-miR-23b-3p
 
 
-0.368
 
 
38.6
 
 
 
 
ANKRD13A
 
 
hsa-miR-22-3p
 
 
-0.478
 
 
50.3
 
 
 
 
ANKRD29
 
 
hsa-miR-132-3p
 
 
-0.47
 
 
18.8
 
 
 
 
ANKRD40
 
 
hsa-miR-27a-3p
 
 
-0.35
 
 
15.5
 
 
 
 
ANP32A
 
 
hsa-miR-132-3p
 
 
-0.374
 
 
145.8
 
 
 
 
ANP32B
 
 
hsa-miR-34a-5p
 
 
-0.29
 
 
207.9
 
 
 
 
ANPEP
 
 
hsa-miR-125a-5p
 
 
-0.372
 
 
12.0
 
 
 
 
ANTXR2
 
 
hsa-miR-29a-3p
 
 
-0.347
 
 
15.2
 
 
 
 
AP3S2
 
 
hsa-miR-34a-5p
 
 
-0.413
 
 
12.0
 
 
 
 
APH1A
 
 
hsa-miR-34a-5p
 
 
-0.304
 
 
75.6
 
 
 
 
APOO
 
 
hsa-miR-181b-5p
 
 
-0.5
 
 
10.9
 
 
 
 
APOO
 
 
hsa-miR-181a-5p
 
 
-0.5
 
 
10.9
 
 
 
 
ARF4
 
 
hsa-miR-152-3p
 
 
-0.487
 
 
69.1
 
 
 
 
ARF4
 
 
hsa-miR-148b-3p
 
 
-0.487
 
 
69.1
 
 
 
 
ARF5
 
 
hsa-miR-29a-3p
 
 
-0.491
 
 
96.1
 
 
 
 
ARF6
 
 
hsa-miR-181b-5p
 
 
-0.33
 
 
57.9
 
 
 
 
ARF6
 
 
hsa-miR-181a-5p
 
 
-0.33
 
 
57.9
 
 
 
 
ARFGEF1
 
 
hsa-miR-148b-3p
 
 
-0.306
 
 
14.4
 
 
 
 
ARFGEF1
 
 
hsa-miR-152-3p
 
 
-0.306
 
 
14.4
 
 
 
 
ARFIP1
 
 
hsa-miR-152-3p
 
 
-0.474
 
 
14.1
 
 
 
 
ARFIP1
 
 
hsa-miR-148b-3p
 
 
-0.474
 
 
14.1
 
 
 
 
ARHGAP1
 
 
hsa-miR-34a-5p
 
 
-0.369
 
 
28.7
 
 
 
 
ARID1B
 
 
hsa-miR-29a-3p
 
 
-0.342
 
 
16.8
 
 
 
 
ARL4A
 
 
hsa-miR-29a-3p
 
 
-0.386
 
 
17.4
 
 
 
 
ARL6IP1
 
 
hsa-miR-148b-3p
 
 
-0.579
 
 
124.0
 
 
 
 
ARL6IP1
 
 
hsa-miR-152-3p
 
 
-0.579
 
 
124.0
 
 
 
 
ARL8B
 
 
hsa-miR-152-3p
 
 
-0.337
 
 
36.2
 
 
 
 
ARL8B
 
 
hsa-miR-148b-3p
 
 
-0.337
 
 
36.2
 
 
 
 
ARMCX3
 
 
hsa-miR-425-5p
 
 
-0.325
 
 
20.4
 
 
 
 
ARPC5
 
 
hsa-miR-22-3p
 
 
-0.679
 
 
148.6
 
 
 
 
ARPP19
 
 
hsa-miR-29a-3p
 
 
-0.371
 
 
45.1
 
 
 
 
ARPP19
 
 
hsa-miR-34a-5p
 
 
-0.497
 
 
45.1
 
 
 
 
ARRB1
 
 
hsa-miR-22-3p
 
 
-0.616
 
 
14.3
 
 
 
 
ARRDC3
 
 
hsa-miR-152-3p
 
 
-0.305
 
 
27.5
 
 
 
 
ARRDC3
 
 
hsa-miR-148b-3p
 
 
-0.305
 
 
27.5
 
 
 
 
ARRDC3
 
 
hsa-miR-29a-3p
 
 
-0.402
 
 
27.5
 
 
 
 
ASAH1
 
 
hsa-miR-27a-3p
 
 
-0.398
 
 
61.7
 
 
 
 
ASF1A
 
 
hsa-miR-23a-3p
 
 
-0.506
 
 
14.9
 
 
 
 
ASF1A
 
 
hsa-miR-23b-3p
 
 
-0.506
 
 
14.9
 
 
 
 
ASPH
 
 
hsa-miR-152-3p
 
 
-0.391
 
 
18.8
 
 
 
 
ASPH
 
 
hsa-miR-148b-3p
 
 
-0.391
 
 
18.8
 
 
 
 
ATG4B
 
 
hsa-miR-34a-5p
 
 
-0.58
 
 
12.4
 
 
 
 
ATMIN
 
 
hsa-miR-34a-5p
 
 
-0.409
 
 
13.0
 
 
 
 
ATP2B4
 
 
hsa-miR-152-3p
 
 
-0.337
 
 
12.0
 
 
 
 
ATP2B4
 
 
hsa-miR-148b-3p
 
 
-0.337
 
 
12.0
 
 
 
 
ATP5MC3
 
 
hsa-miR-425-5p
 
 
-0.328
 
 
118.6
 
 
 
 
ATP5MC3
 
 
hsa-miR-29a-3p
 
 
-0.33
 
 
118.6
 
 
 
 
ATP5MC3
 
 
hsa-miR-27a-3p
 
 
-0.416
 
 
118.6
 
 
 
 
ATP6AP2
 
 
hsa-miR-148b-3p
 
 
-0.592
 
 
63.1
 
 
 
 
ATP6AP2
 
 
hsa-miR-152-3p
 
 
-0.592
 
 
63.1
 
 
 
 
ATP6AP2
 
 
hsa-miR-148b-3p
 
 
-0.668
 
 
63.1
 
 
 
 
ATP6AP2
 
 
hsa-miR-152-3p
 
 
-0.668
 
 
63.1
 
 
 
 
ATP6V1E1
 
 
hsa-miR-23a-3p
 
 
-0.294
 
 
82.9
 
 
 
 
ATP6V1E1
 
 
hsa-miR-23b-3p
 
 
-0.294
 
 
82.9
 
 
 
 
ATP8A1
 
 
hsa-miR-22-3p
 
 
-0.468
 
 
14.0
 
 
 
 
ATXN10
 
 
hsa-miR-27a-3p
 
 
-0.295
 
 
45.2
 
 
 
 
AZIN1
 
 
hsa-miR-132-3p
 
 
-0.29
 
 
53.2
 
 
 
 
AZIN1
 
 
hsa-miR-132-3p
 
 
-0.299
 
 
53.2
 
 
 
 
B4GALT3
 
 
hsa-miR-27a-3p
 
 
-0.41
 
 
21.9
 
 
 
 
 
 
 midbrain 
 
 
 
 
target_symbol
 
 
mature_mirna_id
 
 
score
 
 
nTPM
 
 
 
 
 
 
AAGAB
 
 
hsa-miR-34a-5p
 
 
-0.319
 
 
11.5
 
 
 
 
AAR2
 
 
hsa-miR-29a-3p
 
 
-0.327
 
 
11.2
 
 
 
 
ABCC5
 
 
hsa-miR-125a-5p
 
 
-0.288
 
 
27.3
 
 
 
 
ABCD3
 
 
hsa-miR-148b-3p
 
 
-0.388
 
 
17.1
 
 
 
 
ABCD3
 
 
hsa-miR-152-3p
 
 
-0.388
 
 
17.1
 
 
 
 
ABCE1
 
 
hsa-miR-29a-3p
 
 
-0.404
 
 
13.7
 
 
 
 
ABHD17C
 
 
hsa-miR-27a-3p
 
 
-0.435
 
 
11.2
 
 
 
 
ABHD3
 
 
hsa-miR-125a-5p
 
 
-0.453
 
 
12.3
 
 
 
 
ABHD4
 
 
hsa-miR-29a-3p
 
 
-0.312
 
 
18.3
 
 
 
 
ABHD6
 
 
hsa-miR-125a-5p
 
 
-0.501
 
 
21.1
 
 
 
 
ABTB1
 
 
hsa-miR-125a-5p
 
 
-0.519
 
 
18.7
 
 
 
 
ACBD3
 
 
hsa-miR-34a-5p
 
 
-0.415
 
 
10.0
 
 
 
 
ACHE
 
 
hsa-miR-125a-5p
 
 
-0.308
 
 
47.7
 
 
 
 
ACHE
 
 
hsa-miR-125a-5p
 
 
-0.361
 
 
47.7
 
 
 
 
ACSL1
 
 
hsa-miR-34a-5p
 
 
-0.306
 
 
45.6
 
 
 
 
ACSL4
 
 
hsa-miR-34a-5p
 
 
-0.372
 
 
10.6
 
 
 
 
ACSL6
 
 
hsa-miR-125a-5p
 
 
-0.307
 
 
11.1
 
 
 
 
ACTA2
 
 
hsa-miR-27a-3p
 
 
-0.577
 
 
69.3
 
 
 
 
ACTN2
 
 
hsa-miR-23a-3p
 
 
-0.294
 
 
12.2
 
 
 
 
ACTN2
 
 
hsa-miR-29a-3p
 
 
-0.311
 
 
12.2
 
 
 
 
ADAM10
 
 
hsa-miR-152-3p
 
 
-0.348
 
 
11.1
 
 
 
 
ADAM10
 
 
hsa-miR-148b-3p
 
 
-0.356
 
 
11.1
 
 
 
 
ADAP1
 
 
hsa-miR-27a-3p
 
 
-0.302
 
 
85.8
 
 
 
 
ADCY2
 
 
hsa-miR-152-3p
 
 
-0.299
 
 
20.4
 
 
 
 
ADCY2
 
 
hsa-miR-148b-3p
 
 
-0.299
 
 
20.4
 
 
 
 
ADCY3
 
 
hsa-miR-132-3p
 
 
-0.303
 
 
29.4
 
 
 
 
ADGRA3
 
 
hsa-miR-27a-3p
 
 
-0.296
 
 
13.9
 
 
 
 
ADGRF5
 
 
hsa-miR-148b-3p
 
 
-0.354
 
 
13.0
 
 
 
 
ADGRF5
 
 
hsa-miR-152-3p
 
 
-0.354
 
 
13.0
 
 
 
 
ADIPOR2
 
 
hsa-miR-150-5p
 
 
-0.287
 
 
76.2
 
 
 
 
ADIPOR2
 
 
hsa-miR-34a-5p
 
 
-0.33
 
 
76.2
 
 
 
 
ADK
 
 
hsa-miR-34a-5p
 
 
-0.374
 
 
17.6
 
 
 
 
ADO
 
 
hsa-miR-34a-5p
 
 
-0.377
 
 
13.9
 
 
 
 
AGFG1
 
 
hsa-miR-148b-3p
 
 
-0.332
 
 
32.4
 
 
 
 
AGFG1
 
 
hsa-miR-152-3p
 
 
-0.332
 
 
32.4
 
 
 
 
AGGF1
 
 
hsa-miR-125a-5p
 
 
-0.331
 
 
11.3
 
 
 
 
AGPAT3
 
 
hsa-miR-27a-3p
 
 
-0.29
 
 
44.6
 
 
 
 
AGPAT4
 
 
hsa-miR-29a-3p
 
 
-0.376
 
 
22.2
 
 
 
 
AGTRAP
 
 
hsa-miR-34a-5p
 
 
-0.328
 
 
27.0
 
 
 
 
AHCYL2
 
 
hsa-miR-34a-5p
 
 
-0.359
 
 
19.9
 
 
 
 
AIDA
 
 
hsa-miR-22-3p
 
 
-0.316
 
 
32.2
 
 
 
 
AIFM1
 
 
hsa-miR-125a-5p
 
 
-0.3
 
 
12.7
 
 
 
 
AK1
 
 
hsa-miR-29a-3p
 
 
-0.333
 
 
97.2
 
 
 
 
AKAP1
 
 
hsa-miR-148b-3p
 
 
-0.381
 
 
19.0
 
 
 
 
AKAP1
 
 
hsa-miR-152-3p
 
 
-0.381
 
 
19.0
 
 
 
 
AKIRIN1
 
 
hsa-miR-27a-3p
 
 
-0.368
 
 
38.9
 
 
 
 
AKT3
 
 
hsa-miR-29a-3p
 
 
-0.287
 
 
16.3
 
 
 
 
AKT3
 
 
hsa-miR-22-3p
 
 
-0.451
 
 
16.3
 
 
 
 
ALCAM
 
 
hsa-miR-34a-5p
 
 
-0.303
 
 
38.7
 
 
 
 
ALCAM
 
 
hsa-miR-148b-3p
 
 
-0.339
 
 
38.7
 
 
 
 
ALCAM
 
 
hsa-miR-152-3p
 
 
-0.347
 
 
38.7
 
 
 
 
ALDOA
 
 
hsa-miR-34a-5p
 
 
-0.655
 
 
849.0
 
 
 
 
ALPL
 
 
hsa-miR-10a-5p
 
 
-0.32
 
 
11.9
 
 
 
 
AMER2
 
 
hsa-miR-34a-5p
 
 
-0.48
 
 
27.5
 
 
 
 
AMOT
 
 
hsa-miR-29a-3p
 
 
-0.304
 
 
16.8
 
 
 
 
ANK2
 
 
hsa-miR-34a-5p
 
 
-0.317
 
 
14.7
 
 
 
 
ANK3
 
 
hsa-miR-34a-5p
 
 
-0.29
 
 
63.0
 
 
 
 
ANK3
 
 
hsa-miR-34a-5p
 
 
-0.347
 
 
63.0
 
 
 
 
ANKHD1
 
 
hsa-miR-23a-3p
 
 
-0.368
 
 
20.5
 
 
 
 
ANKHD1
 
 
hsa-miR-23b-3p
 
 
-0.368
 
 
20.5
 
 
 
 
ANKRD29
 
 
hsa-miR-132-3p
 
 
-0.47
 
 
11.5
 
 
 
 
ANKRD40
 
 
hsa-miR-27a-3p
 
 
-0.35
 
 
160.4
 
 
 
 
ANP32A
 
 
hsa-miR-132-3p
 
 
-0.374
 
 
54.3
 
 
 
 
ANP32B
 
 
hsa-miR-34a-5p
 
 
-0.29
 
 
216.9
 
 
 
 
AP3S2
 
 
hsa-miR-34a-5p
 
 
-0.413
 
 
15.2
 
 
 
 
APC
 
 
hsa-miR-29a-3p
 
 
-0.413
 
 
21.2
 
 
 
 
APH1A
 
 
hsa-miR-34a-5p
 
 
-0.304
 
 
54.4
 
 
 
 
APOO
 
 
hsa-miR-181b-5p
 
 
-0.5
 
 
44.2
 
 
 
 
APOO
 
 
hsa-miR-181a-5p
 
 
-0.5
 
 
44.2
 
 
 
 
ARF4
 
 
hsa-miR-152-3p
 
 
-0.487
 
 
62.8
 
 
 
 
ARF4
 
 
hsa-miR-148b-3p
 
 
-0.487
 
 
62.8
 
 
 
 
ARF5
 
 
hsa-miR-29a-3p
 
 
-0.491
 
 
120.0
 
 
 
 
ARF6
 
 
hsa-miR-181b-5p
 
 
-0.33
 
 
12.0
 
 
 
 
ARF6
 
 
hsa-miR-181a-5p
 
 
-0.33
 
 
12.0
 
 
 
 
ARHGAP1
 
 
hsa-miR-34a-5p
 
 
-0.369
 
 
34.8
 
 
 
 
ARHGAP21
 
 
hsa-miR-152-3p
 
 
-0.287
 
 
53.0
 
 
 
 
ARHGAP21
 
 
hsa-miR-148b-3p
 
 
-0.287
 
 
53.0
 
 
 
 
ARL6IP1
 
 
hsa-miR-148b-3p
 
 
-0.579
 
 
198.8
 
 
 
 
ARL6IP1
 
 
hsa-miR-152-3p
 
 
-0.579
 
 
198.8
 
 
 
 
ARL8B
 
 
hsa-miR-152-3p
 
 
-0.337
 
 
52.4
 
 
 
 
ARL8B
 
 
hsa-miR-148b-3p
 
 
-0.337
 
 
52.4
 
 
 
 
ARMCX3
 
 
hsa-miR-425-5p
 
 
-0.325
 
 
35.1
 
 
 
 
ARPC5
 
 
hsa-miR-22-3p
 
 
-0.679
 
 
27.1
 
 
 
 
ARPIN-AP3S2
 
 
hsa-miR-34a-5p
 
 
-0.348
 
 
13.5
 
 
 
 
ARPP19
 
 
hsa-miR-29a-3p
 
 
-0.371
 
 
144.2
 
 
 
 
ARPP19
 
 
hsa-miR-34a-5p
 
 
-0.497
 
 
144.2
 
 
 
 
ARRB1
 
 
hsa-miR-22-3p
 
 
-0.616
 
 
27.6
 
 
 
 
ARRDC3
 
 
hsa-miR-152-3p
 
 
-0.305
 
 
13.8
 
 
 
 
ARRDC3
 
 
hsa-miR-148b-3p
 
 
-0.305
 
 
13.8
 
 
 
 
ARRDC3
 
 
hsa-miR-29a-3p
 
 
-0.402
 
 
13.8
 
 
 
 
ASAH1
 
 
hsa-miR-27a-3p
 
 
-0.398
 
 
63.4
 
 
 
 
ASB1
 
 
hsa-miR-34a-5p
 
 
-0.406
 
 
18.2
 
 
 
 
ASF1A
 
 
hsa-miR-23a-3p
 
 
-0.506
 
 
14.8
 
 
 
 
ASF1A
 
 
hsa-miR-23b-3p
 
 
-0.506
 
 
14.8
 
 
 
 
ASPH
 
 
hsa-miR-152-3p
 
 
-0.391
 
 
48.8
 
 
 
 
ASPH
 
 
hsa-miR-148b-3p
 
 
-0.391
 
 
48.8
 
 
 
 
ATCAY
 
 
hsa-miR-10a-5p
 
 
-0.322
 
 
24.7
 
 
 
 
ATG4B
 
 
hsa-miR-34a-5p
 
 
-0.58
 
 
20.8
 
 
 
 
ATMIN
 
 
hsa-miR-34a-5p
 
 
-0.409
 
 
17.9
 
 
 
 
ATP1A2
 
 
hsa-miR-34a-5p
 
 
-0.306
 
 
328.9
 
 
 
 
 
 
 pancreas 
 
 
 
 
target_symbol
 
 
mature_mirna_id
 
 
score
 
 
nTPM
 
 
 
 
 
 
AAGAB
 
 
hsa-miR-34a-5p
 
 
-0.319
 
 
16.0
 
 
 
 
AAR2
 
 
hsa-miR-29a-3p
 
 
-0.327
 
 
17.6
 
 
 
 
ABCC5
 
 
hsa-miR-125a-5p
 
 
-0.288
 
 
12.8
 
 
 
 
ABCD3
 
 
hsa-miR-148b-3p
 
 
-0.388
 
 
27.0
 
 
 
 
ABCD3
 
 
hsa-miR-152-3p
 
 
-0.388
 
 
27.0
 
 
 
 
ABCE1
 
 
hsa-miR-29a-3p
 
 
-0.404
 
 
27.7
 
 
 
 
ABHD3
 
 
hsa-miR-125a-5p
 
 
-0.453
 
 
11.6
 
 
 
 
ABHD4
 
 
hsa-miR-29a-3p
 
 
-0.312
 
 
15.3
 
 
 
 
ABHD5
 
 
hsa-miR-132-3p
 
 
-0.323
 
 
11.4
 
 
 
 
ABHD5
 
 
hsa-miR-27a-3p
 
 
-0.407
 
 
11.4
 
 
 
 
ABTB1
 
 
hsa-miR-125a-5p
 
 
-0.519
 
 
18.1
 
 
 
 
ACBD3
 
 
hsa-miR-34a-5p
 
 
-0.415
 
 
21.4
 
 
 
 
ACSL1
 
 
hsa-miR-34a-5p
 
 
-0.306
 
 
45.3
 
 
 
 
ACSL4
 
 
hsa-miR-34a-5p
 
 
-0.372
 
 
10.9
 
 
 
 
ACTA2
 
 
hsa-miR-27a-3p
 
 
-0.577
 
 
86.3
 
 
 
 
ADAP1
 
 
hsa-miR-27a-3p
 
 
-0.302
 
 
12.7
 
 
 
 
ADCY3
 
 
hsa-miR-132-3p
 
 
-0.303
 
 
15.3
 
 
 
 
ADGRA3
 
 
hsa-miR-27a-3p
 
 
-0.296
 
 
11.8
 
 
 
 
ADIPOR2
 
 
hsa-miR-150-5p
 
 
-0.287
 
 
30.0
 
 
 
 
ADIPOR2
 
 
hsa-miR-34a-5p
 
 
-0.33
 
 
30.0
 
 
 
 
ADK
 
 
hsa-miR-34a-5p
 
 
-0.374
 
 
17.7
 
 
 
 
ADRA2A
 
 
hsa-miR-23a-3p
 
 
-0.371
 
 
28.8
 
 
 
 
ADRA2A
 
 
hsa-miR-23b-3p
 
 
-0.371
 
 
28.8
 
 
 
 
AFG3L2
 
 
hsa-miR-181b-5p
 
 
-0.491
 
 
23.6
 
 
 
 
AFG3L2
 
 
hsa-miR-181a-5p
 
 
-0.491
 
 
23.6
 
 
 
 
AGFG1
 
 
hsa-miR-148b-3p
 
 
-0.332
 
 
33.0
 
 
 
 
AGFG1
 
 
hsa-miR-152-3p
 
 
-0.332
 
 
33.0
 
 
 
 
AGPAT3
 
 
hsa-miR-27a-3p
 
 
-0.29
 
 
20.6
 
 
 
 
AGTRAP
 
 
hsa-miR-34a-5p
 
 
-0.328
 
 
63.1
 
 
 
 
AHCYL2
 
 
hsa-miR-34a-5p
 
 
-0.359
 
 
13.0
 
 
 
 
AIDA
 
 
hsa-miR-22-3p
 
 
-0.316
 
 
30.6
 
 
 
 
AIFM1
 
 
hsa-miR-125a-5p
 
 
-0.3
 
 
20.4
 
 
 
 
AK1
 
 
hsa-miR-29a-3p
 
 
-0.333
 
 
14.0
 
 
 
 
AKAP1
 
 
hsa-miR-148b-3p
 
 
-0.381
 
 
21.5
 
 
 
 
AKAP1
 
 
hsa-miR-152-3p
 
 
-0.381
 
 
21.5
 
 
 
 
AKIRIN1
 
 
hsa-miR-27a-3p
 
 
-0.368
 
 
26.1
 
 
 
 
ALCAM
 
 
hsa-miR-34a-5p
 
 
-0.303
 
 
30.8
 
 
 
 
ALCAM
 
 
hsa-miR-148b-3p
 
 
-0.339
 
 
30.8
 
 
 
 
ALCAM
 
 
hsa-miR-152-3p
 
 
-0.347
 
 
30.8
 
 
 
 
ALDOA
 
 
hsa-miR-34a-5p
 
 
-0.655
 
 
422.3
 
 
 
 
AMMECR1L
 
 
hsa-miR-29a-3p
 
 
-0.42
 
 
10.5
 
 
 
 
ANAPC16
 
 
hsa-miR-125a-5p
 
 
-0.578
 
 
32.2
 
 
 
 
ANK3
 
 
hsa-miR-34a-5p
 
 
-0.29
 
 
10.5
 
 
 
 
ANK3
 
 
hsa-miR-34a-5p
 
 
-0.347
 
 
10.5
 
 
 
 
ANKHD1
 
 
hsa-miR-23a-3p
 
 
-0.368
 
 
57.6
 
 
 
 
ANKHD1
 
 
hsa-miR-23b-3p
 
 
-0.368
 
 
57.6
 
 
 
 
ANKHD1-EIF4EBP3
 
 
hsa-miR-22-3p
 
 
-0.479
 
 
16.1
 
 
 
 
ANKRD13A
 
 
hsa-miR-22-3p
 
 
-0.478
 
 
11.6
 
 
 
 
ANKRD40
 
 
hsa-miR-27a-3p
 
 
-0.35
 
 
18.4
 
 
 
 
ANP32A
 
 
hsa-miR-132-3p
 
 
-0.374
 
 
50.6
 
 
 
 
ANP32B
 
 
hsa-miR-34a-5p
 
 
-0.29
 
 
147.1
 
 
 
 
ANPEP
 
 
hsa-miR-125a-5p
 
 
-0.372
 
 
643.6
 
 
 
 
AP3S2
 
 
hsa-miR-34a-5p
 
 
-0.413
 
 
17.9
 
 
 
 
APH1A
 
 
hsa-miR-34a-5p
 
 
-0.304
 
 
87.5
 
 
 
 
APOO
 
 
hsa-miR-181b-5p
 
 
-0.5
 
 
13.3
 
 
 
 
APOO
 
 
hsa-miR-181a-5p
 
 
-0.5
 
 
13.3
 
 
 
 
APPBP2
 
 
hsa-miR-27a-3p
 
 
-0.378
 
 
10.8
 
 
 
 
ARF4
 
 
hsa-miR-152-3p
 
 
-0.487
 
 
138.3
 
 
 
 
ARF4
 
 
hsa-miR-148b-3p
 
 
-0.487
 
 
138.3
 
 
 
 
ARF5
 
 
hsa-miR-29a-3p
 
 
-0.491
 
 
181.7
 
 
 
 
ARF6
 
 
hsa-miR-181b-5p
 
 
-0.33
 
 
23.2
 
 
 
 
ARF6
 
 
hsa-miR-181a-5p
 
 
-0.33
 
 
23.2
 
 
 
 
ARFGEF1
 
 
hsa-miR-148b-3p
 
 
-0.306
 
 
16.5
 
 
 
 
ARFGEF1
 
 
hsa-miR-152-3p
 
 
-0.306
 
 
16.5
 
 
 
 
ARHGAP1
 
 
hsa-miR-34a-5p
 
 
-0.369
 
 
34.7
 
 
 
 
ARHGAP21
 
 
hsa-miR-152-3p
 
 
-0.287
 
 
13.3
 
 
 
 
ARHGAP21
 
 
hsa-miR-148b-3p
 
 
-0.287
 
 
13.3
 
 
 
 
ARID1B
 
 
hsa-miR-29a-3p
 
 
-0.342
 
 
15.2
 
 
 
 
ARL6IP1
 
 
hsa-miR-148b-3p
 
 
-0.579
 
 
77.1
 
 
 
 
ARL6IP1
 
 
hsa-miR-152-3p
 
 
-0.579
 
 
77.1
 
 
 
 
ARL8B
 
 
hsa-miR-152-3p
 
 
-0.337
 
 
26.7
 
 
 
 
ARL8B
 
 
hsa-miR-148b-3p
 
 
-0.337
 
 
26.7
 
 
 
 
ARMCX3
 
 
hsa-miR-425-5p
 
 
-0.325
 
 
50.5
 
 
 
 
ARPC5
 
 
hsa-miR-22-3p
 
 
-0.679
 
 
48.7
 
 
 
 
ARPIN-AP3S2
 
 
hsa-miR-34a-5p
 
 
-0.348
 
 
19.2
 
 
 
 
ARPP19
 
 
hsa-miR-29a-3p
 
 
-0.371
 
 
43.6
 
 
 
 
ARPP19
 
 
hsa-miR-34a-5p
 
 
-0.497
 
 
43.6
 
 
 
 
ARRB1
 
 
hsa-miR-22-3p
 
 
-0.616
 
 
23.2
 
 
 
 
ARRDC3
 
 
hsa-miR-152-3p
 
 
-0.305
 
 
19.3
 
 
 
 
ARRDC3
 
 
hsa-miR-148b-3p
 
 
-0.305
 
 
19.3
 
 
 
 
ARRDC3
 
 
hsa-miR-29a-3p
 
 
-0.402
 
 
19.3
 
 
 
 
ASAH1
 
 
hsa-miR-27a-3p
 
 
-0.398
 
 
46.8
 
 
 
 
ASPH
 
 
hsa-miR-152-3p
 
 
-0.391
 
 
42.1
 
 
 
 
ASPH
 
 
hsa-miR-148b-3p
 
 
-0.391
 
 
42.1
 
 
 
 
ATG4B
 
 
hsa-miR-34a-5p
 
 
-0.58
 
 
18.2
 
 
 
 
ATMIN
 
 
hsa-miR-34a-5p
 
 
-0.409
 
 
15.2
 
 
 
 
ATP5MC3
 
 
hsa-miR-425-5p
 
 
-0.328
 
 
115.2
 
 
 
 
ATP5MC3
 
 
hsa-miR-29a-3p
 
 
-0.33
 
 
115.2
 
 
 
 
ATP5MC3
 
 
hsa-miR-27a-3p
 
 
-0.416
 
 
115.2
 
 
 
 
ATP6AP2
 
 
hsa-miR-148b-3p
 
 
-0.592
 
 
50.2
 
 
 
 
ATP6AP2
 
 
hsa-miR-152-3p
 
 
-0.592
 
 
50.2
 
 
 
 
ATP6AP2
 
 
hsa-miR-148b-3p
 
 
-0.668
 
 
50.2
 
 
 
 
ATP6AP2
 
 
hsa-miR-152-3p
 
 
-0.668
 
 
50.2
 
 
 
 
ATP6V1E1
 
 
hsa-miR-23a-3p
 
 
-0.294
 
 
59.3
 
 
 
 
ATP6V1E1
 
 
hsa-miR-23b-3p
 
 
-0.294
 
 
59.3
 
 
 
 
ATXN10
 
 
hsa-miR-27a-3p
 
 
-0.295
 
 
54.1
 
 
 
 
ATXN2
 
 
hsa-miR-10a-5p
 
 
-0.362
 
 
13.3
 
 
 
 
AUH
 
 
hsa-miR-23a-3p
 
 
-0.495
 
 
24.0
 
 
 
 
AUH
 
 
hsa-miR-23b-3p
 
 
-0.503
 
 
24.0
 
 
 
 
AUH
 
 
hsa-miR-23a-3p
 
 
-0.512
 
 
24.0
 
 
 
 
 
 
 parathyroid gland 
 
 
 
 
target_symbol
 
 
mature_mirna_id
 
 
score
 
 
nTPM
 
 
 
 
 
 
AAGAB
 
 
hsa-miR-34a-5p
 
 
-0.319
 
 
33.0
 
 
 
 
AAR2
 
 
hsa-miR-29a-3p
 
 
-0.327
 
 
23.9
 
 
 
 
ABCB7
 
 
hsa-miR-148b-3p
 
 
-0.515
 
 
10.3
 
 
 
 
ABCB7
 
 
hsa-miR-152-3p
 
 
-0.515
 
 
10.3
 
 
 
 
ABCC5
 
 
hsa-miR-125a-5p
 
 
-0.288
 
 
22.6
 
 
 
 
ABCD3
 
 
hsa-miR-148b-3p
 
 
-0.388
 
 
27.9
 
 
 
 
ABCD3
 
 
hsa-miR-152-3p
 
 
-0.388
 
 
27.9
 
 
 
 
ABCE1
 
 
hsa-miR-29a-3p
 
 
-0.404
 
 
25.3
 
 
 
 
ABHD3
 
 
hsa-miR-125a-5p
 
 
-0.453
 
 
15.0
 
 
 
 
ABHD4
 
 
hsa-miR-29a-3p
 
 
-0.312
 
 
23.4
 
 
 
 
ABHD5
 
 
hsa-miR-132-3p
 
 
-0.323
 
 
11.5
 
 
 
 
ABHD5
 
 
hsa-miR-27a-3p
 
 
-0.407
 
 
11.5
 
 
 
 
ABRAXAS2
 
 
hsa-miR-34a-5p
 
 
-0.538
 
 
12.0
 
 
 
 
ACBD3
 
 
hsa-miR-34a-5p
 
 
-0.415
 
 
26.3
 
 
 
 
ACSL1
 
 
hsa-miR-34a-5p
 
 
-0.306
 
 
48.9
 
 
 
 
ACSL4
 
 
hsa-miR-34a-5p
 
 
-0.372
 
 
18.4
 
 
 
 
ACSL6
 
 
hsa-miR-125a-5p
 
 
-0.307
 
 
10.6
 
 
 
 
ACTA2
 
 
hsa-miR-27a-3p
 
 
-0.577
 
 
75.8
 
 
 
 
ADCY3
 
 
hsa-miR-132-3p
 
 
-0.303
 
 
16.0
 
 
 
 
ADGRA3
 
 
hsa-miR-27a-3p
 
 
-0.296
 
 
11.5
 
 
 
 
ADGRF5
 
 
hsa-miR-148b-3p
 
 
-0.354
 
 
12.6
 
 
 
 
ADGRF5
 
 
hsa-miR-152-3p
 
 
-0.354
 
 
12.6
 
 
 
 
ADIPOR2
 
 
hsa-miR-150-5p
 
 
-0.287
 
 
15.9
 
 
 
 
ADIPOR2
 
 
hsa-miR-34a-5p
 
 
-0.33
 
 
15.9
 
 
 
 
ADK
 
 
hsa-miR-34a-5p
 
 
-0.374
 
 
41.5
 
 
 
 
ADO
 
 
hsa-miR-34a-5p
 
 
-0.377
 
 
11.6
 
 
 
 
AFG3L2
 
 
hsa-miR-181b-5p
 
 
-0.491
 
 
41.3
 
 
 
 
AFG3L2
 
 
hsa-miR-181a-5p
 
 
-0.491
 
 
41.3
 
 
 
 
AGFG1
 
 
hsa-miR-148b-3p
 
 
-0.332
 
 
39.0
 
 
 
 
AGFG1
 
 
hsa-miR-152-3p
 
 
-0.332
 
 
39.0
 
 
 
 
AGGF1
 
 
hsa-miR-125a-5p
 
 
-0.331
 
 
15.0
 
 
 
 
AGPAT3
 
 
hsa-miR-27a-3p
 
 
-0.29
 
 
23.1
 
 
 
 
AGTRAP
 
 
hsa-miR-34a-5p
 
 
-0.328
 
 
24.1
 
 
 
 
AHCYL2
 
 
hsa-miR-34a-5p
 
 
-0.359
 
 
18.0
 
 
 
 
AIDA
 
 
hsa-miR-22-3p
 
 
-0.316
 
 
30.5
 
 
 
 
AIFM1
 
 
hsa-miR-125a-5p
 
 
-0.3
 
 
27.9
 
 
 
 
AK1
 
 
hsa-miR-29a-3p
 
 
-0.333
 
 
44.1
 
 
 
 
AKAP1
 
 
hsa-miR-148b-3p
 
 
-0.381
 
 
45.1
 
 
 
 
AKAP1
 
 
hsa-miR-152-3p
 
 
-0.381
 
 
45.1
 
 
 
 
AKIRIN1
 
 
hsa-miR-27a-3p
 
 
-0.368
 
 
40.5
 
 
 
 
ALCAM
 
 
hsa-miR-34a-5p
 
 
-0.303
 
 
412.8
 
 
 
 
ALCAM
 
 
hsa-miR-148b-3p
 
 
-0.339
 
 
412.8
 
 
 
 
ALCAM
 
 
hsa-miR-152-3p
 
 
-0.347
 
 
412.8
 
 
 
 
ALDH1A2
 
 
hsa-miR-23b-3p
 
 
-0.346
 
 
28.0
 
 
 
 
ALDH1A2
 
 
hsa-miR-23a-3p
 
 
-0.354
 
 
28.0
 
 
 
 
ALDOA
 
 
hsa-miR-34a-5p
 
 
-0.655
 
 
885.7
 
 
 
 
AMOT
 
 
hsa-miR-29a-3p
 
 
-0.304
 
 
13.8
 
 
 
 
ANAPC16
 
 
hsa-miR-125a-5p
 
 
-0.578
 
 
30.6
 
 
 
 
ANK3
 
 
hsa-miR-34a-5p
 
 
-0.29
 
 
121.9
 
 
 
 
ANK3
 
 
hsa-miR-34a-5p
 
 
-0.347
 
 
121.9
 
 
 
 
ANKHD1
 
 
hsa-miR-23a-3p
 
 
-0.368
 
 
35.6
 
 
 
 
ANKHD1
 
 
hsa-miR-23b-3p
 
 
-0.368
 
 
35.6
 
 
 
 
ANKHD1-EIF4EBP3
 
 
hsa-miR-22-3p
 
 
-0.479
 
 
19.9
 
 
 
 
ANKRD13A
 
 
hsa-miR-22-3p
 
 
-0.478
 
 
16.3
 
 
 
 
ANKRD29
 
 
hsa-miR-132-3p
 
 
-0.47
 
 
23.3
 
 
 
 
ANKRD40
 
 
hsa-miR-27a-3p
 
 
-0.35
 
 
25.3
 
 
 
 
ANP32A
 
 
hsa-miR-132-3p
 
 
-0.374
 
 
57.6
 
 
 
 
ANP32B
 
 
hsa-miR-34a-5p
 
 
-0.29
 
 
64.6
 
 
 
 
ANTXR2
 
 
hsa-miR-29a-3p
 
 
-0.347
 
 
26.2
 
 
 
 
AP3S2
 
 
hsa-miR-34a-5p
 
 
-0.413
 
 
30.5
 
 
 
 
APH1A
 
 
hsa-miR-34a-5p
 
 
-0.304
 
 
103.7
 
 
 
 
APOO
 
 
hsa-miR-181b-5p
 
 
-0.5
 
 
58.1
 
 
 
 
APOO
 
 
hsa-miR-181a-5p
 
 
-0.5
 
 
58.1
 
 
 
 
APPBP2
 
 
hsa-miR-27a-3p
 
 
-0.378
 
 
13.6
 
 
 
 
ARF4
 
 
hsa-miR-152-3p
 
 
-0.487
 
 
95.4
 
 
 
 
ARF4
 
 
hsa-miR-148b-3p
 
 
-0.487
 
 
95.4
 
 
 
 
ARF5
 
 
hsa-miR-29a-3p
 
 
-0.491
 
 
97.9
 
 
 
 
ARF6
 
 
hsa-miR-181b-5p
 
 
-0.33
 
 
19.3
 
 
 
 
ARF6
 
 
hsa-miR-181a-5p
 
 
-0.33
 
 
19.3
 
 
 
 
ARFGEF1
 
 
hsa-miR-148b-3p
 
 
-0.306
 
 
26.3
 
 
 
 
ARFGEF1
 
 
hsa-miR-152-3p
 
 
-0.306
 
 
26.3
 
 
 
 
ARFIP1
 
 
hsa-miR-152-3p
 
 
-0.474
 
 
21.0
 
 
 
 
ARFIP1
 
 
hsa-miR-148b-3p
 
 
-0.474
 
 
21.0
 
 
 
 
ARHGAP1
 
 
hsa-miR-34a-5p
 
 
-0.369
 
 
23.3
 
 
 
 
ARHGAP21
 
 
hsa-miR-152-3p
 
 
-0.287
 
 
13.6
 
 
 
 
ARHGAP21
 
 
hsa-miR-148b-3p
 
 
-0.287
 
 
13.6
 
 
 
 
ARID1B
 
 
hsa-miR-29a-3p
 
 
-0.342
 
 
25.3
 
 
 
 
ARID2
 
 
hsa-miR-132-3p
 
 
-0.304
 
 
11.0
 
 
 
 
ARL6IP1
 
 
hsa-miR-148b-3p
 
 
-0.579
 
 
169.8
 
 
 
 
ARL6IP1
 
 
hsa-miR-152-3p
 
 
-0.579
 
 
169.8
 
 
 
 
ARL8B
 
 
hsa-miR-152-3p
 
 
-0.337
 
 
45.2
 
 
 
 
ARL8B
 
 
hsa-miR-148b-3p
 
 
-0.337
 
 
45.2
 
 
 
 
ARMCX3
 
 
hsa-miR-425-5p
 
 
-0.325
 
 
97.4
 
 
 
 
ARPC5
 
 
hsa-miR-22-3p
 
 
-0.679
 
 
28.1
 
 
 
 
ARPIN-AP3S2
 
 
hsa-miR-34a-5p
 
 
-0.348
 
 
10.2
 
 
 
 
ARPP19
 
 
hsa-miR-29a-3p
 
 
-0.371
 
 
102.5
 
 
 
 
ARPP19
 
 
hsa-miR-34a-5p
 
 
-0.497
 
 
102.5
 
 
 
 
ARRDC3
 
 
hsa-miR-152-3p
 
 
-0.305
 
 
51.0
 
 
 
 
ARRDC3
 
 
hsa-miR-148b-3p
 
 
-0.305
 
 
51.0
 
 
 
 
ARRDC3
 
 
hsa-miR-29a-3p
 
 
-0.402
 
 
51.0
 
 
 
 
ASAH1
 
 
hsa-miR-27a-3p
 
 
-0.398
 
 
110.6
 
 
 
 
ASF1A
 
 
hsa-miR-23a-3p
 
 
-0.506
 
 
14.9
 
 
 
 
ASF1A
 
 
hsa-miR-23b-3p
 
 
-0.506
 
 
14.9
 
 
 
 
ASPH
 
 
hsa-miR-152-3p
 
 
-0.391
 
 
35.7
 
 
 
 
ASPH
 
 
hsa-miR-148b-3p
 
 
-0.391
 
 
35.7
 
 
 
 
ATG4B
 
 
hsa-miR-34a-5p
 
 
-0.58
 
 
11.3
 
 
 
 
ATMIN
 
 
hsa-miR-34a-5p
 
 
-0.409
 
 
25.4
 
 
 
 
ATP2B4
 
 
hsa-miR-152-3p
 
 
-0.337
 
 
11.1
 
 
 
 
ATP2B4
 
 
hsa-miR-148b-3p
 
 
-0.337
 
 
11.1
 
 
 
 
ATP5MC3
 
 
hsa-miR-425-5p
 
 
-0.328
 
 
515.1
 
 
 
 
 
 
 pituitary gland 
 
 
 
 
target_symbol
 
 
mature_mirna_id
 
 
score
 
 
nTPM
 
 
 
 
 
 
AAGAB
 
 
hsa-miR-34a-5p
 
 
-0.319
 
 
15.6
 
 
 
 
ABCC5
 
 
hsa-miR-125a-5p
 
 
-0.288
 
 
41.4
 
 
 
 
ABCD3
 
 
hsa-miR-148b-3p
 
 
-0.388
 
 
12.4
 
 
 
 
ABCD3
 
 
hsa-miR-152-3p
 
 
-0.388
 
 
12.4
 
 
 
 
ABHD3
 
 
hsa-miR-125a-5p
 
 
-0.453
 
 
10.7
 
 
 
 
ABHD4
 
 
hsa-miR-29a-3p
 
 
-0.312
 
 
13.7
 
 
 
 
ABHD6
 
 
hsa-miR-125a-5p
 
 
-0.501
 
 
17.2
 
 
 
 
ABTB1
 
 
hsa-miR-125a-5p
 
 
-0.519
 
 
33.0
 
 
 
 
ACBD3
 
 
hsa-miR-34a-5p
 
 
-0.415
 
 
13.6
 
 
 
 
ACSL1
 
 
hsa-miR-34a-5p
 
 
-0.306
 
 
39.0
 
 
 
 
ACTA2
 
 
hsa-miR-27a-3p
 
 
-0.577
 
 
56.3
 
 
 
 
ADCY3
 
 
hsa-miR-132-3p
 
 
-0.303
 
 
41.4
 
 
 
 
ADGRB3
 
 
hsa-miR-152-3p
 
 
-0.516
 
 
21.3
 
 
 
 
ADGRB3
 
 
hsa-miR-148b-3p
 
 
-0.524
 
 
21.3
 
 
 
 
ADIPOR2
 
 
hsa-miR-150-5p
 
 
-0.287
 
 
25.4
 
 
 
 
ADIPOR2
 
 
hsa-miR-34a-5p
 
 
-0.33
 
 
25.4
 
 
 
 
ADK
 
 
hsa-miR-34a-5p
 
 
-0.374
 
 
14.1
 
 
 
 
AGFG1
 
 
hsa-miR-148b-3p
 
 
-0.332
 
 
16.7
 
 
 
 
AGFG1
 
 
hsa-miR-152-3p
 
 
-0.332
 
 
16.7
 
 
 
 
AGPAT3
 
 
hsa-miR-27a-3p
 
 
-0.29
 
 
13.9
 
 
 
 
AGPAT4
 
 
hsa-miR-29a-3p
 
 
-0.376
 
 
10.5
 
 
 
 
AGTRAP
 
 
hsa-miR-34a-5p
 
 
-0.328
 
 
29.7
 
 
 
 
AHCYL2
 
 
hsa-miR-34a-5p
 
 
-0.359
 
 
19.9
 
 
 
 
AHDC1
 
 
hsa-miR-148b-3p
 
 
-0.413
 
 
18.1
 
 
 
 
AHDC1
 
 
hsa-miR-152-3p
 
 
-0.413
 
 
18.1
 
 
 
 
AIDA
 
 
hsa-miR-22-3p
 
 
-0.316
 
 
20.6
 
 
 
 
AIFM1
 
 
hsa-miR-125a-5p
 
 
-0.3
 
 
11.0
 
 
 
 
AK1
 
 
hsa-miR-29a-3p
 
 
-0.333
 
 
40.7
 
 
 
 
AKAP1
 
 
hsa-miR-148b-3p
 
 
-0.381
 
 
16.5
 
 
 
 
AKAP1
 
 
hsa-miR-152-3p
 
 
-0.381
 
 
16.5
 
 
 
 
AKIRIN1
 
 
hsa-miR-27a-3p
 
 
-0.368
 
 
37.8
 
 
 
 
AKT3
 
 
hsa-miR-29a-3p
 
 
-0.287
 
 
11.4
 
 
 
 
AKT3
 
 
hsa-miR-22-3p
 
 
-0.451
 
 
11.4
 
 
 
 
ALCAM
 
 
hsa-miR-34a-5p
 
 
-0.303
 
 
17.9
 
 
 
 
ALCAM
 
 
hsa-miR-148b-3p
 
 
-0.339
 
 
17.9
 
 
 
 
ALCAM
 
 
hsa-miR-152-3p
 
 
-0.347
 
 
17.9
 
 
 
 
ALDH1A2
 
 
hsa-miR-23b-3p
 
 
-0.346
 
 
14.4
 
 
 
 
ALDH1A2
 
 
hsa-miR-23a-3p
 
 
-0.354
 
 
14.4
 
 
 
 
ALDOA
 
 
hsa-miR-34a-5p
 
 
-0.655
 
 
586.0
 
 
 
 
ANAPC16
 
 
hsa-miR-125a-5p
 
 
-0.578
 
 
16.0
 
 
 
 
ANK3
 
 
hsa-miR-34a-5p
 
 
-0.29
 
 
13.8
 
 
 
 
ANK3
 
 
hsa-miR-34a-5p
 
 
-0.347
 
 
13.8
 
 
 
 
ANKHD1
 
 
hsa-miR-23a-3p
 
 
-0.368
 
 
41.2
 
 
 
 
ANKHD1
 
 
hsa-miR-23b-3p
 
 
-0.368
 
 
41.2
 
 
 
 
ANKHD1-EIF4EBP3
 
 
hsa-miR-22-3p
 
 
-0.479
 
 
10.0
 
 
 
 
ANKRD40
 
 
hsa-miR-27a-3p
 
 
-0.35
 
 
10.1
 
 
 
 
ANP32A
 
 
hsa-miR-132-3p
 
 
-0.374
 
 
26.1
 
 
 
 
ANP32B
 
 
hsa-miR-34a-5p
 
 
-0.29
 
 
64.1
 
 
 
 
APH1A
 
 
hsa-miR-34a-5p
 
 
-0.304
 
 
54.3
 
 
 
 
APOO
 
 
hsa-miR-181b-5p
 
 
-0.5
 
 
28.1
 
 
 
 
APOO
 
 
hsa-miR-181a-5p
 
 
-0.5
 
 
28.1
 
 
 
 
ARF4
 
 
hsa-miR-152-3p
 
 
-0.487
 
 
93.4
 
 
 
 
ARF4
 
 
hsa-miR-148b-3p
 
 
-0.487
 
 
93.4
 
 
 
 
ARF5
 
 
hsa-miR-29a-3p
 
 
-0.491
 
 
111.9
 
 
 
 
ARHGAP1
 
 
hsa-miR-34a-5p
 
 
-0.369
 
 
35.2
 
 
 
 
ARHGAP21
 
 
hsa-miR-152-3p
 
 
-0.287
 
 
11.3
 
 
 
 
ARHGAP21
 
 
hsa-miR-148b-3p
 
 
-0.287
 
 
11.3
 
 
 
 
ARHGAP26
 
 
hsa-miR-34a-5p
 
 
-0.384
 
 
11.6
 
 
 
 
ARHGAP36
 
 
hsa-miR-34a-5p
 
 
-0.367
 
 
166.5
 
 
 
 
ARL6IP1
 
 
hsa-miR-148b-3p
 
 
-0.579
 
 
39.7
 
 
 
 
ARL6IP1
 
 
hsa-miR-152-3p
 
 
-0.579
 
 
39.7
 
 
 
 
ARL8B
 
 
hsa-miR-152-3p
 
 
-0.337
 
 
25.6
 
 
 
 
ARL8B
 
 
hsa-miR-148b-3p
 
 
-0.337
 
 
25.6
 
 
 
 
ARMCX3
 
 
hsa-miR-425-5p
 
 
-0.325
 
 
43.1
 
 
 
 
ARPC5
 
 
hsa-miR-22-3p
 
 
-0.679
 
 
18.4
 
 
 
 
ARPP19
 
 
hsa-miR-29a-3p
 
 
-0.371
 
 
33.0
 
 
 
 
ARPP19
 
 
hsa-miR-34a-5p
 
 
-0.497
 
 
33.0
 
 
 
 
ARRDC3
 
 
hsa-miR-152-3p
 
 
-0.305
 
 
20.2
 
 
 
 
ARRDC3
 
 
hsa-miR-148b-3p
 
 
-0.305
 
 
20.2
 
 
 
 
ARRDC3
 
 
hsa-miR-29a-3p
 
 
-0.402
 
 
20.2
 
 
 
 
ASAH1
 
 
hsa-miR-27a-3p
 
 
-0.398
 
 
75.8
 
 
 
 
ASPH
 
 
hsa-miR-152-3p
 
 
-0.391
 
 
33.8
 
 
 
 
ASPH
 
 
hsa-miR-148b-3p
 
 
-0.391
 
 
33.8
 
 
 
 
ATCAY
 
 
hsa-miR-10a-5p
 
 
-0.322
 
 
24.7
 
 
 
 
ATG4B
 
 
hsa-miR-34a-5p
 
 
-0.58
 
 
23.4
 
 
 
 
ATMIN
 
 
hsa-miR-34a-5p
 
 
-0.409
 
 
12.7
 
 
 
 
ATP1A2
 
 
hsa-miR-34a-5p
 
 
-0.306
 
 
29.2
 
 
 
 
ATP5MC3
 
 
hsa-miR-425-5p
 
 
-0.328
 
 
134.5
 
 
 
 
ATP5MC3
 
 
hsa-miR-29a-3p
 
 
-0.33
 
 
134.5
 
 
 
 
ATP5MC3
 
 
hsa-miR-27a-3p
 
 
-0.416
 
 
134.5
 
 
 
 
ATP6AP2
 
 
hsa-miR-148b-3p
 
 
-0.592
 
 
34.0
 
 
 
 
ATP6AP2
 
 
hsa-miR-152-3p
 
 
-0.592
 
 
34.0
 
 
 
 
ATP6AP2
 
 
hsa-miR-148b-3p
 
 
-0.668
 
 
34.0
 
 
 
 
ATP6AP2
 
 
hsa-miR-152-3p
 
 
-0.668
 
 
34.0
 
 
 
 
ATP6V1E1
 
 
hsa-miR-23a-3p
 
 
-0.294
 
 
116.0
 
 
 
 
ATP6V1E1
 
 
hsa-miR-23b-3p
 
 
-0.294
 
 
116.0
 
 
 
 
ATXN10
 
 
hsa-miR-27a-3p
 
 
-0.295
 
 
51.5
 
 
 
 
ATXN2
 
 
hsa-miR-10a-5p
 
 
-0.362
 
 
21.6
 
 
 
 
AZIN1
 
 
hsa-miR-132-3p
 
 
-0.29
 
 
27.1
 
 
 
 
AZIN1
 
 
hsa-miR-132-3p
 
 
-0.299
 
 
27.1
 
 
 
 
B4GALT2
 
 
hsa-miR-34a-5p
 
 
-0.314
 
 
20.4
 
 
 
 
B4GALT3
 
 
hsa-miR-27a-3p
 
 
-0.41
 
 
31.7
 
 
 
 
B4GALT5
 
 
hsa-miR-148b-3p
 
 
-0.44
 
 
11.9
 
 
 
 
B4GALT5
 
 
hsa-miR-152-3p
 
 
-0.44
 
 
11.9
 
 
 
 
B4GAT1
 
 
hsa-miR-23a-3p
 
 
-0.361
 
 
60.5
 
 
 
 
B4GAT1
 
 
hsa-miR-23b-3p
 
 
-0.361
 
 
60.5
 
 
 
 
BAG5
 
 
hsa-miR-22-3p
 
 
-0.318
 
 
10.5
 
 
 
 
BAK1
 
 
hsa-miR-125a-5p
 
 
-0.402
 
 
11.6
 
 
 
 
BAK1
 
 
hsa-miR-29a-3p
 
 
-0.409
 
 
11.6
 
 
 
 
BAZ1B
 
 
hsa-miR-10a-5p
 
 
-0.309
 
 
18.2
 
 
 
 
 
 
 placenta 
 
 
 
 
target_symbol
 
 
mature_mirna_id
 
 
score
 
 
nTPM
 
 
 
 
 
 
AAGAB
 
 
hsa-miR-34a-5p
 
 
-0.319
 
 
14.8
 
 
 
 
AAR2
 
 
hsa-miR-29a-3p
 
 
-0.327
 
 
16.5
 
 
 
 
ABCB10
 
 
hsa-miR-152-3p
 
 
-0.291
 
 
10.6
 
 
 
 
ABCB10
 
 
hsa-miR-148b-3p
 
 
-0.291
 
 
10.6
 
 
 
 
ABCC5
 
 
hsa-miR-125a-5p
 
 
-0.288
 
 
16.1
 
 
 
 
ABCD3
 
 
hsa-miR-148b-3p
 
 
-0.388
 
 
13.6
 
 
 
 
ABCD3
 
 
hsa-miR-152-3p
 
 
-0.388
 
 
13.6
 
 
 
 
ABCE1
 
 
hsa-miR-29a-3p
 
 
-0.404
 
 
26.9
 
 
 
 
ABHD3
 
 
hsa-miR-125a-5p
 
 
-0.453
 
 
18.6
 
 
 
 
ABHD4
 
 
hsa-miR-29a-3p
 
 
-0.312
 
 
18.5
 
 
 
 
ABTB1
 
 
hsa-miR-125a-5p
 
 
-0.519
 
 
11.5
 
 
 
 
ACBD3
 
 
hsa-miR-34a-5p
 
 
-0.415
 
 
29.8
 
 
 
 
ACSL1
 
 
hsa-miR-34a-5p
 
 
-0.306
 
 
25.4
 
 
 
 
ACSL4
 
 
hsa-miR-34a-5p
 
 
-0.372
 
 
19.4
 
 
 
 
ACTA2
 
 
hsa-miR-27a-3p
 
 
-0.577
 
 
723.7
 
 
 
 
ACVR1
 
 
hsa-miR-152-3p
 
 
-0.397
 
 
12.5
 
 
 
 
ACVR1
 
 
hsa-miR-148b-3p
 
 
-0.397
 
 
12.5
 
 
 
 
ADAM10
 
 
hsa-miR-152-3p
 
 
-0.348
 
 
27.1
 
 
 
 
ADAM10
 
 
hsa-miR-148b-3p
 
 
-0.356
 
 
27.1
 
 
 
 
ADAM17
 
 
hsa-miR-125a-5p
 
 
-0.292
 
 
17.9
 
 
 
 
ADAM17
 
 
hsa-miR-152-3p
 
 
-0.301
 
 
17.9
 
 
 
 
ADAM17
 
 
hsa-miR-148b-3p
 
 
-0.301
 
 
17.9
 
 
 
 
ADAMTS9
 
 
hsa-miR-29a-3p
 
 
-0.305
 
 
29.8
 
 
 
 
ADAMTS9
 
 
hsa-miR-29a-3p
 
 
-0.485
 
 
29.8
 
 
 
 
ADCY3
 
 
hsa-miR-132-3p
 
 
-0.303
 
 
78.3
 
 
 
 
ADGRF5
 
 
hsa-miR-148b-3p
 
 
-0.354
 
 
48.3
 
 
 
 
ADGRF5
 
 
hsa-miR-152-3p
 
 
-0.354
 
 
48.3
 
 
 
 
ADIPOR2
 
 
hsa-miR-150-5p
 
 
-0.287
 
 
12.2
 
 
 
 
ADIPOR2
 
 
hsa-miR-34a-5p
 
 
-0.33
 
 
12.2
 
 
 
 
ADK
 
 
hsa-miR-34a-5p
 
 
-0.374
 
 
25.0
 
 
 
 
AFG3L2
 
 
hsa-miR-181b-5p
 
 
-0.491
 
 
12.5
 
 
 
 
AFG3L2
 
 
hsa-miR-181a-5p
 
 
-0.491
 
 
12.5
 
 
 
 
AGFG1
 
 
hsa-miR-148b-3p
 
 
-0.332
 
 
71.2
 
 
 
 
AGFG1
 
 
hsa-miR-152-3p
 
 
-0.332
 
 
71.2
 
 
 
 
AGGF1
 
 
hsa-miR-125a-5p
 
 
-0.331
 
 
10.3
 
 
 
 
AGPAT3
 
 
hsa-miR-27a-3p
 
 
-0.29
 
 
11.5
 
 
 
 
AGTRAP
 
 
hsa-miR-34a-5p
 
 
-0.328
 
 
20.7
 
 
 
 
AHCYL2
 
 
hsa-miR-34a-5p
 
 
-0.359
 
 
11.3
 
 
 
 
AHR
 
 
hsa-miR-29a-3p
 
 
-0.316
 
 
16.6
 
 
 
 
AIDA
 
 
hsa-miR-22-3p
 
 
-0.316
 
 
44.9
 
 
 
 
AKIRIN1
 
 
hsa-miR-27a-3p
 
 
-0.368
 
 
24.9
 
 
 
 
AKT3
 
 
hsa-miR-29a-3p
 
 
-0.287
 
 
12.3
 
 
 
 
AKT3
 
 
hsa-miR-22-3p
 
 
-0.451
 
 
12.3
 
 
 
 
ALCAM
 
 
hsa-miR-34a-5p
 
 
-0.303
 
 
11.3
 
 
 
 
ALCAM
 
 
hsa-miR-148b-3p
 
 
-0.339
 
 
11.3
 
 
 
 
ALCAM
 
 
hsa-miR-152-3p
 
 
-0.347
 
 
11.3
 
 
 
 
ALDOA
 
 
hsa-miR-34a-5p
 
 
-0.655
 
 
329.7
 
 
 
 
AMOT
 
 
hsa-miR-29a-3p
 
 
-0.304
 
 
19.6
 
 
 
 
ANAPC16
 
 
hsa-miR-125a-5p
 
 
-0.578
 
 
11.0
 
 
 
 
ANK3
 
 
hsa-miR-34a-5p
 
 
-0.29
 
 
14.5
 
 
 
 
ANK3
 
 
hsa-miR-34a-5p
 
 
-0.347
 
 
14.5
 
 
 
 
ANKHD1
 
 
hsa-miR-23a-3p
 
 
-0.368
 
 
26.8
 
 
 
 
ANKHD1
 
 
hsa-miR-23b-3p
 
 
-0.368
 
 
26.8
 
 
 
 
ANKRD13A
 
 
hsa-miR-22-3p
 
 
-0.478
 
 
22.9
 
 
 
 
ANKRD40
 
 
hsa-miR-27a-3p
 
 
-0.35
 
 
21.7
 
 
 
 
ANP32A
 
 
hsa-miR-132-3p
 
 
-0.374
 
 
95.3
 
 
 
 
ANP32B
 
 
hsa-miR-34a-5p
 
 
-0.29
 
 
103.8
 
 
 
 
ANPEP
 
 
hsa-miR-125a-5p
 
 
-0.372
 
 
31.1
 
 
 
 
ANTXR2
 
 
hsa-miR-29a-3p
 
 
-0.347
 
 
84.5
 
 
 
 
AP3S2
 
 
hsa-miR-34a-5p
 
 
-0.413
 
 
17.4
 
 
 
 
APH1A
 
 
hsa-miR-34a-5p
 
 
-0.304
 
 
68.0
 
 
 
 
APOO
 
 
hsa-miR-181b-5p
 
 
-0.5
 
 
10.3
 
 
 
 
APOO
 
 
hsa-miR-181a-5p
 
 
-0.5
 
 
10.3
 
 
 
 
APPBP2
 
 
hsa-miR-27a-3p
 
 
-0.378
 
 
11.5
 
 
 
 
AREG
 
 
hsa-miR-34a-5p
 
 
-0.62
 
 
308.9
 
 
 
 
ARF4
 
 
hsa-miR-152-3p
 
 
-0.487
 
 
170.7
 
 
 
 
ARF4
 
 
hsa-miR-148b-3p
 
 
-0.487
 
 
170.7
 
 
 
 
ARF5
 
 
hsa-miR-29a-3p
 
 
-0.491
 
 
65.2
 
 
 
 
ARF6
 
 
hsa-miR-181b-5p
 
 
-0.33
 
 
34.8
 
 
 
 
ARF6
 
 
hsa-miR-181a-5p
 
 
-0.33
 
 
34.8
 
 
 
 
ARFGEF1
 
 
hsa-miR-148b-3p
 
 
-0.306
 
 
13.6
 
 
 
 
ARFGEF1
 
 
hsa-miR-152-3p
 
 
-0.306
 
 
13.6
 
 
 
 
ARFIP1
 
 
hsa-miR-152-3p
 
 
-0.474
 
 
16.9
 
 
 
 
ARFIP1
 
 
hsa-miR-148b-3p
 
 
-0.474
 
 
16.9
 
 
 
 
ARHGAP1
 
 
hsa-miR-34a-5p
 
 
-0.369
 
 
36.7
 
 
 
 
ARHGAP21
 
 
hsa-miR-152-3p
 
 
-0.287
 
 
15.6
 
 
 
 
ARHGAP21
 
 
hsa-miR-148b-3p
 
 
-0.287
 
 
15.6
 
 
 
 
ARHGAP26
 
 
hsa-miR-34a-5p
 
 
-0.384
 
 
19.6
 
 
 
 
ARID1B
 
 
hsa-miR-29a-3p
 
 
-0.342
 
 
10.4
 
 
 
 
ARL4A
 
 
hsa-miR-29a-3p
 
 
-0.386
 
 
22.2
 
 
 
 
ARL5B
 
 
hsa-miR-29a-3p
 
 
-0.304
 
 
12.3
 
 
 
 
ARL6IP1
 
 
hsa-miR-148b-3p
 
 
-0.579
 
 
84.9
 
 
 
 
ARL6IP1
 
 
hsa-miR-152-3p
 
 
-0.579
 
 
84.9
 
 
 
 
ARL8B
 
 
hsa-miR-152-3p
 
 
-0.337
 
 
46.8
 
 
 
 
ARL8B
 
 
hsa-miR-148b-3p
 
 
-0.337
 
 
46.8
 
 
 
 
ARMCX3
 
 
hsa-miR-425-5p
 
 
-0.325
 
 
42.7
 
 
 
 
ARPC5
 
 
hsa-miR-22-3p
 
 
-0.679
 
 
101.6
 
 
 
 
ARPP19
 
 
hsa-miR-29a-3p
 
 
-0.371
 
 
53.5
 
 
 
 
ARPP19
 
 
hsa-miR-34a-5p
 
 
-0.497
 
 
53.5
 
 
 
 
ARRB1
 
 
hsa-miR-22-3p
 
 
-0.616
 
 
23.6
 
 
 
 
ARRDC3
 
 
hsa-miR-152-3p
 
 
-0.305
 
 
72.1
 
 
 
 
ARRDC3
 
 
hsa-miR-148b-3p
 
 
-0.305
 
 
72.1
 
 
 
 
ARRDC3
 
 
hsa-miR-29a-3p
 
 
-0.402
 
 
72.1
 
 
 
 
ARSB
 
 
hsa-miR-29a-3p
 
 
-0.36
 
 
10.8
 
 
 
 
ASAH1
 
 
hsa-miR-27a-3p
 
 
-0.398
 
 
114.2
 
 
 
 
ASPH
 
 
hsa-miR-152-3p
 
 
-0.391
 
 
44.6
 
 
 
 
ASPH
 
 
hsa-miR-148b-3p
 
 
-0.391
 
 
44.6
 
 
 
 
ATMIN
 
 
hsa-miR-34a-5p
 
 
-0.409
 
 
19.2
 
 
 
 
ATP10D
 
 
hsa-miR-125a-5p
 
 
-0.375
 
 
14.0
 
 
 
 
ATP2B4
 
 
hsa-miR-152-3p
 
 
-0.337
 
 
38.2
 
 
 
 
 
 
 prostate 
 
 
 
 
target_symbol
 
 
mature_mirna_id
 
 
score
 
 
nTPM
 
 
 
 
 
 
AAGAB
 
 
hsa-miR-34a-5p
 
 
-0.319
 
 
17.6
 
 
 
 
AAR2
 
 
hsa-miR-29a-3p
 
 
-0.327
 
 
18.7
 
 
 
 
ABCC5
 
 
hsa-miR-125a-5p
 
 
-0.288
 
 
27.9
 
 
 
 
ABCD3
 
 
hsa-miR-148b-3p
 
 
-0.388
 
 
20.2
 
 
 
 
ABCD3
 
 
hsa-miR-152-3p
 
 
-0.388
 
 
20.2
 
 
 
 
ABCE1
 
 
hsa-miR-29a-3p
 
 
-0.404
 
 
18.2
 
 
 
 
ABHD17C
 
 
hsa-miR-27a-3p
 
 
-0.435
 
 
17.0
 
 
 
 
ABHD3
 
 
hsa-miR-125a-5p
 
 
-0.453
 
 
10.4
 
 
 
 
ABHD4
 
 
hsa-miR-29a-3p
 
 
-0.312
 
 
20.4
 
 
 
 
ABTB1
 
 
hsa-miR-125a-5p
 
 
-0.519
 
 
33.0
 
 
 
 
ACBD3
 
 
hsa-miR-34a-5p
 
 
-0.415
 
 
21.4
 
 
 
 
ACSL1
 
 
hsa-miR-34a-5p
 
 
-0.306
 
 
75.4
 
 
 
 
ACSL4
 
 
hsa-miR-34a-5p
 
 
-0.372
 
 
12.3
 
 
 
 
ACTA2
 
 
hsa-miR-27a-3p
 
 
-0.577
 
 
2476.6
 
 
 
 
ACTN2
 
 
hsa-miR-23a-3p
 
 
-0.294
 
 
14.6
 
 
 
 
ACTN2
 
 
hsa-miR-29a-3p
 
 
-0.311
 
 
14.6
 
 
 
 
ADAM10
 
 
hsa-miR-152-3p
 
 
-0.348
 
 
23.6
 
 
 
 
ADAM10
 
 
hsa-miR-148b-3p
 
 
-0.356
 
 
23.6
 
 
 
 
ADCY3
 
 
hsa-miR-132-3p
 
 
-0.303
 
 
38.0
 
 
 
 
ADGRA3
 
 
hsa-miR-27a-3p
 
 
-0.296
 
 
11.3
 
 
 
 
ADIPOR2
 
 
hsa-miR-150-5p
 
 
-0.287
 
 
19.8
 
 
 
 
ADIPOR2
 
 
hsa-miR-34a-5p
 
 
-0.33
 
 
19.8
 
 
 
 
ADK
 
 
hsa-miR-34a-5p
 
 
-0.374
 
 
14.0
 
 
 
 
AFG3L2
 
 
hsa-miR-181b-5p
 
 
-0.491
 
 
13.2
 
 
 
 
AFG3L2
 
 
hsa-miR-181a-5p
 
 
-0.491
 
 
13.2
 
 
 
 
AGFG1
 
 
hsa-miR-148b-3p
 
 
-0.332
 
 
19.4
 
 
 
 
AGFG1
 
 
hsa-miR-152-3p
 
 
-0.332
 
 
19.4
 
 
 
 
AGPAT3
 
 
hsa-miR-27a-3p
 
 
-0.29
 
 
12.9
 
 
 
 
AGTRAP
 
 
hsa-miR-34a-5p
 
 
-0.328
 
 
75.3
 
 
 
 
AHDC1
 
 
hsa-miR-148b-3p
 
 
-0.413
 
 
11.3
 
 
 
 
AHDC1
 
 
hsa-miR-152-3p
 
 
-0.413
 
 
11.3
 
 
 
 
AHR
 
 
hsa-miR-29a-3p
 
 
-0.316
 
 
10.3
 
 
 
 
AIDA
 
 
hsa-miR-22-3p
 
 
-0.316
 
 
32.9
 
 
 
 
AIFM1
 
 
hsa-miR-125a-5p
 
 
-0.3
 
 
10.1
 
 
 
 
AK1
 
 
hsa-miR-29a-3p
 
 
-0.333
 
 
16.0
 
 
 
 
AKAP1
 
 
hsa-miR-148b-3p
 
 
-0.381
 
 
33.3
 
 
 
 
AKAP1
 
 
hsa-miR-152-3p
 
 
-0.381
 
 
33.3
 
 
 
 
AKIRIN1
 
 
hsa-miR-27a-3p
 
 
-0.368
 
 
34.0
 
 
 
 
AKT3
 
 
hsa-miR-29a-3p
 
 
-0.287
 
 
13.8
 
 
 
 
AKT3
 
 
hsa-miR-22-3p
 
 
-0.451
 
 
13.8
 
 
 
 
ALCAM
 
 
hsa-miR-34a-5p
 
 
-0.303
 
 
30.7
 
 
 
 
ALCAM
 
 
hsa-miR-148b-3p
 
 
-0.339
 
 
30.7
 
 
 
 
ALCAM
 
 
hsa-miR-152-3p
 
 
-0.347
 
 
30.7
 
 
 
 
ALDH1A2
 
 
hsa-miR-23b-3p
 
 
-0.346
 
 
19.7
 
 
 
 
ALDH1A2
 
 
hsa-miR-23a-3p
 
 
-0.354
 
 
19.7
 
 
 
 
ALDOA
 
 
hsa-miR-34a-5p
 
 
-0.655
 
 
529.6
 
 
 
 
ANAPC16
 
 
hsa-miR-125a-5p
 
 
-0.578
 
 
19.8
 
 
 
 
ANK3
 
 
hsa-miR-34a-5p
 
 
-0.29
 
 
18.4
 
 
 
 
ANK3
 
 
hsa-miR-34a-5p
 
 
-0.347
 
 
18.4
 
 
 
 
ANKHD1
 
 
hsa-miR-23a-3p
 
 
-0.368
 
 
34.8
 
 
 
 
ANKHD1
 
 
hsa-miR-23b-3p
 
 
-0.368
 
 
34.8
 
 
 
 
ANKHD1-EIF4EBP3
 
 
hsa-miR-22-3p
 
 
-0.479
 
 
11.9
 
 
 
 
ANKRD13A
 
 
hsa-miR-22-3p
 
 
-0.478
 
 
16.8
 
 
 
 
ANKRD40
 
 
hsa-miR-27a-3p
 
 
-0.35
 
 
17.2
 
 
 
 
ANP32A
 
 
hsa-miR-132-3p
 
 
-0.374
 
 
70.7
 
 
 
 
ANP32B
 
 
hsa-miR-34a-5p
 
 
-0.29
 
 
93.4
 
 
 
 
ANPEP
 
 
hsa-miR-125a-5p
 
 
-0.372
 
 
88.3
 
 
 
 
ANTXR2
 
 
hsa-miR-29a-3p
 
 
-0.347
 
 
103.3
 
 
 
 
AP3S2
 
 
hsa-miR-34a-5p
 
 
-0.413
 
 
14.3
 
 
 
 
APH1A
 
 
hsa-miR-34a-5p
 
 
-0.304
 
 
58.2
 
 
 
 
APOO
 
 
hsa-miR-181b-5p
 
 
-0.5
 
 
12.8
 
 
 
 
APOO
 
 
hsa-miR-181a-5p
 
 
-0.5
 
 
12.8
 
 
 
 
APPBP2
 
 
hsa-miR-27a-3p
 
 
-0.378
 
 
12.8
 
 
 
 
ARF4
 
 
hsa-miR-152-3p
 
 
-0.487
 
 
129.9
 
 
 
 
ARF4
 
 
hsa-miR-148b-3p
 
 
-0.487
 
 
129.9
 
 
 
 
ARF5
 
 
hsa-miR-29a-3p
 
 
-0.491
 
 
107.0
 
 
 
 
ARF6
 
 
hsa-miR-181b-5p
 
 
-0.33
 
 
30.5
 
 
 
 
ARF6
 
 
hsa-miR-181a-5p
 
 
-0.33
 
 
30.5
 
 
 
 
ARFGEF1
 
 
hsa-miR-148b-3p
 
 
-0.306
 
 
12.2
 
 
 
 
ARFGEF1
 
 
hsa-miR-152-3p
 
 
-0.306
 
 
12.2
 
 
 
 
ARFIP1
 
 
hsa-miR-152-3p
 
 
-0.474
 
 
19.8
 
 
 
 
ARFIP1
 
 
hsa-miR-148b-3p
 
 
-0.474
 
 
19.8
 
 
 
 
ARHGAP1
 
 
hsa-miR-34a-5p
 
 
-0.369
 
 
60.9
 
 
 
 
ARHGAP21
 
 
hsa-miR-152-3p
 
 
-0.287
 
 
10.2
 
 
 
 
ARHGAP21
 
 
hsa-miR-148b-3p
 
 
-0.287
 
 
10.2
 
 
 
 
ARID1B
 
 
hsa-miR-29a-3p
 
 
-0.342
 
 
11.6
 
 
 
 
ARL4A
 
 
hsa-miR-29a-3p
 
 
-0.386
 
 
19.8
 
 
 
 
ARL6IP1
 
 
hsa-miR-148b-3p
 
 
-0.579
 
 
71.3
 
 
 
 
ARL6IP1
 
 
hsa-miR-152-3p
 
 
-0.579
 
 
71.3
 
 
 
 
ARL8B
 
 
hsa-miR-152-3p
 
 
-0.337
 
 
42.2
 
 
 
 
ARL8B
 
 
hsa-miR-148b-3p
 
 
-0.337
 
 
42.2
 
 
 
 
ARMCX3
 
 
hsa-miR-425-5p
 
 
-0.325
 
 
41.8
 
 
 
 
ARPC5
 
 
hsa-miR-22-3p
 
 
-0.679
 
 
43.1
 
 
 
 
ARPIN-AP3S2
 
 
hsa-miR-34a-5p
 
 
-0.348
 
 
10.1
 
 
 
 
ARPP19
 
 
hsa-miR-29a-3p
 
 
-0.371
 
 
37.0
 
 
 
 
ARPP19
 
 
hsa-miR-34a-5p
 
 
-0.497
 
 
37.0
 
 
 
 
ARRB1
 
 
hsa-miR-22-3p
 
 
-0.616
 
 
15.2
 
 
 
 
ARRDC3
 
 
hsa-miR-152-3p
 
 
-0.305
 
 
28.5
 
 
 
 
ARRDC3
 
 
hsa-miR-148b-3p
 
 
-0.305
 
 
28.5
 
 
 
 
ARRDC3
 
 
hsa-miR-29a-3p
 
 
-0.402
 
 
28.5
 
 
 
 
ASAH1
 
 
hsa-miR-27a-3p
 
 
-0.398
 
 
79.1
 
 
 
 
ASPH
 
 
hsa-miR-152-3p
 
 
-0.391
 
 
44.4
 
 
 
 
ASPH
 
 
hsa-miR-148b-3p
 
 
-0.391
 
 
44.4
 
 
 
 
ATG4B
 
 
hsa-miR-34a-5p
 
 
-0.58
 
 
25.1
 
 
 
 
ATMIN
 
 
hsa-miR-34a-5p
 
 
-0.409
 
 
16.5
 
 
 
 
ATP1A2
 
 
hsa-miR-34a-5p
 
 
-0.306
 
 
26.7
 
 
 
 
ATP2B4
 
 
hsa-miR-152-3p
 
 
-0.337
 
 
31.1
 
 
 
 
ATP2B4
 
 
hsa-miR-148b-3p
 
 
-0.337
 
 
31.1
 
 
 
 
ATP5MC3
 
 
hsa-miR-425-5p
 
 
-0.328
 
 
106.2
 
 
 
 
ATP5MC3
 
 
hsa-miR-29a-3p
 
 
-0.33
 
 
106.2
 
 
 
 
 
 
 rectum 
 
 
 
 
target_symbol
 
 
mature_mirna_id
 
 
score
 
 
nTPM
 
 
 
 
 
 
AAGAB
 
 
hsa-miR-34a-5p
 
 
-0.319
 
 
31.7
 
 
 
 
AAR2
 
 
hsa-miR-29a-3p
 
 
-0.327
 
 
16.3
 
 
 
 
ABCB10
 
 
hsa-miR-152-3p
 
 
-0.291
 
 
10.6
 
 
 
 
ABCB10
 
 
hsa-miR-148b-3p
 
 
-0.291
 
 
10.6
 
 
 
 
ABCC5
 
 
hsa-miR-125a-5p
 
 
-0.288
 
 
20.8
 
 
 
 
ABCD3
 
 
hsa-miR-148b-3p
 
 
-0.388
 
 
37.8
 
 
 
 
ABCD3
 
 
hsa-miR-152-3p
 
 
-0.388
 
 
37.8
 
 
 
 
ABCE1
 
 
hsa-miR-29a-3p
 
 
-0.404
 
 
26.0
 
 
 
 
ABHD17C
 
 
hsa-miR-27a-3p
 
 
-0.435
 
 
52.8
 
 
 
 
ABHD3
 
 
hsa-miR-125a-5p
 
 
-0.453
 
 
61.7
 
 
 
 
ABHD4
 
 
hsa-miR-29a-3p
 
 
-0.312
 
 
14.1
 
 
 
 
ABHD6
 
 
hsa-miR-125a-5p
 
 
-0.501
 
 
10.4
 
 
 
 
ACBD3
 
 
hsa-miR-34a-5p
 
 
-0.415
 
 
23.9
 
 
 
 
ACSL1
 
 
hsa-miR-34a-5p
 
 
-0.306
 
 
13.6
 
 
 
 
ACSL4
 
 
hsa-miR-34a-5p
 
 
-0.372
 
 
12.1
 
 
 
 
ACTA2
 
 
hsa-miR-27a-3p
 
 
-0.577
 
 
814.5
 
 
 
 
ADAM10
 
 
hsa-miR-152-3p
 
 
-0.348
 
 
16.1
 
 
 
 
ADAM10
 
 
hsa-miR-148b-3p
 
 
-0.356
 
 
16.1
 
 
 
 
ADAP1
 
 
hsa-miR-27a-3p
 
 
-0.302
 
 
21.3
 
 
 
 
ADCY3
 
 
hsa-miR-132-3p
 
 
-0.303
 
 
14.9
 
 
 
 
ADGRA3
 
 
hsa-miR-27a-3p
 
 
-0.296
 
 
16.1
 
 
 
 
ADIPOR2
 
 
hsa-miR-150-5p
 
 
-0.287
 
 
16.7
 
 
 
 
ADIPOR2
 
 
hsa-miR-34a-5p
 
 
-0.33
 
 
16.7
 
 
 
 
ADK
 
 
hsa-miR-34a-5p
 
 
-0.374
 
 
18.3
 
 
 
 
AFG3L2
 
 
hsa-miR-181b-5p
 
 
-0.491
 
 
28.8
 
 
 
 
AFG3L2
 
 
hsa-miR-181a-5p
 
 
-0.491
 
 
28.8
 
 
 
 
AGFG1
 
 
hsa-miR-148b-3p
 
 
-0.332
 
 
31.0
 
 
 
 
AGFG1
 
 
hsa-miR-152-3p
 
 
-0.332
 
 
31.0
 
 
 
 
AGGF1
 
 
hsa-miR-125a-5p
 
 
-0.331
 
 
10.9
 
 
 
 
AGPAT3
 
 
hsa-miR-27a-3p
 
 
-0.29
 
 
11.1
 
 
 
 
AHCYL2
 
 
hsa-miR-34a-5p
 
 
-0.359
 
 
166.5
 
 
 
 
AHR
 
 
hsa-miR-29a-3p
 
 
-0.316
 
 
15.3
 
 
 
 
AIDA
 
 
hsa-miR-22-3p
 
 
-0.316
 
 
34.0
 
 
 
 
AIFM1
 
 
hsa-miR-125a-5p
 
 
-0.3
 
 
14.9
 
 
 
 
AK1
 
 
hsa-miR-29a-3p
 
 
-0.333
 
 
37.1
 
 
 
 
AKAP1
 
 
hsa-miR-148b-3p
 
 
-0.381
 
 
45.9
 
 
 
 
AKAP1
 
 
hsa-miR-152-3p
 
 
-0.381
 
 
45.9
 
 
 
 
AKIRIN1
 
 
hsa-miR-27a-3p
 
 
-0.368
 
 
34.0
 
 
 
 
ALCAM
 
 
hsa-miR-34a-5p
 
 
-0.303
 
 
13.6
 
 
 
 
ALCAM
 
 
hsa-miR-148b-3p
 
 
-0.339
 
 
13.6
 
 
 
 
ALCAM
 
 
hsa-miR-152-3p
 
 
-0.347
 
 
13.6
 
 
 
 
ALDOA
 
 
hsa-miR-34a-5p
 
 
-0.655
 
 
375.6
 
 
 
 
ANAPC16
 
 
hsa-miR-125a-5p
 
 
-0.578
 
 
16.4
 
 
 
 
ANK3
 
 
hsa-miR-34a-5p
 
 
-0.29
 
 
19.3
 
 
 
 
ANK3
 
 
hsa-miR-34a-5p
 
 
-0.347
 
 
19.3
 
 
 
 
ANKHD1
 
 
hsa-miR-23a-3p
 
 
-0.368
 
 
31.1
 
 
 
 
ANKHD1
 
 
hsa-miR-23b-3p
 
 
-0.368
 
 
31.1
 
 
 
 
ANKHD1-EIF4EBP3
 
 
hsa-miR-22-3p
 
 
-0.479
 
 
11.2
 
 
 
 
ANKRD13A
 
 
hsa-miR-22-3p
 
 
-0.478
 
 
21.0
 
 
 
 
ANKRD40
 
 
hsa-miR-27a-3p
 
 
-0.35
 
 
15.3
 
 
 
 
ANP32A
 
 
hsa-miR-132-3p
 
 
-0.374
 
 
83.5
 
 
 
 
ANP32B
 
 
hsa-miR-34a-5p
 
 
-0.29
 
 
111.5
 
 
 
 
ANPEP
 
 
hsa-miR-125a-5p
 
 
-0.372
 
 
56.3
 
 
 
 
ANTXR2
 
 
hsa-miR-29a-3p
 
 
-0.347
 
 
40.1
 
 
 
 
AP3S2
 
 
hsa-miR-34a-5p
 
 
-0.413
 
 
18.0
 
 
 
 
APH1A
 
 
hsa-miR-34a-5p
 
 
-0.304
 
 
89.6
 
 
 
 
APOO
 
 
hsa-miR-181b-5p
 
 
-0.5
 
 
17.6
 
 
 
 
APOO
 
 
hsa-miR-181a-5p
 
 
-0.5
 
 
17.6
 
 
 
 
AREG
 
 
hsa-miR-34a-5p
 
 
-0.62
 
 
17.8
 
 
 
 
ARF4
 
 
hsa-miR-152-3p
 
 
-0.487
 
 
185.2
 
 
 
 
ARF4
 
 
hsa-miR-148b-3p
 
 
-0.487
 
 
185.2
 
 
 
 
ARF5
 
 
hsa-miR-29a-3p
 
 
-0.491
 
 
57.6
 
 
 
 
ARF6
 
 
hsa-miR-181b-5p
 
 
-0.33
 
 
50.6
 
 
 
 
ARF6
 
 
hsa-miR-181a-5p
 
 
-0.33
 
 
50.6
 
 
 
 
ARFGEF1
 
 
hsa-miR-148b-3p
 
 
-0.306
 
 
16.1
 
 
 
 
ARFGEF1
 
 
hsa-miR-152-3p
 
 
-0.306
 
 
16.1
 
 
 
 
ARFIP1
 
 
hsa-miR-152-3p
 
 
-0.474
 
 
26.2
 
 
 
 
ARFIP1
 
 
hsa-miR-148b-3p
 
 
-0.474
 
 
26.2
 
 
 
 
ARHGAP1
 
 
hsa-miR-34a-5p
 
 
-0.369
 
 
30.6
 
 
 
 
ARHGAP21
 
 
hsa-miR-152-3p
 
 
-0.287
 
 
17.4
 
 
 
 
ARHGAP21
 
 
hsa-miR-148b-3p
 
 
-0.287
 
 
17.4
 
 
 
 
ARHGAP26
 
 
hsa-miR-34a-5p
 
 
-0.384
 
 
11.9
 
 
 
 
ARID1B
 
 
hsa-miR-29a-3p
 
 
-0.342
 
 
11.4
 
 
 
 
ARL4A
 
 
hsa-miR-29a-3p
 
 
-0.386
 
 
32.6
 
 
 
 
ARL6IP1
 
 
hsa-miR-148b-3p
 
 
-0.579
 
 
116.0
 
 
 
 
ARL6IP1
 
 
hsa-miR-152-3p
 
 
-0.579
 
 
116.0
 
 
 
 
ARL8B
 
 
hsa-miR-152-3p
 
 
-0.337
 
 
32.7
 
 
 
 
ARL8B
 
 
hsa-miR-148b-3p
 
 
-0.337
 
 
32.7
 
 
 
 
ARMCX3
 
 
hsa-miR-425-5p
 
 
-0.325
 
 
20.5
 
 
 
 
ARPC5
 
 
hsa-miR-22-3p
 
 
-0.679
 
 
64.7
 
 
 
 
ARPIN-AP3S2
 
 
hsa-miR-34a-5p
 
 
-0.348
 
 
14.1
 
 
 
 
ARPP19
 
 
hsa-miR-29a-3p
 
 
-0.371
 
 
33.6
 
 
 
 
ARPP19
 
 
hsa-miR-34a-5p
 
 
-0.497
 
 
33.6
 
 
 
 
ARRB1
 
 
hsa-miR-22-3p
 
 
-0.616
 
 
21.2
 
 
 
 
ARRDC3
 
 
hsa-miR-152-3p
 
 
-0.305
 
 
25.8
 
 
 
 
ARRDC3
 
 
hsa-miR-148b-3p
 
 
-0.305
 
 
25.8
 
 
 
 
ARRDC3
 
 
hsa-miR-29a-3p
 
 
-0.402
 
 
25.8
 
 
 
 
ASAH1
 
 
hsa-miR-27a-3p
 
 
-0.398
 
 
81.7
 
 
 
 
ASAP2
 
 
hsa-miR-29a-3p
 
 
-0.348
 
 
15.1
 
 
 
 
ASF1A
 
 
hsa-miR-23a-3p
 
 
-0.506
 
 
10.2
 
 
 
 
ASF1A
 
 
hsa-miR-23b-3p
 
 
-0.506
 
 
10.2
 
 
 
 
ASPH
 
 
hsa-miR-152-3p
 
 
-0.391
 
 
77.9
 
 
 
 
ASPH
 
 
hsa-miR-148b-3p
 
 
-0.391
 
 
77.9
 
 
 
 
ATMIN
 
 
hsa-miR-34a-5p
 
 
-0.409
 
 
12.8
 
 
 
 
ATOH1
 
 
hsa-miR-22-3p
 
 
-0.566
 
 
17.3
 
 
 
 
ATP2B4
 
 
hsa-miR-152-3p
 
 
-0.337
 
 
28.6
 
 
 
 
ATP2B4
 
 
hsa-miR-148b-3p
 
 
-0.337
 
 
28.6
 
 
 
 
ATP5MC3
 
 
hsa-miR-425-5p
 
 
-0.328
 
 
295.3
 
 
 
 
ATP5MC3
 
 
hsa-miR-29a-3p
 
 
-0.33
 
 
295.3
 
 
 
 
ATP5MC3
 
 
hsa-miR-27a-3p
 
 
-0.416
 
 
295.3
 
 
 
 
 
 
 retina 
 
 
 
 
target_symbol
 
 
mature_mirna_id
 
 
score
 
 
nTPM
 
 
 
 
 
 
AAGAB
 
 
hsa-miR-34a-5p
 
 
-0.319
 
 
20.5
 
 
 
 
ABCC5
 
 
hsa-miR-125a-5p
 
 
-0.288
 
 
116.9
 
 
 
 
ABCD3
 
 
hsa-miR-148b-3p
 
 
-0.388
 
 
24.4
 
 
 
 
ABCD3
 
 
hsa-miR-152-3p
 
 
-0.388
 
 
24.4
 
 
 
 
ABCE1
 
 
hsa-miR-29a-3p
 
 
-0.404
 
 
13.3
 
 
 
 
ABHD17C
 
 
hsa-miR-27a-3p
 
 
-0.435
 
 
15.6
 
 
 
 
ABHD4
 
 
hsa-miR-29a-3p
 
 
-0.312
 
 
21.0
 
 
 
 
ACBD3
 
 
hsa-miR-34a-5p
 
 
-0.415
 
 
14.9
 
 
 
 
ACSL1
 
 
hsa-miR-34a-5p
 
 
-0.306
 
 
22.4
 
 
 
 
ACSL4
 
 
hsa-miR-34a-5p
 
 
-0.372
 
 
20.7
 
 
 
 
ACSL6
 
 
hsa-miR-125a-5p
 
 
-0.307
 
 
42.4
 
 
 
 
ADD2
 
 
hsa-miR-34a-5p
 
 
-0.305
 
 
14.9
 
 
 
 
ADIPOR2
 
 
hsa-miR-150-5p
 
 
-0.287
 
 
12.1
 
 
 
 
ADIPOR2
 
 
hsa-miR-34a-5p
 
 
-0.33
 
 
12.1
 
 
 
 
ADK
 
 
hsa-miR-34a-5p
 
 
-0.374
 
 
14.9
 
 
 
 
AGFG1
 
 
hsa-miR-148b-3p
 
 
-0.332
 
 
44.2
 
 
 
 
AGFG1
 
 
hsa-miR-152-3p
 
 
-0.332
 
 
44.2
 
 
 
 
AGGF1
 
 
hsa-miR-125a-5p
 
 
-0.331
 
 
20.6
 
 
 
 
AGO4
 
 
hsa-miR-34a-5p
 
 
-0.306
 
 
16.7
 
 
 
 
AGPAT3
 
 
hsa-miR-27a-3p
 
 
-0.29
 
 
57.5
 
 
 
 
AHCYL2
 
 
hsa-miR-34a-5p
 
 
-0.359
 
 
53.5
 
 
 
 
AIDA
 
 
hsa-miR-22-3p
 
 
-0.316
 
 
18.0
 
 
 
 
AK1
 
 
hsa-miR-29a-3p
 
 
-0.333
 
 
27.1
 
 
 
 
AKAP1
 
 
hsa-miR-148b-3p
 
 
-0.381
 
 
32.2
 
 
 
 
AKAP1
 
 
hsa-miR-152-3p
 
 
-0.381
 
 
32.2
 
 
 
 
AKIRIN1
 
 
hsa-miR-27a-3p
 
 
-0.368
 
 
34.7
 
 
 
 
AKT3
 
 
hsa-miR-29a-3p
 
 
-0.287
 
 
15.2
 
 
 
 
AKT3
 
 
hsa-miR-22-3p
 
 
-0.451
 
 
15.2
 
 
 
 
ALDOA
 
 
hsa-miR-34a-5p
 
 
-0.655
 
 
431.9
 
 
 
 
ALPL
 
 
hsa-miR-10a-5p
 
 
-0.32
 
 
17.1
 
 
 
 
AMER2
 
 
hsa-miR-34a-5p
 
 
-0.48
 
 
23.9
 
 
 
 
ANAPC16
 
 
hsa-miR-125a-5p
 
 
-0.578
 
 
18.8
 
 
 
 
ANK2
 
 
hsa-miR-34a-5p
 
 
-0.317
 
 
118.1
 
 
 
 
ANK3
 
 
hsa-miR-34a-5p
 
 
-0.29
 
 
115.3
 
 
 
 
ANK3
 
 
hsa-miR-34a-5p
 
 
-0.347
 
 
115.3
 
 
 
 
ANKHD1
 
 
hsa-miR-23a-3p
 
 
-0.368
 
 
37.5
 
 
 
 
ANKHD1
 
 
hsa-miR-23b-3p
 
 
-0.368
 
 
37.5
 
 
 
 
ANKHD1-EIF4EBP3
 
 
hsa-miR-22-3p
 
 
-0.479
 
 
11.0
 
 
 
 
ANKRD13C
 
 
hsa-miR-29a-3p
 
 
-0.411
 
 
12.3
 
 
 
 
ANKRD33B
 
 
hsa-miR-125a-5p
 
 
-0.389
 
 
47.3
 
 
 
 
ANKRD40
 
 
hsa-miR-27a-3p
 
 
-0.35
 
 
19.2
 
 
 
 
ANP32A
 
 
hsa-miR-132-3p
 
 
-0.374
 
 
98.2
 
 
 
 
ANP32B
 
 
hsa-miR-34a-5p
 
 
-0.29
 
 
42.8
 
 
 
 
AP3S2
 
 
hsa-miR-34a-5p
 
 
-0.413
 
 
40.7
 
 
 
 
APC
 
 
hsa-miR-29a-3p
 
 
-0.413
 
 
18.5
 
 
 
 
APH1A
 
 
hsa-miR-34a-5p
 
 
-0.304
 
 
22.7
 
 
 
 
APOO
 
 
hsa-miR-181b-5p
 
 
-0.5
 
 
48.9
 
 
 
 
APOO
 
 
hsa-miR-181a-5p
 
 
-0.5
 
 
48.9
 
 
 
 
APPBP2
 
 
hsa-miR-27a-3p
 
 
-0.378
 
 
16.0
 
 
 
 
ARF4
 
 
hsa-miR-152-3p
 
 
-0.487
 
 
50.0
 
 
 
 
ARF4
 
 
hsa-miR-148b-3p
 
 
-0.487
 
 
50.0
 
 
 
 
ARF5
 
 
hsa-miR-29a-3p
 
 
-0.491
 
 
21.3
 
 
 
 
ARF6
 
 
hsa-miR-181b-5p
 
 
-0.33
 
 
13.4
 
 
 
 
ARF6
 
 
hsa-miR-181a-5p
 
 
-0.33
 
 
13.4
 
 
 
 
ARFGEF1
 
 
hsa-miR-148b-3p
 
 
-0.306
 
 
33.8
 
 
 
 
ARFGEF1
 
 
hsa-miR-152-3p
 
 
-0.306
 
 
33.8
 
 
 
 
ARFIP1
 
 
hsa-miR-152-3p
 
 
-0.474
 
 
18.7
 
 
 
 
ARFIP1
 
 
hsa-miR-148b-3p
 
 
-0.474
 
 
18.7
 
 
 
 
ARHGAP21
 
 
hsa-miR-152-3p
 
 
-0.287
 
 
37.0
 
 
 
 
ARHGAP21
 
 
hsa-miR-148b-3p
 
 
-0.287
 
 
37.0
 
 
 
 
ARHGAP26
 
 
hsa-miR-34a-5p
 
 
-0.384
 
 
16.0
 
 
 
 
ARID1B
 
 
hsa-miR-29a-3p
 
 
-0.342
 
 
10.7
 
 
 
 
ARID2
 
 
hsa-miR-132-3p
 
 
-0.304
 
 
12.3
 
 
 
 
ARL6IP1
 
 
hsa-miR-148b-3p
 
 
-0.579
 
 
292.6
 
 
 
 
ARL6IP1
 
 
hsa-miR-152-3p
 
 
-0.579
 
 
292.6
 
 
 
 
ARL8B
 
 
hsa-miR-152-3p
 
 
-0.337
 
 
31.7
 
 
 
 
ARL8B
 
 
hsa-miR-148b-3p
 
 
-0.337
 
 
31.7
 
 
 
 
ARMCX3
 
 
hsa-miR-425-5p
 
 
-0.325
 
 
17.6
 
 
 
 
ARPIN-AP3S2
 
 
hsa-miR-34a-5p
 
 
-0.348
 
 
10.1
 
 
 
 
ARPP19
 
 
hsa-miR-29a-3p
 
 
-0.371
 
 
53.1
 
 
 
 
ARPP19
 
 
hsa-miR-34a-5p
 
 
-0.497
 
 
53.1
 
 
 
 
ARRDC3
 
 
hsa-miR-152-3p
 
 
-0.305
 
 
69.6
 
 
 
 
ARRDC3
 
 
hsa-miR-148b-3p
 
 
-0.305
 
 
69.6
 
 
 
 
ARRDC3
 
 
hsa-miR-29a-3p
 
 
-0.402
 
 
69.6
 
 
 
 
ASAH1
 
 
hsa-miR-27a-3p
 
 
-0.398
 
 
73.6
 
 
 
 
ASF1A
 
 
hsa-miR-23a-3p
 
 
-0.506
 
 
10.6
 
 
 
 
ASF1A
 
 
hsa-miR-23b-3p
 
 
-0.506
 
 
10.6
 
 
 
 
ASPH
 
 
hsa-miR-152-3p
 
 
-0.391
 
 
201.9
 
 
 
 
ASPH
 
 
hsa-miR-148b-3p
 
 
-0.391
 
 
201.9
 
 
 
 
ATCAY
 
 
hsa-miR-10a-5p
 
 
-0.322
 
 
16.3
 
 
 
 
ATP1A2
 
 
hsa-miR-34a-5p
 
 
-0.306
 
 
54.2
 
 
 
 
ATP2B4
 
 
hsa-miR-152-3p
 
 
-0.337
 
 
39.4
 
 
 
 
ATP2B4
 
 
hsa-miR-148b-3p
 
 
-0.337
 
 
39.4
 
 
 
 
ATP5MC3
 
 
hsa-miR-425-5p
 
 
-0.328
 
 
117.1
 
 
 
 
ATP5MC3
 
 
hsa-miR-29a-3p
 
 
-0.33
 
 
117.1
 
 
 
 
ATP5MC3
 
 
hsa-miR-27a-3p
 
 
-0.416
 
 
117.1
 
 
 
 
ATP6AP2
 
 
hsa-miR-148b-3p
 
 
-0.592
 
 
92.0
 
 
 
 
ATP6AP2
 
 
hsa-miR-152-3p
 
 
-0.592
 
 
92.0
 
 
 
 
ATP6AP2
 
 
hsa-miR-148b-3p
 
 
-0.668
 
 
92.0
 
 
 
 
ATP6AP2
 
 
hsa-miR-152-3p
 
 
-0.668
 
 
92.0
 
 
 
 
ATP6V1E1
 
 
hsa-miR-23a-3p
 
 
-0.294
 
 
119.0
 
 
 
 
ATP6V1E1
 
 
hsa-miR-23b-3p
 
 
-0.294
 
 
119.0
 
 
 
 
ATP8A1
 
 
hsa-miR-22-3p
 
 
-0.468
 
 
17.9
 
 
 
 
ATXN10
 
 
hsa-miR-27a-3p
 
 
-0.295
 
 
51.0
 
 
 
 
ATXN2
 
 
hsa-miR-10a-5p
 
 
-0.362
 
 
14.6
 
 
 
 
AUH
 
 
hsa-miR-23a-3p
 
 
-0.495
 
 
19.8
 
 
 
 
AUH
 
 
hsa-miR-23b-3p
 
 
-0.503
 
 
19.8
 
 
 
 
AUH
 
 
hsa-miR-23a-3p
 
 
-0.512
 
 
19.8
 
 
 
 
AUH
 
 
hsa-miR-23b-3p
 
 
-0.512
 
 
19.8
 
 
 
 
AZIN1
 
 
hsa-miR-132-3p
 
 
-0.29
 
 
52.2
 
 
 
 
 
 
 salivary gland 
 
 
 
 
target_symbol
 
 
mature_mirna_id
 
 
score
 
 
nTPM
 
 
 
 
 
 
AAGAB
 
 
hsa-miR-34a-5p
 
 
-0.319
 
 
14.5
 
 
 
 
AAR2
 
 
hsa-miR-29a-3p
 
 
-0.327
 
 
17.0
 
 
 
 
ABCC5
 
 
hsa-miR-125a-5p
 
 
-0.288
 
 
18.2
 
 
 
 
ABCD3
 
 
hsa-miR-148b-3p
 
 
-0.388
 
 
16.5
 
 
 
 
ABCD3
 
 
hsa-miR-152-3p
 
 
-0.388
 
 
16.5
 
 
 
 
ABCE1
 
 
hsa-miR-29a-3p
 
 
-0.404
 
 
22.7
 
 
 
 
ABHD17C
 
 
hsa-miR-27a-3p
 
 
-0.435
 
 
22.3
 
 
 
 
ABHD3
 
 
hsa-miR-125a-5p
 
 
-0.453
 
 
13.7
 
 
 
 
ABHD4
 
 
hsa-miR-29a-3p
 
 
-0.312
 
 
17.2
 
 
 
 
ABHD5
 
 
hsa-miR-132-3p
 
 
-0.323
 
 
19.4
 
 
 
 
ABHD5
 
 
hsa-miR-27a-3p
 
 
-0.407
 
 
19.4
 
 
 
 
ABTB1
 
 
hsa-miR-125a-5p
 
 
-0.519
 
 
25.2
 
 
 
 
ACBD3
 
 
hsa-miR-34a-5p
 
 
-0.415
 
 
22.7
 
 
 
 
ACSL1
 
 
hsa-miR-34a-5p
 
 
-0.306
 
 
82.2
 
 
 
 
ACTA2
 
 
hsa-miR-27a-3p
 
 
-0.577
 
 
390.9
 
 
 
 
ACTN2
 
 
hsa-miR-23a-3p
 
 
-0.294
 
 
18.4
 
 
 
 
ACTN2
 
 
hsa-miR-29a-3p
 
 
-0.311
 
 
18.4
 
 
 
 
ADCY3
 
 
hsa-miR-132-3p
 
 
-0.303
 
 
20.4
 
 
 
 
ADIPOR2
 
 
hsa-miR-150-5p
 
 
-0.287
 
 
21.2
 
 
 
 
ADIPOR2
 
 
hsa-miR-34a-5p
 
 
-0.33
 
 
21.2
 
 
 
 
ADK
 
 
hsa-miR-34a-5p
 
 
-0.374
 
 
18.2
 
 
 
 
AFG3L2
 
 
hsa-miR-181b-5p
 
 
-0.491
 
 
15.5
 
 
 
 
AFG3L2
 
 
hsa-miR-181a-5p
 
 
-0.491
 
 
15.5
 
 
 
 
AGFG1
 
 
hsa-miR-148b-3p
 
 
-0.332
 
 
26.3
 
 
 
 
AGFG1
 
 
hsa-miR-152-3p
 
 
-0.332
 
 
26.3
 
 
 
 
AGPAT3
 
 
hsa-miR-27a-3p
 
 
-0.29
 
 
28.8
 
 
 
 
AGTRAP
 
 
hsa-miR-34a-5p
 
 
-0.328
 
 
36.7
 
 
 
 
AHCYL2
 
 
hsa-miR-34a-5p
 
 
-0.359
 
 
28.8
 
 
 
 
AHDC1
 
 
hsa-miR-148b-3p
 
 
-0.413
 
 
16.0
 
 
 
 
AHDC1
 
 
hsa-miR-152-3p
 
 
-0.413
 
 
16.0
 
 
 
 
AIDA
 
 
hsa-miR-22-3p
 
 
-0.316
 
 
26.4
 
 
 
 
AIFM1
 
 
hsa-miR-125a-5p
 
 
-0.3
 
 
17.0
 
 
 
 
AK1
 
 
hsa-miR-29a-3p
 
 
-0.333
 
 
38.2
 
 
 
 
AKAP1
 
 
hsa-miR-148b-3p
 
 
-0.381
 
 
31.6
 
 
 
 
AKAP1
 
 
hsa-miR-152-3p
 
 
-0.381
 
 
31.6
 
 
 
 
AKIRIN1
 
 
hsa-miR-27a-3p
 
 
-0.368
 
 
22.9
 
 
 
 
ALCAM
 
 
hsa-miR-34a-5p
 
 
-0.303
 
 
31.9
 
 
 
 
ALCAM
 
 
hsa-miR-148b-3p
 
 
-0.339
 
 
31.9
 
 
 
 
ALCAM
 
 
hsa-miR-152-3p
 
 
-0.347
 
 
31.9
 
 
 
 
ALDOA
 
 
hsa-miR-34a-5p
 
 
-0.655
 
 
1038.5
 
 
 
 
ANAPC16
 
 
hsa-miR-125a-5p
 
 
-0.578
 
 
29.9
 
 
 
 
ANK3
 
 
hsa-miR-34a-5p
 
 
-0.29
 
 
39.6
 
 
 
 
ANK3
 
 
hsa-miR-34a-5p
 
 
-0.347
 
 
39.6
 
 
 
 
ANKHD1
 
 
hsa-miR-23a-3p
 
 
-0.368
 
 
40.4
 
 
 
 
ANKHD1
 
 
hsa-miR-23b-3p
 
 
-0.368
 
 
40.4
 
 
 
 
ANKHD1-EIF4EBP3
 
 
hsa-miR-22-3p
 
 
-0.479
 
 
12.2
 
 
 
 
ANKRD13A
 
 
hsa-miR-22-3p
 
 
-0.478
 
 
25.9
 
 
 
 
ANKRD40
 
 
hsa-miR-27a-3p
 
 
-0.35
 
 
14.2
 
 
 
 
ANP32A
 
 
hsa-miR-132-3p
 
 
-0.374
 
 
50.8
 
 
 
 
ANP32B
 
 
hsa-miR-34a-5p
 
 
-0.29
 
 
104.9
 
 
 
 
ANPEP
 
 
hsa-miR-125a-5p
 
 
-0.372
 
 
38.3
 
 
 
 
ANTXR2
 
 
hsa-miR-29a-3p
 
 
-0.347
 
 
11.4
 
 
 
 
AP3S2
 
 
hsa-miR-34a-5p
 
 
-0.413
 
 
12.7
 
 
 
 
APH1A
 
 
hsa-miR-34a-5p
 
 
-0.304
 
 
87.9
 
 
 
 
APOO
 
 
hsa-miR-181b-5p
 
 
-0.5
 
 
18.5
 
 
 
 
APOO
 
 
hsa-miR-181a-5p
 
 
-0.5
 
 
18.5
 
 
 
 
AREG
 
 
hsa-miR-34a-5p
 
 
-0.62
 
 
11.3
 
 
 
 
ARF4
 
 
hsa-miR-152-3p
 
 
-0.487
 
 
171.4
 
 
 
 
ARF4
 
 
hsa-miR-148b-3p
 
 
-0.487
 
 
171.4
 
 
 
 
ARF5
 
 
hsa-miR-29a-3p
 
 
-0.491
 
 
110.8
 
 
 
 
ARF6
 
 
hsa-miR-181b-5p
 
 
-0.33
 
 
33.7
 
 
 
 
ARF6
 
 
hsa-miR-181a-5p
 
 
-0.33
 
 
33.7
 
 
 
 
ARFGEF1
 
 
hsa-miR-148b-3p
 
 
-0.306
 
 
13.9
 
 
 
 
ARFGEF1
 
 
hsa-miR-152-3p
 
 
-0.306
 
 
13.9
 
 
 
 
ARFIP1
 
 
hsa-miR-152-3p
 
 
-0.474
 
 
12.9
 
 
 
 
ARFIP1
 
 
hsa-miR-148b-3p
 
 
-0.474
 
 
12.9
 
 
 
 
ARHGAP1
 
 
hsa-miR-34a-5p
 
 
-0.369
 
 
43.3
 
 
 
 
ARHGAP21
 
 
hsa-miR-152-3p
 
 
-0.287
 
 
23.1
 
 
 
 
ARHGAP21
 
 
hsa-miR-148b-3p
 
 
-0.287
 
 
23.1
 
 
 
 
ARHGAP26
 
 
hsa-miR-34a-5p
 
 
-0.384
 
 
13.1
 
 
 
 
ARID1B
 
 
hsa-miR-29a-3p
 
 
-0.342
 
 
18.1
 
 
 
 
ARL4A
 
 
hsa-miR-29a-3p
 
 
-0.386
 
 
81.5
 
 
 
 
ARL6IP1
 
 
hsa-miR-148b-3p
 
 
-0.579
 
 
79.4
 
 
 
 
ARL6IP1
 
 
hsa-miR-152-3p
 
 
-0.579
 
 
79.4
 
 
 
 
ARL8B
 
 
hsa-miR-152-3p
 
 
-0.337
 
 
35.3
 
 
 
 
ARL8B
 
 
hsa-miR-148b-3p
 
 
-0.337
 
 
35.3
 
 
 
 
ARMCX3
 
 
hsa-miR-425-5p
 
 
-0.325
 
 
56.1
 
 
 
 
ARPC5
 
 
hsa-miR-22-3p
 
 
-0.679
 
 
43.2
 
 
 
 
ARPP19
 
 
hsa-miR-29a-3p
 
 
-0.371
 
 
28.7
 
 
 
 
ARPP19
 
 
hsa-miR-34a-5p
 
 
-0.497
 
 
28.7
 
 
 
 
ARRB1
 
 
hsa-miR-22-3p
 
 
-0.616
 
 
11.9
 
 
 
 
ARRDC3
 
 
hsa-miR-152-3p
 
 
-0.305
 
 
60.1
 
 
 
 
ARRDC3
 
 
hsa-miR-148b-3p
 
 
-0.305
 
 
60.1
 
 
 
 
ARRDC3
 
 
hsa-miR-29a-3p
 
 
-0.402
 
 
60.1
 
 
 
 
ASAH1
 
 
hsa-miR-27a-3p
 
 
-0.398
 
 
61.4
 
 
 
 
ASF1A
 
 
hsa-miR-23a-3p
 
 
-0.506
 
 
10.1
 
 
 
 
ASF1A
 
 
hsa-miR-23b-3p
 
 
-0.506
 
 
10.1
 
 
 
 
ASPH
 
 
hsa-miR-152-3p
 
 
-0.391
 
 
95.6
 
 
 
 
ASPH
 
 
hsa-miR-148b-3p
 
 
-0.391
 
 
95.6
 
 
 
 
ATG4B
 
 
hsa-miR-34a-5p
 
 
-0.58
 
 
16.0
 
 
 
 
ATMIN
 
 
hsa-miR-34a-5p
 
 
-0.409
 
 
12.0
 
 
 
 
ATP1A2
 
 
hsa-miR-34a-5p
 
 
-0.306
 
 
17.5
 
 
 
 
ATP5MC3
 
 
hsa-miR-425-5p
 
 
-0.328
 
 
173.5
 
 
 
 
ATP5MC3
 
 
hsa-miR-29a-3p
 
 
-0.33
 
 
173.5
 
 
 
 
ATP5MC3
 
 
hsa-miR-27a-3p
 
 
-0.416
 
 
173.5
 
 
 
 
ATP6AP2
 
 
hsa-miR-148b-3p
 
 
-0.592
 
 
39.6
 
 
 
 
ATP6AP2
 
 
hsa-miR-152-3p
 
 
-0.592
 
 
39.6
 
 
 
 
ATP6AP2
 
 
hsa-miR-148b-3p
 
 
-0.668
 
 
39.6
 
 
 
 
ATP6AP2
 
 
hsa-miR-152-3p
 
 
-0.668
 
 
39.6
 
 
 
 
ATP6V1E1
 
 
hsa-miR-23a-3p
 
 
-0.294
 
 
83.2
 
 
 
 
 
 
 seminal vesicle 
 
 
 
 
target_symbol
 
 
mature_mirna_id
 
 
score
 
 
nTPM
 
 
 
 
 
 
AAGAB
 
 
hsa-miR-34a-5p
 
 
-0.319
 
 
19.3
 
 
 
 
AAR2
 
 
hsa-miR-29a-3p
 
 
-0.327
 
 
19.9
 
 
 
 
ABCC5
 
 
hsa-miR-125a-5p
 
 
-0.288
 
 
12.9
 
 
 
 
ABCD3
 
 
hsa-miR-148b-3p
 
 
-0.388
 
 
25.5
 
 
 
 
ABCD3
 
 
hsa-miR-152-3p
 
 
-0.388
 
 
25.5
 
 
 
 
ABCE1
 
 
hsa-miR-29a-3p
 
 
-0.404
 
 
15.9
 
 
 
 
ABHD4
 
 
hsa-miR-29a-3p
 
 
-0.312
 
 
34.6
 
 
 
 
ABHD6
 
 
hsa-miR-125a-5p
 
 
-0.501
 
 
13.5
 
 
 
 
ABTB1
 
 
hsa-miR-125a-5p
 
 
-0.519
 
 
12.8
 
 
 
 
ACBD3
 
 
hsa-miR-34a-5p
 
 
-0.415
 
 
15.4
 
 
 
 
ACSL1
 
 
hsa-miR-34a-5p
 
 
-0.306
 
 
26.5
 
 
 
 
ACSL4
 
 
hsa-miR-34a-5p
 
 
-0.372
 
 
14.0
 
 
 
 
ACSL6
 
 
hsa-miR-125a-5p
 
 
-0.307
 
 
17.0
 
 
 
 
ACTA2
 
 
hsa-miR-27a-3p
 
 
-0.577
 
 
5569.5
 
 
 
 
ACVR1
 
 
hsa-miR-152-3p
 
 
-0.397
 
 
11.3
 
 
 
 
ACVR1
 
 
hsa-miR-148b-3p
 
 
-0.397
 
 
11.3
 
 
 
 
ADAM10
 
 
hsa-miR-152-3p
 
 
-0.348
 
 
11.5
 
 
 
 
ADAM10
 
 
hsa-miR-148b-3p
 
 
-0.356
 
 
11.5
 
 
 
 
ADCY3
 
 
hsa-miR-132-3p
 
 
-0.303
 
 
31.0
 
 
 
 
ADGRA3
 
 
hsa-miR-27a-3p
 
 
-0.296
 
 
10.8
 
 
 
 
ADIPOR2
 
 
hsa-miR-150-5p
 
 
-0.287
 
 
11.2
 
 
 
 
ADIPOR2
 
 
hsa-miR-34a-5p
 
 
-0.33
 
 
11.2
 
 
 
 
ADK
 
 
hsa-miR-34a-5p
 
 
-0.374
 
 
15.1
 
 
 
 
AFG3L2
 
 
hsa-miR-181b-5p
 
 
-0.491
 
 
11.8
 
 
 
 
AFG3L2
 
 
hsa-miR-181a-5p
 
 
-0.491
 
 
11.8
 
 
 
 
AGFG1
 
 
hsa-miR-148b-3p
 
 
-0.332
 
 
22.0
 
 
 
 
AGFG1
 
 
hsa-miR-152-3p
 
 
-0.332
 
 
22.0
 
 
 
 
AGPAT3
 
 
hsa-miR-27a-3p
 
 
-0.29
 
 
10.9
 
 
 
 
AGTRAP
 
 
hsa-miR-34a-5p
 
 
-0.328
 
 
19.8
 
 
 
 
AHR
 
 
hsa-miR-29a-3p
 
 
-0.316
 
 
10.6
 
 
 
 
AIDA
 
 
hsa-miR-22-3p
 
 
-0.316
 
 
39.7
 
 
 
 
AK1
 
 
hsa-miR-29a-3p
 
 
-0.333
 
 
33.8
 
 
 
 
AKAP1
 
 
hsa-miR-148b-3p
 
 
-0.381
 
 
23.3
 
 
 
 
AKAP1
 
 
hsa-miR-152-3p
 
 
-0.381
 
 
23.3
 
 
 
 
AKIRIN1
 
 
hsa-miR-27a-3p
 
 
-0.368
 
 
33.7
 
 
 
 
AKT3
 
 
hsa-miR-29a-3p
 
 
-0.287
 
 
14.7
 
 
 
 
AKT3
 
 
hsa-miR-22-3p
 
 
-0.451
 
 
14.7
 
 
 
 
ALCAM
 
 
hsa-miR-34a-5p
 
 
-0.303
 
 
37.3
 
 
 
 
ALCAM
 
 
hsa-miR-148b-3p
 
 
-0.339
 
 
37.3
 
 
 
 
ALCAM
 
 
hsa-miR-152-3p
 
 
-0.347
 
 
37.3
 
 
 
 
ALDH1A2
 
 
hsa-miR-23b-3p
 
 
-0.346
 
 
50.2
 
 
 
 
ALDH1A2
 
 
hsa-miR-23a-3p
 
 
-0.354
 
 
50.2
 
 
 
 
ALDOA
 
 
hsa-miR-34a-5p
 
 
-0.655
 
 
561.7
 
 
 
 
AMOT
 
 
hsa-miR-29a-3p
 
 
-0.304
 
 
24.6
 
 
 
 
ANAPC16
 
 
hsa-miR-125a-5p
 
 
-0.578
 
 
20.1
 
 
 
 
ANK2
 
 
hsa-miR-34a-5p
 
 
-0.317
 
 
10.2
 
 
 
 
ANK3
 
 
hsa-miR-34a-5p
 
 
-0.29
 
 
37.0
 
 
 
 
ANK3
 
 
hsa-miR-34a-5p
 
 
-0.347
 
 
37.0
 
 
 
 
ANKHD1
 
 
hsa-miR-23a-3p
 
 
-0.368
 
 
26.1
 
 
 
 
ANKHD1
 
 
hsa-miR-23b-3p
 
 
-0.368
 
 
26.1
 
 
 
 
ANKRD13A
 
 
hsa-miR-22-3p
 
 
-0.478
 
 
23.1
 
 
 
 
ANKRD40
 
 
hsa-miR-27a-3p
 
 
-0.35
 
 
23.3
 
 
 
 
ANP32A
 
 
hsa-miR-132-3p
 
 
-0.374
 
 
62.9
 
 
 
 
ANP32B
 
 
hsa-miR-34a-5p
 
 
-0.29
 
 
67.2
 
 
 
 
ANTXR2
 
 
hsa-miR-29a-3p
 
 
-0.347
 
 
85.0
 
 
 
 
AP3S2
 
 
hsa-miR-34a-5p
 
 
-0.413
 
 
20.1
 
 
 
 
APH1A
 
 
hsa-miR-34a-5p
 
 
-0.304
 
 
64.2
 
 
 
 
APOO
 
 
hsa-miR-181b-5p
 
 
-0.5
 
 
14.1
 
 
 
 
APOO
 
 
hsa-miR-181a-5p
 
 
-0.5
 
 
14.1
 
 
 
 
ARF4
 
 
hsa-miR-152-3p
 
 
-0.487
 
 
125.1
 
 
 
 
ARF4
 
 
hsa-miR-148b-3p
 
 
-0.487
 
 
125.1
 
 
 
 
ARF5
 
 
hsa-miR-29a-3p
 
 
-0.491
 
 
67.1
 
 
 
 
ARF6
 
 
hsa-miR-181b-5p
 
 
-0.33
 
 
18.1
 
 
 
 
ARF6
 
 
hsa-miR-181a-5p
 
 
-0.33
 
 
18.1
 
 
 
 
ARFGEF1
 
 
hsa-miR-148b-3p
 
 
-0.306
 
 
11.4
 
 
 
 
ARFGEF1
 
 
hsa-miR-152-3p
 
 
-0.306
 
 
11.4
 
 
 
 
ARFIP1
 
 
hsa-miR-152-3p
 
 
-0.474
 
 
16.2
 
 
 
 
ARFIP1
 
 
hsa-miR-148b-3p
 
 
-0.474
 
 
16.2
 
 
 
 
ARHGAP1
 
 
hsa-miR-34a-5p
 
 
-0.369
 
 
58.9
 
 
 
 
ARHGAP21
 
 
hsa-miR-152-3p
 
 
-0.287
 
 
10.8
 
 
 
 
ARHGAP21
 
 
hsa-miR-148b-3p
 
 
-0.287
 
 
10.8
 
 
 
 
ARID1B
 
 
hsa-miR-29a-3p
 
 
-0.342
 
 
11.6
 
 
 
 
ARL4A
 
 
hsa-miR-29a-3p
 
 
-0.386
 
 
15.5
 
 
 
 
ARL6IP1
 
 
hsa-miR-148b-3p
 
 
-0.579
 
 
66.4
 
 
 
 
ARL6IP1
 
 
hsa-miR-152-3p
 
 
-0.579
 
 
66.4
 
 
 
 
ARL8B
 
 
hsa-miR-152-3p
 
 
-0.337
 
 
39.4
 
 
 
 
ARL8B
 
 
hsa-miR-148b-3p
 
 
-0.337
 
 
39.4
 
 
 
 
ARMCX3
 
 
hsa-miR-425-5p
 
 
-0.325
 
 
44.1
 
 
 
 
ARPC5
 
 
hsa-miR-22-3p
 
 
-0.679
 
 
33.8
 
 
 
 
ARPP19
 
 
hsa-miR-29a-3p
 
 
-0.371
 
 
38.5
 
 
 
 
ARPP19
 
 
hsa-miR-34a-5p
 
 
-0.497
 
 
38.5
 
 
 
 
ARRDC3
 
 
hsa-miR-152-3p
 
 
-0.305
 
 
22.9
 
 
 
 
ARRDC3
 
 
hsa-miR-148b-3p
 
 
-0.305
 
 
22.9
 
 
 
 
ARRDC3
 
 
hsa-miR-29a-3p
 
 
-0.402
 
 
22.9
 
 
 
 
ASAH1
 
 
hsa-miR-27a-3p
 
 
-0.398
 
 
73.4
 
 
 
 
ASF1A
 
 
hsa-miR-23a-3p
 
 
-0.506
 
 
10.4
 
 
 
 
ASF1A
 
 
hsa-miR-23b-3p
 
 
-0.506
 
 
10.4
 
 
 
 
ASPH
 
 
hsa-miR-152-3p
 
 
-0.391
 
 
65.8
 
 
 
 
ASPH
 
 
hsa-miR-148b-3p
 
 
-0.391
 
 
65.8
 
 
 
 
ATMIN
 
 
hsa-miR-34a-5p
 
 
-0.409
 
 
13.9
 
 
 
 
ATP1A2
 
 
hsa-miR-34a-5p
 
 
-0.306
 
 
26.9
 
 
 
 
ATP2B4
 
 
hsa-miR-152-3p
 
 
-0.337
 
 
66.2
 
 
 
 
ATP2B4
 
 
hsa-miR-148b-3p
 
 
-0.337
 
 
66.2
 
 
 
 
ATP5MC3
 
 
hsa-miR-425-5p
 
 
-0.328
 
 
107.3
 
 
 
 
ATP5MC3
 
 
hsa-miR-29a-3p
 
 
-0.33
 
 
107.3
 
 
 
 
ATP5MC3
 
 
hsa-miR-27a-3p
 
 
-0.416
 
 
107.3
 
 
 
 
ATP6AP2
 
 
hsa-miR-148b-3p
 
 
-0.592
 
 
92.2
 
 
 
 
ATP6AP2
 
 
hsa-miR-152-3p
 
 
-0.592
 
 
92.2
 
 
 
 
ATP6AP2
 
 
hsa-miR-148b-3p
 
 
-0.668
 
 
92.2
 
 
 
 
ATP6AP2
 
 
hsa-miR-152-3p
 
 
-0.668
 
 
92.2
 
 
 
 
 
 
 skeletal muscle 
 
 
 
 
target_symbol
 
 
mature_mirna_id
 
 
score
 
 
nTPM
 
 
 
 
 
 
AAGAB
 
 
hsa-miR-34a-5p
 
 
-0.319
 
 
30.0
 
 
 
 
AAR2
 
 
hsa-miR-29a-3p
 
 
-0.327
 
 
22.2
 
 
 
 
ABCB10
 
 
hsa-miR-152-3p
 
 
-0.291
 
 
12.0
 
 
 
 
ABCB10
 
 
hsa-miR-148b-3p
 
 
-0.291
 
 
12.0
 
 
 
 
ABCB6
 
 
hsa-miR-29a-3p
 
 
-0.48
 
 
26.6
 
 
 
 
ABCB7
 
 
hsa-miR-148b-3p
 
 
-0.515
 
 
29.2
 
 
 
 
ABCB7
 
 
hsa-miR-152-3p
 
 
-0.515
 
 
29.2
 
 
 
 
ABCC5
 
 
hsa-miR-125a-5p
 
 
-0.288
 
 
118.1
 
 
 
 
ABCD3
 
 
hsa-miR-148b-3p
 
 
-0.388
 
 
22.0
 
 
 
 
ABCD3
 
 
hsa-miR-152-3p
 
 
-0.388
 
 
22.0
 
 
 
 
ABCE1
 
 
hsa-miR-29a-3p
 
 
-0.404
 
 
27.0
 
 
 
 
ABHD18
 
 
hsa-miR-29a-3p
 
 
-0.321
 
 
10.1
 
 
 
 
ABHD18
 
 
hsa-miR-29a-3p
 
 
-0.34
 
 
10.1
 
 
 
 
ABHD3
 
 
hsa-miR-125a-5p
 
 
-0.453
 
 
13.6
 
 
 
 
ABHD4
 
 
hsa-miR-29a-3p
 
 
-0.312
 
 
26.2
 
 
 
 
ABHD5
 
 
hsa-miR-132-3p
 
 
-0.323
 
 
12.3
 
 
 
 
ABHD5
 
 
hsa-miR-27a-3p
 
 
-0.407
 
 
12.3
 
 
 
 
ABRAXAS2
 
 
hsa-miR-34a-5p
 
 
-0.538
 
 
17.8
 
 
 
 
ABTB1
 
 
hsa-miR-125a-5p
 
 
-0.519
 
 
44.0
 
 
 
 
ACBD3
 
 
hsa-miR-34a-5p
 
 
-0.415
 
 
20.2
 
 
 
 
ACHE
 
 
hsa-miR-125a-5p
 
 
-0.308
 
 
152.9
 
 
 
 
ACHE
 
 
hsa-miR-125a-5p
 
 
-0.361
 
 
152.9
 
 
 
 
ACSL1
 
 
hsa-miR-34a-5p
 
 
-0.306
 
 
697.4
 
 
 
 
ACSL4
 
 
hsa-miR-34a-5p
 
 
-0.372
 
 
39.3
 
 
 
 
ACTA2
 
 
hsa-miR-27a-3p
 
 
-0.577
 
 
194.7
 
 
 
 
ACTN2
 
 
hsa-miR-23a-3p
 
 
-0.294
 
 
1961.6
 
 
 
 
ACTN2
 
 
hsa-miR-29a-3p
 
 
-0.311
 
 
1961.6
 
 
 
 
ACVR1
 
 
hsa-miR-152-3p
 
 
-0.397
 
 
15.5
 
 
 
 
ACVR1
 
 
hsa-miR-148b-3p
 
 
-0.397
 
 
15.5
 
 
 
 
ACVR2A
 
 
hsa-miR-125a-5p
 
 
-0.301
 
 
16.4
 
 
 
 
ADCY2
 
 
hsa-miR-152-3p
 
 
-0.299
 
 
64.1
 
 
 
 
ADCY2
 
 
hsa-miR-148b-3p
 
 
-0.299
 
 
64.1
 
 
 
 
ADCY3
 
 
hsa-miR-132-3p
 
 
-0.303
 
 
14.1
 
 
 
 
ADGRA3
 
 
hsa-miR-27a-3p
 
 
-0.296
 
 
15.9
 
 
 
 
ADGRF5
 
 
hsa-miR-148b-3p
 
 
-0.354
 
 
29.0
 
 
 
 
ADGRF5
 
 
hsa-miR-152-3p
 
 
-0.354
 
 
29.0
 
 
 
 
ADIPOR2
 
 
hsa-miR-150-5p
 
 
-0.287
 
 
41.4
 
 
 
 
ADIPOR2
 
 
hsa-miR-34a-5p
 
 
-0.33
 
 
41.4
 
 
 
 
ADK
 
 
hsa-miR-34a-5p
 
 
-0.374
 
 
38.8
 
 
 
 
ADO
 
 
hsa-miR-34a-5p
 
 
-0.377
 
 
10.6
 
 
 
 
AFG3L2
 
 
hsa-miR-181b-5p
 
 
-0.491
 
 
96.6
 
 
 
 
AFG3L2
 
 
hsa-miR-181a-5p
 
 
-0.491
 
 
96.6
 
 
 
 
AGFG1
 
 
hsa-miR-148b-3p
 
 
-0.332
 
 
31.6
 
 
 
 
AGFG1
 
 
hsa-miR-152-3p
 
 
-0.332
 
 
31.6
 
 
 
 
AGGF1
 
 
hsa-miR-125a-5p
 
 
-0.331
 
 
12.2
 
 
 
 
AGMAT
 
 
hsa-miR-22-3p
 
 
-0.294
 
 
32.1
 
 
 
 
AGO2
 
 
hsa-miR-132-3p
 
 
-0.406
 
 
16.4
 
 
 
 
AGO2
 
 
hsa-miR-27a-3p
 
 
-0.486
 
 
16.4
 
 
 
 
AGO2
 
 
hsa-miR-23a-3p
 
 
-0.576
 
 
16.4
 
 
 
 
AGO2
 
 
hsa-miR-23b-3p
 
 
-0.587
 
 
16.4
 
 
 
 
AGO2
 
 
hsa-miR-27a-3p
 
 
-0.646
 
 
16.4
 
 
 
 
AGO2
 
 
hsa-miR-125a-5p
 
 
-0.811
 
 
16.4
 
 
 
 
AGO4
 
 
hsa-miR-34a-5p
 
 
-0.306
 
 
12.4
 
 
 
 
AGPAT3
 
 
hsa-miR-27a-3p
 
 
-0.29
 
 
56.6
 
 
 
 
AGTRAP
 
 
hsa-miR-34a-5p
 
 
-0.328
 
 
50.5
 
 
 
 
AHCYL2
 
 
hsa-miR-34a-5p
 
 
-0.359
 
 
35.0
 
 
 
 
AHDC1
 
 
hsa-miR-148b-3p
 
 
-0.413
 
 
29.2
 
 
 
 
AHDC1
 
 
hsa-miR-152-3p
 
 
-0.413
 
 
29.2
 
 
 
 
AIDA
 
 
hsa-miR-22-3p
 
 
-0.316
 
 
26.6
 
 
 
 
AIFM1
 
 
hsa-miR-125a-5p
 
 
-0.3
 
 
58.6
 
 
 
 
AK1
 
 
hsa-miR-29a-3p
 
 
-0.333
 
 
1262.8
 
 
 
 
AKAP1
 
 
hsa-miR-148b-3p
 
 
-0.381
 
 
194.7
 
 
 
 
AKAP1
 
 
hsa-miR-152-3p
 
 
-0.381
 
 
194.7
 
 
 
 
AKIRIN1
 
 
hsa-miR-27a-3p
 
 
-0.368
 
 
28.7
 
 
 
 
ALDH1A2
 
 
hsa-miR-23b-3p
 
 
-0.346
 
 
10.0
 
 
 
 
ALDH1A2
 
 
hsa-miR-23a-3p
 
 
-0.354
 
 
10.0
 
 
 
 
ALDOA
 
 
hsa-miR-34a-5p
 
 
-0.655
 
 
25093.7
 
 
 
 
AMOT
 
 
hsa-miR-29a-3p
 
 
-0.304
 
 
37.2
 
 
 
 
ANAPC16
 
 
hsa-miR-125a-5p
 
 
-0.578
 
 
57.2
 
 
 
 
ANK2
 
 
hsa-miR-34a-5p
 
 
-0.317
 
 
120.2
 
 
 
 
ANK3
 
 
hsa-miR-34a-5p
 
 
-0.29
 
 
75.3
 
 
 
 
ANK3
 
 
hsa-miR-34a-5p
 
 
-0.347
 
 
75.3
 
 
 
 
ANKHD1
 
 
hsa-miR-23a-3p
 
 
-0.368
 
 
67.6
 
 
 
 
ANKHD1
 
 
hsa-miR-23b-3p
 
 
-0.368
 
 
67.6
 
 
 
 
ANKHD1-EIF4EBP3
 
 
hsa-miR-22-3p
 
 
-0.479
 
 
10.4
 
 
 
 
ANKRD40
 
 
hsa-miR-27a-3p
 
 
-0.35
 
 
100.2
 
 
 
 
ANP32A
 
 
hsa-miR-132-3p
 
 
-0.374
 
 
57.9
 
 
 
 
ANP32B
 
 
hsa-miR-34a-5p
 
 
-0.29
 
 
103.8
 
 
 
 
ANTXR2
 
 
hsa-miR-29a-3p
 
 
-0.347
 
 
12.3
 
 
 
 
AP3S2
 
 
hsa-miR-34a-5p
 
 
-0.413
 
 
42.0
 
 
 
 
APH1A
 
 
hsa-miR-34a-5p
 
 
-0.304
 
 
56.0
 
 
 
 
APOO
 
 
hsa-miR-181b-5p
 
 
-0.5
 
 
43.1
 
 
 
 
APOO
 
 
hsa-miR-181a-5p
 
 
-0.5
 
 
43.1
 
 
 
 
AREG
 
 
hsa-miR-34a-5p
 
 
-0.62
 
 
17.7
 
 
 
 
ARF4
 
 
hsa-miR-152-3p
 
 
-0.487
 
 
59.5
 
 
 
 
ARF4
 
 
hsa-miR-148b-3p
 
 
-0.487
 
 
59.5
 
 
 
 
ARF5
 
 
hsa-miR-29a-3p
 
 
-0.491
 
 
145.7
 
 
 
 
ARF6
 
 
hsa-miR-181b-5p
 
 
-0.33
 
 
21.4
 
 
 
 
ARF6
 
 
hsa-miR-181a-5p
 
 
-0.33
 
 
21.4
 
 
 
 
ARFGEF1
 
 
hsa-miR-148b-3p
 
 
-0.306
 
 
12.2
 
 
 
 
ARFGEF1
 
 
hsa-miR-152-3p
 
 
-0.306
 
 
12.2
 
 
 
 
ARHGAP1
 
 
hsa-miR-34a-5p
 
 
-0.369
 
 
28.5
 
 
 
 
ARHGAP21
 
 
hsa-miR-152-3p
 
 
-0.287
 
 
15.7
 
 
 
 
ARHGAP21
 
 
hsa-miR-148b-3p
 
 
-0.287
 
 
15.7
 
 
 
 
ARHGAP26
 
 
hsa-miR-34a-5p
 
 
-0.384
 
 
11.5
 
 
 
 
ARID1B
 
 
hsa-miR-29a-3p
 
 
-0.342
 
 
30.4
 
 
 
 
ARL6IP1
 
 
hsa-miR-148b-3p
 
 
-0.579
 
 
21.6
 
 
 
 
ARL6IP1
 
 
hsa-miR-152-3p
 
 
-0.579
 
 
21.6
 
 
 
 
ARL8B
 
 
hsa-miR-152-3p
 
 
-0.337
 
 
98.5
 
 
 
 
ARL8B
 
 
hsa-miR-148b-3p
 
 
-0.337
 
 
98.5
 
 
 
 
 
 
 skin 
 
 
 
 
target_symbol
 
 
mature_mirna_id
 
 
score
 
 
nTPM
 
 
 
 
 
 
AAGAB
 
 
hsa-miR-34a-5p
 
 
-0.319
 
 
11.7
 
 
 
 
AAR2
 
 
hsa-miR-29a-3p
 
 
-0.327
 
 
24.6
 
 
 
 
ABCB7
 
 
hsa-miR-148b-3p
 
 
-0.515
 
 
12.7
 
 
 
 
ABCB7
 
 
hsa-miR-152-3p
 
 
-0.515
 
 
12.7
 
 
 
 
ABCC5
 
 
hsa-miR-125a-5p
 
 
-0.288
 
 
22.7
 
 
 
 
ABCD3
 
 
hsa-miR-148b-3p
 
 
-0.388
 
 
33.6
 
 
 
 
ABCD3
 
 
hsa-miR-152-3p
 
 
-0.388
 
 
33.6
 
 
 
 
ABCE1
 
 
hsa-miR-29a-3p
 
 
-0.404
 
 
23.8
 
 
 
 
ABHD17C
 
 
hsa-miR-27a-3p
 
 
-0.435
 
 
17.9
 
 
 
 
ABHD4
 
 
hsa-miR-29a-3p
 
 
-0.312
 
 
20.2
 
 
 
 
ABHD5
 
 
hsa-miR-132-3p
 
 
-0.323
 
 
29.7
 
 
 
 
ABHD5
 
 
hsa-miR-27a-3p
 
 
-0.407
 
 
29.7
 
 
 
 
ABHD6
 
 
hsa-miR-125a-5p
 
 
-0.501
 
 
24.1
 
 
 
 
ABRAXAS2
 
 
hsa-miR-34a-5p
 
 
-0.538
 
 
11.0
 
 
 
 
ABTB1
 
 
hsa-miR-125a-5p
 
 
-0.519
 
 
25.3
 
 
 
 
ACBD3
 
 
hsa-miR-34a-5p
 
 
-0.415
 
 
18.1
 
 
 
 
ACSL1
 
 
hsa-miR-34a-5p
 
 
-0.306
 
 
164.9
 
 
 
 
ACTA2
 
 
hsa-miR-27a-3p
 
 
-0.577
 
 
331.5
 
 
 
 
ACVR2A
 
 
hsa-miR-125a-5p
 
 
-0.301
 
 
18.1
 
 
 
 
ADAM10
 
 
hsa-miR-152-3p
 
 
-0.348
 
 
14.8
 
 
 
 
ADAM10
 
 
hsa-miR-148b-3p
 
 
-0.356
 
 
14.8
 
 
 
 
ADCY3
 
 
hsa-miR-132-3p
 
 
-0.303
 
 
36.0
 
 
 
 
ADIPOR2
 
 
hsa-miR-150-5p
 
 
-0.287
 
 
30.8
 
 
 
 
ADIPOR2
 
 
hsa-miR-34a-5p
 
 
-0.33
 
 
30.8
 
 
 
 
ADK
 
 
hsa-miR-34a-5p
 
 
-0.374
 
 
32.7
 
 
 
 
ADRA2A
 
 
hsa-miR-23a-3p
 
 
-0.371
 
 
10.2
 
 
 
 
ADRA2A
 
 
hsa-miR-23b-3p
 
 
-0.371
 
 
10.2
 
 
 
 
AFG3L2
 
 
hsa-miR-181b-5p
 
 
-0.491
 
 
15.1
 
 
 
 
AFG3L2
 
 
hsa-miR-181a-5p
 
 
-0.491
 
 
15.1
 
 
 
 
AGFG1
 
 
hsa-miR-148b-3p
 
 
-0.332
 
 
34.3
 
 
 
 
AGFG1
 
 
hsa-miR-152-3p
 
 
-0.332
 
 
34.3
 
 
 
 
AGGF1
 
 
hsa-miR-125a-5p
 
 
-0.331
 
 
11.8
 
 
 
 
AGO4
 
 
hsa-miR-34a-5p
 
 
-0.306
 
 
18.9
 
 
 
 
AGPAT3
 
 
hsa-miR-27a-3p
 
 
-0.29
 
 
23.2
 
 
 
 
AGTRAP
 
 
hsa-miR-34a-5p
 
 
-0.328
 
 
36.2
 
 
 
 
AHCYL2
 
 
hsa-miR-34a-5p
 
 
-0.359
 
 
22.9
 
 
 
 
AHDC1
 
 
hsa-miR-148b-3p
 
 
-0.413
 
 
29.8
 
 
 
 
AHDC1
 
 
hsa-miR-152-3p
 
 
-0.413
 
 
29.8
 
 
 
 
AHR
 
 
hsa-miR-29a-3p
 
 
-0.316
 
 
20.7
 
 
 
 
AIDA
 
 
hsa-miR-22-3p
 
 
-0.316
 
 
25.1
 
 
 
 
AIFM1
 
 
hsa-miR-125a-5p
 
 
-0.3
 
 
10.3
 
 
 
 
AK1
 
 
hsa-miR-29a-3p
 
 
-0.333
 
 
17.4
 
 
 
 
AKAP1
 
 
hsa-miR-148b-3p
 
 
-0.381
 
 
26.4
 
 
 
 
AKAP1
 
 
hsa-miR-152-3p
 
 
-0.381
 
 
26.4
 
 
 
 
AKIRIN1
 
 
hsa-miR-27a-3p
 
 
-0.368
 
 
16.7
 
 
 
 
ALCAM
 
 
hsa-miR-34a-5p
 
 
-0.303
 
 
10.3
 
 
 
 
ALCAM
 
 
hsa-miR-148b-3p
 
 
-0.339
 
 
10.3
 
 
 
 
ALCAM
 
 
hsa-miR-152-3p
 
 
-0.347
 
 
10.3
 
 
 
 
ALDOA
 
 
hsa-miR-34a-5p
 
 
-0.655
 
 
770.0
 
 
 
 
ANAPC16
 
 
hsa-miR-125a-5p
 
 
-0.578
 
 
14.6
 
 
 
 
ANK3
 
 
hsa-miR-34a-5p
 
 
-0.29
 
 
37.1
 
 
 
 
ANK3
 
 
hsa-miR-34a-5p
 
 
-0.347
 
 
37.1
 
 
 
 
ANKHD1
 
 
hsa-miR-23a-3p
 
 
-0.368
 
 
48.3
 
 
 
 
ANKHD1
 
 
hsa-miR-23b-3p
 
 
-0.368
 
 
48.3
 
 
 
 
ANKHD1-EIF4EBP3
 
 
hsa-miR-22-3p
 
 
-0.479
 
 
15.0
 
 
 
 
ANKRD13A
 
 
hsa-miR-22-3p
 
 
-0.478
 
 
20.5
 
 
 
 
ANKRD40
 
 
hsa-miR-27a-3p
 
 
-0.35
 
 
13.5
 
 
 
 
ANP32A
 
 
hsa-miR-132-3p
 
 
-0.374
 
 
34.1
 
 
 
 
ANP32B
 
 
hsa-miR-34a-5p
 
 
-0.29
 
 
140.0
 
 
 
 
ANPEP
 
 
hsa-miR-125a-5p
 
 
-0.372
 
 
29.7
 
 
 
 
ANTXR2
 
 
hsa-miR-29a-3p
 
 
-0.347
 
 
13.8
 
 
 
 
APH1A
 
 
hsa-miR-34a-5p
 
 
-0.304
 
 
113.8
 
 
 
 
AREG
 
 
hsa-miR-34a-5p
 
 
-0.62
 
 
14.9
 
 
 
 
ARF4
 
 
hsa-miR-152-3p
 
 
-0.487
 
 
61.3
 
 
 
 
ARF4
 
 
hsa-miR-148b-3p
 
 
-0.487
 
 
61.3
 
 
 
 
ARF5
 
 
hsa-miR-29a-3p
 
 
-0.491
 
 
215.0
 
 
 
 
ARF6
 
 
hsa-miR-181b-5p
 
 
-0.33
 
 
50.6
 
 
 
 
ARF6
 
 
hsa-miR-181a-5p
 
 
-0.33
 
 
50.6
 
 
 
 
ARFGEF1
 
 
hsa-miR-148b-3p
 
 
-0.306
 
 
12.9
 
 
 
 
ARFGEF1
 
 
hsa-miR-152-3p
 
 
-0.306
 
 
12.9
 
 
 
 
ARFIP1
 
 
hsa-miR-152-3p
 
 
-0.474
 
 
16.4
 
 
 
 
ARFIP1
 
 
hsa-miR-148b-3p
 
 
-0.474
 
 
16.4
 
 
 
 
ARHGAP1
 
 
hsa-miR-34a-5p
 
 
-0.369
 
 
61.6
 
 
 
 
ARID1B
 
 
hsa-miR-29a-3p
 
 
-0.342
 
 
21.3
 
 
 
 
ARL4A
 
 
hsa-miR-29a-3p
 
 
-0.386
 
 
85.3
 
 
 
 
ARL6IP1
 
 
hsa-miR-148b-3p
 
 
-0.579
 
 
62.4
 
 
 
 
ARL6IP1
 
 
hsa-miR-152-3p
 
 
-0.579
 
 
62.4
 
 
 
 
ARL8B
 
 
hsa-miR-152-3p
 
 
-0.337
 
 
64.4
 
 
 
 
ARL8B
 
 
hsa-miR-148b-3p
 
 
-0.337
 
 
64.4
 
 
 
 
ARMCX3
 
 
hsa-miR-425-5p
 
 
-0.325
 
 
10.4
 
 
 
 
ARPC5
 
 
hsa-miR-22-3p
 
 
-0.679
 
 
40.3
 
 
 
 
ARPP19
 
 
hsa-miR-29a-3p
 
 
-0.371
 
 
28.9
 
 
 
 
ARPP19
 
 
hsa-miR-34a-5p
 
 
-0.497
 
 
28.9
 
 
 
 
ARRB1
 
 
hsa-miR-22-3p
 
 
-0.616
 
 
15.9
 
 
 
 
ARRDC3
 
 
hsa-miR-152-3p
 
 
-0.305
 
 
38.6
 
 
 
 
ARRDC3
 
 
hsa-miR-148b-3p
 
 
-0.305
 
 
38.6
 
 
 
 
ARRDC3
 
 
hsa-miR-29a-3p
 
 
-0.402
 
 
38.6
 
 
 
 
ASAH1
 
 
hsa-miR-27a-3p
 
 
-0.398
 
 
49.7
 
 
 
 
ASAP2
 
 
hsa-miR-29a-3p
 
 
-0.348
 
 
12.3
 
 
 
 
ASF1A
 
 
hsa-miR-23a-3p
 
 
-0.506
 
 
11.0
 
 
 
 
ASF1A
 
 
hsa-miR-23b-3p
 
 
-0.506
 
 
11.0
 
 
 
 
ASPH
 
 
hsa-miR-152-3p
 
 
-0.391
 
 
33.3
 
 
 
 
ASPH
 
 
hsa-miR-148b-3p
 
 
-0.391
 
 
33.3
 
 
 
 
ASPHD2
 
 
hsa-miR-34a-5p
 
 
-0.354
 
 
10.4
 
 
 
 
ATG4B
 
 
hsa-miR-34a-5p
 
 
-0.58
 
 
18.6
 
 
 
 
ATMIN
 
 
hsa-miR-34a-5p
 
 
-0.409
 
 
16.6
 
 
 
 
ATP2B4
 
 
hsa-miR-152-3p
 
 
-0.337
 
 
51.7
 
 
 
 
ATP2B4
 
 
hsa-miR-148b-3p
 
 
-0.337
 
 
51.7
 
 
 
 
ATP5MC3
 
 
hsa-miR-425-5p
 
 
-0.328
 
 
162.4
 
 
 
 
ATP5MC3
 
 
hsa-miR-29a-3p
 
 
-0.33
 
 
162.4
 
 
 
 
 
 
 small intestine 
 
 
 
 
target_symbol
 
 
mature_mirna_id
 
 
score
 
 
nTPM
 
 
 
 
 
 
AAGAB
 
 
hsa-miR-34a-5p
 
 
-0.319
 
 
18.4
 
 
 
 
AAR2
 
 
hsa-miR-29a-3p
 
 
-0.327
 
 
18.1
 
 
 
 
ABCB10
 
 
hsa-miR-152-3p
 
 
-0.291
 
 
13.1
 
 
 
 
ABCB10
 
 
hsa-miR-148b-3p
 
 
-0.291
 
 
13.1
 
 
 
 
ABCB7
 
 
hsa-miR-148b-3p
 
 
-0.515
 
 
11.2
 
 
 
 
ABCB7
 
 
hsa-miR-152-3p
 
 
-0.515
 
 
11.2
 
 
 
 
ABCC5
 
 
hsa-miR-125a-5p
 
 
-0.288
 
 
22.9
 
 
 
 
ABCD3
 
 
hsa-miR-148b-3p
 
 
-0.388
 
 
43.4
 
 
 
 
ABCD3
 
 
hsa-miR-152-3p
 
 
-0.388
 
 
43.4
 
 
 
 
ABCE1
 
 
hsa-miR-29a-3p
 
 
-0.404
 
 
16.7
 
 
 
 
ABHD17C
 
 
hsa-miR-27a-3p
 
 
-0.435
 
 
28.8
 
 
 
 
ABHD3
 
 
hsa-miR-125a-5p
 
 
-0.453
 
 
53.0
 
 
 
 
ABHD4
 
 
hsa-miR-29a-3p
 
 
-0.312
 
 
18.9
 
 
 
 
ABHD6
 
 
hsa-miR-125a-5p
 
 
-0.501
 
 
49.2
 
 
 
 
ABRAXAS2
 
 
hsa-miR-34a-5p
 
 
-0.538
 
 
10.1
 
 
 
 
ABTB1
 
 
hsa-miR-125a-5p
 
 
-0.519
 
 
31.9
 
 
 
 
ACBD3
 
 
hsa-miR-34a-5p
 
 
-0.415
 
 
16.5
 
 
 
 
ACSL1
 
 
hsa-miR-34a-5p
 
 
-0.306
 
 
25.0
 
 
 
 
ACSL4
 
 
hsa-miR-34a-5p
 
 
-0.372
 
 
12.1
 
 
 
 
ACTA2
 
 
hsa-miR-27a-3p
 
 
-0.577
 
 
677.1
 
 
 
 
ADA
 
 
hsa-miR-29a-3p
 
 
-0.376
 
 
15.5
 
 
 
 
ADAM10
 
 
hsa-miR-152-3p
 
 
-0.348
 
 
15.8
 
 
 
 
ADAM10
 
 
hsa-miR-148b-3p
 
 
-0.356
 
 
15.8
 
 
 
 
ADAP1
 
 
hsa-miR-27a-3p
 
 
-0.302
 
 
25.6
 
 
 
 
ADCY3
 
 
hsa-miR-132-3p
 
 
-0.303
 
 
31.8
 
 
 
 
ADIPOR2
 
 
hsa-miR-150-5p
 
 
-0.287
 
 
31.4
 
 
 
 
ADIPOR2
 
 
hsa-miR-34a-5p
 
 
-0.33
 
 
31.4
 
 
 
 
ADK
 
 
hsa-miR-34a-5p
 
 
-0.374
 
 
11.7
 
 
 
 
AFG3L2
 
 
hsa-miR-181b-5p
 
 
-0.491
 
 
34.0
 
 
 
 
AFG3L2
 
 
hsa-miR-181a-5p
 
 
-0.491
 
 
34.0
 
 
 
 
AGFG1
 
 
hsa-miR-148b-3p
 
 
-0.332
 
 
33.9
 
 
 
 
AGFG1
 
 
hsa-miR-152-3p
 
 
-0.332
 
 
33.9
 
 
 
 
AGGF1
 
 
hsa-miR-125a-5p
 
 
-0.331
 
 
12.5
 
 
 
 
AGMAT
 
 
hsa-miR-22-3p
 
 
-0.294
 
 
12.6
 
 
 
 
AGPAT3
 
 
hsa-miR-27a-3p
 
 
-0.29
 
 
49.3
 
 
 
 
AGTRAP
 
 
hsa-miR-34a-5p
 
 
-0.328
 
 
23.9
 
 
 
 
AHCYL2
 
 
hsa-miR-34a-5p
 
 
-0.359
 
 
36.4
 
 
 
 
AHR
 
 
hsa-miR-29a-3p
 
 
-0.316
 
 
13.6
 
 
 
 
AIDA
 
 
hsa-miR-22-3p
 
 
-0.316
 
 
27.3
 
 
 
 
AIFM1
 
 
hsa-miR-125a-5p
 
 
-0.3
 
 
28.2
 
 
 
 
AK1
 
 
hsa-miR-29a-3p
 
 
-0.333
 
 
15.6
 
 
 
 
AKAP1
 
 
hsa-miR-148b-3p
 
 
-0.381
 
 
52.5
 
 
 
 
AKAP1
 
 
hsa-miR-152-3p
 
 
-0.381
 
 
52.5
 
 
 
 
AKIRIN1
 
 
hsa-miR-27a-3p
 
 
-0.368
 
 
25.6
 
 
 
 
ALCAM
 
 
hsa-miR-34a-5p
 
 
-0.303
 
 
16.4
 
 
 
 
ALCAM
 
 
hsa-miR-148b-3p
 
 
-0.339
 
 
16.4
 
 
 
 
ALCAM
 
 
hsa-miR-152-3p
 
 
-0.347
 
 
16.4
 
 
 
 
ALDOA
 
 
hsa-miR-34a-5p
 
 
-0.655
 
 
568.6
 
 
 
 
ANAPC16
 
 
hsa-miR-125a-5p
 
 
-0.578
 
 
19.0
 
 
 
 
ANK3
 
 
hsa-miR-34a-5p
 
 
-0.29
 
 
25.5
 
 
 
 
ANK3
 
 
hsa-miR-34a-5p
 
 
-0.347
 
 
25.5
 
 
 
 
ANKHD1
 
 
hsa-miR-23a-3p
 
 
-0.368
 
 
44.2
 
 
 
 
ANKHD1
 
 
hsa-miR-23b-3p
 
 
-0.368
 
 
44.2
 
 
 
 
ANKHD1-EIF4EBP3
 
 
hsa-miR-22-3p
 
 
-0.479
 
 
11.5
 
 
 
 
ANKRD13A
 
 
hsa-miR-22-3p
 
 
-0.478
 
 
15.8
 
 
 
 
ANKRD40
 
 
hsa-miR-27a-3p
 
 
-0.35
 
 
15.4
 
 
 
 
ANP32A
 
 
hsa-miR-132-3p
 
 
-0.374
 
 
72.4
 
 
 
 
ANP32B
 
 
hsa-miR-34a-5p
 
 
-0.29
 
 
119.5
 
 
 
 
ANPEP
 
 
hsa-miR-125a-5p
 
 
-0.372
 
 
1857.1
 
 
 
 
ANTXR2
 
 
hsa-miR-29a-3p
 
 
-0.347
 
 
35.6
 
 
 
 
APH1A
 
 
hsa-miR-34a-5p
 
 
-0.304
 
 
76.3
 
 
 
 
APOO
 
 
hsa-miR-181b-5p
 
 
-0.5
 
 
11.3
 
 
 
 
APOO
 
 
hsa-miR-181a-5p
 
 
-0.5
 
 
11.3
 
 
 
 
AQP11
 
 
hsa-miR-27a-3p
 
 
-0.666
 
 
23.9
 
 
 
 
ARF4
 
 
hsa-miR-152-3p
 
 
-0.487
 
 
102.8
 
 
 
 
ARF4
 
 
hsa-miR-148b-3p
 
 
-0.487
 
 
102.8
 
 
 
 
ARF5
 
 
hsa-miR-29a-3p
 
 
-0.491
 
 
103.1
 
 
 
 
ARF6
 
 
hsa-miR-181b-5p
 
 
-0.33
 
 
48.2
 
 
 
 
ARF6
 
 
hsa-miR-181a-5p
 
 
-0.33
 
 
48.2
 
 
 
 
ARFGEF1
 
 
hsa-miR-148b-3p
 
 
-0.306
 
 
16.5
 
 
 
 
ARFGEF1
 
 
hsa-miR-152-3p
 
 
-0.306
 
 
16.5
 
 
 
 
ARFIP1
 
 
hsa-miR-152-3p
 
 
-0.474
 
 
20.0
 
 
 
 
ARFIP1
 
 
hsa-miR-148b-3p
 
 
-0.474
 
 
20.0
 
 
 
 
ARHGAP1
 
 
hsa-miR-34a-5p
 
 
-0.369
 
 
44.5
 
 
 
 
ARHGAP21
 
 
hsa-miR-152-3p
 
 
-0.287
 
 
15.2
 
 
 
 
ARHGAP21
 
 
hsa-miR-148b-3p
 
 
-0.287
 
 
15.2
 
 
 
 
ARHGAP26
 
 
hsa-miR-34a-5p
 
 
-0.384
 
 
10.6
 
 
 
 
ARID1B
 
 
hsa-miR-29a-3p
 
 
-0.342
 
 
14.4
 
 
 
 
ARL4A
 
 
hsa-miR-29a-3p
 
 
-0.386
 
 
59.0
 
 
 
 
ARL6IP1
 
 
hsa-miR-148b-3p
 
 
-0.579
 
 
116.2
 
 
 
 
ARL6IP1
 
 
hsa-miR-152-3p
 
 
-0.579
 
 
116.2
 
 
 
 
ARL8B
 
 
hsa-miR-152-3p
 
 
-0.337
 
 
32.3
 
 
 
 
ARL8B
 
 
hsa-miR-148b-3p
 
 
-0.337
 
 
32.3
 
 
 
 
ARMCX3
 
 
hsa-miR-425-5p
 
 
-0.325
 
 
19.2
 
 
 
 
ARPC5
 
 
hsa-miR-22-3p
 
 
-0.679
 
 
110.5
 
 
 
 
ARPP19
 
 
hsa-miR-29a-3p
 
 
-0.371
 
 
28.8
 
 
 
 
ARPP19
 
 
hsa-miR-34a-5p
 
 
-0.497
 
 
28.8
 
 
 
 
ARRB1
 
 
hsa-miR-22-3p
 
 
-0.616
 
 
25.6
 
 
 
 
ARRDC3
 
 
hsa-miR-152-3p
 
 
-0.305
 
 
15.8
 
 
 
 
ARRDC3
 
 
hsa-miR-148b-3p
 
 
-0.305
 
 
15.8
 
 
 
 
ARRDC3
 
 
hsa-miR-29a-3p
 
 
-0.402
 
 
15.8
 
 
 
 
ASAH1
 
 
hsa-miR-27a-3p
 
 
-0.398
 
 
53.4
 
 
 
 
ASAP2
 
 
hsa-miR-29a-3p
 
 
-0.348
 
 
12.8
 
 
 
 
ASPH
 
 
hsa-miR-152-3p
 
 
-0.391
 
 
42.6
 
 
 
 
ASPH
 
 
hsa-miR-148b-3p
 
 
-0.391
 
 
42.6
 
 
 
 
ATG4B
 
 
hsa-miR-34a-5p
 
 
-0.58
 
 
19.9
 
 
 
 
ATMIN
 
 
hsa-miR-34a-5p
 
 
-0.409
 
 
12.2
 
 
 
 
ATOH1
 
 
hsa-miR-22-3p
 
 
-0.566
 
 
18.9
 
 
 
 
ATP1A2
 
 
hsa-miR-34a-5p
 
 
-0.306
 
 
15.0
 
 
 
 
ATP2B4
 
 
hsa-miR-152-3p
 
 
-0.337
 
 
13.8
 
 
 
 
 
 
 smooth muscle 
 
 
 
 
target_symbol
 
 
mature_mirna_id
 
 
score
 
 
nTPM
 
 
 
 
 
 
AAGAB
 
 
hsa-miR-34a-5p
 
 
-0.319
 
 
19.8
 
 
 
 
AAR2
 
 
hsa-miR-29a-3p
 
 
-0.327
 
 
26.1
 
 
 
 
ABCC5
 
 
hsa-miR-125a-5p
 
 
-0.288
 
 
15.0
 
 
 
 
ABCD3
 
 
hsa-miR-148b-3p
 
 
-0.388
 
 
16.6
 
 
 
 
ABCD3
 
 
hsa-miR-152-3p
 
 
-0.388
 
 
16.6
 
 
 
 
ABCE1
 
 
hsa-miR-29a-3p
 
 
-0.404
 
 
27.0
 
 
 
 
ABHD4
 
 
hsa-miR-29a-3p
 
 
-0.312
 
 
25.0
 
 
 
 
ACBD3
 
 
hsa-miR-34a-5p
 
 
-0.415
 
 
20.3
 
 
 
 
ACSL1
 
 
hsa-miR-34a-5p
 
 
-0.306
 
 
38.5
 
 
 
 
ACSL4
 
 
hsa-miR-34a-5p
 
 
-0.372
 
 
19.9
 
 
 
 
ACTA2
 
 
hsa-miR-27a-3p
 
 
-0.577
 
 
7564.0
 
 
 
 
ACVR1
 
 
hsa-miR-152-3p
 
 
-0.397
 
 
26.8
 
 
 
 
ACVR1
 
 
hsa-miR-148b-3p
 
 
-0.397
 
 
26.8
 
 
 
 
ADAM10
 
 
hsa-miR-152-3p
 
 
-0.348
 
 
12.4
 
 
 
 
ADAM10
 
 
hsa-miR-148b-3p
 
 
-0.356
 
 
12.4
 
 
 
 
ADAMTS9
 
 
hsa-miR-29a-3p
 
 
-0.305
 
 
11.3
 
 
 
 
ADAMTS9
 
 
hsa-miR-29a-3p
 
 
-0.485
 
 
11.3
 
 
 
 
ADCY3
 
 
hsa-miR-132-3p
 
 
-0.303
 
 
33.0
 
 
 
 
ADGRA3
 
 
hsa-miR-27a-3p
 
 
-0.296
 
 
13.1
 
 
 
 
ADGRF5
 
 
hsa-miR-148b-3p
 
 
-0.354
 
 
20.6
 
 
 
 
ADGRF5
 
 
hsa-miR-152-3p
 
 
-0.354
 
 
20.6
 
 
 
 
ADIPOR2
 
 
hsa-miR-150-5p
 
 
-0.287
 
 
11.2
 
 
 
 
ADIPOR2
 
 
hsa-miR-34a-5p
 
 
-0.33
 
 
11.2
 
 
 
 
ADK
 
 
hsa-miR-34a-5p
 
 
-0.374
 
 
13.8
 
 
 
 
AFG3L2
 
 
hsa-miR-181b-5p
 
 
-0.491
 
 
14.9
 
 
 
 
AFG3L2
 
 
hsa-miR-181a-5p
 
 
-0.491
 
 
14.9
 
 
 
 
AGFG1
 
 
hsa-miR-148b-3p
 
 
-0.332
 
 
36.7
 
 
 
 
AGFG1
 
 
hsa-miR-152-3p
 
 
-0.332
 
 
36.7
 
 
 
 
AGTRAP
 
 
hsa-miR-34a-5p
 
 
-0.328
 
 
18.9
 
 
 
 
AHCYL2
 
 
hsa-miR-34a-5p
 
 
-0.359
 
 
11.0
 
 
 
 
AHR
 
 
hsa-miR-29a-3p
 
 
-0.316
 
 
14.2
 
 
 
 
AIDA
 
 
hsa-miR-22-3p
 
 
-0.316
 
 
50.6
 
 
 
 
AK1
 
 
hsa-miR-29a-3p
 
 
-0.333
 
 
20.4
 
 
 
 
AKAP1
 
 
hsa-miR-148b-3p
 
 
-0.381
 
 
24.6
 
 
 
 
AKAP1
 
 
hsa-miR-152-3p
 
 
-0.381
 
 
24.6
 
 
 
 
AKIRIN1
 
 
hsa-miR-27a-3p
 
 
-0.368
 
 
39.2
 
 
 
 
AKT3
 
 
hsa-miR-29a-3p
 
 
-0.287
 
 
14.7
 
 
 
 
AKT3
 
 
hsa-miR-22-3p
 
 
-0.451
 
 
14.7
 
 
 
 
ALCAM
 
 
hsa-miR-34a-5p
 
 
-0.303
 
 
15.2
 
 
 
 
ALCAM
 
 
hsa-miR-148b-3p
 
 
-0.339
 
 
15.2
 
 
 
 
ALCAM
 
 
hsa-miR-152-3p
 
 
-0.347
 
 
15.2
 
 
 
 
ALDH1A2
 
 
hsa-miR-23b-3p
 
 
-0.346
 
 
29.8
 
 
 
 
ALDH1A2
 
 
hsa-miR-23a-3p
 
 
-0.354
 
 
29.8
 
 
 
 
ALDOA
 
 
hsa-miR-34a-5p
 
 
-0.655
 
 
433.8
 
 
 
 
ANAPC16
 
 
hsa-miR-125a-5p
 
 
-0.578
 
 
16.4
 
 
 
 
ANK2
 
 
hsa-miR-34a-5p
 
 
-0.317
 
 
20.3
 
 
 
 
ANK3
 
 
hsa-miR-34a-5p
 
 
-0.29
 
 
12.2
 
 
 
 
ANK3
 
 
hsa-miR-34a-5p
 
 
-0.347
 
 
12.2
 
 
 
 
ANKHD1
 
 
hsa-miR-23a-3p
 
 
-0.368
 
 
24.3
 
 
 
 
ANKHD1
 
 
hsa-miR-23b-3p
 
 
-0.368
 
 
24.3
 
 
 
 
ANKRD13A
 
 
hsa-miR-22-3p
 
 
-0.478
 
 
22.8
 
 
 
 
ANKRD29
 
 
hsa-miR-132-3p
 
 
-0.47
 
 
10.9
 
 
 
 
ANKRD40
 
 
hsa-miR-27a-3p
 
 
-0.35
 
 
26.1
 
 
 
 
ANP32A
 
 
hsa-miR-132-3p
 
 
-0.374
 
 
53.2
 
 
 
 
ANP32B
 
 
hsa-miR-34a-5p
 
 
-0.29
 
 
72.9
 
 
 
 
ANPEP
 
 
hsa-miR-125a-5p
 
 
-0.372
 
 
27.8
 
 
 
 
ANTXR2
 
 
hsa-miR-29a-3p
 
 
-0.347
 
 
101.2
 
 
 
 
AP3S2
 
 
hsa-miR-34a-5p
 
 
-0.413
 
 
16.7
 
 
 
 
APH1A
 
 
hsa-miR-34a-5p
 
 
-0.304
 
 
51.2
 
 
 
 
APOO
 
 
hsa-miR-181b-5p
 
 
-0.5
 
 
11.2
 
 
 
 
APOO
 
 
hsa-miR-181a-5p
 
 
-0.5
 
 
11.2
 
 
 
 
APPBP2
 
 
hsa-miR-27a-3p
 
 
-0.378
 
 
11.0
 
 
 
 
ARF4
 
 
hsa-miR-152-3p
 
 
-0.487
 
 
157.9
 
 
 
 
ARF4
 
 
hsa-miR-148b-3p
 
 
-0.487
 
 
157.9
 
 
 
 
ARF5
 
 
hsa-miR-29a-3p
 
 
-0.491
 
 
62.0
 
 
 
 
ARF6
 
 
hsa-miR-181b-5p
 
 
-0.33
 
 
22.2
 
 
 
 
ARF6
 
 
hsa-miR-181a-5p
 
 
-0.33
 
 
22.2
 
 
 
 
ARFGEF1
 
 
hsa-miR-148b-3p
 
 
-0.306
 
 
10.1
 
 
 
 
ARFGEF1
 
 
hsa-miR-152-3p
 
 
-0.306
 
 
10.1
 
 
 
 
ARFIP1
 
 
hsa-miR-152-3p
 
 
-0.474
 
 
18.5
 
 
 
 
ARFIP1
 
 
hsa-miR-148b-3p
 
 
-0.474
 
 
18.5
 
 
 
 
ARHGAP1
 
 
hsa-miR-34a-5p
 
 
-0.369
 
 
75.2
 
 
 
 
ARHGAP21
 
 
hsa-miR-152-3p
 
 
-0.287
 
 
16.5
 
 
 
 
ARHGAP21
 
 
hsa-miR-148b-3p
 
 
-0.287
 
 
16.5
 
 
 
 
ARID1B
 
 
hsa-miR-29a-3p
 
 
-0.342
 
 
14.1
 
 
 
 
ARL4A
 
 
hsa-miR-29a-3p
 
 
-0.386
 
 
23.4
 
 
 
 
ARL6IP1
 
 
hsa-miR-148b-3p
 
 
-0.579
 
 
64.5
 
 
 
 
ARL6IP1
 
 
hsa-miR-152-3p
 
 
-0.579
 
 
64.5
 
 
 
 
ARL8B
 
 
hsa-miR-152-3p
 
 
-0.337
 
 
43.9
 
 
 
 
ARL8B
 
 
hsa-miR-148b-3p
 
 
-0.337
 
 
43.9
 
 
 
 
ARMCX3
 
 
hsa-miR-425-5p
 
 
-0.325
 
 
33.9
 
 
 
 
ARPC5
 
 
hsa-miR-22-3p
 
 
-0.679
 
 
73.7
 
 
 
 
ARPP19
 
 
hsa-miR-29a-3p
 
 
-0.371
 
 
54.2
 
 
 
 
ARPP19
 
 
hsa-miR-34a-5p
 
 
-0.497
 
 
54.2
 
 
 
 
ARRB1
 
 
hsa-miR-22-3p
 
 
-0.616
 
 
20.3
 
 
 
 
ARRDC3
 
 
hsa-miR-152-3p
 
 
-0.305
 
 
30.8
 
 
 
 
ARRDC3
 
 
hsa-miR-148b-3p
 
 
-0.305
 
 
30.8
 
 
 
 
ARRDC3
 
 
hsa-miR-29a-3p
 
 
-0.402
 
 
30.8
 
 
 
 
ARSB
 
 
hsa-miR-29a-3p
 
 
-0.36
 
 
13.1
 
 
 
 
ASAH1
 
 
hsa-miR-27a-3p
 
 
-0.398
 
 
57.6
 
 
 
 
ASAP2
 
 
hsa-miR-29a-3p
 
 
-0.348
 
 
13.0
 
 
 
 
ASPH
 
 
hsa-miR-152-3p
 
 
-0.391
 
 
62.4
 
 
 
 
ASPH
 
 
hsa-miR-148b-3p
 
 
-0.391
 
 
62.4
 
 
 
 
ATMIN
 
 
hsa-miR-34a-5p
 
 
-0.409
 
 
12.8
 
 
 
 
ATP2B4
 
 
hsa-miR-152-3p
 
 
-0.337
 
 
164.7
 
 
 
 
ATP2B4
 
 
hsa-miR-148b-3p
 
 
-0.337
 
 
164.7
 
 
 
 
ATP5MC3
 
 
hsa-miR-425-5p
 
 
-0.328
 
 
113.6
 
 
 
 
ATP5MC3
 
 
hsa-miR-29a-3p
 
 
-0.33
 
 
113.6
 
 
 
 
ATP5MC3
 
 
hsa-miR-27a-3p
 
 
-0.416
 
 
113.6
 
 
 
 
ATP6AP2
 
 
hsa-miR-148b-3p
 
 
-0.592
 
 
116.5
 
 
 
 
 
 
 spleen 
 
 
 
 
target_symbol
 
 
mature_mirna_id
 
 
score
 
 
nTPM
 
 
 
 
 
 
AAGAB
 
 
hsa-miR-34a-5p
 
 
-0.319
 
 
14.5
 
 
 
 
AAR2
 
 
hsa-miR-29a-3p
 
 
-0.327
 
 
17.2
 
 
 
 
ABCB7
 
 
hsa-miR-148b-3p
 
 
-0.515
 
 
10.6
 
 
 
 
ABCB7
 
 
hsa-miR-152-3p
 
 
-0.515
 
 
10.6
 
 
 
 
ABCC5
 
 
hsa-miR-125a-5p
 
 
-0.288
 
 
38.3
 
 
 
 
ABCD3
 
 
hsa-miR-148b-3p
 
 
-0.388
 
 
10.3
 
 
 
 
ABCD3
 
 
hsa-miR-152-3p
 
 
-0.388
 
 
10.3
 
 
 
 
ABCE1
 
 
hsa-miR-29a-3p
 
 
-0.404
 
 
24.9
 
 
 
 
ABHD3
 
 
hsa-miR-125a-5p
 
 
-0.453
 
 
22.6
 
 
 
 
ABHD4
 
 
hsa-miR-29a-3p
 
 
-0.312
 
 
15.7
 
 
 
 
ABHD6
 
 
hsa-miR-125a-5p
 
 
-0.501
 
 
33.0
 
 
 
 
ABTB1
 
 
hsa-miR-125a-5p
 
 
-0.519
 
 
94.6
 
 
 
 
ACBD3
 
 
hsa-miR-34a-5p
 
 
-0.415
 
 
16.7
 
 
 
 
ACSL1
 
 
hsa-miR-34a-5p
 
 
-0.306
 
 
129.8
 
 
 
 
ACSL4
 
 
hsa-miR-34a-5p
 
 
-0.372
 
 
19.4
 
 
 
 
ACTA2
 
 
hsa-miR-27a-3p
 
 
-0.577
 
 
427.6
 
 
 
 
ADA
 
 
hsa-miR-29a-3p
 
 
-0.376
 
 
17.0
 
 
 
 
ADAM10
 
 
hsa-miR-152-3p
 
 
-0.348
 
 
27.3
 
 
 
 
ADAM10
 
 
hsa-miR-148b-3p
 
 
-0.356
 
 
27.3
 
 
 
 
ADAP1
 
 
hsa-miR-27a-3p
 
 
-0.302
 
 
16.5
 
 
 
 
ADCY3
 
 
hsa-miR-132-3p
 
 
-0.303
 
 
28.8
 
 
 
 
ADGRF5
 
 
hsa-miR-148b-3p
 
 
-0.354
 
 
38.8
 
 
 
 
ADGRF5
 
 
hsa-miR-152-3p
 
 
-0.354
 
 
38.8
 
 
 
 
ADIPOR2
 
 
hsa-miR-150-5p
 
 
-0.287
 
 
19.2
 
 
 
 
ADIPOR2
 
 
hsa-miR-34a-5p
 
 
-0.33
 
 
19.2
 
 
 
 
ADK
 
 
hsa-miR-34a-5p
 
 
-0.374
 
 
14.2
 
 
 
 
ADRA2A
 
 
hsa-miR-23a-3p
 
 
-0.371
 
 
13.1
 
 
 
 
ADRA2A
 
 
hsa-miR-23b-3p
 
 
-0.371
 
 
13.1
 
 
 
 
AFG3L2
 
 
hsa-miR-181b-5p
 
 
-0.491
 
 
10.9
 
 
 
 
AFG3L2
 
 
hsa-miR-181a-5p
 
 
-0.491
 
 
10.9
 
 
 
 
AGFG1
 
 
hsa-miR-148b-3p
 
 
-0.332
 
 
44.5
 
 
 
 
AGFG1
 
 
hsa-miR-152-3p
 
 
-0.332
 
 
44.5
 
 
 
 
AGGF1
 
 
hsa-miR-125a-5p
 
 
-0.331
 
 
11.9
 
 
 
 
AGO4
 
 
hsa-miR-34a-5p
 
 
-0.306
 
 
13.3
 
 
 
 
AGPAT3
 
 
hsa-miR-27a-3p
 
 
-0.29
 
 
18.3
 
 
 
 
AGTRAP
 
 
hsa-miR-34a-5p
 
 
-0.328
 
 
79.5
 
 
 
 
AHDC1
 
 
hsa-miR-148b-3p
 
 
-0.413
 
 
15.7
 
 
 
 
AHDC1
 
 
hsa-miR-152-3p
 
 
-0.413
 
 
15.7
 
 
 
 
AHR
 
 
hsa-miR-29a-3p
 
 
-0.316
 
 
17.6
 
 
 
 
AIDA
 
 
hsa-miR-22-3p
 
 
-0.316
 
 
35.3
 
 
 
 
AIFM1
 
 
hsa-miR-125a-5p
 
 
-0.3
 
 
12.9
 
 
 
 
AK1
 
 
hsa-miR-29a-3p
 
 
-0.333
 
 
31.5
 
 
 
 
AKAP1
 
 
hsa-miR-148b-3p
 
 
-0.381
 
 
11.0
 
 
 
 
AKAP1
 
 
hsa-miR-152-3p
 
 
-0.381
 
 
11.0
 
 
 
 
AKIRIN1
 
 
hsa-miR-27a-3p
 
 
-0.368
 
 
29.9
 
 
 
 
ALDOA
 
 
hsa-miR-34a-5p
 
 
-0.655
 
 
495.5
 
 
 
 
ALPL
 
 
hsa-miR-10a-5p
 
 
-0.32
 
 
44.5
 
 
 
 
ANAPC16
 
 
hsa-miR-125a-5p
 
 
-0.578
 
 
21.2
 
 
 
 
ANK2
 
 
hsa-miR-34a-5p
 
 
-0.317
 
 
22.5
 
 
 
 
ANK3
 
 
hsa-miR-34a-5p
 
 
-0.29
 
 
10.5
 
 
 
 
ANK3
 
 
hsa-miR-34a-5p
 
 
-0.347
 
 
10.5
 
 
 
 
ANKHD1
 
 
hsa-miR-23a-3p
 
 
-0.368
 
 
46.1
 
 
 
 
ANKHD1
 
 
hsa-miR-23b-3p
 
 
-0.368
 
 
46.1
 
 
 
 
ANKHD1-EIF4EBP3
 
 
hsa-miR-22-3p
 
 
-0.479
 
 
15.6
 
 
 
 
ANKRD13A
 
 
hsa-miR-22-3p
 
 
-0.478
 
 
30.9
 
 
 
 
ANKRD40
 
 
hsa-miR-27a-3p
 
 
-0.35
 
 
19.3
 
 
 
 
ANP32A
 
 
hsa-miR-132-3p
 
 
-0.374
 
 
80.5
 
 
 
 
ANP32B
 
 
hsa-miR-34a-5p
 
 
-0.29
 
 
117.6
 
 
 
 
ANPEP
 
 
hsa-miR-125a-5p
 
 
-0.372
 
 
24.0
 
 
 
 
ANTXR2
 
 
hsa-miR-29a-3p
 
 
-0.347
 
 
17.3
 
 
 
 
AP3S2
 
 
hsa-miR-34a-5p
 
 
-0.413
 
 
11.9
 
 
 
 
APH1A
 
 
hsa-miR-34a-5p
 
 
-0.304
 
 
94.3
 
 
 
 
APPBP2
 
 
hsa-miR-27a-3p
 
 
-0.378
 
 
11.3
 
 
 
 
ARF4
 
 
hsa-miR-152-3p
 
 
-0.487
 
 
60.0
 
 
 
 
ARF4
 
 
hsa-miR-148b-3p
 
 
-0.487
 
 
60.0
 
 
 
 
ARF5
 
 
hsa-miR-29a-3p
 
 
-0.491
 
 
116.2
 
 
 
 
ARF6
 
 
hsa-miR-181b-5p
 
 
-0.33
 
 
47.5
 
 
 
 
ARF6
 
 
hsa-miR-181a-5p
 
 
-0.33
 
 
47.5
 
 
 
 
ARFGEF1
 
 
hsa-miR-148b-3p
 
 
-0.306
 
 
13.0
 
 
 
 
ARFGEF1
 
 
hsa-miR-152-3p
 
 
-0.306
 
 
13.0
 
 
 
 
ARFIP1
 
 
hsa-miR-152-3p
 
 
-0.474
 
 
17.3
 
 
 
 
ARFIP1
 
 
hsa-miR-148b-3p
 
 
-0.474
 
 
17.3
 
 
 
 
ARHGAP1
 
 
hsa-miR-34a-5p
 
 
-0.369
 
 
55.1
 
 
 
 
ARHGAP26
 
 
hsa-miR-34a-5p
 
 
-0.384
 
 
16.3
 
 
 
 
ARID1B
 
 
hsa-miR-29a-3p
 
 
-0.342
 
 
17.2
 
 
 
 
ARL4A
 
 
hsa-miR-29a-3p
 
 
-0.386
 
 
20.6
 
 
 
 
ARL6IP1
 
 
hsa-miR-148b-3p
 
 
-0.579
 
 
68.6
 
 
 
 
ARL6IP1
 
 
hsa-miR-152-3p
 
 
-0.579
 
 
68.6
 
 
 
 
ARL8B
 
 
hsa-miR-152-3p
 
 
-0.337
 
 
32.0
 
 
 
 
ARL8B
 
 
hsa-miR-148b-3p
 
 
-0.337
 
 
32.0
 
 
 
 
ARMCX3
 
 
hsa-miR-425-5p
 
 
-0.325
 
 
38.9
 
 
 
 
ARPC5
 
 
hsa-miR-22-3p
 
 
-0.679
 
 
113.8
 
 
 
 
ARPP19
 
 
hsa-miR-29a-3p
 
 
-0.371
 
 
41.2
 
 
 
 
ARPP19
 
 
hsa-miR-34a-5p
 
 
-0.497
 
 
41.2
 
 
 
 
ARRB1
 
 
hsa-miR-22-3p
 
 
-0.616
 
 
37.8
 
 
 
 
ARRDC3
 
 
hsa-miR-152-3p
 
 
-0.305
 
 
35.0
 
 
 
 
ARRDC3
 
 
hsa-miR-148b-3p
 
 
-0.305
 
 
35.0
 
 
 
 
ARRDC3
 
 
hsa-miR-29a-3p
 
 
-0.402
 
 
35.0
 
 
 
 
ASAH1
 
 
hsa-miR-27a-3p
 
 
-0.398
 
 
80.4
 
 
 
 
ASF1A
 
 
hsa-miR-23a-3p
 
 
-0.506
 
 
10.9
 
 
 
 
ASF1A
 
 
hsa-miR-23b-3p
 
 
-0.506
 
 
10.9
 
 
 
 
ASPH
 
 
hsa-miR-152-3p
 
 
-0.391
 
 
26.8
 
 
 
 
ASPH
 
 
hsa-miR-148b-3p
 
 
-0.391
 
 
26.8
 
 
 
 
ATG4B
 
 
hsa-miR-34a-5p
 
 
-0.58
 
 
21.8
 
 
 
 
ATMIN
 
 
hsa-miR-34a-5p
 
 
-0.409
 
 
12.1
 
 
 
 
ATP2B4
 
 
hsa-miR-152-3p
 
 
-0.337
 
 
11.5
 
 
 
 
ATP2B4
 
 
hsa-miR-148b-3p
 
 
-0.337
 
 
11.5
 
 
 
 
ATP5MC3
 
 
hsa-miR-425-5p
 
 
-0.328
 
 
126.3
 
 
 
 
ATP5MC3
 
 
hsa-miR-29a-3p
 
 
-0.33
 
 
126.3
 
 
 
 
ATP5MC3
 
 
hsa-miR-27a-3p
 
 
-0.416
 
 
126.3
 
 
 
 
 
 
 testis 
 
 
 
 
target_symbol
 
 
mature_mirna_id
 
 
score
 
 
nTPM
 
 
 
 
 
 
AAGAB
 
 
hsa-miR-34a-5p
 
 
-0.319
 
 
20.5
 
 
 
 
AAR2
 
 
hsa-miR-29a-3p
 
 
-0.327
 
 
15.4
 
 
 
 
ABCB6
 
 
hsa-miR-29a-3p
 
 
-0.48
 
 
15.2
 
 
 
 
ABCC5
 
 
hsa-miR-125a-5p
 
 
-0.288
 
 
17.7
 
 
 
 
ABCD3
 
 
hsa-miR-148b-3p
 
 
-0.388
 
 
15.3
 
 
 
 
ABCD3
 
 
hsa-miR-152-3p
 
 
-0.388
 
 
15.3
 
 
 
 
ABCE1
 
 
hsa-miR-29a-3p
 
 
-0.404
 
 
24.7
 
 
 
 
ABHD4
 
 
hsa-miR-29a-3p
 
 
-0.312
 
 
39.1
 
 
 
 
ACBD3
 
 
hsa-miR-34a-5p
 
 
-0.415
 
 
17.6
 
 
 
 
ACSL1
 
 
hsa-miR-34a-5p
 
 
-0.306
 
 
40.4
 
 
 
 
ACSL4
 
 
hsa-miR-34a-5p
 
 
-0.372
 
 
23.3
 
 
 
 
ACSL6
 
 
hsa-miR-125a-5p
 
 
-0.307
 
 
19.3
 
 
 
 
ACTA2
 
 
hsa-miR-27a-3p
 
 
-0.577
 
 
258.1
 
 
 
 
ADIPOR2
 
 
hsa-miR-150-5p
 
 
-0.287
 
 
26.9
 
 
 
 
ADIPOR2
 
 
hsa-miR-34a-5p
 
 
-0.33
 
 
26.9
 
 
 
 
ADK
 
 
hsa-miR-34a-5p
 
 
-0.374
 
 
14.4
 
 
 
 
ADO
 
 
hsa-miR-34a-5p
 
 
-0.377
 
 
35.2
 
 
 
 
AFG3L2
 
 
hsa-miR-181b-5p
 
 
-0.491
 
 
10.5
 
 
 
 
AFG3L2
 
 
hsa-miR-181a-5p
 
 
-0.491
 
 
10.5
 
 
 
 
AGFG1
 
 
hsa-miR-148b-3p
 
 
-0.332
 
 
86.7
 
 
 
 
AGFG1
 
 
hsa-miR-152-3p
 
 
-0.332
 
 
86.7
 
 
 
 
AGGF1
 
 
hsa-miR-125a-5p
 
 
-0.331
 
 
15.2
 
 
 
 
AGPAT3
 
 
hsa-miR-27a-3p
 
 
-0.29
 
 
25.0
 
 
 
 
AGTRAP
 
 
hsa-miR-34a-5p
 
 
-0.328
 
 
23.7
 
 
 
 
AIDA
 
 
hsa-miR-22-3p
 
 
-0.316
 
 
41.4
 
 
 
 
AIFM1
 
 
hsa-miR-125a-5p
 
 
-0.3
 
 
21.3
 
 
 
 
AK1
 
 
hsa-miR-29a-3p
 
 
-0.333
 
 
64.3
 
 
 
 
AKAP1
 
 
hsa-miR-148b-3p
 
 
-0.381
 
 
108.8
 
 
 
 
AKAP1
 
 
hsa-miR-152-3p
 
 
-0.381
 
 
108.8
 
 
 
 
AKIRIN1
 
 
hsa-miR-27a-3p
 
 
-0.368
 
 
78.8
 
 
 
 
ALDH1A2
 
 
hsa-miR-23b-3p
 
 
-0.346
 
 
53.9
 
 
 
 
ALDH1A2
 
 
hsa-miR-23a-3p
 
 
-0.354
 
 
53.9
 
 
 
 
ALDOA
 
 
hsa-miR-34a-5p
 
 
-0.655
 
 
199.3
 
 
 
 
ANKHD1
 
 
hsa-miR-23a-3p
 
 
-0.368
 
 
29.3
 
 
 
 
ANKHD1
 
 
hsa-miR-23b-3p
 
 
-0.368
 
 
29.3
 
 
 
 
ANKHD1-EIF4EBP3
 
 
hsa-miR-22-3p
 
 
-0.479
 
 
11.4
 
 
 
 
ANKRD13A
 
 
hsa-miR-22-3p
 
 
-0.478
 
 
56.9
 
 
 
 
ANP32A
 
 
hsa-miR-132-3p
 
 
-0.374
 
 
19.4
 
 
 
 
ANP32B
 
 
hsa-miR-34a-5p
 
 
-0.29
 
 
56.3
 
 
 
 
AP3S2
 
 
hsa-miR-34a-5p
 
 
-0.413
 
 
11.6
 
 
 
 
APH1A
 
 
hsa-miR-34a-5p
 
 
-0.304
 
 
33.4
 
 
 
 
APOO
 
 
hsa-miR-181b-5p
 
 
-0.5
 
 
15.7
 
 
 
 
APOO
 
 
hsa-miR-181a-5p
 
 
-0.5
 
 
15.7
 
 
 
 
APPBP2
 
 
hsa-miR-27a-3p
 
 
-0.378
 
 
21.9
 
 
 
 
ARF4
 
 
hsa-miR-152-3p
 
 
-0.487
 
 
94.2
 
 
 
 
ARF4
 
 
hsa-miR-148b-3p
 
 
-0.487
 
 
94.2
 
 
 
 
ARF5
 
 
hsa-miR-29a-3p
 
 
-0.491
 
 
89.2
 
 
 
 
ARF6
 
 
hsa-miR-181b-5p
 
 
-0.33
 
 
15.0
 
 
 
 
ARF6
 
 
hsa-miR-181a-5p
 
 
-0.33
 
 
15.0
 
 
 
 
ARFGEF1
 
 
hsa-miR-148b-3p
 
 
-0.306
 
 
26.5
 
 
 
 
ARFGEF1
 
 
hsa-miR-152-3p
 
 
-0.306
 
 
26.5
 
 
 
 
ARFIP1
 
 
hsa-miR-152-3p
 
 
-0.474
 
 
11.3
 
 
 
 
ARFIP1
 
 
hsa-miR-148b-3p
 
 
-0.474
 
 
11.3
 
 
 
 
ARHGAP1
 
 
hsa-miR-34a-5p
 
 
-0.369
 
 
15.3
 
 
 
 
ARHGAP21
 
 
hsa-miR-152-3p
 
 
-0.287
 
 
13.5
 
 
 
 
ARHGAP21
 
 
hsa-miR-148b-3p
 
 
-0.287
 
 
13.5
 
 
 
 
ARHGAP26
 
 
hsa-miR-34a-5p
 
 
-0.384
 
 
15.3
 
 
 
 
ARID2
 
 
hsa-miR-132-3p
 
 
-0.304
 
 
24.3
 
 
 
 
ARL4A
 
 
hsa-miR-29a-3p
 
 
-0.386
 
 
181.2
 
 
 
 
ARL6IP1
 
 
hsa-miR-148b-3p
 
 
-0.579
 
 
121.4
 
 
 
 
ARL6IP1
 
 
hsa-miR-152-3p
 
 
-0.579
 
 
121.4
 
 
 
 
ARL8B
 
 
hsa-miR-152-3p
 
 
-0.337
 
 
30.1
 
 
 
 
ARL8B
 
 
hsa-miR-148b-3p
 
 
-0.337
 
 
30.1
 
 
 
 
ARMCX3
 
 
hsa-miR-425-5p
 
 
-0.325
 
 
12.0
 
 
 
 
ARPC5
 
 
hsa-miR-22-3p
 
 
-0.679
 
 
35.9
 
 
 
 
ARPIN-AP3S2
 
 
hsa-miR-34a-5p
 
 
-0.348
 
 
11.1
 
 
 
 
ARPP19
 
 
hsa-miR-29a-3p
 
 
-0.371
 
 
79.0
 
 
 
 
ARPP19
 
 
hsa-miR-34a-5p
 
 
-0.497
 
 
79.0
 
 
 
 
ARRDC3
 
 
hsa-miR-152-3p
 
 
-0.305
 
 
15.5
 
 
 
 
ARRDC3
 
 
hsa-miR-148b-3p
 
 
-0.305
 
 
15.5
 
 
 
 
ARRDC3
 
 
hsa-miR-29a-3p
 
 
-0.402
 
 
15.5
 
 
 
 
ASAH1
 
 
hsa-miR-27a-3p
 
 
-0.398
 
 
17.0
 
 
 
 
ASAP2
 
 
hsa-miR-29a-3p
 
 
-0.348
 
 
43.7
 
 
 
 
ASB1
 
 
hsa-miR-34a-5p
 
 
-0.406
 
 
10.2
 
 
 
 
ASPH
 
 
hsa-miR-152-3p
 
 
-0.391
 
 
15.3
 
 
 
 
ASPH
 
 
hsa-miR-148b-3p
 
 
-0.391
 
 
15.3
 
 
 
 
ATG4B
 
 
hsa-miR-34a-5p
 
 
-0.58
 
 
22.6
 
 
 
 
ATMIN
 
 
hsa-miR-34a-5p
 
 
-0.409
 
 
21.1
 
 
 
 
ATP2B4
 
 
hsa-miR-152-3p
 
 
-0.337
 
 
25.4
 
 
 
 
ATP2B4
 
 
hsa-miR-148b-3p
 
 
-0.337
 
 
25.4
 
 
 
 
ATP5MC3
 
 
hsa-miR-425-5p
 
 
-0.328
 
 
60.2
 
 
 
 
ATP5MC3
 
 
hsa-miR-29a-3p
 
 
-0.33
 
 
60.2
 
 
 
 
ATP5MC3
 
 
hsa-miR-27a-3p
 
 
-0.416
 
 
60.2
 
 
 
 
ATP6AP2
 
 
hsa-miR-148b-3p
 
 
-0.592
 
 
37.7
 
 
 
 
ATP6AP2
 
 
hsa-miR-152-3p
 
 
-0.592
 
 
37.7
 
 
 
 
ATP6AP2
 
 
hsa-miR-148b-3p
 
 
-0.668
 
 
37.7
 
 
 
 
ATP6AP2
 
 
hsa-miR-152-3p
 
 
-0.668
 
 
37.7
 
 
 
 
ATP6V1E1
 
 
hsa-miR-23a-3p
 
 
-0.294
 
 
62.9
 
 
 
 
ATP6V1E1
 
 
hsa-miR-23b-3p
 
 
-0.294
 
 
62.9
 
 
 
 
ATXN10
 
 
hsa-miR-27a-3p
 
 
-0.295
 
 
53.5
 
 
 
 
ATXN2
 
 
hsa-miR-10a-5p
 
 
-0.362
 
 
23.4
 
 
 
 
AXL
 
 
hsa-miR-34a-5p
 
 
-0.512
 
 
17.4
 
 
 
 
AZIN1
 
 
hsa-miR-132-3p
 
 
-0.29
 
 
32.4
 
 
 
 
AZIN1
 
 
hsa-miR-132-3p
 
 
-0.299
 
 
32.4
 
 
 
 
B4GALT2
 
 
hsa-miR-34a-5p
 
 
-0.314
 
 
17.6
 
 
 
 
B4GALT3
 
 
hsa-miR-27a-3p
 
 
-0.41
 
 
18.8
 
 
 
 
B4GALT5
 
 
hsa-miR-148b-3p
 
 
-0.44
 
 
20.8
 
 
 
 
B4GALT5
 
 
hsa-miR-152-3p
 
 
-0.44
 
 
20.8
 
 
 
 
BAG5
 
 
hsa-miR-22-3p
 
 
-0.318
 
 
119.4
 
 
 
 
BAZ1B
 
 
hsa-miR-10a-5p
 
 
-0.309
 
 
16.1
 
 
 
 
 
 
 thymus 
 
 
 
 
target_symbol
 
 
mature_mirna_id
 
 
score
 
 
nTPM
 
 
 
 
 
 
AAGAB
 
 
hsa-miR-34a-5p
 
 
-0.319
 
 
27.8
 
 
 
 
AAR2
 
 
hsa-miR-29a-3p
 
 
-0.327
 
 
24.4
 
 
 
 
ABCB7
 
 
hsa-miR-148b-3p
 
 
-0.515
 
 
13.0
 
 
 
 
ABCB7
 
 
hsa-miR-152-3p
 
 
-0.515
 
 
13.0
 
 
 
 
ABCC5
 
 
hsa-miR-125a-5p
 
 
-0.288
 
 
14.2
 
 
 
 
ABCD3
 
 
hsa-miR-148b-3p
 
 
-0.388
 
 
44.2
 
 
 
 
ABCD3
 
 
hsa-miR-152-3p
 
 
-0.388
 
 
44.2
 
 
 
 
ABCE1
 
 
hsa-miR-29a-3p
 
 
-0.404
 
 
31.9
 
 
 
 
ABHD3
 
 
hsa-miR-125a-5p
 
 
-0.453
 
 
20.0
 
 
 
 
ABHD4
 
 
hsa-miR-29a-3p
 
 
-0.312
 
 
10.3
 
 
 
 
ABRAXAS2
 
 
hsa-miR-34a-5p
 
 
-0.538
 
 
12.0
 
 
 
 
ABTB1
 
 
hsa-miR-125a-5p
 
 
-0.519
 
 
20.1
 
 
 
 
ACBD3
 
 
hsa-miR-34a-5p
 
 
-0.415
 
 
16.8
 
 
 
 
ACSL1
 
 
hsa-miR-34a-5p
 
 
-0.306
 
 
10.7
 
 
 
 
ACSL4
 
 
hsa-miR-34a-5p
 
 
-0.372
 
 
18.3
 
 
 
 
ACTA2
 
 
hsa-miR-27a-3p
 
 
-0.577
 
 
52.7
 
 
 
 
ADA
 
 
hsa-miR-29a-3p
 
 
-0.376
 
 
228.3
 
 
 
 
ADAM10
 
 
hsa-miR-152-3p
 
 
-0.348
 
 
38.8
 
 
 
 
ADAM10
 
 
hsa-miR-148b-3p
 
 
-0.356
 
 
38.8
 
 
 
 
ADCY3
 
 
hsa-miR-132-3p
 
 
-0.303
 
 
10.7
 
 
 
 
ADIPOR2
 
 
hsa-miR-150-5p
 
 
-0.287
 
 
11.9
 
 
 
 
ADIPOR2
 
 
hsa-miR-34a-5p
 
 
-0.33
 
 
11.9
 
 
 
 
ADK
 
 
hsa-miR-34a-5p
 
 
-0.374
 
 
25.2
 
 
 
 
AFG3L2
 
 
hsa-miR-181b-5p
 
 
-0.491
 
 
12.1
 
 
 
 
AFG3L2
 
 
hsa-miR-181a-5p
 
 
-0.491
 
 
12.1
 
 
 
 
AGFG1
 
 
hsa-miR-148b-3p
 
 
-0.332
 
 
45.3
 
 
 
 
AGFG1
 
 
hsa-miR-152-3p
 
 
-0.332
 
 
45.3
 
 
 
 
AGGF1
 
 
hsa-miR-125a-5p
 
 
-0.331
 
 
13.4
 
 
 
 
AGPAT3
 
 
hsa-miR-27a-3p
 
 
-0.29
 
 
14.4
 
 
 
 
AGTRAP
 
 
hsa-miR-34a-5p
 
 
-0.328
 
 
10.5
 
 
 
 
AIDA
 
 
hsa-miR-22-3p
 
 
-0.316
 
 
31.5
 
 
 
 
AIFM1
 
 
hsa-miR-125a-5p
 
 
-0.3
 
 
17.2
 
 
 
 
AKAP1
 
 
hsa-miR-148b-3p
 
 
-0.381
 
 
10.6
 
 
 
 
AKAP1
 
 
hsa-miR-152-3p
 
 
-0.381
 
 
10.6
 
 
 
 
AKIRIN1
 
 
hsa-miR-27a-3p
 
 
-0.368
 
 
38.6
 
 
 
 
ALCAM
 
 
hsa-miR-34a-5p
 
 
-0.303
 
 
13.3
 
 
 
 
ALCAM
 
 
hsa-miR-148b-3p
 
 
-0.339
 
 
13.3
 
 
 
 
ALCAM
 
 
hsa-miR-152-3p
 
 
-0.347
 
 
13.3
 
 
 
 
ALDOA
 
 
hsa-miR-34a-5p
 
 
-0.655
 
 
259.9
 
 
 
 
ANAPC16
 
 
hsa-miR-125a-5p
 
 
-0.578
 
 
29.7
 
 
 
 
ANKHD1
 
 
hsa-miR-23a-3p
 
 
-0.368
 
 
45.5
 
 
 
 
ANKHD1
 
 
hsa-miR-23b-3p
 
 
-0.368
 
 
45.5
 
 
 
 
ANKHD1-EIF4EBP3
 
 
hsa-miR-22-3p
 
 
-0.479
 
 
13.4
 
 
 
 
ANKRD13A
 
 
hsa-miR-22-3p
 
 
-0.478
 
 
26.0
 
 
 
 
ANKRD40
 
 
hsa-miR-27a-3p
 
 
-0.35
 
 
15.8
 
 
 
 
ANP32A
 
 
hsa-miR-132-3p
 
 
-0.374
 
 
189.1
 
 
 
 
ANP32B
 
 
hsa-miR-34a-5p
 
 
-0.29
 
 
252.8
 
 
 
 
ANTXR2
 
 
hsa-miR-29a-3p
 
 
-0.347
 
 
10.0
 
 
 
 
AP3S2
 
 
hsa-miR-34a-5p
 
 
-0.413
 
 
18.7
 
 
 
 
APH1A
 
 
hsa-miR-34a-5p
 
 
-0.304
 
 
67.9
 
 
 
 
APOO
 
 
hsa-miR-181b-5p
 
 
-0.5
 
 
13.0
 
 
 
 
APOO
 
 
hsa-miR-181a-5p
 
 
-0.5
 
 
13.0
 
 
 
 
ARF4
 
 
hsa-miR-152-3p
 
 
-0.487
 
 
69.9
 
 
 
 
ARF4
 
 
hsa-miR-148b-3p
 
 
-0.487
 
 
69.9
 
 
 
 
ARF5
 
 
hsa-miR-29a-3p
 
 
-0.491
 
 
89.3
 
 
 
 
ARF6
 
 
hsa-miR-181b-5p
 
 
-0.33
 
 
41.8
 
 
 
 
ARF6
 
 
hsa-miR-181a-5p
 
 
-0.33
 
 
41.8
 
 
 
 
ARFGEF1
 
 
hsa-miR-148b-3p
 
 
-0.306
 
 
20.7
 
 
 
 
ARFGEF1
 
 
hsa-miR-152-3p
 
 
-0.306
 
 
20.7
 
 
 
 
ARFIP1
 
 
hsa-miR-152-3p
 
 
-0.474
 
 
11.2
 
 
 
 
ARFIP1
 
 
hsa-miR-148b-3p
 
 
-0.474
 
 
11.2
 
 
 
 
ARHGAP1
 
 
hsa-miR-34a-5p
 
 
-0.369
 
 
21.6
 
 
 
 
ARHGAP21
 
 
hsa-miR-152-3p
 
 
-0.287
 
 
10.5
 
 
 
 
ARHGAP21
 
 
hsa-miR-148b-3p
 
 
-0.287
 
 
10.5
 
 
 
 
ARHGAP26
 
 
hsa-miR-34a-5p
 
 
-0.384
 
 
12.8
 
 
 
 
ARID1B
 
 
hsa-miR-29a-3p
 
 
-0.342
 
 
23.3
 
 
 
 
ARID2
 
 
hsa-miR-132-3p
 
 
-0.304
 
 
17.5
 
 
 
 
ARL4A
 
 
hsa-miR-29a-3p
 
 
-0.386
 
 
21.1
 
 
 
 
ARL5B
 
 
hsa-miR-29a-3p
 
 
-0.304
 
 
12.6
 
 
 
 
ARL6IP1
 
 
hsa-miR-148b-3p
 
 
-0.579
 
 
147.6
 
 
 
 
ARL6IP1
 
 
hsa-miR-152-3p
 
 
-0.579
 
 
147.6
 
 
 
 
ARL8B
 
 
hsa-miR-152-3p
 
 
-0.337
 
 
33.3
 
 
 
 
ARL8B
 
 
hsa-miR-148b-3p
 
 
-0.337
 
 
33.3
 
 
 
 
ARMCX3
 
 
hsa-miR-425-5p
 
 
-0.325
 
 
18.7
 
 
 
 
ARPC5
 
 
hsa-miR-22-3p
 
 
-0.679
 
 
121.6
 
 
 
 
ARPP19
 
 
hsa-miR-29a-3p
 
 
-0.371
 
 
49.2
 
 
 
 
ARPP19
 
 
hsa-miR-34a-5p
 
 
-0.497
 
 
49.2
 
 
 
 
ARRDC3
 
 
hsa-miR-152-3p
 
 
-0.305
 
 
26.1
 
 
 
 
ARRDC3
 
 
hsa-miR-148b-3p
 
 
-0.305
 
 
26.1
 
 
 
 
ARRDC3
 
 
hsa-miR-29a-3p
 
 
-0.402
 
 
26.1
 
 
 
 
ASAH1
 
 
hsa-miR-27a-3p
 
 
-0.398
 
 
24.2
 
 
 
 
ASF1A
 
 
hsa-miR-23a-3p
 
 
-0.506
 
 
22.7
 
 
 
 
ASF1A
 
 
hsa-miR-23b-3p
 
 
-0.506
 
 
22.7
 
 
 
 
ASPH
 
 
hsa-miR-152-3p
 
 
-0.391
 
 
15.5
 
 
 
 
ASPH
 
 
hsa-miR-148b-3p
 
 
-0.391
 
 
15.5
 
 
 
 
ATG4B
 
 
hsa-miR-34a-5p
 
 
-0.58
 
 
15.0
 
 
 
 
ATMIN
 
 
hsa-miR-34a-5p
 
 
-0.409
 
 
14.6
 
 
 
 
ATP2B4
 
 
hsa-miR-152-3p
 
 
-0.337
 
 
20.2
 
 
 
 
ATP2B4
 
 
hsa-miR-148b-3p
 
 
-0.337
 
 
20.2
 
 
 
 
ATP5MC3
 
 
hsa-miR-425-5p
 
 
-0.328
 
 
175.6
 
 
 
 
ATP5MC3
 
 
hsa-miR-29a-3p
 
 
-0.33
 
 
175.6
 
 
 
 
ATP5MC3
 
 
hsa-miR-27a-3p
 
 
-0.416
 
 
175.6
 
 
 
 
ATP6AP2
 
 
hsa-miR-148b-3p
 
 
-0.592
 
 
67.0
 
 
 
 
ATP6AP2
 
 
hsa-miR-152-3p
 
 
-0.592
 
 
67.0
 
 
 
 
ATP6AP2
 
 
hsa-miR-148b-3p
 
 
-0.668
 
 
67.0
 
 
 
 
ATP6AP2
 
 
hsa-miR-152-3p
 
 
-0.668
 
 
67.0
 
 
 
 
ATP6V1E1
 
 
hsa-miR-23a-3p
 
 
-0.294
 
 
100.8
 
 
 
 
ATP6V1E1
 
 
hsa-miR-23b-3p
 
 
-0.294
 
 
100.8
 
 
 
 
ATXN10
 
 
hsa-miR-27a-3p
 
 
-0.295
 
 
61.3
 
 
 
 
ATXN2
 
 
hsa-miR-10a-5p
 
 
-0.362
 
 
11.9
 
 
 
 
 
 
 thyroid gland 
 
 
 
 
target_symbol
 
 
mature_mirna_id
 
 
score
 
 
nTPM
 
 
 
 
 
 
AAGAB
 
 
hsa-miR-34a-5p
 
 
-0.319
 
 
30.9
 
 
 
 
AAR2
 
 
hsa-miR-29a-3p
 
 
-0.327
 
 
16.7
 
 
 
 
ABCC5
 
 
hsa-miR-125a-5p
 
 
-0.288
 
 
13.7
 
 
 
 
ABCD3
 
 
hsa-miR-148b-3p
 
 
-0.388
 
 
25.8
 
 
 
 
ABCD3
 
 
hsa-miR-152-3p
 
 
-0.388
 
 
25.8
 
 
 
 
ABCE1
 
 
hsa-miR-29a-3p
 
 
-0.404
 
 
25.1
 
 
 
 
ABHD3
 
 
hsa-miR-125a-5p
 
 
-0.453
 
 
21.4
 
 
 
 
ABHD4
 
 
hsa-miR-29a-3p
 
 
-0.312
 
 
22.8
 
 
 
 
ABHD5
 
 
hsa-miR-132-3p
 
 
-0.323
 
 
14.4
 
 
 
 
ABHD5
 
 
hsa-miR-27a-3p
 
 
-0.407
 
 
14.4
 
 
 
 
ABRAXAS2
 
 
hsa-miR-34a-5p
 
 
-0.538
 
 
11.9
 
 
 
 
ABTB1
 
 
hsa-miR-125a-5p
 
 
-0.519
 
 
23.0
 
 
 
 
ACBD3
 
 
hsa-miR-34a-5p
 
 
-0.415
 
 
26.9
 
 
 
 
ACSL1
 
 
hsa-miR-34a-5p
 
 
-0.306
 
 
44.4
 
 
 
 
ACSL4
 
 
hsa-miR-34a-5p
 
 
-0.372
 
 
17.2
 
 
 
 
ACTA2
 
 
hsa-miR-27a-3p
 
 
-0.577
 
 
297.7
 
 
 
 
ACVR1
 
 
hsa-miR-152-3p
 
 
-0.397
 
 
28.6
 
 
 
 
ACVR1
 
 
hsa-miR-148b-3p
 
 
-0.397
 
 
28.6
 
 
 
 
ADAM10
 
 
hsa-miR-152-3p
 
 
-0.348
 
 
26.7
 
 
 
 
ADAM10
 
 
hsa-miR-148b-3p
 
 
-0.356
 
 
26.7
 
 
 
 
ADCY3
 
 
hsa-miR-132-3p
 
 
-0.303
 
 
35.2
 
 
 
 
ADGRA3
 
 
hsa-miR-27a-3p
 
 
-0.296
 
 
23.6
 
 
 
 
ADGRF5
 
 
hsa-miR-148b-3p
 
 
-0.354
 
 
30.7
 
 
 
 
ADGRF5
 
 
hsa-miR-152-3p
 
 
-0.354
 
 
30.7
 
 
 
 
ADIPOR2
 
 
hsa-miR-150-5p
 
 
-0.287
 
 
26.8
 
 
 
 
ADIPOR2
 
 
hsa-miR-34a-5p
 
 
-0.33
 
 
26.8
 
 
 
 
ADK
 
 
hsa-miR-34a-5p
 
 
-0.374
 
 
35.7
 
 
 
 
ADO
 
 
hsa-miR-34a-5p
 
 
-0.377
 
 
11.2
 
 
 
 
AFG3L2
 
 
hsa-miR-181b-5p
 
 
-0.491
 
 
24.7
 
 
 
 
AFG3L2
 
 
hsa-miR-181a-5p
 
 
-0.491
 
 
24.7
 
 
 
 
AGFG1
 
 
hsa-miR-148b-3p
 
 
-0.332
 
 
32.1
 
 
 
 
AGFG1
 
 
hsa-miR-152-3p
 
 
-0.332
 
 
32.1
 
 
 
 
AGGF1
 
 
hsa-miR-125a-5p
 
 
-0.331
 
 
10.3
 
 
 
 
AGPAT3
 
 
hsa-miR-27a-3p
 
 
-0.29
 
 
20.1
 
 
 
 
AGTRAP
 
 
hsa-miR-34a-5p
 
 
-0.328
 
 
38.5
 
 
 
 
AHCYL2
 
 
hsa-miR-34a-5p
 
 
-0.359
 
 
39.9
 
 
 
 
AHDC1
 
 
hsa-miR-148b-3p
 
 
-0.413
 
 
19.9
 
 
 
 
AHDC1
 
 
hsa-miR-152-3p
 
 
-0.413
 
 
19.9
 
 
 
 
AHR
 
 
hsa-miR-29a-3p
 
 
-0.316
 
 
11.8
 
 
 
 
AIDA
 
 
hsa-miR-22-3p
 
 
-0.316
 
 
35.1
 
 
 
 
AIFM1
 
 
hsa-miR-125a-5p
 
 
-0.3
 
 
10.7
 
 
 
 
AK1
 
 
hsa-miR-29a-3p
 
 
-0.333
 
 
42.1
 
 
 
 
AKAP1
 
 
hsa-miR-148b-3p
 
 
-0.381
 
 
35.0
 
 
 
 
AKAP1
 
 
hsa-miR-152-3p
 
 
-0.381
 
 
35.0
 
 
 
 
AKIRIN1
 
 
hsa-miR-27a-3p
 
 
-0.368
 
 
33.8
 
 
 
 
AKT3
 
 
hsa-miR-29a-3p
 
 
-0.287
 
 
13.0
 
 
 
 
AKT3
 
 
hsa-miR-22-3p
 
 
-0.451
 
 
13.0
 
 
 
 
ALCAM
 
 
hsa-miR-34a-5p
 
 
-0.303
 
 
58.4
 
 
 
 
ALCAM
 
 
hsa-miR-148b-3p
 
 
-0.339
 
 
58.4
 
 
 
 
ALCAM
 
 
hsa-miR-152-3p
 
 
-0.347
 
 
58.4
 
 
 
 
ALDOA
 
 
hsa-miR-34a-5p
 
 
-0.655
 
 
510.5
 
 
 
 
ANAPC16
 
 
hsa-miR-125a-5p
 
 
-0.578
 
 
21.8
 
 
 
 
ANK3
 
 
hsa-miR-34a-5p
 
 
-0.29
 
 
35.3
 
 
 
 
ANK3
 
 
hsa-miR-34a-5p
 
 
-0.347
 
 
35.3
 
 
 
 
ANKHD1
 
 
hsa-miR-23a-3p
 
 
-0.368
 
 
44.4
 
 
 
 
ANKHD1
 
 
hsa-miR-23b-3p
 
 
-0.368
 
 
44.4
 
 
 
 
ANKRD13A
 
 
hsa-miR-22-3p
 
 
-0.478
 
 
26.4
 
 
 
 
ANKRD40
 
 
hsa-miR-27a-3p
 
 
-0.35
 
 
15.8
 
 
 
 
ANP32A
 
 
hsa-miR-132-3p
 
 
-0.374
 
 
68.9
 
 
 
 
ANP32B
 
 
hsa-miR-34a-5p
 
 
-0.29
 
 
92.0
 
 
 
 
AP1S3
 
 
hsa-miR-181b-5p
 
 
-0.383
 
 
12.8
 
 
 
 
AP1S3
 
 
hsa-miR-181a-5p
 
 
-0.383
 
 
12.8
 
 
 
 
AP3S2
 
 
hsa-miR-34a-5p
 
 
-0.413
 
 
24.5
 
 
 
 
APH1A
 
 
hsa-miR-34a-5p
 
 
-0.304
 
 
89.7
 
 
 
 
APOO
 
 
hsa-miR-181b-5p
 
 
-0.5
 
 
19.0
 
 
 
 
APOO
 
 
hsa-miR-181a-5p
 
 
-0.5
 
 
19.0
 
 
 
 
APPBP2
 
 
hsa-miR-27a-3p
 
 
-0.378
 
 
12.0
 
 
 
 
ARF4
 
 
hsa-miR-152-3p
 
 
-0.487
 
 
134.8
 
 
 
 
ARF4
 
 
hsa-miR-148b-3p
 
 
-0.487
 
 
134.8
 
 
 
 
ARF5
 
 
hsa-miR-29a-3p
 
 
-0.491
 
 
88.3
 
 
 
 
ARF6
 
 
hsa-miR-181b-5p
 
 
-0.33
 
 
20.1
 
 
 
 
ARF6
 
 
hsa-miR-181a-5p
 
 
-0.33
 
 
20.1
 
 
 
 
ARFGEF1
 
 
hsa-miR-148b-3p
 
 
-0.306
 
 
20.9
 
 
 
 
ARFGEF1
 
 
hsa-miR-152-3p
 
 
-0.306
 
 
20.9
 
 
 
 
ARFIP1
 
 
hsa-miR-152-3p
 
 
-0.474
 
 
18.8
 
 
 
 
ARFIP1
 
 
hsa-miR-148b-3p
 
 
-0.474
 
 
18.8
 
 
 
 
ARHGAP1
 
 
hsa-miR-34a-5p
 
 
-0.369
 
 
42.2
 
 
 
 
ARHGAP21
 
 
hsa-miR-152-3p
 
 
-0.287
 
 
12.0
 
 
 
 
ARHGAP21
 
 
hsa-miR-148b-3p
 
 
-0.287
 
 
12.0
 
 
 
 
ARID1B
 
 
hsa-miR-29a-3p
 
 
-0.342
 
 
19.6
 
 
 
 
ARL4A
 
 
hsa-miR-29a-3p
 
 
-0.386
 
 
18.8
 
 
 
 
ARL6IP1
 
 
hsa-miR-148b-3p
 
 
-0.579
 
 
105.4
 
 
 
 
ARL6IP1
 
 
hsa-miR-152-3p
 
 
-0.579
 
 
105.4
 
 
 
 
ARL8B
 
 
hsa-miR-152-3p
 
 
-0.337
 
 
58.1
 
 
 
 
ARL8B
 
 
hsa-miR-148b-3p
 
 
-0.337
 
 
58.1
 
 
 
 
ARMCX3
 
 
hsa-miR-425-5p
 
 
-0.325
 
 
46.7
 
 
 
 
ARPC5
 
 
hsa-miR-22-3p
 
 
-0.679
 
 
43.0
 
 
 
 
ARPIN-AP3S2
 
 
hsa-miR-34a-5p
 
 
-0.348
 
 
10.3
 
 
 
 
ARPP19
 
 
hsa-miR-29a-3p
 
 
-0.371
 
 
53.7
 
 
 
 
ARPP19
 
 
hsa-miR-34a-5p
 
 
-0.497
 
 
53.7
 
 
 
 
ARRDC3
 
 
hsa-miR-152-3p
 
 
-0.305
 
 
78.7
 
 
 
 
ARRDC3
 
 
hsa-miR-148b-3p
 
 
-0.305
 
 
78.7
 
 
 
 
ARRDC3
 
 
hsa-miR-29a-3p
 
 
-0.402
 
 
78.7
 
 
 
 
ASAH1
 
 
hsa-miR-27a-3p
 
 
-0.398
 
 
305.0
 
 
 
 
ASAP2
 
 
hsa-miR-29a-3p
 
 
-0.348
 
 
17.9
 
 
 
 
ASF1A
 
 
hsa-miR-23a-3p
 
 
-0.506
 
 
14.1
 
 
 
 
ASF1A
 
 
hsa-miR-23b-3p
 
 
-0.506
 
 
14.1
 
 
 
 
ASPH
 
 
hsa-miR-152-3p
 
 
-0.391
 
 
90.3
 
 
 
 
ASPH
 
 
hsa-miR-148b-3p
 
 
-0.391
 
 
90.3
 
 
 
 
ATG4B
 
 
hsa-miR-34a-5p
 
 
-0.58
 
 
20.4
 
 
 
 
 
 
 tongue 
 
 
 
 
target_symbol
 
 
mature_mirna_id
 
 
score
 
 
nTPM
 
 
 
 
 
 
AAGAB
 
 
hsa-miR-34a-5p
 
 
-0.319
 
 
21.5
 
 
 
 
AAR2
 
 
hsa-miR-29a-3p
 
 
-0.327
 
 
19.3
 
 
 
 
ABCB10
 
 
hsa-miR-152-3p
 
 
-0.291
 
 
10.2
 
 
 
 
ABCB10
 
 
hsa-miR-148b-3p
 
 
-0.291
 
 
10.2
 
 
 
 
ABCB7
 
 
hsa-miR-148b-3p
 
 
-0.515
 
 
20.8
 
 
 
 
ABCB7
 
 
hsa-miR-152-3p
 
 
-0.515
 
 
20.8
 
 
 
 
ABCC5
 
 
hsa-miR-125a-5p
 
 
-0.288
 
 
37.6
 
 
 
 
ABCD3
 
 
hsa-miR-148b-3p
 
 
-0.388
 
 
27.9
 
 
 
 
ABCD3
 
 
hsa-miR-152-3p
 
 
-0.388
 
 
27.9
 
 
 
 
ABCE1
 
 
hsa-miR-29a-3p
 
 
-0.404
 
 
28.5
 
 
 
 
ABHD18
 
 
hsa-miR-29a-3p
 
 
-0.321
 
 
10.9
 
 
 
 
ABHD18
 
 
hsa-miR-29a-3p
 
 
-0.34
 
 
10.9
 
 
 
 
ABHD4
 
 
hsa-miR-29a-3p
 
 
-0.312
 
 
27.0
 
 
 
 
ABHD5
 
 
hsa-miR-132-3p
 
 
-0.323
 
 
17.3
 
 
 
 
ABHD5
 
 
hsa-miR-27a-3p
 
 
-0.407
 
 
17.3
 
 
 
 
ABRAXAS2
 
 
hsa-miR-34a-5p
 
 
-0.538
 
 
15.2
 
 
 
 
ABTB1
 
 
hsa-miR-125a-5p
 
 
-0.519
 
 
24.5
 
 
 
 
ACBD3
 
 
hsa-miR-34a-5p
 
 
-0.415
 
 
19.0
 
 
 
 
ACHE
 
 
hsa-miR-125a-5p
 
 
-0.308
 
 
44.9
 
 
 
 
ACHE
 
 
hsa-miR-125a-5p
 
 
-0.361
 
 
44.9
 
 
 
 
ACSL1
 
 
hsa-miR-34a-5p
 
 
-0.306
 
 
397.3
 
 
 
 
ACSL4
 
 
hsa-miR-34a-5p
 
 
-0.372
 
 
15.8
 
 
 
 
ACTA2
 
 
hsa-miR-27a-3p
 
 
-0.577
 
 
200.0
 
 
 
 
ACTN2
 
 
hsa-miR-23a-3p
 
 
-0.294
 
 
1195.2
 
 
 
 
ACTN2
 
 
hsa-miR-29a-3p
 
 
-0.311
 
 
1195.2
 
 
 
 
ADCY2
 
 
hsa-miR-152-3p
 
 
-0.299
 
 
28.8
 
 
 
 
ADCY2
 
 
hsa-miR-148b-3p
 
 
-0.299
 
 
28.8
 
 
 
 
ADCY3
 
 
hsa-miR-132-3p
 
 
-0.303
 
 
19.0
 
 
 
 
ADGRA3
 
 
hsa-miR-27a-3p
 
 
-0.296
 
 
10.7
 
 
 
 
ADGRF5
 
 
hsa-miR-148b-3p
 
 
-0.354
 
 
66.5
 
 
 
 
ADGRF5
 
 
hsa-miR-152-3p
 
 
-0.354
 
 
66.5
 
 
 
 
ADIPOR2
 
 
hsa-miR-150-5p
 
 
-0.287
 
 
18.5
 
 
 
 
ADIPOR2
 
 
hsa-miR-34a-5p
 
 
-0.33
 
 
18.5
 
 
 
 
ADK
 
 
hsa-miR-34a-5p
 
 
-0.374
 
 
44.0
 
 
 
 
ADO
 
 
hsa-miR-34a-5p
 
 
-0.377
 
 
16.2
 
 
 
 
AFG3L2
 
 
hsa-miR-181b-5p
 
 
-0.491
 
 
92.6
 
 
 
 
AFG3L2
 
 
hsa-miR-181a-5p
 
 
-0.491
 
 
92.6
 
 
 
 
AGFG1
 
 
hsa-miR-148b-3p
 
 
-0.332
 
 
36.1
 
 
 
 
AGFG1
 
 
hsa-miR-152-3p
 
 
-0.332
 
 
36.1
 
 
 
 
AGGF1
 
 
hsa-miR-125a-5p
 
 
-0.331
 
 
10.4
 
 
 
 
AGO4
 
 
hsa-miR-34a-5p
 
 
-0.306
 
 
11.1
 
 
 
 
AGPAT3
 
 
hsa-miR-27a-3p
 
 
-0.29
 
 
46.9
 
 
 
 
AGTRAP
 
 
hsa-miR-34a-5p
 
 
-0.328
 
 
11.6
 
 
 
 
AHCYL2
 
 
hsa-miR-34a-5p
 
 
-0.359
 
 
25.9
 
 
 
 
AHDC1
 
 
hsa-miR-148b-3p
 
 
-0.413
 
 
14.5
 
 
 
 
AHDC1
 
 
hsa-miR-152-3p
 
 
-0.413
 
 
14.5
 
 
 
 
AIDA
 
 
hsa-miR-22-3p
 
 
-0.316
 
 
46.5
 
 
 
 
AIFM1
 
 
hsa-miR-125a-5p
 
 
-0.3
 
 
23.4
 
 
 
 
AK1
 
 
hsa-miR-29a-3p
 
 
-0.333
 
 
730.8
 
 
 
 
AKAP1
 
 
hsa-miR-148b-3p
 
 
-0.381
 
 
128.4
 
 
 
 
AKAP1
 
 
hsa-miR-152-3p
 
 
-0.381
 
 
128.4
 
 
 
 
AKIRIN1
 
 
hsa-miR-27a-3p
 
 
-0.368
 
 
28.3
 
 
 
 
AKT3
 
 
hsa-miR-29a-3p
 
 
-0.287
 
 
10.7
 
 
 
 
AKT3
 
 
hsa-miR-22-3p
 
 
-0.451
 
 
10.7
 
 
 
 
ALDOA
 
 
hsa-miR-34a-5p
 
 
-0.655
 
 
8554.2
 
 
 
 
AMER1
 
 
hsa-miR-29a-3p
 
 
-0.343
 
 
14.3
 
 
 
 
AMOT
 
 
hsa-miR-29a-3p
 
 
-0.304
 
 
51.2
 
 
 
 
ANAPC16
 
 
hsa-miR-125a-5p
 
 
-0.578
 
 
38.9
 
 
 
 
ANK2
 
 
hsa-miR-34a-5p
 
 
-0.317
 
 
89.8
 
 
 
 
ANK3
 
 
hsa-miR-34a-5p
 
 
-0.29
 
 
88.2
 
 
 
 
ANK3
 
 
hsa-miR-34a-5p
 
 
-0.347
 
 
88.2
 
 
 
 
ANKHD1
 
 
hsa-miR-23a-3p
 
 
-0.368
 
 
26.1
 
 
 
 
ANKHD1
 
 
hsa-miR-23b-3p
 
 
-0.368
 
 
26.1
 
 
 
 
ANKRD40
 
 
hsa-miR-27a-3p
 
 
-0.35
 
 
78.6
 
 
 
 
ANP32A
 
 
hsa-miR-132-3p
 
 
-0.374
 
 
60.2
 
 
 
 
ANP32B
 
 
hsa-miR-34a-5p
 
 
-0.29
 
 
47.3
 
 
 
 
ANTXR2
 
 
hsa-miR-29a-3p
 
 
-0.347
 
 
20.3
 
 
 
 
AP3S2
 
 
hsa-miR-34a-5p
 
 
-0.413
 
 
42.1
 
 
 
 
APC
 
 
hsa-miR-29a-3p
 
 
-0.413
 
 
11.0
 
 
 
 
APH1A
 
 
hsa-miR-34a-5p
 
 
-0.304
 
 
59.0
 
 
 
 
APOO
 
 
hsa-miR-181b-5p
 
 
-0.5
 
 
68.2
 
 
 
 
APOO
 
 
hsa-miR-181a-5p
 
 
-0.5
 
 
68.2
 
 
 
 
ARF4
 
 
hsa-miR-152-3p
 
 
-0.487
 
 
64.9
 
 
 
 
ARF4
 
 
hsa-miR-148b-3p
 
 
-0.487
 
 
64.9
 
 
 
 
ARF5
 
 
hsa-miR-29a-3p
 
 
-0.491
 
 
94.7
 
 
 
 
ARF6
 
 
hsa-miR-181b-5p
 
 
-0.33
 
 
15.8
 
 
 
 
ARF6
 
 
hsa-miR-181a-5p
 
 
-0.33
 
 
15.8
 
 
 
 
ARFGEF1
 
 
hsa-miR-148b-3p
 
 
-0.306
 
 
13.0
 
 
 
 
ARFGEF1
 
 
hsa-miR-152-3p
 
 
-0.306
 
 
13.0
 
 
 
 
ARHGAP1
 
 
hsa-miR-34a-5p
 
 
-0.369
 
 
19.1
 
 
 
 
ARHGAP21
 
 
hsa-miR-152-3p
 
 
-0.287
 
 
21.5
 
 
 
 
ARHGAP21
 
 
hsa-miR-148b-3p
 
 
-0.287
 
 
21.5
 
 
 
 
ARID1B
 
 
hsa-miR-29a-3p
 
 
-0.342
 
 
22.6
 
 
 
 
ARL6IP1
 
 
hsa-miR-148b-3p
 
 
-0.579
 
 
48.1
 
 
 
 
ARL6IP1
 
 
hsa-miR-152-3p
 
 
-0.579
 
 
48.1
 
 
 
 
ARL8B
 
 
hsa-miR-152-3p
 
 
-0.337
 
 
56.0
 
 
 
 
ARL8B
 
 
hsa-miR-148b-3p
 
 
-0.337
 
 
56.0
 
 
 
 
ARMCX3
 
 
hsa-miR-425-5p
 
 
-0.325
 
 
59.4
 
 
 
 
ARPC5
 
 
hsa-miR-22-3p
 
 
-0.679
 
 
21.0
 
 
 
 
ARPIN-AP3S2
 
 
hsa-miR-34a-5p
 
 
-0.348
 
 
14.1
 
 
 
 
ARPP19
 
 
hsa-miR-29a-3p
 
 
-0.371
 
 
158.9
 
 
 
 
ARPP19
 
 
hsa-miR-34a-5p
 
 
-0.497
 
 
158.9
 
 
 
 
ARRB1
 
 
hsa-miR-22-3p
 
 
-0.616
 
 
10.1
 
 
 
 
ARRDC3
 
 
hsa-miR-152-3p
 
 
-0.305
 
 
41.9
 
 
 
 
ARRDC3
 
 
hsa-miR-148b-3p
 
 
-0.305
 
 
41.9
 
 
 
 
ARRDC3
 
 
hsa-miR-29a-3p
 
 
-0.402
 
 
41.9
 
 
 
 
ASAH1
 
 
hsa-miR-27a-3p
 
 
-0.398
 
 
65.9
 
 
 
 
ASB1
 
 
hsa-miR-34a-5p
 
 
-0.406
 
 
21.7
 
 
 
 
ASB11
 
 
hsa-miR-27a-3p
 
 
-0.353
 
 
57.4
 
 
 
 
ASF1A
 
 
hsa-miR-23a-3p
 
 
-0.506
 
 
15.1
 
 
 
 
 
 
 tonsil 
 
 
 
 
target_symbol
 
 
mature_mirna_id
 
 
score
 
 
nTPM
 
 
 
 
 
 
AAGAB
 
 
hsa-miR-34a-5p
 
 
-0.319
 
 
24.3
 
 
 
 
AAR2
 
 
hsa-miR-29a-3p
 
 
-0.327
 
 
18.7
 
 
 
 
ABCB7
 
 
hsa-miR-148b-3p
 
 
-0.515
 
 
10.4
 
 
 
 
ABCB7
 
 
hsa-miR-152-3p
 
 
-0.515
 
 
10.4
 
 
 
 
ABCC5
 
 
hsa-miR-125a-5p
 
 
-0.288
 
 
11.1
 
 
 
 
ABCD3
 
 
hsa-miR-148b-3p
 
 
-0.388
 
 
19.9
 
 
 
 
ABCD3
 
 
hsa-miR-152-3p
 
 
-0.388
 
 
19.9
 
 
 
 
ABCE1
 
 
hsa-miR-29a-3p
 
 
-0.404
 
 
39.4
 
 
 
 
ABHD17C
 
 
hsa-miR-27a-3p
 
 
-0.435
 
 
21.6
 
 
 
 
ABHD3
 
 
hsa-miR-125a-5p
 
 
-0.453
 
 
24.2
 
 
 
 
ABHD4
 
 
hsa-miR-29a-3p
 
 
-0.312
 
 
15.2
 
 
 
 
ABRAXAS2
 
 
hsa-miR-34a-5p
 
 
-0.538
 
 
12.5
 
 
 
 
ACBD3
 
 
hsa-miR-34a-5p
 
 
-0.415
 
 
24.0
 
 
 
 
ACSL1
 
 
hsa-miR-34a-5p
 
 
-0.306
 
 
20.1
 
 
 
 
ACSL4
 
 
hsa-miR-34a-5p
 
 
-0.372
 
 
16.0
 
 
 
 
ACTA2
 
 
hsa-miR-27a-3p
 
 
-0.577
 
 
45.3
 
 
 
 
ADA
 
 
hsa-miR-29a-3p
 
 
-0.376
 
 
23.2
 
 
 
 
ADAM10
 
 
hsa-miR-152-3p
 
 
-0.348
 
 
15.3
 
 
 
 
ADAM10
 
 
hsa-miR-148b-3p
 
 
-0.356
 
 
15.3
 
 
 
 
ADAM17
 
 
hsa-miR-125a-5p
 
 
-0.292
 
 
11.1
 
 
 
 
ADAM17
 
 
hsa-miR-152-3p
 
 
-0.301
 
 
11.1
 
 
 
 
ADAM17
 
 
hsa-miR-148b-3p
 
 
-0.301
 
 
11.1
 
 
 
 
ADCY3
 
 
hsa-miR-132-3p
 
 
-0.303
 
 
16.9
 
 
 
 
ADIPOR2
 
 
hsa-miR-150-5p
 
 
-0.287
 
 
11.8
 
 
 
 
ADIPOR2
 
 
hsa-miR-34a-5p
 
 
-0.33
 
 
11.8
 
 
 
 
ADK
 
 
hsa-miR-34a-5p
 
 
-0.374
 
 
32.3
 
 
 
 
ADRA2A
 
 
hsa-miR-23a-3p
 
 
-0.371
 
 
12.3
 
 
 
 
ADRA2A
 
 
hsa-miR-23b-3p
 
 
-0.371
 
 
12.3
 
 
 
 
AFG3L2
 
 
hsa-miR-181b-5p
 
 
-0.491
 
 
16.2
 
 
 
 
AFG3L2
 
 
hsa-miR-181a-5p
 
 
-0.491
 
 
16.2
 
 
 
 
AGFG1
 
 
hsa-miR-148b-3p
 
 
-0.332
 
 
49.9
 
 
 
 
AGFG1
 
 
hsa-miR-152-3p
 
 
-0.332
 
 
49.9
 
 
 
 
AGGF1
 
 
hsa-miR-125a-5p
 
 
-0.331
 
 
13.2
 
 
 
 
AGTRAP
 
 
hsa-miR-34a-5p
 
 
-0.328
 
 
12.5
 
 
 
 
AHR
 
 
hsa-miR-29a-3p
 
 
-0.316
 
 
18.6
 
 
 
 
AIDA
 
 
hsa-miR-22-3p
 
 
-0.316
 
 
49.6
 
 
 
 
AIFM1
 
 
hsa-miR-125a-5p
 
 
-0.3
 
 
10.3
 
 
 
 
AK1
 
 
hsa-miR-29a-3p
 
 
-0.333
 
 
11.8
 
 
 
 
AKIRIN1
 
 
hsa-miR-27a-3p
 
 
-0.368
 
 
39.4
 
 
 
 
ALCAM
 
 
hsa-miR-34a-5p
 
 
-0.303
 
 
13.6
 
 
 
 
ALCAM
 
 
hsa-miR-148b-3p
 
 
-0.339
 
 
13.6
 
 
 
 
ALCAM
 
 
hsa-miR-152-3p
 
 
-0.347
 
 
13.6
 
 
 
 
ALDOA
 
 
hsa-miR-34a-5p
 
 
-0.655
 
 
447.4
 
 
 
 
ANAPC16
 
 
hsa-miR-125a-5p
 
 
-0.578
 
 
23.8
 
 
 
 
ANKHD1
 
 
hsa-miR-23a-3p
 
 
-0.368
 
 
38.8
 
 
 
 
ANKHD1
 
 
hsa-miR-23b-3p
 
 
-0.368
 
 
38.8
 
 
 
 
ANKHD1-EIF4EBP3
 
 
hsa-miR-22-3p
 
 
-0.479
 
 
12.6
 
 
 
 
ANKRD13A
 
 
hsa-miR-22-3p
 
 
-0.478
 
 
62.4
 
 
 
 
ANKRD29
 
 
hsa-miR-132-3p
 
 
-0.47
 
 
13.4
 
 
 
 
ANKRD40
 
 
hsa-miR-27a-3p
 
 
-0.35
 
 
13.5
 
 
 
 
ANP32A
 
 
hsa-miR-132-3p
 
 
-0.374
 
 
132.1
 
 
 
 
ANP32B
 
 
hsa-miR-34a-5p
 
 
-0.29
 
 
232.6
 
 
 
 
ANTXR2
 
 
hsa-miR-29a-3p
 
 
-0.347
 
 
15.1
 
 
 
 
AP3S2
 
 
hsa-miR-34a-5p
 
 
-0.413
 
 
16.4
 
 
 
 
APH1A
 
 
hsa-miR-34a-5p
 
 
-0.304
 
 
85.0
 
 
 
 
APOO
 
 
hsa-miR-181b-5p
 
 
-0.5
 
 
10.8
 
 
 
 
APOO
 
 
hsa-miR-181a-5p
 
 
-0.5
 
 
10.8
 
 
 
 
ARF4
 
 
hsa-miR-152-3p
 
 
-0.487
 
 
91.2
 
 
 
 
ARF4
 
 
hsa-miR-148b-3p
 
 
-0.487
 
 
91.2
 
 
 
 
ARF5
 
 
hsa-miR-29a-3p
 
 
-0.491
 
 
69.9
 
 
 
 
ARF6
 
 
hsa-miR-181b-5p
 
 
-0.33
 
 
64.2
 
 
 
 
ARF6
 
 
hsa-miR-181a-5p
 
 
-0.33
 
 
64.2
 
 
 
 
ARFGEF1
 
 
hsa-miR-148b-3p
 
 
-0.306
 
 
16.2
 
 
 
 
ARFGEF1
 
 
hsa-miR-152-3p
 
 
-0.306
 
 
16.2
 
 
 
 
ARFIP1
 
 
hsa-miR-152-3p
 
 
-0.474
 
 
13.9
 
 
 
 
ARFIP1
 
 
hsa-miR-148b-3p
 
 
-0.474
 
 
13.9
 
 
 
 
ARHGAP1
 
 
hsa-miR-34a-5p
 
 
-0.369
 
 
32.4
 
 
 
 
ARHGAP26
 
 
hsa-miR-34a-5p
 
 
-0.384
 
 
11.4
 
 
 
 
ARID1B
 
 
hsa-miR-29a-3p
 
 
-0.342
 
 
17.5
 
 
 
 
ARL4A
 
 
hsa-miR-29a-3p
 
 
-0.386
 
 
14.9
 
 
 
 
ARL6IP1
 
 
hsa-miR-148b-3p
 
 
-0.579
 
 
106.2
 
 
 
 
ARL6IP1
 
 
hsa-miR-152-3p
 
 
-0.579
 
 
106.2
 
 
 
 
ARL8B
 
 
hsa-miR-152-3p
 
 
-0.337
 
 
44.5
 
 
 
 
ARL8B
 
 
hsa-miR-148b-3p
 
 
-0.337
 
 
44.5
 
 
 
 
ARMCX3
 
 
hsa-miR-425-5p
 
 
-0.325
 
 
20.2
 
 
 
 
ARPC5
 
 
hsa-miR-22-3p
 
 
-0.679
 
 
123.0
 
 
 
 
ARPP19
 
 
hsa-miR-29a-3p
 
 
-0.371
 
 
48.4
 
 
 
 
ARPP19
 
 
hsa-miR-34a-5p
 
 
-0.497
 
 
48.4
 
 
 
 
ARRDC3
 
 
hsa-miR-152-3p
 
 
-0.305
 
 
26.4
 
 
 
 
ARRDC3
 
 
hsa-miR-148b-3p
 
 
-0.305
 
 
26.4
 
 
 
 
ARRDC3
 
 
hsa-miR-29a-3p
 
 
-0.402
 
 
26.4
 
 
 
 
ASAH1
 
 
hsa-miR-27a-3p
 
 
-0.398
 
 
54.1
 
 
 
 
ASF1A
 
 
hsa-miR-23a-3p
 
 
-0.506
 
 
14.1
 
 
 
 
ASF1A
 
 
hsa-miR-23b-3p
 
 
-0.506
 
 
14.1
 
 
 
 
ASPH
 
 
hsa-miR-152-3p
 
 
-0.391
 
 
26.4
 
 
 
 
ASPH
 
 
hsa-miR-148b-3p
 
 
-0.391
 
 
26.4
 
 
 
 
ATMIN
 
 
hsa-miR-34a-5p
 
 
-0.409
 
 
14.1
 
 
 
 
ATP2B4
 
 
hsa-miR-152-3p
 
 
-0.337
 
 
18.2
 
 
 
 
ATP2B4
 
 
hsa-miR-148b-3p
 
 
-0.337
 
 
18.2
 
 
 
 
ATP5MC3
 
 
hsa-miR-425-5p
 
 
-0.328
 
 
164.6
 
 
 
 
ATP5MC3
 
 
hsa-miR-29a-3p
 
 
-0.33
 
 
164.6
 
 
 
 
ATP5MC3
 
 
hsa-miR-27a-3p
 
 
-0.416
 
 
164.6
 
 
 
 
ATP6AP2
 
 
hsa-miR-148b-3p
 
 
-0.592
 
 
67.9
 
 
 
 
ATP6AP2
 
 
hsa-miR-152-3p
 
 
-0.592
 
 
67.9
 
 
 
 
ATP6AP2
 
 
hsa-miR-148b-3p
 
 
-0.668
 
 
67.9
 
 
 
 
ATP6AP2
 
 
hsa-miR-152-3p
 
 
-0.668
 
 
67.9
 
 
 
 
ATP6V1E1
 
 
hsa-miR-23a-3p
 
 
-0.294
 
 
102.6
 
 
 
 
ATP6V1E1
 
 
hsa-miR-23b-3p
 
 
-0.294
 
 
102.6
 
 
 
 
ATP8A1
 
 
hsa-miR-22-3p
 
 
-0.468
 
 
11.0
 
 
 
 
ATXN10
 
 
hsa-miR-27a-3p
 
 
-0.295
 
 
48.3
 
 
 
 
 
 
 urinary bladder 
 
 
 
 
target_symbol
 
 
mature_mirna_id
 
 
score
 
 
nTPM
 
 
 
 
 
 
AAGAB
 
 
hsa-miR-34a-5p
 
 
-0.319
 
 
25.7
 
 
 
 
AAR2
 
 
hsa-miR-29a-3p
 
 
-0.327
 
 
25.2
 
 
 
 
ABCB6
 
 
hsa-miR-29a-3p
 
 
-0.48
 
 
13.5
 
 
 
 
ABCB7
 
 
hsa-miR-148b-3p
 
 
-0.515
 
 
11.0
 
 
 
 
ABCB7
 
 
hsa-miR-152-3p
 
 
-0.515
 
 
11.0
 
 
 
 
ABCC5
 
 
hsa-miR-125a-5p
 
 
-0.288
 
 
38.3
 
 
 
 
ABCD3
 
 
hsa-miR-148b-3p
 
 
-0.388
 
 
21.4
 
 
 
 
ABCD3
 
 
hsa-miR-152-3p
 
 
-0.388
 
 
21.4
 
 
 
 
ABCE1
 
 
hsa-miR-29a-3p
 
 
-0.404
 
 
37.8
 
 
 
 
ABHD17C
 
 
hsa-miR-27a-3p
 
 
-0.435
 
 
26.8
 
 
 
 
ABHD3
 
 
hsa-miR-125a-5p
 
 
-0.453
 
 
14.3
 
 
 
 
ABHD4
 
 
hsa-miR-29a-3p
 
 
-0.312
 
 
28.1
 
 
 
 
ABHD5
 
 
hsa-miR-132-3p
 
 
-0.323
 
 
15.5
 
 
 
 
ABHD5
 
 
hsa-miR-27a-3p
 
 
-0.407
 
 
15.5
 
 
 
 
ABTB1
 
 
hsa-miR-125a-5p
 
 
-0.519
 
 
36.6
 
 
 
 
ACBD3
 
 
hsa-miR-34a-5p
 
 
-0.415
 
 
25.2
 
 
 
 
ACER2
 
 
hsa-miR-125a-5p
 
 
-0.338
 
 
36.7
 
 
 
 
ACSL1
 
 
hsa-miR-34a-5p
 
 
-0.306
 
 
59.5
 
 
 
 
ACSL4
 
 
hsa-miR-34a-5p
 
 
-0.372
 
 
62.0
 
 
 
 
ACTA2
 
 
hsa-miR-27a-3p
 
 
-0.577
 
 
3161.7
 
 
 
 
ACVR1
 
 
hsa-miR-152-3p
 
 
-0.397
 
 
13.3
 
 
 
 
ACVR1
 
 
hsa-miR-148b-3p
 
 
-0.397
 
 
13.3
 
 
 
 
ADAM10
 
 
hsa-miR-152-3p
 
 
-0.348
 
 
26.2
 
 
 
 
ADAM10
 
 
hsa-miR-148b-3p
 
 
-0.356
 
 
26.2
 
 
 
 
ADAMTS9
 
 
hsa-miR-29a-3p
 
 
-0.305
 
 
20.9
 
 
 
 
ADAMTS9
 
 
hsa-miR-29a-3p
 
 
-0.485
 
 
20.9
 
 
 
 
ADCY3
 
 
hsa-miR-132-3p
 
 
-0.303
 
 
34.2
 
 
 
 
ADGRF5
 
 
hsa-miR-148b-3p
 
 
-0.354
 
 
10.6
 
 
 
 
ADGRF5
 
 
hsa-miR-152-3p
 
 
-0.354
 
 
10.6
 
 
 
 
ADIPOR2
 
 
hsa-miR-150-5p
 
 
-0.287
 
 
21.3
 
 
 
 
ADIPOR2
 
 
hsa-miR-34a-5p
 
 
-0.33
 
 
21.3
 
 
 
 
ADK
 
 
hsa-miR-34a-5p
 
 
-0.374
 
 
22.9
 
 
 
 
ADORA2B
 
 
hsa-miR-27a-3p
 
 
-0.638
 
 
15.6
 
 
 
 
ADRA2A
 
 
hsa-miR-23a-3p
 
 
-0.371
 
 
14.8
 
 
 
 
ADRA2A
 
 
hsa-miR-23b-3p
 
 
-0.371
 
 
14.8
 
 
 
 
AFG3L2
 
 
hsa-miR-181b-5p
 
 
-0.491
 
 
18.0
 
 
 
 
AFG3L2
 
 
hsa-miR-181a-5p
 
 
-0.491
 
 
18.0
 
 
 
 
AGFG1
 
 
hsa-miR-148b-3p
 
 
-0.332
 
 
61.2
 
 
 
 
AGFG1
 
 
hsa-miR-152-3p
 
 
-0.332
 
 
61.2
 
 
 
 
AGPAT3
 
 
hsa-miR-27a-3p
 
 
-0.29
 
 
14.4
 
 
 
 
AGTRAP
 
 
hsa-miR-34a-5p
 
 
-0.328
 
 
31.7
 
 
 
 
AHCYL2
 
 
hsa-miR-34a-5p
 
 
-0.359
 
 
15.0
 
 
 
 
AHDC1
 
 
hsa-miR-148b-3p
 
 
-0.413
 
 
14.8
 
 
 
 
AHDC1
 
 
hsa-miR-152-3p
 
 
-0.413
 
 
14.8
 
 
 
 
AHR
 
 
hsa-miR-29a-3p
 
 
-0.316
 
 
46.3
 
 
 
 
AIDA
 
 
hsa-miR-22-3p
 
 
-0.316
 
 
44.1
 
 
 
 
AIFM1
 
 
hsa-miR-125a-5p
 
 
-0.3
 
 
13.1
 
 
 
 
AK1
 
 
hsa-miR-29a-3p
 
 
-0.333
 
 
22.9
 
 
 
 
AKAP1
 
 
hsa-miR-148b-3p
 
 
-0.381
 
 
47.8
 
 
 
 
AKAP1
 
 
hsa-miR-152-3p
 
 
-0.381
 
 
47.8
 
 
 
 
AKIRIN1
 
 
hsa-miR-27a-3p
 
 
-0.368
 
 
38.4
 
 
 
 
AKT3
 
 
hsa-miR-29a-3p
 
 
-0.287
 
 
14.4
 
 
 
 
AKT3
 
 
hsa-miR-22-3p
 
 
-0.451
 
 
14.4
 
 
 
 
ALCAM
 
 
hsa-miR-34a-5p
 
 
-0.303
 
 
23.9
 
 
 
 
ALCAM
 
 
hsa-miR-148b-3p
 
 
-0.339
 
 
23.9
 
 
 
 
ALCAM
 
 
hsa-miR-152-3p
 
 
-0.347
 
 
23.9
 
 
 
 
ALDOA
 
 
hsa-miR-34a-5p
 
 
-0.655
 
 
917.9
 
 
 
 
ANAPC16
 
 
hsa-miR-125a-5p
 
 
-0.578
 
 
20.8
 
 
 
 
ANKHD1
 
 
hsa-miR-23a-3p
 
 
-0.368
 
 
34.3
 
 
 
 
ANKHD1
 
 
hsa-miR-23b-3p
 
 
-0.368
 
 
34.3
 
 
 
 
ANKHD1-EIF4EBP3
 
 
hsa-miR-22-3p
 
 
-0.479
 
 
11.2
 
 
 
 
ANKRD13A
 
 
hsa-miR-22-3p
 
 
-0.478
 
 
24.2
 
 
 
 
ANKRD40
 
 
hsa-miR-27a-3p
 
 
-0.35
 
 
22.8
 
 
 
 
ANP32A
 
 
hsa-miR-132-3p
 
 
-0.374
 
 
71.6
 
 
 
 
ANP32B
 
 
hsa-miR-34a-5p
 
 
-0.29
 
 
122.0
 
 
 
 
ANPEP
 
 
hsa-miR-125a-5p
 
 
-0.372
 
 
15.7
 
 
 
 
ANTXR2
 
 
hsa-miR-29a-3p
 
 
-0.347
 
 
82.6
 
 
 
 
AP3S2
 
 
hsa-miR-34a-5p
 
 
-0.413
 
 
14.3
 
 
 
 
APH1A
 
 
hsa-miR-34a-5p
 
 
-0.304
 
 
72.7
 
 
 
 
APOO
 
 
hsa-miR-181b-5p
 
 
-0.5
 
 
10.5
 
 
 
 
APOO
 
 
hsa-miR-181a-5p
 
 
-0.5
 
 
10.5
 
 
 
 
AREG
 
 
hsa-miR-34a-5p
 
 
-0.62
 
 
135.5
 
 
 
 
ARF4
 
 
hsa-miR-152-3p
 
 
-0.487
 
 
190.1
 
 
 
 
ARF4
 
 
hsa-miR-148b-3p
 
 
-0.487
 
 
190.1
 
 
 
 
ARF5
 
 
hsa-miR-29a-3p
 
 
-0.491
 
 
95.3
 
 
 
 
ARF6
 
 
hsa-miR-181b-5p
 
 
-0.33
 
 
43.6
 
 
 
 
ARF6
 
 
hsa-miR-181a-5p
 
 
-0.33
 
 
43.6
 
 
 
 
ARFGEF1
 
 
hsa-miR-148b-3p
 
 
-0.306
 
 
18.4
 
 
 
 
ARFGEF1
 
 
hsa-miR-152-3p
 
 
-0.306
 
 
18.4
 
 
 
 
ARFIP1
 
 
hsa-miR-152-3p
 
 
-0.474
 
 
22.0
 
 
 
 
ARFIP1
 
 
hsa-miR-148b-3p
 
 
-0.474
 
 
22.0
 
 
 
 
ARHGAP1
 
 
hsa-miR-34a-5p
 
 
-0.369
 
 
106.2
 
 
 
 
ARHGAP21
 
 
hsa-miR-152-3p
 
 
-0.287
 
 
12.9
 
 
 
 
ARHGAP21
 
 
hsa-miR-148b-3p
 
 
-0.287
 
 
12.9
 
 
 
 
ARHGAP26
 
 
hsa-miR-34a-5p
 
 
-0.384
 
 
11.4
 
 
 
 
ARID1B
 
 
hsa-miR-29a-3p
 
 
-0.342
 
 
13.9
 
 
 
 
ARL4A
 
 
hsa-miR-29a-3p
 
 
-0.386
 
 
20.6
 
 
 
 
ARL5B
 
 
hsa-miR-29a-3p
 
 
-0.304
 
 
11.8
 
 
 
 
ARL6IP1
 
 
hsa-miR-148b-3p
 
 
-0.579
 
 
124.0
 
 
 
 
ARL6IP1
 
 
hsa-miR-152-3p
 
 
-0.579
 
 
124.0
 
 
 
 
ARL8B
 
 
hsa-miR-152-3p
 
 
-0.337
 
 
46.5
 
 
 
 
ARL8B
 
 
hsa-miR-148b-3p
 
 
-0.337
 
 
46.5
 
 
 
 
ARMCX3
 
 
hsa-miR-425-5p
 
 
-0.325
 
 
29.8
 
 
 
 
ARPC5
 
 
hsa-miR-22-3p
 
 
-0.679
 
 
71.0
 
 
 
 
ARPP19
 
 
hsa-miR-29a-3p
 
 
-0.371
 
 
51.7
 
 
 
 
ARPP19
 
 
hsa-miR-34a-5p
 
 
-0.497
 
 
51.7
 
 
 
 
ARRB1
 
 
hsa-miR-22-3p
 
 
-0.616
 
 
15.3
 
 
 
 
ARRDC3
 
 
hsa-miR-152-3p
 
 
-0.305
 
 
53.2
 
 
 
 
ARRDC3
 
 
hsa-miR-148b-3p
 
 
-0.305
 
 
53.2
 
 
 
 
ARRDC3
 
 
hsa-miR-29a-3p
 
 
-0.402
 
 
53.2
 
 
 
 
 
 
 vagina 
 
 
 
 
target_symbol
 
 
mature_mirna_id
 
 
score
 
 
nTPM
 
 
 
 
 
 
AAGAB
 
 
hsa-miR-34a-5p
 
 
-0.319
 
 
11.9
 
 
 
 
AAR2
 
 
hsa-miR-29a-3p
 
 
-0.327
 
 
15.0
 
 
 
 
ABCB7
 
 
hsa-miR-148b-3p
 
 
-0.515
 
 
10.8
 
 
 
 
ABCB7
 
 
hsa-miR-152-3p
 
 
-0.515
 
 
10.8
 
 
 
 
ABCC5
 
 
hsa-miR-125a-5p
 
 
-0.288
 
 
35.3
 
 
 
 
ABCD3
 
 
hsa-miR-148b-3p
 
 
-0.388
 
 
14.9
 
 
 
 
ABCD3
 
 
hsa-miR-152-3p
 
 
-0.388
 
 
14.9
 
 
 
 
ABCE1
 
 
hsa-miR-29a-3p
 
 
-0.404
 
 
15.6
 
 
 
 
ABHD17C
 
 
hsa-miR-27a-3p
 
 
-0.435
 
 
21.1
 
 
 
 
ABHD3
 
 
hsa-miR-125a-5p
 
 
-0.453
 
 
12.7
 
 
 
 
ABHD4
 
 
hsa-miR-29a-3p
 
 
-0.312
 
 
28.3
 
 
 
 
ABHD5
 
 
hsa-miR-132-3p
 
 
-0.323
 
 
11.9
 
 
 
 
ABHD5
 
 
hsa-miR-27a-3p
 
 
-0.407
 
 
11.9
 
 
 
 
ABTB1
 
 
hsa-miR-125a-5p
 
 
-0.519
 
 
32.4
 
 
 
 
ACBD3
 
 
hsa-miR-34a-5p
 
 
-0.415
 
 
19.2
 
 
 
 
ACSL1
 
 
hsa-miR-34a-5p
 
 
-0.306
 
 
56.5
 
 
 
 
ACTA2
 
 
hsa-miR-27a-3p
 
 
-0.577
 
 
1234.9
 
 
 
 
ADAM10
 
 
hsa-miR-152-3p
 
 
-0.348
 
 
10.3
 
 
 
 
ADAM10
 
 
hsa-miR-148b-3p
 
 
-0.356
 
 
10.3
 
 
 
 
ADCY3
 
 
hsa-miR-132-3p
 
 
-0.303
 
 
46.2
 
 
 
 
ADIPOR2
 
 
hsa-miR-150-5p
 
 
-0.287
 
 
28.4
 
 
 
 
ADIPOR2
 
 
hsa-miR-34a-5p
 
 
-0.33
 
 
28.4
 
 
 
 
ADK
 
 
hsa-miR-34a-5p
 
 
-0.374
 
 
23.3
 
 
 
 
ADRA2A
 
 
hsa-miR-23a-3p
 
 
-0.371
 
 
22.2
 
 
 
 
ADRA2A
 
 
hsa-miR-23b-3p
 
 
-0.371
 
 
22.2
 
 
 
 
AGFG1
 
 
hsa-miR-148b-3p
 
 
-0.332
 
 
31.5
 
 
 
 
AGFG1
 
 
hsa-miR-152-3p
 
 
-0.332
 
 
31.5
 
 
 
 
AGPAT3
 
 
hsa-miR-27a-3p
 
 
-0.29
 
 
14.4
 
 
 
 
AGTRAP
 
 
hsa-miR-34a-5p
 
 
-0.328
 
 
41.9
 
 
 
 
AHDC1
 
 
hsa-miR-148b-3p
 
 
-0.413
 
 
23.0
 
 
 
 
AHDC1
 
 
hsa-miR-152-3p
 
 
-0.413
 
 
23.0
 
 
 
 
AHR
 
 
hsa-miR-29a-3p
 
 
-0.316
 
 
13.8
 
 
 
 
AIDA
 
 
hsa-miR-22-3p
 
 
-0.316
 
 
30.5
 
 
 
 
AK1
 
 
hsa-miR-29a-3p
 
 
-0.333
 
 
24.0
 
 
 
 
AKAP1
 
 
hsa-miR-148b-3p
 
 
-0.381
 
 
24.4
 
 
 
 
AKAP1
 
 
hsa-miR-152-3p
 
 
-0.381
 
 
24.4
 
 
 
 
AKIRIN1
 
 
hsa-miR-27a-3p
 
 
-0.368
 
 
17.7
 
 
 
 
AKT3
 
 
hsa-miR-29a-3p
 
 
-0.287
 
 
12.8
 
 
 
 
AKT3
 
 
hsa-miR-22-3p
 
 
-0.451
 
 
12.8
 
 
 
 
ALDH1A2
 
 
hsa-miR-23b-3p
 
 
-0.346
 
 
13.2
 
 
 
 
ALDH1A2
 
 
hsa-miR-23a-3p
 
 
-0.354
 
 
13.2
 
 
 
 
ALDOA
 
 
hsa-miR-34a-5p
 
 
-0.655
 
 
910.0
 
 
 
 
ANAPC16
 
 
hsa-miR-125a-5p
 
 
-0.578
 
 
14.7
 
 
 
 
ANK3
 
 
hsa-miR-34a-5p
 
 
-0.29
 
 
13.5
 
 
 
 
ANK3
 
 
hsa-miR-34a-5p
 
 
-0.347
 
 
13.5
 
 
 
 
ANKHD1
 
 
hsa-miR-23a-3p
 
 
-0.368
 
 
41.9
 
 
 
 
ANKHD1
 
 
hsa-miR-23b-3p
 
 
-0.368
 
 
41.9
 
 
 
 
ANKHD1-EIF4EBP3
 
 
hsa-miR-22-3p
 
 
-0.479
 
 
10.7
 
 
 
 
ANKRD13A
 
 
hsa-miR-22-3p
 
 
-0.478
 
 
11.8
 
 
 
 
ANKRD29
 
 
hsa-miR-132-3p
 
 
-0.47
 
 
12.3
 
 
 
 
ANKRD40
 
 
hsa-miR-27a-3p
 
 
-0.35
 
 
15.9
 
 
 
 
ANP32A
 
 
hsa-miR-132-3p
 
 
-0.374
 
 
40.8
 
 
 
 
ANP32B
 
 
hsa-miR-34a-5p
 
 
-0.29
 
 
140.4
 
 
 
 
ANPEP
 
 
hsa-miR-125a-5p
 
 
-0.372
 
 
13.0
 
 
 
 
ANTXR2
 
 
hsa-miR-29a-3p
 
 
-0.347
 
 
27.6
 
 
 
 
APH1A
 
 
hsa-miR-34a-5p
 
 
-0.304
 
 
79.7
 
 
 
 
AREG
 
 
hsa-miR-34a-5p
 
 
-0.62
 
 
13.6
 
 
 
 
ARF4
 
 
hsa-miR-152-3p
 
 
-0.487
 
 
85.1
 
 
 
 
ARF4
 
 
hsa-miR-148b-3p
 
 
-0.487
 
 
85.1
 
 
 
 
ARF5
 
 
hsa-miR-29a-3p
 
 
-0.491
 
 
132.3
 
 
 
 
ARF6
 
 
hsa-miR-181b-5p
 
 
-0.33
 
 
41.6
 
 
 
 
ARF6
 
 
hsa-miR-181a-5p
 
 
-0.33
 
 
41.6
 
 
 
 
ARFIP1
 
 
hsa-miR-152-3p
 
 
-0.474
 
 
13.1
 
 
 
 
ARFIP1
 
 
hsa-miR-148b-3p
 
 
-0.474
 
 
13.1
 
 
 
 
ARHGAP1
 
 
hsa-miR-34a-5p
 
 
-0.369
 
 
59.0
 
 
 
 
ARID1B
 
 
hsa-miR-29a-3p
 
 
-0.342
 
 
13.7
 
 
 
 
ARL4A
 
 
hsa-miR-29a-3p
 
 
-0.386
 
 
63.2
 
 
 
 
ARL6IP1
 
 
hsa-miR-148b-3p
 
 
-0.579
 
 
48.0
 
 
 
 
ARL6IP1
 
 
hsa-miR-152-3p
 
 
-0.579
 
 
48.0
 
 
 
 
ARL8B
 
 
hsa-miR-152-3p
 
 
-0.337
 
 
51.8
 
 
 
 
ARL8B
 
 
hsa-miR-148b-3p
 
 
-0.337
 
 
51.8
 
 
 
 
ARMCX3
 
 
hsa-miR-425-5p
 
 
-0.325
 
 
20.2
 
 
 
 
ARPC5
 
 
hsa-miR-22-3p
 
 
-0.679
 
 
49.2
 
 
 
 
ARPP19
 
 
hsa-miR-29a-3p
 
 
-0.371
 
 
27.0
 
 
 
 
ARPP19
 
 
hsa-miR-34a-5p
 
 
-0.497
 
 
27.0
 
 
 
 
ARRB1
 
 
hsa-miR-22-3p
 
 
-0.616
 
 
10.2
 
 
 
 
ARRDC3
 
 
hsa-miR-152-3p
 
 
-0.305
 
 
40.5
 
 
 
 
ARRDC3
 
 
hsa-miR-148b-3p
 
 
-0.305
 
 
40.5
 
 
 
 
ARRDC3
 
 
hsa-miR-29a-3p
 
 
-0.402
 
 
40.5
 
 
 
 
ASAH1
 
 
hsa-miR-27a-3p
 
 
-0.398
 
 
49.3
 
 
 
 
ASPH
 
 
hsa-miR-152-3p
 
 
-0.391
 
 
40.4
 
 
 
 
ASPH
 
 
hsa-miR-148b-3p
 
 
-0.391
 
 
40.4
 
 
 
 
ATG4B
 
 
hsa-miR-34a-5p
 
 
-0.58
 
 
16.3
 
 
 
 
ATMIN
 
 
hsa-miR-34a-5p
 
 
-0.409
 
 
11.4
 
 
 
 
ATP2B4
 
 
hsa-miR-152-3p
 
 
-0.337
 
 
14.3
 
 
 
 
ATP2B4
 
 
hsa-miR-148b-3p
 
 
-0.337
 
 
14.3
 
 
 
 
ATP5MC3
 
 
hsa-miR-425-5p
 
 
-0.328
 
 
160.4
 
 
 
 
ATP5MC3
 
 
hsa-miR-29a-3p
 
 
-0.33
 
 
160.4
 
 
 
 
ATP5MC3
 
 
hsa-miR-27a-3p
 
 
-0.416
 
 
160.4
 
 
 
 
ATP6AP2
 
 
hsa-miR-148b-3p
 
 
-0.592
 
 
44.8
 
 
 
 
ATP6AP2
 
 
hsa-miR-152-3p
 
 
-0.592
 
 
44.8
 
 
 
 
ATP6AP2
 
 
hsa-miR-148b-3p
 
 
-0.668
 
 
44.8
 
 
 
 
ATP6AP2
 
 
hsa-miR-152-3p
 
 
-0.668
 
 
44.8
 
 
 
 
ATP6V1E1
 
 
hsa-miR-23a-3p
 
 
-0.294
 
 
97.4
 
 
 
 
ATP6V1E1
 
 
hsa-miR-23b-3p
 
 
-0.294
 
 
97.4
 
 
 
 
ATXN10
 
 
hsa-miR-27a-3p
 
 
-0.295
 
 
43.9
 
 
 
 
ATXN2
 
 
hsa-miR-10a-5p
 
 
-0.362
 
 
17.8
 
 
 
 
AXL
 
 
hsa-miR-34a-5p
 
 
-0.512
 
 
23.9
 
 
 
 
AZIN1
 
 
hsa-miR-132-3p
 
 
-0.29
 
 
24.5
 
 
 
 
AZIN1
 
 
hsa-miR-132-3p
 
 
-0.299
 
 
24.5
 
 
 
 
 
 
 cerebellum 
 
 
 
 
target_symbol
 
 
mature_mirna_id
 
 
score
 
 
nTPM
 
 
 
 
 
 
AAR2
 
 
hsa-miR-29a-3p
 
 
-0.327
 
 
10.9
 
 
 
 
ABCB10
 
 
hsa-miR-152-3p
 
 
-0.291
 
 
10.6
 
 
 
 
ABCB10
 
 
hsa-miR-148b-3p
 
 
-0.291
 
 
10.6
 
 
 
 
ABCC5
 
 
hsa-miR-125a-5p
 
 
-0.288
 
 
101.0
 
 
 
 
ABCD3
 
 
hsa-miR-148b-3p
 
 
-0.388
 
 
12.8
 
 
 
 
ABCD3
 
 
hsa-miR-152-3p
 
 
-0.388
 
 
12.8
 
 
 
 
ABCE1
 
 
hsa-miR-29a-3p
 
 
-0.404
 
 
12.8
 
 
 
 
ABHD3
 
 
hsa-miR-125a-5p
 
 
-0.453
 
 
13.0
 
 
 
 
ABHD6
 
 
hsa-miR-125a-5p
 
 
-0.501
 
 
24.9
 
 
 
 
ABTB1
 
 
hsa-miR-125a-5p
 
 
-0.519
 
 
100.8
 
 
 
 
ACBD3
 
 
hsa-miR-34a-5p
 
 
-0.415
 
 
11.6
 
 
 
 
ACHE
 
 
hsa-miR-125a-5p
 
 
-0.308
 
 
59.1
 
 
 
 
ACHE
 
 
hsa-miR-125a-5p
 
 
-0.361
 
 
59.1
 
 
 
 
ACSL1
 
 
hsa-miR-34a-5p
 
 
-0.306
 
 
17.4
 
 
 
 
ACSL4
 
 
hsa-miR-34a-5p
 
 
-0.372
 
 
10.1
 
 
 
 
ACSL6
 
 
hsa-miR-125a-5p
 
 
-0.307
 
 
27.8
 
 
 
 
ACTA2
 
 
hsa-miR-27a-3p
 
 
-0.577
 
 
25.2
 
 
 
 
ADAM10
 
 
hsa-miR-152-3p
 
 
-0.348
 
 
15.6
 
 
 
 
ADAM10
 
 
hsa-miR-148b-3p
 
 
-0.356
 
 
15.6
 
 
 
 
ADAP1
 
 
hsa-miR-27a-3p
 
 
-0.302
 
 
43.2
 
 
 
 
ADCY3
 
 
hsa-miR-132-3p
 
 
-0.303
 
 
33.6
 
 
 
 
ADD2
 
 
hsa-miR-34a-5p
 
 
-0.305
 
 
36.1
 
 
 
 
ADGRB3
 
 
hsa-miR-152-3p
 
 
-0.516
 
 
20.8
 
 
 
 
ADGRB3
 
 
hsa-miR-148b-3p
 
 
-0.524
 
 
20.8
 
 
 
 
ADIPOR2
 
 
hsa-miR-150-5p
 
 
-0.287
 
 
18.7
 
 
 
 
ADIPOR2
 
 
hsa-miR-34a-5p
 
 
-0.33
 
 
18.7
 
 
 
 
ADO
 
 
hsa-miR-34a-5p
 
 
-0.377
 
 
12.4
 
 
 
 
AFG3L2
 
 
hsa-miR-181b-5p
 
 
-0.491
 
 
11.5
 
 
 
 
AFG3L2
 
 
hsa-miR-181a-5p
 
 
-0.491
 
 
11.5
 
 
 
 
AGFG1
 
 
hsa-miR-148b-3p
 
 
-0.332
 
 
22.4
 
 
 
 
AGFG1
 
 
hsa-miR-152-3p
 
 
-0.332
 
 
22.4
 
 
 
 
AGPAT3
 
 
hsa-miR-27a-3p
 
 
-0.29
 
 
27.6
 
 
 
 
AHCYL2
 
 
hsa-miR-34a-5p
 
 
-0.359
 
 
11.8
 
 
 
 
AHDC1
 
 
hsa-miR-148b-3p
 
 
-0.413
 
 
32.0
 
 
 
 
AHDC1
 
 
hsa-miR-152-3p
 
 
-0.413
 
 
32.0
 
 
 
 
AIDA
 
 
hsa-miR-22-3p
 
 
-0.316
 
 
24.2
 
 
 
 
AK1
 
 
hsa-miR-29a-3p
 
 
-0.333
 
 
47.4
 
 
 
 
AKAIN1
 
 
hsa-miR-29a-3p
 
 
-0.52
 
 
40.8
 
 
 
 
AKAP1
 
 
hsa-miR-148b-3p
 
 
-0.381
 
 
30.6
 
 
 
 
AKAP1
 
 
hsa-miR-152-3p
 
 
-0.381
 
 
30.6
 
 
 
 
AKIRIN1
 
 
hsa-miR-27a-3p
 
 
-0.368
 
 
20.5
 
 
 
 
AKT3
 
 
hsa-miR-29a-3p
 
 
-0.287
 
 
23.2
 
 
 
 
AKT3
 
 
hsa-miR-22-3p
 
 
-0.451
 
 
23.2
 
 
 
 
ALDOA
 
 
hsa-miR-34a-5p
 
 
-0.655
 
 
717.2
 
 
 
 
AMER2
 
 
hsa-miR-34a-5p
 
 
-0.48
 
 
21.3
 
 
 
 
ANAPC16
 
 
hsa-miR-125a-5p
 
 
-0.578
 
 
13.9
 
 
 
 
ANGEL2
 
 
hsa-miR-132-3p
 
 
-0.357
 
 
12.7
 
 
 
 
ANGEL2
 
 
hsa-miR-22-3p
 
 
-0.601
 
 
12.7
 
 
 
 
ANK2
 
 
hsa-miR-34a-5p
 
 
-0.317
 
 
47.8
 
 
 
 
ANK3
 
 
hsa-miR-34a-5p
 
 
-0.29
 
 
126.1
 
 
 
 
ANK3
 
 
hsa-miR-34a-5p
 
 
-0.347
 
 
126.1
 
 
 
 
ANKHD1
 
 
hsa-miR-23a-3p
 
 
-0.368
 
 
38.6
 
 
 
 
ANKHD1
 
 
hsa-miR-23b-3p
 
 
-0.368
 
 
38.6
 
 
 
 
ANKRD13B
 
 
hsa-miR-29a-3p
 
 
-0.421
 
 
44.3
 
 
 
 
ANKRD13C
 
 
hsa-miR-29a-3p
 
 
-0.411
 
 
12.9
 
 
 
 
ANKRD40
 
 
hsa-miR-27a-3p
 
 
-0.35
 
 
31.7
 
 
 
 
ANP32A
 
 
hsa-miR-132-3p
 
 
-0.374
 
 
71.1
 
 
 
 
ANP32B
 
 
hsa-miR-34a-5p
 
 
-0.29
 
 
49.5
 
 
 
 
AP3S2
 
 
hsa-miR-34a-5p
 
 
-0.413
 
 
13.1
 
 
 
 
APC
 
 
hsa-miR-29a-3p
 
 
-0.413
 
 
12.2
 
 
 
 
APH1A
 
 
hsa-miR-34a-5p
 
 
-0.304
 
 
36.6
 
 
 
 
APOO
 
 
hsa-miR-181b-5p
 
 
-0.5
 
 
32.8
 
 
 
 
APOO
 
 
hsa-miR-181a-5p
 
 
-0.5
 
 
32.8
 
 
 
 
APPBP2
 
 
hsa-miR-27a-3p
 
 
-0.378
 
 
14.6
 
 
 
 
ARF4
 
 
hsa-miR-152-3p
 
 
-0.487
 
 
50.2
 
 
 
 
ARF4
 
 
hsa-miR-148b-3p
 
 
-0.487
 
 
50.2
 
 
 
 
ARF5
 
 
hsa-miR-29a-3p
 
 
-0.491
 
 
167.9
 
 
 
 
ARF6
 
 
hsa-miR-181b-5p
 
 
-0.33
 
 
11.0
 
 
 
 
ARF6
 
 
hsa-miR-181a-5p
 
 
-0.33
 
 
11.0
 
 
 
 
ARHGAP1
 
 
hsa-miR-34a-5p
 
 
-0.369
 
 
19.6
 
 
 
 
ARHGAP21
 
 
hsa-miR-152-3p
 
 
-0.287
 
 
50.0
 
 
 
 
ARHGAP21
 
 
hsa-miR-148b-3p
 
 
-0.287
 
 
50.0
 
 
 
 
ARHGAP26
 
 
hsa-miR-34a-5p
 
 
-0.384
 
 
17.4
 
 
 
 
ARID1B
 
 
hsa-miR-29a-3p
 
 
-0.342
 
 
12.5
 
 
 
 
ARL6IP1
 
 
hsa-miR-148b-3p
 
 
-0.579
 
 
234.0
 
 
 
 
ARL6IP1
 
 
hsa-miR-152-3p
 
 
-0.579
 
 
234.0
 
 
 
 
ARL8B
 
 
hsa-miR-152-3p
 
 
-0.337
 
 
50.7
 
 
 
 
ARL8B
 
 
hsa-miR-148b-3p
 
 
-0.337
 
 
50.7
 
 
 
 
ARMCX3
 
 
hsa-miR-425-5p
 
 
-0.325
 
 
24.0
 
 
 
 
ARPC5
 
 
hsa-miR-22-3p
 
 
-0.679
 
 
16.1
 
 
 
 
ARPP19
 
 
hsa-miR-29a-3p
 
 
-0.371
 
 
66.7
 
 
 
 
ARPP19
 
 
hsa-miR-34a-5p
 
 
-0.497
 
 
66.7
 
 
 
 
ARRB1
 
 
hsa-miR-22-3p
 
 
-0.616
 
 
26.7
 
 
 
 
ARRDC3
 
 
hsa-miR-152-3p
 
 
-0.305
 
 
17.8
 
 
 
 
ARRDC3
 
 
hsa-miR-148b-3p
 
 
-0.305
 
 
17.8
 
 
 
 
ARRDC3
 
 
hsa-miR-29a-3p
 
 
-0.402
 
 
17.8
 
 
 
 
ASAH1
 
 
hsa-miR-27a-3p
 
 
-0.398
 
 
32.2
 
 
 
 
ASAP2
 
 
hsa-miR-29a-3p
 
 
-0.348
 
 
10.8
 
 
 
 
ASF1A
 
 
hsa-miR-23a-3p
 
 
-0.506
 
 
12.6
 
 
 
 
ASF1A
 
 
hsa-miR-23b-3p
 
 
-0.506
 
 
12.6
 
 
 
 
ASPH
 
 
hsa-miR-152-3p
 
 
-0.391
 
 
80.5
 
 
 
 
ASPH
 
 
hsa-miR-148b-3p
 
 
-0.391
 
 
80.5
 
 
 
 
ASPHD2
 
 
hsa-miR-34a-5p
 
 
-0.354
 
 
18.6
 
 
 
 
ATCAY
 
 
hsa-miR-10a-5p
 
 
-0.322
 
 
31.5
 
 
 
 
ATG4B
 
 
hsa-miR-34a-5p
 
 
-0.58
 
 
38.2
 
 
 
 
ATMIN
 
 
hsa-miR-34a-5p
 
 
-0.409
 
 
18.8
 
 
 
 
ATP1A2
 
 
hsa-miR-34a-5p
 
 
-0.306
 
 
166.3
 
 
 
 
ATP5MC3
 
 
hsa-miR-425-5p
 
 
-0.328
 
 
183.5
 
 
 
 
ATP5MC3
 
 
hsa-miR-29a-3p
 
 
-0.33
 
 
183.5
 
 
 
 
ATP5MC3
 
 
hsa-miR-27a-3p
 
 
-0.416
 
 
183.5
 
 
 
 
 
 
 ovary 
 
 
 
 
target_symbol
 
 
mature_mirna_id
 
 
score
 
 
nTPM
 
 
 
 
 
 
AAR2
 
 
hsa-miR-29a-3p
 
 
-0.327
 
 
22.7
 
 
 
 
ABCB6
 
 
hsa-miR-29a-3p
 
 
-0.48
 
 
12.1
 
 
 
 
ABCB7
 
 
hsa-miR-148b-3p
 
 
-0.515
 
 
11.9
 
 
 
 
ABCB7
 
 
hsa-miR-152-3p
 
 
-0.515
 
 
11.9
 
 
 
 
ABCC5
 
 
hsa-miR-125a-5p
 
 
-0.288
 
 
23.4
 
 
 
 
ABCD3
 
 
hsa-miR-148b-3p
 
 
-0.388
 
 
11.0
 
 
 
 
ABCD3
 
 
hsa-miR-152-3p
 
 
-0.388
 
 
11.0
 
 
 
 
ABCE1
 
 
hsa-miR-29a-3p
 
 
-0.404
 
 
25.9
 
 
 
 
ABHD3
 
 
hsa-miR-125a-5p
 
 
-0.453
 
 
12.0
 
 
 
 
ABHD4
 
 
hsa-miR-29a-3p
 
 
-0.312
 
 
17.9
 
 
 
 
ABRAXAS2
 
 
hsa-miR-34a-5p
 
 
-0.538
 
 
10.0
 
 
 
 
ABTB1
 
 
hsa-miR-125a-5p
 
 
-0.519
 
 
31.5
 
 
 
 
ACBD3
 
 
hsa-miR-34a-5p
 
 
-0.415
 
 
20.0
 
 
 
 
ACSL1
 
 
hsa-miR-34a-5p
 
 
-0.306
 
 
14.6
 
 
 
 
ACSL4
 
 
hsa-miR-34a-5p
 
 
-0.372
 
 
20.0
 
 
 
 
ACTA2
 
 
hsa-miR-27a-3p
 
 
-0.577
 
 
689.2
 
 
 
 
ACVR1
 
 
hsa-miR-152-3p
 
 
-0.397
 
 
17.7
 
 
 
 
ACVR1
 
 
hsa-miR-148b-3p
 
 
-0.397
 
 
17.7
 
 
 
 
ADAM10
 
 
hsa-miR-152-3p
 
 
-0.348
 
 
13.1
 
 
 
 
ADAM10
 
 
hsa-miR-148b-3p
 
 
-0.356
 
 
13.1
 
 
 
 
ADCY3
 
 
hsa-miR-132-3p
 
 
-0.303
 
 
67.7
 
 
 
 
ADIPOR2
 
 
hsa-miR-150-5p
 
 
-0.287
 
 
24.2
 
 
 
 
ADIPOR2
 
 
hsa-miR-34a-5p
 
 
-0.33
 
 
24.2
 
 
 
 
ADK
 
 
hsa-miR-34a-5p
 
 
-0.374
 
 
10.8
 
 
 
 
ADO
 
 
hsa-miR-34a-5p
 
 
-0.377
 
 
11.0
 
 
 
 
ADRA2A
 
 
hsa-miR-23a-3p
 
 
-0.371
 
 
17.5
 
 
 
 
ADRA2A
 
 
hsa-miR-23b-3p
 
 
-0.371
 
 
17.5
 
 
 
 
AFG3L2
 
 
hsa-miR-181b-5p
 
 
-0.491
 
 
12.5
 
 
 
 
AFG3L2
 
 
hsa-miR-181a-5p
 
 
-0.491
 
 
12.5
 
 
 
 
AGFG1
 
 
hsa-miR-148b-3p
 
 
-0.332
 
 
22.9
 
 
 
 
AGFG1
 
 
hsa-miR-152-3p
 
 
-0.332
 
 
22.9
 
 
 
 
AGGF1
 
 
hsa-miR-125a-5p
 
 
-0.331
 
 
10.3
 
 
 
 
AGTRAP
 
 
hsa-miR-34a-5p
 
 
-0.328
 
 
25.1
 
 
 
 
AHDC1
 
 
hsa-miR-148b-3p
 
 
-0.413
 
 
21.5
 
 
 
 
AHDC1
 
 
hsa-miR-152-3p
 
 
-0.413
 
 
21.5
 
 
 
 
AIDA
 
 
hsa-miR-22-3p
 
 
-0.316
 
 
41.9
 
 
 
 
AK1
 
 
hsa-miR-29a-3p
 
 
-0.333
 
 
43.4
 
 
 
 
AKAP1
 
 
hsa-miR-148b-3p
 
 
-0.381
 
 
18.8
 
 
 
 
AKAP1
 
 
hsa-miR-152-3p
 
 
-0.381
 
 
18.8
 
 
 
 
AKIRIN1
 
 
hsa-miR-27a-3p
 
 
-0.368
 
 
18.3
 
 
 
 
AKT3
 
 
hsa-miR-29a-3p
 
 
-0.287
 
 
21.9
 
 
 
 
AKT3
 
 
hsa-miR-22-3p
 
 
-0.451
 
 
21.9
 
 
 
 
ALCAM
 
 
hsa-miR-34a-5p
 
 
-0.303
 
 
33.1
 
 
 
 
ALCAM
 
 
hsa-miR-148b-3p
 
 
-0.339
 
 
33.1
 
 
 
 
ALCAM
 
 
hsa-miR-152-3p
 
 
-0.347
 
 
33.1
 
 
 
 
ALDH1A2
 
 
hsa-miR-23b-3p
 
 
-0.346
 
 
13.5
 
 
 
 
ALDH1A2
 
 
hsa-miR-23a-3p
 
 
-0.354
 
 
13.5
 
 
 
 
ALDOA
 
 
hsa-miR-34a-5p
 
 
-0.655
 
 
580.3
 
 
 
 
ANAPC16
 
 
hsa-miR-125a-5p
 
 
-0.578
 
 
22.3
 
 
 
 
ANK2
 
 
hsa-miR-34a-5p
 
 
-0.317
 
 
28.0
 
 
 
 
ANK3
 
 
hsa-miR-34a-5p
 
 
-0.29
 
 
10.3
 
 
 
 
ANK3
 
 
hsa-miR-34a-5p
 
 
-0.347
 
 
10.3
 
 
 
 
ANKHD1
 
 
hsa-miR-23a-3p
 
 
-0.368
 
 
49.9
 
 
 
 
ANKHD1
 
 
hsa-miR-23b-3p
 
 
-0.368
 
 
49.9
 
 
 
 
ANKRD13A
 
 
hsa-miR-22-3p
 
 
-0.478
 
 
16.1
 
 
 
 
ANKRD29
 
 
hsa-miR-132-3p
 
 
-0.47
 
 
15.0
 
 
 
 
ANKRD40
 
 
hsa-miR-27a-3p
 
 
-0.35
 
 
18.3
 
 
 
 
ANP32A
 
 
hsa-miR-132-3p
 
 
-0.374
 
 
51.4
 
 
 
 
ANP32B
 
 
hsa-miR-34a-5p
 
 
-0.29
 
 
160.5
 
 
 
 
ANTXR2
 
 
hsa-miR-29a-3p
 
 
-0.347
 
 
66.3
 
 
 
 
AP3S2
 
 
hsa-miR-34a-5p
 
 
-0.413
 
 
18.2
 
 
 
 
APH1A
 
 
hsa-miR-34a-5p
 
 
-0.304
 
 
97.8
 
 
 
 
APOO
 
 
hsa-miR-181b-5p
 
 
-0.5
 
 
11.0
 
 
 
 
APOO
 
 
hsa-miR-181a-5p
 
 
-0.5
 
 
11.0
 
 
 
 
APPBP2
 
 
hsa-miR-27a-3p
 
 
-0.378
 
 
17.5
 
 
 
 
AREG
 
 
hsa-miR-34a-5p
 
 
-0.62
 
 
11.4
 
 
 
 
ARF4
 
 
hsa-miR-152-3p
 
 
-0.487
 
 
123.7
 
 
 
 
ARF4
 
 
hsa-miR-148b-3p
 
 
-0.487
 
 
123.7
 
 
 
 
ARF5
 
 
hsa-miR-29a-3p
 
 
-0.491
 
 
81.8
 
 
 
 
ARF6
 
 
hsa-miR-181b-5p
 
 
-0.33
 
 
16.9
 
 
 
 
ARF6
 
 
hsa-miR-181a-5p
 
 
-0.33
 
 
16.9
 
 
 
 
ARFIP1
 
 
hsa-miR-152-3p
 
 
-0.474
 
 
17.2
 
 
 
 
ARFIP1
 
 
hsa-miR-148b-3p
 
 
-0.474
 
 
17.2
 
 
 
 
ARHGAP1
 
 
hsa-miR-34a-5p
 
 
-0.369
 
 
43.6
 
 
 
 
ARHGAP21
 
 
hsa-miR-152-3p
 
 
-0.287
 
 
26.5
 
 
 
 
ARHGAP21
 
 
hsa-miR-148b-3p
 
 
-0.287
 
 
26.5
 
 
 
 
ARID1B
 
 
hsa-miR-29a-3p
 
 
-0.342
 
 
20.5
 
 
 
 
ARL4A
 
 
hsa-miR-29a-3p
 
 
-0.386
 
 
14.3
 
 
 
 
ARL6IP1
 
 
hsa-miR-148b-3p
 
 
-0.579
 
 
97.4
 
 
 
 
ARL6IP1
 
 
hsa-miR-152-3p
 
 
-0.579
 
 
97.4
 
 
 
 
ARL8B
 
 
hsa-miR-152-3p
 
 
-0.337
 
 
35.5
 
 
 
 
ARL8B
 
 
hsa-miR-148b-3p
 
 
-0.337
 
 
35.5
 
 
 
 
ARMCX3
 
 
hsa-miR-425-5p
 
 
-0.325
 
 
36.1
 
 
 
 
ARPC5
 
 
hsa-miR-22-3p
 
 
-0.679
 
 
45.9
 
 
 
 
ARPIN-AP3S2
 
 
hsa-miR-34a-5p
 
 
-0.348
 
 
11.2
 
 
 
 
ARPP19
 
 
hsa-miR-29a-3p
 
 
-0.371
 
 
40.6
 
 
 
 
ARPP19
 
 
hsa-miR-34a-5p
 
 
-0.497
 
 
40.6
 
 
 
 
ARRB1
 
 
hsa-miR-22-3p
 
 
-0.616
 
 
17.2
 
 
 
 
ARRDC3
 
 
hsa-miR-152-3p
 
 
-0.305
 
 
64.7
 
 
 
 
ARRDC3
 
 
hsa-miR-148b-3p
 
 
-0.305
 
 
64.7
 
 
 
 
ARRDC3
 
 
hsa-miR-29a-3p
 
 
-0.402
 
 
64.7
 
 
 
 
ASAH1
 
 
hsa-miR-27a-3p
 
 
-0.398
 
 
45.1
 
 
 
 
ASF1A
 
 
hsa-miR-23a-3p
 
 
-0.506
 
 
18.5
 
 
 
 
ASF1A
 
 
hsa-miR-23b-3p
 
 
-0.506
 
 
18.5
 
 
 
 
ASPH
 
 
hsa-miR-152-3p
 
 
-0.391
 
 
58.7
 
 
 
 
ASPH
 
 
hsa-miR-148b-3p
 
 
-0.391
 
 
58.7
 
 
 
 
ATG4B
 
 
hsa-miR-34a-5p
 
 
-0.58
 
 
18.9
 
 
 
 
ATMIN
 
 
hsa-miR-34a-5p
 
 
-0.409
 
 
12.7
 
 
 
 
ATP1A2
 
 
hsa-miR-34a-5p
 
 
-0.306
 
 
12.4
 
 
 
 
ATP2B4
 
 
hsa-miR-152-3p
 
 
-0.337
 
 
30.9
 
 
 
 
 
 
 spinal cord 
 
 
 
 
target_symbol
 
 
mature_mirna_id
 
 
score
 
 
nTPM
 
 
 
 
 
 
AAR2
 
 
hsa-miR-29a-3p
 
 
-0.327
 
 
14.2
 
 
 
 
ABCC5
 
 
hsa-miR-125a-5p
 
 
-0.288
 
 
25.1
 
 
 
 
ABCD3
 
 
hsa-miR-148b-3p
 
 
-0.388
 
 
20.9
 
 
 
 
ABCD3
 
 
hsa-miR-152-3p
 
 
-0.388
 
 
20.9
 
 
 
 
ABCE1
 
 
hsa-miR-29a-3p
 
 
-0.404
 
 
16.8
 
 
 
 
ABHD4
 
 
hsa-miR-29a-3p
 
 
-0.312
 
 
17.8
 
 
 
 
ABHD5
 
 
hsa-miR-132-3p
 
 
-0.323
 
 
19.5
 
 
 
 
ABHD5
 
 
hsa-miR-27a-3p
 
 
-0.407
 
 
19.5
 
 
 
 
ABHD6
 
 
hsa-miR-125a-5p
 
 
-0.501
 
 
36.2
 
 
 
 
ABTB1
 
 
hsa-miR-125a-5p
 
 
-0.519
 
 
20.4
 
 
 
 
ACBD3
 
 
hsa-miR-34a-5p
 
 
-0.415
 
 
10.8
 
 
 
 
ACER3
 
 
hsa-miR-22-3p
 
 
-0.453
 
 
13.0
 
 
 
 
ACHE
 
 
hsa-miR-125a-5p
 
 
-0.308
 
 
14.4
 
 
 
 
ACHE
 
 
hsa-miR-125a-5p
 
 
-0.361
 
 
14.4
 
 
 
 
ACSL1
 
 
hsa-miR-34a-5p
 
 
-0.306
 
 
94.3
 
 
 
 
ACSL4
 
 
hsa-miR-34a-5p
 
 
-0.372
 
 
12.4
 
 
 
 
ACTA2
 
 
hsa-miR-27a-3p
 
 
-0.577
 
 
47.2
 
 
 
 
ACTN2
 
 
hsa-miR-23a-3p
 
 
-0.294
 
 
14.9
 
 
 
 
ACTN2
 
 
hsa-miR-29a-3p
 
 
-0.311
 
 
14.9
 
 
 
 
ADAM10
 
 
hsa-miR-152-3p
 
 
-0.348
 
 
18.7
 
 
 
 
ADAM10
 
 
hsa-miR-148b-3p
 
 
-0.356
 
 
18.7
 
 
 
 
ADAP1
 
 
hsa-miR-27a-3p
 
 
-0.302
 
 
131.1
 
 
 
 
ADCY2
 
 
hsa-miR-152-3p
 
 
-0.299
 
 
11.4
 
 
 
 
ADCY2
 
 
hsa-miR-148b-3p
 
 
-0.299
 
 
11.4
 
 
 
 
ADCY3
 
 
hsa-miR-132-3p
 
 
-0.303
 
 
26.0
 
 
 
 
ADIPOR2
 
 
hsa-miR-150-5p
 
 
-0.287
 
 
121.3
 
 
 
 
ADIPOR2
 
 
hsa-miR-34a-5p
 
 
-0.33
 
 
121.3
 
 
 
 
ADK
 
 
hsa-miR-34a-5p
 
 
-0.374
 
 
18.9
 
 
 
 
ADO
 
 
hsa-miR-34a-5p
 
 
-0.377
 
 
33.5
 
 
 
 
AGFG1
 
 
hsa-miR-148b-3p
 
 
-0.332
 
 
53.5
 
 
 
 
AGFG1
 
 
hsa-miR-152-3p
 
 
-0.332
 
 
53.5
 
 
 
 
AGPAT3
 
 
hsa-miR-27a-3p
 
 
-0.29
 
 
50.1
 
 
 
 
AGPAT4
 
 
hsa-miR-29a-3p
 
 
-0.376
 
 
53.1
 
 
 
 
AGTRAP
 
 
hsa-miR-34a-5p
 
 
-0.328
 
 
34.7
 
 
 
 
AHCYL2
 
 
hsa-miR-34a-5p
 
 
-0.359
 
 
18.2
 
 
 
 
AIDA
 
 
hsa-miR-22-3p
 
 
-0.316
 
 
29.9
 
 
 
 
AIFM1
 
 
hsa-miR-125a-5p
 
 
-0.3
 
 
10.1
 
 
 
 
AK1
 
 
hsa-miR-29a-3p
 
 
-0.333
 
 
67.3
 
 
 
 
AKAP1
 
 
hsa-miR-148b-3p
 
 
-0.381
 
 
14.0
 
 
 
 
AKAP1
 
 
hsa-miR-152-3p
 
 
-0.381
 
 
14.0
 
 
 
 
AKIRIN1
 
 
hsa-miR-27a-3p
 
 
-0.368
 
 
52.7
 
 
 
 
AKT3
 
 
hsa-miR-29a-3p
 
 
-0.287
 
 
15.8
 
 
 
 
AKT3
 
 
hsa-miR-22-3p
 
 
-0.451
 
 
15.8
 
 
 
 
ALCAM
 
 
hsa-miR-34a-5p
 
 
-0.303
 
 
56.1
 
 
 
 
ALCAM
 
 
hsa-miR-148b-3p
 
 
-0.339
 
 
56.1
 
 
 
 
ALCAM
 
 
hsa-miR-152-3p
 
 
-0.347
 
 
56.1
 
 
 
 
ALDOA
 
 
hsa-miR-34a-5p
 
 
-0.655
 
 
561.2
 
 
 
 
AMER2
 
 
hsa-miR-34a-5p
 
 
-0.48
 
 
28.2
 
 
 
 
ANK2
 
 
hsa-miR-34a-5p
 
 
-0.317
 
 
13.8
 
 
 
 
ANK3
 
 
hsa-miR-34a-5p
 
 
-0.29
 
 
39.5
 
 
 
 
ANK3
 
 
hsa-miR-34a-5p
 
 
-0.347
 
 
39.5
 
 
 
 
ANKHD1
 
 
hsa-miR-23a-3p
 
 
-0.368
 
 
22.6
 
 
 
 
ANKHD1
 
 
hsa-miR-23b-3p
 
 
-0.368
 
 
22.6
 
 
 
 
ANKRD13B
 
 
hsa-miR-29a-3p
 
 
-0.421
 
 
14.7
 
 
 
 
ANKRD40
 
 
hsa-miR-27a-3p
 
 
-0.35
 
 
196.0
 
 
 
 
ANP32A
 
 
hsa-miR-132-3p
 
 
-0.374
 
 
72.8
 
 
 
 
ANP32B
 
 
hsa-miR-34a-5p
 
 
-0.29
 
 
363.5
 
 
 
 
AP3S2
 
 
hsa-miR-34a-5p
 
 
-0.413
 
 
15.3
 
 
 
 
APH1A
 
 
hsa-miR-34a-5p
 
 
-0.304
 
 
50.5
 
 
 
 
APOO
 
 
hsa-miR-181b-5p
 
 
-0.5
 
 
36.2
 
 
 
 
APOO
 
 
hsa-miR-181a-5p
 
 
-0.5
 
 
36.2
 
 
 
 
APPBP2
 
 
hsa-miR-27a-3p
 
 
-0.378
 
 
10.4
 
 
 
 
ARF4
 
 
hsa-miR-152-3p
 
 
-0.487
 
 
69.7
 
 
 
 
ARF4
 
 
hsa-miR-148b-3p
 
 
-0.487
 
 
69.7
 
 
 
 
ARF5
 
 
hsa-miR-29a-3p
 
 
-0.491
 
 
100.3
 
 
 
 
ARF6
 
 
hsa-miR-181b-5p
 
 
-0.33
 
 
13.8
 
 
 
 
ARF6
 
 
hsa-miR-181a-5p
 
 
-0.33
 
 
13.8
 
 
 
 
ARFIP1
 
 
hsa-miR-152-3p
 
 
-0.474
 
 
10.6
 
 
 
 
ARFIP1
 
 
hsa-miR-148b-3p
 
 
-0.474
 
 
10.6
 
 
 
 
ARHGAP1
 
 
hsa-miR-34a-5p
 
 
-0.369
 
 
38.6
 
 
 
 
ARHGAP21
 
 
hsa-miR-152-3p
 
 
-0.287
 
 
74.7
 
 
 
 
ARHGAP21
 
 
hsa-miR-148b-3p
 
 
-0.287
 
 
74.7
 
 
 
 
ARL4A
 
 
hsa-miR-29a-3p
 
 
-0.386
 
 
12.2
 
 
 
 
ARL6IP1
 
 
hsa-miR-148b-3p
 
 
-0.579
 
 
140.2
 
 
 
 
ARL6IP1
 
 
hsa-miR-152-3p
 
 
-0.579
 
 
140.2
 
 
 
 
ARL8B
 
 
hsa-miR-152-3p
 
 
-0.337
 
 
57.8
 
 
 
 
ARL8B
 
 
hsa-miR-148b-3p
 
 
-0.337
 
 
57.8
 
 
 
 
ARMCX3
 
 
hsa-miR-425-5p
 
 
-0.325
 
 
33.1
 
 
 
 
ARPC5
 
 
hsa-miR-22-3p
 
 
-0.679
 
 
56.3
 
 
 
 
ARPIN-AP3S2
 
 
hsa-miR-34a-5p
 
 
-0.348
 
 
15.5
 
 
 
 
ARPP19
 
 
hsa-miR-29a-3p
 
 
-0.371
 
 
170.8
 
 
 
 
ARPP19
 
 
hsa-miR-34a-5p
 
 
-0.497
 
 
170.8
 
 
 
 
ARRDC3
 
 
hsa-miR-152-3p
 
 
-0.305
 
 
23.5
 
 
 
 
ARRDC3
 
 
hsa-miR-148b-3p
 
 
-0.305
 
 
23.5
 
 
 
 
ARRDC3
 
 
hsa-miR-29a-3p
 
 
-0.402
 
 
23.5
 
 
 
 
ASAH1
 
 
hsa-miR-27a-3p
 
 
-0.398
 
 
79.8
 
 
 
 
ASB1
 
 
hsa-miR-34a-5p
 
 
-0.406
 
 
19.6
 
 
 
 
ASF1A
 
 
hsa-miR-23a-3p
 
 
-0.506
 
 
14.8
 
 
 
 
ASF1A
 
 
hsa-miR-23b-3p
 
 
-0.506
 
 
14.8
 
 
 
 
ASPH
 
 
hsa-miR-152-3p
 
 
-0.391
 
 
25.3
 
 
 
 
ASPH
 
 
hsa-miR-148b-3p
 
 
-0.391
 
 
25.3
 
 
 
 
ATCAY
 
 
hsa-miR-10a-5p
 
 
-0.322
 
 
10.1
 
 
 
 
ATG4B
 
 
hsa-miR-34a-5p
 
 
-0.58
 
 
18.9
 
 
 
 
ATMIN
 
 
hsa-miR-34a-5p
 
 
-0.409
 
 
15.4
 
 
 
 
ATP1A2
 
 
hsa-miR-34a-5p
 
 
-0.306
 
 
87.6
 
 
 
 
ATP5MC3
 
 
hsa-miR-425-5p
 
 
-0.328
 
 
137.7
 
 
 
 
ATP5MC3
 
 
hsa-miR-29a-3p
 
 
-0.33
 
 
137.7
 
 
 
 
ATP5MC3
 
 
hsa-miR-27a-3p
 
 
-0.416
 
 
137.7
 
 
 
 
ATP6AP2
 
 
hsa-miR-148b-3p
 
 
-0.592
 
 
55.3
 
 
 
 
ATP6AP2
 
 
hsa-miR-152-3p
 
 
-0.592
 
 
55.3
 
 
 
 
 
 
 
 cd4_ev_vs_cd4_s0_downregulated 
 
 Tissue expression heatmap (log10-transformed) 
   
 
 
 basal ganglia 
 
 
 
 
target_symbol
 
 
mature_mirna_id
 
 
score
 
 
nTPM
 
 
 
 
 
 
ABCB9
 
 
hsa-let-7c-5p
 
 
-0.422
 
 
13.6
 
 
 
 
ABCB9
 
 
hsa-miR-98-5p
 
 
-0.444
 
 
13.6
 
 
 
 
ABHD17C
 
 
hsa-let-7c-5p
 
 
-0.359
 
 
20.2
 
 
 
 
ABHD17C
 
 
hsa-miR-98-5p
 
 
-0.383
 
 
20.2
 
 
 
 
ABHD17C
 
 
hsa-miR-330-3p
 
 
-0.399
 
 
20.2
 
 
 
 
ACHE
 
 
hsa-miR-194-5p
 
 
-0.395
 
 
38.3
 
 
 
 
ACSL6
 
 
hsa-miR-98-5p
 
 
-0.336
 
 
18.7
 
 
 
 
ACSL6
 
 
hsa-let-7c-5p
 
 
-0.336
 
 
18.7
 
 
 
 
ADD3
 
 
hsa-miR-200b-3p
 
 
-0.324
 
 
76.3
 
 
 
 
ADD3
 
 
hsa-miR-18a-5p
 
 
-0.39
 
 
76.3
 
 
 
 
ADNP
 
 
hsa-miR-328-3p
 
 
-0.371
 
 
24.2
 
 
 
 
ADRB1
 
 
hsa-let-7c-5p
 
 
-0.351
 
 
10.0
 
 
 
 
ADRB1
 
 
hsa-miR-98-5p
 
 
-0.367
 
 
10.0
 
 
 
 
AGFG2
 
 
hsa-miR-20a-5p
 
 
-0.365
 
 
19.5
 
 
 
 
AGFG2
 
 
hsa-miR-17-5p
 
 
-0.402
 
 
19.5
 
 
 
 
AHNAK
 
 
hsa-miR-20a-5p
 
 
-0.316
 
 
22.5
 
 
 
 
AHNAK
 
 
hsa-miR-17-5p
 
 
-0.337
 
 
22.5
 
 
 
 
AIFM1
 
 
hsa-miR-98-5p
 
 
-0.314
 
 
12.5
 
 
 
 
AIFM1
 
 
hsa-let-7c-5p
 
 
-0.314
 
 
12.5
 
 
 
 
AK1
 
 
hsa-miR-423-5p
 
 
-0.443
 
 
100.7
 
 
 
 
AKAP6
 
 
hsa-miR-98-5p
 
 
-0.307
 
 
10.5
 
 
 
 
AKAP6
 
 
hsa-let-7c-5p
 
 
-0.307
 
 
10.5
 
 
 
 
ALCAM
 
 
hsa-miR-18a-5p
 
 
-0.341
 
 
37.8
 
 
 
 
ALDH4A1
 
 
hsa-miR-184
 
 
-0.421
 
 
86.2
 
 
 
 
ALG3
 
 
hsa-miR-20a-5p
 
 
-0.559
 
 
10.2
 
 
 
 
ALG3
 
 
hsa-miR-17-5p
 
 
-0.572
 
 
10.2
 
 
 
 
AMT
 
 
hsa-miR-98-5p
 
 
-0.463
 
 
11.4
 
 
 
 
AMT
 
 
hsa-let-7c-5p
 
 
-0.463
 
 
11.4
 
 
 
 
ANGEL2
 
 
hsa-miR-200b-3p
 
 
-0.38
 
 
14.7
 
 
 
 
ANGPTL2
 
 
hsa-miR-423-5p
 
 
-0.379
 
 
10.9
 
 
 
 
ANKFY1
 
 
hsa-miR-155-5p
 
 
-0.315
 
 
13.3
 
 
 
 
ANKRD46
 
 
hsa-miR-98-5p
 
 
-0.389
 
 
22.6
 
 
 
 
ANKRD46
 
 
hsa-let-7c-5p
 
 
-0.389
 
 
22.6
 
 
 
 
ANKRD9
 
 
hsa-miR-20a-5p
 
 
-0.31
 
 
18.5
 
 
 
 
ANKRD9
 
 
hsa-miR-17-5p
 
 
-0.347
 
 
18.5
 
 
 
 
ANP32B
 
 
hsa-miR-200b-3p
 
 
-0.287
 
 
112.9
 
 
 
 
AP1S1
 
 
hsa-miR-98-5p
 
 
-0.374
 
 
116.3
 
 
 
 
AP1S1
 
 
hsa-let-7c-5p
 
 
-0.374
 
 
116.3
 
 
 
 
AP1S2
 
 
hsa-miR-200b-3p
 
 
-0.4
 
 
34.2
 
 
 
 
AP2M1
 
 
hsa-miR-423-5p
 
 
-0.49
 
 
241.4
 
 
 
 
AP5S1
 
 
hsa-miR-744-5p
 
 
-0.354
 
 
10.0
 
 
 
 
APCDD1
 
 
hsa-miR-20a-5p
 
 
-0.404
 
 
30.3
 
 
 
 
APCDD1
 
 
hsa-miR-17-5p
 
 
-0.425
 
 
30.3
 
 
 
 
APOO
 
 
hsa-miR-200b-3p
 
 
-0.361
 
 
38.6
 
 
 
 
ARF4
 
 
hsa-miR-194-5p
 
 
-0.293
 
 
71.0
 
 
 
 
ARF4
 
 
hsa-miR-7-5p
 
 
-0.407
 
 
71.0
 
 
 
 
ARGLU1
 
 
hsa-miR-200b-3p
 
 
-0.29
 
 
19.0
 
 
 
 
ARHGAP1
 
 
hsa-miR-17-5p
 
 
-0.325
 
 
41.0
 
 
 
 
ARHGAP1
 
 
hsa-miR-20a-5p
 
 
-0.339
 
 
41.0
 
 
 
 
ARL1
 
 
hsa-miR-20a-5p
 
 
-0.403
 
 
28.4
 
 
 
 
ARL1
 
 
hsa-miR-17-5p
 
 
-0.432
 
 
28.4
 
 
 
 
ARL4D
 
 
hsa-miR-98-5p
 
 
-0.417
 
 
14.0
 
 
 
 
ARL4D
 
 
hsa-let-7c-5p
 
 
-0.417
 
 
14.0
 
 
 
 
ARL6IP1
 
 
hsa-miR-7-5p
 
 
-0.374
 
 
196.4
 
 
 
 
ARL6IP1
 
 
hsa-miR-328-3p
 
 
-0.494
 
 
196.4
 
 
 
 
ARMC8
 
 
hsa-miR-20a-5p
 
 
-0.357
 
 
13.3
 
 
 
 
ARMC8
 
 
hsa-miR-17-5p
 
 
-0.378
 
 
13.3
 
 
 
 
ARMT1
 
 
hsa-miR-98-5p
 
 
-0.303
 
 
10.6
 
 
 
 
ARMT1
 
 
hsa-let-7c-5p
 
 
-0.303
 
 
10.6
 
 
 
 
ARPP19
 
 
hsa-miR-328-3p
 
 
-0.404
 
 
481.4
 
 
 
 
ARPP19
 
 
hsa-let-7c-5p
 
 
-0.492
 
 
481.4
 
 
 
 
ARPP19
 
 
hsa-miR-98-5p
 
 
-0.5
 
 
481.4
 
 
 
 
ARRDC4
 
 
hsa-miR-98-5p
 
 
-0.329
 
 
10.0
 
 
 
 
ARRDC4
 
 
hsa-let-7c-5p
 
 
-0.329
 
 
10.0
 
 
 
 
ASAP1
 
 
hsa-miR-98-5p
 
 
-0.308
 
 
11.6
 
 
 
 
ASAP1
 
 
hsa-let-7c-5p
 
 
-0.308
 
 
11.6
 
 
 
 
ASB6
 
 
hsa-miR-423-5p
 
 
-0.388
 
 
16.0
 
 
 
 
ASF1A
 
 
hsa-miR-200b-3p
 
 
-0.377
 
 
15.3
 
 
 
 
ATG13
 
 
hsa-miR-423-5p
 
 
-0.3
 
 
38.8
 
 
 
 
ATG16L1
 
 
hsa-miR-20a-5p
 
 
-0.446
 
 
11.5
 
 
 
 
ATG16L1
 
 
hsa-miR-17-5p
 
 
-0.467
 
 
11.5
 
 
 
 
ATOSB
 
 
hsa-miR-328-3p
 
 
-0.358
 
 
20.2
 
 
 
 
ATOSB
 
 
hsa-miR-98-5p
 
 
-0.429
 
 
20.2
 
 
 
 
ATOSB
 
 
hsa-let-7c-5p
 
 
-0.429
 
 
20.2
 
 
 
 
ATP2A2
 
 
hsa-miR-98-5p
 
 
-0.311
 
 
21.1
 
 
 
 
ATP2A2
 
 
hsa-let-7c-5p
 
 
-0.311
 
 
21.1
 
 
 
 
ATP2B2
 
 
hsa-miR-7-5p
 
 
-0.297
 
 
34.6
 
 
 
 
ATP6V1F
 
 
hsa-miR-194-5p
 
 
-0.444
 
 
288.5
 
 
 
 
ATP6V1H
 
 
hsa-miR-194-5p
 
 
-0.652
 
 
75.2
 
 
 
 
ATPAF1
 
 
hsa-miR-98-5p
 
 
-0.498
 
 
88.4
 
 
 
 
ATPAF1
 
 
hsa-let-7c-5p
 
 
-0.498
 
 
88.4
 
 
 
 
AVPI1
 
 
hsa-miR-7-5p
 
 
-0.367
 
 
14.2
 
 
 
 
B3GAT3
 
 
hsa-miR-98-5p
 
 
-0.324
 
 
67.8
 
 
 
 
B3GAT3
 
 
hsa-let-7c-5p
 
 
-0.324
 
 
67.8
 
 
 
 
B4GALT5
 
 
hsa-miR-210-3p
 
 
-0.511
 
 
12.9
 
 
 
 
B4GAT1
 
 
hsa-miR-200b-3p
 
 
-0.382
 
 
152.4
 
 
 
 
B4GAT1
 
 
hsa-miR-98-5p
 
 
-0.47
 
 
152.4
 
 
 
 
B4GAT1
 
 
hsa-let-7c-5p
 
 
-0.47
 
 
152.4
 
 
 
 
BAG6
 
 
hsa-miR-200b-3p
 
 
-0.371
 
 
46.2
 
 
 
 
BAMBI
 
 
hsa-miR-20a-5p
 
 
-0.485
 
 
11.5
 
 
 
 
BAMBI
 
 
hsa-miR-17-5p
 
 
-0.506
 
 
11.5
 
 
 
 
BAP1
 
 
hsa-miR-423-5p
 
 
-0.428
 
 
95.6
 
 
 
 
BCAS3
 
 
hsa-miR-423-5p
 
 
-0.322
 
 
10.8
 
 
 
 
BEGAIN
 
 
hsa-let-7c-5p
 
 
-0.466
 
 
23.9
 
 
 
 
BEGAIN
 
 
hsa-miR-98-5p
 
 
-0.474
 
 
23.9
 
 
 
 
BIN1
 
 
hsa-miR-744-5p
 
 
-0.654
 
 
177.7
 
 
 
 
BIN3
 
 
hsa-miR-98-5p
 
 
-0.427
 
 
10.9
 
 
 
 
BIN3
 
 
hsa-let-7c-5p
 
 
-0.427
 
 
10.9
 
 
 
 
BLOC1S6
 
 
hsa-miR-98-5p
 
 
-0.321
 
 
22.3
 
 
 
 
BLOC1S6
 
 
hsa-let-7c-5p
 
 
-0.321
 
 
22.3
 
 
 
 
 
 
 cerebellum 
 
 
 
 
target_symbol
 
 
mature_mirna_id
 
 
score
 
 
nTPM
 
 
 
 
 
 
ABCB9
 
 
hsa-let-7c-5p
 
 
-0.422
 
 
18.0
 
 
 
 
ABCB9
 
 
hsa-miR-98-5p
 
 
-0.444
 
 
18.0
 
 
 
 
ACHE
 
 
hsa-miR-194-5p
 
 
-0.395
 
 
59.1
 
 
 
 
ACSL6
 
 
hsa-miR-98-5p
 
 
-0.336
 
 
27.8
 
 
 
 
ACSL6
 
 
hsa-let-7c-5p
 
 
-0.336
 
 
27.8
 
 
 
 
ADD3
 
 
hsa-miR-200b-3p
 
 
-0.324
 
 
43.3
 
 
 
 
ADD3
 
 
hsa-miR-18a-5p
 
 
-0.39
 
 
43.3
 
 
 
 
ADNP
 
 
hsa-miR-328-3p
 
 
-0.371
 
 
38.9
 
 
 
 
AGFG2
 
 
hsa-miR-20a-5p
 
 
-0.365
 
 
12.7
 
 
 
 
AGFG2
 
 
hsa-miR-17-5p
 
 
-0.402
 
 
12.7
 
 
 
 
AHNAK
 
 
hsa-miR-20a-5p
 
 
-0.316
 
 
15.7
 
 
 
 
AHNAK
 
 
hsa-miR-17-5p
 
 
-0.337
 
 
15.7
 
 
 
 
AK1
 
 
hsa-miR-423-5p
 
 
-0.443
 
 
47.4
 
 
 
 
ALDH4A1
 
 
hsa-miR-184
 
 
-0.421
 
 
10.9
 
 
 
 
AMT
 
 
hsa-miR-98-5p
 
 
-0.463
 
 
20.1
 
 
 
 
AMT
 
 
hsa-let-7c-5p
 
 
-0.463
 
 
20.1
 
 
 
 
ANGEL2
 
 
hsa-miR-200b-3p
 
 
-0.38
 
 
12.7
 
 
 
 
ANGPTL2
 
 
hsa-miR-423-5p
 
 
-0.379
 
 
18.7
 
 
 
 
ANKFY1
 
 
hsa-miR-155-5p
 
 
-0.315
 
 
15.9
 
 
 
 
ANKRD12
 
 
hsa-miR-7-5p
 
 
-0.37
 
 
13.0
 
 
 
 
ANKRD13C
 
 
hsa-miR-18a-5p
 
 
-0.374
 
 
12.9
 
 
 
 
ANKRD13C
 
 
hsa-miR-20a-5p
 
 
-0.505
 
 
12.9
 
 
 
 
ANKRD13C
 
 
hsa-miR-17-5p
 
 
-0.526
 
 
12.9
 
 
 
 
ANKRD46
 
 
hsa-miR-98-5p
 
 
-0.389
 
 
27.5
 
 
 
 
ANKRD46
 
 
hsa-let-7c-5p
 
 
-0.389
 
 
27.5
 
 
 
 
ANKRD9
 
 
hsa-miR-20a-5p
 
 
-0.31
 
 
22.1
 
 
 
 
ANKRD9
 
 
hsa-miR-17-5p
 
 
-0.347
 
 
22.1
 
 
 
 
ANP32B
 
 
hsa-miR-200b-3p
 
 
-0.287
 
 
49.5
 
 
 
 
AP1S1
 
 
hsa-miR-98-5p
 
 
-0.374
 
 
23.9
 
 
 
 
AP1S1
 
 
hsa-let-7c-5p
 
 
-0.374
 
 
23.9
 
 
 
 
AP1S2
 
 
hsa-miR-200b-3p
 
 
-0.4
 
 
27.6
 
 
 
 
AP2M1
 
 
hsa-miR-423-5p
 
 
-0.49
 
 
209.2
 
 
 
 
APBB3
 
 
hsa-miR-98-5p
 
 
-0.587
 
 
23.6
 
 
 
 
APBB3
 
 
hsa-let-7c-5p
 
 
-0.587
 
 
23.6
 
 
 
 
APOO
 
 
hsa-miR-200b-3p
 
 
-0.361
 
 
32.8
 
 
 
 
ARF4
 
 
hsa-miR-194-5p
 
 
-0.293
 
 
50.2
 
 
 
 
ARF4
 
 
hsa-miR-7-5p
 
 
-0.407
 
 
50.2
 
 
 
 
ARG2
 
 
hsa-miR-98-5p
 
 
-0.468
 
 
16.4
 
 
 
 
ARG2
 
 
hsa-let-7c-5p
 
 
-0.468
 
 
16.4
 
 
 
 
ARGLU1
 
 
hsa-miR-200b-3p
 
 
-0.29
 
 
34.3
 
 
 
 
ARHGAP1
 
 
hsa-miR-17-5p
 
 
-0.325
 
 
19.6
 
 
 
 
ARHGAP1
 
 
hsa-miR-20a-5p
 
 
-0.339
 
 
19.6
 
 
 
 
ARID4A
 
 
hsa-miR-7-5p
 
 
-0.341
 
 
10.7
 
 
 
 
ARID4B
 
 
hsa-miR-20a-5p
 
 
-0.29
 
 
15.1
 
 
 
 
ARL1
 
 
hsa-miR-20a-5p
 
 
-0.403
 
 
20.8
 
 
 
 
ARL1
 
 
hsa-miR-17-5p
 
 
-0.432
 
 
20.8
 
 
 
 
ARL6IP1
 
 
hsa-miR-7-5p
 
 
-0.374
 
 
234.0
 
 
 
 
ARL6IP1
 
 
hsa-miR-328-3p
 
 
-0.494
 
 
234.0
 
 
 
 
ARMC8
 
 
hsa-miR-20a-5p
 
 
-0.357
 
 
35.7
 
 
 
 
ARMC8
 
 
hsa-miR-17-5p
 
 
-0.378
 
 
35.7
 
 
 
 
ARMT1
 
 
hsa-miR-98-5p
 
 
-0.303
 
 
11.5
 
 
 
 
ARMT1
 
 
hsa-let-7c-5p
 
 
-0.303
 
 
11.5
 
 
 
 
ARPP19
 
 
hsa-miR-328-3p
 
 
-0.404
 
 
66.7
 
 
 
 
ARPP19
 
 
hsa-let-7c-5p
 
 
-0.492
 
 
66.7
 
 
 
 
ARPP19
 
 
hsa-miR-98-5p
 
 
-0.5
 
 
66.7
 
 
 
 
ARVCF
 
 
hsa-miR-155-5p
 
 
-0.388
 
 
52.5
 
 
 
 
ASB6
 
 
hsa-miR-423-5p
 
 
-0.388
 
 
21.0
 
 
 
 
ASF1A
 
 
hsa-miR-200b-3p
 
 
-0.377
 
 
12.6
 
 
 
 
ATG13
 
 
hsa-miR-423-5p
 
 
-0.3
 
 
30.5
 
 
 
 
ATG16L1
 
 
hsa-miR-20a-5p
 
 
-0.446
 
 
24.1
 
 
 
 
ATG16L1
 
 
hsa-miR-17-5p
 
 
-0.467
 
 
24.1
 
 
 
 
ATOSB
 
 
hsa-miR-328-3p
 
 
-0.358
 
 
14.6
 
 
 
 
ATOSB
 
 
hsa-miR-98-5p
 
 
-0.429
 
 
14.6
 
 
 
 
ATOSB
 
 
hsa-let-7c-5p
 
 
-0.429
 
 
14.6
 
 
 
 
ATP2A2
 
 
hsa-miR-98-5p
 
 
-0.311
 
 
15.0
 
 
 
 
ATP2A2
 
 
hsa-let-7c-5p
 
 
-0.311
 
 
15.0
 
 
 
 
ATP2B2
 
 
hsa-miR-7-5p
 
 
-0.297
 
 
88.0
 
 
 
 
ATP6V1F
 
 
hsa-miR-194-5p
 
 
-0.444
 
 
212.3
 
 
 
 
ATP6V1H
 
 
hsa-miR-194-5p
 
 
-0.652
 
 
39.4
 
 
 
 
ATP8B2
 
 
hsa-miR-423-5p
 
 
-0.297
 
 
33.8
 
 
 
 
ATPAF1
 
 
hsa-miR-98-5p
 
 
-0.498
 
 
24.4
 
 
 
 
ATPAF1
 
 
hsa-let-7c-5p
 
 
-0.498
 
 
24.4
 
 
 
 
AVPI1
 
 
hsa-miR-7-5p
 
 
-0.367
 
 
16.8
 
 
 
 
B3GAT3
 
 
hsa-miR-98-5p
 
 
-0.324
 
 
70.0
 
 
 
 
B3GAT3
 
 
hsa-let-7c-5p
 
 
-0.324
 
 
70.0
 
 
 
 
B4GALT5
 
 
hsa-miR-210-3p
 
 
-0.511
 
 
20.4
 
 
 
 
B4GAT1
 
 
hsa-miR-200b-3p
 
 
-0.382
 
 
105.2
 
 
 
 
B4GAT1
 
 
hsa-miR-98-5p
 
 
-0.47
 
 
105.2
 
 
 
 
B4GAT1
 
 
hsa-let-7c-5p
 
 
-0.47
 
 
105.2
 
 
 
 
BAG6
 
 
hsa-miR-200b-3p
 
 
-0.371
 
 
68.7
 
 
 
 
BAP1
 
 
hsa-miR-423-5p
 
 
-0.428
 
 
67.9
 
 
 
 
BCAS3
 
 
hsa-miR-423-5p
 
 
-0.322
 
 
11.6
 
 
 
 
BEGAIN
 
 
hsa-let-7c-5p
 
 
-0.466
 
 
53.7
 
 
 
 
BEGAIN
 
 
hsa-miR-98-5p
 
 
-0.474
 
 
53.7
 
 
 
 
BHLHE22
 
 
hsa-miR-18a-5p
 
 
-0.325
 
 
17.1
 
 
 
 
BIN1
 
 
hsa-miR-744-5p
 
 
-0.654
 
 
88.3
 
 
 
 
BLOC1S6
 
 
hsa-miR-98-5p
 
 
-0.321
 
 
18.6
 
 
 
 
BLOC1S6
 
 
hsa-let-7c-5p
 
 
-0.321
 
 
18.6
 
 
 
 
BNIP3L
 
 
hsa-miR-20a-5p
 
 
-0.287
 
 
38.4
 
 
 
 
BRWD1
 
 
hsa-miR-18a-5p
 
 
-0.29
 
 
17.6
 
 
 
 
BTBD10
 
 
hsa-miR-17-5p
 
 
-0.409
 
 
18.9
 
 
 
 
BTBD10
 
 
hsa-miR-20a-5p
 
 
-0.412
 
 
18.9
 
 
 
 
BTF3L4
 
 
hsa-miR-200b-3p
 
 
-0.294
 
 
28.2
 
 
 
 
BTF3L4
 
 
hsa-miR-194-5p
 
 
-0.334
 
 
28.2
 
 
 
 
BTF3L4
 
 
hsa-miR-98-5p
 
 
-0.355
 
 
28.2
 
 
 
 
BTF3L4
 
 
hsa-let-7c-5p
 
 
-0.355
 
 
28.2
 
 
 
 
BZW1
 
 
hsa-miR-98-5p
 
 
-0.354
 
 
31.0
 
 
 
 
BZW1
 
 
hsa-let-7c-5p
 
 
-0.354
 
 
31.0
 
 
 
 
BZW1
 
 
hsa-miR-98-5p
 
 
-0.375
 
 
31.0
 
 
 
 
BZW1
 
 
hsa-let-7c-5p
 
 
-0.375
 
 
31.0
 
 
 
 
 
 
 cerebral cortex 
 
 
 
 
target_symbol
 
 
mature_mirna_id
 
 
score
 
 
nTPM
 
 
 
 
 
 
ABCB9
 
 
hsa-let-7c-5p
 
 
-0.422
 
 
19.5
 
 
 
 
ABCB9
 
 
hsa-miR-98-5p
 
 
-0.444
 
 
19.5
 
 
 
 
ABHD17C
 
 
hsa-let-7c-5p
 
 
-0.359
 
 
16.3
 
 
 
 
ABHD17C
 
 
hsa-miR-98-5p
 
 
-0.383
 
 
16.3
 
 
 
 
ABHD17C
 
 
hsa-miR-330-3p
 
 
-0.399
 
 
16.3
 
 
 
 
ACHE
 
 
hsa-miR-194-5p
 
 
-0.395
 
 
23.4
 
 
 
 
ACSL6
 
 
hsa-miR-98-5p
 
 
-0.336
 
 
29.5
 
 
 
 
ACSL6
 
 
hsa-let-7c-5p
 
 
-0.336
 
 
29.5
 
 
 
 
ADAM9
 
 
hsa-miR-20a-5p
 
 
-0.299
 
 
16.2
 
 
 
 
ADAM9
 
 
hsa-miR-17-5p
 
 
-0.32
 
 
16.2
 
 
 
 
ADD3
 
 
hsa-miR-200b-3p
 
 
-0.324
 
 
89.1
 
 
 
 
ADD3
 
 
hsa-miR-18a-5p
 
 
-0.39
 
 
89.1
 
 
 
 
ADNP
 
 
hsa-miR-328-3p
 
 
-0.371
 
 
26.0
 
 
 
 
AEBP2
 
 
hsa-miR-18a-5p
 
 
-0.376
 
 
13.9
 
 
 
 
AGFG2
 
 
hsa-miR-20a-5p
 
 
-0.365
 
 
14.0
 
 
 
 
AGFG2
 
 
hsa-miR-17-5p
 
 
-0.402
 
 
14.0
 
 
 
 
AHNAK
 
 
hsa-miR-20a-5p
 
 
-0.316
 
 
26.8
 
 
 
 
AHNAK
 
 
hsa-miR-17-5p
 
 
-0.337
 
 
26.8
 
 
 
 
AIFM1
 
 
hsa-miR-98-5p
 
 
-0.314
 
 
10.3
 
 
 
 
AIFM1
 
 
hsa-let-7c-5p
 
 
-0.314
 
 
10.3
 
 
 
 
AK1
 
 
hsa-miR-423-5p
 
 
-0.443
 
 
81.2
 
 
 
 
AKAP6
 
 
hsa-miR-98-5p
 
 
-0.307
 
 
27.4
 
 
 
 
AKAP6
 
 
hsa-let-7c-5p
 
 
-0.307
 
 
27.4
 
 
 
 
ALCAM
 
 
hsa-miR-18a-5p
 
 
-0.341
 
 
54.7
 
 
 
 
ALDH4A1
 
 
hsa-miR-184
 
 
-0.421
 
 
69.8
 
 
 
 
ANGPTL2
 
 
hsa-miR-423-5p
 
 
-0.379
 
 
32.8
 
 
 
 
ANKFY1
 
 
hsa-miR-155-5p
 
 
-0.315
 
 
19.9
 
 
 
 
ANKRD12
 
 
hsa-miR-7-5p
 
 
-0.37
 
 
15.1
 
 
 
 
ANKRD13C
 
 
hsa-miR-18a-5p
 
 
-0.374
 
 
10.2
 
 
 
 
ANKRD13C
 
 
hsa-miR-20a-5p
 
 
-0.505
 
 
10.2
 
 
 
 
ANKRD13C
 
 
hsa-miR-17-5p
 
 
-0.526
 
 
10.2
 
 
 
 
ANKRD46
 
 
hsa-miR-98-5p
 
 
-0.389
 
 
39.5
 
 
 
 
ANKRD46
 
 
hsa-let-7c-5p
 
 
-0.389
 
 
39.5
 
 
 
 
ANKRD9
 
 
hsa-miR-20a-5p
 
 
-0.31
 
 
28.9
 
 
 
 
ANKRD9
 
 
hsa-miR-17-5p
 
 
-0.347
 
 
28.9
 
 
 
 
ANP32B
 
 
hsa-miR-200b-3p
 
 
-0.287
 
 
70.2
 
 
 
 
AP1S1
 
 
hsa-miR-98-5p
 
 
-0.374
 
 
104.9
 
 
 
 
AP1S1
 
 
hsa-let-7c-5p
 
 
-0.374
 
 
104.9
 
 
 
 
AP1S2
 
 
hsa-miR-200b-3p
 
 
-0.4
 
 
33.2
 
 
 
 
AP2M1
 
 
hsa-miR-423-5p
 
 
-0.49
 
 
362.0
 
 
 
 
APBB3
 
 
hsa-miR-98-5p
 
 
-0.587
 
 
15.0
 
 
 
 
APBB3
 
 
hsa-let-7c-5p
 
 
-0.587
 
 
15.0
 
 
 
 
APCDD1
 
 
hsa-miR-20a-5p
 
 
-0.404
 
 
33.1
 
 
 
 
APCDD1
 
 
hsa-miR-17-5p
 
 
-0.425
 
 
33.1
 
 
 
 
APOO
 
 
hsa-miR-200b-3p
 
 
-0.361
 
 
56.2
 
 
 
 
ARF4
 
 
hsa-miR-194-5p
 
 
-0.293
 
 
61.5
 
 
 
 
ARF4
 
 
hsa-miR-7-5p
 
 
-0.407
 
 
61.5
 
 
 
 
ARGLU1
 
 
hsa-miR-200b-3p
 
 
-0.29
 
 
16.2
 
 
 
 
ARHGAP1
 
 
hsa-miR-17-5p
 
 
-0.325
 
 
40.8
 
 
 
 
ARHGAP1
 
 
hsa-miR-20a-5p
 
 
-0.339
 
 
40.8
 
 
 
 
ARID4A
 
 
hsa-miR-7-5p
 
 
-0.341
 
 
13.3
 
 
 
 
ARID4B
 
 
hsa-miR-20a-5p
 
 
-0.29
 
 
11.1
 
 
 
 
ARL1
 
 
hsa-miR-20a-5p
 
 
-0.403
 
 
34.0
 
 
 
 
ARL1
 
 
hsa-miR-17-5p
 
 
-0.432
 
 
34.0
 
 
 
 
ARL4D
 
 
hsa-miR-98-5p
 
 
-0.417
 
 
14.3
 
 
 
 
ARL4D
 
 
hsa-let-7c-5p
 
 
-0.417
 
 
14.3
 
 
 
 
ARL6IP1
 
 
hsa-miR-7-5p
 
 
-0.374
 
 
314.1
 
 
 
 
ARL6IP1
 
 
hsa-miR-328-3p
 
 
-0.494
 
 
314.1
 
 
 
 
ARMC8
 
 
hsa-miR-20a-5p
 
 
-0.357
 
 
21.7
 
 
 
 
ARMC8
 
 
hsa-miR-17-5p
 
 
-0.378
 
 
21.7
 
 
 
 
ARMT1
 
 
hsa-miR-98-5p
 
 
-0.303
 
 
10.1
 
 
 
 
ARMT1
 
 
hsa-let-7c-5p
 
 
-0.303
 
 
10.1
 
 
 
 
ARPP19
 
 
hsa-miR-328-3p
 
 
-0.404
 
 
292.4
 
 
 
 
ARPP19
 
 
hsa-let-7c-5p
 
 
-0.492
 
 
292.4
 
 
 
 
ARPP19
 
 
hsa-miR-98-5p
 
 
-0.5
 
 
292.4
 
 
 
 
ARRDC4
 
 
hsa-miR-98-5p
 
 
-0.329
 
 
12.8
 
 
 
 
ARRDC4
 
 
hsa-let-7c-5p
 
 
-0.329
 
 
12.8
 
 
 
 
ASAP1
 
 
hsa-miR-98-5p
 
 
-0.308
 
 
21.7
 
 
 
 
ASAP1
 
 
hsa-let-7c-5p
 
 
-0.308
 
 
21.7
 
 
 
 
ASB6
 
 
hsa-miR-423-5p
 
 
-0.388
 
 
21.5
 
 
 
 
ASF1A
 
 
hsa-miR-200b-3p
 
 
-0.377
 
 
13.7
 
 
 
 
ATG13
 
 
hsa-miR-423-5p
 
 
-0.3
 
 
41.4
 
 
 
 
ATG16L1
 
 
hsa-miR-20a-5p
 
 
-0.446
 
 
13.9
 
 
 
 
ATG16L1
 
 
hsa-miR-17-5p
 
 
-0.467
 
 
13.9
 
 
 
 
ATOSB
 
 
hsa-miR-328-3p
 
 
-0.358
 
 
21.2
 
 
 
 
ATOSB
 
 
hsa-miR-98-5p
 
 
-0.429
 
 
21.2
 
 
 
 
ATOSB
 
 
hsa-let-7c-5p
 
 
-0.429
 
 
21.2
 
 
 
 
ATP2A2
 
 
hsa-miR-98-5p
 
 
-0.311
 
 
54.4
 
 
 
 
ATP2A2
 
 
hsa-let-7c-5p
 
 
-0.311
 
 
54.4
 
 
 
 
ATP2B2
 
 
hsa-miR-7-5p
 
 
-0.297
 
 
63.7
 
 
 
 
ATP6V1F
 
 
hsa-miR-194-5p
 
 
-0.444
 
 
343.1
 
 
 
 
ATP6V1H
 
 
hsa-miR-194-5p
 
 
-0.652
 
 
87.2
 
 
 
 
ATP8B2
 
 
hsa-miR-423-5p
 
 
-0.297
 
 
10.2
 
 
 
 
ATPAF1
 
 
hsa-miR-98-5p
 
 
-0.498
 
 
74.9
 
 
 
 
ATPAF1
 
 
hsa-let-7c-5p
 
 
-0.498
 
 
74.9
 
 
 
 
AVPI1
 
 
hsa-miR-7-5p
 
 
-0.367
 
 
24.4
 
 
 
 
B3GALT2
 
 
hsa-miR-17-5p
 
 
-0.35
 
 
15.3
 
 
 
 
B3GALT2
 
 
hsa-miR-20a-5p
 
 
-0.364
 
 
15.3
 
 
 
 
B3GAT3
 
 
hsa-miR-98-5p
 
 
-0.324
 
 
63.6
 
 
 
 
B3GAT3
 
 
hsa-let-7c-5p
 
 
-0.324
 
 
63.6
 
 
 
 
B4GALT5
 
 
hsa-miR-210-3p
 
 
-0.511
 
 
22.9
 
 
 
 
B4GAT1
 
 
hsa-miR-200b-3p
 
 
-0.382
 
 
171.8
 
 
 
 
B4GAT1
 
 
hsa-miR-98-5p
 
 
-0.47
 
 
171.8
 
 
 
 
B4GAT1
 
 
hsa-let-7c-5p
 
 
-0.47
 
 
171.8
 
 
 
 
BAG6
 
 
hsa-miR-200b-3p
 
 
-0.371
 
 
66.0
 
 
 
 
BAP1
 
 
hsa-miR-423-5p
 
 
-0.428
 
 
137.9
 
 
 
 
BBX
 
 
hsa-miR-18a-5p
 
 
-0.31
 
 
16.6
 
 
 
 
BCAS3
 
 
hsa-miR-423-5p
 
 
-0.322
 
 
17.2
 
 
 
 
BEGAIN
 
 
hsa-let-7c-5p
 
 
-0.466
 
 
42.8
 
 
 
 
BEGAIN
 
 
hsa-miR-98-5p
 
 
-0.474
 
 
42.8
 
 
 
 
 
 
 hypothalamus 
 
 
 
 
target_symbol
 
 
mature_mirna_id
 
 
score
 
 
nTPM
 
 
 
 
 
 
ABCB9
 
 
hsa-let-7c-5p
 
 
-0.422
 
 
11.9
 
 
 
 
ABCB9
 
 
hsa-miR-98-5p
 
 
-0.444
 
 
11.9
 
 
 
 
ABHD17C
 
 
hsa-let-7c-5p
 
 
-0.359
 
 
11.1
 
 
 
 
ABHD17C
 
 
hsa-miR-98-5p
 
 
-0.383
 
 
11.1
 
 
 
 
ABHD17C
 
 
hsa-miR-330-3p
 
 
-0.399
 
 
11.1
 
 
 
 
ACHE
 
 
hsa-miR-194-5p
 
 
-0.395
 
 
103.9
 
 
 
 
ACSL6
 
 
hsa-miR-98-5p
 
 
-0.336
 
 
11.9
 
 
 
 
ACSL6
 
 
hsa-let-7c-5p
 
 
-0.336
 
 
11.9
 
 
 
 
ADD3
 
 
hsa-miR-200b-3p
 
 
-0.324
 
 
50.9
 
 
 
 
ADD3
 
 
hsa-miR-18a-5p
 
 
-0.39
 
 
50.9
 
 
 
 
ADNP
 
 
hsa-miR-328-3p
 
 
-0.371
 
 
23.7
 
 
 
 
AGFG2
 
 
hsa-miR-20a-5p
 
 
-0.365
 
 
10.8
 
 
 
 
AGFG2
 
 
hsa-miR-17-5p
 
 
-0.402
 
 
10.8
 
 
 
 
AHNAK
 
 
hsa-miR-20a-5p
 
 
-0.316
 
 
17.3
 
 
 
 
AHNAK
 
 
hsa-miR-17-5p
 
 
-0.337
 
 
17.3
 
 
 
 
AK1
 
 
hsa-miR-423-5p
 
 
-0.443
 
 
100.1
 
 
 
 
AKAP6
 
 
hsa-miR-98-5p
 
 
-0.307
 
 
13.6
 
 
 
 
AKAP6
 
 
hsa-let-7c-5p
 
 
-0.307
 
 
13.6
 
 
 
 
ALCAM
 
 
hsa-miR-18a-5p
 
 
-0.341
 
 
36.1
 
 
 
 
ALDH4A1
 
 
hsa-miR-184
 
 
-0.421
 
 
31.5
 
 
 
 
ANKFY1
 
 
hsa-miR-155-5p
 
 
-0.315
 
 
10.7
 
 
 
 
ANKRD46
 
 
hsa-miR-98-5p
 
 
-0.389
 
 
28.7
 
 
 
 
ANKRD46
 
 
hsa-let-7c-5p
 
 
-0.389
 
 
28.7
 
 
 
 
ANKRD9
 
 
hsa-miR-20a-5p
 
 
-0.31
 
 
16.0
 
 
 
 
ANKRD9
 
 
hsa-miR-17-5p
 
 
-0.347
 
 
16.0
 
 
 
 
ANP32B
 
 
hsa-miR-200b-3p
 
 
-0.287
 
 
120.3
 
 
 
 
AP1S1
 
 
hsa-miR-98-5p
 
 
-0.374
 
 
44.3
 
 
 
 
AP1S1
 
 
hsa-let-7c-5p
 
 
-0.374
 
 
44.3
 
 
 
 
AP1S2
 
 
hsa-miR-200b-3p
 
 
-0.4
 
 
41.0
 
 
 
 
AP2M1
 
 
hsa-miR-423-5p
 
 
-0.49
 
 
248.8
 
 
 
 
APBB3
 
 
hsa-miR-98-5p
 
 
-0.587
 
 
11.2
 
 
 
 
APBB3
 
 
hsa-let-7c-5p
 
 
-0.587
 
 
11.2
 
 
 
 
APCDD1
 
 
hsa-miR-20a-5p
 
 
-0.404
 
 
19.2
 
 
 
 
APCDD1
 
 
hsa-miR-17-5p
 
 
-0.425
 
 
19.2
 
 
 
 
APOO
 
 
hsa-miR-200b-3p
 
 
-0.361
 
 
43.8
 
 
 
 
ARF4
 
 
hsa-miR-194-5p
 
 
-0.293
 
 
75.3
 
 
 
 
ARF4
 
 
hsa-miR-7-5p
 
 
-0.407
 
 
75.3
 
 
 
 
ARG2
 
 
hsa-miR-98-5p
 
 
-0.468
 
 
10.7
 
 
 
 
ARG2
 
 
hsa-let-7c-5p
 
 
-0.468
 
 
10.7
 
 
 
 
ARGLU1
 
 
hsa-miR-200b-3p
 
 
-0.29
 
 
12.5
 
 
 
 
ARHGAP1
 
 
hsa-miR-17-5p
 
 
-0.325
 
 
31.0
 
 
 
 
ARHGAP1
 
 
hsa-miR-20a-5p
 
 
-0.339
 
 
31.0
 
 
 
 
ARL1
 
 
hsa-miR-20a-5p
 
 
-0.403
 
 
31.3
 
 
 
 
ARL1
 
 
hsa-miR-17-5p
 
 
-0.432
 
 
31.3
 
 
 
 
ARL6IP1
 
 
hsa-miR-7-5p
 
 
-0.374
 
 
212.8
 
 
 
 
ARL6IP1
 
 
hsa-miR-328-3p
 
 
-0.494
 
 
212.8
 
 
 
 
ARMC8
 
 
hsa-miR-20a-5p
 
 
-0.357
 
 
16.8
 
 
 
 
ARMC8
 
 
hsa-miR-17-5p
 
 
-0.378
 
 
16.8
 
 
 
 
ARPP19
 
 
hsa-miR-328-3p
 
 
-0.404
 
 
103.9
 
 
 
 
ARPP19
 
 
hsa-let-7c-5p
 
 
-0.492
 
 
103.9
 
 
 
 
ARPP19
 
 
hsa-miR-98-5p
 
 
-0.5
 
 
103.9
 
 
 
 
ARRDC4
 
 
hsa-miR-98-5p
 
 
-0.329
 
 
13.4
 
 
 
 
ARRDC4
 
 
hsa-let-7c-5p
 
 
-0.329
 
 
13.4
 
 
 
 
ASAP1
 
 
hsa-miR-98-5p
 
 
-0.308
 
 
11.8
 
 
 
 
ASAP1
 
 
hsa-let-7c-5p
 
 
-0.308
 
 
11.8
 
 
 
 
ASB6
 
 
hsa-miR-423-5p
 
 
-0.388
 
 
11.8
 
 
 
 
ASF1A
 
 
hsa-miR-200b-3p
 
 
-0.377
 
 
13.0
 
 
 
 
ATG13
 
 
hsa-miR-423-5p
 
 
-0.3
 
 
32.3
 
 
 
 
ATOSB
 
 
hsa-miR-328-3p
 
 
-0.358
 
 
19.8
 
 
 
 
ATOSB
 
 
hsa-miR-98-5p
 
 
-0.429
 
 
19.8
 
 
 
 
ATOSB
 
 
hsa-let-7c-5p
 
 
-0.429
 
 
19.8
 
 
 
 
ATP2A2
 
 
hsa-miR-98-5p
 
 
-0.311
 
 
11.9
 
 
 
 
ATP2A2
 
 
hsa-let-7c-5p
 
 
-0.311
 
 
11.9
 
 
 
 
ATP2B2
 
 
hsa-miR-7-5p
 
 
-0.297
 
 
12.0
 
 
 
 
ATP6V1F
 
 
hsa-miR-194-5p
 
 
-0.444
 
 
279.3
 
 
 
 
ATP6V1H
 
 
hsa-miR-194-5p
 
 
-0.652
 
 
47.4
 
 
 
 
ATPAF1
 
 
hsa-miR-98-5p
 
 
-0.498
 
 
52.2
 
 
 
 
ATPAF1
 
 
hsa-let-7c-5p
 
 
-0.498
 
 
52.2
 
 
 
 
AVPI1
 
 
hsa-miR-7-5p
 
 
-0.367
 
 
15.0
 
 
 
 
B3GAT3
 
 
hsa-miR-98-5p
 
 
-0.324
 
 
60.2
 
 
 
 
B3GAT3
 
 
hsa-let-7c-5p
 
 
-0.324
 
 
60.2
 
 
 
 
B4GALT5
 
 
hsa-miR-210-3p
 
 
-0.511
 
 
13.0
 
 
 
 
B4GAT1
 
 
hsa-miR-200b-3p
 
 
-0.382
 
 
207.3
 
 
 
 
B4GAT1
 
 
hsa-miR-98-5p
 
 
-0.47
 
 
207.3
 
 
 
 
B4GAT1
 
 
hsa-let-7c-5p
 
 
-0.47
 
 
207.3
 
 
 
 
BAG6
 
 
hsa-miR-200b-3p
 
 
-0.371
 
 
40.3
 
 
 
 
BAMBI
 
 
hsa-miR-20a-5p
 
 
-0.485
 
 
12.9
 
 
 
 
BAMBI
 
 
hsa-miR-17-5p
 
 
-0.506
 
 
12.9
 
 
 
 
BAP1
 
 
hsa-miR-423-5p
 
 
-0.428
 
 
50.4
 
 
 
 
BEGAIN
 
 
hsa-let-7c-5p
 
 
-0.466
 
 
15.9
 
 
 
 
BEGAIN
 
 
hsa-miR-98-5p
 
 
-0.474
 
 
15.9
 
 
 
 
BIN1
 
 
hsa-miR-744-5p
 
 
-0.654
 
 
127.6
 
 
 
 
BLOC1S6
 
 
hsa-miR-98-5p
 
 
-0.321
 
 
24.8
 
 
 
 
BLOC1S6
 
 
hsa-let-7c-5p
 
 
-0.321
 
 
24.8
 
 
 
 
BNIP3L
 
 
hsa-miR-20a-5p
 
 
-0.287
 
 
41.8
 
 
 
 
BRWD1
 
 
hsa-miR-18a-5p
 
 
-0.29
 
 
39.7
 
 
 
 
BTBD10
 
 
hsa-miR-17-5p
 
 
-0.409
 
 
20.3
 
 
 
 
BTBD10
 
 
hsa-miR-20a-5p
 
 
-0.412
 
 
20.3
 
 
 
 
BTF3L4
 
 
hsa-miR-200b-3p
 
 
-0.294
 
 
33.4
 
 
 
 
BTF3L4
 
 
hsa-miR-194-5p
 
 
-0.334
 
 
33.4
 
 
 
 
BTF3L4
 
 
hsa-miR-98-5p
 
 
-0.355
 
 
33.4
 
 
 
 
BTF3L4
 
 
hsa-let-7c-5p
 
 
-0.355
 
 
33.4
 
 
 
 
BTG3
 
 
hsa-miR-18a-5p
 
 
-0.464
 
 
23.0
 
 
 
 
BTG3
 
 
hsa-miR-17-5p
 
 
-0.668
 
 
23.0
 
 
 
 
BTG3
 
 
hsa-miR-20a-5p
 
 
-0.671
 
 
23.0
 
 
 
 
BZW1
 
 
hsa-miR-98-5p
 
 
-0.354
 
 
32.4
 
 
 
 
BZW1
 
 
hsa-let-7c-5p
 
 
-0.354
 
 
32.4
 
 
 
 
BZW1
 
 
hsa-miR-98-5p
 
 
-0.375
 
 
32.4
 
 
 
 
BZW1
 
 
hsa-let-7c-5p
 
 
-0.375
 
 
32.4
 
 
 
 
C19orf47
 
 
hsa-let-7c-5p
 
 
-0.311
 
 
12.4
 
 
 
 
 
 
 midbrain 
 
 
 
 
target_symbol
 
 
mature_mirna_id
 
 
score
 
 
nTPM
 
 
 
 
 
 
ABCB9
 
 
hsa-let-7c-5p
 
 
-0.422
 
 
10.6
 
 
 
 
ABCB9
 
 
hsa-miR-98-5p
 
 
-0.444
 
 
10.6
 
 
 
 
ABHD17C
 
 
hsa-let-7c-5p
 
 
-0.359
 
 
11.2
 
 
 
 
ABHD17C
 
 
hsa-miR-98-5p
 
 
-0.383
 
 
11.2
 
 
 
 
ABHD17C
 
 
hsa-miR-330-3p
 
 
-0.399
 
 
11.2
 
 
 
 
ACHE
 
 
hsa-miR-194-5p
 
 
-0.395
 
 
47.7
 
 
 
 
ACSL6
 
 
hsa-miR-98-5p
 
 
-0.336
 
 
11.1
 
 
 
 
ACSL6
 
 
hsa-let-7c-5p
 
 
-0.336
 
 
11.1
 
 
 
 
ADD3
 
 
hsa-miR-200b-3p
 
 
-0.324
 
 
69.0
 
 
 
 
ADD3
 
 
hsa-miR-18a-5p
 
 
-0.39
 
 
69.0
 
 
 
 
ADNP
 
 
hsa-miR-328-3p
 
 
-0.371
 
 
21.5
 
 
 
 
AGFG2
 
 
hsa-miR-20a-5p
 
 
-0.365
 
 
12.1
 
 
 
 
AGFG2
 
 
hsa-miR-17-5p
 
 
-0.402
 
 
12.1
 
 
 
 
AHNAK
 
 
hsa-miR-20a-5p
 
 
-0.316
 
 
22.7
 
 
 
 
AHNAK
 
 
hsa-miR-17-5p
 
 
-0.337
 
 
22.7
 
 
 
 
AIFM1
 
 
hsa-miR-98-5p
 
 
-0.314
 
 
12.7
 
 
 
 
AIFM1
 
 
hsa-let-7c-5p
 
 
-0.314
 
 
12.7
 
 
 
 
AK1
 
 
hsa-miR-423-5p
 
 
-0.443
 
 
97.2
 
 
 
 
AKAP6
 
 
hsa-miR-98-5p
 
 
-0.307
 
 
17.4
 
 
 
 
AKAP6
 
 
hsa-let-7c-5p
 
 
-0.307
 
 
17.4
 
 
 
 
ALCAM
 
 
hsa-miR-18a-5p
 
 
-0.341
 
 
38.7
 
 
 
 
ALDH4A1
 
 
hsa-miR-184
 
 
-0.421
 
 
73.5
 
 
 
 
ANGPTL2
 
 
hsa-miR-423-5p
 
 
-0.379
 
 
12.4
 
 
 
 
ANKFY1
 
 
hsa-miR-155-5p
 
 
-0.315
 
 
12.6
 
 
 
 
ANKRD29
 
 
hsa-miR-20a-5p
 
 
-0.359
 
 
11.5
 
 
 
 
ANKRD29
 
 
hsa-miR-17-5p
 
 
-0.372
 
 
11.5
 
 
 
 
ANKRD46
 
 
hsa-miR-98-5p
 
 
-0.389
 
 
24.0
 
 
 
 
ANKRD46
 
 
hsa-let-7c-5p
 
 
-0.389
 
 
24.0
 
 
 
 
ANKRD9
 
 
hsa-miR-20a-5p
 
 
-0.31
 
 
20.0
 
 
 
 
ANKRD9
 
 
hsa-miR-17-5p
 
 
-0.347
 
 
20.0
 
 
 
 
ANP32B
 
 
hsa-miR-200b-3p
 
 
-0.287
 
 
216.9
 
 
 
 
AP1S1
 
 
hsa-miR-98-5p
 
 
-0.374
 
 
35.4
 
 
 
 
AP1S1
 
 
hsa-let-7c-5p
 
 
-0.374
 
 
35.4
 
 
 
 
AP1S2
 
 
hsa-miR-200b-3p
 
 
-0.4
 
 
29.1
 
 
 
 
AP2M1
 
 
hsa-miR-423-5p
 
 
-0.49
 
 
209.2
 
 
 
 
APCDD1
 
 
hsa-miR-20a-5p
 
 
-0.404
 
 
22.5
 
 
 
 
APCDD1
 
 
hsa-miR-17-5p
 
 
-0.425
 
 
22.5
 
 
 
 
APOO
 
 
hsa-miR-200b-3p
 
 
-0.361
 
 
44.2
 
 
 
 
ARF4
 
 
hsa-miR-194-5p
 
 
-0.293
 
 
62.8
 
 
 
 
ARF4
 
 
hsa-miR-7-5p
 
 
-0.407
 
 
62.8
 
 
 
 
ARGLU1
 
 
hsa-miR-200b-3p
 
 
-0.29
 
 
12.2
 
 
 
 
ARHGAP1
 
 
hsa-miR-17-5p
 
 
-0.325
 
 
34.8
 
 
 
 
ARHGAP1
 
 
hsa-miR-20a-5p
 
 
-0.339
 
 
34.8
 
 
 
 
ARL1
 
 
hsa-miR-20a-5p
 
 
-0.403
 
 
27.4
 
 
 
 
ARL1
 
 
hsa-miR-17-5p
 
 
-0.432
 
 
27.4
 
 
 
 
ARL6IP1
 
 
hsa-miR-7-5p
 
 
-0.374
 
 
198.8
 
 
 
 
ARL6IP1
 
 
hsa-miR-328-3p
 
 
-0.494
 
 
198.8
 
 
 
 
ARMC8
 
 
hsa-miR-20a-5p
 
 
-0.357
 
 
14.1
 
 
 
 
ARMC8
 
 
hsa-miR-17-5p
 
 
-0.378
 
 
14.1
 
 
 
 
ARPP19
 
 
hsa-miR-328-3p
 
 
-0.404
 
 
144.2
 
 
 
 
ARPP19
 
 
hsa-let-7c-5p
 
 
-0.492
 
 
144.2
 
 
 
 
ARPP19
 
 
hsa-miR-98-5p
 
 
-0.5
 
 
144.2
 
 
 
 
ARRDC4
 
 
hsa-miR-98-5p
 
 
-0.329
 
 
17.7
 
 
 
 
ARRDC4
 
 
hsa-let-7c-5p
 
 
-0.329
 
 
17.7
 
 
 
 
ASAP1
 
 
hsa-miR-98-5p
 
 
-0.308
 
 
12.8
 
 
 
 
ASAP1
 
 
hsa-let-7c-5p
 
 
-0.308
 
 
12.8
 
 
 
 
ASB6
 
 
hsa-miR-423-5p
 
 
-0.388
 
 
11.3
 
 
 
 
ASF1A
 
 
hsa-miR-200b-3p
 
 
-0.377
 
 
14.8
 
 
 
 
ATG13
 
 
hsa-miR-423-5p
 
 
-0.3
 
 
34.2
 
 
 
 
ATOSB
 
 
hsa-miR-328-3p
 
 
-0.358
 
 
24.1
 
 
 
 
ATOSB
 
 
hsa-miR-98-5p
 
 
-0.429
 
 
24.1
 
 
 
 
ATOSB
 
 
hsa-let-7c-5p
 
 
-0.429
 
 
24.1
 
 
 
 
ATP2A2
 
 
hsa-miR-98-5p
 
 
-0.311
 
 
17.1
 
 
 
 
ATP2A2
 
 
hsa-let-7c-5p
 
 
-0.311
 
 
17.1
 
 
 
 
ATP2B2
 
 
hsa-miR-7-5p
 
 
-0.297
 
 
12.7
 
 
 
 
ATP6V1F
 
 
hsa-miR-194-5p
 
 
-0.444
 
 
256.8
 
 
 
 
ATP6V1H
 
 
hsa-miR-194-5p
 
 
-0.652
 
 
36.2
 
 
 
 
ATPAF1
 
 
hsa-miR-98-5p
 
 
-0.498
 
 
74.9
 
 
 
 
ATPAF1
 
 
hsa-let-7c-5p
 
 
-0.498
 
 
74.9
 
 
 
 
AVPI1
 
 
hsa-miR-7-5p
 
 
-0.367
 
 
18.5
 
 
 
 
B3GAT3
 
 
hsa-miR-98-5p
 
 
-0.324
 
 
58.5
 
 
 
 
B3GAT3
 
 
hsa-let-7c-5p
 
 
-0.324
 
 
58.5
 
 
 
 
B4GALT5
 
 
hsa-miR-210-3p
 
 
-0.511
 
 
10.3
 
 
 
 
B4GAT1
 
 
hsa-miR-200b-3p
 
 
-0.382
 
 
85.6
 
 
 
 
B4GAT1
 
 
hsa-miR-98-5p
 
 
-0.47
 
 
85.6
 
 
 
 
B4GAT1
 
 
hsa-let-7c-5p
 
 
-0.47
 
 
85.6
 
 
 
 
BAG6
 
 
hsa-miR-200b-3p
 
 
-0.371
 
 
41.6
 
 
 
 
BAMBI
 
 
hsa-miR-20a-5p
 
 
-0.485
 
 
16.8
 
 
 
 
BAMBI
 
 
hsa-miR-17-5p
 
 
-0.506
 
 
16.8
 
 
 
 
BAP1
 
 
hsa-miR-423-5p
 
 
-0.428
 
 
58.3
 
 
 
 
BBX
 
 
hsa-miR-18a-5p
 
 
-0.31
 
 
11.6
 
 
 
 
BCAS3
 
 
hsa-miR-423-5p
 
 
-0.322
 
 
10.7
 
 
 
 
BIN1
 
 
hsa-miR-744-5p
 
 
-0.654
 
 
190.1
 
 
 
 
BLOC1S6
 
 
hsa-miR-98-5p
 
 
-0.321
 
 
18.5
 
 
 
 
BLOC1S6
 
 
hsa-let-7c-5p
 
 
-0.321
 
 
18.5
 
 
 
 
BNIP3L
 
 
hsa-miR-20a-5p
 
 
-0.287
 
 
52.9
 
 
 
 
BRWD1
 
 
hsa-miR-18a-5p
 
 
-0.29
 
 
29.1
 
 
 
 
BTBD10
 
 
hsa-miR-17-5p
 
 
-0.409
 
 
15.0
 
 
 
 
BTBD10
 
 
hsa-miR-20a-5p
 
 
-0.412
 
 
15.0
 
 
 
 
BTF3L4
 
 
hsa-miR-200b-3p
 
 
-0.294
 
 
28.8
 
 
 
 
BTF3L4
 
 
hsa-miR-194-5p
 
 
-0.334
 
 
28.8
 
 
 
 
BTF3L4
 
 
hsa-miR-98-5p
 
 
-0.355
 
 
28.8
 
 
 
 
BTF3L4
 
 
hsa-let-7c-5p
 
 
-0.355
 
 
28.8
 
 
 
 
BTG3
 
 
hsa-miR-18a-5p
 
 
-0.464
 
 
18.4
 
 
 
 
BTG3
 
 
hsa-miR-17-5p
 
 
-0.668
 
 
18.4
 
 
 
 
BTG3
 
 
hsa-miR-20a-5p
 
 
-0.671
 
 
18.4
 
 
 
 
BZW1
 
 
hsa-miR-98-5p
 
 
-0.354
 
 
39.5
 
 
 
 
BZW1
 
 
hsa-let-7c-5p
 
 
-0.354
 
 
39.5
 
 
 
 
BZW1
 
 
hsa-miR-98-5p
 
 
-0.375
 
 
39.5
 
 
 
 
BZW1
 
 
hsa-let-7c-5p
 
 
-0.375
 
 
39.5
 
 
 
 
 
 
 pituitary gland 
 
 
 
 
target_symbol
 
 
mature_mirna_id
 
 
score
 
 
nTPM
 
 
 
 
 
 
ABCB9
 
 
hsa-let-7c-5p
 
 
-0.422
 
 
11.0
 
 
 
 
ABCB9
 
 
hsa-miR-98-5p
 
 
-0.444
 
 
11.0
 
 
 
 
ADD3
 
 
hsa-miR-200b-3p
 
 
-0.324
 
 
22.5
 
 
 
 
ADD3
 
 
hsa-miR-18a-5p
 
 
-0.39
 
 
22.5
 
 
 
 
ADNP
 
 
hsa-miR-328-3p
 
 
-0.371
 
 
26.6
 
 
 
 
AGFG2
 
 
hsa-miR-20a-5p
 
 
-0.365
 
 
16.7
 
 
 
 
AGFG2
 
 
hsa-miR-17-5p
 
 
-0.402
 
 
16.7
 
 
 
 
AHNAK
 
 
hsa-miR-20a-5p
 
 
-0.316
 
 
16.0
 
 
 
 
AHNAK
 
 
hsa-miR-17-5p
 
 
-0.337
 
 
16.0
 
 
 
 
AIFM1
 
 
hsa-miR-98-5p
 
 
-0.314
 
 
11.0
 
 
 
 
AIFM1
 
 
hsa-let-7c-5p
 
 
-0.314
 
 
11.0
 
 
 
 
AK1
 
 
hsa-miR-423-5p
 
 
-0.443
 
 
40.7
 
 
 
 
ALCAM
 
 
hsa-miR-18a-5p
 
 
-0.341
 
 
17.9
 
 
 
 
ALG3
 
 
hsa-miR-20a-5p
 
 
-0.559
 
 
14.3
 
 
 
 
ALG3
 
 
hsa-miR-17-5p
 
 
-0.572
 
 
14.3
 
 
 
 
AMT
 
 
hsa-miR-98-5p
 
 
-0.463
 
 
16.0
 
 
 
 
AMT
 
 
hsa-let-7c-5p
 
 
-0.463
 
 
16.0
 
 
 
 
ANKFY1
 
 
hsa-miR-155-5p
 
 
-0.315
 
 
11.8
 
 
 
 
ANP32B
 
 
hsa-miR-200b-3p
 
 
-0.287
 
 
64.1
 
 
 
 
AP1S1
 
 
hsa-miR-98-5p
 
 
-0.374
 
 
31.4
 
 
 
 
AP1S1
 
 
hsa-let-7c-5p
 
 
-0.374
 
 
31.4
 
 
 
 
AP1S2
 
 
hsa-miR-200b-3p
 
 
-0.4
 
 
18.0
 
 
 
 
AP2M1
 
 
hsa-miR-423-5p
 
 
-0.49
 
 
179.3
 
 
 
 
APBB3
 
 
hsa-miR-98-5p
 
 
-0.587
 
 
10.1
 
 
 
 
APBB3
 
 
hsa-let-7c-5p
 
 
-0.587
 
 
10.1
 
 
 
 
APOO
 
 
hsa-miR-200b-3p
 
 
-0.361
 
 
28.1
 
 
 
 
ARF4
 
 
hsa-miR-194-5p
 
 
-0.293
 
 
93.4
 
 
 
 
ARF4
 
 
hsa-miR-7-5p
 
 
-0.407
 
 
93.4
 
 
 
 
ARG2
 
 
hsa-miR-98-5p
 
 
-0.468
 
 
47.4
 
 
 
 
ARG2
 
 
hsa-let-7c-5p
 
 
-0.468
 
 
47.4
 
 
 
 
ARHGAP1
 
 
hsa-miR-17-5p
 
 
-0.325
 
 
35.2
 
 
 
 
ARHGAP1
 
 
hsa-miR-20a-5p
 
 
-0.339
 
 
35.2
 
 
 
 
ARID4B
 
 
hsa-miR-20a-5p
 
 
-0.29
 
 
10.3
 
 
 
 
ARL1
 
 
hsa-miR-20a-5p
 
 
-0.403
 
 
52.7
 
 
 
 
ARL1
 
 
hsa-miR-17-5p
 
 
-0.432
 
 
52.7
 
 
 
 
ARL4D
 
 
hsa-miR-98-5p
 
 
-0.417
 
 
20.8
 
 
 
 
ARL4D
 
 
hsa-let-7c-5p
 
 
-0.417
 
 
20.8
 
 
 
 
ARL6IP1
 
 
hsa-miR-7-5p
 
 
-0.374
 
 
39.7
 
 
 
 
ARL6IP1
 
 
hsa-miR-328-3p
 
 
-0.494
 
 
39.7
 
 
 
 
ARMC8
 
 
hsa-miR-20a-5p
 
 
-0.357
 
 
11.3
 
 
 
 
ARMC8
 
 
hsa-miR-17-5p
 
 
-0.378
 
 
11.3
 
 
 
 
ARPP19
 
 
hsa-miR-328-3p
 
 
-0.404
 
 
33.0
 
 
 
 
ARPP19
 
 
hsa-let-7c-5p
 
 
-0.492
 
 
33.0
 
 
 
 
ARPP19
 
 
hsa-miR-98-5p
 
 
-0.5
 
 
33.0
 
 
 
 
ASB6
 
 
hsa-miR-423-5p
 
 
-0.388
 
 
12.1
 
 
 
 
ATG13
 
 
hsa-miR-423-5p
 
 
-0.3
 
 
55.2
 
 
 
 
ATOSB
 
 
hsa-miR-328-3p
 
 
-0.358
 
 
15.4
 
 
 
 
ATOSB
 
 
hsa-miR-98-5p
 
 
-0.429
 
 
15.4
 
 
 
 
ATOSB
 
 
hsa-let-7c-5p
 
 
-0.429
 
 
15.4
 
 
 
 
ATP2A2
 
 
hsa-miR-98-5p
 
 
-0.311
 
 
13.7
 
 
 
 
ATP2A2
 
 
hsa-let-7c-5p
 
 
-0.311
 
 
13.7
 
 
 
 
ATP6V1F
 
 
hsa-miR-194-5p
 
 
-0.444
 
 
141.5
 
 
 
 
ATP6V1H
 
 
hsa-miR-194-5p
 
 
-0.652
 
 
32.4
 
 
 
 
ATP8B2
 
 
hsa-miR-423-5p
 
 
-0.297
 
 
18.1
 
 
 
 
ATPAF1
 
 
hsa-miR-98-5p
 
 
-0.498
 
 
31.1
 
 
 
 
ATPAF1
 
 
hsa-let-7c-5p
 
 
-0.498
 
 
31.1
 
 
 
 
AVPI1
 
 
hsa-miR-7-5p
 
 
-0.367
 
 
20.2
 
 
 
 
B3GAT3
 
 
hsa-miR-98-5p
 
 
-0.324
 
 
61.5
 
 
 
 
B3GAT3
 
 
hsa-let-7c-5p
 
 
-0.324
 
 
61.5
 
 
 
 
B4GALT5
 
 
hsa-miR-210-3p
 
 
-0.511
 
 
11.9
 
 
 
 
B4GAT1
 
 
hsa-miR-200b-3p
 
 
-0.382
 
 
60.5
 
 
 
 
B4GAT1
 
 
hsa-miR-98-5p
 
 
-0.47
 
 
60.5
 
 
 
 
B4GAT1
 
 
hsa-let-7c-5p
 
 
-0.47
 
 
60.5
 
 
 
 
BAG6
 
 
hsa-miR-200b-3p
 
 
-0.371
 
 
67.0
 
 
 
 
BAP1
 
 
hsa-miR-423-5p
 
 
-0.428
 
 
36.0
 
 
 
 
BEGAIN
 
 
hsa-let-7c-5p
 
 
-0.466
 
 
18.8
 
 
 
 
BEGAIN
 
 
hsa-miR-98-5p
 
 
-0.474
 
 
18.8
 
 
 
 
BIN1
 
 
hsa-miR-744-5p
 
 
-0.654
 
 
39.7
 
 
 
 
BIN3
 
 
hsa-miR-98-5p
 
 
-0.427
 
 
13.2
 
 
 
 
BIN3
 
 
hsa-let-7c-5p
 
 
-0.427
 
 
13.2
 
 
 
 
BLOC1S6
 
 
hsa-miR-98-5p
 
 
-0.321
 
 
19.0
 
 
 
 
BLOC1S6
 
 
hsa-let-7c-5p
 
 
-0.321
 
 
19.0
 
 
 
 
BNIP3L
 
 
hsa-miR-20a-5p
 
 
-0.287
 
 
23.7
 
 
 
 
BRWD1
 
 
hsa-miR-18a-5p
 
 
-0.29
 
 
11.9
 
 
 
 
BTBD10
 
 
hsa-miR-17-5p
 
 
-0.409
 
 
13.7
 
 
 
 
BTBD10
 
 
hsa-miR-20a-5p
 
 
-0.412
 
 
13.7
 
 
 
 
BTF3L4
 
 
hsa-miR-200b-3p
 
 
-0.294
 
 
28.3
 
 
 
 
BTF3L4
 
 
hsa-miR-194-5p
 
 
-0.334
 
 
28.3
 
 
 
 
BTF3L4
 
 
hsa-miR-98-5p
 
 
-0.355
 
 
28.3
 
 
 
 
BTF3L4
 
 
hsa-let-7c-5p
 
 
-0.355
 
 
28.3
 
 
 
 
BTG3
 
 
hsa-miR-18a-5p
 
 
-0.464
 
 
43.7
 
 
 
 
BTG3
 
 
hsa-miR-17-5p
 
 
-0.668
 
 
43.7
 
 
 
 
BTG3
 
 
hsa-miR-20a-5p
 
 
-0.671
 
 
43.7
 
 
 
 
BZW1
 
 
hsa-miR-98-5p
 
 
-0.354
 
 
28.1
 
 
 
 
BZW1
 
 
hsa-let-7c-5p
 
 
-0.354
 
 
28.1
 
 
 
 
BZW1
 
 
hsa-miR-98-5p
 
 
-0.375
 
 
28.1
 
 
 
 
BZW1
 
 
hsa-let-7c-5p
 
 
-0.375
 
 
28.1
 
 
 
 
BZW2
 
 
hsa-miR-98-5p
 
 
-0.397
 
 
10.9
 
 
 
 
BZW2
 
 
hsa-let-7c-5p
 
 
-0.397
 
 
10.9
 
 
 
 
C1QBP
 
 
hsa-miR-330-3p
 
 
-0.412
 
 
78.4
 
 
 
 
C3orf18
 
 
hsa-miR-155-5p
 
 
-0.431
 
 
12.0
 
 
 
 
CAB39
 
 
hsa-miR-155-5p
 
 
-0.312
 
 
16.0
 
 
 
 
CACFD1
 
 
hsa-let-7c-5p
 
 
-0.296
 
 
18.3
 
 
 
 
CALD1
 
 
hsa-miR-20a-5p
 
 
-0.352
 
 
49.0
 
 
 
 
CALD1
 
 
hsa-miR-17-5p
 
 
-0.389
 
 
49.0
 
 
 
 
CAMK2N1
 
 
hsa-miR-17-5p
 
 
-0.367
 
 
33.4
 
 
 
 
CAMK2N1
 
 
hsa-miR-20a-5p
 
 
-0.381
 
 
33.4
 
 
 
 
CAMK2N1
 
 
hsa-miR-18a-5p
 
 
-0.408
 
 
33.4
 
 
 
 
CAMK2N2
 
 
hsa-miR-20a-5p
 
 
-0.399
 
 
36.1
 
 
 
 
CAMK2N2
 
 
hsa-miR-17-5p
 
 
-0.42
 
 
36.1
 
 
 
 
 
 
 testis 
 
 
 
 
target_symbol
 
 
mature_mirna_id
 
 
score
 
 
nTPM
 
 
 
 
 
 
ABCB9
 
 
hsa-let-7c-5p
 
 
-0.422
 
 
17.1
 
 
 
 
ABCB9
 
 
hsa-miR-98-5p
 
 
-0.444
 
 
17.1
 
 
 
 
ACRV1
 
 
hsa-miR-7-5p
 
 
-0.3
 
 
110.6
 
 
 
 
ACSL6
 
 
hsa-miR-98-5p
 
 
-0.336
 
 
19.3
 
 
 
 
ACSL6
 
 
hsa-let-7c-5p
 
 
-0.336
 
 
19.3
 
 
 
 
ACTL7A
 
 
hsa-miR-155-5p
 
 
-0.531
 
 
218.7
 
 
 
 
ADAM9
 
 
hsa-miR-20a-5p
 
 
-0.299
 
 
10.4
 
 
 
 
ADAM9
 
 
hsa-miR-17-5p
 
 
-0.32
 
 
10.4
 
 
 
 
ADD3
 
 
hsa-miR-200b-3p
 
 
-0.324
 
 
48.1
 
 
 
 
ADD3
 
 
hsa-miR-18a-5p
 
 
-0.39
 
 
48.1
 
 
 
 
ADNP
 
 
hsa-miR-328-3p
 
 
-0.371
 
 
37.6
 
 
 
 
AEBP2
 
 
hsa-miR-18a-5p
 
 
-0.376
 
 
19.3
 
 
 
 
AGFG2
 
 
hsa-miR-20a-5p
 
 
-0.365
 
 
11.5
 
 
 
 
AGFG2
 
 
hsa-miR-17-5p
 
 
-0.402
 
 
11.5
 
 
 
 
AHCTF1
 
 
hsa-miR-98-5p
 
 
-0.374
 
 
30.4
 
 
 
 
AHCTF1
 
 
hsa-let-7c-5p
 
 
-0.374
 
 
30.4
 
 
 
 
AHNAK
 
 
hsa-miR-20a-5p
 
 
-0.316
 
 
16.9
 
 
 
 
AHNAK
 
 
hsa-miR-17-5p
 
 
-0.337
 
 
16.9
 
 
 
 
AIFM1
 
 
hsa-miR-98-5p
 
 
-0.314
 
 
21.3
 
 
 
 
AIFM1
 
 
hsa-let-7c-5p
 
 
-0.314
 
 
21.3
 
 
 
 
AK1
 
 
hsa-miR-423-5p
 
 
-0.443
 
 
64.3
 
 
 
 
ALG3
 
 
hsa-miR-20a-5p
 
 
-0.559
 
 
25.2
 
 
 
 
ALG3
 
 
hsa-miR-17-5p
 
 
-0.572
 
 
25.2
 
 
 
 
ALG9
 
 
hsa-miR-7-5p
 
 
-0.302
 
 
12.8
 
 
 
 
ANKRD12
 
 
hsa-miR-7-5p
 
 
-0.37
 
 
25.9
 
 
 
 
ANKRD50
 
 
hsa-miR-20a-5p
 
 
-0.3
 
 
14.1
 
 
 
 
ANKRD9
 
 
hsa-miR-20a-5p
 
 
-0.31
 
 
38.5
 
 
 
 
ANKRD9
 
 
hsa-miR-17-5p
 
 
-0.347
 
 
38.5
 
 
 
 
ANP32B
 
 
hsa-miR-200b-3p
 
 
-0.287
 
 
56.3
 
 
 
 
AP1S1
 
 
hsa-miR-98-5p
 
 
-0.374
 
 
17.8
 
 
 
 
AP1S1
 
 
hsa-let-7c-5p
 
 
-0.374
 
 
17.8
 
 
 
 
AP1S2
 
 
hsa-miR-200b-3p
 
 
-0.4
 
 
21.6
 
 
 
 
AP2M1
 
 
hsa-miR-423-5p
 
 
-0.49
 
 
92.6
 
 
 
 
AP5S1
 
 
hsa-miR-744-5p
 
 
-0.354
 
 
10.7
 
 
 
 
APOO
 
 
hsa-miR-200b-3p
 
 
-0.361
 
 
15.7
 
 
 
 
ARF4
 
 
hsa-miR-194-5p
 
 
-0.293
 
 
94.2
 
 
 
 
ARF4
 
 
hsa-miR-7-5p
 
 
-0.407
 
 
94.2
 
 
 
 
ARGLU1
 
 
hsa-miR-200b-3p
 
 
-0.29
 
 
10.0
 
 
 
 
ARHGAP1
 
 
hsa-miR-17-5p
 
 
-0.325
 
 
15.3
 
 
 
 
ARHGAP1
 
 
hsa-miR-20a-5p
 
 
-0.339
 
 
15.3
 
 
 
 
ARHGAP28
 
 
hsa-miR-98-5p
 
 
-0.47
 
 
72.3
 
 
 
 
ARHGAP28
 
 
hsa-let-7c-5p
 
 
-0.47
 
 
72.3
 
 
 
 
ARID2
 
 
hsa-miR-155-5p
 
 
-0.384
 
 
24.3
 
 
 
 
ARID3A
 
 
hsa-miR-98-5p
 
 
-0.34
 
 
16.1
 
 
 
 
ARID3A
 
 
hsa-let-7c-5p
 
 
-0.34
 
 
16.1
 
 
 
 
ARID3A
 
 
hsa-let-7c-5p
 
 
-0.347
 
 
16.1
 
 
 
 
ARID3A
 
 
hsa-miR-98-5p
 
 
-0.358
 
 
16.1
 
 
 
 
ARID3B
 
 
hsa-miR-98-5p
 
 
-0.31
 
 
21.2
 
 
 
 
ARID3B
 
 
hsa-let-7c-5p
 
 
-0.31
 
 
21.2
 
 
 
 
ARID3B
 
 
hsa-miR-98-5p
 
 
-0.475
 
 
21.2
 
 
 
 
ARID3B
 
 
hsa-let-7c-5p
 
 
-0.486
 
 
21.2
 
 
 
 
ARID4B
 
 
hsa-miR-20a-5p
 
 
-0.29
 
 
16.0
 
 
 
 
ARL1
 
 
hsa-miR-20a-5p
 
 
-0.403
 
 
30.6
 
 
 
 
ARL1
 
 
hsa-miR-17-5p
 
 
-0.432
 
 
30.6
 
 
 
 
ARL4D
 
 
hsa-miR-98-5p
 
 
-0.417
 
 
31.4
 
 
 
 
ARL4D
 
 
hsa-let-7c-5p
 
 
-0.417
 
 
31.4
 
 
 
 
ARL6IP1
 
 
hsa-miR-7-5p
 
 
-0.374
 
 
121.4
 
 
 
 
ARL6IP1
 
 
hsa-miR-328-3p
 
 
-0.494
 
 
121.4
 
 
 
 
ARL6IP6
 
 
hsa-miR-98-5p
 
 
-0.293
 
 
10.2
 
 
 
 
ARL6IP6
 
 
hsa-let-7c-5p
 
 
-0.293
 
 
10.2
 
 
 
 
ARMC8
 
 
hsa-miR-20a-5p
 
 
-0.357
 
 
17.2
 
 
 
 
ARMC8
 
 
hsa-miR-17-5p
 
 
-0.378
 
 
17.2
 
 
 
 
ARMT1
 
 
hsa-miR-98-5p
 
 
-0.303
 
 
13.1
 
 
 
 
ARMT1
 
 
hsa-let-7c-5p
 
 
-0.303
 
 
13.1
 
 
 
 
ARPP19
 
 
hsa-miR-328-3p
 
 
-0.404
 
 
79.0
 
 
 
 
ARPP19
 
 
hsa-let-7c-5p
 
 
-0.492
 
 
79.0
 
 
 
 
ARPP19
 
 
hsa-miR-98-5p
 
 
-0.5
 
 
79.0
 
 
 
 
ARRDC4
 
 
hsa-miR-98-5p
 
 
-0.329
 
 
13.7
 
 
 
 
ARRDC4
 
 
hsa-let-7c-5p
 
 
-0.329
 
 
13.7
 
 
 
 
ASAP1
 
 
hsa-miR-98-5p
 
 
-0.308
 
 
26.5
 
 
 
 
ASAP1
 
 
hsa-let-7c-5p
 
 
-0.308
 
 
26.5
 
 
 
 
ASB6
 
 
hsa-miR-423-5p
 
 
-0.388
 
 
12.3
 
 
 
 
ATAD2
 
 
hsa-miR-17-5p
 
 
-0.304
 
 
17.7
 
 
 
 
ATAD2
 
 
hsa-miR-20a-5p
 
 
-0.318
 
 
17.7
 
 
 
 
ATG13
 
 
hsa-miR-423-5p
 
 
-0.3
 
 
75.0
 
 
 
 
ATG16L1
 
 
hsa-miR-20a-5p
 
 
-0.446
 
 
13.1
 
 
 
 
ATG16L1
 
 
hsa-miR-17-5p
 
 
-0.467
 
 
13.1
 
 
 
 
ATP2A2
 
 
hsa-miR-98-5p
 
 
-0.311
 
 
69.3
 
 
 
 
ATP2A2
 
 
hsa-let-7c-5p
 
 
-0.311
 
 
69.3
 
 
 
 
ATP6V1F
 
 
hsa-miR-194-5p
 
 
-0.444
 
 
205.0
 
 
 
 
ATP6V1H
 
 
hsa-miR-194-5p
 
 
-0.652
 
 
17.3
 
 
 
 
ATP8B2
 
 
hsa-miR-423-5p
 
 
-0.297
 
 
16.0
 
 
 
 
ATPAF1
 
 
hsa-miR-98-5p
 
 
-0.498
 
 
29.2
 
 
 
 
ATPAF1
 
 
hsa-let-7c-5p
 
 
-0.498
 
 
29.2
 
 
 
 
ATXN1L
 
 
hsa-miR-155-5p
 
 
-0.315
 
 
11.4
 
 
 
 
AVPI1
 
 
hsa-miR-7-5p
 
 
-0.367
 
 
16.0
 
 
 
 
B3GAT3
 
 
hsa-miR-98-5p
 
 
-0.324
 
 
26.6
 
 
 
 
B3GAT3
 
 
hsa-let-7c-5p
 
 
-0.324
 
 
26.6
 
 
 
 
B4GALT5
 
 
hsa-miR-210-3p
 
 
-0.511
 
 
20.8
 
 
 
 
BACH1
 
 
hsa-miR-98-5p
 
 
-0.298
 
 
25.4
 
 
 
 
BACH1
 
 
hsa-miR-98-5p
 
 
-0.298
 
 
25.4
 
 
 
 
BACH1
 
 
hsa-let-7c-5p
 
 
-0.306
 
 
25.4
 
 
 
 
BACH1
 
 
hsa-let-7c-5p
 
 
-0.306
 
 
25.4
 
 
 
 
BACH1
 
 
hsa-miR-98-5p
 
 
-0.435
 
 
25.4
 
 
 
 
BACH1
 
 
hsa-miR-98-5p
 
 
-0.435
 
 
25.4
 
 
 
 
BACH1
 
 
hsa-let-7c-5p
 
 
-0.435
 
 
25.4
 
 
 
 
BACH1
 
 
hsa-let-7c-5p
 
 
-0.435
 
 
25.4
 
 
 
 
BAG6
 
 
hsa-miR-200b-3p
 
 
-0.371
 
 
144.0
 
 
 
 
BAP1
 
 
hsa-miR-423-5p
 
 
-0.428
 
 
133.3
 
 
 
 
BBX
 
 
hsa-miR-18a-5p
 
 
-0.31
 
 
54.2
 
 
 
 
 
 
 amygdala 
 
 
 
 
target_symbol
 
 
mature_mirna_id
 
 
score
 
 
nTPM
 
 
 
 
 
 
ABHD17C
 
 
hsa-let-7c-5p
 
 
-0.359
 
 
17.9
 
 
 
 
ABHD17C
 
 
hsa-miR-98-5p
 
 
-0.383
 
 
17.9
 
 
 
 
ABHD17C
 
 
hsa-miR-330-3p
 
 
-0.399
 
 
17.9
 
 
 
 
ACHE
 
 
hsa-miR-194-5p
 
 
-0.395
 
 
14.7
 
 
 
 
ACSL6
 
 
hsa-miR-98-5p
 
 
-0.336
 
 
20.3
 
 
 
 
ACSL6
 
 
hsa-let-7c-5p
 
 
-0.336
 
 
20.3
 
 
 
 
ADD3
 
 
hsa-miR-200b-3p
 
 
-0.324
 
 
107.7
 
 
 
 
ADD3
 
 
hsa-miR-18a-5p
 
 
-0.39
 
 
107.7
 
 
 
 
ADNP
 
 
hsa-miR-328-3p
 
 
-0.371
 
 
23.8
 
 
 
 
AGFG2
 
 
hsa-miR-20a-5p
 
 
-0.365
 
 
13.3
 
 
 
 
AGFG2
 
 
hsa-miR-17-5p
 
 
-0.402
 
 
13.3
 
 
 
 
AHNAK
 
 
hsa-miR-20a-5p
 
 
-0.316
 
 
31.4
 
 
 
 
AHNAK
 
 
hsa-miR-17-5p
 
 
-0.337
 
 
31.4
 
 
 
 
AIFM1
 
 
hsa-miR-98-5p
 
 
-0.314
 
 
10.3
 
 
 
 
AIFM1
 
 
hsa-let-7c-5p
 
 
-0.314
 
 
10.3
 
 
 
 
AK1
 
 
hsa-miR-423-5p
 
 
-0.443
 
 
104.5
 
 
 
 
AKAP6
 
 
hsa-miR-98-5p
 
 
-0.307
 
 
12.4
 
 
 
 
AKAP6
 
 
hsa-let-7c-5p
 
 
-0.307
 
 
12.4
 
 
 
 
ALCAM
 
 
hsa-miR-18a-5p
 
 
-0.341
 
 
31.7
 
 
 
 
ALDH4A1
 
 
hsa-miR-184
 
 
-0.421
 
 
66.7
 
 
 
 
ANGEL2
 
 
hsa-miR-200b-3p
 
 
-0.38
 
 
10.6
 
 
 
 
ANKFY1
 
 
hsa-miR-155-5p
 
 
-0.315
 
 
10.9
 
 
 
 
ANKRD46
 
 
hsa-miR-98-5p
 
 
-0.389
 
 
28.3
 
 
 
 
ANKRD46
 
 
hsa-let-7c-5p
 
 
-0.389
 
 
28.3
 
 
 
 
ANKRD9
 
 
hsa-miR-20a-5p
 
 
-0.31
 
 
26.5
 
 
 
 
ANKRD9
 
 
hsa-miR-17-5p
 
 
-0.347
 
 
26.5
 
 
 
 
ANP32B
 
 
hsa-miR-200b-3p
 
 
-0.287
 
 
123.1
 
 
 
 
AP1S1
 
 
hsa-miR-98-5p
 
 
-0.374
 
 
88.4
 
 
 
 
AP1S1
 
 
hsa-let-7c-5p
 
 
-0.374
 
 
88.4
 
 
 
 
AP1S2
 
 
hsa-miR-200b-3p
 
 
-0.4
 
 
38.7
 
 
 
 
AP2M1
 
 
hsa-miR-423-5p
 
 
-0.49
 
 
223.5
 
 
 
 
APCDD1
 
 
hsa-miR-20a-5p
 
 
-0.404
 
 
28.8
 
 
 
 
APCDD1
 
 
hsa-miR-17-5p
 
 
-0.425
 
 
28.8
 
 
 
 
APOO
 
 
hsa-miR-200b-3p
 
 
-0.361
 
 
45.1
 
 
 
 
ARF4
 
 
hsa-miR-194-5p
 
 
-0.293
 
 
60.1
 
 
 
 
ARF4
 
 
hsa-miR-7-5p
 
 
-0.407
 
 
60.1
 
 
 
 
ARGLU1
 
 
hsa-miR-200b-3p
 
 
-0.29
 
 
14.8
 
 
 
 
ARHGAP1
 
 
hsa-miR-17-5p
 
 
-0.325
 
 
37.0
 
 
 
 
ARHGAP1
 
 
hsa-miR-20a-5p
 
 
-0.339
 
 
37.0
 
 
 
 
ARL1
 
 
hsa-miR-20a-5p
 
 
-0.403
 
 
24.9
 
 
 
 
ARL1
 
 
hsa-miR-17-5p
 
 
-0.432
 
 
24.9
 
 
 
 
ARL6IP1
 
 
hsa-miR-7-5p
 
 
-0.374
 
 
241.3
 
 
 
 
ARL6IP1
 
 
hsa-miR-328-3p
 
 
-0.494
 
 
241.3
 
 
 
 
ARMC8
 
 
hsa-miR-20a-5p
 
 
-0.357
 
 
13.4
 
 
 
 
ARMC8
 
 
hsa-miR-17-5p
 
 
-0.378
 
 
13.4
 
 
 
 
ARPP19
 
 
hsa-miR-328-3p
 
 
-0.404
 
 
178.5
 
 
 
 
ARPP19
 
 
hsa-let-7c-5p
 
 
-0.492
 
 
178.5
 
 
 
 
ARPP19
 
 
hsa-miR-98-5p
 
 
-0.5
 
 
178.5
 
 
 
 
ARRDC4
 
 
hsa-miR-98-5p
 
 
-0.329
 
 
13.2
 
 
 
 
ARRDC4
 
 
hsa-let-7c-5p
 
 
-0.329
 
 
13.2
 
 
 
 
ASAP1
 
 
hsa-miR-98-5p
 
 
-0.308
 
 
11.2
 
 
 
 
ASAP1
 
 
hsa-let-7c-5p
 
 
-0.308
 
 
11.2
 
 
 
 
ASB6
 
 
hsa-miR-423-5p
 
 
-0.388
 
 
13.1
 
 
 
 
ASF1A
 
 
hsa-miR-200b-3p
 
 
-0.377
 
 
14.0
 
 
 
 
ATG13
 
 
hsa-miR-423-5p
 
 
-0.3
 
 
33.9
 
 
 
 
ATOSB
 
 
hsa-miR-328-3p
 
 
-0.358
 
 
20.1
 
 
 
 
ATOSB
 
 
hsa-miR-98-5p
 
 
-0.429
 
 
20.1
 
 
 
 
ATOSB
 
 
hsa-let-7c-5p
 
 
-0.429
 
 
20.1
 
 
 
 
ATP2A2
 
 
hsa-miR-98-5p
 
 
-0.311
 
 
15.8
 
 
 
 
ATP2A2
 
 
hsa-let-7c-5p
 
 
-0.311
 
 
15.8
 
 
 
 
ATP2B2
 
 
hsa-miR-7-5p
 
 
-0.297
 
 
14.3
 
 
 
 
ATP6V1F
 
 
hsa-miR-194-5p
 
 
-0.444
 
 
311.8
 
 
 
 
ATP6V1H
 
 
hsa-miR-194-5p
 
 
-0.652
 
 
66.9
 
 
 
 
ATPAF1
 
 
hsa-miR-98-5p
 
 
-0.498
 
 
86.8
 
 
 
 
ATPAF1
 
 
hsa-let-7c-5p
 
 
-0.498
 
 
86.8
 
 
 
 
AVPI1
 
 
hsa-miR-7-5p
 
 
-0.367
 
 
13.3
 
 
 
 
B3GAT3
 
 
hsa-miR-98-5p
 
 
-0.324
 
 
61.0
 
 
 
 
B3GAT3
 
 
hsa-let-7c-5p
 
 
-0.324
 
 
61.0
 
 
 
 
B4GALT5
 
 
hsa-miR-210-3p
 
 
-0.511
 
 
10.8
 
 
 
 
B4GAT1
 
 
hsa-miR-200b-3p
 
 
-0.382
 
 
107.6
 
 
 
 
B4GAT1
 
 
hsa-miR-98-5p
 
 
-0.47
 
 
107.6
 
 
 
 
B4GAT1
 
 
hsa-let-7c-5p
 
 
-0.47
 
 
107.6
 
 
 
 
BAG6
 
 
hsa-miR-200b-3p
 
 
-0.371
 
 
45.3
 
 
 
 
BAMBI
 
 
hsa-miR-20a-5p
 
 
-0.485
 
 
14.6
 
 
 
 
BAMBI
 
 
hsa-miR-17-5p
 
 
-0.506
 
 
14.6
 
 
 
 
BAP1
 
 
hsa-miR-423-5p
 
 
-0.428
 
 
118.3
 
 
 
 
BEGAIN
 
 
hsa-let-7c-5p
 
 
-0.466
 
 
36.9
 
 
 
 
BEGAIN
 
 
hsa-miR-98-5p
 
 
-0.474
 
 
36.9
 
 
 
 
BHLHE22
 
 
hsa-miR-18a-5p
 
 
-0.325
 
 
14.4
 
 
 
 
BIN1
 
 
hsa-miR-744-5p
 
 
-0.654
 
 
167.4
 
 
 
 
BIN3
 
 
hsa-miR-98-5p
 
 
-0.427
 
 
10.6
 
 
 
 
BIN3
 
 
hsa-let-7c-5p
 
 
-0.427
 
 
10.6
 
 
 
 
BLOC1S6
 
 
hsa-miR-98-5p
 
 
-0.321
 
 
20.1
 
 
 
 
BLOC1S6
 
 
hsa-let-7c-5p
 
 
-0.321
 
 
20.1
 
 
 
 
BNIP3L
 
 
hsa-miR-20a-5p
 
 
-0.287
 
 
49.1
 
 
 
 
BRMS1L
 
 
hsa-miR-20a-5p
 
 
-0.326
 
 
10.3
 
 
 
 
BRMS1L
 
 
hsa-miR-194-5p
 
 
-0.333
 
 
10.3
 
 
 
 
BRMS1L
 
 
hsa-miR-17-5p
 
 
-0.347
 
 
10.3
 
 
 
 
BRWD1
 
 
hsa-miR-18a-5p
 
 
-0.29
 
 
39.8
 
 
 
 
BTBD10
 
 
hsa-miR-17-5p
 
 
-0.409
 
 
17.2
 
 
 
 
BTBD10
 
 
hsa-miR-20a-5p
 
 
-0.412
 
 
17.2
 
 
 
 
BTF3L4
 
 
hsa-miR-200b-3p
 
 
-0.294
 
 
29.6
 
 
 
 
BTF3L4
 
 
hsa-miR-194-5p
 
 
-0.334
 
 
29.6
 
 
 
 
BTF3L4
 
 
hsa-miR-98-5p
 
 
-0.355
 
 
29.6
 
 
 
 
BTF3L4
 
 
hsa-let-7c-5p
 
 
-0.355
 
 
29.6
 
 
 
 
BTG3
 
 
hsa-miR-18a-5p
 
 
-0.464
 
 
17.3
 
 
 
 
BTG3
 
 
hsa-miR-17-5p
 
 
-0.668
 
 
17.3
 
 
 
 
BTG3
 
 
hsa-miR-20a-5p
 
 
-0.671
 
 
17.3
 
 
 
 
BZW1
 
 
hsa-miR-98-5p
 
 
-0.354
 
 
34.3
 
 
 
 
BZW1
 
 
hsa-let-7c-5p
 
 
-0.354
 
 
34.3
 
 
 
 
 
 
 breast 
 
 
 
 
target_symbol
 
 
mature_mirna_id
 
 
score
 
 
nTPM
 
 
 
 
 
 
ABHD17C
 
 
hsa-let-7c-5p
 
 
-0.359
 
 
10.9
 
 
 
 
ABHD17C
 
 
hsa-miR-98-5p
 
 
-0.383
 
 
10.9
 
 
 
 
ABHD17C
 
 
hsa-miR-330-3p
 
 
-0.399
 
 
10.9
 
 
 
 
ABHD5
 
 
hsa-miR-20a-5p
 
 
-0.397
 
 
18.8
 
 
 
 
ABHD5
 
 
hsa-miR-17-5p
 
 
-0.45
 
 
18.8
 
 
 
 
ACTA1
 
 
hsa-miR-155-5p
 
 
-0.595
 
 
15.5
 
 
 
 
ACTC1
 
 
hsa-miR-7-5p
 
 
-0.471
 
 
11.2
 
 
 
 
ADAM9
 
 
hsa-miR-20a-5p
 
 
-0.299
 
 
20.2
 
 
 
 
ADAM9
 
 
hsa-miR-17-5p
 
 
-0.32
 
 
20.2
 
 
 
 
ADD3
 
 
hsa-miR-200b-3p
 
 
-0.324
 
 
83.9
 
 
 
 
ADD3
 
 
hsa-miR-18a-5p
 
 
-0.39
 
 
83.9
 
 
 
 
ADNP
 
 
hsa-miR-328-3p
 
 
-0.371
 
 
30.5
 
 
 
 
ADRB2
 
 
hsa-miR-98-5p
 
 
-0.635
 
 
32.4
 
 
 
 
ADRB2
 
 
hsa-let-7c-5p
 
 
-0.635
 
 
32.4
 
 
 
 
AEBP2
 
 
hsa-miR-18a-5p
 
 
-0.376
 
 
21.9
 
 
 
 
AGFG2
 
 
hsa-miR-20a-5p
 
 
-0.365
 
 
10.5
 
 
 
 
AGFG2
 
 
hsa-miR-17-5p
 
 
-0.402
 
 
10.5
 
 
 
 
AHNAK
 
 
hsa-miR-20a-5p
 
 
-0.316
 
 
178.3
 
 
 
 
AHNAK
 
 
hsa-miR-17-5p
 
 
-0.337
 
 
178.3
 
 
 
 
AIFM1
 
 
hsa-miR-98-5p
 
 
-0.314
 
 
11.7
 
 
 
 
AIFM1
 
 
hsa-let-7c-5p
 
 
-0.314
 
 
11.7
 
 
 
 
AK1
 
 
hsa-miR-423-5p
 
 
-0.443
 
 
20.7
 
 
 
 
ALCAM
 
 
hsa-miR-18a-5p
 
 
-0.341
 
 
76.0
 
 
 
 
ALDH4A1
 
 
hsa-miR-184
 
 
-0.421
 
 
22.5
 
 
 
 
ALG3
 
 
hsa-miR-20a-5p
 
 
-0.559
 
 
18.6
 
 
 
 
ALG3
 
 
hsa-miR-17-5p
 
 
-0.572
 
 
18.6
 
 
 
 
ALG9
 
 
hsa-miR-7-5p
 
 
-0.302
 
 
11.3
 
 
 
 
ANGPTL2
 
 
hsa-miR-423-5p
 
 
-0.379
 
 
69.5
 
 
 
 
ANKFY1
 
 
hsa-miR-155-5p
 
 
-0.315
 
 
11.8
 
 
 
 
ANKRD12
 
 
hsa-miR-7-5p
 
 
-0.37
 
 
12.8
 
 
 
 
ANKRD29
 
 
hsa-miR-20a-5p
 
 
-0.359
 
 
15.0
 
 
 
 
ANKRD29
 
 
hsa-miR-17-5p
 
 
-0.372
 
 
15.0
 
 
 
 
ANKRD46
 
 
hsa-miR-98-5p
 
 
-0.389
 
 
13.6
 
 
 
 
ANKRD46
 
 
hsa-let-7c-5p
 
 
-0.389
 
 
13.6
 
 
 
 
ANP32B
 
 
hsa-miR-200b-3p
 
 
-0.287
 
 
132.4
 
 
 
 
AP1S1
 
 
hsa-miR-98-5p
 
 
-0.374
 
 
17.7
 
 
 
 
AP1S1
 
 
hsa-let-7c-5p
 
 
-0.374
 
 
17.7
 
 
 
 
AP1S2
 
 
hsa-miR-200b-3p
 
 
-0.4
 
 
13.6
 
 
 
 
AP2M1
 
 
hsa-miR-423-5p
 
 
-0.49
 
 
142.3
 
 
 
 
APCDD1
 
 
hsa-miR-20a-5p
 
 
-0.404
 
 
30.9
 
 
 
 
APCDD1
 
 
hsa-miR-17-5p
 
 
-0.425
 
 
30.9
 
 
 
 
APOO
 
 
hsa-miR-200b-3p
 
 
-0.361
 
 
12.5
 
 
 
 
ARF4
 
 
hsa-miR-194-5p
 
 
-0.293
 
 
129.6
 
 
 
 
ARF4
 
 
hsa-miR-7-5p
 
 
-0.407
 
 
129.6
 
 
 
 
ARHGAP1
 
 
hsa-miR-17-5p
 
 
-0.325
 
 
59.0
 
 
 
 
ARHGAP1
 
 
hsa-miR-20a-5p
 
 
-0.339
 
 
59.0
 
 
 
 
ARID4B
 
 
hsa-miR-20a-5p
 
 
-0.29
 
 
12.7
 
 
 
 
ARL1
 
 
hsa-miR-20a-5p
 
 
-0.403
 
 
58.5
 
 
 
 
ARL1
 
 
hsa-miR-17-5p
 
 
-0.432
 
 
58.5
 
 
 
 
ARL6IP1
 
 
hsa-miR-7-5p
 
 
-0.374
 
 
105.7
 
 
 
 
ARL6IP1
 
 
hsa-miR-328-3p
 
 
-0.494
 
 
105.7
 
 
 
 
ARL6IP6
 
 
hsa-miR-98-5p
 
 
-0.293
 
 
12.8
 
 
 
 
ARL6IP6
 
 
hsa-let-7c-5p
 
 
-0.293
 
 
12.8
 
 
 
 
ARMC8
 
 
hsa-miR-20a-5p
 
 
-0.357
 
 
15.4
 
 
 
 
ARMC8
 
 
hsa-miR-17-5p
 
 
-0.378
 
 
15.4
 
 
 
 
ARMT1
 
 
hsa-miR-98-5p
 
 
-0.303
 
 
23.4
 
 
 
 
ARMT1
 
 
hsa-let-7c-5p
 
 
-0.303
 
 
23.4
 
 
 
 
ARPP19
 
 
hsa-miR-328-3p
 
 
-0.404
 
 
43.2
 
 
 
 
ARPP19
 
 
hsa-let-7c-5p
 
 
-0.492
 
 
43.2
 
 
 
 
ARPP19
 
 
hsa-miR-98-5p
 
 
-0.5
 
 
43.2
 
 
 
 
ARRDC4
 
 
hsa-miR-98-5p
 
 
-0.329
 
 
18.6
 
 
 
 
ARRDC4
 
 
hsa-let-7c-5p
 
 
-0.329
 
 
18.6
 
 
 
 
ASAP1
 
 
hsa-miR-98-5p
 
 
-0.308
 
 
22.2
 
 
 
 
ASAP1
 
 
hsa-let-7c-5p
 
 
-0.308
 
 
22.2
 
 
 
 
ASF1A
 
 
hsa-miR-200b-3p
 
 
-0.377
 
 
11.7
 
 
 
 
ASPRV1
 
 
hsa-miR-330-3p
 
 
-0.301
 
 
23.5
 
 
 
 
ATG13
 
 
hsa-miR-423-5p
 
 
-0.3
 
 
28.7
 
 
 
 
ATOSB
 
 
hsa-miR-328-3p
 
 
-0.358
 
 
10.1
 
 
 
 
ATOSB
 
 
hsa-miR-98-5p
 
 
-0.429
 
 
10.1
 
 
 
 
ATOSB
 
 
hsa-let-7c-5p
 
 
-0.429
 
 
10.1
 
 
 
 
ATP2A2
 
 
hsa-miR-98-5p
 
 
-0.311
 
 
28.6
 
 
 
 
ATP2A2
 
 
hsa-let-7c-5p
 
 
-0.311
 
 
28.6
 
 
 
 
ATP6V1F
 
 
hsa-miR-194-5p
 
 
-0.444
 
 
123.3
 
 
 
 
ATP6V1H
 
 
hsa-miR-194-5p
 
 
-0.652
 
 
24.3
 
 
 
 
ATP8B2
 
 
hsa-miR-423-5p
 
 
-0.297
 
 
13.1
 
 
 
 
ATPAF1
 
 
hsa-miR-98-5p
 
 
-0.498
 
 
33.1
 
 
 
 
ATPAF1
 
 
hsa-let-7c-5p
 
 
-0.498
 
 
33.1
 
 
 
 
ATXN1L
 
 
hsa-miR-155-5p
 
 
-0.315
 
 
10.2
 
 
 
 
AVPI1
 
 
hsa-miR-7-5p
 
 
-0.367
 
 
22.2
 
 
 
 
B3GAT3
 
 
hsa-miR-98-5p
 
 
-0.324
 
 
29.8
 
 
 
 
B3GAT3
 
 
hsa-let-7c-5p
 
 
-0.324
 
 
29.8
 
 
 
 
B4GALT5
 
 
hsa-miR-210-3p
 
 
-0.511
 
 
17.9
 
 
 
 
B4GAT1
 
 
hsa-miR-200b-3p
 
 
-0.382
 
 
15.6
 
 
 
 
B4GAT1
 
 
hsa-miR-98-5p
 
 
-0.47
 
 
15.6
 
 
 
 
B4GAT1
 
 
hsa-let-7c-5p
 
 
-0.47
 
 
15.6
 
 
 
 
BACH1
 
 
hsa-miR-98-5p
 
 
-0.298
 
 
12.3
 
 
 
 
BACH1
 
 
hsa-miR-98-5p
 
 
-0.298
 
 
12.3
 
 
 
 
BACH1
 
 
hsa-let-7c-5p
 
 
-0.306
 
 
12.3
 
 
 
 
BACH1
 
 
hsa-let-7c-5p
 
 
-0.306
 
 
12.3
 
 
 
 
BACH1
 
 
hsa-miR-98-5p
 
 
-0.435
 
 
12.3
 
 
 
 
BACH1
 
 
hsa-miR-98-5p
 
 
-0.435
 
 
12.3
 
 
 
 
BACH1
 
 
hsa-let-7c-5p
 
 
-0.435
 
 
12.3
 
 
 
 
BACH1
 
 
hsa-let-7c-5p
 
 
-0.435
 
 
12.3
 
 
 
 
BAG6
 
 
hsa-miR-200b-3p
 
 
-0.371
 
 
67.8
 
 
 
 
BAMBI
 
 
hsa-miR-20a-5p
 
 
-0.485
 
 
22.3
 
 
 
 
BAMBI
 
 
hsa-miR-17-5p
 
 
-0.506
 
 
22.3
 
 
 
 
BAP1
 
 
hsa-miR-423-5p
 
 
-0.428
 
 
40.9
 
 
 
 
BBX
 
 
hsa-miR-18a-5p
 
 
-0.31
 
 
13.5
 
 
 
 
BIN1
 
 
hsa-miR-744-5p
 
 
-0.654
 
 
35.8
 
 
 
 
BIN3
 
 
hsa-miR-98-5p
 
 
-0.427
 
 
19.3
 
 
 
 
 
 
 cervix 
 
 
 
 
target_symbol
 
 
mature_mirna_id
 
 
score
 
 
nTPM
 
 
 
 
 
 
ABHD17C
 
 
hsa-let-7c-5p
 
 
-0.359
 
 
18.1
 
 
 
 
ABHD17C
 
 
hsa-miR-98-5p
 
 
-0.383
 
 
18.1
 
 
 
 
ABHD17C
 
 
hsa-miR-330-3p
 
 
-0.399
 
 
18.1
 
 
 
 
ACP3
 
 
hsa-miR-98-5p
 
 
-0.298
 
 
10.9
 
 
 
 
ACP3
 
 
hsa-let-7c-5p
 
 
-0.298
 
 
10.9
 
 
 
 
ACTC1
 
 
hsa-miR-7-5p
 
 
-0.471
 
 
44.2
 
 
 
 
ADAM9
 
 
hsa-miR-20a-5p
 
 
-0.299
 
 
32.7
 
 
 
 
ADAM9
 
 
hsa-miR-17-5p
 
 
-0.32
 
 
32.7
 
 
 
 
ADD3
 
 
hsa-miR-200b-3p
 
 
-0.324
 
 
54.2
 
 
 
 
ADD3
 
 
hsa-miR-18a-5p
 
 
-0.39
 
 
54.2
 
 
 
 
ADNP
 
 
hsa-miR-328-3p
 
 
-0.371
 
 
35.1
 
 
 
 
ADRB2
 
 
hsa-miR-98-5p
 
 
-0.635
 
 
12.7
 
 
 
 
ADRB2
 
 
hsa-let-7c-5p
 
 
-0.635
 
 
12.7
 
 
 
 
AEBP2
 
 
hsa-miR-18a-5p
 
 
-0.376
 
 
24.2
 
 
 
 
AGFG2
 
 
hsa-miR-20a-5p
 
 
-0.365
 
 
11.6
 
 
 
 
AGFG2
 
 
hsa-miR-17-5p
 
 
-0.402
 
 
11.6
 
 
 
 
AHNAK
 
 
hsa-miR-20a-5p
 
 
-0.316
 
 
181.1
 
 
 
 
AHNAK
 
 
hsa-miR-17-5p
 
 
-0.337
 
 
181.1
 
 
 
 
AIFM1
 
 
hsa-miR-98-5p
 
 
-0.314
 
 
14.8
 
 
 
 
AIFM1
 
 
hsa-let-7c-5p
 
 
-0.314
 
 
14.8
 
 
 
 
AK1
 
 
hsa-miR-423-5p
 
 
-0.443
 
 
35.3
 
 
 
 
ALCAM
 
 
hsa-miR-18a-5p
 
 
-0.341
 
 
26.3
 
 
 
 
ALDH4A1
 
 
hsa-miR-184
 
 
-0.421
 
 
15.0
 
 
 
 
ALG3
 
 
hsa-miR-20a-5p
 
 
-0.559
 
 
18.1
 
 
 
 
ALG3
 
 
hsa-miR-17-5p
 
 
-0.572
 
 
18.1
 
 
 
 
ALG9
 
 
hsa-miR-7-5p
 
 
-0.302
 
 
10.8
 
 
 
 
AMT
 
 
hsa-miR-98-5p
 
 
-0.463
 
 
14.5
 
 
 
 
AMT
 
 
hsa-let-7c-5p
 
 
-0.463
 
 
14.5
 
 
 
 
ANGPTL2
 
 
hsa-miR-423-5p
 
 
-0.379
 
 
46.6
 
 
 
 
ANKFY1
 
 
hsa-miR-155-5p
 
 
-0.315
 
 
13.8
 
 
 
 
ANKRD12
 
 
hsa-miR-7-5p
 
 
-0.37
 
 
10.5
 
 
 
 
ANKRD29
 
 
hsa-miR-20a-5p
 
 
-0.359
 
 
12.2
 
 
 
 
ANKRD29
 
 
hsa-miR-17-5p
 
 
-0.372
 
 
12.2
 
 
 
 
ANKRD46
 
 
hsa-miR-98-5p
 
 
-0.389
 
 
14.1
 
 
 
 
ANKRD46
 
 
hsa-let-7c-5p
 
 
-0.389
 
 
14.1
 
 
 
 
ANKRD50
 
 
hsa-miR-20a-5p
 
 
-0.3
 
 
10.7
 
 
 
 
ANKRD9
 
 
hsa-miR-20a-5p
 
 
-0.31
 
 
10.4
 
 
 
 
ANKRD9
 
 
hsa-miR-17-5p
 
 
-0.347
 
 
10.4
 
 
 
 
ANP32B
 
 
hsa-miR-200b-3p
 
 
-0.287
 
 
143.2
 
 
 
 
AP1S1
 
 
hsa-miR-98-5p
 
 
-0.374
 
 
20.1
 
 
 
 
AP1S1
 
 
hsa-let-7c-5p
 
 
-0.374
 
 
20.1
 
 
 
 
AP1S2
 
 
hsa-miR-200b-3p
 
 
-0.4
 
 
29.6
 
 
 
 
AP2M1
 
 
hsa-miR-423-5p
 
 
-0.49
 
 
174.1
 
 
 
 
APCDD1
 
 
hsa-miR-20a-5p
 
 
-0.404
 
 
33.1
 
 
 
 
APCDD1
 
 
hsa-miR-17-5p
 
 
-0.425
 
 
33.1
 
 
 
 
APOO
 
 
hsa-miR-200b-3p
 
 
-0.361
 
 
12.3
 
 
 
 
ARF4
 
 
hsa-miR-194-5p
 
 
-0.293
 
 
196.1
 
 
 
 
ARF4
 
 
hsa-miR-7-5p
 
 
-0.407
 
 
196.1
 
 
 
 
ARG2
 
 
hsa-miR-98-5p
 
 
-0.468
 
 
16.0
 
 
 
 
ARG2
 
 
hsa-let-7c-5p
 
 
-0.468
 
 
16.0
 
 
 
 
ARHGAP1
 
 
hsa-miR-17-5p
 
 
-0.325
 
 
62.9
 
 
 
 
ARHGAP1
 
 
hsa-miR-20a-5p
 
 
-0.339
 
 
62.9
 
 
 
 
ARID4B
 
 
hsa-miR-20a-5p
 
 
-0.29
 
 
14.1
 
 
 
 
ARL1
 
 
hsa-miR-20a-5p
 
 
-0.403
 
 
81.2
 
 
 
 
ARL1
 
 
hsa-miR-17-5p
 
 
-0.432
 
 
81.2
 
 
 
 
ARL4D
 
 
hsa-miR-98-5p
 
 
-0.417
 
 
27.6
 
 
 
 
ARL4D
 
 
hsa-let-7c-5p
 
 
-0.417
 
 
27.6
 
 
 
 
ARL6IP1
 
 
hsa-miR-7-5p
 
 
-0.374
 
 
74.5
 
 
 
 
ARL6IP1
 
 
hsa-miR-328-3p
 
 
-0.494
 
 
74.5
 
 
 
 
ARMC8
 
 
hsa-miR-20a-5p
 
 
-0.357
 
 
13.8
 
 
 
 
ARMC8
 
 
hsa-miR-17-5p
 
 
-0.378
 
 
13.8
 
 
 
 
ARMT1
 
 
hsa-miR-98-5p
 
 
-0.303
 
 
15.1
 
 
 
 
ARMT1
 
 
hsa-let-7c-5p
 
 
-0.303
 
 
15.1
 
 
 
 
ARPP19
 
 
hsa-miR-328-3p
 
 
-0.404
 
 
35.8
 
 
 
 
ARPP19
 
 
hsa-let-7c-5p
 
 
-0.492
 
 
35.8
 
 
 
 
ARPP19
 
 
hsa-miR-98-5p
 
 
-0.5
 
 
35.8
 
 
 
 
ARRDC4
 
 
hsa-miR-98-5p
 
 
-0.329
 
 
14.3
 
 
 
 
ARRDC4
 
 
hsa-let-7c-5p
 
 
-0.329
 
 
14.3
 
 
 
 
ASAP1
 
 
hsa-miR-98-5p
 
 
-0.308
 
 
15.1
 
 
 
 
ASAP1
 
 
hsa-let-7c-5p
 
 
-0.308
 
 
15.1
 
 
 
 
ASB6
 
 
hsa-miR-423-5p
 
 
-0.388
 
 
10.9
 
 
 
 
ASPRV1
 
 
hsa-miR-330-3p
 
 
-0.301
 
 
24.4
 
 
 
 
ATAD2
 
 
hsa-miR-17-5p
 
 
-0.304
 
 
12.1
 
 
 
 
ATAD2
 
 
hsa-miR-20a-5p
 
 
-0.318
 
 
12.1
 
 
 
 
ATG13
 
 
hsa-miR-423-5p
 
 
-0.3
 
 
27.6
 
 
 
 
ATOSB
 
 
hsa-miR-328-3p
 
 
-0.358
 
 
12.3
 
 
 
 
ATOSB
 
 
hsa-miR-98-5p
 
 
-0.429
 
 
12.3
 
 
 
 
ATOSB
 
 
hsa-let-7c-5p
 
 
-0.429
 
 
12.3
 
 
 
 
ATP2A2
 
 
hsa-miR-98-5p
 
 
-0.311
 
 
40.8
 
 
 
 
ATP2A2
 
 
hsa-let-7c-5p
 
 
-0.311
 
 
40.8
 
 
 
 
ATP6V1F
 
 
hsa-miR-194-5p
 
 
-0.444
 
 
139.3
 
 
 
 
ATP6V1H
 
 
hsa-miR-194-5p
 
 
-0.652
 
 
19.6
 
 
 
 
ATP8B2
 
 
hsa-miR-423-5p
 
 
-0.297
 
 
26.7
 
 
 
 
ATPAF1
 
 
hsa-miR-98-5p
 
 
-0.498
 
 
33.4
 
 
 
 
ATPAF1
 
 
hsa-let-7c-5p
 
 
-0.498
 
 
33.4
 
 
 
 
ATXN1L
 
 
hsa-miR-155-5p
 
 
-0.315
 
 
10.4
 
 
 
 
AVPI1
 
 
hsa-miR-7-5p
 
 
-0.367
 
 
30.7
 
 
 
 
B3GAT3
 
 
hsa-miR-98-5p
 
 
-0.324
 
 
36.2
 
 
 
 
B3GAT3
 
 
hsa-let-7c-5p
 
 
-0.324
 
 
36.2
 
 
 
 
B4GALT5
 
 
hsa-miR-210-3p
 
 
-0.511
 
 
19.9
 
 
 
 
B4GAT1
 
 
hsa-miR-200b-3p
 
 
-0.382
 
 
25.3
 
 
 
 
B4GAT1
 
 
hsa-miR-98-5p
 
 
-0.47
 
 
25.3
 
 
 
 
B4GAT1
 
 
hsa-let-7c-5p
 
 
-0.47
 
 
25.3
 
 
 
 
BACH1
 
 
hsa-miR-98-5p
 
 
-0.298
 
 
17.0
 
 
 
 
BACH1
 
 
hsa-miR-98-5p
 
 
-0.298
 
 
17.0
 
 
 
 
BACH1
 
 
hsa-let-7c-5p
 
 
-0.306
 
 
17.0
 
 
 
 
BACH1
 
 
hsa-let-7c-5p
 
 
-0.306
 
 
17.0
 
 
 
 
BACH1
 
 
hsa-miR-98-5p
 
 
-0.435
 
 
17.0
 
 
 
 
BACH1
 
 
hsa-miR-98-5p
 
 
-0.435
 
 
17.0
 
 
 
 
BACH1
 
 
hsa-let-7c-5p
 
 
-0.435
 
 
17.0
 
 
 
 
 
 
 colon 
 
 
 
 
target_symbol
 
 
mature_mirna_id
 
 
score
 
 
nTPM
 
 
 
 
 
 
ABHD17C
 
 
hsa-let-7c-5p
 
 
-0.359
 
 
61.2
 
 
 
 
ABHD17C
 
 
hsa-miR-98-5p
 
 
-0.383
 
 
61.2
 
 
 
 
ABHD17C
 
 
hsa-miR-330-3p
 
 
-0.399
 
 
61.2
 
 
 
 
ABHD5
 
 
hsa-miR-20a-5p
 
 
-0.397
 
 
11.4
 
 
 
 
ABHD5
 
 
hsa-miR-17-5p
 
 
-0.45
 
 
11.4
 
 
 
 
ADAM9
 
 
hsa-miR-20a-5p
 
 
-0.299
 
 
30.9
 
 
 
 
ADAM9
 
 
hsa-miR-17-5p
 
 
-0.32
 
 
30.9
 
 
 
 
ADD3
 
 
hsa-miR-200b-3p
 
 
-0.324
 
 
54.6
 
 
 
 
ADD3
 
 
hsa-miR-18a-5p
 
 
-0.39
 
 
54.6
 
 
 
 
ADNP
 
 
hsa-miR-328-3p
 
 
-0.371
 
 
32.4
 
 
 
 
AEBP2
 
 
hsa-miR-18a-5p
 
 
-0.376
 
 
17.6
 
 
 
 
AGFG2
 
 
hsa-miR-20a-5p
 
 
-0.365
 
 
17.3
 
 
 
 
AGFG2
 
 
hsa-miR-17-5p
 
 
-0.402
 
 
17.3
 
 
 
 
AHNAK
 
 
hsa-miR-20a-5p
 
 
-0.316
 
 
258.8
 
 
 
 
AHNAK
 
 
hsa-miR-17-5p
 
 
-0.337
 
 
258.8
 
 
 
 
AIFM1
 
 
hsa-miR-98-5p
 
 
-0.314
 
 
23.9
 
 
 
 
AIFM1
 
 
hsa-let-7c-5p
 
 
-0.314
 
 
23.9
 
 
 
 
AK1
 
 
hsa-miR-423-5p
 
 
-0.443
 
 
41.6
 
 
 
 
ALCAM
 
 
hsa-miR-18a-5p
 
 
-0.341
 
 
15.7
 
 
 
 
ALG3
 
 
hsa-miR-20a-5p
 
 
-0.559
 
 
22.6
 
 
 
 
ALG3
 
 
hsa-miR-17-5p
 
 
-0.572
 
 
22.6
 
 
 
 
ALG9
 
 
hsa-miR-7-5p
 
 
-0.302
 
 
10.0
 
 
 
 
AMT
 
 
hsa-miR-98-5p
 
 
-0.463
 
 
14.4
 
 
 
 
AMT
 
 
hsa-let-7c-5p
 
 
-0.463
 
 
14.4
 
 
 
 
ANGPTL2
 
 
hsa-miR-423-5p
 
 
-0.379
 
 
73.4
 
 
 
 
ANKFY1
 
 
hsa-miR-155-5p
 
 
-0.315
 
 
24.5
 
 
 
 
ANKRD12
 
 
hsa-miR-7-5p
 
 
-0.37
 
 
12.2
 
 
 
 
ANKRD9
 
 
hsa-miR-20a-5p
 
 
-0.31
 
 
28.9
 
 
 
 
ANKRD9
 
 
hsa-miR-17-5p
 
 
-0.347
 
 
28.9
 
 
 
 
ANP32B
 
 
hsa-miR-200b-3p
 
 
-0.287
 
 
136.6
 
 
 
 
AP1S1
 
 
hsa-miR-98-5p
 
 
-0.374
 
 
31.0
 
 
 
 
AP1S1
 
 
hsa-let-7c-5p
 
 
-0.374
 
 
31.0
 
 
 
 
AP1S2
 
 
hsa-miR-200b-3p
 
 
-0.4
 
 
38.2
 
 
 
 
AP2M1
 
 
hsa-miR-423-5p
 
 
-0.49
 
 
196.7
 
 
 
 
APCDD1
 
 
hsa-miR-20a-5p
 
 
-0.404
 
 
12.1
 
 
 
 
APCDD1
 
 
hsa-miR-17-5p
 
 
-0.425
 
 
12.1
 
 
 
 
APOO
 
 
hsa-miR-200b-3p
 
 
-0.361
 
 
16.2
 
 
 
 
ARF4
 
 
hsa-miR-194-5p
 
 
-0.293
 
 
148.9
 
 
 
 
ARF4
 
 
hsa-miR-7-5p
 
 
-0.407
 
 
148.9
 
 
 
 
ARGLU1
 
 
hsa-miR-200b-3p
 
 
-0.29
 
 
11.0
 
 
 
 
ARHGAP1
 
 
hsa-miR-17-5p
 
 
-0.325
 
 
59.8
 
 
 
 
ARHGAP1
 
 
hsa-miR-20a-5p
 
 
-0.339
 
 
59.8
 
 
 
 
ARL1
 
 
hsa-miR-20a-5p
 
 
-0.403
 
 
53.0
 
 
 
 
ARL1
 
 
hsa-miR-17-5p
 
 
-0.432
 
 
53.0
 
 
 
 
ARL4D
 
 
hsa-miR-98-5p
 
 
-0.417
 
 
27.1
 
 
 
 
ARL4D
 
 
hsa-let-7c-5p
 
 
-0.417
 
 
27.1
 
 
 
 
ARL6IP1
 
 
hsa-miR-7-5p
 
 
-0.374
 
 
132.0
 
 
 
 
ARL6IP1
 
 
hsa-miR-328-3p
 
 
-0.494
 
 
132.0
 
 
 
 
ARL6IP6
 
 
hsa-miR-98-5p
 
 
-0.293
 
 
12.7
 
 
 
 
ARL6IP6
 
 
hsa-let-7c-5p
 
 
-0.293
 
 
12.7
 
 
 
 
ARMC8
 
 
hsa-miR-20a-5p
 
 
-0.357
 
 
15.5
 
 
 
 
ARMC8
 
 
hsa-miR-17-5p
 
 
-0.378
 
 
15.5
 
 
 
 
ARMT1
 
 
hsa-miR-98-5p
 
 
-0.303
 
 
15.5
 
 
 
 
ARMT1
 
 
hsa-let-7c-5p
 
 
-0.303
 
 
15.5
 
 
 
 
ARPP19
 
 
hsa-miR-328-3p
 
 
-0.404
 
 
35.7
 
 
 
 
ARPP19
 
 
hsa-let-7c-5p
 
 
-0.492
 
 
35.7
 
 
 
 
ARPP19
 
 
hsa-miR-98-5p
 
 
-0.5
 
 
35.7
 
 
 
 
ARRDC4
 
 
hsa-miR-98-5p
 
 
-0.329
 
 
40.4
 
 
 
 
ARRDC4
 
 
hsa-let-7c-5p
 
 
-0.329
 
 
40.4
 
 
 
 
ASAP1
 
 
hsa-miR-98-5p
 
 
-0.308
 
 
12.2
 
 
 
 
ASAP1
 
 
hsa-let-7c-5p
 
 
-0.308
 
 
12.2
 
 
 
 
ASB6
 
 
hsa-miR-423-5p
 
 
-0.388
 
 
10.3
 
 
 
 
ATG13
 
 
hsa-miR-423-5p
 
 
-0.3
 
 
24.9
 
 
 
 
ATG16L1
 
 
hsa-miR-20a-5p
 
 
-0.446
 
 
13.4
 
 
 
 
ATG16L1
 
 
hsa-miR-17-5p
 
 
-0.467
 
 
13.4
 
 
 
 
ATG4A
 
 
hsa-miR-7-5p
 
 
-0.448
 
 
11.4
 
 
 
 
ATOSB
 
 
hsa-miR-328-3p
 
 
-0.358
 
 
14.6
 
 
 
 
ATOSB
 
 
hsa-miR-98-5p
 
 
-0.429
 
 
14.6
 
 
 
 
ATOSB
 
 
hsa-let-7c-5p
 
 
-0.429
 
 
14.6
 
 
 
 
ATP2A2
 
 
hsa-miR-98-5p
 
 
-0.311
 
 
60.2
 
 
 
 
ATP2A2
 
 
hsa-let-7c-5p
 
 
-0.311
 
 
60.2
 
 
 
 
ATP6V1F
 
 
hsa-miR-194-5p
 
 
-0.444
 
 
105.2
 
 
 
 
ATP6V1H
 
 
hsa-miR-194-5p
 
 
-0.652
 
 
22.6
 
 
 
 
ATP8B2
 
 
hsa-miR-423-5p
 
 
-0.297
 
 
16.5
 
 
 
 
ATPAF1
 
 
hsa-miR-98-5p
 
 
-0.498
 
 
49.0
 
 
 
 
ATPAF1
 
 
hsa-let-7c-5p
 
 
-0.498
 
 
49.0
 
 
 
 
AVPI1
 
 
hsa-miR-7-5p
 
 
-0.367
 
 
23.0
 
 
 
 
B3GAT3
 
 
hsa-miR-98-5p
 
 
-0.324
 
 
30.8
 
 
 
 
B3GAT3
 
 
hsa-let-7c-5p
 
 
-0.324
 
 
30.8
 
 
 
 
B4GALT5
 
 
hsa-miR-210-3p
 
 
-0.511
 
 
23.3
 
 
 
 
B4GAT1
 
 
hsa-miR-200b-3p
 
 
-0.382
 
 
29.4
 
 
 
 
B4GAT1
 
 
hsa-miR-98-5p
 
 
-0.47
 
 
29.4
 
 
 
 
B4GAT1
 
 
hsa-let-7c-5p
 
 
-0.47
 
 
29.4
 
 
 
 
BACH1
 
 
hsa-miR-98-5p
 
 
-0.298
 
 
12.5
 
 
 
 
BACH1
 
 
hsa-miR-98-5p
 
 
-0.298
 
 
12.5
 
 
 
 
BACH1
 
 
hsa-let-7c-5p
 
 
-0.306
 
 
12.5
 
 
 
 
BACH1
 
 
hsa-let-7c-5p
 
 
-0.306
 
 
12.5
 
 
 
 
BACH1
 
 
hsa-miR-98-5p
 
 
-0.435
 
 
12.5
 
 
 
 
BACH1
 
 
hsa-miR-98-5p
 
 
-0.435
 
 
12.5
 
 
 
 
BACH1
 
 
hsa-let-7c-5p
 
 
-0.435
 
 
12.5
 
 
 
 
BACH1
 
 
hsa-let-7c-5p
 
 
-0.435
 
 
12.5
 
 
 
 
BAG6
 
 
hsa-miR-200b-3p
 
 
-0.371
 
 
69.9
 
 
 
 
BAP1
 
 
hsa-miR-423-5p
 
 
-0.428
 
 
45.8
 
 
 
 
BBX
 
 
hsa-miR-18a-5p
 
 
-0.31
 
 
10.4
 
 
 
 
BIN1
 
 
hsa-miR-744-5p
 
 
-0.654
 
 
33.8
 
 
 
 
BIN3
 
 
hsa-miR-98-5p
 
 
-0.427
 
 
12.9
 
 
 
 
BIN3
 
 
hsa-let-7c-5p
 
 
-0.427
 
 
12.9
 
 
 
 
BLOC1S6
 
 
hsa-miR-98-5p
 
 
-0.321
 
 
19.9
 
 
 
 
BLOC1S6
 
 
hsa-let-7c-5p
 
 
-0.321
 
 
19.9
 
 
 
 
BNIP2
 
 
hsa-miR-194-5p
 
 
-0.489
 
 
17.3
 
 
 
 
 
 
 duodenum 
 
 
 
 
target_symbol
 
 
mature_mirna_id
 
 
score
 
 
nTPM
 
 
 
 
 
 
ABHD17C
 
 
hsa-let-7c-5p
 
 
-0.359
 
 
21.9
 
 
 
 
ABHD17C
 
 
hsa-miR-98-5p
 
 
-0.383
 
 
21.9
 
 
 
 
ABHD17C
 
 
hsa-miR-330-3p
 
 
-0.399
 
 
21.9
 
 
 
 
ADAM9
 
 
hsa-miR-20a-5p
 
 
-0.299
 
 
23.1
 
 
 
 
ADAM9
 
 
hsa-miR-17-5p
 
 
-0.32
 
 
23.1
 
 
 
 
ADD3
 
 
hsa-miR-200b-3p
 
 
-0.324
 
 
67.7
 
 
 
 
ADD3
 
 
hsa-miR-18a-5p
 
 
-0.39
 
 
67.7
 
 
 
 
ADNP
 
 
hsa-miR-328-3p
 
 
-0.371
 
 
18.9
 
 
 
 
AEBP2
 
 
hsa-miR-18a-5p
 
 
-0.376
 
 
15.1
 
 
 
 
AHNAK
 
 
hsa-miR-20a-5p
 
 
-0.316
 
 
46.1
 
 
 
 
AHNAK
 
 
hsa-miR-17-5p
 
 
-0.337
 
 
46.1
 
 
 
 
AIFM1
 
 
hsa-miR-98-5p
 
 
-0.314
 
 
49.8
 
 
 
 
AIFM1
 
 
hsa-let-7c-5p
 
 
-0.314
 
 
49.8
 
 
 
 
AK1
 
 
hsa-miR-423-5p
 
 
-0.443
 
 
16.7
 
 
 
 
ALCAM
 
 
hsa-miR-18a-5p
 
 
-0.341
 
 
16.0
 
 
 
 
ALG3
 
 
hsa-miR-20a-5p
 
 
-0.559
 
 
15.9
 
 
 
 
ALG3
 
 
hsa-miR-17-5p
 
 
-0.572
 
 
15.9
 
 
 
 
ALG9
 
 
hsa-miR-7-5p
 
 
-0.302
 
 
11.5
 
 
 
 
AMT
 
 
hsa-miR-98-5p
 
 
-0.463
 
 
12.9
 
 
 
 
AMT
 
 
hsa-let-7c-5p
 
 
-0.463
 
 
12.9
 
 
 
 
ANGPTL2
 
 
hsa-miR-423-5p
 
 
-0.379
 
 
14.3
 
 
 
 
ANKFY1
 
 
hsa-miR-155-5p
 
 
-0.315
 
 
10.8
 
 
 
 
ANKRD12
 
 
hsa-miR-7-5p
 
 
-0.37
 
 
12.1
 
 
 
 
ANKRD9
 
 
hsa-miR-20a-5p
 
 
-0.31
 
 
47.3
 
 
 
 
ANKRD9
 
 
hsa-miR-17-5p
 
 
-0.347
 
 
47.3
 
 
 
 
ANP32B
 
 
hsa-miR-200b-3p
 
 
-0.287
 
 
89.5
 
 
 
 
AP1S1
 
 
hsa-miR-98-5p
 
 
-0.374
 
 
36.7
 
 
 
 
AP1S1
 
 
hsa-let-7c-5p
 
 
-0.374
 
 
36.7
 
 
 
 
AP1S2
 
 
hsa-miR-200b-3p
 
 
-0.4
 
 
10.9
 
 
 
 
AP2M1
 
 
hsa-miR-423-5p
 
 
-0.49
 
 
139.0
 
 
 
 
APOO
 
 
hsa-miR-200b-3p
 
 
-0.361
 
 
13.6
 
 
 
 
ARF4
 
 
hsa-miR-194-5p
 
 
-0.293
 
 
116.2
 
 
 
 
ARF4
 
 
hsa-miR-7-5p
 
 
-0.407
 
 
116.2
 
 
 
 
ARHGAP1
 
 
hsa-miR-17-5p
 
 
-0.325
 
 
24.2
 
 
 
 
ARHGAP1
 
 
hsa-miR-20a-5p
 
 
-0.339
 
 
24.2
 
 
 
 
ARL1
 
 
hsa-miR-20a-5p
 
 
-0.403
 
 
45.2
 
 
 
 
ARL1
 
 
hsa-miR-17-5p
 
 
-0.432
 
 
45.2
 
 
 
 
ARL6IP1
 
 
hsa-miR-7-5p
 
 
-0.374
 
 
130.6
 
 
 
 
ARL6IP1
 
 
hsa-miR-328-3p
 
 
-0.494
 
 
130.6
 
 
 
 
ARMC8
 
 
hsa-miR-20a-5p
 
 
-0.357
 
 
11.3
 
 
 
 
ARMC8
 
 
hsa-miR-17-5p
 
 
-0.378
 
 
11.3
 
 
 
 
ARMT1
 
 
hsa-miR-98-5p
 
 
-0.303
 
 
17.8
 
 
 
 
ARMT1
 
 
hsa-let-7c-5p
 
 
-0.303
 
 
17.8
 
 
 
 
ARPP19
 
 
hsa-miR-328-3p
 
 
-0.404
 
 
27.2
 
 
 
 
ARPP19
 
 
hsa-let-7c-5p
 
 
-0.492
 
 
27.2
 
 
 
 
ARPP19
 
 
hsa-miR-98-5p
 
 
-0.5
 
 
27.2
 
 
 
 
ARRDC4
 
 
hsa-miR-98-5p
 
 
-0.329
 
 
14.9
 
 
 
 
ARRDC4
 
 
hsa-let-7c-5p
 
 
-0.329
 
 
14.9
 
 
 
 
ATG13
 
 
hsa-miR-423-5p
 
 
-0.3
 
 
27.5
 
 
 
 
ATG4A
 
 
hsa-miR-7-5p
 
 
-0.448
 
 
12.0
 
 
 
 
ATP2A2
 
 
hsa-miR-98-5p
 
 
-0.311
 
 
44.8
 
 
 
 
ATP2A2
 
 
hsa-let-7c-5p
 
 
-0.311
 
 
44.8
 
 
 
 
ATP6V1F
 
 
hsa-miR-194-5p
 
 
-0.444
 
 
70.2
 
 
 
 
ATP6V1H
 
 
hsa-miR-194-5p
 
 
-0.652
 
 
12.2
 
 
 
 
ATP8B2
 
 
hsa-miR-423-5p
 
 
-0.297
 
 
12.2
 
 
 
 
ATPAF1
 
 
hsa-miR-98-5p
 
 
-0.498
 
 
41.3
 
 
 
 
ATPAF1
 
 
hsa-let-7c-5p
 
 
-0.498
 
 
41.3
 
 
 
 
AVPI1
 
 
hsa-miR-7-5p
 
 
-0.367
 
 
13.2
 
 
 
 
B3GAT3
 
 
hsa-miR-98-5p
 
 
-0.324
 
 
16.1
 
 
 
 
B3GAT3
 
 
hsa-let-7c-5p
 
 
-0.324
 
 
16.1
 
 
 
 
B4GALT5
 
 
hsa-miR-210-3p
 
 
-0.511
 
 
23.8
 
 
 
 
B4GAT1
 
 
hsa-miR-200b-3p
 
 
-0.382
 
 
15.2
 
 
 
 
B4GAT1
 
 
hsa-miR-98-5p
 
 
-0.47
 
 
15.2
 
 
 
 
B4GAT1
 
 
hsa-let-7c-5p
 
 
-0.47
 
 
15.2
 
 
 
 
BACH1
 
 
hsa-miR-98-5p
 
 
-0.298
 
 
15.8
 
 
 
 
BACH1
 
 
hsa-miR-98-5p
 
 
-0.298
 
 
15.8
 
 
 
 
BACH1
 
 
hsa-let-7c-5p
 
 
-0.306
 
 
15.8
 
 
 
 
BACH1
 
 
hsa-let-7c-5p
 
 
-0.306
 
 
15.8
 
 
 
 
BACH1
 
 
hsa-miR-98-5p
 
 
-0.435
 
 
15.8
 
 
 
 
BACH1
 
 
hsa-miR-98-5p
 
 
-0.435
 
 
15.8
 
 
 
 
BACH1
 
 
hsa-let-7c-5p
 
 
-0.435
 
 
15.8
 
 
 
 
BACH1
 
 
hsa-let-7c-5p
 
 
-0.435
 
 
15.8
 
 
 
 
BAG6
 
 
hsa-miR-200b-3p
 
 
-0.371
 
 
36.6
 
 
 
 
BAP1
 
 
hsa-miR-423-5p
 
 
-0.428
 
 
20.3
 
 
 
 
BIN1
 
 
hsa-miR-744-5p
 
 
-0.654
 
 
16.4
 
 
 
 
BLOC1S6
 
 
hsa-miR-98-5p
 
 
-0.321
 
 
19.0
 
 
 
 
BLOC1S6
 
 
hsa-let-7c-5p
 
 
-0.321
 
 
19.0
 
 
 
 
BNIP2
 
 
hsa-miR-194-5p
 
 
-0.489
 
 
14.1
 
 
 
 
BNIP3L
 
 
hsa-miR-20a-5p
 
 
-0.287
 
 
20.9
 
 
 
 
BTBD10
 
 
hsa-miR-17-5p
 
 
-0.409
 
 
12.4
 
 
 
 
BTBD10
 
 
hsa-miR-20a-5p
 
 
-0.412
 
 
12.4
 
 
 
 
BTF3L4
 
 
hsa-miR-200b-3p
 
 
-0.294
 
 
13.6
 
 
 
 
BTF3L4
 
 
hsa-miR-194-5p
 
 
-0.334
 
 
13.6
 
 
 
 
BTF3L4
 
 
hsa-miR-98-5p
 
 
-0.355
 
 
13.6
 
 
 
 
BTF3L4
 
 
hsa-let-7c-5p
 
 
-0.355
 
 
13.6
 
 
 
 
BTG3
 
 
hsa-miR-18a-5p
 
 
-0.464
 
 
15.5
 
 
 
 
BTG3
 
 
hsa-miR-17-5p
 
 
-0.668
 
 
15.5
 
 
 
 
BTG3
 
 
hsa-miR-20a-5p
 
 
-0.671
 
 
15.5
 
 
 
 
BZW1
 
 
hsa-miR-98-5p
 
 
-0.354
 
 
60.8
 
 
 
 
BZW1
 
 
hsa-let-7c-5p
 
 
-0.354
 
 
60.8
 
 
 
 
BZW1
 
 
hsa-miR-98-5p
 
 
-0.375
 
 
60.8
 
 
 
 
BZW1
 
 
hsa-let-7c-5p
 
 
-0.375
 
 
60.8
 
 
 
 
BZW2
 
 
hsa-miR-98-5p
 
 
-0.397
 
 
27.2
 
 
 
 
BZW2
 
 
hsa-let-7c-5p
 
 
-0.397
 
 
27.2
 
 
 
 
C15orf39
 
 
hsa-miR-98-5p
 
 
-0.374
 
 
10.8
 
 
 
 
C15orf39
 
 
hsa-let-7c-5p
 
 
-0.374
 
 
10.8
 
 
 
 
C1GALT1
 
 
hsa-let-7c-5p
 
 
-0.322
 
 
27.7
 
 
 
 
C1GALT1
 
 
hsa-miR-98-5p
 
 
-0.33
 
 
27.7
 
 
 
 
C1QBP
 
 
hsa-miR-330-3p
 
 
-0.412
 
 
90.1
 
 
 
 
C2orf88
 
 
hsa-miR-98-5p
 
 
-0.429
 
 
32.6
 
 
 
 
 
 
 esophagus 
 
 
 
 
target_symbol
 
 
mature_mirna_id
 
 
score
 
 
nTPM
 
 
 
 
 
 
ABHD17C
 
 
hsa-let-7c-5p
 
 
-0.359
 
 
45.1
 
 
 
 
ABHD17C
 
 
hsa-miR-98-5p
 
 
-0.383
 
 
45.1
 
 
 
 
ABHD17C
 
 
hsa-miR-330-3p
 
 
-0.399
 
 
45.1
 
 
 
 
ABHD5
 
 
hsa-miR-20a-5p
 
 
-0.397
 
 
18.4
 
 
 
 
ABHD5
 
 
hsa-miR-17-5p
 
 
-0.45
 
 
18.4
 
 
 
 
ACP3
 
 
hsa-miR-98-5p
 
 
-0.298
 
 
42.9
 
 
 
 
ACP3
 
 
hsa-let-7c-5p
 
 
-0.298
 
 
42.9
 
 
 
 
ACTA1
 
 
hsa-miR-155-5p
 
 
-0.595
 
 
568.9
 
 
 
 
ACTC1
 
 
hsa-miR-7-5p
 
 
-0.471
 
 
21.5
 
 
 
 
ADAM9
 
 
hsa-miR-20a-5p
 
 
-0.299
 
 
22.2
 
 
 
 
ADAM9
 
 
hsa-miR-17-5p
 
 
-0.32
 
 
22.2
 
 
 
 
ADD3
 
 
hsa-miR-200b-3p
 
 
-0.324
 
 
36.6
 
 
 
 
ADD3
 
 
hsa-miR-18a-5p
 
 
-0.39
 
 
36.6
 
 
 
 
ADNP
 
 
hsa-miR-328-3p
 
 
-0.371
 
 
30.5
 
 
 
 
ADRB2
 
 
hsa-miR-98-5p
 
 
-0.635
 
 
14.2
 
 
 
 
ADRB2
 
 
hsa-let-7c-5p
 
 
-0.635
 
 
14.2
 
 
 
 
AEBP2
 
 
hsa-miR-18a-5p
 
 
-0.376
 
 
33.0
 
 
 
 
AGFG2
 
 
hsa-miR-20a-5p
 
 
-0.365
 
 
29.3
 
 
 
 
AGFG2
 
 
hsa-miR-17-5p
 
 
-0.402
 
 
29.3
 
 
 
 
AHNAK
 
 
hsa-miR-20a-5p
 
 
-0.316
 
 
234.9
 
 
 
 
AHNAK
 
 
hsa-miR-17-5p
 
 
-0.337
 
 
234.9
 
 
 
 
AIFM1
 
 
hsa-miR-98-5p
 
 
-0.314
 
 
15.9
 
 
 
 
AIFM1
 
 
hsa-let-7c-5p
 
 
-0.314
 
 
15.9
 
 
 
 
AK1
 
 
hsa-miR-423-5p
 
 
-0.443
 
 
30.1
 
 
 
 
ALDH4A1
 
 
hsa-miR-184
 
 
-0.421
 
 
36.6
 
 
 
 
ALG3
 
 
hsa-miR-20a-5p
 
 
-0.559
 
 
29.4
 
 
 
 
ALG3
 
 
hsa-miR-17-5p
 
 
-0.572
 
 
29.4
 
 
 
 
ALG9
 
 
hsa-miR-7-5p
 
 
-0.302
 
 
10.5
 
 
 
 
ANGPTL2
 
 
hsa-miR-423-5p
 
 
-0.379
 
 
20.3
 
 
 
 
ANKFY1
 
 
hsa-miR-155-5p
 
 
-0.315
 
 
12.9
 
 
 
 
ANKRD12
 
 
hsa-miR-7-5p
 
 
-0.37
 
 
13.6
 
 
 
 
ANKRD46
 
 
hsa-miR-98-5p
 
 
-0.389
 
 
11.9
 
 
 
 
ANKRD46
 
 
hsa-let-7c-5p
 
 
-0.389
 
 
11.9
 
 
 
 
ANKRD9
 
 
hsa-miR-20a-5p
 
 
-0.31
 
 
18.5
 
 
 
 
ANKRD9
 
 
hsa-miR-17-5p
 
 
-0.347
 
 
18.5
 
 
 
 
ANP32B
 
 
hsa-miR-200b-3p
 
 
-0.287
 
 
133.7
 
 
 
 
AP1S1
 
 
hsa-miR-98-5p
 
 
-0.374
 
 
36.5
 
 
 
 
AP1S1
 
 
hsa-let-7c-5p
 
 
-0.374
 
 
36.5
 
 
 
 
AP1S2
 
 
hsa-miR-200b-3p
 
 
-0.4
 
 
19.8
 
 
 
 
AP2M1
 
 
hsa-miR-423-5p
 
 
-0.49
 
 
184.3
 
 
 
 
APCDD1
 
 
hsa-miR-20a-5p
 
 
-0.404
 
 
26.2
 
 
 
 
APCDD1
 
 
hsa-miR-17-5p
 
 
-0.425
 
 
26.2
 
 
 
 
APOO
 
 
hsa-miR-200b-3p
 
 
-0.361
 
 
12.1
 
 
 
 
ARF4
 
 
hsa-miR-194-5p
 
 
-0.293
 
 
102.8
 
 
 
 
ARF4
 
 
hsa-miR-7-5p
 
 
-0.407
 
 
102.8
 
 
 
 
ARG2
 
 
hsa-miR-98-5p
 
 
-0.468
 
 
17.2
 
 
 
 
ARG2
 
 
hsa-let-7c-5p
 
 
-0.468
 
 
17.2
 
 
 
 
ARHGAP1
 
 
hsa-miR-17-5p
 
 
-0.325
 
 
71.8
 
 
 
 
ARHGAP1
 
 
hsa-miR-20a-5p
 
 
-0.339
 
 
71.8
 
 
 
 
ARL1
 
 
hsa-miR-20a-5p
 
 
-0.403
 
 
46.2
 
 
 
 
ARL1
 
 
hsa-miR-17-5p
 
 
-0.432
 
 
46.2
 
 
 
 
ARL4D
 
 
hsa-miR-98-5p
 
 
-0.417
 
 
80.8
 
 
 
 
ARL4D
 
 
hsa-let-7c-5p
 
 
-0.417
 
 
80.8
 
 
 
 
ARL6IP1
 
 
hsa-miR-7-5p
 
 
-0.374
 
 
65.8
 
 
 
 
ARL6IP1
 
 
hsa-miR-328-3p
 
 
-0.494
 
 
65.8
 
 
 
 
ARL6IP6
 
 
hsa-miR-98-5p
 
 
-0.293
 
 
11.7
 
 
 
 
ARL6IP6
 
 
hsa-let-7c-5p
 
 
-0.293
 
 
11.7
 
 
 
 
ARMC8
 
 
hsa-miR-20a-5p
 
 
-0.357
 
 
19.8
 
 
 
 
ARMC8
 
 
hsa-miR-17-5p
 
 
-0.378
 
 
19.8
 
 
 
 
ARMT1
 
 
hsa-miR-98-5p
 
 
-0.303
 
 
11.3
 
 
 
 
ARMT1
 
 
hsa-let-7c-5p
 
 
-0.303
 
 
11.3
 
 
 
 
ARPP19
 
 
hsa-miR-328-3p
 
 
-0.404
 
 
49.0
 
 
 
 
ARPP19
 
 
hsa-let-7c-5p
 
 
-0.492
 
 
49.0
 
 
 
 
ARPP19
 
 
hsa-miR-98-5p
 
 
-0.5
 
 
49.0
 
 
 
 
ASAP1
 
 
hsa-miR-98-5p
 
 
-0.308
 
 
10.0
 
 
 
 
ASAP1
 
 
hsa-let-7c-5p
 
 
-0.308
 
 
10.0
 
 
 
 
ASB6
 
 
hsa-miR-423-5p
 
 
-0.388
 
 
15.0
 
 
 
 
ATG13
 
 
hsa-miR-423-5p
 
 
-0.3
 
 
30.6
 
 
 
 
ATG16L1
 
 
hsa-miR-20a-5p
 
 
-0.446
 
 
11.5
 
 
 
 
ATG16L1
 
 
hsa-miR-17-5p
 
 
-0.467
 
 
11.5
 
 
 
 
ATOSB
 
 
hsa-miR-328-3p
 
 
-0.358
 
 
28.9
 
 
 
 
ATOSB
 
 
hsa-miR-98-5p
 
 
-0.429
 
 
28.9
 
 
 
 
ATOSB
 
 
hsa-let-7c-5p
 
 
-0.429
 
 
28.9
 
 
 
 
ATP2A2
 
 
hsa-miR-98-5p
 
 
-0.311
 
 
170.5
 
 
 
 
ATP2A2
 
 
hsa-let-7c-5p
 
 
-0.311
 
 
170.5
 
 
 
 
ATP6V1F
 
 
hsa-miR-194-5p
 
 
-0.444
 
 
225.3
 
 
 
 
ATP6V1H
 
 
hsa-miR-194-5p
 
 
-0.652
 
 
29.5
 
 
 
 
ATP8B2
 
 
hsa-miR-423-5p
 
 
-0.297
 
 
10.8
 
 
 
 
ATPAF1
 
 
hsa-miR-98-5p
 
 
-0.498
 
 
36.9
 
 
 
 
ATPAF1
 
 
hsa-let-7c-5p
 
 
-0.498
 
 
36.9
 
 
 
 
AVPI1
 
 
hsa-miR-7-5p
 
 
-0.367
 
 
26.7
 
 
 
 
B3GAT3
 
 
hsa-miR-98-5p
 
 
-0.324
 
 
23.2
 
 
 
 
B3GAT3
 
 
hsa-let-7c-5p
 
 
-0.324
 
 
23.2
 
 
 
 
B4GALT5
 
 
hsa-miR-210-3p
 
 
-0.511
 
 
35.5
 
 
 
 
BACH1
 
 
hsa-miR-98-5p
 
 
-0.298
 
 
21.6
 
 
 
 
BACH1
 
 
hsa-miR-98-5p
 
 
-0.298
 
 
21.6
 
 
 
 
BACH1
 
 
hsa-let-7c-5p
 
 
-0.306
 
 
21.6
 
 
 
 
BACH1
 
 
hsa-let-7c-5p
 
 
-0.306
 
 
21.6
 
 
 
 
BACH1
 
 
hsa-miR-98-5p
 
 
-0.435
 
 
21.6
 
 
 
 
BACH1
 
 
hsa-miR-98-5p
 
 
-0.435
 
 
21.6
 
 
 
 
BACH1
 
 
hsa-let-7c-5p
 
 
-0.435
 
 
21.6
 
 
 
 
BACH1
 
 
hsa-let-7c-5p
 
 
-0.435
 
 
21.6
 
 
 
 
BAG6
 
 
hsa-miR-200b-3p
 
 
-0.371
 
 
77.2
 
 
 
 
BAP1
 
 
hsa-miR-423-5p
 
 
-0.428
 
 
57.2
 
 
 
 
BBX
 
 
hsa-miR-18a-5p
 
 
-0.31
 
 
12.1
 
 
 
 
BIN1
 
 
hsa-miR-744-5p
 
 
-0.654
 
 
31.5
 
 
 
 
BIN3
 
 
hsa-miR-98-5p
 
 
-0.427
 
 
17.6
 
 
 
 
BIN3
 
 
hsa-let-7c-5p
 
 
-0.427
 
 
17.6
 
 
 
 
BLOC1S4
 
 
hsa-miR-7-5p
 
 
-0.417
 
 
11.4
 
 
 
 
BLOC1S4
 
 
hsa-miR-7-5p
 
 
-0.527
 
 
11.4
 
 
 
 
 
 
 fallopian tube 
 
 
 
 
target_symbol
 
 
mature_mirna_id
 
 
score
 
 
nTPM
 
 
 
 
 
 
ABHD17C
 
 
hsa-let-7c-5p
 
 
-0.359
 
 
10.3
 
 
 
 
ABHD17C
 
 
hsa-miR-98-5p
 
 
-0.383
 
 
10.3
 
 
 
 
ABHD17C
 
 
hsa-miR-330-3p
 
 
-0.399
 
 
10.3
 
 
 
 
ADAM9
 
 
hsa-miR-20a-5p
 
 
-0.299
 
 
15.9
 
 
 
 
ADAM9
 
 
hsa-miR-17-5p
 
 
-0.32
 
 
15.9
 
 
 
 
ADD3
 
 
hsa-miR-200b-3p
 
 
-0.324
 
 
44.8
 
 
 
 
ADD3
 
 
hsa-miR-18a-5p
 
 
-0.39
 
 
44.8
 
 
 
 
ADNP
 
 
hsa-miR-328-3p
 
 
-0.371
 
 
31.2
 
 
 
 
AEBP2
 
 
hsa-miR-18a-5p
 
 
-0.376
 
 
26.3
 
 
 
 
AGFG2
 
 
hsa-miR-20a-5p
 
 
-0.365
 
 
14.4
 
 
 
 
AGFG2
 
 
hsa-miR-17-5p
 
 
-0.402
 
 
14.4
 
 
 
 
AHNAK
 
 
hsa-miR-20a-5p
 
 
-0.316
 
 
141.9
 
 
 
 
AHNAK
 
 
hsa-miR-17-5p
 
 
-0.337
 
 
141.9
 
 
 
 
AIFM1
 
 
hsa-miR-98-5p
 
 
-0.314
 
 
20.8
 
 
 
 
AIFM1
 
 
hsa-let-7c-5p
 
 
-0.314
 
 
20.8
 
 
 
 
AK1
 
 
hsa-miR-423-5p
 
 
-0.443
 
 
321.8
 
 
 
 
ALCAM
 
 
hsa-miR-18a-5p
 
 
-0.341
 
 
46.2
 
 
 
 
ALG3
 
 
hsa-miR-20a-5p
 
 
-0.559
 
 
14.3
 
 
 
 
ALG3
 
 
hsa-miR-17-5p
 
 
-0.572
 
 
14.3
 
 
 
 
AMT
 
 
hsa-miR-98-5p
 
 
-0.463
 
 
13.9
 
 
 
 
AMT
 
 
hsa-let-7c-5p
 
 
-0.463
 
 
13.9
 
 
 
 
ANGPTL2
 
 
hsa-miR-423-5p
 
 
-0.379
 
 
29.3
 
 
 
 
ANKFY1
 
 
hsa-miR-155-5p
 
 
-0.315
 
 
14.3
 
 
 
 
ANKRD29
 
 
hsa-miR-20a-5p
 
 
-0.359
 
 
14.4
 
 
 
 
ANKRD29
 
 
hsa-miR-17-5p
 
 
-0.372
 
 
14.4
 
 
 
 
ANKRD46
 
 
hsa-miR-98-5p
 
 
-0.389
 
 
10.7
 
 
 
 
ANKRD46
 
 
hsa-let-7c-5p
 
 
-0.389
 
 
10.7
 
 
 
 
ANP32B
 
 
hsa-miR-200b-3p
 
 
-0.287
 
 
117.2
 
 
 
 
AP1S1
 
 
hsa-miR-98-5p
 
 
-0.374
 
 
13.4
 
 
 
 
AP1S1
 
 
hsa-let-7c-5p
 
 
-0.374
 
 
13.4
 
 
 
 
AP1S2
 
 
hsa-miR-200b-3p
 
 
-0.4
 
 
12.6
 
 
 
 
AP2M1
 
 
hsa-miR-423-5p
 
 
-0.49
 
 
147.7
 
 
 
 
APCDD1
 
 
hsa-miR-20a-5p
 
 
-0.404
 
 
30.6
 
 
 
 
APCDD1
 
 
hsa-miR-17-5p
 
 
-0.425
 
 
30.6
 
 
 
 
APOO
 
 
hsa-miR-200b-3p
 
 
-0.361
 
 
19.5
 
 
 
 
ARF4
 
 
hsa-miR-194-5p
 
 
-0.293
 
 
79.6
 
 
 
 
ARF4
 
 
hsa-miR-7-5p
 
 
-0.407
 
 
79.6
 
 
 
 
ARGLU1
 
 
hsa-miR-200b-3p
 
 
-0.29
 
 
10.1
 
 
 
 
ARHGAP1
 
 
hsa-miR-17-5p
 
 
-0.325
 
 
71.5
 
 
 
 
ARHGAP1
 
 
hsa-miR-20a-5p
 
 
-0.339
 
 
71.5
 
 
 
 
ARL1
 
 
hsa-miR-20a-5p
 
 
-0.403
 
 
47.1
 
 
 
 
ARL1
 
 
hsa-miR-17-5p
 
 
-0.432
 
 
47.1
 
 
 
 
ARL4D
 
 
hsa-miR-98-5p
 
 
-0.417
 
 
20.8
 
 
 
 
ARL4D
 
 
hsa-let-7c-5p
 
 
-0.417
 
 
20.8
 
 
 
 
ARL6IP1
 
 
hsa-miR-7-5p
 
 
-0.374
 
 
81.6
 
 
 
 
ARL6IP1
 
 
hsa-miR-328-3p
 
 
-0.494
 
 
81.6
 
 
 
 
ARL6IP6
 
 
hsa-miR-98-5p
 
 
-0.293
 
 
10.8
 
 
 
 
ARL6IP6
 
 
hsa-let-7c-5p
 
 
-0.293
 
 
10.8
 
 
 
 
ARMC8
 
 
hsa-miR-20a-5p
 
 
-0.357
 
 
14.8
 
 
 
 
ARMC8
 
 
hsa-miR-17-5p
 
 
-0.378
 
 
14.8
 
 
 
 
ARMT1
 
 
hsa-miR-98-5p
 
 
-0.303
 
 
11.9
 
 
 
 
ARMT1
 
 
hsa-let-7c-5p
 
 
-0.303
 
 
11.9
 
 
 
 
ARPP19
 
 
hsa-miR-328-3p
 
 
-0.404
 
 
31.4
 
 
 
 
ARPP19
 
 
hsa-let-7c-5p
 
 
-0.492
 
 
31.4
 
 
 
 
ARPP19
 
 
hsa-miR-98-5p
 
 
-0.5
 
 
31.4
 
 
 
 
ARRDC4
 
 
hsa-miR-98-5p
 
 
-0.329
 
 
13.7
 
 
 
 
ARRDC4
 
 
hsa-let-7c-5p
 
 
-0.329
 
 
13.7
 
 
 
 
ASAP1
 
 
hsa-miR-98-5p
 
 
-0.308
 
 
15.1
 
 
 
 
ASAP1
 
 
hsa-let-7c-5p
 
 
-0.308
 
 
15.1
 
 
 
 
ASB6
 
 
hsa-miR-423-5p
 
 
-0.388
 
 
10.5
 
 
 
 
ATG13
 
 
hsa-miR-423-5p
 
 
-0.3
 
 
30.2
 
 
 
 
ATP2A2
 
 
hsa-miR-98-5p
 
 
-0.311
 
 
63.0
 
 
 
 
ATP2A2
 
 
hsa-let-7c-5p
 
 
-0.311
 
 
63.0
 
 
 
 
ATP6V1F
 
 
hsa-miR-194-5p
 
 
-0.444
 
 
92.4
 
 
 
 
ATP6V1H
 
 
hsa-miR-194-5p
 
 
-0.652
 
 
19.8
 
 
 
 
ATP8B2
 
 
hsa-miR-423-5p
 
 
-0.297
 
 
29.0
 
 
 
 
ATPAF1
 
 
hsa-miR-98-5p
 
 
-0.498
 
 
27.2
 
 
 
 
ATPAF1
 
 
hsa-let-7c-5p
 
 
-0.498
 
 
27.2
 
 
 
 
ATXN1L
 
 
hsa-miR-155-5p
 
 
-0.315
 
 
10.2
 
 
 
 
AVPI1
 
 
hsa-miR-7-5p
 
 
-0.367
 
 
21.5
 
 
 
 
B3GAT3
 
 
hsa-miR-98-5p
 
 
-0.324
 
 
34.0
 
 
 
 
B3GAT3
 
 
hsa-let-7c-5p
 
 
-0.324
 
 
34.0
 
 
 
 
B4GALT5
 
 
hsa-miR-210-3p
 
 
-0.511
 
 
17.2
 
 
 
 
B4GAT1
 
 
hsa-miR-200b-3p
 
 
-0.382
 
 
25.4
 
 
 
 
B4GAT1
 
 
hsa-miR-98-5p
 
 
-0.47
 
 
25.4
 
 
 
 
B4GAT1
 
 
hsa-let-7c-5p
 
 
-0.47
 
 
25.4
 
 
 
 
BAG6
 
 
hsa-miR-200b-3p
 
 
-0.371
 
 
62.6
 
 
 
 
BAP1
 
 
hsa-miR-423-5p
 
 
-0.428
 
 
35.3
 
 
 
 
BBX
 
 
hsa-miR-18a-5p
 
 
-0.31
 
 
15.8
 
 
 
 
BCAS3
 
 
hsa-miR-423-5p
 
 
-0.322
 
 
13.0
 
 
 
 
BIN1
 
 
hsa-miR-744-5p
 
 
-0.654
 
 
50.2
 
 
 
 
BIN3
 
 
hsa-miR-98-5p
 
 
-0.427
 
 
14.8
 
 
 
 
BIN3
 
 
hsa-let-7c-5p
 
 
-0.427
 
 
14.8
 
 
 
 
BLOC1S6
 
 
hsa-miR-98-5p
 
 
-0.321
 
 
17.1
 
 
 
 
BLOC1S6
 
 
hsa-let-7c-5p
 
 
-0.321
 
 
17.1
 
 
 
 
BNIP2
 
 
hsa-miR-194-5p
 
 
-0.489
 
 
16.1
 
 
 
 
BNIP3L
 
 
hsa-miR-20a-5p
 
 
-0.287
 
 
47.4
 
 
 
 
BRWD1
 
 
hsa-miR-18a-5p
 
 
-0.29
 
 
12.3
 
 
 
 
BTBD10
 
 
hsa-miR-17-5p
 
 
-0.409
 
 
12.5
 
 
 
 
BTBD10
 
 
hsa-miR-20a-5p
 
 
-0.412
 
 
12.5
 
 
 
 
BTF3L4
 
 
hsa-miR-200b-3p
 
 
-0.294
 
 
34.3
 
 
 
 
BTF3L4
 
 
hsa-miR-194-5p
 
 
-0.334
 
 
34.3
 
 
 
 
BTF3L4
 
 
hsa-miR-98-5p
 
 
-0.355
 
 
34.3
 
 
 
 
BTF3L4
 
 
hsa-let-7c-5p
 
 
-0.355
 
 
34.3
 
 
 
 
BTG3
 
 
hsa-miR-18a-5p
 
 
-0.464
 
 
48.5
 
 
 
 
BTG3
 
 
hsa-miR-17-5p
 
 
-0.668
 
 
48.5
 
 
 
 
BTG3
 
 
hsa-miR-20a-5p
 
 
-0.671
 
 
48.5
 
 
 
 
BZW1
 
 
hsa-miR-98-5p
 
 
-0.354
 
 
50.2
 
 
 
 
BZW1
 
 
hsa-let-7c-5p
 
 
-0.354
 
 
50.2
 
 
 
 
BZW1
 
 
hsa-miR-98-5p
 
 
-0.375
 
 
50.2
 
 
 
 
 
 
 hippocampal formation 
 
 
 
 
target_symbol
 
 
mature_mirna_id
 
 
score
 
 
nTPM
 
 
 
 
 
 
ABHD17C
 
 
hsa-let-7c-5p
 
 
-0.359
 
 
14.4
 
 
 
 
ABHD17C
 
 
hsa-miR-98-5p
 
 
-0.383
 
 
14.4
 
 
 
 
ABHD17C
 
 
hsa-miR-330-3p
 
 
-0.399
 
 
14.4
 
 
 
 
ACHE
 
 
hsa-miR-194-5p
 
 
-0.395
 
 
14.5
 
 
 
 
ACSL6
 
 
hsa-miR-98-5p
 
 
-0.336
 
 
15.3
 
 
 
 
ACSL6
 
 
hsa-let-7c-5p
 
 
-0.336
 
 
15.3
 
 
 
 
ADD3
 
 
hsa-miR-200b-3p
 
 
-0.324
 
 
83.3
 
 
 
 
ADD3
 
 
hsa-miR-18a-5p
 
 
-0.39
 
 
83.3
 
 
 
 
ADNP
 
 
hsa-miR-328-3p
 
 
-0.371
 
 
23.7
 
 
 
 
AGFG2
 
 
hsa-miR-20a-5p
 
 
-0.365
 
 
13.2
 
 
 
 
AGFG2
 
 
hsa-miR-17-5p
 
 
-0.402
 
 
13.2
 
 
 
 
AHNAK
 
 
hsa-miR-20a-5p
 
 
-0.316
 
 
24.2
 
 
 
 
AHNAK
 
 
hsa-miR-17-5p
 
 
-0.337
 
 
24.2
 
 
 
 
AIFM1
 
 
hsa-miR-98-5p
 
 
-0.314
 
 
10.5
 
 
 
 
AIFM1
 
 
hsa-let-7c-5p
 
 
-0.314
 
 
10.5
 
 
 
 
AK1
 
 
hsa-miR-423-5p
 
 
-0.443
 
 
93.4
 
 
 
 
AKAP6
 
 
hsa-miR-98-5p
 
 
-0.307
 
 
12.9
 
 
 
 
AKAP6
 
 
hsa-let-7c-5p
 
 
-0.307
 
 
12.9
 
 
 
 
ALCAM
 
 
hsa-miR-18a-5p
 
 
-0.341
 
 
33.8
 
 
 
 
ALDH4A1
 
 
hsa-miR-184
 
 
-0.421
 
 
39.7
 
 
 
 
ANGEL2
 
 
hsa-miR-200b-3p
 
 
-0.38
 
 
11.0
 
 
 
 
ANGPTL2
 
 
hsa-miR-423-5p
 
 
-0.379
 
 
11.4
 
 
 
 
ANKFY1
 
 
hsa-miR-155-5p
 
 
-0.315
 
 
10.8
 
 
 
 
ANKRD46
 
 
hsa-miR-98-5p
 
 
-0.389
 
 
25.5
 
 
 
 
ANKRD46
 
 
hsa-let-7c-5p
 
 
-0.389
 
 
25.5
 
 
 
 
ANKRD9
 
 
hsa-miR-20a-5p
 
 
-0.31
 
 
23.2
 
 
 
 
ANKRD9
 
 
hsa-miR-17-5p
 
 
-0.347
 
 
23.2
 
 
 
 
ANP32B
 
 
hsa-miR-200b-3p
 
 
-0.287
 
 
139.9
 
 
 
 
AP1S1
 
 
hsa-miR-98-5p
 
 
-0.374
 
 
67.5
 
 
 
 
AP1S1
 
 
hsa-let-7c-5p
 
 
-0.374
 
 
67.5
 
 
 
 
AP1S2
 
 
hsa-miR-200b-3p
 
 
-0.4
 
 
23.2
 
 
 
 
AP2M1
 
 
hsa-miR-423-5p
 
 
-0.49
 
 
225.9
 
 
 
 
APCDD1
 
 
hsa-miR-20a-5p
 
 
-0.404
 
 
28.0
 
 
 
 
APCDD1
 
 
hsa-miR-17-5p
 
 
-0.425
 
 
28.0
 
 
 
 
APOO
 
 
hsa-miR-200b-3p
 
 
-0.361
 
 
45.8
 
 
 
 
ARF4
 
 
hsa-miR-194-5p
 
 
-0.293
 
 
61.3
 
 
 
 
ARF4
 
 
hsa-miR-7-5p
 
 
-0.407
 
 
61.3
 
 
 
 
ARGLU1
 
 
hsa-miR-200b-3p
 
 
-0.29
 
 
16.0
 
 
 
 
ARHGAP1
 
 
hsa-miR-17-5p
 
 
-0.325
 
 
48.9
 
 
 
 
ARHGAP1
 
 
hsa-miR-20a-5p
 
 
-0.339
 
 
48.9
 
 
 
 
ARL1
 
 
hsa-miR-20a-5p
 
 
-0.403
 
 
24.0
 
 
 
 
ARL1
 
 
hsa-miR-17-5p
 
 
-0.432
 
 
24.0
 
 
 
 
ARL6IP1
 
 
hsa-miR-7-5p
 
 
-0.374
 
 
196.8
 
 
 
 
ARL6IP1
 
 
hsa-miR-328-3p
 
 
-0.494
 
 
196.8
 
 
 
 
ARMC8
 
 
hsa-miR-20a-5p
 
 
-0.357
 
 
13.3
 
 
 
 
ARMC8
 
 
hsa-miR-17-5p
 
 
-0.378
 
 
13.3
 
 
 
 
ARPP19
 
 
hsa-miR-328-3p
 
 
-0.404
 
 
120.9
 
 
 
 
ARPP19
 
 
hsa-let-7c-5p
 
 
-0.492
 
 
120.9
 
 
 
 
ARPP19
 
 
hsa-miR-98-5p
 
 
-0.5
 
 
120.9
 
 
 
 
ARRDC4
 
 
hsa-miR-98-5p
 
 
-0.329
 
 
23.9
 
 
 
 
ARRDC4
 
 
hsa-let-7c-5p
 
 
-0.329
 
 
23.9
 
 
 
 
ASAP1
 
 
hsa-miR-98-5p
 
 
-0.308
 
 
10.8
 
 
 
 
ASAP1
 
 
hsa-let-7c-5p
 
 
-0.308
 
 
10.8
 
 
 
 
ASB6
 
 
hsa-miR-423-5p
 
 
-0.388
 
 
14.7
 
 
 
 
ASF1A
 
 
hsa-miR-200b-3p
 
 
-0.377
 
 
11.7
 
 
 
 
ATG13
 
 
hsa-miR-423-5p
 
 
-0.3
 
 
37.4
 
 
 
 
ATOSB
 
 
hsa-miR-328-3p
 
 
-0.358
 
 
22.4
 
 
 
 
ATOSB
 
 
hsa-miR-98-5p
 
 
-0.429
 
 
22.4
 
 
 
 
ATOSB
 
 
hsa-let-7c-5p
 
 
-0.429
 
 
22.4
 
 
 
 
ATP2A2
 
 
hsa-miR-98-5p
 
 
-0.311
 
 
16.6
 
 
 
 
ATP2A2
 
 
hsa-let-7c-5p
 
 
-0.311
 
 
16.6
 
 
 
 
ATP2B2
 
 
hsa-miR-7-5p
 
 
-0.297
 
 
19.0
 
 
 
 
ATP6V1F
 
 
hsa-miR-194-5p
 
 
-0.444
 
 
279.8
 
 
 
 
ATP6V1H
 
 
hsa-miR-194-5p
 
 
-0.652
 
 
63.7
 
 
 
 
ATPAF1
 
 
hsa-miR-98-5p
 
 
-0.498
 
 
54.4
 
 
 
 
ATPAF1
 
 
hsa-let-7c-5p
 
 
-0.498
 
 
54.4
 
 
 
 
AVPI1
 
 
hsa-miR-7-5p
 
 
-0.367
 
 
14.1
 
 
 
 
B3GAT3
 
 
hsa-miR-98-5p
 
 
-0.324
 
 
64.6
 
 
 
 
B3GAT3
 
 
hsa-let-7c-5p
 
 
-0.324
 
 
64.6
 
 
 
 
B4GALT5
 
 
hsa-miR-210-3p
 
 
-0.511
 
 
10.8
 
 
 
 
B4GAT1
 
 
hsa-miR-200b-3p
 
 
-0.382
 
 
117.5
 
 
 
 
B4GAT1
 
 
hsa-miR-98-5p
 
 
-0.47
 
 
117.5
 
 
 
 
B4GAT1
 
 
hsa-let-7c-5p
 
 
-0.47
 
 
117.5
 
 
 
 
BAG6
 
 
hsa-miR-200b-3p
 
 
-0.371
 
 
42.5
 
 
 
 
BAMBI
 
 
hsa-miR-20a-5p
 
 
-0.485
 
 
14.9
 
 
 
 
BAMBI
 
 
hsa-miR-17-5p
 
 
-0.506
 
 
14.9
 
 
 
 
BAP1
 
 
hsa-miR-423-5p
 
 
-0.428
 
 
147.8
 
 
 
 
BBX
 
 
hsa-miR-18a-5p
 
 
-0.31
 
 
10.1
 
 
 
 
BCAS3
 
 
hsa-miR-423-5p
 
 
-0.322
 
 
10.3
 
 
 
 
BEGAIN
 
 
hsa-let-7c-5p
 
 
-0.466
 
 
36.8
 
 
 
 
BEGAIN
 
 
hsa-miR-98-5p
 
 
-0.474
 
 
36.8
 
 
 
 
BHLHE22
 
 
hsa-miR-18a-5p
 
 
-0.325
 
 
22.8
 
 
 
 
BIN1
 
 
hsa-miR-744-5p
 
 
-0.654
 
 
207.9
 
 
 
 
BIN3
 
 
hsa-miR-98-5p
 
 
-0.427
 
 
10.9
 
 
 
 
BIN3
 
 
hsa-let-7c-5p
 
 
-0.427
 
 
10.9
 
 
 
 
BLOC1S6
 
 
hsa-miR-98-5p
 
 
-0.321
 
 
18.3
 
 
 
 
BLOC1S6
 
 
hsa-let-7c-5p
 
 
-0.321
 
 
18.3
 
 
 
 
BNIP3L
 
 
hsa-miR-20a-5p
 
 
-0.287
 
 
54.0
 
 
 
 
BRWD1
 
 
hsa-miR-18a-5p
 
 
-0.29
 
 
33.7
 
 
 
 
BTBD10
 
 
hsa-miR-17-5p
 
 
-0.409
 
 
18.2
 
 
 
 
BTBD10
 
 
hsa-miR-20a-5p
 
 
-0.412
 
 
18.2
 
 
 
 
BTF3L4
 
 
hsa-miR-200b-3p
 
 
-0.294
 
 
28.4
 
 
 
 
BTF3L4
 
 
hsa-miR-194-5p
 
 
-0.334
 
 
28.4
 
 
 
 
BTF3L4
 
 
hsa-miR-98-5p
 
 
-0.355
 
 
28.4
 
 
 
 
BTF3L4
 
 
hsa-let-7c-5p
 
 
-0.355
 
 
28.4
 
 
 
 
BTG3
 
 
hsa-miR-18a-5p
 
 
-0.464
 
 
18.5
 
 
 
 
BTG3
 
 
hsa-miR-17-5p
 
 
-0.668
 
 
18.5
 
 
 
 
BTG3
 
 
hsa-miR-20a-5p
 
 
-0.671
 
 
18.5
 
 
 
 
BZW1
 
 
hsa-miR-98-5p
 
 
-0.354
 
 
34.8
 
 
 
 
BZW1
 
 
hsa-let-7c-5p
 
 
-0.354
 
 
34.8
 
 
 
 
 
 
 kidney 
 
 
 
 
target_symbol
 
 
mature_mirna_id
 
 
score
 
 
nTPM
 
 
 
 
 
 
ABHD17C
 
 
hsa-let-7c-5p
 
 
-0.359
 
 
13.6
 
 
 
 
ABHD17C
 
 
hsa-miR-98-5p
 
 
-0.383
 
 
13.6
 
 
 
 
ABHD17C
 
 
hsa-miR-330-3p
 
 
-0.399
 
 
13.6
 
 
 
 
ABHD5
 
 
hsa-miR-20a-5p
 
 
-0.397
 
 
10.4
 
 
 
 
ABHD5
 
 
hsa-miR-17-5p
 
 
-0.45
 
 
10.4
 
 
 
 
ACP3
 
 
hsa-miR-98-5p
 
 
-0.298
 
 
26.9
 
 
 
 
ACP3
 
 
hsa-let-7c-5p
 
 
-0.298
 
 
26.9
 
 
 
 
ADAM9
 
 
hsa-miR-20a-5p
 
 
-0.299
 
 
32.6
 
 
 
 
ADAM9
 
 
hsa-miR-17-5p
 
 
-0.32
 
 
32.6
 
 
 
 
ADD3
 
 
hsa-miR-200b-3p
 
 
-0.324
 
 
95.9
 
 
 
 
ADD3
 
 
hsa-miR-18a-5p
 
 
-0.39
 
 
95.9
 
 
 
 
ADNP
 
 
hsa-miR-328-3p
 
 
-0.371
 
 
28.2
 
 
 
 
AEBP2
 
 
hsa-miR-18a-5p
 
 
-0.376
 
 
25.8
 
 
 
 
AGFG2
 
 
hsa-miR-20a-5p
 
 
-0.365
 
 
15.9
 
 
 
 
AGFG2
 
 
hsa-miR-17-5p
 
 
-0.402
 
 
15.9
 
 
 
 
AHNAK
 
 
hsa-miR-20a-5p
 
 
-0.316
 
 
55.7
 
 
 
 
AHNAK
 
 
hsa-miR-17-5p
 
 
-0.337
 
 
55.7
 
 
 
 
AIFM1
 
 
hsa-miR-98-5p
 
 
-0.314
 
 
69.0
 
 
 
 
AIFM1
 
 
hsa-let-7c-5p
 
 
-0.314
 
 
69.0
 
 
 
 
AK1
 
 
hsa-miR-423-5p
 
 
-0.443
 
 
37.0
 
 
 
 
ALCAM
 
 
hsa-miR-18a-5p
 
 
-0.341
 
 
20.8
 
 
 
 
ALDH4A1
 
 
hsa-miR-184
 
 
-0.421
 
 
204.2
 
 
 
 
ALG3
 
 
hsa-miR-20a-5p
 
 
-0.559
 
 
17.8
 
 
 
 
ALG3
 
 
hsa-miR-17-5p
 
 
-0.572
 
 
17.8
 
 
 
 
AMT
 
 
hsa-miR-98-5p
 
 
-0.463
 
 
23.7
 
 
 
 
AMT
 
 
hsa-let-7c-5p
 
 
-0.463
 
 
23.7
 
 
 
 
ANGPTL2
 
 
hsa-miR-423-5p
 
 
-0.379
 
 
17.6
 
 
 
 
ANKFY1
 
 
hsa-miR-155-5p
 
 
-0.315
 
 
10.6
 
 
 
 
ANKRD12
 
 
hsa-miR-7-5p
 
 
-0.37
 
 
12.1
 
 
 
 
ANKRD46
 
 
hsa-miR-98-5p
 
 
-0.389
 
 
30.5
 
 
 
 
ANKRD46
 
 
hsa-let-7c-5p
 
 
-0.389
 
 
30.5
 
 
 
 
ANKRD9
 
 
hsa-miR-20a-5p
 
 
-0.31
 
 
22.8
 
 
 
 
ANKRD9
 
 
hsa-miR-17-5p
 
 
-0.347
 
 
22.8
 
 
 
 
ANP32B
 
 
hsa-miR-200b-3p
 
 
-0.287
 
 
90.4
 
 
 
 
AP1S1
 
 
hsa-miR-98-5p
 
 
-0.374
 
 
48.3
 
 
 
 
AP1S1
 
 
hsa-let-7c-5p
 
 
-0.374
 
 
48.3
 
 
 
 
AP1S2
 
 
hsa-miR-200b-3p
 
 
-0.4
 
 
12.1
 
 
 
 
AP2M1
 
 
hsa-miR-423-5p
 
 
-0.49
 
 
165.3
 
 
 
 
AP5S1
 
 
hsa-miR-744-5p
 
 
-0.354
 
 
10.3
 
 
 
 
APOO
 
 
hsa-miR-200b-3p
 
 
-0.361
 
 
43.3
 
 
 
 
ARF4
 
 
hsa-miR-194-5p
 
 
-0.293
 
 
106.7
 
 
 
 
ARF4
 
 
hsa-miR-7-5p
 
 
-0.407
 
 
106.7
 
 
 
 
ARG2
 
 
hsa-miR-98-5p
 
 
-0.468
 
 
66.0
 
 
 
 
ARG2
 
 
hsa-let-7c-5p
 
 
-0.468
 
 
66.0
 
 
 
 
ARHGAP1
 
 
hsa-miR-17-5p
 
 
-0.325
 
 
38.9
 
 
 
 
ARHGAP1
 
 
hsa-miR-20a-5p
 
 
-0.339
 
 
38.9
 
 
 
 
ARHGEF28
 
 
hsa-miR-17-5p
 
 
-0.363
 
 
29.5
 
 
 
 
ARHGEF28
 
 
hsa-miR-20a-5p
 
 
-0.366
 
 
29.5
 
 
 
 
ARL1
 
 
hsa-miR-20a-5p
 
 
-0.403
 
 
71.4
 
 
 
 
ARL1
 
 
hsa-miR-17-5p
 
 
-0.432
 
 
71.4
 
 
 
 
ARL4D
 
 
hsa-miR-98-5p
 
 
-0.417
 
 
35.1
 
 
 
 
ARL4D
 
 
hsa-let-7c-5p
 
 
-0.417
 
 
35.1
 
 
 
 
ARL6IP1
 
 
hsa-miR-7-5p
 
 
-0.374
 
 
140.2
 
 
 
 
ARL6IP1
 
 
hsa-miR-328-3p
 
 
-0.494
 
 
140.2
 
 
 
 
ARMC8
 
 
hsa-miR-20a-5p
 
 
-0.357
 
 
17.6
 
 
 
 
ARMC8
 
 
hsa-miR-17-5p
 
 
-0.378
 
 
17.6
 
 
 
 
ARMT1
 
 
hsa-miR-98-5p
 
 
-0.303
 
 
38.7
 
 
 
 
ARMT1
 
 
hsa-let-7c-5p
 
 
-0.303
 
 
38.7
 
 
 
 
ARPP19
 
 
hsa-miR-328-3p
 
 
-0.404
 
 
52.6
 
 
 
 
ARPP19
 
 
hsa-let-7c-5p
 
 
-0.492
 
 
52.6
 
 
 
 
ARPP19
 
 
hsa-miR-98-5p
 
 
-0.5
 
 
52.6
 
 
 
 
ARRDC4
 
 
hsa-miR-98-5p
 
 
-0.329
 
 
23.9
 
 
 
 
ARRDC4
 
 
hsa-let-7c-5p
 
 
-0.329
 
 
23.9
 
 
 
 
ASAP1
 
 
hsa-miR-98-5p
 
 
-0.308
 
 
10.1
 
 
 
 
ASAP1
 
 
hsa-let-7c-5p
 
 
-0.308
 
 
10.1
 
 
 
 
ASB6
 
 
hsa-miR-423-5p
 
 
-0.388
 
 
15.0
 
 
 
 
ASF1A
 
 
hsa-miR-200b-3p
 
 
-0.377
 
 
11.8
 
 
 
 
ATG13
 
 
hsa-miR-423-5p
 
 
-0.3
 
 
27.2
 
 
 
 
ATG4A
 
 
hsa-miR-7-5p
 
 
-0.448
 
 
13.1
 
 
 
 
ATOSB
 
 
hsa-miR-328-3p
 
 
-0.358
 
 
14.3
 
 
 
 
ATOSB
 
 
hsa-miR-98-5p
 
 
-0.429
 
 
14.3
 
 
 
 
ATOSB
 
 
hsa-let-7c-5p
 
 
-0.429
 
 
14.3
 
 
 
 
ATP2A2
 
 
hsa-miR-98-5p
 
 
-0.311
 
 
32.2
 
 
 
 
ATP2A2
 
 
hsa-let-7c-5p
 
 
-0.311
 
 
32.2
 
 
 
 
ATP6V1F
 
 
hsa-miR-194-5p
 
 
-0.444
 
 
310.1
 
 
 
 
ATP6V1H
 
 
hsa-miR-194-5p
 
 
-0.652
 
 
56.5
 
 
 
 
ATPAF1
 
 
hsa-miR-98-5p
 
 
-0.498
 
 
107.2
 
 
 
 
ATPAF1
 
 
hsa-let-7c-5p
 
 
-0.498
 
 
107.2
 
 
 
 
ATXN1L
 
 
hsa-miR-155-5p
 
 
-0.315
 
 
17.5
 
 
 
 
AVPI1
 
 
hsa-miR-7-5p
 
 
-0.367
 
 
12.6
 
 
 
 
B3GAT3
 
 
hsa-miR-98-5p
 
 
-0.324
 
 
48.1
 
 
 
 
B3GAT3
 
 
hsa-let-7c-5p
 
 
-0.324
 
 
48.1
 
 
 
 
B4GALT5
 
 
hsa-miR-210-3p
 
 
-0.511
 
 
21.3
 
 
 
 
B4GAT1
 
 
hsa-miR-200b-3p
 
 
-0.382
 
 
24.3
 
 
 
 
B4GAT1
 
 
hsa-miR-98-5p
 
 
-0.47
 
 
24.3
 
 
 
 
B4GAT1
 
 
hsa-let-7c-5p
 
 
-0.47
 
 
24.3
 
 
 
 
BACH1
 
 
hsa-miR-98-5p
 
 
-0.298
 
 
14.8
 
 
 
 
BACH1
 
 
hsa-miR-98-5p
 
 
-0.298
 
 
14.8
 
 
 
 
BACH1
 
 
hsa-let-7c-5p
 
 
-0.306
 
 
14.8
 
 
 
 
BACH1
 
 
hsa-let-7c-5p
 
 
-0.306
 
 
14.8
 
 
 
 
BACH1
 
 
hsa-miR-98-5p
 
 
-0.435
 
 
14.8
 
 
 
 
BACH1
 
 
hsa-miR-98-5p
 
 
-0.435
 
 
14.8
 
 
 
 
BACH1
 
 
hsa-let-7c-5p
 
 
-0.435
 
 
14.8
 
 
 
 
BACH1
 
 
hsa-let-7c-5p
 
 
-0.435
 
 
14.8
 
 
 
 
BAG6
 
 
hsa-miR-200b-3p
 
 
-0.371
 
 
69.8
 
 
 
 
BAMBI
 
 
hsa-miR-20a-5p
 
 
-0.485
 
 
18.5
 
 
 
 
BAMBI
 
 
hsa-miR-17-5p
 
 
-0.506
 
 
18.5
 
 
 
 
BAP1
 
 
hsa-miR-423-5p
 
 
-0.428
 
 
57.1
 
 
 
 
BBX
 
 
hsa-miR-18a-5p
 
 
-0.31
 
 
18.2
 
 
 
 
BCAS3
 
 
hsa-miR-423-5p
 
 
-0.322
 
 
10.2
 
 
 
 
 
 
 lung 
 
 
 
 
target_symbol
 
 
mature_mirna_id
 
 
score
 
 
nTPM
 
 
 
 
 
 
ABHD17C
 
 
hsa-let-7c-5p
 
 
-0.359
 
 
18.6
 
 
 
 
ABHD17C
 
 
hsa-miR-98-5p
 
 
-0.383
 
 
18.6
 
 
 
 
ABHD17C
 
 
hsa-miR-330-3p
 
 
-0.399
 
 
18.6
 
 
 
 
ABHD5
 
 
hsa-miR-20a-5p
 
 
-0.397
 
 
11.2
 
 
 
 
ABHD5
 
 
hsa-miR-17-5p
 
 
-0.45
 
 
11.2
 
 
 
 
ADAM9
 
 
hsa-miR-20a-5p
 
 
-0.299
 
 
42.9
 
 
 
 
ADAM9
 
 
hsa-miR-17-5p
 
 
-0.32
 
 
42.9
 
 
 
 
ADAMTS8
 
 
hsa-miR-98-5p
 
 
-0.571
 
 
33.2
 
 
 
 
ADAMTS8
 
 
hsa-let-7c-5p
 
 
-0.571
 
 
33.2
 
 
 
 
ADD3
 
 
hsa-miR-200b-3p
 
 
-0.324
 
 
36.2
 
 
 
 
ADD3
 
 
hsa-miR-18a-5p
 
 
-0.39
 
 
36.2
 
 
 
 
ADNP
 
 
hsa-miR-328-3p
 
 
-0.371
 
 
25.6
 
 
 
 
ADRB1
 
 
hsa-let-7c-5p
 
 
-0.351
 
 
11.4
 
 
 
 
ADRB1
 
 
hsa-miR-98-5p
 
 
-0.367
 
 
11.4
 
 
 
 
ADRB2
 
 
hsa-miR-98-5p
 
 
-0.635
 
 
18.4
 
 
 
 
ADRB2
 
 
hsa-let-7c-5p
 
 
-0.635
 
 
18.4
 
 
 
 
AEBP2
 
 
hsa-miR-18a-5p
 
 
-0.376
 
 
19.5
 
 
 
 
AHNAK
 
 
hsa-miR-20a-5p
 
 
-0.316
 
 
135.8
 
 
 
 
AHNAK
 
 
hsa-miR-17-5p
 
 
-0.337
 
 
135.8
 
 
 
 
AK1
 
 
hsa-miR-423-5p
 
 
-0.443
 
 
70.4
 
 
 
 
ALCAM
 
 
hsa-miR-18a-5p
 
 
-0.341
 
 
69.9
 
 
 
 
ALG3
 
 
hsa-miR-20a-5p
 
 
-0.559
 
 
17.4
 
 
 
 
ALG3
 
 
hsa-miR-17-5p
 
 
-0.572
 
 
17.4
 
 
 
 
ANGPTL2
 
 
hsa-miR-423-5p
 
 
-0.379
 
 
21.5
 
 
 
 
ANKFY1
 
 
hsa-miR-155-5p
 
 
-0.315
 
 
14.4
 
 
 
 
ANKRD12
 
 
hsa-miR-7-5p
 
 
-0.37
 
 
10.9
 
 
 
 
ANKRD29
 
 
hsa-miR-20a-5p
 
 
-0.359
 
 
29.5
 
 
 
 
ANKRD29
 
 
hsa-miR-17-5p
 
 
-0.372
 
 
29.5
 
 
 
 
ANKRD46
 
 
hsa-miR-98-5p
 
 
-0.389
 
 
10.1
 
 
 
 
ANKRD46
 
 
hsa-let-7c-5p
 
 
-0.389
 
 
10.1
 
 
 
 
ANKRD9
 
 
hsa-miR-20a-5p
 
 
-0.31
 
 
15.3
 
 
 
 
ANKRD9
 
 
hsa-miR-17-5p
 
 
-0.347
 
 
15.3
 
 
 
 
ANP32B
 
 
hsa-miR-200b-3p
 
 
-0.287
 
 
116.3
 
 
 
 
AP1S1
 
 
hsa-miR-98-5p
 
 
-0.374
 
 
15.2
 
 
 
 
AP1S1
 
 
hsa-let-7c-5p
 
 
-0.374
 
 
15.2
 
 
 
 
AP1S2
 
 
hsa-miR-200b-3p
 
 
-0.4
 
 
24.0
 
 
 
 
AP2M1
 
 
hsa-miR-423-5p
 
 
-0.49
 
 
146.4
 
 
 
 
APCDD1
 
 
hsa-miR-20a-5p
 
 
-0.404
 
 
12.6
 
 
 
 
APCDD1
 
 
hsa-miR-17-5p
 
 
-0.425
 
 
12.6
 
 
 
 
ARF4
 
 
hsa-miR-194-5p
 
 
-0.293
 
 
116.3
 
 
 
 
ARF4
 
 
hsa-miR-7-5p
 
 
-0.407
 
 
116.3
 
 
 
 
ARHGAP1
 
 
hsa-miR-17-5p
 
 
-0.325
 
 
36.0
 
 
 
 
ARHGAP1
 
 
hsa-miR-20a-5p
 
 
-0.339
 
 
36.0
 
 
 
 
ARID4B
 
 
hsa-miR-20a-5p
 
 
-0.29
 
 
11.3
 
 
 
 
ARL1
 
 
hsa-miR-20a-5p
 
 
-0.403
 
 
34.4
 
 
 
 
ARL1
 
 
hsa-miR-17-5p
 
 
-0.432
 
 
34.4
 
 
 
 
ARL4D
 
 
hsa-miR-98-5p
 
 
-0.417
 
 
16.0
 
 
 
 
ARL4D
 
 
hsa-let-7c-5p
 
 
-0.417
 
 
16.0
 
 
 
 
ARL6IP1
 
 
hsa-miR-7-5p
 
 
-0.374
 
 
75.1
 
 
 
 
ARL6IP1
 
 
hsa-miR-328-3p
 
 
-0.494
 
 
75.1
 
 
 
 
ARL6IP6
 
 
hsa-miR-98-5p
 
 
-0.293
 
 
11.9
 
 
 
 
ARL6IP6
 
 
hsa-let-7c-5p
 
 
-0.293
 
 
11.9
 
 
 
 
ARMC8
 
 
hsa-miR-20a-5p
 
 
-0.357
 
 
15.4
 
 
 
 
ARMC8
 
 
hsa-miR-17-5p
 
 
-0.378
 
 
15.4
 
 
 
 
ARMT1
 
 
hsa-miR-98-5p
 
 
-0.303
 
 
18.4
 
 
 
 
ARMT1
 
 
hsa-let-7c-5p
 
 
-0.303
 
 
18.4
 
 
 
 
ARPP19
 
 
hsa-miR-328-3p
 
 
-0.404
 
 
42.5
 
 
 
 
ARPP19
 
 
hsa-let-7c-5p
 
 
-0.492
 
 
42.5
 
 
 
 
ARPP19
 
 
hsa-miR-98-5p
 
 
-0.5
 
 
42.5
 
 
 
 
ARRDC4
 
 
hsa-miR-98-5p
 
 
-0.329
 
 
18.7
 
 
 
 
ARRDC4
 
 
hsa-let-7c-5p
 
 
-0.329
 
 
18.7
 
 
 
 
ASAP1
 
 
hsa-miR-98-5p
 
 
-0.308
 
 
27.6
 
 
 
 
ASAP1
 
 
hsa-let-7c-5p
 
 
-0.308
 
 
27.6
 
 
 
 
ASF1A
 
 
hsa-miR-200b-3p
 
 
-0.377
 
 
12.6
 
 
 
 
ATG13
 
 
hsa-miR-423-5p
 
 
-0.3
 
 
23.9
 
 
 
 
ATOSB
 
 
hsa-miR-328-3p
 
 
-0.358
 
 
20.8
 
 
 
 
ATOSB
 
 
hsa-miR-98-5p
 
 
-0.429
 
 
20.8
 
 
 
 
ATOSB
 
 
hsa-let-7c-5p
 
 
-0.429
 
 
20.8
 
 
 
 
ATP2A2
 
 
hsa-miR-98-5p
 
 
-0.311
 
 
46.2
 
 
 
 
ATP2A2
 
 
hsa-let-7c-5p
 
 
-0.311
 
 
46.2
 
 
 
 
ATP6V1F
 
 
hsa-miR-194-5p
 
 
-0.444
 
 
142.6
 
 
 
 
ATP6V1H
 
 
hsa-miR-194-5p
 
 
-0.652
 
 
22.1
 
 
 
 
ATP8B2
 
 
hsa-miR-423-5p
 
 
-0.297
 
 
16.5
 
 
 
 
ATPAF1
 
 
hsa-miR-98-5p
 
 
-0.498
 
 
24.6
 
 
 
 
ATPAF1
 
 
hsa-let-7c-5p
 
 
-0.498
 
 
24.6
 
 
 
 
ATXN1L
 
 
hsa-miR-155-5p
 
 
-0.315
 
 
11.3
 
 
 
 
AVPI1
 
 
hsa-miR-7-5p
 
 
-0.367
 
 
23.0
 
 
 
 
B3GAT3
 
 
hsa-miR-98-5p
 
 
-0.324
 
 
32.1
 
 
 
 
B3GAT3
 
 
hsa-let-7c-5p
 
 
-0.324
 
 
32.1
 
 
 
 
B4GALT5
 
 
hsa-miR-210-3p
 
 
-0.511
 
 
26.0
 
 
 
 
B4GAT1
 
 
hsa-miR-200b-3p
 
 
-0.382
 
 
16.9
 
 
 
 
B4GAT1
 
 
hsa-miR-98-5p
 
 
-0.47
 
 
16.9
 
 
 
 
B4GAT1
 
 
hsa-let-7c-5p
 
 
-0.47
 
 
16.9
 
 
 
 
BACH1
 
 
hsa-miR-98-5p
 
 
-0.298
 
 
20.2
 
 
 
 
BACH1
 
 
hsa-miR-98-5p
 
 
-0.298
 
 
20.2
 
 
 
 
BACH1
 
 
hsa-let-7c-5p
 
 
-0.306
 
 
20.2
 
 
 
 
BACH1
 
 
hsa-let-7c-5p
 
 
-0.306
 
 
20.2
 
 
 
 
BACH1
 
 
hsa-miR-98-5p
 
 
-0.435
 
 
20.2
 
 
 
 
BACH1
 
 
hsa-miR-98-5p
 
 
-0.435
 
 
20.2
 
 
 
 
BACH1
 
 
hsa-let-7c-5p
 
 
-0.435
 
 
20.2
 
 
 
 
BACH1
 
 
hsa-let-7c-5p
 
 
-0.435
 
 
20.2
 
 
 
 
BAG6
 
 
hsa-miR-200b-3p
 
 
-0.371
 
 
71.5
 
 
 
 
BAMBI
 
 
hsa-miR-20a-5p
 
 
-0.485
 
 
12.8
 
 
 
 
BAMBI
 
 
hsa-miR-17-5p
 
 
-0.506
 
 
12.8
 
 
 
 
BAP1
 
 
hsa-miR-423-5p
 
 
-0.428
 
 
43.9
 
 
 
 
BBX
 
 
hsa-miR-18a-5p
 
 
-0.31
 
 
12.6
 
 
 
 
BCAS3
 
 
hsa-miR-423-5p
 
 
-0.322
 
 
10.4
 
 
 
 
BIN1
 
 
hsa-miR-744-5p
 
 
-0.654
 
 
21.8
 
 
 
 
BIN3
 
 
hsa-miR-98-5p
 
 
-0.427
 
 
12.4
 
 
 
 
BIN3
 
 
hsa-let-7c-5p
 
 
-0.427
 
 
12.4
 
 
 
 
 
 
 prostate 
 
 
 
 
target_symbol
 
 
mature_mirna_id
 
 
score
 
 
nTPM
 
 
 
 
 
 
ABHD17C
 
 
hsa-let-7c-5p
 
 
-0.359
 
 
17.0
 
 
 
 
ABHD17C
 
 
hsa-miR-98-5p
 
 
-0.383
 
 
17.0
 
 
 
 
ABHD17C
 
 
hsa-miR-330-3p
 
 
-0.399
 
 
17.0
 
 
 
 
ACP3
 
 
hsa-miR-98-5p
 
 
-0.298
 
 
3409.5
 
 
 
 
ACP3
 
 
hsa-let-7c-5p
 
 
-0.298
 
 
3409.5
 
 
 
 
ACTA1
 
 
hsa-miR-155-5p
 
 
-0.595
 
 
152.5
 
 
 
 
ACTC1
 
 
hsa-miR-7-5p
 
 
-0.471
 
 
68.4
 
 
 
 
ADAM9
 
 
hsa-miR-20a-5p
 
 
-0.299
 
 
32.8
 
 
 
 
ADAM9
 
 
hsa-miR-17-5p
 
 
-0.32
 
 
32.8
 
 
 
 
ADD3
 
 
hsa-miR-200b-3p
 
 
-0.324
 
 
30.7
 
 
 
 
ADD3
 
 
hsa-miR-18a-5p
 
 
-0.39
 
 
30.7
 
 
 
 
ADNP
 
 
hsa-miR-328-3p
 
 
-0.371
 
 
33.0
 
 
 
 
AEBP2
 
 
hsa-miR-18a-5p
 
 
-0.376
 
 
23.3
 
 
 
 
AGFG2
 
 
hsa-miR-20a-5p
 
 
-0.365
 
 
12.4
 
 
 
 
AGFG2
 
 
hsa-miR-17-5p
 
 
-0.402
 
 
12.4
 
 
 
 
AHNAK
 
 
hsa-miR-20a-5p
 
 
-0.316
 
 
83.5
 
 
 
 
AHNAK
 
 
hsa-miR-17-5p
 
 
-0.337
 
 
83.5
 
 
 
 
AIFM1
 
 
hsa-miR-98-5p
 
 
-0.314
 
 
10.1
 
 
 
 
AIFM1
 
 
hsa-let-7c-5p
 
 
-0.314
 
 
10.1
 
 
 
 
AK1
 
 
hsa-miR-423-5p
 
 
-0.443
 
 
16.0
 
 
 
 
ALCAM
 
 
hsa-miR-18a-5p
 
 
-0.341
 
 
30.7
 
 
 
 
ALDH4A1
 
 
hsa-miR-184
 
 
-0.421
 
 
27.7
 
 
 
 
ALG3
 
 
hsa-miR-20a-5p
 
 
-0.559
 
 
19.0
 
 
 
 
ALG3
 
 
hsa-miR-17-5p
 
 
-0.572
 
 
19.0
 
 
 
 
AMT
 
 
hsa-miR-98-5p
 
 
-0.463
 
 
15.5
 
 
 
 
AMT
 
 
hsa-let-7c-5p
 
 
-0.463
 
 
15.5
 
 
 
 
ANGPTL2
 
 
hsa-miR-423-5p
 
 
-0.379
 
 
23.5
 
 
 
 
ANKFY1
 
 
hsa-miR-155-5p
 
 
-0.315
 
 
11.5
 
 
 
 
ANKRD12
 
 
hsa-miR-7-5p
 
 
-0.37
 
 
11.4
 
 
 
 
ANKRD46
 
 
hsa-miR-98-5p
 
 
-0.389
 
 
12.6
 
 
 
 
ANKRD46
 
 
hsa-let-7c-5p
 
 
-0.389
 
 
12.6
 
 
 
 
ANKRD9
 
 
hsa-miR-20a-5p
 
 
-0.31
 
 
18.8
 
 
 
 
ANKRD9
 
 
hsa-miR-17-5p
 
 
-0.347
 
 
18.8
 
 
 
 
ANP32B
 
 
hsa-miR-200b-3p
 
 
-0.287
 
 
93.4
 
 
 
 
AP1S1
 
 
hsa-miR-98-5p
 
 
-0.374
 
 
23.9
 
 
 
 
AP1S1
 
 
hsa-let-7c-5p
 
 
-0.374
 
 
23.9
 
 
 
 
AP1S2
 
 
hsa-miR-200b-3p
 
 
-0.4
 
 
10.2
 
 
 
 
AP2M1
 
 
hsa-miR-423-5p
 
 
-0.49
 
 
149.7
 
 
 
 
APCDD1
 
 
hsa-miR-20a-5p
 
 
-0.404
 
 
31.5
 
 
 
 
APCDD1
 
 
hsa-miR-17-5p
 
 
-0.425
 
 
31.5
 
 
 
 
APOO
 
 
hsa-miR-200b-3p
 
 
-0.361
 
 
12.8
 
 
 
 
ARF4
 
 
hsa-miR-194-5p
 
 
-0.293
 
 
129.9
 
 
 
 
ARF4
 
 
hsa-miR-7-5p
 
 
-0.407
 
 
129.9
 
 
 
 
ARG2
 
 
hsa-miR-98-5p
 
 
-0.468
 
 
101.8
 
 
 
 
ARG2
 
 
hsa-let-7c-5p
 
 
-0.468
 
 
101.8
 
 
 
 
ARHGAP1
 
 
hsa-miR-17-5p
 
 
-0.325
 
 
60.9
 
 
 
 
ARHGAP1
 
 
hsa-miR-20a-5p
 
 
-0.339
 
 
60.9
 
 
 
 
ARID4B
 
 
hsa-miR-20a-5p
 
 
-0.29
 
 
13.4
 
 
 
 
ARL1
 
 
hsa-miR-20a-5p
 
 
-0.403
 
 
68.8
 
 
 
 
ARL1
 
 
hsa-miR-17-5p
 
 
-0.432
 
 
68.8
 
 
 
 
ARL4D
 
 
hsa-miR-98-5p
 
 
-0.417
 
 
10.7
 
 
 
 
ARL4D
 
 
hsa-let-7c-5p
 
 
-0.417
 
 
10.7
 
 
 
 
ARL6IP1
 
 
hsa-miR-7-5p
 
 
-0.374
 
 
71.3
 
 
 
 
ARL6IP1
 
 
hsa-miR-328-3p
 
 
-0.494
 
 
71.3
 
 
 
 
ARMC8
 
 
hsa-miR-20a-5p
 
 
-0.357
 
 
15.4
 
 
 
 
ARMC8
 
 
hsa-miR-17-5p
 
 
-0.378
 
 
15.4
 
 
 
 
ARMT1
 
 
hsa-miR-98-5p
 
 
-0.303
 
 
11.5
 
 
 
 
ARMT1
 
 
hsa-let-7c-5p
 
 
-0.303
 
 
11.5
 
 
 
 
ARPP19
 
 
hsa-miR-328-3p
 
 
-0.404
 
 
37.0
 
 
 
 
ARPP19
 
 
hsa-let-7c-5p
 
 
-0.492
 
 
37.0
 
 
 
 
ARPP19
 
 
hsa-miR-98-5p
 
 
-0.5
 
 
37.0
 
 
 
 
ARRDC4
 
 
hsa-miR-98-5p
 
 
-0.329
 
 
10.3
 
 
 
 
ARRDC4
 
 
hsa-let-7c-5p
 
 
-0.329
 
 
10.3
 
 
 
 
ASAP1
 
 
hsa-miR-98-5p
 
 
-0.308
 
 
11.6
 
 
 
 
ASAP1
 
 
hsa-let-7c-5p
 
 
-0.308
 
 
11.6
 
 
 
 
ASB6
 
 
hsa-miR-423-5p
 
 
-0.388
 
 
11.8
 
 
 
 
ATG13
 
 
hsa-miR-423-5p
 
 
-0.3
 
 
29.5
 
 
 
 
ATG16L1
 
 
hsa-miR-20a-5p
 
 
-0.446
 
 
12.5
 
 
 
 
ATG16L1
 
 
hsa-miR-17-5p
 
 
-0.467
 
 
12.5
 
 
 
 
ATOSB
 
 
hsa-miR-328-3p
 
 
-0.358
 
 
12.4
 
 
 
 
ATOSB
 
 
hsa-miR-98-5p
 
 
-0.429
 
 
12.4
 
 
 
 
ATOSB
 
 
hsa-let-7c-5p
 
 
-0.429
 
 
12.4
 
 
 
 
ATP2A2
 
 
hsa-miR-98-5p
 
 
-0.311
 
 
87.6
 
 
 
 
ATP2A2
 
 
hsa-let-7c-5p
 
 
-0.311
 
 
87.6
 
 
 
 
ATP6V1F
 
 
hsa-miR-194-5p
 
 
-0.444
 
 
104.5
 
 
 
 
ATP6V1H
 
 
hsa-miR-194-5p
 
 
-0.652
 
 
28.4
 
 
 
 
ATP8B2
 
 
hsa-miR-423-5p
 
 
-0.297
 
 
18.0
 
 
 
 
ATPAF1
 
 
hsa-miR-98-5p
 
 
-0.498
 
 
35.2
 
 
 
 
ATPAF1
 
 
hsa-let-7c-5p
 
 
-0.498
 
 
35.2
 
 
 
 
AVPI1
 
 
hsa-miR-7-5p
 
 
-0.367
 
 
27.2
 
 
 
 
B3GAT3
 
 
hsa-miR-98-5p
 
 
-0.324
 
 
40.8
 
 
 
 
B3GAT3
 
 
hsa-let-7c-5p
 
 
-0.324
 
 
40.8
 
 
 
 
B4GALT5
 
 
hsa-miR-210-3p
 
 
-0.511
 
 
16.0
 
 
 
 
B4GAT1
 
 
hsa-miR-200b-3p
 
 
-0.382
 
 
17.0
 
 
 
 
B4GAT1
 
 
hsa-miR-98-5p
 
 
-0.47
 
 
17.0
 
 
 
 
B4GAT1
 
 
hsa-let-7c-5p
 
 
-0.47
 
 
17.0
 
 
 
 
BACH1
 
 
hsa-miR-98-5p
 
 
-0.298
 
 
12.5
 
 
 
 
BACH1
 
 
hsa-miR-98-5p
 
 
-0.298
 
 
12.5
 
 
 
 
BACH1
 
 
hsa-let-7c-5p
 
 
-0.306
 
 
12.5
 
 
 
 
BACH1
 
 
hsa-let-7c-5p
 
 
-0.306
 
 
12.5
 
 
 
 
BACH1
 
 
hsa-miR-98-5p
 
 
-0.435
 
 
12.5
 
 
 
 
BACH1
 
 
hsa-miR-98-5p
 
 
-0.435
 
 
12.5
 
 
 
 
BACH1
 
 
hsa-let-7c-5p
 
 
-0.435
 
 
12.5
 
 
 
 
BACH1
 
 
hsa-let-7c-5p
 
 
-0.435
 
 
12.5
 
 
 
 
BAG6
 
 
hsa-miR-200b-3p
 
 
-0.371
 
 
62.8
 
 
 
 
BAP1
 
 
hsa-miR-423-5p
 
 
-0.428
 
 
48.1
 
 
 
 
BBX
 
 
hsa-miR-18a-5p
 
 
-0.31
 
 
21.3
 
 
 
 
BCAS3
 
 
hsa-miR-423-5p
 
 
-0.322
 
 
10.2
 
 
 
 
BIN1
 
 
hsa-miR-744-5p
 
 
-0.654
 
 
52.2
 
 
 
 
BIN3
 
 
hsa-miR-98-5p
 
 
-0.427
 
 
16.4
 
 
 
 
 
 
 rectum 
 
 
 
 
target_symbol
 
 
mature_mirna_id
 
 
score
 
 
nTPM
 
 
 
 
 
 
ABHD17C
 
 
hsa-let-7c-5p
 
 
-0.359
 
 
52.8
 
 
 
 
ABHD17C
 
 
hsa-miR-98-5p
 
 
-0.383
 
 
52.8
 
 
 
 
ABHD17C
 
 
hsa-miR-330-3p
 
 
-0.399
 
 
52.8
 
 
 
 
ADAM9
 
 
hsa-miR-20a-5p
 
 
-0.299
 
 
39.2
 
 
 
 
ADAM9
 
 
hsa-miR-17-5p
 
 
-0.32
 
 
39.2
 
 
 
 
ADD3
 
 
hsa-miR-200b-3p
 
 
-0.324
 
 
62.0
 
 
 
 
ADD3
 
 
hsa-miR-18a-5p
 
 
-0.39
 
 
62.0
 
 
 
 
ADNP
 
 
hsa-miR-328-3p
 
 
-0.371
 
 
22.7
 
 
 
 
AEBP2
 
 
hsa-miR-18a-5p
 
 
-0.376
 
 
19.3
 
 
 
 
AHNAK
 
 
hsa-miR-20a-5p
 
 
-0.316
 
 
90.8
 
 
 
 
AHNAK
 
 
hsa-miR-17-5p
 
 
-0.337
 
 
90.8
 
 
 
 
AIFM1
 
 
hsa-miR-98-5p
 
 
-0.314
 
 
14.9
 
 
 
 
AIFM1
 
 
hsa-let-7c-5p
 
 
-0.314
 
 
14.9
 
 
 
 
AK1
 
 
hsa-miR-423-5p
 
 
-0.443
 
 
37.1
 
 
 
 
ALCAM
 
 
hsa-miR-18a-5p
 
 
-0.341
 
 
13.6
 
 
 
 
ALG3
 
 
hsa-miR-20a-5p
 
 
-0.559
 
 
17.2
 
 
 
 
ALG3
 
 
hsa-miR-17-5p
 
 
-0.572
 
 
17.2
 
 
 
 
AMT
 
 
hsa-miR-98-5p
 
 
-0.463
 
 
11.1
 
 
 
 
AMT
 
 
hsa-let-7c-5p
 
 
-0.463
 
 
11.1
 
 
 
 
ANGPTL2
 
 
hsa-miR-423-5p
 
 
-0.379
 
 
16.8
 
 
 
 
ANKFY1
 
 
hsa-miR-155-5p
 
 
-0.315
 
 
13.1
 
 
 
 
ANKRD12
 
 
hsa-miR-7-5p
 
 
-0.37
 
 
11.3
 
 
 
 
ANP32B
 
 
hsa-miR-200b-3p
 
 
-0.287
 
 
111.5
 
 
 
 
AP1S1
 
 
hsa-miR-98-5p
 
 
-0.374
 
 
21.1
 
 
 
 
AP1S1
 
 
hsa-let-7c-5p
 
 
-0.374
 
 
21.1
 
 
 
 
AP1S2
 
 
hsa-miR-200b-3p
 
 
-0.4
 
 
20.4
 
 
 
 
AP2M1
 
 
hsa-miR-423-5p
 
 
-0.49
 
 
121.5
 
 
 
 
APCDD1
 
 
hsa-miR-20a-5p
 
 
-0.404
 
 
12.4
 
 
 
 
APCDD1
 
 
hsa-miR-17-5p
 
 
-0.425
 
 
12.4
 
 
 
 
APOO
 
 
hsa-miR-200b-3p
 
 
-0.361
 
 
17.6
 
 
 
 
ARF4
 
 
hsa-miR-194-5p
 
 
-0.293
 
 
185.2
 
 
 
 
ARF4
 
 
hsa-miR-7-5p
 
 
-0.407
 
 
185.2
 
 
 
 
ARHGAP1
 
 
hsa-miR-17-5p
 
 
-0.325
 
 
30.6
 
 
 
 
ARHGAP1
 
 
hsa-miR-20a-5p
 
 
-0.339
 
 
30.6
 
 
 
 
ARL1
 
 
hsa-miR-20a-5p
 
 
-0.403
 
 
56.8
 
 
 
 
ARL1
 
 
hsa-miR-17-5p
 
 
-0.432
 
 
56.8
 
 
 
 
ARL6IP1
 
 
hsa-miR-7-5p
 
 
-0.374
 
 
116.0
 
 
 
 
ARL6IP1
 
 
hsa-miR-328-3p
 
 
-0.494
 
 
116.0
 
 
 
 
ARL6IP6
 
 
hsa-miR-98-5p
 
 
-0.293
 
 
13.3
 
 
 
 
ARL6IP6
 
 
hsa-let-7c-5p
 
 
-0.293
 
 
13.3
 
 
 
 
ARMC8
 
 
hsa-miR-20a-5p
 
 
-0.357
 
 
17.2
 
 
 
 
ARMC8
 
 
hsa-miR-17-5p
 
 
-0.378
 
 
17.2
 
 
 
 
ARMT1
 
 
hsa-miR-98-5p
 
 
-0.303
 
 
16.5
 
 
 
 
ARMT1
 
 
hsa-let-7c-5p
 
 
-0.303
 
 
16.5
 
 
 
 
ARPP19
 
 
hsa-miR-328-3p
 
 
-0.404
 
 
33.6
 
 
 
 
ARPP19
 
 
hsa-let-7c-5p
 
 
-0.492
 
 
33.6
 
 
 
 
ARPP19
 
 
hsa-miR-98-5p
 
 
-0.5
 
 
33.6
 
 
 
 
ARRDC4
 
 
hsa-miR-98-5p
 
 
-0.329
 
 
21.6
 
 
 
 
ARRDC4
 
 
hsa-let-7c-5p
 
 
-0.329
 
 
21.6
 
 
 
 
ASF1A
 
 
hsa-miR-200b-3p
 
 
-0.377
 
 
10.2
 
 
 
 
ATG13
 
 
hsa-miR-423-5p
 
 
-0.3
 
 
22.6
 
 
 
 
ATG4A
 
 
hsa-miR-7-5p
 
 
-0.448
 
 
12.3
 
 
 
 
ATP2A2
 
 
hsa-miR-98-5p
 
 
-0.311
 
 
64.9
 
 
 
 
ATP2A2
 
 
hsa-let-7c-5p
 
 
-0.311
 
 
64.9
 
 
 
 
ATP6V1F
 
 
hsa-miR-194-5p
 
 
-0.444
 
 
53.0
 
 
 
 
ATP6V1H
 
 
hsa-miR-194-5p
 
 
-0.652
 
 
19.2
 
 
 
 
ATPAF1
 
 
hsa-miR-98-5p
 
 
-0.498
 
 
50.0
 
 
 
 
ATPAF1
 
 
hsa-let-7c-5p
 
 
-0.498
 
 
50.0
 
 
 
 
AVPI1
 
 
hsa-miR-7-5p
 
 
-0.367
 
 
17.8
 
 
 
 
B4GALT5
 
 
hsa-miR-210-3p
 
 
-0.511
 
 
22.3
 
 
 
 
BACH1
 
 
hsa-miR-98-5p
 
 
-0.298
 
 
12.4
 
 
 
 
BACH1
 
 
hsa-miR-98-5p
 
 
-0.298
 
 
12.4
 
 
 
 
BACH1
 
 
hsa-let-7c-5p
 
 
-0.306
 
 
12.4
 
 
 
 
BACH1
 
 
hsa-let-7c-5p
 
 
-0.306
 
 
12.4
 
 
 
 
BACH1
 
 
hsa-miR-98-5p
 
 
-0.435
 
 
12.4
 
 
 
 
BACH1
 
 
hsa-miR-98-5p
 
 
-0.435
 
 
12.4
 
 
 
 
BACH1
 
 
hsa-let-7c-5p
 
 
-0.435
 
 
12.4
 
 
 
 
BACH1
 
 
hsa-let-7c-5p
 
 
-0.435
 
 
12.4
 
 
 
 
BAG6
 
 
hsa-miR-200b-3p
 
 
-0.371
 
 
23.3
 
 
 
 
BAP1
 
 
hsa-miR-423-5p
 
 
-0.428
 
 
22.6
 
 
 
 
BIN1
 
 
hsa-miR-744-5p
 
 
-0.654
 
 
12.3
 
 
 
 
BLOC1S6
 
 
hsa-miR-98-5p
 
 
-0.321
 
 
20.2
 
 
 
 
BLOC1S6
 
 
hsa-let-7c-5p
 
 
-0.321
 
 
20.2
 
 
 
 
BNIP2
 
 
hsa-miR-194-5p
 
 
-0.489
 
 
19.7
 
 
 
 
BNIP3L
 
 
hsa-miR-20a-5p
 
 
-0.287
 
 
26.5
 
 
 
 
BRWD1
 
 
hsa-miR-18a-5p
 
 
-0.29
 
 
11.5
 
 
 
 
BTBD10
 
 
hsa-miR-17-5p
 
 
-0.409
 
 
19.1
 
 
 
 
BTBD10
 
 
hsa-miR-20a-5p
 
 
-0.412
 
 
19.1
 
 
 
 
BTF3L4
 
 
hsa-miR-200b-3p
 
 
-0.294
 
 
22.2
 
 
 
 
BTF3L4
 
 
hsa-miR-194-5p
 
 
-0.334
 
 
22.2
 
 
 
 
BTF3L4
 
 
hsa-miR-98-5p
 
 
-0.355
 
 
22.2
 
 
 
 
BTF3L4
 
 
hsa-let-7c-5p
 
 
-0.355
 
 
22.2
 
 
 
 
BTG3
 
 
hsa-miR-18a-5p
 
 
-0.464
 
 
25.2
 
 
 
 
BTG3
 
 
hsa-miR-17-5p
 
 
-0.668
 
 
25.2
 
 
 
 
BTG3
 
 
hsa-miR-20a-5p
 
 
-0.671
 
 
25.2
 
 
 
 
BZW1
 
 
hsa-miR-98-5p
 
 
-0.354
 
 
85.0
 
 
 
 
BZW1
 
 
hsa-let-7c-5p
 
 
-0.354
 
 
85.0
 
 
 
 
BZW1
 
 
hsa-miR-98-5p
 
 
-0.375
 
 
85.0
 
 
 
 
BZW1
 
 
hsa-let-7c-5p
 
 
-0.375
 
 
85.0
 
 
 
 
BZW2
 
 
hsa-miR-98-5p
 
 
-0.397
 
 
53.4
 
 
 
 
BZW2
 
 
hsa-let-7c-5p
 
 
-0.397
 
 
53.4
 
 
 
 
C1GALT1
 
 
hsa-let-7c-5p
 
 
-0.322
 
 
16.9
 
 
 
 
C1GALT1
 
 
hsa-miR-98-5p
 
 
-0.33
 
 
16.9
 
 
 
 
C1QBP
 
 
hsa-miR-330-3p
 
 
-0.412
 
 
132.9
 
 
 
 
C2orf88
 
 
hsa-miR-98-5p
 
 
-0.429
 
 
36.2
 
 
 
 
C2orf88
 
 
hsa-let-7c-5p
 
 
-0.429
 
 
36.2
 
 
 
 
C3orf52
 
 
hsa-miR-98-5p
 
 
-0.296
 
 
12.8
 
 
 
 
C3orf52
 
 
hsa-let-7c-5p
 
 
-0.329
 
 
12.8
 
 
 
 
C6orf120
 
 
hsa-miR-20a-5p
 
 
-0.298
 
 
11.9
 
 
 
 
C6orf120
 
 
hsa-miR-17-5p
 
 
-0.319
 
 
11.9
 
 
 
 
 
 
 retina 
 
 
 
 
target_symbol
 
 
mature_mirna_id
 
 
score
 
 
nTPM
 
 
 
 
 
 
ABHD17C
 
 
hsa-let-7c-5p
 
 
-0.359
 
 
15.6
 
 
 
 
ABHD17C
 
 
hsa-miR-98-5p
 
 
-0.383
 
 
15.6
 
 
 
 
ABHD17C
 
 
hsa-miR-330-3p
 
 
-0.399
 
 
15.6
 
 
 
 
ACSL6
 
 
hsa-miR-98-5p
 
 
-0.336
 
 
42.4
 
 
 
 
ACSL6
 
 
hsa-let-7c-5p
 
 
-0.336
 
 
42.4
 
 
 
 
ACTC1
 
 
hsa-miR-7-5p
 
 
-0.471
 
 
29.3
 
 
 
 
ADAM9
 
 
hsa-miR-20a-5p
 
 
-0.299
 
 
17.2
 
 
 
 
ADAM9
 
 
hsa-miR-17-5p
 
 
-0.32
 
 
17.2
 
 
 
 
ADD3
 
 
hsa-miR-200b-3p
 
 
-0.324
 
 
92.0
 
 
 
 
ADD3
 
 
hsa-miR-18a-5p
 
 
-0.39
 
 
92.0
 
 
 
 
ADNP
 
 
hsa-miR-328-3p
 
 
-0.371
 
 
40.4
 
 
 
 
AEBP2
 
 
hsa-miR-18a-5p
 
 
-0.376
 
 
25.6
 
 
 
 
AK1
 
 
hsa-miR-423-5p
 
 
-0.443
 
 
27.1
 
 
 
 
AKAP6
 
 
hsa-miR-98-5p
 
 
-0.307
 
 
12.6
 
 
 
 
AKAP6
 
 
hsa-let-7c-5p
 
 
-0.307
 
 
12.6
 
 
 
 
ALG9
 
 
hsa-miR-7-5p
 
 
-0.302
 
 
11.3
 
 
 
 
ANKFY1
 
 
hsa-miR-155-5p
 
 
-0.315
 
 
12.1
 
 
 
 
ANKRD12
 
 
hsa-miR-7-5p
 
 
-0.37
 
 
27.6
 
 
 
 
ANKRD13C
 
 
hsa-miR-18a-5p
 
 
-0.374
 
 
12.3
 
 
 
 
ANKRD13C
 
 
hsa-miR-20a-5p
 
 
-0.505
 
 
12.3
 
 
 
 
ANKRD13C
 
 
hsa-miR-17-5p
 
 
-0.526
 
 
12.3
 
 
 
 
ANKRD46
 
 
hsa-miR-98-5p
 
 
-0.389
 
 
32.6
 
 
 
 
ANKRD46
 
 
hsa-let-7c-5p
 
 
-0.389
 
 
32.6
 
 
 
 
ANP32B
 
 
hsa-miR-200b-3p
 
 
-0.287
 
 
42.8
 
 
 
 
AP1S1
 
 
hsa-miR-98-5p
 
 
-0.374
 
 
13.5
 
 
 
 
AP1S1
 
 
hsa-let-7c-5p
 
 
-0.374
 
 
13.5
 
 
 
 
AP1S2
 
 
hsa-miR-200b-3p
 
 
-0.4
 
 
37.0
 
 
 
 
AP2M1
 
 
hsa-miR-423-5p
 
 
-0.49
 
 
143.7
 
 
 
 
APOO
 
 
hsa-miR-200b-3p
 
 
-0.361
 
 
48.9
 
 
 
 
ARF4
 
 
hsa-miR-194-5p
 
 
-0.293
 
 
50.0
 
 
 
 
ARF4
 
 
hsa-miR-7-5p
 
 
-0.407
 
 
50.0
 
 
 
 
ARG2
 
 
hsa-miR-98-5p
 
 
-0.468
 
 
20.7
 
 
 
 
ARG2
 
 
hsa-let-7c-5p
 
 
-0.468
 
 
20.7
 
 
 
 
ARID2
 
 
hsa-miR-155-5p
 
 
-0.384
 
 
12.3
 
 
 
 
ARID3B
 
 
hsa-miR-98-5p
 
 
-0.31
 
 
17.4
 
 
 
 
ARID3B
 
 
hsa-let-7c-5p
 
 
-0.31
 
 
17.4
 
 
 
 
ARID3B
 
 
hsa-miR-98-5p
 
 
-0.475
 
 
17.4
 
 
 
 
ARID3B
 
 
hsa-let-7c-5p
 
 
-0.486
 
 
17.4
 
 
 
 
ARID4A
 
 
hsa-miR-7-5p
 
 
-0.341
 
 
13.0
 
 
 
 
ARID4B
 
 
hsa-miR-20a-5p
 
 
-0.29
 
 
13.5
 
 
 
 
ARL1
 
 
hsa-miR-20a-5p
 
 
-0.403
 
 
45.1
 
 
 
 
ARL1
 
 
hsa-miR-17-5p
 
 
-0.432
 
 
45.1
 
 
 
 
ARL4D
 
 
hsa-miR-98-5p
 
 
-0.417
 
 
78.9
 
 
 
 
ARL4D
 
 
hsa-let-7c-5p
 
 
-0.417
 
 
78.9
 
 
 
 
ARL6IP1
 
 
hsa-miR-7-5p
 
 
-0.374
 
 
292.6
 
 
 
 
ARL6IP1
 
 
hsa-miR-328-3p
 
 
-0.494
 
 
292.6
 
 
 
 
ARL6IP6
 
 
hsa-miR-98-5p
 
 
-0.293
 
 
10.5
 
 
 
 
ARL6IP6
 
 
hsa-let-7c-5p
 
 
-0.293
 
 
10.5
 
 
 
 
ARMC8
 
 
hsa-miR-20a-5p
 
 
-0.357
 
 
24.3
 
 
 
 
ARMC8
 
 
hsa-miR-17-5p
 
 
-0.378
 
 
24.3
 
 
 
 
ARMT1
 
 
hsa-miR-98-5p
 
 
-0.303
 
 
15.2
 
 
 
 
ARMT1
 
 
hsa-let-7c-5p
 
 
-0.303
 
 
15.2
 
 
 
 
ARPP19
 
 
hsa-miR-328-3p
 
 
-0.404
 
 
53.1
 
 
 
 
ARPP19
 
 
hsa-let-7c-5p
 
 
-0.492
 
 
53.1
 
 
 
 
ARPP19
 
 
hsa-miR-98-5p
 
 
-0.5
 
 
53.1
 
 
 
 
ASF1A
 
 
hsa-miR-200b-3p
 
 
-0.377
 
 
10.6
 
 
 
 
ATG13
 
 
hsa-miR-423-5p
 
 
-0.3
 
 
43.7
 
 
 
 
ATM
 
 
hsa-miR-18a-5p
 
 
-0.347
 
 
16.4
 
 
 
 
ATP2A2
 
 
hsa-miR-98-5p
 
 
-0.311
 
 
17.7
 
 
 
 
ATP2A2
 
 
hsa-let-7c-5p
 
 
-0.311
 
 
17.7
 
 
 
 
ATP2B2
 
 
hsa-miR-7-5p
 
 
-0.297
 
 
12.5
 
 
 
 
ATP6V1F
 
 
hsa-miR-194-5p
 
 
-0.444
 
 
102.7
 
 
 
 
ATP6V1H
 
 
hsa-miR-194-5p
 
 
-0.652
 
 
34.7
 
 
 
 
ATPAF1
 
 
hsa-miR-98-5p
 
 
-0.498
 
 
65.3
 
 
 
 
ATPAF1
 
 
hsa-let-7c-5p
 
 
-0.498
 
 
65.3
 
 
 
 
AVPI1
 
 
hsa-miR-7-5p
 
 
-0.367
 
 
23.7
 
 
 
 
B3GAT3
 
 
hsa-miR-98-5p
 
 
-0.324
 
 
15.0
 
 
 
 
B3GAT3
 
 
hsa-let-7c-5p
 
 
-0.324
 
 
15.0
 
 
 
 
B4GAT1
 
 
hsa-miR-200b-3p
 
 
-0.382
 
 
42.6
 
 
 
 
B4GAT1
 
 
hsa-miR-98-5p
 
 
-0.47
 
 
42.6
 
 
 
 
B4GAT1
 
 
hsa-let-7c-5p
 
 
-0.47
 
 
42.6
 
 
 
 
BACH1
 
 
hsa-miR-98-5p
 
 
-0.298
 
 
10.9
 
 
 
 
BACH1
 
 
hsa-miR-98-5p
 
 
-0.298
 
 
10.9
 
 
 
 
BACH1
 
 
hsa-let-7c-5p
 
 
-0.306
 
 
10.9
 
 
 
 
BACH1
 
 
hsa-let-7c-5p
 
 
-0.306
 
 
10.9
 
 
 
 
BACH1
 
 
hsa-miR-98-5p
 
 
-0.435
 
 
10.9
 
 
 
 
BACH1
 
 
hsa-miR-98-5p
 
 
-0.435
 
 
10.9
 
 
 
 
BACH1
 
 
hsa-let-7c-5p
 
 
-0.435
 
 
10.9
 
 
 
 
BACH1
 
 
hsa-let-7c-5p
 
 
-0.435
 
 
10.9
 
 
 
 
BAG6
 
 
hsa-miR-200b-3p
 
 
-0.371
 
 
15.1
 
 
 
 
BAMBI
 
 
hsa-miR-20a-5p
 
 
-0.485
 
 
12.4
 
 
 
 
BAMBI
 
 
hsa-miR-17-5p
 
 
-0.506
 
 
12.4
 
 
 
 
BAP1
 
 
hsa-miR-423-5p
 
 
-0.428
 
 
18.7
 
 
 
 
BBX
 
 
hsa-miR-18a-5p
 
 
-0.31
 
 
18.6
 
 
 
 
BCAS3
 
 
hsa-miR-423-5p
 
 
-0.322
 
 
16.0
 
 
 
 
BCAT1
 
 
hsa-miR-98-5p
 
 
-0.394
 
 
13.4
 
 
 
 
BCAT1
 
 
hsa-let-7c-5p
 
 
-0.394
 
 
13.4
 
 
 
 
BIN1
 
 
hsa-miR-744-5p
 
 
-0.654
 
 
49.9
 
 
 
 
BLOC1S6
 
 
hsa-miR-98-5p
 
 
-0.321
 
 
22.7
 
 
 
 
BLOC1S6
 
 
hsa-let-7c-5p
 
 
-0.321
 
 
22.7
 
 
 
 
BNIP2
 
 
hsa-miR-194-5p
 
 
-0.489
 
 
23.8
 
 
 
 
BNIP3L
 
 
hsa-miR-20a-5p
 
 
-0.287
 
 
135.8
 
 
 
 
BRWD1
 
 
hsa-miR-18a-5p
 
 
-0.29
 
 
35.7
 
 
 
 
BTBD10
 
 
hsa-miR-17-5p
 
 
-0.409
 
 
17.4
 
 
 
 
BTBD10
 
 
hsa-miR-20a-5p
 
 
-0.412
 
 
17.4
 
 
 
 
BTF3L4
 
 
hsa-miR-200b-3p
 
 
-0.294
 
 
29.1
 
 
 
 
BTF3L4
 
 
hsa-miR-194-5p
 
 
-0.334
 
 
29.1
 
 
 
 
BTF3L4
 
 
hsa-miR-98-5p
 
 
-0.355
 
 
29.1
 
 
 
 
BTF3L4
 
 
hsa-let-7c-5p
 
 
-0.355
 
 
29.1
 
 
 
 
BZW1
 
 
hsa-miR-98-5p
 
 
-0.354
 
 
42.4
 
 
 
 
 
 
 salivary gland 
 
 
 
 
target_symbol
 
 
mature_mirna_id
 
 
score
 
 
nTPM
 
 
 
 
 
 
ABHD17C
 
 
hsa-let-7c-5p
 
 
-0.359
 
 
22.3
 
 
 
 
ABHD17C
 
 
hsa-miR-98-5p
 
 
-0.383
 
 
22.3
 
 
 
 
ABHD17C
 
 
hsa-miR-330-3p
 
 
-0.399
 
 
22.3
 
 
 
 
ABHD5
 
 
hsa-miR-20a-5p
 
 
-0.397
 
 
19.4
 
 
 
 
ABHD5
 
 
hsa-miR-17-5p
 
 
-0.45
 
 
19.4
 
 
 
 
ACTA1
 
 
hsa-miR-155-5p
 
 
-0.595
 
 
552.8
 
 
 
 
ACTC1
 
 
hsa-miR-7-5p
 
 
-0.471
 
 
41.7
 
 
 
 
ADAM9
 
 
hsa-miR-20a-5p
 
 
-0.299
 
 
39.8
 
 
 
 
ADAM9
 
 
hsa-miR-17-5p
 
 
-0.32
 
 
39.8
 
 
 
 
ADD3
 
 
hsa-miR-200b-3p
 
 
-0.324
 
 
55.8
 
 
 
 
ADD3
 
 
hsa-miR-18a-5p
 
 
-0.39
 
 
55.8
 
 
 
 
ADNP
 
 
hsa-miR-328-3p
 
 
-0.371
 
 
27.9
 
 
 
 
AEBP2
 
 
hsa-miR-18a-5p
 
 
-0.376
 
 
18.1
 
 
 
 
AGFG2
 
 
hsa-miR-20a-5p
 
 
-0.365
 
 
272.4
 
 
 
 
AGFG2
 
 
hsa-miR-17-5p
 
 
-0.402
 
 
272.4
 
 
 
 
AHNAK
 
 
hsa-miR-20a-5p
 
 
-0.316
 
 
118.3
 
 
 
 
AHNAK
 
 
hsa-miR-17-5p
 
 
-0.337
 
 
118.3
 
 
 
 
AIFM1
 
 
hsa-miR-98-5p
 
 
-0.314
 
 
17.0
 
 
 
 
AIFM1
 
 
hsa-let-7c-5p
 
 
-0.314
 
 
17.0
 
 
 
 
AK1
 
 
hsa-miR-423-5p
 
 
-0.443
 
 
38.2
 
 
 
 
ALCAM
 
 
hsa-miR-18a-5p
 
 
-0.341
 
 
31.9
 
 
 
 
ALG3
 
 
hsa-miR-20a-5p
 
 
-0.559
 
 
25.8
 
 
 
 
ALG3
 
 
hsa-miR-17-5p
 
 
-0.572
 
 
25.8
 
 
 
 
ALG9
 
 
hsa-miR-7-5p
 
 
-0.302
 
 
16.6
 
 
 
 
AMT
 
 
hsa-miR-98-5p
 
 
-0.463
 
 
10.0
 
 
 
 
AMT
 
 
hsa-let-7c-5p
 
 
-0.463
 
 
10.0
 
 
 
 
ANGPTL2
 
 
hsa-miR-423-5p
 
 
-0.379
 
 
17.1
 
 
 
 
ANKFY1
 
 
hsa-miR-155-5p
 
 
-0.315
 
 
12.9
 
 
 
 
ANKRD12
 
 
hsa-miR-7-5p
 
 
-0.37
 
 
11.6
 
 
 
 
ANKRD46
 
 
hsa-miR-98-5p
 
 
-0.389
 
 
16.1
 
 
 
 
ANKRD46
 
 
hsa-let-7c-5p
 
 
-0.389
 
 
16.1
 
 
 
 
ANKRD9
 
 
hsa-miR-20a-5p
 
 
-0.31
 
 
25.3
 
 
 
 
ANKRD9
 
 
hsa-miR-17-5p
 
 
-0.347
 
 
25.3
 
 
 
 
ANP32B
 
 
hsa-miR-200b-3p
 
 
-0.287
 
 
104.9
 
 
 
 
AP1S1
 
 
hsa-miR-98-5p
 
 
-0.374
 
 
21.5
 
 
 
 
AP1S1
 
 
hsa-let-7c-5p
 
 
-0.374
 
 
21.5
 
 
 
 
AP2M1
 
 
hsa-miR-423-5p
 
 
-0.49
 
 
146.5
 
 
 
 
APCDD1
 
 
hsa-miR-20a-5p
 
 
-0.404
 
 
10.0
 
 
 
 
APCDD1
 
 
hsa-miR-17-5p
 
 
-0.425
 
 
10.0
 
 
 
 
APOO
 
 
hsa-miR-200b-3p
 
 
-0.361
 
 
18.5
 
 
 
 
ARF4
 
 
hsa-miR-194-5p
 
 
-0.293
 
 
171.4
 
 
 
 
ARF4
 
 
hsa-miR-7-5p
 
 
-0.407
 
 
171.4
 
 
 
 
ARG2
 
 
hsa-miR-98-5p
 
 
-0.468
 
 
18.4
 
 
 
 
ARG2
 
 
hsa-let-7c-5p
 
 
-0.468
 
 
18.4
 
 
 
 
ARHGAP1
 
 
hsa-miR-17-5p
 
 
-0.325
 
 
43.3
 
 
 
 
ARHGAP1
 
 
hsa-miR-20a-5p
 
 
-0.339
 
 
43.3
 
 
 
 
ARHGEF28
 
 
hsa-miR-17-5p
 
 
-0.363
 
 
18.6
 
 
 
 
ARHGEF28
 
 
hsa-miR-20a-5p
 
 
-0.366
 
 
18.6
 
 
 
 
ARID4B
 
 
hsa-miR-20a-5p
 
 
-0.29
 
 
10.8
 
 
 
 
ARL1
 
 
hsa-miR-20a-5p
 
 
-0.403
 
 
69.5
 
 
 
 
ARL1
 
 
hsa-miR-17-5p
 
 
-0.432
 
 
69.5
 
 
 
 
ARL4D
 
 
hsa-miR-98-5p
 
 
-0.417
 
 
16.7
 
 
 
 
ARL4D
 
 
hsa-let-7c-5p
 
 
-0.417
 
 
16.7
 
 
 
 
ARL6IP1
 
 
hsa-miR-7-5p
 
 
-0.374
 
 
79.4
 
 
 
 
ARL6IP1
 
 
hsa-miR-328-3p
 
 
-0.494
 
 
79.4
 
 
 
 
ARMC8
 
 
hsa-miR-20a-5p
 
 
-0.357
 
 
11.8
 
 
 
 
ARMC8
 
 
hsa-miR-17-5p
 
 
-0.378
 
 
11.8
 
 
 
 
ARPP19
 
 
hsa-miR-328-3p
 
 
-0.404
 
 
28.7
 
 
 
 
ARPP19
 
 
hsa-let-7c-5p
 
 
-0.492
 
 
28.7
 
 
 
 
ARPP19
 
 
hsa-miR-98-5p
 
 
-0.5
 
 
28.7
 
 
 
 
ASB6
 
 
hsa-miR-423-5p
 
 
-0.388
 
 
11.9
 
 
 
 
ASF1A
 
 
hsa-miR-200b-3p
 
 
-0.377
 
 
10.1
 
 
 
 
ATG13
 
 
hsa-miR-423-5p
 
 
-0.3
 
 
33.5
 
 
 
 
ATG16L1
 
 
hsa-miR-20a-5p
 
 
-0.446
 
 
11.7
 
 
 
 
ATG16L1
 
 
hsa-miR-17-5p
 
 
-0.467
 
 
11.7
 
 
 
 
ATG4A
 
 
hsa-miR-7-5p
 
 
-0.448
 
 
12.3
 
 
 
 
ATOSB
 
 
hsa-miR-328-3p
 
 
-0.358
 
 
10.9
 
 
 
 
ATOSB
 
 
hsa-miR-98-5p
 
 
-0.429
 
 
10.9
 
 
 
 
ATOSB
 
 
hsa-let-7c-5p
 
 
-0.429
 
 
10.9
 
 
 
 
ATP2A2
 
 
hsa-miR-98-5p
 
 
-0.311
 
 
74.5
 
 
 
 
ATP2A2
 
 
hsa-let-7c-5p
 
 
-0.311
 
 
74.5
 
 
 
 
ATP2B2
 
 
hsa-miR-7-5p
 
 
-0.297
 
 
31.3
 
 
 
 
ATP6V1F
 
 
hsa-miR-194-5p
 
 
-0.444
 
 
129.0
 
 
 
 
ATP6V1H
 
 
hsa-miR-194-5p
 
 
-0.652
 
 
25.8
 
 
 
 
ATPAF1
 
 
hsa-miR-98-5p
 
 
-0.498
 
 
54.3
 
 
 
 
ATPAF1
 
 
hsa-let-7c-5p
 
 
-0.498
 
 
54.3
 
 
 
 
AVPI1
 
 
hsa-miR-7-5p
 
 
-0.367
 
 
23.0
 
 
 
 
B3GAT3
 
 
hsa-miR-98-5p
 
 
-0.324
 
 
28.6
 
 
 
 
B3GAT3
 
 
hsa-let-7c-5p
 
 
-0.324
 
 
28.6
 
 
 
 
B4GALT5
 
 
hsa-miR-210-3p
 
 
-0.511
 
 
18.2
 
 
 
 
B4GAT1
 
 
hsa-miR-200b-3p
 
 
-0.382
 
 
14.9
 
 
 
 
B4GAT1
 
 
hsa-miR-98-5p
 
 
-0.47
 
 
14.9
 
 
 
 
B4GAT1
 
 
hsa-let-7c-5p
 
 
-0.47
 
 
14.9
 
 
 
 
BACE2
 
 
hsa-miR-98-5p
 
 
-0.364
 
 
16.3
 
 
 
 
BACE2
 
 
hsa-let-7c-5p
 
 
-0.364
 
 
16.3
 
 
 
 
BACH1
 
 
hsa-miR-98-5p
 
 
-0.298
 
 
16.4
 
 
 
 
BACH1
 
 
hsa-miR-98-5p
 
 
-0.298
 
 
16.4
 
 
 
 
BACH1
 
 
hsa-let-7c-5p
 
 
-0.306
 
 
16.4
 
 
 
 
BACH1
 
 
hsa-let-7c-5p
 
 
-0.306
 
 
16.4
 
 
 
 
BACH1
 
 
hsa-miR-98-5p
 
 
-0.435
 
 
16.4
 
 
 
 
BACH1
 
 
hsa-miR-98-5p
 
 
-0.435
 
 
16.4
 
 
 
 
BACH1
 
 
hsa-let-7c-5p
 
 
-0.435
 
 
16.4
 
 
 
 
BACH1
 
 
hsa-let-7c-5p
 
 
-0.435
 
 
16.4
 
 
 
 
BAG6
 
 
hsa-miR-200b-3p
 
 
-0.371
 
 
71.2
 
 
 
 
BAP1
 
 
hsa-miR-423-5p
 
 
-0.428
 
 
40.9
 
 
 
 
BBX
 
 
hsa-miR-18a-5p
 
 
-0.31
 
 
12.6
 
 
 
 
BIN1
 
 
hsa-miR-744-5p
 
 
-0.654
 
 
40.9
 
 
 
 
BIN3
 
 
hsa-miR-98-5p
 
 
-0.427
 
 
13.3
 
 
 
 
BIN3
 
 
hsa-let-7c-5p
 
 
-0.427
 
 
13.3
 
 
 
 
BLOC1S6
 
 
hsa-miR-98-5p
 
 
-0.321
 
 
19.5
 
 
 
 
 
 
 skin 
 
 
 
 
target_symbol
 
 
mature_mirna_id
 
 
score
 
 
nTPM
 
 
 
 
 
 
ABHD17C
 
 
hsa-let-7c-5p
 
 
-0.359
 
 
17.9
 
 
 
 
ABHD17C
 
 
hsa-miR-98-5p
 
 
-0.383
 
 
17.9
 
 
 
 
ABHD17C
 
 
hsa-miR-330-3p
 
 
-0.399
 
 
17.9
 
 
 
 
ABHD5
 
 
hsa-miR-20a-5p
 
 
-0.397
 
 
29.7
 
 
 
 
ABHD5
 
 
hsa-miR-17-5p
 
 
-0.45
 
 
29.7
 
 
 
 
ACP3
 
 
hsa-miR-98-5p
 
 
-0.298
 
 
25.5
 
 
 
 
ACP3
 
 
hsa-let-7c-5p
 
 
-0.298
 
 
25.5
 
 
 
 
ACTA1
 
 
hsa-miR-155-5p
 
 
-0.595
 
 
18.7
 
 
 
 
ACTC1
 
 
hsa-miR-7-5p
 
 
-0.471
 
 
14.2
 
 
 
 
ADD3
 
 
hsa-miR-200b-3p
 
 
-0.324
 
 
31.0
 
 
 
 
ADD3
 
 
hsa-miR-18a-5p
 
 
-0.39
 
 
31.0
 
 
 
 
ADNP
 
 
hsa-miR-328-3p
 
 
-0.371
 
 
33.2
 
 
 
 
ADRB2
 
 
hsa-miR-98-5p
 
 
-0.635
 
 
36.9
 
 
 
 
ADRB2
 
 
hsa-let-7c-5p
 
 
-0.635
 
 
36.9
 
 
 
 
AEBP2
 
 
hsa-miR-18a-5p
 
 
-0.376
 
 
27.6
 
 
 
 
AGFG2
 
 
hsa-miR-20a-5p
 
 
-0.365
 
 
24.5
 
 
 
 
AGFG2
 
 
hsa-miR-17-5p
 
 
-0.402
 
 
24.5
 
 
 
 
AHNAK
 
 
hsa-miR-20a-5p
 
 
-0.316
 
 
316.1
 
 
 
 
AHNAK
 
 
hsa-miR-17-5p
 
 
-0.337
 
 
316.1
 
 
 
 
AIFM1
 
 
hsa-miR-98-5p
 
 
-0.314
 
 
10.3
 
 
 
 
AIFM1
 
 
hsa-let-7c-5p
 
 
-0.314
 
 
10.3
 
 
 
 
AK1
 
 
hsa-miR-423-5p
 
 
-0.443
 
 
17.4
 
 
 
 
ALCAM
 
 
hsa-miR-18a-5p
 
 
-0.341
 
 
10.3
 
 
 
 
ALDH4A1
 
 
hsa-miR-184
 
 
-0.421
 
 
18.1
 
 
 
 
ALG3
 
 
hsa-miR-20a-5p
 
 
-0.559
 
 
23.7
 
 
 
 
ALG3
 
 
hsa-miR-17-5p
 
 
-0.572
 
 
23.7
 
 
 
 
ALG9
 
 
hsa-miR-7-5p
 
 
-0.302
 
 
13.9
 
 
 
 
ANGPTL2
 
 
hsa-miR-423-5p
 
 
-0.379
 
 
27.9
 
 
 
 
ANKFY1
 
 
hsa-miR-155-5p
 
 
-0.315
 
 
18.7
 
 
 
 
ANKRD12
 
 
hsa-miR-7-5p
 
 
-0.37
 
 
19.1
 
 
 
 
ANKRD46
 
 
hsa-miR-98-5p
 
 
-0.389
 
 
10.5
 
 
 
 
ANKRD46
 
 
hsa-let-7c-5p
 
 
-0.389
 
 
10.5
 
 
 
 
ANKRD50
 
 
hsa-miR-20a-5p
 
 
-0.3
 
 
10.4
 
 
 
 
ANKRD9
 
 
hsa-miR-20a-5p
 
 
-0.31
 
 
24.3
 
 
 
 
ANKRD9
 
 
hsa-miR-17-5p
 
 
-0.347
 
 
24.3
 
 
 
 
ANP32B
 
 
hsa-miR-200b-3p
 
 
-0.287
 
 
140.0
 
 
 
 
AP1S1
 
 
hsa-miR-98-5p
 
 
-0.374
 
 
40.6
 
 
 
 
AP1S1
 
 
hsa-let-7c-5p
 
 
-0.374
 
 
40.6
 
 
 
 
AP1S2
 
 
hsa-miR-200b-3p
 
 
-0.4
 
 
12.4
 
 
 
 
AP2M1
 
 
hsa-miR-423-5p
 
 
-0.49
 
 
130.1
 
 
 
 
APCDD1
 
 
hsa-miR-20a-5p
 
 
-0.404
 
 
122.7
 
 
 
 
APCDD1
 
 
hsa-miR-17-5p
 
 
-0.425
 
 
122.7
 
 
 
 
ARF4
 
 
hsa-miR-194-5p
 
 
-0.293
 
 
61.3
 
 
 
 
ARF4
 
 
hsa-miR-7-5p
 
 
-0.407
 
 
61.3
 
 
 
 
ARHGAP1
 
 
hsa-miR-17-5p
 
 
-0.325
 
 
61.6
 
 
 
 
ARHGAP1
 
 
hsa-miR-20a-5p
 
 
-0.339
 
 
61.6
 
 
 
 
ARHGEF28
 
 
hsa-miR-17-5p
 
 
-0.363
 
 
14.0
 
 
 
 
ARHGEF28
 
 
hsa-miR-20a-5p
 
 
-0.366
 
 
14.0
 
 
 
 
ARL1
 
 
hsa-miR-20a-5p
 
 
-0.403
 
 
26.2
 
 
 
 
ARL1
 
 
hsa-miR-17-5p
 
 
-0.432
 
 
26.2
 
 
 
 
ARL4D
 
 
hsa-miR-98-5p
 
 
-0.417
 
 
44.0
 
 
 
 
ARL4D
 
 
hsa-let-7c-5p
 
 
-0.417
 
 
44.0
 
 
 
 
ARL6IP1
 
 
hsa-miR-7-5p
 
 
-0.374
 
 
62.4
 
 
 
 
ARL6IP1
 
 
hsa-miR-328-3p
 
 
-0.494
 
 
62.4
 
 
 
 
ARL6IP6
 
 
hsa-miR-98-5p
 
 
-0.293
 
 
23.5
 
 
 
 
ARL6IP6
 
 
hsa-let-7c-5p
 
 
-0.293
 
 
23.5
 
 
 
 
ARMC8
 
 
hsa-miR-20a-5p
 
 
-0.357
 
 
12.0
 
 
 
 
ARMC8
 
 
hsa-miR-17-5p
 
 
-0.378
 
 
12.0
 
 
 
 
ARPP19
 
 
hsa-miR-328-3p
 
 
-0.404
 
 
28.9
 
 
 
 
ARPP19
 
 
hsa-let-7c-5p
 
 
-0.492
 
 
28.9
 
 
 
 
ARPP19
 
 
hsa-miR-98-5p
 
 
-0.5
 
 
28.9
 
 
 
 
ARRDC4
 
 
hsa-miR-98-5p
 
 
-0.329
 
 
33.5
 
 
 
 
ARRDC4
 
 
hsa-let-7c-5p
 
 
-0.329
 
 
33.5
 
 
 
 
ASAP1
 
 
hsa-miR-98-5p
 
 
-0.308
 
 
16.2
 
 
 
 
ASAP1
 
 
hsa-let-7c-5p
 
 
-0.308
 
 
16.2
 
 
 
 
ASB6
 
 
hsa-miR-423-5p
 
 
-0.388
 
 
18.6
 
 
 
 
ASF1A
 
 
hsa-miR-200b-3p
 
 
-0.377
 
 
11.0
 
 
 
 
ASPRV1
 
 
hsa-miR-330-3p
 
 
-0.301
 
 
614.8
 
 
 
 
ATG13
 
 
hsa-miR-423-5p
 
 
-0.3
 
 
37.2
 
 
 
 
ATG16L1
 
 
hsa-miR-20a-5p
 
 
-0.446
 
 
17.2
 
 
 
 
ATG16L1
 
 
hsa-miR-17-5p
 
 
-0.467
 
 
17.2
 
 
 
 
ATOSB
 
 
hsa-miR-328-3p
 
 
-0.358
 
 
14.2
 
 
 
 
ATOSB
 
 
hsa-miR-98-5p
 
 
-0.429
 
 
14.2
 
 
 
 
ATOSB
 
 
hsa-let-7c-5p
 
 
-0.429
 
 
14.2
 
 
 
 
ATP2A2
 
 
hsa-miR-98-5p
 
 
-0.311
 
 
41.6
 
 
 
 
ATP2A2
 
 
hsa-let-7c-5p
 
 
-0.311
 
 
41.6
 
 
 
 
ATP6V1F
 
 
hsa-miR-194-5p
 
 
-0.444
 
 
171.3
 
 
 
 
ATP6V1H
 
 
hsa-miR-194-5p
 
 
-0.652
 
 
26.3
 
 
 
 
ATPAF1
 
 
hsa-miR-98-5p
 
 
-0.498
 
 
18.2
 
 
 
 
ATPAF1
 
 
hsa-let-7c-5p
 
 
-0.498
 
 
18.2
 
 
 
 
ATXN1L
 
 
hsa-miR-155-5p
 
 
-0.315
 
 
11.4
 
 
 
 
AVPI1
 
 
hsa-miR-7-5p
 
 
-0.367
 
 
104.5
 
 
 
 
B3GAT3
 
 
hsa-miR-98-5p
 
 
-0.324
 
 
37.0
 
 
 
 
B3GAT3
 
 
hsa-let-7c-5p
 
 
-0.324
 
 
37.0
 
 
 
 
B4GAT1
 
 
hsa-miR-200b-3p
 
 
-0.382
 
 
11.9
 
 
 
 
B4GAT1
 
 
hsa-miR-98-5p
 
 
-0.47
 
 
11.9
 
 
 
 
B4GAT1
 
 
hsa-let-7c-5p
 
 
-0.47
 
 
11.9
 
 
 
 
BACH1
 
 
hsa-miR-98-5p
 
 
-0.298
 
 
21.9
 
 
 
 
BACH1
 
 
hsa-miR-98-5p
 
 
-0.298
 
 
21.9
 
 
 
 
BACH1
 
 
hsa-let-7c-5p
 
 
-0.306
 
 
21.9
 
 
 
 
BACH1
 
 
hsa-let-7c-5p
 
 
-0.306
 
 
21.9
 
 
 
 
BACH1
 
 
hsa-miR-98-5p
 
 
-0.435
 
 
21.9
 
 
 
 
BACH1
 
 
hsa-miR-98-5p
 
 
-0.435
 
 
21.9
 
 
 
 
BACH1
 
 
hsa-let-7c-5p
 
 
-0.435
 
 
21.9
 
 
 
 
BACH1
 
 
hsa-let-7c-5p
 
 
-0.435
 
 
21.9
 
 
 
 
BAG6
 
 
hsa-miR-200b-3p
 
 
-0.371
 
 
85.5
 
 
 
 
BAP1
 
 
hsa-miR-423-5p
 
 
-0.428
 
 
70.4
 
 
 
 
BCAS3
 
 
hsa-miR-423-5p
 
 
-0.322
 
 
13.5
 
 
 
 
BIN1
 
 
hsa-miR-744-5p
 
 
-0.654
 
 
12.2
 
 
 
 
BIN3
 
 
hsa-miR-98-5p
 
 
-0.427
 
 
15.8
 
 
 
 
 
 
 small intestine 
 
 
 
 
target_symbol
 
 
mature_mirna_id
 
 
score
 
 
nTPM
 
 
 
 
 
 
ABHD17C
 
 
hsa-let-7c-5p
 
 
-0.359
 
 
28.8
 
 
 
 
ABHD17C
 
 
hsa-miR-98-5p
 
 
-0.383
 
 
28.8
 
 
 
 
ABHD17C
 
 
hsa-miR-330-3p
 
 
-0.399
 
 
28.8
 
 
 
 
ADAM9
 
 
hsa-miR-20a-5p
 
 
-0.299
 
 
21.4
 
 
 
 
ADAM9
 
 
hsa-miR-17-5p
 
 
-0.32
 
 
21.4
 
 
 
 
ADD3
 
 
hsa-miR-200b-3p
 
 
-0.324
 
 
80.5
 
 
 
 
ADD3
 
 
hsa-miR-18a-5p
 
 
-0.39
 
 
80.5
 
 
 
 
ADNP
 
 
hsa-miR-328-3p
 
 
-0.371
 
 
30.1
 
 
 
 
AEBP2
 
 
hsa-miR-18a-5p
 
 
-0.376
 
 
15.7
 
 
 
 
AGFG2
 
 
hsa-miR-20a-5p
 
 
-0.365
 
 
12.5
 
 
 
 
AGFG2
 
 
hsa-miR-17-5p
 
 
-0.402
 
 
12.5
 
 
 
 
AHNAK
 
 
hsa-miR-20a-5p
 
 
-0.316
 
 
132.1
 
 
 
 
AHNAK
 
 
hsa-miR-17-5p
 
 
-0.337
 
 
132.1
 
 
 
 
AIFM1
 
 
hsa-miR-98-5p
 
 
-0.314
 
 
28.2
 
 
 
 
AIFM1
 
 
hsa-let-7c-5p
 
 
-0.314
 
 
28.2
 
 
 
 
AK1
 
 
hsa-miR-423-5p
 
 
-0.443
 
 
15.6
 
 
 
 
ALCAM
 
 
hsa-miR-18a-5p
 
 
-0.341
 
 
16.4
 
 
 
 
ALG3
 
 
hsa-miR-20a-5p
 
 
-0.559
 
 
19.2
 
 
 
 
ALG3
 
 
hsa-miR-17-5p
 
 
-0.572
 
 
19.2
 
 
 
 
AMT
 
 
hsa-miR-98-5p
 
 
-0.463
 
 
11.5
 
 
 
 
AMT
 
 
hsa-let-7c-5p
 
 
-0.463
 
 
11.5
 
 
 
 
ANGPTL2
 
 
hsa-miR-423-5p
 
 
-0.379
 
 
27.5
 
 
 
 
ANKFY1
 
 
hsa-miR-155-5p
 
 
-0.315
 
 
13.2
 
 
 
 
ANKRD12
 
 
hsa-miR-7-5p
 
 
-0.37
 
 
12.6
 
 
 
 
ANKRD46
 
 
hsa-miR-98-5p
 
 
-0.389
 
 
10.4
 
 
 
 
ANKRD46
 
 
hsa-let-7c-5p
 
 
-0.389
 
 
10.4
 
 
 
 
ANKRD9
 
 
hsa-miR-20a-5p
 
 
-0.31
 
 
31.1
 
 
 
 
ANKRD9
 
 
hsa-miR-17-5p
 
 
-0.347
 
 
31.1
 
 
 
 
ANP32B
 
 
hsa-miR-200b-3p
 
 
-0.287
 
 
119.5
 
 
 
 
AP1S1
 
 
hsa-miR-98-5p
 
 
-0.374
 
 
29.8
 
 
 
 
AP1S1
 
 
hsa-let-7c-5p
 
 
-0.374
 
 
29.8
 
 
 
 
AP1S2
 
 
hsa-miR-200b-3p
 
 
-0.4
 
 
18.2
 
 
 
 
AP2M1
 
 
hsa-miR-423-5p
 
 
-0.49
 
 
133.7
 
 
 
 
APCDD1
 
 
hsa-miR-20a-5p
 
 
-0.404
 
 
13.7
 
 
 
 
APCDD1
 
 
hsa-miR-17-5p
 
 
-0.425
 
 
13.7
 
 
 
 
APOO
 
 
hsa-miR-200b-3p
 
 
-0.361
 
 
11.3
 
 
 
 
ARF4
 
 
hsa-miR-194-5p
 
 
-0.293
 
 
102.8
 
 
 
 
ARF4
 
 
hsa-miR-7-5p
 
 
-0.407
 
 
102.8
 
 
 
 
ARG2
 
 
hsa-miR-98-5p
 
 
-0.468
 
 
14.4
 
 
 
 
ARG2
 
 
hsa-let-7c-5p
 
 
-0.468
 
 
14.4
 
 
 
 
ARHGAP1
 
 
hsa-miR-17-5p
 
 
-0.325
 
 
44.5
 
 
 
 
ARHGAP1
 
 
hsa-miR-20a-5p
 
 
-0.339
 
 
44.5
 
 
 
 
ARL1
 
 
hsa-miR-20a-5p
 
 
-0.403
 
 
40.7
 
 
 
 
ARL1
 
 
hsa-miR-17-5p
 
 
-0.432
 
 
40.7
 
 
 
 
ARL6IP1
 
 
hsa-miR-7-5p
 
 
-0.374
 
 
116.2
 
 
 
 
ARL6IP1
 
 
hsa-miR-328-3p
 
 
-0.494
 
 
116.2
 
 
 
 
ARMC8
 
 
hsa-miR-20a-5p
 
 
-0.357
 
 
12.8
 
 
 
 
ARMC8
 
 
hsa-miR-17-5p
 
 
-0.378
 
 
12.8
 
 
 
 
ARMT1
 
 
hsa-miR-98-5p
 
 
-0.303
 
 
13.7
 
 
 
 
ARMT1
 
 
hsa-let-7c-5p
 
 
-0.303
 
 
13.7
 
 
 
 
ARPP19
 
 
hsa-miR-328-3p
 
 
-0.404
 
 
28.8
 
 
 
 
ARPP19
 
 
hsa-let-7c-5p
 
 
-0.492
 
 
28.8
 
 
 
 
ARPP19
 
 
hsa-miR-98-5p
 
 
-0.5
 
 
28.8
 
 
 
 
ARRDC4
 
 
hsa-miR-98-5p
 
 
-0.329
 
 
22.0
 
 
 
 
ARRDC4
 
 
hsa-let-7c-5p
 
 
-0.329
 
 
22.0
 
 
 
 
ASAP1
 
 
hsa-miR-98-5p
 
 
-0.308
 
 
11.5
 
 
 
 
ASAP1
 
 
hsa-let-7c-5p
 
 
-0.308
 
 
11.5
 
 
 
 
ASB6
 
 
hsa-miR-423-5p
 
 
-0.388
 
 
11.3
 
 
 
 
ATG13
 
 
hsa-miR-423-5p
 
 
-0.3
 
 
26.3
 
 
 
 
ATG16L1
 
 
hsa-miR-20a-5p
 
 
-0.446
 
 
11.3
 
 
 
 
ATG16L1
 
 
hsa-miR-17-5p
 
 
-0.467
 
 
11.3
 
 
 
 
ATG4A
 
 
hsa-miR-7-5p
 
 
-0.448
 
 
10.7
 
 
 
 
ATOSB
 
 
hsa-miR-328-3p
 
 
-0.358
 
 
10.7
 
 
 
 
ATOSB
 
 
hsa-miR-98-5p
 
 
-0.429
 
 
10.7
 
 
 
 
ATOSB
 
 
hsa-let-7c-5p
 
 
-0.429
 
 
10.7
 
 
 
 
ATP2A2
 
 
hsa-miR-98-5p
 
 
-0.311
 
 
53.2
 
 
 
 
ATP2A2
 
 
hsa-let-7c-5p
 
 
-0.311
 
 
53.2
 
 
 
 
ATP6V1F
 
 
hsa-miR-194-5p
 
 
-0.444
 
 
96.4
 
 
 
 
ATP6V1H
 
 
hsa-miR-194-5p
 
 
-0.652
 
 
14.1
 
 
 
 
ATP8B2
 
 
hsa-miR-423-5p
 
 
-0.297
 
 
16.4
 
 
 
 
ATPAF1
 
 
hsa-miR-98-5p
 
 
-0.498
 
 
42.6
 
 
 
 
ATPAF1
 
 
hsa-let-7c-5p
 
 
-0.498
 
 
42.6
 
 
 
 
AVPI1
 
 
hsa-miR-7-5p
 
 
-0.367
 
 
15.7
 
 
 
 
B3GAT3
 
 
hsa-miR-98-5p
 
 
-0.324
 
 
30.1
 
 
 
 
B3GAT3
 
 
hsa-let-7c-5p
 
 
-0.324
 
 
30.1
 
 
 
 
B4GALT5
 
 
hsa-miR-210-3p
 
 
-0.511
 
 
20.1
 
 
 
 
B4GAT1
 
 
hsa-miR-200b-3p
 
 
-0.382
 
 
14.2
 
 
 
 
B4GAT1
 
 
hsa-miR-98-5p
 
 
-0.47
 
 
14.2
 
 
 
 
B4GAT1
 
 
hsa-let-7c-5p
 
 
-0.47
 
 
14.2
 
 
 
 
BACH1
 
 
hsa-miR-98-5p
 
 
-0.298
 
 
16.3
 
 
 
 
BACH1
 
 
hsa-miR-98-5p
 
 
-0.298
 
 
16.3
 
 
 
 
BACH1
 
 
hsa-let-7c-5p
 
 
-0.306
 
 
16.3
 
 
 
 
BACH1
 
 
hsa-let-7c-5p
 
 
-0.306
 
 
16.3
 
 
 
 
BACH1
 
 
hsa-miR-98-5p
 
 
-0.435
 
 
16.3
 
 
 
 
BACH1
 
 
hsa-miR-98-5p
 
 
-0.435
 
 
16.3
 
 
 
 
BACH1
 
 
hsa-let-7c-5p
 
 
-0.435
 
 
16.3
 
 
 
 
BACH1
 
 
hsa-let-7c-5p
 
 
-0.435
 
 
16.3
 
 
 
 
BAG6
 
 
hsa-miR-200b-3p
 
 
-0.371
 
 
78.1
 
 
 
 
BAP1
 
 
hsa-miR-423-5p
 
 
-0.428
 
 
41.2
 
 
 
 
BIN1
 
 
hsa-miR-744-5p
 
 
-0.654
 
 
29.5
 
 
 
 
BIN3
 
 
hsa-miR-98-5p
 
 
-0.427
 
 
12.8
 
 
 
 
BIN3
 
 
hsa-let-7c-5p
 
 
-0.427
 
 
12.8
 
 
 
 
BLOC1S4
 
 
hsa-miR-7-5p
 
 
-0.417
 
 
11.6
 
 
 
 
BLOC1S4
 
 
hsa-miR-7-5p
 
 
-0.527
 
 
11.6
 
 
 
 
BLOC1S6
 
 
hsa-miR-98-5p
 
 
-0.321
 
 
20.1
 
 
 
 
BLOC1S6
 
 
hsa-let-7c-5p
 
 
-0.321
 
 
20.1
 
 
 
 
BNIP2
 
 
hsa-miR-194-5p
 
 
-0.489
 
 
17.2
 
 
 
 
BNIP3L
 
 
hsa-miR-20a-5p
 
 
-0.287
 
 
26.7
 
 
 
 
BRWD1
 
 
hsa-miR-18a-5p
 
 
-0.29
 
 
10.7
 
 
 
 
BTBD10
 
 
hsa-miR-17-5p
 
 
-0.409
 
 
11.0
 
 
 
 
 
 
 stomach 
 
 
 
 
target_symbol
 
 
mature_mirna_id
 
 
score
 
 
nTPM
 
 
 
 
 
 
ABHD17C
 
 
hsa-let-7c-5p
 
 
-0.359
 
 
34.0
 
 
 
 
ABHD17C
 
 
hsa-miR-98-5p
 
 
-0.383
 
 
34.0
 
 
 
 
ABHD17C
 
 
hsa-miR-330-3p
 
 
-0.399
 
 
34.0
 
 
 
 
ACER2
 
 
hsa-miR-20a-5p
 
 
-0.298
 
 
58.3
 
 
 
 
ACER2
 
 
hsa-miR-17-5p
 
 
-0.319
 
 
58.3
 
 
 
 
ACER2
 
 
hsa-let-7c-5p
 
 
-0.333
 
 
58.3
 
 
 
 
ACER2
 
 
hsa-miR-98-5p
 
 
-0.344
 
 
58.3
 
 
 
 
ACTC1
 
 
hsa-miR-7-5p
 
 
-0.471
 
 
32.8
 
 
 
 
ADAM9
 
 
hsa-miR-20a-5p
 
 
-0.299
 
 
41.8
 
 
 
 
ADAM9
 
 
hsa-miR-17-5p
 
 
-0.32
 
 
41.8
 
 
 
 
ADD3
 
 
hsa-miR-200b-3p
 
 
-0.324
 
 
60.2
 
 
 
 
ADD3
 
 
hsa-miR-18a-5p
 
 
-0.39
 
 
60.2
 
 
 
 
ADNP
 
 
hsa-miR-328-3p
 
 
-0.371
 
 
30.6
 
 
 
 
AEBP2
 
 
hsa-miR-18a-5p
 
 
-0.376
 
 
15.1
 
 
 
 
AGFG2
 
 
hsa-miR-20a-5p
 
 
-0.365
 
 
12.9
 
 
 
 
AGFG2
 
 
hsa-miR-17-5p
 
 
-0.402
 
 
12.9
 
 
 
 
AHNAK
 
 
hsa-miR-20a-5p
 
 
-0.316
 
 
125.8
 
 
 
 
AHNAK
 
 
hsa-miR-17-5p
 
 
-0.337
 
 
125.8
 
 
 
 
AIFM1
 
 
hsa-miR-98-5p
 
 
-0.314
 
 
29.8
 
 
 
 
AIFM1
 
 
hsa-let-7c-5p
 
 
-0.314
 
 
29.8
 
 
 
 
AK1
 
 
hsa-miR-423-5p
 
 
-0.443
 
 
28.6
 
 
 
 
ALCAM
 
 
hsa-miR-18a-5p
 
 
-0.341
 
 
31.6
 
 
 
 
ALDH4A1
 
 
hsa-miR-184
 
 
-0.421
 
 
12.6
 
 
 
 
ALG3
 
 
hsa-miR-20a-5p
 
 
-0.559
 
 
22.0
 
 
 
 
ALG3
 
 
hsa-miR-17-5p
 
 
-0.572
 
 
22.0
 
 
 
 
ALG9
 
 
hsa-miR-7-5p
 
 
-0.302
 
 
11.1
 
 
 
 
AMT
 
 
hsa-miR-98-5p
 
 
-0.463
 
 
13.1
 
 
 
 
AMT
 
 
hsa-let-7c-5p
 
 
-0.463
 
 
13.1
 
 
 
 
ANGPTL2
 
 
hsa-miR-423-5p
 
 
-0.379
 
 
27.7
 
 
 
 
ANKFY1
 
 
hsa-miR-155-5p
 
 
-0.315
 
 
12.2
 
 
 
 
ANKRD12
 
 
hsa-miR-7-5p
 
 
-0.37
 
 
13.7
 
 
 
 
ANKRD29
 
 
hsa-miR-20a-5p
 
 
-0.359
 
 
14.3
 
 
 
 
ANKRD29
 
 
hsa-miR-17-5p
 
 
-0.372
 
 
14.3
 
 
 
 
ANKRD46
 
 
hsa-miR-98-5p
 
 
-0.389
 
 
10.9
 
 
 
 
ANKRD46
 
 
hsa-let-7c-5p
 
 
-0.389
 
 
10.9
 
 
 
 
ANKRD9
 
 
hsa-miR-20a-5p
 
 
-0.31
 
 
21.4
 
 
 
 
ANKRD9
 
 
hsa-miR-17-5p
 
 
-0.347
 
 
21.4
 
 
 
 
ANP32B
 
 
hsa-miR-200b-3p
 
 
-0.287
 
 
125.6
 
 
 
 
AP1S1
 
 
hsa-miR-98-5p
 
 
-0.374
 
 
23.2
 
 
 
 
AP1S1
 
 
hsa-let-7c-5p
 
 
-0.374
 
 
23.2
 
 
 
 
AP1S2
 
 
hsa-miR-200b-3p
 
 
-0.4
 
 
17.5
 
 
 
 
AP2M1
 
 
hsa-miR-423-5p
 
 
-0.49
 
 
133.6
 
 
 
 
APCDD1
 
 
hsa-miR-20a-5p
 
 
-0.404
 
 
22.7
 
 
 
 
APCDD1
 
 
hsa-miR-17-5p
 
 
-0.425
 
 
22.7
 
 
 
 
APOO
 
 
hsa-miR-200b-3p
 
 
-0.361
 
 
17.0
 
 
 
 
ARF4
 
 
hsa-miR-194-5p
 
 
-0.293
 
 
123.5
 
 
 
 
ARF4
 
 
hsa-miR-7-5p
 
 
-0.407
 
 
123.5
 
 
 
 
ARHGAP1
 
 
hsa-miR-17-5p
 
 
-0.325
 
 
46.3
 
 
 
 
ARHGAP1
 
 
hsa-miR-20a-5p
 
 
-0.339
 
 
46.3
 
 
 
 
ARHGEF28
 
 
hsa-miR-17-5p
 
 
-0.363
 
 
20.6
 
 
 
 
ARHGEF28
 
 
hsa-miR-20a-5p
 
 
-0.366
 
 
20.6
 
 
 
 
ARID4B
 
 
hsa-miR-20a-5p
 
 
-0.29
 
 
12.5
 
 
 
 
ARL1
 
 
hsa-miR-20a-5p
 
 
-0.403
 
 
54.2
 
 
 
 
ARL1
 
 
hsa-miR-17-5p
 
 
-0.432
 
 
54.2
 
 
 
 
ARL6IP1
 
 
hsa-miR-7-5p
 
 
-0.374
 
 
118.7
 
 
 
 
ARL6IP1
 
 
hsa-miR-328-3p
 
 
-0.494
 
 
118.7
 
 
 
 
ARMC8
 
 
hsa-miR-20a-5p
 
 
-0.357
 
 
14.5
 
 
 
 
ARMC8
 
 
hsa-miR-17-5p
 
 
-0.378
 
 
14.5
 
 
 
 
ARMT1
 
 
hsa-miR-98-5p
 
 
-0.303
 
 
13.3
 
 
 
 
ARMT1
 
 
hsa-let-7c-5p
 
 
-0.303
 
 
13.3
 
 
 
 
ARPP19
 
 
hsa-miR-328-3p
 
 
-0.404
 
 
36.4
 
 
 
 
ARPP19
 
 
hsa-let-7c-5p
 
 
-0.492
 
 
36.4
 
 
 
 
ARPP19
 
 
hsa-miR-98-5p
 
 
-0.5
 
 
36.4
 
 
 
 
ARRDC4
 
 
hsa-miR-98-5p
 
 
-0.329
 
 
25.2
 
 
 
 
ARRDC4
 
 
hsa-let-7c-5p
 
 
-0.329
 
 
25.2
 
 
 
 
ASB6
 
 
hsa-miR-423-5p
 
 
-0.388
 
 
13.6
 
 
 
 
ASF1A
 
 
hsa-miR-200b-3p
 
 
-0.377
 
 
10.3
 
 
 
 
ATG13
 
 
hsa-miR-423-5p
 
 
-0.3
 
 
33.9
 
 
 
 
ATG16L1
 
 
hsa-miR-20a-5p
 
 
-0.446
 
 
11.5
 
 
 
 
ATG16L1
 
 
hsa-miR-17-5p
 
 
-0.467
 
 
11.5
 
 
 
 
ATG4A
 
 
hsa-miR-7-5p
 
 
-0.448
 
 
11.6
 
 
 
 
ATOSB
 
 
hsa-miR-328-3p
 
 
-0.358
 
 
11.2
 
 
 
 
ATOSB
 
 
hsa-miR-98-5p
 
 
-0.429
 
 
11.2
 
 
 
 
ATOSB
 
 
hsa-let-7c-5p
 
 
-0.429
 
 
11.2
 
 
 
 
ATP2A2
 
 
hsa-miR-98-5p
 
 
-0.311
 
 
44.5
 
 
 
 
ATP2A2
 
 
hsa-let-7c-5p
 
 
-0.311
 
 
44.5
 
 
 
 
ATP6V1F
 
 
hsa-miR-194-5p
 
 
-0.444
 
 
115.3
 
 
 
 
ATP6V1H
 
 
hsa-miR-194-5p
 
 
-0.652
 
 
24.1
 
 
 
 
ATP8B2
 
 
hsa-miR-423-5p
 
 
-0.297
 
 
13.4
 
 
 
 
ATPAF1
 
 
hsa-miR-98-5p
 
 
-0.498
 
 
40.7
 
 
 
 
ATPAF1
 
 
hsa-let-7c-5p
 
 
-0.498
 
 
40.7
 
 
 
 
AVPI1
 
 
hsa-miR-7-5p
 
 
-0.367
 
 
31.1
 
 
 
 
B3GAT3
 
 
hsa-miR-98-5p
 
 
-0.324
 
 
32.0
 
 
 
 
B3GAT3
 
 
hsa-let-7c-5p
 
 
-0.324
 
 
32.0
 
 
 
 
B4GALT5
 
 
hsa-miR-210-3p
 
 
-0.511
 
 
24.3
 
 
 
 
B4GAT1
 
 
hsa-miR-200b-3p
 
 
-0.382
 
 
25.1
 
 
 
 
B4GAT1
 
 
hsa-miR-98-5p
 
 
-0.47
 
 
25.1
 
 
 
 
B4GAT1
 
 
hsa-let-7c-5p
 
 
-0.47
 
 
25.1
 
 
 
 
BACE2
 
 
hsa-miR-98-5p
 
 
-0.364
 
 
21.6
 
 
 
 
BACE2
 
 
hsa-let-7c-5p
 
 
-0.364
 
 
21.6
 
 
 
 
BACH1
 
 
hsa-miR-98-5p
 
 
-0.298
 
 
12.7
 
 
 
 
BACH1
 
 
hsa-miR-98-5p
 
 
-0.298
 
 
12.7
 
 
 
 
BACH1
 
 
hsa-let-7c-5p
 
 
-0.306
 
 
12.7
 
 
 
 
BACH1
 
 
hsa-let-7c-5p
 
 
-0.306
 
 
12.7
 
 
 
 
BACH1
 
 
hsa-miR-98-5p
 
 
-0.435
 
 
12.7
 
 
 
 
BACH1
 
 
hsa-miR-98-5p
 
 
-0.435
 
 
12.7
 
 
 
 
BACH1
 
 
hsa-let-7c-5p
 
 
-0.435
 
 
12.7
 
 
 
 
BACH1
 
 
hsa-let-7c-5p
 
 
-0.435
 
 
12.7
 
 
 
 
BAG6
 
 
hsa-miR-200b-3p
 
 
-0.371
 
 
67.2
 
 
 
 
BAP1
 
 
hsa-miR-423-5p
 
 
-0.428
 
 
45.5
 
 
 
 
 
 
 tonsil 
 
 
 
 
target_symbol
 
 
mature_mirna_id
 
 
score
 
 
nTPM
 
 
 
 
 
 
ABHD17C
 
 
hsa-let-7c-5p
 
 
-0.359
 
 
21.6
 
 
 
 
ABHD17C
 
 
hsa-miR-98-5p
 
 
-0.383
 
 
21.6
 
 
 
 
ABHD17C
 
 
hsa-miR-330-3p
 
 
-0.399
 
 
21.6
 
 
 
 
ADAM9
 
 
hsa-miR-20a-5p
 
 
-0.299
 
 
12.4
 
 
 
 
ADAM9
 
 
hsa-miR-17-5p
 
 
-0.32
 
 
12.4
 
 
 
 
ADD3
 
 
hsa-miR-200b-3p
 
 
-0.324
 
 
61.3
 
 
 
 
ADD3
 
 
hsa-miR-18a-5p
 
 
-0.39
 
 
61.3
 
 
 
 
ADNP
 
 
hsa-miR-328-3p
 
 
-0.371
 
 
33.4
 
 
 
 
AEBP2
 
 
hsa-miR-18a-5p
 
 
-0.376
 
 
30.2
 
 
 
 
AHNAK
 
 
hsa-miR-20a-5p
 
 
-0.316
 
 
59.8
 
 
 
 
AHNAK
 
 
hsa-miR-17-5p
 
 
-0.337
 
 
59.8
 
 
 
 
AIFM1
 
 
hsa-miR-98-5p
 
 
-0.314
 
 
10.3
 
 
 
 
AIFM1
 
 
hsa-let-7c-5p
 
 
-0.314
 
 
10.3
 
 
 
 
AK1
 
 
hsa-miR-423-5p
 
 
-0.443
 
 
11.8
 
 
 
 
ALCAM
 
 
hsa-miR-18a-5p
 
 
-0.341
 
 
13.6
 
 
 
 
ALG3
 
 
hsa-miR-20a-5p
 
 
-0.559
 
 
13.2
 
 
 
 
ALG3
 
 
hsa-miR-17-5p
 
 
-0.572
 
 
13.2
 
 
 
 
ANKFY1
 
 
hsa-miR-155-5p
 
 
-0.315
 
 
12.0
 
 
 
 
ANKRD12
 
 
hsa-miR-7-5p
 
 
-0.37
 
 
17.3
 
 
 
 
ANKRD29
 
 
hsa-miR-20a-5p
 
 
-0.359
 
 
13.4
 
 
 
 
ANKRD29
 
 
hsa-miR-17-5p
 
 
-0.372
 
 
13.4
 
 
 
 
ANKRD46
 
 
hsa-miR-98-5p
 
 
-0.389
 
 
10.3
 
 
 
 
ANKRD46
 
 
hsa-let-7c-5p
 
 
-0.389
 
 
10.3
 
 
 
 
ANP32B
 
 
hsa-miR-200b-3p
 
 
-0.287
 
 
232.6
 
 
 
 
AP1S1
 
 
hsa-miR-98-5p
 
 
-0.374
 
 
12.8
 
 
 
 
AP1S1
 
 
hsa-let-7c-5p
 
 
-0.374
 
 
12.8
 
 
 
 
AP1S2
 
 
hsa-miR-200b-3p
 
 
-0.4
 
 
26.7
 
 
 
 
AP2M1
 
 
hsa-miR-423-5p
 
 
-0.49
 
 
135.8
 
 
 
 
APOO
 
 
hsa-miR-200b-3p
 
 
-0.361
 
 
10.8
 
 
 
 
ARF4
 
 
hsa-miR-194-5p
 
 
-0.293
 
 
91.2
 
 
 
 
ARF4
 
 
hsa-miR-7-5p
 
 
-0.407
 
 
91.2
 
 
 
 
ARHGAP1
 
 
hsa-miR-17-5p
 
 
-0.325
 
 
32.4
 
 
 
 
ARHGAP1
 
 
hsa-miR-20a-5p
 
 
-0.339
 
 
32.4
 
 
 
 
ARID4A
 
 
hsa-miR-7-5p
 
 
-0.341
 
 
10.5
 
 
 
 
ARID4B
 
 
hsa-miR-20a-5p
 
 
-0.29
 
 
15.9
 
 
 
 
ARL1
 
 
hsa-miR-20a-5p
 
 
-0.403
 
 
45.6
 
 
 
 
ARL1
 
 
hsa-miR-17-5p
 
 
-0.432
 
 
45.6
 
 
 
 
ARL6IP1
 
 
hsa-miR-7-5p
 
 
-0.374
 
 
106.2
 
 
 
 
ARL6IP1
 
 
hsa-miR-328-3p
 
 
-0.494
 
 
106.2
 
 
 
 
ARL6IP6
 
 
hsa-miR-98-5p
 
 
-0.293
 
 
18.0
 
 
 
 
ARL6IP6
 
 
hsa-let-7c-5p
 
 
-0.293
 
 
18.0
 
 
 
 
ARMC8
 
 
hsa-miR-20a-5p
 
 
-0.357
 
 
21.4
 
 
 
 
ARMC8
 
 
hsa-miR-17-5p
 
 
-0.378
 
 
21.4
 
 
 
 
ARMT1
 
 
hsa-miR-98-5p
 
 
-0.303
 
 
17.3
 
 
 
 
ARMT1
 
 
hsa-let-7c-5p
 
 
-0.303
 
 
17.3
 
 
 
 
ARPP19
 
 
hsa-miR-328-3p
 
 
-0.404
 
 
48.4
 
 
 
 
ARPP19
 
 
hsa-let-7c-5p
 
 
-0.492
 
 
48.4
 
 
 
 
ARPP19
 
 
hsa-miR-98-5p
 
 
-0.5
 
 
48.4
 
 
 
 
ASAP1
 
 
hsa-miR-98-5p
 
 
-0.308
 
 
17.8
 
 
 
 
ASAP1
 
 
hsa-let-7c-5p
 
 
-0.308
 
 
17.8
 
 
 
 
ASF1A
 
 
hsa-miR-200b-3p
 
 
-0.377
 
 
14.1
 
 
 
 
ATAD2
 
 
hsa-miR-17-5p
 
 
-0.304
 
 
12.0
 
 
 
 
ATAD2
 
 
hsa-miR-20a-5p
 
 
-0.318
 
 
12.0
 
 
 
 
ATG13
 
 
hsa-miR-423-5p
 
 
-0.3
 
 
31.4
 
 
 
 
ATG4A
 
 
hsa-miR-7-5p
 
 
-0.448
 
 
10.4
 
 
 
 
ATM
 
 
hsa-miR-18a-5p
 
 
-0.347
 
 
20.0
 
 
 
 
ATP2A2
 
 
hsa-miR-98-5p
 
 
-0.311
 
 
45.1
 
 
 
 
ATP2A2
 
 
hsa-let-7c-5p
 
 
-0.311
 
 
45.1
 
 
 
 
ATP6V1F
 
 
hsa-miR-194-5p
 
 
-0.444
 
 
97.1
 
 
 
 
ATP6V1H
 
 
hsa-miR-194-5p
 
 
-0.652
 
 
20.6
 
 
 
 
ATP8B2
 
 
hsa-miR-423-5p
 
 
-0.297
 
 
19.0
 
 
 
 
ATPAF1
 
 
hsa-miR-98-5p
 
 
-0.498
 
 
30.0
 
 
 
 
ATPAF1
 
 
hsa-let-7c-5p
 
 
-0.498
 
 
30.0
 
 
 
 
B3GAT3
 
 
hsa-miR-98-5p
 
 
-0.324
 
 
10.3
 
 
 
 
B3GAT3
 
 
hsa-let-7c-5p
 
 
-0.324
 
 
10.3
 
 
 
 
B4GALT5
 
 
hsa-miR-210-3p
 
 
-0.511
 
 
10.8
 
 
 
 
BACH1
 
 
hsa-miR-98-5p
 
 
-0.298
 
 
13.8
 
 
 
 
BACH1
 
 
hsa-miR-98-5p
 
 
-0.298
 
 
13.8
 
 
 
 
BACH1
 
 
hsa-let-7c-5p
 
 
-0.306
 
 
13.8
 
 
 
 
BACH1
 
 
hsa-let-7c-5p
 
 
-0.306
 
 
13.8
 
 
 
 
BACH1
 
 
hsa-miR-98-5p
 
 
-0.435
 
 
13.8
 
 
 
 
BACH1
 
 
hsa-miR-98-5p
 
 
-0.435
 
 
13.8
 
 
 
 
BACH1
 
 
hsa-let-7c-5p
 
 
-0.435
 
 
13.8
 
 
 
 
BACH1
 
 
hsa-let-7c-5p
 
 
-0.435
 
 
13.8
 
 
 
 
BAG6
 
 
hsa-miR-200b-3p
 
 
-0.371
 
 
27.2
 
 
 
 
BAP1
 
 
hsa-miR-423-5p
 
 
-0.428
 
 
26.1
 
 
 
 
BBX
 
 
hsa-miR-18a-5p
 
 
-0.31
 
 
12.8
 
 
 
 
BIN1
 
 
hsa-miR-744-5p
 
 
-0.654
 
 
25.8
 
 
 
 
BIN3
 
 
hsa-miR-98-5p
 
 
-0.427
 
 
10.2
 
 
 
 
BIN3
 
 
hsa-let-7c-5p
 
 
-0.427
 
 
10.2
 
 
 
 
BLOC1S6
 
 
hsa-miR-98-5p
 
 
-0.321
 
 
33.2
 
 
 
 
BLOC1S6
 
 
hsa-let-7c-5p
 
 
-0.321
 
 
33.2
 
 
 
 
BNIP2
 
 
hsa-miR-194-5p
 
 
-0.489
 
 
43.4
 
 
 
 
BNIP3L
 
 
hsa-miR-20a-5p
 
 
-0.287
 
 
48.8
 
 
 
 
BRWD1
 
 
hsa-miR-18a-5p
 
 
-0.29
 
 
14.0
 
 
 
 
BTBD10
 
 
hsa-miR-17-5p
 
 
-0.409
 
 
16.3
 
 
 
 
BTBD10
 
 
hsa-miR-20a-5p
 
 
-0.412
 
 
16.3
 
 
 
 
BTF3L4
 
 
hsa-miR-200b-3p
 
 
-0.294
 
 
38.2
 
 
 
 
BTF3L4
 
 
hsa-miR-194-5p
 
 
-0.334
 
 
38.2
 
 
 
 
BTF3L4
 
 
hsa-miR-98-5p
 
 
-0.355
 
 
38.2
 
 
 
 
BTF3L4
 
 
hsa-let-7c-5p
 
 
-0.355
 
 
38.2
 
 
 
 
BTG3
 
 
hsa-miR-18a-5p
 
 
-0.464
 
 
15.3
 
 
 
 
BTG3
 
 
hsa-miR-17-5p
 
 
-0.668
 
 
15.3
 
 
 
 
BTG3
 
 
hsa-miR-20a-5p
 
 
-0.671
 
 
15.3
 
 
 
 
BZW1
 
 
hsa-miR-98-5p
 
 
-0.354
 
 
102.5
 
 
 
 
BZW1
 
 
hsa-let-7c-5p
 
 
-0.354
 
 
102.5
 
 
 
 
BZW1
 
 
hsa-miR-98-5p
 
 
-0.375
 
 
102.5
 
 
 
 
BZW1
 
 
hsa-let-7c-5p
 
 
-0.375
 
 
102.5
 
 
 
 
BZW2
 
 
hsa-miR-98-5p
 
 
-0.397
 
 
35.9
 
 
 
 
BZW2
 
 
hsa-let-7c-5p
 
 
-0.397
 
 
35.9
 
 
 
 
 
 
 urinary bladder 
 
 
 
 
target_symbol
 
 
mature_mirna_id
 
 
score
 
 
nTPM
 
 
 
 
 
 
ABHD17C
 
 
hsa-let-7c-5p
 
 
-0.359
 
 
26.8
 
 
 
 
ABHD17C
 
 
hsa-miR-98-5p
 
 
-0.383
 
 
26.8
 
 
 
 
ABHD17C
 
 
hsa-miR-330-3p
 
 
-0.399
 
 
26.8
 
 
 
 
ABHD5
 
 
hsa-miR-20a-5p
 
 
-0.397
 
 
15.5
 
 
 
 
ABHD5
 
 
hsa-miR-17-5p
 
 
-0.45
 
 
15.5
 
 
 
 
ACER2
 
 
hsa-miR-20a-5p
 
 
-0.298
 
 
36.7
 
 
 
 
ACER2
 
 
hsa-miR-17-5p
 
 
-0.319
 
 
36.7
 
 
 
 
ACER2
 
 
hsa-let-7c-5p
 
 
-0.333
 
 
36.7
 
 
 
 
ACER2
 
 
hsa-miR-98-5p
 
 
-0.344
 
 
36.7
 
 
 
 
ACTC1
 
 
hsa-miR-7-5p
 
 
-0.471
 
 
667.0
 
 
 
 
ADAM9
 
 
hsa-miR-20a-5p
 
 
-0.299
 
 
52.5
 
 
 
 
ADAM9
 
 
hsa-miR-17-5p
 
 
-0.32
 
 
52.5
 
 
 
 
ADD3
 
 
hsa-miR-200b-3p
 
 
-0.324
 
 
39.7
 
 
 
 
ADD3
 
 
hsa-miR-18a-5p
 
 
-0.39
 
 
39.7
 
 
 
 
ADNP
 
 
hsa-miR-328-3p
 
 
-0.371
 
 
34.8
 
 
 
 
AEBP2
 
 
hsa-miR-18a-5p
 
 
-0.376
 
 
23.3
 
 
 
 
AGFG2
 
 
hsa-miR-20a-5p
 
 
-0.365
 
 
10.3
 
 
 
 
AGFG2
 
 
hsa-miR-17-5p
 
 
-0.402
 
 
10.3
 
 
 
 
AHNAK
 
 
hsa-miR-20a-5p
 
 
-0.316
 
 
145.5
 
 
 
 
AHNAK
 
 
hsa-miR-17-5p
 
 
-0.337
 
 
145.5
 
 
 
 
AIFM1
 
 
hsa-miR-98-5p
 
 
-0.314
 
 
13.1
 
 
 
 
AIFM1
 
 
hsa-let-7c-5p
 
 
-0.314
 
 
13.1
 
 
 
 
AK1
 
 
hsa-miR-423-5p
 
 
-0.443
 
 
22.9
 
 
 
 
ALCAM
 
 
hsa-miR-18a-5p
 
 
-0.341
 
 
23.9
 
 
 
 
ALDH4A1
 
 
hsa-miR-184
 
 
-0.421
 
 
21.0
 
 
 
 
ALG3
 
 
hsa-miR-20a-5p
 
 
-0.559
 
 
20.1
 
 
 
 
ALG3
 
 
hsa-miR-17-5p
 
 
-0.572
 
 
20.1
 
 
 
 
ALG9
 
 
hsa-miR-7-5p
 
 
-0.302
 
 
10.1
 
 
 
 
AMT
 
 
hsa-miR-98-5p
 
 
-0.463
 
 
13.1
 
 
 
 
AMT
 
 
hsa-let-7c-5p
 
 
-0.463
 
 
13.1
 
 
 
 
ANGPTL2
 
 
hsa-miR-423-5p
 
 
-0.379
 
 
55.1
 
 
 
 
ANKFY1
 
 
hsa-miR-155-5p
 
 
-0.315
 
 
14.8
 
 
 
 
ANKRD12
 
 
hsa-miR-7-5p
 
 
-0.37
 
 
12.7
 
 
 
 
ANKRD50
 
 
hsa-miR-20a-5p
 
 
-0.3
 
 
13.3
 
 
 
 
ANKRD9
 
 
hsa-miR-20a-5p
 
 
-0.31
 
 
11.5
 
 
 
 
ANKRD9
 
 
hsa-miR-17-5p
 
 
-0.347
 
 
11.5
 
 
 
 
ANP32B
 
 
hsa-miR-200b-3p
 
 
-0.287
 
 
122.0
 
 
 
 
AP1S1
 
 
hsa-miR-98-5p
 
 
-0.374
 
 
18.6
 
 
 
 
AP1S1
 
 
hsa-let-7c-5p
 
 
-0.374
 
 
18.6
 
 
 
 
AP1S2
 
 
hsa-miR-200b-3p
 
 
-0.4
 
 
24.2
 
 
 
 
AP2M1
 
 
hsa-miR-423-5p
 
 
-0.49
 
 
192.8
 
 
 
 
APCDD1
 
 
hsa-miR-20a-5p
 
 
-0.404
 
 
34.0
 
 
 
 
APCDD1
 
 
hsa-miR-17-5p
 
 
-0.425
 
 
34.0
 
 
 
 
APOO
 
 
hsa-miR-200b-3p
 
 
-0.361
 
 
10.5
 
 
 
 
ARF4
 
 
hsa-miR-194-5p
 
 
-0.293
 
 
190.1
 
 
 
 
ARF4
 
 
hsa-miR-7-5p
 
 
-0.407
 
 
190.1
 
 
 
 
ARG2
 
 
hsa-miR-98-5p
 
 
-0.468
 
 
16.6
 
 
 
 
ARG2
 
 
hsa-let-7c-5p
 
 
-0.468
 
 
16.6
 
 
 
 
ARHGAP1
 
 
hsa-miR-17-5p
 
 
-0.325
 
 
106.2
 
 
 
 
ARHGAP1
 
 
hsa-miR-20a-5p
 
 
-0.339
 
 
106.2
 
 
 
 
ARID4B
 
 
hsa-miR-20a-5p
 
 
-0.29
 
 
12.0
 
 
 
 
ARL1
 
 
hsa-miR-20a-5p
 
 
-0.403
 
 
49.5
 
 
 
 
ARL1
 
 
hsa-miR-17-5p
 
 
-0.432
 
 
49.5
 
 
 
 
ARL4D
 
 
hsa-miR-98-5p
 
 
-0.417
 
 
26.6
 
 
 
 
ARL4D
 
 
hsa-let-7c-5p
 
 
-0.417
 
 
26.6
 
 
 
 
ARL5B
 
 
hsa-miR-155-5p
 
 
-0.355
 
 
11.8
 
 
 
 
ARL6IP1
 
 
hsa-miR-7-5p
 
 
-0.374
 
 
124.0
 
 
 
 
ARL6IP1
 
 
hsa-miR-328-3p
 
 
-0.494
 
 
124.0
 
 
 
 
ARL6IP6
 
 
hsa-miR-98-5p
 
 
-0.293
 
 
12.2
 
 
 
 
ARL6IP6
 
 
hsa-let-7c-5p
 
 
-0.293
 
 
12.2
 
 
 
 
ARMC8
 
 
hsa-miR-20a-5p
 
 
-0.357
 
 
20.8
 
 
 
 
ARMC8
 
 
hsa-miR-17-5p
 
 
-0.378
 
 
20.8
 
 
 
 
ARMT1
 
 
hsa-miR-98-5p
 
 
-0.303
 
 
16.1
 
 
 
 
ARMT1
 
 
hsa-let-7c-5p
 
 
-0.303
 
 
16.1
 
 
 
 
ARPP19
 
 
hsa-miR-328-3p
 
 
-0.404
 
 
51.7
 
 
 
 
ARPP19
 
 
hsa-let-7c-5p
 
 
-0.492
 
 
51.7
 
 
 
 
ARPP19
 
 
hsa-miR-98-5p
 
 
-0.5
 
 
51.7
 
 
 
 
ARRDC4
 
 
hsa-miR-98-5p
 
 
-0.329
 
 
10.9
 
 
 
 
ARRDC4
 
 
hsa-let-7c-5p
 
 
-0.329
 
 
10.9
 
 
 
 
ASAP1
 
 
hsa-miR-98-5p
 
 
-0.308
 
 
14.4
 
 
 
 
ASAP1
 
 
hsa-let-7c-5p
 
 
-0.308
 
 
14.4
 
 
 
 
ATG13
 
 
hsa-miR-423-5p
 
 
-0.3
 
 
26.1
 
 
 
 
ATG16L1
 
 
hsa-miR-20a-5p
 
 
-0.446
 
 
13.3
 
 
 
 
ATG16L1
 
 
hsa-miR-17-5p
 
 
-0.467
 
 
13.3
 
 
 
 
ATOSB
 
 
hsa-miR-328-3p
 
 
-0.358
 
 
10.8
 
 
 
 
ATOSB
 
 
hsa-miR-98-5p
 
 
-0.429
 
 
10.8
 
 
 
 
ATOSB
 
 
hsa-let-7c-5p
 
 
-0.429
 
 
10.8
 
 
 
 
ATP2A2
 
 
hsa-miR-98-5p
 
 
-0.311
 
 
84.9
 
 
 
 
ATP2A2
 
 
hsa-let-7c-5p
 
 
-0.311
 
 
84.9
 
 
 
 
ATP6V1F
 
 
hsa-miR-194-5p
 
 
-0.444
 
 
115.0
 
 
 
 
ATP6V1H
 
 
hsa-miR-194-5p
 
 
-0.652
 
 
23.1
 
 
 
 
ATP8B2
 
 
hsa-miR-423-5p
 
 
-0.297
 
 
17.2
 
 
 
 
ATPAF1
 
 
hsa-miR-98-5p
 
 
-0.498
 
 
26.7
 
 
 
 
ATPAF1
 
 
hsa-let-7c-5p
 
 
-0.498
 
 
26.7
 
 
 
 
AVPI1
 
 
hsa-miR-7-5p
 
 
-0.367
 
 
29.9
 
 
 
 
B3GAT3
 
 
hsa-miR-98-5p
 
 
-0.324
 
 
28.2
 
 
 
 
B3GAT3
 
 
hsa-let-7c-5p
 
 
-0.324
 
 
28.2
 
 
 
 
B4GALT5
 
 
hsa-miR-210-3p
 
 
-0.511
 
 
47.9
 
 
 
 
B4GAT1
 
 
hsa-miR-200b-3p
 
 
-0.382
 
 
21.0
 
 
 
 
B4GAT1
 
 
hsa-miR-98-5p
 
 
-0.47
 
 
21.0
 
 
 
 
B4GAT1
 
 
hsa-let-7c-5p
 
 
-0.47
 
 
21.0
 
 
 
 
BACH1
 
 
hsa-miR-98-5p
 
 
-0.298
 
 
27.7
 
 
 
 
BACH1
 
 
hsa-miR-98-5p
 
 
-0.298
 
 
27.7
 
 
 
 
BACH1
 
 
hsa-let-7c-5p
 
 
-0.306
 
 
27.7
 
 
 
 
BACH1
 
 
hsa-let-7c-5p
 
 
-0.306
 
 
27.7
 
 
 
 
BACH1
 
 
hsa-miR-98-5p
 
 
-0.435
 
 
27.7
 
 
 
 
BACH1
 
 
hsa-miR-98-5p
 
 
-0.435
 
 
27.7
 
 
 
 
BACH1
 
 
hsa-let-7c-5p
 
 
-0.435
 
 
27.7
 
 
 
 
BACH1
 
 
hsa-let-7c-5p
 
 
-0.435
 
 
27.7
 
 
 
 
BAG6
 
 
hsa-miR-200b-3p
 
 
-0.371
 
 
68.7
 
 
 
 
 
 
 vagina 
 
 
 
 
target_symbol
 
 
mature_mirna_id
 
 
score
 
 
nTPM
 
 
 
 
 
 
ABHD17C
 
 
hsa-let-7c-5p
 
 
-0.359
 
 
21.1
 
 
 
 
ABHD17C
 
 
hsa-miR-98-5p
 
 
-0.383
 
 
21.1
 
 
 
 
ABHD17C
 
 
hsa-miR-330-3p
 
 
-0.399
 
 
21.1
 
 
 
 
ABHD5
 
 
hsa-miR-20a-5p
 
 
-0.397
 
 
11.9
 
 
 
 
ABHD5
 
 
hsa-miR-17-5p
 
 
-0.45
 
 
11.9
 
 
 
 
ACP3
 
 
hsa-miR-98-5p
 
 
-0.298
 
 
10.8
 
 
 
 
ACP3
 
 
hsa-let-7c-5p
 
 
-0.298
 
 
10.8
 
 
 
 
ACTC1
 
 
hsa-miR-7-5p
 
 
-0.471
 
 
50.8
 
 
 
 
ADAM9
 
 
hsa-miR-20a-5p
 
 
-0.299
 
 
15.5
 
 
 
 
ADAM9
 
 
hsa-miR-17-5p
 
 
-0.32
 
 
15.5
 
 
 
 
ADD3
 
 
hsa-miR-200b-3p
 
 
-0.324
 
 
37.4
 
 
 
 
ADD3
 
 
hsa-miR-18a-5p
 
 
-0.39
 
 
37.4
 
 
 
 
ADNP
 
 
hsa-miR-328-3p
 
 
-0.371
 
 
30.1
 
 
 
 
ADRB2
 
 
hsa-miR-98-5p
 
 
-0.635
 
 
12.2
 
 
 
 
ADRB2
 
 
hsa-let-7c-5p
 
 
-0.635
 
 
12.2
 
 
 
 
AEBP2
 
 
hsa-miR-18a-5p
 
 
-0.376
 
 
19.2
 
 
 
 
AGFG2
 
 
hsa-miR-20a-5p
 
 
-0.365
 
 
11.5
 
 
 
 
AGFG2
 
 
hsa-miR-17-5p
 
 
-0.402
 
 
11.5
 
 
 
 
AHNAK
 
 
hsa-miR-20a-5p
 
 
-0.316
 
 
200.6
 
 
 
 
AHNAK
 
 
hsa-miR-17-5p
 
 
-0.337
 
 
200.6
 
 
 
 
AK1
 
 
hsa-miR-423-5p
 
 
-0.443
 
 
24.0
 
 
 
 
ALDH4A1
 
 
hsa-miR-184
 
 
-0.421
 
 
16.8
 
 
 
 
ALG3
 
 
hsa-miR-20a-5p
 
 
-0.559
 
 
18.9
 
 
 
 
ALG3
 
 
hsa-miR-17-5p
 
 
-0.572
 
 
18.9
 
 
 
 
ALG9
 
 
hsa-miR-7-5p
 
 
-0.302
 
 
10.2
 
 
 
 
ANGPTL2
 
 
hsa-miR-423-5p
 
 
-0.379
 
 
47.7
 
 
 
 
ANKFY1
 
 
hsa-miR-155-5p
 
 
-0.315
 
 
13.3
 
 
 
 
ANKRD29
 
 
hsa-miR-20a-5p
 
 
-0.359
 
 
12.3
 
 
 
 
ANKRD29
 
 
hsa-miR-17-5p
 
 
-0.372
 
 
12.3
 
 
 
 
ANKRD9
 
 
hsa-miR-20a-5p
 
 
-0.31
 
 
14.6
 
 
 
 
ANKRD9
 
 
hsa-miR-17-5p
 
 
-0.347
 
 
14.6
 
 
 
 
ANP32B
 
 
hsa-miR-200b-3p
 
 
-0.287
 
 
140.4
 
 
 
 
AP1S1
 
 
hsa-miR-98-5p
 
 
-0.374
 
 
20.1
 
 
 
 
AP1S1
 
 
hsa-let-7c-5p
 
 
-0.374
 
 
20.1
 
 
 
 
AP1S2
 
 
hsa-miR-200b-3p
 
 
-0.4
 
 
11.5
 
 
 
 
AP2M1
 
 
hsa-miR-423-5p
 
 
-0.49
 
 
156.1
 
 
 
 
APCDD1
 
 
hsa-miR-20a-5p
 
 
-0.404
 
 
33.2
 
 
 
 
APCDD1
 
 
hsa-miR-17-5p
 
 
-0.425
 
 
33.2
 
 
 
 
ARF4
 
 
hsa-miR-194-5p
 
 
-0.293
 
 
85.1
 
 
 
 
ARF4
 
 
hsa-miR-7-5p
 
 
-0.407
 
 
85.1
 
 
 
 
ARG2
 
 
hsa-miR-98-5p
 
 
-0.468
 
 
11.0
 
 
 
 
ARG2
 
 
hsa-let-7c-5p
 
 
-0.468
 
 
11.0
 
 
 
 
ARHGAP1
 
 
hsa-miR-17-5p
 
 
-0.325
 
 
59.0
 
 
 
 
ARHGAP1
 
 
hsa-miR-20a-5p
 
 
-0.339
 
 
59.0
 
 
 
 
ARID4B
 
 
hsa-miR-20a-5p
 
 
-0.29
 
 
10.0
 
 
 
 
ARL1
 
 
hsa-miR-20a-5p
 
 
-0.403
 
 
34.0
 
 
 
 
ARL1
 
 
hsa-miR-17-5p
 
 
-0.432
 
 
34.0
 
 
 
 
ARL4D
 
 
hsa-miR-98-5p
 
 
-0.417
 
 
38.9
 
 
 
 
ARL4D
 
 
hsa-let-7c-5p
 
 
-0.417
 
 
38.9
 
 
 
 
ARL6IP1
 
 
hsa-miR-7-5p
 
 
-0.374
 
 
48.0
 
 
 
 
ARL6IP1
 
 
hsa-miR-328-3p
 
 
-0.494
 
 
48.0
 
 
 
 
ARMC8
 
 
hsa-miR-20a-5p
 
 
-0.357
 
 
12.4
 
 
 
 
ARMC8
 
 
hsa-miR-17-5p
 
 
-0.378
 
 
12.4
 
 
 
 
ARPP19
 
 
hsa-miR-328-3p
 
 
-0.404
 
 
27.0
 
 
 
 
ARPP19
 
 
hsa-let-7c-5p
 
 
-0.492
 
 
27.0
 
 
 
 
ARPP19
 
 
hsa-miR-98-5p
 
 
-0.5
 
 
27.0
 
 
 
 
ARRDC4
 
 
hsa-miR-98-5p
 
 
-0.329
 
 
10.1
 
 
 
 
ARRDC4
 
 
hsa-let-7c-5p
 
 
-0.329
 
 
10.1
 
 
 
 
ASAP1
 
 
hsa-miR-98-5p
 
 
-0.308
 
 
14.4
 
 
 
 
ASAP1
 
 
hsa-let-7c-5p
 
 
-0.308
 
 
14.4
 
 
 
 
ASB6
 
 
hsa-miR-423-5p
 
 
-0.388
 
 
11.5
 
 
 
 
ATG13
 
 
hsa-miR-423-5p
 
 
-0.3
 
 
27.4
 
 
 
 
ATOSB
 
 
hsa-miR-328-3p
 
 
-0.358
 
 
14.3
 
 
 
 
ATOSB
 
 
hsa-miR-98-5p
 
 
-0.429
 
 
14.3
 
 
 
 
ATOSB
 
 
hsa-let-7c-5p
 
 
-0.429
 
 
14.3
 
 
 
 
ATP2A2
 
 
hsa-miR-98-5p
 
 
-0.311
 
 
40.2
 
 
 
 
ATP2A2
 
 
hsa-let-7c-5p
 
 
-0.311
 
 
40.2
 
 
 
 
ATP6V1F
 
 
hsa-miR-194-5p
 
 
-0.444
 
 
149.6
 
 
 
 
ATP6V1H
 
 
hsa-miR-194-5p
 
 
-0.652
 
 
19.9
 
 
 
 
ATP8B2
 
 
hsa-miR-423-5p
 
 
-0.297
 
 
16.3
 
 
 
 
ATPAF1
 
 
hsa-miR-98-5p
 
 
-0.498
 
 
15.7
 
 
 
 
ATPAF1
 
 
hsa-let-7c-5p
 
 
-0.498
 
 
15.7
 
 
 
 
AVPI1
 
 
hsa-miR-7-5p
 
 
-0.367
 
 
46.2
 
 
 
 
B3GAT3
 
 
hsa-miR-98-5p
 
 
-0.324
 
 
28.4
 
 
 
 
B3GAT3
 
 
hsa-let-7c-5p
 
 
-0.324
 
 
28.4
 
 
 
 
B4GALT5
 
 
hsa-miR-210-3p
 
 
-0.511
 
 
16.5
 
 
 
 
B4GAT1
 
 
hsa-miR-200b-3p
 
 
-0.382
 
 
15.8
 
 
 
 
B4GAT1
 
 
hsa-miR-98-5p
 
 
-0.47
 
 
15.8
 
 
 
 
B4GAT1
 
 
hsa-let-7c-5p
 
 
-0.47
 
 
15.8
 
 
 
 
BACH1
 
 
hsa-miR-98-5p
 
 
-0.298
 
 
12.6
 
 
 
 
BACH1
 
 
hsa-miR-98-5p
 
 
-0.298
 
 
12.6
 
 
 
 
BACH1
 
 
hsa-let-7c-5p
 
 
-0.306
 
 
12.6
 
 
 
 
BACH1
 
 
hsa-let-7c-5p
 
 
-0.306
 
 
12.6
 
 
 
 
BACH1
 
 
hsa-miR-98-5p
 
 
-0.435
 
 
12.6
 
 
 
 
BACH1
 
 
hsa-miR-98-5p
 
 
-0.435
 
 
12.6
 
 
 
 
BACH1
 
 
hsa-let-7c-5p
 
 
-0.435
 
 
12.6
 
 
 
 
BACH1
 
 
hsa-let-7c-5p
 
 
-0.435
 
 
12.6
 
 
 
 
BAG6
 
 
hsa-miR-200b-3p
 
 
-0.371
 
 
64.5
 
 
 
 
BAP1
 
 
hsa-miR-423-5p
 
 
-0.428
 
 
39.4
 
 
 
 
BIN1
 
 
hsa-miR-744-5p
 
 
-0.654
 
 
34.7
 
 
 
 
BIN3
 
 
hsa-miR-98-5p
 
 
-0.427
 
 
13.8
 
 
 
 
BIN3
 
 
hsa-let-7c-5p
 
 
-0.427
 
 
13.8
 
 
 
 
BLOC1S6
 
 
hsa-miR-98-5p
 
 
-0.321
 
 
15.7
 
 
 
 
BLOC1S6
 
 
hsa-let-7c-5p
 
 
-0.321
 
 
15.7
 
 
 
 
BNIP3L
 
 
hsa-miR-20a-5p
 
 
-0.287
 
 
49.0
 
 
 
 
BRWD1
 
 
hsa-miR-18a-5p
 
 
-0.29
 
 
10.9
 
 
 
 
BTBD10
 
 
hsa-miR-17-5p
 
 
-0.409
 
 
17.5
 
 
 
 
BTBD10
 
 
hsa-miR-20a-5p
 
 
-0.412
 
 
17.5
 
 
 
 
BTF3L4
 
 
hsa-miR-200b-3p
 
 
-0.294
 
 
27.9
 
 
 
 
BTF3L4
 
 
hsa-miR-194-5p
 
 
-0.334
 
 
27.9
 
 
 
 
 
 
 adipose tissue 
 
 
 
 
target_symbol
 
 
mature_mirna_id
 
 
score
 
 
nTPM
 
 
 
 
 
 
ABHD5
 
 
hsa-miR-20a-5p
 
 
-0.397
 
 
27.9
 
 
 
 
ABHD5
 
 
hsa-miR-17-5p
 
 
-0.45
 
 
27.9
 
 
 
 
ACTA1
 
 
hsa-miR-155-5p
 
 
-0.595
 
 
13.2
 
 
 
 
ACTC1
 
 
hsa-miR-7-5p
 
 
-0.471
 
 
47.8
 
 
 
 
ACVR1C
 
 
hsa-let-7c-5p
 
 
-0.465
 
 
29.3
 
 
 
 
ACVR1C
 
 
hsa-miR-98-5p
 
 
-0.473
 
 
29.3
 
 
 
 
ADAM9
 
 
hsa-miR-20a-5p
 
 
-0.299
 
 
31.0
 
 
 
 
ADAM9
 
 
hsa-miR-17-5p
 
 
-0.32
 
 
31.0
 
 
 
 
ADD3
 
 
hsa-miR-200b-3p
 
 
-0.324
 
 
54.1
 
 
 
 
ADD3
 
 
hsa-miR-18a-5p
 
 
-0.39
 
 
54.1
 
 
 
 
ADNP
 
 
hsa-miR-328-3p
 
 
-0.371
 
 
25.5
 
 
 
 
ADRB2
 
 
hsa-miR-98-5p
 
 
-0.635
 
 
26.4
 
 
 
 
ADRB2
 
 
hsa-let-7c-5p
 
 
-0.635
 
 
26.4
 
 
 
 
AEBP2
 
 
hsa-miR-18a-5p
 
 
-0.376
 
 
17.8
 
 
 
 
AGFG2
 
 
hsa-miR-20a-5p
 
 
-0.365
 
 
12.0
 
 
 
 
AGFG2
 
 
hsa-miR-17-5p
 
 
-0.402
 
 
12.0
 
 
 
 
AHCTF1
 
 
hsa-miR-98-5p
 
 
-0.374
 
 
11.8
 
 
 
 
AHCTF1
 
 
hsa-let-7c-5p
 
 
-0.374
 
 
11.8
 
 
 
 
AHNAK
 
 
hsa-miR-20a-5p
 
 
-0.316
 
 
230.0
 
 
 
 
AHNAK
 
 
hsa-miR-17-5p
 
 
-0.337
 
 
230.0
 
 
 
 
AIFM1
 
 
hsa-miR-98-5p
 
 
-0.314
 
 
17.4
 
 
 
 
AIFM1
 
 
hsa-let-7c-5p
 
 
-0.314
 
 
17.4
 
 
 
 
AK1
 
 
hsa-miR-423-5p
 
 
-0.443
 
 
30.1
 
 
 
 
ALDH4A1
 
 
hsa-miR-184
 
 
-0.421
 
 
66.5
 
 
 
 
ALG3
 
 
hsa-miR-20a-5p
 
 
-0.559
 
 
20.9
 
 
 
 
ALG3
 
 
hsa-miR-17-5p
 
 
-0.572
 
 
20.9
 
 
 
 
ALG9
 
 
hsa-miR-7-5p
 
 
-0.302
 
 
12.6
 
 
 
 
ANGPTL2
 
 
hsa-miR-423-5p
 
 
-0.379
 
 
104.7
 
 
 
 
ANKFY1
 
 
hsa-miR-155-5p
 
 
-0.315
 
 
12.9
 
 
 
 
ANKRD12
 
 
hsa-miR-7-5p
 
 
-0.37
 
 
14.8
 
 
 
 
ANKRD29
 
 
hsa-miR-20a-5p
 
 
-0.359
 
 
17.0
 
 
 
 
ANKRD29
 
 
hsa-miR-17-5p
 
 
-0.372
 
 
17.0
 
 
 
 
ANKRD46
 
 
hsa-miR-98-5p
 
 
-0.389
 
 
18.0
 
 
 
 
ANKRD46
 
 
hsa-let-7c-5p
 
 
-0.389
 
 
18.0
 
 
 
 
ANKRD9
 
 
hsa-miR-20a-5p
 
 
-0.31
 
 
11.3
 
 
 
 
ANKRD9
 
 
hsa-miR-17-5p
 
 
-0.347
 
 
11.3
 
 
 
 
ANP32B
 
 
hsa-miR-200b-3p
 
 
-0.287
 
 
136.8
 
 
 
 
AP1S1
 
 
hsa-miR-98-5p
 
 
-0.374
 
 
15.1
 
 
 
 
AP1S1
 
 
hsa-let-7c-5p
 
 
-0.374
 
 
15.1
 
 
 
 
AP1S2
 
 
hsa-miR-200b-3p
 
 
-0.4
 
 
26.2
 
 
 
 
AP2M1
 
 
hsa-miR-423-5p
 
 
-0.49
 
 
163.7
 
 
 
 
APCDD1
 
 
hsa-miR-20a-5p
 
 
-0.404
 
 
49.2
 
 
 
 
APCDD1
 
 
hsa-miR-17-5p
 
 
-0.425
 
 
49.2
 
 
 
 
ARF4
 
 
hsa-miR-194-5p
 
 
-0.293
 
 
118.7
 
 
 
 
ARF4
 
 
hsa-miR-7-5p
 
 
-0.407
 
 
118.7
 
 
 
 
ARHGAP1
 
 
hsa-miR-17-5p
 
 
-0.325
 
 
79.6
 
 
 
 
ARHGAP1
 
 
hsa-miR-20a-5p
 
 
-0.339
 
 
79.6
 
 
 
 
ARID4B
 
 
hsa-miR-20a-5p
 
 
-0.29
 
 
10.7
 
 
 
 
ARL1
 
 
hsa-miR-20a-5p
 
 
-0.403
 
 
43.5
 
 
 
 
ARL1
 
 
hsa-miR-17-5p
 
 
-0.432
 
 
43.5
 
 
 
 
ARL4D
 
 
hsa-miR-98-5p
 
 
-0.417
 
 
20.3
 
 
 
 
ARL4D
 
 
hsa-let-7c-5p
 
 
-0.417
 
 
20.3
 
 
 
 
ARL5B
 
 
hsa-miR-155-5p
 
 
-0.355
 
 
10.5
 
 
 
 
ARL6IP1
 
 
hsa-miR-7-5p
 
 
-0.374
 
 
133.2
 
 
 
 
ARL6IP1
 
 
hsa-miR-328-3p
 
 
-0.494
 
 
133.2
 
 
 
 
ARL6IP6
 
 
hsa-miR-98-5p
 
 
-0.293
 
 
11.0
 
 
 
 
ARL6IP6
 
 
hsa-let-7c-5p
 
 
-0.293
 
 
11.0
 
 
 
 
ARMC8
 
 
hsa-miR-20a-5p
 
 
-0.357
 
 
19.5
 
 
 
 
ARMC8
 
 
hsa-miR-17-5p
 
 
-0.378
 
 
19.5
 
 
 
 
ARMT1
 
 
hsa-miR-98-5p
 
 
-0.303
 
 
16.0
 
 
 
 
ARMT1
 
 
hsa-let-7c-5p
 
 
-0.303
 
 
16.0
 
 
 
 
ARPP19
 
 
hsa-miR-328-3p
 
 
-0.404
 
 
57.4
 
 
 
 
ARPP19
 
 
hsa-let-7c-5p
 
 
-0.492
 
 
57.4
 
 
 
 
ARPP19
 
 
hsa-miR-98-5p
 
 
-0.5
 
 
57.4
 
 
 
 
ARRDC4
 
 
hsa-miR-98-5p
 
 
-0.329
 
 
11.8
 
 
 
 
ARRDC4
 
 
hsa-let-7c-5p
 
 
-0.329
 
 
11.8
 
 
 
 
ASAP1
 
 
hsa-miR-98-5p
 
 
-0.308
 
 
32.3
 
 
 
 
ASAP1
 
 
hsa-let-7c-5p
 
 
-0.308
 
 
32.3
 
 
 
 
ASB6
 
 
hsa-miR-423-5p
 
 
-0.388
 
 
10.3
 
 
 
 
ATG13
 
 
hsa-miR-423-5p
 
 
-0.3
 
 
30.3
 
 
 
 
ATOSB
 
 
hsa-miR-328-3p
 
 
-0.358
 
 
13.2
 
 
 
 
ATOSB
 
 
hsa-miR-98-5p
 
 
-0.429
 
 
13.2
 
 
 
 
ATOSB
 
 
hsa-let-7c-5p
 
 
-0.429
 
 
13.2
 
 
 
 
ATP2A2
 
 
hsa-miR-98-5p
 
 
-0.311
 
 
56.6
 
 
 
 
ATP2A2
 
 
hsa-let-7c-5p
 
 
-0.311
 
 
56.6
 
 
 
 
ATP6V1F
 
 
hsa-miR-194-5p
 
 
-0.444
 
 
135.2
 
 
 
 
ATP6V1H
 
 
hsa-miR-194-5p
 
 
-0.652
 
 
21.6
 
 
 
 
ATP8B2
 
 
hsa-miR-423-5p
 
 
-0.297
 
 
15.9
 
 
 
 
ATPAF1
 
 
hsa-miR-98-5p
 
 
-0.498
 
 
81.3
 
 
 
 
ATPAF1
 
 
hsa-let-7c-5p
 
 
-0.498
 
 
81.3
 
 
 
 
ATXN1L
 
 
hsa-miR-155-5p
 
 
-0.315
 
 
13.1
 
 
 
 
AVPI1
 
 
hsa-miR-7-5p
 
 
-0.367
 
 
32.2
 
 
 
 
B3GAT3
 
 
hsa-miR-98-5p
 
 
-0.324
 
 
30.8
 
 
 
 
B3GAT3
 
 
hsa-let-7c-5p
 
 
-0.324
 
 
30.8
 
 
 
 
B4GALT5
 
 
hsa-miR-210-3p
 
 
-0.511
 
 
26.8
 
 
 
 
B4GAT1
 
 
hsa-miR-200b-3p
 
 
-0.382
 
 
16.2
 
 
 
 
B4GAT1
 
 
hsa-miR-98-5p
 
 
-0.47
 
 
16.2
 
 
 
 
B4GAT1
 
 
hsa-let-7c-5p
 
 
-0.47
 
 
16.2
 
 
 
 
BACH1
 
 
hsa-miR-98-5p
 
 
-0.298
 
 
14.8
 
 
 
 
BACH1
 
 
hsa-miR-98-5p
 
 
-0.298
 
 
14.8
 
 
 
 
BACH1
 
 
hsa-let-7c-5p
 
 
-0.306
 
 
14.8
 
 
 
 
BACH1
 
 
hsa-let-7c-5p
 
 
-0.306
 
 
14.8
 
 
 
 
BACH1
 
 
hsa-miR-98-5p
 
 
-0.435
 
 
14.8
 
 
 
 
BACH1
 
 
hsa-miR-98-5p
 
 
-0.435
 
 
14.8
 
 
 
 
BACH1
 
 
hsa-let-7c-5p
 
 
-0.435
 
 
14.8
 
 
 
 
BACH1
 
 
hsa-let-7c-5p
 
 
-0.435
 
 
14.8
 
 
 
 
BAG6
 
 
hsa-miR-200b-3p
 
 
-0.371
 
 
69.2
 
 
 
 
BAP1
 
 
hsa-miR-423-5p
 
 
-0.428
 
 
42.6
 
 
 
 
BBX
 
 
hsa-miR-18a-5p
 
 
-0.31
 
 
13.1
 
 
 
 
BIN1
 
 
hsa-miR-744-5p
 
 
-0.654
 
 
41.5
 
 
 
 
 
 
 bone marrow 
 
 
 
 
target_symbol
 
 
mature_mirna_id
 
 
score
 
 
nTPM
 
 
 
 
 
 
ABHD5
 
 
hsa-miR-20a-5p
 
 
-0.397
 
 
37.5
 
 
 
 
ABHD5
 
 
hsa-miR-17-5p
 
 
-0.45
 
 
37.5
 
 
 
 
ACP3
 
 
hsa-miR-98-5p
 
 
-0.298
 
 
16.5
 
 
 
 
ACP3
 
 
hsa-let-7c-5p
 
 
-0.298
 
 
16.5
 
 
 
 
ACSL6
 
 
hsa-miR-98-5p
 
 
-0.336
 
 
21.4
 
 
 
 
ACSL6
 
 
hsa-let-7c-5p
 
 
-0.336
 
 
21.4
 
 
 
 
ADD3
 
 
hsa-miR-200b-3p
 
 
-0.324
 
 
35.4
 
 
 
 
ADD3
 
 
hsa-miR-18a-5p
 
 
-0.39
 
 
35.4
 
 
 
 
ADNP
 
 
hsa-miR-328-3p
 
 
-0.371
 
 
23.9
 
 
 
 
ADRB2
 
 
hsa-miR-98-5p
 
 
-0.635
 
 
11.8
 
 
 
 
ADRB2
 
 
hsa-let-7c-5p
 
 
-0.635
 
 
11.8
 
 
 
 
AEBP2
 
 
hsa-miR-18a-5p
 
 
-0.376
 
 
19.3
 
 
 
 
AGO2
 
 
hsa-miR-184
 
 
-0.831
 
 
10.7
 
 
 
 
AGO2
 
 
hsa-miR-200b-3p
 
 
-1.125
 
 
10.7
 
 
 
 
AHCTF1
 
 
hsa-miR-98-5p
 
 
-0.374
 
 
16.0
 
 
 
 
AHCTF1
 
 
hsa-let-7c-5p
 
 
-0.374
 
 
16.0
 
 
 
 
AHNAK
 
 
hsa-miR-20a-5p
 
 
-0.316
 
 
41.6
 
 
 
 
AHNAK
 
 
hsa-miR-17-5p
 
 
-0.337
 
 
41.6
 
 
 
 
AIFM1
 
 
hsa-miR-98-5p
 
 
-0.314
 
 
11.2
 
 
 
 
AIFM1
 
 
hsa-let-7c-5p
 
 
-0.314
 
 
11.2
 
 
 
 
AK1
 
 
hsa-miR-423-5p
 
 
-0.443
 
 
27.0
 
 
 
 
ALDH4A1
 
 
hsa-miR-184
 
 
-0.421
 
 
11.1
 
 
 
 
ALG3
 
 
hsa-miR-20a-5p
 
 
-0.559
 
 
14.9
 
 
 
 
ALG3
 
 
hsa-miR-17-5p
 
 
-0.572
 
 
14.9
 
 
 
 
ANKFY1
 
 
hsa-miR-155-5p
 
 
-0.315
 
 
15.5
 
 
 
 
ANKRD12
 
 
hsa-miR-7-5p
 
 
-0.37
 
 
19.4
 
 
 
 
ANKRD9
 
 
hsa-miR-20a-5p
 
 
-0.31
 
 
17.0
 
 
 
 
ANKRD9
 
 
hsa-miR-17-5p
 
 
-0.347
 
 
17.0
 
 
 
 
ANP32B
 
 
hsa-miR-200b-3p
 
 
-0.287
 
 
344.9
 
 
 
 
AP1S2
 
 
hsa-miR-200b-3p
 
 
-0.4
 
 
34.8
 
 
 
 
AP2M1
 
 
hsa-miR-423-5p
 
 
-0.49
 
 
204.6
 
 
 
 
ARF4
 
 
hsa-miR-194-5p
 
 
-0.293
 
 
68.5
 
 
 
 
ARF4
 
 
hsa-miR-7-5p
 
 
-0.407
 
 
68.5
 
 
 
 
ARGLU1
 
 
hsa-miR-200b-3p
 
 
-0.29
 
 
10.4
 
 
 
 
ARHGAP1
 
 
hsa-miR-17-5p
 
 
-0.325
 
 
31.5
 
 
 
 
ARHGAP1
 
 
hsa-miR-20a-5p
 
 
-0.339
 
 
31.5
 
 
 
 
ARID2
 
 
hsa-miR-155-5p
 
 
-0.384
 
 
11.7
 
 
 
 
ARID3B
 
 
hsa-miR-98-5p
 
 
-0.31
 
 
23.2
 
 
 
 
ARID3B
 
 
hsa-let-7c-5p
 
 
-0.31
 
 
23.2
 
 
 
 
ARID3B
 
 
hsa-miR-98-5p
 
 
-0.475
 
 
23.2
 
 
 
 
ARID3B
 
 
hsa-let-7c-5p
 
 
-0.486
 
 
23.2
 
 
 
 
ARID4A
 
 
hsa-miR-7-5p
 
 
-0.341
 
 
32.8
 
 
 
 
ARID4B
 
 
hsa-miR-20a-5p
 
 
-0.29
 
 
31.2
 
 
 
 
ARL1
 
 
hsa-miR-20a-5p
 
 
-0.403
 
 
15.6
 
 
 
 
ARL1
 
 
hsa-miR-17-5p
 
 
-0.432
 
 
15.6
 
 
 
 
ARL5B
 
 
hsa-miR-155-5p
 
 
-0.355
 
 
18.5
 
 
 
 
ARL6IP1
 
 
hsa-miR-7-5p
 
 
-0.374
 
 
197.7
 
 
 
 
ARL6IP1
 
 
hsa-miR-328-3p
 
 
-0.494
 
 
197.7
 
 
 
 
ARL6IP6
 
 
hsa-miR-98-5p
 
 
-0.293
 
 
23.4
 
 
 
 
ARL6IP6
 
 
hsa-let-7c-5p
 
 
-0.293
 
 
23.4
 
 
 
 
ARMC8
 
 
hsa-miR-20a-5p
 
 
-0.357
 
 
57.2
 
 
 
 
ARMC8
 
 
hsa-miR-17-5p
 
 
-0.378
 
 
57.2
 
 
 
 
ARPP19
 
 
hsa-miR-328-3p
 
 
-0.404
 
 
97.5
 
 
 
 
ARPP19
 
 
hsa-let-7c-5p
 
 
-0.492
 
 
97.5
 
 
 
 
ARPP19
 
 
hsa-miR-98-5p
 
 
-0.5
 
 
97.5
 
 
 
 
ARRDC4
 
 
hsa-miR-98-5p
 
 
-0.329
 
 
10.8
 
 
 
 
ARRDC4
 
 
hsa-let-7c-5p
 
 
-0.329
 
 
10.8
 
 
 
 
ASAP1
 
 
hsa-miR-98-5p
 
 
-0.308
 
 
44.7
 
 
 
 
ASAP1
 
 
hsa-let-7c-5p
 
 
-0.308
 
 
44.7
 
 
 
 
ASB6
 
 
hsa-miR-423-5p
 
 
-0.388
 
 
25.3
 
 
 
 
ATAD2
 
 
hsa-miR-17-5p
 
 
-0.304
 
 
25.0
 
 
 
 
ATAD2
 
 
hsa-miR-20a-5p
 
 
-0.318
 
 
25.0
 
 
 
 
ATG13
 
 
hsa-miR-423-5p
 
 
-0.3
 
 
57.1
 
 
 
 
ATG16L1
 
 
hsa-miR-20a-5p
 
 
-0.446
 
 
13.5
 
 
 
 
ATG16L1
 
 
hsa-miR-17-5p
 
 
-0.467
 
 
13.5
 
 
 
 
ATM
 
 
hsa-miR-18a-5p
 
 
-0.347
 
 
11.7
 
 
 
 
ATOSB
 
 
hsa-miR-328-3p
 
 
-0.358
 
 
36.6
 
 
 
 
ATOSB
 
 
hsa-miR-98-5p
 
 
-0.429
 
 
36.6
 
 
 
 
ATOSB
 
 
hsa-let-7c-5p
 
 
-0.429
 
 
36.6
 
 
 
 
ATP2A2
 
 
hsa-miR-98-5p
 
 
-0.311
 
 
43.4
 
 
 
 
ATP2A2
 
 
hsa-let-7c-5p
 
 
-0.311
 
 
43.4
 
 
 
 
ATP6V1F
 
 
hsa-miR-194-5p
 
 
-0.444
 
 
198.4
 
 
 
 
ATP6V1H
 
 
hsa-miR-194-5p
 
 
-0.652
 
 
24.2
 
 
 
 
ATP8B4
 
 
hsa-miR-98-5p
 
 
-0.325
 
 
73.7
 
 
 
 
ATP8B4
 
 
hsa-let-7c-5p
 
 
-0.325
 
 
73.7
 
 
 
 
ATPAF1
 
 
hsa-miR-98-5p
 
 
-0.498
 
 
26.0
 
 
 
 
ATPAF1
 
 
hsa-let-7c-5p
 
 
-0.498
 
 
26.0
 
 
 
 
ATXN1L
 
 
hsa-miR-155-5p
 
 
-0.315
 
 
37.3
 
 
 
 
B3GAT3
 
 
hsa-miR-98-5p
 
 
-0.324
 
 
17.5
 
 
 
 
B3GAT3
 
 
hsa-let-7c-5p
 
 
-0.324
 
 
17.5
 
 
 
 
B4GALT5
 
 
hsa-miR-210-3p
 
 
-0.511
 
 
88.5
 
 
 
 
BACH1
 
 
hsa-miR-98-5p
 
 
-0.298
 
 
84.3
 
 
 
 
BACH1
 
 
hsa-miR-98-5p
 
 
-0.298
 
 
84.3
 
 
 
 
BACH1
 
 
hsa-let-7c-5p
 
 
-0.306
 
 
84.3
 
 
 
 
BACH1
 
 
hsa-let-7c-5p
 
 
-0.306
 
 
84.3
 
 
 
 
BACH1
 
 
hsa-miR-98-5p
 
 
-0.435
 
 
84.3
 
 
 
 
BACH1
 
 
hsa-miR-98-5p
 
 
-0.435
 
 
84.3
 
 
 
 
BACH1
 
 
hsa-let-7c-5p
 
 
-0.435
 
 
84.3
 
 
 
 
BACH1
 
 
hsa-let-7c-5p
 
 
-0.435
 
 
84.3
 
 
 
 
BAG6
 
 
hsa-miR-200b-3p
 
 
-0.371
 
 
24.3
 
 
 
 
BAP1
 
 
hsa-miR-423-5p
 
 
-0.428
 
 
69.8
 
 
 
 
BBX
 
 
hsa-miR-18a-5p
 
 
-0.31
 
 
17.4
 
 
 
 
BIN1
 
 
hsa-miR-744-5p
 
 
-0.654
 
 
11.4
 
 
 
 
BIN3
 
 
hsa-miR-98-5p
 
 
-0.427
 
 
40.9
 
 
 
 
BIN3
 
 
hsa-let-7c-5p
 
 
-0.427
 
 
40.9
 
 
 
 
BLOC1S6
 
 
hsa-miR-98-5p
 
 
-0.321
 
 
54.9
 
 
 
 
BLOC1S6
 
 
hsa-let-7c-5p
 
 
-0.321
 
 
54.9
 
 
 
 
BNIP2
 
 
hsa-miR-194-5p
 
 
-0.489
 
 
122.0
 
 
 
 
BNIP3L
 
 
hsa-miR-20a-5p
 
 
-0.287
 
 
181.7
 
 
 
 
BRWD1
 
 
hsa-miR-18a-5p
 
 
-0.29
 
 
17.6
 
 
 
 
 
 
 gallbladder 
 
 
 
 
target_symbol
 
 
mature_mirna_id
 
 
score
 
 
nTPM
 
 
 
 
 
 
ABHD5
 
 
hsa-miR-20a-5p
 
 
-0.397
 
 
10.2
 
 
 
 
ABHD5
 
 
hsa-miR-17-5p
 
 
-0.45
 
 
10.2
 
 
 
 
ACTC1
 
 
hsa-miR-7-5p
 
 
-0.471
 
 
11.1
 
 
 
 
ADAM9
 
 
hsa-miR-20a-5p
 
 
-0.299
 
 
44.2
 
 
 
 
ADAM9
 
 
hsa-miR-17-5p
 
 
-0.32
 
 
44.2
 
 
 
 
ADD3
 
 
hsa-miR-200b-3p
 
 
-0.324
 
 
67.5
 
 
 
 
ADD3
 
 
hsa-miR-18a-5p
 
 
-0.39
 
 
67.5
 
 
 
 
ADNP
 
 
hsa-miR-328-3p
 
 
-0.371
 
 
20.9
 
 
 
 
AEBP2
 
 
hsa-miR-18a-5p
 
 
-0.376
 
 
20.4
 
 
 
 
AGFG2
 
 
hsa-miR-20a-5p
 
 
-0.365
 
 
10.4
 
 
 
 
AGFG2
 
 
hsa-miR-17-5p
 
 
-0.402
 
 
10.4
 
 
 
 
AHNAK
 
 
hsa-miR-20a-5p
 
 
-0.316
 
 
70.2
 
 
 
 
AHNAK
 
 
hsa-miR-17-5p
 
 
-0.337
 
 
70.2
 
 
 
 
AK1
 
 
hsa-miR-423-5p
 
 
-0.443
 
 
21.1
 
 
 
 
ALCAM
 
 
hsa-miR-18a-5p
 
 
-0.341
 
 
21.9
 
 
 
 
ALG3
 
 
hsa-miR-20a-5p
 
 
-0.559
 
 
10.8
 
 
 
 
ALG3
 
 
hsa-miR-17-5p
 
 
-0.572
 
 
10.8
 
 
 
 
ANGPTL2
 
 
hsa-miR-423-5p
 
 
-0.379
 
 
62.7
 
 
 
 
ANKFY1
 
 
hsa-miR-155-5p
 
 
-0.315
 
 
11.1
 
 
 
 
ANKRD12
 
 
hsa-miR-7-5p
 
 
-0.37
 
 
13.7
 
 
 
 
ANP32B
 
 
hsa-miR-200b-3p
 
 
-0.287
 
 
56.6
 
 
 
 
AP1S1
 
 
hsa-miR-98-5p
 
 
-0.374
 
 
12.0
 
 
 
 
AP1S1
 
 
hsa-let-7c-5p
 
 
-0.374
 
 
12.0
 
 
 
 
AP1S2
 
 
hsa-miR-200b-3p
 
 
-0.4
 
 
18.5
 
 
 
 
AP2M1
 
 
hsa-miR-423-5p
 
 
-0.49
 
 
139.3
 
 
 
 
APCDD1
 
 
hsa-miR-20a-5p
 
 
-0.404
 
 
20.5
 
 
 
 
APCDD1
 
 
hsa-miR-17-5p
 
 
-0.425
 
 
20.5
 
 
 
 
ARF4
 
 
hsa-miR-194-5p
 
 
-0.293
 
 
145.1
 
 
 
 
ARF4
 
 
hsa-miR-7-5p
 
 
-0.407
 
 
145.1
 
 
 
 
ARHGAP1
 
 
hsa-miR-17-5p
 
 
-0.325
 
 
47.5
 
 
 
 
ARHGAP1
 
 
hsa-miR-20a-5p
 
 
-0.339
 
 
47.5
 
 
 
 
ARID4B
 
 
hsa-miR-20a-5p
 
 
-0.29
 
 
12.3
 
 
 
 
ARL1
 
 
hsa-miR-20a-5p
 
 
-0.403
 
 
40.9
 
 
 
 
ARL1
 
 
hsa-miR-17-5p
 
 
-0.432
 
 
40.9
 
 
 
 
ARL5B
 
 
hsa-miR-155-5p
 
 
-0.355
 
 
13.2
 
 
 
 
ARL6IP1
 
 
hsa-miR-7-5p
 
 
-0.374
 
 
123.8
 
 
 
 
ARL6IP1
 
 
hsa-miR-328-3p
 
 
-0.494
 
 
123.8
 
 
 
 
ARMC8
 
 
hsa-miR-20a-5p
 
 
-0.357
 
 
14.3
 
 
 
 
ARMC8
 
 
hsa-miR-17-5p
 
 
-0.378
 
 
14.3
 
 
 
 
ARMT1
 
 
hsa-miR-98-5p
 
 
-0.303
 
 
10.6
 
 
 
 
ARMT1
 
 
hsa-let-7c-5p
 
 
-0.303
 
 
10.6
 
 
 
 
ARPP19
 
 
hsa-miR-328-3p
 
 
-0.404
 
 
36.7
 
 
 
 
ARPP19
 
 
hsa-let-7c-5p
 
 
-0.492
 
 
36.7
 
 
 
 
ARPP19
 
 
hsa-miR-98-5p
 
 
-0.5
 
 
36.7
 
 
 
 
ARRDC4
 
 
hsa-miR-98-5p
 
 
-0.329
 
 
16.1
 
 
 
 
ARRDC4
 
 
hsa-let-7c-5p
 
 
-0.329
 
 
16.1
 
 
 
 
ASAP1
 
 
hsa-miR-98-5p
 
 
-0.308
 
 
13.5
 
 
 
 
ASAP1
 
 
hsa-let-7c-5p
 
 
-0.308
 
 
13.5
 
 
 
 
ATG13
 
 
hsa-miR-423-5p
 
 
-0.3
 
 
27.7
 
 
 
 
ATOSB
 
 
hsa-miR-328-3p
 
 
-0.358
 
 
11.0
 
 
 
 
ATOSB
 
 
hsa-miR-98-5p
 
 
-0.429
 
 
11.0
 
 
 
 
ATOSB
 
 
hsa-let-7c-5p
 
 
-0.429
 
 
11.0
 
 
 
 
ATP2A2
 
 
hsa-miR-98-5p
 
 
-0.311
 
 
56.7
 
 
 
 
ATP2A2
 
 
hsa-let-7c-5p
 
 
-0.311
 
 
56.7
 
 
 
 
ATP6V1F
 
 
hsa-miR-194-5p
 
 
-0.444
 
 
97.7
 
 
 
 
ATP6V1H
 
 
hsa-miR-194-5p
 
 
-0.652
 
 
19.4
 
 
 
 
ATP8B2
 
 
hsa-miR-423-5p
 
 
-0.297
 
 
18.0
 
 
 
 
ATPAF1
 
 
hsa-miR-98-5p
 
 
-0.498
 
 
28.6
 
 
 
 
ATPAF1
 
 
hsa-let-7c-5p
 
 
-0.498
 
 
28.6
 
 
 
 
B3GALT2
 
 
hsa-miR-17-5p
 
 
-0.35
 
 
14.1
 
 
 
 
B3GALT2
 
 
hsa-miR-20a-5p
 
 
-0.364
 
 
14.1
 
 
 
 
B3GAT3
 
 
hsa-miR-98-5p
 
 
-0.324
 
 
14.5
 
 
 
 
B3GAT3
 
 
hsa-let-7c-5p
 
 
-0.324
 
 
14.5
 
 
 
 
B4GALT5
 
 
hsa-miR-210-3p
 
 
-0.511
 
 
29.5
 
 
 
 
BACH1
 
 
hsa-miR-98-5p
 
 
-0.298
 
 
24.3
 
 
 
 
BACH1
 
 
hsa-miR-98-5p
 
 
-0.298
 
 
24.3
 
 
 
 
BACH1
 
 
hsa-let-7c-5p
 
 
-0.306
 
 
24.3
 
 
 
 
BACH1
 
 
hsa-let-7c-5p
 
 
-0.306
 
 
24.3
 
 
 
 
BACH1
 
 
hsa-miR-98-5p
 
 
-0.435
 
 
24.3
 
 
 
 
BACH1
 
 
hsa-miR-98-5p
 
 
-0.435
 
 
24.3
 
 
 
 
BACH1
 
 
hsa-let-7c-5p
 
 
-0.435
 
 
24.3
 
 
 
 
BACH1
 
 
hsa-let-7c-5p
 
 
-0.435
 
 
24.3
 
 
 
 
BAG6
 
 
hsa-miR-200b-3p
 
 
-0.371
 
 
15.3
 
 
 
 
BAP1
 
 
hsa-miR-423-5p
 
 
-0.428
 
 
24.0
 
 
 
 
BBX
 
 
hsa-miR-18a-5p
 
 
-0.31
 
 
15.7
 
 
 
 
BIN1
 
 
hsa-miR-744-5p
 
 
-0.654
 
 
21.2
 
 
 
 
BIN3
 
 
hsa-miR-98-5p
 
 
-0.427
 
 
11.2
 
 
 
 
BIN3
 
 
hsa-let-7c-5p
 
 
-0.427
 
 
11.2
 
 
 
 
BLOC1S6
 
 
hsa-miR-98-5p
 
 
-0.321
 
 
24.8
 
 
 
 
BLOC1S6
 
 
hsa-let-7c-5p
 
 
-0.321
 
 
24.8
 
 
 
 
BNIP2
 
 
hsa-miR-194-5p
 
 
-0.489
 
 
23.1
 
 
 
 
BNIP3L
 
 
hsa-miR-20a-5p
 
 
-0.287
 
 
51.3
 
 
 
 
BRWD1
 
 
hsa-miR-18a-5p
 
 
-0.29
 
 
10.3
 
 
 
 
BTBD10
 
 
hsa-miR-17-5p
 
 
-0.409
 
 
14.2
 
 
 
 
BTBD10
 
 
hsa-miR-20a-5p
 
 
-0.412
 
 
14.2
 
 
 
 
BTF3L4
 
 
hsa-miR-200b-3p
 
 
-0.294
 
 
22.4
 
 
 
 
BTF3L4
 
 
hsa-miR-194-5p
 
 
-0.334
 
 
22.4
 
 
 
 
BTF3L4
 
 
hsa-miR-98-5p
 
 
-0.355
 
 
22.4
 
 
 
 
BTF3L4
 
 
hsa-let-7c-5p
 
 
-0.355
 
 
22.4
 
 
 
 
BTG3
 
 
hsa-miR-18a-5p
 
 
-0.464
 
 
21.4
 
 
 
 
BTG3
 
 
hsa-miR-17-5p
 
 
-0.668
 
 
21.4
 
 
 
 
BTG3
 
 
hsa-miR-20a-5p
 
 
-0.671
 
 
21.4
 
 
 
 
BZW1
 
 
hsa-miR-98-5p
 
 
-0.354
 
 
87.2
 
 
 
 
BZW1
 
 
hsa-let-7c-5p
 
 
-0.354
 
 
87.2
 
 
 
 
BZW1
 
 
hsa-miR-98-5p
 
 
-0.375
 
 
87.2
 
 
 
 
BZW1
 
 
hsa-let-7c-5p
 
 
-0.375
 
 
87.2
 
 
 
 
BZW2
 
 
hsa-miR-98-5p
 
 
-0.397
 
 
16.1
 
 
 
 
BZW2
 
 
hsa-let-7c-5p
 
 
-0.397
 
 
16.1
 
 
 
 
C1GALT1
 
 
hsa-let-7c-5p
 
 
-0.322
 
 
15.6
 
 
 
 
C1GALT1
 
 
hsa-miR-98-5p
 
 
-0.33
 
 
15.6
 
 
 
 
 
 
 heart muscle 
 
 
 
 
target_symbol
 
 
mature_mirna_id
 
 
score
 
 
nTPM
 
 
 
 
 
 
ABHD5
 
 
hsa-miR-20a-5p
 
 
-0.397
 
 
10.6
 
 
 
 
ABHD5
 
 
hsa-miR-17-5p
 
 
-0.45
 
 
10.6
 
 
 
 
ABTB2
 
 
hsa-miR-18a-5p
 
 
-0.287
 
 
12.3
 
 
 
 
ACHE
 
 
hsa-miR-194-5p
 
 
-0.395
 
 
11.4
 
 
 
 
ACTA1
 
 
hsa-miR-155-5p
 
 
-0.595
 
 
6223.8
 
 
 
 
ACTC1
 
 
hsa-miR-7-5p
 
 
-0.471
 
 
10840.1
 
 
 
 
ADAM9
 
 
hsa-miR-20a-5p
 
 
-0.299
 
 
26.6
 
 
 
 
ADAM9
 
 
hsa-miR-17-5p
 
 
-0.32
 
 
26.6
 
 
 
 
ADD3
 
 
hsa-miR-200b-3p
 
 
-0.324
 
 
56.1
 
 
 
 
ADD3
 
 
hsa-miR-18a-5p
 
 
-0.39
 
 
56.1
 
 
 
 
ADNP
 
 
hsa-miR-328-3p
 
 
-0.371
 
 
22.3
 
 
 
 
ADRB1
 
 
hsa-let-7c-5p
 
 
-0.351
 
 
20.3
 
 
 
 
ADRB1
 
 
hsa-miR-98-5p
 
 
-0.367
 
 
20.3
 
 
 
 
AEBP2
 
 
hsa-miR-18a-5p
 
 
-0.376
 
 
16.5
 
 
 
 
AHNAK
 
 
hsa-miR-20a-5p
 
 
-0.316
 
 
110.3
 
 
 
 
AHNAK
 
 
hsa-miR-17-5p
 
 
-0.337
 
 
110.3
 
 
 
 
AIFM1
 
 
hsa-miR-98-5p
 
 
-0.314
 
 
74.9
 
 
 
 
AIFM1
 
 
hsa-let-7c-5p
 
 
-0.314
 
 
74.9
 
 
 
 
AK1
 
 
hsa-miR-423-5p
 
 
-0.443
 
 
313.1
 
 
 
 
AKAP6
 
 
hsa-miR-98-5p
 
 
-0.307
 
 
51.1
 
 
 
 
AKAP6
 
 
hsa-let-7c-5p
 
 
-0.307
 
 
51.1
 
 
 
 
ALDH4A1
 
 
hsa-miR-184
 
 
-0.421
 
 
30.9
 
 
 
 
ALG3
 
 
hsa-miR-20a-5p
 
 
-0.559
 
 
18.7
 
 
 
 
ALG3
 
 
hsa-miR-17-5p
 
 
-0.572
 
 
18.7
 
 
 
 
AMT
 
 
hsa-miR-98-5p
 
 
-0.463
 
 
23.5
 
 
 
 
AMT
 
 
hsa-let-7c-5p
 
 
-0.463
 
 
23.5
 
 
 
 
ANGPTL2
 
 
hsa-miR-423-5p
 
 
-0.379
 
 
64.9
 
 
 
 
ANKFY1
 
 
hsa-miR-155-5p
 
 
-0.315
 
 
10.9
 
 
 
 
ANKRD12
 
 
hsa-miR-7-5p
 
 
-0.37
 
 
11.1
 
 
 
 
ANKRD46
 
 
hsa-miR-98-5p
 
 
-0.389
 
 
18.8
 
 
 
 
ANKRD46
 
 
hsa-let-7c-5p
 
 
-0.389
 
 
18.8
 
 
 
 
ANKRD9
 
 
hsa-miR-20a-5p
 
 
-0.31
 
 
104.9
 
 
 
 
ANKRD9
 
 
hsa-miR-17-5p
 
 
-0.347
 
 
104.9
 
 
 
 
ANP32B
 
 
hsa-miR-200b-3p
 
 
-0.287
 
 
87.4
 
 
 
 
AP1S1
 
 
hsa-miR-98-5p
 
 
-0.374
 
 
12.9
 
 
 
 
AP1S1
 
 
hsa-let-7c-5p
 
 
-0.374
 
 
12.9
 
 
 
 
AP1S2
 
 
hsa-miR-200b-3p
 
 
-0.4
 
 
69.2
 
 
 
 
AP2M1
 
 
hsa-miR-423-5p
 
 
-0.49
 
 
317.7
 
 
 
 
AP5S1
 
 
hsa-miR-744-5p
 
 
-0.354
 
 
10.7
 
 
 
 
APCDD1
 
 
hsa-miR-20a-5p
 
 
-0.404
 
 
30.9
 
 
 
 
APCDD1
 
 
hsa-miR-17-5p
 
 
-0.425
 
 
30.9
 
 
 
 
APOO
 
 
hsa-miR-200b-3p
 
 
-0.361
 
 
61.9
 
 
 
 
ARF4
 
 
hsa-miR-194-5p
 
 
-0.293
 
 
106.8
 
 
 
 
ARF4
 
 
hsa-miR-7-5p
 
 
-0.407
 
 
106.8
 
 
 
 
ARGLU1
 
 
hsa-miR-200b-3p
 
 
-0.29
 
 
11.7
 
 
 
 
ARHGAP1
 
 
hsa-miR-17-5p
 
 
-0.325
 
 
105.1
 
 
 
 
ARHGAP1
 
 
hsa-miR-20a-5p
 
 
-0.339
 
 
105.1
 
 
 
 
ARL1
 
 
hsa-miR-20a-5p
 
 
-0.403
 
 
61.3
 
 
 
 
ARL1
 
 
hsa-miR-17-5p
 
 
-0.432
 
 
61.3
 
 
 
 
ARL4D
 
 
hsa-miR-98-5p
 
 
-0.417
 
 
12.5
 
 
 
 
ARL4D
 
 
hsa-let-7c-5p
 
 
-0.417
 
 
12.5
 
 
 
 
ARL6IP1
 
 
hsa-miR-7-5p
 
 
-0.374
 
 
30.1
 
 
 
 
ARL6IP1
 
 
hsa-miR-328-3p
 
 
-0.494
 
 
30.1
 
 
 
 
ARMC8
 
 
hsa-miR-20a-5p
 
 
-0.357
 
 
16.3
 
 
 
 
ARMC8
 
 
hsa-miR-17-5p
 
 
-0.378
 
 
16.3
 
 
 
 
ARMT1
 
 
hsa-miR-98-5p
 
 
-0.303
 
 
15.7
 
 
 
 
ARMT1
 
 
hsa-let-7c-5p
 
 
-0.303
 
 
15.7
 
 
 
 
ARPP19
 
 
hsa-miR-328-3p
 
 
-0.404
 
 
76.2
 
 
 
 
ARPP19
 
 
hsa-let-7c-5p
 
 
-0.492
 
 
76.2
 
 
 
 
ARPP19
 
 
hsa-miR-98-5p
 
 
-0.5
 
 
76.2
 
 
 
 
ARRDC4
 
 
hsa-miR-98-5p
 
 
-0.329
 
 
11.3
 
 
 
 
ARRDC4
 
 
hsa-let-7c-5p
 
 
-0.329
 
 
11.3
 
 
 
 
ASAP1
 
 
hsa-miR-98-5p
 
 
-0.308
 
 
22.3
 
 
 
 
ASAP1
 
 
hsa-let-7c-5p
 
 
-0.308
 
 
22.3
 
 
 
 
ASF1A
 
 
hsa-miR-200b-3p
 
 
-0.377
 
 
12.5
 
 
 
 
ATG13
 
 
hsa-miR-423-5p
 
 
-0.3
 
 
32.3
 
 
 
 
ATG16L1
 
 
hsa-miR-20a-5p
 
 
-0.446
 
 
10.8
 
 
 
 
ATG16L1
 
 
hsa-miR-17-5p
 
 
-0.467
 
 
10.8
 
 
 
 
ATG4A
 
 
hsa-miR-7-5p
 
 
-0.448
 
 
11.7
 
 
 
 
ATOSB
 
 
hsa-miR-328-3p
 
 
-0.358
 
 
18.9
 
 
 
 
ATOSB
 
 
hsa-miR-98-5p
 
 
-0.429
 
 
18.9
 
 
 
 
ATOSB
 
 
hsa-let-7c-5p
 
 
-0.429
 
 
18.9
 
 
 
 
ATP2A2
 
 
hsa-miR-98-5p
 
 
-0.311
 
 
941.9
 
 
 
 
ATP2A2
 
 
hsa-let-7c-5p
 
 
-0.311
 
 
941.9
 
 
 
 
ATP6V1F
 
 
hsa-miR-194-5p
 
 
-0.444
 
 
181.8
 
 
 
 
ATP6V1H
 
 
hsa-miR-194-5p
 
 
-0.652
 
 
29.2
 
 
 
 
ATP8B2
 
 
hsa-miR-423-5p
 
 
-0.297
 
 
14.0
 
 
 
 
ATPAF1
 
 
hsa-miR-98-5p
 
 
-0.498
 
 
249.4
 
 
 
 
ATPAF1
 
 
hsa-let-7c-5p
 
 
-0.498
 
 
249.4
 
 
 
 
AVPI1
 
 
hsa-miR-7-5p
 
 
-0.367
 
 
22.9
 
 
 
 
B3GALT2
 
 
hsa-miR-17-5p
 
 
-0.35
 
 
19.7
 
 
 
 
B3GALT2
 
 
hsa-miR-20a-5p
 
 
-0.364
 
 
19.7
 
 
 
 
B3GAT3
 
 
hsa-miR-98-5p
 
 
-0.324
 
 
25.1
 
 
 
 
B3GAT3
 
 
hsa-let-7c-5p
 
 
-0.324
 
 
25.1
 
 
 
 
B4GALT5
 
 
hsa-miR-210-3p
 
 
-0.511
 
 
19.2
 
 
 
 
B4GAT1
 
 
hsa-miR-200b-3p
 
 
-0.382
 
 
43.6
 
 
 
 
B4GAT1
 
 
hsa-miR-98-5p
 
 
-0.47
 
 
43.6
 
 
 
 
B4GAT1
 
 
hsa-let-7c-5p
 
 
-0.47
 
 
43.6
 
 
 
 
BACH1
 
 
hsa-miR-98-5p
 
 
-0.298
 
 
12.3
 
 
 
 
BACH1
 
 
hsa-miR-98-5p
 
 
-0.298
 
 
12.3
 
 
 
 
BACH1
 
 
hsa-let-7c-5p
 
 
-0.306
 
 
12.3
 
 
 
 
BACH1
 
 
hsa-let-7c-5p
 
 
-0.306
 
 
12.3
 
 
 
 
BACH1
 
 
hsa-miR-98-5p
 
 
-0.435
 
 
12.3
 
 
 
 
BACH1
 
 
hsa-miR-98-5p
 
 
-0.435
 
 
12.3
 
 
 
 
BACH1
 
 
hsa-let-7c-5p
 
 
-0.435
 
 
12.3
 
 
 
 
BACH1
 
 
hsa-let-7c-5p
 
 
-0.435
 
 
12.3
 
 
 
 
BAG6
 
 
hsa-miR-200b-3p
 
 
-0.371
 
 
101.3
 
 
 
 
BAMBI
 
 
hsa-miR-20a-5p
 
 
-0.485
 
 
47.0
 
 
 
 
BAMBI
 
 
hsa-miR-17-5p
 
 
-0.506
 
 
47.0
 
 
 
 
BAP1
 
 
hsa-miR-423-5p
 
 
-0.428
 
 
54.4
 
 
 
 
 
 
 liver 
 
 
 
 
target_symbol
 
 
mature_mirna_id
 
 
score
 
 
nTPM
 
 
 
 
 
 
ABHD5
 
 
hsa-miR-20a-5p
 
 
-0.397
 
 
20.3
 
 
 
 
ABHD5
 
 
hsa-miR-17-5p
 
 
-0.45
 
 
20.3
 
 
 
 
ABTB2
 
 
hsa-miR-18a-5p
 
 
-0.287
 
 
10.8
 
 
 
 
ADAM9
 
 
hsa-miR-20a-5p
 
 
-0.299
 
 
12.7
 
 
 
 
ADAM9
 
 
hsa-miR-17-5p
 
 
-0.32
 
 
12.7
 
 
 
 
ADD3
 
 
hsa-miR-200b-3p
 
 
-0.324
 
 
26.1
 
 
 
 
ADD3
 
 
hsa-miR-18a-5p
 
 
-0.39
 
 
26.1
 
 
 
 
ADNP
 
 
hsa-miR-328-3p
 
 
-0.371
 
 
28.4
 
 
 
 
ADRB2
 
 
hsa-miR-98-5p
 
 
-0.635
 
 
13.3
 
 
 
 
ADRB2
 
 
hsa-let-7c-5p
 
 
-0.635
 
 
13.3
 
 
 
 
AEBP2
 
 
hsa-miR-18a-5p
 
 
-0.376
 
 
18.9
 
 
 
 
AGFG2
 
 
hsa-miR-20a-5p
 
 
-0.365
 
 
51.0
 
 
 
 
AGFG2
 
 
hsa-miR-17-5p
 
 
-0.402
 
 
51.0
 
 
 
 
AHCTF1
 
 
hsa-miR-98-5p
 
 
-0.374
 
 
10.2
 
 
 
 
AHCTF1
 
 
hsa-let-7c-5p
 
 
-0.374
 
 
10.2
 
 
 
 
AHNAK
 
 
hsa-miR-20a-5p
 
 
-0.316
 
 
56.2
 
 
 
 
AHNAK
 
 
hsa-miR-17-5p
 
 
-0.337
 
 
56.2
 
 
 
 
AIFM1
 
 
hsa-miR-98-5p
 
 
-0.314
 
 
77.0
 
 
 
 
AIFM1
 
 
hsa-let-7c-5p
 
 
-0.314
 
 
77.0
 
 
 
 
AK1
 
 
hsa-miR-423-5p
 
 
-0.443
 
 
35.8
 
 
 
 
ALCAM
 
 
hsa-miR-18a-5p
 
 
-0.341
 
 
44.8
 
 
 
 
ALDH4A1
 
 
hsa-miR-184
 
 
-0.421
 
 
438.6
 
 
 
 
ALG3
 
 
hsa-miR-20a-5p
 
 
-0.559
 
 
54.4
 
 
 
 
ALG3
 
 
hsa-miR-17-5p
 
 
-0.572
 
 
54.4
 
 
 
 
ALG9
 
 
hsa-miR-7-5p
 
 
-0.302
 
 
12.5
 
 
 
 
AMT
 
 
hsa-miR-98-5p
 
 
-0.463
 
 
52.5
 
 
 
 
AMT
 
 
hsa-let-7c-5p
 
 
-0.463
 
 
52.5
 
 
 
 
ANGPTL2
 
 
hsa-miR-423-5p
 
 
-0.379
 
 
13.8
 
 
 
 
ANKFY1
 
 
hsa-miR-155-5p
 
 
-0.315
 
 
13.0
 
 
 
 
ANKRD12
 
 
hsa-miR-7-5p
 
 
-0.37
 
 
12.9
 
 
 
 
ANKRD46
 
 
hsa-miR-98-5p
 
 
-0.389
 
 
38.9
 
 
 
 
ANKRD46
 
 
hsa-let-7c-5p
 
 
-0.389
 
 
38.9
 
 
 
 
ANKRD9
 
 
hsa-miR-20a-5p
 
 
-0.31
 
 
15.7
 
 
 
 
ANKRD9
 
 
hsa-miR-17-5p
 
 
-0.347
 
 
15.7
 
 
 
 
ANP32B
 
 
hsa-miR-200b-3p
 
 
-0.287
 
 
96.8
 
 
 
 
AP1S1
 
 
hsa-miR-98-5p
 
 
-0.374
 
 
39.1
 
 
 
 
AP1S1
 
 
hsa-let-7c-5p
 
 
-0.374
 
 
39.1
 
 
 
 
AP1S2
 
 
hsa-miR-200b-3p
 
 
-0.4
 
 
14.8
 
 
 
 
AP2M1
 
 
hsa-miR-423-5p
 
 
-0.49
 
 
124.0
 
 
 
 
AP5S1
 
 
hsa-miR-744-5p
 
 
-0.354
 
 
13.9
 
 
 
 
APOO
 
 
hsa-miR-200b-3p
 
 
-0.361
 
 
17.0
 
 
 
 
ARF4
 
 
hsa-miR-194-5p
 
 
-0.293
 
 
161.3
 
 
 
 
ARF4
 
 
hsa-miR-7-5p
 
 
-0.407
 
 
161.3
 
 
 
 
ARHGAP1
 
 
hsa-miR-17-5p
 
 
-0.325
 
 
44.2
 
 
 
 
ARHGAP1
 
 
hsa-miR-20a-5p
 
 
-0.339
 
 
44.2
 
 
 
 
ARID4A
 
 
hsa-miR-7-5p
 
 
-0.341
 
 
17.5
 
 
 
 
ARID4B
 
 
hsa-miR-20a-5p
 
 
-0.29
 
 
11.1
 
 
 
 
ARL1
 
 
hsa-miR-20a-5p
 
 
-0.403
 
 
64.0
 
 
 
 
ARL1
 
 
hsa-miR-17-5p
 
 
-0.432
 
 
64.0
 
 
 
 
ARL4D
 
 
hsa-miR-98-5p
 
 
-0.417
 
 
55.4
 
 
 
 
ARL4D
 
 
hsa-let-7c-5p
 
 
-0.417
 
 
55.4
 
 
 
 
ARL5B
 
 
hsa-miR-155-5p
 
 
-0.355
 
 
27.9
 
 
 
 
ARL6IP1
 
 
hsa-miR-7-5p
 
 
-0.374
 
 
336.2
 
 
 
 
ARL6IP1
 
 
hsa-miR-328-3p
 
 
-0.494
 
 
336.2
 
 
 
 
ARMC8
 
 
hsa-miR-20a-5p
 
 
-0.357
 
 
15.8
 
 
 
 
ARMC8
 
 
hsa-miR-17-5p
 
 
-0.378
 
 
15.8
 
 
 
 
ARMT1
 
 
hsa-miR-98-5p
 
 
-0.303
 
 
35.2
 
 
 
 
ARMT1
 
 
hsa-let-7c-5p
 
 
-0.303
 
 
35.2
 
 
 
 
ARPP19
 
 
hsa-miR-328-3p
 
 
-0.404
 
 
39.5
 
 
 
 
ARPP19
 
 
hsa-let-7c-5p
 
 
-0.492
 
 
39.5
 
 
 
 
ARPP19
 
 
hsa-miR-98-5p
 
 
-0.5
 
 
39.5
 
 
 
 
ARRDC4
 
 
hsa-miR-98-5p
 
 
-0.329
 
 
28.3
 
 
 
 
ARRDC4
 
 
hsa-let-7c-5p
 
 
-0.329
 
 
28.3
 
 
 
 
ASB6
 
 
hsa-miR-423-5p
 
 
-0.388
 
 
10.5
 
 
 
 
ASF1A
 
 
hsa-miR-200b-3p
 
 
-0.377
 
 
11.3
 
 
 
 
ATG13
 
 
hsa-miR-423-5p
 
 
-0.3
 
 
41.2
 
 
 
 
ATG16L1
 
 
hsa-miR-20a-5p
 
 
-0.446
 
 
14.9
 
 
 
 
ATG16L1
 
 
hsa-miR-17-5p
 
 
-0.467
 
 
14.9
 
 
 
 
ATG4A
 
 
hsa-miR-7-5p
 
 
-0.448
 
 
16.9
 
 
 
 
ATOSB
 
 
hsa-miR-328-3p
 
 
-0.358
 
 
10.9
 
 
 
 
ATOSB
 
 
hsa-miR-98-5p
 
 
-0.429
 
 
10.9
 
 
 
 
ATOSB
 
 
hsa-let-7c-5p
 
 
-0.429
 
 
10.9
 
 
 
 
ATP2A2
 
 
hsa-miR-98-5p
 
 
-0.311
 
 
49.3
 
 
 
 
ATP2A2
 
 
hsa-let-7c-5p
 
 
-0.311
 
 
49.3
 
 
 
 
ATP2B2
 
 
hsa-miR-7-5p
 
 
-0.297
 
 
30.4
 
 
 
 
ATP6V1F
 
 
hsa-miR-194-5p
 
 
-0.444
 
 
84.5
 
 
 
 
ATP6V1H
 
 
hsa-miR-194-5p
 
 
-0.652
 
 
17.9
 
 
 
 
ATPAF1
 
 
hsa-miR-98-5p
 
 
-0.498
 
 
101.4
 
 
 
 
ATPAF1
 
 
hsa-let-7c-5p
 
 
-0.498
 
 
101.4
 
 
 
 
AVPI1
 
 
hsa-miR-7-5p
 
 
-0.367
 
 
47.4
 
 
 
 
B3GAT3
 
 
hsa-miR-98-5p
 
 
-0.324
 
 
53.3
 
 
 
 
B3GAT3
 
 
hsa-let-7c-5p
 
 
-0.324
 
 
53.3
 
 
 
 
B4GALT5
 
 
hsa-miR-210-3p
 
 
-0.511
 
 
24.1
 
 
 
 
B4GAT1
 
 
hsa-miR-200b-3p
 
 
-0.382
 
 
18.5
 
 
 
 
B4GAT1
 
 
hsa-miR-98-5p
 
 
-0.47
 
 
18.5
 
 
 
 
B4GAT1
 
 
hsa-let-7c-5p
 
 
-0.47
 
 
18.5
 
 
 
 
BACH1
 
 
hsa-miR-98-5p
 
 
-0.298
 
 
28.5
 
 
 
 
BACH1
 
 
hsa-miR-98-5p
 
 
-0.298
 
 
28.5
 
 
 
 
BACH1
 
 
hsa-let-7c-5p
 
 
-0.306
 
 
28.5
 
 
 
 
BACH1
 
 
hsa-let-7c-5p
 
 
-0.306
 
 
28.5
 
 
 
 
BACH1
 
 
hsa-miR-98-5p
 
 
-0.435
 
 
28.5
 
 
 
 
BACH1
 
 
hsa-miR-98-5p
 
 
-0.435
 
 
28.5
 
 
 
 
BACH1
 
 
hsa-let-7c-5p
 
 
-0.435
 
 
28.5
 
 
 
 
BACH1
 
 
hsa-let-7c-5p
 
 
-0.435
 
 
28.5
 
 
 
 
BAG6
 
 
hsa-miR-200b-3p
 
 
-0.371
 
 
72.7
 
 
 
 
BAMBI
 
 
hsa-miR-20a-5p
 
 
-0.485
 
 
22.4
 
 
 
 
BAMBI
 
 
hsa-miR-17-5p
 
 
-0.506
 
 
22.4
 
 
 
 
BAP1
 
 
hsa-miR-423-5p
 
 
-0.428
 
 
63.0
 
 
 
 
BCAS3
 
 
hsa-miR-423-5p
 
 
-0.322
 
 
11.6
 
 
 
 
BIN1
 
 
hsa-miR-744-5p
 
 
-0.654
 
 
33.9
 
 
 
 
 
 
 pancreas 
 
 
 
 
target_symbol
 
 
mature_mirna_id
 
 
score
 
 
nTPM
 
 
 
 
 
 
ABHD5
 
 
hsa-miR-20a-5p
 
 
-0.397
 
 
11.4
 
 
 
 
ABHD5
 
 
hsa-miR-17-5p
 
 
-0.45
 
 
11.4
 
 
 
 
ACTC1
 
 
hsa-miR-7-5p
 
 
-0.471
 
 
12.1
 
 
 
 
ADD3
 
 
hsa-miR-200b-3p
 
 
-0.324
 
 
64.0
 
 
 
 
ADD3
 
 
hsa-miR-18a-5p
 
 
-0.39
 
 
64.0
 
 
 
 
ADNP
 
 
hsa-miR-328-3p
 
 
-0.371
 
 
23.5
 
 
 
 
AEBP2
 
 
hsa-miR-18a-5p
 
 
-0.376
 
 
16.4
 
 
 
 
AGFG2
 
 
hsa-miR-20a-5p
 
 
-0.365
 
 
17.7
 
 
 
 
AGFG2
 
 
hsa-miR-17-5p
 
 
-0.402
 
 
17.7
 
 
 
 
AHNAK
 
 
hsa-miR-20a-5p
 
 
-0.316
 
 
28.6
 
 
 
 
AHNAK
 
 
hsa-miR-17-5p
 
 
-0.337
 
 
28.6
 
 
 
 
AIFM1
 
 
hsa-miR-98-5p
 
 
-0.314
 
 
20.4
 
 
 
 
AIFM1
 
 
hsa-let-7c-5p
 
 
-0.314
 
 
20.4
 
 
 
 
AK1
 
 
hsa-miR-423-5p
 
 
-0.443
 
 
14.0
 
 
 
 
ALCAM
 
 
hsa-miR-18a-5p
 
 
-0.341
 
 
30.8
 
 
 
 
ALG3
 
 
hsa-miR-20a-5p
 
 
-0.559
 
 
35.7
 
 
 
 
ALG3
 
 
hsa-miR-17-5p
 
 
-0.572
 
 
35.7
 
 
 
 
ALG9
 
 
hsa-miR-7-5p
 
 
-0.302
 
 
18.1
 
 
 
 
AMT
 
 
hsa-miR-98-5p
 
 
-0.463
 
 
21.5
 
 
 
 
AMT
 
 
hsa-let-7c-5p
 
 
-0.463
 
 
21.5
 
 
 
 
ANKRD12
 
 
hsa-miR-7-5p
 
 
-0.37
 
 
11.2
 
 
 
 
ANKRD46
 
 
hsa-miR-98-5p
 
 
-0.389
 
 
17.5
 
 
 
 
ANKRD46
 
 
hsa-let-7c-5p
 
 
-0.389
 
 
17.5
 
 
 
 
ANKRD9
 
 
hsa-miR-20a-5p
 
 
-0.31
 
 
10.7
 
 
 
 
ANKRD9
 
 
hsa-miR-17-5p
 
 
-0.347
 
 
10.7
 
 
 
 
ANP32B
 
 
hsa-miR-200b-3p
 
 
-0.287
 
 
147.1
 
 
 
 
AP1S1
 
 
hsa-miR-98-5p
 
 
-0.374
 
 
23.9
 
 
 
 
AP1S1
 
 
hsa-let-7c-5p
 
 
-0.374
 
 
23.9
 
 
 
 
AP2M1
 
 
hsa-miR-423-5p
 
 
-0.49
 
 
94.0
 
 
 
 
APOO
 
 
hsa-miR-200b-3p
 
 
-0.361
 
 
13.3
 
 
 
 
ARF4
 
 
hsa-miR-194-5p
 
 
-0.293
 
 
138.3
 
 
 
 
ARF4
 
 
hsa-miR-7-5p
 
 
-0.407
 
 
138.3
 
 
 
 
ARHGAP1
 
 
hsa-miR-17-5p
 
 
-0.325
 
 
34.7
 
 
 
 
ARHGAP1
 
 
hsa-miR-20a-5p
 
 
-0.339
 
 
34.7
 
 
 
 
ARHGEF28
 
 
hsa-miR-17-5p
 
 
-0.363
 
 
19.9
 
 
 
 
ARHGEF28
 
 
hsa-miR-20a-5p
 
 
-0.366
 
 
19.9
 
 
 
 
ARID4B
 
 
hsa-miR-20a-5p
 
 
-0.29
 
 
10.7
 
 
 
 
ARL1
 
 
hsa-miR-20a-5p
 
 
-0.403
 
 
41.8
 
 
 
 
ARL1
 
 
hsa-miR-17-5p
 
 
-0.432
 
 
41.8
 
 
 
 
ARL6IP1
 
 
hsa-miR-7-5p
 
 
-0.374
 
 
77.1
 
 
 
 
ARL6IP1
 
 
hsa-miR-328-3p
 
 
-0.494
 
 
77.1
 
 
 
 
ARMC8
 
 
hsa-miR-20a-5p
 
 
-0.357
 
 
13.4
 
 
 
 
ARMC8
 
 
hsa-miR-17-5p
 
 
-0.378
 
 
13.4
 
 
 
 
ARPP19
 
 
hsa-miR-328-3p
 
 
-0.404
 
 
43.6
 
 
 
 
ARPP19
 
 
hsa-let-7c-5p
 
 
-0.492
 
 
43.6
 
 
 
 
ARPP19
 
 
hsa-miR-98-5p
 
 
-0.5
 
 
43.6
 
 
 
 
ARRDC4
 
 
hsa-miR-98-5p
 
 
-0.329
 
 
32.8
 
 
 
 
ARRDC4
 
 
hsa-let-7c-5p
 
 
-0.329
 
 
32.8
 
 
 
 
ASB6
 
 
hsa-miR-423-5p
 
 
-0.388
 
 
13.0
 
 
 
 
ATG13
 
 
hsa-miR-423-5p
 
 
-0.3
 
 
42.4
 
 
 
 
ATG4A
 
 
hsa-miR-7-5p
 
 
-0.448
 
 
16.3
 
 
 
 
ATP2A2
 
 
hsa-miR-98-5p
 
 
-0.311
 
 
36.0
 
 
 
 
ATP2A2
 
 
hsa-let-7c-5p
 
 
-0.311
 
 
36.0
 
 
 
 
ATP6V1F
 
 
hsa-miR-194-5p
 
 
-0.444
 
 
100.6
 
 
 
 
ATP6V1H
 
 
hsa-miR-194-5p
 
 
-0.652
 
 
15.9
 
 
 
 
ATPAF1
 
 
hsa-miR-98-5p
 
 
-0.498
 
 
54.8
 
 
 
 
ATPAF1
 
 
hsa-let-7c-5p
 
 
-0.498
 
 
54.8
 
 
 
 
ATXN1L
 
 
hsa-miR-155-5p
 
 
-0.315
 
 
11.8
 
 
 
 
B3GAT3
 
 
hsa-miR-98-5p
 
 
-0.324
 
 
33.7
 
 
 
 
B3GAT3
 
 
hsa-let-7c-5p
 
 
-0.324
 
 
33.7
 
 
 
 
B4GALT5
 
 
hsa-miR-210-3p
 
 
-0.511
 
 
11.8
 
 
 
 
B4GAT1
 
 
hsa-miR-200b-3p
 
 
-0.382
 
 
24.7
 
 
 
 
B4GAT1
 
 
hsa-miR-98-5p
 
 
-0.47
 
 
24.7
 
 
 
 
B4GAT1
 
 
hsa-let-7c-5p
 
 
-0.47
 
 
24.7
 
 
 
 
BACH1
 
 
hsa-miR-98-5p
 
 
-0.298
 
 
16.2
 
 
 
 
BACH1
 
 
hsa-miR-98-5p
 
 
-0.298
 
 
16.2
 
 
 
 
BACH1
 
 
hsa-let-7c-5p
 
 
-0.306
 
 
16.2
 
 
 
 
BACH1
 
 
hsa-let-7c-5p
 
 
-0.306
 
 
16.2
 
 
 
 
BACH1
 
 
hsa-miR-98-5p
 
 
-0.435
 
 
16.2
 
 
 
 
BACH1
 
 
hsa-miR-98-5p
 
 
-0.435
 
 
16.2
 
 
 
 
BACH1
 
 
hsa-let-7c-5p
 
 
-0.435
 
 
16.2
 
 
 
 
BACH1
 
 
hsa-let-7c-5p
 
 
-0.435
 
 
16.2
 
 
 
 
BAG6
 
 
hsa-miR-200b-3p
 
 
-0.371
 
 
70.4
 
 
 
 
BAP1
 
 
hsa-miR-423-5p
 
 
-0.428
 
 
50.5
 
 
 
 
BBX
 
 
hsa-miR-18a-5p
 
 
-0.31
 
 
11.6
 
 
 
 
BCAT1
 
 
hsa-miR-98-5p
 
 
-0.394
 
 
54.8
 
 
 
 
BCAT1
 
 
hsa-let-7c-5p
 
 
-0.394
 
 
54.8
 
 
 
 
BEGAIN
 
 
hsa-let-7c-5p
 
 
-0.466
 
 
22.6
 
 
 
 
BEGAIN
 
 
hsa-miR-98-5p
 
 
-0.474
 
 
22.6
 
 
 
 
BIN1
 
 
hsa-miR-744-5p
 
 
-0.654
 
 
20.4
 
 
 
 
BIN3
 
 
hsa-miR-98-5p
 
 
-0.427
 
 
11.6
 
 
 
 
BIN3
 
 
hsa-let-7c-5p
 
 
-0.427
 
 
11.6
 
 
 
 
BLOC1S6
 
 
hsa-miR-98-5p
 
 
-0.321
 
 
19.3
 
 
 
 
BLOC1S6
 
 
hsa-let-7c-5p
 
 
-0.321
 
 
19.3
 
 
 
 
BNIP3L
 
 
hsa-miR-20a-5p
 
 
-0.287
 
 
19.0
 
 
 
 
BRWD1
 
 
hsa-miR-18a-5p
 
 
-0.29
 
 
18.8
 
 
 
 
BTF3L4
 
 
hsa-miR-200b-3p
 
 
-0.294
 
 
36.1
 
 
 
 
BTF3L4
 
 
hsa-miR-194-5p
 
 
-0.334
 
 
36.1
 
 
 
 
BTF3L4
 
 
hsa-miR-98-5p
 
 
-0.355
 
 
36.1
 
 
 
 
BTF3L4
 
 
hsa-let-7c-5p
 
 
-0.355
 
 
36.1
 
 
 
 
BTG3
 
 
hsa-miR-18a-5p
 
 
-0.464
 
 
19.8
 
 
 
 
BTG3
 
 
hsa-miR-17-5p
 
 
-0.668
 
 
19.8
 
 
 
 
BTG3
 
 
hsa-miR-20a-5p
 
 
-0.671
 
 
19.8
 
 
 
 
BZW1
 
 
hsa-miR-98-5p
 
 
-0.354
 
 
59.0
 
 
 
 
BZW1
 
 
hsa-let-7c-5p
 
 
-0.354
 
 
59.0
 
 
 
 
BZW1
 
 
hsa-miR-98-5p
 
 
-0.375
 
 
59.0
 
 
 
 
BZW1
 
 
hsa-let-7c-5p
 
 
-0.375
 
 
59.0
 
 
 
 
BZW2
 
 
hsa-miR-98-5p
 
 
-0.397
 
 
31.2
 
 
 
 
BZW2
 
 
hsa-let-7c-5p
 
 
-0.397
 
 
31.2
 
 
 
 
C15orf39
 
 
hsa-miR-98-5p
 
 
-0.374
 
 
13.7
 
 
 
 
 
 
 parathyroid gland 
 
 
 
 
target_symbol
 
 
mature_mirna_id
 
 
score
 
 
nTPM
 
 
 
 
 
 
ABHD5
 
 
hsa-miR-20a-5p
 
 
-0.397
 
 
11.5
 
 
 
 
ABHD5
 
 
hsa-miR-17-5p
 
 
-0.45
 
 
11.5
 
 
 
 
ACSL6
 
 
hsa-miR-98-5p
 
 
-0.336
 
 
10.6
 
 
 
 
ACSL6
 
 
hsa-let-7c-5p
 
 
-0.336
 
 
10.6
 
 
 
 
ADAM9
 
 
hsa-miR-20a-5p
 
 
-0.299
 
 
49.4
 
 
 
 
ADAM9
 
 
hsa-miR-17-5p
 
 
-0.32
 
 
49.4
 
 
 
 
ADD3
 
 
hsa-miR-200b-3p
 
 
-0.324
 
 
17.6
 
 
 
 
ADD3
 
 
hsa-miR-18a-5p
 
 
-0.39
 
 
17.6
 
 
 
 
ADNP
 
 
hsa-miR-328-3p
 
 
-0.371
 
 
34.0
 
 
 
 
AEBP2
 
 
hsa-miR-18a-5p
 
 
-0.376
 
 
28.1
 
 
 
 
AHCTF1
 
 
hsa-miR-98-5p
 
 
-0.374
 
 
12.3
 
 
 
 
AHCTF1
 
 
hsa-let-7c-5p
 
 
-0.374
 
 
12.3
 
 
 
 
AHNAK
 
 
hsa-miR-20a-5p
 
 
-0.316
 
 
24.7
 
 
 
 
AHNAK
 
 
hsa-miR-17-5p
 
 
-0.337
 
 
24.7
 
 
 
 
AIFM1
 
 
hsa-miR-98-5p
 
 
-0.314
 
 
27.9
 
 
 
 
AIFM1
 
 
hsa-let-7c-5p
 
 
-0.314
 
 
27.9
 
 
 
 
AK1
 
 
hsa-miR-423-5p
 
 
-0.443
 
 
44.1
 
 
 
 
ALCAM
 
 
hsa-miR-18a-5p
 
 
-0.341
 
 
412.8
 
 
 
 
ALDH4A1
 
 
hsa-miR-184
 
 
-0.421
 
 
21.6
 
 
 
 
ALG3
 
 
hsa-miR-20a-5p
 
 
-0.559
 
 
21.7
 
 
 
 
ALG3
 
 
hsa-miR-17-5p
 
 
-0.572
 
 
21.7
 
 
 
 
ALG9
 
 
hsa-miR-7-5p
 
 
-0.302
 
 
12.0
 
 
 
 
AMT
 
 
hsa-miR-98-5p
 
 
-0.463
 
 
13.3
 
 
 
 
AMT
 
 
hsa-let-7c-5p
 
 
-0.463
 
 
13.3
 
 
 
 
ANKFY1
 
 
hsa-miR-155-5p
 
 
-0.315
 
 
17.9
 
 
 
 
ANKRD29
 
 
hsa-miR-20a-5p
 
 
-0.359
 
 
23.3
 
 
 
 
ANKRD29
 
 
hsa-miR-17-5p
 
 
-0.372
 
 
23.3
 
 
 
 
ANKRD46
 
 
hsa-miR-98-5p
 
 
-0.389
 
 
15.1
 
 
 
 
ANKRD46
 
 
hsa-let-7c-5p
 
 
-0.389
 
 
15.1
 
 
 
 
ANP32B
 
 
hsa-miR-200b-3p
 
 
-0.287
 
 
64.6
 
 
 
 
AP1S1
 
 
hsa-miR-98-5p
 
 
-0.374
 
 
21.4
 
 
 
 
AP1S1
 
 
hsa-let-7c-5p
 
 
-0.374
 
 
21.4
 
 
 
 
AP1S2
 
 
hsa-miR-200b-3p
 
 
-0.4
 
 
15.6
 
 
 
 
AP2M1
 
 
hsa-miR-423-5p
 
 
-0.49
 
 
195.8
 
 
 
 
APOO
 
 
hsa-miR-200b-3p
 
 
-0.361
 
 
58.1
 
 
 
 
ARF4
 
 
hsa-miR-194-5p
 
 
-0.293
 
 
95.4
 
 
 
 
ARF4
 
 
hsa-miR-7-5p
 
 
-0.407
 
 
95.4
 
 
 
 
ARG2
 
 
hsa-miR-98-5p
 
 
-0.468
 
 
206.5
 
 
 
 
ARG2
 
 
hsa-let-7c-5p
 
 
-0.468
 
 
206.5
 
 
 
 
ARHGAP1
 
 
hsa-miR-17-5p
 
 
-0.325
 
 
23.3
 
 
 
 
ARHGAP1
 
 
hsa-miR-20a-5p
 
 
-0.339
 
 
23.3
 
 
 
 
ARID2
 
 
hsa-miR-155-5p
 
 
-0.384
 
 
11.0
 
 
 
 
ARID4B
 
 
hsa-miR-20a-5p
 
 
-0.29
 
 
12.8
 
 
 
 
ARL1
 
 
hsa-miR-20a-5p
 
 
-0.403
 
 
86.8
 
 
 
 
ARL1
 
 
hsa-miR-17-5p
 
 
-0.432
 
 
86.8
 
 
 
 
ARL6IP1
 
 
hsa-miR-7-5p
 
 
-0.374
 
 
169.8
 
 
 
 
ARL6IP1
 
 
hsa-miR-328-3p
 
 
-0.494
 
 
169.8
 
 
 
 
ARMC8
 
 
hsa-miR-20a-5p
 
 
-0.357
 
 
20.0
 
 
 
 
ARMC8
 
 
hsa-miR-17-5p
 
 
-0.378
 
 
20.0
 
 
 
 
ARMT1
 
 
hsa-miR-98-5p
 
 
-0.303
 
 
10.9
 
 
 
 
ARMT1
 
 
hsa-let-7c-5p
 
 
-0.303
 
 
10.9
 
 
 
 
ARPP19
 
 
hsa-miR-328-3p
 
 
-0.404
 
 
102.5
 
 
 
 
ARPP19
 
 
hsa-let-7c-5p
 
 
-0.492
 
 
102.5
 
 
 
 
ARPP19
 
 
hsa-miR-98-5p
 
 
-0.5
 
 
102.5
 
 
 
 
ASAP1
 
 
hsa-miR-98-5p
 
 
-0.308
 
 
16.8
 
 
 
 
ASAP1
 
 
hsa-let-7c-5p
 
 
-0.308
 
 
16.8
 
 
 
 
ASF1A
 
 
hsa-miR-200b-3p
 
 
-0.377
 
 
14.9
 
 
 
 
ATG13
 
 
hsa-miR-423-5p
 
 
-0.3
 
 
35.4
 
 
 
 
ATG4A
 
 
hsa-miR-7-5p
 
 
-0.448
 
 
11.7
 
 
 
 
ATOSB
 
 
hsa-miR-328-3p
 
 
-0.358
 
 
10.5
 
 
 
 
ATOSB
 
 
hsa-miR-98-5p
 
 
-0.429
 
 
10.5
 
 
 
 
ATOSB
 
 
hsa-let-7c-5p
 
 
-0.429
 
 
10.5
 
 
 
 
ATP2A2
 
 
hsa-miR-98-5p
 
 
-0.311
 
 
52.7
 
 
 
 
ATP2A2
 
 
hsa-let-7c-5p
 
 
-0.311
 
 
52.7
 
 
 
 
ATP6V1F
 
 
hsa-miR-194-5p
 
 
-0.444
 
 
153.3
 
 
 
 
ATP6V1H
 
 
hsa-miR-194-5p
 
 
-0.652
 
 
44.8
 
 
 
 
ATP8B2
 
 
hsa-miR-423-5p
 
 
-0.297
 
 
10.4
 
 
 
 
ATPAF1
 
 
hsa-miR-98-5p
 
 
-0.498
 
 
129.7
 
 
 
 
ATPAF1
 
 
hsa-let-7c-5p
 
 
-0.498
 
 
129.7
 
 
 
 
ATXN1L
 
 
hsa-miR-155-5p
 
 
-0.315
 
 
15.5
 
 
 
 
B3GAT3
 
 
hsa-miR-98-5p
 
 
-0.324
 
 
18.1
 
 
 
 
B3GAT3
 
 
hsa-let-7c-5p
 
 
-0.324
 
 
18.1
 
 
 
 
B4GALT5
 
 
hsa-miR-210-3p
 
 
-0.511
 
 
10.6
 
 
 
 
B4GAT1
 
 
hsa-miR-200b-3p
 
 
-0.382
 
 
21.3
 
 
 
 
B4GAT1
 
 
hsa-miR-98-5p
 
 
-0.47
 
 
21.3
 
 
 
 
B4GAT1
 
 
hsa-let-7c-5p
 
 
-0.47
 
 
21.3
 
 
 
 
BAG6
 
 
hsa-miR-200b-3p
 
 
-0.371
 
 
23.6
 
 
 
 
BAP1
 
 
hsa-miR-423-5p
 
 
-0.428
 
 
37.8
 
 
 
 
BBX
 
 
hsa-miR-18a-5p
 
 
-0.31
 
 
43.6
 
 
 
 
BCAS3
 
 
hsa-miR-423-5p
 
 
-0.322
 
 
13.6
 
 
 
 
BIN1
 
 
hsa-miR-744-5p
 
 
-0.654
 
 
18.4
 
 
 
 
BIN3
 
 
hsa-miR-98-5p
 
 
-0.427
 
 
11.8
 
 
 
 
BIN3
 
 
hsa-let-7c-5p
 
 
-0.427
 
 
11.8
 
 
 
 
BLOC1S6
 
 
hsa-miR-98-5p
 
 
-0.321
 
 
15.8
 
 
 
 
BLOC1S6
 
 
hsa-let-7c-5p
 
 
-0.321
 
 
15.8
 
 
 
 
BNIP2
 
 
hsa-miR-194-5p
 
 
-0.489
 
 
14.7
 
 
 
 
BNIP3L
 
 
hsa-miR-20a-5p
 
 
-0.287
 
 
51.8
 
 
 
 
BRWD1
 
 
hsa-miR-18a-5p
 
 
-0.29
 
 
12.3
 
 
 
 
BTBD10
 
 
hsa-miR-17-5p
 
 
-0.409
 
 
12.4
 
 
 
 
BTBD10
 
 
hsa-miR-20a-5p
 
 
-0.412
 
 
12.4
 
 
 
 
BTBD9
 
 
hsa-miR-98-5p
 
 
-0.311
 
 
12.9
 
 
 
 
BTBD9
 
 
hsa-let-7c-5p
 
 
-0.311
 
 
12.9
 
 
 
 
BTF3L4
 
 
hsa-miR-200b-3p
 
 
-0.294
 
 
39.5
 
 
 
 
BTF3L4
 
 
hsa-miR-194-5p
 
 
-0.334
 
 
39.5
 
 
 
 
BTF3L4
 
 
hsa-miR-98-5p
 
 
-0.355
 
 
39.5
 
 
 
 
BTF3L4
 
 
hsa-let-7c-5p
 
 
-0.355
 
 
39.5
 
 
 
 
BTG3
 
 
hsa-miR-18a-5p
 
 
-0.464
 
 
33.2
 
 
 
 
BTG3
 
 
hsa-miR-17-5p
 
 
-0.668
 
 
33.2
 
 
 
 
BTG3
 
 
hsa-miR-20a-5p
 
 
-0.671
 
 
33.2
 
 
 
 
BZW1
 
 
hsa-miR-98-5p
 
 
-0.354
 
 
68.1
 
 
 
 
 
 
 skeletal muscle 
 
 
 
 
target_symbol
 
 
mature_mirna_id
 
 
score
 
 
nTPM
 
 
 
 
 
 
ABHD5
 
 
hsa-miR-20a-5p
 
 
-0.397
 
 
12.3
 
 
 
 
ABHD5
 
 
hsa-miR-17-5p
 
 
-0.45
 
 
12.3
 
 
 
 
ABTB2
 
 
hsa-miR-18a-5p
 
 
-0.287
 
 
15.8
 
 
 
 
ACHE
 
 
hsa-miR-194-5p
 
 
-0.395
 
 
152.9
 
 
 
 
ACTA1
 
 
hsa-miR-155-5p
 
 
-0.595
 
 
123974.2
 
 
 
 
ACTC1
 
 
hsa-miR-7-5p
 
 
-0.471
 
 
519.7
 
 
 
 
ADAM9
 
 
hsa-miR-20a-5p
 
 
-0.299
 
 
16.2
 
 
 
 
ADAM9
 
 
hsa-miR-17-5p
 
 
-0.32
 
 
16.2
 
 
 
 
ADNP
 
 
hsa-miR-328-3p
 
 
-0.371
 
 
33.8
 
 
 
 
ADRB2
 
 
hsa-miR-98-5p
 
 
-0.635
 
 
10.9
 
 
 
 
ADRB2
 
 
hsa-let-7c-5p
 
 
-0.635
 
 
10.9
 
 
 
 
AEBP2
 
 
hsa-miR-18a-5p
 
 
-0.376
 
 
13.0
 
 
 
 
AGO2
 
 
hsa-miR-184
 
 
-0.831
 
 
16.4
 
 
 
 
AGO2
 
 
hsa-miR-200b-3p
 
 
-1.125
 
 
16.4
 
 
 
 
AHCTF1
 
 
hsa-miR-98-5p
 
 
-0.374
 
 
10.0
 
 
 
 
AHCTF1
 
 
hsa-let-7c-5p
 
 
-0.374
 
 
10.0
 
 
 
 
AHNAK
 
 
hsa-miR-20a-5p
 
 
-0.316
 
 
178.0
 
 
 
 
AHNAK
 
 
hsa-miR-17-5p
 
 
-0.337
 
 
178.0
 
 
 
 
AIFM1
 
 
hsa-miR-98-5p
 
 
-0.314
 
 
58.6
 
 
 
 
AIFM1
 
 
hsa-let-7c-5p
 
 
-0.314
 
 
58.6
 
 
 
 
AK1
 
 
hsa-miR-423-5p
 
 
-0.443
 
 
1262.8
 
 
 
 
AKAP6
 
 
hsa-miR-98-5p
 
 
-0.307
 
 
44.4
 
 
 
 
AKAP6
 
 
hsa-let-7c-5p
 
 
-0.307
 
 
44.4
 
 
 
 
ALDH4A1
 
 
hsa-miR-184
 
 
-0.421
 
 
69.5
 
 
 
 
ALG3
 
 
hsa-miR-20a-5p
 
 
-0.559
 
 
29.0
 
 
 
 
ALG3
 
 
hsa-miR-17-5p
 
 
-0.572
 
 
29.0
 
 
 
 
ANGPTL2
 
 
hsa-miR-423-5p
 
 
-0.379
 
 
55.7
 
 
 
 
ANKFY1
 
 
hsa-miR-155-5p
 
 
-0.315
 
 
13.2
 
 
 
 
ANKRD12
 
 
hsa-miR-7-5p
 
 
-0.37
 
 
25.1
 
 
 
 
ANKRD46
 
 
hsa-miR-98-5p
 
 
-0.389
 
 
58.3
 
 
 
 
ANKRD46
 
 
hsa-let-7c-5p
 
 
-0.389
 
 
58.3
 
 
 
 
ANKRD9
 
 
hsa-miR-20a-5p
 
 
-0.31
 
 
148.8
 
 
 
 
ANKRD9
 
 
hsa-miR-17-5p
 
 
-0.347
 
 
148.8
 
 
 
 
ANP32B
 
 
hsa-miR-200b-3p
 
 
-0.287
 
 
103.8
 
 
 
 
AP1S2
 
 
hsa-miR-200b-3p
 
 
-0.4
 
 
65.3
 
 
 
 
AP2M1
 
 
hsa-miR-423-5p
 
 
-0.49
 
 
149.2
 
 
 
 
AP5S1
 
 
hsa-miR-744-5p
 
 
-0.354
 
 
17.9
 
 
 
 
APOO
 
 
hsa-miR-200b-3p
 
 
-0.361
 
 
43.1
 
 
 
 
ARF4
 
 
hsa-miR-194-5p
 
 
-0.293
 
 
59.5
 
 
 
 
ARF4
 
 
hsa-miR-7-5p
 
 
-0.407
 
 
59.5
 
 
 
 
ARG2
 
 
hsa-miR-98-5p
 
 
-0.468
 
 
37.4
 
 
 
 
ARG2
 
 
hsa-let-7c-5p
 
 
-0.468
 
 
37.4
 
 
 
 
ARGLU1
 
 
hsa-miR-200b-3p
 
 
-0.29
 
 
12.3
 
 
 
 
ARHGAP1
 
 
hsa-miR-17-5p
 
 
-0.325
 
 
28.5
 
 
 
 
ARHGAP1
 
 
hsa-miR-20a-5p
 
 
-0.339
 
 
28.5
 
 
 
 
ARL1
 
 
hsa-miR-20a-5p
 
 
-0.403
 
 
48.7
 
 
 
 
ARL1
 
 
hsa-miR-17-5p
 
 
-0.432
 
 
48.7
 
 
 
 
ARL6IP1
 
 
hsa-miR-7-5p
 
 
-0.374
 
 
21.6
 
 
 
 
ARL6IP1
 
 
hsa-miR-328-3p
 
 
-0.494
 
 
21.6
 
 
 
 
ARL6IP6
 
 
hsa-miR-98-5p
 
 
-0.293
 
 
53.9
 
 
 
 
ARL6IP6
 
 
hsa-let-7c-5p
 
 
-0.293
 
 
53.9
 
 
 
 
ARMC8
 
 
hsa-miR-20a-5p
 
 
-0.357
 
 
36.6
 
 
 
 
ARMC8
 
 
hsa-miR-17-5p
 
 
-0.378
 
 
36.6
 
 
 
 
ARMT1
 
 
hsa-miR-98-5p
 
 
-0.303
 
 
17.8
 
 
 
 
ARMT1
 
 
hsa-let-7c-5p
 
 
-0.303
 
 
17.8
 
 
 
 
ARPP19
 
 
hsa-miR-328-3p
 
 
-0.404
 
 
225.3
 
 
 
 
ARPP19
 
 
hsa-let-7c-5p
 
 
-0.492
 
 
225.3
 
 
 
 
ARPP19
 
 
hsa-miR-98-5p
 
 
-0.5
 
 
225.3
 
 
 
 
ARRDC4
 
 
hsa-miR-98-5p
 
 
-0.329
 
 
13.8
 
 
 
 
ARRDC4
 
 
hsa-let-7c-5p
 
 
-0.329
 
 
13.8
 
 
 
 
ASAP1
 
 
hsa-miR-98-5p
 
 
-0.308
 
 
12.4
 
 
 
 
ASAP1
 
 
hsa-let-7c-5p
 
 
-0.308
 
 
12.4
 
 
 
 
ASB14
 
 
hsa-miR-423-5p
 
 
-0.434
 
 
26.3
 
 
 
 
ASF1A
 
 
hsa-miR-200b-3p
 
 
-0.377
 
 
15.6
 
 
 
 
ATG13
 
 
hsa-miR-423-5p
 
 
-0.3
 
 
21.5
 
 
 
 
ATG16L1
 
 
hsa-miR-20a-5p
 
 
-0.446
 
 
20.1
 
 
 
 
ATG16L1
 
 
hsa-miR-17-5p
 
 
-0.467
 
 
20.1
 
 
 
 
ATG4A
 
 
hsa-miR-7-5p
 
 
-0.448
 
 
18.0
 
 
 
 
ATOSB
 
 
hsa-miR-328-3p
 
 
-0.358
 
 
13.5
 
 
 
 
ATOSB
 
 
hsa-miR-98-5p
 
 
-0.429
 
 
13.5
 
 
 
 
ATOSB
 
 
hsa-let-7c-5p
 
 
-0.429
 
 
13.5
 
 
 
 
ATP2A2
 
 
hsa-miR-98-5p
 
 
-0.311
 
 
2675.7
 
 
 
 
ATP2A2
 
 
hsa-let-7c-5p
 
 
-0.311
 
 
2675.7
 
 
 
 
ATP2B2
 
 
hsa-miR-7-5p
 
 
-0.297
 
 
35.1
 
 
 
 
ATP6V1F
 
 
hsa-miR-194-5p
 
 
-0.444
 
 
162.2
 
 
 
 
ATP6V1H
 
 
hsa-miR-194-5p
 
 
-0.652
 
 
28.2
 
 
 
 
ATPAF1
 
 
hsa-miR-98-5p
 
 
-0.498
 
 
260.2
 
 
 
 
ATPAF1
 
 
hsa-let-7c-5p
 
 
-0.498
 
 
260.2
 
 
 
 
AVPI1
 
 
hsa-miR-7-5p
 
 
-0.367
 
 
13.0
 
 
 
 
B3GAT3
 
 
hsa-miR-98-5p
 
 
-0.324
 
 
15.7
 
 
 
 
B3GAT3
 
 
hsa-let-7c-5p
 
 
-0.324
 
 
15.7
 
 
 
 
B4GALT5
 
 
hsa-miR-210-3p
 
 
-0.511
 
 
63.3
 
 
 
 
B4GAT1
 
 
hsa-miR-200b-3p
 
 
-0.382
 
 
27.1
 
 
 
 
B4GAT1
 
 
hsa-miR-98-5p
 
 
-0.47
 
 
27.1
 
 
 
 
B4GAT1
 
 
hsa-let-7c-5p
 
 
-0.47
 
 
27.1
 
 
 
 
BACH1
 
 
hsa-miR-98-5p
 
 
-0.298
 
 
24.7
 
 
 
 
BACH1
 
 
hsa-miR-98-5p
 
 
-0.298
 
 
24.7
 
 
 
 
BACH1
 
 
hsa-let-7c-5p
 
 
-0.306
 
 
24.7
 
 
 
 
BACH1
 
 
hsa-let-7c-5p
 
 
-0.306
 
 
24.7
 
 
 
 
BACH1
 
 
hsa-miR-98-5p
 
 
-0.435
 
 
24.7
 
 
 
 
BACH1
 
 
hsa-miR-98-5p
 
 
-0.435
 
 
24.7
 
 
 
 
BACH1
 
 
hsa-let-7c-5p
 
 
-0.435
 
 
24.7
 
 
 
 
BACH1
 
 
hsa-let-7c-5p
 
 
-0.435
 
 
24.7
 
 
 
 
BAG6
 
 
hsa-miR-200b-3p
 
 
-0.371
 
 
200.5
 
 
 
 
BAP1
 
 
hsa-miR-423-5p
 
 
-0.428
 
 
70.6
 
 
 
 
BCAS3
 
 
hsa-miR-423-5p
 
 
-0.322
 
 
16.4
 
 
 
 
BIN1
 
 
hsa-miR-744-5p
 
 
-0.654
 
 
1986.0
 
 
 
 
BIN3
 
 
hsa-miR-98-5p
 
 
-0.427
 
 
12.7
 
 
 
 
BIN3
 
 
hsa-let-7c-5p
 
 
-0.427
 
 
12.7
 
 
 
 
BLOC1S4
 
 
hsa-miR-7-5p
 
 
-0.417
 
 
11.1
 
 
 
 
 
 
 spinal cord 
 
 
 
 
target_symbol
 
 
mature_mirna_id
 
 
score
 
 
nTPM
 
 
 
 
 
 
ABHD5
 
 
hsa-miR-20a-5p
 
 
-0.397
 
 
19.5
 
 
 
 
ABHD5
 
 
hsa-miR-17-5p
 
 
-0.45
 
 
19.5
 
 
 
 
ABTB2
 
 
hsa-miR-18a-5p
 
 
-0.287
 
 
21.6
 
 
 
 
ACHE
 
 
hsa-miR-194-5p
 
 
-0.395
 
 
14.4
 
 
 
 
ADAM9
 
 
hsa-miR-20a-5p
 
 
-0.299
 
 
11.3
 
 
 
 
ADAM9
 
 
hsa-miR-17-5p
 
 
-0.32
 
 
11.3
 
 
 
 
ADD3
 
 
hsa-miR-200b-3p
 
 
-0.324
 
 
66.3
 
 
 
 
ADD3
 
 
hsa-miR-18a-5p
 
 
-0.39
 
 
66.3
 
 
 
 
ADNP
 
 
hsa-miR-328-3p
 
 
-0.371
 
 
22.3
 
 
 
 
AHNAK
 
 
hsa-miR-20a-5p
 
 
-0.316
 
 
27.2
 
 
 
 
AHNAK
 
 
hsa-miR-17-5p
 
 
-0.337
 
 
27.2
 
 
 
 
AIFM1
 
 
hsa-miR-98-5p
 
 
-0.314
 
 
10.1
 
 
 
 
AIFM1
 
 
hsa-let-7c-5p
 
 
-0.314
 
 
10.1
 
 
 
 
AK1
 
 
hsa-miR-423-5p
 
 
-0.443
 
 
67.3
 
 
 
 
AKAP6
 
 
hsa-miR-98-5p
 
 
-0.307
 
 
13.3
 
 
 
 
AKAP6
 
 
hsa-let-7c-5p
 
 
-0.307
 
 
13.3
 
 
 
 
ALCAM
 
 
hsa-miR-18a-5p
 
 
-0.341
 
 
56.1
 
 
 
 
ALDH4A1
 
 
hsa-miR-184
 
 
-0.421
 
 
11.4
 
 
 
 
ALG3
 
 
hsa-miR-20a-5p
 
 
-0.559
 
 
10.8
 
 
 
 
ALG3
 
 
hsa-miR-17-5p
 
 
-0.572
 
 
10.8
 
 
 
 
ANGPTL2
 
 
hsa-miR-423-5p
 
 
-0.379
 
 
15.5
 
 
 
 
ANKFY1
 
 
hsa-miR-155-5p
 
 
-0.315
 
 
14.2
 
 
 
 
ANKRD46
 
 
hsa-miR-98-5p
 
 
-0.389
 
 
24.6
 
 
 
 
ANKRD46
 
 
hsa-let-7c-5p
 
 
-0.389
 
 
24.6
 
 
 
 
ANP32B
 
 
hsa-miR-200b-3p
 
 
-0.287
 
 
363.5
 
 
 
 
AP1S1
 
 
hsa-miR-98-5p
 
 
-0.374
 
 
28.5
 
 
 
 
AP1S1
 
 
hsa-let-7c-5p
 
 
-0.374
 
 
28.5
 
 
 
 
AP1S2
 
 
hsa-miR-200b-3p
 
 
-0.4
 
 
33.3
 
 
 
 
AP2M1
 
 
hsa-miR-423-5p
 
 
-0.49
 
 
162.9
 
 
 
 
APCDD1
 
 
hsa-miR-20a-5p
 
 
-0.404
 
 
14.7
 
 
 
 
APCDD1
 
 
hsa-miR-17-5p
 
 
-0.425
 
 
14.7
 
 
 
 
APOO
 
 
hsa-miR-200b-3p
 
 
-0.361
 
 
36.2
 
 
 
 
ARF4
 
 
hsa-miR-194-5p
 
 
-0.293
 
 
69.7
 
 
 
 
ARF4
 
 
hsa-miR-7-5p
 
 
-0.407
 
 
69.7
 
 
 
 
ARGLU1
 
 
hsa-miR-200b-3p
 
 
-0.29
 
 
11.3
 
 
 
 
ARHGAP1
 
 
hsa-miR-17-5p
 
 
-0.325
 
 
38.6
 
 
 
 
ARHGAP1
 
 
hsa-miR-20a-5p
 
 
-0.339
 
 
38.6
 
 
 
 
ARID4B
 
 
hsa-miR-20a-5p
 
 
-0.29
 
 
12.3
 
 
 
 
ARL1
 
 
hsa-miR-20a-5p
 
 
-0.403
 
 
26.5
 
 
 
 
ARL1
 
 
hsa-miR-17-5p
 
 
-0.432
 
 
26.5
 
 
 
 
ARL6IP1
 
 
hsa-miR-7-5p
 
 
-0.374
 
 
140.2
 
 
 
 
ARL6IP1
 
 
hsa-miR-328-3p
 
 
-0.494
 
 
140.2
 
 
 
 
ARMC8
 
 
hsa-miR-20a-5p
 
 
-0.357
 
 
15.5
 
 
 
 
ARMC8
 
 
hsa-miR-17-5p
 
 
-0.378
 
 
15.5
 
 
 
 
ARPP19
 
 
hsa-miR-328-3p
 
 
-0.404
 
 
170.8
 
 
 
 
ARPP19
 
 
hsa-let-7c-5p
 
 
-0.492
 
 
170.8
 
 
 
 
ARPP19
 
 
hsa-miR-98-5p
 
 
-0.5
 
 
170.8
 
 
 
 
ARRDC4
 
 
hsa-miR-98-5p
 
 
-0.329
 
 
75.7
 
 
 
 
ARRDC4
 
 
hsa-let-7c-5p
 
 
-0.329
 
 
75.7
 
 
 
 
ASAP1
 
 
hsa-miR-98-5p
 
 
-0.308
 
 
11.0
 
 
 
 
ASAP1
 
 
hsa-let-7c-5p
 
 
-0.308
 
 
11.0
 
 
 
 
ASF1A
 
 
hsa-miR-200b-3p
 
 
-0.377
 
 
14.8
 
 
 
 
ATG13
 
 
hsa-miR-423-5p
 
 
-0.3
 
 
32.4
 
 
 
 
ATG16L1
 
 
hsa-miR-20a-5p
 
 
-0.446
 
 
10.3
 
 
 
 
ATG16L1
 
 
hsa-miR-17-5p
 
 
-0.467
 
 
10.3
 
 
 
 
ATOSB
 
 
hsa-miR-328-3p
 
 
-0.358
 
 
30.9
 
 
 
 
ATOSB
 
 
hsa-miR-98-5p
 
 
-0.429
 
 
30.9
 
 
 
 
ATOSB
 
 
hsa-let-7c-5p
 
 
-0.429
 
 
30.9
 
 
 
 
ATP2A2
 
 
hsa-miR-98-5p
 
 
-0.311
 
 
19.0
 
 
 
 
ATP2A2
 
 
hsa-let-7c-5p
 
 
-0.311
 
 
19.0
 
 
 
 
ATP6V1F
 
 
hsa-miR-194-5p
 
 
-0.444
 
 
198.6
 
 
 
 
ATP6V1H
 
 
hsa-miR-194-5p
 
 
-0.652
 
 
26.3
 
 
 
 
ATPAF1
 
 
hsa-miR-98-5p
 
 
-0.498
 
 
50.4
 
 
 
 
ATPAF1
 
 
hsa-let-7c-5p
 
 
-0.498
 
 
50.4
 
 
 
 
AVPI1
 
 
hsa-miR-7-5p
 
 
-0.367
 
 
33.9
 
 
 
 
B3GAT3
 
 
hsa-miR-98-5p
 
 
-0.324
 
 
62.6
 
 
 
 
B3GAT3
 
 
hsa-let-7c-5p
 
 
-0.324
 
 
62.6
 
 
 
 
B4GAT1
 
 
hsa-miR-200b-3p
 
 
-0.382
 
 
48.7
 
 
 
 
B4GAT1
 
 
hsa-miR-98-5p
 
 
-0.47
 
 
48.7
 
 
 
 
B4GAT1
 
 
hsa-let-7c-5p
 
 
-0.47
 
 
48.7
 
 
 
 
BAG6
 
 
hsa-miR-200b-3p
 
 
-0.371
 
 
41.5
 
 
 
 
BAMBI
 
 
hsa-miR-20a-5p
 
 
-0.485
 
 
23.0
 
 
 
 
BAMBI
 
 
hsa-miR-17-5p
 
 
-0.506
 
 
23.0
 
 
 
 
BAP1
 
 
hsa-miR-423-5p
 
 
-0.428
 
 
34.3
 
 
 
 
BBX
 
 
hsa-miR-18a-5p
 
 
-0.31
 
 
17.3
 
 
 
 
BCAS3
 
 
hsa-miR-423-5p
 
 
-0.322
 
 
11.6
 
 
 
 
BIN1
 
 
hsa-miR-744-5p
 
 
-0.654
 
 
197.8
 
 
 
 
BLOC1S6
 
 
hsa-miR-98-5p
 
 
-0.321
 
 
15.5
 
 
 
 
BLOC1S6
 
 
hsa-let-7c-5p
 
 
-0.321
 
 
15.5
 
 
 
 
BNIP3L
 
 
hsa-miR-20a-5p
 
 
-0.287
 
 
78.0
 
 
 
 
BRWD1
 
 
hsa-miR-18a-5p
 
 
-0.29
 
 
20.1
 
 
 
 
BTBD10
 
 
hsa-miR-17-5p
 
 
-0.409
 
 
15.3
 
 
 
 
BTBD10
 
 
hsa-miR-20a-5p
 
 
-0.412
 
 
15.3
 
 
 
 
BTF3L4
 
 
hsa-miR-200b-3p
 
 
-0.294
 
 
36.6
 
 
 
 
BTF3L4
 
 
hsa-miR-194-5p
 
 
-0.334
 
 
36.6
 
 
 
 
BTF3L4
 
 
hsa-miR-98-5p
 
 
-0.355
 
 
36.6
 
 
 
 
BTF3L4
 
 
hsa-let-7c-5p
 
 
-0.355
 
 
36.6
 
 
 
 
BTG3
 
 
hsa-miR-18a-5p
 
 
-0.464
 
 
27.3
 
 
 
 
BTG3
 
 
hsa-miR-17-5p
 
 
-0.668
 
 
27.3
 
 
 
 
BTG3
 
 
hsa-miR-20a-5p
 
 
-0.671
 
 
27.3
 
 
 
 
BZW1
 
 
hsa-miR-98-5p
 
 
-0.354
 
 
46.3
 
 
 
 
BZW1
 
 
hsa-let-7c-5p
 
 
-0.354
 
 
46.3
 
 
 
 
BZW1
 
 
hsa-miR-98-5p
 
 
-0.375
 
 
46.3
 
 
 
 
BZW1
 
 
hsa-let-7c-5p
 
 
-0.375
 
 
46.3
 
 
 
 
C19orf47
 
 
hsa-let-7c-5p
 
 
-0.311
 
 
14.3
 
 
 
 
C19orf47
 
 
hsa-miR-98-5p
 
 
-0.322
 
 
14.3
 
 
 
 
C1GALT1
 
 
hsa-let-7c-5p
 
 
-0.322
 
 
27.9
 
 
 
 
C1GALT1
 
 
hsa-miR-98-5p
 
 
-0.33
 
 
27.9
 
 
 
 
C1QBP
 
 
hsa-miR-330-3p
 
 
-0.412
 
 
74.8
 
 
 
 
C21orf91
 
 
hsa-miR-194-5p
 
 
-0.293
 
 
21.9
 
 
 
 
 
 
 thyroid gland 
 
 
 
 
target_symbol
 
 
mature_mirna_id
 
 
score
 
 
nTPM
 
 
 
 
 
 
ABHD5
 
 
hsa-miR-20a-5p
 
 
-0.397
 
 
14.4
 
 
 
 
ABHD5
 
 
hsa-miR-17-5p
 
 
-0.45
 
 
14.4
 
 
 
 
ACTA1
 
 
hsa-miR-155-5p
 
 
-0.595
 
 
11.1
 
 
 
 
ADAM9
 
 
hsa-miR-20a-5p
 
 
-0.299
 
 
37.4
 
 
 
 
ADAM9
 
 
hsa-miR-17-5p
 
 
-0.32
 
 
37.4
 
 
 
 
ADD3
 
 
hsa-miR-200b-3p
 
 
-0.324
 
 
82.3
 
 
 
 
ADD3
 
 
hsa-miR-18a-5p
 
 
-0.39
 
 
82.3
 
 
 
 
ADNP
 
 
hsa-miR-328-3p
 
 
-0.371
 
 
27.8
 
 
 
 
AEBP2
 
 
hsa-miR-18a-5p
 
 
-0.376
 
 
28.8
 
 
 
 
AGFG2
 
 
hsa-miR-20a-5p
 
 
-0.365
 
 
10.2
 
 
 
 
AGFG2
 
 
hsa-miR-17-5p
 
 
-0.402
 
 
10.2
 
 
 
 
AHCTF1
 
 
hsa-miR-98-5p
 
 
-0.374
 
 
12.9
 
 
 
 
AHCTF1
 
 
hsa-let-7c-5p
 
 
-0.374
 
 
12.9
 
 
 
 
AHNAK
 
 
hsa-miR-20a-5p
 
 
-0.316
 
 
61.4
 
 
 
 
AHNAK
 
 
hsa-miR-17-5p
 
 
-0.337
 
 
61.4
 
 
 
 
AIFM1
 
 
hsa-miR-98-5p
 
 
-0.314
 
 
10.7
 
 
 
 
AIFM1
 
 
hsa-let-7c-5p
 
 
-0.314
 
 
10.7
 
 
 
 
AK1
 
 
hsa-miR-423-5p
 
 
-0.443
 
 
42.1
 
 
 
 
ALCAM
 
 
hsa-miR-18a-5p
 
 
-0.341
 
 
58.4
 
 
 
 
ALG3
 
 
hsa-miR-20a-5p
 
 
-0.559
 
 
17.5
 
 
 
 
ALG3
 
 
hsa-miR-17-5p
 
 
-0.572
 
 
17.5
 
 
 
 
ALG9
 
 
hsa-miR-7-5p
 
 
-0.302
 
 
11.5
 
 
 
 
ANGPTL2
 
 
hsa-miR-423-5p
 
 
-0.379
 
 
15.2
 
 
 
 
ANKFY1
 
 
hsa-miR-155-5p
 
 
-0.315
 
 
13.5
 
 
 
 
ANKRD12
 
 
hsa-miR-7-5p
 
 
-0.37
 
 
15.3
 
 
 
 
ANKRD46
 
 
hsa-miR-98-5p
 
 
-0.389
 
 
18.8
 
 
 
 
ANKRD46
 
 
hsa-let-7c-5p
 
 
-0.389
 
 
18.8
 
 
 
 
ANKRD9
 
 
hsa-miR-20a-5p
 
 
-0.31
 
 
10.7
 
 
 
 
ANKRD9
 
 
hsa-miR-17-5p
 
 
-0.347
 
 
10.7
 
 
 
 
ANP32B
 
 
hsa-miR-200b-3p
 
 
-0.287
 
 
92.0
 
 
 
 
AP1S1
 
 
hsa-miR-98-5p
 
 
-0.374
 
 
15.7
 
 
 
 
AP1S1
 
 
hsa-let-7c-5p
 
 
-0.374
 
 
15.7
 
 
 
 
AP1S2
 
 
hsa-miR-200b-3p
 
 
-0.4
 
 
11.4
 
 
 
 
AP2M1
 
 
hsa-miR-423-5p
 
 
-0.49
 
 
123.2
 
 
 
 
APOO
 
 
hsa-miR-200b-3p
 
 
-0.361
 
 
19.0
 
 
 
 
ARF4
 
 
hsa-miR-194-5p
 
 
-0.293
 
 
134.8
 
 
 
 
ARF4
 
 
hsa-miR-7-5p
 
 
-0.407
 
 
134.8
 
 
 
 
ARG2
 
 
hsa-miR-98-5p
 
 
-0.468
 
 
97.9
 
 
 
 
ARG2
 
 
hsa-let-7c-5p
 
 
-0.468
 
 
97.9
 
 
 
 
ARHGAP1
 
 
hsa-miR-17-5p
 
 
-0.325
 
 
42.2
 
 
 
 
ARHGAP1
 
 
hsa-miR-20a-5p
 
 
-0.339
 
 
42.2
 
 
 
 
ARHGEF28
 
 
hsa-miR-17-5p
 
 
-0.363
 
 
12.7
 
 
 
 
ARHGEF28
 
 
hsa-miR-20a-5p
 
 
-0.366
 
 
12.7
 
 
 
 
ARID4B
 
 
hsa-miR-20a-5p
 
 
-0.29
 
 
15.9
 
 
 
 
ARL1
 
 
hsa-miR-20a-5p
 
 
-0.403
 
 
79.3
 
 
 
 
ARL1
 
 
hsa-miR-17-5p
 
 
-0.432
 
 
79.3
 
 
 
 
ARL4D
 
 
hsa-miR-98-5p
 
 
-0.417
 
 
35.9
 
 
 
 
ARL4D
 
 
hsa-let-7c-5p
 
 
-0.417
 
 
35.9
 
 
 
 
ARL6IP1
 
 
hsa-miR-7-5p
 
 
-0.374
 
 
105.4
 
 
 
 
ARL6IP1
 
 
hsa-miR-328-3p
 
 
-0.494
 
 
105.4
 
 
 
 
ARMC8
 
 
hsa-miR-20a-5p
 
 
-0.357
 
 
20.0
 
 
 
 
ARMC8
 
 
hsa-miR-17-5p
 
 
-0.378
 
 
20.0
 
 
 
 
ARMT1
 
 
hsa-miR-98-5p
 
 
-0.303
 
 
25.9
 
 
 
 
ARMT1
 
 
hsa-let-7c-5p
 
 
-0.303
 
 
25.9
 
 
 
 
ARPP19
 
 
hsa-miR-328-3p
 
 
-0.404
 
 
53.7
 
 
 
 
ARPP19
 
 
hsa-let-7c-5p
 
 
-0.492
 
 
53.7
 
 
 
 
ARPP19
 
 
hsa-miR-98-5p
 
 
-0.5
 
 
53.7
 
 
 
 
ARRDC4
 
 
hsa-miR-98-5p
 
 
-0.329
 
 
16.2
 
 
 
 
ARRDC4
 
 
hsa-let-7c-5p
 
 
-0.329
 
 
16.2
 
 
 
 
ARVCF
 
 
hsa-miR-155-5p
 
 
-0.388
 
 
14.8
 
 
 
 
ASAP1
 
 
hsa-miR-98-5p
 
 
-0.308
 
 
15.1
 
 
 
 
ASAP1
 
 
hsa-let-7c-5p
 
 
-0.308
 
 
15.1
 
 
 
 
ASB6
 
 
hsa-miR-423-5p
 
 
-0.388
 
 
10.3
 
 
 
 
ASF1A
 
 
hsa-miR-200b-3p
 
 
-0.377
 
 
14.1
 
 
 
 
ATG13
 
 
hsa-miR-423-5p
 
 
-0.3
 
 
34.5
 
 
 
 
ATG16L1
 
 
hsa-miR-20a-5p
 
 
-0.446
 
 
11.5
 
 
 
 
ATG16L1
 
 
hsa-miR-17-5p
 
 
-0.467
 
 
11.5
 
 
 
 
ATG4A
 
 
hsa-miR-7-5p
 
 
-0.448
 
 
10.7
 
 
 
 
ATOSB
 
 
hsa-miR-328-3p
 
 
-0.358
 
 
11.1
 
 
 
 
ATOSB
 
 
hsa-miR-98-5p
 
 
-0.429
 
 
11.1
 
 
 
 
ATOSB
 
 
hsa-let-7c-5p
 
 
-0.429
 
 
11.1
 
 
 
 
ATP2A2
 
 
hsa-miR-98-5p
 
 
-0.311
 
 
68.6
 
 
 
 
ATP2A2
 
 
hsa-let-7c-5p
 
 
-0.311
 
 
68.6
 
 
 
 
ATP6V1F
 
 
hsa-miR-194-5p
 
 
-0.444
 
 
85.4
 
 
 
 
ATP6V1H
 
 
hsa-miR-194-5p
 
 
-0.652
 
 
49.0
 
 
 
 
ATP8B2
 
 
hsa-miR-423-5p
 
 
-0.297
 
 
14.8
 
 
 
 
ATPAF1
 
 
hsa-miR-98-5p
 
 
-0.498
 
 
39.3
 
 
 
 
ATPAF1
 
 
hsa-let-7c-5p
 
 
-0.498
 
 
39.3
 
 
 
 
ATXN1L
 
 
hsa-miR-155-5p
 
 
-0.315
 
 
11.1
 
 
 
 
B3GAT3
 
 
hsa-miR-98-5p
 
 
-0.324
 
 
33.1
 
 
 
 
B3GAT3
 
 
hsa-let-7c-5p
 
 
-0.324
 
 
33.1
 
 
 
 
B4GALT5
 
 
hsa-miR-210-3p
 
 
-0.511
 
 
10.8
 
 
 
 
B4GAT1
 
 
hsa-miR-200b-3p
 
 
-0.382
 
 
20.5
 
 
 
 
B4GAT1
 
 
hsa-miR-98-5p
 
 
-0.47
 
 
20.5
 
 
 
 
B4GAT1
 
 
hsa-let-7c-5p
 
 
-0.47
 
 
20.5
 
 
 
 
BACH1
 
 
hsa-miR-98-5p
 
 
-0.298
 
 
11.8
 
 
 
 
BACH1
 
 
hsa-miR-98-5p
 
 
-0.298
 
 
11.8
 
 
 
 
BACH1
 
 
hsa-let-7c-5p
 
 
-0.306
 
 
11.8
 
 
 
 
BACH1
 
 
hsa-let-7c-5p
 
 
-0.306
 
 
11.8
 
 
 
 
BACH1
 
 
hsa-miR-98-5p
 
 
-0.435
 
 
11.8
 
 
 
 
BACH1
 
 
hsa-miR-98-5p
 
 
-0.435
 
 
11.8
 
 
 
 
BACH1
 
 
hsa-let-7c-5p
 
 
-0.435
 
 
11.8
 
 
 
 
BACH1
 
 
hsa-let-7c-5p
 
 
-0.435
 
 
11.8
 
 
 
 
BAG6
 
 
hsa-miR-200b-3p
 
 
-0.371
 
 
74.6
 
 
 
 
BAP1
 
 
hsa-miR-423-5p
 
 
-0.428
 
 
46.7
 
 
 
 
BBX
 
 
hsa-miR-18a-5p
 
 
-0.31
 
 
24.5
 
 
 
 
BIN1
 
 
hsa-miR-744-5p
 
 
-0.654
 
 
27.3
 
 
 
 
BIN3
 
 
hsa-miR-98-5p
 
 
-0.427
 
 
14.4
 
 
 
 
BIN3
 
 
hsa-let-7c-5p
 
 
-0.427
 
 
14.4
 
 
 
 
BLOC1S6
 
 
hsa-miR-98-5p
 
 
-0.321
 
 
41.6
 
 
 
 
 
 
 tongue 
 
 
 
 
target_symbol
 
 
mature_mirna_id
 
 
score
 
 
nTPM
 
 
 
 
 
 
ABHD5
 
 
hsa-miR-20a-5p
 
 
-0.397
 
 
17.3
 
 
 
 
ABHD5
 
 
hsa-miR-17-5p
 
 
-0.45
 
 
17.3
 
 
 
 
ACHE
 
 
hsa-miR-194-5p
 
 
-0.395
 
 
44.9
 
 
 
 
ACTA1
 
 
hsa-miR-155-5p
 
 
-0.595
 
 
19457.9
 
 
 
 
ACTC1
 
 
hsa-miR-7-5p
 
 
-0.471
 
 
182.1
 
 
 
 
ADAM9
 
 
hsa-miR-20a-5p
 
 
-0.299
 
 
23.3
 
 
 
 
ADAM9
 
 
hsa-miR-17-5p
 
 
-0.32
 
 
23.3
 
 
 
 
ADD3
 
 
hsa-miR-200b-3p
 
 
-0.324
 
 
23.9
 
 
 
 
ADD3
 
 
hsa-miR-18a-5p
 
 
-0.39
 
 
23.9
 
 
 
 
ADNP
 
 
hsa-miR-328-3p
 
 
-0.371
 
 
26.5
 
 
 
 
AEBP2
 
 
hsa-miR-18a-5p
 
 
-0.376
 
 
18.4
 
 
 
 
AHCTF1
 
 
hsa-miR-98-5p
 
 
-0.374
 
 
14.7
 
 
 
 
AHCTF1
 
 
hsa-let-7c-5p
 
 
-0.374
 
 
14.7
 
 
 
 
AHNAK
 
 
hsa-miR-20a-5p
 
 
-0.316
 
 
160.9
 
 
 
 
AHNAK
 
 
hsa-miR-17-5p
 
 
-0.337
 
 
160.9
 
 
 
 
AIFM1
 
 
hsa-miR-98-5p
 
 
-0.314
 
 
23.4
 
 
 
 
AIFM1
 
 
hsa-let-7c-5p
 
 
-0.314
 
 
23.4
 
 
 
 
AK1
 
 
hsa-miR-423-5p
 
 
-0.443
 
 
730.8
 
 
 
 
AKAP6
 
 
hsa-miR-98-5p
 
 
-0.307
 
 
47.4
 
 
 
 
AKAP6
 
 
hsa-let-7c-5p
 
 
-0.307
 
 
47.4
 
 
 
 
ALDH4A1
 
 
hsa-miR-184
 
 
-0.421
 
 
69.9
 
 
 
 
ALG3
 
 
hsa-miR-20a-5p
 
 
-0.559
 
 
13.8
 
 
 
 
ALG3
 
 
hsa-miR-17-5p
 
 
-0.572
 
 
13.8
 
 
 
 
ANGPTL2
 
 
hsa-miR-423-5p
 
 
-0.379
 
 
34.2
 
 
 
 
ANKFY1
 
 
hsa-miR-155-5p
 
 
-0.315
 
 
10.6
 
 
 
 
ANKRD12
 
 
hsa-miR-7-5p
 
 
-0.37
 
 
11.5
 
 
 
 
ANKRD46
 
 
hsa-miR-98-5p
 
 
-0.389
 
 
33.8
 
 
 
 
ANKRD46
 
 
hsa-let-7c-5p
 
 
-0.389
 
 
33.8
 
 
 
 
ANKRD9
 
 
hsa-miR-20a-5p
 
 
-0.31
 
 
24.6
 
 
 
 
ANKRD9
 
 
hsa-miR-17-5p
 
 
-0.347
 
 
24.6
 
 
 
 
ANP32B
 
 
hsa-miR-200b-3p
 
 
-0.287
 
 
47.3
 
 
 
 
AP1S2
 
 
hsa-miR-200b-3p
 
 
-0.4
 
 
87.3
 
 
 
 
AP2M1
 
 
hsa-miR-423-5p
 
 
-0.49
 
 
161.0
 
 
 
 
APCDD1
 
 
hsa-miR-20a-5p
 
 
-0.404
 
 
17.3
 
 
 
 
APCDD1
 
 
hsa-miR-17-5p
 
 
-0.425
 
 
17.3
 
 
 
 
APOO
 
 
hsa-miR-200b-3p
 
 
-0.361
 
 
68.2
 
 
 
 
ARF4
 
 
hsa-miR-194-5p
 
 
-0.293
 
 
64.9
 
 
 
 
ARF4
 
 
hsa-miR-7-5p
 
 
-0.407
 
 
64.9
 
 
 
 
ARG2
 
 
hsa-miR-98-5p
 
 
-0.468
 
 
49.2
 
 
 
 
ARG2
 
 
hsa-let-7c-5p
 
 
-0.468
 
 
49.2
 
 
 
 
ARHGAP1
 
 
hsa-miR-17-5p
 
 
-0.325
 
 
19.1
 
 
 
 
ARHGAP1
 
 
hsa-miR-20a-5p
 
 
-0.339
 
 
19.1
 
 
 
 
ARL1
 
 
hsa-miR-20a-5p
 
 
-0.403
 
 
53.0
 
 
 
 
ARL1
 
 
hsa-miR-17-5p
 
 
-0.432
 
 
53.0
 
 
 
 
ARL6IP1
 
 
hsa-miR-7-5p
 
 
-0.374
 
 
48.1
 
 
 
 
ARL6IP1
 
 
hsa-miR-328-3p
 
 
-0.494
 
 
48.1
 
 
 
 
ARMC8
 
 
hsa-miR-20a-5p
 
 
-0.357
 
 
32.4
 
 
 
 
ARMC8
 
 
hsa-miR-17-5p
 
 
-0.378
 
 
32.4
 
 
 
 
ARMT1
 
 
hsa-miR-98-5p
 
 
-0.303
 
 
21.1
 
 
 
 
ARMT1
 
 
hsa-let-7c-5p
 
 
-0.303
 
 
21.1
 
 
 
 
ARPP19
 
 
hsa-miR-328-3p
 
 
-0.404
 
 
158.9
 
 
 
 
ARPP19
 
 
hsa-let-7c-5p
 
 
-0.492
 
 
158.9
 
 
 
 
ARPP19
 
 
hsa-miR-98-5p
 
 
-0.5
 
 
158.9
 
 
 
 
ASB14
 
 
hsa-miR-423-5p
 
 
-0.434
 
 
16.8
 
 
 
 
ASF1A
 
 
hsa-miR-200b-3p
 
 
-0.377
 
 
15.1
 
 
 
 
ATG13
 
 
hsa-miR-423-5p
 
 
-0.3
 
 
30.5
 
 
 
 
ATG4A
 
 
hsa-miR-7-5p
 
 
-0.448
 
 
17.2
 
 
 
 
ATOSB
 
 
hsa-miR-328-3p
 
 
-0.358
 
 
10.9
 
 
 
 
ATOSB
 
 
hsa-miR-98-5p
 
 
-0.429
 
 
10.9
 
 
 
 
ATOSB
 
 
hsa-let-7c-5p
 
 
-0.429
 
 
10.9
 
 
 
 
ATP2A2
 
 
hsa-miR-98-5p
 
 
-0.311
 
 
2586.2
 
 
 
 
ATP2A2
 
 
hsa-let-7c-5p
 
 
-0.311
 
 
2586.2
 
 
 
 
ATP6V1F
 
 
hsa-miR-194-5p
 
 
-0.444
 
 
144.3
 
 
 
 
ATP6V1H
 
 
hsa-miR-194-5p
 
 
-0.652
 
 
25.5
 
 
 
 
ATPAF1
 
 
hsa-miR-98-5p
 
 
-0.498
 
 
295.7
 
 
 
 
ATPAF1
 
 
hsa-let-7c-5p
 
 
-0.498
 
 
295.7
 
 
 
 
ATXN1L
 
 
hsa-miR-155-5p
 
 
-0.315
 
 
12.7
 
 
 
 
B4GALT5
 
 
hsa-miR-210-3p
 
 
-0.511
 
 
22.7
 
 
 
 
B4GAT1
 
 
hsa-miR-200b-3p
 
 
-0.382
 
 
17.9
 
 
 
 
B4GAT1
 
 
hsa-miR-98-5p
 
 
-0.47
 
 
17.9
 
 
 
 
B4GAT1
 
 
hsa-let-7c-5p
 
 
-0.47
 
 
17.9
 
 
 
 
BACH1
 
 
hsa-miR-98-5p
 
 
-0.298
 
 
11.9
 
 
 
 
BACH1
 
 
hsa-miR-98-5p
 
 
-0.298
 
 
11.9
 
 
 
 
BACH1
 
 
hsa-let-7c-5p
 
 
-0.306
 
 
11.9
 
 
 
 
BACH1
 
 
hsa-let-7c-5p
 
 
-0.306
 
 
11.9
 
 
 
 
BACH1
 
 
hsa-miR-98-5p
 
 
-0.435
 
 
11.9
 
 
 
 
BACH1
 
 
hsa-miR-98-5p
 
 
-0.435
 
 
11.9
 
 
 
 
BACH1
 
 
hsa-let-7c-5p
 
 
-0.435
 
 
11.9
 
 
 
 
BACH1
 
 
hsa-let-7c-5p
 
 
-0.435
 
 
11.9
 
 
 
 
BAG6
 
 
hsa-miR-200b-3p
 
 
-0.371
 
 
48.6
 
 
 
 
BAP1
 
 
hsa-miR-423-5p
 
 
-0.428
 
 
40.8
 
 
 
 
BBX
 
 
hsa-miR-18a-5p
 
 
-0.31
 
 
15.1
 
 
 
 
BIN1
 
 
hsa-miR-744-5p
 
 
-0.654
 
 
751.1
 
 
 
 
BLOC1S6
 
 
hsa-miR-98-5p
 
 
-0.321
 
 
21.7
 
 
 
 
BLOC1S6
 
 
hsa-let-7c-5p
 
 
-0.321
 
 
21.7
 
 
 
 
BMP5
 
 
hsa-miR-98-5p
 
 
-0.313
 
 
11.4
 
 
 
 
BMP5
 
 
hsa-let-7c-5p
 
 
-0.337
 
 
11.4
 
 
 
 
BNIP2
 
 
hsa-miR-194-5p
 
 
-0.489
 
 
36.3
 
 
 
 
BNIP3L
 
 
hsa-miR-20a-5p
 
 
-0.287
 
 
29.6
 
 
 
 
BRMS1L
 
 
hsa-miR-20a-5p
 
 
-0.326
 
 
12.8
 
 
 
 
BRMS1L
 
 
hsa-miR-194-5p
 
 
-0.333
 
 
12.8
 
 
 
 
BRMS1L
 
 
hsa-miR-17-5p
 
 
-0.347
 
 
12.8
 
 
 
 
BRWD1
 
 
hsa-miR-18a-5p
 
 
-0.29
 
 
22.4
 
 
 
 
BTBD10
 
 
hsa-miR-17-5p
 
 
-0.409
 
 
17.1
 
 
 
 
BTBD10
 
 
hsa-miR-20a-5p
 
 
-0.412
 
 
17.1
 
 
 
 
BTF3L4
 
 
hsa-miR-200b-3p
 
 
-0.294
 
 
55.0
 
 
 
 
BTF3L4
 
 
hsa-miR-194-5p
 
 
-0.334
 
 
55.0
 
 
 
 
BTF3L4
 
 
hsa-miR-98-5p
 
 
-0.355
 
 
55.0
 
 
 
 
BTF3L4
 
 
hsa-let-7c-5p
 
 
-0.355
 
 
55.0
 
 
 
 
BZW1
 
 
hsa-miR-98-5p
 
 
-0.354
 
 
70.0
 
 
 
 
 
 
 adrenal gland 
 
 
 
 
target_symbol
 
 
mature_mirna_id
 
 
score
 
 
nTPM
 
 
 
 
 
 
ABTB2
 
 
hsa-miR-18a-5p
 
 
-0.287
 
 
14.0
 
 
 
 
ADAM9
 
 
hsa-miR-20a-5p
 
 
-0.299
 
 
19.6
 
 
 
 
ADAM9
 
 
hsa-miR-17-5p
 
 
-0.32
 
 
19.6
 
 
 
 
ADD3
 
 
hsa-miR-200b-3p
 
 
-0.324
 
 
20.5
 
 
 
 
ADD3
 
 
hsa-miR-18a-5p
 
 
-0.39
 
 
20.5
 
 
 
 
ADNP
 
 
hsa-miR-328-3p
 
 
-0.371
 
 
29.8
 
 
 
 
AEBP2
 
 
hsa-miR-18a-5p
 
 
-0.376
 
 
25.6
 
 
 
 
AGFG2
 
 
hsa-miR-20a-5p
 
 
-0.365
 
 
33.6
 
 
 
 
AGFG2
 
 
hsa-miR-17-5p
 
 
-0.402
 
 
33.6
 
 
 
 
AHNAK
 
 
hsa-miR-20a-5p
 
 
-0.316
 
 
26.5
 
 
 
 
AHNAK
 
 
hsa-miR-17-5p
 
 
-0.337
 
 
26.5
 
 
 
 
AIFM1
 
 
hsa-miR-98-5p
 
 
-0.314
 
 
50.1
 
 
 
 
AIFM1
 
 
hsa-let-7c-5p
 
 
-0.314
 
 
50.1
 
 
 
 
AK1
 
 
hsa-miR-423-5p
 
 
-0.443
 
 
40.4
 
 
 
 
ALCAM
 
 
hsa-miR-18a-5p
 
 
-0.341
 
 
30.9
 
 
 
 
ALDH4A1
 
 
hsa-miR-184
 
 
-0.421
 
 
16.2
 
 
 
 
ALG3
 
 
hsa-miR-20a-5p
 
 
-0.559
 
 
30.6
 
 
 
 
ALG3
 
 
hsa-miR-17-5p
 
 
-0.572
 
 
30.6
 
 
 
 
AMT
 
 
hsa-miR-98-5p
 
 
-0.463
 
 
15.0
 
 
 
 
AMT
 
 
hsa-let-7c-5p
 
 
-0.463
 
 
15.0
 
 
 
 
ANGPTL2
 
 
hsa-miR-423-5p
 
 
-0.379
 
 
10.9
 
 
 
 
ANKFY1
 
 
hsa-miR-155-5p
 
 
-0.315
 
 
15.3
 
 
 
 
ANKRD46
 
 
hsa-miR-98-5p
 
 
-0.389
 
 
36.5
 
 
 
 
ANKRD46
 
 
hsa-let-7c-5p
 
 
-0.389
 
 
36.5
 
 
 
 
ANKRD9
 
 
hsa-miR-20a-5p
 
 
-0.31
 
 
20.4
 
 
 
 
ANKRD9
 
 
hsa-miR-17-5p
 
 
-0.347
 
 
20.4
 
 
 
 
ANP32B
 
 
hsa-miR-200b-3p
 
 
-0.287
 
 
72.7
 
 
 
 
AP1S1
 
 
hsa-miR-98-5p
 
 
-0.374
 
 
25.7
 
 
 
 
AP1S1
 
 
hsa-let-7c-5p
 
 
-0.374
 
 
25.7
 
 
 
 
AP1S2
 
 
hsa-miR-200b-3p
 
 
-0.4
 
 
19.2
 
 
 
 
AP2M1
 
 
hsa-miR-423-5p
 
 
-0.49
 
 
306.6
 
 
 
 
AP5S1
 
 
hsa-miR-744-5p
 
 
-0.354
 
 
10.8
 
 
 
 
APCDD1
 
 
hsa-miR-20a-5p
 
 
-0.404
 
 
13.4
 
 
 
 
APCDD1
 
 
hsa-miR-17-5p
 
 
-0.425
 
 
13.4
 
 
 
 
APOO
 
 
hsa-miR-200b-3p
 
 
-0.361
 
 
22.6
 
 
 
 
ARF4
 
 
hsa-miR-194-5p
 
 
-0.293
 
 
151.0
 
 
 
 
ARF4
 
 
hsa-miR-7-5p
 
 
-0.407
 
 
151.0
 
 
 
 
ARHGAP1
 
 
hsa-miR-17-5p
 
 
-0.325
 
 
46.3
 
 
 
 
ARHGAP1
 
 
hsa-miR-20a-5p
 
 
-0.339
 
 
46.3
 
 
 
 
ARHGEF28
 
 
hsa-miR-17-5p
 
 
-0.363
 
 
23.1
 
 
 
 
ARHGEF28
 
 
hsa-miR-20a-5p
 
 
-0.366
 
 
23.1
 
 
 
 
ARID4B
 
 
hsa-miR-20a-5p
 
 
-0.29
 
 
10.2
 
 
 
 
ARL1
 
 
hsa-miR-20a-5p
 
 
-0.403
 
 
62.3
 
 
 
 
ARL1
 
 
hsa-miR-17-5p
 
 
-0.432
 
 
62.3
 
 
 
 
ARL5B
 
 
hsa-miR-155-5p
 
 
-0.355
 
 
19.6
 
 
 
 
ARL6IP1
 
 
hsa-miR-7-5p
 
 
-0.374
 
 
161.7
 
 
 
 
ARL6IP1
 
 
hsa-miR-328-3p
 
 
-0.494
 
 
161.7
 
 
 
 
ARMC8
 
 
hsa-miR-20a-5p
 
 
-0.357
 
 
13.5
 
 
 
 
ARMC8
 
 
hsa-miR-17-5p
 
 
-0.378
 
 
13.5
 
 
 
 
ARMT1
 
 
hsa-miR-98-5p
 
 
-0.303
 
 
15.1
 
 
 
 
ARMT1
 
 
hsa-let-7c-5p
 
 
-0.303
 
 
15.1
 
 
 
 
ARPP19
 
 
hsa-miR-328-3p
 
 
-0.404
 
 
48.8
 
 
 
 
ARPP19
 
 
hsa-let-7c-5p
 
 
-0.492
 
 
48.8
 
 
 
 
ARPP19
 
 
hsa-miR-98-5p
 
 
-0.5
 
 
48.8
 
 
 
 
ARRDC4
 
 
hsa-miR-98-5p
 
 
-0.329
 
 
11.5
 
 
 
 
ARRDC4
 
 
hsa-let-7c-5p
 
 
-0.329
 
 
11.5
 
 
 
 
ASAP1
 
 
hsa-miR-98-5p
 
 
-0.308
 
 
20.2
 
 
 
 
ASAP1
 
 
hsa-let-7c-5p
 
 
-0.308
 
 
20.2
 
 
 
 
ASB6
 
 
hsa-miR-423-5p
 
 
-0.388
 
 
18.8
 
 
 
 
ATG13
 
 
hsa-miR-423-5p
 
 
-0.3
 
 
37.5
 
 
 
 
ATOSB
 
 
hsa-miR-328-3p
 
 
-0.358
 
 
14.2
 
 
 
 
ATOSB
 
 
hsa-miR-98-5p
 
 
-0.429
 
 
14.2
 
 
 
 
ATOSB
 
 
hsa-let-7c-5p
 
 
-0.429
 
 
14.2
 
 
 
 
ATP2A2
 
 
hsa-miR-98-5p
 
 
-0.311
 
 
46.8
 
 
 
 
ATP2A2
 
 
hsa-let-7c-5p
 
 
-0.311
 
 
46.8
 
 
 
 
ATP6V1F
 
 
hsa-miR-194-5p
 
 
-0.444
 
 
160.7
 
 
 
 
ATP6V1H
 
 
hsa-miR-194-5p
 
 
-0.652
 
 
36.8
 
 
 
 
ATPAF1
 
 
hsa-miR-98-5p
 
 
-0.498
 
 
98.1
 
 
 
 
ATPAF1
 
 
hsa-let-7c-5p
 
 
-0.498
 
 
98.1
 
 
 
 
AVPI1
 
 
hsa-miR-7-5p
 
 
-0.367
 
 
46.2
 
 
 
 
B3GAT3
 
 
hsa-miR-98-5p
 
 
-0.324
 
 
33.4
 
 
 
 
B3GAT3
 
 
hsa-let-7c-5p
 
 
-0.324
 
 
33.4
 
 
 
 
B4GALT5
 
 
hsa-miR-210-3p
 
 
-0.511
 
 
44.9
 
 
 
 
B4GAT1
 
 
hsa-miR-200b-3p
 
 
-0.382
 
 
39.1
 
 
 
 
B4GAT1
 
 
hsa-miR-98-5p
 
 
-0.47
 
 
39.1
 
 
 
 
B4GAT1
 
 
hsa-let-7c-5p
 
 
-0.47
 
 
39.1
 
 
 
 
BACH1
 
 
hsa-miR-98-5p
 
 
-0.298
 
 
11.4
 
 
 
 
BACH1
 
 
hsa-miR-98-5p
 
 
-0.298
 
 
11.4
 
 
 
 
BACH1
 
 
hsa-let-7c-5p
 
 
-0.306
 
 
11.4
 
 
 
 
BACH1
 
 
hsa-let-7c-5p
 
 
-0.306
 
 
11.4
 
 
 
 
BACH1
 
 
hsa-miR-98-5p
 
 
-0.435
 
 
11.4
 
 
 
 
BACH1
 
 
hsa-miR-98-5p
 
 
-0.435
 
 
11.4
 
 
 
 
BACH1
 
 
hsa-let-7c-5p
 
 
-0.435
 
 
11.4
 
 
 
 
BACH1
 
 
hsa-let-7c-5p
 
 
-0.435
 
 
11.4
 
 
 
 
BAG6
 
 
hsa-miR-200b-3p
 
 
-0.371
 
 
79.5
 
 
 
 
BAMBI
 
 
hsa-miR-20a-5p
 
 
-0.485
 
 
41.7
 
 
 
 
BAMBI
 
 
hsa-miR-17-5p
 
 
-0.506
 
 
41.7
 
 
 
 
BAP1
 
 
hsa-miR-423-5p
 
 
-0.428
 
 
62.0
 
 
 
 
BBX
 
 
hsa-miR-18a-5p
 
 
-0.31
 
 
23.6
 
 
 
 
BCAS3
 
 
hsa-miR-423-5p
 
 
-0.322
 
 
10.9
 
 
 
 
BIN1
 
 
hsa-miR-744-5p
 
 
-0.654
 
 
88.9
 
 
 
 
BIN3
 
 
hsa-miR-98-5p
 
 
-0.427
 
 
30.6
 
 
 
 
BIN3
 
 
hsa-let-7c-5p
 
 
-0.427
 
 
30.6
 
 
 
 
BLOC1S4
 
 
hsa-miR-7-5p
 
 
-0.417
 
 
11.8
 
 
 
 
BLOC1S4
 
 
hsa-miR-7-5p
 
 
-0.527
 
 
11.8
 
 
 
 
BLOC1S6
 
 
hsa-miR-98-5p
 
 
-0.321
 
 
29.4
 
 
 
 
BLOC1S6
 
 
hsa-let-7c-5p
 
 
-0.321
 
 
29.4
 
 
 
 
BNIP2
 
 
hsa-miR-194-5p
 
 
-0.489
 
 
19.7
 
 
 
 
BNIP3L
 
 
hsa-miR-20a-5p
 
 
-0.287
 
 
50.4
 
 
 
 
BRWD1
 
 
hsa-miR-18a-5p
 
 
-0.29
 
 
17.8
 
 
 
 
 
 
 epididymis 
 
 
 
 
target_symbol
 
 
mature_mirna_id
 
 
score
 
 
nTPM
 
 
 
 
 
 
ACP3
 
 
hsa-miR-98-5p
 
 
-0.298
 
 
16.3
 
 
 
 
ACP3
 
 
hsa-let-7c-5p
 
 
-0.298
 
 
16.3
 
 
 
 
ADAM9
 
 
hsa-miR-20a-5p
 
 
-0.299
 
 
14.3
 
 
 
 
ADAM9
 
 
hsa-miR-17-5p
 
 
-0.32
 
 
14.3
 
 
 
 
ADD3
 
 
hsa-miR-200b-3p
 
 
-0.324
 
 
58.9
 
 
 
 
ADD3
 
 
hsa-miR-18a-5p
 
 
-0.39
 
 
58.9
 
 
 
 
ADNP
 
 
hsa-miR-328-3p
 
 
-0.371
 
 
39.7
 
 
 
 
AEBP2
 
 
hsa-miR-18a-5p
 
 
-0.376
 
 
19.0
 
 
 
 
AHNAK
 
 
hsa-miR-20a-5p
 
 
-0.316
 
 
48.9
 
 
 
 
AHNAK
 
 
hsa-miR-17-5p
 
 
-0.337
 
 
48.9
 
 
 
 
AK1
 
 
hsa-miR-423-5p
 
 
-0.443
 
 
27.0
 
 
 
 
ALCAM
 
 
hsa-miR-18a-5p
 
 
-0.341
 
 
33.4
 
 
 
 
ALG3
 
 
hsa-miR-20a-5p
 
 
-0.559
 
 
11.8
 
 
 
 
ALG3
 
 
hsa-miR-17-5p
 
 
-0.572
 
 
11.8
 
 
 
 
ALG9
 
 
hsa-miR-7-5p
 
 
-0.302
 
 
10.3
 
 
 
 
ANGPTL2
 
 
hsa-miR-423-5p
 
 
-0.379
 
 
12.9
 
 
 
 
ANKFY1
 
 
hsa-miR-155-5p
 
 
-0.315
 
 
10.0
 
 
 
 
ANKRD46
 
 
hsa-miR-98-5p
 
 
-0.389
 
 
15.4
 
 
 
 
ANKRD46
 
 
hsa-let-7c-5p
 
 
-0.389
 
 
15.4
 
 
 
 
ANP32B
 
 
hsa-miR-200b-3p
 
 
-0.287
 
 
98.9
 
 
 
 
AP1S1
 
 
hsa-miR-98-5p
 
 
-0.374
 
 
16.0
 
 
 
 
AP1S1
 
 
hsa-let-7c-5p
 
 
-0.374
 
 
16.0
 
 
 
 
AP1S2
 
 
hsa-miR-200b-3p
 
 
-0.4
 
 
621.5
 
 
 
 
AP2M1
 
 
hsa-miR-423-5p
 
 
-0.49
 
 
89.1
 
 
 
 
APCDD1
 
 
hsa-miR-20a-5p
 
 
-0.404
 
 
14.6
 
 
 
 
APCDD1
 
 
hsa-miR-17-5p
 
 
-0.425
 
 
14.6
 
 
 
 
APOO
 
 
hsa-miR-200b-3p
 
 
-0.361
 
 
18.5
 
 
 
 
ARF4
 
 
hsa-miR-194-5p
 
 
-0.293
 
 
214.1
 
 
 
 
ARF4
 
 
hsa-miR-7-5p
 
 
-0.407
 
 
214.1
 
 
 
 
ARHGAP1
 
 
hsa-miR-17-5p
 
 
-0.325
 
 
20.3
 
 
 
 
ARHGAP1
 
 
hsa-miR-20a-5p
 
 
-0.339
 
 
20.3
 
 
 
 
ARID4B
 
 
hsa-miR-20a-5p
 
 
-0.29
 
 
13.6
 
 
 
 
ARL1
 
 
hsa-miR-20a-5p
 
 
-0.403
 
 
67.1
 
 
 
 
ARL1
 
 
hsa-miR-17-5p
 
 
-0.432
 
 
67.1
 
 
 
 
ARL6IP1
 
 
hsa-miR-7-5p
 
 
-0.374
 
 
116.0
 
 
 
 
ARL6IP1
 
 
hsa-miR-328-3p
 
 
-0.494
 
 
116.0
 
 
 
 
ARMC8
 
 
hsa-miR-20a-5p
 
 
-0.357
 
 
11.5
 
 
 
 
ARMC8
 
 
hsa-miR-17-5p
 
 
-0.378
 
 
11.5
 
 
 
 
ARMT1
 
 
hsa-miR-98-5p
 
 
-0.303
 
 
15.2
 
 
 
 
ARMT1
 
 
hsa-let-7c-5p
 
 
-0.303
 
 
15.2
 
 
 
 
ARPP19
 
 
hsa-miR-328-3p
 
 
-0.404
 
 
34.2
 
 
 
 
ARPP19
 
 
hsa-let-7c-5p
 
 
-0.492
 
 
34.2
 
 
 
 
ARPP19
 
 
hsa-miR-98-5p
 
 
-0.5
 
 
34.2
 
 
 
 
ARRDC4
 
 
hsa-miR-98-5p
 
 
-0.329
 
 
14.7
 
 
 
 
ARRDC4
 
 
hsa-let-7c-5p
 
 
-0.329
 
 
14.7
 
 
 
 
ASAP1
 
 
hsa-miR-98-5p
 
 
-0.308
 
 
11.5
 
 
 
 
ASAP1
 
 
hsa-let-7c-5p
 
 
-0.308
 
 
11.5
 
 
 
 
ASF1A
 
 
hsa-miR-200b-3p
 
 
-0.377
 
 
12.3
 
 
 
 
ATG13
 
 
hsa-miR-423-5p
 
 
-0.3
 
 
30.5
 
 
 
 
ATG4A
 
 
hsa-miR-7-5p
 
 
-0.448
 
 
10.9
 
 
 
 
ATP2A2
 
 
hsa-miR-98-5p
 
 
-0.311
 
 
36.9
 
 
 
 
ATP2A2
 
 
hsa-let-7c-5p
 
 
-0.311
 
 
36.9
 
 
 
 
ATP6V1F
 
 
hsa-miR-194-5p
 
 
-0.444
 
 
58.2
 
 
 
 
ATP6V1H
 
 
hsa-miR-194-5p
 
 
-0.652
 
 
31.1
 
 
 
 
ATP8B2
 
 
hsa-miR-423-5p
 
 
-0.297
 
 
17.4
 
 
 
 
ATPAF1
 
 
hsa-miR-98-5p
 
 
-0.498
 
 
33.7
 
 
 
 
ATPAF1
 
 
hsa-let-7c-5p
 
 
-0.498
 
 
33.7
 
 
 
 
B3GAT3
 
 
hsa-miR-98-5p
 
 
-0.324
 
 
11.2
 
 
 
 
B3GAT3
 
 
hsa-let-7c-5p
 
 
-0.324
 
 
11.2
 
 
 
 
B4GAT1
 
 
hsa-miR-200b-3p
 
 
-0.382
 
 
14.3
 
 
 
 
B4GAT1
 
 
hsa-miR-98-5p
 
 
-0.47
 
 
14.3
 
 
 
 
B4GAT1
 
 
hsa-let-7c-5p
 
 
-0.47
 
 
14.3
 
 
 
 
BAG6
 
 
hsa-miR-200b-3p
 
 
-0.371
 
 
24.9
 
 
 
 
BAP1
 
 
hsa-miR-423-5p
 
 
-0.428
 
 
21.9
 
 
 
 
BBX
 
 
hsa-miR-18a-5p
 
 
-0.31
 
 
36.4
 
 
 
 
BCAS3
 
 
hsa-miR-423-5p
 
 
-0.322
 
 
11.6
 
 
 
 
BIN1
 
 
hsa-miR-744-5p
 
 
-0.654
 
 
15.8
 
 
 
 
BLOC1S6
 
 
hsa-miR-98-5p
 
 
-0.321
 
 
17.3
 
 
 
 
BLOC1S6
 
 
hsa-let-7c-5p
 
 
-0.321
 
 
17.3
 
 
 
 
BNIP2
 
 
hsa-miR-194-5p
 
 
-0.489
 
 
16.6
 
 
 
 
BNIP3L
 
 
hsa-miR-20a-5p
 
 
-0.287
 
 
62.5
 
 
 
 
BRWD1
 
 
hsa-miR-18a-5p
 
 
-0.29
 
 
21.4
 
 
 
 
BTBD10
 
 
hsa-miR-17-5p
 
 
-0.409
 
 
11.3
 
 
 
 
BTBD10
 
 
hsa-miR-20a-5p
 
 
-0.412
 
 
11.3
 
 
 
 
BTF3L4
 
 
hsa-miR-200b-3p
 
 
-0.294
 
 
28.9
 
 
 
 
BTF3L4
 
 
hsa-miR-194-5p
 
 
-0.334
 
 
28.9
 
 
 
 
BTF3L4
 
 
hsa-miR-98-5p
 
 
-0.355
 
 
28.9
 
 
 
 
BTF3L4
 
 
hsa-let-7c-5p
 
 
-0.355
 
 
28.9
 
 
 
 
BTG3
 
 
hsa-miR-18a-5p
 
 
-0.464
 
 
104.1
 
 
 
 
BTG3
 
 
hsa-miR-17-5p
 
 
-0.668
 
 
104.1
 
 
 
 
BTG3
 
 
hsa-miR-20a-5p
 
 
-0.671
 
 
104.1
 
 
 
 
BZW1
 
 
hsa-miR-98-5p
 
 
-0.354
 
 
55.1
 
 
 
 
BZW1
 
 
hsa-let-7c-5p
 
 
-0.354
 
 
55.1
 
 
 
 
BZW1
 
 
hsa-miR-98-5p
 
 
-0.375
 
 
55.1
 
 
 
 
BZW1
 
 
hsa-let-7c-5p
 
 
-0.375
 
 
55.1
 
 
 
 
C1QBP
 
 
hsa-miR-330-3p
 
 
-0.412
 
 
64.4
 
 
 
 
CAB39
 
 
hsa-miR-155-5p
 
 
-0.312
 
 
33.0
 
 
 
 
CALD1
 
 
hsa-miR-20a-5p
 
 
-0.352
 
 
142.4
 
 
 
 
CALD1
 
 
hsa-miR-17-5p
 
 
-0.389
 
 
142.4
 
 
 
 
CAMK2N1
 
 
hsa-miR-17-5p
 
 
-0.367
 
 
36.0
 
 
 
 
CAMK2N1
 
 
hsa-miR-20a-5p
 
 
-0.381
 
 
36.0
 
 
 
 
CAMK2N1
 
 
hsa-miR-18a-5p
 
 
-0.408
 
 
36.0
 
 
 
 
CAPN3
 
 
hsa-miR-98-5p
 
 
-0.317
 
 
154.5
 
 
 
 
CAPN3
 
 
hsa-let-7c-5p
 
 
-0.317
 
 
154.5
 
 
 
 
CAPZA1
 
 
hsa-miR-7-5p
 
 
-0.351
 
 
58.0
 
 
 
 
CARHSP1
 
 
hsa-miR-155-5p
 
 
-0.451
 
 
12.7
 
 
 
 
CBX5
 
 
hsa-let-7c-5p
 
 
-0.307
 
 
14.1
 
 
 
 
CBX5
 
 
hsa-miR-98-5p
 
 
-0.315
 
 
14.1
 
 
 
 
CBX5
 
 
hsa-miR-98-5p
 
 
-0.472
 
 
14.1
 
 
 
 
CBX5
 
 
hsa-let-7c-5p
 
 
-0.472
 
 
14.1
 
 
 
 
 
 
 placenta 
 
 
 
 
target_symbol
 
 
mature_mirna_id
 
 
score
 
 
nTPM
 
 
 
 
 
 
ACP3
 
 
hsa-miR-98-5p
 
 
-0.298
 
 
10.1
 
 
 
 
ACP3
 
 
hsa-let-7c-5p
 
 
-0.298
 
 
10.1
 
 
 
 
ADAM9
 
 
hsa-miR-20a-5p
 
 
-0.299
 
 
44.6
 
 
 
 
ADAM9
 
 
hsa-miR-17-5p
 
 
-0.32
 
 
44.6
 
 
 
 
ADD3
 
 
hsa-miR-200b-3p
 
 
-0.324
 
 
34.4
 
 
 
 
ADD3
 
 
hsa-miR-18a-5p
 
 
-0.39
 
 
34.4
 
 
 
 
ADNP
 
 
hsa-miR-328-3p
 
 
-0.371
 
 
23.8
 
 
 
 
ADRB1
 
 
hsa-let-7c-5p
 
 
-0.351
 
 
27.4
 
 
 
 
ADRB1
 
 
hsa-miR-98-5p
 
 
-0.367
 
 
27.4
 
 
 
 
AEBP2
 
 
hsa-miR-18a-5p
 
 
-0.376
 
 
41.3
 
 
 
 
AHNAK
 
 
hsa-miR-20a-5p
 
 
-0.316
 
 
91.4
 
 
 
 
AHNAK
 
 
hsa-miR-17-5p
 
 
-0.337
 
 
91.4
 
 
 
 
ALCAM
 
 
hsa-miR-18a-5p
 
 
-0.341
 
 
11.3
 
 
 
 
ALDH4A1
 
 
hsa-miR-184
 
 
-0.421
 
 
12.4
 
 
 
 
ALG3
 
 
hsa-miR-20a-5p
 
 
-0.559
 
 
18.4
 
 
 
 
ALG3
 
 
hsa-miR-17-5p
 
 
-0.572
 
 
18.4
 
 
 
 
ANGPTL2
 
 
hsa-miR-423-5p
 
 
-0.379
 
 
20.1
 
 
 
 
ANKFY1
 
 
hsa-miR-155-5p
 
 
-0.315
 
 
13.3
 
 
 
 
ANKRD12
 
 
hsa-miR-7-5p
 
 
-0.37
 
 
13.3
 
 
 
 
ANKRD46
 
 
hsa-miR-98-5p
 
 
-0.389
 
 
19.5
 
 
 
 
ANKRD46
 
 
hsa-let-7c-5p
 
 
-0.389
 
 
19.5
 
 
 
 
ANKRD50
 
 
hsa-miR-20a-5p
 
 
-0.3
 
 
13.9
 
 
 
 
ANP32B
 
 
hsa-miR-200b-3p
 
 
-0.287
 
 
103.8
 
 
 
 
AP1S1
 
 
hsa-miR-98-5p
 
 
-0.374
 
 
14.9
 
 
 
 
AP1S1
 
 
hsa-let-7c-5p
 
 
-0.374
 
 
14.9
 
 
 
 
AP1S2
 
 
hsa-miR-200b-3p
 
 
-0.4
 
 
39.0
 
 
 
 
AP2M1
 
 
hsa-miR-423-5p
 
 
-0.49
 
 
204.3
 
 
 
 
APCDD1
 
 
hsa-miR-20a-5p
 
 
-0.404
 
 
25.2
 
 
 
 
APCDD1
 
 
hsa-miR-17-5p
 
 
-0.425
 
 
25.2
 
 
 
 
APOO
 
 
hsa-miR-200b-3p
 
 
-0.361
 
 
10.3
 
 
 
 
ARF4
 
 
hsa-miR-194-5p
 
 
-0.293
 
 
170.7
 
 
 
 
ARF4
 
 
hsa-miR-7-5p
 
 
-0.407
 
 
170.7
 
 
 
 
ARHGAP1
 
 
hsa-miR-17-5p
 
 
-0.325
 
 
36.7
 
 
 
 
ARHGAP1
 
 
hsa-miR-20a-5p
 
 
-0.339
 
 
36.7
 
 
 
 
ARHGAP28
 
 
hsa-miR-98-5p
 
 
-0.47
 
 
24.3
 
 
 
 
ARHGAP28
 
 
hsa-let-7c-5p
 
 
-0.47
 
 
24.3
 
 
 
 
ARID3A
 
 
hsa-miR-98-5p
 
 
-0.34
 
 
22.0
 
 
 
 
ARID3A
 
 
hsa-let-7c-5p
 
 
-0.34
 
 
22.0
 
 
 
 
ARID3A
 
 
hsa-let-7c-5p
 
 
-0.347
 
 
22.0
 
 
 
 
ARID3A
 
 
hsa-miR-98-5p
 
 
-0.358
 
 
22.0
 
 
 
 
ARID3B
 
 
hsa-miR-98-5p
 
 
-0.31
 
 
14.7
 
 
 
 
ARID3B
 
 
hsa-let-7c-5p
 
 
-0.31
 
 
14.7
 
 
 
 
ARID3B
 
 
hsa-miR-98-5p
 
 
-0.475
 
 
14.7
 
 
 
 
ARID3B
 
 
hsa-let-7c-5p
 
 
-0.486
 
 
14.7
 
 
 
 
ARID4B
 
 
hsa-miR-20a-5p
 
 
-0.29
 
 
12.0
 
 
 
 
ARL1
 
 
hsa-miR-20a-5p
 
 
-0.403
 
 
48.3
 
 
 
 
ARL1
 
 
hsa-miR-17-5p
 
 
-0.432
 
 
48.3
 
 
 
 
ARL5B
 
 
hsa-miR-155-5p
 
 
-0.355
 
 
12.3
 
 
 
 
ARL6IP1
 
 
hsa-miR-7-5p
 
 
-0.374
 
 
84.9
 
 
 
 
ARL6IP1
 
 
hsa-miR-328-3p
 
 
-0.494
 
 
84.9
 
 
 
 
ARL6IP6
 
 
hsa-miR-98-5p
 
 
-0.293
 
 
26.8
 
 
 
 
ARL6IP6
 
 
hsa-let-7c-5p
 
 
-0.293
 
 
26.8
 
 
 
 
ARMC8
 
 
hsa-miR-20a-5p
 
 
-0.357
 
 
18.5
 
 
 
 
ARMC8
 
 
hsa-miR-17-5p
 
 
-0.378
 
 
18.5
 
 
 
 
ARMT1
 
 
hsa-miR-98-5p
 
 
-0.303
 
 
20.8
 
 
 
 
ARMT1
 
 
hsa-let-7c-5p
 
 
-0.303
 
 
20.8
 
 
 
 
ARPP19
 
 
hsa-miR-328-3p
 
 
-0.404
 
 
53.5
 
 
 
 
ARPP19
 
 
hsa-let-7c-5p
 
 
-0.492
 
 
53.5
 
 
 
 
ARPP19
 
 
hsa-miR-98-5p
 
 
-0.5
 
 
53.5
 
 
 
 
ARRDC4
 
 
hsa-miR-98-5p
 
 
-0.329
 
 
17.2
 
 
 
 
ARRDC4
 
 
hsa-let-7c-5p
 
 
-0.329
 
 
17.2
 
 
 
 
ASAP1
 
 
hsa-miR-98-5p
 
 
-0.308
 
 
25.9
 
 
 
 
ASAP1
 
 
hsa-let-7c-5p
 
 
-0.308
 
 
25.9
 
 
 
 
ATG13
 
 
hsa-miR-423-5p
 
 
-0.3
 
 
20.5
 
 
 
 
ATP2A2
 
 
hsa-miR-98-5p
 
 
-0.311
 
 
52.0
 
 
 
 
ATP2A2
 
 
hsa-let-7c-5p
 
 
-0.311
 
 
52.0
 
 
 
 
ATP6V1F
 
 
hsa-miR-194-5p
 
 
-0.444
 
 
98.2
 
 
 
 
ATP6V1H
 
 
hsa-miR-194-5p
 
 
-0.652
 
 
22.4
 
 
 
 
ATP8B2
 
 
hsa-miR-423-5p
 
 
-0.297
 
 
14.9
 
 
 
 
ATPAF1
 
 
hsa-miR-98-5p
 
 
-0.498
 
 
34.1
 
 
 
 
ATPAF1
 
 
hsa-let-7c-5p
 
 
-0.498
 
 
34.1
 
 
 
 
ATXN1L
 
 
hsa-miR-155-5p
 
 
-0.315
 
 
10.1
 
 
 
 
AVPI1
 
 
hsa-miR-7-5p
 
 
-0.367
 
 
15.9
 
 
 
 
B3GAT3
 
 
hsa-miR-98-5p
 
 
-0.324
 
 
18.0
 
 
 
 
B3GAT3
 
 
hsa-let-7c-5p
 
 
-0.324
 
 
18.0
 
 
 
 
B4GALT5
 
 
hsa-miR-210-3p
 
 
-0.511
 
 
25.6
 
 
 
 
B4GAT1
 
 
hsa-miR-200b-3p
 
 
-0.382
 
 
21.1
 
 
 
 
B4GAT1
 
 
hsa-miR-98-5p
 
 
-0.47
 
 
21.1
 
 
 
 
B4GAT1
 
 
hsa-let-7c-5p
 
 
-0.47
 
 
21.1
 
 
 
 
BACH1
 
 
hsa-miR-98-5p
 
 
-0.298
 
 
47.5
 
 
 
 
BACH1
 
 
hsa-miR-98-5p
 
 
-0.298
 
 
47.5
 
 
 
 
BACH1
 
 
hsa-let-7c-5p
 
 
-0.306
 
 
47.5
 
 
 
 
BACH1
 
 
hsa-let-7c-5p
 
 
-0.306
 
 
47.5
 
 
 
 
BACH1
 
 
hsa-miR-98-5p
 
 
-0.435
 
 
47.5
 
 
 
 
BACH1
 
 
hsa-miR-98-5p
 
 
-0.435
 
 
47.5
 
 
 
 
BACH1
 
 
hsa-let-7c-5p
 
 
-0.435
 
 
47.5
 
 
 
 
BACH1
 
 
hsa-let-7c-5p
 
 
-0.435
 
 
47.5
 
 
 
 
BAG6
 
 
hsa-miR-200b-3p
 
 
-0.371
 
 
21.5
 
 
 
 
BAMBI
 
 
hsa-miR-20a-5p
 
 
-0.485
 
 
36.9
 
 
 
 
BAMBI
 
 
hsa-miR-17-5p
 
 
-0.506
 
 
36.9
 
 
 
 
BAP1
 
 
hsa-miR-423-5p
 
 
-0.428
 
 
23.1
 
 
 
 
BBX
 
 
hsa-miR-18a-5p
 
 
-0.31
 
 
22.7
 
 
 
 
BCAT1
 
 
hsa-miR-98-5p
 
 
-0.394
 
 
14.3
 
 
 
 
BCAT1
 
 
hsa-let-7c-5p
 
 
-0.394
 
 
14.3
 
 
 
 
BIN1
 
 
hsa-miR-744-5p
 
 
-0.654
 
 
22.1
 
 
 
 
BLOC1S6
 
 
hsa-miR-98-5p
 
 
-0.321
 
 
24.7
 
 
 
 
BLOC1S6
 
 
hsa-let-7c-5p
 
 
-0.321
 
 
24.7
 
 
 
 
BMP5
 
 
hsa-miR-98-5p
 
 
-0.313
 
 
66.9
 
 
 
 
BMP5
 
 
hsa-let-7c-5p
 
 
-0.337
 
 
66.9
 
 
 
 
BNIP2
 
 
hsa-miR-194-5p
 
 
-0.489
 
 
37.9
 
 
 
 
 
 
 seminal vesicle 
 
 
 
 
target_symbol
 
 
mature_mirna_id
 
 
score
 
 
nTPM
 
 
 
 
 
 
ACSL6
 
 
hsa-miR-98-5p
 
 
-0.336
 
 
17.0
 
 
 
 
ACSL6
 
 
hsa-let-7c-5p
 
 
-0.336
 
 
17.0
 
 
 
 
ACTC1
 
 
hsa-miR-7-5p
 
 
-0.471
 
 
506.6
 
 
 
 
ADAM9
 
 
hsa-miR-20a-5p
 
 
-0.299
 
 
32.8
 
 
 
 
ADAM9
 
 
hsa-miR-17-5p
 
 
-0.32
 
 
32.8
 
 
 
 
ADD3
 
 
hsa-miR-200b-3p
 
 
-0.324
 
 
30.0
 
 
 
 
ADD3
 
 
hsa-miR-18a-5p
 
 
-0.39
 
 
30.0
 
 
 
 
ADNP
 
 
hsa-miR-328-3p
 
 
-0.371
 
 
28.1
 
 
 
 
AEBP2
 
 
hsa-miR-18a-5p
 
 
-0.376
 
 
28.8
 
 
 
 
AGFG2
 
 
hsa-miR-20a-5p
 
 
-0.365
 
 
12.7
 
 
 
 
AGFG2
 
 
hsa-miR-17-5p
 
 
-0.402
 
 
12.7
 
 
 
 
AHNAK
 
 
hsa-miR-20a-5p
 
 
-0.316
 
 
99.6
 
 
 
 
AHNAK
 
 
hsa-miR-17-5p
 
 
-0.337
 
 
99.6
 
 
 
 
AK1
 
 
hsa-miR-423-5p
 
 
-0.443
 
 
33.8
 
 
 
 
ALCAM
 
 
hsa-miR-18a-5p
 
 
-0.341
 
 
37.3
 
 
 
 
ALDH4A1
 
 
hsa-miR-184
 
 
-0.421
 
 
25.6
 
 
 
 
ALG3
 
 
hsa-miR-20a-5p
 
 
-0.559
 
 
12.9
 
 
 
 
ALG3
 
 
hsa-miR-17-5p
 
 
-0.572
 
 
12.9
 
 
 
 
ALG9
 
 
hsa-miR-7-5p
 
 
-0.302
 
 
10.6
 
 
 
 
ANGPTL2
 
 
hsa-miR-423-5p
 
 
-0.379
 
 
25.5
 
 
 
 
ANKFY1
 
 
hsa-miR-155-5p
 
 
-0.315
 
 
11.5
 
 
 
 
ANKRD12
 
 
hsa-miR-7-5p
 
 
-0.37
 
 
10.0
 
 
 
 
ANKRD46
 
 
hsa-miR-98-5p
 
 
-0.389
 
 
12.4
 
 
 
 
ANKRD46
 
 
hsa-let-7c-5p
 
 
-0.389
 
 
12.4
 
 
 
 
ANP32B
 
 
hsa-miR-200b-3p
 
 
-0.287
 
 
67.2
 
 
 
 
AP1S2
 
 
hsa-miR-200b-3p
 
 
-0.4
 
 
28.5
 
 
 
 
AP2M1
 
 
hsa-miR-423-5p
 
 
-0.49
 
 
196.7
 
 
 
 
APCDD1
 
 
hsa-miR-20a-5p
 
 
-0.404
 
 
13.3
 
 
 
 
APCDD1
 
 
hsa-miR-17-5p
 
 
-0.425
 
 
13.3
 
 
 
 
APOO
 
 
hsa-miR-200b-3p
 
 
-0.361
 
 
14.1
 
 
 
 
ARF4
 
 
hsa-miR-194-5p
 
 
-0.293
 
 
125.1
 
 
 
 
ARF4
 
 
hsa-miR-7-5p
 
 
-0.407
 
 
125.1
 
 
 
 
ARHGAP1
 
 
hsa-miR-17-5p
 
 
-0.325
 
 
58.9
 
 
 
 
ARHGAP1
 
 
hsa-miR-20a-5p
 
 
-0.339
 
 
58.9
 
 
 
 
ARID4B
 
 
hsa-miR-20a-5p
 
 
-0.29
 
 
10.2
 
 
 
 
ARL1
 
 
hsa-miR-20a-5p
 
 
-0.403
 
 
65.0
 
 
 
 
ARL1
 
 
hsa-miR-17-5p
 
 
-0.432
 
 
65.0
 
 
 
 
ARL4D
 
 
hsa-miR-98-5p
 
 
-0.417
 
 
14.3
 
 
 
 
ARL4D
 
 
hsa-let-7c-5p
 
 
-0.417
 
 
14.3
 
 
 
 
ARL6IP1
 
 
hsa-miR-7-5p
 
 
-0.374
 
 
66.4
 
 
 
 
ARL6IP1
 
 
hsa-miR-328-3p
 
 
-0.494
 
 
66.4
 
 
 
 
ARMC8
 
 
hsa-miR-20a-5p
 
 
-0.357
 
 
16.5
 
 
 
 
ARMC8
 
 
hsa-miR-17-5p
 
 
-0.378
 
 
16.5
 
 
 
 
ARMT1
 
 
hsa-miR-98-5p
 
 
-0.303
 
 
10.5
 
 
 
 
ARMT1
 
 
hsa-let-7c-5p
 
 
-0.303
 
 
10.5
 
 
 
 
ARPP19
 
 
hsa-miR-328-3p
 
 
-0.404
 
 
38.5
 
 
 
 
ARPP19
 
 
hsa-let-7c-5p
 
 
-0.492
 
 
38.5
 
 
 
 
ARPP19
 
 
hsa-miR-98-5p
 
 
-0.5
 
 
38.5
 
 
 
 
ARRDC4
 
 
hsa-miR-98-5p
 
 
-0.329
 
 
15.4
 
 
 
 
ARRDC4
 
 
hsa-let-7c-5p
 
 
-0.329
 
 
15.4
 
 
 
 
ASAP1
 
 
hsa-miR-98-5p
 
 
-0.308
 
 
10.7
 
 
 
 
ASAP1
 
 
hsa-let-7c-5p
 
 
-0.308
 
 
10.7
 
 
 
 
ASF1A
 
 
hsa-miR-200b-3p
 
 
-0.377
 
 
10.4
 
 
 
 
ATG13
 
 
hsa-miR-423-5p
 
 
-0.3
 
 
24.0
 
 
 
 
ATP2A2
 
 
hsa-miR-98-5p
 
 
-0.311
 
 
66.0
 
 
 
 
ATP2A2
 
 
hsa-let-7c-5p
 
 
-0.311
 
 
66.0
 
 
 
 
ATP6V1F
 
 
hsa-miR-194-5p
 
 
-0.444
 
 
76.8
 
 
 
 
ATP6V1H
 
 
hsa-miR-194-5p
 
 
-0.652
 
 
25.7
 
 
 
 
ATP8B2
 
 
hsa-miR-423-5p
 
 
-0.297
 
 
21.1
 
 
 
 
ATPAF1
 
 
hsa-miR-98-5p
 
 
-0.498
 
 
31.8
 
 
 
 
ATPAF1
 
 
hsa-let-7c-5p
 
 
-0.498
 
 
31.8
 
 
 
 
AVPI1
 
 
hsa-miR-7-5p
 
 
-0.367
 
 
15.6
 
 
 
 
B3GAT3
 
 
hsa-miR-98-5p
 
 
-0.324
 
 
17.2
 
 
 
 
B3GAT3
 
 
hsa-let-7c-5p
 
 
-0.324
 
 
17.2
 
 
 
 
B4GALT5
 
 
hsa-miR-210-3p
 
 
-0.511
 
 
20.7
 
 
 
 
B4GAT1
 
 
hsa-miR-200b-3p
 
 
-0.382
 
 
21.2
 
 
 
 
B4GAT1
 
 
hsa-miR-98-5p
 
 
-0.47
 
 
21.2
 
 
 
 
B4GAT1
 
 
hsa-let-7c-5p
 
 
-0.47
 
 
21.2
 
 
 
 
BAG6
 
 
hsa-miR-200b-3p
 
 
-0.371
 
 
12.5
 
 
 
 
BAMBI
 
 
hsa-miR-20a-5p
 
 
-0.485
 
 
15.7
 
 
 
 
BAMBI
 
 
hsa-miR-17-5p
 
 
-0.506
 
 
15.7
 
 
 
 
BAP1
 
 
hsa-miR-423-5p
 
 
-0.428
 
 
21.2
 
 
 
 
BBX
 
 
hsa-miR-18a-5p
 
 
-0.31
 
 
29.7
 
 
 
 
BIN1
 
 
hsa-miR-744-5p
 
 
-0.654
 
 
22.7
 
 
 
 
BLOC1S6
 
 
hsa-miR-98-5p
 
 
-0.321
 
 
19.2
 
 
 
 
BLOC1S6
 
 
hsa-let-7c-5p
 
 
-0.321
 
 
19.2
 
 
 
 
BNIP2
 
 
hsa-miR-194-5p
 
 
-0.489
 
 
25.9
 
 
 
 
BNIP3L
 
 
hsa-miR-20a-5p
 
 
-0.287
 
 
60.7
 
 
 
 
BRWD1
 
 
hsa-miR-18a-5p
 
 
-0.29
 
 
10.8
 
 
 
 
BTBD10
 
 
hsa-miR-17-5p
 
 
-0.409
 
 
16.4
 
 
 
 
BTBD10
 
 
hsa-miR-20a-5p
 
 
-0.412
 
 
16.4
 
 
 
 
BTF3L4
 
 
hsa-miR-200b-3p
 
 
-0.294
 
 
23.0
 
 
 
 
BTF3L4
 
 
hsa-miR-194-5p
 
 
-0.334
 
 
23.0
 
 
 
 
BTF3L4
 
 
hsa-miR-98-5p
 
 
-0.355
 
 
23.0
 
 
 
 
BTF3L4
 
 
hsa-let-7c-5p
 
 
-0.355
 
 
23.0
 
 
 
 
BTG3
 
 
hsa-miR-18a-5p
 
 
-0.464
 
 
19.3
 
 
 
 
BTG3
 
 
hsa-miR-17-5p
 
 
-0.668
 
 
19.3
 
 
 
 
BTG3
 
 
hsa-miR-20a-5p
 
 
-0.671
 
 
19.3
 
 
 
 
BZW1
 
 
hsa-miR-98-5p
 
 
-0.354
 
 
58.1
 
 
 
 
BZW1
 
 
hsa-let-7c-5p
 
 
-0.354
 
 
58.1
 
 
 
 
BZW1
 
 
hsa-miR-98-5p
 
 
-0.375
 
 
58.1
 
 
 
 
BZW1
 
 
hsa-let-7c-5p
 
 
-0.375
 
 
58.1
 
 
 
 
BZW2
 
 
hsa-miR-98-5p
 
 
-0.397
 
 
32.3
 
 
 
 
BZW2
 
 
hsa-let-7c-5p
 
 
-0.397
 
 
32.3
 
 
 
 
C1QBP
 
 
hsa-miR-330-3p
 
 
-0.412
 
 
68.8
 
 
 
 
C6orf120
 
 
hsa-miR-20a-5p
 
 
-0.298
 
 
10.2
 
 
 
 
C6orf120
 
 
hsa-miR-17-5p
 
 
-0.319
 
 
10.2
 
 
 
 
CAB39
 
 
hsa-miR-155-5p
 
 
-0.312
 
 
28.9
 
 
 
 
CALD1
 
 
hsa-miR-20a-5p
 
 
-0.352
 
 
736.2
 
 
 
 
CALD1
 
 
hsa-miR-17-5p
 
 
-0.389
 
 
736.2
 
 
 
 
 
 
 blood vessel 
 
 
 
 
target_symbol
 
 
mature_mirna_id
 
 
score
 
 
nTPM
 
 
 
 
 
 
ACTA1
 
 
hsa-miR-155-5p
 
 
-0.595
 
 
77.9
 
 
 
 
ACTC1
 
 
hsa-miR-7-5p
 
 
-0.471
 
 
388.9
 
 
 
 
ADAM9
 
 
hsa-miR-20a-5p
 
 
-0.299
 
 
36.8
 
 
 
 
ADAM9
 
 
hsa-miR-17-5p
 
 
-0.32
 
 
36.8
 
 
 
 
ADAMTS8
 
 
hsa-miR-98-5p
 
 
-0.571
 
 
12.7
 
 
 
 
ADAMTS8
 
 
hsa-let-7c-5p
 
 
-0.571
 
 
12.7
 
 
 
 
ADD3
 
 
hsa-miR-200b-3p
 
 
-0.324
 
 
34.0
 
 
 
 
ADD3
 
 
hsa-miR-18a-5p
 
 
-0.39
 
 
34.0
 
 
 
 
ADNP
 
 
hsa-miR-328-3p
 
 
-0.371
 
 
32.1
 
 
 
 
AEBP2
 
 
hsa-miR-18a-5p
 
 
-0.376
 
 
36.5
 
 
 
 
AGFG2
 
 
hsa-miR-20a-5p
 
 
-0.365
 
 
11.4
 
 
 
 
AGFG2
 
 
hsa-miR-17-5p
 
 
-0.402
 
 
11.4
 
 
 
 
AHCTF1
 
 
hsa-miR-98-5p
 
 
-0.374
 
 
10.0
 
 
 
 
AHCTF1
 
 
hsa-let-7c-5p
 
 
-0.374
 
 
10.0
 
 
 
 
AHNAK
 
 
hsa-miR-20a-5p
 
 
-0.316
 
 
225.6
 
 
 
 
AHNAK
 
 
hsa-miR-17-5p
 
 
-0.337
 
 
225.6
 
 
 
 
AK1
 
 
hsa-miR-423-5p
 
 
-0.443
 
 
58.4
 
 
 
 
AKAP6
 
 
hsa-miR-98-5p
 
 
-0.307
 
 
15.0
 
 
 
 
AKAP6
 
 
hsa-let-7c-5p
 
 
-0.307
 
 
15.0
 
 
 
 
ALCAM
 
 
hsa-miR-18a-5p
 
 
-0.341
 
 
85.5
 
 
 
 
ALDH4A1
 
 
hsa-miR-184
 
 
-0.421
 
 
15.0
 
 
 
 
ALG3
 
 
hsa-miR-20a-5p
 
 
-0.559
 
 
20.2
 
 
 
 
ALG3
 
 
hsa-miR-17-5p
 
 
-0.572
 
 
20.2
 
 
 
 
ALG9
 
 
hsa-miR-7-5p
 
 
-0.302
 
 
12.3
 
 
 
 
AMT
 
 
hsa-miR-98-5p
 
 
-0.463
 
 
11.4
 
 
 
 
AMT
 
 
hsa-let-7c-5p
 
 
-0.463
 
 
11.4
 
 
 
 
ANGPTL2
 
 
hsa-miR-423-5p
 
 
-0.379
 
 
31.4
 
 
 
 
ANKFY1
 
 
hsa-miR-155-5p
 
 
-0.315
 
 
13.2
 
 
 
 
ANKRD12
 
 
hsa-miR-7-5p
 
 
-0.37
 
 
11.6
 
 
 
 
ANKRD9
 
 
hsa-miR-20a-5p
 
 
-0.31
 
 
10.4
 
 
 
 
ANKRD9
 
 
hsa-miR-17-5p
 
 
-0.347
 
 
10.4
 
 
 
 
ANP32B
 
 
hsa-miR-200b-3p
 
 
-0.287
 
 
163.8
 
 
 
 
AP1S1
 
 
hsa-miR-98-5p
 
 
-0.374
 
 
13.8
 
 
 
 
AP1S1
 
 
hsa-let-7c-5p
 
 
-0.374
 
 
13.8
 
 
 
 
AP1S2
 
 
hsa-miR-200b-3p
 
 
-0.4
 
 
25.7
 
 
 
 
AP2M1
 
 
hsa-miR-423-5p
 
 
-0.49
 
 
213.7
 
 
 
 
APCDD1
 
 
hsa-miR-20a-5p
 
 
-0.404
 
 
23.7
 
 
 
 
APCDD1
 
 
hsa-miR-17-5p
 
 
-0.425
 
 
23.7
 
 
 
 
ARF4
 
 
hsa-miR-194-5p
 
 
-0.293
 
 
151.4
 
 
 
 
ARF4
 
 
hsa-miR-7-5p
 
 
-0.407
 
 
151.4
 
 
 
 
ARGLU1
 
 
hsa-miR-200b-3p
 
 
-0.29
 
 
10.0
 
 
 
 
ARHGAP1
 
 
hsa-miR-17-5p
 
 
-0.325
 
 
119.0
 
 
 
 
ARHGAP1
 
 
hsa-miR-20a-5p
 
 
-0.339
 
 
119.0
 
 
 
 
ARHGEF28
 
 
hsa-miR-17-5p
 
 
-0.363
 
 
10.7
 
 
 
 
ARHGEF28
 
 
hsa-miR-20a-5p
 
 
-0.366
 
 
10.7
 
 
 
 
ARID4B
 
 
hsa-miR-20a-5p
 
 
-0.29
 
 
12.5
 
 
 
 
ARL1
 
 
hsa-miR-20a-5p
 
 
-0.403
 
 
48.7
 
 
 
 
ARL1
 
 
hsa-miR-17-5p
 
 
-0.432
 
 
48.7
 
 
 
 
ARL4D
 
 
hsa-miR-98-5p
 
 
-0.417
 
 
12.9
 
 
 
 
ARL4D
 
 
hsa-let-7c-5p
 
 
-0.417
 
 
12.9
 
 
 
 
ARL6IP1
 
 
hsa-miR-7-5p
 
 
-0.374
 
 
52.2
 
 
 
 
ARL6IP1
 
 
hsa-miR-328-3p
 
 
-0.494
 
 
52.2
 
 
 
 
ARMC8
 
 
hsa-miR-20a-5p
 
 
-0.357
 
 
23.5
 
 
 
 
ARMC8
 
 
hsa-miR-17-5p
 
 
-0.378
 
 
23.5
 
 
 
 
ARPP19
 
 
hsa-miR-328-3p
 
 
-0.404
 
 
47.3
 
 
 
 
ARPP19
 
 
hsa-let-7c-5p
 
 
-0.492
 
 
47.3
 
 
 
 
ARPP19
 
 
hsa-miR-98-5p
 
 
-0.5
 
 
47.3
 
 
 
 
ARVCF
 
 
hsa-miR-155-5p
 
 
-0.388
 
 
15.5
 
 
 
 
ASAP1
 
 
hsa-miR-98-5p
 
 
-0.308
 
 
18.4
 
 
 
 
ASAP1
 
 
hsa-let-7c-5p
 
 
-0.308
 
 
18.4
 
 
 
 
ASB6
 
 
hsa-miR-423-5p
 
 
-0.388
 
 
11.7
 
 
 
 
ATG13
 
 
hsa-miR-423-5p
 
 
-0.3
 
 
31.6
 
 
 
 
ATG16L1
 
 
hsa-miR-20a-5p
 
 
-0.446
 
 
12.7
 
 
 
 
ATG16L1
 
 
hsa-miR-17-5p
 
 
-0.467
 
 
12.7
 
 
 
 
ATOSB
 
 
hsa-miR-328-3p
 
 
-0.358
 
 
26.7
 
 
 
 
ATOSB
 
 
hsa-miR-98-5p
 
 
-0.429
 
 
26.7
 
 
 
 
ATOSB
 
 
hsa-let-7c-5p
 
 
-0.429
 
 
26.7
 
 
 
 
ATP2A2
 
 
hsa-miR-98-5p
 
 
-0.311
 
 
84.6
 
 
 
 
ATP2A2
 
 
hsa-let-7c-5p
 
 
-0.311
 
 
84.6
 
 
 
 
ATP6V1F
 
 
hsa-miR-194-5p
 
 
-0.444
 
 
127.6
 
 
 
 
ATP6V1H
 
 
hsa-miR-194-5p
 
 
-0.652
 
 
25.7
 
 
 
 
ATP8B2
 
 
hsa-miR-423-5p
 
 
-0.297
 
 
41.9
 
 
 
 
ATPAF1
 
 
hsa-miR-98-5p
 
 
-0.498
 
 
30.6
 
 
 
 
ATPAF1
 
 
hsa-let-7c-5p
 
 
-0.498
 
 
30.6
 
 
 
 
ATXN1L
 
 
hsa-miR-155-5p
 
 
-0.315
 
 
12.5
 
 
 
 
AVPI1
 
 
hsa-miR-7-5p
 
 
-0.367
 
 
57.0
 
 
 
 
B3GAT3
 
 
hsa-miR-98-5p
 
 
-0.324
 
 
38.8
 
 
 
 
B3GAT3
 
 
hsa-let-7c-5p
 
 
-0.324
 
 
38.8
 
 
 
 
B4GALT5
 
 
hsa-miR-210-3p
 
 
-0.511
 
 
15.5
 
 
 
 
B4GAT1
 
 
hsa-miR-200b-3p
 
 
-0.382
 
 
18.5
 
 
 
 
B4GAT1
 
 
hsa-miR-98-5p
 
 
-0.47
 
 
18.5
 
 
 
 
B4GAT1
 
 
hsa-let-7c-5p
 
 
-0.47
 
 
18.5
 
 
 
 
BACE2
 
 
hsa-miR-98-5p
 
 
-0.364
 
 
11.5
 
 
 
 
BACE2
 
 
hsa-let-7c-5p
 
 
-0.364
 
 
11.5
 
 
 
 
BACH1
 
 
hsa-miR-98-5p
 
 
-0.298
 
 
10.9
 
 
 
 
BACH1
 
 
hsa-miR-98-5p
 
 
-0.298
 
 
10.9
 
 
 
 
BACH1
 
 
hsa-let-7c-5p
 
 
-0.306
 
 
10.9
 
 
 
 
BACH1
 
 
hsa-let-7c-5p
 
 
-0.306
 
 
10.9
 
 
 
 
BACH1
 
 
hsa-miR-98-5p
 
 
-0.435
 
 
10.9
 
 
 
 
BACH1
 
 
hsa-miR-98-5p
 
 
-0.435
 
 
10.9
 
 
 
 
BACH1
 
 
hsa-let-7c-5p
 
 
-0.435
 
 
10.9
 
 
 
 
BACH1
 
 
hsa-let-7c-5p
 
 
-0.435
 
 
10.9
 
 
 
 
BAG6
 
 
hsa-miR-200b-3p
 
 
-0.371
 
 
74.1
 
 
 
 
BAMBI
 
 
hsa-miR-20a-5p
 
 
-0.485
 
 
25.4
 
 
 
 
BAMBI
 
 
hsa-miR-17-5p
 
 
-0.506
 
 
25.4
 
 
 
 
BAP1
 
 
hsa-miR-423-5p
 
 
-0.428
 
 
47.2
 
 
 
 
BBX
 
 
hsa-miR-18a-5p
 
 
-0.31
 
 
15.7
 
 
 
 
BCAS3
 
 
hsa-miR-423-5p
 
 
-0.322
 
 
16.8
 
 
 
 
BIN1
 
 
hsa-miR-744-5p
 
 
-0.654
 
 
22.6
 
 
 
 
BIN3
 
 
hsa-miR-98-5p
 
 
-0.427
 
 
15.5
 
 
 
 
 
 
 smooth muscle 
 
 
 
 
target_symbol
 
 
mature_mirna_id
 
 
score
 
 
nTPM
 
 
 
 
 
 
ACTA1
 
 
hsa-miR-155-5p
 
 
-0.595
 
 
61.8
 
 
 
 
ADAM9
 
 
hsa-miR-20a-5p
 
 
-0.299
 
 
48.2
 
 
 
 
ADAM9
 
 
hsa-miR-17-5p
 
 
-0.32
 
 
48.2
 
 
 
 
ADD3
 
 
hsa-miR-200b-3p
 
 
-0.324
 
 
22.3
 
 
 
 
ADD3
 
 
hsa-miR-18a-5p
 
 
-0.39
 
 
22.3
 
 
 
 
ADNP
 
 
hsa-miR-328-3p
 
 
-0.371
 
 
27.7
 
 
 
 
AEBP2
 
 
hsa-miR-18a-5p
 
 
-0.376
 
 
29.0
 
 
 
 
AHNAK
 
 
hsa-miR-20a-5p
 
 
-0.316
 
 
105.6
 
 
 
 
AHNAK
 
 
hsa-miR-17-5p
 
 
-0.337
 
 
105.6
 
 
 
 
AK1
 
 
hsa-miR-423-5p
 
 
-0.443
 
 
20.4
 
 
 
 
ALCAM
 
 
hsa-miR-18a-5p
 
 
-0.341
 
 
15.2
 
 
 
 
ALG3
 
 
hsa-miR-20a-5p
 
 
-0.559
 
 
15.3
 
 
 
 
ALG3
 
 
hsa-miR-17-5p
 
 
-0.572
 
 
15.3
 
 
 
 
ALG9
 
 
hsa-miR-7-5p
 
 
-0.302
 
 
10.4
 
 
 
 
ANGPTL2
 
 
hsa-miR-423-5p
 
 
-0.379
 
 
59.2
 
 
 
 
ANKFY1
 
 
hsa-miR-155-5p
 
 
-0.315
 
 
13.8
 
 
 
 
ANKRD29
 
 
hsa-miR-20a-5p
 
 
-0.359
 
 
10.9
 
 
 
 
ANKRD29
 
 
hsa-miR-17-5p
 
 
-0.372
 
 
10.9
 
 
 
 
ANKRD46
 
 
hsa-miR-98-5p
 
 
-0.389
 
 
11.6
 
 
 
 
ANKRD46
 
 
hsa-let-7c-5p
 
 
-0.389
 
 
11.6
 
 
 
 
ANP32B
 
 
hsa-miR-200b-3p
 
 
-0.287
 
 
72.9
 
 
 
 
AP1S1
 
 
hsa-miR-98-5p
 
 
-0.374
 
 
11.9
 
 
 
 
AP1S1
 
 
hsa-let-7c-5p
 
 
-0.374
 
 
11.9
 
 
 
 
AP1S2
 
 
hsa-miR-200b-3p
 
 
-0.4
 
 
70.4
 
 
 
 
AP2M1
 
 
hsa-miR-423-5p
 
 
-0.49
 
 
220.7
 
 
 
 
APOO
 
 
hsa-miR-200b-3p
 
 
-0.361
 
 
11.2
 
 
 
 
ARF4
 
 
hsa-miR-194-5p
 
 
-0.293
 
 
157.9
 
 
 
 
ARF4
 
 
hsa-miR-7-5p
 
 
-0.407
 
 
157.9
 
 
 
 
ARHGAP1
 
 
hsa-miR-17-5p
 
 
-0.325
 
 
75.2
 
 
 
 
ARHGAP1
 
 
hsa-miR-20a-5p
 
 
-0.339
 
 
75.2
 
 
 
 
ARL1
 
 
hsa-miR-20a-5p
 
 
-0.403
 
 
55.7
 
 
 
 
ARL1
 
 
hsa-miR-17-5p
 
 
-0.432
 
 
55.7
 
 
 
 
ARL4D
 
 
hsa-miR-98-5p
 
 
-0.417
 
 
13.8
 
 
 
 
ARL4D
 
 
hsa-let-7c-5p
 
 
-0.417
 
 
13.8
 
 
 
 
ARL6IP1
 
 
hsa-miR-7-5p
 
 
-0.374
 
 
64.5
 
 
 
 
ARL6IP1
 
 
hsa-miR-328-3p
 
 
-0.494
 
 
64.5
 
 
 
 
ARL6IP6
 
 
hsa-miR-98-5p
 
 
-0.293
 
 
10.8
 
 
 
 
ARL6IP6
 
 
hsa-let-7c-5p
 
 
-0.293
 
 
10.8
 
 
 
 
ARMC8
 
 
hsa-miR-20a-5p
 
 
-0.357
 
 
22.8
 
 
 
 
ARMC8
 
 
hsa-miR-17-5p
 
 
-0.378
 
 
22.8
 
 
 
 
ARMT1
 
 
hsa-miR-98-5p
 
 
-0.303
 
 
13.9
 
 
 
 
ARMT1
 
 
hsa-let-7c-5p
 
 
-0.303
 
 
13.9
 
 
 
 
ARPP19
 
 
hsa-miR-328-3p
 
 
-0.404
 
 
54.2
 
 
 
 
ARPP19
 
 
hsa-let-7c-5p
 
 
-0.492
 
 
54.2
 
 
 
 
ARPP19
 
 
hsa-miR-98-5p
 
 
-0.5
 
 
54.2
 
 
 
 
ARRDC4
 
 
hsa-miR-98-5p
 
 
-0.329
 
 
16.9
 
 
 
 
ARRDC4
 
 
hsa-let-7c-5p
 
 
-0.329
 
 
16.9
 
 
 
 
ASAP1
 
 
hsa-miR-98-5p
 
 
-0.308
 
 
16.7
 
 
 
 
ASAP1
 
 
hsa-let-7c-5p
 
 
-0.308
 
 
16.7
 
 
 
 
ATG13
 
 
hsa-miR-423-5p
 
 
-0.3
 
 
23.2
 
 
 
 
ATOSB
 
 
hsa-miR-328-3p
 
 
-0.358
 
 
10.3
 
 
 
 
ATOSB
 
 
hsa-miR-98-5p
 
 
-0.429
 
 
10.3
 
 
 
 
ATOSB
 
 
hsa-let-7c-5p
 
 
-0.429
 
 
10.3
 
 
 
 
ATP2A2
 
 
hsa-miR-98-5p
 
 
-0.311
 
 
69.4
 
 
 
 
ATP2A2
 
 
hsa-let-7c-5p
 
 
-0.311
 
 
69.4
 
 
 
 
ATP6V1F
 
 
hsa-miR-194-5p
 
 
-0.444
 
 
66.5
 
 
 
 
ATP6V1H
 
 
hsa-miR-194-5p
 
 
-0.652
 
 
22.5
 
 
 
 
ATP8B2
 
 
hsa-miR-423-5p
 
 
-0.297
 
 
39.0
 
 
 
 
ATPAF1
 
 
hsa-miR-98-5p
 
 
-0.498
 
 
30.4
 
 
 
 
ATPAF1
 
 
hsa-let-7c-5p
 
 
-0.498
 
 
30.4
 
 
 
 
ATXN1L
 
 
hsa-miR-155-5p
 
 
-0.315
 
 
12.0
 
 
 
 
B3GAT3
 
 
hsa-miR-98-5p
 
 
-0.324
 
 
13.1
 
 
 
 
B3GAT3
 
 
hsa-let-7c-5p
 
 
-0.324
 
 
13.1
 
 
 
 
B4GALT5
 
 
hsa-miR-210-3p
 
 
-0.511
 
 
35.1
 
 
 
 
B4GAT1
 
 
hsa-miR-200b-3p
 
 
-0.382
 
 
13.1
 
 
 
 
B4GAT1
 
 
hsa-miR-98-5p
 
 
-0.47
 
 
13.1
 
 
 
 
B4GAT1
 
 
hsa-let-7c-5p
 
 
-0.47
 
 
13.1
 
 
 
 
BACH1
 
 
hsa-miR-98-5p
 
 
-0.298
 
 
14.9
 
 
 
 
BACH1
 
 
hsa-miR-98-5p
 
 
-0.298
 
 
14.9
 
 
 
 
BACH1
 
 
hsa-let-7c-5p
 
 
-0.306
 
 
14.9
 
 
 
 
BACH1
 
 
hsa-let-7c-5p
 
 
-0.306
 
 
14.9
 
 
 
 
BACH1
 
 
hsa-miR-98-5p
 
 
-0.435
 
 
14.9
 
 
 
 
BACH1
 
 
hsa-miR-98-5p
 
 
-0.435
 
 
14.9
 
 
 
 
BACH1
 
 
hsa-let-7c-5p
 
 
-0.435
 
 
14.9
 
 
 
 
BACH1
 
 
hsa-let-7c-5p
 
 
-0.435
 
 
14.9
 
 
 
 
BAG6
 
 
hsa-miR-200b-3p
 
 
-0.371
 
 
16.7
 
 
 
 
BAMBI
 
 
hsa-miR-20a-5p
 
 
-0.485
 
 
11.4
 
 
 
 
BAMBI
 
 
hsa-miR-17-5p
 
 
-0.506
 
 
11.4
 
 
 
 
BAP1
 
 
hsa-miR-423-5p
 
 
-0.428
 
 
22.2
 
 
 
 
BBX
 
 
hsa-miR-18a-5p
 
 
-0.31
 
 
15.4
 
 
 
 
BIN1
 
 
hsa-miR-744-5p
 
 
-0.654
 
 
18.3
 
 
 
 
BLOC1S6
 
 
hsa-miR-98-5p
 
 
-0.321
 
 
22.7
 
 
 
 
BLOC1S6
 
 
hsa-let-7c-5p
 
 
-0.321
 
 
22.7
 
 
 
 
BNIP2
 
 
hsa-miR-194-5p
 
 
-0.489
 
 
36.1
 
 
 
 
BNIP3L
 
 
hsa-miR-20a-5p
 
 
-0.287
 
 
47.9
 
 
 
 
BRWD1
 
 
hsa-miR-18a-5p
 
 
-0.29
 
 
10.4
 
 
 
 
BTBD10
 
 
hsa-miR-17-5p
 
 
-0.409
 
 
19.8
 
 
 
 
BTBD10
 
 
hsa-miR-20a-5p
 
 
-0.412
 
 
19.8
 
 
 
 
BTF3L4
 
 
hsa-miR-200b-3p
 
 
-0.294
 
 
32.5
 
 
 
 
BTF3L4
 
 
hsa-miR-194-5p
 
 
-0.334
 
 
32.5
 
 
 
 
BTF3L4
 
 
hsa-miR-98-5p
 
 
-0.355
 
 
32.5
 
 
 
 
BTF3L4
 
 
hsa-let-7c-5p
 
 
-0.355
 
 
32.5
 
 
 
 
BTG3
 
 
hsa-miR-18a-5p
 
 
-0.464
 
 
26.6
 
 
 
 
BTG3
 
 
hsa-miR-17-5p
 
 
-0.668
 
 
26.6
 
 
 
 
BTG3
 
 
hsa-miR-20a-5p
 
 
-0.671
 
 
26.6
 
 
 
 
BZW1
 
 
hsa-miR-98-5p
 
 
-0.354
 
 
74.7
 
 
 
 
BZW1
 
 
hsa-let-7c-5p
 
 
-0.354
 
 
74.7
 
 
 
 
BZW1
 
 
hsa-miR-98-5p
 
 
-0.375
 
 
74.7
 
 
 
 
BZW1
 
 
hsa-let-7c-5p
 
 
-0.375
 
 
74.7
 
 
 
 
BZW2
 
 
hsa-miR-98-5p
 
 
-0.397
 
 
36.4
 
 
 
 
 
 
 appendix 
 
 
 
 
target_symbol
 
 
mature_mirna_id
 
 
score
 
 
nTPM
 
 
 
 
 
 
ACTC1
 
 
hsa-miR-7-5p
 
 
-0.471
 
 
23.3
 
 
 
 
ADAM9
 
 
hsa-miR-20a-5p
 
 
-0.299
 
 
27.3
 
 
 
 
ADAM9
 
 
hsa-miR-17-5p
 
 
-0.32
 
 
27.3
 
 
 
 
ADD3
 
 
hsa-miR-200b-3p
 
 
-0.324
 
 
36.6
 
 
 
 
ADD3
 
 
hsa-miR-18a-5p
 
 
-0.39
 
 
36.6
 
 
 
 
ADNP
 
 
hsa-miR-328-3p
 
 
-0.371
 
 
19.0
 
 
 
 
AEBP2
 
 
hsa-miR-18a-5p
 
 
-0.376
 
 
18.9
 
 
 
 
AHNAK
 
 
hsa-miR-20a-5p
 
 
-0.316
 
 
41.0
 
 
 
 
AHNAK
 
 
hsa-miR-17-5p
 
 
-0.337
 
 
41.0
 
 
 
 
AIFM1
 
 
hsa-miR-98-5p
 
 
-0.314
 
 
16.6
 
 
 
 
AIFM1
 
 
hsa-let-7c-5p
 
 
-0.314
 
 
16.6
 
 
 
 
AK1
 
 
hsa-miR-423-5p
 
 
-0.443
 
 
10.8
 
 
 
 
ALCAM
 
 
hsa-miR-18a-5p
 
 
-0.341
 
 
11.5
 
 
 
 
ALG3
 
 
hsa-miR-20a-5p
 
 
-0.559
 
 
14.1
 
 
 
 
ALG3
 
 
hsa-miR-17-5p
 
 
-0.572
 
 
14.1
 
 
 
 
ANGPTL2
 
 
hsa-miR-423-5p
 
 
-0.379
 
 
31.6
 
 
 
 
ANKFY1
 
 
hsa-miR-155-5p
 
 
-0.315
 
 
12.7
 
 
 
 
ANKRD12
 
 
hsa-miR-7-5p
 
 
-0.37
 
 
19.4
 
 
 
 
ANP32B
 
 
hsa-miR-200b-3p
 
 
-0.287
 
 
117.0
 
 
 
 
AP1S1
 
 
hsa-miR-98-5p
 
 
-0.374
 
 
13.1
 
 
 
 
AP1S1
 
 
hsa-let-7c-5p
 
 
-0.374
 
 
13.1
 
 
 
 
AP1S2
 
 
hsa-miR-200b-3p
 
 
-0.4
 
 
33.8
 
 
 
 
AP2M1
 
 
hsa-miR-423-5p
 
 
-0.49
 
 
146.3
 
 
 
 
ARF4
 
 
hsa-miR-194-5p
 
 
-0.293
 
 
101.3
 
 
 
 
ARF4
 
 
hsa-miR-7-5p
 
 
-0.407
 
 
101.3
 
 
 
 
ARHGAP1
 
 
hsa-miR-17-5p
 
 
-0.325
 
 
35.8
 
 
 
 
ARHGAP1
 
 
hsa-miR-20a-5p
 
 
-0.339
 
 
35.8
 
 
 
 
ARID4A
 
 
hsa-miR-7-5p
 
 
-0.341
 
 
10.5
 
 
 
 
ARID4B
 
 
hsa-miR-20a-5p
 
 
-0.29
 
 
13.3
 
 
 
 
ARL1
 
 
hsa-miR-20a-5p
 
 
-0.403
 
 
37.7
 
 
 
 
ARL1
 
 
hsa-miR-17-5p
 
 
-0.432
 
 
37.7
 
 
 
 
ARL6IP1
 
 
hsa-miR-7-5p
 
 
-0.374
 
 
73.3
 
 
 
 
ARL6IP1
 
 
hsa-miR-328-3p
 
 
-0.494
 
 
73.3
 
 
 
 
ARL6IP6
 
 
hsa-miR-98-5p
 
 
-0.293
 
 
10.9
 
 
 
 
ARL6IP6
 
 
hsa-let-7c-5p
 
 
-0.293
 
 
10.9
 
 
 
 
ARMC8
 
 
hsa-miR-20a-5p
 
 
-0.357
 
 
17.0
 
 
 
 
ARMC8
 
 
hsa-miR-17-5p
 
 
-0.378
 
 
17.0
 
 
 
 
ARMT1
 
 
hsa-miR-98-5p
 
 
-0.303
 
 
14.4
 
 
 
 
ARMT1
 
 
hsa-let-7c-5p
 
 
-0.303
 
 
14.4
 
 
 
 
ARPP19
 
 
hsa-miR-328-3p
 
 
-0.404
 
 
36.4
 
 
 
 
ARPP19
 
 
hsa-let-7c-5p
 
 
-0.492
 
 
36.4
 
 
 
 
ARPP19
 
 
hsa-miR-98-5p
 
 
-0.5
 
 
36.4
 
 
 
 
ARRDC4
 
 
hsa-miR-98-5p
 
 
-0.329
 
 
15.6
 
 
 
 
ARRDC4
 
 
hsa-let-7c-5p
 
 
-0.329
 
 
15.6
 
 
 
 
ASAP1
 
 
hsa-miR-98-5p
 
 
-0.308
 
 
11.4
 
 
 
 
ASAP1
 
 
hsa-let-7c-5p
 
 
-0.308
 
 
11.4
 
 
 
 
ATG13
 
 
hsa-miR-423-5p
 
 
-0.3
 
 
24.6
 
 
 
 
ATM
 
 
hsa-miR-18a-5p
 
 
-0.347
 
 
13.7
 
 
 
 
ATOSB
 
 
hsa-miR-328-3p
 
 
-0.358
 
 
11.5
 
 
 
 
ATOSB
 
 
hsa-miR-98-5p
 
 
-0.429
 
 
11.5
 
 
 
 
ATOSB
 
 
hsa-let-7c-5p
 
 
-0.429
 
 
11.5
 
 
 
 
ATP2A2
 
 
hsa-miR-98-5p
 
 
-0.311
 
 
54.5
 
 
 
 
ATP2A2
 
 
hsa-let-7c-5p
 
 
-0.311
 
 
54.5
 
 
 
 
ATP6V1F
 
 
hsa-miR-194-5p
 
 
-0.444
 
 
100.1
 
 
 
 
ATP6V1H
 
 
hsa-miR-194-5p
 
 
-0.652
 
 
15.0
 
 
 
 
ATP8B2
 
 
hsa-miR-423-5p
 
 
-0.297
 
 
21.9
 
 
 
 
ATPAF1
 
 
hsa-miR-98-5p
 
 
-0.498
 
 
26.8
 
 
 
 
ATPAF1
 
 
hsa-let-7c-5p
 
 
-0.498
 
 
26.8
 
 
 
 
B3GAT3
 
 
hsa-miR-98-5p
 
 
-0.324
 
 
16.3
 
 
 
 
B3GAT3
 
 
hsa-let-7c-5p
 
 
-0.324
 
 
16.3
 
 
 
 
B4GALT5
 
 
hsa-miR-210-3p
 
 
-0.511
 
 
20.5
 
 
 
 
BACH1
 
 
hsa-miR-98-5p
 
 
-0.298
 
 
26.1
 
 
 
 
BACH1
 
 
hsa-miR-98-5p
 
 
-0.298
 
 
26.1
 
 
 
 
BACH1
 
 
hsa-let-7c-5p
 
 
-0.306
 
 
26.1
 
 
 
 
BACH1
 
 
hsa-let-7c-5p
 
 
-0.306
 
 
26.1
 
 
 
 
BACH1
 
 
hsa-miR-98-5p
 
 
-0.435
 
 
26.1
 
 
 
 
BACH1
 
 
hsa-miR-98-5p
 
 
-0.435
 
 
26.1
 
 
 
 
BACH1
 
 
hsa-let-7c-5p
 
 
-0.435
 
 
26.1
 
 
 
 
BACH1
 
 
hsa-let-7c-5p
 
 
-0.435
 
 
26.1
 
 
 
 
BAG6
 
 
hsa-miR-200b-3p
 
 
-0.371
 
 
18.2
 
 
 
 
BAP1
 
 
hsa-miR-423-5p
 
 
-0.428
 
 
23.6
 
 
 
 
BBX
 
 
hsa-miR-18a-5p
 
 
-0.31
 
 
12.0
 
 
 
 
BIN1
 
 
hsa-miR-744-5p
 
 
-0.654
 
 
24.3
 
 
 
 
BIN3
 
 
hsa-miR-98-5p
 
 
-0.427
 
 
10.4
 
 
 
 
BIN3
 
 
hsa-let-7c-5p
 
 
-0.427
 
 
10.4
 
 
 
 
BLOC1S6
 
 
hsa-miR-98-5p
 
 
-0.321
 
 
29.4
 
 
 
 
BLOC1S6
 
 
hsa-let-7c-5p
 
 
-0.321
 
 
29.4
 
 
 
 
BNIP2
 
 
hsa-miR-194-5p
 
 
-0.489
 
 
36.4
 
 
 
 
BNIP3L
 
 
hsa-miR-20a-5p
 
 
-0.287
 
 
35.1
 
 
 
 
BTBD10
 
 
hsa-miR-17-5p
 
 
-0.409
 
 
12.9
 
 
 
 
BTBD10
 
 
hsa-miR-20a-5p
 
 
-0.412
 
 
12.9
 
 
 
 
BTF3L4
 
 
hsa-miR-200b-3p
 
 
-0.294
 
 
28.6
 
 
 
 
BTF3L4
 
 
hsa-miR-194-5p
 
 
-0.334
 
 
28.6
 
 
 
 
BTF3L4
 
 
hsa-miR-98-5p
 
 
-0.355
 
 
28.6
 
 
 
 
BTF3L4
 
 
hsa-let-7c-5p
 
 
-0.355
 
 
28.6
 
 
 
 
BTG3
 
 
hsa-miR-18a-5p
 
 
-0.464
 
 
13.2
 
 
 
 
BTG3
 
 
hsa-miR-17-5p
 
 
-0.668
 
 
13.2
 
 
 
 
BTG3
 
 
hsa-miR-20a-5p
 
 
-0.671
 
 
13.2
 
 
 
 
BZW1
 
 
hsa-miR-98-5p
 
 
-0.354
 
 
91.9
 
 
 
 
BZW1
 
 
hsa-let-7c-5p
 
 
-0.354
 
 
91.9
 
 
 
 
BZW1
 
 
hsa-miR-98-5p
 
 
-0.375
 
 
91.9
 
 
 
 
BZW1
 
 
hsa-let-7c-5p
 
 
-0.375
 
 
91.9
 
 
 
 
BZW2
 
 
hsa-miR-98-5p
 
 
-0.397
 
 
25.1
 
 
 
 
BZW2
 
 
hsa-let-7c-5p
 
 
-0.397
 
 
25.1
 
 
 
 
C15orf39
 
 
hsa-miR-98-5p
 
 
-0.374
 
 
12.0
 
 
 
 
C15orf39
 
 
hsa-let-7c-5p
 
 
-0.374
 
 
12.0
 
 
 
 
C1GALT1
 
 
hsa-let-7c-5p
 
 
-0.322
 
 
13.4
 
 
 
 
C1GALT1
 
 
hsa-miR-98-5p
 
 
-0.33
 
 
13.4
 
 
 
 
C1QBP
 
 
hsa-miR-330-3p
 
 
-0.412
 
 
79.2
 
 
 
 
C6orf120
 
 
hsa-miR-20a-5p
 
 
-0.298
 
 
12.4
 
 
 
 
 
 
 endometrium 
 
 
 
 
target_symbol
 
 
mature_mirna_id
 
 
score
 
 
nTPM
 
 
 
 
 
 
ACTC1
 
 
hsa-miR-7-5p
 
 
-0.471
 
 
17.7
 
 
 
 
ADAM9
 
 
hsa-miR-20a-5p
 
 
-0.299
 
 
21.6
 
 
 
 
ADAM9
 
 
hsa-miR-17-5p
 
 
-0.32
 
 
21.6
 
 
 
 
ADD3
 
 
hsa-miR-200b-3p
 
 
-0.324
 
 
23.3
 
 
 
 
ADD3
 
 
hsa-miR-18a-5p
 
 
-0.39
 
 
23.3
 
 
 
 
ADNP
 
 
hsa-miR-328-3p
 
 
-0.371
 
 
33.6
 
 
 
 
AEBP2
 
 
hsa-miR-18a-5p
 
 
-0.376
 
 
31.8
 
 
 
 
AGFG2
 
 
hsa-miR-20a-5p
 
 
-0.365
 
 
20.0
 
 
 
 
AGFG2
 
 
hsa-miR-17-5p
 
 
-0.402
 
 
20.0
 
 
 
 
AHNAK
 
 
hsa-miR-20a-5p
 
 
-0.316
 
 
187.4
 
 
 
 
AHNAK
 
 
hsa-miR-17-5p
 
 
-0.337
 
 
187.4
 
 
 
 
AIFM1
 
 
hsa-miR-98-5p
 
 
-0.314
 
 
10.6
 
 
 
 
AIFM1
 
 
hsa-let-7c-5p
 
 
-0.314
 
 
10.6
 
 
 
 
AK1
 
 
hsa-miR-423-5p
 
 
-0.443
 
 
16.9
 
 
 
 
ALCAM
 
 
hsa-miR-18a-5p
 
 
-0.341
 
 
14.4
 
 
 
 
ALDH4A1
 
 
hsa-miR-184
 
 
-0.421
 
 
10.1
 
 
 
 
ALG3
 
 
hsa-miR-20a-5p
 
 
-0.559
 
 
15.9
 
 
 
 
ALG3
 
 
hsa-miR-17-5p
 
 
-0.572
 
 
15.9
 
 
 
 
ALG9
 
 
hsa-miR-7-5p
 
 
-0.302
 
 
10.7
 
 
 
 
AMT
 
 
hsa-miR-98-5p
 
 
-0.463
 
 
10.0
 
 
 
 
AMT
 
 
hsa-let-7c-5p
 
 
-0.463
 
 
10.0
 
 
 
 
ANGPTL2
 
 
hsa-miR-423-5p
 
 
-0.379
 
 
59.2
 
 
 
 
ANKFY1
 
 
hsa-miR-155-5p
 
 
-0.315
 
 
17.2
 
 
 
 
ANKRD12
 
 
hsa-miR-7-5p
 
 
-0.37
 
 
11.1
 
 
 
 
ANKRD29
 
 
hsa-miR-20a-5p
 
 
-0.359
 
 
14.7
 
 
 
 
ANKRD29
 
 
hsa-miR-17-5p
 
 
-0.372
 
 
14.7
 
 
 
 
ANKRD46
 
 
hsa-miR-98-5p
 
 
-0.389
 
 
11.8
 
 
 
 
ANKRD46
 
 
hsa-let-7c-5p
 
 
-0.389
 
 
11.8
 
 
 
 
ANP32B
 
 
hsa-miR-200b-3p
 
 
-0.287
 
 
111.3
 
 
 
 
AP1S1
 
 
hsa-miR-98-5p
 
 
-0.374
 
 
16.1
 
 
 
 
AP1S1
 
 
hsa-let-7c-5p
 
 
-0.374
 
 
16.1
 
 
 
 
AP1S2
 
 
hsa-miR-200b-3p
 
 
-0.4
 
 
47.9
 
 
 
 
AP2M1
 
 
hsa-miR-423-5p
 
 
-0.49
 
 
176.4
 
 
 
 
APCDD1
 
 
hsa-miR-20a-5p
 
 
-0.404
 
 
32.5
 
 
 
 
APCDD1
 
 
hsa-miR-17-5p
 
 
-0.425
 
 
32.5
 
 
 
 
ARF4
 
 
hsa-miR-194-5p
 
 
-0.293
 
 
113.9
 
 
 
 
ARF4
 
 
hsa-miR-7-5p
 
 
-0.407
 
 
113.9
 
 
 
 
ARGLU1
 
 
hsa-miR-200b-3p
 
 
-0.29
 
 
11.6
 
 
 
 
ARHGAP1
 
 
hsa-miR-17-5p
 
 
-0.325
 
 
81.8
 
 
 
 
ARHGAP1
 
 
hsa-miR-20a-5p
 
 
-0.339
 
 
81.8
 
 
 
 
ARID4B
 
 
hsa-miR-20a-5p
 
 
-0.29
 
 
14.9
 
 
 
 
ARL1
 
 
hsa-miR-20a-5p
 
 
-0.403
 
 
47.9
 
 
 
 
ARL1
 
 
hsa-miR-17-5p
 
 
-0.432
 
 
47.9
 
 
 
 
ARL4D
 
 
hsa-miR-98-5p
 
 
-0.417
 
 
19.2
 
 
 
 
ARL4D
 
 
hsa-let-7c-5p
 
 
-0.417
 
 
19.2
 
 
 
 
ARL6IP1
 
 
hsa-miR-7-5p
 
 
-0.374
 
 
66.4
 
 
 
 
ARL6IP1
 
 
hsa-miR-328-3p
 
 
-0.494
 
 
66.4
 
 
 
 
ARL6IP6
 
 
hsa-miR-98-5p
 
 
-0.293
 
 
11.0
 
 
 
 
ARL6IP6
 
 
hsa-let-7c-5p
 
 
-0.293
 
 
11.0
 
 
 
 
ARMC8
 
 
hsa-miR-20a-5p
 
 
-0.357
 
 
16.5
 
 
 
 
ARMC8
 
 
hsa-miR-17-5p
 
 
-0.378
 
 
16.5
 
 
 
 
ARMT1
 
 
hsa-miR-98-5p
 
 
-0.303
 
 
15.0
 
 
 
 
ARMT1
 
 
hsa-let-7c-5p
 
 
-0.303
 
 
15.0
 
 
 
 
ARPP19
 
 
hsa-miR-328-3p
 
 
-0.404
 
 
44.7
 
 
 
 
ARPP19
 
 
hsa-let-7c-5p
 
 
-0.492
 
 
44.7
 
 
 
 
ARPP19
 
 
hsa-miR-98-5p
 
 
-0.5
 
 
44.7
 
 
 
 
ARRDC4
 
 
hsa-miR-98-5p
 
 
-0.329
 
 
13.7
 
 
 
 
ARRDC4
 
 
hsa-let-7c-5p
 
 
-0.329
 
 
13.7
 
 
 
 
ASAP1
 
 
hsa-miR-98-5p
 
 
-0.308
 
 
17.2
 
 
 
 
ASAP1
 
 
hsa-let-7c-5p
 
 
-0.308
 
 
17.2
 
 
 
 
ASB6
 
 
hsa-miR-423-5p
 
 
-0.388
 
 
11.1
 
 
 
 
ATG13
 
 
hsa-miR-423-5p
 
 
-0.3
 
 
25.5
 
 
 
 
ATG16L1
 
 
hsa-miR-20a-5p
 
 
-0.446
 
 
11.3
 
 
 
 
ATG16L1
 
 
hsa-miR-17-5p
 
 
-0.467
 
 
11.3
 
 
 
 
ATP2A2
 
 
hsa-miR-98-5p
 
 
-0.311
 
 
43.7
 
 
 
 
ATP2A2
 
 
hsa-let-7c-5p
 
 
-0.311
 
 
43.7
 
 
 
 
ATP6V1F
 
 
hsa-miR-194-5p
 
 
-0.444
 
 
89.8
 
 
 
 
ATP6V1H
 
 
hsa-miR-194-5p
 
 
-0.652
 
 
20.4
 
 
 
 
ATP8B2
 
 
hsa-miR-423-5p
 
 
-0.297
 
 
36.1
 
 
 
 
ATPAF1
 
 
hsa-miR-98-5p
 
 
-0.498
 
 
23.6
 
 
 
 
ATPAF1
 
 
hsa-let-7c-5p
 
 
-0.498
 
 
23.6
 
 
 
 
ATXN1L
 
 
hsa-miR-155-5p
 
 
-0.315
 
 
11.6
 
 
 
 
AVPI1
 
 
hsa-miR-7-5p
 
 
-0.367
 
 
12.2
 
 
 
 
B3GAT3
 
 
hsa-miR-98-5p
 
 
-0.324
 
 
33.5
 
 
 
 
B3GAT3
 
 
hsa-let-7c-5p
 
 
-0.324
 
 
33.5
 
 
 
 
B4GALT5
 
 
hsa-miR-210-3p
 
 
-0.511
 
 
22.0
 
 
 
 
B4GAT1
 
 
hsa-miR-200b-3p
 
 
-0.382
 
 
28.7
 
 
 
 
B4GAT1
 
 
hsa-miR-98-5p
 
 
-0.47
 
 
28.7
 
 
 
 
B4GAT1
 
 
hsa-let-7c-5p
 
 
-0.47
 
 
28.7
 
 
 
 
BACH1
 
 
hsa-miR-98-5p
 
 
-0.298
 
 
18.4
 
 
 
 
BACH1
 
 
hsa-miR-98-5p
 
 
-0.298
 
 
18.4
 
 
 
 
BACH1
 
 
hsa-let-7c-5p
 
 
-0.306
 
 
18.4
 
 
 
 
BACH1
 
 
hsa-let-7c-5p
 
 
-0.306
 
 
18.4
 
 
 
 
BACH1
 
 
hsa-miR-98-5p
 
 
-0.435
 
 
18.4
 
 
 
 
BACH1
 
 
hsa-miR-98-5p
 
 
-0.435
 
 
18.4
 
 
 
 
BACH1
 
 
hsa-let-7c-5p
 
 
-0.435
 
 
18.4
 
 
 
 
BACH1
 
 
hsa-let-7c-5p
 
 
-0.435
 
 
18.4
 
 
 
 
BAG6
 
 
hsa-miR-200b-3p
 
 
-0.371
 
 
68.3
 
 
 
 
BAMBI
 
 
hsa-miR-20a-5p
 
 
-0.485
 
 
16.8
 
 
 
 
BAMBI
 
 
hsa-miR-17-5p
 
 
-0.506
 
 
16.8
 
 
 
 
BAP1
 
 
hsa-miR-423-5p
 
 
-0.428
 
 
39.3
 
 
 
 
BBX
 
 
hsa-miR-18a-5p
 
 
-0.31
 
 
17.7
 
 
 
 
BIN1
 
 
hsa-miR-744-5p
 
 
-0.654
 
 
39.4
 
 
 
 
BIN3
 
 
hsa-miR-98-5p
 
 
-0.427
 
 
11.7
 
 
 
 
BIN3
 
 
hsa-let-7c-5p
 
 
-0.427
 
 
11.7
 
 
 
 
BLOC1S4
 
 
hsa-miR-7-5p
 
 
-0.417
 
 
10.2
 
 
 
 
BLOC1S4
 
 
hsa-miR-7-5p
 
 
-0.527
 
 
10.2
 
 
 
 
BLOC1S6
 
 
hsa-miR-98-5p
 
 
-0.321
 
 
28.8
 
 
 
 
BLOC1S6
 
 
hsa-let-7c-5p
 
 
-0.321
 
 
28.8
 
 
 
 
BNIP2
 
 
hsa-miR-194-5p
 
 
-0.489
 
 
32.6
 
 
 
 
 
 
 thymus 
 
 
 
 
target_symbol
 
 
mature_mirna_id
 
 
score
 
 
nTPM
 
 
 
 
 
 
ACTC1
 
 
hsa-miR-7-5p
 
 
-0.471
 
 
10.5
 
 
 
 
ADD3
 
 
hsa-miR-200b-3p
 
 
-0.324
 
 
81.5
 
 
 
 
ADD3
 
 
hsa-miR-18a-5p
 
 
-0.39
 
 
81.5
 
 
 
 
ADNP
 
 
hsa-miR-328-3p
 
 
-0.371
 
 
41.7
 
 
 
 
AEBP2
 
 
hsa-miR-18a-5p
 
 
-0.376
 
 
27.2
 
 
 
 
AHCTF1
 
 
hsa-miR-98-5p
 
 
-0.374
 
 
11.4
 
 
 
 
AHCTF1
 
 
hsa-let-7c-5p
 
 
-0.374
 
 
11.4
 
 
 
 
AHNAK
 
 
hsa-miR-20a-5p
 
 
-0.316
 
 
26.0
 
 
 
 
AHNAK
 
 
hsa-miR-17-5p
 
 
-0.337
 
 
26.0
 
 
 
 
AIFM1
 
 
hsa-miR-98-5p
 
 
-0.314
 
 
17.2
 
 
 
 
AIFM1
 
 
hsa-let-7c-5p
 
 
-0.314
 
 
17.2
 
 
 
 
ALCAM
 
 
hsa-miR-18a-5p
 
 
-0.341
 
 
13.3
 
 
 
 
ALDH4A1
 
 
hsa-miR-184
 
 
-0.421
 
 
10.9
 
 
 
 
ALG3
 
 
hsa-miR-20a-5p
 
 
-0.559
 
 
11.4
 
 
 
 
ALG3
 
 
hsa-miR-17-5p
 
 
-0.572
 
 
11.4
 
 
 
 
ANKFY1
 
 
hsa-miR-155-5p
 
 
-0.315
 
 
18.9
 
 
 
 
ANKRD12
 
 
hsa-miR-7-5p
 
 
-0.37
 
 
10.7
 
 
 
 
ANKRD46
 
 
hsa-miR-98-5p
 
 
-0.389
 
 
14.3
 
 
 
 
ANKRD46
 
 
hsa-let-7c-5p
 
 
-0.389
 
 
14.3
 
 
 
 
ANP32B
 
 
hsa-miR-200b-3p
 
 
-0.287
 
 
252.8
 
 
 
 
AP1S1
 
 
hsa-miR-98-5p
 
 
-0.374
 
 
17.8
 
 
 
 
AP1S1
 
 
hsa-let-7c-5p
 
 
-0.374
 
 
17.8
 
 
 
 
AP1S2
 
 
hsa-miR-200b-3p
 
 
-0.4
 
 
26.6
 
 
 
 
AP2M1
 
 
hsa-miR-423-5p
 
 
-0.49
 
 
137.3
 
 
 
 
APOO
 
 
hsa-miR-200b-3p
 
 
-0.361
 
 
13.0
 
 
 
 
ARF4
 
 
hsa-miR-194-5p
 
 
-0.293
 
 
69.9
 
 
 
 
ARF4
 
 
hsa-miR-7-5p
 
 
-0.407
 
 
69.9
 
 
 
 
ARHGAP1
 
 
hsa-miR-17-5p
 
 
-0.325
 
 
21.6
 
 
 
 
ARHGAP1
 
 
hsa-miR-20a-5p
 
 
-0.339
 
 
21.6
 
 
 
 
ARID2
 
 
hsa-miR-155-5p
 
 
-0.384
 
 
17.5
 
 
 
 
ARID4A
 
 
hsa-miR-7-5p
 
 
-0.341
 
 
13.5
 
 
 
 
ARID4B
 
 
hsa-miR-20a-5p
 
 
-0.29
 
 
19.9
 
 
 
 
ARL1
 
 
hsa-miR-20a-5p
 
 
-0.403
 
 
30.6
 
 
 
 
ARL1
 
 
hsa-miR-17-5p
 
 
-0.432
 
 
30.6
 
 
 
 
ARL5B
 
 
hsa-miR-155-5p
 
 
-0.355
 
 
12.6
 
 
 
 
ARL6IP1
 
 
hsa-miR-7-5p
 
 
-0.374
 
 
147.6
 
 
 
 
ARL6IP1
 
 
hsa-miR-328-3p
 
 
-0.494
 
 
147.6
 
 
 
 
ARL6IP6
 
 
hsa-miR-98-5p
 
 
-0.293
 
 
20.0
 
 
 
 
ARL6IP6
 
 
hsa-let-7c-5p
 
 
-0.293
 
 
20.0
 
 
 
 
ARMC8
 
 
hsa-miR-20a-5p
 
 
-0.357
 
 
20.7
 
 
 
 
ARMC8
 
 
hsa-miR-17-5p
 
 
-0.378
 
 
20.7
 
 
 
 
ARMT1
 
 
hsa-miR-98-5p
 
 
-0.303
 
 
14.5
 
 
 
 
ARMT1
 
 
hsa-let-7c-5p
 
 
-0.303
 
 
14.5
 
 
 
 
ARPP19
 
 
hsa-miR-328-3p
 
 
-0.404
 
 
49.2
 
 
 
 
ARPP19
 
 
hsa-let-7c-5p
 
 
-0.492
 
 
49.2
 
 
 
 
ARPP19
 
 
hsa-miR-98-5p
 
 
-0.5
 
 
49.2
 
 
 
 
ASAP1
 
 
hsa-miR-98-5p
 
 
-0.308
 
 
14.9
 
 
 
 
ASAP1
 
 
hsa-let-7c-5p
 
 
-0.308
 
 
14.9
 
 
 
 
ASB6
 
 
hsa-miR-423-5p
 
 
-0.388
 
 
10.4
 
 
 
 
ASF1A
 
 
hsa-miR-200b-3p
 
 
-0.377
 
 
22.7
 
 
 
 
ATAD2
 
 
hsa-miR-17-5p
 
 
-0.304
 
 
26.3
 
 
 
 
ATAD2
 
 
hsa-miR-20a-5p
 
 
-0.318
 
 
26.3
 
 
 
 
ATG13
 
 
hsa-miR-423-5p
 
 
-0.3
 
 
37.8
 
 
 
 
ATG4A
 
 
hsa-miR-7-5p
 
 
-0.448
 
 
10.5
 
 
 
 
ATM
 
 
hsa-miR-18a-5p
 
 
-0.347
 
 
14.3
 
 
 
 
ATP2A2
 
 
hsa-miR-98-5p
 
 
-0.311
 
 
33.8
 
 
 
 
ATP2A2
 
 
hsa-let-7c-5p
 
 
-0.311
 
 
33.8
 
 
 
 
ATP6V1F
 
 
hsa-miR-194-5p
 
 
-0.444
 
 
111.4
 
 
 
 
ATP6V1H
 
 
hsa-miR-194-5p
 
 
-0.652
 
 
24.8
 
 
 
 
ATP8B2
 
 
hsa-miR-423-5p
 
 
-0.297
 
 
17.6
 
 
 
 
ATPAF1
 
 
hsa-miR-98-5p
 
 
-0.498
 
 
41.4
 
 
 
 
ATPAF1
 
 
hsa-let-7c-5p
 
 
-0.498
 
 
41.4
 
 
 
 
ATXN1L
 
 
hsa-miR-155-5p
 
 
-0.315
 
 
11.9
 
 
 
 
B3GAT3
 
 
hsa-miR-98-5p
 
 
-0.324
 
 
17.5
 
 
 
 
B3GAT3
 
 
hsa-let-7c-5p
 
 
-0.324
 
 
17.5
 
 
 
 
BACH1
 
 
hsa-miR-98-5p
 
 
-0.298
 
 
13.2
 
 
 
 
BACH1
 
 
hsa-miR-98-5p
 
 
-0.298
 
 
13.2
 
 
 
 
BACH1
 
 
hsa-let-7c-5p
 
 
-0.306
 
 
13.2
 
 
 
 
BACH1
 
 
hsa-let-7c-5p
 
 
-0.306
 
 
13.2
 
 
 
 
BACH1
 
 
hsa-miR-98-5p
 
 
-0.435
 
 
13.2
 
 
 
 
BACH1
 
 
hsa-miR-98-5p
 
 
-0.435
 
 
13.2
 
 
 
 
BACH1
 
 
hsa-let-7c-5p
 
 
-0.435
 
 
13.2
 
 
 
 
BACH1
 
 
hsa-let-7c-5p
 
 
-0.435
 
 
13.2
 
 
 
 
BAG6
 
 
hsa-miR-200b-3p
 
 
-0.371
 
 
22.6
 
 
 
 
BAP1
 
 
hsa-miR-423-5p
 
 
-0.428
 
 
30.9
 
 
 
 
BBX
 
 
hsa-miR-18a-5p
 
 
-0.31
 
 
15.2
 
 
 
 
BIN1
 
 
hsa-miR-744-5p
 
 
-0.654
 
 
17.1
 
 
 
 
BIN3
 
 
hsa-miR-98-5p
 
 
-0.427
 
 
12.4
 
 
 
 
BIN3
 
 
hsa-let-7c-5p
 
 
-0.427
 
 
12.4
 
 
 
 
BLOC1S6
 
 
hsa-miR-98-5p
 
 
-0.321
 
 
23.5
 
 
 
 
BLOC1S6
 
 
hsa-let-7c-5p
 
 
-0.321
 
 
23.5
 
 
 
 
BNIP2
 
 
hsa-miR-194-5p
 
 
-0.489
 
 
40.4
 
 
 
 
BNIP3L
 
 
hsa-miR-20a-5p
 
 
-0.287
 
 
37.1
 
 
 
 
BRWD1
 
 
hsa-miR-18a-5p
 
 
-0.29
 
 
13.9
 
 
 
 
BTBD10
 
 
hsa-miR-17-5p
 
 
-0.409
 
 
17.3
 
 
 
 
BTBD10
 
 
hsa-miR-20a-5p
 
 
-0.412
 
 
17.3
 
 
 
 
BTF3L4
 
 
hsa-miR-200b-3p
 
 
-0.294
 
 
45.8
 
 
 
 
BTF3L4
 
 
hsa-miR-194-5p
 
 
-0.334
 
 
45.8
 
 
 
 
BTF3L4
 
 
hsa-miR-98-5p
 
 
-0.355
 
 
45.8
 
 
 
 
BTF3L4
 
 
hsa-let-7c-5p
 
 
-0.355
 
 
45.8
 
 
 
 
BTG3
 
 
hsa-miR-18a-5p
 
 
-0.464
 
 
25.5
 
 
 
 
BTG3
 
 
hsa-miR-17-5p
 
 
-0.668
 
 
25.5
 
 
 
 
BTG3
 
 
hsa-miR-20a-5p
 
 
-0.671
 
 
25.5
 
 
 
 
BZW1
 
 
hsa-miR-98-5p
 
 
-0.354
 
 
114.6
 
 
 
 
BZW1
 
 
hsa-let-7c-5p
 
 
-0.354
 
 
114.6
 
 
 
 
BZW1
 
 
hsa-miR-98-5p
 
 
-0.375
 
 
114.6
 
 
 
 
BZW1
 
 
hsa-let-7c-5p
 
 
-0.375
 
 
114.6
 
 
 
 
BZW2
 
 
hsa-miR-98-5p
 
 
-0.397
 
 
30.7
 
 
 
 
BZW2
 
 
hsa-let-7c-5p
 
 
-0.397
 
 
30.7
 
 
 
 
C1GALT1
 
 
hsa-let-7c-5p
 
 
-0.322
 
 
13.3
 
 
 
 
 
 
 choroid plexus 
 
 
 
 
target_symbol
 
 
mature_mirna_id
 
 
score
 
 
nTPM
 
 
 
 
 
 
ADAM9
 
 
hsa-miR-20a-5p
 
 
-0.299
 
 
14.9
 
 
 
 
ADAM9
 
 
hsa-miR-17-5p
 
 
-0.32
 
 
14.9
 
 
 
 
ADD3
 
 
hsa-miR-200b-3p
 
 
-0.324
 
 
27.0
 
 
 
 
ADD3
 
 
hsa-miR-18a-5p
 
 
-0.39
 
 
27.0
 
 
 
 
ADNP
 
 
hsa-miR-328-3p
 
 
-0.371
 
 
24.5
 
 
 
 
AHNAK
 
 
hsa-miR-20a-5p
 
 
-0.316
 
 
26.0
 
 
 
 
AHNAK
 
 
hsa-miR-17-5p
 
 
-0.337
 
 
26.0
 
 
 
 
AIFM1
 
 
hsa-miR-98-5p
 
 
-0.314
 
 
37.9
 
 
 
 
AIFM1
 
 
hsa-let-7c-5p
 
 
-0.314
 
 
37.9
 
 
 
 
AK1
 
 
hsa-miR-423-5p
 
 
-0.443
 
 
522.7
 
 
 
 
ALDH4A1
 
 
hsa-miR-184
 
 
-0.421
 
 
121.5
 
 
 
 
ALG3
 
 
hsa-miR-20a-5p
 
 
-0.559
 
 
25.2
 
 
 
 
ALG3
 
 
hsa-miR-17-5p
 
 
-0.572
 
 
25.2
 
 
 
 
AMT
 
 
hsa-miR-98-5p
 
 
-0.463
 
 
33.4
 
 
 
 
AMT
 
 
hsa-let-7c-5p
 
 
-0.463
 
 
33.4
 
 
 
 
ANGPTL2
 
 
hsa-miR-423-5p
 
 
-0.379
 
 
20.8
 
 
 
 
ANKFY1
 
 
hsa-miR-155-5p
 
 
-0.315
 
 
10.2
 
 
 
 
ANKRD46
 
 
hsa-miR-98-5p
 
 
-0.389
 
 
11.0
 
 
 
 
ANKRD46
 
 
hsa-let-7c-5p
 
 
-0.389
 
 
11.0
 
 
 
 
ANKRD9
 
 
hsa-miR-20a-5p
 
 
-0.31
 
 
15.2
 
 
 
 
ANKRD9
 
 
hsa-miR-17-5p
 
 
-0.347
 
 
15.2
 
 
 
 
ANP32B
 
 
hsa-miR-200b-3p
 
 
-0.287
 
 
148.4
 
 
 
 
AP1S1
 
 
hsa-miR-98-5p
 
 
-0.374
 
 
10.3
 
 
 
 
AP1S1
 
 
hsa-let-7c-5p
 
 
-0.374
 
 
10.3
 
 
 
 
AP1S2
 
 
hsa-miR-200b-3p
 
 
-0.4
 
 
22.4
 
 
 
 
AP2M1
 
 
hsa-miR-423-5p
 
 
-0.49
 
 
204.8
 
 
 
 
APOO
 
 
hsa-miR-200b-3p
 
 
-0.361
 
 
102.5
 
 
 
 
ARF4
 
 
hsa-miR-194-5p
 
 
-0.293
 
 
115.4
 
 
 
 
ARF4
 
 
hsa-miR-7-5p
 
 
-0.407
 
 
115.4
 
 
 
 
ARG2
 
 
hsa-miR-98-5p
 
 
-0.468
 
 
49.7
 
 
 
 
ARG2
 
 
hsa-let-7c-5p
 
 
-0.468
 
 
49.7
 
 
 
 
ARHGAP1
 
 
hsa-miR-17-5p
 
 
-0.325
 
 
26.4
 
 
 
 
ARHGAP1
 
 
hsa-miR-20a-5p
 
 
-0.339
 
 
26.4
 
 
 
 
ARL1
 
 
hsa-miR-20a-5p
 
 
-0.403
 
 
84.0
 
 
 
 
ARL1
 
 
hsa-miR-17-5p
 
 
-0.432
 
 
84.0
 
 
 
 
ARL4D
 
 
hsa-miR-98-5p
 
 
-0.417
 
 
15.4
 
 
 
 
ARL4D
 
 
hsa-let-7c-5p
 
 
-0.417
 
 
15.4
 
 
 
 
ARL6IP1
 
 
hsa-miR-7-5p
 
 
-0.374
 
 
185.0
 
 
 
 
ARL6IP1
 
 
hsa-miR-328-3p
 
 
-0.494
 
 
185.0
 
 
 
 
ARMC8
 
 
hsa-miR-20a-5p
 
 
-0.357
 
 
20.8
 
 
 
 
ARMC8
 
 
hsa-miR-17-5p
 
 
-0.378
 
 
20.8
 
 
 
 
ARPP19
 
 
hsa-miR-328-3p
 
 
-0.404
 
 
32.4
 
 
 
 
ARPP19
 
 
hsa-let-7c-5p
 
 
-0.492
 
 
32.4
 
 
 
 
ARPP19
 
 
hsa-miR-98-5p
 
 
-0.5
 
 
32.4
 
 
 
 
ASB6
 
 
hsa-miR-423-5p
 
 
-0.388
 
 
15.8
 
 
 
 
ATG13
 
 
hsa-miR-423-5p
 
 
-0.3
 
 
47.9
 
 
 
 
ATG16L1
 
 
hsa-miR-20a-5p
 
 
-0.446
 
 
21.8
 
 
 
 
ATG16L1
 
 
hsa-miR-17-5p
 
 
-0.467
 
 
21.8
 
 
 
 
ATG4A
 
 
hsa-miR-7-5p
 
 
-0.448
 
 
16.6
 
 
 
 
ATOSB
 
 
hsa-miR-328-3p
 
 
-0.358
 
 
16.6
 
 
 
 
ATOSB
 
 
hsa-miR-98-5p
 
 
-0.429
 
 
16.6
 
 
 
 
ATOSB
 
 
hsa-let-7c-5p
 
 
-0.429
 
 
16.6
 
 
 
 
ATP2A2
 
 
hsa-miR-98-5p
 
 
-0.311
 
 
29.6
 
 
 
 
ATP2A2
 
 
hsa-let-7c-5p
 
 
-0.311
 
 
29.6
 
 
 
 
ATP2B2
 
 
hsa-miR-7-5p
 
 
-0.297
 
 
61.0
 
 
 
 
ATP6V1F
 
 
hsa-miR-194-5p
 
 
-0.444
 
 
245.3
 
 
 
 
ATP6V1H
 
 
hsa-miR-194-5p
 
 
-0.652
 
 
32.0
 
 
 
 
ATP8B2
 
 
hsa-miR-423-5p
 
 
-0.297
 
 
11.6
 
 
 
 
ATPAF1
 
 
hsa-miR-98-5p
 
 
-0.498
 
 
168.0
 
 
 
 
ATPAF1
 
 
hsa-let-7c-5p
 
 
-0.498
 
 
168.0
 
 
 
 
AVPI1
 
 
hsa-miR-7-5p
 
 
-0.367
 
 
20.0
 
 
 
 
B3GAT3
 
 
hsa-miR-98-5p
 
 
-0.324
 
 
87.1
 
 
 
 
B3GAT3
 
 
hsa-let-7c-5p
 
 
-0.324
 
 
87.1
 
 
 
 
B4GAT1
 
 
hsa-miR-200b-3p
 
 
-0.382
 
 
93.8
 
 
 
 
B4GAT1
 
 
hsa-miR-98-5p
 
 
-0.47
 
 
93.8
 
 
 
 
B4GAT1
 
 
hsa-let-7c-5p
 
 
-0.47
 
 
93.8
 
 
 
 
BAG6
 
 
hsa-miR-200b-3p
 
 
-0.371
 
 
11.6
 
 
 
 
BAMBI
 
 
hsa-miR-20a-5p
 
 
-0.485
 
 
37.8
 
 
 
 
BAMBI
 
 
hsa-miR-17-5p
 
 
-0.506
 
 
37.8
 
 
 
 
BAP1
 
 
hsa-miR-423-5p
 
 
-0.428
 
 
30.6
 
 
 
 
BIN1
 
 
hsa-miR-744-5p
 
 
-0.654
 
 
43.9
 
 
 
 
BIN3
 
 
hsa-miR-98-5p
 
 
-0.427
 
 
33.7
 
 
 
 
BIN3
 
 
hsa-let-7c-5p
 
 
-0.427
 
 
33.7
 
 
 
 
BLOC1S4
 
 
hsa-miR-7-5p
 
 
-0.417
 
 
10.3
 
 
 
 
BLOC1S4
 
 
hsa-miR-7-5p
 
 
-0.527
 
 
10.3
 
 
 
 
BLOC1S6
 
 
hsa-miR-98-5p
 
 
-0.321
 
 
14.9
 
 
 
 
BLOC1S6
 
 
hsa-let-7c-5p
 
 
-0.321
 
 
14.9
 
 
 
 
BNIP3L
 
 
hsa-miR-20a-5p
 
 
-0.287
 
 
46.7
 
 
 
 
BRWD1
 
 
hsa-miR-18a-5p
 
 
-0.29
 
 
12.9
 
 
 
 
BTBD10
 
 
hsa-miR-17-5p
 
 
-0.409
 
 
10.3
 
 
 
 
BTBD10
 
 
hsa-miR-20a-5p
 
 
-0.412
 
 
10.3
 
 
 
 
BTF3L4
 
 
hsa-miR-200b-3p
 
 
-0.294
 
 
26.9
 
 
 
 
BTF3L4
 
 
hsa-miR-194-5p
 
 
-0.334
 
 
26.9
 
 
 
 
BTF3L4
 
 
hsa-miR-98-5p
 
 
-0.355
 
 
26.9
 
 
 
 
BTF3L4
 
 
hsa-let-7c-5p
 
 
-0.355
 
 
26.9
 
 
 
 
BTG3
 
 
hsa-miR-18a-5p
 
 
-0.464
 
 
25.9
 
 
 
 
BTG3
 
 
hsa-miR-17-5p
 
 
-0.668
 
 
25.9
 
 
 
 
BTG3
 
 
hsa-miR-20a-5p
 
 
-0.671
 
 
25.9
 
 
 
 
BZW1
 
 
hsa-miR-98-5p
 
 
-0.354
 
 
50.7
 
 
 
 
BZW1
 
 
hsa-let-7c-5p
 
 
-0.354
 
 
50.7
 
 
 
 
BZW1
 
 
hsa-miR-98-5p
 
 
-0.375
 
 
50.7
 
 
 
 
BZW1
 
 
hsa-let-7c-5p
 
 
-0.375
 
 
50.7
 
 
 
 
BZW2
 
 
hsa-miR-98-5p
 
 
-0.397
 
 
19.4
 
 
 
 
BZW2
 
 
hsa-let-7c-5p
 
 
-0.397
 
 
19.4
 
 
 
 
C1GALT1
 
 
hsa-let-7c-5p
 
 
-0.322
 
 
22.9
 
 
 
 
C1GALT1
 
 
hsa-miR-98-5p
 
 
-0.33
 
 
22.9
 
 
 
 
C1QBP
 
 
hsa-miR-330-3p
 
 
-0.412
 
 
247.2
 
 
 
 
C3orf18
 
 
hsa-miR-155-5p
 
 
-0.431
 
 
27.1
 
 
 
 
CAB39
 
 
hsa-miR-155-5p
 
 
-0.312
 
 
22.5
 
 
 
 
CALD1
 
 
hsa-miR-20a-5p
 
 
-0.352
 
 
32.6
 
 
 
 
 
 
 ovary 
 
 
 
 
target_symbol
 
 
mature_mirna_id
 
 
score
 
 
nTPM
 
 
 
 
 
 
ADAM9
 
 
hsa-miR-20a-5p
 
 
-0.299
 
 
16.9
 
 
 
 
ADAM9
 
 
hsa-miR-17-5p
 
 
-0.32
 
 
16.9
 
 
 
 
ADD3
 
 
hsa-miR-200b-3p
 
 
-0.324
 
 
49.3
 
 
 
 
ADD3
 
 
hsa-miR-18a-5p
 
 
-0.39
 
 
49.3
 
 
 
 
ADNP
 
 
hsa-miR-328-3p
 
 
-0.371
 
 
36.2
 
 
 
 
ADRB3
 
 
hsa-let-7c-5p
 
 
-0.415
 
 
12.8
 
 
 
 
ADRB3
 
 
hsa-miR-98-5p
 
 
-0.423
 
 
12.8
 
 
 
 
AEBP2
 
 
hsa-miR-18a-5p
 
 
-0.376
 
 
31.1
 
 
 
 
AGFG2
 
 
hsa-miR-20a-5p
 
 
-0.365
 
 
28.5
 
 
 
 
AGFG2
 
 
hsa-miR-17-5p
 
 
-0.402
 
 
28.5
 
 
 
 
AHCTF1
 
 
hsa-miR-98-5p
 
 
-0.374
 
 
13.1
 
 
 
 
AHCTF1
 
 
hsa-let-7c-5p
 
 
-0.374
 
 
13.1
 
 
 
 
AHNAK
 
 
hsa-miR-20a-5p
 
 
-0.316
 
 
105.9
 
 
 
 
AHNAK
 
 
hsa-miR-17-5p
 
 
-0.337
 
 
105.9
 
 
 
 
AK1
 
 
hsa-miR-423-5p
 
 
-0.443
 
 
43.4
 
 
 
 
ALCAM
 
 
hsa-miR-18a-5p
 
 
-0.341
 
 
33.1
 
 
 
 
ALG3
 
 
hsa-miR-20a-5p
 
 
-0.559
 
 
12.1
 
 
 
 
ALG3
 
 
hsa-miR-17-5p
 
 
-0.572
 
 
12.1
 
 
 
 
ALG9
 
 
hsa-miR-7-5p
 
 
-0.302
 
 
11.1
 
 
 
 
AMT
 
 
hsa-miR-98-5p
 
 
-0.463
 
 
16.7
 
 
 
 
AMT
 
 
hsa-let-7c-5p
 
 
-0.463
 
 
16.7
 
 
 
 
ANGPTL2
 
 
hsa-miR-423-5p
 
 
-0.379
 
 
49.6
 
 
 
 
ANKFY1
 
 
hsa-miR-155-5p
 
 
-0.315
 
 
16.2
 
 
 
 
ANKRD12
 
 
hsa-miR-7-5p
 
 
-0.37
 
 
10.6
 
 
 
 
ANKRD29
 
 
hsa-miR-20a-5p
 
 
-0.359
 
 
15.0
 
 
 
 
ANKRD29
 
 
hsa-miR-17-5p
 
 
-0.372
 
 
15.0
 
 
 
 
ANKRD46
 
 
hsa-miR-98-5p
 
 
-0.389
 
 
19.3
 
 
 
 
ANKRD46
 
 
hsa-let-7c-5p
 
 
-0.389
 
 
19.3
 
 
 
 
ANP32B
 
 
hsa-miR-200b-3p
 
 
-0.287
 
 
160.5
 
 
 
 
AP1S1
 
 
hsa-miR-98-5p
 
 
-0.374
 
 
22.2
 
 
 
 
AP1S1
 
 
hsa-let-7c-5p
 
 
-0.374
 
 
22.2
 
 
 
 
AP1S2
 
 
hsa-miR-200b-3p
 
 
-0.4
 
 
13.5
 
 
 
 
AP2M1
 
 
hsa-miR-423-5p
 
 
-0.49
 
 
168.4
 
 
 
 
APCDD1
 
 
hsa-miR-20a-5p
 
 
-0.404
 
 
28.1
 
 
 
 
APCDD1
 
 
hsa-miR-17-5p
 
 
-0.425
 
 
28.1
 
 
 
 
APOO
 
 
hsa-miR-200b-3p
 
 
-0.361
 
 
11.0
 
 
 
 
ARF4
 
 
hsa-miR-194-5p
 
 
-0.293
 
 
123.7
 
 
 
 
ARF4
 
 
hsa-miR-7-5p
 
 
-0.407
 
 
123.7
 
 
 
 
ARG2
 
 
hsa-miR-98-5p
 
 
-0.468
 
 
18.7
 
 
 
 
ARG2
 
 
hsa-let-7c-5p
 
 
-0.468
 
 
18.7
 
 
 
 
ARGLU1
 
 
hsa-miR-200b-3p
 
 
-0.29
 
 
14.7
 
 
 
 
ARHGAP1
 
 
hsa-miR-17-5p
 
 
-0.325
 
 
43.6
 
 
 
 
ARHGAP1
 
 
hsa-miR-20a-5p
 
 
-0.339
 
 
43.6
 
 
 
 
ARHGEF28
 
 
hsa-miR-17-5p
 
 
-0.363
 
 
18.8
 
 
 
 
ARHGEF28
 
 
hsa-miR-20a-5p
 
 
-0.366
 
 
18.8
 
 
 
 
ARID4A
 
 
hsa-miR-7-5p
 
 
-0.341
 
 
10.1
 
 
 
 
ARID4B
 
 
hsa-miR-20a-5p
 
 
-0.29
 
 
14.0
 
 
 
 
ARL1
 
 
hsa-miR-20a-5p
 
 
-0.403
 
 
63.4
 
 
 
 
ARL1
 
 
hsa-miR-17-5p
 
 
-0.432
 
 
63.4
 
 
 
 
ARL4D
 
 
hsa-miR-98-5p
 
 
-0.417
 
 
20.3
 
 
 
 
ARL4D
 
 
hsa-let-7c-5p
 
 
-0.417
 
 
20.3
 
 
 
 
ARL6IP1
 
 
hsa-miR-7-5p
 
 
-0.374
 
 
97.4
 
 
 
 
ARL6IP1
 
 
hsa-miR-328-3p
 
 
-0.494
 
 
97.4
 
 
 
 
ARL6IP6
 
 
hsa-miR-98-5p
 
 
-0.293
 
 
11.9
 
 
 
 
ARL6IP6
 
 
hsa-let-7c-5p
 
 
-0.293
 
 
11.9
 
 
 
 
ARMC8
 
 
hsa-miR-20a-5p
 
 
-0.357
 
 
14.0
 
 
 
 
ARMC8
 
 
hsa-miR-17-5p
 
 
-0.378
 
 
14.0
 
 
 
 
ARMT1
 
 
hsa-miR-98-5p
 
 
-0.303
 
 
15.5
 
 
 
 
ARMT1
 
 
hsa-let-7c-5p
 
 
-0.303
 
 
15.5
 
 
 
 
ARPP19
 
 
hsa-miR-328-3p
 
 
-0.404
 
 
40.6
 
 
 
 
ARPP19
 
 
hsa-let-7c-5p
 
 
-0.492
 
 
40.6
 
 
 
 
ARPP19
 
 
hsa-miR-98-5p
 
 
-0.5
 
 
40.6
 
 
 
 
ASAP1
 
 
hsa-miR-98-5p
 
 
-0.308
 
 
16.8
 
 
 
 
ASAP1
 
 
hsa-let-7c-5p
 
 
-0.308
 
 
16.8
 
 
 
 
ASF1A
 
 
hsa-miR-200b-3p
 
 
-0.377
 
 
18.5
 
 
 
 
ATG13
 
 
hsa-miR-423-5p
 
 
-0.3
 
 
27.6
 
 
 
 
ATP2A2
 
 
hsa-miR-98-5p
 
 
-0.311
 
 
34.9
 
 
 
 
ATP2A2
 
 
hsa-let-7c-5p
 
 
-0.311
 
 
34.9
 
 
 
 
ATP6V1F
 
 
hsa-miR-194-5p
 
 
-0.444
 
 
99.6
 
 
 
 
ATP6V1H
 
 
hsa-miR-194-5p
 
 
-0.652
 
 
20.6
 
 
 
 
ATP8B2
 
 
hsa-miR-423-5p
 
 
-0.297
 
 
29.1
 
 
 
 
ATPAF1
 
 
hsa-miR-98-5p
 
 
-0.498
 
 
33.9
 
 
 
 
ATPAF1
 
 
hsa-let-7c-5p
 
 
-0.498
 
 
33.9
 
 
 
 
ATXN1L
 
 
hsa-miR-155-5p
 
 
-0.315
 
 
10.8
 
 
 
 
AVPI1
 
 
hsa-miR-7-5p
 
 
-0.367
 
 
85.9
 
 
 
 
B3GAT3
 
 
hsa-miR-98-5p
 
 
-0.324
 
 
35.4
 
 
 
 
B3GAT3
 
 
hsa-let-7c-5p
 
 
-0.324
 
 
35.4
 
 
 
 
B4GALT5
 
 
hsa-miR-210-3p
 
 
-0.511
 
 
19.0
 
 
 
 
B4GAT1
 
 
hsa-miR-200b-3p
 
 
-0.382
 
 
33.5
 
 
 
 
B4GAT1
 
 
hsa-miR-98-5p
 
 
-0.47
 
 
33.5
 
 
 
 
B4GAT1
 
 
hsa-let-7c-5p
 
 
-0.47
 
 
33.5
 
 
 
 
BACH1
 
 
hsa-miR-98-5p
 
 
-0.298
 
 
12.0
 
 
 
 
BACH1
 
 
hsa-miR-98-5p
 
 
-0.298
 
 
12.0
 
 
 
 
BACH1
 
 
hsa-let-7c-5p
 
 
-0.306
 
 
12.0
 
 
 
 
BACH1
 
 
hsa-let-7c-5p
 
 
-0.306
 
 
12.0
 
 
 
 
BACH1
 
 
hsa-miR-98-5p
 
 
-0.435
 
 
12.0
 
 
 
 
BACH1
 
 
hsa-miR-98-5p
 
 
-0.435
 
 
12.0
 
 
 
 
BACH1
 
 
hsa-let-7c-5p
 
 
-0.435
 
 
12.0
 
 
 
 
BACH1
 
 
hsa-let-7c-5p
 
 
-0.435
 
 
12.0
 
 
 
 
BAG6
 
 
hsa-miR-200b-3p
 
 
-0.371
 
 
73.9
 
 
 
 
BAMBI
 
 
hsa-miR-20a-5p
 
 
-0.485
 
 
58.6
 
 
 
 
BAMBI
 
 
hsa-miR-17-5p
 
 
-0.506
 
 
58.6
 
 
 
 
BAP1
 
 
hsa-miR-423-5p
 
 
-0.428
 
 
41.8
 
 
 
 
BBX
 
 
hsa-miR-18a-5p
 
 
-0.31
 
 
20.8
 
 
 
 
BIN1
 
 
hsa-miR-744-5p
 
 
-0.654
 
 
32.3
 
 
 
 
BIN3
 
 
hsa-miR-98-5p
 
 
-0.427
 
 
11.4
 
 
 
 
BIN3
 
 
hsa-let-7c-5p
 
 
-0.427
 
 
11.4
 
 
 
 
BLOC1S4
 
 
hsa-miR-7-5p
 
 
-0.417
 
 
12.4
 
 
 
 
BLOC1S4
 
 
hsa-miR-7-5p
 
 
-0.527
 
 
12.4
 
 
 
 
BLOC1S6
 
 
hsa-miR-98-5p
 
 
-0.321
 
 
33.7
 
 
 
 
 
 
 spleen 
 
 
 
 
target_symbol
 
 
mature_mirna_id
 
 
score
 
 
nTPM
 
 
 
 
 
 
ADAM9
 
 
hsa-miR-20a-5p
 
 
-0.299
 
 
22.6
 
 
 
 
ADAM9
 
 
hsa-miR-17-5p
 
 
-0.32
 
 
22.6
 
 
 
 
ADD3
 
 
hsa-miR-200b-3p
 
 
-0.324
 
 
37.7
 
 
 
 
ADD3
 
 
hsa-miR-18a-5p
 
 
-0.39
 
 
37.7
 
 
 
 
ADNP
 
 
hsa-miR-328-3p
 
 
-0.371
 
 
34.9
 
 
 
 
AEBP2
 
 
hsa-miR-18a-5p
 
 
-0.376
 
 
21.8
 
 
 
 
AGFG2
 
 
hsa-miR-20a-5p
 
 
-0.365
 
 
27.6
 
 
 
 
AGFG2
 
 
hsa-miR-17-5p
 
 
-0.402
 
 
27.6
 
 
 
 
AHNAK
 
 
hsa-miR-20a-5p
 
 
-0.316
 
 
56.3
 
 
 
 
AHNAK
 
 
hsa-miR-17-5p
 
 
-0.337
 
 
56.3
 
 
 
 
AIFM1
 
 
hsa-miR-98-5p
 
 
-0.314
 
 
12.9
 
 
 
 
AIFM1
 
 
hsa-let-7c-5p
 
 
-0.314
 
 
12.9
 
 
 
 
AK1
 
 
hsa-miR-423-5p
 
 
-0.443
 
 
31.5
 
 
 
 
ALG3
 
 
hsa-miR-20a-5p
 
 
-0.559
 
 
19.8
 
 
 
 
ALG3
 
 
hsa-miR-17-5p
 
 
-0.572
 
 
19.8
 
 
 
 
ANGPTL2
 
 
hsa-miR-423-5p
 
 
-0.379
 
 
46.4
 
 
 
 
ANKFY1
 
 
hsa-miR-155-5p
 
 
-0.315
 
 
14.2
 
 
 
 
ANKRD12
 
 
hsa-miR-7-5p
 
 
-0.37
 
 
18.8
 
 
 
 
ANKRD46
 
 
hsa-miR-98-5p
 
 
-0.389
 
 
10.4
 
 
 
 
ANKRD46
 
 
hsa-let-7c-5p
 
 
-0.389
 
 
10.4
 
 
 
 
ANP32B
 
 
hsa-miR-200b-3p
 
 
-0.287
 
 
117.6
 
 
 
 
AP1S1
 
 
hsa-miR-98-5p
 
 
-0.374
 
 
20.9
 
 
 
 
AP1S1
 
 
hsa-let-7c-5p
 
 
-0.374
 
 
20.9
 
 
 
 
AP1S2
 
 
hsa-miR-200b-3p
 
 
-0.4
 
 
39.7
 
 
 
 
AP2M1
 
 
hsa-miR-423-5p
 
 
-0.49
 
 
129.7
 
 
 
 
ARF4
 
 
hsa-miR-194-5p
 
 
-0.293
 
 
60.0
 
 
 
 
ARF4
 
 
hsa-miR-7-5p
 
 
-0.407
 
 
60.0
 
 
 
 
ARGLU1
 
 
hsa-miR-200b-3p
 
 
-0.29
 
 
10.1
 
 
 
 
ARHGAP1
 
 
hsa-miR-17-5p
 
 
-0.325
 
 
55.1
 
 
 
 
ARHGAP1
 
 
hsa-miR-20a-5p
 
 
-0.339
 
 
55.1
 
 
 
 
ARHGAP28
 
 
hsa-miR-98-5p
 
 
-0.47
 
 
11.9
 
 
 
 
ARHGAP28
 
 
hsa-let-7c-5p
 
 
-0.47
 
 
11.9
 
 
 
 
ARID4A
 
 
hsa-miR-7-5p
 
 
-0.341
 
 
13.2
 
 
 
 
ARID4B
 
 
hsa-miR-20a-5p
 
 
-0.29
 
 
17.3
 
 
 
 
ARL1
 
 
hsa-miR-20a-5p
 
 
-0.403
 
 
27.2
 
 
 
 
ARL1
 
 
hsa-miR-17-5p
 
 
-0.432
 
 
27.2
 
 
 
 
ARL4D
 
 
hsa-miR-98-5p
 
 
-0.417
 
 
16.1
 
 
 
 
ARL4D
 
 
hsa-let-7c-5p
 
 
-0.417
 
 
16.1
 
 
 
 
ARL6IP1
 
 
hsa-miR-7-5p
 
 
-0.374
 
 
68.6
 
 
 
 
ARL6IP1
 
 
hsa-miR-328-3p
 
 
-0.494
 
 
68.6
 
 
 
 
ARL6IP6
 
 
hsa-miR-98-5p
 
 
-0.293
 
 
13.7
 
 
 
 
ARL6IP6
 
 
hsa-let-7c-5p
 
 
-0.293
 
 
13.7
 
 
 
 
ARMC8
 
 
hsa-miR-20a-5p
 
 
-0.357
 
 
14.9
 
 
 
 
ARMC8
 
 
hsa-miR-17-5p
 
 
-0.378
 
 
14.9
 
 
 
 
ARMT1
 
 
hsa-miR-98-5p
 
 
-0.303
 
 
12.0
 
 
 
 
ARMT1
 
 
hsa-let-7c-5p
 
 
-0.303
 
 
12.0
 
 
 
 
ARPP19
 
 
hsa-miR-328-3p
 
 
-0.404
 
 
41.2
 
 
 
 
ARPP19
 
 
hsa-let-7c-5p
 
 
-0.492
 
 
41.2
 
 
 
 
ARPP19
 
 
hsa-miR-98-5p
 
 
-0.5
 
 
41.2
 
 
 
 
ARVCF
 
 
hsa-miR-155-5p
 
 
-0.388
 
 
10.3
 
 
 
 
ASAP1
 
 
hsa-miR-98-5p
 
 
-0.308
 
 
28.1
 
 
 
 
ASAP1
 
 
hsa-let-7c-5p
 
 
-0.308
 
 
28.1
 
 
 
 
ASB6
 
 
hsa-miR-423-5p
 
 
-0.388
 
 
17.1
 
 
 
 
ASF1A
 
 
hsa-miR-200b-3p
 
 
-0.377
 
 
10.9
 
 
 
 
ATG13
 
 
hsa-miR-423-5p
 
 
-0.3
 
 
34.5
 
 
 
 
ATG16L1
 
 
hsa-miR-20a-5p
 
 
-0.446
 
 
11.4
 
 
 
 
ATG16L1
 
 
hsa-miR-17-5p
 
 
-0.467
 
 
11.4
 
 
 
 
ATM
 
 
hsa-miR-18a-5p
 
 
-0.347
 
 
17.7
 
 
 
 
ATOSB
 
 
hsa-miR-328-3p
 
 
-0.358
 
 
26.6
 
 
 
 
ATOSB
 
 
hsa-miR-98-5p
 
 
-0.429
 
 
26.6
 
 
 
 
ATOSB
 
 
hsa-let-7c-5p
 
 
-0.429
 
 
26.6
 
 
 
 
ATP2A2
 
 
hsa-miR-98-5p
 
 
-0.311
 
 
28.8
 
 
 
 
ATP2A2
 
 
hsa-let-7c-5p
 
 
-0.311
 
 
28.8
 
 
 
 
ATP6V1F
 
 
hsa-miR-194-5p
 
 
-0.444
 
 
120.8
 
 
 
 
ATP6V1H
 
 
hsa-miR-194-5p
 
 
-0.652
 
 
16.6
 
 
 
 
ATP8B2
 
 
hsa-miR-423-5p
 
 
-0.297
 
 
18.8
 
 
 
 
ATPAF1
 
 
hsa-miR-98-5p
 
 
-0.498
 
 
23.6
 
 
 
 
ATPAF1
 
 
hsa-let-7c-5p
 
 
-0.498
 
 
23.6
 
 
 
 
ATXN1L
 
 
hsa-miR-155-5p
 
 
-0.315
 
 
11.1
 
 
 
 
AVPI1
 
 
hsa-miR-7-5p
 
 
-0.367
 
 
20.8
 
 
 
 
B3GAT3
 
 
hsa-miR-98-5p
 
 
-0.324
 
 
44.2
 
 
 
 
B3GAT3
 
 
hsa-let-7c-5p
 
 
-0.324
 
 
44.2
 
 
 
 
B4GALT5
 
 
hsa-miR-210-3p
 
 
-0.511
 
 
26.0
 
 
 
 
B4GAT1
 
 
hsa-miR-200b-3p
 
 
-0.382
 
 
10.0
 
 
 
 
B4GAT1
 
 
hsa-miR-98-5p
 
 
-0.47
 
 
10.0
 
 
 
 
B4GAT1
 
 
hsa-let-7c-5p
 
 
-0.47
 
 
10.0
 
 
 
 
BACH1
 
 
hsa-miR-98-5p
 
 
-0.298
 
 
19.4
 
 
 
 
BACH1
 
 
hsa-miR-98-5p
 
 
-0.298
 
 
19.4
 
 
 
 
BACH1
 
 
hsa-let-7c-5p
 
 
-0.306
 
 
19.4
 
 
 
 
BACH1
 
 
hsa-let-7c-5p
 
 
-0.306
 
 
19.4
 
 
 
 
BACH1
 
 
hsa-miR-98-5p
 
 
-0.435
 
 
19.4
 
 
 
 
BACH1
 
 
hsa-miR-98-5p
 
 
-0.435
 
 
19.4
 
 
 
 
BACH1
 
 
hsa-let-7c-5p
 
 
-0.435
 
 
19.4
 
 
 
 
BACH1
 
 
hsa-let-7c-5p
 
 
-0.435
 
 
19.4
 
 
 
 
BAG6
 
 
hsa-miR-200b-3p
 
 
-0.371
 
 
79.0
 
 
 
 
BAMBI
 
 
hsa-miR-20a-5p
 
 
-0.485
 
 
19.4
 
 
 
 
BAMBI
 
 
hsa-miR-17-5p
 
 
-0.506
 
 
19.4
 
 
 
 
BAP1
 
 
hsa-miR-423-5p
 
 
-0.428
 
 
52.2
 
 
 
 
BBX
 
 
hsa-miR-18a-5p
 
 
-0.31
 
 
17.8
 
 
 
 
BIN1
 
 
hsa-miR-744-5p
 
 
-0.654
 
 
52.9
 
 
 
 
BIN3
 
 
hsa-miR-98-5p
 
 
-0.427
 
 
14.4
 
 
 
 
BIN3
 
 
hsa-let-7c-5p
 
 
-0.427
 
 
14.4
 
 
 
 
BLOC1S4
 
 
hsa-miR-7-5p
 
 
-0.417
 
 
13.5
 
 
 
 
BLOC1S4
 
 
hsa-miR-7-5p
 
 
-0.527
 
 
13.5
 
 
 
 
BLOC1S6
 
 
hsa-miR-98-5p
 
 
-0.321
 
 
26.3
 
 
 
 
BLOC1S6
 
 
hsa-let-7c-5p
 
 
-0.321
 
 
26.3
 
 
 
 
BNIP2
 
 
hsa-miR-194-5p
 
 
-0.489
 
 
39.0
 
 
 
 
BNIP3L
 
 
hsa-miR-20a-5p
 
 
-0.287
 
 
38.3
 
 
 
 
BRWD1
 
 
hsa-miR-18a-5p
 
 
-0.29
 
 
10.3
 
 
 
 
BTBD10
 
 
hsa-miR-17-5p
 
 
-0.409
 
 
11.8
 
 
 
 
 
 
 lymph node 
 
 
 
 
target_symbol
 
 
mature_mirna_id
 
 
score
 
 
nTPM
 
 
 
 
 
 
ADD3
 
 
hsa-miR-200b-3p
 
 
-0.324
 
 
66.3
 
 
 
 
ADD3
 
 
hsa-miR-18a-5p
 
 
-0.39
 
 
66.3
 
 
 
 
ADNP
 
 
hsa-miR-328-3p
 
 
-0.371
 
 
27.0
 
 
 
 
AEBP2
 
 
hsa-miR-18a-5p
 
 
-0.376
 
 
25.9
 
 
 
 
AHNAK
 
 
hsa-miR-20a-5p
 
 
-0.316
 
 
34.4
 
 
 
 
AHNAK
 
 
hsa-miR-17-5p
 
 
-0.337
 
 
34.4
 
 
 
 
AICDA
 
 
hsa-miR-155-5p
 
 
-0.389
 
 
10.5
 
 
 
 
AIFM1
 
 
hsa-miR-98-5p
 
 
-0.314
 
 
15.1
 
 
 
 
AIFM1
 
 
hsa-let-7c-5p
 
 
-0.314
 
 
15.1
 
 
 
 
ALG3
 
 
hsa-miR-20a-5p
 
 
-0.559
 
 
12.2
 
 
 
 
ALG3
 
 
hsa-miR-17-5p
 
 
-0.572
 
 
12.2
 
 
 
 
ANKFY1
 
 
hsa-miR-155-5p
 
 
-0.315
 
 
13.1
 
 
 
 
ANKRD12
 
 
hsa-miR-7-5p
 
 
-0.37
 
 
20.9
 
 
 
 
ANKRD29
 
 
hsa-miR-20a-5p
 
 
-0.359
 
 
18.8
 
 
 
 
ANKRD29
 
 
hsa-miR-17-5p
 
 
-0.372
 
 
18.8
 
 
 
 
ANP32B
 
 
hsa-miR-200b-3p
 
 
-0.287
 
 
207.9
 
 
 
 
AP1S1
 
 
hsa-miR-98-5p
 
 
-0.374
 
 
11.1
 
 
 
 
AP1S1
 
 
hsa-let-7c-5p
 
 
-0.374
 
 
11.1
 
 
 
 
AP1S2
 
 
hsa-miR-200b-3p
 
 
-0.4
 
 
30.3
 
 
 
 
AP2M1
 
 
hsa-miR-423-5p
 
 
-0.49
 
 
126.9
 
 
 
 
APOO
 
 
hsa-miR-200b-3p
 
 
-0.361
 
 
10.9
 
 
 
 
ARF4
 
 
hsa-miR-194-5p
 
 
-0.293
 
 
69.1
 
 
 
 
ARF4
 
 
hsa-miR-7-5p
 
 
-0.407
 
 
69.1
 
 
 
 
ARHGAP1
 
 
hsa-miR-17-5p
 
 
-0.325
 
 
28.7
 
 
 
 
ARHGAP1
 
 
hsa-miR-20a-5p
 
 
-0.339
 
 
28.7
 
 
 
 
ARID4A
 
 
hsa-miR-7-5p
 
 
-0.341
 
 
14.0
 
 
 
 
ARID4B
 
 
hsa-miR-20a-5p
 
 
-0.29
 
 
19.6
 
 
 
 
ARL1
 
 
hsa-miR-20a-5p
 
 
-0.403
 
 
31.0
 
 
 
 
ARL1
 
 
hsa-miR-17-5p
 
 
-0.432
 
 
31.0
 
 
 
 
ARL6IP1
 
 
hsa-miR-7-5p
 
 
-0.374
 
 
124.0
 
 
 
 
ARL6IP1
 
 
hsa-miR-328-3p
 
 
-0.494
 
 
124.0
 
 
 
 
ARL6IP6
 
 
hsa-miR-98-5p
 
 
-0.293
 
 
17.4
 
 
 
 
ARL6IP6
 
 
hsa-let-7c-5p
 
 
-0.293
 
 
17.4
 
 
 
 
ARMC8
 
 
hsa-miR-20a-5p
 
 
-0.357
 
 
19.8
 
 
 
 
ARMC8
 
 
hsa-miR-17-5p
 
 
-0.378
 
 
19.8
 
 
 
 
ARMT1
 
 
hsa-miR-98-5p
 
 
-0.303
 
 
19.7
 
 
 
 
ARMT1
 
 
hsa-let-7c-5p
 
 
-0.303
 
 
19.7
 
 
 
 
ARPP19
 
 
hsa-miR-328-3p
 
 
-0.404
 
 
45.1
 
 
 
 
ARPP19
 
 
hsa-let-7c-5p
 
 
-0.492
 
 
45.1
 
 
 
 
ARPP19
 
 
hsa-miR-98-5p
 
 
-0.5
 
 
45.1
 
 
 
 
ASAP1
 
 
hsa-miR-98-5p
 
 
-0.308
 
 
16.5
 
 
 
 
ASAP1
 
 
hsa-let-7c-5p
 
 
-0.308
 
 
16.5
 
 
 
 
ASB6
 
 
hsa-miR-423-5p
 
 
-0.388
 
 
10.7
 
 
 
 
ASF1A
 
 
hsa-miR-200b-3p
 
 
-0.377
 
 
14.9
 
 
 
 
ATAD2
 
 
hsa-miR-17-5p
 
 
-0.304
 
 
13.4
 
 
 
 
ATAD2
 
 
hsa-miR-20a-5p
 
 
-0.318
 
 
13.4
 
 
 
 
ATG13
 
 
hsa-miR-423-5p
 
 
-0.3
 
 
29.5
 
 
 
 
ATM
 
 
hsa-miR-18a-5p
 
 
-0.347
 
 
25.8
 
 
 
 
ATP2A2
 
 
hsa-miR-98-5p
 
 
-0.311
 
 
33.7
 
 
 
 
ATP2A2
 
 
hsa-let-7c-5p
 
 
-0.311
 
 
33.7
 
 
 
 
ATP6V1F
 
 
hsa-miR-194-5p
 
 
-0.444
 
 
130.8
 
 
 
 
ATP6V1H
 
 
hsa-miR-194-5p
 
 
-0.652
 
 
20.0
 
 
 
 
ATP8B2
 
 
hsa-miR-423-5p
 
 
-0.297
 
 
24.4
 
 
 
 
ATPAF1
 
 
hsa-miR-98-5p
 
 
-0.498
 
 
30.3
 
 
 
 
ATPAF1
 
 
hsa-let-7c-5p
 
 
-0.498
 
 
30.3
 
 
 
 
B3GAT3
 
 
hsa-miR-98-5p
 
 
-0.324
 
 
24.9
 
 
 
 
B3GAT3
 
 
hsa-let-7c-5p
 
 
-0.324
 
 
24.9
 
 
 
 
BACH1
 
 
hsa-miR-98-5p
 
 
-0.298
 
 
15.7
 
 
 
 
BACH1
 
 
hsa-miR-98-5p
 
 
-0.298
 
 
15.7
 
 
 
 
BACH1
 
 
hsa-let-7c-5p
 
 
-0.306
 
 
15.7
 
 
 
 
BACH1
 
 
hsa-let-7c-5p
 
 
-0.306
 
 
15.7
 
 
 
 
BACH1
 
 
hsa-miR-98-5p
 
 
-0.435
 
 
15.7
 
 
 
 
BACH1
 
 
hsa-miR-98-5p
 
 
-0.435
 
 
15.7
 
 
 
 
BACH1
 
 
hsa-let-7c-5p
 
 
-0.435
 
 
15.7
 
 
 
 
BACH1
 
 
hsa-let-7c-5p
 
 
-0.435
 
 
15.7
 
 
 
 
BAG6
 
 
hsa-miR-200b-3p
 
 
-0.371
 
 
24.3
 
 
 
 
BAP1
 
 
hsa-miR-423-5p
 
 
-0.428
 
 
27.6
 
 
 
 
BBX
 
 
hsa-miR-18a-5p
 
 
-0.31
 
 
13.0
 
 
 
 
BIN1
 
 
hsa-miR-744-5p
 
 
-0.654
 
 
38.4
 
 
 
 
BIN3
 
 
hsa-miR-98-5p
 
 
-0.427
 
 
12.8
 
 
 
 
BIN3
 
 
hsa-let-7c-5p
 
 
-0.427
 
 
12.8
 
 
 
 
BLOC1S4
 
 
hsa-miR-7-5p
 
 
-0.417
 
 
11.4
 
 
 
 
BLOC1S4
 
 
hsa-miR-7-5p
 
 
-0.527
 
 
11.4
 
 
 
 
BLOC1S6
 
 
hsa-miR-98-5p
 
 
-0.321
 
 
39.2
 
 
 
 
BLOC1S6
 
 
hsa-let-7c-5p
 
 
-0.321
 
 
39.2
 
 
 
 
BNIP2
 
 
hsa-miR-194-5p
 
 
-0.489
 
 
42.5
 
 
 
 
BNIP3L
 
 
hsa-miR-20a-5p
 
 
-0.287
 
 
36.9
 
 
 
 
BRWD1
 
 
hsa-miR-18a-5p
 
 
-0.29
 
 
14.7
 
 
 
 
BTBD10
 
 
hsa-miR-17-5p
 
 
-0.409
 
 
14.7
 
 
 
 
BTBD10
 
 
hsa-miR-20a-5p
 
 
-0.412
 
 
14.7
 
 
 
 
BTF3L4
 
 
hsa-miR-200b-3p
 
 
-0.294
 
 
34.2
 
 
 
 
BTF3L4
 
 
hsa-miR-194-5p
 
 
-0.334
 
 
34.2
 
 
 
 
BTF3L4
 
 
hsa-miR-98-5p
 
 
-0.355
 
 
34.2
 
 
 
 
BTF3L4
 
 
hsa-let-7c-5p
 
 
-0.355
 
 
34.2
 
 
 
 
BTG3
 
 
hsa-miR-18a-5p
 
 
-0.464
 
 
13.0
 
 
 
 
BTG3
 
 
hsa-miR-17-5p
 
 
-0.668
 
 
13.0
 
 
 
 
BTG3
 
 
hsa-miR-20a-5p
 
 
-0.671
 
 
13.0
 
 
 
 
BZW1
 
 
hsa-miR-98-5p
 
 
-0.354
 
 
91.4
 
 
 
 
BZW1
 
 
hsa-let-7c-5p
 
 
-0.354
 
 
91.4
 
 
 
 
BZW1
 
 
hsa-miR-98-5p
 
 
-0.375
 
 
91.4
 
 
 
 
BZW1
 
 
hsa-let-7c-5p
 
 
-0.375
 
 
91.4
 
 
 
 
BZW2
 
 
hsa-miR-98-5p
 
 
-0.397
 
 
28.0
 
 
 
 
BZW2
 
 
hsa-let-7c-5p
 
 
-0.397
 
 
28.0
 
 
 
 
C15orf39
 
 
hsa-miR-98-5p
 
 
-0.374
 
 
10.2
 
 
 
 
C15orf39
 
 
hsa-let-7c-5p
 
 
-0.374
 
 
10.2
 
 
 
 
C1GALT1
 
 
hsa-let-7c-5p
 
 
-0.322
 
 
17.8
 
 
 
 
C1GALT1
 
 
hsa-miR-98-5p
 
 
-0.33
 
 
17.8
 
 
 
 
C1QBP
 
 
hsa-miR-330-3p
 
 
-0.412
 
 
108.0
 
 
 
 
C21orf91
 
 
hsa-miR-194-5p
 
 
-0.293
 
 
14.4
 
 
 
 
C6orf120
 
 
hsa-miR-20a-5p
 
 
-0.298
 
 
14.2
 
 
 
 
 
 
 
 cd4_ev_vs_cd4_s1_upregulated 
 
 Tissue expression heatmap (log10-transformed) 
   
 
 
 liver 
 
 
 
 
target_symbol
 
 
mature_mirna_id
 
 
score
 
 
nTPM
 
 
 
 
 
 
A1BG
 
 
hsa-miR-23a-3p
 
 
-0.434
 
 
565.1
 
 
 
 
A1BG
 
 
hsa-miR-23b-3p
 
 
-0.434
 
 
565.1
 
 
 
 
AAR2
 
 
hsa-miR-29a-3p
 
 
-0.327
 
 
22.0
 
 
 
 
ABCB6
 
 
hsa-miR-29a-3p
 
 
-0.48
 
 
12.7
 
 
 
 
ABCE1
 
 
hsa-miR-29a-3p
 
 
-0.404
 
 
37.1
 
 
 
 
ABHD17B
 
 
hsa-miR-486-5p
 
 
-0.348
 
 
11.3
 
 
 
 
ABHD4
 
 
hsa-miR-29a-3p
 
 
-0.312
 
 
24.2
 
 
 
 
ABHD5
 
 
hsa-miR-132-3p
 
 
-0.323
 
 
20.3
 
 
 
 
ACTR2
 
 
hsa-miR-15b-5p
 
 
-0.293
 
 
101.7
 
 
 
 
ACTR3C
 
 
hsa-miR-30b-5p
 
 
-0.448
 
 
18.8
 
 
 
 
ACVR1
 
 
hsa-miR-30b-5p
 
 
-0.29
 
 
15.3
 
 
 
 
ADAM9
 
 
hsa-miR-30b-5p
 
 
-0.312
 
 
12.7
 
 
 
 
ADGRA3
 
 
hsa-miR-30b-5p
 
 
-0.304
 
 
54.5
 
 
 
 
ADIPOR2
 
 
hsa-miR-150-5p
 
 
-0.287
 
 
77.7
 
 
 
 
ADRB2
 
 
hsa-miR-15b-5p
 
 
-0.29
 
 
13.3
 
 
 
 
AFG3L2
 
 
hsa-miR-181b-5p
 
 
-0.491
 
 
25.9
 
 
 
 
AFG3L2
 
 
hsa-miR-181a-5p
 
 
-0.491
 
 
25.9
 
 
 
 
AHR
 
 
hsa-miR-29a-3p
 
 
-0.316
 
 
42.0
 
 
 
 
AK1
 
 
hsa-miR-29a-3p
 
 
-0.333
 
 
35.8
 
 
 
 
AK4
 
 
hsa-miR-15b-5p
 
 
-0.316
 
 
95.6
 
 
 
 
ALAD
 
 
hsa-miR-15b-5p
 
 
-0.446
 
 
196.1
 
 
 
 
ANGPTL2
 
 
hsa-miR-221-3p
 
 
-0.369
 
 
13.8
 
 
 
 
ANKFY1
 
 
hsa-miR-155-5p
 
 
-0.315
 
 
13.0
 
 
 
 
ANKHD1
 
 
hsa-miR-23a-3p
 
 
-0.368
 
 
51.5
 
 
 
 
ANKHD1
 
 
hsa-miR-23b-3p
 
 
-0.368
 
 
51.5
 
 
 
 
ANKHD1
 
 
hsa-miR-30b-5p
 
 
-0.405
 
 
51.5
 
 
 
 
ANKRA2
 
 
hsa-miR-30b-5p
 
 
-0.354
 
 
10.1
 
 
 
 
ANKRA2
 
 
hsa-miR-30b-5p
 
 
-0.472
 
 
10.1
 
 
 
 
ANKRD17
 
 
hsa-miR-30b-5p
 
 
-0.336
 
 
19.3
 
 
 
 
ANP32A
 
 
hsa-miR-132-3p
 
 
-0.374
 
 
71.3
 
 
 
 
ANP32E
 
 
hsa-miR-501-3p
 
 
-0.331
 
 
27.1
 
 
 
 
ANTXR2
 
 
hsa-miR-29a-3p
 
 
-0.347
 
 
32.2
 
 
 
 
ANXA11
 
 
hsa-miR-15b-5p
 
 
-0.463
 
 
46.7
 
 
 
 
AP2A1
 
 
hsa-miR-15b-5p
 
 
-0.333
 
 
48.9
 
 
 
 
AP2B1
 
 
hsa-miR-15b-5p
 
 
-0.334
 
 
32.8
 
 
 
 
AP3S1
 
 
hsa-miR-30b-5p
 
 
-0.293
 
 
69.8
 
 
 
 
APOO
 
 
hsa-miR-181b-5p
 
 
-0.5
 
 
17.0
 
 
 
 
APOO
 
 
hsa-miR-181a-5p
 
 
-0.5
 
 
17.0
 
 
 
 
APP
 
 
hsa-miR-15b-5p
 
 
-0.298
 
 
251.5
 
 
 
 
AQP11
 
 
hsa-miR-15b-5p
 
 
-0.769
 
 
15.1
 
 
 
 
AQP3
 
 
hsa-miR-874-3p
 
 
-0.48
 
 
117.4
 
 
 
 
ARF4
 
 
hsa-miR-30b-5p
 
 
-0.289
 
 
161.3
 
 
 
 
ARF4
 
 
hsa-miR-221-3p
 
 
-0.553
 
 
161.3
 
 
 
 
ARF5
 
 
hsa-miR-29a-3p
 
 
-0.491
 
 
153.7
 
 
 
 
ARF6
 
 
hsa-miR-181b-5p
 
 
-0.33
 
 
45.4
 
 
 
 
ARF6
 
 
hsa-miR-181a-5p
 
 
-0.33
 
 
45.4
 
 
 
 
ARHGAP5
 
 
hsa-miR-486-5p
 
 
-0.3
 
 
29.8
 
 
 
 
ARID4B
 
 
hsa-miR-486-5p
 
 
-0.367
 
 
11.1
 
 
 
 
ARL2
 
 
hsa-miR-15b-5p
 
 
-0.374
 
 
22.7
 
 
 
 
ARL2
 
 
hsa-miR-15b-5p
 
 
-0.442
 
 
22.7
 
 
 
 
ARL4A
 
 
hsa-miR-29a-3p
 
 
-0.386
 
 
14.6
 
 
 
 
ARL4A
 
 
hsa-miR-30b-5p
 
 
-0.448
 
 
14.6
 
 
 
 
ARL5B
 
 
hsa-miR-29a-3p
 
 
-0.304
 
 
27.9
 
 
 
 
ARL5B
 
 
hsa-miR-155-5p
 
 
-0.355
 
 
27.9
 
 
 
 
ARMC8
 
 
hsa-miR-486-5p
 
 
-0.321
 
 
15.8
 
 
 
 
ARMCX3
 
 
hsa-miR-425-5p
 
 
-0.325
 
 
22.5
 
 
 
 
ARNT
 
 
hsa-miR-221-3p
 
 
-0.465
 
 
40.1
 
 
 
 
ARPP19
 
 
hsa-miR-15b-5p
 
 
-0.346
 
 
39.5
 
 
 
 
ARPP19
 
 
hsa-miR-29a-3p
 
 
-0.371
 
 
39.5
 
 
 
 
ARRDC3
 
 
hsa-miR-29a-3p
 
 
-0.402
 
 
71.0
 
 
 
 
ASB6
 
 
hsa-miR-874-3p
 
 
-0.368
 
 
10.5
 
 
 
 
ASF1A
 
 
hsa-miR-23a-3p
 
 
-0.506
 
 
11.3
 
 
 
 
ASF1A
 
 
hsa-miR-23b-3p
 
 
-0.506
 
 
11.3
 
 
 
 
ASH1L
 
 
hsa-miR-15b-5p
 
 
-0.325
 
 
12.8
 
 
 
 
ASPH
 
 
hsa-miR-221-3p
 
 
-0.287
 
 
69.9
 
 
 
 
ATF3
 
 
hsa-miR-874-3p
 
 
-0.288
 
 
130.6
 
 
 
 
ATG5
 
 
hsa-miR-30b-5p
 
 
-0.488
 
 
16.5
 
 
 
 
ATG9A
 
 
hsa-miR-15b-5p
 
 
-0.333
 
 
28.6
 
 
 
 
ATOSA
 
 
hsa-miR-221-3p
 
 
-0.495
 
 
35.4
 
 
 
 
ATP5MC3
 
 
hsa-miR-425-5p
 
 
-0.328
 
 
371.4
 
 
 
 
ATP5MC3
 
 
hsa-miR-29a-3p
 
 
-0.33
 
 
371.4
 
 
 
 
ATP6V1E1
 
 
hsa-miR-23a-3p
 
 
-0.294
 
 
80.0
 
 
 
 
ATP6V1E1
 
 
hsa-miR-23b-3p
 
 
-0.294
 
 
80.0
 
 
 
 
ATPAF1
 
 
hsa-miR-361-5p
 
 
-0.362
 
 
101.4
 
 
 
 
ATXN2
 
 
hsa-miR-15b-5p
 
 
-0.392
 
 
19.6
 
 
 
 
AUH
 
 
hsa-miR-23a-3p
 
 
-0.495
 
 
24.7
 
 
 
 
AUH
 
 
hsa-miR-23b-3p
 
 
-0.503
 
 
24.7
 
 
 
 
AUH
 
 
hsa-miR-23a-3p
 
 
-0.512
 
 
24.7
 
 
 
 
AUH
 
 
hsa-miR-23b-3p
 
 
-0.512
 
 
24.7
 
 
 
 
AZIN1
 
 
hsa-miR-132-3p
 
 
-0.29
 
 
71.0
 
 
 
 
AZIN1
 
 
hsa-miR-132-3p
 
 
-0.299
 
 
71.0
 
 
 
 
B4GALT5
 
 
hsa-miR-501-3p
 
 
-0.408
 
 
24.1
 
 
 
 
B4GAT1
 
 
hsa-miR-23a-3p
 
 
-0.361
 
 
18.5
 
 
 
 
B4GAT1
 
 
hsa-miR-23b-3p
 
 
-0.361
 
 
18.5
 
 
 
 
BAG4
 
 
hsa-miR-181b-5p
 
 
-0.337
 
 
15.7
 
 
 
 
BAG4
 
 
hsa-miR-181a-5p
 
 
-0.337
 
 
15.7
 
 
 
 
BAK1
 
 
hsa-miR-29a-3p
 
 
-0.409
 
 
10.9
 
 
 
 
BCL2L11
 
 
hsa-miR-221-3p
 
 
-0.423
 
 
12.9
 
 
 
 
BCL2L2
 
 
hsa-miR-29a-3p
 
 
-0.312
 
 
21.5
 
 
 
 
BDH1
 
 
hsa-miR-874-3p
 
 
-0.644
 
 
302.0
 
 
 
 
BECN1
 
 
hsa-miR-30b-5p
 
 
-0.452
 
 
40.8
 
 
 
 
BET1
 
 
hsa-miR-23a-3p
 
 
-0.402
 
 
29.8
 
 
 
 
BET1
 
 
hsa-miR-23b-3p
 
 
-0.418
 
 
29.8
 
 
 
 
BFAR
 
 
hsa-miR-15b-5p
 
 
-0.352
 
 
22.0
 
 
 
 
BHLHE40
 
 
hsa-miR-181b-5p
 
 
-0.408
 
 
194.1
 
 
 
 
BHLHE40
 
 
hsa-miR-181a-5p
 
 
-0.408
 
 
194.1
 
 
 
 
BLCAP
 
 
hsa-miR-23a-3p
 
 
-0.601
 
 
99.5
 
 
 
 
BLCAP
 
 
hsa-miR-23b-3p
 
 
-0.601
 
 
99.5
 
 
 
 
BLMH
 
 
hsa-miR-29a-3p
 
 
-0.393
 
 
13.1
 
 
 
 
BLOC1S6
 
 
hsa-miR-181b-5p
 
 
-0.393
 
 
25.1
 
 
 
 
 
 
 adipose tissue 
 
 
 
 
target_symbol
 
 
mature_mirna_id
 
 
score
 
 
nTPM
 
 
 
 
 
 
AAR2
 
 
hsa-miR-29a-3p
 
 
-0.327
 
 
21.1
 
 
 
 
ABCE1
 
 
hsa-miR-29a-3p
 
 
-0.404
 
 
28.1
 
 
 
 
ABHD4
 
 
hsa-miR-29a-3p
 
 
-0.312
 
 
24.3
 
 
 
 
ABHD5
 
 
hsa-miR-132-3p
 
 
-0.323
 
 
27.9
 
 
 
 
ACTA1
 
 
hsa-miR-155-5p
 
 
-0.595
 
 
13.2
 
 
 
 
ACTC1
 
 
hsa-miR-30b-5p
 
 
-0.543
 
 
47.8
 
 
 
 
ACTR2
 
 
hsa-miR-15b-5p
 
 
-0.293
 
 
105.6
 
 
 
 
ACVR1
 
 
hsa-miR-30b-5p
 
 
-0.29
 
 
12.6
 
 
 
 
ADA
 
 
hsa-miR-29a-3p
 
 
-0.376
 
 
16.5
 
 
 
 
ADAM9
 
 
hsa-miR-30b-5p
 
 
-0.312
 
 
31.0
 
 
 
 
ADAMTS10
 
 
hsa-miR-29a-3p
 
 
-0.516
 
 
13.8
 
 
 
 
ADAMTS9
 
 
hsa-miR-29a-3p
 
 
-0.305
 
 
39.1
 
 
 
 
ADAMTS9
 
 
hsa-miR-29a-3p
 
 
-0.485
 
 
39.1
 
 
 
 
ADCY3
 
 
hsa-miR-132-3p
 
 
-0.303
 
 
90.0
 
 
 
 
ADIPOR2
 
 
hsa-miR-150-5p
 
 
-0.287
 
 
53.2
 
 
 
 
ADRA2A
 
 
hsa-miR-23a-3p
 
 
-0.371
 
 
46.9
 
 
 
 
ADRA2A
 
 
hsa-miR-23b-3p
 
 
-0.371
 
 
46.9
 
 
 
 
ADRB2
 
 
hsa-miR-15b-5p
 
 
-0.29
 
 
26.4
 
 
 
 
AFG3L2
 
 
hsa-miR-181b-5p
 
 
-0.491
 
 
17.1
 
 
 
 
AFG3L2
 
 
hsa-miR-181a-5p
 
 
-0.491
 
 
17.1
 
 
 
 
AHR
 
 
hsa-miR-29a-3p
 
 
-0.316
 
 
21.0
 
 
 
 
AK1
 
 
hsa-miR-29a-3p
 
 
-0.333
 
 
30.1
 
 
 
 
AK4
 
 
hsa-miR-15b-5p
 
 
-0.316
 
 
22.8
 
 
 
 
AKT3
 
 
hsa-miR-29a-3p
 
 
-0.287
 
 
16.9
 
 
 
 
AKT3
 
 
hsa-miR-15b-5p
 
 
-0.301
 
 
16.9
 
 
 
 
ALAD
 
 
hsa-miR-15b-5p
 
 
-0.446
 
 
51.3
 
 
 
 
ALDH1A2
 
 
hsa-miR-23b-3p
 
 
-0.346
 
 
20.6
 
 
 
 
ALDH1A2
 
 
hsa-miR-23a-3p
 
 
-0.354
 
 
20.6
 
 
 
 
AMOTL1
 
 
hsa-miR-15b-5p
 
 
-0.319
 
 
14.5
 
 
 
 
ANGPTL2
 
 
hsa-miR-221-3p
 
 
-0.369
 
 
104.7
 
 
 
 
ANKFY1
 
 
hsa-miR-155-5p
 
 
-0.315
 
 
12.9
 
 
 
 
ANKHD1
 
 
hsa-miR-23a-3p
 
 
-0.368
 
 
37.3
 
 
 
 
ANKHD1
 
 
hsa-miR-23b-3p
 
 
-0.368
 
 
37.3
 
 
 
 
ANKHD1
 
 
hsa-miR-30b-5p
 
 
-0.405
 
 
37.3
 
 
 
 
ANKRD17
 
 
hsa-miR-30b-5p
 
 
-0.336
 
 
12.8
 
 
 
 
ANKRD29
 
 
hsa-miR-132-3p
 
 
-0.47
 
 
17.0
 
 
 
 
ANP32A
 
 
hsa-miR-132-3p
 
 
-0.374
 
 
47.7
 
 
 
 
ANP32E
 
 
hsa-miR-501-3p
 
 
-0.331
 
 
18.8
 
 
 
 
ANTXR2
 
 
hsa-miR-29a-3p
 
 
-0.347
 
 
57.1
 
 
 
 
ANXA11
 
 
hsa-miR-15b-5p
 
 
-0.463
 
 
130.8
 
 
 
 
AP2A1
 
 
hsa-miR-15b-5p
 
 
-0.333
 
 
44.9
 
 
 
 
AP2B1
 
 
hsa-miR-15b-5p
 
 
-0.334
 
 
43.1
 
 
 
 
AP3S1
 
 
hsa-miR-30b-5p
 
 
-0.293
 
 
73.6
 
 
 
 
APP
 
 
hsa-miR-15b-5p
 
 
-0.298
 
 
230.6
 
 
 
 
AQP3
 
 
hsa-miR-874-3p
 
 
-0.48
 
 
10.5
 
 
 
 
ARF4
 
 
hsa-miR-30b-5p
 
 
-0.289
 
 
118.7
 
 
 
 
ARF4
 
 
hsa-miR-221-3p
 
 
-0.553
 
 
118.7
 
 
 
 
ARF5
 
 
hsa-miR-29a-3p
 
 
-0.491
 
 
109.3
 
 
 
 
ARF6
 
 
hsa-miR-181b-5p
 
 
-0.33
 
 
30.0
 
 
 
 
ARF6
 
 
hsa-miR-181a-5p
 
 
-0.33
 
 
30.0
 
 
 
 
ARHGAP5
 
 
hsa-miR-486-5p
 
 
-0.3
 
 
22.2
 
 
 
 
ARID1B
 
 
hsa-miR-29a-3p
 
 
-0.342
 
 
14.1
 
 
 
 
ARID4B
 
 
hsa-miR-486-5p
 
 
-0.367
 
 
10.7
 
 
 
 
ARL2
 
 
hsa-miR-15b-5p
 
 
-0.374
 
 
125.8
 
 
 
 
ARL2
 
 
hsa-miR-15b-5p
 
 
-0.442
 
 
125.8
 
 
 
 
ARL4A
 
 
hsa-miR-29a-3p
 
 
-0.386
 
 
30.8
 
 
 
 
ARL4A
 
 
hsa-miR-30b-5p
 
 
-0.448
 
 
30.8
 
 
 
 
ARL5B
 
 
hsa-miR-29a-3p
 
 
-0.304
 
 
10.5
 
 
 
 
ARL5B
 
 
hsa-miR-155-5p
 
 
-0.355
 
 
10.5
 
 
 
 
ARMC8
 
 
hsa-miR-486-5p
 
 
-0.321
 
 
19.5
 
 
 
 
ARMCX2
 
 
hsa-miR-15b-5p
 
 
-0.344
 
 
15.0
 
 
 
 
ARMCX3
 
 
hsa-miR-425-5p
 
 
-0.325
 
 
32.7
 
 
 
 
ARNT
 
 
hsa-miR-221-3p
 
 
-0.465
 
 
25.2
 
 
 
 
ARPP19
 
 
hsa-miR-15b-5p
 
 
-0.346
 
 
57.4
 
 
 
 
ARPP19
 
 
hsa-miR-29a-3p
 
 
-0.371
 
 
57.4
 
 
 
 
ARRDC3
 
 
hsa-miR-29a-3p
 
 
-0.402
 
 
34.5
 
 
 
 
ASAP2
 
 
hsa-miR-29a-3p
 
 
-0.348
 
 
11.6
 
 
 
 
ASB6
 
 
hsa-miR-874-3p
 
 
-0.368
 
 
10.3
 
 
 
 
ASH1L
 
 
hsa-miR-15b-5p
 
 
-0.325
 
 
19.3
 
 
 
 
ASPH
 
 
hsa-miR-221-3p
 
 
-0.287
 
 
362.0
 
 
 
 
ATF3
 
 
hsa-miR-874-3p
 
 
-0.288
 
 
168.3
 
 
 
 
ATG9A
 
 
hsa-miR-15b-5p
 
 
-0.333
 
 
24.0
 
 
 
 
ATOSA
 
 
hsa-miR-221-3p
 
 
-0.495
 
 
29.6
 
 
 
 
ATP5MC3
 
 
hsa-miR-425-5p
 
 
-0.328
 
 
189.1
 
 
 
 
ATP5MC3
 
 
hsa-miR-29a-3p
 
 
-0.33
 
 
189.1
 
 
 
 
ATP6V1E1
 
 
hsa-miR-23a-3p
 
 
-0.294
 
 
119.7
 
 
 
 
ATP6V1E1
 
 
hsa-miR-23b-3p
 
 
-0.294
 
 
119.7
 
 
 
 
ATPAF1
 
 
hsa-miR-361-5p
 
 
-0.362
 
 
81.3
 
 
 
 
ATXN1L
 
 
hsa-miR-155-5p
 
 
-0.315
 
 
13.1
 
 
 
 
ATXN2
 
 
hsa-miR-15b-5p
 
 
-0.392
 
 
19.0
 
 
 
 
AUH
 
 
hsa-miR-23a-3p
 
 
-0.495
 
 
16.1
 
 
 
 
AUH
 
 
hsa-miR-23b-3p
 
 
-0.503
 
 
16.1
 
 
 
 
AUH
 
 
hsa-miR-23a-3p
 
 
-0.512
 
 
16.1
 
 
 
 
AUH
 
 
hsa-miR-23b-3p
 
 
-0.512
 
 
16.1
 
 
 
 
AZIN1
 
 
hsa-miR-132-3p
 
 
-0.29
 
 
56.6
 
 
 
 
AZIN1
 
 
hsa-miR-132-3p
 
 
-0.299
 
 
56.6
 
 
 
 
B3GNT5
 
 
hsa-miR-30b-5p
 
 
-0.326
 
 
11.3
 
 
 
 
B4GALT5
 
 
hsa-miR-501-3p
 
 
-0.408
 
 
26.8
 
 
 
 
B4GAT1
 
 
hsa-miR-23a-3p
 
 
-0.361
 
 
16.2
 
 
 
 
B4GAT1
 
 
hsa-miR-23b-3p
 
 
-0.361
 
 
16.2
 
 
 
 
BCL2
 
 
hsa-miR-342-3p
 
 
-0.428
 
 
12.0
 
 
 
 
BCL2L2
 
 
hsa-miR-29a-3p
 
 
-0.312
 
 
44.8
 
 
 
 
BECN1
 
 
hsa-miR-30b-5p
 
 
-0.452
 
 
42.5
 
 
 
 
BET1
 
 
hsa-miR-23a-3p
 
 
-0.402
 
 
17.0
 
 
 
 
BET1
 
 
hsa-miR-23b-3p
 
 
-0.418
 
 
17.0
 
 
 
 
BFAR
 
 
hsa-miR-15b-5p
 
 
-0.352
 
 
35.1
 
 
 
 
BHLHE40
 
 
hsa-miR-181b-5p
 
 
-0.408
 
 
162.3
 
 
 
 
BHLHE40
 
 
hsa-miR-181a-5p
 
 
-0.408
 
 
162.3
 
 
 
 
BICDL1
 
 
hsa-miR-221-3p
 
 
-0.447
 
 
19.3
 
 
 
 
BLCAP
 
 
hsa-miR-23a-3p
 
 
-0.601
 
 
63.7
 
 
 
 
 
 
 adrenal gland 
 
 
 
 
target_symbol
 
 
mature_mirna_id
 
 
score
 
 
nTPM
 
 
 
 
 
 
AAR2
 
 
hsa-miR-29a-3p
 
 
-0.327
 
 
24.5
 
 
 
 
ABCE1
 
 
hsa-miR-29a-3p
 
 
-0.404
 
 
20.3
 
 
 
 
ABHD4
 
 
hsa-miR-29a-3p
 
 
-0.312
 
 
29.6
 
 
 
 
ACTR2
 
 
hsa-miR-15b-5p
 
 
-0.293
 
 
66.7
 
 
 
 
ACTR3C
 
 
hsa-miR-30b-5p
 
 
-0.448
 
 
10.1
 
 
 
 
ADAM9
 
 
hsa-miR-30b-5p
 
 
-0.312
 
 
19.6
 
 
 
 
ADCY3
 
 
hsa-miR-132-3p
 
 
-0.303
 
 
16.9
 
 
 
 
ADIPOR2
 
 
hsa-miR-150-5p
 
 
-0.287
 
 
69.8
 
 
 
 
AFG3L2
 
 
hsa-miR-181b-5p
 
 
-0.491
 
 
12.9
 
 
 
 
AFG3L2
 
 
hsa-miR-181a-5p
 
 
-0.491
 
 
12.9
 
 
 
 
AHR
 
 
hsa-miR-29a-3p
 
 
-0.316
 
 
14.2
 
 
 
 
AK1
 
 
hsa-miR-29a-3p
 
 
-0.333
 
 
40.4
 
 
 
 
ALAD
 
 
hsa-miR-15b-5p
 
 
-0.446
 
 
148.4
 
 
 
 
ANGPTL2
 
 
hsa-miR-221-3p
 
 
-0.369
 
 
10.9
 
 
 
 
ANKFY1
 
 
hsa-miR-155-5p
 
 
-0.315
 
 
15.3
 
 
 
 
ANKHD1
 
 
hsa-miR-23a-3p
 
 
-0.368
 
 
41.3
 
 
 
 
ANKHD1
 
 
hsa-miR-23b-3p
 
 
-0.368
 
 
41.3
 
 
 
 
ANKHD1
 
 
hsa-miR-30b-5p
 
 
-0.405
 
 
41.3
 
 
 
 
ANKRA2
 
 
hsa-miR-30b-5p
 
 
-0.354
 
 
10.7
 
 
 
 
ANKRA2
 
 
hsa-miR-30b-5p
 
 
-0.472
 
 
10.7
 
 
 
 
ANKRD17
 
 
hsa-miR-30b-5p
 
 
-0.336
 
 
12.7
 
 
 
 
ANP32A
 
 
hsa-miR-132-3p
 
 
-0.374
 
 
32.7
 
 
 
 
ANP32E
 
 
hsa-miR-501-3p
 
 
-0.331
 
 
28.7
 
 
 
 
ANTXR2
 
 
hsa-miR-29a-3p
 
 
-0.347
 
 
28.3
 
 
 
 
ANXA11
 
 
hsa-miR-15b-5p
 
 
-0.463
 
 
28.3
 
 
 
 
AP2A1
 
 
hsa-miR-15b-5p
 
 
-0.333
 
 
83.5
 
 
 
 
AP2B1
 
 
hsa-miR-15b-5p
 
 
-0.334
 
 
39.3
 
 
 
 
AP3S1
 
 
hsa-miR-30b-5p
 
 
-0.293
 
 
42.4
 
 
 
 
APOO
 
 
hsa-miR-181b-5p
 
 
-0.5
 
 
22.6
 
 
 
 
APOO
 
 
hsa-miR-181a-5p
 
 
-0.5
 
 
22.6
 
 
 
 
APP
 
 
hsa-miR-15b-5p
 
 
-0.298
 
 
422.0
 
 
 
 
AQP11
 
 
hsa-miR-15b-5p
 
 
-0.769
 
 
14.1
 
 
 
 
ARF4
 
 
hsa-miR-30b-5p
 
 
-0.289
 
 
151.0
 
 
 
 
ARF4
 
 
hsa-miR-221-3p
 
 
-0.553
 
 
151.0
 
 
 
 
ARF5
 
 
hsa-miR-29a-3p
 
 
-0.491
 
 
102.3
 
 
 
 
ARF6
 
 
hsa-miR-181b-5p
 
 
-0.33
 
 
24.5
 
 
 
 
ARF6
 
 
hsa-miR-181a-5p
 
 
-0.33
 
 
24.5
 
 
 
 
ARHGAP5
 
 
hsa-miR-486-5p
 
 
-0.3
 
 
25.8
 
 
 
 
ARID1B
 
 
hsa-miR-29a-3p
 
 
-0.342
 
 
10.7
 
 
 
 
ARID4B
 
 
hsa-miR-486-5p
 
 
-0.367
 
 
10.2
 
 
 
 
ARL2
 
 
hsa-miR-15b-5p
 
 
-0.374
 
 
94.8
 
 
 
 
ARL2
 
 
hsa-miR-15b-5p
 
 
-0.442
 
 
94.8
 
 
 
 
ARL5B
 
 
hsa-miR-29a-3p
 
 
-0.304
 
 
19.6
 
 
 
 
ARL5B
 
 
hsa-miR-155-5p
 
 
-0.355
 
 
19.6
 
 
 
 
ARMC8
 
 
hsa-miR-486-5p
 
 
-0.321
 
 
13.5
 
 
 
 
ARMCX2
 
 
hsa-miR-15b-5p
 
 
-0.344
 
 
42.3
 
 
 
 
ARMCX3
 
 
hsa-miR-425-5p
 
 
-0.325
 
 
55.6
 
 
 
 
ARNT
 
 
hsa-miR-221-3p
 
 
-0.465
 
 
19.8
 
 
 
 
ARPP19
 
 
hsa-miR-15b-5p
 
 
-0.346
 
 
48.8
 
 
 
 
ARPP19
 
 
hsa-miR-29a-3p
 
 
-0.371
 
 
48.8
 
 
 
 
ARRDC3
 
 
hsa-miR-29a-3p
 
 
-0.402
 
 
75.7
 
 
 
 
ASB3
 
 
hsa-miR-30b-5p
 
 
-0.418
 
 
10.8
 
 
 
 
ASB3
 
 
hsa-miR-30b-5p
 
 
-0.418
 
 
10.8
 
 
 
 
ASB6
 
 
hsa-miR-874-3p
 
 
-0.368
 
 
18.8
 
 
 
 
ASH1L
 
 
hsa-miR-15b-5p
 
 
-0.325
 
 
16.2
 
 
 
 
ASPH
 
 
hsa-miR-221-3p
 
 
-0.287
 
 
150.8
 
 
 
 
ATF3
 
 
hsa-miR-874-3p
 
 
-0.288
 
 
77.8
 
 
 
 
ATG5
 
 
hsa-miR-30b-5p
 
 
-0.488
 
 
15.5
 
 
 
 
ATG9A
 
 
hsa-miR-15b-5p
 
 
-0.333
 
 
25.3
 
 
 
 
ATOSA
 
 
hsa-miR-221-3p
 
 
-0.495
 
 
17.4
 
 
 
 
ATP5MC3
 
 
hsa-miR-425-5p
 
 
-0.328
 
 
190.4
 
 
 
 
ATP5MC3
 
 
hsa-miR-29a-3p
 
 
-0.33
 
 
190.4
 
 
 
 
ATP6V1E1
 
 
hsa-miR-23a-3p
 
 
-0.294
 
 
163.9
 
 
 
 
ATP6V1E1
 
 
hsa-miR-23b-3p
 
 
-0.294
 
 
163.9
 
 
 
 
ATPAF1
 
 
hsa-miR-361-5p
 
 
-0.362
 
 
98.1
 
 
 
 
ATXN2
 
 
hsa-miR-15b-5p
 
 
-0.392
 
 
24.4
 
 
 
 
AUH
 
 
hsa-miR-23a-3p
 
 
-0.495
 
 
39.5
 
 
 
 
AUH
 
 
hsa-miR-23b-3p
 
 
-0.503
 
 
39.5
 
 
 
 
AUH
 
 
hsa-miR-23a-3p
 
 
-0.512
 
 
39.5
 
 
 
 
AUH
 
 
hsa-miR-23b-3p
 
 
-0.512
 
 
39.5
 
 
 
 
AXIN2
 
 
hsa-miR-15b-5p
 
 
-0.3
 
 
10.1
 
 
 
 
AZIN1
 
 
hsa-miR-132-3p
 
 
-0.29
 
 
44.5
 
 
 
 
AZIN1
 
 
hsa-miR-132-3p
 
 
-0.299
 
 
44.5
 
 
 
 
B4GALT5
 
 
hsa-miR-501-3p
 
 
-0.408
 
 
44.9
 
 
 
 
B4GAT1
 
 
hsa-miR-23a-3p
 
 
-0.361
 
 
39.1
 
 
 
 
B4GAT1
 
 
hsa-miR-23b-3p
 
 
-0.361
 
 
39.1
 
 
 
 
BAK1
 
 
hsa-miR-29a-3p
 
 
-0.409
 
 
27.9
 
 
 
 
BCL2L2
 
 
hsa-miR-29a-3p
 
 
-0.312
 
 
37.7
 
 
 
 
BECN1
 
 
hsa-miR-30b-5p
 
 
-0.452
 
 
39.2
 
 
 
 
BET1
 
 
hsa-miR-23a-3p
 
 
-0.402
 
 
26.6
 
 
 
 
BET1
 
 
hsa-miR-23b-3p
 
 
-0.418
 
 
26.6
 
 
 
 
BFAR
 
 
hsa-miR-15b-5p
 
 
-0.352
 
 
23.6
 
 
 
 
BHLHE40
 
 
hsa-miR-181b-5p
 
 
-0.408
 
 
109.5
 
 
 
 
BHLHE40
 
 
hsa-miR-181a-5p
 
 
-0.408
 
 
109.5
 
 
 
 
BLCAP
 
 
hsa-miR-23a-3p
 
 
-0.601
 
 
55.2
 
 
 
 
BLCAP
 
 
hsa-miR-23b-3p
 
 
-0.601
 
 
55.2
 
 
 
 
BLMH
 
 
hsa-miR-29a-3p
 
 
-0.393
 
 
13.1
 
 
 
 
BLOC1S6
 
 
hsa-miR-181b-5p
 
 
-0.393
 
 
29.4
 
 
 
 
BLOC1S6
 
 
hsa-miR-181a-5p
 
 
-0.393
 
 
29.4
 
 
 
 
BOLA3
 
 
hsa-miR-15b-5p
 
 
-0.363
 
 
41.4
 
 
 
 
BOLA3
 
 
hsa-miR-15b-5p
 
 
-0.386
 
 
41.4
 
 
 
 
BOLA3
 
 
hsa-miR-374b-5p
 
 
-0.486
 
 
41.4
 
 
 
 
BORCS6
 
 
hsa-miR-15b-5p
 
 
-0.347
 
 
11.6
 
 
 
 
BRI3
 
 
hsa-miR-132-3p
 
 
-0.385
 
 
142.2
 
 
 
 
BTAF1
 
 
hsa-miR-486-5p
 
 
-0.288
 
 
11.3
 
 
 
 
BTAF1
 
 
hsa-miR-15b-5p
 
 
-0.496
 
 
11.3
 
 
 
 
BTF3
 
 
hsa-miR-15b-5p
 
 
-0.344
 
 
426.2
 
 
 
 
BTF3
 
 
hsa-miR-361-5p
 
 
-0.551
 
 
426.2
 
 
 
 
BTG2
 
 
hsa-miR-29a-3p
 
 
-0.32
 
 
231.0
 
 
 
 
BTN2A1
 
 
hsa-miR-342-3p
 
 
-0.31
 
 
17.2
 
 
 
 
 
 
 amygdala 
 
 
 
 
target_symbol
 
 
mature_mirna_id
 
 
score
 
 
nTPM
 
 
 
 
 
 
AAR2
 
 
hsa-miR-29a-3p
 
 
-0.327
 
 
10.0
 
 
 
 
ABCE1
 
 
hsa-miR-29a-3p
 
 
-0.404
 
 
11.9
 
 
 
 
ABHD17B
 
 
hsa-miR-486-5p
 
 
-0.348
 
 
12.7
 
 
 
 
ABHD4
 
 
hsa-miR-29a-3p
 
 
-0.312
 
 
16.0
 
 
 
 
ACTR2
 
 
hsa-miR-15b-5p
 
 
-0.293
 
 
53.7
 
 
 
 
ADCY3
 
 
hsa-miR-132-3p
 
 
-0.303
 
 
27.0
 
 
 
 
ADGRA3
 
 
hsa-miR-30b-5p
 
 
-0.304
 
 
19.9
 
 
 
 
ADIPOR2
 
 
hsa-miR-150-5p
 
 
-0.287
 
 
43.7
 
 
 
 
AGPAT4
 
 
hsa-miR-29a-3p
 
 
-0.376
 
 
10.0
 
 
 
 
AK1
 
 
hsa-miR-29a-3p
 
 
-0.333
 
 
104.5
 
 
 
 
AK4
 
 
hsa-miR-15b-5p
 
 
-0.316
 
 
19.8
 
 
 
 
AKT3
 
 
hsa-miR-29a-3p
 
 
-0.287
 
 
17.4
 
 
 
 
AKT3
 
 
hsa-miR-15b-5p
 
 
-0.301
 
 
17.4
 
 
 
 
ALAD
 
 
hsa-miR-15b-5p
 
 
-0.446
 
 
58.3
 
 
 
 
AMOT
 
 
hsa-miR-29a-3p
 
 
-0.304
 
 
18.6
 
 
 
 
ANGEL2
 
 
hsa-miR-132-3p
 
 
-0.357
 
 
10.6
 
 
 
 
ANKFY1
 
 
hsa-miR-155-5p
 
 
-0.315
 
 
10.9
 
 
 
 
ANKHD1
 
 
hsa-miR-23a-3p
 
 
-0.368
 
 
18.9
 
 
 
 
ANKHD1
 
 
hsa-miR-23b-3p
 
 
-0.368
 
 
18.9
 
 
 
 
ANKHD1
 
 
hsa-miR-30b-5p
 
 
-0.405
 
 
18.9
 
 
 
 
ANKRD17
 
 
hsa-miR-30b-5p
 
 
-0.336
 
 
10.0
 
 
 
 
ANLN
 
 
hsa-miR-15b-5p
 
 
-0.334
 
 
32.3
 
 
 
 
ANP32A
 
 
hsa-miR-132-3p
 
 
-0.374
 
 
49.8
 
 
 
 
ANP32E
 
 
hsa-miR-501-3p
 
 
-0.331
 
 
38.5
 
 
 
 
ANXA11
 
 
hsa-miR-15b-5p
 
 
-0.463
 
 
35.8
 
 
 
 
AP2A1
 
 
hsa-miR-15b-5p
 
 
-0.333
 
 
49.9
 
 
 
 
AP2B1
 
 
hsa-miR-15b-5p
 
 
-0.334
 
 
69.3
 
 
 
 
AP3S1
 
 
hsa-miR-30b-5p
 
 
-0.293
 
 
82.5
 
 
 
 
APC
 
 
hsa-miR-29a-3p
 
 
-0.413
 
 
38.7
 
 
 
 
APLN
 
 
hsa-miR-15b-5p
 
 
-0.395
 
 
15.8
 
 
 
 
APOO
 
 
hsa-miR-181b-5p
 
 
-0.5
 
 
45.1
 
 
 
 
APOO
 
 
hsa-miR-181a-5p
 
 
-0.5
 
 
45.1
 
 
 
 
APP
 
 
hsa-miR-15b-5p
 
 
-0.298
 
 
396.2
 
 
 
 
ARF4
 
 
hsa-miR-30b-5p
 
 
-0.289
 
 
60.1
 
 
 
 
ARF4
 
 
hsa-miR-221-3p
 
 
-0.553
 
 
60.1
 
 
 
 
ARF5
 
 
hsa-miR-29a-3p
 
 
-0.491
 
 
176.5
 
 
 
 
ARHGAP5
 
 
hsa-miR-486-5p
 
 
-0.3
 
 
27.1
 
 
 
 
ARL2
 
 
hsa-miR-15b-5p
 
 
-0.374
 
 
171.9
 
 
 
 
ARL2
 
 
hsa-miR-15b-5p
 
 
-0.442
 
 
171.9
 
 
 
 
ARL3
 
 
hsa-miR-15b-5p
 
 
-0.507
 
 
26.9
 
 
 
 
ARL4A
 
 
hsa-miR-29a-3p
 
 
-0.386
 
 
14.1
 
 
 
 
ARL4A
 
 
hsa-miR-30b-5p
 
 
-0.448
 
 
14.1
 
 
 
 
ARMC8
 
 
hsa-miR-486-5p
 
 
-0.321
 
 
13.4
 
 
 
 
ARMCX2
 
 
hsa-miR-15b-5p
 
 
-0.344
 
 
20.2
 
 
 
 
ARMCX3
 
 
hsa-miR-425-5p
 
 
-0.325
 
 
32.9
 
 
 
 
ARPP19
 
 
hsa-miR-15b-5p
 
 
-0.346
 
 
178.5
 
 
 
 
ARPP19
 
 
hsa-miR-29a-3p
 
 
-0.371
 
 
178.5
 
 
 
 
ARPP21
 
 
hsa-miR-15b-5p
 
 
-0.342
 
 
38.3
 
 
 
 
ARPP21
 
 
hsa-miR-501-3p
 
 
-0.546
 
 
38.3
 
 
 
 
ASB3
 
 
hsa-miR-30b-5p
 
 
-0.418
 
 
20.8
 
 
 
 
ASB3
 
 
hsa-miR-30b-5p
 
 
-0.418
 
 
20.8
 
 
 
 
ASB6
 
 
hsa-miR-874-3p
 
 
-0.368
 
 
13.1
 
 
 
 
ASF1A
 
 
hsa-miR-23a-3p
 
 
-0.506
 
 
14.0
 
 
 
 
ASF1A
 
 
hsa-miR-23b-3p
 
 
-0.506
 
 
14.0
 
 
 
 
ASH1L
 
 
hsa-miR-15b-5p
 
 
-0.325
 
 
21.0
 
 
 
 
ASPH
 
 
hsa-miR-221-3p
 
 
-0.287
 
 
62.6
 
 
 
 
ATF3
 
 
hsa-miR-874-3p
 
 
-0.288
 
 
15.6
 
 
 
 
ATG9A
 
 
hsa-miR-15b-5p
 
 
-0.333
 
 
22.8
 
 
 
 
ATP5MC3
 
 
hsa-miR-425-5p
 
 
-0.328
 
 
183.1
 
 
 
 
ATP5MC3
 
 
hsa-miR-29a-3p
 
 
-0.33
 
 
183.1
 
 
 
 
ATP6V1E1
 
 
hsa-miR-23a-3p
 
 
-0.294
 
 
227.8
 
 
 
 
ATP6V1E1
 
 
hsa-miR-23b-3p
 
 
-0.294
 
 
227.8
 
 
 
 
ATPAF1
 
 
hsa-miR-361-5p
 
 
-0.362
 
 
86.8
 
 
 
 
ATXN2
 
 
hsa-miR-15b-5p
 
 
-0.392
 
 
21.8
 
 
 
 
AUH
 
 
hsa-miR-23a-3p
 
 
-0.495
 
 
13.0
 
 
 
 
AUH
 
 
hsa-miR-23b-3p
 
 
-0.503
 
 
13.0
 
 
 
 
AUH
 
 
hsa-miR-23a-3p
 
 
-0.512
 
 
13.0
 
 
 
 
AUH
 
 
hsa-miR-23b-3p
 
 
-0.512
 
 
13.0
 
 
 
 
AZIN1
 
 
hsa-miR-132-3p
 
 
-0.29
 
 
37.2
 
 
 
 
AZIN1
 
 
hsa-miR-132-3p
 
 
-0.299
 
 
37.2
 
 
 
 
B4GALT5
 
 
hsa-miR-501-3p
 
 
-0.408
 
 
10.8
 
 
 
 
B4GAT1
 
 
hsa-miR-23a-3p
 
 
-0.361
 
 
107.6
 
 
 
 
B4GAT1
 
 
hsa-miR-23b-3p
 
 
-0.361
 
 
107.6
 
 
 
 
BCL2L2
 
 
hsa-miR-29a-3p
 
 
-0.312
 
 
85.7
 
 
 
 
BDH1
 
 
hsa-miR-874-3p
 
 
-0.644
 
 
20.7
 
 
 
 
BECN1
 
 
hsa-miR-30b-5p
 
 
-0.452
 
 
30.8
 
 
 
 
BEND6
 
 
hsa-miR-361-5p
 
 
-0.305
 
 
18.2
 
 
 
 
BET1
 
 
hsa-miR-23a-3p
 
 
-0.402
 
 
11.0
 
 
 
 
BET1
 
 
hsa-miR-23b-3p
 
 
-0.418
 
 
11.0
 
 
 
 
BFAR
 
 
hsa-miR-15b-5p
 
 
-0.352
 
 
11.0
 
 
 
 
BHLHE40
 
 
hsa-miR-181b-5p
 
 
-0.408
 
 
40.6
 
 
 
 
BHLHE40
 
 
hsa-miR-181a-5p
 
 
-0.408
 
 
40.6
 
 
 
 
BLCAP
 
 
hsa-miR-23a-3p
 
 
-0.601
 
 
126.2
 
 
 
 
BLCAP
 
 
hsa-miR-23b-3p
 
 
-0.601
 
 
126.2
 
 
 
 
BLMH
 
 
hsa-miR-29a-3p
 
 
-0.393
 
 
10.7
 
 
 
 
BLOC1S6
 
 
hsa-miR-181b-5p
 
 
-0.393
 
 
20.1
 
 
 
 
BLOC1S6
 
 
hsa-miR-181a-5p
 
 
-0.393
 
 
20.1
 
 
 
 
BOLA3
 
 
hsa-miR-15b-5p
 
 
-0.363
 
 
61.7
 
 
 
 
BOLA3
 
 
hsa-miR-15b-5p
 
 
-0.386
 
 
61.7
 
 
 
 
BOLA3
 
 
hsa-miR-374b-5p
 
 
-0.486
 
 
61.7
 
 
 
 
BRI3
 
 
hsa-miR-132-3p
 
 
-0.385
 
 
51.8
 
 
 
 
BTF3
 
 
hsa-miR-15b-5p
 
 
-0.344
 
 
276.0
 
 
 
 
BTF3
 
 
hsa-miR-361-5p
 
 
-0.551
 
 
276.0
 
 
 
 
BTG2
 
 
hsa-miR-29a-3p
 
 
-0.32
 
 
39.0
 
 
 
 
BTRC
 
 
hsa-miR-15b-5p
 
 
-0.302
 
 
12.4
 
 
 
 
BTRC
 
 
hsa-miR-15b-5p
 
 
-0.358
 
 
12.4
 
 
 
 
BZW1
 
 
hsa-miR-15b-5p
 
 
-0.367
 
 
34.3
 
 
 
 
C11orf24
 
 
hsa-miR-15b-5p
 
 
-0.403
 
 
10.6
 
 
 
 
C11orf68
 
 
hsa-miR-15b-5p
 
 
-0.29
 
 
48.9
 
 
 
 
C19orf47
 
 
hsa-miR-132-3p
 
 
-0.322
 
 
13.3
 
 
 
 
 
 
 appendix 
 
 
 
 
target_symbol
 
 
mature_mirna_id
 
 
score
 
 
nTPM
 
 
 
 
 
 
AAR2
 
 
hsa-miR-29a-3p
 
 
-0.327
 
 
13.4
 
 
 
 
ABCE1
 
 
hsa-miR-29a-3p
 
 
-0.404
 
 
31.7
 
 
 
 
ACTC1
 
 
hsa-miR-30b-5p
 
 
-0.543
 
 
23.3
 
 
 
 
ACTR2
 
 
hsa-miR-15b-5p
 
 
-0.293
 
 
202.6
 
 
 
 
ADA
 
 
hsa-miR-29a-3p
 
 
-0.376
 
 
15.1
 
 
 
 
ADAM19
 
 
hsa-miR-874-3p
 
 
-0.374
 
 
22.7
 
 
 
 
ADAM9
 
 
hsa-miR-30b-5p
 
 
-0.312
 
 
27.3
 
 
 
 
ADAMTS9
 
 
hsa-miR-29a-3p
 
 
-0.305
 
 
11.5
 
 
 
 
ADAMTS9
 
 
hsa-miR-29a-3p
 
 
-0.485
 
 
11.5
 
 
 
 
ADCY3
 
 
hsa-miR-132-3p
 
 
-0.303
 
 
16.3
 
 
 
 
ADIPOR2
 
 
hsa-miR-150-5p
 
 
-0.287
 
 
10.0
 
 
 
 
AFG3L2
 
 
hsa-miR-181b-5p
 
 
-0.491
 
 
12.5
 
 
 
 
AFG3L2
 
 
hsa-miR-181a-5p
 
 
-0.491
 
 
12.5
 
 
 
 
AHR
 
 
hsa-miR-29a-3p
 
 
-0.316
 
 
24.3
 
 
 
 
AK1
 
 
hsa-miR-29a-3p
 
 
-0.333
 
 
10.8
 
 
 
 
ALAD
 
 
hsa-miR-15b-5p
 
 
-0.446
 
 
15.7
 
 
 
 
ANGPTL2
 
 
hsa-miR-221-3p
 
 
-0.369
 
 
31.6
 
 
 
 
ANKFY1
 
 
hsa-miR-155-5p
 
 
-0.315
 
 
12.7
 
 
 
 
ANKHD1
 
 
hsa-miR-23a-3p
 
 
-0.368
 
 
34.9
 
 
 
 
ANKHD1
 
 
hsa-miR-23b-3p
 
 
-0.368
 
 
34.9
 
 
 
 
ANKHD1
 
 
hsa-miR-30b-5p
 
 
-0.405
 
 
34.9
 
 
 
 
ANKRD17
 
 
hsa-miR-30b-5p
 
 
-0.336
 
 
12.9
 
 
 
 
ANP32A
 
 
hsa-miR-132-3p
 
 
-0.374
 
 
82.3
 
 
 
 
ANP32E
 
 
hsa-miR-501-3p
 
 
-0.331
 
 
36.7
 
 
 
 
ANTXR2
 
 
hsa-miR-29a-3p
 
 
-0.347
 
 
33.4
 
 
 
 
ANXA11
 
 
hsa-miR-15b-5p
 
 
-0.463
 
 
93.8
 
 
 
 
AP2A1
 
 
hsa-miR-15b-5p
 
 
-0.333
 
 
23.7
 
 
 
 
AP2B1
 
 
hsa-miR-15b-5p
 
 
-0.334
 
 
36.0
 
 
 
 
AP3S1
 
 
hsa-miR-30b-5p
 
 
-0.293
 
 
36.6
 
 
 
 
APP
 
 
hsa-miR-15b-5p
 
 
-0.298
 
 
146.0
 
 
 
 
AQP3
 
 
hsa-miR-874-3p
 
 
-0.48
 
 
15.8
 
 
 
 
ARF4
 
 
hsa-miR-30b-5p
 
 
-0.289
 
 
101.3
 
 
 
 
ARF4
 
 
hsa-miR-221-3p
 
 
-0.553
 
 
101.3
 
 
 
 
ARF5
 
 
hsa-miR-29a-3p
 
 
-0.491
 
 
68.5
 
 
 
 
ARF6
 
 
hsa-miR-181b-5p
 
 
-0.33
 
 
41.0
 
 
 
 
ARF6
 
 
hsa-miR-181a-5p
 
 
-0.33
 
 
41.0
 
 
 
 
ARHGAP5
 
 
hsa-miR-486-5p
 
 
-0.3
 
 
18.6
 
 
 
 
ARID1B
 
 
hsa-miR-29a-3p
 
 
-0.342
 
 
13.6
 
 
 
 
ARID4B
 
 
hsa-miR-486-5p
 
 
-0.367
 
 
13.3
 
 
 
 
ARL2
 
 
hsa-miR-15b-5p
 
 
-0.374
 
 
38.3
 
 
 
 
ARL2
 
 
hsa-miR-15b-5p
 
 
-0.442
 
 
38.3
 
 
 
 
ARL4A
 
 
hsa-miR-29a-3p
 
 
-0.386
 
 
15.8
 
 
 
 
ARL4A
 
 
hsa-miR-30b-5p
 
 
-0.448
 
 
15.8
 
 
 
 
ARMC8
 
 
hsa-miR-486-5p
 
 
-0.321
 
 
17.0
 
 
 
 
ARMCX2
 
 
hsa-miR-15b-5p
 
 
-0.344
 
 
10.8
 
 
 
 
ARMCX3
 
 
hsa-miR-425-5p
 
 
-0.325
 
 
23.0
 
 
 
 
ARNT
 
 
hsa-miR-221-3p
 
 
-0.465
 
 
17.1
 
 
 
 
ARPP19
 
 
hsa-miR-15b-5p
 
 
-0.346
 
 
36.4
 
 
 
 
ARPP19
 
 
hsa-miR-29a-3p
 
 
-0.371
 
 
36.4
 
 
 
 
ARRDC3
 
 
hsa-miR-29a-3p
 
 
-0.402
 
 
25.3
 
 
 
 
ASPH
 
 
hsa-miR-221-3p
 
 
-0.287
 
 
47.6
 
 
 
 
ATF3
 
 
hsa-miR-874-3p
 
 
-0.288
 
 
59.7
 
 
 
 
ATG9A
 
 
hsa-miR-15b-5p
 
 
-0.333
 
 
16.3
 
 
 
 
ATOSA
 
 
hsa-miR-221-3p
 
 
-0.495
 
 
12.5
 
 
 
 
ATP5MC3
 
 
hsa-miR-425-5p
 
 
-0.328
 
 
123.3
 
 
 
 
ATP5MC3
 
 
hsa-miR-29a-3p
 
 
-0.33
 
 
123.3
 
 
 
 
ATP6V1E1
 
 
hsa-miR-23a-3p
 
 
-0.294
 
 
64.3
 
 
 
 
ATP6V1E1
 
 
hsa-miR-23b-3p
 
 
-0.294
 
 
64.3
 
 
 
 
ATPAF1
 
 
hsa-miR-361-5p
 
 
-0.362
 
 
26.8
 
 
 
 
ATXN2
 
 
hsa-miR-15b-5p
 
 
-0.392
 
 
10.6
 
 
 
 
AZIN1
 
 
hsa-miR-132-3p
 
 
-0.29
 
 
44.7
 
 
 
 
AZIN1
 
 
hsa-miR-132-3p
 
 
-0.299
 
 
44.7
 
 
 
 
B3GNT5
 
 
hsa-miR-30b-5p
 
 
-0.326
 
 
13.0
 
 
 
 
B4GALT5
 
 
hsa-miR-501-3p
 
 
-0.408
 
 
20.5
 
 
 
 
BAK1
 
 
hsa-miR-29a-3p
 
 
-0.409
 
 
19.5
 
 
 
 
BCL2L11
 
 
hsa-miR-221-3p
 
 
-0.423
 
 
16.0
 
 
 
 
BCL2L2
 
 
hsa-miR-29a-3p
 
 
-0.312
 
 
23.5
 
 
 
 
BECN1
 
 
hsa-miR-30b-5p
 
 
-0.452
 
 
39.5
 
 
 
 
BET1
 
 
hsa-miR-23a-3p
 
 
-0.402
 
 
15.4
 
 
 
 
BET1
 
 
hsa-miR-23b-3p
 
 
-0.418
 
 
15.4
 
 
 
 
BFAR
 
 
hsa-miR-15b-5p
 
 
-0.352
 
 
13.9
 
 
 
 
BHLHE40
 
 
hsa-miR-181b-5p
 
 
-0.408
 
 
50.0
 
 
 
 
BHLHE40
 
 
hsa-miR-181a-5p
 
 
-0.408
 
 
50.0
 
 
 
 
BLCAP
 
 
hsa-miR-23a-3p
 
 
-0.601
 
 
25.7
 
 
 
 
BLCAP
 
 
hsa-miR-23b-3p
 
 
-0.601
 
 
25.7
 
 
 
 
BLMH
 
 
hsa-miR-29a-3p
 
 
-0.393
 
 
17.2
 
 
 
 
BLOC1S6
 
 
hsa-miR-181b-5p
 
 
-0.393
 
 
29.4
 
 
 
 
BLOC1S6
 
 
hsa-miR-181a-5p
 
 
-0.393
 
 
29.4
 
 
 
 
BMF
 
 
hsa-miR-29a-3p
 
 
-0.41
 
 
11.8
 
 
 
 
BMF
 
 
hsa-miR-221-3p
 
 
-0.453
 
 
11.8
 
 
 
 
BOLA3
 
 
hsa-miR-15b-5p
 
 
-0.363
 
 
21.2
 
 
 
 
BOLA3
 
 
hsa-miR-15b-5p
 
 
-0.386
 
 
21.2
 
 
 
 
BOLA3
 
 
hsa-miR-374b-5p
 
 
-0.486
 
 
21.2
 
 
 
 
BRI3
 
 
hsa-miR-132-3p
 
 
-0.385
 
 
34.9
 
 
 
 
BTAF1
 
 
hsa-miR-486-5p
 
 
-0.288
 
 
16.0
 
 
 
 
BTAF1
 
 
hsa-miR-15b-5p
 
 
-0.496
 
 
16.0
 
 
 
 
BTF3
 
 
hsa-miR-15b-5p
 
 
-0.344
 
 
352.3
 
 
 
 
BTF3
 
 
hsa-miR-361-5p
 
 
-0.551
 
 
352.3
 
 
 
 
BTG2
 
 
hsa-miR-29a-3p
 
 
-0.32
 
 
117.3
 
 
 
 
BTLA
 
 
hsa-miR-15b-5p
 
 
-0.306
 
 
10.6
 
 
 
 
BTLA
 
 
hsa-miR-15b-5p
 
 
-0.316
 
 
10.6
 
 
 
 
BTN2A1
 
 
hsa-miR-342-3p
 
 
-0.31
 
 
11.1
 
 
 
 
BZW1
 
 
hsa-miR-15b-5p
 
 
-0.367
 
 
91.9
 
 
 
 
C11orf24
 
 
hsa-miR-15b-5p
 
 
-0.403
 
 
19.5
 
 
 
 
C11orf68
 
 
hsa-miR-15b-5p
 
 
-0.29
 
 
14.1
 
 
 
 
C5orf15
 
 
hsa-miR-29a-3p
 
 
-0.392
 
 
33.6
 
 
 
 
C6orf120
 
 
hsa-miR-221-3p
 
 
-0.438
 
 
12.4
 
 
 
 
C6orf62
 
 
hsa-miR-181b-5p
 
 
-0.334
 
 
108.5
 
 
 
 
C6orf62
 
 
hsa-miR-181a-5p
 
 
-0.334
 
 
108.5
 
 
 
 
C6orf62
 
 
hsa-miR-23a-3p
 
 
-0.507
 
 
108.5
 
 
 
 
 
 
 basal ganglia 
 
 
 
 
target_symbol
 
 
mature_mirna_id
 
 
score
 
 
nTPM
 
 
 
 
 
 
AAR2
 
 
hsa-miR-29a-3p
 
 
-0.327
 
 
11.5
 
 
 
 
ABCE1
 
 
hsa-miR-29a-3p
 
 
-0.404
 
 
11.5
 
 
 
 
ABHD17B
 
 
hsa-miR-486-5p
 
 
-0.348
 
 
13.8
 
 
 
 
ABHD4
 
 
hsa-miR-29a-3p
 
 
-0.312
 
 
15.4
 
 
 
 
ACTN2
 
 
hsa-miR-23a-3p
 
 
-0.294
 
 
36.8
 
 
 
 
ACTN2
 
 
hsa-miR-29a-3p
 
 
-0.311
 
 
36.8
 
 
 
 
ACTR2
 
 
hsa-miR-15b-5p
 
 
-0.293
 
 
55.9
 
 
 
 
ADCY3
 
 
hsa-miR-132-3p
 
 
-0.303
 
 
57.0
 
 
 
 
ADGRA3
 
 
hsa-miR-30b-5p
 
 
-0.304
 
 
18.8
 
 
 
 
ADIPOR2
 
 
hsa-miR-150-5p
 
 
-0.287
 
 
50.5
 
 
 
 
AGPAT4
 
 
hsa-miR-29a-3p
 
 
-0.376
 
 
10.7
 
 
 
 
AK1
 
 
hsa-miR-29a-3p
 
 
-0.333
 
 
100.7
 
 
 
 
AK4
 
 
hsa-miR-15b-5p
 
 
-0.316
 
 
19.2
 
 
 
 
AKAP5
 
 
hsa-miR-29a-3p
 
 
-0.298
 
 
17.6
 
 
 
 
AKT3
 
 
hsa-miR-29a-3p
 
 
-0.287
 
 
15.0
 
 
 
 
AKT3
 
 
hsa-miR-15b-5p
 
 
-0.301
 
 
15.0
 
 
 
 
ALAD
 
 
hsa-miR-15b-5p
 
 
-0.446
 
 
57.8
 
 
 
 
AMOT
 
 
hsa-miR-29a-3p
 
 
-0.304
 
 
18.8
 
 
 
 
ANGEL2
 
 
hsa-miR-132-3p
 
 
-0.357
 
 
14.7
 
 
 
 
ANGPTL2
 
 
hsa-miR-221-3p
 
 
-0.369
 
 
10.9
 
 
 
 
ANKFY1
 
 
hsa-miR-155-5p
 
 
-0.315
 
 
13.3
 
 
 
 
ANKHD1
 
 
hsa-miR-23a-3p
 
 
-0.368
 
 
20.4
 
 
 
 
ANKHD1
 
 
hsa-miR-23b-3p
 
 
-0.368
 
 
20.4
 
 
 
 
ANKHD1
 
 
hsa-miR-30b-5p
 
 
-0.405
 
 
20.4
 
 
 
 
ANKRD13B
 
 
hsa-miR-29a-3p
 
 
-0.421
 
 
12.3
 
 
 
 
ANKRD17
 
 
hsa-miR-30b-5p
 
 
-0.336
 
 
11.2
 
 
 
 
ANLN
 
 
hsa-miR-15b-5p
 
 
-0.334
 
 
27.5
 
 
 
 
ANO3
 
 
hsa-miR-15b-5p
 
 
-0.357
 
 
58.4
 
 
 
 
ANP32A
 
 
hsa-miR-132-3p
 
 
-0.374
 
 
53.9
 
 
 
 
ANP32E
 
 
hsa-miR-501-3p
 
 
-0.331
 
 
38.3
 
 
 
 
ANXA11
 
 
hsa-miR-15b-5p
 
 
-0.463
 
 
35.8
 
 
 
 
AP2A1
 
 
hsa-miR-15b-5p
 
 
-0.333
 
 
40.7
 
 
 
 
AP2B1
 
 
hsa-miR-15b-5p
 
 
-0.334
 
 
72.3
 
 
 
 
AP3S1
 
 
hsa-miR-30b-5p
 
 
-0.293
 
 
79.6
 
 
 
 
APC
 
 
hsa-miR-29a-3p
 
 
-0.413
 
 
15.7
 
 
 
 
APLN
 
 
hsa-miR-15b-5p
 
 
-0.395
 
 
10.2
 
 
 
 
APOO
 
 
hsa-miR-181b-5p
 
 
-0.5
 
 
38.6
 
 
 
 
APOO
 
 
hsa-miR-181a-5p
 
 
-0.5
 
 
38.6
 
 
 
 
APP
 
 
hsa-miR-15b-5p
 
 
-0.298
 
 
458.2
 
 
 
 
ARF4
 
 
hsa-miR-30b-5p
 
 
-0.289
 
 
71.0
 
 
 
 
ARF4
 
 
hsa-miR-221-3p
 
 
-0.553
 
 
71.0
 
 
 
 
ARF5
 
 
hsa-miR-29a-3p
 
 
-0.491
 
 
195.0
 
 
 
 
ARF6
 
 
hsa-miR-181b-5p
 
 
-0.33
 
 
10.2
 
 
 
 
ARF6
 
 
hsa-miR-181a-5p
 
 
-0.33
 
 
10.2
 
 
 
 
ARHGAP5
 
 
hsa-miR-486-5p
 
 
-0.3
 
 
22.9
 
 
 
 
ARL2
 
 
hsa-miR-15b-5p
 
 
-0.374
 
 
286.3
 
 
 
 
ARL2
 
 
hsa-miR-15b-5p
 
 
-0.442
 
 
286.3
 
 
 
 
ARL3
 
 
hsa-miR-15b-5p
 
 
-0.507
 
 
31.8
 
 
 
 
ARL4A
 
 
hsa-miR-29a-3p
 
 
-0.386
 
 
10.5
 
 
 
 
ARL4A
 
 
hsa-miR-30b-5p
 
 
-0.448
 
 
10.5
 
 
 
 
ARMC8
 
 
hsa-miR-486-5p
 
 
-0.321
 
 
13.3
 
 
 
 
ARMCX2
 
 
hsa-miR-15b-5p
 
 
-0.344
 
 
31.3
 
 
 
 
ARMCX3
 
 
hsa-miR-425-5p
 
 
-0.325
 
 
33.9
 
 
 
 
ARPP19
 
 
hsa-miR-15b-5p
 
 
-0.346
 
 
481.4
 
 
 
 
ARPP19
 
 
hsa-miR-29a-3p
 
 
-0.371
 
 
481.4
 
 
 
 
ARPP21
 
 
hsa-miR-15b-5p
 
 
-0.342
 
 
746.4
 
 
 
 
ARPP21
 
 
hsa-miR-501-3p
 
 
-0.546
 
 
746.4
 
 
 
 
ARRDC3
 
 
hsa-miR-29a-3p
 
 
-0.402
 
 
10.6
 
 
 
 
ASB3
 
 
hsa-miR-30b-5p
 
 
-0.418
 
 
20.9
 
 
 
 
ASB3
 
 
hsa-miR-30b-5p
 
 
-0.418
 
 
20.9
 
 
 
 
ASB6
 
 
hsa-miR-874-3p
 
 
-0.368
 
 
16.0
 
 
 
 
ASF1A
 
 
hsa-miR-23a-3p
 
 
-0.506
 
 
15.3
 
 
 
 
ASF1A
 
 
hsa-miR-23b-3p
 
 
-0.506
 
 
15.3
 
 
 
 
ASH1L
 
 
hsa-miR-15b-5p
 
 
-0.325
 
 
23.9
 
 
 
 
ASPH
 
 
hsa-miR-221-3p
 
 
-0.287
 
 
62.4
 
 
 
 
ATF3
 
 
hsa-miR-874-3p
 
 
-0.288
 
 
24.6
 
 
 
 
ATG9A
 
 
hsa-miR-15b-5p
 
 
-0.333
 
 
27.8
 
 
 
 
ATP5MC3
 
 
hsa-miR-425-5p
 
 
-0.328
 
 
198.3
 
 
 
 
ATP5MC3
 
 
hsa-miR-29a-3p
 
 
-0.33
 
 
198.3
 
 
 
 
ATP6V1E1
 
 
hsa-miR-23a-3p
 
 
-0.294
 
 
242.5
 
 
 
 
ATP6V1E1
 
 
hsa-miR-23b-3p
 
 
-0.294
 
 
242.5
 
 
 
 
ATPAF1
 
 
hsa-miR-361-5p
 
 
-0.362
 
 
88.4
 
 
 
 
ATXN2
 
 
hsa-miR-15b-5p
 
 
-0.392
 
 
21.8
 
 
 
 
AUH
 
 
hsa-miR-23a-3p
 
 
-0.495
 
 
18.1
 
 
 
 
AUH
 
 
hsa-miR-23b-3p
 
 
-0.503
 
 
18.1
 
 
 
 
AUH
 
 
hsa-miR-23a-3p
 
 
-0.512
 
 
18.1
 
 
 
 
AUH
 
 
hsa-miR-23b-3p
 
 
-0.512
 
 
18.1
 
 
 
 
AZIN1
 
 
hsa-miR-132-3p
 
 
-0.29
 
 
29.3
 
 
 
 
AZIN1
 
 
hsa-miR-132-3p
 
 
-0.299
 
 
29.3
 
 
 
 
B4GALT5
 
 
hsa-miR-501-3p
 
 
-0.408
 
 
12.9
 
 
 
 
B4GAT1
 
 
hsa-miR-23a-3p
 
 
-0.361
 
 
152.4
 
 
 
 
B4GAT1
 
 
hsa-miR-23b-3p
 
 
-0.361
 
 
152.4
 
 
 
 
BAG4
 
 
hsa-miR-181b-5p
 
 
-0.337
 
 
10.9
 
 
 
 
BAG4
 
 
hsa-miR-181a-5p
 
 
-0.337
 
 
10.9
 
 
 
 
BCL2L2
 
 
hsa-miR-29a-3p
 
 
-0.312
 
 
76.9
 
 
 
 
BCL7A
 
 
hsa-miR-15b-5p
 
 
-0.336
 
 
12.4
 
 
 
 
BCL7A
 
 
hsa-miR-501-3p
 
 
-0.339
 
 
12.4
 
 
 
 
BDH1
 
 
hsa-miR-874-3p
 
 
-0.644
 
 
28.9
 
 
 
 
BECN1
 
 
hsa-miR-30b-5p
 
 
-0.452
 
 
30.5
 
 
 
 
BEND6
 
 
hsa-miR-361-5p
 
 
-0.305
 
 
16.2
 
 
 
 
BET1
 
 
hsa-miR-23a-3p
 
 
-0.402
 
 
12.0
 
 
 
 
BET1
 
 
hsa-miR-23b-3p
 
 
-0.418
 
 
12.0
 
 
 
 
BFAR
 
 
hsa-miR-15b-5p
 
 
-0.352
 
 
12.7
 
 
 
 
BHLHE40
 
 
hsa-miR-181b-5p
 
 
-0.408
 
 
47.4
 
 
 
 
BHLHE40
 
 
hsa-miR-181a-5p
 
 
-0.408
 
 
47.4
 
 
 
 
BLCAP
 
 
hsa-miR-23a-3p
 
 
-0.601
 
 
310.5
 
 
 
 
BLCAP
 
 
hsa-miR-23b-3p
 
 
-0.601
 
 
310.5
 
 
 
 
BLMH
 
 
hsa-miR-29a-3p
 
 
-0.393
 
 
12.3
 
 
 
 
BLOC1S6
 
 
hsa-miR-181b-5p
 
 
-0.393
 
 
22.3
 
 
 
 
BLOC1S6
 
 
hsa-miR-181a-5p
 
 
-0.393
 
 
22.3
 
 
 
 
 
 
 blood vessel 
 
 
 
 
target_symbol
 
 
mature_mirna_id
 
 
score
 
 
nTPM
 
 
 
 
 
 
AAR2
 
 
hsa-miR-29a-3p
 
 
-0.327
 
 
28.2
 
 
 
 
ABCE1
 
 
hsa-miR-29a-3p
 
 
-0.404
 
 
15.1
 
 
 
 
ABHD4
 
 
hsa-miR-29a-3p
 
 
-0.312
 
 
38.3
 
 
 
 
ACTA1
 
 
hsa-miR-155-5p
 
 
-0.595
 
 
77.9
 
 
 
 
ACTC1
 
 
hsa-miR-30b-5p
 
 
-0.543
 
 
388.9
 
 
 
 
ACTN2
 
 
hsa-miR-23a-3p
 
 
-0.294
 
 
12.2
 
 
 
 
ACTN2
 
 
hsa-miR-29a-3p
 
 
-0.311
 
 
12.2
 
 
 
 
ACTR2
 
 
hsa-miR-15b-5p
 
 
-0.293
 
 
77.0
 
 
 
 
ADAM9
 
 
hsa-miR-30b-5p
 
 
-0.312
 
 
36.8
 
 
 
 
ADAMTS10
 
 
hsa-miR-29a-3p
 
 
-0.516
 
 
20.0
 
 
 
 
ADAMTS7
 
 
hsa-miR-29a-3p
 
 
-0.427
 
 
14.2
 
 
 
 
ADAMTS9
 
 
hsa-miR-29a-3p
 
 
-0.305
 
 
18.0
 
 
 
 
ADAMTS9
 
 
hsa-miR-29a-3p
 
 
-0.485
 
 
18.0
 
 
 
 
ADCY3
 
 
hsa-miR-132-3p
 
 
-0.303
 
 
43.2
 
 
 
 
ADIPOR2
 
 
hsa-miR-150-5p
 
 
-0.287
 
 
23.0
 
 
 
 
AHR
 
 
hsa-miR-29a-3p
 
 
-0.316
 
 
15.2
 
 
 
 
AK1
 
 
hsa-miR-29a-3p
 
 
-0.333
 
 
58.4
 
 
 
 
AK4
 
 
hsa-miR-15b-5p
 
 
-0.316
 
 
25.4
 
 
 
 
AKT3
 
 
hsa-miR-29a-3p
 
 
-0.287
 
 
34.5
 
 
 
 
AKT3
 
 
hsa-miR-15b-5p
 
 
-0.301
 
 
34.5
 
 
 
 
ALAD
 
 
hsa-miR-15b-5p
 
 
-0.446
 
 
22.2
 
 
 
 
AMOTL1
 
 
hsa-miR-15b-5p
 
 
-0.319
 
 
18.9
 
 
 
 
ANGPTL2
 
 
hsa-miR-221-3p
 
 
-0.369
 
 
31.4
 
 
 
 
ANKFY1
 
 
hsa-miR-155-5p
 
 
-0.315
 
 
13.2
 
 
 
 
ANKHD1
 
 
hsa-miR-23a-3p
 
 
-0.368
 
 
40.7
 
 
 
 
ANKHD1
 
 
hsa-miR-23b-3p
 
 
-0.368
 
 
40.7
 
 
 
 
ANKHD1
 
 
hsa-miR-30b-5p
 
 
-0.405
 
 
40.7
 
 
 
 
ANKRD17
 
 
hsa-miR-30b-5p
 
 
-0.336
 
 
13.9
 
 
 
 
ANP32A
 
 
hsa-miR-132-3p
 
 
-0.374
 
 
51.8
 
 
 
 
ANP32E
 
 
hsa-miR-501-3p
 
 
-0.331
 
 
23.8
 
 
 
 
ANTXR2
 
 
hsa-miR-29a-3p
 
 
-0.347
 
 
50.3
 
 
 
 
ANXA11
 
 
hsa-miR-15b-5p
 
 
-0.463
 
 
150.6
 
 
 
 
AP2A1
 
 
hsa-miR-15b-5p
 
 
-0.333
 
 
48.0
 
 
 
 
AP2B1
 
 
hsa-miR-15b-5p
 
 
-0.334
 
 
41.4
 
 
 
 
AP3S1
 
 
hsa-miR-30b-5p
 
 
-0.293
 
 
112.6
 
 
 
 
APLN
 
 
hsa-miR-15b-5p
 
 
-0.395
 
 
10.7
 
 
 
 
APP
 
 
hsa-miR-15b-5p
 
 
-0.298
 
 
341.0
 
 
 
 
ARF4
 
 
hsa-miR-30b-5p
 
 
-0.289
 
 
151.4
 
 
 
 
ARF4
 
 
hsa-miR-221-3p
 
 
-0.553
 
 
151.4
 
 
 
 
ARF5
 
 
hsa-miR-29a-3p
 
 
-0.491
 
 
115.2
 
 
 
 
ARF6
 
 
hsa-miR-181b-5p
 
 
-0.33
 
 
22.6
 
 
 
 
ARF6
 
 
hsa-miR-181a-5p
 
 
-0.33
 
 
22.6
 
 
 
 
ARHGAP5
 
 
hsa-miR-486-5p
 
 
-0.3
 
 
18.0
 
 
 
 
ARID1B
 
 
hsa-miR-29a-3p
 
 
-0.342
 
 
13.6
 
 
 
 
ARID4B
 
 
hsa-miR-486-5p
 
 
-0.367
 
 
12.5
 
 
 
 
ARL2
 
 
hsa-miR-15b-5p
 
 
-0.374
 
 
155.0
 
 
 
 
ARL2
 
 
hsa-miR-15b-5p
 
 
-0.442
 
 
155.0
 
 
 
 
ARL3
 
 
hsa-miR-15b-5p
 
 
-0.507
 
 
12.7
 
 
 
 
ARL4A
 
 
hsa-miR-29a-3p
 
 
-0.386
 
 
19.2
 
 
 
 
ARL4A
 
 
hsa-miR-30b-5p
 
 
-0.448
 
 
19.2
 
 
 
 
ARMC8
 
 
hsa-miR-486-5p
 
 
-0.321
 
 
23.5
 
 
 
 
ARMCX2
 
 
hsa-miR-15b-5p
 
 
-0.344
 
 
27.0
 
 
 
 
ARMCX3
 
 
hsa-miR-425-5p
 
 
-0.325
 
 
32.6
 
 
 
 
ARNT
 
 
hsa-miR-221-3p
 
 
-0.465
 
 
26.6
 
 
 
 
ARPP19
 
 
hsa-miR-15b-5p
 
 
-0.346
 
 
47.3
 
 
 
 
ARPP19
 
 
hsa-miR-29a-3p
 
 
-0.371
 
 
47.3
 
 
 
 
ARRDC3
 
 
hsa-miR-29a-3p
 
 
-0.402
 
 
25.4
 
 
 
 
ARVCF
 
 
hsa-miR-155-5p
 
 
-0.388
 
 
15.5
 
 
 
 
ASAP2
 
 
hsa-miR-29a-3p
 
 
-0.348
 
 
16.3
 
 
 
 
ASB6
 
 
hsa-miR-874-3p
 
 
-0.368
 
 
11.7
 
 
 
 
ASH1L
 
 
hsa-miR-15b-5p
 
 
-0.325
 
 
27.4
 
 
 
 
ASPH
 
 
hsa-miR-221-3p
 
 
-0.287
 
 
72.5
 
 
 
 
ATF3
 
 
hsa-miR-874-3p
 
 
-0.288
 
 
137.2
 
 
 
 
ATG5
 
 
hsa-miR-30b-5p
 
 
-0.488
 
 
12.7
 
 
 
 
ATG9A
 
 
hsa-miR-15b-5p
 
 
-0.333
 
 
23.4
 
 
 
 
ATOSA
 
 
hsa-miR-221-3p
 
 
-0.495
 
 
10.4
 
 
 
 
ATP5MC3
 
 
hsa-miR-425-5p
 
 
-0.328
 
 
129.6
 
 
 
 
ATP5MC3
 
 
hsa-miR-29a-3p
 
 
-0.33
 
 
129.6
 
 
 
 
ATP6V1E1
 
 
hsa-miR-23a-3p
 
 
-0.294
 
 
186.7
 
 
 
 
ATP6V1E1
 
 
hsa-miR-23b-3p
 
 
-0.294
 
 
186.7
 
 
 
 
ATPAF1
 
 
hsa-miR-361-5p
 
 
-0.362
 
 
30.6
 
 
 
 
ATXN1L
 
 
hsa-miR-155-5p
 
 
-0.315
 
 
12.5
 
 
 
 
ATXN2
 
 
hsa-miR-15b-5p
 
 
-0.392
 
 
25.1
 
 
 
 
AZIN1
 
 
hsa-miR-132-3p
 
 
-0.29
 
 
27.2
 
 
 
 
AZIN1
 
 
hsa-miR-132-3p
 
 
-0.299
 
 
27.2
 
 
 
 
B4GALT5
 
 
hsa-miR-501-3p
 
 
-0.408
 
 
15.5
 
 
 
 
B4GAT1
 
 
hsa-miR-23a-3p
 
 
-0.361
 
 
18.5
 
 
 
 
B4GAT1
 
 
hsa-miR-23b-3p
 
 
-0.361
 
 
18.5
 
 
 
 
BCL2L2
 
 
hsa-miR-29a-3p
 
 
-0.312
 
 
64.8
 
 
 
 
BCOR
 
 
hsa-miR-425-5p
 
 
-0.346
 
 
17.0
 
 
 
 
BECN1
 
 
hsa-miR-30b-5p
 
 
-0.452
 
 
48.7
 
 
 
 
BET1
 
 
hsa-miR-23a-3p
 
 
-0.402
 
 
11.0
 
 
 
 
BET1
 
 
hsa-miR-23b-3p
 
 
-0.418
 
 
11.0
 
 
 
 
BFAR
 
 
hsa-miR-15b-5p
 
 
-0.352
 
 
33.0
 
 
 
 
BHLHE40
 
 
hsa-miR-181b-5p
 
 
-0.408
 
 
237.8
 
 
 
 
BHLHE40
 
 
hsa-miR-181a-5p
 
 
-0.408
 
 
237.8
 
 
 
 
BLCAP
 
 
hsa-miR-23a-3p
 
 
-0.601
 
 
34.9
 
 
 
 
BLCAP
 
 
hsa-miR-23b-3p
 
 
-0.601
 
 
34.9
 
 
 
 
BLMH
 
 
hsa-miR-29a-3p
 
 
-0.393
 
 
21.6
 
 
 
 
BLOC1S6
 
 
hsa-miR-181b-5p
 
 
-0.393
 
 
21.2
 
 
 
 
BLOC1S6
 
 
hsa-miR-181a-5p
 
 
-0.393
 
 
21.2
 
 
 
 
BOLA3
 
 
hsa-miR-15b-5p
 
 
-0.363
 
 
35.8
 
 
 
 
BOLA3
 
 
hsa-miR-15b-5p
 
 
-0.386
 
 
35.8
 
 
 
 
BOLA3
 
 
hsa-miR-374b-5p
 
 
-0.486
 
 
35.8
 
 
 
 
BORCS6
 
 
hsa-miR-15b-5p
 
 
-0.347
 
 
10.3
 
 
 
 
BRI3
 
 
hsa-miR-132-3p
 
 
-0.385
 
 
115.1
 
 
 
 
BTAF1
 
 
hsa-miR-486-5p
 
 
-0.288
 
 
19.0
 
 
 
 
BTAF1
 
 
hsa-miR-15b-5p
 
 
-0.496
 
 
19.0
 
 
 
 
BTF3
 
 
hsa-miR-15b-5p
 
 
-0.344
 
 
687.7
 
 
 
 
BTF3
 
 
hsa-miR-361-5p
 
 
-0.551
 
 
687.7
 
 
 
 
 
 
 bone marrow 
 
 
 
 
target_symbol
 
 
mature_mirna_id
 
 
score
 
 
nTPM
 
 
 
 
 
 
AAR2
 
 
hsa-miR-29a-3p
 
 
-0.327
 
 
21.0
 
 
 
 
ABCE1
 
 
hsa-miR-29a-3p
 
 
-0.404
 
 
25.7
 
 
 
 
ABHD17B
 
 
hsa-miR-486-5p
 
 
-0.348
 
 
18.0
 
 
 
 
ABHD4
 
 
hsa-miR-29a-3p
 
 
-0.312
 
 
12.9
 
 
 
 
ABHD5
 
 
hsa-miR-132-3p
 
 
-0.323
 
 
37.5
 
 
 
 
ACTR2
 
 
hsa-miR-15b-5p
 
 
-0.293
 
 
138.4
 
 
 
 
ADA
 
 
hsa-miR-29a-3p
 
 
-0.376
 
 
14.3
 
 
 
 
ADCY3
 
 
hsa-miR-132-3p
 
 
-0.303
 
 
16.7
 
 
 
 
ADRB2
 
 
hsa-miR-15b-5p
 
 
-0.29
 
 
11.8
 
 
 
 
AFG3L2
 
 
hsa-miR-181b-5p
 
 
-0.491
 
 
23.4
 
 
 
 
AFG3L2
 
 
hsa-miR-181a-5p
 
 
-0.491
 
 
23.4
 
 
 
 
AGO2
 
 
hsa-miR-132-3p
 
 
-0.406
 
 
10.7
 
 
 
 
AGO2
 
 
hsa-miR-23a-3p
 
 
-0.576
 
 
10.7
 
 
 
 
AGO2
 
 
hsa-miR-23b-3p
 
 
-0.587
 
 
10.7
 
 
 
 
AGO2
 
 
hsa-miR-30b-5p
 
 
-1.034
 
 
10.7
 
 
 
 
AGO4
 
 
hsa-miR-221-3p
 
 
-0.321
 
 
19.2
 
 
 
 
AHR
 
 
hsa-miR-29a-3p
 
 
-0.316
 
 
41.5
 
 
 
 
AK1
 
 
hsa-miR-29a-3p
 
 
-0.333
 
 
27.0
 
 
 
 
ALAD
 
 
hsa-miR-15b-5p
 
 
-0.446
 
 
77.5
 
 
 
 
ANKFY1
 
 
hsa-miR-155-5p
 
 
-0.315
 
 
15.5
 
 
 
 
ANKHD1
 
 
hsa-miR-23a-3p
 
 
-0.368
 
 
69.9
 
 
 
 
ANKHD1
 
 
hsa-miR-23b-3p
 
 
-0.368
 
 
69.9
 
 
 
 
ANKHD1
 
 
hsa-miR-30b-5p
 
 
-0.405
 
 
69.9
 
 
 
 
ANKRA2
 
 
hsa-miR-30b-5p
 
 
-0.354
 
 
13.2
 
 
 
 
ANKRA2
 
 
hsa-miR-30b-5p
 
 
-0.472
 
 
13.2
 
 
 
 
ANLN
 
 
hsa-miR-15b-5p
 
 
-0.334
 
 
34.3
 
 
 
 
ANP32A
 
 
hsa-miR-132-3p
 
 
-0.374
 
 
129.5
 
 
 
 
ANP32E
 
 
hsa-miR-501-3p
 
 
-0.331
 
 
71.4
 
 
 
 
ANTXR2
 
 
hsa-miR-29a-3p
 
 
-0.347
 
 
17.7
 
 
 
 
ANXA11
 
 
hsa-miR-15b-5p
 
 
-0.463
 
 
182.2
 
 
 
 
AP2A1
 
 
hsa-miR-15b-5p
 
 
-0.333
 
 
51.3
 
 
 
 
AP2B1
 
 
hsa-miR-15b-5p
 
 
-0.334
 
 
46.4
 
 
 
 
AP3S1
 
 
hsa-miR-30b-5p
 
 
-0.293
 
 
125.9
 
 
 
 
APP
 
 
hsa-miR-15b-5p
 
 
-0.298
 
 
119.8
 
 
 
 
AQP3
 
 
hsa-miR-874-3p
 
 
-0.48
 
 
18.6
 
 
 
 
ARF4
 
 
hsa-miR-30b-5p
 
 
-0.289
 
 
68.5
 
 
 
 
ARF4
 
 
hsa-miR-221-3p
 
 
-0.553
 
 
68.5
 
 
 
 
ARF5
 
 
hsa-miR-29a-3p
 
 
-0.491
 
 
135.0
 
 
 
 
ARF6
 
 
hsa-miR-181b-5p
 
 
-0.33
 
 
78.0
 
 
 
 
ARF6
 
 
hsa-miR-181a-5p
 
 
-0.33
 
 
78.0
 
 
 
 
ARHGAP5
 
 
hsa-miR-486-5p
 
 
-0.3
 
 
10.3
 
 
 
 
ARID1B
 
 
hsa-miR-29a-3p
 
 
-0.342
 
 
27.3
 
 
 
 
ARID2
 
 
hsa-miR-132-3p
 
 
-0.304
 
 
11.7
 
 
 
 
ARID2
 
 
hsa-miR-155-5p
 
 
-0.384
 
 
11.7
 
 
 
 
ARID4B
 
 
hsa-miR-486-5p
 
 
-0.367
 
 
31.2
 
 
 
 
ARL2
 
 
hsa-miR-15b-5p
 
 
-0.374
 
 
48.4
 
 
 
 
ARL2
 
 
hsa-miR-15b-5p
 
 
-0.442
 
 
48.4
 
 
 
 
ARL4A
 
 
hsa-miR-29a-3p
 
 
-0.386
 
 
92.7
 
 
 
 
ARL4A
 
 
hsa-miR-30b-5p
 
 
-0.448
 
 
92.7
 
 
 
 
ARL5B
 
 
hsa-miR-29a-3p
 
 
-0.304
 
 
18.5
 
 
 
 
ARL5B
 
 
hsa-miR-155-5p
 
 
-0.355
 
 
18.5
 
 
 
 
ARMC8
 
 
hsa-miR-486-5p
 
 
-0.321
 
 
57.2
 
 
 
 
ARMCX3
 
 
hsa-miR-425-5p
 
 
-0.325
 
 
13.9
 
 
 
 
ARNT
 
 
hsa-miR-221-3p
 
 
-0.465
 
 
16.2
 
 
 
 
ARPP19
 
 
hsa-miR-15b-5p
 
 
-0.346
 
 
97.5
 
 
 
 
ARPP19
 
 
hsa-miR-29a-3p
 
 
-0.371
 
 
97.5
 
 
 
 
ARRDC3
 
 
hsa-miR-29a-3p
 
 
-0.402
 
 
158.3
 
 
 
 
ASB3
 
 
hsa-miR-30b-5p
 
 
-0.418
 
 
13.9
 
 
 
 
ASB3
 
 
hsa-miR-30b-5p
 
 
-0.418
 
 
13.9
 
 
 
 
ASB6
 
 
hsa-miR-874-3p
 
 
-0.368
 
 
25.3
 
 
 
 
ASH1L
 
 
hsa-miR-15b-5p
 
 
-0.325
 
 
11.2
 
 
 
 
ASPH
 
 
hsa-miR-221-3p
 
 
-0.287
 
 
15.8
 
 
 
 
ATF3
 
 
hsa-miR-874-3p
 
 
-0.288
 
 
42.6
 
 
 
 
ATG9A
 
 
hsa-miR-15b-5p
 
 
-0.333
 
 
42.2
 
 
 
 
ATOSA
 
 
hsa-miR-221-3p
 
 
-0.495
 
 
20.2
 
 
 
 
ATP5MC3
 
 
hsa-miR-425-5p
 
 
-0.328
 
 
133.0
 
 
 
 
ATP5MC3
 
 
hsa-miR-29a-3p
 
 
-0.33
 
 
133.0
 
 
 
 
ATP6V1E1
 
 
hsa-miR-23a-3p
 
 
-0.294
 
 
85.4
 
 
 
 
ATP6V1E1
 
 
hsa-miR-23b-3p
 
 
-0.294
 
 
85.4
 
 
 
 
ATPAF1
 
 
hsa-miR-361-5p
 
 
-0.362
 
 
26.0
 
 
 
 
ATXN1L
 
 
hsa-miR-155-5p
 
 
-0.315
 
 
37.3
 
 
 
 
AZIN1
 
 
hsa-miR-132-3p
 
 
-0.29
 
 
123.6
 
 
 
 
AZIN1
 
 
hsa-miR-132-3p
 
 
-0.299
 
 
123.6
 
 
 
 
B3GNT5
 
 
hsa-miR-30b-5p
 
 
-0.326
 
 
72.3
 
 
 
 
B4GALT5
 
 
hsa-miR-501-3p
 
 
-0.408
 
 
88.5
 
 
 
 
BAG4
 
 
hsa-miR-181b-5p
 
 
-0.337
 
 
27.0
 
 
 
 
BAG4
 
 
hsa-miR-181a-5p
 
 
-0.337
 
 
27.0
 
 
 
 
BAK1
 
 
hsa-miR-29a-3p
 
 
-0.409
 
 
26.1
 
 
 
 
BCL2L11
 
 
hsa-miR-221-3p
 
 
-0.423
 
 
37.8
 
 
 
 
BCL2L2
 
 
hsa-miR-29a-3p
 
 
-0.312
 
 
12.9
 
 
 
 
BCOR
 
 
hsa-miR-425-5p
 
 
-0.346
 
 
20.9
 
 
 
 
BECN1
 
 
hsa-miR-30b-5p
 
 
-0.452
 
 
47.8
 
 
 
 
BFAR
 
 
hsa-miR-15b-5p
 
 
-0.352
 
 
16.2
 
 
 
 
BHLHE40
 
 
hsa-miR-181b-5p
 
 
-0.408
 
 
177.7
 
 
 
 
BHLHE40
 
 
hsa-miR-181a-5p
 
 
-0.408
 
 
177.7
 
 
 
 
BLCAP
 
 
hsa-miR-23a-3p
 
 
-0.601
 
 
33.9
 
 
 
 
BLCAP
 
 
hsa-miR-23b-3p
 
 
-0.601
 
 
33.9
 
 
 
 
BLMH
 
 
hsa-miR-29a-3p
 
 
-0.393
 
 
17.1
 
 
 
 
BLOC1S6
 
 
hsa-miR-181b-5p
 
 
-0.393
 
 
54.9
 
 
 
 
BLOC1S6
 
 
hsa-miR-181a-5p
 
 
-0.393
 
 
54.9
 
 
 
 
BMF
 
 
hsa-miR-29a-3p
 
 
-0.41
 
 
31.1
 
 
 
 
BMF
 
 
hsa-miR-221-3p
 
 
-0.453
 
 
31.1
 
 
 
 
BOLA3
 
 
hsa-miR-15b-5p
 
 
-0.363
 
 
38.4
 
 
 
 
BOLA3
 
 
hsa-miR-15b-5p
 
 
-0.386
 
 
38.4
 
 
 
 
BOLA3
 
 
hsa-miR-374b-5p
 
 
-0.486
 
 
38.4
 
 
 
 
BRI3
 
 
hsa-miR-132-3p
 
 
-0.385
 
 
89.5
 
 
 
 
BTAF1
 
 
hsa-miR-486-5p
 
 
-0.288
 
 
25.4
 
 
 
 
BTAF1
 
 
hsa-miR-15b-5p
 
 
-0.496
 
 
25.4
 
 
 
 
BTF3
 
 
hsa-miR-15b-5p
 
 
-0.344
 
 
811.7
 
 
 
 
BTF3
 
 
hsa-miR-361-5p
 
 
-0.551
 
 
811.7
 
 
 
 
 
 
 breast 
 
 
 
 
target_symbol
 
 
mature_mirna_id
 
 
score
 
 
nTPM
 
 
 
 
 
 
AAR2
 
 
hsa-miR-29a-3p
 
 
-0.327
 
 
20.3
 
 
 
 
ABCE1
 
 
hsa-miR-29a-3p
 
 
-0.404
 
 
30.9
 
 
 
 
ABHD4
 
 
hsa-miR-29a-3p
 
 
-0.312
 
 
18.7
 
 
 
 
ABHD5
 
 
hsa-miR-132-3p
 
 
-0.323
 
 
18.8
 
 
 
 
ACTA1
 
 
hsa-miR-155-5p
 
 
-0.595
 
 
15.5
 
 
 
 
ACTC1
 
 
hsa-miR-30b-5p
 
 
-0.543
 
 
11.2
 
 
 
 
ACTR2
 
 
hsa-miR-15b-5p
 
 
-0.293
 
 
72.1
 
 
 
 
ACVR1
 
 
hsa-miR-30b-5p
 
 
-0.29
 
 
11.1
 
 
 
 
ADA
 
 
hsa-miR-29a-3p
 
 
-0.376
 
 
11.4
 
 
 
 
ADAM9
 
 
hsa-miR-30b-5p
 
 
-0.312
 
 
20.2
 
 
 
 
ADAMTS9
 
 
hsa-miR-29a-3p
 
 
-0.305
 
 
19.0
 
 
 
 
ADAMTS9
 
 
hsa-miR-29a-3p
 
 
-0.485
 
 
19.0
 
 
 
 
ADCY3
 
 
hsa-miR-132-3p
 
 
-0.303
 
 
72.4
 
 
 
 
ADGRA3
 
 
hsa-miR-30b-5p
 
 
-0.304
 
 
10.2
 
 
 
 
ADIPOR2
 
 
hsa-miR-150-5p
 
 
-0.287
 
 
44.0
 
 
 
 
ADRA2A
 
 
hsa-miR-23a-3p
 
 
-0.371
 
 
31.1
 
 
 
 
ADRA2A
 
 
hsa-miR-23b-3p
 
 
-0.371
 
 
31.1
 
 
 
 
ADRB2
 
 
hsa-miR-15b-5p
 
 
-0.29
 
 
32.4
 
 
 
 
AFG3L2
 
 
hsa-miR-181b-5p
 
 
-0.491
 
 
15.5
 
 
 
 
AFG3L2
 
 
hsa-miR-181a-5p
 
 
-0.491
 
 
15.5
 
 
 
 
AHR
 
 
hsa-miR-29a-3p
 
 
-0.316
 
 
18.4
 
 
 
 
AK1
 
 
hsa-miR-29a-3p
 
 
-0.333
 
 
20.7
 
 
 
 
AK4
 
 
hsa-miR-15b-5p
 
 
-0.316
 
 
24.4
 
 
 
 
AKT3
 
 
hsa-miR-29a-3p
 
 
-0.287
 
 
13.8
 
 
 
 
AKT3
 
 
hsa-miR-15b-5p
 
 
-0.301
 
 
13.8
 
 
 
 
ALAD
 
 
hsa-miR-15b-5p
 
 
-0.446
 
 
21.7
 
 
 
 
AMOTL1
 
 
hsa-miR-15b-5p
 
 
-0.319
 
 
14.0
 
 
 
 
ANGPTL2
 
 
hsa-miR-221-3p
 
 
-0.369
 
 
69.5
 
 
 
 
ANKFY1
 
 
hsa-miR-155-5p
 
 
-0.315
 
 
11.8
 
 
 
 
ANKHD1
 
 
hsa-miR-23a-3p
 
 
-0.368
 
 
37.8
 
 
 
 
ANKHD1
 
 
hsa-miR-23b-3p
 
 
-0.368
 
 
37.8
 
 
 
 
ANKHD1
 
 
hsa-miR-30b-5p
 
 
-0.405
 
 
37.8
 
 
 
 
ANKRD17
 
 
hsa-miR-30b-5p
 
 
-0.336
 
 
18.7
 
 
 
 
ANKRD29
 
 
hsa-miR-132-3p
 
 
-0.47
 
 
15.0
 
 
 
 
ANP32A
 
 
hsa-miR-132-3p
 
 
-0.374
 
 
66.9
 
 
 
 
ANP32E
 
 
hsa-miR-501-3p
 
 
-0.331
 
 
16.9
 
 
 
 
ANTXR2
 
 
hsa-miR-29a-3p
 
 
-0.347
 
 
38.4
 
 
 
 
ANXA11
 
 
hsa-miR-15b-5p
 
 
-0.463
 
 
101.9
 
 
 
 
AP2A1
 
 
hsa-miR-15b-5p
 
 
-0.333
 
 
39.7
 
 
 
 
AP2B1
 
 
hsa-miR-15b-5p
 
 
-0.334
 
 
54.6
 
 
 
 
AP3S1
 
 
hsa-miR-30b-5p
 
 
-0.293
 
 
56.3
 
 
 
 
APOO
 
 
hsa-miR-181b-5p
 
 
-0.5
 
 
12.5
 
 
 
 
APOO
 
 
hsa-miR-181a-5p
 
 
-0.5
 
 
12.5
 
 
 
 
APP
 
 
hsa-miR-15b-5p
 
 
-0.298
 
 
392.7
 
 
 
 
AQP3
 
 
hsa-miR-874-3p
 
 
-0.48
 
 
34.4
 
 
 
 
ARF4
 
 
hsa-miR-30b-5p
 
 
-0.289
 
 
129.6
 
 
 
 
ARF4
 
 
hsa-miR-221-3p
 
 
-0.553
 
 
129.6
 
 
 
 
ARF5
 
 
hsa-miR-29a-3p
 
 
-0.491
 
 
108.8
 
 
 
 
ARF6
 
 
hsa-miR-181b-5p
 
 
-0.33
 
 
31.6
 
 
 
 
ARF6
 
 
hsa-miR-181a-5p
 
 
-0.33
 
 
31.6
 
 
 
 
ARHGAP5
 
 
hsa-miR-486-5p
 
 
-0.3
 
 
25.6
 
 
 
 
ARID1B
 
 
hsa-miR-29a-3p
 
 
-0.342
 
 
11.7
 
 
 
 
ARID4B
 
 
hsa-miR-486-5p
 
 
-0.367
 
 
12.7
 
 
 
 
ARL2
 
 
hsa-miR-15b-5p
 
 
-0.374
 
 
113.6
 
 
 
 
ARL2
 
 
hsa-miR-15b-5p
 
 
-0.442
 
 
113.6
 
 
 
 
ARL4A
 
 
hsa-miR-29a-3p
 
 
-0.386
 
 
51.6
 
 
 
 
ARL4A
 
 
hsa-miR-30b-5p
 
 
-0.448
 
 
51.6
 
 
 
 
ARMC8
 
 
hsa-miR-486-5p
 
 
-0.321
 
 
15.4
 
 
 
 
ARMCX2
 
 
hsa-miR-15b-5p
 
 
-0.344
 
 
16.4
 
 
 
 
ARMCX3
 
 
hsa-miR-425-5p
 
 
-0.325
 
 
30.1
 
 
 
 
ARNT
 
 
hsa-miR-221-3p
 
 
-0.465
 
 
22.2
 
 
 
 
ARPP19
 
 
hsa-miR-15b-5p
 
 
-0.346
 
 
43.2
 
 
 
 
ARPP19
 
 
hsa-miR-29a-3p
 
 
-0.371
 
 
43.2
 
 
 
 
ARRDC3
 
 
hsa-miR-29a-3p
 
 
-0.402
 
 
88.5
 
 
 
 
ASF1A
 
 
hsa-miR-23a-3p
 
 
-0.506
 
 
11.7
 
 
 
 
ASF1A
 
 
hsa-miR-23b-3p
 
 
-0.506
 
 
11.7
 
 
 
 
ASH1L
 
 
hsa-miR-15b-5p
 
 
-0.325
 
 
18.5
 
 
 
 
ASPH
 
 
hsa-miR-221-3p
 
 
-0.287
 
 
99.5
 
 
 
 
ATF3
 
 
hsa-miR-874-3p
 
 
-0.288
 
 
97.6
 
 
 
 
ATG5
 
 
hsa-miR-30b-5p
 
 
-0.488
 
 
14.4
 
 
 
 
ATG9A
 
 
hsa-miR-15b-5p
 
 
-0.333
 
 
20.5
 
 
 
 
ATOSA
 
 
hsa-miR-221-3p
 
 
-0.495
 
 
25.6
 
 
 
 
ATP5MC3
 
 
hsa-miR-425-5p
 
 
-0.328
 
 
159.0
 
 
 
 
ATP5MC3
 
 
hsa-miR-29a-3p
 
 
-0.33
 
 
159.0
 
 
 
 
ATP6V1E1
 
 
hsa-miR-23a-3p
 
 
-0.294
 
 
110.8
 
 
 
 
ATP6V1E1
 
 
hsa-miR-23b-3p
 
 
-0.294
 
 
110.8
 
 
 
 
ATPAF1
 
 
hsa-miR-361-5p
 
 
-0.362
 
 
33.1
 
 
 
 
ATXN1L
 
 
hsa-miR-155-5p
 
 
-0.315
 
 
10.2
 
 
 
 
ATXN2
 
 
hsa-miR-15b-5p
 
 
-0.392
 
 
16.9
 
 
 
 
AUH
 
 
hsa-miR-23a-3p
 
 
-0.495
 
 
14.9
 
 
 
 
AUH
 
 
hsa-miR-23b-3p
 
 
-0.503
 
 
14.9
 
 
 
 
AUH
 
 
hsa-miR-23a-3p
 
 
-0.512
 
 
14.9
 
 
 
 
AUH
 
 
hsa-miR-23b-3p
 
 
-0.512
 
 
14.9
 
 
 
 
AZIN1
 
 
hsa-miR-132-3p
 
 
-0.29
 
 
53.1
 
 
 
 
AZIN1
 
 
hsa-miR-132-3p
 
 
-0.299
 
 
53.1
 
 
 
 
B4GALT5
 
 
hsa-miR-501-3p
 
 
-0.408
 
 
17.9
 
 
 
 
B4GAT1
 
 
hsa-miR-23a-3p
 
 
-0.361
 
 
15.6
 
 
 
 
B4GAT1
 
 
hsa-miR-23b-3p
 
 
-0.361
 
 
15.6
 
 
 
 
BCL2L11
 
 
hsa-miR-221-3p
 
 
-0.423
 
 
11.8
 
 
 
 
BCL2L2
 
 
hsa-miR-29a-3p
 
 
-0.312
 
 
36.1
 
 
 
 
BECN1
 
 
hsa-miR-30b-5p
 
 
-0.452
 
 
41.1
 
 
 
 
BET1
 
 
hsa-miR-23a-3p
 
 
-0.402
 
 
21.8
 
 
 
 
BET1
 
 
hsa-miR-23b-3p
 
 
-0.418
 
 
21.8
 
 
 
 
BFAR
 
 
hsa-miR-15b-5p
 
 
-0.352
 
 
31.8
 
 
 
 
BHLHE40
 
 
hsa-miR-181b-5p
 
 
-0.408
 
 
211.1
 
 
 
 
BHLHE40
 
 
hsa-miR-181a-5p
 
 
-0.408
 
 
211.1
 
 
 
 
BLCAP
 
 
hsa-miR-23a-3p
 
 
-0.601
 
 
58.6
 
 
 
 
BLCAP
 
 
hsa-miR-23b-3p
 
 
-0.601
 
 
58.6
 
 
 
 
BLMH
 
 
hsa-miR-29a-3p
 
 
-0.393
 
 
19.1
 
 
 
 
BLOC1S6
 
 
hsa-miR-181b-5p
 
 
-0.393
 
 
23.9
 
 
 
 
 
 
 cerebellum 
 
 
 
 
target_symbol
 
 
mature_mirna_id
 
 
score
 
 
nTPM
 
 
 
 
 
 
AAR2
 
 
hsa-miR-29a-3p
 
 
-0.327
 
 
10.9
 
 
 
 
ABCE1
 
 
hsa-miR-29a-3p
 
 
-0.404
 
 
12.8
 
 
 
 
ABHD17B
 
 
hsa-miR-486-5p
 
 
-0.348
 
 
11.2
 
 
 
 
ACTR2
 
 
hsa-miR-15b-5p
 
 
-0.293
 
 
54.0
 
 
 
 
ADCY3
 
 
hsa-miR-132-3p
 
 
-0.303
 
 
33.6
 
 
 
 
ADIPOR2
 
 
hsa-miR-150-5p
 
 
-0.287
 
 
18.7
 
 
 
 
AFG3L2
 
 
hsa-miR-181b-5p
 
 
-0.491
 
 
11.5
 
 
 
 
AFG3L2
 
 
hsa-miR-181a-5p
 
 
-0.491
 
 
11.5
 
 
 
 
AK1
 
 
hsa-miR-29a-3p
 
 
-0.333
 
 
47.4
 
 
 
 
AK4
 
 
hsa-miR-15b-5p
 
 
-0.316
 
 
14.5
 
 
 
 
AKAIN1
 
 
hsa-miR-29a-3p
 
 
-0.52
 
 
40.8
 
 
 
 
AKT3
 
 
hsa-miR-29a-3p
 
 
-0.287
 
 
23.2
 
 
 
 
AKT3
 
 
hsa-miR-15b-5p
 
 
-0.301
 
 
23.2
 
 
 
 
ALAD
 
 
hsa-miR-15b-5p
 
 
-0.446
 
 
17.9
 
 
 
 
ANGEL2
 
 
hsa-miR-132-3p
 
 
-0.357
 
 
12.7
 
 
 
 
ANGPTL2
 
 
hsa-miR-221-3p
 
 
-0.369
 
 
18.7
 
 
 
 
ANKFY1
 
 
hsa-miR-155-5p
 
 
-0.315
 
 
15.9
 
 
 
 
ANKHD1
 
 
hsa-miR-23a-3p
 
 
-0.368
 
 
38.6
 
 
 
 
ANKHD1
 
 
hsa-miR-23b-3p
 
 
-0.368
 
 
38.6
 
 
 
 
ANKHD1
 
 
hsa-miR-30b-5p
 
 
-0.405
 
 
38.6
 
 
 
 
ANKRD13B
 
 
hsa-miR-29a-3p
 
 
-0.421
 
 
44.3
 
 
 
 
ANKRD13C
 
 
hsa-miR-29a-3p
 
 
-0.411
 
 
12.9
 
 
 
 
ANKRD17
 
 
hsa-miR-30b-5p
 
 
-0.336
 
 
16.2
 
 
 
 
ANP32A
 
 
hsa-miR-132-3p
 
 
-0.374
 
 
71.1
 
 
 
 
ANP32E
 
 
hsa-miR-501-3p
 
 
-0.331
 
 
58.6
 
 
 
 
ANXA11
 
 
hsa-miR-15b-5p
 
 
-0.463
 
 
22.2
 
 
 
 
AP2A1
 
 
hsa-miR-15b-5p
 
 
-0.333
 
 
67.6
 
 
 
 
AP2B1
 
 
hsa-miR-15b-5p
 
 
-0.334
 
 
63.1
 
 
 
 
AP3S1
 
 
hsa-miR-30b-5p
 
 
-0.293
 
 
52.4
 
 
 
 
APC
 
 
hsa-miR-29a-3p
 
 
-0.413
 
 
12.2
 
 
 
 
APOO
 
 
hsa-miR-181b-5p
 
 
-0.5
 
 
32.8
 
 
 
 
APOO
 
 
hsa-miR-181a-5p
 
 
-0.5
 
 
32.8
 
 
 
 
APP
 
 
hsa-miR-15b-5p
 
 
-0.298
 
 
316.4
 
 
 
 
ARF4
 
 
hsa-miR-30b-5p
 
 
-0.289
 
 
50.2
 
 
 
 
ARF4
 
 
hsa-miR-221-3p
 
 
-0.553
 
 
50.2
 
 
 
 
ARF5
 
 
hsa-miR-29a-3p
 
 
-0.491
 
 
167.9
 
 
 
 
ARF6
 
 
hsa-miR-181b-5p
 
 
-0.33
 
 
11.0
 
 
 
 
ARF6
 
 
hsa-miR-181a-5p
 
 
-0.33
 
 
11.0
 
 
 
 
ARHGAP5
 
 
hsa-miR-486-5p
 
 
-0.3
 
 
20.0
 
 
 
 
ARID1B
 
 
hsa-miR-29a-3p
 
 
-0.342
 
 
12.5
 
 
 
 
ARID4B
 
 
hsa-miR-486-5p
 
 
-0.367
 
 
15.1
 
 
 
 
ARL2
 
 
hsa-miR-15b-5p
 
 
-0.374
 
 
72.8
 
 
 
 
ARL2
 
 
hsa-miR-15b-5p
 
 
-0.442
 
 
72.8
 
 
 
 
ARL3
 
 
hsa-miR-15b-5p
 
 
-0.507
 
 
25.4
 
 
 
 
ARMC8
 
 
hsa-miR-486-5p
 
 
-0.321
 
 
35.7
 
 
 
 
ARMCX2
 
 
hsa-miR-15b-5p
 
 
-0.344
 
 
20.7
 
 
 
 
ARMCX3
 
 
hsa-miR-425-5p
 
 
-0.325
 
 
24.0
 
 
 
 
ARNT
 
 
hsa-miR-221-3p
 
 
-0.465
 
 
19.0
 
 
 
 
ARPP19
 
 
hsa-miR-15b-5p
 
 
-0.346
 
 
66.7
 
 
 
 
ARPP19
 
 
hsa-miR-29a-3p
 
 
-0.371
 
 
66.7
 
 
 
 
ARPP21
 
 
hsa-miR-15b-5p
 
 
-0.342
 
 
42.2
 
 
 
 
ARPP21
 
 
hsa-miR-501-3p
 
 
-0.546
 
 
42.2
 
 
 
 
ARRDC3
 
 
hsa-miR-29a-3p
 
 
-0.402
 
 
17.8
 
 
 
 
ARVCF
 
 
hsa-miR-155-5p
 
 
-0.388
 
 
52.5
 
 
 
 
ASAP2
 
 
hsa-miR-29a-3p
 
 
-0.348
 
 
10.8
 
 
 
 
ASB6
 
 
hsa-miR-874-3p
 
 
-0.368
 
 
21.0
 
 
 
 
ASF1A
 
 
hsa-miR-23a-3p
 
 
-0.506
 
 
12.6
 
 
 
 
ASF1A
 
 
hsa-miR-23b-3p
 
 
-0.506
 
 
12.6
 
 
 
 
ASH1L
 
 
hsa-miR-15b-5p
 
 
-0.325
 
 
34.5
 
 
 
 
ASPH
 
 
hsa-miR-221-3p
 
 
-0.287
 
 
80.5
 
 
 
 
ATG9A
 
 
hsa-miR-15b-5p
 
 
-0.333
 
 
44.0
 
 
 
 
ATOSA
 
 
hsa-miR-221-3p
 
 
-0.495
 
 
11.0
 
 
 
 
ATP5MC3
 
 
hsa-miR-425-5p
 
 
-0.328
 
 
183.5
 
 
 
 
ATP5MC3
 
 
hsa-miR-29a-3p
 
 
-0.33
 
 
183.5
 
 
 
 
ATP6V1E1
 
 
hsa-miR-23a-3p
 
 
-0.294
 
 
190.9
 
 
 
 
ATP6V1E1
 
 
hsa-miR-23b-3p
 
 
-0.294
 
 
190.9
 
 
 
 
ATPAF1
 
 
hsa-miR-361-5p
 
 
-0.362
 
 
24.4
 
 
 
 
ATXN2
 
 
hsa-miR-15b-5p
 
 
-0.392
 
 
28.2
 
 
 
 
AUH
 
 
hsa-miR-23a-3p
 
 
-0.495
 
 
16.6
 
 
 
 
AUH
 
 
hsa-miR-23b-3p
 
 
-0.503
 
 
16.6
 
 
 
 
AUH
 
 
hsa-miR-23a-3p
 
 
-0.512
 
 
16.6
 
 
 
 
AUH
 
 
hsa-miR-23b-3p
 
 
-0.512
 
 
16.6
 
 
 
 
AZIN1
 
 
hsa-miR-132-3p
 
 
-0.29
 
 
31.3
 
 
 
 
AZIN1
 
 
hsa-miR-132-3p
 
 
-0.299
 
 
31.3
 
 
 
 
B4GALT5
 
 
hsa-miR-501-3p
 
 
-0.408
 
 
20.4
 
 
 
 
B4GAT1
 
 
hsa-miR-23a-3p
 
 
-0.361
 
 
105.2
 
 
 
 
B4GAT1
 
 
hsa-miR-23b-3p
 
 
-0.361
 
 
105.2
 
 
 
 
BAG4
 
 
hsa-miR-181b-5p
 
 
-0.337
 
 
14.8
 
 
 
 
BAG4
 
 
hsa-miR-181a-5p
 
 
-0.337
 
 
14.8
 
 
 
 
BCL2L2
 
 
hsa-miR-29a-3p
 
 
-0.312
 
 
53.9
 
 
 
 
BCL7A
 
 
hsa-miR-15b-5p
 
 
-0.336
 
 
17.6
 
 
 
 
BCL7A
 
 
hsa-miR-501-3p
 
 
-0.339
 
 
17.6
 
 
 
 
BDH1
 
 
hsa-miR-874-3p
 
 
-0.644
 
 
48.5
 
 
 
 
BECN1
 
 
hsa-miR-30b-5p
 
 
-0.452
 
 
24.5
 
 
 
 
BEND6
 
 
hsa-miR-361-5p
 
 
-0.305
 
 
44.7
 
 
 
 
BFAR
 
 
hsa-miR-15b-5p
 
 
-0.352
 
 
12.8
 
 
 
 
BHLHE40
 
 
hsa-miR-181b-5p
 
 
-0.408
 
 
18.1
 
 
 
 
BHLHE40
 
 
hsa-miR-181a-5p
 
 
-0.408
 
 
18.1
 
 
 
 
BICDL1
 
 
hsa-miR-221-3p
 
 
-0.447
 
 
32.0
 
 
 
 
BLCAP
 
 
hsa-miR-23a-3p
 
 
-0.601
 
 
93.5
 
 
 
 
BLCAP
 
 
hsa-miR-23b-3p
 
 
-0.601
 
 
93.5
 
 
 
 
BLMH
 
 
hsa-miR-29a-3p
 
 
-0.393
 
 
18.4
 
 
 
 
BLOC1S6
 
 
hsa-miR-181b-5p
 
 
-0.393
 
 
18.6
 
 
 
 
BLOC1S6
 
 
hsa-miR-181a-5p
 
 
-0.393
 
 
18.6
 
 
 
 
BOLA3
 
 
hsa-miR-15b-5p
 
 
-0.363
 
 
34.7
 
 
 
 
BOLA3
 
 
hsa-miR-15b-5p
 
 
-0.386
 
 
34.7
 
 
 
 
BOLA3
 
 
hsa-miR-374b-5p
 
 
-0.486
 
 
34.7
 
 
 
 
BRI3
 
 
hsa-miR-132-3p
 
 
-0.385
 
 
23.3
 
 
 
 
BTAF1
 
 
hsa-miR-486-5p
 
 
-0.288
 
 
10.7
 
 
 
 
BTAF1
 
 
hsa-miR-15b-5p
 
 
-0.496
 
 
10.7
 
 
 
 
 
 
 cerebral cortex 
 
 
 
 
target_symbol
 
 
mature_mirna_id
 
 
score
 
 
nTPM
 
 
 
 
 
 
AAR2
 
 
hsa-miR-29a-3p
 
 
-0.327
 
 
10.6
 
 
 
 
ABCE1
 
 
hsa-miR-29a-3p
 
 
-0.404
 
 
21.8
 
 
 
 
ABHD17B
 
 
hsa-miR-486-5p
 
 
-0.348
 
 
17.0
 
 
 
 
ABHD4
 
 
hsa-miR-29a-3p
 
 
-0.312
 
 
14.6
 
 
 
 
ACTN2
 
 
hsa-miR-23a-3p
 
 
-0.294
 
 
14.9
 
 
 
 
ACTN2
 
 
hsa-miR-29a-3p
 
 
-0.311
 
 
14.9
 
 
 
 
ACTR2
 
 
hsa-miR-15b-5p
 
 
-0.293
 
 
115.2
 
 
 
 
ADAM9
 
 
hsa-miR-30b-5p
 
 
-0.312
 
 
16.2
 
 
 
 
ADAMTS10
 
 
hsa-miR-29a-3p
 
 
-0.516
 
 
10.8
 
 
 
 
ADCY3
 
 
hsa-miR-132-3p
 
 
-0.303
 
 
37.4
 
 
 
 
ADGRA3
 
 
hsa-miR-30b-5p
 
 
-0.304
 
 
17.8
 
 
 
 
ADIPOR2
 
 
hsa-miR-150-5p
 
 
-0.287
 
 
29.8
 
 
 
 
AFG3L2
 
 
hsa-miR-181b-5p
 
 
-0.491
 
 
17.1
 
 
 
 
AFG3L2
 
 
hsa-miR-181a-5p
 
 
-0.491
 
 
17.1
 
 
 
 
AGPAT4
 
 
hsa-miR-29a-3p
 
 
-0.376
 
 
13.7
 
 
 
 
AK1
 
 
hsa-miR-29a-3p
 
 
-0.333
 
 
81.2
 
 
 
 
AK4
 
 
hsa-miR-15b-5p
 
 
-0.316
 
 
19.8
 
 
 
 
AKAP5
 
 
hsa-miR-29a-3p
 
 
-0.298
 
 
12.9
 
 
 
 
AKT3
 
 
hsa-miR-29a-3p
 
 
-0.287
 
 
23.7
 
 
 
 
AKT3
 
 
hsa-miR-15b-5p
 
 
-0.301
 
 
23.7
 
 
 
 
ALAD
 
 
hsa-miR-15b-5p
 
 
-0.446
 
 
40.0
 
 
 
 
AMOT
 
 
hsa-miR-29a-3p
 
 
-0.304
 
 
17.9
 
 
 
 
ANGPTL2
 
 
hsa-miR-221-3p
 
 
-0.369
 
 
32.8
 
 
 
 
ANKFY1
 
 
hsa-miR-155-5p
 
 
-0.315
 
 
19.9
 
 
 
 
ANKHD1
 
 
hsa-miR-23a-3p
 
 
-0.368
 
 
22.1
 
 
 
 
ANKHD1
 
 
hsa-miR-23b-3p
 
 
-0.368
 
 
22.1
 
 
 
 
ANKHD1
 
 
hsa-miR-30b-5p
 
 
-0.405
 
 
22.1
 
 
 
 
ANKRD13B
 
 
hsa-miR-29a-3p
 
 
-0.421
 
 
17.2
 
 
 
 
ANKRD13C
 
 
hsa-miR-29a-3p
 
 
-0.411
 
 
10.2
 
 
 
 
ANKRD17
 
 
hsa-miR-30b-5p
 
 
-0.336
 
 
15.6
 
 
 
 
ANLN
 
 
hsa-miR-15b-5p
 
 
-0.334
 
 
47.6
 
 
 
 
ANP32A
 
 
hsa-miR-132-3p
 
 
-0.374
 
 
64.2
 
 
 
 
ANP32E
 
 
hsa-miR-501-3p
 
 
-0.331
 
 
33.9
 
 
 
 
ANXA11
 
 
hsa-miR-15b-5p
 
 
-0.463
 
 
45.1
 
 
 
 
AP2A1
 
 
hsa-miR-15b-5p
 
 
-0.333
 
 
78.9
 
 
 
 
AP2B1
 
 
hsa-miR-15b-5p
 
 
-0.334
 
 
106.7
 
 
 
 
AP3S1
 
 
hsa-miR-30b-5p
 
 
-0.293
 
 
82.8
 
 
 
 
APC
 
 
hsa-miR-29a-3p
 
 
-0.413
 
 
34.5
 
 
 
 
APLN
 
 
hsa-miR-15b-5p
 
 
-0.395
 
 
14.5
 
 
 
 
APOO
 
 
hsa-miR-181b-5p
 
 
-0.5
 
 
56.2
 
 
 
 
APOO
 
 
hsa-miR-181a-5p
 
 
-0.5
 
 
56.2
 
 
 
 
APP
 
 
hsa-miR-15b-5p
 
 
-0.298
 
 
669.1
 
 
 
 
ARF4
 
 
hsa-miR-30b-5p
 
 
-0.289
 
 
61.5
 
 
 
 
ARF4
 
 
hsa-miR-221-3p
 
 
-0.553
 
 
61.5
 
 
 
 
ARF5
 
 
hsa-miR-29a-3p
 
 
-0.491
 
 
222.4
 
 
 
 
ARF6
 
 
hsa-miR-181b-5p
 
 
-0.33
 
 
13.0
 
 
 
 
ARF6
 
 
hsa-miR-181a-5p
 
 
-0.33
 
 
13.0
 
 
 
 
ARHGAP5
 
 
hsa-miR-486-5p
 
 
-0.3
 
 
48.8
 
 
 
 
ARID1B
 
 
hsa-miR-29a-3p
 
 
-0.342
 
 
12.2
 
 
 
 
ARID4B
 
 
hsa-miR-486-5p
 
 
-0.367
 
 
11.1
 
 
 
 
ARL2
 
 
hsa-miR-15b-5p
 
 
-0.374
 
 
152.3
 
 
 
 
ARL2
 
 
hsa-miR-15b-5p
 
 
-0.442
 
 
152.3
 
 
 
 
ARL3
 
 
hsa-miR-15b-5p
 
 
-0.507
 
 
30.4
 
 
 
 
ARL4A
 
 
hsa-miR-29a-3p
 
 
-0.386
 
 
23.3
 
 
 
 
ARL4A
 
 
hsa-miR-30b-5p
 
 
-0.448
 
 
23.3
 
 
 
 
ARMC8
 
 
hsa-miR-486-5p
 
 
-0.321
 
 
21.7
 
 
 
 
ARMCX2
 
 
hsa-miR-15b-5p
 
 
-0.344
 
 
27.9
 
 
 
 
ARMCX3
 
 
hsa-miR-425-5p
 
 
-0.325
 
 
42.1
 
 
 
 
ARPP19
 
 
hsa-miR-15b-5p
 
 
-0.346
 
 
292.4
 
 
 
 
ARPP19
 
 
hsa-miR-29a-3p
 
 
-0.371
 
 
292.4
 
 
 
 
ARPP21
 
 
hsa-miR-15b-5p
 
 
-0.342
 
 
68.9
 
 
 
 
ARPP21
 
 
hsa-miR-501-3p
 
 
-0.546
 
 
68.9
 
 
 
 
ARRDC3
 
 
hsa-miR-29a-3p
 
 
-0.402
 
 
15.6
 
 
 
 
ASAP2
 
 
hsa-miR-29a-3p
 
 
-0.348
 
 
13.8
 
 
 
 
ASB3
 
 
hsa-miR-30b-5p
 
 
-0.418
 
 
19.6
 
 
 
 
ASB3
 
 
hsa-miR-30b-5p
 
 
-0.418
 
 
19.6
 
 
 
 
ASB6
 
 
hsa-miR-874-3p
 
 
-0.368
 
 
21.5
 
 
 
 
ASF1A
 
 
hsa-miR-23a-3p
 
 
-0.506
 
 
13.7
 
 
 
 
ASF1A
 
 
hsa-miR-23b-3p
 
 
-0.506
 
 
13.7
 
 
 
 
ASH1L
 
 
hsa-miR-15b-5p
 
 
-0.325
 
 
26.4
 
 
 
 
ASPH
 
 
hsa-miR-221-3p
 
 
-0.287
 
 
76.3
 
 
 
 
ATF3
 
 
hsa-miR-874-3p
 
 
-0.288
 
 
13.9
 
 
 
 
ATG9A
 
 
hsa-miR-15b-5p
 
 
-0.333
 
 
41.4
 
 
 
 
ATP5MC3
 
 
hsa-miR-425-5p
 
 
-0.328
 
 
240.0
 
 
 
 
ATP5MC3
 
 
hsa-miR-29a-3p
 
 
-0.33
 
 
240.0
 
 
 
 
ATP6V1E1
 
 
hsa-miR-23a-3p
 
 
-0.294
 
 
326.2
 
 
 
 
ATP6V1E1
 
 
hsa-miR-23b-3p
 
 
-0.294
 
 
326.2
 
 
 
 
ATPAF1
 
 
hsa-miR-361-5p
 
 
-0.362
 
 
74.9
 
 
 
 
ATXN1
 
 
hsa-miR-221-3p
 
 
-0.289
 
 
12.1
 
 
 
 
ATXN2
 
 
hsa-miR-15b-5p
 
 
-0.392
 
 
26.3
 
 
 
 
AUH
 
 
hsa-miR-23a-3p
 
 
-0.495
 
 
19.3
 
 
 
 
AUH
 
 
hsa-miR-23b-3p
 
 
-0.503
 
 
19.3
 
 
 
 
AUH
 
 
hsa-miR-23a-3p
 
 
-0.512
 
 
19.3
 
 
 
 
AUH
 
 
hsa-miR-23b-3p
 
 
-0.512
 
 
19.3
 
 
 
 
AZIN1
 
 
hsa-miR-132-3p
 
 
-0.29
 
 
76.8
 
 
 
 
AZIN1
 
 
hsa-miR-132-3p
 
 
-0.299
 
 
76.8
 
 
 
 
B4GALT5
 
 
hsa-miR-501-3p
 
 
-0.408
 
 
22.9
 
 
 
 
B4GAT1
 
 
hsa-miR-23a-3p
 
 
-0.361
 
 
171.8
 
 
 
 
B4GAT1
 
 
hsa-miR-23b-3p
 
 
-0.361
 
 
171.8
 
 
 
 
BAG4
 
 
hsa-miR-181b-5p
 
 
-0.337
 
 
16.5
 
 
 
 
BAG4
 
 
hsa-miR-181a-5p
 
 
-0.337
 
 
16.5
 
 
 
 
BCL2L2
 
 
hsa-miR-29a-3p
 
 
-0.312
 
 
92.0
 
 
 
 
BCL7A
 
 
hsa-miR-15b-5p
 
 
-0.336
 
 
15.4
 
 
 
 
BCL7A
 
 
hsa-miR-501-3p
 
 
-0.339
 
 
15.4
 
 
 
 
BDH1
 
 
hsa-miR-874-3p
 
 
-0.644
 
 
28.0
 
 
 
 
BECN1
 
 
hsa-miR-30b-5p
 
 
-0.452
 
 
35.8
 
 
 
 
BEND6
 
 
hsa-miR-361-5p
 
 
-0.305
 
 
29.5
 
 
 
 
BET1
 
 
hsa-miR-23a-3p
 
 
-0.402
 
 
10.4
 
 
 
 
BET1
 
 
hsa-miR-23b-3p
 
 
-0.418
 
 
10.4
 
 
 
 
BFAR
 
 
hsa-miR-15b-5p
 
 
-0.352
 
 
12.0
 
 
 
 
 
 
 cervix 
 
 
 
 
target_symbol
 
 
mature_mirna_id
 
 
score
 
 
nTPM
 
 
 
 
 
 
AAR2
 
 
hsa-miR-29a-3p
 
 
-0.327
 
 
19.6
 
 
 
 
ABCE1
 
 
hsa-miR-29a-3p
 
 
-0.404
 
 
19.5
 
 
 
 
ABHD4
 
 
hsa-miR-29a-3p
 
 
-0.312
 
 
27.3
 
 
 
 
ACTC1
 
 
hsa-miR-30b-5p
 
 
-0.543
 
 
44.2
 
 
 
 
ACTR2
 
 
hsa-miR-15b-5p
 
 
-0.293
 
 
76.5
 
 
 
 
ACVR1
 
 
hsa-miR-30b-5p
 
 
-0.29
 
 
20.6
 
 
 
 
ADAM9
 
 
hsa-miR-30b-5p
 
 
-0.312
 
 
32.7
 
 
 
 
ADAMTS10
 
 
hsa-miR-29a-3p
 
 
-0.516
 
 
11.2
 
 
 
 
ADAMTS9
 
 
hsa-miR-29a-3p
 
 
-0.305
 
 
11.0
 
 
 
 
ADAMTS9
 
 
hsa-miR-29a-3p
 
 
-0.485
 
 
11.0
 
 
 
 
ADCY3
 
 
hsa-miR-132-3p
 
 
-0.303
 
 
60.4
 
 
 
 
ADGRA3
 
 
hsa-miR-30b-5p
 
 
-0.304
 
 
10.2
 
 
 
 
ADIPOR2
 
 
hsa-miR-150-5p
 
 
-0.287
 
 
25.7
 
 
 
 
ADRA2A
 
 
hsa-miR-23a-3p
 
 
-0.371
 
 
35.0
 
 
 
 
ADRA2A
 
 
hsa-miR-23b-3p
 
 
-0.371
 
 
35.0
 
 
 
 
ADRB2
 
 
hsa-miR-15b-5p
 
 
-0.29
 
 
12.7
 
 
 
 
AFG3L2
 
 
hsa-miR-181b-5p
 
 
-0.491
 
 
14.0
 
 
 
 
AFG3L2
 
 
hsa-miR-181a-5p
 
 
-0.491
 
 
14.0
 
 
 
 
AHR
 
 
hsa-miR-29a-3p
 
 
-0.316
 
 
13.6
 
 
 
 
AK1
 
 
hsa-miR-29a-3p
 
 
-0.333
 
 
35.3
 
 
 
 
AKT3
 
 
hsa-miR-29a-3p
 
 
-0.287
 
 
15.6
 
 
 
 
AKT3
 
 
hsa-miR-15b-5p
 
 
-0.301
 
 
15.6
 
 
 
 
ALAD
 
 
hsa-miR-15b-5p
 
 
-0.446
 
 
18.9
 
 
 
 
ALDH1A2
 
 
hsa-miR-23b-3p
 
 
-0.346
 
 
49.1
 
 
 
 
ALDH1A2
 
 
hsa-miR-23a-3p
 
 
-0.354
 
 
49.1
 
 
 
 
ALOX12
 
 
hsa-miR-15b-5p
 
 
-0.443
 
 
78.6
 
 
 
 
AMOTL1
 
 
hsa-miR-15b-5p
 
 
-0.319
 
 
12.0
 
 
 
 
ANGPTL2
 
 
hsa-miR-221-3p
 
 
-0.369
 
 
46.6
 
 
 
 
ANKFY1
 
 
hsa-miR-155-5p
 
 
-0.315
 
 
13.8
 
 
 
 
ANKHD1
 
 
hsa-miR-23a-3p
 
 
-0.368
 
 
41.2
 
 
 
 
ANKHD1
 
 
hsa-miR-23b-3p
 
 
-0.368
 
 
41.2
 
 
 
 
ANKHD1
 
 
hsa-miR-30b-5p
 
 
-0.405
 
 
41.2
 
 
 
 
ANKRD17
 
 
hsa-miR-30b-5p
 
 
-0.336
 
 
15.8
 
 
 
 
ANKRD29
 
 
hsa-miR-132-3p
 
 
-0.47
 
 
12.2
 
 
 
 
ANP32A
 
 
hsa-miR-132-3p
 
 
-0.374
 
 
50.7
 
 
 
 
ANP32E
 
 
hsa-miR-501-3p
 
 
-0.331
 
 
19.3
 
 
 
 
ANTXR2
 
 
hsa-miR-29a-3p
 
 
-0.347
 
 
49.9
 
 
 
 
ANXA11
 
 
hsa-miR-15b-5p
 
 
-0.463
 
 
89.1
 
 
 
 
AP2A1
 
 
hsa-miR-15b-5p
 
 
-0.333
 
 
42.3
 
 
 
 
AP2B1
 
 
hsa-miR-15b-5p
 
 
-0.334
 
 
50.8
 
 
 
 
AP3S1
 
 
hsa-miR-30b-5p
 
 
-0.293
 
 
65.9
 
 
 
 
APOO
 
 
hsa-miR-181b-5p
 
 
-0.5
 
 
12.3
 
 
 
 
APOO
 
 
hsa-miR-181a-5p
 
 
-0.5
 
 
12.3
 
 
 
 
APP
 
 
hsa-miR-15b-5p
 
 
-0.298
 
 
239.9
 
 
 
 
AQP3
 
 
hsa-miR-874-3p
 
 
-0.48
 
 
331.4
 
 
 
 
ARF4
 
 
hsa-miR-30b-5p
 
 
-0.289
 
 
196.1
 
 
 
 
ARF4
 
 
hsa-miR-221-3p
 
 
-0.553
 
 
196.1
 
 
 
 
ARF5
 
 
hsa-miR-29a-3p
 
 
-0.491
 
 
129.6
 
 
 
 
ARF6
 
 
hsa-miR-181b-5p
 
 
-0.33
 
 
31.8
 
 
 
 
ARF6
 
 
hsa-miR-181a-5p
 
 
-0.33
 
 
31.8
 
 
 
 
ARHGAP5
 
 
hsa-miR-486-5p
 
 
-0.3
 
 
24.7
 
 
 
 
ARID1B
 
 
hsa-miR-29a-3p
 
 
-0.342
 
 
15.1
 
 
 
 
ARID4B
 
 
hsa-miR-486-5p
 
 
-0.367
 
 
14.1
 
 
 
 
ARL2
 
 
hsa-miR-15b-5p
 
 
-0.374
 
 
102.1
 
 
 
 
ARL2
 
 
hsa-miR-15b-5p
 
 
-0.442
 
 
102.1
 
 
 
 
ARL3
 
 
hsa-miR-15b-5p
 
 
-0.507
 
 
12.6
 
 
 
 
ARL4A
 
 
hsa-miR-29a-3p
 
 
-0.386
 
 
46.9
 
 
 
 
ARL4A
 
 
hsa-miR-30b-5p
 
 
-0.448
 
 
46.9
 
 
 
 
ARMC8
 
 
hsa-miR-486-5p
 
 
-0.321
 
 
13.8
 
 
 
 
ARMCX2
 
 
hsa-miR-15b-5p
 
 
-0.344
 
 
31.5
 
 
 
 
ARMCX3
 
 
hsa-miR-425-5p
 
 
-0.325
 
 
42.3
 
 
 
 
ARNT
 
 
hsa-miR-221-3p
 
 
-0.465
 
 
27.1
 
 
 
 
ARPP19
 
 
hsa-miR-15b-5p
 
 
-0.346
 
 
35.8
 
 
 
 
ARPP19
 
 
hsa-miR-29a-3p
 
 
-0.371
 
 
35.8
 
 
 
 
ARRDC3
 
 
hsa-miR-29a-3p
 
 
-0.402
 
 
42.0
 
 
 
 
ASAP2
 
 
hsa-miR-29a-3p
 
 
-0.348
 
 
10.8
 
 
 
 
ASB6
 
 
hsa-miR-874-3p
 
 
-0.368
 
 
10.9
 
 
 
 
ASH1L
 
 
hsa-miR-15b-5p
 
 
-0.325
 
 
19.2
 
 
 
 
ASPH
 
 
hsa-miR-221-3p
 
 
-0.287
 
 
60.9
 
 
 
 
ATF3
 
 
hsa-miR-874-3p
 
 
-0.288
 
 
99.3
 
 
 
 
ATG5
 
 
hsa-miR-30b-5p
 
 
-0.488
 
 
14.4
 
 
 
 
ATG9A
 
 
hsa-miR-15b-5p
 
 
-0.333
 
 
20.6
 
 
 
 
ATOSA
 
 
hsa-miR-221-3p
 
 
-0.495
 
 
10.7
 
 
 
 
ATP5MC3
 
 
hsa-miR-425-5p
 
 
-0.328
 
 
141.6
 
 
 
 
ATP5MC3
 
 
hsa-miR-29a-3p
 
 
-0.33
 
 
141.6
 
 
 
 
ATP6V1E1
 
 
hsa-miR-23a-3p
 
 
-0.294
 
 
99.8
 
 
 
 
ATP6V1E1
 
 
hsa-miR-23b-3p
 
 
-0.294
 
 
99.8
 
 
 
 
ATPAF1
 
 
hsa-miR-361-5p
 
 
-0.362
 
 
33.4
 
 
 
 
ATXN1L
 
 
hsa-miR-155-5p
 
 
-0.315
 
 
10.4
 
 
 
 
ATXN2
 
 
hsa-miR-15b-5p
 
 
-0.392
 
 
19.5
 
 
 
 
AUH
 
 
hsa-miR-23a-3p
 
 
-0.495
 
 
12.8
 
 
 
 
AUH
 
 
hsa-miR-23b-3p
 
 
-0.503
 
 
12.8
 
 
 
 
AUH
 
 
hsa-miR-23a-3p
 
 
-0.512
 
 
12.8
 
 
 
 
AUH
 
 
hsa-miR-23b-3p
 
 
-0.512
 
 
12.8
 
 
 
 
AXIN2
 
 
hsa-miR-15b-5p
 
 
-0.3
 
 
16.1
 
 
 
 
AZIN1
 
 
hsa-miR-132-3p
 
 
-0.29
 
 
50.9
 
 
 
 
AZIN1
 
 
hsa-miR-132-3p
 
 
-0.299
 
 
50.9
 
 
 
 
B4GALT5
 
 
hsa-miR-501-3p
 
 
-0.408
 
 
19.9
 
 
 
 
B4GAT1
 
 
hsa-miR-23a-3p
 
 
-0.361
 
 
25.3
 
 
 
 
B4GAT1
 
 
hsa-miR-23b-3p
 
 
-0.361
 
 
25.3
 
 
 
 
BAK1
 
 
hsa-miR-29a-3p
 
 
-0.409
 
 
10.0
 
 
 
 
BCL2L2
 
 
hsa-miR-29a-3p
 
 
-0.312
 
 
30.9
 
 
 
 
BDH1
 
 
hsa-miR-874-3p
 
 
-0.644
 
 
10.5
 
 
 
 
BECN1
 
 
hsa-miR-30b-5p
 
 
-0.452
 
 
33.6
 
 
 
 
BET1
 
 
hsa-miR-23a-3p
 
 
-0.402
 
 
21.5
 
 
 
 
BET1
 
 
hsa-miR-23b-3p
 
 
-0.418
 
 
21.5
 
 
 
 
BFAR
 
 
hsa-miR-15b-5p
 
 
-0.352
 
 
23.8
 
 
 
 
BHLHE40
 
 
hsa-miR-181b-5p
 
 
-0.408
 
 
244.8
 
 
 
 
BHLHE40
 
 
hsa-miR-181a-5p
 
 
-0.408
 
 
244.8
 
 
 
 
BICDL1
 
 
hsa-miR-221-3p
 
 
-0.447
 
 
11.0
 
 
 
 
 
 
 choroid plexus 
 
 
 
 
target_symbol
 
 
mature_mirna_id
 
 
score
 
 
nTPM
 
 
 
 
 
 
AAR2
 
 
hsa-miR-29a-3p
 
 
-0.327
 
 
27.3
 
 
 
 
ABHD4
 
 
hsa-miR-29a-3p
 
 
-0.312
 
 
31.8
 
 
 
 
ACTN2
 
 
hsa-miR-23a-3p
 
 
-0.294
 
 
16.8
 
 
 
 
ACTN2
 
 
hsa-miR-29a-3p
 
 
-0.311
 
 
16.8
 
 
 
 
ACTR2
 
 
hsa-miR-15b-5p
 
 
-0.293
 
 
35.9
 
 
 
 
ACTR3C
 
 
hsa-miR-30b-5p
 
 
-0.448
 
 
14.9
 
 
 
 
ADAM9
 
 
hsa-miR-30b-5p
 
 
-0.312
 
 
14.9
 
 
 
 
ADCY3
 
 
hsa-miR-132-3p
 
 
-0.303
 
 
17.0
 
 
 
 
ADGRA3
 
 
hsa-miR-30b-5p
 
 
-0.304
 
 
23.6
 
 
 
 
ADIPOR2
 
 
hsa-miR-150-5p
 
 
-0.287
 
 
17.0
 
 
 
 
AK1
 
 
hsa-miR-29a-3p
 
 
-0.333
 
 
522.7
 
 
 
 
AK4
 
 
hsa-miR-15b-5p
 
 
-0.316
 
 
13.8
 
 
 
 
ALAD
 
 
hsa-miR-15b-5p
 
 
-0.446
 
 
29.1
 
 
 
 
ANGPTL2
 
 
hsa-miR-221-3p
 
 
-0.369
 
 
20.8
 
 
 
 
ANKFY1
 
 
hsa-miR-155-5p
 
 
-0.315
 
 
10.2
 
 
 
 
ANKHD1
 
 
hsa-miR-23a-3p
 
 
-0.368
 
 
42.2
 
 
 
 
ANKHD1
 
 
hsa-miR-23b-3p
 
 
-0.368
 
 
42.2
 
 
 
 
ANKHD1
 
 
hsa-miR-30b-5p
 
 
-0.405
 
 
42.2
 
 
 
 
ANKRD17
 
 
hsa-miR-30b-5p
 
 
-0.336
 
 
13.8
 
 
 
 
ANP32A
 
 
hsa-miR-132-3p
 
 
-0.374
 
 
28.9
 
 
 
 
ANP32E
 
 
hsa-miR-501-3p
 
 
-0.331
 
 
23.6
 
 
 
 
ANXA11
 
 
hsa-miR-15b-5p
 
 
-0.463
 
 
96.6
 
 
 
 
AP2A1
 
 
hsa-miR-15b-5p
 
 
-0.333
 
 
20.9
 
 
 
 
AP2B1
 
 
hsa-miR-15b-5p
 
 
-0.334
 
 
83.9
 
 
 
 
AP3S1
 
 
hsa-miR-30b-5p
 
 
-0.293
 
 
22.5
 
 
 
 
APOO
 
 
hsa-miR-181b-5p
 
 
-0.5
 
 
102.5
 
 
 
 
APOO
 
 
hsa-miR-181a-5p
 
 
-0.5
 
 
102.5
 
 
 
 
APP
 
 
hsa-miR-15b-5p
 
 
-0.298
 
 
394.3
 
 
 
 
ARF4
 
 
hsa-miR-30b-5p
 
 
-0.289
 
 
115.4
 
 
 
 
ARF4
 
 
hsa-miR-221-3p
 
 
-0.553
 
 
115.4
 
 
 
 
ARF5
 
 
hsa-miR-29a-3p
 
 
-0.491
 
 
140.5
 
 
 
 
ARF6
 
 
hsa-miR-181b-5p
 
 
-0.33
 
 
20.9
 
 
 
 
ARF6
 
 
hsa-miR-181a-5p
 
 
-0.33
 
 
20.9
 
 
 
 
ARL2
 
 
hsa-miR-15b-5p
 
 
-0.374
 
 
135.4
 
 
 
 
ARL2
 
 
hsa-miR-15b-5p
 
 
-0.442
 
 
135.4
 
 
 
 
ARL3
 
 
hsa-miR-15b-5p
 
 
-0.507
 
 
27.9
 
 
 
 
ARL4A
 
 
hsa-miR-29a-3p
 
 
-0.386
 
 
29.1
 
 
 
 
ARL4A
 
 
hsa-miR-30b-5p
 
 
-0.448
 
 
29.1
 
 
 
 
ARMC8
 
 
hsa-miR-486-5p
 
 
-0.321
 
 
20.8
 
 
 
 
ARMCX2
 
 
hsa-miR-15b-5p
 
 
-0.344
 
 
19.0
 
 
 
 
ARMCX3
 
 
hsa-miR-425-5p
 
 
-0.325
 
 
25.1
 
 
 
 
ARNT
 
 
hsa-miR-221-3p
 
 
-0.465
 
 
16.9
 
 
 
 
ARPP19
 
 
hsa-miR-15b-5p
 
 
-0.346
 
 
32.4
 
 
 
 
ARPP19
 
 
hsa-miR-29a-3p
 
 
-0.371
 
 
32.4
 
 
 
 
ARRDC3
 
 
hsa-miR-29a-3p
 
 
-0.402
 
 
24.1
 
 
 
 
ASB3
 
 
hsa-miR-30b-5p
 
 
-0.418
 
 
14.1
 
 
 
 
ASB3
 
 
hsa-miR-30b-5p
 
 
-0.418
 
 
14.1
 
 
 
 
ASB6
 
 
hsa-miR-874-3p
 
 
-0.368
 
 
15.8
 
 
 
 
ASPH
 
 
hsa-miR-221-3p
 
 
-0.287
 
 
79.9
 
 
 
 
ATF3
 
 
hsa-miR-874-3p
 
 
-0.288
 
 
42.4
 
 
 
 
ATG9A
 
 
hsa-miR-15b-5p
 
 
-0.333
 
 
10.2
 
 
 
 
ATOSA
 
 
hsa-miR-221-3p
 
 
-0.495
 
 
19.0
 
 
 
 
ATP5MC3
 
 
hsa-miR-425-5p
 
 
-0.328
 
 
264.7
 
 
 
 
ATP5MC3
 
 
hsa-miR-29a-3p
 
 
-0.33
 
 
264.7
 
 
 
 
ATP6V1E1
 
 
hsa-miR-23a-3p
 
 
-0.294
 
 
285.9
 
 
 
 
ATP6V1E1
 
 
hsa-miR-23b-3p
 
 
-0.294
 
 
285.9
 
 
 
 
ATPAF1
 
 
hsa-miR-361-5p
 
 
-0.362
 
 
168.0
 
 
 
 
ATXN2
 
 
hsa-miR-15b-5p
 
 
-0.392
 
 
17.3
 
 
 
 
AUH
 
 
hsa-miR-23a-3p
 
 
-0.495
 
 
34.6
 
 
 
 
AUH
 
 
hsa-miR-23b-3p
 
 
-0.503
 
 
34.6
 
 
 
 
AUH
 
 
hsa-miR-23a-3p
 
 
-0.512
 
 
34.6
 
 
 
 
AUH
 
 
hsa-miR-23b-3p
 
 
-0.512
 
 
34.6
 
 
 
 
AZIN1
 
 
hsa-miR-132-3p
 
 
-0.29
 
 
28.1
 
 
 
 
AZIN1
 
 
hsa-miR-132-3p
 
 
-0.299
 
 
28.1
 
 
 
 
B4GAT1
 
 
hsa-miR-23a-3p
 
 
-0.361
 
 
93.8
 
 
 
 
B4GAT1
 
 
hsa-miR-23b-3p
 
 
-0.361
 
 
93.8
 
 
 
 
BAK1
 
 
hsa-miR-29a-3p
 
 
-0.409
 
 
16.1
 
 
 
 
BCL2L2
 
 
hsa-miR-29a-3p
 
 
-0.312
 
 
22.4
 
 
 
 
BDH1
 
 
hsa-miR-874-3p
 
 
-0.644
 
 
39.2
 
 
 
 
BECN1
 
 
hsa-miR-30b-5p
 
 
-0.452
 
 
42.2
 
 
 
 
BET1
 
 
hsa-miR-23a-3p
 
 
-0.402
 
 
13.1
 
 
 
 
BET1
 
 
hsa-miR-23b-3p
 
 
-0.418
 
 
13.1
 
 
 
 
BFAR
 
 
hsa-miR-15b-5p
 
 
-0.352
 
 
21.5
 
 
 
 
BHLHE40
 
 
hsa-miR-181b-5p
 
 
-0.408
 
 
103.5
 
 
 
 
BHLHE40
 
 
hsa-miR-181a-5p
 
 
-0.408
 
 
103.5
 
 
 
 
BLCAP
 
 
hsa-miR-23a-3p
 
 
-0.601
 
 
138.8
 
 
 
 
BLCAP
 
 
hsa-miR-23b-3p
 
 
-0.601
 
 
138.8
 
 
 
 
BLMH
 
 
hsa-miR-29a-3p
 
 
-0.393
 
 
19.7
 
 
 
 
BLOC1S6
 
 
hsa-miR-181b-5p
 
 
-0.393
 
 
14.9
 
 
 
 
BLOC1S6
 
 
hsa-miR-181a-5p
 
 
-0.393
 
 
14.9
 
 
 
 
BOLA3
 
 
hsa-miR-15b-5p
 
 
-0.363
 
 
49.2
 
 
 
 
BOLA3
 
 
hsa-miR-15b-5p
 
 
-0.386
 
 
49.2
 
 
 
 
BOLA3
 
 
hsa-miR-374b-5p
 
 
-0.486
 
 
49.2
 
 
 
 
BRI3
 
 
hsa-miR-132-3p
 
 
-0.385
 
 
50.2
 
 
 
 
BTF3
 
 
hsa-miR-15b-5p
 
 
-0.344
 
 
321.4
 
 
 
 
BTF3
 
 
hsa-miR-361-5p
 
 
-0.551
 
 
321.4
 
 
 
 
BTG2
 
 
hsa-miR-29a-3p
 
 
-0.32
 
 
39.9
 
 
 
 
BTN2A1
 
 
hsa-miR-342-3p
 
 
-0.31
 
 
11.4
 
 
 
 
BZW1
 
 
hsa-miR-15b-5p
 
 
-0.367
 
 
50.7
 
 
 
 
C11orf24
 
 
hsa-miR-15b-5p
 
 
-0.403
 
 
13.2
 
 
 
 
C11orf68
 
 
hsa-miR-15b-5p
 
 
-0.29
 
 
41.2
 
 
 
 
C3orf18
 
 
hsa-miR-155-5p
 
 
-0.431
 
 
27.1
 
 
 
 
C3orf18
 
 
hsa-miR-30b-5p
 
 
-0.49
 
 
27.1
 
 
 
 
C5orf15
 
 
hsa-miR-29a-3p
 
 
-0.392
 
 
37.1
 
 
 
 
C6orf62
 
 
hsa-miR-181b-5p
 
 
-0.334
 
 
62.6
 
 
 
 
C6orf62
 
 
hsa-miR-181a-5p
 
 
-0.334
 
 
62.6
 
 
 
 
C6orf62
 
 
hsa-miR-23a-3p
 
 
-0.507
 
 
62.6
 
 
 
 
C6orf62
 
 
hsa-miR-23b-3p
 
 
-0.507
 
 
62.6
 
 
 
 
CA12
 
 
hsa-miR-342-3p
 
 
-0.294
 
 
145.1
 
 
 
 
CA2
 
 
hsa-miR-23a-3p
 
 
-0.46
 
 
944.3
 
 
 
 
 
 
 colon 
 
 
 
 
target_symbol
 
 
mature_mirna_id
 
 
score
 
 
nTPM
 
 
 
 
 
 
AAR2
 
 
hsa-miR-29a-3p
 
 
-0.327
 
 
25.8
 
 
 
 
ABCE1
 
 
hsa-miR-29a-3p
 
 
-0.404
 
 
21.3
 
 
 
 
ABHD4
 
 
hsa-miR-29a-3p
 
 
-0.312
 
 
20.3
 
 
 
 
ABHD5
 
 
hsa-miR-132-3p
 
 
-0.323
 
 
11.4
 
 
 
 
ACTR2
 
 
hsa-miR-15b-5p
 
 
-0.293
 
 
132.3
 
 
 
 
ADAM19
 
 
hsa-miR-874-3p
 
 
-0.374
 
 
22.3
 
 
 
 
ADAM9
 
 
hsa-miR-30b-5p
 
 
-0.312
 
 
30.9
 
 
 
 
ADCY3
 
 
hsa-miR-132-3p
 
 
-0.303
 
 
26.7
 
 
 
 
ADGRA3
 
 
hsa-miR-30b-5p
 
 
-0.304
 
 
16.0
 
 
 
 
ADIPOR2
 
 
hsa-miR-150-5p
 
 
-0.287
 
 
31.0
 
 
 
 
AFG3L2
 
 
hsa-miR-181b-5p
 
 
-0.491
 
 
38.7
 
 
 
 
AFG3L2
 
 
hsa-miR-181a-5p
 
 
-0.491
 
 
38.7
 
 
 
 
AHR
 
 
hsa-miR-29a-3p
 
 
-0.316
 
 
12.9
 
 
 
 
AK1
 
 
hsa-miR-29a-3p
 
 
-0.333
 
 
41.6
 
 
 
 
AK4
 
 
hsa-miR-15b-5p
 
 
-0.316
 
 
17.9
 
 
 
 
AKT3
 
 
hsa-miR-29a-3p
 
 
-0.287
 
 
20.3
 
 
 
 
AKT3
 
 
hsa-miR-15b-5p
 
 
-0.301
 
 
20.3
 
 
 
 
ALAD
 
 
hsa-miR-15b-5p
 
 
-0.446
 
 
24.1
 
 
 
 
AMOTL1
 
 
hsa-miR-15b-5p
 
 
-0.319
 
 
34.0
 
 
 
 
ANGPTL2
 
 
hsa-miR-221-3p
 
 
-0.369
 
 
73.4
 
 
 
 
ANKFY1
 
 
hsa-miR-155-5p
 
 
-0.315
 
 
24.5
 
 
 
 
ANKHD1
 
 
hsa-miR-23a-3p
 
 
-0.368
 
 
38.3
 
 
 
 
ANKHD1
 
 
hsa-miR-23b-3p
 
 
-0.368
 
 
38.3
 
 
 
 
ANKHD1
 
 
hsa-miR-30b-5p
 
 
-0.405
 
 
38.3
 
 
 
 
ANKRD17
 
 
hsa-miR-30b-5p
 
 
-0.336
 
 
14.9
 
 
 
 
ANP32A
 
 
hsa-miR-132-3p
 
 
-0.374
 
 
75.8
 
 
 
 
ANP32E
 
 
hsa-miR-501-3p
 
 
-0.331
 
 
18.6
 
 
 
 
ANTXR2
 
 
hsa-miR-29a-3p
 
 
-0.347
 
 
83.9
 
 
 
 
ANXA11
 
 
hsa-miR-15b-5p
 
 
-0.463
 
 
122.1
 
 
 
 
AP2A1
 
 
hsa-miR-15b-5p
 
 
-0.333
 
 
40.3
 
 
 
 
AP2B1
 
 
hsa-miR-15b-5p
 
 
-0.334
 
 
63.4
 
 
 
 
AP3S1
 
 
hsa-miR-30b-5p
 
 
-0.293
 
 
56.5
 
 
 
 
APOO
 
 
hsa-miR-181b-5p
 
 
-0.5
 
 
16.2
 
 
 
 
APOO
 
 
hsa-miR-181a-5p
 
 
-0.5
 
 
16.2
 
 
 
 
APP
 
 
hsa-miR-15b-5p
 
 
-0.298
 
 
322.5
 
 
 
 
ARF4
 
 
hsa-miR-30b-5p
 
 
-0.289
 
 
148.9
 
 
 
 
ARF4
 
 
hsa-miR-221-3p
 
 
-0.553
 
 
148.9
 
 
 
 
ARF5
 
 
hsa-miR-29a-3p
 
 
-0.491
 
 
119.1
 
 
 
 
ARF6
 
 
hsa-miR-181b-5p
 
 
-0.33
 
 
50.4
 
 
 
 
ARF6
 
 
hsa-miR-181a-5p
 
 
-0.33
 
 
50.4
 
 
 
 
ARHGAP5
 
 
hsa-miR-486-5p
 
 
-0.3
 
 
27.7
 
 
 
 
ARID1B
 
 
hsa-miR-29a-3p
 
 
-0.342
 
 
11.7
 
 
 
 
ARL2
 
 
hsa-miR-15b-5p
 
 
-0.374
 
 
122.8
 
 
 
 
ARL2
 
 
hsa-miR-15b-5p
 
 
-0.442
 
 
122.8
 
 
 
 
ARL3
 
 
hsa-miR-15b-5p
 
 
-0.507
 
 
10.7
 
 
 
 
ARL4A
 
 
hsa-miR-29a-3p
 
 
-0.386
 
 
38.1
 
 
 
 
ARL4A
 
 
hsa-miR-30b-5p
 
 
-0.448
 
 
38.1
 
 
 
 
ARMC8
 
 
hsa-miR-486-5p
 
 
-0.321
 
 
15.5
 
 
 
 
ARMCX2
 
 
hsa-miR-15b-5p
 
 
-0.344
 
 
22.4
 
 
 
 
ARMCX3
 
 
hsa-miR-425-5p
 
 
-0.325
 
 
27.7
 
 
 
 
ARNT
 
 
hsa-miR-221-3p
 
 
-0.465
 
 
18.8
 
 
 
 
ARPP19
 
 
hsa-miR-15b-5p
 
 
-0.346
 
 
35.7
 
 
 
 
ARPP19
 
 
hsa-miR-29a-3p
 
 
-0.371
 
 
35.7
 
 
 
 
ARRDC3
 
 
hsa-miR-29a-3p
 
 
-0.402
 
 
18.9
 
 
 
 
ASAP2
 
 
hsa-miR-29a-3p
 
 
-0.348
 
 
16.6
 
 
 
 
ASB6
 
 
hsa-miR-874-3p
 
 
-0.368
 
 
10.3
 
 
 
 
ASH1L
 
 
hsa-miR-15b-5p
 
 
-0.325
 
 
23.9
 
 
 
 
ASPH
 
 
hsa-miR-221-3p
 
 
-0.287
 
 
72.8
 
 
 
 
ATF3
 
 
hsa-miR-874-3p
 
 
-0.288
 
 
65.7
 
 
 
 
ATG5
 
 
hsa-miR-30b-5p
 
 
-0.488
 
 
14.8
 
 
 
 
ATG9A
 
 
hsa-miR-15b-5p
 
 
-0.333
 
 
26.8
 
 
 
 
ATOSA
 
 
hsa-miR-221-3p
 
 
-0.495
 
 
13.6
 
 
 
 
ATP5MC3
 
 
hsa-miR-425-5p
 
 
-0.328
 
 
331.6
 
 
 
 
ATP5MC3
 
 
hsa-miR-29a-3p
 
 
-0.33
 
 
331.6
 
 
 
 
ATP6V1E1
 
 
hsa-miR-23a-3p
 
 
-0.294
 
 
126.9
 
 
 
 
ATP6V1E1
 
 
hsa-miR-23b-3p
 
 
-0.294
 
 
126.9
 
 
 
 
ATPAF1
 
 
hsa-miR-361-5p
 
 
-0.362
 
 
49.0
 
 
 
 
ATXN2
 
 
hsa-miR-15b-5p
 
 
-0.392
 
 
22.4
 
 
 
 
AUH
 
 
hsa-miR-23a-3p
 
 
-0.495
 
 
16.1
 
 
 
 
AUH
 
 
hsa-miR-23b-3p
 
 
-0.503
 
 
16.1
 
 
 
 
AUH
 
 
hsa-miR-23a-3p
 
 
-0.512
 
 
16.1
 
 
 
 
AUH
 
 
hsa-miR-23b-3p
 
 
-0.512
 
 
16.1
 
 
 
 
AXIN2
 
 
hsa-miR-15b-5p
 
 
-0.3
 
 
11.6
 
 
 
 
AZIN1
 
 
hsa-miR-132-3p
 
 
-0.29
 
 
45.9
 
 
 
 
AZIN1
 
 
hsa-miR-132-3p
 
 
-0.299
 
 
45.9
 
 
 
 
B3GNT5
 
 
hsa-miR-30b-5p
 
 
-0.326
 
 
21.2
 
 
 
 
B4GALT5
 
 
hsa-miR-501-3p
 
 
-0.408
 
 
23.3
 
 
 
 
B4GAT1
 
 
hsa-miR-23a-3p
 
 
-0.361
 
 
29.4
 
 
 
 
B4GAT1
 
 
hsa-miR-23b-3p
 
 
-0.361
 
 
29.4
 
 
 
 
BAK1
 
 
hsa-miR-29a-3p
 
 
-0.409
 
 
30.4
 
 
 
 
BCL2L11
 
 
hsa-miR-221-3p
 
 
-0.423
 
 
10.9
 
 
 
 
BCL2L2
 
 
hsa-miR-29a-3p
 
 
-0.312
 
 
84.2
 
 
 
 
BDH1
 
 
hsa-miR-874-3p
 
 
-0.644
 
 
40.4
 
 
 
 
BECN1
 
 
hsa-miR-30b-5p
 
 
-0.452
 
 
65.9
 
 
 
 
BET1
 
 
hsa-miR-23a-3p
 
 
-0.402
 
 
14.8
 
 
 
 
BET1
 
 
hsa-miR-23b-3p
 
 
-0.418
 
 
14.8
 
 
 
 
BFAR
 
 
hsa-miR-15b-5p
 
 
-0.352
 
 
26.5
 
 
 
 
BHLHE40
 
 
hsa-miR-181b-5p
 
 
-0.408
 
 
73.3
 
 
 
 
BHLHE40
 
 
hsa-miR-181a-5p
 
 
-0.408
 
 
73.3
 
 
 
 
BLCAP
 
 
hsa-miR-23a-3p
 
 
-0.601
 
 
54.1
 
 
 
 
BLCAP
 
 
hsa-miR-23b-3p
 
 
-0.601
 
 
54.1
 
 
 
 
BLMH
 
 
hsa-miR-29a-3p
 
 
-0.393
 
 
18.2
 
 
 
 
BLOC1S6
 
 
hsa-miR-181b-5p
 
 
-0.393
 
 
19.9
 
 
 
 
BLOC1S6
 
 
hsa-miR-181a-5p
 
 
-0.393
 
 
19.9
 
 
 
 
BOLA3
 
 
hsa-miR-15b-5p
 
 
-0.363
 
 
43.5
 
 
 
 
BOLA3
 
 
hsa-miR-15b-5p
 
 
-0.386
 
 
43.5
 
 
 
 
BOLA3
 
 
hsa-miR-374b-5p
 
 
-0.486
 
 
43.5
 
 
 
 
BORCS6
 
 
hsa-miR-15b-5p
 
 
-0.347
 
 
11.0
 
 
 
 
BRI3
 
 
hsa-miR-132-3p
 
 
-0.385
 
 
81.4
 
 
 
 
BTAF1
 
 
hsa-miR-486-5p
 
 
-0.288
 
 
16.8
 
 
 
 
 
 
 duodenum 
 
 
 
 
target_symbol
 
 
mature_mirna_id
 
 
score
 
 
nTPM
 
 
 
 
 
 
AAR2
 
 
hsa-miR-29a-3p
 
 
-0.327
 
 
14.2
 
 
 
 
ABCE1
 
 
hsa-miR-29a-3p
 
 
-0.404
 
 
19.0
 
 
 
 
ABHD17B
 
 
hsa-miR-486-5p
 
 
-0.348
 
 
15.0
 
 
 
 
ABHD4
 
 
hsa-miR-29a-3p
 
 
-0.312
 
 
15.6
 
 
 
 
ACTR2
 
 
hsa-miR-15b-5p
 
 
-0.293
 
 
98.7
 
 
 
 
ADA
 
 
hsa-miR-29a-3p
 
 
-0.376
 
 
454.0
 
 
 
 
ADAM9
 
 
hsa-miR-30b-5p
 
 
-0.312
 
 
23.1
 
 
 
 
ADCY3
 
 
hsa-miR-132-3p
 
 
-0.303
 
 
10.3
 
 
 
 
ADIPOR2
 
 
hsa-miR-150-5p
 
 
-0.287
 
 
20.9
 
 
 
 
AFG3L2
 
 
hsa-miR-181b-5p
 
 
-0.491
 
 
48.3
 
 
 
 
AFG3L2
 
 
hsa-miR-181a-5p
 
 
-0.491
 
 
48.3
 
 
 
 
AHR
 
 
hsa-miR-29a-3p
 
 
-0.316
 
 
15.4
 
 
 
 
AK1
 
 
hsa-miR-29a-3p
 
 
-0.333
 
 
16.7
 
 
 
 
ALAD
 
 
hsa-miR-15b-5p
 
 
-0.446
 
 
27.7
 
 
 
 
ANGPTL2
 
 
hsa-miR-221-3p
 
 
-0.369
 
 
14.3
 
 
 
 
ANKFY1
 
 
hsa-miR-155-5p
 
 
-0.315
 
 
10.8
 
 
 
 
ANKHD1
 
 
hsa-miR-23a-3p
 
 
-0.368
 
 
29.8
 
 
 
 
ANKHD1
 
 
hsa-miR-23b-3p
 
 
-0.368
 
 
29.8
 
 
 
 
ANKHD1
 
 
hsa-miR-30b-5p
 
 
-0.405
 
 
29.8
 
 
 
 
ANKRD17
 
 
hsa-miR-30b-5p
 
 
-0.336
 
 
13.9
 
 
 
 
ANP32A
 
 
hsa-miR-132-3p
 
 
-0.374
 
 
66.5
 
 
 
 
ANP32E
 
 
hsa-miR-501-3p
 
 
-0.331
 
 
21.1
 
 
 
 
ANTXR2
 
 
hsa-miR-29a-3p
 
 
-0.347
 
 
21.6
 
 
 
 
ANXA11
 
 
hsa-miR-15b-5p
 
 
-0.463
 
 
129.7
 
 
 
 
AP2A1
 
 
hsa-miR-15b-5p
 
 
-0.333
 
 
23.3
 
 
 
 
AP2B1
 
 
hsa-miR-15b-5p
 
 
-0.334
 
 
31.5
 
 
 
 
AP3S1
 
 
hsa-miR-30b-5p
 
 
-0.293
 
 
58.2
 
 
 
 
APOO
 
 
hsa-miR-181b-5p
 
 
-0.5
 
 
13.6
 
 
 
 
APOO
 
 
hsa-miR-181a-5p
 
 
-0.5
 
 
13.6
 
 
 
 
APP
 
 
hsa-miR-15b-5p
 
 
-0.298
 
 
204.9
 
 
 
 
AQP11
 
 
hsa-miR-15b-5p
 
 
-0.769
 
 
27.0
 
 
 
 
AQP3
 
 
hsa-miR-874-3p
 
 
-0.48
 
 
69.1
 
 
 
 
ARF4
 
 
hsa-miR-30b-5p
 
 
-0.289
 
 
116.2
 
 
 
 
ARF4
 
 
hsa-miR-221-3p
 
 
-0.553
 
 
116.2
 
 
 
 
ARF5
 
 
hsa-miR-29a-3p
 
 
-0.491
 
 
86.9
 
 
 
 
ARF6
 
 
hsa-miR-181b-5p
 
 
-0.33
 
 
43.6
 
 
 
 
ARF6
 
 
hsa-miR-181a-5p
 
 
-0.33
 
 
43.6
 
 
 
 
ARHGAP5
 
 
hsa-miR-486-5p
 
 
-0.3
 
 
33.3
 
 
 
 
ARID1B
 
 
hsa-miR-29a-3p
 
 
-0.342
 
 
13.4
 
 
 
 
ARL2
 
 
hsa-miR-15b-5p
 
 
-0.374
 
 
49.6
 
 
 
 
ARL2
 
 
hsa-miR-15b-5p
 
 
-0.442
 
 
49.6
 
 
 
 
ARL4A
 
 
hsa-miR-29a-3p
 
 
-0.386
 
 
28.1
 
 
 
 
ARL4A
 
 
hsa-miR-30b-5p
 
 
-0.448
 
 
28.1
 
 
 
 
ARMC8
 
 
hsa-miR-486-5p
 
 
-0.321
 
 
11.3
 
 
 
 
ARMCX3
 
 
hsa-miR-425-5p
 
 
-0.325
 
 
19.0
 
 
 
 
ARNT
 
 
hsa-miR-221-3p
 
 
-0.465
 
 
14.1
 
 
 
 
ARPP19
 
 
hsa-miR-15b-5p
 
 
-0.346
 
 
27.2
 
 
 
 
ARPP19
 
 
hsa-miR-29a-3p
 
 
-0.371
 
 
27.2
 
 
 
 
ASAP2
 
 
hsa-miR-29a-3p
 
 
-0.348
 
 
13.0
 
 
 
 
ASPH
 
 
hsa-miR-221-3p
 
 
-0.287
 
 
42.9
 
 
 
 
ATF3
 
 
hsa-miR-874-3p
 
 
-0.288
 
 
19.7
 
 
 
 
ATG5
 
 
hsa-miR-30b-5p
 
 
-0.488
 
 
12.0
 
 
 
 
ATG9A
 
 
hsa-miR-15b-5p
 
 
-0.333
 
 
29.6
 
 
 
 
ATOSA
 
 
hsa-miR-221-3p
 
 
-0.495
 
 
10.6
 
 
 
 
ATP5MC3
 
 
hsa-miR-425-5p
 
 
-0.328
 
 
355.5
 
 
 
 
ATP5MC3
 
 
hsa-miR-29a-3p
 
 
-0.33
 
 
355.5
 
 
 
 
ATP6V1E1
 
 
hsa-miR-23a-3p
 
 
-0.294
 
 
53.2
 
 
 
 
ATP6V1E1
 
 
hsa-miR-23b-3p
 
 
-0.294
 
 
53.2
 
 
 
 
ATPAF1
 
 
hsa-miR-361-5p
 
 
-0.362
 
 
41.3
 
 
 
 
ATXN2
 
 
hsa-miR-15b-5p
 
 
-0.392
 
 
10.5
 
 
 
 
AUH
 
 
hsa-miR-23a-3p
 
 
-0.495
 
 
22.4
 
 
 
 
AUH
 
 
hsa-miR-23b-3p
 
 
-0.503
 
 
22.4
 
 
 
 
AUH
 
 
hsa-miR-23a-3p
 
 
-0.512
 
 
22.4
 
 
 
 
AUH
 
 
hsa-miR-23b-3p
 
 
-0.512
 
 
22.4
 
 
 
 
AZIN1
 
 
hsa-miR-132-3p
 
 
-0.29
 
 
61.2
 
 
 
 
AZIN1
 
 
hsa-miR-132-3p
 
 
-0.299
 
 
61.2
 
 
 
 
B3GNT5
 
 
hsa-miR-30b-5p
 
 
-0.326
 
 
36.0
 
 
 
 
B4GALT5
 
 
hsa-miR-501-3p
 
 
-0.408
 
 
23.8
 
 
 
 
B4GAT1
 
 
hsa-miR-23a-3p
 
 
-0.361
 
 
15.2
 
 
 
 
B4GAT1
 
 
hsa-miR-23b-3p
 
 
-0.361
 
 
15.2
 
 
 
 
BAK1
 
 
hsa-miR-29a-3p
 
 
-0.409
 
 
44.2
 
 
 
 
BCL2L2
 
 
hsa-miR-29a-3p
 
 
-0.312
 
 
25.7
 
 
 
 
BDH1
 
 
hsa-miR-874-3p
 
 
-0.644
 
 
16.3
 
 
 
 
BECN1
 
 
hsa-miR-30b-5p
 
 
-0.452
 
 
53.2
 
 
 
 
BET1
 
 
hsa-miR-23a-3p
 
 
-0.402
 
 
20.6
 
 
 
 
BET1
 
 
hsa-miR-23b-3p
 
 
-0.418
 
 
20.6
 
 
 
 
BFAR
 
 
hsa-miR-15b-5p
 
 
-0.352
 
 
18.6
 
 
 
 
BHLHE40
 
 
hsa-miR-181b-5p
 
 
-0.408
 
 
29.0
 
 
 
 
BHLHE40
 
 
hsa-miR-181a-5p
 
 
-0.408
 
 
29.0
 
 
 
 
BLCAP
 
 
hsa-miR-23a-3p
 
 
-0.601
 
 
48.1
 
 
 
 
BLCAP
 
 
hsa-miR-23b-3p
 
 
-0.601
 
 
48.1
 
 
 
 
BLMH
 
 
hsa-miR-29a-3p
 
 
-0.393
 
 
11.7
 
 
 
 
BLOC1S6
 
 
hsa-miR-181b-5p
 
 
-0.393
 
 
19.0
 
 
 
 
BLOC1S6
 
 
hsa-miR-181a-5p
 
 
-0.393
 
 
19.0
 
 
 
 
BMF
 
 
hsa-miR-29a-3p
 
 
-0.41
 
 
20.0
 
 
 
 
BMF
 
 
hsa-miR-221-3p
 
 
-0.453
 
 
20.0
 
 
 
 
BOLA3
 
 
hsa-miR-15b-5p
 
 
-0.363
 
 
31.7
 
 
 
 
BOLA3
 
 
hsa-miR-15b-5p
 
 
-0.386
 
 
31.7
 
 
 
 
BOLA3
 
 
hsa-miR-374b-5p
 
 
-0.486
 
 
31.7
 
 
 
 
BRI3
 
 
hsa-miR-132-3p
 
 
-0.385
 
 
63.7
 
 
 
 
BTAF1
 
 
hsa-miR-486-5p
 
 
-0.288
 
 
12.8
 
 
 
 
BTAF1
 
 
hsa-miR-15b-5p
 
 
-0.496
 
 
12.8
 
 
 
 
BTF3
 
 
hsa-miR-15b-5p
 
 
-0.344
 
 
433.1
 
 
 
 
BTF3
 
 
hsa-miR-361-5p
 
 
-0.551
 
 
433.1
 
 
 
 
BTG2
 
 
hsa-miR-29a-3p
 
 
-0.32
 
 
83.2
 
 
 
 
BZW1
 
 
hsa-miR-15b-5p
 
 
-0.367
 
 
60.8
 
 
 
 
C11orf24
 
 
hsa-miR-15b-5p
 
 
-0.403
 
 
50.6
 
 
 
 
C11orf68
 
 
hsa-miR-15b-5p
 
 
-0.29
 
 
16.2
 
 
 
 
C2orf88
 
 
hsa-miR-29a-3p
 
 
-0.32
 
 
32.6
 
 
 
 
C5orf15
 
 
hsa-miR-29a-3p
 
 
-0.392
 
 
45.8
 
 
 
 
 
 
 endometrium 
 
 
 
 
target_symbol
 
 
mature_mirna_id
 
 
score
 
 
nTPM
 
 
 
 
 
 
AAR2
 
 
hsa-miR-29a-3p
 
 
-0.327
 
 
23.5
 
 
 
 
ABCE1
 
 
hsa-miR-29a-3p
 
 
-0.404
 
 
22.8
 
 
 
 
ABHD4
 
 
hsa-miR-29a-3p
 
 
-0.312
 
 
26.6
 
 
 
 
ACTC1
 
 
hsa-miR-30b-5p
 
 
-0.543
 
 
17.7
 
 
 
 
ACTR2
 
 
hsa-miR-15b-5p
 
 
-0.293
 
 
65.5
 
 
 
 
ACVR1
 
 
hsa-miR-30b-5p
 
 
-0.29
 
 
15.9
 
 
 
 
ADAM9
 
 
hsa-miR-30b-5p
 
 
-0.312
 
 
21.6
 
 
 
 
ADAMTS10
 
 
hsa-miR-29a-3p
 
 
-0.516
 
 
10.1
 
 
 
 
ADAMTS9
 
 
hsa-miR-29a-3p
 
 
-0.305
 
 
12.4
 
 
 
 
ADAMTS9
 
 
hsa-miR-29a-3p
 
 
-0.485
 
 
12.4
 
 
 
 
ADCY3
 
 
hsa-miR-132-3p
 
 
-0.303
 
 
52.0
 
 
 
 
ADGRA3
 
 
hsa-miR-30b-5p
 
 
-0.304
 
 
14.1
 
 
 
 
ADIPOR2
 
 
hsa-miR-150-5p
 
 
-0.287
 
 
24.5
 
 
 
 
AFG3L2
 
 
hsa-miR-181b-5p
 
 
-0.491
 
 
12.4
 
 
 
 
AFG3L2
 
 
hsa-miR-181a-5p
 
 
-0.491
 
 
12.4
 
 
 
 
AK1
 
 
hsa-miR-29a-3p
 
 
-0.333
 
 
16.9
 
 
 
 
AKT3
 
 
hsa-miR-29a-3p
 
 
-0.287
 
 
19.5
 
 
 
 
AKT3
 
 
hsa-miR-15b-5p
 
 
-0.301
 
 
19.5
 
 
 
 
ALAD
 
 
hsa-miR-15b-5p
 
 
-0.446
 
 
16.4
 
 
 
 
ALDH1A2
 
 
hsa-miR-23b-3p
 
 
-0.346
 
 
76.9
 
 
 
 
ALDH1A2
 
 
hsa-miR-23a-3p
 
 
-0.354
 
 
76.9
 
 
 
 
AMOTL1
 
 
hsa-miR-15b-5p
 
 
-0.319
 
 
13.3
 
 
 
 
ANGPTL2
 
 
hsa-miR-221-3p
 
 
-0.369
 
 
59.2
 
 
 
 
ANKFY1
 
 
hsa-miR-155-5p
 
 
-0.315
 
 
17.2
 
 
 
 
ANKHD1
 
 
hsa-miR-23a-3p
 
 
-0.368
 
 
40.5
 
 
 
 
ANKHD1
 
 
hsa-miR-23b-3p
 
 
-0.368
 
 
40.5
 
 
 
 
ANKHD1
 
 
hsa-miR-30b-5p
 
 
-0.405
 
 
40.5
 
 
 
 
ANKRA2
 
 
hsa-miR-30b-5p
 
 
-0.354
 
 
11.6
 
 
 
 
ANKRA2
 
 
hsa-miR-30b-5p
 
 
-0.472
 
 
11.6
 
 
 
 
ANKRD17
 
 
hsa-miR-30b-5p
 
 
-0.336
 
 
13.7
 
 
 
 
ANKRD29
 
 
hsa-miR-132-3p
 
 
-0.47
 
 
14.7
 
 
 
 
ANP32A
 
 
hsa-miR-132-3p
 
 
-0.374
 
 
55.3
 
 
 
 
ANP32E
 
 
hsa-miR-501-3p
 
 
-0.331
 
 
24.6
 
 
 
 
ANTXR2
 
 
hsa-miR-29a-3p
 
 
-0.347
 
 
89.2
 
 
 
 
ANXA11
 
 
hsa-miR-15b-5p
 
 
-0.463
 
 
67.4
 
 
 
 
AP2A1
 
 
hsa-miR-15b-5p
 
 
-0.333
 
 
53.6
 
 
 
 
AP2B1
 
 
hsa-miR-15b-5p
 
 
-0.334
 
 
39.0
 
 
 
 
AP3S1
 
 
hsa-miR-30b-5p
 
 
-0.293
 
 
55.6
 
 
 
 
APP
 
 
hsa-miR-15b-5p
 
 
-0.298
 
 
254.0
 
 
 
 
AQP3
 
 
hsa-miR-874-3p
 
 
-0.48
 
 
10.3
 
 
 
 
ARF4
 
 
hsa-miR-30b-5p
 
 
-0.289
 
 
113.9
 
 
 
 
ARF4
 
 
hsa-miR-221-3p
 
 
-0.553
 
 
113.9
 
 
 
 
ARF5
 
 
hsa-miR-29a-3p
 
 
-0.491
 
 
111.9
 
 
 
 
ARF6
 
 
hsa-miR-181b-5p
 
 
-0.33
 
 
19.5
 
 
 
 
ARF6
 
 
hsa-miR-181a-5p
 
 
-0.33
 
 
19.5
 
 
 
 
ARHGAP5
 
 
hsa-miR-486-5p
 
 
-0.3
 
 
26.2
 
 
 
 
ARID1B
 
 
hsa-miR-29a-3p
 
 
-0.342
 
 
15.0
 
 
 
 
ARID4B
 
 
hsa-miR-486-5p
 
 
-0.367
 
 
14.9
 
 
 
 
ARL2
 
 
hsa-miR-15b-5p
 
 
-0.374
 
 
124.7
 
 
 
 
ARL2
 
 
hsa-miR-15b-5p
 
 
-0.442
 
 
124.7
 
 
 
 
ARL3
 
 
hsa-miR-15b-5p
 
 
-0.507
 
 
11.8
 
 
 
 
ARL4A
 
 
hsa-miR-29a-3p
 
 
-0.386
 
 
17.7
 
 
 
 
ARL4A
 
 
hsa-miR-30b-5p
 
 
-0.448
 
 
17.7
 
 
 
 
ARMC8
 
 
hsa-miR-486-5p
 
 
-0.321
 
 
16.5
 
 
 
 
ARMCX2
 
 
hsa-miR-15b-5p
 
 
-0.344
 
 
28.6
 
 
 
 
ARMCX3
 
 
hsa-miR-425-5p
 
 
-0.325
 
 
38.3
 
 
 
 
ARNT
 
 
hsa-miR-221-3p
 
 
-0.465
 
 
25.7
 
 
 
 
ARPP19
 
 
hsa-miR-15b-5p
 
 
-0.346
 
 
44.7
 
 
 
 
ARPP19
 
 
hsa-miR-29a-3p
 
 
-0.371
 
 
44.7
 
 
 
 
ARRDC3
 
 
hsa-miR-29a-3p
 
 
-0.402
 
 
38.0
 
 
 
 
ASAP2
 
 
hsa-miR-29a-3p
 
 
-0.348
 
 
12.0
 
 
 
 
ASB3
 
 
hsa-miR-30b-5p
 
 
-0.418
 
 
10.7
 
 
 
 
ASB3
 
 
hsa-miR-30b-5p
 
 
-0.418
 
 
10.7
 
 
 
 
ASB6
 
 
hsa-miR-874-3p
 
 
-0.368
 
 
11.1
 
 
 
 
ASH1L
 
 
hsa-miR-15b-5p
 
 
-0.325
 
 
20.7
 
 
 
 
ASPH
 
 
hsa-miR-221-3p
 
 
-0.287
 
 
45.6
 
 
 
 
ATF3
 
 
hsa-miR-874-3p
 
 
-0.288
 
 
101.3
 
 
 
 
ATG5
 
 
hsa-miR-30b-5p
 
 
-0.488
 
 
14.0
 
 
 
 
ATG9A
 
 
hsa-miR-15b-5p
 
 
-0.333
 
 
24.3
 
 
 
 
ATOSA
 
 
hsa-miR-221-3p
 
 
-0.495
 
 
15.0
 
 
 
 
ATP5MC3
 
 
hsa-miR-425-5p
 
 
-0.328
 
 
118.3
 
 
 
 
ATP5MC3
 
 
hsa-miR-29a-3p
 
 
-0.33
 
 
118.3
 
 
 
 
ATP6V1E1
 
 
hsa-miR-23a-3p
 
 
-0.294
 
 
106.1
 
 
 
 
ATP6V1E1
 
 
hsa-miR-23b-3p
 
 
-0.294
 
 
106.1
 
 
 
 
ATPAF1
 
 
hsa-miR-361-5p
 
 
-0.362
 
 
23.6
 
 
 
 
ATXN1L
 
 
hsa-miR-155-5p
 
 
-0.315
 
 
11.6
 
 
 
 
ATXN2
 
 
hsa-miR-15b-5p
 
 
-0.392
 
 
21.0
 
 
 
 
AXIN2
 
 
hsa-miR-15b-5p
 
 
-0.3
 
 
23.2
 
 
 
 
AZIN1
 
 
hsa-miR-132-3p
 
 
-0.29
 
 
41.0
 
 
 
 
AZIN1
 
 
hsa-miR-132-3p
 
 
-0.299
 
 
41.0
 
 
 
 
B4GALT5
 
 
hsa-miR-501-3p
 
 
-0.408
 
 
22.0
 
 
 
 
B4GAT1
 
 
hsa-miR-23a-3p
 
 
-0.361
 
 
28.7
 
 
 
 
B4GAT1
 
 
hsa-miR-23b-3p
 
 
-0.361
 
 
28.7
 
 
 
 
BCL2
 
 
hsa-miR-342-3p
 
 
-0.428
 
 
10.9
 
 
 
 
BCL2L2
 
 
hsa-miR-29a-3p
 
 
-0.312
 
 
40.2
 
 
 
 
BECN1
 
 
hsa-miR-30b-5p
 
 
-0.452
 
 
29.5
 
 
 
 
BET1
 
 
hsa-miR-23a-3p
 
 
-0.402
 
 
17.5
 
 
 
 
BET1
 
 
hsa-miR-23b-3p
 
 
-0.418
 
 
17.5
 
 
 
 
BFAR
 
 
hsa-miR-15b-5p
 
 
-0.352
 
 
24.5
 
 
 
 
BHLHE40
 
 
hsa-miR-181b-5p
 
 
-0.408
 
 
63.3
 
 
 
 
BHLHE40
 
 
hsa-miR-181a-5p
 
 
-0.408
 
 
63.3
 
 
 
 
BLCAP
 
 
hsa-miR-23a-3p
 
 
-0.601
 
 
32.3
 
 
 
 
BLCAP
 
 
hsa-miR-23b-3p
 
 
-0.601
 
 
32.3
 
 
 
 
BLMH
 
 
hsa-miR-29a-3p
 
 
-0.393
 
 
19.7
 
 
 
 
BLOC1S6
 
 
hsa-miR-181b-5p
 
 
-0.393
 
 
28.8
 
 
 
 
BLOC1S6
 
 
hsa-miR-181a-5p
 
 
-0.393
 
 
28.8
 
 
 
 
BMF
 
 
hsa-miR-29a-3p
 
 
-0.41
 
 
14.8
 
 
 
 
BMF
 
 
hsa-miR-221-3p
 
 
-0.453
 
 
14.8
 
 
 
 
BOLA3
 
 
hsa-miR-15b-5p
 
 
-0.363
 
 
26.2
 
 
 
 
BOLA3
 
 
hsa-miR-15b-5p
 
 
-0.386
 
 
26.2
 
 
 
 
 
 
 epididymis 
 
 
 
 
target_symbol
 
 
mature_mirna_id
 
 
score
 
 
nTPM
 
 
 
 
 
 
AAR2
 
 
hsa-miR-29a-3p
 
 
-0.327
 
 
23.5
 
 
 
 
ABCE1
 
 
hsa-miR-29a-3p
 
 
-0.404
 
 
15.1
 
 
 
 
ABHD4
 
 
hsa-miR-29a-3p
 
 
-0.312
 
 
28.3
 
 
 
 
ACTR2
 
 
hsa-miR-15b-5p
 
 
-0.293
 
 
60.8
 
 
 
 
ACVR1
 
 
hsa-miR-30b-5p
 
 
-0.29
 
 
10.8
 
 
 
 
ADAM9
 
 
hsa-miR-30b-5p
 
 
-0.312
 
 
14.3
 
 
 
 
ADCY3
 
 
hsa-miR-132-3p
 
 
-0.303
 
 
19.3
 
 
 
 
AK1
 
 
hsa-miR-29a-3p
 
 
-0.333
 
 
27.0
 
 
 
 
ALAD
 
 
hsa-miR-15b-5p
 
 
-0.446
 
 
25.1
 
 
 
 
AMOT
 
 
hsa-miR-29a-3p
 
 
-0.304
 
 
137.7
 
 
 
 
ANGPTL2
 
 
hsa-miR-221-3p
 
 
-0.369
 
 
12.9
 
 
 
 
ANKFY1
 
 
hsa-miR-155-5p
 
 
-0.315
 
 
10.0
 
 
 
 
ANKHD1
 
 
hsa-miR-23a-3p
 
 
-0.368
 
 
45.6
 
 
 
 
ANKHD1
 
 
hsa-miR-23b-3p
 
 
-0.368
 
 
45.6
 
 
 
 
ANKHD1
 
 
hsa-miR-30b-5p
 
 
-0.405
 
 
45.6
 
 
 
 
ANKRA2
 
 
hsa-miR-30b-5p
 
 
-0.354
 
 
12.3
 
 
 
 
ANKRA2
 
 
hsa-miR-30b-5p
 
 
-0.472
 
 
12.3
 
 
 
 
ANKRD17
 
 
hsa-miR-30b-5p
 
 
-0.336
 
 
17.5
 
 
 
 
ANO3
 
 
hsa-miR-15b-5p
 
 
-0.357
 
 
54.2
 
 
 
 
ANP32A
 
 
hsa-miR-132-3p
 
 
-0.374
 
 
47.9
 
 
 
 
ANP32E
 
 
hsa-miR-501-3p
 
 
-0.331
 
 
32.5
 
 
 
 
ANTXR2
 
 
hsa-miR-29a-3p
 
 
-0.347
 
 
20.7
 
 
 
 
ANXA11
 
 
hsa-miR-15b-5p
 
 
-0.463
 
 
101.5
 
 
 
 
AP1S3
 
 
hsa-miR-181b-5p
 
 
-0.383
 
 
45.7
 
 
 
 
AP1S3
 
 
hsa-miR-181a-5p
 
 
-0.383
 
 
45.7
 
 
 
 
AP2B1
 
 
hsa-miR-15b-5p
 
 
-0.334
 
 
66.7
 
 
 
 
AP3S1
 
 
hsa-miR-30b-5p
 
 
-0.293
 
 
107.2
 
 
 
 
APOO
 
 
hsa-miR-181b-5p
 
 
-0.5
 
 
18.5
 
 
 
 
APOO
 
 
hsa-miR-181a-5p
 
 
-0.5
 
 
18.5
 
 
 
 
APP
 
 
hsa-miR-15b-5p
 
 
-0.298
 
 
214.2
 
 
 
 
ARF4
 
 
hsa-miR-30b-5p
 
 
-0.289
 
 
214.1
 
 
 
 
ARF4
 
 
hsa-miR-221-3p
 
 
-0.553
 
 
214.1
 
 
 
 
ARF5
 
 
hsa-miR-29a-3p
 
 
-0.491
 
 
107.7
 
 
 
 
ARF6
 
 
hsa-miR-181b-5p
 
 
-0.33
 
 
27.6
 
 
 
 
ARF6
 
 
hsa-miR-181a-5p
 
 
-0.33
 
 
27.6
 
 
 
 
ARHGAP5
 
 
hsa-miR-486-5p
 
 
-0.3
 
 
23.7
 
 
 
 
ARID1B
 
 
hsa-miR-29a-3p
 
 
-0.342
 
 
10.3
 
 
 
 
ARID4B
 
 
hsa-miR-486-5p
 
 
-0.367
 
 
13.6
 
 
 
 
ARL2
 
 
hsa-miR-15b-5p
 
 
-0.374
 
 
26.6
 
 
 
 
ARL2
 
 
hsa-miR-15b-5p
 
 
-0.442
 
 
26.6
 
 
 
 
ARL3
 
 
hsa-miR-15b-5p
 
 
-0.507
 
 
10.0
 
 
 
 
ARL4A
 
 
hsa-miR-29a-3p
 
 
-0.386
 
 
13.0
 
 
 
 
ARL4A
 
 
hsa-miR-30b-5p
 
 
-0.448
 
 
13.0
 
 
 
 
ARMC8
 
 
hsa-miR-486-5p
 
 
-0.321
 
 
11.5
 
 
 
 
ARMCX2
 
 
hsa-miR-15b-5p
 
 
-0.344
 
 
146.2
 
 
 
 
ARMCX3
 
 
hsa-miR-425-5p
 
 
-0.325
 
 
99.4
 
 
 
 
ARNT
 
 
hsa-miR-221-3p
 
 
-0.465
 
 
14.0
 
 
 
 
ARPP19
 
 
hsa-miR-15b-5p
 
 
-0.346
 
 
34.2
 
 
 
 
ARPP19
 
 
hsa-miR-29a-3p
 
 
-0.371
 
 
34.2
 
 
 
 
ARRDC3
 
 
hsa-miR-29a-3p
 
 
-0.402
 
 
25.6
 
 
 
 
ASB3
 
 
hsa-miR-30b-5p
 
 
-0.418
 
 
13.0
 
 
 
 
ASB3
 
 
hsa-miR-30b-5p
 
 
-0.418
 
 
13.0
 
 
 
 
ASF1A
 
 
hsa-miR-23a-3p
 
 
-0.506
 
 
12.3
 
 
 
 
ASF1A
 
 
hsa-miR-23b-3p
 
 
-0.506
 
 
12.3
 
 
 
 
ASH1L
 
 
hsa-miR-15b-5p
 
 
-0.325
 
 
19.6
 
 
 
 
ASPH
 
 
hsa-miR-221-3p
 
 
-0.287
 
 
42.3
 
 
 
 
ATG5
 
 
hsa-miR-30b-5p
 
 
-0.488
 
 
15.7
 
 
 
 
ATG9A
 
 
hsa-miR-15b-5p
 
 
-0.333
 
 
13.4
 
 
 
 
ATP5MC3
 
 
hsa-miR-425-5p
 
 
-0.328
 
 
119.2
 
 
 
 
ATP5MC3
 
 
hsa-miR-29a-3p
 
 
-0.33
 
 
119.2
 
 
 
 
ATP6V1E1
 
 
hsa-miR-23a-3p
 
 
-0.294
 
 
128.2
 
 
 
 
ATP6V1E1
 
 
hsa-miR-23b-3p
 
 
-0.294
 
 
128.2
 
 
 
 
ATPAF1
 
 
hsa-miR-361-5p
 
 
-0.362
 
 
33.7
 
 
 
 
ATXN2
 
 
hsa-miR-15b-5p
 
 
-0.392
 
 
13.1
 
 
 
 
AUH
 
 
hsa-miR-23a-3p
 
 
-0.495
 
 
13.4
 
 
 
 
AUH
 
 
hsa-miR-23b-3p
 
 
-0.503
 
 
13.4
 
 
 
 
AUH
 
 
hsa-miR-23a-3p
 
 
-0.512
 
 
13.4
 
 
 
 
AUH
 
 
hsa-miR-23b-3p
 
 
-0.512
 
 
13.4
 
 
 
 
AZIN1
 
 
hsa-miR-132-3p
 
 
-0.29
 
 
28.4
 
 
 
 
AZIN1
 
 
hsa-miR-132-3p
 
 
-0.299
 
 
28.4
 
 
 
 
B4GAT1
 
 
hsa-miR-23a-3p
 
 
-0.361
 
 
14.3
 
 
 
 
B4GAT1
 
 
hsa-miR-23b-3p
 
 
-0.361
 
 
14.3
 
 
 
 
BCL2L2
 
 
hsa-miR-29a-3p
 
 
-0.312
 
 
29.0
 
 
 
 
BECN1
 
 
hsa-miR-30b-5p
 
 
-0.452
 
 
45.1
 
 
 
 
BET1
 
 
hsa-miR-23a-3p
 
 
-0.402
 
 
32.4
 
 
 
 
BET1
 
 
hsa-miR-23b-3p
 
 
-0.418
 
 
32.4
 
 
 
 
BFAR
 
 
hsa-miR-15b-5p
 
 
-0.352
 
 
13.7
 
 
 
 
BHLHE40
 
 
hsa-miR-181b-5p
 
 
-0.408
 
 
32.6
 
 
 
 
BHLHE40
 
 
hsa-miR-181a-5p
 
 
-0.408
 
 
32.6
 
 
 
 
BICDL1
 
 
hsa-miR-221-3p
 
 
-0.447
 
 
23.0
 
 
 
 
BLCAP
 
 
hsa-miR-23a-3p
 
 
-0.601
 
 
49.5
 
 
 
 
BLCAP
 
 
hsa-miR-23b-3p
 
 
-0.601
 
 
49.5
 
 
 
 
BLMH
 
 
hsa-miR-29a-3p
 
 
-0.393
 
 
20.0
 
 
 
 
BLOC1S6
 
 
hsa-miR-181b-5p
 
 
-0.393
 
 
17.3
 
 
 
 
BLOC1S6
 
 
hsa-miR-181a-5p
 
 
-0.393
 
 
17.3
 
 
 
 
BOLA3
 
 
hsa-miR-15b-5p
 
 
-0.363
 
 
23.7
 
 
 
 
BOLA3
 
 
hsa-miR-15b-5p
 
 
-0.386
 
 
23.7
 
 
 
 
BOLA3
 
 
hsa-miR-374b-5p
 
 
-0.486
 
 
23.7
 
 
 
 
BRI3
 
 
hsa-miR-132-3p
 
 
-0.385
 
 
30.0
 
 
 
 
BTAF1
 
 
hsa-miR-486-5p
 
 
-0.288
 
 
16.9
 
 
 
 
BTAF1
 
 
hsa-miR-15b-5p
 
 
-0.496
 
 
16.9
 
 
 
 
BTF3
 
 
hsa-miR-15b-5p
 
 
-0.344
 
 
398.2
 
 
 
 
BTF3
 
 
hsa-miR-361-5p
 
 
-0.551
 
 
398.2
 
 
 
 
BTG2
 
 
hsa-miR-29a-3p
 
 
-0.32
 
 
39.2
 
 
 
 
BZW1
 
 
hsa-miR-15b-5p
 
 
-0.367
 
 
55.1
 
 
 
 
C11orf68
 
 
hsa-miR-15b-5p
 
 
-0.29
 
 
36.8
 
 
 
 
C2orf42
 
 
hsa-miR-15b-5p
 
 
-0.376
 
 
10.3
 
 
 
 
C5orf15
 
 
hsa-miR-29a-3p
 
 
-0.392
 
 
32.4
 
 
 
 
C6orf62
 
 
hsa-miR-181b-5p
 
 
-0.334
 
 
95.1
 
 
 
 
C6orf62
 
 
hsa-miR-181a-5p
 
 
-0.334
 
 
95.1
 
 
 
 
 
 
 esophagus 
 
 
 
 
target_symbol
 
 
mature_mirna_id
 
 
score
 
 
nTPM
 
 
 
 
 
 
AAR2
 
 
hsa-miR-29a-3p
 
 
-0.327
 
 
16.7
 
 
 
 
ABCE1
 
 
hsa-miR-29a-3p
 
 
-0.404
 
 
33.0
 
 
 
 
ABHD17B
 
 
hsa-miR-486-5p
 
 
-0.348
 
 
12.2
 
 
 
 
ABHD4
 
 
hsa-miR-29a-3p
 
 
-0.312
 
 
40.8
 
 
 
 
ABHD5
 
 
hsa-miR-132-3p
 
 
-0.323
 
 
18.4
 
 
 
 
ACTA1
 
 
hsa-miR-155-5p
 
 
-0.595
 
 
568.9
 
 
 
 
ACTC1
 
 
hsa-miR-30b-5p
 
 
-0.543
 
 
21.5
 
 
 
 
ACTN2
 
 
hsa-miR-23a-3p
 
 
-0.294
 
 
19.6
 
 
 
 
ACTN2
 
 
hsa-miR-29a-3p
 
 
-0.311
 
 
19.6
 
 
 
 
ACTR2
 
 
hsa-miR-15b-5p
 
 
-0.293
 
 
103.7
 
 
 
 
ADAM9
 
 
hsa-miR-30b-5p
 
 
-0.312
 
 
22.2
 
 
 
 
ADCY3
 
 
hsa-miR-132-3p
 
 
-0.303
 
 
29.3
 
 
 
 
ADIPOR2
 
 
hsa-miR-150-5p
 
 
-0.287
 
 
44.0
 
 
 
 
ADRB2
 
 
hsa-miR-15b-5p
 
 
-0.29
 
 
14.2
 
 
 
 
AFG3L2
 
 
hsa-miR-181b-5p
 
 
-0.491
 
 
25.7
 
 
 
 
AFG3L2
 
 
hsa-miR-181a-5p
 
 
-0.491
 
 
25.7
 
 
 
 
AHR
 
 
hsa-miR-29a-3p
 
 
-0.316
 
 
16.2
 
 
 
 
AK1
 
 
hsa-miR-29a-3p
 
 
-0.333
 
 
30.1
 
 
 
 
AK4
 
 
hsa-miR-15b-5p
 
 
-0.316
 
 
22.0
 
 
 
 
ALAD
 
 
hsa-miR-15b-5p
 
 
-0.446
 
 
34.3
 
 
 
 
ALOX12
 
 
hsa-miR-15b-5p
 
 
-0.443
 
 
47.5
 
 
 
 
AMOTL1
 
 
hsa-miR-15b-5p
 
 
-0.319
 
 
28.5
 
 
 
 
ANGPTL2
 
 
hsa-miR-221-3p
 
 
-0.369
 
 
20.3
 
 
 
 
ANKFY1
 
 
hsa-miR-155-5p
 
 
-0.315
 
 
12.9
 
 
 
 
ANKHD1
 
 
hsa-miR-23a-3p
 
 
-0.368
 
 
42.1
 
 
 
 
ANKHD1
 
 
hsa-miR-23b-3p
 
 
-0.368
 
 
42.1
 
 
 
 
ANKHD1
 
 
hsa-miR-30b-5p
 
 
-0.405
 
 
42.1
 
 
 
 
ANKRD17
 
 
hsa-miR-30b-5p
 
 
-0.336
 
 
13.6
 
 
 
 
ANP32A
 
 
hsa-miR-132-3p
 
 
-0.374
 
 
61.9
 
 
 
 
ANP32E
 
 
hsa-miR-501-3p
 
 
-0.331
 
 
17.5
 
 
 
 
ANTXR2
 
 
hsa-miR-29a-3p
 
 
-0.347
 
 
23.0
 
 
 
 
ANXA11
 
 
hsa-miR-15b-5p
 
 
-0.463
 
 
200.5
 
 
 
 
AP2A1
 
 
hsa-miR-15b-5p
 
 
-0.333
 
 
45.1
 
 
 
 
AP2B1
 
 
hsa-miR-15b-5p
 
 
-0.334
 
 
23.5
 
 
 
 
AP3S1
 
 
hsa-miR-30b-5p
 
 
-0.293
 
 
75.8
 
 
 
 
APOO
 
 
hsa-miR-181b-5p
 
 
-0.5
 
 
12.1
 
 
 
 
APOO
 
 
hsa-miR-181a-5p
 
 
-0.5
 
 
12.1
 
 
 
 
APP
 
 
hsa-miR-15b-5p
 
 
-0.298
 
 
163.0
 
 
 
 
AQP3
 
 
hsa-miR-874-3p
 
 
-0.48
 
 
1457.5
 
 
 
 
ARF4
 
 
hsa-miR-30b-5p
 
 
-0.289
 
 
102.8
 
 
 
 
ARF4
 
 
hsa-miR-221-3p
 
 
-0.553
 
 
102.8
 
 
 
 
ARF5
 
 
hsa-miR-29a-3p
 
 
-0.491
 
 
248.7
 
 
 
 
ARF6
 
 
hsa-miR-181b-5p
 
 
-0.33
 
 
81.9
 
 
 
 
ARF6
 
 
hsa-miR-181a-5p
 
 
-0.33
 
 
81.9
 
 
 
 
ARHGAP5
 
 
hsa-miR-486-5p
 
 
-0.3
 
 
62.5
 
 
 
 
ARID1B
 
 
hsa-miR-29a-3p
 
 
-0.342
 
 
13.7
 
 
 
 
ARL2
 
 
hsa-miR-15b-5p
 
 
-0.374
 
 
68.0
 
 
 
 
ARL2
 
 
hsa-miR-15b-5p
 
 
-0.442
 
 
68.0
 
 
 
 
ARL4A
 
 
hsa-miR-29a-3p
 
 
-0.386
 
 
56.3
 
 
 
 
ARL4A
 
 
hsa-miR-30b-5p
 
 
-0.448
 
 
56.3
 
 
 
 
ARMC8
 
 
hsa-miR-486-5p
 
 
-0.321
 
 
19.8
 
 
 
 
ARMCX3
 
 
hsa-miR-425-5p
 
 
-0.325
 
 
17.0
 
 
 
 
ARNT
 
 
hsa-miR-221-3p
 
 
-0.465
 
 
18.0
 
 
 
 
ARPP19
 
 
hsa-miR-15b-5p
 
 
-0.346
 
 
49.0
 
 
 
 
ARPP19
 
 
hsa-miR-29a-3p
 
 
-0.371
 
 
49.0
 
 
 
 
ARRDC3
 
 
hsa-miR-29a-3p
 
 
-0.402
 
 
36.4
 
 
 
 
ASB6
 
 
hsa-miR-874-3p
 
 
-0.368
 
 
15.0
 
 
 
 
ASH1L
 
 
hsa-miR-15b-5p
 
 
-0.325
 
 
16.5
 
 
 
 
ASPH
 
 
hsa-miR-221-3p
 
 
-0.287
 
 
66.3
 
 
 
 
ATF3
 
 
hsa-miR-874-3p
 
 
-0.288
 
 
120.8
 
 
 
 
ATG9A
 
 
hsa-miR-15b-5p
 
 
-0.333
 
 
33.8
 
 
 
 
ATOSA
 
 
hsa-miR-221-3p
 
 
-0.495
 
 
25.7
 
 
 
 
ATP5MC3
 
 
hsa-miR-425-5p
 
 
-0.328
 
 
293.6
 
 
 
 
ATP5MC3
 
 
hsa-miR-29a-3p
 
 
-0.33
 
 
293.6
 
 
 
 
ATP6V1E1
 
 
hsa-miR-23a-3p
 
 
-0.294
 
 
118.2
 
 
 
 
ATP6V1E1
 
 
hsa-miR-23b-3p
 
 
-0.294
 
 
118.2
 
 
 
 
ATPAF1
 
 
hsa-miR-361-5p
 
 
-0.362
 
 
36.9
 
 
 
 
ATXN2
 
 
hsa-miR-15b-5p
 
 
-0.392
 
 
13.9
 
 
 
 
AZIN1
 
 
hsa-miR-132-3p
 
 
-0.29
 
 
48.8
 
 
 
 
AZIN1
 
 
hsa-miR-132-3p
 
 
-0.299
 
 
48.8
 
 
 
 
B3GNT5
 
 
hsa-miR-30b-5p
 
 
-0.326
 
 
35.3
 
 
 
 
B4GALT5
 
 
hsa-miR-501-3p
 
 
-0.408
 
 
35.5
 
 
 
 
BAK1
 
 
hsa-miR-29a-3p
 
 
-0.409
 
 
32.4
 
 
 
 
BCL2L2
 
 
hsa-miR-29a-3p
 
 
-0.312
 
 
46.2
 
 
 
 
BDH1
 
 
hsa-miR-874-3p
 
 
-0.644
 
 
43.7
 
 
 
 
BECN1
 
 
hsa-miR-30b-5p
 
 
-0.452
 
 
46.5
 
 
 
 
BET1
 
 
hsa-miR-23a-3p
 
 
-0.402
 
 
11.5
 
 
 
 
BET1
 
 
hsa-miR-23b-3p
 
 
-0.418
 
 
11.5
 
 
 
 
BFAR
 
 
hsa-miR-15b-5p
 
 
-0.352
 
 
17.8
 
 
 
 
BHLHE40
 
 
hsa-miR-181b-5p
 
 
-0.408
 
 
372.4
 
 
 
 
BHLHE40
 
 
hsa-miR-181a-5p
 
 
-0.408
 
 
372.4
 
 
 
 
BICDL1
 
 
hsa-miR-221-3p
 
 
-0.447
 
 
28.8
 
 
 
 
BLCAP
 
 
hsa-miR-23a-3p
 
 
-0.601
 
 
84.6
 
 
 
 
BLCAP
 
 
hsa-miR-23b-3p
 
 
-0.601
 
 
84.6
 
 
 
 
BLMH
 
 
hsa-miR-29a-3p
 
 
-0.393
 
 
27.6
 
 
 
 
BLOC1S6
 
 
hsa-miR-181b-5p
 
 
-0.393
 
 
19.8
 
 
 
 
BLOC1S6
 
 
hsa-miR-181a-5p
 
 
-0.393
 
 
19.8
 
 
 
 
BOLA3
 
 
hsa-miR-15b-5p
 
 
-0.363
 
 
33.7
 
 
 
 
BOLA3
 
 
hsa-miR-15b-5p
 
 
-0.386
 
 
33.7
 
 
 
 
BOLA3
 
 
hsa-miR-374b-5p
 
 
-0.486
 
 
33.7
 
 
 
 
BORCS6
 
 
hsa-miR-15b-5p
 
 
-0.347
 
 
14.9
 
 
 
 
BRI3
 
 
hsa-miR-132-3p
 
 
-0.385
 
 
58.3
 
 
 
 
BTAF1
 
 
hsa-miR-486-5p
 
 
-0.288
 
 
14.2
 
 
 
 
BTAF1
 
 
hsa-miR-15b-5p
 
 
-0.496
 
 
14.2
 
 
 
 
BTF3
 
 
hsa-miR-15b-5p
 
 
-0.344
 
 
704.5
 
 
 
 
BTF3
 
 
hsa-miR-361-5p
 
 
-0.551
 
 
704.5
 
 
 
 
BTG2
 
 
hsa-miR-29a-3p
 
 
-0.32
 
 
294.8
 
 
 
 
BZW1
 
 
hsa-miR-15b-5p
 
 
-0.367
 
 
113.4
 
 
 
 
C11orf24
 
 
hsa-miR-15b-5p
 
 
-0.403
 
 
14.9
 
 
 
 
C11orf68
 
 
hsa-miR-15b-5p
 
 
-0.29
 
 
28.8
 
 
 
 
 
 
 fallopian tube 
 
 
 
 
target_symbol
 
 
mature_mirna_id
 
 
score
 
 
nTPM
 
 
 
 
 
 
AAR2
 
 
hsa-miR-29a-3p
 
 
-0.327
 
 
21.8
 
 
 
 
ABCE1
 
 
hsa-miR-29a-3p
 
 
-0.404
 
 
21.1
 
 
 
 
ABHD4
 
 
hsa-miR-29a-3p
 
 
-0.312
 
 
18.8
 
 
 
 
ACTR2
 
 
hsa-miR-15b-5p
 
 
-0.293
 
 
50.3
 
 
 
 
ACVR1
 
 
hsa-miR-30b-5p
 
 
-0.29
 
 
13.2
 
 
 
 
ADAM9
 
 
hsa-miR-30b-5p
 
 
-0.312
 
 
15.9
 
 
 
 
ADAMTS9
 
 
hsa-miR-29a-3p
 
 
-0.305
 
 
10.3
 
 
 
 
ADAMTS9
 
 
hsa-miR-29a-3p
 
 
-0.485
 
 
10.3
 
 
 
 
ADCY3
 
 
hsa-miR-132-3p
 
 
-0.303
 
 
52.6
 
 
 
 
ADGRA3
 
 
hsa-miR-30b-5p
 
 
-0.304
 
 
20.3
 
 
 
 
ADIPOR2
 
 
hsa-miR-150-5p
 
 
-0.287
 
 
23.2
 
 
 
 
AFG3L2
 
 
hsa-miR-181b-5p
 
 
-0.491
 
 
12.9
 
 
 
 
AFG3L2
 
 
hsa-miR-181a-5p
 
 
-0.491
 
 
12.9
 
 
 
 
AK1
 
 
hsa-miR-29a-3p
 
 
-0.333
 
 
321.8
 
 
 
 
AKT3
 
 
hsa-miR-29a-3p
 
 
-0.287
 
 
15.1
 
 
 
 
AKT3
 
 
hsa-miR-15b-5p
 
 
-0.301
 
 
15.1
 
 
 
 
ALAD
 
 
hsa-miR-15b-5p
 
 
-0.446
 
 
14.7
 
 
 
 
ALDH1A2
 
 
hsa-miR-23b-3p
 
 
-0.346
 
 
70.1
 
 
 
 
ALDH1A2
 
 
hsa-miR-23a-3p
 
 
-0.354
 
 
70.1
 
 
 
 
AMOT
 
 
hsa-miR-29a-3p
 
 
-0.304
 
 
11.3
 
 
 
 
AMOTL1
 
 
hsa-miR-15b-5p
 
 
-0.319
 
 
12.5
 
 
 
 
ANGPTL2
 
 
hsa-miR-221-3p
 
 
-0.369
 
 
29.3
 
 
 
 
ANKFY1
 
 
hsa-miR-155-5p
 
 
-0.315
 
 
14.3
 
 
 
 
ANKHD1
 
 
hsa-miR-23a-3p
 
 
-0.368
 
 
37.7
 
 
 
 
ANKHD1
 
 
hsa-miR-23b-3p
 
 
-0.368
 
 
37.7
 
 
 
 
ANKHD1
 
 
hsa-miR-30b-5p
 
 
-0.405
 
 
37.7
 
 
 
 
ANKRA2
 
 
hsa-miR-30b-5p
 
 
-0.354
 
 
10.8
 
 
 
 
ANKRA2
 
 
hsa-miR-30b-5p
 
 
-0.472
 
 
10.8
 
 
 
 
ANKRD17
 
 
hsa-miR-30b-5p
 
 
-0.336
 
 
17.1
 
 
 
 
ANKRD29
 
 
hsa-miR-132-3p
 
 
-0.47
 
 
14.4
 
 
 
 
ANP32A
 
 
hsa-miR-132-3p
 
 
-0.374
 
 
70.6
 
 
 
 
ANP32E
 
 
hsa-miR-501-3p
 
 
-0.331
 
 
19.9
 
 
 
 
ANTXR2
 
 
hsa-miR-29a-3p
 
 
-0.347
 
 
42.2
 
 
 
 
ANXA11
 
 
hsa-miR-15b-5p
 
 
-0.463
 
 
102.6
 
 
 
 
AP2A1
 
 
hsa-miR-15b-5p
 
 
-0.333
 
 
44.6
 
 
 
 
AP2B1
 
 
hsa-miR-15b-5p
 
 
-0.334
 
 
43.9
 
 
 
 
AP3S1
 
 
hsa-miR-30b-5p
 
 
-0.293
 
 
44.1
 
 
 
 
APOO
 
 
hsa-miR-181b-5p
 
 
-0.5
 
 
19.5
 
 
 
 
APOO
 
 
hsa-miR-181a-5p
 
 
-0.5
 
 
19.5
 
 
 
 
APP
 
 
hsa-miR-15b-5p
 
 
-0.298
 
 
289.3
 
 
 
 
AQP3
 
 
hsa-miR-874-3p
 
 
-0.48
 
 
23.9
 
 
 
 
ARF4
 
 
hsa-miR-30b-5p
 
 
-0.289
 
 
79.6
 
 
 
 
ARF4
 
 
hsa-miR-221-3p
 
 
-0.553
 
 
79.6
 
 
 
 
ARF5
 
 
hsa-miR-29a-3p
 
 
-0.491
 
 
100.8
 
 
 
 
ARF6
 
 
hsa-miR-181b-5p
 
 
-0.33
 
 
26.6
 
 
 
 
ARF6
 
 
hsa-miR-181a-5p
 
 
-0.33
 
 
26.6
 
 
 
 
ARHGAP5
 
 
hsa-miR-486-5p
 
 
-0.3
 
 
21.8
 
 
 
 
ARID1B
 
 
hsa-miR-29a-3p
 
 
-0.342
 
 
16.6
 
 
 
 
ARL2
 
 
hsa-miR-15b-5p
 
 
-0.374
 
 
96.8
 
 
 
 
ARL2
 
 
hsa-miR-15b-5p
 
 
-0.442
 
 
96.8
 
 
 
 
ARL3
 
 
hsa-miR-15b-5p
 
 
-0.507
 
 
14.3
 
 
 
 
ARL4A
 
 
hsa-miR-29a-3p
 
 
-0.386
 
 
26.0
 
 
 
 
ARL4A
 
 
hsa-miR-30b-5p
 
 
-0.448
 
 
26.0
 
 
 
 
ARMC8
 
 
hsa-miR-486-5p
 
 
-0.321
 
 
14.8
 
 
 
 
ARMCX2
 
 
hsa-miR-15b-5p
 
 
-0.344
 
 
26.8
 
 
 
 
ARMCX3
 
 
hsa-miR-425-5p
 
 
-0.325
 
 
25.7
 
 
 
 
ARNT
 
 
hsa-miR-221-3p
 
 
-0.465
 
 
23.5
 
 
 
 
ARPP19
 
 
hsa-miR-15b-5p
 
 
-0.346
 
 
31.4
 
 
 
 
ARPP19
 
 
hsa-miR-29a-3p
 
 
-0.371
 
 
31.4
 
 
 
 
ARRDC3
 
 
hsa-miR-29a-3p
 
 
-0.402
 
 
23.4
 
 
 
 
ASAP2
 
 
hsa-miR-29a-3p
 
 
-0.348
 
 
10.1
 
 
 
 
ASB6
 
 
hsa-miR-874-3p
 
 
-0.368
 
 
10.5
 
 
 
 
ASH1L
 
 
hsa-miR-15b-5p
 
 
-0.325
 
 
17.7
 
 
 
 
ASPH
 
 
hsa-miR-221-3p
 
 
-0.287
 
 
45.0
 
 
 
 
ATF3
 
 
hsa-miR-874-3p
 
 
-0.288
 
 
153.6
 
 
 
 
ATG5
 
 
hsa-miR-30b-5p
 
 
-0.488
 
 
13.8
 
 
 
 
ATG9A
 
 
hsa-miR-15b-5p
 
 
-0.333
 
 
19.5
 
 
 
 
ATOSA
 
 
hsa-miR-221-3p
 
 
-0.495
 
 
14.8
 
 
 
 
ATP5MC3
 
 
hsa-miR-425-5p
 
 
-0.328
 
 
110.4
 
 
 
 
ATP5MC3
 
 
hsa-miR-29a-3p
 
 
-0.33
 
 
110.4
 
 
 
 
ATP6V1E1
 
 
hsa-miR-23a-3p
 
 
-0.294
 
 
95.5
 
 
 
 
ATP6V1E1
 
 
hsa-miR-23b-3p
 
 
-0.294
 
 
95.5
 
 
 
 
ATPAF1
 
 
hsa-miR-361-5p
 
 
-0.362
 
 
27.2
 
 
 
 
ATXN1L
 
 
hsa-miR-155-5p
 
 
-0.315
 
 
10.2
 
 
 
 
ATXN2
 
 
hsa-miR-15b-5p
 
 
-0.392
 
 
19.4
 
 
 
 
AUH
 
 
hsa-miR-23a-3p
 
 
-0.495
 
 
11.7
 
 
 
 
AUH
 
 
hsa-miR-23b-3p
 
 
-0.503
 
 
11.7
 
 
 
 
AUH
 
 
hsa-miR-23a-3p
 
 
-0.512
 
 
11.7
 
 
 
 
AUH
 
 
hsa-miR-23b-3p
 
 
-0.512
 
 
11.7
 
 
 
 
AXIN2
 
 
hsa-miR-15b-5p
 
 
-0.3
 
 
17.5
 
 
 
 
AZIN1
 
 
hsa-miR-132-3p
 
 
-0.29
 
 
59.5
 
 
 
 
AZIN1
 
 
hsa-miR-132-3p
 
 
-0.299
 
 
59.5
 
 
 
 
B4GALT5
 
 
hsa-miR-501-3p
 
 
-0.408
 
 
17.2
 
 
 
 
B4GAT1
 
 
hsa-miR-23a-3p
 
 
-0.361
 
 
25.4
 
 
 
 
B4GAT1
 
 
hsa-miR-23b-3p
 
 
-0.361
 
 
25.4
 
 
 
 
BCL2
 
 
hsa-miR-342-3p
 
 
-0.428
 
 
11.1
 
 
 
 
BCL2L2
 
 
hsa-miR-29a-3p
 
 
-0.312
 
 
36.3
 
 
 
 
BECN1
 
 
hsa-miR-30b-5p
 
 
-0.452
 
 
34.0
 
 
 
 
BET1
 
 
hsa-miR-23a-3p
 
 
-0.402
 
 
17.4
 
 
 
 
BET1
 
 
hsa-miR-23b-3p
 
 
-0.418
 
 
17.4
 
 
 
 
BFAR
 
 
hsa-miR-15b-5p
 
 
-0.352
 
 
22.6
 
 
 
 
BHLHE40
 
 
hsa-miR-181b-5p
 
 
-0.408
 
 
77.8
 
 
 
 
BHLHE40
 
 
hsa-miR-181a-5p
 
 
-0.408
 
 
77.8
 
 
 
 
BLCAP
 
 
hsa-miR-23a-3p
 
 
-0.601
 
 
132.7
 
 
 
 
BLCAP
 
 
hsa-miR-23b-3p
 
 
-0.601
 
 
132.7
 
 
 
 
BLMH
 
 
hsa-miR-29a-3p
 
 
-0.393
 
 
15.6
 
 
 
 
BLOC1S6
 
 
hsa-miR-181b-5p
 
 
-0.393
 
 
17.1
 
 
 
 
BLOC1S6
 
 
hsa-miR-181a-5p
 
 
-0.393
 
 
17.1
 
 
 
 
BMF
 
 
hsa-miR-29a-3p
 
 
-0.41
 
 
11.2
 
 
 
 
BMF
 
 
hsa-miR-221-3p
 
 
-0.453
 
 
11.2
 
 
 
 
 
 
 gallbladder 
 
 
 
 
target_symbol
 
 
mature_mirna_id
 
 
score
 
 
nTPM
 
 
 
 
 
 
AAR2
 
 
hsa-miR-29a-3p
 
 
-0.327
 
 
15.5
 
 
 
 
ABCE1
 
 
hsa-miR-29a-3p
 
 
-0.404
 
 
21.0
 
 
 
 
ABHD4
 
 
hsa-miR-29a-3p
 
 
-0.312
 
 
25.4
 
 
 
 
ABHD5
 
 
hsa-miR-132-3p
 
 
-0.323
 
 
10.2
 
 
 
 
ACTC1
 
 
hsa-miR-30b-5p
 
 
-0.543
 
 
11.1
 
 
 
 
ACTR2
 
 
hsa-miR-15b-5p
 
 
-0.293
 
 
84.9
 
 
 
 
ACVR1
 
 
hsa-miR-30b-5p
 
 
-0.29
 
 
14.1
 
 
 
 
ADAM9
 
 
hsa-miR-30b-5p
 
 
-0.312
 
 
44.2
 
 
 
 
ADCY3
 
 
hsa-miR-132-3p
 
 
-0.303
 
 
21.6
 
 
 
 
ADRA2A
 
 
hsa-miR-23a-3p
 
 
-0.371
 
 
18.7
 
 
 
 
ADRA2A
 
 
hsa-miR-23b-3p
 
 
-0.371
 
 
18.7
 
 
 
 
AFG3L2
 
 
hsa-miR-181b-5p
 
 
-0.491
 
 
10.6
 
 
 
 
AFG3L2
 
 
hsa-miR-181a-5p
 
 
-0.491
 
 
10.6
 
 
 
 
AHR
 
 
hsa-miR-29a-3p
 
 
-0.316
 
 
31.5
 
 
 
 
AK1
 
 
hsa-miR-29a-3p
 
 
-0.333
 
 
21.1
 
 
 
 
AK4
 
 
hsa-miR-15b-5p
 
 
-0.316
 
 
12.5
 
 
 
 
ALAD
 
 
hsa-miR-15b-5p
 
 
-0.446
 
 
25.7
 
 
 
 
ALDH1A2
 
 
hsa-miR-23b-3p
 
 
-0.346
 
 
22.5
 
 
 
 
ALDH1A2
 
 
hsa-miR-23a-3p
 
 
-0.354
 
 
22.5
 
 
 
 
AMOTL1
 
 
hsa-miR-15b-5p
 
 
-0.319
 
 
12.5
 
 
 
 
ANGPTL2
 
 
hsa-miR-221-3p
 
 
-0.369
 
 
62.7
 
 
 
 
ANKFY1
 
 
hsa-miR-155-5p
 
 
-0.315
 
 
11.1
 
 
 
 
ANKHD1
 
 
hsa-miR-23a-3p
 
 
-0.368
 
 
23.4
 
 
 
 
ANKHD1
 
 
hsa-miR-23b-3p
 
 
-0.368
 
 
23.4
 
 
 
 
ANKHD1
 
 
hsa-miR-30b-5p
 
 
-0.405
 
 
23.4
 
 
 
 
ANKRD17
 
 
hsa-miR-30b-5p
 
 
-0.336
 
 
11.6
 
 
 
 
ANP32A
 
 
hsa-miR-132-3p
 
 
-0.374
 
 
56.0
 
 
 
 
ANP32E
 
 
hsa-miR-501-3p
 
 
-0.331
 
 
18.9
 
 
 
 
ANTXR2
 
 
hsa-miR-29a-3p
 
 
-0.347
 
 
51.4
 
 
 
 
ANXA11
 
 
hsa-miR-15b-5p
 
 
-0.463
 
 
79.6
 
 
 
 
AP2A1
 
 
hsa-miR-15b-5p
 
 
-0.333
 
 
18.3
 
 
 
 
AP2B1
 
 
hsa-miR-15b-5p
 
 
-0.334
 
 
39.7
 
 
 
 
AP3S1
 
 
hsa-miR-30b-5p
 
 
-0.293
 
 
46.1
 
 
 
 
APP
 
 
hsa-miR-15b-5p
 
 
-0.298
 
 
321.7
 
 
 
 
ARF4
 
 
hsa-miR-30b-5p
 
 
-0.289
 
 
145.1
 
 
 
 
ARF4
 
 
hsa-miR-221-3p
 
 
-0.553
 
 
145.1
 
 
 
 
ARF5
 
 
hsa-miR-29a-3p
 
 
-0.491
 
 
76.6
 
 
 
 
ARF6
 
 
hsa-miR-181b-5p
 
 
-0.33
 
 
30.6
 
 
 
 
ARF6
 
 
hsa-miR-181a-5p
 
 
-0.33
 
 
30.6
 
 
 
 
ARHGAP5
 
 
hsa-miR-486-5p
 
 
-0.3
 
 
30.8
 
 
 
 
ARID1B
 
 
hsa-miR-29a-3p
 
 
-0.342
 
 
12.6
 
 
 
 
ARID4B
 
 
hsa-miR-486-5p
 
 
-0.367
 
 
12.3
 
 
 
 
ARL2
 
 
hsa-miR-15b-5p
 
 
-0.374
 
 
45.0
 
 
 
 
ARL2
 
 
hsa-miR-15b-5p
 
 
-0.442
 
 
45.0
 
 
 
 
ARL4A
 
 
hsa-miR-29a-3p
 
 
-0.386
 
 
18.0
 
 
 
 
ARL4A
 
 
hsa-miR-30b-5p
 
 
-0.448
 
 
18.0
 
 
 
 
ARL5B
 
 
hsa-miR-29a-3p
 
 
-0.304
 
 
13.2
 
 
 
 
ARL5B
 
 
hsa-miR-155-5p
 
 
-0.355
 
 
13.2
 
 
 
 
ARMC8
 
 
hsa-miR-486-5p
 
 
-0.321
 
 
14.3
 
 
 
 
ARMCX2
 
 
hsa-miR-15b-5p
 
 
-0.344
 
 
19.3
 
 
 
 
ARMCX3
 
 
hsa-miR-425-5p
 
 
-0.325
 
 
31.9
 
 
 
 
ARNT
 
 
hsa-miR-221-3p
 
 
-0.465
 
 
19.2
 
 
 
 
ARPP19
 
 
hsa-miR-15b-5p
 
 
-0.346
 
 
36.7
 
 
 
 
ARPP19
 
 
hsa-miR-29a-3p
 
 
-0.371
 
 
36.7
 
 
 
 
ARRDC3
 
 
hsa-miR-29a-3p
 
 
-0.402
 
 
36.6
 
 
 
 
ASPH
 
 
hsa-miR-221-3p
 
 
-0.287
 
 
56.5
 
 
 
 
ATF3
 
 
hsa-miR-874-3p
 
 
-0.288
 
 
126.2
 
 
 
 
ATG9A
 
 
hsa-miR-15b-5p
 
 
-0.333
 
 
20.4
 
 
 
 
ATOSA
 
 
hsa-miR-221-3p
 
 
-0.495
 
 
11.0
 
 
 
 
ATP5MC3
 
 
hsa-miR-425-5p
 
 
-0.328
 
 
70.9
 
 
 
 
ATP5MC3
 
 
hsa-miR-29a-3p
 
 
-0.33
 
 
70.9
 
 
 
 
ATP6V1E1
 
 
hsa-miR-23a-3p
 
 
-0.294
 
 
79.5
 
 
 
 
ATP6V1E1
 
 
hsa-miR-23b-3p
 
 
-0.294
 
 
79.5
 
 
 
 
ATPAF1
 
 
hsa-miR-361-5p
 
 
-0.362
 
 
28.6
 
 
 
 
ATXN2
 
 
hsa-miR-15b-5p
 
 
-0.392
 
 
11.2
 
 
 
 
AUH
 
 
hsa-miR-23a-3p
 
 
-0.495
 
 
10.1
 
 
 
 
AUH
 
 
hsa-miR-23b-3p
 
 
-0.503
 
 
10.1
 
 
 
 
AUH
 
 
hsa-miR-23a-3p
 
 
-0.512
 
 
10.1
 
 
 
 
AUH
 
 
hsa-miR-23b-3p
 
 
-0.512
 
 
10.1
 
 
 
 
AZIN1
 
 
hsa-miR-132-3p
 
 
-0.29
 
 
40.1
 
 
 
 
AZIN1
 
 
hsa-miR-132-3p
 
 
-0.299
 
 
40.1
 
 
 
 
B3GNT5
 
 
hsa-miR-30b-5p
 
 
-0.326
 
 
19.9
 
 
 
 
B4GALT5
 
 
hsa-miR-501-3p
 
 
-0.408
 
 
29.5
 
 
 
 
BCL2L11
 
 
hsa-miR-221-3p
 
 
-0.423
 
 
11.1
 
 
 
 
BCL2L2
 
 
hsa-miR-29a-3p
 
 
-0.312
 
 
21.1
 
 
 
 
BECN1
 
 
hsa-miR-30b-5p
 
 
-0.452
 
 
32.2
 
 
 
 
BET1
 
 
hsa-miR-23a-3p
 
 
-0.402
 
 
12.5
 
 
 
 
BET1
 
 
hsa-miR-23b-3p
 
 
-0.418
 
 
12.5
 
 
 
 
BFAR
 
 
hsa-miR-15b-5p
 
 
-0.352
 
 
16.2
 
 
 
 
BHLHE40
 
 
hsa-miR-181b-5p
 
 
-0.408
 
 
94.0
 
 
 
 
BHLHE40
 
 
hsa-miR-181a-5p
 
 
-0.408
 
 
94.0
 
 
 
 
BLCAP
 
 
hsa-miR-23a-3p
 
 
-0.601
 
 
27.2
 
 
 
 
BLCAP
 
 
hsa-miR-23b-3p
 
 
-0.601
 
 
27.2
 
 
 
 
BLMH
 
 
hsa-miR-29a-3p
 
 
-0.393
 
 
17.2
 
 
 
 
BLOC1S6
 
 
hsa-miR-181b-5p
 
 
-0.393
 
 
24.8
 
 
 
 
BLOC1S6
 
 
hsa-miR-181a-5p
 
 
-0.393
 
 
24.8
 
 
 
 
BMF
 
 
hsa-miR-29a-3p
 
 
-0.41
 
 
11.4
 
 
 
 
BMF
 
 
hsa-miR-221-3p
 
 
-0.453
 
 
11.4
 
 
 
 
BOLA3
 
 
hsa-miR-15b-5p
 
 
-0.363
 
 
11.2
 
 
 
 
BOLA3
 
 
hsa-miR-15b-5p
 
 
-0.386
 
 
11.2
 
 
 
 
BOLA3
 
 
hsa-miR-374b-5p
 
 
-0.486
 
 
11.2
 
 
 
 
BRI3
 
 
hsa-miR-132-3p
 
 
-0.385
 
 
42.4
 
 
 
 
BTAF1
 
 
hsa-miR-486-5p
 
 
-0.288
 
 
12.0
 
 
 
 
BTAF1
 
 
hsa-miR-15b-5p
 
 
-0.496
 
 
12.0
 
 
 
 
BTF3
 
 
hsa-miR-15b-5p
 
 
-0.344
 
 
352.7
 
 
 
 
BTF3
 
 
hsa-miR-361-5p
 
 
-0.551
 
 
352.7
 
 
 
 
BTG2
 
 
hsa-miR-29a-3p
 
 
-0.32
 
 
148.1
 
 
 
 
BZW1
 
 
hsa-miR-15b-5p
 
 
-0.367
 
 
87.2
 
 
 
 
C11orf24
 
 
hsa-miR-15b-5p
 
 
-0.403
 
 
16.5
 
 
 
 
C11orf68
 
 
hsa-miR-15b-5p
 
 
-0.29
 
 
19.4
 
 
 
 
 
 
 heart muscle 
 
 
 
 
target_symbol
 
 
mature_mirna_id
 
 
score
 
 
nTPM
 
 
 
 
 
 
AAR2
 
 
hsa-miR-29a-3p
 
 
-0.327
 
 
17.9
 
 
 
 
ABCE1
 
 
hsa-miR-29a-3p
 
 
-0.404
 
 
23.3
 
 
 
 
ABHD18
 
 
hsa-miR-29a-3p
 
 
-0.321
 
 
12.6
 
 
 
 
ABHD18
 
 
hsa-miR-29a-3p
 
 
-0.34
 
 
12.6
 
 
 
 
ABHD4
 
 
hsa-miR-29a-3p
 
 
-0.312
 
 
21.4
 
 
 
 
ABHD5
 
 
hsa-miR-132-3p
 
 
-0.323
 
 
10.6
 
 
 
 
ACTA1
 
 
hsa-miR-155-5p
 
 
-0.595
 
 
6223.8
 
 
 
 
ACTC1
 
 
hsa-miR-30b-5p
 
 
-0.543
 
 
10840.1
 
 
 
 
ACTN2
 
 
hsa-miR-23a-3p
 
 
-0.294
 
 
695.9
 
 
 
 
ACTN2
 
 
hsa-miR-29a-3p
 
 
-0.311
 
 
695.9
 
 
 
 
ACTR2
 
 
hsa-miR-15b-5p
 
 
-0.293
 
 
60.7
 
 
 
 
ACVR1
 
 
hsa-miR-30b-5p
 
 
-0.29
 
 
14.3
 
 
 
 
ADA
 
 
hsa-miR-29a-3p
 
 
-0.376
 
 
18.4
 
 
 
 
ADAM19
 
 
hsa-miR-874-3p
 
 
-0.374
 
 
23.8
 
 
 
 
ADAM9
 
 
hsa-miR-30b-5p
 
 
-0.312
 
 
26.6
 
 
 
 
ADAMTS7
 
 
hsa-miR-29a-3p
 
 
-0.427
 
 
28.7
 
 
 
 
ADAMTS9
 
 
hsa-miR-29a-3p
 
 
-0.305
 
 
14.2
 
 
 
 
ADAMTS9
 
 
hsa-miR-29a-3p
 
 
-0.485
 
 
14.2
 
 
 
 
ADCY3
 
 
hsa-miR-132-3p
 
 
-0.303
 
 
35.0
 
 
 
 
ADGRA3
 
 
hsa-miR-30b-5p
 
 
-0.304
 
 
10.1
 
 
 
 
ADIPOR2
 
 
hsa-miR-150-5p
 
 
-0.287
 
 
31.9
 
 
 
 
AFG3L2
 
 
hsa-miR-181b-5p
 
 
-0.491
 
 
48.7
 
 
 
 
AFG3L2
 
 
hsa-miR-181a-5p
 
 
-0.491
 
 
48.7
 
 
 
 
AK1
 
 
hsa-miR-29a-3p
 
 
-0.333
 
 
313.1
 
 
 
 
AK4
 
 
hsa-miR-15b-5p
 
 
-0.316
 
 
47.4
 
 
 
 
AKT3
 
 
hsa-miR-29a-3p
 
 
-0.287
 
 
10.6
 
 
 
 
AKT3
 
 
hsa-miR-15b-5p
 
 
-0.301
 
 
10.6
 
 
 
 
ALAD
 
 
hsa-miR-15b-5p
 
 
-0.446
 
 
50.8
 
 
 
 
ALDH1A2
 
 
hsa-miR-23b-3p
 
 
-0.346
 
 
27.5
 
 
 
 
ALDH1A2
 
 
hsa-miR-23a-3p
 
 
-0.354
 
 
27.5
 
 
 
 
AMOTL1
 
 
hsa-miR-15b-5p
 
 
-0.319
 
 
17.9
 
 
 
 
ANGPTL2
 
 
hsa-miR-221-3p
 
 
-0.369
 
 
64.9
 
 
 
 
ANKFY1
 
 
hsa-miR-155-5p
 
 
-0.315
 
 
10.9
 
 
 
 
ANKHD1
 
 
hsa-miR-23a-3p
 
 
-0.368
 
 
34.0
 
 
 
 
ANKHD1
 
 
hsa-miR-23b-3p
 
 
-0.368
 
 
34.0
 
 
 
 
ANKHD1
 
 
hsa-miR-30b-5p
 
 
-0.405
 
 
34.0
 
 
 
 
ANKRD17
 
 
hsa-miR-30b-5p
 
 
-0.336
 
 
13.6
 
 
 
 
ANP32A
 
 
hsa-miR-132-3p
 
 
-0.374
 
 
119.7
 
 
 
 
ANP32E
 
 
hsa-miR-501-3p
 
 
-0.331
 
 
32.9
 
 
 
 
ANTXR2
 
 
hsa-miR-29a-3p
 
 
-0.347
 
 
26.5
 
 
 
 
ANXA11
 
 
hsa-miR-15b-5p
 
 
-0.463
 
 
221.3
 
 
 
 
AP2A1
 
 
hsa-miR-15b-5p
 
 
-0.333
 
 
49.3
 
 
 
 
AP2B1
 
 
hsa-miR-15b-5p
 
 
-0.334
 
 
63.3
 
 
 
 
AP3S1
 
 
hsa-miR-30b-5p
 
 
-0.293
 
 
53.4
 
 
 
 
APLN
 
 
hsa-miR-15b-5p
 
 
-0.395
 
 
12.4
 
 
 
 
APOO
 
 
hsa-miR-181b-5p
 
 
-0.5
 
 
61.9
 
 
 
 
APOO
 
 
hsa-miR-181a-5p
 
 
-0.5
 
 
61.9
 
 
 
 
APP
 
 
hsa-miR-15b-5p
 
 
-0.298
 
 
356.3
 
 
 
 
AQP3
 
 
hsa-miR-874-3p
 
 
-0.48
 
 
33.0
 
 
 
 
ARF4
 
 
hsa-miR-30b-5p
 
 
-0.289
 
 
106.8
 
 
 
 
ARF4
 
 
hsa-miR-221-3p
 
 
-0.553
 
 
106.8
 
 
 
 
ARF5
 
 
hsa-miR-29a-3p
 
 
-0.491
 
 
127.9
 
 
 
 
ARF6
 
 
hsa-miR-181b-5p
 
 
-0.33
 
 
15.7
 
 
 
 
ARF6
 
 
hsa-miR-181a-5p
 
 
-0.33
 
 
15.7
 
 
 
 
ARHGAP5
 
 
hsa-miR-486-5p
 
 
-0.3
 
 
59.2
 
 
 
 
ARID1B
 
 
hsa-miR-29a-3p
 
 
-0.342
 
 
12.9
 
 
 
 
ARL2
 
 
hsa-miR-15b-5p
 
 
-0.374
 
 
169.4
 
 
 
 
ARL2
 
 
hsa-miR-15b-5p
 
 
-0.442
 
 
169.4
 
 
 
 
ARL3
 
 
hsa-miR-15b-5p
 
 
-0.507
 
 
12.8
 
 
 
 
ARL4A
 
 
hsa-miR-29a-3p
 
 
-0.386
 
 
14.4
 
 
 
 
ARL4A
 
 
hsa-miR-30b-5p
 
 
-0.448
 
 
14.4
 
 
 
 
ARMC8
 
 
hsa-miR-486-5p
 
 
-0.321
 
 
16.3
 
 
 
 
ARMCX2
 
 
hsa-miR-15b-5p
 
 
-0.344
 
 
31.1
 
 
 
 
ARMCX3
 
 
hsa-miR-425-5p
 
 
-0.325
 
 
47.8
 
 
 
 
ARNT
 
 
hsa-miR-221-3p
 
 
-0.465
 
 
20.3
 
 
 
 
ARPP19
 
 
hsa-miR-15b-5p
 
 
-0.346
 
 
76.2
 
 
 
 
ARPP19
 
 
hsa-miR-29a-3p
 
 
-0.371
 
 
76.2
 
 
 
 
ARRDC3
 
 
hsa-miR-29a-3p
 
 
-0.402
 
 
23.2
 
 
 
 
ASF1A
 
 
hsa-miR-23a-3p
 
 
-0.506
 
 
12.5
 
 
 
 
ASF1A
 
 
hsa-miR-23b-3p
 
 
-0.506
 
 
12.5
 
 
 
 
ASH1L
 
 
hsa-miR-15b-5p
 
 
-0.325
 
 
18.5
 
 
 
 
ASPH
 
 
hsa-miR-221-3p
 
 
-0.287
 
 
100.1
 
 
 
 
ATF3
 
 
hsa-miR-874-3p
 
 
-0.288
 
 
112.5
 
 
 
 
ATG9A
 
 
hsa-miR-15b-5p
 
 
-0.333
 
 
48.2
 
 
 
 
ATOSA
 
 
hsa-miR-221-3p
 
 
-0.495
 
 
13.6
 
 
 
 
ATP5MC3
 
 
hsa-miR-425-5p
 
 
-0.328
 
 
1149.4
 
 
 
 
ATP5MC3
 
 
hsa-miR-29a-3p
 
 
-0.33
 
 
1149.4
 
 
 
 
ATP6V1E1
 
 
hsa-miR-23a-3p
 
 
-0.294
 
 
214.3
 
 
 
 
ATP6V1E1
 
 
hsa-miR-23b-3p
 
 
-0.294
 
 
214.3
 
 
 
 
ATPAF1
 
 
hsa-miR-361-5p
 
 
-0.362
 
 
249.4
 
 
 
 
ATXN2
 
 
hsa-miR-15b-5p
 
 
-0.392
 
 
22.3
 
 
 
 
AUH
 
 
hsa-miR-23a-3p
 
 
-0.495
 
 
36.4
 
 
 
 
AUH
 
 
hsa-miR-23b-3p
 
 
-0.503
 
 
36.4
 
 
 
 
AUH
 
 
hsa-miR-23a-3p
 
 
-0.512
 
 
36.4
 
 
 
 
AUH
 
 
hsa-miR-23b-3p
 
 
-0.512
 
 
36.4
 
 
 
 
AZIN1
 
 
hsa-miR-132-3p
 
 
-0.29
 
 
47.7
 
 
 
 
AZIN1
 
 
hsa-miR-132-3p
 
 
-0.299
 
 
47.7
 
 
 
 
B4GALT5
 
 
hsa-miR-501-3p
 
 
-0.408
 
 
19.2
 
 
 
 
B4GAT1
 
 
hsa-miR-23a-3p
 
 
-0.361
 
 
43.6
 
 
 
 
B4GAT1
 
 
hsa-miR-23b-3p
 
 
-0.361
 
 
43.6
 
 
 
 
BAK1
 
 
hsa-miR-29a-3p
 
 
-0.409
 
 
21.7
 
 
 
 
BCL2L2
 
 
hsa-miR-29a-3p
 
 
-0.312
 
 
36.7
 
 
 
 
BDH1
 
 
hsa-miR-874-3p
 
 
-0.644
 
 
48.7
 
 
 
 
BECN1
 
 
hsa-miR-30b-5p
 
 
-0.452
 
 
55.1
 
 
 
 
BET1
 
 
hsa-miR-23a-3p
 
 
-0.402
 
 
13.3
 
 
 
 
BET1
 
 
hsa-miR-23b-3p
 
 
-0.418
 
 
13.3
 
 
 
 
BFAR
 
 
hsa-miR-15b-5p
 
 
-0.352
 
 
23.9
 
 
 
 
BHLHE40
 
 
hsa-miR-181b-5p
 
 
-0.408
 
 
166.2
 
 
 
 
BHLHE40
 
 
hsa-miR-181a-5p
 
 
-0.408
 
 
166.2
 
 
 
 
BLCAP
 
 
hsa-miR-23a-3p
 
 
-0.601
 
 
73.6
 
 
 
 
 
 
 hippocampal formation 
 
 
 
 
target_symbol
 
 
mature_mirna_id
 
 
score
 
 
nTPM
 
 
 
 
 
 
AAR2
 
 
hsa-miR-29a-3p
 
 
-0.327
 
 
10.6
 
 
 
 
ABCE1
 
 
hsa-miR-29a-3p
 
 
-0.404
 
 
13.9
 
 
 
 
ABHD17B
 
 
hsa-miR-486-5p
 
 
-0.348
 
 
18.9
 
 
 
 
ABHD4
 
 
hsa-miR-29a-3p
 
 
-0.312
 
 
14.2
 
 
 
 
ACTN2
 
 
hsa-miR-23a-3p
 
 
-0.294
 
 
10.1
 
 
 
 
ACTN2
 
 
hsa-miR-29a-3p
 
 
-0.311
 
 
10.1
 
 
 
 
ACTR2
 
 
hsa-miR-15b-5p
 
 
-0.293
 
 
54.7
 
 
 
 
ADCY3
 
 
hsa-miR-132-3p
 
 
-0.303
 
 
30.1
 
 
 
 
ADGRA3
 
 
hsa-miR-30b-5p
 
 
-0.304
 
 
12.8
 
 
 
 
ADIPOR2
 
 
hsa-miR-150-5p
 
 
-0.287
 
 
57.6
 
 
 
 
AGPAT4
 
 
hsa-miR-29a-3p
 
 
-0.376
 
 
16.8
 
 
 
 
AK1
 
 
hsa-miR-29a-3p
 
 
-0.333
 
 
93.4
 
 
 
 
AK4
 
 
hsa-miR-15b-5p
 
 
-0.316
 
 
13.3
 
 
 
 
AKT3
 
 
hsa-miR-29a-3p
 
 
-0.287
 
 
17.7
 
 
 
 
AKT3
 
 
hsa-miR-15b-5p
 
 
-0.301
 
 
17.7
 
 
 
 
ALAD
 
 
hsa-miR-15b-5p
 
 
-0.446
 
 
60.3
 
 
 
 
AMOT
 
 
hsa-miR-29a-3p
 
 
-0.304
 
 
12.0
 
 
 
 
ANGEL2
 
 
hsa-miR-132-3p
 
 
-0.357
 
 
11.0
 
 
 
 
ANGPTL2
 
 
hsa-miR-221-3p
 
 
-0.369
 
 
11.4
 
 
 
 
ANKFY1
 
 
hsa-miR-155-5p
 
 
-0.315
 
 
10.8
 
 
 
 
ANKHD1
 
 
hsa-miR-23a-3p
 
 
-0.368
 
 
18.9
 
 
 
 
ANKHD1
 
 
hsa-miR-23b-3p
 
 
-0.368
 
 
18.9
 
 
 
 
ANKHD1
 
 
hsa-miR-30b-5p
 
 
-0.405
 
 
18.9
 
 
 
 
ANKRD13B
 
 
hsa-miR-29a-3p
 
 
-0.421
 
 
10.6
 
 
 
 
ANKRD17
 
 
hsa-miR-30b-5p
 
 
-0.336
 
 
10.3
 
 
 
 
ANLN
 
 
hsa-miR-15b-5p
 
 
-0.334
 
 
54.9
 
 
 
 
ANP32A
 
 
hsa-miR-132-3p
 
 
-0.374
 
 
49.8
 
 
 
 
ANP32E
 
 
hsa-miR-501-3p
 
 
-0.331
 
 
27.9
 
 
 
 
ANXA11
 
 
hsa-miR-15b-5p
 
 
-0.463
 
 
30.8
 
 
 
 
AP2A1
 
 
hsa-miR-15b-5p
 
 
-0.333
 
 
44.7
 
 
 
 
AP2B1
 
 
hsa-miR-15b-5p
 
 
-0.334
 
 
69.4
 
 
 
 
AP3S1
 
 
hsa-miR-30b-5p
 
 
-0.293
 
 
86.3
 
 
 
 
APC
 
 
hsa-miR-29a-3p
 
 
-0.413
 
 
34.3
 
 
 
 
APLN
 
 
hsa-miR-15b-5p
 
 
-0.395
 
 
14.1
 
 
 
 
APOO
 
 
hsa-miR-181b-5p
 
 
-0.5
 
 
45.8
 
 
 
 
APOO
 
 
hsa-miR-181a-5p
 
 
-0.5
 
 
45.8
 
 
 
 
APP
 
 
hsa-miR-15b-5p
 
 
-0.298
 
 
466.6
 
 
 
 
ARF4
 
 
hsa-miR-30b-5p
 
 
-0.289
 
 
61.3
 
 
 
 
ARF4
 
 
hsa-miR-221-3p
 
 
-0.553
 
 
61.3
 
 
 
 
ARF5
 
 
hsa-miR-29a-3p
 
 
-0.491
 
 
168.1
 
 
 
 
ARF6
 
 
hsa-miR-181b-5p
 
 
-0.33
 
 
10.0
 
 
 
 
ARF6
 
 
hsa-miR-181a-5p
 
 
-0.33
 
 
10.0
 
 
 
 
ARHGAP5
 
 
hsa-miR-486-5p
 
 
-0.3
 
 
22.7
 
 
 
 
ARL2
 
 
hsa-miR-15b-5p
 
 
-0.374
 
 
164.1
 
 
 
 
ARL2
 
 
hsa-miR-15b-5p
 
 
-0.442
 
 
164.1
 
 
 
 
ARL3
 
 
hsa-miR-15b-5p
 
 
-0.507
 
 
21.1
 
 
 
 
ARL4A
 
 
hsa-miR-29a-3p
 
 
-0.386
 
 
12.2
 
 
 
 
ARL4A
 
 
hsa-miR-30b-5p
 
 
-0.448
 
 
12.2
 
 
 
 
ARMC8
 
 
hsa-miR-486-5p
 
 
-0.321
 
 
13.3
 
 
 
 
ARMCX2
 
 
hsa-miR-15b-5p
 
 
-0.344
 
 
21.8
 
 
 
 
ARMCX3
 
 
hsa-miR-425-5p
 
 
-0.325
 
 
33.7
 
 
 
 
ARPP19
 
 
hsa-miR-15b-5p
 
 
-0.346
 
 
120.9
 
 
 
 
ARPP19
 
 
hsa-miR-29a-3p
 
 
-0.371
 
 
120.9
 
 
 
 
ARPP21
 
 
hsa-miR-15b-5p
 
 
-0.342
 
 
19.5
 
 
 
 
ARPP21
 
 
hsa-miR-501-3p
 
 
-0.546
 
 
19.5
 
 
 
 
ARRDC3
 
 
hsa-miR-29a-3p
 
 
-0.402
 
 
11.5
 
 
 
 
ASB3
 
 
hsa-miR-30b-5p
 
 
-0.418
 
 
16.2
 
 
 
 
ASB3
 
 
hsa-miR-30b-5p
 
 
-0.418
 
 
16.2
 
 
 
 
ASB6
 
 
hsa-miR-874-3p
 
 
-0.368
 
 
14.7
 
 
 
 
ASF1A
 
 
hsa-miR-23a-3p
 
 
-0.506
 
 
11.7
 
 
 
 
ASF1A
 
 
hsa-miR-23b-3p
 
 
-0.506
 
 
11.7
 
 
 
 
ASH1L
 
 
hsa-miR-15b-5p
 
 
-0.325
 
 
21.0
 
 
 
 
ASPH
 
 
hsa-miR-221-3p
 
 
-0.287
 
 
45.5
 
 
 
 
ATF3
 
 
hsa-miR-874-3p
 
 
-0.288
 
 
21.8
 
 
 
 
ATG9A
 
 
hsa-miR-15b-5p
 
 
-0.333
 
 
25.0
 
 
 
 
ATP5MC3
 
 
hsa-miR-425-5p
 
 
-0.328
 
 
174.9
 
 
 
 
ATP5MC3
 
 
hsa-miR-29a-3p
 
 
-0.33
 
 
174.9
 
 
 
 
ATP6V1E1
 
 
hsa-miR-23a-3p
 
 
-0.294
 
 
228.9
 
 
 
 
ATP6V1E1
 
 
hsa-miR-23b-3p
 
 
-0.294
 
 
228.9
 
 
 
 
ATPAF1
 
 
hsa-miR-361-5p
 
 
-0.362
 
 
54.4
 
 
 
 
ATXN2
 
 
hsa-miR-15b-5p
 
 
-0.392
 
 
22.7
 
 
 
 
AUH
 
 
hsa-miR-23a-3p
 
 
-0.495
 
 
13.6
 
 
 
 
AUH
 
 
hsa-miR-23b-3p
 
 
-0.503
 
 
13.6
 
 
 
 
AUH
 
 
hsa-miR-23a-3p
 
 
-0.512
 
 
13.6
 
 
 
 
AUH
 
 
hsa-miR-23b-3p
 
 
-0.512
 
 
13.6
 
 
 
 
AZIN1
 
 
hsa-miR-132-3p
 
 
-0.29
 
 
33.7
 
 
 
 
AZIN1
 
 
hsa-miR-132-3p
 
 
-0.299
 
 
33.7
 
 
 
 
B4GALT5
 
 
hsa-miR-501-3p
 
 
-0.408
 
 
10.8
 
 
 
 
B4GAT1
 
 
hsa-miR-23a-3p
 
 
-0.361
 
 
117.5
 
 
 
 
B4GAT1
 
 
hsa-miR-23b-3p
 
 
-0.361
 
 
117.5
 
 
 
 
BCL2L2
 
 
hsa-miR-29a-3p
 
 
-0.312
 
 
80.2
 
 
 
 
BDH1
 
 
hsa-miR-874-3p
 
 
-0.644
 
 
19.7
 
 
 
 
BECN1
 
 
hsa-miR-30b-5p
 
 
-0.452
 
 
27.9
 
 
 
 
BEND6
 
 
hsa-miR-361-5p
 
 
-0.305
 
 
17.2
 
 
 
 
BET1
 
 
hsa-miR-23a-3p
 
 
-0.402
 
 
10.5
 
 
 
 
BET1
 
 
hsa-miR-23b-3p
 
 
-0.418
 
 
10.5
 
 
 
 
BFAR
 
 
hsa-miR-15b-5p
 
 
-0.352
 
 
11.8
 
 
 
 
BHLHE40
 
 
hsa-miR-181b-5p
 
 
-0.408
 
 
38.8
 
 
 
 
BHLHE40
 
 
hsa-miR-181a-5p
 
 
-0.408
 
 
38.8
 
 
 
 
BICDL1
 
 
hsa-miR-221-3p
 
 
-0.447
 
 
11.0
 
 
 
 
BLCAP
 
 
hsa-miR-23a-3p
 
 
-0.601
 
 
112.6
 
 
 
 
BLCAP
 
 
hsa-miR-23b-3p
 
 
-0.601
 
 
112.6
 
 
 
 
BLMH
 
 
hsa-miR-29a-3p
 
 
-0.393
 
 
11.9
 
 
 
 
BLOC1S6
 
 
hsa-miR-181b-5p
 
 
-0.393
 
 
18.3
 
 
 
 
BLOC1S6
 
 
hsa-miR-181a-5p
 
 
-0.393
 
 
18.3
 
 
 
 
BOLA3
 
 
hsa-miR-15b-5p
 
 
-0.363
 
 
49.5
 
 
 
 
BOLA3
 
 
hsa-miR-15b-5p
 
 
-0.386
 
 
49.5
 
 
 
 
BOLA3
 
 
hsa-miR-374b-5p
 
 
-0.486
 
 
49.5
 
 
 
 
BRI3
 
 
hsa-miR-132-3p
 
 
-0.385
 
 
45.2
 
 
 
 
BTF3
 
 
hsa-miR-15b-5p
 
 
-0.344
 
 
258.6
 
 
 
 
 
 
 kidney 
 
 
 
 
target_symbol
 
 
mature_mirna_id
 
 
score
 
 
nTPM
 
 
 
 
 
 
AAR2
 
 
hsa-miR-29a-3p
 
 
-0.327
 
 
19.2
 
 
 
 
ABCE1
 
 
hsa-miR-29a-3p
 
 
-0.404
 
 
22.2
 
 
 
 
ABHD17B
 
 
hsa-miR-486-5p
 
 
-0.348
 
 
10.9
 
 
 
 
ABHD4
 
 
hsa-miR-29a-3p
 
 
-0.312
 
 
26.4
 
 
 
 
ABHD5
 
 
hsa-miR-132-3p
 
 
-0.323
 
 
10.4
 
 
 
 
ACTR2
 
 
hsa-miR-15b-5p
 
 
-0.293
 
 
79.9
 
 
 
 
ACTR3C
 
 
hsa-miR-30b-5p
 
 
-0.448
 
 
11.1
 
 
 
 
ACVR1
 
 
hsa-miR-30b-5p
 
 
-0.29
 
 
15.0
 
 
 
 
ADAM9
 
 
hsa-miR-30b-5p
 
 
-0.312
 
 
32.6
 
 
 
 
ADCY3
 
 
hsa-miR-132-3p
 
 
-0.303
 
 
27.1
 
 
 
 
ADIPOR2
 
 
hsa-miR-150-5p
 
 
-0.287
 
 
20.9
 
 
 
 
AFG3L2
 
 
hsa-miR-181b-5p
 
 
-0.491
 
 
36.4
 
 
 
 
AFG3L2
 
 
hsa-miR-181a-5p
 
 
-0.491
 
 
36.4
 
 
 
 
AK1
 
 
hsa-miR-29a-3p
 
 
-0.333
 
 
37.0
 
 
 
 
AK4
 
 
hsa-miR-15b-5p
 
 
-0.316
 
 
169.3
 
 
 
 
ALAD
 
 
hsa-miR-15b-5p
 
 
-0.446
 
 
68.5
 
 
 
 
ALDH1A2
 
 
hsa-miR-23b-3p
 
 
-0.346
 
 
19.1
 
 
 
 
ALDH1A2
 
 
hsa-miR-23a-3p
 
 
-0.354
 
 
19.1
 
 
 
 
ANGPTL2
 
 
hsa-miR-221-3p
 
 
-0.369
 
 
17.6
 
 
 
 
ANKFY1
 
 
hsa-miR-155-5p
 
 
-0.315
 
 
10.6
 
 
 
 
ANKHD1
 
 
hsa-miR-23a-3p
 
 
-0.368
 
 
34.2
 
 
 
 
ANKHD1
 
 
hsa-miR-23b-3p
 
 
-0.368
 
 
34.2
 
 
 
 
ANKHD1
 
 
hsa-miR-30b-5p
 
 
-0.405
 
 
34.2
 
 
 
 
ANKRD17
 
 
hsa-miR-30b-5p
 
 
-0.336
 
 
18.3
 
 
 
 
ANP32A
 
 
hsa-miR-132-3p
 
 
-0.374
 
 
70.0
 
 
 
 
ANP32E
 
 
hsa-miR-501-3p
 
 
-0.331
 
 
28.8
 
 
 
 
ANTXR2
 
 
hsa-miR-29a-3p
 
 
-0.347
 
 
16.0
 
 
 
 
ANXA11
 
 
hsa-miR-15b-5p
 
 
-0.463
 
 
127.2
 
 
 
 
AP2A1
 
 
hsa-miR-15b-5p
 
 
-0.333
 
 
31.7
 
 
 
 
AP2B1
 
 
hsa-miR-15b-5p
 
 
-0.334
 
 
64.4
 
 
 
 
AP3S1
 
 
hsa-miR-30b-5p
 
 
-0.293
 
 
38.1
 
 
 
 
APOO
 
 
hsa-miR-181b-5p
 
 
-0.5
 
 
43.3
 
 
 
 
APOO
 
 
hsa-miR-181a-5p
 
 
-0.5
 
 
43.3
 
 
 
 
APP
 
 
hsa-miR-15b-5p
 
 
-0.298
 
 
464.8
 
 
 
 
AQP3
 
 
hsa-miR-874-3p
 
 
-0.48
 
 
151.2
 
 
 
 
ARF4
 
 
hsa-miR-30b-5p
 
 
-0.289
 
 
106.7
 
 
 
 
ARF4
 
 
hsa-miR-221-3p
 
 
-0.553
 
 
106.7
 
 
 
 
ARF5
 
 
hsa-miR-29a-3p
 
 
-0.491
 
 
140.7
 
 
 
 
ARF6
 
 
hsa-miR-181b-5p
 
 
-0.33
 
 
29.7
 
 
 
 
ARF6
 
 
hsa-miR-181a-5p
 
 
-0.33
 
 
29.7
 
 
 
 
ARHGAP5
 
 
hsa-miR-486-5p
 
 
-0.3
 
 
40.2
 
 
 
 
ARID1B
 
 
hsa-miR-29a-3p
 
 
-0.342
 
 
10.7
 
 
 
 
ARL2
 
 
hsa-miR-15b-5p
 
 
-0.374
 
 
162.2
 
 
 
 
ARL2
 
 
hsa-miR-15b-5p
 
 
-0.442
 
 
162.2
 
 
 
 
ARL3
 
 
hsa-miR-15b-5p
 
 
-0.507
 
 
15.3
 
 
 
 
ARMC8
 
 
hsa-miR-486-5p
 
 
-0.321
 
 
17.6
 
 
 
 
ARMCX2
 
 
hsa-miR-15b-5p
 
 
-0.344
 
 
26.3
 
 
 
 
ARMCX3
 
 
hsa-miR-425-5p
 
 
-0.325
 
 
34.8
 
 
 
 
ARNT
 
 
hsa-miR-221-3p
 
 
-0.465
 
 
16.6
 
 
 
 
ARPP19
 
 
hsa-miR-15b-5p
 
 
-0.346
 
 
52.6
 
 
 
 
ARPP19
 
 
hsa-miR-29a-3p
 
 
-0.371
 
 
52.6
 
 
 
 
ARRDC3
 
 
hsa-miR-29a-3p
 
 
-0.402
 
 
49.3
 
 
 
 
ARSB
 
 
hsa-miR-29a-3p
 
 
-0.36
 
 
19.9
 
 
 
 
ASAP2
 
 
hsa-miR-29a-3p
 
 
-0.348
 
 
22.2
 
 
 
 
ASB6
 
 
hsa-miR-874-3p
 
 
-0.368
 
 
15.0
 
 
 
 
ASF1A
 
 
hsa-miR-23a-3p
 
 
-0.506
 
 
11.8
 
 
 
 
ASF1A
 
 
hsa-miR-23b-3p
 
 
-0.506
 
 
11.8
 
 
 
 
ASH1L
 
 
hsa-miR-15b-5p
 
 
-0.325
 
 
13.2
 
 
 
 
ASPH
 
 
hsa-miR-221-3p
 
 
-0.287
 
 
55.2
 
 
 
 
ATF3
 
 
hsa-miR-874-3p
 
 
-0.288
 
 
81.3
 
 
 
 
ATG5
 
 
hsa-miR-30b-5p
 
 
-0.488
 
 
13.5
 
 
 
 
ATG9A
 
 
hsa-miR-15b-5p
 
 
-0.333
 
 
19.7
 
 
 
 
ATOSA
 
 
hsa-miR-221-3p
 
 
-0.495
 
 
10.0
 
 
 
 
ATP5MC3
 
 
hsa-miR-425-5p
 
 
-0.328
 
 
416.1
 
 
 
 
ATP5MC3
 
 
hsa-miR-29a-3p
 
 
-0.33
 
 
416.1
 
 
 
 
ATP6V1E1
 
 
hsa-miR-23a-3p
 
 
-0.294
 
 
214.9
 
 
 
 
ATP6V1E1
 
 
hsa-miR-23b-3p
 
 
-0.294
 
 
214.9
 
 
 
 
ATPAF1
 
 
hsa-miR-361-5p
 
 
-0.362
 
 
107.2
 
 
 
 
ATXN1L
 
 
hsa-miR-155-5p
 
 
-0.315
 
 
17.5
 
 
 
 
ATXN2
 
 
hsa-miR-15b-5p
 
 
-0.392
 
 
16.8
 
 
 
 
AUH
 
 
hsa-miR-23a-3p
 
 
-0.495
 
 
58.7
 
 
 
 
AUH
 
 
hsa-miR-23b-3p
 
 
-0.503
 
 
58.7
 
 
 
 
AUH
 
 
hsa-miR-23a-3p
 
 
-0.512
 
 
58.7
 
 
 
 
AUH
 
 
hsa-miR-23b-3p
 
 
-0.512
 
 
58.7
 
 
 
 
AZIN1
 
 
hsa-miR-132-3p
 
 
-0.29
 
 
36.3
 
 
 
 
AZIN1
 
 
hsa-miR-132-3p
 
 
-0.299
 
 
36.3
 
 
 
 
B4GALT5
 
 
hsa-miR-501-3p
 
 
-0.408
 
 
21.3
 
 
 
 
B4GAT1
 
 
hsa-miR-23a-3p
 
 
-0.361
 
 
24.3
 
 
 
 
B4GAT1
 
 
hsa-miR-23b-3p
 
 
-0.361
 
 
24.3
 
 
 
 
BAK1
 
 
hsa-miR-29a-3p
 
 
-0.409
 
 
20.0
 
 
 
 
BCL2L11
 
 
hsa-miR-221-3p
 
 
-0.423
 
 
10.8
 
 
 
 
BCL2L2
 
 
hsa-miR-29a-3p
 
 
-0.312
 
 
37.4
 
 
 
 
BCL7A
 
 
hsa-miR-15b-5p
 
 
-0.336
 
 
12.7
 
 
 
 
BCL7A
 
 
hsa-miR-501-3p
 
 
-0.339
 
 
12.7
 
 
 
 
BDH1
 
 
hsa-miR-874-3p
 
 
-0.644
 
 
21.7
 
 
 
 
BECN1
 
 
hsa-miR-30b-5p
 
 
-0.452
 
 
44.7
 
 
 
 
BET1
 
 
hsa-miR-23a-3p
 
 
-0.402
 
 
22.7
 
 
 
 
BET1
 
 
hsa-miR-23b-3p
 
 
-0.418
 
 
22.7
 
 
 
 
BFAR
 
 
hsa-miR-15b-5p
 
 
-0.352
 
 
24.5
 
 
 
 
BHLHE40
 
 
hsa-miR-181b-5p
 
 
-0.408
 
 
155.1
 
 
 
 
BHLHE40
 
 
hsa-miR-181a-5p
 
 
-0.408
 
 
155.1
 
 
 
 
BICDL1
 
 
hsa-miR-221-3p
 
 
-0.447
 
 
112.8
 
 
 
 
BLCAP
 
 
hsa-miR-23a-3p
 
 
-0.601
 
 
62.5
 
 
 
 
BLCAP
 
 
hsa-miR-23b-3p
 
 
-0.601
 
 
62.5
 
 
 
 
BLMH
 
 
hsa-miR-29a-3p
 
 
-0.393
 
 
20.6
 
 
 
 
BLOC1S6
 
 
hsa-miR-181b-5p
 
 
-0.393
 
 
22.6
 
 
 
 
BLOC1S6
 
 
hsa-miR-181a-5p
 
 
-0.393
 
 
22.6
 
 
 
 
BMF
 
 
hsa-miR-29a-3p
 
 
-0.41
 
 
10.2
 
 
 
 
BMF
 
 
hsa-miR-221-3p
 
 
-0.453
 
 
10.2
 
 
 
 
BOLA3
 
 
hsa-miR-15b-5p
 
 
-0.363
 
 
70.9
 
 
 
 
 
 
 lung 
 
 
 
 
target_symbol
 
 
mature_mirna_id
 
 
score
 
 
nTPM
 
 
 
 
 
 
AAR2
 
 
hsa-miR-29a-3p
 
 
-0.327
 
 
18.1
 
 
 
 
ABCE1
 
 
hsa-miR-29a-3p
 
 
-0.404
 
 
17.4
 
 
 
 
ABHD4
 
 
hsa-miR-29a-3p
 
 
-0.312
 
 
18.5
 
 
 
 
ABHD5
 
 
hsa-miR-132-3p
 
 
-0.323
 
 
11.2
 
 
 
 
ACTR2
 
 
hsa-miR-15b-5p
 
 
-0.293
 
 
105.4
 
 
 
 
ACVR1
 
 
hsa-miR-30b-5p
 
 
-0.29
 
 
12.2
 
 
 
 
ADAM19
 
 
hsa-miR-874-3p
 
 
-0.374
 
 
12.8
 
 
 
 
ADAM9
 
 
hsa-miR-30b-5p
 
 
-0.312
 
 
42.9
 
 
 
 
ADAMTS9
 
 
hsa-miR-29a-3p
 
 
-0.305
 
 
13.3
 
 
 
 
ADAMTS9
 
 
hsa-miR-29a-3p
 
 
-0.485
 
 
13.3
 
 
 
 
ADCY3
 
 
hsa-miR-132-3p
 
 
-0.303
 
 
39.8
 
 
 
 
ADIPOR2
 
 
hsa-miR-150-5p
 
 
-0.287
 
 
42.5
 
 
 
 
ADRB2
 
 
hsa-miR-15b-5p
 
 
-0.29
 
 
18.4
 
 
 
 
AFG3L2
 
 
hsa-miR-181b-5p
 
 
-0.491
 
 
12.4
 
 
 
 
AFG3L2
 
 
hsa-miR-181a-5p
 
 
-0.491
 
 
12.4
 
 
 
 
AGO4
 
 
hsa-miR-221-3p
 
 
-0.321
 
 
10.5
 
 
 
 
AHR
 
 
hsa-miR-29a-3p
 
 
-0.316
 
 
35.5
 
 
 
 
AK1
 
 
hsa-miR-29a-3p
 
 
-0.333
 
 
70.4
 
 
 
 
AKT3
 
 
hsa-miR-29a-3p
 
 
-0.287
 
 
13.9
 
 
 
 
AKT3
 
 
hsa-miR-15b-5p
 
 
-0.301
 
 
13.9
 
 
 
 
ALAD
 
 
hsa-miR-15b-5p
 
 
-0.446
 
 
19.4
 
 
 
 
AMOTL1
 
 
hsa-miR-15b-5p
 
 
-0.319
 
 
14.9
 
 
 
 
ANGPTL2
 
 
hsa-miR-221-3p
 
 
-0.369
 
 
21.5
 
 
 
 
ANKFY1
 
 
hsa-miR-155-5p
 
 
-0.315
 
 
14.4
 
 
 
 
ANKHD1
 
 
hsa-miR-23a-3p
 
 
-0.368
 
 
38.2
 
 
 
 
ANKHD1
 
 
hsa-miR-23b-3p
 
 
-0.368
 
 
38.2
 
 
 
 
ANKHD1
 
 
hsa-miR-30b-5p
 
 
-0.405
 
 
38.2
 
 
 
 
ANKRD17
 
 
hsa-miR-30b-5p
 
 
-0.336
 
 
13.4
 
 
 
 
ANKRD29
 
 
hsa-miR-132-3p
 
 
-0.47
 
 
29.5
 
 
 
 
ANP32A
 
 
hsa-miR-132-3p
 
 
-0.374
 
 
66.9
 
 
 
 
ANP32E
 
 
hsa-miR-501-3p
 
 
-0.331
 
 
21.7
 
 
 
 
ANTXR2
 
 
hsa-miR-29a-3p
 
 
-0.347
 
 
23.3
 
 
 
 
ANXA11
 
 
hsa-miR-15b-5p
 
 
-0.463
 
 
103.2
 
 
 
 
AP2A1
 
 
hsa-miR-15b-5p
 
 
-0.333
 
 
35.7
 
 
 
 
AP2B1
 
 
hsa-miR-15b-5p
 
 
-0.334
 
 
49.6
 
 
 
 
AP3S1
 
 
hsa-miR-30b-5p
 
 
-0.293
 
 
40.1
 
 
 
 
APLN
 
 
hsa-miR-15b-5p
 
 
-0.395
 
 
12.7
 
 
 
 
APP
 
 
hsa-miR-15b-5p
 
 
-0.298
 
 
255.6
 
 
 
 
AQP3
 
 
hsa-miR-874-3p
 
 
-0.48
 
 
107.5
 
 
 
 
ARF4
 
 
hsa-miR-30b-5p
 
 
-0.289
 
 
116.3
 
 
 
 
ARF4
 
 
hsa-miR-221-3p
 
 
-0.553
 
 
116.3
 
 
 
 
ARF5
 
 
hsa-miR-29a-3p
 
 
-0.491
 
 
100.1
 
 
 
 
ARF6
 
 
hsa-miR-181b-5p
 
 
-0.33
 
 
29.7
 
 
 
 
ARF6
 
 
hsa-miR-181a-5p
 
 
-0.33
 
 
29.7
 
 
 
 
ARHGAP5
 
 
hsa-miR-486-5p
 
 
-0.3
 
 
26.5
 
 
 
 
ARID1B
 
 
hsa-miR-29a-3p
 
 
-0.342
 
 
13.4
 
 
 
 
ARID4B
 
 
hsa-miR-486-5p
 
 
-0.367
 
 
11.3
 
 
 
 
ARL2
 
 
hsa-miR-15b-5p
 
 
-0.374
 
 
91.1
 
 
 
 
ARL2
 
 
hsa-miR-15b-5p
 
 
-0.442
 
 
91.1
 
 
 
 
ARL4A
 
 
hsa-miR-29a-3p
 
 
-0.386
 
 
18.9
 
 
 
 
ARL4A
 
 
hsa-miR-30b-5p
 
 
-0.448
 
 
18.9
 
 
 
 
ARMC8
 
 
hsa-miR-486-5p
 
 
-0.321
 
 
15.4
 
 
 
 
ARMCX2
 
 
hsa-miR-15b-5p
 
 
-0.344
 
 
14.8
 
 
 
 
ARMCX3
 
 
hsa-miR-425-5p
 
 
-0.325
 
 
26.1
 
 
 
 
ARNT
 
 
hsa-miR-221-3p
 
 
-0.465
 
 
23.3
 
 
 
 
ARPP19
 
 
hsa-miR-15b-5p
 
 
-0.346
 
 
42.5
 
 
 
 
ARPP19
 
 
hsa-miR-29a-3p
 
 
-0.371
 
 
42.5
 
 
 
 
ARRDC3
 
 
hsa-miR-29a-3p
 
 
-0.402
 
 
42.0
 
 
 
 
ASF1A
 
 
hsa-miR-23a-3p
 
 
-0.506
 
 
12.6
 
 
 
 
ASF1A
 
 
hsa-miR-23b-3p
 
 
-0.506
 
 
12.6
 
 
 
 
ASH1L
 
 
hsa-miR-15b-5p
 
 
-0.325
 
 
15.4
 
 
 
 
ASPH
 
 
hsa-miR-221-3p
 
 
-0.287
 
 
69.4
 
 
 
 
ATF3
 
 
hsa-miR-874-3p
 
 
-0.288
 
 
109.3
 
 
 
 
ATG5
 
 
hsa-miR-30b-5p
 
 
-0.488
 
 
10.2
 
 
 
 
ATG9A
 
 
hsa-miR-15b-5p
 
 
-0.333
 
 
24.4
 
 
 
 
ATOSA
 
 
hsa-miR-221-3p
 
 
-0.495
 
 
12.4
 
 
 
 
ATP5MC3
 
 
hsa-miR-425-5p
 
 
-0.328
 
 
114.1
 
 
 
 
ATP5MC3
 
 
hsa-miR-29a-3p
 
 
-0.33
 
 
114.1
 
 
 
 
ATP6V1E1
 
 
hsa-miR-23a-3p
 
 
-0.294
 
 
110.6
 
 
 
 
ATP6V1E1
 
 
hsa-miR-23b-3p
 
 
-0.294
 
 
110.6
 
 
 
 
ATPAF1
 
 
hsa-miR-361-5p
 
 
-0.362
 
 
24.6
 
 
 
 
ATXN1L
 
 
hsa-miR-155-5p
 
 
-0.315
 
 
11.3
 
 
 
 
ATXN2
 
 
hsa-miR-15b-5p
 
 
-0.392
 
 
18.4
 
 
 
 
AXIN2
 
 
hsa-miR-15b-5p
 
 
-0.3
 
 
15.9
 
 
 
 
AZIN1
 
 
hsa-miR-132-3p
 
 
-0.29
 
 
37.1
 
 
 
 
AZIN1
 
 
hsa-miR-132-3p
 
 
-0.299
 
 
37.1
 
 
 
 
B3GNT5
 
 
hsa-miR-30b-5p
 
 
-0.326
 
 
14.1
 
 
 
 
B4GALT5
 
 
hsa-miR-501-3p
 
 
-0.408
 
 
26.0
 
 
 
 
B4GAT1
 
 
hsa-miR-23a-3p
 
 
-0.361
 
 
16.9
 
 
 
 
B4GAT1
 
 
hsa-miR-23b-3p
 
 
-0.361
 
 
16.9
 
 
 
 
BAK1
 
 
hsa-miR-29a-3p
 
 
-0.409
 
 
12.9
 
 
 
 
BCL2L2
 
 
hsa-miR-29a-3p
 
 
-0.312
 
 
34.4
 
 
 
 
BECN1
 
 
hsa-miR-30b-5p
 
 
-0.452
 
 
37.3
 
 
 
 
BET1
 
 
hsa-miR-23a-3p
 
 
-0.402
 
 
13.1
 
 
 
 
BET1
 
 
hsa-miR-23b-3p
 
 
-0.418
 
 
13.1
 
 
 
 
BFAR
 
 
hsa-miR-15b-5p
 
 
-0.352
 
 
34.5
 
 
 
 
BHLHE40
 
 
hsa-miR-181b-5p
 
 
-0.408
 
 
117.3
 
 
 
 
BHLHE40
 
 
hsa-miR-181a-5p
 
 
-0.408
 
 
117.3
 
 
 
 
BLCAP
 
 
hsa-miR-23a-3p
 
 
-0.601
 
 
36.4
 
 
 
 
BLCAP
 
 
hsa-miR-23b-3p
 
 
-0.601
 
 
36.4
 
 
 
 
BLMH
 
 
hsa-miR-29a-3p
 
 
-0.393
 
 
15.6
 
 
 
 
BLOC1S6
 
 
hsa-miR-181b-5p
 
 
-0.393
 
 
19.2
 
 
 
 
BLOC1S6
 
 
hsa-miR-181a-5p
 
 
-0.393
 
 
19.2
 
 
 
 
BMF
 
 
hsa-miR-29a-3p
 
 
-0.41
 
 
12.0
 
 
 
 
BMF
 
 
hsa-miR-221-3p
 
 
-0.453
 
 
12.0
 
 
 
 
BOLA3
 
 
hsa-miR-15b-5p
 
 
-0.363
 
 
25.0
 
 
 
 
BOLA3
 
 
hsa-miR-15b-5p
 
 
-0.386
 
 
25.0
 
 
 
 
BOLA3
 
 
hsa-miR-374b-5p
 
 
-0.486
 
 
25.0
 
 
 
 
BPIFA1
 
 
hsa-miR-15b-5p
 
 
-0.396
 
 
18.6
 
 
 
 
BRI3
 
 
hsa-miR-132-3p
 
 
-0.385
 
 
148.8
 
 
 
 
 
 
 lymph node 
 
 
 
 
target_symbol
 
 
mature_mirna_id
 
 
score
 
 
nTPM
 
 
 
 
 
 
AAR2
 
 
hsa-miR-29a-3p
 
 
-0.327
 
 
16.4
 
 
 
 
ABCE1
 
 
hsa-miR-29a-3p
 
 
-0.404
 
 
37.8
 
 
 
 
ABHD17B
 
 
hsa-miR-486-5p
 
 
-0.348
 
 
10.2
 
 
 
 
ACTR2
 
 
hsa-miR-15b-5p
 
 
-0.293
 
 
229.6
 
 
 
 
ADA
 
 
hsa-miR-29a-3p
 
 
-0.376
 
 
27.8
 
 
 
 
ADAM19
 
 
hsa-miR-874-3p
 
 
-0.374
 
 
27.1
 
 
 
 
ADCY3
 
 
hsa-miR-132-3p
 
 
-0.303
 
 
17.7
 
 
 
 
ADRA2A
 
 
hsa-miR-23a-3p
 
 
-0.371
 
 
10.5
 
 
 
 
ADRA2A
 
 
hsa-miR-23b-3p
 
 
-0.371
 
 
10.5
 
 
 
 
AFG3L2
 
 
hsa-miR-181b-5p
 
 
-0.491
 
 
12.0
 
 
 
 
AFG3L2
 
 
hsa-miR-181a-5p
 
 
-0.491
 
 
12.0
 
 
 
 
AHR
 
 
hsa-miR-29a-3p
 
 
-0.316
 
 
14.4
 
 
 
 
AICDA
 
 
hsa-miR-155-5p
 
 
-0.389
 
 
10.5
 
 
 
 
ALAD
 
 
hsa-miR-15b-5p
 
 
-0.446
 
 
14.6
 
 
 
 
ANKFY1
 
 
hsa-miR-155-5p
 
 
-0.315
 
 
13.1
 
 
 
 
ANKHD1
 
 
hsa-miR-23a-3p
 
 
-0.368
 
 
38.6
 
 
 
 
ANKHD1
 
 
hsa-miR-23b-3p
 
 
-0.368
 
 
38.6
 
 
 
 
ANKHD1
 
 
hsa-miR-30b-5p
 
 
-0.405
 
 
38.6
 
 
 
 
ANKRD17
 
 
hsa-miR-30b-5p
 
 
-0.336
 
 
14.1
 
 
 
 
ANKRD29
 
 
hsa-miR-132-3p
 
 
-0.47
 
 
18.8
 
 
 
 
ANP32A
 
 
hsa-miR-132-3p
 
 
-0.374
 
 
145.8
 
 
 
 
ANP32E
 
 
hsa-miR-501-3p
 
 
-0.331
 
 
66.3
 
 
 
 
ANTXR2
 
 
hsa-miR-29a-3p
 
 
-0.347
 
 
15.2
 
 
 
 
ANXA11
 
 
hsa-miR-15b-5p
 
 
-0.463
 
 
76.0
 
 
 
 
AP2A1
 
 
hsa-miR-15b-5p
 
 
-0.333
 
 
18.3
 
 
 
 
AP2B1
 
 
hsa-miR-15b-5p
 
 
-0.334
 
 
36.1
 
 
 
 
AP3S1
 
 
hsa-miR-30b-5p
 
 
-0.293
 
 
60.4
 
 
 
 
APOO
 
 
hsa-miR-181b-5p
 
 
-0.5
 
 
10.9
 
 
 
 
APOO
 
 
hsa-miR-181a-5p
 
 
-0.5
 
 
10.9
 
 
 
 
APP
 
 
hsa-miR-15b-5p
 
 
-0.298
 
 
68.3
 
 
 
 
AQP3
 
 
hsa-miR-874-3p
 
 
-0.48
 
 
15.1
 
 
 
 
ARF4
 
 
hsa-miR-30b-5p
 
 
-0.289
 
 
69.1
 
 
 
 
ARF4
 
 
hsa-miR-221-3p
 
 
-0.553
 
 
69.1
 
 
 
 
ARF5
 
 
hsa-miR-29a-3p
 
 
-0.491
 
 
96.1
 
 
 
 
ARF6
 
 
hsa-miR-181b-5p
 
 
-0.33
 
 
57.9
 
 
 
 
ARF6
 
 
hsa-miR-181a-5p
 
 
-0.33
 
 
57.9
 
 
 
 
ARHGAP5
 
 
hsa-miR-486-5p
 
 
-0.3
 
 
13.7
 
 
 
 
ARID1B
 
 
hsa-miR-29a-3p
 
 
-0.342
 
 
16.8
 
 
 
 
ARID4B
 
 
hsa-miR-486-5p
 
 
-0.367
 
 
19.6
 
 
 
 
ARL2
 
 
hsa-miR-15b-5p
 
 
-0.374
 
 
35.3
 
 
 
 
ARL2
 
 
hsa-miR-15b-5p
 
 
-0.442
 
 
35.3
 
 
 
 
ARL4A
 
 
hsa-miR-29a-3p
 
 
-0.386
 
 
17.4
 
 
 
 
ARL4A
 
 
hsa-miR-30b-5p
 
 
-0.448
 
 
17.4
 
 
 
 
ARMC8
 
 
hsa-miR-486-5p
 
 
-0.321
 
 
19.8
 
 
 
 
ARMCX3
 
 
hsa-miR-425-5p
 
 
-0.325
 
 
20.4
 
 
 
 
ARNT
 
 
hsa-miR-221-3p
 
 
-0.465
 
 
16.3
 
 
 
 
ARPP19
 
 
hsa-miR-15b-5p
 
 
-0.346
 
 
45.1
 
 
 
 
ARPP19
 
 
hsa-miR-29a-3p
 
 
-0.371
 
 
45.1
 
 
 
 
ARRDC3
 
 
hsa-miR-29a-3p
 
 
-0.402
 
 
27.5
 
 
 
 
ASB3
 
 
hsa-miR-30b-5p
 
 
-0.418
 
 
10.2
 
 
 
 
ASB3
 
 
hsa-miR-30b-5p
 
 
-0.418
 
 
10.2
 
 
 
 
ASB6
 
 
hsa-miR-874-3p
 
 
-0.368
 
 
10.7
 
 
 
 
ASF1A
 
 
hsa-miR-23a-3p
 
 
-0.506
 
 
14.9
 
 
 
 
ASF1A
 
 
hsa-miR-23b-3p
 
 
-0.506
 
 
14.9
 
 
 
 
ASH1L
 
 
hsa-miR-15b-5p
 
 
-0.325
 
 
10.2
 
 
 
 
ASPH
 
 
hsa-miR-221-3p
 
 
-0.287
 
 
18.8
 
 
 
 
ATF3
 
 
hsa-miR-874-3p
 
 
-0.288
 
 
19.4
 
 
 
 
ATG9A
 
 
hsa-miR-15b-5p
 
 
-0.333
 
 
18.2
 
 
 
 
ATOSA
 
 
hsa-miR-221-3p
 
 
-0.495
 
 
21.9
 
 
 
 
ATP5MC3
 
 
hsa-miR-425-5p
 
 
-0.328
 
 
118.6
 
 
 
 
ATP5MC3
 
 
hsa-miR-29a-3p
 
 
-0.33
 
 
118.6
 
 
 
 
ATP6V1E1
 
 
hsa-miR-23a-3p
 
 
-0.294
 
 
82.9
 
 
 
 
ATP6V1E1
 
 
hsa-miR-23b-3p
 
 
-0.294
 
 
82.9
 
 
 
 
ATPAF1
 
 
hsa-miR-361-5p
 
 
-0.362
 
 
30.3
 
 
 
 
AZIN1
 
 
hsa-miR-132-3p
 
 
-0.29
 
 
53.2
 
 
 
 
AZIN1
 
 
hsa-miR-132-3p
 
 
-0.299
 
 
53.2
 
 
 
 
BACH2
 
 
hsa-miR-29a-3p
 
 
-0.294
 
 
16.3
 
 
 
 
BAK1
 
 
hsa-miR-29a-3p
 
 
-0.409
 
 
20.6
 
 
 
 
BCL2
 
 
hsa-miR-342-3p
 
 
-0.428
 
 
13.7
 
 
 
 
BCL2L11
 
 
hsa-miR-221-3p
 
 
-0.423
 
 
23.7
 
 
 
 
BCL7A
 
 
hsa-miR-15b-5p
 
 
-0.336
 
 
15.1
 
 
 
 
BCL7A
 
 
hsa-miR-501-3p
 
 
-0.339
 
 
15.1
 
 
 
 
BCOR
 
 
hsa-miR-425-5p
 
 
-0.346
 
 
14.0
 
 
 
 
BECN1
 
 
hsa-miR-30b-5p
 
 
-0.452
 
 
44.8
 
 
 
 
BET1
 
 
hsa-miR-23a-3p
 
 
-0.402
 
 
22.7
 
 
 
 
BET1
 
 
hsa-miR-23b-3p
 
 
-0.418
 
 
22.7
 
 
 
 
BFAR
 
 
hsa-miR-15b-5p
 
 
-0.352
 
 
19.3
 
 
 
 
BHLHE40
 
 
hsa-miR-181b-5p
 
 
-0.408
 
 
48.9
 
 
 
 
BHLHE40
 
 
hsa-miR-181a-5p
 
 
-0.408
 
 
48.9
 
 
 
 
BICDL1
 
 
hsa-miR-221-3p
 
 
-0.447
 
 
15.6
 
 
 
 
BLCAP
 
 
hsa-miR-23a-3p
 
 
-0.601
 
 
37.4
 
 
 
 
BLCAP
 
 
hsa-miR-23b-3p
 
 
-0.601
 
 
37.4
 
 
 
 
BLMH
 
 
hsa-miR-29a-3p
 
 
-0.393
 
 
24.3
 
 
 
 
BLOC1S6
 
 
hsa-miR-181b-5p
 
 
-0.393
 
 
39.2
 
 
 
 
BLOC1S6
 
 
hsa-miR-181a-5p
 
 
-0.393
 
 
39.2
 
 
 
 
BMF
 
 
hsa-miR-29a-3p
 
 
-0.41
 
 
12.8
 
 
 
 
BMF
 
 
hsa-miR-221-3p
 
 
-0.453
 
 
12.8
 
 
 
 
BOLA3
 
 
hsa-miR-15b-5p
 
 
-0.363
 
 
20.7
 
 
 
 
BOLA3
 
 
hsa-miR-15b-5p
 
 
-0.386
 
 
20.7
 
 
 
 
BOLA3
 
 
hsa-miR-374b-5p
 
 
-0.486
 
 
20.7
 
 
 
 
BRI3
 
 
hsa-miR-132-3p
 
 
-0.385
 
 
30.8
 
 
 
 
BTAF1
 
 
hsa-miR-486-5p
 
 
-0.288
 
 
25.1
 
 
 
 
BTAF1
 
 
hsa-miR-15b-5p
 
 
-0.496
 
 
25.1
 
 
 
 
BTF3
 
 
hsa-miR-15b-5p
 
 
-0.344
 
 
602.2
 
 
 
 
BTF3
 
 
hsa-miR-361-5p
 
 
-0.551
 
 
602.2
 
 
 
 
BTG2
 
 
hsa-miR-29a-3p
 
 
-0.32
 
 
114.1
 
 
 
 
BTLA
 
 
hsa-miR-15b-5p
 
 
-0.306
 
 
24.7
 
 
 
 
BTLA
 
 
hsa-miR-15b-5p
 
 
-0.316
 
 
24.7
 
 
 
 
BTN2A1
 
 
hsa-miR-342-3p
 
 
-0.31
 
 
13.2
 
 
 
 
BZW1
 
 
hsa-miR-15b-5p
 
 
-0.367
 
 
91.4
 
 
 
 
 
 
 midbrain 
 
 
 
 
target_symbol
 
 
mature_mirna_id
 
 
score
 
 
nTPM
 
 
 
 
 
 
AAR2
 
 
hsa-miR-29a-3p
 
 
-0.327
 
 
11.2
 
 
 
 
ABCE1
 
 
hsa-miR-29a-3p
 
 
-0.404
 
 
13.7
 
 
 
 
ABHD17B
 
 
hsa-miR-486-5p
 
 
-0.348
 
 
16.6
 
 
 
 
ABHD4
 
 
hsa-miR-29a-3p
 
 
-0.312
 
 
18.3
 
 
 
 
ACTN2
 
 
hsa-miR-23a-3p
 
 
-0.294
 
 
12.2
 
 
 
 
ACTN2
 
 
hsa-miR-29a-3p
 
 
-0.311
 
 
12.2
 
 
 
 
ACTR2
 
 
hsa-miR-15b-5p
 
 
-0.293
 
 
53.1
 
 
 
 
ADCY3
 
 
hsa-miR-132-3p
 
 
-0.303
 
 
29.4
 
 
 
 
ADGRA3
 
 
hsa-miR-30b-5p
 
 
-0.304
 
 
13.9
 
 
 
 
ADIPOR2
 
 
hsa-miR-150-5p
 
 
-0.287
 
 
76.2
 
 
 
 
AGPAT4
 
 
hsa-miR-29a-3p
 
 
-0.376
 
 
22.2
 
 
 
 
AK1
 
 
hsa-miR-29a-3p
 
 
-0.333
 
 
97.2
 
 
 
 
AK4
 
 
hsa-miR-15b-5p
 
 
-0.316
 
 
11.3
 
 
 
 
AKT3
 
 
hsa-miR-29a-3p
 
 
-0.287
 
 
16.3
 
 
 
 
AKT3
 
 
hsa-miR-15b-5p
 
 
-0.301
 
 
16.3
 
 
 
 
ALAD
 
 
hsa-miR-15b-5p
 
 
-0.446
 
 
105.2
 
 
 
 
AMOT
 
 
hsa-miR-29a-3p
 
 
-0.304
 
 
16.8
 
 
 
 
ANGPTL2
 
 
hsa-miR-221-3p
 
 
-0.369
 
 
12.4
 
 
 
 
ANKFY1
 
 
hsa-miR-155-5p
 
 
-0.315
 
 
12.6
 
 
 
 
ANKHD1
 
 
hsa-miR-23a-3p
 
 
-0.368
 
 
20.5
 
 
 
 
ANKHD1
 
 
hsa-miR-23b-3p
 
 
-0.368
 
 
20.5
 
 
 
 
ANKHD1
 
 
hsa-miR-30b-5p
 
 
-0.405
 
 
20.5
 
 
 
 
ANKRD29
 
 
hsa-miR-132-3p
 
 
-0.47
 
 
11.5
 
 
 
 
ANLN
 
 
hsa-miR-15b-5p
 
 
-0.334
 
 
72.6
 
 
 
 
ANP32A
 
 
hsa-miR-132-3p
 
 
-0.374
 
 
54.3
 
 
 
 
ANP32E
 
 
hsa-miR-501-3p
 
 
-0.331
 
 
35.7
 
 
 
 
ANXA11
 
 
hsa-miR-15b-5p
 
 
-0.463
 
 
20.2
 
 
 
 
AP2A1
 
 
hsa-miR-15b-5p
 
 
-0.333
 
 
39.4
 
 
 
 
AP2B1
 
 
hsa-miR-15b-5p
 
 
-0.334
 
 
69.9
 
 
 
 
AP3S1
 
 
hsa-miR-30b-5p
 
 
-0.293
 
 
69.9
 
 
 
 
APC
 
 
hsa-miR-29a-3p
 
 
-0.413
 
 
21.2
 
 
 
 
APLN
 
 
hsa-miR-15b-5p
 
 
-0.395
 
 
24.6
 
 
 
 
APOO
 
 
hsa-miR-181b-5p
 
 
-0.5
 
 
44.2
 
 
 
 
APOO
 
 
hsa-miR-181a-5p
 
 
-0.5
 
 
44.2
 
 
 
 
APP
 
 
hsa-miR-15b-5p
 
 
-0.298
 
 
448.8
 
 
 
 
ARF4
 
 
hsa-miR-30b-5p
 
 
-0.289
 
 
62.8
 
 
 
 
ARF4
 
 
hsa-miR-221-3p
 
 
-0.553
 
 
62.8
 
 
 
 
ARF5
 
 
hsa-miR-29a-3p
 
 
-0.491
 
 
120.0
 
 
 
 
ARF6
 
 
hsa-miR-181b-5p
 
 
-0.33
 
 
12.0
 
 
 
 
ARF6
 
 
hsa-miR-181a-5p
 
 
-0.33
 
 
12.0
 
 
 
 
ARHGAP5
 
 
hsa-miR-486-5p
 
 
-0.3
 
 
21.3
 
 
 
 
ARL2
 
 
hsa-miR-15b-5p
 
 
-0.374
 
 
173.0
 
 
 
 
ARL2
 
 
hsa-miR-15b-5p
 
 
-0.442
 
 
173.0
 
 
 
 
ARL3
 
 
hsa-miR-15b-5p
 
 
-0.507
 
 
22.1
 
 
 
 
ARMC8
 
 
hsa-miR-486-5p
 
 
-0.321
 
 
14.1
 
 
 
 
ARMCX2
 
 
hsa-miR-15b-5p
 
 
-0.344
 
 
18.6
 
 
 
 
ARMCX3
 
 
hsa-miR-425-5p
 
 
-0.325
 
 
35.1
 
 
 
 
ARPP19
 
 
hsa-miR-15b-5p
 
 
-0.346
 
 
144.2
 
 
 
 
ARPP19
 
 
hsa-miR-29a-3p
 
 
-0.371
 
 
144.2
 
 
 
 
ARPP21
 
 
hsa-miR-15b-5p
 
 
-0.342
 
 
30.5
 
 
 
 
ARPP21
 
 
hsa-miR-501-3p
 
 
-0.546
 
 
30.5
 
 
 
 
ARRDC3
 
 
hsa-miR-29a-3p
 
 
-0.402
 
 
13.8
 
 
 
 
ASB3
 
 
hsa-miR-30b-5p
 
 
-0.418
 
 
21.9
 
 
 
 
ASB3
 
 
hsa-miR-30b-5p
 
 
-0.418
 
 
21.9
 
 
 
 
ASB6
 
 
hsa-miR-874-3p
 
 
-0.368
 
 
11.3
 
 
 
 
ASF1A
 
 
hsa-miR-23a-3p
 
 
-0.506
 
 
14.8
 
 
 
 
ASF1A
 
 
hsa-miR-23b-3p
 
 
-0.506
 
 
14.8
 
 
 
 
ASH1L
 
 
hsa-miR-15b-5p
 
 
-0.325
 
 
18.6
 
 
 
 
ASPH
 
 
hsa-miR-221-3p
 
 
-0.287
 
 
48.8
 
 
 
 
ATF3
 
 
hsa-miR-874-3p
 
 
-0.288
 
 
20.5
 
 
 
 
ATG9A
 
 
hsa-miR-15b-5p
 
 
-0.333
 
 
20.2
 
 
 
 
ATP5MC3
 
 
hsa-miR-425-5p
 
 
-0.328
 
 
209.2
 
 
 
 
ATP5MC3
 
 
hsa-miR-29a-3p
 
 
-0.33
 
 
209.2
 
 
 
 
ATP6V1E1
 
 
hsa-miR-23a-3p
 
 
-0.294
 
 
210.7
 
 
 
 
ATP6V1E1
 
 
hsa-miR-23b-3p
 
 
-0.294
 
 
210.7
 
 
 
 
ATPAF1
 
 
hsa-miR-361-5p
 
 
-0.362
 
 
74.9
 
 
 
 
ATXN2
 
 
hsa-miR-15b-5p
 
 
-0.392
 
 
24.2
 
 
 
 
AUH
 
 
hsa-miR-23a-3p
 
 
-0.495
 
 
16.6
 
 
 
 
AUH
 
 
hsa-miR-23b-3p
 
 
-0.503
 
 
16.6
 
 
 
 
AUH
 
 
hsa-miR-23a-3p
 
 
-0.512
 
 
16.6
 
 
 
 
AUH
 
 
hsa-miR-23b-3p
 
 
-0.512
 
 
16.6
 
 
 
 
AZIN1
 
 
hsa-miR-132-3p
 
 
-0.29
 
 
33.7
 
 
 
 
AZIN1
 
 
hsa-miR-132-3p
 
 
-0.299
 
 
33.7
 
 
 
 
B4GALT5
 
 
hsa-miR-501-3p
 
 
-0.408
 
 
10.3
 
 
 
 
B4GAT1
 
 
hsa-miR-23a-3p
 
 
-0.361
 
 
85.6
 
 
 
 
B4GAT1
 
 
hsa-miR-23b-3p
 
 
-0.361
 
 
85.6
 
 
 
 
BCL2L2
 
 
hsa-miR-29a-3p
 
 
-0.312
 
 
103.0
 
 
 
 
BDH1
 
 
hsa-miR-874-3p
 
 
-0.644
 
 
18.3
 
 
 
 
BECN1
 
 
hsa-miR-30b-5p
 
 
-0.452
 
 
30.4
 
 
 
 
BEND6
 
 
hsa-miR-361-5p
 
 
-0.305
 
 
16.5
 
 
 
 
BET1
 
 
hsa-miR-23a-3p
 
 
-0.402
 
 
10.6
 
 
 
 
BET1
 
 
hsa-miR-23b-3p
 
 
-0.418
 
 
10.6
 
 
 
 
BFAR
 
 
hsa-miR-15b-5p
 
 
-0.352
 
 
13.1
 
 
 
 
BHLHE40
 
 
hsa-miR-181b-5p
 
 
-0.408
 
 
41.1
 
 
 
 
BHLHE40
 
 
hsa-miR-181a-5p
 
 
-0.408
 
 
41.1
 
 
 
 
BLCAP
 
 
hsa-miR-23a-3p
 
 
-0.601
 
 
88.1
 
 
 
 
BLCAP
 
 
hsa-miR-23b-3p
 
 
-0.601
 
 
88.1
 
 
 
 
BLMH
 
 
hsa-miR-29a-3p
 
 
-0.393
 
 
12.4
 
 
 
 
BLOC1S6
 
 
hsa-miR-181b-5p
 
 
-0.393
 
 
18.5
 
 
 
 
BLOC1S6
 
 
hsa-miR-181a-5p
 
 
-0.393
 
 
18.5
 
 
 
 
BOLA3
 
 
hsa-miR-15b-5p
 
 
-0.363
 
 
63.8
 
 
 
 
BOLA3
 
 
hsa-miR-15b-5p
 
 
-0.386
 
 
63.8
 
 
 
 
BOLA3
 
 
hsa-miR-374b-5p
 
 
-0.486
 
 
63.8
 
 
 
 
BRI3
 
 
hsa-miR-132-3p
 
 
-0.385
 
 
54.4
 
 
 
 
BTF3
 
 
hsa-miR-15b-5p
 
 
-0.344
 
 
316.9
 
 
 
 
BTF3
 
 
hsa-miR-361-5p
 
 
-0.551
 
 
316.9
 
 
 
 
BTG2
 
 
hsa-miR-29a-3p
 
 
-0.32
 
 
46.8
 
 
 
 
BTN2A1
 
 
hsa-miR-342-3p
 
 
-0.31
 
 
11.2
 
 
 
 
BTRC
 
 
hsa-miR-15b-5p
 
 
-0.302
 
 
10.7
 
 
 
 
BTRC
 
 
hsa-miR-15b-5p
 
 
-0.358
 
 
10.7
 
 
 
 
 
 
 ovary 
 
 
 
 
target_symbol
 
 
mature_mirna_id
 
 
score
 
 
nTPM
 
 
 
 
 
 
AAR2
 
 
hsa-miR-29a-3p
 
 
-0.327
 
 
22.7
 
 
 
 
ABCB6
 
 
hsa-miR-29a-3p
 
 
-0.48
 
 
12.1
 
 
 
 
ABCE1
 
 
hsa-miR-29a-3p
 
 
-0.404
 
 
25.9
 
 
 
 
ABHD4
 
 
hsa-miR-29a-3p
 
 
-0.312
 
 
17.9
 
 
 
 
ACTR2
 
 
hsa-miR-15b-5p
 
 
-0.293
 
 
47.8
 
 
 
 
ACVR1
 
 
hsa-miR-30b-5p
 
 
-0.29
 
 
17.7
 
 
 
 
ADAM9
 
 
hsa-miR-30b-5p
 
 
-0.312
 
 
16.9
 
 
 
 
ADCY3
 
 
hsa-miR-132-3p
 
 
-0.303
 
 
67.7
 
 
 
 
ADIPOR2
 
 
hsa-miR-150-5p
 
 
-0.287
 
 
24.2
 
 
 
 
ADRA2A
 
 
hsa-miR-23a-3p
 
 
-0.371
 
 
17.5
 
 
 
 
ADRA2A
 
 
hsa-miR-23b-3p
 
 
-0.371
 
 
17.5
 
 
 
 
AFG3L2
 
 
hsa-miR-181b-5p
 
 
-0.491
 
 
12.5
 
 
 
 
AFG3L2
 
 
hsa-miR-181a-5p
 
 
-0.491
 
 
12.5
 
 
 
 
AK1
 
 
hsa-miR-29a-3p
 
 
-0.333
 
 
43.4
 
 
 
 
AKT3
 
 
hsa-miR-29a-3p
 
 
-0.287
 
 
21.9
 
 
 
 
AKT3
 
 
hsa-miR-15b-5p
 
 
-0.301
 
 
21.9
 
 
 
 
ALAD
 
 
hsa-miR-15b-5p
 
 
-0.446
 
 
34.6
 
 
 
 
ALDH1A2
 
 
hsa-miR-23b-3p
 
 
-0.346
 
 
13.5
 
 
 
 
ALDH1A2
 
 
hsa-miR-23a-3p
 
 
-0.354
 
 
13.5
 
 
 
 
ANGPTL2
 
 
hsa-miR-221-3p
 
 
-0.369
 
 
49.6
 
 
 
 
ANKFY1
 
 
hsa-miR-155-5p
 
 
-0.315
 
 
16.2
 
 
 
 
ANKHD1
 
 
hsa-miR-23a-3p
 
 
-0.368
 
 
49.9
 
 
 
 
ANKHD1
 
 
hsa-miR-23b-3p
 
 
-0.368
 
 
49.9
 
 
 
 
ANKHD1
 
 
hsa-miR-30b-5p
 
 
-0.405
 
 
49.9
 
 
 
 
ANKRA2
 
 
hsa-miR-30b-5p
 
 
-0.354
 
 
11.1
 
 
 
 
ANKRA2
 
 
hsa-miR-30b-5p
 
 
-0.472
 
 
11.1
 
 
 
 
ANKRD17
 
 
hsa-miR-30b-5p
 
 
-0.336
 
 
15.3
 
 
 
 
ANKRD29
 
 
hsa-miR-132-3p
 
 
-0.47
 
 
15.0
 
 
 
 
ANP32A
 
 
hsa-miR-132-3p
 
 
-0.374
 
 
51.4
 
 
 
 
ANP32E
 
 
hsa-miR-501-3p
 
 
-0.331
 
 
20.6
 
 
 
 
ANTXR2
 
 
hsa-miR-29a-3p
 
 
-0.347
 
 
66.3
 
 
 
 
ANXA11
 
 
hsa-miR-15b-5p
 
 
-0.463
 
 
68.2
 
 
 
 
AP2A1
 
 
hsa-miR-15b-5p
 
 
-0.333
 
 
54.6
 
 
 
 
AP2B1
 
 
hsa-miR-15b-5p
 
 
-0.334
 
 
50.8
 
 
 
 
AP3S1
 
 
hsa-miR-30b-5p
 
 
-0.293
 
 
82.7
 
 
 
 
APOO
 
 
hsa-miR-181b-5p
 
 
-0.5
 
 
11.0
 
 
 
 
APOO
 
 
hsa-miR-181a-5p
 
 
-0.5
 
 
11.0
 
 
 
 
APP
 
 
hsa-miR-15b-5p
 
 
-0.298
 
 
401.1
 
 
 
 
AQP3
 
 
hsa-miR-874-3p
 
 
-0.48
 
 
11.4
 
 
 
 
ARF4
 
 
hsa-miR-30b-5p
 
 
-0.289
 
 
123.7
 
 
 
 
ARF4
 
 
hsa-miR-221-3p
 
 
-0.553
 
 
123.7
 
 
 
 
ARF5
 
 
hsa-miR-29a-3p
 
 
-0.491
 
 
81.8
 
 
 
 
ARF6
 
 
hsa-miR-181b-5p
 
 
-0.33
 
 
16.9
 
 
 
 
ARF6
 
 
hsa-miR-181a-5p
 
 
-0.33
 
 
16.9
 
 
 
 
ARHGAP5
 
 
hsa-miR-486-5p
 
 
-0.3
 
 
33.0
 
 
 
 
ARID1B
 
 
hsa-miR-29a-3p
 
 
-0.342
 
 
20.5
 
 
 
 
ARID4B
 
 
hsa-miR-486-5p
 
 
-0.367
 
 
14.0
 
 
 
 
ARL2
 
 
hsa-miR-15b-5p
 
 
-0.374
 
 
95.1
 
 
 
 
ARL2
 
 
hsa-miR-15b-5p
 
 
-0.442
 
 
95.1
 
 
 
 
ARL3
 
 
hsa-miR-15b-5p
 
 
-0.507
 
 
16.3
 
 
 
 
ARL4A
 
 
hsa-miR-29a-3p
 
 
-0.386
 
 
14.3
 
 
 
 
ARL4A
 
 
hsa-miR-30b-5p
 
 
-0.448
 
 
14.3
 
 
 
 
ARMC8
 
 
hsa-miR-486-5p
 
 
-0.321
 
 
14.0
 
 
 
 
ARMCX2
 
 
hsa-miR-15b-5p
 
 
-0.344
 
 
40.5
 
 
 
 
ARMCX3
 
 
hsa-miR-425-5p
 
 
-0.325
 
 
36.1
 
 
 
 
ARNT
 
 
hsa-miR-221-3p
 
 
-0.465
 
 
32.5
 
 
 
 
ARPP19
 
 
hsa-miR-15b-5p
 
 
-0.346
 
 
40.6
 
 
 
 
ARPP19
 
 
hsa-miR-29a-3p
 
 
-0.371
 
 
40.6
 
 
 
 
ARRDC3
 
 
hsa-miR-29a-3p
 
 
-0.402
 
 
64.7
 
 
 
 
ASB3
 
 
hsa-miR-30b-5p
 
 
-0.418
 
 
12.1
 
 
 
 
ASB3
 
 
hsa-miR-30b-5p
 
 
-0.418
 
 
12.1
 
 
 
 
ASF1A
 
 
hsa-miR-23a-3p
 
 
-0.506
 
 
18.5
 
 
 
 
ASF1A
 
 
hsa-miR-23b-3p
 
 
-0.506
 
 
18.5
 
 
 
 
ASH1L
 
 
hsa-miR-15b-5p
 
 
-0.325
 
 
24.5
 
 
 
 
ASPH
 
 
hsa-miR-221-3p
 
 
-0.287
 
 
58.7
 
 
 
 
ATF3
 
 
hsa-miR-874-3p
 
 
-0.288
 
 
34.3
 
 
 
 
ATG5
 
 
hsa-miR-30b-5p
 
 
-0.488
 
 
14.0
 
 
 
 
ATG9A
 
 
hsa-miR-15b-5p
 
 
-0.333
 
 
20.5
 
 
 
 
ATOSA
 
 
hsa-miR-221-3p
 
 
-0.495
 
 
10.4
 
 
 
 
ATP5MC3
 
 
hsa-miR-425-5p
 
 
-0.328
 
 
107.0
 
 
 
 
ATP5MC3
 
 
hsa-miR-29a-3p
 
 
-0.33
 
 
107.0
 
 
 
 
ATP6V1E1
 
 
hsa-miR-23a-3p
 
 
-0.294
 
 
85.8
 
 
 
 
ATP6V1E1
 
 
hsa-miR-23b-3p
 
 
-0.294
 
 
85.8
 
 
 
 
ATPAF1
 
 
hsa-miR-361-5p
 
 
-0.362
 
 
33.9
 
 
 
 
ATXN1L
 
 
hsa-miR-155-5p
 
 
-0.315
 
 
10.8
 
 
 
 
ATXN2
 
 
hsa-miR-15b-5p
 
 
-0.392
 
 
25.4
 
 
 
 
AUH
 
 
hsa-miR-23a-3p
 
 
-0.495
 
 
12.6
 
 
 
 
AUH
 
 
hsa-miR-23b-3p
 
 
-0.503
 
 
12.6
 
 
 
 
AUH
 
 
hsa-miR-23a-3p
 
 
-0.512
 
 
12.6
 
 
 
 
AUH
 
 
hsa-miR-23b-3p
 
 
-0.512
 
 
12.6
 
 
 
 
AXIN2
 
 
hsa-miR-15b-5p
 
 
-0.3
 
 
13.9
 
 
 
 
AZIN1
 
 
hsa-miR-132-3p
 
 
-0.29
 
 
37.6
 
 
 
 
AZIN1
 
 
hsa-miR-132-3p
 
 
-0.299
 
 
37.6
 
 
 
 
B4GALT5
 
 
hsa-miR-501-3p
 
 
-0.408
 
 
19.0
 
 
 
 
B4GAT1
 
 
hsa-miR-23a-3p
 
 
-0.361
 
 
33.5
 
 
 
 
B4GAT1
 
 
hsa-miR-23b-3p
 
 
-0.361
 
 
33.5
 
 
 
 
BCL2L2
 
 
hsa-miR-29a-3p
 
 
-0.312
 
 
32.0
 
 
 
 
BCOR
 
 
hsa-miR-425-5p
 
 
-0.346
 
 
11.8
 
 
 
 
BECN1
 
 
hsa-miR-30b-5p
 
 
-0.452
 
 
27.4
 
 
 
 
BET1
 
 
hsa-miR-23a-3p
 
 
-0.402
 
 
26.0
 
 
 
 
BET1
 
 
hsa-miR-23b-3p
 
 
-0.418
 
 
26.0
 
 
 
 
BFAR
 
 
hsa-miR-15b-5p
 
 
-0.352
 
 
27.7
 
 
 
 
BHLHE40
 
 
hsa-miR-181b-5p
 
 
-0.408
 
 
79.3
 
 
 
 
BHLHE40
 
 
hsa-miR-181a-5p
 
 
-0.408
 
 
79.3
 
 
 
 
BLCAP
 
 
hsa-miR-23a-3p
 
 
-0.601
 
 
39.5
 
 
 
 
BLCAP
 
 
hsa-miR-23b-3p
 
 
-0.601
 
 
39.5
 
 
 
 
BLMH
 
 
hsa-miR-29a-3p
 
 
-0.393
 
 
17.2
 
 
 
 
BLOC1S6
 
 
hsa-miR-181b-5p
 
 
-0.393
 
 
33.7
 
 
 
 
BLOC1S6
 
 
hsa-miR-181a-5p
 
 
-0.393
 
 
33.7
 
 
 
 
BMF
 
 
hsa-miR-29a-3p
 
 
-0.41
 
 
11.0
 
 
 
 
 
 
 pancreas 
 
 
 
 
target_symbol
 
 
mature_mirna_id
 
 
score
 
 
nTPM
 
 
 
 
 
 
AAR2
 
 
hsa-miR-29a-3p
 
 
-0.327
 
 
17.6
 
 
 
 
ABCE1
 
 
hsa-miR-29a-3p
 
 
-0.404
 
 
27.7
 
 
 
 
ABHD4
 
 
hsa-miR-29a-3p
 
 
-0.312
 
 
15.3
 
 
 
 
ABHD5
 
 
hsa-miR-132-3p
 
 
-0.323
 
 
11.4
 
 
 
 
ACTC1
 
 
hsa-miR-30b-5p
 
 
-0.543
 
 
12.1
 
 
 
 
ACTR2
 
 
hsa-miR-15b-5p
 
 
-0.293
 
 
57.4
 
 
 
 
ACTR3C
 
 
hsa-miR-30b-5p
 
 
-0.448
 
 
12.4
 
 
 
 
ADCY3
 
 
hsa-miR-132-3p
 
 
-0.303
 
 
15.3
 
 
 
 
ADGRA3
 
 
hsa-miR-30b-5p
 
 
-0.304
 
 
11.8
 
 
 
 
ADIPOR2
 
 
hsa-miR-150-5p
 
 
-0.287
 
 
30.0
 
 
 
 
ADRA2A
 
 
hsa-miR-23a-3p
 
 
-0.371
 
 
28.8
 
 
 
 
ADRA2A
 
 
hsa-miR-23b-3p
 
 
-0.371
 
 
28.8
 
 
 
 
AFG3L2
 
 
hsa-miR-181b-5p
 
 
-0.491
 
 
23.6
 
 
 
 
AFG3L2
 
 
hsa-miR-181a-5p
 
 
-0.491
 
 
23.6
 
 
 
 
AK1
 
 
hsa-miR-29a-3p
 
 
-0.333
 
 
14.0
 
 
 
 
ALAD
 
 
hsa-miR-15b-5p
 
 
-0.446
 
 
45.8
 
 
 
 
AMMECR1L
 
 
hsa-miR-29a-3p
 
 
-0.42
 
 
10.5
 
 
 
 
AMOTL1
 
 
hsa-miR-15b-5p
 
 
-0.319
 
 
14.7
 
 
 
 
ANKHD1
 
 
hsa-miR-23a-3p
 
 
-0.368
 
 
57.6
 
 
 
 
ANKHD1
 
 
hsa-miR-23b-3p
 
 
-0.368
 
 
57.6
 
 
 
 
ANKHD1
 
 
hsa-miR-30b-5p
 
 
-0.405
 
 
57.6
 
 
 
 
ANKRD17
 
 
hsa-miR-30b-5p
 
 
-0.336
 
 
17.0
 
 
 
 
ANP32A
 
 
hsa-miR-132-3p
 
 
-0.374
 
 
50.6
 
 
 
 
ANXA11
 
 
hsa-miR-15b-5p
 
 
-0.463
 
 
88.5
 
 
 
 
AP2A1
 
 
hsa-miR-15b-5p
 
 
-0.333
 
 
32.4
 
 
 
 
AP2B1
 
 
hsa-miR-15b-5p
 
 
-0.334
 
 
31.1
 
 
 
 
AP3S1
 
 
hsa-miR-30b-5p
 
 
-0.293
 
 
66.7
 
 
 
 
APOO
 
 
hsa-miR-181b-5p
 
 
-0.5
 
 
13.3
 
 
 
 
APOO
 
 
hsa-miR-181a-5p
 
 
-0.5
 
 
13.3
 
 
 
 
APP
 
 
hsa-miR-15b-5p
 
 
-0.298
 
 
305.2
 
 
 
 
AQP3
 
 
hsa-miR-874-3p
 
 
-0.48
 
 
41.8
 
 
 
 
ARF4
 
 
hsa-miR-30b-5p
 
 
-0.289
 
 
138.3
 
 
 
 
ARF4
 
 
hsa-miR-221-3p
 
 
-0.553
 
 
138.3
 
 
 
 
ARF5
 
 
hsa-miR-29a-3p
 
 
-0.491
 
 
181.7
 
 
 
 
ARF6
 
 
hsa-miR-181b-5p
 
 
-0.33
 
 
23.2
 
 
 
 
ARF6
 
 
hsa-miR-181a-5p
 
 
-0.33
 
 
23.2
 
 
 
 
ARHGAP5
 
 
hsa-miR-486-5p
 
 
-0.3
 
 
28.2
 
 
 
 
ARID1B
 
 
hsa-miR-29a-3p
 
 
-0.342
 
 
15.2
 
 
 
 
ARID4B
 
 
hsa-miR-486-5p
 
 
-0.367
 
 
10.7
 
 
 
 
ARL2
 
 
hsa-miR-15b-5p
 
 
-0.374
 
 
74.9
 
 
 
 
ARL2
 
 
hsa-miR-15b-5p
 
 
-0.442
 
 
74.9
 
 
 
 
ARMC8
 
 
hsa-miR-486-5p
 
 
-0.321
 
 
13.4
 
 
 
 
ARMCX2
 
 
hsa-miR-15b-5p
 
 
-0.344
 
 
16.6
 
 
 
 
ARMCX3
 
 
hsa-miR-425-5p
 
 
-0.325
 
 
50.5
 
 
 
 
ARNT
 
 
hsa-miR-221-3p
 
 
-0.465
 
 
27.0
 
 
 
 
ARPP19
 
 
hsa-miR-15b-5p
 
 
-0.346
 
 
43.6
 
 
 
 
ARPP19
 
 
hsa-miR-29a-3p
 
 
-0.371
 
 
43.6
 
 
 
 
ARRDC3
 
 
hsa-miR-29a-3p
 
 
-0.402
 
 
19.3
 
 
 
 
ASB6
 
 
hsa-miR-874-3p
 
 
-0.368
 
 
13.0
 
 
 
 
ASH1L
 
 
hsa-miR-15b-5p
 
 
-0.325
 
 
16.5
 
 
 
 
ASPH
 
 
hsa-miR-221-3p
 
 
-0.287
 
 
42.1
 
 
 
 
ATF3
 
 
hsa-miR-874-3p
 
 
-0.288
 
 
63.3
 
 
 
 
ATG5
 
 
hsa-miR-30b-5p
 
 
-0.488
 
 
10.4
 
 
 
 
ATG9A
 
 
hsa-miR-15b-5p
 
 
-0.333
 
 
26.5
 
 
 
 
ATOSA
 
 
hsa-miR-221-3p
 
 
-0.495
 
 
19.2
 
 
 
 
ATP5MC3
 
 
hsa-miR-425-5p
 
 
-0.328
 
 
115.2
 
 
 
 
ATP5MC3
 
 
hsa-miR-29a-3p
 
 
-0.33
 
 
115.2
 
 
 
 
ATP6V1E1
 
 
hsa-miR-23a-3p
 
 
-0.294
 
 
59.3
 
 
 
 
ATP6V1E1
 
 
hsa-miR-23b-3p
 
 
-0.294
 
 
59.3
 
 
 
 
ATPAF1
 
 
hsa-miR-361-5p
 
 
-0.362
 
 
54.8
 
 
 
 
ATXN1L
 
 
hsa-miR-155-5p
 
 
-0.315
 
 
11.8
 
 
 
 
ATXN2
 
 
hsa-miR-15b-5p
 
 
-0.392
 
 
13.3
 
 
 
 
AUH
 
 
hsa-miR-23a-3p
 
 
-0.495
 
 
24.0
 
 
 
 
AUH
 
 
hsa-miR-23b-3p
 
 
-0.503
 
 
24.0
 
 
 
 
AUH
 
 
hsa-miR-23a-3p
 
 
-0.512
 
 
24.0
 
 
 
 
AUH
 
 
hsa-miR-23b-3p
 
 
-0.512
 
 
24.0
 
 
 
 
AZIN1
 
 
hsa-miR-132-3p
 
 
-0.29
 
 
127.3
 
 
 
 
AZIN1
 
 
hsa-miR-132-3p
 
 
-0.299
 
 
127.3
 
 
 
 
B4GALT5
 
 
hsa-miR-501-3p
 
 
-0.408
 
 
11.8
 
 
 
 
B4GAT1
 
 
hsa-miR-23a-3p
 
 
-0.361
 
 
24.7
 
 
 
 
B4GAT1
 
 
hsa-miR-23b-3p
 
 
-0.361
 
 
24.7
 
 
 
 
BAK1
 
 
hsa-miR-29a-3p
 
 
-0.409
 
 
12.1
 
 
 
 
BCL2L11
 
 
hsa-miR-221-3p
 
 
-0.423
 
 
12.8
 
 
 
 
BCL2L2
 
 
hsa-miR-29a-3p
 
 
-0.312
 
 
54.1
 
 
 
 
BCOR
 
 
hsa-miR-425-5p
 
 
-0.346
 
 
10.6
 
 
 
 
BECN1
 
 
hsa-miR-30b-5p
 
 
-0.452
 
 
37.3
 
 
 
 
BET1
 
 
hsa-miR-23a-3p
 
 
-0.402
 
 
21.4
 
 
 
 
BET1
 
 
hsa-miR-23b-3p
 
 
-0.418
 
 
21.4
 
 
 
 
BFAR
 
 
hsa-miR-15b-5p
 
 
-0.352
 
 
44.3
 
 
 
 
BHLHE40
 
 
hsa-miR-181b-5p
 
 
-0.408
 
 
75.3
 
 
 
 
BHLHE40
 
 
hsa-miR-181a-5p
 
 
-0.408
 
 
75.3
 
 
 
 
BLCAP
 
 
hsa-miR-23a-3p
 
 
-0.601
 
 
26.5
 
 
 
 
BLCAP
 
 
hsa-miR-23b-3p
 
 
-0.601
 
 
26.5
 
 
 
 
BLMH
 
 
hsa-miR-29a-3p
 
 
-0.393
 
 
26.1
 
 
 
 
BLOC1S6
 
 
hsa-miR-181b-5p
 
 
-0.393
 
 
19.3
 
 
 
 
BLOC1S6
 
 
hsa-miR-181a-5p
 
 
-0.393
 
 
19.3
 
 
 
 
BOLA3
 
 
hsa-miR-15b-5p
 
 
-0.363
 
 
42.1
 
 
 
 
BOLA3
 
 
hsa-miR-15b-5p
 
 
-0.386
 
 
42.1
 
 
 
 
BOLA3
 
 
hsa-miR-374b-5p
 
 
-0.486
 
 
42.1
 
 
 
 
BORCS6
 
 
hsa-miR-15b-5p
 
 
-0.347
 
 
20.2
 
 
 
 
BRI3
 
 
hsa-miR-132-3p
 
 
-0.385
 
 
123.0
 
 
 
 
BTAF1
 
 
hsa-miR-486-5p
 
 
-0.288
 
 
15.0
 
 
 
 
BTAF1
 
 
hsa-miR-15b-5p
 
 
-0.496
 
 
15.0
 
 
 
 
BTF3
 
 
hsa-miR-15b-5p
 
 
-0.344
 
 
580.1
 
 
 
 
BTF3
 
 
hsa-miR-361-5p
 
 
-0.551
 
 
580.1
 
 
 
 
BTG2
 
 
hsa-miR-29a-3p
 
 
-0.32
 
 
461.2
 
 
 
 
BZW1
 
 
hsa-miR-15b-5p
 
 
-0.367
 
 
59.0
 
 
 
 
C11orf24
 
 
hsa-miR-15b-5p
 
 
-0.403
 
 
25.9
 
 
 
 
C11orf68
 
 
hsa-miR-15b-5p
 
 
-0.29
 
 
26.1
 
 
 
 
C2orf88
 
 
hsa-miR-29a-3p
 
 
-0.32
 
 
12.0
 
 
 
 
 
 
 parathyroid gland 
 
 
 
 
target_symbol
 
 
mature_mirna_id
 
 
score
 
 
nTPM
 
 
 
 
 
 
AAR2
 
 
hsa-miR-29a-3p
 
 
-0.327
 
 
23.9
 
 
 
 
ABCE1
 
 
hsa-miR-29a-3p
 
 
-0.404
 
 
25.3
 
 
 
 
ABHD4
 
 
hsa-miR-29a-3p
 
 
-0.312
 
 
23.4
 
 
 
 
ABHD5
 
 
hsa-miR-132-3p
 
 
-0.323
 
 
11.5
 
 
 
 
ACTR2
 
 
hsa-miR-15b-5p
 
 
-0.293
 
 
58.1
 
 
 
 
ACTR3C
 
 
hsa-miR-30b-5p
 
 
-0.448
 
 
53.3
 
 
 
 
ADAM9
 
 
hsa-miR-30b-5p
 
 
-0.312
 
 
49.4
 
 
 
 
ADCY3
 
 
hsa-miR-132-3p
 
 
-0.303
 
 
16.0
 
 
 
 
ADGRA3
 
 
hsa-miR-30b-5p
 
 
-0.304
 
 
11.5
 
 
 
 
ADIPOR2
 
 
hsa-miR-150-5p
 
 
-0.287
 
 
15.9
 
 
 
 
AFG3L2
 
 
hsa-miR-181b-5p
 
 
-0.491
 
 
41.3
 
 
 
 
AFG3L2
 
 
hsa-miR-181a-5p
 
 
-0.491
 
 
41.3
 
 
 
 
AK1
 
 
hsa-miR-29a-3p
 
 
-0.333
 
 
44.1
 
 
 
 
AK4
 
 
hsa-miR-15b-5p
 
 
-0.316
 
 
16.7
 
 
 
 
ALAD
 
 
hsa-miR-15b-5p
 
 
-0.446
 
 
68.8
 
 
 
 
ALDH1A2
 
 
hsa-miR-23b-3p
 
 
-0.346
 
 
28.0
 
 
 
 
ALDH1A2
 
 
hsa-miR-23a-3p
 
 
-0.354
 
 
28.0
 
 
 
 
AMOT
 
 
hsa-miR-29a-3p
 
 
-0.304
 
 
13.8
 
 
 
 
AMOTL1
 
 
hsa-miR-15b-5p
 
 
-0.319
 
 
13.0
 
 
 
 
ANKFY1
 
 
hsa-miR-155-5p
 
 
-0.315
 
 
17.9
 
 
 
 
ANKHD1
 
 
hsa-miR-23a-3p
 
 
-0.368
 
 
35.6
 
 
 
 
ANKHD1
 
 
hsa-miR-23b-3p
 
 
-0.368
 
 
35.6
 
 
 
 
ANKHD1
 
 
hsa-miR-30b-5p
 
 
-0.405
 
 
35.6
 
 
 
 
ANKRD17
 
 
hsa-miR-30b-5p
 
 
-0.336
 
 
23.0
 
 
 
 
ANKRD29
 
 
hsa-miR-132-3p
 
 
-0.47
 
 
23.3
 
 
 
 
ANP32A
 
 
hsa-miR-132-3p
 
 
-0.374
 
 
57.6
 
 
 
 
ANP32E
 
 
hsa-miR-501-3p
 
 
-0.331
 
 
31.1
 
 
 
 
ANTXR2
 
 
hsa-miR-29a-3p
 
 
-0.347
 
 
26.2
 
 
 
 
ANXA11
 
 
hsa-miR-15b-5p
 
 
-0.463
 
 
76.9
 
 
 
 
AP2A1
 
 
hsa-miR-15b-5p
 
 
-0.333
 
 
12.9
 
 
 
 
AP2B1
 
 
hsa-miR-15b-5p
 
 
-0.334
 
 
118.9
 
 
 
 
AP3S1
 
 
hsa-miR-30b-5p
 
 
-0.293
 
 
23.4
 
 
 
 
APOO
 
 
hsa-miR-181b-5p
 
 
-0.5
 
 
58.1
 
 
 
 
APOO
 
 
hsa-miR-181a-5p
 
 
-0.5
 
 
58.1
 
 
 
 
APP
 
 
hsa-miR-15b-5p
 
 
-0.298
 
 
259.7
 
 
 
 
ARF4
 
 
hsa-miR-30b-5p
 
 
-0.289
 
 
95.4
 
 
 
 
ARF4
 
 
hsa-miR-221-3p
 
 
-0.553
 
 
95.4
 
 
 
 
ARF5
 
 
hsa-miR-29a-3p
 
 
-0.491
 
 
97.9
 
 
 
 
ARF6
 
 
hsa-miR-181b-5p
 
 
-0.33
 
 
19.3
 
 
 
 
ARF6
 
 
hsa-miR-181a-5p
 
 
-0.33
 
 
19.3
 
 
 
 
ARHGAP5
 
 
hsa-miR-486-5p
 
 
-0.3
 
 
45.3
 
 
 
 
ARID1B
 
 
hsa-miR-29a-3p
 
 
-0.342
 
 
25.3
 
 
 
 
ARID2
 
 
hsa-miR-132-3p
 
 
-0.304
 
 
11.0
 
 
 
 
ARID2
 
 
hsa-miR-155-5p
 
 
-0.384
 
 
11.0
 
 
 
 
ARID4B
 
 
hsa-miR-486-5p
 
 
-0.367
 
 
12.8
 
 
 
 
ARL2
 
 
hsa-miR-15b-5p
 
 
-0.374
 
 
61.4
 
 
 
 
ARL2
 
 
hsa-miR-15b-5p
 
 
-0.442
 
 
61.4
 
 
 
 
ARL3
 
 
hsa-miR-15b-5p
 
 
-0.507
 
 
18.6
 
 
 
 
ARMC8
 
 
hsa-miR-486-5p
 
 
-0.321
 
 
20.0
 
 
 
 
ARMCX2
 
 
hsa-miR-15b-5p
 
 
-0.344
 
 
67.0
 
 
 
 
ARMCX3
 
 
hsa-miR-425-5p
 
 
-0.325
 
 
97.4
 
 
 
 
ARNT
 
 
hsa-miR-221-3p
 
 
-0.465
 
 
18.4
 
 
 
 
ARPP19
 
 
hsa-miR-15b-5p
 
 
-0.346
 
 
102.5
 
 
 
 
ARPP19
 
 
hsa-miR-29a-3p
 
 
-0.371
 
 
102.5
 
 
 
 
ARRDC3
 
 
hsa-miR-29a-3p
 
 
-0.402
 
 
51.0
 
 
 
 
ASF1A
 
 
hsa-miR-23a-3p
 
 
-0.506
 
 
14.9
 
 
 
 
ASF1A
 
 
hsa-miR-23b-3p
 
 
-0.506
 
 
14.9
 
 
 
 
ASH1L
 
 
hsa-miR-15b-5p
 
 
-0.325
 
 
17.1
 
 
 
 
ASPH
 
 
hsa-miR-221-3p
 
 
-0.287
 
 
35.7
 
 
 
 
ATF3
 
 
hsa-miR-874-3p
 
 
-0.288
 
 
45.8
 
 
 
 
ATG5
 
 
hsa-miR-30b-5p
 
 
-0.488
 
 
11.1
 
 
 
 
ATG9A
 
 
hsa-miR-15b-5p
 
 
-0.333
 
 
29.3
 
 
 
 
ATOSA
 
 
hsa-miR-221-3p
 
 
-0.495
 
 
38.4
 
 
 
 
ATP5MC3
 
 
hsa-miR-425-5p
 
 
-0.328
 
 
515.1
 
 
 
 
ATP5MC3
 
 
hsa-miR-29a-3p
 
 
-0.33
 
 
515.1
 
 
 
 
ATP6V1E1
 
 
hsa-miR-23a-3p
 
 
-0.294
 
 
225.3
 
 
 
 
ATP6V1E1
 
 
hsa-miR-23b-3p
 
 
-0.294
 
 
225.3
 
 
 
 
ATPAF1
 
 
hsa-miR-361-5p
 
 
-0.362
 
 
129.7
 
 
 
 
ATXN1L
 
 
hsa-miR-155-5p
 
 
-0.315
 
 
15.5
 
 
 
 
ATXN2
 
 
hsa-miR-15b-5p
 
 
-0.392
 
 
17.5
 
 
 
 
AUH
 
 
hsa-miR-23a-3p
 
 
-0.495
 
 
42.7
 
 
 
 
AUH
 
 
hsa-miR-23b-3p
 
 
-0.503
 
 
42.7
 
 
 
 
AUH
 
 
hsa-miR-23a-3p
 
 
-0.512
 
 
42.7
 
 
 
 
AUH
 
 
hsa-miR-23b-3p
 
 
-0.512
 
 
42.7
 
 
 
 
AZIN1
 
 
hsa-miR-132-3p
 
 
-0.29
 
 
35.6
 
 
 
 
AZIN1
 
 
hsa-miR-132-3p
 
 
-0.299
 
 
35.6
 
 
 
 
B4GALT5
 
 
hsa-miR-501-3p
 
 
-0.408
 
 
10.6
 
 
 
 
B4GAT1
 
 
hsa-miR-23a-3p
 
 
-0.361
 
 
21.3
 
 
 
 
B4GAT1
 
 
hsa-miR-23b-3p
 
 
-0.361
 
 
21.3
 
 
 
 
BAK1
 
 
hsa-miR-29a-3p
 
 
-0.409
 
 
12.6
 
 
 
 
BCL2
 
 
hsa-miR-342-3p
 
 
-0.428
 
 
12.3
 
 
 
 
BCL2L11
 
 
hsa-miR-221-3p
 
 
-0.423
 
 
17.6
 
 
 
 
BCL2L2
 
 
hsa-miR-29a-3p
 
 
-0.312
 
 
50.0
 
 
 
 
BCOR
 
 
hsa-miR-425-5p
 
 
-0.346
 
 
13.2
 
 
 
 
BDH1
 
 
hsa-miR-874-3p
 
 
-0.644
 
 
14.6
 
 
 
 
BECN1
 
 
hsa-miR-30b-5p
 
 
-0.452
 
 
39.2
 
 
 
 
BET1
 
 
hsa-miR-23a-3p
 
 
-0.402
 
 
18.3
 
 
 
 
BET1
 
 
hsa-miR-23b-3p
 
 
-0.418
 
 
18.3
 
 
 
 
BFAR
 
 
hsa-miR-15b-5p
 
 
-0.352
 
 
15.2
 
 
 
 
BHLHE40
 
 
hsa-miR-181b-5p
 
 
-0.408
 
 
63.5
 
 
 
 
BHLHE40
 
 
hsa-miR-181a-5p
 
 
-0.408
 
 
63.5
 
 
 
 
BLCAP
 
 
hsa-miR-23a-3p
 
 
-0.601
 
 
47.8
 
 
 
 
BLCAP
 
 
hsa-miR-23b-3p
 
 
-0.601
 
 
47.8
 
 
 
 
BLMH
 
 
hsa-miR-29a-3p
 
 
-0.393
 
 
20.4
 
 
 
 
BLOC1S6
 
 
hsa-miR-181b-5p
 
 
-0.393
 
 
15.8
 
 
 
 
BLOC1S6
 
 
hsa-miR-181a-5p
 
 
-0.393
 
 
15.8
 
 
 
 
BOLA3
 
 
hsa-miR-15b-5p
 
 
-0.363
 
 
52.6
 
 
 
 
BOLA3
 
 
hsa-miR-15b-5p
 
 
-0.386
 
 
52.6
 
 
 
 
BOLA3
 
 
hsa-miR-374b-5p
 
 
-0.486
 
 
52.6
 
 
 
 
BRI3
 
 
hsa-miR-132-3p
 
 
-0.385
 
 
22.6
 
 
 
 
 
 
 placenta 
 
 
 
 
target_symbol
 
 
mature_mirna_id
 
 
score
 
 
nTPM
 
 
 
 
 
 
AAR2
 
 
hsa-miR-29a-3p
 
 
-0.327
 
 
16.5
 
 
 
 
ABCE1
 
 
hsa-miR-29a-3p
 
 
-0.404
 
 
26.9
 
 
 
 
ABHD4
 
 
hsa-miR-29a-3p
 
 
-0.312
 
 
18.5
 
 
 
 
ACTR2
 
 
hsa-miR-15b-5p
 
 
-0.293
 
 
102.8
 
 
 
 
ACVR1
 
 
hsa-miR-30b-5p
 
 
-0.29
 
 
12.5
 
 
 
 
ADAM19
 
 
hsa-miR-874-3p
 
 
-0.374
 
 
12.3
 
 
 
 
ADAM9
 
 
hsa-miR-30b-5p
 
 
-0.312
 
 
44.6
 
 
 
 
ADAMTS9
 
 
hsa-miR-29a-3p
 
 
-0.305
 
 
29.8
 
 
 
 
ADAMTS9
 
 
hsa-miR-29a-3p
 
 
-0.485
 
 
29.8
 
 
 
 
ADCY3
 
 
hsa-miR-132-3p
 
 
-0.303
 
 
78.3
 
 
 
 
ADIPOR2
 
 
hsa-miR-150-5p
 
 
-0.287
 
 
12.2
 
 
 
 
AFG3L2
 
 
hsa-miR-181b-5p
 
 
-0.491
 
 
12.5
 
 
 
 
AFG3L2
 
 
hsa-miR-181a-5p
 
 
-0.491
 
 
12.5
 
 
 
 
AHR
 
 
hsa-miR-29a-3p
 
 
-0.316
 
 
16.6
 
 
 
 
AKT3
 
 
hsa-miR-29a-3p
 
 
-0.287
 
 
12.3
 
 
 
 
AKT3
 
 
hsa-miR-15b-5p
 
 
-0.301
 
 
12.3
 
 
 
 
ALAD
 
 
hsa-miR-15b-5p
 
 
-0.446
 
 
23.4
 
 
 
 
AMOT
 
 
hsa-miR-29a-3p
 
 
-0.304
 
 
19.6
 
 
 
 
AMOTL1
 
 
hsa-miR-15b-5p
 
 
-0.319
 
 
22.6
 
 
 
 
ANGPTL2
 
 
hsa-miR-221-3p
 
 
-0.369
 
 
20.1
 
 
 
 
ANKFY1
 
 
hsa-miR-155-5p
 
 
-0.315
 
 
13.3
 
 
 
 
ANKHD1
 
 
hsa-miR-23a-3p
 
 
-0.368
 
 
26.8
 
 
 
 
ANKHD1
 
 
hsa-miR-23b-3p
 
 
-0.368
 
 
26.8
 
 
 
 
ANKHD1
 
 
hsa-miR-30b-5p
 
 
-0.405
 
 
26.8
 
 
 
 
ANKRD17
 
 
hsa-miR-30b-5p
 
 
-0.336
 
 
17.9
 
 
 
 
ANP32A
 
 
hsa-miR-132-3p
 
 
-0.374
 
 
95.3
 
 
 
 
ANP32E
 
 
hsa-miR-501-3p
 
 
-0.331
 
 
20.3
 
 
 
 
ANTXR2
 
 
hsa-miR-29a-3p
 
 
-0.347
 
 
84.5
 
 
 
 
ANXA11
 
 
hsa-miR-15b-5p
 
 
-0.463
 
 
85.5
 
 
 
 
AP2A1
 
 
hsa-miR-15b-5p
 
 
-0.333
 
 
29.6
 
 
 
 
AP2B1
 
 
hsa-miR-15b-5p
 
 
-0.334
 
 
62.7
 
 
 
 
AP3S1
 
 
hsa-miR-30b-5p
 
 
-0.293
 
 
56.4
 
 
 
 
APLN
 
 
hsa-miR-15b-5p
 
 
-0.395
 
 
50.8
 
 
 
 
APOO
 
 
hsa-miR-181b-5p
 
 
-0.5
 
 
10.3
 
 
 
 
APOO
 
 
hsa-miR-181a-5p
 
 
-0.5
 
 
10.3
 
 
 
 
APP
 
 
hsa-miR-15b-5p
 
 
-0.298
 
 
417.2
 
 
 
 
ARF4
 
 
hsa-miR-30b-5p
 
 
-0.289
 
 
170.7
 
 
 
 
ARF4
 
 
hsa-miR-221-3p
 
 
-0.553
 
 
170.7
 
 
 
 
ARF5
 
 
hsa-miR-29a-3p
 
 
-0.491
 
 
65.2
 
 
 
 
ARF6
 
 
hsa-miR-181b-5p
 
 
-0.33
 
 
34.8
 
 
 
 
ARF6
 
 
hsa-miR-181a-5p
 
 
-0.33
 
 
34.8
 
 
 
 
ARHGAP42
 
 
hsa-miR-221-3p
 
 
-0.304
 
 
14.1
 
 
 
 
ARHGAP5
 
 
hsa-miR-486-5p
 
 
-0.3
 
 
35.0
 
 
 
 
ARID1B
 
 
hsa-miR-29a-3p
 
 
-0.342
 
 
10.4
 
 
 
 
ARID4B
 
 
hsa-miR-486-5p
 
 
-0.367
 
 
12.0
 
 
 
 
ARL2
 
 
hsa-miR-15b-5p
 
 
-0.374
 
 
52.7
 
 
 
 
ARL2
 
 
hsa-miR-15b-5p
 
 
-0.442
 
 
52.7
 
 
 
 
ARL4A
 
 
hsa-miR-29a-3p
 
 
-0.386
 
 
22.2
 
 
 
 
ARL4A
 
 
hsa-miR-30b-5p
 
 
-0.448
 
 
22.2
 
 
 
 
ARL5B
 
 
hsa-miR-29a-3p
 
 
-0.304
 
 
12.3
 
 
 
 
ARL5B
 
 
hsa-miR-155-5p
 
 
-0.355
 
 
12.3
 
 
 
 
ARMC8
 
 
hsa-miR-486-5p
 
 
-0.321
 
 
18.5
 
 
 
 
ARMCX2
 
 
hsa-miR-15b-5p
 
 
-0.344
 
 
24.7
 
 
 
 
ARMCX3
 
 
hsa-miR-425-5p
 
 
-0.325
 
 
42.7
 
 
 
 
ARNT
 
 
hsa-miR-221-3p
 
 
-0.465
 
 
29.3
 
 
 
 
ARPP19
 
 
hsa-miR-15b-5p
 
 
-0.346
 
 
53.5
 
 
 
 
ARPP19
 
 
hsa-miR-29a-3p
 
 
-0.371
 
 
53.5
 
 
 
 
ARRDC3
 
 
hsa-miR-29a-3p
 
 
-0.402
 
 
72.1
 
 
 
 
ARSB
 
 
hsa-miR-29a-3p
 
 
-0.36
 
 
10.8
 
 
 
 
ASB3
 
 
hsa-miR-30b-5p
 
 
-0.418
 
 
10.7
 
 
 
 
ASB3
 
 
hsa-miR-30b-5p
 
 
-0.418
 
 
10.7
 
 
 
 
ASPH
 
 
hsa-miR-221-3p
 
 
-0.287
 
 
44.6
 
 
 
 
ATF3
 
 
hsa-miR-874-3p
 
 
-0.288
 
 
62.2
 
 
 
 
ATG5
 
 
hsa-miR-30b-5p
 
 
-0.488
 
 
12.4
 
 
 
 
ATG9A
 
 
hsa-miR-15b-5p
 
 
-0.333
 
 
28.8
 
 
 
 
ATP5MC3
 
 
hsa-miR-425-5p
 
 
-0.328
 
 
95.9
 
 
 
 
ATP5MC3
 
 
hsa-miR-29a-3p
 
 
-0.33
 
 
95.9
 
 
 
 
ATP6V1E1
 
 
hsa-miR-23a-3p
 
 
-0.294
 
 
106.9
 
 
 
 
ATP6V1E1
 
 
hsa-miR-23b-3p
 
 
-0.294
 
 
106.9
 
 
 
 
ATPAF1
 
 
hsa-miR-361-5p
 
 
-0.362
 
 
34.1
 
 
 
 
ATXN1L
 
 
hsa-miR-155-5p
 
 
-0.315
 
 
10.1
 
 
 
 
ATXN2
 
 
hsa-miR-15b-5p
 
 
-0.392
 
 
15.3
 
 
 
 
AUH
 
 
hsa-miR-23a-3p
 
 
-0.495
 
 
15.1
 
 
 
 
AUH
 
 
hsa-miR-23b-3p
 
 
-0.503
 
 
15.1
 
 
 
 
AUH
 
 
hsa-miR-23a-3p
 
 
-0.512
 
 
15.1
 
 
 
 
AUH
 
 
hsa-miR-23b-3p
 
 
-0.512
 
 
15.1
 
 
 
 
AXIN2
 
 
hsa-miR-15b-5p
 
 
-0.3
 
 
17.3
 
 
 
 
AZIN1
 
 
hsa-miR-132-3p
 
 
-0.29
 
 
76.3
 
 
 
 
AZIN1
 
 
hsa-miR-132-3p
 
 
-0.299
 
 
76.3
 
 
 
 
B3GNT5
 
 
hsa-miR-30b-5p
 
 
-0.326
 
 
13.1
 
 
 
 
B4GALT5
 
 
hsa-miR-501-3p
 
 
-0.408
 
 
25.6
 
 
 
 
B4GAT1
 
 
hsa-miR-23a-3p
 
 
-0.361
 
 
21.1
 
 
 
 
B4GAT1
 
 
hsa-miR-23b-3p
 
 
-0.361
 
 
21.1
 
 
 
 
BAG4
 
 
hsa-miR-181b-5p
 
 
-0.337
 
 
10.7
 
 
 
 
BAG4
 
 
hsa-miR-181a-5p
 
 
-0.337
 
 
10.7
 
 
 
 
BAK1
 
 
hsa-miR-29a-3p
 
 
-0.409
 
 
10.4
 
 
 
 
BCL2L2
 
 
hsa-miR-29a-3p
 
 
-0.312
 
 
22.1
 
 
 
 
BCOR
 
 
hsa-miR-425-5p
 
 
-0.346
 
 
11.6
 
 
 
 
BECN1
 
 
hsa-miR-30b-5p
 
 
-0.452
 
 
24.6
 
 
 
 
BET1
 
 
hsa-miR-23a-3p
 
 
-0.402
 
 
34.3
 
 
 
 
BET1
 
 
hsa-miR-23b-3p
 
 
-0.418
 
 
34.3
 
 
 
 
BFAR
 
 
hsa-miR-15b-5p
 
 
-0.352
 
 
23.8
 
 
 
 
BHLHE40
 
 
hsa-miR-181b-5p
 
 
-0.408
 
 
67.8
 
 
 
 
BHLHE40
 
 
hsa-miR-181a-5p
 
 
-0.408
 
 
67.8
 
 
 
 
BLCAP
 
 
hsa-miR-23a-3p
 
 
-0.601
 
 
71.4
 
 
 
 
BLCAP
 
 
hsa-miR-23b-3p
 
 
-0.601
 
 
71.4
 
 
 
 
BLMH
 
 
hsa-miR-29a-3p
 
 
-0.393
 
 
25.8
 
 
 
 
BLOC1S6
 
 
hsa-miR-181b-5p
 
 
-0.393
 
 
24.7
 
 
 
 
BLOC1S6
 
 
hsa-miR-181a-5p
 
 
-0.393
 
 
24.7
 
 
 
 
BOLA3
 
 
hsa-miR-15b-5p
 
 
-0.363
 
 
13.3
 
 
 
 
 
 
 prostate 
 
 
 
 
target_symbol
 
 
mature_mirna_id
 
 
score
 
 
nTPM
 
 
 
 
 
 
AAR2
 
 
hsa-miR-29a-3p
 
 
-0.327
 
 
18.7
 
 
 
 
ABCE1
 
 
hsa-miR-29a-3p
 
 
-0.404
 
 
18.2
 
 
 
 
ABHD4
 
 
hsa-miR-29a-3p
 
 
-0.312
 
 
20.4
 
 
 
 
ACTA1
 
 
hsa-miR-155-5p
 
 
-0.595
 
 
152.5
 
 
 
 
ACTC1
 
 
hsa-miR-30b-5p
 
 
-0.543
 
 
68.4
 
 
 
 
ACTN2
 
 
hsa-miR-23a-3p
 
 
-0.294
 
 
14.6
 
 
 
 
ACTN2
 
 
hsa-miR-29a-3p
 
 
-0.311
 
 
14.6
 
 
 
 
ACTR2
 
 
hsa-miR-15b-5p
 
 
-0.293
 
 
67.2
 
 
 
 
ADAM9
 
 
hsa-miR-30b-5p
 
 
-0.312
 
 
32.8
 
 
 
 
ADCY3
 
 
hsa-miR-132-3p
 
 
-0.303
 
 
38.0
 
 
 
 
ADGRA3
 
 
hsa-miR-30b-5p
 
 
-0.304
 
 
11.3
 
 
 
 
ADIPOR2
 
 
hsa-miR-150-5p
 
 
-0.287
 
 
19.8
 
 
 
 
AFG3L2
 
 
hsa-miR-181b-5p
 
 
-0.491
 
 
13.2
 
 
 
 
AFG3L2
 
 
hsa-miR-181a-5p
 
 
-0.491
 
 
13.2
 
 
 
 
AHR
 
 
hsa-miR-29a-3p
 
 
-0.316
 
 
10.3
 
 
 
 
AK1
 
 
hsa-miR-29a-3p
 
 
-0.333
 
 
16.0
 
 
 
 
AK4
 
 
hsa-miR-15b-5p
 
 
-0.316
 
 
14.9
 
 
 
 
AKT3
 
 
hsa-miR-29a-3p
 
 
-0.287
 
 
13.8
 
 
 
 
AKT3
 
 
hsa-miR-15b-5p
 
 
-0.301
 
 
13.8
 
 
 
 
ALAD
 
 
hsa-miR-15b-5p
 
 
-0.446
 
 
32.0
 
 
 
 
ALDH1A2
 
 
hsa-miR-23b-3p
 
 
-0.346
 
 
19.7
 
 
 
 
ALDH1A2
 
 
hsa-miR-23a-3p
 
 
-0.354
 
 
19.7
 
 
 
 
AMOTL1
 
 
hsa-miR-15b-5p
 
 
-0.319
 
 
30.4
 
 
 
 
ANGPTL2
 
 
hsa-miR-221-3p
 
 
-0.369
 
 
23.5
 
 
 
 
ANKFY1
 
 
hsa-miR-155-5p
 
 
-0.315
 
 
11.5
 
 
 
 
ANKHD1
 
 
hsa-miR-23a-3p
 
 
-0.368
 
 
34.8
 
 
 
 
ANKHD1
 
 
hsa-miR-23b-3p
 
 
-0.368
 
 
34.8
 
 
 
 
ANKHD1
 
 
hsa-miR-30b-5p
 
 
-0.405
 
 
34.8
 
 
 
 
ANKRA2
 
 
hsa-miR-30b-5p
 
 
-0.354
 
 
11.6
 
 
 
 
ANKRA2
 
 
hsa-miR-30b-5p
 
 
-0.472
 
 
11.6
 
 
 
 
ANKRD17
 
 
hsa-miR-30b-5p
 
 
-0.336
 
 
14.2
 
 
 
 
ANP32A
 
 
hsa-miR-132-3p
 
 
-0.374
 
 
70.7
 
 
 
 
ANP32E
 
 
hsa-miR-501-3p
 
 
-0.331
 
 
15.2
 
 
 
 
ANTXR2
 
 
hsa-miR-29a-3p
 
 
-0.347
 
 
103.3
 
 
 
 
ANXA11
 
 
hsa-miR-15b-5p
 
 
-0.463
 
 
94.1
 
 
 
 
AP2A1
 
 
hsa-miR-15b-5p
 
 
-0.333
 
 
31.4
 
 
 
 
AP2B1
 
 
hsa-miR-15b-5p
 
 
-0.334
 
 
38.3
 
 
 
 
AP3S1
 
 
hsa-miR-30b-5p
 
 
-0.293
 
 
40.9
 
 
 
 
APOO
 
 
hsa-miR-181b-5p
 
 
-0.5
 
 
12.8
 
 
 
 
APOO
 
 
hsa-miR-181a-5p
 
 
-0.5
 
 
12.8
 
 
 
 
APP
 
 
hsa-miR-15b-5p
 
 
-0.298
 
 
232.5
 
 
 
 
AQP3
 
 
hsa-miR-874-3p
 
 
-0.48
 
 
81.8
 
 
 
 
ARF4
 
 
hsa-miR-30b-5p
 
 
-0.289
 
 
129.9
 
 
 
 
ARF4
 
 
hsa-miR-221-3p
 
 
-0.553
 
 
129.9
 
 
 
 
ARF5
 
 
hsa-miR-29a-3p
 
 
-0.491
 
 
107.0
 
 
 
 
ARF6
 
 
hsa-miR-181b-5p
 
 
-0.33
 
 
30.5
 
 
 
 
ARF6
 
 
hsa-miR-181a-5p
 
 
-0.33
 
 
30.5
 
 
 
 
ARHGAP5
 
 
hsa-miR-486-5p
 
 
-0.3
 
 
28.6
 
 
 
 
ARID1B
 
 
hsa-miR-29a-3p
 
 
-0.342
 
 
11.6
 
 
 
 
ARID4B
 
 
hsa-miR-486-5p
 
 
-0.367
 
 
13.4
 
 
 
 
ARL2
 
 
hsa-miR-15b-5p
 
 
-0.374
 
 
84.5
 
 
 
 
ARL2
 
 
hsa-miR-15b-5p
 
 
-0.442
 
 
84.5
 
 
 
 
ARL4A
 
 
hsa-miR-29a-3p
 
 
-0.386
 
 
19.8
 
 
 
 
ARL4A
 
 
hsa-miR-30b-5p
 
 
-0.448
 
 
19.8
 
 
 
 
ARMC8
 
 
hsa-miR-486-5p
 
 
-0.321
 
 
15.4
 
 
 
 
ARMCX2
 
 
hsa-miR-15b-5p
 
 
-0.344
 
 
26.1
 
 
 
 
ARMCX3
 
 
hsa-miR-425-5p
 
 
-0.325
 
 
41.8
 
 
 
 
ARNT
 
 
hsa-miR-221-3p
 
 
-0.465
 
 
15.5
 
 
 
 
ARPP19
 
 
hsa-miR-15b-5p
 
 
-0.346
 
 
37.0
 
 
 
 
ARPP19
 
 
hsa-miR-29a-3p
 
 
-0.371
 
 
37.0
 
 
 
 
ARRDC3
 
 
hsa-miR-29a-3p
 
 
-0.402
 
 
28.5
 
 
 
 
ASB3
 
 
hsa-miR-30b-5p
 
 
-0.418
 
 
12.1
 
 
 
 
ASB3
 
 
hsa-miR-30b-5p
 
 
-0.418
 
 
12.1
 
 
 
 
ASB6
 
 
hsa-miR-874-3p
 
 
-0.368
 
 
11.8
 
 
 
 
ASH1L
 
 
hsa-miR-15b-5p
 
 
-0.325
 
 
17.4
 
 
 
 
ASPH
 
 
hsa-miR-221-3p
 
 
-0.287
 
 
44.4
 
 
 
 
ATF3
 
 
hsa-miR-874-3p
 
 
-0.288
 
 
108.8
 
 
 
 
ATG5
 
 
hsa-miR-30b-5p
 
 
-0.488
 
 
14.3
 
 
 
 
ATG9A
 
 
hsa-miR-15b-5p
 
 
-0.333
 
 
22.6
 
 
 
 
ATOSA
 
 
hsa-miR-221-3p
 
 
-0.495
 
 
12.7
 
 
 
 
ATP5MC3
 
 
hsa-miR-425-5p
 
 
-0.328
 
 
106.2
 
 
 
 
ATP5MC3
 
 
hsa-miR-29a-3p
 
 
-0.33
 
 
106.2
 
 
 
 
ATP6V1E1
 
 
hsa-miR-23a-3p
 
 
-0.294
 
 
89.0
 
 
 
 
ATP6V1E1
 
 
hsa-miR-23b-3p
 
 
-0.294
 
 
89.0
 
 
 
 
ATPAF1
 
 
hsa-miR-361-5p
 
 
-0.362
 
 
35.2
 
 
 
 
ATXN2
 
 
hsa-miR-15b-5p
 
 
-0.392
 
 
18.8
 
 
 
 
AUH
 
 
hsa-miR-23a-3p
 
 
-0.495
 
 
26.5
 
 
 
 
AUH
 
 
hsa-miR-23b-3p
 
 
-0.503
 
 
26.5
 
 
 
 
AUH
 
 
hsa-miR-23a-3p
 
 
-0.512
 
 
26.5
 
 
 
 
AUH
 
 
hsa-miR-23b-3p
 
 
-0.512
 
 
26.5
 
 
 
 
AXIN2
 
 
hsa-miR-15b-5p
 
 
-0.3
 
 
13.1
 
 
 
 
AZIN1
 
 
hsa-miR-132-3p
 
 
-0.29
 
 
48.5
 
 
 
 
AZIN1
 
 
hsa-miR-132-3p
 
 
-0.299
 
 
48.5
 
 
 
 
B4GALT5
 
 
hsa-miR-501-3p
 
 
-0.408
 
 
16.0
 
 
 
 
B4GAT1
 
 
hsa-miR-23a-3p
 
 
-0.361
 
 
17.0
 
 
 
 
B4GAT1
 
 
hsa-miR-23b-3p
 
 
-0.361
 
 
17.0
 
 
 
 
BAK1
 
 
hsa-miR-29a-3p
 
 
-0.409
 
 
10.5
 
 
 
 
BCL2L2
 
 
hsa-miR-29a-3p
 
 
-0.312
 
 
45.5
 
 
 
 
BCOR
 
 
hsa-miR-425-5p
 
 
-0.346
 
 
10.5
 
 
 
 
BDH1
 
 
hsa-miR-874-3p
 
 
-0.644
 
 
14.7
 
 
 
 
BECN1
 
 
hsa-miR-30b-5p
 
 
-0.452
 
 
39.7
 
 
 
 
BET1
 
 
hsa-miR-23a-3p
 
 
-0.402
 
 
19.5
 
 
 
 
BET1
 
 
hsa-miR-23b-3p
 
 
-0.418
 
 
19.5
 
 
 
 
BFAR
 
 
hsa-miR-15b-5p
 
 
-0.352
 
 
20.0
 
 
 
 
BHLHE40
 
 
hsa-miR-181b-5p
 
 
-0.408
 
 
103.6
 
 
 
 
BHLHE40
 
 
hsa-miR-181a-5p
 
 
-0.408
 
 
103.6
 
 
 
 
BLCAP
 
 
hsa-miR-23a-3p
 
 
-0.601
 
 
58.3
 
 
 
 
BLCAP
 
 
hsa-miR-23b-3p
 
 
-0.601
 
 
58.3
 
 
 
 
BLMH
 
 
hsa-miR-29a-3p
 
 
-0.393
 
 
14.0
 
 
 
 
BLOC1S6
 
 
hsa-miR-181b-5p
 
 
-0.393
 
 
34.2
 
 
 
 
 
 
 rectum 
 
 
 
 
target_symbol
 
 
mature_mirna_id
 
 
score
 
 
nTPM
 
 
 
 
 
 
AAR2
 
 
hsa-miR-29a-3p
 
 
-0.327
 
 
16.3
 
 
 
 
ABCE1
 
 
hsa-miR-29a-3p
 
 
-0.404
 
 
26.0
 
 
 
 
ABHD4
 
 
hsa-miR-29a-3p
 
 
-0.312
 
 
14.1
 
 
 
 
ACTR2
 
 
hsa-miR-15b-5p
 
 
-0.293
 
 
130.5
 
 
 
 
ADAM9
 
 
hsa-miR-30b-5p
 
 
-0.312
 
 
39.2
 
 
 
 
ADCY3
 
 
hsa-miR-132-3p
 
 
-0.303
 
 
14.9
 
 
 
 
ADGRA3
 
 
hsa-miR-30b-5p
 
 
-0.304
 
 
16.1
 
 
 
 
ADIPOR2
 
 
hsa-miR-150-5p
 
 
-0.287
 
 
16.7
 
 
 
 
AFG3L2
 
 
hsa-miR-181b-5p
 
 
-0.491
 
 
28.8
 
 
 
 
AFG3L2
 
 
hsa-miR-181a-5p
 
 
-0.491
 
 
28.8
 
 
 
 
AHR
 
 
hsa-miR-29a-3p
 
 
-0.316
 
 
15.3
 
 
 
 
AK1
 
 
hsa-miR-29a-3p
 
 
-0.333
 
 
37.1
 
 
 
 
AK4
 
 
hsa-miR-15b-5p
 
 
-0.316
 
 
12.8
 
 
 
 
ALAD
 
 
hsa-miR-15b-5p
 
 
-0.446
 
 
23.0
 
 
 
 
ANGPTL2
 
 
hsa-miR-221-3p
 
 
-0.369
 
 
16.8
 
 
 
 
ANKFY1
 
 
hsa-miR-155-5p
 
 
-0.315
 
 
13.1
 
 
 
 
ANKHD1
 
 
hsa-miR-23a-3p
 
 
-0.368
 
 
31.1
 
 
 
 
ANKHD1
 
 
hsa-miR-23b-3p
 
 
-0.368
 
 
31.1
 
 
 
 
ANKHD1
 
 
hsa-miR-30b-5p
 
 
-0.405
 
 
31.1
 
 
 
 
ANKRD17
 
 
hsa-miR-30b-5p
 
 
-0.336
 
 
17.8
 
 
 
 
ANP32A
 
 
hsa-miR-132-3p
 
 
-0.374
 
 
83.5
 
 
 
 
ANP32E
 
 
hsa-miR-501-3p
 
 
-0.331
 
 
17.1
 
 
 
 
ANTXR2
 
 
hsa-miR-29a-3p
 
 
-0.347
 
 
40.1
 
 
 
 
ANXA11
 
 
hsa-miR-15b-5p
 
 
-0.463
 
 
113.3
 
 
 
 
AP2B1
 
 
hsa-miR-15b-5p
 
 
-0.334
 
 
78.9
 
 
 
 
AP3S1
 
 
hsa-miR-30b-5p
 
 
-0.293
 
 
56.4
 
 
 
 
APOO
 
 
hsa-miR-181b-5p
 
 
-0.5
 
 
17.6
 
 
 
 
APOO
 
 
hsa-miR-181a-5p
 
 
-0.5
 
 
17.6
 
 
 
 
APP
 
 
hsa-miR-15b-5p
 
 
-0.298
 
 
296.0
 
 
 
 
ARF4
 
 
hsa-miR-30b-5p
 
 
-0.289
 
 
185.2
 
 
 
 
ARF4
 
 
hsa-miR-221-3p
 
 
-0.553
 
 
185.2
 
 
 
 
ARF5
 
 
hsa-miR-29a-3p
 
 
-0.491
 
 
57.6
 
 
 
 
ARF6
 
 
hsa-miR-181b-5p
 
 
-0.33
 
 
50.6
 
 
 
 
ARF6
 
 
hsa-miR-181a-5p
 
 
-0.33
 
 
50.6
 
 
 
 
ARHGAP5
 
 
hsa-miR-486-5p
 
 
-0.3
 
 
24.8
 
 
 
 
ARID1B
 
 
hsa-miR-29a-3p
 
 
-0.342
 
 
11.4
 
 
 
 
ARL2
 
 
hsa-miR-15b-5p
 
 
-0.374
 
 
30.9
 
 
 
 
ARL2
 
 
hsa-miR-15b-5p
 
 
-0.442
 
 
30.9
 
 
 
 
ARL4A
 
 
hsa-miR-29a-3p
 
 
-0.386
 
 
32.6
 
 
 
 
ARL4A
 
 
hsa-miR-30b-5p
 
 
-0.448
 
 
32.6
 
 
 
 
ARMC8
 
 
hsa-miR-486-5p
 
 
-0.321
 
 
17.2
 
 
 
 
ARMCX3
 
 
hsa-miR-425-5p
 
 
-0.325
 
 
20.5
 
 
 
 
ARNT
 
 
hsa-miR-221-3p
 
 
-0.465
 
 
19.9
 
 
 
 
ARPP19
 
 
hsa-miR-15b-5p
 
 
-0.346
 
 
33.6
 
 
 
 
ARPP19
 
 
hsa-miR-29a-3p
 
 
-0.371
 
 
33.6
 
 
 
 
ARRDC3
 
 
hsa-miR-29a-3p
 
 
-0.402
 
 
25.8
 
 
 
 
ASAP2
 
 
hsa-miR-29a-3p
 
 
-0.348
 
 
15.1
 
 
 
 
ASF1A
 
 
hsa-miR-23a-3p
 
 
-0.506
 
 
10.2
 
 
 
 
ASF1A
 
 
hsa-miR-23b-3p
 
 
-0.506
 
 
10.2
 
 
 
 
ASH1L
 
 
hsa-miR-15b-5p
 
 
-0.325
 
 
10.8
 
 
 
 
ASPH
 
 
hsa-miR-221-3p
 
 
-0.287
 
 
77.9
 
 
 
 
ATF3
 
 
hsa-miR-874-3p
 
 
-0.288
 
 
56.3
 
 
 
 
ATG5
 
 
hsa-miR-30b-5p
 
 
-0.488
 
 
14.3
 
 
 
 
ATG9A
 
 
hsa-miR-15b-5p
 
 
-0.333
 
 
20.5
 
 
 
 
ATOSA
 
 
hsa-miR-221-3p
 
 
-0.495
 
 
15.5
 
 
 
 
ATP5MC3
 
 
hsa-miR-425-5p
 
 
-0.328
 
 
295.3
 
 
 
 
ATP5MC3
 
 
hsa-miR-29a-3p
 
 
-0.33
 
 
295.3
 
 
 
 
ATP6V1E1
 
 
hsa-miR-23a-3p
 
 
-0.294
 
 
93.7
 
 
 
 
ATP6V1E1
 
 
hsa-miR-23b-3p
 
 
-0.294
 
 
93.7
 
 
 
 
ATPAF1
 
 
hsa-miR-361-5p
 
 
-0.362
 
 
50.0
 
 
 
 
ATXN2
 
 
hsa-miR-15b-5p
 
 
-0.392
 
 
10.9
 
 
 
 
AUH
 
 
hsa-miR-23a-3p
 
 
-0.495
 
 
16.1
 
 
 
 
AUH
 
 
hsa-miR-23b-3p
 
 
-0.503
 
 
16.1
 
 
 
 
AUH
 
 
hsa-miR-23a-3p
 
 
-0.512
 
 
16.1
 
 
 
 
AUH
 
 
hsa-miR-23b-3p
 
 
-0.512
 
 
16.1
 
 
 
 
AXIN2
 
 
hsa-miR-15b-5p
 
 
-0.3
 
 
13.7
 
 
 
 
AZIN1
 
 
hsa-miR-132-3p
 
 
-0.29
 
 
49.7
 
 
 
 
AZIN1
 
 
hsa-miR-132-3p
 
 
-0.299
 
 
49.7
 
 
 
 
B3GNT5
 
 
hsa-miR-30b-5p
 
 
-0.326
 
 
18.4
 
 
 
 
B4GALT5
 
 
hsa-miR-501-3p
 
 
-0.408
 
 
22.3
 
 
 
 
BAK1
 
 
hsa-miR-29a-3p
 
 
-0.409
 
 
23.4
 
 
 
 
BCL2L11
 
 
hsa-miR-221-3p
 
 
-0.423
 
 
11.2
 
 
 
 
BCL2L2
 
 
hsa-miR-29a-3p
 
 
-0.312
 
 
30.3
 
 
 
 
BDH1
 
 
hsa-miR-874-3p
 
 
-0.644
 
 
18.6
 
 
 
 
BECN1
 
 
hsa-miR-30b-5p
 
 
-0.452
 
 
60.8
 
 
 
 
BET1
 
 
hsa-miR-23a-3p
 
 
-0.402
 
 
16.0
 
 
 
 
BET1
 
 
hsa-miR-23b-3p
 
 
-0.418
 
 
16.0
 
 
 
 
BFAR
 
 
hsa-miR-15b-5p
 
 
-0.352
 
 
14.4
 
 
 
 
BHLHE40
 
 
hsa-miR-181b-5p
 
 
-0.408
 
 
44.6
 
 
 
 
BHLHE40
 
 
hsa-miR-181a-5p
 
 
-0.408
 
 
44.6
 
 
 
 
BLCAP
 
 
hsa-miR-23a-3p
 
 
-0.601
 
 
49.0
 
 
 
 
BLCAP
 
 
hsa-miR-23b-3p
 
 
-0.601
 
 
49.0
 
 
 
 
BLMH
 
 
hsa-miR-29a-3p
 
 
-0.393
 
 
14.5
 
 
 
 
BLOC1S6
 
 
hsa-miR-181b-5p
 
 
-0.393
 
 
20.2
 
 
 
 
BLOC1S6
 
 
hsa-miR-181a-5p
 
 
-0.393
 
 
20.2
 
 
 
 
BOLA3
 
 
hsa-miR-15b-5p
 
 
-0.363
 
 
35.3
 
 
 
 
BOLA3
 
 
hsa-miR-15b-5p
 
 
-0.386
 
 
35.3
 
 
 
 
BOLA3
 
 
hsa-miR-374b-5p
 
 
-0.486
 
 
35.3
 
 
 
 
BRI3
 
 
hsa-miR-132-3p
 
 
-0.385
 
 
33.8
 
 
 
 
BTAF1
 
 
hsa-miR-486-5p
 
 
-0.288
 
 
12.5
 
 
 
 
BTAF1
 
 
hsa-miR-15b-5p
 
 
-0.496
 
 
12.5
 
 
 
 
BTF3
 
 
hsa-miR-15b-5p
 
 
-0.344
 
 
506.7
 
 
 
 
BTF3
 
 
hsa-miR-361-5p
 
 
-0.551
 
 
506.7
 
 
 
 
BTG2
 
 
hsa-miR-29a-3p
 
 
-0.32
 
 
193.5
 
 
 
 
BTN2A1
 
 
hsa-miR-342-3p
 
 
-0.31
 
 
10.6
 
 
 
 
BZW1
 
 
hsa-miR-15b-5p
 
 
-0.367
 
 
85.0
 
 
 
 
C11orf24
 
 
hsa-miR-15b-5p
 
 
-0.403
 
 
16.3
 
 
 
 
C11orf68
 
 
hsa-miR-15b-5p
 
 
-0.29
 
 
14.7
 
 
 
 
C2orf88
 
 
hsa-miR-29a-3p
 
 
-0.32
 
 
36.2
 
 
 
 
C5orf15
 
 
hsa-miR-29a-3p
 
 
-0.392
 
 
31.1
 
 
 
 
 
 
 salivary gland 
 
 
 
 
target_symbol
 
 
mature_mirna_id
 
 
score
 
 
nTPM
 
 
 
 
 
 
AAR2
 
 
hsa-miR-29a-3p
 
 
-0.327
 
 
17.0
 
 
 
 
ABCE1
 
 
hsa-miR-29a-3p
 
 
-0.404
 
 
22.7
 
 
 
 
ABHD4
 
 
hsa-miR-29a-3p
 
 
-0.312
 
 
17.2
 
 
 
 
ABHD5
 
 
hsa-miR-132-3p
 
 
-0.323
 
 
19.4
 
 
 
 
ACTA1
 
 
hsa-miR-155-5p
 
 
-0.595
 
 
552.8
 
 
 
 
ACTC1
 
 
hsa-miR-30b-5p
 
 
-0.543
 
 
41.7
 
 
 
 
ACTN2
 
 
hsa-miR-23a-3p
 
 
-0.294
 
 
18.4
 
 
 
 
ACTN2
 
 
hsa-miR-29a-3p
 
 
-0.311
 
 
18.4
 
 
 
 
ACTR2
 
 
hsa-miR-15b-5p
 
 
-0.293
 
 
60.3
 
 
 
 
ADAM9
 
 
hsa-miR-30b-5p
 
 
-0.312
 
 
39.8
 
 
 
 
ADCY3
 
 
hsa-miR-132-3p
 
 
-0.303
 
 
20.4
 
 
 
 
ADIPOR2
 
 
hsa-miR-150-5p
 
 
-0.287
 
 
21.2
 
 
 
 
AFG3L2
 
 
hsa-miR-181b-5p
 
 
-0.491
 
 
15.5
 
 
 
 
AFG3L2
 
 
hsa-miR-181a-5p
 
 
-0.491
 
 
15.5
 
 
 
 
AK1
 
 
hsa-miR-29a-3p
 
 
-0.333
 
 
38.2
 
 
 
 
ALAD
 
 
hsa-miR-15b-5p
 
 
-0.446
 
 
92.9
 
 
 
 
ALOX12
 
 
hsa-miR-15b-5p
 
 
-0.443
 
 
17.3
 
 
 
 
AMOTL1
 
 
hsa-miR-15b-5p
 
 
-0.319
 
 
10.4
 
 
 
 
ANGPTL2
 
 
hsa-miR-221-3p
 
 
-0.369
 
 
17.1
 
 
 
 
ANKFY1
 
 
hsa-miR-155-5p
 
 
-0.315
 
 
12.9
 
 
 
 
ANKHD1
 
 
hsa-miR-23a-3p
 
 
-0.368
 
 
40.4
 
 
 
 
ANKHD1
 
 
hsa-miR-23b-3p
 
 
-0.368
 
 
40.4
 
 
 
 
ANKHD1
 
 
hsa-miR-30b-5p
 
 
-0.405
 
 
40.4
 
 
 
 
ANKRD17
 
 
hsa-miR-30b-5p
 
 
-0.336
 
 
15.7
 
 
 
 
ANP32A
 
 
hsa-miR-132-3p
 
 
-0.374
 
 
50.8
 
 
 
 
ANP32E
 
 
hsa-miR-501-3p
 
 
-0.331
 
 
12.1
 
 
 
 
ANTXR2
 
 
hsa-miR-29a-3p
 
 
-0.347
 
 
11.4
 
 
 
 
ANXA11
 
 
hsa-miR-15b-5p
 
 
-0.463
 
 
87.6
 
 
 
 
AP2A1
 
 
hsa-miR-15b-5p
 
 
-0.333
 
 
27.6
 
 
 
 
AP2B1
 
 
hsa-miR-15b-5p
 
 
-0.334
 
 
39.5
 
 
 
 
AP3S1
 
 
hsa-miR-30b-5p
 
 
-0.293
 
 
56.0
 
 
 
 
APOO
 
 
hsa-miR-181b-5p
 
 
-0.5
 
 
18.5
 
 
 
 
APOO
 
 
hsa-miR-181a-5p
 
 
-0.5
 
 
18.5
 
 
 
 
APP
 
 
hsa-miR-15b-5p
 
 
-0.298
 
 
256.4
 
 
 
 
AQP3
 
 
hsa-miR-874-3p
 
 
-0.48
 
 
376.5
 
 
 
 
ARF4
 
 
hsa-miR-30b-5p
 
 
-0.289
 
 
171.4
 
 
 
 
ARF4
 
 
hsa-miR-221-3p
 
 
-0.553
 
 
171.4
 
 
 
 
ARF5
 
 
hsa-miR-29a-3p
 
 
-0.491
 
 
110.8
 
 
 
 
ARF6
 
 
hsa-miR-181b-5p
 
 
-0.33
 
 
33.7
 
 
 
 
ARF6
 
 
hsa-miR-181a-5p
 
 
-0.33
 
 
33.7
 
 
 
 
ARHGAP5
 
 
hsa-miR-486-5p
 
 
-0.3
 
 
56.3
 
 
 
 
ARID1B
 
 
hsa-miR-29a-3p
 
 
-0.342
 
 
18.1
 
 
 
 
ARID4B
 
 
hsa-miR-486-5p
 
 
-0.367
 
 
10.8
 
 
 
 
ARL2
 
 
hsa-miR-15b-5p
 
 
-0.374
 
 
67.7
 
 
 
 
ARL2
 
 
hsa-miR-15b-5p
 
 
-0.442
 
 
67.7
 
 
 
 
ARL4A
 
 
hsa-miR-29a-3p
 
 
-0.386
 
 
81.5
 
 
 
 
ARL4A
 
 
hsa-miR-30b-5p
 
 
-0.448
 
 
81.5
 
 
 
 
ARMC8
 
 
hsa-miR-486-5p
 
 
-0.321
 
 
11.8
 
 
 
 
ARMCX2
 
 
hsa-miR-15b-5p
 
 
-0.344
 
 
42.0
 
 
 
 
ARMCX3
 
 
hsa-miR-425-5p
 
 
-0.325
 
 
56.1
 
 
 
 
ARNT
 
 
hsa-miR-221-3p
 
 
-0.465
 
 
15.0
 
 
 
 
ARPP19
 
 
hsa-miR-15b-5p
 
 
-0.346
 
 
28.7
 
 
 
 
ARPP19
 
 
hsa-miR-29a-3p
 
 
-0.371
 
 
28.7
 
 
 
 
ARRDC3
 
 
hsa-miR-29a-3p
 
 
-0.402
 
 
60.1
 
 
 
 
ASB6
 
 
hsa-miR-874-3p
 
 
-0.368
 
 
11.9
 
 
 
 
ASF1A
 
 
hsa-miR-23a-3p
 
 
-0.506
 
 
10.1
 
 
 
 
ASF1A
 
 
hsa-miR-23b-3p
 
 
-0.506
 
 
10.1
 
 
 
 
ASH1L
 
 
hsa-miR-15b-5p
 
 
-0.325
 
 
17.7
 
 
 
 
ASPH
 
 
hsa-miR-221-3p
 
 
-0.287
 
 
95.6
 
 
 
 
ATF3
 
 
hsa-miR-874-3p
 
 
-0.288
 
 
34.0
 
 
 
 
ATG9A
 
 
hsa-miR-15b-5p
 
 
-0.333
 
 
22.5
 
 
 
 
ATOSA
 
 
hsa-miR-221-3p
 
 
-0.495
 
 
14.6
 
 
 
 
ATP5MC3
 
 
hsa-miR-425-5p
 
 
-0.328
 
 
173.5
 
 
 
 
ATP5MC3
 
 
hsa-miR-29a-3p
 
 
-0.33
 
 
173.5
 
 
 
 
ATP6V1E1
 
 
hsa-miR-23a-3p
 
 
-0.294
 
 
83.2
 
 
 
 
ATP6V1E1
 
 
hsa-miR-23b-3p
 
 
-0.294
 
 
83.2
 
 
 
 
ATPAF1
 
 
hsa-miR-361-5p
 
 
-0.362
 
 
54.3
 
 
 
 
ATXN2
 
 
hsa-miR-15b-5p
 
 
-0.392
 
 
14.4
 
 
 
 
AUH
 
 
hsa-miR-23a-3p
 
 
-0.495
 
 
24.6
 
 
 
 
AUH
 
 
hsa-miR-23b-3p
 
 
-0.503
 
 
24.6
 
 
 
 
AUH
 
 
hsa-miR-23a-3p
 
 
-0.512
 
 
24.6
 
 
 
 
AUH
 
 
hsa-miR-23b-3p
 
 
-0.512
 
 
24.6
 
 
 
 
AZIN1
 
 
hsa-miR-132-3p
 
 
-0.29
 
 
44.6
 
 
 
 
AZIN1
 
 
hsa-miR-132-3p
 
 
-0.299
 
 
44.6
 
 
 
 
B4GALT5
 
 
hsa-miR-501-3p
 
 
-0.408
 
 
18.2
 
 
 
 
B4GAT1
 
 
hsa-miR-23a-3p
 
 
-0.361
 
 
14.9
 
 
 
 
B4GAT1
 
 
hsa-miR-23b-3p
 
 
-0.361
 
 
14.9
 
 
 
 
BAK1
 
 
hsa-miR-29a-3p
 
 
-0.409
 
 
11.3
 
 
 
 
BCL2L2
 
 
hsa-miR-29a-3p
 
 
-0.312
 
 
48.3
 
 
 
 
BCL2L2-PABPN1
 
 
hsa-miR-425-5p
 
 
-0.321
 
 
16.1
 
 
 
 
BDH1
 
 
hsa-miR-874-3p
 
 
-0.644
 
 
22.2
 
 
 
 
BECN1
 
 
hsa-miR-30b-5p
 
 
-0.452
 
 
34.9
 
 
 
 
BET1
 
 
hsa-miR-23a-3p
 
 
-0.402
 
 
24.7
 
 
 
 
BET1
 
 
hsa-miR-23b-3p
 
 
-0.418
 
 
24.7
 
 
 
 
BFAR
 
 
hsa-miR-15b-5p
 
 
-0.352
 
 
22.1
 
 
 
 
BHLHE40
 
 
hsa-miR-181b-5p
 
 
-0.408
 
 
123.4
 
 
 
 
BHLHE40
 
 
hsa-miR-181a-5p
 
 
-0.408
 
 
123.4
 
 
 
 
BLCAP
 
 
hsa-miR-23a-3p
 
 
-0.601
 
 
55.7
 
 
 
 
BLCAP
 
 
hsa-miR-23b-3p
 
 
-0.601
 
 
55.7
 
 
 
 
BLMH
 
 
hsa-miR-29a-3p
 
 
-0.393
 
 
15.3
 
 
 
 
BLOC1S6
 
 
hsa-miR-181b-5p
 
 
-0.393
 
 
19.5
 
 
 
 
BLOC1S6
 
 
hsa-miR-181a-5p
 
 
-0.393
 
 
19.5
 
 
 
 
BMF
 
 
hsa-miR-29a-3p
 
 
-0.41
 
 
10.0
 
 
 
 
BMF
 
 
hsa-miR-221-3p
 
 
-0.453
 
 
10.0
 
 
 
 
BOLA3
 
 
hsa-miR-15b-5p
 
 
-0.363
 
 
37.3
 
 
 
 
BOLA3
 
 
hsa-miR-15b-5p
 
 
-0.386
 
 
37.3
 
 
 
 
BOLA3
 
 
hsa-miR-374b-5p
 
 
-0.486
 
 
37.3
 
 
 
 
BRI3
 
 
hsa-miR-132-3p
 
 
-0.385
 
 
83.9
 
 
 
 
BTAF1
 
 
hsa-miR-486-5p
 
 
-0.288
 
 
15.9
 
 
 
 
BTAF1
 
 
hsa-miR-15b-5p
 
 
-0.496
 
 
15.9
 
 
 
 
 
 
 seminal vesicle 
 
 
 
 
target_symbol
 
 
mature_mirna_id
 
 
score
 
 
nTPM
 
 
 
 
 
 
AAR2
 
 
hsa-miR-29a-3p
 
 
-0.327
 
 
19.9
 
 
 
 
ABCE1
 
 
hsa-miR-29a-3p
 
 
-0.404
 
 
15.9
 
 
 
 
ABHD4
 
 
hsa-miR-29a-3p
 
 
-0.312
 
 
34.6
 
 
 
 
ACTC1
 
 
hsa-miR-30b-5p
 
 
-0.543
 
 
506.6
 
 
 
 
ACTR2
 
 
hsa-miR-15b-5p
 
 
-0.293
 
 
57.7
 
 
 
 
ACVR1
 
 
hsa-miR-30b-5p
 
 
-0.29
 
 
11.3
 
 
 
 
ADAM9
 
 
hsa-miR-30b-5p
 
 
-0.312
 
 
32.8
 
 
 
 
ADCY3
 
 
hsa-miR-132-3p
 
 
-0.303
 
 
31.0
 
 
 
 
ADGRA3
 
 
hsa-miR-30b-5p
 
 
-0.304
 
 
10.8
 
 
 
 
ADIPOR2
 
 
hsa-miR-150-5p
 
 
-0.287
 
 
11.2
 
 
 
 
AFG3L2
 
 
hsa-miR-181b-5p
 
 
-0.491
 
 
11.8
 
 
 
 
AFG3L2
 
 
hsa-miR-181a-5p
 
 
-0.491
 
 
11.8
 
 
 
 
AHR
 
 
hsa-miR-29a-3p
 
 
-0.316
 
 
10.6
 
 
 
 
AK1
 
 
hsa-miR-29a-3p
 
 
-0.333
 
 
33.8
 
 
 
 
AK4
 
 
hsa-miR-15b-5p
 
 
-0.316
 
 
11.8
 
 
 
 
AKT3
 
 
hsa-miR-29a-3p
 
 
-0.287
 
 
14.7
 
 
 
 
AKT3
 
 
hsa-miR-15b-5p
 
 
-0.301
 
 
14.7
 
 
 
 
ALAD
 
 
hsa-miR-15b-5p
 
 
-0.446
 
 
22.8
 
 
 
 
ALDH1A2
 
 
hsa-miR-23b-3p
 
 
-0.346
 
 
50.2
 
 
 
 
ALDH1A2
 
 
hsa-miR-23a-3p
 
 
-0.354
 
 
50.2
 
 
 
 
AMOT
 
 
hsa-miR-29a-3p
 
 
-0.304
 
 
24.6
 
 
 
 
AMOTL1
 
 
hsa-miR-15b-5p
 
 
-0.319
 
 
21.7
 
 
 
 
ANGPTL2
 
 
hsa-miR-221-3p
 
 
-0.369
 
 
25.5
 
 
 
 
ANKFY1
 
 
hsa-miR-155-5p
 
 
-0.315
 
 
11.5
 
 
 
 
ANKHD1
 
 
hsa-miR-23a-3p
 
 
-0.368
 
 
26.1
 
 
 
 
ANKHD1
 
 
hsa-miR-23b-3p
 
 
-0.368
 
 
26.1
 
 
 
 
ANKHD1
 
 
hsa-miR-30b-5p
 
 
-0.405
 
 
26.1
 
 
 
 
ANKRD17
 
 
hsa-miR-30b-5p
 
 
-0.336
 
 
13.0
 
 
 
 
ANP32A
 
 
hsa-miR-132-3p
 
 
-0.374
 
 
62.9
 
 
 
 
ANP32E
 
 
hsa-miR-501-3p
 
 
-0.331
 
 
23.1
 
 
 
 
ANTXR2
 
 
hsa-miR-29a-3p
 
 
-0.347
 
 
85.0
 
 
 
 
ANXA11
 
 
hsa-miR-15b-5p
 
 
-0.463
 
 
77.1
 
 
 
 
AP2A1
 
 
hsa-miR-15b-5p
 
 
-0.333
 
 
17.5
 
 
 
 
AP2B1
 
 
hsa-miR-15b-5p
 
 
-0.334
 
 
47.2
 
 
 
 
AP3S1
 
 
hsa-miR-30b-5p
 
 
-0.293
 
 
80.7
 
 
 
 
APOO
 
 
hsa-miR-181b-5p
 
 
-0.5
 
 
14.1
 
 
 
 
APOO
 
 
hsa-miR-181a-5p
 
 
-0.5
 
 
14.1
 
 
 
 
APP
 
 
hsa-miR-15b-5p
 
 
-0.298
 
 
315.9
 
 
 
 
AQP3
 
 
hsa-miR-874-3p
 
 
-0.48
 
 
34.7
 
 
 
 
ARF4
 
 
hsa-miR-30b-5p
 
 
-0.289
 
 
125.1
 
 
 
 
ARF4
 
 
hsa-miR-221-3p
 
 
-0.553
 
 
125.1
 
 
 
 
ARF5
 
 
hsa-miR-29a-3p
 
 
-0.491
 
 
67.1
 
 
 
 
ARF6
 
 
hsa-miR-181b-5p
 
 
-0.33
 
 
18.1
 
 
 
 
ARF6
 
 
hsa-miR-181a-5p
 
 
-0.33
 
 
18.1
 
 
 
 
ARHGAP5
 
 
hsa-miR-486-5p
 
 
-0.3
 
 
27.6
 
 
 
 
ARID1B
 
 
hsa-miR-29a-3p
 
 
-0.342
 
 
11.6
 
 
 
 
ARID4B
 
 
hsa-miR-486-5p
 
 
-0.367
 
 
10.2
 
 
 
 
ARL2
 
 
hsa-miR-15b-5p
 
 
-0.374
 
 
62.7
 
 
 
 
ARL2
 
 
hsa-miR-15b-5p
 
 
-0.442
 
 
62.7
 
 
 
 
ARL4A
 
 
hsa-miR-29a-3p
 
 
-0.386
 
 
15.5
 
 
 
 
ARL4A
 
 
hsa-miR-30b-5p
 
 
-0.448
 
 
15.5
 
 
 
 
ARMC8
 
 
hsa-miR-486-5p
 
 
-0.321
 
 
16.5
 
 
 
 
ARMCX2
 
 
hsa-miR-15b-5p
 
 
-0.344
 
 
64.0
 
 
 
 
ARMCX3
 
 
hsa-miR-425-5p
 
 
-0.325
 
 
44.1
 
 
 
 
ARNT
 
 
hsa-miR-221-3p
 
 
-0.465
 
 
16.6
 
 
 
 
ARPP19
 
 
hsa-miR-15b-5p
 
 
-0.346
 
 
38.5
 
 
 
 
ARPP19
 
 
hsa-miR-29a-3p
 
 
-0.371
 
 
38.5
 
 
 
 
ARRDC3
 
 
hsa-miR-29a-3p
 
 
-0.402
 
 
22.9
 
 
 
 
ASF1A
 
 
hsa-miR-23a-3p
 
 
-0.506
 
 
10.4
 
 
 
 
ASF1A
 
 
hsa-miR-23b-3p
 
 
-0.506
 
 
10.4
 
 
 
 
ASH1L
 
 
hsa-miR-15b-5p
 
 
-0.325
 
 
14.5
 
 
 
 
ASPH
 
 
hsa-miR-221-3p
 
 
-0.287
 
 
65.8
 
 
 
 
ATF3
 
 
hsa-miR-874-3p
 
 
-0.288
 
 
22.7
 
 
 
 
ATG5
 
 
hsa-miR-30b-5p
 
 
-0.488
 
 
12.3
 
 
 
 
ATG9A
 
 
hsa-miR-15b-5p
 
 
-0.333
 
 
21.4
 
 
 
 
ATOSA
 
 
hsa-miR-221-3p
 
 
-0.495
 
 
14.4
 
 
 
 
ATP5MC3
 
 
hsa-miR-425-5p
 
 
-0.328
 
 
107.3
 
 
 
 
ATP5MC3
 
 
hsa-miR-29a-3p
 
 
-0.33
 
 
107.3
 
 
 
 
ATP6V1E1
 
 
hsa-miR-23a-3p
 
 
-0.294
 
 
119.1
 
 
 
 
ATP6V1E1
 
 
hsa-miR-23b-3p
 
 
-0.294
 
 
119.1
 
 
 
 
ATPAF1
 
 
hsa-miR-361-5p
 
 
-0.362
 
 
31.8
 
 
 
 
ATXN2
 
 
hsa-miR-15b-5p
 
 
-0.392
 
 
14.7
 
 
 
 
AUH
 
 
hsa-miR-23a-3p
 
 
-0.495
 
 
13.9
 
 
 
 
AUH
 
 
hsa-miR-23b-3p
 
 
-0.503
 
 
13.9
 
 
 
 
AUH
 
 
hsa-miR-23a-3p
 
 
-0.512
 
 
13.9
 
 
 
 
AUH
 
 
hsa-miR-23b-3p
 
 
-0.512
 
 
13.9
 
 
 
 
AZIN1
 
 
hsa-miR-132-3p
 
 
-0.29
 
 
46.0
 
 
 
 
AZIN1
 
 
hsa-miR-132-3p
 
 
-0.299
 
 
46.0
 
 
 
 
B4GALT5
 
 
hsa-miR-501-3p
 
 
-0.408
 
 
20.7
 
 
 
 
B4GAT1
 
 
hsa-miR-23a-3p
 
 
-0.361
 
 
21.2
 
 
 
 
B4GAT1
 
 
hsa-miR-23b-3p
 
 
-0.361
 
 
21.2
 
 
 
 
BCL2L2
 
 
hsa-miR-29a-3p
 
 
-0.312
 
 
43.2
 
 
 
 
BCL2L2-PABPN1
 
 
hsa-miR-425-5p
 
 
-0.321
 
 
13.8
 
 
 
 
BECN1
 
 
hsa-miR-30b-5p
 
 
-0.452
 
 
38.9
 
 
 
 
BET1
 
 
hsa-miR-23a-3p
 
 
-0.402
 
 
19.9
 
 
 
 
BET1
 
 
hsa-miR-23b-3p
 
 
-0.418
 
 
19.9
 
 
 
 
BFAR
 
 
hsa-miR-15b-5p
 
 
-0.352
 
 
12.8
 
 
 
 
BHLHE40
 
 
hsa-miR-181b-5p
 
 
-0.408
 
 
30.6
 
 
 
 
BHLHE40
 
 
hsa-miR-181a-5p
 
 
-0.408
 
 
30.6
 
 
 
 
BLCAP
 
 
hsa-miR-23a-3p
 
 
-0.601
 
 
53.1
 
 
 
 
BLCAP
 
 
hsa-miR-23b-3p
 
 
-0.601
 
 
53.1
 
 
 
 
BLMH
 
 
hsa-miR-29a-3p
 
 
-0.393
 
 
13.6
 
 
 
 
BLOC1S6
 
 
hsa-miR-181b-5p
 
 
-0.393
 
 
19.2
 
 
 
 
BLOC1S6
 
 
hsa-miR-181a-5p
 
 
-0.393
 
 
19.2
 
 
 
 
BOLA3
 
 
hsa-miR-15b-5p
 
 
-0.363
 
 
22.2
 
 
 
 
BOLA3
 
 
hsa-miR-15b-5p
 
 
-0.386
 
 
22.2
 
 
 
 
BOLA3
 
 
hsa-miR-374b-5p
 
 
-0.486
 
 
22.2
 
 
 
 
BRI3
 
 
hsa-miR-132-3p
 
 
-0.385
 
 
24.9
 
 
 
 
BTAF1
 
 
hsa-miR-486-5p
 
 
-0.288
 
 
10.1
 
 
 
 
BTAF1
 
 
hsa-miR-15b-5p
 
 
-0.496
 
 
10.1
 
 
 
 
 
 
 skeletal muscle 
 
 
 
 
target_symbol
 
 
mature_mirna_id
 
 
score
 
 
nTPM
 
 
 
 
 
 
AAR2
 
 
hsa-miR-29a-3p
 
 
-0.327
 
 
22.2
 
 
 
 
ABCB6
 
 
hsa-miR-29a-3p
 
 
-0.48
 
 
26.6
 
 
 
 
ABCE1
 
 
hsa-miR-29a-3p
 
 
-0.404
 
 
27.0
 
 
 
 
ABHD17B
 
 
hsa-miR-486-5p
 
 
-0.348
 
 
12.5
 
 
 
 
ABHD18
 
 
hsa-miR-29a-3p
 
 
-0.321
 
 
10.1
 
 
 
 
ABHD18
 
 
hsa-miR-29a-3p
 
 
-0.34
 
 
10.1
 
 
 
 
ABHD4
 
 
hsa-miR-29a-3p
 
 
-0.312
 
 
26.2
 
 
 
 
ABHD5
 
 
hsa-miR-132-3p
 
 
-0.323
 
 
12.3
 
 
 
 
ACTA1
 
 
hsa-miR-155-5p
 
 
-0.595
 
 
123974.2
 
 
 
 
ACTC1
 
 
hsa-miR-30b-5p
 
 
-0.543
 
 
519.7
 
 
 
 
ACTN2
 
 
hsa-miR-23a-3p
 
 
-0.294
 
 
1961.6
 
 
 
 
ACTN2
 
 
hsa-miR-29a-3p
 
 
-0.311
 
 
1961.6
 
 
 
 
ACTR2
 
 
hsa-miR-15b-5p
 
 
-0.293
 
 
56.5
 
 
 
 
ACVR1
 
 
hsa-miR-30b-5p
 
 
-0.29
 
 
15.5
 
 
 
 
ADAM9
 
 
hsa-miR-30b-5p
 
 
-0.312
 
 
16.2
 
 
 
 
ADCY3
 
 
hsa-miR-132-3p
 
 
-0.303
 
 
14.1
 
 
 
 
ADGRA3
 
 
hsa-miR-30b-5p
 
 
-0.304
 
 
15.9
 
 
 
 
ADIPOR2
 
 
hsa-miR-150-5p
 
 
-0.287
 
 
41.4
 
 
 
 
ADRB2
 
 
hsa-miR-15b-5p
 
 
-0.29
 
 
10.9
 
 
 
 
AFG3L2
 
 
hsa-miR-181b-5p
 
 
-0.491
 
 
96.6
 
 
 
 
AFG3L2
 
 
hsa-miR-181a-5p
 
 
-0.491
 
 
96.6
 
 
 
 
AGO2
 
 
hsa-miR-132-3p
 
 
-0.406
 
 
16.4
 
 
 
 
AGO2
 
 
hsa-miR-23a-3p
 
 
-0.576
 
 
16.4
 
 
 
 
AGO2
 
 
hsa-miR-23b-3p
 
 
-0.587
 
 
16.4
 
 
 
 
AGO2
 
 
hsa-miR-30b-5p
 
 
-1.034
 
 
16.4
 
 
 
 
AGO4
 
 
hsa-miR-221-3p
 
 
-0.321
 
 
12.4
 
 
 
 
AK1
 
 
hsa-miR-29a-3p
 
 
-0.333
 
 
1262.8
 
 
 
 
ALAD
 
 
hsa-miR-15b-5p
 
 
-0.446
 
 
26.9
 
 
 
 
ALDH1A2
 
 
hsa-miR-23b-3p
 
 
-0.346
 
 
10.0
 
 
 
 
ALDH1A2
 
 
hsa-miR-23a-3p
 
 
-0.354
 
 
10.0
 
 
 
 
AMOT
 
 
hsa-miR-29a-3p
 
 
-0.304
 
 
37.2
 
 
 
 
AMOTL1
 
 
hsa-miR-15b-5p
 
 
-0.319
 
 
104.6
 
 
 
 
ANGPTL2
 
 
hsa-miR-221-3p
 
 
-0.369
 
 
55.7
 
 
 
 
ANKFY1
 
 
hsa-miR-155-5p
 
 
-0.315
 
 
13.2
 
 
 
 
ANKHD1
 
 
hsa-miR-23a-3p
 
 
-0.368
 
 
67.6
 
 
 
 
ANKHD1
 
 
hsa-miR-23b-3p
 
 
-0.368
 
 
67.6
 
 
 
 
ANKHD1
 
 
hsa-miR-30b-5p
 
 
-0.405
 
 
67.6
 
 
 
 
ANKRD17
 
 
hsa-miR-30b-5p
 
 
-0.336
 
 
21.8
 
 
 
 
ANP32A
 
 
hsa-miR-132-3p
 
 
-0.374
 
 
57.9
 
 
 
 
ANP32E
 
 
hsa-miR-501-3p
 
 
-0.331
 
 
46.6
 
 
 
 
ANTXR2
 
 
hsa-miR-29a-3p
 
 
-0.347
 
 
12.3
 
 
 
 
ANXA11
 
 
hsa-miR-15b-5p
 
 
-0.463
 
 
177.1
 
 
 
 
AP2A1
 
 
hsa-miR-15b-5p
 
 
-0.333
 
 
85.4
 
 
 
 
AP2B1
 
 
hsa-miR-15b-5p
 
 
-0.334
 
 
49.8
 
 
 
 
AP3S1
 
 
hsa-miR-30b-5p
 
 
-0.293
 
 
25.5
 
 
 
 
APOO
 
 
hsa-miR-181b-5p
 
 
-0.5
 
 
43.1
 
 
 
 
APOO
 
 
hsa-miR-181a-5p
 
 
-0.5
 
 
43.1
 
 
 
 
APP
 
 
hsa-miR-15b-5p
 
 
-0.298
 
 
99.7
 
 
 
 
ARF4
 
 
hsa-miR-30b-5p
 
 
-0.289
 
 
59.5
 
 
 
 
ARF4
 
 
hsa-miR-221-3p
 
 
-0.553
 
 
59.5
 
 
 
 
ARF5
 
 
hsa-miR-29a-3p
 
 
-0.491
 
 
145.7
 
 
 
 
ARF6
 
 
hsa-miR-181b-5p
 
 
-0.33
 
 
21.4
 
 
 
 
ARF6
 
 
hsa-miR-181a-5p
 
 
-0.33
 
 
21.4
 
 
 
 
ARHGAP5
 
 
hsa-miR-486-5p
 
 
-0.3
 
 
11.9
 
 
 
 
ARID1B
 
 
hsa-miR-29a-3p
 
 
-0.342
 
 
30.4
 
 
 
 
ARL2
 
 
hsa-miR-15b-5p
 
 
-0.374
 
 
216.4
 
 
 
 
ARL2
 
 
hsa-miR-15b-5p
 
 
-0.442
 
 
216.4
 
 
 
 
ARMC8
 
 
hsa-miR-486-5p
 
 
-0.321
 
 
36.6
 
 
 
 
ARMCX3
 
 
hsa-miR-425-5p
 
 
-0.325
 
 
28.7
 
 
 
 
ARNT
 
 
hsa-miR-221-3p
 
 
-0.465
 
 
30.3
 
 
 
 
ARPP19
 
 
hsa-miR-15b-5p
 
 
-0.346
 
 
225.3
 
 
 
 
ARPP19
 
 
hsa-miR-29a-3p
 
 
-0.371
 
 
225.3
 
 
 
 
ARPP21
 
 
hsa-miR-15b-5p
 
 
-0.342
 
 
48.5
 
 
 
 
ARPP21
 
 
hsa-miR-501-3p
 
 
-0.546
 
 
48.5
 
 
 
 
ARRDC3
 
 
hsa-miR-29a-3p
 
 
-0.402
 
 
31.8
 
 
 
 
ASF1A
 
 
hsa-miR-23a-3p
 
 
-0.506
 
 
15.6
 
 
 
 
ASF1A
 
 
hsa-miR-23b-3p
 
 
-0.506
 
 
15.6
 
 
 
 
ASH1L
 
 
hsa-miR-15b-5p
 
 
-0.325
 
 
33.7
 
 
 
 
ASPH
 
 
hsa-miR-221-3p
 
 
-0.287
 
 
331.0
 
 
 
 
ATF3
 
 
hsa-miR-874-3p
 
 
-0.288
 
 
30.3
 
 
 
 
ATG5
 
 
hsa-miR-30b-5p
 
 
-0.488
 
 
12.0
 
 
 
 
ATG9A
 
 
hsa-miR-15b-5p
 
 
-0.333
 
 
136.4
 
 
 
 
ATOSA
 
 
hsa-miR-221-3p
 
 
-0.495
 
 
42.5
 
 
 
 
ATP1B4
 
 
hsa-miR-29a-3p
 
 
-0.439
 
 
52.1
 
 
 
 
ATP5MC3
 
 
hsa-miR-425-5p
 
 
-0.328
 
 
728.4
 
 
 
 
ATP5MC3
 
 
hsa-miR-29a-3p
 
 
-0.33
 
 
728.4
 
 
 
 
ATP6V1E1
 
 
hsa-miR-23a-3p
 
 
-0.294
 
 
233.8
 
 
 
 
ATP6V1E1
 
 
hsa-miR-23b-3p
 
 
-0.294
 
 
233.8
 
 
 
 
ATPAF1
 
 
hsa-miR-361-5p
 
 
-0.362
 
 
260.2
 
 
 
 
ATXN1
 
 
hsa-miR-221-3p
 
 
-0.289
 
 
13.6
 
 
 
 
ATXN2
 
 
hsa-miR-15b-5p
 
 
-0.392
 
 
26.5
 
 
 
 
AUH
 
 
hsa-miR-23a-3p
 
 
-0.495
 
 
22.7
 
 
 
 
AUH
 
 
hsa-miR-23b-3p
 
 
-0.503
 
 
22.7
 
 
 
 
AUH
 
 
hsa-miR-23a-3p
 
 
-0.512
 
 
22.7
 
 
 
 
AUH
 
 
hsa-miR-23b-3p
 
 
-0.512
 
 
22.7
 
 
 
 
AXIN2
 
 
hsa-miR-15b-5p
 
 
-0.3
 
 
13.7
 
 
 
 
AZIN1
 
 
hsa-miR-132-3p
 
 
-0.29
 
 
38.7
 
 
 
 
AZIN1
 
 
hsa-miR-132-3p
 
 
-0.299
 
 
38.7
 
 
 
 
B4GALT5
 
 
hsa-miR-501-3p
 
 
-0.408
 
 
63.3
 
 
 
 
B4GAT1
 
 
hsa-miR-23a-3p
 
 
-0.361
 
 
27.1
 
 
 
 
B4GAT1
 
 
hsa-miR-23b-3p
 
 
-0.361
 
 
27.1
 
 
 
 
BAG4
 
 
hsa-miR-181b-5p
 
 
-0.337
 
 
13.5
 
 
 
 
BAG4
 
 
hsa-miR-181a-5p
 
 
-0.337
 
 
13.5
 
 
 
 
BAK1
 
 
hsa-miR-29a-3p
 
 
-0.409
 
 
17.7
 
 
 
 
BBC3
 
 
hsa-miR-221-3p
 
 
-0.375
 
 
10.1
 
 
 
 
BCL2
 
 
hsa-miR-342-3p
 
 
-0.428
 
 
15.5
 
 
 
 
BCL2L11
 
 
hsa-miR-221-3p
 
 
-0.423
 
 
11.0
 
 
 
 
BCL2L2
 
 
hsa-miR-29a-3p
 
 
-0.312
 
 
23.6
 
 
 
 
BCL7A
 
 
hsa-miR-15b-5p
 
 
-0.336
 
 
11.4
 
 
 
 
BCL7A
 
 
hsa-miR-501-3p
 
 
-0.339
 
 
11.4
 
 
 
 
 
 
 skin 
 
 
 
 
target_symbol
 
 
mature_mirna_id
 
 
score
 
 
nTPM
 
 
 
 
 
 
AAR2
 
 
hsa-miR-29a-3p
 
 
-0.327
 
 
24.6
 
 
 
 
ABCE1
 
 
hsa-miR-29a-3p
 
 
-0.404
 
 
23.8
 
 
 
 
ABHD4
 
 
hsa-miR-29a-3p
 
 
-0.312
 
 
20.2
 
 
 
 
ABHD5
 
 
hsa-miR-132-3p
 
 
-0.323
 
 
29.7
 
 
 
 
ACTA1
 
 
hsa-miR-155-5p
 
 
-0.595
 
 
18.7
 
 
 
 
ACTC1
 
 
hsa-miR-30b-5p
 
 
-0.543
 
 
14.2
 
 
 
 
ACTR2
 
 
hsa-miR-15b-5p
 
 
-0.293
 
 
65.5
 
 
 
 
ADCY3
 
 
hsa-miR-132-3p
 
 
-0.303
 
 
36.0
 
 
 
 
ADIPOR2
 
 
hsa-miR-150-5p
 
 
-0.287
 
 
30.8
 
 
 
 
ADRA2A
 
 
hsa-miR-23a-3p
 
 
-0.371
 
 
10.2
 
 
 
 
ADRA2A
 
 
hsa-miR-23b-3p
 
 
-0.371
 
 
10.2
 
 
 
 
ADRB2
 
 
hsa-miR-15b-5p
 
 
-0.29
 
 
36.9
 
 
 
 
AFG3L2
 
 
hsa-miR-181b-5p
 
 
-0.491
 
 
15.1
 
 
 
 
AFG3L2
 
 
hsa-miR-181a-5p
 
 
-0.491
 
 
15.1
 
 
 
 
AGO4
 
 
hsa-miR-221-3p
 
 
-0.321
 
 
18.9
 
 
 
 
AHR
 
 
hsa-miR-29a-3p
 
 
-0.316
 
 
20.7
 
 
 
 
AK1
 
 
hsa-miR-29a-3p
 
 
-0.333
 
 
17.4
 
 
 
 
AK4
 
 
hsa-miR-15b-5p
 
 
-0.316
 
 
25.7
 
 
 
 
ALAD
 
 
hsa-miR-15b-5p
 
 
-0.446
 
 
36.8
 
 
 
 
ALOX12
 
 
hsa-miR-15b-5p
 
 
-0.443
 
 
20.5
 
 
 
 
AMOTL1
 
 
hsa-miR-15b-5p
 
 
-0.319
 
 
20.5
 
 
 
 
ANGPTL2
 
 
hsa-miR-221-3p
 
 
-0.369
 
 
27.9
 
 
 
 
ANKFY1
 
 
hsa-miR-155-5p
 
 
-0.315
 
 
18.7
 
 
 
 
ANKHD1
 
 
hsa-miR-23a-3p
 
 
-0.368
 
 
48.3
 
 
 
 
ANKHD1
 
 
hsa-miR-23b-3p
 
 
-0.368
 
 
48.3
 
 
 
 
ANKHD1
 
 
hsa-miR-30b-5p
 
 
-0.405
 
 
48.3
 
 
 
 
ANKRD17
 
 
hsa-miR-30b-5p
 
 
-0.336
 
 
16.2
 
 
 
 
ANLN
 
 
hsa-miR-15b-5p
 
 
-0.334
 
 
12.9
 
 
 
 
ANP32A
 
 
hsa-miR-132-3p
 
 
-0.374
 
 
34.1
 
 
 
 
ANP32E
 
 
hsa-miR-501-3p
 
 
-0.331
 
 
20.4
 
 
 
 
ANTXR2
 
 
hsa-miR-29a-3p
 
 
-0.347
 
 
13.8
 
 
 
 
ANXA11
 
 
hsa-miR-15b-5p
 
 
-0.463
 
 
70.8
 
 
 
 
AP2A1
 
 
hsa-miR-15b-5p
 
 
-0.333
 
 
52.1
 
 
 
 
AP2B1
 
 
hsa-miR-15b-5p
 
 
-0.334
 
 
27.9
 
 
 
 
AP3S1
 
 
hsa-miR-30b-5p
 
 
-0.293
 
 
57.3
 
 
 
 
APP
 
 
hsa-miR-15b-5p
 
 
-0.298
 
 
204.7
 
 
 
 
AQP3
 
 
hsa-miR-874-3p
 
 
-0.48
 
 
554.4
 
 
 
 
ARF4
 
 
hsa-miR-30b-5p
 
 
-0.289
 
 
61.3
 
 
 
 
ARF4
 
 
hsa-miR-221-3p
 
 
-0.553
 
 
61.3
 
 
 
 
ARF5
 
 
hsa-miR-29a-3p
 
 
-0.491
 
 
215.0
 
 
 
 
ARF6
 
 
hsa-miR-181b-5p
 
 
-0.33
 
 
50.6
 
 
 
 
ARF6
 
 
hsa-miR-181a-5p
 
 
-0.33
 
 
50.6
 
 
 
 
ARHGAP5
 
 
hsa-miR-486-5p
 
 
-0.3
 
 
29.1
 
 
 
 
ARID1B
 
 
hsa-miR-29a-3p
 
 
-0.342
 
 
21.3
 
 
 
 
ARL2
 
 
hsa-miR-15b-5p
 
 
-0.374
 
 
96.0
 
 
 
 
ARL2
 
 
hsa-miR-15b-5p
 
 
-0.442
 
 
96.0
 
 
 
 
ARL4A
 
 
hsa-miR-29a-3p
 
 
-0.386
 
 
85.3
 
 
 
 
ARL4A
 
 
hsa-miR-30b-5p
 
 
-0.448
 
 
85.3
 
 
 
 
ARMC8
 
 
hsa-miR-486-5p
 
 
-0.321
 
 
12.0
 
 
 
 
ARMCX3
 
 
hsa-miR-425-5p
 
 
-0.325
 
 
10.4
 
 
 
 
ARNT
 
 
hsa-miR-221-3p
 
 
-0.465
 
 
23.3
 
 
 
 
ARPP19
 
 
hsa-miR-15b-5p
 
 
-0.346
 
 
28.9
 
 
 
 
ARPP19
 
 
hsa-miR-29a-3p
 
 
-0.371
 
 
28.9
 
 
 
 
ARRDC3
 
 
hsa-miR-29a-3p
 
 
-0.402
 
 
38.6
 
 
 
 
ASAP2
 
 
hsa-miR-29a-3p
 
 
-0.348
 
 
12.3
 
 
 
 
ASB6
 
 
hsa-miR-874-3p
 
 
-0.368
 
 
18.6
 
 
 
 
ASF1A
 
 
hsa-miR-23a-3p
 
 
-0.506
 
 
11.0
 
 
 
 
ASF1A
 
 
hsa-miR-23b-3p
 
 
-0.506
 
 
11.0
 
 
 
 
ASH1L
 
 
hsa-miR-15b-5p
 
 
-0.325
 
 
20.9
 
 
 
 
ASPH
 
 
hsa-miR-221-3p
 
 
-0.287
 
 
33.3
 
 
 
 
ATF3
 
 
hsa-miR-874-3p
 
 
-0.288
 
 
151.7
 
 
 
 
ATG9A
 
 
hsa-miR-15b-5p
 
 
-0.333
 
 
34.0
 
 
 
 
ATOSA
 
 
hsa-miR-221-3p
 
 
-0.495
 
 
10.0
 
 
 
 
ATP5MC3
 
 
hsa-miR-425-5p
 
 
-0.328
 
 
162.4
 
 
 
 
ATP5MC3
 
 
hsa-miR-29a-3p
 
 
-0.33
 
 
162.4
 
 
 
 
ATP6V1E1
 
 
hsa-miR-23a-3p
 
 
-0.294
 
 
120.3
 
 
 
 
ATP6V1E1
 
 
hsa-miR-23b-3p
 
 
-0.294
 
 
120.3
 
 
 
 
ATPAF1
 
 
hsa-miR-361-5p
 
 
-0.362
 
 
18.2
 
 
 
 
ATXN1L
 
 
hsa-miR-155-5p
 
 
-0.315
 
 
11.4
 
 
 
 
ATXN2
 
 
hsa-miR-15b-5p
 
 
-0.392
 
 
15.1
 
 
 
 
AXIN2
 
 
hsa-miR-15b-5p
 
 
-0.3
 
 
14.1
 
 
 
 
AZIN1
 
 
hsa-miR-132-3p
 
 
-0.29
 
 
30.1
 
 
 
 
AZIN1
 
 
hsa-miR-132-3p
 
 
-0.299
 
 
30.1
 
 
 
 
B4GAT1
 
 
hsa-miR-23a-3p
 
 
-0.361
 
 
11.9
 
 
 
 
B4GAT1
 
 
hsa-miR-23b-3p
 
 
-0.361
 
 
11.9
 
 
 
 
BAK1
 
 
hsa-miR-29a-3p
 
 
-0.409
 
 
17.5
 
 
 
 
BCL2L2
 
 
hsa-miR-29a-3p
 
 
-0.312
 
 
45.4
 
 
 
 
BDH1
 
 
hsa-miR-874-3p
 
 
-0.644
 
 
26.5
 
 
 
 
BECN1
 
 
hsa-miR-30b-5p
 
 
-0.452
 
 
34.4
 
 
 
 
BET1
 
 
hsa-miR-23a-3p
 
 
-0.402
 
 
13.0
 
 
 
 
BET1
 
 
hsa-miR-23b-3p
 
 
-0.418
 
 
13.0
 
 
 
 
BFAR
 
 
hsa-miR-15b-5p
 
 
-0.352
 
 
23.9
 
 
 
 
BHLHE40
 
 
hsa-miR-181b-5p
 
 
-0.408
 
 
217.6
 
 
 
 
BHLHE40
 
 
hsa-miR-181a-5p
 
 
-0.408
 
 
217.6
 
 
 
 
BICDL1
 
 
hsa-miR-221-3p
 
 
-0.447
 
 
50.4
 
 
 
 
BLCAP
 
 
hsa-miR-23a-3p
 
 
-0.601
 
 
83.4
 
 
 
 
BLCAP
 
 
hsa-miR-23b-3p
 
 
-0.601
 
 
83.4
 
 
 
 
BLMH
 
 
hsa-miR-29a-3p
 
 
-0.393
 
 
128.4
 
 
 
 
BLOC1S6
 
 
hsa-miR-181b-5p
 
 
-0.393
 
 
17.2
 
 
 
 
BLOC1S6
 
 
hsa-miR-181a-5p
 
 
-0.393
 
 
17.2
 
 
 
 
BOLA3
 
 
hsa-miR-15b-5p
 
 
-0.363
 
 
17.1
 
 
 
 
BOLA3
 
 
hsa-miR-15b-5p
 
 
-0.386
 
 
17.1
 
 
 
 
BOLA3
 
 
hsa-miR-374b-5p
 
 
-0.486
 
 
17.1
 
 
 
 
BORCS6
 
 
hsa-miR-15b-5p
 
 
-0.347
 
 
16.2
 
 
 
 
BRI3
 
 
hsa-miR-132-3p
 
 
-0.385
 
 
73.3
 
 
 
 
BTAF1
 
 
hsa-miR-486-5p
 
 
-0.288
 
 
39.9
 
 
 
 
BTAF1
 
 
hsa-miR-15b-5p
 
 
-0.496
 
 
39.9
 
 
 
 
BTF3
 
 
hsa-miR-15b-5p
 
 
-0.344
 
 
748.8
 
 
 
 
BTF3
 
 
hsa-miR-361-5p
 
 
-0.551
 
 
748.8
 
 
 
 
BTG2
 
 
hsa-miR-29a-3p
 
 
-0.32
 
 
130.3
 
 
 
 
 
 
 small intestine 
 
 
 
 
target_symbol
 
 
mature_mirna_id
 
 
score
 
 
nTPM
 
 
 
 
 
 
AAR2
 
 
hsa-miR-29a-3p
 
 
-0.327
 
 
18.1
 
 
 
 
ABCE1
 
 
hsa-miR-29a-3p
 
 
-0.404
 
 
16.7
 
 
 
 
ABHD17B
 
 
hsa-miR-486-5p
 
 
-0.348
 
 
12.6
 
 
 
 
ABHD4
 
 
hsa-miR-29a-3p
 
 
-0.312
 
 
18.9
 
 
 
 
ACTR2
 
 
hsa-miR-15b-5p
 
 
-0.293
 
 
111.3
 
 
 
 
ADA
 
 
hsa-miR-29a-3p
 
 
-0.376
 
 
15.5
 
 
 
 
ADAM19
 
 
hsa-miR-874-3p
 
 
-0.374
 
 
17.9
 
 
 
 
ADAM9
 
 
hsa-miR-30b-5p
 
 
-0.312
 
 
21.4
 
 
 
 
ADCY3
 
 
hsa-miR-132-3p
 
 
-0.303
 
 
31.8
 
 
 
 
ADIPOR2
 
 
hsa-miR-150-5p
 
 
-0.287
 
 
31.4
 
 
 
 
AFG3L2
 
 
hsa-miR-181b-5p
 
 
-0.491
 
 
34.0
 
 
 
 
AFG3L2
 
 
hsa-miR-181a-5p
 
 
-0.491
 
 
34.0
 
 
 
 
AHR
 
 
hsa-miR-29a-3p
 
 
-0.316
 
 
13.6
 
 
 
 
AK1
 
 
hsa-miR-29a-3p
 
 
-0.333
 
 
15.6
 
 
 
 
ALAD
 
 
hsa-miR-15b-5p
 
 
-0.446
 
 
25.1
 
 
 
 
ANGPTL2
 
 
hsa-miR-221-3p
 
 
-0.369
 
 
27.5
 
 
 
 
ANKFY1
 
 
hsa-miR-155-5p
 
 
-0.315
 
 
13.2
 
 
 
 
ANKHD1
 
 
hsa-miR-23a-3p
 
 
-0.368
 
 
44.2
 
 
 
 
ANKHD1
 
 
hsa-miR-23b-3p
 
 
-0.368
 
 
44.2
 
 
 
 
ANKHD1
 
 
hsa-miR-30b-5p
 
 
-0.405
 
 
44.2
 
 
 
 
ANKRD17
 
 
hsa-miR-30b-5p
 
 
-0.336
 
 
14.3
 
 
 
 
ANP32A
 
 
hsa-miR-132-3p
 
 
-0.374
 
 
72.4
 
 
 
 
ANP32E
 
 
hsa-miR-501-3p
 
 
-0.331
 
 
24.2
 
 
 
 
ANTXR2
 
 
hsa-miR-29a-3p
 
 
-0.347
 
 
35.6
 
 
 
 
ANXA11
 
 
hsa-miR-15b-5p
 
 
-0.463
 
 
129.1
 
 
 
 
AP2A1
 
 
hsa-miR-15b-5p
 
 
-0.333
 
 
32.5
 
 
 
 
AP2B1
 
 
hsa-miR-15b-5p
 
 
-0.334
 
 
36.5
 
 
 
 
AP3S1
 
 
hsa-miR-30b-5p
 
 
-0.293
 
 
76.7
 
 
 
 
APOO
 
 
hsa-miR-181b-5p
 
 
-0.5
 
 
11.3
 
 
 
 
APOO
 
 
hsa-miR-181a-5p
 
 
-0.5
 
 
11.3
 
 
 
 
APP
 
 
hsa-miR-15b-5p
 
 
-0.298
 
 
184.2
 
 
 
 
AQP11
 
 
hsa-miR-15b-5p
 
 
-0.769
 
 
23.9
 
 
 
 
AQP3
 
 
hsa-miR-874-3p
 
 
-0.48
 
 
94.7
 
 
 
 
ARF4
 
 
hsa-miR-30b-5p
 
 
-0.289
 
 
102.8
 
 
 
 
ARF4
 
 
hsa-miR-221-3p
 
 
-0.553
 
 
102.8
 
 
 
 
ARF5
 
 
hsa-miR-29a-3p
 
 
-0.491
 
 
103.1
 
 
 
 
ARF6
 
 
hsa-miR-181b-5p
 
 
-0.33
 
 
48.2
 
 
 
 
ARF6
 
 
hsa-miR-181a-5p
 
 
-0.33
 
 
48.2
 
 
 
 
ARHGAP5
 
 
hsa-miR-486-5p
 
 
-0.3
 
 
36.7
 
 
 
 
ARID1B
 
 
hsa-miR-29a-3p
 
 
-0.342
 
 
14.4
 
 
 
 
ARL2
 
 
hsa-miR-15b-5p
 
 
-0.374
 
 
72.6
 
 
 
 
ARL2
 
 
hsa-miR-15b-5p
 
 
-0.442
 
 
72.6
 
 
 
 
ARL4A
 
 
hsa-miR-29a-3p
 
 
-0.386
 
 
59.0
 
 
 
 
ARL4A
 
 
hsa-miR-30b-5p
 
 
-0.448
 
 
59.0
 
 
 
 
ARMC8
 
 
hsa-miR-486-5p
 
 
-0.321
 
 
12.8
 
 
 
 
ARMCX2
 
 
hsa-miR-15b-5p
 
 
-0.344
 
 
11.5
 
 
 
 
ARMCX3
 
 
hsa-miR-425-5p
 
 
-0.325
 
 
19.2
 
 
 
 
ARNT
 
 
hsa-miR-221-3p
 
 
-0.465
 
 
16.2
 
 
 
 
ARPP19
 
 
hsa-miR-15b-5p
 
 
-0.346
 
 
28.8
 
 
 
 
ARPP19
 
 
hsa-miR-29a-3p
 
 
-0.371
 
 
28.8
 
 
 
 
ARRDC3
 
 
hsa-miR-29a-3p
 
 
-0.402
 
 
15.8
 
 
 
 
ASAP2
 
 
hsa-miR-29a-3p
 
 
-0.348
 
 
12.8
 
 
 
 
ASB6
 
 
hsa-miR-874-3p
 
 
-0.368
 
 
11.3
 
 
 
 
ASH1L
 
 
hsa-miR-15b-5p
 
 
-0.325
 
 
17.1
 
 
 
 
ASPH
 
 
hsa-miR-221-3p
 
 
-0.287
 
 
42.6
 
 
 
 
ATF3
 
 
hsa-miR-874-3p
 
 
-0.288
 
 
42.8
 
 
 
 
ATG5
 
 
hsa-miR-30b-5p
 
 
-0.488
 
 
12.3
 
 
 
 
ATG9A
 
 
hsa-miR-15b-5p
 
 
-0.333
 
 
27.9
 
 
 
 
ATOSA
 
 
hsa-miR-221-3p
 
 
-0.495
 
 
11.7
 
 
 
 
ATP5MC3
 
 
hsa-miR-425-5p
 
 
-0.328
 
 
279.4
 
 
 
 
ATP5MC3
 
 
hsa-miR-29a-3p
 
 
-0.33
 
 
279.4
 
 
 
 
ATP6V1E1
 
 
hsa-miR-23a-3p
 
 
-0.294
 
 
83.3
 
 
 
 
ATP6V1E1
 
 
hsa-miR-23b-3p
 
 
-0.294
 
 
83.3
 
 
 
 
ATPAF1
 
 
hsa-miR-361-5p
 
 
-0.362
 
 
42.6
 
 
 
 
ATXN2
 
 
hsa-miR-15b-5p
 
 
-0.392
 
 
14.3
 
 
 
 
AUH
 
 
hsa-miR-23a-3p
 
 
-0.495
 
 
18.9
 
 
 
 
AUH
 
 
hsa-miR-23b-3p
 
 
-0.503
 
 
18.9
 
 
 
 
AUH
 
 
hsa-miR-23a-3p
 
 
-0.512
 
 
18.9
 
 
 
 
AUH
 
 
hsa-miR-23b-3p
 
 
-0.512
 
 
18.9
 
 
 
 
AZIN1
 
 
hsa-miR-132-3p
 
 
-0.29
 
 
74.7
 
 
 
 
AZIN1
 
 
hsa-miR-132-3p
 
 
-0.299
 
 
74.7
 
 
 
 
B3GNT5
 
 
hsa-miR-30b-5p
 
 
-0.326
 
 
39.6
 
 
 
 
B4GALT5
 
 
hsa-miR-501-3p
 
 
-0.408
 
 
20.1
 
 
 
 
B4GAT1
 
 
hsa-miR-23a-3p
 
 
-0.361
 
 
14.2
 
 
 
 
B4GAT1
 
 
hsa-miR-23b-3p
 
 
-0.361
 
 
14.2
 
 
 
 
BAK1
 
 
hsa-miR-29a-3p
 
 
-0.409
 
 
34.9
 
 
 
 
BCL2L11
 
 
hsa-miR-221-3p
 
 
-0.423
 
 
11.3
 
 
 
 
BCL2L2
 
 
hsa-miR-29a-3p
 
 
-0.312
 
 
34.6
 
 
 
 
BDH1
 
 
hsa-miR-874-3p
 
 
-0.644
 
 
25.0
 
 
 
 
BECN1
 
 
hsa-miR-30b-5p
 
 
-0.452
 
 
47.2
 
 
 
 
BET1
 
 
hsa-miR-23a-3p
 
 
-0.402
 
 
22.0
 
 
 
 
BET1
 
 
hsa-miR-23b-3p
 
 
-0.418
 
 
22.0
 
 
 
 
BFAR
 
 
hsa-miR-15b-5p
 
 
-0.352
 
 
24.6
 
 
 
 
BHLHE40
 
 
hsa-miR-181b-5p
 
 
-0.408
 
 
45.0
 
 
 
 
BHLHE40
 
 
hsa-miR-181a-5p
 
 
-0.408
 
 
45.0
 
 
 
 
BLCAP
 
 
hsa-miR-23a-3p
 
 
-0.601
 
 
46.3
 
 
 
 
BLCAP
 
 
hsa-miR-23b-3p
 
 
-0.601
 
 
46.3
 
 
 
 
BLMH
 
 
hsa-miR-29a-3p
 
 
-0.393
 
 
17.8
 
 
 
 
BLOC1S6
 
 
hsa-miR-181b-5p
 
 
-0.393
 
 
20.1
 
 
 
 
BLOC1S6
 
 
hsa-miR-181a-5p
 
 
-0.393
 
 
20.1
 
 
 
 
BMF
 
 
hsa-miR-29a-3p
 
 
-0.41
 
 
22.2
 
 
 
 
BMF
 
 
hsa-miR-221-3p
 
 
-0.453
 
 
22.2
 
 
 
 
BOLA3
 
 
hsa-miR-15b-5p
 
 
-0.363
 
 
27.2
 
 
 
 
BOLA3
 
 
hsa-miR-15b-5p
 
 
-0.386
 
 
27.2
 
 
 
 
BOLA3
 
 
hsa-miR-374b-5p
 
 
-0.486
 
 
27.2
 
 
 
 
BORCS6
 
 
hsa-miR-15b-5p
 
 
-0.347
 
 
11.7
 
 
 
 
BRI3
 
 
hsa-miR-132-3p
 
 
-0.385
 
 
73.7
 
 
 
 
BTAF1
 
 
hsa-miR-486-5p
 
 
-0.288
 
 
18.1
 
 
 
 
BTAF1
 
 
hsa-miR-15b-5p
 
 
-0.496
 
 
18.1
 
 
 
 
BTF3
 
 
hsa-miR-15b-5p
 
 
-0.344
 
 
427.5
 
 
 
 
 
 
 smooth muscle 
 
 
 
 
target_symbol
 
 
mature_mirna_id
 
 
score
 
 
nTPM
 
 
 
 
 
 
AAR2
 
 
hsa-miR-29a-3p
 
 
-0.327
 
 
26.1
 
 
 
 
ABCE1
 
 
hsa-miR-29a-3p
 
 
-0.404
 
 
27.0
 
 
 
 
ABHD4
 
 
hsa-miR-29a-3p
 
 
-0.312
 
 
25.0
 
 
 
 
ACTA1
 
 
hsa-miR-155-5p
 
 
-0.595
 
 
61.8
 
 
 
 
ACTR2
 
 
hsa-miR-15b-5p
 
 
-0.293
 
 
96.0
 
 
 
 
ACVR1
 
 
hsa-miR-30b-5p
 
 
-0.29
 
 
26.8
 
 
 
 
ADAM19
 
 
hsa-miR-874-3p
 
 
-0.374
 
 
15.3
 
 
 
 
ADAM9
 
 
hsa-miR-30b-5p
 
 
-0.312
 
 
48.2
 
 
 
 
ADAMTS9
 
 
hsa-miR-29a-3p
 
 
-0.305
 
 
11.3
 
 
 
 
ADAMTS9
 
 
hsa-miR-29a-3p
 
 
-0.485
 
 
11.3
 
 
 
 
ADCY3
 
 
hsa-miR-132-3p
 
 
-0.303
 
 
33.0
 
 
 
 
ADGRA3
 
 
hsa-miR-30b-5p
 
 
-0.304
 
 
13.1
 
 
 
 
ADIPOR2
 
 
hsa-miR-150-5p
 
 
-0.287
 
 
11.2
 
 
 
 
AFG3L2
 
 
hsa-miR-181b-5p
 
 
-0.491
 
 
14.9
 
 
 
 
AFG3L2
 
 
hsa-miR-181a-5p
 
 
-0.491
 
 
14.9
 
 
 
 
AHR
 
 
hsa-miR-29a-3p
 
 
-0.316
 
 
14.2
 
 
 
 
AK1
 
 
hsa-miR-29a-3p
 
 
-0.333
 
 
20.4
 
 
 
 
AK4
 
 
hsa-miR-15b-5p
 
 
-0.316
 
 
19.6
 
 
 
 
AKT3
 
 
hsa-miR-29a-3p
 
 
-0.287
 
 
14.7
 
 
 
 
AKT3
 
 
hsa-miR-15b-5p
 
 
-0.301
 
 
14.7
 
 
 
 
ALAD
 
 
hsa-miR-15b-5p
 
 
-0.446
 
 
16.4
 
 
 
 
ALDH1A2
 
 
hsa-miR-23b-3p
 
 
-0.346
 
 
29.8
 
 
 
 
ALDH1A2
 
 
hsa-miR-23a-3p
 
 
-0.354
 
 
29.8
 
 
 
 
AMOTL1
 
 
hsa-miR-15b-5p
 
 
-0.319
 
 
19.2
 
 
 
 
ANGPTL2
 
 
hsa-miR-221-3p
 
 
-0.369
 
 
59.2
 
 
 
 
ANKFY1
 
 
hsa-miR-155-5p
 
 
-0.315
 
 
13.8
 
 
 
 
ANKHD1
 
 
hsa-miR-23a-3p
 
 
-0.368
 
 
24.3
 
 
 
 
ANKHD1
 
 
hsa-miR-23b-3p
 
 
-0.368
 
 
24.3
 
 
 
 
ANKHD1
 
 
hsa-miR-30b-5p
 
 
-0.405
 
 
24.3
 
 
 
 
ANKRD17
 
 
hsa-miR-30b-5p
 
 
-0.336
 
 
15.3
 
 
 
 
ANKRD29
 
 
hsa-miR-132-3p
 
 
-0.47
 
 
10.9
 
 
 
 
ANLN
 
 
hsa-miR-15b-5p
 
 
-0.334
 
 
13.9
 
 
 
 
ANP32A
 
 
hsa-miR-132-3p
 
 
-0.374
 
 
53.2
 
 
 
 
ANP32E
 
 
hsa-miR-501-3p
 
 
-0.331
 
 
19.6
 
 
 
 
ANTXR2
 
 
hsa-miR-29a-3p
 
 
-0.347
 
 
101.2
 
 
 
 
ANXA11
 
 
hsa-miR-15b-5p
 
 
-0.463
 
 
80.2
 
 
 
 
AP2A1
 
 
hsa-miR-15b-5p
 
 
-0.333
 
 
20.0
 
 
 
 
AP2B1
 
 
hsa-miR-15b-5p
 
 
-0.334
 
 
52.3
 
 
 
 
AP3S1
 
 
hsa-miR-30b-5p
 
 
-0.293
 
 
59.9
 
 
 
 
APOO
 
 
hsa-miR-181b-5p
 
 
-0.5
 
 
11.2
 
 
 
 
APOO
 
 
hsa-miR-181a-5p
 
 
-0.5
 
 
11.2
 
 
 
 
APP
 
 
hsa-miR-15b-5p
 
 
-0.298
 
 
353.7
 
 
 
 
ARF4
 
 
hsa-miR-30b-5p
 
 
-0.289
 
 
157.9
 
 
 
 
ARF4
 
 
hsa-miR-221-3p
 
 
-0.553
 
 
157.9
 
 
 
 
ARF5
 
 
hsa-miR-29a-3p
 
 
-0.491
 
 
62.0
 
 
 
 
ARF6
 
 
hsa-miR-181b-5p
 
 
-0.33
 
 
22.2
 
 
 
 
ARF6
 
 
hsa-miR-181a-5p
 
 
-0.33
 
 
22.2
 
 
 
 
ARHGAP5
 
 
hsa-miR-486-5p
 
 
-0.3
 
 
22.0
 
 
 
 
ARID1B
 
 
hsa-miR-29a-3p
 
 
-0.342
 
 
14.1
 
 
 
 
ARL2
 
 
hsa-miR-15b-5p
 
 
-0.374
 
 
56.9
 
 
 
 
ARL2
 
 
hsa-miR-15b-5p
 
 
-0.442
 
 
56.9
 
 
 
 
ARL4A
 
 
hsa-miR-29a-3p
 
 
-0.386
 
 
23.4
 
 
 
 
ARL4A
 
 
hsa-miR-30b-5p
 
 
-0.448
 
 
23.4
 
 
 
 
ARMC8
 
 
hsa-miR-486-5p
 
 
-0.321
 
 
22.8
 
 
 
 
ARMCX2
 
 
hsa-miR-15b-5p
 
 
-0.344
 
 
30.1
 
 
 
 
ARMCX3
 
 
hsa-miR-425-5p
 
 
-0.325
 
 
33.9
 
 
 
 
ARNT
 
 
hsa-miR-221-3p
 
 
-0.465
 
 
27.1
 
 
 
 
ARPP19
 
 
hsa-miR-15b-5p
 
 
-0.346
 
 
54.2
 
 
 
 
ARPP19
 
 
hsa-miR-29a-3p
 
 
-0.371
 
 
54.2
 
 
 
 
ARRDC3
 
 
hsa-miR-29a-3p
 
 
-0.402
 
 
30.8
 
 
 
 
ARSB
 
 
hsa-miR-29a-3p
 
 
-0.36
 
 
13.1
 
 
 
 
ASAP2
 
 
hsa-miR-29a-3p
 
 
-0.348
 
 
13.0
 
 
 
 
ASH1L
 
 
hsa-miR-15b-5p
 
 
-0.325
 
 
11.9
 
 
 
 
ASPH
 
 
hsa-miR-221-3p
 
 
-0.287
 
 
62.4
 
 
 
 
ATF3
 
 
hsa-miR-874-3p
 
 
-0.288
 
 
291.8
 
 
 
 
ATG5
 
 
hsa-miR-30b-5p
 
 
-0.488
 
 
13.2
 
 
 
 
ATG9A
 
 
hsa-miR-15b-5p
 
 
-0.333
 
 
26.1
 
 
 
 
ATOSA
 
 
hsa-miR-221-3p
 
 
-0.495
 
 
11.6
 
 
 
 
ATP5MC3
 
 
hsa-miR-425-5p
 
 
-0.328
 
 
113.6
 
 
 
 
ATP5MC3
 
 
hsa-miR-29a-3p
 
 
-0.33
 
 
113.6
 
 
 
 
ATP6V1E1
 
 
hsa-miR-23a-3p
 
 
-0.294
 
 
112.9
 
 
 
 
ATP6V1E1
 
 
hsa-miR-23b-3p
 
 
-0.294
 
 
112.9
 
 
 
 
ATPAF1
 
 
hsa-miR-361-5p
 
 
-0.362
 
 
30.4
 
 
 
 
ATXN1L
 
 
hsa-miR-155-5p
 
 
-0.315
 
 
12.0
 
 
 
 
ATXN2
 
 
hsa-miR-15b-5p
 
 
-0.392
 
 
17.1
 
 
 
 
AXIN2
 
 
hsa-miR-15b-5p
 
 
-0.3
 
 
16.2
 
 
 
 
AZIN1
 
 
hsa-miR-132-3p
 
 
-0.29
 
 
41.1
 
 
 
 
AZIN1
 
 
hsa-miR-132-3p
 
 
-0.299
 
 
41.1
 
 
 
 
B4GALT5
 
 
hsa-miR-501-3p
 
 
-0.408
 
 
35.1
 
 
 
 
B4GAT1
 
 
hsa-miR-23a-3p
 
 
-0.361
 
 
13.1
 
 
 
 
B4GAT1
 
 
hsa-miR-23b-3p
 
 
-0.361
 
 
13.1
 
 
 
 
BAK1
 
 
hsa-miR-29a-3p
 
 
-0.409
 
 
12.6
 
 
 
 
BCL2L2
 
 
hsa-miR-29a-3p
 
 
-0.312
 
 
39.0
 
 
 
 
BECN1
 
 
hsa-miR-30b-5p
 
 
-0.452
 
 
41.5
 
 
 
 
BET1
 
 
hsa-miR-23a-3p
 
 
-0.402
 
 
14.2
 
 
 
 
BET1
 
 
hsa-miR-23b-3p
 
 
-0.418
 
 
14.2
 
 
 
 
BFAR
 
 
hsa-miR-15b-5p
 
 
-0.352
 
 
16.3
 
 
 
 
BHLHE40
 
 
hsa-miR-181b-5p
 
 
-0.408
 
 
60.9
 
 
 
 
BHLHE40
 
 
hsa-miR-181a-5p
 
 
-0.408
 
 
60.9
 
 
 
 
BLCAP
 
 
hsa-miR-23a-3p
 
 
-0.601
 
 
31.7
 
 
 
 
BLCAP
 
 
hsa-miR-23b-3p
 
 
-0.601
 
 
31.7
 
 
 
 
BLMH
 
 
hsa-miR-29a-3p
 
 
-0.393
 
 
20.9
 
 
 
 
BLOC1S6
 
 
hsa-miR-181b-5p
 
 
-0.393
 
 
22.7
 
 
 
 
BLOC1S6
 
 
hsa-miR-181a-5p
 
 
-0.393
 
 
22.7
 
 
 
 
BOLA3
 
 
hsa-miR-15b-5p
 
 
-0.363
 
 
21.1
 
 
 
 
BOLA3
 
 
hsa-miR-15b-5p
 
 
-0.386
 
 
21.1
 
 
 
 
BOLA3
 
 
hsa-miR-374b-5p
 
 
-0.486
 
 
21.1
 
 
 
 
BRI3
 
 
hsa-miR-132-3p
 
 
-0.385
 
 
31.4
 
 
 
 
BTAF1
 
 
hsa-miR-486-5p
 
 
-0.288
 
 
11.0
 
 
 
 
BTAF1
 
 
hsa-miR-15b-5p
 
 
-0.496
 
 
11.0
 
 
 
 
 
 
 spinal cord 
 
 
 
 
target_symbol
 
 
mature_mirna_id
 
 
score
 
 
nTPM
 
 
 
 
 
 
AAR2
 
 
hsa-miR-29a-3p
 
 
-0.327
 
 
14.2
 
 
 
 
ABCE1
 
 
hsa-miR-29a-3p
 
 
-0.404
 
 
16.8
 
 
 
 
ABHD17B
 
 
hsa-miR-486-5p
 
 
-0.348
 
 
26.2
 
 
 
 
ABHD4
 
 
hsa-miR-29a-3p
 
 
-0.312
 
 
17.8
 
 
 
 
ABHD5
 
 
hsa-miR-132-3p
 
 
-0.323
 
 
19.5
 
 
 
 
ACTN2
 
 
hsa-miR-23a-3p
 
 
-0.294
 
 
14.9
 
 
 
 
ACTN2
 
 
hsa-miR-29a-3p
 
 
-0.311
 
 
14.9
 
 
 
 
ACTR2
 
 
hsa-miR-15b-5p
 
 
-0.293
 
 
65.2
 
 
 
 
ADAM9
 
 
hsa-miR-30b-5p
 
 
-0.312
 
 
11.3
 
 
 
 
ADCY3
 
 
hsa-miR-132-3p
 
 
-0.303
 
 
26.0
 
 
 
 
ADIPOR2
 
 
hsa-miR-150-5p
 
 
-0.287
 
 
121.3
 
 
 
 
AGPAT4
 
 
hsa-miR-29a-3p
 
 
-0.376
 
 
53.1
 
 
 
 
AK1
 
 
hsa-miR-29a-3p
 
 
-0.333
 
 
67.3
 
 
 
 
AK4
 
 
hsa-miR-15b-5p
 
 
-0.316
 
 
11.3
 
 
 
 
AKT3
 
 
hsa-miR-29a-3p
 
 
-0.287
 
 
15.8
 
 
 
 
AKT3
 
 
hsa-miR-15b-5p
 
 
-0.301
 
 
15.8
 
 
 
 
ALAD
 
 
hsa-miR-15b-5p
 
 
-0.446
 
 
110.8
 
 
 
 
ANGPTL2
 
 
hsa-miR-221-3p
 
 
-0.369
 
 
15.5
 
 
 
 
ANKFY1
 
 
hsa-miR-155-5p
 
 
-0.315
 
 
14.2
 
 
 
 
ANKHD1
 
 
hsa-miR-23a-3p
 
 
-0.368
 
 
22.6
 
 
 
 
ANKHD1
 
 
hsa-miR-23b-3p
 
 
-0.368
 
 
22.6
 
 
 
 
ANKHD1
 
 
hsa-miR-30b-5p
 
 
-0.405
 
 
22.6
 
 
 
 
ANKRD13B
 
 
hsa-miR-29a-3p
 
 
-0.421
 
 
14.7
 
 
 
 
ANLN
 
 
hsa-miR-15b-5p
 
 
-0.334
 
 
163.3
 
 
 
 
ANP32A
 
 
hsa-miR-132-3p
 
 
-0.374
 
 
72.8
 
 
 
 
ANP32E
 
 
hsa-miR-501-3p
 
 
-0.331
 
 
32.2
 
 
 
 
ANXA11
 
 
hsa-miR-15b-5p
 
 
-0.463
 
 
19.0
 
 
 
 
AP2A1
 
 
hsa-miR-15b-5p
 
 
-0.333
 
 
32.5
 
 
 
 
AP2B1
 
 
hsa-miR-15b-5p
 
 
-0.334
 
 
75.2
 
 
 
 
AP3S1
 
 
hsa-miR-30b-5p
 
 
-0.293
 
 
64.2
 
 
 
 
APLN
 
 
hsa-miR-15b-5p
 
 
-0.395
 
 
29.4
 
 
 
 
APOO
 
 
hsa-miR-181b-5p
 
 
-0.5
 
 
36.2
 
 
 
 
APOO
 
 
hsa-miR-181a-5p
 
 
-0.5
 
 
36.2
 
 
 
 
APP
 
 
hsa-miR-15b-5p
 
 
-0.298
 
 
535.7
 
 
 
 
ARF4
 
 
hsa-miR-30b-5p
 
 
-0.289
 
 
69.7
 
 
 
 
ARF4
 
 
hsa-miR-221-3p
 
 
-0.553
 
 
69.7
 
 
 
 
ARF5
 
 
hsa-miR-29a-3p
 
 
-0.491
 
 
100.3
 
 
 
 
ARF6
 
 
hsa-miR-181b-5p
 
 
-0.33
 
 
13.8
 
 
 
 
ARF6
 
 
hsa-miR-181a-5p
 
 
-0.33
 
 
13.8
 
 
 
 
ARHGAP5
 
 
hsa-miR-486-5p
 
 
-0.3
 
 
21.0
 
 
 
 
ARID4B
 
 
hsa-miR-486-5p
 
 
-0.367
 
 
12.3
 
 
 
 
ARL2
 
 
hsa-miR-15b-5p
 
 
-0.374
 
 
234.0
 
 
 
 
ARL2
 
 
hsa-miR-15b-5p
 
 
-0.442
 
 
234.0
 
 
 
 
ARL3
 
 
hsa-miR-15b-5p
 
 
-0.507
 
 
16.0
 
 
 
 
ARL4A
 
 
hsa-miR-29a-3p
 
 
-0.386
 
 
12.2
 
 
 
 
ARL4A
 
 
hsa-miR-30b-5p
 
 
-0.448
 
 
12.2
 
 
 
 
ARMC8
 
 
hsa-miR-486-5p
 
 
-0.321
 
 
15.5
 
 
 
 
ARMCX2
 
 
hsa-miR-15b-5p
 
 
-0.344
 
 
15.1
 
 
 
 
ARMCX3
 
 
hsa-miR-425-5p
 
 
-0.325
 
 
33.1
 
 
 
 
ARPP19
 
 
hsa-miR-15b-5p
 
 
-0.346
 
 
170.8
 
 
 
 
ARPP19
 
 
hsa-miR-29a-3p
 
 
-0.371
 
 
170.8
 
 
 
 
ARRDC3
 
 
hsa-miR-29a-3p
 
 
-0.402
 
 
23.5
 
 
 
 
ASB3
 
 
hsa-miR-30b-5p
 
 
-0.418
 
 
11.7
 
 
 
 
ASB3
 
 
hsa-miR-30b-5p
 
 
-0.418
 
 
11.7
 
 
 
 
ASF1A
 
 
hsa-miR-23a-3p
 
 
-0.506
 
 
14.8
 
 
 
 
ASF1A
 
 
hsa-miR-23b-3p
 
 
-0.506
 
 
14.8
 
 
 
 
ASH1L
 
 
hsa-miR-15b-5p
 
 
-0.325
 
 
17.5
 
 
 
 
ASPH
 
 
hsa-miR-221-3p
 
 
-0.287
 
 
25.3
 
 
 
 
ATF3
 
 
hsa-miR-874-3p
 
 
-0.288
 
 
33.6
 
 
 
 
ATG9A
 
 
hsa-miR-15b-5p
 
 
-0.333
 
 
25.8
 
 
 
 
ATP5MC3
 
 
hsa-miR-425-5p
 
 
-0.328
 
 
137.7
 
 
 
 
ATP5MC3
 
 
hsa-miR-29a-3p
 
 
-0.33
 
 
137.7
 
 
 
 
ATP6V1E1
 
 
hsa-miR-23a-3p
 
 
-0.294
 
 
235.9
 
 
 
 
ATP6V1E1
 
 
hsa-miR-23b-3p
 
 
-0.294
 
 
235.9
 
 
 
 
ATPAF1
 
 
hsa-miR-361-5p
 
 
-0.362
 
 
50.4
 
 
 
 
ATXN2
 
 
hsa-miR-15b-5p
 
 
-0.392
 
 
27.6
 
 
 
 
AUH
 
 
hsa-miR-23a-3p
 
 
-0.495
 
 
14.9
 
 
 
 
AUH
 
 
hsa-miR-23b-3p
 
 
-0.503
 
 
14.9
 
 
 
 
AUH
 
 
hsa-miR-23a-3p
 
 
-0.512
 
 
14.9
 
 
 
 
AUH
 
 
hsa-miR-23b-3p
 
 
-0.512
 
 
14.9
 
 
 
 
AZIN1
 
 
hsa-miR-132-3p
 
 
-0.29
 
 
40.4
 
 
 
 
AZIN1
 
 
hsa-miR-132-3p
 
 
-0.299
 
 
40.4
 
 
 
 
B4GAT1
 
 
hsa-miR-23a-3p
 
 
-0.361
 
 
48.7
 
 
 
 
B4GAT1
 
 
hsa-miR-23b-3p
 
 
-0.361
 
 
48.7
 
 
 
 
BCL2L2
 
 
hsa-miR-29a-3p
 
 
-0.312
 
 
103.7
 
 
 
 
BCL2L2-PABPN1
 
 
hsa-miR-425-5p
 
 
-0.321
 
 
10.9
 
 
 
 
BDH1
 
 
hsa-miR-874-3p
 
 
-0.644
 
 
11.2
 
 
 
 
BECN1
 
 
hsa-miR-30b-5p
 
 
-0.452
 
 
29.1
 
 
 
 
BEND6
 
 
hsa-miR-361-5p
 
 
-0.305
 
 
11.4
 
 
 
 
BET1
 
 
hsa-miR-23a-3p
 
 
-0.402
 
 
10.7
 
 
 
 
BET1
 
 
hsa-miR-23b-3p
 
 
-0.418
 
 
10.7
 
 
 
 
BFAR
 
 
hsa-miR-15b-5p
 
 
-0.352
 
 
16.2
 
 
 
 
BHLHE40
 
 
hsa-miR-181b-5p
 
 
-0.408
 
 
46.8
 
 
 
 
BHLHE40
 
 
hsa-miR-181a-5p
 
 
-0.408
 
 
46.8
 
 
 
 
BLCAP
 
 
hsa-miR-23a-3p
 
 
-0.601
 
 
55.9
 
 
 
 
BLCAP
 
 
hsa-miR-23b-3p
 
 
-0.601
 
 
55.9
 
 
 
 
BLMH
 
 
hsa-miR-29a-3p
 
 
-0.393
 
 
18.5
 
 
 
 
BLOC1S6
 
 
hsa-miR-181b-5p
 
 
-0.393
 
 
15.5
 
 
 
 
BLOC1S6
 
 
hsa-miR-181a-5p
 
 
-0.393
 
 
15.5
 
 
 
 
BOLA3
 
 
hsa-miR-15b-5p
 
 
-0.363
 
 
45.8
 
 
 
 
BOLA3
 
 
hsa-miR-15b-5p
 
 
-0.386
 
 
45.8
 
 
 
 
BOLA3
 
 
hsa-miR-374b-5p
 
 
-0.486
 
 
45.8
 
 
 
 
BRI3
 
 
hsa-miR-132-3p
 
 
-0.385
 
 
42.6
 
 
 
 
BTF3
 
 
hsa-miR-15b-5p
 
 
-0.344
 
 
358.9
 
 
 
 
BTF3
 
 
hsa-miR-361-5p
 
 
-0.551
 
 
358.9
 
 
 
 
BTG2
 
 
hsa-miR-29a-3p
 
 
-0.32
 
 
41.8
 
 
 
 
BTN2A1
 
 
hsa-miR-342-3p
 
 
-0.31
 
 
14.3
 
 
 
 
BZW1
 
 
hsa-miR-15b-5p
 
 
-0.367
 
 
46.3
 
 
 
 
C11orf24
 
 
hsa-miR-15b-5p
 
 
-0.403
 
 
25.5
 
 
 
 
C11orf68
 
 
hsa-miR-15b-5p
 
 
-0.29
 
 
34.5
 
 
 
 
 
 
 spleen 
 
 
 
 
target_symbol
 
 
mature_mirna_id
 
 
score
 
 
nTPM
 
 
 
 
 
 
AAR2
 
 
hsa-miR-29a-3p
 
 
-0.327
 
 
17.2
 
 
 
 
ABCE1
 
 
hsa-miR-29a-3p
 
 
-0.404
 
 
24.9
 
 
 
 
ABHD4
 
 
hsa-miR-29a-3p
 
 
-0.312
 
 
15.7
 
 
 
 
ACTR2
 
 
hsa-miR-15b-5p
 
 
-0.293
 
 
161.4
 
 
 
 
ADA
 
 
hsa-miR-29a-3p
 
 
-0.376
 
 
17.0
 
 
 
 
ADAM19
 
 
hsa-miR-874-3p
 
 
-0.374
 
 
21.6
 
 
 
 
ADAM9
 
 
hsa-miR-30b-5p
 
 
-0.312
 
 
22.6
 
 
 
 
ADCY3
 
 
hsa-miR-132-3p
 
 
-0.303
 
 
28.8
 
 
 
 
ADIPOR2
 
 
hsa-miR-150-5p
 
 
-0.287
 
 
19.2
 
 
 
 
ADRA2A
 
 
hsa-miR-23a-3p
 
 
-0.371
 
 
13.1
 
 
 
 
ADRA2A
 
 
hsa-miR-23b-3p
 
 
-0.371
 
 
13.1
 
 
 
 
AFG3L2
 
 
hsa-miR-181b-5p
 
 
-0.491
 
 
10.9
 
 
 
 
AFG3L2
 
 
hsa-miR-181a-5p
 
 
-0.491
 
 
10.9
 
 
 
 
AGO4
 
 
hsa-miR-221-3p
 
 
-0.321
 
 
13.3
 
 
 
 
AHR
 
 
hsa-miR-29a-3p
 
 
-0.316
 
 
17.6
 
 
 
 
AK1
 
 
hsa-miR-29a-3p
 
 
-0.333
 
 
31.5
 
 
 
 
ALAD
 
 
hsa-miR-15b-5p
 
 
-0.446
 
 
12.8
 
 
 
 
ANGPTL2
 
 
hsa-miR-221-3p
 
 
-0.369
 
 
46.4
 
 
 
 
ANKFY1
 
 
hsa-miR-155-5p
 
 
-0.315
 
 
14.2
 
 
 
 
ANKHD1
 
 
hsa-miR-23a-3p
 
 
-0.368
 
 
46.1
 
 
 
 
ANKHD1
 
 
hsa-miR-23b-3p
 
 
-0.368
 
 
46.1
 
 
 
 
ANKHD1
 
 
hsa-miR-30b-5p
 
 
-0.405
 
 
46.1
 
 
 
 
ANKRD17
 
 
hsa-miR-30b-5p
 
 
-0.336
 
 
14.3
 
 
 
 
ANP32A
 
 
hsa-miR-132-3p
 
 
-0.374
 
 
80.5
 
 
 
 
ANP32E
 
 
hsa-miR-501-3p
 
 
-0.331
 
 
31.2
 
 
 
 
ANTXR2
 
 
hsa-miR-29a-3p
 
 
-0.347
 
 
17.3
 
 
 
 
ANXA11
 
 
hsa-miR-15b-5p
 
 
-0.463
 
 
73.6
 
 
 
 
AP2A1
 
 
hsa-miR-15b-5p
 
 
-0.333
 
 
45.6
 
 
 
 
AP2B1
 
 
hsa-miR-15b-5p
 
 
-0.334
 
 
39.6
 
 
 
 
AP3S1
 
 
hsa-miR-30b-5p
 
 
-0.293
 
 
36.7
 
 
 
 
APP
 
 
hsa-miR-15b-5p
 
 
-0.298
 
 
140.4
 
 
 
 
AQP3
 
 
hsa-miR-874-3p
 
 
-0.48
 
 
103.8
 
 
 
 
ARF4
 
 
hsa-miR-30b-5p
 
 
-0.289
 
 
60.0
 
 
 
 
ARF4
 
 
hsa-miR-221-3p
 
 
-0.553
 
 
60.0
 
 
 
 
ARF5
 
 
hsa-miR-29a-3p
 
 
-0.491
 
 
116.2
 
 
 
 
ARF6
 
 
hsa-miR-181b-5p
 
 
-0.33
 
 
47.5
 
 
 
 
ARF6
 
 
hsa-miR-181a-5p
 
 
-0.33
 
 
47.5
 
 
 
 
ARHGAP5
 
 
hsa-miR-486-5p
 
 
-0.3
 
 
40.5
 
 
 
 
ARID1B
 
 
hsa-miR-29a-3p
 
 
-0.342
 
 
17.2
 
 
 
 
ARID4B
 
 
hsa-miR-486-5p
 
 
-0.367
 
 
17.3
 
 
 
 
ARL2
 
 
hsa-miR-15b-5p
 
 
-0.374
 
 
63.8
 
 
 
 
ARL2
 
 
hsa-miR-15b-5p
 
 
-0.442
 
 
63.8
 
 
 
 
ARL4A
 
 
hsa-miR-29a-3p
 
 
-0.386
 
 
20.6
 
 
 
 
ARL4A
 
 
hsa-miR-30b-5p
 
 
-0.448
 
 
20.6
 
 
 
 
ARMC8
 
 
hsa-miR-486-5p
 
 
-0.321
 
 
14.9
 
 
 
 
ARMCX2
 
 
hsa-miR-15b-5p
 
 
-0.344
 
 
19.0
 
 
 
 
ARMCX3
 
 
hsa-miR-425-5p
 
 
-0.325
 
 
38.9
 
 
 
 
ARNT
 
 
hsa-miR-221-3p
 
 
-0.465
 
 
18.4
 
 
 
 
ARPP19
 
 
hsa-miR-15b-5p
 
 
-0.346
 
 
41.2
 
 
 
 
ARPP19
 
 
hsa-miR-29a-3p
 
 
-0.371
 
 
41.2
 
 
 
 
ARRDC3
 
 
hsa-miR-29a-3p
 
 
-0.402
 
 
35.0
 
 
 
 
ARVCF
 
 
hsa-miR-155-5p
 
 
-0.388
 
 
10.3
 
 
 
 
ASB3
 
 
hsa-miR-30b-5p
 
 
-0.418
 
 
12.1
 
 
 
 
ASB3
 
 
hsa-miR-30b-5p
 
 
-0.418
 
 
12.1
 
 
 
 
ASB6
 
 
hsa-miR-874-3p
 
 
-0.368
 
 
17.1
 
 
 
 
ASF1A
 
 
hsa-miR-23a-3p
 
 
-0.506
 
 
10.9
 
 
 
 
ASF1A
 
 
hsa-miR-23b-3p
 
 
-0.506
 
 
10.9
 
 
 
 
ASH1L
 
 
hsa-miR-15b-5p
 
 
-0.325
 
 
13.9
 
 
 
 
ASPH
 
 
hsa-miR-221-3p
 
 
-0.287
 
 
26.8
 
 
 
 
ATF3
 
 
hsa-miR-874-3p
 
 
-0.288
 
 
23.0
 
 
 
 
ATG9A
 
 
hsa-miR-15b-5p
 
 
-0.333
 
 
22.4
 
 
 
 
ATOSA
 
 
hsa-miR-221-3p
 
 
-0.495
 
 
12.9
 
 
 
 
ATP5MC3
 
 
hsa-miR-425-5p
 
 
-0.328
 
 
126.3
 
 
 
 
ATP5MC3
 
 
hsa-miR-29a-3p
 
 
-0.33
 
 
126.3
 
 
 
 
ATP6V1E1
 
 
hsa-miR-23a-3p
 
 
-0.294
 
 
99.8
 
 
 
 
ATP6V1E1
 
 
hsa-miR-23b-3p
 
 
-0.294
 
 
99.8
 
 
 
 
ATPAF1
 
 
hsa-miR-361-5p
 
 
-0.362
 
 
23.6
 
 
 
 
ATXN1L
 
 
hsa-miR-155-5p
 
 
-0.315
 
 
11.1
 
 
 
 
ATXN2
 
 
hsa-miR-15b-5p
 
 
-0.392
 
 
13.5
 
 
 
 
AZIN1
 
 
hsa-miR-132-3p
 
 
-0.29
 
 
33.1
 
 
 
 
AZIN1
 
 
hsa-miR-132-3p
 
 
-0.299
 
 
33.1
 
 
 
 
B4GALT5
 
 
hsa-miR-501-3p
 
 
-0.408
 
 
26.0
 
 
 
 
B4GAT1
 
 
hsa-miR-23a-3p
 
 
-0.361
 
 
10.0
 
 
 
 
B4GAT1
 
 
hsa-miR-23b-3p
 
 
-0.361
 
 
10.0
 
 
 
 
BAK1
 
 
hsa-miR-29a-3p
 
 
-0.409
 
 
28.6
 
 
 
 
BCL2
 
 
hsa-miR-342-3p
 
 
-0.428
 
 
16.9
 
 
 
 
BCL2L11
 
 
hsa-miR-221-3p
 
 
-0.423
 
 
15.1
 
 
 
 
BCL2L2
 
 
hsa-miR-29a-3p
 
 
-0.312
 
 
21.4
 
 
 
 
BECN1
 
 
hsa-miR-30b-5p
 
 
-0.452
 
 
37.5
 
 
 
 
BET1
 
 
hsa-miR-23a-3p
 
 
-0.402
 
 
15.7
 
 
 
 
BET1
 
 
hsa-miR-23b-3p
 
 
-0.418
 
 
15.7
 
 
 
 
BFAR
 
 
hsa-miR-15b-5p
 
 
-0.352
 
 
22.0
 
 
 
 
BHLHE40
 
 
hsa-miR-181b-5p
 
 
-0.408
 
 
28.2
 
 
 
 
BHLHE40
 
 
hsa-miR-181a-5p
 
 
-0.408
 
 
28.2
 
 
 
 
BLCAP
 
 
hsa-miR-23a-3p
 
 
-0.601
 
 
45.7
 
 
 
 
BLCAP
 
 
hsa-miR-23b-3p
 
 
-0.601
 
 
45.7
 
 
 
 
BLMH
 
 
hsa-miR-29a-3p
 
 
-0.393
 
 
20.2
 
 
 
 
BLOC1S6
 
 
hsa-miR-181b-5p
 
 
-0.393
 
 
26.3
 
 
 
 
BLOC1S6
 
 
hsa-miR-181a-5p
 
 
-0.393
 
 
26.3
 
 
 
 
BMF
 
 
hsa-miR-29a-3p
 
 
-0.41
 
 
17.8
 
 
 
 
BMF
 
 
hsa-miR-221-3p
 
 
-0.453
 
 
17.8
 
 
 
 
BOLA3
 
 
hsa-miR-15b-5p
 
 
-0.363
 
 
23.2
 
 
 
 
BOLA3
 
 
hsa-miR-15b-5p
 
 
-0.386
 
 
23.2
 
 
 
 
BOLA3
 
 
hsa-miR-374b-5p
 
 
-0.486
 
 
23.2
 
 
 
 
BORCS6
 
 
hsa-miR-15b-5p
 
 
-0.347
 
 
15.9
 
 
 
 
BRI3
 
 
hsa-miR-132-3p
 
 
-0.385
 
 
105.7
 
 
 
 
BTAF1
 
 
hsa-miR-486-5p
 
 
-0.288
 
 
17.6
 
 
 
 
BTAF1
 
 
hsa-miR-15b-5p
 
 
-0.496
 
 
17.6
 
 
 
 
BTF3
 
 
hsa-miR-15b-5p
 
 
-0.344
 
 
377.1
 
 
 
 
BTF3
 
 
hsa-miR-361-5p
 
 
-0.551
 
 
377.1
 
 
 
 
 
 
 stomach 
 
 
 
 
target_symbol
 
 
mature_mirna_id
 
 
score
 
 
nTPM
 
 
 
 
 
 
AAR2
 
 
hsa-miR-29a-3p
 
 
-0.327
 
 
21.7
 
 
 
 
ABCE1
 
 
hsa-miR-29a-3p
 
 
-0.404
 
 
19.0
 
 
 
 
ABHD4
 
 
hsa-miR-29a-3p
 
 
-0.312
 
 
26.1
 
 
 
 
ACTC1
 
 
hsa-miR-30b-5p
 
 
-0.543
 
 
32.8
 
 
 
 
ACTR2
 
 
hsa-miR-15b-5p
 
 
-0.293
 
 
100.4
 
 
 
 
ACVR1
 
 
hsa-miR-30b-5p
 
 
-0.29
 
 
11.0
 
 
 
 
ADA
 
 
hsa-miR-29a-3p
 
 
-0.376
 
 
44.4
 
 
 
 
ADAM9
 
 
hsa-miR-30b-5p
 
 
-0.312
 
 
41.8
 
 
 
 
ADCY3
 
 
hsa-miR-132-3p
 
 
-0.303
 
 
23.8
 
 
 
 
ADIPOR2
 
 
hsa-miR-150-5p
 
 
-0.287
 
 
42.5
 
 
 
 
AFG3L2
 
 
hsa-miR-181b-5p
 
 
-0.491
 
 
26.3
 
 
 
 
AFG3L2
 
 
hsa-miR-181a-5p
 
 
-0.491
 
 
26.3
 
 
 
 
AHR
 
 
hsa-miR-29a-3p
 
 
-0.316
 
 
23.8
 
 
 
 
AK1
 
 
hsa-miR-29a-3p
 
 
-0.333
 
 
28.6
 
 
 
 
AK4
 
 
hsa-miR-15b-5p
 
 
-0.316
 
 
14.0
 
 
 
 
ALAD
 
 
hsa-miR-15b-5p
 
 
-0.446
 
 
49.1
 
 
 
 
AMOTL1
 
 
hsa-miR-15b-5p
 
 
-0.319
 
 
11.8
 
 
 
 
ANGPTL2
 
 
hsa-miR-221-3p
 
 
-0.369
 
 
27.7
 
 
 
 
ANKFY1
 
 
hsa-miR-155-5p
 
 
-0.315
 
 
12.2
 
 
 
 
ANKHD1
 
 
hsa-miR-23a-3p
 
 
-0.368
 
 
39.4
 
 
 
 
ANKHD1
 
 
hsa-miR-23b-3p
 
 
-0.368
 
 
39.4
 
 
 
 
ANKHD1
 
 
hsa-miR-30b-5p
 
 
-0.405
 
 
39.4
 
 
 
 
ANKRD17
 
 
hsa-miR-30b-5p
 
 
-0.336
 
 
14.8
 
 
 
 
ANKRD29
 
 
hsa-miR-132-3p
 
 
-0.47
 
 
14.3
 
 
 
 
ANP32A
 
 
hsa-miR-132-3p
 
 
-0.374
 
 
74.4
 
 
 
 
ANP32E
 
 
hsa-miR-501-3p
 
 
-0.331
 
 
16.3
 
 
 
 
ANTXR2
 
 
hsa-miR-29a-3p
 
 
-0.347
 
 
46.6
 
 
 
 
ANXA11
 
 
hsa-miR-15b-5p
 
 
-0.463
 
 
131.4
 
 
 
 
AP2A1
 
 
hsa-miR-15b-5p
 
 
-0.333
 
 
34.8
 
 
 
 
AP2B1
 
 
hsa-miR-15b-5p
 
 
-0.334
 
 
45.1
 
 
 
 
AP3S1
 
 
hsa-miR-30b-5p
 
 
-0.293
 
 
42.9
 
 
 
 
APOO
 
 
hsa-miR-181b-5p
 
 
-0.5
 
 
17.0
 
 
 
 
APOO
 
 
hsa-miR-181a-5p
 
 
-0.5
 
 
17.0
 
 
 
 
APP
 
 
hsa-miR-15b-5p
 
 
-0.298
 
 
197.5
 
 
 
 
AQP3
 
 
hsa-miR-874-3p
 
 
-0.48
 
 
33.6
 
 
 
 
ARF4
 
 
hsa-miR-30b-5p
 
 
-0.289
 
 
123.5
 
 
 
 
ARF4
 
 
hsa-miR-221-3p
 
 
-0.553
 
 
123.5
 
 
 
 
ARF5
 
 
hsa-miR-29a-3p
 
 
-0.491
 
 
126.0
 
 
 
 
ARF6
 
 
hsa-miR-181b-5p
 
 
-0.33
 
 
44.4
 
 
 
 
ARF6
 
 
hsa-miR-181a-5p
 
 
-0.33
 
 
44.4
 
 
 
 
ARHGAP5
 
 
hsa-miR-486-5p
 
 
-0.3
 
 
27.8
 
 
 
 
ARID1B
 
 
hsa-miR-29a-3p
 
 
-0.342
 
 
11.1
 
 
 
 
ARID4B
 
 
hsa-miR-486-5p
 
 
-0.367
 
 
12.5
 
 
 
 
ARL2
 
 
hsa-miR-15b-5p
 
 
-0.374
 
 
85.3
 
 
 
 
ARL2
 
 
hsa-miR-15b-5p
 
 
-0.442
 
 
85.3
 
 
 
 
ARL4A
 
 
hsa-miR-29a-3p
 
 
-0.386
 
 
24.0
 
 
 
 
ARL4A
 
 
hsa-miR-30b-5p
 
 
-0.448
 
 
24.0
 
 
 
 
ARMC8
 
 
hsa-miR-486-5p
 
 
-0.321
 
 
14.5
 
 
 
 
ARMCX2
 
 
hsa-miR-15b-5p
 
 
-0.344
 
 
14.4
 
 
 
 
ARMCX3
 
 
hsa-miR-425-5p
 
 
-0.325
 
 
26.2
 
 
 
 
ARNT
 
 
hsa-miR-221-3p
 
 
-0.465
 
 
17.9
 
 
 
 
ARPP19
 
 
hsa-miR-15b-5p
 
 
-0.346
 
 
36.4
 
 
 
 
ARPP19
 
 
hsa-miR-29a-3p
 
 
-0.371
 
 
36.4
 
 
 
 
ARRDC3
 
 
hsa-miR-29a-3p
 
 
-0.402
 
 
24.3
 
 
 
 
ASAP2
 
 
hsa-miR-29a-3p
 
 
-0.348
 
 
22.9
 
 
 
 
ASB6
 
 
hsa-miR-874-3p
 
 
-0.368
 
 
13.6
 
 
 
 
ASF1A
 
 
hsa-miR-23a-3p
 
 
-0.506
 
 
10.3
 
 
 
 
ASF1A
 
 
hsa-miR-23b-3p
 
 
-0.506
 
 
10.3
 
 
 
 
ASH1L
 
 
hsa-miR-15b-5p
 
 
-0.325
 
 
17.1
 
 
 
 
ASPH
 
 
hsa-miR-221-3p
 
 
-0.287
 
 
96.9
 
 
 
 
ATF3
 
 
hsa-miR-874-3p
 
 
-0.288
 
 
103.9
 
 
 
 
ATG5
 
 
hsa-miR-30b-5p
 
 
-0.488
 
 
11.4
 
 
 
 
ATG9A
 
 
hsa-miR-15b-5p
 
 
-0.333
 
 
24.0
 
 
 
 
ATOSA
 
 
hsa-miR-221-3p
 
 
-0.495
 
 
14.2
 
 
 
 
ATP5MC3
 
 
hsa-miR-425-5p
 
 
-0.328
 
 
258.5
 
 
 
 
ATP5MC3
 
 
hsa-miR-29a-3p
 
 
-0.33
 
 
258.5
 
 
 
 
ATP6V1E1
 
 
hsa-miR-23a-3p
 
 
-0.294
 
 
101.7
 
 
 
 
ATP6V1E1
 
 
hsa-miR-23b-3p
 
 
-0.294
 
 
101.7
 
 
 
 
ATPAF1
 
 
hsa-miR-361-5p
 
 
-0.362
 
 
40.7
 
 
 
 
ATXN2
 
 
hsa-miR-15b-5p
 
 
-0.392
 
 
16.1
 
 
 
 
AUH
 
 
hsa-miR-23a-3p
 
 
-0.495
 
 
21.4
 
 
 
 
AUH
 
 
hsa-miR-23b-3p
 
 
-0.503
 
 
21.4
 
 
 
 
AUH
 
 
hsa-miR-23a-3p
 
 
-0.512
 
 
21.4
 
 
 
 
AUH
 
 
hsa-miR-23b-3p
 
 
-0.512
 
 
21.4
 
 
 
 
AXIN2
 
 
hsa-miR-15b-5p
 
 
-0.3
 
 
12.9
 
 
 
 
AZIN1
 
 
hsa-miR-132-3p
 
 
-0.29
 
 
48.8
 
 
 
 
AZIN1
 
 
hsa-miR-132-3p
 
 
-0.299
 
 
48.8
 
 
 
 
B3GNT5
 
 
hsa-miR-30b-5p
 
 
-0.326
 
 
10.7
 
 
 
 
B4GALT5
 
 
hsa-miR-501-3p
 
 
-0.408
 
 
24.3
 
 
 
 
B4GAT1
 
 
hsa-miR-23a-3p
 
 
-0.361
 
 
25.1
 
 
 
 
B4GAT1
 
 
hsa-miR-23b-3p
 
 
-0.361
 
 
25.1
 
 
 
 
BAK1
 
 
hsa-miR-29a-3p
 
 
-0.409
 
 
20.7
 
 
 
 
BCL2L2
 
 
hsa-miR-29a-3p
 
 
-0.312
 
 
52.5
 
 
 
 
BDH1
 
 
hsa-miR-874-3p
 
 
-0.644
 
 
15.2
 
 
 
 
BECN1
 
 
hsa-miR-30b-5p
 
 
-0.452
 
 
50.1
 
 
 
 
BET1
 
 
hsa-miR-23a-3p
 
 
-0.402
 
 
18.8
 
 
 
 
BET1
 
 
hsa-miR-23b-3p
 
 
-0.418
 
 
18.8
 
 
 
 
BFAR
 
 
hsa-miR-15b-5p
 
 
-0.352
 
 
27.6
 
 
 
 
BHLHE40
 
 
hsa-miR-181b-5p
 
 
-0.408
 
 
47.9
 
 
 
 
BHLHE40
 
 
hsa-miR-181a-5p
 
 
-0.408
 
 
47.9
 
 
 
 
BICDL1
 
 
hsa-miR-221-3p
 
 
-0.447
 
 
14.9
 
 
 
 
BLCAP
 
 
hsa-miR-23a-3p
 
 
-0.601
 
 
37.8
 
 
 
 
BLCAP
 
 
hsa-miR-23b-3p
 
 
-0.601
 
 
37.8
 
 
 
 
BLMH
 
 
hsa-miR-29a-3p
 
 
-0.393
 
 
15.8
 
 
 
 
BLOC1S6
 
 
hsa-miR-181b-5p
 
 
-0.393
 
 
18.9
 
 
 
 
BLOC1S6
 
 
hsa-miR-181a-5p
 
 
-0.393
 
 
18.9
 
 
 
 
BOLA3
 
 
hsa-miR-15b-5p
 
 
-0.363
 
 
43.1
 
 
 
 
BOLA3
 
 
hsa-miR-15b-5p
 
 
-0.386
 
 
43.1
 
 
 
 
BOLA3
 
 
hsa-miR-374b-5p
 
 
-0.486
 
 
43.1
 
 
 
 
BORCS6
 
 
hsa-miR-15b-5p
 
 
-0.347
 
 
11.2
 
 
 
 
 
 
 testis 
 
 
 
 
target_symbol
 
 
mature_mirna_id
 
 
score
 
 
nTPM
 
 
 
 
 
 
AAR2
 
 
hsa-miR-29a-3p
 
 
-0.327
 
 
15.4
 
 
 
 
ABCB6
 
 
hsa-miR-29a-3p
 
 
-0.48
 
 
15.2
 
 
 
 
ABCE1
 
 
hsa-miR-29a-3p
 
 
-0.404
 
 
24.7
 
 
 
 
ABHD17B
 
 
hsa-miR-486-5p
 
 
-0.348
 
 
10.4
 
 
 
 
ABHD4
 
 
hsa-miR-29a-3p
 
 
-0.312
 
 
39.1
 
 
 
 
ACTL7A
 
 
hsa-miR-155-5p
 
 
-0.531
 
 
218.7
 
 
 
 
ACTR2
 
 
hsa-miR-15b-5p
 
 
-0.293
 
 
37.7
 
 
 
 
ADAM9
 
 
hsa-miR-30b-5p
 
 
-0.312
 
 
10.4
 
 
 
 
ADIPOR2
 
 
hsa-miR-150-5p
 
 
-0.287
 
 
26.9
 
 
 
 
AFG3L2
 
 
hsa-miR-181b-5p
 
 
-0.491
 
 
10.5
 
 
 
 
AFG3L2
 
 
hsa-miR-181a-5p
 
 
-0.491
 
 
10.5
 
 
 
 
AK1
 
 
hsa-miR-29a-3p
 
 
-0.333
 
 
64.3
 
 
 
 
AK4
 
 
hsa-miR-15b-5p
 
 
-0.316
 
 
10.1
 
 
 
 
ALAD
 
 
hsa-miR-15b-5p
 
 
-0.446
 
 
12.6
 
 
 
 
ALDH1A2
 
 
hsa-miR-23b-3p
 
 
-0.346
 
 
53.9
 
 
 
 
ALDH1A2
 
 
hsa-miR-23a-3p
 
 
-0.354
 
 
53.9
 
 
 
 
AMOTL1
 
 
hsa-miR-15b-5p
 
 
-0.319
 
 
11.0
 
 
 
 
ANKHD1
 
 
hsa-miR-23a-3p
 
 
-0.368
 
 
29.3
 
 
 
 
ANKHD1
 
 
hsa-miR-23b-3p
 
 
-0.368
 
 
29.3
 
 
 
 
ANKHD1
 
 
hsa-miR-30b-5p
 
 
-0.405
 
 
29.3
 
 
 
 
ANKRD17
 
 
hsa-miR-30b-5p
 
 
-0.336
 
 
10.4
 
 
 
 
ANLN
 
 
hsa-miR-15b-5p
 
 
-0.334
 
 
11.6
 
 
 
 
ANP32A
 
 
hsa-miR-132-3p
 
 
-0.374
 
 
19.4
 
 
 
 
ANXA11
 
 
hsa-miR-15b-5p
 
 
-0.463
 
 
22.9
 
 
 
 
AP2A1
 
 
hsa-miR-15b-5p
 
 
-0.333
 
 
85.4
 
 
 
 
AP2B1
 
 
hsa-miR-15b-5p
 
 
-0.334
 
 
87.4
 
 
 
 
AP3S1
 
 
hsa-miR-30b-5p
 
 
-0.293
 
 
53.5
 
 
 
 
APOO
 
 
hsa-miR-181b-5p
 
 
-0.5
 
 
15.7
 
 
 
 
APOO
 
 
hsa-miR-181a-5p
 
 
-0.5
 
 
15.7
 
 
 
 
APP
 
 
hsa-miR-15b-5p
 
 
-0.298
 
 
146.3
 
 
 
 
ARF4
 
 
hsa-miR-30b-5p
 
 
-0.289
 
 
94.2
 
 
 
 
ARF4
 
 
hsa-miR-221-3p
 
 
-0.553
 
 
94.2
 
 
 
 
ARF5
 
 
hsa-miR-29a-3p
 
 
-0.491
 
 
89.2
 
 
 
 
ARF6
 
 
hsa-miR-181b-5p
 
 
-0.33
 
 
15.0
 
 
 
 
ARF6
 
 
hsa-miR-181a-5p
 
 
-0.33
 
 
15.0
 
 
 
 
ARHGAP5
 
 
hsa-miR-486-5p
 
 
-0.3
 
 
43.5
 
 
 
 
ARID2
 
 
hsa-miR-132-3p
 
 
-0.304
 
 
24.3
 
 
 
 
ARID2
 
 
hsa-miR-155-5p
 
 
-0.384
 
 
24.3
 
 
 
 
ARID4B
 
 
hsa-miR-486-5p
 
 
-0.367
 
 
16.0
 
 
 
 
ARL2
 
 
hsa-miR-15b-5p
 
 
-0.374
 
 
115.7
 
 
 
 
ARL2
 
 
hsa-miR-15b-5p
 
 
-0.442
 
 
115.7
 
 
 
 
ARL3
 
 
hsa-miR-15b-5p
 
 
-0.507
 
 
10.4
 
 
 
 
ARL4A
 
 
hsa-miR-29a-3p
 
 
-0.386
 
 
181.2
 
 
 
 
ARL4A
 
 
hsa-miR-30b-5p
 
 
-0.448
 
 
181.2
 
 
 
 
ARMC12
 
 
hsa-miR-15b-5p
 
 
-0.485
 
 
58.4
 
 
 
 
ARMC8
 
 
hsa-miR-486-5p
 
 
-0.321
 
 
17.2
 
 
 
 
ARMCX3
 
 
hsa-miR-425-5p
 
 
-0.325
 
 
12.0
 
 
 
 
ARNT
 
 
hsa-miR-221-3p
 
 
-0.465
 
 
16.2
 
 
 
 
ARPP19
 
 
hsa-miR-15b-5p
 
 
-0.346
 
 
79.0
 
 
 
 
ARPP19
 
 
hsa-miR-29a-3p
 
 
-0.371
 
 
79.0
 
 
 
 
ARRDC3
 
 
hsa-miR-29a-3p
 
 
-0.402
 
 
15.5
 
 
 
 
ASAP2
 
 
hsa-miR-29a-3p
 
 
-0.348
 
 
43.7
 
 
 
 
ASB3
 
 
hsa-miR-30b-5p
 
 
-0.418
 
 
10.6
 
 
 
 
ASB3
 
 
hsa-miR-30b-5p
 
 
-0.418
 
 
10.6
 
 
 
 
ASB6
 
 
hsa-miR-874-3p
 
 
-0.368
 
 
12.3
 
 
 
 
ASH1L
 
 
hsa-miR-15b-5p
 
 
-0.325
 
 
11.0
 
 
 
 
ASPH
 
 
hsa-miR-221-3p
 
 
-0.287
 
 
15.3
 
 
 
 
ATG9A
 
 
hsa-miR-15b-5p
 
 
-0.333
 
 
62.2
 
 
 
 
ATOSA
 
 
hsa-miR-221-3p
 
 
-0.495
 
 
17.8
 
 
 
 
ATP5MC3
 
 
hsa-miR-425-5p
 
 
-0.328
 
 
60.2
 
 
 
 
ATP5MC3
 
 
hsa-miR-29a-3p
 
 
-0.33
 
 
60.2
 
 
 
 
ATP6V1E1
 
 
hsa-miR-23a-3p
 
 
-0.294
 
 
62.9
 
 
 
 
ATP6V1E1
 
 
hsa-miR-23b-3p
 
 
-0.294
 
 
62.9
 
 
 
 
ATPAF1
 
 
hsa-miR-361-5p
 
 
-0.362
 
 
29.2
 
 
 
 
ATXN1L
 
 
hsa-miR-155-5p
 
 
-0.315
 
 
11.4
 
 
 
 
ATXN2
 
 
hsa-miR-15b-5p
 
 
-0.392
 
 
23.4
 
 
 
 
AZIN1
 
 
hsa-miR-132-3p
 
 
-0.29
 
 
32.4
 
 
 
 
AZIN1
 
 
hsa-miR-132-3p
 
 
-0.299
 
 
32.4
 
 
 
 
B4GALT5
 
 
hsa-miR-501-3p
 
 
-0.408
 
 
20.8
 
 
 
 
BCL2L2
 
 
hsa-miR-29a-3p
 
 
-0.312
 
 
26.0
 
 
 
 
BCL2L2-PABPN1
 
 
hsa-miR-425-5p
 
 
-0.321
 
 
11.0
 
 
 
 
BECN1
 
 
hsa-miR-30b-5p
 
 
-0.452
 
 
22.7
 
 
 
 
BET1
 
 
hsa-miR-23a-3p
 
 
-0.402
 
 
13.0
 
 
 
 
BET1
 
 
hsa-miR-23b-3p
 
 
-0.418
 
 
13.0
 
 
 
 
BFAR
 
 
hsa-miR-15b-5p
 
 
-0.352
 
 
15.3
 
 
 
 
BHLHE40
 
 
hsa-miR-181b-5p
 
 
-0.408
 
 
14.9
 
 
 
 
BHLHE40
 
 
hsa-miR-181a-5p
 
 
-0.408
 
 
14.9
 
 
 
 
BICDL1
 
 
hsa-miR-221-3p
 
 
-0.447
 
 
27.2
 
 
 
 
BLCAP
 
 
hsa-miR-23a-3p
 
 
-0.601
 
 
29.6
 
 
 
 
BLCAP
 
 
hsa-miR-23b-3p
 
 
-0.601
 
 
29.6
 
 
 
 
BLMH
 
 
hsa-miR-29a-3p
 
 
-0.393
 
 
15.5
 
 
 
 
BLOC1S6
 
 
hsa-miR-181b-5p
 
 
-0.393
 
 
15.9
 
 
 
 
BLOC1S6
 
 
hsa-miR-181a-5p
 
 
-0.393
 
 
15.9
 
 
 
 
BOLA3
 
 
hsa-miR-15b-5p
 
 
-0.363
 
 
17.2
 
 
 
 
BOLA3
 
 
hsa-miR-15b-5p
 
 
-0.386
 
 
17.2
 
 
 
 
BOLA3
 
 
hsa-miR-374b-5p
 
 
-0.486
 
 
17.2
 
 
 
 
BOLL
 
 
hsa-miR-132-3p
 
 
-0.4
 
 
45.7
 
 
 
 
BRI3
 
 
hsa-miR-132-3p
 
 
-0.385
 
 
47.9
 
 
 
 
BTAF1
 
 
hsa-miR-486-5p
 
 
-0.288
 
 
14.0
 
 
 
 
BTAF1
 
 
hsa-miR-15b-5p
 
 
-0.496
 
 
14.0
 
 
 
 
BTF3
 
 
hsa-miR-15b-5p
 
 
-0.344
 
 
169.6
 
 
 
 
BTF3
 
 
hsa-miR-361-5p
 
 
-0.551
 
 
169.6
 
 
 
 
BTN2A1
 
 
hsa-miR-342-3p
 
 
-0.31
 
 
11.4
 
 
 
 
BTRC
 
 
hsa-miR-15b-5p
 
 
-0.302
 
 
12.9
 
 
 
 
BTRC
 
 
hsa-miR-15b-5p
 
 
-0.358
 
 
12.9
 
 
 
 
BZW1
 
 
hsa-miR-15b-5p
 
 
-0.367
 
 
159.6
 
 
 
 
C11orf24
 
 
hsa-miR-15b-5p
 
 
-0.403
 
 
14.0
 
 
 
 
C11orf68
 
 
hsa-miR-15b-5p
 
 
-0.29
 
 
52.0
 
 
 
 
C16orf87
 
 
hsa-miR-29a-3p
 
 
-0.353
 
 
17.2
 
 
 
 
C16orf87
 
 
hsa-miR-181b-5p
 
 
-0.54
 
 
17.2
 
 
 
 
 
 
 thymus 
 
 
 
 
target_symbol
 
 
mature_mirna_id
 
 
score
 
 
nTPM
 
 
 
 
 
 
AAR2
 
 
hsa-miR-29a-3p
 
 
-0.327
 
 
24.4
 
 
 
 
ABCE1
 
 
hsa-miR-29a-3p
 
 
-0.404
 
 
31.9
 
 
 
 
ABHD17B
 
 
hsa-miR-486-5p
 
 
-0.348
 
 
30.8
 
 
 
 
ABHD4
 
 
hsa-miR-29a-3p
 
 
-0.312
 
 
10.3
 
 
 
 
ACTC1
 
 
hsa-miR-30b-5p
 
 
-0.543
 
 
10.5
 
 
 
 
ACTR2
 
 
hsa-miR-15b-5p
 
 
-0.293
 
 
169.2
 
 
 
 
ADA
 
 
hsa-miR-29a-3p
 
 
-0.376
 
 
228.3
 
 
 
 
ADCY3
 
 
hsa-miR-132-3p
 
 
-0.303
 
 
10.7
 
 
 
 
ADIPOR2
 
 
hsa-miR-150-5p
 
 
-0.287
 
 
11.9
 
 
 
 
AFG3L2
 
 
hsa-miR-181b-5p
 
 
-0.491
 
 
12.1
 
 
 
 
AFG3L2
 
 
hsa-miR-181a-5p
 
 
-0.491
 
 
12.1
 
 
 
 
ALAD
 
 
hsa-miR-15b-5p
 
 
-0.446
 
 
18.9
 
 
 
 
AMOTL1
 
 
hsa-miR-15b-5p
 
 
-0.319
 
 
26.0
 
 
 
 
ANKFY1
 
 
hsa-miR-155-5p
 
 
-0.315
 
 
18.9
 
 
 
 
ANKHD1
 
 
hsa-miR-23a-3p
 
 
-0.368
 
 
45.5
 
 
 
 
ANKHD1
 
 
hsa-miR-23b-3p
 
 
-0.368
 
 
45.5
 
 
 
 
ANKHD1
 
 
hsa-miR-30b-5p
 
 
-0.405
 
 
45.5
 
 
 
 
ANKRD17
 
 
hsa-miR-30b-5p
 
 
-0.336
 
 
25.3
 
 
 
 
ANLN
 
 
hsa-miR-15b-5p
 
 
-0.334
 
 
14.9
 
 
 
 
ANP32A
 
 
hsa-miR-132-3p
 
 
-0.374
 
 
189.1
 
 
 
 
ANP32E
 
 
hsa-miR-501-3p
 
 
-0.331
 
 
82.1
 
 
 
 
ANTXR2
 
 
hsa-miR-29a-3p
 
 
-0.347
 
 
10.0
 
 
 
 
ANXA11
 
 
hsa-miR-15b-5p
 
 
-0.463
 
 
93.3
 
 
 
 
AP2A1
 
 
hsa-miR-15b-5p
 
 
-0.333
 
 
14.3
 
 
 
 
AP2B1
 
 
hsa-miR-15b-5p
 
 
-0.334
 
 
55.6
 
 
 
 
AP3S1
 
 
hsa-miR-30b-5p
 
 
-0.293
 
 
38.3
 
 
 
 
APOO
 
 
hsa-miR-181b-5p
 
 
-0.5
 
 
13.0
 
 
 
 
APOO
 
 
hsa-miR-181a-5p
 
 
-0.5
 
 
13.0
 
 
 
 
APP
 
 
hsa-miR-15b-5p
 
 
-0.298
 
 
143.9
 
 
 
 
AQP3
 
 
hsa-miR-874-3p
 
 
-0.48
 
 
217.7
 
 
 
 
ARF4
 
 
hsa-miR-30b-5p
 
 
-0.289
 
 
69.9
 
 
 
 
ARF4
 
 
hsa-miR-221-3p
 
 
-0.553
 
 
69.9
 
 
 
 
ARF5
 
 
hsa-miR-29a-3p
 
 
-0.491
 
 
89.3
 
 
 
 
ARF6
 
 
hsa-miR-181b-5p
 
 
-0.33
 
 
41.8
 
 
 
 
ARF6
 
 
hsa-miR-181a-5p
 
 
-0.33
 
 
41.8
 
 
 
 
ARHGAP5
 
 
hsa-miR-486-5p
 
 
-0.3
 
 
17.1
 
 
 
 
ARID1B
 
 
hsa-miR-29a-3p
 
 
-0.342
 
 
23.3
 
 
 
 
ARID2
 
 
hsa-miR-132-3p
 
 
-0.304
 
 
17.5
 
 
 
 
ARID2
 
 
hsa-miR-155-5p
 
 
-0.384
 
 
17.5
 
 
 
 
ARID4B
 
 
hsa-miR-486-5p
 
 
-0.367
 
 
19.9
 
 
 
 
ARL2
 
 
hsa-miR-15b-5p
 
 
-0.374
 
 
31.2
 
 
 
 
ARL2
 
 
hsa-miR-15b-5p
 
 
-0.442
 
 
31.2
 
 
 
 
ARL4A
 
 
hsa-miR-29a-3p
 
 
-0.386
 
 
21.1
 
 
 
 
ARL4A
 
 
hsa-miR-30b-5p
 
 
-0.448
 
 
21.1
 
 
 
 
ARL5B
 
 
hsa-miR-29a-3p
 
 
-0.304
 
 
12.6
 
 
 
 
ARL5B
 
 
hsa-miR-155-5p
 
 
-0.355
 
 
12.6
 
 
 
 
ARMC8
 
 
hsa-miR-486-5p
 
 
-0.321
 
 
20.7
 
 
 
 
ARMCX3
 
 
hsa-miR-425-5p
 
 
-0.325
 
 
18.7
 
 
 
 
ARNT
 
 
hsa-miR-221-3p
 
 
-0.465
 
 
19.0
 
 
 
 
ARPP19
 
 
hsa-miR-15b-5p
 
 
-0.346
 
 
49.2
 
 
 
 
ARPP19
 
 
hsa-miR-29a-3p
 
 
-0.371
 
 
49.2
 
 
 
 
ARPP21
 
 
hsa-miR-15b-5p
 
 
-0.342
 
 
141.6
 
 
 
 
ARPP21
 
 
hsa-miR-501-3p
 
 
-0.546
 
 
141.6
 
 
 
 
ARRDC3
 
 
hsa-miR-29a-3p
 
 
-0.402
 
 
26.1
 
 
 
 
ASB3
 
 
hsa-miR-30b-5p
 
 
-0.418
 
 
16.0
 
 
 
 
ASB3
 
 
hsa-miR-30b-5p
 
 
-0.418
 
 
16.0
 
 
 
 
ASB6
 
 
hsa-miR-874-3p
 
 
-0.368
 
 
10.4
 
 
 
 
ASF1A
 
 
hsa-miR-23a-3p
 
 
-0.506
 
 
22.7
 
 
 
 
ASF1A
 
 
hsa-miR-23b-3p
 
 
-0.506
 
 
22.7
 
 
 
 
ASH1L
 
 
hsa-miR-15b-5p
 
 
-0.325
 
 
11.3
 
 
 
 
ASPH
 
 
hsa-miR-221-3p
 
 
-0.287
 
 
15.5
 
 
 
 
ATF3
 
 
hsa-miR-874-3p
 
 
-0.288
 
 
79.1
 
 
 
 
ATG9A
 
 
hsa-miR-15b-5p
 
 
-0.333
 
 
14.6
 
 
 
 
ATOSA
 
 
hsa-miR-221-3p
 
 
-0.495
 
 
16.1
 
 
 
 
ATP5MC3
 
 
hsa-miR-425-5p
 
 
-0.328
 
 
175.6
 
 
 
 
ATP5MC3
 
 
hsa-miR-29a-3p
 
 
-0.33
 
 
175.6
 
 
 
 
ATP6V1E1
 
 
hsa-miR-23a-3p
 
 
-0.294
 
 
100.8
 
 
 
 
ATP6V1E1
 
 
hsa-miR-23b-3p
 
 
-0.294
 
 
100.8
 
 
 
 
ATPAF1
 
 
hsa-miR-361-5p
 
 
-0.362
 
 
41.4
 
 
 
 
ATXN1L
 
 
hsa-miR-155-5p
 
 
-0.315
 
 
11.9
 
 
 
 
ATXN2
 
 
hsa-miR-15b-5p
 
 
-0.392
 
 
11.9
 
 
 
 
AXIN2
 
 
hsa-miR-15b-5p
 
 
-0.3
 
 
16.1
 
 
 
 
AZIN1
 
 
hsa-miR-132-3p
 
 
-0.29
 
 
52.8
 
 
 
 
AZIN1
 
 
hsa-miR-132-3p
 
 
-0.299
 
 
52.8
 
 
 
 
BACH2
 
 
hsa-miR-29a-3p
 
 
-0.294
 
 
12.2
 
 
 
 
BAK1
 
 
hsa-miR-29a-3p
 
 
-0.409
 
 
17.9
 
 
 
 
BCL2
 
 
hsa-miR-342-3p
 
 
-0.428
 
 
17.0
 
 
 
 
BCL2L11
 
 
hsa-miR-221-3p
 
 
-0.423
 
 
18.0
 
 
 
 
BCL2L2
 
 
hsa-miR-29a-3p
 
 
-0.312
 
 
10.0
 
 
 
 
BCL7A
 
 
hsa-miR-15b-5p
 
 
-0.336
 
 
23.0
 
 
 
 
BCL7A
 
 
hsa-miR-501-3p
 
 
-0.339
 
 
23.0
 
 
 
 
BCOR
 
 
hsa-miR-425-5p
 
 
-0.346
 
 
24.0
 
 
 
 
BECN1
 
 
hsa-miR-30b-5p
 
 
-0.452
 
 
38.6
 
 
 
 
BET1
 
 
hsa-miR-23a-3p
 
 
-0.402
 
 
18.3
 
 
 
 
BET1
 
 
hsa-miR-23b-3p
 
 
-0.418
 
 
18.3
 
 
 
 
BFAR
 
 
hsa-miR-15b-5p
 
 
-0.352
 
 
19.8
 
 
 
 
BHLHE40
 
 
hsa-miR-181b-5p
 
 
-0.408
 
 
38.0
 
 
 
 
BHLHE40
 
 
hsa-miR-181a-5p
 
 
-0.408
 
 
38.0
 
 
 
 
BICDL1
 
 
hsa-miR-221-3p
 
 
-0.447
 
 
12.0
 
 
 
 
BLCAP
 
 
hsa-miR-23a-3p
 
 
-0.601
 
 
38.8
 
 
 
 
BLCAP
 
 
hsa-miR-23b-3p
 
 
-0.601
 
 
38.8
 
 
 
 
BLMH
 
 
hsa-miR-29a-3p
 
 
-0.393
 
 
33.8
 
 
 
 
BLOC1S6
 
 
hsa-miR-181b-5p
 
 
-0.393
 
 
23.5
 
 
 
 
BLOC1S6
 
 
hsa-miR-181a-5p
 
 
-0.393
 
 
23.5
 
 
 
 
BMF
 
 
hsa-miR-29a-3p
 
 
-0.41
 
 
70.7
 
 
 
 
BMF
 
 
hsa-miR-221-3p
 
 
-0.453
 
 
70.7
 
 
 
 
BOLA3
 
 
hsa-miR-15b-5p
 
 
-0.363
 
 
23.1
 
 
 
 
BOLA3
 
 
hsa-miR-15b-5p
 
 
-0.386
 
 
23.1
 
 
 
 
BOLA3
 
 
hsa-miR-374b-5p
 
 
-0.486
 
 
23.1
 
 
 
 
BRI3
 
 
hsa-miR-132-3p
 
 
-0.385
 
 
13.2
 
 
 
 
 
 
 thyroid gland 
 
 
 
 
target_symbol
 
 
mature_mirna_id
 
 
score
 
 
nTPM
 
 
 
 
 
 
AAR2
 
 
hsa-miR-29a-3p
 
 
-0.327
 
 
16.7
 
 
 
 
ABCE1
 
 
hsa-miR-29a-3p
 
 
-0.404
 
 
25.1
 
 
 
 
ABHD4
 
 
hsa-miR-29a-3p
 
 
-0.312
 
 
22.8
 
 
 
 
ABHD5
 
 
hsa-miR-132-3p
 
 
-0.323
 
 
14.4
 
 
 
 
ACTA1
 
 
hsa-miR-155-5p
 
 
-0.595
 
 
11.1
 
 
 
 
ACTR2
 
 
hsa-miR-15b-5p
 
 
-0.293
 
 
68.0
 
 
 
 
ACVR1
 
 
hsa-miR-30b-5p
 
 
-0.29
 
 
28.6
 
 
 
 
ADAM9
 
 
hsa-miR-30b-5p
 
 
-0.312
 
 
37.4
 
 
 
 
ADCY3
 
 
hsa-miR-132-3p
 
 
-0.303
 
 
35.2
 
 
 
 
ADGRA3
 
 
hsa-miR-30b-5p
 
 
-0.304
 
 
23.6
 
 
 
 
ADIPOR2
 
 
hsa-miR-150-5p
 
 
-0.287
 
 
26.8
 
 
 
 
AFG3L2
 
 
hsa-miR-181b-5p
 
 
-0.491
 
 
24.7
 
 
 
 
AFG3L2
 
 
hsa-miR-181a-5p
 
 
-0.491
 
 
24.7
 
 
 
 
AHR
 
 
hsa-miR-29a-3p
 
 
-0.316
 
 
11.8
 
 
 
 
AK1
 
 
hsa-miR-29a-3p
 
 
-0.333
 
 
42.1
 
 
 
 
AKT3
 
 
hsa-miR-29a-3p
 
 
-0.287
 
 
13.0
 
 
 
 
AKT3
 
 
hsa-miR-15b-5p
 
 
-0.301
 
 
13.0
 
 
 
 
ALAD
 
 
hsa-miR-15b-5p
 
 
-0.446
 
 
24.2
 
 
 
 
ANGPTL2
 
 
hsa-miR-221-3p
 
 
-0.369
 
 
15.2
 
 
 
 
ANKFY1
 
 
hsa-miR-155-5p
 
 
-0.315
 
 
13.5
 
 
 
 
ANKHD1
 
 
hsa-miR-23a-3p
 
 
-0.368
 
 
44.4
 
 
 
 
ANKHD1
 
 
hsa-miR-23b-3p
 
 
-0.368
 
 
44.4
 
 
 
 
ANKHD1
 
 
hsa-miR-30b-5p
 
 
-0.405
 
 
44.4
 
 
 
 
ANKRA2
 
 
hsa-miR-30b-5p
 
 
-0.354
 
 
13.4
 
 
 
 
ANKRA2
 
 
hsa-miR-30b-5p
 
 
-0.472
 
 
13.4
 
 
 
 
ANKRD17
 
 
hsa-miR-30b-5p
 
 
-0.336
 
 
16.8
 
 
 
 
ANP32A
 
 
hsa-miR-132-3p
 
 
-0.374
 
 
68.9
 
 
 
 
ANP32E
 
 
hsa-miR-501-3p
 
 
-0.331
 
 
28.5
 
 
 
 
ANXA11
 
 
hsa-miR-15b-5p
 
 
-0.463
 
 
58.5
 
 
 
 
AP1S3
 
 
hsa-miR-181b-5p
 
 
-0.383
 
 
12.8
 
 
 
 
AP1S3
 
 
hsa-miR-181a-5p
 
 
-0.383
 
 
12.8
 
 
 
 
AP2A1
 
 
hsa-miR-15b-5p
 
 
-0.333
 
 
29.3
 
 
 
 
AP2B1
 
 
hsa-miR-15b-5p
 
 
-0.334
 
 
61.0
 
 
 
 
AP3S1
 
 
hsa-miR-30b-5p
 
 
-0.293
 
 
46.1
 
 
 
 
APOO
 
 
hsa-miR-181b-5p
 
 
-0.5
 
 
19.0
 
 
 
 
APOO
 
 
hsa-miR-181a-5p
 
 
-0.5
 
 
19.0
 
 
 
 
APP
 
 
hsa-miR-15b-5p
 
 
-0.298
 
 
434.2
 
 
 
 
ARF4
 
 
hsa-miR-30b-5p
 
 
-0.289
 
 
134.8
 
 
 
 
ARF4
 
 
hsa-miR-221-3p
 
 
-0.553
 
 
134.8
 
 
 
 
ARF5
 
 
hsa-miR-29a-3p
 
 
-0.491
 
 
88.3
 
 
 
 
ARF6
 
 
hsa-miR-181b-5p
 
 
-0.33
 
 
20.1
 
 
 
 
ARF6
 
 
hsa-miR-181a-5p
 
 
-0.33
 
 
20.1
 
 
 
 
ARHGAP5
 
 
hsa-miR-486-5p
 
 
-0.3
 
 
64.3
 
 
 
 
ARID1B
 
 
hsa-miR-29a-3p
 
 
-0.342
 
 
19.6
 
 
 
 
ARID4B
 
 
hsa-miR-486-5p
 
 
-0.367
 
 
15.9
 
 
 
 
ARL2
 
 
hsa-miR-15b-5p
 
 
-0.374
 
 
86.5
 
 
 
 
ARL2
 
 
hsa-miR-15b-5p
 
 
-0.442
 
 
86.5
 
 
 
 
ARL3
 
 
hsa-miR-15b-5p
 
 
-0.507
 
 
10.7
 
 
 
 
ARL4A
 
 
hsa-miR-29a-3p
 
 
-0.386
 
 
18.8
 
 
 
 
ARL4A
 
 
hsa-miR-30b-5p
 
 
-0.448
 
 
18.8
 
 
 
 
ARMC8
 
 
hsa-miR-486-5p
 
 
-0.321
 
 
20.0
 
 
 
 
ARMCX2
 
 
hsa-miR-15b-5p
 
 
-0.344
 
 
55.9
 
 
 
 
ARMCX3
 
 
hsa-miR-425-5p
 
 
-0.325
 
 
46.7
 
 
 
 
ARNT
 
 
hsa-miR-221-3p
 
 
-0.465
 
 
18.4
 
 
 
 
ARPP19
 
 
hsa-miR-15b-5p
 
 
-0.346
 
 
53.7
 
 
 
 
ARPP19
 
 
hsa-miR-29a-3p
 
 
-0.371
 
 
53.7
 
 
 
 
ARRDC3
 
 
hsa-miR-29a-3p
 
 
-0.402
 
 
78.7
 
 
 
 
ARVCF
 
 
hsa-miR-155-5p
 
 
-0.388
 
 
14.8
 
 
 
 
ASAP2
 
 
hsa-miR-29a-3p
 
 
-0.348
 
 
17.9
 
 
 
 
ASB3
 
 
hsa-miR-30b-5p
 
 
-0.418
 
 
10.8
 
 
 
 
ASB3
 
 
hsa-miR-30b-5p
 
 
-0.418
 
 
10.8
 
 
 
 
ASB6
 
 
hsa-miR-874-3p
 
 
-0.368
 
 
10.3
 
 
 
 
ASF1A
 
 
hsa-miR-23a-3p
 
 
-0.506
 
 
14.1
 
 
 
 
ASF1A
 
 
hsa-miR-23b-3p
 
 
-0.506
 
 
14.1
 
 
 
 
ASH1L
 
 
hsa-miR-15b-5p
 
 
-0.325
 
 
17.4
 
 
 
 
ASPH
 
 
hsa-miR-221-3p
 
 
-0.287
 
 
90.3
 
 
 
 
ATF3
 
 
hsa-miR-874-3p
 
 
-0.288
 
 
169.7
 
 
 
 
ATG5
 
 
hsa-miR-30b-5p
 
 
-0.488
 
 
16.4
 
 
 
 
ATG9A
 
 
hsa-miR-15b-5p
 
 
-0.333
 
 
18.6
 
 
 
 
ATOSA
 
 
hsa-miR-221-3p
 
 
-0.495
 
 
23.3
 
 
 
 
ATP5MC3
 
 
hsa-miR-425-5p
 
 
-0.328
 
 
129.3
 
 
 
 
ATP5MC3
 
 
hsa-miR-29a-3p
 
 
-0.33
 
 
129.3
 
 
 
 
ATP6V1E1
 
 
hsa-miR-23a-3p
 
 
-0.294
 
 
94.4
 
 
 
 
ATP6V1E1
 
 
hsa-miR-23b-3p
 
 
-0.294
 
 
94.4
 
 
 
 
ATPAF1
 
 
hsa-miR-361-5p
 
 
-0.362
 
 
39.3
 
 
 
 
ATXN1L
 
 
hsa-miR-155-5p
 
 
-0.315
 
 
11.1
 
 
 
 
ATXN2
 
 
hsa-miR-15b-5p
 
 
-0.392
 
 
16.2
 
 
 
 
AUH
 
 
hsa-miR-23a-3p
 
 
-0.495
 
 
19.7
 
 
 
 
AUH
 
 
hsa-miR-23b-3p
 
 
-0.503
 
 
19.7
 
 
 
 
AUH
 
 
hsa-miR-23a-3p
 
 
-0.512
 
 
19.7
 
 
 
 
AUH
 
 
hsa-miR-23b-3p
 
 
-0.512
 
 
19.7
 
 
 
 
AXIN2
 
 
hsa-miR-15b-5p
 
 
-0.3
 
 
11.6
 
 
 
 
AZIN1
 
 
hsa-miR-132-3p
 
 
-0.29
 
 
62.4
 
 
 
 
AZIN1
 
 
hsa-miR-132-3p
 
 
-0.299
 
 
62.4
 
 
 
 
B4GALT5
 
 
hsa-miR-501-3p
 
 
-0.408
 
 
10.8
 
 
 
 
B4GAT1
 
 
hsa-miR-23a-3p
 
 
-0.361
 
 
20.5
 
 
 
 
B4GAT1
 
 
hsa-miR-23b-3p
 
 
-0.361
 
 
20.5
 
 
 
 
BCL2
 
 
hsa-miR-342-3p
 
 
-0.428
 
 
22.4
 
 
 
 
BCL2L11
 
 
hsa-miR-221-3p
 
 
-0.423
 
 
18.5
 
 
 
 
BCL2L2
 
 
hsa-miR-29a-3p
 
 
-0.312
 
 
34.3
 
 
 
 
BCOR
 
 
hsa-miR-425-5p
 
 
-0.346
 
 
14.6
 
 
 
 
BDH1
 
 
hsa-miR-874-3p
 
 
-0.644
 
 
15.7
 
 
 
 
BECN1
 
 
hsa-miR-30b-5p
 
 
-0.452
 
 
34.4
 
 
 
 
BET1
 
 
hsa-miR-23a-3p
 
 
-0.402
 
 
20.7
 
 
 
 
BET1
 
 
hsa-miR-23b-3p
 
 
-0.418
 
 
20.7
 
 
 
 
BFAR
 
 
hsa-miR-15b-5p
 
 
-0.352
 
 
21.9
 
 
 
 
BHLHE40
 
 
hsa-miR-181b-5p
 
 
-0.408
 
 
88.0
 
 
 
 
BHLHE40
 
 
hsa-miR-181a-5p
 
 
-0.408
 
 
88.0
 
 
 
 
BICDL1
 
 
hsa-miR-221-3p
 
 
-0.447
 
 
18.4
 
 
 
 
BLCAP
 
 
hsa-miR-23a-3p
 
 
-0.601
 
 
44.2
 
 
 
 
 
 
 tongue 
 
 
 
 
target_symbol
 
 
mature_mirna_id
 
 
score
 
 
nTPM
 
 
 
 
 
 
AAR2
 
 
hsa-miR-29a-3p
 
 
-0.327
 
 
19.3
 
 
 
 
ABCE1
 
 
hsa-miR-29a-3p
 
 
-0.404
 
 
28.5
 
 
 
 
ABHD18
 
 
hsa-miR-29a-3p
 
 
-0.321
 
 
10.9
 
 
 
 
ABHD18
 
 
hsa-miR-29a-3p
 
 
-0.34
 
 
10.9
 
 
 
 
ABHD4
 
 
hsa-miR-29a-3p
 
 
-0.312
 
 
27.0
 
 
 
 
ABHD5
 
 
hsa-miR-132-3p
 
 
-0.323
 
 
17.3
 
 
 
 
ACTA1
 
 
hsa-miR-155-5p
 
 
-0.595
 
 
19457.9
 
 
 
 
ACTC1
 
 
hsa-miR-30b-5p
 
 
-0.543
 
 
182.1
 
 
 
 
ACTN2
 
 
hsa-miR-23a-3p
 
 
-0.294
 
 
1195.2
 
 
 
 
ACTN2
 
 
hsa-miR-29a-3p
 
 
-0.311
 
 
1195.2
 
 
 
 
ACTR2
 
 
hsa-miR-15b-5p
 
 
-0.293
 
 
60.6
 
 
 
 
ADAM9
 
 
hsa-miR-30b-5p
 
 
-0.312
 
 
23.3
 
 
 
 
ADCY3
 
 
hsa-miR-132-3p
 
 
-0.303
 
 
19.0
 
 
 
 
ADGRA3
 
 
hsa-miR-30b-5p
 
 
-0.304
 
 
10.7
 
 
 
 
ADIPOR2
 
 
hsa-miR-150-5p
 
 
-0.287
 
 
18.5
 
 
 
 
AFG3L2
 
 
hsa-miR-181b-5p
 
 
-0.491
 
 
92.6
 
 
 
 
AFG3L2
 
 
hsa-miR-181a-5p
 
 
-0.491
 
 
92.6
 
 
 
 
AGO4
 
 
hsa-miR-221-3p
 
 
-0.321
 
 
11.1
 
 
 
 
AK1
 
 
hsa-miR-29a-3p
 
 
-0.333
 
 
730.8
 
 
 
 
AK4
 
 
hsa-miR-15b-5p
 
 
-0.316
 
 
35.9
 
 
 
 
AKT3
 
 
hsa-miR-29a-3p
 
 
-0.287
 
 
10.7
 
 
 
 
AKT3
 
 
hsa-miR-15b-5p
 
 
-0.301
 
 
10.7
 
 
 
 
ALAD
 
 
hsa-miR-15b-5p
 
 
-0.446
 
 
22.3
 
 
 
 
AMER1
 
 
hsa-miR-29a-3p
 
 
-0.343
 
 
14.3
 
 
 
 
AMOT
 
 
hsa-miR-29a-3p
 
 
-0.304
 
 
51.2
 
 
 
 
AMOTL1
 
 
hsa-miR-15b-5p
 
 
-0.319
 
 
66.9
 
 
 
 
ANGPTL2
 
 
hsa-miR-221-3p
 
 
-0.369
 
 
34.2
 
 
 
 
ANKFY1
 
 
hsa-miR-155-5p
 
 
-0.315
 
 
10.6
 
 
 
 
ANKHD1
 
 
hsa-miR-23a-3p
 
 
-0.368
 
 
26.1
 
 
 
 
ANKHD1
 
 
hsa-miR-23b-3p
 
 
-0.368
 
 
26.1
 
 
 
 
ANKHD1
 
 
hsa-miR-30b-5p
 
 
-0.405
 
 
26.1
 
 
 
 
ANKRD17
 
 
hsa-miR-30b-5p
 
 
-0.336
 
 
24.4
 
 
 
 
ANP32A
 
 
hsa-miR-132-3p
 
 
-0.374
 
 
60.2
 
 
 
 
ANP32E
 
 
hsa-miR-501-3p
 
 
-0.331
 
 
24.3
 
 
 
 
ANTXR2
 
 
hsa-miR-29a-3p
 
 
-0.347
 
 
20.3
 
 
 
 
ANXA11
 
 
hsa-miR-15b-5p
 
 
-0.463
 
 
155.0
 
 
 
 
AP2A1
 
 
hsa-miR-15b-5p
 
 
-0.333
 
 
24.6
 
 
 
 
AP2B1
 
 
hsa-miR-15b-5p
 
 
-0.334
 
 
57.0
 
 
 
 
AP3S1
 
 
hsa-miR-30b-5p
 
 
-0.293
 
 
32.1
 
 
 
 
APC
 
 
hsa-miR-29a-3p
 
 
-0.413
 
 
11.0
 
 
 
 
APOO
 
 
hsa-miR-181b-5p
 
 
-0.5
 
 
68.2
 
 
 
 
APOO
 
 
hsa-miR-181a-5p
 
 
-0.5
 
 
68.2
 
 
 
 
APP
 
 
hsa-miR-15b-5p
 
 
-0.298
 
 
161.6
 
 
 
 
AQP3
 
 
hsa-miR-874-3p
 
 
-0.48
 
 
25.0
 
 
 
 
ARF4
 
 
hsa-miR-30b-5p
 
 
-0.289
 
 
64.9
 
 
 
 
ARF4
 
 
hsa-miR-221-3p
 
 
-0.553
 
 
64.9
 
 
 
 
ARF5
 
 
hsa-miR-29a-3p
 
 
-0.491
 
 
94.7
 
 
 
 
ARF6
 
 
hsa-miR-181b-5p
 
 
-0.33
 
 
15.8
 
 
 
 
ARF6
 
 
hsa-miR-181a-5p
 
 
-0.33
 
 
15.8
 
 
 
 
ARHGAP5
 
 
hsa-miR-486-5p
 
 
-0.3
 
 
23.1
 
 
 
 
ARID1B
 
 
hsa-miR-29a-3p
 
 
-0.342
 
 
22.6
 
 
 
 
ARL2
 
 
hsa-miR-15b-5p
 
 
-0.374
 
 
147.4
 
 
 
 
ARL2
 
 
hsa-miR-15b-5p
 
 
-0.442
 
 
147.4
 
 
 
 
ARMC8
 
 
hsa-miR-486-5p
 
 
-0.321
 
 
32.4
 
 
 
 
ARMCX3
 
 
hsa-miR-425-5p
 
 
-0.325
 
 
59.4
 
 
 
 
ARNT
 
 
hsa-miR-221-3p
 
 
-0.465
 
 
26.3
 
 
 
 
ARPP19
 
 
hsa-miR-15b-5p
 
 
-0.346
 
 
158.9
 
 
 
 
ARPP19
 
 
hsa-miR-29a-3p
 
 
-0.371
 
 
158.9
 
 
 
 
ARPP21
 
 
hsa-miR-15b-5p
 
 
-0.342
 
 
41.9
 
 
 
 
ARPP21
 
 
hsa-miR-501-3p
 
 
-0.546
 
 
41.9
 
 
 
 
ARRDC3
 
 
hsa-miR-29a-3p
 
 
-0.402
 
 
41.9
 
 
 
 
ASF1A
 
 
hsa-miR-23a-3p
 
 
-0.506
 
 
15.1
 
 
 
 
ASF1A
 
 
hsa-miR-23b-3p
 
 
-0.506
 
 
15.1
 
 
 
 
ASH1L
 
 
hsa-miR-15b-5p
 
 
-0.325
 
 
22.7
 
 
 
 
ASPH
 
 
hsa-miR-221-3p
 
 
-0.287
 
 
155.4
 
 
 
 
ATF3
 
 
hsa-miR-874-3p
 
 
-0.288
 
 
28.0
 
 
 
 
ATG5
 
 
hsa-miR-30b-5p
 
 
-0.488
 
 
14.2
 
 
 
 
ATG9A
 
 
hsa-miR-15b-5p
 
 
-0.333
 
 
58.5
 
 
 
 
ATOSA
 
 
hsa-miR-221-3p
 
 
-0.495
 
 
23.7
 
 
 
 
ATP1B4
 
 
hsa-miR-29a-3p
 
 
-0.439
 
 
20.1
 
 
 
 
ATP5MC3
 
 
hsa-miR-425-5p
 
 
-0.328
 
 
1014.6
 
 
 
 
ATP5MC3
 
 
hsa-miR-29a-3p
 
 
-0.33
 
 
1014.6
 
 
 
 
ATP6V1E1
 
 
hsa-miR-23a-3p
 
 
-0.294
 
 
166.7
 
 
 
 
ATP6V1E1
 
 
hsa-miR-23b-3p
 
 
-0.294
 
 
166.7
 
 
 
 
ATPAF1
 
 
hsa-miR-361-5p
 
 
-0.362
 
 
295.7
 
 
 
 
ATXN1
 
 
hsa-miR-221-3p
 
 
-0.289
 
 
13.9
 
 
 
 
ATXN1L
 
 
hsa-miR-155-5p
 
 
-0.315
 
 
12.7
 
 
 
 
ATXN2
 
 
hsa-miR-15b-5p
 
 
-0.392
 
 
19.4
 
 
 
 
AUH
 
 
hsa-miR-23a-3p
 
 
-0.495
 
 
27.3
 
 
 
 
AUH
 
 
hsa-miR-23b-3p
 
 
-0.503
 
 
27.3
 
 
 
 
AUH
 
 
hsa-miR-23a-3p
 
 
-0.512
 
 
27.3
 
 
 
 
AUH
 
 
hsa-miR-23b-3p
 
 
-0.512
 
 
27.3
 
 
 
 
AZIN1
 
 
hsa-miR-132-3p
 
 
-0.29
 
 
28.4
 
 
 
 
AZIN1
 
 
hsa-miR-132-3p
 
 
-0.299
 
 
28.4
 
 
 
 
B4GALT5
 
 
hsa-miR-501-3p
 
 
-0.408
 
 
22.7
 
 
 
 
B4GAT1
 
 
hsa-miR-23a-3p
 
 
-0.361
 
 
17.9
 
 
 
 
B4GAT1
 
 
hsa-miR-23b-3p
 
 
-0.361
 
 
17.9
 
 
 
 
BAK1
 
 
hsa-miR-29a-3p
 
 
-0.409
 
 
16.9
 
 
 
 
BCL2L2
 
 
hsa-miR-29a-3p
 
 
-0.312
 
 
18.6
 
 
 
 
BDH1
 
 
hsa-miR-874-3p
 
 
-0.644
 
 
21.3
 
 
 
 
BECN1
 
 
hsa-miR-30b-5p
 
 
-0.452
 
 
72.4
 
 
 
 
BFAR
 
 
hsa-miR-15b-5p
 
 
-0.352
 
 
24.9
 
 
 
 
BHLHE40
 
 
hsa-miR-181b-5p
 
 
-0.408
 
 
43.7
 
 
 
 
BHLHE40
 
 
hsa-miR-181a-5p
 
 
-0.408
 
 
43.7
 
 
 
 
BLCAP
 
 
hsa-miR-23a-3p
 
 
-0.601
 
 
112.2
 
 
 
 
BLCAP
 
 
hsa-miR-23b-3p
 
 
-0.601
 
 
112.2
 
 
 
 
BLMH
 
 
hsa-miR-29a-3p
 
 
-0.393
 
 
17.7
 
 
 
 
BLOC1S6
 
 
hsa-miR-181b-5p
 
 
-0.393
 
 
21.7
 
 
 
 
BLOC1S6
 
 
hsa-miR-181a-5p
 
 
-0.393
 
 
21.7
 
 
 
 
BOLA3
 
 
hsa-miR-15b-5p
 
 
-0.363
 
 
162.0
 
 
 
 
 
 
 tonsil 
 
 
 
 
target_symbol
 
 
mature_mirna_id
 
 
score
 
 
nTPM
 
 
 
 
 
 
AAR2
 
 
hsa-miR-29a-3p
 
 
-0.327
 
 
18.7
 
 
 
 
ABCE1
 
 
hsa-miR-29a-3p
 
 
-0.404
 
 
39.4
 
 
 
 
ABHD4
 
 
hsa-miR-29a-3p
 
 
-0.312
 
 
15.2
 
 
 
 
ACTR2
 
 
hsa-miR-15b-5p
 
 
-0.293
 
 
239.6
 
 
 
 
ADA
 
 
hsa-miR-29a-3p
 
 
-0.376
 
 
23.2
 
 
 
 
ADAM19
 
 
hsa-miR-874-3p
 
 
-0.374
 
 
28.3
 
 
 
 
ADAM9
 
 
hsa-miR-30b-5p
 
 
-0.312
 
 
12.4
 
 
 
 
ADCY3
 
 
hsa-miR-132-3p
 
 
-0.303
 
 
16.9
 
 
 
 
ADIPOR2
 
 
hsa-miR-150-5p
 
 
-0.287
 
 
11.8
 
 
 
 
ADRA2A
 
 
hsa-miR-23a-3p
 
 
-0.371
 
 
12.3
 
 
 
 
ADRA2A
 
 
hsa-miR-23b-3p
 
 
-0.371
 
 
12.3
 
 
 
 
AFG3L2
 
 
hsa-miR-181b-5p
 
 
-0.491
 
 
16.2
 
 
 
 
AFG3L2
 
 
hsa-miR-181a-5p
 
 
-0.491
 
 
16.2
 
 
 
 
AHR
 
 
hsa-miR-29a-3p
 
 
-0.316
 
 
18.6
 
 
 
 
AK1
 
 
hsa-miR-29a-3p
 
 
-0.333
 
 
11.8
 
 
 
 
ALAD
 
 
hsa-miR-15b-5p
 
 
-0.446
 
 
17.5
 
 
 
 
ANKFY1
 
 
hsa-miR-155-5p
 
 
-0.315
 
 
12.0
 
 
 
 
ANKHD1
 
 
hsa-miR-23a-3p
 
 
-0.368
 
 
38.8
 
 
 
 
ANKHD1
 
 
hsa-miR-23b-3p
 
 
-0.368
 
 
38.8
 
 
 
 
ANKHD1
 
 
hsa-miR-30b-5p
 
 
-0.405
 
 
38.8
 
 
 
 
ANKRD17
 
 
hsa-miR-30b-5p
 
 
-0.336
 
 
16.5
 
 
 
 
ANKRD29
 
 
hsa-miR-132-3p
 
 
-0.47
 
 
13.4
 
 
 
 
ANP32A
 
 
hsa-miR-132-3p
 
 
-0.374
 
 
132.1
 
 
 
 
ANP32E
 
 
hsa-miR-501-3p
 
 
-0.331
 
 
58.2
 
 
 
 
ANTXR2
 
 
hsa-miR-29a-3p
 
 
-0.347
 
 
15.1
 
 
 
 
ANXA11
 
 
hsa-miR-15b-5p
 
 
-0.463
 
 
95.3
 
 
 
 
AP2A1
 
 
hsa-miR-15b-5p
 
 
-0.333
 
 
12.3
 
 
 
 
AP2B1
 
 
hsa-miR-15b-5p
 
 
-0.334
 
 
42.7
 
 
 
 
AP3S1
 
 
hsa-miR-30b-5p
 
 
-0.293
 
 
57.3
 
 
 
 
APOO
 
 
hsa-miR-181b-5p
 
 
-0.5
 
 
10.8
 
 
 
 
APOO
 
 
hsa-miR-181a-5p
 
 
-0.5
 
 
10.8
 
 
 
 
APP
 
 
hsa-miR-15b-5p
 
 
-0.298
 
 
69.8
 
 
 
 
AQP3
 
 
hsa-miR-874-3p
 
 
-0.48
 
 
161.9
 
 
 
 
ARF4
 
 
hsa-miR-30b-5p
 
 
-0.289
 
 
91.2
 
 
 
 
ARF4
 
 
hsa-miR-221-3p
 
 
-0.553
 
 
91.2
 
 
 
 
ARF5
 
 
hsa-miR-29a-3p
 
 
-0.491
 
 
69.9
 
 
 
 
ARF6
 
 
hsa-miR-181b-5p
 
 
-0.33
 
 
64.2
 
 
 
 
ARF6
 
 
hsa-miR-181a-5p
 
 
-0.33
 
 
64.2
 
 
 
 
ARHGAP5
 
 
hsa-miR-486-5p
 
 
-0.3
 
 
17.4
 
 
 
 
ARID1B
 
 
hsa-miR-29a-3p
 
 
-0.342
 
 
17.5
 
 
 
 
ARID4B
 
 
hsa-miR-486-5p
 
 
-0.367
 
 
15.9
 
 
 
 
ARL2
 
 
hsa-miR-15b-5p
 
 
-0.374
 
 
27.2
 
 
 
 
ARL2
 
 
hsa-miR-15b-5p
 
 
-0.442
 
 
27.2
 
 
 
 
ARL4A
 
 
hsa-miR-29a-3p
 
 
-0.386
 
 
14.9
 
 
 
 
ARL4A
 
 
hsa-miR-30b-5p
 
 
-0.448
 
 
14.9
 
 
 
 
ARMC8
 
 
hsa-miR-486-5p
 
 
-0.321
 
 
21.4
 
 
 
 
ARMCX3
 
 
hsa-miR-425-5p
 
 
-0.325
 
 
20.2
 
 
 
 
ARNT
 
 
hsa-miR-221-3p
 
 
-0.465
 
 
21.0
 
 
 
 
ARPP19
 
 
hsa-miR-15b-5p
 
 
-0.346
 
 
48.4
 
 
 
 
ARPP19
 
 
hsa-miR-29a-3p
 
 
-0.371
 
 
48.4
 
 
 
 
ARRDC3
 
 
hsa-miR-29a-3p
 
 
-0.402
 
 
26.4
 
 
 
 
ASB3
 
 
hsa-miR-30b-5p
 
 
-0.418
 
 
11.5
 
 
 
 
ASB3
 
 
hsa-miR-30b-5p
 
 
-0.418
 
 
11.5
 
 
 
 
ASF1A
 
 
hsa-miR-23a-3p
 
 
-0.506
 
 
14.1
 
 
 
 
ASF1A
 
 
hsa-miR-23b-3p
 
 
-0.506
 
 
14.1
 
 
 
 
ASPH
 
 
hsa-miR-221-3p
 
 
-0.287
 
 
26.4
 
 
 
 
ATG9A
 
 
hsa-miR-15b-5p
 
 
-0.333
 
 
19.8
 
 
 
 
ATOSA
 
 
hsa-miR-221-3p
 
 
-0.495
 
 
27.6
 
 
 
 
ATP5MC3
 
 
hsa-miR-425-5p
 
 
-0.328
 
 
164.6
 
 
 
 
ATP5MC3
 
 
hsa-miR-29a-3p
 
 
-0.33
 
 
164.6
 
 
 
 
ATP6V1E1
 
 
hsa-miR-23a-3p
 
 
-0.294
 
 
102.6
 
 
 
 
ATP6V1E1
 
 
hsa-miR-23b-3p
 
 
-0.294
 
 
102.6
 
 
 
 
ATPAF1
 
 
hsa-miR-361-5p
 
 
-0.362
 
 
30.0
 
 
 
 
AZIN1
 
 
hsa-miR-132-3p
 
 
-0.29
 
 
55.9
 
 
 
 
AZIN1
 
 
hsa-miR-132-3p
 
 
-0.299
 
 
55.9
 
 
 
 
B4GALT5
 
 
hsa-miR-501-3p
 
 
-0.408
 
 
10.8
 
 
 
 
BACH2
 
 
hsa-miR-29a-3p
 
 
-0.294
 
 
12.7
 
 
 
 
BAK1
 
 
hsa-miR-29a-3p
 
 
-0.409
 
 
21.9
 
 
 
 
BCL2
 
 
hsa-miR-342-3p
 
 
-0.428
 
 
13.7
 
 
 
 
BCL2L11
 
 
hsa-miR-221-3p
 
 
-0.423
 
 
21.7
 
 
 
 
BCL2L2
 
 
hsa-miR-29a-3p
 
 
-0.312
 
 
15.7
 
 
 
 
BCL7A
 
 
hsa-miR-15b-5p
 
 
-0.336
 
 
14.4
 
 
 
 
BCL7A
 
 
hsa-miR-501-3p
 
 
-0.339
 
 
14.4
 
 
 
 
BCOR
 
 
hsa-miR-425-5p
 
 
-0.346
 
 
11.7
 
 
 
 
BECN1
 
 
hsa-miR-30b-5p
 
 
-0.452
 
 
52.6
 
 
 
 
BET1
 
 
hsa-miR-23a-3p
 
 
-0.402
 
 
19.7
 
 
 
 
BET1
 
 
hsa-miR-23b-3p
 
 
-0.418
 
 
19.7
 
 
 
 
BFAR
 
 
hsa-miR-15b-5p
 
 
-0.352
 
 
16.4
 
 
 
 
BHLHE40
 
 
hsa-miR-181b-5p
 
 
-0.408
 
 
47.5
 
 
 
 
BHLHE40
 
 
hsa-miR-181a-5p
 
 
-0.408
 
 
47.5
 
 
 
 
BLCAP
 
 
hsa-miR-23a-3p
 
 
-0.601
 
 
49.7
 
 
 
 
BLCAP
 
 
hsa-miR-23b-3p
 
 
-0.601
 
 
49.7
 
 
 
 
BLMH
 
 
hsa-miR-29a-3p
 
 
-0.393
 
 
25.3
 
 
 
 
BLOC1S6
 
 
hsa-miR-181b-5p
 
 
-0.393
 
 
33.2
 
 
 
 
BLOC1S6
 
 
hsa-miR-181a-5p
 
 
-0.393
 
 
33.2
 
 
 
 
BMF
 
 
hsa-miR-29a-3p
 
 
-0.41
 
 
22.0
 
 
 
 
BMF
 
 
hsa-miR-221-3p
 
 
-0.453
 
 
22.0
 
 
 
 
BOLA3
 
 
hsa-miR-15b-5p
 
 
-0.363
 
 
20.2
 
 
 
 
BOLA3
 
 
hsa-miR-15b-5p
 
 
-0.386
 
 
20.2
 
 
 
 
BOLA3
 
 
hsa-miR-374b-5p
 
 
-0.486
 
 
20.2
 
 
 
 
BRI3
 
 
hsa-miR-132-3p
 
 
-0.385
 
 
17.1
 
 
 
 
BTAF1
 
 
hsa-miR-486-5p
 
 
-0.288
 
 
20.0
 
 
 
 
BTAF1
 
 
hsa-miR-15b-5p
 
 
-0.496
 
 
20.0
 
 
 
 
BTF3
 
 
hsa-miR-15b-5p
 
 
-0.344
 
 
710.3
 
 
 
 
BTF3
 
 
hsa-miR-361-5p
 
 
-0.551
 
 
710.3
 
 
 
 
BTG2
 
 
hsa-miR-29a-3p
 
 
-0.32
 
 
123.9
 
 
 
 
BTLA
 
 
hsa-miR-15b-5p
 
 
-0.306
 
 
26.8
 
 
 
 
BTLA
 
 
hsa-miR-15b-5p
 
 
-0.316
 
 
26.8
 
 
 
 
BTN2A1
 
 
hsa-miR-342-3p
 
 
-0.31
 
 
10.8
 
 
 
 
BZW1
 
 
hsa-miR-15b-5p
 
 
-0.367
 
 
102.5
 
 
 
 
 
 
 urinary bladder 
 
 
 
 
target_symbol
 
 
mature_mirna_id
 
 
score
 
 
nTPM
 
 
 
 
 
 
AAR2
 
 
hsa-miR-29a-3p
 
 
-0.327
 
 
25.2
 
 
 
 
ABCB6
 
 
hsa-miR-29a-3p
 
 
-0.48
 
 
13.5
 
 
 
 
ABCE1
 
 
hsa-miR-29a-3p
 
 
-0.404
 
 
37.8
 
 
 
 
ABHD17B
 
 
hsa-miR-486-5p
 
 
-0.348
 
 
12.1
 
 
 
 
ABHD4
 
 
hsa-miR-29a-3p
 
 
-0.312
 
 
28.1
 
 
 
 
ABHD5
 
 
hsa-miR-132-3p
 
 
-0.323
 
 
15.5
 
 
 
 
ACTC1
 
 
hsa-miR-30b-5p
 
 
-0.543
 
 
667.0
 
 
 
 
ACTR2
 
 
hsa-miR-15b-5p
 
 
-0.293
 
 
119.8
 
 
 
 
ACVR1
 
 
hsa-miR-30b-5p
 
 
-0.29
 
 
13.3
 
 
 
 
ADAM19
 
 
hsa-miR-874-3p
 
 
-0.374
 
 
15.9
 
 
 
 
ADAM9
 
 
hsa-miR-30b-5p
 
 
-0.312
 
 
52.5
 
 
 
 
ADAMTS9
 
 
hsa-miR-29a-3p
 
 
-0.305
 
 
20.9
 
 
 
 
ADAMTS9
 
 
hsa-miR-29a-3p
 
 
-0.485
 
 
20.9
 
 
 
 
ADCY3
 
 
hsa-miR-132-3p
 
 
-0.303
 
 
34.2
 
 
 
 
ADIPOR2
 
 
hsa-miR-150-5p
 
 
-0.287
 
 
21.3
 
 
 
 
ADRA2A
 
 
hsa-miR-23a-3p
 
 
-0.371
 
 
14.8
 
 
 
 
ADRA2A
 
 
hsa-miR-23b-3p
 
 
-0.371
 
 
14.8
 
 
 
 
AFG3L2
 
 
hsa-miR-181b-5p
 
 
-0.491
 
 
18.0
 
 
 
 
AFG3L2
 
 
hsa-miR-181a-5p
 
 
-0.491
 
 
18.0
 
 
 
 
AHR
 
 
hsa-miR-29a-3p
 
 
-0.316
 
 
46.3
 
 
 
 
AK1
 
 
hsa-miR-29a-3p
 
 
-0.333
 
 
22.9
 
 
 
 
AK4
 
 
hsa-miR-15b-5p
 
 
-0.316
 
 
16.4
 
 
 
 
AKT3
 
 
hsa-miR-29a-3p
 
 
-0.287
 
 
14.4
 
 
 
 
AKT3
 
 
hsa-miR-15b-5p
 
 
-0.301
 
 
14.4
 
 
 
 
ALAD
 
 
hsa-miR-15b-5p
 
 
-0.446
 
 
20.6
 
 
 
 
AMOTL1
 
 
hsa-miR-15b-5p
 
 
-0.319
 
 
26.4
 
 
 
 
ANGPTL2
 
 
hsa-miR-221-3p
 
 
-0.369
 
 
55.1
 
 
 
 
ANKFY1
 
 
hsa-miR-155-5p
 
 
-0.315
 
 
14.8
 
 
 
 
ANKHD1
 
 
hsa-miR-23a-3p
 
 
-0.368
 
 
34.3
 
 
 
 
ANKHD1
 
 
hsa-miR-23b-3p
 
 
-0.368
 
 
34.3
 
 
 
 
ANKHD1
 
 
hsa-miR-30b-5p
 
 
-0.405
 
 
34.3
 
 
 
 
ANKRD17
 
 
hsa-miR-30b-5p
 
 
-0.336
 
 
16.5
 
 
 
 
ANP32A
 
 
hsa-miR-132-3p
 
 
-0.374
 
 
71.6
 
 
 
 
ANP32E
 
 
hsa-miR-501-3p
 
 
-0.331
 
 
20.4
 
 
 
 
ANTXR2
 
 
hsa-miR-29a-3p
 
 
-0.347
 
 
82.6
 
 
 
 
ANXA11
 
 
hsa-miR-15b-5p
 
 
-0.463
 
 
138.5
 
 
 
 
AP2A1
 
 
hsa-miR-15b-5p
 
 
-0.333
 
 
43.6
 
 
 
 
AP2B1
 
 
hsa-miR-15b-5p
 
 
-0.334
 
 
44.1
 
 
 
 
AP3S1
 
 
hsa-miR-30b-5p
 
 
-0.293
 
 
56.3
 
 
 
 
APOO
 
 
hsa-miR-181b-5p
 
 
-0.5
 
 
10.5
 
 
 
 
APOO
 
 
hsa-miR-181a-5p
 
 
-0.5
 
 
10.5
 
 
 
 
APP
 
 
hsa-miR-15b-5p
 
 
-0.298
 
 
275.5
 
 
 
 
AQP3
 
 
hsa-miR-874-3p
 
 
-0.48
 
 
642.4
 
 
 
 
ARF4
 
 
hsa-miR-30b-5p
 
 
-0.289
 
 
190.1
 
 
 
 
ARF4
 
 
hsa-miR-221-3p
 
 
-0.553
 
 
190.1
 
 
 
 
ARF5
 
 
hsa-miR-29a-3p
 
 
-0.491
 
 
95.3
 
 
 
 
ARF6
 
 
hsa-miR-181b-5p
 
 
-0.33
 
 
43.6
 
 
 
 
ARF6
 
 
hsa-miR-181a-5p
 
 
-0.33
 
 
43.6
 
 
 
 
ARHGAP5
 
 
hsa-miR-486-5p
 
 
-0.3
 
 
30.7
 
 
 
 
ARID1B
 
 
hsa-miR-29a-3p
 
 
-0.342
 
 
13.9
 
 
 
 
ARID4B
 
 
hsa-miR-486-5p
 
 
-0.367
 
 
12.0
 
 
 
 
ARL2
 
 
hsa-miR-15b-5p
 
 
-0.374
 
 
110.3
 
 
 
 
ARL2
 
 
hsa-miR-15b-5p
 
 
-0.442
 
 
110.3
 
 
 
 
ARL4A
 
 
hsa-miR-29a-3p
 
 
-0.386
 
 
20.6
 
 
 
 
ARL4A
 
 
hsa-miR-30b-5p
 
 
-0.448
 
 
20.6
 
 
 
 
ARL5B
 
 
hsa-miR-29a-3p
 
 
-0.304
 
 
11.8
 
 
 
 
ARL5B
 
 
hsa-miR-155-5p
 
 
-0.355
 
 
11.8
 
 
 
 
ARMC8
 
 
hsa-miR-486-5p
 
 
-0.321
 
 
20.8
 
 
 
 
ARMCX2
 
 
hsa-miR-15b-5p
 
 
-0.344
 
 
24.0
 
 
 
 
ARMCX3
 
 
hsa-miR-425-5p
 
 
-0.325
 
 
29.8
 
 
 
 
ARNT
 
 
hsa-miR-221-3p
 
 
-0.465
 
 
21.2
 
 
 
 
ARPP19
 
 
hsa-miR-15b-5p
 
 
-0.346
 
 
51.7
 
 
 
 
ARPP19
 
 
hsa-miR-29a-3p
 
 
-0.371
 
 
51.7
 
 
 
 
ARRDC3
 
 
hsa-miR-29a-3p
 
 
-0.402
 
 
53.2
 
 
 
 
ASH1L
 
 
hsa-miR-15b-5p
 
 
-0.325
 
 
21.3
 
 
 
 
ASPH
 
 
hsa-miR-221-3p
 
 
-0.287
 
 
103.6
 
 
 
 
ATF3
 
 
hsa-miR-874-3p
 
 
-0.288
 
 
261.7
 
 
 
 
ATG5
 
 
hsa-miR-30b-5p
 
 
-0.488
 
 
11.9
 
 
 
 
ATG9A
 
 
hsa-miR-15b-5p
 
 
-0.333
 
 
23.7
 
 
 
 
ATOSA
 
 
hsa-miR-221-3p
 
 
-0.495
 
 
11.7
 
 
 
 
ATP5MC3
 
 
hsa-miR-425-5p
 
 
-0.328
 
 
160.0
 
 
 
 
ATP5MC3
 
 
hsa-miR-29a-3p
 
 
-0.33
 
 
160.0
 
 
 
 
ATP6V1E1
 
 
hsa-miR-23a-3p
 
 
-0.294
 
 
118.0
 
 
 
 
ATP6V1E1
 
 
hsa-miR-23b-3p
 
 
-0.294
 
 
118.0
 
 
 
 
ATPAF1
 
 
hsa-miR-361-5p
 
 
-0.362
 
 
26.7
 
 
 
 
ATXN2
 
 
hsa-miR-15b-5p
 
 
-0.392
 
 
19.6
 
 
 
 
AXIN2
 
 
hsa-miR-15b-5p
 
 
-0.3
 
 
11.3
 
 
 
 
AZIN1
 
 
hsa-miR-132-3p
 
 
-0.29
 
 
56.7
 
 
 
 
AZIN1
 
 
hsa-miR-132-3p
 
 
-0.299
 
 
56.7
 
 
 
 
B3GNT5
 
 
hsa-miR-30b-5p
 
 
-0.326
 
 
22.5
 
 
 
 
B4GALT5
 
 
hsa-miR-501-3p
 
 
-0.408
 
 
47.9
 
 
 
 
B4GAT1
 
 
hsa-miR-23a-3p
 
 
-0.361
 
 
21.0
 
 
 
 
B4GAT1
 
 
hsa-miR-23b-3p
 
 
-0.361
 
 
21.0
 
 
 
 
BAK1
 
 
hsa-miR-29a-3p
 
 
-0.409
 
 
20.4
 
 
 
 
BCL2L2
 
 
hsa-miR-29a-3p
 
 
-0.312
 
 
54.9
 
 
 
 
BECN1
 
 
hsa-miR-30b-5p
 
 
-0.452
 
 
46.0
 
 
 
 
BET1
 
 
hsa-miR-23a-3p
 
 
-0.402
 
 
16.5
 
 
 
 
BET1
 
 
hsa-miR-23b-3p
 
 
-0.418
 
 
16.5
 
 
 
 
BFAR
 
 
hsa-miR-15b-5p
 
 
-0.352
 
 
27.0
 
 
 
 
BHLHE40
 
 
hsa-miR-181b-5p
 
 
-0.408
 
 
160.4
 
 
 
 
BHLHE40
 
 
hsa-miR-181a-5p
 
 
-0.408
 
 
160.4
 
 
 
 
BLCAP
 
 
hsa-miR-23a-3p
 
 
-0.601
 
 
71.7
 
 
 
 
BLCAP
 
 
hsa-miR-23b-3p
 
 
-0.601
 
 
71.7
 
 
 
 
BLMH
 
 
hsa-miR-29a-3p
 
 
-0.393
 
 
18.0
 
 
 
 
BLOC1S6
 
 
hsa-miR-181b-5p
 
 
-0.393
 
 
26.7
 
 
 
 
BLOC1S6
 
 
hsa-miR-181a-5p
 
 
-0.393
 
 
26.7
 
 
 
 
BOLA3
 
 
hsa-miR-15b-5p
 
 
-0.363
 
 
24.3
 
 
 
 
BOLA3
 
 
hsa-miR-15b-5p
 
 
-0.386
 
 
24.3
 
 
 
 
BOLA3
 
 
hsa-miR-374b-5p
 
 
-0.486
 
 
24.3
 
 
 
 
BRI3
 
 
hsa-miR-132-3p
 
 
-0.385
 
 
78.8
 
 
 
 
 
 
 vagina 
 
 
 
 
target_symbol
 
 
mature_mirna_id
 
 
score
 
 
nTPM
 
 
 
 
 
 
AAR2
 
 
hsa-miR-29a-3p
 
 
-0.327
 
 
15.0
 
 
 
 
ABCE1
 
 
hsa-miR-29a-3p
 
 
-0.404
 
 
15.6
 
 
 
 
ABHD4
 
 
hsa-miR-29a-3p
 
 
-0.312
 
 
28.3
 
 
 
 
ABHD5
 
 
hsa-miR-132-3p
 
 
-0.323
 
 
11.9
 
 
 
 
ACTC1
 
 
hsa-miR-30b-5p
 
 
-0.543
 
 
50.8
 
 
 
 
ACTR2
 
 
hsa-miR-15b-5p
 
 
-0.293
 
 
44.4
 
 
 
 
ADAM9
 
 
hsa-miR-30b-5p
 
 
-0.312
 
 
15.5
 
 
 
 
ADCY3
 
 
hsa-miR-132-3p
 
 
-0.303
 
 
46.2
 
 
 
 
ADIPOR2
 
 
hsa-miR-150-5p
 
 
-0.287
 
 
28.4
 
 
 
 
ADRA2A
 
 
hsa-miR-23a-3p
 
 
-0.371
 
 
22.2
 
 
 
 
ADRA2A
 
 
hsa-miR-23b-3p
 
 
-0.371
 
 
22.2
 
 
 
 
ADRB2
 
 
hsa-miR-15b-5p
 
 
-0.29
 
 
12.2
 
 
 
 
AHR
 
 
hsa-miR-29a-3p
 
 
-0.316
 
 
13.8
 
 
 
 
AK1
 
 
hsa-miR-29a-3p
 
 
-0.333
 
 
24.0
 
 
 
 
AK4
 
 
hsa-miR-15b-5p
 
 
-0.316
 
 
11.8
 
 
 
 
AKT3
 
 
hsa-miR-29a-3p
 
 
-0.287
 
 
12.8
 
 
 
 
AKT3
 
 
hsa-miR-15b-5p
 
 
-0.301
 
 
12.8
 
 
 
 
ALAD
 
 
hsa-miR-15b-5p
 
 
-0.446
 
 
19.3
 
 
 
 
ALDH1A2
 
 
hsa-miR-23b-3p
 
 
-0.346
 
 
13.2
 
 
 
 
ALDH1A2
 
 
hsa-miR-23a-3p
 
 
-0.354
 
 
13.2
 
 
 
 
ALOX12
 
 
hsa-miR-15b-5p
 
 
-0.443
 
 
95.8
 
 
 
 
AMOTL1
 
 
hsa-miR-15b-5p
 
 
-0.319
 
 
13.1
 
 
 
 
ANGPTL2
 
 
hsa-miR-221-3p
 
 
-0.369
 
 
47.7
 
 
 
 
ANKFY1
 
 
hsa-miR-155-5p
 
 
-0.315
 
 
13.3
 
 
 
 
ANKHD1
 
 
hsa-miR-23a-3p
 
 
-0.368
 
 
41.9
 
 
 
 
ANKHD1
 
 
hsa-miR-23b-3p
 
 
-0.368
 
 
41.9
 
 
 
 
ANKHD1
 
 
hsa-miR-30b-5p
 
 
-0.405
 
 
41.9
 
 
 
 
ANKRD17
 
 
hsa-miR-30b-5p
 
 
-0.336
 
 
12.3
 
 
 
 
ANKRD29
 
 
hsa-miR-132-3p
 
 
-0.47
 
 
12.3
 
 
 
 
ANP32A
 
 
hsa-miR-132-3p
 
 
-0.374
 
 
40.8
 
 
 
 
ANP32E
 
 
hsa-miR-501-3p
 
 
-0.331
 
 
15.5
 
 
 
 
ANTXR2
 
 
hsa-miR-29a-3p
 
 
-0.347
 
 
27.6
 
 
 
 
ANXA11
 
 
hsa-miR-15b-5p
 
 
-0.463
 
 
98.4
 
 
 
 
AP2A1
 
 
hsa-miR-15b-5p
 
 
-0.333
 
 
41.4
 
 
 
 
AP2B1
 
 
hsa-miR-15b-5p
 
 
-0.334
 
 
32.4
 
 
 
 
AP3S1
 
 
hsa-miR-30b-5p
 
 
-0.293
 
 
63.7
 
 
 
 
APP
 
 
hsa-miR-15b-5p
 
 
-0.298
 
 
142.4
 
 
 
 
AQP3
 
 
hsa-miR-874-3p
 
 
-0.48
 
 
505.0
 
 
 
 
ARF4
 
 
hsa-miR-30b-5p
 
 
-0.289
 
 
85.1
 
 
 
 
ARF4
 
 
hsa-miR-221-3p
 
 
-0.553
 
 
85.1
 
 
 
 
ARF5
 
 
hsa-miR-29a-3p
 
 
-0.491
 
 
132.3
 
 
 
 
ARF6
 
 
hsa-miR-181b-5p
 
 
-0.33
 
 
41.6
 
 
 
 
ARF6
 
 
hsa-miR-181a-5p
 
 
-0.33
 
 
41.6
 
 
 
 
ARHGAP5
 
 
hsa-miR-486-5p
 
 
-0.3
 
 
13.4
 
 
 
 
ARID1B
 
 
hsa-miR-29a-3p
 
 
-0.342
 
 
13.7
 
 
 
 
ARID4B
 
 
hsa-miR-486-5p
 
 
-0.367
 
 
10.0
 
 
 
 
ARL2
 
 
hsa-miR-15b-5p
 
 
-0.374
 
 
93.4
 
 
 
 
ARL2
 
 
hsa-miR-15b-5p
 
 
-0.442
 
 
93.4
 
 
 
 
ARL4A
 
 
hsa-miR-29a-3p
 
 
-0.386
 
 
63.2
 
 
 
 
ARL4A
 
 
hsa-miR-30b-5p
 
 
-0.448
 
 
63.2
 
 
 
 
ARMC8
 
 
hsa-miR-486-5p
 
 
-0.321
 
 
12.4
 
 
 
 
ARMCX2
 
 
hsa-miR-15b-5p
 
 
-0.344
 
 
18.0
 
 
 
 
ARMCX3
 
 
hsa-miR-425-5p
 
 
-0.325
 
 
20.2
 
 
 
 
ARNT
 
 
hsa-miR-221-3p
 
 
-0.465
 
 
23.6
 
 
 
 
ARPP19
 
 
hsa-miR-15b-5p
 
 
-0.346
 
 
27.0
 
 
 
 
ARPP19
 
 
hsa-miR-29a-3p
 
 
-0.371
 
 
27.0
 
 
 
 
ARRDC3
 
 
hsa-miR-29a-3p
 
 
-0.402
 
 
40.5
 
 
 
 
ASB6
 
 
hsa-miR-874-3p
 
 
-0.368
 
 
11.5
 
 
 
 
ASH1L
 
 
hsa-miR-15b-5p
 
 
-0.325
 
 
18.0
 
 
 
 
ASPH
 
 
hsa-miR-221-3p
 
 
-0.287
 
 
40.4
 
 
 
 
ATF3
 
 
hsa-miR-874-3p
 
 
-0.288
 
 
53.8
 
 
 
 
ATG9A
 
 
hsa-miR-15b-5p
 
 
-0.333
 
 
23.6
 
 
 
 
ATOSA
 
 
hsa-miR-221-3p
 
 
-0.495
 
 
10.0
 
 
 
 
ATP5MC3
 
 
hsa-miR-425-5p
 
 
-0.328
 
 
160.4
 
 
 
 
ATP5MC3
 
 
hsa-miR-29a-3p
 
 
-0.33
 
 
160.4
 
 
 
 
ATP6V1E1
 
 
hsa-miR-23a-3p
 
 
-0.294
 
 
97.4
 
 
 
 
ATP6V1E1
 
 
hsa-miR-23b-3p
 
 
-0.294
 
 
97.4
 
 
 
 
ATPAF1
 
 
hsa-miR-361-5p
 
 
-0.362
 
 
15.7
 
 
 
 
ATXN2
 
 
hsa-miR-15b-5p
 
 
-0.392
 
 
17.8
 
 
 
 
AZIN1
 
 
hsa-miR-132-3p
 
 
-0.29
 
 
24.5
 
 
 
 
AZIN1
 
 
hsa-miR-132-3p
 
 
-0.299
 
 
24.5
 
 
 
 
B3GNT5
 
 
hsa-miR-30b-5p
 
 
-0.326
 
 
13.3
 
 
 
 
B4GALT5
 
 
hsa-miR-501-3p
 
 
-0.408
 
 
16.5
 
 
 
 
B4GAT1
 
 
hsa-miR-23a-3p
 
 
-0.361
 
 
15.8
 
 
 
 
B4GAT1
 
 
hsa-miR-23b-3p
 
 
-0.361
 
 
15.8
 
 
 
 
BAK1
 
 
hsa-miR-29a-3p
 
 
-0.409
 
 
11.6
 
 
 
 
BCL2L2
 
 
hsa-miR-29a-3p
 
 
-0.312
 
 
35.4
 
 
 
 
BDH1
 
 
hsa-miR-874-3p
 
 
-0.644
 
 
13.3
 
 
 
 
BECN1
 
 
hsa-miR-30b-5p
 
 
-0.452
 
 
28.4
 
 
 
 
BET1
 
 
hsa-miR-23a-3p
 
 
-0.402
 
 
11.3
 
 
 
 
BET1
 
 
hsa-miR-23b-3p
 
 
-0.418
 
 
11.3
 
 
 
 
BFAR
 
 
hsa-miR-15b-5p
 
 
-0.352
 
 
23.3
 
 
 
 
BHLHE40
 
 
hsa-miR-181b-5p
 
 
-0.408
 
 
291.4
 
 
 
 
BHLHE40
 
 
hsa-miR-181a-5p
 
 
-0.408
 
 
291.4
 
 
 
 
BICDL1
 
 
hsa-miR-221-3p
 
 
-0.447
 
 
17.1
 
 
 
 
BLCAP
 
 
hsa-miR-23a-3p
 
 
-0.601
 
 
58.9
 
 
 
 
BLCAP
 
 
hsa-miR-23b-3p
 
 
-0.601
 
 
58.9
 
 
 
 
BLMH
 
 
hsa-miR-29a-3p
 
 
-0.393
 
 
20.2
 
 
 
 
BLOC1S6
 
 
hsa-miR-181b-5p
 
 
-0.393
 
 
15.7
 
 
 
 
BLOC1S6
 
 
hsa-miR-181a-5p
 
 
-0.393
 
 
15.7
 
 
 
 
BOLA3
 
 
hsa-miR-15b-5p
 
 
-0.363
 
 
25.9
 
 
 
 
BOLA3
 
 
hsa-miR-15b-5p
 
 
-0.386
 
 
25.9
 
 
 
 
BOLA3
 
 
hsa-miR-374b-5p
 
 
-0.486
 
 
25.9
 
 
 
 
BRI3
 
 
hsa-miR-132-3p
 
 
-0.385
 
 
63.7
 
 
 
 
BTAF1
 
 
hsa-miR-486-5p
 
 
-0.288
 
 
16.3
 
 
 
 
BTAF1
 
 
hsa-miR-15b-5p
 
 
-0.496
 
 
16.3
 
 
 
 
BTF3
 
 
hsa-miR-15b-5p
 
 
-0.344
 
 
608.5
 
 
 
 
BTF3
 
 
hsa-miR-361-5p
 
 
-0.551
 
 
608.5
 
 
 
 
BTG2
 
 
hsa-miR-29a-3p
 
 
-0.32
 
 
73.7
 
 
 
 
BTN2A1
 
 
hsa-miR-342-3p
 
 
-0.31
 
 
13.4
 
 
 
 
 
 
 hypothalamus 
 
 
 
 
target_symbol
 
 
mature_mirna_id
 
 
score
 
 
nTPM
 
 
 
 
 
 
ABCE1
 
 
hsa-miR-29a-3p
 
 
-0.404
 
 
12.9
 
 
 
 
ABHD17B
 
 
hsa-miR-486-5p
 
 
-0.348
 
 
11.0
 
 
 
 
ABHD4
 
 
hsa-miR-29a-3p
 
 
-0.312
 
 
13.5
 
 
 
 
ACTR2
 
 
hsa-miR-15b-5p
 
 
-0.293
 
 
50.9
 
 
 
 
ADCY3
 
 
hsa-miR-132-3p
 
 
-0.303
 
 
32.1
 
 
 
 
ADGRA3
 
 
hsa-miR-30b-5p
 
 
-0.304
 
 
10.8
 
 
 
 
ADIPOR2
 
 
hsa-miR-150-5p
 
 
-0.287
 
 
40.5
 
 
 
 
AGPAT4
 
 
hsa-miR-29a-3p
 
 
-0.376
 
 
14.0
 
 
 
 
AK1
 
 
hsa-miR-29a-3p
 
 
-0.333
 
 
100.1
 
 
 
 
AK4
 
 
hsa-miR-15b-5p
 
 
-0.316
 
 
12.0
 
 
 
 
AKT3
 
 
hsa-miR-29a-3p
 
 
-0.287
 
 
14.5
 
 
 
 
AKT3
 
 
hsa-miR-15b-5p
 
 
-0.301
 
 
14.5
 
 
 
 
ALAD
 
 
hsa-miR-15b-5p
 
 
-0.446
 
 
54.5
 
 
 
 
AMOT
 
 
hsa-miR-29a-3p
 
 
-0.304
 
 
11.9
 
 
 
 
ANKFY1
 
 
hsa-miR-155-5p
 
 
-0.315
 
 
10.7
 
 
 
 
ANKHD1
 
 
hsa-miR-23a-3p
 
 
-0.368
 
 
17.8
 
 
 
 
ANKHD1
 
 
hsa-miR-23b-3p
 
 
-0.368
 
 
17.8
 
 
 
 
ANKHD1
 
 
hsa-miR-30b-5p
 
 
-0.405
 
 
17.8
 
 
 
 
ANKRD17
 
 
hsa-miR-30b-5p
 
 
-0.336
 
 
11.3
 
 
 
 
ANLN
 
 
hsa-miR-15b-5p
 
 
-0.334
 
 
30.4
 
 
 
 
ANP32A
 
 
hsa-miR-132-3p
 
 
-0.374
 
 
37.9
 
 
 
 
ANP32E
 
 
hsa-miR-501-3p
 
 
-0.331
 
 
33.1
 
 
 
 
ANXA11
 
 
hsa-miR-15b-5p
 
 
-0.463
 
 
13.4
 
 
 
 
AP2A1
 
 
hsa-miR-15b-5p
 
 
-0.333
 
 
44.0
 
 
 
 
AP2B1
 
 
hsa-miR-15b-5p
 
 
-0.334
 
 
68.4
 
 
 
 
AP3S1
 
 
hsa-miR-30b-5p
 
 
-0.293
 
 
61.9
 
 
 
 
APC
 
 
hsa-miR-29a-3p
 
 
-0.413
 
 
11.2
 
 
 
 
APLN
 
 
hsa-miR-15b-5p
 
 
-0.395
 
 
17.3
 
 
 
 
APOO
 
 
hsa-miR-181b-5p
 
 
-0.5
 
 
43.8
 
 
 
 
APOO
 
 
hsa-miR-181a-5p
 
 
-0.5
 
 
43.8
 
 
 
 
APP
 
 
hsa-miR-15b-5p
 
 
-0.298
 
 
437.7
 
 
 
 
ARF4
 
 
hsa-miR-30b-5p
 
 
-0.289
 
 
75.3
 
 
 
 
ARF4
 
 
hsa-miR-221-3p
 
 
-0.553
 
 
75.3
 
 
 
 
ARF5
 
 
hsa-miR-29a-3p
 
 
-0.491
 
 
134.0
 
 
 
 
ARF6
 
 
hsa-miR-181b-5p
 
 
-0.33
 
 
10.2
 
 
 
 
ARF6
 
 
hsa-miR-181a-5p
 
 
-0.33
 
 
10.2
 
 
 
 
ARHGAP5
 
 
hsa-miR-486-5p
 
 
-0.3
 
 
17.7
 
 
 
 
ARL2
 
 
hsa-miR-15b-5p
 
 
-0.374
 
 
156.1
 
 
 
 
ARL2
 
 
hsa-miR-15b-5p
 
 
-0.442
 
 
156.1
 
 
 
 
ARL3
 
 
hsa-miR-15b-5p
 
 
-0.507
 
 
23.1
 
 
 
 
ARMC8
 
 
hsa-miR-486-5p
 
 
-0.321
 
 
16.8
 
 
 
 
ARMCX2
 
 
hsa-miR-15b-5p
 
 
-0.344
 
 
28.0
 
 
 
 
ARMCX3
 
 
hsa-miR-425-5p
 
 
-0.325
 
 
32.9
 
 
 
 
ARPP19
 
 
hsa-miR-15b-5p
 
 
-0.346
 
 
103.9
 
 
 
 
ARPP19
 
 
hsa-miR-29a-3p
 
 
-0.371
 
 
103.9
 
 
 
 
ARPP21
 
 
hsa-miR-15b-5p
 
 
-0.342
 
 
24.9
 
 
 
 
ARPP21
 
 
hsa-miR-501-3p
 
 
-0.546
 
 
24.9
 
 
 
 
ARRDC3
 
 
hsa-miR-29a-3p
 
 
-0.402
 
 
10.3
 
 
 
 
ASB3
 
 
hsa-miR-30b-5p
 
 
-0.418
 
 
17.6
 
 
 
 
ASB3
 
 
hsa-miR-30b-5p
 
 
-0.418
 
 
17.6
 
 
 
 
ASB6
 
 
hsa-miR-874-3p
 
 
-0.368
 
 
11.8
 
 
 
 
ASF1A
 
 
hsa-miR-23a-3p
 
 
-0.506
 
 
13.0
 
 
 
 
ASF1A
 
 
hsa-miR-23b-3p
 
 
-0.506
 
 
13.0
 
 
 
 
ASH1L
 
 
hsa-miR-15b-5p
 
 
-0.325
 
 
19.0
 
 
 
 
ASPH
 
 
hsa-miR-221-3p
 
 
-0.287
 
 
51.0
 
 
 
 
ATF3
 
 
hsa-miR-874-3p
 
 
-0.288
 
 
21.0
 
 
 
 
ATG9A
 
 
hsa-miR-15b-5p
 
 
-0.333
 
 
21.3
 
 
 
 
ATP5MC3
 
 
hsa-miR-425-5p
 
 
-0.328
 
 
193.1
 
 
 
 
ATP5MC3
 
 
hsa-miR-29a-3p
 
 
-0.33
 
 
193.1
 
 
 
 
ATP6V1E1
 
 
hsa-miR-23a-3p
 
 
-0.294
 
 
217.4
 
 
 
 
ATP6V1E1
 
 
hsa-miR-23b-3p
 
 
-0.294
 
 
217.4
 
 
 
 
ATPAF1
 
 
hsa-miR-361-5p
 
 
-0.362
 
 
52.2
 
 
 
 
ATXN2
 
 
hsa-miR-15b-5p
 
 
-0.392
 
 
24.5
 
 
 
 
AUH
 
 
hsa-miR-23a-3p
 
 
-0.495
 
 
18.2
 
 
 
 
AUH
 
 
hsa-miR-23b-3p
 
 
-0.503
 
 
18.2
 
 
 
 
AUH
 
 
hsa-miR-23a-3p
 
 
-0.512
 
 
18.2
 
 
 
 
AUH
 
 
hsa-miR-23b-3p
 
 
-0.512
 
 
18.2
 
 
 
 
AZIN1
 
 
hsa-miR-132-3p
 
 
-0.29
 
 
32.4
 
 
 
 
AZIN1
 
 
hsa-miR-132-3p
 
 
-0.299
 
 
32.4
 
 
 
 
B4GALT5
 
 
hsa-miR-501-3p
 
 
-0.408
 
 
13.0
 
 
 
 
B4GAT1
 
 
hsa-miR-23a-3p
 
 
-0.361
 
 
207.3
 
 
 
 
B4GAT1
 
 
hsa-miR-23b-3p
 
 
-0.361
 
 
207.3
 
 
 
 
BAG4
 
 
hsa-miR-181b-5p
 
 
-0.337
 
 
10.8
 
 
 
 
BAG4
 
 
hsa-miR-181a-5p
 
 
-0.337
 
 
10.8
 
 
 
 
BCL2L2
 
 
hsa-miR-29a-3p
 
 
-0.312
 
 
75.6
 
 
 
 
BDH1
 
 
hsa-miR-874-3p
 
 
-0.644
 
 
18.1
 
 
 
 
BECN1
 
 
hsa-miR-30b-5p
 
 
-0.452
 
 
26.1
 
 
 
 
BEND6
 
 
hsa-miR-361-5p
 
 
-0.305
 
 
15.8
 
 
 
 
BET1
 
 
hsa-miR-23a-3p
 
 
-0.402
 
 
13.4
 
 
 
 
BET1
 
 
hsa-miR-23b-3p
 
 
-0.418
 
 
13.4
 
 
 
 
BFAR
 
 
hsa-miR-15b-5p
 
 
-0.352
 
 
12.7
 
 
 
 
BHLHE40
 
 
hsa-miR-181b-5p
 
 
-0.408
 
 
34.7
 
 
 
 
BHLHE40
 
 
hsa-miR-181a-5p
 
 
-0.408
 
 
34.7
 
 
 
 
BICDL1
 
 
hsa-miR-221-3p
 
 
-0.447
 
 
15.9
 
 
 
 
BLCAP
 
 
hsa-miR-23a-3p
 
 
-0.601
 
 
183.0
 
 
 
 
BLCAP
 
 
hsa-miR-23b-3p
 
 
-0.601
 
 
183.0
 
 
 
 
BLMH
 
 
hsa-miR-29a-3p
 
 
-0.393
 
 
11.4
 
 
 
 
BLOC1S6
 
 
hsa-miR-181b-5p
 
 
-0.393
 
 
24.8
 
 
 
 
BLOC1S6
 
 
hsa-miR-181a-5p
 
 
-0.393
 
 
24.8
 
 
 
 
BOLA3
 
 
hsa-miR-15b-5p
 
 
-0.363
 
 
57.1
 
 
 
 
BOLA3
 
 
hsa-miR-15b-5p
 
 
-0.386
 
 
57.1
 
 
 
 
BOLA3
 
 
hsa-miR-374b-5p
 
 
-0.486
 
 
57.1
 
 
 
 
BRI3
 
 
hsa-miR-132-3p
 
 
-0.385
 
 
47.7
 
 
 
 
BTF3
 
 
hsa-miR-15b-5p
 
 
-0.344
 
 
272.2
 
 
 
 
BTF3
 
 
hsa-miR-361-5p
 
 
-0.551
 
 
272.2
 
 
 
 
BTG2
 
 
hsa-miR-29a-3p
 
 
-0.32
 
 
43.2
 
 
 
 
BTN2A1
 
 
hsa-miR-342-3p
 
 
-0.31
 
 
12.6
 
 
 
 
BTRC
 
 
hsa-miR-15b-5p
 
 
-0.302
 
 
14.2
 
 
 
 
BTRC
 
 
hsa-miR-15b-5p
 
 
-0.358
 
 
14.2
 
 
 
 
BZW1
 
 
hsa-miR-15b-5p
 
 
-0.367
 
 
32.4
 
 
 
 
 
 
 retina 
 
 
 
 
target_symbol
 
 
mature_mirna_id
 
 
score
 
 
nTPM
 
 
 
 
 
 
ABCE1
 
 
hsa-miR-29a-3p
 
 
-0.404
 
 
13.3
 
 
 
 
ABHD17B
 
 
hsa-miR-486-5p
 
 
-0.348
 
 
23.6
 
 
 
 
ABHD4
 
 
hsa-miR-29a-3p
 
 
-0.312
 
 
21.0
 
 
 
 
ACTC1
 
 
hsa-miR-30b-5p
 
 
-0.543
 
 
29.3
 
 
 
 
ACTR2
 
 
hsa-miR-15b-5p
 
 
-0.293
 
 
60.2
 
 
 
 
ADAM19
 
 
hsa-miR-874-3p
 
 
-0.374
 
 
21.0
 
 
 
 
ADAM9
 
 
hsa-miR-30b-5p
 
 
-0.312
 
 
17.2
 
 
 
 
ADIPOR2
 
 
hsa-miR-150-5p
 
 
-0.287
 
 
12.1
 
 
 
 
AGO4
 
 
hsa-miR-221-3p
 
 
-0.321
 
 
16.7
 
 
 
 
AK1
 
 
hsa-miR-29a-3p
 
 
-0.333
 
 
27.1
 
 
 
 
AK4
 
 
hsa-miR-15b-5p
 
 
-0.316
 
 
19.7
 
 
 
 
AKT3
 
 
hsa-miR-29a-3p
 
 
-0.287
 
 
15.2
 
 
 
 
AKT3
 
 
hsa-miR-15b-5p
 
 
-0.301
 
 
15.2
 
 
 
 
ALAD
 
 
hsa-miR-15b-5p
 
 
-0.446
 
 
17.6
 
 
 
 
ANKFY1
 
 
hsa-miR-155-5p
 
 
-0.315
 
 
12.1
 
 
 
 
ANKHD1
 
 
hsa-miR-23a-3p
 
 
-0.368
 
 
37.5
 
 
 
 
ANKHD1
 
 
hsa-miR-23b-3p
 
 
-0.368
 
 
37.5
 
 
 
 
ANKHD1
 
 
hsa-miR-30b-5p
 
 
-0.405
 
 
37.5
 
 
 
 
ANKRD13C
 
 
hsa-miR-29a-3p
 
 
-0.411
 
 
12.3
 
 
 
 
ANKRD17
 
 
hsa-miR-30b-5p
 
 
-0.336
 
 
33.3
 
 
 
 
ANP32A
 
 
hsa-miR-132-3p
 
 
-0.374
 
 
98.2
 
 
 
 
ANP32E
 
 
hsa-miR-501-3p
 
 
-0.331
 
 
91.3
 
 
 
 
AP2A1
 
 
hsa-miR-15b-5p
 
 
-0.333
 
 
12.4
 
 
 
 
AP2B1
 
 
hsa-miR-15b-5p
 
 
-0.334
 
 
70.5
 
 
 
 
AP3S1
 
 
hsa-miR-30b-5p
 
 
-0.293
 
 
31.5
 
 
 
 
APC
 
 
hsa-miR-29a-3p
 
 
-0.413
 
 
18.5
 
 
 
 
APOO
 
 
hsa-miR-181b-5p
 
 
-0.5
 
 
48.9
 
 
 
 
APOO
 
 
hsa-miR-181a-5p
 
 
-0.5
 
 
48.9
 
 
 
 
APP
 
 
hsa-miR-15b-5p
 
 
-0.298
 
 
189.1
 
 
 
 
ARF4
 
 
hsa-miR-30b-5p
 
 
-0.289
 
 
50.0
 
 
 
 
ARF4
 
 
hsa-miR-221-3p
 
 
-0.553
 
 
50.0
 
 
 
 
ARF5
 
 
hsa-miR-29a-3p
 
 
-0.491
 
 
21.3
 
 
 
 
ARF6
 
 
hsa-miR-181b-5p
 
 
-0.33
 
 
13.4
 
 
 
 
ARF6
 
 
hsa-miR-181a-5p
 
 
-0.33
 
 
13.4
 
 
 
 
ARHGAP42
 
 
hsa-miR-221-3p
 
 
-0.304
 
 
29.2
 
 
 
 
ARHGAP5
 
 
hsa-miR-486-5p
 
 
-0.3
 
 
53.6
 
 
 
 
ARID1B
 
 
hsa-miR-29a-3p
 
 
-0.342
 
 
10.7
 
 
 
 
ARID2
 
 
hsa-miR-132-3p
 
 
-0.304
 
 
12.3
 
 
 
 
ARID2
 
 
hsa-miR-155-5p
 
 
-0.384
 
 
12.3
 
 
 
 
ARID4B
 
 
hsa-miR-486-5p
 
 
-0.367
 
 
13.5
 
 
 
 
ARL2
 
 
hsa-miR-15b-5p
 
 
-0.374
 
 
40.5
 
 
 
 
ARL2
 
 
hsa-miR-15b-5p
 
 
-0.442
 
 
40.5
 
 
 
 
ARL3
 
 
hsa-miR-15b-5p
 
 
-0.507
 
 
89.3
 
 
 
 
ARMC8
 
 
hsa-miR-486-5p
 
 
-0.321
 
 
24.3
 
 
 
 
ARMCX2
 
 
hsa-miR-15b-5p
 
 
-0.344
 
 
14.2
 
 
 
 
ARMCX3
 
 
hsa-miR-425-5p
 
 
-0.325
 
 
17.6
 
 
 
 
ARNT
 
 
hsa-miR-221-3p
 
 
-0.465
 
 
36.3
 
 
 
 
ARPP19
 
 
hsa-miR-15b-5p
 
 
-0.346
 
 
53.1
 
 
 
 
ARPP19
 
 
hsa-miR-29a-3p
 
 
-0.371
 
 
53.1
 
 
 
 
ARRDC3
 
 
hsa-miR-29a-3p
 
 
-0.402
 
 
69.6
 
 
 
 
ASB3
 
 
hsa-miR-30b-5p
 
 
-0.418
 
 
21.2
 
 
 
 
ASB3
 
 
hsa-miR-30b-5p
 
 
-0.418
 
 
21.2
 
 
 
 
ASF1A
 
 
hsa-miR-23a-3p
 
 
-0.506
 
 
10.6
 
 
 
 
ASF1A
 
 
hsa-miR-23b-3p
 
 
-0.506
 
 
10.6
 
 
 
 
ASH1L
 
 
hsa-miR-15b-5p
 
 
-0.325
 
 
24.2
 
 
 
 
ASPH
 
 
hsa-miR-221-3p
 
 
-0.287
 
 
201.9
 
 
 
 
ATF3
 
 
hsa-miR-874-3p
 
 
-0.288
 
 
15.0
 
 
 
 
ATG9A
 
 
hsa-miR-15b-5p
 
 
-0.333
 
 
22.8
 
 
 
 
ATOSA
 
 
hsa-miR-221-3p
 
 
-0.495
 
 
16.0
 
 
 
 
ATP5MC3
 
 
hsa-miR-425-5p
 
 
-0.328
 
 
117.1
 
 
 
 
ATP5MC3
 
 
hsa-miR-29a-3p
 
 
-0.33
 
 
117.1
 
 
 
 
ATP6V1E1
 
 
hsa-miR-23a-3p
 
 
-0.294
 
 
119.0
 
 
 
 
ATP6V1E1
 
 
hsa-miR-23b-3p
 
 
-0.294
 
 
119.0
 
 
 
 
ATPAF1
 
 
hsa-miR-361-5p
 
 
-0.362
 
 
65.3
 
 
 
 
ATXN1
 
 
hsa-miR-221-3p
 
 
-0.289
 
 
12.9
 
 
 
 
ATXN2
 
 
hsa-miR-15b-5p
 
 
-0.392
 
 
14.6
 
 
 
 
AUH
 
 
hsa-miR-23a-3p
 
 
-0.495
 
 
19.8
 
 
 
 
AUH
 
 
hsa-miR-23b-3p
 
 
-0.503
 
 
19.8
 
 
 
 
AUH
 
 
hsa-miR-23a-3p
 
 
-0.512
 
 
19.8
 
 
 
 
AUH
 
 
hsa-miR-23b-3p
 
 
-0.512
 
 
19.8
 
 
 
 
AZIN1
 
 
hsa-miR-132-3p
 
 
-0.29
 
 
52.2
 
 
 
 
AZIN1
 
 
hsa-miR-132-3p
 
 
-0.299
 
 
52.2
 
 
 
 
B4GAT1
 
 
hsa-miR-23a-3p
 
 
-0.361
 
 
42.6
 
 
 
 
B4GAT1
 
 
hsa-miR-23b-3p
 
 
-0.361
 
 
42.6
 
 
 
 
BCL2L11
 
 
hsa-miR-221-3p
 
 
-0.423
 
 
11.9
 
 
 
 
BCL2L2
 
 
hsa-miR-29a-3p
 
 
-0.312
 
 
44.1
 
 
 
 
BCOR
 
 
hsa-miR-425-5p
 
 
-0.346
 
 
13.1
 
 
 
 
BECN1
 
 
hsa-miR-30b-5p
 
 
-0.452
 
 
24.5
 
 
 
 
BEND6
 
 
hsa-miR-361-5p
 
 
-0.305
 
 
10.9
 
 
 
 
BET1
 
 
hsa-miR-23a-3p
 
 
-0.402
 
 
12.3
 
 
 
 
BET1
 
 
hsa-miR-23b-3p
 
 
-0.418
 
 
12.3
 
 
 
 
BFAR
 
 
hsa-miR-15b-5p
 
 
-0.352
 
 
18.0
 
 
 
 
BHLHE40
 
 
hsa-miR-181b-5p
 
 
-0.408
 
 
109.8
 
 
 
 
BHLHE40
 
 
hsa-miR-181a-5p
 
 
-0.408
 
 
109.8
 
 
 
 
BICDL1
 
 
hsa-miR-221-3p
 
 
-0.447
 
 
39.5
 
 
 
 
BLCAP
 
 
hsa-miR-23a-3p
 
 
-0.601
 
 
65.7
 
 
 
 
BLCAP
 
 
hsa-miR-23b-3p
 
 
-0.601
 
 
65.7
 
 
 
 
BLMH
 
 
hsa-miR-29a-3p
 
 
-0.393
 
 
13.7
 
 
 
 
BLOC1S6
 
 
hsa-miR-181b-5p
 
 
-0.393
 
 
22.7
 
 
 
 
BLOC1S6
 
 
hsa-miR-181a-5p
 
 
-0.393
 
 
22.7
 
 
 
 
BOLA3
 
 
hsa-miR-15b-5p
 
 
-0.363
 
 
33.1
 
 
 
 
BOLA3
 
 
hsa-miR-15b-5p
 
 
-0.386
 
 
33.1
 
 
 
 
BOLA3
 
 
hsa-miR-374b-5p
 
 
-0.486
 
 
33.1
 
 
 
 
BRI3
 
 
hsa-miR-132-3p
 
 
-0.385
 
 
10.1
 
 
 
 
BTAF1
 
 
hsa-miR-486-5p
 
 
-0.288
 
 
27.2
 
 
 
 
BTAF1
 
 
hsa-miR-15b-5p
 
 
-0.496
 
 
27.2
 
 
 
 
BTF3
 
 
hsa-miR-15b-5p
 
 
-0.344
 
 
170.2
 
 
 
 
BTF3
 
 
hsa-miR-361-5p
 
 
-0.551
 
 
170.2
 
 
 
 
BTG2
 
 
hsa-miR-29a-3p
 
 
-0.32
 
 
141.6
 
 
 
 
BTRC
 
 
hsa-miR-15b-5p
 
 
-0.302
 
 
20.0
 
 
 
 
 
 
 pituitary gland 
 
 
 
 
target_symbol
 
 
mature_mirna_id
 
 
score
 
 
nTPM
 
 
 
 
 
 
ABHD4
 
 
hsa-miR-29a-3p
 
 
-0.312
 
 
13.7
 
 
 
 
ACTR2
 
 
hsa-miR-15b-5p
 
 
-0.293
 
 
23.5
 
 
 
 
ADCY3
 
 
hsa-miR-132-3p
 
 
-0.303
 
 
41.4
 
 
 
 
ADIPOR2
 
 
hsa-miR-150-5p
 
 
-0.287
 
 
25.4
 
 
 
 
AGPAT4
 
 
hsa-miR-29a-3p
 
 
-0.376
 
 
10.5
 
 
 
 
AK1
 
 
hsa-miR-29a-3p
 
 
-0.333
 
 
40.7
 
 
 
 
AKT3
 
 
hsa-miR-29a-3p
 
 
-0.287
 
 
11.4
 
 
 
 
AKT3
 
 
hsa-miR-15b-5p
 
 
-0.301
 
 
11.4
 
 
 
 
ALAD
 
 
hsa-miR-15b-5p
 
 
-0.446
 
 
16.3
 
 
 
 
ALDH1A2
 
 
hsa-miR-23b-3p
 
 
-0.346
 
 
14.4
 
 
 
 
ALDH1A2
 
 
hsa-miR-23a-3p
 
 
-0.354
 
 
14.4
 
 
 
 
AMOTL1
 
 
hsa-miR-15b-5p
 
 
-0.319
 
 
13.5
 
 
 
 
ANKFY1
 
 
hsa-miR-155-5p
 
 
-0.315
 
 
11.8
 
 
 
 
ANKHD1
 
 
hsa-miR-23a-3p
 
 
-0.368
 
 
41.2
 
 
 
 
ANKHD1
 
 
hsa-miR-23b-3p
 
 
-0.368
 
 
41.2
 
 
 
 
ANKHD1
 
 
hsa-miR-30b-5p
 
 
-0.405
 
 
41.2
 
 
 
 
ANKRD17
 
 
hsa-miR-30b-5p
 
 
-0.336
 
 
13.4
 
 
 
 
ANP32A
 
 
hsa-miR-132-3p
 
 
-0.374
 
 
26.1
 
 
 
 
ANP32E
 
 
hsa-miR-501-3p
 
 
-0.331
 
 
15.4
 
 
 
 
ANXA11
 
 
hsa-miR-15b-5p
 
 
-0.463
 
 
25.4
 
 
 
 
AP2A1
 
 
hsa-miR-15b-5p
 
 
-0.333
 
 
33.0
 
 
 
 
AP2B1
 
 
hsa-miR-15b-5p
 
 
-0.334
 
 
51.7
 
 
 
 
AP3S1
 
 
hsa-miR-30b-5p
 
 
-0.293
 
 
47.0
 
 
 
 
APOO
 
 
hsa-miR-181b-5p
 
 
-0.5
 
 
28.1
 
 
 
 
APOO
 
 
hsa-miR-181a-5p
 
 
-0.5
 
 
28.1
 
 
 
 
APP
 
 
hsa-miR-15b-5p
 
 
-0.298
 
 
144.3
 
 
 
 
ARF4
 
 
hsa-miR-30b-5p
 
 
-0.289
 
 
93.4
 
 
 
 
ARF4
 
 
hsa-miR-221-3p
 
 
-0.553
 
 
93.4
 
 
 
 
ARF5
 
 
hsa-miR-29a-3p
 
 
-0.491
 
 
111.9
 
 
 
 
ARID4B
 
 
hsa-miR-486-5p
 
 
-0.367
 
 
10.3
 
 
 
 
ARL2
 
 
hsa-miR-15b-5p
 
 
-0.374
 
 
68.9
 
 
 
 
ARL2
 
 
hsa-miR-15b-5p
 
 
-0.442
 
 
68.9
 
 
 
 
ARL3
 
 
hsa-miR-15b-5p
 
 
-0.507
 
 
19.9
 
 
 
 
ARMC12
 
 
hsa-miR-15b-5p
 
 
-0.485
 
 
11.0
 
 
 
 
ARMC8
 
 
hsa-miR-486-5p
 
 
-0.321
 
 
11.3
 
 
 
 
ARMCX2
 
 
hsa-miR-15b-5p
 
 
-0.344
 
 
70.4
 
 
 
 
ARMCX3
 
 
hsa-miR-425-5p
 
 
-0.325
 
 
43.1
 
 
 
 
ARNT
 
 
hsa-miR-221-3p
 
 
-0.465
 
 
12.2
 
 
 
 
ARPP19
 
 
hsa-miR-15b-5p
 
 
-0.346
 
 
33.0
 
 
 
 
ARPP19
 
 
hsa-miR-29a-3p
 
 
-0.371
 
 
33.0
 
 
 
 
ARPP21
 
 
hsa-miR-15b-5p
 
 
-0.342
 
 
12.2
 
 
 
 
ARPP21
 
 
hsa-miR-501-3p
 
 
-0.546
 
 
12.2
 
 
 
 
ARRDC3
 
 
hsa-miR-29a-3p
 
 
-0.402
 
 
20.2
 
 
 
 
ASB3
 
 
hsa-miR-30b-5p
 
 
-0.418
 
 
13.9
 
 
 
 
ASB3
 
 
hsa-miR-30b-5p
 
 
-0.418
 
 
13.9
 
 
 
 
ASB6
 
 
hsa-miR-874-3p
 
 
-0.368
 
 
12.1
 
 
 
 
ASH1L
 
 
hsa-miR-15b-5p
 
 
-0.325
 
 
17.6
 
 
 
 
ASPH
 
 
hsa-miR-221-3p
 
 
-0.287
 
 
33.8
 
 
 
 
ATF3
 
 
hsa-miR-874-3p
 
 
-0.288
 
 
43.7
 
 
 
 
ATG9A
 
 
hsa-miR-15b-5p
 
 
-0.333
 
 
18.7
 
 
 
 
ATP5MC3
 
 
hsa-miR-425-5p
 
 
-0.328
 
 
134.5
 
 
 
 
ATP5MC3
 
 
hsa-miR-29a-3p
 
 
-0.33
 
 
134.5
 
 
 
 
ATP6V1E1
 
 
hsa-miR-23a-3p
 
 
-0.294
 
 
116.0
 
 
 
 
ATP6V1E1
 
 
hsa-miR-23b-3p
 
 
-0.294
 
 
116.0
 
 
 
 
ATPAF1
 
 
hsa-miR-361-5p
 
 
-0.362
 
 
31.1
 
 
 
 
ATXN2
 
 
hsa-miR-15b-5p
 
 
-0.392
 
 
21.6
 
 
 
 
AZIN1
 
 
hsa-miR-132-3p
 
 
-0.29
 
 
27.1
 
 
 
 
AZIN1
 
 
hsa-miR-132-3p
 
 
-0.299
 
 
27.1
 
 
 
 
B4GALT5
 
 
hsa-miR-501-3p
 
 
-0.408
 
 
11.9
 
 
 
 
B4GAT1
 
 
hsa-miR-23a-3p
 
 
-0.361
 
 
60.5
 
 
 
 
B4GAT1
 
 
hsa-miR-23b-3p
 
 
-0.361
 
 
60.5
 
 
 
 
BAK1
 
 
hsa-miR-29a-3p
 
 
-0.409
 
 
11.6
 
 
 
 
BCL2L2
 
 
hsa-miR-29a-3p
 
 
-0.312
 
 
39.0
 
 
 
 
BDH1
 
 
hsa-miR-874-3p
 
 
-0.644
 
 
11.4
 
 
 
 
BECN1
 
 
hsa-miR-30b-5p
 
 
-0.452
 
 
19.6
 
 
 
 
BET1
 
 
hsa-miR-23a-3p
 
 
-0.402
 
 
17.3
 
 
 
 
BET1
 
 
hsa-miR-23b-3p
 
 
-0.418
 
 
17.3
 
 
 
 
BFAR
 
 
hsa-miR-15b-5p
 
 
-0.352
 
 
16.5
 
 
 
 
BHLHE40
 
 
hsa-miR-181b-5p
 
 
-0.408
 
 
99.3
 
 
 
 
BHLHE40
 
 
hsa-miR-181a-5p
 
 
-0.408
 
 
99.3
 
 
 
 
BICDL1
 
 
hsa-miR-221-3p
 
 
-0.447
 
 
63.8
 
 
 
 
BLCAP
 
 
hsa-miR-23a-3p
 
 
-0.601
 
 
145.4
 
 
 
 
BLCAP
 
 
hsa-miR-23b-3p
 
 
-0.601
 
 
145.4
 
 
 
 
BLOC1S6
 
 
hsa-miR-181b-5p
 
 
-0.393
 
 
19.0
 
 
 
 
BLOC1S6
 
 
hsa-miR-181a-5p
 
 
-0.393
 
 
19.0
 
 
 
 
BOLA3
 
 
hsa-miR-15b-5p
 
 
-0.363
 
 
31.4
 
 
 
 
BOLA3
 
 
hsa-miR-15b-5p
 
 
-0.386
 
 
31.4
 
 
 
 
BOLA3
 
 
hsa-miR-374b-5p
 
 
-0.486
 
 
31.4
 
 
 
 
BPIFA1
 
 
hsa-miR-15b-5p
 
 
-0.396
 
 
12.3
 
 
 
 
BRI3
 
 
hsa-miR-132-3p
 
 
-0.385
 
 
79.3
 
 
 
 
BTAF1
 
 
hsa-miR-486-5p
 
 
-0.288
 
 
11.4
 
 
 
 
BTAF1
 
 
hsa-miR-15b-5p
 
 
-0.496
 
 
11.4
 
 
 
 
BTF3
 
 
hsa-miR-15b-5p
 
 
-0.344
 
 
276.0
 
 
 
 
BTF3
 
 
hsa-miR-361-5p
 
 
-0.551
 
 
276.0
 
 
 
 
BTG2
 
 
hsa-miR-29a-3p
 
 
-0.32
 
 
204.0
 
 
 
 
BTN2A1
 
 
hsa-miR-342-3p
 
 
-0.31
 
 
11.8
 
 
 
 
BZW1
 
 
hsa-miR-15b-5p
 
 
-0.367
 
 
28.1
 
 
 
 
C11orf24
 
 
hsa-miR-15b-5p
 
 
-0.403
 
 
13.8
 
 
 
 
C11orf68
 
 
hsa-miR-15b-5p
 
 
-0.29
 
 
23.2
 
 
 
 
C3orf18
 
 
hsa-miR-155-5p
 
 
-0.431
 
 
12.0
 
 
 
 
C3orf18
 
 
hsa-miR-30b-5p
 
 
-0.49
 
 
12.0
 
 
 
 
C5orf15
 
 
hsa-miR-29a-3p
 
 
-0.392
 
 
20.2
 
 
 
 
C6orf62
 
 
hsa-miR-181b-5p
 
 
-0.334
 
 
60.8
 
 
 
 
C6orf62
 
 
hsa-miR-181a-5p
 
 
-0.334
 
 
60.8
 
 
 
 
C6orf62
 
 
hsa-miR-23a-3p
 
 
-0.507
 
 
60.8
 
 
 
 
C6orf62
 
 
hsa-miR-23b-3p
 
 
-0.507
 
 
60.8
 
 
 
 
C9orf72
 
 
hsa-miR-29a-3p
 
 
-0.322
 
 
15.1
 
 
 
 
C9orf72
 
 
hsa-miR-30b-5p
 
 
-0.358
 
 
15.1
 
 
 
 
CA12
 
 
hsa-miR-342-3p
 
 
-0.294
 
 
10.5
 
 
 
 
CAB39
 
 
hsa-miR-155-5p
 
 
-0.312
 
 
16.0
 
 
 
 
 
 
 
 cd4_ev_vs_cd4_s1_downregulated 
 
 Tissue expression heatmap (log10-transformed) 
   
 
 
 adipose tissue 
 
 
 
 
target_symbol
 
 
mature_mirna_id
 
 
score
 
 
nTPM
 
 
 
 
 
 
ABCB7
 
 
hsa-miR-1-3p
 
 
-0.365
 
 
12.4
 
 
 
 
ABHD2
 
 
hsa-miR-1-3p
 
 
-0.291
 
 
17.3
 
 
 
 
ABHD3
 
 
hsa-miR-130b-3p
 
 
-0.356
 
 
16.0
 
 
 
 
ACBD3
 
 
hsa-miR-130b-3p
 
 
-0.292
 
 
20.3
 
 
 
 
ACBD5
 
 
hsa-miR-130b-3p
 
 
-0.494
 
 
13.7
 
 
 
 
ACSL4
 
 
hsa-miR-130b-3p
 
 
-0.301
 
 
38.3
 
 
 
 
ACTC1
 
 
hsa-miR-7-5p
 
 
-0.471
 
 
47.8
 
 
 
 
ACVR1
 
 
hsa-miR-130b-3p
 
 
-0.44
 
 
12.6
 
 
 
 
ACVR1C
 
 
hsa-let-7e-5p
 
 
-0.457
 
 
29.3
 
 
 
 
ADAR
 
 
hsa-miR-1-3p
 
 
-0.35
 
 
36.0
 
 
 
 
ADPGK
 
 
hsa-miR-1-3p
 
 
-0.393
 
 
12.7
 
 
 
 
ADRB2
 
 
hsa-let-7e-5p
 
 
-0.635
 
 
26.4
 
 
 
 
AHCTF1
 
 
hsa-let-7e-5p
 
 
-0.374
 
 
11.8
 
 
 
 
AIFM1
 
 
hsa-let-7e-5p
 
 
-0.314
 
 
17.4
 
 
 
 
AKIRIN2
 
 
hsa-miR-130b-3p
 
 
-0.315
 
 
30.8
 
 
 
 
ALDH4A1
 
 
hsa-miR-184
 
 
-0.421
 
 
66.5
 
 
 
 
ALG9
 
 
hsa-miR-7-5p
 
 
-0.302
 
 
12.6
 
 
 
 
ANKIB1
 
 
hsa-miR-130b-3p
 
 
-0.329
 
 
11.9
 
 
 
 
ANKRD12
 
 
hsa-miR-7-5p
 
 
-0.37
 
 
14.8
 
 
 
 
ANKRD29
 
 
hsa-miR-1-3p
 
 
-0.426
 
 
17.0
 
 
 
 
ANKRD46
 
 
hsa-let-7e-5p
 
 
-0.389
 
 
18.0
 
 
 
 
ANP32B
 
 
hsa-miR-1-3p
 
 
-0.287
 
 
136.8
 
 
 
 
ANXA2
 
 
hsa-miR-1-3p
 
 
-0.581
 
 
854.1
 
 
 
 
ANXA4
 
 
hsa-miR-1-3p
 
 
-0.539
 
 
46.4
 
 
 
 
AP1S1
 
 
hsa-miR-1-3p
 
 
-0.355
 
 
15.1
 
 
 
 
AP1S1
 
 
hsa-let-7e-5p
 
 
-0.374
 
 
15.1
 
 
 
 
APCDD1
 
 
hsa-miR-130b-3p
 
 
-0.304
 
 
49.2
 
 
 
 
APPL1
 
 
hsa-miR-130b-3p
 
 
-0.347
 
 
14.6
 
 
 
 
ARCN1
 
 
hsa-miR-1-3p
 
 
-0.468
 
 
67.9
 
 
 
 
ARF4
 
 
hsa-miR-7-5p
 
 
-0.407
 
 
118.7
 
 
 
 
ARFGEF1
 
 
hsa-miR-130b-3p
 
 
-0.302
 
 
10.7
 
 
 
 
ARHGAP1
 
 
hsa-miR-130b-3p
 
 
-0.376
 
 
79.6
 
 
 
 
ARHGAP24
 
 
hsa-miR-130b-3p
 
 
-0.287
 
 
13.7
 
 
 
 
ARL4D
 
 
hsa-let-7e-5p
 
 
-0.417
 
 
20.3
 
 
 
 
ARL6IP1
 
 
hsa-miR-130b-3p
 
 
-0.289
 
 
133.2
 
 
 
 
ARL6IP1
 
 
hsa-miR-7-5p
 
 
-0.374
 
 
133.2
 
 
 
 
ARL6IP6
 
 
hsa-let-7e-5p
 
 
-0.305
 
 
11.0
 
 
 
 
ARMT1
 
 
hsa-let-7e-5p
 
 
-0.303
 
 
16.0
 
 
 
 
ARPC3
 
 
hsa-miR-1-3p
 
 
-0.606
 
 
203.1
 
 
 
 
ARPP19
 
 
hsa-let-7e-5p
 
 
-0.492
 
 
57.4
 
 
 
 
ARRDC4
 
 
hsa-let-7e-5p
 
 
-0.329
 
 
11.8
 
 
 
 
ASAP1
 
 
hsa-let-7e-5p
 
 
-0.296
 
 
32.3
 
 
 
 
ASH2L
 
 
hsa-miR-1-3p
 
 
-0.462
 
 
27.4
 
 
 
 
ATF2
 
 
hsa-miR-1-3p
 
 
-0.328
 
 
15.3
 
 
 
 
ATOSB
 
 
hsa-let-7e-5p
 
 
-0.429
 
 
13.2
 
 
 
 
ATP2A2
 
 
hsa-let-7e-5p
 
 
-0.311
 
 
56.6
 
 
 
 
ATPAF1
 
 
hsa-let-7e-5p
 
 
-0.498
 
 
81.3
 
 
 
 
AVPI1
 
 
hsa-miR-7-5p
 
 
-0.367
 
 
32.2
 
 
 
 
AXL
 
 
hsa-miR-1-3p
 
 
-0.304
 
 
41.8
 
 
 
 
AZIN1
 
 
hsa-miR-1-3p
 
 
-0.438
 
 
56.6
 
 
 
 
B3GAT3
 
 
hsa-let-7e-5p
 
 
-0.324
 
 
30.8
 
 
 
 
B4GAT1
 
 
hsa-let-7e-5p
 
 
-0.47
 
 
16.2
 
 
 
 
BACH1
 
 
hsa-let-7e-5p
 
 
-0.298
 
 
14.8
 
 
 
 
BACH1
 
 
hsa-let-7e-5p
 
 
-0.298
 
 
14.8
 
 
 
 
BACH1
 
 
hsa-let-7e-5p
 
 
-0.435
 
 
14.8
 
 
 
 
BACH1
 
 
hsa-let-7e-5p
 
 
-0.435
 
 
14.8
 
 
 
 
BAZ2A
 
 
hsa-miR-100-5p
 
 
-0.29
 
 
26.9
 
 
 
 
BAZ2A
 
 
hsa-miR-100-5p
 
 
-0.421
 
 
26.9
 
 
 
 
BCAP29
 
 
hsa-let-7e-5p
 
 
-0.289
 
 
45.2
 
 
 
 
BET1
 
 
hsa-miR-1-3p
 
 
-0.413
 
 
17.0
 
 
 
 
BHLHE40
 
 
hsa-miR-130b-3p
 
 
-0.351
 
 
162.3
 
 
 
 
BIN3
 
 
hsa-let-7e-5p
 
 
-0.427
 
 
19.4
 
 
 
 
BLCAP
 
 
hsa-miR-130b-3p
 
 
-0.342
 
 
63.7
 
 
 
 
BLOC1S6
 
 
hsa-let-7e-5p
 
 
-0.321
 
 
28.2
 
 
 
 
BMPR2
 
 
hsa-miR-130b-3p
 
 
-0.33
 
 
15.1
 
 
 
 
BMPR2
 
 
hsa-miR-100-5p
 
 
-0.355
 
 
15.1
 
 
 
 
BPNT1
 
 
hsa-miR-1-3p
 
 
-0.329
 
 
15.2
 
 
 
 
BRIX1
 
 
hsa-miR-1-3p
 
 
-0.334
 
 
19.4
 
 
 
 
BSCL2
 
 
hsa-miR-1-3p
 
 
-0.471
 
 
21.1
 
 
 
 
BTAF1
 
 
hsa-miR-130b-3p
 
 
-0.291
 
 
16.7
 
 
 
 
BTF3L4
 
 
hsa-let-7e-5p
 
 
-0.355
 
 
41.8
 
 
 
 
BTF3L4
 
 
hsa-miR-130b-3p
 
 
-0.359
 
 
41.8
 
 
 
 
BTG1
 
 
hsa-miR-130b-3p
 
 
-0.565
 
 
17.1
 
 
 
 
BZW1
 
 
hsa-let-7e-5p
 
 
-0.354
 
 
122.3
 
 
 
 
BZW1
 
 
hsa-let-7e-5p
 
 
-0.375
 
 
122.3
 
 
 
 
BZW2
 
 
hsa-let-7e-5p
 
 
-0.408
 
 
27.0
 
 
 
 
C15orf39
 
 
hsa-let-7e-5p
 
 
-0.374
 
 
14.1
 
 
 
 
C1GALT1
 
 
hsa-let-7e-5p
 
 
-0.322
 
 
12.1
 
 
 
 
C6orf120
 
 
hsa-miR-1-3p
 
 
-0.288
 
 
17.1
 
 
 
 
C8orf58
 
 
hsa-let-7e-5p
 
 
-0.612
 
 
10.5
 
 
 
 
CALM2
 
 
hsa-miR-130b-3p
 
 
-0.584
 
 
477.3
 
 
 
 
CAMSAP2
 
 
hsa-miR-130b-3p
 
 
-0.297
 
 
14.5
 
 
 
 
CANT1
 
 
hsa-let-7e-5p
 
 
-0.402
 
 
14.4
 
 
 
 
CAPN3
 
 
hsa-let-7e-5p
 
 
-0.317
 
 
13.6
 
 
 
 
CAPZA1
 
 
hsa-miR-7-5p
 
 
-0.351
 
 
69.0
 
 
 
 
CASD1
 
 
hsa-miR-130b-3p
 
 
-0.305
 
 
10.4
 
 
 
 
CASP3
 
 
hsa-let-7e-5p
 
 
-0.495
 
 
12.6
 
 
 
 
CCDC71L
 
 
hsa-let-7e-5p
 
 
-0.432
 
 
17.0
 
 
 
 
CCNC
 
 
hsa-miR-1-3p
 
 
-0.336
 
 
29.4
 
 
 
 
CCNL2
 
 
hsa-miR-28-5p
 
 
-0.448
 
 
55.4
 
 
 
 
CCNT2
 
 
hsa-miR-7-5p
 
 
-0.349
 
 
12.8
 
 
 
 
CCNY
 
 
hsa-let-7e-5p
 
 
-0.334
 
 
26.3
 
 
 
 
CCNY
 
 
hsa-miR-130b-3p
 
 
-0.372
 
 
26.3
 
 
 
 
CCT4
 
 
hsa-miR-7-5p
 
 
-0.37
 
 
66.9
 
 
 
 
CCT6A
 
 
hsa-miR-130b-3p
 
 
-0.382
 
 
56.1
 
 
 
 
CD164
 
 
hsa-miR-1-3p
 
 
-0.315
 
 
244.9
 
 
 
 
CD2AP
 
 
hsa-miR-130b-3p
 
 
-0.302
 
 
13.1
 
 
 
 
CD2AP
 
 
hsa-miR-1-3p
 
 
-0.479
 
 
13.1
 
 
 
 
CD69
 
 
hsa-miR-130b-3p
 
 
-0.451
 
 
13.4
 
 
 
 
CDC34
 
 
hsa-let-7e-5p
 
 
-0.321
 
 
35.6
 
 
 
 
 
 
 adrenal gland 
 
 
 
 
target_symbol
 
 
mature_mirna_id
 
 
score
 
 
nTPM
 
 
 
 
 
 
ABCB7
 
 
hsa-miR-1-3p
 
 
-0.365
 
 
12.5
 
 
 
 
ABHD2
 
 
hsa-miR-1-3p
 
 
-0.291
 
 
34.3
 
 
 
 
ACBD3
 
 
hsa-miR-130b-3p
 
 
-0.292
 
 
16.0
 
 
 
 
ACBD5
 
 
hsa-miR-130b-3p
 
 
-0.494
 
 
23.7
 
 
 
 
ACSL4
 
 
hsa-miR-130b-3p
 
 
-0.301
 
 
34.9
 
 
 
 
ADAR
 
 
hsa-miR-1-3p
 
 
-0.35
 
 
34.4
 
 
 
 
AIFM1
 
 
hsa-let-7e-5p
 
 
-0.314
 
 
50.1
 
 
 
 
AKIRIN2
 
 
hsa-miR-130b-3p
 
 
-0.315
 
 
33.1
 
 
 
 
ALDH4A1
 
 
hsa-miR-184
 
 
-0.421
 
 
16.2
 
 
 
 
AMT
 
 
hsa-let-7e-5p
 
 
-0.463
 
 
15.0
 
 
 
 
ANKIB1
 
 
hsa-miR-130b-3p
 
 
-0.329
 
 
10.1
 
 
 
 
ANKRD46
 
 
hsa-let-7e-5p
 
 
-0.389
 
 
36.5
 
 
 
 
ANP32B
 
 
hsa-miR-1-3p
 
 
-0.287
 
 
72.7
 
 
 
 
ANXA2
 
 
hsa-miR-1-3p
 
 
-0.581
 
 
321.0
 
 
 
 
ANXA4
 
 
hsa-miR-1-3p
 
 
-0.539
 
 
20.5
 
 
 
 
AP1AR
 
 
hsa-miR-100-5p
 
 
-0.804
 
 
14.8
 
 
 
 
AP1S1
 
 
hsa-miR-1-3p
 
 
-0.355
 
 
25.7
 
 
 
 
AP1S1
 
 
hsa-let-7e-5p
 
 
-0.374
 
 
25.7
 
 
 
 
APCDD1
 
 
hsa-miR-130b-3p
 
 
-0.304
 
 
13.4
 
 
 
 
APPL1
 
 
hsa-miR-130b-3p
 
 
-0.347
 
 
11.0
 
 
 
 
ARCN1
 
 
hsa-miR-1-3p
 
 
-0.468
 
 
61.8
 
 
 
 
ARF4
 
 
hsa-miR-7-5p
 
 
-0.407
 
 
151.0
 
 
 
 
ARHGAP1
 
 
hsa-miR-130b-3p
 
 
-0.376
 
 
46.3
 
 
 
 
ARHGAP12
 
 
hsa-miR-130b-3p
 
 
-0.287
 
 
11.8
 
 
 
 
ARL6IP1
 
 
hsa-miR-130b-3p
 
 
-0.289
 
 
161.7
 
 
 
 
ARL6IP1
 
 
hsa-miR-7-5p
 
 
-0.374
 
 
161.7
 
 
 
 
ARMT1
 
 
hsa-let-7e-5p
 
 
-0.303
 
 
15.1
 
 
 
 
ARPC3
 
 
hsa-miR-1-3p
 
 
-0.606
 
 
208.3
 
 
 
 
ARPP19
 
 
hsa-let-7e-5p
 
 
-0.492
 
 
48.8
 
 
 
 
ARRDC4
 
 
hsa-let-7e-5p
 
 
-0.329
 
 
11.5
 
 
 
 
AS3MT
 
 
hsa-miR-28-5p
 
 
-0.479
 
 
260.6
 
 
 
 
ASAP1
 
 
hsa-let-7e-5p
 
 
-0.296
 
 
20.2
 
 
 
 
ASH2L
 
 
hsa-miR-1-3p
 
 
-0.462
 
 
19.1
 
 
 
 
ATF2
 
 
hsa-miR-1-3p
 
 
-0.328
 
 
19.1
 
 
 
 
ATOSB
 
 
hsa-let-7e-5p
 
 
-0.429
 
 
14.2
 
 
 
 
ATP2A2
 
 
hsa-let-7e-5p
 
 
-0.311
 
 
46.8
 
 
 
 
ATPAF1
 
 
hsa-let-7e-5p
 
 
-0.498
 
 
98.1
 
 
 
 
AVPI1
 
 
hsa-miR-7-5p
 
 
-0.367
 
 
46.2
 
 
 
 
AXL
 
 
hsa-miR-1-3p
 
 
-0.304
 
 
17.7
 
 
 
 
AZIN1
 
 
hsa-miR-1-3p
 
 
-0.438
 
 
44.5
 
 
 
 
B3GAT3
 
 
hsa-let-7e-5p
 
 
-0.324
 
 
33.4
 
 
 
 
B4GAT1
 
 
hsa-let-7e-5p
 
 
-0.47
 
 
39.1
 
 
 
 
BACH1
 
 
hsa-let-7e-5p
 
 
-0.298
 
 
11.4
 
 
 
 
BACH1
 
 
hsa-let-7e-5p
 
 
-0.298
 
 
11.4
 
 
 
 
BACH1
 
 
hsa-let-7e-5p
 
 
-0.435
 
 
11.4
 
 
 
 
BACH1
 
 
hsa-let-7e-5p
 
 
-0.435
 
 
11.4
 
 
 
 
BAHD1
 
 
hsa-miR-130b-3p
 
 
-0.294
 
 
20.8
 
 
 
 
BAZ2A
 
 
hsa-miR-100-5p
 
 
-0.29
 
 
22.2
 
 
 
 
BAZ2A
 
 
hsa-miR-100-5p
 
 
-0.421
 
 
22.2
 
 
 
 
BCAP29
 
 
hsa-let-7e-5p
 
 
-0.289
 
 
25.1
 
 
 
 
BET1
 
 
hsa-miR-1-3p
 
 
-0.413
 
 
26.6
 
 
 
 
BHLHE40
 
 
hsa-miR-130b-3p
 
 
-0.351
 
 
109.5
 
 
 
 
BIN3
 
 
hsa-let-7e-5p
 
 
-0.427
 
 
30.6
 
 
 
 
BLCAP
 
 
hsa-miR-130b-3p
 
 
-0.342
 
 
55.2
 
 
 
 
BLOC1S4
 
 
hsa-miR-7-5p
 
 
-0.417
 
 
11.8
 
 
 
 
BLOC1S4
 
 
hsa-miR-7-5p
 
 
-0.527
 
 
11.8
 
 
 
 
BLOC1S6
 
 
hsa-let-7e-5p
 
 
-0.321
 
 
29.4
 
 
 
 
BLTP3B
 
 
hsa-miR-1-3p
 
 
-0.29
 
 
10.3
 
 
 
 
BMPR2
 
 
hsa-miR-130b-3p
 
 
-0.33
 
 
17.0
 
 
 
 
BMPR2
 
 
hsa-miR-100-5p
 
 
-0.355
 
 
17.0
 
 
 
 
BPNT1
 
 
hsa-miR-1-3p
 
 
-0.329
 
 
19.5
 
 
 
 
BRIX1
 
 
hsa-miR-1-3p
 
 
-0.334
 
 
16.6
 
 
 
 
BSCL2
 
 
hsa-miR-1-3p
 
 
-0.471
 
 
88.4
 
 
 
 
BTAF1
 
 
hsa-miR-130b-3p
 
 
-0.291
 
 
11.3
 
 
 
 
BTF3L4
 
 
hsa-let-7e-5p
 
 
-0.355
 
 
49.0
 
 
 
 
BTF3L4
 
 
hsa-miR-130b-3p
 
 
-0.359
 
 
49.0
 
 
 
 
BZW1
 
 
hsa-let-7e-5p
 
 
-0.354
 
 
121.8
 
 
 
 
BZW1
 
 
hsa-let-7e-5p
 
 
-0.375
 
 
121.8
 
 
 
 
BZW2
 
 
hsa-let-7e-5p
 
 
-0.408
 
 
18.5
 
 
 
 
C15orf39
 
 
hsa-let-7e-5p
 
 
-0.374
 
 
14.8
 
 
 
 
C2orf69
 
 
hsa-miR-1-3p
 
 
-0.414
 
 
13.3
 
 
 
 
C6orf120
 
 
hsa-miR-1-3p
 
 
-0.288
 
 
16.2
 
 
 
 
CAAP1
 
 
hsa-miR-1-3p
 
 
-0.48
 
 
19.2
 
 
 
 
CACFD1
 
 
hsa-let-7e-5p
 
 
-0.296
 
 
10.8
 
 
 
 
CALM2
 
 
hsa-miR-130b-3p
 
 
-0.584
 
 
614.9
 
 
 
 
CAMSAP2
 
 
hsa-miR-130b-3p
 
 
-0.297
 
 
15.3
 
 
 
 
CANT1
 
 
hsa-let-7e-5p
 
 
-0.402
 
 
21.8
 
 
 
 
CAPN3
 
 
hsa-let-7e-5p
 
 
-0.317
 
 
10.1
 
 
 
 
CAPZA1
 
 
hsa-miR-7-5p
 
 
-0.351
 
 
47.8
 
 
 
 
CASD1
 
 
hsa-miR-130b-3p
 
 
-0.305
 
 
23.9
 
 
 
 
CASP3
 
 
hsa-let-7e-5p
 
 
-0.495
 
 
11.3
 
 
 
 
CBX5
 
 
hsa-let-7e-5p
 
 
-0.307
 
 
12.4
 
 
 
 
CBX5
 
 
hsa-let-7e-5p
 
 
-0.472
 
 
12.4
 
 
 
 
CCNC
 
 
hsa-miR-1-3p
 
 
-0.336
 
 
30.6
 
 
 
 
CCNL2
 
 
hsa-miR-28-5p
 
 
-0.448
 
 
64.0
 
 
 
 
CCNT2
 
 
hsa-miR-7-5p
 
 
-0.349
 
 
11.5
 
 
 
 
CCNY
 
 
hsa-let-7e-5p
 
 
-0.334
 
 
19.9
 
 
 
 
CCNY
 
 
hsa-miR-130b-3p
 
 
-0.372
 
 
19.9
 
 
 
 
CCT4
 
 
hsa-miR-7-5p
 
 
-0.37
 
 
66.7
 
 
 
 
CCT6A
 
 
hsa-miR-130b-3p
 
 
-0.382
 
 
59.0
 
 
 
 
CD164
 
 
hsa-miR-1-3p
 
 
-0.315
 
 
116.7
 
 
 
 
CDC34
 
 
hsa-let-7e-5p
 
 
-0.321
 
 
38.1
 
 
 
 
CDC34
 
 
hsa-let-7e-5p
 
 
-0.555
 
 
38.1
 
 
 
 
CDC42SE1
 
 
hsa-miR-1-3p
 
 
-0.36
 
 
48.7
 
 
 
 
CDK4
 
 
hsa-miR-1-3p
 
 
-0.338
 
 
88.1
 
 
 
 
CDKN1A
 
 
hsa-let-7e-5p
 
 
-0.434
 
 
104.6
 
 
 
 
CDKN2AIP
 
 
hsa-miR-28-5p
 
 
-0.419
 
 
11.0
 
 
 
 
CDV3
 
 
hsa-let-7e-5p
 
 
-0.453
 
 
63.6
 
 
 
 
CEBPD
 
 
hsa-let-7e-5p
 
 
-0.309
 
 
139.8
 
 
 
 
CERS2
 
 
hsa-miR-1-3p
 
 
-0.287
 
 
259.4
 
 
 
 
 
 
 blood vessel 
 
 
 
 
target_symbol
 
 
mature_mirna_id
 
 
score
 
 
nTPM
 
 
 
 
 
 
ABCB7
 
 
hsa-miR-1-3p
 
 
-0.365
 
 
12.3
 
 
 
 
ABHD2
 
 
hsa-miR-1-3p
 
 
-0.291
 
 
30.5
 
 
 
 
ABHD3
 
 
hsa-miR-130b-3p
 
 
-0.356
 
 
10.2
 
 
 
 
ACBD3
 
 
hsa-miR-130b-3p
 
 
-0.292
 
 
19.2
 
 
 
 
ACSL4
 
 
hsa-miR-130b-3p
 
 
-0.301
 
 
15.9
 
 
 
 
ACTC1
 
 
hsa-miR-7-5p
 
 
-0.471
 
 
388.9
 
 
 
 
ADAMTS8
 
 
hsa-let-7e-5p
 
 
-0.571
 
 
12.7
 
 
 
 
ADAR
 
 
hsa-miR-1-3p
 
 
-0.35
 
 
32.0
 
 
 
 
AHCTF1
 
 
hsa-let-7e-5p
 
 
-0.374
 
 
10.0
 
 
 
 
AKAP6
 
 
hsa-let-7e-5p
 
 
-0.307
 
 
15.0
 
 
 
 
AKIRIN2
 
 
hsa-miR-130b-3p
 
 
-0.315
 
 
22.1
 
 
 
 
ALDH4A1
 
 
hsa-miR-184
 
 
-0.421
 
 
15.0
 
 
 
 
ALG9
 
 
hsa-miR-7-5p
 
 
-0.302
 
 
12.3
 
 
 
 
AMT
 
 
hsa-let-7e-5p
 
 
-0.463
 
 
11.4
 
 
 
 
ANKIB1
 
 
hsa-miR-130b-3p
 
 
-0.329
 
 
11.2
 
 
 
 
ANKRD12
 
 
hsa-miR-7-5p
 
 
-0.37
 
 
11.6
 
 
 
 
ANP32B
 
 
hsa-miR-1-3p
 
 
-0.287
 
 
163.8
 
 
 
 
ANXA2
 
 
hsa-miR-1-3p
 
 
-0.581
 
 
711.1
 
 
 
 
ANXA4
 
 
hsa-miR-1-3p
 
 
-0.539
 
 
78.7
 
 
 
 
AP1S1
 
 
hsa-miR-1-3p
 
 
-0.355
 
 
13.8
 
 
 
 
AP1S1
 
 
hsa-let-7e-5p
 
 
-0.374
 
 
13.8
 
 
 
 
APCDD1
 
 
hsa-miR-130b-3p
 
 
-0.304
 
 
23.7
 
 
 
 
APPL1
 
 
hsa-miR-130b-3p
 
 
-0.347
 
 
12.6
 
 
 
 
ARCN1
 
 
hsa-miR-1-3p
 
 
-0.468
 
 
87.0
 
 
 
 
ARF4
 
 
hsa-miR-7-5p
 
 
-0.407
 
 
151.4
 
 
 
 
ARHGAP1
 
 
hsa-miR-130b-3p
 
 
-0.376
 
 
119.0
 
 
 
 
ARHGAP24
 
 
hsa-miR-130b-3p
 
 
-0.287
 
 
17.3
 
 
 
 
ARL4D
 
 
hsa-let-7e-5p
 
 
-0.417
 
 
12.9
 
 
 
 
ARL6IP1
 
 
hsa-miR-130b-3p
 
 
-0.289
 
 
52.2
 
 
 
 
ARL6IP1
 
 
hsa-miR-7-5p
 
 
-0.374
 
 
52.2
 
 
 
 
ARPC3
 
 
hsa-miR-1-3p
 
 
-0.606
 
 
209.2
 
 
 
 
ARPP19
 
 
hsa-let-7e-5p
 
 
-0.492
 
 
47.3
 
 
 
 
ASAP1
 
 
hsa-let-7e-5p
 
 
-0.296
 
 
18.4
 
 
 
 
ASH2L
 
 
hsa-miR-1-3p
 
 
-0.462
 
 
19.1
 
 
 
 
ATF2
 
 
hsa-miR-1-3p
 
 
-0.328
 
 
18.8
 
 
 
 
ATG16L1
 
 
hsa-miR-130b-3p
 
 
-0.407
 
 
12.7
 
 
 
 
ATOSB
 
 
hsa-let-7e-5p
 
 
-0.429
 
 
26.7
 
 
 
 
ATP2A2
 
 
hsa-let-7e-5p
 
 
-0.311
 
 
84.6
 
 
 
 
ATPAF1
 
 
hsa-let-7e-5p
 
 
-0.498
 
 
30.6
 
 
 
 
AVPI1
 
 
hsa-miR-7-5p
 
 
-0.367
 
 
57.0
 
 
 
 
AXL
 
 
hsa-miR-1-3p
 
 
-0.304
 
 
72.4
 
 
 
 
AZIN1
 
 
hsa-miR-1-3p
 
 
-0.438
 
 
27.2
 
 
 
 
B3GAT3
 
 
hsa-let-7e-5p
 
 
-0.324
 
 
38.8
 
 
 
 
B4GAT1
 
 
hsa-let-7e-5p
 
 
-0.47
 
 
18.5
 
 
 
 
BACE2
 
 
hsa-let-7e-5p
 
 
-0.364
 
 
11.5
 
 
 
 
BACH1
 
 
hsa-let-7e-5p
 
 
-0.298
 
 
10.9
 
 
 
 
BACH1
 
 
hsa-let-7e-5p
 
 
-0.298
 
 
10.9
 
 
 
 
BACH1
 
 
hsa-let-7e-5p
 
 
-0.435
 
 
10.9
 
 
 
 
BACH1
 
 
hsa-let-7e-5p
 
 
-0.435
 
 
10.9
 
 
 
 
BAHD1
 
 
hsa-miR-130b-3p
 
 
-0.294
 
 
10.9
 
 
 
 
BAZ2A
 
 
hsa-miR-100-5p
 
 
-0.29
 
 
25.1
 
 
 
 
BAZ2A
 
 
hsa-miR-100-5p
 
 
-0.421
 
 
25.1
 
 
 
 
BCAP29
 
 
hsa-let-7e-5p
 
 
-0.289
 
 
48.5
 
 
 
 
BET1
 
 
hsa-miR-1-3p
 
 
-0.413
 
 
11.0
 
 
 
 
BHLHE40
 
 
hsa-miR-130b-3p
 
 
-0.351
 
 
237.8
 
 
 
 
BIN3
 
 
hsa-let-7e-5p
 
 
-0.427
 
 
15.5
 
 
 
 
BLCAP
 
 
hsa-miR-130b-3p
 
 
-0.342
 
 
34.9
 
 
 
 
BLOC1S6
 
 
hsa-let-7e-5p
 
 
-0.321
 
 
21.2
 
 
 
 
BLTP3B
 
 
hsa-miR-1-3p
 
 
-0.29
 
 
12.6
 
 
 
 
BMPR2
 
 
hsa-miR-130b-3p
 
 
-0.33
 
 
16.9
 
 
 
 
BMPR2
 
 
hsa-miR-100-5p
 
 
-0.355
 
 
16.9
 
 
 
 
BPNT1
 
 
hsa-miR-1-3p
 
 
-0.329
 
 
13.6
 
 
 
 
BRIX1
 
 
hsa-miR-1-3p
 
 
-0.334
 
 
15.7
 
 
 
 
BSCL2
 
 
hsa-miR-1-3p
 
 
-0.471
 
 
19.5
 
 
 
 
BTAF1
 
 
hsa-miR-130b-3p
 
 
-0.291
 
 
19.0
 
 
 
 
BTF3L4
 
 
hsa-let-7e-5p
 
 
-0.355
 
 
27.9
 
 
 
 
BTF3L4
 
 
hsa-miR-130b-3p
 
 
-0.359
 
 
27.9
 
 
 
 
BZW1
 
 
hsa-let-7e-5p
 
 
-0.354
 
 
72.0
 
 
 
 
BZW1
 
 
hsa-let-7e-5p
 
 
-0.375
 
 
72.0
 
 
 
 
BZW2
 
 
hsa-let-7e-5p
 
 
-0.408
 
 
19.3
 
 
 
 
C15orf39
 
 
hsa-let-7e-5p
 
 
-0.374
 
 
28.4
 
 
 
 
C19orf47
 
 
hsa-let-7e-5p
 
 
-0.311
 
 
13.6
 
 
 
 
C8orf58
 
 
hsa-let-7e-5p
 
 
-0.612
 
 
11.3
 
 
 
 
CACFD1
 
 
hsa-let-7e-5p
 
 
-0.296
 
 
11.6
 
 
 
 
CALM2
 
 
hsa-miR-130b-3p
 
 
-0.584
 
 
710.8
 
 
 
 
CAMSAP2
 
 
hsa-miR-130b-3p
 
 
-0.297
 
 
17.4
 
 
 
 
CANT1
 
 
hsa-let-7e-5p
 
 
-0.402
 
 
16.6
 
 
 
 
CAPN3
 
 
hsa-let-7e-5p
 
 
-0.317
 
 
16.3
 
 
 
 
CAPZA1
 
 
hsa-miR-7-5p
 
 
-0.351
 
 
44.0
 
 
 
 
CASP3
 
 
hsa-let-7e-5p
 
 
-0.495
 
 
10.6
 
 
 
 
CBX5
 
 
hsa-let-7e-5p
 
 
-0.307
 
 
10.4
 
 
 
 
CBX5
 
 
hsa-let-7e-5p
 
 
-0.472
 
 
10.4
 
 
 
 
CCNC
 
 
hsa-miR-1-3p
 
 
-0.336
 
 
17.6
 
 
 
 
CCNL2
 
 
hsa-miR-28-5p
 
 
-0.448
 
 
52.3
 
 
 
 
CCNT2
 
 
hsa-miR-7-5p
 
 
-0.349
 
 
12.4
 
 
 
 
CCNY
 
 
hsa-let-7e-5p
 
 
-0.334
 
 
25.6
 
 
 
 
CCNY
 
 
hsa-miR-130b-3p
 
 
-0.372
 
 
25.6
 
 
 
 
CCT4
 
 
hsa-miR-7-5p
 
 
-0.37
 
 
67.9
 
 
 
 
CCT6A
 
 
hsa-miR-130b-3p
 
 
-0.382
 
 
59.5
 
 
 
 
CD164
 
 
hsa-miR-1-3p
 
 
-0.315
 
 
112.3
 
 
 
 
CDC34
 
 
hsa-let-7e-5p
 
 
-0.321
 
 
41.8
 
 
 
 
CDC34
 
 
hsa-let-7e-5p
 
 
-0.555
 
 
41.8
 
 
 
 
CDC42SE1
 
 
hsa-miR-1-3p
 
 
-0.36
 
 
60.3
 
 
 
 
CDK14
 
 
hsa-miR-1-3p
 
 
-0.326
 
 
15.9
 
 
 
 
CDK4
 
 
hsa-miR-1-3p
 
 
-0.338
 
 
60.4
 
 
 
 
CDKN1A
 
 
hsa-let-7e-5p
 
 
-0.434
 
 
339.5
 
 
 
 
CDKN2AIP
 
 
hsa-miR-28-5p
 
 
-0.419
 
 
12.7
 
 
 
 
CDV3
 
 
hsa-let-7e-5p
 
 
-0.453
 
 
61.1
 
 
 
 
CEBPD
 
 
hsa-let-7e-5p
 
 
-0.309
 
 
474.6
 
 
 
 
CERCAM
 
 
hsa-let-7e-5p
 
 
-0.344
 
 
58.6
 
 
 
 
 
 
 bone marrow 
 
 
 
 
target_symbol
 
 
mature_mirna_id
 
 
score
 
 
nTPM
 
 
 
 
 
 
ABCB7
 
 
hsa-miR-1-3p
 
 
-0.365
 
 
10.5
 
 
 
 
ABHD2
 
 
hsa-miR-1-3p
 
 
-0.291
 
 
33.0
 
 
 
 
ABHD3
 
 
hsa-miR-130b-3p
 
 
-0.356
 
 
16.6
 
 
 
 
ABRAXAS2
 
 
hsa-miR-130b-3p
 
 
-0.468
 
 
11.6
 
 
 
 
ACBD3
 
 
hsa-miR-130b-3p
 
 
-0.292
 
 
24.4
 
 
 
 
ACBD5
 
 
hsa-miR-130b-3p
 
 
-0.494
 
 
17.0
 
 
 
 
ACP3
 
 
hsa-let-7e-5p
 
 
-0.298
 
 
16.5
 
 
 
 
ACSL4
 
 
hsa-miR-130b-3p
 
 
-0.301
 
 
11.1
 
 
 
 
ACSL6
 
 
hsa-let-7e-5p
 
 
-0.336
 
 
21.4
 
 
 
 
ADAR
 
 
hsa-miR-1-3p
 
 
-0.35
 
 
44.6
 
 
 
 
ADPGK
 
 
hsa-miR-1-3p
 
 
-0.393
 
 
25.5
 
 
 
 
ADRB2
 
 
hsa-let-7e-5p
 
 
-0.635
 
 
11.8
 
 
 
 
AGO2
 
 
hsa-miR-184
 
 
-0.831
 
 
10.7
 
 
 
 
AGO2
 
 
hsa-miR-100-5p
 
 
-0.884
 
 
10.7
 
 
 
 
AHCTF1
 
 
hsa-let-7e-5p
 
 
-0.374
 
 
16.0
 
 
 
 
AIFM1
 
 
hsa-let-7e-5p
 
 
-0.314
 
 
11.2
 
 
 
 
AKIRIN2
 
 
hsa-miR-130b-3p
 
 
-0.315
 
 
66.5
 
 
 
 
ALDH4A1
 
 
hsa-miR-184
 
 
-0.421
 
 
11.1
 
 
 
 
ANKRD12
 
 
hsa-miR-7-5p
 
 
-0.37
 
 
19.4
 
 
 
 
ANP32B
 
 
hsa-miR-1-3p
 
 
-0.287
 
 
344.9
 
 
 
 
ANXA2
 
 
hsa-miR-1-3p
 
 
-0.581
 
 
143.0
 
 
 
 
ANXA4
 
 
hsa-miR-1-3p
 
 
-0.539
 
 
29.0
 
 
 
 
APPL1
 
 
hsa-miR-130b-3p
 
 
-0.347
 
 
14.3
 
 
 
 
ARCN1
 
 
hsa-miR-1-3p
 
 
-0.468
 
 
28.7
 
 
 
 
ARF4
 
 
hsa-miR-7-5p
 
 
-0.407
 
 
68.5
 
 
 
 
ARFGEF1
 
 
hsa-miR-130b-3p
 
 
-0.302
 
 
26.0
 
 
 
 
ARHGAP1
 
 
hsa-miR-130b-3p
 
 
-0.376
 
 
31.5
 
 
 
 
ARHGAP24
 
 
hsa-miR-130b-3p
 
 
-0.287
 
 
17.7
 
 
 
 
ARID2
 
 
hsa-miR-1-3p
 
 
-0.294
 
 
11.7
 
 
 
 
ARID3B
 
 
hsa-let-7e-5p
 
 
-0.31
 
 
23.2
 
 
 
 
ARID3B
 
 
hsa-let-7e-5p
 
 
-0.486
 
 
23.2
 
 
 
 
ARID4A
 
 
hsa-miR-7-5p
 
 
-0.341
 
 
32.8
 
 
 
 
ARL6IP1
 
 
hsa-miR-130b-3p
 
 
-0.289
 
 
197.7
 
 
 
 
ARL6IP1
 
 
hsa-miR-7-5p
 
 
-0.374
 
 
197.7
 
 
 
 
ARL6IP6
 
 
hsa-let-7e-5p
 
 
-0.305
 
 
23.4
 
 
 
 
ARPC3
 
 
hsa-miR-1-3p
 
 
-0.606
 
 
730.5
 
 
 
 
ARPP19
 
 
hsa-let-7e-5p
 
 
-0.492
 
 
97.5
 
 
 
 
ARRDC4
 
 
hsa-let-7e-5p
 
 
-0.329
 
 
10.8
 
 
 
 
ASAP1
 
 
hsa-let-7e-5p
 
 
-0.296
 
 
44.7
 
 
 
 
ASH2L
 
 
hsa-miR-1-3p
 
 
-0.462
 
 
30.3
 
 
 
 
ATF2
 
 
hsa-miR-1-3p
 
 
-0.328
 
 
14.8
 
 
 
 
ATG16L1
 
 
hsa-miR-130b-3p
 
 
-0.407
 
 
13.5
 
 
 
 
ATOSB
 
 
hsa-let-7e-5p
 
 
-0.429
 
 
36.6
 
 
 
 
ATP2A2
 
 
hsa-let-7e-5p
 
 
-0.311
 
 
43.4
 
 
 
 
ATP8B4
 
 
hsa-let-7e-5p
 
 
-0.325
 
 
73.7
 
 
 
 
ATPAF1
 
 
hsa-let-7e-5p
 
 
-0.498
 
 
26.0
 
 
 
 
AZIN1
 
 
hsa-miR-1-3p
 
 
-0.438
 
 
123.6
 
 
 
 
B3GAT3
 
 
hsa-let-7e-5p
 
 
-0.324
 
 
17.5
 
 
 
 
BACH1
 
 
hsa-let-7e-5p
 
 
-0.298
 
 
84.3
 
 
 
 
BACH1
 
 
hsa-let-7e-5p
 
 
-0.298
 
 
84.3
 
 
 
 
BACH1
 
 
hsa-let-7e-5p
 
 
-0.435
 
 
84.3
 
 
 
 
BACH1
 
 
hsa-let-7e-5p
 
 
-0.435
 
 
84.3
 
 
 
 
BAG4
 
 
hsa-miR-1-3p
 
 
-0.378
 
 
27.0
 
 
 
 
BAZ2A
 
 
hsa-miR-100-5p
 
 
-0.29
 
 
62.3
 
 
 
 
BAZ2A
 
 
hsa-miR-100-5p
 
 
-0.421
 
 
62.3
 
 
 
 
BCAP29
 
 
hsa-let-7e-5p
 
 
-0.289
 
 
16.8
 
 
 
 
BHLHE40
 
 
hsa-miR-130b-3p
 
 
-0.351
 
 
177.7
 
 
 
 
BIN3
 
 
hsa-let-7e-5p
 
 
-0.427
 
 
40.9
 
 
 
 
BLCAP
 
 
hsa-miR-130b-3p
 
 
-0.342
 
 
33.9
 
 
 
 
BLOC1S6
 
 
hsa-let-7e-5p
 
 
-0.321
 
 
54.9
 
 
 
 
BLTP3B
 
 
hsa-miR-1-3p
 
 
-0.29
 
 
31.0
 
 
 
 
BRIX1
 
 
hsa-miR-1-3p
 
 
-0.334
 
 
34.4
 
 
 
 
BSCL2
 
 
hsa-miR-1-3p
 
 
-0.471
 
 
10.7
 
 
 
 
BTAF1
 
 
hsa-miR-130b-3p
 
 
-0.291
 
 
25.4
 
 
 
 
BTF3L4
 
 
hsa-let-7e-5p
 
 
-0.355
 
 
57.6
 
 
 
 
BTF3L4
 
 
hsa-miR-130b-3p
 
 
-0.359
 
 
57.6
 
 
 
 
BTG1
 
 
hsa-miR-130b-3p
 
 
-0.565
 
 
88.7
 
 
 
 
BZW1
 
 
hsa-let-7e-5p
 
 
-0.354
 
 
207.1
 
 
 
 
BZW1
 
 
hsa-let-7e-5p
 
 
-0.375
 
 
207.1
 
 
 
 
BZW2
 
 
hsa-let-7e-5p
 
 
-0.408
 
 
43.1
 
 
 
 
C15orf39
 
 
hsa-let-7e-5p
 
 
-0.374
 
 
58.4
 
 
 
 
C1GALT1
 
 
hsa-let-7e-5p
 
 
-0.322
 
 
35.6
 
 
 
 
C2orf69
 
 
hsa-miR-1-3p
 
 
-0.414
 
 
20.9
 
 
 
 
CALM2
 
 
hsa-miR-130b-3p
 
 
-0.584
 
 
226.3
 
 
 
 
CANT1
 
 
hsa-let-7e-5p
 
 
-0.402
 
 
18.0
 
 
 
 
CAPN3
 
 
hsa-let-7e-5p
 
 
-0.317
 
 
45.0
 
 
 
 
CAPZA1
 
 
hsa-miR-7-5p
 
 
-0.351
 
 
147.7
 
 
 
 
CASP3
 
 
hsa-let-7e-5p
 
 
-0.495
 
 
13.3
 
 
 
 
CBFB
 
 
hsa-miR-130b-3p
 
 
-0.498
 
 
11.0
 
 
 
 
CBX5
 
 
hsa-let-7e-5p
 
 
-0.307
 
 
16.2
 
 
 
 
CBX5
 
 
hsa-let-7e-5p
 
 
-0.472
 
 
16.2
 
 
 
 
CCDC43
 
 
hsa-miR-7-5p
 
 
-0.331
 
 
19.6
 
 
 
 
CCL7
 
 
hsa-let-7e-5p
 
 
-0.718
 
 
24.4
 
 
 
 
CCNC
 
 
hsa-miR-1-3p
 
 
-0.336
 
 
31.1
 
 
 
 
CCNJ
 
 
hsa-let-7e-5p
 
 
-0.531
 
 
10.9
 
 
 
 
CCNL2
 
 
hsa-miR-28-5p
 
 
-0.448
 
 
28.2
 
 
 
 
CCNT2
 
 
hsa-miR-7-5p
 
 
-0.349
 
 
12.7
 
 
 
 
CCNY
 
 
hsa-let-7e-5p
 
 
-0.334
 
 
33.9
 
 
 
 
CCNY
 
 
hsa-miR-130b-3p
 
 
-0.372
 
 
33.9
 
 
 
 
CCR7
 
 
hsa-let-7e-5p
 
 
-0.482
 
 
14.3
 
 
 
 
CCSAP
 
 
hsa-miR-1-3p
 
 
-0.381
 
 
12.2
 
 
 
 
CCT4
 
 
hsa-miR-7-5p
 
 
-0.37
 
 
61.3
 
 
 
 
CCT6A
 
 
hsa-miR-130b-3p
 
 
-0.382
 
 
64.5
 
 
 
 
CD164
 
 
hsa-miR-1-3p
 
 
-0.315
 
 
201.8
 
 
 
 
CD2AP
 
 
hsa-miR-130b-3p
 
 
-0.302
 
 
13.5
 
 
 
 
CD2AP
 
 
hsa-miR-1-3p
 
 
-0.479
 
 
13.5
 
 
 
 
CD69
 
 
hsa-miR-130b-3p
 
 
-0.451
 
 
159.6
 
 
 
 
CDC34
 
 
hsa-let-7e-5p
 
 
-0.321
 
 
32.6
 
 
 
 
CDC34
 
 
hsa-let-7e-5p
 
 
-0.555
 
 
32.6
 
 
 
 
CDC42SE1
 
 
hsa-miR-1-3p
 
 
-0.36
 
 
77.0
 
 
 
 
 
 
 breast 
 
 
 
 
target_symbol
 
 
mature_mirna_id
 
 
score
 
 
nTPM
 
 
 
 
 
 
ABCB7
 
 
hsa-miR-1-3p
 
 
-0.365
 
 
11.6
 
 
 
 
ABHD17C
 
 
hsa-let-7e-5p
 
 
-0.359
 
 
10.9
 
 
 
 
ABHD2
 
 
hsa-miR-1-3p
 
 
-0.291
 
 
21.1
 
 
 
 
ABHD3
 
 
hsa-miR-130b-3p
 
 
-0.356
 
 
14.7
 
 
 
 
ABRAXAS2
 
 
hsa-miR-130b-3p
 
 
-0.468
 
 
11.4
 
 
 
 
ACBD3
 
 
hsa-miR-130b-3p
 
 
-0.292
 
 
22.8
 
 
 
 
ACBD5
 
 
hsa-miR-130b-3p
 
 
-0.494
 
 
11.5
 
 
 
 
ACSL4
 
 
hsa-miR-130b-3p
 
 
-0.301
 
 
16.3
 
 
 
 
ACTC1
 
 
hsa-miR-7-5p
 
 
-0.471
 
 
11.2
 
 
 
 
ACVR1
 
 
hsa-miR-130b-3p
 
 
-0.44
 
 
11.1
 
 
 
 
ADAR
 
 
hsa-miR-1-3p
 
 
-0.35
 
 
36.7
 
 
 
 
ADGRA3
 
 
hsa-miR-1-3p
 
 
-0.351
 
 
10.2
 
 
 
 
ADPGK
 
 
hsa-miR-1-3p
 
 
-0.393
 
 
12.3
 
 
 
 
ADRB2
 
 
hsa-let-7e-5p
 
 
-0.635
 
 
32.4
 
 
 
 
AIFM1
 
 
hsa-let-7e-5p
 
 
-0.314
 
 
11.7
 
 
 
 
AKIRIN2
 
 
hsa-miR-130b-3p
 
 
-0.315
 
 
23.1
 
 
 
 
ALDH4A1
 
 
hsa-miR-184
 
 
-0.421
 
 
22.5
 
 
 
 
ALG11
 
 
hsa-miR-1-3p
 
 
-0.317
 
 
11.3
 
 
 
 
ALG9
 
 
hsa-miR-7-5p
 
 
-0.302
 
 
11.3
 
 
 
 
ANKIB1
 
 
hsa-miR-130b-3p
 
 
-0.329
 
 
11.5
 
 
 
 
ANKRD12
 
 
hsa-miR-7-5p
 
 
-0.37
 
 
12.8
 
 
 
 
ANKRD29
 
 
hsa-miR-1-3p
 
 
-0.426
 
 
15.0
 
 
 
 
ANKRD46
 
 
hsa-let-7e-5p
 
 
-0.389
 
 
13.6
 
 
 
 
ANP32B
 
 
hsa-miR-1-3p
 
 
-0.287
 
 
132.4
 
 
 
 
ANXA2
 
 
hsa-miR-1-3p
 
 
-0.581
 
 
637.5
 
 
 
 
ANXA4
 
 
hsa-miR-1-3p
 
 
-0.539
 
 
43.0
 
 
 
 
AP1AR
 
 
hsa-miR-100-5p
 
 
-0.804
 
 
15.9
 
 
 
 
AP1S1
 
 
hsa-miR-1-3p
 
 
-0.355
 
 
17.7
 
 
 
 
AP1S1
 
 
hsa-let-7e-5p
 
 
-0.374
 
 
17.7
 
 
 
 
APCDD1
 
 
hsa-miR-130b-3p
 
 
-0.304
 
 
30.9
 
 
 
 
APPL1
 
 
hsa-miR-130b-3p
 
 
-0.347
 
 
17.7
 
 
 
 
ARCN1
 
 
hsa-miR-1-3p
 
 
-0.468
 
 
61.8
 
 
 
 
ARF4
 
 
hsa-miR-7-5p
 
 
-0.407
 
 
129.6
 
 
 
 
ARFGEF1
 
 
hsa-miR-130b-3p
 
 
-0.302
 
 
12.7
 
 
 
 
ARHGAP1
 
 
hsa-miR-130b-3p
 
 
-0.376
 
 
59.0
 
 
 
 
ARHGAP12
 
 
hsa-miR-130b-3p
 
 
-0.287
 
 
15.4
 
 
 
 
ARL6IP1
 
 
hsa-miR-130b-3p
 
 
-0.289
 
 
105.7
 
 
 
 
ARL6IP1
 
 
hsa-miR-7-5p
 
 
-0.374
 
 
105.7
 
 
 
 
ARL6IP6
 
 
hsa-let-7e-5p
 
 
-0.305
 
 
12.8
 
 
 
 
ARMT1
 
 
hsa-let-7e-5p
 
 
-0.303
 
 
23.4
 
 
 
 
ARPC3
 
 
hsa-miR-1-3p
 
 
-0.606
 
 
199.9
 
 
 
 
ARPP19
 
 
hsa-let-7e-5p
 
 
-0.492
 
 
43.2
 
 
 
 
ARRDC4
 
 
hsa-let-7e-5p
 
 
-0.329
 
 
18.6
 
 
 
 
ASAP1
 
 
hsa-let-7e-5p
 
 
-0.296
 
 
22.2
 
 
 
 
ASH2L
 
 
hsa-miR-1-3p
 
 
-0.462
 
 
20.3
 
 
 
 
ATF2
 
 
hsa-miR-1-3p
 
 
-0.328
 
 
17.8
 
 
 
 
ATOSB
 
 
hsa-let-7e-5p
 
 
-0.429
 
 
10.1
 
 
 
 
ATP2A2
 
 
hsa-let-7e-5p
 
 
-0.311
 
 
28.6
 
 
 
 
ATPAF1
 
 
hsa-let-7e-5p
 
 
-0.498
 
 
33.1
 
 
 
 
AVPI1
 
 
hsa-miR-7-5p
 
 
-0.367
 
 
22.2
 
 
 
 
AXL
 
 
hsa-miR-1-3p
 
 
-0.304
 
 
25.8
 
 
 
 
AZIN1
 
 
hsa-miR-1-3p
 
 
-0.438
 
 
53.1
 
 
 
 
B3GAT3
 
 
hsa-let-7e-5p
 
 
-0.324
 
 
29.8
 
 
 
 
B4GAT1
 
 
hsa-let-7e-5p
 
 
-0.47
 
 
15.6
 
 
 
 
BACH1
 
 
hsa-let-7e-5p
 
 
-0.298
 
 
12.3
 
 
 
 
BACH1
 
 
hsa-let-7e-5p
 
 
-0.298
 
 
12.3
 
 
 
 
BACH1
 
 
hsa-let-7e-5p
 
 
-0.435
 
 
12.3
 
 
 
 
BACH1
 
 
hsa-let-7e-5p
 
 
-0.435
 
 
12.3
 
 
 
 
BAZ2A
 
 
hsa-miR-100-5p
 
 
-0.29
 
 
25.2
 
 
 
 
BAZ2A
 
 
hsa-miR-100-5p
 
 
-0.421
 
 
25.2
 
 
 
 
BCAP29
 
 
hsa-let-7e-5p
 
 
-0.289
 
 
23.7
 
 
 
 
BET1
 
 
hsa-miR-1-3p
 
 
-0.413
 
 
21.8
 
 
 
 
BHLHE40
 
 
hsa-miR-130b-3p
 
 
-0.351
 
 
211.1
 
 
 
 
BIN3
 
 
hsa-let-7e-5p
 
 
-0.427
 
 
19.3
 
 
 
 
BLCAP
 
 
hsa-miR-130b-3p
 
 
-0.342
 
 
58.6
 
 
 
 
BLOC1S6
 
 
hsa-let-7e-5p
 
 
-0.321
 
 
23.9
 
 
 
 
BMPR2
 
 
hsa-miR-130b-3p
 
 
-0.33
 
 
10.7
 
 
 
 
BMPR2
 
 
hsa-miR-100-5p
 
 
-0.355
 
 
10.7
 
 
 
 
BPNT1
 
 
hsa-miR-1-3p
 
 
-0.329
 
 
19.0
 
 
 
 
BRIX1
 
 
hsa-miR-1-3p
 
 
-0.334
 
 
23.1
 
 
 
 
BSCL2
 
 
hsa-miR-1-3p
 
 
-0.471
 
 
22.3
 
 
 
 
BTAF1
 
 
hsa-miR-130b-3p
 
 
-0.291
 
 
15.3
 
 
 
 
BTF3L4
 
 
hsa-let-7e-5p
 
 
-0.355
 
 
40.8
 
 
 
 
BTF3L4
 
 
hsa-miR-130b-3p
 
 
-0.359
 
 
40.8
 
 
 
 
BTG1
 
 
hsa-miR-130b-3p
 
 
-0.565
 
 
14.4
 
 
 
 
BZW1
 
 
hsa-let-7e-5p
 
 
-0.354
 
 
83.1
 
 
 
 
BZW1
 
 
hsa-let-7e-5p
 
 
-0.375
 
 
83.1
 
 
 
 
BZW2
 
 
hsa-let-7e-5p
 
 
-0.408
 
 
46.4
 
 
 
 
C15orf39
 
 
hsa-let-7e-5p
 
 
-0.374
 
 
13.1
 
 
 
 
C1GALT1
 
 
hsa-let-7e-5p
 
 
-0.322
 
 
15.5
 
 
 
 
C6orf120
 
 
hsa-miR-1-3p
 
 
-0.288
 
 
13.6
 
 
 
 
C8orf58
 
 
hsa-let-7e-5p
 
 
-0.612
 
 
10.3
 
 
 
 
CAAP1
 
 
hsa-miR-1-3p
 
 
-0.48
 
 
10.1
 
 
 
 
CALM2
 
 
hsa-miR-130b-3p
 
 
-0.584
 
 
642.6
 
 
 
 
CAMSAP2
 
 
hsa-miR-130b-3p
 
 
-0.297
 
 
11.5
 
 
 
 
CANT1
 
 
hsa-let-7e-5p
 
 
-0.402
 
 
23.7
 
 
 
 
CAPN3
 
 
hsa-let-7e-5p
 
 
-0.317
 
 
14.5
 
 
 
 
CAPZA1
 
 
hsa-miR-7-5p
 
 
-0.351
 
 
52.2
 
 
 
 
CASD1
 
 
hsa-miR-130b-3p
 
 
-0.305
 
 
14.7
 
 
 
 
CASP3
 
 
hsa-let-7e-5p
 
 
-0.495
 
 
12.5
 
 
 
 
CBX5
 
 
hsa-let-7e-5p
 
 
-0.307
 
 
12.9
 
 
 
 
CBX5
 
 
hsa-let-7e-5p
 
 
-0.472
 
 
12.9
 
 
 
 
CCDC43
 
 
hsa-miR-7-5p
 
 
-0.331
 
 
11.7
 
 
 
 
CCNC
 
 
hsa-miR-1-3p
 
 
-0.336
 
 
44.3
 
 
 
 
CCNL2
 
 
hsa-miR-28-5p
 
 
-0.448
 
 
61.0
 
 
 
 
CCNT2
 
 
hsa-miR-7-5p
 
 
-0.349
 
 
12.5
 
 
 
 
CCNY
 
 
hsa-let-7e-5p
 
 
-0.334
 
 
21.0
 
 
 
 
CCNY
 
 
hsa-miR-130b-3p
 
 
-0.372
 
 
21.0
 
 
 
 
CCT4
 
 
hsa-miR-7-5p
 
 
-0.37
 
 
84.3
 
 
 
 
CCT6A
 
 
hsa-miR-130b-3p
 
 
-0.382
 
 
57.1
 
 
 
 
 
 
 cervix 
 
 
 
 
target_symbol
 
 
mature_mirna_id
 
 
score
 
 
nTPM
 
 
 
 
 
 
ABCB7
 
 
hsa-miR-1-3p
 
 
-0.365
 
 
10.8
 
 
 
 
ABHD17C
 
 
hsa-let-7e-5p
 
 
-0.359
 
 
18.1
 
 
 
 
ABHD2
 
 
hsa-miR-1-3p
 
 
-0.291
 
 
18.3
 
 
 
 
ABHD3
 
 
hsa-miR-130b-3p
 
 
-0.356
 
 
10.5
 
 
 
 
ACBD3
 
 
hsa-miR-130b-3p
 
 
-0.292
 
 
25.9
 
 
 
 
ACBD5
 
 
hsa-miR-130b-3p
 
 
-0.494
 
 
12.5
 
 
 
 
ACP3
 
 
hsa-let-7e-5p
 
 
-0.298
 
 
10.9
 
 
 
 
ACSL4
 
 
hsa-miR-130b-3p
 
 
-0.301
 
 
43.6
 
 
 
 
ACTC1
 
 
hsa-miR-7-5p
 
 
-0.471
 
 
44.2
 
 
 
 
ACVR1
 
 
hsa-miR-130b-3p
 
 
-0.44
 
 
20.6
 
 
 
 
ADAM12
 
 
hsa-miR-130b-3p
 
 
-0.362
 
 
12.4
 
 
 
 
ADAR
 
 
hsa-miR-1-3p
 
 
-0.35
 
 
42.9
 
 
 
 
ADGRA3
 
 
hsa-miR-1-3p
 
 
-0.351
 
 
10.2
 
 
 
 
ADRB2
 
 
hsa-let-7e-5p
 
 
-0.635
 
 
12.7
 
 
 
 
AIFM1
 
 
hsa-let-7e-5p
 
 
-0.314
 
 
14.8
 
 
 
 
AKIRIN2
 
 
hsa-miR-130b-3p
 
 
-0.315
 
 
19.6
 
 
 
 
ALDH4A1
 
 
hsa-miR-184
 
 
-0.421
 
 
15.0
 
 
 
 
ALG11
 
 
hsa-miR-1-3p
 
 
-0.317
 
 
11.3
 
 
 
 
ALG9
 
 
hsa-miR-7-5p
 
 
-0.302
 
 
10.8
 
 
 
 
AMT
 
 
hsa-let-7e-5p
 
 
-0.463
 
 
14.5
 
 
 
 
ANKIB1
 
 
hsa-miR-130b-3p
 
 
-0.329
 
 
11.9
 
 
 
 
ANKRD12
 
 
hsa-miR-7-5p
 
 
-0.37
 
 
10.5
 
 
 
 
ANKRD29
 
 
hsa-miR-1-3p
 
 
-0.426
 
 
12.2
 
 
 
 
ANKRD46
 
 
hsa-let-7e-5p
 
 
-0.389
 
 
14.1
 
 
 
 
ANP32B
 
 
hsa-miR-1-3p
 
 
-0.287
 
 
143.2
 
 
 
 
ANXA2
 
 
hsa-miR-1-3p
 
 
-0.581
 
 
1111.3
 
 
 
 
ANXA4
 
 
hsa-miR-1-3p
 
 
-0.539
 
 
58.6
 
 
 
 
AP1S1
 
 
hsa-miR-1-3p
 
 
-0.355
 
 
20.1
 
 
 
 
AP1S1
 
 
hsa-let-7e-5p
 
 
-0.374
 
 
20.1
 
 
 
 
APCDD1
 
 
hsa-miR-130b-3p
 
 
-0.304
 
 
33.1
 
 
 
 
APPL1
 
 
hsa-miR-130b-3p
 
 
-0.347
 
 
16.1
 
 
 
 
ARCN1
 
 
hsa-miR-1-3p
 
 
-0.468
 
 
70.2
 
 
 
 
ARF4
 
 
hsa-miR-7-5p
 
 
-0.407
 
 
196.1
 
 
 
 
ARFGEF1
 
 
hsa-miR-130b-3p
 
 
-0.302
 
 
13.3
 
 
 
 
ARG2
 
 
hsa-let-7e-5p
 
 
-0.468
 
 
16.0
 
 
 
 
ARHGAP1
 
 
hsa-miR-130b-3p
 
 
-0.376
 
 
62.9
 
 
 
 
ARHGAP12
 
 
hsa-miR-130b-3p
 
 
-0.287
 
 
12.4
 
 
 
 
ARHGEF4
 
 
hsa-miR-130b-3p
 
 
-0.377
 
 
16.4
 
 
 
 
ARL4D
 
 
hsa-let-7e-5p
 
 
-0.417
 
 
27.6
 
 
 
 
ARL6IP1
 
 
hsa-miR-130b-3p
 
 
-0.289
 
 
74.5
 
 
 
 
ARL6IP1
 
 
hsa-miR-7-5p
 
 
-0.374
 
 
74.5
 
 
 
 
ARMT1
 
 
hsa-let-7e-5p
 
 
-0.303
 
 
15.1
 
 
 
 
ARPC3
 
 
hsa-miR-1-3p
 
 
-0.606
 
 
199.6
 
 
 
 
ARPP19
 
 
hsa-let-7e-5p
 
 
-0.492
 
 
35.8
 
 
 
 
ARRDC4
 
 
hsa-let-7e-5p
 
 
-0.329
 
 
14.3
 
 
 
 
ASAP1
 
 
hsa-let-7e-5p
 
 
-0.296
 
 
15.1
 
 
 
 
ASH2L
 
 
hsa-miR-1-3p
 
 
-0.462
 
 
18.7
 
 
 
 
ATF2
 
 
hsa-miR-1-3p
 
 
-0.328
 
 
24.1
 
 
 
 
ATOSB
 
 
hsa-let-7e-5p
 
 
-0.429
 
 
12.3
 
 
 
 
ATP2A2
 
 
hsa-let-7e-5p
 
 
-0.311
 
 
40.8
 
 
 
 
ATPAF1
 
 
hsa-let-7e-5p
 
 
-0.498
 
 
33.4
 
 
 
 
AVPI1
 
 
hsa-miR-7-5p
 
 
-0.367
 
 
30.7
 
 
 
 
AXL
 
 
hsa-miR-1-3p
 
 
-0.304
 
 
30.0
 
 
 
 
AZIN1
 
 
hsa-miR-1-3p
 
 
-0.438
 
 
50.9
 
 
 
 
B3GAT3
 
 
hsa-let-7e-5p
 
 
-0.324
 
 
36.2
 
 
 
 
B4GAT1
 
 
hsa-let-7e-5p
 
 
-0.47
 
 
25.3
 
 
 
 
BACH1
 
 
hsa-let-7e-5p
 
 
-0.298
 
 
17.0
 
 
 
 
BACH1
 
 
hsa-let-7e-5p
 
 
-0.298
 
 
17.0
 
 
 
 
BACH1
 
 
hsa-let-7e-5p
 
 
-0.435
 
 
17.0
 
 
 
 
BACH1
 
 
hsa-let-7e-5p
 
 
-0.435
 
 
17.0
 
 
 
 
BAHD1
 
 
hsa-miR-130b-3p
 
 
-0.294
 
 
10.9
 
 
 
 
BAZ2A
 
 
hsa-miR-100-5p
 
 
-0.29
 
 
25.9
 
 
 
 
BAZ2A
 
 
hsa-miR-100-5p
 
 
-0.421
 
 
25.9
 
 
 
 
BCAP29
 
 
hsa-let-7e-5p
 
 
-0.289
 
 
47.0
 
 
 
 
BET1
 
 
hsa-miR-1-3p
 
 
-0.413
 
 
21.5
 
 
 
 
BHLHE40
 
 
hsa-miR-130b-3p
 
 
-0.351
 
 
244.8
 
 
 
 
BIN3
 
 
hsa-let-7e-5p
 
 
-0.427
 
 
13.1
 
 
 
 
BLCAP
 
 
hsa-miR-130b-3p
 
 
-0.342
 
 
54.0
 
 
 
 
BLOC1S6
 
 
hsa-let-7e-5p
 
 
-0.321
 
 
24.2
 
 
 
 
BMPR2
 
 
hsa-miR-130b-3p
 
 
-0.33
 
 
15.5
 
 
 
 
BMPR2
 
 
hsa-miR-100-5p
 
 
-0.355
 
 
15.5
 
 
 
 
BPNT1
 
 
hsa-miR-1-3p
 
 
-0.329
 
 
17.0
 
 
 
 
BRIX1
 
 
hsa-miR-1-3p
 
 
-0.334
 
 
15.4
 
 
 
 
BSCL2
 
 
hsa-miR-1-3p
 
 
-0.471
 
 
35.6
 
 
 
 
BTAF1
 
 
hsa-miR-130b-3p
 
 
-0.291
 
 
16.0
 
 
 
 
BTF3L4
 
 
hsa-let-7e-5p
 
 
-0.355
 
 
29.5
 
 
 
 
BTF3L4
 
 
hsa-miR-130b-3p
 
 
-0.359
 
 
29.5
 
 
 
 
BZW1
 
 
hsa-let-7e-5p
 
 
-0.354
 
 
61.6
 
 
 
 
BZW1
 
 
hsa-let-7e-5p
 
 
-0.375
 
 
61.6
 
 
 
 
BZW2
 
 
hsa-let-7e-5p
 
 
-0.408
 
 
22.5
 
 
 
 
C15orf39
 
 
hsa-let-7e-5p
 
 
-0.374
 
 
12.8
 
 
 
 
C1GALT1
 
 
hsa-let-7e-5p
 
 
-0.322
 
 
17.4
 
 
 
 
C2orf88
 
 
hsa-let-7e-5p
 
 
-0.429
 
 
45.4
 
 
 
 
C6orf120
 
 
hsa-miR-1-3p
 
 
-0.288
 
 
10.4
 
 
 
 
CALM2
 
 
hsa-miR-130b-3p
 
 
-0.584
 
 
395.0
 
 
 
 
CAMSAP2
 
 
hsa-miR-130b-3p
 
 
-0.297
 
 
13.3
 
 
 
 
CANT1
 
 
hsa-let-7e-5p
 
 
-0.402
 
 
30.3
 
 
 
 
CAPN3
 
 
hsa-let-7e-5p
 
 
-0.317
 
 
18.4
 
 
 
 
CAPZA1
 
 
hsa-miR-7-5p
 
 
-0.351
 
 
54.6
 
 
 
 
CASD1
 
 
hsa-miR-130b-3p
 
 
-0.305
 
 
10.6
 
 
 
 
CASP3
 
 
hsa-let-7e-5p
 
 
-0.495
 
 
11.5
 
 
 
 
CBX5
 
 
hsa-let-7e-5p
 
 
-0.307
 
 
10.2
 
 
 
 
CBX5
 
 
hsa-let-7e-5p
 
 
-0.472
 
 
10.2
 
 
 
 
CCNC
 
 
hsa-miR-1-3p
 
 
-0.336
 
 
28.0
 
 
 
 
CCNL2
 
 
hsa-miR-28-5p
 
 
-0.448
 
 
73.9
 
 
 
 
CCNT2
 
 
hsa-miR-7-5p
 
 
-0.349
 
 
13.9
 
 
 
 
CCNY
 
 
hsa-let-7e-5p
 
 
-0.334
 
 
20.5
 
 
 
 
CCNY
 
 
hsa-miR-130b-3p
 
 
-0.372
 
 
20.5
 
 
 
 
CCT4
 
 
hsa-miR-7-5p
 
 
-0.37
 
 
65.9
 
 
 
 
CCT6A
 
 
hsa-miR-130b-3p
 
 
-0.382
 
 
46.2
 
 
 
 
 
 
 choroid plexus 
 
 
 
 
target_symbol
 
 
mature_mirna_id
 
 
score
 
 
nTPM
 
 
 
 
 
 
ABCB7
 
 
hsa-miR-1-3p
 
 
-0.365
 
 
15.2
 
 
 
 
ABHD2
 
 
hsa-miR-1-3p
 
 
-0.291
 
 
64.8
 
 
 
 
ABHD3
 
 
hsa-miR-130b-3p
 
 
-0.356
 
 
24.1
 
 
 
 
ACBD3
 
 
hsa-miR-130b-3p
 
 
-0.292
 
 
12.2
 
 
 
 
ACBD5
 
 
hsa-miR-130b-3p
 
 
-0.494
 
 
15.7
 
 
 
 
ADAR
 
 
hsa-miR-1-3p
 
 
-0.35
 
 
26.5
 
 
 
 
ADGRA3
 
 
hsa-miR-1-3p
 
 
-0.351
 
 
23.6
 
 
 
 
ADPGK
 
 
hsa-miR-1-3p
 
 
-0.393
 
 
12.6
 
 
 
 
AIFM1
 
 
hsa-let-7e-5p
 
 
-0.314
 
 
37.9
 
 
 
 
AKIRIN2
 
 
hsa-miR-130b-3p
 
 
-0.315
 
 
23.7
 
 
 
 
ALDH4A1
 
 
hsa-miR-184
 
 
-0.421
 
 
121.5
 
 
 
 
AMT
 
 
hsa-let-7e-5p
 
 
-0.463
 
 
33.4
 
 
 
 
ANKRD46
 
 
hsa-let-7e-5p
 
 
-0.389
 
 
11.0
 
 
 
 
ANP32B
 
 
hsa-miR-1-3p
 
 
-0.287
 
 
148.4
 
 
 
 
ANXA2
 
 
hsa-miR-1-3p
 
 
-0.581
 
 
399.0
 
 
 
 
ANXA4
 
 
hsa-miR-1-3p
 
 
-0.539
 
 
152.5
 
 
 
 
AP1S1
 
 
hsa-miR-1-3p
 
 
-0.355
 
 
10.3
 
 
 
 
AP1S1
 
 
hsa-let-7e-5p
 
 
-0.374
 
 
10.3
 
 
 
 
ARCN1
 
 
hsa-miR-1-3p
 
 
-0.468
 
 
83.5
 
 
 
 
ARF4
 
 
hsa-miR-7-5p
 
 
-0.407
 
 
115.4
 
 
 
 
ARG2
 
 
hsa-let-7e-5p
 
 
-0.468
 
 
49.7
 
 
 
 
ARHGAP1
 
 
hsa-miR-130b-3p
 
 
-0.376
 
 
26.4
 
 
 
 
ARHGAP12
 
 
hsa-miR-130b-3p
 
 
-0.287
 
 
10.3
 
 
 
 
ARHGEF4
 
 
hsa-miR-130b-3p
 
 
-0.377
 
 
13.6
 
 
 
 
ARL4D
 
 
hsa-let-7e-5p
 
 
-0.417
 
 
15.4
 
 
 
 
ARL6IP1
 
 
hsa-miR-130b-3p
 
 
-0.289
 
 
185.0
 
 
 
 
ARL6IP1
 
 
hsa-miR-7-5p
 
 
-0.374
 
 
185.0
 
 
 
 
ARPC3
 
 
hsa-miR-1-3p
 
 
-0.606
 
 
210.8
 
 
 
 
ARPP19
 
 
hsa-let-7e-5p
 
 
-0.492
 
 
32.4
 
 
 
 
AS3MT
 
 
hsa-miR-28-5p
 
 
-0.479
 
 
11.9
 
 
 
 
ATG16L1
 
 
hsa-miR-130b-3p
 
 
-0.407
 
 
21.8
 
 
 
 
ATG4A
 
 
hsa-miR-7-5p
 
 
-0.448
 
 
16.6
 
 
 
 
ATOSB
 
 
hsa-let-7e-5p
 
 
-0.429
 
 
16.6
 
 
 
 
ATP2A2
 
 
hsa-let-7e-5p
 
 
-0.311
 
 
29.6
 
 
 
 
ATP2B2
 
 
hsa-miR-7-5p
 
 
-0.297
 
 
61.0
 
 
 
 
ATP2B2
 
 
hsa-miR-130b-3p
 
 
-0.314
 
 
61.0
 
 
 
 
ATPAF1
 
 
hsa-let-7e-5p
 
 
-0.498
 
 
168.0
 
 
 
 
AVPI1
 
 
hsa-miR-7-5p
 
 
-0.367
 
 
20.0
 
 
 
 
AZIN1
 
 
hsa-miR-1-3p
 
 
-0.438
 
 
28.1
 
 
 
 
B3GAT3
 
 
hsa-let-7e-5p
 
 
-0.324
 
 
87.1
 
 
 
 
B4GAT1
 
 
hsa-let-7e-5p
 
 
-0.47
 
 
93.8
 
 
 
 
BCAP29
 
 
hsa-let-7e-5p
 
 
-0.289
 
 
12.5
 
 
 
 
BET1
 
 
hsa-miR-1-3p
 
 
-0.413
 
 
13.1
 
 
 
 
BHLHE40
 
 
hsa-miR-130b-3p
 
 
-0.351
 
 
103.5
 
 
 
 
BIN3
 
 
hsa-let-7e-5p
 
 
-0.427
 
 
33.7
 
 
 
 
BLCAP
 
 
hsa-miR-130b-3p
 
 
-0.342
 
 
138.8
 
 
 
 
BLOC1S4
 
 
hsa-miR-7-5p
 
 
-0.417
 
 
10.3
 
 
 
 
BLOC1S4
 
 
hsa-miR-7-5p
 
 
-0.527
 
 
10.3
 
 
 
 
BLOC1S6
 
 
hsa-let-7e-5p
 
 
-0.321
 
 
14.9
 
 
 
 
BLTP3B
 
 
hsa-miR-1-3p
 
 
-0.29
 
 
10.3
 
 
 
 
BRIX1
 
 
hsa-miR-1-3p
 
 
-0.334
 
 
14.1
 
 
 
 
BSCL2
 
 
hsa-miR-1-3p
 
 
-0.471
 
 
156.7
 
 
 
 
BTF3L4
 
 
hsa-let-7e-5p
 
 
-0.355
 
 
26.9
 
 
 
 
BTF3L4
 
 
hsa-miR-130b-3p
 
 
-0.359
 
 
26.9
 
 
 
 
BZW1
 
 
hsa-let-7e-5p
 
 
-0.354
 
 
50.7
 
 
 
 
BZW1
 
 
hsa-let-7e-5p
 
 
-0.375
 
 
50.7
 
 
 
 
BZW2
 
 
hsa-let-7e-5p
 
 
-0.408
 
 
19.4
 
 
 
 
C1GALT1
 
 
hsa-let-7e-5p
 
 
-0.322
 
 
22.9
 
 
 
 
CAAP1
 
 
hsa-miR-1-3p
 
 
-0.48
 
 
11.5
 
 
 
 
CALM2
 
 
hsa-miR-130b-3p
 
 
-0.584
 
 
389.3
 
 
 
 
CANT1
 
 
hsa-let-7e-5p
 
 
-0.402
 
 
11.0
 
 
 
 
CAPN3
 
 
hsa-let-7e-5p
 
 
-0.317
 
 
17.5
 
 
 
 
CAPZA1
 
 
hsa-miR-7-5p
 
 
-0.351
 
 
36.9
 
 
 
 
CBX5
 
 
hsa-let-7e-5p
 
 
-0.307
 
 
112.0
 
 
 
 
CBX5
 
 
hsa-let-7e-5p
 
 
-0.472
 
 
112.0
 
 
 
 
CCNC
 
 
hsa-miR-1-3p
 
 
-0.336
 
 
33.9
 
 
 
 
CCNL2
 
 
hsa-miR-28-5p
 
 
-0.448
 
 
18.9
 
 
 
 
CCNY
 
 
hsa-let-7e-5p
 
 
-0.334
 
 
13.3
 
 
 
 
CCNY
 
 
hsa-miR-130b-3p
 
 
-0.372
 
 
13.3
 
 
 
 
CCT4
 
 
hsa-miR-7-5p
 
 
-0.37
 
 
61.9
 
 
 
 
CCT6A
 
 
hsa-miR-130b-3p
 
 
-0.382
 
 
46.5
 
 
 
 
CD164
 
 
hsa-miR-1-3p
 
 
-0.315
 
 
46.0
 
 
 
 
CDC34
 
 
hsa-let-7e-5p
 
 
-0.321
 
 
42.2
 
 
 
 
CDC34
 
 
hsa-let-7e-5p
 
 
-0.555
 
 
42.2
 
 
 
 
CDC42SE1
 
 
hsa-miR-1-3p
 
 
-0.36
 
 
31.3
 
 
 
 
CDK4
 
 
hsa-miR-1-3p
 
 
-0.338
 
 
44.0
 
 
 
 
CDKN1A
 
 
hsa-let-7e-5p
 
 
-0.434
 
 
300.9
 
 
 
 
CDV3
 
 
hsa-let-7e-5p
 
 
-0.453
 
 
57.1
 
 
 
 
CEBPD
 
 
hsa-let-7e-5p
 
 
-0.309
 
 
20.5
 
 
 
 
CERCAM
 
 
hsa-let-7e-5p
 
 
-0.344
 
 
29.3
 
 
 
 
CERS2
 
 
hsa-miR-1-3p
 
 
-0.287
 
 
150.4
 
 
 
 
CGGBP1
 
 
hsa-miR-7-5p
 
 
-0.465
 
 
26.7
 
 
 
 
CHMP3
 
 
hsa-miR-130b-3p
 
 
-0.521
 
 
21.3
 
 
 
 
CHMP4B
 
 
hsa-miR-130b-3p
 
 
-0.292
 
 
101.4
 
 
 
 
CKAP4
 
 
hsa-miR-7-5p
 
 
-0.443
 
 
35.3
 
 
 
 
CLASP2
 
 
hsa-let-7e-5p
 
 
-0.344
 
 
17.7
 
 
 
 
CLCN3
 
 
hsa-miR-1-3p
 
 
-0.339
 
 
13.5
 
 
 
 
CLCN3
 
 
hsa-miR-130b-3p
 
 
-0.384
 
 
13.5
 
 
 
 
CLDN12
 
 
hsa-let-7e-5p
 
 
-0.511
 
 
10.5
 
 
 
 
CLIP1
 
 
hsa-miR-130b-3p
 
 
-0.452
 
 
13.4
 
 
 
 
CLTC
 
 
hsa-miR-1-3p
 
 
-0.39
 
 
82.2
 
 
 
 
CMPK1
 
 
hsa-miR-130b-3p
 
 
-0.384
 
 
37.5
 
 
 
 
CNIH1
 
 
hsa-miR-130b-3p
 
 
-0.335
 
 
83.2
 
 
 
 
CNN3
 
 
hsa-miR-1-3p
 
 
-0.414
 
 
159.0
 
 
 
 
CNN3
 
 
hsa-miR-7-5p
 
 
-0.489
 
 
159.0
 
 
 
 
CNOT7
 
 
hsa-miR-130b-3p
 
 
-0.318
 
 
27.0
 
 
 
 
CNOT8
 
 
hsa-miR-7-5p
 
 
-0.329
 
 
48.9
 
 
 
 
CNPPD1
 
 
hsa-miR-7-5p
 
 
-0.541
 
 
24.2
 
 
 
 
COL1A2
 
 
hsa-let-7e-5p
 
 
-0.499
 
 
10.7
 
 
 
 
COL9A3
 
 
hsa-miR-130b-3p
 
 
-0.379
 
 
17.0
 
 
 
 
 
 
 colon 
 
 
 
 
target_symbol
 
 
mature_mirna_id
 
 
score
 
 
nTPM
 
 
 
 
 
 
ABCB7
 
 
hsa-miR-1-3p
 
 
-0.365
 
 
13.4
 
 
 
 
ABHD17C
 
 
hsa-let-7e-5p
 
 
-0.359
 
 
61.2
 
 
 
 
ABHD2
 
 
hsa-miR-1-3p
 
 
-0.291
 
 
19.8
 
 
 
 
ABHD3
 
 
hsa-miR-130b-3p
 
 
-0.356
 
 
59.5
 
 
 
 
ACBD3
 
 
hsa-miR-130b-3p
 
 
-0.292
 
 
21.8
 
 
 
 
ACBD5
 
 
hsa-miR-130b-3p
 
 
-0.494
 
 
19.0
 
 
 
 
ACSL4
 
 
hsa-miR-130b-3p
 
 
-0.301
 
 
11.3
 
 
 
 
ADAR
 
 
hsa-miR-1-3p
 
 
-0.35
 
 
39.7
 
 
 
 
ADGRA3
 
 
hsa-miR-1-3p
 
 
-0.351
 
 
16.0
 
 
 
 
ADPGK
 
 
hsa-miR-1-3p
 
 
-0.393
 
 
12.3
 
 
 
 
AIFM1
 
 
hsa-let-7e-5p
 
 
-0.314
 
 
23.9
 
 
 
 
AKIRIN2
 
 
hsa-miR-130b-3p
 
 
-0.315
 
 
17.5
 
 
 
 
ALG11
 
 
hsa-miR-1-3p
 
 
-0.317
 
 
10.3
 
 
 
 
ALG9
 
 
hsa-miR-7-5p
 
 
-0.302
 
 
10.0
 
 
 
 
AMT
 
 
hsa-let-7e-5p
 
 
-0.463
 
 
14.4
 
 
 
 
ANKIB1
 
 
hsa-miR-130b-3p
 
 
-0.329
 
 
12.1
 
 
 
 
ANKRD12
 
 
hsa-miR-7-5p
 
 
-0.37
 
 
12.2
 
 
 
 
ANP32B
 
 
hsa-miR-1-3p
 
 
-0.287
 
 
136.6
 
 
 
 
ANXA2
 
 
hsa-miR-1-3p
 
 
-0.581
 
 
591.2
 
 
 
 
ANXA4
 
 
hsa-miR-1-3p
 
 
-0.539
 
 
95.4
 
 
 
 
AP1AR
 
 
hsa-miR-100-5p
 
 
-0.804
 
 
16.9
 
 
 
 
AP1S1
 
 
hsa-miR-1-3p
 
 
-0.355
 
 
31.0
 
 
 
 
AP1S1
 
 
hsa-let-7e-5p
 
 
-0.374
 
 
31.0
 
 
 
 
APCDD1
 
 
hsa-miR-130b-3p
 
 
-0.304
 
 
12.1
 
 
 
 
APPL1
 
 
hsa-miR-130b-3p
 
 
-0.347
 
 
13.4
 
 
 
 
ARCN1
 
 
hsa-miR-1-3p
 
 
-0.468
 
 
58.5
 
 
 
 
ARF4
 
 
hsa-miR-7-5p
 
 
-0.407
 
 
148.9
 
 
 
 
ARFGEF1
 
 
hsa-miR-130b-3p
 
 
-0.302
 
 
18.0
 
 
 
 
ARHGAP1
 
 
hsa-miR-130b-3p
 
 
-0.376
 
 
59.8
 
 
 
 
ARHGAP12
 
 
hsa-miR-130b-3p
 
 
-0.287
 
 
14.7
 
 
 
 
ARL4D
 
 
hsa-let-7e-5p
 
 
-0.417
 
 
27.1
 
 
 
 
ARL6IP1
 
 
hsa-miR-130b-3p
 
 
-0.289
 
 
132.0
 
 
 
 
ARL6IP1
 
 
hsa-miR-7-5p
 
 
-0.374
 
 
132.0
 
 
 
 
ARL6IP6
 
 
hsa-let-7e-5p
 
 
-0.305
 
 
12.7
 
 
 
 
ARMT1
 
 
hsa-let-7e-5p
 
 
-0.303
 
 
15.5
 
 
 
 
ARPC3
 
 
hsa-miR-1-3p
 
 
-0.606
 
 
303.4
 
 
 
 
ARPP19
 
 
hsa-let-7e-5p
 
 
-0.492
 
 
35.7
 
 
 
 
ARRDC4
 
 
hsa-let-7e-5p
 
 
-0.329
 
 
40.4
 
 
 
 
ASAP1
 
 
hsa-let-7e-5p
 
 
-0.296
 
 
12.2
 
 
 
 
ASH2L
 
 
hsa-miR-1-3p
 
 
-0.462
 
 
21.4
 
 
 
 
ATF2
 
 
hsa-miR-1-3p
 
 
-0.328
 
 
13.9
 
 
 
 
ATG16L1
 
 
hsa-miR-130b-3p
 
 
-0.407
 
 
13.4
 
 
 
 
ATG4A
 
 
hsa-miR-7-5p
 
 
-0.448
 
 
11.4
 
 
 
 
ATOSB
 
 
hsa-let-7e-5p
 
 
-0.429
 
 
14.6
 
 
 
 
ATP2A2
 
 
hsa-let-7e-5p
 
 
-0.311
 
 
60.2
 
 
 
 
ATPAF1
 
 
hsa-let-7e-5p
 
 
-0.498
 
 
49.0
 
 
 
 
AVPI1
 
 
hsa-miR-7-5p
 
 
-0.367
 
 
23.0
 
 
 
 
AXL
 
 
hsa-miR-1-3p
 
 
-0.304
 
 
40.1
 
 
 
 
AZIN1
 
 
hsa-miR-1-3p
 
 
-0.438
 
 
45.9
 
 
 
 
B3GAT3
 
 
hsa-let-7e-5p
 
 
-0.324
 
 
30.8
 
 
 
 
B4GAT1
 
 
hsa-let-7e-5p
 
 
-0.47
 
 
29.4
 
 
 
 
BACH1
 
 
hsa-let-7e-5p
 
 
-0.298
 
 
12.5
 
 
 
 
BACH1
 
 
hsa-let-7e-5p
 
 
-0.298
 
 
12.5
 
 
 
 
BACH1
 
 
hsa-let-7e-5p
 
 
-0.435
 
 
12.5
 
 
 
 
BACH1
 
 
hsa-let-7e-5p
 
 
-0.435
 
 
12.5
 
 
 
 
BAHD1
 
 
hsa-miR-130b-3p
 
 
-0.294
 
 
11.5
 
 
 
 
BAZ2A
 
 
hsa-miR-100-5p
 
 
-0.29
 
 
25.3
 
 
 
 
BAZ2A
 
 
hsa-miR-100-5p
 
 
-0.421
 
 
25.3
 
 
 
 
BCAP29
 
 
hsa-let-7e-5p
 
 
-0.289
 
 
31.3
 
 
 
 
BET1
 
 
hsa-miR-1-3p
 
 
-0.413
 
 
14.8
 
 
 
 
BHLHE40
 
 
hsa-miR-130b-3p
 
 
-0.351
 
 
73.3
 
 
 
 
BIN3
 
 
hsa-let-7e-5p
 
 
-0.427
 
 
12.9
 
 
 
 
BLCAP
 
 
hsa-miR-130b-3p
 
 
-0.342
 
 
54.1
 
 
 
 
BLOC1S6
 
 
hsa-let-7e-5p
 
 
-0.321
 
 
19.9
 
 
 
 
BMPR2
 
 
hsa-miR-130b-3p
 
 
-0.33
 
 
10.5
 
 
 
 
BMPR2
 
 
hsa-miR-100-5p
 
 
-0.355
 
 
10.5
 
 
 
 
BPNT1
 
 
hsa-miR-1-3p
 
 
-0.329
 
 
35.3
 
 
 
 
BRI3BP
 
 
hsa-miR-1-3p
 
 
-0.293
 
 
14.5
 
 
 
 
BRIX1
 
 
hsa-miR-1-3p
 
 
-0.334
 
 
14.6
 
 
 
 
BSCL2
 
 
hsa-miR-1-3p
 
 
-0.471
 
 
21.8
 
 
 
 
BTAF1
 
 
hsa-miR-130b-3p
 
 
-0.291
 
 
16.8
 
 
 
 
BTF3L4
 
 
hsa-let-7e-5p
 
 
-0.355
 
 
22.9
 
 
 
 
BTF3L4
 
 
hsa-miR-130b-3p
 
 
-0.359
 
 
22.9
 
 
 
 
BZW1
 
 
hsa-let-7e-5p
 
 
-0.354
 
 
92.2
 
 
 
 
BZW1
 
 
hsa-let-7e-5p
 
 
-0.375
 
 
92.2
 
 
 
 
BZW2
 
 
hsa-let-7e-5p
 
 
-0.408
 
 
47.9
 
 
 
 
C15orf39
 
 
hsa-let-7e-5p
 
 
-0.374
 
 
10.3
 
 
 
 
C1GALT1
 
 
hsa-let-7e-5p
 
 
-0.322
 
 
14.3
 
 
 
 
C2orf88
 
 
hsa-let-7e-5p
 
 
-0.429
 
 
33.8
 
 
 
 
C6orf120
 
 
hsa-miR-1-3p
 
 
-0.288
 
 
12.9
 
 
 
 
CAAP1
 
 
hsa-miR-1-3p
 
 
-0.48
 
 
17.4
 
 
 
 
CACFD1
 
 
hsa-let-7e-5p
 
 
-0.296
 
 
18.7
 
 
 
 
CALM2
 
 
hsa-miR-130b-3p
 
 
-0.584
 
 
640.7
 
 
 
 
CAMSAP2
 
 
hsa-miR-130b-3p
 
 
-0.297
 
 
10.0
 
 
 
 
CANT1
 
 
hsa-let-7e-5p
 
 
-0.402
 
 
83.6
 
 
 
 
CAPN3
 
 
hsa-let-7e-5p
 
 
-0.317
 
 
14.6
 
 
 
 
CAPZA1
 
 
hsa-miR-7-5p
 
 
-0.351
 
 
80.7
 
 
 
 
CASD1
 
 
hsa-miR-130b-3p
 
 
-0.305
 
 
15.5
 
 
 
 
CASP3
 
 
hsa-let-7e-5p
 
 
-0.495
 
 
29.8
 
 
 
 
CASP5
 
 
hsa-let-7e-5p
 
 
-0.379
 
 
14.6
 
 
 
 
CCDC43
 
 
hsa-miR-7-5p
 
 
-0.331
 
 
11.3
 
 
 
 
CCNC
 
 
hsa-miR-1-3p
 
 
-0.336
 
 
29.0
 
 
 
 
CCNL2
 
 
hsa-miR-28-5p
 
 
-0.448
 
 
60.7
 
 
 
 
CCNT2
 
 
hsa-miR-7-5p
 
 
-0.349
 
 
12.1
 
 
 
 
CCNY
 
 
hsa-let-7e-5p
 
 
-0.334
 
 
24.9
 
 
 
 
CCNY
 
 
hsa-miR-130b-3p
 
 
-0.372
 
 
24.9
 
 
 
 
CCT4
 
 
hsa-miR-7-5p
 
 
-0.37
 
 
63.9
 
 
 
 
CCT6A
 
 
hsa-miR-130b-3p
 
 
-0.382
 
 
50.1
 
 
 
 
CD164
 
 
hsa-miR-1-3p
 
 
-0.315
 
 
248.4
 
 
 
 
CD2AP
 
 
hsa-miR-130b-3p
 
 
-0.302
 
 
37.5
 
 
 
 
 
 
 duodenum 
 
 
 
 
target_symbol
 
 
mature_mirna_id
 
 
score
 
 
nTPM
 
 
 
 
 
 
ABCB7
 
 
hsa-miR-1-3p
 
 
-0.365
 
 
15.1
 
 
 
 
ABHD17C
 
 
hsa-let-7e-5p
 
 
-0.359
 
 
21.9
 
 
 
 
ABHD2
 
 
hsa-miR-1-3p
 
 
-0.291
 
 
45.1
 
 
 
 
ABHD3
 
 
hsa-miR-130b-3p
 
 
-0.356
 
 
41.5
 
 
 
 
ACBD3
 
 
hsa-miR-130b-3p
 
 
-0.292
 
 
16.5
 
 
 
 
ACBD5
 
 
hsa-miR-130b-3p
 
 
-0.494
 
 
37.5
 
 
 
 
ACSL4
 
 
hsa-miR-130b-3p
 
 
-0.301
 
 
13.8
 
 
 
 
ADAR
 
 
hsa-miR-1-3p
 
 
-0.35
 
 
43.7
 
 
 
 
AIFM1
 
 
hsa-let-7e-5p
 
 
-0.314
 
 
49.8
 
 
 
 
AKIRIN2
 
 
hsa-miR-130b-3p
 
 
-0.315
 
 
12.0
 
 
 
 
ALG9
 
 
hsa-miR-7-5p
 
 
-0.302
 
 
11.5
 
 
 
 
AMT
 
 
hsa-let-7e-5p
 
 
-0.463
 
 
12.9
 
 
 
 
ANKIB1
 
 
hsa-miR-130b-3p
 
 
-0.329
 
 
10.5
 
 
 
 
ANKRD12
 
 
hsa-miR-7-5p
 
 
-0.37
 
 
12.1
 
 
 
 
ANP32B
 
 
hsa-miR-1-3p
 
 
-0.287
 
 
89.5
 
 
 
 
ANXA2
 
 
hsa-miR-1-3p
 
 
-0.581
 
 
471.7
 
 
 
 
ANXA4
 
 
hsa-miR-1-3p
 
 
-0.539
 
 
255.4
 
 
 
 
AP1AR
 
 
hsa-miR-100-5p
 
 
-0.804
 
 
20.9
 
 
 
 
AP1S1
 
 
hsa-miR-1-3p
 
 
-0.355
 
 
36.7
 
 
 
 
AP1S1
 
 
hsa-let-7e-5p
 
 
-0.374
 
 
36.7
 
 
 
 
APPL1
 
 
hsa-miR-130b-3p
 
 
-0.347
 
 
11.9
 
 
 
 
ARCN1
 
 
hsa-miR-1-3p
 
 
-0.468
 
 
55.7
 
 
 
 
ARF4
 
 
hsa-miR-7-5p
 
 
-0.407
 
 
116.2
 
 
 
 
ARFGEF1
 
 
hsa-miR-130b-3p
 
 
-0.302
 
 
15.9
 
 
 
 
ARHGAP1
 
 
hsa-miR-130b-3p
 
 
-0.376
 
 
24.2
 
 
 
 
ARHGAP12
 
 
hsa-miR-130b-3p
 
 
-0.287
 
 
14.5
 
 
 
 
ARL6IP1
 
 
hsa-miR-130b-3p
 
 
-0.289
 
 
130.6
 
 
 
 
ARL6IP1
 
 
hsa-miR-7-5p
 
 
-0.374
 
 
130.6
 
 
 
 
ARMT1
 
 
hsa-let-7e-5p
 
 
-0.303
 
 
17.8
 
 
 
 
ARPC3
 
 
hsa-miR-1-3p
 
 
-0.606
 
 
244.0
 
 
 
 
ARPP19
 
 
hsa-let-7e-5p
 
 
-0.492
 
 
27.2
 
 
 
 
ARRDC4
 
 
hsa-let-7e-5p
 
 
-0.329
 
 
14.9
 
 
 
 
ASH2L
 
 
hsa-miR-1-3p
 
 
-0.462
 
 
17.2
 
 
 
 
ATF2
 
 
hsa-miR-1-3p
 
 
-0.328
 
 
12.2
 
 
 
 
ATG4A
 
 
hsa-miR-7-5p
 
 
-0.448
 
 
12.0
 
 
 
 
ATP2A2
 
 
hsa-let-7e-5p
 
 
-0.311
 
 
44.8
 
 
 
 
ATPAF1
 
 
hsa-let-7e-5p
 
 
-0.498
 
 
41.3
 
 
 
 
AVPI1
 
 
hsa-miR-7-5p
 
 
-0.367
 
 
13.2
 
 
 
 
AXL
 
 
hsa-miR-1-3p
 
 
-0.304
 
 
11.0
 
 
 
 
AZIN1
 
 
hsa-miR-1-3p
 
 
-0.438
 
 
61.2
 
 
 
 
B3GAT3
 
 
hsa-let-7e-5p
 
 
-0.324
 
 
16.1
 
 
 
 
B4GAT1
 
 
hsa-let-7e-5p
 
 
-0.47
 
 
15.2
 
 
 
 
BACH1
 
 
hsa-let-7e-5p
 
 
-0.298
 
 
15.8
 
 
 
 
BACH1
 
 
hsa-let-7e-5p
 
 
-0.298
 
 
15.8
 
 
 
 
BACH1
 
 
hsa-let-7e-5p
 
 
-0.435
 
 
15.8
 
 
 
 
BACH1
 
 
hsa-let-7e-5p
 
 
-0.435
 
 
15.8
 
 
 
 
BAZ2A
 
 
hsa-miR-100-5p
 
 
-0.29
 
 
23.9
 
 
 
 
BAZ2A
 
 
hsa-miR-100-5p
 
 
-0.421
 
 
23.9
 
 
 
 
BCAP29
 
 
hsa-let-7e-5p
 
 
-0.289
 
 
15.5
 
 
 
 
BET1
 
 
hsa-miR-1-3p
 
 
-0.413
 
 
20.6
 
 
 
 
BHLHE40
 
 
hsa-miR-130b-3p
 
 
-0.351
 
 
29.0
 
 
 
 
BLCAP
 
 
hsa-miR-130b-3p
 
 
-0.342
 
 
48.1
 
 
 
 
BLOC1S6
 
 
hsa-let-7e-5p
 
 
-0.321
 
 
19.0
 
 
 
 
BPNT1
 
 
hsa-miR-1-3p
 
 
-0.329
 
 
41.2
 
 
 
 
BRI3BP
 
 
hsa-miR-1-3p
 
 
-0.293
 
 
19.6
 
 
 
 
BRIX1
 
 
hsa-miR-1-3p
 
 
-0.334
 
 
11.7
 
 
 
 
BTAF1
 
 
hsa-miR-130b-3p
 
 
-0.291
 
 
12.8
 
 
 
 
BTF3L4
 
 
hsa-let-7e-5p
 
 
-0.355
 
 
13.6
 
 
 
 
BTF3L4
 
 
hsa-miR-130b-3p
 
 
-0.359
 
 
13.6
 
 
 
 
BZW1
 
 
hsa-let-7e-5p
 
 
-0.354
 
 
60.8
 
 
 
 
BZW1
 
 
hsa-let-7e-5p
 
 
-0.375
 
 
60.8
 
 
 
 
BZW2
 
 
hsa-let-7e-5p
 
 
-0.408
 
 
27.2
 
 
 
 
C15orf39
 
 
hsa-let-7e-5p
 
 
-0.374
 
 
10.8
 
 
 
 
C1GALT1
 
 
hsa-let-7e-5p
 
 
-0.322
 
 
27.7
 
 
 
 
C2orf88
 
 
hsa-let-7e-5p
 
 
-0.429
 
 
32.6
 
 
 
 
C6orf120
 
 
hsa-miR-1-3p
 
 
-0.288
 
 
13.9
 
 
 
 
CAAP1
 
 
hsa-miR-1-3p
 
 
-0.48
 
 
16.4
 
 
 
 
CALM2
 
 
hsa-miR-130b-3p
 
 
-0.584
 
 
516.7
 
 
 
 
CANT1
 
 
hsa-let-7e-5p
 
 
-0.402
 
 
48.8
 
 
 
 
CAPZA1
 
 
hsa-miR-7-5p
 
 
-0.351
 
 
63.9
 
 
 
 
CARNMT1
 
 
hsa-let-7e-5p
 
 
-0.307
 
 
12.0
 
 
 
 
CASP3
 
 
hsa-let-7e-5p
 
 
-0.495
 
 
47.5
 
 
 
 
CCDC43
 
 
hsa-miR-7-5p
 
 
-0.331
 
 
11.0
 
 
 
 
CCNC
 
 
hsa-miR-1-3p
 
 
-0.336
 
 
40.2
 
 
 
 
CCNL2
 
 
hsa-miR-28-5p
 
 
-0.448
 
 
33.4
 
 
 
 
CCNY
 
 
hsa-let-7e-5p
 
 
-0.334
 
 
20.2
 
 
 
 
CCNY
 
 
hsa-miR-130b-3p
 
 
-0.372
 
 
20.2
 
 
 
 
CCT4
 
 
hsa-miR-7-5p
 
 
-0.37
 
 
43.1
 
 
 
 
CCT6A
 
 
hsa-miR-130b-3p
 
 
-0.382
 
 
50.6
 
 
 
 
CD164
 
 
hsa-miR-1-3p
 
 
-0.315
 
 
237.3
 
 
 
 
CD2AP
 
 
hsa-miR-130b-3p
 
 
-0.302
 
 
53.6
 
 
 
 
CD2AP
 
 
hsa-miR-1-3p
 
 
-0.479
 
 
53.6
 
 
 
 
CDC34
 
 
hsa-let-7e-5p
 
 
-0.321
 
 
16.8
 
 
 
 
CDC34
 
 
hsa-let-7e-5p
 
 
-0.555
 
 
16.8
 
 
 
 
CDC42SE1
 
 
hsa-miR-1-3p
 
 
-0.36
 
 
18.8
 
 
 
 
CDK4
 
 
hsa-miR-1-3p
 
 
-0.338
 
 
37.8
 
 
 
 
CDKN1A
 
 
hsa-let-7e-5p
 
 
-0.434
 
 
108.4
 
 
 
 
CDKN2AIP
 
 
hsa-miR-28-5p
 
 
-0.419
 
 
17.9
 
 
 
 
CDV3
 
 
hsa-let-7e-5p
 
 
-0.453
 
 
53.7
 
 
 
 
CEBPD
 
 
hsa-let-7e-5p
 
 
-0.309
 
 
25.1
 
 
 
 
CEMIP2
 
 
hsa-let-7e-5p
 
 
-0.453
 
 
25.6
 
 
 
 
CERS2
 
 
hsa-miR-1-3p
 
 
-0.287
 
 
88.9
 
 
 
 
CGGBP1
 
 
hsa-miR-7-5p
 
 
-0.465
 
 
33.5
 
 
 
 
CHMP3
 
 
hsa-miR-130b-3p
 
 
-0.521
 
 
59.4
 
 
 
 
CHMP4B
 
 
hsa-miR-130b-3p
 
 
-0.292
 
 
96.9
 
 
 
 
CHUK
 
 
hsa-let-7e-5p
 
 
-0.327
 
 
12.1
 
 
 
 
CITED2
 
 
hsa-miR-1-3p
 
 
-0.37
 
 
34.5
 
 
 
 
CKAP4
 
 
hsa-miR-7-5p
 
 
-0.443
 
 
18.2
 
 
 
 
CLCN3
 
 
hsa-miR-1-3p
 
 
-0.339
 
 
23.4
 
 
 
 
CLCN3
 
 
hsa-miR-130b-3p
 
 
-0.384
 
 
23.4
 
 
 
 
 
 
 endometrium 
 
 
 
 
target_symbol
 
 
mature_mirna_id
 
 
score
 
 
nTPM
 
 
 
 
 
 
ABCB7
 
 
hsa-miR-1-3p
 
 
-0.365
 
 
10.5
 
 
 
 
ABHD2
 
 
hsa-miR-1-3p
 
 
-0.291
 
 
19.1
 
 
 
 
ACBD3
 
 
hsa-miR-130b-3p
 
 
-0.292
 
 
21.4
 
 
 
 
ACSL4
 
 
hsa-miR-130b-3p
 
 
-0.301
 
 
17.4
 
 
 
 
ACTC1
 
 
hsa-miR-7-5p
 
 
-0.471
 
 
17.7
 
 
 
 
ACVR1
 
 
hsa-miR-130b-3p
 
 
-0.44
 
 
15.9
 
 
 
 
ADAR
 
 
hsa-miR-1-3p
 
 
-0.35
 
 
46.2
 
 
 
 
ADGRA3
 
 
hsa-miR-1-3p
 
 
-0.351
 
 
14.1
 
 
 
 
AIFM1
 
 
hsa-let-7e-5p
 
 
-0.314
 
 
10.6
 
 
 
 
AKIRIN2
 
 
hsa-miR-130b-3p
 
 
-0.315
 
 
20.8
 
 
 
 
ALDH4A1
 
 
hsa-miR-184
 
 
-0.421
 
 
10.1
 
 
 
 
ALG11
 
 
hsa-miR-1-3p
 
 
-0.317
 
 
10.3
 
 
 
 
ALG9
 
 
hsa-miR-7-5p
 
 
-0.302
 
 
10.7
 
 
 
 
AMT
 
 
hsa-let-7e-5p
 
 
-0.463
 
 
10.0
 
 
 
 
ANKIB1
 
 
hsa-miR-130b-3p
 
 
-0.329
 
 
11.2
 
 
 
 
ANKRD12
 
 
hsa-miR-7-5p
 
 
-0.37
 
 
11.1
 
 
 
 
ANKRD29
 
 
hsa-miR-1-3p
 
 
-0.426
 
 
14.7
 
 
 
 
ANKRD46
 
 
hsa-let-7e-5p
 
 
-0.389
 
 
11.8
 
 
 
 
ANP32B
 
 
hsa-miR-1-3p
 
 
-0.287
 
 
111.3
 
 
 
 
ANXA2
 
 
hsa-miR-1-3p
 
 
-0.581
 
 
257.8
 
 
 
 
ANXA4
 
 
hsa-miR-1-3p
 
 
-0.539
 
 
47.7
 
 
 
 
AP1AR
 
 
hsa-miR-100-5p
 
 
-0.804
 
 
10.6
 
 
 
 
AP1S1
 
 
hsa-miR-1-3p
 
 
-0.355
 
 
16.1
 
 
 
 
AP1S1
 
 
hsa-let-7e-5p
 
 
-0.374
 
 
16.1
 
 
 
 
APCDD1
 
 
hsa-miR-130b-3p
 
 
-0.304
 
 
32.5
 
 
 
 
APPL1
 
 
hsa-miR-130b-3p
 
 
-0.347
 
 
16.0
 
 
 
 
ARCN1
 
 
hsa-miR-1-3p
 
 
-0.468
 
 
62.7
 
 
 
 
ARF4
 
 
hsa-miR-7-5p
 
 
-0.407
 
 
113.9
 
 
 
 
ARHGAP1
 
 
hsa-miR-130b-3p
 
 
-0.376
 
 
81.8
 
 
 
 
ARL4D
 
 
hsa-let-7e-5p
 
 
-0.417
 
 
19.2
 
 
 
 
ARL6IP1
 
 
hsa-miR-130b-3p
 
 
-0.289
 
 
66.4
 
 
 
 
ARL6IP1
 
 
hsa-miR-7-5p
 
 
-0.374
 
 
66.4
 
 
 
 
ARL6IP6
 
 
hsa-let-7e-5p
 
 
-0.305
 
 
11.0
 
 
 
 
ARMT1
 
 
hsa-let-7e-5p
 
 
-0.303
 
 
15.0
 
 
 
 
ARPC3
 
 
hsa-miR-1-3p
 
 
-0.606
 
 
142.0
 
 
 
 
ARPP19
 
 
hsa-let-7e-5p
 
 
-0.492
 
 
44.7
 
 
 
 
ARRDC4
 
 
hsa-let-7e-5p
 
 
-0.329
 
 
13.7
 
 
 
 
ASAP1
 
 
hsa-let-7e-5p
 
 
-0.296
 
 
17.2
 
 
 
 
ASH2L
 
 
hsa-miR-1-3p
 
 
-0.462
 
 
20.7
 
 
 
 
ATF2
 
 
hsa-miR-1-3p
 
 
-0.328
 
 
20.4
 
 
 
 
ATG16L1
 
 
hsa-miR-130b-3p
 
 
-0.407
 
 
11.3
 
 
 
 
ATP2A2
 
 
hsa-let-7e-5p
 
 
-0.311
 
 
43.7
 
 
 
 
ATPAF1
 
 
hsa-let-7e-5p
 
 
-0.498
 
 
23.6
 
 
 
 
AVPI1
 
 
hsa-miR-7-5p
 
 
-0.367
 
 
12.2
 
 
 
 
AXL
 
 
hsa-miR-1-3p
 
 
-0.304
 
 
31.2
 
 
 
 
AZIN1
 
 
hsa-miR-1-3p
 
 
-0.438
 
 
41.0
 
 
 
 
B3GAT3
 
 
hsa-let-7e-5p
 
 
-0.324
 
 
33.5
 
 
 
 
B4GAT1
 
 
hsa-let-7e-5p
 
 
-0.47
 
 
28.7
 
 
 
 
BACH1
 
 
hsa-let-7e-5p
 
 
-0.298
 
 
18.4
 
 
 
 
BACH1
 
 
hsa-let-7e-5p
 
 
-0.298
 
 
18.4
 
 
 
 
BACH1
 
 
hsa-let-7e-5p
 
 
-0.435
 
 
18.4
 
 
 
 
BACH1
 
 
hsa-let-7e-5p
 
 
-0.435
 
 
18.4
 
 
 
 
BAHD1
 
 
hsa-miR-130b-3p
 
 
-0.294
 
 
12.0
 
 
 
 
BAZ2A
 
 
hsa-miR-100-5p
 
 
-0.29
 
 
24.8
 
 
 
 
BAZ2A
 
 
hsa-miR-100-5p
 
 
-0.421
 
 
24.8
 
 
 
 
BCAP29
 
 
hsa-let-7e-5p
 
 
-0.289
 
 
41.4
 
 
 
 
BET1
 
 
hsa-miR-1-3p
 
 
-0.413
 
 
17.5
 
 
 
 
BHLHE40
 
 
hsa-miR-130b-3p
 
 
-0.351
 
 
63.3
 
 
 
 
BIN3
 
 
hsa-let-7e-5p
 
 
-0.427
 
 
11.7
 
 
 
 
BLCAP
 
 
hsa-miR-130b-3p
 
 
-0.342
 
 
32.3
 
 
 
 
BLOC1S4
 
 
hsa-miR-7-5p
 
 
-0.417
 
 
10.2
 
 
 
 
BLOC1S4
 
 
hsa-miR-7-5p
 
 
-0.527
 
 
10.2
 
 
 
 
BLOC1S6
 
 
hsa-let-7e-5p
 
 
-0.321
 
 
28.8
 
 
 
 
BMPR2
 
 
hsa-miR-130b-3p
 
 
-0.33
 
 
14.3
 
 
 
 
BMPR2
 
 
hsa-miR-100-5p
 
 
-0.355
 
 
14.3
 
 
 
 
BPNT1
 
 
hsa-miR-1-3p
 
 
-0.329
 
 
13.0
 
 
 
 
BRIX1
 
 
hsa-miR-1-3p
 
 
-0.334
 
 
16.3
 
 
 
 
BSCL2
 
 
hsa-miR-1-3p
 
 
-0.471
 
 
25.2
 
 
 
 
BTAF1
 
 
hsa-miR-130b-3p
 
 
-0.291
 
 
17.3
 
 
 
 
BTF3L4
 
 
hsa-let-7e-5p
 
 
-0.355
 
 
34.0
 
 
 
 
BTF3L4
 
 
hsa-miR-130b-3p
 
 
-0.359
 
 
34.0
 
 
 
 
BZW1
 
 
hsa-let-7e-5p
 
 
-0.354
 
 
62.3
 
 
 
 
BZW1
 
 
hsa-let-7e-5p
 
 
-0.375
 
 
62.3
 
 
 
 
BZW2
 
 
hsa-let-7e-5p
 
 
-0.408
 
 
18.0
 
 
 
 
C15orf39
 
 
hsa-let-7e-5p
 
 
-0.374
 
 
14.0
 
 
 
 
C2orf88
 
 
hsa-let-7e-5p
 
 
-0.429
 
 
14.8
 
 
 
 
CALM2
 
 
hsa-miR-130b-3p
 
 
-0.584
 
 
457.1
 
 
 
 
CAMSAP2
 
 
hsa-miR-130b-3p
 
 
-0.297
 
 
15.6
 
 
 
 
CANT1
 
 
hsa-let-7e-5p
 
 
-0.402
 
 
14.8
 
 
 
 
CAPN3
 
 
hsa-let-7e-5p
 
 
-0.317
 
 
12.3
 
 
 
 
CAPZA1
 
 
hsa-miR-7-5p
 
 
-0.351
 
 
40.3
 
 
 
 
CASD1
 
 
hsa-miR-130b-3p
 
 
-0.305
 
 
13.2
 
 
 
 
CASP3
 
 
hsa-let-7e-5p
 
 
-0.495
 
 
10.7
 
 
 
 
CCNC
 
 
hsa-miR-1-3p
 
 
-0.336
 
 
20.5
 
 
 
 
CCNL2
 
 
hsa-miR-28-5p
 
 
-0.448
 
 
72.8
 
 
 
 
CCNT2
 
 
hsa-miR-7-5p
 
 
-0.349
 
 
13.7
 
 
 
 
CCNY
 
 
hsa-let-7e-5p
 
 
-0.334
 
 
24.0
 
 
 
 
CCNY
 
 
hsa-miR-130b-3p
 
 
-0.372
 
 
24.0
 
 
 
 
CCT4
 
 
hsa-miR-7-5p
 
 
-0.37
 
 
67.4
 
 
 
 
CCT6A
 
 
hsa-miR-130b-3p
 
 
-0.382
 
 
51.7
 
 
 
 
CD164
 
 
hsa-miR-1-3p
 
 
-0.315
 
 
109.5
 
 
 
 
CD2AP
 
 
hsa-miR-130b-3p
 
 
-0.302
 
 
11.2
 
 
 
 
CD2AP
 
 
hsa-miR-1-3p
 
 
-0.479
 
 
11.2
 
 
 
 
CDC34
 
 
hsa-let-7e-5p
 
 
-0.321
 
 
35.3
 
 
 
 
CDC34
 
 
hsa-let-7e-5p
 
 
-0.555
 
 
35.3
 
 
 
 
CDC42SE1
 
 
hsa-miR-1-3p
 
 
-0.36
 
 
33.8
 
 
 
 
CDK4
 
 
hsa-miR-1-3p
 
 
-0.338
 
 
91.5
 
 
 
 
CDKN1A
 
 
hsa-let-7e-5p
 
 
-0.434
 
 
61.1
 
 
 
 
CDKN2AIP
 
 
hsa-miR-28-5p
 
 
-0.419
 
 
14.7
 
 
 
 
CDV3
 
 
hsa-let-7e-5p
 
 
-0.453
 
 
55.1
 
 
 
 
 
 
 esophagus 
 
 
 
 
target_symbol
 
 
mature_mirna_id
 
 
score
 
 
nTPM
 
 
 
 
 
 
ABCB7
 
 
hsa-miR-1-3p
 
 
-0.365
 
 
11.1
 
 
 
 
ABHD17C
 
 
hsa-let-7e-5p
 
 
-0.359
 
 
45.1
 
 
 
 
ABHD2
 
 
hsa-miR-1-3p
 
 
-0.291
 
 
16.3
 
 
 
 
ABHD3
 
 
hsa-miR-130b-3p
 
 
-0.356
 
 
17.3
 
 
 
 
ACBD3
 
 
hsa-miR-130b-3p
 
 
-0.292
 
 
29.0
 
 
 
 
ACBD5
 
 
hsa-miR-130b-3p
 
 
-0.494
 
 
11.3
 
 
 
 
ACP3
 
 
hsa-let-7e-5p
 
 
-0.298
 
 
42.9
 
 
 
 
ACSL4
 
 
hsa-miR-130b-3p
 
 
-0.301
 
 
14.6
 
 
 
 
ACTC1
 
 
hsa-miR-7-5p
 
 
-0.471
 
 
21.5
 
 
 
 
ADAR
 
 
hsa-miR-1-3p
 
 
-0.35
 
 
32.8
 
 
 
 
ADRB2
 
 
hsa-let-7e-5p
 
 
-0.635
 
 
14.2
 
 
 
 
AIFM1
 
 
hsa-let-7e-5p
 
 
-0.314
 
 
15.9
 
 
 
 
AKIRIN2
 
 
hsa-miR-130b-3p
 
 
-0.315
 
 
29.6
 
 
 
 
ALDH4A1
 
 
hsa-miR-184
 
 
-0.421
 
 
36.6
 
 
 
 
ALG9
 
 
hsa-miR-7-5p
 
 
-0.302
 
 
10.5
 
 
 
 
ANKIB1
 
 
hsa-miR-130b-3p
 
 
-0.329
 
 
11.1
 
 
 
 
ANKRD12
 
 
hsa-miR-7-5p
 
 
-0.37
 
 
13.6
 
 
 
 
ANKRD46
 
 
hsa-let-7e-5p
 
 
-0.389
 
 
11.9
 
 
 
 
ANP32B
 
 
hsa-miR-1-3p
 
 
-0.287
 
 
133.7
 
 
 
 
ANXA2
 
 
hsa-miR-1-3p
 
 
-0.581
 
 
1962.5
 
 
 
 
ANXA4
 
 
hsa-miR-1-3p
 
 
-0.539
 
 
40.8
 
 
 
 
AP1AR
 
 
hsa-miR-100-5p
 
 
-0.804
 
 
12.5
 
 
 
 
AP1S1
 
 
hsa-miR-1-3p
 
 
-0.355
 
 
36.5
 
 
 
 
AP1S1
 
 
hsa-let-7e-5p
 
 
-0.374
 
 
36.5
 
 
 
 
APCDD1
 
 
hsa-miR-130b-3p
 
 
-0.304
 
 
26.2
 
 
 
 
APPL1
 
 
hsa-miR-130b-3p
 
 
-0.347
 
 
15.9
 
 
 
 
ARCN1
 
 
hsa-miR-1-3p
 
 
-0.468
 
 
66.7
 
 
 
 
ARF4
 
 
hsa-miR-7-5p
 
 
-0.407
 
 
102.8
 
 
 
 
ARFGEF1
 
 
hsa-miR-130b-3p
 
 
-0.302
 
 
14.2
 
 
 
 
ARG2
 
 
hsa-let-7e-5p
 
 
-0.468
 
 
17.2
 
 
 
 
ARHGAP1
 
 
hsa-miR-130b-3p
 
 
-0.376
 
 
71.8
 
 
 
 
ARHGEF4
 
 
hsa-miR-130b-3p
 
 
-0.377
 
 
31.8
 
 
 
 
ARL4D
 
 
hsa-let-7e-5p
 
 
-0.417
 
 
80.8
 
 
 
 
ARL6IP1
 
 
hsa-miR-130b-3p
 
 
-0.289
 
 
65.8
 
 
 
 
ARL6IP1
 
 
hsa-miR-7-5p
 
 
-0.374
 
 
65.8
 
 
 
 
ARL6IP6
 
 
hsa-let-7e-5p
 
 
-0.305
 
 
11.7
 
 
 
 
ARMT1
 
 
hsa-let-7e-5p
 
 
-0.303
 
 
11.3
 
 
 
 
ARPC3
 
 
hsa-miR-1-3p
 
 
-0.606
 
 
381.2
 
 
 
 
ARPP19
 
 
hsa-let-7e-5p
 
 
-0.492
 
 
49.0
 
 
 
 
ASAP1
 
 
hsa-let-7e-5p
 
 
-0.296
 
 
10.0
 
 
 
 
ASH2L
 
 
hsa-miR-1-3p
 
 
-0.462
 
 
22.9
 
 
 
 
ATF2
 
 
hsa-miR-1-3p
 
 
-0.328
 
 
13.0
 
 
 
 
ATG16L1
 
 
hsa-miR-130b-3p
 
 
-0.407
 
 
11.5
 
 
 
 
ATOSB
 
 
hsa-let-7e-5p
 
 
-0.429
 
 
28.9
 
 
 
 
ATP2A2
 
 
hsa-let-7e-5p
 
 
-0.311
 
 
170.5
 
 
 
 
ATPAF1
 
 
hsa-let-7e-5p
 
 
-0.498
 
 
36.9
 
 
 
 
AVPI1
 
 
hsa-miR-7-5p
 
 
-0.367
 
 
26.7
 
 
 
 
AXL
 
 
hsa-miR-1-3p
 
 
-0.304
 
 
16.4
 
 
 
 
AZIN1
 
 
hsa-miR-1-3p
 
 
-0.438
 
 
48.8
 
 
 
 
B3GAT3
 
 
hsa-let-7e-5p
 
 
-0.324
 
 
23.2
 
 
 
 
BACH1
 
 
hsa-let-7e-5p
 
 
-0.298
 
 
21.6
 
 
 
 
BACH1
 
 
hsa-let-7e-5p
 
 
-0.298
 
 
21.6
 
 
 
 
BACH1
 
 
hsa-let-7e-5p
 
 
-0.435
 
 
21.6
 
 
 
 
BACH1
 
 
hsa-let-7e-5p
 
 
-0.435
 
 
21.6
 
 
 
 
BAHD1
 
 
hsa-miR-130b-3p
 
 
-0.294
 
 
11.4
 
 
 
 
BAZ2A
 
 
hsa-miR-100-5p
 
 
-0.29
 
 
29.0
 
 
 
 
BAZ2A
 
 
hsa-miR-100-5p
 
 
-0.421
 
 
29.0
 
 
 
 
BCAP29
 
 
hsa-let-7e-5p
 
 
-0.289
 
 
32.4
 
 
 
 
BET1
 
 
hsa-miR-1-3p
 
 
-0.413
 
 
11.5
 
 
 
 
BHLHE40
 
 
hsa-miR-130b-3p
 
 
-0.351
 
 
372.4
 
 
 
 
BIN3
 
 
hsa-let-7e-5p
 
 
-0.427
 
 
17.6
 
 
 
 
BLCAP
 
 
hsa-miR-130b-3p
 
 
-0.342
 
 
84.6
 
 
 
 
BLOC1S4
 
 
hsa-miR-7-5p
 
 
-0.417
 
 
11.4
 
 
 
 
BLOC1S4
 
 
hsa-miR-7-5p
 
 
-0.527
 
 
11.4
 
 
 
 
BLOC1S6
 
 
hsa-let-7e-5p
 
 
-0.321
 
 
19.8
 
 
 
 
BPNT1
 
 
hsa-miR-1-3p
 
 
-0.329
 
 
13.8
 
 
 
 
BRIX1
 
 
hsa-miR-1-3p
 
 
-0.334
 
 
19.8
 
 
 
 
BSCL2
 
 
hsa-miR-1-3p
 
 
-0.471
 
 
22.0
 
 
 
 
BTAF1
 
 
hsa-miR-130b-3p
 
 
-0.291
 
 
14.2
 
 
 
 
BTF3L4
 
 
hsa-let-7e-5p
 
 
-0.355
 
 
33.3
 
 
 
 
BTF3L4
 
 
hsa-miR-130b-3p
 
 
-0.359
 
 
33.3
 
 
 
 
BZW1
 
 
hsa-let-7e-5p
 
 
-0.354
 
 
113.4
 
 
 
 
BZW1
 
 
hsa-let-7e-5p
 
 
-0.375
 
 
113.4
 
 
 
 
BZW2
 
 
hsa-let-7e-5p
 
 
-0.408
 
 
40.0
 
 
 
 
C15orf39
 
 
hsa-let-7e-5p
 
 
-0.374
 
 
19.7
 
 
 
 
C19orf47
 
 
hsa-let-7e-5p
 
 
-0.311
 
 
11.8
 
 
 
 
C1GALT1
 
 
hsa-let-7e-5p
 
 
-0.322
 
 
22.4
 
 
 
 
C6orf120
 
 
hsa-miR-1-3p
 
 
-0.288
 
 
10.4
 
 
 
 
CAAP1
 
 
hsa-miR-1-3p
 
 
-0.48
 
 
10.6
 
 
 
 
CALM2
 
 
hsa-miR-130b-3p
 
 
-0.584
 
 
344.3
 
 
 
 
CAMSAP2
 
 
hsa-miR-130b-3p
 
 
-0.297
 
 
10.7
 
 
 
 
CANT1
 
 
hsa-let-7e-5p
 
 
-0.402
 
 
42.0
 
 
 
 
CAPN3
 
 
hsa-let-7e-5p
 
 
-0.317
 
 
15.1
 
 
 
 
CAPZA1
 
 
hsa-miR-7-5p
 
 
-0.351
 
 
119.9
 
 
 
 
CASP3
 
 
hsa-let-7e-5p
 
 
-0.495
 
 
25.0
 
 
 
 
CCDC43
 
 
hsa-miR-7-5p
 
 
-0.331
 
 
11.2
 
 
 
 
CCNC
 
 
hsa-miR-1-3p
 
 
-0.336
 
 
38.8
 
 
 
 
CCNL2
 
 
hsa-miR-28-5p
 
 
-0.448
 
 
53.8
 
 
 
 
CCNT2
 
 
hsa-miR-7-5p
 
 
-0.349
 
 
12.1
 
 
 
 
CCNY
 
 
hsa-let-7e-5p
 
 
-0.334
 
 
19.2
 
 
 
 
CCNY
 
 
hsa-miR-130b-3p
 
 
-0.372
 
 
19.2
 
 
 
 
CCT4
 
 
hsa-miR-7-5p
 
 
-0.37
 
 
75.8
 
 
 
 
CCT6A
 
 
hsa-miR-130b-3p
 
 
-0.382
 
 
69.7
 
 
 
 
CD164
 
 
hsa-miR-1-3p
 
 
-0.315
 
 
89.3
 
 
 
 
CD2AP
 
 
hsa-miR-130b-3p
 
 
-0.302
 
 
18.1
 
 
 
 
CD2AP
 
 
hsa-miR-1-3p
 
 
-0.479
 
 
18.1
 
 
 
 
CD69
 
 
hsa-miR-130b-3p
 
 
-0.451
 
 
11.2
 
 
 
 
CDC34
 
 
hsa-let-7e-5p
 
 
-0.321
 
 
42.6
 
 
 
 
CDC34
 
 
hsa-let-7e-5p
 
 
-0.555
 
 
42.6
 
 
 
 
CDC42SE1
 
 
hsa-miR-1-3p
 
 
-0.36
 
 
96.8
 
 
 
 
 
 
 fallopian tube 
 
 
 
 
target_symbol
 
 
mature_mirna_id
 
 
score
 
 
nTPM
 
 
 
 
 
 
ABCB7
 
 
hsa-miR-1-3p
 
 
-0.365
 
 
11.3
 
 
 
 
ABHD17C
 
 
hsa-let-7e-5p
 
 
-0.359
 
 
10.3
 
 
 
 
ABHD2
 
 
hsa-miR-1-3p
 
 
-0.291
 
 
22.1
 
 
 
 
ACBD3
 
 
hsa-miR-130b-3p
 
 
-0.292
 
 
16.8
 
 
 
 
ACBD5
 
 
hsa-miR-130b-3p
 
 
-0.494
 
 
11.3
 
 
 
 
ACSL4
 
 
hsa-miR-130b-3p
 
 
-0.301
 
 
12.4
 
 
 
 
ACVR1
 
 
hsa-miR-130b-3p
 
 
-0.44
 
 
13.2
 
 
 
 
ADAR
 
 
hsa-miR-1-3p
 
 
-0.35
 
 
47.3
 
 
 
 
ADGRA3
 
 
hsa-miR-1-3p
 
 
-0.351
 
 
20.3
 
 
 
 
ADPGK
 
 
hsa-miR-1-3p
 
 
-0.393
 
 
13.1
 
 
 
 
AIFM1
 
 
hsa-let-7e-5p
 
 
-0.314
 
 
20.8
 
 
 
 
AKIRIN2
 
 
hsa-miR-130b-3p
 
 
-0.315
 
 
18.4
 
 
 
 
ALG11
 
 
hsa-miR-1-3p
 
 
-0.317
 
 
10.1
 
 
 
 
AMT
 
 
hsa-let-7e-5p
 
 
-0.463
 
 
13.9
 
 
 
 
ANKRD29
 
 
hsa-miR-1-3p
 
 
-0.426
 
 
14.4
 
 
 
 
ANKRD46
 
 
hsa-let-7e-5p
 
 
-0.389
 
 
10.7
 
 
 
 
ANP32B
 
 
hsa-miR-1-3p
 
 
-0.287
 
 
117.2
 
 
 
 
ANXA2
 
 
hsa-miR-1-3p
 
 
-0.581
 
 
365.5
 
 
 
 
ANXA4
 
 
hsa-miR-1-3p
 
 
-0.539
 
 
64.5
 
 
 
 
AP1S1
 
 
hsa-miR-1-3p
 
 
-0.355
 
 
13.4
 
 
 
 
AP1S1
 
 
hsa-let-7e-5p
 
 
-0.374
 
 
13.4
 
 
 
 
APCDD1
 
 
hsa-miR-130b-3p
 
 
-0.304
 
 
30.6
 
 
 
 
APPL1
 
 
hsa-miR-130b-3p
 
 
-0.347
 
 
11.1
 
 
 
 
ARCN1
 
 
hsa-miR-1-3p
 
 
-0.468
 
 
56.3
 
 
 
 
ARF4
 
 
hsa-miR-7-5p
 
 
-0.407
 
 
79.6
 
 
 
 
ARFGEF1
 
 
hsa-miR-130b-3p
 
 
-0.302
 
 
11.1
 
 
 
 
ARHGAP1
 
 
hsa-miR-130b-3p
 
 
-0.376
 
 
71.5
 
 
 
 
ARL4D
 
 
hsa-let-7e-5p
 
 
-0.417
 
 
20.8
 
 
 
 
ARL6IP1
 
 
hsa-miR-130b-3p
 
 
-0.289
 
 
81.6
 
 
 
 
ARL6IP1
 
 
hsa-miR-7-5p
 
 
-0.374
 
 
81.6
 
 
 
 
ARL6IP6
 
 
hsa-let-7e-5p
 
 
-0.305
 
 
10.8
 
 
 
 
ARMT1
 
 
hsa-let-7e-5p
 
 
-0.303
 
 
11.9
 
 
 
 
ARPC3
 
 
hsa-miR-1-3p
 
 
-0.606
 
 
152.6
 
 
 
 
ARPP19
 
 
hsa-let-7e-5p
 
 
-0.492
 
 
31.4
 
 
 
 
ARRDC4
 
 
hsa-let-7e-5p
 
 
-0.329
 
 
13.7
 
 
 
 
ASAP1
 
 
hsa-let-7e-5p
 
 
-0.296
 
 
15.1
 
 
 
 
ASH2L
 
 
hsa-miR-1-3p
 
 
-0.462
 
 
19.1
 
 
 
 
ATF2
 
 
hsa-miR-1-3p
 
 
-0.328
 
 
17.0
 
 
 
 
ATP2A2
 
 
hsa-let-7e-5p
 
 
-0.311
 
 
63.0
 
 
 
 
ATPAF1
 
 
hsa-let-7e-5p
 
 
-0.498
 
 
27.2
 
 
 
 
AVPI1
 
 
hsa-miR-7-5p
 
 
-0.367
 
 
21.5
 
 
 
 
AXL
 
 
hsa-miR-1-3p
 
 
-0.304
 
 
23.6
 
 
 
 
AZIN1
 
 
hsa-miR-1-3p
 
 
-0.438
 
 
59.5
 
 
 
 
B3GAT3
 
 
hsa-let-7e-5p
 
 
-0.324
 
 
34.0
 
 
 
 
B4GAT1
 
 
hsa-let-7e-5p
 
 
-0.47
 
 
25.4
 
 
 
 
BAHD1
 
 
hsa-miR-130b-3p
 
 
-0.294
 
 
13.6
 
 
 
 
BAZ2A
 
 
hsa-miR-100-5p
 
 
-0.29
 
 
23.4
 
 
 
 
BAZ2A
 
 
hsa-miR-100-5p
 
 
-0.421
 
 
23.4
 
 
 
 
BCAP29
 
 
hsa-let-7e-5p
 
 
-0.289
 
 
17.1
 
 
 
 
BET1
 
 
hsa-miR-1-3p
 
 
-0.413
 
 
17.4
 
 
 
 
BHLHE40
 
 
hsa-miR-130b-3p
 
 
-0.351
 
 
77.8
 
 
 
 
BIN3
 
 
hsa-let-7e-5p
 
 
-0.427
 
 
14.8
 
 
 
 
BLCAP
 
 
hsa-miR-130b-3p
 
 
-0.342
 
 
132.7
 
 
 
 
BLOC1S6
 
 
hsa-let-7e-5p
 
 
-0.321
 
 
17.1
 
 
 
 
BPNT1
 
 
hsa-miR-1-3p
 
 
-0.329
 
 
16.6
 
 
 
 
BRIX1
 
 
hsa-miR-1-3p
 
 
-0.334
 
 
15.1
 
 
 
 
BSCL2
 
 
hsa-miR-1-3p
 
 
-0.471
 
 
58.9
 
 
 
 
BTAF1
 
 
hsa-miR-130b-3p
 
 
-0.291
 
 
20.1
 
 
 
 
BTF3L4
 
 
hsa-let-7e-5p
 
 
-0.355
 
 
34.3
 
 
 
 
BTF3L4
 
 
hsa-miR-130b-3p
 
 
-0.359
 
 
34.3
 
 
 
 
BZW1
 
 
hsa-let-7e-5p
 
 
-0.354
 
 
50.2
 
 
 
 
BZW1
 
 
hsa-let-7e-5p
 
 
-0.375
 
 
50.2
 
 
 
 
BZW2
 
 
hsa-let-7e-5p
 
 
-0.408
 
 
19.0
 
 
 
 
C15orf39
 
 
hsa-let-7e-5p
 
 
-0.374
 
 
15.2
 
 
 
 
C1GALT1
 
 
hsa-let-7e-5p
 
 
-0.322
 
 
15.3
 
 
 
 
C2orf88
 
 
hsa-let-7e-5p
 
 
-0.429
 
 
19.7
 
 
 
 
CALM2
 
 
hsa-miR-130b-3p
 
 
-0.584
 
 
495.4
 
 
 
 
CANT1
 
 
hsa-let-7e-5p
 
 
-0.402
 
 
17.1
 
 
 
 
CAPN3
 
 
hsa-let-7e-5p
 
 
-0.317
 
 
15.7
 
 
 
 
CAPZA1
 
 
hsa-miR-7-5p
 
 
-0.351
 
 
40.4
 
 
 
 
CASD1
 
 
hsa-miR-130b-3p
 
 
-0.305
 
 
10.3
 
 
 
 
CBX5
 
 
hsa-let-7e-5p
 
 
-0.307
 
 
15.0
 
 
 
 
CBX5
 
 
hsa-let-7e-5p
 
 
-0.472
 
 
15.0
 
 
 
 
CCDC113
 
 
hsa-miR-7-5p
 
 
-0.324
 
 
29.4
 
 
 
 
CCNC
 
 
hsa-miR-1-3p
 
 
-0.336
 
 
17.8
 
 
 
 
CCNL2
 
 
hsa-miR-28-5p
 
 
-0.448
 
 
70.5
 
 
 
 
CCNT2
 
 
hsa-miR-7-5p
 
 
-0.349
 
 
11.6
 
 
 
 
CCNY
 
 
hsa-let-7e-5p
 
 
-0.334
 
 
20.9
 
 
 
 
CCNY
 
 
hsa-miR-130b-3p
 
 
-0.372
 
 
20.9
 
 
 
 
CCT4
 
 
hsa-miR-7-5p
 
 
-0.37
 
 
70.0
 
 
 
 
CCT6A
 
 
hsa-miR-130b-3p
 
 
-0.382
 
 
48.4
 
 
 
 
CD164
 
 
hsa-miR-1-3p
 
 
-0.315
 
 
117.4
 
 
 
 
CD2AP
 
 
hsa-miR-130b-3p
 
 
-0.302
 
 
13.3
 
 
 
 
CD2AP
 
 
hsa-miR-1-3p
 
 
-0.479
 
 
13.3
 
 
 
 
CDC34
 
 
hsa-let-7e-5p
 
 
-0.321
 
 
38.1
 
 
 
 
CDC34
 
 
hsa-let-7e-5p
 
 
-0.555
 
 
38.1
 
 
 
 
CDC42SE1
 
 
hsa-miR-1-3p
 
 
-0.36
 
 
43.0
 
 
 
 
CDK14
 
 
hsa-miR-1-3p
 
 
-0.326
 
 
11.5
 
 
 
 
CDK4
 
 
hsa-miR-1-3p
 
 
-0.338
 
 
70.8
 
 
 
 
CDKN1A
 
 
hsa-let-7e-5p
 
 
-0.434
 
 
168.3
 
 
 
 
CDKN2AIP
 
 
hsa-miR-28-5p
 
 
-0.419
 
 
11.1
 
 
 
 
CDV3
 
 
hsa-let-7e-5p
 
 
-0.453
 
 
41.3
 
 
 
 
CEBPD
 
 
hsa-let-7e-5p
 
 
-0.309
 
 
257.4
 
 
 
 
CERCAM
 
 
hsa-let-7e-5p
 
 
-0.344
 
 
29.3
 
 
 
 
CERS2
 
 
hsa-miR-1-3p
 
 
-0.287
 
 
88.4
 
 
 
 
CGGBP1
 
 
hsa-miR-7-5p
 
 
-0.465
 
 
31.5
 
 
 
 
CHMP3
 
 
hsa-miR-130b-3p
 
 
-0.521
 
 
48.8
 
 
 
 
CHMP4B
 
 
hsa-miR-130b-3p
 
 
-0.292
 
 
83.0
 
 
 
 
CHRD
 
 
hsa-let-7e-5p
 
 
-0.303
 
 
14.1
 
 
 
 
CITED2
 
 
hsa-miR-1-3p
 
 
-0.37
 
 
133.3
 
 
 
 
 
 
 heart muscle 
 
 
 
 
target_symbol
 
 
mature_mirna_id
 
 
score
 
 
nTPM
 
 
 
 
 
 
ABCB7
 
 
hsa-miR-1-3p
 
 
-0.365
 
 
20.0
 
 
 
 
ABHD2
 
 
hsa-miR-1-3p
 
 
-0.291
 
 
33.8
 
 
 
 
ACBD3
 
 
hsa-miR-130b-3p
 
 
-0.292
 
 
20.1
 
 
 
 
ACBD5
 
 
hsa-miR-130b-3p
 
 
-0.494
 
 
37.7
 
 
 
 
ACSL4
 
 
hsa-miR-130b-3p
 
 
-0.301
 
 
26.9
 
 
 
 
ACTC1
 
 
hsa-miR-7-5p
 
 
-0.471
 
 
10840.1
 
 
 
 
ACVR1
 
 
hsa-miR-130b-3p
 
 
-0.44
 
 
14.3
 
 
 
 
ADAR
 
 
hsa-miR-1-3p
 
 
-0.35
 
 
27.6
 
 
 
 
ADGRA3
 
 
hsa-miR-1-3p
 
 
-0.351
 
 
10.1
 
 
 
 
ADRB1
 
 
hsa-let-7e-5p
 
 
-0.367
 
 
20.3
 
 
 
 
AIFM1
 
 
hsa-let-7e-5p
 
 
-0.314
 
 
74.9
 
 
 
 
AKAP6
 
 
hsa-let-7e-5p
 
 
-0.307
 
 
51.1
 
 
 
 
AKIRIN2
 
 
hsa-miR-130b-3p
 
 
-0.315
 
 
27.5
 
 
 
 
ALDH4A1
 
 
hsa-miR-184
 
 
-0.421
 
 
30.9
 
 
 
 
AMT
 
 
hsa-let-7e-5p
 
 
-0.463
 
 
23.5
 
 
 
 
ANKRD12
 
 
hsa-miR-7-5p
 
 
-0.37
 
 
11.1
 
 
 
 
ANKRD46
 
 
hsa-let-7e-5p
 
 
-0.389
 
 
18.8
 
 
 
 
ANP32B
 
 
hsa-miR-1-3p
 
 
-0.287
 
 
87.4
 
 
 
 
ANXA2
 
 
hsa-miR-1-3p
 
 
-0.581
 
 
347.2
 
 
 
 
ANXA4
 
 
hsa-miR-1-3p
 
 
-0.539
 
 
25.4
 
 
 
 
AP1S1
 
 
hsa-miR-1-3p
 
 
-0.355
 
 
12.9
 
 
 
 
AP1S1
 
 
hsa-let-7e-5p
 
 
-0.374
 
 
12.9
 
 
 
 
APCDD1
 
 
hsa-miR-130b-3p
 
 
-0.304
 
 
30.9
 
 
 
 
APPL1
 
 
hsa-miR-130b-3p
 
 
-0.347
 
 
23.1
 
 
 
 
ARCN1
 
 
hsa-miR-1-3p
 
 
-0.468
 
 
68.9
 
 
 
 
ARF4
 
 
hsa-miR-7-5p
 
 
-0.407
 
 
106.8
 
 
 
 
ARHGAP1
 
 
hsa-miR-130b-3p
 
 
-0.376
 
 
105.1
 
 
 
 
ARHGAP12
 
 
hsa-miR-130b-3p
 
 
-0.287
 
 
11.1
 
 
 
 
ARHGAP24
 
 
hsa-miR-130b-3p
 
 
-0.287
 
 
25.6
 
 
 
 
ARL4D
 
 
hsa-let-7e-5p
 
 
-0.417
 
 
12.5
 
 
 
 
ARL6IP1
 
 
hsa-miR-130b-3p
 
 
-0.289
 
 
30.1
 
 
 
 
ARL6IP1
 
 
hsa-miR-7-5p
 
 
-0.374
 
 
30.1
 
 
 
 
ARMT1
 
 
hsa-let-7e-5p
 
 
-0.303
 
 
15.7
 
 
 
 
ARPC3
 
 
hsa-miR-1-3p
 
 
-0.606
 
 
143.2
 
 
 
 
ARPP19
 
 
hsa-let-7e-5p
 
 
-0.492
 
 
76.2
 
 
 
 
ARRDC4
 
 
hsa-let-7e-5p
 
 
-0.329
 
 
11.3
 
 
 
 
AS3MT
 
 
hsa-miR-28-5p
 
 
-0.479
 
 
24.0
 
 
 
 
ASAP1
 
 
hsa-let-7e-5p
 
 
-0.296
 
 
22.3
 
 
 
 
ASH2L
 
 
hsa-miR-1-3p
 
 
-0.462
 
 
20.0
 
 
 
 
ATF2
 
 
hsa-miR-1-3p
 
 
-0.328
 
 
11.3
 
 
 
 
ATG16L1
 
 
hsa-miR-130b-3p
 
 
-0.407
 
 
10.8
 
 
 
 
ATG4A
 
 
hsa-miR-7-5p
 
 
-0.448
 
 
11.7
 
 
 
 
ATOSB
 
 
hsa-let-7e-5p
 
 
-0.429
 
 
18.9
 
 
 
 
ATP2A2
 
 
hsa-let-7e-5p
 
 
-0.311
 
 
941.9
 
 
 
 
ATPAF1
 
 
hsa-let-7e-5p
 
 
-0.498
 
 
249.4
 
 
 
 
AVPI1
 
 
hsa-miR-7-5p
 
 
-0.367
 
 
22.9
 
 
 
 
AXL
 
 
hsa-miR-1-3p
 
 
-0.304
 
 
25.8
 
 
 
 
AZIN1
 
 
hsa-miR-1-3p
 
 
-0.438
 
 
47.7
 
 
 
 
B3GAT3
 
 
hsa-let-7e-5p
 
 
-0.324
 
 
25.1
 
 
 
 
B4GAT1
 
 
hsa-let-7e-5p
 
 
-0.47
 
 
43.6
 
 
 
 
BACH1
 
 
hsa-let-7e-5p
 
 
-0.298
 
 
12.3
 
 
 
 
BACH1
 
 
hsa-let-7e-5p
 
 
-0.298
 
 
12.3
 
 
 
 
BACH1
 
 
hsa-let-7e-5p
 
 
-0.435
 
 
12.3
 
 
 
 
BACH1
 
 
hsa-let-7e-5p
 
 
-0.435
 
 
12.3
 
 
 
 
BAHD1
 
 
hsa-miR-130b-3p
 
 
-0.294
 
 
13.7
 
 
 
 
BAZ2A
 
 
hsa-miR-100-5p
 
 
-0.29
 
 
19.8
 
 
 
 
BAZ2A
 
 
hsa-miR-100-5p
 
 
-0.421
 
 
19.8
 
 
 
 
BCAP29
 
 
hsa-let-7e-5p
 
 
-0.289
 
 
57.5
 
 
 
 
BET1
 
 
hsa-miR-1-3p
 
 
-0.413
 
 
13.3
 
 
 
 
BHLHE40
 
 
hsa-miR-130b-3p
 
 
-0.351
 
 
166.2
 
 
 
 
BIN3
 
 
hsa-let-7e-5p
 
 
-0.427
 
 
13.1
 
 
 
 
BLCAP
 
 
hsa-miR-130b-3p
 
 
-0.342
 
 
73.6
 
 
 
 
BLOC1S6
 
 
hsa-let-7e-5p
 
 
-0.321
 
 
23.5
 
 
 
 
BMPR2
 
 
hsa-miR-130b-3p
 
 
-0.33
 
 
16.2
 
 
 
 
BMPR2
 
 
hsa-miR-100-5p
 
 
-0.355
 
 
16.2
 
 
 
 
BPNT1
 
 
hsa-miR-1-3p
 
 
-0.329
 
 
16.5
 
 
 
 
BRIX1
 
 
hsa-miR-1-3p
 
 
-0.334
 
 
13.6
 
 
 
 
BSCL2
 
 
hsa-miR-1-3p
 
 
-0.471
 
 
16.4
 
 
 
 
BTAF1
 
 
hsa-miR-130b-3p
 
 
-0.291
 
 
11.8
 
 
 
 
BTF3L4
 
 
hsa-let-7e-5p
 
 
-0.355
 
 
37.6
 
 
 
 
BTF3L4
 
 
hsa-miR-130b-3p
 
 
-0.359
 
 
37.6
 
 
 
 
BZW1
 
 
hsa-let-7e-5p
 
 
-0.354
 
 
53.4
 
 
 
 
BZW1
 
 
hsa-let-7e-5p
 
 
-0.375
 
 
53.4
 
 
 
 
BZW2
 
 
hsa-let-7e-5p
 
 
-0.408
 
 
248.4
 
 
 
 
C15orf39
 
 
hsa-let-7e-5p
 
 
-0.374
 
 
13.8
 
 
 
 
C19orf47
 
 
hsa-let-7e-5p
 
 
-0.311
 
 
164.2
 
 
 
 
C1GALT1
 
 
hsa-let-7e-5p
 
 
-0.322
 
 
11.8
 
 
 
 
C2orf88
 
 
hsa-let-7e-5p
 
 
-0.429
 
 
11.0
 
 
 
 
CACFD1
 
 
hsa-let-7e-5p
 
 
-0.296
 
 
10.5
 
 
 
 
CALM2
 
 
hsa-miR-130b-3p
 
 
-0.584
 
 
433.6
 
 
 
 
CAMSAP2
 
 
hsa-miR-130b-3p
 
 
-0.297
 
 
10.6
 
 
 
 
CANT1
 
 
hsa-let-7e-5p
 
 
-0.402
 
 
15.5
 
 
 
 
CAPN3
 
 
hsa-let-7e-5p
 
 
-0.317
 
 
16.9
 
 
 
 
CAPZA1
 
 
hsa-miR-7-5p
 
 
-0.351
 
 
31.7
 
 
 
 
CASD1
 
 
hsa-miR-130b-3p
 
 
-0.305
 
 
16.0
 
 
 
 
CASP3
 
 
hsa-let-7e-5p
 
 
-0.495
 
 
12.7
 
 
 
 
CBFA2T3
 
 
hsa-let-7e-5p
 
 
-0.305
 
 
16.4
 
 
 
 
CBX5
 
 
hsa-let-7e-5p
 
 
-0.307
 
 
18.9
 
 
 
 
CBX5
 
 
hsa-let-7e-5p
 
 
-0.472
 
 
18.9
 
 
 
 
CCDC43
 
 
hsa-miR-7-5p
 
 
-0.331
 
 
19.1
 
 
 
 
CCNC
 
 
hsa-miR-1-3p
 
 
-0.336
 
 
22.1
 
 
 
 
CCNL2
 
 
hsa-miR-28-5p
 
 
-0.448
 
 
45.6
 
 
 
 
CCNT2
 
 
hsa-miR-7-5p
 
 
-0.349
 
 
11.0
 
 
 
 
CCNY
 
 
hsa-let-7e-5p
 
 
-0.334
 
 
28.5
 
 
 
 
CCNY
 
 
hsa-miR-130b-3p
 
 
-0.372
 
 
28.5
 
 
 
 
CCT4
 
 
hsa-miR-7-5p
 
 
-0.37
 
 
65.0
 
 
 
 
CCT6A
 
 
hsa-miR-130b-3p
 
 
-0.382
 
 
56.1
 
 
 
 
CD164
 
 
hsa-miR-1-3p
 
 
-0.315
 
 
85.9
 
 
 
 
CDC34
 
 
hsa-let-7e-5p
 
 
-0.321
 
 
81.4
 
 
 
 
CDC34
 
 
hsa-let-7e-5p
 
 
-0.555
 
 
81.4
 
 
 
 
 
 
 liver 
 
 
 
 
target_symbol
 
 
mature_mirna_id
 
 
score
 
 
nTPM
 
 
 
 
 
 
ABCB7
 
 
hsa-miR-1-3p
 
 
-0.365
 
 
14.1
 
 
 
 
ABHD2
 
 
hsa-miR-1-3p
 
 
-0.291
 
 
128.7
 
 
 
 
ABHD3
 
 
hsa-miR-130b-3p
 
 
-0.356
 
 
36.7
 
 
 
 
ABRAXAS2
 
 
hsa-miR-130b-3p
 
 
-0.468
 
 
12.8
 
 
 
 
ACBD3
 
 
hsa-miR-130b-3p
 
 
-0.292
 
 
22.7
 
 
 
 
ACBD5
 
 
hsa-miR-130b-3p
 
 
-0.494
 
 
69.0
 
 
 
 
ACSL4
 
 
hsa-miR-130b-3p
 
 
-0.301
 
 
12.3
 
 
 
 
ACVR1
 
 
hsa-miR-130b-3p
 
 
-0.44
 
 
15.3
 
 
 
 
ADAR
 
 
hsa-miR-1-3p
 
 
-0.35
 
 
52.5
 
 
 
 
ADGRA3
 
 
hsa-miR-1-3p
 
 
-0.351
 
 
54.5
 
 
 
 
ADPGK
 
 
hsa-miR-1-3p
 
 
-0.393
 
 
14.1
 
 
 
 
ADRB2
 
 
hsa-let-7e-5p
 
 
-0.635
 
 
13.3
 
 
 
 
AHCTF1
 
 
hsa-let-7e-5p
 
 
-0.374
 
 
10.2
 
 
 
 
AIFM1
 
 
hsa-let-7e-5p
 
 
-0.314
 
 
77.0
 
 
 
 
AKIRIN2
 
 
hsa-miR-130b-3p
 
 
-0.315
 
 
29.2
 
 
 
 
ALDH4A1
 
 
hsa-miR-184
 
 
-0.421
 
 
438.6
 
 
 
 
ALG11
 
 
hsa-miR-1-3p
 
 
-0.317
 
 
17.9
 
 
 
 
ALG9
 
 
hsa-miR-7-5p
 
 
-0.302
 
 
12.5
 
 
 
 
AMT
 
 
hsa-let-7e-5p
 
 
-0.463
 
 
52.5
 
 
 
 
ANKRD12
 
 
hsa-miR-7-5p
 
 
-0.37
 
 
12.9
 
 
 
 
ANKRD46
 
 
hsa-let-7e-5p
 
 
-0.389
 
 
38.9
 
 
 
 
ANP32B
 
 
hsa-miR-1-3p
 
 
-0.287
 
 
96.8
 
 
 
 
ANXA2
 
 
hsa-miR-1-3p
 
 
-0.581
 
 
173.2
 
 
 
 
ANXA4
 
 
hsa-miR-1-3p
 
 
-0.539
 
 
113.0
 
 
 
 
AP1AR
 
 
hsa-miR-100-5p
 
 
-0.804
 
 
19.1
 
 
 
 
AP1S1
 
 
hsa-miR-1-3p
 
 
-0.355
 
 
39.1
 
 
 
 
AP1S1
 
 
hsa-let-7e-5p
 
 
-0.374
 
 
39.1
 
 
 
 
APPL1
 
 
hsa-miR-130b-3p
 
 
-0.347
 
 
12.0
 
 
 
 
ARCN1
 
 
hsa-miR-1-3p
 
 
-0.468
 
 
83.4
 
 
 
 
ARF4
 
 
hsa-miR-7-5p
 
 
-0.407
 
 
161.3
 
 
 
 
ARFGEF1
 
 
hsa-miR-130b-3p
 
 
-0.302
 
 
14.2
 
 
 
 
ARHGAP1
 
 
hsa-miR-130b-3p
 
 
-0.376
 
 
44.2
 
 
 
 
ARHGAP12
 
 
hsa-miR-130b-3p
 
 
-0.287
 
 
15.3
 
 
 
 
ARID4A
 
 
hsa-miR-7-5p
 
 
-0.341
 
 
17.5
 
 
 
 
ARL4D
 
 
hsa-let-7e-5p
 
 
-0.417
 
 
55.4
 
 
 
 
ARL6IP1
 
 
hsa-miR-130b-3p
 
 
-0.289
 
 
336.2
 
 
 
 
ARL6IP1
 
 
hsa-miR-7-5p
 
 
-0.374
 
 
336.2
 
 
 
 
ARMT1
 
 
hsa-let-7e-5p
 
 
-0.303
 
 
35.2
 
 
 
 
ARPC3
 
 
hsa-miR-1-3p
 
 
-0.606
 
 
245.0
 
 
 
 
ARPP19
 
 
hsa-let-7e-5p
 
 
-0.492
 
 
39.5
 
 
 
 
ARRDC4
 
 
hsa-let-7e-5p
 
 
-0.329
 
 
28.3
 
 
 
 
AS3MT
 
 
hsa-miR-28-5p
 
 
-0.479
 
 
44.3
 
 
 
 
ASH2L
 
 
hsa-miR-1-3p
 
 
-0.462
 
 
22.2
 
 
 
 
ATF2
 
 
hsa-miR-1-3p
 
 
-0.328
 
 
24.7
 
 
 
 
ATG16L1
 
 
hsa-miR-130b-3p
 
 
-0.407
 
 
14.9
 
 
 
 
ATG4A
 
 
hsa-miR-7-5p
 
 
-0.448
 
 
16.9
 
 
 
 
ATOSB
 
 
hsa-let-7e-5p
 
 
-0.429
 
 
10.9
 
 
 
 
ATP2A2
 
 
hsa-let-7e-5p
 
 
-0.311
 
 
49.3
 
 
 
 
ATP2B2
 
 
hsa-miR-7-5p
 
 
-0.297
 
 
30.4
 
 
 
 
ATP2B2
 
 
hsa-miR-130b-3p
 
 
-0.314
 
 
30.4
 
 
 
 
ATPAF1
 
 
hsa-let-7e-5p
 
 
-0.498
 
 
101.4
 
 
 
 
AVPI1
 
 
hsa-miR-7-5p
 
 
-0.367
 
 
47.4
 
 
 
 
AXL
 
 
hsa-miR-1-3p
 
 
-0.304
 
 
14.0
 
 
 
 
AZIN1
 
 
hsa-miR-1-3p
 
 
-0.438
 
 
71.0
 
 
 
 
B3GAT3
 
 
hsa-let-7e-5p
 
 
-0.324
 
 
53.3
 
 
 
 
B4GAT1
 
 
hsa-let-7e-5p
 
 
-0.47
 
 
18.5
 
 
 
 
BACH1
 
 
hsa-let-7e-5p
 
 
-0.298
 
 
28.5
 
 
 
 
BACH1
 
 
hsa-let-7e-5p
 
 
-0.298
 
 
28.5
 
 
 
 
BACH1
 
 
hsa-let-7e-5p
 
 
-0.435
 
 
28.5
 
 
 
 
BACH1
 
 
hsa-let-7e-5p
 
 
-0.435
 
 
28.5
 
 
 
 
BAG4
 
 
hsa-miR-1-3p
 
 
-0.378
 
 
15.7
 
 
 
 
BAHD1
 
 
hsa-miR-130b-3p
 
 
-0.294
 
 
11.0
 
 
 
 
BAZ2A
 
 
hsa-miR-100-5p
 
 
-0.29
 
 
20.5
 
 
 
 
BAZ2A
 
 
hsa-miR-100-5p
 
 
-0.421
 
 
20.5
 
 
 
 
BCAP29
 
 
hsa-let-7e-5p
 
 
-0.289
 
 
31.8
 
 
 
 
BET1
 
 
hsa-miR-1-3p
 
 
-0.413
 
 
29.8
 
 
 
 
BHLHE40
 
 
hsa-miR-130b-3p
 
 
-0.351
 
 
194.1
 
 
 
 
BIN3
 
 
hsa-let-7e-5p
 
 
-0.427
 
 
14.7
 
 
 
 
BLCAP
 
 
hsa-miR-130b-3p
 
 
-0.342
 
 
99.5
 
 
 
 
BLOC1S4
 
 
hsa-miR-7-5p
 
 
-0.417
 
 
11.6
 
 
 
 
BLOC1S4
 
 
hsa-miR-7-5p
 
 
-0.527
 
 
11.6
 
 
 
 
BLOC1S6
 
 
hsa-let-7e-5p
 
 
-0.321
 
 
25.1
 
 
 
 
BLTP3B
 
 
hsa-miR-1-3p
 
 
-0.29
 
 
23.0
 
 
 
 
BMPR2
 
 
hsa-miR-130b-3p
 
 
-0.33
 
 
12.0
 
 
 
 
BMPR2
 
 
hsa-miR-100-5p
 
 
-0.355
 
 
12.0
 
 
 
 
BPNT1
 
 
hsa-miR-1-3p
 
 
-0.329
 
 
23.3
 
 
 
 
BRIX1
 
 
hsa-miR-1-3p
 
 
-0.334
 
 
17.2
 
 
 
 
BSCL2
 
 
hsa-miR-1-3p
 
 
-0.471
 
 
34.2
 
 
 
 
BTAF1
 
 
hsa-miR-130b-3p
 
 
-0.291
 
 
12.8
 
 
 
 
BTF3L4
 
 
hsa-let-7e-5p
 
 
-0.355
 
 
38.1
 
 
 
 
BTF3L4
 
 
hsa-miR-130b-3p
 
 
-0.359
 
 
38.1
 
 
 
 
BZW1
 
 
hsa-let-7e-5p
 
 
-0.354
 
 
212.2
 
 
 
 
BZW1
 
 
hsa-let-7e-5p
 
 
-0.375
 
 
212.2
 
 
 
 
BZW2
 
 
hsa-let-7e-5p
 
 
-0.408
 
 
17.2
 
 
 
 
C1GALT1
 
 
hsa-let-7e-5p
 
 
-0.322
 
 
20.5
 
 
 
 
C6orf120
 
 
hsa-miR-1-3p
 
 
-0.288
 
 
23.1
 
 
 
 
CAAP1
 
 
hsa-miR-1-3p
 
 
-0.48
 
 
22.3
 
 
 
 
CACFD1
 
 
hsa-let-7e-5p
 
 
-0.296
 
 
56.6
 
 
 
 
CALM2
 
 
hsa-miR-130b-3p
 
 
-0.584
 
 
460.6
 
 
 
 
CANT1
 
 
hsa-let-7e-5p
 
 
-0.402
 
 
16.0
 
 
 
 
CAPN3
 
 
hsa-let-7e-5p
 
 
-0.317
 
 
41.4
 
 
 
 
CAPZA1
 
 
hsa-miR-7-5p
 
 
-0.351
 
 
68.4
 
 
 
 
CARNMT1
 
 
hsa-let-7e-5p
 
 
-0.307
 
 
16.0
 
 
 
 
CASP3
 
 
hsa-let-7e-5p
 
 
-0.495
 
 
19.8
 
 
 
 
CCDC126
 
 
hsa-miR-130b-3p
 
 
-0.591
 
 
10.9
 
 
 
 
CCDC71L
 
 
hsa-let-7e-5p
 
 
-0.432
 
 
14.3
 
 
 
 
CCNC
 
 
hsa-miR-1-3p
 
 
-0.336
 
 
69.7
 
 
 
 
CCNL2
 
 
hsa-miR-28-5p
 
 
-0.448
 
 
76.1
 
 
 
 
CCNT2
 
 
hsa-miR-7-5p
 
 
-0.349
 
 
17.0
 
 
 
 
CCNY
 
 
hsa-let-7e-5p
 
 
-0.334
 
 
29.0
 
 
 
 
 
 
 lymph node 
 
 
 
 
target_symbol
 
 
mature_mirna_id
 
 
score
 
 
nTPM
 
 
 
 
 
 
ABCB7
 
 
hsa-miR-1-3p
 
 
-0.365
 
 
11.2
 
 
 
 
ABHD2
 
 
hsa-miR-1-3p
 
 
-0.291
 
 
13.7
 
 
 
 
ABHD3
 
 
hsa-miR-130b-3p
 
 
-0.356
 
 
27.9
 
 
 
 
ABRAXAS2
 
 
hsa-miR-130b-3p
 
 
-0.468
 
 
11.7
 
 
 
 
ACBD3
 
 
hsa-miR-130b-3p
 
 
-0.292
 
 
19.9
 
 
 
 
ACBD5
 
 
hsa-miR-130b-3p
 
 
-0.494
 
 
13.8
 
 
 
 
ACSL4
 
 
hsa-miR-130b-3p
 
 
-0.301
 
 
20.7
 
 
 
 
ADAR
 
 
hsa-miR-1-3p
 
 
-0.35
 
 
61.8
 
 
 
 
ADPGK
 
 
hsa-miR-1-3p
 
 
-0.393
 
 
15.8
 
 
 
 
AIFM1
 
 
hsa-let-7e-5p
 
 
-0.314
 
 
15.1
 
 
 
 
AKIRIN2
 
 
hsa-miR-130b-3p
 
 
-0.315
 
 
26.6
 
 
 
 
ANKIB1
 
 
hsa-miR-130b-3p
 
 
-0.329
 
 
12.4
 
 
 
 
ANKRD12
 
 
hsa-miR-7-5p
 
 
-0.37
 
 
20.9
 
 
 
 
ANKRD29
 
 
hsa-miR-1-3p
 
 
-0.426
 
 
18.8
 
 
 
 
ANP32B
 
 
hsa-miR-1-3p
 
 
-0.287
 
 
207.9
 
 
 
 
ANXA2
 
 
hsa-miR-1-3p
 
 
-0.581
 
 
187.6
 
 
 
 
ANXA4
 
 
hsa-miR-1-3p
 
 
-0.539
 
 
19.8
 
 
 
 
AP1AR
 
 
hsa-miR-100-5p
 
 
-0.804
 
 
11.3
 
 
 
 
AP1S1
 
 
hsa-miR-1-3p
 
 
-0.355
 
 
11.1
 
 
 
 
AP1S1
 
 
hsa-let-7e-5p
 
 
-0.374
 
 
11.1
 
 
 
 
APPL1
 
 
hsa-miR-130b-3p
 
 
-0.347
 
 
15.6
 
 
 
 
ARCN1
 
 
hsa-miR-1-3p
 
 
-0.468
 
 
49.7
 
 
 
 
ARF4
 
 
hsa-miR-7-5p
 
 
-0.407
 
 
69.1
 
 
 
 
ARFGEF1
 
 
hsa-miR-130b-3p
 
 
-0.302
 
 
14.4
 
 
 
 
ARHGAP1
 
 
hsa-miR-130b-3p
 
 
-0.376
 
 
28.7
 
 
 
 
ARID4A
 
 
hsa-miR-7-5p
 
 
-0.341
 
 
14.0
 
 
 
 
ARL6IP1
 
 
hsa-miR-130b-3p
 
 
-0.289
 
 
124.0
 
 
 
 
ARL6IP1
 
 
hsa-miR-7-5p
 
 
-0.374
 
 
124.0
 
 
 
 
ARL6IP6
 
 
hsa-let-7e-5p
 
 
-0.305
 
 
17.4
 
 
 
 
ARMT1
 
 
hsa-let-7e-5p
 
 
-0.303
 
 
19.7
 
 
 
 
ARPC3
 
 
hsa-miR-1-3p
 
 
-0.606
 
 
540.4
 
 
 
 
ARPP19
 
 
hsa-let-7e-5p
 
 
-0.492
 
 
45.1
 
 
 
 
ASAP1
 
 
hsa-let-7e-5p
 
 
-0.296
 
 
16.5
 
 
 
 
ASH2L
 
 
hsa-miR-1-3p
 
 
-0.462
 
 
26.6
 
 
 
 
ATF2
 
 
hsa-miR-1-3p
 
 
-0.328
 
 
17.9
 
 
 
 
ATP2A2
 
 
hsa-let-7e-5p
 
 
-0.311
 
 
33.7
 
 
 
 
ATPAF1
 
 
hsa-let-7e-5p
 
 
-0.498
 
 
30.3
 
 
 
 
AZIN1
 
 
hsa-miR-1-3p
 
 
-0.438
 
 
53.2
 
 
 
 
B3GAT3
 
 
hsa-let-7e-5p
 
 
-0.324
 
 
24.9
 
 
 
 
BACH1
 
 
hsa-let-7e-5p
 
 
-0.298
 
 
15.7
 
 
 
 
BACH1
 
 
hsa-let-7e-5p
 
 
-0.298
 
 
15.7
 
 
 
 
BACH1
 
 
hsa-let-7e-5p
 
 
-0.435
 
 
15.7
 
 
 
 
BACH1
 
 
hsa-let-7e-5p
 
 
-0.435
 
 
15.7
 
 
 
 
BAZ2A
 
 
hsa-miR-100-5p
 
 
-0.29
 
 
31.2
 
 
 
 
BAZ2A
 
 
hsa-miR-100-5p
 
 
-0.421
 
 
31.2
 
 
 
 
BCAP29
 
 
hsa-let-7e-5p
 
 
-0.289
 
 
15.9
 
 
 
 
BET1
 
 
hsa-miR-1-3p
 
 
-0.413
 
 
22.7
 
 
 
 
BHLHE40
 
 
hsa-miR-130b-3p
 
 
-0.351
 
 
48.9
 
 
 
 
BIN3
 
 
hsa-let-7e-5p
 
 
-0.427
 
 
12.8
 
 
 
 
BLCAP
 
 
hsa-miR-130b-3p
 
 
-0.342
 
 
37.4
 
 
 
 
BLOC1S4
 
 
hsa-miR-7-5p
 
 
-0.417
 
 
11.4
 
 
 
 
BLOC1S4
 
 
hsa-miR-7-5p
 
 
-0.527
 
 
11.4
 
 
 
 
BLOC1S6
 
 
hsa-let-7e-5p
 
 
-0.321
 
 
39.2
 
 
 
 
BPNT1
 
 
hsa-miR-1-3p
 
 
-0.329
 
 
31.9
 
 
 
 
BRI3BP
 
 
hsa-miR-1-3p
 
 
-0.293
 
 
12.9
 
 
 
 
BRIX1
 
 
hsa-miR-1-3p
 
 
-0.334
 
 
24.4
 
 
 
 
BSCL2
 
 
hsa-miR-1-3p
 
 
-0.471
 
 
14.0
 
 
 
 
BTAF1
 
 
hsa-miR-130b-3p
 
 
-0.291
 
 
25.1
 
 
 
 
BTF3L4
 
 
hsa-let-7e-5p
 
 
-0.355
 
 
34.2
 
 
 
 
BTF3L4
 
 
hsa-miR-130b-3p
 
 
-0.359
 
 
34.2
 
 
 
 
BTG1
 
 
hsa-miR-130b-3p
 
 
-0.565
 
 
22.3
 
 
 
 
BZW1
 
 
hsa-let-7e-5p
 
 
-0.354
 
 
91.4
 
 
 
 
BZW1
 
 
hsa-let-7e-5p
 
 
-0.375
 
 
91.4
 
 
 
 
BZW2
 
 
hsa-let-7e-5p
 
 
-0.408
 
 
28.0
 
 
 
 
C15orf39
 
 
hsa-let-7e-5p
 
 
-0.374
 
 
10.2
 
 
 
 
C1GALT1
 
 
hsa-let-7e-5p
 
 
-0.322
 
 
17.8
 
 
 
 
C6orf120
 
 
hsa-miR-1-3p
 
 
-0.288
 
 
14.2
 
 
 
 
CAAP1
 
 
hsa-miR-1-3p
 
 
-0.48
 
 
13.7
 
 
 
 
CALM2
 
 
hsa-miR-130b-3p
 
 
-0.584
 
 
399.9
 
 
 
 
CANT1
 
 
hsa-let-7e-5p
 
 
-0.402
 
 
14.3
 
 
 
 
CAPZA1
 
 
hsa-miR-7-5p
 
 
-0.351
 
 
143.9
 
 
 
 
CASD1
 
 
hsa-miR-130b-3p
 
 
-0.305
 
 
11.7
 
 
 
 
CASP3
 
 
hsa-let-7e-5p
 
 
-0.495
 
 
35.8
 
 
 
 
CBFB
 
 
hsa-miR-130b-3p
 
 
-0.498
 
 
15.3
 
 
 
 
CBX5
 
 
hsa-let-7e-5p
 
 
-0.307
 
 
17.6
 
 
 
 
CBX5
 
 
hsa-let-7e-5p
 
 
-0.472
 
 
17.6
 
 
 
 
CCDC43
 
 
hsa-miR-7-5p
 
 
-0.331
 
 
12.6
 
 
 
 
CCNC
 
 
hsa-miR-1-3p
 
 
-0.336
 
 
37.0
 
 
 
 
CCNL2
 
 
hsa-miR-28-5p
 
 
-0.448
 
 
29.6
 
 
 
 
CCNY
 
 
hsa-let-7e-5p
 
 
-0.334
 
 
21.9
 
 
 
 
CCNY
 
 
hsa-miR-130b-3p
 
 
-0.372
 
 
21.9
 
 
 
 
CCR7
 
 
hsa-let-7e-5p
 
 
-0.482
 
 
78.7
 
 
 
 
CCT4
 
 
hsa-miR-7-5p
 
 
-0.37
 
 
76.1
 
 
 
 
CCT6A
 
 
hsa-miR-130b-3p
 
 
-0.382
 
 
66.0
 
 
 
 
CD164
 
 
hsa-miR-1-3p
 
 
-0.315
 
 
129.0
 
 
 
 
CD2AP
 
 
hsa-miR-130b-3p
 
 
-0.302
 
 
14.9
 
 
 
 
CD2AP
 
 
hsa-miR-1-3p
 
 
-0.479
 
 
14.9
 
 
 
 
CD69
 
 
hsa-miR-130b-3p
 
 
-0.451
 
 
88.3
 
 
 
 
CD86
 
 
hsa-let-7e-5p
 
 
-0.323
 
 
27.0
 
 
 
 
CDC34
 
 
hsa-let-7e-5p
 
 
-0.321
 
 
11.7
 
 
 
 
CDC34
 
 
hsa-let-7e-5p
 
 
-0.555
 
 
11.7
 
 
 
 
CDC42SE1
 
 
hsa-miR-1-3p
 
 
-0.36
 
 
79.0
 
 
 
 
CDCA8
 
 
hsa-let-7e-5p
 
 
-0.342
 
 
18.1
 
 
 
 
CDK14
 
 
hsa-miR-1-3p
 
 
-0.326
 
 
11.5
 
 
 
 
CDK4
 
 
hsa-miR-1-3p
 
 
-0.338
 
 
62.3
 
 
 
 
CDKN1A
 
 
hsa-let-7e-5p
 
 
-0.434
 
 
16.1
 
 
 
 
CDKN2AIP
 
 
hsa-miR-28-5p
 
 
-0.419
 
 
18.5
 
 
 
 
CDV3
 
 
hsa-let-7e-5p
 
 
-0.453
 
 
147.0
 
 
 
 
CEMIP2
 
 
hsa-let-7e-5p
 
 
-0.453
 
 
21.6
 
 
 
 
CEP55
 
 
hsa-miR-130b-3p
 
 
-0.304
 
 
13.7
 
 
 
 
 
 
 ovary 
 
 
 
 
target_symbol
 
 
mature_mirna_id
 
 
score
 
 
nTPM
 
 
 
 
 
 
ABCB7
 
 
hsa-miR-1-3p
 
 
-0.365
 
 
11.9
 
 
 
 
ABHD2
 
 
hsa-miR-1-3p
 
 
-0.291
 
 
16.8
 
 
 
 
ABHD3
 
 
hsa-miR-130b-3p
 
 
-0.356
 
 
12.0
 
 
 
 
ABRAXAS2
 
 
hsa-miR-130b-3p
 
 
-0.468
 
 
10.0
 
 
 
 
ACBD3
 
 
hsa-miR-130b-3p
 
 
-0.292
 
 
20.0
 
 
 
 
ACSL4
 
 
hsa-miR-130b-3p
 
 
-0.301
 
 
20.0
 
 
 
 
ACVR1
 
 
hsa-miR-130b-3p
 
 
-0.44
 
 
17.7
 
 
 
 
ADAR
 
 
hsa-miR-1-3p
 
 
-0.35
 
 
47.5
 
 
 
 
ADPGK
 
 
hsa-miR-1-3p
 
 
-0.393
 
 
10.5
 
 
 
 
ADRB3
 
 
hsa-let-7e-5p
 
 
-0.415
 
 
12.8
 
 
 
 
AHCTF1
 
 
hsa-let-7e-5p
 
 
-0.374
 
 
13.1
 
 
 
 
AKIRIN2
 
 
hsa-miR-130b-3p
 
 
-0.315
 
 
22.8
 
 
 
 
ALG11
 
 
hsa-miR-1-3p
 
 
-0.317
 
 
10.1
 
 
 
 
ALG9
 
 
hsa-miR-7-5p
 
 
-0.302
 
 
11.1
 
 
 
 
AMT
 
 
hsa-let-7e-5p
 
 
-0.463
 
 
16.7
 
 
 
 
ANKIB1
 
 
hsa-miR-130b-3p
 
 
-0.329
 
 
12.5
 
 
 
 
ANKRD12
 
 
hsa-miR-7-5p
 
 
-0.37
 
 
10.6
 
 
 
 
ANKRD29
 
 
hsa-miR-1-3p
 
 
-0.426
 
 
15.0
 
 
 
 
ANKRD46
 
 
hsa-let-7e-5p
 
 
-0.389
 
 
19.3
 
 
 
 
ANP32B
 
 
hsa-miR-1-3p
 
 
-0.287
 
 
160.5
 
 
 
 
ANXA2
 
 
hsa-miR-1-3p
 
 
-0.581
 
 
222.6
 
 
 
 
ANXA4
 
 
hsa-miR-1-3p
 
 
-0.539
 
 
39.0
 
 
 
 
AP1AR
 
 
hsa-miR-100-5p
 
 
-0.804
 
 
10.0
 
 
 
 
AP1S1
 
 
hsa-miR-1-3p
 
 
-0.355
 
 
22.2
 
 
 
 
AP1S1
 
 
hsa-let-7e-5p
 
 
-0.374
 
 
22.2
 
 
 
 
APCDD1
 
 
hsa-miR-130b-3p
 
 
-0.304
 
 
28.1
 
 
 
 
APPL1
 
 
hsa-miR-130b-3p
 
 
-0.347
 
 
19.2
 
 
 
 
ARCN1
 
 
hsa-miR-1-3p
 
 
-0.468
 
 
59.3
 
 
 
 
ARF4
 
 
hsa-miR-7-5p
 
 
-0.407
 
 
123.7
 
 
 
 
ARG2
 
 
hsa-let-7e-5p
 
 
-0.468
 
 
18.7
 
 
 
 
ARHGAP1
 
 
hsa-miR-130b-3p
 
 
-0.376
 
 
43.6
 
 
 
 
ARHGAP12
 
 
hsa-miR-130b-3p
 
 
-0.287
 
 
11.9
 
 
 
 
ARID4A
 
 
hsa-miR-7-5p
 
 
-0.341
 
 
10.1
 
 
 
 
ARL4D
 
 
hsa-let-7e-5p
 
 
-0.417
 
 
20.3
 
 
 
 
ARL6IP1
 
 
hsa-miR-130b-3p
 
 
-0.289
 
 
97.4
 
 
 
 
ARL6IP1
 
 
hsa-miR-7-5p
 
 
-0.374
 
 
97.4
 
 
 
 
ARL6IP6
 
 
hsa-let-7e-5p
 
 
-0.305
 
 
11.9
 
 
 
 
ARMT1
 
 
hsa-let-7e-5p
 
 
-0.303
 
 
15.5
 
 
 
 
ARPC3
 
 
hsa-miR-1-3p
 
 
-0.606
 
 
145.5
 
 
 
 
ARPP19
 
 
hsa-let-7e-5p
 
 
-0.492
 
 
40.6
 
 
 
 
ASAP1
 
 
hsa-let-7e-5p
 
 
-0.296
 
 
16.8
 
 
 
 
ASH2L
 
 
hsa-miR-1-3p
 
 
-0.462
 
 
28.3
 
 
 
 
ATF2
 
 
hsa-miR-1-3p
 
 
-0.328
 
 
23.0
 
 
 
 
ATP2A2
 
 
hsa-let-7e-5p
 
 
-0.311
 
 
34.9
 
 
 
 
ATPAF1
 
 
hsa-let-7e-5p
 
 
-0.498
 
 
33.9
 
 
 
 
AVPI1
 
 
hsa-miR-7-5p
 
 
-0.367
 
 
85.9
 
 
 
 
AXL
 
 
hsa-miR-1-3p
 
 
-0.304
 
 
22.7
 
 
 
 
AZIN1
 
 
hsa-miR-1-3p
 
 
-0.438
 
 
37.6
 
 
 
 
B3GAT3
 
 
hsa-let-7e-5p
 
 
-0.324
 
 
35.4
 
 
 
 
B4GAT1
 
 
hsa-let-7e-5p
 
 
-0.47
 
 
33.5
 
 
 
 
BACH1
 
 
hsa-let-7e-5p
 
 
-0.298
 
 
12.0
 
 
 
 
BACH1
 
 
hsa-let-7e-5p
 
 
-0.298
 
 
12.0
 
 
 
 
BACH1
 
 
hsa-let-7e-5p
 
 
-0.435
 
 
12.0
 
 
 
 
BACH1
 
 
hsa-let-7e-5p
 
 
-0.435
 
 
12.0
 
 
 
 
BAHD1
 
 
hsa-miR-130b-3p
 
 
-0.294
 
 
20.4
 
 
 
 
BAZ2A
 
 
hsa-miR-100-5p
 
 
-0.29
 
 
30.7
 
 
 
 
BAZ2A
 
 
hsa-miR-100-5p
 
 
-0.421
 
 
30.7
 
 
 
 
BCAP29
 
 
hsa-let-7e-5p
 
 
-0.289
 
 
22.2
 
 
 
 
BET1
 
 
hsa-miR-1-3p
 
 
-0.413
 
 
26.0
 
 
 
 
BHLHE40
 
 
hsa-miR-130b-3p
 
 
-0.351
 
 
79.3
 
 
 
 
BIN3
 
 
hsa-let-7e-5p
 
 
-0.427
 
 
11.4
 
 
 
 
BLCAP
 
 
hsa-miR-130b-3p
 
 
-0.342
 
 
39.5
 
 
 
 
BLOC1S4
 
 
hsa-miR-7-5p
 
 
-0.417
 
 
12.4
 
 
 
 
BLOC1S4
 
 
hsa-miR-7-5p
 
 
-0.527
 
 
12.4
 
 
 
 
BLOC1S6
 
 
hsa-let-7e-5p
 
 
-0.321
 
 
33.7
 
 
 
 
BMPR2
 
 
hsa-miR-130b-3p
 
 
-0.33
 
 
11.2
 
 
 
 
BMPR2
 
 
hsa-miR-100-5p
 
 
-0.355
 
 
11.2
 
 
 
 
BPNT1
 
 
hsa-miR-1-3p
 
 
-0.329
 
 
16.8
 
 
 
 
BRIX1
 
 
hsa-miR-1-3p
 
 
-0.334
 
 
18.1
 
 
 
 
BSCL2
 
 
hsa-miR-1-3p
 
 
-0.471
 
 
27.8
 
 
 
 
BTAF1
 
 
hsa-miR-130b-3p
 
 
-0.291
 
 
21.0
 
 
 
 
BTF3L4
 
 
hsa-let-7e-5p
 
 
-0.355
 
 
61.0
 
 
 
 
BTF3L4
 
 
hsa-miR-130b-3p
 
 
-0.359
 
 
61.0
 
 
 
 
BZW1
 
 
hsa-let-7e-5p
 
 
-0.354
 
 
60.3
 
 
 
 
BZW1
 
 
hsa-let-7e-5p
 
 
-0.375
 
 
60.3
 
 
 
 
BZW2
 
 
hsa-let-7e-5p
 
 
-0.408
 
 
18.7
 
 
 
 
C15orf39
 
 
hsa-let-7e-5p
 
 
-0.374
 
 
17.4
 
 
 
 
C6orf120
 
 
hsa-miR-1-3p
 
 
-0.288
 
 
13.6
 
 
 
 
CACFD1
 
 
hsa-let-7e-5p
 
 
-0.296
 
 
10.2
 
 
 
 
CALM2
 
 
hsa-miR-130b-3p
 
 
-0.584
 
 
578.9
 
 
 
 
CAMSAP2
 
 
hsa-miR-130b-3p
 
 
-0.297
 
 
17.2
 
 
 
 
CANT1
 
 
hsa-let-7e-5p
 
 
-0.402
 
 
13.8
 
 
 
 
CAPN3
 
 
hsa-let-7e-5p
 
 
-0.317
 
 
17.3
 
 
 
 
CAPZA1
 
 
hsa-miR-7-5p
 
 
-0.351
 
 
42.8
 
 
 
 
CASD1
 
 
hsa-miR-130b-3p
 
 
-0.305
 
 
14.5
 
 
 
 
CBX5
 
 
hsa-let-7e-5p
 
 
-0.307
 
 
15.1
 
 
 
 
CBX5
 
 
hsa-let-7e-5p
 
 
-0.472
 
 
15.1
 
 
 
 
CCNC
 
 
hsa-miR-1-3p
 
 
-0.336
 
 
19.0
 
 
 
 
CCNL2
 
 
hsa-miR-28-5p
 
 
-0.448
 
 
80.5
 
 
 
 
CCNT2
 
 
hsa-miR-7-5p
 
 
-0.349
 
 
16.6
 
 
 
 
CCNY
 
 
hsa-let-7e-5p
 
 
-0.334
 
 
25.0
 
 
 
 
CCNY
 
 
hsa-miR-130b-3p
 
 
-0.372
 
 
25.0
 
 
 
 
CCT4
 
 
hsa-miR-7-5p
 
 
-0.37
 
 
109.2
 
 
 
 
CCT6A
 
 
hsa-miR-130b-3p
 
 
-0.382
 
 
43.9
 
 
 
 
CD164
 
 
hsa-miR-1-3p
 
 
-0.315
 
 
129.2
 
 
 
 
CDC34
 
 
hsa-let-7e-5p
 
 
-0.321
 
 
36.7
 
 
 
 
CDC34
 
 
hsa-let-7e-5p
 
 
-0.555
 
 
36.7
 
 
 
 
CDC42SE1
 
 
hsa-miR-1-3p
 
 
-0.36
 
 
42.3
 
 
 
 
CDK14
 
 
hsa-miR-1-3p
 
 
-0.326
 
 
13.9
 
 
 
 
CDK4
 
 
hsa-miR-1-3p
 
 
-0.338
 
 
114.8
 
 
 
 
 
 
 parathyroid gland 
 
 
 
 
target_symbol
 
 
mature_mirna_id
 
 
score
 
 
nTPM
 
 
 
 
 
 
ABCB7
 
 
hsa-miR-1-3p
 
 
-0.365
 
 
10.3
 
 
 
 
ABHD2
 
 
hsa-miR-1-3p
 
 
-0.291
 
 
17.3
 
 
 
 
ABHD3
 
 
hsa-miR-130b-3p
 
 
-0.356
 
 
15.0
 
 
 
 
ABRAXAS2
 
 
hsa-miR-130b-3p
 
 
-0.468
 
 
12.0
 
 
 
 
ACBD3
 
 
hsa-miR-130b-3p
 
 
-0.292
 
 
26.3
 
 
 
 
ACBD5
 
 
hsa-miR-130b-3p
 
 
-0.494
 
 
29.3
 
 
 
 
ACSL4
 
 
hsa-miR-130b-3p
 
 
-0.301
 
 
18.4
 
 
 
 
ACSL6
 
 
hsa-let-7e-5p
 
 
-0.336
 
 
10.6
 
 
 
 
ADAR
 
 
hsa-miR-1-3p
 
 
-0.35
 
 
59.0
 
 
 
 
ADGRA3
 
 
hsa-miR-1-3p
 
 
-0.351
 
 
11.5
 
 
 
 
AHCTF1
 
 
hsa-let-7e-5p
 
 
-0.374
 
 
12.3
 
 
 
 
AIFM1
 
 
hsa-let-7e-5p
 
 
-0.314
 
 
27.9
 
 
 
 
AKIRIN2
 
 
hsa-miR-130b-3p
 
 
-0.315
 
 
16.7
 
 
 
 
ALDH4A1
 
 
hsa-miR-184
 
 
-0.421
 
 
21.6
 
 
 
 
ALG11
 
 
hsa-miR-1-3p
 
 
-0.317
 
 
16.1
 
 
 
 
ALG9
 
 
hsa-miR-7-5p
 
 
-0.302
 
 
12.0
 
 
 
 
AMT
 
 
hsa-let-7e-5p
 
 
-0.463
 
 
13.3
 
 
 
 
ANKIB1
 
 
hsa-miR-130b-3p
 
 
-0.329
 
 
22.5
 
 
 
 
ANKRD29
 
 
hsa-miR-1-3p
 
 
-0.426
 
 
23.3
 
 
 
 
ANKRD46
 
 
hsa-let-7e-5p
 
 
-0.389
 
 
15.1
 
 
 
 
ANP32B
 
 
hsa-miR-1-3p
 
 
-0.287
 
 
64.6
 
 
 
 
ANXA2
 
 
hsa-miR-1-3p
 
 
-0.581
 
 
323.7
 
 
 
 
ANXA4
 
 
hsa-miR-1-3p
 
 
-0.539
 
 
73.3
 
 
 
 
AP1AR
 
 
hsa-miR-100-5p
 
 
-0.804
 
 
38.5
 
 
 
 
AP1S1
 
 
hsa-miR-1-3p
 
 
-0.355
 
 
21.4
 
 
 
 
AP1S1
 
 
hsa-let-7e-5p
 
 
-0.374
 
 
21.4
 
 
 
 
APPL1
 
 
hsa-miR-130b-3p
 
 
-0.347
 
 
23.6
 
 
 
 
ARCN1
 
 
hsa-miR-1-3p
 
 
-0.468
 
 
57.6
 
 
 
 
ARF4
 
 
hsa-miR-7-5p
 
 
-0.407
 
 
95.4
 
 
 
 
ARFGEF1
 
 
hsa-miR-130b-3p
 
 
-0.302
 
 
26.3
 
 
 
 
ARG2
 
 
hsa-let-7e-5p
 
 
-0.468
 
 
206.5
 
 
 
 
ARHGAP1
 
 
hsa-miR-130b-3p
 
 
-0.376
 
 
23.3
 
 
 
 
ARHGAP12
 
 
hsa-miR-130b-3p
 
 
-0.287
 
 
18.4
 
 
 
 
ARID2
 
 
hsa-miR-1-3p
 
 
-0.294
 
 
11.0
 
 
 
 
ARL6IP1
 
 
hsa-miR-130b-3p
 
 
-0.289
 
 
169.8
 
 
 
 
ARL6IP1
 
 
hsa-miR-7-5p
 
 
-0.374
 
 
169.8
 
 
 
 
ARMT1
 
 
hsa-let-7e-5p
 
 
-0.303
 
 
10.9
 
 
 
 
ARPC3
 
 
hsa-miR-1-3p
 
 
-0.606
 
 
164.2
 
 
 
 
ARPP19
 
 
hsa-let-7e-5p
 
 
-0.492
 
 
102.5
 
 
 
 
AS3MT
 
 
hsa-miR-28-5p
 
 
-0.479
 
 
53.2
 
 
 
 
ASAP1
 
 
hsa-let-7e-5p
 
 
-0.296
 
 
16.8
 
 
 
 
ASH2L
 
 
hsa-miR-1-3p
 
 
-0.462
 
 
23.4
 
 
 
 
ATF2
 
 
hsa-miR-1-3p
 
 
-0.328
 
 
21.2
 
 
 
 
ATG4A
 
 
hsa-miR-7-5p
 
 
-0.448
 
 
11.7
 
 
 
 
ATOSB
 
 
hsa-let-7e-5p
 
 
-0.429
 
 
10.5
 
 
 
 
ATP2A2
 
 
hsa-let-7e-5p
 
 
-0.311
 
 
52.7
 
 
 
 
ATPAF1
 
 
hsa-let-7e-5p
 
 
-0.498
 
 
129.7
 
 
 
 
AXL
 
 
hsa-miR-1-3p
 
 
-0.304
 
 
10.0
 
 
 
 
AZIN1
 
 
hsa-miR-1-3p
 
 
-0.438
 
 
35.6
 
 
 
 
B3GAT3
 
 
hsa-let-7e-5p
 
 
-0.324
 
 
18.1
 
 
 
 
B4GAT1
 
 
hsa-let-7e-5p
 
 
-0.47
 
 
21.3
 
 
 
 
BAHD1
 
 
hsa-miR-130b-3p
 
 
-0.294
 
 
12.2
 
 
 
 
BAZ2A
 
 
hsa-miR-100-5p
 
 
-0.29
 
 
31.4
 
 
 
 
BAZ2A
 
 
hsa-miR-100-5p
 
 
-0.421
 
 
31.4
 
 
 
 
BCAP29
 
 
hsa-let-7e-5p
 
 
-0.289
 
 
32.8
 
 
 
 
BET1
 
 
hsa-miR-1-3p
 
 
-0.413
 
 
18.3
 
 
 
 
BHLHE40
 
 
hsa-miR-130b-3p
 
 
-0.351
 
 
63.5
 
 
 
 
BIN3
 
 
hsa-let-7e-5p
 
 
-0.427
 
 
11.8
 
 
 
 
BLCAP
 
 
hsa-miR-130b-3p
 
 
-0.342
 
 
47.8
 
 
 
 
BLOC1S6
 
 
hsa-let-7e-5p
 
 
-0.321
 
 
15.8
 
 
 
 
BLTP3B
 
 
hsa-miR-1-3p
 
 
-0.29
 
 
12.8
 
 
 
 
BMPR2
 
 
hsa-miR-130b-3p
 
 
-0.33
 
 
12.6
 
 
 
 
BMPR2
 
 
hsa-miR-100-5p
 
 
-0.355
 
 
12.6
 
 
 
 
BPNT1
 
 
hsa-miR-1-3p
 
 
-0.329
 
 
20.2
 
 
 
 
BRIX1
 
 
hsa-miR-1-3p
 
 
-0.334
 
 
17.1
 
 
 
 
BSCL2
 
 
hsa-miR-1-3p
 
 
-0.471
 
 
57.1
 
 
 
 
BTAF1
 
 
hsa-miR-130b-3p
 
 
-0.291
 
 
11.8
 
 
 
 
BTBD9
 
 
hsa-let-7e-5p
 
 
-0.311
 
 
12.9
 
 
 
 
BTF3L4
 
 
hsa-let-7e-5p
 
 
-0.355
 
 
39.5
 
 
 
 
BTF3L4
 
 
hsa-miR-130b-3p
 
 
-0.359
 
 
39.5
 
 
 
 
BZW1
 
 
hsa-let-7e-5p
 
 
-0.354
 
 
68.1
 
 
 
 
BZW1
 
 
hsa-let-7e-5p
 
 
-0.375
 
 
68.1
 
 
 
 
C15orf39
 
 
hsa-let-7e-5p
 
 
-0.374
 
 
14.8
 
 
 
 
C1GALT1
 
 
hsa-let-7e-5p
 
 
-0.322
 
 
43.0
 
 
 
 
C2orf88
 
 
hsa-let-7e-5p
 
 
-0.429
 
 
33.0
 
 
 
 
C6orf120
 
 
hsa-miR-1-3p
 
 
-0.288
 
 
10.6
 
 
 
 
CAAP1
 
 
hsa-miR-1-3p
 
 
-0.48
 
 
17.7
 
 
 
 
CALM2
 
 
hsa-miR-130b-3p
 
 
-0.584
 
 
311.8
 
 
 
 
CAMSAP2
 
 
hsa-miR-130b-3p
 
 
-0.297
 
 
21.3
 
 
 
 
CANT1
 
 
hsa-let-7e-5p
 
 
-0.402
 
 
23.0
 
 
 
 
CAPZA1
 
 
hsa-miR-7-5p
 
 
-0.351
 
 
37.2
 
 
 
 
CARNMT1
 
 
hsa-let-7e-5p
 
 
-0.307
 
 
10.6
 
 
 
 
CASD1
 
 
hsa-miR-130b-3p
 
 
-0.305
 
 
13.0
 
 
 
 
CBX5
 
 
hsa-let-7e-5p
 
 
-0.307
 
 
24.1
 
 
 
 
CBX5
 
 
hsa-let-7e-5p
 
 
-0.472
 
 
24.1
 
 
 
 
CCDC126
 
 
hsa-miR-130b-3p
 
 
-0.591
 
 
11.0
 
 
 
 
CCDC43
 
 
hsa-miR-7-5p
 
 
-0.331
 
 
14.4
 
 
 
 
CCNC
 
 
hsa-miR-1-3p
 
 
-0.336
 
 
44.4
 
 
 
 
CCNL2
 
 
hsa-miR-28-5p
 
 
-0.448
 
 
29.4
 
 
 
 
CCNY
 
 
hsa-let-7e-5p
 
 
-0.334
 
 
16.8
 
 
 
 
CCNY
 
 
hsa-miR-130b-3p
 
 
-0.372
 
 
16.8
 
 
 
 
CCT4
 
 
hsa-miR-7-5p
 
 
-0.37
 
 
59.0
 
 
 
 
CCT6A
 
 
hsa-miR-130b-3p
 
 
-0.382
 
 
65.8
 
 
 
 
CD164
 
 
hsa-miR-1-3p
 
 
-0.315
 
 
205.9
 
 
 
 
CD2AP
 
 
hsa-miR-130b-3p
 
 
-0.302
 
 
33.5
 
 
 
 
CD2AP
 
 
hsa-miR-1-3p
 
 
-0.479
 
 
33.5
 
 
 
 
CDC34
 
 
hsa-let-7e-5p
 
 
-0.321
 
 
17.6
 
 
 
 
CDC34
 
 
hsa-let-7e-5p
 
 
-0.555
 
 
17.6
 
 
 
 
CDC42SE1
 
 
hsa-miR-1-3p
 
 
-0.36
 
 
67.2
 
 
 
 
CDK4
 
 
hsa-miR-1-3p
 
 
-0.338
 
 
59.6
 
 
 
 
 
 
 skeletal muscle 
 
 
 
 
target_symbol
 
 
mature_mirna_id
 
 
score
 
 
nTPM
 
 
 
 
 
 
ABCB7
 
 
hsa-miR-1-3p
 
 
-0.365
 
 
29.2
 
 
 
 
ABHD2
 
 
hsa-miR-1-3p
 
 
-0.291
 
 
21.1
 
 
 
 
ABHD3
 
 
hsa-miR-130b-3p
 
 
-0.356
 
 
13.6
 
 
 
 
ABRAXAS2
 
 
hsa-miR-130b-3p
 
 
-0.468
 
 
17.8
 
 
 
 
ACBD3
 
 
hsa-miR-130b-3p
 
 
-0.292
 
 
20.2
 
 
 
 
ACBD5
 
 
hsa-miR-130b-3p
 
 
-0.494
 
 
29.1
 
 
 
 
ACSL4
 
 
hsa-miR-130b-3p
 
 
-0.301
 
 
39.3
 
 
 
 
ACTC1
 
 
hsa-miR-7-5p
 
 
-0.471
 
 
519.7
 
 
 
 
ACVR1
 
 
hsa-miR-130b-3p
 
 
-0.44
 
 
15.5
 
 
 
 
ADAR
 
 
hsa-miR-1-3p
 
 
-0.35
 
 
13.6
 
 
 
 
ADGRA3
 
 
hsa-miR-1-3p
 
 
-0.351
 
 
15.9
 
 
 
 
ADRB2
 
 
hsa-let-7e-5p
 
 
-0.635
 
 
10.9
 
 
 
 
AGO2
 
 
hsa-miR-184
 
 
-0.831
 
 
16.4
 
 
 
 
AGO2
 
 
hsa-miR-100-5p
 
 
-0.884
 
 
16.4
 
 
 
 
AHCTF1
 
 
hsa-let-7e-5p
 
 
-0.374
 
 
10.0
 
 
 
 
AIFM1
 
 
hsa-let-7e-5p
 
 
-0.314
 
 
58.6
 
 
 
 
AKAP6
 
 
hsa-let-7e-5p
 
 
-0.307
 
 
44.4
 
 
 
 
AKIRIN2
 
 
hsa-miR-130b-3p
 
 
-0.315
 
 
85.6
 
 
 
 
ALDH4A1
 
 
hsa-miR-184
 
 
-0.421
 
 
69.5
 
 
 
 
ANKRD12
 
 
hsa-miR-7-5p
 
 
-0.37
 
 
25.1
 
 
 
 
ANKRD46
 
 
hsa-let-7e-5p
 
 
-0.389
 
 
58.3
 
 
 
 
ANP32B
 
 
hsa-miR-1-3p
 
 
-0.287
 
 
103.8
 
 
 
 
ANXA2
 
 
hsa-miR-1-3p
 
 
-0.581
 
 
265.9
 
 
 
 
ANXA4
 
 
hsa-miR-1-3p
 
 
-0.539
 
 
16.6
 
 
 
 
APPL1
 
 
hsa-miR-130b-3p
 
 
-0.347
 
 
41.3
 
 
 
 
ARCN1
 
 
hsa-miR-1-3p
 
 
-0.468
 
 
45.8
 
 
 
 
ARF4
 
 
hsa-miR-7-5p
 
 
-0.407
 
 
59.5
 
 
 
 
ARFGEF1
 
 
hsa-miR-130b-3p
 
 
-0.302
 
 
12.2
 
 
 
 
ARG2
 
 
hsa-let-7e-5p
 
 
-0.468
 
 
37.4
 
 
 
 
ARHGAP1
 
 
hsa-miR-130b-3p
 
 
-0.376
 
 
28.5
 
 
 
 
ARL6IP1
 
 
hsa-miR-130b-3p
 
 
-0.289
 
 
21.6
 
 
 
 
ARL6IP1
 
 
hsa-miR-7-5p
 
 
-0.374
 
 
21.6
 
 
 
 
ARL6IP6
 
 
hsa-let-7e-5p
 
 
-0.305
 
 
53.9
 
 
 
 
ARMT1
 
 
hsa-let-7e-5p
 
 
-0.303
 
 
17.8
 
 
 
 
ARPC3
 
 
hsa-miR-1-3p
 
 
-0.606
 
 
110.4
 
 
 
 
ARPP19
 
 
hsa-let-7e-5p
 
 
-0.492
 
 
225.3
 
 
 
 
ARRDC4
 
 
hsa-let-7e-5p
 
 
-0.329
 
 
13.8
 
 
 
 
ASAP1
 
 
hsa-let-7e-5p
 
 
-0.296
 
 
12.4
 
 
 
 
ASH2L
 
 
hsa-miR-1-3p
 
 
-0.462
 
 
30.4
 
 
 
 
ATF2
 
 
hsa-miR-1-3p
 
 
-0.328
 
 
11.7
 
 
 
 
ATG16L1
 
 
hsa-miR-130b-3p
 
 
-0.407
 
 
20.1
 
 
 
 
ATG4A
 
 
hsa-miR-7-5p
 
 
-0.448
 
 
18.0
 
 
 
 
ATOSB
 
 
hsa-let-7e-5p
 
 
-0.429
 
 
13.5
 
 
 
 
ATP2A2
 
 
hsa-let-7e-5p
 
 
-0.311
 
 
2675.7
 
 
 
 
ATP2B2
 
 
hsa-miR-7-5p
 
 
-0.297
 
 
35.1
 
 
 
 
ATP2B2
 
 
hsa-miR-130b-3p
 
 
-0.314
 
 
35.1
 
 
 
 
ATPAF1
 
 
hsa-let-7e-5p
 
 
-0.498
 
 
260.2
 
 
 
 
ATXN1
 
 
hsa-miR-130b-3p
 
 
-0.321
 
 
13.6
 
 
 
 
AVPI1
 
 
hsa-miR-7-5p
 
 
-0.367
 
 
13.0
 
 
 
 
AZIN1
 
 
hsa-miR-1-3p
 
 
-0.438
 
 
38.7
 
 
 
 
B3GAT3
 
 
hsa-let-7e-5p
 
 
-0.324
 
 
15.7
 
 
 
 
B4GAT1
 
 
hsa-let-7e-5p
 
 
-0.47
 
 
27.1
 
 
 
 
BACH1
 
 
hsa-let-7e-5p
 
 
-0.298
 
 
24.7
 
 
 
 
BACH1
 
 
hsa-let-7e-5p
 
 
-0.298
 
 
24.7
 
 
 
 
BACH1
 
 
hsa-let-7e-5p
 
 
-0.435
 
 
24.7
 
 
 
 
BACH1
 
 
hsa-let-7e-5p
 
 
-0.435
 
 
24.7
 
 
 
 
BAG4
 
 
hsa-miR-1-3p
 
 
-0.378
 
 
13.5
 
 
 
 
BAHD1
 
 
hsa-miR-130b-3p
 
 
-0.294
 
 
11.9
 
 
 
 
BAZ2A
 
 
hsa-miR-100-5p
 
 
-0.29
 
 
26.0
 
 
 
 
BAZ2A
 
 
hsa-miR-100-5p
 
 
-0.421
 
 
26.0
 
 
 
 
BCAP29
 
 
hsa-let-7e-5p
 
 
-0.289
 
 
28.3
 
 
 
 
BHLHE40
 
 
hsa-miR-130b-3p
 
 
-0.351
 
 
269.9
 
 
 
 
BIN3
 
 
hsa-let-7e-5p
 
 
-0.427
 
 
12.7
 
 
 
 
BLCAP
 
 
hsa-miR-130b-3p
 
 
-0.342
 
 
452.1
 
 
 
 
BLOC1S4
 
 
hsa-miR-7-5p
 
 
-0.417
 
 
11.1
 
 
 
 
BLOC1S4
 
 
hsa-miR-7-5p
 
 
-0.527
 
 
11.1
 
 
 
 
BLOC1S6
 
 
hsa-let-7e-5p
 
 
-0.321
 
 
22.1
 
 
 
 
BLTP3B
 
 
hsa-miR-1-3p
 
 
-0.29
 
 
18.9
 
 
 
 
BRIX1
 
 
hsa-miR-1-3p
 
 
-0.334
 
 
13.0
 
 
 
 
BSCL2
 
 
hsa-miR-1-3p
 
 
-0.471
 
 
26.9
 
 
 
 
BTAF1
 
 
hsa-miR-130b-3p
 
 
-0.291
 
 
11.9
 
 
 
 
BTF3L4
 
 
hsa-let-7e-5p
 
 
-0.355
 
 
65.0
 
 
 
 
BTF3L4
 
 
hsa-miR-130b-3p
 
 
-0.359
 
 
65.0
 
 
 
 
BZW1
 
 
hsa-let-7e-5p
 
 
-0.354
 
 
93.0
 
 
 
 
BZW1
 
 
hsa-let-7e-5p
 
 
-0.375
 
 
93.0
 
 
 
 
BZW2
 
 
hsa-let-7e-5p
 
 
-0.408
 
 
344.4
 
 
 
 
C19orf47
 
 
hsa-let-7e-5p
 
 
-0.311
 
 
335.4
 
 
 
 
C1orf21
 
 
hsa-miR-7-5p
 
 
-0.373
 
 
36.2
 
 
 
 
C2orf88
 
 
hsa-let-7e-5p
 
 
-0.429
 
 
12.6
 
 
 
 
C5orf22
 
 
hsa-miR-7-5p
 
 
-0.516
 
 
13.3
 
 
 
 
C6orf120
 
 
hsa-miR-1-3p
 
 
-0.288
 
 
12.4
 
 
 
 
CALM2
 
 
hsa-miR-130b-3p
 
 
-0.584
 
 
261.2
 
 
 
 
CANT1
 
 
hsa-let-7e-5p
 
 
-0.402
 
 
13.4
 
 
 
 
CAPN3
 
 
hsa-let-7e-5p
 
 
-0.317
 
 
848.7
 
 
 
 
CAPZA1
 
 
hsa-miR-7-5p
 
 
-0.351
 
 
31.0
 
 
 
 
CASD1
 
 
hsa-miR-130b-3p
 
 
-0.305
 
 
13.2
 
 
 
 
CASP3
 
 
hsa-let-7e-5p
 
 
-0.495
 
 
13.2
 
 
 
 
CBFA2T3
 
 
hsa-let-7e-5p
 
 
-0.305
 
 
13.1
 
 
 
 
CBFB
 
 
hsa-miR-130b-3p
 
 
-0.498
 
 
14.5
 
 
 
 
CBX5
 
 
hsa-let-7e-5p
 
 
-0.307
 
 
12.9
 
 
 
 
CBX5
 
 
hsa-let-7e-5p
 
 
-0.472
 
 
12.9
 
 
 
 
CCDC43
 
 
hsa-miR-7-5p
 
 
-0.331
 
 
68.8
 
 
 
 
CCNC
 
 
hsa-miR-1-3p
 
 
-0.336
 
 
20.6
 
 
 
 
CCNL2
 
 
hsa-miR-28-5p
 
 
-0.448
 
 
39.4
 
 
 
 
CCNY
 
 
hsa-let-7e-5p
 
 
-0.334
 
 
21.2
 
 
 
 
CCNY
 
 
hsa-miR-130b-3p
 
 
-0.372
 
 
21.2
 
 
 
 
CCT4
 
 
hsa-miR-7-5p
 
 
-0.37
 
 
97.4
 
 
 
 
CCT6A
 
 
hsa-miR-130b-3p
 
 
-0.382
 
 
125.4
 
 
 
 
CD164
 
 
hsa-miR-1-3p
 
 
-0.315
 
 
87.5
 
 
 
 
CDC34
 
 
hsa-let-7e-5p
 
 
-0.321
 
 
107.6
 
 
 
 
 
 
 skin 
 
 
 
 
target_symbol
 
 
mature_mirna_id
 
 
score
 
 
nTPM
 
 
 
 
 
 
ABCB7
 
 
hsa-miR-1-3p
 
 
-0.365
 
 
12.7
 
 
 
 
ABHD17C
 
 
hsa-let-7e-5p
 
 
-0.359
 
 
17.9
 
 
 
 
ABHD2
 
 
hsa-miR-1-3p
 
 
-0.291
 
 
12.5
 
 
 
 
ABRAXAS2
 
 
hsa-miR-130b-3p
 
 
-0.468
 
 
11.0
 
 
 
 
ACBD3
 
 
hsa-miR-130b-3p
 
 
-0.292
 
 
18.1
 
 
 
 
ACBD5
 
 
hsa-miR-130b-3p
 
 
-0.494
 
 
16.3
 
 
 
 
ACP3
 
 
hsa-let-7e-5p
 
 
-0.298
 
 
25.5
 
 
 
 
ACTC1
 
 
hsa-miR-7-5p
 
 
-0.471
 
 
14.2
 
 
 
 
ADAR
 
 
hsa-miR-1-3p
 
 
-0.35
 
 
31.7
 
 
 
 
ADRB2
 
 
hsa-let-7e-5p
 
 
-0.635
 
 
36.9
 
 
 
 
AIFM1
 
 
hsa-let-7e-5p
 
 
-0.314
 
 
10.3
 
 
 
 
AKIRIN2
 
 
hsa-miR-130b-3p
 
 
-0.315
 
 
20.2
 
 
 
 
ALDH4A1
 
 
hsa-miR-184
 
 
-0.421
 
 
18.1
 
 
 
 
ALG9
 
 
hsa-miR-7-5p
 
 
-0.302
 
 
13.9
 
 
 
 
ANKIB1
 
 
hsa-miR-130b-3p
 
 
-0.329
 
 
13.6
 
 
 
 
ANKRD12
 
 
hsa-miR-7-5p
 
 
-0.37
 
 
19.1
 
 
 
 
ANKRD46
 
 
hsa-let-7e-5p
 
 
-0.389
 
 
10.5
 
 
 
 
ANP32B
 
 
hsa-miR-1-3p
 
 
-0.287
 
 
140.0
 
 
 
 
ANXA2
 
 
hsa-miR-1-3p
 
 
-0.581
 
 
1033.9
 
 
 
 
ANXA4
 
 
hsa-miR-1-3p
 
 
-0.539
 
 
57.6
 
 
 
 
AP1AR
 
 
hsa-miR-100-5p
 
 
-0.804
 
 
13.4
 
 
 
 
AP1S1
 
 
hsa-miR-1-3p
 
 
-0.355
 
 
40.6
 
 
 
 
AP1S1
 
 
hsa-let-7e-5p
 
 
-0.374
 
 
40.6
 
 
 
 
APCDD1
 
 
hsa-miR-130b-3p
 
 
-0.304
 
 
122.7
 
 
 
 
APPL1
 
 
hsa-miR-130b-3p
 
 
-0.347
 
 
19.0
 
 
 
 
ARCN1
 
 
hsa-miR-1-3p
 
 
-0.468
 
 
55.2
 
 
 
 
ARF4
 
 
hsa-miR-7-5p
 
 
-0.407
 
 
61.3
 
 
 
 
ARFGEF1
 
 
hsa-miR-130b-3p
 
 
-0.302
 
 
12.9
 
 
 
 
ARHGAP1
 
 
hsa-miR-130b-3p
 
 
-0.376
 
 
61.6
 
 
 
 
ARHGEF4
 
 
hsa-miR-130b-3p
 
 
-0.377
 
 
37.8
 
 
 
 
ARL4D
 
 
hsa-let-7e-5p
 
 
-0.417
 
 
44.0
 
 
 
 
ARL6IP1
 
 
hsa-miR-130b-3p
 
 
-0.289
 
 
62.4
 
 
 
 
ARL6IP1
 
 
hsa-miR-7-5p
 
 
-0.374
 
 
62.4
 
 
 
 
ARL6IP6
 
 
hsa-let-7e-5p
 
 
-0.305
 
 
23.5
 
 
 
 
ARPC3
 
 
hsa-miR-1-3p
 
 
-0.606
 
 
161.9
 
 
 
 
ARPP19
 
 
hsa-let-7e-5p
 
 
-0.492
 
 
28.9
 
 
 
 
ARRDC4
 
 
hsa-let-7e-5p
 
 
-0.329
 
 
33.5
 
 
 
 
ASAP1
 
 
hsa-let-7e-5p
 
 
-0.296
 
 
16.2
 
 
 
 
ASH2L
 
 
hsa-miR-1-3p
 
 
-0.462
 
 
19.7
 
 
 
 
ATG16L1
 
 
hsa-miR-130b-3p
 
 
-0.407
 
 
17.2
 
 
 
 
ATOSB
 
 
hsa-let-7e-5p
 
 
-0.429
 
 
14.2
 
 
 
 
ATP2A2
 
 
hsa-let-7e-5p
 
 
-0.311
 
 
41.6
 
 
 
 
ATPAF1
 
 
hsa-let-7e-5p
 
 
-0.498
 
 
18.2
 
 
 
 
AVPI1
 
 
hsa-miR-7-5p
 
 
-0.367
 
 
104.5
 
 
 
 
AXL
 
 
hsa-miR-1-3p
 
 
-0.304
 
 
18.9
 
 
 
 
AZIN1
 
 
hsa-miR-1-3p
 
 
-0.438
 
 
30.1
 
 
 
 
B3GAT3
 
 
hsa-let-7e-5p
 
 
-0.324
 
 
37.0
 
 
 
 
B4GAT1
 
 
hsa-let-7e-5p
 
 
-0.47
 
 
11.9
 
 
 
 
BACH1
 
 
hsa-let-7e-5p
 
 
-0.298
 
 
21.9
 
 
 
 
BACH1
 
 
hsa-let-7e-5p
 
 
-0.298
 
 
21.9
 
 
 
 
BACH1
 
 
hsa-let-7e-5p
 
 
-0.435
 
 
21.9
 
 
 
 
BACH1
 
 
hsa-let-7e-5p
 
 
-0.435
 
 
21.9
 
 
 
 
BAHD1
 
 
hsa-miR-130b-3p
 
 
-0.294
 
 
10.4
 
 
 
 
BAZ2A
 
 
hsa-miR-100-5p
 
 
-0.29
 
 
27.8
 
 
 
 
BAZ2A
 
 
hsa-miR-100-5p
 
 
-0.421
 
 
27.8
 
 
 
 
BCAP29
 
 
hsa-let-7e-5p
 
 
-0.289
 
 
23.3
 
 
 
 
BET1
 
 
hsa-miR-1-3p
 
 
-0.413
 
 
13.0
 
 
 
 
BHLHE40
 
 
hsa-miR-130b-3p
 
 
-0.351
 
 
217.6
 
 
 
 
BIN3
 
 
hsa-let-7e-5p
 
 
-0.427
 
 
15.8
 
 
 
 
BLCAP
 
 
hsa-miR-130b-3p
 
 
-0.342
 
 
83.4
 
 
 
 
BLOC1S6
 
 
hsa-let-7e-5p
 
 
-0.321
 
 
17.2
 
 
 
 
BPNT1
 
 
hsa-miR-1-3p
 
 
-0.329
 
 
10.2
 
 
 
 
BRIX1
 
 
hsa-miR-1-3p
 
 
-0.334
 
 
14.9
 
 
 
 
BSCL2
 
 
hsa-miR-1-3p
 
 
-0.471
 
 
22.1
 
 
 
 
BTAF1
 
 
hsa-miR-130b-3p
 
 
-0.291
 
 
39.9
 
 
 
 
BTF3L4
 
 
hsa-let-7e-5p
 
 
-0.355
 
 
33.3
 
 
 
 
BTF3L4
 
 
hsa-miR-130b-3p
 
 
-0.359
 
 
33.3
 
 
 
 
BTG1
 
 
hsa-miR-130b-3p
 
 
-0.565
 
 
21.1
 
 
 
 
BZW1
 
 
hsa-let-7e-5p
 
 
-0.354
 
 
45.2
 
 
 
 
BZW1
 
 
hsa-let-7e-5p
 
 
-0.375
 
 
45.2
 
 
 
 
BZW2
 
 
hsa-let-7e-5p
 
 
-0.408
 
 
52.4
 
 
 
 
C15orf39
 
 
hsa-let-7e-5p
 
 
-0.374
 
 
14.5
 
 
 
 
C19orf47
 
 
hsa-let-7e-5p
 
 
-0.311
 
 
11.4
 
 
 
 
C1orf21
 
 
hsa-miR-7-5p
 
 
-0.373
 
 
13.4
 
 
 
 
C3orf52
 
 
hsa-let-7e-5p
 
 
-0.296
 
 
25.9
 
 
 
 
C8orf58
 
 
hsa-let-7e-5p
 
 
-0.612
 
 
12.9
 
 
 
 
CALM2
 
 
hsa-miR-130b-3p
 
 
-0.584
 
 
293.2
 
 
 
 
CAMSAP2
 
 
hsa-miR-130b-3p
 
 
-0.297
 
 
13.3
 
 
 
 
CANT1
 
 
hsa-let-7e-5p
 
 
-0.402
 
 
13.6
 
 
 
 
CAPN3
 
 
hsa-let-7e-5p
 
 
-0.317
 
 
51.8
 
 
 
 
CAPZA1
 
 
hsa-miR-7-5p
 
 
-0.351
 
 
87.3
 
 
 
 
CASP3
 
 
hsa-let-7e-5p
 
 
-0.495
 
 
12.6
 
 
 
 
CBX5
 
 
hsa-let-7e-5p
 
 
-0.307
 
 
16.3
 
 
 
 
CBX5
 
 
hsa-let-7e-5p
 
 
-0.472
 
 
16.3
 
 
 
 
CCDC43
 
 
hsa-miR-7-5p
 
 
-0.331
 
 
10.7
 
 
 
 
CCNC
 
 
hsa-miR-1-3p
 
 
-0.336
 
 
30.7
 
 
 
 
CCNL2
 
 
hsa-miR-28-5p
 
 
-0.448
 
 
65.5
 
 
 
 
CCNT2
 
 
hsa-miR-7-5p
 
 
-0.349
 
 
13.5
 
 
 
 
CCNY
 
 
hsa-let-7e-5p
 
 
-0.334
 
 
17.4
 
 
 
 
CCNY
 
 
hsa-miR-130b-3p
 
 
-0.372
 
 
17.4
 
 
 
 
CCSAP
 
 
hsa-miR-1-3p
 
 
-0.381
 
 
10.5
 
 
 
 
CCT4
 
 
hsa-miR-7-5p
 
 
-0.37
 
 
66.3
 
 
 
 
CCT6A
 
 
hsa-miR-130b-3p
 
 
-0.382
 
 
56.0
 
 
 
 
CD164
 
 
hsa-miR-1-3p
 
 
-0.315
 
 
84.2
 
 
 
 
CD2AP
 
 
hsa-miR-130b-3p
 
 
-0.302
 
 
17.0
 
 
 
 
CD2AP
 
 
hsa-miR-1-3p
 
 
-0.479
 
 
17.0
 
 
 
 
CDC34
 
 
hsa-let-7e-5p
 
 
-0.321
 
 
53.5
 
 
 
 
CDC34
 
 
hsa-let-7e-5p
 
 
-0.555
 
 
53.5
 
 
 
 
CDC42SE1
 
 
hsa-miR-1-3p
 
 
-0.36
 
 
60.0
 
 
 
 
CDK4
 
 
hsa-miR-1-3p
 
 
-0.338
 
 
61.0
 
 
 
 
 
 
 small intestine 
 
 
 
 
target_symbol
 
 
mature_mirna_id
 
 
score
 
 
nTPM
 
 
 
 
 
 
ABCB7
 
 
hsa-miR-1-3p
 
 
-0.365
 
 
11.2
 
 
 
 
ABHD17C
 
 
hsa-let-7e-5p
 
 
-0.359
 
 
28.8
 
 
 
 
ABHD2
 
 
hsa-miR-1-3p
 
 
-0.291
 
 
33.8
 
 
 
 
ABHD3
 
 
hsa-miR-130b-3p
 
 
-0.356
 
 
53.0
 
 
 
 
ABRAXAS2
 
 
hsa-miR-130b-3p
 
 
-0.468
 
 
10.1
 
 
 
 
ACBD3
 
 
hsa-miR-130b-3p
 
 
-0.292
 
 
16.5
 
 
 
 
ACBD5
 
 
hsa-miR-130b-3p
 
 
-0.494
 
 
36.9
 
 
 
 
ACSL4
 
 
hsa-miR-130b-3p
 
 
-0.301
 
 
12.1
 
 
 
 
ADAR
 
 
hsa-miR-1-3p
 
 
-0.35
 
 
46.7
 
 
 
 
ADPGK
 
 
hsa-miR-1-3p
 
 
-0.393
 
 
11.6
 
 
 
 
AIFM1
 
 
hsa-let-7e-5p
 
 
-0.314
 
 
28.2
 
 
 
 
AKIRIN2
 
 
hsa-miR-130b-3p
 
 
-0.315
 
 
20.2
 
 
 
 
AMT
 
 
hsa-let-7e-5p
 
 
-0.463
 
 
11.5
 
 
 
 
ANKIB1
 
 
hsa-miR-130b-3p
 
 
-0.329
 
 
11.3
 
 
 
 
ANKRD12
 
 
hsa-miR-7-5p
 
 
-0.37
 
 
12.6
 
 
 
 
ANKRD46
 
 
hsa-let-7e-5p
 
 
-0.389
 
 
10.4
 
 
 
 
ANP32B
 
 
hsa-miR-1-3p
 
 
-0.287
 
 
119.5
 
 
 
 
ANXA2
 
 
hsa-miR-1-3p
 
 
-0.581
 
 
458.7
 
 
 
 
ANXA4
 
 
hsa-miR-1-3p
 
 
-0.539
 
 
218.8
 
 
 
 
AP1AR
 
 
hsa-miR-100-5p
 
 
-0.804
 
 
22.4
 
 
 
 
AP1S1
 
 
hsa-miR-1-3p
 
 
-0.355
 
 
29.8
 
 
 
 
AP1S1
 
 
hsa-let-7e-5p
 
 
-0.374
 
 
29.8
 
 
 
 
APCDD1
 
 
hsa-miR-130b-3p
 
 
-0.304
 
 
13.7
 
 
 
 
APPL1
 
 
hsa-miR-130b-3p
 
 
-0.347
 
 
13.8
 
 
 
 
ARCN1
 
 
hsa-miR-1-3p
 
 
-0.468
 
 
60.4
 
 
 
 
ARF4
 
 
hsa-miR-7-5p
 
 
-0.407
 
 
102.8
 
 
 
 
ARFGEF1
 
 
hsa-miR-130b-3p
 
 
-0.302
 
 
16.5
 
 
 
 
ARG2
 
 
hsa-let-7e-5p
 
 
-0.468
 
 
14.4
 
 
 
 
ARHGAP1
 
 
hsa-miR-130b-3p
 
 
-0.376
 
 
44.5
 
 
 
 
ARHGAP12
 
 
hsa-miR-130b-3p
 
 
-0.287
 
 
14.9
 
 
 
 
ARL6IP1
 
 
hsa-miR-130b-3p
 
 
-0.289
 
 
116.2
 
 
 
 
ARL6IP1
 
 
hsa-miR-7-5p
 
 
-0.374
 
 
116.2
 
 
 
 
ARMT1
 
 
hsa-let-7e-5p
 
 
-0.303
 
 
13.7
 
 
 
 
ARPC3
 
 
hsa-miR-1-3p
 
 
-0.606
 
 
243.0
 
 
 
 
ARPP19
 
 
hsa-let-7e-5p
 
 
-0.492
 
 
28.8
 
 
 
 
ARRDC4
 
 
hsa-let-7e-5p
 
 
-0.329
 
 
22.0
 
 
 
 
ASAP1
 
 
hsa-let-7e-5p
 
 
-0.296
 
 
11.5
 
 
 
 
ASH2L
 
 
hsa-miR-1-3p
 
 
-0.462
 
 
17.5
 
 
 
 
ATF2
 
 
hsa-miR-1-3p
 
 
-0.328
 
 
12.8
 
 
 
 
ATG16L1
 
 
hsa-miR-130b-3p
 
 
-0.407
 
 
11.3
 
 
 
 
ATG4A
 
 
hsa-miR-7-5p
 
 
-0.448
 
 
10.7
 
 
 
 
ATOSB
 
 
hsa-let-7e-5p
 
 
-0.429
 
 
10.7
 
 
 
 
ATP2A2
 
 
hsa-let-7e-5p
 
 
-0.311
 
 
53.2
 
 
 
 
ATPAF1
 
 
hsa-let-7e-5p
 
 
-0.498
 
 
42.6
 
 
 
 
AVPI1
 
 
hsa-miR-7-5p
 
 
-0.367
 
 
15.7
 
 
 
 
AXL
 
 
hsa-miR-1-3p
 
 
-0.304
 
 
18.5
 
 
 
 
AZIN1
 
 
hsa-miR-1-3p
 
 
-0.438
 
 
74.7
 
 
 
 
B3GAT3
 
 
hsa-let-7e-5p
 
 
-0.324
 
 
30.1
 
 
 
 
B4GAT1
 
 
hsa-let-7e-5p
 
 
-0.47
 
 
14.2
 
 
 
 
BACH1
 
 
hsa-let-7e-5p
 
 
-0.298
 
 
16.3
 
 
 
 
BACH1
 
 
hsa-let-7e-5p
 
 
-0.298
 
 
16.3
 
 
 
 
BACH1
 
 
hsa-let-7e-5p
 
 
-0.435
 
 
16.3
 
 
 
 
BACH1
 
 
hsa-let-7e-5p
 
 
-0.435
 
 
16.3
 
 
 
 
BAHD1
 
 
hsa-miR-130b-3p
 
 
-0.294
 
 
11.4
 
 
 
 
BAZ2A
 
 
hsa-miR-100-5p
 
 
-0.29
 
 
28.7
 
 
 
 
BAZ2A
 
 
hsa-miR-100-5p
 
 
-0.421
 
 
28.7
 
 
 
 
BCAP29
 
 
hsa-let-7e-5p
 
 
-0.289
 
 
17.9
 
 
 
 
BET1
 
 
hsa-miR-1-3p
 
 
-0.413
 
 
22.0
 
 
 
 
BHLHE40
 
 
hsa-miR-130b-3p
 
 
-0.351
 
 
45.0
 
 
 
 
BIN3
 
 
hsa-let-7e-5p
 
 
-0.427
 
 
12.8
 
 
 
 
BLCAP
 
 
hsa-miR-130b-3p
 
 
-0.342
 
 
46.3
 
 
 
 
BLOC1S4
 
 
hsa-miR-7-5p
 
 
-0.417
 
 
11.6
 
 
 
 
BLOC1S4
 
 
hsa-miR-7-5p
 
 
-0.527
 
 
11.6
 
 
 
 
BLOC1S6
 
 
hsa-let-7e-5p
 
 
-0.321
 
 
20.1
 
 
 
 
BPNT1
 
 
hsa-miR-1-3p
 
 
-0.329
 
 
43.0
 
 
 
 
BRI3BP
 
 
hsa-miR-1-3p
 
 
-0.293
 
 
17.7
 
 
 
 
BRIX1
 
 
hsa-miR-1-3p
 
 
-0.334
 
 
12.0
 
 
 
 
BSCL2
 
 
hsa-miR-1-3p
 
 
-0.471
 
 
14.4
 
 
 
 
BTAF1
 
 
hsa-miR-130b-3p
 
 
-0.291
 
 
18.1
 
 
 
 
BTF3L4
 
 
hsa-let-7e-5p
 
 
-0.355
 
 
17.1
 
 
 
 
BTF3L4
 
 
hsa-miR-130b-3p
 
 
-0.359
 
 
17.1
 
 
 
 
BTG1
 
 
hsa-miR-130b-3p
 
 
-0.565
 
 
10.1
 
 
 
 
BZW1
 
 
hsa-let-7e-5p
 
 
-0.354
 
 
67.2
 
 
 
 
BZW1
 
 
hsa-let-7e-5p
 
 
-0.375
 
 
67.2
 
 
 
 
BZW2
 
 
hsa-let-7e-5p
 
 
-0.408
 
 
28.2
 
 
 
 
C1GALT1
 
 
hsa-let-7e-5p
 
 
-0.322
 
 
18.1
 
 
 
 
C2orf88
 
 
hsa-let-7e-5p
 
 
-0.429
 
 
41.8
 
 
 
 
C6orf120
 
 
hsa-miR-1-3p
 
 
-0.288
 
 
13.3
 
 
 
 
C9orf40
 
 
hsa-let-7e-5p
 
 
-0.809
 
 
10.7
 
 
 
 
CAAP1
 
 
hsa-miR-1-3p
 
 
-0.48
 
 
12.0
 
 
 
 
CACFD1
 
 
hsa-let-7e-5p
 
 
-0.296
 
 
14.3
 
 
 
 
CALM2
 
 
hsa-miR-130b-3p
 
 
-0.584
 
 
584.4
 
 
 
 
CANT1
 
 
hsa-let-7e-5p
 
 
-0.402
 
 
52.3
 
 
 
 
CAPN3
 
 
hsa-let-7e-5p
 
 
-0.317
 
 
15.6
 
 
 
 
CAPZA1
 
 
hsa-miR-7-5p
 
 
-0.351
 
 
66.7
 
 
 
 
CARNMT1
 
 
hsa-let-7e-5p
 
 
-0.307
 
 
11.6
 
 
 
 
CASP3
 
 
hsa-let-7e-5p
 
 
-0.495
 
 
36.3
 
 
 
 
CASP5
 
 
hsa-let-7e-5p
 
 
-0.379
 
 
15.8
 
 
 
 
CBFA2T3
 
 
hsa-let-7e-5p
 
 
-0.305
 
 
10.0
 
 
 
 
CCNC
 
 
hsa-miR-1-3p
 
 
-0.336
 
 
32.9
 
 
 
 
CCNL2
 
 
hsa-miR-28-5p
 
 
-0.448
 
 
65.4
 
 
 
 
CCNT2
 
 
hsa-miR-7-5p
 
 
-0.349
 
 
13.5
 
 
 
 
CCNY
 
 
hsa-let-7e-5p
 
 
-0.334
 
 
20.9
 
 
 
 
CCNY
 
 
hsa-miR-130b-3p
 
 
-0.372
 
 
20.9
 
 
 
 
CCR7
 
 
hsa-let-7e-5p
 
 
-0.482
 
 
19.7
 
 
 
 
CCT4
 
 
hsa-miR-7-5p
 
 
-0.37
 
 
48.3
 
 
 
 
CCT6A
 
 
hsa-miR-130b-3p
 
 
-0.382
 
 
49.9
 
 
 
 
CD164
 
 
hsa-miR-1-3p
 
 
-0.315
 
 
261.5
 
 
 
 
CD2AP
 
 
hsa-miR-130b-3p
 
 
-0.302
 
 
56.0
 
 
 
 
CD2AP
 
 
hsa-miR-1-3p
 
 
-0.479
 
 
56.0
 
 
 
 
 
 
 spleen 
 
 
 
 
target_symbol
 
 
mature_mirna_id
 
 
score
 
 
nTPM
 
 
 
 
 
 
ABCB7
 
 
hsa-miR-1-3p
 
 
-0.365
 
 
10.6
 
 
 
 
ABHD2
 
 
hsa-miR-1-3p
 
 
-0.291
 
 
23.2
 
 
 
 
ABHD3
 
 
hsa-miR-130b-3p
 
 
-0.356
 
 
22.6
 
 
 
 
ACBD3
 
 
hsa-miR-130b-3p
 
 
-0.292
 
 
16.7
 
 
 
 
ACBD5
 
 
hsa-miR-130b-3p
 
 
-0.494
 
 
13.9
 
 
 
 
ACSL4
 
 
hsa-miR-130b-3p
 
 
-0.301
 
 
19.4
 
 
 
 
ADAR
 
 
hsa-miR-1-3p
 
 
-0.35
 
 
69.3
 
 
 
 
ADPGK
 
 
hsa-miR-1-3p
 
 
-0.393
 
 
17.9
 
 
 
 
AIFM1
 
 
hsa-let-7e-5p
 
 
-0.314
 
 
12.9
 
 
 
 
AKIRIN2
 
 
hsa-miR-130b-3p
 
 
-0.315
 
 
43.9
 
 
 
 
ANKIB1
 
 
hsa-miR-130b-3p
 
 
-0.329
 
 
11.7
 
 
 
 
ANKRD12
 
 
hsa-miR-7-5p
 
 
-0.37
 
 
18.8
 
 
 
 
ANKRD46
 
 
hsa-let-7e-5p
 
 
-0.389
 
 
10.4
 
 
 
 
ANP32B
 
 
hsa-miR-1-3p
 
 
-0.287
 
 
117.6
 
 
 
 
ANXA2
 
 
hsa-miR-1-3p
 
 
-0.581
 
 
271.7
 
 
 
 
ANXA4
 
 
hsa-miR-1-3p
 
 
-0.539
 
 
22.8
 
 
 
 
AP1AR
 
 
hsa-miR-100-5p
 
 
-0.804
 
 
12.4
 
 
 
 
AP1S1
 
 
hsa-miR-1-3p
 
 
-0.355
 
 
20.9
 
 
 
 
AP1S1
 
 
hsa-let-7e-5p
 
 
-0.374
 
 
20.9
 
 
 
 
APPL1
 
 
hsa-miR-130b-3p
 
 
-0.347
 
 
14.9
 
 
 
 
ARCN1
 
 
hsa-miR-1-3p
 
 
-0.468
 
 
53.9
 
 
 
 
ARF4
 
 
hsa-miR-7-5p
 
 
-0.407
 
 
60.0
 
 
 
 
ARFGEF1
 
 
hsa-miR-130b-3p
 
 
-0.302
 
 
13.0
 
 
 
 
ARHGAP1
 
 
hsa-miR-130b-3p
 
 
-0.376
 
 
55.1
 
 
 
 
ARHGAP12
 
 
hsa-miR-130b-3p
 
 
-0.287
 
 
16.8
 
 
 
 
ARHGAP24
 
 
hsa-miR-130b-3p
 
 
-0.287
 
 
15.5
 
 
 
 
ARHGAP28
 
 
hsa-let-7e-5p
 
 
-0.486
 
 
11.9
 
 
 
 
ARID4A
 
 
hsa-miR-7-5p
 
 
-0.341
 
 
13.2
 
 
 
 
ARL4D
 
 
hsa-let-7e-5p
 
 
-0.417
 
 
16.1
 
 
 
 
ARL6IP1
 
 
hsa-miR-130b-3p
 
 
-0.289
 
 
68.6
 
 
 
 
ARL6IP1
 
 
hsa-miR-7-5p
 
 
-0.374
 
 
68.6
 
 
 
 
ARL6IP6
 
 
hsa-let-7e-5p
 
 
-0.305
 
 
13.7
 
 
 
 
ARMT1
 
 
hsa-let-7e-5p
 
 
-0.303
 
 
12.0
 
 
 
 
ARPC3
 
 
hsa-miR-1-3p
 
 
-0.606
 
 
376.6
 
 
 
 
ARPP19
 
 
hsa-let-7e-5p
 
 
-0.492
 
 
41.2
 
 
 
 
AS3MT
 
 
hsa-miR-28-5p
 
 
-0.479
 
 
16.2
 
 
 
 
ASAP1
 
 
hsa-let-7e-5p
 
 
-0.296
 
 
28.1
 
 
 
 
ASH2L
 
 
hsa-miR-1-3p
 
 
-0.462
 
 
18.2
 
 
 
 
ATF2
 
 
hsa-miR-1-3p
 
 
-0.328
 
 
16.6
 
 
 
 
ATG16L1
 
 
hsa-miR-130b-3p
 
 
-0.407
 
 
11.4
 
 
 
 
ATOSB
 
 
hsa-let-7e-5p
 
 
-0.429
 
 
26.6
 
 
 
 
ATP2A2
 
 
hsa-let-7e-5p
 
 
-0.311
 
 
28.8
 
 
 
 
ATPAF1
 
 
hsa-let-7e-5p
 
 
-0.498
 
 
23.6
 
 
 
 
AVPI1
 
 
hsa-miR-7-5p
 
 
-0.367
 
 
20.8
 
 
 
 
AXL
 
 
hsa-miR-1-3p
 
 
-0.304
 
 
35.4
 
 
 
 
AZIN1
 
 
hsa-miR-1-3p
 
 
-0.438
 
 
33.1
 
 
 
 
B3GAT3
 
 
hsa-let-7e-5p
 
 
-0.324
 
 
44.2
 
 
 
 
B4GAT1
 
 
hsa-let-7e-5p
 
 
-0.47
 
 
10.0
 
 
 
 
BACH1
 
 
hsa-let-7e-5p
 
 
-0.298
 
 
19.4
 
 
 
 
BACH1
 
 
hsa-let-7e-5p
 
 
-0.298
 
 
19.4
 
 
 
 
BACH1
 
 
hsa-let-7e-5p
 
 
-0.435
 
 
19.4
 
 
 
 
BACH1
 
 
hsa-let-7e-5p
 
 
-0.435
 
 
19.4
 
 
 
 
BAHD1
 
 
hsa-miR-130b-3p
 
 
-0.294
 
 
11.9
 
 
 
 
BAZ2A
 
 
hsa-miR-100-5p
 
 
-0.29
 
 
33.2
 
 
 
 
BAZ2A
 
 
hsa-miR-100-5p
 
 
-0.421
 
 
33.2
 
 
 
 
BCAP29
 
 
hsa-let-7e-5p
 
 
-0.289
 
 
17.6
 
 
 
 
BET1
 
 
hsa-miR-1-3p
 
 
-0.413
 
 
15.7
 
 
 
 
BHLHE40
 
 
hsa-miR-130b-3p
 
 
-0.351
 
 
28.2
 
 
 
 
BIN3
 
 
hsa-let-7e-5p
 
 
-0.427
 
 
14.4
 
 
 
 
BLCAP
 
 
hsa-miR-130b-3p
 
 
-0.342
 
 
45.7
 
 
 
 
BLOC1S4
 
 
hsa-miR-7-5p
 
 
-0.417
 
 
13.5
 
 
 
 
BLOC1S4
 
 
hsa-miR-7-5p
 
 
-0.527
 
 
13.5
 
 
 
 
BLOC1S6
 
 
hsa-let-7e-5p
 
 
-0.321
 
 
26.3
 
 
 
 
BMPR2
 
 
hsa-miR-130b-3p
 
 
-0.33
 
 
11.5
 
 
 
 
BMPR2
 
 
hsa-miR-100-5p
 
 
-0.355
 
 
11.5
 
 
 
 
BPNT1
 
 
hsa-miR-1-3p
 
 
-0.329
 
 
13.7
 
 
 
 
BRIX1
 
 
hsa-miR-1-3p
 
 
-0.334
 
 
13.3
 
 
 
 
BSCL2
 
 
hsa-miR-1-3p
 
 
-0.471
 
 
13.7
 
 
 
 
BTAF1
 
 
hsa-miR-130b-3p
 
 
-0.291
 
 
17.6
 
 
 
 
BTF3L4
 
 
hsa-let-7e-5p
 
 
-0.355
 
 
26.5
 
 
 
 
BTF3L4
 
 
hsa-miR-130b-3p
 
 
-0.359
 
 
26.5
 
 
 
 
BTG1
 
 
hsa-miR-130b-3p
 
 
-0.565
 
 
22.1
 
 
 
 
BZW1
 
 
hsa-let-7e-5p
 
 
-0.354
 
 
59.1
 
 
 
 
BZW1
 
 
hsa-let-7e-5p
 
 
-0.375
 
 
59.1
 
 
 
 
BZW2
 
 
hsa-let-7e-5p
 
 
-0.408
 
 
16.1
 
 
 
 
C15orf39
 
 
hsa-let-7e-5p
 
 
-0.374
 
 
24.1
 
 
 
 
C1GALT1
 
 
hsa-let-7e-5p
 
 
-0.322
 
 
14.6
 
 
 
 
C6orf120
 
 
hsa-miR-1-3p
 
 
-0.288
 
 
12.2
 
 
 
 
C8orf58
 
 
hsa-let-7e-5p
 
 
-0.612
 
 
24.5
 
 
 
 
CALM2
 
 
hsa-miR-130b-3p
 
 
-0.584
 
 
605.2
 
 
 
 
CANT1
 
 
hsa-let-7e-5p
 
 
-0.402
 
 
24.4
 
 
 
 
CAPN3
 
 
hsa-let-7e-5p
 
 
-0.317
 
 
23.6
 
 
 
 
CAPZA1
 
 
hsa-miR-7-5p
 
 
-0.351
 
 
99.1
 
 
 
 
CASD1
 
 
hsa-miR-130b-3p
 
 
-0.305
 
 
10.0
 
 
 
 
CASP3
 
 
hsa-let-7e-5p
 
 
-0.495
 
 
19.4
 
 
 
 
CBFA2T3
 
 
hsa-let-7e-5p
 
 
-0.305
 
 
17.3
 
 
 
 
CBFB
 
 
hsa-miR-130b-3p
 
 
-0.498
 
 
12.9
 
 
 
 
CBX5
 
 
hsa-let-7e-5p
 
 
-0.307
 
 
10.1
 
 
 
 
CBX5
 
 
hsa-let-7e-5p
 
 
-0.472
 
 
10.1
 
 
 
 
CCNC
 
 
hsa-miR-1-3p
 
 
-0.336
 
 
22.7
 
 
 
 
CCNL2
 
 
hsa-miR-28-5p
 
 
-0.448
 
 
47.6
 
 
 
 
CCNT2
 
 
hsa-miR-7-5p
 
 
-0.349
 
 
14.5
 
 
 
 
CCNY
 
 
hsa-let-7e-5p
 
 
-0.334
 
 
23.0
 
 
 
 
CCNY
 
 
hsa-miR-130b-3p
 
 
-0.372
 
 
23.0
 
 
 
 
CCR7
 
 
hsa-let-7e-5p
 
 
-0.482
 
 
27.0
 
 
 
 
CCT4
 
 
hsa-miR-7-5p
 
 
-0.37
 
 
55.2
 
 
 
 
CCT6A
 
 
hsa-miR-130b-3p
 
 
-0.382
 
 
44.4
 
 
 
 
CD164
 
 
hsa-miR-1-3p
 
 
-0.315
 
 
114.1
 
 
 
 
CD2AP
 
 
hsa-miR-130b-3p
 
 
-0.302
 
 
12.9
 
 
 
 
CD2AP
 
 
hsa-miR-1-3p
 
 
-0.479
 
 
12.9
 
 
 
 
 
 
 stomach 
 
 
 
 
target_symbol
 
 
mature_mirna_id
 
 
score
 
 
nTPM
 
 
 
 
 
 
ABCB7
 
 
hsa-miR-1-3p
 
 
-0.365
 
 
11.6
 
 
 
 
ABHD17C
 
 
hsa-let-7e-5p
 
 
-0.359
 
 
34.0
 
 
 
 
ABHD2
 
 
hsa-miR-1-3p
 
 
-0.291
 
 
68.9
 
 
 
 
ABHD3
 
 
hsa-miR-130b-3p
 
 
-0.356
 
 
33.6
 
 
 
 
ACBD3
 
 
hsa-miR-130b-3p
 
 
-0.292
 
 
29.9
 
 
 
 
ACBD5
 
 
hsa-miR-130b-3p
 
 
-0.494
 
 
19.8
 
 
 
 
ACER2
 
 
hsa-let-7e-5p
 
 
-0.333
 
 
58.3
 
 
 
 
ACSL4
 
 
hsa-miR-130b-3p
 
 
-0.301
 
 
17.6
 
 
 
 
ACTC1
 
 
hsa-miR-7-5p
 
 
-0.471
 
 
32.8
 
 
 
 
ACVR1
 
 
hsa-miR-130b-3p
 
 
-0.44
 
 
11.0
 
 
 
 
ADAR
 
 
hsa-miR-1-3p
 
 
-0.35
 
 
38.0
 
 
 
 
ADPGK
 
 
hsa-miR-1-3p
 
 
-0.393
 
 
13.3
 
 
 
 
AIFM1
 
 
hsa-let-7e-5p
 
 
-0.314
 
 
29.8
 
 
 
 
AKIRIN2
 
 
hsa-miR-130b-3p
 
 
-0.315
 
 
14.8
 
 
 
 
ALDH4A1
 
 
hsa-miR-184
 
 
-0.421
 
 
12.6
 
 
 
 
ALG11
 
 
hsa-miR-1-3p
 
 
-0.317
 
 
11.1
 
 
 
 
ALG9
 
 
hsa-miR-7-5p
 
 
-0.302
 
 
11.1
 
 
 
 
AMT
 
 
hsa-let-7e-5p
 
 
-0.463
 
 
13.1
 
 
 
 
ANKIB1
 
 
hsa-miR-130b-3p
 
 
-0.329
 
 
11.0
 
 
 
 
ANKRD12
 
 
hsa-miR-7-5p
 
 
-0.37
 
 
13.7
 
 
 
 
ANKRD29
 
 
hsa-miR-1-3p
 
 
-0.426
 
 
14.3
 
 
 
 
ANKRD46
 
 
hsa-let-7e-5p
 
 
-0.389
 
 
10.9
 
 
 
 
ANP32B
 
 
hsa-miR-1-3p
 
 
-0.287
 
 
125.6
 
 
 
 
ANXA2
 
 
hsa-miR-1-3p
 
 
-0.581
 
 
357.3
 
 
 
 
ANXA4
 
 
hsa-miR-1-3p
 
 
-0.539
 
 
102.1
 
 
 
 
AP1AR
 
 
hsa-miR-100-5p
 
 
-0.804
 
 
12.3
 
 
 
 
AP1S1
 
 
hsa-miR-1-3p
 
 
-0.355
 
 
23.2
 
 
 
 
AP1S1
 
 
hsa-let-7e-5p
 
 
-0.374
 
 
23.2
 
 
 
 
APCDD1
 
 
hsa-miR-130b-3p
 
 
-0.304
 
 
22.7
 
 
 
 
APPL1
 
 
hsa-miR-130b-3p
 
 
-0.347
 
 
14.3
 
 
 
 
ARCN1
 
 
hsa-miR-1-3p
 
 
-0.468
 
 
64.3
 
 
 
 
ARF4
 
 
hsa-miR-7-5p
 
 
-0.407
 
 
123.5
 
 
 
 
ARFGEF1
 
 
hsa-miR-130b-3p
 
 
-0.302
 
 
18.5
 
 
 
 
ARHGAP1
 
 
hsa-miR-130b-3p
 
 
-0.376
 
 
46.3
 
 
 
 
ARHGAP12
 
 
hsa-miR-130b-3p
 
 
-0.287
 
 
27.8
 
 
 
 
ARHGAP24
 
 
hsa-miR-130b-3p
 
 
-0.287
 
 
24.6
 
 
 
 
ARL6IP1
 
 
hsa-miR-130b-3p
 
 
-0.289
 
 
118.7
 
 
 
 
ARL6IP1
 
 
hsa-miR-7-5p
 
 
-0.374
 
 
118.7
 
 
 
 
ARMT1
 
 
hsa-let-7e-5p
 
 
-0.303
 
 
13.3
 
 
 
 
ARPC3
 
 
hsa-miR-1-3p
 
 
-0.606
 
 
248.3
 
 
 
 
ARPP19
 
 
hsa-let-7e-5p
 
 
-0.492
 
 
36.4
 
 
 
 
ARRDC4
 
 
hsa-let-7e-5p
 
 
-0.329
 
 
25.2
 
 
 
 
ASH2L
 
 
hsa-miR-1-3p
 
 
-0.462
 
 
20.9
 
 
 
 
ATF2
 
 
hsa-miR-1-3p
 
 
-0.328
 
 
13.5
 
 
 
 
ATG16L1
 
 
hsa-miR-130b-3p
 
 
-0.407
 
 
11.5
 
 
 
 
ATG4A
 
 
hsa-miR-7-5p
 
 
-0.448
 
 
11.6
 
 
 
 
ATOSB
 
 
hsa-let-7e-5p
 
 
-0.429
 
 
11.2
 
 
 
 
ATP2A2
 
 
hsa-let-7e-5p
 
 
-0.311
 
 
44.5
 
 
 
 
ATPAF1
 
 
hsa-let-7e-5p
 
 
-0.498
 
 
40.7
 
 
 
 
AVPI1
 
 
hsa-miR-7-5p
 
 
-0.367
 
 
31.1
 
 
 
 
AXL
 
 
hsa-miR-1-3p
 
 
-0.304
 
 
20.2
 
 
 
 
AZIN1
 
 
hsa-miR-1-3p
 
 
-0.438
 
 
48.8
 
 
 
 
B3GAT3
 
 
hsa-let-7e-5p
 
 
-0.324
 
 
32.0
 
 
 
 
B4GAT1
 
 
hsa-let-7e-5p
 
 
-0.47
 
 
25.1
 
 
 
 
BACE2
 
 
hsa-let-7e-5p
 
 
-0.364
 
 
21.6
 
 
 
 
BACH1
 
 
hsa-let-7e-5p
 
 
-0.298
 
 
12.7
 
 
 
 
BACH1
 
 
hsa-let-7e-5p
 
 
-0.298
 
 
12.7
 
 
 
 
BACH1
 
 
hsa-let-7e-5p
 
 
-0.435
 
 
12.7
 
 
 
 
BACH1
 
 
hsa-let-7e-5p
 
 
-0.435
 
 
12.7
 
 
 
 
BAHD1
 
 
hsa-miR-130b-3p
 
 
-0.294
 
 
12.1
 
 
 
 
BAZ2A
 
 
hsa-miR-100-5p
 
 
-0.29
 
 
24.7
 
 
 
 
BAZ2A
 
 
hsa-miR-100-5p
 
 
-0.421
 
 
24.7
 
 
 
 
BCAP29
 
 
hsa-let-7e-5p
 
 
-0.289
 
 
19.2
 
 
 
 
BET1
 
 
hsa-miR-1-3p
 
 
-0.413
 
 
18.8
 
 
 
 
BHLHE40
 
 
hsa-miR-130b-3p
 
 
-0.351
 
 
47.9
 
 
 
 
BIN3
 
 
hsa-let-7e-5p
 
 
-0.427
 
 
14.3
 
 
 
 
BLCAP
 
 
hsa-miR-130b-3p
 
 
-0.342
 
 
37.8
 
 
 
 
BLOC1S6
 
 
hsa-let-7e-5p
 
 
-0.321
 
 
18.9
 
 
 
 
BPNT1
 
 
hsa-miR-1-3p
 
 
-0.329
 
 
18.6
 
 
 
 
BRIX1
 
 
hsa-miR-1-3p
 
 
-0.334
 
 
12.7
 
 
 
 
BSCL2
 
 
hsa-miR-1-3p
 
 
-0.471
 
 
31.8
 
 
 
 
BTAF1
 
 
hsa-miR-130b-3p
 
 
-0.291
 
 
15.2
 
 
 
 
BTF3L4
 
 
hsa-let-7e-5p
 
 
-0.355
 
 
26.1
 
 
 
 
BTF3L4
 
 
hsa-miR-130b-3p
 
 
-0.359
 
 
26.1
 
 
 
 
BTG1
 
 
hsa-miR-130b-3p
 
 
-0.565
 
 
13.2
 
 
 
 
BZW1
 
 
hsa-let-7e-5p
 
 
-0.354
 
 
65.1
 
 
 
 
BZW1
 
 
hsa-let-7e-5p
 
 
-0.375
 
 
65.1
 
 
 
 
BZW2
 
 
hsa-let-7e-5p
 
 
-0.408
 
 
47.3
 
 
 
 
C1GALT1
 
 
hsa-let-7e-5p
 
 
-0.322
 
 
21.1
 
 
 
 
C3orf52
 
 
hsa-let-7e-5p
 
 
-0.296
 
 
12.9
 
 
 
 
C6orf120
 
 
hsa-miR-1-3p
 
 
-0.288
 
 
12.5
 
 
 
 
CAAP1
 
 
hsa-miR-1-3p
 
 
-0.48
 
 
10.6
 
 
 
 
CACFD1
 
 
hsa-let-7e-5p
 
 
-0.296
 
 
26.9
 
 
 
 
CALM2
 
 
hsa-miR-130b-3p
 
 
-0.584
 
 
368.3
 
 
 
 
CANT1
 
 
hsa-let-7e-5p
 
 
-0.402
 
 
48.2
 
 
 
 
CAPZA1
 
 
hsa-miR-7-5p
 
 
-0.351
 
 
67.0
 
 
 
 
CASP3
 
 
hsa-let-7e-5p
 
 
-0.495
 
 
26.1
 
 
 
 
CBFB
 
 
hsa-miR-130b-3p
 
 
-0.498
 
 
14.2
 
 
 
 
CCDC43
 
 
hsa-miR-7-5p
 
 
-0.331
 
 
10.6
 
 
 
 
CCNC
 
 
hsa-miR-1-3p
 
 
-0.336
 
 
39.3
 
 
 
 
CCNL2
 
 
hsa-miR-28-5p
 
 
-0.448
 
 
61.5
 
 
 
 
CCNT2
 
 
hsa-miR-7-5p
 
 
-0.349
 
 
13.8
 
 
 
 
CCNY
 
 
hsa-let-7e-5p
 
 
-0.334
 
 
22.7
 
 
 
 
CCNY
 
 
hsa-miR-130b-3p
 
 
-0.372
 
 
22.7
 
 
 
 
CCT4
 
 
hsa-miR-7-5p
 
 
-0.37
 
 
64.3
 
 
 
 
CCT6A
 
 
hsa-miR-130b-3p
 
 
-0.382
 
 
52.5
 
 
 
 
CD164
 
 
hsa-miR-1-3p
 
 
-0.315
 
 
257.6
 
 
 
 
CD2AP
 
 
hsa-miR-130b-3p
 
 
-0.302
 
 
38.1
 
 
 
 
CD2AP
 
 
hsa-miR-1-3p
 
 
-0.479
 
 
38.1
 
 
 
 
CD69
 
 
hsa-miR-130b-3p
 
 
-0.451
 
 
11.1
 
 
 
 
 
 
 thymus 
 
 
 
 
target_symbol
 
 
mature_mirna_id
 
 
score
 
 
nTPM
 
 
 
 
 
 
ABCB7
 
 
hsa-miR-1-3p
 
 
-0.365
 
 
13.0
 
 
 
 
ABHD2
 
 
hsa-miR-1-3p
 
 
-0.291
 
 
14.5
 
 
 
 
ABHD3
 
 
hsa-miR-130b-3p
 
 
-0.356
 
 
20.0
 
 
 
 
ABRAXAS2
 
 
hsa-miR-130b-3p
 
 
-0.468
 
 
12.0
 
 
 
 
ACBD3
 
 
hsa-miR-130b-3p
 
 
-0.292
 
 
16.8
 
 
 
 
ACBD5
 
 
hsa-miR-130b-3p
 
 
-0.494
 
 
11.4
 
 
 
 
ACSL4
 
 
hsa-miR-130b-3p
 
 
-0.301
 
 
18.3
 
 
 
 
ACTC1
 
 
hsa-miR-7-5p
 
 
-0.471
 
 
10.5
 
 
 
 
ADAR
 
 
hsa-miR-1-3p
 
 
-0.35
 
 
79.5
 
 
 
 
ADPGK
 
 
hsa-miR-1-3p
 
 
-0.393
 
 
19.6
 
 
 
 
AHCTF1
 
 
hsa-let-7e-5p
 
 
-0.374
 
 
11.4
 
 
 
 
AIFM1
 
 
hsa-let-7e-5p
 
 
-0.314
 
 
17.2
 
 
 
 
AKIRIN2
 
 
hsa-miR-130b-3p
 
 
-0.315
 
 
29.1
 
 
 
 
ALDH4A1
 
 
hsa-miR-184
 
 
-0.421
 
 
10.9
 
 
 
 
ANKIB1
 
 
hsa-miR-130b-3p
 
 
-0.329
 
 
13.5
 
 
 
 
ANKRD12
 
 
hsa-miR-7-5p
 
 
-0.37
 
 
10.7
 
 
 
 
ANKRD46
 
 
hsa-let-7e-5p
 
 
-0.389
 
 
14.3
 
 
 
 
ANP32B
 
 
hsa-miR-1-3p
 
 
-0.287
 
 
252.8
 
 
 
 
ANXA2
 
 
hsa-miR-1-3p
 
 
-0.581
 
 
176.1
 
 
 
 
ANXA4
 
 
hsa-miR-1-3p
 
 
-0.539
 
 
21.5
 
 
 
 
AP1AR
 
 
hsa-miR-100-5p
 
 
-0.804
 
 
10.2
 
 
 
 
AP1S1
 
 
hsa-miR-1-3p
 
 
-0.355
 
 
17.8
 
 
 
 
AP1S1
 
 
hsa-let-7e-5p
 
 
-0.374
 
 
17.8
 
 
 
 
APPL1
 
 
hsa-miR-130b-3p
 
 
-0.347
 
 
17.6
 
 
 
 
ARCN1
 
 
hsa-miR-1-3p
 
 
-0.468
 
 
54.4
 
 
 
 
ARF4
 
 
hsa-miR-7-5p
 
 
-0.407
 
 
69.9
 
 
 
 
ARFGEF1
 
 
hsa-miR-130b-3p
 
 
-0.302
 
 
20.7
 
 
 
 
ARHGAP1
 
 
hsa-miR-130b-3p
 
 
-0.376
 
 
21.6
 
 
 
 
ARID2
 
 
hsa-miR-1-3p
 
 
-0.294
 
 
17.5
 
 
 
 
ARID4A
 
 
hsa-miR-7-5p
 
 
-0.341
 
 
13.5
 
 
 
 
ARL6IP1
 
 
hsa-miR-130b-3p
 
 
-0.289
 
 
147.6
 
 
 
 
ARL6IP1
 
 
hsa-miR-7-5p
 
 
-0.374
 
 
147.6
 
 
 
 
ARL6IP6
 
 
hsa-let-7e-5p
 
 
-0.305
 
 
20.0
 
 
 
 
ARMT1
 
 
hsa-let-7e-5p
 
 
-0.303
 
 
14.5
 
 
 
 
ARPC3
 
 
hsa-miR-1-3p
 
 
-0.606
 
 
319.7
 
 
 
 
ARPP19
 
 
hsa-let-7e-5p
 
 
-0.492
 
 
49.2
 
 
 
 
ASAP1
 
 
hsa-let-7e-5p
 
 
-0.296
 
 
14.9
 
 
 
 
ASH2L
 
 
hsa-miR-1-3p
 
 
-0.462
 
 
31.4
 
 
 
 
ATF2
 
 
hsa-miR-1-3p
 
 
-0.328
 
 
26.3
 
 
 
 
ATG4A
 
 
hsa-miR-7-5p
 
 
-0.448
 
 
10.5
 
 
 
 
ATP2A2
 
 
hsa-let-7e-5p
 
 
-0.311
 
 
33.8
 
 
 
 
ATPAF1
 
 
hsa-let-7e-5p
 
 
-0.498
 
 
41.4
 
 
 
 
AZIN1
 
 
hsa-miR-1-3p
 
 
-0.438
 
 
52.8
 
 
 
 
B3GAT3
 
 
hsa-let-7e-5p
 
 
-0.324
 
 
17.5
 
 
 
 
BACH1
 
 
hsa-let-7e-5p
 
 
-0.298
 
 
13.2
 
 
 
 
BACH1
 
 
hsa-let-7e-5p
 
 
-0.298
 
 
13.2
 
 
 
 
BACH1
 
 
hsa-let-7e-5p
 
 
-0.435
 
 
13.2
 
 
 
 
BACH1
 
 
hsa-let-7e-5p
 
 
-0.435
 
 
13.2
 
 
 
 
BAHD1
 
 
hsa-miR-130b-3p
 
 
-0.294
 
 
10.8
 
 
 
 
BAZ2A
 
 
hsa-miR-100-5p
 
 
-0.29
 
 
47.1
 
 
 
 
BAZ2A
 
 
hsa-miR-100-5p
 
 
-0.421
 
 
47.1
 
 
 
 
BCAP29
 
 
hsa-let-7e-5p
 
 
-0.289
 
 
12.4
 
 
 
 
BET1
 
 
hsa-miR-1-3p
 
 
-0.413
 
 
18.3
 
 
 
 
BHLHE40
 
 
hsa-miR-130b-3p
 
 
-0.351
 
 
38.0
 
 
 
 
BIN3
 
 
hsa-let-7e-5p
 
 
-0.427
 
 
12.4
 
 
 
 
BLCAP
 
 
hsa-miR-130b-3p
 
 
-0.342
 
 
38.8
 
 
 
 
BLOC1S6
 
 
hsa-let-7e-5p
 
 
-0.321
 
 
23.5
 
 
 
 
BPNT1
 
 
hsa-miR-1-3p
 
 
-0.329
 
 
16.4
 
 
 
 
BRIX1
 
 
hsa-miR-1-3p
 
 
-0.334
 
 
19.3
 
 
 
 
BSCL2
 
 
hsa-miR-1-3p
 
 
-0.471
 
 
13.0
 
 
 
 
BTAF1
 
 
hsa-miR-130b-3p
 
 
-0.291
 
 
17.2
 
 
 
 
BTF3L4
 
 
hsa-let-7e-5p
 
 
-0.355
 
 
45.8
 
 
 
 
BTF3L4
 
 
hsa-miR-130b-3p
 
 
-0.359
 
 
45.8
 
 
 
 
BTG1
 
 
hsa-miR-130b-3p
 
 
-0.565
 
 
18.7
 
 
 
 
BZW1
 
 
hsa-let-7e-5p
 
 
-0.354
 
 
114.6
 
 
 
 
BZW1
 
 
hsa-let-7e-5p
 
 
-0.375
 
 
114.6
 
 
 
 
BZW2
 
 
hsa-let-7e-5p
 
 
-0.408
 
 
30.7
 
 
 
 
C1GALT1
 
 
hsa-let-7e-5p
 
 
-0.322
 
 
13.3
 
 
 
 
C6orf120
 
 
hsa-miR-1-3p
 
 
-0.288
 
 
10.0
 
 
 
 
CAAP1
 
 
hsa-miR-1-3p
 
 
-0.48
 
 
13.2
 
 
 
 
CALM2
 
 
hsa-miR-130b-3p
 
 
-0.584
 
 
531.8
 
 
 
 
CANT1
 
 
hsa-let-7e-5p
 
 
-0.402
 
 
13.7
 
 
 
 
CAPZA1
 
 
hsa-miR-7-5p
 
 
-0.351
 
 
110.1
 
 
 
 
CASD1
 
 
hsa-miR-130b-3p
 
 
-0.305
 
 
10.5
 
 
 
 
CASP3
 
 
hsa-let-7e-5p
 
 
-0.495
 
 
33.6
 
 
 
 
CBFA2T3
 
 
hsa-let-7e-5p
 
 
-0.305
 
 
27.4
 
 
 
 
CBFB
 
 
hsa-miR-130b-3p
 
 
-0.498
 
 
12.5
 
 
 
 
CBX5
 
 
hsa-let-7e-5p
 
 
-0.307
 
 
28.9
 
 
 
 
CBX5
 
 
hsa-let-7e-5p
 
 
-0.472
 
 
28.9
 
 
 
 
CCDC43
 
 
hsa-miR-7-5p
 
 
-0.331
 
 
12.8
 
 
 
 
CCNC
 
 
hsa-miR-1-3p
 
 
-0.336
 
 
29.9
 
 
 
 
CCNF
 
 
hsa-let-7e-5p
 
 
-0.474
 
 
14.1
 
 
 
 
CCNL2
 
 
hsa-miR-28-5p
 
 
-0.448
 
 
28.7
 
 
 
 
CCNY
 
 
hsa-let-7e-5p
 
 
-0.334
 
 
21.0
 
 
 
 
CCNY
 
 
hsa-miR-130b-3p
 
 
-0.372
 
 
21.0
 
 
 
 
CCR7
 
 
hsa-let-7e-5p
 
 
-0.482
 
 
54.9
 
 
 
 
CCSAP
 
 
hsa-miR-1-3p
 
 
-0.381
 
 
13.1
 
 
 
 
CCT4
 
 
hsa-miR-7-5p
 
 
-0.37
 
 
77.6
 
 
 
 
CCT6A
 
 
hsa-miR-130b-3p
 
 
-0.382
 
 
58.5
 
 
 
 
CD164
 
 
hsa-miR-1-3p
 
 
-0.315
 
 
123.1
 
 
 
 
CD2AP
 
 
hsa-miR-130b-3p
 
 
-0.302
 
 
31.7
 
 
 
 
CD2AP
 
 
hsa-miR-1-3p
 
 
-0.479
 
 
31.7
 
 
 
 
CD69
 
 
hsa-miR-130b-3p
 
 
-0.451
 
 
53.2
 
 
 
 
CDC34
 
 
hsa-let-7e-5p
 
 
-0.321
 
 
15.3
 
 
 
 
CDC34
 
 
hsa-let-7e-5p
 
 
-0.555
 
 
15.3
 
 
 
 
CDC42SE1
 
 
hsa-miR-1-3p
 
 
-0.36
 
 
52.6
 
 
 
 
CDCA8
 
 
hsa-let-7e-5p
 
 
-0.342
 
 
36.1
 
 
 
 
CDK4
 
 
hsa-miR-1-3p
 
 
-0.338
 
 
63.0
 
 
 
 
CDKN1A
 
 
hsa-let-7e-5p
 
 
-0.434
 
 
55.8
 
 
 
 
CDKN2AIP
 
 
hsa-miR-28-5p
 
 
-0.419
 
 
22.4
 
 
 
 
 
 
 tongue 
 
 
 
 
target_symbol
 
 
mature_mirna_id
 
 
score
 
 
nTPM
 
 
 
 
 
 
ABCB7
 
 
hsa-miR-1-3p
 
 
-0.365
 
 
20.8
 
 
 
 
ABHD2
 
 
hsa-miR-1-3p
 
 
-0.291
 
 
43.2
 
 
 
 
ABRAXAS2
 
 
hsa-miR-130b-3p
 
 
-0.468
 
 
15.2
 
 
 
 
ACBD3
 
 
hsa-miR-130b-3p
 
 
-0.292
 
 
19.0
 
 
 
 
ACBD5
 
 
hsa-miR-130b-3p
 
 
-0.494
 
 
34.2
 
 
 
 
ACSL4
 
 
hsa-miR-130b-3p
 
 
-0.301
 
 
15.8
 
 
 
 
ACTC1
 
 
hsa-miR-7-5p
 
 
-0.471
 
 
182.1
 
 
 
 
ADAR
 
 
hsa-miR-1-3p
 
 
-0.35
 
 
25.8
 
 
 
 
ADGRA3
 
 
hsa-miR-1-3p
 
 
-0.351
 
 
10.7
 
 
 
 
AHCTF1
 
 
hsa-let-7e-5p
 
 
-0.374
 
 
14.7
 
 
 
 
AIFM1
 
 
hsa-let-7e-5p
 
 
-0.314
 
 
23.4
 
 
 
 
AKAP6
 
 
hsa-let-7e-5p
 
 
-0.307
 
 
47.4
 
 
 
 
AKIRIN2
 
 
hsa-miR-130b-3p
 
 
-0.315
 
 
39.8
 
 
 
 
ALDH4A1
 
 
hsa-miR-184
 
 
-0.421
 
 
69.9
 
 
 
 
ANKRD12
 
 
hsa-miR-7-5p
 
 
-0.37
 
 
11.5
 
 
 
 
ANKRD46
 
 
hsa-let-7e-5p
 
 
-0.389
 
 
33.8
 
 
 
 
ANP32B
 
 
hsa-miR-1-3p
 
 
-0.287
 
 
47.3
 
 
 
 
ANXA2
 
 
hsa-miR-1-3p
 
 
-0.581
 
 
192.2
 
 
 
 
ANXA4
 
 
hsa-miR-1-3p
 
 
-0.539
 
 
19.1
 
 
 
 
APCDD1
 
 
hsa-miR-130b-3p
 
 
-0.304
 
 
17.3
 
 
 
 
APPL1
 
 
hsa-miR-130b-3p
 
 
-0.347
 
 
24.8
 
 
 
 
ARCN1
 
 
hsa-miR-1-3p
 
 
-0.468
 
 
51.2
 
 
 
 
ARF4
 
 
hsa-miR-7-5p
 
 
-0.407
 
 
64.9
 
 
 
 
ARFGEF1
 
 
hsa-miR-130b-3p
 
 
-0.302
 
 
13.0
 
 
 
 
ARG2
 
 
hsa-let-7e-5p
 
 
-0.468
 
 
49.2
 
 
 
 
ARHGAP1
 
 
hsa-miR-130b-3p
 
 
-0.376
 
 
19.1
 
 
 
 
ARL6IP1
 
 
hsa-miR-130b-3p
 
 
-0.289
 
 
48.1
 
 
 
 
ARL6IP1
 
 
hsa-miR-7-5p
 
 
-0.374
 
 
48.1
 
 
 
 
ARMT1
 
 
hsa-let-7e-5p
 
 
-0.303
 
 
21.1
 
 
 
 
ARPC3
 
 
hsa-miR-1-3p
 
 
-0.606
 
 
95.5
 
 
 
 
ARPP19
 
 
hsa-let-7e-5p
 
 
-0.492
 
 
158.9
 
 
 
 
AS3MT
 
 
hsa-miR-28-5p
 
 
-0.479
 
 
12.5
 
 
 
 
ASH2L
 
 
hsa-miR-1-3p
 
 
-0.462
 
 
26.1
 
 
 
 
ATF2
 
 
hsa-miR-1-3p
 
 
-0.328
 
 
20.1
 
 
 
 
ATG4A
 
 
hsa-miR-7-5p
 
 
-0.448
 
 
17.2
 
 
 
 
ATOSB
 
 
hsa-let-7e-5p
 
 
-0.429
 
 
10.9
 
 
 
 
ATP2A2
 
 
hsa-let-7e-5p
 
 
-0.311
 
 
2586.2
 
 
 
 
ATPAF1
 
 
hsa-let-7e-5p
 
 
-0.498
 
 
295.7
 
 
 
 
ATXN1
 
 
hsa-miR-130b-3p
 
 
-0.321
 
 
13.9
 
 
 
 
AXL
 
 
hsa-miR-1-3p
 
 
-0.304
 
 
10.6
 
 
 
 
AZIN1
 
 
hsa-miR-1-3p
 
 
-0.438
 
 
28.4
 
 
 
 
B4GAT1
 
 
hsa-let-7e-5p
 
 
-0.47
 
 
17.9
 
 
 
 
BACH1
 
 
hsa-let-7e-5p
 
 
-0.298
 
 
11.9
 
 
 
 
BACH1
 
 
hsa-let-7e-5p
 
 
-0.298
 
 
11.9
 
 
 
 
BACH1
 
 
hsa-let-7e-5p
 
 
-0.435
 
 
11.9
 
 
 
 
BACH1
 
 
hsa-let-7e-5p
 
 
-0.435
 
 
11.9
 
 
 
 
BAZ2A
 
 
hsa-miR-100-5p
 
 
-0.29
 
 
29.9
 
 
 
 
BAZ2A
 
 
hsa-miR-100-5p
 
 
-0.421
 
 
29.9
 
 
 
 
BCAP29
 
 
hsa-let-7e-5p
 
 
-0.289
 
 
30.7
 
 
 
 
BHLHE40
 
 
hsa-miR-130b-3p
 
 
-0.351
 
 
43.7
 
 
 
 
BLCAP
 
 
hsa-miR-130b-3p
 
 
-0.342
 
 
112.2
 
 
 
 
BLOC1S6
 
 
hsa-let-7e-5p
 
 
-0.321
 
 
21.7
 
 
 
 
BLTP3B
 
 
hsa-miR-1-3p
 
 
-0.29
 
 
13.6
 
 
 
 
BMP5
 
 
hsa-let-7e-5p
 
 
-0.337
 
 
11.4
 
 
 
 
BMPR2
 
 
hsa-miR-130b-3p
 
 
-0.33
 
 
15.0
 
 
 
 
BMPR2
 
 
hsa-miR-100-5p
 
 
-0.355
 
 
15.0
 
 
 
 
BPNT1
 
 
hsa-miR-1-3p
 
 
-0.329
 
 
10.5
 
 
 
 
BRIX1
 
 
hsa-miR-1-3p
 
 
-0.334
 
 
11.3
 
 
 
 
BSCL2
 
 
hsa-miR-1-3p
 
 
-0.471
 
 
14.3
 
 
 
 
BTF3L4
 
 
hsa-let-7e-5p
 
 
-0.355
 
 
55.0
 
 
 
 
BTF3L4
 
 
hsa-miR-130b-3p
 
 
-0.359
 
 
55.0
 
 
 
 
BZW1
 
 
hsa-let-7e-5p
 
 
-0.354
 
 
70.0
 
 
 
 
BZW1
 
 
hsa-let-7e-5p
 
 
-0.375
 
 
70.0
 
 
 
 
BZW2
 
 
hsa-let-7e-5p
 
 
-0.408
 
 
241.7
 
 
 
 
C19orf47
 
 
hsa-let-7e-5p
 
 
-0.311
 
 
128.4
 
 
 
 
C1GALT1
 
 
hsa-let-7e-5p
 
 
-0.322
 
 
10.5
 
 
 
 
C1orf21
 
 
hsa-miR-7-5p
 
 
-0.373
 
 
19.3
 
 
 
 
C2orf88
 
 
hsa-let-7e-5p
 
 
-0.429
 
 
27.3
 
 
 
 
C5orf22
 
 
hsa-miR-7-5p
 
 
-0.516
 
 
14.1
 
 
 
 
C6orf120
 
 
hsa-miR-1-3p
 
 
-0.288
 
 
10.5
 
 
 
 
CALM2
 
 
hsa-miR-130b-3p
 
 
-0.584
 
 
239.7
 
 
 
 
CAMSAP2
 
 
hsa-miR-130b-3p
 
 
-0.297
 
 
12.6
 
 
 
 
CANT1
 
 
hsa-let-7e-5p
 
 
-0.402
 
 
11.5
 
 
 
 
CAPN3
 
 
hsa-let-7e-5p
 
 
-0.317
 
 
352.2
 
 
 
 
CAPZA1
 
 
hsa-miR-7-5p
 
 
-0.351
 
 
20.7
 
 
 
 
CASD1
 
 
hsa-miR-130b-3p
 
 
-0.305
 
 
18.2
 
 
 
 
CASP3
 
 
hsa-let-7e-5p
 
 
-0.495
 
 
12.2
 
 
 
 
CCDC43
 
 
hsa-miR-7-5p
 
 
-0.331
 
 
39.8
 
 
 
 
CCNC
 
 
hsa-miR-1-3p
 
 
-0.336
 
 
16.5
 
 
 
 
CCNL2
 
 
hsa-miR-28-5p
 
 
-0.448
 
 
16.9
 
 
 
 
CCNY
 
 
hsa-let-7e-5p
 
 
-0.334
 
 
21.4
 
 
 
 
CCNY
 
 
hsa-miR-130b-3p
 
 
-0.372
 
 
21.4
 
 
 
 
CCT4
 
 
hsa-miR-7-5p
 
 
-0.37
 
 
69.1
 
 
 
 
CCT6A
 
 
hsa-miR-130b-3p
 
 
-0.382
 
 
80.7
 
 
 
 
CD164
 
 
hsa-miR-1-3p
 
 
-0.315
 
 
85.7
 
 
 
 
CDC34
 
 
hsa-let-7e-5p
 
 
-0.321
 
 
58.9
 
 
 
 
CDC34
 
 
hsa-let-7e-5p
 
 
-0.555
 
 
58.9
 
 
 
 
CDC42SE1
 
 
hsa-miR-1-3p
 
 
-0.36
 
 
18.0
 
 
 
 
CDIN1
 
 
hsa-let-7e-5p
 
 
-0.401
 
 
32.2
 
 
 
 
CDK4
 
 
hsa-miR-1-3p
 
 
-0.338
 
 
49.2
 
 
 
 
CDKN1A
 
 
hsa-let-7e-5p
 
 
-0.434
 
 
25.1
 
 
 
 
CDKN2AIP
 
 
hsa-miR-28-5p
 
 
-0.419
 
 
13.3
 
 
 
 
CDV3
 
 
hsa-let-7e-5p
 
 
-0.453
 
 
144.5
 
 
 
 
CEBPD
 
 
hsa-let-7e-5p
 
 
-0.309
 
 
20.2
 
 
 
 
CEP85
 
 
hsa-miR-100-5p
 
 
-0.328
 
 
61.6
 
 
 
 
CERS2
 
 
hsa-miR-1-3p
 
 
-0.287
 
 
53.7
 
 
 
 
CGGBP1
 
 
hsa-miR-7-5p
 
 
-0.465
 
 
44.5
 
 
 
 
CHMP3
 
 
hsa-miR-130b-3p
 
 
-0.521
 
 
75.5
 
 
 
 
CHMP4B
 
 
hsa-miR-130b-3p
 
 
-0.292
 
 
123.8
 
 
 
 
CHUK
 
 
hsa-let-7e-5p
 
 
-0.327
 
 
10.9
 
 
 
 
 
 
 tonsil 
 
 
 
 
target_symbol
 
 
mature_mirna_id
 
 
score
 
 
nTPM
 
 
 
 
 
 
ABCB7
 
 
hsa-miR-1-3p
 
 
-0.365
 
 
10.4
 
 
 
 
ABHD17C
 
 
hsa-let-7e-5p
 
 
-0.359
 
 
21.6
 
 
 
 
ABHD2
 
 
hsa-miR-1-3p
 
 
-0.291
 
 
15.1
 
 
 
 
ABHD3
 
 
hsa-miR-130b-3p
 
 
-0.356
 
 
24.2
 
 
 
 
ABRAXAS2
 
 
hsa-miR-130b-3p
 
 
-0.468
 
 
12.5
 
 
 
 
ACBD3
 
 
hsa-miR-130b-3p
 
 
-0.292
 
 
24.0
 
 
 
 
ACBD5
 
 
hsa-miR-130b-3p
 
 
-0.494
 
 
11.9
 
 
 
 
ACSL4
 
 
hsa-miR-130b-3p
 
 
-0.301
 
 
16.0
 
 
 
 
ADAR
 
 
hsa-miR-1-3p
 
 
-0.35
 
 
60.9
 
 
 
 
ADPGK
 
 
hsa-miR-1-3p
 
 
-0.393
 
 
22.6
 
 
 
 
AIFM1
 
 
hsa-let-7e-5p
 
 
-0.314
 
 
10.3
 
 
 
 
AKIRIN2
 
 
hsa-miR-130b-3p
 
 
-0.315
 
 
28.4
 
 
 
 
ANKIB1
 
 
hsa-miR-130b-3p
 
 
-0.329
 
 
10.9
 
 
 
 
ANKRD12
 
 
hsa-miR-7-5p
 
 
-0.37
 
 
17.3
 
 
 
 
ANKRD29
 
 
hsa-miR-1-3p
 
 
-0.426
 
 
13.4
 
 
 
 
ANKRD46
 
 
hsa-let-7e-5p
 
 
-0.389
 
 
10.3
 
 
 
 
ANP32B
 
 
hsa-miR-1-3p
 
 
-0.287
 
 
232.6
 
 
 
 
ANXA2
 
 
hsa-miR-1-3p
 
 
-0.581
 
 
507.1
 
 
 
 
ANXA4
 
 
hsa-miR-1-3p
 
 
-0.539
 
 
22.5
 
 
 
 
AP1AR
 
 
hsa-miR-100-5p
 
 
-0.804
 
 
11.3
 
 
 
 
AP1S1
 
 
hsa-miR-1-3p
 
 
-0.355
 
 
12.8
 
 
 
 
AP1S1
 
 
hsa-let-7e-5p
 
 
-0.374
 
 
12.8
 
 
 
 
APPL1
 
 
hsa-miR-130b-3p
 
 
-0.347
 
 
18.4
 
 
 
 
ARCN1
 
 
hsa-miR-1-3p
 
 
-0.468
 
 
57.2
 
 
 
 
ARF4
 
 
hsa-miR-7-5p
 
 
-0.407
 
 
91.2
 
 
 
 
ARFGEF1
 
 
hsa-miR-130b-3p
 
 
-0.302
 
 
16.2
 
 
 
 
ARHGAP1
 
 
hsa-miR-130b-3p
 
 
-0.376
 
 
32.4
 
 
 
 
ARHGAP24
 
 
hsa-miR-130b-3p
 
 
-0.287
 
 
14.5
 
 
 
 
ARID4A
 
 
hsa-miR-7-5p
 
 
-0.341
 
 
10.5
 
 
 
 
ARL6IP1
 
 
hsa-miR-130b-3p
 
 
-0.289
 
 
106.2
 
 
 
 
ARL6IP1
 
 
hsa-miR-7-5p
 
 
-0.374
 
 
106.2
 
 
 
 
ARL6IP6
 
 
hsa-let-7e-5p
 
 
-0.305
 
 
18.0
 
 
 
 
ARMT1
 
 
hsa-let-7e-5p
 
 
-0.303
 
 
17.3
 
 
 
 
ARPC3
 
 
hsa-miR-1-3p
 
 
-0.606
 
 
537.0
 
 
 
 
ARPP19
 
 
hsa-let-7e-5p
 
 
-0.492
 
 
48.4
 
 
 
 
ASAP1
 
 
hsa-let-7e-5p
 
 
-0.296
 
 
17.8
 
 
 
 
ASH2L
 
 
hsa-miR-1-3p
 
 
-0.462
 
 
26.7
 
 
 
 
ATF2
 
 
hsa-miR-1-3p
 
 
-0.328
 
 
18.7
 
 
 
 
ATG4A
 
 
hsa-miR-7-5p
 
 
-0.448
 
 
10.4
 
 
 
 
ATP2A2
 
 
hsa-let-7e-5p
 
 
-0.311
 
 
45.1
 
 
 
 
ATPAF1
 
 
hsa-let-7e-5p
 
 
-0.498
 
 
30.0
 
 
 
 
AZIN1
 
 
hsa-miR-1-3p
 
 
-0.438
 
 
55.9
 
 
 
 
B3GAT3
 
 
hsa-let-7e-5p
 
 
-0.324
 
 
10.3
 
 
 
 
BACH1
 
 
hsa-let-7e-5p
 
 
-0.298
 
 
13.8
 
 
 
 
BACH1
 
 
hsa-let-7e-5p
 
 
-0.298
 
 
13.8
 
 
 
 
BACH1
 
 
hsa-let-7e-5p
 
 
-0.435
 
 
13.8
 
 
 
 
BACH1
 
 
hsa-let-7e-5p
 
 
-0.435
 
 
13.8
 
 
 
 
BAZ2A
 
 
hsa-miR-100-5p
 
 
-0.29
 
 
28.7
 
 
 
 
BAZ2A
 
 
hsa-miR-100-5p
 
 
-0.421
 
 
28.7
 
 
 
 
BCAP29
 
 
hsa-let-7e-5p
 
 
-0.289
 
 
15.2
 
 
 
 
BET1
 
 
hsa-miR-1-3p
 
 
-0.413
 
 
19.7
 
 
 
 
BHLHE40
 
 
hsa-miR-130b-3p
 
 
-0.351
 
 
47.5
 
 
 
 
BIN3
 
 
hsa-let-7e-5p
 
 
-0.427
 
 
10.2
 
 
 
 
BLCAP
 
 
hsa-miR-130b-3p
 
 
-0.342
 
 
49.7
 
 
 
 
BLOC1S6
 
 
hsa-let-7e-5p
 
 
-0.321
 
 
33.2
 
 
 
 
BPNT1
 
 
hsa-miR-1-3p
 
 
-0.329
 
 
41.3
 
 
 
 
BRI3BP
 
 
hsa-miR-1-3p
 
 
-0.293
 
 
15.2
 
 
 
 
BRIX1
 
 
hsa-miR-1-3p
 
 
-0.334
 
 
24.2
 
 
 
 
BTAF1
 
 
hsa-miR-130b-3p
 
 
-0.291
 
 
20.0
 
 
 
 
BTF3L4
 
 
hsa-let-7e-5p
 
 
-0.355
 
 
38.2
 
 
 
 
BTF3L4
 
 
hsa-miR-130b-3p
 
 
-0.359
 
 
38.2
 
 
 
 
BTG1
 
 
hsa-miR-130b-3p
 
 
-0.565
 
 
17.1
 
 
 
 
BZW1
 
 
hsa-let-7e-5p
 
 
-0.354
 
 
102.5
 
 
 
 
BZW1
 
 
hsa-let-7e-5p
 
 
-0.375
 
 
102.5
 
 
 
 
BZW2
 
 
hsa-let-7e-5p
 
 
-0.408
 
 
35.9
 
 
 
 
C1GALT1
 
 
hsa-let-7e-5p
 
 
-0.322
 
 
13.2
 
 
 
 
C6orf120
 
 
hsa-miR-1-3p
 
 
-0.288
 
 
12.1
 
 
 
 
CAAP1
 
 
hsa-miR-1-3p
 
 
-0.48
 
 
14.2
 
 
 
 
CALM2
 
 
hsa-miR-130b-3p
 
 
-0.584
 
 
437.6
 
 
 
 
CANT1
 
 
hsa-let-7e-5p
 
 
-0.402
 
 
14.2
 
 
 
 
CAPZA1
 
 
hsa-miR-7-5p
 
 
-0.351
 
 
175.8
 
 
 
 
CASD1
 
 
hsa-miR-130b-3p
 
 
-0.305
 
 
10.1
 
 
 
 
CASP3
 
 
hsa-let-7e-5p
 
 
-0.495
 
 
36.6
 
 
 
 
CBFB
 
 
hsa-miR-130b-3p
 
 
-0.498
 
 
11.1
 
 
 
 
CBX5
 
 
hsa-let-7e-5p
 
 
-0.307
 
 
20.1
 
 
 
 
CBX5
 
 
hsa-let-7e-5p
 
 
-0.472
 
 
20.1
 
 
 
 
CCDC43
 
 
hsa-miR-7-5p
 
 
-0.331
 
 
13.6
 
 
 
 
CCNC
 
 
hsa-miR-1-3p
 
 
-0.336
 
 
40.6
 
 
 
 
CCNL2
 
 
hsa-miR-28-5p
 
 
-0.448
 
 
25.9
 
 
 
 
CCNY
 
 
hsa-let-7e-5p
 
 
-0.334
 
 
19.7
 
 
 
 
CCNY
 
 
hsa-miR-130b-3p
 
 
-0.372
 
 
19.7
 
 
 
 
CCR7
 
 
hsa-let-7e-5p
 
 
-0.482
 
 
78.1
 
 
 
 
CCT4
 
 
hsa-miR-7-5p
 
 
-0.37
 
 
86.2
 
 
 
 
CCT6A
 
 
hsa-miR-130b-3p
 
 
-0.382
 
 
77.0
 
 
 
 
CD164
 
 
hsa-miR-1-3p
 
 
-0.315
 
 
115.3
 
 
 
 
CD2AP
 
 
hsa-miR-130b-3p
 
 
-0.302
 
 
16.3
 
 
 
 
CD2AP
 
 
hsa-miR-1-3p
 
 
-0.479
 
 
16.3
 
 
 
 
CD69
 
 
hsa-miR-130b-3p
 
 
-0.451
 
 
56.3
 
 
 
 
CD86
 
 
hsa-let-7e-5p
 
 
-0.323
 
 
20.6
 
 
 
 
CDC42SE1
 
 
hsa-miR-1-3p
 
 
-0.36
 
 
79.3
 
 
 
 
CDCA8
 
 
hsa-let-7e-5p
 
 
-0.342
 
 
21.2
 
 
 
 
CDK14
 
 
hsa-miR-1-3p
 
 
-0.326
 
 
12.1
 
 
 
 
CDK4
 
 
hsa-miR-1-3p
 
 
-0.338
 
 
66.2
 
 
 
 
CDKN1A
 
 
hsa-let-7e-5p
 
 
-0.434
 
 
20.4
 
 
 
 
CDKN2AIP
 
 
hsa-miR-28-5p
 
 
-0.419
 
 
17.6
 
 
 
 
CDV3
 
 
hsa-let-7e-5p
 
 
-0.453
 
 
151.5
 
 
 
 
CEMIP2
 
 
hsa-let-7e-5p
 
 
-0.453
 
 
19.4
 
 
 
 
CEP55
 
 
hsa-miR-130b-3p
 
 
-0.304
 
 
14.5
 
 
 
 
CERS2
 
 
hsa-miR-1-3p
 
 
-0.287
 
 
65.4
 
 
 
 
CGGBP1
 
 
hsa-miR-7-5p
 
 
-0.465
 
 
58.0
 
 
 
 
 
 
 urinary bladder 
 
 
 
 
target_symbol
 
 
mature_mirna_id
 
 
score
 
 
nTPM
 
 
 
 
 
 
ABCB7
 
 
hsa-miR-1-3p
 
 
-0.365
 
 
11.0
 
 
 
 
ABHD17C
 
 
hsa-let-7e-5p
 
 
-0.359
 
 
26.8
 
 
 
 
ABHD2
 
 
hsa-miR-1-3p
 
 
-0.291
 
 
29.1
 
 
 
 
ABHD3
 
 
hsa-miR-130b-3p
 
 
-0.356
 
 
14.3
 
 
 
 
ACBD3
 
 
hsa-miR-130b-3p
 
 
-0.292
 
 
25.2
 
 
 
 
ACBD5
 
 
hsa-miR-130b-3p
 
 
-0.494
 
 
12.6
 
 
 
 
ACER2
 
 
hsa-let-7e-5p
 
 
-0.333
 
 
36.7
 
 
 
 
ACSL4
 
 
hsa-miR-130b-3p
 
 
-0.301
 
 
62.0
 
 
 
 
ACTC1
 
 
hsa-miR-7-5p
 
 
-0.471
 
 
667.0
 
 
 
 
ACVR1
 
 
hsa-miR-130b-3p
 
 
-0.44
 
 
13.3
 
 
 
 
ADAR
 
 
hsa-miR-1-3p
 
 
-0.35
 
 
47.2
 
 
 
 
ADPGK
 
 
hsa-miR-1-3p
 
 
-0.393
 
 
16.4
 
 
 
 
AIFM1
 
 
hsa-let-7e-5p
 
 
-0.314
 
 
13.1
 
 
 
 
AKIRIN2
 
 
hsa-miR-130b-3p
 
 
-0.315
 
 
19.4
 
 
 
 
ALDH4A1
 
 
hsa-miR-184
 
 
-0.421
 
 
21.0
 
 
 
 
ALG9
 
 
hsa-miR-7-5p
 
 
-0.302
 
 
10.1
 
 
 
 
AMT
 
 
hsa-let-7e-5p
 
 
-0.463
 
 
13.1
 
 
 
 
ANKIB1
 
 
hsa-miR-130b-3p
 
 
-0.329
 
 
12.3
 
 
 
 
ANKRD12
 
 
hsa-miR-7-5p
 
 
-0.37
 
 
12.7
 
 
 
 
ANP32B
 
 
hsa-miR-1-3p
 
 
-0.287
 
 
122.0
 
 
 
 
ANXA2
 
 
hsa-miR-1-3p
 
 
-0.581
 
 
785.1
 
 
 
 
ANXA4
 
 
hsa-miR-1-3p
 
 
-0.539
 
 
57.3
 
 
 
 
AP1AR
 
 
hsa-miR-100-5p
 
 
-0.804
 
 
12.2
 
 
 
 
AP1S1
 
 
hsa-miR-1-3p
 
 
-0.355
 
 
18.6
 
 
 
 
AP1S1
 
 
hsa-let-7e-5p
 
 
-0.374
 
 
18.6
 
 
 
 
APCDD1
 
 
hsa-miR-130b-3p
 
 
-0.304
 
 
34.0
 
 
 
 
APPL1
 
 
hsa-miR-130b-3p
 
 
-0.347
 
 
12.5
 
 
 
 
ARCN1
 
 
hsa-miR-1-3p
 
 
-0.468
 
 
61.6
 
 
 
 
ARF4
 
 
hsa-miR-7-5p
 
 
-0.407
 
 
190.1
 
 
 
 
ARFGEF1
 
 
hsa-miR-130b-3p
 
 
-0.302
 
 
18.4
 
 
 
 
ARG2
 
 
hsa-let-7e-5p
 
 
-0.468
 
 
16.6
 
 
 
 
ARHGAP1
 
 
hsa-miR-130b-3p
 
 
-0.376
 
 
106.2
 
 
 
 
ARHGAP12
 
 
hsa-miR-130b-3p
 
 
-0.287
 
 
10.1
 
 
 
 
ARL4D
 
 
hsa-let-7e-5p
 
 
-0.417
 
 
26.6
 
 
 
 
ARL6IP1
 
 
hsa-miR-130b-3p
 
 
-0.289
 
 
124.0
 
 
 
 
ARL6IP1
 
 
hsa-miR-7-5p
 
 
-0.374
 
 
124.0
 
 
 
 
ARL6IP6
 
 
hsa-let-7e-5p
 
 
-0.305
 
 
12.2
 
 
 
 
ARMT1
 
 
hsa-let-7e-5p
 
 
-0.303
 
 
16.1
 
 
 
 
ARPC3
 
 
hsa-miR-1-3p
 
 
-0.606
 
 
332.6
 
 
 
 
ARPP19
 
 
hsa-let-7e-5p
 
 
-0.492
 
 
51.7
 
 
 
 
ARRDC4
 
 
hsa-let-7e-5p
 
 
-0.329
 
 
10.9
 
 
 
 
ASAP1
 
 
hsa-let-7e-5p
 
 
-0.296
 
 
14.4
 
 
 
 
ASH2L
 
 
hsa-miR-1-3p
 
 
-0.462
 
 
17.3
 
 
 
 
ATF2
 
 
hsa-miR-1-3p
 
 
-0.328
 
 
16.7
 
 
 
 
ATG16L1
 
 
hsa-miR-130b-3p
 
 
-0.407
 
 
13.3
 
 
 
 
ATOSB
 
 
hsa-let-7e-5p
 
 
-0.429
 
 
10.8
 
 
 
 
ATP2A2
 
 
hsa-let-7e-5p
 
 
-0.311
 
 
84.9
 
 
 
 
ATPAF1
 
 
hsa-let-7e-5p
 
 
-0.498
 
 
26.7
 
 
 
 
AVPI1
 
 
hsa-miR-7-5p
 
 
-0.367
 
 
29.9
 
 
 
 
AXL
 
 
hsa-miR-1-3p
 
 
-0.304
 
 
28.8
 
 
 
 
AZIN1
 
 
hsa-miR-1-3p
 
 
-0.438
 
 
56.7
 
 
 
 
B3GAT3
 
 
hsa-let-7e-5p
 
 
-0.324
 
 
28.2
 
 
 
 
B4GAT1
 
 
hsa-let-7e-5p
 
 
-0.47
 
 
21.0
 
 
 
 
BACH1
 
 
hsa-let-7e-5p
 
 
-0.298
 
 
27.7
 
 
 
 
BACH1
 
 
hsa-let-7e-5p
 
 
-0.298
 
 
27.7
 
 
 
 
BACH1
 
 
hsa-let-7e-5p
 
 
-0.435
 
 
27.7
 
 
 
 
BACH1
 
 
hsa-let-7e-5p
 
 
-0.435
 
 
27.7
 
 
 
 
BAHD1
 
 
hsa-miR-130b-3p
 
 
-0.294
 
 
12.1
 
 
 
 
BAZ2A
 
 
hsa-miR-100-5p
 
 
-0.29
 
 
23.9
 
 
 
 
BAZ2A
 
 
hsa-miR-100-5p
 
 
-0.421
 
 
23.9
 
 
 
 
BCAP29
 
 
hsa-let-7e-5p
 
 
-0.289
 
 
29.3
 
 
 
 
BET1
 
 
hsa-miR-1-3p
 
 
-0.413
 
 
16.5
 
 
 
 
BHLHE40
 
 
hsa-miR-130b-3p
 
 
-0.351
 
 
160.4
 
 
 
 
BIN3
 
 
hsa-let-7e-5p
 
 
-0.427
 
 
13.2
 
 
 
 
BLCAP
 
 
hsa-miR-130b-3p
 
 
-0.342
 
 
71.7
 
 
 
 
BLOC1S6
 
 
hsa-let-7e-5p
 
 
-0.321
 
 
26.7
 
 
 
 
BMPR2
 
 
hsa-miR-130b-3p
 
 
-0.33
 
 
14.5
 
 
 
 
BMPR2
 
 
hsa-miR-100-5p
 
 
-0.355
 
 
14.5
 
 
 
 
BPNT1
 
 
hsa-miR-1-3p
 
 
-0.329
 
 
14.8
 
 
 
 
BRIX1
 
 
hsa-miR-1-3p
 
 
-0.334
 
 
28.1
 
 
 
 
BSCL2
 
 
hsa-miR-1-3p
 
 
-0.471
 
 
15.6
 
 
 
 
BTAF1
 
 
hsa-miR-130b-3p
 
 
-0.291
 
 
16.7
 
 
 
 
BTF3L4
 
 
hsa-let-7e-5p
 
 
-0.355
 
 
39.5
 
 
 
 
BTF3L4
 
 
hsa-miR-130b-3p
 
 
-0.359
 
 
39.5
 
 
 
 
BZW1
 
 
hsa-let-7e-5p
 
 
-0.354
 
 
153.5
 
 
 
 
BZW1
 
 
hsa-let-7e-5p
 
 
-0.375
 
 
153.5
 
 
 
 
BZW2
 
 
hsa-let-7e-5p
 
 
-0.408
 
 
55.5
 
 
 
 
C15orf39
 
 
hsa-let-7e-5p
 
 
-0.374
 
 
14.3
 
 
 
 
C1GALT1
 
 
hsa-let-7e-5p
 
 
-0.322
 
 
12.1
 
 
 
 
C1orf21
 
 
hsa-miR-7-5p
 
 
-0.373
 
 
10.7
 
 
 
 
C6orf120
 
 
hsa-miR-1-3p
 
 
-0.288
 
 
11.8
 
 
 
 
CACFD1
 
 
hsa-let-7e-5p
 
 
-0.296
 
 
11.0
 
 
 
 
CALM2
 
 
hsa-miR-130b-3p
 
 
-0.584
 
 
428.9
 
 
 
 
CAMSAP2
 
 
hsa-miR-130b-3p
 
 
-0.297
 
 
13.9
 
 
 
 
CANT1
 
 
hsa-let-7e-5p
 
 
-0.402
 
 
15.6
 
 
 
 
CAPN3
 
 
hsa-let-7e-5p
 
 
-0.317
 
 
13.4
 
 
 
 
CAPZA1
 
 
hsa-miR-7-5p
 
 
-0.351
 
 
90.9
 
 
 
 
CASP3
 
 
hsa-let-7e-5p
 
 
-0.495
 
 
22.0
 
 
 
 
CCDC43
 
 
hsa-miR-7-5p
 
 
-0.331
 
 
11.1
 
 
 
 
CCDC71L
 
 
hsa-let-7e-5p
 
 
-0.432
 
 
18.0
 
 
 
 
CCNC
 
 
hsa-miR-1-3p
 
 
-0.336
 
 
32.4
 
 
 
 
CCNL2
 
 
hsa-miR-28-5p
 
 
-0.448
 
 
61.8
 
 
 
 
CCNT2
 
 
hsa-miR-7-5p
 
 
-0.349
 
 
12.8
 
 
 
 
CCNY
 
 
hsa-let-7e-5p
 
 
-0.334
 
 
22.2
 
 
 
 
CCNY
 
 
hsa-miR-130b-3p
 
 
-0.372
 
 
22.2
 
 
 
 
CCT4
 
 
hsa-miR-7-5p
 
 
-0.37
 
 
63.9
 
 
 
 
CCT6A
 
 
hsa-miR-130b-3p
 
 
-0.382
 
 
68.3
 
 
 
 
CD164
 
 
hsa-miR-1-3p
 
 
-0.315
 
 
144.2
 
 
 
 
CD2AP
 
 
hsa-miR-130b-3p
 
 
-0.302
 
 
28.2
 
 
 
 
CD2AP
 
 
hsa-miR-1-3p
 
 
-0.479
 
 
28.2
 
 
 
 
 
 
 vagina 
 
 
 
 
target_symbol
 
 
mature_mirna_id
 
 
score
 
 
nTPM
 
 
 
 
 
 
ABCB7
 
 
hsa-miR-1-3p
 
 
-0.365
 
 
10.8
 
 
 
 
ABHD17C
 
 
hsa-let-7e-5p
 
 
-0.359
 
 
21.1
 
 
 
 
ABHD3
 
 
hsa-miR-130b-3p
 
 
-0.356
 
 
12.7
 
 
 
 
ACBD3
 
 
hsa-miR-130b-3p
 
 
-0.292
 
 
19.2
 
 
 
 
ACP3
 
 
hsa-let-7e-5p
 
 
-0.298
 
 
10.8
 
 
 
 
ACTC1
 
 
hsa-miR-7-5p
 
 
-0.471
 
 
50.8
 
 
 
 
ADAR
 
 
hsa-miR-1-3p
 
 
-0.35
 
 
23.9
 
 
 
 
ADRB2
 
 
hsa-let-7e-5p
 
 
-0.635
 
 
12.2
 
 
 
 
AKIRIN2
 
 
hsa-miR-130b-3p
 
 
-0.315
 
 
22.7
 
 
 
 
ALDH4A1
 
 
hsa-miR-184
 
 
-0.421
 
 
16.8
 
 
 
 
ALG9
 
 
hsa-miR-7-5p
 
 
-0.302
 
 
10.2
 
 
 
 
ANKRD29
 
 
hsa-miR-1-3p
 
 
-0.426
 
 
12.3
 
 
 
 
ANP32B
 
 
hsa-miR-1-3p
 
 
-0.287
 
 
140.4
 
 
 
 
ANXA2
 
 
hsa-miR-1-3p
 
 
-0.581
 
 
1143.4
 
 
 
 
ANXA4
 
 
hsa-miR-1-3p
 
 
-0.539
 
 
48.4
 
 
 
 
AP1S1
 
 
hsa-miR-1-3p
 
 
-0.355
 
 
20.1
 
 
 
 
AP1S1
 
 
hsa-let-7e-5p
 
 
-0.374
 
 
20.1
 
 
 
 
APCDD1
 
 
hsa-miR-130b-3p
 
 
-0.304
 
 
33.2
 
 
 
 
APPL1
 
 
hsa-miR-130b-3p
 
 
-0.347
 
 
11.1
 
 
 
 
ARCN1
 
 
hsa-miR-1-3p
 
 
-0.468
 
 
55.9
 
 
 
 
ARF4
 
 
hsa-miR-7-5p
 
 
-0.407
 
 
85.1
 
 
 
 
ARG2
 
 
hsa-let-7e-5p
 
 
-0.468
 
 
11.0
 
 
 
 
ARHGAP1
 
 
hsa-miR-130b-3p
 
 
-0.376
 
 
59.0
 
 
 
 
ARHGEF4
 
 
hsa-miR-130b-3p
 
 
-0.377
 
 
18.8
 
 
 
 
ARL4D
 
 
hsa-let-7e-5p
 
 
-0.417
 
 
38.9
 
 
 
 
ARL6IP1
 
 
hsa-miR-130b-3p
 
 
-0.289
 
 
48.0
 
 
 
 
ARL6IP1
 
 
hsa-miR-7-5p
 
 
-0.374
 
 
48.0
 
 
 
 
ARPC3
 
 
hsa-miR-1-3p
 
 
-0.606
 
 
217.0
 
 
 
 
ARPP19
 
 
hsa-let-7e-5p
 
 
-0.492
 
 
27.0
 
 
 
 
ARRDC4
 
 
hsa-let-7e-5p
 
 
-0.329
 
 
10.1
 
 
 
 
ASAP1
 
 
hsa-let-7e-5p
 
 
-0.296
 
 
14.4
 
 
 
 
ASH2L
 
 
hsa-miR-1-3p
 
 
-0.462
 
 
15.4
 
 
 
 
ATF2
 
 
hsa-miR-1-3p
 
 
-0.328
 
 
12.3
 
 
 
 
ATOSB
 
 
hsa-let-7e-5p
 
 
-0.429
 
 
14.3
 
 
 
 
ATP2A2
 
 
hsa-let-7e-5p
 
 
-0.311
 
 
40.2
 
 
 
 
ATPAF1
 
 
hsa-let-7e-5p
 
 
-0.498
 
 
15.7
 
 
 
 
AVPI1
 
 
hsa-miR-7-5p
 
 
-0.367
 
 
46.2
 
 
 
 
AXL
 
 
hsa-miR-1-3p
 
 
-0.304
 
 
23.9
 
 
 
 
AZIN1
 
 
hsa-miR-1-3p
 
 
-0.438
 
 
24.5
 
 
 
 
B3GAT3
 
 
hsa-let-7e-5p
 
 
-0.324
 
 
28.4
 
 
 
 
B4GAT1
 
 
hsa-let-7e-5p
 
 
-0.47
 
 
15.8
 
 
 
 
BACH1
 
 
hsa-let-7e-5p
 
 
-0.298
 
 
12.6
 
 
 
 
BACH1
 
 
hsa-let-7e-5p
 
 
-0.298
 
 
12.6
 
 
 
 
BACH1
 
 
hsa-let-7e-5p
 
 
-0.435
 
 
12.6
 
 
 
 
BACH1
 
 
hsa-let-7e-5p
 
 
-0.435
 
 
12.6
 
 
 
 
BAZ2A
 
 
hsa-miR-100-5p
 
 
-0.29
 
 
29.0
 
 
 
 
BAZ2A
 
 
hsa-miR-100-5p
 
 
-0.421
 
 
29.0
 
 
 
 
BCAP29
 
 
hsa-let-7e-5p
 
 
-0.289
 
 
25.4
 
 
 
 
BET1
 
 
hsa-miR-1-3p
 
 
-0.413
 
 
11.3
 
 
 
 
BHLHE40
 
 
hsa-miR-130b-3p
 
 
-0.351
 
 
291.4
 
 
 
 
BIN3
 
 
hsa-let-7e-5p
 
 
-0.427
 
 
13.8
 
 
 
 
BLCAP
 
 
hsa-miR-130b-3p
 
 
-0.342
 
 
58.9
 
 
 
 
BLOC1S6
 
 
hsa-let-7e-5p
 
 
-0.321
 
 
15.7
 
 
 
 
BRIX1
 
 
hsa-miR-1-3p
 
 
-0.334
 
 
15.5
 
 
 
 
BSCL2
 
 
hsa-miR-1-3p
 
 
-0.471
 
 
22.2
 
 
 
 
BTAF1
 
 
hsa-miR-130b-3p
 
 
-0.291
 
 
16.3
 
 
 
 
BTF3L4
 
 
hsa-let-7e-5p
 
 
-0.355
 
 
27.9
 
 
 
 
BTF3L4
 
 
hsa-miR-130b-3p
 
 
-0.359
 
 
27.9
 
 
 
 
BTG1
 
 
hsa-miR-130b-3p
 
 
-0.565
 
 
10.6
 
 
 
 
BZW1
 
 
hsa-let-7e-5p
 
 
-0.354
 
 
58.1
 
 
 
 
BZW1
 
 
hsa-let-7e-5p
 
 
-0.375
 
 
58.1
 
 
 
 
BZW2
 
 
hsa-let-7e-5p
 
 
-0.408
 
 
27.5
 
 
 
 
C15orf39
 
 
hsa-let-7e-5p
 
 
-0.374
 
 
13.6
 
 
 
 
C2orf88
 
 
hsa-let-7e-5p
 
 
-0.429
 
 
11.1
 
 
 
 
CALM2
 
 
hsa-miR-130b-3p
 
 
-0.584
 
 
305.1
 
 
 
 
CAMSAP2
 
 
hsa-miR-130b-3p
 
 
-0.297
 
 
11.7
 
 
 
 
CANT1
 
 
hsa-let-7e-5p
 
 
-0.402
 
 
20.5
 
 
 
 
CAPN3
 
 
hsa-let-7e-5p
 
 
-0.317
 
 
16.2
 
 
 
 
CAPZA1
 
 
hsa-miR-7-5p
 
 
-0.351
 
 
66.5
 
 
 
 
CASP3
 
 
hsa-let-7e-5p
 
 
-0.495
 
 
11.4
 
 
 
 
CCNC
 
 
hsa-miR-1-3p
 
 
-0.336
 
 
19.6
 
 
 
 
CCNL2
 
 
hsa-miR-28-5p
 
 
-0.448
 
 
65.1
 
 
 
 
CCNT2
 
 
hsa-miR-7-5p
 
 
-0.349
 
 
15.5
 
 
 
 
CCNY
 
 
hsa-let-7e-5p
 
 
-0.334
 
 
15.1
 
 
 
 
CCNY
 
 
hsa-miR-130b-3p
 
 
-0.372
 
 
15.1
 
 
 
 
CCT4
 
 
hsa-miR-7-5p
 
 
-0.37
 
 
62.5
 
 
 
 
CCT6A
 
 
hsa-miR-130b-3p
 
 
-0.382
 
 
48.6
 
 
 
 
CD164
 
 
hsa-miR-1-3p
 
 
-0.315
 
 
70.3
 
 
 
 
CDC34
 
 
hsa-let-7e-5p
 
 
-0.321
 
 
35.6
 
 
 
 
CDC34
 
 
hsa-let-7e-5p
 
 
-0.555
 
 
35.6
 
 
 
 
CDC42SE1
 
 
hsa-miR-1-3p
 
 
-0.36
 
 
63.5
 
 
 
 
CDK4
 
 
hsa-miR-1-3p
 
 
-0.338
 
 
62.1
 
 
 
 
CDKN1A
 
 
hsa-let-7e-5p
 
 
-0.434
 
 
142.2
 
 
 
 
CDKN2AIP
 
 
hsa-miR-28-5p
 
 
-0.419
 
 
11.5
 
 
 
 
CDV3
 
 
hsa-let-7e-5p
 
 
-0.453
 
 
42.4
 
 
 
 
CEBPD
 
 
hsa-let-7e-5p
 
 
-0.309
 
 
251.2
 
 
 
 
CERCAM
 
 
hsa-let-7e-5p
 
 
-0.344
 
 
34.4
 
 
 
 
CERS2
 
 
hsa-miR-1-3p
 
 
-0.287
 
 
63.0
 
 
 
 
CGGBP1
 
 
hsa-miR-7-5p
 
 
-0.465
 
 
27.5
 
 
 
 
CHMP3
 
 
hsa-miR-130b-3p
 
 
-0.521
 
 
48.8
 
 
 
 
CHMP4B
 
 
hsa-miR-130b-3p
 
 
-0.292
 
 
105.4
 
 
 
 
CHRD
 
 
hsa-let-7e-5p
 
 
-0.303
 
 
14.9
 
 
 
 
CITED2
 
 
hsa-miR-1-3p
 
 
-0.37
 
 
54.2
 
 
 
 
CKAP4
 
 
hsa-miR-7-5p
 
 
-0.443
 
 
39.1
 
 
 
 
CLCN3
 
 
hsa-miR-1-3p
 
 
-0.339
 
 
10.7
 
 
 
 
CLCN3
 
 
hsa-miR-130b-3p
 
 
-0.384
 
 
10.7
 
 
 
 
CLIP1
 
 
hsa-miR-130b-3p
 
 
-0.452
 
 
33.1
 
 
 
 
CLTC
 
 
hsa-miR-1-3p
 
 
-0.39
 
 
51.7
 
 
 
 
CMPK1
 
 
hsa-miR-130b-3p
 
 
-0.384
 
 
60.3
 
 
 
 
CNIH1
 
 
hsa-miR-130b-3p
 
 
-0.335
 
 
38.0
 
 
 
 
 
 
 basal ganglia 
 
 
 
 
target_symbol
 
 
mature_mirna_id
 
 
score
 
 
nTPM
 
 
 
 
 
 
ABCB9
 
 
hsa-let-7e-5p
 
 
-0.422
 
 
13.6
 
 
 
 
ABHD17C
 
 
hsa-let-7e-5p
 
 
-0.359
 
 
20.2
 
 
 
 
ABHD2
 
 
hsa-miR-1-3p
 
 
-0.291
 
 
17.5
 
 
 
 
ABHD3
 
 
hsa-miR-130b-3p
 
 
-0.356
 
 
11.4
 
 
 
 
ACBD5
 
 
hsa-miR-130b-3p
 
 
-0.494
 
 
26.0
 
 
 
 
ACSL6
 
 
hsa-let-7e-5p
 
 
-0.336
 
 
18.7
 
 
 
 
ADAR
 
 
hsa-miR-1-3p
 
 
-0.35
 
 
35.3
 
 
 
 
ADGRA3
 
 
hsa-miR-1-3p
 
 
-0.351
 
 
18.8
 
 
 
 
ADGRB3
 
 
hsa-miR-130b-3p
 
 
-0.308
 
 
21.1
 
 
 
 
ADRB1
 
 
hsa-let-7e-5p
 
 
-0.367
 
 
10.0
 
 
 
 
AIFM1
 
 
hsa-let-7e-5p
 
 
-0.314
 
 
12.5
 
 
 
 
AKAP6
 
 
hsa-let-7e-5p
 
 
-0.307
 
 
10.5
 
 
 
 
AKIRIN2
 
 
hsa-miR-130b-3p
 
 
-0.315
 
 
31.9
 
 
 
 
ALDH4A1
 
 
hsa-miR-184
 
 
-0.421
 
 
86.2
 
 
 
 
AMT
 
 
hsa-let-7e-5p
 
 
-0.463
 
 
11.4
 
 
 
 
ANKIB1
 
 
hsa-miR-130b-3p
 
 
-0.329
 
 
14.3
 
 
 
 
ANKRD46
 
 
hsa-let-7e-5p
 
 
-0.389
 
 
22.6
 
 
 
 
ANP32B
 
 
hsa-miR-1-3p
 
 
-0.287
 
 
112.9
 
 
 
 
ANXA2
 
 
hsa-miR-1-3p
 
 
-0.581
 
 
46.5
 
 
 
 
ANXA4
 
 
hsa-miR-1-3p
 
 
-0.539
 
 
16.9
 
 
 
 
AP1AR
 
 
hsa-miR-100-5p
 
 
-0.804
 
 
13.0
 
 
 
 
AP1S1
 
 
hsa-miR-1-3p
 
 
-0.355
 
 
116.3
 
 
 
 
AP1S1
 
 
hsa-let-7e-5p
 
 
-0.374
 
 
116.3
 
 
 
 
APCDD1
 
 
hsa-miR-130b-3p
 
 
-0.304
 
 
30.3
 
 
 
 
ARCN1
 
 
hsa-miR-1-3p
 
 
-0.468
 
 
46.0
 
 
 
 
ARF4
 
 
hsa-miR-7-5p
 
 
-0.407
 
 
71.0
 
 
 
 
ARHGAP1
 
 
hsa-miR-130b-3p
 
 
-0.376
 
 
41.0
 
 
 
 
ARHGEF4
 
 
hsa-miR-130b-3p
 
 
-0.377
 
 
79.5
 
 
 
 
ARL4D
 
 
hsa-let-7e-5p
 
 
-0.417
 
 
14.0
 
 
 
 
ARL6IP1
 
 
hsa-miR-130b-3p
 
 
-0.289
 
 
196.4
 
 
 
 
ARL6IP1
 
 
hsa-miR-7-5p
 
 
-0.374
 
 
196.4
 
 
 
 
ARMT1
 
 
hsa-let-7e-5p
 
 
-0.303
 
 
10.6
 
 
 
 
ARPC3
 
 
hsa-miR-1-3p
 
 
-0.606
 
 
197.6
 
 
 
 
ARPP19
 
 
hsa-let-7e-5p
 
 
-0.492
 
 
481.4
 
 
 
 
ARRDC4
 
 
hsa-let-7e-5p
 
 
-0.329
 
 
10.0
 
 
 
 
ASAP1
 
 
hsa-let-7e-5p
 
 
-0.296
 
 
11.6
 
 
 
 
ASH2L
 
 
hsa-miR-1-3p
 
 
-0.462
 
 
17.0
 
 
 
 
ATF2
 
 
hsa-miR-1-3p
 
 
-0.328
 
 
14.8
 
 
 
 
ATG16L1
 
 
hsa-miR-130b-3p
 
 
-0.407
 
 
11.5
 
 
 
 
ATOSB
 
 
hsa-let-7e-5p
 
 
-0.429
 
 
20.2
 
 
 
 
ATP2A2
 
 
hsa-let-7e-5p
 
 
-0.311
 
 
21.1
 
 
 
 
ATP2B2
 
 
hsa-miR-7-5p
 
 
-0.297
 
 
34.6
 
 
 
 
ATP2B2
 
 
hsa-miR-130b-3p
 
 
-0.314
 
 
34.6
 
 
 
 
ATPAF1
 
 
hsa-let-7e-5p
 
 
-0.498
 
 
88.4
 
 
 
 
AVPI1
 
 
hsa-miR-7-5p
 
 
-0.367
 
 
14.2
 
 
 
 
AXL
 
 
hsa-miR-1-3p
 
 
-0.304
 
 
17.5
 
 
 
 
AZIN1
 
 
hsa-miR-1-3p
 
 
-0.438
 
 
29.3
 
 
 
 
B3GAT3
 
 
hsa-let-7e-5p
 
 
-0.324
 
 
67.8
 
 
 
 
B4GAT1
 
 
hsa-let-7e-5p
 
 
-0.47
 
 
152.4
 
 
 
 
BAG4
 
 
hsa-miR-1-3p
 
 
-0.378
 
 
10.9
 
 
 
 
BCAP29
 
 
hsa-let-7e-5p
 
 
-0.289
 
 
16.0
 
 
 
 
BEGAIN
 
 
hsa-miR-28-5p
 
 
-0.325
 
 
23.9
 
 
 
 
BEGAIN
 
 
hsa-let-7e-5p
 
 
-0.466
 
 
23.9
 
 
 
 
BET1
 
 
hsa-miR-1-3p
 
 
-0.413
 
 
12.0
 
 
 
 
BHLHE40
 
 
hsa-miR-130b-3p
 
 
-0.351
 
 
47.4
 
 
 
 
BIN3
 
 
hsa-let-7e-5p
 
 
-0.427
 
 
10.9
 
 
 
 
BLCAP
 
 
hsa-miR-130b-3p
 
 
-0.342
 
 
310.5
 
 
 
 
BLOC1S6
 
 
hsa-let-7e-5p
 
 
-0.321
 
 
22.3
 
 
 
 
BLTP3B
 
 
hsa-miR-1-3p
 
 
-0.29
 
 
11.5
 
 
 
 
BMPR2
 
 
hsa-miR-130b-3p
 
 
-0.33
 
 
12.6
 
 
 
 
BMPR2
 
 
hsa-miR-100-5p
 
 
-0.355
 
 
12.6
 
 
 
 
BPNT1
 
 
hsa-miR-1-3p
 
 
-0.329
 
 
11.9
 
 
 
 
BRIX1
 
 
hsa-miR-1-3p
 
 
-0.334
 
 
11.4
 
 
 
 
BSCL2
 
 
hsa-miR-1-3p
 
 
-0.471
 
 
226.5
 
 
 
 
BTBD9
 
 
hsa-let-7e-5p
 
 
-0.311
 
 
12.3
 
 
 
 
BTF3L4
 
 
hsa-let-7e-5p
 
 
-0.355
 
 
27.3
 
 
 
 
BTF3L4
 
 
hsa-miR-130b-3p
 
 
-0.359
 
 
27.3
 
 
 
 
BZW1
 
 
hsa-let-7e-5p
 
 
-0.354
 
 
31.9
 
 
 
 
BZW1
 
 
hsa-let-7e-5p
 
 
-0.375
 
 
31.9
 
 
 
 
BZW2
 
 
hsa-let-7e-5p
 
 
-0.408
 
 
14.1
 
 
 
 
C19orf47
 
 
hsa-let-7e-5p
 
 
-0.311
 
 
10.9
 
 
 
 
CACFD1
 
 
hsa-let-7e-5p
 
 
-0.296
 
 
10.2
 
 
 
 
CACNB4
 
 
hsa-miR-7-5p
 
 
-0.372
 
 
12.9
 
 
 
 
CACNG4
 
 
hsa-let-7e-5p
 
 
-0.33
 
 
58.1
 
 
 
 
CACNG7
 
 
hsa-miR-7-5p
 
 
-0.421
 
 
42.2
 
 
 
 
CADM2
 
 
hsa-let-7e-5p
 
 
-0.294
 
 
18.3
 
 
 
 
CALM2
 
 
hsa-miR-130b-3p
 
 
-0.584
 
 
1078.8
 
 
 
 
CAMSAP2
 
 
hsa-miR-130b-3p
 
 
-0.297
 
 
16.8
 
 
 
 
CAPN3
 
 
hsa-let-7e-5p
 
 
-0.317
 
 
125.8
 
 
 
 
CAPZA1
 
 
hsa-miR-7-5p
 
 
-0.351
 
 
32.0
 
 
 
 
CASD1
 
 
hsa-miR-130b-3p
 
 
-0.305
 
 
10.8
 
 
 
 
CASP3
 
 
hsa-let-7e-5p
 
 
-0.495
 
 
10.0
 
 
 
 
CBX5
 
 
hsa-let-7e-5p
 
 
-0.307
 
 
17.3
 
 
 
 
CBX5
 
 
hsa-let-7e-5p
 
 
-0.472
 
 
17.3
 
 
 
 
CCDC43
 
 
hsa-miR-7-5p
 
 
-0.331
 
 
11.6
 
 
 
 
CCNC
 
 
hsa-miR-1-3p
 
 
-0.336
 
 
16.4
 
 
 
 
CCNL2
 
 
hsa-miR-28-5p
 
 
-0.448
 
 
19.4
 
 
 
 
CCNY
 
 
hsa-let-7e-5p
 
 
-0.334
 
 
34.7
 
 
 
 
CCNY
 
 
hsa-miR-130b-3p
 
 
-0.372
 
 
34.7
 
 
 
 
CCSAP
 
 
hsa-miR-1-3p
 
 
-0.381
 
 
16.0
 
 
 
 
CCT4
 
 
hsa-miR-7-5p
 
 
-0.37
 
 
48.5
 
 
 
 
CCT6A
 
 
hsa-miR-130b-3p
 
 
-0.382
 
 
44.4
 
 
 
 
CD164
 
 
hsa-miR-1-3p
 
 
-0.315
 
 
35.6
 
 
 
 
CDC34
 
 
hsa-let-7e-5p
 
 
-0.321
 
 
48.3
 
 
 
 
CDC34
 
 
hsa-let-7e-5p
 
 
-0.555
 
 
48.3
 
 
 
 
CDC42SE1
 
 
hsa-miR-1-3p
 
 
-0.36
 
 
22.7
 
 
 
 
CDK14
 
 
hsa-miR-1-3p
 
 
-0.326
 
 
10.3
 
 
 
 
CDK4
 
 
hsa-miR-1-3p
 
 
-0.338
 
 
34.2
 
 
 
 
CDKN1A
 
 
hsa-let-7e-5p
 
 
-0.434
 
 
35.1
 
 
 
 
CDKN2AIP
 
 
hsa-miR-28-5p
 
 
-0.419
 
 
10.3
 
 
 
 
 
 
 cerebellum 
 
 
 
 
target_symbol
 
 
mature_mirna_id
 
 
score
 
 
nTPM
 
 
 
 
 
 
ABCB9
 
 
hsa-let-7e-5p
 
 
-0.422
 
 
18.0
 
 
 
 
ABHD2
 
 
hsa-miR-1-3p
 
 
-0.291
 
 
19.8
 
 
 
 
ABHD3
 
 
hsa-miR-130b-3p
 
 
-0.356
 
 
13.0
 
 
 
 
ACBD3
 
 
hsa-miR-130b-3p
 
 
-0.292
 
 
11.6
 
 
 
 
ACBD5
 
 
hsa-miR-130b-3p
 
 
-0.494
 
 
13.3
 
 
 
 
ACSL4
 
 
hsa-miR-130b-3p
 
 
-0.301
 
 
10.1
 
 
 
 
ACSL6
 
 
hsa-let-7e-5p
 
 
-0.336
 
 
27.8
 
 
 
 
ADAR
 
 
hsa-miR-1-3p
 
 
-0.35
 
 
30.8
 
 
 
 
ADGRB3
 
 
hsa-miR-130b-3p
 
 
-0.308
 
 
20.8
 
 
 
 
AKIRIN2
 
 
hsa-miR-130b-3p
 
 
-0.315
 
 
25.9
 
 
 
 
ALDH4A1
 
 
hsa-miR-184
 
 
-0.421
 
 
10.9
 
 
 
 
AMT
 
 
hsa-let-7e-5p
 
 
-0.463
 
 
20.1
 
 
 
 
ANKRD12
 
 
hsa-miR-7-5p
 
 
-0.37
 
 
13.0
 
 
 
 
ANKRD13C
 
 
hsa-miR-130b-3p
 
 
-0.33
 
 
12.9
 
 
 
 
ANKRD46
 
 
hsa-let-7e-5p
 
 
-0.389
 
 
27.5
 
 
 
 
ANP32B
 
 
hsa-miR-1-3p
 
 
-0.287
 
 
49.5
 
 
 
 
ANXA2
 
 
hsa-miR-1-3p
 
 
-0.581
 
 
15.9
 
 
 
 
AP1AR
 
 
hsa-miR-100-5p
 
 
-0.804
 
 
13.2
 
 
 
 
AP1S1
 
 
hsa-miR-1-3p
 
 
-0.355
 
 
23.9
 
 
 
 
AP1S1
 
 
hsa-let-7e-5p
 
 
-0.374
 
 
23.9
 
 
 
 
APBB3
 
 
hsa-let-7e-5p
 
 
-0.587
 
 
23.6
 
 
 
 
APPL1
 
 
hsa-miR-130b-3p
 
 
-0.347
 
 
11.8
 
 
 
 
ARCN1
 
 
hsa-miR-1-3p
 
 
-0.468
 
 
53.3
 
 
 
 
ARF4
 
 
hsa-miR-7-5p
 
 
-0.407
 
 
50.2
 
 
 
 
ARG2
 
 
hsa-let-7e-5p
 
 
-0.468
 
 
16.4
 
 
 
 
ARHGAP1
 
 
hsa-miR-130b-3p
 
 
-0.376
 
 
19.6
 
 
 
 
ARHGEF4
 
 
hsa-miR-130b-3p
 
 
-0.377
 
 
16.7
 
 
 
 
ARID4A
 
 
hsa-miR-7-5p
 
 
-0.341
 
 
10.7
 
 
 
 
ARL6IP1
 
 
hsa-miR-130b-3p
 
 
-0.289
 
 
234.0
 
 
 
 
ARL6IP1
 
 
hsa-miR-7-5p
 
 
-0.374
 
 
234.0
 
 
 
 
ARMT1
 
 
hsa-let-7e-5p
 
 
-0.303
 
 
11.5
 
 
 
 
ARPC3
 
 
hsa-miR-1-3p
 
 
-0.606
 
 
87.0
 
 
 
 
ARPP19
 
 
hsa-let-7e-5p
 
 
-0.492
 
 
66.7
 
 
 
 
AS3MT
 
 
hsa-miR-28-5p
 
 
-0.479
 
 
12.0
 
 
 
 
ASH2L
 
 
hsa-miR-1-3p
 
 
-0.462
 
 
20.0
 
 
 
 
ATF2
 
 
hsa-miR-1-3p
 
 
-0.328
 
 
26.7
 
 
 
 
ATG16L1
 
 
hsa-miR-130b-3p
 
 
-0.407
 
 
24.1
 
 
 
 
ATOSB
 
 
hsa-let-7e-5p
 
 
-0.429
 
 
14.6
 
 
 
 
ATP2A2
 
 
hsa-let-7e-5p
 
 
-0.311
 
 
15.0
 
 
 
 
ATP2B2
 
 
hsa-miR-7-5p
 
 
-0.297
 
 
88.0
 
 
 
 
ATP2B2
 
 
hsa-miR-130b-3p
 
 
-0.314
 
 
88.0
 
 
 
 
ATPAF1
 
 
hsa-let-7e-5p
 
 
-0.498
 
 
24.4
 
 
 
 
AVPI1
 
 
hsa-miR-7-5p
 
 
-0.367
 
 
16.8
 
 
 
 
AZIN1
 
 
hsa-miR-1-3p
 
 
-0.438
 
 
31.3
 
 
 
 
B3GAT3
 
 
hsa-let-7e-5p
 
 
-0.324
 
 
70.0
 
 
 
 
B4GAT1
 
 
hsa-let-7e-5p
 
 
-0.47
 
 
105.2
 
 
 
 
BAG4
 
 
hsa-miR-1-3p
 
 
-0.378
 
 
14.8
 
 
 
 
BAZ2A
 
 
hsa-miR-100-5p
 
 
-0.29
 
 
26.6
 
 
 
 
BAZ2A
 
 
hsa-miR-100-5p
 
 
-0.421
 
 
26.6
 
 
 
 
BEGAIN
 
 
hsa-miR-28-5p
 
 
-0.325
 
 
53.7
 
 
 
 
BEGAIN
 
 
hsa-let-7e-5p
 
 
-0.466
 
 
53.7
 
 
 
 
BHLHE40
 
 
hsa-miR-130b-3p
 
 
-0.351
 
 
18.1
 
 
 
 
BLCAP
 
 
hsa-miR-130b-3p
 
 
-0.342
 
 
93.5
 
 
 
 
BLOC1S6
 
 
hsa-let-7e-5p
 
 
-0.321
 
 
18.6
 
 
 
 
BRIX1
 
 
hsa-miR-1-3p
 
 
-0.334
 
 
13.6
 
 
 
 
BSCL2
 
 
hsa-miR-1-3p
 
 
-0.471
 
 
153.5
 
 
 
 
BTAF1
 
 
hsa-miR-130b-3p
 
 
-0.291
 
 
10.7
 
 
 
 
BTF3L4
 
 
hsa-let-7e-5p
 
 
-0.355
 
 
28.2
 
 
 
 
BTF3L4
 
 
hsa-miR-130b-3p
 
 
-0.359
 
 
28.2
 
 
 
 
BTG1
 
 
hsa-miR-130b-3p
 
 
-0.565
 
 
10.5
 
 
 
 
BZW1
 
 
hsa-let-7e-5p
 
 
-0.354
 
 
31.0
 
 
 
 
BZW1
 
 
hsa-let-7e-5p
 
 
-0.375
 
 
31.0
 
 
 
 
C1orf21
 
 
hsa-miR-7-5p
 
 
-0.373
 
 
14.9
 
 
 
 
C8orf58
 
 
hsa-let-7e-5p
 
 
-0.612
 
 
15.2
 
 
 
 
CACFD1
 
 
hsa-let-7e-5p
 
 
-0.296
 
 
12.1
 
 
 
 
CACNB4
 
 
hsa-miR-7-5p
 
 
-0.372
 
 
24.9
 
 
 
 
CACNG7
 
 
hsa-miR-7-5p
 
 
-0.421
 
 
63.8
 
 
 
 
CADM2
 
 
hsa-let-7e-5p
 
 
-0.294
 
 
16.0
 
 
 
 
CALM2
 
 
hsa-miR-130b-3p
 
 
-0.584
 
 
475.1
 
 
 
 
CAMSAP2
 
 
hsa-miR-130b-3p
 
 
-0.297
 
 
22.3
 
 
 
 
CAPN3
 
 
hsa-let-7e-5p
 
 
-0.317
 
 
19.1
 
 
 
 
CAPZA1
 
 
hsa-miR-7-5p
 
 
-0.351
 
 
29.2
 
 
 
 
CASD1
 
 
hsa-miR-130b-3p
 
 
-0.305
 
 
12.4
 
 
 
 
CASP3
 
 
hsa-let-7e-5p
 
 
-0.495
 
 
20.1
 
 
 
 
CBFA2T3
 
 
hsa-let-7e-5p
 
 
-0.305
 
 
36.7
 
 
 
 
CBX5
 
 
hsa-let-7e-5p
 
 
-0.307
 
 
15.7
 
 
 
 
CBX5
 
 
hsa-let-7e-5p
 
 
-0.472
 
 
15.7
 
 
 
 
CCDC43
 
 
hsa-miR-7-5p
 
 
-0.331
 
 
12.5
 
 
 
 
CCNC
 
 
hsa-miR-1-3p
 
 
-0.336
 
 
25.7
 
 
 
 
CCNL2
 
 
hsa-miR-28-5p
 
 
-0.448
 
 
84.5
 
 
 
 
CCNT2
 
 
hsa-miR-7-5p
 
 
-0.349
 
 
20.8
 
 
 
 
CCNY
 
 
hsa-let-7e-5p
 
 
-0.334
 
 
12.0
 
 
 
 
CCNY
 
 
hsa-miR-130b-3p
 
 
-0.372
 
 
12.0
 
 
 
 
CCSAP
 
 
hsa-miR-1-3p
 
 
-0.381
 
 
29.8
 
 
 
 
CCT4
 
 
hsa-miR-7-5p
 
 
-0.37
 
 
64.4
 
 
 
 
CCT6A
 
 
hsa-miR-130b-3p
 
 
-0.382
 
 
58.7
 
 
 
 
CD164
 
 
hsa-miR-1-3p
 
 
-0.315
 
 
22.8
 
 
 
 
CDC34
 
 
hsa-let-7e-5p
 
 
-0.321
 
 
66.6
 
 
 
 
CDC34
 
 
hsa-let-7e-5p
 
 
-0.555
 
 
66.6
 
 
 
 
CDC42SE1
 
 
hsa-miR-1-3p
 
 
-0.36
 
 
25.6
 
 
 
 
CDH22
 
 
hsa-let-7e-5p
 
 
-0.32
 
 
44.9
 
 
 
 
CDK14
 
 
hsa-miR-1-3p
 
 
-0.326
 
 
20.2
 
 
 
 
CDK4
 
 
hsa-miR-1-3p
 
 
-0.338
 
 
29.7
 
 
 
 
CDKN1A
 
 
hsa-let-7e-5p
 
 
-0.434
 
 
14.6
 
 
 
 
CDKN2AIP
 
 
hsa-miR-28-5p
 
 
-0.419
 
 
14.1
 
 
 
 
CDV3
 
 
hsa-let-7e-5p
 
 
-0.453
 
 
21.2
 
 
 
 
CDYL2
 
 
hsa-miR-100-5p
 
 
-0.312
 
 
13.2
 
 
 
 
CEBPD
 
 
hsa-let-7e-5p
 
 
-0.309
 
 
15.9
 
 
 
 
CECR2
 
 
hsa-let-7e-5p
 
 
-0.356
 
 
11.1
 
 
 
 
CERCAM
 
 
hsa-let-7e-5p
 
 
-0.344
 
 
52.4
 
 
 
 
 
 
 cerebral cortex 
 
 
 
 
target_symbol
 
 
mature_mirna_id
 
 
score
 
 
nTPM
 
 
 
 
 
 
ABCB9
 
 
hsa-let-7e-5p
 
 
-0.422
 
 
19.5
 
 
 
 
ABHD17C
 
 
hsa-let-7e-5p
 
 
-0.359
 
 
16.3
 
 
 
 
ABHD2
 
 
hsa-miR-1-3p
 
 
-0.291
 
 
34.2
 
 
 
 
ACBD3
 
 
hsa-miR-130b-3p
 
 
-0.292
 
 
15.8
 
 
 
 
ACBD5
 
 
hsa-miR-130b-3p
 
 
-0.494
 
 
31.3
 
 
 
 
ACSL4
 
 
hsa-miR-130b-3p
 
 
-0.301
 
 
27.8
 
 
 
 
ACSL6
 
 
hsa-let-7e-5p
 
 
-0.336
 
 
29.5
 
 
 
 
ADAR
 
 
hsa-miR-1-3p
 
 
-0.35
 
 
52.6
 
 
 
 
ADGRA3
 
 
hsa-miR-1-3p
 
 
-0.351
 
 
17.8
 
 
 
 
ADGRB3
 
 
hsa-miR-130b-3p
 
 
-0.308
 
 
28.2
 
 
 
 
AIFM1
 
 
hsa-let-7e-5p
 
 
-0.314
 
 
10.3
 
 
 
 
AKAP6
 
 
hsa-let-7e-5p
 
 
-0.307
 
 
27.4
 
 
 
 
AKIRIN2
 
 
hsa-miR-130b-3p
 
 
-0.315
 
 
33.4
 
 
 
 
ALDH4A1
 
 
hsa-miR-184
 
 
-0.421
 
 
69.8
 
 
 
 
ANKIB1
 
 
hsa-miR-130b-3p
 
 
-0.329
 
 
19.3
 
 
 
 
ANKRD12
 
 
hsa-miR-7-5p
 
 
-0.37
 
 
15.1
 
 
 
 
ANKRD13C
 
 
hsa-miR-130b-3p
 
 
-0.33
 
 
10.2
 
 
 
 
ANKRD46
 
 
hsa-let-7e-5p
 
 
-0.389
 
 
39.5
 
 
 
 
ANP32B
 
 
hsa-miR-1-3p
 
 
-0.287
 
 
70.2
 
 
 
 
ANXA2
 
 
hsa-miR-1-3p
 
 
-0.581
 
 
41.9
 
 
 
 
ANXA4
 
 
hsa-miR-1-3p
 
 
-0.539
 
 
12.2
 
 
 
 
AP1AR
 
 
hsa-miR-100-5p
 
 
-0.804
 
 
12.2
 
 
 
 
AP1S1
 
 
hsa-miR-1-3p
 
 
-0.355
 
 
104.9
 
 
 
 
AP1S1
 
 
hsa-let-7e-5p
 
 
-0.374
 
 
104.9
 
 
 
 
APBB3
 
 
hsa-let-7e-5p
 
 
-0.587
 
 
15.0
 
 
 
 
APCDD1
 
 
hsa-miR-130b-3p
 
 
-0.304
 
 
33.1
 
 
 
 
APPL1
 
 
hsa-miR-130b-3p
 
 
-0.347
 
 
15.1
 
 
 
 
ARCN1
 
 
hsa-miR-1-3p
 
 
-0.468
 
 
47.6
 
 
 
 
ARF4
 
 
hsa-miR-7-5p
 
 
-0.407
 
 
61.5
 
 
 
 
ARHGAP1
 
 
hsa-miR-130b-3p
 
 
-0.376
 
 
40.8
 
 
 
 
ARHGAP12
 
 
hsa-miR-130b-3p
 
 
-0.287
 
 
14.7
 
 
 
 
ARHGEF4
 
 
hsa-miR-130b-3p
 
 
-0.377
 
 
93.3
 
 
 
 
ARID4A
 
 
hsa-miR-7-5p
 
 
-0.341
 
 
13.3
 
 
 
 
ARL4D
 
 
hsa-let-7e-5p
 
 
-0.417
 
 
14.3
 
 
 
 
ARL6IP1
 
 
hsa-miR-130b-3p
 
 
-0.289
 
 
314.1
 
 
 
 
ARL6IP1
 
 
hsa-miR-7-5p
 
 
-0.374
 
 
314.1
 
 
 
 
ARMT1
 
 
hsa-let-7e-5p
 
 
-0.303
 
 
10.1
 
 
 
 
ARPC3
 
 
hsa-miR-1-3p
 
 
-0.606
 
 
171.6
 
 
 
 
ARPP19
 
 
hsa-let-7e-5p
 
 
-0.492
 
 
292.4
 
 
 
 
ARRDC4
 
 
hsa-let-7e-5p
 
 
-0.329
 
 
12.8
 
 
 
 
ASAP1
 
 
hsa-let-7e-5p
 
 
-0.296
 
 
21.7
 
 
 
 
ASH2L
 
 
hsa-miR-1-3p
 
 
-0.462
 
 
21.5
 
 
 
 
ATF2
 
 
hsa-miR-1-3p
 
 
-0.328
 
 
23.3
 
 
 
 
ATG16L1
 
 
hsa-miR-130b-3p
 
 
-0.407
 
 
13.9
 
 
 
 
ATOSB
 
 
hsa-let-7e-5p
 
 
-0.429
 
 
21.2
 
 
 
 
ATP2A2
 
 
hsa-let-7e-5p
 
 
-0.311
 
 
54.4
 
 
 
 
ATP2B2
 
 
hsa-miR-7-5p
 
 
-0.297
 
 
63.7
 
 
 
 
ATP2B2
 
 
hsa-miR-130b-3p
 
 
-0.314
 
 
63.7
 
 
 
 
ATPAF1
 
 
hsa-let-7e-5p
 
 
-0.498
 
 
74.9
 
 
 
 
ATXN1
 
 
hsa-miR-130b-3p
 
 
-0.321
 
 
12.1
 
 
 
 
AVPI1
 
 
hsa-miR-7-5p
 
 
-0.367
 
 
24.4
 
 
 
 
AXL
 
 
hsa-miR-1-3p
 
 
-0.304
 
 
14.7
 
 
 
 
AZIN1
 
 
hsa-miR-1-3p
 
 
-0.438
 
 
76.8
 
 
 
 
B3GAT3
 
 
hsa-let-7e-5p
 
 
-0.324
 
 
63.6
 
 
 
 
B4GAT1
 
 
hsa-let-7e-5p
 
 
-0.47
 
 
171.8
 
 
 
 
BAG4
 
 
hsa-miR-1-3p
 
 
-0.378
 
 
16.5
 
 
 
 
BAHD1
 
 
hsa-miR-130b-3p
 
 
-0.294
 
 
10.6
 
 
 
 
BAZ2A
 
 
hsa-miR-100-5p
 
 
-0.29
 
 
13.9
 
 
 
 
BAZ2A
 
 
hsa-miR-100-5p
 
 
-0.421
 
 
13.9
 
 
 
 
BCAP29
 
 
hsa-let-7e-5p
 
 
-0.289
 
 
33.6
 
 
 
 
BEGAIN
 
 
hsa-miR-28-5p
 
 
-0.325
 
 
42.8
 
 
 
 
BEGAIN
 
 
hsa-let-7e-5p
 
 
-0.466
 
 
42.8
 
 
 
 
BET1
 
 
hsa-miR-1-3p
 
 
-0.413
 
 
10.4
 
 
 
 
BHLHE40
 
 
hsa-miR-130b-3p
 
 
-0.351
 
 
59.9
 
 
 
 
BIN3
 
 
hsa-let-7e-5p
 
 
-0.427
 
 
10.8
 
 
 
 
BLCAP
 
 
hsa-miR-130b-3p
 
 
-0.342
 
 
139.8
 
 
 
 
BLOC1S6
 
 
hsa-let-7e-5p
 
 
-0.321
 
 
30.5
 
 
 
 
BLTP3B
 
 
hsa-miR-1-3p
 
 
-0.29
 
 
18.6
 
 
 
 
BMPR2
 
 
hsa-miR-130b-3p
 
 
-0.33
 
 
20.6
 
 
 
 
BMPR2
 
 
hsa-miR-100-5p
 
 
-0.355
 
 
20.6
 
 
 
 
BPNT1
 
 
hsa-miR-1-3p
 
 
-0.329
 
 
12.2
 
 
 
 
BRIX1
 
 
hsa-miR-1-3p
 
 
-0.334
 
 
11.5
 
 
 
 
BSCL2
 
 
hsa-miR-1-3p
 
 
-0.471
 
 
228.0
 
 
 
 
BTBD9
 
 
hsa-let-7e-5p
 
 
-0.311
 
 
12.7
 
 
 
 
BTF3L4
 
 
hsa-let-7e-5p
 
 
-0.355
 
 
37.5
 
 
 
 
BTF3L4
 
 
hsa-miR-130b-3p
 
 
-0.359
 
 
37.5
 
 
 
 
BZW1
 
 
hsa-let-7e-5p
 
 
-0.354
 
 
64.1
 
 
 
 
BZW1
 
 
hsa-let-7e-5p
 
 
-0.375
 
 
64.1
 
 
 
 
BZW2
 
 
hsa-let-7e-5p
 
 
-0.408
 
 
16.2
 
 
 
 
C19orf47
 
 
hsa-let-7e-5p
 
 
-0.311
 
 
14.5
 
 
 
 
C1GALT1
 
 
hsa-let-7e-5p
 
 
-0.322
 
 
12.5
 
 
 
 
C1orf21
 
 
hsa-miR-7-5p
 
 
-0.373
 
 
17.9
 
 
 
 
C2orf69
 
 
hsa-miR-1-3p
 
 
-0.414
 
 
12.9
 
 
 
 
C6orf120
 
 
hsa-miR-1-3p
 
 
-0.288
 
 
12.8
 
 
 
 
CACNB4
 
 
hsa-miR-7-5p
 
 
-0.372
 
 
16.9
 
 
 
 
CACNG4
 
 
hsa-let-7e-5p
 
 
-0.33
 
 
24.4
 
 
 
 
CACNG7
 
 
hsa-miR-7-5p
 
 
-0.421
 
 
78.7
 
 
 
 
CADM2
 
 
hsa-let-7e-5p
 
 
-0.294
 
 
48.5
 
 
 
 
CALM2
 
 
hsa-miR-130b-3p
 
 
-0.584
 
 
1193.7
 
 
 
 
CAMSAP2
 
 
hsa-miR-130b-3p
 
 
-0.297
 
 
36.5
 
 
 
 
CAPN3
 
 
hsa-let-7e-5p
 
 
-0.317
 
 
48.1
 
 
 
 
CAPZA1
 
 
hsa-miR-7-5p
 
 
-0.351
 
 
33.3
 
 
 
 
CASD1
 
 
hsa-miR-130b-3p
 
 
-0.305
 
 
21.8
 
 
 
 
CASP3
 
 
hsa-let-7e-5p
 
 
-0.495
 
 
11.2
 
 
 
 
CBX5
 
 
hsa-let-7e-5p
 
 
-0.307
 
 
25.0
 
 
 
 
CBX5
 
 
hsa-let-7e-5p
 
 
-0.472
 
 
25.0
 
 
 
 
CCDC43
 
 
hsa-miR-7-5p
 
 
-0.331
 
 
12.9
 
 
 
 
CCNC
 
 
hsa-miR-1-3p
 
 
-0.336
 
 
18.8
 
 
 
 
CCNL2
 
 
hsa-miR-28-5p
 
 
-0.448
 
 
31.1
 
 
 
 
CCNY
 
 
hsa-let-7e-5p
 
 
-0.334
 
 
38.1
 
 
 
 
 
 
 hypothalamus 
 
 
 
 
target_symbol
 
 
mature_mirna_id
 
 
score
 
 
nTPM
 
 
 
 
 
 
ABCB9
 
 
hsa-let-7e-5p
 
 
-0.422
 
 
11.9
 
 
 
 
ABHD17C
 
 
hsa-let-7e-5p
 
 
-0.359
 
 
11.1
 
 
 
 
ABHD2
 
 
hsa-miR-1-3p
 
 
-0.291
 
 
12.1
 
 
 
 
ACBD5
 
 
hsa-miR-130b-3p
 
 
-0.494
 
 
25.5
 
 
 
 
ACSL4
 
 
hsa-miR-130b-3p
 
 
-0.301
 
 
13.7
 
 
 
 
ACSL6
 
 
hsa-let-7e-5p
 
 
-0.336
 
 
11.9
 
 
 
 
ADAR
 
 
hsa-miR-1-3p
 
 
-0.35
 
 
39.1
 
 
 
 
ADGRA3
 
 
hsa-miR-1-3p
 
 
-0.351
 
 
10.8
 
 
 
 
ADGRB3
 
 
hsa-miR-130b-3p
 
 
-0.308
 
 
16.0
 
 
 
 
AKAP6
 
 
hsa-let-7e-5p
 
 
-0.307
 
 
13.6
 
 
 
 
AKIRIN2
 
 
hsa-miR-130b-3p
 
 
-0.315
 
 
29.8
 
 
 
 
ALDH4A1
 
 
hsa-miR-184
 
 
-0.421
 
 
31.5
 
 
 
 
ANKIB1
 
 
hsa-miR-130b-3p
 
 
-0.329
 
 
14.5
 
 
 
 
ANKRD46
 
 
hsa-let-7e-5p
 
 
-0.389
 
 
28.7
 
 
 
 
ANP32B
 
 
hsa-miR-1-3p
 
 
-0.287
 
 
120.3
 
 
 
 
ANXA2
 
 
hsa-miR-1-3p
 
 
-0.581
 
 
63.6
 
 
 
 
ANXA4
 
 
hsa-miR-1-3p
 
 
-0.539
 
 
11.9
 
 
 
 
AP1S1
 
 
hsa-miR-1-3p
 
 
-0.355
 
 
44.3
 
 
 
 
AP1S1
 
 
hsa-let-7e-5p
 
 
-0.374
 
 
44.3
 
 
 
 
APBB3
 
 
hsa-let-7e-5p
 
 
-0.587
 
 
11.2
 
 
 
 
APCDD1
 
 
hsa-miR-130b-3p
 
 
-0.304
 
 
19.2
 
 
 
 
ARCN1
 
 
hsa-miR-1-3p
 
 
-0.468
 
 
49.3
 
 
 
 
ARF4
 
 
hsa-miR-7-5p
 
 
-0.407
 
 
75.3
 
 
 
 
ARG2
 
 
hsa-let-7e-5p
 
 
-0.468
 
 
10.7
 
 
 
 
ARHGAP1
 
 
hsa-miR-130b-3p
 
 
-0.376
 
 
31.0
 
 
 
 
ARHGAP12
 
 
hsa-miR-130b-3p
 
 
-0.287
 
 
12.7
 
 
 
 
ARHGEF4
 
 
hsa-miR-130b-3p
 
 
-0.377
 
 
41.1
 
 
 
 
ARL6IP1
 
 
hsa-miR-130b-3p
 
 
-0.289
 
 
212.8
 
 
 
 
ARL6IP1
 
 
hsa-miR-7-5p
 
 
-0.374
 
 
212.8
 
 
 
 
ARPC3
 
 
hsa-miR-1-3p
 
 
-0.606
 
 
163.1
 
 
 
 
ARPP19
 
 
hsa-let-7e-5p
 
 
-0.492
 
 
103.9
 
 
 
 
ARRDC4
 
 
hsa-let-7e-5p
 
 
-0.329
 
 
13.4
 
 
 
 
ASAP1
 
 
hsa-let-7e-5p
 
 
-0.296
 
 
11.8
 
 
 
 
ASH2L
 
 
hsa-miR-1-3p
 
 
-0.462
 
 
18.7
 
 
 
 
ATF2
 
 
hsa-miR-1-3p
 
 
-0.328
 
 
18.6
 
 
 
 
ATOSB
 
 
hsa-let-7e-5p
 
 
-0.429
 
 
19.8
 
 
 
 
ATP2A2
 
 
hsa-let-7e-5p
 
 
-0.311
 
 
11.9
 
 
 
 
ATP2B2
 
 
hsa-miR-7-5p
 
 
-0.297
 
 
12.0
 
 
 
 
ATP2B2
 
 
hsa-miR-130b-3p
 
 
-0.314
 
 
12.0
 
 
 
 
ATPAF1
 
 
hsa-let-7e-5p
 
 
-0.498
 
 
52.2
 
 
 
 
AVPI1
 
 
hsa-miR-7-5p
 
 
-0.367
 
 
15.0
 
 
 
 
AZIN1
 
 
hsa-miR-1-3p
 
 
-0.438
 
 
32.4
 
 
 
 
B3GAT3
 
 
hsa-let-7e-5p
 
 
-0.324
 
 
60.2
 
 
 
 
B4GAT1
 
 
hsa-let-7e-5p
 
 
-0.47
 
 
207.3
 
 
 
 
BAG4
 
 
hsa-miR-1-3p
 
 
-0.378
 
 
10.8
 
 
 
 
BCAP29
 
 
hsa-let-7e-5p
 
 
-0.289
 
 
16.4
 
 
 
 
BEGAIN
 
 
hsa-miR-28-5p
 
 
-0.325
 
 
15.9
 
 
 
 
BEGAIN
 
 
hsa-let-7e-5p
 
 
-0.466
 
 
15.9
 
 
 
 
BET1
 
 
hsa-miR-1-3p
 
 
-0.413
 
 
13.4
 
 
 
 
BHLHE40
 
 
hsa-miR-130b-3p
 
 
-0.351
 
 
34.7
 
 
 
 
BLCAP
 
 
hsa-miR-130b-3p
 
 
-0.342
 
 
183.0
 
 
 
 
BLOC1S6
 
 
hsa-let-7e-5p
 
 
-0.321
 
 
24.8
 
 
 
 
BMPR2
 
 
hsa-miR-130b-3p
 
 
-0.33
 
 
11.9
 
 
 
 
BMPR2
 
 
hsa-miR-100-5p
 
 
-0.355
 
 
11.9
 
 
 
 
BRIX1
 
 
hsa-miR-1-3p
 
 
-0.334
 
 
12.5
 
 
 
 
BSCL2
 
 
hsa-miR-1-3p
 
 
-0.471
 
 
274.6
 
 
 
 
BTF3L4
 
 
hsa-let-7e-5p
 
 
-0.355
 
 
33.4
 
 
 
 
BTF3L4
 
 
hsa-miR-130b-3p
 
 
-0.359
 
 
33.4
 
 
 
 
BZW1
 
 
hsa-let-7e-5p
 
 
-0.354
 
 
32.4
 
 
 
 
BZW1
 
 
hsa-let-7e-5p
 
 
-0.375
 
 
32.4
 
 
 
 
C19orf47
 
 
hsa-let-7e-5p
 
 
-0.311
 
 
12.4
 
 
 
 
CACNG4
 
 
hsa-let-7e-5p
 
 
-0.33
 
 
28.6
 
 
 
 
CACNG7
 
 
hsa-miR-7-5p
 
 
-0.421
 
 
38.3
 
 
 
 
CADM2
 
 
hsa-let-7e-5p
 
 
-0.294
 
 
11.9
 
 
 
 
CALM2
 
 
hsa-miR-130b-3p
 
 
-0.584
 
 
1070.8
 
 
 
 
CAMSAP2
 
 
hsa-miR-130b-3p
 
 
-0.297
 
 
16.6
 
 
 
 
CAPN3
 
 
hsa-let-7e-5p
 
 
-0.317
 
 
74.2
 
 
 
 
CAPZA1
 
 
hsa-miR-7-5p
 
 
-0.351
 
 
42.3
 
 
 
 
CASD1
 
 
hsa-miR-130b-3p
 
 
-0.305
 
 
13.5
 
 
 
 
CBX5
 
 
hsa-let-7e-5p
 
 
-0.307
 
 
20.5
 
 
 
 
CBX5
 
 
hsa-let-7e-5p
 
 
-0.472
 
 
20.5
 
 
 
 
CCNC
 
 
hsa-miR-1-3p
 
 
-0.336
 
 
18.5
 
 
 
 
CCNL2
 
 
hsa-miR-28-5p
 
 
-0.448
 
 
18.2
 
 
 
 
CCNY
 
 
hsa-let-7e-5p
 
 
-0.334
 
 
22.8
 
 
 
 
CCNY
 
 
hsa-miR-130b-3p
 
 
-0.372
 
 
22.8
 
 
 
 
CCT4
 
 
hsa-miR-7-5p
 
 
-0.37
 
 
49.8
 
 
 
 
CCT6A
 
 
hsa-miR-130b-3p
 
 
-0.382
 
 
50.6
 
 
 
 
CD164
 
 
hsa-miR-1-3p
 
 
-0.315
 
 
44.5
 
 
 
 
CDC34
 
 
hsa-let-7e-5p
 
 
-0.321
 
 
45.1
 
 
 
 
CDC34
 
 
hsa-let-7e-5p
 
 
-0.555
 
 
45.1
 
 
 
 
CDC42SE1
 
 
hsa-miR-1-3p
 
 
-0.36
 
 
26.3
 
 
 
 
CDK14
 
 
hsa-miR-1-3p
 
 
-0.326
 
 
13.8
 
 
 
 
CDK4
 
 
hsa-miR-1-3p
 
 
-0.338
 
 
30.8
 
 
 
 
CDKN1A
 
 
hsa-let-7e-5p
 
 
-0.434
 
 
37.8
 
 
 
 
CDV3
 
 
hsa-let-7e-5p
 
 
-0.453
 
 
32.9
 
 
 
 
CEBPD
 
 
hsa-let-7e-5p
 
 
-0.309
 
 
59.6
 
 
 
 
CERCAM
 
 
hsa-let-7e-5p
 
 
-0.344
 
 
182.5
 
 
 
 
CERS2
 
 
hsa-miR-1-3p
 
 
-0.287
 
 
68.3
 
 
 
 
CGGBP1
 
 
hsa-miR-7-5p
 
 
-0.465
 
 
18.1
 
 
 
 
CHMP3
 
 
hsa-miR-130b-3p
 
 
-0.521
 
 
39.8
 
 
 
 
CHMP4B
 
 
hsa-miR-130b-3p
 
 
-0.292
 
 
120.3
 
 
 
 
CHST1
 
 
hsa-miR-130b-3p
 
 
-0.288
 
 
21.9
 
 
 
 
CHST1
 
 
hsa-miR-130b-3p
 
 
-0.484
 
 
21.9
 
 
 
 
CITED2
 
 
hsa-miR-1-3p
 
 
-0.37
 
 
14.6
 
 
 
 
CKAP4
 
 
hsa-miR-7-5p
 
 
-0.443
 
 
11.9
 
 
 
 
CLASP2
 
 
hsa-let-7e-5p
 
 
-0.344
 
 
36.2
 
 
 
 
CLCN3
 
 
hsa-miR-1-3p
 
 
-0.339
 
 
15.5
 
 
 
 
CLCN3
 
 
hsa-miR-130b-3p
 
 
-0.384
 
 
15.5
 
 
 
 
CLDN11
 
 
hsa-miR-100-5p
 
 
-0.42
 
 
19.0
 
 
 
 
CLDN12
 
 
hsa-let-7e-5p
 
 
-0.511
 
 
11.7
 
 
 
 
 
 
 midbrain 
 
 
 
 
target_symbol
 
 
mature_mirna_id
 
 
score
 
 
nTPM
 
 
 
 
 
 
ABCB9
 
 
hsa-let-7e-5p
 
 
-0.422
 
 
10.6
 
 
 
 
ABHD17C
 
 
hsa-let-7e-5p
 
 
-0.359
 
 
11.2
 
 
 
 
ABHD2
 
 
hsa-miR-1-3p
 
 
-0.291
 
 
14.1
 
 
 
 
ABHD3
 
 
hsa-miR-130b-3p
 
 
-0.356
 
 
12.3
 
 
 
 
ACBD3
 
 
hsa-miR-130b-3p
 
 
-0.292
 
 
10.0
 
 
 
 
ACBD5
 
 
hsa-miR-130b-3p
 
 
-0.494
 
 
34.8
 
 
 
 
ACSL4
 
 
hsa-miR-130b-3p
 
 
-0.301
 
 
10.6
 
 
 
 
ACSL6
 
 
hsa-let-7e-5p
 
 
-0.336
 
 
11.1
 
 
 
 
ADAR
 
 
hsa-miR-1-3p
 
 
-0.35
 
 
28.9
 
 
 
 
ADGRA3
 
 
hsa-miR-1-3p
 
 
-0.351
 
 
13.9
 
 
 
 
AIFM1
 
 
hsa-let-7e-5p
 
 
-0.314
 
 
12.7
 
 
 
 
AKAP6
 
 
hsa-let-7e-5p
 
 
-0.307
 
 
17.4
 
 
 
 
AKIRIN2
 
 
hsa-miR-130b-3p
 
 
-0.315
 
 
28.8
 
 
 
 
ALDH4A1
 
 
hsa-miR-184
 
 
-0.421
 
 
73.5
 
 
 
 
ANKIB1
 
 
hsa-miR-130b-3p
 
 
-0.329
 
 
19.4
 
 
 
 
ANKRD29
 
 
hsa-miR-1-3p
 
 
-0.426
 
 
11.5
 
 
 
 
ANKRD46
 
 
hsa-let-7e-5p
 
 
-0.389
 
 
24.0
 
 
 
 
ANP32B
 
 
hsa-miR-1-3p
 
 
-0.287
 
 
216.9
 
 
 
 
ANXA2
 
 
hsa-miR-1-3p
 
 
-0.581
 
 
63.8
 
 
 
 
ANXA4
 
 
hsa-miR-1-3p
 
 
-0.539
 
 
20.9
 
 
 
 
AP1AR
 
 
hsa-miR-100-5p
 
 
-0.804
 
 
10.1
 
 
 
 
AP1S1
 
 
hsa-miR-1-3p
 
 
-0.355
 
 
35.4
 
 
 
 
AP1S1
 
 
hsa-let-7e-5p
 
 
-0.374
 
 
35.4
 
 
 
 
APCDD1
 
 
hsa-miR-130b-3p
 
 
-0.304
 
 
22.5
 
 
 
 
ARCN1
 
 
hsa-miR-1-3p
 
 
-0.468
 
 
48.9
 
 
 
 
ARF4
 
 
hsa-miR-7-5p
 
 
-0.407
 
 
62.8
 
 
 
 
ARHGAP1
 
 
hsa-miR-130b-3p
 
 
-0.376
 
 
34.8
 
 
 
 
ARHGAP12
 
 
hsa-miR-130b-3p
 
 
-0.287
 
 
13.4
 
 
 
 
ARHGEF4
 
 
hsa-miR-130b-3p
 
 
-0.377
 
 
35.7
 
 
 
 
ARL6IP1
 
 
hsa-miR-130b-3p
 
 
-0.289
 
 
198.8
 
 
 
 
ARL6IP1
 
 
hsa-miR-7-5p
 
 
-0.374
 
 
198.8
 
 
 
 
ARPC3
 
 
hsa-miR-1-3p
 
 
-0.606
 
 
176.5
 
 
 
 
ARPP19
 
 
hsa-let-7e-5p
 
 
-0.492
 
 
144.2
 
 
 
 
ARRDC4
 
 
hsa-let-7e-5p
 
 
-0.329
 
 
17.7
 
 
 
 
ASAP1
 
 
hsa-let-7e-5p
 
 
-0.296
 
 
12.8
 
 
 
 
ASH2L
 
 
hsa-miR-1-3p
 
 
-0.462
 
 
16.5
 
 
 
 
ATF2
 
 
hsa-miR-1-3p
 
 
-0.328
 
 
15.2
 
 
 
 
ATOSB
 
 
hsa-let-7e-5p
 
 
-0.429
 
 
24.1
 
 
 
 
ATP2A2
 
 
hsa-let-7e-5p
 
 
-0.311
 
 
17.1
 
 
 
 
ATP2B2
 
 
hsa-miR-7-5p
 
 
-0.297
 
 
12.7
 
 
 
 
ATP2B2
 
 
hsa-miR-130b-3p
 
 
-0.314
 
 
12.7
 
 
 
 
ATPAF1
 
 
hsa-let-7e-5p
 
 
-0.498
 
 
74.9
 
 
 
 
AVPI1
 
 
hsa-miR-7-5p
 
 
-0.367
 
 
18.5
 
 
 
 
AXL
 
 
hsa-miR-1-3p
 
 
-0.304
 
 
11.2
 
 
 
 
AZIN1
 
 
hsa-miR-1-3p
 
 
-0.438
 
 
33.7
 
 
 
 
B3GAT3
 
 
hsa-let-7e-5p
 
 
-0.324
 
 
58.5
 
 
 
 
B4GAT1
 
 
hsa-let-7e-5p
 
 
-0.47
 
 
85.6
 
 
 
 
BCAP29
 
 
hsa-let-7e-5p
 
 
-0.289
 
 
19.1
 
 
 
 
BET1
 
 
hsa-miR-1-3p
 
 
-0.413
 
 
10.6
 
 
 
 
BHLHE40
 
 
hsa-miR-130b-3p
 
 
-0.351
 
 
41.1
 
 
 
 
BLCAP
 
 
hsa-miR-130b-3p
 
 
-0.342
 
 
88.1
 
 
 
 
BLOC1S6
 
 
hsa-let-7e-5p
 
 
-0.321
 
 
18.5
 
 
 
 
BRIX1
 
 
hsa-miR-1-3p
 
 
-0.334
 
 
13.7
 
 
 
 
BSCL2
 
 
hsa-miR-1-3p
 
 
-0.471
 
 
109.8
 
 
 
 
BTF3L4
 
 
hsa-let-7e-5p
 
 
-0.355
 
 
28.8
 
 
 
 
BTF3L4
 
 
hsa-miR-130b-3p
 
 
-0.359
 
 
28.8
 
 
 
 
BZW1
 
 
hsa-let-7e-5p
 
 
-0.354
 
 
39.5
 
 
 
 
BZW1
 
 
hsa-let-7e-5p
 
 
-0.375
 
 
39.5
 
 
 
 
C19orf47
 
 
hsa-let-7e-5p
 
 
-0.311
 
 
13.9
 
 
 
 
C1GALT1
 
 
hsa-let-7e-5p
 
 
-0.322
 
 
13.7
 
 
 
 
C2orf88
 
 
hsa-let-7e-5p
 
 
-0.429
 
 
10.0
 
 
 
 
CACFD1
 
 
hsa-let-7e-5p
 
 
-0.296
 
 
10.3
 
 
 
 
CACNG4
 
 
hsa-let-7e-5p
 
 
-0.33
 
 
17.0
 
 
 
 
CACNG7
 
 
hsa-miR-7-5p
 
 
-0.421
 
 
43.9
 
 
 
 
CADM2
 
 
hsa-let-7e-5p
 
 
-0.294
 
 
16.8
 
 
 
 
CALM2
 
 
hsa-miR-130b-3p
 
 
-0.584
 
 
1156.5
 
 
 
 
CAMSAP2
 
 
hsa-miR-130b-3p
 
 
-0.297
 
 
14.7
 
 
 
 
CAPN3
 
 
hsa-let-7e-5p
 
 
-0.317
 
 
184.7
 
 
 
 
CAPZA1
 
 
hsa-miR-7-5p
 
 
-0.351
 
 
43.8
 
 
 
 
CBX5
 
 
hsa-let-7e-5p
 
 
-0.307
 
 
16.9
 
 
 
 
CBX5
 
 
hsa-let-7e-5p
 
 
-0.472
 
 
16.9
 
 
 
 
CCDC43
 
 
hsa-miR-7-5p
 
 
-0.331
 
 
11.2
 
 
 
 
CCNC
 
 
hsa-miR-1-3p
 
 
-0.336
 
 
14.6
 
 
 
 
CCNL2
 
 
hsa-miR-28-5p
 
 
-0.448
 
 
19.3
 
 
 
 
CCNY
 
 
hsa-let-7e-5p
 
 
-0.334
 
 
28.8
 
 
 
 
CCNY
 
 
hsa-miR-130b-3p
 
 
-0.372
 
 
28.8
 
 
 
 
CCT4
 
 
hsa-miR-7-5p
 
 
-0.37
 
 
50.4
 
 
 
 
CCT6A
 
 
hsa-miR-130b-3p
 
 
-0.382
 
 
47.8
 
 
 
 
CD164
 
 
hsa-miR-1-3p
 
 
-0.315
 
 
53.3
 
 
 
 
CDC34
 
 
hsa-let-7e-5p
 
 
-0.321
 
 
46.8
 
 
 
 
CDC34
 
 
hsa-let-7e-5p
 
 
-0.555
 
 
46.8
 
 
 
 
CDC42SE1
 
 
hsa-miR-1-3p
 
 
-0.36
 
 
31.7
 
 
 
 
CDK14
 
 
hsa-miR-1-3p
 
 
-0.326
 
 
14.9
 
 
 
 
CDK4
 
 
hsa-miR-1-3p
 
 
-0.338
 
 
36.7
 
 
 
 
CDKN1A
 
 
hsa-let-7e-5p
 
 
-0.434
 
 
54.4
 
 
 
 
CDV3
 
 
hsa-let-7e-5p
 
 
-0.453
 
 
42.0
 
 
 
 
CEBPD
 
 
hsa-let-7e-5p
 
 
-0.309
 
 
69.5
 
 
 
 
CERCAM
 
 
hsa-let-7e-5p
 
 
-0.344
 
 
324.4
 
 
 
 
CERS2
 
 
hsa-miR-1-3p
 
 
-0.287
 
 
120.2
 
 
 
 
CGGBP1
 
 
hsa-miR-7-5p
 
 
-0.465
 
 
18.4
 
 
 
 
CHMP3
 
 
hsa-miR-130b-3p
 
 
-0.521
 
 
45.4
 
 
 
 
CHMP4B
 
 
hsa-miR-130b-3p
 
 
-0.292
 
 
130.6
 
 
 
 
CHST1
 
 
hsa-miR-130b-3p
 
 
-0.288
 
 
19.4
 
 
 
 
CHST1
 
 
hsa-miR-130b-3p
 
 
-0.484
 
 
19.4
 
 
 
 
CLASP2
 
 
hsa-let-7e-5p
 
 
-0.344
 
 
37.4
 
 
 
 
CLCN3
 
 
hsa-miR-1-3p
 
 
-0.339
 
 
19.7
 
 
 
 
CLCN3
 
 
hsa-miR-130b-3p
 
 
-0.384
 
 
19.7
 
 
 
 
CLDN11
 
 
hsa-miR-100-5p
 
 
-0.42
 
 
42.3
 
 
 
 
CLIP1
 
 
hsa-miR-130b-3p
 
 
-0.452
 
 
15.5
 
 
 
 
CLTC
 
 
hsa-miR-1-3p
 
 
-0.39
 
 
83.9
 
 
 
 
 
 
 pituitary gland 
 
 
 
 
target_symbol
 
 
mature_mirna_id
 
 
score
 
 
nTPM
 
 
 
 
 
 
ABCB9
 
 
hsa-let-7e-5p
 
 
-0.422
 
 
11.0
 
 
 
 
ABHD2
 
 
hsa-miR-1-3p
 
 
-0.291
 
 
13.6
 
 
 
 
ABHD3
 
 
hsa-miR-130b-3p
 
 
-0.356
 
 
10.7
 
 
 
 
ACBD3
 
 
hsa-miR-130b-3p
 
 
-0.292
 
 
13.6
 
 
 
 
ADAR
 
 
hsa-miR-1-3p
 
 
-0.35
 
 
32.6
 
 
 
 
ADGRB3
 
 
hsa-miR-130b-3p
 
 
-0.308
 
 
21.3
 
 
 
 
AIFM1
 
 
hsa-let-7e-5p
 
 
-0.314
 
 
11.0
 
 
 
 
AKIRIN2
 
 
hsa-miR-130b-3p
 
 
-0.315
 
 
21.2
 
 
 
 
AMT
 
 
hsa-let-7e-5p
 
 
-0.463
 
 
16.0
 
 
 
 
ANP32B
 
 
hsa-miR-1-3p
 
 
-0.287
 
 
64.1
 
 
 
 
ANXA2
 
 
hsa-miR-1-3p
 
 
-0.581
 
 
144.0
 
 
 
 
ANXA4
 
 
hsa-miR-1-3p
 
 
-0.539
 
 
11.3
 
 
 
 
AP1S1
 
 
hsa-miR-1-3p
 
 
-0.355
 
 
31.4
 
 
 
 
AP1S1
 
 
hsa-let-7e-5p
 
 
-0.374
 
 
31.4
 
 
 
 
APBB3
 
 
hsa-let-7e-5p
 
 
-0.587
 
 
10.1
 
 
 
 
ARCN1
 
 
hsa-miR-1-3p
 
 
-0.468
 
 
84.2
 
 
 
 
ARF4
 
 
hsa-miR-7-5p
 
 
-0.407
 
 
93.4
 
 
 
 
ARG2
 
 
hsa-let-7e-5p
 
 
-0.468
 
 
47.4
 
 
 
 
ARHGAP1
 
 
hsa-miR-130b-3p
 
 
-0.376
 
 
35.2
 
 
 
 
ARHGEF4
 
 
hsa-miR-130b-3p
 
 
-0.377
 
 
15.3
 
 
 
 
ARL4D
 
 
hsa-let-7e-5p
 
 
-0.417
 
 
20.8
 
 
 
 
ARL6IP1
 
 
hsa-miR-130b-3p
 
 
-0.289
 
 
39.7
 
 
 
 
ARL6IP1
 
 
hsa-miR-7-5p
 
 
-0.374
 
 
39.7
 
 
 
 
ARPC3
 
 
hsa-miR-1-3p
 
 
-0.606
 
 
169.4
 
 
 
 
ARPP19
 
 
hsa-let-7e-5p
 
 
-0.492
 
 
33.0
 
 
 
 
ASH2L
 
 
hsa-miR-1-3p
 
 
-0.462
 
 
11.5
 
 
 
 
ATF2
 
 
hsa-miR-1-3p
 
 
-0.328
 
 
13.0
 
 
 
 
ATOSB
 
 
hsa-let-7e-5p
 
 
-0.429
 
 
15.4
 
 
 
 
ATP2A2
 
 
hsa-let-7e-5p
 
 
-0.311
 
 
13.7
 
 
 
 
ATPAF1
 
 
hsa-let-7e-5p
 
 
-0.498
 
 
31.1
 
 
 
 
AVPI1
 
 
hsa-miR-7-5p
 
 
-0.367
 
 
20.2
 
 
 
 
AZIN1
 
 
hsa-miR-1-3p
 
 
-0.438
 
 
27.1
 
 
 
 
B3GAT3
 
 
hsa-let-7e-5p
 
 
-0.324
 
 
61.5
 
 
 
 
B4GAT1
 
 
hsa-let-7e-5p
 
 
-0.47
 
 
60.5
 
 
 
 
BAHD1
 
 
hsa-miR-130b-3p
 
 
-0.294
 
 
12.8
 
 
 
 
BAZ2A
 
 
hsa-miR-100-5p
 
 
-0.29
 
 
25.8
 
 
 
 
BAZ2A
 
 
hsa-miR-100-5p
 
 
-0.421
 
 
25.8
 
 
 
 
BEGAIN
 
 
hsa-miR-28-5p
 
 
-0.325
 
 
18.8
 
 
 
 
BEGAIN
 
 
hsa-let-7e-5p
 
 
-0.466
 
 
18.8
 
 
 
 
BET1
 
 
hsa-miR-1-3p
 
 
-0.413
 
 
17.3
 
 
 
 
BHLHE40
 
 
hsa-miR-130b-3p
 
 
-0.351
 
 
99.3
 
 
 
 
BIN3
 
 
hsa-let-7e-5p
 
 
-0.427
 
 
13.2
 
 
 
 
BLCAP
 
 
hsa-miR-130b-3p
 
 
-0.342
 
 
145.4
 
 
 
 
BLOC1S6
 
 
hsa-let-7e-5p
 
 
-0.321
 
 
19.0
 
 
 
 
BPNT1
 
 
hsa-miR-1-3p
 
 
-0.329
 
 
10.6
 
 
 
 
BRIX1
 
 
hsa-miR-1-3p
 
 
-0.334
 
 
11.8
 
 
 
 
BSCL2
 
 
hsa-miR-1-3p
 
 
-0.471
 
 
219.0
 
 
 
 
BTAF1
 
 
hsa-miR-130b-3p
 
 
-0.291
 
 
11.4
 
 
 
 
BTF3L4
 
 
hsa-let-7e-5p
 
 
-0.355
 
 
28.3
 
 
 
 
BTF3L4
 
 
hsa-miR-130b-3p
 
 
-0.359
 
 
28.3
 
 
 
 
BZW1
 
 
hsa-let-7e-5p
 
 
-0.354
 
 
28.1
 
 
 
 
BZW1
 
 
hsa-let-7e-5p
 
 
-0.375
 
 
28.1
 
 
 
 
BZW2
 
 
hsa-let-7e-5p
 
 
-0.408
 
 
10.9
 
 
 
 
CACFD1
 
 
hsa-let-7e-5p
 
 
-0.296
 
 
18.3
 
 
 
 
CALM2
 
 
hsa-miR-130b-3p
 
 
-0.584
 
 
372.4
 
 
 
 
CANT1
 
 
hsa-let-7e-5p
 
 
-0.402
 
 
16.3
 
 
 
 
CAPN3
 
 
hsa-let-7e-5p
 
 
-0.317
 
 
14.0
 
 
 
 
CAPZA1
 
 
hsa-miR-7-5p
 
 
-0.351
 
 
26.6
 
 
 
 
CBX5
 
 
hsa-let-7e-5p
 
 
-0.307
 
 
18.3
 
 
 
 
CBX5
 
 
hsa-let-7e-5p
 
 
-0.472
 
 
18.3
 
 
 
 
CCNC
 
 
hsa-miR-1-3p
 
 
-0.336
 
 
22.0
 
 
 
 
CCNL2
 
 
hsa-miR-28-5p
 
 
-0.448
 
 
56.8
 
 
 
 
CCNT2
 
 
hsa-miR-7-5p
 
 
-0.349
 
 
14.2
 
 
 
 
CCNY
 
 
hsa-let-7e-5p
 
 
-0.334
 
 
13.7
 
 
 
 
CCNY
 
 
hsa-miR-130b-3p
 
 
-0.372
 
 
13.7
 
 
 
 
CCT4
 
 
hsa-miR-7-5p
 
 
-0.37
 
 
45.4
 
 
 
 
CCT6A
 
 
hsa-miR-130b-3p
 
 
-0.382
 
 
39.3
 
 
 
 
CD164
 
 
hsa-miR-1-3p
 
 
-0.315
 
 
83.3
 
 
 
 
CDC34
 
 
hsa-let-7e-5p
 
 
-0.321
 
 
44.1
 
 
 
 
CDC34
 
 
hsa-let-7e-5p
 
 
-0.555
 
 
44.1
 
 
 
 
CDC42SE1
 
 
hsa-miR-1-3p
 
 
-0.36
 
 
37.5
 
 
 
 
CDK4
 
 
hsa-miR-1-3p
 
 
-0.338
 
 
69.4
 
 
 
 
CDKN1A
 
 
hsa-let-7e-5p
 
 
-0.434
 
 
104.4
 
 
 
 
CDKN2AIP
 
 
hsa-miR-28-5p
 
 
-0.419
 
 
10.8
 
 
 
 
CDV3
 
 
hsa-let-7e-5p
 
 
-0.453
 
 
37.0
 
 
 
 
CEBPD
 
 
hsa-let-7e-5p
 
 
-0.309
 
 
212.0
 
 
 
 
CERCAM
 
 
hsa-let-7e-5p
 
 
-0.344
 
 
115.4
 
 
 
 
CERS2
 
 
hsa-miR-1-3p
 
 
-0.287
 
 
76.8
 
 
 
 
CGGBP1
 
 
hsa-miR-7-5p
 
 
-0.465
 
 
22.4
 
 
 
 
CHMP3
 
 
hsa-miR-130b-3p
 
 
-0.521
 
 
31.4
 
 
 
 
CHMP4B
 
 
hsa-miR-130b-3p
 
 
-0.292
 
 
73.1
 
 
 
 
CITED2
 
 
hsa-miR-1-3p
 
 
-0.37
 
 
15.5
 
 
 
 
CKAP4
 
 
hsa-miR-7-5p
 
 
-0.443
 
 
19.1
 
 
 
 
CLASP2
 
 
hsa-let-7e-5p
 
 
-0.344
 
 
20.4
 
 
 
 
CLCN3
 
 
hsa-miR-1-3p
 
 
-0.339
 
 
11.8
 
 
 
 
CLCN3
 
 
hsa-miR-130b-3p
 
 
-0.384
 
 
11.8
 
 
 
 
CLDN12
 
 
hsa-let-7e-5p
 
 
-0.511
 
 
21.0
 
 
 
 
CLIP1
 
 
hsa-miR-130b-3p
 
 
-0.452
 
 
17.2
 
 
 
 
CLTC
 
 
hsa-miR-1-3p
 
 
-0.39
 
 
51.3
 
 
 
 
CMPK1
 
 
hsa-miR-130b-3p
 
 
-0.384
 
 
30.6
 
 
 
 
CNIH1
 
 
hsa-miR-130b-3p
 
 
-0.335
 
 
39.0
 
 
 
 
CNN3
 
 
hsa-miR-1-3p
 
 
-0.414
 
 
105.8
 
 
 
 
CNN3
 
 
hsa-miR-7-5p
 
 
-0.489
 
 
105.8
 
 
 
 
CNOT7
 
 
hsa-miR-130b-3p
 
 
-0.318
 
 
23.6
 
 
 
 
CNOT8
 
 
hsa-miR-7-5p
 
 
-0.329
 
 
18.0
 
 
 
 
CNPPD1
 
 
hsa-miR-7-5p
 
 
-0.541
 
 
23.9
 
 
 
 
COL1A2
 
 
hsa-let-7e-5p
 
 
-0.499
 
 
33.9
 
 
 
 
COL3A1
 
 
hsa-let-7e-5p
 
 
-0.474
 
 
12.8
 
 
 
 
COL9A3
 
 
hsa-miR-130b-3p
 
 
-0.379
 
 
13.2
 
 
 
 
CORO1C
 
 
hsa-miR-1-3p
 
 
-0.476
 
 
21.4
 
 
 
 
 
 
 testis 
 
 
 
 
target_symbol
 
 
mature_mirna_id
 
 
score
 
 
nTPM
 
 
 
 
 
 
ABCB9
 
 
hsa-let-7e-5p
 
 
-0.422
 
 
17.1
 
 
 
 
ABHD2
 
 
hsa-miR-1-3p
 
 
-0.291
 
 
48.6
 
 
 
 
ACBD3
 
 
hsa-miR-130b-3p
 
 
-0.292
 
 
17.6
 
 
 
 
ACBD5
 
 
hsa-miR-130b-3p
 
 
-0.494
 
 
18.0
 
 
 
 
ACRV1
 
 
hsa-miR-7-5p
 
 
-0.3
 
 
110.6
 
 
 
 
ACSL4
 
 
hsa-miR-130b-3p
 
 
-0.301
 
 
23.3
 
 
 
 
ACSL6
 
 
hsa-let-7e-5p
 
 
-0.336
 
 
19.3
 
 
 
 
ADAR
 
 
hsa-miR-1-3p
 
 
-0.35
 
 
32.3
 
 
 
 
AHCTF1
 
 
hsa-let-7e-5p
 
 
-0.374
 
 
30.4
 
 
 
 
AIFM1
 
 
hsa-let-7e-5p
 
 
-0.314
 
 
21.3
 
 
 
 
AKIRIN2
 
 
hsa-miR-130b-3p
 
 
-0.315
 
 
21.4
 
 
 
 
ALG9
 
 
hsa-miR-7-5p
 
 
-0.302
 
 
12.8
 
 
 
 
ANKIB1
 
 
hsa-miR-130b-3p
 
 
-0.329
 
 
19.4
 
 
 
 
ANKRD12
 
 
hsa-miR-7-5p
 
 
-0.37
 
 
25.9
 
 
 
 
ANP32B
 
 
hsa-miR-1-3p
 
 
-0.287
 
 
56.3
 
 
 
 
ANXA2
 
 
hsa-miR-1-3p
 
 
-0.581
 
 
108.4
 
 
 
 
AP1AR
 
 
hsa-miR-100-5p
 
 
-0.804
 
 
13.6
 
 
 
 
AP1S1
 
 
hsa-miR-1-3p
 
 
-0.355
 
 
17.8
 
 
 
 
AP1S1
 
 
hsa-let-7e-5p
 
 
-0.374
 
 
17.8
 
 
 
 
ARCN1
 
 
hsa-miR-1-3p
 
 
-0.468
 
 
42.4
 
 
 
 
ARF4
 
 
hsa-miR-7-5p
 
 
-0.407
 
 
94.2
 
 
 
 
ARFGEF1
 
 
hsa-miR-130b-3p
 
 
-0.302
 
 
26.5
 
 
 
 
ARHGAP1
 
 
hsa-miR-130b-3p
 
 
-0.376
 
 
15.3
 
 
 
 
ARHGAP28
 
 
hsa-let-7e-5p
 
 
-0.486
 
 
72.3
 
 
 
 
ARID2
 
 
hsa-miR-1-3p
 
 
-0.294
 
 
24.3
 
 
 
 
ARID3A
 
 
hsa-let-7e-5p
 
 
-0.347
 
 
16.1
 
 
 
 
ARID3A
 
 
hsa-let-7e-5p
 
 
-0.348
 
 
16.1
 
 
 
 
ARID3B
 
 
hsa-let-7e-5p
 
 
-0.31
 
 
21.2
 
 
 
 
ARID3B
 
 
hsa-let-7e-5p
 
 
-0.486
 
 
21.2
 
 
 
 
ARL4D
 
 
hsa-let-7e-5p
 
 
-0.417
 
 
31.4
 
 
 
 
ARL6IP1
 
 
hsa-miR-130b-3p
 
 
-0.289
 
 
121.4
 
 
 
 
ARL6IP1
 
 
hsa-miR-7-5p
 
 
-0.374
 
 
121.4
 
 
 
 
ARL6IP6
 
 
hsa-let-7e-5p
 
 
-0.305
 
 
10.2
 
 
 
 
ARMT1
 
 
hsa-let-7e-5p
 
 
-0.303
 
 
13.1
 
 
 
 
ARPC3
 
 
hsa-miR-1-3p
 
 
-0.606
 
 
99.9
 
 
 
 
ARPP19
 
 
hsa-let-7e-5p
 
 
-0.492
 
 
79.0
 
 
 
 
ARRDC4
 
 
hsa-let-7e-5p
 
 
-0.329
 
 
13.7
 
 
 
 
ASAP1
 
 
hsa-let-7e-5p
 
 
-0.296
 
 
26.5
 
 
 
 
ASH2L
 
 
hsa-miR-1-3p
 
 
-0.462
 
 
38.9
 
 
 
 
ATF2
 
 
hsa-miR-1-3p
 
 
-0.328
 
 
17.8
 
 
 
 
ATG16L1
 
 
hsa-miR-130b-3p
 
 
-0.407
 
 
13.1
 
 
 
 
ATP2A2
 
 
hsa-let-7e-5p
 
 
-0.311
 
 
69.3
 
 
 
 
ATPAF1
 
 
hsa-let-7e-5p
 
 
-0.498
 
 
29.2
 
 
 
 
AVPI1
 
 
hsa-miR-7-5p
 
 
-0.367
 
 
16.0
 
 
 
 
AXL
 
 
hsa-miR-1-3p
 
 
-0.304
 
 
17.4
 
 
 
 
AZIN1
 
 
hsa-miR-1-3p
 
 
-0.438
 
 
32.4
 
 
 
 
B3GAT3
 
 
hsa-let-7e-5p
 
 
-0.324
 
 
26.6
 
 
 
 
BACH1
 
 
hsa-let-7e-5p
 
 
-0.298
 
 
25.4
 
 
 
 
BACH1
 
 
hsa-let-7e-5p
 
 
-0.298
 
 
25.4
 
 
 
 
BACH1
 
 
hsa-let-7e-5p
 
 
-0.435
 
 
25.4
 
 
 
 
BACH1
 
 
hsa-let-7e-5p
 
 
-0.435
 
 
25.4
 
 
 
 
BAHD1
 
 
hsa-miR-130b-3p
 
 
-0.294
 
 
17.8
 
 
 
 
BAZ2A
 
 
hsa-miR-100-5p
 
 
-0.29
 
 
44.2
 
 
 
 
BAZ2A
 
 
hsa-miR-100-5p
 
 
-0.421
 
 
44.2
 
 
 
 
BCAP29
 
 
hsa-let-7e-5p
 
 
-0.289
 
 
65.9
 
 
 
 
BET1
 
 
hsa-miR-1-3p
 
 
-0.413
 
 
13.0
 
 
 
 
BHLHE40
 
 
hsa-miR-130b-3p
 
 
-0.351
 
 
14.9
 
 
 
 
BIN3
 
 
hsa-let-7e-5p
 
 
-0.427
 
 
13.0
 
 
 
 
BLCAP
 
 
hsa-miR-130b-3p
 
 
-0.342
 
 
29.6
 
 
 
 
BLOC1S6
 
 
hsa-let-7e-5p
 
 
-0.321
 
 
15.9
 
 
 
 
BLTP3B
 
 
hsa-miR-1-3p
 
 
-0.29
 
 
13.1
 
 
 
 
BPNT1
 
 
hsa-miR-1-3p
 
 
-0.329
 
 
13.8
 
 
 
 
BRIX1
 
 
hsa-miR-1-3p
 
 
-0.334
 
 
12.8
 
 
 
 
BSCL2
 
 
hsa-miR-1-3p
 
 
-0.471
 
 
169.3
 
 
 
 
BTAF1
 
 
hsa-miR-130b-3p
 
 
-0.291
 
 
14.0
 
 
 
 
BTF3L4
 
 
hsa-let-7e-5p
 
 
-0.355
 
 
31.8
 
 
 
 
BTF3L4
 
 
hsa-miR-130b-3p
 
 
-0.359
 
 
31.8
 
 
 
 
BZW1
 
 
hsa-let-7e-5p
 
 
-0.354
 
 
159.6
 
 
 
 
BZW1
 
 
hsa-let-7e-5p
 
 
-0.375
 
 
159.6
 
 
 
 
BZW2
 
 
hsa-let-7e-5p
 
 
-0.408
 
 
10.9
 
 
 
 
C15orf39
 
 
hsa-let-7e-5p
 
 
-0.374
 
 
69.4
 
 
 
 
C19orf47
 
 
hsa-let-7e-5p
 
 
-0.311
 
 
37.1
 
 
 
 
C2orf69
 
 
hsa-miR-1-3p
 
 
-0.414
 
 
13.7
 
 
 
 
C2orf88
 
 
hsa-let-7e-5p
 
 
-0.429
 
 
68.6
 
 
 
 
C8orf58
 
 
hsa-let-7e-5p
 
 
-0.612
 
 
15.8
 
 
 
 
C9orf40
 
 
hsa-let-7e-5p
 
 
-0.809
 
 
11.0
 
 
 
 
CAAP1
 
 
hsa-miR-1-3p
 
 
-0.48
 
 
11.7
 
 
 
 
CACFD1
 
 
hsa-let-7e-5p
 
 
-0.296
 
 
23.0
 
 
 
 
CALM2
 
 
hsa-miR-130b-3p
 
 
-0.584
 
 
377.7
 
 
 
 
CAMSAP2
 
 
hsa-miR-130b-3p
 
 
-0.297
 
 
14.2
 
 
 
 
CANT1
 
 
hsa-let-7e-5p
 
 
-0.402
 
 
19.3
 
 
 
 
CAPN3
 
 
hsa-let-7e-5p
 
 
-0.317
 
 
29.0
 
 
 
 
CAPZA1
 
 
hsa-miR-7-5p
 
 
-0.351
 
 
66.5
 
 
 
 
CARNMT1
 
 
hsa-let-7e-5p
 
 
-0.307
 
 
15.3
 
 
 
 
CASD1
 
 
hsa-miR-130b-3p
 
 
-0.305
 
 
12.3
 
 
 
 
CBX2
 
 
hsa-let-7e-5p
 
 
-0.313
 
 
12.3
 
 
 
 
CBX5
 
 
hsa-let-7e-5p
 
 
-0.307
 
 
23.7
 
 
 
 
CBX5
 
 
hsa-let-7e-5p
 
 
-0.472
 
 
23.7
 
 
 
 
CCDC113
 
 
hsa-miR-7-5p
 
 
-0.324
 
 
10.4
 
 
 
 
CCDC126
 
 
hsa-miR-130b-3p
 
 
-0.591
 
 
32.5
 
 
 
 
CCNC
 
 
hsa-miR-1-3p
 
 
-0.336
 
 
15.9
 
 
 
 
CCNL2
 
 
hsa-miR-28-5p
 
 
-0.448
 
 
23.3
 
 
 
 
CCNT2
 
 
hsa-miR-7-5p
 
 
-0.349
 
 
10.6
 
 
 
 
CCNY
 
 
hsa-let-7e-5p
 
 
-0.334
 
 
73.8
 
 
 
 
CCNY
 
 
hsa-miR-130b-3p
 
 
-0.372
 
 
73.8
 
 
 
 
CCT4
 
 
hsa-miR-7-5p
 
 
-0.37
 
 
72.5
 
 
 
 
CCT6A
 
 
hsa-miR-130b-3p
 
 
-0.382
 
 
47.1
 
 
 
 
CD164
 
 
hsa-miR-1-3p
 
 
-0.315
 
 
106.2
 
 
 
 
CDC34
 
 
hsa-let-7e-5p
 
 
-0.321
 
 
100.9
 
 
 
 
CDC34
 
 
hsa-let-7e-5p
 
 
-0.555
 
 
100.9
 
 
 
 
 
 
 amygdala 
 
 
 
 
target_symbol
 
 
mature_mirna_id
 
 
score
 
 
nTPM
 
 
 
 
 
 
ABHD17C
 
 
hsa-let-7e-5p
 
 
-0.359
 
 
17.9
 
 
 
 
ABHD2
 
 
hsa-miR-1-3p
 
 
-0.291
 
 
18.0
 
 
 
 
ABHD3
 
 
hsa-miR-130b-3p
 
 
-0.356
 
 
10.3
 
 
 
 
ACBD5
 
 
hsa-miR-130b-3p
 
 
-0.494
 
 
30.0
 
 
 
 
ACSL6
 
 
hsa-let-7e-5p
 
 
-0.336
 
 
20.3
 
 
 
 
ADAR
 
 
hsa-miR-1-3p
 
 
-0.35
 
 
30.2
 
 
 
 
ADGRA3
 
 
hsa-miR-1-3p
 
 
-0.351
 
 
19.9
 
 
 
 
ADGRB3
 
 
hsa-miR-130b-3p
 
 
-0.308
 
 
12.8
 
 
 
 
AIFM1
 
 
hsa-let-7e-5p
 
 
-0.314
 
 
10.3
 
 
 
 
AKAP6
 
 
hsa-let-7e-5p
 
 
-0.307
 
 
12.4
 
 
 
 
AKIRIN2
 
 
hsa-miR-130b-3p
 
 
-0.315
 
 
33.1
 
 
 
 
ALDH4A1
 
 
hsa-miR-184
 
 
-0.421
 
 
66.7
 
 
 
 
ANKIB1
 
 
hsa-miR-130b-3p
 
 
-0.329
 
 
15.2
 
 
 
 
ANKRD46
 
 
hsa-let-7e-5p
 
 
-0.389
 
 
28.3
 
 
 
 
ANP32B
 
 
hsa-miR-1-3p
 
 
-0.287
 
 
123.1
 
 
 
 
ANXA2
 
 
hsa-miR-1-3p
 
 
-0.581
 
 
34.4
 
 
 
 
ANXA4
 
 
hsa-miR-1-3p
 
 
-0.539
 
 
15.5
 
 
 
 
AP1S1
 
 
hsa-miR-1-3p
 
 
-0.355
 
 
88.4
 
 
 
 
AP1S1
 
 
hsa-let-7e-5p
 
 
-0.374
 
 
88.4
 
 
 
 
APCDD1
 
 
hsa-miR-130b-3p
 
 
-0.304
 
 
28.8
 
 
 
 
ARCN1
 
 
hsa-miR-1-3p
 
 
-0.468
 
 
40.8
 
 
 
 
ARF4
 
 
hsa-miR-7-5p
 
 
-0.407
 
 
60.1
 
 
 
 
ARHGAP1
 
 
hsa-miR-130b-3p
 
 
-0.376
 
 
37.0
 
 
 
 
ARHGEF4
 
 
hsa-miR-130b-3p
 
 
-0.377
 
 
80.2
 
 
 
 
ARL6IP1
 
 
hsa-miR-130b-3p
 
 
-0.289
 
 
241.3
 
 
 
 
ARL6IP1
 
 
hsa-miR-7-5p
 
 
-0.374
 
 
241.3
 
 
 
 
ARPC3
 
 
hsa-miR-1-3p
 
 
-0.606
 
 
187.7
 
 
 
 
ARPP19
 
 
hsa-let-7e-5p
 
 
-0.492
 
 
178.5
 
 
 
 
ARRDC4
 
 
hsa-let-7e-5p
 
 
-0.329
 
 
13.2
 
 
 
 
ASAP1
 
 
hsa-let-7e-5p
 
 
-0.296
 
 
11.2
 
 
 
 
ASH2L
 
 
hsa-miR-1-3p
 
 
-0.462
 
 
17.5
 
 
 
 
ATF2
 
 
hsa-miR-1-3p
 
 
-0.328
 
 
16.3
 
 
 
 
ATOSB
 
 
hsa-let-7e-5p
 
 
-0.429
 
 
20.1
 
 
 
 
ATP2A2
 
 
hsa-let-7e-5p
 
 
-0.311
 
 
15.8
 
 
 
 
ATP2B2
 
 
hsa-miR-7-5p
 
 
-0.297
 
 
14.3
 
 
 
 
ATP2B2
 
 
hsa-miR-130b-3p
 
 
-0.314
 
 
14.3
 
 
 
 
ATPAF1
 
 
hsa-let-7e-5p
 
 
-0.498
 
 
86.8
 
 
 
 
AVPI1
 
 
hsa-miR-7-5p
 
 
-0.367
 
 
13.3
 
 
 
 
AXL
 
 
hsa-miR-1-3p
 
 
-0.304
 
 
12.9
 
 
 
 
AZIN1
 
 
hsa-miR-1-3p
 
 
-0.438
 
 
37.2
 
 
 
 
B3GAT3
 
 
hsa-let-7e-5p
 
 
-0.324
 
 
61.0
 
 
 
 
B4GAT1
 
 
hsa-let-7e-5p
 
 
-0.47
 
 
107.6
 
 
 
 
BCAP29
 
 
hsa-let-7e-5p
 
 
-0.289
 
 
16.2
 
 
 
 
BEGAIN
 
 
hsa-miR-28-5p
 
 
-0.325
 
 
36.9
 
 
 
 
BEGAIN
 
 
hsa-let-7e-5p
 
 
-0.466
 
 
36.9
 
 
 
 
BET1
 
 
hsa-miR-1-3p
 
 
-0.413
 
 
11.0
 
 
 
 
BHLHE40
 
 
hsa-miR-130b-3p
 
 
-0.351
 
 
40.6
 
 
 
 
BIN3
 
 
hsa-let-7e-5p
 
 
-0.427
 
 
10.6
 
 
 
 
BLCAP
 
 
hsa-miR-130b-3p
 
 
-0.342
 
 
126.2
 
 
 
 
BLOC1S6
 
 
hsa-let-7e-5p
 
 
-0.321
 
 
20.1
 
 
 
 
BMPR2
 
 
hsa-miR-130b-3p
 
 
-0.33
 
 
10.7
 
 
 
 
BMPR2
 
 
hsa-miR-100-5p
 
 
-0.355
 
 
10.7
 
 
 
 
BRIX1
 
 
hsa-miR-1-3p
 
 
-0.334
 
 
11.3
 
 
 
 
BSCL2
 
 
hsa-miR-1-3p
 
 
-0.471
 
 
131.2
 
 
 
 
BTF3L4
 
 
hsa-let-7e-5p
 
 
-0.355
 
 
29.6
 
 
 
 
BTF3L4
 
 
hsa-miR-130b-3p
 
 
-0.359
 
 
29.6
 
 
 
 
BZW1
 
 
hsa-let-7e-5p
 
 
-0.354
 
 
34.3
 
 
 
 
BZW1
 
 
hsa-let-7e-5p
 
 
-0.375
 
 
34.3
 
 
 
 
C19orf47
 
 
hsa-let-7e-5p
 
 
-0.311
 
 
13.3
 
 
 
 
C1orf21
 
 
hsa-miR-7-5p
 
 
-0.373
 
 
13.5
 
 
 
 
CACFD1
 
 
hsa-let-7e-5p
 
 
-0.296
 
 
10.5
 
 
 
 
CACNG4
 
 
hsa-let-7e-5p
 
 
-0.33
 
 
35.0
 
 
 
 
CACNG7
 
 
hsa-miR-7-5p
 
 
-0.421
 
 
63.9
 
 
 
 
CADM2
 
 
hsa-let-7e-5p
 
 
-0.294
 
 
18.3
 
 
 
 
CALM2
 
 
hsa-miR-130b-3p
 
 
-0.584
 
 
1267.2
 
 
 
 
CAMSAP2
 
 
hsa-miR-130b-3p
 
 
-0.297
 
 
17.2
 
 
 
 
CAPN3
 
 
hsa-let-7e-5p
 
 
-0.317
 
 
74.7
 
 
 
 
CAPZA1
 
 
hsa-miR-7-5p
 
 
-0.351
 
 
34.6
 
 
 
 
CASP3
 
 
hsa-let-7e-5p
 
 
-0.495
 
 
10.5
 
 
 
 
CBX5
 
 
hsa-let-7e-5p
 
 
-0.307
 
 
18.6
 
 
 
 
CBX5
 
 
hsa-let-7e-5p
 
 
-0.472
 
 
18.6
 
 
 
 
CCDC43
 
 
hsa-miR-7-5p
 
 
-0.331
 
 
12.7
 
 
 
 
CCNC
 
 
hsa-miR-1-3p
 
 
-0.336
 
 
15.3
 
 
 
 
CCNL2
 
 
hsa-miR-28-5p
 
 
-0.448
 
 
17.7
 
 
 
 
CCNY
 
 
hsa-let-7e-5p
 
 
-0.334
 
 
36.8
 
 
 
 
CCNY
 
 
hsa-miR-130b-3p
 
 
-0.372
 
 
36.8
 
 
 
 
CCSAP
 
 
hsa-miR-1-3p
 
 
-0.381
 
 
13.5
 
 
 
 
CCT4
 
 
hsa-miR-7-5p
 
 
-0.37
 
 
45.3
 
 
 
 
CCT6A
 
 
hsa-miR-130b-3p
 
 
-0.382
 
 
42.2
 
 
 
 
CD164
 
 
hsa-miR-1-3p
 
 
-0.315
 
 
33.0
 
 
 
 
CDC34
 
 
hsa-let-7e-5p
 
 
-0.321
 
 
53.0
 
 
 
 
CDC34
 
 
hsa-let-7e-5p
 
 
-0.555
 
 
53.0
 
 
 
 
CDC42SE1
 
 
hsa-miR-1-3p
 
 
-0.36
 
 
23.8
 
 
 
 
CDH22
 
 
hsa-let-7e-5p
 
 
-0.32
 
 
12.8
 
 
 
 
CDK14
 
 
hsa-miR-1-3p
 
 
-0.326
 
 
17.8
 
 
 
 
CDK4
 
 
hsa-miR-1-3p
 
 
-0.338
 
 
31.9
 
 
 
 
CDKN1A
 
 
hsa-let-7e-5p
 
 
-0.434
 
 
34.5
 
 
 
 
CDV3
 
 
hsa-let-7e-5p
 
 
-0.453
 
 
33.8
 
 
 
 
CEBPD
 
 
hsa-let-7e-5p
 
 
-0.309
 
 
46.8
 
 
 
 
CERCAM
 
 
hsa-let-7e-5p
 
 
-0.344
 
 
203.9
 
 
 
 
CERS2
 
 
hsa-miR-1-3p
 
 
-0.287
 
 
66.8
 
 
 
 
CGGBP1
 
 
hsa-miR-7-5p
 
 
-0.465
 
 
18.1
 
 
 
 
CHMP3
 
 
hsa-miR-130b-3p
 
 
-0.521
 
 
52.4
 
 
 
 
CHMP4B
 
 
hsa-miR-130b-3p
 
 
-0.292
 
 
139.8
 
 
 
 
CHST1
 
 
hsa-miR-130b-3p
 
 
-0.288
 
 
32.2
 
 
 
 
CHST1
 
 
hsa-miR-130b-3p
 
 
-0.484
 
 
32.2
 
 
 
 
CKAP4
 
 
hsa-miR-7-5p
 
 
-0.443
 
 
10.7
 
 
 
 
CLASP2
 
 
hsa-let-7e-5p
 
 
-0.344
 
 
34.8
 
 
 
 
CLCN3
 
 
hsa-miR-1-3p
 
 
-0.339
 
 
16.2
 
 
 
 
CLCN3
 
 
hsa-miR-130b-3p
 
 
-0.384
 
 
16.2
 
 
 
 
 
 
 hippocampal formation 
 
 
 
 
target_symbol
 
 
mature_mirna_id
 
 
score
 
 
nTPM
 
 
 
 
 
 
ABHD17C
 
 
hsa-let-7e-5p
 
 
-0.359
 
 
14.4
 
 
 
 
ABHD2
 
 
hsa-miR-1-3p
 
 
-0.291
 
 
14.7
 
 
 
 
ACBD5
 
 
hsa-miR-130b-3p
 
 
-0.494
 
 
26.6
 
 
 
 
ACSL4
 
 
hsa-miR-130b-3p
 
 
-0.301
 
 
12.3
 
 
 
 
ACSL6
 
 
hsa-let-7e-5p
 
 
-0.336
 
 
15.3
 
 
 
 
ADAR
 
 
hsa-miR-1-3p
 
 
-0.35
 
 
27.1
 
 
 
 
ADGRA3
 
 
hsa-miR-1-3p
 
 
-0.351
 
 
12.8
 
 
 
 
ADGRB3
 
 
hsa-miR-130b-3p
 
 
-0.308
 
 
12.8
 
 
 
 
AIFM1
 
 
hsa-let-7e-5p
 
 
-0.314
 
 
10.5
 
 
 
 
AKAP6
 
 
hsa-let-7e-5p
 
 
-0.307
 
 
12.9
 
 
 
 
AKIRIN2
 
 
hsa-miR-130b-3p
 
 
-0.315
 
 
31.7
 
 
 
 
ALDH4A1
 
 
hsa-miR-184
 
 
-0.421
 
 
39.7
 
 
 
 
ANKIB1
 
 
hsa-miR-130b-3p
 
 
-0.329
 
 
16.1
 
 
 
 
ANKRD46
 
 
hsa-let-7e-5p
 
 
-0.389
 
 
25.5
 
 
 
 
ANP32B
 
 
hsa-miR-1-3p
 
 
-0.287
 
 
139.9
 
 
 
 
ANXA2
 
 
hsa-miR-1-3p
 
 
-0.581
 
 
46.0
 
 
 
 
ANXA4
 
 
hsa-miR-1-3p
 
 
-0.539
 
 
15.5
 
 
 
 
AP1S1
 
 
hsa-miR-1-3p
 
 
-0.355
 
 
67.5
 
 
 
 
AP1S1
 
 
hsa-let-7e-5p
 
 
-0.374
 
 
67.5
 
 
 
 
APCDD1
 
 
hsa-miR-130b-3p
 
 
-0.304
 
 
28.0
 
 
 
 
ARCN1
 
 
hsa-miR-1-3p
 
 
-0.468
 
 
46.9
 
 
 
 
ARF4
 
 
hsa-miR-7-5p
 
 
-0.407
 
 
61.3
 
 
 
 
ARHGAP1
 
 
hsa-miR-130b-3p
 
 
-0.376
 
 
48.9
 
 
 
 
ARHGEF4
 
 
hsa-miR-130b-3p
 
 
-0.377
 
 
96.3
 
 
 
 
ARL6IP1
 
 
hsa-miR-130b-3p
 
 
-0.289
 
 
196.8
 
 
 
 
ARL6IP1
 
 
hsa-miR-7-5p
 
 
-0.374
 
 
196.8
 
 
 
 
ARPC3
 
 
hsa-miR-1-3p
 
 
-0.606
 
 
183.3
 
 
 
 
ARPP19
 
 
hsa-let-7e-5p
 
 
-0.492
 
 
120.9
 
 
 
 
ARRDC4
 
 
hsa-let-7e-5p
 
 
-0.329
 
 
23.9
 
 
 
 
ASAP1
 
 
hsa-let-7e-5p
 
 
-0.296
 
 
10.8
 
 
 
 
ASH2L
 
 
hsa-miR-1-3p
 
 
-0.462
 
 
16.8
 
 
 
 
ATF2
 
 
hsa-miR-1-3p
 
 
-0.328
 
 
16.3
 
 
 
 
ATOSB
 
 
hsa-let-7e-5p
 
 
-0.429
 
 
22.4
 
 
 
 
ATP2A2
 
 
hsa-let-7e-5p
 
 
-0.311
 
 
16.6
 
 
 
 
ATP2B2
 
 
hsa-miR-7-5p
 
 
-0.297
 
 
19.0
 
 
 
 
ATP2B2
 
 
hsa-miR-130b-3p
 
 
-0.314
 
 
19.0
 
 
 
 
ATPAF1
 
 
hsa-let-7e-5p
 
 
-0.498
 
 
54.4
 
 
 
 
AVPI1
 
 
hsa-miR-7-5p
 
 
-0.367
 
 
14.1
 
 
 
 
AZIN1
 
 
hsa-miR-1-3p
 
 
-0.438
 
 
33.7
 
 
 
 
B3GAT3
 
 
hsa-let-7e-5p
 
 
-0.324
 
 
64.6
 
 
 
 
B4GAT1
 
 
hsa-let-7e-5p
 
 
-0.47
 
 
117.5
 
 
 
 
BAZ2A
 
 
hsa-miR-100-5p
 
 
-0.29
 
 
10.1
 
 
 
 
BAZ2A
 
 
hsa-miR-100-5p
 
 
-0.421
 
 
10.1
 
 
 
 
BCAP29
 
 
hsa-let-7e-5p
 
 
-0.289
 
 
16.3
 
 
 
 
BEGAIN
 
 
hsa-miR-28-5p
 
 
-0.325
 
 
36.8
 
 
 
 
BEGAIN
 
 
hsa-let-7e-5p
 
 
-0.466
 
 
36.8
 
 
 
 
BET1
 
 
hsa-miR-1-3p
 
 
-0.413
 
 
10.5
 
 
 
 
BHLHE40
 
 
hsa-miR-130b-3p
 
 
-0.351
 
 
38.8
 
 
 
 
BIN3
 
 
hsa-let-7e-5p
 
 
-0.427
 
 
10.9
 
 
 
 
BLCAP
 
 
hsa-miR-130b-3p
 
 
-0.342
 
 
112.6
 
 
 
 
BLOC1S6
 
 
hsa-let-7e-5p
 
 
-0.321
 
 
18.3
 
 
 
 
BLTP3B
 
 
hsa-miR-1-3p
 
 
-0.29
 
 
10.9
 
 
 
 
BRIX1
 
 
hsa-miR-1-3p
 
 
-0.334
 
 
12.3
 
 
 
 
BSCL2
 
 
hsa-miR-1-3p
 
 
-0.471
 
 
133.4
 
 
 
 
BTF3L4
 
 
hsa-let-7e-5p
 
 
-0.355
 
 
28.4
 
 
 
 
BTF3L4
 
 
hsa-miR-130b-3p
 
 
-0.359
 
 
28.4
 
 
 
 
BZW1
 
 
hsa-let-7e-5p
 
 
-0.354
 
 
34.8
 
 
 
 
BZW1
 
 
hsa-let-7e-5p
 
 
-0.375
 
 
34.8
 
 
 
 
C19orf47
 
 
hsa-let-7e-5p
 
 
-0.311
 
 
14.0
 
 
 
 
C1GALT1
 
 
hsa-let-7e-5p
 
 
-0.322
 
 
11.6
 
 
 
 
C1orf21
 
 
hsa-miR-7-5p
 
 
-0.373
 
 
10.3
 
 
 
 
CACNG4
 
 
hsa-let-7e-5p
 
 
-0.33
 
 
24.4
 
 
 
 
CACNG7
 
 
hsa-miR-7-5p
 
 
-0.421
 
 
60.1
 
 
 
 
CADM2
 
 
hsa-let-7e-5p
 
 
-0.294
 
 
17.6
 
 
 
 
CALM2
 
 
hsa-miR-130b-3p
 
 
-0.584
 
 
1305.6
 
 
 
 
CAMSAP2
 
 
hsa-miR-130b-3p
 
 
-0.297
 
 
17.9
 
 
 
 
CAPN3
 
 
hsa-let-7e-5p
 
 
-0.317
 
 
121.1
 
 
 
 
CAPZA1
 
 
hsa-miR-7-5p
 
 
-0.351
 
 
34.0
 
 
 
 
CBX5
 
 
hsa-let-7e-5p
 
 
-0.307
 
 
16.0
 
 
 
 
CBX5
 
 
hsa-let-7e-5p
 
 
-0.472
 
 
16.0
 
 
 
 
CCDC43
 
 
hsa-miR-7-5p
 
 
-0.331
 
 
12.2
 
 
 
 
CCNC
 
 
hsa-miR-1-3p
 
 
-0.336
 
 
15.1
 
 
 
 
CCNL2
 
 
hsa-miR-28-5p
 
 
-0.448
 
 
20.7
 
 
 
 
CCNY
 
 
hsa-let-7e-5p
 
 
-0.334
 
 
33.9
 
 
 
 
CCNY
 
 
hsa-miR-130b-3p
 
 
-0.372
 
 
33.9
 
 
 
 
CCSAP
 
 
hsa-miR-1-3p
 
 
-0.381
 
 
14.0
 
 
 
 
CCT4
 
 
hsa-miR-7-5p
 
 
-0.37
 
 
45.6
 
 
 
 
CCT6A
 
 
hsa-miR-130b-3p
 
 
-0.382
 
 
43.6
 
 
 
 
CD164
 
 
hsa-miR-1-3p
 
 
-0.315
 
 
36.4
 
 
 
 
CDC34
 
 
hsa-let-7e-5p
 
 
-0.321
 
 
52.2
 
 
 
 
CDC34
 
 
hsa-let-7e-5p
 
 
-0.555
 
 
52.2
 
 
 
 
CDC42SE1
 
 
hsa-miR-1-3p
 
 
-0.36
 
 
26.6
 
 
 
 
CDK14
 
 
hsa-miR-1-3p
 
 
-0.326
 
 
19.4
 
 
 
 
CDK4
 
 
hsa-miR-1-3p
 
 
-0.338
 
 
29.9
 
 
 
 
CDKN1A
 
 
hsa-let-7e-5p
 
 
-0.434
 
 
35.2
 
 
 
 
CDV3
 
 
hsa-let-7e-5p
 
 
-0.453
 
 
33.9
 
 
 
 
CEBPD
 
 
hsa-let-7e-5p
 
 
-0.309
 
 
62.1
 
 
 
 
CERCAM
 
 
hsa-let-7e-5p
 
 
-0.344
 
 
359.8
 
 
 
 
CERS2
 
 
hsa-miR-1-3p
 
 
-0.287
 
 
95.6
 
 
 
 
CGGBP1
 
 
hsa-miR-7-5p
 
 
-0.465
 
 
18.3
 
 
 
 
CHMP3
 
 
hsa-miR-130b-3p
 
 
-0.521
 
 
42.0
 
 
 
 
CHMP4B
 
 
hsa-miR-130b-3p
 
 
-0.292
 
 
120.6
 
 
 
 
CHRD
 
 
hsa-let-7e-5p
 
 
-0.303
 
 
10.7
 
 
 
 
CHST1
 
 
hsa-miR-130b-3p
 
 
-0.288
 
 
27.5
 
 
 
 
CHST1
 
 
hsa-miR-130b-3p
 
 
-0.484
 
 
27.5
 
 
 
 
CLASP2
 
 
hsa-let-7e-5p
 
 
-0.344
 
 
39.8
 
 
 
 
CLCN3
 
 
hsa-miR-1-3p
 
 
-0.339
 
 
16.0
 
 
 
 
CLCN3
 
 
hsa-miR-130b-3p
 
 
-0.384
 
 
16.0
 
 
 
 
CLDN11
 
 
hsa-miR-100-5p
 
 
-0.42
 
 
41.1
 
 
 
 
CLIP1
 
 
hsa-miR-130b-3p
 
 
-0.452
 
 
16.7
 
 
 
 
 
 
 kidney 
 
 
 
 
target_symbol
 
 
mature_mirna_id
 
 
score
 
 
nTPM
 
 
 
 
 
 
ABHD17C
 
 
hsa-let-7e-5p
 
 
-0.359
 
 
13.6
 
 
 
 
ABHD2
 
 
hsa-miR-1-3p
 
 
-0.291
 
 
20.9
 
 
 
 
ABHD3
 
 
hsa-miR-130b-3p
 
 
-0.356
 
 
13.5
 
 
 
 
ABRAXAS2
 
 
hsa-miR-130b-3p
 
 
-0.468
 
 
11.5
 
 
 
 
ACBD3
 
 
hsa-miR-130b-3p
 
 
-0.292
 
 
18.8
 
 
 
 
ACBD5
 
 
hsa-miR-130b-3p
 
 
-0.494
 
 
34.9
 
 
 
 
ACP3
 
 
hsa-let-7e-5p
 
 
-0.298
 
 
26.9
 
 
 
 
ACSL4
 
 
hsa-miR-130b-3p
 
 
-0.301
 
 
42.0
 
 
 
 
ACVR1
 
 
hsa-miR-130b-3p
 
 
-0.44
 
 
15.0
 
 
 
 
ADAR
 
 
hsa-miR-1-3p
 
 
-0.35
 
 
48.3
 
 
 
 
ADPGK
 
 
hsa-miR-1-3p
 
 
-0.393
 
 
14.3
 
 
 
 
AIFM1
 
 
hsa-let-7e-5p
 
 
-0.314
 
 
69.0
 
 
 
 
AKIRIN2
 
 
hsa-miR-130b-3p
 
 
-0.315
 
 
17.3
 
 
 
 
ALDH4A1
 
 
hsa-miR-184
 
 
-0.421
 
 
204.2
 
 
 
 
ALG11
 
 
hsa-miR-1-3p
 
 
-0.317
 
 
13.7
 
 
 
 
AMT
 
 
hsa-let-7e-5p
 
 
-0.463
 
 
23.7
 
 
 
 
ANKIB1
 
 
hsa-miR-130b-3p
 
 
-0.329
 
 
23.9
 
 
 
 
ANKRD12
 
 
hsa-miR-7-5p
 
 
-0.37
 
 
12.1
 
 
 
 
ANKRD46
 
 
hsa-let-7e-5p
 
 
-0.389
 
 
30.5
 
 
 
 
ANP32B
 
 
hsa-miR-1-3p
 
 
-0.287
 
 
90.4
 
 
 
 
ANXA2
 
 
hsa-miR-1-3p
 
 
-0.581
 
 
294.3
 
 
 
 
ANXA4
 
 
hsa-miR-1-3p
 
 
-0.539
 
 
97.1
 
 
 
 
AP1AR
 
 
hsa-miR-100-5p
 
 
-0.804
 
 
20.6
 
 
 
 
AP1S1
 
 
hsa-miR-1-3p
 
 
-0.355
 
 
48.3
 
 
 
 
AP1S1
 
 
hsa-let-7e-5p
 
 
-0.374
 
 
48.3
 
 
 
 
APPL1
 
 
hsa-miR-130b-3p
 
 
-0.347
 
 
15.4
 
 
 
 
ARCN1
 
 
hsa-miR-1-3p
 
 
-0.468
 
 
52.9
 
 
 
 
ARF4
 
 
hsa-miR-7-5p
 
 
-0.407
 
 
106.7
 
 
 
 
ARFGEF1
 
 
hsa-miR-130b-3p
 
 
-0.302
 
 
21.3
 
 
 
 
ARG2
 
 
hsa-let-7e-5p
 
 
-0.468
 
 
66.0
 
 
 
 
ARHGAP1
 
 
hsa-miR-130b-3p
 
 
-0.376
 
 
38.9
 
 
 
 
ARHGAP12
 
 
hsa-miR-130b-3p
 
 
-0.287
 
 
13.7
 
 
 
 
ARHGAP24
 
 
hsa-miR-130b-3p
 
 
-0.287
 
 
93.2
 
 
 
 
ARL4D
 
 
hsa-let-7e-5p
 
 
-0.417
 
 
35.1
 
 
 
 
ARL6IP1
 
 
hsa-miR-130b-3p
 
 
-0.289
 
 
140.2
 
 
 
 
ARL6IP1
 
 
hsa-miR-7-5p
 
 
-0.374
 
 
140.2
 
 
 
 
ARMT1
 
 
hsa-let-7e-5p
 
 
-0.303
 
 
38.7
 
 
 
 
ARPC3
 
 
hsa-miR-1-3p
 
 
-0.606
 
 
228.3
 
 
 
 
ARPP19
 
 
hsa-let-7e-5p
 
 
-0.492
 
 
52.6
 
 
 
 
ARRDC4
 
 
hsa-let-7e-5p
 
 
-0.329
 
 
23.9
 
 
 
 
AS3MT
 
 
hsa-miR-28-5p
 
 
-0.479
 
 
14.4
 
 
 
 
ASAP1
 
 
hsa-let-7e-5p
 
 
-0.296
 
 
10.1
 
 
 
 
ASH2L
 
 
hsa-miR-1-3p
 
 
-0.462
 
 
21.9
 
 
 
 
ATF2
 
 
hsa-miR-1-3p
 
 
-0.328
 
 
20.3
 
 
 
 
ATG4A
 
 
hsa-miR-7-5p
 
 
-0.448
 
 
13.1
 
 
 
 
ATOSB
 
 
hsa-let-7e-5p
 
 
-0.429
 
 
14.3
 
 
 
 
ATP2A2
 
 
hsa-let-7e-5p
 
 
-0.311
 
 
32.2
 
 
 
 
ATPAF1
 
 
hsa-let-7e-5p
 
 
-0.498
 
 
107.2
 
 
 
 
AVPI1
 
 
hsa-miR-7-5p
 
 
-0.367
 
 
12.6
 
 
 
 
AZIN1
 
 
hsa-miR-1-3p
 
 
-0.438
 
 
36.3
 
 
 
 
B3GAT3
 
 
hsa-let-7e-5p
 
 
-0.324
 
 
48.1
 
 
 
 
B4GAT1
 
 
hsa-let-7e-5p
 
 
-0.47
 
 
24.3
 
 
 
 
BACH1
 
 
hsa-let-7e-5p
 
 
-0.298
 
 
14.8
 
 
 
 
BACH1
 
 
hsa-let-7e-5p
 
 
-0.298
 
 
14.8
 
 
 
 
BACH1
 
 
hsa-let-7e-5p
 
 
-0.435
 
 
14.8
 
 
 
 
BACH1
 
 
hsa-let-7e-5p
 
 
-0.435
 
 
14.8
 
 
 
 
BAHD1
 
 
hsa-miR-130b-3p
 
 
-0.294
 
 
12.6
 
 
 
 
BAZ2A
 
 
hsa-miR-100-5p
 
 
-0.29
 
 
18.9
 
 
 
 
BAZ2A
 
 
hsa-miR-100-5p
 
 
-0.421
 
 
18.9
 
 
 
 
BCAP29
 
 
hsa-let-7e-5p
 
 
-0.289
 
 
19.2
 
 
 
 
BET1
 
 
hsa-miR-1-3p
 
 
-0.413
 
 
22.7
 
 
 
 
BHLHE40
 
 
hsa-miR-130b-3p
 
 
-0.351
 
 
155.1
 
 
 
 
BIN3
 
 
hsa-let-7e-5p
 
 
-0.427
 
 
17.8
 
 
 
 
BLCAP
 
 
hsa-miR-130b-3p
 
 
-0.342
 
 
62.5
 
 
 
 
BLOC1S6
 
 
hsa-let-7e-5p
 
 
-0.321
 
 
22.6
 
 
 
 
BLTP3B
 
 
hsa-miR-1-3p
 
 
-0.29
 
 
11.4
 
 
 
 
BMPR2
 
 
hsa-miR-130b-3p
 
 
-0.33
 
 
12.2
 
 
 
 
BMPR2
 
 
hsa-miR-100-5p
 
 
-0.355
 
 
12.2
 
 
 
 
BPNT1
 
 
hsa-miR-1-3p
 
 
-0.329
 
 
29.2
 
 
 
 
BRIX1
 
 
hsa-miR-1-3p
 
 
-0.334
 
 
14.1
 
 
 
 
BSCL2
 
 
hsa-miR-1-3p
 
 
-0.471
 
 
51.2
 
 
 
 
BTF3L4
 
 
hsa-let-7e-5p
 
 
-0.355
 
 
32.7
 
 
 
 
BTF3L4
 
 
hsa-miR-130b-3p
 
 
-0.359
 
 
32.7
 
 
 
 
BZW1
 
 
hsa-let-7e-5p
 
 
-0.354
 
 
82.6
 
 
 
 
BZW1
 
 
hsa-let-7e-5p
 
 
-0.375
 
 
82.6
 
 
 
 
BZW2
 
 
hsa-let-7e-5p
 
 
-0.408
 
 
25.1
 
 
 
 
C1GALT1
 
 
hsa-let-7e-5p
 
 
-0.322
 
 
28.8
 
 
 
 
C6orf120
 
 
hsa-miR-1-3p
 
 
-0.288
 
 
15.1
 
 
 
 
C8orf58
 
 
hsa-let-7e-5p
 
 
-0.612
 
 
12.6
 
 
 
 
CAAP1
 
 
hsa-miR-1-3p
 
 
-0.48
 
 
15.2
 
 
 
 
CACFD1
 
 
hsa-let-7e-5p
 
 
-0.296
 
 
28.4
 
 
 
 
CALM2
 
 
hsa-miR-130b-3p
 
 
-0.584
 
 
407.3
 
 
 
 
CAMSAP2
 
 
hsa-miR-130b-3p
 
 
-0.297
 
 
11.3
 
 
 
 
CANT1
 
 
hsa-let-7e-5p
 
 
-0.402
 
 
14.3
 
 
 
 
CAPN3
 
 
hsa-let-7e-5p
 
 
-0.317
 
 
87.1
 
 
 
 
CAPZA1
 
 
hsa-miR-7-5p
 
 
-0.351
 
 
38.4
 
 
 
 
CARNMT1
 
 
hsa-let-7e-5p
 
 
-0.307
 
 
11.4
 
 
 
 
CASD1
 
 
hsa-miR-130b-3p
 
 
-0.305
 
 
11.6
 
 
 
 
CBX5
 
 
hsa-let-7e-5p
 
 
-0.307
 
 
16.1
 
 
 
 
CBX5
 
 
hsa-let-7e-5p
 
 
-0.472
 
 
16.1
 
 
 
 
CCDC43
 
 
hsa-miR-7-5p
 
 
-0.331
 
 
13.3
 
 
 
 
CCNC
 
 
hsa-miR-1-3p
 
 
-0.336
 
 
50.7
 
 
 
 
CCNL2
 
 
hsa-miR-28-5p
 
 
-0.448
 
 
47.6
 
 
 
 
CCNY
 
 
hsa-let-7e-5p
 
 
-0.334
 
 
23.2
 
 
 
 
CCNY
 
 
hsa-miR-130b-3p
 
 
-0.372
 
 
23.2
 
 
 
 
CCT4
 
 
hsa-miR-7-5p
 
 
-0.37
 
 
68.6
 
 
 
 
CCT6A
 
 
hsa-miR-130b-3p
 
 
-0.382
 
 
68.3
 
 
 
 
CD164
 
 
hsa-miR-1-3p
 
 
-0.315
 
 
278.4
 
 
 
 
CD2AP
 
 
hsa-miR-130b-3p
 
 
-0.302
 
 
31.8
 
 
 
 
CD2AP
 
 
hsa-miR-1-3p
 
 
-0.479
 
 
31.8
 
 
 
 
 
 
 lung 
 
 
 
 
target_symbol
 
 
mature_mirna_id
 
 
score
 
 
nTPM
 
 
 
 
 
 
ABHD17C
 
 
hsa-let-7e-5p
 
 
-0.359
 
 
18.6
 
 
 
 
ABHD2
 
 
hsa-miR-1-3p
 
 
-0.291
 
 
42.7
 
 
 
 
ABHD3
 
 
hsa-miR-130b-3p
 
 
-0.356
 
 
14.8
 
 
 
 
ACBD3
 
 
hsa-miR-130b-3p
 
 
-0.292
 
 
21.1
 
 
 
 
ACBD5
 
 
hsa-miR-130b-3p
 
 
-0.494
 
 
13.9
 
 
 
 
ACSL4
 
 
hsa-miR-130b-3p
 
 
-0.301
 
 
35.4
 
 
 
 
ACVR1
 
 
hsa-miR-130b-3p
 
 
-0.44
 
 
12.2
 
 
 
 
ADAMTS8
 
 
hsa-let-7e-5p
 
 
-0.571
 
 
33.2
 
 
 
 
ADAR
 
 
hsa-miR-1-3p
 
 
-0.35
 
 
51.5
 
 
 
 
ADPGK
 
 
hsa-miR-1-3p
 
 
-0.393
 
 
22.9
 
 
 
 
ADRB1
 
 
hsa-let-7e-5p
 
 
-0.367
 
 
11.4
 
 
 
 
ADRB2
 
 
hsa-let-7e-5p
 
 
-0.635
 
 
18.4
 
 
 
 
AKIRIN2
 
 
hsa-miR-130b-3p
 
 
-0.315
 
 
29.6
 
 
 
 
ANKIB1
 
 
hsa-miR-130b-3p
 
 
-0.329
 
 
10.4
 
 
 
 
ANKRD12
 
 
hsa-miR-7-5p
 
 
-0.37
 
 
10.9
 
 
 
 
ANKRD29
 
 
hsa-miR-1-3p
 
 
-0.426
 
 
29.5
 
 
 
 
ANKRD46
 
 
hsa-let-7e-5p
 
 
-0.389
 
 
10.1
 
 
 
 
ANP32B
 
 
hsa-miR-1-3p
 
 
-0.287
 
 
116.3
 
 
 
 
ANXA2
 
 
hsa-miR-1-3p
 
 
-0.581
 
 
827.4
 
 
 
 
ANXA4
 
 
hsa-miR-1-3p
 
 
-0.539
 
 
76.9
 
 
 
 
AP1S1
 
 
hsa-miR-1-3p
 
 
-0.355
 
 
15.2
 
 
 
 
AP1S1
 
 
hsa-let-7e-5p
 
 
-0.374
 
 
15.2
 
 
 
 
APCDD1
 
 
hsa-miR-130b-3p
 
 
-0.304
 
 
12.6
 
 
 
 
APPL1
 
 
hsa-miR-130b-3p
 
 
-0.347
 
 
12.8
 
 
 
 
ARCN1
 
 
hsa-miR-1-3p
 
 
-0.468
 
 
57.0
 
 
 
 
ARF4
 
 
hsa-miR-7-5p
 
 
-0.407
 
 
116.3
 
 
 
 
ARFGEF1
 
 
hsa-miR-130b-3p
 
 
-0.302
 
 
13.0
 
 
 
 
ARHGAP1
 
 
hsa-miR-130b-3p
 
 
-0.376
 
 
36.0
 
 
 
 
ARHGAP12
 
 
hsa-miR-130b-3p
 
 
-0.287
 
 
11.6
 
 
 
 
ARHGAP24
 
 
hsa-miR-130b-3p
 
 
-0.287
 
 
14.8
 
 
 
 
ARL4D
 
 
hsa-let-7e-5p
 
 
-0.417
 
 
16.0
 
 
 
 
ARL6IP1
 
 
hsa-miR-130b-3p
 
 
-0.289
 
 
75.1
 
 
 
 
ARL6IP1
 
 
hsa-miR-7-5p
 
 
-0.374
 
 
75.1
 
 
 
 
ARL6IP6
 
 
hsa-let-7e-5p
 
 
-0.305
 
 
11.9
 
 
 
 
ARMT1
 
 
hsa-let-7e-5p
 
 
-0.303
 
 
18.4
 
 
 
 
ARPC3
 
 
hsa-miR-1-3p
 
 
-0.606
 
 
249.4
 
 
 
 
ARPP19
 
 
hsa-let-7e-5p
 
 
-0.492
 
 
42.5
 
 
 
 
ARRDC4
 
 
hsa-let-7e-5p
 
 
-0.329
 
 
18.7
 
 
 
 
ASAP1
 
 
hsa-let-7e-5p
 
 
-0.296
 
 
27.6
 
 
 
 
ASH2L
 
 
hsa-miR-1-3p
 
 
-0.462
 
 
17.5
 
 
 
 
ATF2
 
 
hsa-miR-1-3p
 
 
-0.328
 
 
14.3
 
 
 
 
ATOSB
 
 
hsa-let-7e-5p
 
 
-0.429
 
 
20.8
 
 
 
 
ATP2A2
 
 
hsa-let-7e-5p
 
 
-0.311
 
 
46.2
 
 
 
 
ATPAF1
 
 
hsa-let-7e-5p
 
 
-0.498
 
 
24.6
 
 
 
 
AVPI1
 
 
hsa-miR-7-5p
 
 
-0.367
 
 
23.0
 
 
 
 
AXL
 
 
hsa-miR-1-3p
 
 
-0.304
 
 
24.4
 
 
 
 
AZIN1
 
 
hsa-miR-1-3p
 
 
-0.438
 
 
37.1
 
 
 
 
B3GAT3
 
 
hsa-let-7e-5p
 
 
-0.324
 
 
32.1
 
 
 
 
B4GAT1
 
 
hsa-let-7e-5p
 
 
-0.47
 
 
16.9
 
 
 
 
BACH1
 
 
hsa-let-7e-5p
 
 
-0.298
 
 
20.2
 
 
 
 
BACH1
 
 
hsa-let-7e-5p
 
 
-0.298
 
 
20.2
 
 
 
 
BACH1
 
 
hsa-let-7e-5p
 
 
-0.435
 
 
20.2
 
 
 
 
BACH1
 
 
hsa-let-7e-5p
 
 
-0.435
 
 
20.2
 
 
 
 
BAHD1
 
 
hsa-miR-130b-3p
 
 
-0.294
 
 
11.8
 
 
 
 
BAZ2A
 
 
hsa-miR-100-5p
 
 
-0.29
 
 
33.3
 
 
 
 
BAZ2A
 
 
hsa-miR-100-5p
 
 
-0.421
 
 
33.3
 
 
 
 
BCAP29
 
 
hsa-let-7e-5p
 
 
-0.289
 
 
24.4
 
 
 
 
BET1
 
 
hsa-miR-1-3p
 
 
-0.413
 
 
13.1
 
 
 
 
BHLHE40
 
 
hsa-miR-130b-3p
 
 
-0.351
 
 
117.3
 
 
 
 
BIN3
 
 
hsa-let-7e-5p
 
 
-0.427
 
 
12.4
 
 
 
 
BLCAP
 
 
hsa-miR-130b-3p
 
 
-0.342
 
 
36.4
 
 
 
 
BLOC1S6
 
 
hsa-let-7e-5p
 
 
-0.321
 
 
19.2
 
 
 
 
BMP5
 
 
hsa-let-7e-5p
 
 
-0.337
 
 
12.2
 
 
 
 
BMPR2
 
 
hsa-miR-130b-3p
 
 
-0.33
 
 
21.8
 
 
 
 
BMPR2
 
 
hsa-miR-100-5p
 
 
-0.355
 
 
21.8
 
 
 
 
BPNT1
 
 
hsa-miR-1-3p
 
 
-0.329
 
 
16.6
 
 
 
 
BRIX1
 
 
hsa-miR-1-3p
 
 
-0.334
 
 
12.2
 
 
 
 
BSCL2
 
 
hsa-miR-1-3p
 
 
-0.471
 
 
23.1
 
 
 
 
BTAF1
 
 
hsa-miR-130b-3p
 
 
-0.291
 
 
15.8
 
 
 
 
BTF3L4
 
 
hsa-let-7e-5p
 
 
-0.355
 
 
24.8
 
 
 
 
BTF3L4
 
 
hsa-miR-130b-3p
 
 
-0.359
 
 
24.8
 
 
 
 
BZW1
 
 
hsa-let-7e-5p
 
 
-0.354
 
 
69.3
 
 
 
 
BZW1
 
 
hsa-let-7e-5p
 
 
-0.375
 
 
69.3
 
 
 
 
BZW2
 
 
hsa-let-7e-5p
 
 
-0.408
 
 
20.9
 
 
 
 
C15orf39
 
 
hsa-let-7e-5p
 
 
-0.374
 
 
14.5
 
 
 
 
C1GALT1
 
 
hsa-let-7e-5p
 
 
-0.322
 
 
14.2
 
 
 
 
C8orf58
 
 
hsa-let-7e-5p
 
 
-0.612
 
 
10.1
 
 
 
 
CACFD1
 
 
hsa-let-7e-5p
 
 
-0.296
 
 
12.3
 
 
 
 
CALM2
 
 
hsa-miR-130b-3p
 
 
-0.584
 
 
476.3
 
 
 
 
CAMSAP2
 
 
hsa-miR-130b-3p
 
 
-0.297
 
 
12.0
 
 
 
 
CANT1
 
 
hsa-let-7e-5p
 
 
-0.402
 
 
14.8
 
 
 
 
CAPN3
 
 
hsa-let-7e-5p
 
 
-0.317
 
 
23.0
 
 
 
 
CAPZA1
 
 
hsa-miR-7-5p
 
 
-0.351
 
 
64.7
 
 
 
 
CASD1
 
 
hsa-miR-130b-3p
 
 
-0.305
 
 
10.7
 
 
 
 
CASP3
 
 
hsa-let-7e-5p
 
 
-0.495
 
 
10.4
 
 
 
 
CBFA2T3
 
 
hsa-let-7e-5p
 
 
-0.305
 
 
10.6
 
 
 
 
CCNC
 
 
hsa-miR-1-3p
 
 
-0.336
 
 
23.6
 
 
 
 
CCNL2
 
 
hsa-miR-28-5p
 
 
-0.448
 
 
50.7
 
 
 
 
CCNT2
 
 
hsa-miR-7-5p
 
 
-0.349
 
 
14.0
 
 
 
 
CCNY
 
 
hsa-let-7e-5p
 
 
-0.334
 
 
24.4
 
 
 
 
CCNY
 
 
hsa-miR-130b-3p
 
 
-0.372
 
 
24.4
 
 
 
 
CCT4
 
 
hsa-miR-7-5p
 
 
-0.37
 
 
49.0
 
 
 
 
CCT6A
 
 
hsa-miR-130b-3p
 
 
-0.382
 
 
42.2
 
 
 
 
CD164
 
 
hsa-miR-1-3p
 
 
-0.315
 
 
114.9
 
 
 
 
CD2AP
 
 
hsa-miR-130b-3p
 
 
-0.302
 
 
16.2
 
 
 
 
CD2AP
 
 
hsa-miR-1-3p
 
 
-0.479
 
 
16.2
 
 
 
 
CD69
 
 
hsa-miR-130b-3p
 
 
-0.451
 
 
21.3
 
 
 
 
CD86
 
 
hsa-let-7e-5p
 
 
-0.323
 
 
17.6
 
 
 
 
CDC34
 
 
hsa-let-7e-5p
 
 
-0.321
 
 
33.2
 
 
 
 
CDC34
 
 
hsa-let-7e-5p
 
 
-0.555
 
 
33.2
 
 
 
 
 
 
 prostate 
 
 
 
 
target_symbol
 
 
mature_mirna_id
 
 
score
 
 
nTPM
 
 
 
 
 
 
ABHD17C
 
 
hsa-let-7e-5p
 
 
-0.359
 
 
17.0
 
 
 
 
ABHD2
 
 
hsa-miR-1-3p
 
 
-0.291
 
 
41.5
 
 
 
 
ABHD3
 
 
hsa-miR-130b-3p
 
 
-0.356
 
 
10.4
 
 
 
 
ACBD3
 
 
hsa-miR-130b-3p
 
 
-0.292
 
 
21.4
 
 
 
 
ACBD5
 
 
hsa-miR-130b-3p
 
 
-0.494
 
 
14.3
 
 
 
 
ACP3
 
 
hsa-let-7e-5p
 
 
-0.298
 
 
3409.5
 
 
 
 
ACSL4
 
 
hsa-miR-130b-3p
 
 
-0.301
 
 
12.3
 
 
 
 
ACTC1
 
 
hsa-miR-7-5p
 
 
-0.471
 
 
68.4
 
 
 
 
ADAR
 
 
hsa-miR-1-3p
 
 
-0.35
 
 
38.8
 
 
 
 
ADGRA3
 
 
hsa-miR-1-3p
 
 
-0.351
 
 
11.3
 
 
 
 
ADPGK
 
 
hsa-miR-1-3p
 
 
-0.393
 
 
15.9
 
 
 
 
AIFM1
 
 
hsa-let-7e-5p
 
 
-0.314
 
 
10.1
 
 
 
 
AKIRIN2
 
 
hsa-miR-130b-3p
 
 
-0.315
 
 
15.2
 
 
 
 
ALDH4A1
 
 
hsa-miR-184
 
 
-0.421
 
 
27.7
 
 
 
 
ALG11
 
 
hsa-miR-1-3p
 
 
-0.317
 
 
11.4
 
 
 
 
AMT
 
 
hsa-let-7e-5p
 
 
-0.463
 
 
15.5
 
 
 
 
ANKRD12
 
 
hsa-miR-7-5p
 
 
-0.37
 
 
11.4
 
 
 
 
ANKRD46
 
 
hsa-let-7e-5p
 
 
-0.389
 
 
12.6
 
 
 
 
ANP32B
 
 
hsa-miR-1-3p
 
 
-0.287
 
 
93.4
 
 
 
 
ANXA2
 
 
hsa-miR-1-3p
 
 
-0.581
 
 
353.5
 
 
 
 
ANXA4
 
 
hsa-miR-1-3p
 
 
-0.539
 
 
43.5
 
 
 
 
AP1S1
 
 
hsa-miR-1-3p
 
 
-0.355
 
 
23.9
 
 
 
 
AP1S1
 
 
hsa-let-7e-5p
 
 
-0.374
 
 
23.9
 
 
 
 
APCDD1
 
 
hsa-miR-130b-3p
 
 
-0.304
 
 
31.5
 
 
 
 
APPL1
 
 
hsa-miR-130b-3p
 
 
-0.347
 
 
14.1
 
 
 
 
ARCN1
 
 
hsa-miR-1-3p
 
 
-0.468
 
 
62.7
 
 
 
 
ARF4
 
 
hsa-miR-7-5p
 
 
-0.407
 
 
129.9
 
 
 
 
ARFGEF1
 
 
hsa-miR-130b-3p
 
 
-0.302
 
 
12.2
 
 
 
 
ARG2
 
 
hsa-let-7e-5p
 
 
-0.468
 
 
101.8
 
 
 
 
ARHGAP1
 
 
hsa-miR-130b-3p
 
 
-0.376
 
 
60.9
 
 
 
 
ARHGAP12
 
 
hsa-miR-130b-3p
 
 
-0.287
 
 
10.9
 
 
 
 
ARL4D
 
 
hsa-let-7e-5p
 
 
-0.417
 
 
10.7
 
 
 
 
ARL6IP1
 
 
hsa-miR-130b-3p
 
 
-0.289
 
 
71.3
 
 
 
 
ARL6IP1
 
 
hsa-miR-7-5p
 
 
-0.374
 
 
71.3
 
 
 
 
ARMT1
 
 
hsa-let-7e-5p
 
 
-0.303
 
 
11.5
 
 
 
 
ARPC3
 
 
hsa-miR-1-3p
 
 
-0.606
 
 
156.5
 
 
 
 
ARPP19
 
 
hsa-let-7e-5p
 
 
-0.492
 
 
37.0
 
 
 
 
ARRDC4
 
 
hsa-let-7e-5p
 
 
-0.329
 
 
10.3
 
 
 
 
ASAP1
 
 
hsa-let-7e-5p
 
 
-0.296
 
 
11.6
 
 
 
 
ASH2L
 
 
hsa-miR-1-3p
 
 
-0.462
 
 
18.9
 
 
 
 
ATF2
 
 
hsa-miR-1-3p
 
 
-0.328
 
 
17.1
 
 
 
 
ATG16L1
 
 
hsa-miR-130b-3p
 
 
-0.407
 
 
12.5
 
 
 
 
ATOSB
 
 
hsa-let-7e-5p
 
 
-0.429
 
 
12.4
 
 
 
 
ATP2A2
 
 
hsa-let-7e-5p
 
 
-0.311
 
 
87.6
 
 
 
 
ATPAF1
 
 
hsa-let-7e-5p
 
 
-0.498
 
 
35.2
 
 
 
 
AVPI1
 
 
hsa-miR-7-5p
 
 
-0.367
 
 
27.2
 
 
 
 
AXL
 
 
hsa-miR-1-3p
 
 
-0.304
 
 
17.8
 
 
 
 
AZIN1
 
 
hsa-miR-1-3p
 
 
-0.438
 
 
48.5
 
 
 
 
B3GAT3
 
 
hsa-let-7e-5p
 
 
-0.324
 
 
40.8
 
 
 
 
B4GAT1
 
 
hsa-let-7e-5p
 
 
-0.47
 
 
17.0
 
 
 
 
BACH1
 
 
hsa-let-7e-5p
 
 
-0.298
 
 
12.5
 
 
 
 
BACH1
 
 
hsa-let-7e-5p
 
 
-0.298
 
 
12.5
 
 
 
 
BACH1
 
 
hsa-let-7e-5p
 
 
-0.435
 
 
12.5
 
 
 
 
BACH1
 
 
hsa-let-7e-5p
 
 
-0.435
 
 
12.5
 
 
 
 
BAZ2A
 
 
hsa-miR-100-5p
 
 
-0.29
 
 
21.4
 
 
 
 
BAZ2A
 
 
hsa-miR-100-5p
 
 
-0.421
 
 
21.4
 
 
 
 
BCAP29
 
 
hsa-let-7e-5p
 
 
-0.289
 
 
30.7
 
 
 
 
BET1
 
 
hsa-miR-1-3p
 
 
-0.413
 
 
19.5
 
 
 
 
BHLHE40
 
 
hsa-miR-130b-3p
 
 
-0.351
 
 
103.6
 
 
 
 
BIN3
 
 
hsa-let-7e-5p
 
 
-0.427
 
 
16.4
 
 
 
 
BLCAP
 
 
hsa-miR-130b-3p
 
 
-0.342
 
 
58.3
 
 
 
 
BLOC1S6
 
 
hsa-let-7e-5p
 
 
-0.321
 
 
34.2
 
 
 
 
BMP5
 
 
hsa-let-7e-5p
 
 
-0.337
 
 
13.2
 
 
 
 
BMPR2
 
 
hsa-miR-130b-3p
 
 
-0.33
 
 
12.9
 
 
 
 
BMPR2
 
 
hsa-miR-100-5p
 
 
-0.355
 
 
12.9
 
 
 
 
BPNT1
 
 
hsa-miR-1-3p
 
 
-0.329
 
 
12.9
 
 
 
 
BRIX1
 
 
hsa-miR-1-3p
 
 
-0.334
 
 
14.6
 
 
 
 
BSCL2
 
 
hsa-miR-1-3p
 
 
-0.471
 
 
31.4
 
 
 
 
BTAF1
 
 
hsa-miR-130b-3p
 
 
-0.291
 
 
13.7
 
 
 
 
BTF3L4
 
 
hsa-let-7e-5p
 
 
-0.355
 
 
27.4
 
 
 
 
BTF3L4
 
 
hsa-miR-130b-3p
 
 
-0.359
 
 
27.4
 
 
 
 
BZW1
 
 
hsa-let-7e-5p
 
 
-0.354
 
 
93.5
 
 
 
 
BZW1
 
 
hsa-let-7e-5p
 
 
-0.375
 
 
93.5
 
 
 
 
BZW2
 
 
hsa-let-7e-5p
 
 
-0.408
 
 
18.0
 
 
 
 
C6orf120
 
 
hsa-miR-1-3p
 
 
-0.288
 
 
10.1
 
 
 
 
CAAP1
 
 
hsa-miR-1-3p
 
 
-0.48
 
 
10.0
 
 
 
 
CACFD1
 
 
hsa-let-7e-5p
 
 
-0.296
 
 
12.8
 
 
 
 
CACNG4
 
 
hsa-let-7e-5p
 
 
-0.33
 
 
11.0
 
 
 
 
CALM2
 
 
hsa-miR-130b-3p
 
 
-0.584
 
 
420.8
 
 
 
 
CAMSAP2
 
 
hsa-miR-130b-3p
 
 
-0.297
 
 
10.5
 
 
 
 
CANT1
 
 
hsa-let-7e-5p
 
 
-0.402
 
 
67.4
 
 
 
 
CAPN3
 
 
hsa-let-7e-5p
 
 
-0.317
 
 
17.5
 
 
 
 
CAPZA1
 
 
hsa-miR-7-5p
 
 
-0.351
 
 
39.8
 
 
 
 
CASD1
 
 
hsa-miR-130b-3p
 
 
-0.305
 
 
13.0
 
 
 
 
CBX5
 
 
hsa-let-7e-5p
 
 
-0.307
 
 
10.8
 
 
 
 
CBX5
 
 
hsa-let-7e-5p
 
 
-0.472
 
 
10.8
 
 
 
 
CCDC43
 
 
hsa-miR-7-5p
 
 
-0.331
 
 
11.2
 
 
 
 
CCNC
 
 
hsa-miR-1-3p
 
 
-0.336
 
 
32.5
 
 
 
 
CCNL2
 
 
hsa-miR-28-5p
 
 
-0.448
 
 
58.2
 
 
 
 
CCNT2
 
 
hsa-miR-7-5p
 
 
-0.349
 
 
12.5
 
 
 
 
CCNY
 
 
hsa-let-7e-5p
 
 
-0.334
 
 
17.2
 
 
 
 
CCNY
 
 
hsa-miR-130b-3p
 
 
-0.372
 
 
17.2
 
 
 
 
CCT4
 
 
hsa-miR-7-5p
 
 
-0.37
 
 
54.2
 
 
 
 
CCT6A
 
 
hsa-miR-130b-3p
 
 
-0.382
 
 
43.1
 
 
 
 
CD164
 
 
hsa-miR-1-3p
 
 
-0.315
 
 
137.1
 
 
 
 
CD2AP
 
 
hsa-miR-130b-3p
 
 
-0.302
 
 
18.5
 
 
 
 
CD2AP
 
 
hsa-miR-1-3p
 
 
-0.479
 
 
18.5
 
 
 
 
CDC34
 
 
hsa-let-7e-5p
 
 
-0.321
 
 
38.3
 
 
 
 
CDC34
 
 
hsa-let-7e-5p
 
 
-0.555
 
 
38.3
 
 
 
 
CDC42SE1
 
 
hsa-miR-1-3p
 
 
-0.36
 
 
54.0
 
 
 
 
 
 
 rectum 
 
 
 
 
target_symbol
 
 
mature_mirna_id
 
 
score
 
 
nTPM
 
 
 
 
 
 
ABHD17C
 
 
hsa-let-7e-5p
 
 
-0.359
 
 
52.8
 
 
 
 
ABHD2
 
 
hsa-miR-1-3p
 
 
-0.291
 
 
17.8
 
 
 
 
ABHD3
 
 
hsa-miR-130b-3p
 
 
-0.356
 
 
61.7
 
 
 
 
ACBD3
 
 
hsa-miR-130b-3p
 
 
-0.292
 
 
23.9
 
 
 
 
ACBD5
 
 
hsa-miR-130b-3p
 
 
-0.494
 
 
19.7
 
 
 
 
ACSL4
 
 
hsa-miR-130b-3p
 
 
-0.301
 
 
12.1
 
 
 
 
ADAR
 
 
hsa-miR-1-3p
 
 
-0.35
 
 
41.8
 
 
 
 
ADGRA3
 
 
hsa-miR-1-3p
 
 
-0.351
 
 
16.1
 
 
 
 
ADPGK
 
 
hsa-miR-1-3p
 
 
-0.393
 
 
16.2
 
 
 
 
AIFM1
 
 
hsa-let-7e-5p
 
 
-0.314
 
 
14.9
 
 
 
 
AKIRIN2
 
 
hsa-miR-130b-3p
 
 
-0.315
 
 
11.7
 
 
 
 
ALG11
 
 
hsa-miR-1-3p
 
 
-0.317
 
 
11.1
 
 
 
 
AMT
 
 
hsa-let-7e-5p
 
 
-0.463
 
 
11.1
 
 
 
 
ANKIB1
 
 
hsa-miR-130b-3p
 
 
-0.329
 
 
12.0
 
 
 
 
ANKRD12
 
 
hsa-miR-7-5p
 
 
-0.37
 
 
11.3
 
 
 
 
ANP32B
 
 
hsa-miR-1-3p
 
 
-0.287
 
 
111.5
 
 
 
 
ANXA2
 
 
hsa-miR-1-3p
 
 
-0.581
 
 
569.2
 
 
 
 
ANXA4
 
 
hsa-miR-1-3p
 
 
-0.539
 
 
86.7
 
 
 
 
AP1AR
 
 
hsa-miR-100-5p
 
 
-0.804
 
 
15.3
 
 
 
 
AP1S1
 
 
hsa-miR-1-3p
 
 
-0.355
 
 
21.1
 
 
 
 
AP1S1
 
 
hsa-let-7e-5p
 
 
-0.374
 
 
21.1
 
 
 
 
APCDD1
 
 
hsa-miR-130b-3p
 
 
-0.304
 
 
12.4
 
 
 
 
APPL1
 
 
hsa-miR-130b-3p
 
 
-0.347
 
 
13.1
 
 
 
 
ARCN1
 
 
hsa-miR-1-3p
 
 
-0.468
 
 
62.8
 
 
 
 
ARF4
 
 
hsa-miR-7-5p
 
 
-0.407
 
 
185.2
 
 
 
 
ARFGEF1
 
 
hsa-miR-130b-3p
 
 
-0.302
 
 
16.1
 
 
 
 
ARHGAP1
 
 
hsa-miR-130b-3p
 
 
-0.376
 
 
30.6
 
 
 
 
ARHGAP12
 
 
hsa-miR-130b-3p
 
 
-0.287
 
 
13.8
 
 
 
 
ARL6IP1
 
 
hsa-miR-130b-3p
 
 
-0.289
 
 
116.0
 
 
 
 
ARL6IP1
 
 
hsa-miR-7-5p
 
 
-0.374
 
 
116.0
 
 
 
 
ARL6IP6
 
 
hsa-let-7e-5p
 
 
-0.305
 
 
13.3
 
 
 
 
ARMT1
 
 
hsa-let-7e-5p
 
 
-0.303
 
 
16.5
 
 
 
 
ARPC3
 
 
hsa-miR-1-3p
 
 
-0.606
 
 
322.9
 
 
 
 
ARPP19
 
 
hsa-let-7e-5p
 
 
-0.492
 
 
33.6
 
 
 
 
ARRDC4
 
 
hsa-let-7e-5p
 
 
-0.329
 
 
21.6
 
 
 
 
ASH2L
 
 
hsa-miR-1-3p
 
 
-0.462
 
 
21.2
 
 
 
 
ATF2
 
 
hsa-miR-1-3p
 
 
-0.328
 
 
14.6
 
 
 
 
ATG4A
 
 
hsa-miR-7-5p
 
 
-0.448
 
 
12.3
 
 
 
 
ATP2A2
 
 
hsa-let-7e-5p
 
 
-0.311
 
 
64.9
 
 
 
 
ATPAF1
 
 
hsa-let-7e-5p
 
 
-0.498
 
 
50.0
 
 
 
 
AVPI1
 
 
hsa-miR-7-5p
 
 
-0.367
 
 
17.8
 
 
 
 
AXL
 
 
hsa-miR-1-3p
 
 
-0.304
 
 
11.8
 
 
 
 
AZIN1
 
 
hsa-miR-1-3p
 
 
-0.438
 
 
49.7
 
 
 
 
BACH1
 
 
hsa-let-7e-5p
 
 
-0.298
 
 
12.4
 
 
 
 
BACH1
 
 
hsa-let-7e-5p
 
 
-0.298
 
 
12.4
 
 
 
 
BACH1
 
 
hsa-let-7e-5p
 
 
-0.435
 
 
12.4
 
 
 
 
BACH1
 
 
hsa-let-7e-5p
 
 
-0.435
 
 
12.4
 
 
 
 
BAZ2A
 
 
hsa-miR-100-5p
 
 
-0.29
 
 
24.9
 
 
 
 
BAZ2A
 
 
hsa-miR-100-5p
 
 
-0.421
 
 
24.9
 
 
 
 
BCAP29
 
 
hsa-let-7e-5p
 
 
-0.289
 
 
30.4
 
 
 
 
BET1
 
 
hsa-miR-1-3p
 
 
-0.413
 
 
16.0
 
 
 
 
BHLHE40
 
 
hsa-miR-130b-3p
 
 
-0.351
 
 
44.6
 
 
 
 
BLCAP
 
 
hsa-miR-130b-3p
 
 
-0.342
 
 
49.0
 
 
 
 
BLOC1S6
 
 
hsa-let-7e-5p
 
 
-0.321
 
 
20.2
 
 
 
 
BPNT1
 
 
hsa-miR-1-3p
 
 
-0.329
 
 
30.3
 
 
 
 
BRI3BP
 
 
hsa-miR-1-3p
 
 
-0.293
 
 
14.3
 
 
 
 
BRIX1
 
 
hsa-miR-1-3p
 
 
-0.334
 
 
16.0
 
 
 
 
BSCL2
 
 
hsa-miR-1-3p
 
 
-0.471
 
 
11.9
 
 
 
 
BTAF1
 
 
hsa-miR-130b-3p
 
 
-0.291
 
 
12.5
 
 
 
 
BTF3L4
 
 
hsa-let-7e-5p
 
 
-0.355
 
 
22.2
 
 
 
 
BTF3L4
 
 
hsa-miR-130b-3p
 
 
-0.359
 
 
22.2
 
 
 
 
BZW1
 
 
hsa-let-7e-5p
 
 
-0.354
 
 
85.0
 
 
 
 
BZW1
 
 
hsa-let-7e-5p
 
 
-0.375
 
 
85.0
 
 
 
 
BZW2
 
 
hsa-let-7e-5p
 
 
-0.408
 
 
53.4
 
 
 
 
C1GALT1
 
 
hsa-let-7e-5p
 
 
-0.322
 
 
16.9
 
 
 
 
C2orf88
 
 
hsa-let-7e-5p
 
 
-0.429
 
 
36.2
 
 
 
 
C3orf52
 
 
hsa-let-7e-5p
 
 
-0.296
 
 
12.8
 
 
 
 
C6orf120
 
 
hsa-miR-1-3p
 
 
-0.288
 
 
11.9
 
 
 
 
CAAP1
 
 
hsa-miR-1-3p
 
 
-0.48
 
 
18.4
 
 
 
 
CALM2
 
 
hsa-miR-130b-3p
 
 
-0.584
 
 
663.0
 
 
 
 
CANT1
 
 
hsa-let-7e-5p
 
 
-0.402
 
 
77.7
 
 
 
 
CAPZA1
 
 
hsa-miR-7-5p
 
 
-0.351
 
 
89.2
 
 
 
 
CASD1
 
 
hsa-miR-130b-3p
 
 
-0.305
 
 
18.1
 
 
 
 
CASP3
 
 
hsa-let-7e-5p
 
 
-0.495
 
 
28.1
 
 
 
 
CASP5
 
 
hsa-let-7e-5p
 
 
-0.379
 
 
12.1
 
 
 
 
CBX5
 
 
hsa-let-7e-5p
 
 
-0.307
 
 
10.2
 
 
 
 
CBX5
 
 
hsa-let-7e-5p
 
 
-0.472
 
 
10.2
 
 
 
 
CCDC43
 
 
hsa-miR-7-5p
 
 
-0.331
 
 
14.0
 
 
 
 
CCNC
 
 
hsa-miR-1-3p
 
 
-0.336
 
 
28.5
 
 
 
 
CCNL2
 
 
hsa-miR-28-5p
 
 
-0.448
 
 
21.2
 
 
 
 
CCNY
 
 
hsa-let-7e-5p
 
 
-0.334
 
 
18.8
 
 
 
 
CCNY
 
 
hsa-miR-130b-3p
 
 
-0.372
 
 
18.8
 
 
 
 
CCT4
 
 
hsa-miR-7-5p
 
 
-0.37
 
 
55.3
 
 
 
 
CCT6A
 
 
hsa-miR-130b-3p
 
 
-0.382
 
 
57.4
 
 
 
 
CD164
 
 
hsa-miR-1-3p
 
 
-0.315
 
 
254.1
 
 
 
 
CD2AP
 
 
hsa-miR-130b-3p
 
 
-0.302
 
 
34.7
 
 
 
 
CD2AP
 
 
hsa-miR-1-3p
 
 
-0.479
 
 
34.7
 
 
 
 
CD69
 
 
hsa-miR-130b-3p
 
 
-0.451
 
 
20.1
 
 
 
 
CDC34
 
 
hsa-let-7e-5p
 
 
-0.321
 
 
10.8
 
 
 
 
CDC34
 
 
hsa-let-7e-5p
 
 
-0.555
 
 
10.8
 
 
 
 
CDC42SE1
 
 
hsa-miR-1-3p
 
 
-0.36
 
 
31.7
 
 
 
 
CDCA8
 
 
hsa-let-7e-5p
 
 
-0.342
 
 
10.6
 
 
 
 
CDK4
 
 
hsa-miR-1-3p
 
 
-0.338
 
 
59.3
 
 
 
 
CDKN1A
 
 
hsa-let-7e-5p
 
 
-0.434
 
 
101.7
 
 
 
 
CDKN2AIP
 
 
hsa-miR-28-5p
 
 
-0.419
 
 
12.8
 
 
 
 
CDV3
 
 
hsa-let-7e-5p
 
 
-0.453
 
 
65.2
 
 
 
 
CEBPD
 
 
hsa-let-7e-5p
 
 
-0.309
 
 
11.4
 
 
 
 
CEMIP2
 
 
hsa-let-7e-5p
 
 
-0.453
 
 
26.3
 
 
 
 
CERS2
 
 
hsa-miR-1-3p
 
 
-0.287
 
 
83.1
 
 
 
 
CGGBP1
 
 
hsa-miR-7-5p
 
 
-0.465
 
 
37.3
 
 
 
 
 
 
 retina 
 
 
 
 
target_symbol
 
 
mature_mirna_id
 
 
score
 
 
nTPM
 
 
 
 
 
 
ABHD17C
 
 
hsa-let-7e-5p
 
 
-0.359
 
 
15.6
 
 
 
 
ABHD2
 
 
hsa-miR-1-3p
 
 
-0.291
 
 
13.4
 
 
 
 
ACBD3
 
 
hsa-miR-130b-3p
 
 
-0.292
 
 
14.9
 
 
 
 
ACBD5
 
 
hsa-miR-130b-3p
 
 
-0.494
 
 
33.5
 
 
 
 
ACSL4
 
 
hsa-miR-130b-3p
 
 
-0.301
 
 
20.7
 
 
 
 
ACSL6
 
 
hsa-let-7e-5p
 
 
-0.336
 
 
42.4
 
 
 
 
ACTC1
 
 
hsa-miR-7-5p
 
 
-0.471
 
 
29.3
 
 
 
 
ADAR
 
 
hsa-miR-1-3p
 
 
-0.35
 
 
44.4
 
 
 
 
AKAP6
 
 
hsa-let-7e-5p
 
 
-0.307
 
 
12.6
 
 
 
 
AKIRIN2
 
 
hsa-miR-130b-3p
 
 
-0.315
 
 
27.5
 
 
 
 
ALG9
 
 
hsa-miR-7-5p
 
 
-0.302
 
 
11.3
 
 
 
 
ANKRD12
 
 
hsa-miR-7-5p
 
 
-0.37
 
 
27.6
 
 
 
 
ANKRD13C
 
 
hsa-miR-130b-3p
 
 
-0.33
 
 
12.3
 
 
 
 
ANKRD46
 
 
hsa-let-7e-5p
 
 
-0.389
 
 
32.6
 
 
 
 
ANP32B
 
 
hsa-miR-1-3p
 
 
-0.287
 
 
42.8
 
 
 
 
ANXA2
 
 
hsa-miR-1-3p
 
 
-0.581
 
 
27.9
 
 
 
 
ANXA4
 
 
hsa-miR-1-3p
 
 
-0.539
 
 
27.9
 
 
 
 
AP1S1
 
 
hsa-miR-1-3p
 
 
-0.355
 
 
13.5
 
 
 
 
AP1S1
 
 
hsa-let-7e-5p
 
 
-0.374
 
 
13.5
 
 
 
 
APPL1
 
 
hsa-miR-130b-3p
 
 
-0.347
 
 
12.5
 
 
 
 
ARCN1
 
 
hsa-miR-1-3p
 
 
-0.468
 
 
34.7
 
 
 
 
ARF4
 
 
hsa-miR-7-5p
 
 
-0.407
 
 
50.0
 
 
 
 
ARFGEF1
 
 
hsa-miR-130b-3p
 
 
-0.302
 
 
33.8
 
 
 
 
ARG2
 
 
hsa-let-7e-5p
 
 
-0.468
 
 
20.7
 
 
 
 
ARHGAP12
 
 
hsa-miR-130b-3p
 
 
-0.287
 
 
16.4
 
 
 
 
ARID2
 
 
hsa-miR-1-3p
 
 
-0.294
 
 
12.3
 
 
 
 
ARID3B
 
 
hsa-let-7e-5p
 
 
-0.31
 
 
17.4
 
 
 
 
ARID3B
 
 
hsa-let-7e-5p
 
 
-0.486
 
 
17.4
 
 
 
 
ARID4A
 
 
hsa-miR-7-5p
 
 
-0.341
 
 
13.0
 
 
 
 
ARL4D
 
 
hsa-let-7e-5p
 
 
-0.417
 
 
78.9
 
 
 
 
ARL6IP1
 
 
hsa-miR-130b-3p
 
 
-0.289
 
 
292.6
 
 
 
 
ARL6IP1
 
 
hsa-miR-7-5p
 
 
-0.374
 
 
292.6
 
 
 
 
ARL6IP6
 
 
hsa-let-7e-5p
 
 
-0.305
 
 
10.5
 
 
 
 
ARMT1
 
 
hsa-let-7e-5p
 
 
-0.303
 
 
15.2
 
 
 
 
ARPC3
 
 
hsa-miR-1-3p
 
 
-0.606
 
 
56.0
 
 
 
 
ARPP19
 
 
hsa-let-7e-5p
 
 
-0.492
 
 
53.1
 
 
 
 
ASH2L
 
 
hsa-miR-1-3p
 
 
-0.462
 
 
20.5
 
 
 
 
ATF2
 
 
hsa-miR-1-3p
 
 
-0.328
 
 
19.6
 
 
 
 
ATP2A2
 
 
hsa-let-7e-5p
 
 
-0.311
 
 
17.7
 
 
 
 
ATP2B2
 
 
hsa-miR-7-5p
 
 
-0.297
 
 
12.5
 
 
 
 
ATP2B2
 
 
hsa-miR-130b-3p
 
 
-0.314
 
 
12.5
 
 
 
 
ATPAF1
 
 
hsa-let-7e-5p
 
 
-0.498
 
 
65.3
 
 
 
 
ATXN1
 
 
hsa-miR-130b-3p
 
 
-0.321
 
 
12.9
 
 
 
 
AVPI1
 
 
hsa-miR-7-5p
 
 
-0.367
 
 
23.7
 
 
 
 
AZIN1
 
 
hsa-miR-1-3p
 
 
-0.438
 
 
52.2
 
 
 
 
B3GAT3
 
 
hsa-let-7e-5p
 
 
-0.324
 
 
15.0
 
 
 
 
B4GAT1
 
 
hsa-let-7e-5p
 
 
-0.47
 
 
42.6
 
 
 
 
BACH1
 
 
hsa-let-7e-5p
 
 
-0.298
 
 
10.9
 
 
 
 
BACH1
 
 
hsa-let-7e-5p
 
 
-0.298
 
 
10.9
 
 
 
 
BACH1
 
 
hsa-let-7e-5p
 
 
-0.435
 
 
10.9
 
 
 
 
BACH1
 
 
hsa-let-7e-5p
 
 
-0.435
 
 
10.9
 
 
 
 
BAZ2A
 
 
hsa-miR-100-5p
 
 
-0.29
 
 
45.3
 
 
 
 
BAZ2A
 
 
hsa-miR-100-5p
 
 
-0.421
 
 
45.3
 
 
 
 
BCAT1
 
 
hsa-let-7e-5p
 
 
-0.394
 
 
13.4
 
 
 
 
BET1
 
 
hsa-miR-1-3p
 
 
-0.413
 
 
12.3
 
 
 
 
BHLHE40
 
 
hsa-miR-130b-3p
 
 
-0.351
 
 
109.8
 
 
 
 
BLCAP
 
 
hsa-miR-130b-3p
 
 
-0.342
 
 
65.7
 
 
 
 
BLOC1S6
 
 
hsa-let-7e-5p
 
 
-0.321
 
 
22.7
 
 
 
 
BLTP3B
 
 
hsa-miR-1-3p
 
 
-0.29
 
 
13.5
 
 
 
 
BMPR2
 
 
hsa-miR-130b-3p
 
 
-0.33
 
 
17.0
 
 
 
 
BMPR2
 
 
hsa-miR-100-5p
 
 
-0.355
 
 
17.0
 
 
 
 
BPNT1
 
 
hsa-miR-1-3p
 
 
-0.329
 
 
14.4
 
 
 
 
BRIX1
 
 
hsa-miR-1-3p
 
 
-0.334
 
 
10.7
 
 
 
 
BSCL2
 
 
hsa-miR-1-3p
 
 
-0.471
 
 
52.4
 
 
 
 
BTAF1
 
 
hsa-miR-130b-3p
 
 
-0.291
 
 
27.2
 
 
 
 
BTF3L4
 
 
hsa-let-7e-5p
 
 
-0.355
 
 
29.1
 
 
 
 
BTF3L4
 
 
hsa-miR-130b-3p
 
 
-0.359
 
 
29.1
 
 
 
 
BZW1
 
 
hsa-let-7e-5p
 
 
-0.354
 
 
42.4
 
 
 
 
BZW1
 
 
hsa-let-7e-5p
 
 
-0.375
 
 
42.4
 
 
 
 
BZW2
 
 
hsa-let-7e-5p
 
 
-0.408
 
 
80.5
 
 
 
 
C1GALT1
 
 
hsa-let-7e-5p
 
 
-0.322
 
 
21.8
 
 
 
 
C6orf120
 
 
hsa-miR-1-3p
 
 
-0.288
 
 
21.6
 
 
 
 
CACNG7
 
 
hsa-miR-7-5p
 
 
-0.421
 
 
15.8
 
 
 
 
CADM2
 
 
hsa-let-7e-5p
 
 
-0.294
 
 
87.9
 
 
 
 
CALM2
 
 
hsa-miR-130b-3p
 
 
-0.584
 
 
532.0
 
 
 
 
CAMSAP2
 
 
hsa-miR-130b-3p
 
 
-0.297
 
 
24.2
 
 
 
 
CAPZA1
 
 
hsa-miR-7-5p
 
 
-0.351
 
 
34.4
 
 
 
 
CASD1
 
 
hsa-miR-130b-3p
 
 
-0.305
 
 
20.4
 
 
 
 
CBX5
 
 
hsa-let-7e-5p
 
 
-0.307
 
 
21.2
 
 
 
 
CBX5
 
 
hsa-let-7e-5p
 
 
-0.472
 
 
21.2
 
 
 
 
CCDC43
 
 
hsa-miR-7-5p
 
 
-0.331
 
 
17.1
 
 
 
 
CCNC
 
 
hsa-miR-1-3p
 
 
-0.336
 
 
25.0
 
 
 
 
CCNL2
 
 
hsa-miR-28-5p
 
 
-0.448
 
 
19.6
 
 
 
 
CCNT2
 
 
hsa-miR-7-5p
 
 
-0.349
 
 
12.3
 
 
 
 
CCNY
 
 
hsa-let-7e-5p
 
 
-0.334
 
 
17.9
 
 
 
 
CCNY
 
 
hsa-miR-130b-3p
 
 
-0.372
 
 
17.9
 
 
 
 
CCSAP
 
 
hsa-miR-1-3p
 
 
-0.381
 
 
23.9
 
 
 
 
CCT4
 
 
hsa-miR-7-5p
 
 
-0.37
 
 
52.6
 
 
 
 
CCT6A
 
 
hsa-miR-130b-3p
 
 
-0.382
 
 
44.2
 
 
 
 
CD164
 
 
hsa-miR-1-3p
 
 
-0.315
 
 
33.7
 
 
 
 
CDC34
 
 
hsa-let-7e-5p
 
 
-0.321
 
 
26.2
 
 
 
 
CDC34
 
 
hsa-let-7e-5p
 
 
-0.555
 
 
26.2
 
 
 
 
CDC42SE1
 
 
hsa-miR-1-3p
 
 
-0.36
 
 
19.1
 
 
 
 
CDK14
 
 
hsa-miR-1-3p
 
 
-0.326
 
 
16.1
 
 
 
 
CDK4
 
 
hsa-miR-1-3p
 
 
-0.338
 
 
13.5
 
 
 
 
CDKN1A
 
 
hsa-let-7e-5p
 
 
-0.434
 
 
27.1
 
 
 
 
CDKN2AIP
 
 
hsa-miR-28-5p
 
 
-0.419
 
 
12.1
 
 
 
 
CDV3
 
 
hsa-let-7e-5p
 
 
-0.453
 
 
45.1
 
 
 
 
CEBPD
 
 
hsa-let-7e-5p
 
 
-0.309
 
 
15.7
 
 
 
 
CECR2
 
 
hsa-let-7e-5p
 
 
-0.356
 
 
11.8
 
 
 
 
 
 
 salivary gland 
 
 
 
 
target_symbol
 
 
mature_mirna_id
 
 
score
 
 
nTPM
 
 
 
 
 
 
ABHD17C
 
 
hsa-let-7e-5p
 
 
-0.359
 
 
22.3
 
 
 
 
ABHD2
 
 
hsa-miR-1-3p
 
 
-0.291
 
 
12.4
 
 
 
 
ABHD3
 
 
hsa-miR-130b-3p
 
 
-0.356
 
 
13.7
 
 
 
 
ACBD3
 
 
hsa-miR-130b-3p
 
 
-0.292
 
 
22.7
 
 
 
 
ACTC1
 
 
hsa-miR-7-5p
 
 
-0.471
 
 
41.7
 
 
 
 
ADAR
 
 
hsa-miR-1-3p
 
 
-0.35
 
 
56.0
 
 
 
 
ADPGK
 
 
hsa-miR-1-3p
 
 
-0.393
 
 
12.2
 
 
 
 
AIFM1
 
 
hsa-let-7e-5p
 
 
-0.314
 
 
17.0
 
 
 
 
AKIRIN2
 
 
hsa-miR-130b-3p
 
 
-0.315
 
 
21.8
 
 
 
 
ALG11
 
 
hsa-miR-1-3p
 
 
-0.317
 
 
10.2
 
 
 
 
ALG9
 
 
hsa-miR-7-5p
 
 
-0.302
 
 
16.6
 
 
 
 
AMT
 
 
hsa-let-7e-5p
 
 
-0.463
 
 
10.0
 
 
 
 
ANKIB1
 
 
hsa-miR-130b-3p
 
 
-0.329
 
 
13.1
 
 
 
 
ANKRD12
 
 
hsa-miR-7-5p
 
 
-0.37
 
 
11.6
 
 
 
 
ANKRD46
 
 
hsa-let-7e-5p
 
 
-0.389
 
 
16.1
 
 
 
 
ANP32B
 
 
hsa-miR-1-3p
 
 
-0.287
 
 
104.9
 
 
 
 
ANXA2
 
 
hsa-miR-1-3p
 
 
-0.581
 
 
800.6
 
 
 
 
ANXA4
 
 
hsa-miR-1-3p
 
 
-0.539
 
 
36.7
 
 
 
 
AP1AR
 
 
hsa-miR-100-5p
 
 
-0.804
 
 
11.4
 
 
 
 
AP1S1
 
 
hsa-miR-1-3p
 
 
-0.355
 
 
21.5
 
 
 
 
AP1S1
 
 
hsa-let-7e-5p
 
 
-0.374
 
 
21.5
 
 
 
 
APCDD1
 
 
hsa-miR-130b-3p
 
 
-0.304
 
 
10.0
 
 
 
 
APPL1
 
 
hsa-miR-130b-3p
 
 
-0.347
 
 
14.3
 
 
 
 
ARCN1
 
 
hsa-miR-1-3p
 
 
-0.468
 
 
75.0
 
 
 
 
ARF4
 
 
hsa-miR-7-5p
 
 
-0.407
 
 
171.4
 
 
 
 
ARFGEF1
 
 
hsa-miR-130b-3p
 
 
-0.302
 
 
13.9
 
 
 
 
ARG2
 
 
hsa-let-7e-5p
 
 
-0.468
 
 
18.4
 
 
 
 
ARHGAP1
 
 
hsa-miR-130b-3p
 
 
-0.376
 
 
43.3
 
 
 
 
ARHGAP12
 
 
hsa-miR-130b-3p
 
 
-0.287
 
 
15.7
 
 
 
 
ARL4D
 
 
hsa-let-7e-5p
 
 
-0.417
 
 
16.7
 
 
 
 
ARL6IP1
 
 
hsa-miR-130b-3p
 
 
-0.289
 
 
79.4
 
 
 
 
ARL6IP1
 
 
hsa-miR-7-5p
 
 
-0.374
 
 
79.4
 
 
 
 
ARPC3
 
 
hsa-miR-1-3p
 
 
-0.606
 
 
210.5
 
 
 
 
ARPP19
 
 
hsa-let-7e-5p
 
 
-0.492
 
 
28.7
 
 
 
 
ASH2L
 
 
hsa-miR-1-3p
 
 
-0.462
 
 
19.9
 
 
 
 
ATF2
 
 
hsa-miR-1-3p
 
 
-0.328
 
 
16.9
 
 
 
 
ATG16L1
 
 
hsa-miR-130b-3p
 
 
-0.407
 
 
11.7
 
 
 
 
ATG4A
 
 
hsa-miR-7-5p
 
 
-0.448
 
 
12.3
 
 
 
 
ATOSB
 
 
hsa-let-7e-5p
 
 
-0.429
 
 
10.9
 
 
 
 
ATP2A2
 
 
hsa-let-7e-5p
 
 
-0.311
 
 
74.5
 
 
 
 
ATP2B2
 
 
hsa-miR-7-5p
 
 
-0.297
 
 
31.3
 
 
 
 
ATP2B2
 
 
hsa-miR-130b-3p
 
 
-0.314
 
 
31.3
 
 
 
 
ATPAF1
 
 
hsa-let-7e-5p
 
 
-0.498
 
 
54.3
 
 
 
 
AVPI1
 
 
hsa-miR-7-5p
 
 
-0.367
 
 
23.0
 
 
 
 
AZIN1
 
 
hsa-miR-1-3p
 
 
-0.438
 
 
44.6
 
 
 
 
B3GAT3
 
 
hsa-let-7e-5p
 
 
-0.324
 
 
28.6
 
 
 
 
B4GAT1
 
 
hsa-let-7e-5p
 
 
-0.47
 
 
14.9
 
 
 
 
BACE2
 
 
hsa-let-7e-5p
 
 
-0.364
 
 
16.3
 
 
 
 
BACH1
 
 
hsa-let-7e-5p
 
 
-0.298
 
 
16.4
 
 
 
 
BACH1
 
 
hsa-let-7e-5p
 
 
-0.298
 
 
16.4
 
 
 
 
BACH1
 
 
hsa-let-7e-5p
 
 
-0.435
 
 
16.4
 
 
 
 
BACH1
 
 
hsa-let-7e-5p
 
 
-0.435
 
 
16.4
 
 
 
 
BAHD1
 
 
hsa-miR-130b-3p
 
 
-0.294
 
 
12.0
 
 
 
 
BAZ2A
 
 
hsa-miR-100-5p
 
 
-0.29
 
 
23.8
 
 
 
 
BAZ2A
 
 
hsa-miR-100-5p
 
 
-0.421
 
 
23.8
 
 
 
 
BCAP29
 
 
hsa-let-7e-5p
 
 
-0.289
 
 
13.9
 
 
 
 
BET1
 
 
hsa-miR-1-3p
 
 
-0.413
 
 
24.7
 
 
 
 
BHLHE40
 
 
hsa-miR-130b-3p
 
 
-0.351
 
 
123.4
 
 
 
 
BIN3
 
 
hsa-let-7e-5p
 
 
-0.427
 
 
13.3
 
 
 
 
BLCAP
 
 
hsa-miR-130b-3p
 
 
-0.342
 
 
55.7
 
 
 
 
BLOC1S6
 
 
hsa-let-7e-5p
 
 
-0.321
 
 
19.5
 
 
 
 
BPNT1
 
 
hsa-miR-1-3p
 
 
-0.329
 
 
16.4
 
 
 
 
BRIX1
 
 
hsa-miR-1-3p
 
 
-0.334
 
 
13.1
 
 
 
 
BSCL2
 
 
hsa-miR-1-3p
 
 
-0.471
 
 
61.6
 
 
 
 
BTAF1
 
 
hsa-miR-130b-3p
 
 
-0.291
 
 
15.9
 
 
 
 
BTF3L4
 
 
hsa-let-7e-5p
 
 
-0.355
 
 
28.2
 
 
 
 
BTF3L4
 
 
hsa-miR-130b-3p
 
 
-0.359
 
 
28.2
 
 
 
 
BZW1
 
 
hsa-let-7e-5p
 
 
-0.354
 
 
55.2
 
 
 
 
BZW1
 
 
hsa-let-7e-5p
 
 
-0.375
 
 
55.2
 
 
 
 
BZW2
 
 
hsa-let-7e-5p
 
 
-0.408
 
 
24.9
 
 
 
 
C15orf39
 
 
hsa-let-7e-5p
 
 
-0.374
 
 
13.6
 
 
 
 
C19orf47
 
 
hsa-let-7e-5p
 
 
-0.311
 
 
12.5
 
 
 
 
C1GALT1
 
 
hsa-let-7e-5p
 
 
-0.322
 
 
22.0
 
 
 
 
C2orf88
 
 
hsa-let-7e-5p
 
 
-0.429
 
 
13.2
 
 
 
 
C6orf120
 
 
hsa-miR-1-3p
 
 
-0.288
 
 
12.1
 
 
 
 
CAAP1
 
 
hsa-miR-1-3p
 
 
-0.48
 
 
10.5
 
 
 
 
CACFD1
 
 
hsa-let-7e-5p
 
 
-0.296
 
 
15.2
 
 
 
 
CALM2
 
 
hsa-miR-130b-3p
 
 
-0.584
 
 
462.3
 
 
 
 
CANT1
 
 
hsa-let-7e-5p
 
 
-0.402
 
 
59.0
 
 
 
 
CAPN3
 
 
hsa-let-7e-5p
 
 
-0.317
 
 
19.7
 
 
 
 
CAPZA1
 
 
hsa-miR-7-5p
 
 
-0.351
 
 
46.6
 
 
 
 
CASD1
 
 
hsa-miR-130b-3p
 
 
-0.305
 
 
19.1
 
 
 
 
CASP3
 
 
hsa-let-7e-5p
 
 
-0.495
 
 
12.4
 
 
 
 
CCNC
 
 
hsa-miR-1-3p
 
 
-0.336
 
 
66.4
 
 
 
 
CCNL2
 
 
hsa-miR-28-5p
 
 
-0.448
 
 
69.6
 
 
 
 
CCNT2
 
 
hsa-miR-7-5p
 
 
-0.349
 
 
12.3
 
 
 
 
CCNY
 
 
hsa-let-7e-5p
 
 
-0.334
 
 
16.1
 
 
 
 
CCNY
 
 
hsa-miR-130b-3p
 
 
-0.372
 
 
16.1
 
 
 
 
CCT4
 
 
hsa-miR-7-5p
 
 
-0.37
 
 
51.2
 
 
 
 
CCT6A
 
 
hsa-miR-130b-3p
 
 
-0.382
 
 
46.2
 
 
 
 
CD164
 
 
hsa-miR-1-3p
 
 
-0.315
 
 
286.3
 
 
 
 
CD2AP
 
 
hsa-miR-130b-3p
 
 
-0.302
 
 
16.0
 
 
 
 
CD2AP
 
 
hsa-miR-1-3p
 
 
-0.479
 
 
16.0
 
 
 
 
CDC34
 
 
hsa-let-7e-5p
 
 
-0.321
 
 
35.2
 
 
 
 
CDC34
 
 
hsa-let-7e-5p
 
 
-0.555
 
 
35.2
 
 
 
 
CDC42SE1
 
 
hsa-miR-1-3p
 
 
-0.36
 
 
53.6
 
 
 
 
CDK4
 
 
hsa-miR-1-3p
 
 
-0.338
 
 
51.1
 
 
 
 
CDKN1A
 
 
hsa-let-7e-5p
 
 
-0.434
 
 
79.2
 
 
 
 
CDKN2AIP
 
 
hsa-miR-28-5p
 
 
-0.419
 
 
12.0
 
 
 
 
CDV3
 
 
hsa-let-7e-5p
 
 
-0.453
 
 
45.1
 
 
 
 
 
 
 appendix 
 
 
 
 
target_symbol
 
 
mature_mirna_id
 
 
score
 
 
nTPM
 
 
 
 
 
 
ABHD2
 
 
hsa-miR-1-3p
 
 
-0.291
 
 
19.1
 
 
 
 
ABHD3
 
 
hsa-miR-130b-3p
 
 
-0.356
 
 
23.7
 
 
 
 
ACBD3
 
 
hsa-miR-130b-3p
 
 
-0.292
 
 
19.2
 
 
 
 
ACBD5
 
 
hsa-miR-130b-3p
 
 
-0.494
 
 
12.7
 
 
 
 
ACSL4
 
 
hsa-miR-130b-3p
 
 
-0.301
 
 
34.5
 
 
 
 
ACTC1
 
 
hsa-miR-7-5p
 
 
-0.471
 
 
23.3
 
 
 
 
ADAR
 
 
hsa-miR-1-3p
 
 
-0.35
 
 
54.1
 
 
 
 
ADPGK
 
 
hsa-miR-1-3p
 
 
-0.393
 
 
20.1
 
 
 
 
AIFM1
 
 
hsa-let-7e-5p
 
 
-0.314
 
 
16.6
 
 
 
 
AKIRIN2
 
 
hsa-miR-130b-3p
 
 
-0.315
 
 
22.4
 
 
 
 
ANKRD12
 
 
hsa-miR-7-5p
 
 
-0.37
 
 
19.4
 
 
 
 
ANP32B
 
 
hsa-miR-1-3p
 
 
-0.287
 
 
117.0
 
 
 
 
ANXA2
 
 
hsa-miR-1-3p
 
 
-0.581
 
 
435.5
 
 
 
 
ANXA4
 
 
hsa-miR-1-3p
 
 
-0.539
 
 
31.7
 
 
 
 
AP1AR
 
 
hsa-miR-100-5p
 
 
-0.804
 
 
11.9
 
 
 
 
AP1S1
 
 
hsa-miR-1-3p
 
 
-0.355
 
 
13.1
 
 
 
 
AP1S1
 
 
hsa-let-7e-5p
 
 
-0.374
 
 
13.1
 
 
 
 
APPL1
 
 
hsa-miR-130b-3p
 
 
-0.347
 
 
14.0
 
 
 
 
ARCN1
 
 
hsa-miR-1-3p
 
 
-0.468
 
 
50.7
 
 
 
 
ARF4
 
 
hsa-miR-7-5p
 
 
-0.407
 
 
101.3
 
 
 
 
ARFGEF1
 
 
hsa-miR-130b-3p
 
 
-0.302
 
 
13.8
 
 
 
 
ARHGAP1
 
 
hsa-miR-130b-3p
 
 
-0.376
 
 
35.8
 
 
 
 
ARID4A
 
 
hsa-miR-7-5p
 
 
-0.341
 
 
10.5
 
 
 
 
ARL6IP1
 
 
hsa-miR-130b-3p
 
 
-0.289
 
 
73.3
 
 
 
 
ARL6IP1
 
 
hsa-miR-7-5p
 
 
-0.374
 
 
73.3
 
 
 
 
ARL6IP6
 
 
hsa-let-7e-5p
 
 
-0.305
 
 
10.9
 
 
 
 
ARMT1
 
 
hsa-let-7e-5p
 
 
-0.303
 
 
14.4
 
 
 
 
ARPC3
 
 
hsa-miR-1-3p
 
 
-0.606
 
 
344.9
 
 
 
 
ARPP19
 
 
hsa-let-7e-5p
 
 
-0.492
 
 
36.4
 
 
 
 
ARRDC4
 
 
hsa-let-7e-5p
 
 
-0.329
 
 
15.6
 
 
 
 
ASAP1
 
 
hsa-let-7e-5p
 
 
-0.296
 
 
11.4
 
 
 
 
ASH2L
 
 
hsa-miR-1-3p
 
 
-0.462
 
 
17.4
 
 
 
 
ATF2
 
 
hsa-miR-1-3p
 
 
-0.328
 
 
14.4
 
 
 
 
ATOSB
 
 
hsa-let-7e-5p
 
 
-0.429
 
 
11.5
 
 
 
 
ATP2A2
 
 
hsa-let-7e-5p
 
 
-0.311
 
 
54.5
 
 
 
 
ATPAF1
 
 
hsa-let-7e-5p
 
 
-0.498
 
 
26.8
 
 
 
 
AXL
 
 
hsa-miR-1-3p
 
 
-0.304
 
 
14.6
 
 
 
 
AZIN1
 
 
hsa-miR-1-3p
 
 
-0.438
 
 
44.7
 
 
 
 
B3GAT3
 
 
hsa-let-7e-5p
 
 
-0.324
 
 
16.3
 
 
 
 
BACH1
 
 
hsa-let-7e-5p
 
 
-0.298
 
 
26.1
 
 
 
 
BACH1
 
 
hsa-let-7e-5p
 
 
-0.298
 
 
26.1
 
 
 
 
BACH1
 
 
hsa-let-7e-5p
 
 
-0.435
 
 
26.1
 
 
 
 
BACH1
 
 
hsa-let-7e-5p
 
 
-0.435
 
 
26.1
 
 
 
 
BAZ2A
 
 
hsa-miR-100-5p
 
 
-0.29
 
 
29.1
 
 
 
 
BAZ2A
 
 
hsa-miR-100-5p
 
 
-0.421
 
 
29.1
 
 
 
 
BCAP29
 
 
hsa-let-7e-5p
 
 
-0.289
 
 
21.6
 
 
 
 
BET1
 
 
hsa-miR-1-3p
 
 
-0.413
 
 
15.4
 
 
 
 
BHLHE40
 
 
hsa-miR-130b-3p
 
 
-0.351
 
 
50.0
 
 
 
 
BIN3
 
 
hsa-let-7e-5p
 
 
-0.427
 
 
10.4
 
 
 
 
BLCAP
 
 
hsa-miR-130b-3p
 
 
-0.342
 
 
25.7
 
 
 
 
BLOC1S6
 
 
hsa-let-7e-5p
 
 
-0.321
 
 
29.4
 
 
 
 
BPNT1
 
 
hsa-miR-1-3p
 
 
-0.329
 
 
17.2
 
 
 
 
BRIX1
 
 
hsa-miR-1-3p
 
 
-0.334
 
 
16.6
 
 
 
 
BTAF1
 
 
hsa-miR-130b-3p
 
 
-0.291
 
 
16.0
 
 
 
 
BTF3L4
 
 
hsa-let-7e-5p
 
 
-0.355
 
 
28.6
 
 
 
 
BTF3L4
 
 
hsa-miR-130b-3p
 
 
-0.359
 
 
28.6
 
 
 
 
BTG1
 
 
hsa-miR-130b-3p
 
 
-0.565
 
 
18.7
 
 
 
 
BZW1
 
 
hsa-let-7e-5p
 
 
-0.354
 
 
91.9
 
 
 
 
BZW1
 
 
hsa-let-7e-5p
 
 
-0.375
 
 
91.9
 
 
 
 
BZW2
 
 
hsa-let-7e-5p
 
 
-0.408
 
 
25.1
 
 
 
 
C15orf39
 
 
hsa-let-7e-5p
 
 
-0.374
 
 
12.0
 
 
 
 
C1GALT1
 
 
hsa-let-7e-5p
 
 
-0.322
 
 
13.4
 
 
 
 
C6orf120
 
 
hsa-miR-1-3p
 
 
-0.288
 
 
12.4
 
 
 
 
CALM2
 
 
hsa-miR-130b-3p
 
 
-0.584
 
 
315.5
 
 
 
 
CANT1
 
 
hsa-let-7e-5p
 
 
-0.402
 
 
23.3
 
 
 
 
CAPZA1
 
 
hsa-miR-7-5p
 
 
-0.351
 
 
108.8
 
 
 
 
CASP3
 
 
hsa-let-7e-5p
 
 
-0.495
 
 
22.0
 
 
 
 
CASP5
 
 
hsa-let-7e-5p
 
 
-0.379
 
 
13.8
 
 
 
 
CBFB
 
 
hsa-miR-130b-3p
 
 
-0.498
 
 
16.3
 
 
 
 
CBX5
 
 
hsa-let-7e-5p
 
 
-0.307
 
 
11.9
 
 
 
 
CBX5
 
 
hsa-let-7e-5p
 
 
-0.472
 
 
11.9
 
 
 
 
CCDC43
 
 
hsa-miR-7-5p
 
 
-0.331
 
 
10.8
 
 
 
 
CCNC
 
 
hsa-miR-1-3p
 
 
-0.336
 
 
25.8
 
 
 
 
CCNL2
 
 
hsa-miR-28-5p
 
 
-0.448
 
 
27.6
 
 
 
 
CCNY
 
 
hsa-let-7e-5p
 
 
-0.334
 
 
20.0
 
 
 
 
CCNY
 
 
hsa-miR-130b-3p
 
 
-0.372
 
 
20.0
 
 
 
 
CCR7
 
 
hsa-let-7e-5p
 
 
-0.482
 
 
63.4
 
 
 
 
CCT4
 
 
hsa-miR-7-5p
 
 
-0.37
 
 
56.5
 
 
 
 
CCT6A
 
 
hsa-miR-130b-3p
 
 
-0.382
 
 
63.9
 
 
 
 
CD164
 
 
hsa-miR-1-3p
 
 
-0.315
 
 
112.4
 
 
 
 
CD2AP
 
 
hsa-miR-130b-3p
 
 
-0.302
 
 
14.2
 
 
 
 
CD2AP
 
 
hsa-miR-1-3p
 
 
-0.479
 
 
14.2
 
 
 
 
CD69
 
 
hsa-miR-130b-3p
 
 
-0.451
 
 
45.4
 
 
 
 
CD86
 
 
hsa-let-7e-5p
 
 
-0.323
 
 
32.5
 
 
 
 
CDC34
 
 
hsa-let-7e-5p
 
 
-0.321
 
 
12.5
 
 
 
 
CDC34
 
 
hsa-let-7e-5p
 
 
-0.555
 
 
12.5
 
 
 
 
CDC42SE1
 
 
hsa-miR-1-3p
 
 
-0.36
 
 
60.2
 
 
 
 
CDK4
 
 
hsa-miR-1-3p
 
 
-0.338
 
 
49.8
 
 
 
 
CDKN1A
 
 
hsa-let-7e-5p
 
 
-0.434
 
 
58.6
 
 
 
 
CDKN2AIP
 
 
hsa-miR-28-5p
 
 
-0.419
 
 
11.2
 
 
 
 
CDV3
 
 
hsa-let-7e-5p
 
 
-0.453
 
 
104.7
 
 
 
 
CEBPD
 
 
hsa-let-7e-5p
 
 
-0.309
 
 
58.7
 
 
 
 
CEMIP2
 
 
hsa-let-7e-5p
 
 
-0.453
 
 
23.4
 
 
 
 
CERCAM
 
 
hsa-let-7e-5p
 
 
-0.344
 
 
11.6
 
 
 
 
CERS2
 
 
hsa-miR-1-3p
 
 
-0.287
 
 
50.1
 
 
 
 
CGGBP1
 
 
hsa-miR-7-5p
 
 
-0.465
 
 
43.2
 
 
 
 
CHMP3
 
 
hsa-miR-130b-3p
 
 
-0.521
 
 
38.7
 
 
 
 
CHMP4B
 
 
hsa-miR-130b-3p
 
 
-0.292
 
 
71.5
 
 
 
 
CHUK
 
 
hsa-let-7e-5p
 
 
-0.327
 
 
11.1
 
 
 
 
CITED2
 
 
hsa-miR-1-3p
 
 
-0.37
 
 
46.9
 
 
 
 
 
 
 epididymis 
 
 
 
 
target_symbol
 
 
mature_mirna_id
 
 
score
 
 
nTPM
 
 
 
 
 
 
ABHD2
 
 
hsa-miR-1-3p
 
 
-0.291
 
 
53.0
 
 
 
 
ACBD3
 
 
hsa-miR-130b-3p
 
 
-0.292
 
 
19.4
 
 
 
 
ACP3
 
 
hsa-let-7e-5p
 
 
-0.298
 
 
16.3
 
 
 
 
ACSL4
 
 
hsa-miR-130b-3p
 
 
-0.301
 
 
10.2
 
 
 
 
ACVR1
 
 
hsa-miR-130b-3p
 
 
-0.44
 
 
10.8
 
 
 
 
ADAR
 
 
hsa-miR-1-3p
 
 
-0.35
 
 
24.9
 
 
 
 
ADPGK
 
 
hsa-miR-1-3p
 
 
-0.393
 
 
14.7
 
 
 
 
AKIRIN2
 
 
hsa-miR-130b-3p
 
 
-0.315
 
 
22.5
 
 
 
 
ALG9
 
 
hsa-miR-7-5p
 
 
-0.302
 
 
10.3
 
 
 
 
ANKIB1
 
 
hsa-miR-130b-3p
 
 
-0.329
 
 
15.0
 
 
 
 
ANKRD46
 
 
hsa-let-7e-5p
 
 
-0.389
 
 
15.4
 
 
 
 
ANP32B
 
 
hsa-miR-1-3p
 
 
-0.287
 
 
98.9
 
 
 
 
ANXA2
 
 
hsa-miR-1-3p
 
 
-0.581
 
 
252.4
 
 
 
 
ANXA4
 
 
hsa-miR-1-3p
 
 
-0.539
 
 
28.7
 
 
 
 
AP1S1
 
 
hsa-miR-1-3p
 
 
-0.355
 
 
16.0
 
 
 
 
AP1S1
 
 
hsa-let-7e-5p
 
 
-0.374
 
 
16.0
 
 
 
 
APCDD1
 
 
hsa-miR-130b-3p
 
 
-0.304
 
 
14.6
 
 
 
 
APPL1
 
 
hsa-miR-130b-3p
 
 
-0.347
 
 
10.0
 
 
 
 
ARCN1
 
 
hsa-miR-1-3p
 
 
-0.468
 
 
104.7
 
 
 
 
ARF4
 
 
hsa-miR-7-5p
 
 
-0.407
 
 
214.1
 
 
 
 
ARFGEF1
 
 
hsa-miR-130b-3p
 
 
-0.302
 
 
10.4
 
 
 
 
ARHGAP1
 
 
hsa-miR-130b-3p
 
 
-0.376
 
 
20.3
 
 
 
 
ARHGAP12
 
 
hsa-miR-130b-3p
 
 
-0.287
 
 
10.2
 
 
 
 
ARL6IP1
 
 
hsa-miR-130b-3p
 
 
-0.289
 
 
116.0
 
 
 
 
ARL6IP1
 
 
hsa-miR-7-5p
 
 
-0.374
 
 
116.0
 
 
 
 
ARMT1
 
 
hsa-let-7e-5p
 
 
-0.303
 
 
15.2
 
 
 
 
ARPC3
 
 
hsa-miR-1-3p
 
 
-0.606
 
 
281.5
 
 
 
 
ARPP19
 
 
hsa-let-7e-5p
 
 
-0.492
 
 
34.2
 
 
 
 
ARRDC4
 
 
hsa-let-7e-5p
 
 
-0.329
 
 
14.7
 
 
 
 
ASAP1
 
 
hsa-let-7e-5p
 
 
-0.296
 
 
11.5
 
 
 
 
ASH2L
 
 
hsa-miR-1-3p
 
 
-0.462
 
 
16.2
 
 
 
 
ATF2
 
 
hsa-miR-1-3p
 
 
-0.328
 
 
20.4
 
 
 
 
ATG4A
 
 
hsa-miR-7-5p
 
 
-0.448
 
 
10.9
 
 
 
 
ATP2A2
 
 
hsa-let-7e-5p
 
 
-0.311
 
 
36.9
 
 
 
 
ATPAF1
 
 
hsa-let-7e-5p
 
 
-0.498
 
 
33.7
 
 
 
 
AZIN1
 
 
hsa-miR-1-3p
 
 
-0.438
 
 
28.4
 
 
 
 
B3GAT3
 
 
hsa-let-7e-5p
 
 
-0.324
 
 
11.2
 
 
 
 
B4GAT1
 
 
hsa-let-7e-5p
 
 
-0.47
 
 
14.3
 
 
 
 
BAZ2A
 
 
hsa-miR-100-5p
 
 
-0.29
 
 
21.6
 
 
 
 
BAZ2A
 
 
hsa-miR-100-5p
 
 
-0.421
 
 
21.6
 
 
 
 
BCAP29
 
 
hsa-let-7e-5p
 
 
-0.289
 
 
18.0
 
 
 
 
BET1
 
 
hsa-miR-1-3p
 
 
-0.413
 
 
32.4
 
 
 
 
BHLHE40
 
 
hsa-miR-130b-3p
 
 
-0.351
 
 
32.6
 
 
 
 
BLCAP
 
 
hsa-miR-130b-3p
 
 
-0.342
 
 
49.5
 
 
 
 
BLOC1S6
 
 
hsa-let-7e-5p
 
 
-0.321
 
 
17.3
 
 
 
 
BPNT1
 
 
hsa-miR-1-3p
 
 
-0.329
 
 
12.2
 
 
 
 
BRIX1
 
 
hsa-miR-1-3p
 
 
-0.334
 
 
16.8
 
 
 
 
BSCL2
 
 
hsa-miR-1-3p
 
 
-0.471
 
 
143.1
 
 
 
 
BTAF1
 
 
hsa-miR-130b-3p
 
 
-0.291
 
 
16.9
 
 
 
 
BTF3L4
 
 
hsa-let-7e-5p
 
 
-0.355
 
 
28.9
 
 
 
 
BTF3L4
 
 
hsa-miR-130b-3p
 
 
-0.359
 
 
28.9
 
 
 
 
BZW1
 
 
hsa-let-7e-5p
 
 
-0.354
 
 
55.1
 
 
 
 
BZW1
 
 
hsa-let-7e-5p
 
 
-0.375
 
 
55.1
 
 
 
 
CAAP1
 
 
hsa-miR-1-3p
 
 
-0.48
 
 
16.0
 
 
 
 
CALM2
 
 
hsa-miR-130b-3p
 
 
-0.584
 
 
520.7
 
 
 
 
CAPN3
 
 
hsa-let-7e-5p
 
 
-0.317
 
 
154.5
 
 
 
 
CAPZA1
 
 
hsa-miR-7-5p
 
 
-0.351
 
 
58.0
 
 
 
 
CASD1
 
 
hsa-miR-130b-3p
 
 
-0.305
 
 
13.6
 
 
 
 
CBX5
 
 
hsa-let-7e-5p
 
 
-0.307
 
 
14.1
 
 
 
 
CBX5
 
 
hsa-let-7e-5p
 
 
-0.472
 
 
14.1
 
 
 
 
CCDC43
 
 
hsa-miR-7-5p
 
 
-0.331
 
 
10.3
 
 
 
 
CCNC
 
 
hsa-miR-1-3p
 
 
-0.336
 
 
55.4
 
 
 
 
CCNL2
 
 
hsa-miR-28-5p
 
 
-0.448
 
 
28.7
 
 
 
 
CCNY
 
 
hsa-let-7e-5p
 
 
-0.334
 
 
25.4
 
 
 
 
CCNY
 
 
hsa-miR-130b-3p
 
 
-0.372
 
 
25.4
 
 
 
 
CCT4
 
 
hsa-miR-7-5p
 
 
-0.37
 
 
48.0
 
 
 
 
CCT6A
 
 
hsa-miR-130b-3p
 
 
-0.382
 
 
37.2
 
 
 
 
CD164
 
 
hsa-miR-1-3p
 
 
-0.315
 
 
152.9
 
 
 
 
CD2AP
 
 
hsa-miR-130b-3p
 
 
-0.302
 
 
12.5
 
 
 
 
CD2AP
 
 
hsa-miR-1-3p
 
 
-0.479
 
 
12.5
 
 
 
 
CDC34
 
 
hsa-let-7e-5p
 
 
-0.321
 
 
13.3
 
 
 
 
CDC34
 
 
hsa-let-7e-5p
 
 
-0.555
 
 
13.3
 
 
 
 
CDC42SE1
 
 
hsa-miR-1-3p
 
 
-0.36
 
 
25.3
 
 
 
 
CDK4
 
 
hsa-miR-1-3p
 
 
-0.338
 
 
60.6
 
 
 
 
CDKN1A
 
 
hsa-let-7e-5p
 
 
-0.434
 
 
38.8
 
 
 
 
CDKN2AIP
 
 
hsa-miR-28-5p
 
 
-0.419
 
 
11.7
 
 
 
 
CDV3
 
 
hsa-let-7e-5p
 
 
-0.453
 
 
50.7
 
 
 
 
CERS2
 
 
hsa-miR-1-3p
 
 
-0.287
 
 
84.6
 
 
 
 
CGGBP1
 
 
hsa-miR-7-5p
 
 
-0.465
 
 
30.9
 
 
 
 
CHMP3
 
 
hsa-miR-130b-3p
 
 
-0.521
 
 
53.7
 
 
 
 
CHMP4B
 
 
hsa-miR-130b-3p
 
 
-0.292
 
 
55.3
 
 
 
 
CITED2
 
 
hsa-miR-1-3p
 
 
-0.37
 
 
36.5
 
 
 
 
CKAP4
 
 
hsa-miR-7-5p
 
 
-0.443
 
 
55.9
 
 
 
 
CLASP2
 
 
hsa-let-7e-5p
 
 
-0.344
 
 
10.4
 
 
 
 
CLCN3
 
 
hsa-miR-1-3p
 
 
-0.339
 
 
22.5
 
 
 
 
CLCN3
 
 
hsa-miR-130b-3p
 
 
-0.384
 
 
22.5
 
 
 
 
CLCN5
 
 
hsa-miR-130b-3p
 
 
-0.306
 
 
22.3
 
 
 
 
CLDN12
 
 
hsa-let-7e-5p
 
 
-0.511
 
 
18.7
 
 
 
 
CLIP1
 
 
hsa-miR-130b-3p
 
 
-0.452
 
 
16.9
 
 
 
 
CLTC
 
 
hsa-miR-1-3p
 
 
-0.39
 
 
90.1
 
 
 
 
CMPK1
 
 
hsa-miR-130b-3p
 
 
-0.384
 
 
41.6
 
 
 
 
CNIH1
 
 
hsa-miR-130b-3p
 
 
-0.335
 
 
47.9
 
 
 
 
CNN3
 
 
hsa-miR-1-3p
 
 
-0.414
 
 
67.0
 
 
 
 
CNN3
 
 
hsa-miR-7-5p
 
 
-0.489
 
 
67.0
 
 
 
 
CNOT7
 
 
hsa-miR-130b-3p
 
 
-0.318
 
 
42.5
 
 
 
 
CNOT8
 
 
hsa-miR-7-5p
 
 
-0.329
 
 
34.9
 
 
 
 
CNPPD1
 
 
hsa-miR-7-5p
 
 
-0.541
 
 
35.2
 
 
 
 
COIL
 
 
hsa-let-7e-5p
 
 
-0.618
 
 
10.9
 
 
 
 
COL1A2
 
 
hsa-let-7e-5p
 
 
-0.499
 
 
58.2
 
 
 
 
COL2A1
 
 
hsa-miR-7-5p
 
 
-0.345
 
 
18.0
 
 
 
 
 
 
 gallbladder 
 
 
 
 
target_symbol
 
 
mature_mirna_id
 
 
score
 
 
nTPM
 
 
 
 
 
 
ABHD2
 
 
hsa-miR-1-3p
 
 
-0.291
 
 
44.4
 
 
 
 
ABHD3
 
 
hsa-miR-130b-3p
 
 
-0.356
 
 
19.5
 
 
 
 
ACBD3
 
 
hsa-miR-130b-3p
 
 
-0.292
 
 
22.0
 
 
 
 
ACBD5
 
 
hsa-miR-130b-3p
 
 
-0.494
 
 
14.5
 
 
 
 
ACSL4
 
 
hsa-miR-130b-3p
 
 
-0.301
 
 
25.8
 
 
 
 
ACTC1
 
 
hsa-miR-7-5p
 
 
-0.471
 
 
11.1
 
 
 
 
ACVR1
 
 
hsa-miR-130b-3p
 
 
-0.44
 
 
14.1
 
 
 
 
ADAR
 
 
hsa-miR-1-3p
 
 
-0.35
 
 
42.7
 
 
 
 
AKIRIN2
 
 
hsa-miR-130b-3p
 
 
-0.315
 
 
14.3
 
 
 
 
ANKIB1
 
 
hsa-miR-130b-3p
 
 
-0.329
 
 
13.9
 
 
 
 
ANKRD12
 
 
hsa-miR-7-5p
 
 
-0.37
 
 
13.7
 
 
 
 
ANP32B
 
 
hsa-miR-1-3p
 
 
-0.287
 
 
56.6
 
 
 
 
ANXA2
 
 
hsa-miR-1-3p
 
 
-0.581
 
 
425.2
 
 
 
 
ANXA4
 
 
hsa-miR-1-3p
 
 
-0.539
 
 
535.2
 
 
 
 
AP1AR
 
 
hsa-miR-100-5p
 
 
-0.804
 
 
12.0
 
 
 
 
AP1S1
 
 
hsa-miR-1-3p
 
 
-0.355
 
 
12.0
 
 
 
 
AP1S1
 
 
hsa-let-7e-5p
 
 
-0.374
 
 
12.0
 
 
 
 
APCDD1
 
 
hsa-miR-130b-3p
 
 
-0.304
 
 
20.5
 
 
 
 
APPL1
 
 
hsa-miR-130b-3p
 
 
-0.347
 
 
12.2
 
 
 
 
ARCN1
 
 
hsa-miR-1-3p
 
 
-0.468
 
 
48.3
 
 
 
 
ARF4
 
 
hsa-miR-7-5p
 
 
-0.407
 
 
145.1
 
 
 
 
ARFGEF1
 
 
hsa-miR-130b-3p
 
 
-0.302
 
 
11.7
 
 
 
 
ARHGAP1
 
 
hsa-miR-130b-3p
 
 
-0.376
 
 
47.5
 
 
 
 
ARHGAP12
 
 
hsa-miR-130b-3p
 
 
-0.287
 
 
14.3
 
 
 
 
ARHGAP24
 
 
hsa-miR-130b-3p
 
 
-0.287
 
 
10.2
 
 
 
 
ARL6IP1
 
 
hsa-miR-130b-3p
 
 
-0.289
 
 
123.8
 
 
 
 
ARL6IP1
 
 
hsa-miR-7-5p
 
 
-0.374
 
 
123.8
 
 
 
 
ARMT1
 
 
hsa-let-7e-5p
 
 
-0.303
 
 
10.6
 
 
 
 
ARPC3
 
 
hsa-miR-1-3p
 
 
-0.606
 
 
237.2
 
 
 
 
ARPP19
 
 
hsa-let-7e-5p
 
 
-0.492
 
 
36.7
 
 
 
 
ARRDC4
 
 
hsa-let-7e-5p
 
 
-0.329
 
 
16.1
 
 
 
 
ASAP1
 
 
hsa-let-7e-5p
 
 
-0.296
 
 
13.5
 
 
 
 
ASH2L
 
 
hsa-miR-1-3p
 
 
-0.462
 
 
16.3
 
 
 
 
ATF2
 
 
hsa-miR-1-3p
 
 
-0.328
 
 
16.5
 
 
 
 
ATOSB
 
 
hsa-let-7e-5p
 
 
-0.429
 
 
11.0
 
 
 
 
ATP2A2
 
 
hsa-let-7e-5p
 
 
-0.311
 
 
56.7
 
 
 
 
ATPAF1
 
 
hsa-let-7e-5p
 
 
-0.498
 
 
28.6
 
 
 
 
AXL
 
 
hsa-miR-1-3p
 
 
-0.304
 
 
20.9
 
 
 
 
AZIN1
 
 
hsa-miR-1-3p
 
 
-0.438
 
 
40.1
 
 
 
 
B3GAT3
 
 
hsa-let-7e-5p
 
 
-0.324
 
 
14.5
 
 
 
 
BACH1
 
 
hsa-let-7e-5p
 
 
-0.298
 
 
24.3
 
 
 
 
BACH1
 
 
hsa-let-7e-5p
 
 
-0.298
 
 
24.3
 
 
 
 
BACH1
 
 
hsa-let-7e-5p
 
 
-0.435
 
 
24.3
 
 
 
 
BACH1
 
 
hsa-let-7e-5p
 
 
-0.435
 
 
24.3
 
 
 
 
BAZ2A
 
 
hsa-miR-100-5p
 
 
-0.29
 
 
19.6
 
 
 
 
BAZ2A
 
 
hsa-miR-100-5p
 
 
-0.421
 
 
19.6
 
 
 
 
BCAP29
 
 
hsa-let-7e-5p
 
 
-0.289
 
 
21.7
 
 
 
 
BET1
 
 
hsa-miR-1-3p
 
 
-0.413
 
 
12.5
 
 
 
 
BHLHE40
 
 
hsa-miR-130b-3p
 
 
-0.351
 
 
94.0
 
 
 
 
BIN3
 
 
hsa-let-7e-5p
 
 
-0.427
 
 
11.2
 
 
 
 
BLCAP
 
 
hsa-miR-130b-3p
 
 
-0.342
 
 
27.2
 
 
 
 
BLOC1S6
 
 
hsa-let-7e-5p
 
 
-0.321
 
 
24.8
 
 
 
 
BMPR2
 
 
hsa-miR-130b-3p
 
 
-0.33
 
 
12.7
 
 
 
 
BMPR2
 
 
hsa-miR-100-5p
 
 
-0.355
 
 
12.7
 
 
 
 
BPNT1
 
 
hsa-miR-1-3p
 
 
-0.329
 
 
19.9
 
 
 
 
BRIX1
 
 
hsa-miR-1-3p
 
 
-0.334
 
 
11.5
 
 
 
 
BSCL2
 
 
hsa-miR-1-3p
 
 
-0.471
 
 
19.7
 
 
 
 
BTAF1
 
 
hsa-miR-130b-3p
 
 
-0.291
 
 
12.0
 
 
 
 
BTF3L4
 
 
hsa-let-7e-5p
 
 
-0.355
 
 
22.4
 
 
 
 
BTF3L4
 
 
hsa-miR-130b-3p
 
 
-0.359
 
 
22.4
 
 
 
 
BZW1
 
 
hsa-let-7e-5p
 
 
-0.354
 
 
87.2
 
 
 
 
BZW1
 
 
hsa-let-7e-5p
 
 
-0.375
 
 
87.2
 
 
 
 
BZW2
 
 
hsa-let-7e-5p
 
 
-0.408
 
 
16.1
 
 
 
 
C1GALT1
 
 
hsa-let-7e-5p
 
 
-0.322
 
 
15.6
 
 
 
 
C6orf120
 
 
hsa-miR-1-3p
 
 
-0.288
 
 
10.4
 
 
 
 
CALM2
 
 
hsa-miR-130b-3p
 
 
-0.584
 
 
366.3
 
 
 
 
CAMSAP2
 
 
hsa-miR-130b-3p
 
 
-0.297
 
 
11.8
 
 
 
 
CANT1
 
 
hsa-let-7e-5p
 
 
-0.402
 
 
23.6
 
 
 
 
CAPZA1
 
 
hsa-miR-7-5p
 
 
-0.351
 
 
55.4
 
 
 
 
CASP3
 
 
hsa-let-7e-5p
 
 
-0.495
 
 
15.7
 
 
 
 
CCNC
 
 
hsa-miR-1-3p
 
 
-0.336
 
 
39.5
 
 
 
 
CCNL2
 
 
hsa-miR-28-5p
 
 
-0.448
 
 
24.6
 
 
 
 
CCNY
 
 
hsa-let-7e-5p
 
 
-0.334
 
 
21.3
 
 
 
 
CCNY
 
 
hsa-miR-130b-3p
 
 
-0.372
 
 
21.3
 
 
 
 
CCT4
 
 
hsa-miR-7-5p
 
 
-0.37
 
 
40.4
 
 
 
 
CCT6A
 
 
hsa-miR-130b-3p
 
 
-0.382
 
 
38.0
 
 
 
 
CD164
 
 
hsa-miR-1-3p
 
 
-0.315
 
 
149.5
 
 
 
 
CD2AP
 
 
hsa-miR-130b-3p
 
 
-0.302
 
 
14.1
 
 
 
 
CD2AP
 
 
hsa-miR-1-3p
 
 
-0.479
 
 
14.1
 
 
 
 
CD69
 
 
hsa-miR-130b-3p
 
 
-0.451
 
 
25.6
 
 
 
 
CD86
 
 
hsa-let-7e-5p
 
 
-0.323
 
 
13.4
 
 
 
 
CDC42SE1
 
 
hsa-miR-1-3p
 
 
-0.36
 
 
34.8
 
 
 
 
CDK14
 
 
hsa-miR-1-3p
 
 
-0.326
 
 
11.4
 
 
 
 
CDK4
 
 
hsa-miR-1-3p
 
 
-0.338
 
 
53.5
 
 
 
 
CDKN1A
 
 
hsa-let-7e-5p
 
 
-0.434
 
 
124.3
 
 
 
 
CDKN2AIP
 
 
hsa-miR-28-5p
 
 
-0.419
 
 
12.1
 
 
 
 
CDV3
 
 
hsa-let-7e-5p
 
 
-0.453
 
 
70.6
 
 
 
 
CEBPD
 
 
hsa-let-7e-5p
 
 
-0.309
 
 
13.8
 
 
 
 
CEMIP2
 
 
hsa-let-7e-5p
 
 
-0.453
 
 
38.9
 
 
 
 
CERCAM
 
 
hsa-let-7e-5p
 
 
-0.344
 
 
29.2
 
 
 
 
CERS2
 
 
hsa-miR-1-3p
 
 
-0.287
 
 
91.0
 
 
 
 
CGGBP1
 
 
hsa-miR-7-5p
 
 
-0.465
 
 
35.0
 
 
 
 
CHMP3
 
 
hsa-miR-130b-3p
 
 
-0.521
 
 
51.4
 
 
 
 
CHMP4B
 
 
hsa-miR-130b-3p
 
 
-0.292
 
 
75.6
 
 
 
 
CITED2
 
 
hsa-miR-1-3p
 
 
-0.37
 
 
30.6
 
 
 
 
CKAP4
 
 
hsa-miR-7-5p
 
 
-0.443
 
 
24.6
 
 
 
 
CLASP2
 
 
hsa-let-7e-5p
 
 
-0.344
 
 
16.8
 
 
 
 
CLCN3
 
 
hsa-miR-1-3p
 
 
-0.339
 
 
28.8
 
 
 
 
CLCN3
 
 
hsa-miR-130b-3p
 
 
-0.384
 
 
28.8
 
 
 
 
CLDN12
 
 
hsa-let-7e-5p
 
 
-0.511
 
 
17.8
 
 
 
 
 
 
 pancreas 
 
 
 
 
target_symbol
 
 
mature_mirna_id
 
 
score
 
 
nTPM
 
 
 
 
 
 
ABHD2
 
 
hsa-miR-1-3p
 
 
-0.291
 
 
54.6
 
 
 
 
ABHD3
 
 
hsa-miR-130b-3p
 
 
-0.356
 
 
11.6
 
 
 
 
ACBD3
 
 
hsa-miR-130b-3p
 
 
-0.292
 
 
21.4
 
 
 
 
ACBD5
 
 
hsa-miR-130b-3p
 
 
-0.494
 
 
10.5
 
 
 
 
ACSL4
 
 
hsa-miR-130b-3p
 
 
-0.301
 
 
10.9
 
 
 
 
ACTC1
 
 
hsa-miR-7-5p
 
 
-0.471
 
 
12.1
 
 
 
 
ADAR
 
 
hsa-miR-1-3p
 
 
-0.35
 
 
38.3
 
 
 
 
ADGRA3
 
 
hsa-miR-1-3p
 
 
-0.351
 
 
11.8
 
 
 
 
ADPGK
 
 
hsa-miR-1-3p
 
 
-0.393
 
 
11.7
 
 
 
 
AIFM1
 
 
hsa-let-7e-5p
 
 
-0.314
 
 
20.4
 
 
 
 
AKIRIN2
 
 
hsa-miR-130b-3p
 
 
-0.315
 
 
11.7
 
 
 
 
ALG9
 
 
hsa-miR-7-5p
 
 
-0.302
 
 
18.1
 
 
 
 
AMT
 
 
hsa-let-7e-5p
 
 
-0.463
 
 
21.5
 
 
 
 
ANKIB1
 
 
hsa-miR-130b-3p
 
 
-0.329
 
 
11.5
 
 
 
 
ANKRD12
 
 
hsa-miR-7-5p
 
 
-0.37
 
 
11.2
 
 
 
 
ANKRD46
 
 
hsa-let-7e-5p
 
 
-0.389
 
 
17.5
 
 
 
 
ANP32B
 
 
hsa-miR-1-3p
 
 
-0.287
 
 
147.1
 
 
 
 
ANXA2
 
 
hsa-miR-1-3p
 
 
-0.581
 
 
291.3
 
 
 
 
ANXA4
 
 
hsa-miR-1-3p
 
 
-0.539
 
 
336.5
 
 
 
 
AP1S1
 
 
hsa-miR-1-3p
 
 
-0.355
 
 
23.9
 
 
 
 
AP1S1
 
 
hsa-let-7e-5p
 
 
-0.374
 
 
23.9
 
 
 
 
APPL1
 
 
hsa-miR-130b-3p
 
 
-0.347
 
 
27.9
 
 
 
 
ARCN1
 
 
hsa-miR-1-3p
 
 
-0.468
 
 
109.0
 
 
 
 
ARF4
 
 
hsa-miR-7-5p
 
 
-0.407
 
 
138.3
 
 
 
 
ARFGEF1
 
 
hsa-miR-130b-3p
 
 
-0.302
 
 
16.5
 
 
 
 
ARHGAP1
 
 
hsa-miR-130b-3p
 
 
-0.376
 
 
34.7
 
 
 
 
ARHGAP12
 
 
hsa-miR-130b-3p
 
 
-0.287
 
 
20.1
 
 
 
 
ARHGAP24
 
 
hsa-miR-130b-3p
 
 
-0.287
 
 
28.4
 
 
 
 
ARHGEF4
 
 
hsa-miR-130b-3p
 
 
-0.377
 
 
16.7
 
 
 
 
ARL6IP1
 
 
hsa-miR-130b-3p
 
 
-0.289
 
 
77.1
 
 
 
 
ARL6IP1
 
 
hsa-miR-7-5p
 
 
-0.374
 
 
77.1
 
 
 
 
ARPC3
 
 
hsa-miR-1-3p
 
 
-0.606
 
 
177.5
 
 
 
 
ARPP19
 
 
hsa-let-7e-5p
 
 
-0.492
 
 
43.6
 
 
 
 
ARRDC4
 
 
hsa-let-7e-5p
 
 
-0.329
 
 
32.8
 
 
 
 
AS3MT
 
 
hsa-miR-28-5p
 
 
-0.479
 
 
10.1
 
 
 
 
ASH2L
 
 
hsa-miR-1-3p
 
 
-0.462
 
 
16.9
 
 
 
 
ATF2
 
 
hsa-miR-1-3p
 
 
-0.328
 
 
16.9
 
 
 
 
ATG4A
 
 
hsa-miR-7-5p
 
 
-0.448
 
 
16.3
 
 
 
 
ATP2A2
 
 
hsa-let-7e-5p
 
 
-0.311
 
 
36.0
 
 
 
 
ATPAF1
 
 
hsa-let-7e-5p
 
 
-0.498
 
 
54.8
 
 
 
 
AZIN1
 
 
hsa-miR-1-3p
 
 
-0.438
 
 
127.3
 
 
 
 
B3GAT3
 
 
hsa-let-7e-5p
 
 
-0.324
 
 
33.7
 
 
 
 
B4GAT1
 
 
hsa-let-7e-5p
 
 
-0.47
 
 
24.7
 
 
 
 
BACH1
 
 
hsa-let-7e-5p
 
 
-0.298
 
 
16.2
 
 
 
 
BACH1
 
 
hsa-let-7e-5p
 
 
-0.298
 
 
16.2
 
 
 
 
BACH1
 
 
hsa-let-7e-5p
 
 
-0.435
 
 
16.2
 
 
 
 
BACH1
 
 
hsa-let-7e-5p
 
 
-0.435
 
 
16.2
 
 
 
 
BAHD1
 
 
hsa-miR-130b-3p
 
 
-0.294
 
 
13.2
 
 
 
 
BAZ2A
 
 
hsa-miR-100-5p
 
 
-0.29
 
 
29.1
 
 
 
 
BAZ2A
 
 
hsa-miR-100-5p
 
 
-0.421
 
 
29.1
 
 
 
 
BCAP29
 
 
hsa-let-7e-5p
 
 
-0.289
 
 
16.5
 
 
 
 
BCAT1
 
 
hsa-let-7e-5p
 
 
-0.394
 
 
54.8
 
 
 
 
BEGAIN
 
 
hsa-miR-28-5p
 
 
-0.325
 
 
22.6
 
 
 
 
BEGAIN
 
 
hsa-let-7e-5p
 
 
-0.466
 
 
22.6
 
 
 
 
BET1
 
 
hsa-miR-1-3p
 
 
-0.413
 
 
21.4
 
 
 
 
BHLHE40
 
 
hsa-miR-130b-3p
 
 
-0.351
 
 
75.3
 
 
 
 
BIN3
 
 
hsa-let-7e-5p
 
 
-0.427
 
 
11.6
 
 
 
 
BLCAP
 
 
hsa-miR-130b-3p
 
 
-0.342
 
 
26.5
 
 
 
 
BLOC1S6
 
 
hsa-let-7e-5p
 
 
-0.321
 
 
19.3
 
 
 
 
BRIX1
 
 
hsa-miR-1-3p
 
 
-0.334
 
 
13.0
 
 
 
 
BSCL2
 
 
hsa-miR-1-3p
 
 
-0.471
 
 
75.1
 
 
 
 
BTAF1
 
 
hsa-miR-130b-3p
 
 
-0.291
 
 
15.0
 
 
 
 
BTF3L4
 
 
hsa-let-7e-5p
 
 
-0.355
 
 
36.1
 
 
 
 
BTF3L4
 
 
hsa-miR-130b-3p
 
 
-0.359
 
 
36.1
 
 
 
 
BTG1
 
 
hsa-miR-130b-3p
 
 
-0.565
 
 
18.5
 
 
 
 
BZW1
 
 
hsa-let-7e-5p
 
 
-0.354
 
 
59.0
 
 
 
 
BZW1
 
 
hsa-let-7e-5p
 
 
-0.375
 
 
59.0
 
 
 
 
BZW2
 
 
hsa-let-7e-5p
 
 
-0.408
 
 
31.2
 
 
 
 
C15orf39
 
 
hsa-let-7e-5p
 
 
-0.374
 
 
13.7
 
 
 
 
C2orf88
 
 
hsa-let-7e-5p
 
 
-0.429
 
 
12.0
 
 
 
 
C3orf52
 
 
hsa-let-7e-5p
 
 
-0.296
 
 
17.1
 
 
 
 
C5orf22
 
 
hsa-miR-7-5p
 
 
-0.516
 
 
10.4
 
 
 
 
C6orf120
 
 
hsa-miR-1-3p
 
 
-0.288
 
 
12.1
 
 
 
 
CAAP1
 
 
hsa-miR-1-3p
 
 
-0.48
 
 
10.2
 
 
 
 
CACFD1
 
 
hsa-let-7e-5p
 
 
-0.296
 
 
18.2
 
 
 
 
CALM2
 
 
hsa-miR-130b-3p
 
 
-0.584
 
 
208.7
 
 
 
 
CANT1
 
 
hsa-let-7e-5p
 
 
-0.402
 
 
18.2
 
 
 
 
CAPZA1
 
 
hsa-miR-7-5p
 
 
-0.351
 
 
43.4
 
 
 
 
CASP3
 
 
hsa-let-7e-5p
 
 
-0.495
 
 
11.6
 
 
 
 
CBFA2T3
 
 
hsa-let-7e-5p
 
 
-0.305
 
 
25.8
 
 
 
 
CCNC
 
 
hsa-miR-1-3p
 
 
-0.336
 
 
59.0
 
 
 
 
CCNL2
 
 
hsa-miR-28-5p
 
 
-0.448
 
 
79.7
 
 
 
 
CCNT2
 
 
hsa-miR-7-5p
 
 
-0.349
 
 
18.7
 
 
 
 
CCNY
 
 
hsa-let-7e-5p
 
 
-0.334
 
 
19.4
 
 
 
 
CCNY
 
 
hsa-miR-130b-3p
 
 
-0.372
 
 
19.4
 
 
 
 
CCT4
 
 
hsa-miR-7-5p
 
 
-0.37
 
 
93.4
 
 
 
 
CCT6A
 
 
hsa-miR-130b-3p
 
 
-0.382
 
 
61.8
 
 
 
 
CD164
 
 
hsa-miR-1-3p
 
 
-0.315
 
 
117.1
 
 
 
 
CD2AP
 
 
hsa-miR-130b-3p
 
 
-0.302
 
 
19.1
 
 
 
 
CD2AP
 
 
hsa-miR-1-3p
 
 
-0.479
 
 
19.1
 
 
 
 
CDC34
 
 
hsa-let-7e-5p
 
 
-0.321
 
 
60.2
 
 
 
 
CDC34
 
 
hsa-let-7e-5p
 
 
-0.555
 
 
60.2
 
 
 
 
CDC42SE1
 
 
hsa-miR-1-3p
 
 
-0.36
 
 
35.5
 
 
 
 
CDK14
 
 
hsa-miR-1-3p
 
 
-0.326
 
 
14.3
 
 
 
 
CDK4
 
 
hsa-miR-1-3p
 
 
-0.338
 
 
72.8
 
 
 
 
CDKN1A
 
 
hsa-let-7e-5p
 
 
-0.434
 
 
123.1
 
 
 
 
CDKN2AIP
 
 
hsa-miR-28-5p
 
 
-0.419
 
 
17.4
 
 
 
 
CDV3
 
 
hsa-let-7e-5p
 
 
-0.453
 
 
76.9
 
 
 
 
CEBPD
 
 
hsa-let-7e-5p
 
 
-0.309
 
 
147.6
 
 
 
 
CEMIP2
 
 
hsa-let-7e-5p
 
 
-0.453
 
 
18.2
 
 
 
 
 
 
 placenta 
 
 
 
 
target_symbol
 
 
mature_mirna_id
 
 
score
 
 
nTPM
 
 
 
 
 
 
ABHD2
 
 
hsa-miR-1-3p
 
 
-0.291
 
 
10.4
 
 
 
 
ABHD3
 
 
hsa-miR-130b-3p
 
 
-0.356
 
 
18.6
 
 
 
 
ACBD3
 
 
hsa-miR-130b-3p
 
 
-0.292
 
 
29.8
 
 
 
 
ACBD5
 
 
hsa-miR-130b-3p
 
 
-0.494
 
 
12.6
 
 
 
 
ACP3
 
 
hsa-let-7e-5p
 
 
-0.298
 
 
10.1
 
 
 
 
ACSL4
 
 
hsa-miR-130b-3p
 
 
-0.301
 
 
19.4
 
 
 
 
ACVR1
 
 
hsa-miR-130b-3p
 
 
-0.44
 
 
12.5
 
 
 
 
ADAM12
 
 
hsa-miR-130b-3p
 
 
-0.362
 
 
126.8
 
 
 
 
ADAR
 
 
hsa-miR-1-3p
 
 
-0.35
 
 
43.7
 
 
 
 
ADPGK
 
 
hsa-miR-1-3p
 
 
-0.393
 
 
21.8
 
 
 
 
ADRB1
 
 
hsa-let-7e-5p
 
 
-0.367
 
 
27.4
 
 
 
 
AKIRIN2
 
 
hsa-miR-130b-3p
 
 
-0.315
 
 
17.4
 
 
 
 
ALDH4A1
 
 
hsa-miR-184
 
 
-0.421
 
 
12.4
 
 
 
 
ALG11
 
 
hsa-miR-1-3p
 
 
-0.317
 
 
12.0
 
 
 
 
ANKIB1
 
 
hsa-miR-130b-3p
 
 
-0.329
 
 
11.9
 
 
 
 
ANKRD12
 
 
hsa-miR-7-5p
 
 
-0.37
 
 
13.3
 
 
 
 
ANKRD46
 
 
hsa-let-7e-5p
 
 
-0.389
 
 
19.5
 
 
 
 
ANP32B
 
 
hsa-miR-1-3p
 
 
-0.287
 
 
103.8
 
 
 
 
ANXA2
 
 
hsa-miR-1-3p
 
 
-0.581
 
 
610.8
 
 
 
 
ANXA4
 
 
hsa-miR-1-3p
 
 
-0.539
 
 
49.5
 
 
 
 
AP1AR
 
 
hsa-miR-100-5p
 
 
-0.804
 
 
11.2
 
 
 
 
AP1S1
 
 
hsa-miR-1-3p
 
 
-0.355
 
 
14.9
 
 
 
 
AP1S1
 
 
hsa-let-7e-5p
 
 
-0.374
 
 
14.9
 
 
 
 
APCDD1
 
 
hsa-miR-130b-3p
 
 
-0.304
 
 
25.2
 
 
 
 
APPL1
 
 
hsa-miR-130b-3p
 
 
-0.347
 
 
13.5
 
 
 
 
ARCN1
 
 
hsa-miR-1-3p
 
 
-0.468
 
 
51.0
 
 
 
 
ARF4
 
 
hsa-miR-7-5p
 
 
-0.407
 
 
170.7
 
 
 
 
ARFGEF1
 
 
hsa-miR-130b-3p
 
 
-0.302
 
 
13.6
 
 
 
 
ARHGAP1
 
 
hsa-miR-130b-3p
 
 
-0.376
 
 
36.7
 
 
 
 
ARHGAP12
 
 
hsa-miR-130b-3p
 
 
-0.287
 
 
10.2
 
 
 
 
ARHGAP24
 
 
hsa-miR-130b-3p
 
 
-0.287
 
 
21.0
 
 
 
 
ARHGAP28
 
 
hsa-let-7e-5p
 
 
-0.486
 
 
24.3
 
 
 
 
ARID3A
 
 
hsa-let-7e-5p
 
 
-0.347
 
 
22.0
 
 
 
 
ARID3A
 
 
hsa-let-7e-5p
 
 
-0.348
 
 
22.0
 
 
 
 
ARID3B
 
 
hsa-let-7e-5p
 
 
-0.31
 
 
14.7
 
 
 
 
ARID3B
 
 
hsa-let-7e-5p
 
 
-0.486
 
 
14.7
 
 
 
 
ARL6IP1
 
 
hsa-miR-130b-3p
 
 
-0.289
 
 
84.9
 
 
 
 
ARL6IP1
 
 
hsa-miR-7-5p
 
 
-0.374
 
 
84.9
 
 
 
 
ARL6IP6
 
 
hsa-let-7e-5p
 
 
-0.305
 
 
26.8
 
 
 
 
ARMT1
 
 
hsa-let-7e-5p
 
 
-0.303
 
 
20.8
 
 
 
 
ARPC3
 
 
hsa-miR-1-3p
 
 
-0.606
 
 
259.7
 
 
 
 
ARPP19
 
 
hsa-let-7e-5p
 
 
-0.492
 
 
53.5
 
 
 
 
ARRDC4
 
 
hsa-let-7e-5p
 
 
-0.329
 
 
17.2
 
 
 
 
ASAP1
 
 
hsa-let-7e-5p
 
 
-0.296
 
 
25.9
 
 
 
 
ASH2L
 
 
hsa-miR-1-3p
 
 
-0.462
 
 
20.9
 
 
 
 
ATF2
 
 
hsa-miR-1-3p
 
 
-0.328
 
 
19.1
 
 
 
 
ATP2A2
 
 
hsa-let-7e-5p
 
 
-0.311
 
 
52.0
 
 
 
 
ATPAF1
 
 
hsa-let-7e-5p
 
 
-0.498
 
 
34.1
 
 
 
 
AVPI1
 
 
hsa-miR-7-5p
 
 
-0.367
 
 
15.9
 
 
 
 
AXL
 
 
hsa-miR-1-3p
 
 
-0.304
 
 
32.0
 
 
 
 
AZIN1
 
 
hsa-miR-1-3p
 
 
-0.438
 
 
76.3
 
 
 
 
B3GAT3
 
 
hsa-let-7e-5p
 
 
-0.324
 
 
18.0
 
 
 
 
B4GAT1
 
 
hsa-let-7e-5p
 
 
-0.47
 
 
21.1
 
 
 
 
BACH1
 
 
hsa-let-7e-5p
 
 
-0.298
 
 
47.5
 
 
 
 
BACH1
 
 
hsa-let-7e-5p
 
 
-0.298
 
 
47.5
 
 
 
 
BACH1
 
 
hsa-let-7e-5p
 
 
-0.435
 
 
47.5
 
 
 
 
BACH1
 
 
hsa-let-7e-5p
 
 
-0.435
 
 
47.5
 
 
 
 
BAG4
 
 
hsa-miR-1-3p
 
 
-0.378
 
 
10.7
 
 
 
 
BAZ2A
 
 
hsa-miR-100-5p
 
 
-0.29
 
 
33.2
 
 
 
 
BAZ2A
 
 
hsa-miR-100-5p
 
 
-0.421
 
 
33.2
 
 
 
 
BCAP29
 
 
hsa-let-7e-5p
 
 
-0.289
 
 
25.6
 
 
 
 
BCAT1
 
 
hsa-let-7e-5p
 
 
-0.394
 
 
14.3
 
 
 
 
BET1
 
 
hsa-miR-1-3p
 
 
-0.413
 
 
34.3
 
 
 
 
BHLHE40
 
 
hsa-miR-130b-3p
 
 
-0.351
 
 
67.8
 
 
 
 
BLCAP
 
 
hsa-miR-130b-3p
 
 
-0.342
 
 
71.4
 
 
 
 
BLOC1S6
 
 
hsa-let-7e-5p
 
 
-0.321
 
 
24.7
 
 
 
 
BMP5
 
 
hsa-let-7e-5p
 
 
-0.337
 
 
66.9
 
 
 
 
BMPR2
 
 
hsa-miR-130b-3p
 
 
-0.33
 
 
21.8
 
 
 
 
BMPR2
 
 
hsa-miR-100-5p
 
 
-0.355
 
 
21.8
 
 
 
 
BPNT1
 
 
hsa-miR-1-3p
 
 
-0.329
 
 
12.3
 
 
 
 
BRIX1
 
 
hsa-miR-1-3p
 
 
-0.334
 
 
23.9
 
 
 
 
BTAF1
 
 
hsa-miR-130b-3p
 
 
-0.291
 
 
10.9
 
 
 
 
BTF3L4
 
 
hsa-let-7e-5p
 
 
-0.355
 
 
35.1
 
 
 
 
BTF3L4
 
 
hsa-miR-130b-3p
 
 
-0.359
 
 
35.1
 
 
 
 
BZW1
 
 
hsa-let-7e-5p
 
 
-0.354
 
 
98.8
 
 
 
 
BZW1
 
 
hsa-let-7e-5p
 
 
-0.375
 
 
98.8
 
 
 
 
BZW2
 
 
hsa-let-7e-5p
 
 
-0.408
 
 
75.6
 
 
 
 
C15orf39
 
 
hsa-let-7e-5p
 
 
-0.374
 
 
14.3
 
 
 
 
C1GALT1
 
 
hsa-let-7e-5p
 
 
-0.322
 
 
25.3
 
 
 
 
C6orf120
 
 
hsa-miR-1-3p
 
 
-0.288
 
 
14.8
 
 
 
 
C8orf58
 
 
hsa-let-7e-5p
 
 
-0.612
 
 
15.5
 
 
 
 
CALM2
 
 
hsa-miR-130b-3p
 
 
-0.584
 
 
440.1
 
 
 
 
CAMSAP2
 
 
hsa-miR-130b-3p
 
 
-0.297
 
 
17.8
 
 
 
 
CANT1
 
 
hsa-let-7e-5p
 
 
-0.402
 
 
24.4
 
 
 
 
CAPZA1
 
 
hsa-miR-7-5p
 
 
-0.351
 
 
80.3
 
 
 
 
CASD1
 
 
hsa-miR-130b-3p
 
 
-0.305
 
 
11.6
 
 
 
 
CASP3
 
 
hsa-let-7e-5p
 
 
-0.495
 
 
23.2
 
 
 
 
CBX5
 
 
hsa-let-7e-5p
 
 
-0.307
 
 
11.5
 
 
 
 
CBX5
 
 
hsa-let-7e-5p
 
 
-0.472
 
 
11.5
 
 
 
 
CCDC43
 
 
hsa-miR-7-5p
 
 
-0.331
 
 
13.1
 
 
 
 
CCNC
 
 
hsa-miR-1-3p
 
 
-0.336
 
 
33.5
 
 
 
 
CCNL2
 
 
hsa-miR-28-5p
 
 
-0.448
 
 
31.9
 
 
 
 
CCNY
 
 
hsa-let-7e-5p
 
 
-0.334
 
 
36.4
 
 
 
 
CCNY
 
 
hsa-miR-130b-3p
 
 
-0.372
 
 
36.4
 
 
 
 
CCSAP
 
 
hsa-miR-1-3p
 
 
-0.381
 
 
12.3
 
 
 
 
CCT4
 
 
hsa-miR-7-5p
 
 
-0.37
 
 
61.9
 
 
 
 
CCT6A
 
 
hsa-miR-130b-3p
 
 
-0.382
 
 
54.0
 
 
 
 
CD164
 
 
hsa-miR-1-3p
 
 
-0.315
 
 
178.7
 
 
 
 
CD2AP
 
 
hsa-miR-130b-3p
 
 
-0.302
 
 
20.3
 
 
 
 
CD2AP
 
 
hsa-miR-1-3p
 
 
-0.479
 
 
20.3
 
 
 
 
 
 
 seminal vesicle 
 
 
 
 
target_symbol
 
 
mature_mirna_id
 
 
score
 
 
nTPM
 
 
 
 
 
 
ABHD2
 
 
hsa-miR-1-3p
 
 
-0.291
 
 
17.5
 
 
 
 
ACBD3
 
 
hsa-miR-130b-3p
 
 
-0.292
 
 
15.4
 
 
 
 
ACBD5
 
 
hsa-miR-130b-3p
 
 
-0.494
 
 
10.8
 
 
 
 
ACSL4
 
 
hsa-miR-130b-3p
 
 
-0.301
 
 
14.0
 
 
 
 
ACSL6
 
 
hsa-let-7e-5p
 
 
-0.336
 
 
17.0
 
 
 
 
ACTC1
 
 
hsa-miR-7-5p
 
 
-0.471
 
 
506.6
 
 
 
 
ACVR1
 
 
hsa-miR-130b-3p
 
 
-0.44
 
 
11.3
 
 
 
 
ADAR
 
 
hsa-miR-1-3p
 
 
-0.35
 
 
40.5
 
 
 
 
ADGRA3
 
 
hsa-miR-1-3p
 
 
-0.351
 
 
10.8
 
 
 
 
AKIRIN2
 
 
hsa-miR-130b-3p
 
 
-0.315
 
 
11.0
 
 
 
 
ALDH4A1
 
 
hsa-miR-184
 
 
-0.421
 
 
25.6
 
 
 
 
ALG11
 
 
hsa-miR-1-3p
 
 
-0.317
 
 
10.3
 
 
 
 
ALG9
 
 
hsa-miR-7-5p
 
 
-0.302
 
 
10.6
 
 
 
 
ANKIB1
 
 
hsa-miR-130b-3p
 
 
-0.329
 
 
10.5
 
 
 
 
ANKRD12
 
 
hsa-miR-7-5p
 
 
-0.37
 
 
10.0
 
 
 
 
ANKRD46
 
 
hsa-let-7e-5p
 
 
-0.389
 
 
12.4
 
 
 
 
ANP32B
 
 
hsa-miR-1-3p
 
 
-0.287
 
 
67.2
 
 
 
 
ANXA2
 
 
hsa-miR-1-3p
 
 
-0.581
 
 
364.0
 
 
 
 
ANXA4
 
 
hsa-miR-1-3p
 
 
-0.539
 
 
49.1
 
 
 
 
APCDD1
 
 
hsa-miR-130b-3p
 
 
-0.304
 
 
13.3
 
 
 
 
APPL1
 
 
hsa-miR-130b-3p
 
 
-0.347
 
 
13.5
 
 
 
 
ARCN1
 
 
hsa-miR-1-3p
 
 
-0.468
 
 
58.6
 
 
 
 
ARF4
 
 
hsa-miR-7-5p
 
 
-0.407
 
 
125.1
 
 
 
 
ARFGEF1
 
 
hsa-miR-130b-3p
 
 
-0.302
 
 
11.4
 
 
 
 
ARHGAP1
 
 
hsa-miR-130b-3p
 
 
-0.376
 
 
58.9
 
 
 
 
ARL4D
 
 
hsa-let-7e-5p
 
 
-0.417
 
 
14.3
 
 
 
 
ARL6IP1
 
 
hsa-miR-130b-3p
 
 
-0.289
 
 
66.4
 
 
 
 
ARL6IP1
 
 
hsa-miR-7-5p
 
 
-0.374
 
 
66.4
 
 
 
 
ARMT1
 
 
hsa-let-7e-5p
 
 
-0.303
 
 
10.5
 
 
 
 
ARPC3
 
 
hsa-miR-1-3p
 
 
-0.606
 
 
143.2
 
 
 
 
ARPP19
 
 
hsa-let-7e-5p
 
 
-0.492
 
 
38.5
 
 
 
 
ARRDC4
 
 
hsa-let-7e-5p
 
 
-0.329
 
 
15.4
 
 
 
 
ASAP1
 
 
hsa-let-7e-5p
 
 
-0.296
 
 
10.7
 
 
 
 
ASH2L
 
 
hsa-miR-1-3p
 
 
-0.462
 
 
19.9
 
 
 
 
ATF2
 
 
hsa-miR-1-3p
 
 
-0.328
 
 
16.1
 
 
 
 
ATP2A2
 
 
hsa-let-7e-5p
 
 
-0.311
 
 
66.0
 
 
 
 
ATPAF1
 
 
hsa-let-7e-5p
 
 
-0.498
 
 
31.8
 
 
 
 
AVPI1
 
 
hsa-miR-7-5p
 
 
-0.367
 
 
15.6
 
 
 
 
AXL
 
 
hsa-miR-1-3p
 
 
-0.304
 
 
23.1
 
 
 
 
AZIN1
 
 
hsa-miR-1-3p
 
 
-0.438
 
 
46.0
 
 
 
 
B3GAT3
 
 
hsa-let-7e-5p
 
 
-0.324
 
 
17.2
 
 
 
 
B4GAT1
 
 
hsa-let-7e-5p
 
 
-0.47
 
 
21.2
 
 
 
 
BAZ2A
 
 
hsa-miR-100-5p
 
 
-0.29
 
 
19.8
 
 
 
 
BAZ2A
 
 
hsa-miR-100-5p
 
 
-0.421
 
 
19.8
 
 
 
 
BCAP29
 
 
hsa-let-7e-5p
 
 
-0.289
 
 
24.0
 
 
 
 
BET1
 
 
hsa-miR-1-3p
 
 
-0.413
 
 
19.9
 
 
 
 
BHLHE40
 
 
hsa-miR-130b-3p
 
 
-0.351
 
 
30.6
 
 
 
 
BLCAP
 
 
hsa-miR-130b-3p
 
 
-0.342
 
 
53.1
 
 
 
 
BLOC1S6
 
 
hsa-let-7e-5p
 
 
-0.321
 
 
19.2
 
 
 
 
BMPR2
 
 
hsa-miR-130b-3p
 
 
-0.33
 
 
10.5
 
 
 
 
BMPR2
 
 
hsa-miR-100-5p
 
 
-0.355
 
 
10.5
 
 
 
 
BPNT1
 
 
hsa-miR-1-3p
 
 
-0.329
 
 
11.2
 
 
 
 
BRIX1
 
 
hsa-miR-1-3p
 
 
-0.334
 
 
13.6
 
 
 
 
BSCL2
 
 
hsa-miR-1-3p
 
 
-0.471
 
 
27.8
 
 
 
 
BTAF1
 
 
hsa-miR-130b-3p
 
 
-0.291
 
 
10.1
 
 
 
 
BTF3L4
 
 
hsa-let-7e-5p
 
 
-0.355
 
 
23.0
 
 
 
 
BTF3L4
 
 
hsa-miR-130b-3p
 
 
-0.359
 
 
23.0
 
 
 
 
BZW1
 
 
hsa-let-7e-5p
 
 
-0.354
 
 
58.1
 
 
 
 
BZW1
 
 
hsa-let-7e-5p
 
 
-0.375
 
 
58.1
 
 
 
 
BZW2
 
 
hsa-let-7e-5p
 
 
-0.408
 
 
32.3
 
 
 
 
C6orf120
 
 
hsa-miR-1-3p
 
 
-0.288
 
 
10.2
 
 
 
 
CALM2
 
 
hsa-miR-130b-3p
 
 
-0.584
 
 
528.5
 
 
 
 
CAMSAP2
 
 
hsa-miR-130b-3p
 
 
-0.297
 
 
12.6
 
 
 
 
CANT1
 
 
hsa-let-7e-5p
 
 
-0.402
 
 
30.5
 
 
 
 
CAPZA1
 
 
hsa-miR-7-5p
 
 
-0.351
 
 
35.2
 
 
 
 
CBX5
 
 
hsa-let-7e-5p
 
 
-0.307
 
 
11.1
 
 
 
 
CBX5
 
 
hsa-let-7e-5p
 
 
-0.472
 
 
11.1
 
 
 
 
CCNC
 
 
hsa-miR-1-3p
 
 
-0.336
 
 
21.9
 
 
 
 
CCNL2
 
 
hsa-miR-28-5p
 
 
-0.448
 
 
29.7
 
 
 
 
CCNY
 
 
hsa-let-7e-5p
 
 
-0.334
 
 
19.0
 
 
 
 
CCNY
 
 
hsa-miR-130b-3p
 
 
-0.372
 
 
19.0
 
 
 
 
CCT4
 
 
hsa-miR-7-5p
 
 
-0.37
 
 
54.1
 
 
 
 
CCT6A
 
 
hsa-miR-130b-3p
 
 
-0.382
 
 
47.3
 
 
 
 
CD164
 
 
hsa-miR-1-3p
 
 
-0.315
 
 
106.2
 
 
 
 
CD2AP
 
 
hsa-miR-130b-3p
 
 
-0.302
 
 
12.8
 
 
 
 
CD2AP
 
 
hsa-miR-1-3p
 
 
-0.479
 
 
12.8
 
 
 
 
CDC34
 
 
hsa-let-7e-5p
 
 
-0.321
 
 
18.2
 
 
 
 
CDC34
 
 
hsa-let-7e-5p
 
 
-0.555
 
 
18.2
 
 
 
 
CDC42SE1
 
 
hsa-miR-1-3p
 
 
-0.36
 
 
31.3
 
 
 
 
CDK4
 
 
hsa-miR-1-3p
 
 
-0.338
 
 
51.0
 
 
 
 
CDKN1A
 
 
hsa-let-7e-5p
 
 
-0.434
 
 
27.7
 
 
 
 
CDKN2AIP
 
 
hsa-miR-28-5p
 
 
-0.419
 
 
12.2
 
 
 
 
CDV3
 
 
hsa-let-7e-5p
 
 
-0.453
 
 
44.8
 
 
 
 
CEBPD
 
 
hsa-let-7e-5p
 
 
-0.309
 
 
18.3
 
 
 
 
CERCAM
 
 
hsa-let-7e-5p
 
 
-0.344
 
 
21.4
 
 
 
 
CERS2
 
 
hsa-miR-1-3p
 
 
-0.287
 
 
77.8
 
 
 
 
CGGBP1
 
 
hsa-miR-7-5p
 
 
-0.465
 
 
33.2
 
 
 
 
CHMP3
 
 
hsa-miR-130b-3p
 
 
-0.521
 
 
58.3
 
 
 
 
CHMP4B
 
 
hsa-miR-130b-3p
 
 
-0.292
 
 
77.7
 
 
 
 
CITED2
 
 
hsa-miR-1-3p
 
 
-0.37
 
 
40.4
 
 
 
 
CKAP4
 
 
hsa-miR-7-5p
 
 
-0.443
 
 
34.7
 
 
 
 
CLASP2
 
 
hsa-let-7e-5p
 
 
-0.344
 
 
19.6
 
 
 
 
CLCN3
 
 
hsa-miR-1-3p
 
 
-0.339
 
 
15.9
 
 
 
 
CLCN3
 
 
hsa-miR-130b-3p
 
 
-0.384
 
 
15.9
 
 
 
 
CLDN12
 
 
hsa-let-7e-5p
 
 
-0.511
 
 
11.7
 
 
 
 
CLIP1
 
 
hsa-miR-130b-3p
 
 
-0.452
 
 
41.8
 
 
 
 
CLTC
 
 
hsa-miR-1-3p
 
 
-0.39
 
 
70.7
 
 
 
 
CMPK1
 
 
hsa-miR-130b-3p
 
 
-0.384
 
 
48.9
 
 
 
 
CNIH1
 
 
hsa-miR-130b-3p
 
 
-0.335
 
 
53.7
 
 
 
 
CNN3
 
 
hsa-miR-1-3p
 
 
-0.414
 
 
125.9
 
 
 
 
 
 
 smooth muscle 
 
 
 
 
target_symbol
 
 
mature_mirna_id
 
 
score
 
 
nTPM
 
 
 
 
 
 
ABHD2
 
 
hsa-miR-1-3p
 
 
-0.291
 
 
21.0
 
 
 
 
ACBD3
 
 
hsa-miR-130b-3p
 
 
-0.292
 
 
20.3
 
 
 
 
ACSL4
 
 
hsa-miR-130b-3p
 
 
-0.301
 
 
19.9
 
 
 
 
ACVR1
 
 
hsa-miR-130b-3p
 
 
-0.44
 
 
26.8
 
 
 
 
ADAR
 
 
hsa-miR-1-3p
 
 
-0.35
 
 
52.8
 
 
 
 
ADGRA3
 
 
hsa-miR-1-3p
 
 
-0.351
 
 
13.1
 
 
 
 
ADPGK
 
 
hsa-miR-1-3p
 
 
-0.393
 
 
11.1
 
 
 
 
AKIRIN2
 
 
hsa-miR-130b-3p
 
 
-0.315
 
 
15.3
 
 
 
 
ALG11
 
 
hsa-miR-1-3p
 
 
-0.317
 
 
10.7
 
 
 
 
ALG9
 
 
hsa-miR-7-5p
 
 
-0.302
 
 
10.4
 
 
 
 
ANKIB1
 
 
hsa-miR-130b-3p
 
 
-0.329
 
 
12.7
 
 
 
 
ANKRD29
 
 
hsa-miR-1-3p
 
 
-0.426
 
 
10.9
 
 
 
 
ANKRD46
 
 
hsa-let-7e-5p
 
 
-0.389
 
 
11.6
 
 
 
 
ANP32B
 
 
hsa-miR-1-3p
 
 
-0.287
 
 
72.9
 
 
 
 
ANXA2
 
 
hsa-miR-1-3p
 
 
-0.581
 
 
397.2
 
 
 
 
ANXA4
 
 
hsa-miR-1-3p
 
 
-0.539
 
 
48.5
 
 
 
 
AP1S1
 
 
hsa-miR-1-3p
 
 
-0.355
 
 
11.9
 
 
 
 
AP1S1
 
 
hsa-let-7e-5p
 
 
-0.374
 
 
11.9
 
 
 
 
APPL1
 
 
hsa-miR-130b-3p
 
 
-0.347
 
 
14.3
 
 
 
 
ARCN1
 
 
hsa-miR-1-3p
 
 
-0.468
 
 
66.1
 
 
 
 
ARF4
 
 
hsa-miR-7-5p
 
 
-0.407
 
 
157.9
 
 
 
 
ARFGEF1
 
 
hsa-miR-130b-3p
 
 
-0.302
 
 
10.1
 
 
 
 
ARHGAP1
 
 
hsa-miR-130b-3p
 
 
-0.376
 
 
75.2
 
 
 
 
ARHGAP24
 
 
hsa-miR-130b-3p
 
 
-0.287
 
 
13.1
 
 
 
 
ARL4D
 
 
hsa-let-7e-5p
 
 
-0.417
 
 
13.8
 
 
 
 
ARL6IP1
 
 
hsa-miR-130b-3p
 
 
-0.289
 
 
64.5
 
 
 
 
ARL6IP1
 
 
hsa-miR-7-5p
 
 
-0.374
 
 
64.5
 
 
 
 
ARL6IP6
 
 
hsa-let-7e-5p
 
 
-0.305
 
 
10.8
 
 
 
 
ARMT1
 
 
hsa-let-7e-5p
 
 
-0.303
 
 
13.9
 
 
 
 
ARPC3
 
 
hsa-miR-1-3p
 
 
-0.606
 
 
192.8
 
 
 
 
ARPP19
 
 
hsa-let-7e-5p
 
 
-0.492
 
 
54.2
 
 
 
 
ARRDC4
 
 
hsa-let-7e-5p
 
 
-0.329
 
 
16.9
 
 
 
 
ASAP1
 
 
hsa-let-7e-5p
 
 
-0.296
 
 
16.7
 
 
 
 
ASH2L
 
 
hsa-miR-1-3p
 
 
-0.462
 
 
22.6
 
 
 
 
ATF2
 
 
hsa-miR-1-3p
 
 
-0.328
 
 
16.2
 
 
 
 
ATOSB
 
 
hsa-let-7e-5p
 
 
-0.429
 
 
10.3
 
 
 
 
ATP2A2
 
 
hsa-let-7e-5p
 
 
-0.311
 
 
69.4
 
 
 
 
ATPAF1
 
 
hsa-let-7e-5p
 
 
-0.498
 
 
30.4
 
 
 
 
AXL
 
 
hsa-miR-1-3p
 
 
-0.304
 
 
34.7
 
 
 
 
AZIN1
 
 
hsa-miR-1-3p
 
 
-0.438
 
 
41.1
 
 
 
 
B3GAT3
 
 
hsa-let-7e-5p
 
 
-0.324
 
 
13.1
 
 
 
 
B4GAT1
 
 
hsa-let-7e-5p
 
 
-0.47
 
 
13.1
 
 
 
 
BACH1
 
 
hsa-let-7e-5p
 
 
-0.298
 
 
14.9
 
 
 
 
BACH1
 
 
hsa-let-7e-5p
 
 
-0.298
 
 
14.9
 
 
 
 
BACH1
 
 
hsa-let-7e-5p
 
 
-0.435
 
 
14.9
 
 
 
 
BACH1
 
 
hsa-let-7e-5p
 
 
-0.435
 
 
14.9
 
 
 
 
BAZ2A
 
 
hsa-miR-100-5p
 
 
-0.29
 
 
21.6
 
 
 
 
BAZ2A
 
 
hsa-miR-100-5p
 
 
-0.421
 
 
21.6
 
 
 
 
BCAP29
 
 
hsa-let-7e-5p
 
 
-0.289
 
 
36.5
 
 
 
 
BET1
 
 
hsa-miR-1-3p
 
 
-0.413
 
 
14.2
 
 
 
 
BHLHE40
 
 
hsa-miR-130b-3p
 
 
-0.351
 
 
60.9
 
 
 
 
BLCAP
 
 
hsa-miR-130b-3p
 
 
-0.342
 
 
31.7
 
 
 
 
BLOC1S6
 
 
hsa-let-7e-5p
 
 
-0.321
 
 
22.7
 
 
 
 
BMPR2
 
 
hsa-miR-130b-3p
 
 
-0.33
 
 
15.4
 
 
 
 
BMPR2
 
 
hsa-miR-100-5p
 
 
-0.355
 
 
15.4
 
 
 
 
BPNT1
 
 
hsa-miR-1-3p
 
 
-0.329
 
 
17.0
 
 
 
 
BRIX1
 
 
hsa-miR-1-3p
 
 
-0.334
 
 
15.0
 
 
 
 
BSCL2
 
 
hsa-miR-1-3p
 
 
-0.471
 
 
12.2
 
 
 
 
BTAF1
 
 
hsa-miR-130b-3p
 
 
-0.291
 
 
11.0
 
 
 
 
BTF3L4
 
 
hsa-let-7e-5p
 
 
-0.355
 
 
32.5
 
 
 
 
BTF3L4
 
 
hsa-miR-130b-3p
 
 
-0.359
 
 
32.5
 
 
 
 
BZW1
 
 
hsa-let-7e-5p
 
 
-0.354
 
 
74.7
 
 
 
 
BZW1
 
 
hsa-let-7e-5p
 
 
-0.375
 
 
74.7
 
 
 
 
BZW2
 
 
hsa-let-7e-5p
 
 
-0.408
 
 
36.4
 
 
 
 
C6orf120
 
 
hsa-miR-1-3p
 
 
-0.288
 
 
10.6
 
 
 
 
CALM2
 
 
hsa-miR-130b-3p
 
 
-0.584
 
 
592.7
 
 
 
 
CAMSAP2
 
 
hsa-miR-130b-3p
 
 
-0.297
 
 
15.1
 
 
 
 
CANT1
 
 
hsa-let-7e-5p
 
 
-0.402
 
 
14.5
 
 
 
 
CAPZA1
 
 
hsa-miR-7-5p
 
 
-0.351
 
 
59.7
 
 
 
 
CASD1
 
 
hsa-miR-130b-3p
 
 
-0.305
 
 
10.2
 
 
 
 
CASP3
 
 
hsa-let-7e-5p
 
 
-0.495
 
 
12.5
 
 
 
 
CBX5
 
 
hsa-let-7e-5p
 
 
-0.307
 
 
10.1
 
 
 
 
CBX5
 
 
hsa-let-7e-5p
 
 
-0.472
 
 
10.1
 
 
 
 
CCDC43
 
 
hsa-miR-7-5p
 
 
-0.331
 
 
10.0
 
 
 
 
CCNC
 
 
hsa-miR-1-3p
 
 
-0.336
 
 
20.5
 
 
 
 
CCNL2
 
 
hsa-miR-28-5p
 
 
-0.448
 
 
24.6
 
 
 
 
CCNY
 
 
hsa-let-7e-5p
 
 
-0.334
 
 
24.0
 
 
 
 
CCNY
 
 
hsa-miR-130b-3p
 
 
-0.372
 
 
24.0
 
 
 
 
CCT4
 
 
hsa-miR-7-5p
 
 
-0.37
 
 
67.2
 
 
 
 
CCT6A
 
 
hsa-miR-130b-3p
 
 
-0.382
 
 
58.0
 
 
 
 
CD164
 
 
hsa-miR-1-3p
 
 
-0.315
 
 
108.4
 
 
 
 
CD2AP
 
 
hsa-miR-130b-3p
 
 
-0.302
 
 
10.2
 
 
 
 
CD2AP
 
 
hsa-miR-1-3p
 
 
-0.479
 
 
10.2
 
 
 
 
CD69
 
 
hsa-miR-130b-3p
 
 
-0.451
 
 
13.3
 
 
 
 
CDC34
 
 
hsa-let-7e-5p
 
 
-0.321
 
 
17.3
 
 
 
 
CDC34
 
 
hsa-let-7e-5p
 
 
-0.555
 
 
17.3
 
 
 
 
CDC42SE1
 
 
hsa-miR-1-3p
 
 
-0.36
 
 
34.8
 
 
 
 
CDK4
 
 
hsa-miR-1-3p
 
 
-0.338
 
 
75.0
 
 
 
 
CDKN1A
 
 
hsa-let-7e-5p
 
 
-0.434
 
 
106.7
 
 
 
 
CDV3
 
 
hsa-let-7e-5p
 
 
-0.453
 
 
68.4
 
 
 
 
CEBPD
 
 
hsa-let-7e-5p
 
 
-0.309
 
 
20.2
 
 
 
 
CEMIP2
 
 
hsa-let-7e-5p
 
 
-0.453
 
 
10.9
 
 
 
 
CERCAM
 
 
hsa-let-7e-5p
 
 
-0.344
 
 
44.2
 
 
 
 
CERS2
 
 
hsa-miR-1-3p
 
 
-0.287
 
 
92.6
 
 
 
 
CGGBP1
 
 
hsa-miR-7-5p
 
 
-0.465
 
 
41.4
 
 
 
 
CHMP3
 
 
hsa-miR-130b-3p
 
 
-0.521
 
 
68.5
 
 
 
 
CHMP4B
 
 
hsa-miR-130b-3p
 
 
-0.292
 
 
71.3
 
 
 
 
CHUK
 
 
hsa-let-7e-5p
 
 
-0.327
 
 
11.0
 
 
 
 
CITED2
 
 
hsa-miR-1-3p
 
 
-0.37
 
 
76.5
 
 
 
 
CKAP4
 
 
hsa-miR-7-5p
 
 
-0.443
 
 
43.6
 
 
 
 
 
 
 thyroid gland 
 
 
 
 
target_symbol
 
 
mature_mirna_id
 
 
score
 
 
nTPM
 
 
 
 
 
 
ABHD2
 
 
hsa-miR-1-3p
 
 
-0.291
 
 
16.0
 
 
 
 
ABHD3
 
 
hsa-miR-130b-3p
 
 
-0.356
 
 
21.4
 
 
 
 
ABRAXAS2
 
 
hsa-miR-130b-3p
 
 
-0.468
 
 
11.9
 
 
 
 
ACBD3
 
 
hsa-miR-130b-3p
 
 
-0.292
 
 
26.9
 
 
 
 
ACBD5
 
 
hsa-miR-130b-3p
 
 
-0.494
 
 
26.0
 
 
 
 
ACSL4
 
 
hsa-miR-130b-3p
 
 
-0.301
 
 
17.2
 
 
 
 
ACVR1
 
 
hsa-miR-130b-3p
 
 
-0.44
 
 
28.6
 
 
 
 
ADAR
 
 
hsa-miR-1-3p
 
 
-0.35
 
 
44.2
 
 
 
 
ADGRA3
 
 
hsa-miR-1-3p
 
 
-0.351
 
 
23.6
 
 
 
 
ADPGK
 
 
hsa-miR-1-3p
 
 
-0.393
 
 
11.3
 
 
 
 
AHCTF1
 
 
hsa-let-7e-5p
 
 
-0.374
 
 
12.9
 
 
 
 
AIFM1
 
 
hsa-let-7e-5p
 
 
-0.314
 
 
10.7
 
 
 
 
AKIRIN2
 
 
hsa-miR-130b-3p
 
 
-0.315
 
 
15.5
 
 
 
 
ALG11
 
 
hsa-miR-1-3p
 
 
-0.317
 
 
15.7
 
 
 
 
ALG9
 
 
hsa-miR-7-5p
 
 
-0.302
 
 
11.5
 
 
 
 
ANKIB1
 
 
hsa-miR-130b-3p
 
 
-0.329
 
 
16.1
 
 
 
 
ANKRD12
 
 
hsa-miR-7-5p
 
 
-0.37
 
 
15.3
 
 
 
 
ANKRD46
 
 
hsa-let-7e-5p
 
 
-0.389
 
 
18.8
 
 
 
 
ANP32B
 
 
hsa-miR-1-3p
 
 
-0.287
 
 
92.0
 
 
 
 
ANXA2
 
 
hsa-miR-1-3p
 
 
-0.581
 
 
309.0
 
 
 
 
ANXA4
 
 
hsa-miR-1-3p
 
 
-0.539
 
 
40.9
 
 
 
 
AP1AR
 
 
hsa-miR-100-5p
 
 
-0.804
 
 
10.1
 
 
 
 
AP1S1
 
 
hsa-miR-1-3p
 
 
-0.355
 
 
15.7
 
 
 
 
AP1S1
 
 
hsa-let-7e-5p
 
 
-0.374
 
 
15.7
 
 
 
 
APPL1
 
 
hsa-miR-130b-3p
 
 
-0.347
 
 
18.5
 
 
 
 
ARCN1
 
 
hsa-miR-1-3p
 
 
-0.468
 
 
60.9
 
 
 
 
ARF4
 
 
hsa-miR-7-5p
 
 
-0.407
 
 
134.8
 
 
 
 
ARFGEF1
 
 
hsa-miR-130b-3p
 
 
-0.302
 
 
20.9
 
 
 
 
ARG2
 
 
hsa-let-7e-5p
 
 
-0.468
 
 
97.9
 
 
 
 
ARHGAP1
 
 
hsa-miR-130b-3p
 
 
-0.376
 
 
42.2
 
 
 
 
ARHGAP12
 
 
hsa-miR-130b-3p
 
 
-0.287
 
 
15.0
 
 
 
 
ARHGAP24
 
 
hsa-miR-130b-3p
 
 
-0.287
 
 
26.1
 
 
 
 
ARL4D
 
 
hsa-let-7e-5p
 
 
-0.417
 
 
35.9
 
 
 
 
ARL6IP1
 
 
hsa-miR-130b-3p
 
 
-0.289
 
 
105.4
 
 
 
 
ARL6IP1
 
 
hsa-miR-7-5p
 
 
-0.374
 
 
105.4
 
 
 
 
ARMT1
 
 
hsa-let-7e-5p
 
 
-0.303
 
 
25.9
 
 
 
 
ARPC3
 
 
hsa-miR-1-3p
 
 
-0.606
 
 
172.4
 
 
 
 
ARPP19
 
 
hsa-let-7e-5p
 
 
-0.492
 
 
53.7
 
 
 
 
ARRDC4
 
 
hsa-let-7e-5p
 
 
-0.329
 
 
16.2
 
 
 
 
ASAP1
 
 
hsa-let-7e-5p
 
 
-0.296
 
 
15.1
 
 
 
 
ASH2L
 
 
hsa-miR-1-3p
 
 
-0.462
 
 
19.8
 
 
 
 
ATF2
 
 
hsa-miR-1-3p
 
 
-0.328
 
 
29.6
 
 
 
 
ATG16L1
 
 
hsa-miR-130b-3p
 
 
-0.407
 
 
11.5
 
 
 
 
ATG4A
 
 
hsa-miR-7-5p
 
 
-0.448
 
 
10.7
 
 
 
 
ATOSB
 
 
hsa-let-7e-5p
 
 
-0.429
 
 
11.1
 
 
 
 
ATP2A2
 
 
hsa-let-7e-5p
 
 
-0.311
 
 
68.6
 
 
 
 
ATPAF1
 
 
hsa-let-7e-5p
 
 
-0.498
 
 
39.3
 
 
 
 
AXL
 
 
hsa-miR-1-3p
 
 
-0.304
 
 
15.7
 
 
 
 
AZIN1
 
 
hsa-miR-1-3p
 
 
-0.438
 
 
62.4
 
 
 
 
B3GAT3
 
 
hsa-let-7e-5p
 
 
-0.324
 
 
33.1
 
 
 
 
B4GAT1
 
 
hsa-let-7e-5p
 
 
-0.47
 
 
20.5
 
 
 
 
BACH1
 
 
hsa-let-7e-5p
 
 
-0.298
 
 
11.8
 
 
 
 
BACH1
 
 
hsa-let-7e-5p
 
 
-0.298
 
 
11.8
 
 
 
 
BACH1
 
 
hsa-let-7e-5p
 
 
-0.435
 
 
11.8
 
 
 
 
BACH1
 
 
hsa-let-7e-5p
 
 
-0.435
 
 
11.8
 
 
 
 
BAHD1
 
 
hsa-miR-130b-3p
 
 
-0.294
 
 
15.6
 
 
 
 
BAZ2A
 
 
hsa-miR-100-5p
 
 
-0.29
 
 
25.4
 
 
 
 
BAZ2A
 
 
hsa-miR-100-5p
 
 
-0.421
 
 
25.4
 
 
 
 
BCAP29
 
 
hsa-let-7e-5p
 
 
-0.289
 
 
81.1
 
 
 
 
BET1
 
 
hsa-miR-1-3p
 
 
-0.413
 
 
20.7
 
 
 
 
BHLHE40
 
 
hsa-miR-130b-3p
 
 
-0.351
 
 
88.0
 
 
 
 
BIN3
 
 
hsa-let-7e-5p
 
 
-0.427
 
 
14.4
 
 
 
 
BLCAP
 
 
hsa-miR-130b-3p
 
 
-0.342
 
 
44.2
 
 
 
 
BLOC1S6
 
 
hsa-let-7e-5p
 
 
-0.321
 
 
41.6
 
 
 
 
BLTP3B
 
 
hsa-miR-1-3p
 
 
-0.29
 
 
10.2
 
 
 
 
BMPR2
 
 
hsa-miR-130b-3p
 
 
-0.33
 
 
13.2
 
 
 
 
BMPR2
 
 
hsa-miR-100-5p
 
 
-0.355
 
 
13.2
 
 
 
 
BPNT1
 
 
hsa-miR-1-3p
 
 
-0.329
 
 
19.5
 
 
 
 
BRIX1
 
 
hsa-miR-1-3p
 
 
-0.334
 
 
14.6
 
 
 
 
BSCL2
 
 
hsa-miR-1-3p
 
 
-0.471
 
 
33.1
 
 
 
 
BTAF1
 
 
hsa-miR-130b-3p
 
 
-0.291
 
 
14.3
 
 
 
 
BTF3L4
 
 
hsa-let-7e-5p
 
 
-0.355
 
 
40.3
 
 
 
 
BTF3L4
 
 
hsa-miR-130b-3p
 
 
-0.359
 
 
40.3
 
 
 
 
BZW1
 
 
hsa-let-7e-5p
 
 
-0.354
 
 
69.6
 
 
 
 
BZW1
 
 
hsa-let-7e-5p
 
 
-0.375
 
 
69.6
 
 
 
 
BZW2
 
 
hsa-let-7e-5p
 
 
-0.408
 
 
34.2
 
 
 
 
C15orf39
 
 
hsa-let-7e-5p
 
 
-0.374
 
 
10.6
 
 
 
 
C1GALT1
 
 
hsa-let-7e-5p
 
 
-0.322
 
 
11.8
 
 
 
 
CAAP1
 
 
hsa-miR-1-3p
 
 
-0.48
 
 
25.7
 
 
 
 
CACFD1
 
 
hsa-let-7e-5p
 
 
-0.296
 
 
16.5
 
 
 
 
CALM2
 
 
hsa-miR-130b-3p
 
 
-0.584
 
 
415.1
 
 
 
 
CAMSAP2
 
 
hsa-miR-130b-3p
 
 
-0.297
 
 
18.5
 
 
 
 
CANT1
 
 
hsa-let-7e-5p
 
 
-0.402
 
 
12.7
 
 
 
 
CAPN3
 
 
hsa-let-7e-5p
 
 
-0.317
 
 
12.0
 
 
 
 
CAPZA1
 
 
hsa-miR-7-5p
 
 
-0.351
 
 
45.0
 
 
 
 
CARNMT1
 
 
hsa-let-7e-5p
 
 
-0.307
 
 
12.7
 
 
 
 
CASD1
 
 
hsa-miR-130b-3p
 
 
-0.305
 
 
15.0
 
 
 
 
CBX5
 
 
hsa-let-7e-5p
 
 
-0.307
 
 
22.4
 
 
 
 
CBX5
 
 
hsa-let-7e-5p
 
 
-0.472
 
 
22.4
 
 
 
 
CCDC43
 
 
hsa-miR-7-5p
 
 
-0.331
 
 
12.7
 
 
 
 
CCNC
 
 
hsa-miR-1-3p
 
 
-0.336
 
 
56.7
 
 
 
 
CCNL2
 
 
hsa-miR-28-5p
 
 
-0.448
 
 
74.9
 
 
 
 
CCNT2
 
 
hsa-miR-7-5p
 
 
-0.349
 
 
13.7
 
 
 
 
CCNY
 
 
hsa-let-7e-5p
 
 
-0.334
 
 
27.8
 
 
 
 
CCNY
 
 
hsa-miR-130b-3p
 
 
-0.372
 
 
27.8
 
 
 
 
CCT4
 
 
hsa-miR-7-5p
 
 
-0.37
 
 
61.1
 
 
 
 
CCT6A
 
 
hsa-miR-130b-3p
 
 
-0.382
 
 
47.1
 
 
 
 
CD164
 
 
hsa-miR-1-3p
 
 
-0.315
 
 
337.3
 
 
 
 
CD2AP
 
 
hsa-miR-130b-3p
 
 
-0.302
 
 
17.0
 
 
 
 
CD2AP
 
 
hsa-miR-1-3p
 
 
-0.479
 
 
17.0
 
 
 
 
 
 
 spinal cord 
 
 
 
 
target_symbol
 
 
mature_mirna_id
 
 
score
 
 
nTPM
 
 
 
 
 
 
ACBD3
 
 
hsa-miR-130b-3p
 
 
-0.292
 
 
10.8
 
 
 
 
ACBD5
 
 
hsa-miR-130b-3p
 
 
-0.494
 
 
38.7
 
 
 
 
ACSL4
 
 
hsa-miR-130b-3p
 
 
-0.301
 
 
12.4
 
 
 
 
ADAR
 
 
hsa-miR-1-3p
 
 
-0.35
 
 
24.2
 
 
 
 
AIFM1
 
 
hsa-let-7e-5p
 
 
-0.314
 
 
10.1
 
 
 
 
AKAP6
 
 
hsa-let-7e-5p
 
 
-0.307
 
 
13.3
 
 
 
 
AKIRIN2
 
 
hsa-miR-130b-3p
 
 
-0.315
 
 
29.7
 
 
 
 
ALDH4A1
 
 
hsa-miR-184
 
 
-0.421
 
 
11.4
 
 
 
 
ANKIB1
 
 
hsa-miR-130b-3p
 
 
-0.329
 
 
17.8
 
 
 
 
ANKRD46
 
 
hsa-let-7e-5p
 
 
-0.389
 
 
24.6
 
 
 
 
ANP32B
 
 
hsa-miR-1-3p
 
 
-0.287
 
 
363.5
 
 
 
 
ANXA2
 
 
hsa-miR-1-3p
 
 
-0.581
 
 
100.8
 
 
 
 
ANXA4
 
 
hsa-miR-1-3p
 
 
-0.539
 
 
25.4
 
 
 
 
AP1S1
 
 
hsa-miR-1-3p
 
 
-0.355
 
 
28.5
 
 
 
 
AP1S1
 
 
hsa-let-7e-5p
 
 
-0.374
 
 
28.5
 
 
 
 
APCDD1
 
 
hsa-miR-130b-3p
 
 
-0.304
 
 
14.7
 
 
 
 
ARCN1
 
 
hsa-miR-1-3p
 
 
-0.468
 
 
58.6
 
 
 
 
ARF4
 
 
hsa-miR-7-5p
 
 
-0.407
 
 
69.7
 
 
 
 
ARHGAP1
 
 
hsa-miR-130b-3p
 
 
-0.376
 
 
38.6
 
 
 
 
ARHGAP12
 
 
hsa-miR-130b-3p
 
 
-0.287
 
 
24.6
 
 
 
 
ARHGEF4
 
 
hsa-miR-130b-3p
 
 
-0.377
 
 
26.2
 
 
 
 
ARL6IP1
 
 
hsa-miR-130b-3p
 
 
-0.289
 
 
140.2
 
 
 
 
ARL6IP1
 
 
hsa-miR-7-5p
 
 
-0.374
 
 
140.2
 
 
 
 
ARPC3
 
 
hsa-miR-1-3p
 
 
-0.606
 
 
217.0
 
 
 
 
ARPP19
 
 
hsa-let-7e-5p
 
 
-0.492
 
 
170.8
 
 
 
 
ARRDC4
 
 
hsa-let-7e-5p
 
 
-0.329
 
 
75.7
 
 
 
 
ASAP1
 
 
hsa-let-7e-5p
 
 
-0.296
 
 
11.0
 
 
 
 
ASH2L
 
 
hsa-miR-1-3p
 
 
-0.462
 
 
17.1
 
 
 
 
ATF2
 
 
hsa-miR-1-3p
 
 
-0.328
 
 
16.0
 
 
 
 
ATG16L1
 
 
hsa-miR-130b-3p
 
 
-0.407
 
 
10.3
 
 
 
 
ATOSB
 
 
hsa-let-7e-5p
 
 
-0.429
 
 
30.9
 
 
 
 
ATP2A2
 
 
hsa-let-7e-5p
 
 
-0.311
 
 
19.0
 
 
 
 
ATPAF1
 
 
hsa-let-7e-5p
 
 
-0.498
 
 
50.4
 
 
 
 
AVPI1
 
 
hsa-miR-7-5p
 
 
-0.367
 
 
33.9
 
 
 
 
AZIN1
 
 
hsa-miR-1-3p
 
 
-0.438
 
 
40.4
 
 
 
 
B3GAT3
 
 
hsa-let-7e-5p
 
 
-0.324
 
 
62.6
 
 
 
 
B4GAT1
 
 
hsa-let-7e-5p
 
 
-0.47
 
 
48.7
 
 
 
 
BAZ2A
 
 
hsa-miR-100-5p
 
 
-0.29
 
 
10.5
 
 
 
 
BAZ2A
 
 
hsa-miR-100-5p
 
 
-0.421
 
 
10.5
 
 
 
 
BCAP29
 
 
hsa-let-7e-5p
 
 
-0.289
 
 
26.8
 
 
 
 
BET1
 
 
hsa-miR-1-3p
 
 
-0.413
 
 
10.7
 
 
 
 
BHLHE40
 
 
hsa-miR-130b-3p
 
 
-0.351
 
 
46.8
 
 
 
 
BLCAP
 
 
hsa-miR-130b-3p
 
 
-0.342
 
 
55.9
 
 
 
 
BLOC1S6
 
 
hsa-let-7e-5p
 
 
-0.321
 
 
15.5
 
 
 
 
BPNT1
 
 
hsa-miR-1-3p
 
 
-0.329
 
 
14.9
 
 
 
 
BRIX1
 
 
hsa-miR-1-3p
 
 
-0.334
 
 
16.9
 
 
 
 
BSCL2
 
 
hsa-miR-1-3p
 
 
-0.471
 
 
44.3
 
 
 
 
BTF3L4
 
 
hsa-let-7e-5p
 
 
-0.355
 
 
36.6
 
 
 
 
BTF3L4
 
 
hsa-miR-130b-3p
 
 
-0.359
 
 
36.6
 
 
 
 
BZW1
 
 
hsa-let-7e-5p
 
 
-0.354
 
 
46.3
 
 
 
 
BZW1
 
 
hsa-let-7e-5p
 
 
-0.375
 
 
46.3
 
 
 
 
C19orf47
 
 
hsa-let-7e-5p
 
 
-0.311
 
 
14.3
 
 
 
 
C1GALT1
 
 
hsa-let-7e-5p
 
 
-0.322
 
 
27.9
 
 
 
 
C2orf88
 
 
hsa-let-7e-5p
 
 
-0.429
 
 
14.8
 
 
 
 
CACFD1
 
 
hsa-let-7e-5p
 
 
-0.296
 
 
13.3
 
 
 
 
CACNG7
 
 
hsa-miR-7-5p
 
 
-0.421
 
 
40.0
 
 
 
 
CADM2
 
 
hsa-let-7e-5p
 
 
-0.294
 
 
10.6
 
 
 
 
CALM2
 
 
hsa-miR-130b-3p
 
 
-0.584
 
 
1254.5
 
 
 
 
CAMSAP2
 
 
hsa-miR-130b-3p
 
 
-0.297
 
 
15.1
 
 
 
 
CANT1
 
 
hsa-let-7e-5p
 
 
-0.402
 
 
15.9
 
 
 
 
CAPN3
 
 
hsa-let-7e-5p
 
 
-0.317
 
 
318.3
 
 
 
 
CAPZA1
 
 
hsa-miR-7-5p
 
 
-0.351
 
 
51.3
 
 
 
 
CBX5
 
 
hsa-let-7e-5p
 
 
-0.307
 
 
18.6
 
 
 
 
CBX5
 
 
hsa-let-7e-5p
 
 
-0.472
 
 
18.6
 
 
 
 
CCDC43
 
 
hsa-miR-7-5p
 
 
-0.331
 
 
14.1
 
 
 
 
CCNC
 
 
hsa-miR-1-3p
 
 
-0.336
 
 
13.5
 
 
 
 
CCNL2
 
 
hsa-miR-28-5p
 
 
-0.448
 
 
21.4
 
 
 
 
CCNT2
 
 
hsa-miR-7-5p
 
 
-0.349
 
 
11.2
 
 
 
 
CCNY
 
 
hsa-let-7e-5p
 
 
-0.334
 
 
32.4
 
 
 
 
CCNY
 
 
hsa-miR-130b-3p
 
 
-0.372
 
 
32.4
 
 
 
 
CCSAP
 
 
hsa-miR-1-3p
 
 
-0.381
 
 
11.5
 
 
 
 
CCT4
 
 
hsa-miR-7-5p
 
 
-0.37
 
 
63.9
 
 
 
 
CCT6A
 
 
hsa-miR-130b-3p
 
 
-0.382
 
 
54.3
 
 
 
 
CD164
 
 
hsa-miR-1-3p
 
 
-0.315
 
 
87.8
 
 
 
 
CDC34
 
 
hsa-let-7e-5p
 
 
-0.321
 
 
39.3
 
 
 
 
CDC34
 
 
hsa-let-7e-5p
 
 
-0.555
 
 
39.3
 
 
 
 
CDC42SE1
 
 
hsa-miR-1-3p
 
 
-0.36
 
 
39.2
 
 
 
 
CDK4
 
 
hsa-miR-1-3p
 
 
-0.338
 
 
34.9
 
 
 
 
CDKN1A
 
 
hsa-let-7e-5p
 
 
-0.434
 
 
49.5
 
 
 
 
CDV3
 
 
hsa-let-7e-5p
 
 
-0.453
 
 
47.4
 
 
 
 
CEBPD
 
 
hsa-let-7e-5p
 
 
-0.309
 
 
90.6
 
 
 
 
CERCAM
 
 
hsa-let-7e-5p
 
 
-0.344
 
 
436.8
 
 
 
 
CERS2
 
 
hsa-miR-1-3p
 
 
-0.287
 
 
289.9
 
 
 
 
CGGBP1
 
 
hsa-miR-7-5p
 
 
-0.465
 
 
21.8
 
 
 
 
CHMP3
 
 
hsa-miR-130b-3p
 
 
-0.521
 
 
43.7
 
 
 
 
CHMP4B
 
 
hsa-miR-130b-3p
 
 
-0.292
 
 
109.7
 
 
 
 
CLASP2
 
 
hsa-let-7e-5p
 
 
-0.344
 
 
56.4
 
 
 
 
CLCN3
 
 
hsa-miR-1-3p
 
 
-0.339
 
 
26.8
 
 
 
 
CLCN3
 
 
hsa-miR-130b-3p
 
 
-0.384
 
 
26.8
 
 
 
 
CLDN11
 
 
hsa-miR-100-5p
 
 
-0.42
 
 
119.5
 
 
 
 
CLIP1
 
 
hsa-miR-130b-3p
 
 
-0.452
 
 
12.5
 
 
 
 
CLTC
 
 
hsa-miR-1-3p
 
 
-0.39
 
 
80.9
 
 
 
 
CMPK1
 
 
hsa-miR-130b-3p
 
 
-0.384
 
 
69.0
 
 
 
 
CNIH1
 
 
hsa-miR-130b-3p
 
 
-0.335
 
 
44.0
 
 
 
 
CNN3
 
 
hsa-miR-1-3p
 
 
-0.414
 
 
113.7
 
 
 
 
CNN3
 
 
hsa-miR-7-5p
 
 
-0.489
 
 
113.7
 
 
 
 
CNOT7
 
 
hsa-miR-130b-3p
 
 
-0.318
 
 
16.9
 
 
 
 
CNOT8
 
 
hsa-miR-7-5p
 
 
-0.329
 
 
35.2
 
 
 
 
CNPPD1
 
 
hsa-miR-7-5p
 
 
-0.541
 
 
51.9
 
 
 
 
COL9A3
 
 
hsa-miR-130b-3p
 
 
-0.379
 
 
70.9
 
 
 
 
 
 
 
 cd4_s0_vs_cd4_s1_upregulated 
 
 Tissue expression heatmap (log10-transformed) 
   
 
 
 adipose tissue 
 
 
 
 
target_symbol
 
 
mature_mirna_id
 
 
score
 
 
nTPM
 
 
 
 
 
 
AARS1
 
 
hsa-miR-92a-3p
 
 
-0.458
 
 
36.2
 
 
 
 
ABHD5
 
 
hsa-miR-20a-5p
 
 
-0.397
 
 
27.9
 
 
 
 
ABHD5
 
 
hsa-miR-93-5p
 
 
-0.402
 
 
27.9
 
 
 
 
ABHD5
 
 
hsa-miR-17-5p
 
 
-0.45
 
 
27.9
 
 
 
 
ACOX1
 
 
hsa-miR-92a-3p
 
 
-0.321
 
 
26.2
 
 
 
 
ACTA1
 
 
hsa-miR-155-5p
 
 
-0.595
 
 
13.2
 
 
 
 
ACTC1
 
 
hsa-miR-92a-3p
 
 
-0.361
 
 
47.8
 
 
 
 
ACTC1
 
 
hsa-miR-7-5p
 
 
-0.471
 
 
47.8
 
 
 
 
ACVR1C
 
 
hsa-let-7c-5p
 
 
-0.465
 
 
29.3
 
 
 
 
ADAM9
 
 
hsa-miR-20a-5p
 
 
-0.299
 
 
31.0
 
 
 
 
ADAM9
 
 
hsa-miR-17-5p
 
 
-0.32
 
 
31.0
 
 
 
 
ADAM9
 
 
hsa-miR-93-5p
 
 
-0.32
 
 
31.0
 
 
 
 
ADCY3
 
 
hsa-miR-92a-3p
 
 
-0.381
 
 
90.0
 
 
 
 
ADNP
 
 
hsa-miR-328-3p
 
 
-0.371
 
 
25.5
 
 
 
 
ADRB2
 
 
hsa-let-7c-5p
 
 
-0.635
 
 
26.4
 
 
 
 
AGFG1
 
 
hsa-miR-103a-3p
 
 
-0.389
 
 
64.8
 
 
 
 
AGFG2
 
 
hsa-miR-20a-5p
 
 
-0.365
 
 
12.0
 
 
 
 
AGFG2
 
 
hsa-miR-93-5p
 
 
-0.378
 
 
12.0
 
 
 
 
AGFG2
 
 
hsa-miR-17-5p
 
 
-0.402
 
 
12.0
 
 
 
 
AHCTF1
 
 
hsa-let-7c-5p
 
 
-0.374
 
 
11.8
 
 
 
 
AHNAK
 
 
hsa-miR-20a-5p
 
 
-0.316
 
 
230.0
 
 
 
 
AHNAK
 
 
hsa-miR-17-5p
 
 
-0.337
 
 
230.0
 
 
 
 
AHNAK
 
 
hsa-miR-93-5p
 
 
-0.337
 
 
230.0
 
 
 
 
AIFM1
 
 
hsa-let-7c-5p
 
 
-0.314
 
 
17.4
 
 
 
 
AKAP10
 
 
hsa-miR-339-5p
 
 
-0.302
 
 
10.8
 
 
 
 
AKIRIN2
 
 
hsa-miR-103a-3p
 
 
-0.299
 
 
30.8
 
 
 
 
ALAD
 
 
hsa-miR-103a-3p
 
 
-0.385
 
 
51.3
 
 
 
 
ALG3
 
 
hsa-miR-20a-5p
 
 
-0.559
 
 
20.9
 
 
 
 
ALG3
 
 
hsa-miR-93-5p
 
 
-0.564
 
 
20.9
 
 
 
 
ALG3
 
 
hsa-miR-17-5p
 
 
-0.572
 
 
20.9
 
 
 
 
ALG9
 
 
hsa-miR-7-5p
 
 
-0.302
 
 
12.6
 
 
 
 
ALKAL2
 
 
hsa-miR-92a-3p
 
 
-0.608
 
 
28.4
 
 
 
 
ANGPTL2
 
 
hsa-miR-221-3p
 
 
-0.369
 
 
104.7
 
 
 
 
ANKFY1
 
 
hsa-miR-155-5p
 
 
-0.315
 
 
12.9
 
 
 
 
ANKRD12
 
 
hsa-miR-7-5p
 
 
-0.37
 
 
14.8
 
 
 
 
ANKRD29
 
 
hsa-miR-20a-5p
 
 
-0.359
 
 
17.0
 
 
 
 
ANKRD29
 
 
hsa-miR-17-5p
 
 
-0.372
 
 
17.0
 
 
 
 
ANKRD29
 
 
hsa-miR-93-5p
 
 
-0.372
 
 
17.0
 
 
 
 
ANKRD46
 
 
hsa-let-7c-5p
 
 
-0.389
 
 
18.0
 
 
 
 
ANKRD9
 
 
hsa-miR-20a-5p
 
 
-0.31
 
 
11.3
 
 
 
 
ANKRD9
 
 
hsa-miR-93-5p
 
 
-0.331
 
 
11.3
 
 
 
 
ANKRD9
 
 
hsa-miR-17-5p
 
 
-0.347
 
 
11.3
 
 
 
 
ANP32E
 
 
hsa-miR-92a-3p
 
 
-0.329
 
 
18.8
 
 
 
 
AP1S1
 
 
hsa-let-7c-5p
 
 
-0.374
 
 
15.1
 
 
 
 
APCDD1
 
 
hsa-miR-20a-5p
 
 
-0.404
 
 
49.2
 
 
 
 
APCDD1
 
 
hsa-miR-17-5p
 
 
-0.425
 
 
49.2
 
 
 
 
APCDD1
 
 
hsa-miR-93-5p
 
 
-0.425
 
 
49.2
 
 
 
 
APPL1
 
 
hsa-miR-92a-3p
 
 
-0.375
 
 
14.6
 
 
 
 
AQP3
 
 
hsa-miR-874-3p
 
 
-0.48
 
 
10.5
 
 
 
 
ARF4
 
 
hsa-miR-7-5p
 
 
-0.407
 
 
118.7
 
 
 
 
ARF4
 
 
hsa-miR-221-3p
 
 
-0.553
 
 
118.7
 
 
 
 
ARHGAP1
 
 
hsa-miR-17-5p
 
 
-0.325
 
 
79.6
 
 
 
 
ARHGAP1
 
 
hsa-miR-93-5p
 
 
-0.325
 
 
79.6
 
 
 
 
ARHGAP1
 
 
hsa-miR-20a-5p
 
 
-0.339
 
 
79.6
 
 
 
 
ARHGAP5
 
 
hsa-miR-486-5p
 
 
-0.3
 
 
22.2
 
 
 
 
ARID4B
 
 
hsa-miR-20a-5p
 
 
-0.29
 
 
10.7
 
 
 
 
ARID4B
 
 
hsa-miR-486-5p
 
 
-0.367
 
 
10.7
 
 
 
 
ARIH2
 
 
hsa-miR-103a-3p
 
 
-0.307
 
 
19.8
 
 
 
 
ARL1
 
 
hsa-miR-20a-5p
 
 
-0.403
 
 
43.5
 
 
 
 
ARL1
 
 
hsa-miR-93-5p
 
 
-0.424
 
 
43.5
 
 
 
 
ARL1
 
 
hsa-miR-17-5p
 
 
-0.432
 
 
43.5
 
 
 
 
ARL4D
 
 
hsa-let-7c-5p
 
 
-0.417
 
 
20.3
 
 
 
 
ARL5B
 
 
hsa-miR-92a-3p
 
 
-0.331
 
 
10.5
 
 
 
 
ARL5B
 
 
hsa-miR-155-5p
 
 
-0.355
 
 
10.5
 
 
 
 
ARL6IP1
 
 
hsa-miR-7-5p
 
 
-0.374
 
 
133.2
 
 
 
 
ARL6IP1
 
 
hsa-miR-328-3p
 
 
-0.494
 
 
133.2
 
 
 
 
ARL6IP6
 
 
hsa-let-7c-5p
 
 
-0.293
 
 
11.0
 
 
 
 
ARL8A
 
 
hsa-miR-103a-3p
 
 
-0.38
 
 
39.7
 
 
 
 
ARMC1
 
 
hsa-miR-92a-3p
 
 
-0.322
 
 
15.5
 
 
 
 
ARMC1
 
 
hsa-miR-103a-3p
 
 
-0.525
 
 
15.5
 
 
 
 
ARMC8
 
 
hsa-miR-486-5p
 
 
-0.321
 
 
19.5
 
 
 
 
ARMC8
 
 
hsa-miR-20a-5p
 
 
-0.357
 
 
19.5
 
 
 
 
ARMC8
 
 
hsa-miR-93-5p
 
 
-0.362
 
 
19.5
 
 
 
 
ARMC8
 
 
hsa-miR-17-5p
 
 
-0.378
 
 
19.5
 
 
 
 
ARMC8
 
 
hsa-miR-103a-3p
 
 
-0.412
 
 
19.5
 
 
 
 
ARMT1
 
 
hsa-let-7c-5p
 
 
-0.303
 
 
16.0
 
 
 
 
ARNT
 
 
hsa-miR-103a-3p
 
 
-0.422
 
 
25.2
 
 
 
 
ARNT
 
 
hsa-miR-221-3p
 
 
-0.465
 
 
25.2
 
 
 
 
ARPP19
 
 
hsa-miR-328-3p
 
 
-0.404
 
 
57.4
 
 
 
 
ARPP19
 
 
hsa-let-7c-5p
 
 
-0.492
 
 
57.4
 
 
 
 
ARRDC3
 
 
hsa-miR-92a-3p
 
 
-0.458
 
 
34.5
 
 
 
 
ARRDC4
 
 
hsa-let-7c-5p
 
 
-0.329
 
 
11.8
 
 
 
 
ASAP1
 
 
hsa-let-7c-5p
 
 
-0.308
 
 
32.3
 
 
 
 
ASB6
 
 
hsa-miR-874-3p
 
 
-0.368
 
 
10.3
 
 
 
 
ASPH
 
 
hsa-miR-221-3p
 
 
-0.287
 
 
362.0
 
 
 
 
ASPH
 
 
hsa-miR-92a-3p
 
 
-0.416
 
 
362.0
 
 
 
 
ASPN
 
 
hsa-miR-92a-3p
 
 
-0.422
 
 
27.7
 
 
 
 
ATF3
 
 
hsa-miR-874-3p
 
 
-0.288
 
 
168.3
 
 
 
 
ATL2
 
 
hsa-miR-103a-3p
 
 
-0.489
 
 
16.4
 
 
 
 
ATOSA
 
 
hsa-miR-221-3p
 
 
-0.495
 
 
29.6
 
 
 
 
ATOSB
 
 
hsa-miR-328-3p
 
 
-0.358
 
 
13.2
 
 
 
 
ATOSB
 
 
hsa-let-7c-5p
 
 
-0.429
 
 
13.2
 
 
 
 
ATP2A2
 
 
hsa-let-7c-5p
 
 
-0.311
 
 
56.6
 
 
 
 
ATP6V1F
 
 
hsa-miR-185-5p
 
 
-0.765
 
 
135.2
 
 
 
 
ATPAF1
 
 
hsa-let-7c-5p
 
 
-0.498
 
 
81.3
 
 
 
 
ATXN1L
 
 
hsa-miR-155-5p
 
 
-0.315
 
 
13.1
 
 
 
 
AVPI1
 
 
hsa-miR-7-5p
 
 
-0.367
 
 
32.2
 
 
 
 
B3GAT3
 
 
hsa-let-7c-5p
 
 
-0.324
 
 
30.8
 
 
 
 
B4GALT5
 
 
hsa-miR-210-3p
 
 
-0.511
 
 
26.8
 
 
 
 
B4GAT1
 
 
hsa-let-7c-5p
 
 
-0.47
 
 
16.2
 
 
 
 
 
 
 adrenal gland 
 
 
 
 
target_symbol
 
 
mature_mirna_id
 
 
score
 
 
nTPM
 
 
 
 
 
 
AARS1
 
 
hsa-miR-92a-3p
 
 
-0.458
 
 
42.6
 
 
 
 
ACOX1
 
 
hsa-miR-92a-3p
 
 
-0.321
 
 
17.7
 
 
 
 
ADAM9
 
 
hsa-miR-20a-5p
 
 
-0.299
 
 
19.6
 
 
 
 
ADAM9
 
 
hsa-miR-17-5p
 
 
-0.32
 
 
19.6
 
 
 
 
ADAM9
 
 
hsa-miR-93-5p
 
 
-0.32
 
 
19.6
 
 
 
 
ADCY3
 
 
hsa-miR-92a-3p
 
 
-0.381
 
 
16.9
 
 
 
 
ADNP
 
 
hsa-miR-328-3p
 
 
-0.371
 
 
29.8
 
 
 
 
AGFG1
 
 
hsa-miR-103a-3p
 
 
-0.389
 
 
37.2
 
 
 
 
AGFG2
 
 
hsa-miR-20a-5p
 
 
-0.365
 
 
33.6
 
 
 
 
AGFG2
 
 
hsa-miR-93-5p
 
 
-0.378
 
 
33.6
 
 
 
 
AGFG2
 
 
hsa-miR-17-5p
 
 
-0.402
 
 
33.6
 
 
 
 
AHNAK
 
 
hsa-miR-20a-5p
 
 
-0.316
 
 
26.5
 
 
 
 
AHNAK
 
 
hsa-miR-17-5p
 
 
-0.337
 
 
26.5
 
 
 
 
AHNAK
 
 
hsa-miR-93-5p
 
 
-0.337
 
 
26.5
 
 
 
 
AIFM1
 
 
hsa-let-7c-5p
 
 
-0.314
 
 
50.1
 
 
 
 
AKAP10
 
 
hsa-miR-339-5p
 
 
-0.302
 
 
12.6
 
 
 
 
AKIRIN2
 
 
hsa-miR-103a-3p
 
 
-0.299
 
 
33.1
 
 
 
 
ALAD
 
 
hsa-miR-103a-3p
 
 
-0.385
 
 
148.4
 
 
 
 
ALG3
 
 
hsa-miR-20a-5p
 
 
-0.559
 
 
30.6
 
 
 
 
ALG3
 
 
hsa-miR-93-5p
 
 
-0.564
 
 
30.6
 
 
 
 
ALG3
 
 
hsa-miR-17-5p
 
 
-0.572
 
 
30.6
 
 
 
 
ALKAL2
 
 
hsa-miR-92a-3p
 
 
-0.608
 
 
212.1
 
 
 
 
AMT
 
 
hsa-let-7c-5p
 
 
-0.463
 
 
15.0
 
 
 
 
ANGPTL2
 
 
hsa-miR-221-3p
 
 
-0.369
 
 
10.9
 
 
 
 
ANKFY1
 
 
hsa-miR-155-5p
 
 
-0.315
 
 
15.3
 
 
 
 
ANKRD46
 
 
hsa-let-7c-5p
 
 
-0.389
 
 
36.5
 
 
 
 
ANKRD9
 
 
hsa-miR-20a-5p
 
 
-0.31
 
 
20.4
 
 
 
 
ANKRD9
 
 
hsa-miR-93-5p
 
 
-0.331
 
 
20.4
 
 
 
 
ANKRD9
 
 
hsa-miR-17-5p
 
 
-0.347
 
 
20.4
 
 
 
 
ANP32E
 
 
hsa-miR-92a-3p
 
 
-0.329
 
 
28.7
 
 
 
 
AP1AR
 
 
hsa-miR-92a-3p
 
 
-0.325
 
 
14.8
 
 
 
 
AP1S1
 
 
hsa-let-7c-5p
 
 
-0.374
 
 
25.7
 
 
 
 
AP5S1
 
 
hsa-miR-744-5p
 
 
-0.354
 
 
10.8
 
 
 
 
APCDD1
 
 
hsa-miR-20a-5p
 
 
-0.404
 
 
13.4
 
 
 
 
APCDD1
 
 
hsa-miR-17-5p
 
 
-0.425
 
 
13.4
 
 
 
 
APCDD1
 
 
hsa-miR-93-5p
 
 
-0.425
 
 
13.4
 
 
 
 
APPL1
 
 
hsa-miR-92a-3p
 
 
-0.375
 
 
11.0
 
 
 
 
AQP11
 
 
hsa-miR-103a-3p
 
 
-0.517
 
 
14.1
 
 
 
 
ARF4
 
 
hsa-miR-7-5p
 
 
-0.407
 
 
151.0
 
 
 
 
ARF4
 
 
hsa-miR-221-3p
 
 
-0.553
 
 
151.0
 
 
 
 
ARHGAP1
 
 
hsa-miR-17-5p
 
 
-0.325
 
 
46.3
 
 
 
 
ARHGAP1
 
 
hsa-miR-93-5p
 
 
-0.325
 
 
46.3
 
 
 
 
ARHGAP1
 
 
hsa-miR-20a-5p
 
 
-0.339
 
 
46.3
 
 
 
 
ARHGAP5
 
 
hsa-miR-486-5p
 
 
-0.3
 
 
25.8
 
 
 
 
ARHGEF28
 
 
hsa-miR-93-5p
 
 
-0.33
 
 
23.1
 
 
 
 
ARHGEF28
 
 
hsa-miR-17-5p
 
 
-0.363
 
 
23.1
 
 
 
 
ARHGEF28
 
 
hsa-miR-20a-5p
 
 
-0.366
 
 
23.1
 
 
 
 
ARID4B
 
 
hsa-miR-20a-5p
 
 
-0.29
 
 
10.2
 
 
 
 
ARID4B
 
 
hsa-miR-486-5p
 
 
-0.367
 
 
10.2
 
 
 
 
ARIH2
 
 
hsa-miR-103a-3p
 
 
-0.307
 
 
22.0
 
 
 
 
ARL1
 
 
hsa-miR-20a-5p
 
 
-0.403
 
 
62.3
 
 
 
 
ARL1
 
 
hsa-miR-93-5p
 
 
-0.424
 
 
62.3
 
 
 
 
ARL1
 
 
hsa-miR-17-5p
 
 
-0.432
 
 
62.3
 
 
 
 
ARL5B
 
 
hsa-miR-92a-3p
 
 
-0.331
 
 
19.6
 
 
 
 
ARL5B
 
 
hsa-miR-155-5p
 
 
-0.355
 
 
19.6
 
 
 
 
ARL6IP1
 
 
hsa-miR-7-5p
 
 
-0.374
 
 
161.7
 
 
 
 
ARL6IP1
 
 
hsa-miR-328-3p
 
 
-0.494
 
 
161.7
 
 
 
 
ARL8A
 
 
hsa-miR-103a-3p
 
 
-0.38
 
 
38.6
 
 
 
 
ARMC1
 
 
hsa-miR-92a-3p
 
 
-0.322
 
 
14.5
 
 
 
 
ARMC1
 
 
hsa-miR-103a-3p
 
 
-0.525
 
 
14.5
 
 
 
 
ARMC8
 
 
hsa-miR-486-5p
 
 
-0.321
 
 
13.5
 
 
 
 
ARMC8
 
 
hsa-miR-20a-5p
 
 
-0.357
 
 
13.5
 
 
 
 
ARMC8
 
 
hsa-miR-93-5p
 
 
-0.362
 
 
13.5
 
 
 
 
ARMC8
 
 
hsa-miR-17-5p
 
 
-0.378
 
 
13.5
 
 
 
 
ARMC8
 
 
hsa-miR-103a-3p
 
 
-0.412
 
 
13.5
 
 
 
 
ARMT1
 
 
hsa-let-7c-5p
 
 
-0.303
 
 
15.1
 
 
 
 
ARNT
 
 
hsa-miR-103a-3p
 
 
-0.422
 
 
19.8
 
 
 
 
ARNT
 
 
hsa-miR-221-3p
 
 
-0.465
 
 
19.8
 
 
 
 
ARPP19
 
 
hsa-miR-328-3p
 
 
-0.404
 
 
48.8
 
 
 
 
ARPP19
 
 
hsa-let-7c-5p
 
 
-0.492
 
 
48.8
 
 
 
 
ARRDC3
 
 
hsa-miR-92a-3p
 
 
-0.458
 
 
75.7
 
 
 
 
ARRDC4
 
 
hsa-let-7c-5p
 
 
-0.329
 
 
11.5
 
 
 
 
ASAP1
 
 
hsa-let-7c-5p
 
 
-0.308
 
 
20.2
 
 
 
 
ASB6
 
 
hsa-miR-874-3p
 
 
-0.368
 
 
18.8
 
 
 
 
ASPH
 
 
hsa-miR-221-3p
 
 
-0.287
 
 
150.8
 
 
 
 
ASPH
 
 
hsa-miR-92a-3p
 
 
-0.416
 
 
150.8
 
 
 
 
ATF3
 
 
hsa-miR-874-3p
 
 
-0.288
 
 
77.8
 
 
 
 
ATL2
 
 
hsa-miR-103a-3p
 
 
-0.489
 
 
30.6
 
 
 
 
ATOSA
 
 
hsa-miR-221-3p
 
 
-0.495
 
 
17.4
 
 
 
 
ATOSB
 
 
hsa-miR-328-3p
 
 
-0.358
 
 
14.2
 
 
 
 
ATOSB
 
 
hsa-let-7c-5p
 
 
-0.429
 
 
14.2
 
 
 
 
ATP2A2
 
 
hsa-let-7c-5p
 
 
-0.311
 
 
46.8
 
 
 
 
ATP6V1F
 
 
hsa-miR-185-5p
 
 
-0.765
 
 
160.7
 
 
 
 
ATPAF1
 
 
hsa-let-7c-5p
 
 
-0.498
 
 
98.1
 
 
 
 
AVPI1
 
 
hsa-miR-7-5p
 
 
-0.367
 
 
46.2
 
 
 
 
B3GAT3
 
 
hsa-let-7c-5p
 
 
-0.324
 
 
33.4
 
 
 
 
B4GALT5
 
 
hsa-miR-210-3p
 
 
-0.511
 
 
44.9
 
 
 
 
B4GAT1
 
 
hsa-let-7c-5p
 
 
-0.47
 
 
39.1
 
 
 
 
BACE1
 
 
hsa-miR-339-5p
 
 
-0.371
 
 
13.1
 
 
 
 
BACH1
 
 
hsa-let-7c-5p
 
 
-0.306
 
 
11.4
 
 
 
 
BACH1
 
 
hsa-let-7c-5p
 
 
-0.306
 
 
11.4
 
 
 
 
BACH1
 
 
hsa-let-7c-5p
 
 
-0.435
 
 
11.4
 
 
 
 
BACH1
 
 
hsa-let-7c-5p
 
 
-0.435
 
 
11.4
 
 
 
 
BAK1
 
 
hsa-miR-103a-3p
 
 
-0.364
 
 
27.9
 
 
 
 
BAMBI
 
 
hsa-miR-20a-5p
 
 
-0.485
 
 
41.7
 
 
 
 
BAMBI
 
 
hsa-miR-17-5p
 
 
-0.506
 
 
41.7
 
 
 
 
BAMBI
 
 
hsa-miR-93-5p
 
 
-0.506
 
 
41.7
 
 
 
 
BCAT2
 
 
hsa-miR-103a-3p
 
 
-0.364
 
 
79.9
 
 
 
 
BCAT2
 
 
hsa-miR-92a-3p
 
 
-0.609
 
 
79.9
 
 
 
 
BIN1
 
 
hsa-miR-744-5p
 
 
-0.654
 
 
88.9
 
 
 
 
 
 
 amygdala 
 
 
 
 
target_symbol
 
 
mature_mirna_id
 
 
score
 
 
nTPM
 
 
 
 
 
 
AARS1
 
 
hsa-miR-92a-3p
 
 
-0.458
 
 
47.9
 
 
 
 
ABHD17B
 
 
hsa-miR-486-5p
 
 
-0.348
 
 
12.7
 
 
 
 
ABHD17C
 
 
hsa-let-7c-5p
 
 
-0.359
 
 
17.9
 
 
 
 
ABHD17C
 
 
hsa-miR-330-3p
 
 
-0.399
 
 
17.9
 
 
 
 
ACOX1
 
 
hsa-miR-92a-3p
 
 
-0.321
 
 
12.5
 
 
 
 
ACSL6
 
 
hsa-let-7c-5p
 
 
-0.336
 
 
20.3
 
 
 
 
ADCY3
 
 
hsa-miR-92a-3p
 
 
-0.381
 
 
27.0
 
 
 
 
ADNP
 
 
hsa-miR-328-3p
 
 
-0.371
 
 
23.8
 
 
 
 
AGFG1
 
 
hsa-miR-103a-3p
 
 
-0.389
 
 
25.1
 
 
 
 
AGFG2
 
 
hsa-miR-20a-5p
 
 
-0.365
 
 
13.3
 
 
 
 
AGFG2
 
 
hsa-miR-93-5p
 
 
-0.378
 
 
13.3
 
 
 
 
AGFG2
 
 
hsa-miR-17-5p
 
 
-0.402
 
 
13.3
 
 
 
 
AHNAK
 
 
hsa-miR-20a-5p
 
 
-0.316
 
 
31.4
 
 
 
 
AHNAK
 
 
hsa-miR-17-5p
 
 
-0.337
 
 
31.4
 
 
 
 
AHNAK
 
 
hsa-miR-93-5p
 
 
-0.337
 
 
31.4
 
 
 
 
AIFM1
 
 
hsa-let-7c-5p
 
 
-0.314
 
 
10.3
 
 
 
 
AKAP6
 
 
hsa-let-7c-5p
 
 
-0.307
 
 
12.4
 
 
 
 
AKIRIN2
 
 
hsa-miR-103a-3p
 
 
-0.299
 
 
33.1
 
 
 
 
ALAD
 
 
hsa-miR-103a-3p
 
 
-0.385
 
 
58.3
 
 
 
 
ANKFY1
 
 
hsa-miR-155-5p
 
 
-0.315
 
 
10.9
 
 
 
 
ANKRD46
 
 
hsa-let-7c-5p
 
 
-0.389
 
 
28.3
 
 
 
 
ANKRD9
 
 
hsa-miR-20a-5p
 
 
-0.31
 
 
26.5
 
 
 
 
ANKRD9
 
 
hsa-miR-93-5p
 
 
-0.331
 
 
26.5
 
 
 
 
ANKRD9
 
 
hsa-miR-17-5p
 
 
-0.347
 
 
26.5
 
 
 
 
ANP32E
 
 
hsa-miR-92a-3p
 
 
-0.329
 
 
38.5
 
 
 
 
AP1S1
 
 
hsa-let-7c-5p
 
 
-0.374
 
 
88.4
 
 
 
 
APCDD1
 
 
hsa-miR-20a-5p
 
 
-0.404
 
 
28.8
 
 
 
 
APCDD1
 
 
hsa-miR-17-5p
 
 
-0.425
 
 
28.8
 
 
 
 
APCDD1
 
 
hsa-miR-93-5p
 
 
-0.425
 
 
28.8
 
 
 
 
ARF4
 
 
hsa-miR-7-5p
 
 
-0.407
 
 
60.1
 
 
 
 
ARF4
 
 
hsa-miR-221-3p
 
 
-0.553
 
 
60.1
 
 
 
 
ARHGAP1
 
 
hsa-miR-17-5p
 
 
-0.325
 
 
37.0
 
 
 
 
ARHGAP1
 
 
hsa-miR-93-5p
 
 
-0.325
 
 
37.0
 
 
 
 
ARHGAP1
 
 
hsa-miR-20a-5p
 
 
-0.339
 
 
37.0
 
 
 
 
ARHGAP5
 
 
hsa-miR-486-5p
 
 
-0.3
 
 
27.1
 
 
 
 
ARIH2
 
 
hsa-miR-103a-3p
 
 
-0.307
 
 
17.1
 
 
 
 
ARL1
 
 
hsa-miR-20a-5p
 
 
-0.403
 
 
24.9
 
 
 
 
ARL1
 
 
hsa-miR-93-5p
 
 
-0.424
 
 
24.9
 
 
 
 
ARL1
 
 
hsa-miR-17-5p
 
 
-0.432
 
 
24.9
 
 
 
 
ARL6IP1
 
 
hsa-miR-7-5p
 
 
-0.374
 
 
241.3
 
 
 
 
ARL6IP1
 
 
hsa-miR-328-3p
 
 
-0.494
 
 
241.3
 
 
 
 
ARL8A
 
 
hsa-miR-103a-3p
 
 
-0.38
 
 
68.0
 
 
 
 
ARMC1
 
 
hsa-miR-92a-3p
 
 
-0.322
 
 
17.1
 
 
 
 
ARMC1
 
 
hsa-miR-103a-3p
 
 
-0.525
 
 
17.1
 
 
 
 
ARMC8
 
 
hsa-miR-486-5p
 
 
-0.321
 
 
13.4
 
 
 
 
ARMC8
 
 
hsa-miR-20a-5p
 
 
-0.357
 
 
13.4
 
 
 
 
ARMC8
 
 
hsa-miR-93-5p
 
 
-0.362
 
 
13.4
 
 
 
 
ARMC8
 
 
hsa-miR-17-5p
 
 
-0.378
 
 
13.4
 
 
 
 
ARMC8
 
 
hsa-miR-103a-3p
 
 
-0.412
 
 
13.4
 
 
 
 
ARPP19
 
 
hsa-miR-328-3p
 
 
-0.404
 
 
178.5
 
 
 
 
ARPP19
 
 
hsa-let-7c-5p
 
 
-0.492
 
 
178.5
 
 
 
 
ARRDC4
 
 
hsa-let-7c-5p
 
 
-0.329
 
 
13.2
 
 
 
 
ASAP1
 
 
hsa-let-7c-5p
 
 
-0.308
 
 
11.2
 
 
 
 
ASB6
 
 
hsa-miR-874-3p
 
 
-0.368
 
 
13.1
 
 
 
 
ASPH
 
 
hsa-miR-221-3p
 
 
-0.287
 
 
62.6
 
 
 
 
ASPH
 
 
hsa-miR-92a-3p
 
 
-0.416
 
 
62.6
 
 
 
 
ATF3
 
 
hsa-miR-874-3p
 
 
-0.288
 
 
15.6
 
 
 
 
ATL2
 
 
hsa-miR-103a-3p
 
 
-0.489
 
 
10.7
 
 
 
 
ATOSB
 
 
hsa-miR-328-3p
 
 
-0.358
 
 
20.1
 
 
 
 
ATOSB
 
 
hsa-let-7c-5p
 
 
-0.429
 
 
20.1
 
 
 
 
ATP1B2
 
 
hsa-miR-103a-3p
 
 
-0.6
 
 
246.5
 
 
 
 
ATP2A2
 
 
hsa-let-7c-5p
 
 
-0.311
 
 
15.8
 
 
 
 
ATP2B2
 
 
hsa-miR-7-5p
 
 
-0.297
 
 
14.3
 
 
 
 
ATP6V1F
 
 
hsa-miR-185-5p
 
 
-0.765
 
 
311.8
 
 
 
 
ATPAF1
 
 
hsa-let-7c-5p
 
 
-0.498
 
 
86.8
 
 
 
 
AVPI1
 
 
hsa-miR-7-5p
 
 
-0.367
 
 
13.3
 
 
 
 
B3GAT3
 
 
hsa-let-7c-5p
 
 
-0.324
 
 
61.0
 
 
 
 
B4GALT5
 
 
hsa-miR-210-3p
 
 
-0.511
 
 
10.8
 
 
 
 
B4GAT1
 
 
hsa-let-7c-5p
 
 
-0.47
 
 
107.6
 
 
 
 
BACE1
 
 
hsa-miR-339-5p
 
 
-0.371
 
 
22.0
 
 
 
 
BAMBI
 
 
hsa-miR-20a-5p
 
 
-0.485
 
 
14.6
 
 
 
 
BAMBI
 
 
hsa-miR-17-5p
 
 
-0.506
 
 
14.6
 
 
 
 
BAMBI
 
 
hsa-miR-93-5p
 
 
-0.506
 
 
14.6
 
 
 
 
BCAT2
 
 
hsa-miR-103a-3p
 
 
-0.364
 
 
16.4
 
 
 
 
BCAT2
 
 
hsa-miR-92a-3p
 
 
-0.609
 
 
16.4
 
 
 
 
BDH1
 
 
hsa-miR-874-3p
 
 
-0.644
 
 
20.7
 
 
 
 
BEGAIN
 
 
hsa-let-7c-5p
 
 
-0.466
 
 
36.9
 
 
 
 
BIN1
 
 
hsa-miR-744-5p
 
 
-0.654
 
 
167.4
 
 
 
 
BIN3
 
 
hsa-let-7c-5p
 
 
-0.427
 
 
10.6
 
 
 
 
BLOC1S6
 
 
hsa-let-7c-5p
 
 
-0.321
 
 
20.1
 
 
 
 
BLTP1
 
 
hsa-miR-92a-3p
 
 
-0.462
 
 
18.1
 
 
 
 
BNIP3L
 
 
hsa-miR-20a-5p
 
 
-0.287
 
 
49.1
 
 
 
 
BOLA3
 
 
hsa-miR-103a-3p
 
 
-0.362
 
 
61.7
 
 
 
 
BRMS1L
 
 
hsa-miR-92a-3p
 
 
-0.306
 
 
10.3
 
 
 
 
BRMS1L
 
 
hsa-miR-20a-5p
 
 
-0.326
 
 
10.3
 
 
 
 
BRMS1L
 
 
hsa-miR-17-5p
 
 
-0.347
 
 
10.3
 
 
 
 
BRMS1L
 
 
hsa-miR-93-5p
 
 
-0.347
 
 
10.3
 
 
 
 
BRMS1L
 
 
hsa-miR-339-5p
 
 
-0.41
 
 
10.3
 
 
 
 
BSDC1
 
 
hsa-miR-92a-3p
 
 
-0.362
 
 
25.7
 
 
 
 
BTBD10
 
 
hsa-miR-17-5p
 
 
-0.409
 
 
17.2
 
 
 
 
BTBD10
 
 
hsa-miR-93-5p
 
 
-0.409
 
 
17.2
 
 
 
 
BTBD10
 
 
hsa-miR-20a-5p
 
 
-0.412
 
 
17.2
 
 
 
 
BTF3L4
 
 
hsa-let-7c-5p
 
 
-0.355
 
 
29.6
 
 
 
 
BTG2
 
 
hsa-miR-92a-3p
 
 
-0.314
 
 
39.0
 
 
 
 
BTG2
 
 
hsa-miR-92a-3p
 
 
-0.402
 
 
39.0
 
 
 
 
BTG3
 
 
hsa-miR-17-5p
 
 
-0.668
 
 
17.3
 
 
 
 
BTG3
 
 
hsa-miR-93-5p
 
 
-0.668
 
 
17.3
 
 
 
 
BTG3
 
 
hsa-miR-20a-5p
 
 
-0.671
 
 
17.3
 
 
 
 
BZW1
 
 
hsa-let-7c-5p
 
 
-0.354
 
 
34.3
 
 
 
 
BZW1
 
 
hsa-let-7c-5p
 
 
-0.375
 
 
34.3
 
 
 
 
 
 
 appendix 
 
 
 
 
target_symbol
 
 
mature_mirna_id
 
 
score
 
 
nTPM
 
 
 
 
 
 
AARS1
 
 
hsa-miR-92a-3p
 
 
-0.458
 
 
31.6
 
 
 
 
ACTC1
 
 
hsa-miR-92a-3p
 
 
-0.361
 
 
23.3
 
 
 
 
ACTC1
 
 
hsa-miR-7-5p
 
 
-0.471
 
 
23.3
 
 
 
 
ADAM19
 
 
hsa-miR-874-3p
 
 
-0.374
 
 
22.7
 
 
 
 
ADAM9
 
 
hsa-miR-20a-5p
 
 
-0.299
 
 
27.3
 
 
 
 
ADAM9
 
 
hsa-miR-17-5p
 
 
-0.32
 
 
27.3
 
 
 
 
ADAM9
 
 
hsa-miR-93-5p
 
 
-0.32
 
 
27.3
 
 
 
 
ADCY3
 
 
hsa-miR-92a-3p
 
 
-0.381
 
 
16.3
 
 
 
 
ADCYAP1
 
 
hsa-miR-103a-3p
 
 
-0.443
 
 
10.3
 
 
 
 
ADNP
 
 
hsa-miR-328-3p
 
 
-0.371
 
 
19.0
 
 
 
 
AGFG1
 
 
hsa-miR-103a-3p
 
 
-0.389
 
 
45.7
 
 
 
 
AHNAK
 
 
hsa-miR-20a-5p
 
 
-0.316
 
 
41.0
 
 
 
 
AHNAK
 
 
hsa-miR-17-5p
 
 
-0.337
 
 
41.0
 
 
 
 
AHNAK
 
 
hsa-miR-93-5p
 
 
-0.337
 
 
41.0
 
 
 
 
AIFM1
 
 
hsa-let-7c-5p
 
 
-0.314
 
 
16.6
 
 
 
 
AKAP10
 
 
hsa-miR-339-5p
 
 
-0.302
 
 
11.6
 
 
 
 
AKIRIN2
 
 
hsa-miR-103a-3p
 
 
-0.299
 
 
22.4
 
 
 
 
ALAD
 
 
hsa-miR-103a-3p
 
 
-0.385
 
 
15.7
 
 
 
 
ALG3
 
 
hsa-miR-20a-5p
 
 
-0.559
 
 
14.1
 
 
 
 
ALG3
 
 
hsa-miR-93-5p
 
 
-0.564
 
 
14.1
 
 
 
 
ALG3
 
 
hsa-miR-17-5p
 
 
-0.572
 
 
14.1
 
 
 
 
ANGPTL2
 
 
hsa-miR-221-3p
 
 
-0.369
 
 
31.6
 
 
 
 
ANKFY1
 
 
hsa-miR-155-5p
 
 
-0.315
 
 
12.7
 
 
 
 
ANKRD12
 
 
hsa-miR-7-5p
 
 
-0.37
 
 
19.4
 
 
 
 
ANKRD44
 
 
hsa-miR-92a-3p
 
 
-0.324
 
 
23.2
 
 
 
 
ANP32E
 
 
hsa-miR-92a-3p
 
 
-0.329
 
 
36.7
 
 
 
 
AP1AR
 
 
hsa-miR-92a-3p
 
 
-0.325
 
 
11.9
 
 
 
 
AP1S1
 
 
hsa-let-7c-5p
 
 
-0.374
 
 
13.1
 
 
 
 
AP4B1
 
 
hsa-miR-378a-3p
 
 
-0.62
 
 
10.4
 
 
 
 
APPL1
 
 
hsa-miR-92a-3p
 
 
-0.375
 
 
14.0
 
 
 
 
AQP3
 
 
hsa-miR-874-3p
 
 
-0.48
 
 
15.8
 
 
 
 
ARF4
 
 
hsa-miR-7-5p
 
 
-0.407
 
 
101.3
 
 
 
 
ARF4
 
 
hsa-miR-221-3p
 
 
-0.553
 
 
101.3
 
 
 
 
ARHGAP1
 
 
hsa-miR-17-5p
 
 
-0.325
 
 
35.8
 
 
 
 
ARHGAP1
 
 
hsa-miR-93-5p
 
 
-0.325
 
 
35.8
 
 
 
 
ARHGAP1
 
 
hsa-miR-20a-5p
 
 
-0.339
 
 
35.8
 
 
 
 
ARHGAP5
 
 
hsa-miR-486-5p
 
 
-0.3
 
 
18.6
 
 
 
 
ARID4A
 
 
hsa-miR-7-5p
 
 
-0.341
 
 
10.5
 
 
 
 
ARID4B
 
 
hsa-miR-20a-5p
 
 
-0.29
 
 
13.3
 
 
 
 
ARID4B
 
 
hsa-miR-486-5p
 
 
-0.367
 
 
13.3
 
 
 
 
ARIH2
 
 
hsa-miR-103a-3p
 
 
-0.307
 
 
15.1
 
 
 
 
ARL1
 
 
hsa-miR-20a-5p
 
 
-0.403
 
 
37.7
 
 
 
 
ARL1
 
 
hsa-miR-93-5p
 
 
-0.424
 
 
37.7
 
 
 
 
ARL1
 
 
hsa-miR-17-5p
 
 
-0.432
 
 
37.7
 
 
 
 
ARL6IP1
 
 
hsa-miR-7-5p
 
 
-0.374
 
 
73.3
 
 
 
 
ARL6IP1
 
 
hsa-miR-328-3p
 
 
-0.494
 
 
73.3
 
 
 
 
ARL6IP6
 
 
hsa-let-7c-5p
 
 
-0.293
 
 
10.9
 
 
 
 
ARL8A
 
 
hsa-miR-103a-3p
 
 
-0.38
 
 
23.6
 
 
 
 
ARMC1
 
 
hsa-miR-92a-3p
 
 
-0.322
 
 
12.8
 
 
 
 
ARMC1
 
 
hsa-miR-103a-3p
 
 
-0.525
 
 
12.8
 
 
 
 
ARMC8
 
 
hsa-miR-486-5p
 
 
-0.321
 
 
17.0
 
 
 
 
ARMC8
 
 
hsa-miR-20a-5p
 
 
-0.357
 
 
17.0
 
 
 
 
ARMC8
 
 
hsa-miR-93-5p
 
 
-0.362
 
 
17.0
 
 
 
 
ARMC8
 
 
hsa-miR-17-5p
 
 
-0.378
 
 
17.0
 
 
 
 
ARMC8
 
 
hsa-miR-103a-3p
 
 
-0.412
 
 
17.0
 
 
 
 
ARMT1
 
 
hsa-let-7c-5p
 
 
-0.303
 
 
14.4
 
 
 
 
ARNT
 
 
hsa-miR-103a-3p
 
 
-0.422
 
 
17.1
 
 
 
 
ARNT
 
 
hsa-miR-221-3p
 
 
-0.465
 
 
17.1
 
 
 
 
ARPP19
 
 
hsa-miR-328-3p
 
 
-0.404
 
 
36.4
 
 
 
 
ARPP19
 
 
hsa-let-7c-5p
 
 
-0.492
 
 
36.4
 
 
 
 
ARRDC3
 
 
hsa-miR-92a-3p
 
 
-0.458
 
 
25.3
 
 
 
 
ARRDC4
 
 
hsa-let-7c-5p
 
 
-0.329
 
 
15.6
 
 
 
 
ASAP1
 
 
hsa-let-7c-5p
 
 
-0.308
 
 
11.4
 
 
 
 
ASPH
 
 
hsa-miR-221-3p
 
 
-0.287
 
 
47.6
 
 
 
 
ASPH
 
 
hsa-miR-92a-3p
 
 
-0.416
 
 
47.6
 
 
 
 
ATF3
 
 
hsa-miR-874-3p
 
 
-0.288
 
 
59.7
 
 
 
 
ATL2
 
 
hsa-miR-103a-3p
 
 
-0.489
 
 
16.0
 
 
 
 
ATOSA
 
 
hsa-miR-221-3p
 
 
-0.495
 
 
12.5
 
 
 
 
ATOSB
 
 
hsa-miR-328-3p
 
 
-0.358
 
 
11.5
 
 
 
 
ATOSB
 
 
hsa-let-7c-5p
 
 
-0.429
 
 
11.5
 
 
 
 
ATP2A2
 
 
hsa-let-7c-5p
 
 
-0.311
 
 
54.5
 
 
 
 
ATP6V1F
 
 
hsa-miR-185-5p
 
 
-0.765
 
 
100.1
 
 
 
 
ATPAF1
 
 
hsa-let-7c-5p
 
 
-0.498
 
 
26.8
 
 
 
 
B3GAT3
 
 
hsa-let-7c-5p
 
 
-0.324
 
 
16.3
 
 
 
 
B4GALT5
 
 
hsa-miR-210-3p
 
 
-0.511
 
 
20.5
 
 
 
 
BACH1
 
 
hsa-let-7c-5p
 
 
-0.306
 
 
26.1
 
 
 
 
BACH1
 
 
hsa-let-7c-5p
 
 
-0.306
 
 
26.1
 
 
 
 
BACH1
 
 
hsa-let-7c-5p
 
 
-0.435
 
 
26.1
 
 
 
 
BACH1
 
 
hsa-let-7c-5p
 
 
-0.435
 
 
26.1
 
 
 
 
BAK1
 
 
hsa-miR-103a-3p
 
 
-0.364
 
 
19.5
 
 
 
 
BCAT2
 
 
hsa-miR-103a-3p
 
 
-0.364
 
 
10.8
 
 
 
 
BCAT2
 
 
hsa-miR-92a-3p
 
 
-0.609
 
 
10.8
 
 
 
 
BCL2L11
 
 
hsa-miR-339-5p
 
 
-0.306
 
 
16.0
 
 
 
 
BCL2L11
 
 
hsa-miR-92a-3p
 
 
-0.394
 
 
16.0
 
 
 
 
BCL2L11
 
 
hsa-miR-221-3p
 
 
-0.423
 
 
16.0
 
 
 
 
BIN1
 
 
hsa-miR-744-5p
 
 
-0.654
 
 
24.3
 
 
 
 
BIN3
 
 
hsa-let-7c-5p
 
 
-0.427
 
 
10.4
 
 
 
 
BLOC1S6
 
 
hsa-let-7c-5p
 
 
-0.321
 
 
29.4
 
 
 
 
BMF
 
 
hsa-miR-221-3p
 
 
-0.453
 
 
11.8
 
 
 
 
BNIP3L
 
 
hsa-miR-20a-5p
 
 
-0.287
 
 
35.1
 
 
 
 
BOLA3
 
 
hsa-miR-103a-3p
 
 
-0.362
 
 
21.2
 
 
 
 
BSDC1
 
 
hsa-miR-92a-3p
 
 
-0.362
 
 
16.4
 
 
 
 
BTAF1
 
 
hsa-miR-486-5p
 
 
-0.288
 
 
16.0
 
 
 
 
BTBD10
 
 
hsa-miR-17-5p
 
 
-0.409
 
 
12.9
 
 
 
 
BTBD10
 
 
hsa-miR-93-5p
 
 
-0.409
 
 
12.9
 
 
 
 
BTBD10
 
 
hsa-miR-20a-5p
 
 
-0.412
 
 
12.9
 
 
 
 
BTF3L4
 
 
hsa-let-7c-5p
 
 
-0.355
 
 
28.6
 
 
 
 
BTG2
 
 
hsa-miR-92a-3p
 
 
-0.314
 
 
117.3
 
 
 
 
BTG2
 
 
hsa-miR-92a-3p
 
 
-0.402
 
 
117.3
 
 
 
 
BTG3
 
 
hsa-miR-17-5p
 
 
-0.668
 
 
13.2
 
 
 
 
 
 
 basal ganglia 
 
 
 
 
target_symbol
 
 
mature_mirna_id
 
 
score
 
 
nTPM
 
 
 
 
 
 
AARS1
 
 
hsa-miR-92a-3p
 
 
-0.458
 
 
60.2
 
 
 
 
ABCB9
 
 
hsa-let-7c-5p
 
 
-0.422
 
 
13.6
 
 
 
 
ABHD17B
 
 
hsa-miR-486-5p
 
 
-0.348
 
 
13.8
 
 
 
 
ABHD17C
 
 
hsa-let-7c-5p
 
 
-0.359
 
 
20.2
 
 
 
 
ABHD17C
 
 
hsa-miR-330-3p
 
 
-0.399
 
 
20.2
 
 
 
 
ACOX1
 
 
hsa-miR-92a-3p
 
 
-0.321
 
 
15.1
 
 
 
 
ACSL6
 
 
hsa-let-7c-5p
 
 
-0.336
 
 
18.7
 
 
 
 
ADCY3
 
 
hsa-miR-92a-3p
 
 
-0.381
 
 
57.0
 
 
 
 
ADNP
 
 
hsa-miR-328-3p
 
 
-0.371
 
 
24.2
 
 
 
 
ADRB1
 
 
hsa-let-7c-5p
 
 
-0.351
 
 
10.0
 
 
 
 
ADRB1
 
 
hsa-miR-92a-3p
 
 
-0.478
 
 
10.0
 
 
 
 
AGFG1
 
 
hsa-miR-103a-3p
 
 
-0.389
 
 
27.0
 
 
 
 
AGFG2
 
 
hsa-miR-20a-5p
 
 
-0.365
 
 
19.5
 
 
 
 
AGFG2
 
 
hsa-miR-93-5p
 
 
-0.378
 
 
19.5
 
 
 
 
AGFG2
 
 
hsa-miR-17-5p
 
 
-0.402
 
 
19.5
 
 
 
 
AHNAK
 
 
hsa-miR-20a-5p
 
 
-0.316
 
 
22.5
 
 
 
 
AHNAK
 
 
hsa-miR-17-5p
 
 
-0.337
 
 
22.5
 
 
 
 
AHNAK
 
 
hsa-miR-93-5p
 
 
-0.337
 
 
22.5
 
 
 
 
AIFM1
 
 
hsa-let-7c-5p
 
 
-0.314
 
 
12.5
 
 
 
 
AKAP6
 
 
hsa-let-7c-5p
 
 
-0.307
 
 
10.5
 
 
 
 
AKIRIN2
 
 
hsa-miR-103a-3p
 
 
-0.299
 
 
31.9
 
 
 
 
ALAD
 
 
hsa-miR-103a-3p
 
 
-0.385
 
 
57.8
 
 
 
 
ALG3
 
 
hsa-miR-20a-5p
 
 
-0.559
 
 
10.2
 
 
 
 
ALG3
 
 
hsa-miR-93-5p
 
 
-0.564
 
 
10.2
 
 
 
 
ALG3
 
 
hsa-miR-17-5p
 
 
-0.572
 
 
10.2
 
 
 
 
ALKAL2
 
 
hsa-miR-92a-3p
 
 
-0.608
 
 
17.9
 
 
 
 
AMT
 
 
hsa-let-7c-5p
 
 
-0.463
 
 
11.4
 
 
 
 
ANGPTL2
 
 
hsa-miR-221-3p
 
 
-0.369
 
 
10.9
 
 
 
 
ANKFY1
 
 
hsa-miR-155-5p
 
 
-0.315
 
 
13.3
 
 
 
 
ANKRD46
 
 
hsa-let-7c-5p
 
 
-0.389
 
 
22.6
 
 
 
 
ANKRD9
 
 
hsa-miR-20a-5p
 
 
-0.31
 
 
18.5
 
 
 
 
ANKRD9
 
 
hsa-miR-93-5p
 
 
-0.331
 
 
18.5
 
 
 
 
ANKRD9
 
 
hsa-miR-17-5p
 
 
-0.347
 
 
18.5
 
 
 
 
ANP32E
 
 
hsa-miR-92a-3p
 
 
-0.329
 
 
38.3
 
 
 
 
AP1AR
 
 
hsa-miR-92a-3p
 
 
-0.325
 
 
13.0
 
 
 
 
AP1S1
 
 
hsa-let-7c-5p
 
 
-0.374
 
 
116.3
 
 
 
 
AP4B1
 
 
hsa-miR-378a-3p
 
 
-0.62
 
 
11.6
 
 
 
 
AP5S1
 
 
hsa-miR-744-5p
 
 
-0.354
 
 
10.0
 
 
 
 
APCDD1
 
 
hsa-miR-20a-5p
 
 
-0.404
 
 
30.3
 
 
 
 
APCDD1
 
 
hsa-miR-17-5p
 
 
-0.425
 
 
30.3
 
 
 
 
APCDD1
 
 
hsa-miR-93-5p
 
 
-0.425
 
 
30.3
 
 
 
 
ARF4
 
 
hsa-miR-7-5p
 
 
-0.407
 
 
71.0
 
 
 
 
ARF4
 
 
hsa-miR-221-3p
 
 
-0.553
 
 
71.0
 
 
 
 
ARHGAP1
 
 
hsa-miR-17-5p
 
 
-0.325
 
 
41.0
 
 
 
 
ARHGAP1
 
 
hsa-miR-93-5p
 
 
-0.325
 
 
41.0
 
 
 
 
ARHGAP1
 
 
hsa-miR-20a-5p
 
 
-0.339
 
 
41.0
 
 
 
 
ARHGAP5
 
 
hsa-miR-486-5p
 
 
-0.3
 
 
22.9
 
 
 
 
ARIH2
 
 
hsa-miR-103a-3p
 
 
-0.307
 
 
19.4
 
 
 
 
ARL1
 
 
hsa-miR-20a-5p
 
 
-0.403
 
 
28.4
 
 
 
 
ARL1
 
 
hsa-miR-93-5p
 
 
-0.424
 
 
28.4
 
 
 
 
ARL1
 
 
hsa-miR-17-5p
 
 
-0.432
 
 
28.4
 
 
 
 
ARL4D
 
 
hsa-let-7c-5p
 
 
-0.417
 
 
14.0
 
 
 
 
ARL6IP1
 
 
hsa-miR-7-5p
 
 
-0.374
 
 
196.4
 
 
 
 
ARL6IP1
 
 
hsa-miR-328-3p
 
 
-0.494
 
 
196.4
 
 
 
 
ARL8A
 
 
hsa-miR-103a-3p
 
 
-0.38
 
 
74.0
 
 
 
 
ARMC1
 
 
hsa-miR-92a-3p
 
 
-0.322
 
 
17.4
 
 
 
 
ARMC1
 
 
hsa-miR-103a-3p
 
 
-0.525
 
 
17.4
 
 
 
 
ARMC8
 
 
hsa-miR-486-5p
 
 
-0.321
 
 
13.3
 
 
 
 
ARMC8
 
 
hsa-miR-20a-5p
 
 
-0.357
 
 
13.3
 
 
 
 
ARMC8
 
 
hsa-miR-93-5p
 
 
-0.362
 
 
13.3
 
 
 
 
ARMC8
 
 
hsa-miR-17-5p
 
 
-0.378
 
 
13.3
 
 
 
 
ARMC8
 
 
hsa-miR-103a-3p
 
 
-0.412
 
 
13.3
 
 
 
 
ARMT1
 
 
hsa-let-7c-5p
 
 
-0.303
 
 
10.6
 
 
 
 
ARPP19
 
 
hsa-miR-328-3p
 
 
-0.404
 
 
481.4
 
 
 
 
ARPP19
 
 
hsa-let-7c-5p
 
 
-0.492
 
 
481.4
 
 
 
 
ARRDC3
 
 
hsa-miR-92a-3p
 
 
-0.458
 
 
10.6
 
 
 
 
ARRDC4
 
 
hsa-let-7c-5p
 
 
-0.329
 
 
10.0
 
 
 
 
ASAP1
 
 
hsa-let-7c-5p
 
 
-0.308
 
 
11.6
 
 
 
 
ASB6
 
 
hsa-miR-874-3p
 
 
-0.368
 
 
16.0
 
 
 
 
ASPH
 
 
hsa-miR-221-3p
 
 
-0.287
 
 
62.4
 
 
 
 
ASPH
 
 
hsa-miR-92a-3p
 
 
-0.416
 
 
62.4
 
 
 
 
ATF3
 
 
hsa-miR-874-3p
 
 
-0.288
 
 
24.6
 
 
 
 
ATG16L1
 
 
hsa-miR-20a-5p
 
 
-0.446
 
 
11.5
 
 
 
 
ATG16L1
 
 
hsa-miR-93-5p
 
 
-0.451
 
 
11.5
 
 
 
 
ATG16L1
 
 
hsa-miR-17-5p
 
 
-0.467
 
 
11.5
 
 
 
 
ATL2
 
 
hsa-miR-103a-3p
 
 
-0.489
 
 
18.9
 
 
 
 
ATOSB
 
 
hsa-miR-328-3p
 
 
-0.358
 
 
20.2
 
 
 
 
ATOSB
 
 
hsa-let-7c-5p
 
 
-0.429
 
 
20.2
 
 
 
 
ATP1B2
 
 
hsa-miR-103a-3p
 
 
-0.6
 
 
290.0
 
 
 
 
ATP2A2
 
 
hsa-let-7c-5p
 
 
-0.311
 
 
21.1
 
 
 
 
ATP2B2
 
 
hsa-miR-7-5p
 
 
-0.297
 
 
34.6
 
 
 
 
ATP6V1F
 
 
hsa-miR-185-5p
 
 
-0.765
 
 
288.5
 
 
 
 
ATPAF1
 
 
hsa-let-7c-5p
 
 
-0.498
 
 
88.4
 
 
 
 
AVPI1
 
 
hsa-miR-7-5p
 
 
-0.367
 
 
14.2
 
 
 
 
B3GAT3
 
 
hsa-let-7c-5p
 
 
-0.324
 
 
67.8
 
 
 
 
B4GALT5
 
 
hsa-miR-210-3p
 
 
-0.511
 
 
12.9
 
 
 
 
B4GAT1
 
 
hsa-let-7c-5p
 
 
-0.47
 
 
152.4
 
 
 
 
BACE1
 
 
hsa-miR-339-5p
 
 
-0.371
 
 
22.4
 
 
 
 
BAMBI
 
 
hsa-miR-20a-5p
 
 
-0.485
 
 
11.5
 
 
 
 
BAMBI
 
 
hsa-miR-17-5p
 
 
-0.506
 
 
11.5
 
 
 
 
BAMBI
 
 
hsa-miR-93-5p
 
 
-0.506
 
 
11.5
 
 
 
 
BCAT2
 
 
hsa-miR-103a-3p
 
 
-0.364
 
 
17.0
 
 
 
 
BCAT2
 
 
hsa-miR-92a-3p
 
 
-0.609
 
 
17.0
 
 
 
 
BDH1
 
 
hsa-miR-874-3p
 
 
-0.644
 
 
28.9
 
 
 
 
BEGAIN
 
 
hsa-let-7c-5p
 
 
-0.466
 
 
23.9
 
 
 
 
BIN1
 
 
hsa-miR-744-5p
 
 
-0.654
 
 
177.7
 
 
 
 
BIN3
 
 
hsa-let-7c-5p
 
 
-0.427
 
 
10.9
 
 
 
 
BLOC1S6
 
 
hsa-let-7c-5p
 
 
-0.321
 
 
22.3
 
 
 
 
BLTP1
 
 
hsa-miR-92a-3p
 
 
-0.462
 
 
22.7
 
 
 
 
BNIP3L
 
 
hsa-miR-20a-5p
 
 
-0.287
 
 
44.6
 
 
 
 
 
 
 blood vessel 
 
 
 
 
target_symbol
 
 
mature_mirna_id
 
 
score
 
 
nTPM
 
 
 
 
 
 
AARS1
 
 
hsa-miR-92a-3p
 
 
-0.458
 
 
38.1
 
 
 
 
ACTA1
 
 
hsa-miR-155-5p
 
 
-0.595
 
 
77.9
 
 
 
 
ACTC1
 
 
hsa-miR-92a-3p
 
 
-0.361
 
 
388.9
 
 
 
 
ACTC1
 
 
hsa-miR-7-5p
 
 
-0.471
 
 
388.9
 
 
 
 
ADAM9
 
 
hsa-miR-20a-5p
 
 
-0.299
 
 
36.8
 
 
 
 
ADAM9
 
 
hsa-miR-17-5p
 
 
-0.32
 
 
36.8
 
 
 
 
ADAM9
 
 
hsa-miR-93-5p
 
 
-0.32
 
 
36.8
 
 
 
 
ADAMTS8
 
 
hsa-let-7c-5p
 
 
-0.571
 
 
12.7
 
 
 
 
ADCY3
 
 
hsa-miR-92a-3p
 
 
-0.381
 
 
43.2
 
 
 
 
ADNP
 
 
hsa-miR-328-3p
 
 
-0.371
 
 
32.1
 
 
 
 
AGFG1
 
 
hsa-miR-103a-3p
 
 
-0.389
 
 
37.3
 
 
 
 
AGFG2
 
 
hsa-miR-20a-5p
 
 
-0.365
 
 
11.4
 
 
 
 
AGFG2
 
 
hsa-miR-93-5p
 
 
-0.378
 
 
11.4
 
 
 
 
AGFG2
 
 
hsa-miR-17-5p
 
 
-0.402
 
 
11.4
 
 
 
 
AHCTF1
 
 
hsa-let-7c-5p
 
 
-0.374
 
 
10.0
 
 
 
 
AHNAK
 
 
hsa-miR-20a-5p
 
 
-0.316
 
 
225.6
 
 
 
 
AHNAK
 
 
hsa-miR-17-5p
 
 
-0.337
 
 
225.6
 
 
 
 
AHNAK
 
 
hsa-miR-93-5p
 
 
-0.337
 
 
225.6
 
 
 
 
AKAP10
 
 
hsa-miR-339-5p
 
 
-0.302
 
 
11.9
 
 
 
 
AKAP6
 
 
hsa-let-7c-5p
 
 
-0.307
 
 
15.0
 
 
 
 
AKIRIN2
 
 
hsa-miR-103a-3p
 
 
-0.299
 
 
22.1
 
 
 
 
ALAD
 
 
hsa-miR-103a-3p
 
 
-0.385
 
 
22.2
 
 
 
 
ALG3
 
 
hsa-miR-20a-5p
 
 
-0.559
 
 
20.2
 
 
 
 
ALG3
 
 
hsa-miR-93-5p
 
 
-0.564
 
 
20.2
 
 
 
 
ALG3
 
 
hsa-miR-17-5p
 
 
-0.572
 
 
20.2
 
 
 
 
ALG9
 
 
hsa-miR-7-5p
 
 
-0.302
 
 
12.3
 
 
 
 
ALKAL2
 
 
hsa-miR-92a-3p
 
 
-0.608
 
 
43.6
 
 
 
 
AMT
 
 
hsa-let-7c-5p
 
 
-0.463
 
 
11.4
 
 
 
 
ANGPTL2
 
 
hsa-miR-221-3p
 
 
-0.369
 
 
31.4
 
 
 
 
ANKFY1
 
 
hsa-miR-155-5p
 
 
-0.315
 
 
13.2
 
 
 
 
ANKRD12
 
 
hsa-miR-7-5p
 
 
-0.37
 
 
11.6
 
 
 
 
ANKRD44
 
 
hsa-miR-92a-3p
 
 
-0.324
 
 
10.5
 
 
 
 
ANKRD9
 
 
hsa-miR-20a-5p
 
 
-0.31
 
 
10.4
 
 
 
 
ANKRD9
 
 
hsa-miR-93-5p
 
 
-0.331
 
 
10.4
 
 
 
 
ANKRD9
 
 
hsa-miR-17-5p
 
 
-0.347
 
 
10.4
 
 
 
 
ANP32E
 
 
hsa-miR-92a-3p
 
 
-0.329
 
 
23.8
 
 
 
 
AP1S1
 
 
hsa-let-7c-5p
 
 
-0.374
 
 
13.8
 
 
 
 
APCDD1
 
 
hsa-miR-20a-5p
 
 
-0.404
 
 
23.7
 
 
 
 
APCDD1
 
 
hsa-miR-17-5p
 
 
-0.425
 
 
23.7
 
 
 
 
APCDD1
 
 
hsa-miR-93-5p
 
 
-0.425
 
 
23.7
 
 
 
 
APPL1
 
 
hsa-miR-92a-3p
 
 
-0.375
 
 
12.6
 
 
 
 
ARF4
 
 
hsa-miR-7-5p
 
 
-0.407
 
 
151.4
 
 
 
 
ARF4
 
 
hsa-miR-221-3p
 
 
-0.553
 
 
151.4
 
 
 
 
ARHGAP1
 
 
hsa-miR-17-5p
 
 
-0.325
 
 
119.0
 
 
 
 
ARHGAP1
 
 
hsa-miR-93-5p
 
 
-0.325
 
 
119.0
 
 
 
 
ARHGAP1
 
 
hsa-miR-20a-5p
 
 
-0.339
 
 
119.0
 
 
 
 
ARHGAP5
 
 
hsa-miR-486-5p
 
 
-0.3
 
 
18.0
 
 
 
 
ARHGEF28
 
 
hsa-miR-93-5p
 
 
-0.33
 
 
10.7
 
 
 
 
ARHGEF28
 
 
hsa-miR-17-5p
 
 
-0.363
 
 
10.7
 
 
 
 
ARHGEF28
 
 
hsa-miR-20a-5p
 
 
-0.366
 
 
10.7
 
 
 
 
ARID4B
 
 
hsa-miR-20a-5p
 
 
-0.29
 
 
12.5
 
 
 
 
ARID4B
 
 
hsa-miR-486-5p
 
 
-0.367
 
 
12.5
 
 
 
 
ARIH2
 
 
hsa-miR-103a-3p
 
 
-0.307
 
 
23.5
 
 
 
 
ARL1
 
 
hsa-miR-20a-5p
 
 
-0.403
 
 
48.7
 
 
 
 
ARL1
 
 
hsa-miR-93-5p
 
 
-0.424
 
 
48.7
 
 
 
 
ARL1
 
 
hsa-miR-17-5p
 
 
-0.432
 
 
48.7
 
 
 
 
ARL4D
 
 
hsa-let-7c-5p
 
 
-0.417
 
 
12.9
 
 
 
 
ARL6IP1
 
 
hsa-miR-7-5p
 
 
-0.374
 
 
52.2
 
 
 
 
ARL6IP1
 
 
hsa-miR-328-3p
 
 
-0.494
 
 
52.2
 
 
 
 
ARL8A
 
 
hsa-miR-103a-3p
 
 
-0.38
 
 
42.8
 
 
 
 
ARMC1
 
 
hsa-miR-92a-3p
 
 
-0.322
 
 
15.7
 
 
 
 
ARMC1
 
 
hsa-miR-103a-3p
 
 
-0.525
 
 
15.7
 
 
 
 
ARMC8
 
 
hsa-miR-486-5p
 
 
-0.321
 
 
23.5
 
 
 
 
ARMC8
 
 
hsa-miR-20a-5p
 
 
-0.357
 
 
23.5
 
 
 
 
ARMC8
 
 
hsa-miR-93-5p
 
 
-0.362
 
 
23.5
 
 
 
 
ARMC8
 
 
hsa-miR-17-5p
 
 
-0.378
 
 
23.5
 
 
 
 
ARMC8
 
 
hsa-miR-103a-3p
 
 
-0.412
 
 
23.5
 
 
 
 
ARNT
 
 
hsa-miR-103a-3p
 
 
-0.422
 
 
26.6
 
 
 
 
ARNT
 
 
hsa-miR-221-3p
 
 
-0.465
 
 
26.6
 
 
 
 
ARPP19
 
 
hsa-miR-328-3p
 
 
-0.404
 
 
47.3
 
 
 
 
ARPP19
 
 
hsa-let-7c-5p
 
 
-0.492
 
 
47.3
 
 
 
 
ARRDC3
 
 
hsa-miR-92a-3p
 
 
-0.458
 
 
25.4
 
 
 
 
ARVCF
 
 
hsa-miR-155-5p
 
 
-0.388
 
 
15.5
 
 
 
 
ASAP1
 
 
hsa-let-7c-5p
 
 
-0.308
 
 
18.4
 
 
 
 
ASB6
 
 
hsa-miR-874-3p
 
 
-0.368
 
 
11.7
 
 
 
 
ASPH
 
 
hsa-miR-221-3p
 
 
-0.287
 
 
72.5
 
 
 
 
ASPH
 
 
hsa-miR-92a-3p
 
 
-0.416
 
 
72.5
 
 
 
 
ASPN
 
 
hsa-miR-92a-3p
 
 
-0.422
 
 
129.3
 
 
 
 
ATF3
 
 
hsa-miR-874-3p
 
 
-0.288
 
 
137.2
 
 
 
 
ATG16L1
 
 
hsa-miR-20a-5p
 
 
-0.446
 
 
12.7
 
 
 
 
ATG16L1
 
 
hsa-miR-93-5p
 
 
-0.451
 
 
12.7
 
 
 
 
ATG16L1
 
 
hsa-miR-17-5p
 
 
-0.467
 
 
12.7
 
 
 
 
ATL2
 
 
hsa-miR-103a-3p
 
 
-0.489
 
 
21.0
 
 
 
 
ATOSA
 
 
hsa-miR-221-3p
 
 
-0.495
 
 
10.4
 
 
 
 
ATOSB
 
 
hsa-miR-328-3p
 
 
-0.358
 
 
26.7
 
 
 
 
ATOSB
 
 
hsa-let-7c-5p
 
 
-0.429
 
 
26.7
 
 
 
 
ATP2A2
 
 
hsa-let-7c-5p
 
 
-0.311
 
 
84.6
 
 
 
 
ATP6V1F
 
 
hsa-miR-185-5p
 
 
-0.765
 
 
127.6
 
 
 
 
ATPAF1
 
 
hsa-let-7c-5p
 
 
-0.498
 
 
30.6
 
 
 
 
ATXN1L
 
 
hsa-miR-155-5p
 
 
-0.315
 
 
12.5
 
 
 
 
AVPI1
 
 
hsa-miR-7-5p
 
 
-0.367
 
 
57.0
 
 
 
 
B3GAT3
 
 
hsa-let-7c-5p
 
 
-0.324
 
 
38.8
 
 
 
 
B4GALT5
 
 
hsa-miR-210-3p
 
 
-0.511
 
 
15.5
 
 
 
 
B4GAT1
 
 
hsa-let-7c-5p
 
 
-0.47
 
 
18.5
 
 
 
 
BACE1
 
 
hsa-miR-339-5p
 
 
-0.371
 
 
16.1
 
 
 
 
BACE2
 
 
hsa-let-7c-5p
 
 
-0.364
 
 
11.5
 
 
 
 
BACH1
 
 
hsa-let-7c-5p
 
 
-0.306
 
 
10.9
 
 
 
 
BACH1
 
 
hsa-let-7c-5p
 
 
-0.306
 
 
10.9
 
 
 
 
BACH1
 
 
hsa-let-7c-5p
 
 
-0.435
 
 
10.9
 
 
 
 
BACH1
 
 
hsa-let-7c-5p
 
 
-0.435
 
 
10.9
 
 
 
 
 
 
 breast 
 
 
 
 
target_symbol
 
 
mature_mirna_id
 
 
score
 
 
nTPM
 
 
 
 
 
 
AARS1
 
 
hsa-miR-92a-3p
 
 
-0.458
 
 
32.1
 
 
 
 
ABHD17C
 
 
hsa-let-7c-5p
 
 
-0.359
 
 
10.9
 
 
 
 
ABHD17C
 
 
hsa-miR-330-3p
 
 
-0.399
 
 
10.9
 
 
 
 
ABHD5
 
 
hsa-miR-20a-5p
 
 
-0.397
 
 
18.8
 
 
 
 
ABHD5
 
 
hsa-miR-93-5p
 
 
-0.402
 
 
18.8
 
 
 
 
ABHD5
 
 
hsa-miR-17-5p
 
 
-0.45
 
 
18.8
 
 
 
 
ACOX1
 
 
hsa-miR-92a-3p
 
 
-0.321
 
 
17.1
 
 
 
 
ACTA1
 
 
hsa-miR-155-5p
 
 
-0.595
 
 
15.5
 
 
 
 
ACTC1
 
 
hsa-miR-92a-3p
 
 
-0.361
 
 
11.2
 
 
 
 
ACTC1
 
 
hsa-miR-7-5p
 
 
-0.471
 
 
11.2
 
 
 
 
ADAM9
 
 
hsa-miR-20a-5p
 
 
-0.299
 
 
20.2
 
 
 
 
ADAM9
 
 
hsa-miR-17-5p
 
 
-0.32
 
 
20.2
 
 
 
 
ADAM9
 
 
hsa-miR-93-5p
 
 
-0.32
 
 
20.2
 
 
 
 
ADCY3
 
 
hsa-miR-92a-3p
 
 
-0.381
 
 
72.4
 
 
 
 
ADNP
 
 
hsa-miR-328-3p
 
 
-0.371
 
 
30.5
 
 
 
 
ADRB2
 
 
hsa-let-7c-5p
 
 
-0.635
 
 
32.4
 
 
 
 
AGFG1
 
 
hsa-miR-103a-3p
 
 
-0.389
 
 
48.8
 
 
 
 
AGFG2
 
 
hsa-miR-20a-5p
 
 
-0.365
 
 
10.5
 
 
 
 
AGFG2
 
 
hsa-miR-93-5p
 
 
-0.378
 
 
10.5
 
 
 
 
AGFG2
 
 
hsa-miR-17-5p
 
 
-0.402
 
 
10.5
 
 
 
 
AHNAK
 
 
hsa-miR-20a-5p
 
 
-0.316
 
 
178.3
 
 
 
 
AHNAK
 
 
hsa-miR-17-5p
 
 
-0.337
 
 
178.3
 
 
 
 
AHNAK
 
 
hsa-miR-93-5p
 
 
-0.337
 
 
178.3
 
 
 
 
AIFM1
 
 
hsa-let-7c-5p
 
 
-0.314
 
 
11.7
 
 
 
 
AKAP10
 
 
hsa-miR-339-5p
 
 
-0.302
 
 
11.5
 
 
 
 
AKIRIN2
 
 
hsa-miR-103a-3p
 
 
-0.299
 
 
23.1
 
 
 
 
ALAD
 
 
hsa-miR-103a-3p
 
 
-0.385
 
 
21.7
 
 
 
 
ALG3
 
 
hsa-miR-20a-5p
 
 
-0.559
 
 
18.6
 
 
 
 
ALG3
 
 
hsa-miR-93-5p
 
 
-0.564
 
 
18.6
 
 
 
 
ALG3
 
 
hsa-miR-17-5p
 
 
-0.572
 
 
18.6
 
 
 
 
ALG9
 
 
hsa-miR-7-5p
 
 
-0.302
 
 
11.3
 
 
 
 
ALKAL2
 
 
hsa-miR-92a-3p
 
 
-0.608
 
 
27.8
 
 
 
 
ANGPTL2
 
 
hsa-miR-221-3p
 
 
-0.369
 
 
69.5
 
 
 
 
ANKFY1
 
 
hsa-miR-155-5p
 
 
-0.315
 
 
11.8
 
 
 
 
ANKRD12
 
 
hsa-miR-7-5p
 
 
-0.37
 
 
12.8
 
 
 
 
ANKRD29
 
 
hsa-miR-20a-5p
 
 
-0.359
 
 
15.0
 
 
 
 
ANKRD29
 
 
hsa-miR-17-5p
 
 
-0.372
 
 
15.0
 
 
 
 
ANKRD29
 
 
hsa-miR-93-5p
 
 
-0.372
 
 
15.0
 
 
 
 
ANKRD46
 
 
hsa-let-7c-5p
 
 
-0.389
 
 
13.6
 
 
 
 
ANP32E
 
 
hsa-miR-92a-3p
 
 
-0.329
 
 
16.9
 
 
 
 
AP1AR
 
 
hsa-miR-92a-3p
 
 
-0.325
 
 
15.9
 
 
 
 
AP1S1
 
 
hsa-let-7c-5p
 
 
-0.374
 
 
17.7
 
 
 
 
APCDD1
 
 
hsa-miR-20a-5p
 
 
-0.404
 
 
30.9
 
 
 
 
APCDD1
 
 
hsa-miR-17-5p
 
 
-0.425
 
 
30.9
 
 
 
 
APCDD1
 
 
hsa-miR-93-5p
 
 
-0.425
 
 
30.9
 
 
 
 
APPL1
 
 
hsa-miR-92a-3p
 
 
-0.375
 
 
17.7
 
 
 
 
AQP3
 
 
hsa-miR-874-3p
 
 
-0.48
 
 
34.4
 
 
 
 
ARF4
 
 
hsa-miR-7-5p
 
 
-0.407
 
 
129.6
 
 
 
 
ARF4
 
 
hsa-miR-221-3p
 
 
-0.553
 
 
129.6
 
 
 
 
ARHGAP1
 
 
hsa-miR-17-5p
 
 
-0.325
 
 
59.0
 
 
 
 
ARHGAP1
 
 
hsa-miR-93-5p
 
 
-0.325
 
 
59.0
 
 
 
 
ARHGAP1
 
 
hsa-miR-20a-5p
 
 
-0.339
 
 
59.0
 
 
 
 
ARHGAP5
 
 
hsa-miR-486-5p
 
 
-0.3
 
 
25.6
 
 
 
 
ARID4B
 
 
hsa-miR-20a-5p
 
 
-0.29
 
 
12.7
 
 
 
 
ARID4B
 
 
hsa-miR-486-5p
 
 
-0.367
 
 
12.7
 
 
 
 
ARIH2
 
 
hsa-miR-103a-3p
 
 
-0.307
 
 
19.2
 
 
 
 
ARL1
 
 
hsa-miR-20a-5p
 
 
-0.403
 
 
58.5
 
 
 
 
ARL1
 
 
hsa-miR-93-5p
 
 
-0.424
 
 
58.5
 
 
 
 
ARL1
 
 
hsa-miR-17-5p
 
 
-0.432
 
 
58.5
 
 
 
 
ARL6IP1
 
 
hsa-miR-7-5p
 
 
-0.374
 
 
105.7
 
 
 
 
ARL6IP1
 
 
hsa-miR-328-3p
 
 
-0.494
 
 
105.7
 
 
 
 
ARL6IP6
 
 
hsa-let-7c-5p
 
 
-0.293
 
 
12.8
 
 
 
 
ARL8A
 
 
hsa-miR-103a-3p
 
 
-0.38
 
 
36.1
 
 
 
 
ARMC1
 
 
hsa-miR-92a-3p
 
 
-0.322
 
 
17.2
 
 
 
 
ARMC1
 
 
hsa-miR-103a-3p
 
 
-0.525
 
 
17.2
 
 
 
 
ARMC8
 
 
hsa-miR-486-5p
 
 
-0.321
 
 
15.4
 
 
 
 
ARMC8
 
 
hsa-miR-20a-5p
 
 
-0.357
 
 
15.4
 
 
 
 
ARMC8
 
 
hsa-miR-93-5p
 
 
-0.362
 
 
15.4
 
 
 
 
ARMC8
 
 
hsa-miR-17-5p
 
 
-0.378
 
 
15.4
 
 
 
 
ARMC8
 
 
hsa-miR-103a-3p
 
 
-0.412
 
 
15.4
 
 
 
 
ARMT1
 
 
hsa-let-7c-5p
 
 
-0.303
 
 
23.4
 
 
 
 
ARNT
 
 
hsa-miR-103a-3p
 
 
-0.422
 
 
22.2
 
 
 
 
ARNT
 
 
hsa-miR-221-3p
 
 
-0.465
 
 
22.2
 
 
 
 
ARPP19
 
 
hsa-miR-328-3p
 
 
-0.404
 
 
43.2
 
 
 
 
ARPP19
 
 
hsa-let-7c-5p
 
 
-0.492
 
 
43.2
 
 
 
 
ARRDC3
 
 
hsa-miR-92a-3p
 
 
-0.458
 
 
88.5
 
 
 
 
ARRDC4
 
 
hsa-let-7c-5p
 
 
-0.329
 
 
18.6
 
 
 
 
ASAP1
 
 
hsa-let-7c-5p
 
 
-0.308
 
 
22.2
 
 
 
 
ASPH
 
 
hsa-miR-221-3p
 
 
-0.287
 
 
99.5
 
 
 
 
ASPH
 
 
hsa-miR-92a-3p
 
 
-0.416
 
 
99.5
 
 
 
 
ASPN
 
 
hsa-miR-92a-3p
 
 
-0.422
 
 
11.1
 
 
 
 
ASPRV1
 
 
hsa-miR-330-3p
 
 
-0.301
 
 
23.5
 
 
 
 
ATF3
 
 
hsa-miR-874-3p
 
 
-0.288
 
 
97.6
 
 
 
 
ATL2
 
 
hsa-miR-103a-3p
 
 
-0.489
 
 
38.9
 
 
 
 
ATOSA
 
 
hsa-miR-221-3p
 
 
-0.495
 
 
25.6
 
 
 
 
ATOSB
 
 
hsa-miR-328-3p
 
 
-0.358
 
 
10.1
 
 
 
 
ATOSB
 
 
hsa-let-7c-5p
 
 
-0.429
 
 
10.1
 
 
 
 
ATP2A2
 
 
hsa-let-7c-5p
 
 
-0.311
 
 
28.6
 
 
 
 
ATP6V1F
 
 
hsa-miR-185-5p
 
 
-0.765
 
 
123.3
 
 
 
 
ATPAF1
 
 
hsa-let-7c-5p
 
 
-0.498
 
 
33.1
 
 
 
 
ATXN1L
 
 
hsa-miR-155-5p
 
 
-0.315
 
 
10.2
 
 
 
 
AVPI1
 
 
hsa-miR-7-5p
 
 
-0.367
 
 
22.2
 
 
 
 
B3GAT3
 
 
hsa-let-7c-5p
 
 
-0.324
 
 
29.8
 
 
 
 
B4GALT5
 
 
hsa-miR-210-3p
 
 
-0.511
 
 
17.9
 
 
 
 
B4GAT1
 
 
hsa-let-7c-5p
 
 
-0.47
 
 
15.6
 
 
 
 
BACH1
 
 
hsa-let-7c-5p
 
 
-0.306
 
 
12.3
 
 
 
 
BACH1
 
 
hsa-let-7c-5p
 
 
-0.306
 
 
12.3
 
 
 
 
BACH1
 
 
hsa-let-7c-5p
 
 
-0.435
 
 
12.3
 
 
 
 
BACH1
 
 
hsa-let-7c-5p
 
 
-0.435
 
 
12.3
 
 
 
 
BAMBI
 
 
hsa-miR-20a-5p
 
 
-0.485
 
 
22.3
 
 
 
 
 
 
 cerebellum 
 
 
 
 
target_symbol
 
 
mature_mirna_id
 
 
score
 
 
nTPM
 
 
 
 
 
 
AARS1
 
 
hsa-miR-92a-3p
 
 
-0.458
 
 
60.7
 
 
 
 
ABCB9
 
 
hsa-let-7c-5p
 
 
-0.422
 
 
18.0
 
 
 
 
ABHD17B
 
 
hsa-miR-486-5p
 
 
-0.348
 
 
11.2
 
 
 
 
ACSL6
 
 
hsa-let-7c-5p
 
 
-0.336
 
 
27.8
 
 
 
 
ADCY3
 
 
hsa-miR-92a-3p
 
 
-0.381
 
 
33.6
 
 
 
 
ADNP
 
 
hsa-miR-328-3p
 
 
-0.371
 
 
38.9
 
 
 
 
AGFG1
 
 
hsa-miR-103a-3p
 
 
-0.389
 
 
22.4
 
 
 
 
AGFG2
 
 
hsa-miR-20a-5p
 
 
-0.365
 
 
12.7
 
 
 
 
AGFG2
 
 
hsa-miR-93-5p
 
 
-0.378
 
 
12.7
 
 
 
 
AGFG2
 
 
hsa-miR-17-5p
 
 
-0.402
 
 
12.7
 
 
 
 
AHNAK
 
 
hsa-miR-20a-5p
 
 
-0.316
 
 
15.7
 
 
 
 
AHNAK
 
 
hsa-miR-17-5p
 
 
-0.337
 
 
15.7
 
 
 
 
AHNAK
 
 
hsa-miR-93-5p
 
 
-0.337
 
 
15.7
 
 
 
 
AKAP10
 
 
hsa-miR-339-5p
 
 
-0.302
 
 
10.0
 
 
 
 
AKIRIN2
 
 
hsa-miR-103a-3p
 
 
-0.299
 
 
25.9
 
 
 
 
ALAD
 
 
hsa-miR-103a-3p
 
 
-0.385
 
 
17.9
 
 
 
 
ALS2
 
 
hsa-miR-378a-3p
 
 
-0.335
 
 
21.3
 
 
 
 
AMT
 
 
hsa-let-7c-5p
 
 
-0.463
 
 
20.1
 
 
 
 
ANGPTL2
 
 
hsa-miR-221-3p
 
 
-0.369
 
 
18.7
 
 
 
 
ANK1
 
 
hsa-miR-103a-3p
 
 
-0.321
 
 
48.2
 
 
 
 
ANKFY1
 
 
hsa-miR-155-5p
 
 
-0.315
 
 
15.9
 
 
 
 
ANKRD12
 
 
hsa-miR-7-5p
 
 
-0.37
 
 
13.0
 
 
 
 
ANKRD13C
 
 
hsa-miR-20a-5p
 
 
-0.505
 
 
12.9
 
 
 
 
ANKRD13C
 
 
hsa-miR-17-5p
 
 
-0.526
 
 
12.9
 
 
 
 
ANKRD13C
 
 
hsa-miR-93-5p
 
 
-0.526
 
 
12.9
 
 
 
 
ANKRD46
 
 
hsa-let-7c-5p
 
 
-0.389
 
 
27.5
 
 
 
 
ANKRD9
 
 
hsa-miR-20a-5p
 
 
-0.31
 
 
22.1
 
 
 
 
ANKRD9
 
 
hsa-miR-93-5p
 
 
-0.331
 
 
22.1
 
 
 
 
ANKRD9
 
 
hsa-miR-17-5p
 
 
-0.347
 
 
22.1
 
 
 
 
ANP32E
 
 
hsa-miR-92a-3p
 
 
-0.329
 
 
58.6
 
 
 
 
AP1AR
 
 
hsa-miR-92a-3p
 
 
-0.325
 
 
13.2
 
 
 
 
AP1S1
 
 
hsa-let-7c-5p
 
 
-0.374
 
 
23.9
 
 
 
 
AP4B1
 
 
hsa-miR-378a-3p
 
 
-0.62
 
 
20.4
 
 
 
 
APBB3
 
 
hsa-let-7c-5p
 
 
-0.587
 
 
23.6
 
 
 
 
APPL1
 
 
hsa-miR-92a-3p
 
 
-0.375
 
 
11.8
 
 
 
 
ARF4
 
 
hsa-miR-7-5p
 
 
-0.407
 
 
50.2
 
 
 
 
ARF4
 
 
hsa-miR-221-3p
 
 
-0.553
 
 
50.2
 
 
 
 
ARG2
 
 
hsa-let-7c-5p
 
 
-0.468
 
 
16.4
 
 
 
 
ARHGAP1
 
 
hsa-miR-17-5p
 
 
-0.325
 
 
19.6
 
 
 
 
ARHGAP1
 
 
hsa-miR-93-5p
 
 
-0.325
 
 
19.6
 
 
 
 
ARHGAP1
 
 
hsa-miR-20a-5p
 
 
-0.339
 
 
19.6
 
 
 
 
ARHGAP5
 
 
hsa-miR-486-5p
 
 
-0.3
 
 
20.0
 
 
 
 
ARID4A
 
 
hsa-miR-7-5p
 
 
-0.341
 
 
10.7
 
 
 
 
ARID4B
 
 
hsa-miR-20a-5p
 
 
-0.29
 
 
15.1
 
 
 
 
ARID4B
 
 
hsa-miR-486-5p
 
 
-0.367
 
 
15.1
 
 
 
 
ARIH2
 
 
hsa-miR-103a-3p
 
 
-0.307
 
 
27.6
 
 
 
 
ARL1
 
 
hsa-miR-20a-5p
 
 
-0.403
 
 
20.8
 
 
 
 
ARL1
 
 
hsa-miR-93-5p
 
 
-0.424
 
 
20.8
 
 
 
 
ARL1
 
 
hsa-miR-17-5p
 
 
-0.432
 
 
20.8
 
 
 
 
ARL6IP1
 
 
hsa-miR-7-5p
 
 
-0.374
 
 
234.0
 
 
 
 
ARL6IP1
 
 
hsa-miR-328-3p
 
 
-0.494
 
 
234.0
 
 
 
 
ARL8A
 
 
hsa-miR-103a-3p
 
 
-0.38
 
 
55.9
 
 
 
 
ARMC1
 
 
hsa-miR-92a-3p
 
 
-0.322
 
 
20.5
 
 
 
 
ARMC1
 
 
hsa-miR-103a-3p
 
 
-0.525
 
 
20.5
 
 
 
 
ARMC8
 
 
hsa-miR-486-5p
 
 
-0.321
 
 
35.7
 
 
 
 
ARMC8
 
 
hsa-miR-20a-5p
 
 
-0.357
 
 
35.7
 
 
 
 
ARMC8
 
 
hsa-miR-93-5p
 
 
-0.362
 
 
35.7
 
 
 
 
ARMC8
 
 
hsa-miR-17-5p
 
 
-0.378
 
 
35.7
 
 
 
 
ARMC8
 
 
hsa-miR-103a-3p
 
 
-0.412
 
 
35.7
 
 
 
 
ARMT1
 
 
hsa-let-7c-5p
 
 
-0.303
 
 
11.5
 
 
 
 
ARNT
 
 
hsa-miR-103a-3p
 
 
-0.422
 
 
19.0
 
 
 
 
ARNT
 
 
hsa-miR-221-3p
 
 
-0.465
 
 
19.0
 
 
 
 
ARPP19
 
 
hsa-miR-328-3p
 
 
-0.404
 
 
66.7
 
 
 
 
ARPP19
 
 
hsa-let-7c-5p
 
 
-0.492
 
 
66.7
 
 
 
 
ARRDC3
 
 
hsa-miR-92a-3p
 
 
-0.458
 
 
17.8
 
 
 
 
ARVCF
 
 
hsa-miR-155-5p
 
 
-0.388
 
 
52.5
 
 
 
 
ASB6
 
 
hsa-miR-874-3p
 
 
-0.368
 
 
21.0
 
 
 
 
ASPH
 
 
hsa-miR-221-3p
 
 
-0.287
 
 
80.5
 
 
 
 
ASPH
 
 
hsa-miR-92a-3p
 
 
-0.416
 
 
80.5
 
 
 
 
ATG16L1
 
 
hsa-miR-20a-5p
 
 
-0.446
 
 
24.1
 
 
 
 
ATG16L1
 
 
hsa-miR-93-5p
 
 
-0.451
 
 
24.1
 
 
 
 
ATG16L1
 
 
hsa-miR-17-5p
 
 
-0.467
 
 
24.1
 
 
 
 
ATL2
 
 
hsa-miR-103a-3p
 
 
-0.489
 
 
21.7
 
 
 
 
ATOSA
 
 
hsa-miR-221-3p
 
 
-0.495
 
 
11.0
 
 
 
 
ATOSB
 
 
hsa-miR-328-3p
 
 
-0.358
 
 
14.6
 
 
 
 
ATOSB
 
 
hsa-let-7c-5p
 
 
-0.429
 
 
14.6
 
 
 
 
ATP1B2
 
 
hsa-miR-103a-3p
 
 
-0.6
 
 
261.0
 
 
 
 
ATP2A2
 
 
hsa-let-7c-5p
 
 
-0.311
 
 
15.0
 
 
 
 
ATP2B2
 
 
hsa-miR-7-5p
 
 
-0.297
 
 
88.0
 
 
 
 
ATP6V1F
 
 
hsa-miR-185-5p
 
 
-0.765
 
 
212.3
 
 
 
 
ATPAF1
 
 
hsa-let-7c-5p
 
 
-0.498
 
 
24.4
 
 
 
 
AVPI1
 
 
hsa-miR-7-5p
 
 
-0.367
 
 
16.8
 
 
 
 
B3GAT3
 
 
hsa-let-7c-5p
 
 
-0.324
 
 
70.0
 
 
 
 
B4GALT5
 
 
hsa-miR-210-3p
 
 
-0.511
 
 
20.4
 
 
 
 
B4GAT1
 
 
hsa-let-7c-5p
 
 
-0.47
 
 
105.2
 
 
 
 
BACE1
 
 
hsa-miR-339-5p
 
 
-0.371
 
 
16.3
 
 
 
 
BCAT2
 
 
hsa-miR-103a-3p
 
 
-0.364
 
 
34.8
 
 
 
 
BCAT2
 
 
hsa-miR-92a-3p
 
 
-0.609
 
 
34.8
 
 
 
 
BDH1
 
 
hsa-miR-874-3p
 
 
-0.644
 
 
48.5
 
 
 
 
BEGAIN
 
 
hsa-let-7c-5p
 
 
-0.466
 
 
53.7
 
 
 
 
BICDL1
 
 
hsa-miR-221-3p
 
 
-0.447
 
 
32.0
 
 
 
 
BIN1
 
 
hsa-miR-744-5p
 
 
-0.654
 
 
88.3
 
 
 
 
BLOC1S6
 
 
hsa-let-7c-5p
 
 
-0.321
 
 
18.6
 
 
 
 
BLTP1
 
 
hsa-miR-92a-3p
 
 
-0.462
 
 
22.7
 
 
 
 
BNIP3L
 
 
hsa-miR-20a-5p
 
 
-0.287
 
 
38.4
 
 
 
 
BOLA3
 
 
hsa-miR-103a-3p
 
 
-0.362
 
 
34.7
 
 
 
 
BSDC1
 
 
hsa-miR-92a-3p
 
 
-0.362
 
 
40.0
 
 
 
 
BTAF1
 
 
hsa-miR-486-5p
 
 
-0.288
 
 
10.7
 
 
 
 
BTBD10
 
 
hsa-miR-17-5p
 
 
-0.409
 
 
18.9
 
 
 
 
BTBD10
 
 
hsa-miR-93-5p
 
 
-0.409
 
 
18.9
 
 
 
 
 
 
 cerebral cortex 
 
 
 
 
target_symbol
 
 
mature_mirna_id
 
 
score
 
 
nTPM
 
 
 
 
 
 
AARS1
 
 
hsa-miR-92a-3p
 
 
-0.458
 
 
89.1
 
 
 
 
ABCB9
 
 
hsa-let-7c-5p
 
 
-0.422
 
 
19.5
 
 
 
 
ABHD17B
 
 
hsa-miR-486-5p
 
 
-0.348
 
 
17.0
 
 
 
 
ABHD17C
 
 
hsa-let-7c-5p
 
 
-0.359
 
 
16.3
 
 
 
 
ABHD17C
 
 
hsa-miR-330-3p
 
 
-0.399
 
 
16.3
 
 
 
 
ACOX1
 
 
hsa-miR-92a-3p
 
 
-0.321
 
 
12.8
 
 
 
 
ACSL6
 
 
hsa-let-7c-5p
 
 
-0.336
 
 
29.5
 
 
 
 
ADAM9
 
 
hsa-miR-20a-5p
 
 
-0.299
 
 
16.2
 
 
 
 
ADAM9
 
 
hsa-miR-17-5p
 
 
-0.32
 
 
16.2
 
 
 
 
ADAM9
 
 
hsa-miR-93-5p
 
 
-0.32
 
 
16.2
 
 
 
 
ADCY3
 
 
hsa-miR-92a-3p
 
 
-0.381
 
 
37.4
 
 
 
 
ADNP
 
 
hsa-miR-328-3p
 
 
-0.371
 
 
26.0
 
 
 
 
AGFG1
 
 
hsa-miR-103a-3p
 
 
-0.389
 
 
33.6
 
 
 
 
AGFG2
 
 
hsa-miR-20a-5p
 
 
-0.365
 
 
14.0
 
 
 
 
AGFG2
 
 
hsa-miR-93-5p
 
 
-0.378
 
 
14.0
 
 
 
 
AGFG2
 
 
hsa-miR-17-5p
 
 
-0.402
 
 
14.0
 
 
 
 
AHNAK
 
 
hsa-miR-20a-5p
 
 
-0.316
 
 
26.8
 
 
 
 
AHNAK
 
 
hsa-miR-17-5p
 
 
-0.337
 
 
26.8
 
 
 
 
AHNAK
 
 
hsa-miR-93-5p
 
 
-0.337
 
 
26.8
 
 
 
 
AIFM1
 
 
hsa-let-7c-5p
 
 
-0.314
 
 
10.3
 
 
 
 
AKAP10
 
 
hsa-miR-339-5p
 
 
-0.302
 
 
12.2
 
 
 
 
AKAP6
 
 
hsa-let-7c-5p
 
 
-0.307
 
 
27.4
 
 
 
 
AKIRIN2
 
 
hsa-miR-103a-3p
 
 
-0.299
 
 
33.4
 
 
 
 
ALAD
 
 
hsa-miR-103a-3p
 
 
-0.385
 
 
40.0
 
 
 
 
ANGPTL2
 
 
hsa-miR-221-3p
 
 
-0.369
 
 
32.8
 
 
 
 
ANKFY1
 
 
hsa-miR-155-5p
 
 
-0.315
 
 
19.9
 
 
 
 
ANKRD12
 
 
hsa-miR-7-5p
 
 
-0.37
 
 
15.1
 
 
 
 
ANKRD13C
 
 
hsa-miR-20a-5p
 
 
-0.505
 
 
10.2
 
 
 
 
ANKRD13C
 
 
hsa-miR-17-5p
 
 
-0.526
 
 
10.2
 
 
 
 
ANKRD13C
 
 
hsa-miR-93-5p
 
 
-0.526
 
 
10.2
 
 
 
 
ANKRD46
 
 
hsa-let-7c-5p
 
 
-0.389
 
 
39.5
 
 
 
 
ANKRD9
 
 
hsa-miR-20a-5p
 
 
-0.31
 
 
28.9
 
 
 
 
ANKRD9
 
 
hsa-miR-93-5p
 
 
-0.331
 
 
28.9
 
 
 
 
ANKRD9
 
 
hsa-miR-17-5p
 
 
-0.347
 
 
28.9
 
 
 
 
ANP32E
 
 
hsa-miR-92a-3p
 
 
-0.329
 
 
33.9
 
 
 
 
AP1AR
 
 
hsa-miR-92a-3p
 
 
-0.325
 
 
12.2
 
 
 
 
AP1S1
 
 
hsa-let-7c-5p
 
 
-0.374
 
 
104.9
 
 
 
 
AP4B1
 
 
hsa-miR-378a-3p
 
 
-0.62
 
 
10.6
 
 
 
 
APBB3
 
 
hsa-let-7c-5p
 
 
-0.587
 
 
15.0
 
 
 
 
APCDD1
 
 
hsa-miR-20a-5p
 
 
-0.404
 
 
33.1
 
 
 
 
APCDD1
 
 
hsa-miR-17-5p
 
 
-0.425
 
 
33.1
 
 
 
 
APCDD1
 
 
hsa-miR-93-5p
 
 
-0.425
 
 
33.1
 
 
 
 
APPL1
 
 
hsa-miR-92a-3p
 
 
-0.375
 
 
15.1
 
 
 
 
ARF4
 
 
hsa-miR-7-5p
 
 
-0.407
 
 
61.5
 
 
 
 
ARF4
 
 
hsa-miR-221-3p
 
 
-0.553
 
 
61.5
 
 
 
 
ARHGAP1
 
 
hsa-miR-17-5p
 
 
-0.325
 
 
40.8
 
 
 
 
ARHGAP1
 
 
hsa-miR-93-5p
 
 
-0.325
 
 
40.8
 
 
 
 
ARHGAP1
 
 
hsa-miR-20a-5p
 
 
-0.339
 
 
40.8
 
 
 
 
ARHGAP5
 
 
hsa-miR-486-5p
 
 
-0.3
 
 
48.8
 
 
 
 
ARID4A
 
 
hsa-miR-7-5p
 
 
-0.341
 
 
13.3
 
 
 
 
ARID4B
 
 
hsa-miR-20a-5p
 
 
-0.29
 
 
11.1
 
 
 
 
ARID4B
 
 
hsa-miR-486-5p
 
 
-0.367
 
 
11.1
 
 
 
 
ARIH2
 
 
hsa-miR-103a-3p
 
 
-0.307
 
 
19.9
 
 
 
 
ARL1
 
 
hsa-miR-20a-5p
 
 
-0.403
 
 
34.0
 
 
 
 
ARL1
 
 
hsa-miR-93-5p
 
 
-0.424
 
 
34.0
 
 
 
 
ARL1
 
 
hsa-miR-17-5p
 
 
-0.432
 
 
34.0
 
 
 
 
ARL4D
 
 
hsa-let-7c-5p
 
 
-0.417
 
 
14.3
 
 
 
 
ARL6IP1
 
 
hsa-miR-7-5p
 
 
-0.374
 
 
314.1
 
 
 
 
ARL6IP1
 
 
hsa-miR-328-3p
 
 
-0.494
 
 
314.1
 
 
 
 
ARL8A
 
 
hsa-miR-103a-3p
 
 
-0.38
 
 
75.4
 
 
 
 
ARMC1
 
 
hsa-miR-92a-3p
 
 
-0.322
 
 
19.6
 
 
 
 
ARMC1
 
 
hsa-miR-103a-3p
 
 
-0.525
 
 
19.6
 
 
 
 
ARMC8
 
 
hsa-miR-486-5p
 
 
-0.321
 
 
21.7
 
 
 
 
ARMC8
 
 
hsa-miR-20a-5p
 
 
-0.357
 
 
21.7
 
 
 
 
ARMC8
 
 
hsa-miR-93-5p
 
 
-0.362
 
 
21.7
 
 
 
 
ARMC8
 
 
hsa-miR-17-5p
 
 
-0.378
 
 
21.7
 
 
 
 
ARMC8
 
 
hsa-miR-103a-3p
 
 
-0.412
 
 
21.7
 
 
 
 
ARMT1
 
 
hsa-let-7c-5p
 
 
-0.303
 
 
10.1
 
 
 
 
ARPP19
 
 
hsa-miR-328-3p
 
 
-0.404
 
 
292.4
 
 
 
 
ARPP19
 
 
hsa-let-7c-5p
 
 
-0.492
 
 
292.4
 
 
 
 
ARRDC3
 
 
hsa-miR-92a-3p
 
 
-0.458
 
 
15.6
 
 
 
 
ARRDC4
 
 
hsa-let-7c-5p
 
 
-0.329
 
 
12.8
 
 
 
 
ASAP1
 
 
hsa-let-7c-5p
 
 
-0.308
 
 
21.7
 
 
 
 
ASB6
 
 
hsa-miR-874-3p
 
 
-0.368
 
 
21.5
 
 
 
 
ASPH
 
 
hsa-miR-221-3p
 
 
-0.287
 
 
76.3
 
 
 
 
ASPH
 
 
hsa-miR-92a-3p
 
 
-0.416
 
 
76.3
 
 
 
 
ATF3
 
 
hsa-miR-874-3p
 
 
-0.288
 
 
13.9
 
 
 
 
ATG16L1
 
 
hsa-miR-20a-5p
 
 
-0.446
 
 
13.9
 
 
 
 
ATG16L1
 
 
hsa-miR-93-5p
 
 
-0.451
 
 
13.9
 
 
 
 
ATG16L1
 
 
hsa-miR-17-5p
 
 
-0.467
 
 
13.9
 
 
 
 
ATL2
 
 
hsa-miR-103a-3p
 
 
-0.489
 
 
14.6
 
 
 
 
ATOSB
 
 
hsa-miR-328-3p
 
 
-0.358
 
 
21.2
 
 
 
 
ATOSB
 
 
hsa-let-7c-5p
 
 
-0.429
 
 
21.2
 
 
 
 
ATP1B2
 
 
hsa-miR-103a-3p
 
 
-0.6
 
 
257.0
 
 
 
 
ATP2A2
 
 
hsa-let-7c-5p
 
 
-0.311
 
 
54.4
 
 
 
 
ATP2B2
 
 
hsa-miR-7-5p
 
 
-0.297
 
 
63.7
 
 
 
 
ATP6V1F
 
 
hsa-miR-185-5p
 
 
-0.765
 
 
343.1
 
 
 
 
ATPAF1
 
 
hsa-let-7c-5p
 
 
-0.498
 
 
74.9
 
 
 
 
ATXN1
 
 
hsa-miR-221-3p
 
 
-0.289
 
 
12.1
 
 
 
 
AVPI1
 
 
hsa-miR-7-5p
 
 
-0.367
 
 
24.4
 
 
 
 
B3GALT2
 
 
hsa-miR-17-5p
 
 
-0.35
 
 
15.3
 
 
 
 
B3GALT2
 
 
hsa-miR-93-5p
 
 
-0.35
 
 
15.3
 
 
 
 
B3GALT2
 
 
hsa-miR-20a-5p
 
 
-0.364
 
 
15.3
 
 
 
 
B3GALT2
 
 
hsa-miR-92a-3p
 
 
-0.44
 
 
15.3
 
 
 
 
B3GAT3
 
 
hsa-let-7c-5p
 
 
-0.324
 
 
63.6
 
 
 
 
B4GALT5
 
 
hsa-miR-210-3p
 
 
-0.511
 
 
22.9
 
 
 
 
B4GAT1
 
 
hsa-let-7c-5p
 
 
-0.47
 
 
171.8
 
 
 
 
BACE1
 
 
hsa-miR-339-5p
 
 
-0.371
 
 
45.5
 
 
 
 
BCAT2
 
 
hsa-miR-103a-3p
 
 
-0.364
 
 
18.5
 
 
 
 
BCAT2
 
 
hsa-miR-92a-3p
 
 
-0.609
 
 
18.5
 
 
 
 
 
 
 cervix 
 
 
 
 
target_symbol
 
 
mature_mirna_id
 
 
score
 
 
nTPM
 
 
 
 
 
 
AARS1
 
 
hsa-miR-92a-3p
 
 
-0.458
 
 
34.1
 
 
 
 
ABHD17C
 
 
hsa-let-7c-5p
 
 
-0.359
 
 
18.1
 
 
 
 
ABHD17C
 
 
hsa-miR-330-3p
 
 
-0.399
 
 
18.1
 
 
 
 
ACOX1
 
 
hsa-miR-92a-3p
 
 
-0.321
 
 
12.8
 
 
 
 
ACP3
 
 
hsa-let-7c-5p
 
 
-0.298
 
 
10.9
 
 
 
 
ACTC1
 
 
hsa-miR-92a-3p
 
 
-0.361
 
 
44.2
 
 
 
 
ACTC1
 
 
hsa-miR-7-5p
 
 
-0.471
 
 
44.2
 
 
 
 
ADAM9
 
 
hsa-miR-20a-5p
 
 
-0.299
 
 
32.7
 
 
 
 
ADAM9
 
 
hsa-miR-17-5p
 
 
-0.32
 
 
32.7
 
 
 
 
ADAM9
 
 
hsa-miR-93-5p
 
 
-0.32
 
 
32.7
 
 
 
 
ADCY3
 
 
hsa-miR-92a-3p
 
 
-0.381
 
 
60.4
 
 
 
 
ADNP
 
 
hsa-miR-328-3p
 
 
-0.371
 
 
35.1
 
 
 
 
ADRB2
 
 
hsa-let-7c-5p
 
 
-0.635
 
 
12.7
 
 
 
 
AGFG1
 
 
hsa-miR-103a-3p
 
 
-0.389
 
 
27.5
 
 
 
 
AGFG2
 
 
hsa-miR-20a-5p
 
 
-0.365
 
 
11.6
 
 
 
 
AGFG2
 
 
hsa-miR-93-5p
 
 
-0.378
 
 
11.6
 
 
 
 
AGFG2
 
 
hsa-miR-17-5p
 
 
-0.402
 
 
11.6
 
 
 
 
AHNAK
 
 
hsa-miR-20a-5p
 
 
-0.316
 
 
181.1
 
 
 
 
AHNAK
 
 
hsa-miR-17-5p
 
 
-0.337
 
 
181.1
 
 
 
 
AHNAK
 
 
hsa-miR-93-5p
 
 
-0.337
 
 
181.1
 
 
 
 
AIFM1
 
 
hsa-let-7c-5p
 
 
-0.314
 
 
14.8
 
 
 
 
AJUBA
 
 
hsa-miR-103a-3p
 
 
-0.448
 
 
14.5
 
 
 
 
AKAP10
 
 
hsa-miR-339-5p
 
 
-0.302
 
 
10.8
 
 
 
 
AKIRIN2
 
 
hsa-miR-103a-3p
 
 
-0.299
 
 
19.6
 
 
 
 
ALAD
 
 
hsa-miR-103a-3p
 
 
-0.385
 
 
18.9
 
 
 
 
ALG3
 
 
hsa-miR-20a-5p
 
 
-0.559
 
 
18.1
 
 
 
 
ALG3
 
 
hsa-miR-93-5p
 
 
-0.564
 
 
18.1
 
 
 
 
ALG3
 
 
hsa-miR-17-5p
 
 
-0.572
 
 
18.1
 
 
 
 
ALG9
 
 
hsa-miR-7-5p
 
 
-0.302
 
 
10.8
 
 
 
 
AMT
 
 
hsa-let-7c-5p
 
 
-0.463
 
 
14.5
 
 
 
 
ANGPTL2
 
 
hsa-miR-221-3p
 
 
-0.369
 
 
46.6
 
 
 
 
ANKFY1
 
 
hsa-miR-155-5p
 
 
-0.315
 
 
13.8
 
 
 
 
ANKRD12
 
 
hsa-miR-7-5p
 
 
-0.37
 
 
10.5
 
 
 
 
ANKRD29
 
 
hsa-miR-20a-5p
 
 
-0.359
 
 
12.2
 
 
 
 
ANKRD29
 
 
hsa-miR-17-5p
 
 
-0.372
 
 
12.2
 
 
 
 
ANKRD29
 
 
hsa-miR-93-5p
 
 
-0.372
 
 
12.2
 
 
 
 
ANKRD46
 
 
hsa-let-7c-5p
 
 
-0.389
 
 
14.1
 
 
 
 
ANKRD50
 
 
hsa-miR-20a-5p
 
 
-0.3
 
 
10.7
 
 
 
 
ANKRD9
 
 
hsa-miR-20a-5p
 
 
-0.31
 
 
10.4
 
 
 
 
ANKRD9
 
 
hsa-miR-93-5p
 
 
-0.331
 
 
10.4
 
 
 
 
ANKRD9
 
 
hsa-miR-17-5p
 
 
-0.347
 
 
10.4
 
 
 
 
ANP32E
 
 
hsa-miR-92a-3p
 
 
-0.329
 
 
19.3
 
 
 
 
AP1S1
 
 
hsa-let-7c-5p
 
 
-0.374
 
 
20.1
 
 
 
 
AP4B1
 
 
hsa-miR-378a-3p
 
 
-0.62
 
 
11.8
 
 
 
 
APCDD1
 
 
hsa-miR-20a-5p
 
 
-0.404
 
 
33.1
 
 
 
 
APCDD1
 
 
hsa-miR-17-5p
 
 
-0.425
 
 
33.1
 
 
 
 
APCDD1
 
 
hsa-miR-93-5p
 
 
-0.425
 
 
33.1
 
 
 
 
APPL1
 
 
hsa-miR-92a-3p
 
 
-0.375
 
 
16.1
 
 
 
 
AQP3
 
 
hsa-miR-874-3p
 
 
-0.48
 
 
331.4
 
 
 
 
ARF4
 
 
hsa-miR-7-5p
 
 
-0.407
 
 
196.1
 
 
 
 
ARF4
 
 
hsa-miR-221-3p
 
 
-0.553
 
 
196.1
 
 
 
 
ARG2
 
 
hsa-let-7c-5p
 
 
-0.468
 
 
16.0
 
 
 
 
ARHGAP1
 
 
hsa-miR-17-5p
 
 
-0.325
 
 
62.9
 
 
 
 
ARHGAP1
 
 
hsa-miR-93-5p
 
 
-0.325
 
 
62.9
 
 
 
 
ARHGAP1
 
 
hsa-miR-20a-5p
 
 
-0.339
 
 
62.9
 
 
 
 
ARHGAP5
 
 
hsa-miR-486-5p
 
 
-0.3
 
 
24.7
 
 
 
 
ARID4B
 
 
hsa-miR-20a-5p
 
 
-0.29
 
 
14.1
 
 
 
 
ARID4B
 
 
hsa-miR-486-5p
 
 
-0.367
 
 
14.1
 
 
 
 
ARIH2
 
 
hsa-miR-103a-3p
 
 
-0.307
 
 
20.7
 
 
 
 
ARL1
 
 
hsa-miR-20a-5p
 
 
-0.403
 
 
81.2
 
 
 
 
ARL1
 
 
hsa-miR-93-5p
 
 
-0.424
 
 
81.2
 
 
 
 
ARL1
 
 
hsa-miR-17-5p
 
 
-0.432
 
 
81.2
 
 
 
 
ARL4D
 
 
hsa-let-7c-5p
 
 
-0.417
 
 
27.6
 
 
 
 
ARL6IP1
 
 
hsa-miR-7-5p
 
 
-0.374
 
 
74.5
 
 
 
 
ARL6IP1
 
 
hsa-miR-328-3p
 
 
-0.494
 
 
74.5
 
 
 
 
ARL8A
 
 
hsa-miR-103a-3p
 
 
-0.38
 
 
32.9
 
 
 
 
ARMC1
 
 
hsa-miR-92a-3p
 
 
-0.322
 
 
14.3
 
 
 
 
ARMC1
 
 
hsa-miR-103a-3p
 
 
-0.525
 
 
14.3
 
 
 
 
ARMC8
 
 
hsa-miR-486-5p
 
 
-0.321
 
 
13.8
 
 
 
 
ARMC8
 
 
hsa-miR-20a-5p
 
 
-0.357
 
 
13.8
 
 
 
 
ARMC8
 
 
hsa-miR-93-5p
 
 
-0.362
 
 
13.8
 
 
 
 
ARMC8
 
 
hsa-miR-17-5p
 
 
-0.378
 
 
13.8
 
 
 
 
ARMC8
 
 
hsa-miR-103a-3p
 
 
-0.412
 
 
13.8
 
 
 
 
ARMT1
 
 
hsa-let-7c-5p
 
 
-0.303
 
 
15.1
 
 
 
 
ARNT
 
 
hsa-miR-103a-3p
 
 
-0.422
 
 
27.1
 
 
 
 
ARNT
 
 
hsa-miR-221-3p
 
 
-0.465
 
 
27.1
 
 
 
 
ARPP19
 
 
hsa-miR-328-3p
 
 
-0.404
 
 
35.8
 
 
 
 
ARPP19
 
 
hsa-let-7c-5p
 
 
-0.492
 
 
35.8
 
 
 
 
ARRDC3
 
 
hsa-miR-92a-3p
 
 
-0.458
 
 
42.0
 
 
 
 
ARRDC4
 
 
hsa-let-7c-5p
 
 
-0.329
 
 
14.3
 
 
 
 
ASAP1
 
 
hsa-let-7c-5p
 
 
-0.308
 
 
15.1
 
 
 
 
ASB6
 
 
hsa-miR-874-3p
 
 
-0.368
 
 
10.9
 
 
 
 
ASPH
 
 
hsa-miR-221-3p
 
 
-0.287
 
 
60.9
 
 
 
 
ASPH
 
 
hsa-miR-92a-3p
 
 
-0.416
 
 
60.9
 
 
 
 
ASPN
 
 
hsa-miR-92a-3p
 
 
-0.422
 
 
38.4
 
 
 
 
ASPRV1
 
 
hsa-miR-330-3p
 
 
-0.301
 
 
24.4
 
 
 
 
ATAD2
 
 
hsa-miR-17-5p
 
 
-0.304
 
 
12.1
 
 
 
 
ATAD2
 
 
hsa-miR-20a-5p
 
 
-0.318
 
 
12.1
 
 
 
 
ATF3
 
 
hsa-miR-874-3p
 
 
-0.288
 
 
99.3
 
 
 
 
ATL2
 
 
hsa-miR-103a-3p
 
 
-0.489
 
 
24.3
 
 
 
 
ATOSA
 
 
hsa-miR-221-3p
 
 
-0.495
 
 
10.7
 
 
 
 
ATOSB
 
 
hsa-miR-328-3p
 
 
-0.358
 
 
12.3
 
 
 
 
ATOSB
 
 
hsa-let-7c-5p
 
 
-0.429
 
 
12.3
 
 
 
 
ATP1B2
 
 
hsa-miR-103a-3p
 
 
-0.6
 
 
14.1
 
 
 
 
ATP2A2
 
 
hsa-let-7c-5p
 
 
-0.311
 
 
40.8
 
 
 
 
ATP6V1F
 
 
hsa-miR-185-5p
 
 
-0.765
 
 
139.3
 
 
 
 
ATPAF1
 
 
hsa-let-7c-5p
 
 
-0.498
 
 
33.4
 
 
 
 
ATXN1L
 
 
hsa-miR-155-5p
 
 
-0.315
 
 
10.4
 
 
 
 
AVPI1
 
 
hsa-miR-7-5p
 
 
-0.367
 
 
30.7
 
 
 
 
B3GAT3
 
 
hsa-let-7c-5p
 
 
-0.324
 
 
36.2
 
 
 
 
 
 
 choroid plexus 
 
 
 
 
target_symbol
 
 
mature_mirna_id
 
 
score
 
 
nTPM
 
 
 
 
 
 
AARS1
 
 
hsa-miR-92a-3p
 
 
-0.458
 
 
70.8
 
 
 
 
ACOX1
 
 
hsa-miR-92a-3p
 
 
-0.321
 
 
13.5
 
 
 
 
ADAM9
 
 
hsa-miR-20a-5p
 
 
-0.299
 
 
14.9
 
 
 
 
ADAM9
 
 
hsa-miR-17-5p
 
 
-0.32
 
 
14.9
 
 
 
 
ADAM9
 
 
hsa-miR-93-5p
 
 
-0.32
 
 
14.9
 
 
 
 
ADCY3
 
 
hsa-miR-92a-3p
 
 
-0.381
 
 
17.0
 
 
 
 
ADNP
 
 
hsa-miR-328-3p
 
 
-0.371
 
 
24.5
 
 
 
 
AGFG1
 
 
hsa-miR-103a-3p
 
 
-0.389
 
 
23.3
 
 
 
 
AHNAK
 
 
hsa-miR-20a-5p
 
 
-0.316
 
 
26.0
 
 
 
 
AHNAK
 
 
hsa-miR-17-5p
 
 
-0.337
 
 
26.0
 
 
 
 
AHNAK
 
 
hsa-miR-93-5p
 
 
-0.337
 
 
26.0
 
 
 
 
AIFM1
 
 
hsa-let-7c-5p
 
 
-0.314
 
 
37.9
 
 
 
 
AKAP10
 
 
hsa-miR-339-5p
 
 
-0.302
 
 
13.0
 
 
 
 
AKIRIN2
 
 
hsa-miR-103a-3p
 
 
-0.299
 
 
23.7
 
 
 
 
ALAD
 
 
hsa-miR-103a-3p
 
 
-0.385
 
 
29.1
 
 
 
 
ALG3
 
 
hsa-miR-20a-5p
 
 
-0.559
 
 
25.2
 
 
 
 
ALG3
 
 
hsa-miR-93-5p
 
 
-0.564
 
 
25.2
 
 
 
 
ALG3
 
 
hsa-miR-17-5p
 
 
-0.572
 
 
25.2
 
 
 
 
AMT
 
 
hsa-let-7c-5p
 
 
-0.463
 
 
33.4
 
 
 
 
ANGPTL2
 
 
hsa-miR-221-3p
 
 
-0.369
 
 
20.8
 
 
 
 
ANKFY1
 
 
hsa-miR-155-5p
 
 
-0.315
 
 
10.2
 
 
 
 
ANKRD46
 
 
hsa-let-7c-5p
 
 
-0.389
 
 
11.0
 
 
 
 
ANKRD9
 
 
hsa-miR-20a-5p
 
 
-0.31
 
 
15.2
 
 
 
 
ANKRD9
 
 
hsa-miR-93-5p
 
 
-0.331
 
 
15.2
 
 
 
 
ANKRD9
 
 
hsa-miR-17-5p
 
 
-0.347
 
 
15.2
 
 
 
 
ANP32E
 
 
hsa-miR-92a-3p
 
 
-0.329
 
 
23.6
 
 
 
 
AP1S1
 
 
hsa-let-7c-5p
 
 
-0.374
 
 
10.3
 
 
 
 
ARF4
 
 
hsa-miR-7-5p
 
 
-0.407
 
 
115.4
 
 
 
 
ARF4
 
 
hsa-miR-221-3p
 
 
-0.553
 
 
115.4
 
 
 
 
ARG2
 
 
hsa-let-7c-5p
 
 
-0.468
 
 
49.7
 
 
 
 
ARHGAP1
 
 
hsa-miR-17-5p
 
 
-0.325
 
 
26.4
 
 
 
 
ARHGAP1
 
 
hsa-miR-93-5p
 
 
-0.325
 
 
26.4
 
 
 
 
ARHGAP1
 
 
hsa-miR-20a-5p
 
 
-0.339
 
 
26.4
 
 
 
 
ARIH2
 
 
hsa-miR-103a-3p
 
 
-0.307
 
 
32.2
 
 
 
 
ARL1
 
 
hsa-miR-20a-5p
 
 
-0.403
 
 
84.0
 
 
 
 
ARL1
 
 
hsa-miR-93-5p
 
 
-0.424
 
 
84.0
 
 
 
 
ARL1
 
 
hsa-miR-17-5p
 
 
-0.432
 
 
84.0
 
 
 
 
ARL4D
 
 
hsa-let-7c-5p
 
 
-0.417
 
 
15.4
 
 
 
 
ARL6IP1
 
 
hsa-miR-7-5p
 
 
-0.374
 
 
185.0
 
 
 
 
ARL6IP1
 
 
hsa-miR-328-3p
 
 
-0.494
 
 
185.0
 
 
 
 
ARL8A
 
 
hsa-miR-103a-3p
 
 
-0.38
 
 
29.6
 
 
 
 
ARMC8
 
 
hsa-miR-486-5p
 
 
-0.321
 
 
20.8
 
 
 
 
ARMC8
 
 
hsa-miR-20a-5p
 
 
-0.357
 
 
20.8
 
 
 
 
ARMC8
 
 
hsa-miR-93-5p
 
 
-0.362
 
 
20.8
 
 
 
 
ARMC8
 
 
hsa-miR-17-5p
 
 
-0.378
 
 
20.8
 
 
 
 
ARMC8
 
 
hsa-miR-103a-3p
 
 
-0.412
 
 
20.8
 
 
 
 
ARNT
 
 
hsa-miR-103a-3p
 
 
-0.422
 
 
16.9
 
 
 
 
ARNT
 
 
hsa-miR-221-3p
 
 
-0.465
 
 
16.9
 
 
 
 
ARPP19
 
 
hsa-miR-328-3p
 
 
-0.404
 
 
32.4
 
 
 
 
ARPP19
 
 
hsa-let-7c-5p
 
 
-0.492
 
 
32.4
 
 
 
 
ARRDC3
 
 
hsa-miR-92a-3p
 
 
-0.458
 
 
24.1
 
 
 
 
ASB6
 
 
hsa-miR-874-3p
 
 
-0.368
 
 
15.8
 
 
 
 
ASPH
 
 
hsa-miR-221-3p
 
 
-0.287
 
 
79.9
 
 
 
 
ASPH
 
 
hsa-miR-92a-3p
 
 
-0.416
 
 
79.9
 
 
 
 
ATF3
 
 
hsa-miR-874-3p
 
 
-0.288
 
 
42.4
 
 
 
 
ATG16L1
 
 
hsa-miR-20a-5p
 
 
-0.446
 
 
21.8
 
 
 
 
ATG16L1
 
 
hsa-miR-93-5p
 
 
-0.451
 
 
21.8
 
 
 
 
ATG16L1
 
 
hsa-miR-17-5p
 
 
-0.467
 
 
21.8
 
 
 
 
ATG4A
 
 
hsa-miR-7-5p
 
 
-0.448
 
 
16.6
 
 
 
 
ATL2
 
 
hsa-miR-103a-3p
 
 
-0.489
 
 
10.4
 
 
 
 
ATOSA
 
 
hsa-miR-221-3p
 
 
-0.495
 
 
19.0
 
 
 
 
ATOSB
 
 
hsa-miR-328-3p
 
 
-0.358
 
 
16.6
 
 
 
 
ATOSB
 
 
hsa-let-7c-5p
 
 
-0.429
 
 
16.6
 
 
 
 
ATP1B2
 
 
hsa-miR-103a-3p
 
 
-0.6
 
 
47.2
 
 
 
 
ATP2A2
 
 
hsa-let-7c-5p
 
 
-0.311
 
 
29.6
 
 
 
 
ATP2B2
 
 
hsa-miR-7-5p
 
 
-0.297
 
 
61.0
 
 
 
 
ATP6V1F
 
 
hsa-miR-185-5p
 
 
-0.765
 
 
245.3
 
 
 
 
ATPAF1
 
 
hsa-let-7c-5p
 
 
-0.498
 
 
168.0
 
 
 
 
AVPI1
 
 
hsa-miR-7-5p
 
 
-0.367
 
 
20.0
 
 
 
 
B3GAT3
 
 
hsa-let-7c-5p
 
 
-0.324
 
 
87.1
 
 
 
 
B4GAT1
 
 
hsa-let-7c-5p
 
 
-0.47
 
 
93.8
 
 
 
 
BACE1
 
 
hsa-miR-339-5p
 
 
-0.371
 
 
16.5
 
 
 
 
BAK1
 
 
hsa-miR-103a-3p
 
 
-0.364
 
 
16.1
 
 
 
 
BAMBI
 
 
hsa-miR-20a-5p
 
 
-0.485
 
 
37.8
 
 
 
 
BAMBI
 
 
hsa-miR-17-5p
 
 
-0.506
 
 
37.8
 
 
 
 
BAMBI
 
 
hsa-miR-93-5p
 
 
-0.506
 
 
37.8
 
 
 
 
BCAT2
 
 
hsa-miR-103a-3p
 
 
-0.364
 
 
200.2
 
 
 
 
BCAT2
 
 
hsa-miR-92a-3p
 
 
-0.609
 
 
200.2
 
 
 
 
BDH1
 
 
hsa-miR-874-3p
 
 
-0.644
 
 
39.2
 
 
 
 
BIN1
 
 
hsa-miR-744-5p
 
 
-0.654
 
 
43.9
 
 
 
 
BIN3
 
 
hsa-let-7c-5p
 
 
-0.427
 
 
33.7
 
 
 
 
BLOC1S4
 
 
hsa-miR-7-5p
 
 
-0.417
 
 
10.3
 
 
 
 
BLOC1S4
 
 
hsa-miR-7-5p
 
 
-0.527
 
 
10.3
 
 
 
 
BLOC1S6
 
 
hsa-let-7c-5p
 
 
-0.321
 
 
14.9
 
 
 
 
BLTP1
 
 
hsa-miR-92a-3p
 
 
-0.462
 
 
32.1
 
 
 
 
BNIP3L
 
 
hsa-miR-20a-5p
 
 
-0.287
 
 
46.7
 
 
 
 
BOLA3
 
 
hsa-miR-103a-3p
 
 
-0.362
 
 
49.2
 
 
 
 
BSDC1
 
 
hsa-miR-92a-3p
 
 
-0.362
 
 
16.7
 
 
 
 
BTBD10
 
 
hsa-miR-17-5p
 
 
-0.409
 
 
10.3
 
 
 
 
BTBD10
 
 
hsa-miR-93-5p
 
 
-0.409
 
 
10.3
 
 
 
 
BTBD10
 
 
hsa-miR-20a-5p
 
 
-0.412
 
 
10.3
 
 
 
 
BTF3L4
 
 
hsa-let-7c-5p
 
 
-0.355
 
 
26.9
 
 
 
 
BTG2
 
 
hsa-miR-92a-3p
 
 
-0.314
 
 
39.9
 
 
 
 
BTG2
 
 
hsa-miR-92a-3p
 
 
-0.402
 
 
39.9
 
 
 
 
BTG3
 
 
hsa-miR-17-5p
 
 
-0.668
 
 
25.9
 
 
 
 
BTG3
 
 
hsa-miR-93-5p
 
 
-0.668
 
 
25.9
 
 
 
 
BTG3
 
 
hsa-miR-20a-5p
 
 
-0.671
 
 
25.9
 
 
 
 
BZW1
 
 
hsa-let-7c-5p
 
 
-0.354
 
 
50.7
 
 
 
 
BZW1
 
 
hsa-let-7c-5p
 
 
-0.375
 
 
50.7
 
 
 
 
BZW2
 
 
hsa-let-7c-5p
 
 
-0.397
 
 
19.4
 
 
 
 
 
 
 colon 
 
 
 
 
target_symbol
 
 
mature_mirna_id
 
 
score
 
 
nTPM
 
 
 
 
 
 
AARS1
 
 
hsa-miR-92a-3p
 
 
-0.458
 
 
37.1
 
 
 
 
ABHD17C
 
 
hsa-let-7c-5p
 
 
-0.359
 
 
61.2
 
 
 
 
ABHD17C
 
 
hsa-miR-330-3p
 
 
-0.399
 
 
61.2
 
 
 
 
ABHD5
 
 
hsa-miR-20a-5p
 
 
-0.397
 
 
11.4
 
 
 
 
ABHD5
 
 
hsa-miR-93-5p
 
 
-0.402
 
 
11.4
 
 
 
 
ABHD5
 
 
hsa-miR-17-5p
 
 
-0.45
 
 
11.4
 
 
 
 
ACOX1
 
 
hsa-miR-92a-3p
 
 
-0.321
 
 
23.4
 
 
 
 
ADAM19
 
 
hsa-miR-874-3p
 
 
-0.374
 
 
22.3
 
 
 
 
ADAM9
 
 
hsa-miR-20a-5p
 
 
-0.299
 
 
30.9
 
 
 
 
ADAM9
 
 
hsa-miR-17-5p
 
 
-0.32
 
 
30.9
 
 
 
 
ADAM9
 
 
hsa-miR-93-5p
 
 
-0.32
 
 
30.9
 
 
 
 
ADCY3
 
 
hsa-miR-92a-3p
 
 
-0.381
 
 
26.7
 
 
 
 
ADNP
 
 
hsa-miR-328-3p
 
 
-0.371
 
 
32.4
 
 
 
 
AGFG1
 
 
hsa-miR-103a-3p
 
 
-0.389
 
 
30.2
 
 
 
 
AGFG2
 
 
hsa-miR-20a-5p
 
 
-0.365
 
 
17.3
 
 
 
 
AGFG2
 
 
hsa-miR-93-5p
 
 
-0.378
 
 
17.3
 
 
 
 
AGFG2
 
 
hsa-miR-17-5p
 
 
-0.402
 
 
17.3
 
 
 
 
AHNAK
 
 
hsa-miR-20a-5p
 
 
-0.316
 
 
258.8
 
 
 
 
AHNAK
 
 
hsa-miR-17-5p
 
 
-0.337
 
 
258.8
 
 
 
 
AHNAK
 
 
hsa-miR-93-5p
 
 
-0.337
 
 
258.8
 
 
 
 
AIFM1
 
 
hsa-let-7c-5p
 
 
-0.314
 
 
23.9
 
 
 
 
AKAP10
 
 
hsa-miR-339-5p
 
 
-0.302
 
 
10.7
 
 
 
 
AKIRIN2
 
 
hsa-miR-103a-3p
 
 
-0.299
 
 
17.5
 
 
 
 
ALAD
 
 
hsa-miR-103a-3p
 
 
-0.385
 
 
24.1
 
 
 
 
ALG3
 
 
hsa-miR-20a-5p
 
 
-0.559
 
 
22.6
 
 
 
 
ALG3
 
 
hsa-miR-93-5p
 
 
-0.564
 
 
22.6
 
 
 
 
ALG3
 
 
hsa-miR-17-5p
 
 
-0.572
 
 
22.6
 
 
 
 
ALG9
 
 
hsa-miR-7-5p
 
 
-0.302
 
 
10.0
 
 
 
 
AMT
 
 
hsa-let-7c-5p
 
 
-0.463
 
 
14.4
 
 
 
 
ANGPTL2
 
 
hsa-miR-221-3p
 
 
-0.369
 
 
73.4
 
 
 
 
ANKFY1
 
 
hsa-miR-155-5p
 
 
-0.315
 
 
24.5
 
 
 
 
ANKRD12
 
 
hsa-miR-7-5p
 
 
-0.37
 
 
12.2
 
 
 
 
ANKRD9
 
 
hsa-miR-20a-5p
 
 
-0.31
 
 
28.9
 
 
 
 
ANKRD9
 
 
hsa-miR-93-5p
 
 
-0.331
 
 
28.9
 
 
 
 
ANKRD9
 
 
hsa-miR-17-5p
 
 
-0.347
 
 
28.9
 
 
 
 
ANP32E
 
 
hsa-miR-92a-3p
 
 
-0.329
 
 
18.6
 
 
 
 
AP1AR
 
 
hsa-miR-92a-3p
 
 
-0.325
 
 
16.9
 
 
 
 
AP1S1
 
 
hsa-let-7c-5p
 
 
-0.374
 
 
31.0
 
 
 
 
AP4B1
 
 
hsa-miR-378a-3p
 
 
-0.62
 
 
11.3
 
 
 
 
APCDD1
 
 
hsa-miR-20a-5p
 
 
-0.404
 
 
12.1
 
 
 
 
APCDD1
 
 
hsa-miR-17-5p
 
 
-0.425
 
 
12.1
 
 
 
 
APCDD1
 
 
hsa-miR-93-5p
 
 
-0.425
 
 
12.1
 
 
 
 
APPL1
 
 
hsa-miR-92a-3p
 
 
-0.375
 
 
13.4
 
 
 
 
ARF4
 
 
hsa-miR-7-5p
 
 
-0.407
 
 
148.9
 
 
 
 
ARF4
 
 
hsa-miR-221-3p
 
 
-0.553
 
 
148.9
 
 
 
 
ARHGAP1
 
 
hsa-miR-17-5p
 
 
-0.325
 
 
59.8
 
 
 
 
ARHGAP1
 
 
hsa-miR-93-5p
 
 
-0.325
 
 
59.8
 
 
 
 
ARHGAP1
 
 
hsa-miR-20a-5p
 
 
-0.339
 
 
59.8
 
 
 
 
ARHGAP5
 
 
hsa-miR-486-5p
 
 
-0.3
 
 
27.7
 
 
 
 
ARIH2
 
 
hsa-miR-103a-3p
 
 
-0.307
 
 
28.0
 
 
 
 
ARL1
 
 
hsa-miR-20a-5p
 
 
-0.403
 
 
53.0
 
 
 
 
ARL1
 
 
hsa-miR-93-5p
 
 
-0.424
 
 
53.0
 
 
 
 
ARL1
 
 
hsa-miR-17-5p
 
 
-0.432
 
 
53.0
 
 
 
 
ARL4D
 
 
hsa-let-7c-5p
 
 
-0.417
 
 
27.1
 
 
 
 
ARL6IP1
 
 
hsa-miR-7-5p
 
 
-0.374
 
 
132.0
 
 
 
 
ARL6IP1
 
 
hsa-miR-328-3p
 
 
-0.494
 
 
132.0
 
 
 
 
ARL6IP6
 
 
hsa-let-7c-5p
 
 
-0.293
 
 
12.7
 
 
 
 
ARL8A
 
 
hsa-miR-103a-3p
 
 
-0.38
 
 
34.3
 
 
 
 
ARMC1
 
 
hsa-miR-92a-3p
 
 
-0.322
 
 
15.3
 
 
 
 
ARMC1
 
 
hsa-miR-103a-3p
 
 
-0.525
 
 
15.3
 
 
 
 
ARMC8
 
 
hsa-miR-486-5p
 
 
-0.321
 
 
15.5
 
 
 
 
ARMC8
 
 
hsa-miR-20a-5p
 
 
-0.357
 
 
15.5
 
 
 
 
ARMC8
 
 
hsa-miR-93-5p
 
 
-0.362
 
 
15.5
 
 
 
 
ARMC8
 
 
hsa-miR-17-5p
 
 
-0.378
 
 
15.5
 
 
 
 
ARMC8
 
 
hsa-miR-103a-3p
 
 
-0.412
 
 
15.5
 
 
 
 
ARMT1
 
 
hsa-let-7c-5p
 
 
-0.303
 
 
15.5
 
 
 
 
ARNT
 
 
hsa-miR-103a-3p
 
 
-0.422
 
 
18.8
 
 
 
 
ARNT
 
 
hsa-miR-221-3p
 
 
-0.465
 
 
18.8
 
 
 
 
ARPP19
 
 
hsa-miR-328-3p
 
 
-0.404
 
 
35.7
 
 
 
 
ARPP19
 
 
hsa-let-7c-5p
 
 
-0.492
 
 
35.7
 
 
 
 
ARRDC3
 
 
hsa-miR-92a-3p
 
 
-0.458
 
 
18.9
 
 
 
 
ARRDC4
 
 
hsa-let-7c-5p
 
 
-0.329
 
 
40.4
 
 
 
 
ASAP1
 
 
hsa-let-7c-5p
 
 
-0.308
 
 
12.2
 
 
 
 
ASB6
 
 
hsa-miR-874-3p
 
 
-0.368
 
 
10.3
 
 
 
 
ASPH
 
 
hsa-miR-221-3p
 
 
-0.287
 
 
72.8
 
 
 
 
ASPH
 
 
hsa-miR-92a-3p
 
 
-0.416
 
 
72.8
 
 
 
 
ASPN
 
 
hsa-miR-92a-3p
 
 
-0.422
 
 
25.3
 
 
 
 
ATF3
 
 
hsa-miR-874-3p
 
 
-0.288
 
 
65.7
 
 
 
 
ATG16L1
 
 
hsa-miR-20a-5p
 
 
-0.446
 
 
13.4
 
 
 
 
ATG16L1
 
 
hsa-miR-93-5p
 
 
-0.451
 
 
13.4
 
 
 
 
ATG16L1
 
 
hsa-miR-17-5p
 
 
-0.467
 
 
13.4
 
 
 
 
ATG4A
 
 
hsa-miR-7-5p
 
 
-0.448
 
 
11.4
 
 
 
 
ATL2
 
 
hsa-miR-103a-3p
 
 
-0.489
 
 
44.6
 
 
 
 
ATOSA
 
 
hsa-miR-221-3p
 
 
-0.495
 
 
13.6
 
 
 
 
ATOSB
 
 
hsa-miR-328-3p
 
 
-0.358
 
 
14.6
 
 
 
 
ATOSB
 
 
hsa-let-7c-5p
 
 
-0.429
 
 
14.6
 
 
 
 
ATP2A2
 
 
hsa-let-7c-5p
 
 
-0.311
 
 
60.2
 
 
 
 
ATP6V1F
 
 
hsa-miR-185-5p
 
 
-0.765
 
 
105.2
 
 
 
 
ATPAF1
 
 
hsa-let-7c-5p
 
 
-0.498
 
 
49.0
 
 
 
 
AURKA
 
 
hsa-miR-92a-3p
 
 
-0.358
 
 
10.1
 
 
 
 
AVPI1
 
 
hsa-miR-7-5p
 
 
-0.367
 
 
23.0
 
 
 
 
B3GAT3
 
 
hsa-let-7c-5p
 
 
-0.324
 
 
30.8
 
 
 
 
B4GALT5
 
 
hsa-miR-210-3p
 
 
-0.511
 
 
23.3
 
 
 
 
B4GAT1
 
 
hsa-let-7c-5p
 
 
-0.47
 
 
29.4
 
 
 
 
BACE1
 
 
hsa-miR-339-5p
 
 
-0.371
 
 
10.0
 
 
 
 
BACH1
 
 
hsa-let-7c-5p
 
 
-0.306
 
 
12.5
 
 
 
 
BACH1
 
 
hsa-let-7c-5p
 
 
-0.306
 
 
12.5
 
 
 
 
BACH1
 
 
hsa-let-7c-5p
 
 
-0.435
 
 
12.5
 
 
 
 
BACH1
 
 
hsa-let-7c-5p
 
 
-0.435
 
 
12.5
 
 
 
 
BAK1
 
 
hsa-miR-103a-3p
 
 
-0.364
 
 
30.4
 
 
 
 
 
 
 duodenum 
 
 
 
 
target_symbol
 
 
mature_mirna_id
 
 
score
 
 
nTPM
 
 
 
 
 
 
AARS1
 
 
hsa-miR-92a-3p
 
 
-0.458
 
 
24.5
 
 
 
 
ABHD17B
 
 
hsa-miR-486-5p
 
 
-0.348
 
 
15.0
 
 
 
 
ABHD17C
 
 
hsa-let-7c-5p
 
 
-0.359
 
 
21.9
 
 
 
 
ABHD17C
 
 
hsa-miR-330-3p
 
 
-0.399
 
 
21.9
 
 
 
 
ACOX1
 
 
hsa-miR-92a-3p
 
 
-0.321
 
 
34.1
 
 
 
 
ADAM9
 
 
hsa-miR-20a-5p
 
 
-0.299
 
 
23.1
 
 
 
 
ADAM9
 
 
hsa-miR-17-5p
 
 
-0.32
 
 
23.1
 
 
 
 
ADAM9
 
 
hsa-miR-93-5p
 
 
-0.32
 
 
23.1
 
 
 
 
ADCY3
 
 
hsa-miR-92a-3p
 
 
-0.381
 
 
10.3
 
 
 
 
ADNP
 
 
hsa-miR-328-3p
 
 
-0.371
 
 
18.9
 
 
 
 
AGFG1
 
 
hsa-miR-103a-3p
 
 
-0.389
 
 
40.8
 
 
 
 
AHNAK
 
 
hsa-miR-20a-5p
 
 
-0.316
 
 
46.1
 
 
 
 
AHNAK
 
 
hsa-miR-17-5p
 
 
-0.337
 
 
46.1
 
 
 
 
AHNAK
 
 
hsa-miR-93-5p
 
 
-0.337
 
 
46.1
 
 
 
 
AIFM1
 
 
hsa-let-7c-5p
 
 
-0.314
 
 
49.8
 
 
 
 
AKIRIN2
 
 
hsa-miR-103a-3p
 
 
-0.299
 
 
12.0
 
 
 
 
ALAD
 
 
hsa-miR-103a-3p
 
 
-0.385
 
 
27.7
 
 
 
 
ALG3
 
 
hsa-miR-20a-5p
 
 
-0.559
 
 
15.9
 
 
 
 
ALG3
 
 
hsa-miR-93-5p
 
 
-0.564
 
 
15.9
 
 
 
 
ALG3
 
 
hsa-miR-17-5p
 
 
-0.572
 
 
15.9
 
 
 
 
ALG9
 
 
hsa-miR-7-5p
 
 
-0.302
 
 
11.5
 
 
 
 
AMT
 
 
hsa-let-7c-5p
 
 
-0.463
 
 
12.9
 
 
 
 
ANGPTL2
 
 
hsa-miR-221-3p
 
 
-0.369
 
 
14.3
 
 
 
 
ANKFY1
 
 
hsa-miR-155-5p
 
 
-0.315
 
 
10.8
 
 
 
 
ANKRD12
 
 
hsa-miR-7-5p
 
 
-0.37
 
 
12.1
 
 
 
 
ANKRD9
 
 
hsa-miR-20a-5p
 
 
-0.31
 
 
47.3
 
 
 
 
ANKRD9
 
 
hsa-miR-93-5p
 
 
-0.331
 
 
47.3
 
 
 
 
ANKRD9
 
 
hsa-miR-17-5p
 
 
-0.347
 
 
47.3
 
 
 
 
ANP32E
 
 
hsa-miR-92a-3p
 
 
-0.329
 
 
21.1
 
 
 
 
AP1AR
 
 
hsa-miR-92a-3p
 
 
-0.325
 
 
20.9
 
 
 
 
AP1S1
 
 
hsa-let-7c-5p
 
 
-0.374
 
 
36.7
 
 
 
 
APPL1
 
 
hsa-miR-92a-3p
 
 
-0.375
 
 
11.9
 
 
 
 
AQP11
 
 
hsa-miR-103a-3p
 
 
-0.517
 
 
27.0
 
 
 
 
AQP3
 
 
hsa-miR-874-3p
 
 
-0.48
 
 
69.1
 
 
 
 
ARF4
 
 
hsa-miR-7-5p
 
 
-0.407
 
 
116.2
 
 
 
 
ARF4
 
 
hsa-miR-221-3p
 
 
-0.553
 
 
116.2
 
 
 
 
ARHGAP1
 
 
hsa-miR-17-5p
 
 
-0.325
 
 
24.2
 
 
 
 
ARHGAP1
 
 
hsa-miR-93-5p
 
 
-0.325
 
 
24.2
 
 
 
 
ARHGAP1
 
 
hsa-miR-20a-5p
 
 
-0.339
 
 
24.2
 
 
 
 
ARHGAP5
 
 
hsa-miR-486-5p
 
 
-0.3
 
 
33.3
 
 
 
 
ARIH2
 
 
hsa-miR-103a-3p
 
 
-0.307
 
 
11.0
 
 
 
 
ARL1
 
 
hsa-miR-20a-5p
 
 
-0.403
 
 
45.2
 
 
 
 
ARL1
 
 
hsa-miR-93-5p
 
 
-0.424
 
 
45.2
 
 
 
 
ARL1
 
 
hsa-miR-17-5p
 
 
-0.432
 
 
45.2
 
 
 
 
ARL6IP1
 
 
hsa-miR-7-5p
 
 
-0.374
 
 
130.6
 
 
 
 
ARL6IP1
 
 
hsa-miR-328-3p
 
 
-0.494
 
 
130.6
 
 
 
 
ARL8A
 
 
hsa-miR-103a-3p
 
 
-0.38
 
 
20.3
 
 
 
 
ARMC1
 
 
hsa-miR-92a-3p
 
 
-0.322
 
 
14.6
 
 
 
 
ARMC1
 
 
hsa-miR-103a-3p
 
 
-0.525
 
 
14.6
 
 
 
 
ARMC8
 
 
hsa-miR-486-5p
 
 
-0.321
 
 
11.3
 
 
 
 
ARMC8
 
 
hsa-miR-20a-5p
 
 
-0.357
 
 
11.3
 
 
 
 
ARMC8
 
 
hsa-miR-93-5p
 
 
-0.362
 
 
11.3
 
 
 
 
ARMC8
 
 
hsa-miR-17-5p
 
 
-0.378
 
 
11.3
 
 
 
 
ARMC8
 
 
hsa-miR-103a-3p
 
 
-0.412
 
 
11.3
 
 
 
 
ARMT1
 
 
hsa-let-7c-5p
 
 
-0.303
 
 
17.8
 
 
 
 
ARNT
 
 
hsa-miR-103a-3p
 
 
-0.422
 
 
14.1
 
 
 
 
ARNT
 
 
hsa-miR-221-3p
 
 
-0.465
 
 
14.1
 
 
 
 
ARPP19
 
 
hsa-miR-328-3p
 
 
-0.404
 
 
27.2
 
 
 
 
ARPP19
 
 
hsa-let-7c-5p
 
 
-0.492
 
 
27.2
 
 
 
 
ARRDC4
 
 
hsa-let-7c-5p
 
 
-0.329
 
 
14.9
 
 
 
 
ASPH
 
 
hsa-miR-221-3p
 
 
-0.287
 
 
42.9
 
 
 
 
ASPH
 
 
hsa-miR-92a-3p
 
 
-0.416
 
 
42.9
 
 
 
 
ATF3
 
 
hsa-miR-874-3p
 
 
-0.288
 
 
19.7
 
 
 
 
ATG4A
 
 
hsa-miR-7-5p
 
 
-0.448
 
 
12.0
 
 
 
 
ATL2
 
 
hsa-miR-103a-3p
 
 
-0.489
 
 
31.7
 
 
 
 
ATOSA
 
 
hsa-miR-221-3p
 
 
-0.495
 
 
10.6
 
 
 
 
ATP2A2
 
 
hsa-let-7c-5p
 
 
-0.311
 
 
44.8
 
 
 
 
ATP6V1F
 
 
hsa-miR-185-5p
 
 
-0.765
 
 
70.2
 
 
 
 
ATPAF1
 
 
hsa-let-7c-5p
 
 
-0.498
 
 
41.3
 
 
 
 
AURKA
 
 
hsa-miR-92a-3p
 
 
-0.358
 
 
11.2
 
 
 
 
AVPI1
 
 
hsa-miR-7-5p
 
 
-0.367
 
 
13.2
 
 
 
 
B3GAT3
 
 
hsa-let-7c-5p
 
 
-0.324
 
 
16.1
 
 
 
 
B4GALT5
 
 
hsa-miR-210-3p
 
 
-0.511
 
 
23.8
 
 
 
 
B4GAT1
 
 
hsa-let-7c-5p
 
 
-0.47
 
 
15.2
 
 
 
 
BACH1
 
 
hsa-let-7c-5p
 
 
-0.306
 
 
15.8
 
 
 
 
BACH1
 
 
hsa-let-7c-5p
 
 
-0.306
 
 
15.8
 
 
 
 
BACH1
 
 
hsa-let-7c-5p
 
 
-0.435
 
 
15.8
 
 
 
 
BACH1
 
 
hsa-let-7c-5p
 
 
-0.435
 
 
15.8
 
 
 
 
BAK1
 
 
hsa-miR-103a-3p
 
 
-0.364
 
 
44.2
 
 
 
 
BCAT2
 
 
hsa-miR-103a-3p
 
 
-0.364
 
 
13.9
 
 
 
 
BCAT2
 
 
hsa-miR-92a-3p
 
 
-0.609
 
 
13.9
 
 
 
 
BDH1
 
 
hsa-miR-874-3p
 
 
-0.644
 
 
16.3
 
 
 
 
BIN1
 
 
hsa-miR-744-5p
 
 
-0.654
 
 
16.4
 
 
 
 
BLOC1S6
 
 
hsa-let-7c-5p
 
 
-0.321
 
 
19.0
 
 
 
 
BLTP1
 
 
hsa-miR-92a-3p
 
 
-0.462
 
 
11.2
 
 
 
 
BMF
 
 
hsa-miR-221-3p
 
 
-0.453
 
 
20.0
 
 
 
 
BNIP3L
 
 
hsa-miR-20a-5p
 
 
-0.287
 
 
20.9
 
 
 
 
BOLA3
 
 
hsa-miR-103a-3p
 
 
-0.362
 
 
31.7
 
 
 
 
BSDC1
 
 
hsa-miR-92a-3p
 
 
-0.362
 
 
23.8
 
 
 
 
BTAF1
 
 
hsa-miR-486-5p
 
 
-0.288
 
 
12.8
 
 
 
 
BTBD10
 
 
hsa-miR-17-5p
 
 
-0.409
 
 
12.4
 
 
 
 
BTBD10
 
 
hsa-miR-93-5p
 
 
-0.409
 
 
12.4
 
 
 
 
BTBD10
 
 
hsa-miR-20a-5p
 
 
-0.412
 
 
12.4
 
 
 
 
BTF3L4
 
 
hsa-let-7c-5p
 
 
-0.355
 
 
13.6
 
 
 
 
BTG2
 
 
hsa-miR-92a-3p
 
 
-0.314
 
 
83.2
 
 
 
 
BTG2
 
 
hsa-miR-92a-3p
 
 
-0.402
 
 
83.2
 
 
 
 
BTG3
 
 
hsa-miR-17-5p
 
 
-0.668
 
 
15.5
 
 
 
 
BTG3
 
 
hsa-miR-93-5p
 
 
-0.668
 
 
15.5
 
 
 
 
BTG3
 
 
hsa-miR-20a-5p
 
 
-0.671
 
 
15.5
 
 
 
 
BZW1
 
 
hsa-let-7c-5p
 
 
-0.354
 
 
60.8
 
 
 
 
 
 
 endometrium 
 
 
 
 
target_symbol
 
 
mature_mirna_id
 
 
score
 
 
nTPM
 
 
 
 
 
 
AARS1
 
 
hsa-miR-92a-3p
 
 
-0.458
 
 
39.3
 
 
 
 
ACTC1
 
 
hsa-miR-92a-3p
 
 
-0.361
 
 
17.7
 
 
 
 
ACTC1
 
 
hsa-miR-7-5p
 
 
-0.471
 
 
17.7
 
 
 
 
ADAM9
 
 
hsa-miR-20a-5p
 
 
-0.299
 
 
21.6
 
 
 
 
ADAM9
 
 
hsa-miR-17-5p
 
 
-0.32
 
 
21.6
 
 
 
 
ADAM9
 
 
hsa-miR-93-5p
 
 
-0.32
 
 
21.6
 
 
 
 
ADAMTSL1
 
 
hsa-miR-92a-3p
 
 
-0.296
 
 
12.5
 
 
 
 
ADCY3
 
 
hsa-miR-92a-3p
 
 
-0.381
 
 
52.0
 
 
 
 
ADNP
 
 
hsa-miR-328-3p
 
 
-0.371
 
 
33.6
 
 
 
 
AGFG1
 
 
hsa-miR-103a-3p
 
 
-0.389
 
 
23.1
 
 
 
 
AGFG2
 
 
hsa-miR-20a-5p
 
 
-0.365
 
 
20.0
 
 
 
 
AGFG2
 
 
hsa-miR-93-5p
 
 
-0.378
 
 
20.0
 
 
 
 
AGFG2
 
 
hsa-miR-17-5p
 
 
-0.402
 
 
20.0
 
 
 
 
AHNAK
 
 
hsa-miR-20a-5p
 
 
-0.316
 
 
187.4
 
 
 
 
AHNAK
 
 
hsa-miR-17-5p
 
 
-0.337
 
 
187.4
 
 
 
 
AHNAK
 
 
hsa-miR-93-5p
 
 
-0.337
 
 
187.4
 
 
 
 
AIFM1
 
 
hsa-let-7c-5p
 
 
-0.314
 
 
10.6
 
 
 
 
AJUBA
 
 
hsa-miR-103a-3p
 
 
-0.448
 
 
11.2
 
 
 
 
AKAP10
 
 
hsa-miR-339-5p
 
 
-0.302
 
 
11.7
 
 
 
 
AKIRIN2
 
 
hsa-miR-103a-3p
 
 
-0.299
 
 
20.8
 
 
 
 
ALAD
 
 
hsa-miR-103a-3p
 
 
-0.385
 
 
16.4
 
 
 
 
ALG3
 
 
hsa-miR-20a-5p
 
 
-0.559
 
 
15.9
 
 
 
 
ALG3
 
 
hsa-miR-93-5p
 
 
-0.564
 
 
15.9
 
 
 
 
ALG3
 
 
hsa-miR-17-5p
 
 
-0.572
 
 
15.9
 
 
 
 
ALG9
 
 
hsa-miR-7-5p
 
 
-0.302
 
 
10.7
 
 
 
 
ALKAL2
 
 
hsa-miR-92a-3p
 
 
-0.608
 
 
32.6
 
 
 
 
AMT
 
 
hsa-let-7c-5p
 
 
-0.463
 
 
10.0
 
 
 
 
ANGPTL2
 
 
hsa-miR-221-3p
 
 
-0.369
 
 
59.2
 
 
 
 
ANKFY1
 
 
hsa-miR-155-5p
 
 
-0.315
 
 
17.2
 
 
 
 
ANKRD12
 
 
hsa-miR-7-5p
 
 
-0.37
 
 
11.1
 
 
 
 
ANKRD29
 
 
hsa-miR-20a-5p
 
 
-0.359
 
 
14.7
 
 
 
 
ANKRD29
 
 
hsa-miR-17-5p
 
 
-0.372
 
 
14.7
 
 
 
 
ANKRD29
 
 
hsa-miR-93-5p
 
 
-0.372
 
 
14.7
 
 
 
 
ANKRD46
 
 
hsa-let-7c-5p
 
 
-0.389
 
 
11.8
 
 
 
 
ANP32E
 
 
hsa-miR-92a-3p
 
 
-0.329
 
 
24.6
 
 
 
 
AP1AR
 
 
hsa-miR-92a-3p
 
 
-0.325
 
 
10.6
 
 
 
 
AP1S1
 
 
hsa-let-7c-5p
 
 
-0.374
 
 
16.1
 
 
 
 
AP4B1
 
 
hsa-miR-378a-3p
 
 
-0.62
 
 
10.1
 
 
 
 
APCDD1
 
 
hsa-miR-20a-5p
 
 
-0.404
 
 
32.5
 
 
 
 
APCDD1
 
 
hsa-miR-17-5p
 
 
-0.425
 
 
32.5
 
 
 
 
APCDD1
 
 
hsa-miR-93-5p
 
 
-0.425
 
 
32.5
 
 
 
 
APPL1
 
 
hsa-miR-92a-3p
 
 
-0.375
 
 
16.0
 
 
 
 
AQP3
 
 
hsa-miR-874-3p
 
 
-0.48
 
 
10.3
 
 
 
 
ARF4
 
 
hsa-miR-7-5p
 
 
-0.407
 
 
113.9
 
 
 
 
ARF4
 
 
hsa-miR-221-3p
 
 
-0.553
 
 
113.9
 
 
 
 
ARHGAP1
 
 
hsa-miR-17-5p
 
 
-0.325
 
 
81.8
 
 
 
 
ARHGAP1
 
 
hsa-miR-93-5p
 
 
-0.325
 
 
81.8
 
 
 
 
ARHGAP1
 
 
hsa-miR-20a-5p
 
 
-0.339
 
 
81.8
 
 
 
 
ARHGAP5
 
 
hsa-miR-486-5p
 
 
-0.3
 
 
26.2
 
 
 
 
ARID4B
 
 
hsa-miR-20a-5p
 
 
-0.29
 
 
14.9
 
 
 
 
ARID4B
 
 
hsa-miR-486-5p
 
 
-0.367
 
 
14.9
 
 
 
 
ARIH2
 
 
hsa-miR-103a-3p
 
 
-0.307
 
 
22.7
 
 
 
 
ARL1
 
 
hsa-miR-20a-5p
 
 
-0.403
 
 
47.9
 
 
 
 
ARL1
 
 
hsa-miR-93-5p
 
 
-0.424
 
 
47.9
 
 
 
 
ARL1
 
 
hsa-miR-17-5p
 
 
-0.432
 
 
47.9
 
 
 
 
ARL4D
 
 
hsa-let-7c-5p
 
 
-0.417
 
 
19.2
 
 
 
 
ARL6IP1
 
 
hsa-miR-7-5p
 
 
-0.374
 
 
66.4
 
 
 
 
ARL6IP1
 
 
hsa-miR-328-3p
 
 
-0.494
 
 
66.4
 
 
 
 
ARL6IP6
 
 
hsa-let-7c-5p
 
 
-0.293
 
 
11.0
 
 
 
 
ARL8A
 
 
hsa-miR-103a-3p
 
 
-0.38
 
 
32.8
 
 
 
 
ARMC1
 
 
hsa-miR-92a-3p
 
 
-0.322
 
 
17.4
 
 
 
 
ARMC1
 
 
hsa-miR-103a-3p
 
 
-0.525
 
 
17.4
 
 
 
 
ARMC8
 
 
hsa-miR-486-5p
 
 
-0.321
 
 
16.5
 
 
 
 
ARMC8
 
 
hsa-miR-20a-5p
 
 
-0.357
 
 
16.5
 
 
 
 
ARMC8
 
 
hsa-miR-93-5p
 
 
-0.362
 
 
16.5
 
 
 
 
ARMC8
 
 
hsa-miR-17-5p
 
 
-0.378
 
 
16.5
 
 
 
 
ARMC8
 
 
hsa-miR-103a-3p
 
 
-0.412
 
 
16.5
 
 
 
 
ARMT1
 
 
hsa-let-7c-5p
 
 
-0.303
 
 
15.0
 
 
 
 
ARNT
 
 
hsa-miR-103a-3p
 
 
-0.422
 
 
25.7
 
 
 
 
ARNT
 
 
hsa-miR-221-3p
 
 
-0.465
 
 
25.7
 
 
 
 
ARPP19
 
 
hsa-miR-328-3p
 
 
-0.404
 
 
44.7
 
 
 
 
ARPP19
 
 
hsa-let-7c-5p
 
 
-0.492
 
 
44.7
 
 
 
 
ARRDC3
 
 
hsa-miR-92a-3p
 
 
-0.458
 
 
38.0
 
 
 
 
ARRDC4
 
 
hsa-let-7c-5p
 
 
-0.329
 
 
13.7
 
 
 
 
ASAP1
 
 
hsa-let-7c-5p
 
 
-0.308
 
 
17.2
 
 
 
 
ASB6
 
 
hsa-miR-874-3p
 
 
-0.368
 
 
11.1
 
 
 
 
ASPH
 
 
hsa-miR-221-3p
 
 
-0.287
 
 
45.6
 
 
 
 
ASPH
 
 
hsa-miR-92a-3p
 
 
-0.416
 
 
45.6
 
 
 
 
ASPN
 
 
hsa-miR-92a-3p
 
 
-0.422
 
 
65.1
 
 
 
 
ATF3
 
 
hsa-miR-874-3p
 
 
-0.288
 
 
101.3
 
 
 
 
ATG16L1
 
 
hsa-miR-20a-5p
 
 
-0.446
 
 
11.3
 
 
 
 
ATG16L1
 
 
hsa-miR-93-5p
 
 
-0.451
 
 
11.3
 
 
 
 
ATG16L1
 
 
hsa-miR-17-5p
 
 
-0.467
 
 
11.3
 
 
 
 
ATL2
 
 
hsa-miR-103a-3p
 
 
-0.489
 
 
31.3
 
 
 
 
ATOSA
 
 
hsa-miR-221-3p
 
 
-0.495
 
 
15.0
 
 
 
 
ATP2A2
 
 
hsa-let-7c-5p
 
 
-0.311
 
 
43.7
 
 
 
 
ATP6V1F
 
 
hsa-miR-185-5p
 
 
-0.765
 
 
89.8
 
 
 
 
ATPAF1
 
 
hsa-let-7c-5p
 
 
-0.498
 
 
23.6
 
 
 
 
ATXN1L
 
 
hsa-miR-155-5p
 
 
-0.315
 
 
11.6
 
 
 
 
AVPI1
 
 
hsa-miR-7-5p
 
 
-0.367
 
 
12.2
 
 
 
 
B3GAT3
 
 
hsa-let-7c-5p
 
 
-0.324
 
 
33.5
 
 
 
 
B4GALT5
 
 
hsa-miR-210-3p
 
 
-0.511
 
 
22.0
 
 
 
 
B4GAT1
 
 
hsa-let-7c-5p
 
 
-0.47
 
 
28.7
 
 
 
 
BACE1
 
 
hsa-miR-339-5p
 
 
-0.371
 
 
15.6
 
 
 
 
BACH1
 
 
hsa-let-7c-5p
 
 
-0.306
 
 
18.4
 
 
 
 
BACH1
 
 
hsa-let-7c-5p
 
 
-0.306
 
 
18.4
 
 
 
 
BACH1
 
 
hsa-let-7c-5p
 
 
-0.435
 
 
18.4
 
 
 
 
BACH1
 
 
hsa-let-7c-5p
 
 
-0.435
 
 
18.4
 
 
 
 
BAMBI
 
 
hsa-miR-20a-5p
 
 
-0.485
 
 
16.8
 
 
 
 
BAMBI
 
 
hsa-miR-17-5p
 
 
-0.506
 
 
16.8
 
 
 
 
 
 
 epididymis 
 
 
 
 
target_symbol
 
 
mature_mirna_id
 
 
score
 
 
nTPM
 
 
 
 
 
 
AARS1
 
 
hsa-miR-92a-3p
 
 
-0.458
 
 
46.8
 
 
 
 
ACP3
 
 
hsa-let-7c-5p
 
 
-0.298
 
 
16.3
 
 
 
 
ADAM9
 
 
hsa-miR-20a-5p
 
 
-0.299
 
 
14.3
 
 
 
 
ADAM9
 
 
hsa-miR-17-5p
 
 
-0.32
 
 
14.3
 
 
 
 
ADAM9
 
 
hsa-miR-93-5p
 
 
-0.32
 
 
14.3
 
 
 
 
ADCY3
 
 
hsa-miR-92a-3p
 
 
-0.381
 
 
19.3
 
 
 
 
ADNP
 
 
hsa-miR-328-3p
 
 
-0.371
 
 
39.7
 
 
 
 
AGFG1
 
 
hsa-miR-103a-3p
 
 
-0.389
 
 
18.8
 
 
 
 
AHNAK
 
 
hsa-miR-20a-5p
 
 
-0.316
 
 
48.9
 
 
 
 
AHNAK
 
 
hsa-miR-17-5p
 
 
-0.337
 
 
48.9
 
 
 
 
AHNAK
 
 
hsa-miR-93-5p
 
 
-0.337
 
 
48.9
 
 
 
 
AKIRIN2
 
 
hsa-miR-103a-3p
 
 
-0.299
 
 
22.5
 
 
 
 
ALAD
 
 
hsa-miR-103a-3p
 
 
-0.385
 
 
25.1
 
 
 
 
ALG3
 
 
hsa-miR-20a-5p
 
 
-0.559
 
 
11.8
 
 
 
 
ALG3
 
 
hsa-miR-93-5p
 
 
-0.564
 
 
11.8
 
 
 
 
ALG3
 
 
hsa-miR-17-5p
 
 
-0.572
 
 
11.8
 
 
 
 
ALG9
 
 
hsa-miR-7-5p
 
 
-0.302
 
 
10.3
 
 
 
 
ALKAL2
 
 
hsa-miR-92a-3p
 
 
-0.608
 
 
12.6
 
 
 
 
ANGPTL2
 
 
hsa-miR-221-3p
 
 
-0.369
 
 
12.9
 
 
 
 
ANKFY1
 
 
hsa-miR-155-5p
 
 
-0.315
 
 
10.0
 
 
 
 
ANKRD46
 
 
hsa-let-7c-5p
 
 
-0.389
 
 
15.4
 
 
 
 
ANP32E
 
 
hsa-miR-92a-3p
 
 
-0.329
 
 
32.5
 
 
 
 
AP1S1
 
 
hsa-let-7c-5p
 
 
-0.374
 
 
16.0
 
 
 
 
AP4B1
 
 
hsa-miR-378a-3p
 
 
-0.62
 
 
13.4
 
 
 
 
APCDD1
 
 
hsa-miR-20a-5p
 
 
-0.404
 
 
14.6
 
 
 
 
APCDD1
 
 
hsa-miR-17-5p
 
 
-0.425
 
 
14.6
 
 
 
 
APCDD1
 
 
hsa-miR-93-5p
 
 
-0.425
 
 
14.6
 
 
 
 
APPL1
 
 
hsa-miR-92a-3p
 
 
-0.375
 
 
10.0
 
 
 
 
ARF4
 
 
hsa-miR-7-5p
 
 
-0.407
 
 
214.1
 
 
 
 
ARF4
 
 
hsa-miR-221-3p
 
 
-0.553
 
 
214.1
 
 
 
 
ARHGAP1
 
 
hsa-miR-17-5p
 
 
-0.325
 
 
20.3
 
 
 
 
ARHGAP1
 
 
hsa-miR-93-5p
 
 
-0.325
 
 
20.3
 
 
 
 
ARHGAP1
 
 
hsa-miR-20a-5p
 
 
-0.339
 
 
20.3
 
 
 
 
ARHGAP5
 
 
hsa-miR-486-5p
 
 
-0.3
 
 
23.7
 
 
 
 
ARID4B
 
 
hsa-miR-20a-5p
 
 
-0.29
 
 
13.6
 
 
 
 
ARID4B
 
 
hsa-miR-486-5p
 
 
-0.367
 
 
13.6
 
 
 
 
ARIH2
 
 
hsa-miR-103a-3p
 
 
-0.307
 
 
31.9
 
 
 
 
ARL1
 
 
hsa-miR-20a-5p
 
 
-0.403
 
 
67.1
 
 
 
 
ARL1
 
 
hsa-miR-93-5p
 
 
-0.424
 
 
67.1
 
 
 
 
ARL1
 
 
hsa-miR-17-5p
 
 
-0.432
 
 
67.1
 
 
 
 
ARL6IP1
 
 
hsa-miR-7-5p
 
 
-0.374
 
 
116.0
 
 
 
 
ARL6IP1
 
 
hsa-miR-328-3p
 
 
-0.494
 
 
116.0
 
 
 
 
ARL8A
 
 
hsa-miR-103a-3p
 
 
-0.38
 
 
15.9
 
 
 
 
ARMC1
 
 
hsa-miR-92a-3p
 
 
-0.322
 
 
15.5
 
 
 
 
ARMC1
 
 
hsa-miR-103a-3p
 
 
-0.525
 
 
15.5
 
 
 
 
ARMC8
 
 
hsa-miR-486-5p
 
 
-0.321
 
 
11.5
 
 
 
 
ARMC8
 
 
hsa-miR-20a-5p
 
 
-0.357
 
 
11.5
 
 
 
 
ARMC8
 
 
hsa-miR-93-5p
 
 
-0.362
 
 
11.5
 
 
 
 
ARMC8
 
 
hsa-miR-17-5p
 
 
-0.378
 
 
11.5
 
 
 
 
ARMC8
 
 
hsa-miR-103a-3p
 
 
-0.412
 
 
11.5
 
 
 
 
ARMT1
 
 
hsa-let-7c-5p
 
 
-0.303
 
 
15.2
 
 
 
 
ARNT
 
 
hsa-miR-103a-3p
 
 
-0.422
 
 
14.0
 
 
 
 
ARNT
 
 
hsa-miR-221-3p
 
 
-0.465
 
 
14.0
 
 
 
 
ARPP19
 
 
hsa-miR-328-3p
 
 
-0.404
 
 
34.2
 
 
 
 
ARPP19
 
 
hsa-let-7c-5p
 
 
-0.492
 
 
34.2
 
 
 
 
ARRDC3
 
 
hsa-miR-92a-3p
 
 
-0.458
 
 
25.6
 
 
 
 
ARRDC4
 
 
hsa-let-7c-5p
 
 
-0.329
 
 
14.7
 
 
 
 
ASAP1
 
 
hsa-let-7c-5p
 
 
-0.308
 
 
11.5
 
 
 
 
ASPH
 
 
hsa-miR-221-3p
 
 
-0.287
 
 
42.3
 
 
 
 
ASPH
 
 
hsa-miR-92a-3p
 
 
-0.416
 
 
42.3
 
 
 
 
ATG4A
 
 
hsa-miR-7-5p
 
 
-0.448
 
 
10.9
 
 
 
 
ATL2
 
 
hsa-miR-103a-3p
 
 
-0.489
 
 
18.3
 
 
 
 
ATP1B2
 
 
hsa-miR-103a-3p
 
 
-0.6
 
 
16.4
 
 
 
 
ATP2A2
 
 
hsa-let-7c-5p
 
 
-0.311
 
 
36.9
 
 
 
 
ATP6V1F
 
 
hsa-miR-185-5p
 
 
-0.765
 
 
58.2
 
 
 
 
ATPAF1
 
 
hsa-let-7c-5p
 
 
-0.498
 
 
33.7
 
 
 
 
B3GAT3
 
 
hsa-let-7c-5p
 
 
-0.324
 
 
11.2
 
 
 
 
B4GAT1
 
 
hsa-let-7c-5p
 
 
-0.47
 
 
14.3
 
 
 
 
BICDL1
 
 
hsa-miR-221-3p
 
 
-0.447
 
 
23.0
 
 
 
 
BIN1
 
 
hsa-miR-744-5p
 
 
-0.654
 
 
15.8
 
 
 
 
BLOC1S6
 
 
hsa-let-7c-5p
 
 
-0.321
 
 
17.3
 
 
 
 
BLTP1
 
 
hsa-miR-92a-3p
 
 
-0.462
 
 
17.9
 
 
 
 
BNIP3L
 
 
hsa-miR-20a-5p
 
 
-0.287
 
 
62.5
 
 
 
 
BOLA3
 
 
hsa-miR-103a-3p
 
 
-0.362
 
 
23.7
 
 
 
 
BSDC1
 
 
hsa-miR-92a-3p
 
 
-0.362
 
 
23.5
 
 
 
 
BTAF1
 
 
hsa-miR-486-5p
 
 
-0.288
 
 
16.9
 
 
 
 
BTBD10
 
 
hsa-miR-17-5p
 
 
-0.409
 
 
11.3
 
 
 
 
BTBD10
 
 
hsa-miR-93-5p
 
 
-0.409
 
 
11.3
 
 
 
 
BTBD10
 
 
hsa-miR-20a-5p
 
 
-0.412
 
 
11.3
 
 
 
 
BTF3L4
 
 
hsa-let-7c-5p
 
 
-0.355
 
 
28.9
 
 
 
 
BTG2
 
 
hsa-miR-92a-3p
 
 
-0.314
 
 
39.2
 
 
 
 
BTG2
 
 
hsa-miR-92a-3p
 
 
-0.402
 
 
39.2
 
 
 
 
BTG3
 
 
hsa-miR-17-5p
 
 
-0.668
 
 
104.1
 
 
 
 
BTG3
 
 
hsa-miR-93-5p
 
 
-0.668
 
 
104.1
 
 
 
 
BTG3
 
 
hsa-miR-20a-5p
 
 
-0.671
 
 
104.1
 
 
 
 
BZW1
 
 
hsa-let-7c-5p
 
 
-0.354
 
 
55.1
 
 
 
 
BZW1
 
 
hsa-let-7c-5p
 
 
-0.375
 
 
55.1
 
 
 
 
C1QBP
 
 
hsa-miR-330-3p
 
 
-0.412
 
 
64.4
 
 
 
 
C1orf210
 
 
hsa-miR-378a-3p
 
 
-0.307
 
 
12.2
 
 
 
 
C2CD5
 
 
hsa-miR-103a-3p
 
 
-0.475
 
 
15.0
 
 
 
 
C6orf62
 
 
hsa-miR-92a-3p
 
 
-0.326
 
 
95.1
 
 
 
 
CAB39
 
 
hsa-miR-155-5p
 
 
-0.312
 
 
33.0
 
 
 
 
CAB39
 
 
hsa-miR-103a-3p
 
 
-0.532
 
 
33.0
 
 
 
 
CALD1
 
 
hsa-miR-20a-5p
 
 
-0.352
 
 
142.4
 
 
 
 
CALD1
 
 
hsa-miR-93-5p
 
 
-0.373
 
 
142.4
 
 
 
 
CALD1
 
 
hsa-miR-17-5p
 
 
-0.389
 
 
142.4
 
 
 
 
CAMK2N1
 
 
hsa-miR-17-5p
 
 
-0.367
 
 
36.0
 
 
 
 
CAMK2N1
 
 
hsa-miR-20a-5p
 
 
-0.381
 
 
36.0
 
 
 
 
CAMK2N1
 
 
hsa-miR-93-5p
 
 
-0.389
 
 
36.0
 
 
 
 
CAND1
 
 
hsa-miR-92a-3p
 
 
-0.347
 
 
26.2
 
 
 
 
 
 
 esophagus 
 
 
 
 
target_symbol
 
 
mature_mirna_id
 
 
score
 
 
nTPM
 
 
 
 
 
 
AARS1
 
 
hsa-miR-92a-3p
 
 
-0.458
 
 
22.4
 
 
 
 
ABHD17B
 
 
hsa-miR-486-5p
 
 
-0.348
 
 
12.2
 
 
 
 
ABHD17C
 
 
hsa-let-7c-5p
 
 
-0.359
 
 
45.1
 
 
 
 
ABHD17C
 
 
hsa-miR-330-3p
 
 
-0.399
 
 
45.1
 
 
 
 
ABHD5
 
 
hsa-miR-20a-5p
 
 
-0.397
 
 
18.4
 
 
 
 
ABHD5
 
 
hsa-miR-93-5p
 
 
-0.402
 
 
18.4
 
 
 
 
ABHD5
 
 
hsa-miR-17-5p
 
 
-0.45
 
 
18.4
 
 
 
 
ACOX1
 
 
hsa-miR-92a-3p
 
 
-0.321
 
 
30.5
 
 
 
 
ACP3
 
 
hsa-let-7c-5p
 
 
-0.298
 
 
42.9
 
 
 
 
ACTA1
 
 
hsa-miR-155-5p
 
 
-0.595
 
 
568.9
 
 
 
 
ACTC1
 
 
hsa-miR-92a-3p
 
 
-0.361
 
 
21.5
 
 
 
 
ACTC1
 
 
hsa-miR-7-5p
 
 
-0.471
 
 
21.5
 
 
 
 
ADAM9
 
 
hsa-miR-20a-5p
 
 
-0.299
 
 
22.2
 
 
 
 
ADAM9
 
 
hsa-miR-17-5p
 
 
-0.32
 
 
22.2
 
 
 
 
ADAM9
 
 
hsa-miR-93-5p
 
 
-0.32
 
 
22.2
 
 
 
 
ADCY3
 
 
hsa-miR-92a-3p
 
 
-0.381
 
 
29.3
 
 
 
 
ADNP
 
 
hsa-miR-328-3p
 
 
-0.371
 
 
30.5
 
 
 
 
ADRB2
 
 
hsa-let-7c-5p
 
 
-0.635
 
 
14.2
 
 
 
 
AGFG1
 
 
hsa-miR-103a-3p
 
 
-0.389
 
 
44.3
 
 
 
 
AGFG2
 
 
hsa-miR-20a-5p
 
 
-0.365
 
 
29.3
 
 
 
 
AGFG2
 
 
hsa-miR-93-5p
 
 
-0.378
 
 
29.3
 
 
 
 
AGFG2
 
 
hsa-miR-17-5p
 
 
-0.402
 
 
29.3
 
 
 
 
AHNAK
 
 
hsa-miR-20a-5p
 
 
-0.316
 
 
234.9
 
 
 
 
AHNAK
 
 
hsa-miR-17-5p
 
 
-0.337
 
 
234.9
 
 
 
 
AHNAK
 
 
hsa-miR-93-5p
 
 
-0.337
 
 
234.9
 
 
 
 
AIFM1
 
 
hsa-let-7c-5p
 
 
-0.314
 
 
15.9
 
 
 
 
AJUBA
 
 
hsa-miR-103a-3p
 
 
-0.448
 
 
17.4
 
 
 
 
AKIRIN2
 
 
hsa-miR-103a-3p
 
 
-0.299
 
 
29.6
 
 
 
 
ALAD
 
 
hsa-miR-103a-3p
 
 
-0.385
 
 
34.3
 
 
 
 
ALG3
 
 
hsa-miR-20a-5p
 
 
-0.559
 
 
29.4
 
 
 
 
ALG3
 
 
hsa-miR-93-5p
 
 
-0.564
 
 
29.4
 
 
 
 
ALG3
 
 
hsa-miR-17-5p
 
 
-0.572
 
 
29.4
 
 
 
 
ALG9
 
 
hsa-miR-7-5p
 
 
-0.302
 
 
10.5
 
 
 
 
ANGPTL2
 
 
hsa-miR-221-3p
 
 
-0.369
 
 
20.3
 
 
 
 
ANKFY1
 
 
hsa-miR-155-5p
 
 
-0.315
 
 
12.9
 
 
 
 
ANKRD12
 
 
hsa-miR-7-5p
 
 
-0.37
 
 
13.6
 
 
 
 
ANKRD46
 
 
hsa-let-7c-5p
 
 
-0.389
 
 
11.9
 
 
 
 
ANKRD9
 
 
hsa-miR-20a-5p
 
 
-0.31
 
 
18.5
 
 
 
 
ANKRD9
 
 
hsa-miR-93-5p
 
 
-0.331
 
 
18.5
 
 
 
 
ANKRD9
 
 
hsa-miR-17-5p
 
 
-0.347
 
 
18.5
 
 
 
 
ANP32E
 
 
hsa-miR-92a-3p
 
 
-0.329
 
 
17.5
 
 
 
 
AP1AR
 
 
hsa-miR-92a-3p
 
 
-0.325
 
 
12.5
 
 
 
 
AP1S1
 
 
hsa-let-7c-5p
 
 
-0.374
 
 
36.5
 
 
 
 
AP4B1
 
 
hsa-miR-378a-3p
 
 
-0.62
 
 
11.6
 
 
 
 
APCDD1
 
 
hsa-miR-20a-5p
 
 
-0.404
 
 
26.2
 
 
 
 
APCDD1
 
 
hsa-miR-17-5p
 
 
-0.425
 
 
26.2
 
 
 
 
APCDD1
 
 
hsa-miR-93-5p
 
 
-0.425
 
 
26.2
 
 
 
 
APPL1
 
 
hsa-miR-92a-3p
 
 
-0.375
 
 
15.9
 
 
 
 
AQP3
 
 
hsa-miR-874-3p
 
 
-0.48
 
 
1457.5
 
 
 
 
ARF4
 
 
hsa-miR-7-5p
 
 
-0.407
 
 
102.8
 
 
 
 
ARF4
 
 
hsa-miR-221-3p
 
 
-0.553
 
 
102.8
 
 
 
 
ARG2
 
 
hsa-let-7c-5p
 
 
-0.468
 
 
17.2
 
 
 
 
ARHGAP1
 
 
hsa-miR-17-5p
 
 
-0.325
 
 
71.8
 
 
 
 
ARHGAP1
 
 
hsa-miR-93-5p
 
 
-0.325
 
 
71.8
 
 
 
 
ARHGAP1
 
 
hsa-miR-20a-5p
 
 
-0.339
 
 
71.8
 
 
 
 
ARHGAP5
 
 
hsa-miR-486-5p
 
 
-0.3
 
 
62.5
 
 
 
 
ARIH2
 
 
hsa-miR-103a-3p
 
 
-0.307
 
 
18.8
 
 
 
 
ARL1
 
 
hsa-miR-20a-5p
 
 
-0.403
 
 
46.2
 
 
 
 
ARL1
 
 
hsa-miR-93-5p
 
 
-0.424
 
 
46.2
 
 
 
 
ARL1
 
 
hsa-miR-17-5p
 
 
-0.432
 
 
46.2
 
 
 
 
ARL4D
 
 
hsa-let-7c-5p
 
 
-0.417
 
 
80.8
 
 
 
 
ARL6IP1
 
 
hsa-miR-7-5p
 
 
-0.374
 
 
65.8
 
 
 
 
ARL6IP1
 
 
hsa-miR-328-3p
 
 
-0.494
 
 
65.8
 
 
 
 
ARL6IP6
 
 
hsa-let-7c-5p
 
 
-0.293
 
 
11.7
 
 
 
 
ARL8A
 
 
hsa-miR-103a-3p
 
 
-0.38
 
 
31.2
 
 
 
 
ARMC1
 
 
hsa-miR-92a-3p
 
 
-0.322
 
 
15.8
 
 
 
 
ARMC1
 
 
hsa-miR-103a-3p
 
 
-0.525
 
 
15.8
 
 
 
 
ARMC8
 
 
hsa-miR-486-5p
 
 
-0.321
 
 
19.8
 
 
 
 
ARMC8
 
 
hsa-miR-20a-5p
 
 
-0.357
 
 
19.8
 
 
 
 
ARMC8
 
 
hsa-miR-93-5p
 
 
-0.362
 
 
19.8
 
 
 
 
ARMC8
 
 
hsa-miR-17-5p
 
 
-0.378
 
 
19.8
 
 
 
 
ARMC8
 
 
hsa-miR-103a-3p
 
 
-0.412
 
 
19.8
 
 
 
 
ARMT1
 
 
hsa-let-7c-5p
 
 
-0.303
 
 
11.3
 
 
 
 
ARNT
 
 
hsa-miR-103a-3p
 
 
-0.422
 
 
18.0
 
 
 
 
ARNT
 
 
hsa-miR-221-3p
 
 
-0.465
 
 
18.0
 
 
 
 
ARPP19
 
 
hsa-miR-328-3p
 
 
-0.404
 
 
49.0
 
 
 
 
ARPP19
 
 
hsa-let-7c-5p
 
 
-0.492
 
 
49.0
 
 
 
 
ARRDC3
 
 
hsa-miR-92a-3p
 
 
-0.458
 
 
36.4
 
 
 
 
ASAP1
 
 
hsa-let-7c-5p
 
 
-0.308
 
 
10.0
 
 
 
 
ASB6
 
 
hsa-miR-874-3p
 
 
-0.368
 
 
15.0
 
 
 
 
ASPH
 
 
hsa-miR-221-3p
 
 
-0.287
 
 
66.3
 
 
 
 
ASPH
 
 
hsa-miR-92a-3p
 
 
-0.416
 
 
66.3
 
 
 
 
ATF3
 
 
hsa-miR-874-3p
 
 
-0.288
 
 
120.8
 
 
 
 
ATG16L1
 
 
hsa-miR-20a-5p
 
 
-0.446
 
 
11.5
 
 
 
 
ATG16L1
 
 
hsa-miR-93-5p
 
 
-0.451
 
 
11.5
 
 
 
 
ATG16L1
 
 
hsa-miR-17-5p
 
 
-0.467
 
 
11.5
 
 
 
 
ATL2
 
 
hsa-miR-103a-3p
 
 
-0.489
 
 
39.4
 
 
 
 
ATOSA
 
 
hsa-miR-221-3p
 
 
-0.495
 
 
25.7
 
 
 
 
ATOSB
 
 
hsa-miR-328-3p
 
 
-0.358
 
 
28.9
 
 
 
 
ATOSB
 
 
hsa-let-7c-5p
 
 
-0.429
 
 
28.9
 
 
 
 
ATP2A2
 
 
hsa-let-7c-5p
 
 
-0.311
 
 
170.5
 
 
 
 
ATP6V1F
 
 
hsa-miR-185-5p
 
 
-0.765
 
 
225.3
 
 
 
 
ATPAF1
 
 
hsa-let-7c-5p
 
 
-0.498
 
 
36.9
 
 
 
 
AVPI1
 
 
hsa-miR-7-5p
 
 
-0.367
 
 
26.7
 
 
 
 
B3GAT3
 
 
hsa-let-7c-5p
 
 
-0.324
 
 
23.2
 
 
 
 
B4GALT5
 
 
hsa-miR-210-3p
 
 
-0.511
 
 
35.5
 
 
 
 
BACE1
 
 
hsa-miR-339-5p
 
 
-0.371
 
 
12.3
 
 
 
 
BACH1
 
 
hsa-let-7c-5p
 
 
-0.306
 
 
21.6
 
 
 
 
BACH1
 
 
hsa-let-7c-5p
 
 
-0.306
 
 
21.6
 
 
 
 
BACH1
 
 
hsa-let-7c-5p
 
 
-0.435
 
 
21.6
 
 
 
 
 
 
 fallopian tube 
 
 
 
 
target_symbol
 
 
mature_mirna_id
 
 
score
 
 
nTPM
 
 
 
 
 
 
AARS1
 
 
hsa-miR-92a-3p
 
 
-0.458
 
 
33.8
 
 
 
 
ABHD17C
 
 
hsa-let-7c-5p
 
 
-0.359
 
 
10.3
 
 
 
 
ABHD17C
 
 
hsa-miR-330-3p
 
 
-0.399
 
 
10.3
 
 
 
 
ADAM9
 
 
hsa-miR-20a-5p
 
 
-0.299
 
 
15.9
 
 
 
 
ADAM9
 
 
hsa-miR-17-5p
 
 
-0.32
 
 
15.9
 
 
 
 
ADAM9
 
 
hsa-miR-93-5p
 
 
-0.32
 
 
15.9
 
 
 
 
ADCY3
 
 
hsa-miR-92a-3p
 
 
-0.381
 
 
52.6
 
 
 
 
ADNP
 
 
hsa-miR-328-3p
 
 
-0.371
 
 
31.2
 
 
 
 
AGFG1
 
 
hsa-miR-103a-3p
 
 
-0.389
 
 
23.3
 
 
 
 
AGFG2
 
 
hsa-miR-20a-5p
 
 
-0.365
 
 
14.4
 
 
 
 
AGFG2
 
 
hsa-miR-93-5p
 
 
-0.378
 
 
14.4
 
 
 
 
AGFG2
 
 
hsa-miR-17-5p
 
 
-0.402
 
 
14.4
 
 
 
 
AHNAK
 
 
hsa-miR-20a-5p
 
 
-0.316
 
 
141.9
 
 
 
 
AHNAK
 
 
hsa-miR-17-5p
 
 
-0.337
 
 
141.9
 
 
 
 
AHNAK
 
 
hsa-miR-93-5p
 
 
-0.337
 
 
141.9
 
 
 
 
AIFM1
 
 
hsa-let-7c-5p
 
 
-0.314
 
 
20.8
 
 
 
 
AKIRIN2
 
 
hsa-miR-103a-3p
 
 
-0.299
 
 
18.4
 
 
 
 
ALAD
 
 
hsa-miR-103a-3p
 
 
-0.385
 
 
14.7
 
 
 
 
ALG3
 
 
hsa-miR-20a-5p
 
 
-0.559
 
 
14.3
 
 
 
 
ALG3
 
 
hsa-miR-93-5p
 
 
-0.564
 
 
14.3
 
 
 
 
ALG3
 
 
hsa-miR-17-5p
 
 
-0.572
 
 
14.3
 
 
 
 
ALKAL2
 
 
hsa-miR-92a-3p
 
 
-0.608
 
 
16.2
 
 
 
 
AMT
 
 
hsa-let-7c-5p
 
 
-0.463
 
 
13.9
 
 
 
 
ANGPTL2
 
 
hsa-miR-221-3p
 
 
-0.369
 
 
29.3
 
 
 
 
ANKFY1
 
 
hsa-miR-155-5p
 
 
-0.315
 
 
14.3
 
 
 
 
ANKRD29
 
 
hsa-miR-20a-5p
 
 
-0.359
 
 
14.4
 
 
 
 
ANKRD29
 
 
hsa-miR-17-5p
 
 
-0.372
 
 
14.4
 
 
 
 
ANKRD29
 
 
hsa-miR-93-5p
 
 
-0.372
 
 
14.4
 
 
 
 
ANKRD46
 
 
hsa-let-7c-5p
 
 
-0.389
 
 
10.7
 
 
 
 
ANP32E
 
 
hsa-miR-92a-3p
 
 
-0.329
 
 
19.9
 
 
 
 
AP1S1
 
 
hsa-let-7c-5p
 
 
-0.374
 
 
13.4
 
 
 
 
AP4B1
 
 
hsa-miR-378a-3p
 
 
-0.62
 
 
11.8
 
 
 
 
APCDD1
 
 
hsa-miR-20a-5p
 
 
-0.404
 
 
30.6
 
 
 
 
APCDD1
 
 
hsa-miR-17-5p
 
 
-0.425
 
 
30.6
 
 
 
 
APCDD1
 
 
hsa-miR-93-5p
 
 
-0.425
 
 
30.6
 
 
 
 
APPL1
 
 
hsa-miR-92a-3p
 
 
-0.375
 
 
11.1
 
 
 
 
AQP3
 
 
hsa-miR-874-3p
 
 
-0.48
 
 
23.9
 
 
 
 
ARF4
 
 
hsa-miR-7-5p
 
 
-0.407
 
 
79.6
 
 
 
 
ARF4
 
 
hsa-miR-221-3p
 
 
-0.553
 
 
79.6
 
 
 
 
ARHGAP1
 
 
hsa-miR-17-5p
 
 
-0.325
 
 
71.5
 
 
 
 
ARHGAP1
 
 
hsa-miR-93-5p
 
 
-0.325
 
 
71.5
 
 
 
 
ARHGAP1
 
 
hsa-miR-20a-5p
 
 
-0.339
 
 
71.5
 
 
 
 
ARHGAP5
 
 
hsa-miR-486-5p
 
 
-0.3
 
 
21.8
 
 
 
 
ARIH2
 
 
hsa-miR-103a-3p
 
 
-0.307
 
 
20.9
 
 
 
 
ARL1
 
 
hsa-miR-20a-5p
 
 
-0.403
 
 
47.1
 
 
 
 
ARL1
 
 
hsa-miR-93-5p
 
 
-0.424
 
 
47.1
 
 
 
 
ARL1
 
 
hsa-miR-17-5p
 
 
-0.432
 
 
47.1
 
 
 
 
ARL4D
 
 
hsa-let-7c-5p
 
 
-0.417
 
 
20.8
 
 
 
 
ARL6IP1
 
 
hsa-miR-7-5p
 
 
-0.374
 
 
81.6
 
 
 
 
ARL6IP1
 
 
hsa-miR-328-3p
 
 
-0.494
 
 
81.6
 
 
 
 
ARL6IP6
 
 
hsa-let-7c-5p
 
 
-0.293
 
 
10.8
 
 
 
 
ARL8A
 
 
hsa-miR-103a-3p
 
 
-0.38
 
 
32.5
 
 
 
 
ARMC1
 
 
hsa-miR-92a-3p
 
 
-0.322
 
 
12.4
 
 
 
 
ARMC1
 
 
hsa-miR-103a-3p
 
 
-0.525
 
 
12.4
 
 
 
 
ARMC8
 
 
hsa-miR-486-5p
 
 
-0.321
 
 
14.8
 
 
 
 
ARMC8
 
 
hsa-miR-20a-5p
 
 
-0.357
 
 
14.8
 
 
 
 
ARMC8
 
 
hsa-miR-93-5p
 
 
-0.362
 
 
14.8
 
 
 
 
ARMC8
 
 
hsa-miR-17-5p
 
 
-0.378
 
 
14.8
 
 
 
 
ARMC8
 
 
hsa-miR-103a-3p
 
 
-0.412
 
 
14.8
 
 
 
 
ARMT1
 
 
hsa-let-7c-5p
 
 
-0.303
 
 
11.9
 
 
 
 
ARNT
 
 
hsa-miR-103a-3p
 
 
-0.422
 
 
23.5
 
 
 
 
ARNT
 
 
hsa-miR-221-3p
 
 
-0.465
 
 
23.5
 
 
 
 
ARPP19
 
 
hsa-miR-328-3p
 
 
-0.404
 
 
31.4
 
 
 
 
ARPP19
 
 
hsa-let-7c-5p
 
 
-0.492
 
 
31.4
 
 
 
 
ARRDC3
 
 
hsa-miR-92a-3p
 
 
-0.458
 
 
23.4
 
 
 
 
ARRDC4
 
 
hsa-let-7c-5p
 
 
-0.329
 
 
13.7
 
 
 
 
ASAP1
 
 
hsa-let-7c-5p
 
 
-0.308
 
 
15.1
 
 
 
 
ASB6
 
 
hsa-miR-874-3p
 
 
-0.368
 
 
10.5
 
 
 
 
ASPH
 
 
hsa-miR-221-3p
 
 
-0.287
 
 
45.0
 
 
 
 
ASPH
 
 
hsa-miR-92a-3p
 
 
-0.416
 
 
45.0
 
 
 
 
ASPN
 
 
hsa-miR-92a-3p
 
 
-0.422
 
 
20.7
 
 
 
 
ATF3
 
 
hsa-miR-874-3p
 
 
-0.288
 
 
153.6
 
 
 
 
ATL2
 
 
hsa-miR-103a-3p
 
 
-0.489
 
 
24.8
 
 
 
 
ATOSA
 
 
hsa-miR-221-3p
 
 
-0.495
 
 
14.8
 
 
 
 
ATP1B2
 
 
hsa-miR-103a-3p
 
 
-0.6
 
 
14.2
 
 
 
 
ATP2A2
 
 
hsa-let-7c-5p
 
 
-0.311
 
 
63.0
 
 
 
 
ATP6V1F
 
 
hsa-miR-185-5p
 
 
-0.765
 
 
92.4
 
 
 
 
ATPAF1
 
 
hsa-let-7c-5p
 
 
-0.498
 
 
27.2
 
 
 
 
ATXN1L
 
 
hsa-miR-155-5p
 
 
-0.315
 
 
10.2
 
 
 
 
AVPI1
 
 
hsa-miR-7-5p
 
 
-0.367
 
 
21.5
 
 
 
 
B3GAT3
 
 
hsa-let-7c-5p
 
 
-0.324
 
 
34.0
 
 
 
 
B4GALT5
 
 
hsa-miR-210-3p
 
 
-0.511
 
 
17.2
 
 
 
 
B4GAT1
 
 
hsa-let-7c-5p
 
 
-0.47
 
 
25.4
 
 
 
 
BACE1
 
 
hsa-miR-339-5p
 
 
-0.371
 
 
13.7
 
 
 
 
BCAT2
 
 
hsa-miR-103a-3p
 
 
-0.364
 
 
27.1
 
 
 
 
BCAT2
 
 
hsa-miR-92a-3p
 
 
-0.609
 
 
27.1
 
 
 
 
BIN1
 
 
hsa-miR-744-5p
 
 
-0.654
 
 
50.2
 
 
 
 
BIN3
 
 
hsa-let-7c-5p
 
 
-0.427
 
 
14.8
 
 
 
 
BLOC1S6
 
 
hsa-let-7c-5p
 
 
-0.321
 
 
17.1
 
 
 
 
BLTP1
 
 
hsa-miR-92a-3p
 
 
-0.462
 
 
20.5
 
 
 
 
BMF
 
 
hsa-miR-221-3p
 
 
-0.453
 
 
11.2
 
 
 
 
BNIP3L
 
 
hsa-miR-20a-5p
 
 
-0.287
 
 
47.4
 
 
 
 
BOLA3
 
 
hsa-miR-103a-3p
 
 
-0.362
 
 
22.8
 
 
 
 
BSDC1
 
 
hsa-miR-92a-3p
 
 
-0.362
 
 
26.9
 
 
 
 
BTAF1
 
 
hsa-miR-486-5p
 
 
-0.288
 
 
20.1
 
 
 
 
BTBD10
 
 
hsa-miR-17-5p
 
 
-0.409
 
 
12.5
 
 
 
 
BTBD10
 
 
hsa-miR-93-5p
 
 
-0.409
 
 
12.5
 
 
 
 
BTBD10
 
 
hsa-miR-20a-5p
 
 
-0.412
 
 
12.5
 
 
 
 
BTF3L4
 
 
hsa-let-7c-5p
 
 
-0.355
 
 
34.3
 
 
 
 
BTG2
 
 
hsa-miR-92a-3p
 
 
-0.314
 
 
240.6
 
 
 
 
 
 
 gallbladder 
 
 
 
 
target_symbol
 
 
mature_mirna_id
 
 
score
 
 
nTPM
 
 
 
 
 
 
AARS1
 
 
hsa-miR-92a-3p
 
 
-0.458
 
 
20.9
 
 
 
 
ABHD5
 
 
hsa-miR-20a-5p
 
 
-0.397
 
 
10.2
 
 
 
 
ABHD5
 
 
hsa-miR-93-5p
 
 
-0.402
 
 
10.2
 
 
 
 
ABHD5
 
 
hsa-miR-17-5p
 
 
-0.45
 
 
10.2
 
 
 
 
ACTC1
 
 
hsa-miR-92a-3p
 
 
-0.361
 
 
11.1
 
 
 
 
ACTC1
 
 
hsa-miR-7-5p
 
 
-0.471
 
 
11.1
 
 
 
 
ADAM9
 
 
hsa-miR-20a-5p
 
 
-0.299
 
 
44.2
 
 
 
 
ADAM9
 
 
hsa-miR-17-5p
 
 
-0.32
 
 
44.2
 
 
 
 
ADAM9
 
 
hsa-miR-93-5p
 
 
-0.32
 
 
44.2
 
 
 
 
ADCY3
 
 
hsa-miR-92a-3p
 
 
-0.381
 
 
21.6
 
 
 
 
ADNP
 
 
hsa-miR-328-3p
 
 
-0.371
 
 
20.9
 
 
 
 
AGFG1
 
 
hsa-miR-103a-3p
 
 
-0.389
 
 
36.5
 
 
 
 
AGFG2
 
 
hsa-miR-20a-5p
 
 
-0.365
 
 
10.4
 
 
 
 
AGFG2
 
 
hsa-miR-93-5p
 
 
-0.378
 
 
10.4
 
 
 
 
AGFG2
 
 
hsa-miR-17-5p
 
 
-0.402
 
 
10.4
 
 
 
 
AHNAK
 
 
hsa-miR-20a-5p
 
 
-0.316
 
 
70.2
 
 
 
 
AHNAK
 
 
hsa-miR-17-5p
 
 
-0.337
 
 
70.2
 
 
 
 
AHNAK
 
 
hsa-miR-93-5p
 
 
-0.337
 
 
70.2
 
 
 
 
AKAP10
 
 
hsa-miR-339-5p
 
 
-0.302
 
 
10.0
 
 
 
 
AKIRIN2
 
 
hsa-miR-103a-3p
 
 
-0.299
 
 
14.3
 
 
 
 
ALAD
 
 
hsa-miR-103a-3p
 
 
-0.385
 
 
25.7
 
 
 
 
ALG3
 
 
hsa-miR-20a-5p
 
 
-0.559
 
 
10.8
 
 
 
 
ALG3
 
 
hsa-miR-93-5p
 
 
-0.564
 
 
10.8
 
 
 
 
ALG3
 
 
hsa-miR-17-5p
 
 
-0.572
 
 
10.8
 
 
 
 
ALKAL2
 
 
hsa-miR-92a-3p
 
 
-0.608
 
 
10.1
 
 
 
 
ANGPTL2
 
 
hsa-miR-221-3p
 
 
-0.369
 
 
62.7
 
 
 
 
ANKFY1
 
 
hsa-miR-155-5p
 
 
-0.315
 
 
11.1
 
 
 
 
ANKRD12
 
 
hsa-miR-7-5p
 
 
-0.37
 
 
13.7
 
 
 
 
ANP32E
 
 
hsa-miR-92a-3p
 
 
-0.329
 
 
18.9
 
 
 
 
AP1AR
 
 
hsa-miR-92a-3p
 
 
-0.325
 
 
12.0
 
 
 
 
AP1S1
 
 
hsa-let-7c-5p
 
 
-0.374
 
 
12.0
 
 
 
 
APCDD1
 
 
hsa-miR-20a-5p
 
 
-0.404
 
 
20.5
 
 
 
 
APCDD1
 
 
hsa-miR-17-5p
 
 
-0.425
 
 
20.5
 
 
 
 
APCDD1
 
 
hsa-miR-93-5p
 
 
-0.425
 
 
20.5
 
 
 
 
APPL1
 
 
hsa-miR-92a-3p
 
 
-0.375
 
 
12.2
 
 
 
 
ARF4
 
 
hsa-miR-7-5p
 
 
-0.407
 
 
145.1
 
 
 
 
ARF4
 
 
hsa-miR-221-3p
 
 
-0.553
 
 
145.1
 
 
 
 
ARHGAP1
 
 
hsa-miR-17-5p
 
 
-0.325
 
 
47.5
 
 
 
 
ARHGAP1
 
 
hsa-miR-93-5p
 
 
-0.325
 
 
47.5
 
 
 
 
ARHGAP1
 
 
hsa-miR-20a-5p
 
 
-0.339
 
 
47.5
 
 
 
 
ARHGAP5
 
 
hsa-miR-486-5p
 
 
-0.3
 
 
30.8
 
 
 
 
ARID4B
 
 
hsa-miR-20a-5p
 
 
-0.29
 
 
12.3
 
 
 
 
ARID4B
 
 
hsa-miR-486-5p
 
 
-0.367
 
 
12.3
 
 
 
 
ARIH2
 
 
hsa-miR-103a-3p
 
 
-0.307
 
 
14.0
 
 
 
 
ARL1
 
 
hsa-miR-20a-5p
 
 
-0.403
 
 
40.9
 
 
 
 
ARL1
 
 
hsa-miR-93-5p
 
 
-0.424
 
 
40.9
 
 
 
 
ARL1
 
 
hsa-miR-17-5p
 
 
-0.432
 
 
40.9
 
 
 
 
ARL5B
 
 
hsa-miR-92a-3p
 
 
-0.331
 
 
13.2
 
 
 
 
ARL5B
 
 
hsa-miR-155-5p
 
 
-0.355
 
 
13.2
 
 
 
 
ARL6IP1
 
 
hsa-miR-7-5p
 
 
-0.374
 
 
123.8
 
 
 
 
ARL6IP1
 
 
hsa-miR-328-3p
 
 
-0.494
 
 
123.8
 
 
 
 
ARL8A
 
 
hsa-miR-103a-3p
 
 
-0.38
 
 
20.1
 
 
 
 
ARMC1
 
 
hsa-miR-92a-3p
 
 
-0.322
 
 
11.1
 
 
 
 
ARMC1
 
 
hsa-miR-103a-3p
 
 
-0.525
 
 
11.1
 
 
 
 
ARMC8
 
 
hsa-miR-486-5p
 
 
-0.321
 
 
14.3
 
 
 
 
ARMC8
 
 
hsa-miR-20a-5p
 
 
-0.357
 
 
14.3
 
 
 
 
ARMC8
 
 
hsa-miR-93-5p
 
 
-0.362
 
 
14.3
 
 
 
 
ARMC8
 
 
hsa-miR-17-5p
 
 
-0.378
 
 
14.3
 
 
 
 
ARMC8
 
 
hsa-miR-103a-3p
 
 
-0.412
 
 
14.3
 
 
 
 
ARMT1
 
 
hsa-let-7c-5p
 
 
-0.303
 
 
10.6
 
 
 
 
ARNT
 
 
hsa-miR-103a-3p
 
 
-0.422
 
 
19.2
 
 
 
 
ARNT
 
 
hsa-miR-221-3p
 
 
-0.465
 
 
19.2
 
 
 
 
ARPP19
 
 
hsa-miR-328-3p
 
 
-0.404
 
 
36.7
 
 
 
 
ARPP19
 
 
hsa-let-7c-5p
 
 
-0.492
 
 
36.7
 
 
 
 
ARRDC3
 
 
hsa-miR-92a-3p
 
 
-0.458
 
 
36.6
 
 
 
 
ARRDC4
 
 
hsa-let-7c-5p
 
 
-0.329
 
 
16.1
 
 
 
 
ASAP1
 
 
hsa-let-7c-5p
 
 
-0.308
 
 
13.5
 
 
 
 
ASPH
 
 
hsa-miR-221-3p
 
 
-0.287
 
 
56.5
 
 
 
 
ASPH
 
 
hsa-miR-92a-3p
 
 
-0.416
 
 
56.5
 
 
 
 
ASPN
 
 
hsa-miR-92a-3p
 
 
-0.422
 
 
61.7
 
 
 
 
ATF3
 
 
hsa-miR-874-3p
 
 
-0.288
 
 
126.2
 
 
 
 
ATL2
 
 
hsa-miR-103a-3p
 
 
-0.489
 
 
17.4
 
 
 
 
ATOSA
 
 
hsa-miR-221-3p
 
 
-0.495
 
 
11.0
 
 
 
 
ATOSB
 
 
hsa-miR-328-3p
 
 
-0.358
 
 
11.0
 
 
 
 
ATOSB
 
 
hsa-let-7c-5p
 
 
-0.429
 
 
11.0
 
 
 
 
ATP2A2
 
 
hsa-let-7c-5p
 
 
-0.311
 
 
56.7
 
 
 
 
ATP6V1F
 
 
hsa-miR-185-5p
 
 
-0.765
 
 
97.7
 
 
 
 
ATPAF1
 
 
hsa-let-7c-5p
 
 
-0.498
 
 
28.6
 
 
 
 
B3GALT2
 
 
hsa-miR-17-5p
 
 
-0.35
 
 
14.1
 
 
 
 
B3GALT2
 
 
hsa-miR-93-5p
 
 
-0.35
 
 
14.1
 
 
 
 
B3GALT2
 
 
hsa-miR-20a-5p
 
 
-0.364
 
 
14.1
 
 
 
 
B3GALT2
 
 
hsa-miR-92a-3p
 
 
-0.44
 
 
14.1
 
 
 
 
B3GAT3
 
 
hsa-let-7c-5p
 
 
-0.324
 
 
14.5
 
 
 
 
B4GALT5
 
 
hsa-miR-210-3p
 
 
-0.511
 
 
29.5
 
 
 
 
BACE1
 
 
hsa-miR-339-5p
 
 
-0.371
 
 
17.4
 
 
 
 
BACH1
 
 
hsa-let-7c-5p
 
 
-0.306
 
 
24.3
 
 
 
 
BACH1
 
 
hsa-let-7c-5p
 
 
-0.306
 
 
24.3
 
 
 
 
BACH1
 
 
hsa-let-7c-5p
 
 
-0.435
 
 
24.3
 
 
 
 
BACH1
 
 
hsa-let-7c-5p
 
 
-0.435
 
 
24.3
 
 
 
 
BCAT2
 
 
hsa-miR-103a-3p
 
 
-0.364
 
 
10.0
 
 
 
 
BCAT2
 
 
hsa-miR-92a-3p
 
 
-0.609
 
 
10.0
 
 
 
 
BCL2L11
 
 
hsa-miR-339-5p
 
 
-0.306
 
 
11.1
 
 
 
 
BCL2L11
 
 
hsa-miR-92a-3p
 
 
-0.394
 
 
11.1
 
 
 
 
BCL2L11
 
 
hsa-miR-221-3p
 
 
-0.423
 
 
11.1
 
 
 
 
BIN1
 
 
hsa-miR-744-5p
 
 
-0.654
 
 
21.2
 
 
 
 
BIN3
 
 
hsa-let-7c-5p
 
 
-0.427
 
 
11.2
 
 
 
 
BLOC1S6
 
 
hsa-let-7c-5p
 
 
-0.321
 
 
24.8
 
 
 
 
BLTP1
 
 
hsa-miR-92a-3p
 
 
-0.462
 
 
12.0
 
 
 
 
BMF
 
 
hsa-miR-221-3p
 
 
-0.453
 
 
11.4
 
 
 
 
BMP2
 
 
hsa-miR-378a-3p
 
 
-0.481
 
 
15.1
 
 
 
 
 
 
 heart muscle 
 
 
 
 
target_symbol
 
 
mature_mirna_id
 
 
score
 
 
nTPM
 
 
 
 
 
 
AARS1
 
 
hsa-miR-92a-3p
 
 
-0.458
 
 
37.0
 
 
 
 
ABHD5
 
 
hsa-miR-20a-5p
 
 
-0.397
 
 
10.6
 
 
 
 
ABHD5
 
 
hsa-miR-93-5p
 
 
-0.402
 
 
10.6
 
 
 
 
ABHD5
 
 
hsa-miR-17-5p
 
 
-0.45
 
 
10.6
 
 
 
 
ACOX1
 
 
hsa-miR-92a-3p
 
 
-0.321
 
 
21.9
 
 
 
 
ACTA1
 
 
hsa-miR-155-5p
 
 
-0.595
 
 
6223.8
 
 
 
 
ACTC1
 
 
hsa-miR-92a-3p
 
 
-0.361
 
 
10840.1
 
 
 
 
ACTC1
 
 
hsa-miR-7-5p
 
 
-0.471
 
 
10840.1
 
 
 
 
ADAM19
 
 
hsa-miR-874-3p
 
 
-0.374
 
 
23.8
 
 
 
 
ADAM9
 
 
hsa-miR-20a-5p
 
 
-0.299
 
 
26.6
 
 
 
 
ADAM9
 
 
hsa-miR-17-5p
 
 
-0.32
 
 
26.6
 
 
 
 
ADAM9
 
 
hsa-miR-93-5p
 
 
-0.32
 
 
26.6
 
 
 
 
ADCY3
 
 
hsa-miR-92a-3p
 
 
-0.381
 
 
35.0
 
 
 
 
ADNP
 
 
hsa-miR-328-3p
 
 
-0.371
 
 
22.3
 
 
 
 
ADRB1
 
 
hsa-let-7c-5p
 
 
-0.351
 
 
20.3
 
 
 
 
ADRB1
 
 
hsa-miR-92a-3p
 
 
-0.478
 
 
20.3
 
 
 
 
AGFG1
 
 
hsa-miR-103a-3p
 
 
-0.389
 
 
33.8
 
 
 
 
AHNAK
 
 
hsa-miR-20a-5p
 
 
-0.316
 
 
110.3
 
 
 
 
AHNAK
 
 
hsa-miR-17-5p
 
 
-0.337
 
 
110.3
 
 
 
 
AHNAK
 
 
hsa-miR-93-5p
 
 
-0.337
 
 
110.3
 
 
 
 
AIFM1
 
 
hsa-let-7c-5p
 
 
-0.314
 
 
74.9
 
 
 
 
AKAP6
 
 
hsa-let-7c-5p
 
 
-0.307
 
 
51.1
 
 
 
 
AKIRIN2
 
 
hsa-miR-103a-3p
 
 
-0.299
 
 
27.5
 
 
 
 
ALAD
 
 
hsa-miR-103a-3p
 
 
-0.385
 
 
50.8
 
 
 
 
ALG3
 
 
hsa-miR-20a-5p
 
 
-0.559
 
 
18.7
 
 
 
 
ALG3
 
 
hsa-miR-93-5p
 
 
-0.564
 
 
18.7
 
 
 
 
ALG3
 
 
hsa-miR-17-5p
 
 
-0.572
 
 
18.7
 
 
 
 
AMT
 
 
hsa-let-7c-5p
 
 
-0.463
 
 
23.5
 
 
 
 
ANGPTL2
 
 
hsa-miR-221-3p
 
 
-0.369
 
 
64.9
 
 
 
 
ANK1
 
 
hsa-miR-103a-3p
 
 
-0.321
 
 
93.7
 
 
 
 
ANKFY1
 
 
hsa-miR-155-5p
 
 
-0.315
 
 
10.9
 
 
 
 
ANKRD12
 
 
hsa-miR-7-5p
 
 
-0.37
 
 
11.1
 
 
 
 
ANKRD46
 
 
hsa-let-7c-5p
 
 
-0.389
 
 
18.8
 
 
 
 
ANKRD9
 
 
hsa-miR-20a-5p
 
 
-0.31
 
 
104.9
 
 
 
 
ANKRD9
 
 
hsa-miR-93-5p
 
 
-0.331
 
 
104.9
 
 
 
 
ANKRD9
 
 
hsa-miR-17-5p
 
 
-0.347
 
 
104.9
 
 
 
 
ANP32E
 
 
hsa-miR-92a-3p
 
 
-0.329
 
 
32.9
 
 
 
 
AP1S1
 
 
hsa-let-7c-5p
 
 
-0.374
 
 
12.9
 
 
 
 
AP4B1
 
 
hsa-miR-378a-3p
 
 
-0.62
 
 
10.7
 
 
 
 
AP5S1
 
 
hsa-miR-744-5p
 
 
-0.354
 
 
10.7
 
 
 
 
APCDD1
 
 
hsa-miR-20a-5p
 
 
-0.404
 
 
30.9
 
 
 
 
APCDD1
 
 
hsa-miR-17-5p
 
 
-0.425
 
 
30.9
 
 
 
 
APCDD1
 
 
hsa-miR-93-5p
 
 
-0.425
 
 
30.9
 
 
 
 
APPL1
 
 
hsa-miR-92a-3p
 
 
-0.375
 
 
23.1
 
 
 
 
AQP3
 
 
hsa-miR-874-3p
 
 
-0.48
 
 
33.0
 
 
 
 
ARF4
 
 
hsa-miR-7-5p
 
 
-0.407
 
 
106.8
 
 
 
 
ARF4
 
 
hsa-miR-221-3p
 
 
-0.553
 
 
106.8
 
 
 
 
ARHGAP1
 
 
hsa-miR-17-5p
 
 
-0.325
 
 
105.1
 
 
 
 
ARHGAP1
 
 
hsa-miR-93-5p
 
 
-0.325
 
 
105.1
 
 
 
 
ARHGAP1
 
 
hsa-miR-20a-5p
 
 
-0.339
 
 
105.1
 
 
 
 
ARHGAP5
 
 
hsa-miR-486-5p
 
 
-0.3
 
 
59.2
 
 
 
 
ARIH2
 
 
hsa-miR-103a-3p
 
 
-0.307
 
 
50.8
 
 
 
 
ARL1
 
 
hsa-miR-20a-5p
 
 
-0.403
 
 
61.3
 
 
 
 
ARL1
 
 
hsa-miR-93-5p
 
 
-0.424
 
 
61.3
 
 
 
 
ARL1
 
 
hsa-miR-17-5p
 
 
-0.432
 
 
61.3
 
 
 
 
ARL4D
 
 
hsa-let-7c-5p
 
 
-0.417
 
 
12.5
 
 
 
 
ARL6IP1
 
 
hsa-miR-7-5p
 
 
-0.374
 
 
30.1
 
 
 
 
ARL6IP1
 
 
hsa-miR-328-3p
 
 
-0.494
 
 
30.1
 
 
 
 
ARL8A
 
 
hsa-miR-103a-3p
 
 
-0.38
 
 
42.1
 
 
 
 
ARMC1
 
 
hsa-miR-92a-3p
 
 
-0.322
 
 
20.2
 
 
 
 
ARMC1
 
 
hsa-miR-103a-3p
 
 
-0.525
 
 
20.2
 
 
 
 
ARMC8
 
 
hsa-miR-486-5p
 
 
-0.321
 
 
16.3
 
 
 
 
ARMC8
 
 
hsa-miR-20a-5p
 
 
-0.357
 
 
16.3
 
 
 
 
ARMC8
 
 
hsa-miR-93-5p
 
 
-0.362
 
 
16.3
 
 
 
 
ARMC8
 
 
hsa-miR-17-5p
 
 
-0.378
 
 
16.3
 
 
 
 
ARMC8
 
 
hsa-miR-103a-3p
 
 
-0.412
 
 
16.3
 
 
 
 
ARMT1
 
 
hsa-let-7c-5p
 
 
-0.303
 
 
15.7
 
 
 
 
ARNT
 
 
hsa-miR-103a-3p
 
 
-0.422
 
 
20.3
 
 
 
 
ARNT
 
 
hsa-miR-221-3p
 
 
-0.465
 
 
20.3
 
 
 
 
ARPP19
 
 
hsa-miR-328-3p
 
 
-0.404
 
 
76.2
 
 
 
 
ARPP19
 
 
hsa-let-7c-5p
 
 
-0.492
 
 
76.2
 
 
 
 
ARRDC3
 
 
hsa-miR-92a-3p
 
 
-0.458
 
 
23.2
 
 
 
 
ARRDC4
 
 
hsa-let-7c-5p
 
 
-0.329
 
 
11.3
 
 
 
 
ASAP1
 
 
hsa-let-7c-5p
 
 
-0.308
 
 
22.3
 
 
 
 
ASPH
 
 
hsa-miR-221-3p
 
 
-0.287
 
 
100.1
 
 
 
 
ASPH
 
 
hsa-miR-92a-3p
 
 
-0.416
 
 
100.1
 
 
 
 
ASPN
 
 
hsa-miR-92a-3p
 
 
-0.422
 
 
89.2
 
 
 
 
ATF3
 
 
hsa-miR-874-3p
 
 
-0.288
 
 
112.5
 
 
 
 
ATG16L1
 
 
hsa-miR-20a-5p
 
 
-0.446
 
 
10.8
 
 
 
 
ATG16L1
 
 
hsa-miR-93-5p
 
 
-0.451
 
 
10.8
 
 
 
 
ATG16L1
 
 
hsa-miR-17-5p
 
 
-0.467
 
 
10.8
 
 
 
 
ATG4A
 
 
hsa-miR-7-5p
 
 
-0.448
 
 
11.7
 
 
 
 
ATL2
 
 
hsa-miR-103a-3p
 
 
-0.489
 
 
14.5
 
 
 
 
ATOSA
 
 
hsa-miR-221-3p
 
 
-0.495
 
 
13.6
 
 
 
 
ATOSB
 
 
hsa-miR-328-3p
 
 
-0.358
 
 
18.9
 
 
 
 
ATOSB
 
 
hsa-let-7c-5p
 
 
-0.429
 
 
18.9
 
 
 
 
ATP2A2
 
 
hsa-let-7c-5p
 
 
-0.311
 
 
941.9
 
 
 
 
ATP6V1F
 
 
hsa-miR-185-5p
 
 
-0.765
 
 
181.8
 
 
 
 
ATPAF1
 
 
hsa-let-7c-5p
 
 
-0.498
 
 
249.4
 
 
 
 
AVPI1
 
 
hsa-miR-7-5p
 
 
-0.367
 
 
22.9
 
 
 
 
B3GALT2
 
 
hsa-miR-17-5p
 
 
-0.35
 
 
19.7
 
 
 
 
B3GALT2
 
 
hsa-miR-93-5p
 
 
-0.35
 
 
19.7
 
 
 
 
B3GALT2
 
 
hsa-miR-20a-5p
 
 
-0.364
 
 
19.7
 
 
 
 
B3GALT2
 
 
hsa-miR-92a-3p
 
 
-0.44
 
 
19.7
 
 
 
 
B3GAT3
 
 
hsa-let-7c-5p
 
 
-0.324
 
 
25.1
 
 
 
 
B4GALT5
 
 
hsa-miR-210-3p
 
 
-0.511
 
 
19.2
 
 
 
 
B4GAT1
 
 
hsa-let-7c-5p
 
 
-0.47
 
 
43.6
 
 
 
 
BACE1
 
 
hsa-miR-339-5p
 
 
-0.371
 
 
10.1
 
 
 
 
BACH1
 
 
hsa-let-7c-5p
 
 
-0.306
 
 
12.3
 
 
 
 
BACH1
 
 
hsa-let-7c-5p
 
 
-0.306
 
 
12.3
 
 
 
 
 
 
 hippocampal formation 
 
 
 
 
target_symbol
 
 
mature_mirna_id
 
 
score
 
 
nTPM
 
 
 
 
 
 
AARS1
 
 
hsa-miR-92a-3p
 
 
-0.458
 
 
48.1
 
 
 
 
ABHD17B
 
 
hsa-miR-486-5p
 
 
-0.348
 
 
18.9
 
 
 
 
ABHD17C
 
 
hsa-let-7c-5p
 
 
-0.359
 
 
14.4
 
 
 
 
ABHD17C
 
 
hsa-miR-330-3p
 
 
-0.399
 
 
14.4
 
 
 
 
ACOX1
 
 
hsa-miR-92a-3p
 
 
-0.321
 
 
10.5
 
 
 
 
ACSL6
 
 
hsa-let-7c-5p
 
 
-0.336
 
 
15.3
 
 
 
 
ADCY3
 
 
hsa-miR-92a-3p
 
 
-0.381
 
 
30.1
 
 
 
 
ADNP
 
 
hsa-miR-328-3p
 
 
-0.371
 
 
23.7
 
 
 
 
AGFG1
 
 
hsa-miR-103a-3p
 
 
-0.389
 
 
29.8
 
 
 
 
AGFG2
 
 
hsa-miR-20a-5p
 
 
-0.365
 
 
13.2
 
 
 
 
AGFG2
 
 
hsa-miR-93-5p
 
 
-0.378
 
 
13.2
 
 
 
 
AGFG2
 
 
hsa-miR-17-5p
 
 
-0.402
 
 
13.2
 
 
 
 
AHNAK
 
 
hsa-miR-20a-5p
 
 
-0.316
 
 
24.2
 
 
 
 
AHNAK
 
 
hsa-miR-17-5p
 
 
-0.337
 
 
24.2
 
 
 
 
AHNAK
 
 
hsa-miR-93-5p
 
 
-0.337
 
 
24.2
 
 
 
 
AIFM1
 
 
hsa-let-7c-5p
 
 
-0.314
 
 
10.5
 
 
 
 
AKAP6
 
 
hsa-let-7c-5p
 
 
-0.307
 
 
12.9
 
 
 
 
AKIRIN2
 
 
hsa-miR-103a-3p
 
 
-0.299
 
 
31.7
 
 
 
 
ALAD
 
 
hsa-miR-103a-3p
 
 
-0.385
 
 
60.3
 
 
 
 
ANGPTL2
 
 
hsa-miR-221-3p
 
 
-0.369
 
 
11.4
 
 
 
 
ANKFY1
 
 
hsa-miR-155-5p
 
 
-0.315
 
 
10.8
 
 
 
 
ANKRD46
 
 
hsa-let-7c-5p
 
 
-0.389
 
 
25.5
 
 
 
 
ANKRD9
 
 
hsa-miR-20a-5p
 
 
-0.31
 
 
23.2
 
 
 
 
ANKRD9
 
 
hsa-miR-93-5p
 
 
-0.331
 
 
23.2
 
 
 
 
ANKRD9
 
 
hsa-miR-17-5p
 
 
-0.347
 
 
23.2
 
 
 
 
ANP32E
 
 
hsa-miR-92a-3p
 
 
-0.329
 
 
27.9
 
 
 
 
AP1S1
 
 
hsa-let-7c-5p
 
 
-0.374
 
 
67.5
 
 
 
 
AP4B1
 
 
hsa-miR-378a-3p
 
 
-0.62
 
 
10.1
 
 
 
 
APCDD1
 
 
hsa-miR-20a-5p
 
 
-0.404
 
 
28.0
 
 
 
 
APCDD1
 
 
hsa-miR-17-5p
 
 
-0.425
 
 
28.0
 
 
 
 
APCDD1
 
 
hsa-miR-93-5p
 
 
-0.425
 
 
28.0
 
 
 
 
ARF4
 
 
hsa-miR-7-5p
 
 
-0.407
 
 
61.3
 
 
 
 
ARF4
 
 
hsa-miR-221-3p
 
 
-0.553
 
 
61.3
 
 
 
 
ARHGAP1
 
 
hsa-miR-17-5p
 
 
-0.325
 
 
48.9
 
 
 
 
ARHGAP1
 
 
hsa-miR-93-5p
 
 
-0.325
 
 
48.9
 
 
 
 
ARHGAP1
 
 
hsa-miR-20a-5p
 
 
-0.339
 
 
48.9
 
 
 
 
ARHGAP5
 
 
hsa-miR-486-5p
 
 
-0.3
 
 
22.7
 
 
 
 
ARIH2
 
 
hsa-miR-103a-3p
 
 
-0.307
 
 
19.7
 
 
 
 
ARL1
 
 
hsa-miR-20a-5p
 
 
-0.403
 
 
24.0
 
 
 
 
ARL1
 
 
hsa-miR-93-5p
 
 
-0.424
 
 
24.0
 
 
 
 
ARL1
 
 
hsa-miR-17-5p
 
 
-0.432
 
 
24.0
 
 
 
 
ARL6IP1
 
 
hsa-miR-7-5p
 
 
-0.374
 
 
196.8
 
 
 
 
ARL6IP1
 
 
hsa-miR-328-3p
 
 
-0.494
 
 
196.8
 
 
 
 
ARL8A
 
 
hsa-miR-103a-3p
 
 
-0.38
 
 
89.3
 
 
 
 
ARMC1
 
 
hsa-miR-92a-3p
 
 
-0.322
 
 
18.1
 
 
 
 
ARMC1
 
 
hsa-miR-103a-3p
 
 
-0.525
 
 
18.1
 
 
 
 
ARMC8
 
 
hsa-miR-486-5p
 
 
-0.321
 
 
13.3
 
 
 
 
ARMC8
 
 
hsa-miR-20a-5p
 
 
-0.357
 
 
13.3
 
 
 
 
ARMC8
 
 
hsa-miR-93-5p
 
 
-0.362
 
 
13.3
 
 
 
 
ARMC8
 
 
hsa-miR-17-5p
 
 
-0.378
 
 
13.3
 
 
 
 
ARMC8
 
 
hsa-miR-103a-3p
 
 
-0.412
 
 
13.3
 
 
 
 
ARPP19
 
 
hsa-miR-328-3p
 
 
-0.404
 
 
120.9
 
 
 
 
ARPP19
 
 
hsa-let-7c-5p
 
 
-0.492
 
 
120.9
 
 
 
 
ARRDC3
 
 
hsa-miR-92a-3p
 
 
-0.458
 
 
11.5
 
 
 
 
ARRDC4
 
 
hsa-let-7c-5p
 
 
-0.329
 
 
23.9
 
 
 
 
ASAP1
 
 
hsa-let-7c-5p
 
 
-0.308
 
 
10.8
 
 
 
 
ASB6
 
 
hsa-miR-874-3p
 
 
-0.368
 
 
14.7
 
 
 
 
ASPH
 
 
hsa-miR-221-3p
 
 
-0.287
 
 
45.5
 
 
 
 
ASPH
 
 
hsa-miR-92a-3p
 
 
-0.416
 
 
45.5
 
 
 
 
ATF3
 
 
hsa-miR-874-3p
 
 
-0.288
 
 
21.8
 
 
 
 
ATOSB
 
 
hsa-miR-328-3p
 
 
-0.358
 
 
22.4
 
 
 
 
ATOSB
 
 
hsa-let-7c-5p
 
 
-0.429
 
 
22.4
 
 
 
 
ATP1B2
 
 
hsa-miR-103a-3p
 
 
-0.6
 
 
142.7
 
 
 
 
ATP2A2
 
 
hsa-let-7c-5p
 
 
-0.311
 
 
16.6
 
 
 
 
ATP2B2
 
 
hsa-miR-7-5p
 
 
-0.297
 
 
19.0
 
 
 
 
ATP6V1F
 
 
hsa-miR-185-5p
 
 
-0.765
 
 
279.8
 
 
 
 
ATPAF1
 
 
hsa-let-7c-5p
 
 
-0.498
 
 
54.4
 
 
 
 
AVPI1
 
 
hsa-miR-7-5p
 
 
-0.367
 
 
14.1
 
 
 
 
B3GAT3
 
 
hsa-let-7c-5p
 
 
-0.324
 
 
64.6
 
 
 
 
B4GALT5
 
 
hsa-miR-210-3p
 
 
-0.511
 
 
10.8
 
 
 
 
B4GAT1
 
 
hsa-let-7c-5p
 
 
-0.47
 
 
117.5
 
 
 
 
BACE1
 
 
hsa-miR-339-5p
 
 
-0.371
 
 
31.7
 
 
 
 
BAMBI
 
 
hsa-miR-20a-5p
 
 
-0.485
 
 
14.9
 
 
 
 
BAMBI
 
 
hsa-miR-17-5p
 
 
-0.506
 
 
14.9
 
 
 
 
BAMBI
 
 
hsa-miR-93-5p
 
 
-0.506
 
 
14.9
 
 
 
 
BCAT2
 
 
hsa-miR-103a-3p
 
 
-0.364
 
 
15.7
 
 
 
 
BCAT2
 
 
hsa-miR-92a-3p
 
 
-0.609
 
 
15.7
 
 
 
 
BDH1
 
 
hsa-miR-874-3p
 
 
-0.644
 
 
19.7
 
 
 
 
BEGAIN
 
 
hsa-let-7c-5p
 
 
-0.466
 
 
36.8
 
 
 
 
BICDL1
 
 
hsa-miR-221-3p
 
 
-0.447
 
 
11.0
 
 
 
 
BIN1
 
 
hsa-miR-744-5p
 
 
-0.654
 
 
207.9
 
 
 
 
BIN3
 
 
hsa-let-7c-5p
 
 
-0.427
 
 
10.9
 
 
 
 
BLOC1S6
 
 
hsa-let-7c-5p
 
 
-0.321
 
 
18.3
 
 
 
 
BLTP1
 
 
hsa-miR-92a-3p
 
 
-0.462
 
 
17.7
 
 
 
 
BNIP3L
 
 
hsa-miR-20a-5p
 
 
-0.287
 
 
54.0
 
 
 
 
BOLA3
 
 
hsa-miR-103a-3p
 
 
-0.362
 
 
49.5
 
 
 
 
BSDC1
 
 
hsa-miR-92a-3p
 
 
-0.362
 
 
25.1
 
 
 
 
BTBD10
 
 
hsa-miR-17-5p
 
 
-0.409
 
 
18.2
 
 
 
 
BTBD10
 
 
hsa-miR-93-5p
 
 
-0.409
 
 
18.2
 
 
 
 
BTBD10
 
 
hsa-miR-20a-5p
 
 
-0.412
 
 
18.2
 
 
 
 
BTF3L4
 
 
hsa-let-7c-5p
 
 
-0.355
 
 
28.4
 
 
 
 
BTG2
 
 
hsa-miR-92a-3p
 
 
-0.314
 
 
41.2
 
 
 
 
BTG2
 
 
hsa-miR-92a-3p
 
 
-0.402
 
 
41.2
 
 
 
 
BTG3
 
 
hsa-miR-17-5p
 
 
-0.668
 
 
18.5
 
 
 
 
BTG3
 
 
hsa-miR-93-5p
 
 
-0.668
 
 
18.5
 
 
 
 
BTG3
 
 
hsa-miR-20a-5p
 
 
-0.671
 
 
18.5
 
 
 
 
BZW1
 
 
hsa-let-7c-5p
 
 
-0.354
 
 
34.8
 
 
 
 
BZW1
 
 
hsa-let-7c-5p
 
 
-0.375
 
 
34.8
 
 
 
 
C11orf24
 
 
hsa-miR-92a-3p
 
 
-0.35
 
 
15.6
 
 
 
 
C19orf47
 
 
hsa-let-7c-5p
 
 
-0.311
 
 
14.0
 
 
 
 
 
 
 hypothalamus 
 
 
 
 
target_symbol
 
 
mature_mirna_id
 
 
score
 
 
nTPM
 
 
 
 
 
 
AARS1
 
 
hsa-miR-92a-3p
 
 
-0.458
 
 
54.4
 
 
 
 
ABCB9
 
 
hsa-let-7c-5p
 
 
-0.422
 
 
11.9
 
 
 
 
ABHD17B
 
 
hsa-miR-486-5p
 
 
-0.348
 
 
11.0
 
 
 
 
ABHD17C
 
 
hsa-let-7c-5p
 
 
-0.359
 
 
11.1
 
 
 
 
ABHD17C
 
 
hsa-miR-330-3p
 
 
-0.399
 
 
11.1
 
 
 
 
ACOX1
 
 
hsa-miR-92a-3p
 
 
-0.321
 
 
11.5
 
 
 
 
ACSL6
 
 
hsa-let-7c-5p
 
 
-0.336
 
 
11.9
 
 
 
 
ADCY3
 
 
hsa-miR-92a-3p
 
 
-0.381
 
 
32.1
 
 
 
 
ADCYAP1
 
 
hsa-miR-103a-3p
 
 
-0.443
 
 
11.5
 
 
 
 
ADNP
 
 
hsa-miR-328-3p
 
 
-0.371
 
 
23.7
 
 
 
 
AGFG1
 
 
hsa-miR-103a-3p
 
 
-0.389
 
 
26.0
 
 
 
 
AGFG2
 
 
hsa-miR-20a-5p
 
 
-0.365
 
 
10.8
 
 
 
 
AGFG2
 
 
hsa-miR-93-5p
 
 
-0.378
 
 
10.8
 
 
 
 
AGFG2
 
 
hsa-miR-17-5p
 
 
-0.402
 
 
10.8
 
 
 
 
AHNAK
 
 
hsa-miR-20a-5p
 
 
-0.316
 
 
17.3
 
 
 
 
AHNAK
 
 
hsa-miR-17-5p
 
 
-0.337
 
 
17.3
 
 
 
 
AHNAK
 
 
hsa-miR-93-5p
 
 
-0.337
 
 
17.3
 
 
 
 
AKAP6
 
 
hsa-let-7c-5p
 
 
-0.307
 
 
13.6
 
 
 
 
AKIRIN2
 
 
hsa-miR-103a-3p
 
 
-0.299
 
 
29.8
 
 
 
 
ALAD
 
 
hsa-miR-103a-3p
 
 
-0.385
 
 
54.5
 
 
 
 
ANKFY1
 
 
hsa-miR-155-5p
 
 
-0.315
 
 
10.7
 
 
 
 
ANKRD46
 
 
hsa-let-7c-5p
 
 
-0.389
 
 
28.7
 
 
 
 
ANKRD9
 
 
hsa-miR-20a-5p
 
 
-0.31
 
 
16.0
 
 
 
 
ANKRD9
 
 
hsa-miR-93-5p
 
 
-0.331
 
 
16.0
 
 
 
 
ANKRD9
 
 
hsa-miR-17-5p
 
 
-0.347
 
 
16.0
 
 
 
 
ANP32E
 
 
hsa-miR-92a-3p
 
 
-0.329
 
 
33.1
 
 
 
 
AP1S1
 
 
hsa-let-7c-5p
 
 
-0.374
 
 
44.3
 
 
 
 
AP4B1
 
 
hsa-miR-378a-3p
 
 
-0.62
 
 
10.0
 
 
 
 
APBB3
 
 
hsa-let-7c-5p
 
 
-0.587
 
 
11.2
 
 
 
 
APCDD1
 
 
hsa-miR-20a-5p
 
 
-0.404
 
 
19.2
 
 
 
 
APCDD1
 
 
hsa-miR-17-5p
 
 
-0.425
 
 
19.2
 
 
 
 
APCDD1
 
 
hsa-miR-93-5p
 
 
-0.425
 
 
19.2
 
 
 
 
ARF4
 
 
hsa-miR-7-5p
 
 
-0.407
 
 
75.3
 
 
 
 
ARF4
 
 
hsa-miR-221-3p
 
 
-0.553
 
 
75.3
 
 
 
 
ARG2
 
 
hsa-let-7c-5p
 
 
-0.468
 
 
10.7
 
 
 
 
ARHGAP1
 
 
hsa-miR-17-5p
 
 
-0.325
 
 
31.0
 
 
 
 
ARHGAP1
 
 
hsa-miR-93-5p
 
 
-0.325
 
 
31.0
 
 
 
 
ARHGAP1
 
 
hsa-miR-20a-5p
 
 
-0.339
 
 
31.0
 
 
 
 
ARHGAP5
 
 
hsa-miR-486-5p
 
 
-0.3
 
 
17.7
 
 
 
 
ARIH2
 
 
hsa-miR-103a-3p
 
 
-0.307
 
 
18.5
 
 
 
 
ARL1
 
 
hsa-miR-20a-5p
 
 
-0.403
 
 
31.3
 
 
 
 
ARL1
 
 
hsa-miR-93-5p
 
 
-0.424
 
 
31.3
 
 
 
 
ARL1
 
 
hsa-miR-17-5p
 
 
-0.432
 
 
31.3
 
 
 
 
ARL6IP1
 
 
hsa-miR-7-5p
 
 
-0.374
 
 
212.8
 
 
 
 
ARL6IP1
 
 
hsa-miR-328-3p
 
 
-0.494
 
 
212.8
 
 
 
 
ARL8A
 
 
hsa-miR-103a-3p
 
 
-0.38
 
 
79.8
 
 
 
 
ARMC1
 
 
hsa-miR-92a-3p
 
 
-0.322
 
 
16.6
 
 
 
 
ARMC1
 
 
hsa-miR-103a-3p
 
 
-0.525
 
 
16.6
 
 
 
 
ARMC8
 
 
hsa-miR-486-5p
 
 
-0.321
 
 
16.8
 
 
 
 
ARMC8
 
 
hsa-miR-20a-5p
 
 
-0.357
 
 
16.8
 
 
 
 
ARMC8
 
 
hsa-miR-93-5p
 
 
-0.362
 
 
16.8
 
 
 
 
ARMC8
 
 
hsa-miR-17-5p
 
 
-0.378
 
 
16.8
 
 
 
 
ARMC8
 
 
hsa-miR-103a-3p
 
 
-0.412
 
 
16.8
 
 
 
 
ARPP19
 
 
hsa-miR-328-3p
 
 
-0.404
 
 
103.9
 
 
 
 
ARPP19
 
 
hsa-let-7c-5p
 
 
-0.492
 
 
103.9
 
 
 
 
ARRDC3
 
 
hsa-miR-92a-3p
 
 
-0.458
 
 
10.3
 
 
 
 
ARRDC4
 
 
hsa-let-7c-5p
 
 
-0.329
 
 
13.4
 
 
 
 
ASAP1
 
 
hsa-let-7c-5p
 
 
-0.308
 
 
11.8
 
 
 
 
ASB6
 
 
hsa-miR-874-3p
 
 
-0.368
 
 
11.8
 
 
 
 
ASPH
 
 
hsa-miR-221-3p
 
 
-0.287
 
 
51.0
 
 
 
 
ASPH
 
 
hsa-miR-92a-3p
 
 
-0.416
 
 
51.0
 
 
 
 
ATF3
 
 
hsa-miR-874-3p
 
 
-0.288
 
 
21.0
 
 
 
 
ATL2
 
 
hsa-miR-103a-3p
 
 
-0.489
 
 
10.2
 
 
 
 
ATOSB
 
 
hsa-miR-328-3p
 
 
-0.358
 
 
19.8
 
 
 
 
ATOSB
 
 
hsa-let-7c-5p
 
 
-0.429
 
 
19.8
 
 
 
 
ATP1B2
 
 
hsa-miR-103a-3p
 
 
-0.6
 
 
140.2
 
 
 
 
ATP2A2
 
 
hsa-let-7c-5p
 
 
-0.311
 
 
11.9
 
 
 
 
ATP2B2
 
 
hsa-miR-7-5p
 
 
-0.297
 
 
12.0
 
 
 
 
ATP6V1F
 
 
hsa-miR-185-5p
 
 
-0.765
 
 
279.3
 
 
 
 
ATPAF1
 
 
hsa-let-7c-5p
 
 
-0.498
 
 
52.2
 
 
 
 
AVPI1
 
 
hsa-miR-7-5p
 
 
-0.367
 
 
15.0
 
 
 
 
B3GAT3
 
 
hsa-let-7c-5p
 
 
-0.324
 
 
60.2
 
 
 
 
B4GALT5
 
 
hsa-miR-210-3p
 
 
-0.511
 
 
13.0
 
 
 
 
B4GAT1
 
 
hsa-let-7c-5p
 
 
-0.47
 
 
207.3
 
 
 
 
BACE1
 
 
hsa-miR-339-5p
 
 
-0.371
 
 
23.0
 
 
 
 
BAMBI
 
 
hsa-miR-20a-5p
 
 
-0.485
 
 
12.9
 
 
 
 
BAMBI
 
 
hsa-miR-17-5p
 
 
-0.506
 
 
12.9
 
 
 
 
BAMBI
 
 
hsa-miR-93-5p
 
 
-0.506
 
 
12.9
 
 
 
 
BCAT2
 
 
hsa-miR-103a-3p
 
 
-0.364
 
 
16.1
 
 
 
 
BCAT2
 
 
hsa-miR-92a-3p
 
 
-0.609
 
 
16.1
 
 
 
 
BDH1
 
 
hsa-miR-874-3p
 
 
-0.644
 
 
18.1
 
 
 
 
BEGAIN
 
 
hsa-let-7c-5p
 
 
-0.466
 
 
15.9
 
 
 
 
BICDL1
 
 
hsa-miR-221-3p
 
 
-0.447
 
 
15.9
 
 
 
 
BIN1
 
 
hsa-miR-744-5p
 
 
-0.654
 
 
127.6
 
 
 
 
BLOC1S6
 
 
hsa-let-7c-5p
 
 
-0.321
 
 
24.8
 
 
 
 
BLTP1
 
 
hsa-miR-92a-3p
 
 
-0.462
 
 
14.0
 
 
 
 
BNIP3L
 
 
hsa-miR-20a-5p
 
 
-0.287
 
 
41.8
 
 
 
 
BOLA3
 
 
hsa-miR-103a-3p
 
 
-0.362
 
 
57.1
 
 
 
 
BSDC1
 
 
hsa-miR-92a-3p
 
 
-0.362
 
 
23.0
 
 
 
 
BTBD10
 
 
hsa-miR-17-5p
 
 
-0.409
 
 
20.3
 
 
 
 
BTBD10
 
 
hsa-miR-93-5p
 
 
-0.409
 
 
20.3
 
 
 
 
BTBD10
 
 
hsa-miR-20a-5p
 
 
-0.412
 
 
20.3
 
 
 
 
BTF3L4
 
 
hsa-let-7c-5p
 
 
-0.355
 
 
33.4
 
 
 
 
BTG2
 
 
hsa-miR-92a-3p
 
 
-0.314
 
 
43.2
 
 
 
 
BTG2
 
 
hsa-miR-92a-3p
 
 
-0.402
 
 
43.2
 
 
 
 
BTG3
 
 
hsa-miR-17-5p
 
 
-0.668
 
 
23.0
 
 
 
 
BTG3
 
 
hsa-miR-93-5p
 
 
-0.668
 
 
23.0
 
 
 
 
BTG3
 
 
hsa-miR-20a-5p
 
 
-0.671
 
 
23.0
 
 
 
 
BZW1
 
 
hsa-let-7c-5p
 
 
-0.354
 
 
32.4
 
 
 
 
BZW1
 
 
hsa-let-7c-5p
 
 
-0.375
 
 
32.4
 
 
 
 
 
 
 kidney 
 
 
 
 
target_symbol
 
 
mature_mirna_id
 
 
score
 
 
nTPM
 
 
 
 
 
 
AARS1
 
 
hsa-miR-92a-3p
 
 
-0.458
 
 
40.8
 
 
 
 
ABHD17B
 
 
hsa-miR-486-5p
 
 
-0.348
 
 
10.9
 
 
 
 
ABHD17C
 
 
hsa-let-7c-5p
 
 
-0.359
 
 
13.6
 
 
 
 
ABHD17C
 
 
hsa-miR-330-3p
 
 
-0.399
 
 
13.6
 
 
 
 
ABHD5
 
 
hsa-miR-20a-5p
 
 
-0.397
 
 
10.4
 
 
 
 
ABHD5
 
 
hsa-miR-93-5p
 
 
-0.402
 
 
10.4
 
 
 
 
ABHD5
 
 
hsa-miR-17-5p
 
 
-0.45
 
 
10.4
 
 
 
 
ACOX1
 
 
hsa-miR-92a-3p
 
 
-0.321
 
 
22.4
 
 
 
 
ACP3
 
 
hsa-let-7c-5p
 
 
-0.298
 
 
26.9
 
 
 
 
ADAM9
 
 
hsa-miR-20a-5p
 
 
-0.299
 
 
32.6
 
 
 
 
ADAM9
 
 
hsa-miR-17-5p
 
 
-0.32
 
 
32.6
 
 
 
 
ADAM9
 
 
hsa-miR-93-5p
 
 
-0.32
 
 
32.6
 
 
 
 
ADCY3
 
 
hsa-miR-92a-3p
 
 
-0.381
 
 
27.1
 
 
 
 
ADNP
 
 
hsa-miR-328-3p
 
 
-0.371
 
 
28.2
 
 
 
 
AGFG1
 
 
hsa-miR-103a-3p
 
 
-0.389
 
 
30.6
 
 
 
 
AGFG2
 
 
hsa-miR-20a-5p
 
 
-0.365
 
 
15.9
 
 
 
 
AGFG2
 
 
hsa-miR-93-5p
 
 
-0.378
 
 
15.9
 
 
 
 
AGFG2
 
 
hsa-miR-17-5p
 
 
-0.402
 
 
15.9
 
 
 
 
AHNAK
 
 
hsa-miR-20a-5p
 
 
-0.316
 
 
55.7
 
 
 
 
AHNAK
 
 
hsa-miR-17-5p
 
 
-0.337
 
 
55.7
 
 
 
 
AHNAK
 
 
hsa-miR-93-5p
 
 
-0.337
 
 
55.7
 
 
 
 
AIFM1
 
 
hsa-let-7c-5p
 
 
-0.314
 
 
69.0
 
 
 
 
AJUBA
 
 
hsa-miR-103a-3p
 
 
-0.448
 
 
16.5
 
 
 
 
AKAP10
 
 
hsa-miR-339-5p
 
 
-0.302
 
 
12.2
 
 
 
 
AKIRIN2
 
 
hsa-miR-103a-3p
 
 
-0.299
 
 
17.3
 
 
 
 
ALAD
 
 
hsa-miR-103a-3p
 
 
-0.385
 
 
68.5
 
 
 
 
ALG3
 
 
hsa-miR-20a-5p
 
 
-0.559
 
 
17.8
 
 
 
 
ALG3
 
 
hsa-miR-93-5p
 
 
-0.564
 
 
17.8
 
 
 
 
ALG3
 
 
hsa-miR-17-5p
 
 
-0.572
 
 
17.8
 
 
 
 
ALKAL2
 
 
hsa-miR-92a-3p
 
 
-0.608
 
 
20.1
 
 
 
 
AMT
 
 
hsa-let-7c-5p
 
 
-0.463
 
 
23.7
 
 
 
 
ANGPTL2
 
 
hsa-miR-221-3p
 
 
-0.369
 
 
17.6
 
 
 
 
ANKFY1
 
 
hsa-miR-155-5p
 
 
-0.315
 
 
10.6
 
 
 
 
ANKRD12
 
 
hsa-miR-7-5p
 
 
-0.37
 
 
12.1
 
 
 
 
ANKRD46
 
 
hsa-let-7c-5p
 
 
-0.389
 
 
30.5
 
 
 
 
ANKRD9
 
 
hsa-miR-20a-5p
 
 
-0.31
 
 
22.8
 
 
 
 
ANKRD9
 
 
hsa-miR-93-5p
 
 
-0.331
 
 
22.8
 
 
 
 
ANKRD9
 
 
hsa-miR-17-5p
 
 
-0.347
 
 
22.8
 
 
 
 
ANP32E
 
 
hsa-miR-92a-3p
 
 
-0.329
 
 
28.8
 
 
 
 
AP1AR
 
 
hsa-miR-92a-3p
 
 
-0.325
 
 
20.6
 
 
 
 
AP1S1
 
 
hsa-let-7c-5p
 
 
-0.374
 
 
48.3
 
 
 
 
AP5S1
 
 
hsa-miR-744-5p
 
 
-0.354
 
 
10.3
 
 
 
 
APPL1
 
 
hsa-miR-92a-3p
 
 
-0.375
 
 
15.4
 
 
 
 
AQP3
 
 
hsa-miR-874-3p
 
 
-0.48
 
 
151.2
 
 
 
 
ARF4
 
 
hsa-miR-7-5p
 
 
-0.407
 
 
106.7
 
 
 
 
ARF4
 
 
hsa-miR-221-3p
 
 
-0.553
 
 
106.7
 
 
 
 
ARG2
 
 
hsa-let-7c-5p
 
 
-0.468
 
 
66.0
 
 
 
 
ARHGAP1
 
 
hsa-miR-17-5p
 
 
-0.325
 
 
38.9
 
 
 
 
ARHGAP1
 
 
hsa-miR-93-5p
 
 
-0.325
 
 
38.9
 
 
 
 
ARHGAP1
 
 
hsa-miR-20a-5p
 
 
-0.339
 
 
38.9
 
 
 
 
ARHGAP5
 
 
hsa-miR-486-5p
 
 
-0.3
 
 
40.2
 
 
 
 
ARHGEF28
 
 
hsa-miR-93-5p
 
 
-0.33
 
 
29.5
 
 
 
 
ARHGEF28
 
 
hsa-miR-17-5p
 
 
-0.363
 
 
29.5
 
 
 
 
ARHGEF28
 
 
hsa-miR-20a-5p
 
 
-0.366
 
 
29.5
 
 
 
 
ARIH2
 
 
hsa-miR-103a-3p
 
 
-0.307
 
 
21.0
 
 
 
 
ARL1
 
 
hsa-miR-20a-5p
 
 
-0.403
 
 
71.4
 
 
 
 
ARL1
 
 
hsa-miR-93-5p
 
 
-0.424
 
 
71.4
 
 
 
 
ARL1
 
 
hsa-miR-17-5p
 
 
-0.432
 
 
71.4
 
 
 
 
ARL4D
 
 
hsa-let-7c-5p
 
 
-0.417
 
 
35.1
 
 
 
 
ARL6IP1
 
 
hsa-miR-7-5p
 
 
-0.374
 
 
140.2
 
 
 
 
ARL6IP1
 
 
hsa-miR-328-3p
 
 
-0.494
 
 
140.2
 
 
 
 
ARL8A
 
 
hsa-miR-103a-3p
 
 
-0.38
 
 
32.8
 
 
 
 
ARMC1
 
 
hsa-miR-92a-3p
 
 
-0.322
 
 
20.6
 
 
 
 
ARMC1
 
 
hsa-miR-103a-3p
 
 
-0.525
 
 
20.6
 
 
 
 
ARMC8
 
 
hsa-miR-486-5p
 
 
-0.321
 
 
17.6
 
 
 
 
ARMC8
 
 
hsa-miR-20a-5p
 
 
-0.357
 
 
17.6
 
 
 
 
ARMC8
 
 
hsa-miR-93-5p
 
 
-0.362
 
 
17.6
 
 
 
 
ARMC8
 
 
hsa-miR-17-5p
 
 
-0.378
 
 
17.6
 
 
 
 
ARMC8
 
 
hsa-miR-103a-3p
 
 
-0.412
 
 
17.6
 
 
 
 
ARMT1
 
 
hsa-let-7c-5p
 
 
-0.303
 
 
38.7
 
 
 
 
ARNT
 
 
hsa-miR-103a-3p
 
 
-0.422
 
 
16.6
 
 
 
 
ARNT
 
 
hsa-miR-221-3p
 
 
-0.465
 
 
16.6
 
 
 
 
ARPP19
 
 
hsa-miR-328-3p
 
 
-0.404
 
 
52.6
 
 
 
 
ARPP19
 
 
hsa-let-7c-5p
 
 
-0.492
 
 
52.6
 
 
 
 
ARRDC3
 
 
hsa-miR-92a-3p
 
 
-0.458
 
 
49.3
 
 
 
 
ARRDC4
 
 
hsa-let-7c-5p
 
 
-0.329
 
 
23.9
 
 
 
 
ASAP1
 
 
hsa-let-7c-5p
 
 
-0.308
 
 
10.1
 
 
 
 
ASB6
 
 
hsa-miR-874-3p
 
 
-0.368
 
 
15.0
 
 
 
 
ASPH
 
 
hsa-miR-221-3p
 
 
-0.287
 
 
55.2
 
 
 
 
ASPH
 
 
hsa-miR-92a-3p
 
 
-0.416
 
 
55.2
 
 
 
 
ASPN
 
 
hsa-miR-92a-3p
 
 
-0.422
 
 
10.5
 
 
 
 
ATF3
 
 
hsa-miR-874-3p
 
 
-0.288
 
 
81.3
 
 
 
 
ATG4A
 
 
hsa-miR-7-5p
 
 
-0.448
 
 
13.1
 
 
 
 
ATL2
 
 
hsa-miR-103a-3p
 
 
-0.489
 
 
26.2
 
 
 
 
ATOSA
 
 
hsa-miR-221-3p
 
 
-0.495
 
 
10.0
 
 
 
 
ATOSB
 
 
hsa-miR-328-3p
 
 
-0.358
 
 
14.3
 
 
 
 
ATOSB
 
 
hsa-let-7c-5p
 
 
-0.429
 
 
14.3
 
 
 
 
ATP2A2
 
 
hsa-let-7c-5p
 
 
-0.311
 
 
32.2
 
 
 
 
ATP6V1F
 
 
hsa-miR-185-5p
 
 
-0.765
 
 
310.1
 
 
 
 
ATPAF1
 
 
hsa-let-7c-5p
 
 
-0.498
 
 
107.2
 
 
 
 
ATXN1L
 
 
hsa-miR-155-5p
 
 
-0.315
 
 
17.5
 
 
 
 
AVPI1
 
 
hsa-miR-7-5p
 
 
-0.367
 
 
12.6
 
 
 
 
B3GAT3
 
 
hsa-let-7c-5p
 
 
-0.324
 
 
48.1
 
 
 
 
B4GALT5
 
 
hsa-miR-210-3p
 
 
-0.511
 
 
21.3
 
 
 
 
B4GAT1
 
 
hsa-let-7c-5p
 
 
-0.47
 
 
24.3
 
 
 
 
BACE1
 
 
hsa-miR-339-5p
 
 
-0.371
 
 
13.3
 
 
 
 
BACH1
 
 
hsa-let-7c-5p
 
 
-0.306
 
 
14.8
 
 
 
 
BACH1
 
 
hsa-let-7c-5p
 
 
-0.306
 
 
14.8
 
 
 
 
BACH1
 
 
hsa-let-7c-5p
 
 
-0.435
 
 
14.8
 
 
 
 
BACH1
 
 
hsa-let-7c-5p
 
 
-0.435
 
 
14.8
 
 
 
 
 
 
 liver 
 
 
 
 
target_symbol
 
 
mature_mirna_id
 
 
score
 
 
nTPM
 
 
 
 
 
 
AARS1
 
 
hsa-miR-92a-3p
 
 
-0.458
 
 
60.8
 
 
 
 
ABHD17B
 
 
hsa-miR-486-5p
 
 
-0.348
 
 
11.3
 
 
 
 
ABHD5
 
 
hsa-miR-20a-5p
 
 
-0.397
 
 
20.3
 
 
 
 
ABHD5
 
 
hsa-miR-93-5p
 
 
-0.402
 
 
20.3
 
 
 
 
ABHD5
 
 
hsa-miR-17-5p
 
 
-0.45
 
 
20.3
 
 
 
 
ACOX1
 
 
hsa-miR-92a-3p
 
 
-0.321
 
 
113.3
 
 
 
 
ADAM9
 
 
hsa-miR-20a-5p
 
 
-0.299
 
 
12.7
 
 
 
 
ADAM9
 
 
hsa-miR-17-5p
 
 
-0.32
 
 
12.7
 
 
 
 
ADAM9
 
 
hsa-miR-93-5p
 
 
-0.32
 
 
12.7
 
 
 
 
ADNP
 
 
hsa-miR-328-3p
 
 
-0.371
 
 
28.4
 
 
 
 
ADRB2
 
 
hsa-let-7c-5p
 
 
-0.635
 
 
13.3
 
 
 
 
AGFG1
 
 
hsa-miR-103a-3p
 
 
-0.389
 
 
33.8
 
 
 
 
AGFG2
 
 
hsa-miR-20a-5p
 
 
-0.365
 
 
51.0
 
 
 
 
AGFG2
 
 
hsa-miR-93-5p
 
 
-0.378
 
 
51.0
 
 
 
 
AGFG2
 
 
hsa-miR-17-5p
 
 
-0.402
 
 
51.0
 
 
 
 
AHCTF1
 
 
hsa-let-7c-5p
 
 
-0.374
 
 
10.2
 
 
 
 
AHNAK
 
 
hsa-miR-20a-5p
 
 
-0.316
 
 
56.2
 
 
 
 
AHNAK
 
 
hsa-miR-17-5p
 
 
-0.337
 
 
56.2
 
 
 
 
AHNAK
 
 
hsa-miR-93-5p
 
 
-0.337
 
 
56.2
 
 
 
 
AIFM1
 
 
hsa-let-7c-5p
 
 
-0.314
 
 
77.0
 
 
 
 
AJUBA
 
 
hsa-miR-103a-3p
 
 
-0.448
 
 
40.5
 
 
 
 
AKIRIN2
 
 
hsa-miR-103a-3p
 
 
-0.299
 
 
29.2
 
 
 
 
ALAD
 
 
hsa-miR-103a-3p
 
 
-0.385
 
 
196.1
 
 
 
 
ALG3
 
 
hsa-miR-20a-5p
 
 
-0.559
 
 
54.4
 
 
 
 
ALG3
 
 
hsa-miR-93-5p
 
 
-0.564
 
 
54.4
 
 
 
 
ALG3
 
 
hsa-miR-17-5p
 
 
-0.572
 
 
54.4
 
 
 
 
ALG9
 
 
hsa-miR-7-5p
 
 
-0.302
 
 
12.5
 
 
 
 
AMT
 
 
hsa-let-7c-5p
 
 
-0.463
 
 
52.5
 
 
 
 
ANGPTL2
 
 
hsa-miR-221-3p
 
 
-0.369
 
 
13.8
 
 
 
 
ANKFY1
 
 
hsa-miR-155-5p
 
 
-0.315
 
 
13.0
 
 
 
 
ANKRD12
 
 
hsa-miR-7-5p
 
 
-0.37
 
 
12.9
 
 
 
 
ANKRD46
 
 
hsa-let-7c-5p
 
 
-0.389
 
 
38.9
 
 
 
 
ANKRD9
 
 
hsa-miR-20a-5p
 
 
-0.31
 
 
15.7
 
 
 
 
ANKRD9
 
 
hsa-miR-93-5p
 
 
-0.331
 
 
15.7
 
 
 
 
ANKRD9
 
 
hsa-miR-17-5p
 
 
-0.347
 
 
15.7
 
 
 
 
ANP32E
 
 
hsa-miR-92a-3p
 
 
-0.329
 
 
27.1
 
 
 
 
AP1AR
 
 
hsa-miR-92a-3p
 
 
-0.325
 
 
19.1
 
 
 
 
AP1S1
 
 
hsa-let-7c-5p
 
 
-0.374
 
 
39.1
 
 
 
 
AP5S1
 
 
hsa-miR-744-5p
 
 
-0.354
 
 
13.9
 
 
 
 
APPL1
 
 
hsa-miR-92a-3p
 
 
-0.375
 
 
12.0
 
 
 
 
AQP11
 
 
hsa-miR-103a-3p
 
 
-0.517
 
 
15.1
 
 
 
 
AQP3
 
 
hsa-miR-874-3p
 
 
-0.48
 
 
117.4
 
 
 
 
ARF4
 
 
hsa-miR-7-5p
 
 
-0.407
 
 
161.3
 
 
 
 
ARF4
 
 
hsa-miR-221-3p
 
 
-0.553
 
 
161.3
 
 
 
 
ARHGAP1
 
 
hsa-miR-17-5p
 
 
-0.325
 
 
44.2
 
 
 
 
ARHGAP1
 
 
hsa-miR-93-5p
 
 
-0.325
 
 
44.2
 
 
 
 
ARHGAP1
 
 
hsa-miR-20a-5p
 
 
-0.339
 
 
44.2
 
 
 
 
ARHGAP5
 
 
hsa-miR-486-5p
 
 
-0.3
 
 
29.8
 
 
 
 
ARID4A
 
 
hsa-miR-7-5p
 
 
-0.341
 
 
17.5
 
 
 
 
ARID4B
 
 
hsa-miR-20a-5p
 
 
-0.29
 
 
11.1
 
 
 
 
ARID4B
 
 
hsa-miR-486-5p
 
 
-0.367
 
 
11.1
 
 
 
 
ARIH2
 
 
hsa-miR-103a-3p
 
 
-0.307
 
 
20.4
 
 
 
 
ARL1
 
 
hsa-miR-20a-5p
 
 
-0.403
 
 
64.0
 
 
 
 
ARL1
 
 
hsa-miR-93-5p
 
 
-0.424
 
 
64.0
 
 
 
 
ARL1
 
 
hsa-miR-17-5p
 
 
-0.432
 
 
64.0
 
 
 
 
ARL4D
 
 
hsa-let-7c-5p
 
 
-0.417
 
 
55.4
 
 
 
 
ARL5B
 
 
hsa-miR-92a-3p
 
 
-0.331
 
 
27.9
 
 
 
 
ARL5B
 
 
hsa-miR-155-5p
 
 
-0.355
 
 
27.9
 
 
 
 
ARL6IP1
 
 
hsa-miR-7-5p
 
 
-0.374
 
 
336.2
 
 
 
 
ARL6IP1
 
 
hsa-miR-328-3p
 
 
-0.494
 
 
336.2
 
 
 
 
ARL8A
 
 
hsa-miR-103a-3p
 
 
-0.38
 
 
42.7
 
 
 
 
ARMC1
 
 
hsa-miR-92a-3p
 
 
-0.322
 
 
21.7
 
 
 
 
ARMC1
 
 
hsa-miR-103a-3p
 
 
-0.525
 
 
21.7
 
 
 
 
ARMC8
 
 
hsa-miR-486-5p
 
 
-0.321
 
 
15.8
 
 
 
 
ARMC8
 
 
hsa-miR-20a-5p
 
 
-0.357
 
 
15.8
 
 
 
 
ARMC8
 
 
hsa-miR-93-5p
 
 
-0.362
 
 
15.8
 
 
 
 
ARMC8
 
 
hsa-miR-17-5p
 
 
-0.378
 
 
15.8
 
 
 
 
ARMC8
 
 
hsa-miR-103a-3p
 
 
-0.412
 
 
15.8
 
 
 
 
ARMT1
 
 
hsa-let-7c-5p
 
 
-0.303
 
 
35.2
 
 
 
 
ARNT
 
 
hsa-miR-103a-3p
 
 
-0.422
 
 
40.1
 
 
 
 
ARNT
 
 
hsa-miR-221-3p
 
 
-0.465
 
 
40.1
 
 
 
 
ARPP19
 
 
hsa-miR-328-3p
 
 
-0.404
 
 
39.5
 
 
 
 
ARPP19
 
 
hsa-let-7c-5p
 
 
-0.492
 
 
39.5
 
 
 
 
ARRDC3
 
 
hsa-miR-92a-3p
 
 
-0.458
 
 
71.0
 
 
 
 
ARRDC4
 
 
hsa-let-7c-5p
 
 
-0.329
 
 
28.3
 
 
 
 
ASB6
 
 
hsa-miR-874-3p
 
 
-0.368
 
 
10.5
 
 
 
 
ASPH
 
 
hsa-miR-221-3p
 
 
-0.287
 
 
69.9
 
 
 
 
ASPH
 
 
hsa-miR-92a-3p
 
 
-0.416
 
 
69.9
 
 
 
 
ASPN
 
 
hsa-miR-92a-3p
 
 
-0.422
 
 
38.4
 
 
 
 
ATF3
 
 
hsa-miR-874-3p
 
 
-0.288
 
 
130.6
 
 
 
 
ATG16L1
 
 
hsa-miR-20a-5p
 
 
-0.446
 
 
14.9
 
 
 
 
ATG16L1
 
 
hsa-miR-93-5p
 
 
-0.451
 
 
14.9
 
 
 
 
ATG16L1
 
 
hsa-miR-17-5p
 
 
-0.467
 
 
14.9
 
 
 
 
ATG4A
 
 
hsa-miR-7-5p
 
 
-0.448
 
 
16.9
 
 
 
 
ATL2
 
 
hsa-miR-103a-3p
 
 
-0.489
 
 
37.3
 
 
 
 
ATOSA
 
 
hsa-miR-221-3p
 
 
-0.495
 
 
35.4
 
 
 
 
ATOSB
 
 
hsa-miR-328-3p
 
 
-0.358
 
 
10.9
 
 
 
 
ATOSB
 
 
hsa-let-7c-5p
 
 
-0.429
 
 
10.9
 
 
 
 
ATP2A2
 
 
hsa-let-7c-5p
 
 
-0.311
 
 
49.3
 
 
 
 
ATP2B2
 
 
hsa-miR-7-5p
 
 
-0.297
 
 
30.4
 
 
 
 
ATP6V1F
 
 
hsa-miR-185-5p
 
 
-0.765
 
 
84.5
 
 
 
 
ATPAF1
 
 
hsa-let-7c-5p
 
 
-0.498
 
 
101.4
 
 
 
 
AVPI1
 
 
hsa-miR-7-5p
 
 
-0.367
 
 
47.4
 
 
 
 
B3GAT3
 
 
hsa-let-7c-5p
 
 
-0.324
 
 
53.3
 
 
 
 
B4GALT5
 
 
hsa-miR-210-3p
 
 
-0.511
 
 
24.1
 
 
 
 
B4GAT1
 
 
hsa-let-7c-5p
 
 
-0.47
 
 
18.5
 
 
 
 
BACE1
 
 
hsa-miR-339-5p
 
 
-0.371
 
 
23.1
 
 
 
 
BACH1
 
 
hsa-let-7c-5p
 
 
-0.306
 
 
28.5
 
 
 
 
BACH1
 
 
hsa-let-7c-5p
 
 
-0.306
 
 
28.5
 
 
 
 
BACH1
 
 
hsa-let-7c-5p
 
 
-0.435
 
 
28.5
 
 
 
 
 
 
 lung 
 
 
 
 
target_symbol
 
 
mature_mirna_id
 
 
score
 
 
nTPM
 
 
 
 
 
 
AARS1
 
 
hsa-miR-92a-3p
 
 
-0.458
 
 
34.7
 
 
 
 
ABHD17C
 
 
hsa-let-7c-5p
 
 
-0.359
 
 
18.6
 
 
 
 
ABHD17C
 
 
hsa-miR-330-3p
 
 
-0.399
 
 
18.6
 
 
 
 
ABHD5
 
 
hsa-miR-20a-5p
 
 
-0.397
 
 
11.2
 
 
 
 
ABHD5
 
 
hsa-miR-93-5p
 
 
-0.402
 
 
11.2
 
 
 
 
ABHD5
 
 
hsa-miR-17-5p
 
 
-0.45
 
 
11.2
 
 
 
 
ACOX1
 
 
hsa-miR-92a-3p
 
 
-0.321
 
 
11.0
 
 
 
 
ADAM19
 
 
hsa-miR-874-3p
 
 
-0.374
 
 
12.8
 
 
 
 
ADAM9
 
 
hsa-miR-20a-5p
 
 
-0.299
 
 
42.9
 
 
 
 
ADAM9
 
 
hsa-miR-17-5p
 
 
-0.32
 
 
42.9
 
 
 
 
ADAM9
 
 
hsa-miR-93-5p
 
 
-0.32
 
 
42.9
 
 
 
 
ADAMTS8
 
 
hsa-let-7c-5p
 
 
-0.571
 
 
33.2
 
 
 
 
ADCY3
 
 
hsa-miR-92a-3p
 
 
-0.381
 
 
39.8
 
 
 
 
ADNP
 
 
hsa-miR-328-3p
 
 
-0.371
 
 
25.6
 
 
 
 
ADRB1
 
 
hsa-let-7c-5p
 
 
-0.351
 
 
11.4
 
 
 
 
ADRB1
 
 
hsa-miR-92a-3p
 
 
-0.478
 
 
11.4
 
 
 
 
ADRB2
 
 
hsa-let-7c-5p
 
 
-0.635
 
 
18.4
 
 
 
 
AGFG1
 
 
hsa-miR-103a-3p
 
 
-0.389
 
 
51.7
 
 
 
 
AGO4
 
 
hsa-miR-103a-3p
 
 
-0.32
 
 
10.5
 
 
 
 
AGO4
 
 
hsa-miR-221-3p
 
 
-0.321
 
 
10.5
 
 
 
 
AHNAK
 
 
hsa-miR-20a-5p
 
 
-0.316
 
 
135.8
 
 
 
 
AHNAK
 
 
hsa-miR-17-5p
 
 
-0.337
 
 
135.8
 
 
 
 
AHNAK
 
 
hsa-miR-93-5p
 
 
-0.337
 
 
135.8
 
 
 
 
AJUBA
 
 
hsa-miR-103a-3p
 
 
-0.448
 
 
14.4
 
 
 
 
AKAP10
 
 
hsa-miR-339-5p
 
 
-0.302
 
 
10.3
 
 
 
 
AKIRIN2
 
 
hsa-miR-103a-3p
 
 
-0.299
 
 
29.6
 
 
 
 
ALAD
 
 
hsa-miR-103a-3p
 
 
-0.385
 
 
19.4
 
 
 
 
ALG3
 
 
hsa-miR-20a-5p
 
 
-0.559
 
 
17.4
 
 
 
 
ALG3
 
 
hsa-miR-93-5p
 
 
-0.564
 
 
17.4
 
 
 
 
ALG3
 
 
hsa-miR-17-5p
 
 
-0.572
 
 
17.4
 
 
 
 
ALKAL2
 
 
hsa-miR-92a-3p
 
 
-0.608
 
 
33.6
 
 
 
 
ANGPTL2
 
 
hsa-miR-221-3p
 
 
-0.369
 
 
21.5
 
 
 
 
ANKFY1
 
 
hsa-miR-155-5p
 
 
-0.315
 
 
14.4
 
 
 
 
ANKRD12
 
 
hsa-miR-7-5p
 
 
-0.37
 
 
10.9
 
 
 
 
ANKRD29
 
 
hsa-miR-20a-5p
 
 
-0.359
 
 
29.5
 
 
 
 
ANKRD29
 
 
hsa-miR-17-5p
 
 
-0.372
 
 
29.5
 
 
 
 
ANKRD29
 
 
hsa-miR-93-5p
 
 
-0.372
 
 
29.5
 
 
 
 
ANKRD44
 
 
hsa-miR-92a-3p
 
 
-0.324
 
 
11.0
 
 
 
 
ANKRD46
 
 
hsa-let-7c-5p
 
 
-0.389
 
 
10.1
 
 
 
 
ANKRD9
 
 
hsa-miR-20a-5p
 
 
-0.31
 
 
15.3
 
 
 
 
ANKRD9
 
 
hsa-miR-93-5p
 
 
-0.331
 
 
15.3
 
 
 
 
ANKRD9
 
 
hsa-miR-17-5p
 
 
-0.347
 
 
15.3
 
 
 
 
ANP32E
 
 
hsa-miR-92a-3p
 
 
-0.329
 
 
21.7
 
 
 
 
AP1S1
 
 
hsa-let-7c-5p
 
 
-0.374
 
 
15.2
 
 
 
 
APCDD1
 
 
hsa-miR-20a-5p
 
 
-0.404
 
 
12.6
 
 
 
 
APCDD1
 
 
hsa-miR-17-5p
 
 
-0.425
 
 
12.6
 
 
 
 
APCDD1
 
 
hsa-miR-93-5p
 
 
-0.425
 
 
12.6
 
 
 
 
APPL1
 
 
hsa-miR-92a-3p
 
 
-0.375
 
 
12.8
 
 
 
 
AQP3
 
 
hsa-miR-874-3p
 
 
-0.48
 
 
107.5
 
 
 
 
ARF4
 
 
hsa-miR-7-5p
 
 
-0.407
 
 
116.3
 
 
 
 
ARF4
 
 
hsa-miR-221-3p
 
 
-0.553
 
 
116.3
 
 
 
 
ARHGAP1
 
 
hsa-miR-17-5p
 
 
-0.325
 
 
36.0
 
 
 
 
ARHGAP1
 
 
hsa-miR-93-5p
 
 
-0.325
 
 
36.0
 
 
 
 
ARHGAP1
 
 
hsa-miR-20a-5p
 
 
-0.339
 
 
36.0
 
 
 
 
ARHGAP5
 
 
hsa-miR-486-5p
 
 
-0.3
 
 
26.5
 
 
 
 
ARID4B
 
 
hsa-miR-20a-5p
 
 
-0.29
 
 
11.3
 
 
 
 
ARID4B
 
 
hsa-miR-486-5p
 
 
-0.367
 
 
11.3
 
 
 
 
ARIH2
 
 
hsa-miR-103a-3p
 
 
-0.307
 
 
19.0
 
 
 
 
ARL1
 
 
hsa-miR-20a-5p
 
 
-0.403
 
 
34.4
 
 
 
 
ARL1
 
 
hsa-miR-93-5p
 
 
-0.424
 
 
34.4
 
 
 
 
ARL1
 
 
hsa-miR-17-5p
 
 
-0.432
 
 
34.4
 
 
 
 
ARL4D
 
 
hsa-let-7c-5p
 
 
-0.417
 
 
16.0
 
 
 
 
ARL6IP1
 
 
hsa-miR-7-5p
 
 
-0.374
 
 
75.1
 
 
 
 
ARL6IP1
 
 
hsa-miR-328-3p
 
 
-0.494
 
 
75.1
 
 
 
 
ARL6IP6
 
 
hsa-let-7c-5p
 
 
-0.293
 
 
11.9
 
 
 
 
ARL8A
 
 
hsa-miR-103a-3p
 
 
-0.38
 
 
44.5
 
 
 
 
ARMC1
 
 
hsa-miR-92a-3p
 
 
-0.322
 
 
11.7
 
 
 
 
ARMC1
 
 
hsa-miR-103a-3p
 
 
-0.525
 
 
11.7
 
 
 
 
ARMC8
 
 
hsa-miR-486-5p
 
 
-0.321
 
 
15.4
 
 
 
 
ARMC8
 
 
hsa-miR-20a-5p
 
 
-0.357
 
 
15.4
 
 
 
 
ARMC8
 
 
hsa-miR-93-5p
 
 
-0.362
 
 
15.4
 
 
 
 
ARMC8
 
 
hsa-miR-17-5p
 
 
-0.378
 
 
15.4
 
 
 
 
ARMC8
 
 
hsa-miR-103a-3p
 
 
-0.412
 
 
15.4
 
 
 
 
ARMT1
 
 
hsa-let-7c-5p
 
 
-0.303
 
 
18.4
 
 
 
 
ARNT
 
 
hsa-miR-103a-3p
 
 
-0.422
 
 
23.3
 
 
 
 
ARNT
 
 
hsa-miR-221-3p
 
 
-0.465
 
 
23.3
 
 
 
 
ARPP19
 
 
hsa-miR-328-3p
 
 
-0.404
 
 
42.5
 
 
 
 
ARPP19
 
 
hsa-let-7c-5p
 
 
-0.492
 
 
42.5
 
 
 
 
ARRDC3
 
 
hsa-miR-92a-3p
 
 
-0.458
 
 
42.0
 
 
 
 
ARRDC4
 
 
hsa-let-7c-5p
 
 
-0.329
 
 
18.7
 
 
 
 
ASAP1
 
 
hsa-let-7c-5p
 
 
-0.308
 
 
27.6
 
 
 
 
ASPH
 
 
hsa-miR-221-3p
 
 
-0.287
 
 
69.4
 
 
 
 
ASPH
 
 
hsa-miR-92a-3p
 
 
-0.416
 
 
69.4
 
 
 
 
ASPN
 
 
hsa-miR-92a-3p
 
 
-0.422
 
 
13.5
 
 
 
 
ATF3
 
 
hsa-miR-874-3p
 
 
-0.288
 
 
109.3
 
 
 
 
ATL2
 
 
hsa-miR-103a-3p
 
 
-0.489
 
 
21.6
 
 
 
 
ATOSA
 
 
hsa-miR-221-3p
 
 
-0.495
 
 
12.4
 
 
 
 
ATOSB
 
 
hsa-miR-328-3p
 
 
-0.358
 
 
20.8
 
 
 
 
ATOSB
 
 
hsa-let-7c-5p
 
 
-0.429
 
 
20.8
 
 
 
 
ATP2A2
 
 
hsa-let-7c-5p
 
 
-0.311
 
 
46.2
 
 
 
 
ATP6V1F
 
 
hsa-miR-185-5p
 
 
-0.765
 
 
142.6
 
 
 
 
ATPAF1
 
 
hsa-let-7c-5p
 
 
-0.498
 
 
24.6
 
 
 
 
ATXN1L
 
 
hsa-miR-155-5p
 
 
-0.315
 
 
11.3
 
 
 
 
AVPI1
 
 
hsa-miR-7-5p
 
 
-0.367
 
 
23.0
 
 
 
 
B3GAT3
 
 
hsa-let-7c-5p
 
 
-0.324
 
 
32.1
 
 
 
 
B4GALT5
 
 
hsa-miR-210-3p
 
 
-0.511
 
 
26.0
 
 
 
 
B4GAT1
 
 
hsa-let-7c-5p
 
 
-0.47
 
 
16.9
 
 
 
 
BACE1
 
 
hsa-miR-339-5p
 
 
-0.371
 
 
13.1
 
 
 
 
BACH1
 
 
hsa-let-7c-5p
 
 
-0.306
 
 
20.2
 
 
 
 
BACH1
 
 
hsa-let-7c-5p
 
 
-0.306
 
 
20.2
 
 
 
 
 
 
 lymph node 
 
 
 
 
target_symbol
 
 
mature_mirna_id
 
 
score
 
 
nTPM
 
 
 
 
 
 
AARS1
 
 
hsa-miR-92a-3p
 
 
-0.458
 
 
28.1
 
 
 
 
ABHD17B
 
 
hsa-miR-486-5p
 
 
-0.348
 
 
10.2
 
 
 
 
ADAM19
 
 
hsa-miR-874-3p
 
 
-0.374
 
 
27.1
 
 
 
 
ADCY3
 
 
hsa-miR-92a-3p
 
 
-0.381
 
 
17.7
 
 
 
 
ADNP
 
 
hsa-miR-328-3p
 
 
-0.371
 
 
27.0
 
 
 
 
AGFG1
 
 
hsa-miR-103a-3p
 
 
-0.389
 
 
44.0
 
 
 
 
AHNAK
 
 
hsa-miR-20a-5p
 
 
-0.316
 
 
34.4
 
 
 
 
AHNAK
 
 
hsa-miR-17-5p
 
 
-0.337
 
 
34.4
 
 
 
 
AHNAK
 
 
hsa-miR-93-5p
 
 
-0.337
 
 
34.4
 
 
 
 
AICDA
 
 
hsa-miR-155-5p
 
 
-0.389
 
 
10.5
 
 
 
 
AIFM1
 
 
hsa-let-7c-5p
 
 
-0.314
 
 
15.1
 
 
 
 
AKAP10
 
 
hsa-miR-339-5p
 
 
-0.302
 
 
15.0
 
 
 
 
AKIRIN2
 
 
hsa-miR-103a-3p
 
 
-0.299
 
 
26.6
 
 
 
 
ALAD
 
 
hsa-miR-103a-3p
 
 
-0.385
 
 
14.6
 
 
 
 
ALG3
 
 
hsa-miR-20a-5p
 
 
-0.559
 
 
12.2
 
 
 
 
ALG3
 
 
hsa-miR-93-5p
 
 
-0.564
 
 
12.2
 
 
 
 
ALG3
 
 
hsa-miR-17-5p
 
 
-0.572
 
 
12.2
 
 
 
 
ANKFY1
 
 
hsa-miR-155-5p
 
 
-0.315
 
 
13.1
 
 
 
 
ANKRD12
 
 
hsa-miR-7-5p
 
 
-0.37
 
 
20.9
 
 
 
 
ANKRD29
 
 
hsa-miR-20a-5p
 
 
-0.359
 
 
18.8
 
 
 
 
ANKRD29
 
 
hsa-miR-17-5p
 
 
-0.372
 
 
18.8
 
 
 
 
ANKRD29
 
 
hsa-miR-93-5p
 
 
-0.372
 
 
18.8
 
 
 
 
ANKRD44
 
 
hsa-miR-92a-3p
 
 
-0.324
 
 
34.2
 
 
 
 
ANP32E
 
 
hsa-miR-92a-3p
 
 
-0.329
 
 
66.3
 
 
 
 
AP1AR
 
 
hsa-miR-92a-3p
 
 
-0.325
 
 
11.3
 
 
 
 
AP1S1
 
 
hsa-let-7c-5p
 
 
-0.374
 
 
11.1
 
 
 
 
AP4B1
 
 
hsa-miR-378a-3p
 
 
-0.62
 
 
13.4
 
 
 
 
APPL1
 
 
hsa-miR-92a-3p
 
 
-0.375
 
 
15.6
 
 
 
 
AQP3
 
 
hsa-miR-874-3p
 
 
-0.48
 
 
15.1
 
 
 
 
ARF4
 
 
hsa-miR-7-5p
 
 
-0.407
 
 
69.1
 
 
 
 
ARF4
 
 
hsa-miR-221-3p
 
 
-0.553
 
 
69.1
 
 
 
 
ARHGAP1
 
 
hsa-miR-17-5p
 
 
-0.325
 
 
28.7
 
 
 
 
ARHGAP1
 
 
hsa-miR-93-5p
 
 
-0.325
 
 
28.7
 
 
 
 
ARHGAP1
 
 
hsa-miR-20a-5p
 
 
-0.339
 
 
28.7
 
 
 
 
ARHGAP5
 
 
hsa-miR-486-5p
 
 
-0.3
 
 
13.7
 
 
 
 
ARID4A
 
 
hsa-miR-7-5p
 
 
-0.341
 
 
14.0
 
 
 
 
ARID4B
 
 
hsa-miR-20a-5p
 
 
-0.29
 
 
19.6
 
 
 
 
ARID4B
 
 
hsa-miR-486-5p
 
 
-0.367
 
 
19.6
 
 
 
 
ARIH2
 
 
hsa-miR-103a-3p
 
 
-0.307
 
 
19.3
 
 
 
 
ARL1
 
 
hsa-miR-20a-5p
 
 
-0.403
 
 
31.0
 
 
 
 
ARL1
 
 
hsa-miR-93-5p
 
 
-0.424
 
 
31.0
 
 
 
 
ARL1
 
 
hsa-miR-17-5p
 
 
-0.432
 
 
31.0
 
 
 
 
ARL6IP1
 
 
hsa-miR-7-5p
 
 
-0.374
 
 
124.0
 
 
 
 
ARL6IP1
 
 
hsa-miR-328-3p
 
 
-0.494
 
 
124.0
 
 
 
 
ARL6IP6
 
 
hsa-let-7c-5p
 
 
-0.293
 
 
17.4
 
 
 
 
ARL8A
 
 
hsa-miR-103a-3p
 
 
-0.38
 
 
19.9
 
 
 
 
ARMC1
 
 
hsa-miR-92a-3p
 
 
-0.322
 
 
18.0
 
 
 
 
ARMC1
 
 
hsa-miR-103a-3p
 
 
-0.525
 
 
18.0
 
 
 
 
ARMC8
 
 
hsa-miR-486-5p
 
 
-0.321
 
 
19.8
 
 
 
 
ARMC8
 
 
hsa-miR-20a-5p
 
 
-0.357
 
 
19.8
 
 
 
 
ARMC8
 
 
hsa-miR-93-5p
 
 
-0.362
 
 
19.8
 
 
 
 
ARMC8
 
 
hsa-miR-17-5p
 
 
-0.378
 
 
19.8
 
 
 
 
ARMC8
 
 
hsa-miR-103a-3p
 
 
-0.412
 
 
19.8
 
 
 
 
ARMT1
 
 
hsa-let-7c-5p
 
 
-0.303
 
 
19.7
 
 
 
 
ARNT
 
 
hsa-miR-103a-3p
 
 
-0.422
 
 
16.3
 
 
 
 
ARNT
 
 
hsa-miR-221-3p
 
 
-0.465
 
 
16.3
 
 
 
 
ARPP19
 
 
hsa-miR-328-3p
 
 
-0.404
 
 
45.1
 
 
 
 
ARPP19
 
 
hsa-let-7c-5p
 
 
-0.492
 
 
45.1
 
 
 
 
ARRDC3
 
 
hsa-miR-92a-3p
 
 
-0.458
 
 
27.5
 
 
 
 
ASAP1
 
 
hsa-let-7c-5p
 
 
-0.308
 
 
16.5
 
 
 
 
ASB6
 
 
hsa-miR-874-3p
 
 
-0.368
 
 
10.7
 
 
 
 
ASPH
 
 
hsa-miR-221-3p
 
 
-0.287
 
 
18.8
 
 
 
 
ASPH
 
 
hsa-miR-92a-3p
 
 
-0.416
 
 
18.8
 
 
 
 
ATAD2
 
 
hsa-miR-17-5p
 
 
-0.304
 
 
13.4
 
 
 
 
ATAD2
 
 
hsa-miR-20a-5p
 
 
-0.318
 
 
13.4
 
 
 
 
ATF3
 
 
hsa-miR-874-3p
 
 
-0.288
 
 
19.4
 
 
 
 
ATL2
 
 
hsa-miR-103a-3p
 
 
-0.489
 
 
17.9
 
 
 
 
ATOSA
 
 
hsa-miR-221-3p
 
 
-0.495
 
 
21.9
 
 
 
 
ATP2A2
 
 
hsa-let-7c-5p
 
 
-0.311
 
 
33.7
 
 
 
 
ATP6V1F
 
 
hsa-miR-185-5p
 
 
-0.765
 
 
130.8
 
 
 
 
ATPAF1
 
 
hsa-let-7c-5p
 
 
-0.498
 
 
30.3
 
 
 
 
AURKA
 
 
hsa-miR-92a-3p
 
 
-0.358
 
 
19.5
 
 
 
 
B3GAT3
 
 
hsa-let-7c-5p
 
 
-0.324
 
 
24.9
 
 
 
 
BACH1
 
 
hsa-let-7c-5p
 
 
-0.306
 
 
15.7
 
 
 
 
BACH1
 
 
hsa-let-7c-5p
 
 
-0.306
 
 
15.7
 
 
 
 
BACH1
 
 
hsa-let-7c-5p
 
 
-0.435
 
 
15.7
 
 
 
 
BACH1
 
 
hsa-let-7c-5p
 
 
-0.435
 
 
15.7
 
 
 
 
BAK1
 
 
hsa-miR-103a-3p
 
 
-0.364
 
 
20.6
 
 
 
 
BCAT2
 
 
hsa-miR-103a-3p
 
 
-0.364
 
 
13.1
 
 
 
 
BCAT2
 
 
hsa-miR-92a-3p
 
 
-0.609
 
 
13.1
 
 
 
 
BCL2L11
 
 
hsa-miR-339-5p
 
 
-0.306
 
 
23.7
 
 
 
 
BCL2L11
 
 
hsa-miR-92a-3p
 
 
-0.394
 
 
23.7
 
 
 
 
BCL2L11
 
 
hsa-miR-221-3p
 
 
-0.423
 
 
23.7
 
 
 
 
BICDL1
 
 
hsa-miR-221-3p
 
 
-0.447
 
 
15.6
 
 
 
 
BIN1
 
 
hsa-miR-744-5p
 
 
-0.654
 
 
38.4
 
 
 
 
BIN3
 
 
hsa-let-7c-5p
 
 
-0.427
 
 
12.8
 
 
 
 
BLOC1S4
 
 
hsa-miR-7-5p
 
 
-0.417
 
 
11.4
 
 
 
 
BLOC1S4
 
 
hsa-miR-7-5p
 
 
-0.527
 
 
11.4
 
 
 
 
BLOC1S6
 
 
hsa-let-7c-5p
 
 
-0.321
 
 
39.2
 
 
 
 
BLTP1
 
 
hsa-miR-92a-3p
 
 
-0.462
 
 
11.1
 
 
 
 
BMF
 
 
hsa-miR-221-3p
 
 
-0.453
 
 
12.8
 
 
 
 
BNIP3L
 
 
hsa-miR-20a-5p
 
 
-0.287
 
 
36.9
 
 
 
 
BOLA3
 
 
hsa-miR-103a-3p
 
 
-0.362
 
 
20.7
 
 
 
 
BSDC1
 
 
hsa-miR-92a-3p
 
 
-0.362
 
 
21.0
 
 
 
 
BTAF1
 
 
hsa-miR-486-5p
 
 
-0.288
 
 
25.1
 
 
 
 
BTBD10
 
 
hsa-miR-17-5p
 
 
-0.409
 
 
14.7
 
 
 
 
BTBD10
 
 
hsa-miR-93-5p
 
 
-0.409
 
 
14.7
 
 
 
 
BTBD10
 
 
hsa-miR-20a-5p
 
 
-0.412
 
 
14.7
 
 
 
 
BTF3L4
 
 
hsa-let-7c-5p
 
 
-0.355
 
 
34.2
 
 
 
 
BTG2
 
 
hsa-miR-92a-3p
 
 
-0.314
 
 
114.1
 
 
 
 
 
 
 midbrain 
 
 
 
 
target_symbol
 
 
mature_mirna_id
 
 
score
 
 
nTPM
 
 
 
 
 
 
AARS1
 
 
hsa-miR-92a-3p
 
 
-0.458
 
 
58.4
 
 
 
 
ABCB9
 
 
hsa-let-7c-5p
 
 
-0.422
 
 
10.6
 
 
 
 
ABHD17B
 
 
hsa-miR-486-5p
 
 
-0.348
 
 
16.6
 
 
 
 
ABHD17C
 
 
hsa-let-7c-5p
 
 
-0.359
 
 
11.2
 
 
 
 
ABHD17C
 
 
hsa-miR-330-3p
 
 
-0.399
 
 
11.2
 
 
 
 
ACOX1
 
 
hsa-miR-92a-3p
 
 
-0.321
 
 
12.2
 
 
 
 
ACSL6
 
 
hsa-let-7c-5p
 
 
-0.336
 
 
11.1
 
 
 
 
ADCY3
 
 
hsa-miR-92a-3p
 
 
-0.381
 
 
29.4
 
 
 
 
ADNP
 
 
hsa-miR-328-3p
 
 
-0.371
 
 
21.5
 
 
 
 
AGFG1
 
 
hsa-miR-103a-3p
 
 
-0.389
 
 
32.4
 
 
 
 
AGFG2
 
 
hsa-miR-20a-5p
 
 
-0.365
 
 
12.1
 
 
 
 
AGFG2
 
 
hsa-miR-93-5p
 
 
-0.378
 
 
12.1
 
 
 
 
AGFG2
 
 
hsa-miR-17-5p
 
 
-0.402
 
 
12.1
 
 
 
 
AHNAK
 
 
hsa-miR-20a-5p
 
 
-0.316
 
 
22.7
 
 
 
 
AHNAK
 
 
hsa-miR-17-5p
 
 
-0.337
 
 
22.7
 
 
 
 
AHNAK
 
 
hsa-miR-93-5p
 
 
-0.337
 
 
22.7
 
 
 
 
AIFM1
 
 
hsa-let-7c-5p
 
 
-0.314
 
 
12.7
 
 
 
 
AKAP6
 
 
hsa-let-7c-5p
 
 
-0.307
 
 
17.4
 
 
 
 
AKIRIN2
 
 
hsa-miR-103a-3p
 
 
-0.299
 
 
28.8
 
 
 
 
ALAD
 
 
hsa-miR-103a-3p
 
 
-0.385
 
 
105.2
 
 
 
 
ANGPTL2
 
 
hsa-miR-221-3p
 
 
-0.369
 
 
12.4
 
 
 
 
ANK1
 
 
hsa-miR-103a-3p
 
 
-0.321
 
 
14.7
 
 
 
 
ANKFY1
 
 
hsa-miR-155-5p
 
 
-0.315
 
 
12.6
 
 
 
 
ANKRD29
 
 
hsa-miR-20a-5p
 
 
-0.359
 
 
11.5
 
 
 
 
ANKRD29
 
 
hsa-miR-17-5p
 
 
-0.372
 
 
11.5
 
 
 
 
ANKRD29
 
 
hsa-miR-93-5p
 
 
-0.372
 
 
11.5
 
 
 
 
ANKRD46
 
 
hsa-let-7c-5p
 
 
-0.389
 
 
24.0
 
 
 
 
ANKRD9
 
 
hsa-miR-20a-5p
 
 
-0.31
 
 
20.0
 
 
 
 
ANKRD9
 
 
hsa-miR-93-5p
 
 
-0.331
 
 
20.0
 
 
 
 
ANKRD9
 
 
hsa-miR-17-5p
 
 
-0.347
 
 
20.0
 
 
 
 
ANP32E
 
 
hsa-miR-92a-3p
 
 
-0.329
 
 
35.7
 
 
 
 
AP1AR
 
 
hsa-miR-92a-3p
 
 
-0.325
 
 
10.1
 
 
 
 
AP1S1
 
 
hsa-let-7c-5p
 
 
-0.374
 
 
35.4
 
 
 
 
APCDD1
 
 
hsa-miR-20a-5p
 
 
-0.404
 
 
22.5
 
 
 
 
APCDD1
 
 
hsa-miR-17-5p
 
 
-0.425
 
 
22.5
 
 
 
 
APCDD1
 
 
hsa-miR-93-5p
 
 
-0.425
 
 
22.5
 
 
 
 
ARF4
 
 
hsa-miR-7-5p
 
 
-0.407
 
 
62.8
 
 
 
 
ARF4
 
 
hsa-miR-221-3p
 
 
-0.553
 
 
62.8
 
 
 
 
ARHGAP1
 
 
hsa-miR-17-5p
 
 
-0.325
 
 
34.8
 
 
 
 
ARHGAP1
 
 
hsa-miR-93-5p
 
 
-0.325
 
 
34.8
 
 
 
 
ARHGAP1
 
 
hsa-miR-20a-5p
 
 
-0.339
 
 
34.8
 
 
 
 
ARHGAP5
 
 
hsa-miR-486-5p
 
 
-0.3
 
 
21.3
 
 
 
 
ARIH2
 
 
hsa-miR-103a-3p
 
 
-0.307
 
 
21.8
 
 
 
 
ARL1
 
 
hsa-miR-20a-5p
 
 
-0.403
 
 
27.4
 
 
 
 
ARL1
 
 
hsa-miR-93-5p
 
 
-0.424
 
 
27.4
 
 
 
 
ARL1
 
 
hsa-miR-17-5p
 
 
-0.432
 
 
27.4
 
 
 
 
ARL6IP1
 
 
hsa-miR-7-5p
 
 
-0.374
 
 
198.8
 
 
 
 
ARL6IP1
 
 
hsa-miR-328-3p
 
 
-0.494
 
 
198.8
 
 
 
 
ARL8A
 
 
hsa-miR-103a-3p
 
 
-0.38
 
 
98.8
 
 
 
 
ARMC1
 
 
hsa-miR-92a-3p
 
 
-0.322
 
 
17.2
 
 
 
 
ARMC1
 
 
hsa-miR-103a-3p
 
 
-0.525
 
 
17.2
 
 
 
 
ARMC8
 
 
hsa-miR-486-5p
 
 
-0.321
 
 
14.1
 
 
 
 
ARMC8
 
 
hsa-miR-20a-5p
 
 
-0.357
 
 
14.1
 
 
 
 
ARMC8
 
 
hsa-miR-93-5p
 
 
-0.362
 
 
14.1
 
 
 
 
ARMC8
 
 
hsa-miR-17-5p
 
 
-0.378
 
 
14.1
 
 
 
 
ARMC8
 
 
hsa-miR-103a-3p
 
 
-0.412
 
 
14.1
 
 
 
 
ARPP19
 
 
hsa-miR-328-3p
 
 
-0.404
 
 
144.2
 
 
 
 
ARPP19
 
 
hsa-let-7c-5p
 
 
-0.492
 
 
144.2
 
 
 
 
ARRDC3
 
 
hsa-miR-92a-3p
 
 
-0.458
 
 
13.8
 
 
 
 
ARRDC4
 
 
hsa-let-7c-5p
 
 
-0.329
 
 
17.7
 
 
 
 
ASAP1
 
 
hsa-let-7c-5p
 
 
-0.308
 
 
12.8
 
 
 
 
ASB6
 
 
hsa-miR-874-3p
 
 
-0.368
 
 
11.3
 
 
 
 
ASPH
 
 
hsa-miR-221-3p
 
 
-0.287
 
 
48.8
 
 
 
 
ASPH
 
 
hsa-miR-92a-3p
 
 
-0.416
 
 
48.8
 
 
 
 
ATF3
 
 
hsa-miR-874-3p
 
 
-0.288
 
 
20.5
 
 
 
 
ATOSB
 
 
hsa-miR-328-3p
 
 
-0.358
 
 
24.1
 
 
 
 
ATOSB
 
 
hsa-let-7c-5p
 
 
-0.429
 
 
24.1
 
 
 
 
ATP1B2
 
 
hsa-miR-103a-3p
 
 
-0.6
 
 
199.0
 
 
 
 
ATP2A2
 
 
hsa-let-7c-5p
 
 
-0.311
 
 
17.1
 
 
 
 
ATP2B2
 
 
hsa-miR-7-5p
 
 
-0.297
 
 
12.7
 
 
 
 
ATP6V1F
 
 
hsa-miR-185-5p
 
 
-0.765
 
 
256.8
 
 
 
 
ATPAF1
 
 
hsa-let-7c-5p
 
 
-0.498
 
 
74.9
 
 
 
 
AVPI1
 
 
hsa-miR-7-5p
 
 
-0.367
 
 
18.5
 
 
 
 
B3GAT3
 
 
hsa-let-7c-5p
 
 
-0.324
 
 
58.5
 
 
 
 
B4GALT5
 
 
hsa-miR-210-3p
 
 
-0.511
 
 
10.3
 
 
 
 
B4GAT1
 
 
hsa-let-7c-5p
 
 
-0.47
 
 
85.6
 
 
 
 
BACE1
 
 
hsa-miR-339-5p
 
 
-0.371
 
 
48.1
 
 
 
 
BAMBI
 
 
hsa-miR-20a-5p
 
 
-0.485
 
 
16.8
 
 
 
 
BAMBI
 
 
hsa-miR-17-5p
 
 
-0.506
 
 
16.8
 
 
 
 
BAMBI
 
 
hsa-miR-93-5p
 
 
-0.506
 
 
16.8
 
 
 
 
BCAT2
 
 
hsa-miR-103a-3p
 
 
-0.364
 
 
16.5
 
 
 
 
BCAT2
 
 
hsa-miR-92a-3p
 
 
-0.609
 
 
16.5
 
 
 
 
BCL2L13
 
 
hsa-miR-103a-3p
 
 
-0.302
 
 
10.0
 
 
 
 
BDH1
 
 
hsa-miR-874-3p
 
 
-0.644
 
 
18.3
 
 
 
 
BIN1
 
 
hsa-miR-744-5p
 
 
-0.654
 
 
190.1
 
 
 
 
BLOC1S6
 
 
hsa-let-7c-5p
 
 
-0.321
 
 
18.5
 
 
 
 
BLTP1
 
 
hsa-miR-92a-3p
 
 
-0.462
 
 
19.4
 
 
 
 
BNIP3L
 
 
hsa-miR-20a-5p
 
 
-0.287
 
 
52.9
 
 
 
 
BOLA3
 
 
hsa-miR-103a-3p
 
 
-0.362
 
 
63.8
 
 
 
 
BSDC1
 
 
hsa-miR-92a-3p
 
 
-0.362
 
 
23.3
 
 
 
 
BTBD10
 
 
hsa-miR-17-5p
 
 
-0.409
 
 
15.0
 
 
 
 
BTBD10
 
 
hsa-miR-93-5p
 
 
-0.409
 
 
15.0
 
 
 
 
BTBD10
 
 
hsa-miR-20a-5p
 
 
-0.412
 
 
15.0
 
 
 
 
BTF3L4
 
 
hsa-let-7c-5p
 
 
-0.355
 
 
28.8
 
 
 
 
BTG2
 
 
hsa-miR-92a-3p
 
 
-0.314
 
 
46.8
 
 
 
 
BTG2
 
 
hsa-miR-92a-3p
 
 
-0.402
 
 
46.8
 
 
 
 
BTG3
 
 
hsa-miR-17-5p
 
 
-0.668
 
 
18.4
 
 
 
 
BTG3
 
 
hsa-miR-93-5p
 
 
-0.668
 
 
18.4
 
 
 
 
BTG3
 
 
hsa-miR-20a-5p
 
 
-0.671
 
 
18.4
 
 
 
 
BZW1
 
 
hsa-let-7c-5p
 
 
-0.354
 
 
39.5
 
 
 
 
 
 
 ovary 
 
 
 
 
target_symbol
 
 
mature_mirna_id
 
 
score
 
 
nTPM
 
 
 
 
 
 
AARS1
 
 
hsa-miR-92a-3p
 
 
-0.458
 
 
27.3
 
 
 
 
ADAM9
 
 
hsa-miR-20a-5p
 
 
-0.299
 
 
16.9
 
 
 
 
ADAM9
 
 
hsa-miR-17-5p
 
 
-0.32
 
 
16.9
 
 
 
 
ADAM9
 
 
hsa-miR-93-5p
 
 
-0.32
 
 
16.9
 
 
 
 
ADCY3
 
 
hsa-miR-92a-3p
 
 
-0.381
 
 
67.7
 
 
 
 
ADNP
 
 
hsa-miR-328-3p
 
 
-0.371
 
 
36.2
 
 
 
 
ADRB3
 
 
hsa-let-7c-5p
 
 
-0.415
 
 
12.8
 
 
 
 
AGFG1
 
 
hsa-miR-103a-3p
 
 
-0.389
 
 
22.9
 
 
 
 
AGFG2
 
 
hsa-miR-20a-5p
 
 
-0.365
 
 
28.5
 
 
 
 
AGFG2
 
 
hsa-miR-93-5p
 
 
-0.378
 
 
28.5
 
 
 
 
AGFG2
 
 
hsa-miR-17-5p
 
 
-0.402
 
 
28.5
 
 
 
 
AHCTF1
 
 
hsa-let-7c-5p
 
 
-0.374
 
 
13.1
 
 
 
 
AHNAK
 
 
hsa-miR-20a-5p
 
 
-0.316
 
 
105.9
 
 
 
 
AHNAK
 
 
hsa-miR-17-5p
 
 
-0.337
 
 
105.9
 
 
 
 
AHNAK
 
 
hsa-miR-93-5p
 
 
-0.337
 
 
105.9
 
 
 
 
AJUBA
 
 
hsa-miR-103a-3p
 
 
-0.448
 
 
11.2
 
 
 
 
AKAP10
 
 
hsa-miR-339-5p
 
 
-0.302
 
 
12.1
 
 
 
 
AKIRIN2
 
 
hsa-miR-103a-3p
 
 
-0.299
 
 
22.8
 
 
 
 
ALAD
 
 
hsa-miR-103a-3p
 
 
-0.385
 
 
34.6
 
 
 
 
ALG3
 
 
hsa-miR-20a-5p
 
 
-0.559
 
 
12.1
 
 
 
 
ALG3
 
 
hsa-miR-93-5p
 
 
-0.564
 
 
12.1
 
 
 
 
ALG3
 
 
hsa-miR-17-5p
 
 
-0.572
 
 
12.1
 
 
 
 
ALG9
 
 
hsa-miR-7-5p
 
 
-0.302
 
 
11.1
 
 
 
 
ALKAL2
 
 
hsa-miR-92a-3p
 
 
-0.608
 
 
72.6
 
 
 
 
AMT
 
 
hsa-let-7c-5p
 
 
-0.463
 
 
16.7
 
 
 
 
ANGPTL2
 
 
hsa-miR-221-3p
 
 
-0.369
 
 
49.6
 
 
 
 
ANKFY1
 
 
hsa-miR-155-5p
 
 
-0.315
 
 
16.2
 
 
 
 
ANKRD12
 
 
hsa-miR-7-5p
 
 
-0.37
 
 
10.6
 
 
 
 
ANKRD29
 
 
hsa-miR-20a-5p
 
 
-0.359
 
 
15.0
 
 
 
 
ANKRD29
 
 
hsa-miR-17-5p
 
 
-0.372
 
 
15.0
 
 
 
 
ANKRD29
 
 
hsa-miR-93-5p
 
 
-0.372
 
 
15.0
 
 
 
 
ANKRD46
 
 
hsa-let-7c-5p
 
 
-0.389
 
 
19.3
 
 
 
 
ANP32E
 
 
hsa-miR-92a-3p
 
 
-0.329
 
 
20.6
 
 
 
 
AP1AR
 
 
hsa-miR-92a-3p
 
 
-0.325
 
 
10.0
 
 
 
 
AP1S1
 
 
hsa-let-7c-5p
 
 
-0.374
 
 
22.2
 
 
 
 
AP4B1
 
 
hsa-miR-378a-3p
 
 
-0.62
 
 
13.3
 
 
 
 
APCDD1
 
 
hsa-miR-20a-5p
 
 
-0.404
 
 
28.1
 
 
 
 
APCDD1
 
 
hsa-miR-17-5p
 
 
-0.425
 
 
28.1
 
 
 
 
APCDD1
 
 
hsa-miR-93-5p
 
 
-0.425
 
 
28.1
 
 
 
 
APPL1
 
 
hsa-miR-92a-3p
 
 
-0.375
 
 
19.2
 
 
 
 
AQP3
 
 
hsa-miR-874-3p
 
 
-0.48
 
 
11.4
 
 
 
 
ARF4
 
 
hsa-miR-7-5p
 
 
-0.407
 
 
123.7
 
 
 
 
ARF4
 
 
hsa-miR-221-3p
 
 
-0.553
 
 
123.7
 
 
 
 
ARG2
 
 
hsa-let-7c-5p
 
 
-0.468
 
 
18.7
 
 
 
 
ARHGAP1
 
 
hsa-miR-17-5p
 
 
-0.325
 
 
43.6
 
 
 
 
ARHGAP1
 
 
hsa-miR-93-5p
 
 
-0.325
 
 
43.6
 
 
 
 
ARHGAP1
 
 
hsa-miR-20a-5p
 
 
-0.339
 
 
43.6
 
 
 
 
ARHGAP5
 
 
hsa-miR-486-5p
 
 
-0.3
 
 
33.0
 
 
 
 
ARHGEF28
 
 
hsa-miR-93-5p
 
 
-0.33
 
 
18.8
 
 
 
 
ARHGEF28
 
 
hsa-miR-17-5p
 
 
-0.363
 
 
18.8
 
 
 
 
ARHGEF28
 
 
hsa-miR-20a-5p
 
 
-0.366
 
 
18.8
 
 
 
 
ARID4A
 
 
hsa-miR-7-5p
 
 
-0.341
 
 
10.1
 
 
 
 
ARID4B
 
 
hsa-miR-20a-5p
 
 
-0.29
 
 
14.0
 
 
 
 
ARID4B
 
 
hsa-miR-486-5p
 
 
-0.367
 
 
14.0
 
 
 
 
ARIH2
 
 
hsa-miR-103a-3p
 
 
-0.307
 
 
19.4
 
 
 
 
ARL1
 
 
hsa-miR-20a-5p
 
 
-0.403
 
 
63.4
 
 
 
 
ARL1
 
 
hsa-miR-93-5p
 
 
-0.424
 
 
63.4
 
 
 
 
ARL1
 
 
hsa-miR-17-5p
 
 
-0.432
 
 
63.4
 
 
 
 
ARL4D
 
 
hsa-let-7c-5p
 
 
-0.417
 
 
20.3
 
 
 
 
ARL6IP1
 
 
hsa-miR-7-5p
 
 
-0.374
 
 
97.4
 
 
 
 
ARL6IP1
 
 
hsa-miR-328-3p
 
 
-0.494
 
 
97.4
 
 
 
 
ARL6IP6
 
 
hsa-let-7c-5p
 
 
-0.293
 
 
11.9
 
 
 
 
ARL8A
 
 
hsa-miR-103a-3p
 
 
-0.38
 
 
33.7
 
 
 
 
ARMC1
 
 
hsa-miR-92a-3p
 
 
-0.322
 
 
16.7
 
 
 
 
ARMC1
 
 
hsa-miR-103a-3p
 
 
-0.525
 
 
16.7
 
 
 
 
ARMC8
 
 
hsa-miR-486-5p
 
 
-0.321
 
 
14.0
 
 
 
 
ARMC8
 
 
hsa-miR-20a-5p
 
 
-0.357
 
 
14.0
 
 
 
 
ARMC8
 
 
hsa-miR-93-5p
 
 
-0.362
 
 
14.0
 
 
 
 
ARMC8
 
 
hsa-miR-17-5p
 
 
-0.378
 
 
14.0
 
 
 
 
ARMC8
 
 
hsa-miR-103a-3p
 
 
-0.412
 
 
14.0
 
 
 
 
ARMT1
 
 
hsa-let-7c-5p
 
 
-0.303
 
 
15.5
 
 
 
 
ARNT
 
 
hsa-miR-103a-3p
 
 
-0.422
 
 
32.5
 
 
 
 
ARNT
 
 
hsa-miR-221-3p
 
 
-0.465
 
 
32.5
 
 
 
 
ARPP19
 
 
hsa-miR-328-3p
 
 
-0.404
 
 
40.6
 
 
 
 
ARPP19
 
 
hsa-let-7c-5p
 
 
-0.492
 
 
40.6
 
 
 
 
ARRDC3
 
 
hsa-miR-92a-3p
 
 
-0.458
 
 
64.7
 
 
 
 
ASAP1
 
 
hsa-let-7c-5p
 
 
-0.308
 
 
16.8
 
 
 
 
ASPH
 
 
hsa-miR-221-3p
 
 
-0.287
 
 
58.7
 
 
 
 
ASPH
 
 
hsa-miR-92a-3p
 
 
-0.416
 
 
58.7
 
 
 
 
ATF3
 
 
hsa-miR-874-3p
 
 
-0.288
 
 
34.3
 
 
 
 
ATL2
 
 
hsa-miR-103a-3p
 
 
-0.489
 
 
32.7
 
 
 
 
ATOSA
 
 
hsa-miR-221-3p
 
 
-0.495
 
 
10.4
 
 
 
 
ATP1B2
 
 
hsa-miR-103a-3p
 
 
-0.6
 
 
21.8
 
 
 
 
ATP2A2
 
 
hsa-let-7c-5p
 
 
-0.311
 
 
34.9
 
 
 
 
ATP6V1F
 
 
hsa-miR-185-5p
 
 
-0.765
 
 
99.6
 
 
 
 
ATPAF1
 
 
hsa-let-7c-5p
 
 
-0.498
 
 
33.9
 
 
 
 
ATXN1L
 
 
hsa-miR-155-5p
 
 
-0.315
 
 
10.8
 
 
 
 
AVPI1
 
 
hsa-miR-7-5p
 
 
-0.367
 
 
85.9
 
 
 
 
B3GAT3
 
 
hsa-let-7c-5p
 
 
-0.324
 
 
35.4
 
 
 
 
B4GALT5
 
 
hsa-miR-210-3p
 
 
-0.511
 
 
19.0
 
 
 
 
B4GAT1
 
 
hsa-let-7c-5p
 
 
-0.47
 
 
33.5
 
 
 
 
BACE1
 
 
hsa-miR-339-5p
 
 
-0.371
 
 
21.8
 
 
 
 
BACH1
 
 
hsa-let-7c-5p
 
 
-0.306
 
 
12.0
 
 
 
 
BACH1
 
 
hsa-let-7c-5p
 
 
-0.306
 
 
12.0
 
 
 
 
BACH1
 
 
hsa-let-7c-5p
 
 
-0.435
 
 
12.0
 
 
 
 
BACH1
 
 
hsa-let-7c-5p
 
 
-0.435
 
 
12.0
 
 
 
 
BAMBI
 
 
hsa-miR-20a-5p
 
 
-0.485
 
 
58.6
 
 
 
 
BAMBI
 
 
hsa-miR-17-5p
 
 
-0.506
 
 
58.6
 
 
 
 
BAMBI
 
 
hsa-miR-93-5p
 
 
-0.506
 
 
58.6
 
 
 
 
BCAT2
 
 
hsa-miR-103a-3p
 
 
-0.364
 
 
51.3
 
 
 
 
 
 
 pancreas 
 
 
 
 
target_symbol
 
 
mature_mirna_id
 
 
score
 
 
nTPM
 
 
 
 
 
 
AARS1
 
 
hsa-miR-92a-3p
 
 
-0.458
 
 
71.6
 
 
 
 
ABHD5
 
 
hsa-miR-20a-5p
 
 
-0.397
 
 
11.4
 
 
 
 
ABHD5
 
 
hsa-miR-93-5p
 
 
-0.402
 
 
11.4
 
 
 
 
ABHD5
 
 
hsa-miR-17-5p
 
 
-0.45
 
 
11.4
 
 
 
 
ACOX1
 
 
hsa-miR-92a-3p
 
 
-0.321
 
 
26.8
 
 
 
 
ACTC1
 
 
hsa-miR-92a-3p
 
 
-0.361
 
 
12.1
 
 
 
 
ACTC1
 
 
hsa-miR-7-5p
 
 
-0.471
 
 
12.1
 
 
 
 
ADCY3
 
 
hsa-miR-92a-3p
 
 
-0.381
 
 
15.3
 
 
 
 
ADNP
 
 
hsa-miR-328-3p
 
 
-0.371
 
 
23.5
 
 
 
 
AGFG1
 
 
hsa-miR-103a-3p
 
 
-0.389
 
 
33.0
 
 
 
 
AGFG2
 
 
hsa-miR-20a-5p
 
 
-0.365
 
 
17.7
 
 
 
 
AGFG2
 
 
hsa-miR-93-5p
 
 
-0.378
 
 
17.7
 
 
 
 
AGFG2
 
 
hsa-miR-17-5p
 
 
-0.402
 
 
17.7
 
 
 
 
AHNAK
 
 
hsa-miR-20a-5p
 
 
-0.316
 
 
28.6
 
 
 
 
AHNAK
 
 
hsa-miR-17-5p
 
 
-0.337
 
 
28.6
 
 
 
 
AHNAK
 
 
hsa-miR-93-5p
 
 
-0.337
 
 
28.6
 
 
 
 
AIFM1
 
 
hsa-let-7c-5p
 
 
-0.314
 
 
20.4
 
 
 
 
AJUBA
 
 
hsa-miR-103a-3p
 
 
-0.448
 
 
10.9
 
 
 
 
AKIRIN2
 
 
hsa-miR-103a-3p
 
 
-0.299
 
 
11.7
 
 
 
 
ALAD
 
 
hsa-miR-103a-3p
 
 
-0.385
 
 
45.8
 
 
 
 
ALG3
 
 
hsa-miR-20a-5p
 
 
-0.559
 
 
35.7
 
 
 
 
ALG3
 
 
hsa-miR-93-5p
 
 
-0.564
 
 
35.7
 
 
 
 
ALG3
 
 
hsa-miR-17-5p
 
 
-0.572
 
 
35.7
 
 
 
 
ALG9
 
 
hsa-miR-7-5p
 
 
-0.302
 
 
18.1
 
 
 
 
ALKAL2
 
 
hsa-miR-92a-3p
 
 
-0.608
 
 
62.6
 
 
 
 
AMT
 
 
hsa-let-7c-5p
 
 
-0.463
 
 
21.5
 
 
 
 
ANKRD12
 
 
hsa-miR-7-5p
 
 
-0.37
 
 
11.2
 
 
 
 
ANKRD46
 
 
hsa-let-7c-5p
 
 
-0.389
 
 
17.5
 
 
 
 
ANKRD9
 
 
hsa-miR-20a-5p
 
 
-0.31
 
 
10.7
 
 
 
 
ANKRD9
 
 
hsa-miR-93-5p
 
 
-0.331
 
 
10.7
 
 
 
 
ANKRD9
 
 
hsa-miR-17-5p
 
 
-0.347
 
 
10.7
 
 
 
 
AP1S1
 
 
hsa-let-7c-5p
 
 
-0.374
 
 
23.9
 
 
 
 
AP4B1
 
 
hsa-miR-378a-3p
 
 
-0.62
 
 
13.3
 
 
 
 
APPL1
 
 
hsa-miR-92a-3p
 
 
-0.375
 
 
27.9
 
 
 
 
AQP3
 
 
hsa-miR-874-3p
 
 
-0.48
 
 
41.8
 
 
 
 
ARF4
 
 
hsa-miR-7-5p
 
 
-0.407
 
 
138.3
 
 
 
 
ARF4
 
 
hsa-miR-221-3p
 
 
-0.553
 
 
138.3
 
 
 
 
ARHGAP1
 
 
hsa-miR-17-5p
 
 
-0.325
 
 
34.7
 
 
 
 
ARHGAP1
 
 
hsa-miR-93-5p
 
 
-0.325
 
 
34.7
 
 
 
 
ARHGAP1
 
 
hsa-miR-20a-5p
 
 
-0.339
 
 
34.7
 
 
 
 
ARHGAP5
 
 
hsa-miR-486-5p
 
 
-0.3
 
 
28.2
 
 
 
 
ARHGEF28
 
 
hsa-miR-93-5p
 
 
-0.33
 
 
19.9
 
 
 
 
ARHGEF28
 
 
hsa-miR-17-5p
 
 
-0.363
 
 
19.9
 
 
 
 
ARHGEF28
 
 
hsa-miR-20a-5p
 
 
-0.366
 
 
19.9
 
 
 
 
ARID4B
 
 
hsa-miR-20a-5p
 
 
-0.29
 
 
10.7
 
 
 
 
ARID4B
 
 
hsa-miR-486-5p
 
 
-0.367
 
 
10.7
 
 
 
 
ARIH2
 
 
hsa-miR-103a-3p
 
 
-0.307
 
 
23.5
 
 
 
 
ARL1
 
 
hsa-miR-20a-5p
 
 
-0.403
 
 
41.8
 
 
 
 
ARL1
 
 
hsa-miR-93-5p
 
 
-0.424
 
 
41.8
 
 
 
 
ARL1
 
 
hsa-miR-17-5p
 
 
-0.432
 
 
41.8
 
 
 
 
ARL6IP1
 
 
hsa-miR-7-5p
 
 
-0.374
 
 
77.1
 
 
 
 
ARL6IP1
 
 
hsa-miR-328-3p
 
 
-0.494
 
 
77.1
 
 
 
 
ARL8A
 
 
hsa-miR-103a-3p
 
 
-0.38
 
 
26.7
 
 
 
 
ARMC1
 
 
hsa-miR-92a-3p
 
 
-0.322
 
 
14.1
 
 
 
 
ARMC1
 
 
hsa-miR-103a-3p
 
 
-0.525
 
 
14.1
 
 
 
 
ARMC8
 
 
hsa-miR-486-5p
 
 
-0.321
 
 
13.4
 
 
 
 
ARMC8
 
 
hsa-miR-20a-5p
 
 
-0.357
 
 
13.4
 
 
 
 
ARMC8
 
 
hsa-miR-93-5p
 
 
-0.362
 
 
13.4
 
 
 
 
ARMC8
 
 
hsa-miR-17-5p
 
 
-0.378
 
 
13.4
 
 
 
 
ARMC8
 
 
hsa-miR-103a-3p
 
 
-0.412
 
 
13.4
 
 
 
 
ARNT
 
 
hsa-miR-103a-3p
 
 
-0.422
 
 
27.0
 
 
 
 
ARNT
 
 
hsa-miR-221-3p
 
 
-0.465
 
 
27.0
 
 
 
 
ARPP19
 
 
hsa-miR-328-3p
 
 
-0.404
 
 
43.6
 
 
 
 
ARPP19
 
 
hsa-let-7c-5p
 
 
-0.492
 
 
43.6
 
 
 
 
ARRDC3
 
 
hsa-miR-92a-3p
 
 
-0.458
 
 
19.3
 
 
 
 
ARRDC4
 
 
hsa-let-7c-5p
 
 
-0.329
 
 
32.8
 
 
 
 
ASB6
 
 
hsa-miR-874-3p
 
 
-0.368
 
 
13.0
 
 
 
 
ASPH
 
 
hsa-miR-221-3p
 
 
-0.287
 
 
42.1
 
 
 
 
ASPH
 
 
hsa-miR-92a-3p
 
 
-0.416
 
 
42.1
 
 
 
 
ASPN
 
 
hsa-miR-92a-3p
 
 
-0.422
 
 
12.6
 
 
 
 
ATF3
 
 
hsa-miR-874-3p
 
 
-0.288
 
 
63.3
 
 
 
 
ATG4A
 
 
hsa-miR-7-5p
 
 
-0.448
 
 
16.3
 
 
 
 
ATL2
 
 
hsa-miR-103a-3p
 
 
-0.489
 
 
14.3
 
 
 
 
ATOSA
 
 
hsa-miR-221-3p
 
 
-0.495
 
 
19.2
 
 
 
 
ATP2A2
 
 
hsa-let-7c-5p
 
 
-0.311
 
 
36.0
 
 
 
 
ATP6V1F
 
 
hsa-miR-185-5p
 
 
-0.765
 
 
100.6
 
 
 
 
ATPAF1
 
 
hsa-let-7c-5p
 
 
-0.498
 
 
54.8
 
 
 
 
ATXN1L
 
 
hsa-miR-155-5p
 
 
-0.315
 
 
11.8
 
 
 
 
B3GAT3
 
 
hsa-let-7c-5p
 
 
-0.324
 
 
33.7
 
 
 
 
B4GALT5
 
 
hsa-miR-210-3p
 
 
-0.511
 
 
11.8
 
 
 
 
B4GAT1
 
 
hsa-let-7c-5p
 
 
-0.47
 
 
24.7
 
 
 
 
BACE1
 
 
hsa-miR-339-5p
 
 
-0.371
 
 
243.6
 
 
 
 
BACH1
 
 
hsa-let-7c-5p
 
 
-0.306
 
 
16.2
 
 
 
 
BACH1
 
 
hsa-let-7c-5p
 
 
-0.306
 
 
16.2
 
 
 
 
BACH1
 
 
hsa-let-7c-5p
 
 
-0.435
 
 
16.2
 
 
 
 
BACH1
 
 
hsa-let-7c-5p
 
 
-0.435
 
 
16.2
 
 
 
 
BAK1
 
 
hsa-miR-103a-3p
 
 
-0.364
 
 
12.1
 
 
 
 
BCAT1
 
 
hsa-let-7c-5p
 
 
-0.394
 
 
54.8
 
 
 
 
BCAT2
 
 
hsa-miR-103a-3p
 
 
-0.364
 
 
73.6
 
 
 
 
BCAT2
 
 
hsa-miR-92a-3p
 
 
-0.609
 
 
73.6
 
 
 
 
BCL2L11
 
 
hsa-miR-339-5p
 
 
-0.306
 
 
12.8
 
 
 
 
BCL2L11
 
 
hsa-miR-92a-3p
 
 
-0.394
 
 
12.8
 
 
 
 
BCL2L11
 
 
hsa-miR-221-3p
 
 
-0.423
 
 
12.8
 
 
 
 
BCL2L13
 
 
hsa-miR-103a-3p
 
 
-0.302
 
 
10.9
 
 
 
 
BEGAIN
 
 
hsa-let-7c-5p
 
 
-0.466
 
 
22.6
 
 
 
 
BIN1
 
 
hsa-miR-744-5p
 
 
-0.654
 
 
20.4
 
 
 
 
BIN3
 
 
hsa-let-7c-5p
 
 
-0.427
 
 
11.6
 
 
 
 
BLOC1S6
 
 
hsa-let-7c-5p
 
 
-0.321
 
 
19.3
 
 
 
 
BLTP1
 
 
hsa-miR-92a-3p
 
 
-0.462
 
 
13.7
 
 
 
 
BNIP3L
 
 
hsa-miR-20a-5p
 
 
-0.287
 
 
19.0
 
 
 
 
 
 
 parathyroid gland 
 
 
 
 
target_symbol
 
 
mature_mirna_id
 
 
score
 
 
nTPM
 
 
 
 
 
 
AARS1
 
 
hsa-miR-92a-3p
 
 
-0.458
 
 
70.5
 
 
 
 
ABHD5
 
 
hsa-miR-20a-5p
 
 
-0.397
 
 
11.5
 
 
 
 
ABHD5
 
 
hsa-miR-93-5p
 
 
-0.402
 
 
11.5
 
 
 
 
ABHD5
 
 
hsa-miR-17-5p
 
 
-0.45
 
 
11.5
 
 
 
 
ACOX1
 
 
hsa-miR-92a-3p
 
 
-0.321
 
 
11.6
 
 
 
 
ACSL6
 
 
hsa-let-7c-5p
 
 
-0.336
 
 
10.6
 
 
 
 
ADAM9
 
 
hsa-miR-20a-5p
 
 
-0.299
 
 
49.4
 
 
 
 
ADAM9
 
 
hsa-miR-17-5p
 
 
-0.32
 
 
49.4
 
 
 
 
ADAM9
 
 
hsa-miR-93-5p
 
 
-0.32
 
 
49.4
 
 
 
 
ADCY3
 
 
hsa-miR-92a-3p
 
 
-0.381
 
 
16.0
 
 
 
 
ADNP
 
 
hsa-miR-328-3p
 
 
-0.371
 
 
34.0
 
 
 
 
AGFG1
 
 
hsa-miR-103a-3p
 
 
-0.389
 
 
39.0
 
 
 
 
AHCTF1
 
 
hsa-let-7c-5p
 
 
-0.374
 
 
12.3
 
 
 
 
AHNAK
 
 
hsa-miR-20a-5p
 
 
-0.316
 
 
24.7
 
 
 
 
AHNAK
 
 
hsa-miR-17-5p
 
 
-0.337
 
 
24.7
 
 
 
 
AHNAK
 
 
hsa-miR-93-5p
 
 
-0.337
 
 
24.7
 
 
 
 
AIFM1
 
 
hsa-let-7c-5p
 
 
-0.314
 
 
27.9
 
 
 
 
AKAP10
 
 
hsa-miR-339-5p
 
 
-0.302
 
 
14.2
 
 
 
 
AKIRIN2
 
 
hsa-miR-103a-3p
 
 
-0.299
 
 
16.7
 
 
 
 
ALAD
 
 
hsa-miR-103a-3p
 
 
-0.385
 
 
68.8
 
 
 
 
ALG3
 
 
hsa-miR-20a-5p
 
 
-0.559
 
 
21.7
 
 
 
 
ALG3
 
 
hsa-miR-93-5p
 
 
-0.564
 
 
21.7
 
 
 
 
ALG3
 
 
hsa-miR-17-5p
 
 
-0.572
 
 
21.7
 
 
 
 
ALG9
 
 
hsa-miR-7-5p
 
 
-0.302
 
 
12.0
 
 
 
 
AMT
 
 
hsa-let-7c-5p
 
 
-0.463
 
 
13.3
 
 
 
 
ANK1
 
 
hsa-miR-103a-3p
 
 
-0.321
 
 
46.9
 
 
 
 
ANKFY1
 
 
hsa-miR-155-5p
 
 
-0.315
 
 
17.9
 
 
 
 
ANKRD29
 
 
hsa-miR-20a-5p
 
 
-0.359
 
 
23.3
 
 
 
 
ANKRD29
 
 
hsa-miR-17-5p
 
 
-0.372
 
 
23.3
 
 
 
 
ANKRD29
 
 
hsa-miR-93-5p
 
 
-0.372
 
 
23.3
 
 
 
 
ANKRD46
 
 
hsa-let-7c-5p
 
 
-0.389
 
 
15.1
 
 
 
 
ANP32E
 
 
hsa-miR-92a-3p
 
 
-0.329
 
 
31.1
 
 
 
 
AP1AR
 
 
hsa-miR-92a-3p
 
 
-0.325
 
 
38.5
 
 
 
 
AP1S1
 
 
hsa-let-7c-5p
 
 
-0.374
 
 
21.4
 
 
 
 
APPL1
 
 
hsa-miR-92a-3p
 
 
-0.375
 
 
23.6
 
 
 
 
ARF4
 
 
hsa-miR-7-5p
 
 
-0.407
 
 
95.4
 
 
 
 
ARF4
 
 
hsa-miR-221-3p
 
 
-0.553
 
 
95.4
 
 
 
 
ARG2
 
 
hsa-let-7c-5p
 
 
-0.468
 
 
206.5
 
 
 
 
ARHGAP1
 
 
hsa-miR-17-5p
 
 
-0.325
 
 
23.3
 
 
 
 
ARHGAP1
 
 
hsa-miR-93-5p
 
 
-0.325
 
 
23.3
 
 
 
 
ARHGAP1
 
 
hsa-miR-20a-5p
 
 
-0.339
 
 
23.3
 
 
 
 
ARHGAP5
 
 
hsa-miR-486-5p
 
 
-0.3
 
 
45.3
 
 
 
 
ARID2
 
 
hsa-miR-155-5p
 
 
-0.384
 
 
11.0
 
 
 
 
ARID2
 
 
hsa-miR-103a-3p
 
 
-0.415
 
 
11.0
 
 
 
 
ARID4B
 
 
hsa-miR-20a-5p
 
 
-0.29
 
 
12.8
 
 
 
 
ARID4B
 
 
hsa-miR-486-5p
 
 
-0.367
 
 
12.8
 
 
 
 
ARIH2
 
 
hsa-miR-103a-3p
 
 
-0.307
 
 
23.0
 
 
 
 
ARL1
 
 
hsa-miR-20a-5p
 
 
-0.403
 
 
86.8
 
 
 
 
ARL1
 
 
hsa-miR-93-5p
 
 
-0.424
 
 
86.8
 
 
 
 
ARL1
 
 
hsa-miR-17-5p
 
 
-0.432
 
 
86.8
 
 
 
 
ARL6IP1
 
 
hsa-miR-7-5p
 
 
-0.374
 
 
169.8
 
 
 
 
ARL6IP1
 
 
hsa-miR-328-3p
 
 
-0.494
 
 
169.8
 
 
 
 
ARL8A
 
 
hsa-miR-103a-3p
 
 
-0.38
 
 
24.7
 
 
 
 
ARMC1
 
 
hsa-miR-92a-3p
 
 
-0.322
 
 
19.7
 
 
 
 
ARMC1
 
 
hsa-miR-103a-3p
 
 
-0.525
 
 
19.7
 
 
 
 
ARMC8
 
 
hsa-miR-486-5p
 
 
-0.321
 
 
20.0
 
 
 
 
ARMC8
 
 
hsa-miR-20a-5p
 
 
-0.357
 
 
20.0
 
 
 
 
ARMC8
 
 
hsa-miR-93-5p
 
 
-0.362
 
 
20.0
 
 
 
 
ARMC8
 
 
hsa-miR-17-5p
 
 
-0.378
 
 
20.0
 
 
 
 
ARMC8
 
 
hsa-miR-103a-3p
 
 
-0.412
 
 
20.0
 
 
 
 
ARMT1
 
 
hsa-let-7c-5p
 
 
-0.303
 
 
10.9
 
 
 
 
ARNT
 
 
hsa-miR-103a-3p
 
 
-0.422
 
 
18.4
 
 
 
 
ARNT
 
 
hsa-miR-221-3p
 
 
-0.465
 
 
18.4
 
 
 
 
ARPP19
 
 
hsa-miR-328-3p
 
 
-0.404
 
 
102.5
 
 
 
 
ARPP19
 
 
hsa-let-7c-5p
 
 
-0.492
 
 
102.5
 
 
 
 
ARRDC3
 
 
hsa-miR-92a-3p
 
 
-0.458
 
 
51.0
 
 
 
 
ASAP1
 
 
hsa-let-7c-5p
 
 
-0.308
 
 
16.8
 
 
 
 
ASPH
 
 
hsa-miR-221-3p
 
 
-0.287
 
 
35.7
 
 
 
 
ASPH
 
 
hsa-miR-92a-3p
 
 
-0.416
 
 
35.7
 
 
 
 
ATF3
 
 
hsa-miR-874-3p
 
 
-0.288
 
 
45.8
 
 
 
 
ATG4A
 
 
hsa-miR-7-5p
 
 
-0.448
 
 
11.7
 
 
 
 
ATL2
 
 
hsa-miR-103a-3p
 
 
-0.489
 
 
14.9
 
 
 
 
ATOSA
 
 
hsa-miR-221-3p
 
 
-0.495
 
 
38.4
 
 
 
 
ATOSB
 
 
hsa-miR-328-3p
 
 
-0.358
 
 
10.5
 
 
 
 
ATOSB
 
 
hsa-let-7c-5p
 
 
-0.429
 
 
10.5
 
 
 
 
ATP2A2
 
 
hsa-let-7c-5p
 
 
-0.311
 
 
52.7
 
 
 
 
ATP6V1F
 
 
hsa-miR-185-5p
 
 
-0.765
 
 
153.3
 
 
 
 
ATPAF1
 
 
hsa-let-7c-5p
 
 
-0.498
 
 
129.7
 
 
 
 
ATXN1L
 
 
hsa-miR-155-5p
 
 
-0.315
 
 
15.5
 
 
 
 
B3GAT3
 
 
hsa-let-7c-5p
 
 
-0.324
 
 
18.1
 
 
 
 
B4GALT5
 
 
hsa-miR-210-3p
 
 
-0.511
 
 
10.6
 
 
 
 
B4GAT1
 
 
hsa-let-7c-5p
 
 
-0.47
 
 
21.3
 
 
 
 
BACE1
 
 
hsa-miR-339-5p
 
 
-0.371
 
 
17.1
 
 
 
 
BAK1
 
 
hsa-miR-103a-3p
 
 
-0.364
 
 
12.6
 
 
 
 
BCAT2
 
 
hsa-miR-103a-3p
 
 
-0.364
 
 
18.9
 
 
 
 
BCAT2
 
 
hsa-miR-92a-3p
 
 
-0.609
 
 
18.9
 
 
 
 
BCL2L11
 
 
hsa-miR-339-5p
 
 
-0.306
 
 
17.6
 
 
 
 
BCL2L11
 
 
hsa-miR-92a-3p
 
 
-0.394
 
 
17.6
 
 
 
 
BCL2L11
 
 
hsa-miR-221-3p
 
 
-0.423
 
 
17.6
 
 
 
 
BCL2L13
 
 
hsa-miR-103a-3p
 
 
-0.302
 
 
13.9
 
 
 
 
BDH1
 
 
hsa-miR-874-3p
 
 
-0.644
 
 
14.6
 
 
 
 
BIN1
 
 
hsa-miR-744-5p
 
 
-0.654
 
 
18.4
 
 
 
 
BIN3
 
 
hsa-let-7c-5p
 
 
-0.427
 
 
11.8
 
 
 
 
BLOC1S6
 
 
hsa-let-7c-5p
 
 
-0.321
 
 
15.8
 
 
 
 
BLTP1
 
 
hsa-miR-92a-3p
 
 
-0.462
 
 
39.7
 
 
 
 
BNIP3L
 
 
hsa-miR-20a-5p
 
 
-0.287
 
 
51.8
 
 
 
 
BOLA3
 
 
hsa-miR-103a-3p
 
 
-0.362
 
 
52.6
 
 
 
 
BSDC1
 
 
hsa-miR-92a-3p
 
 
-0.362
 
 
39.8
 
 
 
 
BTAF1
 
 
hsa-miR-486-5p
 
 
-0.288
 
 
11.8
 
 
 
 
BTBD10
 
 
hsa-miR-17-5p
 
 
-0.409
 
 
12.4
 
 
 
 
 
 
 pituitary gland 
 
 
 
 
target_symbol
 
 
mature_mirna_id
 
 
score
 
 
nTPM
 
 
 
 
 
 
AARS1
 
 
hsa-miR-92a-3p
 
 
-0.458
 
 
34.8
 
 
 
 
ABCB9
 
 
hsa-let-7c-5p
 
 
-0.422
 
 
11.0
 
 
 
 
ACOX1
 
 
hsa-miR-92a-3p
 
 
-0.321
 
 
12.6
 
 
 
 
ADCY3
 
 
hsa-miR-92a-3p
 
 
-0.381
 
 
41.4
 
 
 
 
ADNP
 
 
hsa-miR-328-3p
 
 
-0.371
 
 
26.6
 
 
 
 
AGFG1
 
 
hsa-miR-103a-3p
 
 
-0.389
 
 
16.7
 
 
 
 
AGFG2
 
 
hsa-miR-20a-5p
 
 
-0.365
 
 
16.7
 
 
 
 
AGFG2
 
 
hsa-miR-93-5p
 
 
-0.378
 
 
16.7
 
 
 
 
AGFG2
 
 
hsa-miR-17-5p
 
 
-0.402
 
 
16.7
 
 
 
 
AHNAK
 
 
hsa-miR-20a-5p
 
 
-0.316
 
 
16.0
 
 
 
 
AHNAK
 
 
hsa-miR-17-5p
 
 
-0.337
 
 
16.0
 
 
 
 
AHNAK
 
 
hsa-miR-93-5p
 
 
-0.337
 
 
16.0
 
 
 
 
AIFM1
 
 
hsa-let-7c-5p
 
 
-0.314
 
 
11.0
 
 
 
 
AKIRIN2
 
 
hsa-miR-103a-3p
 
 
-0.299
 
 
21.2
 
 
 
 
ALAD
 
 
hsa-miR-103a-3p
 
 
-0.385
 
 
16.3
 
 
 
 
ALG3
 
 
hsa-miR-20a-5p
 
 
-0.559
 
 
14.3
 
 
 
 
ALG3
 
 
hsa-miR-93-5p
 
 
-0.564
 
 
14.3
 
 
 
 
ALG3
 
 
hsa-miR-17-5p
 
 
-0.572
 
 
14.3
 
 
 
 
AMT
 
 
hsa-let-7c-5p
 
 
-0.463
 
 
16.0
 
 
 
 
ANKFY1
 
 
hsa-miR-155-5p
 
 
-0.315
 
 
11.8
 
 
 
 
ANP32E
 
 
hsa-miR-92a-3p
 
 
-0.329
 
 
15.4
 
 
 
 
AP1S1
 
 
hsa-let-7c-5p
 
 
-0.374
 
 
31.4
 
 
 
 
AP4B1
 
 
hsa-miR-378a-3p
 
 
-0.62
 
 
13.4
 
 
 
 
APBB3
 
 
hsa-let-7c-5p
 
 
-0.587
 
 
10.1
 
 
 
 
ARF4
 
 
hsa-miR-7-5p
 
 
-0.407
 
 
93.4
 
 
 
 
ARF4
 
 
hsa-miR-221-3p
 
 
-0.553
 
 
93.4
 
 
 
 
ARG2
 
 
hsa-let-7c-5p
 
 
-0.468
 
 
47.4
 
 
 
 
ARHGAP1
 
 
hsa-miR-17-5p
 
 
-0.325
 
 
35.2
 
 
 
 
ARHGAP1
 
 
hsa-miR-93-5p
 
 
-0.325
 
 
35.2
 
 
 
 
ARHGAP1
 
 
hsa-miR-20a-5p
 
 
-0.339
 
 
35.2
 
 
 
 
ARID4B
 
 
hsa-miR-20a-5p
 
 
-0.29
 
 
10.3
 
 
 
 
ARID4B
 
 
hsa-miR-486-5p
 
 
-0.367
 
 
10.3
 
 
 
 
ARIH2
 
 
hsa-miR-103a-3p
 
 
-0.307
 
 
25.4
 
 
 
 
ARL1
 
 
hsa-miR-20a-5p
 
 
-0.403
 
 
52.7
 
 
 
 
ARL1
 
 
hsa-miR-93-5p
 
 
-0.424
 
 
52.7
 
 
 
 
ARL1
 
 
hsa-miR-17-5p
 
 
-0.432
 
 
52.7
 
 
 
 
ARL4D
 
 
hsa-let-7c-5p
 
 
-0.417
 
 
20.8
 
 
 
 
ARL6IP1
 
 
hsa-miR-7-5p
 
 
-0.374
 
 
39.7
 
 
 
 
ARL6IP1
 
 
hsa-miR-328-3p
 
 
-0.494
 
 
39.7
 
 
 
 
ARL8A
 
 
hsa-miR-103a-3p
 
 
-0.38
 
 
30.4
 
 
 
 
ARMC8
 
 
hsa-miR-486-5p
 
 
-0.321
 
 
11.3
 
 
 
 
ARMC8
 
 
hsa-miR-20a-5p
 
 
-0.357
 
 
11.3
 
 
 
 
ARMC8
 
 
hsa-miR-93-5p
 
 
-0.362
 
 
11.3
 
 
 
 
ARMC8
 
 
hsa-miR-17-5p
 
 
-0.378
 
 
11.3
 
 
 
 
ARMC8
 
 
hsa-miR-103a-3p
 
 
-0.412
 
 
11.3
 
 
 
 
ARNT
 
 
hsa-miR-103a-3p
 
 
-0.422
 
 
12.2
 
 
 
 
ARNT
 
 
hsa-miR-221-3p
 
 
-0.465
 
 
12.2
 
 
 
 
ARPP19
 
 
hsa-miR-328-3p
 
 
-0.404
 
 
33.0
 
 
 
 
ARPP19
 
 
hsa-let-7c-5p
 
 
-0.492
 
 
33.0
 
 
 
 
ARRDC3
 
 
hsa-miR-92a-3p
 
 
-0.458
 
 
20.2
 
 
 
 
ASB6
 
 
hsa-miR-874-3p
 
 
-0.368
 
 
12.1
 
 
 
 
ASPH
 
 
hsa-miR-221-3p
 
 
-0.287
 
 
33.8
 
 
 
 
ASPH
 
 
hsa-miR-92a-3p
 
 
-0.416
 
 
33.8
 
 
 
 
ATF3
 
 
hsa-miR-874-3p
 
 
-0.288
 
 
43.7
 
 
 
 
ATL2
 
 
hsa-miR-103a-3p
 
 
-0.489
 
 
12.2
 
 
 
 
ATOSB
 
 
hsa-miR-328-3p
 
 
-0.358
 
 
15.4
 
 
 
 
ATOSB
 
 
hsa-let-7c-5p
 
 
-0.429
 
 
15.4
 
 
 
 
ATP1B2
 
 
hsa-miR-103a-3p
 
 
-0.6
 
 
40.7
 
 
 
 
ATP2A2
 
 
hsa-let-7c-5p
 
 
-0.311
 
 
13.7
 
 
 
 
ATP6V1F
 
 
hsa-miR-185-5p
 
 
-0.765
 
 
141.5
 
 
 
 
ATPAF1
 
 
hsa-let-7c-5p
 
 
-0.498
 
 
31.1
 
 
 
 
AVPI1
 
 
hsa-miR-7-5p
 
 
-0.367
 
 
20.2
 
 
 
 
B3GAT3
 
 
hsa-let-7c-5p
 
 
-0.324
 
 
61.5
 
 
 
 
B4GALT5
 
 
hsa-miR-210-3p
 
 
-0.511
 
 
11.9
 
 
 
 
B4GAT1
 
 
hsa-let-7c-5p
 
 
-0.47
 
 
60.5
 
 
 
 
BAK1
 
 
hsa-miR-103a-3p
 
 
-0.364
 
 
11.6
 
 
 
 
BCAT2
 
 
hsa-miR-103a-3p
 
 
-0.364
 
 
29.9
 
 
 
 
BCAT2
 
 
hsa-miR-92a-3p
 
 
-0.609
 
 
29.9
 
 
 
 
BDH1
 
 
hsa-miR-874-3p
 
 
-0.644
 
 
11.4
 
 
 
 
BEGAIN
 
 
hsa-let-7c-5p
 
 
-0.466
 
 
18.8
 
 
 
 
BICDL1
 
 
hsa-miR-221-3p
 
 
-0.447
 
 
63.8
 
 
 
 
BIN1
 
 
hsa-miR-744-5p
 
 
-0.654
 
 
39.7
 
 
 
 
BIN3
 
 
hsa-let-7c-5p
 
 
-0.427
 
 
13.2
 
 
 
 
BLOC1S6
 
 
hsa-let-7c-5p
 
 
-0.321
 
 
19.0
 
 
 
 
BLTP1
 
 
hsa-miR-92a-3p
 
 
-0.462
 
 
25.8
 
 
 
 
BNIP3L
 
 
hsa-miR-20a-5p
 
 
-0.287
 
 
23.7
 
 
 
 
BOLA3
 
 
hsa-miR-103a-3p
 
 
-0.362
 
 
31.4
 
 
 
 
BSDC1
 
 
hsa-miR-92a-3p
 
 
-0.362
 
 
27.0
 
 
 
 
BTAF1
 
 
hsa-miR-486-5p
 
 
-0.288
 
 
11.4
 
 
 
 
BTBD10
 
 
hsa-miR-17-5p
 
 
-0.409
 
 
13.7
 
 
 
 
BTBD10
 
 
hsa-miR-93-5p
 
 
-0.409
 
 
13.7
 
 
 
 
BTBD10
 
 
hsa-miR-20a-5p
 
 
-0.412
 
 
13.7
 
 
 
 
BTF3L4
 
 
hsa-let-7c-5p
 
 
-0.355
 
 
28.3
 
 
 
 
BTG2
 
 
hsa-miR-92a-3p
 
 
-0.314
 
 
204.0
 
 
 
 
BTG2
 
 
hsa-miR-92a-3p
 
 
-0.402
 
 
204.0
 
 
 
 
BTG3
 
 
hsa-miR-17-5p
 
 
-0.668
 
 
43.7
 
 
 
 
BTG3
 
 
hsa-miR-93-5p
 
 
-0.668
 
 
43.7
 
 
 
 
BTG3
 
 
hsa-miR-20a-5p
 
 
-0.671
 
 
43.7
 
 
 
 
BZW1
 
 
hsa-let-7c-5p
 
 
-0.354
 
 
28.1
 
 
 
 
BZW1
 
 
hsa-let-7c-5p
 
 
-0.375
 
 
28.1
 
 
 
 
BZW2
 
 
hsa-let-7c-5p
 
 
-0.397
 
 
10.9
 
 
 
 
C11orf24
 
 
hsa-miR-92a-3p
 
 
-0.35
 
 
13.8
 
 
 
 
C1QBP
 
 
hsa-miR-330-3p
 
 
-0.412
 
 
78.4
 
 
 
 
C2CD5
 
 
hsa-miR-103a-3p
 
 
-0.475
 
 
19.3
 
 
 
 
C3orf18
 
 
hsa-miR-155-5p
 
 
-0.431
 
 
12.0
 
 
 
 
C6orf62
 
 
hsa-miR-92a-3p
 
 
-0.326
 
 
60.8
 
 
 
 
CAB39
 
 
hsa-miR-155-5p
 
 
-0.312
 
 
16.0
 
 
 
 
CAB39
 
 
hsa-miR-103a-3p
 
 
-0.532
 
 
16.0
 
 
 
 
CACFD1
 
 
hsa-let-7c-5p
 
 
-0.296
 
 
18.3
 
 
 
 
CALD1
 
 
hsa-miR-20a-5p
 
 
-0.352
 
 
49.0
 
 
 
 
 
 
 placenta 
 
 
 
 
target_symbol
 
 
mature_mirna_id
 
 
score
 
 
nTPM
 
 
 
 
 
 
AARS1
 
 
hsa-miR-92a-3p
 
 
-0.458
 
 
33.5
 
 
 
 
ACP3
 
 
hsa-let-7c-5p
 
 
-0.298
 
 
10.1
 
 
 
 
ADAM19
 
 
hsa-miR-874-3p
 
 
-0.374
 
 
12.3
 
 
 
 
ADAM9
 
 
hsa-miR-20a-5p
 
 
-0.299
 
 
44.6
 
 
 
 
ADAM9
 
 
hsa-miR-17-5p
 
 
-0.32
 
 
44.6
 
 
 
 
ADAM9
 
 
hsa-miR-93-5p
 
 
-0.32
 
 
44.6
 
 
 
 
ADCY3
 
 
hsa-miR-92a-3p
 
 
-0.381
 
 
78.3
 
 
 
 
ADNP
 
 
hsa-miR-328-3p
 
 
-0.371
 
 
23.8
 
 
 
 
ADRB1
 
 
hsa-let-7c-5p
 
 
-0.351
 
 
27.4
 
 
 
 
ADRB1
 
 
hsa-miR-92a-3p
 
 
-0.478
 
 
27.4
 
 
 
 
AGFG1
 
 
hsa-miR-103a-3p
 
 
-0.389
 
 
71.2
 
 
 
 
AHNAK
 
 
hsa-miR-20a-5p
 
 
-0.316
 
 
91.4
 
 
 
 
AHNAK
 
 
hsa-miR-17-5p
 
 
-0.337
 
 
91.4
 
 
 
 
AHNAK
 
 
hsa-miR-93-5p
 
 
-0.337
 
 
91.4
 
 
 
 
AJUBA
 
 
hsa-miR-103a-3p
 
 
-0.448
 
 
18.1
 
 
 
 
AKAP10
 
 
hsa-miR-339-5p
 
 
-0.302
 
 
11.4
 
 
 
 
AKIRIN2
 
 
hsa-miR-103a-3p
 
 
-0.299
 
 
17.4
 
 
 
 
ALAD
 
 
hsa-miR-103a-3p
 
 
-0.385
 
 
23.4
 
 
 
 
ALG3
 
 
hsa-miR-20a-5p
 
 
-0.559
 
 
18.4
 
 
 
 
ALG3
 
 
hsa-miR-93-5p
 
 
-0.564
 
 
18.4
 
 
 
 
ALG3
 
 
hsa-miR-17-5p
 
 
-0.572
 
 
18.4
 
 
 
 
ANGPTL2
 
 
hsa-miR-221-3p
 
 
-0.369
 
 
20.1
 
 
 
 
ANKFY1
 
 
hsa-miR-155-5p
 
 
-0.315
 
 
13.3
 
 
 
 
ANKRD12
 
 
hsa-miR-7-5p
 
 
-0.37
 
 
13.3
 
 
 
 
ANKRD46
 
 
hsa-let-7c-5p
 
 
-0.389
 
 
19.5
 
 
 
 
ANKRD50
 
 
hsa-miR-20a-5p
 
 
-0.3
 
 
13.9
 
 
 
 
ANP32E
 
 
hsa-miR-92a-3p
 
 
-0.329
 
 
20.3
 
 
 
 
AP1AR
 
 
hsa-miR-92a-3p
 
 
-0.325
 
 
11.2
 
 
 
 
AP1S1
 
 
hsa-let-7c-5p
 
 
-0.374
 
 
14.9
 
 
 
 
APCDD1
 
 
hsa-miR-20a-5p
 
 
-0.404
 
 
25.2
 
 
 
 
APCDD1
 
 
hsa-miR-17-5p
 
 
-0.425
 
 
25.2
 
 
 
 
APCDD1
 
 
hsa-miR-93-5p
 
 
-0.425
 
 
25.2
 
 
 
 
APPL1
 
 
hsa-miR-92a-3p
 
 
-0.375
 
 
13.5
 
 
 
 
ARF4
 
 
hsa-miR-7-5p
 
 
-0.407
 
 
170.7
 
 
 
 
ARF4
 
 
hsa-miR-221-3p
 
 
-0.553
 
 
170.7
 
 
 
 
ARHGAP1
 
 
hsa-miR-17-5p
 
 
-0.325
 
 
36.7
 
 
 
 
ARHGAP1
 
 
hsa-miR-93-5p
 
 
-0.325
 
 
36.7
 
 
 
 
ARHGAP1
 
 
hsa-miR-20a-5p
 
 
-0.339
 
 
36.7
 
 
 
 
ARHGAP28
 
 
hsa-let-7c-5p
 
 
-0.47
 
 
24.3
 
 
 
 
ARHGAP42
 
 
hsa-miR-221-3p
 
 
-0.304
 
 
14.1
 
 
 
 
ARHGAP5
 
 
hsa-miR-486-5p
 
 
-0.3
 
 
35.0
 
 
 
 
ARID3A
 
 
hsa-let-7c-5p
 
 
-0.34
 
 
22.0
 
 
 
 
ARID3A
 
 
hsa-let-7c-5p
 
 
-0.347
 
 
22.0
 
 
 
 
ARID3B
 
 
hsa-let-7c-5p
 
 
-0.31
 
 
14.7
 
 
 
 
ARID3B
 
 
hsa-let-7c-5p
 
 
-0.486
 
 
14.7
 
 
 
 
ARID4B
 
 
hsa-miR-20a-5p
 
 
-0.29
 
 
12.0
 
 
 
 
ARID4B
 
 
hsa-miR-486-5p
 
 
-0.367
 
 
12.0
 
 
 
 
ARIH2
 
 
hsa-miR-103a-3p
 
 
-0.307
 
 
17.3
 
 
 
 
ARL1
 
 
hsa-miR-20a-5p
 
 
-0.403
 
 
48.3
 
 
 
 
ARL1
 
 
hsa-miR-93-5p
 
 
-0.424
 
 
48.3
 
 
 
 
ARL1
 
 
hsa-miR-17-5p
 
 
-0.432
 
 
48.3
 
 
 
 
ARL5B
 
 
hsa-miR-92a-3p
 
 
-0.331
 
 
12.3
 
 
 
 
ARL5B
 
 
hsa-miR-155-5p
 
 
-0.355
 
 
12.3
 
 
 
 
ARL6IP1
 
 
hsa-miR-7-5p
 
 
-0.374
 
 
84.9
 
 
 
 
ARL6IP1
 
 
hsa-miR-328-3p
 
 
-0.494
 
 
84.9
 
 
 
 
ARL6IP6
 
 
hsa-let-7c-5p
 
 
-0.293
 
 
26.8
 
 
 
 
ARL8A
 
 
hsa-miR-103a-3p
 
 
-0.38
 
 
29.2
 
 
 
 
ARMC1
 
 
hsa-miR-92a-3p
 
 
-0.322
 
 
14.1
 
 
 
 
ARMC1
 
 
hsa-miR-103a-3p
 
 
-0.525
 
 
14.1
 
 
 
 
ARMC8
 
 
hsa-miR-486-5p
 
 
-0.321
 
 
18.5
 
 
 
 
ARMC8
 
 
hsa-miR-20a-5p
 
 
-0.357
 
 
18.5
 
 
 
 
ARMC8
 
 
hsa-miR-93-5p
 
 
-0.362
 
 
18.5
 
 
 
 
ARMC8
 
 
hsa-miR-17-5p
 
 
-0.378
 
 
18.5
 
 
 
 
ARMC8
 
 
hsa-miR-103a-3p
 
 
-0.412
 
 
18.5
 
 
 
 
ARMT1
 
 
hsa-let-7c-5p
 
 
-0.303
 
 
20.8
 
 
 
 
ARNT
 
 
hsa-miR-103a-3p
 
 
-0.422
 
 
29.3
 
 
 
 
ARNT
 
 
hsa-miR-221-3p
 
 
-0.465
 
 
29.3
 
 
 
 
ARPP19
 
 
hsa-miR-328-3p
 
 
-0.404
 
 
53.5
 
 
 
 
ARPP19
 
 
hsa-let-7c-5p
 
 
-0.492
 
 
53.5
 
 
 
 
ARRDC3
 
 
hsa-miR-92a-3p
 
 
-0.458
 
 
72.1
 
 
 
 
ARRDC4
 
 
hsa-let-7c-5p
 
 
-0.329
 
 
17.2
 
 
 
 
ASAP1
 
 
hsa-let-7c-5p
 
 
-0.308
 
 
25.9
 
 
 
 
ASPH
 
 
hsa-miR-221-3p
 
 
-0.287
 
 
44.6
 
 
 
 
ASPH
 
 
hsa-miR-92a-3p
 
 
-0.416
 
 
44.6
 
 
 
 
ATF3
 
 
hsa-miR-874-3p
 
 
-0.288
 
 
62.2
 
 
 
 
ATL2
 
 
hsa-miR-103a-3p
 
 
-0.489
 
 
15.8
 
 
 
 
ATP2A2
 
 
hsa-let-7c-5p
 
 
-0.311
 
 
52.0
 
 
 
 
ATP6V1F
 
 
hsa-miR-185-5p
 
 
-0.765
 
 
98.2
 
 
 
 
ATPAF1
 
 
hsa-let-7c-5p
 
 
-0.498
 
 
34.1
 
 
 
 
ATXN1L
 
 
hsa-miR-155-5p
 
 
-0.315
 
 
10.1
 
 
 
 
AVPI1
 
 
hsa-miR-7-5p
 
 
-0.367
 
 
15.9
 
 
 
 
B3GAT3
 
 
hsa-let-7c-5p
 
 
-0.324
 
 
18.0
 
 
 
 
B4GALT5
 
 
hsa-miR-210-3p
 
 
-0.511
 
 
25.6
 
 
 
 
B4GAT1
 
 
hsa-let-7c-5p
 
 
-0.47
 
 
21.1
 
 
 
 
BACE1
 
 
hsa-miR-339-5p
 
 
-0.371
 
 
12.6
 
 
 
 
BACH1
 
 
hsa-let-7c-5p
 
 
-0.306
 
 
47.5
 
 
 
 
BACH1
 
 
hsa-let-7c-5p
 
 
-0.306
 
 
47.5
 
 
 
 
BACH1
 
 
hsa-let-7c-5p
 
 
-0.435
 
 
47.5
 
 
 
 
BACH1
 
 
hsa-let-7c-5p
 
 
-0.435
 
 
47.5
 
 
 
 
BAK1
 
 
hsa-miR-103a-3p
 
 
-0.364
 
 
10.4
 
 
 
 
BAMBI
 
 
hsa-miR-20a-5p
 
 
-0.485
 
 
36.9
 
 
 
 
BAMBI
 
 
hsa-miR-17-5p
 
 
-0.506
 
 
36.9
 
 
 
 
BAMBI
 
 
hsa-miR-93-5p
 
 
-0.506
 
 
36.9
 
 
 
 
BCAT1
 
 
hsa-let-7c-5p
 
 
-0.394
 
 
14.3
 
 
 
 
BCAT2
 
 
hsa-miR-103a-3p
 
 
-0.364
 
 
11.6
 
 
 
 
BCAT2
 
 
hsa-miR-92a-3p
 
 
-0.609
 
 
11.6
 
 
 
 
BCORL1
 
 
hsa-miR-423-3p
 
 
-0.606
 
 
10.6
 
 
 
 
BIN1
 
 
hsa-miR-744-5p
 
 
-0.654
 
 
22.1
 
 
 
 
BLOC1S6
 
 
hsa-let-7c-5p
 
 
-0.321
 
 
24.7
 
 
 
 
BMP2
 
 
hsa-miR-378a-3p
 
 
-0.481
 
 
10.8
 
 
 
 
 
 
 prostate 
 
 
 
 
target_symbol
 
 
mature_mirna_id
 
 
score
 
 
nTPM
 
 
 
 
 
 
AARS1
 
 
hsa-miR-92a-3p
 
 
-0.458
 
 
34.0
 
 
 
 
ABHD17C
 
 
hsa-let-7c-5p
 
 
-0.359
 
 
17.0
 
 
 
 
ABHD17C
 
 
hsa-miR-330-3p
 
 
-0.399
 
 
17.0
 
 
 
 
ACOX1
 
 
hsa-miR-92a-3p
 
 
-0.321
 
 
13.0
 
 
 
 
ACP3
 
 
hsa-let-7c-5p
 
 
-0.298
 
 
3409.5
 
 
 
 
ACTA1
 
 
hsa-miR-155-5p
 
 
-0.595
 
 
152.5
 
 
 
 
ACTC1
 
 
hsa-miR-92a-3p
 
 
-0.361
 
 
68.4
 
 
 
 
ACTC1
 
 
hsa-miR-7-5p
 
 
-0.471
 
 
68.4
 
 
 
 
ADAM9
 
 
hsa-miR-20a-5p
 
 
-0.299
 
 
32.8
 
 
 
 
ADAM9
 
 
hsa-miR-17-5p
 
 
-0.32
 
 
32.8
 
 
 
 
ADAM9
 
 
hsa-miR-93-5p
 
 
-0.32
 
 
32.8
 
 
 
 
ADCY3
 
 
hsa-miR-92a-3p
 
 
-0.381
 
 
38.0
 
 
 
 
ADNP
 
 
hsa-miR-328-3p
 
 
-0.371
 
 
33.0
 
 
 
 
AGFG1
 
 
hsa-miR-103a-3p
 
 
-0.389
 
 
19.4
 
 
 
 
AGFG2
 
 
hsa-miR-20a-5p
 
 
-0.365
 
 
12.4
 
 
 
 
AGFG2
 
 
hsa-miR-93-5p
 
 
-0.378
 
 
12.4
 
 
 
 
AGFG2
 
 
hsa-miR-17-5p
 
 
-0.402
 
 
12.4
 
 
 
 
AHNAK
 
 
hsa-miR-20a-5p
 
 
-0.316
 
 
83.5
 
 
 
 
AHNAK
 
 
hsa-miR-17-5p
 
 
-0.337
 
 
83.5
 
 
 
 
AHNAK
 
 
hsa-miR-93-5p
 
 
-0.337
 
 
83.5
 
 
 
 
AIFM1
 
 
hsa-let-7c-5p
 
 
-0.314
 
 
10.1
 
 
 
 
AKAP10
 
 
hsa-miR-339-5p
 
 
-0.302
 
 
10.2
 
 
 
 
AKIRIN2
 
 
hsa-miR-103a-3p
 
 
-0.299
 
 
15.2
 
 
 
 
ALAD
 
 
hsa-miR-103a-3p
 
 
-0.385
 
 
32.0
 
 
 
 
ALG3
 
 
hsa-miR-20a-5p
 
 
-0.559
 
 
19.0
 
 
 
 
ALG3
 
 
hsa-miR-93-5p
 
 
-0.564
 
 
19.0
 
 
 
 
ALG3
 
 
hsa-miR-17-5p
 
 
-0.572
 
 
19.0
 
 
 
 
AMT
 
 
hsa-let-7c-5p
 
 
-0.463
 
 
15.5
 
 
 
 
ANGPTL2
 
 
hsa-miR-221-3p
 
 
-0.369
 
 
23.5
 
 
 
 
ANKFY1
 
 
hsa-miR-155-5p
 
 
-0.315
 
 
11.5
 
 
 
 
ANKRD12
 
 
hsa-miR-7-5p
 
 
-0.37
 
 
11.4
 
 
 
 
ANKRD46
 
 
hsa-let-7c-5p
 
 
-0.389
 
 
12.6
 
 
 
 
ANKRD9
 
 
hsa-miR-20a-5p
 
 
-0.31
 
 
18.8
 
 
 
 
ANKRD9
 
 
hsa-miR-93-5p
 
 
-0.331
 
 
18.8
 
 
 
 
ANKRD9
 
 
hsa-miR-17-5p
 
 
-0.347
 
 
18.8
 
 
 
 
ANP32E
 
 
hsa-miR-92a-3p
 
 
-0.329
 
 
15.2
 
 
 
 
AP1S1
 
 
hsa-let-7c-5p
 
 
-0.374
 
 
23.9
 
 
 
 
APCDD1
 
 
hsa-miR-20a-5p
 
 
-0.404
 
 
31.5
 
 
 
 
APCDD1
 
 
hsa-miR-17-5p
 
 
-0.425
 
 
31.5
 
 
 
 
APCDD1
 
 
hsa-miR-93-5p
 
 
-0.425
 
 
31.5
 
 
 
 
APPL1
 
 
hsa-miR-92a-3p
 
 
-0.375
 
 
14.1
 
 
 
 
AQP3
 
 
hsa-miR-874-3p
 
 
-0.48
 
 
81.8
 
 
 
 
ARF4
 
 
hsa-miR-7-5p
 
 
-0.407
 
 
129.9
 
 
 
 
ARF4
 
 
hsa-miR-221-3p
 
 
-0.553
 
 
129.9
 
 
 
 
ARG2
 
 
hsa-let-7c-5p
 
 
-0.468
 
 
101.8
 
 
 
 
ARHGAP1
 
 
hsa-miR-17-5p
 
 
-0.325
 
 
60.9
 
 
 
 
ARHGAP1
 
 
hsa-miR-93-5p
 
 
-0.325
 
 
60.9
 
 
 
 
ARHGAP1
 
 
hsa-miR-20a-5p
 
 
-0.339
 
 
60.9
 
 
 
 
ARHGAP5
 
 
hsa-miR-486-5p
 
 
-0.3
 
 
28.6
 
 
 
 
ARID4B
 
 
hsa-miR-20a-5p
 
 
-0.29
 
 
13.4
 
 
 
 
ARID4B
 
 
hsa-miR-486-5p
 
 
-0.367
 
 
13.4
 
 
 
 
ARIH2
 
 
hsa-miR-103a-3p
 
 
-0.307
 
 
22.3
 
 
 
 
ARL1
 
 
hsa-miR-20a-5p
 
 
-0.403
 
 
68.8
 
 
 
 
ARL1
 
 
hsa-miR-93-5p
 
 
-0.424
 
 
68.8
 
 
 
 
ARL1
 
 
hsa-miR-17-5p
 
 
-0.432
 
 
68.8
 
 
 
 
ARL4D
 
 
hsa-let-7c-5p
 
 
-0.417
 
 
10.7
 
 
 
 
ARL6IP1
 
 
hsa-miR-7-5p
 
 
-0.374
 
 
71.3
 
 
 
 
ARL6IP1
 
 
hsa-miR-328-3p
 
 
-0.494
 
 
71.3
 
 
 
 
ARL8A
 
 
hsa-miR-103a-3p
 
 
-0.38
 
 
28.3
 
 
 
 
ARMC1
 
 
hsa-miR-92a-3p
 
 
-0.322
 
 
16.7
 
 
 
 
ARMC1
 
 
hsa-miR-103a-3p
 
 
-0.525
 
 
16.7
 
 
 
 
ARMC8
 
 
hsa-miR-486-5p
 
 
-0.321
 
 
15.4
 
 
 
 
ARMC8
 
 
hsa-miR-20a-5p
 
 
-0.357
 
 
15.4
 
 
 
 
ARMC8
 
 
hsa-miR-93-5p
 
 
-0.362
 
 
15.4
 
 
 
 
ARMC8
 
 
hsa-miR-17-5p
 
 
-0.378
 
 
15.4
 
 
 
 
ARMC8
 
 
hsa-miR-103a-3p
 
 
-0.412
 
 
15.4
 
 
 
 
ARMT1
 
 
hsa-let-7c-5p
 
 
-0.303
 
 
11.5
 
 
 
 
ARNT
 
 
hsa-miR-103a-3p
 
 
-0.422
 
 
15.5
 
 
 
 
ARNT
 
 
hsa-miR-221-3p
 
 
-0.465
 
 
15.5
 
 
 
 
ARPP19
 
 
hsa-miR-328-3p
 
 
-0.404
 
 
37.0
 
 
 
 
ARPP19
 
 
hsa-let-7c-5p
 
 
-0.492
 
 
37.0
 
 
 
 
ARRDC3
 
 
hsa-miR-92a-3p
 
 
-0.458
 
 
28.5
 
 
 
 
ARRDC4
 
 
hsa-let-7c-5p
 
 
-0.329
 
 
10.3
 
 
 
 
ASAP1
 
 
hsa-let-7c-5p
 
 
-0.308
 
 
11.6
 
 
 
 
ASB6
 
 
hsa-miR-874-3p
 
 
-0.368
 
 
11.8
 
 
 
 
ASPH
 
 
hsa-miR-221-3p
 
 
-0.287
 
 
44.4
 
 
 
 
ASPH
 
 
hsa-miR-92a-3p
 
 
-0.416
 
 
44.4
 
 
 
 
ATF3
 
 
hsa-miR-874-3p
 
 
-0.288
 
 
108.8
 
 
 
 
ATG16L1
 
 
hsa-miR-20a-5p
 
 
-0.446
 
 
12.5
 
 
 
 
ATG16L1
 
 
hsa-miR-93-5p
 
 
-0.451
 
 
12.5
 
 
 
 
ATG16L1
 
 
hsa-miR-17-5p
 
 
-0.467
 
 
12.5
 
 
 
 
ATL2
 
 
hsa-miR-103a-3p
 
 
-0.489
 
 
29.5
 
 
 
 
ATOSA
 
 
hsa-miR-221-3p
 
 
-0.495
 
 
12.7
 
 
 
 
ATOSB
 
 
hsa-miR-328-3p
 
 
-0.358
 
 
12.4
 
 
 
 
ATOSB
 
 
hsa-let-7c-5p
 
 
-0.429
 
 
12.4
 
 
 
 
ATP2A2
 
 
hsa-let-7c-5p
 
 
-0.311
 
 
87.6
 
 
 
 
ATP6V1F
 
 
hsa-miR-185-5p
 
 
-0.765
 
 
104.5
 
 
 
 
ATPAF1
 
 
hsa-let-7c-5p
 
 
-0.498
 
 
35.2
 
 
 
 
AVPI1
 
 
hsa-miR-7-5p
 
 
-0.367
 
 
27.2
 
 
 
 
B3GAT3
 
 
hsa-let-7c-5p
 
 
-0.324
 
 
40.8
 
 
 
 
B4GALT5
 
 
hsa-miR-210-3p
 
 
-0.511
 
 
16.0
 
 
 
 
B4GAT1
 
 
hsa-let-7c-5p
 
 
-0.47
 
 
17.0
 
 
 
 
BACE1
 
 
hsa-miR-339-5p
 
 
-0.371
 
 
12.0
 
 
 
 
BACH1
 
 
hsa-let-7c-5p
 
 
-0.306
 
 
12.5
 
 
 
 
BACH1
 
 
hsa-let-7c-5p
 
 
-0.306
 
 
12.5
 
 
 
 
BACH1
 
 
hsa-let-7c-5p
 
 
-0.435
 
 
12.5
 
 
 
 
BACH1
 
 
hsa-let-7c-5p
 
 
-0.435
 
 
12.5
 
 
 
 
BAK1
 
 
hsa-miR-103a-3p
 
 
-0.364
 
 
10.5
 
 
 
 
BCAT2
 
 
hsa-miR-103a-3p
 
 
-0.364
 
 
37.0
 
 
 
 
BCAT2
 
 
hsa-miR-92a-3p
 
 
-0.609
 
 
37.0
 
 
 
 
 
 
 rectum 
 
 
 
 
target_symbol
 
 
mature_mirna_id
 
 
score
 
 
nTPM
 
 
 
 
 
 
AARS1
 
 
hsa-miR-92a-3p
 
 
-0.458
 
 
24.6
 
 
 
 
ABHD17C
 
 
hsa-let-7c-5p
 
 
-0.359
 
 
52.8
 
 
 
 
ABHD17C
 
 
hsa-miR-330-3p
 
 
-0.399
 
 
52.8
 
 
 
 
ACOX1
 
 
hsa-miR-92a-3p
 
 
-0.321
 
 
14.9
 
 
 
 
ADAM9
 
 
hsa-miR-20a-5p
 
 
-0.299
 
 
39.2
 
 
 
 
ADAM9
 
 
hsa-miR-17-5p
 
 
-0.32
 
 
39.2
 
 
 
 
ADAM9
 
 
hsa-miR-93-5p
 
 
-0.32
 
 
39.2
 
 
 
 
ADCY3
 
 
hsa-miR-92a-3p
 
 
-0.381
 
 
14.9
 
 
 
 
ADNP
 
 
hsa-miR-328-3p
 
 
-0.371
 
 
22.7
 
 
 
 
AGFG1
 
 
hsa-miR-103a-3p
 
 
-0.389
 
 
31.0
 
 
 
 
AHNAK
 
 
hsa-miR-20a-5p
 
 
-0.316
 
 
90.8
 
 
 
 
AHNAK
 
 
hsa-miR-17-5p
 
 
-0.337
 
 
90.8
 
 
 
 
AHNAK
 
 
hsa-miR-93-5p
 
 
-0.337
 
 
90.8
 
 
 
 
AIFM1
 
 
hsa-let-7c-5p
 
 
-0.314
 
 
14.9
 
 
 
 
AKAP10
 
 
hsa-miR-339-5p
 
 
-0.302
 
 
10.9
 
 
 
 
AKIRIN2
 
 
hsa-miR-103a-3p
 
 
-0.299
 
 
11.7
 
 
 
 
ALAD
 
 
hsa-miR-103a-3p
 
 
-0.385
 
 
23.0
 
 
 
 
ALG3
 
 
hsa-miR-20a-5p
 
 
-0.559
 
 
17.2
 
 
 
 
ALG3
 
 
hsa-miR-93-5p
 
 
-0.564
 
 
17.2
 
 
 
 
ALG3
 
 
hsa-miR-17-5p
 
 
-0.572
 
 
17.2
 
 
 
 
AMT
 
 
hsa-let-7c-5p
 
 
-0.463
 
 
11.1
 
 
 
 
ANGPTL2
 
 
hsa-miR-221-3p
 
 
-0.369
 
 
16.8
 
 
 
 
ANKFY1
 
 
hsa-miR-155-5p
 
 
-0.315
 
 
13.1
 
 
 
 
ANKRD12
 
 
hsa-miR-7-5p
 
 
-0.37
 
 
11.3
 
 
 
 
ANP32E
 
 
hsa-miR-92a-3p
 
 
-0.329
 
 
17.1
 
 
 
 
AP1AR
 
 
hsa-miR-92a-3p
 
 
-0.325
 
 
15.3
 
 
 
 
AP1S1
 
 
hsa-let-7c-5p
 
 
-0.374
 
 
21.1
 
 
 
 
AP4B1
 
 
hsa-miR-378a-3p
 
 
-0.62
 
 
12.0
 
 
 
 
APCDD1
 
 
hsa-miR-20a-5p
 
 
-0.404
 
 
12.4
 
 
 
 
APCDD1
 
 
hsa-miR-17-5p
 
 
-0.425
 
 
12.4
 
 
 
 
APCDD1
 
 
hsa-miR-93-5p
 
 
-0.425
 
 
12.4
 
 
 
 
APPL1
 
 
hsa-miR-92a-3p
 
 
-0.375
 
 
13.1
 
 
 
 
ARF4
 
 
hsa-miR-7-5p
 
 
-0.407
 
 
185.2
 
 
 
 
ARF4
 
 
hsa-miR-221-3p
 
 
-0.553
 
 
185.2
 
 
 
 
ARHGAP1
 
 
hsa-miR-17-5p
 
 
-0.325
 
 
30.6
 
 
 
 
ARHGAP1
 
 
hsa-miR-93-5p
 
 
-0.325
 
 
30.6
 
 
 
 
ARHGAP1
 
 
hsa-miR-20a-5p
 
 
-0.339
 
 
30.6
 
 
 
 
ARHGAP5
 
 
hsa-miR-486-5p
 
 
-0.3
 
 
24.8
 
 
 
 
ARIH2
 
 
hsa-miR-103a-3p
 
 
-0.307
 
 
16.6
 
 
 
 
ARL1
 
 
hsa-miR-20a-5p
 
 
-0.403
 
 
56.8
 
 
 
 
ARL1
 
 
hsa-miR-93-5p
 
 
-0.424
 
 
56.8
 
 
 
 
ARL1
 
 
hsa-miR-17-5p
 
 
-0.432
 
 
56.8
 
 
 
 
ARL6IP1
 
 
hsa-miR-7-5p
 
 
-0.374
 
 
116.0
 
 
 
 
ARL6IP1
 
 
hsa-miR-328-3p
 
 
-0.494
 
 
116.0
 
 
 
 
ARL6IP6
 
 
hsa-let-7c-5p
 
 
-0.293
 
 
13.3
 
 
 
 
ARL8A
 
 
hsa-miR-103a-3p
 
 
-0.38
 
 
10.2
 
 
 
 
ARMC1
 
 
hsa-miR-92a-3p
 
 
-0.322
 
 
15.6
 
 
 
 
ARMC1
 
 
hsa-miR-103a-3p
 
 
-0.525
 
 
15.6
 
 
 
 
ARMC8
 
 
hsa-miR-486-5p
 
 
-0.321
 
 
17.2
 
 
 
 
ARMC8
 
 
hsa-miR-20a-5p
 
 
-0.357
 
 
17.2
 
 
 
 
ARMC8
 
 
hsa-miR-93-5p
 
 
-0.362
 
 
17.2
 
 
 
 
ARMC8
 
 
hsa-miR-17-5p
 
 
-0.378
 
 
17.2
 
 
 
 
ARMC8
 
 
hsa-miR-103a-3p
 
 
-0.412
 
 
17.2
 
 
 
 
ARMT1
 
 
hsa-let-7c-5p
 
 
-0.303
 
 
16.5
 
 
 
 
ARNT
 
 
hsa-miR-103a-3p
 
 
-0.422
 
 
19.9
 
 
 
 
ARNT
 
 
hsa-miR-221-3p
 
 
-0.465
 
 
19.9
 
 
 
 
ARPP19
 
 
hsa-miR-328-3p
 
 
-0.404
 
 
33.6
 
 
 
 
ARPP19
 
 
hsa-let-7c-5p
 
 
-0.492
 
 
33.6
 
 
 
 
ARRDC3
 
 
hsa-miR-92a-3p
 
 
-0.458
 
 
25.8
 
 
 
 
ARRDC4
 
 
hsa-let-7c-5p
 
 
-0.329
 
 
21.6
 
 
 
 
ASPH
 
 
hsa-miR-221-3p
 
 
-0.287
 
 
77.9
 
 
 
 
ASPH
 
 
hsa-miR-92a-3p
 
 
-0.416
 
 
77.9
 
 
 
 
ASPN
 
 
hsa-miR-92a-3p
 
 
-0.422
 
 
10.6
 
 
 
 
ATF3
 
 
hsa-miR-874-3p
 
 
-0.288
 
 
56.3
 
 
 
 
ATG4A
 
 
hsa-miR-7-5p
 
 
-0.448
 
 
12.3
 
 
 
 
ATL2
 
 
hsa-miR-103a-3p
 
 
-0.489
 
 
25.1
 
 
 
 
ATOSA
 
 
hsa-miR-221-3p
 
 
-0.495
 
 
15.5
 
 
 
 
ATP2A2
 
 
hsa-let-7c-5p
 
 
-0.311
 
 
64.9
 
 
 
 
ATP6V1F
 
 
hsa-miR-185-5p
 
 
-0.765
 
 
53.0
 
 
 
 
ATPAF1
 
 
hsa-let-7c-5p
 
 
-0.498
 
 
50.0
 
 
 
 
AURKA
 
 
hsa-miR-92a-3p
 
 
-0.358
 
 
12.1
 
 
 
 
AVPI1
 
 
hsa-miR-7-5p
 
 
-0.367
 
 
17.8
 
 
 
 
B4GALT5
 
 
hsa-miR-210-3p
 
 
-0.511
 
 
22.3
 
 
 
 
BACH1
 
 
hsa-let-7c-5p
 
 
-0.306
 
 
12.4
 
 
 
 
BACH1
 
 
hsa-let-7c-5p
 
 
-0.306
 
 
12.4
 
 
 
 
BACH1
 
 
hsa-let-7c-5p
 
 
-0.435
 
 
12.4
 
 
 
 
BACH1
 
 
hsa-let-7c-5p
 
 
-0.435
 
 
12.4
 
 
 
 
BAK1
 
 
hsa-miR-103a-3p
 
 
-0.364
 
 
23.4
 
 
 
 
BCAT2
 
 
hsa-miR-103a-3p
 
 
-0.364
 
 
16.3
 
 
 
 
BCAT2
 
 
hsa-miR-92a-3p
 
 
-0.609
 
 
16.3
 
 
 
 
BCL2L11
 
 
hsa-miR-339-5p
 
 
-0.306
 
 
11.2
 
 
 
 
BCL2L11
 
 
hsa-miR-92a-3p
 
 
-0.394
 
 
11.2
 
 
 
 
BCL2L11
 
 
hsa-miR-221-3p
 
 
-0.423
 
 
11.2
 
 
 
 
BDH1
 
 
hsa-miR-874-3p
 
 
-0.644
 
 
18.6
 
 
 
 
BIN1
 
 
hsa-miR-744-5p
 
 
-0.654
 
 
12.3
 
 
 
 
BLOC1S6
 
 
hsa-let-7c-5p
 
 
-0.321
 
 
20.2
 
 
 
 
BLTP1
 
 
hsa-miR-92a-3p
 
 
-0.462
 
 
13.6
 
 
 
 
BMP2
 
 
hsa-miR-378a-3p
 
 
-0.481
 
 
12.8
 
 
 
 
BNIP3L
 
 
hsa-miR-20a-5p
 
 
-0.287
 
 
26.5
 
 
 
 
BOLA3
 
 
hsa-miR-103a-3p
 
 
-0.362
 
 
35.3
 
 
 
 
BSDC1
 
 
hsa-miR-92a-3p
 
 
-0.362
 
 
21.1
 
 
 
 
BTAF1
 
 
hsa-miR-486-5p
 
 
-0.288
 
 
12.5
 
 
 
 
BTBD10
 
 
hsa-miR-17-5p
 
 
-0.409
 
 
19.1
 
 
 
 
BTBD10
 
 
hsa-miR-93-5p
 
 
-0.409
 
 
19.1
 
 
 
 
BTBD10
 
 
hsa-miR-20a-5p
 
 
-0.412
 
 
19.1
 
 
 
 
BTF3L4
 
 
hsa-let-7c-5p
 
 
-0.355
 
 
22.2
 
 
 
 
BTG2
 
 
hsa-miR-92a-3p
 
 
-0.314
 
 
193.5
 
 
 
 
BTG2
 
 
hsa-miR-92a-3p
 
 
-0.402
 
 
193.5
 
 
 
 
BTG3
 
 
hsa-miR-17-5p
 
 
-0.668
 
 
25.2
 
 
 
 
BTG3
 
 
hsa-miR-93-5p
 
 
-0.668
 
 
25.2
 
 
 
 
 
 
 retina 
 
 
 
 
target_symbol
 
 
mature_mirna_id
 
 
score
 
 
nTPM
 
 
 
 
 
 
AARS1
 
 
hsa-miR-92a-3p
 
 
-0.458
 
 
55.1
 
 
 
 
ABHD17B
 
 
hsa-miR-486-5p
 
 
-0.348
 
 
23.6
 
 
 
 
ABHD17C
 
 
hsa-let-7c-5p
 
 
-0.359
 
 
15.6
 
 
 
 
ABHD17C
 
 
hsa-miR-330-3p
 
 
-0.399
 
 
15.6
 
 
 
 
ACOX1
 
 
hsa-miR-92a-3p
 
 
-0.321
 
 
12.5
 
 
 
 
ACSL6
 
 
hsa-let-7c-5p
 
 
-0.336
 
 
42.4
 
 
 
 
ACTC1
 
 
hsa-miR-92a-3p
 
 
-0.361
 
 
29.3
 
 
 
 
ACTC1
 
 
hsa-miR-7-5p
 
 
-0.471
 
 
29.3
 
 
 
 
ADAM19
 
 
hsa-miR-874-3p
 
 
-0.374
 
 
21.0
 
 
 
 
ADAM9
 
 
hsa-miR-20a-5p
 
 
-0.299
 
 
17.2
 
 
 
 
ADAM9
 
 
hsa-miR-17-5p
 
 
-0.32
 
 
17.2
 
 
 
 
ADAM9
 
 
hsa-miR-93-5p
 
 
-0.32
 
 
17.2
 
 
 
 
ADNP
 
 
hsa-miR-328-3p
 
 
-0.371
 
 
40.4
 
 
 
 
AGFG1
 
 
hsa-miR-103a-3p
 
 
-0.389
 
 
44.2
 
 
 
 
AGO4
 
 
hsa-miR-103a-3p
 
 
-0.32
 
 
16.7
 
 
 
 
AGO4
 
 
hsa-miR-221-3p
 
 
-0.321
 
 
16.7
 
 
 
 
AKAP10
 
 
hsa-miR-339-5p
 
 
-0.302
 
 
14.2
 
 
 
 
AKAP6
 
 
hsa-let-7c-5p
 
 
-0.307
 
 
12.6
 
 
 
 
AKIRIN2
 
 
hsa-miR-103a-3p
 
 
-0.299
 
 
27.5
 
 
 
 
ALAD
 
 
hsa-miR-103a-3p
 
 
-0.385
 
 
17.6
 
 
 
 
ALG9
 
 
hsa-miR-7-5p
 
 
-0.302
 
 
11.3
 
 
 
 
ANKFY1
 
 
hsa-miR-155-5p
 
 
-0.315
 
 
12.1
 
 
 
 
ANKRD12
 
 
hsa-miR-7-5p
 
 
-0.37
 
 
27.6
 
 
 
 
ANKRD13C
 
 
hsa-miR-20a-5p
 
 
-0.505
 
 
12.3
 
 
 
 
ANKRD13C
 
 
hsa-miR-17-5p
 
 
-0.526
 
 
12.3
 
 
 
 
ANKRD13C
 
 
hsa-miR-93-5p
 
 
-0.526
 
 
12.3
 
 
 
 
ANKRD46
 
 
hsa-let-7c-5p
 
 
-0.389
 
 
32.6
 
 
 
 
ANP32E
 
 
hsa-miR-92a-3p
 
 
-0.329
 
 
91.3
 
 
 
 
AP1S1
 
 
hsa-let-7c-5p
 
 
-0.374
 
 
13.5
 
 
 
 
AP4B1
 
 
hsa-miR-378a-3p
 
 
-0.62
 
 
18.5
 
 
 
 
APPL1
 
 
hsa-miR-92a-3p
 
 
-0.375
 
 
12.5
 
 
 
 
ARF4
 
 
hsa-miR-7-5p
 
 
-0.407
 
 
50.0
 
 
 
 
ARF4
 
 
hsa-miR-221-3p
 
 
-0.553
 
 
50.0
 
 
 
 
ARG2
 
 
hsa-let-7c-5p
 
 
-0.468
 
 
20.7
 
 
 
 
ARHGAP42
 
 
hsa-miR-221-3p
 
 
-0.304
 
 
29.2
 
 
 
 
ARHGAP5
 
 
hsa-miR-486-5p
 
 
-0.3
 
 
53.6
 
 
 
 
ARID2
 
 
hsa-miR-155-5p
 
 
-0.384
 
 
12.3
 
 
 
 
ARID2
 
 
hsa-miR-103a-3p
 
 
-0.415
 
 
12.3
 
 
 
 
ARID3B
 
 
hsa-let-7c-5p
 
 
-0.31
 
 
17.4
 
 
 
 
ARID3B
 
 
hsa-let-7c-5p
 
 
-0.486
 
 
17.4
 
 
 
 
ARID4A
 
 
hsa-miR-7-5p
 
 
-0.341
 
 
13.0
 
 
 
 
ARID4B
 
 
hsa-miR-20a-5p
 
 
-0.29
 
 
13.5
 
 
 
 
ARID4B
 
 
hsa-miR-486-5p
 
 
-0.367
 
 
13.5
 
 
 
 
ARIH2
 
 
hsa-miR-103a-3p
 
 
-0.307
 
 
30.1
 
 
 
 
ARL1
 
 
hsa-miR-20a-5p
 
 
-0.403
 
 
45.1
 
 
 
 
ARL1
 
 
hsa-miR-93-5p
 
 
-0.424
 
 
45.1
 
 
 
 
ARL1
 
 
hsa-miR-17-5p
 
 
-0.432
 
 
45.1
 
 
 
 
ARL4D
 
 
hsa-let-7c-5p
 
 
-0.417
 
 
78.9
 
 
 
 
ARL6IP1
 
 
hsa-miR-7-5p
 
 
-0.374
 
 
292.6
 
 
 
 
ARL6IP1
 
 
hsa-miR-328-3p
 
 
-0.494
 
 
292.6
 
 
 
 
ARL6IP6
 
 
hsa-let-7c-5p
 
 
-0.293
 
 
10.5
 
 
 
 
ARL8A
 
 
hsa-miR-103a-3p
 
 
-0.38
 
 
17.4
 
 
 
 
ARMC1
 
 
hsa-miR-92a-3p
 
 
-0.322
 
 
11.6
 
 
 
 
ARMC1
 
 
hsa-miR-103a-3p
 
 
-0.525
 
 
11.6
 
 
 
 
ARMC8
 
 
hsa-miR-486-5p
 
 
-0.321
 
 
24.3
 
 
 
 
ARMC8
 
 
hsa-miR-20a-5p
 
 
-0.357
 
 
24.3
 
 
 
 
ARMC8
 
 
hsa-miR-93-5p
 
 
-0.362
 
 
24.3
 
 
 
 
ARMC8
 
 
hsa-miR-17-5p
 
 
-0.378
 
 
24.3
 
 
 
 
ARMC8
 
 
hsa-miR-103a-3p
 
 
-0.412
 
 
24.3
 
 
 
 
ARMT1
 
 
hsa-let-7c-5p
 
 
-0.303
 
 
15.2
 
 
 
 
ARNT
 
 
hsa-miR-103a-3p
 
 
-0.422
 
 
36.3
 
 
 
 
ARNT
 
 
hsa-miR-221-3p
 
 
-0.465
 
 
36.3
 
 
 
 
ARPP19
 
 
hsa-miR-328-3p
 
 
-0.404
 
 
53.1
 
 
 
 
ARPP19
 
 
hsa-let-7c-5p
 
 
-0.492
 
 
53.1
 
 
 
 
ARRDC3
 
 
hsa-miR-92a-3p
 
 
-0.458
 
 
69.6
 
 
 
 
ASPH
 
 
hsa-miR-221-3p
 
 
-0.287
 
 
201.9
 
 
 
 
ASPH
 
 
hsa-miR-92a-3p
 
 
-0.416
 
 
201.9
 
 
 
 
ATF3
 
 
hsa-miR-874-3p
 
 
-0.288
 
 
15.0
 
 
 
 
ATL2
 
 
hsa-miR-103a-3p
 
 
-0.489
 
 
23.5
 
 
 
 
ATOSA
 
 
hsa-miR-221-3p
 
 
-0.495
 
 
16.0
 
 
 
 
ATP1B2
 
 
hsa-miR-103a-3p
 
 
-0.6
 
 
570.1
 
 
 
 
ATP2A2
 
 
hsa-let-7c-5p
 
 
-0.311
 
 
17.7
 
 
 
 
ATP2B2
 
 
hsa-miR-7-5p
 
 
-0.297
 
 
12.5
 
 
 
 
ATP6V1F
 
 
hsa-miR-185-5p
 
 
-0.765
 
 
102.7
 
 
 
 
ATPAF1
 
 
hsa-let-7c-5p
 
 
-0.498
 
 
65.3
 
 
 
 
ATXN1
 
 
hsa-miR-221-3p
 
 
-0.289
 
 
12.9
 
 
 
 
AVPI1
 
 
hsa-miR-7-5p
 
 
-0.367
 
 
23.7
 
 
 
 
B3GAT3
 
 
hsa-let-7c-5p
 
 
-0.324
 
 
15.0
 
 
 
 
B4GAT1
 
 
hsa-let-7c-5p
 
 
-0.47
 
 
42.6
 
 
 
 
BACE1
 
 
hsa-miR-339-5p
 
 
-0.371
 
 
41.4
 
 
 
 
BACH1
 
 
hsa-let-7c-5p
 
 
-0.306
 
 
10.9
 
 
 
 
BACH1
 
 
hsa-let-7c-5p
 
 
-0.306
 
 
10.9
 
 
 
 
BACH1
 
 
hsa-let-7c-5p
 
 
-0.435
 
 
10.9
 
 
 
 
BACH1
 
 
hsa-let-7c-5p
 
 
-0.435
 
 
10.9
 
 
 
 
BAMBI
 
 
hsa-miR-20a-5p
 
 
-0.485
 
 
12.4
 
 
 
 
BAMBI
 
 
hsa-miR-17-5p
 
 
-0.506
 
 
12.4
 
 
 
 
BAMBI
 
 
hsa-miR-93-5p
 
 
-0.506
 
 
12.4
 
 
 
 
BCAT1
 
 
hsa-let-7c-5p
 
 
-0.394
 
 
13.4
 
 
 
 
BCL2L11
 
 
hsa-miR-339-5p
 
 
-0.306
 
 
11.9
 
 
 
 
BCL2L11
 
 
hsa-miR-92a-3p
 
 
-0.394
 
 
11.9
 
 
 
 
BCL2L11
 
 
hsa-miR-221-3p
 
 
-0.423
 
 
11.9
 
 
 
 
BCL2L13
 
 
hsa-miR-103a-3p
 
 
-0.302
 
 
40.6
 
 
 
 
BICDL1
 
 
hsa-miR-221-3p
 
 
-0.447
 
 
39.5
 
 
 
 
BIN1
 
 
hsa-miR-744-5p
 
 
-0.654
 
 
49.9
 
 
 
 
BLOC1S6
 
 
hsa-let-7c-5p
 
 
-0.321
 
 
22.7
 
 
 
 
BLTP1
 
 
hsa-miR-92a-3p
 
 
-0.462
 
 
57.7
 
 
 
 
BNIP3L
 
 
hsa-miR-20a-5p
 
 
-0.287
 
 
135.8
 
 
 
 
BOLA3
 
 
hsa-miR-103a-3p
 
 
-0.362
 
 
33.1
 
 
 
 
BSDC1
 
 
hsa-miR-92a-3p
 
 
-0.362
 
 
20.1
 
 
 
 
BTAF1
 
 
hsa-miR-486-5p
 
 
-0.288
 
 
27.2
 
 
 
 
 
 
 salivary gland 
 
 
 
 
target_symbol
 
 
mature_mirna_id
 
 
score
 
 
nTPM
 
 
 
 
 
 
AARS1
 
 
hsa-miR-92a-3p
 
 
-0.458
 
 
50.3
 
 
 
 
ABHD17C
 
 
hsa-let-7c-5p
 
 
-0.359
 
 
22.3
 
 
 
 
ABHD17C
 
 
hsa-miR-330-3p
 
 
-0.399
 
 
22.3
 
 
 
 
ABHD5
 
 
hsa-miR-20a-5p
 
 
-0.397
 
 
19.4
 
 
 
 
ABHD5
 
 
hsa-miR-93-5p
 
 
-0.402
 
 
19.4
 
 
 
 
ABHD5
 
 
hsa-miR-17-5p
 
 
-0.45
 
 
19.4
 
 
 
 
ACOX1
 
 
hsa-miR-92a-3p
 
 
-0.321
 
 
14.1
 
 
 
 
ACTA1
 
 
hsa-miR-155-5p
 
 
-0.595
 
 
552.8
 
 
 
 
ACTC1
 
 
hsa-miR-92a-3p
 
 
-0.361
 
 
41.7
 
 
 
 
ACTC1
 
 
hsa-miR-7-5p
 
 
-0.471
 
 
41.7
 
 
 
 
ADAM9
 
 
hsa-miR-20a-5p
 
 
-0.299
 
 
39.8
 
 
 
 
ADAM9
 
 
hsa-miR-17-5p
 
 
-0.32
 
 
39.8
 
 
 
 
ADAM9
 
 
hsa-miR-93-5p
 
 
-0.32
 
 
39.8
 
 
 
 
ADCY3
 
 
hsa-miR-92a-3p
 
 
-0.381
 
 
20.4
 
 
 
 
ADNP
 
 
hsa-miR-328-3p
 
 
-0.371
 
 
27.9
 
 
 
 
AGFG1
 
 
hsa-miR-103a-3p
 
 
-0.389
 
 
26.3
 
 
 
 
AGFG2
 
 
hsa-miR-20a-5p
 
 
-0.365
 
 
272.4
 
 
 
 
AGFG2
 
 
hsa-miR-93-5p
 
 
-0.378
 
 
272.4
 
 
 
 
AGFG2
 
 
hsa-miR-17-5p
 
 
-0.402
 
 
272.4
 
 
 
 
AHNAK
 
 
hsa-miR-20a-5p
 
 
-0.316
 
 
118.3
 
 
 
 
AHNAK
 
 
hsa-miR-17-5p
 
 
-0.337
 
 
118.3
 
 
 
 
AHNAK
 
 
hsa-miR-93-5p
 
 
-0.337
 
 
118.3
 
 
 
 
AIFM1
 
 
hsa-let-7c-5p
 
 
-0.314
 
 
17.0
 
 
 
 
AKIRIN2
 
 
hsa-miR-103a-3p
 
 
-0.299
 
 
21.8
 
 
 
 
ALAD
 
 
hsa-miR-103a-3p
 
 
-0.385
 
 
92.9
 
 
 
 
ALG3
 
 
hsa-miR-20a-5p
 
 
-0.559
 
 
25.8
 
 
 
 
ALG3
 
 
hsa-miR-93-5p
 
 
-0.564
 
 
25.8
 
 
 
 
ALG3
 
 
hsa-miR-17-5p
 
 
-0.572
 
 
25.8
 
 
 
 
ALG9
 
 
hsa-miR-7-5p
 
 
-0.302
 
 
16.6
 
 
 
 
AMT
 
 
hsa-let-7c-5p
 
 
-0.463
 
 
10.0
 
 
 
 
ANGPTL2
 
 
hsa-miR-221-3p
 
 
-0.369
 
 
17.1
 
 
 
 
ANKFY1
 
 
hsa-miR-155-5p
 
 
-0.315
 
 
12.9
 
 
 
 
ANKRD12
 
 
hsa-miR-7-5p
 
 
-0.37
 
 
11.6
 
 
 
 
ANKRD46
 
 
hsa-let-7c-5p
 
 
-0.389
 
 
16.1
 
 
 
 
ANKRD9
 
 
hsa-miR-20a-5p
 
 
-0.31
 
 
25.3
 
 
 
 
ANKRD9
 
 
hsa-miR-93-5p
 
 
-0.331
 
 
25.3
 
 
 
 
ANKRD9
 
 
hsa-miR-17-5p
 
 
-0.347
 
 
25.3
 
 
 
 
ANP32E
 
 
hsa-miR-92a-3p
 
 
-0.329
 
 
12.1
 
 
 
 
AP1AR
 
 
hsa-miR-92a-3p
 
 
-0.325
 
 
11.4
 
 
 
 
AP1S1
 
 
hsa-let-7c-5p
 
 
-0.374
 
 
21.5
 
 
 
 
APCDD1
 
 
hsa-miR-20a-5p
 
 
-0.404
 
 
10.0
 
 
 
 
APCDD1
 
 
hsa-miR-17-5p
 
 
-0.425
 
 
10.0
 
 
 
 
APCDD1
 
 
hsa-miR-93-5p
 
 
-0.425
 
 
10.0
 
 
 
 
APPL1
 
 
hsa-miR-92a-3p
 
 
-0.375
 
 
14.3
 
 
 
 
AQP3
 
 
hsa-miR-874-3p
 
 
-0.48
 
 
376.5
 
 
 
 
ARF4
 
 
hsa-miR-7-5p
 
 
-0.407
 
 
171.4
 
 
 
 
ARF4
 
 
hsa-miR-221-3p
 
 
-0.553
 
 
171.4
 
 
 
 
ARG2
 
 
hsa-let-7c-5p
 
 
-0.468
 
 
18.4
 
 
 
 
ARHGAP1
 
 
hsa-miR-17-5p
 
 
-0.325
 
 
43.3
 
 
 
 
ARHGAP1
 
 
hsa-miR-93-5p
 
 
-0.325
 
 
43.3
 
 
 
 
ARHGAP1
 
 
hsa-miR-20a-5p
 
 
-0.339
 
 
43.3
 
 
 
 
ARHGAP5
 
 
hsa-miR-486-5p
 
 
-0.3
 
 
56.3
 
 
 
 
ARHGEF28
 
 
hsa-miR-93-5p
 
 
-0.33
 
 
18.6
 
 
 
 
ARHGEF28
 
 
hsa-miR-17-5p
 
 
-0.363
 
 
18.6
 
 
 
 
ARHGEF28
 
 
hsa-miR-20a-5p
 
 
-0.366
 
 
18.6
 
 
 
 
ARID4B
 
 
hsa-miR-20a-5p
 
 
-0.29
 
 
10.8
 
 
 
 
ARID4B
 
 
hsa-miR-486-5p
 
 
-0.367
 
 
10.8
 
 
 
 
ARIH2
 
 
hsa-miR-103a-3p
 
 
-0.307
 
 
18.1
 
 
 
 
ARL1
 
 
hsa-miR-20a-5p
 
 
-0.403
 
 
69.5
 
 
 
 
ARL1
 
 
hsa-miR-93-5p
 
 
-0.424
 
 
69.5
 
 
 
 
ARL1
 
 
hsa-miR-17-5p
 
 
-0.432
 
 
69.5
 
 
 
 
ARL4D
 
 
hsa-let-7c-5p
 
 
-0.417
 
 
16.7
 
 
 
 
ARL6IP1
 
 
hsa-miR-7-5p
 
 
-0.374
 
 
79.4
 
 
 
 
ARL6IP1
 
 
hsa-miR-328-3p
 
 
-0.494
 
 
79.4
 
 
 
 
ARL8A
 
 
hsa-miR-103a-3p
 
 
-0.38
 
 
29.6
 
 
 
 
ARMC1
 
 
hsa-miR-92a-3p
 
 
-0.322
 
 
14.8
 
 
 
 
ARMC1
 
 
hsa-miR-103a-3p
 
 
-0.525
 
 
14.8
 
 
 
 
ARMC8
 
 
hsa-miR-486-5p
 
 
-0.321
 
 
11.8
 
 
 
 
ARMC8
 
 
hsa-miR-20a-5p
 
 
-0.357
 
 
11.8
 
 
 
 
ARMC8
 
 
hsa-miR-93-5p
 
 
-0.362
 
 
11.8
 
 
 
 
ARMC8
 
 
hsa-miR-17-5p
 
 
-0.378
 
 
11.8
 
 
 
 
ARMC8
 
 
hsa-miR-103a-3p
 
 
-0.412
 
 
11.8
 
 
 
 
ARNT
 
 
hsa-miR-103a-3p
 
 
-0.422
 
 
15.0
 
 
 
 
ARNT
 
 
hsa-miR-221-3p
 
 
-0.465
 
 
15.0
 
 
 
 
ARPP19
 
 
hsa-miR-328-3p
 
 
-0.404
 
 
28.7
 
 
 
 
ARPP19
 
 
hsa-let-7c-5p
 
 
-0.492
 
 
28.7
 
 
 
 
ARRDC3
 
 
hsa-miR-92a-3p
 
 
-0.458
 
 
60.1
 
 
 
 
ASB6
 
 
hsa-miR-874-3p
 
 
-0.368
 
 
11.9
 
 
 
 
ASPH
 
 
hsa-miR-221-3p
 
 
-0.287
 
 
95.6
 
 
 
 
ASPH
 
 
hsa-miR-92a-3p
 
 
-0.416
 
 
95.6
 
 
 
 
ATF3
 
 
hsa-miR-874-3p
 
 
-0.288
 
 
34.0
 
 
 
 
ATG16L1
 
 
hsa-miR-20a-5p
 
 
-0.446
 
 
11.7
 
 
 
 
ATG16L1
 
 
hsa-miR-93-5p
 
 
-0.451
 
 
11.7
 
 
 
 
ATG16L1
 
 
hsa-miR-17-5p
 
 
-0.467
 
 
11.7
 
 
 
 
ATG4A
 
 
hsa-miR-7-5p
 
 
-0.448
 
 
12.3
 
 
 
 
ATL2
 
 
hsa-miR-103a-3p
 
 
-0.489
 
 
48.8
 
 
 
 
ATOSA
 
 
hsa-miR-221-3p
 
 
-0.495
 
 
14.6
 
 
 
 
ATOSB
 
 
hsa-miR-328-3p
 
 
-0.358
 
 
10.9
 
 
 
 
ATOSB
 
 
hsa-let-7c-5p
 
 
-0.429
 
 
10.9
 
 
 
 
ATP2A2
 
 
hsa-let-7c-5p
 
 
-0.311
 
 
74.5
 
 
 
 
ATP2B2
 
 
hsa-miR-7-5p
 
 
-0.297
 
 
31.3
 
 
 
 
ATP6V1F
 
 
hsa-miR-185-5p
 
 
-0.765
 
 
129.0
 
 
 
 
ATPAF1
 
 
hsa-let-7c-5p
 
 
-0.498
 
 
54.3
 
 
 
 
AVPI1
 
 
hsa-miR-7-5p
 
 
-0.367
 
 
23.0
 
 
 
 
B3GAT3
 
 
hsa-let-7c-5p
 
 
-0.324
 
 
28.6
 
 
 
 
B4GALT5
 
 
hsa-miR-210-3p
 
 
-0.511
 
 
18.2
 
 
 
 
B4GAT1
 
 
hsa-let-7c-5p
 
 
-0.47
 
 
14.9
 
 
 
 
BACE1
 
 
hsa-miR-339-5p
 
 
-0.371
 
 
10.2
 
 
 
 
BACE2
 
 
hsa-let-7c-5p
 
 
-0.364
 
 
16.3
 
 
 
 
BACH1
 
 
hsa-let-7c-5p
 
 
-0.306
 
 
16.4
 
 
 
 
 
 
 seminal vesicle 
 
 
 
 
target_symbol
 
 
mature_mirna_id
 
 
score
 
 
nTPM
 
 
 
 
 
 
AARS1
 
 
hsa-miR-92a-3p
 
 
-0.458
 
 
44.5
 
 
 
 
ACSL6
 
 
hsa-let-7c-5p
 
 
-0.336
 
 
17.0
 
 
 
 
ACTC1
 
 
hsa-miR-92a-3p
 
 
-0.361
 
 
506.6
 
 
 
 
ACTC1
 
 
hsa-miR-7-5p
 
 
-0.471
 
 
506.6
 
 
 
 
ADAM9
 
 
hsa-miR-20a-5p
 
 
-0.299
 
 
32.8
 
 
 
 
ADAM9
 
 
hsa-miR-17-5p
 
 
-0.32
 
 
32.8
 
 
 
 
ADAM9
 
 
hsa-miR-93-5p
 
 
-0.32
 
 
32.8
 
 
 
 
ADCY3
 
 
hsa-miR-92a-3p
 
 
-0.381
 
 
31.0
 
 
 
 
ADNP
 
 
hsa-miR-328-3p
 
 
-0.371
 
 
28.1
 
 
 
 
AGFG1
 
 
hsa-miR-103a-3p
 
 
-0.389
 
 
22.0
 
 
 
 
AGFG2
 
 
hsa-miR-20a-5p
 
 
-0.365
 
 
12.7
 
 
 
 
AGFG2
 
 
hsa-miR-93-5p
 
 
-0.378
 
 
12.7
 
 
 
 
AGFG2
 
 
hsa-miR-17-5p
 
 
-0.402
 
 
12.7
 
 
 
 
AHNAK
 
 
hsa-miR-20a-5p
 
 
-0.316
 
 
99.6
 
 
 
 
AHNAK
 
 
hsa-miR-17-5p
 
 
-0.337
 
 
99.6
 
 
 
 
AHNAK
 
 
hsa-miR-93-5p
 
 
-0.337
 
 
99.6
 
 
 
 
AJUBA
 
 
hsa-miR-103a-3p
 
 
-0.448
 
 
14.9
 
 
 
 
AKAP10
 
 
hsa-miR-339-5p
 
 
-0.302
 
 
11.0
 
 
 
 
AKIRIN2
 
 
hsa-miR-103a-3p
 
 
-0.299
 
 
11.0
 
 
 
 
ALAD
 
 
hsa-miR-103a-3p
 
 
-0.385
 
 
22.8
 
 
 
 
ALG3
 
 
hsa-miR-20a-5p
 
 
-0.559
 
 
12.9
 
 
 
 
ALG3
 
 
hsa-miR-93-5p
 
 
-0.564
 
 
12.9
 
 
 
 
ALG3
 
 
hsa-miR-17-5p
 
 
-0.572
 
 
12.9
 
 
 
 
ALG9
 
 
hsa-miR-7-5p
 
 
-0.302
 
 
10.6
 
 
 
 
ANGPTL2
 
 
hsa-miR-221-3p
 
 
-0.369
 
 
25.5
 
 
 
 
ANKFY1
 
 
hsa-miR-155-5p
 
 
-0.315
 
 
11.5
 
 
 
 
ANKRD12
 
 
hsa-miR-7-5p
 
 
-0.37
 
 
10.0
 
 
 
 
ANKRD46
 
 
hsa-let-7c-5p
 
 
-0.389
 
 
12.4
 
 
 
 
ANP32E
 
 
hsa-miR-92a-3p
 
 
-0.329
 
 
23.1
 
 
 
 
APCDD1
 
 
hsa-miR-20a-5p
 
 
-0.404
 
 
13.3
 
 
 
 
APCDD1
 
 
hsa-miR-17-5p
 
 
-0.425
 
 
13.3
 
 
 
 
APCDD1
 
 
hsa-miR-93-5p
 
 
-0.425
 
 
13.3
 
 
 
 
APPL1
 
 
hsa-miR-92a-3p
 
 
-0.375
 
 
13.5
 
 
 
 
AQP3
 
 
hsa-miR-874-3p
 
 
-0.48
 
 
34.7
 
 
 
 
ARF4
 
 
hsa-miR-7-5p
 
 
-0.407
 
 
125.1
 
 
 
 
ARF4
 
 
hsa-miR-221-3p
 
 
-0.553
 
 
125.1
 
 
 
 
ARHGAP1
 
 
hsa-miR-17-5p
 
 
-0.325
 
 
58.9
 
 
 
 
ARHGAP1
 
 
hsa-miR-93-5p
 
 
-0.325
 
 
58.9
 
 
 
 
ARHGAP1
 
 
hsa-miR-20a-5p
 
 
-0.339
 
 
58.9
 
 
 
 
ARHGAP5
 
 
hsa-miR-486-5p
 
 
-0.3
 
 
27.6
 
 
 
 
ARID4B
 
 
hsa-miR-20a-5p
 
 
-0.29
 
 
10.2
 
 
 
 
ARID4B
 
 
hsa-miR-486-5p
 
 
-0.367
 
 
10.2
 
 
 
 
ARIH2
 
 
hsa-miR-103a-3p
 
 
-0.307
 
 
20.6
 
 
 
 
ARL1
 
 
hsa-miR-20a-5p
 
 
-0.403
 
 
65.0
 
 
 
 
ARL1
 
 
hsa-miR-93-5p
 
 
-0.424
 
 
65.0
 
 
 
 
ARL1
 
 
hsa-miR-17-5p
 
 
-0.432
 
 
65.0
 
 
 
 
ARL4D
 
 
hsa-let-7c-5p
 
 
-0.417
 
 
14.3
 
 
 
 
ARL6IP1
 
 
hsa-miR-7-5p
 
 
-0.374
 
 
66.4
 
 
 
 
ARL6IP1
 
 
hsa-miR-328-3p
 
 
-0.494
 
 
66.4
 
 
 
 
ARL8A
 
 
hsa-miR-103a-3p
 
 
-0.38
 
 
18.4
 
 
 
 
ARMC1
 
 
hsa-miR-92a-3p
 
 
-0.322
 
 
16.0
 
 
 
 
ARMC1
 
 
hsa-miR-103a-3p
 
 
-0.525
 
 
16.0
 
 
 
 
ARMC8
 
 
hsa-miR-486-5p
 
 
-0.321
 
 
16.5
 
 
 
 
ARMC8
 
 
hsa-miR-20a-5p
 
 
-0.357
 
 
16.5
 
 
 
 
ARMC8
 
 
hsa-miR-93-5p
 
 
-0.362
 
 
16.5
 
 
 
 
ARMC8
 
 
hsa-miR-17-5p
 
 
-0.378
 
 
16.5
 
 
 
 
ARMC8
 
 
hsa-miR-103a-3p
 
 
-0.412
 
 
16.5
 
 
 
 
ARMT1
 
 
hsa-let-7c-5p
 
 
-0.303
 
 
10.5
 
 
 
 
ARNT
 
 
hsa-miR-103a-3p
 
 
-0.422
 
 
16.6
 
 
 
 
ARNT
 
 
hsa-miR-221-3p
 
 
-0.465
 
 
16.6
 
 
 
 
ARPP19
 
 
hsa-miR-328-3p
 
 
-0.404
 
 
38.5
 
 
 
 
ARPP19
 
 
hsa-let-7c-5p
 
 
-0.492
 
 
38.5
 
 
 
 
ARRDC3
 
 
hsa-miR-92a-3p
 
 
-0.458
 
 
22.9
 
 
 
 
ARRDC4
 
 
hsa-let-7c-5p
 
 
-0.329
 
 
15.4
 
 
 
 
ASAP1
 
 
hsa-let-7c-5p
 
 
-0.308
 
 
10.7
 
 
 
 
ASPH
 
 
hsa-miR-221-3p
 
 
-0.287
 
 
65.8
 
 
 
 
ASPH
 
 
hsa-miR-92a-3p
 
 
-0.416
 
 
65.8
 
 
 
 
ASPN
 
 
hsa-miR-92a-3p
 
 
-0.422
 
 
11.0
 
 
 
 
ATF3
 
 
hsa-miR-874-3p
 
 
-0.288
 
 
22.7
 
 
 
 
ATL2
 
 
hsa-miR-103a-3p
 
 
-0.489
 
 
29.4
 
 
 
 
ATOSA
 
 
hsa-miR-221-3p
 
 
-0.495
 
 
14.4
 
 
 
 
ATP2A2
 
 
hsa-let-7c-5p
 
 
-0.311
 
 
66.0
 
 
 
 
ATP6V1F
 
 
hsa-miR-185-5p
 
 
-0.765
 
 
76.8
 
 
 
 
ATPAF1
 
 
hsa-let-7c-5p
 
 
-0.498
 
 
31.8
 
 
 
 
AVPI1
 
 
hsa-miR-7-5p
 
 
-0.367
 
 
15.6
 
 
 
 
B3GAT3
 
 
hsa-let-7c-5p
 
 
-0.324
 
 
17.2
 
 
 
 
B4GALT5
 
 
hsa-miR-210-3p
 
 
-0.511
 
 
20.7
 
 
 
 
B4GAT1
 
 
hsa-let-7c-5p
 
 
-0.47
 
 
21.2
 
 
 
 
BACE1
 
 
hsa-miR-339-5p
 
 
-0.371
 
 
16.4
 
 
 
 
BAMBI
 
 
hsa-miR-20a-5p
 
 
-0.485
 
 
15.7
 
 
 
 
BAMBI
 
 
hsa-miR-17-5p
 
 
-0.506
 
 
15.7
 
 
 
 
BAMBI
 
 
hsa-miR-93-5p
 
 
-0.506
 
 
15.7
 
 
 
 
BCAT2
 
 
hsa-miR-103a-3p
 
 
-0.364
 
 
16.0
 
 
 
 
BCAT2
 
 
hsa-miR-92a-3p
 
 
-0.609
 
 
16.0
 
 
 
 
BIN1
 
 
hsa-miR-744-5p
 
 
-0.654
 
 
22.7
 
 
 
 
BLOC1S6
 
 
hsa-let-7c-5p
 
 
-0.321
 
 
19.2
 
 
 
 
BLTP1
 
 
hsa-miR-92a-3p
 
 
-0.462
 
 
13.9
 
 
 
 
BNIP3L
 
 
hsa-miR-20a-5p
 
 
-0.287
 
 
60.7
 
 
 
 
BOLA3
 
 
hsa-miR-103a-3p
 
 
-0.362
 
 
22.2
 
 
 
 
BSDC1
 
 
hsa-miR-92a-3p
 
 
-0.362
 
 
22.9
 
 
 
 
BTAF1
 
 
hsa-miR-486-5p
 
 
-0.288
 
 
10.1
 
 
 
 
BTBD10
 
 
hsa-miR-17-5p
 
 
-0.409
 
 
16.4
 
 
 
 
BTBD10
 
 
hsa-miR-93-5p
 
 
-0.409
 
 
16.4
 
 
 
 
BTBD10
 
 
hsa-miR-20a-5p
 
 
-0.412
 
 
16.4
 
 
 
 
BTF3L4
 
 
hsa-let-7c-5p
 
 
-0.355
 
 
23.0
 
 
 
 
BTG2
 
 
hsa-miR-92a-3p
 
 
-0.314
 
 
172.4
 
 
 
 
BTG2
 
 
hsa-miR-92a-3p
 
 
-0.402
 
 
172.4
 
 
 
 
BTG3
 
 
hsa-miR-17-5p
 
 
-0.668
 
 
19.3
 
 
 
 
BTG3
 
 
hsa-miR-93-5p
 
 
-0.668
 
 
19.3
 
 
 
 
BTG3
 
 
hsa-miR-20a-5p
 
 
-0.671
 
 
19.3
 
 
 
 
 
 
 skeletal muscle 
 
 
 
 
target_symbol
 
 
mature_mirna_id
 
 
score
 
 
nTPM
 
 
 
 
 
 
AARS1
 
 
hsa-miR-92a-3p
 
 
-0.458
 
 
54.1
 
 
 
 
ABHD17B
 
 
hsa-miR-486-5p
 
 
-0.348
 
 
12.5
 
 
 
 
ABHD5
 
 
hsa-miR-20a-5p
 
 
-0.397
 
 
12.3
 
 
 
 
ABHD5
 
 
hsa-miR-93-5p
 
 
-0.402
 
 
12.3
 
 
 
 
ABHD5
 
 
hsa-miR-17-5p
 
 
-0.45
 
 
12.3
 
 
 
 
ACOX1
 
 
hsa-miR-92a-3p
 
 
-0.321
 
 
29.2
 
 
 
 
ACTA1
 
 
hsa-miR-155-5p
 
 
-0.595
 
 
123974.2
 
 
 
 
ACTC1
 
 
hsa-miR-92a-3p
 
 
-0.361
 
 
519.7
 
 
 
 
ACTC1
 
 
hsa-miR-7-5p
 
 
-0.471
 
 
519.7
 
 
 
 
ADAM9
 
 
hsa-miR-20a-5p
 
 
-0.299
 
 
16.2
 
 
 
 
ADAM9
 
 
hsa-miR-17-5p
 
 
-0.32
 
 
16.2
 
 
 
 
ADAM9
 
 
hsa-miR-93-5p
 
 
-0.32
 
 
16.2
 
 
 
 
ADCY3
 
 
hsa-miR-92a-3p
 
 
-0.381
 
 
14.1
 
 
 
 
ADNP
 
 
hsa-miR-328-3p
 
 
-0.371
 
 
33.8
 
 
 
 
ADRB2
 
 
hsa-let-7c-5p
 
 
-0.635
 
 
10.9
 
 
 
 
AGFG1
 
 
hsa-miR-103a-3p
 
 
-0.389
 
 
31.6
 
 
 
 
AGO4
 
 
hsa-miR-103a-3p
 
 
-0.32
 
 
12.4
 
 
 
 
AGO4
 
 
hsa-miR-221-3p
 
 
-0.321
 
 
12.4
 
 
 
 
AHCTF1
 
 
hsa-let-7c-5p
 
 
-0.374
 
 
10.0
 
 
 
 
AHNAK
 
 
hsa-miR-20a-5p
 
 
-0.316
 
 
178.0
 
 
 
 
AHNAK
 
 
hsa-miR-17-5p
 
 
-0.337
 
 
178.0
 
 
 
 
AHNAK
 
 
hsa-miR-93-5p
 
 
-0.337
 
 
178.0
 
 
 
 
AIFM1
 
 
hsa-let-7c-5p
 
 
-0.314
 
 
58.6
 
 
 
 
AKAP6
 
 
hsa-let-7c-5p
 
 
-0.307
 
 
44.4
 
 
 
 
AKIRIN2
 
 
hsa-miR-103a-3p
 
 
-0.299
 
 
85.6
 
 
 
 
ALAD
 
 
hsa-miR-103a-3p
 
 
-0.385
 
 
26.9
 
 
 
 
ALG3
 
 
hsa-miR-20a-5p
 
 
-0.559
 
 
29.0
 
 
 
 
ALG3
 
 
hsa-miR-93-5p
 
 
-0.564
 
 
29.0
 
 
 
 
ALG3
 
 
hsa-miR-17-5p
 
 
-0.572
 
 
29.0
 
 
 
 
ANGPTL2
 
 
hsa-miR-221-3p
 
 
-0.369
 
 
55.7
 
 
 
 
ANK1
 
 
hsa-miR-103a-3p
 
 
-0.321
 
 
922.8
 
 
 
 
ANKFY1
 
 
hsa-miR-155-5p
 
 
-0.315
 
 
13.2
 
 
 
 
ANKRD12
 
 
hsa-miR-7-5p
 
 
-0.37
 
 
25.1
 
 
 
 
ANKRD46
 
 
hsa-let-7c-5p
 
 
-0.389
 
 
58.3
 
 
 
 
ANKRD9
 
 
hsa-miR-20a-5p
 
 
-0.31
 
 
148.8
 
 
 
 
ANKRD9
 
 
hsa-miR-93-5p
 
 
-0.331
 
 
148.8
 
 
 
 
ANKRD9
 
 
hsa-miR-17-5p
 
 
-0.347
 
 
148.8
 
 
 
 
ANP32E
 
 
hsa-miR-92a-3p
 
 
-0.329
 
 
46.6
 
 
 
 
AP4B1
 
 
hsa-miR-378a-3p
 
 
-0.62
 
 
11.0
 
 
 
 
AP5S1
 
 
hsa-miR-744-5p
 
 
-0.354
 
 
17.9
 
 
 
 
APPL1
 
 
hsa-miR-92a-3p
 
 
-0.375
 
 
41.3
 
 
 
 
ARF4
 
 
hsa-miR-7-5p
 
 
-0.407
 
 
59.5
 
 
 
 
ARF4
 
 
hsa-miR-221-3p
 
 
-0.553
 
 
59.5
 
 
 
 
ARG2
 
 
hsa-let-7c-5p
 
 
-0.468
 
 
37.4
 
 
 
 
ARHGAP1
 
 
hsa-miR-17-5p
 
 
-0.325
 
 
28.5
 
 
 
 
ARHGAP1
 
 
hsa-miR-93-5p
 
 
-0.325
 
 
28.5
 
 
 
 
ARHGAP1
 
 
hsa-miR-20a-5p
 
 
-0.339
 
 
28.5
 
 
 
 
ARHGAP5
 
 
hsa-miR-486-5p
 
 
-0.3
 
 
11.9
 
 
 
 
ARIH2
 
 
hsa-miR-103a-3p
 
 
-0.307
 
 
94.1
 
 
 
 
ARL1
 
 
hsa-miR-20a-5p
 
 
-0.403
 
 
48.7
 
 
 
 
ARL1
 
 
hsa-miR-93-5p
 
 
-0.424
 
 
48.7
 
 
 
 
ARL1
 
 
hsa-miR-17-5p
 
 
-0.432
 
 
48.7
 
 
 
 
ARL6IP1
 
 
hsa-miR-7-5p
 
 
-0.374
 
 
21.6
 
 
 
 
ARL6IP1
 
 
hsa-miR-328-3p
 
 
-0.494
 
 
21.6
 
 
 
 
ARL6IP6
 
 
hsa-let-7c-5p
 
 
-0.293
 
 
53.9
 
 
 
 
ARL8A
 
 
hsa-miR-103a-3p
 
 
-0.38
 
 
44.5
 
 
 
 
ARMC1
 
 
hsa-miR-92a-3p
 
 
-0.322
 
 
17.9
 
 
 
 
ARMC1
 
 
hsa-miR-103a-3p
 
 
-0.525
 
 
17.9
 
 
 
 
ARMC8
 
 
hsa-miR-486-5p
 
 
-0.321
 
 
36.6
 
 
 
 
ARMC8
 
 
hsa-miR-20a-5p
 
 
-0.357
 
 
36.6
 
 
 
 
ARMC8
 
 
hsa-miR-93-5p
 
 
-0.362
 
 
36.6
 
 
 
 
ARMC8
 
 
hsa-miR-17-5p
 
 
-0.378
 
 
36.6
 
 
 
 
ARMC8
 
 
hsa-miR-103a-3p
 
 
-0.412
 
 
36.6
 
 
 
 
ARMT1
 
 
hsa-let-7c-5p
 
 
-0.303
 
 
17.8
 
 
 
 
ARNT
 
 
hsa-miR-103a-3p
 
 
-0.422
 
 
30.3
 
 
 
 
ARNT
 
 
hsa-miR-221-3p
 
 
-0.465
 
 
30.3
 
 
 
 
ARPP19
 
 
hsa-miR-328-3p
 
 
-0.404
 
 
225.3
 
 
 
 
ARPP19
 
 
hsa-let-7c-5p
 
 
-0.492
 
 
225.3
 
 
 
 
ARRDC3
 
 
hsa-miR-92a-3p
 
 
-0.458
 
 
31.8
 
 
 
 
ARRDC4
 
 
hsa-let-7c-5p
 
 
-0.329
 
 
13.8
 
 
 
 
ASAP1
 
 
hsa-let-7c-5p
 
 
-0.308
 
 
12.4
 
 
 
 
ASPH
 
 
hsa-miR-221-3p
 
 
-0.287
 
 
331.0
 
 
 
 
ASPH
 
 
hsa-miR-92a-3p
 
 
-0.416
 
 
331.0
 
 
 
 
ATF3
 
 
hsa-miR-874-3p
 
 
-0.288
 
 
30.3
 
 
 
 
ATG16L1
 
 
hsa-miR-20a-5p
 
 
-0.446
 
 
20.1
 
 
 
 
ATG16L1
 
 
hsa-miR-93-5p
 
 
-0.451
 
 
20.1
 
 
 
 
ATG16L1
 
 
hsa-miR-17-5p
 
 
-0.467
 
 
20.1
 
 
 
 
ATG4A
 
 
hsa-miR-7-5p
 
 
-0.448
 
 
18.0
 
 
 
 
ATL2
 
 
hsa-miR-103a-3p
 
 
-0.489
 
 
49.2
 
 
 
 
ATOSA
 
 
hsa-miR-221-3p
 
 
-0.495
 
 
42.5
 
 
 
 
ATOSB
 
 
hsa-miR-328-3p
 
 
-0.358
 
 
13.5
 
 
 
 
ATOSB
 
 
hsa-let-7c-5p
 
 
-0.429
 
 
13.5
 
 
 
 
ATP2A2
 
 
hsa-let-7c-5p
 
 
-0.311
 
 
2675.7
 
 
 
 
ATP2B2
 
 
hsa-miR-7-5p
 
 
-0.297
 
 
35.1
 
 
 
 
ATP6V1F
 
 
hsa-miR-185-5p
 
 
-0.765
 
 
162.2
 
 
 
 
ATPAF1
 
 
hsa-let-7c-5p
 
 
-0.498
 
 
260.2
 
 
 
 
ATXN1
 
 
hsa-miR-221-3p
 
 
-0.289
 
 
13.6
 
 
 
 
AVPI1
 
 
hsa-miR-7-5p
 
 
-0.367
 
 
13.0
 
 
 
 
B3GAT3
 
 
hsa-let-7c-5p
 
 
-0.324
 
 
15.7
 
 
 
 
B4GALT5
 
 
hsa-miR-210-3p
 
 
-0.511
 
 
63.3
 
 
 
 
B4GAT1
 
 
hsa-let-7c-5p
 
 
-0.47
 
 
27.1
 
 
 
 
BACE1
 
 
hsa-miR-339-5p
 
 
-0.371
 
 
11.0
 
 
 
 
BACH1
 
 
hsa-let-7c-5p
 
 
-0.306
 
 
24.7
 
 
 
 
BACH1
 
 
hsa-let-7c-5p
 
 
-0.306
 
 
24.7
 
 
 
 
BACH1
 
 
hsa-let-7c-5p
 
 
-0.435
 
 
24.7
 
 
 
 
BACH1
 
 
hsa-let-7c-5p
 
 
-0.435
 
 
24.7
 
 
 
 
BAK1
 
 
hsa-miR-103a-3p
 
 
-0.364
 
 
17.7
 
 
 
 
BBC3
 
 
hsa-miR-221-3p
 
 
-0.375
 
 
10.1
 
 
 
 
BCAT2
 
 
hsa-miR-103a-3p
 
 
-0.364
 
 
55.5
 
 
 
 
BCAT2
 
 
hsa-miR-92a-3p
 
 
-0.609
 
 
55.5
 
 
 
 
 
 
 skin 
 
 
 
 
target_symbol
 
 
mature_mirna_id
 
 
score
 
 
nTPM
 
 
 
 
 
 
AARS1
 
 
hsa-miR-92a-3p
 
 
-0.458
 
 
21.2
 
 
 
 
ABHD17C
 
 
hsa-let-7c-5p
 
 
-0.359
 
 
17.9
 
 
 
 
ABHD17C
 
 
hsa-miR-330-3p
 
 
-0.399
 
 
17.9
 
 
 
 
ABHD5
 
 
hsa-miR-20a-5p
 
 
-0.397
 
 
29.7
 
 
 
 
ABHD5
 
 
hsa-miR-93-5p
 
 
-0.402
 
 
29.7
 
 
 
 
ABHD5
 
 
hsa-miR-17-5p
 
 
-0.45
 
 
29.7
 
 
 
 
ACOX1
 
 
hsa-miR-92a-3p
 
 
-0.321
 
 
22.1
 
 
 
 
ACP3
 
 
hsa-let-7c-5p
 
 
-0.298
 
 
25.5
 
 
 
 
ACTA1
 
 
hsa-miR-155-5p
 
 
-0.595
 
 
18.7
 
 
 
 
ACTC1
 
 
hsa-miR-92a-3p
 
 
-0.361
 
 
14.2
 
 
 
 
ACTC1
 
 
hsa-miR-7-5p
 
 
-0.471
 
 
14.2
 
 
 
 
ADCY3
 
 
hsa-miR-92a-3p
 
 
-0.381
 
 
36.0
 
 
 
 
ADNP
 
 
hsa-miR-328-3p
 
 
-0.371
 
 
33.2
 
 
 
 
ADRB2
 
 
hsa-let-7c-5p
 
 
-0.635
 
 
36.9
 
 
 
 
AGFG1
 
 
hsa-miR-103a-3p
 
 
-0.389
 
 
34.3
 
 
 
 
AGFG2
 
 
hsa-miR-20a-5p
 
 
-0.365
 
 
24.5
 
 
 
 
AGFG2
 
 
hsa-miR-93-5p
 
 
-0.378
 
 
24.5
 
 
 
 
AGFG2
 
 
hsa-miR-17-5p
 
 
-0.402
 
 
24.5
 
 
 
 
AGO4
 
 
hsa-miR-103a-3p
 
 
-0.32
 
 
18.9
 
 
 
 
AGO4
 
 
hsa-miR-221-3p
 
 
-0.321
 
 
18.9
 
 
 
 
AHNAK
 
 
hsa-miR-20a-5p
 
 
-0.316
 
 
316.1
 
 
 
 
AHNAK
 
 
hsa-miR-17-5p
 
 
-0.337
 
 
316.1
 
 
 
 
AHNAK
 
 
hsa-miR-93-5p
 
 
-0.337
 
 
316.1
 
 
 
 
AIFM1
 
 
hsa-let-7c-5p
 
 
-0.314
 
 
10.3
 
 
 
 
AJUBA
 
 
hsa-miR-103a-3p
 
 
-0.448
 
 
58.2
 
 
 
 
AKIRIN2
 
 
hsa-miR-103a-3p
 
 
-0.299
 
 
20.2
 
 
 
 
ALAD
 
 
hsa-miR-103a-3p
 
 
-0.385
 
 
36.8
 
 
 
 
ALG3
 
 
hsa-miR-20a-5p
 
 
-0.559
 
 
23.7
 
 
 
 
ALG3
 
 
hsa-miR-93-5p
 
 
-0.564
 
 
23.7
 
 
 
 
ALG3
 
 
hsa-miR-17-5p
 
 
-0.572
 
 
23.7
 
 
 
 
ALG9
 
 
hsa-miR-7-5p
 
 
-0.302
 
 
13.9
 
 
 
 
ALKAL2
 
 
hsa-miR-92a-3p
 
 
-0.608
 
 
16.9
 
 
 
 
ANGPTL2
 
 
hsa-miR-221-3p
 
 
-0.369
 
 
27.9
 
 
 
 
ANKFY1
 
 
hsa-miR-155-5p
 
 
-0.315
 
 
18.7
 
 
 
 
ANKRD12
 
 
hsa-miR-7-5p
 
 
-0.37
 
 
19.1
 
 
 
 
ANKRD46
 
 
hsa-let-7c-5p
 
 
-0.389
 
 
10.5
 
 
 
 
ANKRD50
 
 
hsa-miR-20a-5p
 
 
-0.3
 
 
10.4
 
 
 
 
ANKRD9
 
 
hsa-miR-20a-5p
 
 
-0.31
 
 
24.3
 
 
 
 
ANKRD9
 
 
hsa-miR-93-5p
 
 
-0.331
 
 
24.3
 
 
 
 
ANKRD9
 
 
hsa-miR-17-5p
 
 
-0.347
 
 
24.3
 
 
 
 
ANP32E
 
 
hsa-miR-92a-3p
 
 
-0.329
 
 
20.4
 
 
 
 
AP1AR
 
 
hsa-miR-92a-3p
 
 
-0.325
 
 
13.4
 
 
 
 
AP1S1
 
 
hsa-let-7c-5p
 
 
-0.374
 
 
40.6
 
 
 
 
AP4B1
 
 
hsa-miR-378a-3p
 
 
-0.62
 
 
10.9
 
 
 
 
APCDD1
 
 
hsa-miR-20a-5p
 
 
-0.404
 
 
122.7
 
 
 
 
APCDD1
 
 
hsa-miR-17-5p
 
 
-0.425
 
 
122.7
 
 
 
 
APCDD1
 
 
hsa-miR-93-5p
 
 
-0.425
 
 
122.7
 
 
 
 
APPL1
 
 
hsa-miR-92a-3p
 
 
-0.375
 
 
19.0
 
 
 
 
AQP3
 
 
hsa-miR-874-3p
 
 
-0.48
 
 
554.4
 
 
 
 
ARF4
 
 
hsa-miR-7-5p
 
 
-0.407
 
 
61.3
 
 
 
 
ARF4
 
 
hsa-miR-221-3p
 
 
-0.553
 
 
61.3
 
 
 
 
ARHGAP1
 
 
hsa-miR-17-5p
 
 
-0.325
 
 
61.6
 
 
 
 
ARHGAP1
 
 
hsa-miR-93-5p
 
 
-0.325
 
 
61.6
 
 
 
 
ARHGAP1
 
 
hsa-miR-20a-5p
 
 
-0.339
 
 
61.6
 
 
 
 
ARHGAP5
 
 
hsa-miR-486-5p
 
 
-0.3
 
 
29.1
 
 
 
 
ARHGEF28
 
 
hsa-miR-93-5p
 
 
-0.33
 
 
14.0
 
 
 
 
ARHGEF28
 
 
hsa-miR-17-5p
 
 
-0.363
 
 
14.0
 
 
 
 
ARHGEF28
 
 
hsa-miR-20a-5p
 
 
-0.366
 
 
14.0
 
 
 
 
ARIH2
 
 
hsa-miR-103a-3p
 
 
-0.307
 
 
15.8
 
 
 
 
ARL1
 
 
hsa-miR-20a-5p
 
 
-0.403
 
 
26.2
 
 
 
 
ARL1
 
 
hsa-miR-93-5p
 
 
-0.424
 
 
26.2
 
 
 
 
ARL1
 
 
hsa-miR-17-5p
 
 
-0.432
 
 
26.2
 
 
 
 
ARL4D
 
 
hsa-let-7c-5p
 
 
-0.417
 
 
44.0
 
 
 
 
ARL6IP1
 
 
hsa-miR-7-5p
 
 
-0.374
 
 
62.4
 
 
 
 
ARL6IP1
 
 
hsa-miR-328-3p
 
 
-0.494
 
 
62.4
 
 
 
 
ARL6IP6
 
 
hsa-let-7c-5p
 
 
-0.293
 
 
23.5
 
 
 
 
ARL8A
 
 
hsa-miR-103a-3p
 
 
-0.38
 
 
35.7
 
 
 
 
ARMC1
 
 
hsa-miR-92a-3p
 
 
-0.322
 
 
10.8
 
 
 
 
ARMC1
 
 
hsa-miR-103a-3p
 
 
-0.525
 
 
10.8
 
 
 
 
ARMC8
 
 
hsa-miR-486-5p
 
 
-0.321
 
 
12.0
 
 
 
 
ARMC8
 
 
hsa-miR-20a-5p
 
 
-0.357
 
 
12.0
 
 
 
 
ARMC8
 
 
hsa-miR-93-5p
 
 
-0.362
 
 
12.0
 
 
 
 
ARMC8
 
 
hsa-miR-17-5p
 
 
-0.378
 
 
12.0
 
 
 
 
ARMC8
 
 
hsa-miR-103a-3p
 
 
-0.412
 
 
12.0
 
 
 
 
ARNT
 
 
hsa-miR-103a-3p
 
 
-0.422
 
 
23.3
 
 
 
 
ARNT
 
 
hsa-miR-221-3p
 
 
-0.465
 
 
23.3
 
 
 
 
ARPP19
 
 
hsa-miR-328-3p
 
 
-0.404
 
 
28.9
 
 
 
 
ARPP19
 
 
hsa-let-7c-5p
 
 
-0.492
 
 
28.9
 
 
 
 
ARRDC3
 
 
hsa-miR-92a-3p
 
 
-0.458
 
 
38.6
 
 
 
 
ARRDC4
 
 
hsa-let-7c-5p
 
 
-0.329
 
 
33.5
 
 
 
 
ASAP1
 
 
hsa-let-7c-5p
 
 
-0.308
 
 
16.2
 
 
 
 
ASB6
 
 
hsa-miR-874-3p
 
 
-0.368
 
 
18.6
 
 
 
 
ASPH
 
 
hsa-miR-221-3p
 
 
-0.287
 
 
33.3
 
 
 
 
ASPH
 
 
hsa-miR-92a-3p
 
 
-0.416
 
 
33.3
 
 
 
 
ASPN
 
 
hsa-miR-92a-3p
 
 
-0.422
 
 
11.4
 
 
 
 
ASPRV1
 
 
hsa-miR-330-3p
 
 
-0.301
 
 
614.8
 
 
 
 
ATF3
 
 
hsa-miR-874-3p
 
 
-0.288
 
 
151.7
 
 
 
 
ATG16L1
 
 
hsa-miR-20a-5p
 
 
-0.446
 
 
17.2
 
 
 
 
ATG16L1
 
 
hsa-miR-93-5p
 
 
-0.451
 
 
17.2
 
 
 
 
ATG16L1
 
 
hsa-miR-17-5p
 
 
-0.467
 
 
17.2
 
 
 
 
ATL2
 
 
hsa-miR-103a-3p
 
 
-0.489
 
 
44.7
 
 
 
 
ATOSA
 
 
hsa-miR-221-3p
 
 
-0.495
 
 
10.0
 
 
 
 
ATOSB
 
 
hsa-miR-328-3p
 
 
-0.358
 
 
14.2
 
 
 
 
ATOSB
 
 
hsa-let-7c-5p
 
 
-0.429
 
 
14.2
 
 
 
 
ATP2A2
 
 
hsa-let-7c-5p
 
 
-0.311
 
 
41.6
 
 
 
 
ATP6V1F
 
 
hsa-miR-185-5p
 
 
-0.765
 
 
171.3
 
 
 
 
ATPAF1
 
 
hsa-let-7c-5p
 
 
-0.498
 
 
18.2
 
 
 
 
ATXN1L
 
 
hsa-miR-155-5p
 
 
-0.315
 
 
11.4
 
 
 
 
ATXN3
 
 
hsa-miR-92a-3p
 
 
-0.391
 
 
12.0
 
 
 
 
AVPI1
 
 
hsa-miR-7-5p
 
 
-0.367
 
 
104.5
 
 
 
 
 
 
 small intestine 
 
 
 
 
target_symbol
 
 
mature_mirna_id
 
 
score
 
 
nTPM
 
 
 
 
 
 
AARS1
 
 
hsa-miR-92a-3p
 
 
-0.458
 
 
24.5
 
 
 
 
ABHD17B
 
 
hsa-miR-486-5p
 
 
-0.348
 
 
12.6
 
 
 
 
ABHD17C
 
 
hsa-let-7c-5p
 
 
-0.359
 
 
28.8
 
 
 
 
ABHD17C
 
 
hsa-miR-330-3p
 
 
-0.399
 
 
28.8
 
 
 
 
ACOX1
 
 
hsa-miR-92a-3p
 
 
-0.321
 
 
29.8
 
 
 
 
ADAM19
 
 
hsa-miR-874-3p
 
 
-0.374
 
 
17.9
 
 
 
 
ADAM9
 
 
hsa-miR-20a-5p
 
 
-0.299
 
 
21.4
 
 
 
 
ADAM9
 
 
hsa-miR-17-5p
 
 
-0.32
 
 
21.4
 
 
 
 
ADAM9
 
 
hsa-miR-93-5p
 
 
-0.32
 
 
21.4
 
 
 
 
ADCY3
 
 
hsa-miR-92a-3p
 
 
-0.381
 
 
31.8
 
 
 
 
ADNP
 
 
hsa-miR-328-3p
 
 
-0.371
 
 
30.1
 
 
 
 
AGFG1
 
 
hsa-miR-103a-3p
 
 
-0.389
 
 
33.9
 
 
 
 
AGFG2
 
 
hsa-miR-20a-5p
 
 
-0.365
 
 
12.5
 
 
 
 
AGFG2
 
 
hsa-miR-93-5p
 
 
-0.378
 
 
12.5
 
 
 
 
AGFG2
 
 
hsa-miR-17-5p
 
 
-0.402
 
 
12.5
 
 
 
 
AHNAK
 
 
hsa-miR-20a-5p
 
 
-0.316
 
 
132.1
 
 
 
 
AHNAK
 
 
hsa-miR-17-5p
 
 
-0.337
 
 
132.1
 
 
 
 
AHNAK
 
 
hsa-miR-93-5p
 
 
-0.337
 
 
132.1
 
 
 
 
AIFM1
 
 
hsa-let-7c-5p
 
 
-0.314
 
 
28.2
 
 
 
 
AKIRIN2
 
 
hsa-miR-103a-3p
 
 
-0.299
 
 
20.2
 
 
 
 
ALAD
 
 
hsa-miR-103a-3p
 
 
-0.385
 
 
25.1
 
 
 
 
ALG3
 
 
hsa-miR-20a-5p
 
 
-0.559
 
 
19.2
 
 
 
 
ALG3
 
 
hsa-miR-93-5p
 
 
-0.564
 
 
19.2
 
 
 
 
ALG3
 
 
hsa-miR-17-5p
 
 
-0.572
 
 
19.2
 
 
 
 
AMT
 
 
hsa-let-7c-5p
 
 
-0.463
 
 
11.5
 
 
 
 
ANGPTL2
 
 
hsa-miR-221-3p
 
 
-0.369
 
 
27.5
 
 
 
 
ANKFY1
 
 
hsa-miR-155-5p
 
 
-0.315
 
 
13.2
 
 
 
 
ANKRD12
 
 
hsa-miR-7-5p
 
 
-0.37
 
 
12.6
 
 
 
 
ANKRD44
 
 
hsa-miR-92a-3p
 
 
-0.324
 
 
13.5
 
 
 
 
ANKRD46
 
 
hsa-let-7c-5p
 
 
-0.389
 
 
10.4
 
 
 
 
ANKRD9
 
 
hsa-miR-20a-5p
 
 
-0.31
 
 
31.1
 
 
 
 
ANKRD9
 
 
hsa-miR-93-5p
 
 
-0.331
 
 
31.1
 
 
 
 
ANKRD9
 
 
hsa-miR-17-5p
 
 
-0.347
 
 
31.1
 
 
 
 
ANP32E
 
 
hsa-miR-92a-3p
 
 
-0.329
 
 
24.2
 
 
 
 
AP1AR
 
 
hsa-miR-92a-3p
 
 
-0.325
 
 
22.4
 
 
 
 
AP1S1
 
 
hsa-let-7c-5p
 
 
-0.374
 
 
29.8
 
 
 
 
AP4B1
 
 
hsa-miR-378a-3p
 
 
-0.62
 
 
12.3
 
 
 
 
APCDD1
 
 
hsa-miR-20a-5p
 
 
-0.404
 
 
13.7
 
 
 
 
APCDD1
 
 
hsa-miR-17-5p
 
 
-0.425
 
 
13.7
 
 
 
 
APCDD1
 
 
hsa-miR-93-5p
 
 
-0.425
 
 
13.7
 
 
 
 
APPL1
 
 
hsa-miR-92a-3p
 
 
-0.375
 
 
13.8
 
 
 
 
AQP11
 
 
hsa-miR-103a-3p
 
 
-0.517
 
 
23.9
 
 
 
 
AQP3
 
 
hsa-miR-874-3p
 
 
-0.48
 
 
94.7
 
 
 
 
ARF4
 
 
hsa-miR-7-5p
 
 
-0.407
 
 
102.8
 
 
 
 
ARF4
 
 
hsa-miR-221-3p
 
 
-0.553
 
 
102.8
 
 
 
 
ARG2
 
 
hsa-let-7c-5p
 
 
-0.468
 
 
14.4
 
 
 
 
ARHGAP1
 
 
hsa-miR-17-5p
 
 
-0.325
 
 
44.5
 
 
 
 
ARHGAP1
 
 
hsa-miR-93-5p
 
 
-0.325
 
 
44.5
 
 
 
 
ARHGAP1
 
 
hsa-miR-20a-5p
 
 
-0.339
 
 
44.5
 
 
 
 
ARHGAP5
 
 
hsa-miR-486-5p
 
 
-0.3
 
 
36.7
 
 
 
 
ARIH2
 
 
hsa-miR-103a-3p
 
 
-0.307
 
 
20.5
 
 
 
 
ARL1
 
 
hsa-miR-20a-5p
 
 
-0.403
 
 
40.7
 
 
 
 
ARL1
 
 
hsa-miR-93-5p
 
 
-0.424
 
 
40.7
 
 
 
 
ARL1
 
 
hsa-miR-17-5p
 
 
-0.432
 
 
40.7
 
 
 
 
ARL6IP1
 
 
hsa-miR-7-5p
 
 
-0.374
 
 
116.2
 
 
 
 
ARL6IP1
 
 
hsa-miR-328-3p
 
 
-0.494
 
 
116.2
 
 
 
 
ARL8A
 
 
hsa-miR-103a-3p
 
 
-0.38
 
 
25.2
 
 
 
 
ARMC1
 
 
hsa-miR-92a-3p
 
 
-0.322
 
 
12.9
 
 
 
 
ARMC1
 
 
hsa-miR-103a-3p
 
 
-0.525
 
 
12.9
 
 
 
 
ARMC8
 
 
hsa-miR-486-5p
 
 
-0.321
 
 
12.8
 
 
 
 
ARMC8
 
 
hsa-miR-20a-5p
 
 
-0.357
 
 
12.8
 
 
 
 
ARMC8
 
 
hsa-miR-93-5p
 
 
-0.362
 
 
12.8
 
 
 
 
ARMC8
 
 
hsa-miR-17-5p
 
 
-0.378
 
 
12.8
 
 
 
 
ARMC8
 
 
hsa-miR-103a-3p
 
 
-0.412
 
 
12.8
 
 
 
 
ARMT1
 
 
hsa-let-7c-5p
 
 
-0.303
 
 
13.7
 
 
 
 
ARNT
 
 
hsa-miR-103a-3p
 
 
-0.422
 
 
16.2
 
 
 
 
ARNT
 
 
hsa-miR-221-3p
 
 
-0.465
 
 
16.2
 
 
 
 
ARPP19
 
 
hsa-miR-328-3p
 
 
-0.404
 
 
28.8
 
 
 
 
ARPP19
 
 
hsa-let-7c-5p
 
 
-0.492
 
 
28.8
 
 
 
 
ARRDC3
 
 
hsa-miR-92a-3p
 
 
-0.458
 
 
15.8
 
 
 
 
ARRDC4
 
 
hsa-let-7c-5p
 
 
-0.329
 
 
22.0
 
 
 
 
ASAP1
 
 
hsa-let-7c-5p
 
 
-0.308
 
 
11.5
 
 
 
 
ASB6
 
 
hsa-miR-874-3p
 
 
-0.368
 
 
11.3
 
 
 
 
ASPH
 
 
hsa-miR-221-3p
 
 
-0.287
 
 
42.6
 
 
 
 
ASPH
 
 
hsa-miR-92a-3p
 
 
-0.416
 
 
42.6
 
 
 
 
ASPN
 
 
hsa-miR-92a-3p
 
 
-0.422
 
 
16.6
 
 
 
 
ATF3
 
 
hsa-miR-874-3p
 
 
-0.288
 
 
42.8
 
 
 
 
ATG16L1
 
 
hsa-miR-20a-5p
 
 
-0.446
 
 
11.3
 
 
 
 
ATG16L1
 
 
hsa-miR-93-5p
 
 
-0.451
 
 
11.3
 
 
 
 
ATG16L1
 
 
hsa-miR-17-5p
 
 
-0.467
 
 
11.3
 
 
 
 
ATG4A
 
 
hsa-miR-7-5p
 
 
-0.448
 
 
10.7
 
 
 
 
ATL2
 
 
hsa-miR-103a-3p
 
 
-0.489
 
 
29.6
 
 
 
 
ATOSA
 
 
hsa-miR-221-3p
 
 
-0.495
 
 
11.7
 
 
 
 
ATOSB
 
 
hsa-miR-328-3p
 
 
-0.358
 
 
10.7
 
 
 
 
ATOSB
 
 
hsa-let-7c-5p
 
 
-0.429
 
 
10.7
 
 
 
 
ATP2A2
 
 
hsa-let-7c-5p
 
 
-0.311
 
 
53.2
 
 
 
 
ATP6V1F
 
 
hsa-miR-185-5p
 
 
-0.765
 
 
96.4
 
 
 
 
ATPAF1
 
 
hsa-let-7c-5p
 
 
-0.498
 
 
42.6
 
 
 
 
AVPI1
 
 
hsa-miR-7-5p
 
 
-0.367
 
 
15.7
 
 
 
 
B3GAT3
 
 
hsa-let-7c-5p
 
 
-0.324
 
 
30.1
 
 
 
 
B4GALT5
 
 
hsa-miR-210-3p
 
 
-0.511
 
 
20.1
 
 
 
 
B4GAT1
 
 
hsa-let-7c-5p
 
 
-0.47
 
 
14.2
 
 
 
 
BACH1
 
 
hsa-let-7c-5p
 
 
-0.306
 
 
16.3
 
 
 
 
BACH1
 
 
hsa-let-7c-5p
 
 
-0.306
 
 
16.3
 
 
 
 
BACH1
 
 
hsa-let-7c-5p
 
 
-0.435
 
 
16.3
 
 
 
 
BACH1
 
 
hsa-let-7c-5p
 
 
-0.435
 
 
16.3
 
 
 
 
BAK1
 
 
hsa-miR-103a-3p
 
 
-0.364
 
 
34.9
 
 
 
 
BCAT2
 
 
hsa-miR-103a-3p
 
 
-0.364
 
 
22.2
 
 
 
 
BCAT2
 
 
hsa-miR-92a-3p
 
 
-0.609
 
 
22.2
 
 
 
 
BCL2L11
 
 
hsa-miR-339-5p
 
 
-0.306
 
 
11.3
 
 
 
 
 
 
 smooth muscle 
 
 
 
 
target_symbol
 
 
mature_mirna_id
 
 
score
 
 
nTPM
 
 
 
 
 
 
AARS1
 
 
hsa-miR-92a-3p
 
 
-0.458
 
 
44.7
 
 
 
 
ACTA1
 
 
hsa-miR-155-5p
 
 
-0.595
 
 
61.8
 
 
 
 
ADAM19
 
 
hsa-miR-874-3p
 
 
-0.374
 
 
15.3
 
 
 
 
ADAM9
 
 
hsa-miR-20a-5p
 
 
-0.299
 
 
48.2
 
 
 
 
ADAM9
 
 
hsa-miR-17-5p
 
 
-0.32
 
 
48.2
 
 
 
 
ADAM9
 
 
hsa-miR-93-5p
 
 
-0.32
 
 
48.2
 
 
 
 
ADAMTSL1
 
 
hsa-miR-92a-3p
 
 
-0.296
 
 
10.4
 
 
 
 
ADCY3
 
 
hsa-miR-92a-3p
 
 
-0.381
 
 
33.0
 
 
 
 
ADNP
 
 
hsa-miR-328-3p
 
 
-0.371
 
 
27.7
 
 
 
 
AGFG1
 
 
hsa-miR-103a-3p
 
 
-0.389
 
 
36.7
 
 
 
 
AHNAK
 
 
hsa-miR-20a-5p
 
 
-0.316
 
 
105.6
 
 
 
 
AHNAK
 
 
hsa-miR-17-5p
 
 
-0.337
 
 
105.6
 
 
 
 
AHNAK
 
 
hsa-miR-93-5p
 
 
-0.337
 
 
105.6
 
 
 
 
AKAP10
 
 
hsa-miR-339-5p
 
 
-0.302
 
 
12.4
 
 
 
 
AKIRIN2
 
 
hsa-miR-103a-3p
 
 
-0.299
 
 
15.3
 
 
 
 
ALAD
 
 
hsa-miR-103a-3p
 
 
-0.385
 
 
16.4
 
 
 
 
ALG3
 
 
hsa-miR-20a-5p
 
 
-0.559
 
 
15.3
 
 
 
 
ALG3
 
 
hsa-miR-93-5p
 
 
-0.564
 
 
15.3
 
 
 
 
ALG3
 
 
hsa-miR-17-5p
 
 
-0.572
 
 
15.3
 
 
 
 
ALG9
 
 
hsa-miR-7-5p
 
 
-0.302
 
 
10.4
 
 
 
 
ANGPTL2
 
 
hsa-miR-221-3p
 
 
-0.369
 
 
59.2
 
 
 
 
ANKFY1
 
 
hsa-miR-155-5p
 
 
-0.315
 
 
13.8
 
 
 
 
ANKRD29
 
 
hsa-miR-20a-5p
 
 
-0.359
 
 
10.9
 
 
 
 
ANKRD29
 
 
hsa-miR-17-5p
 
 
-0.372
 
 
10.9
 
 
 
 
ANKRD29
 
 
hsa-miR-93-5p
 
 
-0.372
 
 
10.9
 
 
 
 
ANKRD46
 
 
hsa-let-7c-5p
 
 
-0.389
 
 
11.6
 
 
 
 
ANP32E
 
 
hsa-miR-92a-3p
 
 
-0.329
 
 
19.6
 
 
 
 
AP1S1
 
 
hsa-let-7c-5p
 
 
-0.374
 
 
11.9
 
 
 
 
APPL1
 
 
hsa-miR-92a-3p
 
 
-0.375
 
 
14.3
 
 
 
 
ARF4
 
 
hsa-miR-7-5p
 
 
-0.407
 
 
157.9
 
 
 
 
ARF4
 
 
hsa-miR-221-3p
 
 
-0.553
 
 
157.9
 
 
 
 
ARHGAP1
 
 
hsa-miR-17-5p
 
 
-0.325
 
 
75.2
 
 
 
 
ARHGAP1
 
 
hsa-miR-93-5p
 
 
-0.325
 
 
75.2
 
 
 
 
ARHGAP1
 
 
hsa-miR-20a-5p
 
 
-0.339
 
 
75.2
 
 
 
 
ARHGAP5
 
 
hsa-miR-486-5p
 
 
-0.3
 
 
22.0
 
 
 
 
ARIH2
 
 
hsa-miR-103a-3p
 
 
-0.307
 
 
22.7
 
 
 
 
ARL1
 
 
hsa-miR-20a-5p
 
 
-0.403
 
 
55.7
 
 
 
 
ARL1
 
 
hsa-miR-93-5p
 
 
-0.424
 
 
55.7
 
 
 
 
ARL1
 
 
hsa-miR-17-5p
 
 
-0.432
 
 
55.7
 
 
 
 
ARL4D
 
 
hsa-let-7c-5p
 
 
-0.417
 
 
13.8
 
 
 
 
ARL6IP1
 
 
hsa-miR-7-5p
 
 
-0.374
 
 
64.5
 
 
 
 
ARL6IP1
 
 
hsa-miR-328-3p
 
 
-0.494
 
 
64.5
 
 
 
 
ARL6IP6
 
 
hsa-let-7c-5p
 
 
-0.293
 
 
10.8
 
 
 
 
ARL8A
 
 
hsa-miR-103a-3p
 
 
-0.38
 
 
20.3
 
 
 
 
ARMC1
 
 
hsa-miR-92a-3p
 
 
-0.322
 
 
20.1
 
 
 
 
ARMC1
 
 
hsa-miR-103a-3p
 
 
-0.525
 
 
20.1
 
 
 
 
ARMC8
 
 
hsa-miR-486-5p
 
 
-0.321
 
 
22.8
 
 
 
 
ARMC8
 
 
hsa-miR-20a-5p
 
 
-0.357
 
 
22.8
 
 
 
 
ARMC8
 
 
hsa-miR-93-5p
 
 
-0.362
 
 
22.8
 
 
 
 
ARMC8
 
 
hsa-miR-17-5p
 
 
-0.378
 
 
22.8
 
 
 
 
ARMC8
 
 
hsa-miR-103a-3p
 
 
-0.412
 
 
22.8
 
 
 
 
ARMT1
 
 
hsa-let-7c-5p
 
 
-0.303
 
 
13.9
 
 
 
 
ARNT
 
 
hsa-miR-103a-3p
 
 
-0.422
 
 
27.1
 
 
 
 
ARNT
 
 
hsa-miR-221-3p
 
 
-0.465
 
 
27.1
 
 
 
 
ARPP19
 
 
hsa-miR-328-3p
 
 
-0.404
 
 
54.2
 
 
 
 
ARPP19
 
 
hsa-let-7c-5p
 
 
-0.492
 
 
54.2
 
 
 
 
ARRDC3
 
 
hsa-miR-92a-3p
 
 
-0.458
 
 
30.8
 
 
 
 
ARRDC4
 
 
hsa-let-7c-5p
 
 
-0.329
 
 
16.9
 
 
 
 
ASAP1
 
 
hsa-let-7c-5p
 
 
-0.308
 
 
16.7
 
 
 
 
ASPH
 
 
hsa-miR-221-3p
 
 
-0.287
 
 
62.4
 
 
 
 
ASPH
 
 
hsa-miR-92a-3p
 
 
-0.416
 
 
62.4
 
 
 
 
ASPN
 
 
hsa-miR-92a-3p
 
 
-0.422
 
 
103.1
 
 
 
 
ATF3
 
 
hsa-miR-874-3p
 
 
-0.288
 
 
291.8
 
 
 
 
ATL2
 
 
hsa-miR-103a-3p
 
 
-0.489
 
 
33.5
 
 
 
 
ATOSA
 
 
hsa-miR-221-3p
 
 
-0.495
 
 
11.6
 
 
 
 
ATOSB
 
 
hsa-miR-328-3p
 
 
-0.358
 
 
10.3
 
 
 
 
ATOSB
 
 
hsa-let-7c-5p
 
 
-0.429
 
 
10.3
 
 
 
 
ATP2A2
 
 
hsa-let-7c-5p
 
 
-0.311
 
 
69.4
 
 
 
 
ATP6V1F
 
 
hsa-miR-185-5p
 
 
-0.765
 
 
66.5
 
 
 
 
ATPAF1
 
 
hsa-let-7c-5p
 
 
-0.498
 
 
30.4
 
 
 
 
ATXN1L
 
 
hsa-miR-155-5p
 
 
-0.315
 
 
12.0
 
 
 
 
B3GAT3
 
 
hsa-let-7c-5p
 
 
-0.324
 
 
13.1
 
 
 
 
B4GALT5
 
 
hsa-miR-210-3p
 
 
-0.511
 
 
35.1
 
 
 
 
B4GAT1
 
 
hsa-let-7c-5p
 
 
-0.47
 
 
13.1
 
 
 
 
BACE1
 
 
hsa-miR-339-5p
 
 
-0.371
 
 
19.5
 
 
 
 
BACH1
 
 
hsa-let-7c-5p
 
 
-0.306
 
 
14.9
 
 
 
 
BACH1
 
 
hsa-let-7c-5p
 
 
-0.306
 
 
14.9
 
 
 
 
BACH1
 
 
hsa-let-7c-5p
 
 
-0.435
 
 
14.9
 
 
 
 
BACH1
 
 
hsa-let-7c-5p
 
 
-0.435
 
 
14.9
 
 
 
 
BAK1
 
 
hsa-miR-103a-3p
 
 
-0.364
 
 
12.6
 
 
 
 
BAMBI
 
 
hsa-miR-20a-5p
 
 
-0.485
 
 
11.4
 
 
 
 
BAMBI
 
 
hsa-miR-17-5p
 
 
-0.506
 
 
11.4
 
 
 
 
BAMBI
 
 
hsa-miR-93-5p
 
 
-0.506
 
 
11.4
 
 
 
 
BCAT2
 
 
hsa-miR-103a-3p
 
 
-0.364
 
 
12.6
 
 
 
 
BCAT2
 
 
hsa-miR-92a-3p
 
 
-0.609
 
 
12.6
 
 
 
 
BIN1
 
 
hsa-miR-744-5p
 
 
-0.654
 
 
18.3
 
 
 
 
BLOC1S6
 
 
hsa-let-7c-5p
 
 
-0.321
 
 
22.7
 
 
 
 
BLTP1
 
 
hsa-miR-92a-3p
 
 
-0.462
 
 
13.4
 
 
 
 
BNIP3L
 
 
hsa-miR-20a-5p
 
 
-0.287
 
 
47.9
 
 
 
 
BOLA3
 
 
hsa-miR-103a-3p
 
 
-0.362
 
 
21.1
 
 
 
 
BSDC1
 
 
hsa-miR-92a-3p
 
 
-0.362
 
 
19.3
 
 
 
 
BTAF1
 
 
hsa-miR-486-5p
 
 
-0.288
 
 
11.0
 
 
 
 
BTBD10
 
 
hsa-miR-17-5p
 
 
-0.409
 
 
19.8
 
 
 
 
BTBD10
 
 
hsa-miR-93-5p
 
 
-0.409
 
 
19.8
 
 
 
 
BTBD10
 
 
hsa-miR-20a-5p
 
 
-0.412
 
 
19.8
 
 
 
 
BTF3L4
 
 
hsa-let-7c-5p
 
 
-0.355
 
 
32.5
 
 
 
 
BTG2
 
 
hsa-miR-92a-3p
 
 
-0.314
 
 
199.6
 
 
 
 
BTG2
 
 
hsa-miR-92a-3p
 
 
-0.402
 
 
199.6
 
 
 
 
BTG3
 
 
hsa-miR-17-5p
 
 
-0.668
 
 
26.6
 
 
 
 
BTG3
 
 
hsa-miR-93-5p
 
 
-0.668
 
 
26.6
 
 
 
 
 
 
 spinal cord 
 
 
 
 
target_symbol
 
 
mature_mirna_id
 
 
score
 
 
nTPM
 
 
 
 
 
 
AARS1
 
 
hsa-miR-92a-3p
 
 
-0.458
 
 
62.9
 
 
 
 
ABHD17B
 
 
hsa-miR-486-5p
 
 
-0.348
 
 
26.2
 
 
 
 
ABHD5
 
 
hsa-miR-20a-5p
 
 
-0.397
 
 
19.5
 
 
 
 
ABHD5
 
 
hsa-miR-93-5p
 
 
-0.402
 
 
19.5
 
 
 
 
ABHD5
 
 
hsa-miR-17-5p
 
 
-0.45
 
 
19.5
 
 
 
 
ADAM9
 
 
hsa-miR-20a-5p
 
 
-0.299
 
 
11.3
 
 
 
 
ADAM9
 
 
hsa-miR-17-5p
 
 
-0.32
 
 
11.3
 
 
 
 
ADAM9
 
 
hsa-miR-93-5p
 
 
-0.32
 
 
11.3
 
 
 
 
ADCY3
 
 
hsa-miR-92a-3p
 
 
-0.381
 
 
26.0
 
 
 
 
ADNP
 
 
hsa-miR-328-3p
 
 
-0.371
 
 
22.3
 
 
 
 
AGFG1
 
 
hsa-miR-103a-3p
 
 
-0.389
 
 
53.5
 
 
 
 
AHNAK
 
 
hsa-miR-20a-5p
 
 
-0.316
 
 
27.2
 
 
 
 
AHNAK
 
 
hsa-miR-17-5p
 
 
-0.337
 
 
27.2
 
 
 
 
AHNAK
 
 
hsa-miR-93-5p
 
 
-0.337
 
 
27.2
 
 
 
 
AIFM1
 
 
hsa-let-7c-5p
 
 
-0.314
 
 
10.1
 
 
 
 
AKAP6
 
 
hsa-let-7c-5p
 
 
-0.307
 
 
13.3
 
 
 
 
AKIRIN2
 
 
hsa-miR-103a-3p
 
 
-0.299
 
 
29.7
 
 
 
 
ALAD
 
 
hsa-miR-103a-3p
 
 
-0.385
 
 
110.8
 
 
 
 
ALG3
 
 
hsa-miR-20a-5p
 
 
-0.559
 
 
10.8
 
 
 
 
ALG3
 
 
hsa-miR-93-5p
 
 
-0.564
 
 
10.8
 
 
 
 
ALG3
 
 
hsa-miR-17-5p
 
 
-0.572
 
 
10.8
 
 
 
 
ALKAL2
 
 
hsa-miR-92a-3p
 
 
-0.608
 
 
12.2
 
 
 
 
ANGPTL2
 
 
hsa-miR-221-3p
 
 
-0.369
 
 
15.5
 
 
 
 
ANKFY1
 
 
hsa-miR-155-5p
 
 
-0.315
 
 
14.2
 
 
 
 
ANKRD46
 
 
hsa-let-7c-5p
 
 
-0.389
 
 
24.6
 
 
 
 
ANP32E
 
 
hsa-miR-92a-3p
 
 
-0.329
 
 
32.2
 
 
 
 
AP1S1
 
 
hsa-let-7c-5p
 
 
-0.374
 
 
28.5
 
 
 
 
APCDD1
 
 
hsa-miR-20a-5p
 
 
-0.404
 
 
14.7
 
 
 
 
APCDD1
 
 
hsa-miR-17-5p
 
 
-0.425
 
 
14.7
 
 
 
 
APCDD1
 
 
hsa-miR-93-5p
 
 
-0.425
 
 
14.7
 
 
 
 
ARF4
 
 
hsa-miR-7-5p
 
 
-0.407
 
 
69.7
 
 
 
 
ARF4
 
 
hsa-miR-221-3p
 
 
-0.553
 
 
69.7
 
 
 
 
ARHGAP1
 
 
hsa-miR-17-5p
 
 
-0.325
 
 
38.6
 
 
 
 
ARHGAP1
 
 
hsa-miR-93-5p
 
 
-0.325
 
 
38.6
 
 
 
 
ARHGAP1
 
 
hsa-miR-20a-5p
 
 
-0.339
 
 
38.6
 
 
 
 
ARHGAP5
 
 
hsa-miR-486-5p
 
 
-0.3
 
 
21.0
 
 
 
 
ARID4B
 
 
hsa-miR-20a-5p
 
 
-0.29
 
 
12.3
 
 
 
 
ARID4B
 
 
hsa-miR-486-5p
 
 
-0.367
 
 
12.3
 
 
 
 
ARIH2
 
 
hsa-miR-103a-3p
 
 
-0.307
 
 
22.5
 
 
 
 
ARL1
 
 
hsa-miR-20a-5p
 
 
-0.403
 
 
26.5
 
 
 
 
ARL1
 
 
hsa-miR-93-5p
 
 
-0.424
 
 
26.5
 
 
 
 
ARL1
 
 
hsa-miR-17-5p
 
 
-0.432
 
 
26.5
 
 
 
 
ARL6IP1
 
 
hsa-miR-7-5p
 
 
-0.374
 
 
140.2
 
 
 
 
ARL6IP1
 
 
hsa-miR-328-3p
 
 
-0.494
 
 
140.2
 
 
 
 
ARL8A
 
 
hsa-miR-103a-3p
 
 
-0.38
 
 
157.0
 
 
 
 
ARMC1
 
 
hsa-miR-92a-3p
 
 
-0.322
 
 
20.3
 
 
 
 
ARMC1
 
 
hsa-miR-103a-3p
 
 
-0.525
 
 
20.3
 
 
 
 
ARMC8
 
 
hsa-miR-486-5p
 
 
-0.321
 
 
15.5
 
 
 
 
ARMC8
 
 
hsa-miR-20a-5p
 
 
-0.357
 
 
15.5
 
 
 
 
ARMC8
 
 
hsa-miR-93-5p
 
 
-0.362
 
 
15.5
 
 
 
 
ARMC8
 
 
hsa-miR-17-5p
 
 
-0.378
 
 
15.5
 
 
 
 
ARMC8
 
 
hsa-miR-103a-3p
 
 
-0.412
 
 
15.5
 
 
 
 
ARPP19
 
 
hsa-miR-328-3p
 
 
-0.404
 
 
170.8
 
 
 
 
ARPP19
 
 
hsa-let-7c-5p
 
 
-0.492
 
 
170.8
 
 
 
 
ARRDC3
 
 
hsa-miR-92a-3p
 
 
-0.458
 
 
23.5
 
 
 
 
ARRDC4
 
 
hsa-let-7c-5p
 
 
-0.329
 
 
75.7
 
 
 
 
ASAP1
 
 
hsa-let-7c-5p
 
 
-0.308
 
 
11.0
 
 
 
 
ASPH
 
 
hsa-miR-221-3p
 
 
-0.287
 
 
25.3
 
 
 
 
ASPH
 
 
hsa-miR-92a-3p
 
 
-0.416
 
 
25.3
 
 
 
 
ATF3
 
 
hsa-miR-874-3p
 
 
-0.288
 
 
33.6
 
 
 
 
ATG16L1
 
 
hsa-miR-20a-5p
 
 
-0.446
 
 
10.3
 
 
 
 
ATG16L1
 
 
hsa-miR-93-5p
 
 
-0.451
 
 
10.3
 
 
 
 
ATG16L1
 
 
hsa-miR-17-5p
 
 
-0.467
 
 
10.3
 
 
 
 
ATOSB
 
 
hsa-miR-328-3p
 
 
-0.358
 
 
30.9
 
 
 
 
ATOSB
 
 
hsa-let-7c-5p
 
 
-0.429
 
 
30.9
 
 
 
 
ATP1B2
 
 
hsa-miR-103a-3p
 
 
-0.6
 
 
65.0
 
 
 
 
ATP2A2
 
 
hsa-let-7c-5p
 
 
-0.311
 
 
19.0
 
 
 
 
ATP6V1F
 
 
hsa-miR-185-5p
 
 
-0.765
 
 
198.6
 
 
 
 
ATPAF1
 
 
hsa-let-7c-5p
 
 
-0.498
 
 
50.4
 
 
 
 
AVPI1
 
 
hsa-miR-7-5p
 
 
-0.367
 
 
33.9
 
 
 
 
B3GAT3
 
 
hsa-let-7c-5p
 
 
-0.324
 
 
62.6
 
 
 
 
B4GAT1
 
 
hsa-let-7c-5p
 
 
-0.47
 
 
48.7
 
 
 
 
BACE1
 
 
hsa-miR-339-5p
 
 
-0.371
 
 
113.1
 
 
 
 
BAMBI
 
 
hsa-miR-20a-5p
 
 
-0.485
 
 
23.0
 
 
 
 
BAMBI
 
 
hsa-miR-17-5p
 
 
-0.506
 
 
23.0
 
 
 
 
BAMBI
 
 
hsa-miR-93-5p
 
 
-0.506
 
 
23.0
 
 
 
 
BCAT2
 
 
hsa-miR-103a-3p
 
 
-0.364
 
 
18.6
 
 
 
 
BCAT2
 
 
hsa-miR-92a-3p
 
 
-0.609
 
 
18.6
 
 
 
 
BDH1
 
 
hsa-miR-874-3p
 
 
-0.644
 
 
11.2
 
 
 
 
BIN1
 
 
hsa-miR-744-5p
 
 
-0.654
 
 
197.8
 
 
 
 
BLOC1S6
 
 
hsa-let-7c-5p
 
 
-0.321
 
 
15.5
 
 
 
 
BLTP1
 
 
hsa-miR-92a-3p
 
 
-0.462
 
 
26.2
 
 
 
 
BNIP3L
 
 
hsa-miR-20a-5p
 
 
-0.287
 
 
78.0
 
 
 
 
BOLA3
 
 
hsa-miR-103a-3p
 
 
-0.362
 
 
45.8
 
 
 
 
BSDC1
 
 
hsa-miR-92a-3p
 
 
-0.362
 
 
25.3
 
 
 
 
BTBD10
 
 
hsa-miR-17-5p
 
 
-0.409
 
 
15.3
 
 
 
 
BTBD10
 
 
hsa-miR-93-5p
 
 
-0.409
 
 
15.3
 
 
 
 
BTBD10
 
 
hsa-miR-20a-5p
 
 
-0.412
 
 
15.3
 
 
 
 
BTF3L4
 
 
hsa-let-7c-5p
 
 
-0.355
 
 
36.6
 
 
 
 
BTG2
 
 
hsa-miR-92a-3p
 
 
-0.314
 
 
41.8
 
 
 
 
BTG2
 
 
hsa-miR-92a-3p
 
 
-0.402
 
 
41.8
 
 
 
 
BTG3
 
 
hsa-miR-17-5p
 
 
-0.668
 
 
27.3
 
 
 
 
BTG3
 
 
hsa-miR-93-5p
 
 
-0.668
 
 
27.3
 
 
 
 
BTG3
 
 
hsa-miR-20a-5p
 
 
-0.671
 
 
27.3
 
 
 
 
BZW1
 
 
hsa-let-7c-5p
 
 
-0.354
 
 
46.3
 
 
 
 
BZW1
 
 
hsa-let-7c-5p
 
 
-0.375
 
 
46.3
 
 
 
 
C11orf24
 
 
hsa-miR-92a-3p
 
 
-0.35
 
 
25.5
 
 
 
 
C19orf47
 
 
hsa-let-7c-5p
 
 
-0.311
 
 
14.3
 
 
 
 
C1GALT1
 
 
hsa-let-7c-5p
 
 
-0.322
 
 
27.9
 
 
 
 
C1QBP
 
 
hsa-miR-330-3p
 
 
-0.412
 
 
74.8
 
 
 
 
 
 
 spleen 
 
 
 
 
target_symbol
 
 
mature_mirna_id
 
 
score
 
 
nTPM
 
 
 
 
 
 
AARS1
 
 
hsa-miR-92a-3p
 
 
-0.458
 
 
27.6
 
 
 
 
ACOX1
 
 
hsa-miR-92a-3p
 
 
-0.321
 
 
10.3
 
 
 
 
ADAM19
 
 
hsa-miR-874-3p
 
 
-0.374
 
 
21.6
 
 
 
 
ADAM9
 
 
hsa-miR-20a-5p
 
 
-0.299
 
 
22.6
 
 
 
 
ADAM9
 
 
hsa-miR-17-5p
 
 
-0.32
 
 
22.6
 
 
 
 
ADAM9
 
 
hsa-miR-93-5p
 
 
-0.32
 
 
22.6
 
 
 
 
ADCY3
 
 
hsa-miR-92a-3p
 
 
-0.381
 
 
28.8
 
 
 
 
ADNP
 
 
hsa-miR-328-3p
 
 
-0.371
 
 
34.9
 
 
 
 
AGFG1
 
 
hsa-miR-103a-3p
 
 
-0.389
 
 
44.5
 
 
 
 
AGFG2
 
 
hsa-miR-20a-5p
 
 
-0.365
 
 
27.6
 
 
 
 
AGFG2
 
 
hsa-miR-93-5p
 
 
-0.378
 
 
27.6
 
 
 
 
AGFG2
 
 
hsa-miR-17-5p
 
 
-0.402
 
 
27.6
 
 
 
 
AGO4
 
 
hsa-miR-103a-3p
 
 
-0.32
 
 
13.3
 
 
 
 
AGO4
 
 
hsa-miR-221-3p
 
 
-0.321
 
 
13.3
 
 
 
 
AHNAK
 
 
hsa-miR-20a-5p
 
 
-0.316
 
 
56.3
 
 
 
 
AHNAK
 
 
hsa-miR-17-5p
 
 
-0.337
 
 
56.3
 
 
 
 
AHNAK
 
 
hsa-miR-93-5p
 
 
-0.337
 
 
56.3
 
 
 
 
AIFM1
 
 
hsa-let-7c-5p
 
 
-0.314
 
 
12.9
 
 
 
 
AKAP10
 
 
hsa-miR-339-5p
 
 
-0.302
 
 
14.1
 
 
 
 
AKIRIN2
 
 
hsa-miR-103a-3p
 
 
-0.299
 
 
43.9
 
 
 
 
ALAD
 
 
hsa-miR-103a-3p
 
 
-0.385
 
 
12.8
 
 
 
 
ALG3
 
 
hsa-miR-20a-5p
 
 
-0.559
 
 
19.8
 
 
 
 
ALG3
 
 
hsa-miR-93-5p
 
 
-0.564
 
 
19.8
 
 
 
 
ALG3
 
 
hsa-miR-17-5p
 
 
-0.572
 
 
19.8
 
 
 
 
ALKAL2
 
 
hsa-miR-92a-3p
 
 
-0.608
 
 
12.2
 
 
 
 
ANGPTL2
 
 
hsa-miR-221-3p
 
 
-0.369
 
 
46.4
 
 
 
 
ANKFY1
 
 
hsa-miR-155-5p
 
 
-0.315
 
 
14.2
 
 
 
 
ANKRD12
 
 
hsa-miR-7-5p
 
 
-0.37
 
 
18.8
 
 
 
 
ANKRD44
 
 
hsa-miR-92a-3p
 
 
-0.324
 
 
21.7
 
 
 
 
ANKRD46
 
 
hsa-let-7c-5p
 
 
-0.389
 
 
10.4
 
 
 
 
ANP32E
 
 
hsa-miR-92a-3p
 
 
-0.329
 
 
31.2
 
 
 
 
AP1AR
 
 
hsa-miR-92a-3p
 
 
-0.325
 
 
12.4
 
 
 
 
AP1S1
 
 
hsa-let-7c-5p
 
 
-0.374
 
 
20.9
 
 
 
 
AP4B1
 
 
hsa-miR-378a-3p
 
 
-0.62
 
 
19.7
 
 
 
 
APPL1
 
 
hsa-miR-92a-3p
 
 
-0.375
 
 
14.9
 
 
 
 
AQP3
 
 
hsa-miR-874-3p
 
 
-0.48
 
 
103.8
 
 
 
 
ARF4
 
 
hsa-miR-7-5p
 
 
-0.407
 
 
60.0
 
 
 
 
ARF4
 
 
hsa-miR-221-3p
 
 
-0.553
 
 
60.0
 
 
 
 
ARHGAP1
 
 
hsa-miR-17-5p
 
 
-0.325
 
 
55.1
 
 
 
 
ARHGAP1
 
 
hsa-miR-93-5p
 
 
-0.325
 
 
55.1
 
 
 
 
ARHGAP1
 
 
hsa-miR-20a-5p
 
 
-0.339
 
 
55.1
 
 
 
 
ARHGAP28
 
 
hsa-let-7c-5p
 
 
-0.47
 
 
11.9
 
 
 
 
ARHGAP5
 
 
hsa-miR-486-5p
 
 
-0.3
 
 
40.5
 
 
 
 
ARID4A
 
 
hsa-miR-7-5p
 
 
-0.341
 
 
13.2
 
 
 
 
ARID4B
 
 
hsa-miR-20a-5p
 
 
-0.29
 
 
17.3
 
 
 
 
ARID4B
 
 
hsa-miR-486-5p
 
 
-0.367
 
 
17.3
 
 
 
 
ARIH2
 
 
hsa-miR-103a-3p
 
 
-0.307
 
 
23.5
 
 
 
 
ARL1
 
 
hsa-miR-20a-5p
 
 
-0.403
 
 
27.2
 
 
 
 
ARL1
 
 
hsa-miR-93-5p
 
 
-0.424
 
 
27.2
 
 
 
 
ARL1
 
 
hsa-miR-17-5p
 
 
-0.432
 
 
27.2
 
 
 
 
ARL4D
 
 
hsa-let-7c-5p
 
 
-0.417
 
 
16.1
 
 
 
 
ARL6IP1
 
 
hsa-miR-7-5p
 
 
-0.374
 
 
68.6
 
 
 
 
ARL6IP1
 
 
hsa-miR-328-3p
 
 
-0.494
 
 
68.6
 
 
 
 
ARL6IP6
 
 
hsa-let-7c-5p
 
 
-0.293
 
 
13.7
 
 
 
 
ARL8A
 
 
hsa-miR-103a-3p
 
 
-0.38
 
 
36.8
 
 
 
 
ARMC1
 
 
hsa-miR-92a-3p
 
 
-0.322
 
 
13.1
 
 
 
 
ARMC1
 
 
hsa-miR-103a-3p
 
 
-0.525
 
 
13.1
 
 
 
 
ARMC8
 
 
hsa-miR-486-5p
 
 
-0.321
 
 
14.9
 
 
 
 
ARMC8
 
 
hsa-miR-20a-5p
 
 
-0.357
 
 
14.9
 
 
 
 
ARMC8
 
 
hsa-miR-93-5p
 
 
-0.362
 
 
14.9
 
 
 
 
ARMC8
 
 
hsa-miR-17-5p
 
 
-0.378
 
 
14.9
 
 
 
 
ARMC8
 
 
hsa-miR-103a-3p
 
 
-0.412
 
 
14.9
 
 
 
 
ARMT1
 
 
hsa-let-7c-5p
 
 
-0.303
 
 
12.0
 
 
 
 
ARNT
 
 
hsa-miR-103a-3p
 
 
-0.422
 
 
18.4
 
 
 
 
ARNT
 
 
hsa-miR-221-3p
 
 
-0.465
 
 
18.4
 
 
 
 
ARPP19
 
 
hsa-miR-328-3p
 
 
-0.404
 
 
41.2
 
 
 
 
ARPP19
 
 
hsa-let-7c-5p
 
 
-0.492
 
 
41.2
 
 
 
 
ARRDC3
 
 
hsa-miR-92a-3p
 
 
-0.458
 
 
35.0
 
 
 
 
ARVCF
 
 
hsa-miR-155-5p
 
 
-0.388
 
 
10.3
 
 
 
 
ASAP1
 
 
hsa-let-7c-5p
 
 
-0.308
 
 
28.1
 
 
 
 
ASB6
 
 
hsa-miR-874-3p
 
 
-0.368
 
 
17.1
 
 
 
 
ASPH
 
 
hsa-miR-221-3p
 
 
-0.287
 
 
26.8
 
 
 
 
ASPH
 
 
hsa-miR-92a-3p
 
 
-0.416
 
 
26.8
 
 
 
 
ATF3
 
 
hsa-miR-874-3p
 
 
-0.288
 
 
23.0
 
 
 
 
ATG16L1
 
 
hsa-miR-20a-5p
 
 
-0.446
 
 
11.4
 
 
 
 
ATG16L1
 
 
hsa-miR-93-5p
 
 
-0.451
 
 
11.4
 
 
 
 
ATG16L1
 
 
hsa-miR-17-5p
 
 
-0.467
 
 
11.4
 
 
 
 
ATL2
 
 
hsa-miR-103a-3p
 
 
-0.489
 
 
18.2
 
 
 
 
ATOSA
 
 
hsa-miR-221-3p
 
 
-0.495
 
 
12.9
 
 
 
 
ATOSB
 
 
hsa-miR-328-3p
 
 
-0.358
 
 
26.6
 
 
 
 
ATOSB
 
 
hsa-let-7c-5p
 
 
-0.429
 
 
26.6
 
 
 
 
ATP2A2
 
 
hsa-let-7c-5p
 
 
-0.311
 
 
28.8
 
 
 
 
ATP6V1F
 
 
hsa-miR-185-5p
 
 
-0.765
 
 
120.8
 
 
 
 
ATPAF1
 
 
hsa-let-7c-5p
 
 
-0.498
 
 
23.6
 
 
 
 
ATXN1L
 
 
hsa-miR-155-5p
 
 
-0.315
 
 
11.1
 
 
 
 
AVPI1
 
 
hsa-miR-7-5p
 
 
-0.367
 
 
20.8
 
 
 
 
B3GAT3
 
 
hsa-let-7c-5p
 
 
-0.324
 
 
44.2
 
 
 
 
B4GALT5
 
 
hsa-miR-210-3p
 
 
-0.511
 
 
26.0
 
 
 
 
B4GAT1
 
 
hsa-let-7c-5p
 
 
-0.47
 
 
10.0
 
 
 
 
BACE1
 
 
hsa-miR-339-5p
 
 
-0.371
 
 
11.2
 
 
 
 
BACH1
 
 
hsa-let-7c-5p
 
 
-0.306
 
 
19.4
 
 
 
 
BACH1
 
 
hsa-let-7c-5p
 
 
-0.306
 
 
19.4
 
 
 
 
BACH1
 
 
hsa-let-7c-5p
 
 
-0.435
 
 
19.4
 
 
 
 
BACH1
 
 
hsa-let-7c-5p
 
 
-0.435
 
 
19.4
 
 
 
 
BAK1
 
 
hsa-miR-103a-3p
 
 
-0.364
 
 
28.6
 
 
 
 
BAMBI
 
 
hsa-miR-20a-5p
 
 
-0.485
 
 
19.4
 
 
 
 
BAMBI
 
 
hsa-miR-17-5p
 
 
-0.506
 
 
19.4
 
 
 
 
BAMBI
 
 
hsa-miR-93-5p
 
 
-0.506
 
 
19.4
 
 
 
 
BCAT2
 
 
hsa-miR-103a-3p
 
 
-0.364
 
 
18.7
 
 
 
 
BCAT2
 
 
hsa-miR-92a-3p
 
 
-0.609
 
 
18.7
 
 
 
 
 
 
 stomach 
 
 
 
 
target_symbol
 
 
mature_mirna_id
 
 
score
 
 
nTPM
 
 
 
 
 
 
AARS1
 
 
hsa-miR-92a-3p
 
 
-0.458
 
 
39.9
 
 
 
 
ABHD17C
 
 
hsa-let-7c-5p
 
 
-0.359
 
 
34.0
 
 
 
 
ABHD17C
 
 
hsa-miR-330-3p
 
 
-0.399
 
 
34.0
 
 
 
 
ACER2
 
 
hsa-miR-20a-5p
 
 
-0.298
 
 
58.3
 
 
 
 
ACER2
 
 
hsa-miR-93-5p
 
 
-0.303
 
 
58.3
 
 
 
 
ACER2
 
 
hsa-miR-17-5p
 
 
-0.319
 
 
58.3
 
 
 
 
ACER2
 
 
hsa-let-7c-5p
 
 
-0.333
 
 
58.3
 
 
 
 
ACOX1
 
 
hsa-miR-92a-3p
 
 
-0.321
 
 
17.5
 
 
 
 
ACTC1
 
 
hsa-miR-92a-3p
 
 
-0.361
 
 
32.8
 
 
 
 
ACTC1
 
 
hsa-miR-7-5p
 
 
-0.471
 
 
32.8
 
 
 
 
ADAM9
 
 
hsa-miR-20a-5p
 
 
-0.299
 
 
41.8
 
 
 
 
ADAM9
 
 
hsa-miR-17-5p
 
 
-0.32
 
 
41.8
 
 
 
 
ADAM9
 
 
hsa-miR-93-5p
 
 
-0.32
 
 
41.8
 
 
 
 
ADCY3
 
 
hsa-miR-92a-3p
 
 
-0.381
 
 
23.8
 
 
 
 
ADNP
 
 
hsa-miR-328-3p
 
 
-0.371
 
 
30.6
 
 
 
 
AGFG1
 
 
hsa-miR-103a-3p
 
 
-0.389
 
 
31.3
 
 
 
 
AGFG2
 
 
hsa-miR-20a-5p
 
 
-0.365
 
 
12.9
 
 
 
 
AGFG2
 
 
hsa-miR-93-5p
 
 
-0.378
 
 
12.9
 
 
 
 
AGFG2
 
 
hsa-miR-17-5p
 
 
-0.402
 
 
12.9
 
 
 
 
AHNAK
 
 
hsa-miR-20a-5p
 
 
-0.316
 
 
125.8
 
 
 
 
AHNAK
 
 
hsa-miR-17-5p
 
 
-0.337
 
 
125.8
 
 
 
 
AHNAK
 
 
hsa-miR-93-5p
 
 
-0.337
 
 
125.8
 
 
 
 
AIFM1
 
 
hsa-let-7c-5p
 
 
-0.314
 
 
29.8
 
 
 
 
AKAP10
 
 
hsa-miR-339-5p
 
 
-0.302
 
 
10.5
 
 
 
 
AKIRIN2
 
 
hsa-miR-103a-3p
 
 
-0.299
 
 
14.8
 
 
 
 
ALAD
 
 
hsa-miR-103a-3p
 
 
-0.385
 
 
49.1
 
 
 
 
ALG3
 
 
hsa-miR-20a-5p
 
 
-0.559
 
 
22.0
 
 
 
 
ALG3
 
 
hsa-miR-93-5p
 
 
-0.564
 
 
22.0
 
 
 
 
ALG3
 
 
hsa-miR-17-5p
 
 
-0.572
 
 
22.0
 
 
 
 
ALG9
 
 
hsa-miR-7-5p
 
 
-0.302
 
 
11.1
 
 
 
 
ALKAL2
 
 
hsa-miR-92a-3p
 
 
-0.608
 
 
16.5
 
 
 
 
AMT
 
 
hsa-let-7c-5p
 
 
-0.463
 
 
13.1
 
 
 
 
ANGPTL2
 
 
hsa-miR-221-3p
 
 
-0.369
 
 
27.7
 
 
 
 
ANKFY1
 
 
hsa-miR-155-5p
 
 
-0.315
 
 
12.2
 
 
 
 
ANKRD12
 
 
hsa-miR-7-5p
 
 
-0.37
 
 
13.7
 
 
 
 
ANKRD29
 
 
hsa-miR-20a-5p
 
 
-0.359
 
 
14.3
 
 
 
 
ANKRD29
 
 
hsa-miR-17-5p
 
 
-0.372
 
 
14.3
 
 
 
 
ANKRD29
 
 
hsa-miR-93-5p
 
 
-0.372
 
 
14.3
 
 
 
 
ANKRD46
 
 
hsa-let-7c-5p
 
 
-0.389
 
 
10.9
 
 
 
 
ANKRD9
 
 
hsa-miR-20a-5p
 
 
-0.31
 
 
21.4
 
 
 
 
ANKRD9
 
 
hsa-miR-93-5p
 
 
-0.331
 
 
21.4
 
 
 
 
ANKRD9
 
 
hsa-miR-17-5p
 
 
-0.347
 
 
21.4
 
 
 
 
ANP32E
 
 
hsa-miR-92a-3p
 
 
-0.329
 
 
16.3
 
 
 
 
AP1AR
 
 
hsa-miR-92a-3p
 
 
-0.325
 
 
12.3
 
 
 
 
AP1S1
 
 
hsa-let-7c-5p
 
 
-0.374
 
 
23.2
 
 
 
 
AP4B1
 
 
hsa-miR-378a-3p
 
 
-0.62
 
 
10.0
 
 
 
 
APCDD1
 
 
hsa-miR-20a-5p
 
 
-0.404
 
 
22.7
 
 
 
 
APCDD1
 
 
hsa-miR-17-5p
 
 
-0.425
 
 
22.7
 
 
 
 
APCDD1
 
 
hsa-miR-93-5p
 
 
-0.425
 
 
22.7
 
 
 
 
APPL1
 
 
hsa-miR-92a-3p
 
 
-0.375
 
 
14.3
 
 
 
 
AQP3
 
 
hsa-miR-874-3p
 
 
-0.48
 
 
33.6
 
 
 
 
ARF4
 
 
hsa-miR-7-5p
 
 
-0.407
 
 
123.5
 
 
 
 
ARF4
 
 
hsa-miR-221-3p
 
 
-0.553
 
 
123.5
 
 
 
 
ARHGAP1
 
 
hsa-miR-17-5p
 
 
-0.325
 
 
46.3
 
 
 
 
ARHGAP1
 
 
hsa-miR-93-5p
 
 
-0.325
 
 
46.3
 
 
 
 
ARHGAP1
 
 
hsa-miR-20a-5p
 
 
-0.339
 
 
46.3
 
 
 
 
ARHGAP5
 
 
hsa-miR-486-5p
 
 
-0.3
 
 
27.8
 
 
 
 
ARHGEF28
 
 
hsa-miR-93-5p
 
 
-0.33
 
 
20.6
 
 
 
 
ARHGEF28
 
 
hsa-miR-17-5p
 
 
-0.363
 
 
20.6
 
 
 
 
ARHGEF28
 
 
hsa-miR-20a-5p
 
 
-0.366
 
 
20.6
 
 
 
 
ARID4B
 
 
hsa-miR-20a-5p
 
 
-0.29
 
 
12.5
 
 
 
 
ARID4B
 
 
hsa-miR-486-5p
 
 
-0.367
 
 
12.5
 
 
 
 
ARIH2
 
 
hsa-miR-103a-3p
 
 
-0.307
 
 
21.8
 
 
 
 
ARL1
 
 
hsa-miR-20a-5p
 
 
-0.403
 
 
54.2
 
 
 
 
ARL1
 
 
hsa-miR-93-5p
 
 
-0.424
 
 
54.2
 
 
 
 
ARL1
 
 
hsa-miR-17-5p
 
 
-0.432
 
 
54.2
 
 
 
 
ARL6IP1
 
 
hsa-miR-7-5p
 
 
-0.374
 
 
118.7
 
 
 
 
ARL6IP1
 
 
hsa-miR-328-3p
 
 
-0.494
 
 
118.7
 
 
 
 
ARL8A
 
 
hsa-miR-103a-3p
 
 
-0.38
 
 
30.5
 
 
 
 
ARMC1
 
 
hsa-miR-92a-3p
 
 
-0.322
 
 
12.9
 
 
 
 
ARMC1
 
 
hsa-miR-103a-3p
 
 
-0.525
 
 
12.9
 
 
 
 
ARMC8
 
 
hsa-miR-486-5p
 
 
-0.321
 
 
14.5
 
 
 
 
ARMC8
 
 
hsa-miR-20a-5p
 
 
-0.357
 
 
14.5
 
 
 
 
ARMC8
 
 
hsa-miR-93-5p
 
 
-0.362
 
 
14.5
 
 
 
 
ARMC8
 
 
hsa-miR-17-5p
 
 
-0.378
 
 
14.5
 
 
 
 
ARMC8
 
 
hsa-miR-103a-3p
 
 
-0.412
 
 
14.5
 
 
 
 
ARMT1
 
 
hsa-let-7c-5p
 
 
-0.303
 
 
13.3
 
 
 
 
ARNT
 
 
hsa-miR-103a-3p
 
 
-0.422
 
 
17.9
 
 
 
 
ARNT
 
 
hsa-miR-221-3p
 
 
-0.465
 
 
17.9
 
 
 
 
ARPP19
 
 
hsa-miR-328-3p
 
 
-0.404
 
 
36.4
 
 
 
 
ARPP19
 
 
hsa-let-7c-5p
 
 
-0.492
 
 
36.4
 
 
 
 
ARRDC3
 
 
hsa-miR-92a-3p
 
 
-0.458
 
 
24.3
 
 
 
 
ARRDC4
 
 
hsa-let-7c-5p
 
 
-0.329
 
 
25.2
 
 
 
 
ASB6
 
 
hsa-miR-874-3p
 
 
-0.368
 
 
13.6
 
 
 
 
ASPH
 
 
hsa-miR-221-3p
 
 
-0.287
 
 
96.9
 
 
 
 
ASPH
 
 
hsa-miR-92a-3p
 
 
-0.416
 
 
96.9
 
 
 
 
ASPN
 
 
hsa-miR-92a-3p
 
 
-0.422
 
 
11.3
 
 
 
 
ATF3
 
 
hsa-miR-874-3p
 
 
-0.288
 
 
103.9
 
 
 
 
ATG16L1
 
 
hsa-miR-20a-5p
 
 
-0.446
 
 
11.5
 
 
 
 
ATG16L1
 
 
hsa-miR-93-5p
 
 
-0.451
 
 
11.5
 
 
 
 
ATG16L1
 
 
hsa-miR-17-5p
 
 
-0.467
 
 
11.5
 
 
 
 
ATG4A
 
 
hsa-miR-7-5p
 
 
-0.448
 
 
11.6
 
 
 
 
ATL2
 
 
hsa-miR-103a-3p
 
 
-0.489
 
 
26.3
 
 
 
 
ATOSA
 
 
hsa-miR-221-3p
 
 
-0.495
 
 
14.2
 
 
 
 
ATOSB
 
 
hsa-miR-328-3p
 
 
-0.358
 
 
11.2
 
 
 
 
ATOSB
 
 
hsa-let-7c-5p
 
 
-0.429
 
 
11.2
 
 
 
 
ATP2A2
 
 
hsa-let-7c-5p
 
 
-0.311
 
 
44.5
 
 
 
 
ATP6V1F
 
 
hsa-miR-185-5p
 
 
-0.765
 
 
115.3
 
 
 
 
ATPAF1
 
 
hsa-let-7c-5p
 
 
-0.498
 
 
40.7
 
 
 
 
AVPI1
 
 
hsa-miR-7-5p
 
 
-0.367
 
 
31.1
 
 
 
 
 
 
 testis 
 
 
 
 
target_symbol
 
 
mature_mirna_id
 
 
score
 
 
nTPM
 
 
 
 
 
 
AARS1
 
 
hsa-miR-92a-3p
 
 
-0.458
 
 
17.3
 
 
 
 
ABCB9
 
 
hsa-let-7c-5p
 
 
-0.422
 
 
17.1
 
 
 
 
ABHD17B
 
 
hsa-miR-486-5p
 
 
-0.348
 
 
10.4
 
 
 
 
ACRV1
 
 
hsa-miR-7-5p
 
 
-0.3
 
 
110.6
 
 
 
 
ACSL6
 
 
hsa-let-7c-5p
 
 
-0.336
 
 
19.3
 
 
 
 
ACTL7A
 
 
hsa-miR-155-5p
 
 
-0.531
 
 
218.7
 
 
 
 
ADAM9
 
 
hsa-miR-20a-5p
 
 
-0.299
 
 
10.4
 
 
 
 
ADAM9
 
 
hsa-miR-17-5p
 
 
-0.32
 
 
10.4
 
 
 
 
ADAM9
 
 
hsa-miR-93-5p
 
 
-0.32
 
 
10.4
 
 
 
 
ADNP
 
 
hsa-miR-328-3p
 
 
-0.371
 
 
37.6
 
 
 
 
AGFG1
 
 
hsa-miR-103a-3p
 
 
-0.389
 
 
86.7
 
 
 
 
AGFG2
 
 
hsa-miR-20a-5p
 
 
-0.365
 
 
11.5
 
 
 
 
AGFG2
 
 
hsa-miR-93-5p
 
 
-0.378
 
 
11.5
 
 
 
 
AGFG2
 
 
hsa-miR-17-5p
 
 
-0.402
 
 
11.5
 
 
 
 
AHCTF1
 
 
hsa-let-7c-5p
 
 
-0.374
 
 
30.4
 
 
 
 
AHNAK
 
 
hsa-miR-20a-5p
 
 
-0.316
 
 
16.9
 
 
 
 
AHNAK
 
 
hsa-miR-17-5p
 
 
-0.337
 
 
16.9
 
 
 
 
AHNAK
 
 
hsa-miR-93-5p
 
 
-0.337
 
 
16.9
 
 
 
 
AIFM1
 
 
hsa-let-7c-5p
 
 
-0.314
 
 
21.3
 
 
 
 
AKAP10
 
 
hsa-miR-339-5p
 
 
-0.302
 
 
13.3
 
 
 
 
AKIRIN2
 
 
hsa-miR-103a-3p
 
 
-0.299
 
 
21.4
 
 
 
 
ALAD
 
 
hsa-miR-103a-3p
 
 
-0.385
 
 
12.6
 
 
 
 
ALG3
 
 
hsa-miR-20a-5p
 
 
-0.559
 
 
25.2
 
 
 
 
ALG3
 
 
hsa-miR-93-5p
 
 
-0.564
 
 
25.2
 
 
 
 
ALG3
 
 
hsa-miR-17-5p
 
 
-0.572
 
 
25.2
 
 
 
 
ALG9
 
 
hsa-miR-7-5p
 
 
-0.302
 
 
12.8
 
 
 
 
ALKAL2
 
 
hsa-miR-92a-3p
 
 
-0.608
 
 
33.1
 
 
 
 
ANKRD12
 
 
hsa-miR-7-5p
 
 
-0.37
 
 
25.9
 
 
 
 
ANKRD50
 
 
hsa-miR-20a-5p
 
 
-0.3
 
 
14.1
 
 
 
 
ANKRD9
 
 
hsa-miR-20a-5p
 
 
-0.31
 
 
38.5
 
 
 
 
ANKRD9
 
 
hsa-miR-93-5p
 
 
-0.331
 
 
38.5
 
 
 
 
ANKRD9
 
 
hsa-miR-17-5p
 
 
-0.347
 
 
38.5
 
 
 
 
AP1AR
 
 
hsa-miR-92a-3p
 
 
-0.325
 
 
13.6
 
 
 
 
AP1S1
 
 
hsa-let-7c-5p
 
 
-0.374
 
 
17.8
 
 
 
 
AP4B1
 
 
hsa-miR-378a-3p
 
 
-0.62
 
 
10.7
 
 
 
 
AP5S1
 
 
hsa-miR-744-5p
 
 
-0.354
 
 
10.7
 
 
 
 
ARF4
 
 
hsa-miR-7-5p
 
 
-0.407
 
 
94.2
 
 
 
 
ARF4
 
 
hsa-miR-221-3p
 
 
-0.553
 
 
94.2
 
 
 
 
ARHGAP1
 
 
hsa-miR-17-5p
 
 
-0.325
 
 
15.3
 
 
 
 
ARHGAP1
 
 
hsa-miR-93-5p
 
 
-0.325
 
 
15.3
 
 
 
 
ARHGAP1
 
 
hsa-miR-20a-5p
 
 
-0.339
 
 
15.3
 
 
 
 
ARHGAP28
 
 
hsa-let-7c-5p
 
 
-0.47
 
 
72.3
 
 
 
 
ARHGAP5
 
 
hsa-miR-486-5p
 
 
-0.3
 
 
43.5
 
 
 
 
ARID2
 
 
hsa-miR-155-5p
 
 
-0.384
 
 
24.3
 
 
 
 
ARID2
 
 
hsa-miR-103a-3p
 
 
-0.415
 
 
24.3
 
 
 
 
ARID3A
 
 
hsa-let-7c-5p
 
 
-0.34
 
 
16.1
 
 
 
 
ARID3A
 
 
hsa-let-7c-5p
 
 
-0.347
 
 
16.1
 
 
 
 
ARID3B
 
 
hsa-let-7c-5p
 
 
-0.31
 
 
21.2
 
 
 
 
ARID3B
 
 
hsa-let-7c-5p
 
 
-0.486
 
 
21.2
 
 
 
 
ARID4B
 
 
hsa-miR-20a-5p
 
 
-0.29
 
 
16.0
 
 
 
 
ARID4B
 
 
hsa-miR-486-5p
 
 
-0.367
 
 
16.0
 
 
 
 
ARIH2
 
 
hsa-miR-103a-3p
 
 
-0.307
 
 
25.2
 
 
 
 
ARL1
 
 
hsa-miR-20a-5p
 
 
-0.403
 
 
30.6
 
 
 
 
ARL1
 
 
hsa-miR-93-5p
 
 
-0.424
 
 
30.6
 
 
 
 
ARL1
 
 
hsa-miR-17-5p
 
 
-0.432
 
 
30.6
 
 
 
 
ARL4D
 
 
hsa-let-7c-5p
 
 
-0.417
 
 
31.4
 
 
 
 
ARL6IP1
 
 
hsa-miR-7-5p
 
 
-0.374
 
 
121.4
 
 
 
 
ARL6IP1
 
 
hsa-miR-328-3p
 
 
-0.494
 
 
121.4
 
 
 
 
ARL6IP6
 
 
hsa-let-7c-5p
 
 
-0.293
 
 
10.2
 
 
 
 
ARL8A
 
 
hsa-miR-103a-3p
 
 
-0.38
 
 
18.8
 
 
 
 
ARMC8
 
 
hsa-miR-486-5p
 
 
-0.321
 
 
17.2
 
 
 
 
ARMC8
 
 
hsa-miR-20a-5p
 
 
-0.357
 
 
17.2
 
 
 
 
ARMC8
 
 
hsa-miR-93-5p
 
 
-0.362
 
 
17.2
 
 
 
 
ARMC8
 
 
hsa-miR-17-5p
 
 
-0.378
 
 
17.2
 
 
 
 
ARMC8
 
 
hsa-miR-103a-3p
 
 
-0.412
 
 
17.2
 
 
 
 
ARMT1
 
 
hsa-let-7c-5p
 
 
-0.303
 
 
13.1
 
 
 
 
ARNT
 
 
hsa-miR-103a-3p
 
 
-0.422
 
 
16.2
 
 
 
 
ARNT
 
 
hsa-miR-221-3p
 
 
-0.465
 
 
16.2
 
 
 
 
ARPP19
 
 
hsa-miR-328-3p
 
 
-0.404
 
 
79.0
 
 
 
 
ARPP19
 
 
hsa-let-7c-5p
 
 
-0.492
 
 
79.0
 
 
 
 
ARRDC3
 
 
hsa-miR-92a-3p
 
 
-0.458
 
 
15.5
 
 
 
 
ARRDC4
 
 
hsa-let-7c-5p
 
 
-0.329
 
 
13.7
 
 
 
 
ASAP1
 
 
hsa-let-7c-5p
 
 
-0.308
 
 
26.5
 
 
 
 
ASB6
 
 
hsa-miR-874-3p
 
 
-0.368
 
 
12.3
 
 
 
 
ASPH
 
 
hsa-miR-221-3p
 
 
-0.287
 
 
15.3
 
 
 
 
ASPH
 
 
hsa-miR-92a-3p
 
 
-0.416
 
 
15.3
 
 
 
 
ATAD2
 
 
hsa-miR-17-5p
 
 
-0.304
 
 
17.7
 
 
 
 
ATAD2
 
 
hsa-miR-20a-5p
 
 
-0.318
 
 
17.7
 
 
 
 
ATG16L1
 
 
hsa-miR-20a-5p
 
 
-0.446
 
 
13.1
 
 
 
 
ATG16L1
 
 
hsa-miR-93-5p
 
 
-0.451
 
 
13.1
 
 
 
 
ATG16L1
 
 
hsa-miR-17-5p
 
 
-0.467
 
 
13.1
 
 
 
 
ATL2
 
 
hsa-miR-103a-3p
 
 
-0.489
 
 
16.7
 
 
 
 
ATOSA
 
 
hsa-miR-221-3p
 
 
-0.495
 
 
17.8
 
 
 
 
ATP2A2
 
 
hsa-let-7c-5p
 
 
-0.311
 
 
69.3
 
 
 
 
ATP6V1F
 
 
hsa-miR-185-5p
 
 
-0.765
 
 
205.0
 
 
 
 
ATPAF1
 
 
hsa-let-7c-5p
 
 
-0.498
 
 
29.2
 
 
 
 
ATXN1L
 
 
hsa-miR-155-5p
 
 
-0.315
 
 
11.4
 
 
 
 
AURKA
 
 
hsa-miR-92a-3p
 
 
-0.358
 
 
28.8
 
 
 
 
AVPI1
 
 
hsa-miR-7-5p
 
 
-0.367
 
 
16.0
 
 
 
 
B3GAT3
 
 
hsa-let-7c-5p
 
 
-0.324
 
 
26.6
 
 
 
 
B4GALT5
 
 
hsa-miR-210-3p
 
 
-0.511
 
 
20.8
 
 
 
 
BACH1
 
 
hsa-let-7c-5p
 
 
-0.306
 
 
25.4
 
 
 
 
BACH1
 
 
hsa-let-7c-5p
 
 
-0.306
 
 
25.4
 
 
 
 
BACH1
 
 
hsa-let-7c-5p
 
 
-0.435
 
 
25.4
 
 
 
 
BACH1
 
 
hsa-let-7c-5p
 
 
-0.435
 
 
25.4
 
 
 
 
BCAT2
 
 
hsa-miR-103a-3p
 
 
-0.364
 
 
19.4
 
 
 
 
BCAT2
 
 
hsa-miR-92a-3p
 
 
-0.609
 
 
19.4
 
 
 
 
BCL2L13
 
 
hsa-miR-103a-3p
 
 
-0.302
 
 
10.0
 
 
 
 
BICDL1
 
 
hsa-miR-221-3p
 
 
-0.447
 
 
27.2
 
 
 
 
BIN1
 
 
hsa-miR-744-5p
 
 
-0.654
 
 
10.0
 
 
 
 
 
 
 thymus 
 
 
 
 
target_symbol
 
 
mature_mirna_id
 
 
score
 
 
nTPM
 
 
 
 
 
 
AARS1
 
 
hsa-miR-92a-3p
 
 
-0.458
 
 
34.0
 
 
 
 
ABHD17B
 
 
hsa-miR-486-5p
 
 
-0.348
 
 
30.8
 
 
 
 
ACTC1
 
 
hsa-miR-92a-3p
 
 
-0.361
 
 
10.5
 
 
 
 
ACTC1
 
 
hsa-miR-7-5p
 
 
-0.471
 
 
10.5
 
 
 
 
ADCY3
 
 
hsa-miR-92a-3p
 
 
-0.381
 
 
10.7
 
 
 
 
ADNP
 
 
hsa-miR-328-3p
 
 
-0.371
 
 
41.7
 
 
 
 
AGFG1
 
 
hsa-miR-103a-3p
 
 
-0.389
 
 
45.3
 
 
 
 
AHCTF1
 
 
hsa-let-7c-5p
 
 
-0.374
 
 
11.4
 
 
 
 
AHNAK
 
 
hsa-miR-20a-5p
 
 
-0.316
 
 
26.0
 
 
 
 
AHNAK
 
 
hsa-miR-17-5p
 
 
-0.337
 
 
26.0
 
 
 
 
AHNAK
 
 
hsa-miR-93-5p
 
 
-0.337
 
 
26.0
 
 
 
 
AIFM1
 
 
hsa-let-7c-5p
 
 
-0.314
 
 
17.2
 
 
 
 
AKAP10
 
 
hsa-miR-339-5p
 
 
-0.302
 
 
13.1
 
 
 
 
AKIRIN2
 
 
hsa-miR-103a-3p
 
 
-0.299
 
 
29.1
 
 
 
 
ALAD
 
 
hsa-miR-103a-3p
 
 
-0.385
 
 
18.9
 
 
 
 
ALG3
 
 
hsa-miR-20a-5p
 
 
-0.559
 
 
11.4
 
 
 
 
ALG3
 
 
hsa-miR-93-5p
 
 
-0.564
 
 
11.4
 
 
 
 
ALG3
 
 
hsa-miR-17-5p
 
 
-0.572
 
 
11.4
 
 
 
 
ANKFY1
 
 
hsa-miR-155-5p
 
 
-0.315
 
 
18.9
 
 
 
 
ANKRD12
 
 
hsa-miR-7-5p
 
 
-0.37
 
 
10.7
 
 
 
 
ANKRD44
 
 
hsa-miR-92a-3p
 
 
-0.324
 
 
26.3
 
 
 
 
ANKRD46
 
 
hsa-let-7c-5p
 
 
-0.389
 
 
14.3
 
 
 
 
ANP32E
 
 
hsa-miR-92a-3p
 
 
-0.329
 
 
82.1
 
 
 
 
AP1AR
 
 
hsa-miR-92a-3p
 
 
-0.325
 
 
10.2
 
 
 
 
AP1S1
 
 
hsa-let-7c-5p
 
 
-0.374
 
 
17.8
 
 
 
 
AP4B1
 
 
hsa-miR-378a-3p
 
 
-0.62
 
 
15.0
 
 
 
 
APPL1
 
 
hsa-miR-92a-3p
 
 
-0.375
 
 
17.6
 
 
 
 
AQP3
 
 
hsa-miR-874-3p
 
 
-0.48
 
 
217.7
 
 
 
 
ARF4
 
 
hsa-miR-7-5p
 
 
-0.407
 
 
69.9
 
 
 
 
ARF4
 
 
hsa-miR-221-3p
 
 
-0.553
 
 
69.9
 
 
 
 
ARHGAP1
 
 
hsa-miR-17-5p
 
 
-0.325
 
 
21.6
 
 
 
 
ARHGAP1
 
 
hsa-miR-93-5p
 
 
-0.325
 
 
21.6
 
 
 
 
ARHGAP1
 
 
hsa-miR-20a-5p
 
 
-0.339
 
 
21.6
 
 
 
 
ARHGAP5
 
 
hsa-miR-486-5p
 
 
-0.3
 
 
17.1
 
 
 
 
ARID2
 
 
hsa-miR-155-5p
 
 
-0.384
 
 
17.5
 
 
 
 
ARID2
 
 
hsa-miR-103a-3p
 
 
-0.415
 
 
17.5
 
 
 
 
ARID4A
 
 
hsa-miR-7-5p
 
 
-0.341
 
 
13.5
 
 
 
 
ARID4B
 
 
hsa-miR-20a-5p
 
 
-0.29
 
 
19.9
 
 
 
 
ARID4B
 
 
hsa-miR-486-5p
 
 
-0.367
 
 
19.9
 
 
 
 
ARIH2
 
 
hsa-miR-103a-3p
 
 
-0.307
 
 
24.3
 
 
 
 
ARL1
 
 
hsa-miR-20a-5p
 
 
-0.403
 
 
30.6
 
 
 
 
ARL1
 
 
hsa-miR-93-5p
 
 
-0.424
 
 
30.6
 
 
 
 
ARL1
 
 
hsa-miR-17-5p
 
 
-0.432
 
 
30.6
 
 
 
 
ARL5B
 
 
hsa-miR-92a-3p
 
 
-0.331
 
 
12.6
 
 
 
 
ARL5B
 
 
hsa-miR-155-5p
 
 
-0.355
 
 
12.6
 
 
 
 
ARL6IP1
 
 
hsa-miR-7-5p
 
 
-0.374
 
 
147.6
 
 
 
 
ARL6IP1
 
 
hsa-miR-328-3p
 
 
-0.494
 
 
147.6
 
 
 
 
ARL6IP6
 
 
hsa-let-7c-5p
 
 
-0.293
 
 
20.0
 
 
 
 
ARL8A
 
 
hsa-miR-103a-3p
 
 
-0.38
 
 
16.8
 
 
 
 
ARMC1
 
 
hsa-miR-92a-3p
 
 
-0.322
 
 
20.8
 
 
 
 
ARMC1
 
 
hsa-miR-103a-3p
 
 
-0.525
 
 
20.8
 
 
 
 
ARMC8
 
 
hsa-miR-486-5p
 
 
-0.321
 
 
20.7
 
 
 
 
ARMC8
 
 
hsa-miR-20a-5p
 
 
-0.357
 
 
20.7
 
 
 
 
ARMC8
 
 
hsa-miR-93-5p
 
 
-0.362
 
 
20.7
 
 
 
 
ARMC8
 
 
hsa-miR-17-5p
 
 
-0.378
 
 
20.7
 
 
 
 
ARMC8
 
 
hsa-miR-103a-3p
 
 
-0.412
 
 
20.7
 
 
 
 
ARMT1
 
 
hsa-let-7c-5p
 
 
-0.303
 
 
14.5
 
 
 
 
ARNT
 
 
hsa-miR-103a-3p
 
 
-0.422
 
 
19.0
 
 
 
 
ARNT
 
 
hsa-miR-221-3p
 
 
-0.465
 
 
19.0
 
 
 
 
ARPP19
 
 
hsa-miR-328-3p
 
 
-0.404
 
 
49.2
 
 
 
 
ARPP19
 
 
hsa-let-7c-5p
 
 
-0.492
 
 
49.2
 
 
 
 
ARRDC3
 
 
hsa-miR-92a-3p
 
 
-0.458
 
 
26.1
 
 
 
 
ASAP1
 
 
hsa-let-7c-5p
 
 
-0.308
 
 
14.9
 
 
 
 
ASB6
 
 
hsa-miR-874-3p
 
 
-0.368
 
 
10.4
 
 
 
 
ASPH
 
 
hsa-miR-221-3p
 
 
-0.287
 
 
15.5
 
 
 
 
ASPH
 
 
hsa-miR-92a-3p
 
 
-0.416
 
 
15.5
 
 
 
 
ATAD2
 
 
hsa-miR-17-5p
 
 
-0.304
 
 
26.3
 
 
 
 
ATAD2
 
 
hsa-miR-20a-5p
 
 
-0.318
 
 
26.3
 
 
 
 
ATF3
 
 
hsa-miR-874-3p
 
 
-0.288
 
 
79.1
 
 
 
 
ATG4A
 
 
hsa-miR-7-5p
 
 
-0.448
 
 
10.5
 
 
 
 
ATL2
 
 
hsa-miR-103a-3p
 
 
-0.489
 
 
26.0
 
 
 
 
ATOSA
 
 
hsa-miR-221-3p
 
 
-0.495
 
 
16.1
 
 
 
 
ATP2A2
 
 
hsa-let-7c-5p
 
 
-0.311
 
 
33.8
 
 
 
 
ATP6V1F
 
 
hsa-miR-185-5p
 
 
-0.765
 
 
111.4
 
 
 
 
ATPAF1
 
 
hsa-let-7c-5p
 
 
-0.498
 
 
41.4
 
 
 
 
ATXN1L
 
 
hsa-miR-155-5p
 
 
-0.315
 
 
11.9
 
 
 
 
AURKA
 
 
hsa-miR-92a-3p
 
 
-0.358
 
 
18.8
 
 
 
 
B3GAT3
 
 
hsa-let-7c-5p
 
 
-0.324
 
 
17.5
 
 
 
 
BACH1
 
 
hsa-let-7c-5p
 
 
-0.306
 
 
13.2
 
 
 
 
BACH1
 
 
hsa-let-7c-5p
 
 
-0.306
 
 
13.2
 
 
 
 
BACH1
 
 
hsa-let-7c-5p
 
 
-0.435
 
 
13.2
 
 
 
 
BACH1
 
 
hsa-let-7c-5p
 
 
-0.435
 
 
13.2
 
 
 
 
BAK1
 
 
hsa-miR-103a-3p
 
 
-0.364
 
 
17.9
 
 
 
 
BCAT2
 
 
hsa-miR-103a-3p
 
 
-0.364
 
 
11.2
 
 
 
 
BCAT2
 
 
hsa-miR-92a-3p
 
 
-0.609
 
 
11.2
 
 
 
 
BCL2L11
 
 
hsa-miR-339-5p
 
 
-0.306
 
 
18.0
 
 
 
 
BCL2L11
 
 
hsa-miR-92a-3p
 
 
-0.394
 
 
18.0
 
 
 
 
BCL2L11
 
 
hsa-miR-221-3p
 
 
-0.423
 
 
18.0
 
 
 
 
BICDL1
 
 
hsa-miR-221-3p
 
 
-0.447
 
 
12.0
 
 
 
 
BIN1
 
 
hsa-miR-744-5p
 
 
-0.654
 
 
17.1
 
 
 
 
BIN3
 
 
hsa-let-7c-5p
 
 
-0.427
 
 
12.4
 
 
 
 
BLOC1S6
 
 
hsa-let-7c-5p
 
 
-0.321
 
 
23.5
 
 
 
 
BLTP1
 
 
hsa-miR-92a-3p
 
 
-0.462
 
 
15.7
 
 
 
 
BMF
 
 
hsa-miR-221-3p
 
 
-0.453
 
 
70.7
 
 
 
 
BNIP3L
 
 
hsa-miR-20a-5p
 
 
-0.287
 
 
37.1
 
 
 
 
BOLA3
 
 
hsa-miR-103a-3p
 
 
-0.362
 
 
23.1
 
 
 
 
BSDC1
 
 
hsa-miR-92a-3p
 
 
-0.362
 
 
18.1
 
 
 
 
BTAF1
 
 
hsa-miR-486-5p
 
 
-0.288
 
 
17.2
 
 
 
 
BTBD10
 
 
hsa-miR-17-5p
 
 
-0.409
 
 
17.3
 
 
 
 
BTBD10
 
 
hsa-miR-93-5p
 
 
-0.409
 
 
17.3
 
 
 
 
 
 
 thyroid gland 
 
 
 
 
target_symbol
 
 
mature_mirna_id
 
 
score
 
 
nTPM
 
 
 
 
 
 
AARS1
 
 
hsa-miR-92a-3p
 
 
-0.458
 
 
62.3
 
 
 
 
ABHD5
 
 
hsa-miR-20a-5p
 
 
-0.397
 
 
14.4
 
 
 
 
ABHD5
 
 
hsa-miR-93-5p
 
 
-0.402
 
 
14.4
 
 
 
 
ABHD5
 
 
hsa-miR-17-5p
 
 
-0.45
 
 
14.4
 
 
 
 
ACOX1
 
 
hsa-miR-92a-3p
 
 
-0.321
 
 
10.1
 
 
 
 
ACTA1
 
 
hsa-miR-155-5p
 
 
-0.595
 
 
11.1
 
 
 
 
ADAM9
 
 
hsa-miR-20a-5p
 
 
-0.299
 
 
37.4
 
 
 
 
ADAM9
 
 
hsa-miR-17-5p
 
 
-0.32
 
 
37.4
 
 
 
 
ADAM9
 
 
hsa-miR-93-5p
 
 
-0.32
 
 
37.4
 
 
 
 
ADCY3
 
 
hsa-miR-92a-3p
 
 
-0.381
 
 
35.2
 
 
 
 
ADNP
 
 
hsa-miR-328-3p
 
 
-0.371
 
 
27.8
 
 
 
 
AGFG1
 
 
hsa-miR-103a-3p
 
 
-0.389
 
 
32.1
 
 
 
 
AGFG2
 
 
hsa-miR-20a-5p
 
 
-0.365
 
 
10.2
 
 
 
 
AGFG2
 
 
hsa-miR-93-5p
 
 
-0.378
 
 
10.2
 
 
 
 
AGFG2
 
 
hsa-miR-17-5p
 
 
-0.402
 
 
10.2
 
 
 
 
AHCTF1
 
 
hsa-let-7c-5p
 
 
-0.374
 
 
12.9
 
 
 
 
AHNAK
 
 
hsa-miR-20a-5p
 
 
-0.316
 
 
61.4
 
 
 
 
AHNAK
 
 
hsa-miR-17-5p
 
 
-0.337
 
 
61.4
 
 
 
 
AHNAK
 
 
hsa-miR-93-5p
 
 
-0.337
 
 
61.4
 
 
 
 
AIFM1
 
 
hsa-let-7c-5p
 
 
-0.314
 
 
10.7
 
 
 
 
AJUBA
 
 
hsa-miR-103a-3p
 
 
-0.448
 
 
10.2
 
 
 
 
AKAP10
 
 
hsa-miR-339-5p
 
 
-0.302
 
 
14.1
 
 
 
 
AKIRIN2
 
 
hsa-miR-103a-3p
 
 
-0.299
 
 
15.5
 
 
 
 
ALAD
 
 
hsa-miR-103a-3p
 
 
-0.385
 
 
24.2
 
 
 
 
ALG3
 
 
hsa-miR-20a-5p
 
 
-0.559
 
 
17.5
 
 
 
 
ALG3
 
 
hsa-miR-93-5p
 
 
-0.564
 
 
17.5
 
 
 
 
ALG3
 
 
hsa-miR-17-5p
 
 
-0.572
 
 
17.5
 
 
 
 
ALG9
 
 
hsa-miR-7-5p
 
 
-0.302
 
 
11.5
 
 
 
 
ALKAL2
 
 
hsa-miR-92a-3p
 
 
-0.608
 
 
10.3
 
 
 
 
ANGPTL2
 
 
hsa-miR-221-3p
 
 
-0.369
 
 
15.2
 
 
 
 
ANKFY1
 
 
hsa-miR-155-5p
 
 
-0.315
 
 
13.5
 
 
 
 
ANKRD12
 
 
hsa-miR-7-5p
 
 
-0.37
 
 
15.3
 
 
 
 
ANKRD46
 
 
hsa-let-7c-5p
 
 
-0.389
 
 
18.8
 
 
 
 
ANKRD9
 
 
hsa-miR-20a-5p
 
 
-0.31
 
 
10.7
 
 
 
 
ANKRD9
 
 
hsa-miR-93-5p
 
 
-0.331
 
 
10.7
 
 
 
 
ANKRD9
 
 
hsa-miR-17-5p
 
 
-0.347
 
 
10.7
 
 
 
 
ANP32E
 
 
hsa-miR-92a-3p
 
 
-0.329
 
 
28.5
 
 
 
 
AP1AR
 
 
hsa-miR-92a-3p
 
 
-0.325
 
 
10.1
 
 
 
 
AP1S1
 
 
hsa-let-7c-5p
 
 
-0.374
 
 
15.7
 
 
 
 
AP4B1
 
 
hsa-miR-378a-3p
 
 
-0.62
 
 
10.7
 
 
 
 
APPL1
 
 
hsa-miR-92a-3p
 
 
-0.375
 
 
18.5
 
 
 
 
ARF4
 
 
hsa-miR-7-5p
 
 
-0.407
 
 
134.8
 
 
 
 
ARF4
 
 
hsa-miR-221-3p
 
 
-0.553
 
 
134.8
 
 
 
 
ARG2
 
 
hsa-let-7c-5p
 
 
-0.468
 
 
97.9
 
 
 
 
ARHGAP1
 
 
hsa-miR-17-5p
 
 
-0.325
 
 
42.2
 
 
 
 
ARHGAP1
 
 
hsa-miR-93-5p
 
 
-0.325
 
 
42.2
 
 
 
 
ARHGAP1
 
 
hsa-miR-20a-5p
 
 
-0.339
 
 
42.2
 
 
 
 
ARHGAP5
 
 
hsa-miR-486-5p
 
 
-0.3
 
 
64.3
 
 
 
 
ARHGEF28
 
 
hsa-miR-93-5p
 
 
-0.33
 
 
12.7
 
 
 
 
ARHGEF28
 
 
hsa-miR-17-5p
 
 
-0.363
 
 
12.7
 
 
 
 
ARHGEF28
 
 
hsa-miR-20a-5p
 
 
-0.366
 
 
12.7
 
 
 
 
ARID4B
 
 
hsa-miR-20a-5p
 
 
-0.29
 
 
15.9
 
 
 
 
ARID4B
 
 
hsa-miR-486-5p
 
 
-0.367
 
 
15.9
 
 
 
 
ARIH2
 
 
hsa-miR-103a-3p
 
 
-0.307
 
 
20.6
 
 
 
 
ARL1
 
 
hsa-miR-20a-5p
 
 
-0.403
 
 
79.3
 
 
 
 
ARL1
 
 
hsa-miR-93-5p
 
 
-0.424
 
 
79.3
 
 
 
 
ARL1
 
 
hsa-miR-17-5p
 
 
-0.432
 
 
79.3
 
 
 
 
ARL4D
 
 
hsa-let-7c-5p
 
 
-0.417
 
 
35.9
 
 
 
 
ARL6IP1
 
 
hsa-miR-7-5p
 
 
-0.374
 
 
105.4
 
 
 
 
ARL6IP1
 
 
hsa-miR-328-3p
 
 
-0.494
 
 
105.4
 
 
 
 
ARL8A
 
 
hsa-miR-103a-3p
 
 
-0.38
 
 
30.9
 
 
 
 
ARMC1
 
 
hsa-miR-92a-3p
 
 
-0.322
 
 
13.8
 
 
 
 
ARMC1
 
 
hsa-miR-103a-3p
 
 
-0.525
 
 
13.8
 
 
 
 
ARMC8
 
 
hsa-miR-486-5p
 
 
-0.321
 
 
20.0
 
 
 
 
ARMC8
 
 
hsa-miR-20a-5p
 
 
-0.357
 
 
20.0
 
 
 
 
ARMC8
 
 
hsa-miR-93-5p
 
 
-0.362
 
 
20.0
 
 
 
 
ARMC8
 
 
hsa-miR-17-5p
 
 
-0.378
 
 
20.0
 
 
 
 
ARMC8
 
 
hsa-miR-103a-3p
 
 
-0.412
 
 
20.0
 
 
 
 
ARMT1
 
 
hsa-let-7c-5p
 
 
-0.303
 
 
25.9
 
 
 
 
ARNT
 
 
hsa-miR-103a-3p
 
 
-0.422
 
 
18.4
 
 
 
 
ARNT
 
 
hsa-miR-221-3p
 
 
-0.465
 
 
18.4
 
 
 
 
ARPP19
 
 
hsa-miR-328-3p
 
 
-0.404
 
 
53.7
 
 
 
 
ARPP19
 
 
hsa-let-7c-5p
 
 
-0.492
 
 
53.7
 
 
 
 
ARRDC3
 
 
hsa-miR-92a-3p
 
 
-0.458
 
 
78.7
 
 
 
 
ARRDC4
 
 
hsa-let-7c-5p
 
 
-0.329
 
 
16.2
 
 
 
 
ARVCF
 
 
hsa-miR-155-5p
 
 
-0.388
 
 
14.8
 
 
 
 
ASAP1
 
 
hsa-let-7c-5p
 
 
-0.308
 
 
15.1
 
 
 
 
ASB6
 
 
hsa-miR-874-3p
 
 
-0.368
 
 
10.3
 
 
 
 
ASPH
 
 
hsa-miR-221-3p
 
 
-0.287
 
 
90.3
 
 
 
 
ASPH
 
 
hsa-miR-92a-3p
 
 
-0.416
 
 
90.3
 
 
 
 
ASPN
 
 
hsa-miR-92a-3p
 
 
-0.422
 
 
10.8
 
 
 
 
ATF3
 
 
hsa-miR-874-3p
 
 
-0.288
 
 
169.7
 
 
 
 
ATG16L1
 
 
hsa-miR-20a-5p
 
 
-0.446
 
 
11.5
 
 
 
 
ATG16L1
 
 
hsa-miR-93-5p
 
 
-0.451
 
 
11.5
 
 
 
 
ATG16L1
 
 
hsa-miR-17-5p
 
 
-0.467
 
 
11.5
 
 
 
 
ATG4A
 
 
hsa-miR-7-5p
 
 
-0.448
 
 
10.7
 
 
 
 
ATL2
 
 
hsa-miR-103a-3p
 
 
-0.489
 
 
51.5
 
 
 
 
ATOSA
 
 
hsa-miR-221-3p
 
 
-0.495
 
 
23.3
 
 
 
 
ATOSB
 
 
hsa-miR-328-3p
 
 
-0.358
 
 
11.1
 
 
 
 
ATOSB
 
 
hsa-let-7c-5p
 
 
-0.429
 
 
11.1
 
 
 
 
ATP2A2
 
 
hsa-let-7c-5p
 
 
-0.311
 
 
68.6
 
 
 
 
ATP6V1F
 
 
hsa-miR-185-5p
 
 
-0.765
 
 
85.4
 
 
 
 
ATPAF1
 
 
hsa-let-7c-5p
 
 
-0.498
 
 
39.3
 
 
 
 
ATXN1L
 
 
hsa-miR-155-5p
 
 
-0.315
 
 
11.1
 
 
 
 
B3GAT3
 
 
hsa-let-7c-5p
 
 
-0.324
 
 
33.1
 
 
 
 
B4GALT5
 
 
hsa-miR-210-3p
 
 
-0.511
 
 
10.8
 
 
 
 
B4GAT1
 
 
hsa-let-7c-5p
 
 
-0.47
 
 
20.5
 
 
 
 
BACE1
 
 
hsa-miR-339-5p
 
 
-0.371
 
 
11.3
 
 
 
 
BACH1
 
 
hsa-let-7c-5p
 
 
-0.306
 
 
11.8
 
 
 
 
BACH1
 
 
hsa-let-7c-5p
 
 
-0.306
 
 
11.8
 
 
 
 
 
 
 tongue 
 
 
 
 
target_symbol
 
 
mature_mirna_id
 
 
score
 
 
nTPM
 
 
 
 
 
 
AARS1
 
 
hsa-miR-92a-3p
 
 
-0.458
 
 
37.1
 
 
 
 
ABHD5
 
 
hsa-miR-20a-5p
 
 
-0.397
 
 
17.3
 
 
 
 
ABHD5
 
 
hsa-miR-93-5p
 
 
-0.402
 
 
17.3
 
 
 
 
ABHD5
 
 
hsa-miR-17-5p
 
 
-0.45
 
 
17.3
 
 
 
 
ACOX1
 
 
hsa-miR-92a-3p
 
 
-0.321
 
 
15.2
 
 
 
 
ACTA1
 
 
hsa-miR-155-5p
 
 
-0.595
 
 
19457.9
 
 
 
 
ACTC1
 
 
hsa-miR-92a-3p
 
 
-0.361
 
 
182.1
 
 
 
 
ACTC1
 
 
hsa-miR-7-5p
 
 
-0.471
 
 
182.1
 
 
 
 
ADAM9
 
 
hsa-miR-20a-5p
 
 
-0.299
 
 
23.3
 
 
 
 
ADAM9
 
 
hsa-miR-17-5p
 
 
-0.32
 
 
23.3
 
 
 
 
ADAM9
 
 
hsa-miR-93-5p
 
 
-0.32
 
 
23.3
 
 
 
 
ADCY3
 
 
hsa-miR-92a-3p
 
 
-0.381
 
 
19.0
 
 
 
 
ADNP
 
 
hsa-miR-328-3p
 
 
-0.371
 
 
26.5
 
 
 
 
AGFG1
 
 
hsa-miR-103a-3p
 
 
-0.389
 
 
36.1
 
 
 
 
AGO4
 
 
hsa-miR-103a-3p
 
 
-0.32
 
 
11.1
 
 
 
 
AGO4
 
 
hsa-miR-221-3p
 
 
-0.321
 
 
11.1
 
 
 
 
AHCTF1
 
 
hsa-let-7c-5p
 
 
-0.374
 
 
14.7
 
 
 
 
AHNAK
 
 
hsa-miR-20a-5p
 
 
-0.316
 
 
160.9
 
 
 
 
AHNAK
 
 
hsa-miR-17-5p
 
 
-0.337
 
 
160.9
 
 
 
 
AHNAK
 
 
hsa-miR-93-5p
 
 
-0.337
 
 
160.9
 
 
 
 
AIFM1
 
 
hsa-let-7c-5p
 
 
-0.314
 
 
23.4
 
 
 
 
AKAP6
 
 
hsa-let-7c-5p
 
 
-0.307
 
 
47.4
 
 
 
 
AKIRIN2
 
 
hsa-miR-103a-3p
 
 
-0.299
 
 
39.8
 
 
 
 
ALAD
 
 
hsa-miR-103a-3p
 
 
-0.385
 
 
22.3
 
 
 
 
ALG3
 
 
hsa-miR-20a-5p
 
 
-0.559
 
 
13.8
 
 
 
 
ALG3
 
 
hsa-miR-93-5p
 
 
-0.564
 
 
13.8
 
 
 
 
ALG3
 
 
hsa-miR-17-5p
 
 
-0.572
 
 
13.8
 
 
 
 
ANGPTL2
 
 
hsa-miR-221-3p
 
 
-0.369
 
 
34.2
 
 
 
 
ANK1
 
 
hsa-miR-103a-3p
 
 
-0.321
 
 
592.2
 
 
 
 
ANKFY1
 
 
hsa-miR-155-5p
 
 
-0.315
 
 
10.6
 
 
 
 
ANKRD12
 
 
hsa-miR-7-5p
 
 
-0.37
 
 
11.5
 
 
 
 
ANKRD46
 
 
hsa-let-7c-5p
 
 
-0.389
 
 
33.8
 
 
 
 
ANKRD9
 
 
hsa-miR-20a-5p
 
 
-0.31
 
 
24.6
 
 
 
 
ANKRD9
 
 
hsa-miR-93-5p
 
 
-0.331
 
 
24.6
 
 
 
 
ANKRD9
 
 
hsa-miR-17-5p
 
 
-0.347
 
 
24.6
 
 
 
 
ANP32E
 
 
hsa-miR-92a-3p
 
 
-0.329
 
 
24.3
 
 
 
 
APCDD1
 
 
hsa-miR-20a-5p
 
 
-0.404
 
 
17.3
 
 
 
 
APCDD1
 
 
hsa-miR-17-5p
 
 
-0.425
 
 
17.3
 
 
 
 
APCDD1
 
 
hsa-miR-93-5p
 
 
-0.425
 
 
17.3
 
 
 
 
APPL1
 
 
hsa-miR-92a-3p
 
 
-0.375
 
 
24.8
 
 
 
 
AQP3
 
 
hsa-miR-874-3p
 
 
-0.48
 
 
25.0
 
 
 
 
ARF4
 
 
hsa-miR-7-5p
 
 
-0.407
 
 
64.9
 
 
 
 
ARF4
 
 
hsa-miR-221-3p
 
 
-0.553
 
 
64.9
 
 
 
 
ARG2
 
 
hsa-let-7c-5p
 
 
-0.468
 
 
49.2
 
 
 
 
ARHGAP1
 
 
hsa-miR-17-5p
 
 
-0.325
 
 
19.1
 
 
 
 
ARHGAP1
 
 
hsa-miR-93-5p
 
 
-0.325
 
 
19.1
 
 
 
 
ARHGAP1
 
 
hsa-miR-20a-5p
 
 
-0.339
 
 
19.1
 
 
 
 
ARHGAP5
 
 
hsa-miR-486-5p
 
 
-0.3
 
 
23.1
 
 
 
 
ARIH2
 
 
hsa-miR-103a-3p
 
 
-0.307
 
 
74.3
 
 
 
 
ARL1
 
 
hsa-miR-20a-5p
 
 
-0.403
 
 
53.0
 
 
 
 
ARL1
 
 
hsa-miR-93-5p
 
 
-0.424
 
 
53.0
 
 
 
 
ARL1
 
 
hsa-miR-17-5p
 
 
-0.432
 
 
53.0
 
 
 
 
ARL6IP1
 
 
hsa-miR-7-5p
 
 
-0.374
 
 
48.1
 
 
 
 
ARL6IP1
 
 
hsa-miR-328-3p
 
 
-0.494
 
 
48.1
 
 
 
 
ARL8A
 
 
hsa-miR-103a-3p
 
 
-0.38
 
 
24.2
 
 
 
 
ARMC1
 
 
hsa-miR-92a-3p
 
 
-0.322
 
 
23.0
 
 
 
 
ARMC1
 
 
hsa-miR-103a-3p
 
 
-0.525
 
 
23.0
 
 
 
 
ARMC8
 
 
hsa-miR-486-5p
 
 
-0.321
 
 
32.4
 
 
 
 
ARMC8
 
 
hsa-miR-20a-5p
 
 
-0.357
 
 
32.4
 
 
 
 
ARMC8
 
 
hsa-miR-93-5p
 
 
-0.362
 
 
32.4
 
 
 
 
ARMC8
 
 
hsa-miR-17-5p
 
 
-0.378
 
 
32.4
 
 
 
 
ARMC8
 
 
hsa-miR-103a-3p
 
 
-0.412
 
 
32.4
 
 
 
 
ARMT1
 
 
hsa-let-7c-5p
 
 
-0.303
 
 
21.1
 
 
 
 
ARNT
 
 
hsa-miR-103a-3p
 
 
-0.422
 
 
26.3
 
 
 
 
ARNT
 
 
hsa-miR-221-3p
 
 
-0.465
 
 
26.3
 
 
 
 
ARPP19
 
 
hsa-miR-328-3p
 
 
-0.404
 
 
158.9
 
 
 
 
ARPP19
 
 
hsa-let-7c-5p
 
 
-0.492
 
 
158.9
 
 
 
 
ARRDC3
 
 
hsa-miR-92a-3p
 
 
-0.458
 
 
41.9
 
 
 
 
ASPH
 
 
hsa-miR-221-3p
 
 
-0.287
 
 
155.4
 
 
 
 
ASPH
 
 
hsa-miR-92a-3p
 
 
-0.416
 
 
155.4
 
 
 
 
ASPN
 
 
hsa-miR-92a-3p
 
 
-0.422
 
 
18.5
 
 
 
 
ATF3
 
 
hsa-miR-874-3p
 
 
-0.288
 
 
28.0
 
 
 
 
ATG4A
 
 
hsa-miR-7-5p
 
 
-0.448
 
 
17.2
 
 
 
 
ATL2
 
 
hsa-miR-103a-3p
 
 
-0.489
 
 
43.2
 
 
 
 
ATOSA
 
 
hsa-miR-221-3p
 
 
-0.495
 
 
23.7
 
 
 
 
ATOSB
 
 
hsa-miR-328-3p
 
 
-0.358
 
 
10.9
 
 
 
 
ATOSB
 
 
hsa-let-7c-5p
 
 
-0.429
 
 
10.9
 
 
 
 
ATP2A2
 
 
hsa-let-7c-5p
 
 
-0.311
 
 
2586.2
 
 
 
 
ATP6V1F
 
 
hsa-miR-185-5p
 
 
-0.765
 
 
144.3
 
 
 
 
ATPAF1
 
 
hsa-let-7c-5p
 
 
-0.498
 
 
295.7
 
 
 
 
ATXN1
 
 
hsa-miR-221-3p
 
 
-0.289
 
 
13.9
 
 
 
 
ATXN1L
 
 
hsa-miR-155-5p
 
 
-0.315
 
 
12.7
 
 
 
 
B4GALT5
 
 
hsa-miR-210-3p
 
 
-0.511
 
 
22.7
 
 
 
 
B4GAT1
 
 
hsa-let-7c-5p
 
 
-0.47
 
 
17.9
 
 
 
 
BACH1
 
 
hsa-let-7c-5p
 
 
-0.306
 
 
11.9
 
 
 
 
BACH1
 
 
hsa-let-7c-5p
 
 
-0.306
 
 
11.9
 
 
 
 
BACH1
 
 
hsa-let-7c-5p
 
 
-0.435
 
 
11.9
 
 
 
 
BACH1
 
 
hsa-let-7c-5p
 
 
-0.435
 
 
11.9
 
 
 
 
BAK1
 
 
hsa-miR-103a-3p
 
 
-0.364
 
 
16.9
 
 
 
 
BCAT2
 
 
hsa-miR-103a-3p
 
 
-0.364
 
 
27.4
 
 
 
 
BCAT2
 
 
hsa-miR-92a-3p
 
 
-0.609
 
 
27.4
 
 
 
 
BCL2L13
 
 
hsa-miR-103a-3p
 
 
-0.302
 
 
37.8
 
 
 
 
BDH1
 
 
hsa-miR-874-3p
 
 
-0.644
 
 
21.3
 
 
 
 
BIN1
 
 
hsa-miR-744-5p
 
 
-0.654
 
 
751.1
 
 
 
 
BLOC1S6
 
 
hsa-let-7c-5p
 
 
-0.321
 
 
21.7
 
 
 
 
BLTP1
 
 
hsa-miR-92a-3p
 
 
-0.462
 
 
31.9
 
 
 
 
BMP5
 
 
hsa-let-7c-5p
 
 
-0.337
 
 
11.4
 
 
 
 
BNIP3L
 
 
hsa-miR-20a-5p
 
 
-0.287
 
 
29.6
 
 
 
 
BOLA3
 
 
hsa-miR-103a-3p
 
 
-0.362
 
 
162.0
 
 
 
 
BRMS1L
 
 
hsa-miR-92a-3p
 
 
-0.306
 
 
12.8
 
 
 
 
 
 
 tonsil 
 
 
 
 
target_symbol
 
 
mature_mirna_id
 
 
score
 
 
nTPM
 
 
 
 
 
 
AARS1
 
 
hsa-miR-92a-3p
 
 
-0.458
 
 
33.3
 
 
 
 
ABHD17C
 
 
hsa-let-7c-5p
 
 
-0.359
 
 
21.6
 
 
 
 
ABHD17C
 
 
hsa-miR-330-3p
 
 
-0.399
 
 
21.6
 
 
 
 
ADAM19
 
 
hsa-miR-874-3p
 
 
-0.374
 
 
28.3
 
 
 
 
ADAM9
 
 
hsa-miR-20a-5p
 
 
-0.299
 
 
12.4
 
 
 
 
ADAM9
 
 
hsa-miR-17-5p
 
 
-0.32
 
 
12.4
 
 
 
 
ADAM9
 
 
hsa-miR-93-5p
 
 
-0.32
 
 
12.4
 
 
 
 
ADCY3
 
 
hsa-miR-92a-3p
 
 
-0.381
 
 
16.9
 
 
 
 
ADNP
 
 
hsa-miR-328-3p
 
 
-0.371
 
 
33.4
 
 
 
 
AGFG1
 
 
hsa-miR-103a-3p
 
 
-0.389
 
 
49.9
 
 
 
 
AHNAK
 
 
hsa-miR-20a-5p
 
 
-0.316
 
 
59.8
 
 
 
 
AHNAK
 
 
hsa-miR-17-5p
 
 
-0.337
 
 
59.8
 
 
 
 
AHNAK
 
 
hsa-miR-93-5p
 
 
-0.337
 
 
59.8
 
 
 
 
AIFM1
 
 
hsa-let-7c-5p
 
 
-0.314
 
 
10.3
 
 
 
 
AKAP10
 
 
hsa-miR-339-5p
 
 
-0.302
 
 
11.7
 
 
 
 
AKIRIN2
 
 
hsa-miR-103a-3p
 
 
-0.299
 
 
28.4
 
 
 
 
ALAD
 
 
hsa-miR-103a-3p
 
 
-0.385
 
 
17.5
 
 
 
 
ALG3
 
 
hsa-miR-20a-5p
 
 
-0.559
 
 
13.2
 
 
 
 
ALG3
 
 
hsa-miR-93-5p
 
 
-0.564
 
 
13.2
 
 
 
 
ALG3
 
 
hsa-miR-17-5p
 
 
-0.572
 
 
13.2
 
 
 
 
ANKFY1
 
 
hsa-miR-155-5p
 
 
-0.315
 
 
12.0
 
 
 
 
ANKRD12
 
 
hsa-miR-7-5p
 
 
-0.37
 
 
17.3
 
 
 
 
ANKRD29
 
 
hsa-miR-20a-5p
 
 
-0.359
 
 
13.4
 
 
 
 
ANKRD29
 
 
hsa-miR-17-5p
 
 
-0.372
 
 
13.4
 
 
 
 
ANKRD29
 
 
hsa-miR-93-5p
 
 
-0.372
 
 
13.4
 
 
 
 
ANKRD44
 
 
hsa-miR-92a-3p
 
 
-0.324
 
 
31.1
 
 
 
 
ANKRD46
 
 
hsa-let-7c-5p
 
 
-0.389
 
 
10.3
 
 
 
 
ANP32E
 
 
hsa-miR-92a-3p
 
 
-0.329
 
 
58.2
 
 
 
 
AP1AR
 
 
hsa-miR-92a-3p
 
 
-0.325
 
 
11.3
 
 
 
 
AP1S1
 
 
hsa-let-7c-5p
 
 
-0.374
 
 
12.8
 
 
 
 
AP4B1
 
 
hsa-miR-378a-3p
 
 
-0.62
 
 
13.0
 
 
 
 
APPL1
 
 
hsa-miR-92a-3p
 
 
-0.375
 
 
18.4
 
 
 
 
AQP3
 
 
hsa-miR-874-3p
 
 
-0.48
 
 
161.9
 
 
 
 
ARF4
 
 
hsa-miR-7-5p
 
 
-0.407
 
 
91.2
 
 
 
 
ARF4
 
 
hsa-miR-221-3p
 
 
-0.553
 
 
91.2
 
 
 
 
ARHGAP1
 
 
hsa-miR-17-5p
 
 
-0.325
 
 
32.4
 
 
 
 
ARHGAP1
 
 
hsa-miR-93-5p
 
 
-0.325
 
 
32.4
 
 
 
 
ARHGAP1
 
 
hsa-miR-20a-5p
 
 
-0.339
 
 
32.4
 
 
 
 
ARHGAP5
 
 
hsa-miR-486-5p
 
 
-0.3
 
 
17.4
 
 
 
 
ARID4A
 
 
hsa-miR-7-5p
 
 
-0.341
 
 
10.5
 
 
 
 
ARID4B
 
 
hsa-miR-20a-5p
 
 
-0.29
 
 
15.9
 
 
 
 
ARID4B
 
 
hsa-miR-486-5p
 
 
-0.367
 
 
15.9
 
 
 
 
ARIH2
 
 
hsa-miR-103a-3p
 
 
-0.307
 
 
20.1
 
 
 
 
ARL1
 
 
hsa-miR-20a-5p
 
 
-0.403
 
 
45.6
 
 
 
 
ARL1
 
 
hsa-miR-93-5p
 
 
-0.424
 
 
45.6
 
 
 
 
ARL1
 
 
hsa-miR-17-5p
 
 
-0.432
 
 
45.6
 
 
 
 
ARL6IP1
 
 
hsa-miR-7-5p
 
 
-0.374
 
 
106.2
 
 
 
 
ARL6IP1
 
 
hsa-miR-328-3p
 
 
-0.494
 
 
106.2
 
 
 
 
ARL6IP6
 
 
hsa-let-7c-5p
 
 
-0.293
 
 
18.0
 
 
 
 
ARL8A
 
 
hsa-miR-103a-3p
 
 
-0.38
 
 
12.4
 
 
 
 
ARMC1
 
 
hsa-miR-92a-3p
 
 
-0.322
 
 
19.0
 
 
 
 
ARMC1
 
 
hsa-miR-103a-3p
 
 
-0.525
 
 
19.0
 
 
 
 
ARMC8
 
 
hsa-miR-486-5p
 
 
-0.321
 
 
21.4
 
 
 
 
ARMC8
 
 
hsa-miR-20a-5p
 
 
-0.357
 
 
21.4
 
 
 
 
ARMC8
 
 
hsa-miR-93-5p
 
 
-0.362
 
 
21.4
 
 
 
 
ARMC8
 
 
hsa-miR-17-5p
 
 
-0.378
 
 
21.4
 
 
 
 
ARMC8
 
 
hsa-miR-103a-3p
 
 
-0.412
 
 
21.4
 
 
 
 
ARMT1
 
 
hsa-let-7c-5p
 
 
-0.303
 
 
17.3
 
 
 
 
ARNT
 
 
hsa-miR-103a-3p
 
 
-0.422
 
 
21.0
 
 
 
 
ARNT
 
 
hsa-miR-221-3p
 
 
-0.465
 
 
21.0
 
 
 
 
ARPP19
 
 
hsa-miR-328-3p
 
 
-0.404
 
 
48.4
 
 
 
 
ARPP19
 
 
hsa-let-7c-5p
 
 
-0.492
 
 
48.4
 
 
 
 
ARRDC3
 
 
hsa-miR-92a-3p
 
 
-0.458
 
 
26.4
 
 
 
 
ASAP1
 
 
hsa-let-7c-5p
 
 
-0.308
 
 
17.8
 
 
 
 
ASPH
 
 
hsa-miR-221-3p
 
 
-0.287
 
 
26.4
 
 
 
 
ASPH
 
 
hsa-miR-92a-3p
 
 
-0.416
 
 
26.4
 
 
 
 
ATAD2
 
 
hsa-miR-17-5p
 
 
-0.304
 
 
12.0
 
 
 
 
ATAD2
 
 
hsa-miR-20a-5p
 
 
-0.318
 
 
12.0
 
 
 
 
ATG4A
 
 
hsa-miR-7-5p
 
 
-0.448
 
 
10.4
 
 
 
 
ATL2
 
 
hsa-miR-103a-3p
 
 
-0.489
 
 
32.9
 
 
 
 
ATOSA
 
 
hsa-miR-221-3p
 
 
-0.495
 
 
27.6
 
 
 
 
ATP2A2
 
 
hsa-let-7c-5p
 
 
-0.311
 
 
45.1
 
 
 
 
ATP6V1F
 
 
hsa-miR-185-5p
 
 
-0.765
 
 
97.1
 
 
 
 
ATPAF1
 
 
hsa-let-7c-5p
 
 
-0.498
 
 
30.0
 
 
 
 
AURKA
 
 
hsa-miR-92a-3p
 
 
-0.358
 
 
20.6
 
 
 
 
B3GAT3
 
 
hsa-let-7c-5p
 
 
-0.324
 
 
10.3
 
 
 
 
B4GALT5
 
 
hsa-miR-210-3p
 
 
-0.511
 
 
10.8
 
 
 
 
BACH1
 
 
hsa-let-7c-5p
 
 
-0.306
 
 
13.8
 
 
 
 
BACH1
 
 
hsa-let-7c-5p
 
 
-0.306
 
 
13.8
 
 
 
 
BACH1
 
 
hsa-let-7c-5p
 
 
-0.435
 
 
13.8
 
 
 
 
BACH1
 
 
hsa-let-7c-5p
 
 
-0.435
 
 
13.8
 
 
 
 
BAK1
 
 
hsa-miR-103a-3p
 
 
-0.364
 
 
21.9
 
 
 
 
BCAT2
 
 
hsa-miR-103a-3p
 
 
-0.364
 
 
15.5
 
 
 
 
BCAT2
 
 
hsa-miR-92a-3p
 
 
-0.609
 
 
15.5
 
 
 
 
BCL2L11
 
 
hsa-miR-339-5p
 
 
-0.306
 
 
21.7
 
 
 
 
BCL2L11
 
 
hsa-miR-92a-3p
 
 
-0.394
 
 
21.7
 
 
 
 
BCL2L11
 
 
hsa-miR-221-3p
 
 
-0.423
 
 
21.7
 
 
 
 
BIN1
 
 
hsa-miR-744-5p
 
 
-0.654
 
 
25.8
 
 
 
 
BIN3
 
 
hsa-let-7c-5p
 
 
-0.427
 
 
10.2
 
 
 
 
BLOC1S6
 
 
hsa-let-7c-5p
 
 
-0.321
 
 
33.2
 
 
 
 
BMF
 
 
hsa-miR-221-3p
 
 
-0.453
 
 
22.0
 
 
 
 
BNIP3L
 
 
hsa-miR-20a-5p
 
 
-0.287
 
 
48.8
 
 
 
 
BOLA3
 
 
hsa-miR-103a-3p
 
 
-0.362
 
 
20.2
 
 
 
 
BSDC1
 
 
hsa-miR-92a-3p
 
 
-0.362
 
 
20.4
 
 
 
 
BTAF1
 
 
hsa-miR-486-5p
 
 
-0.288
 
 
20.0
 
 
 
 
BTBD10
 
 
hsa-miR-17-5p
 
 
-0.409
 
 
16.3
 
 
 
 
BTBD10
 
 
hsa-miR-93-5p
 
 
-0.409
 
 
16.3
 
 
 
 
BTBD10
 
 
hsa-miR-20a-5p
 
 
-0.412
 
 
16.3
 
 
 
 
BTF3L4
 
 
hsa-let-7c-5p
 
 
-0.355
 
 
38.2
 
 
 
 
BTG2
 
 
hsa-miR-92a-3p
 
 
-0.314
 
 
123.9
 
 
 
 
 
 
 urinary bladder 
 
 
 
 
target_symbol
 
 
mature_mirna_id
 
 
score
 
 
nTPM
 
 
 
 
 
 
AARS1
 
 
hsa-miR-92a-3p
 
 
-0.458
 
 
35.4
 
 
 
 
ABHD17B
 
 
hsa-miR-486-5p
 
 
-0.348
 
 
12.1
 
 
 
 
ABHD17C
 
 
hsa-let-7c-5p
 
 
-0.359
 
 
26.8
 
 
 
 
ABHD17C
 
 
hsa-miR-330-3p
 
 
-0.399
 
 
26.8
 
 
 
 
ABHD5
 
 
hsa-miR-20a-5p
 
 
-0.397
 
 
15.5
 
 
 
 
ABHD5
 
 
hsa-miR-93-5p
 
 
-0.402
 
 
15.5
 
 
 
 
ABHD5
 
 
hsa-miR-17-5p
 
 
-0.45
 
 
15.5
 
 
 
 
ACER2
 
 
hsa-miR-20a-5p
 
 
-0.298
 
 
36.7
 
 
 
 
ACER2
 
 
hsa-miR-93-5p
 
 
-0.303
 
 
36.7
 
 
 
 
ACER2
 
 
hsa-miR-17-5p
 
 
-0.319
 
 
36.7
 
 
 
 
ACER2
 
 
hsa-let-7c-5p
 
 
-0.333
 
 
36.7
 
 
 
 
ACOX1
 
 
hsa-miR-92a-3p
 
 
-0.321
 
 
13.7
 
 
 
 
ACTC1
 
 
hsa-miR-92a-3p
 
 
-0.361
 
 
667.0
 
 
 
 
ACTC1
 
 
hsa-miR-7-5p
 
 
-0.471
 
 
667.0
 
 
 
 
ADAM19
 
 
hsa-miR-874-3p
 
 
-0.374
 
 
15.9
 
 
 
 
ADAM9
 
 
hsa-miR-20a-5p
 
 
-0.299
 
 
52.5
 
 
 
 
ADAM9
 
 
hsa-miR-17-5p
 
 
-0.32
 
 
52.5
 
 
 
 
ADAM9
 
 
hsa-miR-93-5p
 
 
-0.32
 
 
52.5
 
 
 
 
ADCY3
 
 
hsa-miR-92a-3p
 
 
-0.381
 
 
34.2
 
 
 
 
ADNP
 
 
hsa-miR-328-3p
 
 
-0.371
 
 
34.8
 
 
 
 
AGFG1
 
 
hsa-miR-103a-3p
 
 
-0.389
 
 
61.2
 
 
 
 
AGFG2
 
 
hsa-miR-20a-5p
 
 
-0.365
 
 
10.3
 
 
 
 
AGFG2
 
 
hsa-miR-93-5p
 
 
-0.378
 
 
10.3
 
 
 
 
AGFG2
 
 
hsa-miR-17-5p
 
 
-0.402
 
 
10.3
 
 
 
 
AHNAK
 
 
hsa-miR-20a-5p
 
 
-0.316
 
 
145.5
 
 
 
 
AHNAK
 
 
hsa-miR-17-5p
 
 
-0.337
 
 
145.5
 
 
 
 
AHNAK
 
 
hsa-miR-93-5p
 
 
-0.337
 
 
145.5
 
 
 
 
AIFM1
 
 
hsa-let-7c-5p
 
 
-0.314
 
 
13.1
 
 
 
 
AKAP10
 
 
hsa-miR-339-5p
 
 
-0.302
 
 
10.9
 
 
 
 
AKIRIN2
 
 
hsa-miR-103a-3p
 
 
-0.299
 
 
19.4
 
 
 
 
ALAD
 
 
hsa-miR-103a-3p
 
 
-0.385
 
 
20.6
 
 
 
 
ALG3
 
 
hsa-miR-20a-5p
 
 
-0.559
 
 
20.1
 
 
 
 
ALG3
 
 
hsa-miR-93-5p
 
 
-0.564
 
 
20.1
 
 
 
 
ALG3
 
 
hsa-miR-17-5p
 
 
-0.572
 
 
20.1
 
 
 
 
ALG9
 
 
hsa-miR-7-5p
 
 
-0.302
 
 
10.1
 
 
 
 
AMT
 
 
hsa-let-7c-5p
 
 
-0.463
 
 
13.1
 
 
 
 
ANGPTL2
 
 
hsa-miR-221-3p
 
 
-0.369
 
 
55.1
 
 
 
 
ANKFY1
 
 
hsa-miR-155-5p
 
 
-0.315
 
 
14.8
 
 
 
 
ANKRD12
 
 
hsa-miR-7-5p
 
 
-0.37
 
 
12.7
 
 
 
 
ANKRD50
 
 
hsa-miR-20a-5p
 
 
-0.3
 
 
13.3
 
 
 
 
ANKRD9
 
 
hsa-miR-20a-5p
 
 
-0.31
 
 
11.5
 
 
 
 
ANKRD9
 
 
hsa-miR-93-5p
 
 
-0.331
 
 
11.5
 
 
 
 
ANKRD9
 
 
hsa-miR-17-5p
 
 
-0.347
 
 
11.5
 
 
 
 
ANP32E
 
 
hsa-miR-92a-3p
 
 
-0.329
 
 
20.4
 
 
 
 
AP1AR
 
 
hsa-miR-92a-3p
 
 
-0.325
 
 
12.2
 
 
 
 
AP1S1
 
 
hsa-let-7c-5p
 
 
-0.374
 
 
18.6
 
 
 
 
APCDD1
 
 
hsa-miR-20a-5p
 
 
-0.404
 
 
34.0
 
 
 
 
APCDD1
 
 
hsa-miR-17-5p
 
 
-0.425
 
 
34.0
 
 
 
 
APCDD1
 
 
hsa-miR-93-5p
 
 
-0.425
 
 
34.0
 
 
 
 
APPL1
 
 
hsa-miR-92a-3p
 
 
-0.375
 
 
12.5
 
 
 
 
AQP3
 
 
hsa-miR-874-3p
 
 
-0.48
 
 
642.4
 
 
 
 
ARF4
 
 
hsa-miR-7-5p
 
 
-0.407
 
 
190.1
 
 
 
 
ARF4
 
 
hsa-miR-221-3p
 
 
-0.553
 
 
190.1
 
 
 
 
ARG2
 
 
hsa-let-7c-5p
 
 
-0.468
 
 
16.6
 
 
 
 
ARHGAP1
 
 
hsa-miR-17-5p
 
 
-0.325
 
 
106.2
 
 
 
 
ARHGAP1
 
 
hsa-miR-93-5p
 
 
-0.325
 
 
106.2
 
 
 
 
ARHGAP1
 
 
hsa-miR-20a-5p
 
 
-0.339
 
 
106.2
 
 
 
 
ARHGAP5
 
 
hsa-miR-486-5p
 
 
-0.3
 
 
30.7
 
 
 
 
ARID4B
 
 
hsa-miR-20a-5p
 
 
-0.29
 
 
12.0
 
 
 
 
ARID4B
 
 
hsa-miR-486-5p
 
 
-0.367
 
 
12.0
 
 
 
 
ARIH2
 
 
hsa-miR-103a-3p
 
 
-0.307
 
 
23.4
 
 
 
 
ARL1
 
 
hsa-miR-20a-5p
 
 
-0.403
 
 
49.5
 
 
 
 
ARL1
 
 
hsa-miR-93-5p
 
 
-0.424
 
 
49.5
 
 
 
 
ARL1
 
 
hsa-miR-17-5p
 
 
-0.432
 
 
49.5
 
 
 
 
ARL4D
 
 
hsa-let-7c-5p
 
 
-0.417
 
 
26.6
 
 
 
 
ARL5B
 
 
hsa-miR-92a-3p
 
 
-0.331
 
 
11.8
 
 
 
 
ARL5B
 
 
hsa-miR-155-5p
 
 
-0.355
 
 
11.8
 
 
 
 
ARL6IP1
 
 
hsa-miR-7-5p
 
 
-0.374
 
 
124.0
 
 
 
 
ARL6IP1
 
 
hsa-miR-328-3p
 
 
-0.494
 
 
124.0
 
 
 
 
ARL6IP6
 
 
hsa-let-7c-5p
 
 
-0.293
 
 
12.2
 
 
 
 
ARL8A
 
 
hsa-miR-103a-3p
 
 
-0.38
 
 
28.6
 
 
 
 
ARMC1
 
 
hsa-miR-92a-3p
 
 
-0.322
 
 
15.7
 
 
 
 
ARMC1
 
 
hsa-miR-103a-3p
 
 
-0.525
 
 
15.7
 
 
 
 
ARMC8
 
 
hsa-miR-486-5p
 
 
-0.321
 
 
20.8
 
 
 
 
ARMC8
 
 
hsa-miR-20a-5p
 
 
-0.357
 
 
20.8
 
 
 
 
ARMC8
 
 
hsa-miR-93-5p
 
 
-0.362
 
 
20.8
 
 
 
 
ARMC8
 
 
hsa-miR-17-5p
 
 
-0.378
 
 
20.8
 
 
 
 
ARMC8
 
 
hsa-miR-103a-3p
 
 
-0.412
 
 
20.8
 
 
 
 
ARMT1
 
 
hsa-let-7c-5p
 
 
-0.303
 
 
16.1
 
 
 
 
ARNT
 
 
hsa-miR-103a-3p
 
 
-0.422
 
 
21.2
 
 
 
 
ARNT
 
 
hsa-miR-221-3p
 
 
-0.465
 
 
21.2
 
 
 
 
ARPP19
 
 
hsa-miR-328-3p
 
 
-0.404
 
 
51.7
 
 
 
 
ARPP19
 
 
hsa-let-7c-5p
 
 
-0.492
 
 
51.7
 
 
 
 
ARRDC3
 
 
hsa-miR-92a-3p
 
 
-0.458
 
 
53.2
 
 
 
 
ARRDC4
 
 
hsa-let-7c-5p
 
 
-0.329
 
 
10.9
 
 
 
 
ASAP1
 
 
hsa-let-7c-5p
 
 
-0.308
 
 
14.4
 
 
 
 
ASPH
 
 
hsa-miR-221-3p
 
 
-0.287
 
 
103.6
 
 
 
 
ASPH
 
 
hsa-miR-92a-3p
 
 
-0.416
 
 
103.6
 
 
 
 
ASPN
 
 
hsa-miR-92a-3p
 
 
-0.422
 
 
12.5
 
 
 
 
ATF3
 
 
hsa-miR-874-3p
 
 
-0.288
 
 
261.7
 
 
 
 
ATG16L1
 
 
hsa-miR-20a-5p
 
 
-0.446
 
 
13.3
 
 
 
 
ATG16L1
 
 
hsa-miR-93-5p
 
 
-0.451
 
 
13.3
 
 
 
 
ATG16L1
 
 
hsa-miR-17-5p
 
 
-0.467
 
 
13.3
 
 
 
 
ATL2
 
 
hsa-miR-103a-3p
 
 
-0.489
 
 
23.2
 
 
 
 
ATOSA
 
 
hsa-miR-221-3p
 
 
-0.495
 
 
11.7
 
 
 
 
ATOSB
 
 
hsa-miR-328-3p
 
 
-0.358
 
 
10.8
 
 
 
 
ATOSB
 
 
hsa-let-7c-5p
 
 
-0.429
 
 
10.8
 
 
 
 
ATP2A2
 
 
hsa-let-7c-5p
 
 
-0.311
 
 
84.9
 
 
 
 
ATP6V1F
 
 
hsa-miR-185-5p
 
 
-0.765
 
 
115.0
 
 
 
 
ATPAF1
 
 
hsa-let-7c-5p
 
 
-0.498
 
 
26.7
 
 
 
 
 
 
 vagina 
 
 
 
 
target_symbol
 
 
mature_mirna_id
 
 
score
 
 
nTPM
 
 
 
 
 
 
AARS1
 
 
hsa-miR-92a-3p
 
 
-0.458
 
 
24.7
 
 
 
 
ABHD17C
 
 
hsa-let-7c-5p
 
 
-0.359
 
 
21.1
 
 
 
 
ABHD17C
 
 
hsa-miR-330-3p
 
 
-0.399
 
 
21.1
 
 
 
 
ABHD5
 
 
hsa-miR-20a-5p
 
 
-0.397
 
 
11.9
 
 
 
 
ABHD5
 
 
hsa-miR-93-5p
 
 
-0.402
 
 
11.9
 
 
 
 
ABHD5
 
 
hsa-miR-17-5p
 
 
-0.45
 
 
11.9
 
 
 
 
ACOX1
 
 
hsa-miR-92a-3p
 
 
-0.321
 
 
12.7
 
 
 
 
ACP3
 
 
hsa-let-7c-5p
 
 
-0.298
 
 
10.8
 
 
 
 
ACTC1
 
 
hsa-miR-92a-3p
 
 
-0.361
 
 
50.8
 
 
 
 
ACTC1
 
 
hsa-miR-7-5p
 
 
-0.471
 
 
50.8
 
 
 
 
ADAM9
 
 
hsa-miR-20a-5p
 
 
-0.299
 
 
15.5
 
 
 
 
ADAM9
 
 
hsa-miR-17-5p
 
 
-0.32
 
 
15.5
 
 
 
 
ADAM9
 
 
hsa-miR-93-5p
 
 
-0.32
 
 
15.5
 
 
 
 
ADCY3
 
 
hsa-miR-92a-3p
 
 
-0.381
 
 
46.2
 
 
 
 
ADNP
 
 
hsa-miR-328-3p
 
 
-0.371
 
 
30.1
 
 
 
 
ADRB2
 
 
hsa-let-7c-5p
 
 
-0.635
 
 
12.2
 
 
 
 
AGFG1
 
 
hsa-miR-103a-3p
 
 
-0.389
 
 
31.5
 
 
 
 
AGFG2
 
 
hsa-miR-20a-5p
 
 
-0.365
 
 
11.5
 
 
 
 
AGFG2
 
 
hsa-miR-93-5p
 
 
-0.378
 
 
11.5
 
 
 
 
AGFG2
 
 
hsa-miR-17-5p
 
 
-0.402
 
 
11.5
 
 
 
 
AHNAK
 
 
hsa-miR-20a-5p
 
 
-0.316
 
 
200.6
 
 
 
 
AHNAK
 
 
hsa-miR-17-5p
 
 
-0.337
 
 
200.6
 
 
 
 
AHNAK
 
 
hsa-miR-93-5p
 
 
-0.337
 
 
200.6
 
 
 
 
AJUBA
 
 
hsa-miR-103a-3p
 
 
-0.448
 
 
15.1
 
 
 
 
AKIRIN2
 
 
hsa-miR-103a-3p
 
 
-0.299
 
 
22.7
 
 
 
 
ALAD
 
 
hsa-miR-103a-3p
 
 
-0.385
 
 
19.3
 
 
 
 
ALG3
 
 
hsa-miR-20a-5p
 
 
-0.559
 
 
18.9
 
 
 
 
ALG3
 
 
hsa-miR-93-5p
 
 
-0.564
 
 
18.9
 
 
 
 
ALG3
 
 
hsa-miR-17-5p
 
 
-0.572
 
 
18.9
 
 
 
 
ALG9
 
 
hsa-miR-7-5p
 
 
-0.302
 
 
10.2
 
 
 
 
ANGPTL2
 
 
hsa-miR-221-3p
 
 
-0.369
 
 
47.7
 
 
 
 
ANKFY1
 
 
hsa-miR-155-5p
 
 
-0.315
 
 
13.3
 
 
 
 
ANKRD29
 
 
hsa-miR-20a-5p
 
 
-0.359
 
 
12.3
 
 
 
 
ANKRD29
 
 
hsa-miR-17-5p
 
 
-0.372
 
 
12.3
 
 
 
 
ANKRD29
 
 
hsa-miR-93-5p
 
 
-0.372
 
 
12.3
 
 
 
 
ANKRD9
 
 
hsa-miR-20a-5p
 
 
-0.31
 
 
14.6
 
 
 
 
ANKRD9
 
 
hsa-miR-93-5p
 
 
-0.331
 
 
14.6
 
 
 
 
ANKRD9
 
 
hsa-miR-17-5p
 
 
-0.347
 
 
14.6
 
 
 
 
ANP32E
 
 
hsa-miR-92a-3p
 
 
-0.329
 
 
15.5
 
 
 
 
AP1S1
 
 
hsa-let-7c-5p
 
 
-0.374
 
 
20.1
 
 
 
 
APCDD1
 
 
hsa-miR-20a-5p
 
 
-0.404
 
 
33.2
 
 
 
 
APCDD1
 
 
hsa-miR-17-5p
 
 
-0.425
 
 
33.2
 
 
 
 
APCDD1
 
 
hsa-miR-93-5p
 
 
-0.425
 
 
33.2
 
 
 
 
APPL1
 
 
hsa-miR-92a-3p
 
 
-0.375
 
 
11.1
 
 
 
 
AQP3
 
 
hsa-miR-874-3p
 
 
-0.48
 
 
505.0
 
 
 
 
ARF4
 
 
hsa-miR-7-5p
 
 
-0.407
 
 
85.1
 
 
 
 
ARF4
 
 
hsa-miR-221-3p
 
 
-0.553
 
 
85.1
 
 
 
 
ARG2
 
 
hsa-let-7c-5p
 
 
-0.468
 
 
11.0
 
 
 
 
ARHGAP1
 
 
hsa-miR-17-5p
 
 
-0.325
 
 
59.0
 
 
 
 
ARHGAP1
 
 
hsa-miR-93-5p
 
 
-0.325
 
 
59.0
 
 
 
 
ARHGAP1
 
 
hsa-miR-20a-5p
 
 
-0.339
 
 
59.0
 
 
 
 
ARHGAP5
 
 
hsa-miR-486-5p
 
 
-0.3
 
 
13.4
 
 
 
 
ARID4B
 
 
hsa-miR-20a-5p
 
 
-0.29
 
 
10.0
 
 
 
 
ARID4B
 
 
hsa-miR-486-5p
 
 
-0.367
 
 
10.0
 
 
 
 
ARIH2
 
 
hsa-miR-103a-3p
 
 
-0.307
 
 
16.6
 
 
 
 
ARL1
 
 
hsa-miR-20a-5p
 
 
-0.403
 
 
34.0
 
 
 
 
ARL1
 
 
hsa-miR-93-5p
 
 
-0.424
 
 
34.0
 
 
 
 
ARL1
 
 
hsa-miR-17-5p
 
 
-0.432
 
 
34.0
 
 
 
 
ARL4D
 
 
hsa-let-7c-5p
 
 
-0.417
 
 
38.9
 
 
 
 
ARL6IP1
 
 
hsa-miR-7-5p
 
 
-0.374
 
 
48.0
 
 
 
 
ARL6IP1
 
 
hsa-miR-328-3p
 
 
-0.494
 
 
48.0
 
 
 
 
ARL8A
 
 
hsa-miR-103a-3p
 
 
-0.38
 
 
32.8
 
 
 
 
ARMC1
 
 
hsa-miR-92a-3p
 
 
-0.322
 
 
10.3
 
 
 
 
ARMC1
 
 
hsa-miR-103a-3p
 
 
-0.525
 
 
10.3
 
 
 
 
ARMC8
 
 
hsa-miR-486-5p
 
 
-0.321
 
 
12.4
 
 
 
 
ARMC8
 
 
hsa-miR-20a-5p
 
 
-0.357
 
 
12.4
 
 
 
 
ARMC8
 
 
hsa-miR-93-5p
 
 
-0.362
 
 
12.4
 
 
 
 
ARMC8
 
 
hsa-miR-17-5p
 
 
-0.378
 
 
12.4
 
 
 
 
ARMC8
 
 
hsa-miR-103a-3p
 
 
-0.412
 
 
12.4
 
 
 
 
ARNT
 
 
hsa-miR-103a-3p
 
 
-0.422
 
 
23.6
 
 
 
 
ARNT
 
 
hsa-miR-221-3p
 
 
-0.465
 
 
23.6
 
 
 
 
ARPP19
 
 
hsa-miR-328-3p
 
 
-0.404
 
 
27.0
 
 
 
 
ARPP19
 
 
hsa-let-7c-5p
 
 
-0.492
 
 
27.0
 
 
 
 
ARRDC3
 
 
hsa-miR-92a-3p
 
 
-0.458
 
 
40.5
 
 
 
 
ARRDC4
 
 
hsa-let-7c-5p
 
 
-0.329
 
 
10.1
 
 
 
 
ASAP1
 
 
hsa-let-7c-5p
 
 
-0.308
 
 
14.4
 
 
 
 
ASB6
 
 
hsa-miR-874-3p
 
 
-0.368
 
 
11.5
 
 
 
 
ASPH
 
 
hsa-miR-221-3p
 
 
-0.287
 
 
40.4
 
 
 
 
ASPH
 
 
hsa-miR-92a-3p
 
 
-0.416
 
 
40.4
 
 
 
 
ATF3
 
 
hsa-miR-874-3p
 
 
-0.288
 
 
53.8
 
 
 
 
ATL2
 
 
hsa-miR-103a-3p
 
 
-0.489
 
 
27.6
 
 
 
 
ATOSA
 
 
hsa-miR-221-3p
 
 
-0.495
 
 
10.0
 
 
 
 
ATOSB
 
 
hsa-miR-328-3p
 
 
-0.358
 
 
14.3
 
 
 
 
ATOSB
 
 
hsa-let-7c-5p
 
 
-0.429
 
 
14.3
 
 
 
 
ATP2A2
 
 
hsa-let-7c-5p
 
 
-0.311
 
 
40.2
 
 
 
 
ATP6V1F
 
 
hsa-miR-185-5p
 
 
-0.765
 
 
149.6
 
 
 
 
ATPAF1
 
 
hsa-let-7c-5p
 
 
-0.498
 
 
15.7
 
 
 
 
AVPI1
 
 
hsa-miR-7-5p
 
 
-0.367
 
 
46.2
 
 
 
 
B3GAT3
 
 
hsa-let-7c-5p
 
 
-0.324
 
 
28.4
 
 
 
 
B4GALT5
 
 
hsa-miR-210-3p
 
 
-0.511
 
 
16.5
 
 
 
 
B4GAT1
 
 
hsa-let-7c-5p
 
 
-0.47
 
 
15.8
 
 
 
 
BACH1
 
 
hsa-let-7c-5p
 
 
-0.306
 
 
12.6
 
 
 
 
BACH1
 
 
hsa-let-7c-5p
 
 
-0.306
 
 
12.6
 
 
 
 
BACH1
 
 
hsa-let-7c-5p
 
 
-0.435
 
 
12.6
 
 
 
 
BACH1
 
 
hsa-let-7c-5p
 
 
-0.435
 
 
12.6
 
 
 
 
BAK1
 
 
hsa-miR-103a-3p
 
 
-0.364
 
 
11.6
 
 
 
 
BCAT2
 
 
hsa-miR-103a-3p
 
 
-0.364
 
 
27.2
 
 
 
 
BCAT2
 
 
hsa-miR-92a-3p
 
 
-0.609
 
 
27.2
 
 
 
 
BDH1
 
 
hsa-miR-874-3p
 
 
-0.644
 
 
13.3
 
 
 
 
BICDL1
 
 
hsa-miR-221-3p
 
 
-0.447
 
 
17.1
 
 
 
 
 
 
 bone marrow 
 
 
 
 
target_symbol
 
 
mature_mirna_id
 
 
score
 
 
nTPM
 
 
 
 
 
 
ABHD17B
 
 
hsa-miR-486-5p
 
 
-0.348
 
 
18.0
 
 
 
 
ABHD5
 
 
hsa-miR-20a-5p
 
 
-0.397
 
 
37.5
 
 
 
 
ABHD5
 
 
hsa-miR-93-5p
 
 
-0.402
 
 
37.5
 
 
 
 
ABHD5
 
 
hsa-miR-17-5p
 
 
-0.45
 
 
37.5
 
 
 
 
ACP3
 
 
hsa-let-7c-5p
 
 
-0.298
 
 
16.5
 
 
 
 
ACSL6
 
 
hsa-let-7c-5p
 
 
-0.336
 
 
21.4
 
 
 
 
ADCY3
 
 
hsa-miR-92a-3p
 
 
-0.381
 
 
16.7
 
 
 
 
ADNP
 
 
hsa-miR-328-3p
 
 
-0.371
 
 
23.9
 
 
 
 
ADRB2
 
 
hsa-let-7c-5p
 
 
-0.635
 
 
11.8
 
 
 
 
AGFG1
 
 
hsa-miR-103a-3p
 
 
-0.389
 
 
146.9
 
 
 
 
AGO4
 
 
hsa-miR-103a-3p
 
 
-0.32
 
 
19.2
 
 
 
 
AGO4
 
 
hsa-miR-221-3p
 
 
-0.321
 
 
19.2
 
 
 
 
AHCTF1
 
 
hsa-let-7c-5p
 
 
-0.374
 
 
16.0
 
 
 
 
AHNAK
 
 
hsa-miR-20a-5p
 
 
-0.316
 
 
41.6
 
 
 
 
AHNAK
 
 
hsa-miR-17-5p
 
 
-0.337
 
 
41.6
 
 
 
 
AHNAK
 
 
hsa-miR-93-5p
 
 
-0.337
 
 
41.6
 
 
 
 
AIFM1
 
 
hsa-let-7c-5p
 
 
-0.314
 
 
11.2
 
 
 
 
AKAP10
 
 
hsa-miR-339-5p
 
 
-0.302
 
 
10.0
 
 
 
 
AKIRIN2
 
 
hsa-miR-103a-3p
 
 
-0.299
 
 
66.5
 
 
 
 
ALAD
 
 
hsa-miR-103a-3p
 
 
-0.385
 
 
77.5
 
 
 
 
ALG3
 
 
hsa-miR-20a-5p
 
 
-0.559
 
 
14.9
 
 
 
 
ALG3
 
 
hsa-miR-93-5p
 
 
-0.564
 
 
14.9
 
 
 
 
ALG3
 
 
hsa-miR-17-5p
 
 
-0.572
 
 
14.9
 
 
 
 
ANK1
 
 
hsa-miR-103a-3p
 
 
-0.321
 
 
94.9
 
 
 
 
ANKFY1
 
 
hsa-miR-155-5p
 
 
-0.315
 
 
15.5
 
 
 
 
ANKRD12
 
 
hsa-miR-7-5p
 
 
-0.37
 
 
19.4
 
 
 
 
ANKRD44
 
 
hsa-miR-92a-3p
 
 
-0.324
 
 
16.4
 
 
 
 
ANKRD9
 
 
hsa-miR-20a-5p
 
 
-0.31
 
 
17.0
 
 
 
 
ANKRD9
 
 
hsa-miR-93-5p
 
 
-0.331
 
 
17.0
 
 
 
 
ANKRD9
 
 
hsa-miR-17-5p
 
 
-0.347
 
 
17.0
 
 
 
 
ANP32E
 
 
hsa-miR-92a-3p
 
 
-0.329
 
 
71.4
 
 
 
 
AP4B1
 
 
hsa-miR-378a-3p
 
 
-0.62
 
 
19.4
 
 
 
 
APPL1
 
 
hsa-miR-92a-3p
 
 
-0.375
 
 
14.3
 
 
 
 
AQP3
 
 
hsa-miR-874-3p
 
 
-0.48
 
 
18.6
 
 
 
 
ARF4
 
 
hsa-miR-7-5p
 
 
-0.407
 
 
68.5
 
 
 
 
ARF4
 
 
hsa-miR-221-3p
 
 
-0.553
 
 
68.5
 
 
 
 
ARHGAP1
 
 
hsa-miR-17-5p
 
 
-0.325
 
 
31.5
 
 
 
 
ARHGAP1
 
 
hsa-miR-93-5p
 
 
-0.325
 
 
31.5
 
 
 
 
ARHGAP1
 
 
hsa-miR-20a-5p
 
 
-0.339
 
 
31.5
 
 
 
 
ARHGAP5
 
 
hsa-miR-486-5p
 
 
-0.3
 
 
10.3
 
 
 
 
ARID2
 
 
hsa-miR-155-5p
 
 
-0.384
 
 
11.7
 
 
 
 
ARID2
 
 
hsa-miR-103a-3p
 
 
-0.415
 
 
11.7
 
 
 
 
ARID3B
 
 
hsa-let-7c-5p
 
 
-0.31
 
 
23.2
 
 
 
 
ARID3B
 
 
hsa-let-7c-5p
 
 
-0.486
 
 
23.2
 
 
 
 
ARID4A
 
 
hsa-miR-7-5p
 
 
-0.341
 
 
32.8
 
 
 
 
ARID4B
 
 
hsa-miR-20a-5p
 
 
-0.29
 
 
31.2
 
 
 
 
ARID4B
 
 
hsa-miR-486-5p
 
 
-0.367
 
 
31.2
 
 
 
 
ARIH2
 
 
hsa-miR-103a-3p
 
 
-0.307
 
 
21.7
 
 
 
 
ARL1
 
 
hsa-miR-20a-5p
 
 
-0.403
 
 
15.6
 
 
 
 
ARL1
 
 
hsa-miR-93-5p
 
 
-0.424
 
 
15.6
 
 
 
 
ARL1
 
 
hsa-miR-17-5p
 
 
-0.432
 
 
15.6
 
 
 
 
ARL5B
 
 
hsa-miR-92a-3p
 
 
-0.331
 
 
18.5
 
 
 
 
ARL5B
 
 
hsa-miR-155-5p
 
 
-0.355
 
 
18.5
 
 
 
 
ARL6IP1
 
 
hsa-miR-7-5p
 
 
-0.374
 
 
197.7
 
 
 
 
ARL6IP1
 
 
hsa-miR-328-3p
 
 
-0.494
 
 
197.7
 
 
 
 
ARL6IP6
 
 
hsa-let-7c-5p
 
 
-0.293
 
 
23.4
 
 
 
 
ARL8A
 
 
hsa-miR-103a-3p
 
 
-0.38
 
 
66.1
 
 
 
 
ARMC1
 
 
hsa-miR-92a-3p
 
 
-0.322
 
 
12.4
 
 
 
 
ARMC1
 
 
hsa-miR-103a-3p
 
 
-0.525
 
 
12.4
 
 
 
 
ARMC8
 
 
hsa-miR-486-5p
 
 
-0.321
 
 
57.2
 
 
 
 
ARMC8
 
 
hsa-miR-20a-5p
 
 
-0.357
 
 
57.2
 
 
 
 
ARMC8
 
 
hsa-miR-93-5p
 
 
-0.362
 
 
57.2
 
 
 
 
ARMC8
 
 
hsa-miR-17-5p
 
 
-0.378
 
 
57.2
 
 
 
 
ARMC8
 
 
hsa-miR-103a-3p
 
 
-0.412
 
 
57.2
 
 
 
 
ARNT
 
 
hsa-miR-103a-3p
 
 
-0.422
 
 
16.2
 
 
 
 
ARNT
 
 
hsa-miR-221-3p
 
 
-0.465
 
 
16.2
 
 
 
 
ARPP19
 
 
hsa-miR-328-3p
 
 
-0.404
 
 
97.5
 
 
 
 
ARPP19
 
 
hsa-let-7c-5p
 
 
-0.492
 
 
97.5
 
 
 
 
ARRDC3
 
 
hsa-miR-92a-3p
 
 
-0.458
 
 
158.3
 
 
 
 
ARRDC4
 
 
hsa-let-7c-5p
 
 
-0.329
 
 
10.8
 
 
 
 
ASAP1
 
 
hsa-let-7c-5p
 
 
-0.308
 
 
44.7
 
 
 
 
ASB6
 
 
hsa-miR-874-3p
 
 
-0.368
 
 
25.3
 
 
 
 
ASPH
 
 
hsa-miR-221-3p
 
 
-0.287
 
 
15.8
 
 
 
 
ASPH
 
 
hsa-miR-92a-3p
 
 
-0.416
 
 
15.8
 
 
 
 
ATAD2
 
 
hsa-miR-17-5p
 
 
-0.304
 
 
25.0
 
 
 
 
ATAD2
 
 
hsa-miR-20a-5p
 
 
-0.318
 
 
25.0
 
 
 
 
ATF3
 
 
hsa-miR-874-3p
 
 
-0.288
 
 
42.6
 
 
 
 
ATG16L1
 
 
hsa-miR-20a-5p
 
 
-0.446
 
 
13.5
 
 
 
 
ATG16L1
 
 
hsa-miR-93-5p
 
 
-0.451
 
 
13.5
 
 
 
 
ATG16L1
 
 
hsa-miR-17-5p
 
 
-0.467
 
 
13.5
 
 
 
 
ATL2
 
 
hsa-miR-103a-3p
 
 
-0.489
 
 
30.6
 
 
 
 
ATOSA
 
 
hsa-miR-221-3p
 
 
-0.495
 
 
20.2
 
 
 
 
ATOSB
 
 
hsa-miR-328-3p
 
 
-0.358
 
 
36.6
 
 
 
 
ATOSB
 
 
hsa-let-7c-5p
 
 
-0.429
 
 
36.6
 
 
 
 
ATP1B2
 
 
hsa-miR-103a-3p
 
 
-0.6
 
 
10.8
 
 
 
 
ATP2A2
 
 
hsa-let-7c-5p
 
 
-0.311
 
 
43.4
 
 
 
 
ATP6V1F
 
 
hsa-miR-185-5p
 
 
-0.765
 
 
198.4
 
 
 
 
ATP8B4
 
 
hsa-let-7c-5p
 
 
-0.325
 
 
73.7
 
 
 
 
ATPAF1
 
 
hsa-let-7c-5p
 
 
-0.498
 
 
26.0
 
 
 
 
ATXN1L
 
 
hsa-miR-155-5p
 
 
-0.315
 
 
37.3
 
 
 
 
AURKA
 
 
hsa-miR-92a-3p
 
 
-0.358
 
 
14.5
 
 
 
 
B3GAT3
 
 
hsa-let-7c-5p
 
 
-0.324
 
 
17.5
 
 
 
 
B4GALT5
 
 
hsa-miR-210-3p
 
 
-0.511
 
 
88.5
 
 
 
 
BACH1
 
 
hsa-let-7c-5p
 
 
-0.306
 
 
84.3
 
 
 
 
BACH1
 
 
hsa-let-7c-5p
 
 
-0.306
 
 
84.3
 
 
 
 
BACH1
 
 
hsa-let-7c-5p
 
 
-0.435
 
 
84.3
 
 
 
 
BACH1
 
 
hsa-let-7c-5p
 
 
-0.435
 
 
84.3
 
 
 
 
BAK1
 
 
hsa-miR-103a-3p
 
 
-0.364
 
 
26.1
 
 
 
 
BCL2L11
 
 
hsa-miR-339-5p
 
 
-0.306
 
 
37.8
 
 
 
 
BCL2L11
 
 
hsa-miR-92a-3p
 
 
-0.394
 
 
37.8
 
 
 
 
 
 
 
 cd4_s0_vs_cd4_s1_downregulated 
 
 Tissue expression heatmap (log10-transformed) 
   
 
 
 stomach 
 
 
 
 
target_symbol
 
 
mature_mirna_id
 
 
score
 
 
nTPM
 
 
 
 
 
 
A4GNT
 
 
hsa-miR-22-3p
 
 
-0.601
 
 
22.3
 
 
 
 
AAGAB
 
 
hsa-miR-34a-5p
 
 
-0.319
 
 
22.5
 
 
 
 
ABCB7
 
 
hsa-miR-152-3p
 
 
-0.515
 
 
11.6
 
 
 
 
ABCD3
 
 
hsa-miR-152-3p
 
 
-0.388
 
 
41.1
 
 
 
 
ABHD17C
 
 
hsa-miR-27a-3p
 
 
-0.435
 
 
34.0
 
 
 
 
ACBD3
 
 
hsa-miR-34a-5p
 
 
-0.415
 
 
29.9
 
 
 
 
ACSL1
 
 
hsa-miR-34a-5p
 
 
-0.306
 
 
50.6
 
 
 
 
ACSL4
 
 
hsa-miR-34a-5p
 
 
-0.372
 
 
17.6
 
 
 
 
ACTA2
 
 
hsa-miR-27a-3p
 
 
-0.577
 
 
1353.4
 
 
 
 
ACVR1
 
 
hsa-miR-152-3p
 
 
-0.397
 
 
11.0
 
 
 
 
ADAM10
 
 
hsa-miR-152-3p
 
 
-0.348
 
 
19.0
 
 
 
 
ADAP1
 
 
hsa-miR-27a-3p
 
 
-0.302
 
 
18.4
 
 
 
 
ADIPOR2
 
 
hsa-miR-34a-5p
 
 
-0.33
 
 
42.5
 
 
 
 
ADK
 
 
hsa-miR-34a-5p
 
 
-0.374
 
 
11.3
 
 
 
 
AFG3L2
 
 
hsa-miR-181a-5p
 
 
-0.491
 
 
26.3
 
 
 
 
AGFG1
 
 
hsa-miR-152-3p
 
 
-0.332
 
 
31.3
 
 
 
 
AGPAT3
 
 
hsa-miR-27a-3p
 
 
-0.29
 
 
16.9
 
 
 
 
AGTRAP
 
 
hsa-miR-34a-5p
 
 
-0.328
 
 
31.9
 
 
 
 
AHCYL2
 
 
hsa-miR-34a-5p
 
 
-0.359
 
 
39.6
 
 
 
 
AHDC1
 
 
hsa-miR-152-3p
 
 
-0.413
 
 
10.8
 
 
 
 
AIDA
 
 
hsa-miR-22-3p
 
 
-0.316
 
 
26.6
 
 
 
 
AKAP1
 
 
hsa-miR-152-3p
 
 
-0.381
 
 
53.0
 
 
 
 
AKIRIN1
 
 
hsa-miR-27a-3p
 
 
-0.368
 
 
31.7
 
 
 
 
ALCAM
 
 
hsa-miR-34a-5p
 
 
-0.303
 
 
31.6
 
 
 
 
ALCAM
 
 
hsa-miR-152-3p
 
 
-0.347
 
 
31.6
 
 
 
 
ALDOA
 
 
hsa-miR-34a-5p
 
 
-0.655
 
 
700.2
 
 
 
 
ANK3
 
 
hsa-miR-34a-5p
 
 
-0.29
 
 
14.2
 
 
 
 
ANK3
 
 
hsa-miR-34a-5p
 
 
-0.347
 
 
14.2
 
 
 
 
ANKHD1-EIF4EBP3
 
 
hsa-miR-22-3p
 
 
-0.479
 
 
11.7
 
 
 
 
ANKRD13A
 
 
hsa-miR-22-3p
 
 
-0.478
 
 
16.1
 
 
 
 
ANKRD40
 
 
hsa-miR-27a-3p
 
 
-0.35
 
 
17.6
 
 
 
 
ANP32B
 
 
hsa-miR-34a-5p
 
 
-0.29
 
 
125.6
 
 
 
 
AP1AR
 
 
hsa-miR-100-5p
 
 
-0.804
 
 
12.3
 
 
 
 
APH1A
 
 
hsa-miR-34a-5p
 
 
-0.304
 
 
90.3
 
 
 
 
APOO
 
 
hsa-miR-181a-5p
 
 
-0.5
 
 
17.0
 
 
 
 
APPBP2
 
 
hsa-miR-27a-3p
 
 
-0.378
 
 
10.1
 
 
 
 
AREG
 
 
hsa-miR-34a-5p
 
 
-0.62
 
 
19.1
 
 
 
 
ARF4
 
 
hsa-miR-152-3p
 
 
-0.487
 
 
123.5
 
 
 
 
ARF6
 
 
hsa-miR-181a-5p
 
 
-0.33
 
 
44.4
 
 
 
 
ARFGEF1
 
 
hsa-miR-152-3p
 
 
-0.306
 
 
18.5
 
 
 
 
ARFIP1
 
 
hsa-miR-320d
 
 
-0.339
 
 
21.5
 
 
 
 
ARFIP1
 
 
hsa-miR-152-3p
 
 
-0.474
 
 
21.5
 
 
 
 
ARHGAP1
 
 
hsa-miR-34a-5p
 
 
-0.369
 
 
46.3
 
 
 
 
ARHGAP21
 
 
hsa-miR-152-3p
 
 
-0.287
 
 
25.5
 
 
 
 
ARHGAP26
 
 
hsa-miR-34a-5p
 
 
-0.384
 
 
14.4
 
 
 
 
ARL6IP1
 
 
hsa-miR-152-3p
 
 
-0.579
 
 
118.7
 
 
 
 
ARL8B
 
 
hsa-miR-152-3p
 
 
-0.337
 
 
32.2
 
 
 
 
ARL8B
 
 
hsa-miR-320d
 
 
-0.376
 
 
32.2
 
 
 
 
ARPC5
 
 
hsa-miR-22-3p
 
 
-0.679
 
 
83.5
 
 
 
 
ARPP19
 
 
hsa-miR-320d
 
 
-0.325
 
 
36.4
 
 
 
 
ARPP19
 
 
hsa-miR-34a-5p
 
 
-0.497
 
 
36.4
 
 
 
 
ARRB1
 
 
hsa-miR-22-3p
 
 
-0.616
 
 
31.7
 
 
 
 
ARRDC3
 
 
hsa-miR-152-3p
 
 
-0.305
 
 
24.3
 
 
 
 
ASAH1
 
 
hsa-miR-27a-3p
 
 
-0.398
 
 
57.7
 
 
 
 
ASH2L
 
 
hsa-miR-320d
 
 
-0.287
 
 
20.9
 
 
 
 
ASPH
 
 
hsa-miR-152-3p
 
 
-0.391
 
 
96.9
 
 
 
 
ATG4B
 
 
hsa-miR-34a-5p
 
 
-0.58
 
 
18.1
 
 
 
 
ATMIN
 
 
hsa-miR-34a-5p
 
 
-0.409
 
 
12.0
 
 
 
 
ATP1A2
 
 
hsa-miR-34a-5p
 
 
-0.306
 
 
15.1
 
 
 
 
ATP2B4
 
 
hsa-miR-152-3p
 
 
-0.337
 
 
14.5
 
 
 
 
ATP5MC3
 
 
hsa-miR-27a-3p
 
 
-0.416
 
 
258.5
 
 
 
 
ATP6AP2
 
 
hsa-miR-152-3p
 
 
-0.592
 
 
46.4
 
 
 
 
ATP6AP2
 
 
hsa-miR-152-3p
 
 
-0.668
 
 
46.4
 
 
 
 
ATP8A1
 
 
hsa-miR-22-3p
 
 
-0.468
 
 
11.8
 
 
 
 
ATXN10
 
 
hsa-miR-27a-3p
 
 
-0.295
 
 
33.1
 
 
 
 
ATXN2
 
 
hsa-miR-10a-5p
 
 
-0.362
 
 
16.1
 
 
 
 
AXL
 
 
hsa-miR-34a-5p
 
 
-0.512
 
 
20.2
 
 
 
 
B3GNT7
 
 
hsa-miR-27a-3p
 
 
-0.355
 
 
33.6
 
 
 
 
B4GALT2
 
 
hsa-miR-34a-5p
 
 
-0.314
 
 
17.7
 
 
 
 
B4GALT3
 
 
hsa-miR-27a-3p
 
 
-0.41
 
 
26.3
 
 
 
 
B4GALT5
 
 
hsa-miR-152-3p
 
 
-0.44
 
 
24.3
 
 
 
 
BAG5
 
 
hsa-miR-22-3p
 
 
-0.318
 
 
14.0
 
 
 
 
BAZ1B
 
 
hsa-miR-10a-5p
 
 
-0.309
 
 
17.8
 
 
 
 
BAZ2A
 
 
hsa-miR-100-5p
 
 
-0.29
 
 
24.7
 
 
 
 
BAZ2A
 
 
hsa-miR-100-5p
 
 
-0.421
 
 
24.7
 
 
 
 
BBX
 
 
hsa-miR-10a-5p
 
 
-0.305
 
 
11.4
 
 
 
 
BCL3
 
 
hsa-miR-27a-3p
 
 
-0.413
 
 
38.8
 
 
 
 
BEX3
 
 
hsa-miR-27a-3p
 
 
-0.454
 
 
98.5
 
 
 
 
BHLHE40
 
 
hsa-miR-181a-5p
 
 
-0.408
 
 
47.9
 
 
 
 
BIN1
 
 
hsa-miR-22-3p
 
 
-0.346
 
 
42.8
 
 
 
 
BLOC1S1
 
 
hsa-miR-22-3p
 
 
-0.346
 
 
161.2
 
 
 
 
BLOC1S6
 
 
hsa-miR-181a-5p
 
 
-0.393
 
 
18.9
 
 
 
 
BLTP1
 
 
hsa-miR-27a-3p
 
 
-0.426
 
 
14.6
 
 
 
 
BMI1
 
 
hsa-miR-27a-3p
 
 
-0.322
 
 
21.2
 
 
 
 
BRPF3
 
 
hsa-miR-34a-5p
 
 
-0.33
 
 
10.1
 
 
 
 
BTAF1
 
 
hsa-miR-152-3p
 
 
-0.401
 
 
15.2
 
 
 
 
BTBD3
 
 
hsa-miR-152-3p
 
 
-0.495
 
 
25.7
 
 
 
 
BTG1
 
 
hsa-miR-22-3p
 
 
-0.468
 
 
13.2
 
 
 
 
BTG2
 
 
hsa-miR-27a-3p
 
 
-0.339
 
 
242.2
 
 
 
 
BZW1
 
 
hsa-miR-27a-3p
 
 
-0.337
 
 
65.1
 
 
 
 
C1GALT1
 
 
hsa-miR-152-3p
 
 
-0.487
 
 
21.1
 
 
 
 
C1orf35
 
 
hsa-miR-152-3p
 
 
-0.511
 
 
13.6
 
 
 
 
C5orf24
 
 
hsa-miR-22-3p
 
 
-0.493
 
 
15.2
 
 
 
 
C6orf120
 
 
hsa-miR-34a-5p
 
 
-0.331
 
 
12.5
 
 
 
 
C6orf62
 
 
hsa-miR-181a-5p
 
 
-0.334
 
 
104.0
 
 
 
 
C8orf88
 
 
hsa-miR-27a-3p
 
 
-0.33
 
 
11.6
 
 
 
 
CACYBP
 
 
hsa-miR-27a-3p
 
 
-0.367
 
 
20.5
 
 
 
 
CADM4
 
 
hsa-miR-34a-5p
 
 
-0.365
 
 
14.4
 
 
 
 
CAMK2N1
 
 
hsa-miR-22-3p
 
 
-0.302
 
 
18.2
 
 
 
 
CAND1
 
 
hsa-miR-27a-3p
 
 
-0.318
 
 
17.2
 
 
 
 
 
 
 adipose tissue 
 
 
 
 
target_symbol
 
 
mature_mirna_id
 
 
score
 
 
nTPM
 
 
 
 
 
 
AAGAB
 
 
hsa-miR-34a-5p
 
 
-0.319
 
 
14.1
 
 
 
 
ABCB7
 
 
hsa-miR-152-3p
 
 
-0.515
 
 
12.4
 
 
 
 
ABCD3
 
 
hsa-miR-152-3p
 
 
-0.388
 
 
15.7
 
 
 
 
ABHD5
 
 
hsa-miR-27a-3p
 
 
-0.407
 
 
27.9
 
 
 
 
ACBD3
 
 
hsa-miR-34a-5p
 
 
-0.415
 
 
20.3
 
 
 
 
ACER3
 
 
hsa-miR-22-3p
 
 
-0.453
 
 
10.2
 
 
 
 
ACSL1
 
 
hsa-miR-34a-5p
 
 
-0.306
 
 
680.0
 
 
 
 
ACSL4
 
 
hsa-miR-34a-5p
 
 
-0.372
 
 
38.3
 
 
 
 
ACTA2
 
 
hsa-miR-27a-3p
 
 
-0.577
 
 
979.1
 
 
 
 
ACVR1
 
 
hsa-miR-152-3p
 
 
-0.397
 
 
12.6
 
 
 
 
ADAM10
 
 
hsa-miR-152-3p
 
 
-0.348
 
 
14.0
 
 
 
 
ADAMTS10
 
 
hsa-miR-27a-3p
 
 
-0.354
 
 
13.8
 
 
 
 
ADGRF5
 
 
hsa-miR-152-3p
 
 
-0.354
 
 
66.6
 
 
 
 
ADIPOR2
 
 
hsa-miR-34a-5p
 
 
-0.33
 
 
53.2
 
 
 
 
ADK
 
 
hsa-miR-34a-5p
 
 
-0.374
 
 
22.4
 
 
 
 
AFG3L2
 
 
hsa-miR-181a-5p
 
 
-0.491
 
 
17.1
 
 
 
 
AGFG1
 
 
hsa-miR-152-3p
 
 
-0.332
 
 
64.8
 
 
 
 
AGPAT3
 
 
hsa-miR-27a-3p
 
 
-0.29
 
 
19.6
 
 
 
 
AGTRAP
 
 
hsa-miR-34a-5p
 
 
-0.328
 
 
41.4
 
 
 
 
AHDC1
 
 
hsa-miR-152-3p
 
 
-0.413
 
 
11.7
 
 
 
 
AIDA
 
 
hsa-miR-22-3p
 
 
-0.316
 
 
67.8
 
 
 
 
AKAP1
 
 
hsa-miR-152-3p
 
 
-0.381
 
 
38.1
 
 
 
 
AKIRIN1
 
 
hsa-miR-27a-3p
 
 
-0.368
 
 
43.1
 
 
 
 
AKT3
 
 
hsa-miR-22-3p
 
 
-0.451
 
 
16.9
 
 
 
 
ALDOA
 
 
hsa-miR-34a-5p
 
 
-0.655
 
 
657.0
 
 
 
 
ALPL
 
 
hsa-miR-10a-5p
 
 
-0.32
 
 
23.7
 
 
 
 
ANK2
 
 
hsa-miR-34a-5p
 
 
-0.317
 
 
23.6
 
 
 
 
ANKRD13A
 
 
hsa-miR-22-3p
 
 
-0.478
 
 
12.3
 
 
 
 
ANKRD40
 
 
hsa-miR-27a-3p
 
 
-0.35
 
 
40.1
 
 
 
 
ANP32B
 
 
hsa-miR-34a-5p
 
 
-0.29
 
 
136.8
 
 
 
 
AP3S2
 
 
hsa-miR-34a-5p
 
 
-0.413
 
 
22.7
 
 
 
 
APH1A
 
 
hsa-miR-34a-5p
 
 
-0.304
 
 
69.8
 
 
 
 
APPBP2
 
 
hsa-miR-27a-3p
 
 
-0.378
 
 
13.9
 
 
 
 
AREG
 
 
hsa-miR-34a-5p
 
 
-0.62
 
 
45.9
 
 
 
 
ARF4
 
 
hsa-miR-152-3p
 
 
-0.487
 
 
118.7
 
 
 
 
ARF6
 
 
hsa-miR-181a-5p
 
 
-0.33
 
 
30.0
 
 
 
 
ARFGEF1
 
 
hsa-miR-152-3p
 
 
-0.306
 
 
10.7
 
 
 
 
ARFIP1
 
 
hsa-miR-320d
 
 
-0.339
 
 
15.8
 
 
 
 
ARFIP1
 
 
hsa-miR-152-3p
 
 
-0.474
 
 
15.8
 
 
 
 
ARHGAP1
 
 
hsa-miR-34a-5p
 
 
-0.369
 
 
79.6
 
 
 
 
ARHGAP21
 
 
hsa-miR-152-3p
 
 
-0.287
 
 
26.1
 
 
 
 
ARHGAP26
 
 
hsa-miR-34a-5p
 
 
-0.384
 
 
11.2
 
 
 
 
ARL6IP1
 
 
hsa-miR-152-3p
 
 
-0.579
 
 
133.2
 
 
 
 
ARL8B
 
 
hsa-miR-152-3p
 
 
-0.337
 
 
39.1
 
 
 
 
ARL8B
 
 
hsa-miR-320d
 
 
-0.376
 
 
39.1
 
 
 
 
ARPC5
 
 
hsa-miR-22-3p
 
 
-0.679
 
 
80.7
 
 
 
 
ARPIN-AP3S2
 
 
hsa-miR-34a-5p
 
 
-0.348
 
 
11.1
 
 
 
 
ARPP19
 
 
hsa-miR-320d
 
 
-0.325
 
 
57.4
 
 
 
 
ARPP19
 
 
hsa-miR-34a-5p
 
 
-0.497
 
 
57.4
 
 
 
 
ARRB1
 
 
hsa-miR-22-3p
 
 
-0.616
 
 
28.8
 
 
 
 
ARRDC3
 
 
hsa-miR-152-3p
 
 
-0.305
 
 
34.5
 
 
 
 
ASAH1
 
 
hsa-miR-27a-3p
 
 
-0.398
 
 
201.2
 
 
 
 
ASH2L
 
 
hsa-miR-320d
 
 
-0.287
 
 
27.4
 
 
 
 
ASPH
 
 
hsa-miR-152-3p
 
 
-0.391
 
 
362.0
 
 
 
 
ATG4B
 
 
hsa-miR-34a-5p
 
 
-0.58
 
 
13.1
 
 
 
 
ATMIN
 
 
hsa-miR-34a-5p
 
 
-0.409
 
 
14.9
 
 
 
 
ATP1A2
 
 
hsa-miR-34a-5p
 
 
-0.306
 
 
35.4
 
 
 
 
ATP2B4
 
 
hsa-miR-152-3p
 
 
-0.337
 
 
55.3
 
 
 
 
ATP5MC3
 
 
hsa-miR-27a-3p
 
 
-0.416
 
 
189.1
 
 
 
 
ATP6AP2
 
 
hsa-miR-152-3p
 
 
-0.592
 
 
76.1
 
 
 
 
ATP6AP2
 
 
hsa-miR-152-3p
 
 
-0.668
 
 
76.1
 
 
 
 
ATXN10
 
 
hsa-miR-27a-3p
 
 
-0.295
 
 
38.9
 
 
 
 
ATXN2
 
 
hsa-miR-10a-5p
 
 
-0.362
 
 
19.0
 
 
 
 
AXL
 
 
hsa-miR-34a-5p
 
 
-0.512
 
 
41.8
 
 
 
 
B4GALT2
 
 
hsa-miR-34a-5p
 
 
-0.314
 
 
22.6
 
 
 
 
B4GALT3
 
 
hsa-miR-27a-3p
 
 
-0.41
 
 
26.9
 
 
 
 
B4GALT5
 
 
hsa-miR-152-3p
 
 
-0.44
 
 
26.8
 
 
 
 
BAG5
 
 
hsa-miR-22-3p
 
 
-0.318
 
 
23.0
 
 
 
 
BAZ1B
 
 
hsa-miR-10a-5p
 
 
-0.309
 
 
21.2
 
 
 
 
BAZ2A
 
 
hsa-miR-100-5p
 
 
-0.29
 
 
26.9
 
 
 
 
BAZ2A
 
 
hsa-miR-100-5p
 
 
-0.421
 
 
26.9
 
 
 
 
BBX
 
 
hsa-miR-10a-5p
 
 
-0.305
 
 
13.1
 
 
 
 
BCL2
 
 
hsa-miR-34a-5p
 
 
-0.289
 
 
12.0
 
 
 
 
BCL2L13
 
 
hsa-miR-34a-5p
 
 
-0.431
 
 
11.0
 
 
 
 
BCL3
 
 
hsa-miR-27a-3p
 
 
-0.413
 
 
69.6
 
 
 
 
BEX3
 
 
hsa-miR-27a-3p
 
 
-0.454
 
 
111.5
 
 
 
 
BHLHE40
 
 
hsa-miR-181a-5p
 
 
-0.408
 
 
162.3
 
 
 
 
BHLHE41
 
 
hsa-miR-152-3p
 
 
-0.442
 
 
10.3
 
 
 
 
BIN1
 
 
hsa-miR-22-3p
 
 
-0.346
 
 
41.5
 
 
 
 
BLOC1S1
 
 
hsa-miR-22-3p
 
 
-0.346
 
 
122.1
 
 
 
 
BLOC1S6
 
 
hsa-miR-181a-5p
 
 
-0.393
 
 
28.2
 
 
 
 
BLTP1
 
 
hsa-miR-27a-3p
 
 
-0.426
 
 
19.6
 
 
 
 
BMI1
 
 
hsa-miR-27a-3p
 
 
-0.322
 
 
27.2
 
 
 
 
BMPR2
 
 
hsa-miR-100-5p
 
 
-0.355
 
 
15.1
 
 
 
 
BTAF1
 
 
hsa-miR-152-3p
 
 
-0.401
 
 
16.7
 
 
 
 
BTBD3
 
 
hsa-miR-152-3p
 
 
-0.495
 
 
10.5
 
 
 
 
BTG1
 
 
hsa-miR-22-3p
 
 
-0.468
 
 
17.1
 
 
 
 
BTG2
 
 
hsa-miR-27a-3p
 
 
-0.339
 
 
257.3
 
 
 
 
BZW1
 
 
hsa-miR-27a-3p
 
 
-0.337
 
 
122.3
 
 
 
 
C1GALT1
 
 
hsa-miR-152-3p
 
 
-0.487
 
 
12.1
 
 
 
 
C1orf35
 
 
hsa-miR-152-3p
 
 
-0.511
 
 
12.8
 
 
 
 
C5orf24
 
 
hsa-miR-22-3p
 
 
-0.493
 
 
22.6
 
 
 
 
C6orf120
 
 
hsa-miR-34a-5p
 
 
-0.331
 
 
17.1
 
 
 
 
C6orf62
 
 
hsa-miR-181a-5p
 
 
-0.334
 
 
103.1
 
 
 
 
CABP1
 
 
hsa-miR-27a-3p
 
 
-0.343
 
 
11.0
 
 
 
 
CACYBP
 
 
hsa-miR-27a-3p
 
 
-0.367
 
 
25.8
 
 
 
 
CAMK2N1
 
 
hsa-miR-22-3p
 
 
-0.302
 
 
19.0
 
 
 
 
CAND1
 
 
hsa-miR-27a-3p
 
 
-0.318
 
 
17.2
 
 
 
 
CAND1
 
 
hsa-miR-152-3p
 
 
-0.44
 
 
17.2
 
 
 
 
CANX
 
 
hsa-miR-152-3p
 
 
-0.434
 
 
112.0
 
 
 
 
 
 
 adrenal gland 
 
 
 
 
target_symbol
 
 
mature_mirna_id
 
 
score
 
 
nTPM
 
 
 
 
 
 
AAGAB
 
 
hsa-miR-34a-5p
 
 
-0.319
 
 
17.7
 
 
 
 
ABCB7
 
 
hsa-miR-152-3p
 
 
-0.515
 
 
12.5
 
 
 
 
ABCD3
 
 
hsa-miR-152-3p
 
 
-0.388
 
 
17.6
 
 
 
 
ACBD3
 
 
hsa-miR-34a-5p
 
 
-0.415
 
 
16.0
 
 
 
 
ACSL1
 
 
hsa-miR-34a-5p
 
 
-0.306
 
 
41.7
 
 
 
 
ACSL4
 
 
hsa-miR-34a-5p
 
 
-0.372
 
 
34.9
 
 
 
 
ACTA2
 
 
hsa-miR-27a-3p
 
 
-0.577
 
 
227.6
 
 
 
 
ADGRF5
 
 
hsa-miR-152-3p
 
 
-0.354
 
 
22.0
 
 
 
 
ADIPOR2
 
 
hsa-miR-34a-5p
 
 
-0.33
 
 
69.8
 
 
 
 
ADK
 
 
hsa-miR-34a-5p
 
 
-0.374
 
 
14.6
 
 
 
 
AFG3L2
 
 
hsa-miR-181a-5p
 
 
-0.491
 
 
12.9
 
 
 
 
AGFG1
 
 
hsa-miR-152-3p
 
 
-0.332
 
 
37.2
 
 
 
 
AGPAT3
 
 
hsa-miR-27a-3p
 
 
-0.29
 
 
19.9
 
 
 
 
AGTRAP
 
 
hsa-miR-34a-5p
 
 
-0.328
 
 
46.3
 
 
 
 
AHDC1
 
 
hsa-miR-152-3p
 
 
-0.413
 
 
23.0
 
 
 
 
AIDA
 
 
hsa-miR-22-3p
 
 
-0.316
 
 
34.1
 
 
 
 
AKAP1
 
 
hsa-miR-152-3p
 
 
-0.381
 
 
14.9
 
 
 
 
AKIRIN1
 
 
hsa-miR-27a-3p
 
 
-0.368
 
 
28.2
 
 
 
 
ALCAM
 
 
hsa-miR-34a-5p
 
 
-0.303
 
 
30.9
 
 
 
 
ALCAM
 
 
hsa-miR-152-3p
 
 
-0.347
 
 
30.9
 
 
 
 
ALDOA
 
 
hsa-miR-34a-5p
 
 
-0.655
 
 
799.7
 
 
 
 
ALPL
 
 
hsa-miR-10a-5p
 
 
-0.32
 
 
57.7
 
 
 
 
ANK2
 
 
hsa-miR-34a-5p
 
 
-0.317
 
 
10.7
 
 
 
 
ANKHD1-EIF4EBP3
 
 
hsa-miR-22-3p
 
 
-0.479
 
 
14.6
 
 
 
 
ANKRD40
 
 
hsa-miR-27a-3p
 
 
-0.35
 
 
18.5
 
 
 
 
ANP32B
 
 
hsa-miR-34a-5p
 
 
-0.29
 
 
72.7
 
 
 
 
AP1AR
 
 
hsa-miR-100-5p
 
 
-0.804
 
 
14.8
 
 
 
 
AP3S2
 
 
hsa-miR-34a-5p
 
 
-0.413
 
 
19.2
 
 
 
 
APH1A
 
 
hsa-miR-34a-5p
 
 
-0.304
 
 
90.1
 
 
 
 
APOO
 
 
hsa-miR-181a-5p
 
 
-0.5
 
 
22.6
 
 
 
 
APPBP2
 
 
hsa-miR-27a-3p
 
 
-0.378
 
 
10.6
 
 
 
 
AQP11
 
 
hsa-miR-27a-3p
 
 
-0.666
 
 
14.1
 
 
 
 
ARF4
 
 
hsa-miR-152-3p
 
 
-0.487
 
 
151.0
 
 
 
 
ARF6
 
 
hsa-miR-181a-5p
 
 
-0.33
 
 
24.5
 
 
 
 
ARFIP1
 
 
hsa-miR-320d
 
 
-0.339
 
 
13.9
 
 
 
 
ARFIP1
 
 
hsa-miR-152-3p
 
 
-0.474
 
 
13.9
 
 
 
 
ARHGAP1
 
 
hsa-miR-34a-5p
 
 
-0.369
 
 
46.3
 
 
 
 
ARHGAP21
 
 
hsa-miR-152-3p
 
 
-0.287
 
 
16.9
 
 
 
 
ARHGAP36
 
 
hsa-miR-34a-5p
 
 
-0.367
 
 
99.5
 
 
 
 
ARL6IP1
 
 
hsa-miR-152-3p
 
 
-0.579
 
 
161.7
 
 
 
 
ARL8B
 
 
hsa-miR-152-3p
 
 
-0.337
 
 
48.3
 
 
 
 
ARL8B
 
 
hsa-miR-320d
 
 
-0.376
 
 
48.3
 
 
 
 
ARPC5
 
 
hsa-miR-22-3p
 
 
-0.679
 
 
46.4
 
 
 
 
ARPIN-AP3S2
 
 
hsa-miR-34a-5p
 
 
-0.348
 
 
11.3
 
 
 
 
ARPP19
 
 
hsa-miR-320d
 
 
-0.325
 
 
48.8
 
 
 
 
ARPP19
 
 
hsa-miR-34a-5p
 
 
-0.497
 
 
48.8
 
 
 
 
ARRDC3
 
 
hsa-miR-152-3p
 
 
-0.305
 
 
75.7
 
 
 
 
AS3MT
 
 
hsa-miR-28-5p
 
 
-0.479
 
 
260.6
 
 
 
 
ASAH1
 
 
hsa-miR-27a-3p
 
 
-0.398
 
 
67.8
 
 
 
 
ASH2L
 
 
hsa-miR-320d
 
 
-0.287
 
 
19.1
 
 
 
 
ASPH
 
 
hsa-miR-152-3p
 
 
-0.391
 
 
150.8
 
 
 
 
ATG4B
 
 
hsa-miR-34a-5p
 
 
-0.58
 
 
19.2
 
 
 
 
ATMIN
 
 
hsa-miR-34a-5p
 
 
-0.409
 
 
12.0
 
 
 
 
ATP2B4
 
 
hsa-miR-152-3p
 
 
-0.337
 
 
23.7
 
 
 
 
ATP5MC3
 
 
hsa-miR-27a-3p
 
 
-0.416
 
 
190.4
 
 
 
 
ATP6AP2
 
 
hsa-miR-152-3p
 
 
-0.592
 
 
67.5
 
 
 
 
ATP6AP2
 
 
hsa-miR-152-3p
 
 
-0.668
 
 
67.5
 
 
 
 
ATXN10
 
 
hsa-miR-27a-3p
 
 
-0.295
 
 
56.3
 
 
 
 
ATXN2
 
 
hsa-miR-10a-5p
 
 
-0.362
 
 
24.4
 
 
 
 
AXL
 
 
hsa-miR-34a-5p
 
 
-0.512
 
 
17.7
 
 
 
 
B3GNT7
 
 
hsa-miR-27a-3p
 
 
-0.355
 
 
13.5
 
 
 
 
B4GALT2
 
 
hsa-miR-34a-5p
 
 
-0.314
 
 
15.3
 
 
 
 
B4GALT3
 
 
hsa-miR-27a-3p
 
 
-0.41
 
 
22.1
 
 
 
 
B4GALT5
 
 
hsa-miR-152-3p
 
 
-0.44
 
 
44.9
 
 
 
 
B4GALT6
 
 
hsa-miR-152-3p
 
 
-0.528
 
 
17.1
 
 
 
 
BAG5
 
 
hsa-miR-22-3p
 
 
-0.318
 
 
10.8
 
 
 
 
BAZ1B
 
 
hsa-miR-10a-5p
 
 
-0.309
 
 
18.3
 
 
 
 
BAZ2A
 
 
hsa-miR-100-5p
 
 
-0.29
 
 
22.2
 
 
 
 
BAZ2A
 
 
hsa-miR-100-5p
 
 
-0.421
 
 
22.2
 
 
 
 
BBX
 
 
hsa-miR-10a-5p
 
 
-0.305
 
 
23.6
 
 
 
 
BCL3
 
 
hsa-miR-27a-3p
 
 
-0.413
 
 
46.2
 
 
 
 
BEX3
 
 
hsa-miR-27a-3p
 
 
-0.454
 
 
360.8
 
 
 
 
BHLHE40
 
 
hsa-miR-181a-5p
 
 
-0.408
 
 
109.5
 
 
 
 
BHLHE41
 
 
hsa-miR-152-3p
 
 
-0.442
 
 
13.8
 
 
 
 
BIN1
 
 
hsa-miR-22-3p
 
 
-0.346
 
 
88.9
 
 
 
 
BLOC1S1
 
 
hsa-miR-22-3p
 
 
-0.346
 
 
165.7
 
 
 
 
BLOC1S6
 
 
hsa-miR-181a-5p
 
 
-0.393
 
 
29.4
 
 
 
 
BLTP1
 
 
hsa-miR-27a-3p
 
 
-0.426
 
 
16.4
 
 
 
 
BMI1
 
 
hsa-miR-27a-3p
 
 
-0.322
 
 
29.3
 
 
 
 
BMPR2
 
 
hsa-miR-100-5p
 
 
-0.355
 
 
17.0
 
 
 
 
BRPF3
 
 
hsa-miR-34a-5p
 
 
-0.33
 
 
11.2
 
 
 
 
BRSK1
 
 
hsa-miR-27a-3p
 
 
-0.372
 
 
11.3
 
 
 
 
BTAF1
 
 
hsa-miR-152-3p
 
 
-0.401
 
 
11.3
 
 
 
 
BTBD3
 
 
hsa-miR-152-3p
 
 
-0.495
 
 
20.8
 
 
 
 
BTG2
 
 
hsa-miR-27a-3p
 
 
-0.339
 
 
231.0
 
 
 
 
BZW1
 
 
hsa-miR-27a-3p
 
 
-0.337
 
 
121.8
 
 
 
 
C1orf35
 
 
hsa-miR-152-3p
 
 
-0.511
 
 
14.2
 
 
 
 
C2orf69
 
 
hsa-miR-181a-5p
 
 
-0.38
 
 
13.3
 
 
 
 
C5orf24
 
 
hsa-miR-22-3p
 
 
-0.493
 
 
24.5
 
 
 
 
C6orf120
 
 
hsa-miR-34a-5p
 
 
-0.331
 
 
16.2
 
 
 
 
C6orf62
 
 
hsa-miR-181a-5p
 
 
-0.334
 
 
77.8
 
 
 
 
CACYBP
 
 
hsa-miR-27a-3p
 
 
-0.367
 
 
22.2
 
 
 
 
CADM4
 
 
hsa-miR-34a-5p
 
 
-0.365
 
 
22.0
 
 
 
 
CAMK2N1
 
 
hsa-miR-22-3p
 
 
-0.302
 
 
58.3
 
 
 
 
CAND1
 
 
hsa-miR-27a-3p
 
 
-0.318
 
 
21.8
 
 
 
 
CAND1
 
 
hsa-miR-152-3p
 
 
-0.44
 
 
21.8
 
 
 
 
CANX
 
 
hsa-miR-152-3p
 
 
-0.434
 
 
97.0
 
 
 
 
CAPN1
 
 
hsa-miR-22-3p
 
 
-0.307
 
 
41.1
 
 
 
 
CAPZA1
 
 
hsa-miR-27a-3p
 
 
-0.325
 
 
47.8
 
 
 
 
CBX4
 
 
hsa-miR-181a-5p
 
 
-0.354
 
 
13.5
 
 
 
 
 
 
 amygdala 
 
 
 
 
target_symbol
 
 
mature_mirna_id
 
 
score
 
 
nTPM
 
 
 
 
 
 
AAGAB
 
 
hsa-miR-34a-5p
 
 
-0.319
 
 
10.9
 
 
 
 
ABCD3
 
 
hsa-miR-152-3p
 
 
-0.388
 
 
16.0
 
 
 
 
ABHD17C
 
 
hsa-miR-27a-3p
 
 
-0.435
 
 
17.9
 
 
 
 
ACSL1
 
 
hsa-miR-34a-5p
 
 
-0.306
 
 
24.5
 
 
 
 
ACTA2
 
 
hsa-miR-27a-3p
 
 
-0.577
 
 
36.7
 
 
 
 
ADAP1
 
 
hsa-miR-27a-3p
 
 
-0.302
 
 
109.3
 
 
 
 
ADCY2
 
 
hsa-miR-152-3p
 
 
-0.299
 
 
28.0
 
 
 
 
ADD2
 
 
hsa-miR-34a-5p
 
 
-0.305
 
 
12.1
 
 
 
 
ADGRA3
 
 
hsa-miR-27a-3p
 
 
-0.296
 
 
19.9
 
 
 
 
ADGRB3
 
 
hsa-miR-152-3p
 
 
-0.516
 
 
12.8
 
 
 
 
ADIPOR2
 
 
hsa-miR-34a-5p
 
 
-0.33
 
 
43.7
 
 
 
 
ADK
 
 
hsa-miR-34a-5p
 
 
-0.374
 
 
16.1
 
 
 
 
AGFG1
 
 
hsa-miR-152-3p
 
 
-0.332
 
 
25.1
 
 
 
 
AGPAT3
 
 
hsa-miR-27a-3p
 
 
-0.29
 
 
41.1
 
 
 
 
AGTRAP
 
 
hsa-miR-34a-5p
 
 
-0.328
 
 
23.7
 
 
 
 
AHCYL2
 
 
hsa-miR-34a-5p
 
 
-0.359
 
 
17.8
 
 
 
 
AHDC1
 
 
hsa-miR-152-3p
 
 
-0.413
 
 
10.1
 
 
 
 
AIDA
 
 
hsa-miR-22-3p
 
 
-0.316
 
 
28.0
 
 
 
 
AKAP1
 
 
hsa-miR-152-3p
 
 
-0.381
 
 
22.8
 
 
 
 
AKIRIN1
 
 
hsa-miR-27a-3p
 
 
-0.368
 
 
32.7
 
 
 
 
AKT3
 
 
hsa-miR-22-3p
 
 
-0.451
 
 
17.4
 
 
 
 
ALCAM
 
 
hsa-miR-34a-5p
 
 
-0.303
 
 
31.7
 
 
 
 
ALCAM
 
 
hsa-miR-152-3p
 
 
-0.347
 
 
31.7
 
 
 
 
ALDOA
 
 
hsa-miR-34a-5p
 
 
-0.655
 
 
853.4
 
 
 
 
ALPL
 
 
hsa-miR-10a-5p
 
 
-0.32
 
 
10.6
 
 
 
 
AMER2
 
 
hsa-miR-34a-5p
 
 
-0.48
 
 
25.2
 
 
 
 
ANGEL2
 
 
hsa-miR-22-3p
 
 
-0.601
 
 
10.6
 
 
 
 
ANK2
 
 
hsa-miR-34a-5p
 
 
-0.317
 
 
17.3
 
 
 
 
ANK3
 
 
hsa-miR-34a-5p
 
 
-0.29
 
 
37.4
 
 
 
 
ANK3
 
 
hsa-miR-34a-5p
 
 
-0.347
 
 
37.4
 
 
 
 
ANKRD40
 
 
hsa-miR-27a-3p
 
 
-0.35
 
 
96.8
 
 
 
 
ANP32B
 
 
hsa-miR-34a-5p
 
 
-0.29
 
 
123.1
 
 
 
 
AP3S2
 
 
hsa-miR-34a-5p
 
 
-0.413
 
 
14.5
 
 
 
 
APH1A
 
 
hsa-miR-34a-5p
 
 
-0.304
 
 
50.4
 
 
 
 
APOO
 
 
hsa-miR-181a-5p
 
 
-0.5
 
 
45.1
 
 
 
 
ARF4
 
 
hsa-miR-152-3p
 
 
-0.487
 
 
60.1
 
 
 
 
ARHGAP1
 
 
hsa-miR-34a-5p
 
 
-0.369
 
 
37.0
 
 
 
 
ARHGAP21
 
 
hsa-miR-152-3p
 
 
-0.287
 
 
40.6
 
 
 
 
ARL6IP1
 
 
hsa-miR-152-3p
 
 
-0.579
 
 
241.3
 
 
 
 
ARL8B
 
 
hsa-miR-152-3p
 
 
-0.337
 
 
53.8
 
 
 
 
ARL8B
 
 
hsa-miR-320d
 
 
-0.376
 
 
53.8
 
 
 
 
ARPC5
 
 
hsa-miR-22-3p
 
 
-0.679
 
 
19.6
 
 
 
 
ARPIN-AP3S2
 
 
hsa-miR-34a-5p
 
 
-0.348
 
 
10.5
 
 
 
 
ARPP19
 
 
hsa-miR-320d
 
 
-0.325
 
 
178.5
 
 
 
 
ARPP19
 
 
hsa-miR-34a-5p
 
 
-0.497
 
 
178.5
 
 
 
 
ARRB1
 
 
hsa-miR-22-3p
 
 
-0.616
 
 
32.5
 
 
 
 
ASAH1
 
 
hsa-miR-27a-3p
 
 
-0.398
 
 
50.6
 
 
 
 
ASB1
 
 
hsa-miR-34a-5p
 
 
-0.406
 
 
16.3
 
 
 
 
ASH2L
 
 
hsa-miR-320d
 
 
-0.287
 
 
17.5
 
 
 
 
ASPH
 
 
hsa-miR-152-3p
 
 
-0.391
 
 
62.6
 
 
 
 
ATCAY
 
 
hsa-miR-10a-5p
 
 
-0.322
 
 
44.9
 
 
 
 
ATG4B
 
 
hsa-miR-34a-5p
 
 
-0.58
 
 
21.3
 
 
 
 
ATMIN
 
 
hsa-miR-34a-5p
 
 
-0.409
 
 
19.5
 
 
 
 
ATP1A2
 
 
hsa-miR-34a-5p
 
 
-0.306
 
 
434.8
 
 
 
 
ATP5MC3
 
 
hsa-miR-27a-3p
 
 
-0.416
 
 
183.1
 
 
 
 
ATP6AP2
 
 
hsa-miR-152-3p
 
 
-0.592
 
 
69.8
 
 
 
 
ATP6AP2
 
 
hsa-miR-152-3p
 
 
-0.668
 
 
69.8
 
 
 
 
ATXN10
 
 
hsa-miR-27a-3p
 
 
-0.295
 
 
52.1
 
 
 
 
ATXN2
 
 
hsa-miR-10a-5p
 
 
-0.362
 
 
21.8
 
 
 
 
AXL
 
 
hsa-miR-34a-5p
 
 
-0.512
 
 
12.9
 
 
 
 
B4GALT2
 
 
hsa-miR-34a-5p
 
 
-0.314
 
 
66.2
 
 
 
 
B4GALT3
 
 
hsa-miR-27a-3p
 
 
-0.41
 
 
13.8
 
 
 
 
B4GALT5
 
 
hsa-miR-152-3p
 
 
-0.44
 
 
10.8
 
 
 
 
BAZ1B
 
 
hsa-miR-10a-5p
 
 
-0.309
 
 
17.0
 
 
 
 
BEGAIN
 
 
hsa-miR-28-5p
 
 
-0.325
 
 
36.9
 
 
 
 
BEX3
 
 
hsa-miR-27a-3p
 
 
-0.454
 
 
543.6
 
 
 
 
BHLHE40
 
 
hsa-miR-181a-5p
 
 
-0.408
 
 
40.6
 
 
 
 
BHLHE41
 
 
hsa-miR-152-3p
 
 
-0.442
 
 
27.2
 
 
 
 
BIN1
 
 
hsa-miR-22-3p
 
 
-0.346
 
 
167.4
 
 
 
 
BLOC1S1
 
 
hsa-miR-22-3p
 
 
-0.346
 
 
261.3
 
 
 
 
BLOC1S6
 
 
hsa-miR-181a-5p
 
 
-0.393
 
 
20.1
 
 
 
 
BLTP1
 
 
hsa-miR-27a-3p
 
 
-0.426
 
 
18.1
 
 
 
 
BMI1
 
 
hsa-miR-27a-3p
 
 
-0.322
 
 
22.4
 
 
 
 
BMPR2
 
 
hsa-miR-100-5p
 
 
-0.355
 
 
10.7
 
 
 
 
BRINP1
 
 
hsa-miR-34a-5p
 
 
-0.612
 
 
28.2
 
 
 
 
BRSK1
 
 
hsa-miR-27a-3p
 
 
-0.372
 
 
124.7
 
 
 
 
BTBD3
 
 
hsa-miR-152-3p
 
 
-0.495
 
 
27.3
 
 
 
 
BTG2
 
 
hsa-miR-27a-3p
 
 
-0.339
 
 
39.0
 
 
 
 
BZW1
 
 
hsa-miR-27a-3p
 
 
-0.337
 
 
34.3
 
 
 
 
C1orf35
 
 
hsa-miR-152-3p
 
 
-0.511
 
 
14.3
 
 
 
 
C5orf24
 
 
hsa-miR-22-3p
 
 
-0.493
 
 
17.3
 
 
 
 
C6orf62
 
 
hsa-miR-181a-5p
 
 
-0.334
 
 
82.1
 
 
 
 
C8orf88
 
 
hsa-miR-27a-3p
 
 
-0.33
 
 
17.2
 
 
 
 
CA10
 
 
hsa-miR-27a-3p
 
 
-0.464
 
 
35.6
 
 
 
 
CABP1
 
 
hsa-miR-27a-3p
 
 
-0.343
 
 
116.4
 
 
 
 
CACNB3
 
 
hsa-miR-34a-5p
 
 
-0.426
 
 
74.6
 
 
 
 
CACYBP
 
 
hsa-miR-27a-3p
 
 
-0.367
 
 
56.4
 
 
 
 
CADM4
 
 
hsa-miR-34a-5p
 
 
-0.365
 
 
119.8
 
 
 
 
CAMK2N1
 
 
hsa-miR-22-3p
 
 
-0.302
 
 
570.4
 
 
 
 
CAND1
 
 
hsa-miR-27a-3p
 
 
-0.318
 
 
16.4
 
 
 
 
CAND1
 
 
hsa-miR-152-3p
 
 
-0.44
 
 
16.4
 
 
 
 
CANX
 
 
hsa-miR-152-3p
 
 
-0.434
 
 
108.0
 
 
 
 
CAPN1
 
 
hsa-miR-22-3p
 
 
-0.307
 
 
25.9
 
 
 
 
CAPZA1
 
 
hsa-miR-27a-3p
 
 
-0.325
 
 
34.6
 
 
 
 
CBX4
 
 
hsa-miR-181a-5p
 
 
-0.354
 
 
12.9
 
 
 
 
CCDC149
 
 
hsa-miR-22-3p
 
 
-0.327
 
 
10.0
 
 
 
 
CCDC149
 
 
hsa-miR-27a-3p
 
 
-0.551
 
 
10.0
 
 
 
 
CCDC28B
 
 
hsa-miR-27a-3p
 
 
-0.314
 
 
21.5
 
 
 
 
CCDC47
 
 
hsa-miR-22-3p
 
 
-0.604
 
 
29.4
 
 
 
 
CCDC6
 
 
hsa-miR-152-3p
 
 
-0.511
 
 
16.2
 
 
 
 
 
 
 appendix 
 
 
 
 
target_symbol
 
 
mature_mirna_id
 
 
score
 
 
nTPM
 
 
 
 
 
 
AAGAB
 
 
hsa-miR-34a-5p
 
 
-0.319
 
 
18.2
 
 
 
 
ABCD3
 
 
hsa-miR-152-3p
 
 
-0.388
 
 
15.3
 
 
 
 
ACBD3
 
 
hsa-miR-34a-5p
 
 
-0.415
 
 
19.2
 
 
 
 
ACSL1
 
 
hsa-miR-34a-5p
 
 
-0.306
 
 
84.1
 
 
 
 
ACSL4
 
 
hsa-miR-34a-5p
 
 
-0.372
 
 
34.5
 
 
 
 
ACTA2
 
 
hsa-miR-27a-3p
 
 
-0.577
 
 
687.0
 
 
 
 
ADAM10
 
 
hsa-miR-152-3p
 
 
-0.348
 
 
17.9
 
 
 
 
ADAP1
 
 
hsa-miR-27a-3p
 
 
-0.302
 
 
10.6
 
 
 
 
ADIPOR2
 
 
hsa-miR-34a-5p
 
 
-0.33
 
 
10.0
 
 
 
 
ADK
 
 
hsa-miR-34a-5p
 
 
-0.374
 
 
15.9
 
 
 
 
AFG3L2
 
 
hsa-miR-181a-5p
 
 
-0.491
 
 
12.5
 
 
 
 
AGFG1
 
 
hsa-miR-152-3p
 
 
-0.332
 
 
45.7
 
 
 
 
AGPAT3
 
 
hsa-miR-27a-3p
 
 
-0.29
 
 
18.1
 
 
 
 
AGTRAP
 
 
hsa-miR-34a-5p
 
 
-0.328
 
 
28.5
 
 
 
 
AHCYL2
 
 
hsa-miR-34a-5p
 
 
-0.359
 
 
14.7
 
 
 
 
AIDA
 
 
hsa-miR-22-3p
 
 
-0.316
 
 
36.9
 
 
 
 
AKAP1
 
 
hsa-miR-152-3p
 
 
-0.381
 
 
16.8
 
 
 
 
AKIRIN1
 
 
hsa-miR-27a-3p
 
 
-0.368
 
 
31.6
 
 
 
 
ALCAM
 
 
hsa-miR-34a-5p
 
 
-0.303
 
 
11.5
 
 
 
 
ALCAM
 
 
hsa-miR-152-3p
 
 
-0.347
 
 
11.5
 
 
 
 
ALDOA
 
 
hsa-miR-34a-5p
 
 
-0.655
 
 
417.5
 
 
 
 
ALPL
 
 
hsa-miR-10a-5p
 
 
-0.32
 
 
26.3
 
 
 
 
ANKRD13A
 
 
hsa-miR-22-3p
 
 
-0.478
 
 
34.4
 
 
 
 
ANKRD40
 
 
hsa-miR-27a-3p
 
 
-0.35
 
 
14.6
 
 
 
 
ANP32B
 
 
hsa-miR-34a-5p
 
 
-0.29
 
 
117.0
 
 
 
 
AP1AR
 
 
hsa-miR-100-5p
 
 
-0.804
 
 
11.9
 
 
 
 
AP3S2
 
 
hsa-miR-34a-5p
 
 
-0.413
 
 
10.5
 
 
 
 
APH1A
 
 
hsa-miR-34a-5p
 
 
-0.304
 
 
57.1
 
 
 
 
AREG
 
 
hsa-miR-34a-5p
 
 
-0.62
 
 
34.6
 
 
 
 
ARF4
 
 
hsa-miR-152-3p
 
 
-0.487
 
 
101.3
 
 
 
 
ARF6
 
 
hsa-miR-181a-5p
 
 
-0.33
 
 
41.0
 
 
 
 
ARFGEF1
 
 
hsa-miR-152-3p
 
 
-0.306
 
 
13.8
 
 
 
 
ARFIP1
 
 
hsa-miR-320d
 
 
-0.339
 
 
14.9
 
 
 
 
ARFIP1
 
 
hsa-miR-152-3p
 
 
-0.474
 
 
14.9
 
 
 
 
ARHGAP1
 
 
hsa-miR-34a-5p
 
 
-0.369
 
 
35.8
 
 
 
 
ARHGAP26
 
 
hsa-miR-34a-5p
 
 
-0.384
 
 
16.7
 
 
 
 
ARL6IP1
 
 
hsa-miR-152-3p
 
 
-0.579
 
 
73.3
 
 
 
 
ARL8B
 
 
hsa-miR-152-3p
 
 
-0.337
 
 
34.1
 
 
 
 
ARL8B
 
 
hsa-miR-320d
 
 
-0.376
 
 
34.1
 
 
 
 
ARPC5
 
 
hsa-miR-22-3p
 
 
-0.679
 
 
139.8
 
 
 
 
ARPP19
 
 
hsa-miR-320d
 
 
-0.325
 
 
36.4
 
 
 
 
ARPP19
 
 
hsa-miR-34a-5p
 
 
-0.497
 
 
36.4
 
 
 
 
ARRB1
 
 
hsa-miR-22-3p
 
 
-0.616
 
 
20.7
 
 
 
 
ARRDC3
 
 
hsa-miR-152-3p
 
 
-0.305
 
 
25.3
 
 
 
 
ASAH1
 
 
hsa-miR-27a-3p
 
 
-0.398
 
 
48.7
 
 
 
 
ASH2L
 
 
hsa-miR-320d
 
 
-0.287
 
 
17.4
 
 
 
 
ASPH
 
 
hsa-miR-152-3p
 
 
-0.391
 
 
47.6
 
 
 
 
ATMIN
 
 
hsa-miR-34a-5p
 
 
-0.409
 
 
10.8
 
 
 
 
ATP2B4
 
 
hsa-miR-152-3p
 
 
-0.337
 
 
24.2
 
 
 
 
ATP5MC3
 
 
hsa-miR-27a-3p
 
 
-0.416
 
 
123.3
 
 
 
 
ATP6AP2
 
 
hsa-miR-152-3p
 
 
-0.592
 
 
57.3
 
 
 
 
ATP6AP2
 
 
hsa-miR-152-3p
 
 
-0.668
 
 
57.3
 
 
 
 
ATXN10
 
 
hsa-miR-27a-3p
 
 
-0.295
 
 
32.7
 
 
 
 
ATXN2
 
 
hsa-miR-10a-5p
 
 
-0.362
 
 
10.6
 
 
 
 
AXL
 
 
hsa-miR-34a-5p
 
 
-0.512
 
 
14.6
 
 
 
 
B4GALT2
 
 
hsa-miR-34a-5p
 
 
-0.314
 
 
12.3
 
 
 
 
B4GALT3
 
 
hsa-miR-27a-3p
 
 
-0.41
 
 
15.1
 
 
 
 
B4GALT5
 
 
hsa-miR-152-3p
 
 
-0.44
 
 
20.5
 
 
 
 
BAG5
 
 
hsa-miR-22-3p
 
 
-0.318
 
 
14.3
 
 
 
 
BAZ1B
 
 
hsa-miR-10a-5p
 
 
-0.309
 
 
20.9
 
 
 
 
BAZ2A
 
 
hsa-miR-100-5p
 
 
-0.29
 
 
29.1
 
 
 
 
BAZ2A
 
 
hsa-miR-100-5p
 
 
-0.421
 
 
29.1
 
 
 
 
BBX
 
 
hsa-miR-10a-5p
 
 
-0.305
 
 
12.0
 
 
 
 
BCL2L11
 
 
hsa-miR-152-3p
 
 
-0.445
 
 
16.0
 
 
 
 
BCL2L11
 
 
hsa-miR-10a-5p
 
 
-0.483
 
 
16.0
 
 
 
 
BCL3
 
 
hsa-miR-27a-3p
 
 
-0.413
 
 
23.6
 
 
 
 
BEX3
 
 
hsa-miR-27a-3p
 
 
-0.454
 
 
37.3
 
 
 
 
BHLHE40
 
 
hsa-miR-181a-5p
 
 
-0.408
 
 
50.0
 
 
 
 
BIN1
 
 
hsa-miR-22-3p
 
 
-0.346
 
 
24.3
 
 
 
 
BLOC1S1
 
 
hsa-miR-22-3p
 
 
-0.346
 
 
78.5
 
 
 
 
BLOC1S6
 
 
hsa-miR-181a-5p
 
 
-0.393
 
 
29.4
 
 
 
 
BMI1
 
 
hsa-miR-27a-3p
 
 
-0.322
 
 
23.6
 
 
 
 
BTAF1
 
 
hsa-miR-152-3p
 
 
-0.401
 
 
16.0
 
 
 
 
BTG1
 
 
hsa-miR-22-3p
 
 
-0.468
 
 
18.7
 
 
 
 
BTG2
 
 
hsa-miR-27a-3p
 
 
-0.339
 
 
117.3
 
 
 
 
BZW1
 
 
hsa-miR-27a-3p
 
 
-0.337
 
 
91.9
 
 
 
 
C1GALT1
 
 
hsa-miR-152-3p
 
 
-0.487
 
 
13.4
 
 
 
 
C5orf24
 
 
hsa-miR-22-3p
 
 
-0.493
 
 
16.8
 
 
 
 
C6orf120
 
 
hsa-miR-34a-5p
 
 
-0.331
 
 
12.4
 
 
 
 
C6orf62
 
 
hsa-miR-181a-5p
 
 
-0.334
 
 
108.5
 
 
 
 
CACYBP
 
 
hsa-miR-27a-3p
 
 
-0.367
 
 
24.0
 
 
 
 
CAND1
 
 
hsa-miR-27a-3p
 
 
-0.318
 
 
18.7
 
 
 
 
CAND1
 
 
hsa-miR-152-3p
 
 
-0.44
 
 
18.7
 
 
 
 
CANX
 
 
hsa-miR-152-3p
 
 
-0.434
 
 
34.2
 
 
 
 
CAPN1
 
 
hsa-miR-22-3p
 
 
-0.307
 
 
48.9
 
 
 
 
CAPZA1
 
 
hsa-miR-27a-3p
 
 
-0.325
 
 
108.8
 
 
 
 
CASP2
 
 
hsa-miR-34a-5p
 
 
-0.382
 
 
13.1
 
 
 
 
CAV2
 
 
hsa-miR-152-3p
 
 
-0.32
 
 
17.7
 
 
 
 
CBFB
 
 
hsa-miR-27a-3p
 
 
-0.302
 
 
16.3
 
 
 
 
CBX4
 
 
hsa-miR-181a-5p
 
 
-0.354
 
 
10.5
 
 
 
 
CCDC47
 
 
hsa-miR-22-3p
 
 
-0.604
 
 
38.2
 
 
 
 
CCDC6
 
 
hsa-miR-152-3p
 
 
-0.511
 
 
13.1
 
 
 
 
CCM2
 
 
hsa-miR-27a-3p
 
 
-0.335
 
 
23.7
 
 
 
 
CCN1
 
 
hsa-miR-22-3p
 
 
-0.601
 
 
26.6
 
 
 
 
CCNC
 
 
hsa-miR-27a-3p
 
 
-0.378
 
 
25.8
 
 
 
 
CCNG1
 
 
hsa-miR-27a-3p
 
 
-0.419
 
 
28.7
 
 
 
 
CCNK
 
 
hsa-miR-181a-5p
 
 
-0.31
 
 
25.1
 
 
 
 
CCNK
 
 
hsa-miR-27a-3p
 
 
-0.519
 
 
25.1
 
 
 
 
CCNL2
 
 
hsa-miR-28-5p
 
 
-0.448
 
 
27.6
 
 
 
 
CCNY
 
 
hsa-miR-27a-3p
 
 
-0.334
 
 
20.0
 
 
 
 
 
 
 basal ganglia 
 
 
 
 
target_symbol
 
 
mature_mirna_id
 
 
score
 
 
nTPM
 
 
 
 
 
 
AAGAB
 
 
hsa-miR-34a-5p
 
 
-0.319
 
 
12.7
 
 
 
 
ABCD3
 
 
hsa-miR-152-3p
 
 
-0.388
 
 
17.4
 
 
 
 
ABHD17C
 
 
hsa-miR-27a-3p
 
 
-0.435
 
 
20.2
 
 
 
 
ACSL1
 
 
hsa-miR-34a-5p
 
 
-0.306
 
 
28.3
 
 
 
 
ACTA2
 
 
hsa-miR-27a-3p
 
 
-0.577
 
 
45.5
 
 
 
 
ADAP1
 
 
hsa-miR-27a-3p
 
 
-0.302
 
 
77.3
 
 
 
 
ADCY2
 
 
hsa-miR-152-3p
 
 
-0.299
 
 
23.7
 
 
 
 
ADD2
 
 
hsa-miR-34a-5p
 
 
-0.305
 
 
16.7
 
 
 
 
ADGRA3
 
 
hsa-miR-27a-3p
 
 
-0.296
 
 
18.8
 
 
 
 
ADGRB3
 
 
hsa-miR-152-3p
 
 
-0.516
 
 
21.1
 
 
 
 
ADGRF5
 
 
hsa-miR-152-3p
 
 
-0.354
 
 
13.3
 
 
 
 
ADIPOR2
 
 
hsa-miR-34a-5p
 
 
-0.33
 
 
50.5
 
 
 
 
ADK
 
 
hsa-miR-34a-5p
 
 
-0.374
 
 
22.3
 
 
 
 
ADO
 
 
hsa-miR-34a-5p
 
 
-0.377
 
 
11.0
 
 
 
 
AGFG1
 
 
hsa-miR-152-3p
 
 
-0.332
 
 
27.0
 
 
 
 
AGPAT3
 
 
hsa-miR-27a-3p
 
 
-0.29
 
 
44.7
 
 
 
 
AGTRAP
 
 
hsa-miR-34a-5p
 
 
-0.328
 
 
21.3
 
 
 
 
AHCYL2
 
 
hsa-miR-34a-5p
 
 
-0.359
 
 
31.1
 
 
 
 
AHDC1
 
 
hsa-miR-152-3p
 
 
-0.413
 
 
12.3
 
 
 
 
AIDA
 
 
hsa-miR-22-3p
 
 
-0.316
 
 
28.2
 
 
 
 
AKAP1
 
 
hsa-miR-152-3p
 
 
-0.381
 
 
26.7
 
 
 
 
AKIRIN1
 
 
hsa-miR-27a-3p
 
 
-0.368
 
 
33.9
 
 
 
 
AKT3
 
 
hsa-miR-22-3p
 
 
-0.451
 
 
15.0
 
 
 
 
ALCAM
 
 
hsa-miR-34a-5p
 
 
-0.303
 
 
37.8
 
 
 
 
ALCAM
 
 
hsa-miR-152-3p
 
 
-0.347
 
 
37.8
 
 
 
 
ALDOA
 
 
hsa-miR-34a-5p
 
 
-0.655
 
 
972.8
 
 
 
 
ALPL
 
 
hsa-miR-10a-5p
 
 
-0.32
 
 
12.3
 
 
 
 
AMER2
 
 
hsa-miR-34a-5p
 
 
-0.48
 
 
31.7
 
 
 
 
ANGEL2
 
 
hsa-miR-22-3p
 
 
-0.601
 
 
14.7
 
 
 
 
ANK2
 
 
hsa-miR-34a-5p
 
 
-0.317
 
 
20.3
 
 
 
 
ANK3
 
 
hsa-miR-34a-5p
 
 
-0.29
 
 
40.8
 
 
 
 
ANK3
 
 
hsa-miR-34a-5p
 
 
-0.347
 
 
40.8
 
 
 
 
ANKRD40
 
 
hsa-miR-27a-3p
 
 
-0.35
 
 
100.5
 
 
 
 
ANP32B
 
 
hsa-miR-34a-5p
 
 
-0.29
 
 
112.9
 
 
 
 
AP1AR
 
 
hsa-miR-100-5p
 
 
-0.804
 
 
13.0
 
 
 
 
AP3S2
 
 
hsa-miR-34a-5p
 
 
-0.413
 
 
15.3
 
 
 
 
APH1A
 
 
hsa-miR-34a-5p
 
 
-0.304
 
 
58.7
 
 
 
 
APOO
 
 
hsa-miR-181a-5p
 
 
-0.5
 
 
38.6
 
 
 
 
ARF4
 
 
hsa-miR-152-3p
 
 
-0.487
 
 
71.0
 
 
 
 
ARF6
 
 
hsa-miR-181a-5p
 
 
-0.33
 
 
10.2
 
 
 
 
ARHGAP1
 
 
hsa-miR-34a-5p
 
 
-0.369
 
 
41.0
 
 
 
 
ARHGAP21
 
 
hsa-miR-152-3p
 
 
-0.287
 
 
37.1
 
 
 
 
ARHGAP26
 
 
hsa-miR-34a-5p
 
 
-0.384
 
 
10.6
 
 
 
 
ARL6IP1
 
 
hsa-miR-152-3p
 
 
-0.579
 
 
196.4
 
 
 
 
ARL8B
 
 
hsa-miR-152-3p
 
 
-0.337
 
 
53.5
 
 
 
 
ARL8B
 
 
hsa-miR-320d
 
 
-0.376
 
 
53.5
 
 
 
 
ARPC5
 
 
hsa-miR-22-3p
 
 
-0.679
 
 
21.4
 
 
 
 
ARPIN-AP3S2
 
 
hsa-miR-34a-5p
 
 
-0.348
 
 
11.8
 
 
 
 
ARPP19
 
 
hsa-miR-320d
 
 
-0.325
 
 
481.4
 
 
 
 
ARPP19
 
 
hsa-miR-34a-5p
 
 
-0.497
 
 
481.4
 
 
 
 
ARRB1
 
 
hsa-miR-22-3p
 
 
-0.616
 
 
40.7
 
 
 
 
ARRDC3
 
 
hsa-miR-152-3p
 
 
-0.305
 
 
10.6
 
 
 
 
ASAH1
 
 
hsa-miR-27a-3p
 
 
-0.398
 
 
51.7
 
 
 
 
ASB1
 
 
hsa-miR-34a-5p
 
 
-0.406
 
 
16.2
 
 
 
 
ASH2L
 
 
hsa-miR-320d
 
 
-0.287
 
 
17.0
 
 
 
 
ASPH
 
 
hsa-miR-152-3p
 
 
-0.391
 
 
62.4
 
 
 
 
ASPHD2
 
 
hsa-miR-34a-5p
 
 
-0.354
 
 
17.8
 
 
 
 
ATCAY
 
 
hsa-miR-10a-5p
 
 
-0.322
 
 
37.7
 
 
 
 
ATG4B
 
 
hsa-miR-34a-5p
 
 
-0.58
 
 
21.2
 
 
 
 
ATMIN
 
 
hsa-miR-34a-5p
 
 
-0.409
 
 
17.6
 
 
 
 
ATP1A2
 
 
hsa-miR-34a-5p
 
 
-0.306
 
 
582.8
 
 
 
 
ATP5MC3
 
 
hsa-miR-27a-3p
 
 
-0.416
 
 
198.3
 
 
 
 
ATP6AP2
 
 
hsa-miR-152-3p
 
 
-0.592
 
 
77.2
 
 
 
 
ATP6AP2
 
 
hsa-miR-152-3p
 
 
-0.668
 
 
77.2
 
 
 
 
ATXN10
 
 
hsa-miR-27a-3p
 
 
-0.295
 
 
56.3
 
 
 
 
ATXN2
 
 
hsa-miR-10a-5p
 
 
-0.362
 
 
21.8
 
 
 
 
AXL
 
 
hsa-miR-34a-5p
 
 
-0.512
 
 
17.5
 
 
 
 
B4GALT2
 
 
hsa-miR-34a-5p
 
 
-0.314
 
 
42.1
 
 
 
 
B4GALT3
 
 
hsa-miR-27a-3p
 
 
-0.41
 
 
17.9
 
 
 
 
B4GALT5
 
 
hsa-miR-152-3p
 
 
-0.44
 
 
12.9
 
 
 
 
BAG4
 
 
hsa-miR-181a-5p
 
 
-0.337
 
 
10.9
 
 
 
 
BAG5
 
 
hsa-miR-22-3p
 
 
-0.318
 
 
10.8
 
 
 
 
BATF3
 
 
hsa-miR-22-3p
 
 
-0.786
 
 
10.3
 
 
 
 
BAZ1B
 
 
hsa-miR-10a-5p
 
 
-0.309
 
 
19.2
 
 
 
 
BEGAIN
 
 
hsa-miR-28-5p
 
 
-0.325
 
 
23.9
 
 
 
 
BEX3
 
 
hsa-miR-27a-3p
 
 
-0.454
 
 
623.6
 
 
 
 
BHLHE40
 
 
hsa-miR-181a-5p
 
 
-0.408
 
 
47.4
 
 
 
 
BHLHE41
 
 
hsa-miR-152-3p
 
 
-0.442
 
 
27.8
 
 
 
 
BIN1
 
 
hsa-miR-22-3p
 
 
-0.346
 
 
177.7
 
 
 
 
BLOC1S1
 
 
hsa-miR-22-3p
 
 
-0.346
 
 
224.9
 
 
 
 
BLOC1S6
 
 
hsa-miR-181a-5p
 
 
-0.393
 
 
22.3
 
 
 
 
BLTP1
 
 
hsa-miR-27a-3p
 
 
-0.426
 
 
22.7
 
 
 
 
BMI1
 
 
hsa-miR-27a-3p
 
 
-0.322
 
 
21.1
 
 
 
 
BMPR2
 
 
hsa-miR-100-5p
 
 
-0.355
 
 
12.6
 
 
 
 
BRINP1
 
 
hsa-miR-34a-5p
 
 
-0.612
 
 
27.3
 
 
 
 
BRSK1
 
 
hsa-miR-27a-3p
 
 
-0.372
 
 
92.7
 
 
 
 
BTBD3
 
 
hsa-miR-152-3p
 
 
-0.495
 
 
16.4
 
 
 
 
BTG2
 
 
hsa-miR-27a-3p
 
 
-0.339
 
 
36.1
 
 
 
 
BZW1
 
 
hsa-miR-27a-3p
 
 
-0.337
 
 
31.9
 
 
 
 
C1orf35
 
 
hsa-miR-152-3p
 
 
-0.511
 
 
16.0
 
 
 
 
C1orf52
 
 
hsa-miR-27a-3p
 
 
-0.591
 
 
13.6
 
 
 
 
C5orf24
 
 
hsa-miR-22-3p
 
 
-0.493
 
 
21.2
 
 
 
 
C6orf62
 
 
hsa-miR-181a-5p
 
 
-0.334
 
 
90.1
 
 
 
 
C8orf88
 
 
hsa-miR-27a-3p
 
 
-0.33
 
 
17.0
 
 
 
 
CABP1
 
 
hsa-miR-27a-3p
 
 
-0.343
 
 
33.2
 
 
 
 
CACNA2D3
 
 
hsa-miR-27a-3p
 
 
-0.561
 
 
16.4
 
 
 
 
CACNB3
 
 
hsa-miR-34a-5p
 
 
-0.426
 
 
42.4
 
 
 
 
CACYBP
 
 
hsa-miR-27a-3p
 
 
-0.367
 
 
59.0
 
 
 
 
CADM4
 
 
hsa-miR-34a-5p
 
 
-0.365
 
 
127.8
 
 
 
 
CAMK2N1
 
 
hsa-miR-22-3p
 
 
-0.302
 
 
203.3
 
 
 
 
 
 
 blood vessel 
 
 
 
 
target_symbol
 
 
mature_mirna_id
 
 
score
 
 
nTPM
 
 
 
 
 
 
AAGAB
 
 
hsa-miR-34a-5p
 
 
-0.319
 
 
11.8
 
 
 
 
ABCB7
 
 
hsa-miR-152-3p
 
 
-0.515
 
 
12.3
 
 
 
 
ABCD3
 
 
hsa-miR-152-3p
 
 
-0.388
 
 
14.4
 
 
 
 
ACBD3
 
 
hsa-miR-34a-5p
 
 
-0.415
 
 
19.2
 
 
 
 
ACSL1
 
 
hsa-miR-34a-5p
 
 
-0.306
 
 
119.9
 
 
 
 
ACSL4
 
 
hsa-miR-34a-5p
 
 
-0.372
 
 
15.9
 
 
 
 
ACTA2
 
 
hsa-miR-27a-3p
 
 
-0.577
 
 
10509.9
 
 
 
 
ADAMTS10
 
 
hsa-miR-27a-3p
 
 
-0.354
 
 
20.0
 
 
 
 
ADGRF5
 
 
hsa-miR-152-3p
 
 
-0.354
 
 
12.6
 
 
 
 
ADIPOR2
 
 
hsa-miR-34a-5p
 
 
-0.33
 
 
23.0
 
 
 
 
ADK
 
 
hsa-miR-34a-5p
 
 
-0.374
 
 
15.2
 
 
 
 
AGFG1
 
 
hsa-miR-152-3p
 
 
-0.332
 
 
37.3
 
 
 
 
AGPAT3
 
 
hsa-miR-27a-3p
 
 
-0.29
 
 
11.3
 
 
 
 
AGTRAP
 
 
hsa-miR-34a-5p
 
 
-0.328
 
 
38.4
 
 
 
 
AHDC1
 
 
hsa-miR-152-3p
 
 
-0.413
 
 
27.7
 
 
 
 
AIDA
 
 
hsa-miR-22-3p
 
 
-0.316
 
 
49.9
 
 
 
 
AKAP1
 
 
hsa-miR-152-3p
 
 
-0.381
 
 
84.5
 
 
 
 
AKIRIN1
 
 
hsa-miR-27a-3p
 
 
-0.368
 
 
32.7
 
 
 
 
AKT3
 
 
hsa-miR-22-3p
 
 
-0.451
 
 
34.5
 
 
 
 
ALCAM
 
 
hsa-miR-34a-5p
 
 
-0.303
 
 
85.5
 
 
 
 
ALCAM
 
 
hsa-miR-152-3p
 
 
-0.347
 
 
85.5
 
 
 
 
ALDOA
 
 
hsa-miR-34a-5p
 
 
-0.655
 
 
986.4
 
 
 
 
ALPL
 
 
hsa-miR-10a-5p
 
 
-0.32
 
 
12.2
 
 
 
 
ANK3
 
 
hsa-miR-34a-5p
 
 
-0.29
 
 
22.9
 
 
 
 
ANK3
 
 
hsa-miR-34a-5p
 
 
-0.347
 
 
22.9
 
 
 
 
ANKRD13A
 
 
hsa-miR-22-3p
 
 
-0.478
 
 
16.9
 
 
 
 
ANKRD40
 
 
hsa-miR-27a-3p
 
 
-0.35
 
 
29.5
 
 
 
 
ANP32B
 
 
hsa-miR-34a-5p
 
 
-0.29
 
 
163.8
 
 
 
 
AP3S2
 
 
hsa-miR-34a-5p
 
 
-0.413
 
 
10.1
 
 
 
 
APH1A
 
 
hsa-miR-34a-5p
 
 
-0.304
 
 
45.6
 
 
 
 
AREG
 
 
hsa-miR-34a-5p
 
 
-0.62
 
 
10.0
 
 
 
 
ARF4
 
 
hsa-miR-152-3p
 
 
-0.487
 
 
151.4
 
 
 
 
ARF6
 
 
hsa-miR-181a-5p
 
 
-0.33
 
 
22.6
 
 
 
 
ARFIP1
 
 
hsa-miR-320d
 
 
-0.339
 
 
14.2
 
 
 
 
ARFIP1
 
 
hsa-miR-152-3p
 
 
-0.474
 
 
14.2
 
 
 
 
ARHGAP1
 
 
hsa-miR-34a-5p
 
 
-0.369
 
 
119.0
 
 
 
 
ARHGAP21
 
 
hsa-miR-152-3p
 
 
-0.287
 
 
14.0
 
 
 
 
ARHGEF26
 
 
hsa-miR-22-3p
 
 
-0.374
 
 
10.5
 
 
 
 
ARL6IP1
 
 
hsa-miR-152-3p
 
 
-0.579
 
 
52.2
 
 
 
 
ARL8B
 
 
hsa-miR-152-3p
 
 
-0.337
 
 
49.5
 
 
 
 
ARL8B
 
 
hsa-miR-320d
 
 
-0.376
 
 
49.5
 
 
 
 
ARPC5
 
 
hsa-miR-22-3p
 
 
-0.679
 
 
68.6
 
 
 
 
ARPP19
 
 
hsa-miR-320d
 
 
-0.325
 
 
47.3
 
 
 
 
ARPP19
 
 
hsa-miR-34a-5p
 
 
-0.497
 
 
47.3
 
 
 
 
ARRB1
 
 
hsa-miR-22-3p
 
 
-0.616
 
 
10.5
 
 
 
 
ARRDC3
 
 
hsa-miR-152-3p
 
 
-0.305
 
 
25.4
 
 
 
 
ASAH1
 
 
hsa-miR-27a-3p
 
 
-0.398
 
 
83.2
 
 
 
 
ASB1
 
 
hsa-miR-34a-5p
 
 
-0.406
 
 
11.1
 
 
 
 
ASH2L
 
 
hsa-miR-320d
 
 
-0.287
 
 
19.1
 
 
 
 
ASPH
 
 
hsa-miR-152-3p
 
 
-0.391
 
 
72.5
 
 
 
 
ATG4B
 
 
hsa-miR-34a-5p
 
 
-0.58
 
 
16.6
 
 
 
 
ATMIN
 
 
hsa-miR-34a-5p
 
 
-0.409
 
 
11.8
 
 
 
 
ATP1A2
 
 
hsa-miR-34a-5p
 
 
-0.306
 
 
72.3
 
 
 
 
ATP2B4
 
 
hsa-miR-152-3p
 
 
-0.337
 
 
24.9
 
 
 
 
ATP5MC3
 
 
hsa-miR-27a-3p
 
 
-0.416
 
 
129.6
 
 
 
 
ATP6AP2
 
 
hsa-miR-152-3p
 
 
-0.592
 
 
93.4
 
 
 
 
ATP6AP2
 
 
hsa-miR-152-3p
 
 
-0.668
 
 
93.4
 
 
 
 
ATXN10
 
 
hsa-miR-27a-3p
 
 
-0.295
 
 
46.0
 
 
 
 
ATXN2
 
 
hsa-miR-10a-5p
 
 
-0.362
 
 
25.1
 
 
 
 
AXL
 
 
hsa-miR-34a-5p
 
 
-0.512
 
 
72.4
 
 
 
 
B4GALT2
 
 
hsa-miR-34a-5p
 
 
-0.314
 
 
27.5
 
 
 
 
B4GALT3
 
 
hsa-miR-27a-3p
 
 
-0.41
 
 
18.2
 
 
 
 
B4GALT5
 
 
hsa-miR-152-3p
 
 
-0.44
 
 
15.5
 
 
 
 
BAG5
 
 
hsa-miR-22-3p
 
 
-0.318
 
 
13.3
 
 
 
 
BAZ1B
 
 
hsa-miR-10a-5p
 
 
-0.309
 
 
22.6
 
 
 
 
BAZ2A
 
 
hsa-miR-100-5p
 
 
-0.29
 
 
25.1
 
 
 
 
BAZ2A
 
 
hsa-miR-100-5p
 
 
-0.421
 
 
25.1
 
 
 
 
BBX
 
 
hsa-miR-10a-5p
 
 
-0.305
 
 
15.7
 
 
 
 
BCL3
 
 
hsa-miR-27a-3p
 
 
-0.413
 
 
32.7
 
 
 
 
BEX3
 
 
hsa-miR-27a-3p
 
 
-0.454
 
 
223.9
 
 
 
 
BHLHE40
 
 
hsa-miR-181a-5p
 
 
-0.408
 
 
237.8
 
 
 
 
BHLHE41
 
 
hsa-miR-152-3p
 
 
-0.442
 
 
18.7
 
 
 
 
BIN1
 
 
hsa-miR-22-3p
 
 
-0.346
 
 
22.6
 
 
 
 
BLOC1S1
 
 
hsa-miR-22-3p
 
 
-0.346
 
 
131.0
 
 
 
 
BLOC1S6
 
 
hsa-miR-181a-5p
 
 
-0.393
 
 
21.2
 
 
 
 
BLTP1
 
 
hsa-miR-27a-3p
 
 
-0.426
 
 
24.8
 
 
 
 
BMI1
 
 
hsa-miR-27a-3p
 
 
-0.322
 
 
27.3
 
 
 
 
BMPR2
 
 
hsa-miR-100-5p
 
 
-0.355
 
 
16.9
 
 
 
 
BRSK1
 
 
hsa-miR-27a-3p
 
 
-0.372
 
 
11.0
 
 
 
 
BTAF1
 
 
hsa-miR-152-3p
 
 
-0.401
 
 
19.0
 
 
 
 
BTG2
 
 
hsa-miR-27a-3p
 
 
-0.339
 
 
265.1
 
 
 
 
BZW1
 
 
hsa-miR-27a-3p
 
 
-0.337
 
 
72.0
 
 
 
 
C1orf35
 
 
hsa-miR-152-3p
 
 
-0.511
 
 
14.8
 
 
 
 
C5orf24
 
 
hsa-miR-22-3p
 
 
-0.493
 
 
29.6
 
 
 
 
C6orf62
 
 
hsa-miR-181a-5p
 
 
-0.334
 
 
47.8
 
 
 
 
C8orf88
 
 
hsa-miR-27a-3p
 
 
-0.33
 
 
24.4
 
 
 
 
CACNB3
 
 
hsa-miR-34a-5p
 
 
-0.426
 
 
16.1
 
 
 
 
CACYBP
 
 
hsa-miR-27a-3p
 
 
-0.367
 
 
25.7
 
 
 
 
CAMK2N1
 
 
hsa-miR-22-3p
 
 
-0.302
 
 
14.9
 
 
 
 
CAND1
 
 
hsa-miR-27a-3p
 
 
-0.318
 
 
18.4
 
 
 
 
CAND1
 
 
hsa-miR-152-3p
 
 
-0.44
 
 
18.4
 
 
 
 
CANX
 
 
hsa-miR-152-3p
 
 
-0.434
 
 
142.5
 
 
 
 
CAPN1
 
 
hsa-miR-22-3p
 
 
-0.307
 
 
54.0
 
 
 
 
CAPZA1
 
 
hsa-miR-27a-3p
 
 
-0.325
 
 
44.0
 
 
 
 
CAV2
 
 
hsa-miR-152-3p
 
 
-0.32
 
 
88.6
 
 
 
 
CCDC149
 
 
hsa-miR-22-3p
 
 
-0.327
 
 
10.7
 
 
 
 
CCDC149
 
 
hsa-miR-27a-3p
 
 
-0.551
 
 
10.7
 
 
 
 
CCDC47
 
 
hsa-miR-22-3p
 
 
-0.604
 
 
31.5
 
 
 
 
CCDC6
 
 
hsa-miR-152-3p
 
 
-0.511
 
 
26.2
 
 
 
 
CCM2
 
 
hsa-miR-27a-3p
 
 
-0.335
 
 
26.8
 
 
 
 
 
 
 bone marrow 
 
 
 
 
target_symbol
 
 
mature_mirna_id
 
 
score
 
 
nTPM
 
 
 
 
 
 
AAGAB
 
 
hsa-miR-34a-5p
 
 
-0.319
 
 
18.7
 
 
 
 
ABCB10
 
 
hsa-miR-152-3p
 
 
-0.291
 
 
56.7
 
 
 
 
ABCB7
 
 
hsa-miR-152-3p
 
 
-0.515
 
 
10.5
 
 
 
 
ABCD3
 
 
hsa-miR-152-3p
 
 
-0.388
 
 
23.6
 
 
 
 
ABHD5
 
 
hsa-miR-27a-3p
 
 
-0.407
 
 
37.5
 
 
 
 
ABRAXAS2
 
 
hsa-miR-34a-5p
 
 
-0.538
 
 
11.6
 
 
 
 
ACBD3
 
 
hsa-miR-34a-5p
 
 
-0.415
 
 
24.4
 
 
 
 
ACSL1
 
 
hsa-miR-34a-5p
 
 
-0.306
 
 
237.5
 
 
 
 
ACSL4
 
 
hsa-miR-34a-5p
 
 
-0.372
 
 
11.1
 
 
 
 
ADAM10
 
 
hsa-miR-152-3p
 
 
-0.348
 
 
17.3
 
 
 
 
ADD2
 
 
hsa-miR-34a-5p
 
 
-0.305
 
 
62.7
 
 
 
 
ADK
 
 
hsa-miR-34a-5p
 
 
-0.374
 
 
27.0
 
 
 
 
AFG3L2
 
 
hsa-miR-181a-5p
 
 
-0.491
 
 
23.4
 
 
 
 
AGFG1
 
 
hsa-miR-152-3p
 
 
-0.332
 
 
146.9
 
 
 
 
AGO2
 
 
hsa-miR-27a-3p
 
 
-0.486
 
 
10.7
 
 
 
 
AGO2
 
 
hsa-miR-27a-3p
 
 
-0.646
 
 
10.7
 
 
 
 
AGO2
 
 
hsa-miR-100-5p
 
 
-0.884
 
 
10.7
 
 
 
 
AGO4
 
 
hsa-miR-34a-5p
 
 
-0.306
 
 
19.2
 
 
 
 
AGPAT3
 
 
hsa-miR-27a-3p
 
 
-0.29
 
 
10.4
 
 
 
 
AGTRAP
 
 
hsa-miR-34a-5p
 
 
-0.328
 
 
125.0
 
 
 
 
AIDA
 
 
hsa-miR-22-3p
 
 
-0.316
 
 
20.7
 
 
 
 
AKIRIN1
 
 
hsa-miR-27a-3p
 
 
-0.368
 
 
72.8
 
 
 
 
ALDOA
 
 
hsa-miR-34a-5p
 
 
-0.655
 
 
691.0
 
 
 
 
ANKRD13A
 
 
hsa-miR-22-3p
 
 
-0.478
 
 
34.0
 
 
 
 
ANP32B
 
 
hsa-miR-34a-5p
 
 
-0.29
 
 
344.9
 
 
 
 
APH1A
 
 
hsa-miR-34a-5p
 
 
-0.304
 
 
74.2
 
 
 
 
AREG
 
 
hsa-miR-34a-5p
 
 
-0.62
 
 
122.6
 
 
 
 
ARF4
 
 
hsa-miR-152-3p
 
 
-0.487
 
 
68.5
 
 
 
 
ARF6
 
 
hsa-miR-181a-5p
 
 
-0.33
 
 
78.0
 
 
 
 
ARFGEF1
 
 
hsa-miR-152-3p
 
 
-0.306
 
 
26.0
 
 
 
 
ARHGAP1
 
 
hsa-miR-34a-5p
 
 
-0.369
 
 
31.5
 
 
 
 
ARHGAP26
 
 
hsa-miR-34a-5p
 
 
-0.384
 
 
34.3
 
 
 
 
ARL6IP1
 
 
hsa-miR-152-3p
 
 
-0.579
 
 
197.7
 
 
 
 
ARL8B
 
 
hsa-miR-152-3p
 
 
-0.337
 
 
56.4
 
 
 
 
ARL8B
 
 
hsa-miR-320d
 
 
-0.376
 
 
56.4
 
 
 
 
ARPC5
 
 
hsa-miR-22-3p
 
 
-0.679
 
 
65.3
 
 
 
 
ARPP19
 
 
hsa-miR-320d
 
 
-0.325
 
 
97.5
 
 
 
 
ARPP19
 
 
hsa-miR-34a-5p
 
 
-0.497
 
 
97.5
 
 
 
 
ARRB1
 
 
hsa-miR-22-3p
 
 
-0.616
 
 
14.8
 
 
 
 
ARRDC3
 
 
hsa-miR-152-3p
 
 
-0.305
 
 
158.3
 
 
 
 
ASAH1
 
 
hsa-miR-27a-3p
 
 
-0.398
 
 
101.8
 
 
 
 
ASH2L
 
 
hsa-miR-320d
 
 
-0.287
 
 
30.3
 
 
 
 
ASPH
 
 
hsa-miR-152-3p
 
 
-0.391
 
 
15.8
 
 
 
 
ATG4B
 
 
hsa-miR-34a-5p
 
 
-0.58
 
 
13.9
 
 
 
 
ATP2B4
 
 
hsa-miR-152-3p
 
 
-0.337
 
 
14.9
 
 
 
 
ATP5MC3
 
 
hsa-miR-27a-3p
 
 
-0.416
 
 
133.0
 
 
 
 
ATP6AP2
 
 
hsa-miR-152-3p
 
 
-0.592
 
 
117.4
 
 
 
 
ATP6AP2
 
 
hsa-miR-152-3p
 
 
-0.668
 
 
117.4
 
 
 
 
ATP8A1
 
 
hsa-miR-22-3p
 
 
-0.468
 
 
22.1
 
 
 
 
ATXN10
 
 
hsa-miR-27a-3p
 
 
-0.295
 
 
39.6
 
 
 
 
B3GNT7
 
 
hsa-miR-27a-3p
 
 
-0.355
 
 
13.4
 
 
 
 
B4GALT3
 
 
hsa-miR-27a-3p
 
 
-0.41
 
 
18.0
 
 
 
 
B4GALT5
 
 
hsa-miR-152-3p
 
 
-0.44
 
 
88.5
 
 
 
 
BAG4
 
 
hsa-miR-181a-5p
 
 
-0.337
 
 
27.0
 
 
 
 
BAG5
 
 
hsa-miR-22-3p
 
 
-0.318
 
 
26.2
 
 
 
 
BAZ1B
 
 
hsa-miR-10a-5p
 
 
-0.309
 
 
23.3
 
 
 
 
BAZ2A
 
 
hsa-miR-100-5p
 
 
-0.29
 
 
62.3
 
 
 
 
BAZ2A
 
 
hsa-miR-100-5p
 
 
-0.421
 
 
62.3
 
 
 
 
BBX
 
 
hsa-miR-10a-5p
 
 
-0.305
 
 
17.4
 
 
 
 
BCL2L11
 
 
hsa-miR-152-3p
 
 
-0.445
 
 
37.8
 
 
 
 
BCL2L11
 
 
hsa-miR-10a-5p
 
 
-0.483
 
 
37.8
 
 
 
 
BCL2L13
 
 
hsa-miR-34a-5p
 
 
-0.431
 
 
10.2
 
 
 
 
BCL3
 
 
hsa-miR-27a-3p
 
 
-0.413
 
 
50.9
 
 
 
 
BEX3
 
 
hsa-miR-27a-3p
 
 
-0.454
 
 
29.3
 
 
 
 
BHLHE40
 
 
hsa-miR-181a-5p
 
 
-0.408
 
 
177.7
 
 
 
 
BIN1
 
 
hsa-miR-22-3p
 
 
-0.346
 
 
11.4
 
 
 
 
BLOC1S1
 
 
hsa-miR-22-3p
 
 
-0.346
 
 
216.6
 
 
 
 
BLOC1S6
 
 
hsa-miR-181a-5p
 
 
-0.393
 
 
54.9
 
 
 
 
BLTP1
 
 
hsa-miR-27a-3p
 
 
-0.426
 
 
36.6
 
 
 
 
BMI1
 
 
hsa-miR-27a-3p
 
 
-0.322
 
 
20.7
 
 
 
 
BRPF3
 
 
hsa-miR-34a-5p
 
 
-0.33
 
 
11.4
 
 
 
 
BTAF1
 
 
hsa-miR-152-3p
 
 
-0.401
 
 
25.4
 
 
 
 
BTBD3
 
 
hsa-miR-152-3p
 
 
-0.495
 
 
11.5
 
 
 
 
BTG1
 
 
hsa-miR-22-3p
 
 
-0.468
 
 
88.7
 
 
 
 
BTG2
 
 
hsa-miR-27a-3p
 
 
-0.339
 
 
292.0
 
 
 
 
BZW1
 
 
hsa-miR-27a-3p
 
 
-0.337
 
 
207.1
 
 
 
 
C16orf87
 
 
hsa-miR-181a-5p
 
 
-0.54
 
 
11.1
 
 
 
 
C1GALT1
 
 
hsa-miR-152-3p
 
 
-0.487
 
 
35.6
 
 
 
 
C1orf35
 
 
hsa-miR-152-3p
 
 
-0.511
 
 
13.4
 
 
 
 
C1orf52
 
 
hsa-miR-27a-3p
 
 
-0.591
 
 
21.7
 
 
 
 
C2orf69
 
 
hsa-miR-181a-5p
 
 
-0.38
 
 
20.9
 
 
 
 
C5orf24
 
 
hsa-miR-22-3p
 
 
-0.493
 
 
23.5
 
 
 
 
C6orf62
 
 
hsa-miR-181a-5p
 
 
-0.334
 
 
111.3
 
 
 
 
C8orf88
 
 
hsa-miR-27a-3p
 
 
-0.33
 
 
10.4
 
 
 
 
CACYBP
 
 
hsa-miR-27a-3p
 
 
-0.367
 
 
31.8
 
 
 
 
CAND1
 
 
hsa-miR-27a-3p
 
 
-0.318
 
 
21.4
 
 
 
 
CAND1
 
 
hsa-miR-152-3p
 
 
-0.44
 
 
21.4
 
 
 
 
CANX
 
 
hsa-miR-152-3p
 
 
-0.434
 
 
70.9
 
 
 
 
CAPN1
 
 
hsa-miR-22-3p
 
 
-0.307
 
 
62.8
 
 
 
 
CAPZA1
 
 
hsa-miR-27a-3p
 
 
-0.325
 
 
147.7
 
 
 
 
CASP2
 
 
hsa-miR-34a-5p
 
 
-0.382
 
 
37.9
 
 
 
 
CBFB
 
 
hsa-miR-27a-3p
 
 
-0.302
 
 
11.0
 
 
 
 
CBLB
 
 
hsa-miR-152-3p
 
 
-0.424
 
 
12.6
 
 
 
 
CBX4
 
 
hsa-miR-181a-5p
 
 
-0.354
 
 
123.0
 
 
 
 
CCDC47
 
 
hsa-miR-22-3p
 
 
-0.604
 
 
27.2
 
 
 
 
CCDC6
 
 
hsa-miR-152-3p
 
 
-0.511
 
 
15.9
 
 
 
 
CCM2
 
 
hsa-miR-27a-3p
 
 
-0.335
 
 
14.7
 
 
 
 
CCNC
 
 
hsa-miR-27a-3p
 
 
-0.378
 
 
31.1
 
 
 
 
CCNE2
 
 
hsa-miR-34a-5p
 
 
-0.553
 
 
34.9
 
 
 
 
CCNG1
 
 
hsa-miR-27a-3p
 
 
-0.419
 
 
36.2
 
 
 
 
 
 
 breast 
 
 
 
 
target_symbol
 
 
mature_mirna_id
 
 
score
 
 
nTPM
 
 
 
 
 
 
AAGAB
 
 
hsa-miR-34a-5p
 
 
-0.319
 
 
21.1
 
 
 
 
ABCB7
 
 
hsa-miR-152-3p
 
 
-0.515
 
 
11.6
 
 
 
 
ABCD3
 
 
hsa-miR-152-3p
 
 
-0.388
 
 
30.8
 
 
 
 
ABHD17C
 
 
hsa-miR-27a-3p
 
 
-0.435
 
 
10.9
 
 
 
 
ABHD5
 
 
hsa-miR-27a-3p
 
 
-0.407
 
 
18.8
 
 
 
 
ABRAXAS2
 
 
hsa-miR-34a-5p
 
 
-0.538
 
 
11.4
 
 
 
 
ACBD3
 
 
hsa-miR-34a-5p
 
 
-0.415
 
 
22.8
 
 
 
 
ACSL1
 
 
hsa-miR-34a-5p
 
 
-0.306
 
 
321.1
 
 
 
 
ACSL4
 
 
hsa-miR-34a-5p
 
 
-0.372
 
 
16.3
 
 
 
 
ACTA2
 
 
hsa-miR-27a-3p
 
 
-0.577
 
 
802.4
 
 
 
 
ACVR1
 
 
hsa-miR-152-3p
 
 
-0.397
 
 
11.1
 
 
 
 
ADAM10
 
 
hsa-miR-152-3p
 
 
-0.348
 
 
14.2
 
 
 
 
ADGRA3
 
 
hsa-miR-27a-3p
 
 
-0.296
 
 
10.2
 
 
 
 
ADGRF5
 
 
hsa-miR-152-3p
 
 
-0.354
 
 
44.5
 
 
 
 
ADIPOR2
 
 
hsa-miR-34a-5p
 
 
-0.33
 
 
44.0
 
 
 
 
ADK
 
 
hsa-miR-34a-5p
 
 
-0.374
 
 
25.7
 
 
 
 
AFG3L2
 
 
hsa-miR-181a-5p
 
 
-0.491
 
 
15.5
 
 
 
 
AGFG1
 
 
hsa-miR-152-3p
 
 
-0.332
 
 
48.8
 
 
 
 
AGPAT3
 
 
hsa-miR-27a-3p
 
 
-0.29
 
 
14.5
 
 
 
 
AGTRAP
 
 
hsa-miR-34a-5p
 
 
-0.328
 
 
41.1
 
 
 
 
AHDC1
 
 
hsa-miR-152-3p
 
 
-0.413
 
 
11.9
 
 
 
 
AIDA
 
 
hsa-miR-22-3p
 
 
-0.316
 
 
39.1
 
 
 
 
AKAP1
 
 
hsa-miR-152-3p
 
 
-0.381
 
 
34.0
 
 
 
 
AKIRIN1
 
 
hsa-miR-27a-3p
 
 
-0.368
 
 
21.6
 
 
 
 
AKT3
 
 
hsa-miR-22-3p
 
 
-0.451
 
 
13.8
 
 
 
 
ALCAM
 
 
hsa-miR-34a-5p
 
 
-0.303
 
 
76.0
 
 
 
 
ALCAM
 
 
hsa-miR-152-3p
 
 
-0.347
 
 
76.0
 
 
 
 
ALDOA
 
 
hsa-miR-34a-5p
 
 
-0.655
 
 
590.2
 
 
 
 
ALPL
 
 
hsa-miR-10a-5p
 
 
-0.32
 
 
21.4
 
 
 
 
ANK2
 
 
hsa-miR-34a-5p
 
 
-0.317
 
 
14.7
 
 
 
 
ANK3
 
 
hsa-miR-34a-5p
 
 
-0.29
 
 
19.6
 
 
 
 
ANK3
 
 
hsa-miR-34a-5p
 
 
-0.347
 
 
19.6
 
 
 
 
ANKRD13A
 
 
hsa-miR-22-3p
 
 
-0.478
 
 
15.4
 
 
 
 
ANKRD40
 
 
hsa-miR-27a-3p
 
 
-0.35
 
 
24.7
 
 
 
 
ANP32B
 
 
hsa-miR-34a-5p
 
 
-0.29
 
 
132.4
 
 
 
 
AP1AR
 
 
hsa-miR-100-5p
 
 
-0.804
 
 
15.9
 
 
 
 
AP3S2
 
 
hsa-miR-34a-5p
 
 
-0.413
 
 
18.1
 
 
 
 
APH1A
 
 
hsa-miR-34a-5p
 
 
-0.304
 
 
91.6
 
 
 
 
APOO
 
 
hsa-miR-181a-5p
 
 
-0.5
 
 
12.5
 
 
 
 
APPBP2
 
 
hsa-miR-27a-3p
 
 
-0.378
 
 
10.6
 
 
 
 
AREG
 
 
hsa-miR-34a-5p
 
 
-0.62
 
 
57.8
 
 
 
 
ARF4
 
 
hsa-miR-152-3p
 
 
-0.487
 
 
129.6
 
 
 
 
ARF6
 
 
hsa-miR-181a-5p
 
 
-0.33
 
 
31.6
 
 
 
 
ARFGEF1
 
 
hsa-miR-152-3p
 
 
-0.306
 
 
12.7
 
 
 
 
ARFIP1
 
 
hsa-miR-320d
 
 
-0.339
 
 
22.0
 
 
 
 
ARFIP1
 
 
hsa-miR-152-3p
 
 
-0.474
 
 
22.0
 
 
 
 
ARHGAP1
 
 
hsa-miR-34a-5p
 
 
-0.369
 
 
59.0
 
 
 
 
ARHGAP21
 
 
hsa-miR-152-3p
 
 
-0.287
 
 
15.1
 
 
 
 
ARHGAP26
 
 
hsa-miR-34a-5p
 
 
-0.384
 
 
11.5
 
 
 
 
ARL6IP1
 
 
hsa-miR-152-3p
 
 
-0.579
 
 
105.7
 
 
 
 
ARL8B
 
 
hsa-miR-152-3p
 
 
-0.337
 
 
39.7
 
 
 
 
ARL8B
 
 
hsa-miR-320d
 
 
-0.376
 
 
39.7
 
 
 
 
ARPC5
 
 
hsa-miR-22-3p
 
 
-0.679
 
 
45.7
 
 
 
 
ARPIN-AP3S2
 
 
hsa-miR-34a-5p
 
 
-0.348
 
 
10.4
 
 
 
 
ARPP19
 
 
hsa-miR-320d
 
 
-0.325
 
 
43.2
 
 
 
 
ARPP19
 
 
hsa-miR-34a-5p
 
 
-0.497
 
 
43.2
 
 
 
 
ARRB1
 
 
hsa-miR-22-3p
 
 
-0.616
 
 
22.5
 
 
 
 
ARRDC3
 
 
hsa-miR-152-3p
 
 
-0.305
 
 
88.5
 
 
 
 
ASAH1
 
 
hsa-miR-27a-3p
 
 
-0.398
 
 
89.9
 
 
 
 
ASH2L
 
 
hsa-miR-320d
 
 
-0.287
 
 
20.3
 
 
 
 
ASPH
 
 
hsa-miR-152-3p
 
 
-0.391
 
 
99.5
 
 
 
 
ATG4B
 
 
hsa-miR-34a-5p
 
 
-0.58
 
 
14.4
 
 
 
 
ATMIN
 
 
hsa-miR-34a-5p
 
 
-0.409
 
 
13.5
 
 
 
 
ATP1A2
 
 
hsa-miR-34a-5p
 
 
-0.306
 
 
27.4
 
 
 
 
ATP2B4
 
 
hsa-miR-152-3p
 
 
-0.337
 
 
15.5
 
 
 
 
ATP5MC3
 
 
hsa-miR-27a-3p
 
 
-0.416
 
 
159.0
 
 
 
 
ATP6AP2
 
 
hsa-miR-152-3p
 
 
-0.592
 
 
89.1
 
 
 
 
ATP6AP2
 
 
hsa-miR-152-3p
 
 
-0.668
 
 
89.1
 
 
 
 
ATXN10
 
 
hsa-miR-27a-3p
 
 
-0.295
 
 
39.0
 
 
 
 
ATXN2
 
 
hsa-miR-10a-5p
 
 
-0.362
 
 
16.9
 
 
 
 
AXL
 
 
hsa-miR-34a-5p
 
 
-0.512
 
 
25.8
 
 
 
 
B4GALT2
 
 
hsa-miR-34a-5p
 
 
-0.314
 
 
19.6
 
 
 
 
B4GALT3
 
 
hsa-miR-27a-3p
 
 
-0.41
 
 
20.8
 
 
 
 
B4GALT5
 
 
hsa-miR-152-3p
 
 
-0.44
 
 
17.9
 
 
 
 
BAG5
 
 
hsa-miR-22-3p
 
 
-0.318
 
 
17.5
 
 
 
 
BAZ1B
 
 
hsa-miR-10a-5p
 
 
-0.309
 
 
15.9
 
 
 
 
BAZ2A
 
 
hsa-miR-100-5p
 
 
-0.29
 
 
25.2
 
 
 
 
BAZ2A
 
 
hsa-miR-100-5p
 
 
-0.421
 
 
25.2
 
 
 
 
BBX
 
 
hsa-miR-10a-5p
 
 
-0.305
 
 
13.5
 
 
 
 
BCL2L11
 
 
hsa-miR-152-3p
 
 
-0.445
 
 
11.8
 
 
 
 
BCL2L11
 
 
hsa-miR-10a-5p
 
 
-0.483
 
 
11.8
 
 
 
 
BCL3
 
 
hsa-miR-27a-3p
 
 
-0.413
 
 
40.0
 
 
 
 
BEX3
 
 
hsa-miR-27a-3p
 
 
-0.454
 
 
115.0
 
 
 
 
BHLHE40
 
 
hsa-miR-181a-5p
 
 
-0.408
 
 
211.1
 
 
 
 
BHLHE41
 
 
hsa-miR-152-3p
 
 
-0.442
 
 
12.9
 
 
 
 
BIN1
 
 
hsa-miR-22-3p
 
 
-0.346
 
 
35.8
 
 
 
 
BLOC1S1
 
 
hsa-miR-22-3p
 
 
-0.346
 
 
110.5
 
 
 
 
BLOC1S6
 
 
hsa-miR-181a-5p
 
 
-0.393
 
 
23.9
 
 
 
 
BLTP1
 
 
hsa-miR-27a-3p
 
 
-0.426
 
 
20.1
 
 
 
 
BMI1
 
 
hsa-miR-27a-3p
 
 
-0.322
 
 
33.7
 
 
 
 
BMPR2
 
 
hsa-miR-100-5p
 
 
-0.355
 
 
10.7
 
 
 
 
BTAF1
 
 
hsa-miR-152-3p
 
 
-0.401
 
 
15.3
 
 
 
 
BTBD3
 
 
hsa-miR-152-3p
 
 
-0.495
 
 
11.8
 
 
 
 
BTG1
 
 
hsa-miR-22-3p
 
 
-0.468
 
 
14.4
 
 
 
 
BTG2
 
 
hsa-miR-27a-3p
 
 
-0.339
 
 
114.8
 
 
 
 
BZW1
 
 
hsa-miR-27a-3p
 
 
-0.337
 
 
83.1
 
 
 
 
C1GALT1
 
 
hsa-miR-152-3p
 
 
-0.487
 
 
15.5
 
 
 
 
C1orf35
 
 
hsa-miR-152-3p
 
 
-0.511
 
 
13.0
 
 
 
 
C5orf24
 
 
hsa-miR-22-3p
 
 
-0.493
 
 
18.9
 
 
 
 
C6orf120
 
 
hsa-miR-34a-5p
 
 
-0.331
 
 
13.6
 
 
 
 
 
 
 cerebral cortex 
 
 
 
 
target_symbol
 
 
mature_mirna_id
 
 
score
 
 
nTPM
 
 
 
 
 
 
AAGAB
 
 
hsa-miR-34a-5p
 
 
-0.319
 
 
20.5
 
 
 
 
ABCD3
 
 
hsa-miR-152-3p
 
 
-0.388
 
 
17.1
 
 
 
 
ABHD17C
 
 
hsa-miR-27a-3p
 
 
-0.435
 
 
16.3
 
 
 
 
ACBD3
 
 
hsa-miR-34a-5p
 
 
-0.415
 
 
15.8
 
 
 
 
ACSL1
 
 
hsa-miR-34a-5p
 
 
-0.306
 
 
19.9
 
 
 
 
ACSL4
 
 
hsa-miR-34a-5p
 
 
-0.372
 
 
27.8
 
 
 
 
ACTA2
 
 
hsa-miR-27a-3p
 
 
-0.577
 
 
56.2
 
 
 
 
ADAM10
 
 
hsa-miR-152-3p
 
 
-0.348
 
 
16.0
 
 
 
 
ADAMTS10
 
 
hsa-miR-27a-3p
 
 
-0.354
 
 
10.8
 
 
 
 
ADAP1
 
 
hsa-miR-27a-3p
 
 
-0.302
 
 
126.8
 
 
 
 
ADCY2
 
 
hsa-miR-152-3p
 
 
-0.299
 
 
35.4
 
 
 
 
ADD2
 
 
hsa-miR-34a-5p
 
 
-0.305
 
 
25.2
 
 
 
 
ADGRA3
 
 
hsa-miR-27a-3p
 
 
-0.296
 
 
17.8
 
 
 
 
ADGRB3
 
 
hsa-miR-152-3p
 
 
-0.516
 
 
28.2
 
 
 
 
ADIPOR2
 
 
hsa-miR-34a-5p
 
 
-0.33
 
 
29.8
 
 
 
 
ADK
 
 
hsa-miR-34a-5p
 
 
-0.374
 
 
16.5
 
 
 
 
ADO
 
 
hsa-miR-34a-5p
 
 
-0.377
 
 
15.9
 
 
 
 
ADORA2B
 
 
hsa-miR-27a-3p
 
 
-0.638
 
 
12.5
 
 
 
 
AFG3L2
 
 
hsa-miR-181a-5p
 
 
-0.491
 
 
17.1
 
 
 
 
AGFG1
 
 
hsa-miR-152-3p
 
 
-0.332
 
 
33.6
 
 
 
 
AGPAT3
 
 
hsa-miR-27a-3p
 
 
-0.29
 
 
38.5
 
 
 
 
AGTRAP
 
 
hsa-miR-34a-5p
 
 
-0.328
 
 
16.4
 
 
 
 
AHCYL2
 
 
hsa-miR-34a-5p
 
 
-0.359
 
 
30.6
 
 
 
 
AHDC1
 
 
hsa-miR-152-3p
 
 
-0.413
 
 
15.5
 
 
 
 
AIDA
 
 
hsa-miR-22-3p
 
 
-0.316
 
 
30.3
 
 
 
 
AKAP1
 
 
hsa-miR-152-3p
 
 
-0.381
 
 
24.9
 
 
 
 
AKIRIN1
 
 
hsa-miR-27a-3p
 
 
-0.368
 
 
38.1
 
 
 
 
AKT3
 
 
hsa-miR-22-3p
 
 
-0.451
 
 
23.7
 
 
 
 
ALCAM
 
 
hsa-miR-34a-5p
 
 
-0.303
 
 
54.7
 
 
 
 
ALCAM
 
 
hsa-miR-152-3p
 
 
-0.347
 
 
54.7
 
 
 
 
ALDOA
 
 
hsa-miR-34a-5p
 
 
-0.655
 
 
1334.6
 
 
 
 
ALPL
 
 
hsa-miR-10a-5p
 
 
-0.32
 
 
13.5
 
 
 
 
AMER2
 
 
hsa-miR-34a-5p
 
 
-0.48
 
 
35.0
 
 
 
 
ANK2
 
 
hsa-miR-34a-5p
 
 
-0.317
 
 
79.8
 
 
 
 
ANK3
 
 
hsa-miR-34a-5p
 
 
-0.29
 
 
51.5
 
 
 
 
ANK3
 
 
hsa-miR-34a-5p
 
 
-0.347
 
 
51.5
 
 
 
 
ANKRD40
 
 
hsa-miR-27a-3p
 
 
-0.35
 
 
54.5
 
 
 
 
ANP32B
 
 
hsa-miR-34a-5p
 
 
-0.29
 
 
70.2
 
 
 
 
AP1AR
 
 
hsa-miR-100-5p
 
 
-0.804
 
 
12.2
 
 
 
 
AP3S2
 
 
hsa-miR-34a-5p
 
 
-0.413
 
 
25.3
 
 
 
 
APH1A
 
 
hsa-miR-34a-5p
 
 
-0.304
 
 
45.1
 
 
 
 
APOO
 
 
hsa-miR-181a-5p
 
 
-0.5
 
 
56.2
 
 
 
 
APPBP2
 
 
hsa-miR-27a-3p
 
 
-0.378
 
 
17.6
 
 
 
 
ARF4
 
 
hsa-miR-152-3p
 
 
-0.487
 
 
61.5
 
 
 
 
ARF6
 
 
hsa-miR-181a-5p
 
 
-0.33
 
 
13.0
 
 
 
 
ARHGAP1
 
 
hsa-miR-34a-5p
 
 
-0.369
 
 
40.8
 
 
 
 
ARHGAP21
 
 
hsa-miR-152-3p
 
 
-0.287
 
 
47.3
 
 
 
 
ARHGAP26
 
 
hsa-miR-34a-5p
 
 
-0.384
 
 
16.8
 
 
 
 
ARL6IP1
 
 
hsa-miR-152-3p
 
 
-0.579
 
 
314.1
 
 
 
 
ARL8B
 
 
hsa-miR-152-3p
 
 
-0.337
 
 
75.1
 
 
 
 
ARL8B
 
 
hsa-miR-320d
 
 
-0.376
 
 
75.1
 
 
 
 
ARPC5
 
 
hsa-miR-22-3p
 
 
-0.679
 
 
53.1
 
 
 
 
ARPIN-AP3S2
 
 
hsa-miR-34a-5p
 
 
-0.348
 
 
13.1
 
 
 
 
ARPP19
 
 
hsa-miR-320d
 
 
-0.325
 
 
292.4
 
 
 
 
ARPP19
 
 
hsa-miR-34a-5p
 
 
-0.497
 
 
292.4
 
 
 
 
ARRB1
 
 
hsa-miR-22-3p
 
 
-0.616
 
 
40.1
 
 
 
 
ARRDC3
 
 
hsa-miR-152-3p
 
 
-0.305
 
 
15.6
 
 
 
 
ASAH1
 
 
hsa-miR-27a-3p
 
 
-0.398
 
 
55.8
 
 
 
 
ASB1
 
 
hsa-miR-34a-5p
 
 
-0.406
 
 
12.8
 
 
 
 
ASH2L
 
 
hsa-miR-320d
 
 
-0.287
 
 
21.5
 
 
 
 
ASIC2
 
 
hsa-miR-34a-5p
 
 
-0.496
 
 
12.7
 
 
 
 
ASPH
 
 
hsa-miR-152-3p
 
 
-0.391
 
 
76.3
 
 
 
 
ATCAY
 
 
hsa-miR-10a-5p
 
 
-0.322
 
 
78.9
 
 
 
 
ATG4B
 
 
hsa-miR-34a-5p
 
 
-0.58
 
 
29.9
 
 
 
 
ATMIN
 
 
hsa-miR-34a-5p
 
 
-0.409
 
 
21.8
 
 
 
 
ATP1A2
 
 
hsa-miR-34a-5p
 
 
-0.306
 
 
440.9
 
 
 
 
ATP2B4
 
 
hsa-miR-152-3p
 
 
-0.337
 
 
35.3
 
 
 
 
ATP5MC3
 
 
hsa-miR-27a-3p
 
 
-0.416
 
 
240.0
 
 
 
 
ATP6AP2
 
 
hsa-miR-152-3p
 
 
-0.592
 
 
109.4
 
 
 
 
ATP6AP2
 
 
hsa-miR-152-3p
 
 
-0.668
 
 
109.4
 
 
 
 
ATP8A1
 
 
hsa-miR-22-3p
 
 
-0.468
 
 
32.7
 
 
 
 
ATXN10
 
 
hsa-miR-27a-3p
 
 
-0.295
 
 
74.2
 
 
 
 
ATXN2
 
 
hsa-miR-10a-5p
 
 
-0.362
 
 
26.3
 
 
 
 
AXL
 
 
hsa-miR-34a-5p
 
 
-0.512
 
 
14.7
 
 
 
 
B4GALT2
 
 
hsa-miR-34a-5p
 
 
-0.314
 
 
63.2
 
 
 
 
B4GALT3
 
 
hsa-miR-27a-3p
 
 
-0.41
 
 
17.7
 
 
 
 
B4GALT5
 
 
hsa-miR-152-3p
 
 
-0.44
 
 
22.9
 
 
 
 
B4GALT6
 
 
hsa-miR-152-3p
 
 
-0.528
 
 
15.0
 
 
 
 
BAG4
 
 
hsa-miR-181a-5p
 
 
-0.337
 
 
16.5
 
 
 
 
BAG5
 
 
hsa-miR-22-3p
 
 
-0.318
 
 
15.1
 
 
 
 
BAZ1B
 
 
hsa-miR-10a-5p
 
 
-0.309
 
 
19.0
 
 
 
 
BAZ2A
 
 
hsa-miR-100-5p
 
 
-0.29
 
 
13.9
 
 
 
 
BAZ2A
 
 
hsa-miR-100-5p
 
 
-0.421
 
 
13.9
 
 
 
 
BBX
 
 
hsa-miR-10a-5p
 
 
-0.305
 
 
16.6
 
 
 
 
BCL2L13
 
 
hsa-miR-34a-5p
 
 
-0.431
 
 
10.0
 
 
 
 
BEGAIN
 
 
hsa-miR-28-5p
 
 
-0.325
 
 
42.8
 
 
 
 
BEX3
 
 
hsa-miR-27a-3p
 
 
-0.454
 
 
629.6
 
 
 
 
BHLHE40
 
 
hsa-miR-181a-5p
 
 
-0.408
 
 
59.9
 
 
 
 
BHLHE41
 
 
hsa-miR-152-3p
 
 
-0.442
 
 
27.2
 
 
 
 
BIN1
 
 
hsa-miR-22-3p
 
 
-0.346
 
 
121.8
 
 
 
 
BLOC1S1
 
 
hsa-miR-22-3p
 
 
-0.346
 
 
215.7
 
 
 
 
BLOC1S6
 
 
hsa-miR-181a-5p
 
 
-0.393
 
 
30.5
 
 
 
 
BLTP1
 
 
hsa-miR-27a-3p
 
 
-0.426
 
 
22.6
 
 
 
 
BMI1
 
 
hsa-miR-27a-3p
 
 
-0.322
 
 
26.7
 
 
 
 
BMPR2
 
 
hsa-miR-100-5p
 
 
-0.355
 
 
20.6
 
 
 
 
BRINP1
 
 
hsa-miR-34a-5p
 
 
-0.612
 
 
37.8
 
 
 
 
BRPF3
 
 
hsa-miR-34a-5p
 
 
-0.33
 
 
10.7
 
 
 
 
BRSK1
 
 
hsa-miR-27a-3p
 
 
-0.372
 
 
191.3
 
 
 
 
BTBD3
 
 
hsa-miR-152-3p
 
 
-0.495
 
 
32.2
 
 
 
 
BTG2
 
 
hsa-miR-27a-3p
 
 
-0.339
 
 
41.9
 
 
 
 
 
 
 cervix 
 
 
 
 
target_symbol
 
 
mature_mirna_id
 
 
score
 
 
nTPM
 
 
 
 
 
 
AAGAB
 
 
hsa-miR-34a-5p
 
 
-0.319
 
 
16.7
 
 
 
 
ABCB7
 
 
hsa-miR-152-3p
 
 
-0.515
 
 
10.8
 
 
 
 
ABCD3
 
 
hsa-miR-152-3p
 
 
-0.388
 
 
21.0
 
 
 
 
ABHD17C
 
 
hsa-miR-27a-3p
 
 
-0.435
 
 
18.1
 
 
 
 
ACBD3
 
 
hsa-miR-34a-5p
 
 
-0.415
 
 
25.9
 
 
 
 
ACSL1
 
 
hsa-miR-34a-5p
 
 
-0.306
 
 
55.5
 
 
 
 
ACSL4
 
 
hsa-miR-34a-5p
 
 
-0.372
 
 
43.6
 
 
 
 
ACTA2
 
 
hsa-miR-27a-3p
 
 
-0.577
 
 
2602.4
 
 
 
 
ACVR1
 
 
hsa-miR-152-3p
 
 
-0.397
 
 
20.6
 
 
 
 
ADAM10
 
 
hsa-miR-152-3p
 
 
-0.348
 
 
15.1
 
 
 
 
ADAMTS10
 
 
hsa-miR-27a-3p
 
 
-0.354
 
 
11.2
 
 
 
 
ADGRA3
 
 
hsa-miR-27a-3p
 
 
-0.296
 
 
10.2
 
 
 
 
ADIPOR2
 
 
hsa-miR-34a-5p
 
 
-0.33
 
 
25.7
 
 
 
 
ADK
 
 
hsa-miR-34a-5p
 
 
-0.374
 
 
20.2
 
 
 
 
AFG3L2
 
 
hsa-miR-181a-5p
 
 
-0.491
 
 
14.0
 
 
 
 
AGFG1
 
 
hsa-miR-152-3p
 
 
-0.332
 
 
27.5
 
 
 
 
AGPAT3
 
 
hsa-miR-27a-3p
 
 
-0.29
 
 
12.1
 
 
 
 
AGTRAP
 
 
hsa-miR-34a-5p
 
 
-0.328
 
 
35.6
 
 
 
 
AHDC1
 
 
hsa-miR-152-3p
 
 
-0.413
 
 
23.6
 
 
 
 
AIDA
 
 
hsa-miR-22-3p
 
 
-0.316
 
 
44.9
 
 
 
 
AKAP1
 
 
hsa-miR-152-3p
 
 
-0.381
 
 
22.6
 
 
 
 
AKIRIN1
 
 
hsa-miR-27a-3p
 
 
-0.368
 
 
20.5
 
 
 
 
AKT3
 
 
hsa-miR-22-3p
 
 
-0.451
 
 
15.6
 
 
 
 
ALCAM
 
 
hsa-miR-34a-5p
 
 
-0.303
 
 
26.3
 
 
 
 
ALCAM
 
 
hsa-miR-152-3p
 
 
-0.347
 
 
26.3
 
 
 
 
ALDOA
 
 
hsa-miR-34a-5p
 
 
-0.655
 
 
694.9
 
 
 
 
ANK2
 
 
hsa-miR-34a-5p
 
 
-0.317
 
 
25.2
 
 
 
 
ANK3
 
 
hsa-miR-34a-5p
 
 
-0.29
 
 
11.0
 
 
 
 
ANK3
 
 
hsa-miR-34a-5p
 
 
-0.347
 
 
11.0
 
 
 
 
ANKRD13A
 
 
hsa-miR-22-3p
 
 
-0.478
 
 
13.2
 
 
 
 
ANKRD40
 
 
hsa-miR-27a-3p
 
 
-0.35
 
 
18.9
 
 
 
 
ANP32B
 
 
hsa-miR-34a-5p
 
 
-0.29
 
 
143.2
 
 
 
 
AP3S2
 
 
hsa-miR-34a-5p
 
 
-0.413
 
 
16.2
 
 
 
 
APH1A
 
 
hsa-miR-34a-5p
 
 
-0.304
 
 
80.9
 
 
 
 
APOO
 
 
hsa-miR-181a-5p
 
 
-0.5
 
 
12.3
 
 
 
 
APPBP2
 
 
hsa-miR-27a-3p
 
 
-0.378
 
 
11.0
 
 
 
 
AREG
 
 
hsa-miR-34a-5p
 
 
-0.62
 
 
18.0
 
 
 
 
ARF4
 
 
hsa-miR-152-3p
 
 
-0.487
 
 
196.1
 
 
 
 
ARF6
 
 
hsa-miR-181a-5p
 
 
-0.33
 
 
31.8
 
 
 
 
ARFGEF1
 
 
hsa-miR-152-3p
 
 
-0.306
 
 
13.3
 
 
 
 
ARFIP1
 
 
hsa-miR-320d
 
 
-0.339
 
 
21.1
 
 
 
 
ARFIP1
 
 
hsa-miR-152-3p
 
 
-0.474
 
 
21.1
 
 
 
 
ARHGAP1
 
 
hsa-miR-34a-5p
 
 
-0.369
 
 
62.9
 
 
 
 
ARHGAP21
 
 
hsa-miR-152-3p
 
 
-0.287
 
 
18.5
 
 
 
 
ARHGAP26
 
 
hsa-miR-34a-5p
 
 
-0.384
 
 
11.2
 
 
 
 
ARL6IP1
 
 
hsa-miR-152-3p
 
 
-0.579
 
 
74.5
 
 
 
 
ARL8B
 
 
hsa-miR-152-3p
 
 
-0.337
 
 
42.9
 
 
 
 
ARL8B
 
 
hsa-miR-320d
 
 
-0.376
 
 
42.9
 
 
 
 
ARPC5
 
 
hsa-miR-22-3p
 
 
-0.679
 
 
45.8
 
 
 
 
ARPP19
 
 
hsa-miR-320d
 
 
-0.325
 
 
35.8
 
 
 
 
ARPP19
 
 
hsa-miR-34a-5p
 
 
-0.497
 
 
35.8
 
 
 
 
ARRB1
 
 
hsa-miR-22-3p
 
 
-0.616
 
 
10.1
 
 
 
 
ARRDC3
 
 
hsa-miR-152-3p
 
 
-0.305
 
 
42.0
 
 
 
 
ASAH1
 
 
hsa-miR-27a-3p
 
 
-0.398
 
 
92.6
 
 
 
 
ASH2L
 
 
hsa-miR-320d
 
 
-0.287
 
 
18.7
 
 
 
 
ASPH
 
 
hsa-miR-152-3p
 
 
-0.391
 
 
60.9
 
 
 
 
ATG4B
 
 
hsa-miR-34a-5p
 
 
-0.58
 
 
20.4
 
 
 
 
ATMIN
 
 
hsa-miR-34a-5p
 
 
-0.409
 
 
12.7
 
 
 
 
ATP1A2
 
 
hsa-miR-34a-5p
 
 
-0.306
 
 
13.5
 
 
 
 
ATP2B4
 
 
hsa-miR-152-3p
 
 
-0.337
 
 
44.8
 
 
 
 
ATP5MC3
 
 
hsa-miR-27a-3p
 
 
-0.416
 
 
141.6
 
 
 
 
ATP6AP2
 
 
hsa-miR-152-3p
 
 
-0.592
 
 
99.7
 
 
 
 
ATP6AP2
 
 
hsa-miR-152-3p
 
 
-0.668
 
 
99.7
 
 
 
 
ATXN10
 
 
hsa-miR-27a-3p
 
 
-0.295
 
 
47.8
 
 
 
 
ATXN2
 
 
hsa-miR-10a-5p
 
 
-0.362
 
 
19.5
 
 
 
 
AXL
 
 
hsa-miR-34a-5p
 
 
-0.512
 
 
30.0
 
 
 
 
B4GALT2
 
 
hsa-miR-34a-5p
 
 
-0.314
 
 
22.2
 
 
 
 
B4GALT3
 
 
hsa-miR-27a-3p
 
 
-0.41
 
 
19.4
 
 
 
 
B4GALT5
 
 
hsa-miR-152-3p
 
 
-0.44
 
 
19.9
 
 
 
 
BAG5
 
 
hsa-miR-22-3p
 
 
-0.318
 
 
13.9
 
 
 
 
BAZ1B
 
 
hsa-miR-10a-5p
 
 
-0.309
 
 
21.1
 
 
 
 
BAZ2A
 
 
hsa-miR-100-5p
 
 
-0.29
 
 
25.9
 
 
 
 
BAZ2A
 
 
hsa-miR-100-5p
 
 
-0.421
 
 
25.9
 
 
 
 
BBX
 
 
hsa-miR-10a-5p
 
 
-0.305
 
 
16.2
 
 
 
 
BCL3
 
 
hsa-miR-27a-3p
 
 
-0.413
 
 
29.2
 
 
 
 
BEX3
 
 
hsa-miR-27a-3p
 
 
-0.454
 
 
168.9
 
 
 
 
BHLHE40
 
 
hsa-miR-181a-5p
 
 
-0.408
 
 
244.8
 
 
 
 
BHLHE41
 
 
hsa-miR-152-3p
 
 
-0.442
 
 
36.5
 
 
 
 
BIN1
 
 
hsa-miR-22-3p
 
 
-0.346
 
 
42.5
 
 
 
 
BLOC1S1
 
 
hsa-miR-22-3p
 
 
-0.346
 
 
100.3
 
 
 
 
BLOC1S6
 
 
hsa-miR-181a-5p
 
 
-0.393
 
 
24.2
 
 
 
 
BLTP1
 
 
hsa-miR-27a-3p
 
 
-0.426
 
 
21.8
 
 
 
 
BMI1
 
 
hsa-miR-27a-3p
 
 
-0.322
 
 
25.9
 
 
 
 
BMPR2
 
 
hsa-miR-100-5p
 
 
-0.355
 
 
15.5
 
 
 
 
BTAF1
 
 
hsa-miR-152-3p
 
 
-0.401
 
 
16.0
 
 
 
 
BTBD3
 
 
hsa-miR-152-3p
 
 
-0.495
 
 
14.2
 
 
 
 
BTG2
 
 
hsa-miR-27a-3p
 
 
-0.339
 
 
95.4
 
 
 
 
BZW1
 
 
hsa-miR-27a-3p
 
 
-0.337
 
 
61.6
 
 
 
 
C1GALT1
 
 
hsa-miR-152-3p
 
 
-0.487
 
 
17.4
 
 
 
 
C1orf35
 
 
hsa-miR-152-3p
 
 
-0.511
 
 
17.8
 
 
 
 
C5orf24
 
 
hsa-miR-22-3p
 
 
-0.493
 
 
19.0
 
 
 
 
C6orf120
 
 
hsa-miR-34a-5p
 
 
-0.331
 
 
10.4
 
 
 
 
C6orf62
 
 
hsa-miR-181a-5p
 
 
-0.334
 
 
57.0
 
 
 
 
CA10
 
 
hsa-miR-27a-3p
 
 
-0.464
 
 
20.5
 
 
 
 
CACNB3
 
 
hsa-miR-34a-5p
 
 
-0.426
 
 
33.5
 
 
 
 
CACYBP
 
 
hsa-miR-27a-3p
 
 
-0.367
 
 
23.0
 
 
 
 
CADM4
 
 
hsa-miR-34a-5p
 
 
-0.365
 
 
14.1
 
 
 
 
CAMK2N1
 
 
hsa-miR-22-3p
 
 
-0.302
 
 
26.9
 
 
 
 
CAND1
 
 
hsa-miR-27a-3p
 
 
-0.318
 
 
20.1
 
 
 
 
CAND1
 
 
hsa-miR-152-3p
 
 
-0.44
 
 
20.1
 
 
 
 
 
 
 choroid plexus 
 
 
 
 
target_symbol
 
 
mature_mirna_id
 
 
score
 
 
nTPM
 
 
 
 
 
 
AAGAB
 
 
hsa-miR-34a-5p
 
 
-0.319
 
 
27.4
 
 
 
 
ABCB7
 
 
hsa-miR-152-3p
 
 
-0.515
 
 
15.2
 
 
 
 
ABCD3
 
 
hsa-miR-152-3p
 
 
-0.388
 
 
13.3
 
 
 
 
ACBD3
 
 
hsa-miR-34a-5p
 
 
-0.415
 
 
12.2
 
 
 
 
ACSL1
 
 
hsa-miR-34a-5p
 
 
-0.306
 
 
57.8
 
 
 
 
ACTA2
 
 
hsa-miR-27a-3p
 
 
-0.577
 
 
76.7
 
 
 
 
ADGRA3
 
 
hsa-miR-27a-3p
 
 
-0.296
 
 
23.6
 
 
 
 
ADIPOR2
 
 
hsa-miR-34a-5p
 
 
-0.33
 
 
17.0
 
 
 
 
ADK
 
 
hsa-miR-34a-5p
 
 
-0.374
 
 
13.6
 
 
 
 
ADORA2B
 
 
hsa-miR-27a-3p
 
 
-0.638
 
 
11.1
 
 
 
 
AGFG1
 
 
hsa-miR-152-3p
 
 
-0.332
 
 
23.3
 
 
 
 
AGPAT3
 
 
hsa-miR-27a-3p
 
 
-0.29
 
 
12.0
 
 
 
 
AGTRAP
 
 
hsa-miR-34a-5p
 
 
-0.328
 
 
114.6
 
 
 
 
AHCYL2
 
 
hsa-miR-34a-5p
 
 
-0.359
 
 
258.9
 
 
 
 
AIDA
 
 
hsa-miR-22-3p
 
 
-0.316
 
 
24.6
 
 
 
 
AKIRIN1
 
 
hsa-miR-27a-3p
 
 
-0.368
 
 
30.6
 
 
 
 
ALDOA
 
 
hsa-miR-34a-5p
 
 
-0.655
 
 
1143.6
 
 
 
 
ALPL
 
 
hsa-miR-10a-5p
 
 
-0.32
 
 
17.0
 
 
 
 
ANKHD1-EIF4EBP3
 
 
hsa-miR-22-3p
 
 
-0.479
 
 
11.6
 
 
 
 
ANKRD40
 
 
hsa-miR-27a-3p
 
 
-0.35
 
 
17.4
 
 
 
 
ANP32B
 
 
hsa-miR-34a-5p
 
 
-0.29
 
 
148.4
 
 
 
 
AP3S2
 
 
hsa-miR-34a-5p
 
 
-0.413
 
 
15.9
 
 
 
 
APH1A
 
 
hsa-miR-34a-5p
 
 
-0.304
 
 
54.0
 
 
 
 
APOO
 
 
hsa-miR-181a-5p
 
 
-0.5
 
 
102.5
 
 
 
 
ARF4
 
 
hsa-miR-152-3p
 
 
-0.487
 
 
115.4
 
 
 
 
ARF6
 
 
hsa-miR-181a-5p
 
 
-0.33
 
 
20.9
 
 
 
 
ARHGAP1
 
 
hsa-miR-34a-5p
 
 
-0.369
 
 
26.4
 
 
 
 
ARHGAP26
 
 
hsa-miR-34a-5p
 
 
-0.384
 
 
14.6
 
 
 
 
ARL6IP1
 
 
hsa-miR-152-3p
 
 
-0.579
 
 
185.0
 
 
 
 
ARL8B
 
 
hsa-miR-152-3p
 
 
-0.337
 
 
19.0
 
 
 
 
ARL8B
 
 
hsa-miR-320d
 
 
-0.376
 
 
19.0
 
 
 
 
ARPC5
 
 
hsa-miR-22-3p
 
 
-0.679
 
 
17.2
 
 
 
 
ARPIN-AP3S2
 
 
hsa-miR-34a-5p
 
 
-0.348
 
 
12.4
 
 
 
 
ARPP19
 
 
hsa-miR-320d
 
 
-0.325
 
 
32.4
 
 
 
 
ARPP19
 
 
hsa-miR-34a-5p
 
 
-0.497
 
 
32.4
 
 
 
 
ARRDC3
 
 
hsa-miR-152-3p
 
 
-0.305
 
 
24.1
 
 
 
 
AS3MT
 
 
hsa-miR-28-5p
 
 
-0.479
 
 
11.9
 
 
 
 
ASAH1
 
 
hsa-miR-27a-3p
 
 
-0.398
 
 
113.7
 
 
 
 
ASPH
 
 
hsa-miR-152-3p
 
 
-0.391
 
 
79.9
 
 
 
 
ATG4B
 
 
hsa-miR-34a-5p
 
 
-0.58
 
 
17.1
 
 
 
 
ATP1A2
 
 
hsa-miR-34a-5p
 
 
-0.306
 
 
41.0
 
 
 
 
ATP5MC3
 
 
hsa-miR-27a-3p
 
 
-0.416
 
 
264.7
 
 
 
 
ATP6AP2
 
 
hsa-miR-152-3p
 
 
-0.592
 
 
85.2
 
 
 
 
ATP6AP2
 
 
hsa-miR-152-3p
 
 
-0.668
 
 
85.2
 
 
 
 
ATXN10
 
 
hsa-miR-27a-3p
 
 
-0.295
 
 
46.3
 
 
 
 
ATXN2
 
 
hsa-miR-10a-5p
 
 
-0.362
 
 
17.3
 
 
 
 
B4GALT2
 
 
hsa-miR-34a-5p
 
 
-0.314
 
 
21.4
 
 
 
 
B4GALT3
 
 
hsa-miR-27a-3p
 
 
-0.41
 
 
19.1
 
 
 
 
BAG5
 
 
hsa-miR-22-3p
 
 
-0.318
 
 
18.0
 
 
 
 
BATF3
 
 
hsa-miR-22-3p
 
 
-0.786
 
 
24.8
 
 
 
 
BAZ1B
 
 
hsa-miR-10a-5p
 
 
-0.309
 
 
12.0
 
 
 
 
BCL3
 
 
hsa-miR-27a-3p
 
 
-0.413
 
 
12.1
 
 
 
 
BEX3
 
 
hsa-miR-27a-3p
 
 
-0.454
 
 
829.7
 
 
 
 
BHLHE40
 
 
hsa-miR-181a-5p
 
 
-0.408
 
 
103.5
 
 
 
 
BIN1
 
 
hsa-miR-22-3p
 
 
-0.346
 
 
43.9
 
 
 
 
BLOC1S1
 
 
hsa-miR-22-3p
 
 
-0.346
 
 
256.2
 
 
 
 
BLOC1S6
 
 
hsa-miR-181a-5p
 
 
-0.393
 
 
14.9
 
 
 
 
BLTP1
 
 
hsa-miR-27a-3p
 
 
-0.426
 
 
32.1
 
 
 
 
BMI1
 
 
hsa-miR-27a-3p
 
 
-0.322
 
 
10.0
 
 
 
 
BRSK1
 
 
hsa-miR-27a-3p
 
 
-0.372
 
 
13.9
 
 
 
 
BTBD3
 
 
hsa-miR-152-3p
 
 
-0.495
 
 
18.2
 
 
 
 
BTG2
 
 
hsa-miR-27a-3p
 
 
-0.339
 
 
39.9
 
 
 
 
BZW1
 
 
hsa-miR-27a-3p
 
 
-0.337
 
 
50.7
 
 
 
 
C1GALT1
 
 
hsa-miR-152-3p
 
 
-0.487
 
 
22.9
 
 
 
 
C1orf35
 
 
hsa-miR-152-3p
 
 
-0.511
 
 
11.0
 
 
 
 
C6orf62
 
 
hsa-miR-181a-5p
 
 
-0.334
 
 
62.6
 
 
 
 
CACYBP
 
 
hsa-miR-27a-3p
 
 
-0.367
 
 
27.7
 
 
 
 
CADM4
 
 
hsa-miR-34a-5p
 
 
-0.365
 
 
60.8
 
 
 
 
CAMK2N1
 
 
hsa-miR-22-3p
 
 
-0.302
 
 
15.0
 
 
 
 
CAND1
 
 
hsa-miR-27a-3p
 
 
-0.318
 
 
15.9
 
 
 
 
CAND1
 
 
hsa-miR-152-3p
 
 
-0.44
 
 
15.9
 
 
 
 
CANX
 
 
hsa-miR-152-3p
 
 
-0.434
 
 
111.7
 
 
 
 
CAPN1
 
 
hsa-miR-22-3p
 
 
-0.307
 
 
21.6
 
 
 
 
CAPZA1
 
 
hsa-miR-27a-3p
 
 
-0.325
 
 
36.9
 
 
 
 
CCDC47
 
 
hsa-miR-22-3p
 
 
-0.604
 
 
79.1
 
 
 
 
CCM2
 
 
hsa-miR-27a-3p
 
 
-0.335
 
 
31.0
 
 
 
 
CCNC
 
 
hsa-miR-27a-3p
 
 
-0.378
 
 
33.9
 
 
 
 
CCNG1
 
 
hsa-miR-27a-3p
 
 
-0.419
 
 
27.3
 
 
 
 
CCNK
 
 
hsa-miR-181a-5p
 
 
-0.31
 
 
11.5
 
 
 
 
CCNK
 
 
hsa-miR-27a-3p
 
 
-0.519
 
 
11.5
 
 
 
 
CCNL2
 
 
hsa-miR-28-5p
 
 
-0.448
 
 
18.9
 
 
 
 
CCNY
 
 
hsa-miR-27a-3p
 
 
-0.334
 
 
13.3
 
 
 
 
CCT6A
 
 
hsa-miR-152-3p
 
 
-0.665
 
 
46.5
 
 
 
 
CDC25B
 
 
hsa-miR-152-3p
 
 
-0.298
 
 
13.6
 
 
 
 
CDIP1
 
 
hsa-miR-27a-3p
 
 
-0.362
 
 
10.5
 
 
 
 
CDIP1
 
 
hsa-miR-34a-5p
 
 
-0.461
 
 
10.5
 
 
 
 
CDR2
 
 
hsa-miR-27a-3p
 
 
-0.367
 
 
35.4
 
 
 
 
CDS1
 
 
hsa-miR-27a-3p
 
 
-0.464
 
 
19.2
 
 
 
 
CDS2
 
 
hsa-miR-27a-3p
 
 
-0.291
 
 
11.0
 
 
 
 
CEP63
 
 
hsa-miR-152-3p
 
 
-0.329
 
 
13.4
 
 
 
 
CERS4
 
 
hsa-miR-34a-5p
 
 
-0.328
 
 
34.2
 
 
 
 
CERS5
 
 
hsa-miR-152-3p
 
 
-0.311
 
 
27.4
 
 
 
 
CFAP36
 
 
hsa-miR-22-3p
 
 
-0.549
 
 
178.7
 
 
 
 
CFL2
 
 
hsa-miR-152-3p
 
 
-0.487
 
 
25.6
 
 
 
 
CHMP1B
 
 
hsa-miR-181a-5p
 
 
-0.341
 
 
44.3
 
 
 
 
CHMP4B
 
 
hsa-miR-152-3p
 
 
-0.36
 
 
101.4
 
 
 
 
CIPC
 
 
hsa-miR-27a-3p
 
 
-0.337
 
 
22.0
 
 
 
 
CKAP4
 
 
hsa-miR-27a-3p
 
 
-0.467
 
 
35.3
 
 
 
 
CLCN3
 
 
hsa-miR-34a-5p
 
 
-0.377
 
 
13.5
 
 
 
 
CLIC4
 
 
hsa-miR-22-3p
 
 
-0.594
 
 
19.2
 
 
 
 
 
 
 colon 
 
 
 
 
target_symbol
 
 
mature_mirna_id
 
 
score
 
 
nTPM
 
 
 
 
 
 
AAGAB
 
 
hsa-miR-34a-5p
 
 
-0.319
 
 
26.1
 
 
 
 
ABCB10
 
 
hsa-miR-152-3p
 
 
-0.291
 
 
10.4
 
 
 
 
ABCB7
 
 
hsa-miR-152-3p
 
 
-0.515
 
 
13.4
 
 
 
 
ABCD3
 
 
hsa-miR-152-3p
 
 
-0.388
 
 
41.8
 
 
 
 
ABHD17C
 
 
hsa-miR-27a-3p
 
 
-0.435
 
 
61.2
 
 
 
 
ABHD5
 
 
hsa-miR-27a-3p
 
 
-0.407
 
 
11.4
 
 
 
 
ACBD3
 
 
hsa-miR-34a-5p
 
 
-0.415
 
 
21.8
 
 
 
 
ACSL1
 
 
hsa-miR-34a-5p
 
 
-0.306
 
 
33.2
 
 
 
 
ACSL4
 
 
hsa-miR-34a-5p
 
 
-0.372
 
 
11.3
 
 
 
 
ACTA2
 
 
hsa-miR-27a-3p
 
 
-0.577
 
 
2601.4
 
 
 
 
ADAM10
 
 
hsa-miR-152-3p
 
 
-0.348
 
 
20.8
 
 
 
 
ADAP1
 
 
hsa-miR-27a-3p
 
 
-0.302
 
 
41.4
 
 
 
 
ADGRA3
 
 
hsa-miR-27a-3p
 
 
-0.296
 
 
16.0
 
 
 
 
ADIPOR2
 
 
hsa-miR-34a-5p
 
 
-0.33
 
 
31.0
 
 
 
 
ADK
 
 
hsa-miR-34a-5p
 
 
-0.374
 
 
16.7
 
 
 
 
AFG3L2
 
 
hsa-miR-181a-5p
 
 
-0.491
 
 
38.7
 
 
 
 
AGFG1
 
 
hsa-miR-152-3p
 
 
-0.332
 
 
30.2
 
 
 
 
AGPAT3
 
 
hsa-miR-27a-3p
 
 
-0.29
 
 
20.6
 
 
 
 
AGTRAP
 
 
hsa-miR-34a-5p
 
 
-0.328
 
 
27.8
 
 
 
 
AHCYL2
 
 
hsa-miR-34a-5p
 
 
-0.359
 
 
147.3
 
 
 
 
AHDC1
 
 
hsa-miR-152-3p
 
 
-0.413
 
 
15.6
 
 
 
 
AIDA
 
 
hsa-miR-22-3p
 
 
-0.316
 
 
47.7
 
 
 
 
AKAP1
 
 
hsa-miR-152-3p
 
 
-0.381
 
 
72.8
 
 
 
 
AKIRIN1
 
 
hsa-miR-27a-3p
 
 
-0.368
 
 
33.0
 
 
 
 
AKT3
 
 
hsa-miR-22-3p
 
 
-0.451
 
 
20.3
 
 
 
 
ALCAM
 
 
hsa-miR-34a-5p
 
 
-0.303
 
 
15.7
 
 
 
 
ALCAM
 
 
hsa-miR-152-3p
 
 
-0.347
 
 
15.7
 
 
 
 
ALDOA
 
 
hsa-miR-34a-5p
 
 
-0.655
 
 
907.5
 
 
 
 
ANK2
 
 
hsa-miR-34a-5p
 
 
-0.317
 
 
18.0
 
 
 
 
ANK3
 
 
hsa-miR-34a-5p
 
 
-0.29
 
 
24.1
 
 
 
 
ANK3
 
 
hsa-miR-34a-5p
 
 
-0.347
 
 
24.1
 
 
 
 
ANKHD1-EIF4EBP3
 
 
hsa-miR-22-3p
 
 
-0.479
 
 
11.6
 
 
 
 
ANKRD13A
 
 
hsa-miR-22-3p
 
 
-0.478
 
 
20.4
 
 
 
 
ANKRD40
 
 
hsa-miR-27a-3p
 
 
-0.35
 
 
29.8
 
 
 
 
ANP32B
 
 
hsa-miR-34a-5p
 
 
-0.29
 
 
136.6
 
 
 
 
AP1AR
 
 
hsa-miR-100-5p
 
 
-0.804
 
 
16.9
 
 
 
 
AP3S2
 
 
hsa-miR-34a-5p
 
 
-0.413
 
 
18.1
 
 
 
 
APH1A
 
 
hsa-miR-34a-5p
 
 
-0.304
 
 
94.2
 
 
 
 
APOO
 
 
hsa-miR-181a-5p
 
 
-0.5
 
 
16.2
 
 
 
 
AREG
 
 
hsa-miR-34a-5p
 
 
-0.62
 
 
17.5
 
 
 
 
ARF4
 
 
hsa-miR-152-3p
 
 
-0.487
 
 
148.9
 
 
 
 
ARF6
 
 
hsa-miR-181a-5p
 
 
-0.33
 
 
50.4
 
 
 
 
ARFGEF1
 
 
hsa-miR-152-3p
 
 
-0.306
 
 
18.0
 
 
 
 
ARFIP1
 
 
hsa-miR-320d
 
 
-0.339
 
 
26.4
 
 
 
 
ARFIP1
 
 
hsa-miR-152-3p
 
 
-0.474
 
 
26.4
 
 
 
 
ARHGAP1
 
 
hsa-miR-34a-5p
 
 
-0.369
 
 
59.8
 
 
 
 
ARHGAP21
 
 
hsa-miR-152-3p
 
 
-0.287
 
 
15.5
 
 
 
 
ARHGAP26
 
 
hsa-miR-34a-5p
 
 
-0.384
 
 
12.1
 
 
 
 
ARHGEF26
 
 
hsa-miR-22-3p
 
 
-0.374
 
 
16.6
 
 
 
 
ARL6IP1
 
 
hsa-miR-152-3p
 
 
-0.579
 
 
132.0
 
 
 
 
ARL8B
 
 
hsa-miR-152-3p
 
 
-0.337
 
 
34.2
 
 
 
 
ARL8B
 
 
hsa-miR-320d
 
 
-0.376
 
 
34.2
 
 
 
 
ARPC5
 
 
hsa-miR-22-3p
 
 
-0.679
 
 
98.1
 
 
 
 
ARPIN-AP3S2
 
 
hsa-miR-34a-5p
 
 
-0.348
 
 
13.7
 
 
 
 
ARPP19
 
 
hsa-miR-320d
 
 
-0.325
 
 
35.7
 
 
 
 
ARPP19
 
 
hsa-miR-34a-5p
 
 
-0.497
 
 
35.7
 
 
 
 
ARRB1
 
 
hsa-miR-22-3p
 
 
-0.616
 
 
24.1
 
 
 
 
ARRDC3
 
 
hsa-miR-152-3p
 
 
-0.305
 
 
18.9
 
 
 
 
ASAH1
 
 
hsa-miR-27a-3p
 
 
-0.398
 
 
79.0
 
 
 
 
ASH2L
 
 
hsa-miR-320d
 
 
-0.287
 
 
21.4
 
 
 
 
ASPH
 
 
hsa-miR-152-3p
 
 
-0.391
 
 
72.8
 
 
 
 
ATG4B
 
 
hsa-miR-34a-5p
 
 
-0.58
 
 
21.1
 
 
 
 
ATMIN
 
 
hsa-miR-34a-5p
 
 
-0.409
 
 
12.1
 
 
 
 
ATOH1
 
 
hsa-miR-22-3p
 
 
-0.566
 
 
16.9
 
 
 
 
ATP1A2
 
 
hsa-miR-34a-5p
 
 
-0.306
 
 
54.1
 
 
 
 
ATP2B4
 
 
hsa-miR-152-3p
 
 
-0.337
 
 
46.5
 
 
 
 
ATP5MC3
 
 
hsa-miR-27a-3p
 
 
-0.416
 
 
331.6
 
 
 
 
ATP6AP2
 
 
hsa-miR-152-3p
 
 
-0.592
 
 
77.7
 
 
 
 
ATP6AP2
 
 
hsa-miR-152-3p
 
 
-0.668
 
 
77.7
 
 
 
 
ATP8A1
 
 
hsa-miR-22-3p
 
 
-0.468
 
 
12.0
 
 
 
 
ATXN10
 
 
hsa-miR-27a-3p
 
 
-0.295
 
 
40.5
 
 
 
 
ATXN2
 
 
hsa-miR-10a-5p
 
 
-0.362
 
 
22.4
 
 
 
 
AXL
 
 
hsa-miR-34a-5p
 
 
-0.512
 
 
40.1
 
 
 
 
B3GNT7
 
 
hsa-miR-27a-3p
 
 
-0.355
 
 
52.7
 
 
 
 
B4GALT2
 
 
hsa-miR-34a-5p
 
 
-0.314
 
 
34.4
 
 
 
 
B4GALT3
 
 
hsa-miR-27a-3p
 
 
-0.41
 
 
18.8
 
 
 
 
B4GALT5
 
 
hsa-miR-152-3p
 
 
-0.44
 
 
23.3
 
 
 
 
BAG5
 
 
hsa-miR-22-3p
 
 
-0.318
 
 
13.8
 
 
 
 
BAZ1B
 
 
hsa-miR-10a-5p
 
 
-0.309
 
 
21.5
 
 
 
 
BAZ2A
 
 
hsa-miR-100-5p
 
 
-0.29
 
 
25.3
 
 
 
 
BAZ2A
 
 
hsa-miR-100-5p
 
 
-0.421
 
 
25.3
 
 
 
 
BBX
 
 
hsa-miR-10a-5p
 
 
-0.305
 
 
10.4
 
 
 
 
BCL2L11
 
 
hsa-miR-152-3p
 
 
-0.445
 
 
10.9
 
 
 
 
BCL2L11
 
 
hsa-miR-10a-5p
 
 
-0.483
 
 
10.9
 
 
 
 
BCL3
 
 
hsa-miR-27a-3p
 
 
-0.413
 
 
43.8
 
 
 
 
BEX3
 
 
hsa-miR-27a-3p
 
 
-0.454
 
 
129.5
 
 
 
 
BHLHE40
 
 
hsa-miR-181a-5p
 
 
-0.408
 
 
73.3
 
 
 
 
BIN1
 
 
hsa-miR-22-3p
 
 
-0.346
 
 
33.8
 
 
 
 
BLOC1S1
 
 
hsa-miR-22-3p
 
 
-0.346
 
 
182.5
 
 
 
 
BLOC1S6
 
 
hsa-miR-181a-5p
 
 
-0.393
 
 
19.9
 
 
 
 
BLTP1
 
 
hsa-miR-27a-3p
 
 
-0.426
 
 
18.9
 
 
 
 
BMI1
 
 
hsa-miR-27a-3p
 
 
-0.322
 
 
24.3
 
 
 
 
BMPR2
 
 
hsa-miR-100-5p
 
 
-0.355
 
 
10.5
 
 
 
 
BRPF3
 
 
hsa-miR-34a-5p
 
 
-0.33
 
 
12.3
 
 
 
 
BRSK1
 
 
hsa-miR-27a-3p
 
 
-0.372
 
 
11.3
 
 
 
 
BTAF1
 
 
hsa-miR-152-3p
 
 
-0.401
 
 
16.8
 
 
 
 
BTBD3
 
 
hsa-miR-152-3p
 
 
-0.495
 
 
20.0
 
 
 
 
BTG2
 
 
hsa-miR-27a-3p
 
 
-0.339
 
 
220.3
 
 
 
 
BZW1
 
 
hsa-miR-27a-3p
 
 
-0.337
 
 
92.2
 
 
 
 
C1GALT1
 
 
hsa-miR-152-3p
 
 
-0.487
 
 
14.3
 
 
 
 
 
 
 duodenum 
 
 
 
 
target_symbol
 
 
mature_mirna_id
 
 
score
 
 
nTPM
 
 
 
 
 
 
AAGAB
 
 
hsa-miR-34a-5p
 
 
-0.319
 
 
14.8
 
 
 
 
ABCB10
 
 
hsa-miR-152-3p
 
 
-0.291
 
 
16.1
 
 
 
 
ABCB7
 
 
hsa-miR-152-3p
 
 
-0.515
 
 
15.1
 
 
 
 
ABCD3
 
 
hsa-miR-152-3p
 
 
-0.388
 
 
42.1
 
 
 
 
ABHD17C
 
 
hsa-miR-27a-3p
 
 
-0.435
 
 
21.9
 
 
 
 
ACBD3
 
 
hsa-miR-34a-5p
 
 
-0.415
 
 
16.5
 
 
 
 
ACSL1
 
 
hsa-miR-34a-5p
 
 
-0.306
 
 
28.3
 
 
 
 
ACSL4
 
 
hsa-miR-34a-5p
 
 
-0.372
 
 
13.8
 
 
 
 
ACTA2
 
 
hsa-miR-27a-3p
 
 
-0.577
 
 
234.0
 
 
 
 
ADAM10
 
 
hsa-miR-152-3p
 
 
-0.348
 
 
14.7
 
 
 
 
ADAP1
 
 
hsa-miR-27a-3p
 
 
-0.302
 
 
18.5
 
 
 
 
ADIPOR2
 
 
hsa-miR-34a-5p
 
 
-0.33
 
 
20.9
 
 
 
 
ADK
 
 
hsa-miR-34a-5p
 
 
-0.374
 
 
15.9
 
 
 
 
AFG3L2
 
 
hsa-miR-181a-5p
 
 
-0.491
 
 
48.3
 
 
 
 
AGFG1
 
 
hsa-miR-152-3p
 
 
-0.332
 
 
40.8
 
 
 
 
AGMAT
 
 
hsa-miR-22-3p
 
 
-0.294
 
 
16.8
 
 
 
 
AGPAT3
 
 
hsa-miR-27a-3p
 
 
-0.29
 
 
42.0
 
 
 
 
AGTRAP
 
 
hsa-miR-34a-5p
 
 
-0.328
 
 
10.5
 
 
 
 
AHCYL2
 
 
hsa-miR-34a-5p
 
 
-0.359
 
 
54.3
 
 
 
 
AIDA
 
 
hsa-miR-22-3p
 
 
-0.316
 
 
23.9
 
 
 
 
AKAP1
 
 
hsa-miR-152-3p
 
 
-0.381
 
 
51.5
 
 
 
 
AKIRIN1
 
 
hsa-miR-27a-3p
 
 
-0.368
 
 
22.8
 
 
 
 
ALCAM
 
 
hsa-miR-34a-5p
 
 
-0.303
 
 
16.0
 
 
 
 
ALCAM
 
 
hsa-miR-152-3p
 
 
-0.347
 
 
16.0
 
 
 
 
ALDOA
 
 
hsa-miR-34a-5p
 
 
-0.655
 
 
486.2
 
 
 
 
ANK3
 
 
hsa-miR-34a-5p
 
 
-0.29
 
 
36.7
 
 
 
 
ANK3
 
 
hsa-miR-34a-5p
 
 
-0.347
 
 
36.7
 
 
 
 
ANKRD13A
 
 
hsa-miR-22-3p
 
 
-0.478
 
 
13.4
 
 
 
 
ANKRD40
 
 
hsa-miR-27a-3p
 
 
-0.35
 
 
11.1
 
 
 
 
ANP32B
 
 
hsa-miR-34a-5p
 
 
-0.29
 
 
89.5
 
 
 
 
AP1AR
 
 
hsa-miR-100-5p
 
 
-0.804
 
 
20.9
 
 
 
 
AP3S2
 
 
hsa-miR-34a-5p
 
 
-0.413
 
 
10.3
 
 
 
 
APH1A
 
 
hsa-miR-34a-5p
 
 
-0.304
 
 
77.4
 
 
 
 
APOO
 
 
hsa-miR-181a-5p
 
 
-0.5
 
 
13.6
 
 
 
 
AQP11
 
 
hsa-miR-27a-3p
 
 
-0.666
 
 
27.0
 
 
 
 
ARF4
 
 
hsa-miR-152-3p
 
 
-0.487
 
 
116.2
 
 
 
 
ARF6
 
 
hsa-miR-181a-5p
 
 
-0.33
 
 
43.6
 
 
 
 
ARFGEF1
 
 
hsa-miR-152-3p
 
 
-0.306
 
 
15.9
 
 
 
 
ARFIP1
 
 
hsa-miR-320d
 
 
-0.339
 
 
21.8
 
 
 
 
ARFIP1
 
 
hsa-miR-152-3p
 
 
-0.474
 
 
21.8
 
 
 
 
ARHGAP1
 
 
hsa-miR-34a-5p
 
 
-0.369
 
 
24.2
 
 
 
 
ARHGAP21
 
 
hsa-miR-152-3p
 
 
-0.287
 
 
12.1
 
 
 
 
ARHGAP26
 
 
hsa-miR-34a-5p
 
 
-0.384
 
 
11.3
 
 
 
 
ARL6IP1
 
 
hsa-miR-152-3p
 
 
-0.579
 
 
130.6
 
 
 
 
ARL8B
 
 
hsa-miR-152-3p
 
 
-0.337
 
 
28.9
 
 
 
 
ARL8B
 
 
hsa-miR-320d
 
 
-0.376
 
 
28.9
 
 
 
 
ARPC5
 
 
hsa-miR-22-3p
 
 
-0.679
 
 
93.6
 
 
 
 
ARPP19
 
 
hsa-miR-320d
 
 
-0.325
 
 
27.2
 
 
 
 
ARPP19
 
 
hsa-miR-34a-5p
 
 
-0.497
 
 
27.2
 
 
 
 
ARRB1
 
 
hsa-miR-22-3p
 
 
-0.616
 
 
20.6
 
 
 
 
ASAH1
 
 
hsa-miR-27a-3p
 
 
-0.398
 
 
51.6
 
 
 
 
ASH2L
 
 
hsa-miR-320d
 
 
-0.287
 
 
17.2
 
 
 
 
ASPH
 
 
hsa-miR-152-3p
 
 
-0.391
 
 
42.9
 
 
 
 
ATG4B
 
 
hsa-miR-34a-5p
 
 
-0.58
 
 
10.7
 
 
 
 
ATMIN
 
 
hsa-miR-34a-5p
 
 
-0.409
 
 
12.1
 
 
 
 
ATOH1
 
 
hsa-miR-22-3p
 
 
-0.566
 
 
10.4
 
 
 
 
ATP2B4
 
 
hsa-miR-152-3p
 
 
-0.337
 
 
11.8
 
 
 
 
ATP5MC3
 
 
hsa-miR-27a-3p
 
 
-0.416
 
 
355.5
 
 
 
 
ATP6AP2
 
 
hsa-miR-152-3p
 
 
-0.592
 
 
30.3
 
 
 
 
ATP6AP2
 
 
hsa-miR-152-3p
 
 
-0.668
 
 
30.3
 
 
 
 
ATXN10
 
 
hsa-miR-27a-3p
 
 
-0.295
 
 
22.4
 
 
 
 
ATXN2
 
 
hsa-miR-10a-5p
 
 
-0.362
 
 
10.5
 
 
 
 
AXL
 
 
hsa-miR-34a-5p
 
 
-0.512
 
 
11.0
 
 
 
 
B3GNT7
 
 
hsa-miR-27a-3p
 
 
-0.355
 
 
23.7
 
 
 
 
B4GALT2
 
 
hsa-miR-34a-5p
 
 
-0.314
 
 
11.8
 
 
 
 
B4GALT3
 
 
hsa-miR-27a-3p
 
 
-0.41
 
 
16.2
 
 
 
 
B4GALT5
 
 
hsa-miR-152-3p
 
 
-0.44
 
 
23.8
 
 
 
 
BAG5
 
 
hsa-miR-22-3p
 
 
-0.318
 
 
11.6
 
 
 
 
BAZ1B
 
 
hsa-miR-10a-5p
 
 
-0.309
 
 
15.1
 
 
 
 
BAZ2A
 
 
hsa-miR-100-5p
 
 
-0.29
 
 
23.9
 
 
 
 
BAZ2A
 
 
hsa-miR-100-5p
 
 
-0.421
 
 
23.9
 
 
 
 
BCL3
 
 
hsa-miR-27a-3p
 
 
-0.413
 
 
30.4
 
 
 
 
BEND7
 
 
hsa-miR-27a-3p
 
 
-0.304
 
 
17.4
 
 
 
 
BEX3
 
 
hsa-miR-27a-3p
 
 
-0.454
 
 
29.3
 
 
 
 
BHLHE40
 
 
hsa-miR-181a-5p
 
 
-0.408
 
 
29.0
 
 
 
 
BIN1
 
 
hsa-miR-22-3p
 
 
-0.346
 
 
16.4
 
 
 
 
BLOC1S1
 
 
hsa-miR-22-3p
 
 
-0.346
 
 
139.3
 
 
 
 
BLOC1S6
 
 
hsa-miR-181a-5p
 
 
-0.393
 
 
19.0
 
 
 
 
BLTP1
 
 
hsa-miR-27a-3p
 
 
-0.426
 
 
11.2
 
 
 
 
BMI1
 
 
hsa-miR-27a-3p
 
 
-0.322
 
 
18.5
 
 
 
 
BRPF3
 
 
hsa-miR-34a-5p
 
 
-0.33
 
 
11.0
 
 
 
 
BTAF1
 
 
hsa-miR-152-3p
 
 
-0.401
 
 
12.8
 
 
 
 
BTBD3
 
 
hsa-miR-152-3p
 
 
-0.495
 
 
10.1
 
 
 
 
BTG2
 
 
hsa-miR-27a-3p
 
 
-0.339
 
 
83.2
 
 
 
 
BZW1
 
 
hsa-miR-27a-3p
 
 
-0.337
 
 
60.8
 
 
 
 
C1GALT1
 
 
hsa-miR-152-3p
 
 
-0.487
 
 
27.7
 
 
 
 
C5orf24
 
 
hsa-miR-22-3p
 
 
-0.493
 
 
20.8
 
 
 
 
C6orf120
 
 
hsa-miR-34a-5p
 
 
-0.331
 
 
13.9
 
 
 
 
C6orf62
 
 
hsa-miR-181a-5p
 
 
-0.334
 
 
85.0
 
 
 
 
CACYBP
 
 
hsa-miR-27a-3p
 
 
-0.367
 
 
14.9
 
 
 
 
CAMK2N1
 
 
hsa-miR-22-3p
 
 
-0.302
 
 
75.8
 
 
 
 
CAND1
 
 
hsa-miR-27a-3p
 
 
-0.318
 
 
18.4
 
 
 
 
CAND1
 
 
hsa-miR-152-3p
 
 
-0.44
 
 
18.4
 
 
 
 
CANX
 
 
hsa-miR-152-3p
 
 
-0.434
 
 
41.6
 
 
 
 
CAPN1
 
 
hsa-miR-22-3p
 
 
-0.307
 
 
75.6
 
 
 
 
CAPZA1
 
 
hsa-miR-27a-3p
 
 
-0.325
 
 
63.9
 
 
 
 
CARMIL1
 
 
hsa-miR-22-3p
 
 
-0.329
 
 
15.2
 
 
 
 
CASP2
 
 
hsa-miR-34a-5p
 
 
-0.382
 
 
10.1
 
 
 
 
CAV2
 
 
hsa-miR-152-3p
 
 
-0.32
 
 
10.6
 
 
 
 
CCDC47
 
 
hsa-miR-22-3p
 
 
-0.604
 
 
79.4
 
 
 
 
 
 
 endometrium 
 
 
 
 
target_symbol
 
 
mature_mirna_id
 
 
score
 
 
nTPM
 
 
 
 
 
 
AAGAB
 
 
hsa-miR-34a-5p
 
 
-0.319
 
 
13.5
 
 
 
 
ABCB7
 
 
hsa-miR-152-3p
 
 
-0.515
 
 
10.5
 
 
 
 
ABCD3
 
 
hsa-miR-152-3p
 
 
-0.388
 
 
13.8
 
 
 
 
ACBD3
 
 
hsa-miR-34a-5p
 
 
-0.415
 
 
21.4
 
 
 
 
ACSL1
 
 
hsa-miR-34a-5p
 
 
-0.306
 
 
36.5
 
 
 
 
ACSL4
 
 
hsa-miR-34a-5p
 
 
-0.372
 
 
17.4
 
 
 
 
ACTA2
 
 
hsa-miR-27a-3p
 
 
-0.577
 
 
5917.5
 
 
 
 
ACVR1
 
 
hsa-miR-152-3p
 
 
-0.397
 
 
15.9
 
 
 
 
ADAM10
 
 
hsa-miR-152-3p
 
 
-0.348
 
 
15.2
 
 
 
 
ADAMTS10
 
 
hsa-miR-27a-3p
 
 
-0.354
 
 
10.1
 
 
 
 
ADGRA3
 
 
hsa-miR-27a-3p
 
 
-0.296
 
 
14.1
 
 
 
 
ADGRF5
 
 
hsa-miR-152-3p
 
 
-0.354
 
 
16.0
 
 
 
 
ADIPOR2
 
 
hsa-miR-34a-5p
 
 
-0.33
 
 
24.5
 
 
 
 
ADK
 
 
hsa-miR-34a-5p
 
 
-0.374
 
 
10.1
 
 
 
 
AFG3L2
 
 
hsa-miR-181a-5p
 
 
-0.491
 
 
12.4
 
 
 
 
AGFG1
 
 
hsa-miR-152-3p
 
 
-0.332
 
 
23.1
 
 
 
 
AGTRAP
 
 
hsa-miR-34a-5p
 
 
-0.328
 
 
23.7
 
 
 
 
AHDC1
 
 
hsa-miR-152-3p
 
 
-0.413
 
 
26.0
 
 
 
 
AIDA
 
 
hsa-miR-22-3p
 
 
-0.316
 
 
33.8
 
 
 
 
AKAP1
 
 
hsa-miR-152-3p
 
 
-0.381
 
 
24.8
 
 
 
 
AKIRIN1
 
 
hsa-miR-27a-3p
 
 
-0.368
 
 
26.8
 
 
 
 
AKT3
 
 
hsa-miR-22-3p
 
 
-0.451
 
 
19.5
 
 
 
 
ALCAM
 
 
hsa-miR-34a-5p
 
 
-0.303
 
 
14.4
 
 
 
 
ALCAM
 
 
hsa-miR-152-3p
 
 
-0.347
 
 
14.4
 
 
 
 
ALDOA
 
 
hsa-miR-34a-5p
 
 
-0.655
 
 
580.3
 
 
 
 
ANK2
 
 
hsa-miR-34a-5p
 
 
-0.317
 
 
13.0
 
 
 
 
ANK3
 
 
hsa-miR-34a-5p
 
 
-0.29
 
 
21.4
 
 
 
 
ANK3
 
 
hsa-miR-34a-5p
 
 
-0.347
 
 
21.4
 
 
 
 
ANKRD13A
 
 
hsa-miR-22-3p
 
 
-0.478
 
 
11.2
 
 
 
 
ANKRD40
 
 
hsa-miR-27a-3p
 
 
-0.35
 
 
22.4
 
 
 
 
ANP32B
 
 
hsa-miR-34a-5p
 
 
-0.29
 
 
111.3
 
 
 
 
AP1AR
 
 
hsa-miR-100-5p
 
 
-0.804
 
 
10.6
 
 
 
 
AP3S2
 
 
hsa-miR-34a-5p
 
 
-0.413
 
 
12.2
 
 
 
 
APH1A
 
 
hsa-miR-34a-5p
 
 
-0.304
 
 
57.8
 
 
 
 
APPBP2
 
 
hsa-miR-27a-3p
 
 
-0.378
 
 
12.0
 
 
 
 
ARF4
 
 
hsa-miR-152-3p
 
 
-0.487
 
 
113.9
 
 
 
 
ARF6
 
 
hsa-miR-181a-5p
 
 
-0.33
 
 
19.5
 
 
 
 
ARFIP1
 
 
hsa-miR-320d
 
 
-0.339
 
 
20.3
 
 
 
 
ARFIP1
 
 
hsa-miR-152-3p
 
 
-0.474
 
 
20.3
 
 
 
 
ARHGAP1
 
 
hsa-miR-34a-5p
 
 
-0.369
 
 
81.8
 
 
 
 
ARHGAP21
 
 
hsa-miR-152-3p
 
 
-0.287
 
 
18.5
 
 
 
 
ARL6IP1
 
 
hsa-miR-152-3p
 
 
-0.579
 
 
66.4
 
 
 
 
ARL8B
 
 
hsa-miR-152-3p
 
 
-0.337
 
 
42.3
 
 
 
 
ARL8B
 
 
hsa-miR-320d
 
 
-0.376
 
 
42.3
 
 
 
 
ARPC5
 
 
hsa-miR-22-3p
 
 
-0.679
 
 
63.7
 
 
 
 
ARPP19
 
 
hsa-miR-320d
 
 
-0.325
 
 
44.7
 
 
 
 
ARPP19
 
 
hsa-miR-34a-5p
 
 
-0.497
 
 
44.7
 
 
 
 
ARRDC3
 
 
hsa-miR-152-3p
 
 
-0.305
 
 
38.0
 
 
 
 
ASAH1
 
 
hsa-miR-27a-3p
 
 
-0.398
 
 
43.1
 
 
 
 
ASB1
 
 
hsa-miR-34a-5p
 
 
-0.406
 
 
10.1
 
 
 
 
ASH2L
 
 
hsa-miR-320d
 
 
-0.287
 
 
20.7
 
 
 
 
ASPH
 
 
hsa-miR-152-3p
 
 
-0.391
 
 
45.6
 
 
 
 
ATG4B
 
 
hsa-miR-34a-5p
 
 
-0.58
 
 
19.1
 
 
 
 
ATMIN
 
 
hsa-miR-34a-5p
 
 
-0.409
 
 
12.9
 
 
 
 
ATP2B4
 
 
hsa-miR-152-3p
 
 
-0.337
 
 
98.2
 
 
 
 
ATP5MC3
 
 
hsa-miR-27a-3p
 
 
-0.416
 
 
118.3
 
 
 
 
ATP6AP2
 
 
hsa-miR-152-3p
 
 
-0.592
 
 
81.0
 
 
 
 
ATP6AP2
 
 
hsa-miR-152-3p
 
 
-0.668
 
 
81.0
 
 
 
 
ATXN10
 
 
hsa-miR-27a-3p
 
 
-0.295
 
 
47.3
 
 
 
 
ATXN2
 
 
hsa-miR-10a-5p
 
 
-0.362
 
 
21.0
 
 
 
 
AXL
 
 
hsa-miR-34a-5p
 
 
-0.512
 
 
31.2
 
 
 
 
B4GALT2
 
 
hsa-miR-34a-5p
 
 
-0.314
 
 
25.1
 
 
 
 
B4GALT3
 
 
hsa-miR-27a-3p
 
 
-0.41
 
 
14.3
 
 
 
 
B4GALT5
 
 
hsa-miR-152-3p
 
 
-0.44
 
 
22.0
 
 
 
 
BAG5
 
 
hsa-miR-22-3p
 
 
-0.318
 
 
15.1
 
 
 
 
BAZ1B
 
 
hsa-miR-10a-5p
 
 
-0.309
 
 
22.9
 
 
 
 
BAZ2A
 
 
hsa-miR-100-5p
 
 
-0.29
 
 
24.8
 
 
 
 
BAZ2A
 
 
hsa-miR-100-5p
 
 
-0.421
 
 
24.8
 
 
 
 
BBX
 
 
hsa-miR-10a-5p
 
 
-0.305
 
 
17.7
 
 
 
 
BCL2
 
 
hsa-miR-34a-5p
 
 
-0.289
 
 
10.9
 
 
 
 
BCL3
 
 
hsa-miR-27a-3p
 
 
-0.413
 
 
25.5
 
 
 
 
BEX3
 
 
hsa-miR-27a-3p
 
 
-0.454
 
 
152.4
 
 
 
 
BHLHE40
 
 
hsa-miR-181a-5p
 
 
-0.408
 
 
63.3
 
 
 
 
BIN1
 
 
hsa-miR-22-3p
 
 
-0.346
 
 
39.4
 
 
 
 
BLOC1S1
 
 
hsa-miR-22-3p
 
 
-0.346
 
 
110.1
 
 
 
 
BLOC1S6
 
 
hsa-miR-181a-5p
 
 
-0.393
 
 
28.8
 
 
 
 
BLTP1
 
 
hsa-miR-27a-3p
 
 
-0.426
 
 
20.8
 
 
 
 
BMI1
 
 
hsa-miR-27a-3p
 
 
-0.322
 
 
28.7
 
 
 
 
BMPR2
 
 
hsa-miR-100-5p
 
 
-0.355
 
 
14.3
 
 
 
 
BRPF3
 
 
hsa-miR-34a-5p
 
 
-0.33
 
 
11.1
 
 
 
 
BTAF1
 
 
hsa-miR-152-3p
 
 
-0.401
 
 
17.3
 
 
 
 
BTBD3
 
 
hsa-miR-152-3p
 
 
-0.495
 
 
17.5
 
 
 
 
BTG2
 
 
hsa-miR-27a-3p
 
 
-0.339
 
 
86.1
 
 
 
 
BZW1
 
 
hsa-miR-27a-3p
 
 
-0.337
 
 
62.3
 
 
 
 
C1orf35
 
 
hsa-miR-152-3p
 
 
-0.511
 
 
15.9
 
 
 
 
C5orf24
 
 
hsa-miR-22-3p
 
 
-0.493
 
 
27.1
 
 
 
 
C6orf62
 
 
hsa-miR-181a-5p
 
 
-0.334
 
 
66.1
 
 
 
 
C8orf88
 
 
hsa-miR-27a-3p
 
 
-0.33
 
 
17.9
 
 
 
 
CACNB3
 
 
hsa-miR-34a-5p
 
 
-0.426
 
 
30.6
 
 
 
 
CACYBP
 
 
hsa-miR-27a-3p
 
 
-0.367
 
 
19.5
 
 
 
 
CADM4
 
 
hsa-miR-34a-5p
 
 
-0.365
 
 
13.4
 
 
 
 
CAMK2N1
 
 
hsa-miR-22-3p
 
 
-0.302
 
 
25.6
 
 
 
 
CAND1
 
 
hsa-miR-27a-3p
 
 
-0.318
 
 
19.9
 
 
 
 
CAND1
 
 
hsa-miR-152-3p
 
 
-0.44
 
 
19.9
 
 
 
 
CANX
 
 
hsa-miR-152-3p
 
 
-0.434
 
 
116.8
 
 
 
 
CAPN1
 
 
hsa-miR-22-3p
 
 
-0.307
 
 
53.8
 
 
 
 
CAPZA1
 
 
hsa-miR-27a-3p
 
 
-0.325
 
 
40.3
 
 
 
 
CAV2
 
 
hsa-miR-152-3p
 
 
-0.32
 
 
33.4
 
 
 
 
CCDC47
 
 
hsa-miR-22-3p
 
 
-0.604
 
 
36.0
 
 
 
 
CCDC6
 
 
hsa-miR-152-3p
 
 
-0.511
 
 
22.0
 
 
 
 
 
 
 epididymis 
 
 
 
 
target_symbol
 
 
mature_mirna_id
 
 
score
 
 
nTPM
 
 
 
 
 
 
AAGAB
 
 
hsa-miR-34a-5p
 
 
-0.319
 
 
24.4
 
 
 
 
ABCD3
 
 
hsa-miR-152-3p
 
 
-0.388
 
 
20.0
 
 
 
 
ACBD3
 
 
hsa-miR-34a-5p
 
 
-0.415
 
 
19.4
 
 
 
 
ACSL1
 
 
hsa-miR-34a-5p
 
 
-0.306
 
 
50.9
 
 
 
 
ACSL4
 
 
hsa-miR-34a-5p
 
 
-0.372
 
 
10.2
 
 
 
 
ACTA2
 
 
hsa-miR-27a-3p
 
 
-0.577
 
 
1284.3
 
 
 
 
ACVR1
 
 
hsa-miR-152-3p
 
 
-0.397
 
 
10.8
 
 
 
 
ADAM17
 
 
hsa-miR-152-3p
 
 
-0.301
 
 
10.4
 
 
 
 
ADK
 
 
hsa-miR-34a-5p
 
 
-0.374
 
 
15.6
 
 
 
 
ADO
 
 
hsa-miR-34a-5p
 
 
-0.377
 
 
12.4
 
 
 
 
AGFG1
 
 
hsa-miR-152-3p
 
 
-0.332
 
 
18.8
 
 
 
 
AGPAT3
 
 
hsa-miR-27a-3p
 
 
-0.29
 
 
11.9
 
 
 
 
AGTRAP
 
 
hsa-miR-34a-5p
 
 
-0.328
 
 
45.1
 
 
 
 
AIDA
 
 
hsa-miR-22-3p
 
 
-0.316
 
 
52.9
 
 
 
 
AKAP1
 
 
hsa-miR-152-3p
 
 
-0.381
 
 
18.6
 
 
 
 
AKIRIN1
 
 
hsa-miR-27a-3p
 
 
-0.368
 
 
39.9
 
 
 
 
ALCAM
 
 
hsa-miR-34a-5p
 
 
-0.303
 
 
33.4
 
 
 
 
ALCAM
 
 
hsa-miR-152-3p
 
 
-0.347
 
 
33.4
 
 
 
 
ALDOA
 
 
hsa-miR-34a-5p
 
 
-0.655
 
 
189.5
 
 
 
 
ANK3
 
 
hsa-miR-34a-5p
 
 
-0.29
 
 
116.7
 
 
 
 
ANK3
 
 
hsa-miR-34a-5p
 
 
-0.347
 
 
116.7
 
 
 
 
ANKHD1-EIF4EBP3
 
 
hsa-miR-22-3p
 
 
-0.479
 
 
18.5
 
 
 
 
ANKRD40
 
 
hsa-miR-27a-3p
 
 
-0.35
 
 
13.0
 
 
 
 
ANP32B
 
 
hsa-miR-34a-5p
 
 
-0.29
 
 
98.9
 
 
 
 
AP1S3
 
 
hsa-miR-181a-5p
 
 
-0.383
 
 
45.7
 
 
 
 
AP3S2
 
 
hsa-miR-34a-5p
 
 
-0.413
 
 
15.3
 
 
 
 
APH1A
 
 
hsa-miR-34a-5p
 
 
-0.304
 
 
79.3
 
 
 
 
APOO
 
 
hsa-miR-181a-5p
 
 
-0.5
 
 
18.5
 
 
 
 
ARF4
 
 
hsa-miR-152-3p
 
 
-0.487
 
 
214.1
 
 
 
 
ARF6
 
 
hsa-miR-181a-5p
 
 
-0.33
 
 
27.6
 
 
 
 
ARFGEF1
 
 
hsa-miR-152-3p
 
 
-0.306
 
 
10.4
 
 
 
 
ARFIP1
 
 
hsa-miR-320d
 
 
-0.339
 
 
19.3
 
 
 
 
ARFIP1
 
 
hsa-miR-152-3p
 
 
-0.474
 
 
19.3
 
 
 
 
ARHGAP1
 
 
hsa-miR-34a-5p
 
 
-0.369
 
 
20.3
 
 
 
 
ARL6IP1
 
 
hsa-miR-152-3p
 
 
-0.579
 
 
116.0
 
 
 
 
ARL8B
 
 
hsa-miR-152-3p
 
 
-0.337
 
 
37.5
 
 
 
 
ARL8B
 
 
hsa-miR-320d
 
 
-0.376
 
 
37.5
 
 
 
 
ARPC5
 
 
hsa-miR-22-3p
 
 
-0.679
 
 
14.8
 
 
 
 
ARPIN-AP3S2
 
 
hsa-miR-34a-5p
 
 
-0.348
 
 
10.7
 
 
 
 
ARPP19
 
 
hsa-miR-320d
 
 
-0.325
 
 
34.2
 
 
 
 
ARPP19
 
 
hsa-miR-34a-5p
 
 
-0.497
 
 
34.2
 
 
 
 
ARRDC3
 
 
hsa-miR-152-3p
 
 
-0.305
 
 
25.6
 
 
 
 
ASAH1
 
 
hsa-miR-27a-3p
 
 
-0.398
 
 
124.2
 
 
 
 
ASH2L
 
 
hsa-miR-320d
 
 
-0.287
 
 
16.2
 
 
 
 
ASPH
 
 
hsa-miR-152-3p
 
 
-0.391
 
 
42.3
 
 
 
 
ATMIN
 
 
hsa-miR-34a-5p
 
 
-0.409
 
 
16.9
 
 
 
 
ATP1A2
 
 
hsa-miR-34a-5p
 
 
-0.306
 
 
11.8
 
 
 
 
ATP2B4
 
 
hsa-miR-152-3p
 
 
-0.337
 
 
18.9
 
 
 
 
ATP5MC3
 
 
hsa-miR-27a-3p
 
 
-0.416
 
 
119.2
 
 
 
 
ATP6AP2
 
 
hsa-miR-152-3p
 
 
-0.592
 
 
86.1
 
 
 
 
ATP6AP2
 
 
hsa-miR-152-3p
 
 
-0.668
 
 
86.1
 
 
 
 
ATP8A1
 
 
hsa-miR-22-3p
 
 
-0.468
 
 
11.5
 
 
 
 
ATXN10
 
 
hsa-miR-27a-3p
 
 
-0.295
 
 
36.8
 
 
 
 
ATXN2
 
 
hsa-miR-10a-5p
 
 
-0.362
 
 
13.1
 
 
 
 
B4GALT3
 
 
hsa-miR-27a-3p
 
 
-0.41
 
 
17.3
 
 
 
 
BAG5
 
 
hsa-miR-22-3p
 
 
-0.318
 
 
11.8
 
 
 
 
BAZ1B
 
 
hsa-miR-10a-5p
 
 
-0.309
 
 
16.7
 
 
 
 
BAZ2A
 
 
hsa-miR-100-5p
 
 
-0.29
 
 
21.6
 
 
 
 
BAZ2A
 
 
hsa-miR-100-5p
 
 
-0.421
 
 
21.6
 
 
 
 
BBX
 
 
hsa-miR-10a-5p
 
 
-0.305
 
 
36.4
 
 
 
 
BEND7
 
 
hsa-miR-27a-3p
 
 
-0.304
 
 
11.8
 
 
 
 
BEX3
 
 
hsa-miR-27a-3p
 
 
-0.454
 
 
471.3
 
 
 
 
BHLHE40
 
 
hsa-miR-181a-5p
 
 
-0.408
 
 
32.6
 
 
 
 
BHLHE41
 
 
hsa-miR-152-3p
 
 
-0.442
 
 
18.2
 
 
 
 
BIN1
 
 
hsa-miR-22-3p
 
 
-0.346
 
 
15.8
 
 
 
 
BLOC1S1
 
 
hsa-miR-22-3p
 
 
-0.346
 
 
147.0
 
 
 
 
BLOC1S6
 
 
hsa-miR-181a-5p
 
 
-0.393
 
 
17.3
 
 
 
 
BLTP1
 
 
hsa-miR-27a-3p
 
 
-0.426
 
 
17.9
 
 
 
 
BMI1
 
 
hsa-miR-27a-3p
 
 
-0.322
 
 
28.1
 
 
 
 
BTAF1
 
 
hsa-miR-152-3p
 
 
-0.401
 
 
16.9
 
 
 
 
BTG2
 
 
hsa-miR-27a-3p
 
 
-0.339
 
 
39.2
 
 
 
 
BZW1
 
 
hsa-miR-27a-3p
 
 
-0.337
 
 
55.1
 
 
 
 
C5orf24
 
 
hsa-miR-22-3p
 
 
-0.493
 
 
14.9
 
 
 
 
C6orf62
 
 
hsa-miR-181a-5p
 
 
-0.334
 
 
95.1
 
 
 
 
C8orf88
 
 
hsa-miR-27a-3p
 
 
-0.33
 
 
12.3
 
 
 
 
CACYBP
 
 
hsa-miR-27a-3p
 
 
-0.367
 
 
23.0
 
 
 
 
CAMK2N1
 
 
hsa-miR-22-3p
 
 
-0.302
 
 
36.0
 
 
 
 
CAND1
 
 
hsa-miR-27a-3p
 
 
-0.318
 
 
26.2
 
 
 
 
CAND1
 
 
hsa-miR-152-3p
 
 
-0.44
 
 
26.2
 
 
 
 
CANX
 
 
hsa-miR-152-3p
 
 
-0.434
 
 
162.1
 
 
 
 
CAPN1
 
 
hsa-miR-22-3p
 
 
-0.307
 
 
25.9
 
 
 
 
CAPZA1
 
 
hsa-miR-27a-3p
 
 
-0.325
 
 
58.0
 
 
 
 
CAV2
 
 
hsa-miR-152-3p
 
 
-0.32
 
 
21.8
 
 
 
 
CCDC47
 
 
hsa-miR-22-3p
 
 
-0.604
 
 
47.4
 
 
 
 
CCDC6
 
 
hsa-miR-152-3p
 
 
-0.511
 
 
15.8
 
 
 
 
CCNC
 
 
hsa-miR-27a-3p
 
 
-0.378
 
 
55.4
 
 
 
 
CCNG1
 
 
hsa-miR-27a-3p
 
 
-0.419
 
 
69.4
 
 
 
 
CCNK
 
 
hsa-miR-181a-5p
 
 
-0.31
 
 
17.7
 
 
 
 
CCNK
 
 
hsa-miR-27a-3p
 
 
-0.519
 
 
17.7
 
 
 
 
CCNL2
 
 
hsa-miR-28-5p
 
 
-0.448
 
 
28.7
 
 
 
 
CCNY
 
 
hsa-miR-27a-3p
 
 
-0.334
 
 
25.4
 
 
 
 
CCT6A
 
 
hsa-miR-152-3p
 
 
-0.665
 
 
37.2
 
 
 
 
CDKN2AIP
 
 
hsa-miR-146a-5p
 
 
-0.293
 
 
11.7
 
 
 
 
CDKN2AIP
 
 
hsa-miR-146b-5p
 
 
-0.293
 
 
11.7
 
 
 
 
CDKN2AIP
 
 
hsa-miR-28-5p
 
 
-0.419
 
 
11.7
 
 
 
 
CDR2
 
 
hsa-miR-27a-3p
 
 
-0.367
 
 
10.0
 
 
 
 
CDS1
 
 
hsa-miR-27a-3p
 
 
-0.464
 
 
27.4
 
 
 
 
CEP63
 
 
hsa-miR-152-3p
 
 
-0.329
 
 
11.9
 
 
 
 
CERS4
 
 
hsa-miR-34a-5p
 
 
-0.328
 
 
58.1
 
 
 
 
CERS5
 
 
hsa-miR-152-3p
 
 
-0.311
 
 
12.3
 
 
 
 
 
 
 esophagus 
 
 
 
 
target_symbol
 
 
mature_mirna_id
 
 
score
 
 
nTPM
 
 
 
 
 
 
AAGAB
 
 
hsa-miR-34a-5p
 
 
-0.319
 
 
19.3
 
 
 
 
ABCB7
 
 
hsa-miR-152-3p
 
 
-0.515
 
 
11.1
 
 
 
 
ABCD3
 
 
hsa-miR-152-3p
 
 
-0.388
 
 
25.0
 
 
 
 
ABHD17C
 
 
hsa-miR-27a-3p
 
 
-0.435
 
 
45.1
 
 
 
 
ABHD5
 
 
hsa-miR-27a-3p
 
 
-0.407
 
 
18.4
 
 
 
 
ACBD3
 
 
hsa-miR-34a-5p
 
 
-0.415
 
 
29.0
 
 
 
 
ACSL1
 
 
hsa-miR-34a-5p
 
 
-0.306
 
 
63.8
 
 
 
 
ACSL4
 
 
hsa-miR-34a-5p
 
 
-0.372
 
 
14.6
 
 
 
 
ACTA2
 
 
hsa-miR-27a-3p
 
 
-0.577
 
 
1416.9
 
 
 
 
ADAM10
 
 
hsa-miR-152-3p
 
 
-0.348
 
 
14.2
 
 
 
 
ADIPOR2
 
 
hsa-miR-34a-5p
 
 
-0.33
 
 
44.0
 
 
 
 
ADK
 
 
hsa-miR-34a-5p
 
 
-0.374
 
 
46.4
 
 
 
 
ADORA2B
 
 
hsa-miR-27a-3p
 
 
-0.638
 
 
10.4
 
 
 
 
AFG3L2
 
 
hsa-miR-181a-5p
 
 
-0.491
 
 
25.7
 
 
 
 
AGFG1
 
 
hsa-miR-152-3p
 
 
-0.332
 
 
44.3
 
 
 
 
AGPAT3
 
 
hsa-miR-27a-3p
 
 
-0.29
 
 
24.6
 
 
 
 
AGTRAP
 
 
hsa-miR-34a-5p
 
 
-0.328
 
 
51.2
 
 
 
 
AHDC1
 
 
hsa-miR-152-3p
 
 
-0.413
 
 
34.3
 
 
 
 
AIDA
 
 
hsa-miR-22-3p
 
 
-0.316
 
 
42.4
 
 
 
 
AKAP1
 
 
hsa-miR-152-3p
 
 
-0.381
 
 
34.2
 
 
 
 
AKIRIN1
 
 
hsa-miR-27a-3p
 
 
-0.368
 
 
27.4
 
 
 
 
ALDOA
 
 
hsa-miR-34a-5p
 
 
-0.655
 
 
1511.5
 
 
 
 
ANK3
 
 
hsa-miR-34a-5p
 
 
-0.29
 
 
15.5
 
 
 
 
ANK3
 
 
hsa-miR-34a-5p
 
 
-0.347
 
 
15.5
 
 
 
 
ANKHD1-EIF4EBP3
 
 
hsa-miR-22-3p
 
 
-0.479
 
 
14.8
 
 
 
 
ANKRD13A
 
 
hsa-miR-22-3p
 
 
-0.478
 
 
29.8
 
 
 
 
ANKRD40
 
 
hsa-miR-27a-3p
 
 
-0.35
 
 
17.3
 
 
 
 
ANP32B
 
 
hsa-miR-34a-5p
 
 
-0.29
 
 
133.7
 
 
 
 
AP1AR
 
 
hsa-miR-100-5p
 
 
-0.804
 
 
12.5
 
 
 
 
AP3S2
 
 
hsa-miR-34a-5p
 
 
-0.413
 
 
19.4
 
 
 
 
APH1A
 
 
hsa-miR-34a-5p
 
 
-0.304
 
 
113.5
 
 
 
 
APOO
 
 
hsa-miR-181a-5p
 
 
-0.5
 
 
12.1
 
 
 
 
AREG
 
 
hsa-miR-34a-5p
 
 
-0.62
 
 
42.1
 
 
 
 
ARF4
 
 
hsa-miR-152-3p
 
 
-0.487
 
 
102.8
 
 
 
 
ARF6
 
 
hsa-miR-181a-5p
 
 
-0.33
 
 
81.9
 
 
 
 
ARFGEF1
 
 
hsa-miR-152-3p
 
 
-0.306
 
 
14.2
 
 
 
 
ARFIP1
 
 
hsa-miR-320d
 
 
-0.339
 
 
19.6
 
 
 
 
ARFIP1
 
 
hsa-miR-152-3p
 
 
-0.474
 
 
19.6
 
 
 
 
ARHGAP1
 
 
hsa-miR-34a-5p
 
 
-0.369
 
 
71.8
 
 
 
 
ARHGAP21
 
 
hsa-miR-152-3p
 
 
-0.287
 
 
10.2
 
 
 
 
ARL6IP1
 
 
hsa-miR-152-3p
 
 
-0.579
 
 
65.8
 
 
 
 
ARL8B
 
 
hsa-miR-152-3p
 
 
-0.337
 
 
71.1
 
 
 
 
ARL8B
 
 
hsa-miR-320d
 
 
-0.376
 
 
71.1
 
 
 
 
ARPC5
 
 
hsa-miR-22-3p
 
 
-0.679
 
 
73.2
 
 
 
 
ARPIN-AP3S2
 
 
hsa-miR-34a-5p
 
 
-0.348
 
 
11.9
 
 
 
 
ARPP19
 
 
hsa-miR-320d
 
 
-0.325
 
 
49.0
 
 
 
 
ARPP19
 
 
hsa-miR-34a-5p
 
 
-0.497
 
 
49.0
 
 
 
 
ARRB1
 
 
hsa-miR-22-3p
 
 
-0.616
 
 
17.9
 
 
 
 
ARRDC3
 
 
hsa-miR-152-3p
 
 
-0.305
 
 
36.4
 
 
 
 
ASAH1
 
 
hsa-miR-27a-3p
 
 
-0.398
 
 
68.8
 
 
 
 
ASH2L
 
 
hsa-miR-320d
 
 
-0.287
 
 
22.9
 
 
 
 
ASPH
 
 
hsa-miR-152-3p
 
 
-0.391
 
 
66.3
 
 
 
 
ATG4B
 
 
hsa-miR-34a-5p
 
 
-0.58
 
 
18.3
 
 
 
 
ATMIN
 
 
hsa-miR-34a-5p
 
 
-0.409
 
 
17.8
 
 
 
 
ATP1A2
 
 
hsa-miR-34a-5p
 
 
-0.306
 
 
31.5
 
 
 
 
ATP2B4
 
 
hsa-miR-152-3p
 
 
-0.337
 
 
30.6
 
 
 
 
ATP5MC3
 
 
hsa-miR-27a-3p
 
 
-0.416
 
 
293.6
 
 
 
 
ATP6AP2
 
 
hsa-miR-152-3p
 
 
-0.592
 
 
65.8
 
 
 
 
ATP6AP2
 
 
hsa-miR-152-3p
 
 
-0.668
 
 
65.8
 
 
 
 
ATXN10
 
 
hsa-miR-27a-3p
 
 
-0.295
 
 
44.8
 
 
 
 
ATXN2
 
 
hsa-miR-10a-5p
 
 
-0.362
 
 
13.9
 
 
 
 
AXL
 
 
hsa-miR-34a-5p
 
 
-0.512
 
 
16.4
 
 
 
 
B4GALT2
 
 
hsa-miR-34a-5p
 
 
-0.314
 
 
26.4
 
 
 
 
B4GALT3
 
 
hsa-miR-27a-3p
 
 
-0.41
 
 
24.3
 
 
 
 
B4GALT5
 
 
hsa-miR-152-3p
 
 
-0.44
 
 
35.5
 
 
 
 
BAG5
 
 
hsa-miR-22-3p
 
 
-0.318
 
 
20.5
 
 
 
 
BAZ1B
 
 
hsa-miR-10a-5p
 
 
-0.309
 
 
16.9
 
 
 
 
BAZ2A
 
 
hsa-miR-100-5p
 
 
-0.29
 
 
29.0
 
 
 
 
BAZ2A
 
 
hsa-miR-100-5p
 
 
-0.421
 
 
29.0
 
 
 
 
BBX
 
 
hsa-miR-10a-5p
 
 
-0.305
 
 
12.1
 
 
 
 
BCL3
 
 
hsa-miR-27a-3p
 
 
-0.413
 
 
74.9
 
 
 
 
BEX3
 
 
hsa-miR-27a-3p
 
 
-0.454
 
 
76.3
 
 
 
 
BHLHE40
 
 
hsa-miR-181a-5p
 
 
-0.408
 
 
372.4
 
 
 
 
BIN1
 
 
hsa-miR-22-3p
 
 
-0.346
 
 
31.5
 
 
 
 
BLOC1S1
 
 
hsa-miR-22-3p
 
 
-0.346
 
 
175.9
 
 
 
 
BLOC1S6
 
 
hsa-miR-181a-5p
 
 
-0.393
 
 
19.8
 
 
 
 
BLTP1
 
 
hsa-miR-27a-3p
 
 
-0.426
 
 
10.8
 
 
 
 
BMI1
 
 
hsa-miR-27a-3p
 
 
-0.322
 
 
20.6
 
 
 
 
BTAF1
 
 
hsa-miR-152-3p
 
 
-0.401
 
 
14.2
 
 
 
 
BTG2
 
 
hsa-miR-27a-3p
 
 
-0.339
 
 
294.8
 
 
 
 
BZW1
 
 
hsa-miR-27a-3p
 
 
-0.337
 
 
113.4
 
 
 
 
C1GALT1
 
 
hsa-miR-152-3p
 
 
-0.487
 
 
22.4
 
 
 
 
C1orf35
 
 
hsa-miR-152-3p
 
 
-0.511
 
 
21.2
 
 
 
 
C5orf24
 
 
hsa-miR-22-3p
 
 
-0.493
 
 
19.7
 
 
 
 
C6orf120
 
 
hsa-miR-34a-5p
 
 
-0.331
 
 
10.4
 
 
 
 
C6orf62
 
 
hsa-miR-181a-5p
 
 
-0.334
 
 
60.3
 
 
 
 
CACNB3
 
 
hsa-miR-34a-5p
 
 
-0.426
 
 
21.0
 
 
 
 
CACYBP
 
 
hsa-miR-27a-3p
 
 
-0.367
 
 
23.4
 
 
 
 
CADM4
 
 
hsa-miR-34a-5p
 
 
-0.365
 
 
19.8
 
 
 
 
CAMK2N1
 
 
hsa-miR-22-3p
 
 
-0.302
 
 
27.0
 
 
 
 
CAND1
 
 
hsa-miR-27a-3p
 
 
-0.318
 
 
21.3
 
 
 
 
CAND1
 
 
hsa-miR-152-3p
 
 
-0.44
 
 
21.3
 
 
 
 
CANX
 
 
hsa-miR-152-3p
 
 
-0.434
 
 
94.4
 
 
 
 
CAPN1
 
 
hsa-miR-22-3p
 
 
-0.307
 
 
267.3
 
 
 
 
CAPZA1
 
 
hsa-miR-27a-3p
 
 
-0.325
 
 
119.9
 
 
 
 
CASP2
 
 
hsa-miR-34a-5p
 
 
-0.382
 
 
14.9
 
 
 
 
CAV2
 
 
hsa-miR-152-3p
 
 
-0.32
 
 
40.8
 
 
 
 
CBX4
 
 
hsa-miR-181a-5p
 
 
-0.354
 
 
19.6
 
 
 
 
CCDC47
 
 
hsa-miR-22-3p
 
 
-0.604
 
 
47.6
 
 
 
 
CCDC6
 
 
hsa-miR-152-3p
 
 
-0.511
 
 
32.0
 
 
 
 
 
 
 fallopian tube 
 
 
 
 
target_symbol
 
 
mature_mirna_id
 
 
score
 
 
nTPM
 
 
 
 
 
 
AAGAB
 
 
hsa-miR-34a-5p
 
 
-0.319
 
 
15.5
 
 
 
 
ABCB7
 
 
hsa-miR-152-3p
 
 
-0.515
 
 
11.3
 
 
 
 
ABCD3
 
 
hsa-miR-152-3p
 
 
-0.388
 
 
15.8
 
 
 
 
ABHD17C
 
 
hsa-miR-27a-3p
 
 
-0.435
 
 
10.3
 
 
 
 
ACBD3
 
 
hsa-miR-34a-5p
 
 
-0.415
 
 
16.8
 
 
 
 
ACSL1
 
 
hsa-miR-34a-5p
 
 
-0.306
 
 
34.0
 
 
 
 
ACSL4
 
 
hsa-miR-34a-5p
 
 
-0.372
 
 
12.4
 
 
 
 
ACTA2
 
 
hsa-miR-27a-3p
 
 
-0.577
 
 
3776.0
 
 
 
 
ACVR1
 
 
hsa-miR-152-3p
 
 
-0.397
 
 
13.2
 
 
 
 
ADAM10
 
 
hsa-miR-152-3p
 
 
-0.348
 
 
13.1
 
 
 
 
ADGRA3
 
 
hsa-miR-27a-3p
 
 
-0.296
 
 
20.3
 
 
 
 
ADGRF5
 
 
hsa-miR-152-3p
 
 
-0.354
 
 
13.6
 
 
 
 
ADIPOR2
 
 
hsa-miR-34a-5p
 
 
-0.33
 
 
23.2
 
 
 
 
ADK
 
 
hsa-miR-34a-5p
 
 
-0.374
 
 
15.5
 
 
 
 
AFG3L2
 
 
hsa-miR-181a-5p
 
 
-0.491
 
 
12.9
 
 
 
 
AGFG1
 
 
hsa-miR-152-3p
 
 
-0.332
 
 
23.3
 
 
 
 
AGPAT3
 
 
hsa-miR-27a-3p
 
 
-0.29
 
 
11.6
 
 
 
 
AGTRAP
 
 
hsa-miR-34a-5p
 
 
-0.328
 
 
45.4
 
 
 
 
AHDC1
 
 
hsa-miR-152-3p
 
 
-0.413
 
 
19.8
 
 
 
 
AIDA
 
 
hsa-miR-22-3p
 
 
-0.316
 
 
29.5
 
 
 
 
AKAP1
 
 
hsa-miR-152-3p
 
 
-0.381
 
 
33.9
 
 
 
 
AKIRIN1
 
 
hsa-miR-27a-3p
 
 
-0.368
 
 
23.3
 
 
 
 
AKT3
 
 
hsa-miR-22-3p
 
 
-0.451
 
 
15.1
 
 
 
 
ALCAM
 
 
hsa-miR-34a-5p
 
 
-0.303
 
 
46.2
 
 
 
 
ALCAM
 
 
hsa-miR-152-3p
 
 
-0.347
 
 
46.2
 
 
 
 
ALDOA
 
 
hsa-miR-34a-5p
 
 
-0.655
 
 
522.8
 
 
 
 
ANK3
 
 
hsa-miR-34a-5p
 
 
-0.29
 
 
17.4
 
 
 
 
ANK3
 
 
hsa-miR-34a-5p
 
 
-0.347
 
 
17.4
 
 
 
 
ANKRD13A
 
 
hsa-miR-22-3p
 
 
-0.478
 
 
11.6
 
 
 
 
ANKRD40
 
 
hsa-miR-27a-3p
 
 
-0.35
 
 
18.4
 
 
 
 
ANP32B
 
 
hsa-miR-34a-5p
 
 
-0.29
 
 
117.2
 
 
 
 
AP3S2
 
 
hsa-miR-34a-5p
 
 
-0.413
 
 
16.2
 
 
 
 
APH1A
 
 
hsa-miR-34a-5p
 
 
-0.304
 
 
78.3
 
 
 
 
APOO
 
 
hsa-miR-181a-5p
 
 
-0.5
 
 
19.5
 
 
 
 
ARF4
 
 
hsa-miR-152-3p
 
 
-0.487
 
 
79.6
 
 
 
 
ARF6
 
 
hsa-miR-181a-5p
 
 
-0.33
 
 
26.6
 
 
 
 
ARFGEF1
 
 
hsa-miR-152-3p
 
 
-0.306
 
 
11.1
 
 
 
 
ARFIP1
 
 
hsa-miR-320d
 
 
-0.339
 
 
17.4
 
 
 
 
ARFIP1
 
 
hsa-miR-152-3p
 
 
-0.474
 
 
17.4
 
 
 
 
ARHGAP1
 
 
hsa-miR-34a-5p
 
 
-0.369
 
 
71.5
 
 
 
 
ARHGAP21
 
 
hsa-miR-152-3p
 
 
-0.287
 
 
16.4
 
 
 
 
ARHGAP26
 
 
hsa-miR-34a-5p
 
 
-0.384
 
 
14.6
 
 
 
 
ARL6IP1
 
 
hsa-miR-152-3p
 
 
-0.579
 
 
81.6
 
 
 
 
ARL8B
 
 
hsa-miR-152-3p
 
 
-0.337
 
 
29.3
 
 
 
 
ARL8B
 
 
hsa-miR-320d
 
 
-0.376
 
 
29.3
 
 
 
 
ARPC5
 
 
hsa-miR-22-3p
 
 
-0.679
 
 
50.4
 
 
 
 
ARPP19
 
 
hsa-miR-320d
 
 
-0.325
 
 
31.4
 
 
 
 
ARPP19
 
 
hsa-miR-34a-5p
 
 
-0.497
 
 
31.4
 
 
 
 
ARRB1
 
 
hsa-miR-22-3p
 
 
-0.616
 
 
15.0
 
 
 
 
ARRDC3
 
 
hsa-miR-152-3p
 
 
-0.305
 
 
23.4
 
 
 
 
ASAH1
 
 
hsa-miR-27a-3p
 
 
-0.398
 
 
66.2
 
 
 
 
ASH2L
 
 
hsa-miR-320d
 
 
-0.287
 
 
19.1
 
 
 
 
ASPH
 
 
hsa-miR-152-3p
 
 
-0.391
 
 
45.0
 
 
 
 
ATG4B
 
 
hsa-miR-34a-5p
 
 
-0.58
 
 
19.3
 
 
 
 
ATMIN
 
 
hsa-miR-34a-5p
 
 
-0.409
 
 
10.8
 
 
 
 
ATP1A2
 
 
hsa-miR-34a-5p
 
 
-0.306
 
 
15.4
 
 
 
 
ATP2B4
 
 
hsa-miR-152-3p
 
 
-0.337
 
 
45.7
 
 
 
 
ATP5MC3
 
 
hsa-miR-27a-3p
 
 
-0.416
 
 
110.4
 
 
 
 
ATP6AP2
 
 
hsa-miR-152-3p
 
 
-0.592
 
 
71.1
 
 
 
 
ATP6AP2
 
 
hsa-miR-152-3p
 
 
-0.668
 
 
71.1
 
 
 
 
ATXN10
 
 
hsa-miR-27a-3p
 
 
-0.295
 
 
52.8
 
 
 
 
ATXN2
 
 
hsa-miR-10a-5p
 
 
-0.362
 
 
19.4
 
 
 
 
AXL
 
 
hsa-miR-34a-5p
 
 
-0.512
 
 
23.6
 
 
 
 
B4GALT2
 
 
hsa-miR-34a-5p
 
 
-0.314
 
 
18.3
 
 
 
 
B4GALT3
 
 
hsa-miR-27a-3p
 
 
-0.41
 
 
17.6
 
 
 
 
B4GALT5
 
 
hsa-miR-152-3p
 
 
-0.44
 
 
17.2
 
 
 
 
BAG5
 
 
hsa-miR-22-3p
 
 
-0.318
 
 
15.0
 
 
 
 
BAZ1B
 
 
hsa-miR-10a-5p
 
 
-0.309
 
 
18.3
 
 
 
 
BAZ2A
 
 
hsa-miR-100-5p
 
 
-0.29
 
 
23.4
 
 
 
 
BAZ2A
 
 
hsa-miR-100-5p
 
 
-0.421
 
 
23.4
 
 
 
 
BBX
 
 
hsa-miR-10a-5p
 
 
-0.305
 
 
15.8
 
 
 
 
BCL2
 
 
hsa-miR-34a-5p
 
 
-0.289
 
 
11.1
 
 
 
 
BCL3
 
 
hsa-miR-27a-3p
 
 
-0.413
 
 
34.8
 
 
 
 
BEND7
 
 
hsa-miR-27a-3p
 
 
-0.304
 
 
12.2
 
 
 
 
BEX3
 
 
hsa-miR-27a-3p
 
 
-0.454
 
 
172.5
 
 
 
 
BHLHE40
 
 
hsa-miR-181a-5p
 
 
-0.408
 
 
77.8
 
 
 
 
BHLHE41
 
 
hsa-miR-152-3p
 
 
-0.442
 
 
42.8
 
 
 
 
BIN1
 
 
hsa-miR-22-3p
 
 
-0.346
 
 
50.2
 
 
 
 
BLOC1S1
 
 
hsa-miR-22-3p
 
 
-0.346
 
 
129.0
 
 
 
 
BLOC1S6
 
 
hsa-miR-181a-5p
 
 
-0.393
 
 
17.1
 
 
 
 
BLTP1
 
 
hsa-miR-27a-3p
 
 
-0.426
 
 
20.5
 
 
 
 
BMI1
 
 
hsa-miR-27a-3p
 
 
-0.322
 
 
21.2
 
 
 
 
BTAF1
 
 
hsa-miR-152-3p
 
 
-0.401
 
 
20.1
 
 
 
 
BTBD3
 
 
hsa-miR-152-3p
 
 
-0.495
 
 
14.4
 
 
 
 
BTG2
 
 
hsa-miR-27a-3p
 
 
-0.339
 
 
240.6
 
 
 
 
BZW1
 
 
hsa-miR-27a-3p
 
 
-0.337
 
 
50.2
 
 
 
 
C1GALT1
 
 
hsa-miR-152-3p
 
 
-0.487
 
 
15.3
 
 
 
 
C1orf35
 
 
hsa-miR-152-3p
 
 
-0.511
 
 
15.3
 
 
 
 
C5orf24
 
 
hsa-miR-22-3p
 
 
-0.493
 
 
15.5
 
 
 
 
C6orf62
 
 
hsa-miR-181a-5p
 
 
-0.334
 
 
72.1
 
 
 
 
C8orf88
 
 
hsa-miR-27a-3p
 
 
-0.33
 
 
11.0
 
 
 
 
CACNB3
 
 
hsa-miR-34a-5p
 
 
-0.426
 
 
18.4
 
 
 
 
CACYBP
 
 
hsa-miR-27a-3p
 
 
-0.367
 
 
17.7
 
 
 
 
CAMK2N1
 
 
hsa-miR-22-3p
 
 
-0.302
 
 
18.8
 
 
 
 
CAND1
 
 
hsa-miR-27a-3p
 
 
-0.318
 
 
18.1
 
 
 
 
CAND1
 
 
hsa-miR-152-3p
 
 
-0.44
 
 
18.1
 
 
 
 
CANX
 
 
hsa-miR-152-3p
 
 
-0.434
 
 
92.6
 
 
 
 
CAPN1
 
 
hsa-miR-22-3p
 
 
-0.307
 
 
86.8
 
 
 
 
CAPZA1
 
 
hsa-miR-27a-3p
 
 
-0.325
 
 
40.4
 
 
 
 
CAV2
 
 
hsa-miR-152-3p
 
 
-0.32
 
 
29.3
 
 
 
 
 
 
 gallbladder 
 
 
 
 
target_symbol
 
 
mature_mirna_id
 
 
score
 
 
nTPM
 
 
 
 
 
 
AAGAB
 
 
hsa-miR-34a-5p
 
 
-0.319
 
 
17.3
 
 
 
 
ABCD3
 
 
hsa-miR-152-3p
 
 
-0.388
 
 
25.2
 
 
 
 
ABHD5
 
 
hsa-miR-27a-3p
 
 
-0.407
 
 
10.2
 
 
 
 
ACBD3
 
 
hsa-miR-34a-5p
 
 
-0.415
 
 
22.0
 
 
 
 
ACSL1
 
 
hsa-miR-34a-5p
 
 
-0.306
 
 
32.1
 
 
 
 
ACSL4
 
 
hsa-miR-34a-5p
 
 
-0.372
 
 
25.8
 
 
 
 
ACTA2
 
 
hsa-miR-27a-3p
 
 
-0.577
 
 
849.5
 
 
 
 
ACVR1
 
 
hsa-miR-152-3p
 
 
-0.397
 
 
14.1
 
 
 
 
ADAM10
 
 
hsa-miR-152-3p
 
 
-0.348
 
 
17.7
 
 
 
 
ADGRF5
 
 
hsa-miR-152-3p
 
 
-0.354
 
 
16.1
 
 
 
 
ADK
 
 
hsa-miR-34a-5p
 
 
-0.374
 
 
10.7
 
 
 
 
AFG3L2
 
 
hsa-miR-181a-5p
 
 
-0.491
 
 
10.6
 
 
 
 
AGFG1
 
 
hsa-miR-152-3p
 
 
-0.332
 
 
36.5
 
 
 
 
AGPAT3
 
 
hsa-miR-27a-3p
 
 
-0.29
 
 
12.9
 
 
 
 
AGTRAP
 
 
hsa-miR-34a-5p
 
 
-0.328
 
 
23.6
 
 
 
 
AHCYL2
 
 
hsa-miR-34a-5p
 
 
-0.359
 
 
52.3
 
 
 
 
AIDA
 
 
hsa-miR-22-3p
 
 
-0.316
 
 
38.3
 
 
 
 
AKAP1
 
 
hsa-miR-152-3p
 
 
-0.381
 
 
16.8
 
 
 
 
AKIRIN1
 
 
hsa-miR-27a-3p
 
 
-0.368
 
 
39.1
 
 
 
 
ALCAM
 
 
hsa-miR-34a-5p
 
 
-0.303
 
 
21.9
 
 
 
 
ALCAM
 
 
hsa-miR-152-3p
 
 
-0.347
 
 
21.9
 
 
 
 
ALDOA
 
 
hsa-miR-34a-5p
 
 
-0.655
 
 
394.6
 
 
 
 
ANK2
 
 
hsa-miR-34a-5p
 
 
-0.317
 
 
18.1
 
 
 
 
ANK3
 
 
hsa-miR-34a-5p
 
 
-0.29
 
 
19.8
 
 
 
 
ANK3
 
 
hsa-miR-34a-5p
 
 
-0.347
 
 
19.8
 
 
 
 
ANKRD13A
 
 
hsa-miR-22-3p
 
 
-0.478
 
 
21.4
 
 
 
 
ANKRD40
 
 
hsa-miR-27a-3p
 
 
-0.35
 
 
15.1
 
 
 
 
ANP32B
 
 
hsa-miR-34a-5p
 
 
-0.29
 
 
56.6
 
 
 
 
AP1AR
 
 
hsa-miR-100-5p
 
 
-0.804
 
 
12.0
 
 
 
 
AP3S2
 
 
hsa-miR-34a-5p
 
 
-0.413
 
 
16.4
 
 
 
 
APH1A
 
 
hsa-miR-34a-5p
 
 
-0.304
 
 
64.7
 
 
 
 
APPBP2
 
 
hsa-miR-27a-3p
 
 
-0.378
 
 
10.9
 
 
 
 
AREG
 
 
hsa-miR-34a-5p
 
 
-0.62
 
 
69.2
 
 
 
 
ARF4
 
 
hsa-miR-152-3p
 
 
-0.487
 
 
145.1
 
 
 
 
ARF6
 
 
hsa-miR-181a-5p
 
 
-0.33
 
 
30.6
 
 
 
 
ARFGEF1
 
 
hsa-miR-152-3p
 
 
-0.306
 
 
11.7
 
 
 
 
ARFIP1
 
 
hsa-miR-320d
 
 
-0.339
 
 
18.7
 
 
 
 
ARFIP1
 
 
hsa-miR-152-3p
 
 
-0.474
 
 
18.7
 
 
 
 
ARHGAP1
 
 
hsa-miR-34a-5p
 
 
-0.369
 
 
47.5
 
 
 
 
ARHGAP21
 
 
hsa-miR-152-3p
 
 
-0.287
 
 
16.7
 
 
 
 
ARHGAP26
 
 
hsa-miR-34a-5p
 
 
-0.384
 
 
13.3
 
 
 
 
ARL6IP1
 
 
hsa-miR-152-3p
 
 
-0.579
 
 
123.8
 
 
 
 
ARL8B
 
 
hsa-miR-152-3p
 
 
-0.337
 
 
36.3
 
 
 
 
ARL8B
 
 
hsa-miR-320d
 
 
-0.376
 
 
36.3
 
 
 
 
ARPC5
 
 
hsa-miR-22-3p
 
 
-0.679
 
 
49.6
 
 
 
 
ARPP19
 
 
hsa-miR-320d
 
 
-0.325
 
 
36.7
 
 
 
 
ARPP19
 
 
hsa-miR-34a-5p
 
 
-0.497
 
 
36.7
 
 
 
 
ARRB1
 
 
hsa-miR-22-3p
 
 
-0.616
 
 
12.1
 
 
 
 
ARRDC3
 
 
hsa-miR-152-3p
 
 
-0.305
 
 
36.6
 
 
 
 
ASAH1
 
 
hsa-miR-27a-3p
 
 
-0.398
 
 
92.8
 
 
 
 
ASH2L
 
 
hsa-miR-320d
 
 
-0.287
 
 
16.3
 
 
 
 
ASPH
 
 
hsa-miR-152-3p
 
 
-0.391
 
 
56.5
 
 
 
 
ATMIN
 
 
hsa-miR-34a-5p
 
 
-0.409
 
 
10.8
 
 
 
 
ATP2B4
 
 
hsa-miR-152-3p
 
 
-0.337
 
 
23.7
 
 
 
 
ATP5MC3
 
 
hsa-miR-27a-3p
 
 
-0.416
 
 
70.9
 
 
 
 
ATP6AP2
 
 
hsa-miR-152-3p
 
 
-0.592
 
 
74.7
 
 
 
 
ATP6AP2
 
 
hsa-miR-152-3p
 
 
-0.668
 
 
74.7
 
 
 
 
ATXN10
 
 
hsa-miR-27a-3p
 
 
-0.295
 
 
37.1
 
 
 
 
ATXN2
 
 
hsa-miR-10a-5p
 
 
-0.362
 
 
11.2
 
 
 
 
AXL
 
 
hsa-miR-34a-5p
 
 
-0.512
 
 
20.9
 
 
 
 
B3GNT7
 
 
hsa-miR-27a-3p
 
 
-0.355
 
 
13.2
 
 
 
 
B4GALT2
 
 
hsa-miR-34a-5p
 
 
-0.314
 
 
13.9
 
 
 
 
B4GALT3
 
 
hsa-miR-27a-3p
 
 
-0.41
 
 
13.5
 
 
 
 
B4GALT5
 
 
hsa-miR-152-3p
 
 
-0.44
 
 
29.5
 
 
 
 
BAG5
 
 
hsa-miR-22-3p
 
 
-0.318
 
 
12.1
 
 
 
 
BAZ1B
 
 
hsa-miR-10a-5p
 
 
-0.309
 
 
15.0
 
 
 
 
BAZ2A
 
 
hsa-miR-100-5p
 
 
-0.29
 
 
19.6
 
 
 
 
BAZ2A
 
 
hsa-miR-100-5p
 
 
-0.421
 
 
19.6
 
 
 
 
BBX
 
 
hsa-miR-10a-5p
 
 
-0.305
 
 
15.7
 
 
 
 
BCL2L11
 
 
hsa-miR-152-3p
 
 
-0.445
 
 
11.1
 
 
 
 
BCL2L11
 
 
hsa-miR-10a-5p
 
 
-0.483
 
 
11.1
 
 
 
 
BCL3
 
 
hsa-miR-27a-3p
 
 
-0.413
 
 
12.5
 
 
 
 
BEX3
 
 
hsa-miR-27a-3p
 
 
-0.454
 
 
72.3
 
 
 
 
BHLHE40
 
 
hsa-miR-181a-5p
 
 
-0.408
 
 
94.0
 
 
 
 
BHLHE41
 
 
hsa-miR-152-3p
 
 
-0.442
 
 
17.1
 
 
 
 
BICC1
 
 
hsa-miR-152-3p
 
 
-0.442
 
 
10.9
 
 
 
 
BIN1
 
 
hsa-miR-22-3p
 
 
-0.346
 
 
21.2
 
 
 
 
BLOC1S1
 
 
hsa-miR-22-3p
 
 
-0.346
 
 
74.6
 
 
 
 
BLOC1S6
 
 
hsa-miR-181a-5p
 
 
-0.393
 
 
24.8
 
 
 
 
BLTP1
 
 
hsa-miR-27a-3p
 
 
-0.426
 
 
12.0
 
 
 
 
BMI1
 
 
hsa-miR-27a-3p
 
 
-0.322
 
 
22.4
 
 
 
 
BMPR2
 
 
hsa-miR-100-5p
 
 
-0.355
 
 
12.7
 
 
 
 
BTAF1
 
 
hsa-miR-152-3p
 
 
-0.401
 
 
12.0
 
 
 
 
BTBD3
 
 
hsa-miR-152-3p
 
 
-0.495
 
 
12.5
 
 
 
 
BTG2
 
 
hsa-miR-27a-3p
 
 
-0.339
 
 
148.1
 
 
 
 
BZW1
 
 
hsa-miR-27a-3p
 
 
-0.337
 
 
87.2
 
 
 
 
C1GALT1
 
 
hsa-miR-152-3p
 
 
-0.487
 
 
15.6
 
 
 
 
C5orf24
 
 
hsa-miR-22-3p
 
 
-0.493
 
 
20.0
 
 
 
 
C6orf120
 
 
hsa-miR-34a-5p
 
 
-0.331
 
 
10.4
 
 
 
 
C6orf62
 
 
hsa-miR-181a-5p
 
 
-0.334
 
 
58.8
 
 
 
 
CACYBP
 
 
hsa-miR-27a-3p
 
 
-0.367
 
 
13.8
 
 
 
 
CAMK2N1
 
 
hsa-miR-22-3p
 
 
-0.302
 
 
25.7
 
 
 
 
CAND1
 
 
hsa-miR-27a-3p
 
 
-0.318
 
 
17.2
 
 
 
 
CAND1
 
 
hsa-miR-152-3p
 
 
-0.44
 
 
17.2
 
 
 
 
CAPN1
 
 
hsa-miR-22-3p
 
 
-0.307
 
 
52.1
 
 
 
 
CAPZA1
 
 
hsa-miR-27a-3p
 
 
-0.325
 
 
55.4
 
 
 
 
CAV2
 
 
hsa-miR-152-3p
 
 
-0.32
 
 
67.6
 
 
 
 
CCDC47
 
 
hsa-miR-22-3p
 
 
-0.604
 
 
44.0
 
 
 
 
CCDC6
 
 
hsa-miR-152-3p
 
 
-0.511
 
 
15.7
 
 
 
 
CCM2
 
 
hsa-miR-27a-3p
 
 
-0.335
 
 
10.8
 
 
 
 
 
 
 heart muscle 
 
 
 
 
target_symbol
 
 
mature_mirna_id
 
 
score
 
 
nTPM
 
 
 
 
 
 
AAGAB
 
 
hsa-miR-34a-5p
 
 
-0.319
 
 
14.8
 
 
 
 
ABCB7
 
 
hsa-miR-152-3p
 
 
-0.515
 
 
20.0
 
 
 
 
ABCD3
 
 
hsa-miR-152-3p
 
 
-0.388
 
 
24.0
 
 
 
 
ABHD5
 
 
hsa-miR-27a-3p
 
 
-0.407
 
 
10.6
 
 
 
 
ACBD3
 
 
hsa-miR-34a-5p
 
 
-0.415
 
 
20.1
 
 
 
 
ACSL1
 
 
hsa-miR-34a-5p
 
 
-0.306
 
 
161.9
 
 
 
 
ACSL4
 
 
hsa-miR-34a-5p
 
 
-0.372
 
 
26.9
 
 
 
 
ACTA2
 
 
hsa-miR-27a-3p
 
 
-0.577
 
 
1015.4
 
 
 
 
ACVR1
 
 
hsa-miR-152-3p
 
 
-0.397
 
 
14.3
 
 
 
 
ADAM10
 
 
hsa-miR-152-3p
 
 
-0.348
 
 
11.3
 
 
 
 
ADGRA3
 
 
hsa-miR-27a-3p
 
 
-0.296
 
 
10.1
 
 
 
 
ADGRF5
 
 
hsa-miR-152-3p
 
 
-0.354
 
 
50.1
 
 
 
 
ADIPOR2
 
 
hsa-miR-34a-5p
 
 
-0.33
 
 
31.9
 
 
 
 
ADK
 
 
hsa-miR-34a-5p
 
 
-0.374
 
 
25.4
 
 
 
 
ADO
 
 
hsa-miR-34a-5p
 
 
-0.377
 
 
13.5
 
 
 
 
AFG3L2
 
 
hsa-miR-181a-5p
 
 
-0.491
 
 
48.7
 
 
 
 
AGFG1
 
 
hsa-miR-152-3p
 
 
-0.332
 
 
33.8
 
 
 
 
AGPAT3
 
 
hsa-miR-27a-3p
 
 
-0.29
 
 
25.4
 
 
 
 
AGTRAP
 
 
hsa-miR-34a-5p
 
 
-0.328
 
 
34.3
 
 
 
 
AHDC1
 
 
hsa-miR-152-3p
 
 
-0.413
 
 
17.4
 
 
 
 
AIDA
 
 
hsa-miR-22-3p
 
 
-0.316
 
 
42.8
 
 
 
 
AKAP1
 
 
hsa-miR-152-3p
 
 
-0.381
 
 
78.5
 
 
 
 
AKIRIN1
 
 
hsa-miR-27a-3p
 
 
-0.368
 
 
36.6
 
 
 
 
AKT3
 
 
hsa-miR-22-3p
 
 
-0.451
 
 
10.6
 
 
 
 
ALDOA
 
 
hsa-miR-34a-5p
 
 
-0.655
 
 
2567.3
 
 
 
 
ALPL
 
 
hsa-miR-10a-5p
 
 
-0.32
 
 
15.8
 
 
 
 
ANK2
 
 
hsa-miR-34a-5p
 
 
-0.317
 
 
73.9
 
 
 
 
ANK3
 
 
hsa-miR-34a-5p
 
 
-0.29
 
 
77.4
 
 
 
 
ANK3
 
 
hsa-miR-34a-5p
 
 
-0.347
 
 
77.4
 
 
 
 
ANKRD13A
 
 
hsa-miR-22-3p
 
 
-0.478
 
 
10.0
 
 
 
 
ANKRD40
 
 
hsa-miR-27a-3p
 
 
-0.35
 
 
25.8
 
 
 
 
ANP32B
 
 
hsa-miR-34a-5p
 
 
-0.29
 
 
87.4
 
 
 
 
AP3S2
 
 
hsa-miR-34a-5p
 
 
-0.413
 
 
28.7
 
 
 
 
APH1A
 
 
hsa-miR-34a-5p
 
 
-0.304
 
 
68.4
 
 
 
 
APOO
 
 
hsa-miR-181a-5p
 
 
-0.5
 
 
61.9
 
 
 
 
AREG
 
 
hsa-miR-34a-5p
 
 
-0.62
 
 
20.3
 
 
 
 
ARF4
 
 
hsa-miR-152-3p
 
 
-0.487
 
 
106.8
 
 
 
 
ARF6
 
 
hsa-miR-181a-5p
 
 
-0.33
 
 
15.7
 
 
 
 
ARHGAP1
 
 
hsa-miR-34a-5p
 
 
-0.369
 
 
105.1
 
 
 
 
ARHGAP21
 
 
hsa-miR-152-3p
 
 
-0.287
 
 
19.7
 
 
 
 
ARHGAP26
 
 
hsa-miR-34a-5p
 
 
-0.384
 
 
14.8
 
 
 
 
ARL6IP1
 
 
hsa-miR-152-3p
 
 
-0.579
 
 
30.1
 
 
 
 
ARL8B
 
 
hsa-miR-152-3p
 
 
-0.337
 
 
51.7
 
 
 
 
ARL8B
 
 
hsa-miR-320d
 
 
-0.376
 
 
51.7
 
 
 
 
ARPC5
 
 
hsa-miR-22-3p
 
 
-0.679
 
 
33.5
 
 
 
 
ARPIN-AP3S2
 
 
hsa-miR-34a-5p
 
 
-0.348
 
 
17.1
 
 
 
 
ARPP19
 
 
hsa-miR-320d
 
 
-0.325
 
 
76.2
 
 
 
 
ARPP19
 
 
hsa-miR-34a-5p
 
 
-0.497
 
 
76.2
 
 
 
 
ARRB1
 
 
hsa-miR-22-3p
 
 
-0.616
 
 
16.5
 
 
 
 
ARRDC3
 
 
hsa-miR-152-3p
 
 
-0.305
 
 
23.2
 
 
 
 
AS3MT
 
 
hsa-miR-28-5p
 
 
-0.479
 
 
24.0
 
 
 
 
ASAH1
 
 
hsa-miR-27a-3p
 
 
-0.398
 
 
537.2
 
 
 
 
ASB1
 
 
hsa-miR-34a-5p
 
 
-0.406
 
 
15.2
 
 
 
 
ASB11
 
 
hsa-miR-27a-3p
 
 
-0.353
 
 
31.3
 
 
 
 
ASH2L
 
 
hsa-miR-320d
 
 
-0.287
 
 
20.0
 
 
 
 
ASPH
 
 
hsa-miR-152-3p
 
 
-0.391
 
 
100.1
 
 
 
 
ATG4B
 
 
hsa-miR-34a-5p
 
 
-0.58
 
 
20.3
 
 
 
 
ATMIN
 
 
hsa-miR-34a-5p
 
 
-0.409
 
 
13.3
 
 
 
 
ATP1A2
 
 
hsa-miR-34a-5p
 
 
-0.306
 
 
136.6
 
 
 
 
ATP2B4
 
 
hsa-miR-152-3p
 
 
-0.337
 
 
56.1
 
 
 
 
ATP5MC3
 
 
hsa-miR-27a-3p
 
 
-0.416
 
 
1149.4
 
 
 
 
ATP6AP2
 
 
hsa-miR-152-3p
 
 
-0.592
 
 
51.6
 
 
 
 
ATP6AP2
 
 
hsa-miR-152-3p
 
 
-0.668
 
 
51.6
 
 
 
 
ATXN10
 
 
hsa-miR-27a-3p
 
 
-0.295
 
 
69.7
 
 
 
 
ATXN2
 
 
hsa-miR-10a-5p
 
 
-0.362
 
 
22.3
 
 
 
 
AXL
 
 
hsa-miR-34a-5p
 
 
-0.512
 
 
25.8
 
 
 
 
B4GALT2
 
 
hsa-miR-34a-5p
 
 
-0.314
 
 
30.9
 
 
 
 
B4GALT3
 
 
hsa-miR-27a-3p
 
 
-0.41
 
 
23.6
 
 
 
 
B4GALT5
 
 
hsa-miR-152-3p
 
 
-0.44
 
 
19.2
 
 
 
 
BAG5
 
 
hsa-miR-22-3p
 
 
-0.318
 
 
12.4
 
 
 
 
BAZ1B
 
 
hsa-miR-10a-5p
 
 
-0.309
 
 
18.3
 
 
 
 
BAZ2A
 
 
hsa-miR-100-5p
 
 
-0.29
 
 
19.8
 
 
 
 
BAZ2A
 
 
hsa-miR-100-5p
 
 
-0.421
 
 
19.8
 
 
 
 
BBX
 
 
hsa-miR-10a-5p
 
 
-0.305
 
 
15.4
 
 
 
 
BCL2L13
 
 
hsa-miR-34a-5p
 
 
-0.431
 
 
14.4
 
 
 
 
BCL3
 
 
hsa-miR-27a-3p
 
 
-0.413
 
 
38.6
 
 
 
 
BEX3
 
 
hsa-miR-27a-3p
 
 
-0.454
 
 
254.6
 
 
 
 
BHLHE40
 
 
hsa-miR-181a-5p
 
 
-0.408
 
 
166.2
 
 
 
 
BHLHE41
 
 
hsa-miR-152-3p
 
 
-0.442
 
 
18.7
 
 
 
 
BIN1
 
 
hsa-miR-22-3p
 
 
-0.346
 
 
15.7
 
 
 
 
BLOC1S1
 
 
hsa-miR-22-3p
 
 
-0.346
 
 
165.5
 
 
 
 
BLOC1S6
 
 
hsa-miR-181a-5p
 
 
-0.393
 
 
23.5
 
 
 
 
BLTP1
 
 
hsa-miR-27a-3p
 
 
-0.426
 
 
14.3
 
 
 
 
BMI1
 
 
hsa-miR-27a-3p
 
 
-0.322
 
 
19.1
 
 
 
 
BMPR2
 
 
hsa-miR-100-5p
 
 
-0.355
 
 
16.2
 
 
 
 
BMT2
 
 
hsa-miR-152-3p
 
 
-0.4
 
 
11.9
 
 
 
 
BRSK1
 
 
hsa-miR-27a-3p
 
 
-0.372
 
 
12.3
 
 
 
 
BTAF1
 
 
hsa-miR-152-3p
 
 
-0.401
 
 
11.8
 
 
 
 
BTBD3
 
 
hsa-miR-152-3p
 
 
-0.495
 
 
13.0
 
 
 
 
BTG2
 
 
hsa-miR-27a-3p
 
 
-0.339
 
 
256.5
 
 
 
 
BZW1
 
 
hsa-miR-27a-3p
 
 
-0.337
 
 
53.4
 
 
 
 
C1GALT1
 
 
hsa-miR-152-3p
 
 
-0.487
 
 
11.8
 
 
 
 
C1orf35
 
 
hsa-miR-152-3p
 
 
-0.511
 
 
11.1
 
 
 
 
C5orf24
 
 
hsa-miR-22-3p
 
 
-0.493
 
 
18.3
 
 
 
 
C6orf62
 
 
hsa-miR-181a-5p
 
 
-0.334
 
 
55.9
 
 
 
 
C8orf88
 
 
hsa-miR-27a-3p
 
 
-0.33
 
 
33.6
 
 
 
 
CABP1
 
 
hsa-miR-27a-3p
 
 
-0.343
 
 
12.6
 
 
 
 
CACYBP
 
 
hsa-miR-27a-3p
 
 
-0.367
 
 
25.7
 
 
 
 
CADM4
 
 
hsa-miR-34a-5p
 
 
-0.365
 
 
11.5
 
 
 
 
CAMK2N1
 
 
hsa-miR-22-3p
 
 
-0.302
 
 
21.2
 
 
 
 
 
 
 hippocampal formation 
 
 
 
 
target_symbol
 
 
mature_mirna_id
 
 
score
 
 
nTPM
 
 
 
 
 
 
AAGAB
 
 
hsa-miR-34a-5p
 
 
-0.319
 
 
10.0
 
 
 
 
ABCD3
 
 
hsa-miR-152-3p
 
 
-0.388
 
 
15.1
 
 
 
 
ABHD17C
 
 
hsa-miR-27a-3p
 
 
-0.435
 
 
14.4
 
 
 
 
ACSL1
 
 
hsa-miR-34a-5p
 
 
-0.306
 
 
39.9
 
 
 
 
ACSL4
 
 
hsa-miR-34a-5p
 
 
-0.372
 
 
12.3
 
 
 
 
ACTA2
 
 
hsa-miR-27a-3p
 
 
-0.577
 
 
32.3
 
 
 
 
ADAP1
 
 
hsa-miR-27a-3p
 
 
-0.302
 
 
123.8
 
 
 
 
ADCY2
 
 
hsa-miR-152-3p
 
 
-0.299
 
 
18.5
 
 
 
 
ADD2
 
 
hsa-miR-34a-5p
 
 
-0.305
 
 
12.8
 
 
 
 
ADGRA3
 
 
hsa-miR-27a-3p
 
 
-0.296
 
 
12.8
 
 
 
 
ADGRB3
 
 
hsa-miR-152-3p
 
 
-0.516
 
 
12.8
 
 
 
 
ADGRF5
 
 
hsa-miR-152-3p
 
 
-0.354
 
 
10.4
 
 
 
 
ADIPOR2
 
 
hsa-miR-34a-5p
 
 
-0.33
 
 
57.6
 
 
 
 
ADK
 
 
hsa-miR-34a-5p
 
 
-0.374
 
 
13.3
 
 
 
 
ADO
 
 
hsa-miR-34a-5p
 
 
-0.377
 
 
11.3
 
 
 
 
AGFG1
 
 
hsa-miR-152-3p
 
 
-0.332
 
 
29.8
 
 
 
 
AGPAT3
 
 
hsa-miR-27a-3p
 
 
-0.29
 
 
37.1
 
 
 
 
AGTRAP
 
 
hsa-miR-34a-5p
 
 
-0.328
 
 
20.9
 
 
 
 
AHCYL2
 
 
hsa-miR-34a-5p
 
 
-0.359
 
 
15.5
 
 
 
 
AIDA
 
 
hsa-miR-22-3p
 
 
-0.316
 
 
22.9
 
 
 
 
AKAP1
 
 
hsa-miR-152-3p
 
 
-0.381
 
 
17.0
 
 
 
 
AKIRIN1
 
 
hsa-miR-27a-3p
 
 
-0.368
 
 
35.2
 
 
 
 
AKT3
 
 
hsa-miR-22-3p
 
 
-0.451
 
 
17.7
 
 
 
 
ALCAM
 
 
hsa-miR-34a-5p
 
 
-0.303
 
 
33.8
 
 
 
 
ALCAM
 
 
hsa-miR-152-3p
 
 
-0.347
 
 
33.8
 
 
 
 
ALDOA
 
 
hsa-miR-34a-5p
 
 
-0.655
 
 
777.3
 
 
 
 
ALPL
 
 
hsa-miR-10a-5p
 
 
-0.32
 
 
13.7
 
 
 
 
AMER2
 
 
hsa-miR-34a-5p
 
 
-0.48
 
 
32.9
 
 
 
 
ANGEL2
 
 
hsa-miR-22-3p
 
 
-0.601
 
 
11.0
 
 
 
 
ANK2
 
 
hsa-miR-34a-5p
 
 
-0.317
 
 
18.2
 
 
 
 
ANK3
 
 
hsa-miR-34a-5p
 
 
-0.29
 
 
51.4
 
 
 
 
ANK3
 
 
hsa-miR-34a-5p
 
 
-0.347
 
 
51.4
 
 
 
 
ANKRD40
 
 
hsa-miR-27a-3p
 
 
-0.35
 
 
105.0
 
 
 
 
ANP32B
 
 
hsa-miR-34a-5p
 
 
-0.29
 
 
139.9
 
 
 
 
AP3S2
 
 
hsa-miR-34a-5p
 
 
-0.413
 
 
12.8
 
 
 
 
APH1A
 
 
hsa-miR-34a-5p
 
 
-0.304
 
 
46.6
 
 
 
 
APOO
 
 
hsa-miR-181a-5p
 
 
-0.5
 
 
45.8
 
 
 
 
ARF4
 
 
hsa-miR-152-3p
 
 
-0.487
 
 
61.3
 
 
 
 
ARF6
 
 
hsa-miR-181a-5p
 
 
-0.33
 
 
10.0
 
 
 
 
ARHGAP1
 
 
hsa-miR-34a-5p
 
 
-0.369
 
 
48.9
 
 
 
 
ARHGAP21
 
 
hsa-miR-152-3p
 
 
-0.287
 
 
50.4
 
 
 
 
ARL6IP1
 
 
hsa-miR-152-3p
 
 
-0.579
 
 
196.8
 
 
 
 
ARL8B
 
 
hsa-miR-152-3p
 
 
-0.337
 
 
53.8
 
 
 
 
ARL8B
 
 
hsa-miR-320d
 
 
-0.376
 
 
53.8
 
 
 
 
ARPC5
 
 
hsa-miR-22-3p
 
 
-0.679
 
 
23.8
 
 
 
 
ARPIN-AP3S2
 
 
hsa-miR-34a-5p
 
 
-0.348
 
 
10.6
 
 
 
 
ARPP19
 
 
hsa-miR-320d
 
 
-0.325
 
 
120.9
 
 
 
 
ARPP19
 
 
hsa-miR-34a-5p
 
 
-0.497
 
 
120.9
 
 
 
 
ARRB1
 
 
hsa-miR-22-3p
 
 
-0.616
 
 
25.3
 
 
 
 
ARRDC3
 
 
hsa-miR-152-3p
 
 
-0.305
 
 
11.5
 
 
 
 
ASAH1
 
 
hsa-miR-27a-3p
 
 
-0.398
 
 
51.2
 
 
 
 
ASB1
 
 
hsa-miR-34a-5p
 
 
-0.406
 
 
15.5
 
 
 
 
ASH2L
 
 
hsa-miR-320d
 
 
-0.287
 
 
16.8
 
 
 
 
ASPH
 
 
hsa-miR-152-3p
 
 
-0.391
 
 
45.5
 
 
 
 
ATCAY
 
 
hsa-miR-10a-5p
 
 
-0.322
 
 
38.9
 
 
 
 
ATG4B
 
 
hsa-miR-34a-5p
 
 
-0.58
 
 
19.9
 
 
 
 
ATMIN
 
 
hsa-miR-34a-5p
 
 
-0.409
 
 
18.7
 
 
 
 
ATP1A2
 
 
hsa-miR-34a-5p
 
 
-0.306
 
 
260.8
 
 
 
 
ATP5MC3
 
 
hsa-miR-27a-3p
 
 
-0.416
 
 
174.9
 
 
 
 
ATP6AP2
 
 
hsa-miR-152-3p
 
 
-0.592
 
 
69.0
 
 
 
 
ATP6AP2
 
 
hsa-miR-152-3p
 
 
-0.668
 
 
69.0
 
 
 
 
ATXN10
 
 
hsa-miR-27a-3p
 
 
-0.295
 
 
53.4
 
 
 
 
ATXN2
 
 
hsa-miR-10a-5p
 
 
-0.362
 
 
22.7
 
 
 
 
B4GALT2
 
 
hsa-miR-34a-5p
 
 
-0.314
 
 
52.5
 
 
 
 
B4GALT3
 
 
hsa-miR-27a-3p
 
 
-0.41
 
 
13.8
 
 
 
 
B4GALT5
 
 
hsa-miR-152-3p
 
 
-0.44
 
 
10.8
 
 
 
 
BAG5
 
 
hsa-miR-22-3p
 
 
-0.318
 
 
10.9
 
 
 
 
BAZ1B
 
 
hsa-miR-10a-5p
 
 
-0.309
 
 
16.8
 
 
 
 
BAZ2A
 
 
hsa-miR-100-5p
 
 
-0.29
 
 
10.1
 
 
 
 
BAZ2A
 
 
hsa-miR-100-5p
 
 
-0.421
 
 
10.1
 
 
 
 
BBX
 
 
hsa-miR-10a-5p
 
 
-0.305
 
 
10.1
 
 
 
 
BEGAIN
 
 
hsa-miR-28-5p
 
 
-0.325
 
 
36.8
 
 
 
 
BEX3
 
 
hsa-miR-27a-3p
 
 
-0.454
 
 
531.1
 
 
 
 
BHLHE40
 
 
hsa-miR-181a-5p
 
 
-0.408
 
 
38.8
 
 
 
 
BHLHE41
 
 
hsa-miR-152-3p
 
 
-0.442
 
 
27.9
 
 
 
 
BIN1
 
 
hsa-miR-22-3p
 
 
-0.346
 
 
207.9
 
 
 
 
BLOC1S1
 
 
hsa-miR-22-3p
 
 
-0.346
 
 
227.5
 
 
 
 
BLOC1S6
 
 
hsa-miR-181a-5p
 
 
-0.393
 
 
18.3
 
 
 
 
BLTP1
 
 
hsa-miR-27a-3p
 
 
-0.426
 
 
17.7
 
 
 
 
BMI1
 
 
hsa-miR-27a-3p
 
 
-0.322
 
 
24.7
 
 
 
 
BRINP1
 
 
hsa-miR-34a-5p
 
 
-0.612
 
 
27.6
 
 
 
 
BRSK1
 
 
hsa-miR-27a-3p
 
 
-0.372
 
 
150.4
 
 
 
 
BTBD3
 
 
hsa-miR-152-3p
 
 
-0.495
 
 
49.8
 
 
 
 
BTG2
 
 
hsa-miR-27a-3p
 
 
-0.339
 
 
41.2
 
 
 
 
BZW1
 
 
hsa-miR-27a-3p
 
 
-0.337
 
 
34.8
 
 
 
 
C1GALT1
 
 
hsa-miR-152-3p
 
 
-0.487
 
 
11.6
 
 
 
 
C1orf35
 
 
hsa-miR-152-3p
 
 
-0.511
 
 
14.2
 
 
 
 
C5orf24
 
 
hsa-miR-22-3p
 
 
-0.493
 
 
18.9
 
 
 
 
C6orf62
 
 
hsa-miR-181a-5p
 
 
-0.334
 
 
69.8
 
 
 
 
C8orf88
 
 
hsa-miR-27a-3p
 
 
-0.33
 
 
16.6
 
 
 
 
CA10
 
 
hsa-miR-27a-3p
 
 
-0.464
 
 
23.0
 
 
 
 
CABP1
 
 
hsa-miR-27a-3p
 
 
-0.343
 
 
132.1
 
 
 
 
CABP7
 
 
hsa-miR-152-3p
 
 
-0.511
 
 
52.8
 
 
 
 
CACNB3
 
 
hsa-miR-34a-5p
 
 
-0.426
 
 
50.1
 
 
 
 
CACYBP
 
 
hsa-miR-27a-3p
 
 
-0.367
 
 
57.0
 
 
 
 
CADM4
 
 
hsa-miR-34a-5p
 
 
-0.365
 
 
125.9
 
 
 
 
CAMK2N1
 
 
hsa-miR-22-3p
 
 
-0.302
 
 
353.4
 
 
 
 
CAND1
 
 
hsa-miR-27a-3p
 
 
-0.318
 
 
16.6
 
 
 
 
CAND1
 
 
hsa-miR-152-3p
 
 
-0.44
 
 
16.6
 
 
 
 
CANX
 
 
hsa-miR-152-3p
 
 
-0.434
 
 
100.3
 
 
 
 
 
 
 hypothalamus 
 
 
 
 
target_symbol
 
 
mature_mirna_id
 
 
score
 
 
nTPM
 
 
 
 
 
 
AAGAB
 
 
hsa-miR-34a-5p
 
 
-0.319
 
 
14.4
 
 
 
 
ABCD3
 
 
hsa-miR-152-3p
 
 
-0.388
 
 
13.5
 
 
 
 
ABHD17C
 
 
hsa-miR-27a-3p
 
 
-0.435
 
 
11.1
 
 
 
 
ACSL1
 
 
hsa-miR-34a-5p
 
 
-0.306
 
 
28.9
 
 
 
 
ACSL4
 
 
hsa-miR-34a-5p
 
 
-0.372
 
 
13.7
 
 
 
 
ACTA2
 
 
hsa-miR-27a-3p
 
 
-0.577
 
 
34.8
 
 
 
 
ADAP1
 
 
hsa-miR-27a-3p
 
 
-0.302
 
 
58.0
 
 
 
 
ADCY2
 
 
hsa-miR-152-3p
 
 
-0.299
 
 
14.0
 
 
 
 
ADD2
 
 
hsa-miR-34a-5p
 
 
-0.305
 
 
12.7
 
 
 
 
ADGRA3
 
 
hsa-miR-27a-3p
 
 
-0.296
 
 
10.8
 
 
 
 
ADGRB3
 
 
hsa-miR-152-3p
 
 
-0.516
 
 
16.0
 
 
 
 
ADIPOR2
 
 
hsa-miR-34a-5p
 
 
-0.33
 
 
40.5
 
 
 
 
ADK
 
 
hsa-miR-34a-5p
 
 
-0.374
 
 
15.8
 
 
 
 
AGFG1
 
 
hsa-miR-152-3p
 
 
-0.332
 
 
26.0
 
 
 
 
AGPAT3
 
 
hsa-miR-27a-3p
 
 
-0.29
 
 
28.2
 
 
 
 
AGTRAP
 
 
hsa-miR-34a-5p
 
 
-0.328
 
 
18.3
 
 
 
 
AHCYL2
 
 
hsa-miR-34a-5p
 
 
-0.359
 
 
16.7
 
 
 
 
AHDC1
 
 
hsa-miR-152-3p
 
 
-0.413
 
 
10.9
 
 
 
 
AIDA
 
 
hsa-miR-22-3p
 
 
-0.316
 
 
30.0
 
 
 
 
AKAP1
 
 
hsa-miR-152-3p
 
 
-0.381
 
 
15.1
 
 
 
 
AKIRIN1
 
 
hsa-miR-27a-3p
 
 
-0.368
 
 
39.9
 
 
 
 
AKT3
 
 
hsa-miR-22-3p
 
 
-0.451
 
 
14.5
 
 
 
 
ALCAM
 
 
hsa-miR-34a-5p
 
 
-0.303
 
 
36.1
 
 
 
 
ALCAM
 
 
hsa-miR-152-3p
 
 
-0.347
 
 
36.1
 
 
 
 
ALDOA
 
 
hsa-miR-34a-5p
 
 
-0.655
 
 
780.8
 
 
 
 
AMER2
 
 
hsa-miR-34a-5p
 
 
-0.48
 
 
16.5
 
 
 
 
ANK2
 
 
hsa-miR-34a-5p
 
 
-0.317
 
 
16.1
 
 
 
 
ANK3
 
 
hsa-miR-34a-5p
 
 
-0.29
 
 
37.6
 
 
 
 
ANK3
 
 
hsa-miR-34a-5p
 
 
-0.347
 
 
37.6
 
 
 
 
ANKRD40
 
 
hsa-miR-27a-3p
 
 
-0.35
 
 
80.8
 
 
 
 
ANP32B
 
 
hsa-miR-34a-5p
 
 
-0.29
 
 
120.3
 
 
 
 
AP3S2
 
 
hsa-miR-34a-5p
 
 
-0.413
 
 
14.6
 
 
 
 
APH1A
 
 
hsa-miR-34a-5p
 
 
-0.304
 
 
40.7
 
 
 
 
APOO
 
 
hsa-miR-181a-5p
 
 
-0.5
 
 
43.8
 
 
 
 
ARF4
 
 
hsa-miR-152-3p
 
 
-0.487
 
 
75.3
 
 
 
 
ARF6
 
 
hsa-miR-181a-5p
 
 
-0.33
 
 
10.2
 
 
 
 
ARHGAP1
 
 
hsa-miR-34a-5p
 
 
-0.369
 
 
31.0
 
 
 
 
ARHGAP21
 
 
hsa-miR-152-3p
 
 
-0.287
 
 
30.5
 
 
 
 
ARHGAP36
 
 
hsa-miR-34a-5p
 
 
-0.367
 
 
77.0
 
 
 
 
ARL6IP1
 
 
hsa-miR-152-3p
 
 
-0.579
 
 
212.8
 
 
 
 
ARL8B
 
 
hsa-miR-152-3p
 
 
-0.337
 
 
54.9
 
 
 
 
ARL8B
 
 
hsa-miR-320d
 
 
-0.376
 
 
54.9
 
 
 
 
ARPC5
 
 
hsa-miR-22-3p
 
 
-0.679
 
 
21.6
 
 
 
 
ARPIN-AP3S2
 
 
hsa-miR-34a-5p
 
 
-0.348
 
 
13.7
 
 
 
 
ARPP19
 
 
hsa-miR-320d
 
 
-0.325
 
 
103.9
 
 
 
 
ARPP19
 
 
hsa-miR-34a-5p
 
 
-0.497
 
 
103.9
 
 
 
 
ARRB1
 
 
hsa-miR-22-3p
 
 
-0.616
 
 
21.9
 
 
 
 
ARRDC3
 
 
hsa-miR-152-3p
 
 
-0.305
 
 
10.3
 
 
 
 
ASAH1
 
 
hsa-miR-27a-3p
 
 
-0.398
 
 
58.0
 
 
 
 
ASB1
 
 
hsa-miR-34a-5p
 
 
-0.406
 
 
13.8
 
 
 
 
ASH2L
 
 
hsa-miR-320d
 
 
-0.287
 
 
18.7
 
 
 
 
ASIC2
 
 
hsa-miR-34a-5p
 
 
-0.496
 
 
10.2
 
 
 
 
ASPH
 
 
hsa-miR-152-3p
 
 
-0.391
 
 
51.0
 
 
 
 
ATCAY
 
 
hsa-miR-10a-5p
 
 
-0.322
 
 
56.2
 
 
 
 
ATG4B
 
 
hsa-miR-34a-5p
 
 
-0.58
 
 
22.5
 
 
 
 
ATMIN
 
 
hsa-miR-34a-5p
 
 
-0.409
 
 
20.4
 
 
 
 
ATP1A2
 
 
hsa-miR-34a-5p
 
 
-0.306
 
 
193.2
 
 
 
 
ATP5MC3
 
 
hsa-miR-27a-3p
 
 
-0.416
 
 
193.1
 
 
 
 
ATP6AP2
 
 
hsa-miR-152-3p
 
 
-0.592
 
 
69.0
 
 
 
 
ATP6AP2
 
 
hsa-miR-152-3p
 
 
-0.668
 
 
69.0
 
 
 
 
ATXN10
 
 
hsa-miR-27a-3p
 
 
-0.295
 
 
58.6
 
 
 
 
ATXN2
 
 
hsa-miR-10a-5p
 
 
-0.362
 
 
24.5
 
 
 
 
B4GALT2
 
 
hsa-miR-34a-5p
 
 
-0.314
 
 
35.9
 
 
 
 
B4GALT3
 
 
hsa-miR-27a-3p
 
 
-0.41
 
 
15.7
 
 
 
 
B4GALT5
 
 
hsa-miR-152-3p
 
 
-0.44
 
 
13.0
 
 
 
 
BAG4
 
 
hsa-miR-181a-5p
 
 
-0.337
 
 
10.8
 
 
 
 
BAG5
 
 
hsa-miR-22-3p
 
 
-0.318
 
 
10.3
 
 
 
 
BAZ1B
 
 
hsa-miR-10a-5p
 
 
-0.309
 
 
17.2
 
 
 
 
BEGAIN
 
 
hsa-miR-28-5p
 
 
-0.325
 
 
15.9
 
 
 
 
BEX3
 
 
hsa-miR-27a-3p
 
 
-0.454
 
 
662.4
 
 
 
 
BHLHE40
 
 
hsa-miR-181a-5p
 
 
-0.408
 
 
34.7
 
 
 
 
BHLHE41
 
 
hsa-miR-152-3p
 
 
-0.442
 
 
27.6
 
 
 
 
BIN1
 
 
hsa-miR-22-3p
 
 
-0.346
 
 
127.6
 
 
 
 
BLOC1S1
 
 
hsa-miR-22-3p
 
 
-0.346
 
 
179.6
 
 
 
 
BLOC1S6
 
 
hsa-miR-181a-5p
 
 
-0.393
 
 
24.8
 
 
 
 
BLTP1
 
 
hsa-miR-27a-3p
 
 
-0.426
 
 
14.0
 
 
 
 
BMI1
 
 
hsa-miR-27a-3p
 
 
-0.322
 
 
21.3
 
 
 
 
BMPR2
 
 
hsa-miR-100-5p
 
 
-0.355
 
 
11.9
 
 
 
 
BRINP1
 
 
hsa-miR-34a-5p
 
 
-0.612
 
 
20.8
 
 
 
 
BRSK1
 
 
hsa-miR-27a-3p
 
 
-0.372
 
 
86.4
 
 
 
 
BTBD3
 
 
hsa-miR-152-3p
 
 
-0.495
 
 
24.8
 
 
 
 
BTG2
 
 
hsa-miR-27a-3p
 
 
-0.339
 
 
43.2
 
 
 
 
BZW1
 
 
hsa-miR-27a-3p
 
 
-0.337
 
 
32.4
 
 
 
 
C1orf35
 
 
hsa-miR-152-3p
 
 
-0.511
 
 
12.9
 
 
 
 
C5orf24
 
 
hsa-miR-22-3p
 
 
-0.493
 
 
18.2
 
 
 
 
C6orf62
 
 
hsa-miR-181a-5p
 
 
-0.334
 
 
72.6
 
 
 
 
C8orf88
 
 
hsa-miR-27a-3p
 
 
-0.33
 
 
10.2
 
 
 
 
CA10
 
 
hsa-miR-27a-3p
 
 
-0.464
 
 
30.4
 
 
 
 
CABP1
 
 
hsa-miR-27a-3p
 
 
-0.343
 
 
23.5
 
 
 
 
CACNB3
 
 
hsa-miR-34a-5p
 
 
-0.426
 
 
32.6
 
 
 
 
CACYBP
 
 
hsa-miR-27a-3p
 
 
-0.367
 
 
59.2
 
 
 
 
CADM4
 
 
hsa-miR-34a-5p
 
 
-0.365
 
 
108.1
 
 
 
 
CAMK2N1
 
 
hsa-miR-22-3p
 
 
-0.302
 
 
175.0
 
 
 
 
CAND1
 
 
hsa-miR-27a-3p
 
 
-0.318
 
 
18.8
 
 
 
 
CAND1
 
 
hsa-miR-152-3p
 
 
-0.44
 
 
18.8
 
 
 
 
CANX
 
 
hsa-miR-152-3p
 
 
-0.434
 
 
136.0
 
 
 
 
CAPN1
 
 
hsa-miR-22-3p
 
 
-0.307
 
 
27.9
 
 
 
 
CAPZA1
 
 
hsa-miR-27a-3p
 
 
-0.325
 
 
42.3
 
 
 
 
CBX4
 
 
hsa-miR-181a-5p
 
 
-0.354
 
 
14.6
 
 
 
 
CCDC28B
 
 
hsa-miR-27a-3p
 
 
-0.314
 
 
14.3
 
 
 
 
 
 
 kidney 
 
 
 
 
target_symbol
 
 
mature_mirna_id
 
 
score
 
 
nTPM
 
 
 
 
 
 
AAGAB
 
 
hsa-miR-34a-5p
 
 
-0.319
 
 
25.5
 
 
 
 
ABCD3
 
 
hsa-miR-152-3p
 
 
-0.388
 
 
55.5
 
 
 
 
ABHD17C
 
 
hsa-miR-27a-3p
 
 
-0.435
 
 
13.6
 
 
 
 
ABHD5
 
 
hsa-miR-27a-3p
 
 
-0.407
 
 
10.4
 
 
 
 
ABRAXAS2
 
 
hsa-miR-34a-5p
 
 
-0.538
 
 
11.5
 
 
 
 
ACBD3
 
 
hsa-miR-34a-5p
 
 
-0.415
 
 
18.8
 
 
 
 
ACSL1
 
 
hsa-miR-34a-5p
 
 
-0.306
 
 
173.2
 
 
 
 
ACSL4
 
 
hsa-miR-34a-5p
 
 
-0.372
 
 
42.0
 
 
 
 
ACTA2
 
 
hsa-miR-27a-3p
 
 
-0.577
 
 
281.9
 
 
 
 
ACVR1
 
 
hsa-miR-152-3p
 
 
-0.397
 
 
15.0
 
 
 
 
ADAM10
 
 
hsa-miR-152-3p
 
 
-0.348
 
 
16.8
 
 
 
 
ADAP1
 
 
hsa-miR-27a-3p
 
 
-0.302
 
 
21.6
 
 
 
 
ADGRF5
 
 
hsa-miR-152-3p
 
 
-0.354
 
 
55.8
 
 
 
 
ADIPOR2
 
 
hsa-miR-34a-5p
 
 
-0.33
 
 
20.9
 
 
 
 
ADK
 
 
hsa-miR-34a-5p
 
 
-0.374
 
 
33.4
 
 
 
 
ADO
 
 
hsa-miR-34a-5p
 
 
-0.377
 
 
10.5
 
 
 
 
AFG3L2
 
 
hsa-miR-181a-5p
 
 
-0.491
 
 
36.4
 
 
 
 
AGFG1
 
 
hsa-miR-152-3p
 
 
-0.332
 
 
30.6
 
 
 
 
AGMAT
 
 
hsa-miR-22-3p
 
 
-0.294
 
 
125.0
 
 
 
 
AGPAT3
 
 
hsa-miR-27a-3p
 
 
-0.29
 
 
49.9
 
 
 
 
AGTRAP
 
 
hsa-miR-34a-5p
 
 
-0.328
 
 
61.8
 
 
 
 
AHCYL2
 
 
hsa-miR-34a-5p
 
 
-0.359
 
 
26.6
 
 
 
 
AHDC1
 
 
hsa-miR-152-3p
 
 
-0.413
 
 
12.2
 
 
 
 
AIDA
 
 
hsa-miR-22-3p
 
 
-0.316
 
 
36.7
 
 
 
 
AKAP1
 
 
hsa-miR-152-3p
 
 
-0.381
 
 
24.5
 
 
 
 
AKIRIN1
 
 
hsa-miR-27a-3p
 
 
-0.368
 
 
40.0
 
 
 
 
ALCAM
 
 
hsa-miR-34a-5p
 
 
-0.303
 
 
20.8
 
 
 
 
ALCAM
 
 
hsa-miR-152-3p
 
 
-0.347
 
 
20.8
 
 
 
 
ALDOA
 
 
hsa-miR-34a-5p
 
 
-0.655
 
 
878.7
 
 
 
 
ALPL
 
 
hsa-miR-10a-5p
 
 
-0.32
 
 
42.7
 
 
 
 
ANK2
 
 
hsa-miR-34a-5p
 
 
-0.317
 
 
76.8
 
 
 
 
ANK3
 
 
hsa-miR-34a-5p
 
 
-0.29
 
 
87.6
 
 
 
 
ANK3
 
 
hsa-miR-34a-5p
 
 
-0.347
 
 
87.6
 
 
 
 
ANKHD1-EIF4EBP3
 
 
hsa-miR-22-3p
 
 
-0.479
 
 
11.4
 
 
 
 
ANKRD13A
 
 
hsa-miR-22-3p
 
 
-0.478
 
 
16.4
 
 
 
 
ANKRD40
 
 
hsa-miR-27a-3p
 
 
-0.35
 
 
16.6
 
 
 
 
ANP32B
 
 
hsa-miR-34a-5p
 
 
-0.29
 
 
90.4
 
 
 
 
AP1AR
 
 
hsa-miR-100-5p
 
 
-0.804
 
 
20.6
 
 
 
 
AP3S2
 
 
hsa-miR-34a-5p
 
 
-0.413
 
 
33.9
 
 
 
 
APH1A
 
 
hsa-miR-34a-5p
 
 
-0.304
 
 
75.0
 
 
 
 
APOO
 
 
hsa-miR-181a-5p
 
 
-0.5
 
 
43.3
 
 
 
 
APPBP2
 
 
hsa-miR-27a-3p
 
 
-0.378
 
 
12.2
 
 
 
 
ARF4
 
 
hsa-miR-152-3p
 
 
-0.487
 
 
106.7
 
 
 
 
ARF6
 
 
hsa-miR-181a-5p
 
 
-0.33
 
 
29.7
 
 
 
 
ARFGEF1
 
 
hsa-miR-152-3p
 
 
-0.306
 
 
21.3
 
 
 
 
ARFIP1
 
 
hsa-miR-320d
 
 
-0.339
 
 
18.5
 
 
 
 
ARFIP1
 
 
hsa-miR-152-3p
 
 
-0.474
 
 
18.5
 
 
 
 
ARHGAP1
 
 
hsa-miR-34a-5p
 
 
-0.369
 
 
38.9
 
 
 
 
ARHGAP21
 
 
hsa-miR-152-3p
 
 
-0.287
 
 
10.2
 
 
 
 
ARL6IP1
 
 
hsa-miR-152-3p
 
 
-0.579
 
 
140.2
 
 
 
 
ARL8B
 
 
hsa-miR-152-3p
 
 
-0.337
 
 
50.2
 
 
 
 
ARL8B
 
 
hsa-miR-320d
 
 
-0.376
 
 
50.2
 
 
 
 
ARPC5
 
 
hsa-miR-22-3p
 
 
-0.679
 
 
36.4
 
 
 
 
ARPIN-AP3S2
 
 
hsa-miR-34a-5p
 
 
-0.348
 
 
14.2
 
 
 
 
ARPP19
 
 
hsa-miR-320d
 
 
-0.325
 
 
52.6
 
 
 
 
ARPP19
 
 
hsa-miR-34a-5p
 
 
-0.497
 
 
52.6
 
 
 
 
ARRB1
 
 
hsa-miR-22-3p
 
 
-0.616
 
 
16.5
 
 
 
 
ARRDC3
 
 
hsa-miR-152-3p
 
 
-0.305
 
 
49.3
 
 
 
 
AS3MT
 
 
hsa-miR-28-5p
 
 
-0.479
 
 
14.4
 
 
 
 
ASAH1
 
 
hsa-miR-27a-3p
 
 
-0.398
 
 
190.4
 
 
 
 
ASH2L
 
 
hsa-miR-320d
 
 
-0.287
 
 
21.9
 
 
 
 
ASPH
 
 
hsa-miR-152-3p
 
 
-0.391
 
 
55.2
 
 
 
 
ATG4B
 
 
hsa-miR-34a-5p
 
 
-0.58
 
 
24.5
 
 
 
 
ATMIN
 
 
hsa-miR-34a-5p
 
 
-0.409
 
 
18.4
 
 
 
 
ATP5MC3
 
 
hsa-miR-27a-3p
 
 
-0.416
 
 
416.1
 
 
 
 
ATP6AP2
 
 
hsa-miR-152-3p
 
 
-0.592
 
 
154.5
 
 
 
 
ATP6AP2
 
 
hsa-miR-152-3p
 
 
-0.668
 
 
154.5
 
 
 
 
ATXN10
 
 
hsa-miR-27a-3p
 
 
-0.295
 
 
49.9
 
 
 
 
ATXN2
 
 
hsa-miR-10a-5p
 
 
-0.362
 
 
16.8
 
 
 
 
B4GALT2
 
 
hsa-miR-34a-5p
 
 
-0.314
 
 
17.9
 
 
 
 
B4GALT3
 
 
hsa-miR-27a-3p
 
 
-0.41
 
 
26.5
 
 
 
 
B4GALT5
 
 
hsa-miR-152-3p
 
 
-0.44
 
 
21.3
 
 
 
 
BAG5
 
 
hsa-miR-22-3p
 
 
-0.318
 
 
14.8
 
 
 
 
BAZ1B
 
 
hsa-miR-10a-5p
 
 
-0.309
 
 
15.7
 
 
 
 
BAZ2A
 
 
hsa-miR-100-5p
 
 
-0.29
 
 
18.9
 
 
 
 
BAZ2A
 
 
hsa-miR-100-5p
 
 
-0.421
 
 
18.9
 
 
 
 
BBX
 
 
hsa-miR-10a-5p
 
 
-0.305
 
 
18.2
 
 
 
 
BCL2L11
 
 
hsa-miR-152-3p
 
 
-0.445
 
 
10.8
 
 
 
 
BCL2L11
 
 
hsa-miR-10a-5p
 
 
-0.483
 
 
10.8
 
 
 
 
BCL2L13
 
 
hsa-miR-34a-5p
 
 
-0.431
 
 
13.1
 
 
 
 
BCL3
 
 
hsa-miR-27a-3p
 
 
-0.413
 
 
42.2
 
 
 
 
BEND7
 
 
hsa-miR-27a-3p
 
 
-0.304
 
 
19.6
 
 
 
 
BEX3
 
 
hsa-miR-27a-3p
 
 
-0.454
 
 
295.5
 
 
 
 
BHLHE40
 
 
hsa-miR-181a-5p
 
 
-0.408
 
 
155.1
 
 
 
 
BICC1
 
 
hsa-miR-152-3p
 
 
-0.442
 
 
26.8
 
 
 
 
BIN1
 
 
hsa-miR-22-3p
 
 
-0.346
 
 
78.7
 
 
 
 
BLOC1S1
 
 
hsa-miR-22-3p
 
 
-0.346
 
 
215.5
 
 
 
 
BLOC1S6
 
 
hsa-miR-181a-5p
 
 
-0.393
 
 
22.6
 
 
 
 
BLTP1
 
 
hsa-miR-27a-3p
 
 
-0.426
 
 
20.4
 
 
 
 
BMI1
 
 
hsa-miR-27a-3p
 
 
-0.322
 
 
24.2
 
 
 
 
BMPR2
 
 
hsa-miR-100-5p
 
 
-0.355
 
 
12.2
 
 
 
 
BRPF3
 
 
hsa-miR-34a-5p
 
 
-0.33
 
 
11.0
 
 
 
 
BTBD3
 
 
hsa-miR-152-3p
 
 
-0.495
 
 
10.9
 
 
 
 
BTG2
 
 
hsa-miR-27a-3p
 
 
-0.339
 
 
123.4
 
 
 
 
BZW1
 
 
hsa-miR-27a-3p
 
 
-0.337
 
 
82.6
 
 
 
 
C1GALT1
 
 
hsa-miR-152-3p
 
 
-0.487
 
 
28.8
 
 
 
 
C1orf35
 
 
hsa-miR-152-3p
 
 
-0.511
 
 
13.6
 
 
 
 
C5orf24
 
 
hsa-miR-22-3p
 
 
-0.493
 
 
38.3
 
 
 
 
C6orf120
 
 
hsa-miR-34a-5p
 
 
-0.331
 
 
15.1
 
 
 
 
C6orf62
 
 
hsa-miR-181a-5p
 
 
-0.334
 
 
86.7
 
 
 
 
 
 
 liver 
 
 
 
 
target_symbol
 
 
mature_mirna_id
 
 
score
 
 
nTPM
 
 
 
 
 
 
AAGAB
 
 
hsa-miR-34a-5p
 
 
-0.319
 
 
16.9
 
 
 
 
ABCB10
 
 
hsa-miR-152-3p
 
 
-0.291
 
 
17.7
 
 
 
 
ABCB7
 
 
hsa-miR-152-3p
 
 
-0.515
 
 
14.1
 
 
 
 
ABCD3
 
 
hsa-miR-152-3p
 
 
-0.388
 
 
118.4
 
 
 
 
ABHD5
 
 
hsa-miR-27a-3p
 
 
-0.407
 
 
20.3
 
 
 
 
ABRAXAS2
 
 
hsa-miR-34a-5p
 
 
-0.538
 
 
12.8
 
 
 
 
ACBD3
 
 
hsa-miR-34a-5p
 
 
-0.415
 
 
22.7
 
 
 
 
ACSL1
 
 
hsa-miR-34a-5p
 
 
-0.306
 
 
1068.9
 
 
 
 
ACSL4
 
 
hsa-miR-34a-5p
 
 
-0.372
 
 
12.3
 
 
 
 
ACTA2
 
 
hsa-miR-27a-3p
 
 
-0.577
 
 
154.5
 
 
 
 
ACVR1
 
 
hsa-miR-152-3p
 
 
-0.397
 
 
15.3
 
 
 
 
ADAM10
 
 
hsa-miR-152-3p
 
 
-0.348
 
 
15.0
 
 
 
 
ADGRA3
 
 
hsa-miR-27a-3p
 
 
-0.296
 
 
54.5
 
 
 
 
ADGRF5
 
 
hsa-miR-152-3p
 
 
-0.354
 
 
12.3
 
 
 
 
ADIPOR2
 
 
hsa-miR-34a-5p
 
 
-0.33
 
 
77.7
 
 
 
 
ADK
 
 
hsa-miR-34a-5p
 
 
-0.374
 
 
141.2
 
 
 
 
AFG3L2
 
 
hsa-miR-181a-5p
 
 
-0.491
 
 
25.9
 
 
 
 
AGFG1
 
 
hsa-miR-152-3p
 
 
-0.332
 
 
33.8
 
 
 
 
AGMAT
 
 
hsa-miR-22-3p
 
 
-0.294
 
 
58.0
 
 
 
 
AGPAT3
 
 
hsa-miR-27a-3p
 
 
-0.29
 
 
50.8
 
 
 
 
AGTRAP
 
 
hsa-miR-34a-5p
 
 
-0.328
 
 
27.6
 
 
 
 
AHSG
 
 
hsa-miR-27a-3p
 
 
-0.29
 
 
5439.8
 
 
 
 
AIDA
 
 
hsa-miR-22-3p
 
 
-0.316
 
 
45.8
 
 
 
 
AKAP1
 
 
hsa-miR-152-3p
 
 
-0.381
 
 
48.4
 
 
 
 
AKIRIN1
 
 
hsa-miR-27a-3p
 
 
-0.368
 
 
49.7
 
 
 
 
ALCAM
 
 
hsa-miR-34a-5p
 
 
-0.303
 
 
44.8
 
 
 
 
ALCAM
 
 
hsa-miR-152-3p
 
 
-0.347
 
 
44.8
 
 
 
 
ALDOA
 
 
hsa-miR-34a-5p
 
 
-0.655
 
 
253.5
 
 
 
 
ALPL
 
 
hsa-miR-10a-5p
 
 
-0.32
 
 
52.2
 
 
 
 
ANK3
 
 
hsa-miR-34a-5p
 
 
-0.29
 
 
15.4
 
 
 
 
ANK3
 
 
hsa-miR-34a-5p
 
 
-0.347
 
 
15.4
 
 
 
 
ANKRD13A
 
 
hsa-miR-22-3p
 
 
-0.478
 
 
14.3
 
 
 
 
ANKRD40
 
 
hsa-miR-27a-3p
 
 
-0.35
 
 
16.6
 
 
 
 
ANP32B
 
 
hsa-miR-34a-5p
 
 
-0.29
 
 
96.8
 
 
 
 
AP1AR
 
 
hsa-miR-100-5p
 
 
-0.804
 
 
19.1
 
 
 
 
AP3S2
 
 
hsa-miR-34a-5p
 
 
-0.413
 
 
37.7
 
 
 
 
APH1A
 
 
hsa-miR-34a-5p
 
 
-0.304
 
 
127.5
 
 
 
 
APOO
 
 
hsa-miR-181a-5p
 
 
-0.5
 
 
17.0
 
 
 
 
APPBP2
 
 
hsa-miR-27a-3p
 
 
-0.378
 
 
10.3
 
 
 
 
AQP11
 
 
hsa-miR-27a-3p
 
 
-0.666
 
 
15.1
 
 
 
 
ARF4
 
 
hsa-miR-152-3p
 
 
-0.487
 
 
161.3
 
 
 
 
ARF6
 
 
hsa-miR-181a-5p
 
 
-0.33
 
 
45.4
 
 
 
 
ARFGEF1
 
 
hsa-miR-152-3p
 
 
-0.306
 
 
14.2
 
 
 
 
ARFIP1
 
 
hsa-miR-320d
 
 
-0.339
 
 
14.4
 
 
 
 
ARFIP1
 
 
hsa-miR-152-3p
 
 
-0.474
 
 
14.4
 
 
 
 
ARHGAP1
 
 
hsa-miR-34a-5p
 
 
-0.369
 
 
44.2
 
 
 
 
ARHGAP21
 
 
hsa-miR-152-3p
 
 
-0.287
 
 
11.9
 
 
 
 
ARHGEF26
 
 
hsa-miR-22-3p
 
 
-0.374
 
 
14.5
 
 
 
 
ARL6IP1
 
 
hsa-miR-152-3p
 
 
-0.579
 
 
336.2
 
 
 
 
ARL8B
 
 
hsa-miR-152-3p
 
 
-0.337
 
 
41.1
 
 
 
 
ARL8B
 
 
hsa-miR-320d
 
 
-0.376
 
 
41.1
 
 
 
 
ARPC5
 
 
hsa-miR-22-3p
 
 
-0.679
 
 
60.1
 
 
 
 
ARPIN-AP3S2
 
 
hsa-miR-34a-5p
 
 
-0.348
 
 
21.4
 
 
 
 
ARPP19
 
 
hsa-miR-320d
 
 
-0.325
 
 
39.5
 
 
 
 
ARPP19
 
 
hsa-miR-34a-5p
 
 
-0.497
 
 
39.5
 
 
 
 
ARRDC3
 
 
hsa-miR-152-3p
 
 
-0.305
 
 
71.0
 
 
 
 
AS3MT
 
 
hsa-miR-28-5p
 
 
-0.479
 
 
44.3
 
 
 
 
ASAH1
 
 
hsa-miR-27a-3p
 
 
-0.398
 
 
56.9
 
 
 
 
ASH2L
 
 
hsa-miR-320d
 
 
-0.287
 
 
22.2
 
 
 
 
ASPH
 
 
hsa-miR-152-3p
 
 
-0.391
 
 
69.9
 
 
 
 
ATG4B
 
 
hsa-miR-34a-5p
 
 
-0.58
 
 
20.6
 
 
 
 
ATMIN
 
 
hsa-miR-34a-5p
 
 
-0.409
 
 
16.8
 
 
 
 
ATP5MC3
 
 
hsa-miR-27a-3p
 
 
-0.416
 
 
371.4
 
 
 
 
ATP6AP2
 
 
hsa-miR-152-3p
 
 
-0.592
 
 
90.5
 
 
 
 
ATP6AP2
 
 
hsa-miR-152-3p
 
 
-0.668
 
 
90.5
 
 
 
 
ATXN10
 
 
hsa-miR-27a-3p
 
 
-0.295
 
 
60.5
 
 
 
 
ATXN2
 
 
hsa-miR-10a-5p
 
 
-0.362
 
 
19.6
 
 
 
 
AXL
 
 
hsa-miR-34a-5p
 
 
-0.512
 
 
14.0
 
 
 
 
B4GALT2
 
 
hsa-miR-34a-5p
 
 
-0.314
 
 
31.3
 
 
 
 
B4GALT3
 
 
hsa-miR-27a-3p
 
 
-0.41
 
 
21.4
 
 
 
 
B4GALT5
 
 
hsa-miR-152-3p
 
 
-0.44
 
 
24.1
 
 
 
 
BAG4
 
 
hsa-miR-181a-5p
 
 
-0.337
 
 
15.7
 
 
 
 
BAG5
 
 
hsa-miR-22-3p
 
 
-0.318
 
 
11.1
 
 
 
 
BAZ1B
 
 
hsa-miR-10a-5p
 
 
-0.309
 
 
22.6
 
 
 
 
BAZ2A
 
 
hsa-miR-100-5p
 
 
-0.29
 
 
20.5
 
 
 
 
BAZ2A
 
 
hsa-miR-100-5p
 
 
-0.421
 
 
20.5
 
 
 
 
BCL2L11
 
 
hsa-miR-152-3p
 
 
-0.445
 
 
12.9
 
 
 
 
BCL2L11
 
 
hsa-miR-10a-5p
 
 
-0.483
 
 
12.9
 
 
 
 
BCL2L13
 
 
hsa-miR-34a-5p
 
 
-0.431
 
 
15.3
 
 
 
 
BCL3
 
 
hsa-miR-27a-3p
 
 
-0.413
 
 
168.5
 
 
 
 
BEX3
 
 
hsa-miR-27a-3p
 
 
-0.454
 
 
86.6
 
 
 
 
BHLHE40
 
 
hsa-miR-181a-5p
 
 
-0.408
 
 
194.1
 
 
 
 
BIN1
 
 
hsa-miR-22-3p
 
 
-0.346
 
 
33.9
 
 
 
 
BLOC1S1
 
 
hsa-miR-22-3p
 
 
-0.346
 
 
217.0
 
 
 
 
BLOC1S6
 
 
hsa-miR-181a-5p
 
 
-0.393
 
 
25.1
 
 
 
 
BLTP1
 
 
hsa-miR-27a-3p
 
 
-0.426
 
 
14.0
 
 
 
 
BMI1
 
 
hsa-miR-27a-3p
 
 
-0.322
 
 
33.3
 
 
 
 
BMPR2
 
 
hsa-miR-100-5p
 
 
-0.355
 
 
12.0
 
 
 
 
BTAF1
 
 
hsa-miR-152-3p
 
 
-0.401
 
 
12.8
 
 
 
 
BTG2
 
 
hsa-miR-27a-3p
 
 
-0.339
 
 
19.4
 
 
 
 
BZW1
 
 
hsa-miR-27a-3p
 
 
-0.337
 
 
212.2
 
 
 
 
C1GALT1
 
 
hsa-miR-152-3p
 
 
-0.487
 
 
20.5
 
 
 
 
C1orf35
 
 
hsa-miR-152-3p
 
 
-0.511
 
 
13.7
 
 
 
 
C5orf24
 
 
hsa-miR-22-3p
 
 
-0.493
 
 
40.4
 
 
 
 
C6orf120
 
 
hsa-miR-34a-5p
 
 
-0.331
 
 
23.1
 
 
 
 
C6orf62
 
 
hsa-miR-181a-5p
 
 
-0.334
 
 
100.4
 
 
 
 
CACYBP
 
 
hsa-miR-27a-3p
 
 
-0.367
 
 
25.9
 
 
 
 
CAMK2N1
 
 
hsa-miR-22-3p
 
 
-0.302
 
 
50.9
 
 
 
 
CAND1
 
 
hsa-miR-27a-3p
 
 
-0.318
 
 
25.6
 
 
 
 
CAND1
 
 
hsa-miR-152-3p
 
 
-0.44
 
 
25.6
 
 
 
 
 
 
 lung 
 
 
 
 
target_symbol
 
 
mature_mirna_id
 
 
score
 
 
nTPM
 
 
 
 
 
 
AAGAB
 
 
hsa-miR-34a-5p
 
 
-0.319
 
 
14.7
 
 
 
 
ABCD3
 
 
hsa-miR-152-3p
 
 
-0.388
 
 
22.0
 
 
 
 
ABHD17C
 
 
hsa-miR-27a-3p
 
 
-0.435
 
 
18.6
 
 
 
 
ABHD5
 
 
hsa-miR-27a-3p
 
 
-0.407
 
 
11.2
 
 
 
 
ACBD3
 
 
hsa-miR-34a-5p
 
 
-0.415
 
 
21.1
 
 
 
 
ACSL1
 
 
hsa-miR-34a-5p
 
 
-0.306
 
 
169.5
 
 
 
 
ACSL4
 
 
hsa-miR-34a-5p
 
 
-0.372
 
 
35.4
 
 
 
 
ACTA2
 
 
hsa-miR-27a-3p
 
 
-0.577
 
 
687.9
 
 
 
 
ACVR1
 
 
hsa-miR-152-3p
 
 
-0.397
 
 
12.2
 
 
 
 
ADAM10
 
 
hsa-miR-152-3p
 
 
-0.348
 
 
19.7
 
 
 
 
ADAM17
 
 
hsa-miR-152-3p
 
 
-0.301
 
 
12.5
 
 
 
 
ADAP1
 
 
hsa-miR-27a-3p
 
 
-0.302
 
 
10.1
 
 
 
 
ADGRF5
 
 
hsa-miR-152-3p
 
 
-0.354
 
 
99.9
 
 
 
 
ADIPOR2
 
 
hsa-miR-34a-5p
 
 
-0.33
 
 
42.5
 
 
 
 
ADK
 
 
hsa-miR-34a-5p
 
 
-0.374
 
 
23.0
 
 
 
 
AFG3L2
 
 
hsa-miR-181a-5p
 
 
-0.491
 
 
12.4
 
 
 
 
AGFG1
 
 
hsa-miR-152-3p
 
 
-0.332
 
 
51.7
 
 
 
 
AGO4
 
 
hsa-miR-34a-5p
 
 
-0.306
 
 
10.5
 
 
 
 
AGPAT3
 
 
hsa-miR-27a-3p
 
 
-0.29
 
 
18.0
 
 
 
 
AGTRAP
 
 
hsa-miR-34a-5p
 
 
-0.328
 
 
58.9
 
 
 
 
AHCYL2
 
 
hsa-miR-34a-5p
 
 
-0.359
 
 
62.0
 
 
 
 
AHDC1
 
 
hsa-miR-152-3p
 
 
-0.413
 
 
11.4
 
 
 
 
AIDA
 
 
hsa-miR-22-3p
 
 
-0.316
 
 
43.6
 
 
 
 
AKAP1
 
 
hsa-miR-152-3p
 
 
-0.381
 
 
18.4
 
 
 
 
AKIRIN1
 
 
hsa-miR-27a-3p
 
 
-0.368
 
 
28.3
 
 
 
 
AKT3
 
 
hsa-miR-22-3p
 
 
-0.451
 
 
13.9
 
 
 
 
ALCAM
 
 
hsa-miR-34a-5p
 
 
-0.303
 
 
69.9
 
 
 
 
ALCAM
 
 
hsa-miR-152-3p
 
 
-0.347
 
 
69.9
 
 
 
 
ALDOA
 
 
hsa-miR-34a-5p
 
 
-0.655
 
 
498.0
 
 
 
 
ALPL
 
 
hsa-miR-10a-5p
 
 
-0.32
 
 
53.5
 
 
 
 
ANK3
 
 
hsa-miR-34a-5p
 
 
-0.29
 
 
22.3
 
 
 
 
ANK3
 
 
hsa-miR-34a-5p
 
 
-0.347
 
 
22.3
 
 
 
 
ANKHD1-EIF4EBP3
 
 
hsa-miR-22-3p
 
 
-0.479
 
 
10.9
 
 
 
 
ANKRD13A
 
 
hsa-miR-22-3p
 
 
-0.478
 
 
25.4
 
 
 
 
ANKRD40
 
 
hsa-miR-27a-3p
 
 
-0.35
 
 
21.2
 
 
 
 
ANP32B
 
 
hsa-miR-34a-5p
 
 
-0.29
 
 
116.3
 
 
 
 
AP3S2
 
 
hsa-miR-34a-5p
 
 
-0.413
 
 
15.3
 
 
 
 
APH1A
 
 
hsa-miR-34a-5p
 
 
-0.304
 
 
78.9
 
 
 
 
AREG
 
 
hsa-miR-34a-5p
 
 
-0.62
 
 
35.6
 
 
 
 
ARF4
 
 
hsa-miR-152-3p
 
 
-0.487
 
 
116.3
 
 
 
 
ARF6
 
 
hsa-miR-181a-5p
 
 
-0.33
 
 
29.7
 
 
 
 
ARFGEF1
 
 
hsa-miR-152-3p
 
 
-0.306
 
 
13.0
 
 
 
 
ARFIP1
 
 
hsa-miR-320d
 
 
-0.339
 
 
15.8
 
 
 
 
ARFIP1
 
 
hsa-miR-152-3p
 
 
-0.474
 
 
15.8
 
 
 
 
ARHGAP1
 
 
hsa-miR-34a-5p
 
 
-0.369
 
 
36.0
 
 
 
 
ARHGAP21
 
 
hsa-miR-152-3p
 
 
-0.287
 
 
20.4
 
 
 
 
ARHGAP26
 
 
hsa-miR-34a-5p
 
 
-0.384
 
 
19.9
 
 
 
 
ARHGEF26
 
 
hsa-miR-22-3p
 
 
-0.374
 
 
15.4
 
 
 
 
ARL6IP1
 
 
hsa-miR-152-3p
 
 
-0.579
 
 
75.1
 
 
 
 
ARL8B
 
 
hsa-miR-152-3p
 
 
-0.337
 
 
41.1
 
 
 
 
ARL8B
 
 
hsa-miR-320d
 
 
-0.376
 
 
41.1
 
 
 
 
ARPC5
 
 
hsa-miR-22-3p
 
 
-0.679
 
 
97.1
 
 
 
 
ARPP19
 
 
hsa-miR-320d
 
 
-0.325
 
 
42.5
 
 
 
 
ARPP19
 
 
hsa-miR-34a-5p
 
 
-0.497
 
 
42.5
 
 
 
 
ARRB1
 
 
hsa-miR-22-3p
 
 
-0.616
 
 
50.0
 
 
 
 
ARRDC3
 
 
hsa-miR-152-3p
 
 
-0.305
 
 
42.0
 
 
 
 
ASAH1
 
 
hsa-miR-27a-3p
 
 
-0.398
 
 
179.4
 
 
 
 
ASH2L
 
 
hsa-miR-320d
 
 
-0.287
 
 
17.5
 
 
 
 
ASPH
 
 
hsa-miR-152-3p
 
 
-0.391
 
 
69.4
 
 
 
 
ATG4B
 
 
hsa-miR-34a-5p
 
 
-0.58
 
 
14.0
 
 
 
 
ATMIN
 
 
hsa-miR-34a-5p
 
 
-0.409
 
 
10.1
 
 
 
 
ATP2B4
 
 
hsa-miR-152-3p
 
 
-0.337
 
 
33.0
 
 
 
 
ATP5MC3
 
 
hsa-miR-27a-3p
 
 
-0.416
 
 
114.1
 
 
 
 
ATP6AP2
 
 
hsa-miR-152-3p
 
 
-0.592
 
 
82.3
 
 
 
 
ATP6AP2
 
 
hsa-miR-152-3p
 
 
-0.668
 
 
82.3
 
 
 
 
ATP8A1
 
 
hsa-miR-22-3p
 
 
-0.468
 
 
11.2
 
 
 
 
ATXN10
 
 
hsa-miR-27a-3p
 
 
-0.295
 
 
36.4
 
 
 
 
ATXN2
 
 
hsa-miR-10a-5p
 
 
-0.362
 
 
18.4
 
 
 
 
AXL
 
 
hsa-miR-34a-5p
 
 
-0.512
 
 
24.4
 
 
 
 
B3GNT7
 
 
hsa-miR-27a-3p
 
 
-0.355
 
 
14.5
 
 
 
 
B4GALT2
 
 
hsa-miR-34a-5p
 
 
-0.314
 
 
16.5
 
 
 
 
B4GALT3
 
 
hsa-miR-27a-3p
 
 
-0.41
 
 
24.2
 
 
 
 
B4GALT5
 
 
hsa-miR-152-3p
 
 
-0.44
 
 
26.0
 
 
 
 
BAG5
 
 
hsa-miR-22-3p
 
 
-0.318
 
 
14.6
 
 
 
 
BAZ1B
 
 
hsa-miR-10a-5p
 
 
-0.309
 
 
19.1
 
 
 
 
BAZ2A
 
 
hsa-miR-100-5p
 
 
-0.29
 
 
33.3
 
 
 
 
BAZ2A
 
 
hsa-miR-100-5p
 
 
-0.421
 
 
33.3
 
 
 
 
BBX
 
 
hsa-miR-10a-5p
 
 
-0.305
 
 
12.6
 
 
 
 
BCL3
 
 
hsa-miR-27a-3p
 
 
-0.413
 
 
94.7
 
 
 
 
BEX3
 
 
hsa-miR-27a-3p
 
 
-0.454
 
 
121.3
 
 
 
 
BHLHE40
 
 
hsa-miR-181a-5p
 
 
-0.408
 
 
117.3
 
 
 
 
BHLHE41
 
 
hsa-miR-152-3p
 
 
-0.442
 
 
10.9
 
 
 
 
BIN1
 
 
hsa-miR-22-3p
 
 
-0.346
 
 
21.8
 
 
 
 
BLOC1S1
 
 
hsa-miR-22-3p
 
 
-0.346
 
 
112.1
 
 
 
 
BLOC1S6
 
 
hsa-miR-181a-5p
 
 
-0.393
 
 
19.2
 
 
 
 
BLTP1
 
 
hsa-miR-27a-3p
 
 
-0.426
 
 
18.0
 
 
 
 
BMI1
 
 
hsa-miR-27a-3p
 
 
-0.322
 
 
22.0
 
 
 
 
BMPR2
 
 
hsa-miR-100-5p
 
 
-0.355
 
 
21.8
 
 
 
 
BTAF1
 
 
hsa-miR-152-3p
 
 
-0.401
 
 
15.8
 
 
 
 
BTBD3
 
 
hsa-miR-152-3p
 
 
-0.495
 
 
11.5
 
 
 
 
BTG2
 
 
hsa-miR-27a-3p
 
 
-0.339
 
 
99.0
 
 
 
 
BZW1
 
 
hsa-miR-27a-3p
 
 
-0.337
 
 
69.3
 
 
 
 
C1GALT1
 
 
hsa-miR-152-3p
 
 
-0.487
 
 
14.2
 
 
 
 
C5orf24
 
 
hsa-miR-22-3p
 
 
-0.493
 
 
21.6
 
 
 
 
C6orf62
 
 
hsa-miR-181a-5p
 
 
-0.334
 
 
90.9
 
 
 
 
CACNB3
 
 
hsa-miR-34a-5p
 
 
-0.426
 
 
11.1
 
 
 
 
CACYBP
 
 
hsa-miR-27a-3p
 
 
-0.367
 
 
22.6
 
 
 
 
CAMK2N1
 
 
hsa-miR-22-3p
 
 
-0.302
 
 
63.8
 
 
 
 
CAND1
 
 
hsa-miR-27a-3p
 
 
-0.318
 
 
15.8
 
 
 
 
CAND1
 
 
hsa-miR-152-3p
 
 
-0.44
 
 
15.8
 
 
 
 
 
 
 lymph node 
 
 
 
 
target_symbol
 
 
mature_mirna_id
 
 
score
 
 
nTPM
 
 
 
 
 
 
AAGAB
 
 
hsa-miR-34a-5p
 
 
-0.319
 
 
18.6
 
 
 
 
ABCB10
 
 
hsa-miR-152-3p
 
 
-0.291
 
 
10.3
 
 
 
 
ABCB7
 
 
hsa-miR-152-3p
 
 
-0.515
 
 
11.2
 
 
 
 
ABCD3
 
 
hsa-miR-152-3p
 
 
-0.388
 
 
12.4
 
 
 
 
ABRAXAS2
 
 
hsa-miR-34a-5p
 
 
-0.538
 
 
11.7
 
 
 
 
ACBD3
 
 
hsa-miR-34a-5p
 
 
-0.415
 
 
19.9
 
 
 
 
ACSL1
 
 
hsa-miR-34a-5p
 
 
-0.306
 
 
17.6
 
 
 
 
ACSL4
 
 
hsa-miR-34a-5p
 
 
-0.372
 
 
20.7
 
 
 
 
ACTA2
 
 
hsa-miR-27a-3p
 
 
-0.577
 
 
162.6
 
 
 
 
ADAM10
 
 
hsa-miR-152-3p
 
 
-0.348
 
 
20.8
 
 
 
 
ADK
 
 
hsa-miR-34a-5p
 
 
-0.374
 
 
21.3
 
 
 
 
AFG3L2
 
 
hsa-miR-181a-5p
 
 
-0.491
 
 
12.0
 
 
 
 
AGFG1
 
 
hsa-miR-152-3p
 
 
-0.332
 
 
44.0
 
 
 
 
AGPAT3
 
 
hsa-miR-27a-3p
 
 
-0.29
 
 
15.3
 
 
 
 
AGTRAP
 
 
hsa-miR-34a-5p
 
 
-0.328
 
 
21.9
 
 
 
 
AHCYL2
 
 
hsa-miR-34a-5p
 
 
-0.359
 
 
10.0
 
 
 
 
AIDA
 
 
hsa-miR-22-3p
 
 
-0.316
 
 
47.9
 
 
 
 
AKAP1
 
 
hsa-miR-152-3p
 
 
-0.381
 
 
10.0
 
 
 
 
AKIRIN1
 
 
hsa-miR-27a-3p
 
 
-0.368
 
 
39.5
 
 
 
 
ALDOA
 
 
hsa-miR-34a-5p
 
 
-0.655
 
 
335.0
 
 
 
 
ANKRD13A
 
 
hsa-miR-22-3p
 
 
-0.478
 
 
50.3
 
 
 
 
ANKRD40
 
 
hsa-miR-27a-3p
 
 
-0.35
 
 
15.5
 
 
 
 
ANP32B
 
 
hsa-miR-34a-5p
 
 
-0.29
 
 
207.9
 
 
 
 
AP1AR
 
 
hsa-miR-100-5p
 
 
-0.804
 
 
11.3
 
 
 
 
AP3S2
 
 
hsa-miR-34a-5p
 
 
-0.413
 
 
12.0
 
 
 
 
APH1A
 
 
hsa-miR-34a-5p
 
 
-0.304
 
 
75.6
 
 
 
 
APOO
 
 
hsa-miR-181a-5p
 
 
-0.5
 
 
10.9
 
 
 
 
ARF4
 
 
hsa-miR-152-3p
 
 
-0.487
 
 
69.1
 
 
 
 
ARF6
 
 
hsa-miR-181a-5p
 
 
-0.33
 
 
57.9
 
 
 
 
ARFGEF1
 
 
hsa-miR-152-3p
 
 
-0.306
 
 
14.4
 
 
 
 
ARFIP1
 
 
hsa-miR-320d
 
 
-0.339
 
 
14.1
 
 
 
 
ARFIP1
 
 
hsa-miR-152-3p
 
 
-0.474
 
 
14.1
 
 
 
 
ARHGAP1
 
 
hsa-miR-34a-5p
 
 
-0.369
 
 
28.7
 
 
 
 
ARL6IP1
 
 
hsa-miR-152-3p
 
 
-0.579
 
 
124.0
 
 
 
 
ARL8B
 
 
hsa-miR-152-3p
 
 
-0.337
 
 
36.2
 
 
 
 
ARL8B
 
 
hsa-miR-320d
 
 
-0.376
 
 
36.2
 
 
 
 
ARPC5
 
 
hsa-miR-22-3p
 
 
-0.679
 
 
148.6
 
 
 
 
ARPP19
 
 
hsa-miR-320d
 
 
-0.325
 
 
45.1
 
 
 
 
ARPP19
 
 
hsa-miR-34a-5p
 
 
-0.497
 
 
45.1
 
 
 
 
ARRB1
 
 
hsa-miR-22-3p
 
 
-0.616
 
 
14.3
 
 
 
 
ARRDC3
 
 
hsa-miR-152-3p
 
 
-0.305
 
 
27.5
 
 
 
 
ASAH1
 
 
hsa-miR-27a-3p
 
 
-0.398
 
 
61.7
 
 
 
 
ASH2L
 
 
hsa-miR-320d
 
 
-0.287
 
 
26.6
 
 
 
 
ASPH
 
 
hsa-miR-152-3p
 
 
-0.391
 
 
18.8
 
 
 
 
ATG4B
 
 
hsa-miR-34a-5p
 
 
-0.58
 
 
12.4
 
 
 
 
ATMIN
 
 
hsa-miR-34a-5p
 
 
-0.409
 
 
13.0
 
 
 
 
ATP2B4
 
 
hsa-miR-152-3p
 
 
-0.337
 
 
12.0
 
 
 
 
ATP5MC3
 
 
hsa-miR-27a-3p
 
 
-0.416
 
 
118.6
 
 
 
 
ATP6AP2
 
 
hsa-miR-152-3p
 
 
-0.592
 
 
63.1
 
 
 
 
ATP6AP2
 
 
hsa-miR-152-3p
 
 
-0.668
 
 
63.1
 
 
 
 
ATP8A1
 
 
hsa-miR-22-3p
 
 
-0.468
 
 
14.0
 
 
 
 
ATXN10
 
 
hsa-miR-27a-3p
 
 
-0.295
 
 
45.2
 
 
 
 
B4GALT3
 
 
hsa-miR-27a-3p
 
 
-0.41
 
 
21.9
 
 
 
 
BAG5
 
 
hsa-miR-22-3p
 
 
-0.318
 
 
16.0
 
 
 
 
BAZ1B
 
 
hsa-miR-10a-5p
 
 
-0.309
 
 
26.0
 
 
 
 
BAZ2A
 
 
hsa-miR-100-5p
 
 
-0.29
 
 
31.2
 
 
 
 
BAZ2A
 
 
hsa-miR-100-5p
 
 
-0.421
 
 
31.2
 
 
 
 
BBX
 
 
hsa-miR-10a-5p
 
 
-0.305
 
 
13.0
 
 
 
 
BCL2
 
 
hsa-miR-34a-5p
 
 
-0.289
 
 
13.7
 
 
 
 
BCL2L11
 
 
hsa-miR-152-3p
 
 
-0.445
 
 
23.7
 
 
 
 
BCL2L11
 
 
hsa-miR-10a-5p
 
 
-0.483
 
 
23.7
 
 
 
 
BCL3
 
 
hsa-miR-27a-3p
 
 
-0.413
 
 
10.9
 
 
 
 
BEX3
 
 
hsa-miR-27a-3p
 
 
-0.454
 
 
27.4
 
 
 
 
BHLHE40
 
 
hsa-miR-181a-5p
 
 
-0.408
 
 
48.9
 
 
 
 
BIN1
 
 
hsa-miR-22-3p
 
 
-0.346
 
 
38.4
 
 
 
 
BLOC1S1
 
 
hsa-miR-22-3p
 
 
-0.346
 
 
103.4
 
 
 
 
BLOC1S6
 
 
hsa-miR-181a-5p
 
 
-0.393
 
 
39.2
 
 
 
 
BLTP1
 
 
hsa-miR-27a-3p
 
 
-0.426
 
 
11.1
 
 
 
 
BMI1
 
 
hsa-miR-27a-3p
 
 
-0.322
 
 
26.0
 
 
 
 
BTAF1
 
 
hsa-miR-152-3p
 
 
-0.401
 
 
25.1
 
 
 
 
BTG1
 
 
hsa-miR-22-3p
 
 
-0.468
 
 
22.3
 
 
 
 
BTG2
 
 
hsa-miR-27a-3p
 
 
-0.339
 
 
114.1
 
 
 
 
BZW1
 
 
hsa-miR-27a-3p
 
 
-0.337
 
 
91.4
 
 
 
 
C1GALT1
 
 
hsa-miR-152-3p
 
 
-0.487
 
 
17.8
 
 
 
 
C1orf52
 
 
hsa-miR-27a-3p
 
 
-0.591
 
 
14.2
 
 
 
 
C5orf24
 
 
hsa-miR-22-3p
 
 
-0.493
 
 
15.3
 
 
 
 
C6orf120
 
 
hsa-miR-34a-5p
 
 
-0.331
 
 
14.2
 
 
 
 
C6orf62
 
 
hsa-miR-181a-5p
 
 
-0.334
 
 
135.0
 
 
 
 
CACYBP
 
 
hsa-miR-27a-3p
 
 
-0.367
 
 
30.5
 
 
 
 
CAND1
 
 
hsa-miR-27a-3p
 
 
-0.318
 
 
25.7
 
 
 
 
CAND1
 
 
hsa-miR-152-3p
 
 
-0.44
 
 
25.7
 
 
 
 
CANX
 
 
hsa-miR-152-3p
 
 
-0.434
 
 
16.7
 
 
 
 
CAPN1
 
 
hsa-miR-22-3p
 
 
-0.307
 
 
53.3
 
 
 
 
CAPZA1
 
 
hsa-miR-27a-3p
 
 
-0.325
 
 
143.9
 
 
 
 
CASP2
 
 
hsa-miR-34a-5p
 
 
-0.382
 
 
20.5
 
 
 
 
CBFB
 
 
hsa-miR-27a-3p
 
 
-0.302
 
 
15.3
 
 
 
 
CBLB
 
 
hsa-miR-152-3p
 
 
-0.424
 
 
13.8
 
 
 
 
CBX4
 
 
hsa-miR-181a-5p
 
 
-0.354
 
 
13.6
 
 
 
 
CCDC28B
 
 
hsa-miR-27a-3p
 
 
-0.314
 
 
13.1
 
 
 
 
CCDC47
 
 
hsa-miR-22-3p
 
 
-0.604
 
 
30.4
 
 
 
 
CCDC6
 
 
hsa-miR-152-3p
 
 
-0.511
 
 
20.0
 
 
 
 
CCDC71
 
 
hsa-miR-152-3p
 
 
-0.369
 
 
12.6
 
 
 
 
CCM2
 
 
hsa-miR-27a-3p
 
 
-0.335
 
 
40.8
 
 
 
 
CCNC
 
 
hsa-miR-27a-3p
 
 
-0.378
 
 
37.0
 
 
 
 
CCNE2
 
 
hsa-miR-34a-5p
 
 
-0.553
 
 
10.2
 
 
 
 
CCNG1
 
 
hsa-miR-27a-3p
 
 
-0.419
 
 
45.8
 
 
 
 
CCNK
 
 
hsa-miR-181a-5p
 
 
-0.31
 
 
26.5
 
 
 
 
CCNK
 
 
hsa-miR-27a-3p
 
 
-0.519
 
 
26.5
 
 
 
 
CCNL2
 
 
hsa-miR-28-5p
 
 
-0.448
 
 
29.6
 
 
 
 
CCNY
 
 
hsa-miR-27a-3p
 
 
-0.334
 
 
21.9
 
 
 
 
 
 
 midbrain 
 
 
 
 
target_symbol
 
 
mature_mirna_id
 
 
score
 
 
nTPM
 
 
 
 
 
 
AAGAB
 
 
hsa-miR-34a-5p
 
 
-0.319
 
 
11.5
 
 
 
 
ABCD3
 
 
hsa-miR-152-3p
 
 
-0.388
 
 
17.1
 
 
 
 
ABHD17C
 
 
hsa-miR-27a-3p
 
 
-0.435
 
 
11.2
 
 
 
 
ACBD3
 
 
hsa-miR-34a-5p
 
 
-0.415
 
 
10.0
 
 
 
 
ACSL1
 
 
hsa-miR-34a-5p
 
 
-0.306
 
 
45.6
 
 
 
 
ACSL4
 
 
hsa-miR-34a-5p
 
 
-0.372
 
 
10.6
 
 
 
 
ACTA2
 
 
hsa-miR-27a-3p
 
 
-0.577
 
 
69.3
 
 
 
 
ADAM10
 
 
hsa-miR-152-3p
 
 
-0.348
 
 
11.1
 
 
 
 
ADAP1
 
 
hsa-miR-27a-3p
 
 
-0.302
 
 
85.8
 
 
 
 
ADCY2
 
 
hsa-miR-152-3p
 
 
-0.299
 
 
20.4
 
 
 
 
ADGRA3
 
 
hsa-miR-27a-3p
 
 
-0.296
 
 
13.9
 
 
 
 
ADGRF5
 
 
hsa-miR-152-3p
 
 
-0.354
 
 
13.0
 
 
 
 
ADIPOR2
 
 
hsa-miR-34a-5p
 
 
-0.33
 
 
76.2
 
 
 
 
ADK
 
 
hsa-miR-34a-5p
 
 
-0.374
 
 
17.6
 
 
 
 
ADO
 
 
hsa-miR-34a-5p
 
 
-0.377
 
 
13.9
 
 
 
 
AGFG1
 
 
hsa-miR-152-3p
 
 
-0.332
 
 
32.4
 
 
 
 
AGPAT3
 
 
hsa-miR-27a-3p
 
 
-0.29
 
 
44.6
 
 
 
 
AGTRAP
 
 
hsa-miR-34a-5p
 
 
-0.328
 
 
27.0
 
 
 
 
AHCYL2
 
 
hsa-miR-34a-5p
 
 
-0.359
 
 
19.9
 
 
 
 
AIDA
 
 
hsa-miR-22-3p
 
 
-0.316
 
 
32.2
 
 
 
 
AKAP1
 
 
hsa-miR-152-3p
 
 
-0.381
 
 
19.0
 
 
 
 
AKIRIN1
 
 
hsa-miR-27a-3p
 
 
-0.368
 
 
38.9
 
 
 
 
AKT3
 
 
hsa-miR-22-3p
 
 
-0.451
 
 
16.3
 
 
 
 
ALCAM
 
 
hsa-miR-34a-5p
 
 
-0.303
 
 
38.7
 
 
 
 
ALCAM
 
 
hsa-miR-152-3p
 
 
-0.347
 
 
38.7
 
 
 
 
ALDOA
 
 
hsa-miR-34a-5p
 
 
-0.655
 
 
849.0
 
 
 
 
ALPL
 
 
hsa-miR-10a-5p
 
 
-0.32
 
 
11.9
 
 
 
 
AMER2
 
 
hsa-miR-34a-5p
 
 
-0.48
 
 
27.5
 
 
 
 
ANK2
 
 
hsa-miR-34a-5p
 
 
-0.317
 
 
14.7
 
 
 
 
ANK3
 
 
hsa-miR-34a-5p
 
 
-0.29
 
 
63.0
 
 
 
 
ANK3
 
 
hsa-miR-34a-5p
 
 
-0.347
 
 
63.0
 
 
 
 
ANKRD40
 
 
hsa-miR-27a-3p
 
 
-0.35
 
 
160.4
 
 
 
 
ANP32B
 
 
hsa-miR-34a-5p
 
 
-0.29
 
 
216.9
 
 
 
 
AP1AR
 
 
hsa-miR-100-5p
 
 
-0.804
 
 
10.1
 
 
 
 
AP3S2
 
 
hsa-miR-34a-5p
 
 
-0.413
 
 
15.2
 
 
 
 
APH1A
 
 
hsa-miR-34a-5p
 
 
-0.304
 
 
54.4
 
 
 
 
APOO
 
 
hsa-miR-181a-5p
 
 
-0.5
 
 
44.2
 
 
 
 
ARF4
 
 
hsa-miR-152-3p
 
 
-0.487
 
 
62.8
 
 
 
 
ARF6
 
 
hsa-miR-181a-5p
 
 
-0.33
 
 
12.0
 
 
 
 
ARHGAP1
 
 
hsa-miR-34a-5p
 
 
-0.369
 
 
34.8
 
 
 
 
ARHGAP21
 
 
hsa-miR-152-3p
 
 
-0.287
 
 
53.0
 
 
 
 
ARL6IP1
 
 
hsa-miR-152-3p
 
 
-0.579
 
 
198.8
 
 
 
 
ARL8B
 
 
hsa-miR-152-3p
 
 
-0.337
 
 
52.4
 
 
 
 
ARL8B
 
 
hsa-miR-320d
 
 
-0.376
 
 
52.4
 
 
 
 
ARPC5
 
 
hsa-miR-22-3p
 
 
-0.679
 
 
27.1
 
 
 
 
ARPIN-AP3S2
 
 
hsa-miR-34a-5p
 
 
-0.348
 
 
13.5
 
 
 
 
ARPP19
 
 
hsa-miR-320d
 
 
-0.325
 
 
144.2
 
 
 
 
ARPP19
 
 
hsa-miR-34a-5p
 
 
-0.497
 
 
144.2
 
 
 
 
ARRB1
 
 
hsa-miR-22-3p
 
 
-0.616
 
 
27.6
 
 
 
 
ARRDC3
 
 
hsa-miR-152-3p
 
 
-0.305
 
 
13.8
 
 
 
 
ASAH1
 
 
hsa-miR-27a-3p
 
 
-0.398
 
 
63.4
 
 
 
 
ASB1
 
 
hsa-miR-34a-5p
 
 
-0.406
 
 
18.2
 
 
 
 
ASH2L
 
 
hsa-miR-320d
 
 
-0.287
 
 
16.5
 
 
 
 
ASPH
 
 
hsa-miR-152-3p
 
 
-0.391
 
 
48.8
 
 
 
 
ATCAY
 
 
hsa-miR-10a-5p
 
 
-0.322
 
 
24.7
 
 
 
 
ATG4B
 
 
hsa-miR-34a-5p
 
 
-0.58
 
 
20.8
 
 
 
 
ATMIN
 
 
hsa-miR-34a-5p
 
 
-0.409
 
 
17.9
 
 
 
 
ATP1A2
 
 
hsa-miR-34a-5p
 
 
-0.306
 
 
328.9
 
 
 
 
ATP5MC3
 
 
hsa-miR-27a-3p
 
 
-0.416
 
 
209.2
 
 
 
 
ATP6AP2
 
 
hsa-miR-152-3p
 
 
-0.592
 
 
65.6
 
 
 
 
ATP6AP2
 
 
hsa-miR-152-3p
 
 
-0.668
 
 
65.6
 
 
 
 
ATXN10
 
 
hsa-miR-27a-3p
 
 
-0.295
 
 
47.1
 
 
 
 
ATXN2
 
 
hsa-miR-10a-5p
 
 
-0.362
 
 
24.2
 
 
 
 
AXL
 
 
hsa-miR-34a-5p
 
 
-0.512
 
 
11.2
 
 
 
 
B4GALT2
 
 
hsa-miR-34a-5p
 
 
-0.314
 
 
37.2
 
 
 
 
B4GALT3
 
 
hsa-miR-27a-3p
 
 
-0.41
 
 
12.5
 
 
 
 
B4GALT5
 
 
hsa-miR-152-3p
 
 
-0.44
 
 
10.3
 
 
 
 
BAG5
 
 
hsa-miR-22-3p
 
 
-0.318
 
 
10.6
 
 
 
 
BAZ1B
 
 
hsa-miR-10a-5p
 
 
-0.309
 
 
19.8
 
 
 
 
BBX
 
 
hsa-miR-10a-5p
 
 
-0.305
 
 
11.6
 
 
 
 
BCL2L13
 
 
hsa-miR-34a-5p
 
 
-0.431
 
 
10.0
 
 
 
 
BEX3
 
 
hsa-miR-27a-3p
 
 
-0.454
 
 
476.6
 
 
 
 
BHLHE40
 
 
hsa-miR-181a-5p
 
 
-0.408
 
 
41.1
 
 
 
 
BHLHE41
 
 
hsa-miR-152-3p
 
 
-0.442
 
 
40.4
 
 
 
 
BIN1
 
 
hsa-miR-22-3p
 
 
-0.346
 
 
190.1
 
 
 
 
BLOC1S1
 
 
hsa-miR-22-3p
 
 
-0.346
 
 
246.9
 
 
 
 
BLOC1S6
 
 
hsa-miR-181a-5p
 
 
-0.393
 
 
18.5
 
 
 
 
BLTP1
 
 
hsa-miR-27a-3p
 
 
-0.426
 
 
19.4
 
 
 
 
BMI1
 
 
hsa-miR-27a-3p
 
 
-0.322
 
 
25.5
 
 
 
 
BRINP1
 
 
hsa-miR-34a-5p
 
 
-0.612
 
 
12.8
 
 
 
 
BRSK1
 
 
hsa-miR-27a-3p
 
 
-0.372
 
 
79.6
 
 
 
 
BTBD3
 
 
hsa-miR-152-3p
 
 
-0.495
 
 
26.0
 
 
 
 
BTG2
 
 
hsa-miR-27a-3p
 
 
-0.339
 
 
46.8
 
 
 
 
BZW1
 
 
hsa-miR-27a-3p
 
 
-0.337
 
 
39.5
 
 
 
 
C1GALT1
 
 
hsa-miR-152-3p
 
 
-0.487
 
 
13.7
 
 
 
 
C1orf35
 
 
hsa-miR-152-3p
 
 
-0.511
 
 
13.7
 
 
 
 
C5orf24
 
 
hsa-miR-22-3p
 
 
-0.493
 
 
17.1
 
 
 
 
C6orf62
 
 
hsa-miR-181a-5p
 
 
-0.334
 
 
94.9
 
 
 
 
CA10
 
 
hsa-miR-27a-3p
 
 
-0.464
 
 
16.5
 
 
 
 
CABP1
 
 
hsa-miR-27a-3p
 
 
-0.343
 
 
22.2
 
 
 
 
CACNB3
 
 
hsa-miR-34a-5p
 
 
-0.426
 
 
23.1
 
 
 
 
CACYBP
 
 
hsa-miR-27a-3p
 
 
-0.367
 
 
53.3
 
 
 
 
CADM4
 
 
hsa-miR-34a-5p
 
 
-0.365
 
 
156.6
 
 
 
 
CAMK2N1
 
 
hsa-miR-22-3p
 
 
-0.302
 
 
198.9
 
 
 
 
CAND1
 
 
hsa-miR-27a-3p
 
 
-0.318
 
 
16.6
 
 
 
 
CAND1
 
 
hsa-miR-152-3p
 
 
-0.44
 
 
16.6
 
 
 
 
CANX
 
 
hsa-miR-152-3p
 
 
-0.434
 
 
130.1
 
 
 
 
CAPN1
 
 
hsa-miR-22-3p
 
 
-0.307
 
 
27.9
 
 
 
 
CAPZA1
 
 
hsa-miR-27a-3p
 
 
-0.325
 
 
43.8
 
 
 
 
CAV2
 
 
hsa-miR-152-3p
 
 
-0.32
 
 
12.0
 
 
 
 
 
 
 pancreas 
 
 
 
 
target_symbol
 
 
mature_mirna_id
 
 
score
 
 
nTPM
 
 
 
 
 
 
AAGAB
 
 
hsa-miR-34a-5p
 
 
-0.319
 
 
16.0
 
 
 
 
ABCD3
 
 
hsa-miR-152-3p
 
 
-0.388
 
 
27.0
 
 
 
 
ABHD5
 
 
hsa-miR-27a-3p
 
 
-0.407
 
 
11.4
 
 
 
 
ACBD3
 
 
hsa-miR-34a-5p
 
 
-0.415
 
 
21.4
 
 
 
 
ACSL1
 
 
hsa-miR-34a-5p
 
 
-0.306
 
 
45.3
 
 
 
 
ACSL4
 
 
hsa-miR-34a-5p
 
 
-0.372
 
 
10.9
 
 
 
 
ACTA2
 
 
hsa-miR-27a-3p
 
 
-0.577
 
 
86.3
 
 
 
 
ADAP1
 
 
hsa-miR-27a-3p
 
 
-0.302
 
 
12.7
 
 
 
 
ADGRA3
 
 
hsa-miR-27a-3p
 
 
-0.296
 
 
11.8
 
 
 
 
ADIPOR2
 
 
hsa-miR-34a-5p
 
 
-0.33
 
 
30.0
 
 
 
 
ADK
 
 
hsa-miR-34a-5p
 
 
-0.374
 
 
17.7
 
 
 
 
AFG3L2
 
 
hsa-miR-181a-5p
 
 
-0.491
 
 
23.6
 
 
 
 
AGFG1
 
 
hsa-miR-152-3p
 
 
-0.332
 
 
33.0
 
 
 
 
AGPAT3
 
 
hsa-miR-27a-3p
 
 
-0.29
 
 
20.6
 
 
 
 
AGTRAP
 
 
hsa-miR-34a-5p
 
 
-0.328
 
 
63.1
 
 
 
 
AHCYL2
 
 
hsa-miR-34a-5p
 
 
-0.359
 
 
13.0
 
 
 
 
AIDA
 
 
hsa-miR-22-3p
 
 
-0.316
 
 
30.6
 
 
 
 
AKAP1
 
 
hsa-miR-152-3p
 
 
-0.381
 
 
21.5
 
 
 
 
AKIRIN1
 
 
hsa-miR-27a-3p
 
 
-0.368
 
 
26.1
 
 
 
 
ALCAM
 
 
hsa-miR-34a-5p
 
 
-0.303
 
 
30.8
 
 
 
 
ALCAM
 
 
hsa-miR-152-3p
 
 
-0.347
 
 
30.8
 
 
 
 
ALDOA
 
 
hsa-miR-34a-5p
 
 
-0.655
 
 
422.3
 
 
 
 
ANK3
 
 
hsa-miR-34a-5p
 
 
-0.29
 
 
10.5
 
 
 
 
ANK3
 
 
hsa-miR-34a-5p
 
 
-0.347
 
 
10.5
 
 
 
 
ANKHD1-EIF4EBP3
 
 
hsa-miR-22-3p
 
 
-0.479
 
 
16.1
 
 
 
 
ANKRD13A
 
 
hsa-miR-22-3p
 
 
-0.478
 
 
11.6
 
 
 
 
ANKRD40
 
 
hsa-miR-27a-3p
 
 
-0.35
 
 
18.4
 
 
 
 
ANP32B
 
 
hsa-miR-34a-5p
 
 
-0.29
 
 
147.1
 
 
 
 
AP3S2
 
 
hsa-miR-34a-5p
 
 
-0.413
 
 
17.9
 
 
 
 
APH1A
 
 
hsa-miR-34a-5p
 
 
-0.304
 
 
87.5
 
 
 
 
APOO
 
 
hsa-miR-181a-5p
 
 
-0.5
 
 
13.3
 
 
 
 
APPBP2
 
 
hsa-miR-27a-3p
 
 
-0.378
 
 
10.8
 
 
 
 
ARF4
 
 
hsa-miR-152-3p
 
 
-0.487
 
 
138.3
 
 
 
 
ARF6
 
 
hsa-miR-181a-5p
 
 
-0.33
 
 
23.2
 
 
 
 
ARFGEF1
 
 
hsa-miR-152-3p
 
 
-0.306
 
 
16.5
 
 
 
 
ARHGAP1
 
 
hsa-miR-34a-5p
 
 
-0.369
 
 
34.7
 
 
 
 
ARHGAP21
 
 
hsa-miR-152-3p
 
 
-0.287
 
 
13.3
 
 
 
 
ARL6IP1
 
 
hsa-miR-152-3p
 
 
-0.579
 
 
77.1
 
 
 
 
ARL8B
 
 
hsa-miR-152-3p
 
 
-0.337
 
 
26.7
 
 
 
 
ARL8B
 
 
hsa-miR-320d
 
 
-0.376
 
 
26.7
 
 
 
 
ARPC5
 
 
hsa-miR-22-3p
 
 
-0.679
 
 
48.7
 
 
 
 
ARPIN-AP3S2
 
 
hsa-miR-34a-5p
 
 
-0.348
 
 
19.2
 
 
 
 
ARPP19
 
 
hsa-miR-320d
 
 
-0.325
 
 
43.6
 
 
 
 
ARPP19
 
 
hsa-miR-34a-5p
 
 
-0.497
 
 
43.6
 
 
 
 
ARRB1
 
 
hsa-miR-22-3p
 
 
-0.616
 
 
23.2
 
 
 
 
ARRDC3
 
 
hsa-miR-152-3p
 
 
-0.305
 
 
19.3
 
 
 
 
AS3MT
 
 
hsa-miR-28-5p
 
 
-0.479
 
 
10.1
 
 
 
 
ASAH1
 
 
hsa-miR-27a-3p
 
 
-0.398
 
 
46.8
 
 
 
 
ASH2L
 
 
hsa-miR-320d
 
 
-0.287
 
 
16.9
 
 
 
 
ASPH
 
 
hsa-miR-152-3p
 
 
-0.391
 
 
42.1
 
 
 
 
ATG4B
 
 
hsa-miR-34a-5p
 
 
-0.58
 
 
18.2
 
 
 
 
ATMIN
 
 
hsa-miR-34a-5p
 
 
-0.409
 
 
15.2
 
 
 
 
ATP5MC3
 
 
hsa-miR-27a-3p
 
 
-0.416
 
 
115.2
 
 
 
 
ATP6AP2
 
 
hsa-miR-152-3p
 
 
-0.592
 
 
50.2
 
 
 
 
ATP6AP2
 
 
hsa-miR-152-3p
 
 
-0.668
 
 
50.2
 
 
 
 
ATXN10
 
 
hsa-miR-27a-3p
 
 
-0.295
 
 
54.1
 
 
 
 
ATXN2
 
 
hsa-miR-10a-5p
 
 
-0.362
 
 
13.3
 
 
 
 
B3GNT7
 
 
hsa-miR-27a-3p
 
 
-0.355
 
 
24.0
 
 
 
 
B4GALT2
 
 
hsa-miR-34a-5p
 
 
-0.314
 
 
40.3
 
 
 
 
B4GALT3
 
 
hsa-miR-27a-3p
 
 
-0.41
 
 
32.7
 
 
 
 
B4GALT5
 
 
hsa-miR-152-3p
 
 
-0.44
 
 
11.8
 
 
 
 
BAG5
 
 
hsa-miR-22-3p
 
 
-0.318
 
 
11.5
 
 
 
 
BAZ1B
 
 
hsa-miR-10a-5p
 
 
-0.309
 
 
19.8
 
 
 
 
BAZ2A
 
 
hsa-miR-100-5p
 
 
-0.29
 
 
29.1
 
 
 
 
BAZ2A
 
 
hsa-miR-100-5p
 
 
-0.421
 
 
29.1
 
 
 
 
BBX
 
 
hsa-miR-10a-5p
 
 
-0.305
 
 
11.6
 
 
 
 
BCL2L11
 
 
hsa-miR-152-3p
 
 
-0.445
 
 
12.8
 
 
 
 
BCL2L11
 
 
hsa-miR-10a-5p
 
 
-0.483
 
 
12.8
 
 
 
 
BCL2L13
 
 
hsa-miR-34a-5p
 
 
-0.431
 
 
10.9
 
 
 
 
BCL3
 
 
hsa-miR-27a-3p
 
 
-0.413
 
 
41.4
 
 
 
 
BEGAIN
 
 
hsa-miR-28-5p
 
 
-0.325
 
 
22.6
 
 
 
 
BEND7
 
 
hsa-miR-27a-3p
 
 
-0.304
 
 
12.3
 
 
 
 
BEX3
 
 
hsa-miR-27a-3p
 
 
-0.454
 
 
113.1
 
 
 
 
BHLHE40
 
 
hsa-miR-181a-5p
 
 
-0.408
 
 
75.3
 
 
 
 
BICC1
 
 
hsa-miR-152-3p
 
 
-0.442
 
 
15.1
 
 
 
 
BIN1
 
 
hsa-miR-22-3p
 
 
-0.346
 
 
20.4
 
 
 
 
BLOC1S1
 
 
hsa-miR-22-3p
 
 
-0.346
 
 
108.1
 
 
 
 
BLOC1S6
 
 
hsa-miR-181a-5p
 
 
-0.393
 
 
19.3
 
 
 
 
BLTP1
 
 
hsa-miR-27a-3p
 
 
-0.426
 
 
13.7
 
 
 
 
BMI1
 
 
hsa-miR-27a-3p
 
 
-0.322
 
 
24.2
 
 
 
 
BRPF3
 
 
hsa-miR-34a-5p
 
 
-0.33
 
 
17.0
 
 
 
 
BTAF1
 
 
hsa-miR-152-3p
 
 
-0.401
 
 
15.0
 
 
 
 
BTBD3
 
 
hsa-miR-152-3p
 
 
-0.495
 
 
12.8
 
 
 
 
BTG1
 
 
hsa-miR-22-3p
 
 
-0.468
 
 
18.5
 
 
 
 
BTG2
 
 
hsa-miR-27a-3p
 
 
-0.339
 
 
461.2
 
 
 
 
BZW1
 
 
hsa-miR-27a-3p
 
 
-0.337
 
 
59.0
 
 
 
 
C1orf35
 
 
hsa-miR-152-3p
 
 
-0.511
 
 
15.1
 
 
 
 
C5orf24
 
 
hsa-miR-22-3p
 
 
-0.493
 
 
12.3
 
 
 
 
C6orf120
 
 
hsa-miR-34a-5p
 
 
-0.331
 
 
12.1
 
 
 
 
C6orf62
 
 
hsa-miR-181a-5p
 
 
-0.334
 
 
114.8
 
 
 
 
CACYBP
 
 
hsa-miR-27a-3p
 
 
-0.367
 
 
16.5
 
 
 
 
CADM4
 
 
hsa-miR-34a-5p
 
 
-0.365
 
 
14.8
 
 
 
 
CAND1
 
 
hsa-miR-27a-3p
 
 
-0.318
 
 
17.7
 
 
 
 
CAND1
 
 
hsa-miR-152-3p
 
 
-0.44
 
 
17.7
 
 
 
 
CANX
 
 
hsa-miR-152-3p
 
 
-0.434
 
 
163.1
 
 
 
 
CAPN1
 
 
hsa-miR-22-3p
 
 
-0.307
 
 
68.8
 
 
 
 
CAPZA1
 
 
hsa-miR-27a-3p
 
 
-0.325
 
 
43.4
 
 
 
 
CAV2
 
 
hsa-miR-152-3p
 
 
-0.32
 
 
26.6
 
 
 
 
CBFA2T3
 
 
hsa-miR-34a-5p
 
 
-0.501
 
 
25.8
 
 
 
 
CBX4
 
 
hsa-miR-181a-5p
 
 
-0.354
 
 
18.0
 
 
 
 
 
 
 parathyroid gland 
 
 
 
 
target_symbol
 
 
mature_mirna_id
 
 
score
 
 
nTPM
 
 
 
 
 
 
AAGAB
 
 
hsa-miR-34a-5p
 
 
-0.319
 
 
33.0
 
 
 
 
ABCB7
 
 
hsa-miR-152-3p
 
 
-0.515
 
 
10.3
 
 
 
 
ABCD3
 
 
hsa-miR-152-3p
 
 
-0.388
 
 
27.9
 
 
 
 
ABHD5
 
 
hsa-miR-27a-3p
 
 
-0.407
 
 
11.5
 
 
 
 
ABRAXAS2
 
 
hsa-miR-34a-5p
 
 
-0.538
 
 
12.0
 
 
 
 
ACBD3
 
 
hsa-miR-34a-5p
 
 
-0.415
 
 
26.3
 
 
 
 
ACSL1
 
 
hsa-miR-34a-5p
 
 
-0.306
 
 
48.9
 
 
 
 
ACSL4
 
 
hsa-miR-34a-5p
 
 
-0.372
 
 
18.4
 
 
 
 
ACTA2
 
 
hsa-miR-27a-3p
 
 
-0.577
 
 
75.8
 
 
 
 
ADGRA3
 
 
hsa-miR-27a-3p
 
 
-0.296
 
 
11.5
 
 
 
 
ADGRF5
 
 
hsa-miR-152-3p
 
 
-0.354
 
 
12.6
 
 
 
 
ADIPOR2
 
 
hsa-miR-34a-5p
 
 
-0.33
 
 
15.9
 
 
 
 
ADK
 
 
hsa-miR-34a-5p
 
 
-0.374
 
 
41.5
 
 
 
 
ADO
 
 
hsa-miR-34a-5p
 
 
-0.377
 
 
11.6
 
 
 
 
AFG3L2
 
 
hsa-miR-181a-5p
 
 
-0.491
 
 
41.3
 
 
 
 
AGFG1
 
 
hsa-miR-152-3p
 
 
-0.332
 
 
39.0
 
 
 
 
AGPAT3
 
 
hsa-miR-27a-3p
 
 
-0.29
 
 
23.1
 
 
 
 
AGTRAP
 
 
hsa-miR-34a-5p
 
 
-0.328
 
 
24.1
 
 
 
 
AHCYL2
 
 
hsa-miR-34a-5p
 
 
-0.359
 
 
18.0
 
 
 
 
AIDA
 
 
hsa-miR-22-3p
 
 
-0.316
 
 
30.5
 
 
 
 
AKAP1
 
 
hsa-miR-152-3p
 
 
-0.381
 
 
45.1
 
 
 
 
AKIRIN1
 
 
hsa-miR-27a-3p
 
 
-0.368
 
 
40.5
 
 
 
 
ALCAM
 
 
hsa-miR-34a-5p
 
 
-0.303
 
 
412.8
 
 
 
 
ALCAM
 
 
hsa-miR-152-3p
 
 
-0.347
 
 
412.8
 
 
 
 
ALDOA
 
 
hsa-miR-34a-5p
 
 
-0.655
 
 
885.7
 
 
 
 
ANK3
 
 
hsa-miR-34a-5p
 
 
-0.29
 
 
121.9
 
 
 
 
ANK3
 
 
hsa-miR-34a-5p
 
 
-0.347
 
 
121.9
 
 
 
 
ANKHD1-EIF4EBP3
 
 
hsa-miR-22-3p
 
 
-0.479
 
 
19.9
 
 
 
 
ANKRD13A
 
 
hsa-miR-22-3p
 
 
-0.478
 
 
16.3
 
 
 
 
ANKRD40
 
 
hsa-miR-27a-3p
 
 
-0.35
 
 
25.3
 
 
 
 
ANP32B
 
 
hsa-miR-34a-5p
 
 
-0.29
 
 
64.6
 
 
 
 
AP1AR
 
 
hsa-miR-100-5p
 
 
-0.804
 
 
38.5
 
 
 
 
AP3S2
 
 
hsa-miR-34a-5p
 
 
-0.413
 
 
30.5
 
 
 
 
APH1A
 
 
hsa-miR-34a-5p
 
 
-0.304
 
 
103.7
 
 
 
 
APOO
 
 
hsa-miR-181a-5p
 
 
-0.5
 
 
58.1
 
 
 
 
APPBP2
 
 
hsa-miR-27a-3p
 
 
-0.378
 
 
13.6
 
 
 
 
ARF4
 
 
hsa-miR-152-3p
 
 
-0.487
 
 
95.4
 
 
 
 
ARF6
 
 
hsa-miR-181a-5p
 
 
-0.33
 
 
19.3
 
 
 
 
ARFGEF1
 
 
hsa-miR-152-3p
 
 
-0.306
 
 
26.3
 
 
 
 
ARFIP1
 
 
hsa-miR-320d
 
 
-0.339
 
 
21.0
 
 
 
 
ARFIP1
 
 
hsa-miR-152-3p
 
 
-0.474
 
 
21.0
 
 
 
 
ARHGAP1
 
 
hsa-miR-34a-5p
 
 
-0.369
 
 
23.3
 
 
 
 
ARHGAP21
 
 
hsa-miR-152-3p
 
 
-0.287
 
 
13.6
 
 
 
 
ARL6IP1
 
 
hsa-miR-152-3p
 
 
-0.579
 
 
169.8
 
 
 
 
ARL8B
 
 
hsa-miR-152-3p
 
 
-0.337
 
 
45.2
 
 
 
 
ARL8B
 
 
hsa-miR-320d
 
 
-0.376
 
 
45.2
 
 
 
 
ARPC5
 
 
hsa-miR-22-3p
 
 
-0.679
 
 
28.1
 
 
 
 
ARPIN-AP3S2
 
 
hsa-miR-34a-5p
 
 
-0.348
 
 
10.2
 
 
 
 
ARPP19
 
 
hsa-miR-320d
 
 
-0.325
 
 
102.5
 
 
 
 
ARPP19
 
 
hsa-miR-34a-5p
 
 
-0.497
 
 
102.5
 
 
 
 
ARRDC3
 
 
hsa-miR-152-3p
 
 
-0.305
 
 
51.0
 
 
 
 
AS3MT
 
 
hsa-miR-28-5p
 
 
-0.479
 
 
53.2
 
 
 
 
ASAH1
 
 
hsa-miR-27a-3p
 
 
-0.398
 
 
110.6
 
 
 
 
ASH2L
 
 
hsa-miR-320d
 
 
-0.287
 
 
23.4
 
 
 
 
ASPH
 
 
hsa-miR-152-3p
 
 
-0.391
 
 
35.7
 
 
 
 
ATG4B
 
 
hsa-miR-34a-5p
 
 
-0.58
 
 
11.3
 
 
 
 
ATMIN
 
 
hsa-miR-34a-5p
 
 
-0.409
 
 
25.4
 
 
 
 
ATP2B4
 
 
hsa-miR-152-3p
 
 
-0.337
 
 
11.1
 
 
 
 
ATP5MC3
 
 
hsa-miR-27a-3p
 
 
-0.416
 
 
515.1
 
 
 
 
ATP6AP2
 
 
hsa-miR-152-3p
 
 
-0.592
 
 
719.4
 
 
 
 
ATP6AP2
 
 
hsa-miR-152-3p
 
 
-0.668
 
 
719.4
 
 
 
 
ATP8A1
 
 
hsa-miR-22-3p
 
 
-0.468
 
 
33.8
 
 
 
 
ATXN10
 
 
hsa-miR-27a-3p
 
 
-0.295
 
 
61.1
 
 
 
 
ATXN2
 
 
hsa-miR-10a-5p
 
 
-0.362
 
 
17.5
 
 
 
 
AXL
 
 
hsa-miR-34a-5p
 
 
-0.512
 
 
10.0
 
 
 
 
B3GNT7
 
 
hsa-miR-27a-3p
 
 
-0.355
 
 
13.4
 
 
 
 
B4GALT2
 
 
hsa-miR-34a-5p
 
 
-0.314
 
 
13.4
 
 
 
 
B4GALT3
 
 
hsa-miR-27a-3p
 
 
-0.41
 
 
20.6
 
 
 
 
B4GALT5
 
 
hsa-miR-152-3p
 
 
-0.44
 
 
10.6
 
 
 
 
BAG5
 
 
hsa-miR-22-3p
 
 
-0.318
 
 
13.2
 
 
 
 
BAZ1B
 
 
hsa-miR-10a-5p
 
 
-0.309
 
 
26.0
 
 
 
 
BAZ2A
 
 
hsa-miR-100-5p
 
 
-0.29
 
 
31.4
 
 
 
 
BAZ2A
 
 
hsa-miR-100-5p
 
 
-0.421
 
 
31.4
 
 
 
 
BBX
 
 
hsa-miR-10a-5p
 
 
-0.305
 
 
43.6
 
 
 
 
BCL2
 
 
hsa-miR-34a-5p
 
 
-0.289
 
 
12.3
 
 
 
 
BCL2L11
 
 
hsa-miR-152-3p
 
 
-0.445
 
 
17.6
 
 
 
 
BCL2L11
 
 
hsa-miR-10a-5p
 
 
-0.483
 
 
17.6
 
 
 
 
BCL2L13
 
 
hsa-miR-34a-5p
 
 
-0.431
 
 
13.9
 
 
 
 
BEND7
 
 
hsa-miR-27a-3p
 
 
-0.304
 
 
15.8
 
 
 
 
BEX3
 
 
hsa-miR-27a-3p
 
 
-0.454
 
 
217.0
 
 
 
 
BHLHE40
 
 
hsa-miR-181a-5p
 
 
-0.408
 
 
63.5
 
 
 
 
BIN1
 
 
hsa-miR-22-3p
 
 
-0.346
 
 
18.4
 
 
 
 
BLOC1S1
 
 
hsa-miR-22-3p
 
 
-0.346
 
 
168.9
 
 
 
 
BLOC1S6
 
 
hsa-miR-181a-5p
 
 
-0.393
 
 
15.8
 
 
 
 
BLTP1
 
 
hsa-miR-27a-3p
 
 
-0.426
 
 
39.7
 
 
 
 
BMI1
 
 
hsa-miR-27a-3p
 
 
-0.322
 
 
53.5
 
 
 
 
BMPR2
 
 
hsa-miR-100-5p
 
 
-0.355
 
 
12.6
 
 
 
 
BRPF3
 
 
hsa-miR-34a-5p
 
 
-0.33
 
 
13.6
 
 
 
 
BRSK1
 
 
hsa-miR-27a-3p
 
 
-0.372
 
 
15.8
 
 
 
 
BTAF1
 
 
hsa-miR-152-3p
 
 
-0.401
 
 
11.8
 
 
 
 
BTBD3
 
 
hsa-miR-152-3p
 
 
-0.495
 
 
10.5
 
 
 
 
BTG2
 
 
hsa-miR-27a-3p
 
 
-0.339
 
 
35.2
 
 
 
 
BZW1
 
 
hsa-miR-27a-3p
 
 
-0.337
 
 
68.1
 
 
 
 
C1GALT1
 
 
hsa-miR-152-3p
 
 
-0.487
 
 
43.0
 
 
 
 
C5orf24
 
 
hsa-miR-22-3p
 
 
-0.493
 
 
25.2
 
 
 
 
C6orf120
 
 
hsa-miR-34a-5p
 
 
-0.331
 
 
10.6
 
 
 
 
C6orf62
 
 
hsa-miR-181a-5p
 
 
-0.334
 
 
66.9
 
 
 
 
CACYBP
 
 
hsa-miR-27a-3p
 
 
-0.367
 
 
19.4
 
 
 
 
CADM4
 
 
hsa-miR-34a-5p
 
 
-0.365
 
 
35.8
 
 
 
 
CAMK2N1
 
 
hsa-miR-22-3p
 
 
-0.302
 
 
39.8
 
 
 
 
 
 
 pituitary gland 
 
 
 
 
target_symbol
 
 
mature_mirna_id
 
 
score
 
 
nTPM
 
 
 
 
 
 
AAGAB
 
 
hsa-miR-34a-5p
 
 
-0.319
 
 
15.6
 
 
 
 
ABCD3
 
 
hsa-miR-152-3p
 
 
-0.388
 
 
12.4
 
 
 
 
ACBD3
 
 
hsa-miR-34a-5p
 
 
-0.415
 
 
13.6
 
 
 
 
ACSL1
 
 
hsa-miR-34a-5p
 
 
-0.306
 
 
39.0
 
 
 
 
ACTA2
 
 
hsa-miR-27a-3p
 
 
-0.577
 
 
56.3
 
 
 
 
ADGRB3
 
 
hsa-miR-152-3p
 
 
-0.516
 
 
21.3
 
 
 
 
ADIPOR2
 
 
hsa-miR-34a-5p
 
 
-0.33
 
 
25.4
 
 
 
 
ADK
 
 
hsa-miR-34a-5p
 
 
-0.374
 
 
14.1
 
 
 
 
AGFG1
 
 
hsa-miR-152-3p
 
 
-0.332
 
 
16.7
 
 
 
 
AGPAT3
 
 
hsa-miR-27a-3p
 
 
-0.29
 
 
13.9
 
 
 
 
AGTRAP
 
 
hsa-miR-34a-5p
 
 
-0.328
 
 
29.7
 
 
 
 
AHCYL2
 
 
hsa-miR-34a-5p
 
 
-0.359
 
 
19.9
 
 
 
 
AHDC1
 
 
hsa-miR-152-3p
 
 
-0.413
 
 
18.1
 
 
 
 
AIDA
 
 
hsa-miR-22-3p
 
 
-0.316
 
 
20.6
 
 
 
 
AKAP1
 
 
hsa-miR-152-3p
 
 
-0.381
 
 
16.5
 
 
 
 
AKIRIN1
 
 
hsa-miR-27a-3p
 
 
-0.368
 
 
37.8
 
 
 
 
AKT3
 
 
hsa-miR-22-3p
 
 
-0.451
 
 
11.4
 
 
 
 
ALCAM
 
 
hsa-miR-34a-5p
 
 
-0.303
 
 
17.9
 
 
 
 
ALCAM
 
 
hsa-miR-152-3p
 
 
-0.347
 
 
17.9
 
 
 
 
ALDOA
 
 
hsa-miR-34a-5p
 
 
-0.655
 
 
586.0
 
 
 
 
ANK3
 
 
hsa-miR-34a-5p
 
 
-0.29
 
 
13.8
 
 
 
 
ANK3
 
 
hsa-miR-34a-5p
 
 
-0.347
 
 
13.8
 
 
 
 
ANKHD1-EIF4EBP3
 
 
hsa-miR-22-3p
 
 
-0.479
 
 
10.0
 
 
 
 
ANKRD40
 
 
hsa-miR-27a-3p
 
 
-0.35
 
 
10.1
 
 
 
 
ANP32B
 
 
hsa-miR-34a-5p
 
 
-0.29
 
 
64.1
 
 
 
 
APH1A
 
 
hsa-miR-34a-5p
 
 
-0.304
 
 
54.3
 
 
 
 
APOO
 
 
hsa-miR-181a-5p
 
 
-0.5
 
 
28.1
 
 
 
 
ARF4
 
 
hsa-miR-152-3p
 
 
-0.487
 
 
93.4
 
 
 
 
ARHGAP1
 
 
hsa-miR-34a-5p
 
 
-0.369
 
 
35.2
 
 
 
 
ARHGAP21
 
 
hsa-miR-152-3p
 
 
-0.287
 
 
11.3
 
 
 
 
ARHGAP26
 
 
hsa-miR-34a-5p
 
 
-0.384
 
 
11.6
 
 
 
 
ARHGAP36
 
 
hsa-miR-34a-5p
 
 
-0.367
 
 
166.5
 
 
 
 
ARL6IP1
 
 
hsa-miR-152-3p
 
 
-0.579
 
 
39.7
 
 
 
 
ARL8B
 
 
hsa-miR-152-3p
 
 
-0.337
 
 
25.6
 
 
 
 
ARL8B
 
 
hsa-miR-320d
 
 
-0.376
 
 
25.6
 
 
 
 
ARPC5
 
 
hsa-miR-22-3p
 
 
-0.679
 
 
18.4
 
 
 
 
ARPP19
 
 
hsa-miR-320d
 
 
-0.325
 
 
33.0
 
 
 
 
ARPP19
 
 
hsa-miR-34a-5p
 
 
-0.497
 
 
33.0
 
 
 
 
ARRDC3
 
 
hsa-miR-152-3p
 
 
-0.305
 
 
20.2
 
 
 
 
ASAH1
 
 
hsa-miR-27a-3p
 
 
-0.398
 
 
75.8
 
 
 
 
ASH2L
 
 
hsa-miR-320d
 
 
-0.287
 
 
11.5
 
 
 
 
ASPH
 
 
hsa-miR-152-3p
 
 
-0.391
 
 
33.8
 
 
 
 
ATCAY
 
 
hsa-miR-10a-5p
 
 
-0.322
 
 
24.7
 
 
 
 
ATG4B
 
 
hsa-miR-34a-5p
 
 
-0.58
 
 
23.4
 
 
 
 
ATMIN
 
 
hsa-miR-34a-5p
 
 
-0.409
 
 
12.7
 
 
 
 
ATP1A2
 
 
hsa-miR-34a-5p
 
 
-0.306
 
 
29.2
 
 
 
 
ATP5MC3
 
 
hsa-miR-27a-3p
 
 
-0.416
 
 
134.5
 
 
 
 
ATP6AP2
 
 
hsa-miR-152-3p
 
 
-0.592
 
 
34.0
 
 
 
 
ATP6AP2
 
 
hsa-miR-152-3p
 
 
-0.668
 
 
34.0
 
 
 
 
ATXN10
 
 
hsa-miR-27a-3p
 
 
-0.295
 
 
51.5
 
 
 
 
ATXN2
 
 
hsa-miR-10a-5p
 
 
-0.362
 
 
21.6
 
 
 
 
B4GALT2
 
 
hsa-miR-34a-5p
 
 
-0.314
 
 
20.4
 
 
 
 
B4GALT3
 
 
hsa-miR-27a-3p
 
 
-0.41
 
 
31.7
 
 
 
 
B4GALT5
 
 
hsa-miR-152-3p
 
 
-0.44
 
 
11.9
 
 
 
 
BAG5
 
 
hsa-miR-22-3p
 
 
-0.318
 
 
10.5
 
 
 
 
BAZ1B
 
 
hsa-miR-10a-5p
 
 
-0.309
 
 
18.2
 
 
 
 
BAZ2A
 
 
hsa-miR-100-5p
 
 
-0.29
 
 
25.8
 
 
 
 
BAZ2A
 
 
hsa-miR-100-5p
 
 
-0.421
 
 
25.8
 
 
 
 
BCL3
 
 
hsa-miR-27a-3p
 
 
-0.413
 
 
29.0
 
 
 
 
BEGAIN
 
 
hsa-miR-28-5p
 
 
-0.325
 
 
18.8
 
 
 
 
BEX3
 
 
hsa-miR-27a-3p
 
 
-0.454
 
 
389.6
 
 
 
 
BHLHE40
 
 
hsa-miR-181a-5p
 
 
-0.408
 
 
99.3
 
 
 
 
BHLHE41
 
 
hsa-miR-152-3p
 
 
-0.442
 
 
15.9
 
 
 
 
BIN1
 
 
hsa-miR-22-3p
 
 
-0.346
 
 
39.7
 
 
 
 
BLOC1S1
 
 
hsa-miR-22-3p
 
 
-0.346
 
 
107.6
 
 
 
 
BLOC1S6
 
 
hsa-miR-181a-5p
 
 
-0.393
 
 
19.0
 
 
 
 
BLTP1
 
 
hsa-miR-27a-3p
 
 
-0.426
 
 
25.8
 
 
 
 
BMI1
 
 
hsa-miR-27a-3p
 
 
-0.322
 
 
22.6
 
 
 
 
BRSK1
 
 
hsa-miR-27a-3p
 
 
-0.372
 
 
49.5
 
 
 
 
BTAF1
 
 
hsa-miR-152-3p
 
 
-0.401
 
 
11.4
 
 
 
 
BTG2
 
 
hsa-miR-27a-3p
 
 
-0.339
 
 
204.0
 
 
 
 
BZW1
 
 
hsa-miR-27a-3p
 
 
-0.337
 
 
28.1
 
 
 
 
C1orf35
 
 
hsa-miR-152-3p
 
 
-0.511
 
 
11.5
 
 
 
 
C2CD4A
 
 
hsa-miR-34a-5p
 
 
-0.396
 
 
12.7
 
 
 
 
C5orf24
 
 
hsa-miR-22-3p
 
 
-0.493
 
 
14.4
 
 
 
 
C6orf62
 
 
hsa-miR-181a-5p
 
 
-0.334
 
 
60.8
 
 
 
 
CACNB3
 
 
hsa-miR-34a-5p
 
 
-0.426
 
 
11.5
 
 
 
 
CACYBP
 
 
hsa-miR-27a-3p
 
 
-0.367
 
 
25.2
 
 
 
 
CADM4
 
 
hsa-miR-34a-5p
 
 
-0.365
 
 
29.7
 
 
 
 
CAMK2N1
 
 
hsa-miR-22-3p
 
 
-0.302
 
 
33.4
 
 
 
 
CAND1
 
 
hsa-miR-27a-3p
 
 
-0.318
 
 
18.1
 
 
 
 
CAND1
 
 
hsa-miR-152-3p
 
 
-0.44
 
 
18.1
 
 
 
 
CANX
 
 
hsa-miR-152-3p
 
 
-0.434
 
 
83.6
 
 
 
 
CAPN1
 
 
hsa-miR-22-3p
 
 
-0.307
 
 
38.7
 
 
 
 
CAPZA1
 
 
hsa-miR-27a-3p
 
 
-0.325
 
 
26.6
 
 
 
 
CBX4
 
 
hsa-miR-181a-5p
 
 
-0.354
 
 
17.9
 
 
 
 
CCDC28B
 
 
hsa-miR-27a-3p
 
 
-0.314
 
 
24.4
 
 
 
 
CCDC47
 
 
hsa-miR-22-3p
 
 
-0.604
 
 
20.7
 
 
 
 
CCDC6
 
 
hsa-miR-152-3p
 
 
-0.511
 
 
11.1
 
 
 
 
CCM2
 
 
hsa-miR-27a-3p
 
 
-0.335
 
 
30.1
 
 
 
 
CCN1
 
 
hsa-miR-22-3p
 
 
-0.601
 
 
23.5
 
 
 
 
CCNC
 
 
hsa-miR-27a-3p
 
 
-0.378
 
 
22.0
 
 
 
 
CCNG1
 
 
hsa-miR-27a-3p
 
 
-0.419
 
 
13.6
 
 
 
 
CCNK
 
 
hsa-miR-181a-5p
 
 
-0.31
 
 
18.2
 
 
 
 
CCNK
 
 
hsa-miR-27a-3p
 
 
-0.519
 
 
18.2
 
 
 
 
CCNL2
 
 
hsa-miR-28-5p
 
 
-0.448
 
 
56.8
 
 
 
 
CCNY
 
 
hsa-miR-27a-3p
 
 
-0.334
 
 
13.7
 
 
 
 
CCT6A
 
 
hsa-miR-152-3p
 
 
-0.665
 
 
39.3
 
 
 
 
CDC25B
 
 
hsa-miR-152-3p
 
 
-0.298
 
 
44.9
 
 
 
 
CDIP1
 
 
hsa-miR-27a-3p
 
 
-0.362
 
 
59.9
 
 
 
 
 
 
 placenta 
 
 
 
 
target_symbol
 
 
mature_mirna_id
 
 
score
 
 
nTPM
 
 
 
 
 
 
AAGAB
 
 
hsa-miR-34a-5p
 
 
-0.319
 
 
14.8
 
 
 
 
ABCB10
 
 
hsa-miR-152-3p
 
 
-0.291
 
 
10.6
 
 
 
 
ABCD3
 
 
hsa-miR-152-3p
 
 
-0.388
 
 
13.6
 
 
 
 
ACBD3
 
 
hsa-miR-34a-5p
 
 
-0.415
 
 
29.8
 
 
 
 
ACSL1
 
 
hsa-miR-34a-5p
 
 
-0.306
 
 
25.4
 
 
 
 
ACSL4
 
 
hsa-miR-34a-5p
 
 
-0.372
 
 
19.4
 
 
 
 
ACTA2
 
 
hsa-miR-27a-3p
 
 
-0.577
 
 
723.7
 
 
 
 
ACVR1
 
 
hsa-miR-152-3p
 
 
-0.397
 
 
12.5
 
 
 
 
ADAM10
 
 
hsa-miR-152-3p
 
 
-0.348
 
 
27.1
 
 
 
 
ADAM17
 
 
hsa-miR-152-3p
 
 
-0.301
 
 
17.9
 
 
 
 
ADGRF5
 
 
hsa-miR-152-3p
 
 
-0.354
 
 
48.3
 
 
 
 
ADIPOR2
 
 
hsa-miR-34a-5p
 
 
-0.33
 
 
12.2
 
 
 
 
ADK
 
 
hsa-miR-34a-5p
 
 
-0.374
 
 
25.0
 
 
 
 
AFG3L2
 
 
hsa-miR-181a-5p
 
 
-0.491
 
 
12.5
 
 
 
 
AGFG1
 
 
hsa-miR-152-3p
 
 
-0.332
 
 
71.2
 
 
 
 
AGPAT3
 
 
hsa-miR-27a-3p
 
 
-0.29
 
 
11.5
 
 
 
 
AGTRAP
 
 
hsa-miR-34a-5p
 
 
-0.328
 
 
20.7
 
 
 
 
AHCYL2
 
 
hsa-miR-34a-5p
 
 
-0.359
 
 
11.3
 
 
 
 
AIDA
 
 
hsa-miR-22-3p
 
 
-0.316
 
 
44.9
 
 
 
 
AKIRIN1
 
 
hsa-miR-27a-3p
 
 
-0.368
 
 
24.9
 
 
 
 
AKT3
 
 
hsa-miR-22-3p
 
 
-0.451
 
 
12.3
 
 
 
 
ALCAM
 
 
hsa-miR-34a-5p
 
 
-0.303
 
 
11.3
 
 
 
 
ALCAM
 
 
hsa-miR-152-3p
 
 
-0.347
 
 
11.3
 
 
 
 
ALDOA
 
 
hsa-miR-34a-5p
 
 
-0.655
 
 
329.7
 
 
 
 
ANK3
 
 
hsa-miR-34a-5p
 
 
-0.29
 
 
14.5
 
 
 
 
ANK3
 
 
hsa-miR-34a-5p
 
 
-0.347
 
 
14.5
 
 
 
 
ANKRD13A
 
 
hsa-miR-22-3p
 
 
-0.478
 
 
22.9
 
 
 
 
ANKRD40
 
 
hsa-miR-27a-3p
 
 
-0.35
 
 
21.7
 
 
 
 
ANP32B
 
 
hsa-miR-34a-5p
 
 
-0.29
 
 
103.8
 
 
 
 
AP1AR
 
 
hsa-miR-100-5p
 
 
-0.804
 
 
11.2
 
 
 
 
AP3S2
 
 
hsa-miR-34a-5p
 
 
-0.413
 
 
17.4
 
 
 
 
APH1A
 
 
hsa-miR-34a-5p
 
 
-0.304
 
 
68.0
 
 
 
 
APOO
 
 
hsa-miR-181a-5p
 
 
-0.5
 
 
10.3
 
 
 
 
APPBP2
 
 
hsa-miR-27a-3p
 
 
-0.378
 
 
11.5
 
 
 
 
AREG
 
 
hsa-miR-34a-5p
 
 
-0.62
 
 
308.9
 
 
 
 
ARF4
 
 
hsa-miR-152-3p
 
 
-0.487
 
 
170.7
 
 
 
 
ARF6
 
 
hsa-miR-181a-5p
 
 
-0.33
 
 
34.8
 
 
 
 
ARFGEF1
 
 
hsa-miR-152-3p
 
 
-0.306
 
 
13.6
 
 
 
 
ARFIP1
 
 
hsa-miR-320d
 
 
-0.339
 
 
16.9
 
 
 
 
ARFIP1
 
 
hsa-miR-152-3p
 
 
-0.474
 
 
16.9
 
 
 
 
ARHGAP1
 
 
hsa-miR-34a-5p
 
 
-0.369
 
 
36.7
 
 
 
 
ARHGAP21
 
 
hsa-miR-152-3p
 
 
-0.287
 
 
15.6
 
 
 
 
ARHGAP26
 
 
hsa-miR-34a-5p
 
 
-0.384
 
 
19.6
 
 
 
 
ARL6IP1
 
 
hsa-miR-152-3p
 
 
-0.579
 
 
84.9
 
 
 
 
ARL8B
 
 
hsa-miR-152-3p
 
 
-0.337
 
 
46.8
 
 
 
 
ARL8B
 
 
hsa-miR-320d
 
 
-0.376
 
 
46.8
 
 
 
 
ARPC5
 
 
hsa-miR-22-3p
 
 
-0.679
 
 
101.6
 
 
 
 
ARPP19
 
 
hsa-miR-320d
 
 
-0.325
 
 
53.5
 
 
 
 
ARPP19
 
 
hsa-miR-34a-5p
 
 
-0.497
 
 
53.5
 
 
 
 
ARRB1
 
 
hsa-miR-22-3p
 
 
-0.616
 
 
23.6
 
 
 
 
ARRDC3
 
 
hsa-miR-152-3p
 
 
-0.305
 
 
72.1
 
 
 
 
ASAH1
 
 
hsa-miR-27a-3p
 
 
-0.398
 
 
114.2
 
 
 
 
ASH2L
 
 
hsa-miR-320d
 
 
-0.287
 
 
20.9
 
 
 
 
ASPH
 
 
hsa-miR-152-3p
 
 
-0.391
 
 
44.6
 
 
 
 
ATMIN
 
 
hsa-miR-34a-5p
 
 
-0.409
 
 
19.2
 
 
 
 
ATP2B4
 
 
hsa-miR-152-3p
 
 
-0.337
 
 
38.2
 
 
 
 
ATP5MC3
 
 
hsa-miR-27a-3p
 
 
-0.416
 
 
95.9
 
 
 
 
ATP6AP2
 
 
hsa-miR-152-3p
 
 
-0.592
 
 
100.4
 
 
 
 
ATP6AP2
 
 
hsa-miR-152-3p
 
 
-0.668
 
 
100.4
 
 
 
 
ATXN10
 
 
hsa-miR-27a-3p
 
 
-0.295
 
 
45.8
 
 
 
 
ATXN2
 
 
hsa-miR-10a-5p
 
 
-0.362
 
 
15.3
 
 
 
 
AXL
 
 
hsa-miR-34a-5p
 
 
-0.512
 
 
32.0
 
 
 
 
B4GALT3
 
 
hsa-miR-27a-3p
 
 
-0.41
 
 
20.4
 
 
 
 
B4GALT5
 
 
hsa-miR-152-3p
 
 
-0.44
 
 
25.6
 
 
 
 
BAG4
 
 
hsa-miR-181a-5p
 
 
-0.337
 
 
10.7
 
 
 
 
BAG5
 
 
hsa-miR-22-3p
 
 
-0.318
 
 
13.6
 
 
 
 
BAZ1B
 
 
hsa-miR-10a-5p
 
 
-0.309
 
 
21.3
 
 
 
 
BAZ2A
 
 
hsa-miR-100-5p
 
 
-0.29
 
 
33.2
 
 
 
 
BAZ2A
 
 
hsa-miR-100-5p
 
 
-0.421
 
 
33.2
 
 
 
 
BBX
 
 
hsa-miR-10a-5p
 
 
-0.305
 
 
22.7
 
 
 
 
BCORL1
 
 
hsa-miR-146a-5p
 
 
-0.287
 
 
10.6
 
 
 
 
BCORL1
 
 
hsa-miR-146b-5p
 
 
-0.287
 
 
10.6
 
 
 
 
BEX3
 
 
hsa-miR-27a-3p
 
 
-0.454
 
 
194.4
 
 
 
 
BHLHE40
 
 
hsa-miR-181a-5p
 
 
-0.408
 
 
67.8
 
 
 
 
BIN1
 
 
hsa-miR-22-3p
 
 
-0.346
 
 
22.1
 
 
 
 
BLOC1S1
 
 
hsa-miR-22-3p
 
 
-0.346
 
 
69.0
 
 
 
 
BLOC1S6
 
 
hsa-miR-181a-5p
 
 
-0.393
 
 
24.7
 
 
 
 
BMI1
 
 
hsa-miR-27a-3p
 
 
-0.322
 
 
27.0
 
 
 
 
BMPR2
 
 
hsa-miR-100-5p
 
 
-0.355
 
 
21.8
 
 
 
 
BTAF1
 
 
hsa-miR-152-3p
 
 
-0.401
 
 
10.9
 
 
 
 
BTBD3
 
 
hsa-miR-152-3p
 
 
-0.495
 
 
39.5
 
 
 
 
BTG2
 
 
hsa-miR-27a-3p
 
 
-0.339
 
 
100.2
 
 
 
 
BZW1
 
 
hsa-miR-27a-3p
 
 
-0.337
 
 
98.8
 
 
 
 
C1GALT1
 
 
hsa-miR-152-3p
 
 
-0.487
 
 
25.3
 
 
 
 
C1orf52
 
 
hsa-miR-27a-3p
 
 
-0.591
 
 
11.4
 
 
 
 
C5orf24
 
 
hsa-miR-22-3p
 
 
-0.493
 
 
26.9
 
 
 
 
C6orf120
 
 
hsa-miR-34a-5p
 
 
-0.331
 
 
14.8
 
 
 
 
C6orf62
 
 
hsa-miR-181a-5p
 
 
-0.334
 
 
82.1
 
 
 
 
CACYBP
 
 
hsa-miR-27a-3p
 
 
-0.367
 
 
21.4
 
 
 
 
CAMK2N1
 
 
hsa-miR-22-3p
 
 
-0.302
 
 
10.9
 
 
 
 
CAND1
 
 
hsa-miR-27a-3p
 
 
-0.318
 
 
21.8
 
 
 
 
CAND1
 
 
hsa-miR-152-3p
 
 
-0.44
 
 
21.8
 
 
 
 
CANX
 
 
hsa-miR-152-3p
 
 
-0.434
 
 
70.1
 
 
 
 
CAPN1
 
 
hsa-miR-22-3p
 
 
-0.307
 
 
52.8
 
 
 
 
CAPZA1
 
 
hsa-miR-27a-3p
 
 
-0.325
 
 
80.3
 
 
 
 
CASP2
 
 
hsa-miR-34a-5p
 
 
-0.382
 
 
11.7
 
 
 
 
CAV2
 
 
hsa-miR-152-3p
 
 
-0.32
 
 
87.6
 
 
 
 
CBX4
 
 
hsa-miR-181a-5p
 
 
-0.354
 
 
14.3
 
 
 
 
CCDC47
 
 
hsa-miR-22-3p
 
 
-0.604
 
 
50.6
 
 
 
 
CCDC6
 
 
hsa-miR-152-3p
 
 
-0.511
 
 
12.4
 
 
 
 
 
 
 prostate 
 
 
 
 
target_symbol
 
 
mature_mirna_id
 
 
score
 
 
nTPM
 
 
 
 
 
 
AAGAB
 
 
hsa-miR-34a-5p
 
 
-0.319
 
 
17.6
 
 
 
 
ABCD3
 
 
hsa-miR-152-3p
 
 
-0.388
 
 
20.2
 
 
 
 
ABHD17C
 
 
hsa-miR-27a-3p
 
 
-0.435
 
 
17.0
 
 
 
 
ACBD3
 
 
hsa-miR-34a-5p
 
 
-0.415
 
 
21.4
 
 
 
 
ACSL1
 
 
hsa-miR-34a-5p
 
 
-0.306
 
 
75.4
 
 
 
 
ACSL4
 
 
hsa-miR-34a-5p
 
 
-0.372
 
 
12.3
 
 
 
 
ACTA2
 
 
hsa-miR-27a-3p
 
 
-0.577
 
 
2476.6
 
 
 
 
ADAM10
 
 
hsa-miR-152-3p
 
 
-0.348
 
 
23.6
 
 
 
 
ADGRA3
 
 
hsa-miR-27a-3p
 
 
-0.296
 
 
11.3
 
 
 
 
ADIPOR2
 
 
hsa-miR-34a-5p
 
 
-0.33
 
 
19.8
 
 
 
 
ADK
 
 
hsa-miR-34a-5p
 
 
-0.374
 
 
14.0
 
 
 
 
AFG3L2
 
 
hsa-miR-181a-5p
 
 
-0.491
 
 
13.2
 
 
 
 
AGFG1
 
 
hsa-miR-152-3p
 
 
-0.332
 
 
19.4
 
 
 
 
AGPAT3
 
 
hsa-miR-27a-3p
 
 
-0.29
 
 
12.9
 
 
 
 
AGTRAP
 
 
hsa-miR-34a-5p
 
 
-0.328
 
 
75.3
 
 
 
 
AHDC1
 
 
hsa-miR-152-3p
 
 
-0.413
 
 
11.3
 
 
 
 
AIDA
 
 
hsa-miR-22-3p
 
 
-0.316
 
 
32.9
 
 
 
 
AKAP1
 
 
hsa-miR-152-3p
 
 
-0.381
 
 
33.3
 
 
 
 
AKIRIN1
 
 
hsa-miR-27a-3p
 
 
-0.368
 
 
34.0
 
 
 
 
AKT3
 
 
hsa-miR-22-3p
 
 
-0.451
 
 
13.8
 
 
 
 
ALCAM
 
 
hsa-miR-34a-5p
 
 
-0.303
 
 
30.7
 
 
 
 
ALCAM
 
 
hsa-miR-152-3p
 
 
-0.347
 
 
30.7
 
 
 
 
ALDOA
 
 
hsa-miR-34a-5p
 
 
-0.655
 
 
529.6
 
 
 
 
ANK3
 
 
hsa-miR-34a-5p
 
 
-0.29
 
 
18.4
 
 
 
 
ANK3
 
 
hsa-miR-34a-5p
 
 
-0.347
 
 
18.4
 
 
 
 
ANKHD1-EIF4EBP3
 
 
hsa-miR-22-3p
 
 
-0.479
 
 
11.9
 
 
 
 
ANKRD13A
 
 
hsa-miR-22-3p
 
 
-0.478
 
 
16.8
 
 
 
 
ANKRD40
 
 
hsa-miR-27a-3p
 
 
-0.35
 
 
17.2
 
 
 
 
ANP32B
 
 
hsa-miR-34a-5p
 
 
-0.29
 
 
93.4
 
 
 
 
AP3S2
 
 
hsa-miR-34a-5p
 
 
-0.413
 
 
14.3
 
 
 
 
APH1A
 
 
hsa-miR-34a-5p
 
 
-0.304
 
 
58.2
 
 
 
 
APOO
 
 
hsa-miR-181a-5p
 
 
-0.5
 
 
12.8
 
 
 
 
APPBP2
 
 
hsa-miR-27a-3p
 
 
-0.378
 
 
12.8
 
 
 
 
ARF4
 
 
hsa-miR-152-3p
 
 
-0.487
 
 
129.9
 
 
 
 
ARF6
 
 
hsa-miR-181a-5p
 
 
-0.33
 
 
30.5
 
 
 
 
ARFGEF1
 
 
hsa-miR-152-3p
 
 
-0.306
 
 
12.2
 
 
 
 
ARFIP1
 
 
hsa-miR-320d
 
 
-0.339
 
 
19.8
 
 
 
 
ARFIP1
 
 
hsa-miR-152-3p
 
 
-0.474
 
 
19.8
 
 
 
 
ARHGAP1
 
 
hsa-miR-34a-5p
 
 
-0.369
 
 
60.9
 
 
 
 
ARHGAP21
 
 
hsa-miR-152-3p
 
 
-0.287
 
 
10.2
 
 
 
 
ARL6IP1
 
 
hsa-miR-152-3p
 
 
-0.579
 
 
71.3
 
 
 
 
ARL8B
 
 
hsa-miR-152-3p
 
 
-0.337
 
 
42.2
 
 
 
 
ARL8B
 
 
hsa-miR-320d
 
 
-0.376
 
 
42.2
 
 
 
 
ARPC5
 
 
hsa-miR-22-3p
 
 
-0.679
 
 
43.1
 
 
 
 
ARPIN-AP3S2
 
 
hsa-miR-34a-5p
 
 
-0.348
 
 
10.1
 
 
 
 
ARPP19
 
 
hsa-miR-320d
 
 
-0.325
 
 
37.0
 
 
 
 
ARPP19
 
 
hsa-miR-34a-5p
 
 
-0.497
 
 
37.0
 
 
 
 
ARRB1
 
 
hsa-miR-22-3p
 
 
-0.616
 
 
15.2
 
 
 
 
ARRDC3
 
 
hsa-miR-152-3p
 
 
-0.305
 
 
28.5
 
 
 
 
ASAH1
 
 
hsa-miR-27a-3p
 
 
-0.398
 
 
79.1
 
 
 
 
ASH2L
 
 
hsa-miR-320d
 
 
-0.287
 
 
18.9
 
 
 
 
ASPH
 
 
hsa-miR-152-3p
 
 
-0.391
 
 
44.4
 
 
 
 
ATG4B
 
 
hsa-miR-34a-5p
 
 
-0.58
 
 
25.1
 
 
 
 
ATMIN
 
 
hsa-miR-34a-5p
 
 
-0.409
 
 
16.5
 
 
 
 
ATP1A2
 
 
hsa-miR-34a-5p
 
 
-0.306
 
 
26.7
 
 
 
 
ATP2B4
 
 
hsa-miR-152-3p
 
 
-0.337
 
 
31.1
 
 
 
 
ATP5MC3
 
 
hsa-miR-27a-3p
 
 
-0.416
 
 
106.2
 
 
 
 
ATP6AP2
 
 
hsa-miR-152-3p
 
 
-0.592
 
 
81.2
 
 
 
 
ATP6AP2
 
 
hsa-miR-152-3p
 
 
-0.668
 
 
81.2
 
 
 
 
ATXN10
 
 
hsa-miR-27a-3p
 
 
-0.295
 
 
43.6
 
 
 
 
ATXN2
 
 
hsa-miR-10a-5p
 
 
-0.362
 
 
18.8
 
 
 
 
AXL
 
 
hsa-miR-34a-5p
 
 
-0.512
 
 
17.8
 
 
 
 
B4GALT2
 
 
hsa-miR-34a-5p
 
 
-0.314
 
 
24.1
 
 
 
 
B4GALT3
 
 
hsa-miR-27a-3p
 
 
-0.41
 
 
20.7
 
 
 
 
B4GALT5
 
 
hsa-miR-152-3p
 
 
-0.44
 
 
16.0
 
 
 
 
BAG5
 
 
hsa-miR-22-3p
 
 
-0.318
 
 
13.1
 
 
 
 
BAZ1B
 
 
hsa-miR-10a-5p
 
 
-0.309
 
 
21.2
 
 
 
 
BAZ2A
 
 
hsa-miR-100-5p
 
 
-0.29
 
 
21.4
 
 
 
 
BAZ2A
 
 
hsa-miR-100-5p
 
 
-0.421
 
 
21.4
 
 
 
 
BBX
 
 
hsa-miR-10a-5p
 
 
-0.305
 
 
21.3
 
 
 
 
BCL3
 
 
hsa-miR-27a-3p
 
 
-0.413
 
 
45.9
 
 
 
 
BEX3
 
 
hsa-miR-27a-3p
 
 
-0.454
 
 
175.3
 
 
 
 
BHLHE40
 
 
hsa-miR-181a-5p
 
 
-0.408
 
 
103.6
 
 
 
 
BIN1
 
 
hsa-miR-22-3p
 
 
-0.346
 
 
52.2
 
 
 
 
BLOC1S1
 
 
hsa-miR-22-3p
 
 
-0.346
 
 
112.8
 
 
 
 
BLOC1S6
 
 
hsa-miR-181a-5p
 
 
-0.393
 
 
34.2
 
 
 
 
BLTP1
 
 
hsa-miR-27a-3p
 
 
-0.426
 
 
14.1
 
 
 
 
BMI1
 
 
hsa-miR-27a-3p
 
 
-0.322
 
 
31.6
 
 
 
 
BMPR2
 
 
hsa-miR-100-5p
 
 
-0.355
 
 
12.9
 
 
 
 
BTAF1
 
 
hsa-miR-152-3p
 
 
-0.401
 
 
13.7
 
 
 
 
BTBD3
 
 
hsa-miR-152-3p
 
 
-0.495
 
 
14.2
 
 
 
 
BTG2
 
 
hsa-miR-27a-3p
 
 
-0.339
 
 
249.1
 
 
 
 
BZW1
 
 
hsa-miR-27a-3p
 
 
-0.337
 
 
93.5
 
 
 
 
C1orf35
 
 
hsa-miR-152-3p
 
 
-0.511
 
 
16.0
 
 
 
 
C5orf24
 
 
hsa-miR-22-3p
 
 
-0.493
 
 
20.2
 
 
 
 
C6orf120
 
 
hsa-miR-34a-5p
 
 
-0.331
 
 
10.1
 
 
 
 
C6orf62
 
 
hsa-miR-181a-5p
 
 
-0.334
 
 
78.9
 
 
 
 
C8orf88
 
 
hsa-miR-27a-3p
 
 
-0.33
 
 
13.0
 
 
 
 
CACNB3
 
 
hsa-miR-34a-5p
 
 
-0.426
 
 
13.0
 
 
 
 
CACYBP
 
 
hsa-miR-27a-3p
 
 
-0.367
 
 
15.3
 
 
 
 
CADM4
 
 
hsa-miR-34a-5p
 
 
-0.365
 
 
18.6
 
 
 
 
CAMK2N1
 
 
hsa-miR-22-3p
 
 
-0.302
 
 
33.6
 
 
 
 
CAND1
 
 
hsa-miR-27a-3p
 
 
-0.318
 
 
21.0
 
 
 
 
CAND1
 
 
hsa-miR-152-3p
 
 
-0.44
 
 
21.0
 
 
 
 
CANX
 
 
hsa-miR-152-3p
 
 
-0.434
 
 
84.4
 
 
 
 
CAPN1
 
 
hsa-miR-22-3p
 
 
-0.307
 
 
60.3
 
 
 
 
CAPZA1
 
 
hsa-miR-27a-3p
 
 
-0.325
 
 
39.8
 
 
 
 
CAV2
 
 
hsa-miR-152-3p
 
 
-0.32
 
 
30.0
 
 
 
 
CCDC149
 
 
hsa-miR-22-3p
 
 
-0.327
 
 
10.7
 
 
 
 
CCDC149
 
 
hsa-miR-27a-3p
 
 
-0.551
 
 
10.7
 
 
 
 
 
 
 rectum 
 
 
 
 
target_symbol
 
 
mature_mirna_id
 
 
score
 
 
nTPM
 
 
 
 
 
 
AAGAB
 
 
hsa-miR-34a-5p
 
 
-0.319
 
 
31.7
 
 
 
 
ABCB10
 
 
hsa-miR-152-3p
 
 
-0.291
 
 
10.6
 
 
 
 
ABCD3
 
 
hsa-miR-152-3p
 
 
-0.388
 
 
37.8
 
 
 
 
ABHD17C
 
 
hsa-miR-27a-3p
 
 
-0.435
 
 
52.8
 
 
 
 
ACBD3
 
 
hsa-miR-34a-5p
 
 
-0.415
 
 
23.9
 
 
 
 
ACSL1
 
 
hsa-miR-34a-5p
 
 
-0.306
 
 
13.6
 
 
 
 
ACSL4
 
 
hsa-miR-34a-5p
 
 
-0.372
 
 
12.1
 
 
 
 
ACTA2
 
 
hsa-miR-27a-3p
 
 
-0.577
 
 
814.5
 
 
 
 
ADAM10
 
 
hsa-miR-152-3p
 
 
-0.348
 
 
16.1
 
 
 
 
ADAP1
 
 
hsa-miR-27a-3p
 
 
-0.302
 
 
21.3
 
 
 
 
ADGRA3
 
 
hsa-miR-27a-3p
 
 
-0.296
 
 
16.1
 
 
 
 
ADIPOR2
 
 
hsa-miR-34a-5p
 
 
-0.33
 
 
16.7
 
 
 
 
ADK
 
 
hsa-miR-34a-5p
 
 
-0.374
 
 
18.3
 
 
 
 
AFG3L2
 
 
hsa-miR-181a-5p
 
 
-0.491
 
 
28.8
 
 
 
 
AGFG1
 
 
hsa-miR-152-3p
 
 
-0.332
 
 
31.0
 
 
 
 
AGPAT3
 
 
hsa-miR-27a-3p
 
 
-0.29
 
 
11.1
 
 
 
 
AHCYL2
 
 
hsa-miR-34a-5p
 
 
-0.359
 
 
166.5
 
 
 
 
AIDA
 
 
hsa-miR-22-3p
 
 
-0.316
 
 
34.0
 
 
 
 
AKAP1
 
 
hsa-miR-152-3p
 
 
-0.381
 
 
45.9
 
 
 
 
AKIRIN1
 
 
hsa-miR-27a-3p
 
 
-0.368
 
 
34.0
 
 
 
 
ALCAM
 
 
hsa-miR-34a-5p
 
 
-0.303
 
 
13.6
 
 
 
 
ALCAM
 
 
hsa-miR-152-3p
 
 
-0.347
 
 
13.6
 
 
 
 
ALDOA
 
 
hsa-miR-34a-5p
 
 
-0.655
 
 
375.6
 
 
 
 
ANK3
 
 
hsa-miR-34a-5p
 
 
-0.29
 
 
19.3
 
 
 
 
ANK3
 
 
hsa-miR-34a-5p
 
 
-0.347
 
 
19.3
 
 
 
 
ANKHD1-EIF4EBP3
 
 
hsa-miR-22-3p
 
 
-0.479
 
 
11.2
 
 
 
 
ANKRD13A
 
 
hsa-miR-22-3p
 
 
-0.478
 
 
21.0
 
 
 
 
ANKRD40
 
 
hsa-miR-27a-3p
 
 
-0.35
 
 
15.3
 
 
 
 
ANP32B
 
 
hsa-miR-34a-5p
 
 
-0.29
 
 
111.5
 
 
 
 
AP1AR
 
 
hsa-miR-100-5p
 
 
-0.804
 
 
15.3
 
 
 
 
AP3S2
 
 
hsa-miR-34a-5p
 
 
-0.413
 
 
18.0
 
 
 
 
APH1A
 
 
hsa-miR-34a-5p
 
 
-0.304
 
 
89.6
 
 
 
 
APOO
 
 
hsa-miR-181a-5p
 
 
-0.5
 
 
17.6
 
 
 
 
AREG
 
 
hsa-miR-34a-5p
 
 
-0.62
 
 
17.8
 
 
 
 
ARF4
 
 
hsa-miR-152-3p
 
 
-0.487
 
 
185.2
 
 
 
 
ARF6
 
 
hsa-miR-181a-5p
 
 
-0.33
 
 
50.6
 
 
 
 
ARFGEF1
 
 
hsa-miR-152-3p
 
 
-0.306
 
 
16.1
 
 
 
 
ARFIP1
 
 
hsa-miR-320d
 
 
-0.339
 
 
26.2
 
 
 
 
ARFIP1
 
 
hsa-miR-152-3p
 
 
-0.474
 
 
26.2
 
 
 
 
ARHGAP1
 
 
hsa-miR-34a-5p
 
 
-0.369
 
 
30.6
 
 
 
 
ARHGAP21
 
 
hsa-miR-152-3p
 
 
-0.287
 
 
17.4
 
 
 
 
ARHGAP26
 
 
hsa-miR-34a-5p
 
 
-0.384
 
 
11.9
 
 
 
 
ARL6IP1
 
 
hsa-miR-152-3p
 
 
-0.579
 
 
116.0
 
 
 
 
ARL8B
 
 
hsa-miR-152-3p
 
 
-0.337
 
 
32.7
 
 
 
 
ARL8B
 
 
hsa-miR-320d
 
 
-0.376
 
 
32.7
 
 
 
 
ARPC5
 
 
hsa-miR-22-3p
 
 
-0.679
 
 
64.7
 
 
 
 
ARPIN-AP3S2
 
 
hsa-miR-34a-5p
 
 
-0.348
 
 
14.1
 
 
 
 
ARPP19
 
 
hsa-miR-320d
 
 
-0.325
 
 
33.6
 
 
 
 
ARPP19
 
 
hsa-miR-34a-5p
 
 
-0.497
 
 
33.6
 
 
 
 
ARRB1
 
 
hsa-miR-22-3p
 
 
-0.616
 
 
21.2
 
 
 
 
ARRDC3
 
 
hsa-miR-152-3p
 
 
-0.305
 
 
25.8
 
 
 
 
ASAH1
 
 
hsa-miR-27a-3p
 
 
-0.398
 
 
81.7
 
 
 
 
ASH2L
 
 
hsa-miR-320d
 
 
-0.287
 
 
21.2
 
 
 
 
ASPH
 
 
hsa-miR-152-3p
 
 
-0.391
 
 
77.9
 
 
 
 
ATMIN
 
 
hsa-miR-34a-5p
 
 
-0.409
 
 
12.8
 
 
 
 
ATOH1
 
 
hsa-miR-22-3p
 
 
-0.566
 
 
17.3
 
 
 
 
ATP2B4
 
 
hsa-miR-152-3p
 
 
-0.337
 
 
28.6
 
 
 
 
ATP5MC3
 
 
hsa-miR-27a-3p
 
 
-0.416
 
 
295.3
 
 
 
 
ATP6AP2
 
 
hsa-miR-152-3p
 
 
-0.592
 
 
83.6
 
 
 
 
ATP6AP2
 
 
hsa-miR-152-3p
 
 
-0.668
 
 
83.6
 
 
 
 
ATP8A1
 
 
hsa-miR-22-3p
 
 
-0.468
 
 
11.0
 
 
 
 
ATXN10
 
 
hsa-miR-27a-3p
 
 
-0.295
 
 
34.1
 
 
 
 
ATXN2
 
 
hsa-miR-10a-5p
 
 
-0.362
 
 
10.9
 
 
 
 
AXL
 
 
hsa-miR-34a-5p
 
 
-0.512
 
 
11.8
 
 
 
 
B3GNT7
 
 
hsa-miR-27a-3p
 
 
-0.355
 
 
66.9
 
 
 
 
B4GALT3
 
 
hsa-miR-27a-3p
 
 
-0.41
 
 
16.4
 
 
 
 
B4GALT5
 
 
hsa-miR-152-3p
 
 
-0.44
 
 
22.3
 
 
 
 
BAG5
 
 
hsa-miR-22-3p
 
 
-0.318
 
 
13.7
 
 
 
 
BAZ1B
 
 
hsa-miR-10a-5p
 
 
-0.309
 
 
19.3
 
 
 
 
BAZ2A
 
 
hsa-miR-100-5p
 
 
-0.29
 
 
24.9
 
 
 
 
BAZ2A
 
 
hsa-miR-100-5p
 
 
-0.421
 
 
24.9
 
 
 
 
BCL2L11
 
 
hsa-miR-152-3p
 
 
-0.445
 
 
11.2
 
 
 
 
BCL2L11
 
 
hsa-miR-10a-5p
 
 
-0.483
 
 
11.2
 
 
 
 
BEX3
 
 
hsa-miR-27a-3p
 
 
-0.454
 
 
49.7
 
 
 
 
BHLHE40
 
 
hsa-miR-181a-5p
 
 
-0.408
 
 
44.6
 
 
 
 
BHLHE41
 
 
hsa-miR-152-3p
 
 
-0.442
 
 
10.4
 
 
 
 
BIN1
 
 
hsa-miR-22-3p
 
 
-0.346
 
 
12.3
 
 
 
 
BLOC1S1
 
 
hsa-miR-22-3p
 
 
-0.346
 
 
161.5
 
 
 
 
BLOC1S6
 
 
hsa-miR-181a-5p
 
 
-0.393
 
 
20.2
 
 
 
 
BLTP1
 
 
hsa-miR-27a-3p
 
 
-0.426
 
 
13.6
 
 
 
 
BMI1
 
 
hsa-miR-27a-3p
 
 
-0.322
 
 
20.1
 
 
 
 
BRPF3
 
 
hsa-miR-34a-5p
 
 
-0.33
 
 
12.1
 
 
 
 
BTAF1
 
 
hsa-miR-152-3p
 
 
-0.401
 
 
12.5
 
 
 
 
BTBD3
 
 
hsa-miR-152-3p
 
 
-0.495
 
 
16.5
 
 
 
 
BTG2
 
 
hsa-miR-27a-3p
 
 
-0.339
 
 
193.5
 
 
 
 
BZW1
 
 
hsa-miR-27a-3p
 
 
-0.337
 
 
85.0
 
 
 
 
C1GALT1
 
 
hsa-miR-152-3p
 
 
-0.487
 
 
16.9
 
 
 
 
C5orf24
 
 
hsa-miR-22-3p
 
 
-0.493
 
 
17.3
 
 
 
 
C6orf120
 
 
hsa-miR-34a-5p
 
 
-0.331
 
 
11.9
 
 
 
 
C6orf62
 
 
hsa-miR-181a-5p
 
 
-0.334
 
 
72.1
 
 
 
 
CA7
 
 
hsa-miR-34a-5p
 
 
-0.523
 
 
45.7
 
 
 
 
CACYBP
 
 
hsa-miR-27a-3p
 
 
-0.367
 
 
19.4
 
 
 
 
CAMK2N1
 
 
hsa-miR-22-3p
 
 
-0.302
 
 
72.4
 
 
 
 
CAND1
 
 
hsa-miR-27a-3p
 
 
-0.318
 
 
19.4
 
 
 
 
CAND1
 
 
hsa-miR-152-3p
 
 
-0.44
 
 
19.4
 
 
 
 
CANX
 
 
hsa-miR-152-3p
 
 
-0.434
 
 
58.8
 
 
 
 
CAPN1
 
 
hsa-miR-22-3p
 
 
-0.307
 
 
54.1
 
 
 
 
CAPZA1
 
 
hsa-miR-27a-3p
 
 
-0.325
 
 
89.2
 
 
 
 
CASP2
 
 
hsa-miR-34a-5p
 
 
-0.382
 
 
11.9
 
 
 
 
CAV2
 
 
hsa-miR-152-3p
 
 
-0.32
 
 
15.5
 
 
 
 
 
 
 retina 
 
 
 
 
target_symbol
 
 
mature_mirna_id
 
 
score
 
 
nTPM
 
 
 
 
 
 
AAGAB
 
 
hsa-miR-34a-5p
 
 
-0.319
 
 
20.5
 
 
 
 
ABCD3
 
 
hsa-miR-152-3p
 
 
-0.388
 
 
24.4
 
 
 
 
ABHD17C
 
 
hsa-miR-27a-3p
 
 
-0.435
 
 
15.6
 
 
 
 
ACBD3
 
 
hsa-miR-34a-5p
 
 
-0.415
 
 
14.9
 
 
 
 
ACSL1
 
 
hsa-miR-34a-5p
 
 
-0.306
 
 
22.4
 
 
 
 
ACSL4
 
 
hsa-miR-34a-5p
 
 
-0.372
 
 
20.7
 
 
 
 
ADD2
 
 
hsa-miR-34a-5p
 
 
-0.305
 
 
14.9
 
 
 
 
ADIPOR2
 
 
hsa-miR-34a-5p
 
 
-0.33
 
 
12.1
 
 
 
 
ADK
 
 
hsa-miR-34a-5p
 
 
-0.374
 
 
14.9
 
 
 
 
AGFG1
 
 
hsa-miR-152-3p
 
 
-0.332
 
 
44.2
 
 
 
 
AGO4
 
 
hsa-miR-34a-5p
 
 
-0.306
 
 
16.7
 
 
 
 
AGPAT3
 
 
hsa-miR-27a-3p
 
 
-0.29
 
 
57.5
 
 
 
 
AHCYL2
 
 
hsa-miR-34a-5p
 
 
-0.359
 
 
53.5
 
 
 
 
AIDA
 
 
hsa-miR-22-3p
 
 
-0.316
 
 
18.0
 
 
 
 
AKAP1
 
 
hsa-miR-152-3p
 
 
-0.381
 
 
32.2
 
 
 
 
AKIRIN1
 
 
hsa-miR-27a-3p
 
 
-0.368
 
 
34.7
 
 
 
 
AKT3
 
 
hsa-miR-22-3p
 
 
-0.451
 
 
15.2
 
 
 
 
ALDOA
 
 
hsa-miR-34a-5p
 
 
-0.655
 
 
431.9
 
 
 
 
ALPL
 
 
hsa-miR-10a-5p
 
 
-0.32
 
 
17.1
 
 
 
 
AMER2
 
 
hsa-miR-34a-5p
 
 
-0.48
 
 
23.9
 
 
 
 
ANK2
 
 
hsa-miR-34a-5p
 
 
-0.317
 
 
118.1
 
 
 
 
ANK3
 
 
hsa-miR-34a-5p
 
 
-0.29
 
 
115.3
 
 
 
 
ANK3
 
 
hsa-miR-34a-5p
 
 
-0.347
 
 
115.3
 
 
 
 
ANKHD1-EIF4EBP3
 
 
hsa-miR-22-3p
 
 
-0.479
 
 
11.0
 
 
 
 
ANKRD40
 
 
hsa-miR-27a-3p
 
 
-0.35
 
 
19.2
 
 
 
 
ANP32B
 
 
hsa-miR-34a-5p
 
 
-0.29
 
 
42.8
 
 
 
 
AP3S2
 
 
hsa-miR-34a-5p
 
 
-0.413
 
 
40.7
 
 
 
 
APH1A
 
 
hsa-miR-34a-5p
 
 
-0.304
 
 
22.7
 
 
 
 
APOO
 
 
hsa-miR-181a-5p
 
 
-0.5
 
 
48.9
 
 
 
 
APPBP2
 
 
hsa-miR-27a-3p
 
 
-0.378
 
 
16.0
 
 
 
 
ARF4
 
 
hsa-miR-152-3p
 
 
-0.487
 
 
50.0
 
 
 
 
ARF6
 
 
hsa-miR-181a-5p
 
 
-0.33
 
 
13.4
 
 
 
 
ARFGEF1
 
 
hsa-miR-152-3p
 
 
-0.306
 
 
33.8
 
 
 
 
ARFIP1
 
 
hsa-miR-320d
 
 
-0.339
 
 
18.7
 
 
 
 
ARFIP1
 
 
hsa-miR-152-3p
 
 
-0.474
 
 
18.7
 
 
 
 
ARHGAP21
 
 
hsa-miR-152-3p
 
 
-0.287
 
 
37.0
 
 
 
 
ARHGAP26
 
 
hsa-miR-34a-5p
 
 
-0.384
 
 
16.0
 
 
 
 
ARL6IP1
 
 
hsa-miR-152-3p
 
 
-0.579
 
 
292.6
 
 
 
 
ARL8B
 
 
hsa-miR-152-3p
 
 
-0.337
 
 
31.7
 
 
 
 
ARL8B
 
 
hsa-miR-320d
 
 
-0.376
 
 
31.7
 
 
 
 
ARPIN-AP3S2
 
 
hsa-miR-34a-5p
 
 
-0.348
 
 
10.1
 
 
 
 
ARPP19
 
 
hsa-miR-320d
 
 
-0.325
 
 
53.1
 
 
 
 
ARPP19
 
 
hsa-miR-34a-5p
 
 
-0.497
 
 
53.1
 
 
 
 
ARRDC3
 
 
hsa-miR-152-3p
 
 
-0.305
 
 
69.6
 
 
 
 
ASAH1
 
 
hsa-miR-27a-3p
 
 
-0.398
 
 
73.6
 
 
 
 
ASH2L
 
 
hsa-miR-320d
 
 
-0.287
 
 
20.5
 
 
 
 
ASPH
 
 
hsa-miR-152-3p
 
 
-0.391
 
 
201.9
 
 
 
 
ATCAY
 
 
hsa-miR-10a-5p
 
 
-0.322
 
 
16.3
 
 
 
 
ATP1A2
 
 
hsa-miR-34a-5p
 
 
-0.306
 
 
54.2
 
 
 
 
ATP2B4
 
 
hsa-miR-152-3p
 
 
-0.337
 
 
39.4
 
 
 
 
ATP5MC3
 
 
hsa-miR-27a-3p
 
 
-0.416
 
 
117.1
 
 
 
 
ATP6AP2
 
 
hsa-miR-152-3p
 
 
-0.592
 
 
92.0
 
 
 
 
ATP6AP2
 
 
hsa-miR-152-3p
 
 
-0.668
 
 
92.0
 
 
 
 
ATP8A1
 
 
hsa-miR-22-3p
 
 
-0.468
 
 
17.9
 
 
 
 
ATXN10
 
 
hsa-miR-27a-3p
 
 
-0.295
 
 
51.0
 
 
 
 
ATXN2
 
 
hsa-miR-10a-5p
 
 
-0.362
 
 
14.6
 
 
 
 
B4GALT6
 
 
hsa-miR-152-3p
 
 
-0.528
 
 
24.7
 
 
 
 
BAG5
 
 
hsa-miR-22-3p
 
 
-0.318
 
 
17.0
 
 
 
 
BAZ1B
 
 
hsa-miR-10a-5p
 
 
-0.309
 
 
26.3
 
 
 
 
BAZ2A
 
 
hsa-miR-100-5p
 
 
-0.29
 
 
45.3
 
 
 
 
BAZ2A
 
 
hsa-miR-100-5p
 
 
-0.421
 
 
45.3
 
 
 
 
BBX
 
 
hsa-miR-10a-5p
 
 
-0.305
 
 
18.6
 
 
 
 
BCL2L11
 
 
hsa-miR-152-3p
 
 
-0.445
 
 
11.9
 
 
 
 
BCL2L11
 
 
hsa-miR-10a-5p
 
 
-0.483
 
 
11.9
 
 
 
 
BCL2L13
 
 
hsa-miR-34a-5p
 
 
-0.431
 
 
40.6
 
 
 
 
BEND7
 
 
hsa-miR-27a-3p
 
 
-0.304
 
 
15.1
 
 
 
 
BEX3
 
 
hsa-miR-27a-3p
 
 
-0.454
 
 
125.9
 
 
 
 
BHLHE40
 
 
hsa-miR-181a-5p
 
 
-0.408
 
 
109.8
 
 
 
 
BHLHE41
 
 
hsa-miR-152-3p
 
 
-0.442
 
 
113.5
 
 
 
 
BIN1
 
 
hsa-miR-22-3p
 
 
-0.346
 
 
49.9
 
 
 
 
BLOC1S1
 
 
hsa-miR-22-3p
 
 
-0.346
 
 
77.5
 
 
 
 
BLOC1S6
 
 
hsa-miR-181a-5p
 
 
-0.393
 
 
22.7
 
 
 
 
BLTP1
 
 
hsa-miR-27a-3p
 
 
-0.426
 
 
57.7
 
 
 
 
BMI1
 
 
hsa-miR-27a-3p
 
 
-0.322
 
 
37.4
 
 
 
 
BMPR2
 
 
hsa-miR-100-5p
 
 
-0.355
 
 
17.0
 
 
 
 
BRSK1
 
 
hsa-miR-27a-3p
 
 
-0.372
 
 
18.9
 
 
 
 
BTAF1
 
 
hsa-miR-152-3p
 
 
-0.401
 
 
27.2
 
 
 
 
BTBD3
 
 
hsa-miR-152-3p
 
 
-0.495
 
 
39.4
 
 
 
 
BTG2
 
 
hsa-miR-27a-3p
 
 
-0.339
 
 
141.6
 
 
 
 
BZW1
 
 
hsa-miR-27a-3p
 
 
-0.337
 
 
42.4
 
 
 
 
C1GALT1
 
 
hsa-miR-152-3p
 
 
-0.487
 
 
21.8
 
 
 
 
C5orf24
 
 
hsa-miR-22-3p
 
 
-0.493
 
 
17.8
 
 
 
 
C6orf120
 
 
hsa-miR-34a-5p
 
 
-0.331
 
 
21.6
 
 
 
 
C6orf62
 
 
hsa-miR-181a-5p
 
 
-0.334
 
 
139.7
 
 
 
 
CA10
 
 
hsa-miR-27a-3p
 
 
-0.464
 
 
41.2
 
 
 
 
CABP1
 
 
hsa-miR-27a-3p
 
 
-0.343
 
 
23.9
 
 
 
 
CACYBP
 
 
hsa-miR-27a-3p
 
 
-0.367
 
 
48.6
 
 
 
 
CADM4
 
 
hsa-miR-34a-5p
 
 
-0.365
 
 
13.4
 
 
 
 
CAMK2N1
 
 
hsa-miR-22-3p
 
 
-0.302
 
 
30.3
 
 
 
 
CAND1
 
 
hsa-miR-27a-3p
 
 
-0.318
 
 
34.7
 
 
 
 
CAND1
 
 
hsa-miR-152-3p
 
 
-0.44
 
 
34.7
 
 
 
 
CANX
 
 
hsa-miR-152-3p
 
 
-0.434
 
 
61.7
 
 
 
 
CAPZA1
 
 
hsa-miR-27a-3p
 
 
-0.325
 
 
34.4
 
 
 
 
CAV2
 
 
hsa-miR-152-3p
 
 
-0.32
 
 
15.5
 
 
 
 
CBX4
 
 
hsa-miR-181a-5p
 
 
-0.354
 
 
11.4
 
 
 
 
CCDC144A
 
 
hsa-miR-152-3p
 
 
-0.292
 
 
11.9
 
 
 
 
CCDC47
 
 
hsa-miR-22-3p
 
 
-0.604
 
 
42.0
 
 
 
 
CCDC6
 
 
hsa-miR-152-3p
 
 
-0.511
 
 
16.9
 
 
 
 
CCNC
 
 
hsa-miR-27a-3p
 
 
-0.378
 
 
25.0
 
 
 
 
CCNG1
 
 
hsa-miR-27a-3p
 
 
-0.419
 
 
19.7
 
 
 
 
 
 
 salivary gland 
 
 
 
 
target_symbol
 
 
mature_mirna_id
 
 
score
 
 
nTPM
 
 
 
 
 
 
AAGAB
 
 
hsa-miR-34a-5p
 
 
-0.319
 
 
14.5
 
 
 
 
ABCD3
 
 
hsa-miR-152-3p
 
 
-0.388
 
 
16.5
 
 
 
 
ABHD17C
 
 
hsa-miR-27a-3p
 
 
-0.435
 
 
22.3
 
 
 
 
ABHD5
 
 
hsa-miR-27a-3p
 
 
-0.407
 
 
19.4
 
 
 
 
ACBD3
 
 
hsa-miR-34a-5p
 
 
-0.415
 
 
22.7
 
 
 
 
ACSL1
 
 
hsa-miR-34a-5p
 
 
-0.306
 
 
82.2
 
 
 
 
ACTA2
 
 
hsa-miR-27a-3p
 
 
-0.577
 
 
390.9
 
 
 
 
ADIPOR2
 
 
hsa-miR-34a-5p
 
 
-0.33
 
 
21.2
 
 
 
 
ADK
 
 
hsa-miR-34a-5p
 
 
-0.374
 
 
18.2
 
 
 
 
AFG3L2
 
 
hsa-miR-181a-5p
 
 
-0.491
 
 
15.5
 
 
 
 
AGFG1
 
 
hsa-miR-152-3p
 
 
-0.332
 
 
26.3
 
 
 
 
AGPAT3
 
 
hsa-miR-27a-3p
 
 
-0.29
 
 
28.8
 
 
 
 
AGTRAP
 
 
hsa-miR-34a-5p
 
 
-0.328
 
 
36.7
 
 
 
 
AHCYL2
 
 
hsa-miR-34a-5p
 
 
-0.359
 
 
28.8
 
 
 
 
AHDC1
 
 
hsa-miR-152-3p
 
 
-0.413
 
 
16.0
 
 
 
 
AIDA
 
 
hsa-miR-22-3p
 
 
-0.316
 
 
26.4
 
 
 
 
AKAP1
 
 
hsa-miR-152-3p
 
 
-0.381
 
 
31.6
 
 
 
 
AKIRIN1
 
 
hsa-miR-27a-3p
 
 
-0.368
 
 
22.9
 
 
 
 
ALCAM
 
 
hsa-miR-34a-5p
 
 
-0.303
 
 
31.9
 
 
 
 
ALCAM
 
 
hsa-miR-152-3p
 
 
-0.347
 
 
31.9
 
 
 
 
ALDOA
 
 
hsa-miR-34a-5p
 
 
-0.655
 
 
1038.5
 
 
 
 
ANK3
 
 
hsa-miR-34a-5p
 
 
-0.29
 
 
39.6
 
 
 
 
ANK3
 
 
hsa-miR-34a-5p
 
 
-0.347
 
 
39.6
 
 
 
 
ANKHD1-EIF4EBP3
 
 
hsa-miR-22-3p
 
 
-0.479
 
 
12.2
 
 
 
 
ANKRD13A
 
 
hsa-miR-22-3p
 
 
-0.478
 
 
25.9
 
 
 
 
ANKRD40
 
 
hsa-miR-27a-3p
 
 
-0.35
 
 
14.2
 
 
 
 
ANP32B
 
 
hsa-miR-34a-5p
 
 
-0.29
 
 
104.9
 
 
 
 
AP1AR
 
 
hsa-miR-100-5p
 
 
-0.804
 
 
11.4
 
 
 
 
AP3S2
 
 
hsa-miR-34a-5p
 
 
-0.413
 
 
12.7
 
 
 
 
APH1A
 
 
hsa-miR-34a-5p
 
 
-0.304
 
 
87.9
 
 
 
 
APOO
 
 
hsa-miR-181a-5p
 
 
-0.5
 
 
18.5
 
 
 
 
AREG
 
 
hsa-miR-34a-5p
 
 
-0.62
 
 
11.3
 
 
 
 
ARF4
 
 
hsa-miR-152-3p
 
 
-0.487
 
 
171.4
 
 
 
 
ARF6
 
 
hsa-miR-181a-5p
 
 
-0.33
 
 
33.7
 
 
 
 
ARFGEF1
 
 
hsa-miR-152-3p
 
 
-0.306
 
 
13.9
 
 
 
 
ARFIP1
 
 
hsa-miR-320d
 
 
-0.339
 
 
12.9
 
 
 
 
ARFIP1
 
 
hsa-miR-152-3p
 
 
-0.474
 
 
12.9
 
 
 
 
ARHGAP1
 
 
hsa-miR-34a-5p
 
 
-0.369
 
 
43.3
 
 
 
 
ARHGAP21
 
 
hsa-miR-152-3p
 
 
-0.287
 
 
23.1
 
 
 
 
ARHGAP26
 
 
hsa-miR-34a-5p
 
 
-0.384
 
 
13.1
 
 
 
 
ARL6IP1
 
 
hsa-miR-152-3p
 
 
-0.579
 
 
79.4
 
 
 
 
ARL8B
 
 
hsa-miR-152-3p
 
 
-0.337
 
 
35.3
 
 
 
 
ARL8B
 
 
hsa-miR-320d
 
 
-0.376
 
 
35.3
 
 
 
 
ARPC5
 
 
hsa-miR-22-3p
 
 
-0.679
 
 
43.2
 
 
 
 
ARPP19
 
 
hsa-miR-320d
 
 
-0.325
 
 
28.7
 
 
 
 
ARPP19
 
 
hsa-miR-34a-5p
 
 
-0.497
 
 
28.7
 
 
 
 
ARRB1
 
 
hsa-miR-22-3p
 
 
-0.616
 
 
11.9
 
 
 
 
ARRDC3
 
 
hsa-miR-152-3p
 
 
-0.305
 
 
60.1
 
 
 
 
ASAH1
 
 
hsa-miR-27a-3p
 
 
-0.398
 
 
61.4
 
 
 
 
ASH2L
 
 
hsa-miR-320d
 
 
-0.287
 
 
19.9
 
 
 
 
ASPH
 
 
hsa-miR-152-3p
 
 
-0.391
 
 
95.6
 
 
 
 
ATG4B
 
 
hsa-miR-34a-5p
 
 
-0.58
 
 
16.0
 
 
 
 
ATMIN
 
 
hsa-miR-34a-5p
 
 
-0.409
 
 
12.0
 
 
 
 
ATP1A2
 
 
hsa-miR-34a-5p
 
 
-0.306
 
 
17.5
 
 
 
 
ATP5MC3
 
 
hsa-miR-27a-3p
 
 
-0.416
 
 
173.5
 
 
 
 
ATP6AP2
 
 
hsa-miR-152-3p
 
 
-0.592
 
 
39.6
 
 
 
 
ATP6AP2
 
 
hsa-miR-152-3p
 
 
-0.668
 
 
39.6
 
 
 
 
ATXN10
 
 
hsa-miR-27a-3p
 
 
-0.295
 
 
40.4
 
 
 
 
ATXN2
 
 
hsa-miR-10a-5p
 
 
-0.362
 
 
14.4
 
 
 
 
B4GALT2
 
 
hsa-miR-34a-5p
 
 
-0.314
 
 
25.7
 
 
 
 
B4GALT3
 
 
hsa-miR-27a-3p
 
 
-0.41
 
 
30.4
 
 
 
 
B4GALT5
 
 
hsa-miR-152-3p
 
 
-0.44
 
 
18.2
 
 
 
 
BAG5
 
 
hsa-miR-22-3p
 
 
-0.318
 
 
15.5
 
 
 
 
BAZ1B
 
 
hsa-miR-10a-5p
 
 
-0.309
 
 
16.1
 
 
 
 
BAZ2A
 
 
hsa-miR-100-5p
 
 
-0.29
 
 
23.8
 
 
 
 
BAZ2A
 
 
hsa-miR-100-5p
 
 
-0.421
 
 
23.8
 
 
 
 
BBX
 
 
hsa-miR-10a-5p
 
 
-0.305
 
 
12.6
 
 
 
 
BCL3
 
 
hsa-miR-27a-3p
 
 
-0.413
 
 
46.3
 
 
 
 
BEND7
 
 
hsa-miR-27a-3p
 
 
-0.304
 
 
11.8
 
 
 
 
BEX3
 
 
hsa-miR-27a-3p
 
 
-0.454
 
 
93.6
 
 
 
 
BHLHE40
 
 
hsa-miR-181a-5p
 
 
-0.408
 
 
123.4
 
 
 
 
BHLHE41
 
 
hsa-miR-152-3p
 
 
-0.442
 
 
17.3
 
 
 
 
BIN1
 
 
hsa-miR-22-3p
 
 
-0.346
 
 
40.9
 
 
 
 
BLOC1S1
 
 
hsa-miR-22-3p
 
 
-0.346
 
 
104.2
 
 
 
 
BLOC1S6
 
 
hsa-miR-181a-5p
 
 
-0.393
 
 
19.5
 
 
 
 
BLTP1
 
 
hsa-miR-27a-3p
 
 
-0.426
 
 
14.6
 
 
 
 
BMI1
 
 
hsa-miR-27a-3p
 
 
-0.322
 
 
18.8
 
 
 
 
BRPF3
 
 
hsa-miR-34a-5p
 
 
-0.33
 
 
14.8
 
 
 
 
BTAF1
 
 
hsa-miR-152-3p
 
 
-0.401
 
 
15.9
 
 
 
 
BTBD3
 
 
hsa-miR-152-3p
 
 
-0.495
 
 
13.1
 
 
 
 
BTG2
 
 
hsa-miR-27a-3p
 
 
-0.339
 
 
70.8
 
 
 
 
BZW1
 
 
hsa-miR-27a-3p
 
 
-0.337
 
 
55.2
 
 
 
 
C1GALT1
 
 
hsa-miR-152-3p
 
 
-0.487
 
 
22.0
 
 
 
 
C1orf35
 
 
hsa-miR-152-3p
 
 
-0.511
 
 
13.7
 
 
 
 
C5orf24
 
 
hsa-miR-22-3p
 
 
-0.493
 
 
17.2
 
 
 
 
C6orf120
 
 
hsa-miR-34a-5p
 
 
-0.331
 
 
12.1
 
 
 
 
C6orf62
 
 
hsa-miR-181a-5p
 
 
-0.334
 
 
88.5
 
 
 
 
CACNB3
 
 
hsa-miR-34a-5p
 
 
-0.426
 
 
12.3
 
 
 
 
CACYBP
 
 
hsa-miR-27a-3p
 
 
-0.367
 
 
17.7
 
 
 
 
CADM4
 
 
hsa-miR-34a-5p
 
 
-0.365
 
 
18.7
 
 
 
 
CAMK2N1
 
 
hsa-miR-22-3p
 
 
-0.302
 
 
28.7
 
 
 
 
CAND1
 
 
hsa-miR-27a-3p
 
 
-0.318
 
 
15.7
 
 
 
 
CAND1
 
 
hsa-miR-152-3p
 
 
-0.44
 
 
15.7
 
 
 
 
CANX
 
 
hsa-miR-152-3p
 
 
-0.434
 
 
107.0
 
 
 
 
CAPN1
 
 
hsa-miR-22-3p
 
 
-0.307
 
 
113.6
 
 
 
 
CAPZA1
 
 
hsa-miR-27a-3p
 
 
-0.325
 
 
46.6
 
 
 
 
CASP2
 
 
hsa-miR-34a-5p
 
 
-0.382
 
 
11.7
 
 
 
 
CAV2
 
 
hsa-miR-152-3p
 
 
-0.32
 
 
23.6
 
 
 
 
CBX4
 
 
hsa-miR-181a-5p
 
 
-0.354
 
 
12.5
 
 
 
 
CCDC149
 
 
hsa-miR-22-3p
 
 
-0.327
 
 
17.0
 
 
 
 
 
 
 seminal vesicle 
 
 
 
 
target_symbol
 
 
mature_mirna_id
 
 
score
 
 
nTPM
 
 
 
 
 
 
AAGAB
 
 
hsa-miR-34a-5p
 
 
-0.319
 
 
19.3
 
 
 
 
ABCD3
 
 
hsa-miR-152-3p
 
 
-0.388
 
 
25.5
 
 
 
 
ACBD3
 
 
hsa-miR-34a-5p
 
 
-0.415
 
 
15.4
 
 
 
 
ACSL1
 
 
hsa-miR-34a-5p
 
 
-0.306
 
 
26.5
 
 
 
 
ACSL4
 
 
hsa-miR-34a-5p
 
 
-0.372
 
 
14.0
 
 
 
 
ACTA2
 
 
hsa-miR-27a-3p
 
 
-0.577
 
 
5569.5
 
 
 
 
ACVR1
 
 
hsa-miR-152-3p
 
 
-0.397
 
 
11.3
 
 
 
 
ADAM10
 
 
hsa-miR-152-3p
 
 
-0.348
 
 
11.5
 
 
 
 
ADGRA3
 
 
hsa-miR-27a-3p
 
 
-0.296
 
 
10.8
 
 
 
 
ADIPOR2
 
 
hsa-miR-34a-5p
 
 
-0.33
 
 
11.2
 
 
 
 
ADK
 
 
hsa-miR-34a-5p
 
 
-0.374
 
 
15.1
 
 
 
 
AFG3L2
 
 
hsa-miR-181a-5p
 
 
-0.491
 
 
11.8
 
 
 
 
AGFG1
 
 
hsa-miR-152-3p
 
 
-0.332
 
 
22.0
 
 
 
 
AGPAT3
 
 
hsa-miR-27a-3p
 
 
-0.29
 
 
10.9
 
 
 
 
AGTRAP
 
 
hsa-miR-34a-5p
 
 
-0.328
 
 
19.8
 
 
 
 
AIDA
 
 
hsa-miR-22-3p
 
 
-0.316
 
 
39.7
 
 
 
 
AKAP1
 
 
hsa-miR-152-3p
 
 
-0.381
 
 
23.3
 
 
 
 
AKIRIN1
 
 
hsa-miR-27a-3p
 
 
-0.368
 
 
33.7
 
 
 
 
AKT3
 
 
hsa-miR-22-3p
 
 
-0.451
 
 
14.7
 
 
 
 
ALCAM
 
 
hsa-miR-34a-5p
 
 
-0.303
 
 
37.3
 
 
 
 
ALCAM
 
 
hsa-miR-152-3p
 
 
-0.347
 
 
37.3
 
 
 
 
ALDOA
 
 
hsa-miR-34a-5p
 
 
-0.655
 
 
561.7
 
 
 
 
ANK2
 
 
hsa-miR-34a-5p
 
 
-0.317
 
 
10.2
 
 
 
 
ANK3
 
 
hsa-miR-34a-5p
 
 
-0.29
 
 
37.0
 
 
 
 
ANK3
 
 
hsa-miR-34a-5p
 
 
-0.347
 
 
37.0
 
 
 
 
ANKRD13A
 
 
hsa-miR-22-3p
 
 
-0.478
 
 
23.1
 
 
 
 
ANKRD40
 
 
hsa-miR-27a-3p
 
 
-0.35
 
 
23.3
 
 
 
 
ANP32B
 
 
hsa-miR-34a-5p
 
 
-0.29
 
 
67.2
 
 
 
 
AP3S2
 
 
hsa-miR-34a-5p
 
 
-0.413
 
 
20.1
 
 
 
 
APH1A
 
 
hsa-miR-34a-5p
 
 
-0.304
 
 
64.2
 
 
 
 
APOO
 
 
hsa-miR-181a-5p
 
 
-0.5
 
 
14.1
 
 
 
 
ARF4
 
 
hsa-miR-152-3p
 
 
-0.487
 
 
125.1
 
 
 
 
ARF6
 
 
hsa-miR-181a-5p
 
 
-0.33
 
 
18.1
 
 
 
 
ARFGEF1
 
 
hsa-miR-152-3p
 
 
-0.306
 
 
11.4
 
 
 
 
ARFIP1
 
 
hsa-miR-320d
 
 
-0.339
 
 
16.2
 
 
 
 
ARFIP1
 
 
hsa-miR-152-3p
 
 
-0.474
 
 
16.2
 
 
 
 
ARHGAP1
 
 
hsa-miR-34a-5p
 
 
-0.369
 
 
58.9
 
 
 
 
ARHGAP21
 
 
hsa-miR-152-3p
 
 
-0.287
 
 
10.8
 
 
 
 
ARL6IP1
 
 
hsa-miR-152-3p
 
 
-0.579
 
 
66.4
 
 
 
 
ARL8B
 
 
hsa-miR-152-3p
 
 
-0.337
 
 
39.4
 
 
 
 
ARL8B
 
 
hsa-miR-320d
 
 
-0.376
 
 
39.4
 
 
 
 
ARPC5
 
 
hsa-miR-22-3p
 
 
-0.679
 
 
33.8
 
 
 
 
ARPP19
 
 
hsa-miR-320d
 
 
-0.325
 
 
38.5
 
 
 
 
ARPP19
 
 
hsa-miR-34a-5p
 
 
-0.497
 
 
38.5
 
 
 
 
ARRDC3
 
 
hsa-miR-152-3p
 
 
-0.305
 
 
22.9
 
 
 
 
ASAH1
 
 
hsa-miR-27a-3p
 
 
-0.398
 
 
73.4
 
 
 
 
ASH2L
 
 
hsa-miR-320d
 
 
-0.287
 
 
19.9
 
 
 
 
ASPH
 
 
hsa-miR-152-3p
 
 
-0.391
 
 
65.8
 
 
 
 
ATMIN
 
 
hsa-miR-34a-5p
 
 
-0.409
 
 
13.9
 
 
 
 
ATP1A2
 
 
hsa-miR-34a-5p
 
 
-0.306
 
 
26.9
 
 
 
 
ATP2B4
 
 
hsa-miR-152-3p
 
 
-0.337
 
 
66.2
 
 
 
 
ATP5MC3
 
 
hsa-miR-27a-3p
 
 
-0.416
 
 
107.3
 
 
 
 
ATP6AP2
 
 
hsa-miR-152-3p
 
 
-0.592
 
 
92.2
 
 
 
 
ATP6AP2
 
 
hsa-miR-152-3p
 
 
-0.668
 
 
92.2
 
 
 
 
ATXN10
 
 
hsa-miR-27a-3p
 
 
-0.295
 
 
38.6
 
 
 
 
ATXN2
 
 
hsa-miR-10a-5p
 
 
-0.362
 
 
14.7
 
 
 
 
AXL
 
 
hsa-miR-34a-5p
 
 
-0.512
 
 
23.1
 
 
 
 
B4GALT2
 
 
hsa-miR-34a-5p
 
 
-0.314
 
 
22.4
 
 
 
 
B4GALT3
 
 
hsa-miR-27a-3p
 
 
-0.41
 
 
11.6
 
 
 
 
B4GALT5
 
 
hsa-miR-152-3p
 
 
-0.44
 
 
20.7
 
 
 
 
BAG5
 
 
hsa-miR-22-3p
 
 
-0.318
 
 
13.4
 
 
 
 
BAZ1B
 
 
hsa-miR-10a-5p
 
 
-0.309
 
 
24.0
 
 
 
 
BAZ2A
 
 
hsa-miR-100-5p
 
 
-0.29
 
 
19.8
 
 
 
 
BAZ2A
 
 
hsa-miR-100-5p
 
 
-0.421
 
 
19.8
 
 
 
 
BBX
 
 
hsa-miR-10a-5p
 
 
-0.305
 
 
29.7
 
 
 
 
BEX3
 
 
hsa-miR-27a-3p
 
 
-0.454
 
 
211.7
 
 
 
 
BHLHE40
 
 
hsa-miR-181a-5p
 
 
-0.408
 
 
30.6
 
 
 
 
BHLHE41
 
 
hsa-miR-152-3p
 
 
-0.442
 
 
13.2
 
 
 
 
BIN1
 
 
hsa-miR-22-3p
 
 
-0.346
 
 
22.7
 
 
 
 
BLOC1S1
 
 
hsa-miR-22-3p
 
 
-0.346
 
 
74.4
 
 
 
 
BLOC1S6
 
 
hsa-miR-181a-5p
 
 
-0.393
 
 
19.2
 
 
 
 
BLTP1
 
 
hsa-miR-27a-3p
 
 
-0.426
 
 
13.9
 
 
 
 
BMI1
 
 
hsa-miR-27a-3p
 
 
-0.322
 
 
24.6
 
 
 
 
BMPR2
 
 
hsa-miR-100-5p
 
 
-0.355
 
 
10.5
 
 
 
 
BRPF3
 
 
hsa-miR-34a-5p
 
 
-0.33
 
 
10.2
 
 
 
 
BTAF1
 
 
hsa-miR-152-3p
 
 
-0.401
 
 
10.1
 
 
 
 
BTBD3
 
 
hsa-miR-152-3p
 
 
-0.495
 
 
10.0
 
 
 
 
BTG2
 
 
hsa-miR-27a-3p
 
 
-0.339
 
 
172.4
 
 
 
 
BZW1
 
 
hsa-miR-27a-3p
 
 
-0.337
 
 
58.1
 
 
 
 
C5orf24
 
 
hsa-miR-22-3p
 
 
-0.493
 
 
17.3
 
 
 
 
C6orf120
 
 
hsa-miR-34a-5p
 
 
-0.331
 
 
10.2
 
 
 
 
C6orf62
 
 
hsa-miR-181a-5p
 
 
-0.334
 
 
71.0
 
 
 
 
C8orf88
 
 
hsa-miR-27a-3p
 
 
-0.33
 
 
22.1
 
 
 
 
CACYBP
 
 
hsa-miR-27a-3p
 
 
-0.367
 
 
17.4
 
 
 
 
CADM4
 
 
hsa-miR-34a-5p
 
 
-0.365
 
 
13.4
 
 
 
 
CAMK2N1
 
 
hsa-miR-22-3p
 
 
-0.302
 
 
38.4
 
 
 
 
CAND1
 
 
hsa-miR-27a-3p
 
 
-0.318
 
 
18.8
 
 
 
 
CAND1
 
 
hsa-miR-152-3p
 
 
-0.44
 
 
18.8
 
 
 
 
CANX
 
 
hsa-miR-152-3p
 
 
-0.434
 
 
16.6
 
 
 
 
CAPN1
 
 
hsa-miR-22-3p
 
 
-0.307
 
 
63.6
 
 
 
 
CAPZA1
 
 
hsa-miR-27a-3p
 
 
-0.325
 
 
35.2
 
 
 
 
CAV2
 
 
hsa-miR-152-3p
 
 
-0.32
 
 
30.5
 
 
 
 
CCDC47
 
 
hsa-miR-22-3p
 
 
-0.604
 
 
49.0
 
 
 
 
CCDC6
 
 
hsa-miR-152-3p
 
 
-0.511
 
 
13.5
 
 
 
 
CCM2
 
 
hsa-miR-27a-3p
 
 
-0.335
 
 
13.6
 
 
 
 
CCNC
 
 
hsa-miR-27a-3p
 
 
-0.378
 
 
21.9
 
 
 
 
CCNG1
 
 
hsa-miR-27a-3p
 
 
-0.419
 
 
52.5
 
 
 
 
CCNK
 
 
hsa-miR-181a-5p
 
 
-0.31
 
 
15.2
 
 
 
 
CCNK
 
 
hsa-miR-27a-3p
 
 
-0.519
 
 
15.2
 
 
 
 
CCNL2
 
 
hsa-miR-28-5p
 
 
-0.448
 
 
29.7
 
 
 
 
 
 
 skeletal muscle 
 
 
 
 
target_symbol
 
 
mature_mirna_id
 
 
score
 
 
nTPM
 
 
 
 
 
 
AAGAB
 
 
hsa-miR-34a-5p
 
 
-0.319
 
 
30.0
 
 
 
 
ABCB10
 
 
hsa-miR-152-3p
 
 
-0.291
 
 
12.0
 
 
 
 
ABCB7
 
 
hsa-miR-152-3p
 
 
-0.515
 
 
29.2
 
 
 
 
ABCD3
 
 
hsa-miR-152-3p
 
 
-0.388
 
 
22.0
 
 
 
 
ABHD5
 
 
hsa-miR-27a-3p
 
 
-0.407
 
 
12.3
 
 
 
 
ABRAXAS2
 
 
hsa-miR-34a-5p
 
 
-0.538
 
 
17.8
 
 
 
 
ACBD3
 
 
hsa-miR-34a-5p
 
 
-0.415
 
 
20.2
 
 
 
 
ACSL1
 
 
hsa-miR-34a-5p
 
 
-0.306
 
 
697.4
 
 
 
 
ACSL4
 
 
hsa-miR-34a-5p
 
 
-0.372
 
 
39.3
 
 
 
 
ACTA2
 
 
hsa-miR-27a-3p
 
 
-0.577
 
 
194.7
 
 
 
 
ACVR1
 
 
hsa-miR-152-3p
 
 
-0.397
 
 
15.5
 
 
 
 
ADCY2
 
 
hsa-miR-152-3p
 
 
-0.299
 
 
64.1
 
 
 
 
ADGRA3
 
 
hsa-miR-27a-3p
 
 
-0.296
 
 
15.9
 
 
 
 
ADGRF5
 
 
hsa-miR-152-3p
 
 
-0.354
 
 
29.0
 
 
 
 
ADIPOR2
 
 
hsa-miR-34a-5p
 
 
-0.33
 
 
41.4
 
 
 
 
ADK
 
 
hsa-miR-34a-5p
 
 
-0.374
 
 
38.8
 
 
 
 
ADO
 
 
hsa-miR-34a-5p
 
 
-0.377
 
 
10.6
 
 
 
 
AFG3L2
 
 
hsa-miR-181a-5p
 
 
-0.491
 
 
96.6
 
 
 
 
AGFG1
 
 
hsa-miR-152-3p
 
 
-0.332
 
 
31.6
 
 
 
 
AGMAT
 
 
hsa-miR-22-3p
 
 
-0.294
 
 
32.1
 
 
 
 
AGO2
 
 
hsa-miR-27a-3p
 
 
-0.486
 
 
16.4
 
 
 
 
AGO2
 
 
hsa-miR-27a-3p
 
 
-0.646
 
 
16.4
 
 
 
 
AGO2
 
 
hsa-miR-100-5p
 
 
-0.884
 
 
16.4
 
 
 
 
AGO4
 
 
hsa-miR-34a-5p
 
 
-0.306
 
 
12.4
 
 
 
 
AGPAT3
 
 
hsa-miR-27a-3p
 
 
-0.29
 
 
56.6
 
 
 
 
AGTRAP
 
 
hsa-miR-34a-5p
 
 
-0.328
 
 
50.5
 
 
 
 
AHCYL2
 
 
hsa-miR-34a-5p
 
 
-0.359
 
 
35.0
 
 
 
 
AHDC1
 
 
hsa-miR-152-3p
 
 
-0.413
 
 
29.2
 
 
 
 
AIDA
 
 
hsa-miR-22-3p
 
 
-0.316
 
 
26.6
 
 
 
 
AKAP1
 
 
hsa-miR-152-3p
 
 
-0.381
 
 
194.7
 
 
 
 
AKIRIN1
 
 
hsa-miR-27a-3p
 
 
-0.368
 
 
28.7
 
 
 
 
ALDOA
 
 
hsa-miR-34a-5p
 
 
-0.655
 
 
25093.7
 
 
 
 
ANK2
 
 
hsa-miR-34a-5p
 
 
-0.317
 
 
120.2
 
 
 
 
ANK3
 
 
hsa-miR-34a-5p
 
 
-0.29
 
 
75.3
 
 
 
 
ANK3
 
 
hsa-miR-34a-5p
 
 
-0.347
 
 
75.3
 
 
 
 
ANKHD1-EIF4EBP3
 
 
hsa-miR-22-3p
 
 
-0.479
 
 
10.4
 
 
 
 
ANKRD40
 
 
hsa-miR-27a-3p
 
 
-0.35
 
 
100.2
 
 
 
 
ANP32B
 
 
hsa-miR-34a-5p
 
 
-0.29
 
 
103.8
 
 
 
 
AP3S2
 
 
hsa-miR-34a-5p
 
 
-0.413
 
 
42.0
 
 
 
 
APH1A
 
 
hsa-miR-34a-5p
 
 
-0.304
 
 
56.0
 
 
 
 
APOO
 
 
hsa-miR-181a-5p
 
 
-0.5
 
 
43.1
 
 
 
 
AREG
 
 
hsa-miR-34a-5p
 
 
-0.62
 
 
17.7
 
 
 
 
ARF4
 
 
hsa-miR-152-3p
 
 
-0.487
 
 
59.5
 
 
 
 
ARF6
 
 
hsa-miR-181a-5p
 
 
-0.33
 
 
21.4
 
 
 
 
ARFGEF1
 
 
hsa-miR-152-3p
 
 
-0.306
 
 
12.2
 
 
 
 
ARHGAP1
 
 
hsa-miR-34a-5p
 
 
-0.369
 
 
28.5
 
 
 
 
ARHGAP21
 
 
hsa-miR-152-3p
 
 
-0.287
 
 
15.7
 
 
 
 
ARHGAP26
 
 
hsa-miR-34a-5p
 
 
-0.384
 
 
11.5
 
 
 
 
ARL6IP1
 
 
hsa-miR-152-3p
 
 
-0.579
 
 
21.6
 
 
 
 
ARL8B
 
 
hsa-miR-152-3p
 
 
-0.337
 
 
98.5
 
 
 
 
ARL8B
 
 
hsa-miR-320d
 
 
-0.376
 
 
98.5
 
 
 
 
ARPC5
 
 
hsa-miR-22-3p
 
 
-0.679
 
 
17.5
 
 
 
 
ARPIN-AP3S2
 
 
hsa-miR-34a-5p
 
 
-0.348
 
 
18.9
 
 
 
 
ARPP19
 
 
hsa-miR-320d
 
 
-0.325
 
 
225.3
 
 
 
 
ARPP19
 
 
hsa-miR-34a-5p
 
 
-0.497
 
 
225.3
 
 
 
 
ARRB1
 
 
hsa-miR-22-3p
 
 
-0.616
 
 
10.7
 
 
 
 
ARRDC3
 
 
hsa-miR-152-3p
 
 
-0.305
 
 
31.8
 
 
 
 
ASAH1
 
 
hsa-miR-27a-3p
 
 
-0.398
 
 
61.6
 
 
 
 
ASB1
 
 
hsa-miR-34a-5p
 
 
-0.406
 
 
19.8
 
 
 
 
ASB11
 
 
hsa-miR-27a-3p
 
 
-0.353
 
 
107.3
 
 
 
 
ASH2L
 
 
hsa-miR-320d
 
 
-0.287
 
 
30.4
 
 
 
 
ASPH
 
 
hsa-miR-152-3p
 
 
-0.391
 
 
331.0
 
 
 
 
ATG4B
 
 
hsa-miR-34a-5p
 
 
-0.58
 
 
20.2
 
 
 
 
ATMIN
 
 
hsa-miR-34a-5p
 
 
-0.409
 
 
15.1
 
 
 
 
ATP1A2
 
 
hsa-miR-34a-5p
 
 
-0.306
 
 
825.7
 
 
 
 
ATP2B4
 
 
hsa-miR-152-3p
 
 
-0.337
 
 
13.5
 
 
 
 
ATP5MC3
 
 
hsa-miR-27a-3p
 
 
-0.416
 
 
728.4
 
 
 
 
ATP6AP2
 
 
hsa-miR-152-3p
 
 
-0.592
 
 
20.5
 
 
 
 
ATP6AP2
 
 
hsa-miR-152-3p
 
 
-0.668
 
 
20.5
 
 
 
 
ATXN10
 
 
hsa-miR-27a-3p
 
 
-0.295
 
 
66.8
 
 
 
 
ATXN2
 
 
hsa-miR-10a-5p
 
 
-0.362
 
 
26.5
 
 
 
 
B4GALT2
 
 
hsa-miR-34a-5p
 
 
-0.314
 
 
49.2
 
 
 
 
B4GALT3
 
 
hsa-miR-27a-3p
 
 
-0.41
 
 
23.4
 
 
 
 
B4GALT5
 
 
hsa-miR-152-3p
 
 
-0.44
 
 
63.3
 
 
 
 
BAG4
 
 
hsa-miR-181a-5p
 
 
-0.337
 
 
13.5
 
 
 
 
BAG5
 
 
hsa-miR-22-3p
 
 
-0.318
 
 
12.8
 
 
 
 
BAZ1B
 
 
hsa-miR-10a-5p
 
 
-0.309
 
 
26.3
 
 
 
 
BAZ2A
 
 
hsa-miR-100-5p
 
 
-0.29
 
 
26.0
 
 
 
 
BAZ2A
 
 
hsa-miR-100-5p
 
 
-0.421
 
 
26.0
 
 
 
 
BCL2
 
 
hsa-miR-34a-5p
 
 
-0.289
 
 
15.5
 
 
 
 
BCL2L11
 
 
hsa-miR-152-3p
 
 
-0.445
 
 
11.0
 
 
 
 
BCL2L11
 
 
hsa-miR-10a-5p
 
 
-0.483
 
 
11.0
 
 
 
 
BCL2L13
 
 
hsa-miR-34a-5p
 
 
-0.431
 
 
37.4
 
 
 
 
BCL3
 
 
hsa-miR-27a-3p
 
 
-0.413
 
 
19.3
 
 
 
 
BEND7
 
 
hsa-miR-27a-3p
 
 
-0.304
 
 
13.9
 
 
 
 
BEX3
 
 
hsa-miR-27a-3p
 
 
-0.454
 
 
18.6
 
 
 
 
BHLHE40
 
 
hsa-miR-181a-5p
 
 
-0.408
 
 
269.9
 
 
 
 
BHLHE41
 
 
hsa-miR-152-3p
 
 
-0.442
 
 
86.6
 
 
 
 
BIN1
 
 
hsa-miR-22-3p
 
 
-0.346
 
 
1986.0
 
 
 
 
BLOC1S1
 
 
hsa-miR-22-3p
 
 
-0.346
 
 
308.2
 
 
 
 
BLOC1S6
 
 
hsa-miR-181a-5p
 
 
-0.393
 
 
22.1
 
 
 
 
BLTP1
 
 
hsa-miR-27a-3p
 
 
-0.426
 
 
38.9
 
 
 
 
BMI1
 
 
hsa-miR-27a-3p
 
 
-0.322
 
 
41.6
 
 
 
 
BRPF3
 
 
hsa-miR-34a-5p
 
 
-0.33
 
 
10.7
 
 
 
 
BTAF1
 
 
hsa-miR-152-3p
 
 
-0.401
 
 
11.9
 
 
 
 
BTBD3
 
 
hsa-miR-152-3p
 
 
-0.495
 
 
14.9
 
 
 
 
BTG2
 
 
hsa-miR-27a-3p
 
 
-0.339
 
 
204.1
 
 
 
 
BZW1
 
 
hsa-miR-27a-3p
 
 
-0.337
 
 
93.0
 
 
 
 
C1orf35
 
 
hsa-miR-152-3p
 
 
-0.511
 
 
10.7
 
 
 
 
C1orf52
 
 
hsa-miR-27a-3p
 
 
-0.591
 
 
15.4
 
 
 
 
 
 
 skin 
 
 
 
 
target_symbol
 
 
mature_mirna_id
 
 
score
 
 
nTPM
 
 
 
 
 
 
AAGAB
 
 
hsa-miR-34a-5p
 
 
-0.319
 
 
11.7
 
 
 
 
ABCB7
 
 
hsa-miR-152-3p
 
 
-0.515
 
 
12.7
 
 
 
 
ABCD3
 
 
hsa-miR-152-3p
 
 
-0.388
 
 
33.6
 
 
 
 
ABHD17C
 
 
hsa-miR-27a-3p
 
 
-0.435
 
 
17.9
 
 
 
 
ABHD5
 
 
hsa-miR-27a-3p
 
 
-0.407
 
 
29.7
 
 
 
 
ABRAXAS2
 
 
hsa-miR-34a-5p
 
 
-0.538
 
 
11.0
 
 
 
 
ACBD3
 
 
hsa-miR-34a-5p
 
 
-0.415
 
 
18.1
 
 
 
 
ACSL1
 
 
hsa-miR-34a-5p
 
 
-0.306
 
 
164.9
 
 
 
 
ACTA2
 
 
hsa-miR-27a-3p
 
 
-0.577
 
 
331.5
 
 
 
 
ADAM10
 
 
hsa-miR-152-3p
 
 
-0.348
 
 
14.8
 
 
 
 
ADIPOR2
 
 
hsa-miR-34a-5p
 
 
-0.33
 
 
30.8
 
 
 
 
ADK
 
 
hsa-miR-34a-5p
 
 
-0.374
 
 
32.7
 
 
 
 
AFG3L2
 
 
hsa-miR-181a-5p
 
 
-0.491
 
 
15.1
 
 
 
 
AGFG1
 
 
hsa-miR-152-3p
 
 
-0.332
 
 
34.3
 
 
 
 
AGO4
 
 
hsa-miR-34a-5p
 
 
-0.306
 
 
18.9
 
 
 
 
AGPAT3
 
 
hsa-miR-27a-3p
 
 
-0.29
 
 
23.2
 
 
 
 
AGTRAP
 
 
hsa-miR-34a-5p
 
 
-0.328
 
 
36.2
 
 
 
 
AHCYL2
 
 
hsa-miR-34a-5p
 
 
-0.359
 
 
22.9
 
 
 
 
AHDC1
 
 
hsa-miR-152-3p
 
 
-0.413
 
 
29.8
 
 
 
 
AIDA
 
 
hsa-miR-22-3p
 
 
-0.316
 
 
25.1
 
 
 
 
AKAP1
 
 
hsa-miR-152-3p
 
 
-0.381
 
 
26.4
 
 
 
 
AKIRIN1
 
 
hsa-miR-27a-3p
 
 
-0.368
 
 
16.7
 
 
 
 
ALCAM
 
 
hsa-miR-34a-5p
 
 
-0.303
 
 
10.3
 
 
 
 
ALCAM
 
 
hsa-miR-152-3p
 
 
-0.347
 
 
10.3
 
 
 
 
ALDOA
 
 
hsa-miR-34a-5p
 
 
-0.655
 
 
770.0
 
 
 
 
ANK3
 
 
hsa-miR-34a-5p
 
 
-0.29
 
 
37.1
 
 
 
 
ANK3
 
 
hsa-miR-34a-5p
 
 
-0.347
 
 
37.1
 
 
 
 
ANKHD1-EIF4EBP3
 
 
hsa-miR-22-3p
 
 
-0.479
 
 
15.0
 
 
 
 
ANKRD13A
 
 
hsa-miR-22-3p
 
 
-0.478
 
 
20.5
 
 
 
 
ANKRD40
 
 
hsa-miR-27a-3p
 
 
-0.35
 
 
13.5
 
 
 
 
ANP32B
 
 
hsa-miR-34a-5p
 
 
-0.29
 
 
140.0
 
 
 
 
AP1AR
 
 
hsa-miR-100-5p
 
 
-0.804
 
 
13.4
 
 
 
 
APH1A
 
 
hsa-miR-34a-5p
 
 
-0.304
 
 
113.8
 
 
 
 
AREG
 
 
hsa-miR-34a-5p
 
 
-0.62
 
 
14.9
 
 
 
 
ARF4
 
 
hsa-miR-152-3p
 
 
-0.487
 
 
61.3
 
 
 
 
ARF6
 
 
hsa-miR-181a-5p
 
 
-0.33
 
 
50.6
 
 
 
 
ARFGEF1
 
 
hsa-miR-152-3p
 
 
-0.306
 
 
12.9
 
 
 
 
ARFIP1
 
 
hsa-miR-320d
 
 
-0.339
 
 
16.4
 
 
 
 
ARFIP1
 
 
hsa-miR-152-3p
 
 
-0.474
 
 
16.4
 
 
 
 
ARHGAP1
 
 
hsa-miR-34a-5p
 
 
-0.369
 
 
61.6
 
 
 
 
ARL6IP1
 
 
hsa-miR-152-3p
 
 
-0.579
 
 
62.4
 
 
 
 
ARL8B
 
 
hsa-miR-152-3p
 
 
-0.337
 
 
64.4
 
 
 
 
ARL8B
 
 
hsa-miR-320d
 
 
-0.376
 
 
64.4
 
 
 
 
ARPC5
 
 
hsa-miR-22-3p
 
 
-0.679
 
 
40.3
 
 
 
 
ARPP19
 
 
hsa-miR-320d
 
 
-0.325
 
 
28.9
 
 
 
 
ARPP19
 
 
hsa-miR-34a-5p
 
 
-0.497
 
 
28.9
 
 
 
 
ARRB1
 
 
hsa-miR-22-3p
 
 
-0.616
 
 
15.9
 
 
 
 
ARRDC3
 
 
hsa-miR-152-3p
 
 
-0.305
 
 
38.6
 
 
 
 
ASAH1
 
 
hsa-miR-27a-3p
 
 
-0.398
 
 
49.7
 
 
 
 
ASH2L
 
 
hsa-miR-320d
 
 
-0.287
 
 
19.7
 
 
 
 
ASPH
 
 
hsa-miR-152-3p
 
 
-0.391
 
 
33.3
 
 
 
 
ASPHD2
 
 
hsa-miR-34a-5p
 
 
-0.354
 
 
10.4
 
 
 
 
ATG4B
 
 
hsa-miR-34a-5p
 
 
-0.58
 
 
18.6
 
 
 
 
ATMIN
 
 
hsa-miR-34a-5p
 
 
-0.409
 
 
16.6
 
 
 
 
ATP2B4
 
 
hsa-miR-152-3p
 
 
-0.337
 
 
51.7
 
 
 
 
ATP5MC3
 
 
hsa-miR-27a-3p
 
 
-0.416
 
 
162.4
 
 
 
 
ATP6AP2
 
 
hsa-miR-152-3p
 
 
-0.592
 
 
68.0
 
 
 
 
ATP6AP2
 
 
hsa-miR-152-3p
 
 
-0.668
 
 
68.0
 
 
 
 
ATP7A
 
 
hsa-miR-152-3p
 
 
-0.292
 
 
11.6
 
 
 
 
ATXN10
 
 
hsa-miR-27a-3p
 
 
-0.295
 
 
33.6
 
 
 
 
ATXN2
 
 
hsa-miR-10a-5p
 
 
-0.362
 
 
15.1
 
 
 
 
AXL
 
 
hsa-miR-34a-5p
 
 
-0.512
 
 
18.9
 
 
 
 
B4GALT2
 
 
hsa-miR-34a-5p
 
 
-0.314
 
 
24.7
 
 
 
 
B4GALT3
 
 
hsa-miR-27a-3p
 
 
-0.41
 
 
23.6
 
 
 
 
BAG5
 
 
hsa-miR-22-3p
 
 
-0.318
 
 
18.9
 
 
 
 
BAZ1B
 
 
hsa-miR-10a-5p
 
 
-0.309
 
 
13.4
 
 
 
 
BAZ2A
 
 
hsa-miR-100-5p
 
 
-0.29
 
 
27.8
 
 
 
 
BAZ2A
 
 
hsa-miR-100-5p
 
 
-0.421
 
 
27.8
 
 
 
 
BCL2L13
 
 
hsa-miR-34a-5p
 
 
-0.431
 
 
11.1
 
 
 
 
BCL3
 
 
hsa-miR-27a-3p
 
 
-0.413
 
 
31.1
 
 
 
 
BEX3
 
 
hsa-miR-27a-3p
 
 
-0.454
 
 
63.6
 
 
 
 
BHLHE40
 
 
hsa-miR-181a-5p
 
 
-0.408
 
 
217.6
 
 
 
 
BIN1
 
 
hsa-miR-22-3p
 
 
-0.346
 
 
12.2
 
 
 
 
BLOC1S1
 
 
hsa-miR-22-3p
 
 
-0.346
 
 
112.1
 
 
 
 
BLOC1S6
 
 
hsa-miR-181a-5p
 
 
-0.393
 
 
17.2
 
 
 
 
BLTP1
 
 
hsa-miR-27a-3p
 
 
-0.426
 
 
15.8
 
 
 
 
BMI1
 
 
hsa-miR-27a-3p
 
 
-0.322
 
 
13.6
 
 
 
 
BRPF3
 
 
hsa-miR-34a-5p
 
 
-0.33
 
 
11.8
 
 
 
 
BTAF1
 
 
hsa-miR-152-3p
 
 
-0.401
 
 
39.9
 
 
 
 
BTBD3
 
 
hsa-miR-152-3p
 
 
-0.495
 
 
19.6
 
 
 
 
BTG1
 
 
hsa-miR-22-3p
 
 
-0.468
 
 
21.1
 
 
 
 
BTG2
 
 
hsa-miR-27a-3p
 
 
-0.339
 
 
130.3
 
 
 
 
BZW1
 
 
hsa-miR-27a-3p
 
 
-0.337
 
 
45.2
 
 
 
 
C1orf35
 
 
hsa-miR-152-3p
 
 
-0.511
 
 
22.4
 
 
 
 
C1orf52
 
 
hsa-miR-27a-3p
 
 
-0.591
 
 
10.9
 
 
 
 
C5orf24
 
 
hsa-miR-22-3p
 
 
-0.493
 
 
18.0
 
 
 
 
C6orf62
 
 
hsa-miR-181a-5p
 
 
-0.334
 
 
88.3
 
 
 
 
CACNB3
 
 
hsa-miR-34a-5p
 
 
-0.426
 
 
10.9
 
 
 
 
CACYBP
 
 
hsa-miR-27a-3p
 
 
-0.367
 
 
17.0
 
 
 
 
CADM4
 
 
hsa-miR-34a-5p
 
 
-0.365
 
 
19.1
 
 
 
 
CAMK2N1
 
 
hsa-miR-22-3p
 
 
-0.302
 
 
11.4
 
 
 
 
CAND1
 
 
hsa-miR-27a-3p
 
 
-0.318
 
 
27.6
 
 
 
 
CAND1
 
 
hsa-miR-152-3p
 
 
-0.44
 
 
27.6
 
 
 
 
CANX
 
 
hsa-miR-152-3p
 
 
-0.434
 
 
71.7
 
 
 
 
CAPN1
 
 
hsa-miR-22-3p
 
 
-0.307
 
 
118.3
 
 
 
 
CAPZA1
 
 
hsa-miR-27a-3p
 
 
-0.325
 
 
87.3
 
 
 
 
CASP2
 
 
hsa-miR-34a-5p
 
 
-0.382
 
 
16.5
 
 
 
 
CAV2
 
 
hsa-miR-152-3p
 
 
-0.32
 
 
44.7
 
 
 
 
CBX4
 
 
hsa-miR-181a-5p
 
 
-0.354
 
 
30.1
 
 
 
 
CCDC47
 
 
hsa-miR-22-3p
 
 
-0.604
 
 
48.7
 
 
 
 
 
 
 small intestine 
 
 
 
 
target_symbol
 
 
mature_mirna_id
 
 
score
 
 
nTPM
 
 
 
 
 
 
AAGAB
 
 
hsa-miR-34a-5p
 
 
-0.319
 
 
18.4
 
 
 
 
ABCB10
 
 
hsa-miR-152-3p
 
 
-0.291
 
 
13.1
 
 
 
 
ABCB7
 
 
hsa-miR-152-3p
 
 
-0.515
 
 
11.2
 
 
 
 
ABCD3
 
 
hsa-miR-152-3p
 
 
-0.388
 
 
43.4
 
 
 
 
ABHD17C
 
 
hsa-miR-27a-3p
 
 
-0.435
 
 
28.8
 
 
 
 
ABRAXAS2
 
 
hsa-miR-34a-5p
 
 
-0.538
 
 
10.1
 
 
 
 
ACBD3
 
 
hsa-miR-34a-5p
 
 
-0.415
 
 
16.5
 
 
 
 
ACSL1
 
 
hsa-miR-34a-5p
 
 
-0.306
 
 
25.0
 
 
 
 
ACSL4
 
 
hsa-miR-34a-5p
 
 
-0.372
 
 
12.1
 
 
 
 
ACTA2
 
 
hsa-miR-27a-3p
 
 
-0.577
 
 
677.1
 
 
 
 
ADAM10
 
 
hsa-miR-152-3p
 
 
-0.348
 
 
15.8
 
 
 
 
ADAP1
 
 
hsa-miR-27a-3p
 
 
-0.302
 
 
25.6
 
 
 
 
ADIPOR2
 
 
hsa-miR-34a-5p
 
 
-0.33
 
 
31.4
 
 
 
 
ADK
 
 
hsa-miR-34a-5p
 
 
-0.374
 
 
11.7
 
 
 
 
AFG3L2
 
 
hsa-miR-181a-5p
 
 
-0.491
 
 
34.0
 
 
 
 
AGFG1
 
 
hsa-miR-152-3p
 
 
-0.332
 
 
33.9
 
 
 
 
AGMAT
 
 
hsa-miR-22-3p
 
 
-0.294
 
 
12.6
 
 
 
 
AGPAT3
 
 
hsa-miR-27a-3p
 
 
-0.29
 
 
49.3
 
 
 
 
AGTRAP
 
 
hsa-miR-34a-5p
 
 
-0.328
 
 
23.9
 
 
 
 
AHCYL2
 
 
hsa-miR-34a-5p
 
 
-0.359
 
 
36.4
 
 
 
 
AIDA
 
 
hsa-miR-22-3p
 
 
-0.316
 
 
27.3
 
 
 
 
AKAP1
 
 
hsa-miR-152-3p
 
 
-0.381
 
 
52.5
 
 
 
 
AKIRIN1
 
 
hsa-miR-27a-3p
 
 
-0.368
 
 
25.6
 
 
 
 
ALCAM
 
 
hsa-miR-34a-5p
 
 
-0.303
 
 
16.4
 
 
 
 
ALCAM
 
 
hsa-miR-152-3p
 
 
-0.347
 
 
16.4
 
 
 
 
ALDOA
 
 
hsa-miR-34a-5p
 
 
-0.655
 
 
568.6
 
 
 
 
ANK3
 
 
hsa-miR-34a-5p
 
 
-0.29
 
 
25.5
 
 
 
 
ANK3
 
 
hsa-miR-34a-5p
 
 
-0.347
 
 
25.5
 
 
 
 
ANKHD1-EIF4EBP3
 
 
hsa-miR-22-3p
 
 
-0.479
 
 
11.5
 
 
 
 
ANKRD13A
 
 
hsa-miR-22-3p
 
 
-0.478
 
 
15.8
 
 
 
 
ANKRD40
 
 
hsa-miR-27a-3p
 
 
-0.35
 
 
15.4
 
 
 
 
ANP32B
 
 
hsa-miR-34a-5p
 
 
-0.29
 
 
119.5
 
 
 
 
AP1AR
 
 
hsa-miR-100-5p
 
 
-0.804
 
 
22.4
 
 
 
 
APH1A
 
 
hsa-miR-34a-5p
 
 
-0.304
 
 
76.3
 
 
 
 
APOO
 
 
hsa-miR-181a-5p
 
 
-0.5
 
 
11.3
 
 
 
 
AQP11
 
 
hsa-miR-27a-3p
 
 
-0.666
 
 
23.9
 
 
 
 
ARF4
 
 
hsa-miR-152-3p
 
 
-0.487
 
 
102.8
 
 
 
 
ARF6
 
 
hsa-miR-181a-5p
 
 
-0.33
 
 
48.2
 
 
 
 
ARFGEF1
 
 
hsa-miR-152-3p
 
 
-0.306
 
 
16.5
 
 
 
 
ARFIP1
 
 
hsa-miR-320d
 
 
-0.339
 
 
20.0
 
 
 
 
ARFIP1
 
 
hsa-miR-152-3p
 
 
-0.474
 
 
20.0
 
 
 
 
ARHGAP1
 
 
hsa-miR-34a-5p
 
 
-0.369
 
 
44.5
 
 
 
 
ARHGAP21
 
 
hsa-miR-152-3p
 
 
-0.287
 
 
15.2
 
 
 
 
ARHGAP26
 
 
hsa-miR-34a-5p
 
 
-0.384
 
 
10.6
 
 
 
 
ARL6IP1
 
 
hsa-miR-152-3p
 
 
-0.579
 
 
116.2
 
 
 
 
ARL8B
 
 
hsa-miR-152-3p
 
 
-0.337
 
 
32.3
 
 
 
 
ARL8B
 
 
hsa-miR-320d
 
 
-0.376
 
 
32.3
 
 
 
 
ARPC5
 
 
hsa-miR-22-3p
 
 
-0.679
 
 
110.5
 
 
 
 
ARPP19
 
 
hsa-miR-320d
 
 
-0.325
 
 
28.8
 
 
 
 
ARPP19
 
 
hsa-miR-34a-5p
 
 
-0.497
 
 
28.8
 
 
 
 
ARRB1
 
 
hsa-miR-22-3p
 
 
-0.616
 
 
25.6
 
 
 
 
ARRDC3
 
 
hsa-miR-152-3p
 
 
-0.305
 
 
15.8
 
 
 
 
ASAH1
 
 
hsa-miR-27a-3p
 
 
-0.398
 
 
53.4
 
 
 
 
ASH2L
 
 
hsa-miR-320d
 
 
-0.287
 
 
17.5
 
 
 
 
ASPH
 
 
hsa-miR-152-3p
 
 
-0.391
 
 
42.6
 
 
 
 
ATG4B
 
 
hsa-miR-34a-5p
 
 
-0.58
 
 
19.9
 
 
 
 
ATMIN
 
 
hsa-miR-34a-5p
 
 
-0.409
 
 
12.2
 
 
 
 
ATOH1
 
 
hsa-miR-22-3p
 
 
-0.566
 
 
18.9
 
 
 
 
ATP1A2
 
 
hsa-miR-34a-5p
 
 
-0.306
 
 
15.0
 
 
 
 
ATP2B4
 
 
hsa-miR-152-3p
 
 
-0.337
 
 
13.8
 
 
 
 
ATP5MC3
 
 
hsa-miR-27a-3p
 
 
-0.416
 
 
279.4
 
 
 
 
ATP6AP2
 
 
hsa-miR-152-3p
 
 
-0.592
 
 
45.0
 
 
 
 
ATP6AP2
 
 
hsa-miR-152-3p
 
 
-0.668
 
 
45.0
 
 
 
 
ATXN10
 
 
hsa-miR-27a-3p
 
 
-0.295
 
 
26.4
 
 
 
 
ATXN2
 
 
hsa-miR-10a-5p
 
 
-0.362
 
 
14.3
 
 
 
 
AXL
 
 
hsa-miR-34a-5p
 
 
-0.512
 
 
18.5
 
 
 
 
B3GNT7
 
 
hsa-miR-27a-3p
 
 
-0.355
 
 
26.8
 
 
 
 
B4GALT2
 
 
hsa-miR-34a-5p
 
 
-0.314
 
 
11.9
 
 
 
 
B4GALT3
 
 
hsa-miR-27a-3p
 
 
-0.41
 
 
17.6
 
 
 
 
B4GALT5
 
 
hsa-miR-152-3p
 
 
-0.44
 
 
20.1
 
 
 
 
BAG5
 
 
hsa-miR-22-3p
 
 
-0.318
 
 
11.3
 
 
 
 
BAZ1B
 
 
hsa-miR-10a-5p
 
 
-0.309
 
 
17.3
 
 
 
 
BAZ2A
 
 
hsa-miR-100-5p
 
 
-0.29
 
 
28.7
 
 
 
 
BAZ2A
 
 
hsa-miR-100-5p
 
 
-0.421
 
 
28.7
 
 
 
 
BCL2L11
 
 
hsa-miR-152-3p
 
 
-0.445
 
 
11.3
 
 
 
 
BCL2L11
 
 
hsa-miR-10a-5p
 
 
-0.483
 
 
11.3
 
 
 
 
BCL3
 
 
hsa-miR-27a-3p
 
 
-0.413
 
 
48.2
 
 
 
 
BEND7
 
 
hsa-miR-27a-3p
 
 
-0.304
 
 
21.1
 
 
 
 
BEX3
 
 
hsa-miR-27a-3p
 
 
-0.454
 
 
59.2
 
 
 
 
BHLHE40
 
 
hsa-miR-181a-5p
 
 
-0.408
 
 
45.0
 
 
 
 
BIN1
 
 
hsa-miR-22-3p
 
 
-0.346
 
 
29.5
 
 
 
 
BLOC1S1
 
 
hsa-miR-22-3p
 
 
-0.346
 
 
151.2
 
 
 
 
BLOC1S6
 
 
hsa-miR-181a-5p
 
 
-0.393
 
 
20.1
 
 
 
 
BLTP1
 
 
hsa-miR-27a-3p
 
 
-0.426
 
 
17.6
 
 
 
 
BMI1
 
 
hsa-miR-27a-3p
 
 
-0.322
 
 
19.6
 
 
 
 
BRPF3
 
 
hsa-miR-34a-5p
 
 
-0.33
 
 
13.0
 
 
 
 
BTAF1
 
 
hsa-miR-152-3p
 
 
-0.401
 
 
18.1
 
 
 
 
BTBD3
 
 
hsa-miR-152-3p
 
 
-0.495
 
 
10.3
 
 
 
 
BTG1
 
 
hsa-miR-22-3p
 
 
-0.468
 
 
10.1
 
 
 
 
BTG2
 
 
hsa-miR-27a-3p
 
 
-0.339
 
 
109.0
 
 
 
 
BZW1
 
 
hsa-miR-27a-3p
 
 
-0.337
 
 
67.2
 
 
 
 
C1GALT1
 
 
hsa-miR-152-3p
 
 
-0.487
 
 
18.1
 
 
 
 
C1orf35
 
 
hsa-miR-152-3p
 
 
-0.511
 
 
11.9
 
 
 
 
C5orf24
 
 
hsa-miR-22-3p
 
 
-0.493
 
 
16.1
 
 
 
 
C6orf120
 
 
hsa-miR-34a-5p
 
 
-0.331
 
 
13.3
 
 
 
 
C6orf62
 
 
hsa-miR-181a-5p
 
 
-0.334
 
 
87.6
 
 
 
 
CACNB3
 
 
hsa-miR-34a-5p
 
 
-0.426
 
 
10.2
 
 
 
 
CACYBP
 
 
hsa-miR-27a-3p
 
 
-0.367
 
 
18.8
 
 
 
 
CAMK2N1
 
 
hsa-miR-22-3p
 
 
-0.302
 
 
81.9
 
 
 
 
CAND1
 
 
hsa-miR-27a-3p
 
 
-0.318
 
 
17.4
 
 
 
 
 
 
 smooth muscle 
 
 
 
 
target_symbol
 
 
mature_mirna_id
 
 
score
 
 
nTPM
 
 
 
 
 
 
AAGAB
 
 
hsa-miR-34a-5p
 
 
-0.319
 
 
19.8
 
 
 
 
ABCD3
 
 
hsa-miR-152-3p
 
 
-0.388
 
 
16.6
 
 
 
 
ACBD3
 
 
hsa-miR-34a-5p
 
 
-0.415
 
 
20.3
 
 
 
 
ACSL1
 
 
hsa-miR-34a-5p
 
 
-0.306
 
 
38.5
 
 
 
 
ACSL4
 
 
hsa-miR-34a-5p
 
 
-0.372
 
 
19.9
 
 
 
 
ACTA2
 
 
hsa-miR-27a-3p
 
 
-0.577
 
 
7564.0
 
 
 
 
ACVR1
 
 
hsa-miR-152-3p
 
 
-0.397
 
 
26.8
 
 
 
 
ADAM10
 
 
hsa-miR-152-3p
 
 
-0.348
 
 
12.4
 
 
 
 
ADGRA3
 
 
hsa-miR-27a-3p
 
 
-0.296
 
 
13.1
 
 
 
 
ADGRF5
 
 
hsa-miR-152-3p
 
 
-0.354
 
 
20.6
 
 
 
 
ADIPOR2
 
 
hsa-miR-34a-5p
 
 
-0.33
 
 
11.2
 
 
 
 
ADK
 
 
hsa-miR-34a-5p
 
 
-0.374
 
 
13.8
 
 
 
 
AFG3L2
 
 
hsa-miR-181a-5p
 
 
-0.491
 
 
14.9
 
 
 
 
AGFG1
 
 
hsa-miR-152-3p
 
 
-0.332
 
 
36.7
 
 
 
 
AGTRAP
 
 
hsa-miR-34a-5p
 
 
-0.328
 
 
18.9
 
 
 
 
AHCYL2
 
 
hsa-miR-34a-5p
 
 
-0.359
 
 
11.0
 
 
 
 
AIDA
 
 
hsa-miR-22-3p
 
 
-0.316
 
 
50.6
 
 
 
 
AKAP1
 
 
hsa-miR-152-3p
 
 
-0.381
 
 
24.6
 
 
 
 
AKIRIN1
 
 
hsa-miR-27a-3p
 
 
-0.368
 
 
39.2
 
 
 
 
AKT3
 
 
hsa-miR-22-3p
 
 
-0.451
 
 
14.7
 
 
 
 
ALCAM
 
 
hsa-miR-34a-5p
 
 
-0.303
 
 
15.2
 
 
 
 
ALCAM
 
 
hsa-miR-152-3p
 
 
-0.347
 
 
15.2
 
 
 
 
ALDOA
 
 
hsa-miR-34a-5p
 
 
-0.655
 
 
433.8
 
 
 
 
ANK2
 
 
hsa-miR-34a-5p
 
 
-0.317
 
 
20.3
 
 
 
 
ANK3
 
 
hsa-miR-34a-5p
 
 
-0.29
 
 
12.2
 
 
 
 
ANK3
 
 
hsa-miR-34a-5p
 
 
-0.347
 
 
12.2
 
 
 
 
ANKRD13A
 
 
hsa-miR-22-3p
 
 
-0.478
 
 
22.8
 
 
 
 
ANKRD40
 
 
hsa-miR-27a-3p
 
 
-0.35
 
 
26.1
 
 
 
 
ANP32B
 
 
hsa-miR-34a-5p
 
 
-0.29
 
 
72.9
 
 
 
 
AP3S2
 
 
hsa-miR-34a-5p
 
 
-0.413
 
 
16.7
 
 
 
 
APH1A
 
 
hsa-miR-34a-5p
 
 
-0.304
 
 
51.2
 
 
 
 
APOO
 
 
hsa-miR-181a-5p
 
 
-0.5
 
 
11.2
 
 
 
 
APPBP2
 
 
hsa-miR-27a-3p
 
 
-0.378
 
 
11.0
 
 
 
 
ARF4
 
 
hsa-miR-152-3p
 
 
-0.487
 
 
157.9
 
 
 
 
ARF6
 
 
hsa-miR-181a-5p
 
 
-0.33
 
 
22.2
 
 
 
 
ARFGEF1
 
 
hsa-miR-152-3p
 
 
-0.306
 
 
10.1
 
 
 
 
ARFIP1
 
 
hsa-miR-320d
 
 
-0.339
 
 
18.5
 
 
 
 
ARFIP1
 
 
hsa-miR-152-3p
 
 
-0.474
 
 
18.5
 
 
 
 
ARHGAP1
 
 
hsa-miR-34a-5p
 
 
-0.369
 
 
75.2
 
 
 
 
ARHGAP21
 
 
hsa-miR-152-3p
 
 
-0.287
 
 
16.5
 
 
 
 
ARL6IP1
 
 
hsa-miR-152-3p
 
 
-0.579
 
 
64.5
 
 
 
 
ARL8B
 
 
hsa-miR-152-3p
 
 
-0.337
 
 
43.9
 
 
 
 
ARL8B
 
 
hsa-miR-320d
 
 
-0.376
 
 
43.9
 
 
 
 
ARPC5
 
 
hsa-miR-22-3p
 
 
-0.679
 
 
73.7
 
 
 
 
ARPP19
 
 
hsa-miR-320d
 
 
-0.325
 
 
54.2
 
 
 
 
ARPP19
 
 
hsa-miR-34a-5p
 
 
-0.497
 
 
54.2
 
 
 
 
ARRB1
 
 
hsa-miR-22-3p
 
 
-0.616
 
 
20.3
 
 
 
 
ARRDC3
 
 
hsa-miR-152-3p
 
 
-0.305
 
 
30.8
 
 
 
 
ASAH1
 
 
hsa-miR-27a-3p
 
 
-0.398
 
 
57.6
 
 
 
 
ASH2L
 
 
hsa-miR-320d
 
 
-0.287
 
 
22.6
 
 
 
 
ASPH
 
 
hsa-miR-152-3p
 
 
-0.391
 
 
62.4
 
 
 
 
ATMIN
 
 
hsa-miR-34a-5p
 
 
-0.409
 
 
12.8
 
 
 
 
ATP2B4
 
 
hsa-miR-152-3p
 
 
-0.337
 
 
164.7
 
 
 
 
ATP5MC3
 
 
hsa-miR-27a-3p
 
 
-0.416
 
 
113.6
 
 
 
 
ATP6AP2
 
 
hsa-miR-152-3p
 
 
-0.592
 
 
116.5
 
 
 
 
ATP6AP2
 
 
hsa-miR-152-3p
 
 
-0.668
 
 
116.5
 
 
 
 
ATXN10
 
 
hsa-miR-27a-3p
 
 
-0.295
 
 
46.9
 
 
 
 
ATXN2
 
 
hsa-miR-10a-5p
 
 
-0.362
 
 
17.1
 
 
 
 
AXL
 
 
hsa-miR-34a-5p
 
 
-0.512
 
 
34.7
 
 
 
 
B4GALT2
 
 
hsa-miR-34a-5p
 
 
-0.314
 
 
24.5
 
 
 
 
B4GALT5
 
 
hsa-miR-152-3p
 
 
-0.44
 
 
35.1
 
 
 
 
BAG5
 
 
hsa-miR-22-3p
 
 
-0.318
 
 
14.1
 
 
 
 
BAZ1B
 
 
hsa-miR-10a-5p
 
 
-0.309
 
 
23.0
 
 
 
 
BAZ2A
 
 
hsa-miR-100-5p
 
 
-0.29
 
 
21.6
 
 
 
 
BAZ2A
 
 
hsa-miR-100-5p
 
 
-0.421
 
 
21.6
 
 
 
 
BBX
 
 
hsa-miR-10a-5p
 
 
-0.305
 
 
15.4
 
 
 
 
BCL3
 
 
hsa-miR-27a-3p
 
 
-0.413
 
 
10.9
 
 
 
 
BEX3
 
 
hsa-miR-27a-3p
 
 
-0.454
 
 
108.7
 
 
 
 
BHLHE40
 
 
hsa-miR-181a-5p
 
 
-0.408
 
 
60.9
 
 
 
 
BIN1
 
 
hsa-miR-22-3p
 
 
-0.346
 
 
18.3
 
 
 
 
BLOC1S1
 
 
hsa-miR-22-3p
 
 
-0.346
 
 
95.8
 
 
 
 
BLOC1S6
 
 
hsa-miR-181a-5p
 
 
-0.393
 
 
22.7
 
 
 
 
BLTP1
 
 
hsa-miR-27a-3p
 
 
-0.426
 
 
13.4
 
 
 
 
BMI1
 
 
hsa-miR-27a-3p
 
 
-0.322
 
 
33.8
 
 
 
 
BMPR2
 
 
hsa-miR-100-5p
 
 
-0.355
 
 
15.4
 
 
 
 
BTAF1
 
 
hsa-miR-152-3p
 
 
-0.401
 
 
11.0
 
 
 
 
BTBD3
 
 
hsa-miR-152-3p
 
 
-0.495
 
 
15.4
 
 
 
 
BTG2
 
 
hsa-miR-27a-3p
 
 
-0.339
 
 
199.6
 
 
 
 
BZW1
 
 
hsa-miR-27a-3p
 
 
-0.337
 
 
74.7
 
 
 
 
C5orf24
 
 
hsa-miR-22-3p
 
 
-0.493
 
 
24.9
 
 
 
 
C6orf120
 
 
hsa-miR-34a-5p
 
 
-0.331
 
 
10.6
 
 
 
 
C6orf62
 
 
hsa-miR-181a-5p
 
 
-0.334
 
 
64.6
 
 
 
 
C8orf88
 
 
hsa-miR-27a-3p
 
 
-0.33
 
 
28.8
 
 
 
 
CACNB3
 
 
hsa-miR-34a-5p
 
 
-0.426
 
 
21.6
 
 
 
 
CACYBP
 
 
hsa-miR-27a-3p
 
 
-0.367
 
 
15.3
 
 
 
 
CAMK2N1
 
 
hsa-miR-22-3p
 
 
-0.302
 
 
21.2
 
 
 
 
CAND1
 
 
hsa-miR-27a-3p
 
 
-0.318
 
 
20.2
 
 
 
 
CAND1
 
 
hsa-miR-152-3p
 
 
-0.44
 
 
20.2
 
 
 
 
CANX
 
 
hsa-miR-152-3p
 
 
-0.434
 
 
79.3
 
 
 
 
CAPN1
 
 
hsa-miR-22-3p
 
 
-0.307
 
 
42.3
 
 
 
 
CAPZA1
 
 
hsa-miR-27a-3p
 
 
-0.325
 
 
59.7
 
 
 
 
CAV2
 
 
hsa-miR-152-3p
 
 
-0.32
 
 
39.9
 
 
 
 
CCDC47
 
 
hsa-miR-22-3p
 
 
-0.604
 
 
39.3
 
 
 
 
CCDC6
 
 
hsa-miR-152-3p
 
 
-0.511
 
 
28.8
 
 
 
 
CCM2
 
 
hsa-miR-27a-3p
 
 
-0.335
 
 
15.8
 
 
 
 
CCN1
 
 
hsa-miR-22-3p
 
 
-0.601
 
 
132.7
 
 
 
 
CCN4
 
 
hsa-miR-27a-3p
 
 
-0.327
 
 
12.7
 
 
 
 
CCNC
 
 
hsa-miR-27a-3p
 
 
-0.378
 
 
20.5
 
 
 
 
CCNG1
 
 
hsa-miR-27a-3p
 
 
-0.419
 
 
48.8
 
 
 
 
CCNK
 
 
hsa-miR-181a-5p
 
 
-0.31
 
 
18.5
 
 
 
 
 
 
 spleen 
 
 
 
 
target_symbol
 
 
mature_mirna_id
 
 
score
 
 
nTPM
 
 
 
 
 
 
AAGAB
 
 
hsa-miR-34a-5p
 
 
-0.319
 
 
14.5
 
 
 
 
ABCB7
 
 
hsa-miR-152-3p
 
 
-0.515
 
 
10.6
 
 
 
 
ABCD3
 
 
hsa-miR-152-3p
 
 
-0.388
 
 
10.3
 
 
 
 
ACBD3
 
 
hsa-miR-34a-5p
 
 
-0.415
 
 
16.7
 
 
 
 
ACSL1
 
 
hsa-miR-34a-5p
 
 
-0.306
 
 
129.8
 
 
 
 
ACSL4
 
 
hsa-miR-34a-5p
 
 
-0.372
 
 
19.4
 
 
 
 
ACTA2
 
 
hsa-miR-27a-3p
 
 
-0.577
 
 
427.6
 
 
 
 
ADAM10
 
 
hsa-miR-152-3p
 
 
-0.348
 
 
27.3
 
 
 
 
ADAP1
 
 
hsa-miR-27a-3p
 
 
-0.302
 
 
16.5
 
 
 
 
ADGRF5
 
 
hsa-miR-152-3p
 
 
-0.354
 
 
38.8
 
 
 
 
ADIPOR2
 
 
hsa-miR-34a-5p
 
 
-0.33
 
 
19.2
 
 
 
 
ADK
 
 
hsa-miR-34a-5p
 
 
-0.374
 
 
14.2
 
 
 
 
AFG3L2
 
 
hsa-miR-181a-5p
 
 
-0.491
 
 
10.9
 
 
 
 
AGFG1
 
 
hsa-miR-152-3p
 
 
-0.332
 
 
44.5
 
 
 
 
AGO4
 
 
hsa-miR-34a-5p
 
 
-0.306
 
 
13.3
 
 
 
 
AGPAT3
 
 
hsa-miR-27a-3p
 
 
-0.29
 
 
18.3
 
 
 
 
AGTRAP
 
 
hsa-miR-34a-5p
 
 
-0.328
 
 
79.5
 
 
 
 
AHDC1
 
 
hsa-miR-152-3p
 
 
-0.413
 
 
15.7
 
 
 
 
AIDA
 
 
hsa-miR-22-3p
 
 
-0.316
 
 
35.3
 
 
 
 
AKAP1
 
 
hsa-miR-152-3p
 
 
-0.381
 
 
11.0
 
 
 
 
AKIRIN1
 
 
hsa-miR-27a-3p
 
 
-0.368
 
 
29.9
 
 
 
 
ALDOA
 
 
hsa-miR-34a-5p
 
 
-0.655
 
 
495.5
 
 
 
 
ALPL
 
 
hsa-miR-10a-5p
 
 
-0.32
 
 
44.5
 
 
 
 
ANK2
 
 
hsa-miR-34a-5p
 
 
-0.317
 
 
22.5
 
 
 
 
ANK3
 
 
hsa-miR-34a-5p
 
 
-0.29
 
 
10.5
 
 
 
 
ANK3
 
 
hsa-miR-34a-5p
 
 
-0.347
 
 
10.5
 
 
 
 
ANKHD1-EIF4EBP3
 
 
hsa-miR-22-3p
 
 
-0.479
 
 
15.6
 
 
 
 
ANKRD13A
 
 
hsa-miR-22-3p
 
 
-0.478
 
 
30.9
 
 
 
 
ANKRD40
 
 
hsa-miR-27a-3p
 
 
-0.35
 
 
19.3
 
 
 
 
ANP32B
 
 
hsa-miR-34a-5p
 
 
-0.29
 
 
117.6
 
 
 
 
AP1AR
 
 
hsa-miR-100-5p
 
 
-0.804
 
 
12.4
 
 
 
 
AP3S2
 
 
hsa-miR-34a-5p
 
 
-0.413
 
 
11.9
 
 
 
 
APH1A
 
 
hsa-miR-34a-5p
 
 
-0.304
 
 
94.3
 
 
 
 
APPBP2
 
 
hsa-miR-27a-3p
 
 
-0.378
 
 
11.3
 
 
 
 
ARF4
 
 
hsa-miR-152-3p
 
 
-0.487
 
 
60.0
 
 
 
 
ARF6
 
 
hsa-miR-181a-5p
 
 
-0.33
 
 
47.5
 
 
 
 
ARFGEF1
 
 
hsa-miR-152-3p
 
 
-0.306
 
 
13.0
 
 
 
 
ARFIP1
 
 
hsa-miR-320d
 
 
-0.339
 
 
17.3
 
 
 
 
ARFIP1
 
 
hsa-miR-152-3p
 
 
-0.474
 
 
17.3
 
 
 
 
ARHGAP1
 
 
hsa-miR-34a-5p
 
 
-0.369
 
 
55.1
 
 
 
 
ARHGAP26
 
 
hsa-miR-34a-5p
 
 
-0.384
 
 
16.3
 
 
 
 
ARL6IP1
 
 
hsa-miR-152-3p
 
 
-0.579
 
 
68.6
 
 
 
 
ARL8B
 
 
hsa-miR-152-3p
 
 
-0.337
 
 
32.0
 
 
 
 
ARL8B
 
 
hsa-miR-320d
 
 
-0.376
 
 
32.0
 
 
 
 
ARPC5
 
 
hsa-miR-22-3p
 
 
-0.679
 
 
113.8
 
 
 
 
ARPP19
 
 
hsa-miR-320d
 
 
-0.325
 
 
41.2
 
 
 
 
ARPP19
 
 
hsa-miR-34a-5p
 
 
-0.497
 
 
41.2
 
 
 
 
ARRB1
 
 
hsa-miR-22-3p
 
 
-0.616
 
 
37.8
 
 
 
 
ARRDC3
 
 
hsa-miR-152-3p
 
 
-0.305
 
 
35.0
 
 
 
 
AS3MT
 
 
hsa-miR-28-5p
 
 
-0.479
 
 
16.2
 
 
 
 
ASAH1
 
 
hsa-miR-27a-3p
 
 
-0.398
 
 
80.4
 
 
 
 
ASH2L
 
 
hsa-miR-320d
 
 
-0.287
 
 
18.2
 
 
 
 
ASPH
 
 
hsa-miR-152-3p
 
 
-0.391
 
 
26.8
 
 
 
 
ATG4B
 
 
hsa-miR-34a-5p
 
 
-0.58
 
 
21.8
 
 
 
 
ATMIN
 
 
hsa-miR-34a-5p
 
 
-0.409
 
 
12.1
 
 
 
 
ATP2B4
 
 
hsa-miR-152-3p
 
 
-0.337
 
 
11.5
 
 
 
 
ATP5MC3
 
 
hsa-miR-27a-3p
 
 
-0.416
 
 
126.3
 
 
 
 
ATP6AP2
 
 
hsa-miR-152-3p
 
 
-0.592
 
 
65.0
 
 
 
 
ATP6AP2
 
 
hsa-miR-152-3p
 
 
-0.668
 
 
65.0
 
 
 
 
ATXN10
 
 
hsa-miR-27a-3p
 
 
-0.295
 
 
32.5
 
 
 
 
ATXN2
 
 
hsa-miR-10a-5p
 
 
-0.362
 
 
13.5
 
 
 
 
AXL
 
 
hsa-miR-34a-5p
 
 
-0.512
 
 
35.4
 
 
 
 
B4GALT3
 
 
hsa-miR-27a-3p
 
 
-0.41
 
 
29.7
 
 
 
 
B4GALT5
 
 
hsa-miR-152-3p
 
 
-0.44
 
 
26.0
 
 
 
 
BAG5
 
 
hsa-miR-22-3p
 
 
-0.318
 
 
17.2
 
 
 
 
BATF3
 
 
hsa-miR-22-3p
 
 
-0.786
 
 
11.3
 
 
 
 
BAZ1B
 
 
hsa-miR-10a-5p
 
 
-0.309
 
 
22.0
 
 
 
 
BAZ2A
 
 
hsa-miR-100-5p
 
 
-0.29
 
 
33.2
 
 
 
 
BAZ2A
 
 
hsa-miR-100-5p
 
 
-0.421
 
 
33.2
 
 
 
 
BBX
 
 
hsa-miR-10a-5p
 
 
-0.305
 
 
17.8
 
 
 
 
BCL2
 
 
hsa-miR-34a-5p
 
 
-0.289
 
 
16.9
 
 
 
 
BCL2L11
 
 
hsa-miR-152-3p
 
 
-0.445
 
 
15.1
 
 
 
 
BCL2L11
 
 
hsa-miR-10a-5p
 
 
-0.483
 
 
15.1
 
 
 
 
BCL3
 
 
hsa-miR-27a-3p
 
 
-0.413
 
 
63.0
 
 
 
 
BEX3
 
 
hsa-miR-27a-3p
 
 
-0.454
 
 
102.4
 
 
 
 
BHLHE40
 
 
hsa-miR-181a-5p
 
 
-0.408
 
 
28.2
 
 
 
 
BIN1
 
 
hsa-miR-22-3p
 
 
-0.346
 
 
52.9
 
 
 
 
BLOC1S1
 
 
hsa-miR-22-3p
 
 
-0.346
 
 
172.7
 
 
 
 
BLOC1S6
 
 
hsa-miR-181a-5p
 
 
-0.393
 
 
26.3
 
 
 
 
BLTP1
 
 
hsa-miR-27a-3p
 
 
-0.426
 
 
15.9
 
 
 
 
BMI1
 
 
hsa-miR-27a-3p
 
 
-0.322
 
 
34.0
 
 
 
 
BMPR2
 
 
hsa-miR-100-5p
 
 
-0.355
 
 
11.5
 
 
 
 
BTAF1
 
 
hsa-miR-152-3p
 
 
-0.401
 
 
17.6
 
 
 
 
BTG1
 
 
hsa-miR-22-3p
 
 
-0.468
 
 
22.1
 
 
 
 
BTG2
 
 
hsa-miR-27a-3p
 
 
-0.339
 
 
103.8
 
 
 
 
BZW1
 
 
hsa-miR-27a-3p
 
 
-0.337
 
 
59.1
 
 
 
 
C1GALT1
 
 
hsa-miR-152-3p
 
 
-0.487
 
 
14.6
 
 
 
 
C1orf35
 
 
hsa-miR-152-3p
 
 
-0.511
 
 
12.6
 
 
 
 
C5orf24
 
 
hsa-miR-22-3p
 
 
-0.493
 
 
15.7
 
 
 
 
C6orf120
 
 
hsa-miR-34a-5p
 
 
-0.331
 
 
12.2
 
 
 
 
C6orf62
 
 
hsa-miR-181a-5p
 
 
-0.334
 
 
147.1
 
 
 
 
CACYBP
 
 
hsa-miR-27a-3p
 
 
-0.367
 
 
23.9
 
 
 
 
CAMK2N1
 
 
hsa-miR-22-3p
 
 
-0.302
 
 
11.4
 
 
 
 
CAND1
 
 
hsa-miR-27a-3p
 
 
-0.318
 
 
18.0
 
 
 
 
CAND1
 
 
hsa-miR-152-3p
 
 
-0.44
 
 
18.0
 
 
 
 
CANX
 
 
hsa-miR-152-3p
 
 
-0.434
 
 
63.9
 
 
 
 
CAPN1
 
 
hsa-miR-22-3p
 
 
-0.307
 
 
48.5
 
 
 
 
CAPZA1
 
 
hsa-miR-27a-3p
 
 
-0.325
 
 
99.1
 
 
 
 
CASP2
 
 
hsa-miR-34a-5p
 
 
-0.382
 
 
18.2
 
 
 
 
CAV2
 
 
hsa-miR-152-3p
 
 
-0.32
 
 
49.2
 
 
 
 
 
 
 testis 
 
 
 
 
target_symbol
 
 
mature_mirna_id
 
 
score
 
 
nTPM
 
 
 
 
 
 
AAGAB
 
 
hsa-miR-34a-5p
 
 
-0.319
 
 
20.5
 
 
 
 
ABCD3
 
 
hsa-miR-152-3p
 
 
-0.388
 
 
15.3
 
 
 
 
ACBD3
 
 
hsa-miR-34a-5p
 
 
-0.415
 
 
17.6
 
 
 
 
ACSL1
 
 
hsa-miR-34a-5p
 
 
-0.306
 
 
40.4
 
 
 
 
ACSL4
 
 
hsa-miR-34a-5p
 
 
-0.372
 
 
23.3
 
 
 
 
ACTA2
 
 
hsa-miR-27a-3p
 
 
-0.577
 
 
258.1
 
 
 
 
ADIPOR2
 
 
hsa-miR-34a-5p
 
 
-0.33
 
 
26.9
 
 
 
 
ADK
 
 
hsa-miR-34a-5p
 
 
-0.374
 
 
14.4
 
 
 
 
ADO
 
 
hsa-miR-34a-5p
 
 
-0.377
 
 
35.2
 
 
 
 
AFG3L2
 
 
hsa-miR-181a-5p
 
 
-0.491
 
 
10.5
 
 
 
 
AGFG1
 
 
hsa-miR-152-3p
 
 
-0.332
 
 
86.7
 
 
 
 
AGPAT3
 
 
hsa-miR-27a-3p
 
 
-0.29
 
 
25.0
 
 
 
 
AGTRAP
 
 
hsa-miR-34a-5p
 
 
-0.328
 
 
23.7
 
 
 
 
AIDA
 
 
hsa-miR-22-3p
 
 
-0.316
 
 
41.4
 
 
 
 
AKAP1
 
 
hsa-miR-152-3p
 
 
-0.381
 
 
108.8
 
 
 
 
AKIRIN1
 
 
hsa-miR-27a-3p
 
 
-0.368
 
 
78.8
 
 
 
 
ALDOA
 
 
hsa-miR-34a-5p
 
 
-0.655
 
 
199.3
 
 
 
 
ANKHD1-EIF4EBP3
 
 
hsa-miR-22-3p
 
 
-0.479
 
 
11.4
 
 
 
 
ANKRD13A
 
 
hsa-miR-22-3p
 
 
-0.478
 
 
56.9
 
 
 
 
ANP32B
 
 
hsa-miR-34a-5p
 
 
-0.29
 
 
56.3
 
 
 
 
AP1AR
 
 
hsa-miR-100-5p
 
 
-0.804
 
 
13.6
 
 
 
 
AP3S2
 
 
hsa-miR-34a-5p
 
 
-0.413
 
 
11.6
 
 
 
 
APH1A
 
 
hsa-miR-34a-5p
 
 
-0.304
 
 
33.4
 
 
 
 
APOO
 
 
hsa-miR-181a-5p
 
 
-0.5
 
 
15.7
 
 
 
 
APPBP2
 
 
hsa-miR-27a-3p
 
 
-0.378
 
 
21.9
 
 
 
 
ARF4
 
 
hsa-miR-152-3p
 
 
-0.487
 
 
94.2
 
 
 
 
ARF6
 
 
hsa-miR-181a-5p
 
 
-0.33
 
 
15.0
 
 
 
 
ARFGEF1
 
 
hsa-miR-152-3p
 
 
-0.306
 
 
26.5
 
 
 
 
ARFIP1
 
 
hsa-miR-320d
 
 
-0.339
 
 
11.3
 
 
 
 
ARFIP1
 
 
hsa-miR-152-3p
 
 
-0.474
 
 
11.3
 
 
 
 
ARHGAP1
 
 
hsa-miR-34a-5p
 
 
-0.369
 
 
15.3
 
 
 
 
ARHGAP21
 
 
hsa-miR-152-3p
 
 
-0.287
 
 
13.5
 
 
 
 
ARHGAP26
 
 
hsa-miR-34a-5p
 
 
-0.384
 
 
15.3
 
 
 
 
ARL6IP1
 
 
hsa-miR-152-3p
 
 
-0.579
 
 
121.4
 
 
 
 
ARL8B
 
 
hsa-miR-152-3p
 
 
-0.337
 
 
30.1
 
 
 
 
ARL8B
 
 
hsa-miR-320d
 
 
-0.376
 
 
30.1
 
 
 
 
ARPC5
 
 
hsa-miR-22-3p
 
 
-0.679
 
 
35.9
 
 
 
 
ARPIN-AP3S2
 
 
hsa-miR-34a-5p
 
 
-0.348
 
 
11.1
 
 
 
 
ARPP19
 
 
hsa-miR-320d
 
 
-0.325
 
 
79.0
 
 
 
 
ARPP19
 
 
hsa-miR-34a-5p
 
 
-0.497
 
 
79.0
 
 
 
 
ARRDC3
 
 
hsa-miR-152-3p
 
 
-0.305
 
 
15.5
 
 
 
 
ASAH1
 
 
hsa-miR-27a-3p
 
 
-0.398
 
 
17.0
 
 
 
 
ASB1
 
 
hsa-miR-34a-5p
 
 
-0.406
 
 
10.2
 
 
 
 
ASH2L
 
 
hsa-miR-320d
 
 
-0.287
 
 
38.9
 
 
 
 
ASPH
 
 
hsa-miR-152-3p
 
 
-0.391
 
 
15.3
 
 
 
 
ATG4B
 
 
hsa-miR-34a-5p
 
 
-0.58
 
 
22.6
 
 
 
 
ATMIN
 
 
hsa-miR-34a-5p
 
 
-0.409
 
 
21.1
 
 
 
 
ATP2B4
 
 
hsa-miR-152-3p
 
 
-0.337
 
 
25.4
 
 
 
 
ATP5MC3
 
 
hsa-miR-27a-3p
 
 
-0.416
 
 
60.2
 
 
 
 
ATP6AP2
 
 
hsa-miR-152-3p
 
 
-0.592
 
 
37.7
 
 
 
 
ATP6AP2
 
 
hsa-miR-152-3p
 
 
-0.668
 
 
37.7
 
 
 
 
ATXN10
 
 
hsa-miR-27a-3p
 
 
-0.295
 
 
53.5
 
 
 
 
ATXN2
 
 
hsa-miR-10a-5p
 
 
-0.362
 
 
23.4
 
 
 
 
AXL
 
 
hsa-miR-34a-5p
 
 
-0.512
 
 
17.4
 
 
 
 
B4GALT2
 
 
hsa-miR-34a-5p
 
 
-0.314
 
 
17.6
 
 
 
 
B4GALT3
 
 
hsa-miR-27a-3p
 
 
-0.41
 
 
18.8
 
 
 
 
B4GALT5
 
 
hsa-miR-152-3p
 
 
-0.44
 
 
20.8
 
 
 
 
BAG5
 
 
hsa-miR-22-3p
 
 
-0.318
 
 
119.4
 
 
 
 
BAZ1B
 
 
hsa-miR-10a-5p
 
 
-0.309
 
 
16.1
 
 
 
 
BAZ2A
 
 
hsa-miR-100-5p
 
 
-0.29
 
 
44.2
 
 
 
 
BAZ2A
 
 
hsa-miR-100-5p
 
 
-0.421
 
 
44.2
 
 
 
 
BBX
 
 
hsa-miR-10a-5p
 
 
-0.305
 
 
54.2
 
 
 
 
BCL2L13
 
 
hsa-miR-34a-5p
 
 
-0.431
 
 
10.0
 
 
 
 
BCL3
 
 
hsa-miR-27a-3p
 
 
-0.413
 
 
10.5
 
 
 
 
BEND7
 
 
hsa-miR-27a-3p
 
 
-0.304
 
 
14.4
 
 
 
 
BEX3
 
 
hsa-miR-27a-3p
 
 
-0.454
 
 
147.1
 
 
 
 
BHLHE40
 
 
hsa-miR-181a-5p
 
 
-0.408
 
 
14.9
 
 
 
 
BIN1
 
 
hsa-miR-22-3p
 
 
-0.346
 
 
10.0
 
 
 
 
BLOC1S1
 
 
hsa-miR-22-3p
 
 
-0.346
 
 
54.5
 
 
 
 
BLOC1S6
 
 
hsa-miR-181a-5p
 
 
-0.393
 
 
15.9
 
 
 
 
BLTP1
 
 
hsa-miR-27a-3p
 
 
-0.426
 
 
18.6
 
 
 
 
BMI1
 
 
hsa-miR-27a-3p
 
 
-0.322
 
 
32.9
 
 
 
 
BRPF3
 
 
hsa-miR-34a-5p
 
 
-0.33
 
 
18.6
 
 
 
 
BRSK1
 
 
hsa-miR-27a-3p
 
 
-0.372
 
 
11.2
 
 
 
 
BTAF1
 
 
hsa-miR-152-3p
 
 
-0.401
 
 
14.0
 
 
 
 
BTBD3
 
 
hsa-miR-152-3p
 
 
-0.495
 
 
13.8
 
 
 
 
BZW1
 
 
hsa-miR-27a-3p
 
 
-0.337
 
 
159.6
 
 
 
 
C16orf87
 
 
hsa-miR-181a-5p
 
 
-0.54
 
 
17.2
 
 
 
 
C1orf52
 
 
hsa-miR-27a-3p
 
 
-0.591
 
 
10.4
 
 
 
 
C2orf69
 
 
hsa-miR-181a-5p
 
 
-0.38
 
 
13.7
 
 
 
 
C5orf24
 
 
hsa-miR-22-3p
 
 
-0.493
 
 
11.6
 
 
 
 
C6orf62
 
 
hsa-miR-181a-5p
 
 
-0.334
 
 
116.8
 
 
 
 
C8orf88
 
 
hsa-miR-27a-3p
 
 
-0.33
 
 
85.6
 
 
 
 
CACYBP
 
 
hsa-miR-27a-3p
 
 
-0.367
 
 
28.5
 
 
 
 
CAND1
 
 
hsa-miR-27a-3p
 
 
-0.318
 
 
26.0
 
 
 
 
CAND1
 
 
hsa-miR-152-3p
 
 
-0.44
 
 
26.0
 
 
 
 
CANX
 
 
hsa-miR-152-3p
 
 
-0.434
 
 
67.8
 
 
 
 
CAPZA1
 
 
hsa-miR-27a-3p
 
 
-0.325
 
 
66.5
 
 
 
 
CARMIL1
 
 
hsa-miR-22-3p
 
 
-0.329
 
 
11.5
 
 
 
 
CATSPERG
 
 
hsa-miR-22-3p
 
 
-0.403
 
 
30.6
 
 
 
 
CAV2
 
 
hsa-miR-152-3p
 
 
-0.32
 
 
14.5
 
 
 
 
CCDC28B
 
 
hsa-miR-27a-3p
 
 
-0.314
 
 
26.9
 
 
 
 
CCDC47
 
 
hsa-miR-22-3p
 
 
-0.604
 
 
28.8
 
 
 
 
CCDC6
 
 
hsa-miR-152-3p
 
 
-0.511
 
 
10.6
 
 
 
 
CCDC71
 
 
hsa-miR-152-3p
 
 
-0.369
 
 
11.7
 
 
 
 
CCM2
 
 
hsa-miR-27a-3p
 
 
-0.335
 
 
29.8
 
 
 
 
CCNC
 
 
hsa-miR-27a-3p
 
 
-0.378
 
 
15.9
 
 
 
 
CCNE2
 
 
hsa-miR-34a-5p
 
 
-0.553
 
 
13.5
 
 
 
 
CCNG1
 
 
hsa-miR-27a-3p
 
 
-0.419
 
 
19.3
 
 
 
 
CCNK
 
 
hsa-miR-181a-5p
 
 
-0.31
 
 
42.4
 
 
 
 
 
 
 thymus 
 
 
 
 
target_symbol
 
 
mature_mirna_id
 
 
score
 
 
nTPM
 
 
 
 
 
 
AAGAB
 
 
hsa-miR-34a-5p
 
 
-0.319
 
 
27.8
 
 
 
 
ABCB7
 
 
hsa-miR-152-3p
 
 
-0.515
 
 
13.0
 
 
 
 
ABCD3
 
 
hsa-miR-152-3p
 
 
-0.388
 
 
44.2
 
 
 
 
ABRAXAS2
 
 
hsa-miR-34a-5p
 
 
-0.538
 
 
12.0
 
 
 
 
ACBD3
 
 
hsa-miR-34a-5p
 
 
-0.415
 
 
16.8
 
 
 
 
ACSL1
 
 
hsa-miR-34a-5p
 
 
-0.306
 
 
10.7
 
 
 
 
ACSL4
 
 
hsa-miR-34a-5p
 
 
-0.372
 
 
18.3
 
 
 
 
ACTA2
 
 
hsa-miR-27a-3p
 
 
-0.577
 
 
52.7
 
 
 
 
ADAM10
 
 
hsa-miR-152-3p
 
 
-0.348
 
 
38.8
 
 
 
 
ADIPOR2
 
 
hsa-miR-34a-5p
 
 
-0.33
 
 
11.9
 
 
 
 
ADK
 
 
hsa-miR-34a-5p
 
 
-0.374
 
 
25.2
 
 
 
 
AFG3L2
 
 
hsa-miR-181a-5p
 
 
-0.491
 
 
12.1
 
 
 
 
AGFG1
 
 
hsa-miR-152-3p
 
 
-0.332
 
 
45.3
 
 
 
 
AGPAT3
 
 
hsa-miR-27a-3p
 
 
-0.29
 
 
14.4
 
 
 
 
AGTRAP
 
 
hsa-miR-34a-5p
 
 
-0.328
 
 
10.5
 
 
 
 
AIDA
 
 
hsa-miR-22-3p
 
 
-0.316
 
 
31.5
 
 
 
 
AKAP1
 
 
hsa-miR-152-3p
 
 
-0.381
 
 
10.6
 
 
 
 
AKIRIN1
 
 
hsa-miR-27a-3p
 
 
-0.368
 
 
38.6
 
 
 
 
ALCAM
 
 
hsa-miR-34a-5p
 
 
-0.303
 
 
13.3
 
 
 
 
ALCAM
 
 
hsa-miR-152-3p
 
 
-0.347
 
 
13.3
 
 
 
 
ALDOA
 
 
hsa-miR-34a-5p
 
 
-0.655
 
 
259.9
 
 
 
 
ANKHD1-EIF4EBP3
 
 
hsa-miR-22-3p
 
 
-0.479
 
 
13.4
 
 
 
 
ANKRD13A
 
 
hsa-miR-22-3p
 
 
-0.478
 
 
26.0
 
 
 
 
ANKRD40
 
 
hsa-miR-27a-3p
 
 
-0.35
 
 
15.8
 
 
 
 
ANP32B
 
 
hsa-miR-34a-5p
 
 
-0.29
 
 
252.8
 
 
 
 
AP1AR
 
 
hsa-miR-100-5p
 
 
-0.804
 
 
10.2
 
 
 
 
AP3S2
 
 
hsa-miR-34a-5p
 
 
-0.413
 
 
18.7
 
 
 
 
APH1A
 
 
hsa-miR-34a-5p
 
 
-0.304
 
 
67.9
 
 
 
 
APOO
 
 
hsa-miR-181a-5p
 
 
-0.5
 
 
13.0
 
 
 
 
ARF4
 
 
hsa-miR-152-3p
 
 
-0.487
 
 
69.9
 
 
 
 
ARF6
 
 
hsa-miR-181a-5p
 
 
-0.33
 
 
41.8
 
 
 
 
ARFGEF1
 
 
hsa-miR-152-3p
 
 
-0.306
 
 
20.7
 
 
 
 
ARFIP1
 
 
hsa-miR-320d
 
 
-0.339
 
 
11.2
 
 
 
 
ARFIP1
 
 
hsa-miR-152-3p
 
 
-0.474
 
 
11.2
 
 
 
 
ARHGAP1
 
 
hsa-miR-34a-5p
 
 
-0.369
 
 
21.6
 
 
 
 
ARHGAP21
 
 
hsa-miR-152-3p
 
 
-0.287
 
 
10.5
 
 
 
 
ARHGAP26
 
 
hsa-miR-34a-5p
 
 
-0.384
 
 
12.8
 
 
 
 
ARL6IP1
 
 
hsa-miR-152-3p
 
 
-0.579
 
 
147.6
 
 
 
 
ARL8B
 
 
hsa-miR-152-3p
 
 
-0.337
 
 
33.3
 
 
 
 
ARL8B
 
 
hsa-miR-320d
 
 
-0.376
 
 
33.3
 
 
 
 
ARPC5
 
 
hsa-miR-22-3p
 
 
-0.679
 
 
121.6
 
 
 
 
ARPP19
 
 
hsa-miR-320d
 
 
-0.325
 
 
49.2
 
 
 
 
ARPP19
 
 
hsa-miR-34a-5p
 
 
-0.497
 
 
49.2
 
 
 
 
ARRDC3
 
 
hsa-miR-152-3p
 
 
-0.305
 
 
26.1
 
 
 
 
ASAH1
 
 
hsa-miR-27a-3p
 
 
-0.398
 
 
24.2
 
 
 
 
ASH2L
 
 
hsa-miR-320d
 
 
-0.287
 
 
31.4
 
 
 
 
ASPH
 
 
hsa-miR-152-3p
 
 
-0.391
 
 
15.5
 
 
 
 
ATG4B
 
 
hsa-miR-34a-5p
 
 
-0.58
 
 
15.0
 
 
 
 
ATMIN
 
 
hsa-miR-34a-5p
 
 
-0.409
 
 
14.6
 
 
 
 
ATP2B4
 
 
hsa-miR-152-3p
 
 
-0.337
 
 
20.2
 
 
 
 
ATP5MC3
 
 
hsa-miR-27a-3p
 
 
-0.416
 
 
175.6
 
 
 
 
ATP6AP2
 
 
hsa-miR-152-3p
 
 
-0.592
 
 
67.0
 
 
 
 
ATP6AP2
 
 
hsa-miR-152-3p
 
 
-0.668
 
 
67.0
 
 
 
 
ATXN10
 
 
hsa-miR-27a-3p
 
 
-0.295
 
 
61.3
 
 
 
 
ATXN2
 
 
hsa-miR-10a-5p
 
 
-0.362
 
 
11.9
 
 
 
 
B4GALT3
 
 
hsa-miR-27a-3p
 
 
-0.41
 
 
15.8
 
 
 
 
BAG5
 
 
hsa-miR-22-3p
 
 
-0.318
 
 
13.9
 
 
 
 
BAZ1B
 
 
hsa-miR-10a-5p
 
 
-0.309
 
 
37.8
 
 
 
 
BAZ2A
 
 
hsa-miR-100-5p
 
 
-0.29
 
 
47.1
 
 
 
 
BAZ2A
 
 
hsa-miR-100-5p
 
 
-0.421
 
 
47.1
 
 
 
 
BBX
 
 
hsa-miR-10a-5p
 
 
-0.305
 
 
15.2
 
 
 
 
BCL2
 
 
hsa-miR-34a-5p
 
 
-0.289
 
 
17.0
 
 
 
 
BCL2L11
 
 
hsa-miR-152-3p
 
 
-0.445
 
 
18.0
 
 
 
 
BCL2L11
 
 
hsa-miR-10a-5p
 
 
-0.483
 
 
18.0
 
 
 
 
BEX3
 
 
hsa-miR-27a-3p
 
 
-0.454
 
 
90.4
 
 
 
 
BHLHE40
 
 
hsa-miR-181a-5p
 
 
-0.408
 
 
38.0
 
 
 
 
BIN1
 
 
hsa-miR-22-3p
 
 
-0.346
 
 
17.1
 
 
 
 
BLOC1S1
 
 
hsa-miR-22-3p
 
 
-0.346
 
 
140.0
 
 
 
 
BLOC1S6
 
 
hsa-miR-181a-5p
 
 
-0.393
 
 
23.5
 
 
 
 
BLTP1
 
 
hsa-miR-27a-3p
 
 
-0.426
 
 
15.7
 
 
 
 
BMI1
 
 
hsa-miR-27a-3p
 
 
-0.322
 
 
23.2
 
 
 
 
BTAF1
 
 
hsa-miR-152-3p
 
 
-0.401
 
 
17.2
 
 
 
 
BTBD3
 
 
hsa-miR-152-3p
 
 
-0.495
 
 
10.0
 
 
 
 
BTG1
 
 
hsa-miR-22-3p
 
 
-0.468
 
 
18.7
 
 
 
 
BTG2
 
 
hsa-miR-27a-3p
 
 
-0.339
 
 
312.3
 
 
 
 
BZW1
 
 
hsa-miR-27a-3p
 
 
-0.337
 
 
114.6
 
 
 
 
C1GALT1
 
 
hsa-miR-152-3p
 
 
-0.487
 
 
13.3
 
 
 
 
C5orf24
 
 
hsa-miR-22-3p
 
 
-0.493
 
 
14.0
 
 
 
 
C6orf120
 
 
hsa-miR-34a-5p
 
 
-0.331
 
 
10.0
 
 
 
 
C6orf62
 
 
hsa-miR-181a-5p
 
 
-0.334
 
 
123.9
 
 
 
 
C8orf88
 
 
hsa-miR-27a-3p
 
 
-0.33
 
 
12.1
 
 
 
 
CACYBP
 
 
hsa-miR-27a-3p
 
 
-0.367
 
 
26.0
 
 
 
 
CAMK4
 
 
hsa-miR-34a-5p
 
 
-0.353
 
 
51.6
 
 
 
 
CAND1
 
 
hsa-miR-27a-3p
 
 
-0.318
 
 
36.1
 
 
 
 
CAND1
 
 
hsa-miR-152-3p
 
 
-0.44
 
 
36.1
 
 
 
 
CAPN1
 
 
hsa-miR-22-3p
 
 
-0.307
 
 
46.0
 
 
 
 
CAPZA1
 
 
hsa-miR-27a-3p
 
 
-0.325
 
 
110.1
 
 
 
 
CASP2
 
 
hsa-miR-34a-5p
 
 
-0.382
 
 
25.6
 
 
 
 
CBFA2T3
 
 
hsa-miR-34a-5p
 
 
-0.501
 
 
27.4
 
 
 
 
CBFB
 
 
hsa-miR-27a-3p
 
 
-0.302
 
 
12.5
 
 
 
 
CBX4
 
 
hsa-miR-181a-5p
 
 
-0.354
 
 
21.5
 
 
 
 
CCDC28B
 
 
hsa-miR-27a-3p
 
 
-0.314
 
 
11.6
 
 
 
 
CCDC47
 
 
hsa-miR-22-3p
 
 
-0.604
 
 
27.6
 
 
 
 
CCDC6
 
 
hsa-miR-152-3p
 
 
-0.511
 
 
11.3
 
 
 
 
CCDC71
 
 
hsa-miR-152-3p
 
 
-0.369
 
 
12.2
 
 
 
 
CCL8
 
 
hsa-miR-181a-5p
 
 
-0.39
 
 
11.7
 
 
 
 
CCM2
 
 
hsa-miR-27a-3p
 
 
-0.335
 
 
42.3
 
 
 
 
CCN1
 
 
hsa-miR-22-3p
 
 
-0.601
 
 
10.4
 
 
 
 
CCNC
 
 
hsa-miR-27a-3p
 
 
-0.378
 
 
29.9
 
 
 
 
CCNE2
 
 
hsa-miR-34a-5p
 
 
-0.553
 
 
14.1
 
 
 
 
 
 
 thyroid gland 
 
 
 
 
target_symbol
 
 
mature_mirna_id
 
 
score
 
 
nTPM
 
 
 
 
 
 
AAGAB
 
 
hsa-miR-34a-5p
 
 
-0.319
 
 
30.9
 
 
 
 
ABCD3
 
 
hsa-miR-152-3p
 
 
-0.388
 
 
25.8
 
 
 
 
ABHD5
 
 
hsa-miR-27a-3p
 
 
-0.407
 
 
14.4
 
 
 
 
ABRAXAS2
 
 
hsa-miR-34a-5p
 
 
-0.538
 
 
11.9
 
 
 
 
ACBD3
 
 
hsa-miR-34a-5p
 
 
-0.415
 
 
26.9
 
 
 
 
ACSL1
 
 
hsa-miR-34a-5p
 
 
-0.306
 
 
44.4
 
 
 
 
ACSL4
 
 
hsa-miR-34a-5p
 
 
-0.372
 
 
17.2
 
 
 
 
ACTA2
 
 
hsa-miR-27a-3p
 
 
-0.577
 
 
297.7
 
 
 
 
ACVR1
 
 
hsa-miR-152-3p
 
 
-0.397
 
 
28.6
 
 
 
 
ADAM10
 
 
hsa-miR-152-3p
 
 
-0.348
 
 
26.7
 
 
 
 
ADGRA3
 
 
hsa-miR-27a-3p
 
 
-0.296
 
 
23.6
 
 
 
 
ADGRF5
 
 
hsa-miR-152-3p
 
 
-0.354
 
 
30.7
 
 
 
 
ADIPOR2
 
 
hsa-miR-34a-5p
 
 
-0.33
 
 
26.8
 
 
 
 
ADK
 
 
hsa-miR-34a-5p
 
 
-0.374
 
 
35.7
 
 
 
 
ADO
 
 
hsa-miR-34a-5p
 
 
-0.377
 
 
11.2
 
 
 
 
AFG3L2
 
 
hsa-miR-181a-5p
 
 
-0.491
 
 
24.7
 
 
 
 
AGFG1
 
 
hsa-miR-152-3p
 
 
-0.332
 
 
32.1
 
 
 
 
AGPAT3
 
 
hsa-miR-27a-3p
 
 
-0.29
 
 
20.1
 
 
 
 
AGTRAP
 
 
hsa-miR-34a-5p
 
 
-0.328
 
 
38.5
 
 
 
 
AHCYL2
 
 
hsa-miR-34a-5p
 
 
-0.359
 
 
39.9
 
 
 
 
AHDC1
 
 
hsa-miR-152-3p
 
 
-0.413
 
 
19.9
 
 
 
 
AIDA
 
 
hsa-miR-22-3p
 
 
-0.316
 
 
35.1
 
 
 
 
AKAP1
 
 
hsa-miR-152-3p
 
 
-0.381
 
 
35.0
 
 
 
 
AKIRIN1
 
 
hsa-miR-27a-3p
 
 
-0.368
 
 
33.8
 
 
 
 
AKT3
 
 
hsa-miR-22-3p
 
 
-0.451
 
 
13.0
 
 
 
 
ALCAM
 
 
hsa-miR-34a-5p
 
 
-0.303
 
 
58.4
 
 
 
 
ALCAM
 
 
hsa-miR-152-3p
 
 
-0.347
 
 
58.4
 
 
 
 
ALDOA
 
 
hsa-miR-34a-5p
 
 
-0.655
 
 
510.5
 
 
 
 
ANK3
 
 
hsa-miR-34a-5p
 
 
-0.29
 
 
35.3
 
 
 
 
ANK3
 
 
hsa-miR-34a-5p
 
 
-0.347
 
 
35.3
 
 
 
 
ANKRD13A
 
 
hsa-miR-22-3p
 
 
-0.478
 
 
26.4
 
 
 
 
ANKRD40
 
 
hsa-miR-27a-3p
 
 
-0.35
 
 
15.8
 
 
 
 
ANP32B
 
 
hsa-miR-34a-5p
 
 
-0.29
 
 
92.0
 
 
 
 
AP1AR
 
 
hsa-miR-100-5p
 
 
-0.804
 
 
10.1
 
 
 
 
AP1S3
 
 
hsa-miR-181a-5p
 
 
-0.383
 
 
12.8
 
 
 
 
AP3S2
 
 
hsa-miR-34a-5p
 
 
-0.413
 
 
24.5
 
 
 
 
APH1A
 
 
hsa-miR-34a-5p
 
 
-0.304
 
 
89.7
 
 
 
 
APOO
 
 
hsa-miR-181a-5p
 
 
-0.5
 
 
19.0
 
 
 
 
APPBP2
 
 
hsa-miR-27a-3p
 
 
-0.378
 
 
12.0
 
 
 
 
ARF4
 
 
hsa-miR-152-3p
 
 
-0.487
 
 
134.8
 
 
 
 
ARF6
 
 
hsa-miR-181a-5p
 
 
-0.33
 
 
20.1
 
 
 
 
ARFGEF1
 
 
hsa-miR-152-3p
 
 
-0.306
 
 
20.9
 
 
 
 
ARFIP1
 
 
hsa-miR-320d
 
 
-0.339
 
 
18.8
 
 
 
 
ARFIP1
 
 
hsa-miR-152-3p
 
 
-0.474
 
 
18.8
 
 
 
 
ARHGAP1
 
 
hsa-miR-34a-5p
 
 
-0.369
 
 
42.2
 
 
 
 
ARHGAP21
 
 
hsa-miR-152-3p
 
 
-0.287
 
 
12.0
 
 
 
 
ARL6IP1
 
 
hsa-miR-152-3p
 
 
-0.579
 
 
105.4
 
 
 
 
ARL8B
 
 
hsa-miR-152-3p
 
 
-0.337
 
 
58.1
 
 
 
 
ARL8B
 
 
hsa-miR-320d
 
 
-0.376
 
 
58.1
 
 
 
 
ARPC5
 
 
hsa-miR-22-3p
 
 
-0.679
 
 
43.0
 
 
 
 
ARPIN-AP3S2
 
 
hsa-miR-34a-5p
 
 
-0.348
 
 
10.3
 
 
 
 
ARPP19
 
 
hsa-miR-320d
 
 
-0.325
 
 
53.7
 
 
 
 
ARPP19
 
 
hsa-miR-34a-5p
 
 
-0.497
 
 
53.7
 
 
 
 
ARRDC3
 
 
hsa-miR-152-3p
 
 
-0.305
 
 
78.7
 
 
 
 
ASAH1
 
 
hsa-miR-27a-3p
 
 
-0.398
 
 
305.0
 
 
 
 
ASH2L
 
 
hsa-miR-320d
 
 
-0.287
 
 
19.8
 
 
 
 
ASPH
 
 
hsa-miR-152-3p
 
 
-0.391
 
 
90.3
 
 
 
 
ATG4B
 
 
hsa-miR-34a-5p
 
 
-0.58
 
 
20.4
 
 
 
 
ATMIN
 
 
hsa-miR-34a-5p
 
 
-0.409
 
 
19.3
 
 
 
 
ATP2B4
 
 
hsa-miR-152-3p
 
 
-0.337
 
 
14.2
 
 
 
 
ATP5MC3
 
 
hsa-miR-27a-3p
 
 
-0.416
 
 
129.3
 
 
 
 
ATP6AP2
 
 
hsa-miR-152-3p
 
 
-0.592
 
 
166.0
 
 
 
 
ATP6AP2
 
 
hsa-miR-152-3p
 
 
-0.668
 
 
166.0
 
 
 
 
ATP8A1
 
 
hsa-miR-22-3p
 
 
-0.468
 
 
37.1
 
 
 
 
ATXN10
 
 
hsa-miR-27a-3p
 
 
-0.295
 
 
59.0
 
 
 
 
ATXN2
 
 
hsa-miR-10a-5p
 
 
-0.362
 
 
16.2
 
 
 
 
AXL
 
 
hsa-miR-34a-5p
 
 
-0.512
 
 
15.7
 
 
 
 
B4GALT2
 
 
hsa-miR-34a-5p
 
 
-0.314
 
 
11.7
 
 
 
 
B4GALT3
 
 
hsa-miR-27a-3p
 
 
-0.41
 
 
21.3
 
 
 
 
B4GALT5
 
 
hsa-miR-152-3p
 
 
-0.44
 
 
10.8
 
 
 
 
BAG5
 
 
hsa-miR-22-3p
 
 
-0.318
 
 
22.1
 
 
 
 
BAZ1B
 
 
hsa-miR-10a-5p
 
 
-0.309
 
 
22.3
 
 
 
 
BAZ2A
 
 
hsa-miR-100-5p
 
 
-0.29
 
 
25.4
 
 
 
 
BAZ2A
 
 
hsa-miR-100-5p
 
 
-0.421
 
 
25.4
 
 
 
 
BBX
 
 
hsa-miR-10a-5p
 
 
-0.305
 
 
24.5
 
 
 
 
BCL2
 
 
hsa-miR-34a-5p
 
 
-0.289
 
 
22.4
 
 
 
 
BCL2L11
 
 
hsa-miR-152-3p
 
 
-0.445
 
 
18.5
 
 
 
 
BCL2L11
 
 
hsa-miR-10a-5p
 
 
-0.483
 
 
18.5
 
 
 
 
BCL3
 
 
hsa-miR-27a-3p
 
 
-0.413
 
 
40.2
 
 
 
 
BEND7
 
 
hsa-miR-27a-3p
 
 
-0.304
 
 
23.1
 
 
 
 
BEX3
 
 
hsa-miR-27a-3p
 
 
-0.454
 
 
279.0
 
 
 
 
BHLHE40
 
 
hsa-miR-181a-5p
 
 
-0.408
 
 
88.0
 
 
 
 
BHLHE41
 
 
hsa-miR-152-3p
 
 
-0.442
 
 
54.2
 
 
 
 
BIN1
 
 
hsa-miR-22-3p
 
 
-0.346
 
 
27.3
 
 
 
 
BLOC1S1
 
 
hsa-miR-22-3p
 
 
-0.346
 
 
116.6
 
 
 
 
BLOC1S6
 
 
hsa-miR-181a-5p
 
 
-0.393
 
 
41.6
 
 
 
 
BLTP1
 
 
hsa-miR-27a-3p
 
 
-0.426
 
 
21.2
 
 
 
 
BMI1
 
 
hsa-miR-27a-3p
 
 
-0.322
 
 
22.3
 
 
 
 
BMPR2
 
 
hsa-miR-100-5p
 
 
-0.355
 
 
13.2
 
 
 
 
BRPF3
 
 
hsa-miR-34a-5p
 
 
-0.33
 
 
11.3
 
 
 
 
BTAF1
 
 
hsa-miR-152-3p
 
 
-0.401
 
 
14.3
 
 
 
 
BTBD3
 
 
hsa-miR-152-3p
 
 
-0.495
 
 
10.8
 
 
 
 
BTG2
 
 
hsa-miR-27a-3p
 
 
-0.339
 
 
188.9
 
 
 
 
BZW1
 
 
hsa-miR-27a-3p
 
 
-0.337
 
 
69.6
 
 
 
 
C1GALT1
 
 
hsa-miR-152-3p
 
 
-0.487
 
 
11.8
 
 
 
 
C1orf35
 
 
hsa-miR-152-3p
 
 
-0.511
 
 
13.7
 
 
 
 
C5orf24
 
 
hsa-miR-22-3p
 
 
-0.493
 
 
27.9
 
 
 
 
C6orf62
 
 
hsa-miR-181a-5p
 
 
-0.334
 
 
80.0
 
 
 
 
C8orf88
 
 
hsa-miR-27a-3p
 
 
-0.33
 
 
10.2
 
 
 
 
CACYBP
 
 
hsa-miR-27a-3p
 
 
-0.367
 
 
15.0
 
 
 
 
 
 
 tongue 
 
 
 
 
target_symbol
 
 
mature_mirna_id
 
 
score
 
 
nTPM
 
 
 
 
 
 
AAGAB
 
 
hsa-miR-34a-5p
 
 
-0.319
 
 
21.5
 
 
 
 
ABCB10
 
 
hsa-miR-152-3p
 
 
-0.291
 
 
10.2
 
 
 
 
ABCB7
 
 
hsa-miR-152-3p
 
 
-0.515
 
 
20.8
 
 
 
 
ABCD3
 
 
hsa-miR-152-3p
 
 
-0.388
 
 
27.9
 
 
 
 
ABHD5
 
 
hsa-miR-27a-3p
 
 
-0.407
 
 
17.3
 
 
 
 
ABRAXAS2
 
 
hsa-miR-34a-5p
 
 
-0.538
 
 
15.2
 
 
 
 
ACBD3
 
 
hsa-miR-34a-5p
 
 
-0.415
 
 
19.0
 
 
 
 
ACSL1
 
 
hsa-miR-34a-5p
 
 
-0.306
 
 
397.3
 
 
 
 
ACSL4
 
 
hsa-miR-34a-5p
 
 
-0.372
 
 
15.8
 
 
 
 
ACTA2
 
 
hsa-miR-27a-3p
 
 
-0.577
 
 
200.0
 
 
 
 
ADCY2
 
 
hsa-miR-152-3p
 
 
-0.299
 
 
28.8
 
 
 
 
ADGRA3
 
 
hsa-miR-27a-3p
 
 
-0.296
 
 
10.7
 
 
 
 
ADGRF5
 
 
hsa-miR-152-3p
 
 
-0.354
 
 
66.5
 
 
 
 
ADIPOR2
 
 
hsa-miR-34a-5p
 
 
-0.33
 
 
18.5
 
 
 
 
ADK
 
 
hsa-miR-34a-5p
 
 
-0.374
 
 
44.0
 
 
 
 
ADO
 
 
hsa-miR-34a-5p
 
 
-0.377
 
 
16.2
 
 
 
 
AFG3L2
 
 
hsa-miR-181a-5p
 
 
-0.491
 
 
92.6
 
 
 
 
AGFG1
 
 
hsa-miR-152-3p
 
 
-0.332
 
 
36.1
 
 
 
 
AGO4
 
 
hsa-miR-34a-5p
 
 
-0.306
 
 
11.1
 
 
 
 
AGPAT3
 
 
hsa-miR-27a-3p
 
 
-0.29
 
 
46.9
 
 
 
 
AGTRAP
 
 
hsa-miR-34a-5p
 
 
-0.328
 
 
11.6
 
 
 
 
AHCYL2
 
 
hsa-miR-34a-5p
 
 
-0.359
 
 
25.9
 
 
 
 
AHDC1
 
 
hsa-miR-152-3p
 
 
-0.413
 
 
14.5
 
 
 
 
AIDA
 
 
hsa-miR-22-3p
 
 
-0.316
 
 
46.5
 
 
 
 
AKAP1
 
 
hsa-miR-152-3p
 
 
-0.381
 
 
128.4
 
 
 
 
AKIRIN1
 
 
hsa-miR-27a-3p
 
 
-0.368
 
 
28.3
 
 
 
 
AKT3
 
 
hsa-miR-22-3p
 
 
-0.451
 
 
10.7
 
 
 
 
ALDOA
 
 
hsa-miR-34a-5p
 
 
-0.655
 
 
8554.2
 
 
 
 
ANK2
 
 
hsa-miR-34a-5p
 
 
-0.317
 
 
89.8
 
 
 
 
ANK3
 
 
hsa-miR-34a-5p
 
 
-0.29
 
 
88.2
 
 
 
 
ANK3
 
 
hsa-miR-34a-5p
 
 
-0.347
 
 
88.2
 
 
 
 
ANKRD40
 
 
hsa-miR-27a-3p
 
 
-0.35
 
 
78.6
 
 
 
 
ANP32B
 
 
hsa-miR-34a-5p
 
 
-0.29
 
 
47.3
 
 
 
 
AP3S2
 
 
hsa-miR-34a-5p
 
 
-0.413
 
 
42.1
 
 
 
 
APH1A
 
 
hsa-miR-34a-5p
 
 
-0.304
 
 
59.0
 
 
 
 
APOO
 
 
hsa-miR-181a-5p
 
 
-0.5
 
 
68.2
 
 
 
 
ARF4
 
 
hsa-miR-152-3p
 
 
-0.487
 
 
64.9
 
 
 
 
ARF6
 
 
hsa-miR-181a-5p
 
 
-0.33
 
 
15.8
 
 
 
 
ARFGEF1
 
 
hsa-miR-152-3p
 
 
-0.306
 
 
13.0
 
 
 
 
ARHGAP1
 
 
hsa-miR-34a-5p
 
 
-0.369
 
 
19.1
 
 
 
 
ARHGAP21
 
 
hsa-miR-152-3p
 
 
-0.287
 
 
21.5
 
 
 
 
ARL6IP1
 
 
hsa-miR-152-3p
 
 
-0.579
 
 
48.1
 
 
 
 
ARL8B
 
 
hsa-miR-152-3p
 
 
-0.337
 
 
56.0
 
 
 
 
ARL8B
 
 
hsa-miR-320d
 
 
-0.376
 
 
56.0
 
 
 
 
ARPC5
 
 
hsa-miR-22-3p
 
 
-0.679
 
 
21.0
 
 
 
 
ARPIN-AP3S2
 
 
hsa-miR-34a-5p
 
 
-0.348
 
 
14.1
 
 
 
 
ARPP19
 
 
hsa-miR-320d
 
 
-0.325
 
 
158.9
 
 
 
 
ARPP19
 
 
hsa-miR-34a-5p
 
 
-0.497
 
 
158.9
 
 
 
 
ARRB1
 
 
hsa-miR-22-3p
 
 
-0.616
 
 
10.1
 
 
 
 
ARRDC3
 
 
hsa-miR-152-3p
 
 
-0.305
 
 
41.9
 
 
 
 
AS3MT
 
 
hsa-miR-28-5p
 
 
-0.479
 
 
12.5
 
 
 
 
ASAH1
 
 
hsa-miR-27a-3p
 
 
-0.398
 
 
65.9
 
 
 
 
ASB1
 
 
hsa-miR-34a-5p
 
 
-0.406
 
 
21.7
 
 
 
 
ASB11
 
 
hsa-miR-27a-3p
 
 
-0.353
 
 
57.4
 
 
 
 
ASH2L
 
 
hsa-miR-320d
 
 
-0.287
 
 
26.1
 
 
 
 
ASPH
 
 
hsa-miR-152-3p
 
 
-0.391
 
 
155.4
 
 
 
 
ATMIN
 
 
hsa-miR-34a-5p
 
 
-0.409
 
 
15.9
 
 
 
 
ATP1A2
 
 
hsa-miR-34a-5p
 
 
-0.306
 
 
446.6
 
 
 
 
ATP2B4
 
 
hsa-miR-152-3p
 
 
-0.337
 
 
25.9
 
 
 
 
ATP5MC3
 
 
hsa-miR-27a-3p
 
 
-0.416
 
 
1014.6
 
 
 
 
ATP6AP2
 
 
hsa-miR-152-3p
 
 
-0.592
 
 
37.6
 
 
 
 
ATP6AP2
 
 
hsa-miR-152-3p
 
 
-0.668
 
 
37.6
 
 
 
 
ATP8A1
 
 
hsa-miR-22-3p
 
 
-0.468
 
 
14.5
 
 
 
 
ATXN10
 
 
hsa-miR-27a-3p
 
 
-0.295
 
 
55.4
 
 
 
 
ATXN2
 
 
hsa-miR-10a-5p
 
 
-0.362
 
 
19.4
 
 
 
 
AXL
 
 
hsa-miR-34a-5p
 
 
-0.512
 
 
10.6
 
 
 
 
B4GALT2
 
 
hsa-miR-34a-5p
 
 
-0.314
 
 
31.0
 
 
 
 
B4GALT3
 
 
hsa-miR-27a-3p
 
 
-0.41
 
 
15.2
 
 
 
 
B4GALT5
 
 
hsa-miR-152-3p
 
 
-0.44
 
 
22.7
 
 
 
 
BAG5
 
 
hsa-miR-22-3p
 
 
-0.318
 
 
12.6
 
 
 
 
BAZ1B
 
 
hsa-miR-10a-5p
 
 
-0.309
 
 
28.7
 
 
 
 
BAZ2A
 
 
hsa-miR-100-5p
 
 
-0.29
 
 
29.9
 
 
 
 
BAZ2A
 
 
hsa-miR-100-5p
 
 
-0.421
 
 
29.9
 
 
 
 
BBX
 
 
hsa-miR-10a-5p
 
 
-0.305
 
 
15.1
 
 
 
 
BCL2L13
 
 
hsa-miR-34a-5p
 
 
-0.431
 
 
37.8
 
 
 
 
BEND7
 
 
hsa-miR-27a-3p
 
 
-0.304
 
 
12.6
 
 
 
 
BEX3
 
 
hsa-miR-27a-3p
 
 
-0.454
 
 
41.5
 
 
 
 
BHLHE40
 
 
hsa-miR-181a-5p
 
 
-0.408
 
 
43.7
 
 
 
 
BHLHE41
 
 
hsa-miR-152-3p
 
 
-0.442
 
 
12.1
 
 
 
 
BIN1
 
 
hsa-miR-22-3p
 
 
-0.346
 
 
751.1
 
 
 
 
BLOC1S1
 
 
hsa-miR-22-3p
 
 
-0.346
 
 
191.8
 
 
 
 
BLOC1S6
 
 
hsa-miR-181a-5p
 
 
-0.393
 
 
21.7
 
 
 
 
BLTP1
 
 
hsa-miR-27a-3p
 
 
-0.426
 
 
31.9
 
 
 
 
BMI1
 
 
hsa-miR-27a-3p
 
 
-0.322
 
 
33.2
 
 
 
 
BMPR2
 
 
hsa-miR-100-5p
 
 
-0.355
 
 
15.0
 
 
 
 
BRPF3
 
 
hsa-miR-34a-5p
 
 
-0.33
 
 
11.4
 
 
 
 
BTBD3
 
 
hsa-miR-152-3p
 
 
-0.495
 
 
12.3
 
 
 
 
BTG2
 
 
hsa-miR-27a-3p
 
 
-0.339
 
 
268.3
 
 
 
 
BZW1
 
 
hsa-miR-27a-3p
 
 
-0.337
 
 
70.0
 
 
 
 
C1GALT1
 
 
hsa-miR-152-3p
 
 
-0.487
 
 
10.5
 
 
 
 
C5orf24
 
 
hsa-miR-22-3p
 
 
-0.493
 
 
24.9
 
 
 
 
C6orf120
 
 
hsa-miR-34a-5p
 
 
-0.331
 
 
10.5
 
 
 
 
C6orf62
 
 
hsa-miR-181a-5p
 
 
-0.334
 
 
54.5
 
 
 
 
CACNA2D3
 
 
hsa-miR-27a-3p
 
 
-0.561
 
 
10.0
 
 
 
 
CACYBP
 
 
hsa-miR-27a-3p
 
 
-0.367
 
 
25.5
 
 
 
 
CAND1
 
 
hsa-miR-27a-3p
 
 
-0.318
 
 
10.6
 
 
 
 
CAND1
 
 
hsa-miR-152-3p
 
 
-0.44
 
 
10.6
 
 
 
 
CANX
 
 
hsa-miR-152-3p
 
 
-0.434
 
 
49.8
 
 
 
 
CAPN1
 
 
hsa-miR-22-3p
 
 
-0.307
 
 
51.5
 
 
 
 
CAPZA1
 
 
hsa-miR-27a-3p
 
 
-0.325
 
 
20.7
 
 
 
 
 
 
 tonsil 
 
 
 
 
target_symbol
 
 
mature_mirna_id
 
 
score
 
 
nTPM
 
 
 
 
 
 
AAGAB
 
 
hsa-miR-34a-5p
 
 
-0.319
 
 
24.3
 
 
 
 
ABCB7
 
 
hsa-miR-152-3p
 
 
-0.515
 
 
10.4
 
 
 
 
ABCD3
 
 
hsa-miR-152-3p
 
 
-0.388
 
 
19.9
 
 
 
 
ABHD17C
 
 
hsa-miR-27a-3p
 
 
-0.435
 
 
21.6
 
 
 
 
ABRAXAS2
 
 
hsa-miR-34a-5p
 
 
-0.538
 
 
12.5
 
 
 
 
ACBD3
 
 
hsa-miR-34a-5p
 
 
-0.415
 
 
24.0
 
 
 
 
ACSL1
 
 
hsa-miR-34a-5p
 
 
-0.306
 
 
20.1
 
 
 
 
ACSL4
 
 
hsa-miR-34a-5p
 
 
-0.372
 
 
16.0
 
 
 
 
ACTA2
 
 
hsa-miR-27a-3p
 
 
-0.577
 
 
45.3
 
 
 
 
ADAM10
 
 
hsa-miR-152-3p
 
 
-0.348
 
 
15.3
 
 
 
 
ADAM17
 
 
hsa-miR-152-3p
 
 
-0.301
 
 
11.1
 
 
 
 
ADIPOR2
 
 
hsa-miR-34a-5p
 
 
-0.33
 
 
11.8
 
 
 
 
ADK
 
 
hsa-miR-34a-5p
 
 
-0.374
 
 
32.3
 
 
 
 
AFG3L2
 
 
hsa-miR-181a-5p
 
 
-0.491
 
 
16.2
 
 
 
 
AGFG1
 
 
hsa-miR-152-3p
 
 
-0.332
 
 
49.9
 
 
 
 
AGTRAP
 
 
hsa-miR-34a-5p
 
 
-0.328
 
 
12.5
 
 
 
 
AIDA
 
 
hsa-miR-22-3p
 
 
-0.316
 
 
49.6
 
 
 
 
AKIRIN1
 
 
hsa-miR-27a-3p
 
 
-0.368
 
 
39.4
 
 
 
 
ALCAM
 
 
hsa-miR-34a-5p
 
 
-0.303
 
 
13.6
 
 
 
 
ALCAM
 
 
hsa-miR-152-3p
 
 
-0.347
 
 
13.6
 
 
 
 
ALDOA
 
 
hsa-miR-34a-5p
 
 
-0.655
 
 
447.4
 
 
 
 
ANKHD1-EIF4EBP3
 
 
hsa-miR-22-3p
 
 
-0.479
 
 
12.6
 
 
 
 
ANKRD13A
 
 
hsa-miR-22-3p
 
 
-0.478
 
 
62.4
 
 
 
 
ANKRD40
 
 
hsa-miR-27a-3p
 
 
-0.35
 
 
13.5
 
 
 
 
ANP32B
 
 
hsa-miR-34a-5p
 
 
-0.29
 
 
232.6
 
 
 
 
AP1AR
 
 
hsa-miR-100-5p
 
 
-0.804
 
 
11.3
 
 
 
 
AP3S2
 
 
hsa-miR-34a-5p
 
 
-0.413
 
 
16.4
 
 
 
 
APH1A
 
 
hsa-miR-34a-5p
 
 
-0.304
 
 
85.0
 
 
 
 
APOO
 
 
hsa-miR-181a-5p
 
 
-0.5
 
 
10.8
 
 
 
 
ARF4
 
 
hsa-miR-152-3p
 
 
-0.487
 
 
91.2
 
 
 
 
ARF6
 
 
hsa-miR-181a-5p
 
 
-0.33
 
 
64.2
 
 
 
 
ARFGEF1
 
 
hsa-miR-152-3p
 
 
-0.306
 
 
16.2
 
 
 
 
ARFIP1
 
 
hsa-miR-320d
 
 
-0.339
 
 
13.9
 
 
 
 
ARFIP1
 
 
hsa-miR-152-3p
 
 
-0.474
 
 
13.9
 
 
 
 
ARHGAP1
 
 
hsa-miR-34a-5p
 
 
-0.369
 
 
32.4
 
 
 
 
ARHGAP26
 
 
hsa-miR-34a-5p
 
 
-0.384
 
 
11.4
 
 
 
 
ARL6IP1
 
 
hsa-miR-152-3p
 
 
-0.579
 
 
106.2
 
 
 
 
ARL8B
 
 
hsa-miR-152-3p
 
 
-0.337
 
 
44.5
 
 
 
 
ARL8B
 
 
hsa-miR-320d
 
 
-0.376
 
 
44.5
 
 
 
 
ARPC5
 
 
hsa-miR-22-3p
 
 
-0.679
 
 
123.0
 
 
 
 
ARPP19
 
 
hsa-miR-320d
 
 
-0.325
 
 
48.4
 
 
 
 
ARPP19
 
 
hsa-miR-34a-5p
 
 
-0.497
 
 
48.4
 
 
 
 
ARRDC3
 
 
hsa-miR-152-3p
 
 
-0.305
 
 
26.4
 
 
 
 
ASAH1
 
 
hsa-miR-27a-3p
 
 
-0.398
 
 
54.1
 
 
 
 
ASH2L
 
 
hsa-miR-320d
 
 
-0.287
 
 
26.7
 
 
 
 
ASPH
 
 
hsa-miR-152-3p
 
 
-0.391
 
 
26.4
 
 
 
 
ATMIN
 
 
hsa-miR-34a-5p
 
 
-0.409
 
 
14.1
 
 
 
 
ATP2B4
 
 
hsa-miR-152-3p
 
 
-0.337
 
 
18.2
 
 
 
 
ATP5MC3
 
 
hsa-miR-27a-3p
 
 
-0.416
 
 
164.6
 
 
 
 
ATP6AP2
 
 
hsa-miR-152-3p
 
 
-0.592
 
 
67.9
 
 
 
 
ATP6AP2
 
 
hsa-miR-152-3p
 
 
-0.668
 
 
67.9
 
 
 
 
ATP8A1
 
 
hsa-miR-22-3p
 
 
-0.468
 
 
11.0
 
 
 
 
ATXN10
 
 
hsa-miR-27a-3p
 
 
-0.295
 
 
48.3
 
 
 
 
B4GALT3
 
 
hsa-miR-27a-3p
 
 
-0.41
 
 
19.4
 
 
 
 
B4GALT5
 
 
hsa-miR-152-3p
 
 
-0.44
 
 
10.8
 
 
 
 
BAG5
 
 
hsa-miR-22-3p
 
 
-0.318
 
 
17.9
 
 
 
 
BAZ1B
 
 
hsa-miR-10a-5p
 
 
-0.309
 
 
27.8
 
 
 
 
BAZ2A
 
 
hsa-miR-100-5p
 
 
-0.29
 
 
28.7
 
 
 
 
BAZ2A
 
 
hsa-miR-100-5p
 
 
-0.421
 
 
28.7
 
 
 
 
BBX
 
 
hsa-miR-10a-5p
 
 
-0.305
 
 
12.8
 
 
 
 
BCL2
 
 
hsa-miR-34a-5p
 
 
-0.289
 
 
13.7
 
 
 
 
BCL2L11
 
 
hsa-miR-152-3p
 
 
-0.445
 
 
21.7
 
 
 
 
BCL2L11
 
 
hsa-miR-10a-5p
 
 
-0.483
 
 
21.7
 
 
 
 
BCL3
 
 
hsa-miR-27a-3p
 
 
-0.413
 
 
11.9
 
 
 
 
BEX3
 
 
hsa-miR-27a-3p
 
 
-0.454
 
 
26.6
 
 
 
 
BHLHE40
 
 
hsa-miR-181a-5p
 
 
-0.408
 
 
47.5
 
 
 
 
BHLHE41
 
 
hsa-miR-152-3p
 
 
-0.442
 
 
16.8
 
 
 
 
BIN1
 
 
hsa-miR-22-3p
 
 
-0.346
 
 
25.8
 
 
 
 
BLOC1S1
 
 
hsa-miR-22-3p
 
 
-0.346
 
 
107.4
 
 
 
 
BLOC1S6
 
 
hsa-miR-181a-5p
 
 
-0.393
 
 
33.2
 
 
 
 
BMI1
 
 
hsa-miR-27a-3p
 
 
-0.322
 
 
25.3
 
 
 
 
BTAF1
 
 
hsa-miR-152-3p
 
 
-0.401
 
 
20.0
 
 
 
 
BTG1
 
 
hsa-miR-22-3p
 
 
-0.468
 
 
17.1
 
 
 
 
BTG2
 
 
hsa-miR-27a-3p
 
 
-0.339
 
 
123.9
 
 
 
 
BZW1
 
 
hsa-miR-27a-3p
 
 
-0.337
 
 
102.5
 
 
 
 
C1GALT1
 
 
hsa-miR-152-3p
 
 
-0.487
 
 
13.2
 
 
 
 
C5orf24
 
 
hsa-miR-22-3p
 
 
-0.493
 
 
13.7
 
 
 
 
C6orf120
 
 
hsa-miR-34a-5p
 
 
-0.331
 
 
12.1
 
 
 
 
C6orf62
 
 
hsa-miR-181a-5p
 
 
-0.334
 
 
133.9
 
 
 
 
CACYBP
 
 
hsa-miR-27a-3p
 
 
-0.367
 
 
27.1
 
 
 
 
CAND1
 
 
hsa-miR-27a-3p
 
 
-0.318
 
 
28.9
 
 
 
 
CAND1
 
 
hsa-miR-152-3p
 
 
-0.44
 
 
28.9
 
 
 
 
CANX
 
 
hsa-miR-152-3p
 
 
-0.434
 
 
15.5
 
 
 
 
CAPN1
 
 
hsa-miR-22-3p
 
 
-0.307
 
 
80.9
 
 
 
 
CAPZA1
 
 
hsa-miR-27a-3p
 
 
-0.325
 
 
175.8
 
 
 
 
CASP2
 
 
hsa-miR-34a-5p
 
 
-0.382
 
 
25.3
 
 
 
 
CBFB
 
 
hsa-miR-27a-3p
 
 
-0.302
 
 
11.1
 
 
 
 
CCDC47
 
 
hsa-miR-22-3p
 
 
-0.604
 
 
37.9
 
 
 
 
CCDC6
 
 
hsa-miR-152-3p
 
 
-0.511
 
 
20.3
 
 
 
 
CCM2
 
 
hsa-miR-27a-3p
 
 
-0.335
 
 
27.8
 
 
 
 
CCNC
 
 
hsa-miR-27a-3p
 
 
-0.378
 
 
40.6
 
 
 
 
CCNG1
 
 
hsa-miR-27a-3p
 
 
-0.419
 
 
44.0
 
 
 
 
CCNK
 
 
hsa-miR-181a-5p
 
 
-0.31
 
 
31.9
 
 
 
 
CCNK
 
 
hsa-miR-27a-3p
 
 
-0.519
 
 
31.9
 
 
 
 
CCNL2
 
 
hsa-miR-28-5p
 
 
-0.448
 
 
25.9
 
 
 
 
CCNY
 
 
hsa-miR-27a-3p
 
 
-0.334
 
 
19.7
 
 
 
 
CCR7
 
 
hsa-miR-320d
 
 
-0.395
 
 
78.1
 
 
 
 
CCT6A
 
 
hsa-miR-152-3p
 
 
-0.665
 
 
77.0
 
 
 
 
CD69
 
 
hsa-miR-181a-5p
 
 
-0.293
 
 
56.3
 
 
 
 
CDC25B
 
 
hsa-miR-152-3p
 
 
-0.298
 
 
30.7
 
 
 
 
 
 
 urinary bladder 
 
 
 
 
target_symbol
 
 
mature_mirna_id
 
 
score
 
 
nTPM
 
 
 
 
 
 
AAGAB
 
 
hsa-miR-34a-5p
 
 
-0.319
 
 
25.7
 
 
 
 
ABCB7
 
 
hsa-miR-152-3p
 
 
-0.515
 
 
11.0
 
 
 
 
ABCD3
 
 
hsa-miR-152-3p
 
 
-0.388
 
 
21.4
 
 
 
 
ABHD17C
 
 
hsa-miR-27a-3p
 
 
-0.435
 
 
26.8
 
 
 
 
ABHD5
 
 
hsa-miR-27a-3p
 
 
-0.407
 
 
15.5
 
 
 
 
ACBD3
 
 
hsa-miR-34a-5p
 
 
-0.415
 
 
25.2
 
 
 
 
ACSL1
 
 
hsa-miR-34a-5p
 
 
-0.306
 
 
59.5
 
 
 
 
ACSL4
 
 
hsa-miR-34a-5p
 
 
-0.372
 
 
62.0
 
 
 
 
ACTA2
 
 
hsa-miR-27a-3p
 
 
-0.577
 
 
3161.7
 
 
 
 
ACVR1
 
 
hsa-miR-152-3p
 
 
-0.397
 
 
13.3
 
 
 
 
ADAM10
 
 
hsa-miR-152-3p
 
 
-0.348
 
 
26.2
 
 
 
 
ADGRF5
 
 
hsa-miR-152-3p
 
 
-0.354
 
 
10.6
 
 
 
 
ADIPOR2
 
 
hsa-miR-34a-5p
 
 
-0.33
 
 
21.3
 
 
 
 
ADK
 
 
hsa-miR-34a-5p
 
 
-0.374
 
 
22.9
 
 
 
 
ADORA2B
 
 
hsa-miR-27a-3p
 
 
-0.638
 
 
15.6
 
 
 
 
AFG3L2
 
 
hsa-miR-181a-5p
 
 
-0.491
 
 
18.0
 
 
 
 
AGFG1
 
 
hsa-miR-152-3p
 
 
-0.332
 
 
61.2
 
 
 
 
AGPAT3
 
 
hsa-miR-27a-3p
 
 
-0.29
 
 
14.4
 
 
 
 
AGTRAP
 
 
hsa-miR-34a-5p
 
 
-0.328
 
 
31.7
 
 
 
 
AHCYL2
 
 
hsa-miR-34a-5p
 
 
-0.359
 
 
15.0
 
 
 
 
AHDC1
 
 
hsa-miR-152-3p
 
 
-0.413
 
 
14.8
 
 
 
 
AIDA
 
 
hsa-miR-22-3p
 
 
-0.316
 
 
44.1
 
 
 
 
AKAP1
 
 
hsa-miR-152-3p
 
 
-0.381
 
 
47.8
 
 
 
 
AKIRIN1
 
 
hsa-miR-27a-3p
 
 
-0.368
 
 
38.4
 
 
 
 
AKT3
 
 
hsa-miR-22-3p
 
 
-0.451
 
 
14.4
 
 
 
 
ALCAM
 
 
hsa-miR-34a-5p
 
 
-0.303
 
 
23.9
 
 
 
 
ALCAM
 
 
hsa-miR-152-3p
 
 
-0.347
 
 
23.9
 
 
 
 
ALDOA
 
 
hsa-miR-34a-5p
 
 
-0.655
 
 
917.9
 
 
 
 
ANKHD1-EIF4EBP3
 
 
hsa-miR-22-3p
 
 
-0.479
 
 
11.2
 
 
 
 
ANKRD13A
 
 
hsa-miR-22-3p
 
 
-0.478
 
 
24.2
 
 
 
 
ANKRD40
 
 
hsa-miR-27a-3p
 
 
-0.35
 
 
22.8
 
 
 
 
ANP32B
 
 
hsa-miR-34a-5p
 
 
-0.29
 
 
122.0
 
 
 
 
AP1AR
 
 
hsa-miR-100-5p
 
 
-0.804
 
 
12.2
 
 
 
 
AP3S2
 
 
hsa-miR-34a-5p
 
 
-0.413
 
 
14.3
 
 
 
 
APH1A
 
 
hsa-miR-34a-5p
 
 
-0.304
 
 
72.7
 
 
 
 
APOO
 
 
hsa-miR-181a-5p
 
 
-0.5
 
 
10.5
 
 
 
 
AREG
 
 
hsa-miR-34a-5p
 
 
-0.62
 
 
135.5
 
 
 
 
ARF4
 
 
hsa-miR-152-3p
 
 
-0.487
 
 
190.1
 
 
 
 
ARF6
 
 
hsa-miR-181a-5p
 
 
-0.33
 
 
43.6
 
 
 
 
ARFGEF1
 
 
hsa-miR-152-3p
 
 
-0.306
 
 
18.4
 
 
 
 
ARFIP1
 
 
hsa-miR-320d
 
 
-0.339
 
 
22.0
 
 
 
 
ARFIP1
 
 
hsa-miR-152-3p
 
 
-0.474
 
 
22.0
 
 
 
 
ARHGAP1
 
 
hsa-miR-34a-5p
 
 
-0.369
 
 
106.2
 
 
 
 
ARHGAP21
 
 
hsa-miR-152-3p
 
 
-0.287
 
 
12.9
 
 
 
 
ARHGAP26
 
 
hsa-miR-34a-5p
 
 
-0.384
 
 
11.4
 
 
 
 
ARL6IP1
 
 
hsa-miR-152-3p
 
 
-0.579
 
 
124.0
 
 
 
 
ARL8B
 
 
hsa-miR-152-3p
 
 
-0.337
 
 
46.5
 
 
 
 
ARL8B
 
 
hsa-miR-320d
 
 
-0.376
 
 
46.5
 
 
 
 
ARPC5
 
 
hsa-miR-22-3p
 
 
-0.679
 
 
71.0
 
 
 
 
ARPP19
 
 
hsa-miR-320d
 
 
-0.325
 
 
51.7
 
 
 
 
ARPP19
 
 
hsa-miR-34a-5p
 
 
-0.497
 
 
51.7
 
 
 
 
ARRB1
 
 
hsa-miR-22-3p
 
 
-0.616
 
 
15.3
 
 
 
 
ARRDC3
 
 
hsa-miR-152-3p
 
 
-0.305
 
 
53.2
 
 
 
 
ASAH1
 
 
hsa-miR-27a-3p
 
 
-0.398
 
 
105.7
 
 
 
 
ASH2L
 
 
hsa-miR-320d
 
 
-0.287
 
 
17.3
 
 
 
 
ASPH
 
 
hsa-miR-152-3p
 
 
-0.391
 
 
103.6
 
 
 
 
ATG4B
 
 
hsa-miR-34a-5p
 
 
-0.58
 
 
18.7
 
 
 
 
ATMIN
 
 
hsa-miR-34a-5p
 
 
-0.409
 
 
13.3
 
 
 
 
ATP1A2
 
 
hsa-miR-34a-5p
 
 
-0.306
 
 
37.3
 
 
 
 
ATP2B4
 
 
hsa-miR-152-3p
 
 
-0.337
 
 
34.9
 
 
 
 
ATP5MC3
 
 
hsa-miR-27a-3p
 
 
-0.416
 
 
160.0
 
 
 
 
ATP6AP2
 
 
hsa-miR-152-3p
 
 
-0.592
 
 
91.4
 
 
 
 
ATP6AP2
 
 
hsa-miR-152-3p
 
 
-0.668
 
 
91.4
 
 
 
 
ATXN10
 
 
hsa-miR-27a-3p
 
 
-0.295
 
 
47.7
 
 
 
 
ATXN2
 
 
hsa-miR-10a-5p
 
 
-0.362
 
 
19.6
 
 
 
 
AXL
 
 
hsa-miR-34a-5p
 
 
-0.512
 
 
28.8
 
 
 
 
B4GALT2
 
 
hsa-miR-34a-5p
 
 
-0.314
 
 
24.6
 
 
 
 
B4GALT3
 
 
hsa-miR-27a-3p
 
 
-0.41
 
 
22.1
 
 
 
 
B4GALT5
 
 
hsa-miR-152-3p
 
 
-0.44
 
 
47.9
 
 
 
 
BAG5
 
 
hsa-miR-22-3p
 
 
-0.318
 
 
14.3
 
 
 
 
BAZ1B
 
 
hsa-miR-10a-5p
 
 
-0.309
 
 
21.9
 
 
 
 
BAZ2A
 
 
hsa-miR-100-5p
 
 
-0.29
 
 
23.9
 
 
 
 
BAZ2A
 
 
hsa-miR-100-5p
 
 
-0.421
 
 
23.9
 
 
 
 
BBX
 
 
hsa-miR-10a-5p
 
 
-0.305
 
 
13.7
 
 
 
 
BCL3
 
 
hsa-miR-27a-3p
 
 
-0.413
 
 
34.9
 
 
 
 
BEX3
 
 
hsa-miR-27a-3p
 
 
-0.454
 
 
126.6
 
 
 
 
BHLHE40
 
 
hsa-miR-181a-5p
 
 
-0.408
 
 
160.4
 
 
 
 
BHLHE41
 
 
hsa-miR-152-3p
 
 
-0.442
 
 
19.9
 
 
 
 
BIN1
 
 
hsa-miR-22-3p
 
 
-0.346
 
 
89.3
 
 
 
 
BLOC1S1
 
 
hsa-miR-22-3p
 
 
-0.346
 
 
126.3
 
 
 
 
BLOC1S6
 
 
hsa-miR-181a-5p
 
 
-0.393
 
 
26.7
 
 
 
 
BLTP1
 
 
hsa-miR-27a-3p
 
 
-0.426
 
 
18.2
 
 
 
 
BMI1
 
 
hsa-miR-27a-3p
 
 
-0.322
 
 
22.2
 
 
 
 
BMPR2
 
 
hsa-miR-100-5p
 
 
-0.355
 
 
14.5
 
 
 
 
BTAF1
 
 
hsa-miR-152-3p
 
 
-0.401
 
 
16.7
 
 
 
 
BTBD3
 
 
hsa-miR-152-3p
 
 
-0.495
 
 
17.2
 
 
 
 
BTG2
 
 
hsa-miR-27a-3p
 
 
-0.339
 
 
261.4
 
 
 
 
BZW1
 
 
hsa-miR-27a-3p
 
 
-0.337
 
 
153.5
 
 
 
 
C1GALT1
 
 
hsa-miR-152-3p
 
 
-0.487
 
 
12.1
 
 
 
 
C1orf35
 
 
hsa-miR-152-3p
 
 
-0.511
 
 
16.3
 
 
 
 
C5orf24
 
 
hsa-miR-22-3p
 
 
-0.493
 
 
20.7
 
 
 
 
C6orf120
 
 
hsa-miR-34a-5p
 
 
-0.331
 
 
11.8
 
 
 
 
C6orf62
 
 
hsa-miR-181a-5p
 
 
-0.334
 
 
69.0
 
 
 
 
C8orf88
 
 
hsa-miR-27a-3p
 
 
-0.33
 
 
16.5
 
 
 
 
CACNB3
 
 
hsa-miR-34a-5p
 
 
-0.426
 
 
12.0
 
 
 
 
CACYBP
 
 
hsa-miR-27a-3p
 
 
-0.367
 
 
23.1
 
 
 
 
CAMK2N1
 
 
hsa-miR-22-3p
 
 
-0.302
 
 
23.0
 
 
 
 
CAND1
 
 
hsa-miR-27a-3p
 
 
-0.318
 
 
21.8
 
 
 
 
CAND1
 
 
hsa-miR-152-3p
 
 
-0.44
 
 
21.8
 
 
 
 
CANX
 
 
hsa-miR-152-3p
 
 
-0.434
 
 
92.3
 
 
 
 
 
 
 vagina 
 
 
 
 
target_symbol
 
 
mature_mirna_id
 
 
score
 
 
nTPM
 
 
 
 
 
 
AAGAB
 
 
hsa-miR-34a-5p
 
 
-0.319
 
 
11.9
 
 
 
 
ABCB7
 
 
hsa-miR-152-3p
 
 
-0.515
 
 
10.8
 
 
 
 
ABCD3
 
 
hsa-miR-152-3p
 
 
-0.388
 
 
14.9
 
 
 
 
ABHD17C
 
 
hsa-miR-27a-3p
 
 
-0.435
 
 
21.1
 
 
 
 
ABHD5
 
 
hsa-miR-27a-3p
 
 
-0.407
 
 
11.9
 
 
 
 
ACBD3
 
 
hsa-miR-34a-5p
 
 
-0.415
 
 
19.2
 
 
 
 
ACSL1
 
 
hsa-miR-34a-5p
 
 
-0.306
 
 
56.5
 
 
 
 
ACTA2
 
 
hsa-miR-27a-3p
 
 
-0.577
 
 
1234.9
 
 
 
 
ADAM10
 
 
hsa-miR-152-3p
 
 
-0.348
 
 
10.3
 
 
 
 
ADIPOR2
 
 
hsa-miR-34a-5p
 
 
-0.33
 
 
28.4
 
 
 
 
ADK
 
 
hsa-miR-34a-5p
 
 
-0.374
 
 
23.3
 
 
 
 
AGFG1
 
 
hsa-miR-152-3p
 
 
-0.332
 
 
31.5
 
 
 
 
AGPAT3
 
 
hsa-miR-27a-3p
 
 
-0.29
 
 
14.4
 
 
 
 
AGTRAP
 
 
hsa-miR-34a-5p
 
 
-0.328
 
 
41.9
 
 
 
 
AHDC1
 
 
hsa-miR-152-3p
 
 
-0.413
 
 
23.0
 
 
 
 
AIDA
 
 
hsa-miR-22-3p
 
 
-0.316
 
 
30.5
 
 
 
 
AKAP1
 
 
hsa-miR-152-3p
 
 
-0.381
 
 
24.4
 
 
 
 
AKIRIN1
 
 
hsa-miR-27a-3p
 
 
-0.368
 
 
17.7
 
 
 
 
AKT3
 
 
hsa-miR-22-3p
 
 
-0.451
 
 
12.8
 
 
 
 
ALDOA
 
 
hsa-miR-34a-5p
 
 
-0.655
 
 
910.0
 
 
 
 
ANK3
 
 
hsa-miR-34a-5p
 
 
-0.29
 
 
13.5
 
 
 
 
ANK3
 
 
hsa-miR-34a-5p
 
 
-0.347
 
 
13.5
 
 
 
 
ANKHD1-EIF4EBP3
 
 
hsa-miR-22-3p
 
 
-0.479
 
 
10.7
 
 
 
 
ANKRD13A
 
 
hsa-miR-22-3p
 
 
-0.478
 
 
11.8
 
 
 
 
ANKRD40
 
 
hsa-miR-27a-3p
 
 
-0.35
 
 
15.9
 
 
 
 
ANP32B
 
 
hsa-miR-34a-5p
 
 
-0.29
 
 
140.4
 
 
 
 
APH1A
 
 
hsa-miR-34a-5p
 
 
-0.304
 
 
79.7
 
 
 
 
AREG
 
 
hsa-miR-34a-5p
 
 
-0.62
 
 
13.6
 
 
 
 
ARF4
 
 
hsa-miR-152-3p
 
 
-0.487
 
 
85.1
 
 
 
 
ARF6
 
 
hsa-miR-181a-5p
 
 
-0.33
 
 
41.6
 
 
 
 
ARFIP1
 
 
hsa-miR-320d
 
 
-0.339
 
 
13.1
 
 
 
 
ARFIP1
 
 
hsa-miR-152-3p
 
 
-0.474
 
 
13.1
 
 
 
 
ARHGAP1
 
 
hsa-miR-34a-5p
 
 
-0.369
 
 
59.0
 
 
 
 
ARL6IP1
 
 
hsa-miR-152-3p
 
 
-0.579
 
 
48.0
 
 
 
 
ARL8B
 
 
hsa-miR-152-3p
 
 
-0.337
 
 
51.8
 
 
 
 
ARL8B
 
 
hsa-miR-320d
 
 
-0.376
 
 
51.8
 
 
 
 
ARPC5
 
 
hsa-miR-22-3p
 
 
-0.679
 
 
49.2
 
 
 
 
ARPP19
 
 
hsa-miR-320d
 
 
-0.325
 
 
27.0
 
 
 
 
ARPP19
 
 
hsa-miR-34a-5p
 
 
-0.497
 
 
27.0
 
 
 
 
ARRB1
 
 
hsa-miR-22-3p
 
 
-0.616
 
 
10.2
 
 
 
 
ARRDC3
 
 
hsa-miR-152-3p
 
 
-0.305
 
 
40.5
 
 
 
 
ASAH1
 
 
hsa-miR-27a-3p
 
 
-0.398
 
 
49.3
 
 
 
 
ASH2L
 
 
hsa-miR-320d
 
 
-0.287
 
 
15.4
 
 
 
 
ASPH
 
 
hsa-miR-152-3p
 
 
-0.391
 
 
40.4
 
 
 
 
ATG4B
 
 
hsa-miR-34a-5p
 
 
-0.58
 
 
16.3
 
 
 
 
ATMIN
 
 
hsa-miR-34a-5p
 
 
-0.409
 
 
11.4
 
 
 
 
ATP2B4
 
 
hsa-miR-152-3p
 
 
-0.337
 
 
14.3
 
 
 
 
ATP5MC3
 
 
hsa-miR-27a-3p
 
 
-0.416
 
 
160.4
 
 
 
 
ATP6AP2
 
 
hsa-miR-152-3p
 
 
-0.592
 
 
44.8
 
 
 
 
ATP6AP2
 
 
hsa-miR-152-3p
 
 
-0.668
 
 
44.8
 
 
 
 
ATXN10
 
 
hsa-miR-27a-3p
 
 
-0.295
 
 
43.9
 
 
 
 
ATXN2
 
 
hsa-miR-10a-5p
 
 
-0.362
 
 
17.8
 
 
 
 
AXL
 
 
hsa-miR-34a-5p
 
 
-0.512
 
 
23.9
 
 
 
 
B4GALT2
 
 
hsa-miR-34a-5p
 
 
-0.314
 
 
21.5
 
 
 
 
B4GALT3
 
 
hsa-miR-27a-3p
 
 
-0.41
 
 
20.9
 
 
 
 
B4GALT5
 
 
hsa-miR-152-3p
 
 
-0.44
 
 
16.5
 
 
 
 
BAG5
 
 
hsa-miR-22-3p
 
 
-0.318
 
 
12.4
 
 
 
 
BAZ1B
 
 
hsa-miR-10a-5p
 
 
-0.309
 
 
16.5
 
 
 
 
BAZ2A
 
 
hsa-miR-100-5p
 
 
-0.29
 
 
29.0
 
 
 
 
BAZ2A
 
 
hsa-miR-100-5p
 
 
-0.421
 
 
29.0
 
 
 
 
BCL3
 
 
hsa-miR-27a-3p
 
 
-0.413
 
 
42.1
 
 
 
 
BEX3
 
 
hsa-miR-27a-3p
 
 
-0.454
 
 
108.6
 
 
 
 
BHLHE40
 
 
hsa-miR-181a-5p
 
 
-0.408
 
 
291.4
 
 
 
 
BIN1
 
 
hsa-miR-22-3p
 
 
-0.346
 
 
34.7
 
 
 
 
BLOC1S1
 
 
hsa-miR-22-3p
 
 
-0.346
 
 
103.3
 
 
 
 
BLOC1S6
 
 
hsa-miR-181a-5p
 
 
-0.393
 
 
15.7
 
 
 
 
BLTP1
 
 
hsa-miR-27a-3p
 
 
-0.426
 
 
16.5
 
 
 
 
BMI1
 
 
hsa-miR-27a-3p
 
 
-0.322
 
 
13.6
 
 
 
 
BTAF1
 
 
hsa-miR-152-3p
 
 
-0.401
 
 
16.3
 
 
 
 
BTG1
 
 
hsa-miR-22-3p
 
 
-0.468
 
 
10.6
 
 
 
 
BTG2
 
 
hsa-miR-27a-3p
 
 
-0.339
 
 
73.7
 
 
 
 
BZW1
 
 
hsa-miR-27a-3p
 
 
-0.337
 
 
58.1
 
 
 
 
C1orf35
 
 
hsa-miR-152-3p
 
 
-0.511
 
 
16.5
 
 
 
 
C5orf24
 
 
hsa-miR-22-3p
 
 
-0.493
 
 
15.6
 
 
 
 
C6orf62
 
 
hsa-miR-181a-5p
 
 
-0.334
 
 
47.4
 
 
 
 
CACNB3
 
 
hsa-miR-34a-5p
 
 
-0.426
 
 
19.2
 
 
 
 
CACYBP
 
 
hsa-miR-27a-3p
 
 
-0.367
 
 
21.5
 
 
 
 
CADM4
 
 
hsa-miR-34a-5p
 
 
-0.365
 
 
13.9
 
 
 
 
CAMK2N1
 
 
hsa-miR-22-3p
 
 
-0.302
 
 
23.6
 
 
 
 
CAND1
 
 
hsa-miR-27a-3p
 
 
-0.318
 
 
18.5
 
 
 
 
CAND1
 
 
hsa-miR-152-3p
 
 
-0.44
 
 
18.5
 
 
 
 
CANX
 
 
hsa-miR-152-3p
 
 
-0.434
 
 
85.8
 
 
 
 
CAPN1
 
 
hsa-miR-22-3p
 
 
-0.307
 
 
112.9
 
 
 
 
CAPZA1
 
 
hsa-miR-27a-3p
 
 
-0.325
 
 
66.5
 
 
 
 
CASP2
 
 
hsa-miR-34a-5p
 
 
-0.382
 
 
11.0
 
 
 
 
CAV2
 
 
hsa-miR-152-3p
 
 
-0.32
 
 
36.5
 
 
 
 
CCDC47
 
 
hsa-miR-22-3p
 
 
-0.604
 
 
28.3
 
 
 
 
CCDC6
 
 
hsa-miR-152-3p
 
 
-0.511
 
 
22.5
 
 
 
 
CCDC71
 
 
hsa-miR-152-3p
 
 
-0.369
 
 
11.2
 
 
 
 
CCM2
 
 
hsa-miR-27a-3p
 
 
-0.335
 
 
19.3
 
 
 
 
CCN1
 
 
hsa-miR-22-3p
 
 
-0.601
 
 
85.1
 
 
 
 
CCNC
 
 
hsa-miR-27a-3p
 
 
-0.378
 
 
19.6
 
 
 
 
CCNG1
 
 
hsa-miR-27a-3p
 
 
-0.419
 
 
32.4
 
 
 
 
CCNK
 
 
hsa-miR-181a-5p
 
 
-0.31
 
 
18.1
 
 
 
 
CCNK
 
 
hsa-miR-27a-3p
 
 
-0.519
 
 
18.1
 
 
 
 
CCNL2
 
 
hsa-miR-28-5p
 
 
-0.448
 
 
65.1
 
 
 
 
CCNY
 
 
hsa-miR-27a-3p
 
 
-0.334
 
 
15.1
 
 
 
 
CCT6A
 
 
hsa-miR-152-3p
 
 
-0.665
 
 
48.6
 
 
 
 
CDC25B
 
 
hsa-miR-152-3p
 
 
-0.298
 
 
30.0
 
 
 
 
CDH11
 
 
hsa-miR-27a-3p
 
 
-0.374
 
 
12.6
 
 
 
 
 
 
 cerebellum 
 
 
 
 
target_symbol
 
 
mature_mirna_id
 
 
score
 
 
nTPM
 
 
 
 
 
 
ABCB10
 
 
hsa-miR-152-3p
 
 
-0.291
 
 
10.6
 
 
 
 
ABCD3
 
 
hsa-miR-152-3p
 
 
-0.388
 
 
12.8
 
 
 
 
ACBD3
 
 
hsa-miR-34a-5p
 
 
-0.415
 
 
11.6
 
 
 
 
ACSL1
 
 
hsa-miR-34a-5p
 
 
-0.306
 
 
17.4
 
 
 
 
ACSL4
 
 
hsa-miR-34a-5p
 
 
-0.372
 
 
10.1
 
 
 
 
ACTA2
 
 
hsa-miR-27a-3p
 
 
-0.577
 
 
25.2
 
 
 
 
ADAM10
 
 
hsa-miR-152-3p
 
 
-0.348
 
 
15.6
 
 
 
 
ADAP1
 
 
hsa-miR-27a-3p
 
 
-0.302
 
 
43.2
 
 
 
 
ADD2
 
 
hsa-miR-34a-5p
 
 
-0.305
 
 
36.1
 
 
 
 
ADGRB3
 
 
hsa-miR-152-3p
 
 
-0.516
 
 
20.8
 
 
 
 
ADIPOR2
 
 
hsa-miR-34a-5p
 
 
-0.33
 
 
18.7
 
 
 
 
ADO
 
 
hsa-miR-34a-5p
 
 
-0.377
 
 
12.4
 
 
 
 
AFG3L2
 
 
hsa-miR-181a-5p
 
 
-0.491
 
 
11.5
 
 
 
 
AGFG1
 
 
hsa-miR-152-3p
 
 
-0.332
 
 
22.4
 
 
 
 
AGPAT3
 
 
hsa-miR-27a-3p
 
 
-0.29
 
 
27.6
 
 
 
 
AHCYL2
 
 
hsa-miR-34a-5p
 
 
-0.359
 
 
11.8
 
 
 
 
AHDC1
 
 
hsa-miR-152-3p
 
 
-0.413
 
 
32.0
 
 
 
 
AIDA
 
 
hsa-miR-22-3p
 
 
-0.316
 
 
24.2
 
 
 
 
AKAP1
 
 
hsa-miR-152-3p
 
 
-0.381
 
 
30.6
 
 
 
 
AKIRIN1
 
 
hsa-miR-27a-3p
 
 
-0.368
 
 
20.5
 
 
 
 
AKT3
 
 
hsa-miR-22-3p
 
 
-0.451
 
 
23.2
 
 
 
 
ALDOA
 
 
hsa-miR-34a-5p
 
 
-0.655
 
 
717.2
 
 
 
 
AMER2
 
 
hsa-miR-34a-5p
 
 
-0.48
 
 
21.3
 
 
 
 
ANGEL2
 
 
hsa-miR-22-3p
 
 
-0.601
 
 
12.7
 
 
 
 
ANK2
 
 
hsa-miR-34a-5p
 
 
-0.317
 
 
47.8
 
 
 
 
ANK3
 
 
hsa-miR-34a-5p
 
 
-0.29
 
 
126.1
 
 
 
 
ANK3
 
 
hsa-miR-34a-5p
 
 
-0.347
 
 
126.1
 
 
 
 
ANKRD40
 
 
hsa-miR-27a-3p
 
 
-0.35
 
 
31.7
 
 
 
 
ANP32B
 
 
hsa-miR-34a-5p
 
 
-0.29
 
 
49.5
 
 
 
 
AP1AR
 
 
hsa-miR-100-5p
 
 
-0.804
 
 
13.2
 
 
 
 
AP3S2
 
 
hsa-miR-34a-5p
 
 
-0.413
 
 
13.1
 
 
 
 
APH1A
 
 
hsa-miR-34a-5p
 
 
-0.304
 
 
36.6
 
 
 
 
APOO
 
 
hsa-miR-181a-5p
 
 
-0.5
 
 
32.8
 
 
 
 
APPBP2
 
 
hsa-miR-27a-3p
 
 
-0.378
 
 
14.6
 
 
 
 
ARF4
 
 
hsa-miR-152-3p
 
 
-0.487
 
 
50.2
 
 
 
 
ARF6
 
 
hsa-miR-181a-5p
 
 
-0.33
 
 
11.0
 
 
 
 
ARHGAP1
 
 
hsa-miR-34a-5p
 
 
-0.369
 
 
19.6
 
 
 
 
ARHGAP21
 
 
hsa-miR-152-3p
 
 
-0.287
 
 
50.0
 
 
 
 
ARHGAP26
 
 
hsa-miR-34a-5p
 
 
-0.384
 
 
17.4
 
 
 
 
ARL6IP1
 
 
hsa-miR-152-3p
 
 
-0.579
 
 
234.0
 
 
 
 
ARL8B
 
 
hsa-miR-152-3p
 
 
-0.337
 
 
50.7
 
 
 
 
ARL8B
 
 
hsa-miR-320d
 
 
-0.376
 
 
50.7
 
 
 
 
ARPC5
 
 
hsa-miR-22-3p
 
 
-0.679
 
 
16.1
 
 
 
 
ARPP19
 
 
hsa-miR-320d
 
 
-0.325
 
 
66.7
 
 
 
 
ARPP19
 
 
hsa-miR-34a-5p
 
 
-0.497
 
 
66.7
 
 
 
 
ARRB1
 
 
hsa-miR-22-3p
 
 
-0.616
 
 
26.7
 
 
 
 
ARRDC3
 
 
hsa-miR-152-3p
 
 
-0.305
 
 
17.8
 
 
 
 
AS3MT
 
 
hsa-miR-28-5p
 
 
-0.479
 
 
12.0
 
 
 
 
ASAH1
 
 
hsa-miR-27a-3p
 
 
-0.398
 
 
32.2
 
 
 
 
ASH2L
 
 
hsa-miR-320d
 
 
-0.287
 
 
20.0
 
 
 
 
ASPH
 
 
hsa-miR-152-3p
 
 
-0.391
 
 
80.5
 
 
 
 
ASPHD2
 
 
hsa-miR-34a-5p
 
 
-0.354
 
 
18.6
 
 
 
 
ATCAY
 
 
hsa-miR-10a-5p
 
 
-0.322
 
 
31.5
 
 
 
 
ATG4B
 
 
hsa-miR-34a-5p
 
 
-0.58
 
 
38.2
 
 
 
 
ATMIN
 
 
hsa-miR-34a-5p
 
 
-0.409
 
 
18.8
 
 
 
 
ATP1A2
 
 
hsa-miR-34a-5p
 
 
-0.306
 
 
166.3
 
 
 
 
ATP5MC3
 
 
hsa-miR-27a-3p
 
 
-0.416
 
 
183.5
 
 
 
 
ATP6AP2
 
 
hsa-miR-152-3p
 
 
-0.592
 
 
52.9
 
 
 
 
ATP6AP2
 
 
hsa-miR-152-3p
 
 
-0.668
 
 
52.9
 
 
 
 
ATXN10
 
 
hsa-miR-27a-3p
 
 
-0.295
 
 
46.5
 
 
 
 
ATXN2
 
 
hsa-miR-10a-5p
 
 
-0.362
 
 
28.2
 
 
 
 
B4GALT2
 
 
hsa-miR-34a-5p
 
 
-0.314
 
 
15.5
 
 
 
 
B4GALT3
 
 
hsa-miR-27a-3p
 
 
-0.41
 
 
16.1
 
 
 
 
B4GALT5
 
 
hsa-miR-152-3p
 
 
-0.44
 
 
20.4
 
 
 
 
B4GALT6
 
 
hsa-miR-152-3p
 
 
-0.528
 
 
16.6
 
 
 
 
BAG4
 
 
hsa-miR-181a-5p
 
 
-0.337
 
 
14.8
 
 
 
 
BAG5
 
 
hsa-miR-22-3p
 
 
-0.318
 
 
17.8
 
 
 
 
BARHL2
 
 
hsa-miR-152-3p
 
 
-0.544
 
 
42.1
 
 
 
 
BAZ1B
 
 
hsa-miR-10a-5p
 
 
-0.309
 
 
24.8
 
 
 
 
BAZ2A
 
 
hsa-miR-100-5p
 
 
-0.29
 
 
26.6
 
 
 
 
BAZ2A
 
 
hsa-miR-100-5p
 
 
-0.421
 
 
26.6
 
 
 
 
BEGAIN
 
 
hsa-miR-28-5p
 
 
-0.325
 
 
53.7
 
 
 
 
BEX3
 
 
hsa-miR-27a-3p
 
 
-0.454
 
 
345.2
 
 
 
 
BHLHE40
 
 
hsa-miR-181a-5p
 
 
-0.408
 
 
18.1
 
 
 
 
BHLHE41
 
 
hsa-miR-152-3p
 
 
-0.442
 
 
31.9
 
 
 
 
BIN1
 
 
hsa-miR-22-3p
 
 
-0.346
 
 
88.3
 
 
 
 
BLOC1S1
 
 
hsa-miR-22-3p
 
 
-0.346
 
 
82.8
 
 
 
 
BLOC1S6
 
 
hsa-miR-181a-5p
 
 
-0.393
 
 
18.6
 
 
 
 
BLTP1
 
 
hsa-miR-27a-3p
 
 
-0.426
 
 
22.7
 
 
 
 
BMI1
 
 
hsa-miR-27a-3p
 
 
-0.322
 
 
22.5
 
 
 
 
BRINP1
 
 
hsa-miR-34a-5p
 
 
-0.612
 
 
33.3
 
 
 
 
BRSK1
 
 
hsa-miR-27a-3p
 
 
-0.372
 
 
111.6
 
 
 
 
BTAF1
 
 
hsa-miR-152-3p
 
 
-0.401
 
 
10.7
 
 
 
 
BTBD3
 
 
hsa-miR-152-3p
 
 
-0.495
 
 
65.8
 
 
 
 
BTG1
 
 
hsa-miR-22-3p
 
 
-0.468
 
 
10.5
 
 
 
 
BTG2
 
 
hsa-miR-27a-3p
 
 
-0.339
 
 
40.6
 
 
 
 
BZW1
 
 
hsa-miR-27a-3p
 
 
-0.337
 
 
31.0
 
 
 
 
C1orf35
 
 
hsa-miR-152-3p
 
 
-0.511
 
 
23.9
 
 
 
 
C5orf24
 
 
hsa-miR-22-3p
 
 
-0.493
 
 
34.2
 
 
 
 
C6orf62
 
 
hsa-miR-181a-5p
 
 
-0.334
 
 
57.4
 
 
 
 
CA10
 
 
hsa-miR-27a-3p
 
 
-0.464
 
 
120.4
 
 
 
 
CABP1
 
 
hsa-miR-27a-3p
 
 
-0.343
 
 
58.8
 
 
 
 
CABP7
 
 
hsa-miR-152-3p
 
 
-0.511
 
 
10.8
 
 
 
 
CACNB3
 
 
hsa-miR-34a-5p
 
 
-0.426
 
 
48.5
 
 
 
 
CACNG2
 
 
hsa-miR-27a-3p
 
 
-0.301
 
 
10.3
 
 
 
 
CACYBP
 
 
hsa-miR-27a-3p
 
 
-0.367
 
 
61.8
 
 
 
 
CADM4
 
 
hsa-miR-34a-5p
 
 
-0.365
 
 
86.9
 
 
 
 
CAMK2N1
 
 
hsa-miR-22-3p
 
 
-0.302
 
 
53.0
 
 
 
 
CAMK4
 
 
hsa-miR-34a-5p
 
 
-0.353
 
 
52.7
 
 
 
 
CAND1
 
 
hsa-miR-27a-3p
 
 
-0.318
 
 
24.5
 
 
 
 
 
 
 ovary 
 
 
 
 
target_symbol
 
 
mature_mirna_id
 
 
score
 
 
nTPM
 
 
 
 
 
 
ABCB7
 
 
hsa-miR-152-3p
 
 
-0.515
 
 
11.9
 
 
 
 
ABCD3
 
 
hsa-miR-152-3p
 
 
-0.388
 
 
11.0
 
 
 
 
ABRAXAS2
 
 
hsa-miR-34a-5p
 
 
-0.538
 
 
10.0
 
 
 
 
ACBD3
 
 
hsa-miR-34a-5p
 
 
-0.415
 
 
20.0
 
 
 
 
ACSL1
 
 
hsa-miR-34a-5p
 
 
-0.306
 
 
14.6
 
 
 
 
ACSL4
 
 
hsa-miR-34a-5p
 
 
-0.372
 
 
20.0
 
 
 
 
ACTA2
 
 
hsa-miR-27a-3p
 
 
-0.577
 
 
689.2
 
 
 
 
ACVR1
 
 
hsa-miR-152-3p
 
 
-0.397
 
 
17.7
 
 
 
 
ADAM10
 
 
hsa-miR-152-3p
 
 
-0.348
 
 
13.1
 
 
 
 
ADIPOR2
 
 
hsa-miR-34a-5p
 
 
-0.33
 
 
24.2
 
 
 
 
ADK
 
 
hsa-miR-34a-5p
 
 
-0.374
 
 
10.8
 
 
 
 
ADO
 
 
hsa-miR-34a-5p
 
 
-0.377
 
 
11.0
 
 
 
 
AFG3L2
 
 
hsa-miR-181a-5p
 
 
-0.491
 
 
12.5
 
 
 
 
AGFG1
 
 
hsa-miR-152-3p
 
 
-0.332
 
 
22.9
 
 
 
 
AGTRAP
 
 
hsa-miR-34a-5p
 
 
-0.328
 
 
25.1
 
 
 
 
AHDC1
 
 
hsa-miR-152-3p
 
 
-0.413
 
 
21.5
 
 
 
 
AIDA
 
 
hsa-miR-22-3p
 
 
-0.316
 
 
41.9
 
 
 
 
AKAP1
 
 
hsa-miR-152-3p
 
 
-0.381
 
 
18.8
 
 
 
 
AKIRIN1
 
 
hsa-miR-27a-3p
 
 
-0.368
 
 
18.3
 
 
 
 
AKT3
 
 
hsa-miR-22-3p
 
 
-0.451
 
 
21.9
 
 
 
 
ALCAM
 
 
hsa-miR-34a-5p
 
 
-0.303
 
 
33.1
 
 
 
 
ALCAM
 
 
hsa-miR-152-3p
 
 
-0.347
 
 
33.1
 
 
 
 
ALDOA
 
 
hsa-miR-34a-5p
 
 
-0.655
 
 
580.3
 
 
 
 
ANK2
 
 
hsa-miR-34a-5p
 
 
-0.317
 
 
28.0
 
 
 
 
ANK3
 
 
hsa-miR-34a-5p
 
 
-0.29
 
 
10.3
 
 
 
 
ANK3
 
 
hsa-miR-34a-5p
 
 
-0.347
 
 
10.3
 
 
 
 
ANKRD13A
 
 
hsa-miR-22-3p
 
 
-0.478
 
 
16.1
 
 
 
 
ANKRD40
 
 
hsa-miR-27a-3p
 
 
-0.35
 
 
18.3
 
 
 
 
ANP32B
 
 
hsa-miR-34a-5p
 
 
-0.29
 
 
160.5
 
 
 
 
AP1AR
 
 
hsa-miR-100-5p
 
 
-0.804
 
 
10.0
 
 
 
 
AP3S2
 
 
hsa-miR-34a-5p
 
 
-0.413
 
 
18.2
 
 
 
 
APH1A
 
 
hsa-miR-34a-5p
 
 
-0.304
 
 
97.8
 
 
 
 
APOO
 
 
hsa-miR-181a-5p
 
 
-0.5
 
 
11.0
 
 
 
 
APPBP2
 
 
hsa-miR-27a-3p
 
 
-0.378
 
 
17.5
 
 
 
 
AREG
 
 
hsa-miR-34a-5p
 
 
-0.62
 
 
11.4
 
 
 
 
ARF4
 
 
hsa-miR-152-3p
 
 
-0.487
 
 
123.7
 
 
 
 
ARF6
 
 
hsa-miR-181a-5p
 
 
-0.33
 
 
16.9
 
 
 
 
ARFIP1
 
 
hsa-miR-320d
 
 
-0.339
 
 
17.2
 
 
 
 
ARFIP1
 
 
hsa-miR-152-3p
 
 
-0.474
 
 
17.2
 
 
 
 
ARHGAP1
 
 
hsa-miR-34a-5p
 
 
-0.369
 
 
43.6
 
 
 
 
ARHGAP21
 
 
hsa-miR-152-3p
 
 
-0.287
 
 
26.5
 
 
 
 
ARL6IP1
 
 
hsa-miR-152-3p
 
 
-0.579
 
 
97.4
 
 
 
 
ARL8B
 
 
hsa-miR-152-3p
 
 
-0.337
 
 
35.5
 
 
 
 
ARL8B
 
 
hsa-miR-320d
 
 
-0.376
 
 
35.5
 
 
 
 
ARPC5
 
 
hsa-miR-22-3p
 
 
-0.679
 
 
45.9
 
 
 
 
ARPIN-AP3S2
 
 
hsa-miR-34a-5p
 
 
-0.348
 
 
11.2
 
 
 
 
ARPP19
 
 
hsa-miR-320d
 
 
-0.325
 
 
40.6
 
 
 
 
ARPP19
 
 
hsa-miR-34a-5p
 
 
-0.497
 
 
40.6
 
 
 
 
ARRB1
 
 
hsa-miR-22-3p
 
 
-0.616
 
 
17.2
 
 
 
 
ARRDC3
 
 
hsa-miR-152-3p
 
 
-0.305
 
 
64.7
 
 
 
 
ASAH1
 
 
hsa-miR-27a-3p
 
 
-0.398
 
 
45.1
 
 
 
 
ASH2L
 
 
hsa-miR-320d
 
 
-0.287
 
 
28.3
 
 
 
 
ASPH
 
 
hsa-miR-152-3p
 
 
-0.391
 
 
58.7
 
 
 
 
ATG4B
 
 
hsa-miR-34a-5p
 
 
-0.58
 
 
18.9
 
 
 
 
ATMIN
 
 
hsa-miR-34a-5p
 
 
-0.409
 
 
12.7
 
 
 
 
ATP1A2
 
 
hsa-miR-34a-5p
 
 
-0.306
 
 
12.4
 
 
 
 
ATP2B4
 
 
hsa-miR-152-3p
 
 
-0.337
 
 
30.9
 
 
 
 
ATP5MC3
 
 
hsa-miR-27a-3p
 
 
-0.416
 
 
107.0
 
 
 
 
ATP6AP2
 
 
hsa-miR-152-3p
 
 
-0.592
 
 
86.3
 
 
 
 
ATP6AP2
 
 
hsa-miR-152-3p
 
 
-0.668
 
 
86.3
 
 
 
 
ATXN10
 
 
hsa-miR-27a-3p
 
 
-0.295
 
 
50.3
 
 
 
 
ATXN2
 
 
hsa-miR-10a-5p
 
 
-0.362
 
 
25.4
 
 
 
 
AXL
 
 
hsa-miR-34a-5p
 
 
-0.512
 
 
22.7
 
 
 
 
B3GNT7
 
 
hsa-miR-27a-3p
 
 
-0.355
 
 
16.8
 
 
 
 
B4GALT2
 
 
hsa-miR-34a-5p
 
 
-0.314
 
 
21.1
 
 
 
 
B4GALT3
 
 
hsa-miR-27a-3p
 
 
-0.41
 
 
28.0
 
 
 
 
B4GALT5
 
 
hsa-miR-152-3p
 
 
-0.44
 
 
19.0
 
 
 
 
BAG5
 
 
hsa-miR-22-3p
 
 
-0.318
 
 
10.0
 
 
 
 
BAZ1B
 
 
hsa-miR-10a-5p
 
 
-0.309
 
 
25.4
 
 
 
 
BAZ2A
 
 
hsa-miR-100-5p
 
 
-0.29
 
 
30.7
 
 
 
 
BAZ2A
 
 
hsa-miR-100-5p
 
 
-0.421
 
 
30.7
 
 
 
 
BBX
 
 
hsa-miR-10a-5p
 
 
-0.305
 
 
20.8
 
 
 
 
BCL3
 
 
hsa-miR-27a-3p
 
 
-0.413
 
 
24.7
 
 
 
 
BEX3
 
 
hsa-miR-27a-3p
 
 
-0.454
 
 
482.6
 
 
 
 
BHLHE40
 
 
hsa-miR-181a-5p
 
 
-0.408
 
 
79.3
 
 
 
 
BICC1
 
 
hsa-miR-152-3p
 
 
-0.442
 
 
17.4
 
 
 
 
BIN1
 
 
hsa-miR-22-3p
 
 
-0.346
 
 
32.3
 
 
 
 
BLOC1S1
 
 
hsa-miR-22-3p
 
 
-0.346
 
 
123.1
 
 
 
 
BLOC1S6
 
 
hsa-miR-181a-5p
 
 
-0.393
 
 
33.7
 
 
 
 
BLTP1
 
 
hsa-miR-27a-3p
 
 
-0.426
 
 
28.4
 
 
 
 
BMI1
 
 
hsa-miR-27a-3p
 
 
-0.322
 
 
27.2
 
 
 
 
BMPR2
 
 
hsa-miR-100-5p
 
 
-0.355
 
 
11.2
 
 
 
 
BTAF1
 
 
hsa-miR-152-3p
 
 
-0.401
 
 
21.0
 
 
 
 
BTBD3
 
 
hsa-miR-152-3p
 
 
-0.495
 
 
13.2
 
 
 
 
BTG2
 
 
hsa-miR-27a-3p
 
 
-0.339
 
 
377.3
 
 
 
 
BZW1
 
 
hsa-miR-27a-3p
 
 
-0.337
 
 
60.3
 
 
 
 
C1orf35
 
 
hsa-miR-152-3p
 
 
-0.511
 
 
18.4
 
 
 
 
C5orf24
 
 
hsa-miR-22-3p
 
 
-0.493
 
 
19.6
 
 
 
 
C6orf120
 
 
hsa-miR-34a-5p
 
 
-0.331
 
 
13.6
 
 
 
 
C6orf62
 
 
hsa-miR-181a-5p
 
 
-0.334
 
 
86.2
 
 
 
 
C8orf88
 
 
hsa-miR-27a-3p
 
 
-0.33
 
 
10.7
 
 
 
 
CACNB3
 
 
hsa-miR-34a-5p
 
 
-0.426
 
 
42.6
 
 
 
 
CACYBP
 
 
hsa-miR-27a-3p
 
 
-0.367
 
 
35.2
 
 
 
 
CADM4
 
 
hsa-miR-34a-5p
 
 
-0.365
 
 
12.7
 
 
 
 
CAMK2N1
 
 
hsa-miR-22-3p
 
 
-0.302
 
 
28.4
 
 
 
 
CAND1
 
 
hsa-miR-27a-3p
 
 
-0.318
 
 
23.6
 
 
 
 
CAND1
 
 
hsa-miR-152-3p
 
 
-0.44
 
 
23.6
 
 
 
 
CANX
 
 
hsa-miR-152-3p
 
 
-0.434
 
 
112.9
 
 
 
 
CAPN1
 
 
hsa-miR-22-3p
 
 
-0.307
 
 
42.4
 
 
 
 
CAPZA1
 
 
hsa-miR-27a-3p
 
 
-0.325
 
 
42.8
 
 
 
 
 
 
 spinal cord 
 
 
 
 
target_symbol
 
 
mature_mirna_id
 
 
score
 
 
nTPM
 
 
 
 
 
 
ABCD3
 
 
hsa-miR-152-3p
 
 
-0.388
 
 
20.9
 
 
 
 
ABHD5
 
 
hsa-miR-27a-3p
 
 
-0.407
 
 
19.5
 
 
 
 
ACBD3
 
 
hsa-miR-34a-5p
 
 
-0.415
 
 
10.8
 
 
 
 
ACER3
 
 
hsa-miR-22-3p
 
 
-0.453
 
 
13.0
 
 
 
 
ACSL1
 
 
hsa-miR-34a-5p
 
 
-0.306
 
 
94.3
 
 
 
 
ACSL4
 
 
hsa-miR-34a-5p
 
 
-0.372
 
 
12.4
 
 
 
 
ACTA2
 
 
hsa-miR-27a-3p
 
 
-0.577
 
 
47.2
 
 
 
 
ADAM10
 
 
hsa-miR-152-3p
 
 
-0.348
 
 
18.7
 
 
 
 
ADAP1
 
 
hsa-miR-27a-3p
 
 
-0.302
 
 
131.1
 
 
 
 
ADCY2
 
 
hsa-miR-152-3p
 
 
-0.299
 
 
11.4
 
 
 
 
ADIPOR2
 
 
hsa-miR-34a-5p
 
 
-0.33
 
 
121.3
 
 
 
 
ADK
 
 
hsa-miR-34a-5p
 
 
-0.374
 
 
18.9
 
 
 
 
ADO
 
 
hsa-miR-34a-5p
 
 
-0.377
 
 
33.5
 
 
 
 
AGFG1
 
 
hsa-miR-152-3p
 
 
-0.332
 
 
53.5
 
 
 
 
AGPAT3
 
 
hsa-miR-27a-3p
 
 
-0.29
 
 
50.1
 
 
 
 
AGTRAP
 
 
hsa-miR-34a-5p
 
 
-0.328
 
 
34.7
 
 
 
 
AHCYL2
 
 
hsa-miR-34a-5p
 
 
-0.359
 
 
18.2
 
 
 
 
AIDA
 
 
hsa-miR-22-3p
 
 
-0.316
 
 
29.9
 
 
 
 
AKAP1
 
 
hsa-miR-152-3p
 
 
-0.381
 
 
14.0
 
 
 
 
AKIRIN1
 
 
hsa-miR-27a-3p
 
 
-0.368
 
 
52.7
 
 
 
 
AKT3
 
 
hsa-miR-22-3p
 
 
-0.451
 
 
15.8
 
 
 
 
ALCAM
 
 
hsa-miR-34a-5p
 
 
-0.303
 
 
56.1
 
 
 
 
ALCAM
 
 
hsa-miR-152-3p
 
 
-0.347
 
 
56.1
 
 
 
 
ALDOA
 
 
hsa-miR-34a-5p
 
 
-0.655
 
 
561.2
 
 
 
 
AMER2
 
 
hsa-miR-34a-5p
 
 
-0.48
 
 
28.2
 
 
 
 
ANK2
 
 
hsa-miR-34a-5p
 
 
-0.317
 
 
13.8
 
 
 
 
ANK3
 
 
hsa-miR-34a-5p
 
 
-0.29
 
 
39.5
 
 
 
 
ANK3
 
 
hsa-miR-34a-5p
 
 
-0.347
 
 
39.5
 
 
 
 
ANKRD40
 
 
hsa-miR-27a-3p
 
 
-0.35
 
 
196.0
 
 
 
 
ANP32B
 
 
hsa-miR-34a-5p
 
 
-0.29
 
 
363.5
 
 
 
 
AP3S2
 
 
hsa-miR-34a-5p
 
 
-0.413
 
 
15.3
 
 
 
 
APH1A
 
 
hsa-miR-34a-5p
 
 
-0.304
 
 
50.5
 
 
 
 
APOO
 
 
hsa-miR-181a-5p
 
 
-0.5
 
 
36.2
 
 
 
 
APPBP2
 
 
hsa-miR-27a-3p
 
 
-0.378
 
 
10.4
 
 
 
 
ARF4
 
 
hsa-miR-152-3p
 
 
-0.487
 
 
69.7
 
 
 
 
ARF6
 
 
hsa-miR-181a-5p
 
 
-0.33
 
 
13.8
 
 
 
 
ARFIP1
 
 
hsa-miR-320d
 
 
-0.339
 
 
10.6
 
 
 
 
ARFIP1
 
 
hsa-miR-152-3p
 
 
-0.474
 
 
10.6
 
 
 
 
ARHGAP1
 
 
hsa-miR-34a-5p
 
 
-0.369
 
 
38.6
 
 
 
 
ARHGAP21
 
 
hsa-miR-152-3p
 
 
-0.287
 
 
74.7
 
 
 
 
ARL6IP1
 
 
hsa-miR-152-3p
 
 
-0.579
 
 
140.2
 
 
 
 
ARL8B
 
 
hsa-miR-152-3p
 
 
-0.337
 
 
57.8
 
 
 
 
ARL8B
 
 
hsa-miR-320d
 
 
-0.376
 
 
57.8
 
 
 
 
ARPC5
 
 
hsa-miR-22-3p
 
 
-0.679
 
 
56.3
 
 
 
 
ARPIN-AP3S2
 
 
hsa-miR-34a-5p
 
 
-0.348
 
 
15.5
 
 
 
 
ARPP19
 
 
hsa-miR-320d
 
 
-0.325
 
 
170.8
 
 
 
 
ARPP19
 
 
hsa-miR-34a-5p
 
 
-0.497
 
 
170.8
 
 
 
 
ARRDC3
 
 
hsa-miR-152-3p
 
 
-0.305
 
 
23.5
 
 
 
 
ASAH1
 
 
hsa-miR-27a-3p
 
 
-0.398
 
 
79.8
 
 
 
 
ASB1
 
 
hsa-miR-34a-5p
 
 
-0.406
 
 
19.6
 
 
 
 
ASH2L
 
 
hsa-miR-320d
 
 
-0.287
 
 
17.1
 
 
 
 
ASPH
 
 
hsa-miR-152-3p
 
 
-0.391
 
 
25.3
 
 
 
 
ATCAY
 
 
hsa-miR-10a-5p
 
 
-0.322
 
 
10.1
 
 
 
 
ATG4B
 
 
hsa-miR-34a-5p
 
 
-0.58
 
 
18.9
 
 
 
 
ATMIN
 
 
hsa-miR-34a-5p
 
 
-0.409
 
 
15.4
 
 
 
 
ATP1A2
 
 
hsa-miR-34a-5p
 
 
-0.306
 
 
87.6
 
 
 
 
ATP5MC3
 
 
hsa-miR-27a-3p
 
 
-0.416
 
 
137.7
 
 
 
 
ATP6AP2
 
 
hsa-miR-152-3p
 
 
-0.592
 
 
55.3
 
 
 
 
ATP6AP2
 
 
hsa-miR-152-3p
 
 
-0.668
 
 
55.3
 
 
 
 
ATXN10
 
 
hsa-miR-27a-3p
 
 
-0.295
 
 
46.2
 
 
 
 
ATXN2
 
 
hsa-miR-10a-5p
 
 
-0.362
 
 
27.6
 
 
 
 
B4GALT2
 
 
hsa-miR-34a-5p
 
 
-0.314
 
 
36.9
 
 
 
 
B4GALT3
 
 
hsa-miR-27a-3p
 
 
-0.41
 
 
10.8
 
 
 
 
BAG5
 
 
hsa-miR-22-3p
 
 
-0.318
 
 
12.4
 
 
 
 
BAZ1B
 
 
hsa-miR-10a-5p
 
 
-0.309
 
 
19.2
 
 
 
 
BAZ2A
 
 
hsa-miR-100-5p
 
 
-0.29
 
 
10.5
 
 
 
 
BAZ2A
 
 
hsa-miR-100-5p
 
 
-0.421
 
 
10.5
 
 
 
 
BBX
 
 
hsa-miR-10a-5p
 
 
-0.305
 
 
17.3
 
 
 
 
BEX3
 
 
hsa-miR-27a-3p
 
 
-0.454
 
 
317.4
 
 
 
 
BHLHE40
 
 
hsa-miR-181a-5p
 
 
-0.408
 
 
46.8
 
 
 
 
BHLHE41
 
 
hsa-miR-152-3p
 
 
-0.442
 
 
74.7
 
 
 
 
BIN1
 
 
hsa-miR-22-3p
 
 
-0.346
 
 
197.8
 
 
 
 
BLOC1S1
 
 
hsa-miR-22-3p
 
 
-0.346
 
 
273.9
 
 
 
 
BLOC1S6
 
 
hsa-miR-181a-5p
 
 
-0.393
 
 
15.5
 
 
 
 
BLTP1
 
 
hsa-miR-27a-3p
 
 
-0.426
 
 
26.2
 
 
 
 
BMI1
 
 
hsa-miR-27a-3p
 
 
-0.322
 
 
33.0
 
 
 
 
BRSK1
 
 
hsa-miR-27a-3p
 
 
-0.372
 
 
44.1
 
 
 
 
BTBD3
 
 
hsa-miR-152-3p
 
 
-0.495
 
 
32.8
 
 
 
 
BTG2
 
 
hsa-miR-27a-3p
 
 
-0.339
 
 
41.8
 
 
 
 
BZW1
 
 
hsa-miR-27a-3p
 
 
-0.337
 
 
46.3
 
 
 
 
C1GALT1
 
 
hsa-miR-152-3p
 
 
-0.487
 
 
27.9
 
 
 
 
C1orf35
 
 
hsa-miR-152-3p
 
 
-0.511
 
 
12.3
 
 
 
 
C5orf24
 
 
hsa-miR-22-3p
 
 
-0.493
 
 
26.2
 
 
 
 
C6orf62
 
 
hsa-miR-181a-5p
 
 
-0.334
 
 
64.9
 
 
 
 
C8orf88
 
 
hsa-miR-27a-3p
 
 
-0.33
 
 
13.9
 
 
 
 
CA10
 
 
hsa-miR-27a-3p
 
 
-0.464
 
 
12.1
 
 
 
 
CABP1
 
 
hsa-miR-27a-3p
 
 
-0.343
 
 
11.0
 
 
 
 
CACYBP
 
 
hsa-miR-27a-3p
 
 
-0.367
 
 
51.6
 
 
 
 
CADM4
 
 
hsa-miR-34a-5p
 
 
-0.365
 
 
203.4
 
 
 
 
CAMK2N1
 
 
hsa-miR-22-3p
 
 
-0.302
 
 
147.9
 
 
 
 
CAND1
 
 
hsa-miR-27a-3p
 
 
-0.318
 
 
15.1
 
 
 
 
CAND1
 
 
hsa-miR-152-3p
 
 
-0.44
 
 
15.1
 
 
 
 
CANX
 
 
hsa-miR-152-3p
 
 
-0.434
 
 
133.1
 
 
 
 
CAPN1
 
 
hsa-miR-22-3p
 
 
-0.307
 
 
15.3
 
 
 
 
CAPZA1
 
 
hsa-miR-27a-3p
 
 
-0.325
 
 
51.3
 
 
 
 
CAV2
 
 
hsa-miR-152-3p
 
 
-0.32
 
 
19.3
 
 
 
 
CBX4
 
 
hsa-miR-181a-5p
 
 
-0.354
 
 
15.7
 
 
 
 
CCDC28B
 
 
hsa-miR-27a-3p
 
 
-0.314
 
 
12.1
 
 
 
 
CCDC47
 
 
hsa-miR-22-3p
 
 
-0.604
 
 
37.5
 
 
 
 
CCM2
 
 
hsa-miR-27a-3p
 
 
-0.335
 
 
46.0
 
 
 
 
 
 
 
 
 cd4_ev_vs_cd4_s0_upregulated - Tissue specific
ORA 
 
 all_genes 
 
 GO_Biological_Process_2025 
 This category comprises Gene Ontology (GO) Biological Process terms
describing coordinated and temporally ordered biological programs such
as cell cycle progression, immune activation, apoptosis,
differentiation, and metabolic regulation. GO Biological Process
annotations are curated by the Gene Ontology Consortium and integrate
experimental evidence, computational inference, and expert review.
Enrichment in this category indicates that the differentially expressed
target genes participate disproportionately in shared biological
programs rather than isolated molecular events. These results provide
high-level functional insight into systemic biological changes and are
particularly useful for interpreting phenotypic consequences of gene
expression alterations. 
 
 
 
 
Term
 
 
Overlap
 
 
P.value
 
 
Adjusted.P.value
 
 
Old.P.value
 
 
Old.Adjusted.P.value
 
 
Odds.Ratio
 
 
Combined.Score
 
 
Genes
 
 
 
 
 
 
Positive Regulation of DNA-templated Transcription ( GO:0045893 )
 
 
262/1274
 
 
0.0000000
 
 
0.0000000
 
 
0
 
 
0
 
 
2.046402
 
 
92.99088
 
 
TCERG1;CCNK;PHB1;CCNC;DCAF6;ZNF609;KAT5;CREB3L1;MYB;RPS6KA1;EPC1;EPC2;HOXA1;HOXA5;IER2;SOX4;HOXA4;MEF2A;ACVR1;DDX17;PDHX;ZNF281;SERPINF2;RFX3;SOX11;EBF4;ISL1;RUNX1;SFRP1;HOXB4;HOXB3;TP53;ZNF395;GATA6;PIK3R1;MED12L;MBTD1;DHX33;PRDM16;ALX1;ZNF268;RREB1;SNX5;ZBED4;JAG1;FZD5;FOXF1;FZD4;SS18L1;LIF;NR2F2;IGF1;INO80;GDF6;ST18;COL1A1;MLLT11;FOSL1;TADA2B;MLLT10;AGO2;HNRNPD;SNAI1;CREBRF;CDKN1C;ARF4;CSRNP1;HNF4G;MIA3;CTCF;MEOX2;AREG;NAT14;PHF8;EDRF1;MED14;SHH;NKRF;HEY2;NCOA1;SS18;FOXD3;SREBF1;FOXD2;ATMIN;TET2;TFEB;TET1;FOS;PROX1;BAZ1B;SIRT1;RGMA;NR5A2;MDFIC;PTRH2;TET3;TFEC;RARA;WAC;RARB;PPARG;NCOA7;RHOQ;NFIX;SATB2;PURB;SERTAD3;ZNF462;CDX2;NFYA;PCGF5;STAT3;GTF2H1;VEGFA;HINFP;PER1;RYBP;NFIA;WT1;CAMK4;CRLF3;FOXA1;CCNT2;PRDM4;CRTC1;CD80;UBP1;RORA;RORB;AHR;TNF;HOXA11;HOXA10;SALL1;POGZ;EP300;PITX2;MAP3K5;EOMES;LMO1;NANOS1;MAP2K1;MLIP;DYRK1B;MITF;MYT1;MORF4L1;MYCN;KAT6B;TBL1XR1;UBE2V1;ERG;NOTCH2;INO80E;NOTCH1;MAX;FOXO3;RLF;GLIS2;NPAS3;BCL2L12;NEUROD1;ZKSCAN3;ZNF304;OTX2;NDP;WNT1;ZBTB18;ESRRA;TFAP2C;AKIRIN1;SMAD4;TGFB2;FOXN4;RFXANK;ESRRG;ESR1;NR4A2;MAVS;MAFB;BCL3;TADA1;NFE2L1;MAP3K12;DDX3X;YWHAB;ONECUT1;PTEN;HMGB2;SIX1;ARRB1;TRIAP1;CDC73;ZIC1;MYBL2;KDM6B;MYOCD;PPP1R12A;CHUK;MSL2;VEZF1;ETV1;ETV4;ARID1B;KLF15;TGFBR1;TNFRSF1A;SENP1;GREM1;CREB1;ELF2;ASPH;TFDP2;IFNG;IRF4;TRAF6;IRF2;IRF5;SOS1;CREB5;HDAC4;PRKAA1;KDM3A;HDAC1;PLAG1;WBP2;CEBPG;LEF1;LRP5;NLRC5;FOXK2;MOSPD1;MLLT3;DLL1;STRN3;TBX21;TP53INP1;E2F3;VHL;RBBP7;MTA2;E2F7;ATOH1;H2AZ1;TCF7L2;FOXJ2;TBX5;KLF4;MEIS2;ACVR2A;USF1;KLF7;REST;KLF6;MEIS1;KLF5;PITHD1;CCM2;ERCC6;TAF5;TAF4
 
 
 
 
Positive Regulation of Transcription by RNA Polymerase II ( GO:0045944 )
 
 
212/983
 
 
0.0000000
 
 
0.0000000
 
 
0
 
 
0
 
 
2.153764
 
 
91.08768
 
 
TCERG1;CCNK;CCNC;DCAF6;ZNF609;KAT5;CREB3L1;MYB;RPS6KA1;EPC1;HOXA1;HOXA5;IER2;SOX4;HOXA4;MEF2A;ACVR1;DDX17;PDHX;SERPINF2;MED8;RFX3;MED9;SOX11;EBF4;ISL1;RUNX1;HOXB4;HOXB3;TP53;ZNF395;MEPCE;GATA6;PIK3R1;MED12L;ALX1;ZNF268;RREB1;ZBED4;JAG1;FZD5;FOXF1;FZD4;SS18L1;LIF;NR2F2;IGF1;INO80;ST18;MLLT10;AGO2;HNRNPD;CREBRF;ARF4;CSRNP1;HNF4G;MIA3;CTCF;MEOX2;AREG;PHF8;MED14;SHH;NKRF;HEY2;NCOA1;SS18;FOXD3;SREBF1;FOXD2;ATMIN;TET2;TFEB;TET1;FOS;PROX1;BAZ1B;SIRT1;MED28;RGMA;NR5A2;PTRH2;TET3;TFEC;RARA;RARB;PPARG;NCOA7;RHOQ;NFIX;SATB2;PURB;ZNF462;CDX2;NFYA;PCGF5;STAT3;VEGFA;HINFP;PER1;NFIA;WT1;CRLF3;FOXA1;CCNT2;PRDM4;CRTC1;UBP1;RORA;RORB;AHR;TNF;HOXA10;SALL1;POGZ;EP300;PITX2;EOMES;LMO1;NANOS1;MLIP;MITF;MYT1;MYCN;TBL1XR1;ERG;NOTCH2;NOTCH1;MAX;FOXO3;RLF;GLIS2;BCL2L12;NEUROD1;ZNF304;OTX2;NDP;ZBTB18;ESRRA;TFAP2C;AKIRIN1;SMAD4;FOXN4;RFXANK;ESRRG;ESR1;NR4A2;MAVS;NFE2L1;DDX3X;YWHAB;ONECUT1;PTEN;HMGB2;SIX1;ARRB1;TRIAP1;CDC73;MYBL2;KDM6B;MYOCD;PPP1R12A;CHUK;VEZF1;ETV1;ETV4;KLF15;TNFRSF1A;SENP1;GREM1;CREB1;TFDP2;IFNG;IRF4;TRAF6;IRF2;IRF5;SOS1;HDAC4;HDAC1;PLAG1;WBP2;CEBPG;LEF1;LRP5;NLRC5;FOXK2;MOSPD1;DLL1;STRN3;TBX21;E2F3;E2F7;ATOH1;H2AZ1;TCF7L2;TAF11;FOXJ2;TBX5;KLF4;MEIS2;ACVR2A;USF1;KLF7;REST;KLF6;MEIS1;KLF5;CCM2;ERCC6;TAF5;TAF4
 
 
 
 
Regulation of Transcription by RNA Polymerase II ( GO:0006357 )
 
 
399/2250
 
 
0.0000000
 
 
0.0000000
 
 
0
 
 
0
 
 
1.730613
 
 
71.03673
 
 
RB1;PHB1;ENO1;BACH2;DCAF6;CREB3L1;MYB;HOXA3;BBX;HOXA1;HOXA5;IER2;HOXA4;DDX17;MEF2A;ZNF287;ZNF283;ZNF281;SERPINF2;MED8;RFX3;SOX11;DICER1;ISL1;BIN1;HOXB4;COMMD3-BMI1;HOXB3;ZSCAN25;HOXB8;CGGBP1;ZSCAN29;HOXB5;ZNF397;ZNF395;ZNF274;HOXD1;GATA6;ZNF23;MTDH;PRDM16;ALX1;ZNF268;HOXC8;NKX2-4;RREB1;ZNF267;ZNF266;JAG1;FZD5;FZD4;NR2F2;INO80;ST18;NR2F6;FOSL1;TADA2B;MLLT10;HNRNPK;AGO2;HNRNPD;SNAI1;HBP1;ZNF136;PHF3;ARF4;TCF24;BHLHE41;CTCF;HIF3A;AREG;PHF8;MECP2;MED14;SHH;NKRF;HEY2;ZNF367;ZNF366;SS18;BATF3;HSBP1;EN2;TET2;TET1;FOS;BAZ1B;SIRT1;NCOR2;PTRH2;PAX9;TET3;RARA;RARB;PPARG;MDFI;ZNF594;ZNF471;NFIX;ZNF470;ZNF518B;ZNF469;TAF9B;ZNF225;ZNF462;PCGF6;PCGF5;USP9X;MBD1;PCGF3;STAT3;ZNF33B;HINFP;NFIA;WT1;FOSB;ZNF699;CRLF3;CCNT2;ZNF691;ZNF570;UBE2D3;ZBTB26;UBP1;UBE2D1;RORA;RORB;AHR;TNF;SALL1;ZFP30;PITX2;ZBTB39;FST;SKIDA1;USP2;ZBTB34;MITF;MYT1;FOXP1;MAF;KAT6B;ERG;NOTCH2;ZNF792;NOTCH1;CBFB;MAX;FOXO3;MACROH2A1;RLF;NPAS3;GLIS2;BCL2L12;NEUROD1;SCRT2;ZKSCAN3;SAFB2;NDN;ZNF304;ZKSCAN4;NDP;ZNF420;ZBTB18;ESRRA;AKIRIN1;BRPF3;RFXANK;FOXN4;ESRRG;MAFB;ZFP62;MAFG;TADA1;MDM4;LHX9;NFE2L1;ONECUT1;PTEN;ARRB1;ZFP82;CDC73;DNMT3B;ZNF644;EMX2;PPP1R12A;CHUK;DNMT3A;VEZF1;TNFRSF1A;TFDP2;TRAF6;SOS1;HDAC4;ZBTB47;HDAC1;PLAG1;WBP2;CEBPG;NLRC5;LRP5;FOXK2;PPM1D;DNAJB1;STOX2;DNAJB4;ZNF624;E2F3;RBBP7;E2F5;E2F7;ZNF740;DRGX;JAZF1;H2AZ1;SEMA4D;FBXL19;KCNIP3;FOXJ2;TBX5;KLF4;TBX4;KLF3;SLC25A37;KLF7;KLF6;KLF5;CCM2;BCOR;TCERG1;CCNK;CCNH;CCNC;IKZF1;IKZF4;IKZF5;ZNF609;KAT5;RPS6KA1;EPC1;EPC2;SOX4;SOX5;ACVR1;PDHX;EBF2;EBF4;THAP1;RUNX1;BCORL1;TP53;PIK3R1;ZBTB5;ZBTB4;MED12L;ZBTB2;MIER1;ZNF821;ZBED4;EGR3;FOXF1;GADD45A;SS18L1;LIF;IGF1;ID1;ID4;CREBRF;ID3;SIM1;RCOR3;SLC2A4RG;CSRNP1;BARHL2;SUV39H2;SUV39H1;HNF4G;MIA3;MEOX2;NCOA1;SREBF1;FOXD3;FOXD2;ATMIN;NOG;TFEB;PROX1;ZHX1;RGMA;NR5A2;TFEC;NCOA7;ZNF780B;VGLL2;RHOQ;SATB1;SATB2;PURB;PURA;ZFP14;MNT;HDX;NFYA;CDX2;VEGFA;PER1;NR6A1;NEDD4;FOXA1;PRDM4;CRTC1;WWC2;PRDM1;HOXA13;LOXL2;AMOT;HOXA11;HOXA10;POGZ;EP300;ARID2;MAGEB10;LMO1;NANOS1;EOMES;MLIP;MYCN;TBL1XR1;OTX2;PRNP;TFAP2C;SMAD4;UBE2I;CBX4;ESR1;NR4A2;MAVS;BHLHE40;JMY;MXD1;BRWD3;DDX3X;YWHAB;HMGB2;SIX1;TRIAP1;ZIC1;SIX3;MYBL2;KDM6B;KLF11;MYOCD;TGIF2;RBM15;KLF13;ETV1;SAFB;ETV4;YWHAZ;ARID1B;KLF15;ETV6;SENP1;GREM1;ELF2;CREB1;IFNG;IRF4;ELF5;IRF2;IRF5;KDM7A;CREB5;KDM3A;LEF1;MOSPD1;DLL1;STRN3;DLL4;TBX21;SAP30L;RALY;VHL;MTA2;ATOH1;TCF7L2;ATP2B4;PEDS1;MAPK14;MEIS2;USF1;ACVR2A;REST;MEIS1;POLE3;PMP22;ERCC6;TAF5;TAF4
 
 
 
 
Negative Regulation of DNA-templated Transcription ( GO:0045892 )
 
 
210/1006
 
 
0.0000000
 
 
0.0000000
 
 
0
 
 
0
 
 
2.061462
 
 
78.42767
 
 
RB1;TCERG1;PHB1;IKZF1;ENO1;IKZF4;BACH2;IKZF5;KAT5;CREB3L1;MYB;EPC1;SOX4;MEF2A;ZNF282;SFMBT1;ZNF281;RFX3;SOX11;DICER1;ISL1;THAP1;RUNX1;SFRP1;BIN1;WDR82;COMMD3-BMI1;BCORL1;TP53;CGGBP1;ZNF397;ZNF274;GATA6;ZBTB5;ZBTB4;ZBTB2;MTDH;MBTD1;MIER1;PRDM16;ALX1;ZNF268;RREB1;GADD45A;NR2F2;NR2F6;HNRNPK;TNFSF4;ID1;ID4;SNAI1;CREBRF;ID3;RCOR3;ZNF136;CDKN1C;SUV39H2;SUV39H1;BHLHE41;CTCF;PTPRK;AREG;MECP2;SHH;SCML2;NKRF;HEY2;ZNF366;FOXD3;BATF3;HSBP1;NOG;TET1;PROX1;SIRT1;ZHX1;CBFA2T3;NCOR2;MDFIC;PAX9;TFEC;RARA;RARB;PPARG;MDFI;NFIX;SATB1;CIPC;PURB;PURA;MNT;BTAF1;ZNF469;TAF9B;PCGF6;CDX2;USP9X;MBD1;STAT3;VEGFA;HINFP;PER1;NR6A1;WT1;NEDD4;RLIM;FOSB;LIMS1;CDK5R1;UBE2D3;ZBTB26;WWC2;UBE2D1;RORB;PRDM1;AHR;TNF;LOXL2;AMOT;SALL1;EP300;PITX2;MAF1;MAGEB10;EOMES;ZBTB39;MLIP;FST;USP2;ZBTB34;MITF;FOXP1;MAF;PSPC1;KAT6B;TBL1XR1;NOTCH1;CBFB;MAX;FOXO3;MACROH2A1;GLIS2;SCRT2;ZKSCAN3;NDN;ZNF304;ZBTB18;PRNP;SMAD4;UBE2I;CBX4;ESR1;NR4A2;CNOT7;MAFB;MAFG;BHLHE40;BCL3;MAP3K10;MDM4;MXD1;NFE2L1;LHX9;ZNF512B;HMGB2;CDC73;YWHAQ;SIX3;DNMT3B;KLF11;RBM15;TGIF2;DNMT3A;YWHAZ;ETV6;CREB1;ELF2;TFDP2;IFNG;TRAF6;IRF2;ZMYND11;HDAC4;HDAC1;LEF1;FOXK2;MOSPD1;PBXIP1;PPM1F;STRN3;DLL4;DNAJB1;TBX21;DNAJB4;SAP30L;RALY;VHL;RBBP7;MTA2;E2F7;JAZF1;TCF7L2;SEMA4D;KCNIP3;KLF4;MEIS2;KLF7;REST;POLE3;BCOR
 
 
 
 
Negative Regulation of Transcription by RNA Polymerase II ( GO:0000122 )
 
 
163/732
 
 
0.0000000
 
 
0.0000000
 
 
0
 
 
0
 
 
2.220188
 
 
79.10559
 
 
RB1;TCERG1;PHB1;UBE2D3;ZBTB26;WWC2;UBE2D1;ENO1;PRDM1;TNF;BACH2;IKZF5;AMOT;LOXL2;SALL1;KAT5;MYB;EPC1;EP300;PITX2;MAGEB10;SOX4;MEF2A;EOMES;ZBTB39;MLIP;ZNF281;FST;USP2;SOX11;MITF;ZBTB34;DICER1;ISL1;RUNX1;THAP1;MAF;BIN1;TBL1XR1;COMMD3-BMI1;BCORL1;TP53;CGGBP1;ZNF274;NOTCH1;CBFB;MAX;GATA6;FOXO3;ZBTB5;MACROH2A1;ZBTB4;MTDH;GLIS2;ZBTB2;SCRT2;PRDM16;MIER1;ZKSCAN3;NDN;ALX1;ZNF304;RREB1;ZNF268;ZBTB18;PRNP;SMAD4;UBE2I;CBX4;GADD45A;NR2F2;ESR1;NR2F6;NR4A2;MAFB;MAFG;ID1;BHLHE40;SNAI1;CREBRF;ID4;ID3;MDM4;MXD1;ZNF136;NFE2L1;SUV39H2;SUV39H1;BHLHE41;HMGB2;CTCF;AREG;CDC73;MECP2;SHH;HEY2;DNMT3B;ZNF366;FOXD3;KLF11;BATF3;RBM15;TGIF2;HSBP1;DNMT3A;NOG;TET1;PROX1;SIRT1;ZHX1;YWHAZ;ETV6;NCOR2;IFNG;TRAF6;TFEC;IRF2;RARA;RARB;PPARG;MDFI;HDAC4;NFIX;HDAC1;SATB1;LEF1;MOSPD1;STRN3;PURB;DLL4;DNAJB1;PURA;MNT;DNAJB4;SAP30L;TBX21;ZNF469;RALY;RBBP7;VHL;TAF9B;MTA2;E2F7;JAZF1;TCF7L2;SEMA4D;CDX2;USP9X;MBD1;KCNIP3;STAT3;KLF4;MEIS2;VEGFA;KLF7;PER1;REST;NR6A1;WT1;NEDD4;POLE3;FOSB;BCOR
 
 
 
 
Positive Regulation of RNA Biosynthetic Process ( GO:1902680 )
 
 
129/555
 
 
0.0000000
 
 
0.0000000
 
 
0
 
 
0
 
 
2.330580
 
 
73.97318
 
 
PHB1;CD80;RORA;AHR;RORB;TNF;HOXA11;SALL1;KAT5;MYB;RPS6KA1;EPC1;EP300;EPC2;SOX4;MAP3K5;ACVR1;EOMES;NANOS1;MAP2K1;ZNF281;RFX3;DYRK1B;MITF;RUNX1;MORF4L1;SFRP1;MYCN;KAT6B;TBL1XR1;UBE2V1;TP53;INO80E;NOTCH1;MAX;GATA6;FOXO3;RLF;GLIS2;NPAS3;NEUROD1;MBTD1;PRDM16;ZKSCAN3;ALX1;OTX2;NDP;WNT1;RREB1;SNX5;SMAD4;FZD4;FOXF1;SS18L1;INO80;NR2F2;IGF1;GDF6;ESR1;COL1A1;MLLT11;TADA2B;MAFB;BCL3;HNRNPD;SNAI1;TADA1;MAP3K12;CDKN1C;HMGB2;SIX1;CTCF;AREG;NAT14;PHF8;EDRF1;MED14;SHH;ZIC1;NCOA1;MYOCD;ATMIN;MSL2;TFEB;FOS;ARID1B;TGFBR1;NR5A2;CREB1;ELF2;ASPH;MDFIC;IRF4;RARA;WAC;PPARG;CREB5;HDAC4;PRKAA1;KDM3A;HDAC1;WBP2;LEF1;LRP5;FOXK2;MLLT3;SERTAD3;TBX21;TP53INP1;RBBP7;VHL;MTA2;TCF7L2;NFYA;STAT3;FOXJ2;GTF2H1;KLF4;TBX5;HINFP;KLF6;REST;RYBP;WT1;PITHD1;CAMK4;TAF5;CRLF3;TAF4
 
 
 
 
Regulation of DNA-templated Transcription ( GO:0006355 )
 
 
364/2139
 
 
0.0000000
 
 
0.0000000
 
 
0
 
 
0
 
 
1.625396
 
 
50.21044
 
 
RB1;PHB1;CCNH;IKZF1;ENO1;IKZF4;BACH2;IKZF5;ZNF609;KAT5;CREB3L1;GPBP1;RPS6KA1;MYB;HOXA3;BBX;EPC1;EPC2;HOXA1;HOXA5;SOX4;SOX5;DDX17;ZNF287;ACVR1;PDHX;ZNF283;ZNF282;SFMBT1;ZNF281;MED8;RFX3;EBF2;EBF4;THAP1;RUNX1;SFRP1;HOXB3;ZSCAN25;HOXB8;TP53;ZSCAN29;HOXB5;ZNF397;ZNF395;ZNF274;HOXD1;IGSF1;GATA6;ZNF23;ZBTB5;ZBTB4;MTDH;MBTD1;PRDM16;MIER1;ALX1;ZNF821;ZNF268;HOXC8;NKX2-4;RREB1;ZNF267;SNX5;ZNF266;EGR3;FOXF1;FZD4;SS18L1;NR2F2;IGF1;INO80;GDF6;FOSL1;COL1A1;MLLT11;TADA2B;MLLT10;HNRNPK;TNFSF4;ID1;HNRNPD;SNAI1;ID4;CREBRF;ID3;HBP1;SIM1;RCOR3;ZNF136;SLC2A4RG;CDKN1C;PHF3;LIN54;CSRNP1;BARHL2;SUV39H2;BTG1;SUV39H1;TCF24;BHLHE41;HNF4G;MIA3;CTCF;PTPRK;HIF3A;MEOX2;NAT14;AREG;PHF8;MECP2;ING5;MED14;SHH;EDRF1;SCML2;NKRF;ZNF280C;HEY2;ZNF367;NCOA1;SREBF1;SS18;FOXD3;FOXD2;BATF3;ATMIN;EN2;TFEB;FOS;PROX1;BAZ1B;ZHX1;CBFA2T3;SIRT1;MLF1;NCOR2;NR5A2;MDFIC;PAX9;TFEC;RARA;WAC;PPARG;NCOA7;ZNF780B;VGLL2;MDFI;ZNF594;ZNF471;NFIX;MYCBP;ZNF470;SATB1;CIPC;ZNF518B;SATB2;PURB;PURA;ZFP14;MNT;SERTAD3;BTAF1;ZNF225;HDX;SRSF10;PCGF6;NFYA;CDX2;PCGF5;MBD1;PCGF3;STAT3;GTF2H1;VEGFA;ZNF33B;HINFP;PER1;NR6A1;RYBP;NFIA;WT1;CAMK4;RLIM;FOSB;ZNF699;CRLF3;LIMS1;CDK5R1;FOXA1;ZNF691;ZNF570;CD80;WWC2;UBP1;RORA;PRDM1;RORB;AHR;TNF;HOXA13;LOXL2;HOXA11;HOXA10;SALL1;ZFP30;EP300;PITX2;ARID2;MAP3K5;NANOS1;EOMES;ZNF562;MAP2K1;SKIDA1;DYRK1B;ATP1B4;MITF;MYT1;FOXP1;MORF4L1;MYCN;MAF;PSPC1;KAT6B;TBL1XR1;UBE2V1;ERG;ZNF792;INO80E;NOTCH1;CBFB;MAX;FOXO3;RLF;NPAS3;GLIS2;NEUROD1;SCRT2;ZKSCAN3;SAFB2;ZNF304;OTX2;ZKSCAN4;NDP;WNT1;ZNF420;ZBTB18;ESRRA;TFAP2C;SMAD4;UBE2I;CBX4;BRPF3;FOXN4;ESRRG;ESR1;NR4A2;CNOT7;MAFB;ZFP62;MAFG;BHLHE40;BCL3;JMY;MAP3K10;TADA1;MDM4;MXD1;LHX9;BRWD3;NFE2L1;MAP3K12;ONECUT1;CHD7;HMGB2;SIX1;ARRB1;ZFP82;ELAVL2;YWHAQ;ZIC1;SIX3;ZNF644;KLF11;MYOCD;EMX2;TGIF2;RBM15;KLF13;DNMT3A;MSL2;ETV1;VEZF1;SAFB;ETV4;ARID1B;KLF15;TGFBR1;ETV6;ELF2;CREB1;TFDP2;ASPH;IFNG;IRF4;ELF5;TRAF6;IRF2;IRF5;ZMYND11;SOS1;KDM7A;CREB5;HDAC4;PRKAA1;KDM3A;ZBTB47;HDAC1;PLAG1;WBP2;CEBPG;LEF1;LRP5;FOXK2;PBXIP1;MLLT3;PPM1F;STRN3;STOX2;TBX21;SAP30L;TP53INP1;ZNF624;E2F3;VHL;RBBP7;E2F5;MTA2;E2F7;ZNF740;DRGX;TCF7L2;FBXL19;FOXJ2;ATP2B4;PEDS1;TBX5;MAPK14;KLF4;TBX4;KLF3;USF1;SLC25A37;KLF7;REST;KLF6;KLF5;PITHD1;PMP22;ERCC6;BCOR;TAF5;TAF4
 
 
 
 
Negative Regulation of Gene Expression ( GO:0010629 )
 
 
85/364
 
 
0.0000000
 
 
0.0000002
 
 
0
 
 
0
 
 
2.319154
 
 
50.15901
 
 
RB1;DDX3Y;TRIM71;SUV39H2;BTG2;DDX3X;PPP1R11;FMR1;SNX12;TNF;ROBO1;MECP2;ENDOU;PAIP2B;CREB3L1;HEY2;NPTN;EPC1;FLOT2;DIS3L2;NANOS1;FBXW7;ZNF281;ADAM10;SOX11;DICER1;SIRT1;FOXP1;PTBP3;SFRP1;MYCN;IFNG;PTRH2;MTF2;MYADM;COMMD3-BMI1;PPARG;TMBIM6;MAPT;EIF4E2;RBM24;DCP2;NOTCH2;HDAC4;DDX6;DNMT1;PRKAA1;NOTCH1;HDAC1;NRXN1;PPM1D;MACROH2A1;ZFP36L2;FXR1;DLL4;PDCD10;TASOR;RPS3;EIF4EBP1;VHL;EXOSC3;PAIP2;MARK1;NDFIP2;TFAP2C;TGFB2;SETDB1;STAT3;ATP2B4;IGF1;KLF4;ESR1;QKI;HINFP;VEGFA;REST;CNOT7;LIN28A;AGO4;WT1;AGO2;SERBP1;HSPA1B;LGR4;ASB1
 
 
 
 
Negative Regulation of RNA Biosynthetic Process ( GO:1902679 )
 
 
102/470
 
 
0.0000000
 
 
0.0000003
 
 
0
 
 
0
 
 
2.114875
 
 
44.86472
 
 
RB1;PHB1;ENO1;AHR;RORB;IKZF1;TNF;IKZF4;LOXL2;SALL1;KAT5;CREB3L1;MYB;EPC1;ZNF282;SFMBT1;ZNF281;RFX3;FOXP1;SFRP1;KAT6B;PSPC1;TP53;ZNF397;NOTCH1;GATA6;ZBTB4;GLIS2;MBTD1;PRDM16;ZKSCAN3;ALX1;ZBTB18;SMAD4;UBE2I;CBX4;NR2F2;HNRNPK;CNOT7;TNFSF4;BCL3;ID1;BHLHE40;ID4;MAP3K10;ID3;MDM4;RCOR3;LHX9;CDKN1C;SUV39H2;SUV39H1;BHLHE41;HMGB2;CTCF;PTPRK;MECP2;SCML2;YWHAQ;NKRF;HEY2;SIX3;RBM15;DNMT3A;PROX1;SIRT1;CBFA2T3;ZHX1;NCOR2;ELF2;TFDP2;MDFIC;IFNG;TRAF6;PAX9;RARA;PPARG;ZMYND11;MDFI;HDAC1;CIPC;LEF1;FOXK2;PBXIP1;PPM1F;STRN3;BTAF1;TBX21;RBBP7;MTA2;TCF7L2;PCGF6;MBD1;KLF4;HINFP;PER1;REST;WT1;RLIM;BCOR;LIMS1;CDK5R1
 
 
 
 
Positive Regulation of Cell Differentiation ( GO:0045597 )
 
 
72/297
 
 
0.0000000
 
 
0.0000007
 
 
0
 
 
0
 
 
2.429655
 
 
49.22906
 
 
RIPOR2;BTG1;LAMC1;AREG;TNF;HOXA11;LOXL2;KAT5;GLIPR2;RPS6KA1;CCN4;CCN2;HIF1AN;ARID2;IL6R;HOXA5;SOX4;ZNF385A;SOX5;ACVR1;MMD;SERPINF2;CTNNBIP1;SOX11;NPNT;ISL1;ARID1B;TGFBR1;TIAM1;SFRP1;NR5A2;CREB1;HOXB4;RAPGEF2;PPARG;IL6ST;SOS1;RBM24;CRB2;PKDCC;LAMA2;ZC4H2;LEF1;LRP5;LPL;NID1;MACROH2A1;ZFP36L1;NEUROD1;BLOC1S6;RAP1A;RRAS;ALX1;VHL;ZNF268;ATOH1;TCF7L2;AKIRIN1;SMAD4;TGFB2;SUCO;IGF1;GDF6;MAPK14;GDF5;ACVR2A;MAPK12;COL1A1;KITLG;REST;SNAI1;FERMT2
 
 
 
 
Positive Regulation of Signal Transduction ( GO:0009967 )
 
 
64/256
 
 
0.0000000
 
 
0.0000014
 
 
0
 
 
0
 
 
2.526872
 
 
49.21579
 
 
ITGB1;PHB1;POGLUT1;CD80;PTEN;TNFAIP3;MIA3;LAMC1;TXNDC15;CDC73;ROBO1;ING5;CDH5;ESM1;SHH;SALL1;NETO1;GRB10;NPTN;RSPO3;NEPRO;RSPO4;ACVR1;ATP6AP2;F3;SIRT1;TGFBR1;SFRP1;RAB34;ULK3;PPARG;UBE2V1;IL6ST;JCAD;KANK1;LAMA2;NRXN1;TUBD1;NID1;DLL1;CDKN2AIP;HSP90B1;ENHO;DLL4;GHRHR;PDCD10;RPS3;WNT1;SNX5;PRNP;TGFB2;JAG1;HOMER1;TSLP;BDNF;STAT3;LIF;IGF1;VEGFA;TSPAN14;AXL;UBE2N;TEK;LIMS1
 
 
 
 
Regulation of Cell Migration ( GO:0030334 )
 
 
101/479
 
 
0.0000000
 
 
0.0000014
 
 
0
 
 
0
 
 
2.036660
 
 
39.49622
 
 
SERPINE1;RORA;LDLRAD4;CLDN1;AMOT;CDH5;DPYSL3;ENPP2;ARID2;TMSB10;ACVR1;PDGFRB;PDGFRA;MAGI2;TPM1;ADAM10;MITF;TIAM1;FRMD5;SFRP1;ADAM17;MYADM;EPHA2;CSF1R;IGSF8;SEMA7A;NOTCH1;CUL5;PIK3R3;STC1;THY1;FOXO3;RTN4;CORO1C;PDCD10;PDGFC;ZNF304;SRGAP2;ZNF268;CARMIL1;JAG1;ARPIN-AP3S2;UBE2I;FZD4;IGF1;CYRIB;COL1A1;STK26;SNAI1;FERMT2;ITGB1;CLIC4;PTEN;MIA3;PTPRK;VSIR;ROBO1;SHH;TMSB4X;NOG;TET1;RHOC;F3;ADRA2A;TGFBR1;PTP4A1;RAP2B;NAV3;CDH11;NUMB;SGK3;SOS1;SGK1;HBEGF;KANK1;HDAC1;LEF1;RNF7;PPM1F;CDC42;PARD6B;CCL8;CCL7;SAP30L;NTF3;TP53INP1;CCL2;STX4;RBBP7;MAP4K4;SEMA4D;SEMA4B;STAT3;SEMA4C;SEMA4F;PPP2R3A;TBX5;VEGFA;TJP1;KIF26A;SPRY2
 
 
 
 
Negative Regulation of Cell Differentiation ( GO:0045596 )
 
 
56/220
 
 
0.0000000
 
 
0.0000057
 
 
0
 
 
0
 
 
2.583676
 
 
46.29988
 
 
RB1;FOXA1;TNFAIP6;PTEN;RORA;LDLRAD4;RORB;IPO7;TNF;CDC73;SHH;SPRED1;MYB;SIX3;ENPP1;NEPRO;SOX4;ADAMTS7;SS18;MYOCD;NOG;SIRT1;TGFBR1;GREM1;SFRP1;NR5A2;IFNG;PPARG;HOXB8;TRIB2;NOTCH1;LRP5;DLL1;RTN4;ZFP36L2;PTHLH;PPP2CA;WNT1;MARK1;WNT10B;JAG1;SEMA4D;CRIM1;TBX5;GDF5;MEIS2;VASN;MEIS1;REST;COL5A1;COL5A2;SPRY2;SPRY1;ANP32B;CNTN4;FERMT2
 
 
 
 
Positive Regulation of Cell-Substrate Junction Organization ( GO:0150117 )
 
 
14/23
 
 
0.0000000
 
 
0.0000093
 
 
0
 
 
0
 
 
11.662366
 
 
202.39158
 
 
NRP1;COL16A1;TSC1;PIK3R1;THY1;PPM1F;VEGFA;SFRP1;TEK;MAPRE2;FERMT2;ARF6;LIMS1;MAP4K4
 
 
 
 
Positive Regulation of Cellular Process ( GO:0048522 )
 
 
120/622
 
 
0.0000000
 
 
0.0000094
 
 
0
 
 
0
 
 
1.824293
 
 
31.49741
 
 
PGAP1;TNF;NCMAP;CCND2;RPS6KA1;CCN4;IL12B;CCN2;CAPN1;PITX2;ARID2;IL6R;SOX4;MFSD2A;TPD52;PDGFRB;PDGFRA;SERPINF2;TPM1;ADAM10;ACSL4;DICER1;TMEM250;ISL1;CDC25B;TIAM1;FRMD5;SFRP1;ADAM17;IL6ST;TP53;CSF1R;NOTCH1;CUL3;TGFA;LPL;TNFRSF11A;NID1;CDKN2AIP;ZFP36L1;PRDX3;NEUROD1;EFNB2;GHRHR;PDCD10;PDGFC;SSR1;WNT1;ZNF268;SRPK2;TGFB2;TSLP;LIF;INO80;IGF1;QKI;FOSL1;ACER2;CNOT7;TNFSF4;ACER3;BCL2;EIF4G2;DDX3X;DDX49;PTEN;LAMC1;AREG;SHH;ESM1;H3-3B;SS18;CNOT6L;IL15;TBC1D8;PROX1;TNFRSF1B;SIRT1;ARID1B;ADRA2A;TGFBR1;GREM1;IFNG;RARA;MAPT;HBEGF;HDAC4;PRKAA1;LAMA2;HDAC1;LEF1;LRP5;TTK;DLL1;PTHLH;CDC42;RPS15A;NTF3;STX4;STX3;VHL;TAF9B;LGI1;NTRK2;VEGFC;NAP1L1;MEIS2;VEGFA;TJP1;KITLG;H3-5;MEIS1;REST;GID8;DNAJA2;FKBP1B;AQP11;CCM2;YPEL3;METAP1
 
 
 
 
Positive Regulation of Cell Migration ( GO:0030335 )
 
 
76/344
 
 
0.0000000
 
 
0.0000119
 
 
0
 
 
0
 
 
2.151581
 
 
36.51602
 
 
ITGB1;NRP1;SCARB1;SPARC;MIA3;RORA;CLDN1;VSIR;CDH5;ZNF609;GLIPR2;ENPP2;CCN4;GFUS;ACVR1;PDGFRB;PDGFRA;SEMA6A;ADAM10;PROX1;RHOC;F3;ADRA2A;TGFBR1;FOXP1;PTP4A1;SHTN1;TIAM1;ADAM17;IFNG;MYADM;NUMB;RAPGEF2;PLAA;SOS1;EPHA2;HBEGF;IGSF8;CSF1R;SEMA7A;NOTCH1;LEF1;PIK3R3;RTN4;PPM1F;CDC42;CCL8;RRAS;CCL7;PDCD10;PDGFC;NTF3;CCL2;STX4;ZNF304;RREB1;ZNF268;MAP4K4;CARMIL1;TGFB2;UBE2I;SEMA4D;FZD4;SEMA4B;STAT3;SEMA4C;SEMA4F;IGF1;VEGFA;COL1A1;TJP1;AGO2;SNAI1;SPRY2;TEK;FERMT2
 
 
 
 
Protein Transport ( GO:0015031 )
 
 
69/308
 
 
0.0000001
 
 
0.0000263
 
 
0
 
 
0
 
 
2.187403
 
 
35.25125
 
 
ARF4;SYS1;TFRC;TMEM167A;ARHGAP1;GCC2;MIA3;RAB22A;SCAMP5;RAB43;ARL5B;CHMP1B;TOM1;ARFIP1;TMED1;TMED7;GOLGA7;TMED5;TRIM23;TMED4;ARRDC3;GOLPH3L;LMTK2;ANK3;SCAMP1;ZDHHC17;COPZ1;ARL4A;RAB34;CANX;MYH9;PREB;PPARG;TSNARE1;BLZF1;STX1A;ARF5;VAMP2;ARF6;NAPB;ZNF395;RAB5B;NECAB3;CALCRL;LMAN2L;SAR1A;STX17;STX16;LRP2;DNAJC27;STX11;HSP90B1;GGA2;SNX27;GRPEL2;RAB24;STX6;STX4;STX3;CTSA;RIC1;CMTM6;VMP1;RAB10;SNX18;RAB14;SNX17;SNX15;PPT1
 
 
 
 
Negative Regulation of Macromolecule Biosynthetic Process ( GO:0010558 )
 
 
50/202
 
 
0.0000002
 
 
0.0000599
 
 
0
 
 
0
 
 
2.484228
 
 
37.85009
 
 
RB1;DDX3Y;DDX3X;SNX12;AREG;TNF;ROBO1;MECP2;CREB3L1;HEY2;GRB10;ENPP1;FLOT2;FBXW7;ZNF281;ADAM10;SOX11;DICER1;SIRT1;FOXP1;SFRP1;MYCN;IFNG;PTRH2;MYADM;PPARG;MAPT;NOTCH2;DNMT1;PRKAA1;NOTCH1;HDAC1;NRXN1;STK39;DLL4;PDCD10;VHL;MARK1;NDFIP2;TGFB2;SETDB1;STAT3;ATP2B4;IGF1;KLF4;ESR1;HINFP;VEGFA;REST;CNOT7
 
 
 
 
Collagen Fibril Organization ( GO:0030199 )
 
 
19/45
 
 
0.0000003
 
 
0.0000607
 
 
0
 
 
0
 
 
5.485130
 
 
83.20198
 
 
COLGALT2;TGFB2;COL11A1;COL12A1;SERPINF2;LOXL1;TGFBR1;LOXL2;COL1A1;GREM1;COL3A1;COL1A2;COL2A1;COL5A1;LOX;COL5A2;PXDN;TLL1;ATP7A
 
 
 
 
Regulation of Gene Expression ( GO:0010468 )
 
 
187/1127
 
 
0.0000007
 
 
0.0001516
 
 
0
 
 
0
 
 
1.524903
 
 
21.65750
 
 
RB1;PHB1;TOR2A;TFRC;PRDM4;ZBTB26;WWC2;RORA;RORB;AHR;TNF;CDH5;CREB3L1;GPBP1;MYB;CCN4;IL12B;CCNL2;PITX2;HOXA5;SOX4;ZNF562;NANOS1;ZBTB39;MAP2K1;ZNF282;FBXW7;ZNF281;RFX3;ADAM10;ATP1B4;ZBTB34;ANK2;SOX11;MITF;DICER1;ANK3;FOXP1;THAP1;SFRP1;MYCN;PSPC1;KAT6B;MYADM;COMMD3-BMI1;UBE2V1;TP53;CGGBP1;ZNF397;NOTCH2;ZNF274;ZNF395;DNMT1;NOTCH1;IGSF1;PIK3R3;VTCN1;ZBTB4;MACROH2A1;ZBTB2;ZFP36L1;SCRT2;CALCR;PDCD10;MIER1;ZNF268;RREB1;CD276;MARK1;ESRRA;SRPK2;NDFIP2;SMAD4;TGFB2;SETDB1;BRPF3;BTN2A1;FOXN4;ESRRG;IGF1;LARP4;ESR1;ST18;TADA2B;CNOT7;MAFB;HNRNPF;BHLHE40;HNRNPD;SNAI1;TADA1;SLC2A4RG;TRIM71;DDX3Y;LIN54;DDX3X;BTG1;ONECUT1;CHD7;SNX12;SIX1;CTCF;AREG;YBX3;VSIR;ELAVL2;ROBO1;MECP2;ING5;SHH;HEY2;ZNF280C;DNMT3B;FLOT2;JARID2;KDM6B;TGIF2;NOG;MSL2;TFEB;PROX1;F3;SIRT1;MLF1;TGFBR1;PTBP3;ACTA2;RNF168;NR5A2;IFNG;PTRH2;IRF2;MTF2;RBM12B;RBM20;PPARG;MAPT;HDAC4;PRKAA1;MYCBP;HDAC1;PLAG1;NRXN1;LEF1;FOXK2;PBXIP1;DLL1;PPM1F;PTHLH;DLL4;NRAS;SERTAD3;TDG;SAP30L;RPS3;CCL2;STX3;VHL;RBBP7;HNRNPA1;SRSF10;RBM38;NTRK2;MOG;NFYA;STAT3;CAVIN4;ATP2B4;ZNF804A;DTNBP1;MAPK14;KLF4;WARS1;VEGFA;HINFP;SLC25A37;PDCL3;PER1;REST;RYBP;NFIA;WT1;DNAJA4;SPRY2;ERCC6;HSPA1B;LIMS1
 
 
 
 
Positive Regulation of Focal Adhesion Assembly ( GO:0051894 )
 
 
11/18
 
 
0.0000009
 
 
0.0001887
 
 
0
 
 
0
 
 
11.767690
 
 
163.97930
 
 
NRP1;SFRP1;COL16A1;TSC1;THY1;TEK;PPM1F;FERMT2;VEGFA;LIMS1;MAP4K4
 
 
 
 
Extracellular Matrix Organization ( GO:0030198 )
 
 
45/182
 
 
0.0000010
 
 
0.0001984
 
 
0
 
 
0
 
 
2.477379
 
 
34.28230
 
 
ITGB1;COLGALT2;COL15A1;COL16A1;COL11A1;COL12A1;LAMC1;NID1;LOXL1;ADAMTS10;CTSS;COL19A1;LOXL2;CREB3L1;ADAMTS17;ATP7A;ADAMTS9;ADAMTS7;DDX18;TGFB2;COL22A1;SERPINF2;ADAM10;NPNT;TGFBR1;COLQ;COL1A1;GREM1;COL3A1;COL1A2;COL2A1;COL4A2;LOX;COL5A1;MMP16;COL4A1;MMP15;COL7A1;COL5A2;PXDN;COL9A1;COL4A5;COL8A1;TLL1;MATN3
 
 
 
 
Regulation of Cell Population Proliferation ( GO:0042127 )
 
 
135/769
 
 
0.0000013
 
 
0.0002526
 
 
0
 
 
0
 
 
1.623357
 
 
22.00007
 
 
PHB1;PGAP1;TFRC;WWC2;ZNF609;CCND2;CAPN1;PITX2;ARID2;IL6R;SOX4;PDGFRB;TPD52;PDGFRA;MAP2K1;MAGI2;ADAM10;P3H1;TSC1;TMEM250;CDC25B;RUNX1;TIAM1;SFRP1;ADAM17;IL6ST;TP53;CSF1R;NOTCH1;CUL3;TGFA;TNFRSF11A;PDS5B;ZFP36L1;PRDX3;EFNB2;GHRHR;PDCD10;PDGFC;SSR1;WNT1;ZNF268;SRPK2;TFAP2C;TGFB2;CADM4;JAG1;TSLP;THAP12;LIF;IGF1;ST18;ACER2;FOSL1;CNOT7;TNFSF4;ACER3;BCL2;MDM4;TRIM71;BTG2;BTG1;PTEN;HMGB2;SIX1;CTCF;PTPRK;AREG;CDC73;ING5;SHH;ESM1;RB1CC1;SIX3;DIS3L2;SS18;KLF11;MYOCD;CNOT6L;IL15;TBC1D8;PROX1;SIRT1;CBFA2T3;ADRA2A;TGFBR1;GREM1;DDAH1;IFNG;ALDH1A2;RARA;RAPGEF2;SGK3;SOS1;SGK1;HBEGF;HDAC4;HDAC1;LRP5;TTK;PPM1D;DLL1;PTHLH;DLL4;RPS15A;ERBB3;CLMN;NTF3;TP53INP1;STX4;STX3;VHL;RBM38;NTRK2;CGREF1;TCF7L2;STAT3;VEGFC;NAP1L1;TBX5;MEIS2;WARS1;VEGFA;TJP1;KITLG;REST;MEIS1;WT1;PRKRA;GID8;DNAJA2;AQP11;CCM2;HSPA1B;LIMS1
 
 
 
 
Protein Ubiquitination ( GO:0016567 )
 
 
75/369
 
 
0.0000017
 
 
0.0003073
 
 
0
 
 
0
 
 
1.931757
 
 
25.71877
 
 
TRIM71;UBE2D2;FBXO28;UBE2D3;UBE2D1;ARRB1;PRPF19;NPEPPS;CDC23;RNF152;SKP1;TRIM23;WSB1;FBXW7;UBE2E3;MSL2;FBXO10;SIRT1;DDB2;RNF168;UBE2R2;TRAF6;UBE2V1;OBI1;MARCHF1;RNF34;ANAPC16;CBFB;CUL5;UHRF2;UBA6;CUL3;ZC4H2;MYCBP2;PELI2;RNF38;FBXO45;ASB11;RNF7;RCHY1;UBE2J1;PCNP;CAND1;RMND5A;TNKS2;RNF138;UBE2NL;MARCHF8;VHL;MARCHF3;MARCHF5;UBE2H;TRIM41;UBE2B;USP9X;SYVN1;DAW1;KLHL2;UBE2G1;UBE2W;NEDD4;KLHL8;OTULIN;RLIM;BCL2;RNF182;UBE2N;BLMH;MDM4;RNF186;RNF141;UBE2K;SPSB4;ASB1;CUL4B
 
 
 
 
Regulation of Apoptotic Process ( GO:0042981 )
 
 
125/704
 
 
0.0000018
 
 
0.0003182
 
 
0
 
 
0
 
 
1.643844
 
 
21.76095
 
 
ANKRD13C;FOXA1;PHB1;TFRC;ARL6IP1;ZFAND6;TNF;LGALS1;CCND2;KAT5;RPS6KA1;PROK2;EPC1;EPC2;CCNL2;PHLDA1;HOXA5;SOX4;MAP3K5;ACTN2;FBXO10;MORF4L1;SFRP1;MELK;BIN1;PPIF;IL6ST;TP53;NOTCH2;ANP32A;GATA6;VTCN1;PIK3R1;FOXO3;RTN4;MTDH;HSP90B1;PRDX3;BCL2L12;NEUROD1;SOCS2;MBTD1;BAG4;BCL2L11;NUAK2;PDCD10;PMAIP1;BMF;WNT1;ZNF268;MCL1;WNT10B;PLK5;EGR3;TSLP;CBX4;GADD45A;PLK2;SIAH2;IGF1;MLLT11;ACER2;HNRNPK;PTPRA;STK26;BCL3;JMY;PPT1;BCL2;MAP3K10;ANP32B;BCL2L2;MAP3K11;HRK;ITGB1;ARF4;RBM25;TNFAIP8;DDX3X;DDX42;TRIAP1;IER3IP1;ING5;SHH;AIFM1;CASP2;TOMM70;KLF11;SIRT1;YWHAZ;GREM1;RNF144B;CREB1;PLSCR3;GAS1;TMBIM6;IRF5;SOS1;SGK1;PRKAA1;HDAC1;LEF1;LRP2;FSTL1;HSPD1;PNMA1;ERBB3;NTF3;RPS3;BAK1;VHL;TAF9B;MAP4K4;SEMA4D;PNMA2;VEGFA;TJP1;REST;RYBP;TCIM;WT1;AXL;SPRY2;TEK;HSPA1B
 
 
 
 
Negative Regulation of Apoptotic Process ( GO:0043066 )
 
 
91/475
 
 
0.0000020
 
 
0.0003362
 
 
0
 
 
0
 
 
1.797769
 
 
23.61554
 
 
RB1;ITGB1;ARF4;TNFAIP8;DDX3X;TFRC;ARL6IP1;ZFAND6;SERPINE1;SIX1;TNFAIP3;TRIAP1;TNF;CDH5;SHH;CCND2;RPS6KA1;PDK4;PROK2;CASP2;PRKCD;ISL1;SIRT1;YWHAZ;GREM1;RNF144B;SFRP1;CREB1;PIK3CA;PTRH2;TYRO3;PPIF;TMBIM6;IL6ST;SOS1;TP53;NOTCH2;PRKAA1;NOTCH1;HDAC1;LEF1;GATA6;PIK3R3;VTCN1;LRP2;PIK3R1;FSTL1;DLL1;MTDH;HSP90B1;HSPD1;PRDX3;BCL2L12;SOCS2;BAG4;NUAK2;ERBB3;PDCD10;NTF3;CCL2;ZNF304;VHL;TAF9B;WNT1;ZNF268;MCL1;MAP4K4;NTRK2;EGR3;TSLP;SEMA4D;CBX4;BDNF;PLK2;SIAH2;IGF1;VEGFA;TJP1;NR4A2;HNRNPK;TCIM;WT1;AXL;BCL3;PPT1;BCL2;SPRY2;ANP32B;TEK;HSPA1B;BCL2L2
 
 
 
 
Central Nervous System Neuron Differentiation ( GO:0021953 )
 
 
18/46
 
 
0.0000020
 
 
0.0003362
 
 
0
 
 
0
 
 
4.822661
 
 
63.20634
 
 
NTRK2;NRXN1;ZC4H2;WNT9B;FOXN4;RORB;PROX1;ISL1;NEUROD1;NR4A2;BRINP1;MAPT;ATP7A;WNT1;MDGA2;CBLN1;METAP2;PALS1
 
 
 
 
Positive Regulation of Cell Population Proliferation ( GO:0008284 )
 
 
92/484
 
 
0.0000024
 
 
0.0003901
 
 
0
 
 
0
 
 
1.780390
 
 
23.00446
 
 
PHB1;PGAP1;PTEN;LAMC1;AREG;TNF;SHH;ESM1;CCND2;RBPMS2;CCN4;CAPN1;PITX2;IL6R;SOX4;TPD52;PDGFRB;SS18;PDGFRA;CNOT6L;IL15;SERPINF2;TBC1D8;ADAM10;SOX11;PROX1;TMEM250;SIRT1;ADRA2A;TGFBR1;CDC25B;FOXP1;GREM1;TIAM1;SFRP1;ADAM17;IFNG;RARA;COMMD3-BMI1;IL6ST;HBEGF;HDAC4;CSF1R;NOTCH1;HDAC1;CUL3;LEF1;LRP5;TGFA;TTK;TNFRSF11A;PIK3R1;DLL1;PTHLH;PRDX3;EFNB2;DLL4;GHRHR;RPS15A;ERBB3;PDCD10;PDGFC;NTF3;SSR1;STX4;ZNF304;STX3;WNT1;ZNF268;SRPK2;NTRK2;TCF7L2;TGFB2;TSLP;LIF;VEGFC;NAP1L1;IGF1;MEIS2;VEGFA;FOSL1;TJP1;KITLG;MEIS1;CNOT7;TNFSF4;GID8;DNAJA2;ACER3;BCL2;AQP11;CCM2
 
 
 
 
Regulation of miRNA Transcription ( GO:1902893 )
 
 
22/66
 
 
0.0000037
 
 
0.0005626
 
 
0
 
 
0
 
 
3.753948
 
 
46.99810
 
 
FOXA1;ZNF512B;NOTCH2;SREBF1;TGFB2;STAT3;FOS;FOXO3;KLF4;AREG;TNF;ESR1;VEGFA;NCOR2;FOSL1;REST;MYCN;WT1;MYB;RARA;PPARG;TP53
 
 
 
 
Chromatin Organization ( GO:0006325 )
 
 
66/323
 
 
0.0000056
 
 
0.0008405
 
 
0
 
 
0
 
 
1.941206
 
 
23.45820
 
 
RB1;FOXA1;SUV39H2;SUV39H1;CHD7;HMGB2;CTCF;CHD1;PHF8;LOXL2;ING5;EPC1;JARID2;ARID2;HMGN2;HMGN3;SKP1;H3-3B;KDM6B;SS18;TET1;BAZ1B;SAFB;SIRT1;ARID1B;NR5A2;KAT6B;WAC;COMMD3-BMI1;TOP1;ASF1A;KDM7A;HDAC4;ZNF274;DNMT1;INO80E;SATB1;HDAC1;SATB2;MACROH2A1;ATAD2B;MIER1;PRDM16;RBBP7;MTA2;MORC2;H2AZ1;PCGF6;SLF2;SETDB1;FBXL19;MTHFR;NAP1L1;INO80;NPM3;ESR1;TADA2B;PER1;REST;RYBP;NASP;POLE3;SNAI1;ANP32B;BCOR;ERCC6
 
 
 
 
Positive Regulation of Cell Motility ( GO:2000147 )
 
 
54/249
 
 
0.0000066
 
 
0.0009464
 
 
0
 
 
0
 
 
2.089805
 
 
24.93747
 
 
ITGB1;RORA;CLDN1;VSIR;CDH5;ACVR1;PDGFRB;PDGFRA;ADAM10;RHOC;F3;ADRA2A;TGFBR1;PTP4A1;TIAM1;ADAM17;MYADM;NUMB;SOS1;EPHA2;HBEGF;IGSF8;CSF1R;SEMA7A;NOTCH1;LEF1;PIK3R3;PPM1F;CDC42;CCL8;CCL7;PDCD10;PDGFC;NTF3;CCL2;STX4;ZNF304;ZNF268;MAP4K4;CARMIL1;UBE2I;SEMA4D;FZD4;SEMA4B;STAT3;SEMA4C;SEMA4F;IGF1;VEGFA;COL1A1;TJP1;SNAI1;SPRY2;FERMT2
 
 
 
 
Myoblast Differentiation ( GO:0045445 )
 
 
11/21
 
 
0.0000070
 
 
0.0009585
 
 
0
 
 
0
 
 
8.235981
 
 
97.75676
 
 
ITGB1;RB1;TCF7L2;MYOCD;REST;JAG1;NOTCH1;SDC1;IGF1;MAPK12;HINFP
 
 
 
 
Chromatin Remodeling ( GO:0006338 )
 
 
56/262
 
 
0.0000071
 
 
0.0009585
 
 
0
 
 
0
 
 
2.051962
 
 
24.33145
 
 
RB1;FOXA1;LIN54;SUV39H2;PHB1;CHD7;CTCF;CHD1;PHF8;KAT5;JARID2;ARID2;SKP1;KDM6B;SS18;USP3;MSL2;TET1;BAZ1B;SIRT1;ARID1B;RNF168;NR5A2;MTF2;WAC;COMMD3-BMI1;TOP1;ASF1A;CGGBP1;KDM7A;HDAC4;ZNF274;INO80E;SATB1;HDAC1;SATB2;MACROH2A1;TDG;MIER1;RBBP7;MTA2;MORC2;SRPK2;PCGF6;FBXL19;INO80;NPM3;ESR1;TADA2B;PER1;MCM3AP;REST;RYBP;POLE3;BCOR;ERCC6
 
 
 
 
Membrane Organization ( GO:0061024 )
 
 
41/173
 
 
0.0000090
 
 
0.0011763
 
 
0
 
 
0
 
 
2.339302
 
 
27.18979
 
 
GLTP;STEEP1;SAR1A;VPS4B;FNBP1L;PIK3C2A;STX11;CORO1C;SNX3;SPAST;SERP1;TMEM43;TTPA;CHMP1B;VPS36;SNAP29;CEP55;VTI1B;SH3GLB1;CAV3;DMPK;GCA;IST1;VPS37C;ANK2;ANK3;VPS37B;TMEM170A;BLTP1;PLEKHA8;PLSCR3;BIN1;TMEM33;VAPB;MYADM;PPT1;CHMP4B;TMCC1;NEMP1;CHMP7;VAMP2
 
 
 
 
Positive Regulation of Macromolecule Biosynthetic Process ( GO:0010557 )
 
 
70/355
 
 
0.0000107
 
 
0.0013700
 
 
0
 
 
0
 
 
1.856821
 
 
21.24505
 
 
PHB1;TOR2A;DDX3X;DYRK2;TFRC;CTCF;AREG;TNF;VSIR;ROBO1;CDH5;DNMT3B;HOXA5;PDGFRB;MAP2K1;NOG;ANK2;SOX11;MITF;ANK3;F3;SIRT1;TGFBR1;ACTA2;MYCN;IFNG;PTRH2;PPARG;SOS1;TP53;DNMT1;NOTCH1;HDAC1;NRXN1;LEF1;NLRC5;PIK3R3;DLL1;PPM1F;DLL4;NRAS;CALCR;PDCD10;RPS3;CCL2;MARK1;SRPK2;NTRK2;SMAD4;TGFB2;STAT3;DTNBP1;ZNF804A;RFXANK;IGF1;KLF4;MAPK14;WARS1;HINFP;VEGFA;PDCL3;SLC25A37;REST;WT1;DNAJA4;SPRY2;ERCC6;HSPA1B;CDK5R1;LIMS1
 
 
 
 
Central Nervous System Neuron Development ( GO:0021954 )
 
 
9/15
 
 
0.0000114
 
 
0.0014100
 
 
0
 
 
0
 
 
11.223896
 
 
127.73410
 
 
NTRK2;PTEN;RAPGEF2;MAPT;ATP7A;B4GALT6;B4GALT5;METAP2;PALS1
 
 
 
 
Regulation of Signal Transduction ( GO:0009966 )
 
 
47/211
 
 
0.0000122
 
 
0.0014100
 
 
0
 
 
0
 
 
2.160023
 
 
24.44311
 
 
NRP1;CCNK;PGAP1;RGS14;HHIP;CD80;GSKIP;ZDHHC7;RORA;PPM1K;PPM1D;DLL1;CDKN2AIP;SNX3;LFNG;SOCS2;UGCG;RGS2;RIC8B;IRAK1;ERBB3;SH3TC2;PPP6R1;ZIC1;OTX2;HIF1AN;VHL;GPR158;ACVR1;PRNP;HOMER1;CAV3;DYNLT1;KCNIP3;NOG;KCNIP4;DTNBP1;ZDHHC17;MDFIC;VSNL1;KIF26A;VEPH1;PXDN;GAS1;ZMYND11;MDFI;KCTD16
 
 
 
 
Positive Regulation of Cell Growth ( GO:0030307 )
 
 
29/107
 
 
0.0000123
 
 
0.0014100
 
 
0
 
 
0
 
 
2.794350
 
 
31.59133
 
 
ITSN2;SEMA7A;DDX3X;DDX49;RASAL1;CDKN2AIP;CDC42;RPS6KA1;TAF9B;MFSD2A;H3-3B;LGI1;TGFB2;SYT1;SEMA4D;BDNF;LIMK1;ADAM10;IGF1;INO80;TGFBR1;SHTN1;ADAM17;SFRP1;H3-5;BCL2;PLAA;MAPT;EIF4G2
 
 
 
 
Regulation of Epithelial to Mesenchymal Transition ( GO:0010717 )
 
 
26/91
 
 
0.0000123
 
 
0.0014100
 
 
0
 
 
0
 
 
3.004703
 
 
33.96703
 
 
FOXA1;CRB2;LEF1;PTEN;LDLRAD4;LOXL2;PPP2CA;SPRED1;GLIPR2;ALX1;MARK1;TCF7L2;SMAD4;TGFB2;TBX5;TGFBR1;QKI;VASN;GREM1;COL1A1;TIAM1;SFRP1;SNAI1;SPRY2;SPRY1;FERMT2
 
 
 
 
Protein Modification Process ( GO:0036211 )
 
 
98/545
 
 
0.0000135
 
 
0.0015075
 
 
0
 
 
0
 
 
1.662235
 
 
18.63773
 
 
GMFB;UBE2D2;UBE2D3;TESK2;LOXL1;LOXL2;PPP1CB;PPP1CC;PPP4R2;AKT3;RPS6KA1;MAP3K9;LARGE1;SEPHS1;USP6;PRKCD;DYRK1B;P3H1;ADAM10;LMTK2;CDC25B;TPST1;LOX;ERG;TRIB2;BRSK1;STK39;FUT4;UGCG;NUAK2;FUT9;ST8SIA4;STK38L;ST3GAL6;TRPM7;MARK3;MARK1;B3GALNT2;SRPK2;UBE2F;NUS1;PTPRN2;UBE2I;DMPK;PLK2;NEK6;PMM2;NEK7;TBCK;MEX3B;STK26;OSTC;MAP3K11;MAP3K12;DYRK2;PTEN;PTPRM;PTPRK;SERP1;TLK2;ZNF367;PPP1R12A;BAZ1B;DUSP9;YWHAZ;CTTNBP2NL;CCNA2;CREB1;MMP15;CTDSP2;RARA;ATG4B;SGK3;SGK1;B4GALT5;PRKAA1;RNF7;PPM1D;PPM1G;PPP2CA;CCL2;MAPK1;MAP4K4;ARFGEF1;PTPN18;CSNK1A1;CDC42BPG;LIMK1;PPP2R3A;COL3A1;SNRK;MGAT4A;CAMK4;NEK10;AQP11;PTPN4;METAP1;METAP2
 
 
 
 
Regulation of Chondrocyte Differentiation ( GO:0032330 )
 
 
12/26
 
 
0.0000142
 
 
0.0015447
 
 
0
 
 
0
 
 
6.418918
 
 
71.65662
 
 
GREM1;PKDCC;NR5A2;MBOAT2;GDF6;GDF5;PTHLH;TGFBR1;HOXA11;LOXL2;ADAMTS7;SOX5
 
 
 
 
Embryonic Digestive Tract Development ( GO:0048566 )
 
 
10/19
 
 
0.0000176
 
 
0.0018603
 
 
0
 
 
0
 
 
8.316112
 
 
91.04469
 
 
PDGFRA;TGFB2;PKDCC;SALL1;FOXF1;RBPMS2;RARB;SOX11;PCSK5;TNF
 
 
 
 
Negative Regulation of Catabolic Process ( GO:0009895 )
 
 
31/120
 
 
0.0000179
 
 
0.0018603
 
 
0
 
 
0
 
 
2.618478
 
 
28.61960
 
 
PHB1;MTMR2;PRKAA1;SNX12;MTMR9;FOXK2;ZDHHC7;HMGCR;TNF;SNX3;ENDOU;DNPEP;ZKSCAN3;TLK2;EP300;STK38L;BMF;VHL;MCL1;RUBCN;RIC1;SGTA;STAT3;AZIN1;ADRA2A;PDCL3;RRAGC;BCL2;MDM4;SOS1;EIF4G2
 
 
 
 
Negative Regulation of Cell Migration ( GO:0030336 )
 
 
42/184
 
 
0.0000186
 
 
0.0018912
 
 
0
 
 
0
 
 
2.227286
 
 
24.25604
 
 
KANK1;CLIC4;MACIR;HDAC1;SERPINE1;PTEN;STC1;PTPRM;MIA3;LDLRAD4;THY1;PTPRK;FOXO3;CORO1C;ROBO1;DLL4;SHH;SAP30L;DPYSL3;TP53INP1;RBBP7;ARID2;SRGAP2;ADAMTS9;DDX18;ARPIN-AP3S2;JAG1;MAGI2;TPM1;NOG;TET1;MITF;NR2F2;TBX5;TGFBR1;SFRP1;COL3A1;RAP2B;NAV3;DNAJA4;STK26;CDH11
 
 
 
 
Negative Regulation of Programmed Cell Death ( GO:0043069 )
 
 
73/382
 
 
0.0000215
 
 
0.0021335
 
 
0
 
 
0
 
 
1.785863
 
 
19.19326
 
 
ARF4;TNFAIP8;DDX3X;ARL6IP1;TFRC;ZFAND6;SLC7A11;TRIAP1;TM7SF3;SHH;CCND2;RPS6KA1;PROK2;CASP2;TMEM109;SIRT1;YWHAZ;GREM1;RNF144B;SFRP1;CREB1;PPIF;TMBIM6;IL6ST;SOS1;TP53;NOTCH2;PRKAA1;HDAC1;LEF1;GATA6;PIK3R1;VTCN1;LRP2;FSTL1;MTDH;HSP90B1;HSPD1;PRDX3;BCL2L12;SOCS2;BAG4;NUAK2;ERBB3;PDCD10;VHL;TAF9B;WNT1;ZNF268;MAP4K4;MCL1;EGR3;TSLP;CBX4;SEMA4D;PLK2;SIAH2;IGF1;VEGFA;TJP1;HNRNPK;TCIM;XPNPEP1;AXL;WT1;BCL3;PPT1;BCL2;SPRY2;ANP32B;TEK;HSPA1B;BCL2L2
 
 
 
 
Protein Polyubiquitination ( GO:0000209 )
 
 
49/228
 
 
0.0000221
 
 
0.0021489
 
 
0
 
 
0
 
 
2.063238
 
 
22.11415
 
 
TRIM71;UBE2D2;FBXO28;UBE2D3;UBE2D1;TNFAIP3;PRPF19;NPEPPS;CDC23;RNF152;SKP1;WSB1;UBE2E3;DDB2;RNF168;UBE2R2;TRAF6;UBE2V1;MARCHF1;RNF34;ANAPC16;CBFB;CUL5;CUL3;MYCBP2;PELI2;FBXO45;ASB11;RNF7;UBE2J1;ZNRF2;RMND5A;TNKS2;UBE2NL;MARCHF8;RNF39;MARCHF5;UBE2H;UBE2B;SYVN1;DAW1;UBE2G1;UBE2W;NEDD4;BCL2;UBE2N;BLMH;RNF186;UBE2K
 
 
 
 
Negative Regulation of Cell Motility ( GO:2000146 )
 
 
34/139
 
 
0.0000251
 
 
0.0023888
 
 
0
 
 
0
 
 
2.435170
 
 
25.79055
 
 
KANK1;CLIC4;HDAC1;SERPINE1;PTEN;STC1;MIA3;LDLRAD4;THY1;PTPRK;FOXO3;ROBO1;SHH;SAP30L;DPYSL3;TP53INP1;RBBP7;ARID2;SRGAP2;ARPIN-AP3S2;JAG1;MAGI2;TPM1;NOG;TET1;MITF;TBX5;TGFBR1;FRMD5;SFRP1;RAP2B;NAV3;STK26;CDH11
 
 
 
 
Regulation of Filopodium Assembly ( GO:0051489 )
 
 
15/40
 
 
0.0000263
 
 
0.0024424
 
 
0
 
 
0
 
 
4.496170
 
 
47.42387
 
 
NRP1;RIPOR2;FMR1;NRXN1;PLPPR5;PRKCD;PIK3R1;FNBP1L;CDC42;GAP43;DPYSL3;PALM;FSCN1;RHOQ;ARF6
 
 
 
 
Sphingolipid Metabolic Process ( GO:0006665 )
 
 
23/79
 
 
0.0000276
 
 
0.0024658
 
 
0
 
 
0
 
 
3.082812
 
 
32.36519
 
 
CERS4;CERS6;ELOVL4;PPM1L;SGMS1;ELOVL2;ELOVL3;ORMDL1;ELOVL6;SGMS2;SGPP1;SMPD3;PLA2G15;ACER2;SGPL1;SPTLC2;ORMDL2;ORMDL3;ENPP2;PPT1;SPTSSB;SPTSSA;ST6GALNAC3
 
 
 
 
pre-miRNA Processing ( GO:0031054 )
 
 
8/13
 
 
0.0000280
 
 
0.0024658
 
 
0
 
 
0
 
 
11.967756
 
 
125.44489
 
 
LIN28B;LIN28A;AGO4;PRKRA;TUT4;AGO2;DROSHA;DICER1
 
 
 
 
Regulation of Autophagy ( GO:0010506 )
 
 
49/230
 
 
0.0000282
 
 
0.0024658
 
 
0
 
 
0
 
 
2.040205
 
 
21.37617
 
 
USP32;PIK3C2A;KAT5;RB1CC1;TLK2;EP300;PIP4K2B;CAPN1;ATP6V1E1;RNF152;TRIM23;SH3GLB1;RUBCN;TFEB;CCNY;RRAGC;IFNG;ULK3;WAC;MAPT;SOS1;PRKAA1;MTMR9;FOXK2;FOXO3;C9ORF72;MTDH;DNPEP;ORMDL3;TP53INP1;ZKSCAN3;UBQLN1;STK38L;BMF;VHL;DRAM2;DCAF12;SNX5;MCL1;CTSA;PLK2;STAT3;RAB39B;SVIP;ACER2;NEDD4;BCL2;EIF4G2;CDK5R1
 
 
 
 
Nervous System Development ( GO:0007399 )
 
 
87/480
 
 
0.0000299
 
 
0.0025673
 
 
0
 
 
0
 
 
1.675563
 
 
17.45548
 
 
TRIM71;ACHE;GMFB;NRSN1;CHD7;PTEN;SLC2A1;MSI1;HAPLN3;RAPGEFL1;NRGN;ROBO1;SHH;DPYSL4;DPYSL5;ZIC1;EP300;JARID2;PCDHAC2;SOX4;CDON;KIRREL3;EMX2;SEMA6A;NOG;SOX11;PROX1;MYT1;TGFBR1;DNM3;NR5A2;NAV3;ALDH1A2;FEZ2;TYRO3;DCX;MAPT;RAPGEF5;NOTCH2;HDAC4;IGSF8;NUMBL;ZC4H2;NRXN1;LRP5;NRXN2;EFNA5;GLIS2;TRAK2;NEUROD1;ERBB3;FLRT3;SLITRK1;BTBD3;PDGFC;NTF3;SLITRK6;ST8SIA4;NDP;NPTX1;ATOH1;LGI1;MPP2;PRNP;MARCKSL1;JAG1;FZD5;MOG;BDNF;STAT3;LIMK1;CRIM1;SEMA4F;NR2F2;NR2F6;VEGFA;CYP26A1;MARCKS;P2RX5;AXL;MAB21L2;PPT1;ATXN10;CNTN4;ERCC6;MDGA2;CBLN1
 
 
 
 
Hemopoiesis ( GO:0030097 )
 
 
27/101
 
 
0.0000316
 
 
0.0026659
 
 
0
 
 
0
 
 
2.740544
 
 
28.39466
 
 
NOTCH2;CSF1R;PBXIP1;DLL1;RASGRP4;ZFP36L2;PRDX3;RRAS;MYB;KIRREL3;WNT10B;TGFB2;JAG1;FST;GLRX5;TET2;KLF3;RUNX1;ETV6;SFRP1;KITLG;MEIS1;SNRK;MELK;COMMD3-BMI1;TEK;TP53
 
 
 
 
Protein O-linked Glycosylation ( GO:0006493 )
 
 
25/91
 
 
0.0000373
 
 
0.0030627
 
 
0
 
 
0
 
 
2.843985
 
 
28.99661
 
 
GALNT12;POMT2;GALNT14;POGLUT2;POGLUT1;GCNT1;TMTC3;GXYLT2;FUT4;FUT9;GCNT3;LARGE1;B3GALNT2;GALNT5;GALNT1;TET2;TET1;POMGNT1;DPM2;B3GNT7;B4GAT1;EOGT;TET3;POC1B-GALNT4;B4GALT5
 
 
 
 
Positive Regulation of Cell Junction Assembly ( GO:1901890 )
 
 
20/65
 
 
0.0000377
 
 
0.0030627
 
 
0
 
 
0
 
 
3.333807
 
 
33.95537
 
 
CNTNAP2;NRP1;COL16A1;BDNF;NRXN1;TSC1;IL1RAP;THY1;TBX5;PPM1F;VEGFA;SFRP1;SLITRK1;IL1RAPL1;SLITRK6;TEK;FERMT2;EPHA2;MAP4K4;LIMS1
 
 
 
 
Positive Regulation of Gene Expression ( GO:0010628 )
 
 
80/436
 
 
0.0000398
 
 
0.0031738
 
 
0
 
 
0
 
 
1.699309
 
 
17.21653
 
 
PHB1;TOR2A;DDX3X;TFRC;FMR1;CTCF;AREG;TNF;TENT4B;VSIR;ROBO1;SCAMP5;CDH5;DNMT3B;HOXA5;MAP2K1;IL15;NOG;SOX11;MITF;ANK2;LARP4B;ANK3;F3;ADRA2A;TGFBR1;ACTA2;NR5A2;MYCN;IFNG;CSDE1;PRR16;RBM20;PPARG;TP53;RBM24;DNMT1;NOTCH1;HDAC1;NRXN1;LEF1;STK39;PIK3R3;PIK3R1;DLL1;PPM1F;FXR1;DLL4;NRAS;CALCR;PDCD10;RPS3;CCL2;MARK1;SRPK2;NTRK2;SMAD4;STAT3;SAMD4A;DTNBP1;ZNF804A;IGF1;LARP4;KLF4;MAPK14;WARS1;HINFP;VEGFA;PDCL3;REST;WT1;DNAJA4;TNFSF4;AGO2;BCL3;HNRNPD;SPRY2;ERCC6;HSPA1B;LIMS1
 
 
 
 
Regulation of Neurogenesis ( GO:0050767 )
 
 
20/66
 
 
0.0000482
 
 
0.0037730
 
 
0
 
 
0
 
 
3.261148
 
 
32.41862
 
 
NUMBL;RBM15;NOTCH1;DYNLT1;RGS14;LEF1;BHLHE41;HMGB2;SOX11;NAP1L1;LRP2;HOOK3;TNF;DLL1;FXR1;REST;IFNG;HEY2;BHLHE40;NUMB
 
 
 
 
Positive Regulation of Notch Signaling Pathway ( GO:0045747 )
 
 
13/33
 
 
0.0000494
 
 
0.0038002
 
 
0
 
 
0
 
 
4.868091
 
 
48.27334
 
 
TGFB2;POGLUT1;JAG1;STAT3;DLL1;ROBO1;DLL4;ENHO;TSPAN14;PDCD10;WNT1;IL6ST;NEPRO
 
 
 
 
Generation of Neurons ( GO:0048699 )
 
 
42/192
 
 
0.0000539
 
 
0.0040770
 
 
0
 
 
0
 
 
2.107533
 
 
20.71472
 
 
BRSK1;NRP1;SIX1;RTN4;SHH;AIFM1;ERBB3;FUT9;SIX3;WNT1;IER2;SOX4;MARK1;WNT10B;NTRK2;MAP2K1;TGFB2;EMX2;FZD5;FZD4;USP9X;EN2;WNT9B;ATP2B4;ACSL4;SOX11;RUNX1;NR4A2;TUBB2A;DDIT4;ID1;IL1RAPL1;ID4;DCX;ID3;RAPGEF2;MAPT;ERCC6;LHX9;PALS1;PAFAH1B1;CDK5R1
 
 
 
 
Positive Regulation of miRNA Transcription ( GO:1902895 )
 
 
16/47
 
 
0.0000569
 
 
0.0042336
 
 
0
 
 
0
 
 
3.868002
 
 
37.80734
 
 
FOXA1;NOTCH2;SREBF1;TGFB2;STAT3;FOS;FOXO3;KLF4;AREG;TNF;FOSL1;MYCN;WT1;MYB;PPARG;TP53
 
 
 
 
miRNA Metabolic Process ( GO:0010586 )
 
 
8/14
 
 
0.0000586
 
 
0.0042908
 
 
0
 
 
0
 
 
9.972564
 
 
97.17709
 
 
DDX17;LIN28B;LIN28A;TUT4;XPO5;DICER1;TENT4B;DIS3L2
 
 
 
 
Intrinsic Apoptotic Signaling Pathway in Response to DNA Damage ( GO:0008630 )
 
 
17/52
 
 
0.0000605
 
 
0.0043565
 
 
0
 
 
0
 
 
3.640789
 
 
35.36291
 
 
DYRK2;TNFRSF1B;SIRT1;UACA;BCL2L11;DDIT4;BCL3;BCL2;CASP2;EP300;TMEM109;CDIP1;BAK1;TP53;BCL2L2;EPHA2;MCL1
 
 
 
 
Neuron Differentiation ( GO:0030182 )
 
 
40/181
 
 
0.0000631
 
 
0.0044739
 
 
0
 
 
0
 
 
2.134553
 
 
20.64197
 
 
FOXA1;BRSK1;RTN4;UGCG;SHH;BRINP1;AIFM1;ERBB3;FUT9;OTX2;PITX2;WNT1;IER2;SOX4;ATOH1;WNT10B;NTRK2;MAP2K1;TGFB2;EMX2;FZD5;FZD4;EN2;WNT9B;ATP2B4;ACSL4;SOX11;TGFBR1;RUNX1;VEGFA;NR4A2;DDIT4;ID1;IL1RAPL1;ID4;PPT1;ID3;ERCC6;LHX9;CDK5R1
 
 
 
 
Cellular Response to Decreased Oxygen Levels ( GO:0036294 )
 
 
22/78
 
 
0.0000695
 
 
0.0048507
 
 
0
 
 
0
 
 
2.947518
 
 
28.21887
 
 
SUV39H2;NOTCH1;SUV39H1;GATA6;RORA;TSC1;HIF3A;ADO;SIRT1;RTN4;ZFP36L1;VEGFA;NPEPPS;SFRP1;CPEB1;TBL2;UBQLN1;PMAIP1;PPARG;MDM4;VHL;TP53
 
 
 
 
Extracellular Structure Organization ( GO:0043062 )
 
 
28/111
 
 
0.0000706
 
 
0.0048515
 
 
0
 
 
0
 
 
2.533657
 
 
24.21694
 
 
ITGB1;COL15A1;COL16A1;COL11A1;ADAMTS10;COL19A1;ADAMTS17;ATP7A;ADAMTS9;ADAMTS7;COL22A1;NPNT;TGFBR1;COLQ;COL3A1;COL1A2;COL2A1;COL4A2;MMP16;COL4A1;MMP15;COL7A1;COL5A2;PXDN;COL9A1;COL4A5;COL8A1;MATN3
 
 
 
 
Membrane Lipid Biosynthetic Process ( GO:0046467 )
 
 
16/48
 
 
0.0000760
 
 
0.0051439
 
 
0
 
 
0
 
 
3.746914
 
 
35.53690
 
 
CERS4;CERS6;PPM1L;SGMS1;ELOVL4;ELOVL2;ELOVL3;ORMDL1;ELOVL6;SGMS2;SPTLC2;ORMDL2;ORMDL3;SPTSSB;ST3GAL6;SPTSSA
 
 
 
 
Ceramide Metabolic Process ( GO:0006672 )
 
 
17/53
 
 
0.0000793
 
 
0.0051958
 
 
0
 
 
0
 
 
3.539454
 
 
33.41932
 
 
CERS4;ASAH1;CERS6;SGMS1;ORMDL1;SGMS2;TLCD3B;PLA2G15;ACER2;SGPL1;SPTLC2;ORMDL2;ORMDL3;ACER3;SPTSSB;SPTSSA;ST6GALNAC3
 
 
 
 
Positive Regulation of miRNA Metabolic Process ( GO:2000630 )
 
 
17/53
 
 
0.0000793
 
 
0.0051958
 
 
0
 
 
0
 
 
3.539454
 
 
33.41932
 
 
FOXA1;NOTCH2;SREBF1;TGFB2;STAT3;FOS;FOXO3;KLF4;AREG;TNF;FOSL1;LIN28B;MYCN;WT1;MYB;PPARG;TP53
 
 
 
 
Sprouting Angiogenesis ( GO:0002040 )
 
 
18/58
 
 
0.0000803
 
 
0.0051958
 
 
0
 
 
0
 
 
3.373562
 
 
31.81211
 
 
NRP1;EGR3;PIK3R3;VEGFC;MIA3;NRARP;DLL1;LOXL2;ROBO1;VEGFA;GREM1;EFNB2;CDH5;ESM1;OTULIN;RSPO3;TEK;E2F7
 
 
 
 
Blood Vessel Endothelial Cell Migration ( GO:0043534 )
 
 
11/26
 
 
0.0000872
 
 
0.0054410
 
 
0
 
 
0
 
 
5.489097
 
 
51.30949
 
 
EFNB2;GREM1;NRP1;CDH5;EGR3;ID1;PIK3R3;MYH9;MIA3;ROBO1;VEGFA
 
 
 
 
Positive Regulation of Filopodium Assembly ( GO:0051491 )
 
 
11/26
 
 
0.0000872
 
 
0.0054410
 
 
0
 
 
0
 
 
5.489097
 
 
51.30949
 
 
CDC42;NRP1;RIPOR2;FMR1;PLPPR5;DPYSL3;PALM;FSCN1;PIK3R1;FNBP1L;RHOQ
 
 
 
 
Positive Regulation of Epithelial Cell Proliferation ( GO:0050679 )
 
 
29/118
 
 
0.0000877
 
 
0.0054410
 
 
0
 
 
0
 
 
2.447447
 
 
22.86196
 
 
NOTCH2;NRP1;HMGB2;TGFA;MIA3;NRARP;LAMC1;CLDN1;TNF;AREG;RTN4;NRAS;ERBB3;AKT3;ZNF304;RREB1;TCF7L2;EGR3;VASH2;SOX11;IGF1;PROX1;F3;SIRT1;TGFBR1;VEGFA;PDCL3;SFRP1;TEK
 
 
 
 
Skeletal System Development ( GO:0001501 )
 
 
36/160
 
 
0.0000975
 
 
0.0058878
 
 
0
 
 
0
 
 
2.182842
 
 
20.16073
 
 
PKDCC;HHIP;CHD7;STC1;TNFRSF11B;HAPLN3;HOXA13;PCSK5;COL19A1;PTHLH;HOXA11;WNT1;ATP7A;SOX5;TGFB2;SLC10A7;AHSG;NOG;RFLNB;P3H1;IGF1;TBX4;TGFBR1;KIAA1217;COL1A1;BMP3;COL1A2;COL2A1;MMP16;PTRH2;OGN;CDH11;ALPL;TLL1;MATN3;FBN1
 
 
 
 
Regulation of Cold-Induced Thermogenesis ( GO:0120161 )
 
 
34/148
 
 
0.0000976
 
 
0.0058878
 
 
0
 
 
0
 
 
2.241768
 
 
20.70234
 
 
RB1;ACHE;KDM3A;NOTCH1;LAMA4;PCTP;ADIPOR2;PDGFC;PRDM16;GRB10;LDLR;MAP2K6;KDM6B;WNT10B;PRKAB2;ACSL1;IL15;ARRDC3;ELOVL3;PLCL2;EBF2;ESRRG;ELOVL6;QKI;VEGFA;ADAM17;GATM;IRF4;NOVA1;SCD;ID1;MFAP2;ALPL;LGR4
 
 
 
 
Regulation of Smooth Muscle Cell Proliferation ( GO:0048660 )
 
 
17/54
 
 
0.0001031
 
 
0.0061396
 
 
0
 
 
0
 
 
3.443598
 
 
31.61064
 
 
PDGFRB;TCF7L2;PHB1;HDAC1;SERPINF2;TNFAIP3;PIK3R1;IGF1;TNF;FOXP1;IFNG;OGN;RBPMS2;CCN4;IL12B;PPARG;IL6R
 
 
 
 
Signal Transduction in Response to DNA Damage ( GO:0042770 )
 
 
22/80
 
 
0.0001052
 
 
0.0061795
 
 
0
 
 
0
 
 
2.845556
 
 
26.06476
 
 
RBM38;USP32;GADD45A;TRIAP1;PRPF19;MAPK14;MAPK12;HINFP;ACER2;KAT5;SESN2;BCL3;CASP2;GRB2;INTS7;MDM4;CEP63;ERCC6;WNT1;TP53;E2F7;MAP2K6
 
 
 
 
Peptidyl-Serine Modification ( GO:0018209 )
 
 
21/75
 
 
0.0001131
 
 
0.0065557
 
 
0
 
 
0
 
 
2.916833
 
 
26.50712
 
 
SRPK2;BRSK1;POGLUT2;POGLUT1;GALNT1;NEK6;PLK2;PRKCD;STK39;LMTK2;MAPK14;TGFBR1;MAPK12;TLK2;MAPK1;MAP3K10;POC1B-GALNT4;TOP1;MARK3;CDK5R1;MAP3K12
 
 
 
 
Artery Morphogenesis ( GO:0048844 )
 
 
12/31
 
 
0.0001175
 
 
0.0067256
 
 
0
 
 
0
 
 
4.728387
 
 
42.78786
 
 
PDGFRB;NRP1;TGFB2;NOTCH1;JAG1;FOXF1;HEY2;NOG;HOXA1;LRP2;TGFBR1;VEGFA
 
 
 
 
Random Inactivation of X Chromosome ( GO:0060816 )
 
 
5/6
 
 
0.0001246
 
 
0.0069818
 
 
0
 
 
0
 
 
37.360169
 
 
335.87988
 
 
HNRNPK;RBM15;PCGF5;PCGF3;RLIM
 
 
 
 
Negative Regulation of Autophagy ( GO:0010507 )
 
 
19/65
 
 
0.0001264
 
 
0.0069818
 
 
0
 
 
0
 
 
3.096770
 
 
27.79586
 
 
CTSA;RUBCN;STAT3;FOXK2;MTMR9;TSC1;DNPEP;PIK3CA;RRAGC;TLK2;ZKSCAN3;BCL2;EP300;STK38L;BMF;VHL;SOS1;EIF4G2;MCL1
 
 
 
 
Negative Regulation of BMP Signaling Pathway ( GO:0030514 )
 
 
15/45
 
 
0.0001267
 
 
0.0069818
 
 
0
 
 
0
 
 
3.745745
 
 
33.61438
 
 
NOTCH1;TNFAIP6;UBE2D3;NOG;CRIM1;UBE2D1;LRP2;LEMD3;GREM1;GREM2;RBPMS2;VWC2L;PPARG;WNT1;FBN1
 
 
 
 
Mitotic Sister Chromatid Cohesion ( GO:0007064 )
 
 
9/19
 
 
0.0001351
 
 
0.0073584
 
 
0
 
 
0
 
 
6.732810
 
 
59.98403
 
 
NAA50;RB1;SGO1;MAU2;STAG2;ESCO1;POGZ;PDS5B;PDS5A
 
 
 
 
Regulation of Notch Signaling Pathway ( GO:0008593 )
 
 
21/76
 
 
0.0001390
 
 
0.0073966
 
 
0
 
 
0
 
 
2.863636
 
 
25.43177
 
 
TGFB2;POGLUT1;JAG1;FBXW7;STAT3;ARRB1;CCNC;NRARP;DLL1;ROBO1;LFNG;DLL4;ENHO;TSPAN14;TCIM;PDCD10;HEY2;HIF1AN;IL6ST;WNT1;NEPRO
 
 
 
 
Negative Regulation of Signal Transduction ( GO:0009968 )
 
 
54/277
 
 
0.0001415
 
 
0.0073966
 
 
0
 
 
0
 
 
1.824474
 
 
16.17042
 
 
RB1;OTUD4;YWHAB;HHIP;TNFAIP3;CCNC;ARRB1;NRARP;TNF;RGS2;SPRED1;CREB3L1;HEY2;GRB10;IL12B;ENPP1;PIP4K2B;HIF1AN;ACVR1;SEMA6A;AHSG;FBXW7;PRKCD;NOG;CTNNBIP1;RPGRIP1L;SFRP1;TYRO3;ULK3;PPARG;TMBIM6;MDFI;KANK1;RGS14;DLL1;GLIS2;SOCS2;DLL4;ERBB3;KCTD21;LEPROTL1;VHL;PTPN18;CADM4;BDNF;NR4A2;MFHAS1;TCIM;KIF26A;NEDD4;BCL2;SPRY2;SPRY1;LGR4
 
 
 
 
Axonogenesis ( GO:0007409 )
 
 
42/200
 
 
0.0001421
 
 
0.0073966
 
 
0
 
 
0
 
 
1.999907
 
 
17.71730
 
 
NOTCH2;NRP1;BRSK1;SEMA7A;NOTCH1;NRXN1;MYCBP2;PTEN;EFNA5;C9ORF72;EFNB2;SHH;PARD6B;DPYSL5;SLITRK1;NPTN;SLITRK6;OTX2;VASP;LGI1;SEMA6A;SEMA4D;USP9X;BDNF;SEMA4B;SEMA4C;SEMA4F;ANK3;ISL1;ZDHHC17;RAB10;AFG3L2;SHTN1;KLF7;PALLD;FEZ2;CNTN4;B4GALT6;SOS1;METAP1;CDK5R1;B4GALT5
 
 
 
 
Cellular Response to Tumor Necrosis Factor ( GO:0071356 )
 
 
23/87
 
 
0.0001425
 
 
0.0073966
 
 
0
 
 
0
 
 
2.696233
 
 
23.87897
 
 
ASAH1;CHUK;ZFAND6;TNFRSF11A;NPNT;TNFRSF1B;FOXO3;TNF;YBX3;SIRT1;ZFP36L2;ST18;TNFRSF1A;ZFP36L1;SFRP1;BAG4;TMSB4X;RPS3;CCL2;TP53;ZNF268;ADAMTS7;LIMS1
 
 
 
 
Neuron Projection Morphogenesis ( GO:0048812 )
 
 
35/157
 
 
0.0001467
 
 
0.0075204
 
 
0
 
 
0
 
 
2.156318
 
 
19.03355
 
 
BRSK1;CNTNAP2;NRN1;CTNND2;NRXN1;PTEN;LRP2;NYAP2;CDC42;PARD6B;ALCAM;FLRT3;SLITRK1;BTBD3;NTF3;SLITRK6;SRGAP2;ATP7A;MAP4K4;MEF2A;PPP1R12A;BDNF;DTNBP1;ANK3;DICER1;ISL1;ZDHHC17;RAB10;AFG3L2;SHTN1;KLF7;CNTN4;SGK1;METAP1;METAP2
 
 
 
 
Protein Glycosylation ( GO:0006486 )
 
 
33/145
 
 
0.0001482
 
 
0.0075204
 
 
0
 
 
0
 
 
2.213991
 
 
19.52036
 
 
GALNT12;GALNT14;POGLUT1;RPN1;FUT4;TMEM165;FUT8;SERP1;FUT9;ST8SIA4;GCNT3;ST3GAL6;LARGE1;B3GALNT2;ARFGEF1;NUS1;GALNT5;GFPT2;GALNT1;GFPT1;PMM2;TET2;TET1;POMGNT1;B3GNT7;OSTC;DOLPP1;MGAT4A;EOGT;TET3;AQP11;POC1B-GALNT4;B4GALT5
 
 
 
 
 
 Reg of Transmembrane Receptor Prot Serine/Threonine Kinase Sgnlng
Pway ( GO:0090101 )
 
 
29/122
 
 
0.0001646
 
 
0.0082571
 
 
0
 
 
0
 
 
2.341646
 
 
20.40057
 
 
NOTCH1;TNFAIP6;HDAC1;IGSF1;UBE2D3;UBE2D1;LDLRAD4;LRP2;SPRED1;SAP30L;PRDM16;RBPMS2;VWC2L;RBBP7;WNT1;FST;MAGI2;NOG;CRIM1;TET1;LEMD3;SIRT1;VASN;GREM1;ADAM17;VEPH1;SPRY2;PPARG;SPRY1
 
 
 
 
Cellular Response to Hypoxia ( GO:0071456 )
 
 
21/77
 
 
0.0001701
 
 
0.0084009
 
 
0
 
 
0
 
 
2.812340
 
 
24.40865
 
 
SUV39H2;NOTCH1;SUV39H1;GATA6;RORA;HIF3A;ADO;SIRT1;RTN4;ZFP36L1;VEGFA;NPEPPS;SFRP1;CPEB1;TBL2;UBQLN1;PMAIP1;PPARG;MDM4;VHL;TP53
 
 
 
 
Blood Vessel Morphogenesis ( GO:0048514 )
 
 
16/51
 
 
0.0001712
 
 
0.0084009
 
 
0
 
 
0
 
 
3.425166
 
 
29.70502
 
 
NRP1;SERPINF2;LRP5;PROX1;TGFBR1;VEGFA;EFNB2;SHH;LOX;COL4A1;WT1;HEY2;ID1;AGGF1;HOXA1;CCM2
 
 
 
 
Positive Regulation of Multicellular Organismal Process ( GO:0051240 )
 
 
73/407
 
 
0.0001757
 
 
0.0085293
 
 
0
 
 
0
 
 
1.649806
 
 
14.26502
 
 
ACHE;SERPINE1;SIX1;ENO1;TNF;LOXL2;SCAMP5;SHH;GLIPR2;GRB10;IL12B;SOX5;ACVR1;NCOA1;KDM6B;SS18;PRKAB2;ACSL1;IL15;FST;SERPINF2;ELOVL3;NOG;RFX3;TET1;EBF2;SOX11;ELOVL6;SIRT1;ETV4;ADRA2A;TGFBR1;TIAM1;VAMP7;IFNG;IRF4;PPARG;B4GALT5;CRB2;PRKAA1;KDM3A;HDAC1;LEF1;LPL;TNFRSF11A;MACROH2A1;ADIPOR2;RTN4;GHRHR;SAP30L;PDGFC;PRDM16;ALX1;OTX2;RBBP7;RREB1;LDLR;TCF7L2;SMAD4;TGFB2;ESRRG;INHBB;VEGFA;COL1A1;GATM;REST;SCD;TNFSF4;AGO2;MFAP2;SNAI1;ALPL;FERMT2
 
 
 
 
External Encapsulating Structure Organization ( GO:0045229 )
 
 
27/111
 
 
0.0001817
 
 
0.0087239
 
 
0
 
 
0
 
 
2.412914
 
 
20.78270
 
 
ITGB1;COL15A1;COL16A1;COL11A1;ADAMTS10;COL19A1;ADAMTS17;ATP7A;ADAMTS9;ADAMTS7;COL22A1;NPNT;COLQ;COL3A1;COL1A2;COL2A1;COL4A2;MMP16;COL4A1;MMP15;COL7A1;COL5A2;PXDN;COL9A1;COL4A5;COL8A1;MATN3
 
 
 
 
Sphingolipid Biosynthetic Process ( GO:0030148 )
 
 
22/83
 
 
0.0001891
 
 
0.0089600
 
 
0
 
 
0
 
 
2.705149
 
 
23.19182
 
 
CERS4;ASAH1;CERS6;ELOVL4;PPM1L;SGMS1;ELOVL2;ELOVL3;ORMDL1;ELOVL6;SGMS2;TLCD3B;FUT4;UGCG;SPTLC2;FUT9;ORMDL2;ORMDL3;SPTSSB;SPTSSA;LARGE1;B4GALT6
 
 
 
 
Neuron Development ( GO:0048666 )
 
 
35/159
 
 
0.0001906
 
 
0.0089600
 
 
0
 
 
0
 
 
2.121297
 
 
18.16918
 
 
RB1;CNTNAP2;BTG2;NRXN1;STMN2;PTPRM;PTPRK;UGCG;BLOC1S6;ATCAY;UNK;BLOC1S1;FLRT3;STMN1;STX3;ATP7A;LGI1;NTRK2;TGFB2;DTNBP1;ETV1;RUNX1;NEDD4;PPT1;RAPGEF2;CNTN4;MAPT;ATXN10;ERCC6;B4GALT6;MAPRE2;PALS1;B4GALT5;CDK5R1;METAP2
 
 
 
 
Regulation of Release of Cytochrome C From Mitochondria ( GO:0090199 )
 
 
13/37
 
 
0.0001945
 
 
0.0090483
 
 
0
 
 
0
 
 
4.055821
 
 
34.65640
 
 
HRK;GHITM;TRIAP1;IGF1;MLLT11;BCL2L11;PLSCR3;PPIF;PMAIP1;BMF;BAK1;SOS1;TP53
 
 
 
 
Neuron Fate Commitment ( GO:0048663 )
 
 
8/16
 
 
0.0002018
 
 
0.0092886
 
 
0
 
 
0
 
 
7.478574
 
 
63.62981
 
 
DLL4;SMAD4;SHH;FOXN4;DLL1;ISL1;TGFBR1;ATOH1
 
 
 
 
Positive Regulation of Ion Transmembrane Transporter Activity ( GO:0032414 )
 
 
9/20
 
 
0.0002200
 
 
0.0100222
 
 
0
 
 
0
 
 
6.120389
 
 
51.54598
 
 
EDNRA;TRPC6;IFNG;STK39;CCL2;ANK2;ANK3;COA8;CTSS
 
 
 
 
Negative Regulation of Multicellular Organismal Process ( GO:0051241 )
 
 
48/243
 
 
0.0002343
 
 
0.0105669
 
 
0
 
 
0
 
 
1.852794
 
 
15.48739
 
 
RB1;FOXA1;ITGB1;NOTCH1;PPP1R11;HDAC1;LAMA4;WWC2;PTEN;STC1;PCTP;LDLRAD4;HMGCR;TNF;ROBO1;LOXL2;CORO1C;PPP2CA;SHH;SPRED1;CHSY1;SAP30L;NPTN;RBBP7;MAP2K6;MARK1;WNT10B;MYOCD;SFMBT1;IL15;ARRDC3;PLCL2;WNT9B;TET1;TBX5;VASN;QKI;BMP3;KLF7;SFRP1;ADAM17;IFNG;NOVA1;ID1;SPRY2;SPRY1;LGR4;ASB1
 
 
 
 
Ras Protein Signal Transduction ( GO:0007265 )
 
 
19/68
 
 
0.0002428
 
 
0.0108401
 
 
0
 
 
0
 
 
2.906676
 
 
24.19331
 
 
RB1;PLK2;RRAS2;IGF1;RAPGEFL1;RASGRP4;CCNA2;SHTN1;NRAS;MRAS;RRAS;GRAP2;RAPGEF2;GRB2;RAPGEF5;SOS1;SOS2;TP53;RALGDS
 
 
 
 
Ventricular Septum Development ( GO:0003281 )
 
 
13/38
 
 
0.0002639
 
 
0.0116682
 
 
0
 
 
0
 
 
3.893367
 
 
32.08055
 
 
SMAD4;TGFB2;NOTCH1;NOG;SOX11;PROX1;TBX5;TGFBR1;ROBO1;SALL1;HEY2;MDM4;SOX4
 
 
 
 
Negative Regulation of Translation ( GO:0017148 )
 
 
25/102
 
 
0.0002732
 
 
0.0117768
 
 
0
 
 
0
 
 
2.436175
 
 
19.98928
 
 
TRIM71;DDX6;BTG2;DDX3X;FMR1;PAIP2B;DNPEP;UNK;EIF4EBP1;RPS3;EIF4EBP2;EIF4EBP3;PAIP2;C8ORF88;NANOS1;QKI;CPEB1;LIN28A;AGO4;WT1;AGO2;SERBP1;EIF4E2;RBM24;TNRC6A
 
 
 
 
Negative Regulation of Apoptotic Signaling Pathway ( GO:2001234 )
 
 
20/74
 
 
0.0002748
 
 
0.0117768
 
 
0
 
 
0
 
 
2.776751
 
 
22.76779
 
 
RB1;ACVR1;TCF7L2;DDX3X;BDNF;SIAH2;GHITM;TRIAP1;IGF1;AREG;TNF;TGFBR1;NR4A2;PPIF;BCL2;TMBIM6;BAK1;SOS1;ZMYND11;SLC25A4
 
 
 
 
 
 Reg of Phosphatidylinositol 3-Kinase/Prot Kinase B Signal
Transduction ( GO:0051897 )
 
 
35/162
 
 
0.0002787
 
 
0.0117768
 
 
0
 
 
0
 
 
2.070832
 
 
16.95071
 
 
ITGB1;CSF1R;PHB1;NRXN1;EFNA5;TNF;RTN4;TXN2;MTDH;CALCR;ERBB3;PDGFC;NTF3;PDGFRB;PDGFRA;NTRK2;TCF7L2;TGFB2;SEMA4D;IGF1;SIRT1;TGFBR1;VEGFA;MFHAS1;LIN28A;PIK3CA;AXL;NEDD4;TYRO3;SPRY2;CCM2;TEK;SOS1;FERMT2;HBEGF
 
 
 
 
Cellular Response to Glucose Starvation ( GO:0042149 )
 
 
14/43
 
 
0.0002796
 
 
0.0117768
 
 
0
 
 
0
 
 
3.615248
 
 
29.58072
 
 
SH3GLB1;PRKAA1;SUV39H1;FOXO3;SIRT1;YWHAZ;NUAK2;TBL2;KAT5;SESN1;SESN2;PMAIP1;HNRNPA1;TP53
 
 
 
 
Positive Regulation of Stress Fiber Assembly ( GO:0051496 )
 
 
14/43
 
 
0.0002796
 
 
0.0117768
 
 
0
 
 
0
 
 
3.615248
 
 
29.58072
 
 
NRP1;CARMIL1;LIMK1;SERPINF2;TPM1;RHOC;PPM1F;TGFBR1;AMOT;CDC42;SFRP1;PXDN;CCN2;FERMT2
 
 
 
 
Ubiquitin-Dependent Protein Catabolic Process ( GO:0006511 )
 
 
79/455
 
 
0.0002837
 
 
0.0118403
 
 
0
 
 
0
 
 
1.586276
 
 
12.95581
 
 
TRIM71;UBXN1;PPP1R11;UBE2D2;UBE2D3;UBE2D1;TNFAIP3;ARRB1;CDC23;KAT5;RNF19A;CHMP1B;SKP1;FBXW4;FBXW7;VPS37C;VPS37B;FBXO10;SIRT1;KCTD5;RNF168;RNF144B;PSMA4;PSMA1;UBE2R2;TBL1XR1;HECW2;CHMP4B;PLAA;CHMP7;PSMD12;RNF34;CUL5;UBA6;CUL3;VPS4B;FBXO45;ASB11;RNF7;RCHY1;SOCS2;PCNP;ZNRF2;RMND5A;KCTD21;TBX21;RNF217;UBQLN1;VHL;KLHL29;NDFIP2;UBE2H;UBE2I;UBE2B;CSNK1A1;USP9X;FEM1B;FBXL19;SIAH2;KLHL28;SYVN1;KLHL2;UBE2G1;PSMB8;CHFR;PSMB9;RNF145;UBE2W;APPBP2;KLHL6;NEDD4;GID8;KLHL8;RLIM;UBE2N;RNF186;UBE2K;SPSB4;ASB1
 
 
 
 
Negative Regulation of Cardiac Muscle Hypertrophy ( GO:0010614 )
 
 
7/13
 
 
0.0002872
 
 
0.0118597
 
 
0
 
 
0
 
 
8.722293
 
 
71.13175
 
 
MLIP;SMAD4;RGS2;NOTCH1;CAV3;ATP2B4;PPARG
 
 
 
 
Cellular Response to Growth Factor Stimulus ( GO:0071363 )
 
 
33/150
 
 
0.0002895
 
 
0.0118597
 
 
0
 
 
0
 
 
2.118771
 
 
17.26224
 
 
NRP1;NOTCH1;HDAC1;STMN2;LRP2;ZFP36L2;ZFP36L1;DLL4;RAP1A;CCL2;SOX5;ADAMTS7;ACVR1;NANOS1;DDX18;SMAD4;SEMA6A;EGR3;PLK5;MAGI2;KLF4;MAPK14;TGFBR1;ACVR2A;VEGFA;SLC4A7;SFRP1;GAS1;RAPGEF2;SPRY2;MAPT;SOS1;LIMS1
 
 
 
 
Regulation of TORC1 Signaling ( GO:1903432 )
 
 
24/97
 
 
0.0003085
 
 
0.0125225
 
 
0
 
 
0
 
 
2.466385
 
 
19.93770
 
 
PRKAA1;SAR1A;USP4;USP32;CSNK1A1;CUL3;UBE2D1;TSC1;C9ORF72;YWHAZ;GPR137C;UBE2W;RRAGC;MAT2A;SESN1;SESN2;TBC1D7;UBE2N;WAC;EP300;SLC38A9;VHL;RNF152;OTUB1
 
 
 
 
Stress Granule Assembly ( GO:0034063 )
 
 
10/25
 
 
0.0003182
 
 
0.0128016
 
 
0
 
 
0
 
 
4.987969
 
 
40.16652
 
 
DAZAP2;DDX6;ATXN2;DDX3X;FMR1;CSDE1;UBAP2L;MAPT;C9ORF72;EIF2S1
 
 
 
 
Regulation of Cell Differentiation ( GO:0045595 )
 
 
41/201
 
 
0.0003233
 
 
0.0128904
 
 
0
 
 
0
 
 
1.926837
 
 
15.48560
 
 
CCNT2;HDAC1;LEF1;SIX1;NREP;AREG;TNF;DLL1;PTHLH;ZFP36L1;PPP2CA;NEUROD1;SHH;RPS6KA1;HEY2;CCN4;FLOT2;CCN2;RBBP7;VHL;ARID2;MTA2;ZNF268;DDX17;SS18;SERPINF2;LIF;MBOAT2;KLF4;ISL1;ARID1B;BLTP1;QKI;TGFBR1;RUNX1;GREM1;TIAM1;REST;BIN1;ANP32B;CDK5R1
 
 
 
 
Positive Regulation of Apoptotic Process ( GO:0043065 )
 
 
58/313
 
 
0.0003326
 
 
0.0130671
 
 
0
 
 
0
 
 
1.713523
 
 
13.72266
 
 
HRK;FOXA1;ITGB1;PHB1;TNFAIP8;BTG1;DDX3X;TNF;ING5;CASP7;LGALS1;AIFM1;CASP2;PHLDA1;HOXA5;SOX4;MAP3K5;NANOS1;KLF11;F3;SIRT1;TGFBR1;SFRP1;MELK;BIN1;PTRH2;PPARG;IRF5;TP53;FOXO3;HSPD1;NEUROD1;BCL2L11;PNMA1;RPS3;PMAIP1;CCL2;BMF;BAK1;ZNF268;MCL1;SRPK2;WNT10B;PRNP;TGFB2;GADD45A;PNMA2;MLLT11;REST;RYBP;WT1;PTPRA;JMY;BCL2;MAP3K10;BCL2L2;MAP3K11;CDK5R1
 
 
 
 
Plasma Membrane Organization ( GO:0007009 )
 
 
20/75
 
 
0.0003336
 
 
0.0130671
 
 
0
 
 
0
 
 
2.726110
 
 
21.82383
 
 
CAV3;VPS4B;ANK2;ANK3;FNBP1L;UGCG;EHD2;SNX18;SERP1;BIN2;PLSCR3;COL5A1;BIN1;CHMP1B;CHMP4B;MYH9;STX4;WHAMM;CHMP7;ARL8B
 
 
 
 
Regulation of Anatomical Structure Morphogenesis ( GO:0022603 )
 
 
29/127
 
 
0.0003417
 
 
0.0131435
 
 
0
 
 
0
 
 
2.221541
 
 
17.73140
 
 
CRB2;BRSK1;POGLUT1;SPARC;TNFAIP3;EFNA5;DLL1;LFNG;PDCD10;ZMYM4;SH3D19;MARCHF5;CDX2;SEMA4D;VASH2;CYRIB;LARP4;TANC2;WARS1;SFRP1;FMNL2;ADGRB3;HECW2;NEDD4;CCM2;MAPT;FERMT2;EPHA2;CDK5R1
 
 
 
 
Negative Regulation of Anoikis ( GO:2000811 )
 
 
8/17
 
 
0.0003417
 
 
0.0131435
 
 
0
 
 
0
 
 
6.647245
 
 
53.05547
 
 
ITGB1;NOTCH1;PIK3CA;PTRH2;PDK4;PIK3R3;ZNF304;MCL1
 
 
 
 
Cellular Response to Nerve Growth Factor Stimulus ( GO:1990090 )
 
 
9/21
 
 
0.0003447
 
 
0.0131435
 
 
0
 
 
0
 
 
5.610038
 
 
44.72808
 
 
NTRK2;RAP1A;BDNF;MAGI2;NTF3;STMN2;RAPGEF2;MAPT;WASF1
 
 
 
 
Regulation of Osteoblast Differentiation ( GO:0045667 )
 
 
23/92
 
 
0.0003474
 
 
0.0131435
 
 
0
 
 
0
 
 
2.500142
 
 
19.91405
 
 
NOTCH1;TNFAIP6;SEMA4D;CTNNBIP1;NOG;PTEN;LRP5;CRIM1;SUCO;SOX11;RORB;IGF1;NPNT;IPO7;ACVR2A;GREM1;REST;CCN4;PPARG;IL6ST;SOS1;IL6R;FERMT2
 
 
 
 
Positive Regulation of Epithelial to Mesenchymal Transition ( GO:0010718 )
 
 
15/49
 
 
0.0003658
 
 
0.0137260
 
 
0
 
 
0
 
 
3.304318
 
 
26.14827
 
 
CRB2;TCF7L2;SMAD4;TGFB2;NOTCH1;JAG1;LEF1;TGFBR1;LOXL2;COL1A1;TIAM1;GLIPR2;SNAI1;ALX1;FERMT2
 
 
 
 
Positive Regulation of Chondrocyte Differentiation ( GO:0032332 )
 
 
6/10
 
 
0.0003733
 
 
0.0138897
 
 
0
 
 
0
 
 
11.210894
 
 
88.48918
 
 
PKDCC;GDF6;GDF5;HOXA11;LOXL2;SOX5
 
 
 
 
Cell Migration Involved in Heart Development ( GO:0060973 )
 
 
5/7
 
 
0.0003934
 
 
0.0141646
 
 
0
 
 
0
 
 
18.679025
 
 
146.45758
 
 
PDGFRB;NOTCH1;FLRT3;PITX2;TBX5
 
 
 
 
Coronary Vasculature Morphogenesis ( GO:0060977 )
 
 
5/7
 
 
0.0003934
 
 
0.0141646
 
 
0
 
 
0
 
 
18.679025
 
 
146.45758
 
 
NRP1;SPRED1;NOTCH1;TGFBR1;VEGFA
 
 
 
 
Negative Regulation of Muscle Hypertrophy ( GO:0014741 )
 
 
5/7
 
 
0.0003934
 
 
0.0141646
 
 
0
 
 
0
 
 
18.679025
 
 
146.45758
 
 
MLIP;RGS2;SMAD4;NOTCH1;CAV3
 
 
 
 
Regulation of Extracellular Matrix Constituent Secretion ( GO:0003330 )
 
 
5/7
 
 
0.0003934
 
 
0.0141646
 
 
0
 
 
0
 
 
18.679025
 
 
146.45758
 
 
NOTCH1;RIC1;IER3IP1;TNFRSF1B;TNFRSF1A
 
 
 
 
Regulation of ERK1 and ERK2 Cascade ( GO:0070372 )
 
 
44/223
 
 
0.0004271
 
 
0.0152549
 
 
0
 
 
0
 
 
1.848712
 
 
14.34335
 
 
NOTCH2;NRP1;CSF1R;PHB1;SEMA7A;NOTCH1;RGS14;NRXN1;HTR2C;ARRB1;TNFRSF11A;SLC30A10;TNF;SPRED1;CALCR;RAP1A;RRAS;GLIPR2;PDGFC;CCN2;PDGFRB;PDGFRA;MAP2K1;SMAD4;SEMA6A;FBXW7;SERPINF2;STYX;IGF1;NPNT;DUSP9;ZDHHC17;YWHAZ;MTURN;VEGFA;MFHAS1;NEK10;RAPGEF2;SPRY2;SPRY1;TEK;FERMT2;EPHA2;MAP3K12
 
 
 
 
Positive Regulation of Cell-Matrix Adhesion ( GO:0001954 )
 
 
14/45
 
 
0.0004739
 
 
0.0167927
 
 
0
 
 
0
 
 
3.381622
 
 
25.88482
 
 
NRP1;COL16A1;LEF1;TSC1;THY1;PPM1F;VEGFA;SFRP1;RRAS;TEK;GFUS;FERMT2;MAP4K4;LIMS1
 
 
 
 
Endoplasmic Reticulum to Golgi Vesicle-Mediated Transport ( GO:0006888 )
 
 
26/112
 
 
0.0005171
 
 
0.0179424
 
 
0
 
 
0
 
 
2.268282
 
 
17.16470
 
 
STEEP1;ARF4;LMAN2L;SAR1A;STX17;CUL3;GOSR2;MIA3;IER3IP1;LMAN1;SPAST;LMAN2;TMED1;WHAMM;TMED7;TMED5;TMED4;TRAPPC8;VAMP7;TRAPPC6B;VAPB;PREB;ERGIC2;SEC22A;ERGIC1;BET1
 
 
 
 
Cell Migration Involved in Sprouting Angiogenesis ( GO:0002042 )
 
 
9/22
 
 
0.0005224
 
 
0.0179424
 
 
0
 
 
0
 
 
5.178203
 
 
39.13208
 
 
EFNB2;GREM1;NRP1;CDH5;EGR3;PIK3R3;MIA3;ROBO1;VEGFA
 
 
 
 
Negative Regulation of Cell Development ( GO:0010721 )
 
 
9/22
 
 
0.0005224
 
 
0.0179424
 
 
0
 
 
0
 
 
5.178203
 
 
39.13208
 
 
REST;NOTCH1;CAV3;DYNLT1;RFLNB;HOOK3;IGF1;TNF;PTHLH
 
 
 
 
Regulation of Anoikis ( GO:2000209 )
 
 
9/22
 
 
0.0005224
 
 
0.0179424
 
 
0
 
 
0
 
 
5.178203
 
 
39.13208
 
 
ITGB1;NOTCH1;PIK3CA;PTRH2;BCL2;PDK4;PIK3R3;ZNF304;MCL1
 
 
 
 
Regulation of Phosphatidylinositol 3-Kinase/Protein Kinase B Signal
Transduction ( GO:0051896 )
 
 
43/219
 
 
0.0005484
 
 
0.0183471
 
 
0
 
 
0
 
 
1.837016
 
 
13.79335
 
 
ITGB1;CSF1R;PHB1;NRXN1;PTEN;EFNA5;TNF;RTN4;MTDH;TXN2;PPP2CA;CALCR;RRAS;ERBB3;PDGFC;NTF3;PIP5K1A;PIP5K1B;PDGFRB;PDGFRA;RUBCN;NTRK2;TCF7L2;TGFB2;SEMA4D;MAGI2;IGF1;SIRT1;TGFBR1;VEGFA;MFHAS1;LIN28A;PIK3CA;AXL;NEDD4;TYRO3;SPRY2;CCM2;TEK;DIPK2A;SOS1;FERMT2;HBEGF
 
 
 
 
Cardiac Atrium Morphogenesis ( GO:0003209 )
 
 
8/18
 
 
0.0005515
 
 
0.0183471
 
 
0
 
 
0
 
 
5.982181
 
 
44.88355
 
 
NOTCH2;DLL4;TGFB2;NOTCH1;NOG;HEY2;PROX1;ISL1
 
 
 
 
Negative Regulation of Translational Initiation ( GO:0045947 )
 
 
8/18
 
 
0.0005515
 
 
0.0183471
 
 
0
 
 
0
 
 
5.982181
 
 
44.88355
 
 
PAIP2B;FMR1;EIF4EBP1;EIF4EBP2;EIF4EBP3;EIF4E2;PAIP2;C8ORF88
 
 
 
 
Positive Regulation of Muscle Cell Differentiation ( GO:0051149 )
 
 
8/18
 
 
0.0005515
 
 
0.0183471
 
 
0
 
 
0
 
 
5.982181
 
 
44.88355
 
 
NOTCH2;MYOCD;NOTCH1;LAMA2;LAMC1;MAPK14;NID1;MAPK12
 
 
 
 
Intracellular Protein Transport ( GO:0006886 )
 
 
59/326
 
 
0.0005547
 
 
0.0183471
 
 
0
 
 
0
 
 
1.664299
 
 
12.47730
 
 
STEEP1;ARF4;PPP1R10;CSE1L;IPO7;RAB22A;RAB43;IPO8;ARL5B;XPO4;XPO5;ARFIP1;TMED1;KPNA5;TMED7;TNPO1;VPS36;TMED5;TNPO3;TRIM23;RAB8B;TMED4;TOMM70;VPS37C;VPS37B;COPZ1;ARL4A;NUP50;TSNARE1;STX1A;ARF5;KPNB1;ARF6;NAPB;RAB5B;STX17;STX16;TIMM9;DNAJC27;STX11;GGA2;LMAN1;POM121C;SNX27;GRPEL2;RAB24;STX6;STX4;E2F3;STX3;CTSA;TIMM8B;RIC1;STAT3;RAB14;SNX17;SNX15;BCL3;SPRN
 
 
 
 
Epithelial to Mesenchymal Transition ( GO:0001837 )
 
 
15/51
 
 
0.0005890
 
 
0.0191655
 
 
0
 
 
0
 
 
3.120390
 
 
23.20679
 
 
DDX17;EOMES;SMAD4;TGFB2;NOTCH1;NOG;LEF1;IGF1;TGFBR1;LOXL2;HEY2;TASOR;SNAI1;SOS1;LIMS1
 
 
 
 
Cellular Response to Vascular Endothelial Growth Factor Stimulus ( GO:0035924 )
 
 
12/36
 
 
0.0005923
 
 
0.0191655
 
 
0
 
 
0
 
 
3.742244
 
 
27.81016
 
 
DLL4;NRP1;NUS1;NOTCH1;EGR3;SEMA6A;PIK3CA;VEGFC;GAS1;SPRY2;MAPK14;VEGFA
 
 
 
 
Negative Regulation of Epithelial to Mesenchymal Transition ( GO:0010719 )
 
 
12/36
 
 
0.0005923
 
 
0.0191655
 
 
0
 
 
0
 
 
3.742244
 
 
27.81016
 
 
FOXA1;PPP2CA;SPRED1;SFRP1;NOG;PTEN;SPRY2;SPRY1;LDLRAD4;TBX5;VASN;MARK1
 
 
 
 
Mitotic Cell Cycle Phase Transition ( GO:0044772 )
 
 
26/113
 
 
0.0005973
 
 
0.0191878
 
 
0
 
 
0
 
 
2.242082
 
 
16.64304
 
 
TRIM71;BRSK1;USP37;CCNJ;CUL5;CUL3;ARPP19;PHF8;CCND2;CDC23;SPAST;ENSA;EIF4EBP1;E2F3;PLK2;CDC25B;HINFP;CCNA2;MELK;CCNE2;CCNY;CCNG1;CHMP4B;CHMP7;CCNJL;CUL4B
 
 
 
 
Negative Regulation of Cellular Process ( GO:0048523 )
 
 
88/531
 
 
0.0006374
 
 
0.0203289
 
 
0
 
 
0
 
 
1.499831
 
 
11.03590
 
 
RB1;RIPOR2;PHB1;BTG2;BTG1;DDX3X;SUV39H1;WWC2;PTEN;MIA3;ENO1;CTCF;PTPRK;TM7SF3;AREG;IPO7;TNF;CDC73;ING5;SHH;RB1CC1;AKT3;SESN2;ENPP1;TMEM109;ARID2;DIS3L2;SS18;KLF11;MAP2K1;MYOCD;MAGI2;P3H1;ADAM10;TSC1;PROX1;SIRT1;CBFA2T3;CTTNBP2NL;FRMD5;SFRP1;DDAH1;ALDH1A2;RAPGEF2;MYH9;PPARG;GMNC;TP53;NOTCH1;PDS5B;PPM1D;DLL1;CDKN2AIP;RTN4;PTHLH;DLL4;SERTAD3;ERBB3;CLMN;TP53INP1;ZKSCAN3;RBBP7;VHL;WNT1;ZNF268;RBM38;CGREF1;SMAD4;TGFB2;SEMA4D;THAP12;OSGIN2;TBX5;ST18;WARS1;REST;CNOT7;XPNPEP1;WT1;PRKRA;PPT1;AQP11;MDM4;CCM2;ANP32B;CRLF3;HSPA1B;SIPA1
 
 
 
 
Negative Regulation of Cellular Response to Growth Factor Stimulus ( GO:0090288 )
 
 
19/73
 
 
0.0006461
 
 
0.0203870
 
 
0
 
 
0
 
 
2.636789
 
 
19.36593
 
 
NOTCH1;CADM4;TNFAIP6;UBE2D3;NOG;ATP2B4;CRIM1;UBE2D1;LRP2;LEMD3;GREM1;CREB3L1;NEDD4;RBPMS2;VWC2L;SPRY2;PPARG;SPRY1;WNT1
 
 
 
 
Negative Regulation of miRNA Metabolic Process ( GO:2000629 )
 
 
10/27
 
 
0.0006575
 
 
0.0203870
 
 
0
 
 
0
 
 
4.400649
 
 
32.24386
 
 
ZNF512B;NCOR2;REST;RARA;PPARG;TENT2;TNF;ESR1;QKI;VEGFA
 
 
 
 
Negative Regulation of Neurogenesis ( GO:0050768 )
 
 
10/27
 
 
0.0006575
 
 
0.0203870
 
 
0
 
 
0
 
 
4.400649
 
 
32.24386
 
 
REST;NOTCH1;DYNLT1;DPYSL5;GORASP1;PTEN;RAPGEF2;THY1;HOOK3;TNF
 
 
 
 
Regulation of Cation Channel Activity ( GO:2001257 )
 
 
10/27
 
 
0.0006575
 
 
0.0203870
 
 
0
 
 
0
 
 
4.400649
 
 
32.24386
 
 
ITGB1;SELENON;EDNRA;JPH3;HOMER1;ASPH;ANK2;ANK3;CTSS;VAMP2
 
 
 
 
Negative Regulation of Response to Stimulus ( GO:0048585 )
 
 
16/57
 
 
0.0006936
 
 
0.0213569
 
 
0
 
 
0
 
 
2.922925
 
 
21.26040
 
 
HHIP;THY1;KLF4;SIRT1;C9ORF72;MACROH2A1;ADRA2A;SOCS2;KLF7;COL3A1;DDAH1;ERBB3;KIF26A;RPS3;AQP11;VHL
 
 
 
 
Negative Regulation of Intracellular Transport ( GO:0032387 )
 
 
6/11
 
 
0.0007390
 
 
0.0224461
 
 
0
 
 
0
 
 
8.968207
 
 
64.66283
 
 
SNX3;PLN;INSIG1;ARHGAP1;SNX12;ARFIP1
 
 
 
 
Progesterone Receptor Signaling Pathway ( GO:0050847 )
 
 
6/11
 
 
0.0007390
 
 
0.0224461
 
 
0
 
 
0
 
 
8.968207
 
 
64.66283
 
 
NCOA1;PHB1;WBP2;NEDD4;MAPK1;ZDHHC7
 
 
 
 
Membraneless Organelle Assembly ( GO:0140694 )
 
 
30/139
 
 
0.0007457
 
 
0.0224975
 
 
0
 
 
0
 
 
2.065811
 
 
14.87625
 
 
DDX6;DAZAP2;DDX3X;FMR1;MOSPD2;ZFYVE1;C9ORF72;AREG;FXR1;ATXN2;POGZ;RPS3;UBAP2L;TMOD1;TMOD3;NEK6;NEK7;INO80;EIF2S1;HAUS1;BIN2;PSPC1;CSDE1;CEP63;MAPT;SUGT1;MAPRE1;MAPRE2;TNRC6A;CDS2
 
 
 
 
Eye Morphogenesis ( GO:0048592 )
 
 
9/23
 
 
0.0007687
 
 
0.0229252
 
 
0
 
 
0
 
 
4.808058
 
 
34.47751
 
 
COL5A1;STAT3;COL5A2;MFAP2;SOX11;PPP2R3A;SOX4;VEGFA;FBN1
 
 
 
 
Negative Regulation of Cold-Induced Thermogenesis ( GO:0120163 )
 
 
14/47
 
 
0.0007727
 
 
0.0229252
 
 
0
 
 
0
 
 
3.176314
 
 
22.76030
 
 
RB1;WNT10B;NOTCH1;IL15;LAMA4;ARRDC3;PLCL2;PCTP;QKI;ADAM17;NOVA1;ID1;LGR4;MAP2K6
 
 
 
 
Regulation of mRNA Splicing, via Spliceosome ( GO:0048024 )
 
 
23/97
 
 
0.0007753
 
 
0.0229252
 
 
0
 
 
0
 
 
2.330550
 
 
16.69201
 
 
DDX17;SRPK2;DAZAP1;RBM25;RBM15;WTAP;FMR1;CELF2;CELF3;MAGOHB;QKI;REST;HNRNPK;NOVA1;RBPMS2;RBM20;RBM7;HNRNPA1;SRSF7;RBM24;SRSF10;SRSF9;SRSF12
 
 
 
 
Negative Regulation of Endothelial Cell Migration ( GO:0010596 )
 
 
12/37
 
 
0.0007833
 
 
0.0230093
 
 
0
 
 
0
 
 
3.592350
 
 
25.69249
 
 
DLL4;MECP2;NOTCH1;GADD45A;DNAJA4;ATP2B4;PTPRM;STC1;PPARG;NR2F2;TNF;ADAMTS9
 
 
 
 
DNA Damage Response ( GO:0006974 )
 
 
72/421
 
 
0.0007955
 
 
0.0232140
 
 
0
 
 
0
 
 
1.555240
 
 
11.09911
 
 
BTG2;CCNK;DYRK2;USP32;FMR1;SUV39H1;KAT5;TLK2;CASP2;KMT5B;ZNF385A;USP47;FBXW7;KMT5C;USP3;PRKCD;USP45;MSL2;PPP4R3A;BAZ1B;VRK3;TNFRSF1B;ZDHHC16;SIRT1;DDB2;FOXP1;RNF168;WAC;CEP63;MAPT;TP53;RBM24;ASF1A;EPHA2;OTUB1;BRSK1;UBA6;FBXO45;PDS5B;PDS5A;MACROH2A1;ZBTB4;UACA;CDKN2AIP;BCL2L11;RPS3;BAK1;WNT1;LDLR;MORC2;MAP2K6;MCL1;PLK5;SLF2;UBE2B;GADD45A;GTF2H1;INO80;MAPK14;MAPK12;HINFP;ACER2;NEDD4;RPA3;BCL3;POLE3;BCL2;NABP1;GRB2;ERCC6;BCL2L2;CUL4B
 
 
 
 
Skin Development ( GO:0043588 )
 
 
18/69
 
 
0.0008617
 
 
0.0248659
 
 
0
 
 
0
 
 
2.644277
 
 
18.65971
 
 
ERRFI1;CLIC4;ASAH1;ST14;JAG1;ABCB6;GATA6;DLL1;COL1A1;UGCG;COL3A1;COL1A2;COL5A1;COL5A2;PIP5K1A;ATP7A;AHDC1;EPHA2
 
 
 
 
Negative Regulation of Chondrocyte Differentiation ( GO:0032331 )
 
 
7/15
 
 
0.0008688
 
 
0.0248659
 
 
0
 
 
0
 
 
6.540978
 
 
46.10360
 
 
GREM1;NR5A2;RFLNB;GDF5;PTHLH;TGFBR1;ADAMTS7
 
 
 
 
Regulation of Establishment of Cell Polarity ( GO:2000114 )
 
 
7/15
 
 
0.0008688
 
 
0.0248659
 
 
0
 
 
0
 
 
6.540978
 
 
46.10360
 
 
ARFGEF1;RAP1B;KANK1;RIPOR2;CDH5;SHTN1;CYRIB
 
 
 
 
Positive Regulation of ERK1 and ERK2 Cascade ( GO:0070374 )
 
 
32/153
 
 
0.0008940
 
 
0.0254259
 
 
0
 
 
0
 
 
1.985342
 
 
13.93663
 
 
NOTCH2;CSF1R;NRP1;PHB1;SEMA7A;NOTCH1;NRXN1;HTR2C;ARRB1;TNFRSF11A;SLC30A10;TNF;CALCR;RAP1A;GLIPR2;PDGFC;CCN2;PDGFRB;PDGFRA;MAP2K1;FBXW7;SERPINF2;IGF1;NPNT;MTURN;VEGFA;MFHAS1;RAPGEF2;SPRY2;TEK;FERMT2;MAP3K12
 
 
 
 
Regulation of Angiogenesis ( GO:0045765 )
 
 
41/211
 
 
0.0009014
 
 
0.0254657
 
 
0
 
 
0
 
 
1.812456
 
 
12.70808
 
 
ITGB1;SPARC;BTG1;SERPINE1;GATA6;PTPRM;TNFAIP3;RTN4;MTDH;AMOT;MECP2;CDH5;SPRED1;RRAS;PDCD10;ZNF304;HOXA5;TGFB2;SEMA6A;VASH2;ATP2B4;FOXJ2;KLF4;ISL1;F3;SIRT1;QKI;WARS1;RUNX1;VEGFA;PDCL3;SFRP1;COL4A2;TSPAN18;AGO2;AGGF1;SPRY2;PPARG;CCM2;TEK;EPHA2
 
 
 
 
Negative Regulation of Protein Polymerization ( GO:0032272 )
 
 
10/28
 
 
0.0009153
 
 
0.0254657
 
 
0
 
 
0
 
 
4.155933
 
 
29.07604
 
 
KANK1;CDH5;TMSB4X;STMN1;PRKCD;STMN2;MYADM;CYRIB;MAPRE1;METAP2
 
 
 
 
Ventricular Septum Morphogenesis ( GO:0060412 )
 
 
10/28
 
 
0.0009153
 
 
0.0254657
 
 
0
 
 
0
 
 
4.155933
 
 
29.07604
 
 
SMAD4;TGFB2;NOTCH1;HEY2;NOG;SOX11;PROX1;SOX4;TGFBR1;ROBO1
 
 
 
 
Regulation of Focal Adhesion Assembly ( GO:0051893 )
 
 
15/53
 
 
0.0009187
 
 
0.0254657
 
 
0
 
 
0
 
 
2.955823
 
 
20.66861
 
 
NRP1;COL16A1;PTEN;TSC1;THY1;EFNA5;PPM1F;CORO1C;VEGFA;SFRP1;PTPRA;TEK;FERMT2;MAP4K4;LIMS1
 
 
 
 
Regulation of Stress Fiber Assembly ( GO:0051492 )
 
 
19/75
 
 
0.0009240
 
 
0.0254657
 
 
0
 
 
0
 
 
2.542329
 
 
17.76287
 
 
NRP1;CARMIL1;SERPINF2;TPM1;LIMK1;TSC1;PIK3R1;RHOC;TGFBR1;PPM1F;AMOT;CDC42;TJP1;SFRP1;STMN1;PXDN;PPFIA1;CCN2;FERMT2
 
 
 
 
Positive Regulation of Neurogenesis ( GO:0050769 )
 
 
17/64
 
 
0.0009527
 
 
0.0259395
 
 
0
 
 
0
 
 
2.709377
 
 
18.84697
 
 
NTRK2;NUMBL;RBM15;RGS14;FMR1;SS18L1;SOX11;NAP1L1;LRP2;ROBO1;VEGFA;FXR1;TIAM1;IFNG;SLITRK1;NPTN;NUMB
 
 
 
 
Cellular Response to Starvation ( GO:0009267 )
 
 
30/141
 
 
0.0009528
 
 
0.0259395
 
 
0
 
 
0
 
 
2.028358
 
 
14.10956
 
 
PRKAA1;SUV39H1;FOXO3;ZFYVE1;NUAK2;KAT5;SESN1;SESN2;PDK4;PMAIP1;MAPK1;HNRNPA1;RNF152;MAP3K5;MFSD2A;SH3GLB1;SREBF1;TFEB;TSC1;INHBB;EIF2S1;SIRT1;YWHAZ;SFRP1;TBL2;RRAGC;STK26;JMY;TBC1D7;TP53
 
 
 
 
Positive Regulation of Developmental Process ( GO:0051094 )
 
 
46/245
 
 
0.0009790
 
 
0.0264917
 
 
0
 
 
0
 
 
1.738004
 
 
12.04264
 
 
PRKAA1;TFRC;HDAC1;FMR1;NRXN1;LEF1;LPL;AREG;TNF;RTN4;CORO1C;NEUROD1;SHH;SAP30L;RPS6KA1;ENPP2;CCN4;KMT5B;OTX2;CCN2;RBBP7;VHL;ARID2;RREB1;ZNF268;SOX5;MARCHF5;REPS1;ACVR1;NCOA1;SS18;FZD4;KMT5C;FST;SERPINF2;TET1;ISL1;SIRT1;ARID1B;TGFBR1;VEGFA;REST;WT1;AGO2;PPARG;B4GALT5
 
 
 
 
Embryonic Skeletal System Morphogenesis ( GO:0048704 )
 
 
11/33
 
 
0.0009956
 
 
0.0266192
 
 
0
 
 
0
 
 
3.741079
 
 
25.85891
 
 
PDGFRA;COL2A1;NOG;HOXA3;HOXB4;SIX1;HOXB3;SOX11;TGFBR1;HOXA11;HOXA4
 
 
 
 
Vasculogenesis ( GO:0001570 )
 
 
11/33
 
 
0.0009956
 
 
0.0266192
 
 
0
 
 
0
 
 
3.741079
 
 
25.85891
 
 
PDGFRB;NRP1;SHH;SPRED1;NOTCH1;WT1;HEY2;AGGF1;CCM2;RTN4;VEGFA
 
 
 
 
Regulation of Endothelial Cell Proliferation ( GO:0001936 )
 
 
21/87
 
 
0.0010078
 
 
0.0267270
 
 
0
 
 
0
 
 
2.384870
 
 
16.45549
 
 
NRP1;SPARC;EGR3;CAV2;HMGB2;VASH2;PTPRM;MIA3;NRARP;PROX1;NR2F2;F3;SIRT1;TGFBR1;VEGFA;PDCL3;NRAS;SLC39A9;AKT3;AGGF1;TEK
 
 
 
 
Regulation of Peptidyl-Tyrosine Phosphorylation ( GO:0050730 )
 
 
13/43
 
 
0.0010116
 
 
0.0267270
 
 
0
 
 
0
 
 
3.243552
 
 
22.36821
 
 
PGAP1;CADM4;IL15;CD80;LIF;EFNA5;VEGFA;PDCL3;SFRP1;CNOT7;RAP2B;ENPP2;CCM2
 
 
 
 
Positive Regulation of Autophagy ( GO:0010508 )
 
 
23/99
 
 
0.0010453
 
 
0.0272552
 
 
0
 
 
0
 
 
2.268962
 
 
15.57289
 
 
SH3GLB1;PRKAA1;PLK2;TFEB;FOXO3;PIK3C2A;C9ORF72;SIRT1;SVIP;MTDH;KAT5;CCNY;IFNG;SESN1;RB1CC1;SESN2;ORMDL3;TP53INP1;WAC;MAP3K7;RNF152;PAFAH1B2;TRIM23
 
 
 
 
Neuron Projection Development ( GO:0031175 )
 
 
40/206
 
 
0.0010482
 
 
0.0272552
 
 
0
 
 
0
 
 
1.810494
 
 
12.42128
 
 
RB1;CNTNAP2;BTG2;CTNND2;NRXN1;STMN2;PTEN;PTPRM;PTPRK;NYAP2;NEUROD1;CDC42;BLOC1S6;ATCAY;GRM7;BLOC1S1;FLRT3;STMN1;NTF3;STX3;SRGAP2;ATP7A;ATOH1;MAP4K4;LGI1;PPP1R12A;BDNF;VASH2;DTNBP1;DICER1;SHTN1;NEDD4;RAPGEF2;MAPT;CNTN4;ATXN10;ERCC6;SGK1;CDK5R1;METAP2
 
 
 
 
Positive Regulation of Protein-Containing Complex Assembly ( GO:0031334 )
 
 
24/105
 
 
0.0010499
 
 
0.0272552
 
 
0
 
 
0
 
 
2.221779
 
 
15.23928
 
 
SH3GLB1;HRK;DDX3X;SLF2;TFRC;NEK7;RHOC;TNF;WARS1;VEGFA;RAP1B;PPP2CA;CDH5;MAVS;BCL2L11;DHX33;IFNG;STMP1;FSCN1;RPS3;BMF;BAK1;TP53;FERMT2
 
 
 
 
Glycoprotein Biosynthetic Process ( GO:0009101 )
 
 
19/76
 
 
0.0010976
 
 
0.0281137
 
 
0
 
 
0
 
 
2.497584
 
 
17.02021
 
 
ARFGEF1;B3GALNT2;NUS1;CSGALNACT2;GCNT1;PMM2;IGF1;FUT4;SERP1;CHST12;FUT9;MGAT4A;CHST10;ST8SIA4;AQP11;ST3GAL6;ATP7A;LARGE1;B4GALT5
 
 
 
 
Negative Regulation of Supramolecular Fiber Organization ( GO:1902904 )
 
 
19/76
 
 
0.0010976
 
 
0.0281137
 
 
0
 
 
0
 
 
2.497584
 
 
17.02021
 
 
ARFGEF1;KANK1;PLEKHH2;SMAD4;ARPIN-AP3S2;CAV3;PRKCD;STMN2;CYRIB;CDH5;NAV3;PIK3CA;TMSB4X;STMN1;MYADM;MAPRE1;LDLR;PFDN6;METAP2
 
 
 
 
Positive Regulation of JUN Kinase Activity ( GO:0043507 )
 
 
9/24
 
 
0.0011019
 
 
0.0281137
 
 
0
 
 
0
 
 
4.487267
 
 
30.56159
 
 
TRAF6;MAP3K10;TNFRSF11A;TNF;MAP3K7;MAP3K11;MAP3K4;MAP3K12;MAP3K5
 
 
 
 
Cellular Response to Oxygen-Containing Compound ( GO:1901701 )
 
 
66/384
 
 
0.0011254
 
 
0.0285513
 
 
0
 
 
0
 
 
1.563329
 
 
10.61437
 
 
CRTC1;CD80;SERPINE1;HMGB2;TNFAIP3;RORA;PTPRK;AHR;RORB;CDC73;CASP7;GRM5;KAT5;SESN1;SESN2;PDK4;PDE4B;EP300;PDGFRA;GPR37;PRKCD;TPM1;WNT9B;TET1;FOS;TNFRSF1B;AFG3L2;SFRP1;CREB1;TRAF6;PXDN;RARA;RAPGEF2;PLAA;IRF5;MAPT;TP53;HCN3;INSIG1;CACNA1A;LPL;HSP90B1;ZFP36L1;PRDX3;RAP1B;BRINP1;RAP1A;GNG2;MAT2A;PABPN1;RPS3;CCL2;H2AZ1;WNT10B;PRNP;TRIM41;FZD4;ATP2B4;IGF1;MAPK14;ESR1;AXL;WT1;TNFSF4;SLC26A6;HCN1
 
 
 
 
Plasma Membrane Bounded Cell Projection Morphogenesis ( GO:0120039 )
 
 
15/54
 
 
0.0011350
 
 
0.0286309
 
 
0
 
 
0
 
 
2.879869
 
 
19.52880
 
 
CNTNAP2;PPP1R12A;BDNF;NRXN1;DTNBP1;DICER1;NYAP2;SHTN1;SNX2;NTF3;ATP7A;SGK1;SRGAP2;WASF1;MAP4K4
 
 
 
 
Regulation of Intracellular Signal Transduction ( GO:1902531 )
 
 
54/302
 
 
0.0012335
 
 
0.0309427
 
 
0
 
 
0
 
 
1.638200
 
 
10.97243
 
 
PGAP1;DOCK3;DYRK2;FAM13B;SETD9;ZFAND6;ITSN1;WWC2;ARHGAP1;PTEN;TNF;CDH5;SPRED1;GRAP2;EP300;ACVR1;USP2;ATP6AP2;SIRT1;MLF1;TIAM1;RRAGC;TRAF4;TRAF6;TRAF5;MAPT;UBE2V1;SGK1;DIPK2A;CSF1R;RNF34;SGMS1;AKAP5;PPM1K;PPM1D;GNA13;ARHGAP21;ARHGAP20;NUAK2;RRAS;PIP5K1A;PIP5K1B;SRGAP2;MAP4K4;MAP2K6;REPS1;STARD13;ARHGAP26;VEGFA;TBCK;BCL3;UBE2N;GRB2;TEK
 
 
 
 
Negative Regulation of Cytoskeleton Organization ( GO:0051494 )
 
 
10/29
 
 
0.0012504
 
 
0.0311900
 
 
0
 
 
0
 
 
3.936976
 
 
26.31591
 
 
KANK1;PLEKHH2;SMAD4;ARPIN-AP3S2;CAV3;PIK3CA;TMSB4X;PRKCD;MYADM;CYRIB
 
 
 
 
Embryonic Organ Morphogenesis ( GO:0048562 )
 
 
13/44
 
 
0.0012824
 
 
0.0318111
 
 
0
 
 
0
 
 
3.138743
 
 
20.90091
 
 
PDGFRA;FOXF1;SIX1;SOX11;NEUROD1;MFAP2;HOXA3;RBPMS2;HOXB4;HOXB3;ALX1;HOXA4;FBN1
 
 
 
 
Proteasomal Protein Catabolic Process ( GO:0010498 )
 
 
64/372
 
 
0.0012922
 
 
0.0318501
 
 
0
 
 
0
 
 
1.564718
 
 
10.40752
 
 
TRIM71;UBXN1;CCDC47;UBE2D3;UBE2D1;ARRB1;PRPF19;KAT5;SKP1;FBXW7;SIRT1;KCTD5;PSMA4;PSMA1;TBL1XR1;CANX;PLAA;MAN1B1;PSMD12;RNF34;CUL5;CUL3;DERL1;FBXO45;ASB11;RNF7;UBE2J1;HSP90B1;PCNP;SOCS2;ERLEC1;ZNRF2;RMND5A;TBX21;UBQLN1;PMAIP1;VHL;KLHL29;UBE2B;CSNK1A1;FEM1B;SIAH2;FBXL19;KLHL28;SGTA;SYVN1;TMUB1;KLHL2;UBE2G1;ERLIN2;PSMB8;PSMB9;TMUB2;RNF145;UBE2W;APPBP2;KLHL6;GID8;KLHL8;UBE2N;RNF186;SPSB4;ASB1;CUL4B
 
 
 
 
Regulation of BMP Signaling Pathway ( GO:0030510 )
 
 
19/77
 
 
0.0012983
 
 
0.0318501
 
 
0
 
 
0
 
 
2.454383
 
 
16.31363
 
 
CRB2;NOTCH1;TNFAIP6;FST;UBE2D3;NOG;CRIM1;UBE2D1;SOX11;LRP2;LEMD3;FSTL1;GDF5;GREM1;CDH5;RBPMS2;VWC2L;PPARG;WNT1
 
 
 
 
Negative Regulation of Blood Vessel Endothelial Cell Migration ( GO:0043537 )
 
 
11/34
 
 
0.0013163
 
 
0.0320460
 
 
0
 
 
0
 
 
3.578220
 
 
23.73422
 
 
DLL4;MECP2;STARD13;NOTCH1;PDCD10;GADD45A;ATP2B4;PPARG;MEOX2;KLF4;TNF
 
 
 
 
Positive Regulation of Neuron Apoptotic Process ( GO:0043525 )
 
 
12/39
 
 
0.0013206
 
 
0.0320460
 
 
0
 
 
0
 
 
3.325872
 
 
22.04944
 
 
NANOS1;SRPK2;PRNP;PHB1;CASP7;TGFB2;FBXW7;CASP2;FOXO3;TNF;MCL1;CDK5R1
 
 
 
 
p38MAPK Cascade ( GO:0038066 )
 
 
6/12
 
 
0.0013303
 
 
0.0321071
 
 
0
 
 
0
 
 
7.473082
 
 
49.48934
 
 
MAPK14;MAP3K7;MAP3K4;MAP2K6;MAP3K5;ZFP36L1
 
 
 
 
Regulation of Cell Growth ( GO:0001558 )
 
 
42/222
 
 
0.0013599
 
 
0.0323830
 
 
0
 
 
0
 
 
1.753265
 
 
11.57220
 
 
RB1;OGFR;PHB1;BTG1;DDX3X;DDX49;TFRC;ENO1;CDKN2AIP;RTN4;SOCS2;CDC42;ING5;SERTAD3;RPS6KA1;SESN2;ENPP1;RBBP7;TAF9B;MFSD2A;H3-3B;LGI1;SMAD4;TGFB2;ADAM10;INO80;OSGIN2;BLTP1;TGFBR1;SFRP1;ADAM17;H3-5;WT1;PPT1;BCL2;SGK3;SGK1;CRLF3;TP53;HSPA1B;SIPA1;EIF4G2
 
 
 
 
Positive Regulation of Canonical Wnt Signaling Pathway ( GO:0090263 )
 
 
21/89
 
 
0.0013739
 
 
0.0323830
 
 
0
 
 
0
 
 
2.314464
 
 
15.25259
 
 
AMER1;USP47;KANK1;DDX3X;UBE2B;USP34;ATP6AP2;GSKIP;NRARP;PPP2R3A;LYPD6;DKK2;COL1A1;SFRP1;TNKS2;TBL1XR1;GID8;RSPO3;RSPO4;SOX4;LGR4
 
 
 
 
Cell Cycle G1/S Phase Transition ( GO:0044843 )
 
 
17/66
 
 
0.0013812
 
 
0.0323830
 
 
0
 
 
0
 
 
2.598495
 
 
17.11051
 
 
TRIM71;USP37;CCNJ;CUL5;CUL3;PLK2;PHF8;HINFP;CCNA2;CCND2;CCNE2;CCNG1;EIF4EBP1;E2F3;MAP3K11;CCNJL;CUL4B
 
 
 
 
Neurotrophin Signaling Pathway ( GO:0038179 )
 
 
7/16
 
 
0.0013875
 
 
0.0323830
 
 
0
 
 
0
 
 
5.813872
 
 
38.25657
 
 
DOCK3;RAP1A;BDNF;MAGI2;DDIT4;NTF3;RAPGEF2
 
 
 
 
Regulation of Establishment or Maintenance of Cell Polarity ( GO:0032878 )
 
 
7/16
 
 
0.0013875
 
 
0.0323830
 
 
0
 
 
0
 
 
5.813872
 
 
38.25657
 
 
RAP1B;KANK1;RIPOR2;CDH5;SHTN1;TEK;CYRIB
 
 
 
 
Regulation of Triglyceride Biosynthetic Process ( GO:0010866 )
 
 
7/16
 
 
0.0013875
 
 
0.0323830
 
 
0
 
 
0
 
 
5.813872
 
 
38.25657
 
 
SCARB1;SREBF1;KAT5;FBXW7;SIRT1;LDLR;MFSD2A
 
 
 
 
Regulation of Alternative mRNA Splicing, via Spliceosome ( GO:0000381 )
 
 
15/55
 
 
0.0013925
 
 
0.0323830
 
 
0
 
 
0
 
 
2.807713
 
 
18.46535
 
 
DDX17;RBM25;RBM15;WTAP;CELF2;FMR1;CELF3;MAGOHB;REST;NOVA1;RBPMS2;RBM7;HNRNPA1;RBM24;SRSF12
 
 
 
 
Response to Endoplasmic Reticulum Stress ( GO:0034976 )
 
 
38/196
 
 
0.0014179
 
 
0.0328038
 
 
0
 
 
0
 
 
1.806330
 
 
11.84690
 
 
HERPUD2;PPP1R15B;CCDC47;TMTC3;DERL1;ASB11;PIK3R1;RNF7;SELENOK;UBE2J1;HSP90B1;SCAMP5;ERLEC1;SERP1;BCL2L11;AIFM1;CREB3L1;UBQLN1;BAK1;MAP3K5;PDIA3;EIF2B5;SGTA;SYVN1;TMUB1;ERLIN2;EIF2S1;TMUB2;RNF145;TBL2;TMEM33;VAPB;CANX;CREBRF;BCL2;MAN1B1;RNF186;TP53
 
 
 
 
Circulatory System Development ( GO:0072359 )
 
 
28/132
 
 
0.0014505
 
 
0.0332128
 
 
0
 
 
0
 
 
2.019634
 
 
13.20001
 
 
CRB2;NOTCH1;PTEN;ECE1;PCSK5;SHH;SALL1;ERBB3;FLRT3;HEY2;SOX4;MEF2A;KDM6B;TGFB2;FBXW7;FOXF1;CACYBP;ZDHHC16;TBX5;ISL1;TGFBR1;COL3A1;PIK3CA;WT1;CCM2;BCOR;TEK;FBN1
 
 
 
 
Protein Localization to Plasma Membrane ( GO:0072659 )
 
 
28/132
 
 
0.0014505
 
 
0.0332128
 
 
0
 
 
0
 
 
2.019634
 
 
13.20001
 
 
DPP10;ZDHHC23;ZDHHC7;TNF;HSP90B1;RAP1A;MAP7;PIP5K1A;FLOT2;GPR158;CAV3;ACTN2;KCNIP3;KCNIP4;ANK2;ANK3;TTC7A;TNFRSF1A;RAB10;EHD2;CACNB3;TSPAN14;RAB34;GORASP1;RAPGEF2;EPHA2;PALS1;ARF6
 
 
 
 
Negative Regulation of Extrinsic Apoptotic Signaling Pathway ( GO:2001237 )
 
 
18/72
 
 
0.0014667
 
 
0.0334120
 
 
0
 
 
0
 
 
2.496946
 
 
16.29195
 
 
TCF7L2;DDX3X;RNF34;EYA3;SIAH2;SERPINE1;TNFAIP3;IGF1;KLF4;AREG;TNF;TGFBR1;SCRT2;PEA15;BCL2;ZMYND11;HSPA1B;MCL1
 
 
 
 
Regulation of Cardiac Muscle Hypertrophy ( GO:0010611 )
 
 
9/25
 
 
0.0015427
 
 
0.0347881
 
 
0
 
 
0
 
 
4.206574
 
 
27.23435
 
 
MEF2A;RGS2;MLIP;SMAD4;NOTCH1;CAV3;ARRB1;IGF1;IL6ST
 
 
 
 
Regulation of Vascular Endothelial Growth Factor Signaling Pathway ( GO:1900746 )
 
 
9/25
 
 
0.0015427
 
 
0.0347881
 
 
0
 
 
0
 
 
4.206574
 
 
27.23435
 
 
ITGB1;CADM4;SEMA6A;IL12B;SPRY2;DLL1;JCAD;ROBO1;VEGFA
 
 
 
 
Positive Regulation of Wnt Signaling Pathway ( GO:0030177 )
 
 
25/114
 
 
0.0015615
 
 
0.0350346
 
 
0
 
 
0
 
 
2.106261
 
 
13.61096
 
 
AMER1;KANK1;DDX3X;USP34;GSKIP;TNFAIP3;NRARP;LYPD6;CDC73;HSP90B1;SALL1;TNKS2;RSPO3;SOX4;RSPO4;USP47;UBE2B;ATP6AP2;PPP2R3A;DKK2;COL1A1;SFRP1;TBL1XR1;GID8;LGR4
 
 
 
 
Intracellular Signaling Cassette ( GO:0141124 )
 
 
61/354
 
 
0.0015901
 
 
0.0354987
 
 
0
 
 
0
 
 
1.567036
 
 
10.09793
 
 
ARHGAP1;PTEN;HTR2C;PIK3C2A;NUDT4;PPP1CB;PPP1CC;EDNRA;PDE4B;EP300;MAP3K7;MAP3K4;MAP3K5;MEF2A;NANOS1;MAP2K4;BATF3;MAP2K1;CHUK;MAGI2;FOS;DUSP9;TIAM1;ADAM17;MAF;CREB1;RRAGC;DDAH1;PIK3CA;TRAF6;TYRO3;RAPGEF2;RAPGEF5;DGKH;CEBPG;PIK3R3;PIK3R1;LRP2;NYAP2;ZFP36L1;NRAS;IRAK1;ERBB3;PABPN1;MAPK1;MAP4K4;MAP2K6;REPS1;SMAD4;SIAH2;STAT3;MAPK12;FOSL1;RCAN1;MAFB;RCAN2;FKBP1B;BCL3;PDE7B;PDE7A;MAP3K11
 
 
 
  
  
 
 
 
 GO_Cellular_Component_2025 
 This category captures Gene Ontology Cellular Component annotations
describing the subcellular localization of gene products, including
organelles, macromolecular complexes, membranes, and specialized
cellular structures. GO Cellular Component terms are curated through
experimental localization studies and integrative annotation pipelines.
Enrichment reveals whether target genes preferentially localize to
specific cellular compartments, suggesting spatial organization of
regulatory effects. These insights are valuable for understanding
compartment-specific processes such as mitochondrial regulation, vesicle
trafficking, nuclear control mechanisms, or membrane-associated
signaling. 
 
 
 
 
Term
 
 
Overlap
 
 
P.value
 
 
Adjusted.P.value
 
 
Old.P.value
 
 
Old.Adjusted.P.value
 
 
Odds.Ratio
 
 
Combined.Score
 
 
Genes
 
 
 
 
 
 
Intracellular Membrane-Bounded Organelle ( GO:0043231 )
 
 
858/5597
 
 
0.0000000
 
 
0.0000000
 
 
0
 
 
0
 
 
1.549324
 
 
72.4174258
 
 
ITSN2;CHIC2;TFRC;ITSN1;TESK2;ZC3H12C;PPP4R2;ZC3H12B;GPBP1;BBX;MEF2A;PRKAB2;ZNF283;ZNF281;RFX3;DICER1;ISL1;CDIP1;CGGBP1;SLBP;MTMR2;MEPCE;GATA6;ZNF23;OAZ2;PIP5K1A;ZNF268;ZNF267;VTI1B;ZNF266;PRPF38A;NR2F2;MAGOHB;NR2F6;CS;PKIB;MLLT10;LAS1L;AGO4;PKIA;AGO2;C19ORF47;SFXN4;FERMT2;LIN54;ARHGAP1;BHLHE41;CTCF;AREG;MECP2;MED14;NKRF;HEY2;SS18;CNOT6L;SLC39A11;FOS;BAZ1B;DUSP9;DYNLL2;SIRT1;LRRC41;DDB2;MED28;PSMA4;VAMP7;PLSCR3;BDH1;PSMA1;PPARG;RAPGEF5;ERGIC2;ERGIC1;MDFI;VAMP2;TRUB1;KANK1;ZNF471;ZNF470;CIPC;GLRA2;MYCT1;BTAF1;SNX27;TMEM65;ZNF225;SRSF10;SRSF11;ZNF462;CENPV;MBD6;SLF2;USP9X;MBD1;STAT3;PSMB8;PSMB9;SNX17;WT1;GID8;SNX15;SERBP1;ZNF699;CRLF3;CCNT2;CSE1L;UBE2D2;UBE2D3;MAP3K7CL;UBE2D1;RORA;RORB;NUDT11;SESN1;KMT5B;KPNA5;PITX2;MAP3K7;NEPRO;KHNYN;FBXW7;IST1;KMT5C;UBE2E3;CTNNBIP1;MYT1;CDC25B;FOXP1;MAF;GMNC;PKP4;SRSF7;KPNB1;MAX;ZC4H2;ISG20L2;STK39;FBXO45;FOXO3;HSP90B1;GLIS2;BCL2L12;EIF1AD;ZKSCAN3;ZNF420;ZBTB18;ODF1;BRPF3;NEK6;NEK7;STYX;RFXANK;FOXN4;ARPC5;QKI;PTPRA;MDM4;NFE2L1;SPRN;CELF2;ONECUT1;CELF3;PTEN;ARRB1;NXT2;CDC73;FUNDC2;PPP6R1;EXO5;ZNF644;EMX2;MSL2;VPS37C;VEZF1;SLC25A15;ARL4A;TMBIM6;TOP1;AHDC1;PRKAA1;UHRF2;ZBTB47;FOXK2;PBXIP1;PRCP;MLLT3;RCHY1;PPM1D;PPM1F;PPM1G;ZNRF2;PARD6B;TP53INP1;ZNF624;E2F3;DRAM2;E2F5;HNRNPA1;H2AZ1;TRIM41;FEM1B;FOXJ2;TBX5;TBX4;SLC25A37;MCM3AP;SNRK;NOVA1;JPT1;CCNK;CCNJ;CCNH;CCNC;IKZF1;LDLRAD4;IFI30;IKZF4;IKZF5;NUDT4;EPC1;CCN2;CCNL2;KCTD19;SFMBT1;FNDC8;THAP1;CYP39A1;CCNY;PREB;BCORL1;CCDC82;TP53;TRIB2;MYCBP2;PIK3R1;ZBTB5;ZBTB4;UBE2J1;MBTD1;DHX33;MIER1;SNX5;ZBED4;CTSA;FOXF1;EAF1;WAPL;LPAR6;ID1;ID4;ID3;GRB2;CALM3;INTS7;SLC2A4RG;CCNJL;CSRNP1;BARHL2;SUV39H2;PPP1R11;PPP1R10;SUV39H1;MIA3;PTPRK;NAT14;RAB22A;CTSS;SCML2;REV3L;SPTAN1;NCOA1;SREBF1;GRIPAP1;ATMIN;STARD3NL;AGTRAP;CACYBP;ZHX1;NR5A2;PDIK1L;FAM193B;NCOA7;ZNF780B;MARCHF1;SATB1;STX16;PCNP;ZFP14;SERTAD3;GPC4;EXOSC3;EPS15;GPC6;MARCHF3;GCH1;PER1;THEMIS2;NEDD4;CCNG1;FOXA1;SCARB1;PRDM4;CRTC1;GEMIN2;PRDM1;LOXL2;TXN2;RGS2;AMMECR1L;CCND2;EP300;SNIP1;ARID2;RGS8;EOMES;PDGFRB;PDGFRA;SLC30A9;BORA;ADAM10;TMEM250;PLA2G15;CCNE2;PSPC1;WDFY1;PLAA;DENND6A;UBE2V1;PPIC;DDX6;UBA6;CACNA1A;PDS5B;PDS5A;IRAK1;MORC2;UBE2F;PRNP;TFAP2C;UBE2H;UBE2I;CAV3;UBE2B;VTA1;HOOK3;BLTP1;UBE2W;NASP;APPBP2;BHLHE40;UBE2N;NABP1;CYCS;MXD1;UBE2K;RERE;DDX3Y;DYRK2;DDX3X;DDX49;NAA40;YWHAB;CHD7;DDX42;HMGB2;DPY30;TNFAIP3;TRIAP1;SOBP;CHD1;YWHAQ;ZIC1;MYBL2;HORMAD2;ZNF385D;ZNF385A;MYOCD;TGIF2;RBM15;ETV1;ETV4;YWHAZ;ETV6;CCNA2;ELF2;CREB1;IRF4;TBC1D5;ELF5;GORASP1;IRF2;RBM20;IRF5;ZMYND11;RBM24;CREB5;PARG;NAA20;RAP1GDS1;RNF7;CLCN3;ATAD2B;CAND1;DNPEP;PABPN1;TNKS2;FAM241B;TBX21;TDG;UBE2NL;FAM241A;DROSHA;RALY;VHL;MTA2;ATOH1;RBM38;NIPSNAP3A;EYA3;TAF11;ZNF804A;ATP2B4;PEDS1;MAPK14;USF1;WARS1;CHFR;MAPK12;CLCN6;REST;TCIM;KATNBL1;POLE3;ERCC6;TAF5;TAF4;RB1;PHB1;GPATCH2;PHAX;ENO1;FNBP1L;GPATCH8;BACH2;DCAF6;GPR174;EVA1A;CREB3L1;MYB;HOXA3;HOXA1;PIP4K2B;AP1S3;HOXA5;IER2;DDX17;PRKCD;MED8;MED9;SOX11;BIN1;TYRO3;COMMD3-BMI1;HOXB3;HOXB8;ASF1A;HOXB5;ZNF397;OSER1;ZNF395;HOXD1;C9ORF72;MTDH;PRDX3;LDHA;PRDM16;MCMBP;ALX1;HOXC8;NKX2-4;RREB1;NAA50;NDFIP2;CDK19;PLK5;PLK2;SLC16A10;INO80;ST18;FOSL1;TADA2B;EHD2;CPEB1;HNRNPK;HNRNPF;HNRNPD;PPT1;SNAI1;MMS19;HBP1;DUS1L;ZNF136;CDKN1C;PHF3;BTG2;BTG1;PHF8;ING5;CSRP2;AIFM1;TMSB4X;PEA15;RBPMS2;PHF24;AP3S2;JARID2;ZNF367;ZNF366;SLC2A14;BATF3;HSBP1;EN2;TET2;TET1;NCOR2;RNF168;TUBB2A;MTF2;TET3;RARA;RARB;ZNF594;ASAH1;NFIX;SGMS1;WDR20;TMEM165;RNF138;PPP1R7;RPS3;LDLR;PCGF6;CSNK1A1;PCGF5;PCGF3;ZNF33B;HINFP;RYBP;NFIA;RLIM;FOSB;HSPA1B;ANKRD13B;DGKD;ZNF570;ATP8A1;GTF2B;CTNND2;FMR1;UBP1;AHR;PPP1CB;PPP1CC;SALL1;ZFP30;SLC39A9;PAPOLG;GRAP2;HMGN2;MAF1;HMGN3;PHLDA1;MAP2K1;USP4;USP3;MAGI2;THOC1;DYRK1B;MITF;VRK3;DGKZ;RAB30;KAT6B;DMWD;ERG;SNRPC;DGKH;IPPK;NOTCH2;BRSK1;ZNF792;CSF1R;RAB5B;DNMT1;PARM1;NOTCH1;CUL5;CUL3;AK1;ABHD5;MACROH2A1;RLF;NEUROD1;BAG4;BAG5;BTBD3;SAFB2;NDN;ZNF304;MCL1;EIF2B5;OSBPL9;ESRRA;AKIRIN1;ESRRG;ADAP1;MOB1A;RCAN1;CNOT7;MAFB;P4HA2;ZFP62;MAFG;RCAN2;BCL3;BCL2;TADA1;LHX9;ZFP82;PIK3C2A;DUSP16;TLK2;DNMT3B;HIF1AN;TNPO1;ADAMTS9;LBR;ELAC2;DNMT3A;PTBP3;PTP4A1;NPEPL1;TFDP2;TRAF4;TRAF6;NOS1AP;POLR1D;HDAC4;RNF34;HDAC1;WBP2;VPS4B;CEBPG;RNF38;NLRC5;TTK;PDHB;DNAJB1;STOX2;RSBN1L;RBBP7;JAZF1;TUB;PTPN18;SEMA4D;KCNIP3;PNRC1;PNRC2;KLF7;KLF6;EVI2A;KLF5;RAB14;MAB21L2;BCOR;TNRC6A;BET1;CUL4B;STEEP1;TCERG1;SPARC;PANK1;ZNF609;FRAT2;KAT5;UBAP2L;SOX4;AGFG1;SKP1;SOX5;PDHX;ESCO1;P3H1;RUNX1;RRAGC;XRN1;BLZF1;IGSF8;NECAB3;ANP32A;DCUN1D4;EIF5A2;C2CD4A;GSKIP;STC1;FXR1;SPAST;ZNHIT6;TRPM7;RALGDS;SRPK2;SETDB1;GADD45A;SIAH2;SS18L1;STAG2;RAET1L;CREBRF;ANP32B;DAZAP2;DAZAP1;UBXN1;MEOX2;PRPF19;SPPL3;XPO4;XPO5;TMED5;H3-3B;STRBP;TFEB;PROX1;CBFA2T3;ZDHHC17;MLF1;PALD1;MDFIC;TFEC;WAC;RGS7BP;MAPT;VGLL2;SGK1;AMER1;MYCBP;PPP2CA;PURB;PURA;FAM184A;GINS2;NFYA;CDX2;PTGES3;NR6A1;PRKRA;AXL;CAMK4;FRA10AC1;CDK5R1;POGK;HAPSTR1;HOXA11;HOXA10;POGZ;MAGEB10;LMO1;NANOS1;PDIA3;MLIP;TSC1;WRNIP1;RSBN1;MAU2;MYCN;TBL1XR1;CMPK1;CHMP4B;ZFP36L2;ZFP36L1;ZMAT2;PMAIP1;OTX2;RPRD1A;CPSF4;CPSF7;SMAD4;CPSF6;CBX4;DNAJC13;ESR1;AZIN1;NR4A2;LIN28B;RALYL;LIN28A;JMY;BLMH;BRWD3;B4GALT2;USP37;ABCD3;USP38;USP32;HSPB8;USP34;SIX1;YBX3;DTWD2;CASP7;SIX3;CASP2;FLOT2;RBM7;KDM6B;USP46;KLF11;RUBCN;USP47;USP48;CAMK1D;USP45;SAFB;KLF15;TGFBR1;SENP1;RABEP2;TRAPPC6B;PALLD;MYH9;KDM7A;ITGB3BP;KDM3A;RGS14;LEF1;POMP;MOSPD1;ABRAXAS2;UACA;STRN3;GNA13;NXF1;RMND5A;SAP30L;PDE6D;S1PR1;MAPK1;REPS1;TCF7L2;WTAP;LIMK1;SGTA;DTNBP1;NAP1L1;H3-5;PITHD1;PMP22;AQP11;SPRY2
 
 
 
 
Nucleus ( GO:0005634 )
 
 
747/4879
 
 
0.0000000
 
 
0.0000000
 
 
0
 
 
0
 
 
1.508732
 
 
58.2937984
 
 
RB1;PHB1;GPATCH2;TESK2;PHAX;ENO1;GPATCH8;BACH2;DCAF6;ZC3H12C;PPP4R2;ZC3H12B;CREB3L1;GPBP1;MYB;HOXA3;BBX;HOXA1;PIP4K2B;HOXA5;IER2;DDX17;MEF2A;PRKAB2;ZNF283;ZNF281;PRKCD;MED8;RFX3;MED9;SOX11;DICER1;ISL1;BIN1;TYRO3;CDIP1;COMMD3-BMI1;HOXB3;HOXB8;CGGBP1;ASF1A;HOXB5;ZNF397;OSER1;SLBP;ZNF395;MTMR2;HOXD1;MEPCE;GATA6;ZNF23;C9ORF72;OAZ2;MTDH;LDHA;PRDM16;PIP5K1A;MCMBP;ALX1;ZNF268;HOXC8;NKX2-4;RREB1;ZNF267;ZNF266;NAA50;CDK19;PRPF38A;PLK5;PLK2;NR2F2;INO80;MAGOHB;ST18;NR2F6;FOSL1;CS;TADA2B;PKIB;EHD2;MLLT10;CPEB1;HNRNPK;LAS1L;AGO4;PKIA;HNRNPF;AGO2;C19ORF47;HNRNPD;PPT1;SNAI1;MMS19;HBP1;DUS1L;ZNF136;FERMT2;CDKN1C;PHF3;LIN54;BTG2;BTG1;BHLHE41;CTCF;AREG;PHF8;MECP2;ING5;MED14;CSRP2;AIFM1;NKRF;TMSB4X;HEY2;RBPMS2;PHF24;JARID2;ZNF367;ZNF366;SS18;SLC2A14;BATF3;CNOT6L;HSBP1;SLC39A11;EN2;TET2;TET1;FOS;BAZ1B;DUSP9;DYNLL2;SIRT1;LRRC41;DDB2;MED28;NCOR2;RNF168;TUBB2A;PSMA4;PLSCR3;PSMA1;MTF2;TET3;RARA;RARB;PPARG;RAPGEF5;ERGIC2;MDFI;TRUB1;KANK1;ZNF594;ASAH1;ZNF471;NFIX;ZNF470;SGMS1;CIPC;WDR20;RNF138;PPP1R7;RPS3;ZNF225;LDLR;SRSF10;SRSF11;ZNF462;CENPV;PCGF6;MBD6;SLF2;CSNK1A1;PCGF5;USP9X;MBD1;PCGF3;STAT3;PSMB8;ZNF33B;PSMB9;HINFP;RYBP;NFIA;WT1;GID8;SERBP1;RLIM;FOSB;ZNF699;CRLF3;HSPA1B;CCNT2;DGKD;ZNF570;GTF2B;CSE1L;UBE2D2;CTNND2;FMR1;UBE2D3;MAP3K7CL;UBP1;UBE2D1;RORA;RORB;AHR;NUDT11;PPP1CB;PPP1CC;SALL1;ZFP30;SLC39A9;PAPOLG;SESN1;GRAP2;KMT5B;KPNA5;HMGN2;PITX2;MAF1;MAP3K7;HMGN3;NEPRO;PHLDA1;MAP2K1;KHNYN;FBXW7;KMT5C;USP4;USP3;UBE2E3;CTNNBIP1;MAGI2;THOC1;DYRK1B;MITF;VRK3;MYT1;DGKZ;CDC25B;FOXP1;MAF;KAT6B;DMWD;GMNC;PKP4;ERG;SNRPC;SRSF7;KPNB1;IPPK;NOTCH2;BRSK1;ZNF792;DNMT1;NOTCH1;CUL5;MAX;ZC4H2;CUL3;AK1;ISG20L2;STK39;FOXO3;MACROH2A1;RLF;HSP90B1;GLIS2;BCL2L12;NEUROD1;EIF1AD;BAG4;BAG5;BTBD3;ZKSCAN3;SAFB2;NDN;ZNF304;ZNF420;MCL1;EIF2B5;ZBTB18;ESRRA;AKIRIN1;ODF1;BRPF3;NEK6;NEK7;STYX;RFXANK;FOXN4;ESRRG;ARPC5;ADAP1;QKI;MOB1A;RCAN1;CNOT7;MAFB;PTPRA;ZFP62;MAFG;RCAN2;BCL3;BCL2;TADA1;MDM4;LHX9;NFE2L1;SPRN;CELF2;ONECUT1;CELF3;PTEN;ARRB1;ZFP82;NXT2;DUSP16;CDC73;FUNDC2;PPP6R1;TLK2;DNMT3B;EXO5;ZNF644;HIF1AN;TNPO1;LBR;EMX2;ELAC2;DNMT3A;MSL2;VEZF1;PTBP3;PTP4A1;SLC25A15;ARL4A;NPEPL1;TFDP2;TRAF4;TRAF6;NOS1AP;POLR1D;TMBIM6;TOP1;AHDC1;HDAC4;PRKAA1;RNF34;UHRF2;ZBTB47;HDAC1;WBP2;VPS4B;CEBPG;RNF38;NLRC5;FOXK2;PBXIP1;TTK;PDHB;MLLT3;RCHY1;PPM1D;PPM1F;PPM1G;DNAJB1;STOX2;PARD6B;RSBN1L;TP53INP1;ZNF624;E2F3;RBBP7;HNRNPA1;JAZF1;H2AZ1;TUB;PTPN18;FEM1B;KCNIP3;FOXJ2;TBX5;TBX4;PNRC1;PNRC2;SLC25A37;KLF7;MCM3AP;SNRK;KLF6;NOVA1;MAB21L2;BCOR;CUL4B;STEEP1;TCERG1;JPT1;CCNK;CCNJ;PANK1;CCNH;CCNC;IKZF1;IKZF4;IKZF5;NUDT4;ZNF609;FRAT2;KAT5;EPC1;UBAP2L;CCNL2;SOX4;SKP1;SOX5;PDHX;KCTD19;ESCO1;SFMBT1;P3H1;FNDC8;THAP1;RUNX1;CCNY;RRAGC;XRN1;PREB;BCORL1;CCDC82;TP53;TRIB2;BLZF1;NECAB3;ANP32A;MYCBP2;DCUN1D4;C2CD4A;GSKIP;STC1;PIK3R1;ZBTB5;ZBTB4;UBE2J1;MBTD1;FXR1;SPAST;DHX33;ZNHIT6;MIER1;TRPM7;RALGDS;ZBED4;SRPK2;SETDB1;FOXF1;GADD45A;SS18L1;WAPL;STAG2;ID1;ID4;CREBRF;ID3;GRB2;CALM3;INTS7;ANP32B;SLC2A4RG;CCNJL;CSRNP1;BARHL2;SUV39H2;DAZAP2;DAZAP1;UBXN1;PPP1R11;PPP1R10;SUV39H1;MIA3;MEOX2;PRPF19;NAT14;SCML2;XPO4;XPO5;REV3L;H3-3B;NCOA1;SREBF1;ATMIN;STRBP;TFEB;CACYBP;PROX1;ZHX1;CBFA2T3;MLF1;PALD1;NR5A2;MDFIC;PDIK1L;TFEC;WAC;FAM193B;NCOA7;ZNF780B;RGS7BP;MAPT;VGLL2;SGK1;MYCBP;SATB1;PCNP;PPP2CA;PURB;PURA;ZFP14;SERTAD3;GPC4;EXOSC3;GPC6;GINS2;NFYA;GCH1;CDX2;PTGES3;PER1;NR6A1;THEMIS2;PRKRA;NEDD4;CAMK4;CCNG1;FRA10AC1;CDK5R1;FOXA1;PRDM4;CRTC1;GEMIN2;POGK;PRDM1;HAPSTR1;LOXL2;TXN2;HOXA11;HOXA10;RGS2;AMMECR1L;CCND2;POGZ;EP300;SNIP1;ARID2;RGS8;MAGEB10;LMO1;NANOS1;EOMES;PDGFRB;PDIA3;PDGFRA;MLIP;SLC30A9;BORA;ADAM10;TSC1;WRNIP1;TMEM250;RSBN1;MAU2;MYCN;CCNE2;PSPC1;TBL1XR1;WDFY1;CMPK1;PLAA;CHMP4B;UBE2V1;DDX6;UBA6;CACNA1A;PDS5B;PDS5A;ZFP36L2;ZFP36L1;IRAK1;ZMAT2;PMAIP1;OTX2;RPRD1A;MORC2;UBE2F;TFAP2C;UBE2H;CPSF7;SMAD4;UBE2I;CPSF6;UBE2B;CBX4;VTA1;ESR1;BLTP1;AZIN1;NR4A2;UBE2W;LIN28B;RALYL;LIN28A;NASP;APPBP2;BHLHE40;JMY;UBE2N;NABP1;CYCS;BLMH;MXD1;UBE2K;BRWD3;RERE;DDX3Y;USP37;DYRK2;DDX3X;DDX49;NAA40;USP38;YWHAB;USP32;HSPB8;CHD7;USP34;DDX42;HMGB2;DPY30;TNFAIP3;SIX1;TRIAP1;SOBP;YBX3;CHD1;DTWD2;CASP7;YWHAQ;ZIC1;SIX3;CASP2;MYBL2;HORMAD2;ZNF385D;RBM7;ZNF385A;KDM6B;USP46;KLF11;USP47;MYOCD;USP48;TGIF2;RBM15;CAMK1D;USP45;ETV1;SAFB;ETV4;YWHAZ;KLF15;TGFBR1;ETV6;SENP1;CCNA2;ELF2;CREB1;IRF4;PALLD;ELF5;IRF2;MYH9;RBM20;IRF5;ZMYND11;RBM24;KDM7A;ITGB3BP;CREB5;KDM3A;PARG;RGS14;NAA20;RAP1GDS1;LEF1;POMP;MOSPD1;ABRAXAS2;RNF7;UACA;ATAD2B;STRN3;GNA13;NXF1;CAND1;DNPEP;RMND5A;PABPN1;TNKS2;TBX21;TDG;SAP30L;UBE2NL;DROSHA;MAPK1;RALY;VHL;MTA2;ATOH1;RBM38;TCF7L2;NIPSNAP3A;WTAP;EYA3;LIMK1;TAF11;SGTA;ZNF804A;DTNBP1;PEDS1;NAP1L1;MAPK14;USF1;WARS1;CHFR;MAPK12;REST;H3-5;TCIM;PITHD1;KATNBL1;POLE3;PMP22;SPRY2;ERCC6;TAF5;TAF4
 
 
 
 
Bounding Membrane of Organelle ( GO:0098588 )
 
 
169/881
 
 
0.0000000
 
 
0.0000000
 
 
0
 
 
0
 
 
1.829160
 
 
42.7197773
 
 
SCARB2;GALNT12;SCARB1;PI4K2B;GALNT14;SYS1;PGAP2;TFRC;PGAP4;HHIP;ZDHHC7;LDLRAD4;ANTXR2;SCAMP5;GOLGA3;ANPEP;TOM1;ARFIP1;AP1S3;LARGE1;MAP3K7;GOLGA7;TRIM23;SLC30A7;SLC30A5;CSGALNACT2;ENTPD7;GOLPH3L;TPST1;ADAM17;RAB30;MALL;BLZF1;NOTCH2;RAB5B;NOTCH1;ABCB6;LMAN2L;MYCBP2;TGFA;CABP1;FUT4;RHOBTB1;CORO1C;APH1A;SNX3;APOO;UGCG;FUT8;RAP1A;SNX2;IRAK1;FUT9;CHST12;PDGFC;CHST10;ST8SIA4;ST3GAL6;WNT1;B3GALNT2;PTPRN2;DMPK;DNAJC13;ARHGAP26;ACER2;EHD2;CNIH1;B4GAT1;ACER3;PI4KA;POC1B-GALNT4;ACBD3;PALS1;ITGB1;B4GALT2;ARF4;B4GALT3;MCFD2;ARHGAP1;STAB2;SLC2A1;ARRB1;ECE1;SLC2A3;IER3IP1;AREG;PCSK5;SPPL3;HS2ST1;LFNG;GLIPR2;CHMP1B;MRC1;TMEM109;TMED7;SNAP29;RAB8B;HS3ST3B1;SREBF1;CHST7;ATP6AP2;VPS37C;ZDHHC13;VPS37B;SSTR3;ZDHHC17;POMGNT1;COPZ1;TNFRSF1A;VAMP7;ASPH;B3GNT7;TBC1D5;GORASP1;TRAF6;NOS1AP;CHST1;ST6GALNAC3;DIPK2A;B4GALT6;ERGIC1;VAMP2;RHOQ;HBEGF;B4GALT5;STX17;SGMS1;STX16;GOSR2;VPS4B;GCNT1;ABHD17C;SGMS2;CLCN3;AGPAT3;TMEM165;GGA2;CDC42;CYTH3;LMAN1;NRAS;PLN;CHSY1;LMAN2;ORMDL3;RAB24;STX6;GCNT3;WHAMM;CACNG2;LDLR;CYTH1;ARFGEF1;GALNT5;SLC35A4;RIC1;GALNT1;C1GALT1;LYSMD3;SVIP;PLEKHA8;RAB10;CLCN6;SNX17;COL7A1;A4GNT;MGAT4A;FKBP1B;BET1
 
 
 
 
Golgi Membrane ( GO:0000139 )
 
 
101/476
 
 
0.0000000
 
 
0.0000003
 
 
0
 
 
0
 
 
2.053310
 
 
40.5370809
 
 
GALNT12;SCARB2;PI4K2B;GALNT14;SYS1;PGAP2;PGAP4;ZDHHC7;LDLRAD4;SCAMP5;GOLGA3;ARFIP1;AP1S3;LARGE1;GOLGA7;TRIM23;SLC30A5;CSGALNACT2;GOLPH3L;TPST1;RAB30;ADAM17;MALL;BLZF1;NOTCH2;NOTCH1;LMAN2L;CABP1;FUT4;APH1A;APOO;UGCG;FUT8;CHST12;FUT9;CHST10;PDGFC;ST8SIA4;ST3GAL6;B3GALNT2;ACER2;B4GAT1;ACER3;POC1B-GALNT4;ACBD3;B4GALT2;ARF4;B4GALT3;SLC2A1;ARRB1;IER3IP1;PCSK5;HS2ST1;LFNG;GLIPR2;TMED7;SNAP29;HS3ST3B1;SREBF1;CHST7;ATP6AP2;ZDHHC13;ZDHHC17;POMGNT1;COPZ1;TNFRSF1A;B3GNT7;GORASP1;CHST1;ST6GALNAC3;B4GALT6;DIPK2A;ERGIC1;B4GALT5;SGMS1;STX16;GOSR2;GCNT1;SGMS2;AGPAT3;CDC42;CYTH3;LMAN1;NRAS;CHSY1;LMAN2;STX6;GCNT3;WHAMM;CYTH1;ARFGEF1;SLC35A4;GALNT5;RIC1;GALNT1;C1GALT1;LYSMD3;PLEKHA8;A4GNT;MGAT4A;BET1
 
 
 
 
Endoplasmic Reticulum Lumen ( GO:0005788 )
 
 
69/289
 
 
0.0000000
 
 
0.0000005
 
 
0
 
 
0
 
 
2.378910
 
 
44.7467624
 
 
POGLUT2;TOR2A;COLGALT2;COL16A1;POGLUT1;FKBP14;COL12A1;TXNDC12;MIA3;LAMC1;SHH;LGALS1;EVA1A;IL12B;ADAMTS7;PDIA3;AHSG;COL25A1;GPX8;P3H1;ADAM10;GPX7;PDIA5;CKAP4;ADAM17;RCN1;COL2A1;COL4A2;ASPH;COL4A1;COL4A4;COL6A2;CANX;DNAJB11;EOGT;COL8A1;COL21A1;COL4A5;COL6A3;MFGE8;MATN3;COL15A1;COL11A1;FSTL1;COL19A1;HSP90B1;ERLEC1;ARSJ;PDGFC;ESD;ARSI;MAPK1;WNT1;GOLM1;BDNF;COL22A1;COL1A1;COL3A1;TSPAN14;COL1A2;COL5A1;P4HA2;COL7A1;MGAT4A;COL5A2;CALU;COL9A1;PNPLA2;FBN1
 
 
 
 
Coated Vesicle ( GO:0030135 )
 
 
25/84
 
 
0.0000082
 
 
0.0005341
 
 
0
 
 
0
 
 
3.182674
 
 
37.2747945
 
 
LMAN2L;STX17;IER3IP1;PIK3C2A;HSPD1;GGA2;SNX3;LMAN1;LMAN2;CLVS2;STX6;TMED1;TMED7;TMED5;TMED4;CLVS1;SCAMP1;SNAP91;SNX18;COL7A1;MALL;NUMB;ERGIC2;ERGIC1;VAMP2
 
 
 
 
Endoplasmic Reticulum Membrane ( GO:0005789 )
 
 
143/872
 
 
0.0000263
 
 
0.0013704
 
 
0
 
 
0
 
 
1.492469
 
 
15.7418489
 
 
SCARB2;STEEP1;PI4K2B;PGAP1;PGAP2;ARL6IP1;PGAP3;GHITM;ZFYVE1;MAP3K7;SLC30A7;PDGFRA;DGAT1;MMGT1;ACSL1;SLC30A5;ACSL6;ACSL4;TMEM170A;PDIA5;CYP39A1;DPM2;SELENON;TYRO3;PREB;NOTCH2;NOTCH1;NECAB3;ABCB6;ABHD4;LMAN2L;INSIG2;INSIG1;RPN1;TGFA;RTN4;HSP90B1;UBE2J1;MTDH;APH1A;APOO;CYB5R4;SGPL1;SSR1;VTI1B;B3GALNT2;NUS1;DMPK;ERLIN2;DNAJC16;CYP26A1;G6PC2;TBL2;G6PC3;CNIH1;NCEH1;OSTC;ACER3;CALU;BCL2;SRPRA;RNF186;NFE2L1;CDS2;POMT2;LMBR1L;MCFD2;CCDC47;MIA3;IER3IP1;AREG;SPPL3;PIGX;JPH3;SERP1;SPTLC2;RB1CC1;TMEM109;TMED7;LBR;SH3D19;CERS4;SREBF1;CERS6;ELOVL4;STARD3NL;ELOVL2;ELOVL3;ATP6AP2;ELOVL6;SLC35G1;RHOC;COPZ1;MBLAC2;VAMP7;ASPH;TMEM33;CANX;NOS1AP;TMBIM6;ERGIC1;MARCHF1;PPM1L;STX17;SGMS1;TMPRSS3;GOSR2;DERL1;MOSPD2;HMGCR;SGMS2;SELENOK;AGPAT3;PTGS1;AGPAT4;CDC42;LMAN1;NRAS;PLN;LMAN2;EMC3;SMIM30;SPTSSB;SPTSSA;MARCHF5;GALNT1;SYVN1;DTNBP1;MBOAT2;ORMDL1;PEDS1;SVIP;SGPP1;VMP1;RAB10;VAPB;SCD;DOLPP1;FKBP1B;ESYT3;PNPLA6;PLP2;PNPLA2
 
 
 
 
RISC Complex ( GO:0016442 )
 
 
8/13
 
 
0.0000280
 
 
0.0013704
 
 
0
 
 
0
 
 
11.967756
 
 
125.4448857
 
 
DDX6;AGO4;PRKRA;AGO2;XPO5;DICER1;DCP2;TNRC6A
 
 
 
 
PTW/PP1 Phosphatase Complex ( GO:0072357 )
 
 
5/6
 
 
0.0001246
 
 
0.0051551
 
 
0
 
 
0
 
 
37.360169
 
 
335.8798792
 
 
PPP1CB;PPP1CC;PPP1R12A;PPP1R10;WDR82
 
 
 
 
Basement Membrane ( GO:0005604 )
 
 
16/50
 
 
0.0001318
 
 
0.0051551
 
 
0
 
 
0
 
 
3.526106
 
 
31.5018570
 
 
COL15A1;COL16A1;LAMA2;LAMA4;COL22A1;LAMC1;NID1;COLQ;LOXL2;COL4A2;COL4A1;COL4A4;COL7A1;PXDN;COL8A1;FBN1
 
 
 
 
Endosome Membrane ( GO:0010008 )
 
 
70/392
 
 
0.0002696
 
 
0.0095822
 
 
0
 
 
0
 
 
1.639954
 
 
13.4782248
 
 
ITGB1;SCARB2;PI4K2B;TFRC;ARHGAP1;SNX12;ECE1;LDLRAD4;ANTXR2;SCAMP5;RAB43;PIP4P2;CHMP1B;MRC1;TOM1;ATP7A;VPS36;MAP3K7;RAB8B;PDIA3;STARD3NL;ATP6AP2;VPS37C;VPS37B;SCAMP1;VAMP7;RAP2B;TBC1D5;TRAF6;ARF6;ARL8B;RAB5B;NOTCH1;MARCHF1;ABCB6;TMEM63B;VPS4B;GOSR2;ABHD6;ABHD17C;SLC30A10;CLCN3;RHOBTB1;TMEM165;CORO1C;GGA2;APH1A;SNX3;RAP1A;SNX2;IRAK1;MARCHF8;LDLR;VTI1B;RAB4A;MON2;FZD5;SLC31A1;CMTM6;DNAJC13;ARHGAP26;RAB10;EHD2;CLCN6;SNX18;RAB14;SNX17;RAB12;CD207;TMEM9B
 
 
 
 
Palmitoyltransferase Complex ( GO:0002178 )
 
 
6/10
 
 
0.0003733
 
 
0.0118315
 
 
0
 
 
0
 
 
11.210894
 
 
88.4891798
 
 
SPTLC2;ORMDL2;ORMDL3;ORMDL1;SPTSSB;SPTSSA
 
 
 
 
Serine Palmitoyltransferase Complex ( GO:0017059 )
 
 
5/7
 
 
0.0003934
 
 
0.0118315
 
 
0
 
 
0
 
 
18.679025
 
 
146.4575803
 
 
SPTLC2;ORMDL2;ORMDL3;SPTSSB;SPTSSA
 
 
 
 
Neuron Projection ( GO:0043005 )
 
 
92/560
 
 
0.0006501
 
 
0.0181574
 
 
0
 
 
0
 
 
1.484694
 
 
10.8951710
 
 
CNTNAP2;SNAP25;NRP1;NRSN1;FMR1;STMN2;PTEN;HTR2C;RORA;SLC6A1;GRM3;TMEM266;GRM5;ALCAM;ATCAY;GRM7;STMN1;PALM;NPTN;ATP7A;RGS8;VSTM5;ZNF385A;KIRREL3;SEMA6A;SLC30A3;MAGI2;ANK2;ANK3;CCSAP;SSTR3;SHTN1;VAMP7;BIN1;PALLD;FEZ2;IL1RAPL1;DCX;RAPGEF2;MAPT;RGS7BP;VAMP2;STX1A;PAFAH1B1;TMEM185A;HCN3;MTMR2;PRKAA1;RGS14;KCNA4;ADCY2;THY1;LRP2;AKAP5;C9ORF72;RTN4;PTGS1;TRAK2;STRN3;CDC42;FXR1;SPAST;BRINP1;CALCR;NTF3;MAP7;STX3;MARK3;MARK1;NTRK2;MPP2;PRNP;TGFB2;HOMER1;SYT1;FZD4;BDNF;PLK2;LIMK1;DTNBP1;RAB39B;CPEB1;PNOC;NAPEPLD;PPT1;ATXN10;CNTN4;KCNK2;METAP1;METAP2;CDK5R1;HCN1
 
 
 
 
Golgi Stack ( GO:0005795 )
 
 
10/31
 
 
0.0022191
 
 
0.0578443
 
 
0
 
 
0
 
 
3.561622
 
 
21.7638500
 
 
RAB30;RAB14;STX16;MGAT4A;RAB34;GCNT1;GOLPH3L;LYSET;ZFYVE1;GOLGA7
 
 
 
 
Postsynaptic Density ( GO:0014069 )
 
 
32/164
 
 
0.0029048
 
 
0.0667899
 
 
0
 
 
0
 
 
1.818753
 
 
10.6240495
 
 
MTMR2;RGS14;CTNND2;FMR1;CABP1;AKAP5;RTN4;ADD2;GRM5;NETO1;SLITRK1;RPL14;RPS3;SRGAP2;CACNG2;FAM81A;NANOS1;PRNP;MPP2;NTRK2;USP6;HOMER1;GRID1;MAGI2;SEMA4C;ATP2B4;DTNBP1;DNM3;LRFN2;GAP43;LRRC7;CDK5R1
 
 
 
 
COPII-coated ER to Golgi Transport Vesicle ( GO:0030134 )
 
 
18/77
 
 
0.0032599
 
 
0.0667899
 
 
0
 
 
0
 
 
2.284691
 
 
13.0822865
 
 
MCFD2;LMAN2L;STX17;SLC30A5;GOSR2;TGFA;IER3IP1;LMAN1;CNIH1;LMAN2;COL7A1;TMED1;TMED7;ERGIC2;TMED5;ERGIC1;TMED4;VTI1B
 
 
 
 
P-body ( GO:0000932 )
 
 
19/83
 
 
0.0032749
 
 
0.0667899
 
 
0
 
 
0
 
 
2.223525
 
 
12.7218253
 
 
TRIM71;DDX6;GARRE1;SAMD4A;C9ORF72;PNRC1;ZFP36L1;PNRC2;MEX3B;PSMA4;CNOT7;LIN28A;FAM184A;TOP1;EIF4E2;DCP1A;DCP2;TNRC6A;DIS3L2
 
 
 
 
Dendrite ( GO:0030425 )
 
 
49/281
 
 
0.0033969
 
 
0.0667899
 
 
0
 
 
0
 
 
1.587061
 
 
9.0222904
 
 
CNTNAP2;FMR1;HTR2C;TMEM266;GRM5;ATCAY;GRM7;RGS8;ATP7A;VSTM5;ZNF385A;KIRREL3;MAGI2;ANK3;BIN1;IL1RAPL1;MAPT;TMEM185A;HCN3;MTMR2;PRKAA1;RGS14;ADCY2;THY1;AKAP5;LRP2;C9ORF72;TRAK2;STRN3;BRINP1;NTF3;MARK3;MARK1;MPP2;PRNP;NTRK2;HOMER1;FZD4;BDNF;PLK2;GABRA3;ZNF804A;PNOC;ATXN10;KCNK2;METAP1;CDK5R1;HCN1;METAP2
 
 
 
 
Focal Adhesion ( GO:0005925 )
 
 
64/387
 
 
0.0034164
 
 
0.0667899
 
 
0
 
 
0
 
 
1.490761
 
 
8.4662985
 
 
ITGB1;SCARB2;NRP1;YWHAB;PPP1CB;PPP1CC;ALCAM;CSRP2;YWHAQ;PPFIA1;FLOT2;CAPN1;PDGFRB;PDIA3;KLF11;MAP2K1;PPP1R12A;ACTN2;RRAS2;ADAM10;YWHAZ;SENP1;ADAM17;PALLD;PXDN;IRF2;NUMB;MYH9;MAPRE1;MAPRE2;ARF6;EPHA2;STX16;THY1;CORO1C;HSP90B1;CNN3;CDC42;CNN2;EFNB2;GNA13;NRAS;RRAS;EPB41L2;RPS3;PIP5K1A;MAPK1;RSU1;MAP4K4;VASP;CAV3;CAV2;ARPC5;RAB10;MARCKS;HNRNPK;PTPRA;ITGA11;PI4KA;TADA1;FERMT2;HSPA1B;LIMS1;SNTB2
 
 
 
 
Glutamatergic Synapse ( GO:0098978 )
 
 
22/102
 
 
0.0035879
 
 
0.0668039
 
 
0
 
 
0
 
 
2.060446
 
 
11.6006808
 
 
GRIPAP1;USP6;NRXN1;LIMK1;ATP2B4;CLCN3;DGKZ;DYNLL2;YWHAZ;EFNB2;TANC1;RAP1A;SNX27;SLITRK1;TRAF6;AGO2;IL1RAPL1;NOS1AP;DLGAP1;STX4;NPTX1;CBLN1
 
 
 
 
Axon ( GO:0030424 )
 
 
39/217
 
 
0.0049721
 
 
0.0823978
 
 
0
 
 
0
 
 
1.644389
 
 
8.7216939
 
 
NRP1;CNTNAP2;HCN3;MTMR2;PRKAA1;FMR1;KCNA4;RORA;LRP2;SLC6A1;GRM3;ALCAM;SPAST;ATCAY;CALCR;GRM7;NTF3;MAP7;NPTN;ATP7A;VSTM5;KIRREL3;NTRK2;TGFB2;SEMA6A;SYT1;BDNF;DTNBP1;CCSAP;SHTN1;BIN1;PALLD;FEZ2;PPT1;MAPT;CNTN4;METAP1;CDK5R1;HCN1
 
 
 
 
RISC-loading Complex ( GO:0070578 )
 
 
4/7
 
 
0.0050770
 
 
0.0823978
 
 
0
 
 
0
 
 
9.957363
 
 
52.6050788
 
 
AGO4;PRKRA;AGO2;DICER1
 
 
 
 
Asymmetric Synapse ( GO:0032279 )
 
 
26/131
 
 
0.0054027
 
 
0.0823978
 
 
0
 
 
0
 
 
1.855819
 
 
9.6889796
 
 
MTMR2;RGS14;CTNND2;FMR1;CABP1;AKAP5;RTN4;ADD2;GRM7;NETO1;RPL14;RPS3;SRGAP2;FAM81A;NANOS1;PRNP;MPP2;NTRK2;HOMER1;MAGI2;SEMA4C;DTNBP1;DNM3;GAP43;LRRC7;CDK5R1
 
 
 
 
Cell-Cell Junction ( GO:0005911 )
 
 
53/316
 
 
0.0054333
 
 
0.0823978
 
 
0
 
 
0
 
 
1.514196
 
 
7.8968471
 
 
CLIC4;CTNND2;PTPRM;PTPRK;CLDN1;PNN;CDH5;FLOT2;KIRREL3;OPALIN;TMOD3;MAGI2;RPGRIP1L;TIAM1;ADAM17;FRMD5;RAP2B;LRRC7;PXDN;CDH11;FSCN1;MYADM;RAPGEF2;PKP4;JCAD;EPHA2;CRB2;NOTCH1;USP53;LIN7A;LIN7C;DLL1;MTDH;CNN3;CNN2;RAP1B;CYTH3;PARD6B;CDH20;CCDC85A;STX3;MPP2;CADM4;JAG1;VEGFA;TJP1;RAB10;RCAN1;TEK;FERMT2;EIF4G2;LIMS1;PALS1
 
 
 
 
Cell-Substrate Junction ( GO:0030055 )
 
 
64/395
 
 
0.0054791
 
 
0.0823978
 
 
0
 
 
0
 
 
1.454058
 
 
7.5710032
 
 
ITGB1;SCARB2;NRP1;YWHAB;PPP1CB;PPP1CC;ALCAM;CSRP2;YWHAQ;PPFIA1;FLOT2;CAPN1;PDGFRB;PDIA3;KLF11;MAP2K1;PPP1R12A;ACTN2;RRAS2;ADAM10;YWHAZ;SENP1;ADAM17;PALLD;PXDN;IRF2;NUMB;MYH9;MAPRE1;MAPRE2;ARF6;EPHA2;STX16;THY1;CORO1C;HSP90B1;CNN3;CDC42;CNN2;EFNB2;GNA13;NRAS;RRAS;EPB41L2;RPS3;PIP5K1A;MAPK1;RSU1;MAP4K4;VASP;CAV3;CAV2;ARPC5;RAB10;MARCKS;HNRNPK;PTPRA;ITGA11;PI4KA;TADA1;FERMT2;HSPA1B;LIMS1;SNTB2
 
 
 
 
Autophagosome ( GO:0005776 )
 
 
18/81
 
 
0.0057604
 
 
0.0832613
 
 
0
 
 
0
 
 
2.139144
 
 
11.0310130
 
 
SH3GLB1;STX17;ZFYVE1;C9ORF72;VMP1;TBC1D5;CHMP1B;RAB24;TP53INP1;JMY;ULK3;UBQLN1;ATG4B;CHMP4B;PIP4K2B;TBC1D12;SNAP29;CHMP7
 
 
 
 
Cytoplasmic Side of Plasma Membrane ( GO:0009898 )
 
 
23/113
 
 
0.0062467
 
 
0.0832613
 
 
0
 
 
0
 
 
1.914484
 
 
9.7173510
 
 
CHUK;PTEN;AKAP5;RASGRP4;PTP4A1;RGS2;NRAS;SNX18;TRAF6;PALM;TRAF5;CHMP4B;MYH9;ESYT3;PKP4;RGS8;SAMD12;PTPN4;CHMP7;SAMD10;SNX5;FERMT2;CYTH1
 
 
 
 
Endoplasmic reticulum-Golgi Intermediate Compartment Membrane ( GO:0033116 )
 
 
13/52
 
 
0.0063791
 
 
0.0832613
 
 
0
 
 
0
 
 
2.493764
 
 
12.6053121
 
 
MCFD2;GOSR2;GALNT1;TGFA;SPPL3;LMAN1;CNIH1;GORASP1;COL7A1;WHAMM;TMED7;BET1;PALS1
 
 
 
 
Rough Endoplasmic Reticulum ( GO:0005791 )
 
 
8/25
 
 
0.0063883
 
 
0.0832613
 
 
0
 
 
0
 
 
3.517532
 
 
17.7750826
 
 
LIN28A;STX17;RAB14;RPN1;SUCO;TMCC1;SPPL3;CKAP4
 
 
 
 
Early Endosome ( GO:0005769 )
 
 
53/320
 
 
0.0069946
 
 
0.0881508
 
 
0
 
 
0
 
 
1.491168
 
 
7.4000882
 
 
PLEKHF2;PI4K2B;NRP1;ANKRD13B;CNTNAP2;PHB1;USP32;SNX12;ECE1;LDLRAD4;RAB22A;ZFYVE26;TOM1;AP3S2;AP1S3;ATP7A;RUBCN;MAP2K1;MMGT1;LMTK2;WDFY1;ARL8B;ASAH1;RAB5B;MARCHF1;PARM1;TMEM63B;SLC30A10;CLCN3;HSPD1;PRDX3;SNX3;GGA2;RAP1A;SNX27;MARCHF8;STX6;MAPK1;LDLR;VTI1B;NTRK2;MON2;RAB4A;FZD5;SLC31A1;CMTM6;DNAJC13;EHD2;RAB14;SNX17;CD207;NAPEPLD;TMEM9B
 
 
 
 
Cyclin-Dependent Protein Kinase Holoenzyme Complex ( GO:0000307 )
 
 
12/47
 
 
0.0072144
 
 
0.0881508
 
 
0
 
 
0
 
 
2.564507
 
 
12.6473234
 
 
CCNA2;CCNK;CCNJ;CCND2;CCNE2;CCNY;CCNH;CCNG1;CCNC;CCNL2;CCNJL;CDK5R1
 
 
 
 
Intracellular Organelle Lumen ( GO:0070013 )
 
 
132/912
 
 
0.0076250
 
 
0.0903444
 
 
0
 
 
0
 
 
1.277378
 
 
6.2289113
 
 
ISCA2;POGLUT2;TOR2A;COL16A1;POGLUT1;SPARC;COL12A1;TXNDC12;YARS2;TXN2;LGALS1;EVA1A;PDK4;IL12B;CAPN1;GOLGA7;NUDT13;PDIA3;PDHX;MLIP;ANGEL2;ADAM10;P3H1;PDIA5;ADAM17;BIN2;COL4A2;MTHFD2;COL4A1;CLPP;BOLA3;COL4A4;DNAJB11;FDX1;EOGT;COL8A1;COL4A5;KPNB1;PSMD12;FOXO3;GNS;HSP90B1;CNN2;PRDX3;BLOC1S1;ARSJ;PDGFC;ESD;ARSI;WNT1;BDNF;ARPC5;HOOK3;COL1A1;CS;COL1A2;COL5A1;P4HA2;OGN;COL5A2;CALU;COL9A1;SDC1;NIT2;FBN1;COLGALT2;DDX3X;TNFAIP6;FKBP14;MRPS10;C1ORF35;MIA3;LAMC1;PCSK5;CTSS;SHH;AUH;MUC13;UQCRFS1;SPTAN1;PGM1;ADAMTS7;DYNLT1;GRID1;AHSG;COL25A1;GLRX5;ELAC2;GPX8;GPX7;CKAP4;RCN1;COL2A1;BDH1;MMP16;COL6A2;CANX;FASTKD5;COL21A1;COL6A3;MFGE8;PAFAH1B2;MATN3;COL15A1;ASAH1;COL11A1;PPM1K;PDHB;FSTL1;COL19A1;HSPD1;ERLEC1;CAND1;RNF138;RPS3;MAPK1;PDSS1;GPC4;GPC6;LDLR;GOLM1;MMAB;COL22A1;MAPK14;WARS2;COL3A1;TSPAN14;COL7A1;MGAT4A;ABCE1;HSPA1B;PNPLA2
 
 
 
 
Pericentric Heterochromatin ( GO:0005721 )
 
 
7/21
 
 
0.0082839
 
 
0.0952650
 
 
0
 
 
0
 
 
3.736429
 
 
17.9103487
 
 
UHRF2;KMT5C;POLE3;SNAI1;IKZF1;BAZ1B;MACROH2A1
 
 
 
 
Collagen-Containing Extracellular Matrix ( GO:0062023 )
 
 
61/383
 
 
0.0094488
 
 
0.1055567
 
 
0
 
 
0
 
 
1.423521
 
 
6.6362639
 
 
ACHE;SPARC;COL16A1;COL12A1;SERPINE1;LAMC1;CTSS;LOXL1;LOXL2;ESM1;SHH;LGALS1;CCN2;ADAMTS9;CDON;AHSG;COL25A1;SERPINF2;P3H1;NPNT;F3;GREM1;SFRP1;COL2A1;COL4A2;LOX;COL4A1;COL4A4;COL6A2;PXDN;COL8A1;COL21A1;COL4A5;COL6A3;MFGE8;MATN3;SEMA7A;COL15A1;LAMA2;COL11A1;LAMA4;NID1;ADAMTS10;COL19A1;HSP90B1;LMAN1;NDP;TGFB2;COL22A1;COLQ;COL1A1;COL3A1;COL1A2;COL5A1;COL7A1;COL5A2;OGN;MFAP2;COL9A1;FBN1;CBLN1
 
 
 
 
H3 Histone Acetyltransferase Complex ( GO:0070775 )
 
 
3/5
 
 
0.0137282
 
 
0.1411018
 
 
0
 
 
0
 
 
11.197926
 
 
48.0200847
 
 
ING5;BRPF3;KAT6B
 
 
 
 
MOZ/MORF Histone Acetyltransferase Complex ( GO:0070776 )
 
 
3/5
 
 
0.0137282
 
 
0.1411018
 
 
0
 
 
0
 
 
11.197926
 
 
48.0200847
 
 
ING5;BRPF3;KAT6B
 
 
 
 
Sex Chromosome ( GO:0000803 )
 
 
3/5
 
 
0.0137282
 
 
0.1411018
 
 
0
 
 
0
 
 
11.197926
 
 
48.0200847
 
 
PCGF5;PCGF3;MACROH2A1
 
 
 
 
Filopodium ( GO:0030175 )
 
 
13/57
 
 
0.0140741
 
 
0.1411018
 
 
0
 
 
0
 
 
2.209754
 
 
9.4211070
 
 
ITGB1;RIPOR2;ACTN2;FMR1;ACTA2;CDC42;SHTN1;GAP43;KITLG;PALM;FSCN1;ARF6;METAP2
 
 
 
 
Recycling Endosome Membrane ( GO:0055038 )
 
 
15/70
 
 
0.0154705
 
 
0.1512239
 
 
0
 
 
0
 
 
2.040232
 
 
8.5053645
 
 
PDIA3;SLC31A1;CMTM6;SCAMP1;SLC30A10;RAB43;SCAMP5;EHD2;SNX18;RAP2B;RAB14;RAB12;RAB8B;ARF6;VTI1B
 
 
 
 
Clathrin-Coated Vesicle ( GO:0030136 )
 
 
17/84
 
 
0.0180530
 
 
0.1721636
 
 
0
 
 
0
 
 
1.898446
 
 
7.6212099
 
 
ECE1;PIK3C2A;SCAMP1;SNAP91;GGA2;SNX3;SNX18;RAB14;RAB12;MALL;NUMB;CLVS2;STX6;ATP7A;VAMP2;RAB8B;CLVS1
 
 
 
 
Early Endosome Membrane ( GO:0031901 )
 
 
23/124
 
 
0.0187129
 
 
0.1742081
 
 
0
 
 
0
 
 
1.704907
 
 
6.7830437
 
 
PI4K2B;RAB5B;RAB4A;MON2;MARCHF1;TMEM63B;FZD5;SLC31A1;CMTM6;SNX12;DNAJC13;LDLRAD4;SLC30A10;SNX3;GGA2;RAB14;CD207;MARCHF8;ATP7A;SNX5;TMEM9B;ARL8B;VTI1B
 
 
 
 
Clathrin-Coated Endocytic Vesicle Membrane ( GO:0030669 )
 
 
14/66
 
 
0.0205920
 
 
0.1791267
 
 
0
 
 
0
 
 
2.013562
 
 
7.8183679
 
 
SCARB2;TFRC;FZD5;SYT1;FZD4;TGFA;RORA;LRP2;AREG;VAMP7;CD207;LDLR;VAMP2;HBEGF
 
 
 
 
Platelet Alpha Granule Lumen ( GO:0031093 )
 
 
14/66
 
 
0.0205920
 
 
0.1791267
 
 
0
 
 
0
 
 
2.013562
 
 
7.8183679
 
 
TGFB2;SPARC;ACTN2;AHSG;SERPINE1;SERPINF2;VEGFC;IGF1;GTPBP2;CYRIB;VEGFA;TMSB4X;SOS1;VTI1B
 
 
 
 
Vesicle ( GO:0031982 )
 
 
38/230
 
 
0.0206156
 
 
0.1791267
 
 
0
 
 
0
 
 
1.483567
 
 
5.7587727
 
 
ITSN2;DYNC1I2;RAB3C;SNAP25;TFRC;NRXN1;COL12A1;ITSN1;ECE1;ATP1A2;PIK3C2A;CLCN3;CORO1C;LFNG;PCMT1;FLOT2;SPTAN1;VTI1B;SLC30A7;RAB4A;SLC6A14;RAB39B;YWHAZ;MYL6;TUBB2A;BIN1;RAB34;STK26;COL6A2;OGN;CHMP4B;COL6A3;CALM3;MAN1B1;MFGE8;VAMP2;HSPA1B;SLC26A6
 
 
 
 
Golgi-associated Vesicle ( GO:0005798 )
 
 
11/48
 
 
0.0221152
 
 
0.1852784
 
 
0
 
 
0
 
 
2.222531
 
 
8.4711544
 
 
CHIC2;RAB14;RAB12;STK26;PI4KA;ZDHHC13;ATP7A;SPPL3;ZDHHC17;RHOQ;RAB8B
 
 
 
 
Cytoplasmic Vesicle Membrane ( GO:0030659 )
 
 
63/415
 
 
0.0222713
 
 
0.1852784
 
 
0
 
 
0
 
 
1.343729
 
 
5.1121577
 
 
ITGB1;SCARB2;SCARB1;TFRC;ARHGAP1;STAB2;ARRB1;ECE1;SLC2A3;ANTXR2;SPPL3;ANPEP;CHMP1B;MRC1;TOM1;AP1S3;MAP3K7;RAB8B;SLC30A5;ATP6AP2;VPS37C;ENTPD7;ZDHHC13;VPS37B;ZDHHC17;VAMP7;TBC1D5;TRAF6;VAMP2;RHOQ;HBEGF;RAB5B;NOTCH1;VPS4B;MYCBP2;ABHD17C;CLCN3;RHOBTB1;TMEM165;CORO1C;GGA2;APH1A;SNX3;ZNRF2;RAP1A;SNX2;IRAK1;RAB24;ORMDL3;CACNG2;WNT1;LDLR;RAB4A;PTPRN2;DNAJC13;ARHGAP26;SVIP;RAB10;EHD2;CLCN6;APPBP2;SNX17;PI4KA
 
 
 
 
Sarcoplasmic Reticulum Membrane ( GO:0033017 )
 
 
8/31
 
 
0.0245460
 
 
0.1920947
 
 
0
 
 
0
 
 
2.599030
 
 
9.6351393
 
 
SLC30A7;JPH3;PLN;DMPK;ASPH;FKBP1B;NOS1AP;TMEM109
 
 
 
 
Glial Cell Projection ( GO:0097386 )
 
 
5/15
 
 
0.0249583
 
 
0.1920947
 
 
0
 
 
0
 
 
3.734110
 
 
13.7809157
 
 
ITGB1;GRM3;FMR1;MAPT;KCNK2
 
 
 
 
Recycling Endosome ( GO:0055037 )
 
 
27/155
 
 
0.0250224
 
 
0.1920947
 
 
0
 
 
0
 
 
1.579505
 
 
5.8251906
 
 
TFRC;AKAP5;SLC30A10;CLCN3;TNF;RAB43;SCAMP5;STX6;TBC1D12;VTI1B;RAB8B;PDIA3;RAB4A;USP6;SLC31A1;CMTM6;LMTK2;SCAMP1;RAB10;EHD2;SNX18;RAP2B;RAB14;RAB12;RELCH;DENND6A;ARF6
 
 
 
 
X Chromosome ( GO:0000805 )
 
 
3/6
 
 
0.0250558
 
 
0.1920947
 
 
0
 
 
0
 
 
7.464860
 
 
27.5203188
 
 
PCGF5;PCGF3;MACROH2A1
 
 
 
 
Bicellular Tight Junction ( GO:0005923 )
 
 
16/81
 
 
0.0264105
 
 
0.1985863
 
 
0
 
 
0
 
 
1.841176
 
 
6.6908257
 
 
USP53;MAGI2;ANK3;CLDN1;YBX3;TGFBR1;MTDH;CYTH3;TJP1;CDH5;PARD6B;RAP2B;CLDN14;CLDN12;RAPGEF2;CYTH1
 
 
 
 
Lysosomal Membrane ( GO:0005765 )
 
 
58/383
 
 
0.0285359
 
 
0.2087234
 
 
0
 
 
0
 
 
1.339042
 
 
4.7624270
 
 
SLC46A3;SCARB2;SCARB1;ATP8A1;RAB3D;ARRB1;ECE1;ZFYVE26;PIP4P2;ANPEP;CHMP1B;SESN2;TOM1;ENPP1;AP1S3;ATP6V1E1;RNF152;SNAP29;BORCS6;TRIM23;STARD3NL;ATP6AP2;TFEB;MITF;TSC1;CKAP4;VAMP7;RRAGC;TBC1D7;CDIP1;CHMP4B;CHMP7;ARL8B;SIDT2;ENPEP;MARCHF1;ABCB6;SAR1A;STX17;ABHD6;PRCP;LRP2;CLCN3;TMEM165;RAP1B;BLOC1S1;SLC38A9;LDLR;VTI1B;CTSA;CMTM6;DNAJC13;VASN;GPR137C;CLCN6;RAB14;PPT1;TMEM9B
 
 
 
 
Membrane Raft ( GO:0045121 )
 
 
29/171
 
 
0.0288262
 
 
0.2087234
 
 
0
 
 
0
 
 
1.529330
 
 
5.4237201
 
 
ITGB1;ADCY2;THY1;TNFRSF11A;AKAP5;TNF;DLL1;ADD2;RAP1B;PPP2CA;SHH;NANOS1;PRNP;CAV3;CAV2;MMAB;ATP2B4;ERLIN2;TNFRSF1B;TGFBR1;TNFRSF1A;ADAM17;RAP2B;MALL;MYADM;PPT1;MAPT;TEK;IL6ST
 
 
 
 
trans-Golgi Network ( GO:0005802 )
 
 
40/251
 
 
0.0304226
 
 
0.2154346
 
 
0
 
 
0
 
 
1.420700
 
 
4.9618951
 
 
PI4K2B;MARCHF1;SYS1;ATP8A1;STX16;CABP7;GCNT1;DPY30;GCC2;PIK3C2A;PCSK5;FUT4;TMEM165;SCAMP5;GGA2;RAB43;ATXN2;ARL5B;SLC39A9;FUT9;STX6;CLVS2;ARFIP1;AP1S3;ATP7A;CLVS1;ARFGEF1;SLC30A6;RIC1;SLC30A5;GOLPH3L;SCAMP1;TJAP1;PLEKHA8;RAB10;RAB30;VAMP7;TRAPPC6B;RELCH;VAMP2
 
 
 
 
Adherens Junction ( GO:0005912 )
 
 
26/151
 
 
0.0312577
 
 
0.2154346
 
 
0
 
 
0
 
 
1.557110
 
 
5.3961478
 
 
NOTCH1;CTNND2;PTPRM;LIN7A;LIN7C;DLL1;CNN3;CYTH3;CDH5;CDH20;CCDC85A;FLOT2;MPP2;JAG1;TMOD3;VEGFA;RAB10;TJP1;FRMD5;LRRC7;CDH11;PKP4;JCAD;EIF4G2;FERMT2;PALS1
 
 
 
 
Tight Junction ( GO:0070160 )
 
 
18/96
 
 
0.0314061
 
 
0.2154346
 
 
0
 
 
0
 
 
1.726295
 
 
5.9742839
 
 
USP53;MAGI2;ANK3;CLDN1;YBX3;TGFBR1;AMOT;MTDH;CYTH3;TJP1;CDH5;PARD6B;RAP2B;CLDN14;CLDN12;RAPGEF2;CYTH1;EPHA2
 
 
 
 
Nuclear Membrane ( GO:0031965 )
 
 
38/239
 
 
0.0352191
 
 
0.2374251
 
 
0
 
 
0
 
 
1.416407
 
 
4.7395365
 
 
NRXN1;GATA6;TXLNG;ENO1;C9ORF72;PHF8;GHRHR;CDH5;TMEM168;SPAST;CCND2;POM121C;TMEM43;CLMN;RB1CC1;EPC1;TNRC18;SEPHS1;LBR;SH3D19;PRNP;TMEM120B;LYPLA1;CENPV;AKIRIN1;DMPK;GCH1;WTAP;OSBPL3;ATP1B4;LEMD3;SIRT1;SENP1;MCM3AP;NUP50;NOS1AP;BCL2;KPNB1
 
 
 
 
Extracellular Membrane-Bounded Organelle ( GO:0065010 )
 
 
12/59
 
 
0.0411334
 
 
0.2725962
 
 
0
 
 
0
 
 
1.908437
 
 
6.0896977
 
 
LFNG;PCMT1;TUBB2A;TFRC;COL6A2;COL12A1;OGN;COL6A3;ATP1A2;MAN1B1;MFGE8;SPTAN1
 
 
 
 
PRC1 Complex ( GO:0035102 )
 
 
5/17
 
 
0.0421368
 
 
0.2745912
 
 
0
 
 
0
 
 
3.111405
 
 
9.8533063
 
 
PCGF6;CBX4;PCGF5;PCGF3;COMMD3-BMI1
 
 
 
 
Apical Junction Complex ( GO:0043296 )
 
 
19/107
 
 
0.0452755
 
 
0.2902082
 
 
0
 
 
0
 
 
1.614901
 
 
4.9981017
 
 
CRB2;USP53;MAGI2;ANK3;CLDN1;YBX3;TGFBR1;AMOT;MTDH;CYTH3;TJP1;CDH5;PARD6B;RAP2B;CLDN14;CLDN12;RAPGEF2;CYTH1;PALS1
 
 
 
 
Nuclear Inner Membrane ( GO:0005637 )
 
 
7/29
 
 
0.0481298
 
 
0.3035284
 
 
0
 
 
0
 
 
2.376648
 
 
7.2104020
 
 
GHRHR;TMEM120B;TMEM43;ATP1B4;LEMD3;LBR;SIRT1
 
 
 
 
trans-Golgi Network Membrane ( GO:0032588 )
 
 
19/108
 
 
0.0490880
 
 
0.3046572
 
 
0
 
 
0
 
 
1.596665
 
 
4.8125720
 
 
SLC30A6;SYS1;MARCHF1;RIC1;CABP7;STX16;SLC30A5;GOLPH3L;SCAMP1;TMEM165;RAB43;SCAMP5;FUT9;CLVS2;STX6;ARFIP1;AP1S3;ATP7A;CLVS1
 
 
 
 
Cell-Cell Contact Zone ( GO:0044291 )
 
 
10/48
 
 
0.0513349
 
 
0.3131449
 
 
0
 
 
0
 
 
1.966365
 
 
5.8388927
 
 
TIAM1;CADM4;RAP2B;TMEM65;PIK3CA;OPALIN;FLOT2;ANK2;PKP4;ANK3
 
 
 
 
Clathrin-Coated Vesicle Membrane ( GO:0030665 )
 
 
16/88
 
 
0.0520573
 
 
0.3131449
 
 
0
 
 
0
 
 
1.661511
 
 
4.9104448
 
 
SCARB2;TFRC;FZD5;SYT1;FZD4;TGFA;RORA;LRP2;AREG;NECAP2;VAMP7;NECAP1;CD207;LDLR;VAMP2;HBEGF
 
 
 
 
Autophagosome Membrane ( GO:0000421 )
 
 
8/36
 
 
0.0555723
 
 
0.3269846
 
 
0
 
 
0
 
 
2.134311
 
 
6.1683100
 
 
SH3GLB1;VMP1;STX17;CHMP1B;JMY;ATG4B;CHMP4B;CHMP7
 
 
 
 
Caveola ( GO:0005901 )
 
 
12/62
 
 
0.0572217
 
 
0.3269846
 
 
0
 
 
0
 
 
1.793625
 
 
5.1312427
 
 
SCARB1;EHD2;ENTPD1;CAV3;CAV2;NOS1AP;CAVIN4;ATP2B4;FLOT2;MAPK1;TFPI;CORO1C
 
 
 
 
Extracellular Vesicle ( GO:1903561 )
 
 
12/62
 
 
0.0572217
 
 
0.3269846
 
 
0
 
 
0
 
 
1.793625
 
 
5.1312427
 
 
LFNG;PCMT1;TUBB2A;TFRC;COL6A2;COL12A1;OGN;COL6A3;ATP1A2;MAN1B1;MFGE8;SPTAN1
 
 
 
 
Phosphatidylinositol 3-Kinase Complex, Class I ( GO:0097651 )
 
 
3/8
 
 
0.0585394
 
 
0.3269846
 
 
0
 
 
0
 
 
4.478408
 
 
12.7099666
 
 
PIK3CA;PIK3R3;PIK3R1
 
 
 
 
Phosphatidylinositol 3-Kinase Complex, Class IA ( GO:0005943 )
 
 
3/8
 
 
0.0585394
 
 
0.3269846
 
 
0
 
 
0
 
 
4.478408
 
 
12.7099666
 
 
PIK3CA;PIK3R3;PIK3R1
 
 
 
 
Cytoplasmic Stress Granule ( GO:0010494 )
 
 
15/84
 
 
0.0667907
 
 
0.3678191
 
 
0
 
 
0
 
 
1.624977
 
 
4.3974987
 
 
DAZAP2;DDX6;DDX3X;FMR1;LARP4B;LARP4;C9ORF72;EIF2S1;QKI;FXR1;ATXN2;HNRNPK;LIN28A;RBPMS2;KPNB1
 
 
 
 
Late Endosome ( GO:0005770 )
 
 
34/222
 
 
0.0686022
 
 
0.3707763
 
 
0
 
 
0
 
 
1.353629
 
 
3.6269551
 
 
SCARB2;ANKRD13B;MARCHF1;PARM1;GOSR2;ABHD6;CLCN3;CTSS;TMEM25;ZFYVE26;PIP4P2;RAP1A;TTPA;CHMP1B;MARCHF8;IL12B;MAPK1;SLC38A9;ATP7A;VPS36;LDLR;ANKRD13A;VTI1B;RUBCN;MAP2K1;STARD3NL;SLC30A3;VTA1;MAGI2;VAMP7;RAB14;RAPGEF2;CHMP4B;CHMP7
 
 
 
 
Mitochondrial Intermembrane Space ( GO:0005758 )
 
 
13/71
 
 
0.0713749
 
 
0.3707763
 
 
0
 
 
0
 
 
1.675031
 
 
4.4217606
 
 
TIMM8B;CLPB;TAFAZZIN;TIMM9;CPOX;TRIAP1;NLN;GATM;AIFM1;BLOC1S1;STMP1;ALPL;CYCS
 
 
 
 
Calcium Channel Complex ( GO:0034704 )
 
 
10/51
 
 
0.0727606
 
 
0.3707763
 
 
0
 
 
0
 
 
1.822174
 
 
4.7751529
 
 
CACNB3;ASPH;PTPRA;SESTD1;FKBP1B;PDE4B;CACNA2D3;CACNA1A;MICU3;CALM3
 
 
 
 
Lytic Vacuole Membrane ( GO:0098852 )
 
 
46/314
 
 
0.0732413
 
 
0.3707763
 
 
0
 
 
0
 
 
1.285426
 
 
3.3600991
 
 
SLC46A3;SCARB2;SCARB1;ENPEP;MARCHF1;ABCB6;SAR1A;STX17;ABHD6;ARRB1;ECE1;LRP2;CLCN3;TMEM165;ZFYVE26;PIP4P2;BLOC1S1;ANPEP;CHMP1B;SESN2;ENPP1;SLC38A9;AP1S3;ATP6V1E1;RNF152;TRIM23;VTI1B;STARD3NL;ATP6AP2;TFEB;MITF;TSC1;DNAJC13;VASN;GPR137C;CLCN6;VAMP7;RRAGC;RAB14;TBC1D7;PPT1;CHMP4B;CHMP7;TMEM9B;ARL8B;SIDT2
 
 
 
 
Endocytic Vesicle ( GO:0030139 )
 
 
31/201
 
 
0.0734612
 
 
0.3707763
 
 
0
 
 
0
 
 
1.364522
 
 
3.5627647
 
 
SCARB2;SCARB1;RAB5B;SPARC;NRXN1;STAB2;LPAR2;ARRB1;CLCN3;CTSS;RAB22A;AMOT;HSP90B1;RAB43;RAB24;FLOT2;SRGAP2;WNT1;CACNG2;RAB8B;PDIA3;ENTPD7;GAPVD1;EHD2;VAMP7;RAB14;RAB34;CD207;RAPGEF2;ARF6;HBEGF
 
 
 
 
BLOC-1 Complex ( GO:0031083 )
 
 
4/14
 
 
0.0739656
 
 
0.3707763
 
 
0
 
 
0
 
 
2.986023
 
 
7.7760670
 
 
SNAP25;BLOC1S6;BLOC1S1;DTNBP1
 
 
 
 
Cul5-RING Ubiquitin Ligase Complex ( GO:0031466 )
 
 
4/14
 
 
0.0739656
 
 
0.3707763
 
 
0
 
 
0
 
 
2.986023
 
 
7.7760670
 
 
SOCS2;CUL5;ASB11;RNF7
 
 
 
 
SCF Ubiquitin Ligase Complex ( GO:0019005 )
 
 
11/58
 
 
0.0753324
 
 
0.3728476
 
 
0
 
 
0
 
 
1.748658
 
 
4.5217586
 
 
FBXW4;USP47;TMEM183A;CUL5;FBXW7;FBXL19;DAW1;FBXO45;FBXL13;SKP1;SPSB4
 
 
 
 
Lysosome ( GO:0005764 )
 
 
74/532
 
 
0.0770189
 
 
0.3764297
 
 
0
 
 
0
 
 
1.211402
 
 
3.1056785
 
 
SLC46A3;SCARB2;SCARB1;ARRB1;ECE1;IFI30;CTSS;ZFYVE26;PIP4P2;ANPEP;CHMP1B;RB1CC1;SESN2;ENPP1;AP1S3;ATP6V1E1;RNF152;TRIM23;USP6;STARD3NL;USP4;PRKCD;ATP6AP2;TFEB;MITF;TSC1;PLA2G15;VAMP7;RRAGC;NPC2;TBC1D7;SIDT1;CHMP4B;CHMP7;ARL8B;SIDT2;ENPEP;ZNF395;ASAH1;CALCRL;MARCHF1;ABCB6;SAR1A;STX17;ABHD6;LRP2;CLCN3;C9ORF72;GNS;TMEM165;TMEM25;SNX2;BLOC1S1;MARCHF8;SLC38A9;GPC4;DRAM2;LDLR;GPC6;MARCHF3;VTI1B;CTSA;DNAJC13;VASN;GPR137C;MFHAS1;CLCN6;RAB14;RAB12;OGN;PPT1;SDC1;ADA;TMEM9B
 
 
 
 
Serine/Threonine Protein Kinase Complex ( GO:1902554 )
 
 
13/72
 
 
0.0781068
 
 
0.3770341
 
 
0
 
 
0
 
 
1.646547
 
 
4.1981643
 
 
CCNJ;CCNH;CCNC;TGFBR1;ACVR2A;CCNA2;PARD6B;CCND2;CCNY;CCNG1;CCNL2;CCNJL;CDK5R1
 
 
 
 
Amphisome Membrane ( GO:1904930 )
 
 
3/9
 
 
0.0802912
 
 
0.3815027
 
 
0
 
 
0
 
 
3.731795
 
 
9.4119428
 
 
CHMP1B;CHMP4B;CHMP7
 
 
 
 
Sarcolemma ( GO:0042383 )
 
 
10/52
 
 
0.0809839
 
 
0.3815027
 
 
0
 
 
0
 
 
1.778688
 
 
4.4707394
 
 
ITGB1;NANOS1;MLIP;CAV3;SGCB;NOS1AP;SLC2A1;DTNBP1;ANK2;ANK3
 
 
 
 
cullin-RING Ubiquitin Ligase Complex ( GO:0031461 )
 
 
33/219
 
 
0.0855279
 
 
0.3981119
 
 
0
 
 
0
 
 
1.327526
 
 
3.2642704
 
 
TMEM183A;ANAPC16;CUL5;CUL3;DCAF12L1;FBXO45;ASB11;RNF7;DCAF6;SOCS2;CDC23;CAND1;ZSWIM6;ZSWIM5;DCAF12;SKP1;FBXW4;KLHL29;USP47;FBXW7;FEM1B;FBXL19;KLHL28;DAW1;KLHL2;FBXL13;KCTD5;DDB2;APPBP2;KLHL6;KLHL8;SPSB4;CUL4B
 
 
 
 
ER to Golgi Transport Vesicle Membrane ( GO:0012507 )
 
 
10/54
 
 
0.0990715
 
 
0.4471068
 
 
0
 
 
0
 
 
1.697645
 
 
3.9248082
 
 
LMAN1;MCFD2;CNIH1;SAR1A;SLC30A5;GOSR2;TGFA;TMED7;SEC23B;VTI1B
 
 
 
 
Clathrin-Coated Endocytic Vesicle ( GO:0045334 )
 
 
14/82
 
 
0.1001417
 
 
0.4471068
 
 
0
 
 
0
 
 
1.538382
 
 
3.5400762
 
 
SCARB2;TFRC;FZD5;SYT1;FZD4;TGFA;RORA;LRP2;AREG;VAMP7;CD207;LDLR;VAMP2;HBEGF
 
 
 
 
CD40 Receptor Complex ( GO:0035631 )
 
 
3/10
 
 
0.1049534
 
 
0.4471068
 
 
0
 
 
0
 
 
3.198500
 
 
7.2101825
 
 
CHUK;TRAF6;TRAF5
 
 
 
 
Neuron Projection Cytoplasm ( GO:0120111 )
 
 
3/10
 
 
0.1049534
 
 
0.4471068
 
 
0
 
 
0
 
 
3.198500
 
 
7.2101825
 
 
FMR1;EIF4EBP2;METAP2
 
 
 
 
Platelet Alpha Granule ( GO:0031091 )
 
 
15/90
 
 
0.1067907
 
 
0.4471068
 
 
0
 
 
0
 
 
1.494468
 
 
3.3429527
 
 
TGFB2;SPARC;ACTN2;AHSG;SERPINE1;SERPINF2;VEGFC;IGF1;GTPBP2;CYRIB;VEGFA;VAMP7;TMSB4X;SOS1;VTI1B
 
 
 
 
Specific Granule Membrane ( GO:0035579 )
 
 
15/90
 
 
0.1067907
 
 
0.4471068
 
 
0
 
 
0
 
 
1.494468
 
 
3.3429527
 
 
SNAP25;PGAP1;ATP8A1;DGAT1;CMTM6;MOSPD2;SLC2A3;SCAMP1;TNFRSF1B;CKAP4;TSPAN14;RAP2B;RAP1A;ORMDL3;TOM1
 
 
 
 
Sarcoplasmic Reticulum ( GO:0016529 )
 
 
9/48
 
 
0.1072947
 
 
0.4471068
 
 
0
 
 
0
 
 
1.723521
 
 
3.8472029
 
 
NANOS1;SLC30A7;PLN;DMPK;ASPH;FKBP1B;NOS1AP;TMEM109;ANK3
 
 
 
 
Hrd1p Ubiquitin Ligase Complex ( GO:0000836 )
 
 
2/5
 
 
0.1095793
 
 
0.4471068
 
 
0
 
 
0
 
 
4.974468
 
 
10.9990773
 
 
SYVN1;UBE2J1
 
 
 
 
Chromocenter ( GO:0010369 )
 
 
2/5
 
 
0.1095793
 
 
0.4471068
 
 
0
 
 
0
 
 
4.974468
 
 
10.9990773
 
 
MBD6;SALL1
 
 
 
 
Neuron Spine ( GO:0044309 )
 
 
2/5
 
 
0.1095793
 
 
0.4471068
 
 
0
 
 
0
 
 
4.974468
 
 
10.9990773
 
 
HOMER1;PALM
 
 
 
 
Protein Phosphatase 4 Complex ( GO:0030289 )
 
 
2/5
 
 
0.1095793
 
 
0.4471068
 
 
0
 
 
0
 
 
4.974468
 
 
10.9990773
 
 
PPP4R2;PPP4R3A
 
 
 
 
mRNA Cleavage and Polyadenylation Specificity Factor Complex ( GO:0005847 )
 
 
5/22
 
 
0.1097756
 
 
0.4471068
 
 
0
 
 
0
 
 
2.195663
 
 
4.8509161
 
 
CPSF4;CPSF7;CPSF6;CSNK1A1;PIP5K1A
 
 
 
 
Specific Granule ( GO:0042581 )
 
 
24/159
 
 
0.1249628
 
 
0.5037162
 
 
0
 
 
0
 
 
1.328967
 
 
2.7639037
 
 
SNAP25;PGAP1;ATP8A1;DGAT1;CMTM6;MOSPD2;ADAM10;SLC2A3;SCAMP1;TNFRSF1B;CLCN3;CKAP4;CNN2;TSPAN14;RAP1A;RAP2B;LRRC7;ORMDL3;TOM1;STX4;STX3;NIT2;SPTAN1;KPNB1
 
 
 
 
Golgi-associated Vesicle Membrane ( GO:0030660 )
 
 
5/23
 
 
0.1273276
 
 
0.5080110
 
 
0
 
 
0
 
 
2.073564
 
 
4.2735993
 
 
PI4KA;ZDHHC13;SPPL3;ZDHHC17;RHOQ
 
 
 
 
Amphisome ( GO:0044753 )
 
 
3/11
 
 
0.1321386
 
 
0.5166620
 
 
0
 
 
0
 
 
2.798529
 
 
5.6639529
 
 
CHMP1B;CHMP4B;CHMP7
 
 
 
 
Filopodium Membrane ( GO:0031527 )
 
 
3/11
 
 
0.1321386
 
 
0.5166620
 
 
0
 
 
0
 
 
2.798529
 
 
5.6639529
 
 
GAP43;PALM;ARF6
 
 
 
 
Ficolin-1-Rich Granule Lumen ( GO:1904813 )
 
 
19/123
 
 
0.1350890
 
 
0.5229684
 
 
0
 
 
0
 
 
1.365208
 
 
2.7329031
 
 
PSMD12;ASAH1;DDX3X;TNFAIP6;DYNLT1;C1ORF35;ARPC5;HOOK3;MAPK14;GNS;CTSS;CAND1;BIN2;MAPK1;CAPN1;KPNB1;PGM1;PAFAH1B2;HSPA1B
 
 
 
 
Vesicle Coat ( GO:0030120 )
 
 
7/37
 
 
0.1401550
 
 
0.5278569
 
 
0
 
 
0
 
 
1.742084
 
 
3.4232059
 
 
NECAP2;NECAP1;SAR1A;TMED7;DIPK2A;SEC23B;COPZ1
 
 
 
 
Azurophil Granule Membrane ( GO:0035577 )
 
 
10/58
 
 
0.1416979
 
 
0.5278569
 
 
0
 
 
0
 
 
1.555821
 
 
3.0401645
 
 
RAP1B;VAMP7;ATP8A1;RAB3D;CMTM6;TOM1;PRCP;DNAJC13;SNAP29;CKAP4
 
 
 
 
Transport Vesicle Membrane ( GO:0030658 )
 
 
10/58
 
 
0.1416979
 
 
0.5278569
 
 
0
 
 
0
 
 
1.555821
 
 
3.0401645
 
 
LMAN1;MCFD2;CNIH1;SYT1;SLC30A5;GOSR2;TGFA;AREG;VAMP2;VTI1B
 
 
 
 
Basolateral Plasma Membrane ( GO:0016323 )
 
 
29/200
 
 
0.1434196
 
 
0.5278569
 
 
0
 
 
0
 
 
1.267864
 
 
2.4621682
 
 
TFRC;SLC2A1;TGFA;LIN7A;LIN7C;CLDN1;SLC7A1;ERBB3;SLC16A6;ENPP1;FLOT2;STX4;ATP7A;LDLR;MPP2;SLC31A1;ABCC5;ANK2;SLC16A10;ANK3;ADRA2A;TJP1;SLC4A7;SLC7A7;LRRC7;NUMB;TEK;SLC22A7;SLC26A6
 
 
 
 
Activin Receptor Complex ( GO:0048179 )
 
 
2/6
 
 
0.1518411
 
 
0.5278569
 
 
0
 
 
0
 
 
3.730639
 
 
7.0319584
 
 
ACVR2A;TGFBR1
 
 
 
 
Apicolateral Plasma Membrane ( GO:0016327 )
 
 
2/6
 
 
0.1518411
 
 
0.5278569
 
 
0
 
 
0
 
 
3.730639
 
 
7.0319584
 
 
MTCL1;NEDD4
 
 
 
 
Astral Microtubule ( GO:0000235 )
 
 
2/6
 
 
0.1518411
 
 
0.5278569
 
 
0
 
 
0
 
 
3.730639
 
 
7.0319584
 
 
FAM161A;PAFAH1B1
 
 
 
 
Flotillin Complex ( GO:0016600 )
 
 
2/6
 
 
0.1518411
 
 
0.5278569
 
 
0
 
 
0
 
 
3.730639
 
 
7.0319584
 
 
FLOT2;CORO1C
 
 
 
 
Insulin-Responsive Compartment ( GO:0032593 )
 
 
2/6
 
 
0.1518411
 
 
0.5278569
 
 
0
 
 
0
 
 
3.730639
 
 
7.0319584
 
 
RAB10;RAB4A
 
 
 
 
Matrix Side of Mitochondrial Inner Membrane ( GO:0099617 )
 
 
2/6
 
 
0.1518411
 
 
0.5278569
 
 
0
 
 
0
 
 
3.730639
 
 
7.0319584
 
 
BDH1;COA8
 
 
 
 
Pyruvate Dehydrogenase Complex ( GO:0045254 )
 
 
2/6
 
 
0.1518411
 
 
0.5278569
 
 
0
 
 
0
 
 
3.730639
 
 
7.0319584
 
 
PDHX;PDHB
 
 
 
 
Secretory Vesicle ( GO:0099503 )
 
 
6/31
 
 
0.1525520
 
 
0.5278569
 
 
0
 
 
0
 
 
1.791607
 
 
3.3686680
 
 
RAB10;DYNLT1;SYT1;RAB3D;DPYSL3;IGF1
 
 
 
 
Endoribonuclease Complex ( GO:1902555 )
 
 
4/18
 
 
0.1555833
 
 
0.5336234
 
 
0
 
 
0
 
 
2.132389
 
 
3.9674683
 
 
AGO4;PRKRA;AGO2;DICER1
 
 
 
 
COPI Vesicle Coat ( GO:0030126 )
 
 
3/12
 
 
0.1614388
 
 
0.5488919
 
 
0
 
 
0
 
 
2.487440
 
 
4.5361683
 
 
DIPK2A;TMED7;COPZ1
 
 
 
 
SAGA-type Complex ( GO:0070461 )
 
 
7/39
 
 
0.1712594
 
 
0.5772622
 
 
0
 
 
0
 
 
1.633018
 
 
2.8815842
 
 
TADA2B;POLE3;TADA1;TAF5;TAF9B;TAF4;MAP3K7
 
 
 
 
Lytic Vacuole ( GO:0000323 )
 
 
33/236
 
 
0.1747834
 
 
0.5841050
 
 
0
 
 
0
 
 
1.215169
 
 
2.1195070
 
 
SCARB1;ASAH1;MARCHF1;CALCRL;LRP2;IFI30;C9ORF72;CTSS;TMEM25;ZFYVE26;SNX2;RB1CC1;MARCHF8;SLC38A9;DRAM2;RNF152;LDLR;MARCHF3;CTSA;USP6;USP4;ATP6AP2;PLA2G15;MFHAS1;RRAGC;RAB14;NPC2;RAB12;PPT1;SIDT1;ADA;ARL8B;SIDT2
 
 
 
 
Voltage-Gated Potassium Channel Complex ( GO:0008076 )
 
 
13/83
 
 
0.1778696
 
 
0.5893815
 
 
0
 
 
0
 
 
1.386935
 
 
2.3948271
 
 
DPP10;CNTNAP2;SNAP25;HCN3;MPP2;KCND3;KCNIP3;KCNIP4;KCNA4;KCNS3;STX1A;VAMP2;HCN1
 
 
 
 
Germ Plasm ( GO:0060293 )
 
 
4/19
 
 
0.1797435
 
 
0.5905859
 
 
0
 
 
0
 
 
1.990117
 
 
3.4154872
 
 
DDX3Y;TDRKH;DDX3X;HELZ
 
 
 
 
ESC/E(Z) Complex ( GO:0035098 )
 
 
3/13
 
 
0.1924441
 
 
0.6198031
 
 
0
 
 
0
 
 
2.238569
 
 
3.6890489
 
 
MTF2;RBBP7;SIRT1
 
 
 
 
Clathrin-Sculpted Gamma-Aminobutyric Acid Transport Vesicle ( GO:0061200 )
 
 
2/7
 
 
0.1965616
 
 
0.6198031
 
 
0
 
 
0
 
 
2.984342
 
 
4.8548663
 
 
SYT1;VAMP2
 
 
 
 
Clathrin-Sculpted Gamma-Aminobutyric Acid Transport Vesicle Membrane ( GO:0061202 )
 
 
2/7
 
 
0.1965616
 
 
0.6198031
 
 
0
 
 
0
 
 
2.984342
 
 
4.8548663
 
 
SYT1;VAMP2
 
 
 
 
cyclin/CDK Positive Transcription Elongation Factor Complex ( GO:0008024 )
 
 
2/7
 
 
0.1965616
 
 
0.6198031
 
 
0
 
 
0
 
 
2.984342
 
 
4.8548663
 
 
RB1;CCNT2
 
 
 
 
Oligosaccharyltransferase Complex A ( GO:0160226 )
 
 
2/7
 
 
0.1965616
 
 
0.6198031
 
 
0
 
 
0
 
 
2.984342
 
 
4.8548663
 
 
OSTC;RPN1
 
 
 
 
Golgi Cisterna ( GO:0031985 )
 
 
8/48
 
 
0.2012915
 
 
0.6296397
 
 
0
 
 
0
 
 
1.493000
 
 
2.3932804
 
 
RAB30;NECAB3;SGMS1;STX16;RAB34;GCNT1;GOLPH3L;LYSET
 
 
 
 
Aggresome ( GO:0016235 )
 
 
6/34
 
 
0.2077300
 
 
0.6446224
 
 
0
 
 
0
 
 
1.599376
 
 
2.5134457
 
 
SLC2A14;PRDM16;UBQLN1;SLC2A3;EPS15;HSPA1B
 
 
 
 
Vacuolar Membrane ( GO:0005774 )
 
 
9/56
 
 
0.2118192
 
 
0.6521361
 
 
0
 
 
0
 
 
1.429506
 
 
2.2186245
 
 
SH3GLB1;VMP1;STX17;CHMP1B;ATP6AP2;JMY;ATG4B;CHMP4B;CHMP7
 
 
 
 
Mitochondrial Outer Membrane ( GO:0005741 )
 
 
24/171
 
 
0.2142002
 
 
0.6543147
 
 
0
 
 
0
 
 
1.219643
 
 
1.8792794
 
 
VAT1;TOMM70;PRNP;DMPK;ACSL1;RMDN3;ACSL4;FOXO3;AGPAT4;FUNDC2;MAVS;NRAS;BCL2L11;STMP1;PTRH2;BCL2;MFN1;PMAIP1;BMF;SFXN2;BAK1;BCL2L2;MCL1;MARCHF5
 
 
 
 
Intracellular Membraneless Organelle ( GO:0043232 )
 
 
164/1310
 
 
0.2222294
 
 
0.6735791
 
 
0
 
 
0
 
 
1.072096
 
 
1.6124800
 
 
SRP19;RIPOR2;GEMIN2;FMR1;GTF2B;RORA;PRDM1;DEUP1;ZFYVE1;HOXA13;CDC14A;TXN2;PPP1CC;GOLGA3;CCND2;SALL1;KAT5;EPC1;KMT5B;NEPRO;PHLDA1;TMOD1;NANOS1;DDX18;SLC30A9;ESCO1;FBXW7;ACTN2;SLC30A5;TMOD3;TPM1;DYRK1B;ACSL4;TSC1;LARP4B;FRMD5;MYCN;CCNY;BIN2;BIN1;WDR82;FSCN1;WDFY1;CMPK1;PKP4;TP53;SRSF9;ZNF397;IPPK;SLBP;INO80E;ABHD4;C2CD4A;PDS5B;ABHD5;PDS5A;MACROH2A1;CDKN2AIP;RHOBTB1;RAP1B;CNN2;FXR1;DHX33;IRAK1;EPB41L2;EIF4EBP2;MARK1;NAA50;SRPK2;WAPL;ABTB1;MFHAS1;LIN28B;CNOT7;STAG2;LIN28A;LAS1L;RCAN2;INTS7;MMS19;ANP32B;POMT2;SNAP25;DYRK2;MTCL1;TUT4;SUV39H1;CHD7;HMGB2;SIX1;CTCF;TENT4B;PHF8;EPB41L4A;SERP1;NKRF;CASP2;HORMAD2;TNPO1;RBM7;ZNF367;BATF3;PPP1R12A;EN2;BAZ1B;CCSAP;DYNLL2;SIRT1;DDX50;ETV4;CKAP4;ETV6;ARL4A;MDFIC;TRAF6;PAX9;RARA;DCX;FAM193B;TOP1;MAPRE1;ERGIC2;PAFAH1B2;KDM7A;KANK1;RGS14;CIPC;NRXN1;TTK;PPM1D;ADD2;TMEM65;PNMA1;RNF138;PPP1R7;SAP30L;RPS3;MAPK1;VHL;E2F5;EXOSC3;RPP14;WASF1;DEDD;ARFGEF1;MPP2;MBD6;CSNK1A1;CDC42BPG;NPM3;HINFP;VMP1;KLF6;TCIM;NOVA1;KATNBL1;SCD;CAPZA1;SNX15;SPRY2;FILIP1;ERCC6;TAF5;PNPLA2
 
 
 
 
ISWI-type Complex ( GO:0031010 )
 
 
3/14
 
 
0.2247564
 
 
0.6759981
 
 
0
 
 
0
 
 
2.034947
 
 
3.0376433
 
 
POLE3;RBBP7;BAZ1B
 
 
 
 
Endocytic Vesicle Membrane ( GO:0030666 )
 
 
23/165
 
 
0.2302059
 
 
0.6871030
 
 
0
 
 
0
 
 
1.209809
 
 
1.7769453
 
 
SCARB2;SCARB1;TFRC;FZD5;SYT1;FZD4;ENTPD7;STAB2;TGFA;RORA;ARRB1;LRP2;AREG;PIP4P2;VAMP7;CD207;STX4;WNT1;ATP7A;CACNG2;LDLR;VAMP2;HBEGF
 
 
 
 
RNA N6-methyladenosine Methyltransferase Complex ( GO:0036396 )
 
 
2/8
 
 
0.2425690
 
 
0.7077945
 
 
0
 
 
0
 
 
2.486811
 
 
3.5224905
 
 
RBM15;WTAP
 
 
 
 
Pseudopodium ( GO:0031143 )
 
 
2/8
 
 
0.2425690
 
 
0.7077945
 
 
0
 
 
0
 
 
2.486811
 
 
3.5224905
 
 
VAMP7;ACTN2
 
 
 
 
Transferase Complex, Transferring Phosphorus-Containing Groups ( GO:0061695 )
 
 
2/8
 
 
0.2425690
 
 
0.7077945
 
 
0
 
 
0
 
 
2.486811
 
 
3.5224905
 
 
NEK10;MAP3K5
 
 
 
 
H4/H2A Histone Acetyltransferase Complex ( GO:0043189 )
 
 
5/29
 
 
0.2539658
 
 
0.7176259
 
 
0
 
 
0
 
 
1.554643
 
 
2.1307250
 
 
MBTD1;MORF4L1;KAT5;EPC1;EPC2
 
 
 
 
NuA4 Histone Acetyltransferase Complex ( GO:0035267 )
 
 
5/29
 
 
0.2539658
 
 
0.7176259
 
 
0
 
 
0
 
 
1.554643
 
 
2.1307250
 
 
MBTD1;MORF4L1;KAT5;EPC1;EPC2
 
 
 
 
Endoplasmic Reticulum Tubular Network ( GO:0071782 )
 
 
5/29
 
 
0.2539658
 
 
0.7176259
 
 
0
 
 
0
 
 
1.554643
 
 
2.1307250
 
 
RAB10;SPAST;ASPH;RTN4;KPNB1
 
 
 
 
COPII Vesicle Coat ( GO:0030127 )
 
 
3/15
 
 
0.2579989
 
 
0.7176259
 
 
0
 
 
0
 
 
1.865263
 
 
2.5270575
 
 
SAR1A;TMED7;SEC23B
 
 
 
 
trans-Golgi Network Transport Vesicle ( GO:0030140 )
 
 
3/15
 
 
0.2579989
 
 
0.7176259
 
 
0
 
 
0
 
 
1.865263
 
 
2.5270575
 
 
RAB14;RAB12;RAB8B
 
 
 
 
Exocytic Vesicle Membrane ( GO:0099501 )
 
 
10/67
 
 
0.2648301
 
 
0.7176259
 
 
0
 
 
0
 
 
1.309495
 
 
1.7398821
 
 
UNC13C;PTPRN2;SYT1;SEMA4C;STX6;DTNBP1;SLC35F1;SYPL2;CLCN3;VAMP2
 
 
 
 
Supramolecular Fiber ( GO:0099512 )
 
 
6/37
 
 
0.2687293
 
 
0.7176259
 
 
0
 
 
0
 
 
1.444352
 
 
1.8979516
 
 
MYH1;MFAP2;MYH9;MYH4;ADAMTS10;FBN1
 
 
 
 
Plasma Membrane Raft ( GO:0044853 )
 
 
12/83
 
 
0.2733300
 
 
0.7176259
 
 
0
 
 
0
 
 
1.261608
 
 
1.6364005
 
 
SCARB1;EHD2;ENTPD1;CAV3;CAV2;NOS1AP;CAVIN4;ATP2B4;FLOT2;MAPK1;TFPI;ADD2
 
 
 
 
Nucleolus ( GO:0005730 )
 
 
101/805
 
 
0.2742784
 
 
0.7176259
 
 
0
 
 
0
 
 
1.072889
 
 
1.3879021
 
 
SRP19;GEMIN2;FMR1;RORA;PRDM1;CDC14A;TXN2;PPP1CC;GOLGA3;CCND2;KAT5;EPC1;KMT5B;NEPRO;PHLDA1;DDX18;FBXW7;SLC30A5;LARP4B;MYCN;CCNY;WDR82;WDFY1;CMPK1;TP53;SRSF9;ZNF397;IPPK;SLBP;INO80E;C2CD4A;MACROH2A1;CDKN2AIP;FXR1;DHX33;NAA50;SRPK2;ABTB1;LIN28B;STAG2;LIN28A;LAS1L;RCAN2;ANP32B;POMT2;TUT4;SUV39H1;CHD7;HMGB2;SIX1;CTCF;TENT4B;PHF8;NKRF;CASP2;TNPO1;RBM7;ZNF367;BATF3;PPP1R12A;EN2;BAZ1B;SIRT1;DDX50;ETV4;ETV6;ARL4A;MDFIC;PAX9;RARA;POLR1D;FAM193B;TOP1;ERGIC2;PAFAH1B2;KDM7A;CIPC;NRXN1;PPM1D;TMEM65;PNMA1;RNF138;SAP30L;RPS3;E2F5;EXOSC3;RPP14;DEDD;ARFGEF1;NPM3;HINFP;VMP1;KLF6;TCIM;NOVA1;KATNBL1;SCD;SNX15;FILIP1;ERCC6;TAF5
 
 
 
 
Protein Serine/Threonine Phosphatase Complex ( GO:0008287 )
 
 
5/30
 
 
0.2776003
 
 
0.7176259
 
 
0
 
 
0
 
 
1.492373
 
 
1.9125849
 
 
PPP2CA;PPP4R2;PTPRA;PPP4R3A;PPP2R3A
 
 
 
 
Nuclear Lumen ( GO:0031981 )
 
 
102/814
 
 
0.2776748
 
 
0.7176259
 
 
0
 
 
0
 
 
1.071305
 
 
1.3726677
 
 
SRP19;GEMIN2;FMR1;RORA;PRDM1;CDC14A;TXN2;PPP1CC;GOLGA3;CCND2;KAT5;EPC1;KMT5B;NEPRO;PHLDA1;DDX18;MLIP;FBXW7;SLC30A5;LARP4B;MYCN;CCNY;WDR82;WDFY1;CMPK1;TP53;SRSF9;ZNF397;IPPK;SLBP;INO80E;C2CD4A;MACROH2A1;CDKN2AIP;FXR1;DHX33;NAA50;SRPK2;ABTB1;LIN28B;STAG2;LIN28A;LAS1L;RCAN2;ANP32B;POMT2;TUT4;SUV39H1;CHD7;HMGB2;SIX1;CTCF;CHD1;TENT4B;PHF8;NKRF;CASP2;TNPO1;RBM7;ZNF367;BATF3;PPP1R12A;EN2;BAZ1B;SIRT1;DDX50;ETV4;ETV6;ARL4A;MDFIC;PAX9;RARA;FAM193B;TOP1;ERGIC2;PAFAH1B2;KDM7A;CIPC;NRXN1;PPM1D;TMEM65;PNMA1;RNF138;SAP30L;RPS3;E2F5;EXOSC3;RPP14;DEDD;ARFGEF1;NPM3;HINFP;VMP1;KLF6;TCIM;NOVA1;KATNBL1;SCD;SNX15;FILIP1;ERCC6;TAF5
 
 
 
 
Late Endosome Membrane ( GO:0031902 )
 
 
14/99
 
 
0.2788038
 
 
0.7176259
 
 
0
 
 
0
 
 
1.229514
 
 
1.5703935
 
 
SCARB2;MARCHF1;STARD3NL;GOSR2;ABHD6;PIP4P2;VAMP7;CHMP1B;MARCHF8;CDIP1;CHMP4B;VPS36;CHMP7;VTI1B
 
 
 
 
Synaptic Vesicle Membrane ( GO:0030672 )
 
 
10/68
 
 
0.2803024
 
 
0.7176259
 
 
0
 
 
0
 
 
1.286844
 
 
1.6367189
 
 
UNC13C;PTPRN2;SYT1;SEMA4C;STX6;DTNBP1;SLC35F1;SYPL2;CLCN3;VAMP2
 
 
 
 
GABA-ergic Synapse ( GO:0098982 )
 
 
4/23
 
 
0.2858670
 
 
0.7176259
 
 
0
 
 
0
 
 
1.570788
 
 
1.9669864
 
 
SLITRK1;NRXN1;ATP2B4;CLCN3
 
 
 
 
Secretory Granule Lumen ( GO:0034774 )
 
 
41/316
 
 
0.2859780
 
 
0.7176259
 
 
0
 
 
0
 
 
1.113691
 
 
1.3941635
 
 
PSMD12;SPARC;DDX3X;SERPINE1;C1ORF35;GNS;CNN2;CAND1;TMSB4X;CDC37L1;MAPK1;FAM3C;SPTAN1;VTI1B;CTSA;VAT1;TGFB2;GCA;DYNLT1;ACTN2;AHSG;IST1;PRKCD;SERPINF2;VEGFC;IGF1;ARPC5;HOOK3;GTPBP2;CYRIB;MAPK14;C6ORF120;VEGFA;BIN2;LRRC7;NPC2;SOS1;NIT2;PAFAH1B2;KPNB1;FTL
 
 
 
 
Azurophil Granule ( GO:0042582 )
 
 
21/155
 
 
0.2863292
 
 
0.7176259
 
 
0
 
 
0
 
 
1.170091
 
 
1.4633313
 
 
CTSA;VAT1;ATP8A1;GCA;IST1;RAB3D;PRKCD;CMTM6;DNAJC13;PRCP;GNS;C6ORF120;CKAP4;RAP1B;VAMP7;NPC2;TOM1;MAPK1;STX3;SNAP29;FTL
 
 
 
 
Mitotic Spindle ( GO:0072686 )
 
 
21/155
 
 
0.2863292
 
 
0.7176259
 
 
0
 
 
0
 
 
1.170091
 
 
1.4633313
 
 
CUL3;HNF4G;CCSAP;CDC14A;WAPL;HAUS1;EML4;CDC42;TBCK;TUBB2A;FAM161A;TFDP2;TBL1XR1;KATNBL1;HECW2;RPS3;MAPK1;KMT5B;PKP4;MAPRE1;ZNF367
 
 
 
 
FNIP-folliculin RagC/D GAP ( GO:1990877 )
 
 
2/9
 
 
0.2889340
 
 
0.7176259
 
 
0
 
 
0
 
 
2.131431
 
 
2.6462927
 
 
RRAGC;SLC38A9
 
 
 
 
Junctional Sarcoplasmic Reticulum Membrane ( GO:0014701 )
 
 
2/9
 
 
0.2889340
 
 
0.7176259
 
 
0
 
 
0
 
 
2.131431
 
 
2.6462927
 
 
JPH3;ASPH
 
 
 
 
Neuromuscular Junction ( GO:0031594 )
 
 
6/38
 
 
0.2899882
 
 
0.7176259
 
 
0
 
 
0
 
 
1.399136
 
 
1.7320119
 
 
ITGB1;MPP2;UNC13C;CAV3;NRXN1;CDK5R1
 
 
 
 
Phagocytic Vesicle ( GO:0045335 )
 
 
14/100
 
 
0.2917194
 
 
0.7176259
 
 
0
 
 
0
 
 
1.215148
 
 
1.4970179
 
 
PDIA3;CLCN3;CTSS;RAB22A;RAB43;SNX3;PIP4P2;VAMP7;RAB14;RAB34;STX4;SRGAP2;ATP7A;RAB8B
 
 
 
 
COPI-coated Vesicle Membrane ( GO:0030663 )
 
 
3/16
 
 
0.2918223
 
 
0.7176259
 
 
0
 
 
0
 
 
1.721683
 
 
2.1204425
 
 
DIPK2A;TMED7;COPZ1
 
 
 
 
Axonal Growth Cone ( GO:0044295 )
 
 
3/16
 
 
0.2918223
 
 
0.7176259
 
 
0
 
 
0
 
 
1.721683
 
 
2.1204425
 
 
SHTN1;FLRT3;NRXN1
 
 
 
 
Clathrin Coat ( GO:0030118 )
 
 
3/16
 
 
0.2918223
 
 
0.7176259
 
 
0
 
 
0
 
 
1.721683
 
 
2.1204425
 
 
NECAP2;NECAP1;EPS15
 
 
 
 
Myosin Filament ( GO:0032982 )
 
 
3/16
 
 
0.2918223
 
 
0.7176259
 
 
0
 
 
0
 
 
1.721683
 
 
2.1204425
 
 
MYH1;MYH9;MYH4
 
 
 
 
Coated Vesicle Membrane ( GO:0030662 )
 
 
8/54
 
 
0.3044419
 
 
0.7393590
 
 
0
 
 
0
 
 
1.297818
 
 
1.5434621
 
 
LMAN1;MCFD2;CNIH1;SLC30A5;GOSR2;TGFA;AREG;VTI1B
 
 
 
 
Tertiary Granule Lumen ( GO:1904724 )
 
 
8/54
 
 
0.3044419
 
 
0.7393590
 
 
0
 
 
0
 
 
1.297818
 
 
1.5434621
 
 
CNN2;ASAH1;TNFAIP6;GOLGA7;SPTAN1;NIT2;CTSS;PGM1
 
 
 
 
Secretory Granule Membrane ( GO:0030667 )
 
 
36/279
 
 
0.3134054
 
 
0.7539598
 
 
0
 
 
0
 
 
1.106308
 
 
1.2836032
 
 
SNAP25;RAB5B;PGAP1;SPARC;ATP8A1;RAB3D;MYCBP2;MOSPD2;SLC2A3;PRCP;RAP1B;NRAS;RAP1A;ANPEP;ORMDL3;RAB24;TOM1;SNAP29;PTPRN2;DGAT1;SLC30A5;ATP6AP2;CMTM6;ADAM10;DNAJC13;SCAMP1;TNFRSF1B;SVIP;CKAP4;RAB10;RCAN1;TSPAN14;RAP2B;VAMP7;RAB14;VAMP2
 
 
 
 
Lysosomal Lumen ( GO:0043202 )
 
 
12/86
 
 
0.3160190
 
 
0.7539598
 
 
0
 
 
0
 
 
1.210255
 
 
1.3941566
 
 
SCARB2;CTSA;ASAH1;NPC2;OGN;PPT1;SDC1;GPC4;IFI30;GNS;GPC6;CTSS
 
 
 
 
Actin Cytoskeleton ( GO:0015629 )
 
 
46/361
 
 
0.3162389
 
 
0.7539598
 
 
0
 
 
0
 
 
1.090673
 
 
1.2556449
 
 
CLIC4;PLEKHH2;GMFB;TMEM63B;SLC2A1;LIMD2;AMOT;CNN3;CORO1C;CNN2;ARHGAP21;ARSJ;CFL2;PCDHA4;STK38L;FLOT2;WASF1;SPTAN1;VASP;TMOD1;PPP1R12A;ACTN2;TPM1;KLHL2;TSC1;ARPC5;NPM3;CTTNBP2NL;ACTA2;ADAM17;MARCKS;BIN2;BIN1;AXL;PALLD;CAPZA1;RARA;FSCN1;MYADM;MYH9;SPRY2;FILIP1;TAF5;TEK;RHOQ;DGKH
 
 
 
 
Postsynaptic Density Membrane ( GO:0098839 )
 
 
7/47
 
 
0.3181896
 
 
0.7540130
 
 
0
 
 
0
 
 
1.305821
 
 
1.4953054
 
 
MPP2;LRFN2;GRM5;SLITRK1;GRID1;ATP2B4;CACNG2
 
 
 
 
Cortical Cytoskeleton ( GO:0030863 )
 
 
9/63
 
 
0.3259079
 
 
0.7585138
 
 
0
 
 
0
 
 
1.243704
 
 
1.3943670
 
 
PLEKHH2;GMFB;ACTN2;CAPZA1;SLC2A1;MYADM;FLOT2;MAPRE1;SPTAN1
 
 
 
 
Protein Phosphatase Type 2A Complex ( GO:0000159 )
 
 
3/17
 
 
0.3259088
 
 
0.7585138
 
 
0
 
 
0
 
 
1.598615
 
 
1.7922677
 
 
PPP2CA;PTPRA;PPP2R3A
 
 
 
 
Transcription Factor TFTC Complex ( GO:0033276 )
 
 
3/17
 
 
0.3259088
 
 
0.7585138
 
 
0
 
 
0
 
 
1.598615
 
 
1.7922677
 
 
TAF5;TAF4;TAF9B
 
 
 
 
Spindle Microtubule ( GO:0005876 )
 
 
10/71
 
 
0.3281138
 
 
0.7591271
 
 
0
 
 
0
 
 
1.223348
 
 
1.3632923
 
 
FAM161A;MTCL1;CHMP1B;CHMP4B;RMDN3;CALM3;MAPRE1;CHMP7;HAUS1;PAFAH1B1
 
 
 
 
Astrocyte Projection ( GO:0097449 )
 
 
2/10
 
 
0.3349292
 
 
0.7682487
 
 
0
 
 
0
 
 
1.864896
 
 
2.0398909
 
 
GRM3;KCNK2
 
 
 
 
Ficolin-1-Rich Granule ( GO:0101002 )
 
 
24/184
 
 
0.3359860
 
 
0.7682487
 
 
0
 
 
0
 
 
1.119714
 
 
1.2212559
 
 
PSMD12;ASAH1;PTPRN2;DDX3X;TNFAIP6;DYNLT1;ATP6AP2;C1ORF35;SLC2A3;PRCP;ARPC5;HOOK3;MAPK14;GNS;CTSS;RCAN1;CAND1;BIN2;MAPK1;CAPN1;KPNB1;PGM1;PAFAH1B2;HSPA1B
 
 
 
 
P Granule ( GO:0043186 )
 
 
4/25
 
 
0.3420054
 
 
0.7729719
 
 
0
 
 
0
 
 
1.421028
 
 
1.5246618
 
 
DDX3Y;TDRKH;DDX3X;HELZ
 
 
 
 
Core Mediator Complex ( GO:0070847 )
 
 
4/25
 
 
0.3420054
 
 
0.7729719
 
 
0
 
 
0
 
 
1.421028
 
 
1.5246618
 
 
MED14;MED8;MED9;MED28
 
 
 
 
Synaptic Membrane ( GO:0097060 )
 
 
6/41
 
 
0.3554158
 
 
0.7907291
 
 
0
 
 
0
 
 
1.278992
 
 
1.3230751
 
 
GABRB2;MTMR2;RIMS4;FLRT3;PALM;ATP2B4
 
 
 
 
Cortical Actin Cytoskeleton ( GO:0030864 )
 
 
7/49
 
 
0.3582615
 
 
0.7907291
 
 
0
 
 
0
 
 
1.243497
 
 
1.2764401
 
 
PLEKHH2;GMFB;ACTN2;SLC2A1;MYADM;FLOT2;SPTAN1
 
 
 
 
Postsynaptic Specialization Membrane ( GO:0099634 )
 
 
8/57
 
 
0.3598545
 
 
0.7907291
 
 
0
 
 
0
 
 
1.218152
 
 
1.2450187
 
 
GABRB2;MPP2;LRFN2;GRM5;GRID1;SLITRK1;ATP2B4;CACNG2
 
 
 
 
GABA-A Receptor Complex ( GO:1902711 )
 
 
3/18
 
 
0.3599738
 
 
0.7907291
 
 
0
 
 
0
 
 
1.491956
 
 
1.5243671
 
 
GABRB2;GABRP;GABRA3
 
 
 
 
Kinetochore Microtubule ( GO:0005828 )
 
 
3/18
 
 
0.3599738
 
 
0.7907291
 
 
0
 
 
0
 
 
1.491956
 
 
1.5243671
 
 
CHMP1B;CHMP4B;CHMP7
 
 
 
 
MLL3/4 Complex ( GO:0044666 )
 
 
2/11
 
 
0.3799938
 
 
0.8031220
 
 
0
 
 
0
 
 
1.657592
 
 
1.6038863
 
 
KDM6B;DPY30
 
 
 
 
U2-type Catalytic Step 1 Spliceosome ( GO:0071006 )
 
 
2/11
 
 
0.3799938
 
 
0.8031220
 
 
0
 
 
0
 
 
1.657592
 
 
1.6038863
 
 
MAGOHB;PRPF19
 
 
 
 
beta-catenin-TCF Complex ( GO:1990907 )
 
 
2/11
 
 
0.3799938
 
 
0.8031220
 
 
0
 
 
0
 
 
1.657592
 
 
1.6038863
 
 
TCF7L2;LEF1
 
 
 
 
Intracellular Vesicle ( GO:0097708 )
 
 
2/11
 
 
0.3799938
 
 
0.8031220
 
 
0
 
 
0
 
 
1.657592
 
 
1.6038863
 
 
ITSN2;ITSN1
 
 
 
 
Microfibril ( GO:0001527 )
 
 
2/11
 
 
0.3799938
 
 
0.8031220
 
 
0
 
 
0
 
 
1.657592
 
 
1.6038863
 
 
MFAP2;FBN1
 
 
 
 
Primary Lysosome ( GO:0005766 )
 
 
2/11
 
 
0.3799938
 
 
0.8031220
 
 
0
 
 
0
 
 
1.657592
 
 
1.6038863
 
 
STX3;GNS
 
 
 
 
Transcription Factor TFIIH Holo Complex ( GO:0005675 )
 
 
2/11
 
 
0.3799938
 
 
0.8031220
 
 
0
 
 
0
 
 
1.657592
 
 
1.6038863
 
 
GTF2H1;MMS19
 
 
 
 
Mitochondrial Membrane ( GO:0031966 )
 
 
73/595
 
 
0.3858377
 
 
0.8110889
 
 
0
 
 
0
 
 
1.044152
 
 
0.9943866
 
 
PHB1;MRPS10;GHITM;MICOS13;FUNDC2;ATCAY;AIFM1;SLC39A9;UQCRFS1;TOMM70;SLC30A9;ACSL1;MRPS25;ACSL4;SDHD;MRPL52;SLC25A16;SLC25A15;AFG3L2;RNF144B;PLSCR3;PTRH2;NDUFS4;MICU3;TMEM126B;SLC25A4;ABCB6;COX16;TAFAZZIN;ABCB7;TIMM9;MRPL17;ATP5MC3;FOXO3;AGPAT4;HSPD1;SLC25A27;APOO;NRAS;BCL2L11;SLC25A28;TMEM65;STMP1;UCP3;GRPEL2;MFN1;RPS3;PMAIP1;BMF;SMIM30;BAK1;MCL1;MARCHF5;DMAC2L;VAT1;PRNP;SLC35A4;DMPK;NDUFA3;UQCC1;RMDN3;UQCC6;TTC19;SLC25A37;MAVS;CYP24A1;BCL2;SFXN4;ALPL;CYCS;SFXN2;BCL2L2;CDS2
 
 
 
 
Multivesicular Body Membrane ( GO:0032585 )
 
 
3/19
 
 
0.3937670
 
 
0.8233310
 
 
0
 
 
0
 
 
1.398629
 
 
1.3035168
 
 
CHMP1B;CHMP4B;CHMP7
 
 
 
 
SUMO Ligase Complex ( GO:0106068 )
 
 
2/12
 
 
0.4237047
 
 
0.8613712
 
 
0
 
 
0
 
 
1.491748
 
 
1.2809913
 
 
SLF2;UBE2I
 
 
 
 
Set1C/COMPASS Complex ( GO:0048188 )
 
 
2/12
 
 
0.4237047
 
 
0.8613712
 
 
0
 
 
0
 
 
1.491748
 
 
1.2809913
 
 
WDR82;DPY30
 
 
 
 
Clathrin Vesicle Coat ( GO:0030125 )
 
 
2/12
 
 
0.4237047
 
 
0.8613712
 
 
0
 
 
0
 
 
1.491748
 
 
1.2809913
 
 
NECAP2;NECAP1
 
 
 
 
Cytoplasmic Side of Lysosomal Membrane ( GO:0098574 )
 
 
2/12
 
 
0.4237047
 
 
0.8613712
 
 
0
 
 
0
 
 
1.491748
 
 
1.2809913
 
 
CDIP1;BORCS6
 
 
 
 
Cul4-RING E3 Ubiquitin Ligase Complex ( GO:0080008 )
 
 
5/36
 
 
0.4242554
 
 
0.8613712
 
 
0
 
 
0
 
 
1.203116
 
 
1.0315756
 
 
DCAF12L1;DCAF12;DCAF6;DDB2;CUL4B
 
 
 
 
Vesicle Membrane ( GO:0012506 )
 
 
10/77
 
 
0.4271793
 
 
0.8613712
 
 
0
 
 
0
 
 
1.113414
 
 
0.9470158
 
 
RAB10;ZNRF2;RAB4A;APPBP2;STK26;ENTPD7;ARRB1;AP1S3;CLCN3;SLC26A6
 
 
 
 
cis-Golgi Network ( GO:0005801 )
 
 
8/61
 
 
0.4349147
 
 
0.8613712
 
 
0
 
 
0
 
 
1.125960
 
 
0.9374799
 
 
RAB30;TRAPPC6B;GORASP1;HOOK3;TMED5;BLZF1;TMEM165;BET1
 
 
 
 
SWI/SNF Complex ( GO:0016514 )
 
 
4/29
 
 
0.4538826
 
 
0.8613712
 
 
0
 
 
0
 
 
1.193393
 
 
0.9426808
 
 
RB1;SS18;ARID2;ARID1B
 
 
 
 
Dendrite Membrane ( GO:0032590 )
 
 
4/29
 
 
0.4538826
 
 
0.8613712
 
 
0
 
 
0
 
 
1.193393
 
 
0.9426808
 
 
MPP2;GABRA3;ATP2B4;THY1
 
 
 
 
Exocytic Vesicle ( GO:0070382 )
 
 
4/29
 
 
0.4538826
 
 
0.8613712
 
 
0
 
 
0
 
 
1.193393
 
 
0.9426808
 
 
RAB10;SYT1;DPYSL3;IGF1
 
 
 
 
Myofibril ( GO:0030016 )
 
 
4/29
 
 
0.4538826
 
 
0.8613712
 
 
0
 
 
0
 
 
1.193393
 
 
0.9426808
 
 
TMOD1;TMOD3;MYH4;MYL12B
 
 
 
 
Cytoplasmic Dynein Complex ( GO:0005868 )
 
 
3/21
 
 
0.4597011
 
 
0.8613712
 
 
0
 
 
0
 
 
1.243085
 
 
0.9660994
 
 
DYNC1I2;DYNLT1;DYNLL2
 
 
 
 
ATAC Complex ( GO:0140672 )
 
 
2/13
 
 
0.4657521
 
 
0.8613712
 
 
0
 
 
0
 
 
1.356057
 
 
1.0361658
 
 
POLE3;MAP3K7
 
 
 
 
 
 
 GO_Molecular_Function_2025 
 GO Molecular Function terms describe the elemental biochemical
activities of gene products, including binding affinities, catalytic
activities, transporter functions, and receptor interactions. These
annotations are curated by the Gene Ontology Consortium based on
experimental data and validated inference. Enrichment in this category
highlights specific molecular activities that are over-represented among
the target genes, offering mechanistic insight into how regulatory
changes may alter protein function or signaling capacity. This category
is especially informative for identifying enzymatic classes, signaling
mediators, or binding modules affected by miRNA regulation. 
 
 
 
 
Term
 
 
Overlap
 
 
P.value
 
 
Adjusted.P.value
 
 
Old.P.value
 
 
Old.Adjusted.P.value
 
 
Odds.Ratio
 
 
Combined.Score
 
 
Genes
 
 
 
 
 
 
Sequence-Specific Double-Stranded DNA Binding ( GO:1990837 )
 
 
151/706
 
 
0.0000000
 
 
0.0000000
 
 
0
 
 
0
 
 
2.098912
 
 
62.037595
 
 
FOXA1;PRDM4;ZBTB26;UBP1;AHR;RORB;PRDM1;TNF;HOXA13;BACH2;HOXA11;HOXA10;CREB3L1;BBX;HOXA1;PKNOX1;PITX2;HOXA5;SOX4;HOXA4;SOX5;EOMES;PDHX;ZNF282;ZNF281;RFX3;SOX11;ISL1;MYT1;FOXP1;RUNX1;MAF;MYCN;TBL1XR1;HOXB4;ERG;HOXB8;TP53;HOXB5;ZSCAN29;ZNF274;HOXD1;MAX;GATA6;ZNF23;FOXO3;MACROH2A1;GLIS2;ZBTB2;NEUROD1;SCRT2;DHX33;PRDM16;ALX1;ZNF821;OTX2;HOXC8;ZBED4;ESRRA;ZBTB18;CPSF4;SMAD4;TFAP2C;EGR3;ESRRG;ESR1;NR2F6;FOSL1;NR4A2;LIN28B;MAFG;BHLHE40;SNAI1;LHX9;BARHL2;SUV39H2;SUV39H1;ONECUT1;BHLHE41;HMGB2;SIX1;MIA3;CTCF;MEOX2;AREG;HEY2;SIX3;ZIC1;MYBL2;ZNF385D;FOXD3;SREBF1;KLF11;BATF3;EMX2;FOXD2;KLF13;TGIF2;ATMIN;EN2;TFEB;ETV1;FOS;PROX1;ETV4;KLF15;SLC25A15;NR5A2;CREB1;ELF2;IRF4;ELF5;PAX9;TFEC;IRF2;RARA;RARB;PPARG;IRF5;CREB5;HDAC4;NFIX;CEBPG;LEF1;FOXK2;HSPD1;TBX21;E2F3;E2F7;ZNF740;ATOH1;DRGX;TCF7L2;CDX2;NFYA;STAT3;FOXJ2;KLF4;KLF3;MEIS2;USF1;HINFP;SLC25A37;PER1;KLF6;REST;KLF5;NR6A1;WT1;FOSB;BCOR
 
 
 
 
Sequence-Specific DNA Binding ( GO:0043565 )
 
 
152/718
 
 
0.0000000
 
 
0.0000000
 
 
0
 
 
0
 
 
2.071352
 
 
59.812722
 
 
FOXA1;PRDM4;ZBTB26;UBP1;RORA;AHR;RORB;PRDM1;HOXA13;BACH2;HOXA11;HOXA10;CREB3L1;BBX;HOXA1;PKNOX1;PITX2;HOXA5;SOX4;HOXA4;MEF2A;EOMES;PDHX;ZNF282;ZNF281;RFX3;ISL1;MYT1;FOXP1;THAP1;MAF;MYCN;HOXB4;ERG;HOXB8;HOXB5;ZSCAN29;ZNF274;CBFB;HOXD1;MAX;GATA6;ZNF23;FOXO3;ZBTB4;GLIS2;ZBTB2;NEUROD1;SCRT2;PRDM16;SAFB2;ZKSCAN3;ALX1;ZNF821;OTX2;HOXC8;ESRRA;ZBTB18;CPSF4;SMAD4;TFAP2C;EGR3;FOXF1;ESRRG;NR2F2;ESR1;NR2F6;FOSL1;NR4A2;LIN28B;MAFB;MAFG;BHLHE40;HNRNPD;SNAI1;BCL2;LHX9;BARHL2;CSRNP1;ONECUT1;BHLHE41;SIX1;MIA3;CTCF;MEOX2;MECP2;HEY2;SIX3;ZIC1;MYBL2;ZNF385D;FOXD3;SREBF1;KLF11;BATF3;EMX2;FOXD2;KLF13;TGIF2;DNMT3A;EN2;TFEB;TET1;ETV1;FOS;PROX1;SAFB;ETV4;KLF15;NR5A2;CREB1;ELF2;IRF4;ELF5;PAX9;TET3;TFEC;IRF2;RARA;RARB;PPARG;IRF5;MAPT;CREB5;NFIX;SATB1;CEBPG;LEF1;FOXK2;TBX21;E2F3;E2F7;ZNF740;ATOH1;DRGX;TCF7L2;CDX2;MBD1;FBXL19;FOXJ2;KLF4;TBX5;KLF3;MEIS2;USF1;SLC25A37;KLF6;KLF5;NR6A1;WT1;FOSB;ERCC6
 
 
 
 
RNA Polymerase II Cis-Regulatory Region Sequence-Specific DNA Binding
( GO:0000978 )
 
 
200/1054
 
 
0.0000000
 
 
0.0000000
 
 
0
 
 
0
 
 
1.815232
 
 
48.014109
 
 
IKZF1;IKZF4;BACH2;IKZF5;CREB3L1;MYB;HOXA3;HOXA1;HOXA5;SOX4;HOXA4;SOX5;MEF2A;ZNF287;PDHX;ZNF283;ZNF281;MED8;RFX3;EBF2;SOX11;EBF4;ISL1;THAP1;RUNX1;HOXB4;HOXB3;ZSCAN25;TP53;HOXB5;ZSCAN29;ZNF397;ZNF274;ZNF395;HOXD1;GATA6;ZNF23;ZBTB4;ZBTB2;PRDM16;ZNF821;NKX2-4;RREB1;EGR3;FOXF1;NR2F2;ST18;NR2F6;FOSL1;SNAI1;HBP1;ZNF136;SLC2A4RG;BHLHE41;HNF4G;MIA3;CTCF;HIF3A;MEOX2;AREG;NKRF;HEY2;ZNF280C;ZNF367;ZNF366;H3-3B;FOXD3;SREBF1;FOXD2;BATF3;EN2;TFEB;TET1;FOS;PROX1;NR5A2;PAX9;TET3;TFEC;RARA;RARB;PPARG;ZNF780B;ZNF471;NFIX;ZNF470;SATB1;SATB2;MNT;ZFP14;HDX;CDX2;NFYA;STAT3;HINFP;PER1;NR6A1;NFIA;WT1;FOSB;ZNF699;FOXA1;ZNF691;ZNF570;PRDM4;ZBTB26;UBP1;RORA;RORB;PRDM1;HOXA13;HOXA11;HOXA10;ZFP30;PITX2;EOMES;ZNF562;ZBTB39;ZBTB34;MITF;MYT1;FOXP1;MAF;MYCN;ERG;ZNF792;MAX;FOXO3;GLIS2;NEUROD1;SCRT2;ZKSCAN3;ZNF304;OTX2;ZKSCAN4;ZNF420;ESRRA;TFAP2C;SMAD4;ESRRG;ESR1;NR4A2;MAFB;MAFG;BHLHE40;MXD1;NFE2L1;ONECUT1;SIX1;ZFP82;ZIC1;SIX3;MYBL2;ZNF644;KDM6B;KLF11;EMX2;KLF13;DNMT3A;ETV1;SAFB;ETV4;KLF15;CREB1;IRF4;IRF2;IRF5;TOP1;CREB5;HDAC4;HDAC1;PLAG1;WBP2;CEBPG;LEF1;NLRC5;FOXK2;TBX21;ZNF624;E2F3;E2F5;E2F7;ATOH1;ZNF740;H2AZ1;TCF7L2;KCNIP3;FOXJ2;PEDS1;TBX5;KLF4;TBX4;KLF3;MEIS2;USF1;SLC25A37;KLF7;REST;KLF6;KLF5
 
 
 
 
Double-Stranded DNA Binding ( GO:0003690 )
 
 
138/651
 
 
0.0000000
 
 
0.0000000
 
 
0
 
 
0
 
 
2.068217
 
 
54.633596
 
 
FOXA1;PRDM4;ZBTB26;UBP1;AHR;RORB;PRDM1;HOXA13;BACH2;HOXA11;HOXA10;CREB3L1;BBX;HOXA1;PKNOX1;PITX2;HOXA5;SOX4;HOXA4;EOMES;PDHX;ZNF282;ZNF281;RFX3;ISL1;MYT1;FOXP1;MAF;MYCN;HOXB4;ERG;HOXB8;CGGBP1;HOXB5;ZSCAN29;ZNF274;HOXD1;MAX;GATA6;ZNF23;FOXO3;MACROH2A1;GLIS2;ZBTB2;NEUROD1;SCRT2;ALX1;ZNF821;OTX2;HOXC8;ESRRA;ZBTB18;CPSF4;TFAP2C;EGR3;ESRRG;ESR1;NR2F6;FOSL1;NR4A2;LIN28B;MAFG;BHLHE40;SNAI1;LHX9;BARHL2;ONECUT1;BHLHE41;HMGB2;SIX1;MIA3;CTCF;MEOX2;MECP2;HEY2;SIX3;ZIC1;MYBL2;ZNF385D;FOXD3;SREBF1;KLF11;BATF3;EMX2;FOXD2;KLF13;TGIF2;EN2;TFEB;ETV1;FOS;PROX1;SAFB;ETV4;KLF15;NR5A2;CREB1;ELF2;IRF4;ELF5;PAX9;TFEC;IRF2;RARA;RARB;PPARG;IRF5;TOP1;MAPT;ZMYND11;CREB5;NFIX;SATB1;CEBPG;LEF1;PURA;TBX21;TDG;RPS3;E2F3;E2F7;ZNF740;ATOH1;DRGX;TCF7L2;CDX2;MBD1;FOXJ2;KLF4;KLF3;MEIS2;USF1;SLC25A37;KLF6;KLF5;NR6A1;WT1;FOSB
 
 
 
 
DNA-binding Transcription Activator Activity, RNA Polymerase II-specific
( GO:0001228 )
 
 
82/318
 
 
0.0000000
 
 
0.0000000
 
 
0
 
 
0
 
 
2.648014
 
 
69.454470
 
 
FOXA1;PRDM4;ONECUT1;HNF4G;UBP1;SIX1;MEOX2;AREG;HOXA10;NKRF;CREB3L1;MYB;SIX3;HOXA1;MYBL2;HOXA5;SOX4;MEF2A;SREBF1;FOXD2;ATMIN;SOX11;MITF;ETV1;VEZF1;FOS;MYT1;ETV4;KLF15;RUNX1;MYCN;CREB1;TFDP2;IRF4;TFEC;IRF2;HOXB3;PPARG;ZNF780B;IRF5;ERG;TP53;NOTCH2;ZNF395;NOTCH1;PLAG1;SATB2;CEBPG;LEF1;FOXK2;FOXO3;RLF;GLIS2;NEUROD1;PRDM16;OTX2;E2F3;RREB1;ZBED4;TCF7L2;SMAD4;TFAP2C;FOXF1;STAT3;FOXJ2;RFXANK;PEDS1;KLF4;TBX5;ESR1;MEIS2;USF1;HINFP;NR4A2;KLF7;KLF6;MEIS1;KLF5;NFIA;WT1;CREBRF;NFE2L1
 
 
 
 
Cis-Regulatory Region Sequence-Specific DNA Binding ( GO:0000987 )
 
 
196/1035
 
 
0.0000000
 
 
0.0000000
 
 
0
 
 
0
 
 
1.809008
 
 
46.576099
 
 
IKZF1;IKZF4;BACH2;IKZF5;MYB;HOXA3;HOXA1;HOXA5;SOX4;HOXA4;SOX5;MEF2A;ZNF287;PDHX;ZNF283;ZNF281;MED8;RFX3;EBF2;SOX11;EBF4;ISL1;THAP1;RUNX1;HOXB4;HOXB3;ZSCAN25;TP53;HOXB5;ZSCAN29;ZNF397;ZNF274;ZNF395;HOXD1;GATA6;ZNF23;ZBTB4;ZBTB2;PRDM16;ZNF821;NKX2-4;RREB1;EGR3;FOXF1;NR2F2;ST18;NR2F6;FOSL1;SNAI1;HBP1;ZNF136;SLC2A4RG;BHLHE41;HNF4G;MIA3;CTCF;HIF3A;MEOX2;AREG;NKRF;HEY2;ZNF280C;ZNF367;ZNF366;H3-3B;FOXD3;SREBF1;FOXD2;BATF3;EN2;TFEB;TET1;FOS;PROX1;NR5A2;PAX9;TET3;TFEC;RARA;RARB;PPARG;ZNF780B;ZNF471;NFIX;ZNF470;SATB1;SATB2;MNT;ZFP14;HDX;CDX2;NFYA;STAT3;HINFP;PER1;NFIA;WT1;FOSB;ZNF699;FOXA1;ZNF691;ZNF570;PRDM4;ZBTB26;UBP1;RORA;RORB;PRDM1;HOXA13;HOXA11;HOXA10;ZFP30;PITX2;EOMES;ZNF562;ZBTB39;ZBTB34;MITF;MYT1;FOXP1;MAF;MYCN;ERG;NOTCH2;ZNF792;NOTCH1;FOXO3;GLIS2;SCRT2;ZKSCAN3;ZNF304;OTX2;ZKSCAN4;ZNF420;TFAP2C;SMAD4;FOXN4;ESR1;NR4A2;MAFB;MAFG;BHLHE40;MXD1;NFE2L1;ONECUT1;HMGB2;SIX1;ZFP82;ZIC1;SIX3;MYBL2;ZNF644;KDM6B;KLF11;EMX2;KLF13;DNMT3A;ETV1;SAFB;ETV4;KLF15;CREB1;IRF4;IRF2;IRF5;TOP1;HDAC4;HDAC1;PLAG1;WBP2;CEBPG;LEF1;NLRC5;FOXK2;TBX21;ZNF624;E2F3;E2F5;E2F7;ZNF740;H2AZ1;TCF7L2;KCNIP3;FOXJ2;PEDS1;TBX5;KLF4;TBX4;KLF3;MEIS2;USF1;SLC25A37;KLF7;REST;KLF6;KLF5
 
 
 
 
RNA Polymerase II Transcription Regulatory Region Sequence-Specific DNA
Binding ( GO:0000977 )
 
 
221/1236
 
 
0.0000000
 
 
0.0000000
 
 
0
 
 
0
 
 
1.687845
 
 
39.169730
 
 
RB1;IKZF1;ENO1;IKZF4;BACH2;IKZF5;MYB;HOXA3;BBX;HOXA1;HOXA5;SOX4;HOXA4;SOX5;MEF2A;ZNF287;PDHX;ZNF283;ZNF281;MED8;RFX3;EBF2;SOX11;EBF4;ISL1;THAP1;RUNX1;HOXB4;TBPL1;HOXB3;ZSCAN25;HOXB8;TP53;HOXB5;ZSCAN29;ZNF397;ZNF274;ZNF395;HOXD1;GATA6;ZNF23;ZBTB5;ZBTB4;ZBTB2;PRDM16;ALX1;ZNF821;HOXC8;NKX2-4;RREB1;ZBED4;ZNF266;EGR3;FOXF1;NR2F2;ST18;NR2F6;FOSL1;SNAI1;CREBRF;HBP1;SIM1;ZNF136;SLC2A4RG;BARHL2;TCF24;BHLHE41;HNF4G;MIA3;CTCF;HIF3A;MEOX2;AREG;NKRF;HEY2;ZNF280C;ZNF367;ZNF366;H3-3B;FOXD3;SREBF1;FOXD2;BATF3;EN2;TFEB;TET1;FOS;PROX1;NR5A2;PAX9;TET3;TFEC;RARA;RARB;PPARG;ZNF780B;ZNF471;NFIX;ZNF470;SATB1;SATB2;PURB;PURA;MNT;ZFP14;RPS3;HDX;CDX2;NFYA;STAT3;HINFP;PER1;NFIA;WT1;FOSB;ZNF699;FOXA1;ZNF691;ZNF570;PRDM4;GTF2B;ZBTB26;UBP1;RORA;RORB;PRDM1;HOXA13;HOXA11;HOXA10;ZFP30;PITX2;EOMES;ZNF562;ZBTB39;ZBTB34;MITF;MYT1;FOXP1;MAF;MYCN;ERG;ZNF792;MAX;FOXO3;MACROH2A1;GLIS2;NPAS3;SCRT2;ZKSCAN3;ZNF304;OTX2;ZKSCAN4;ZNF420;TFAP2C;SMAD4;RFXANK;ESR1;NR4A2;MAFB;MAFG;BHLHE40;MXD1;NFE2L1;LHX9;ZNF512B;ONECUT1;SIX1;ZFP82;ZIC1;SIX3;MYBL2;ZNF644;KDM6B;KLF11;EMX2;KLF13;DNMT3A;VEZF1;ETV1;SAFB;ETV4;KLF15;ETV6;CREB1;TFDP2;IRF4;IRF2;IRF5;TOP1;HDAC4;HDAC1;PLAG1;WBP2;CEBPG;LEF1;NLRC5;FOXK2;STOX2;TBX21;ZNF624;E2F3;E2F5;E2F7;ZNF740;DRGX;H2AZ1;TCF7L2;KCNIP3;FOXJ2;PEDS1;TBX5;KLF4;TBX4;KLF3;MEIS2;USF1;SLC25A37;KLF7;REST;KLF6;KLF5
 
 
 
 
Transcription Cis-Regulatory Region Binding ( GO:0000976 )
 
 
104/548
 
 
0.0000006
 
 
0.0000662
 
 
0
 
 
0
 
 
1.780947
 
 
25.532588
 
 
RB1;FOXA1;RORA;ENO1;AHR;TNF;IKZF5;CREB3L1;BBX;PITX2;SOX4;SOX5;MEF2A;ZNF281;RFX3;SOX11;ISL1;MYT1;FOXP1;RUNX1;TBL1XR1;HOXB8;TP53;NOTCH2;ZNF395;NOTCH1;MAX;GATA6;FOXO3;ZBTB5;MACROH2A1;ZBTB4;GLIS2;NPAS3;PRDM16;ALX1;HOXC8;RREB1;ZBED4;ZNF266;SMAD4;TFAP2C;FOXF1;FOXN4;RFXANK;AGO2;SNAI1;CREBRF;SIM1;LHX9;ZNF512B;BARHL2;SUV39H2;TCF24;SUV39H1;HMGB2;SIX1;MIA3;CTCF;HIF3A;AREG;HEY2;FOXD3;SREBF1;KLF11;ATMIN;TFEB;VEZF1;FOS;PROX1;KLF15;ETV6;NR5A2;TFDP2;IRF2;RARA;PPARG;HDAC4;HDAC1;SATB1;SATB2;LEF1;FOXK2;PURB;STOX2;PURA;MNT;TBX21;RPS3;E2F3;E2F7;DRGX;TCF7L2;CDX2;NFYA;STAT3;KLF4;HINFP;SLC25A37;PER1;REST;WT1;BCOR;TAF4
 
 
 
 
DNA-binding Transcription Factor Binding ( GO:0140297 )
 
 
61/294
 
 
0.0000076
 
 
0.0007569
 
 
0
 
 
0
 
 
1.977382
 
 
23.298646
 
 
RB1;TCERG1;DAZAP2;CRTC1;GTF2B;BHLHE41;MIA3;AHR;AREG;KAT5;HEY2;EP300;PITX2;MEF2A;EOMES;LMO1;MYOCD;RBM15;DNMT3A;EBF4;FOS;ISL1;SIRT1;YWHAZ;RUNX1;CREB1;MDFIC;PPARG;TP53;TRIB2;CGGBP1;HDAC4;UHRF2;MAX;HDAC1;CEBPG;GATA6;TXLNG;PBXIP1;MTDH;PURB;NEUROD1;PURA;PRDM16;RPS3;VHL;MTA2;TCF7L2;NEK6;STAT3;KLF4;TBX5;USF1;REST;CNOT7;NFIA;BCL3;BHLHE40;BCL2;MAP3K10;BCOR
 
 
 
 
DNA-binding Transcription Repressor Activity, RNA Polymerase II-specific
( GO:0001227 )
 
 
47/210
 
 
0.0000107
 
 
0.0009515
 
 
0
 
 
0
 
 
2.173399
 
 
24.881755
 
 
ZNF512B;SATB1;MAX;ZBTB26;BHLHE41;FOXK2;ENO1;CTCF;PRDM1;ZBTB5;FOXO3;ZBTB4;IKZF5;ZBTB2;PURB;PURA;SCRT2;MNT;CREB3L1;TBX21;PRDM16;HEY2;ZKSCAN3;PITX2;ZBTB18;FOXD3;ZBTB39;BATF3;TFAP2C;TGIF2;CDX2;ZNF281;KCNIP3;MITF;ZBTB34;PROX1;THAP1;FOXP1;ETV6;REST;TFEC;BHLHE40;SNAI1;PPARG;MXD1;TP53;NFE2L1
 
 
 
 
Ubiquitin-Like Protein Conjugating Enzyme Activity ( GO:0061650 )
 
 
15/39
 
 
0.0000184
 
 
0.0014933
 
 
0
 
 
0
 
 
4.683777
 
 
51.064256
 
 
UBE2F;UBE2H;UBE2I;UBE2B;UBE2D2;UBE2E3;UBE2D3;UBE2D1;UBE2G1;UBE2J1;UBE2W;UBE2R2;UBE2NL;UBE2N;UBE2K
 
 
 
 
Platelet-Derived Growth Factor Binding ( GO:0048407 )
 
 
7/10
 
 
0.0000278
 
 
0.0020649
 
 
0
 
 
0
 
 
17.447554
 
 
183.046192
 
 
PDGFRB;PDGFRA;COL3A1;COL1A2;COL2A1;COL5A1;COL4A1
 
 
 
 
mRNA Binding ( GO:0003729 )
 
 
61/310
 
 
0.0000410
 
 
0.0028133
 
 
0
 
 
0
 
 
1.848620
 
 
18.674581
 
 
DDX3Y;DAZAP1;RBM25;DDX3X;FMR1;CELF2;CELF3;MSI1;YBX3;ELAVL2;ZC3H12C;BOLL;AUH;ZC3H12B;XPO5;RBPMS2;SNIP1;ZNF385A;DDX17;NANOS1;RBM15;KHNYN;ANGEL2;SECISBP2L;LARP4B;DDX50;CDC40;PTBP3;CSDE1;RBM20;EIF4E2;TP53;SRSF7;RBM24;SLBP;DDX6;IREB2;ZFP36L1;FXR1;PURB;ATXN2;UNK;DHX33;RPS3;HNRNPA1;RBM38;CPSF7;CPSF6;SAMD4A;LARP4;QKI;CPEB1;LIN28B;HNRNPK;LIN28A;CSDC2;NOVA1;AGO2;SERBP1;DCP1A;EIF4G2
 
 
 
 
Kinase Binding ( GO:0019900 )
 
 
85/472
 
 
0.0000466
 
 
0.0029686
 
 
0
 
 
0
 
 
1.661544
 
 
16.572354
 
 
RB1;ITGB1;ERRFI1;CCNK;USP37;BTG1;CCNT2;DGKD;TFRC;WWC2;PTPRK;RND2;PPP1CB;PPP1CC;ZFYVE26;SPRED1;CCND2;RB1CC1;PDGFRB;PRKAB2;PPP1R12A;BORA;PRKCD;ADAM10;ANK2;RHOC;YWHAZ;ADRA2A;CDC25B;CNKSR2;SGO1;ACTA2;CCNA2;TIAM1;SIKE1;FRMD5;MYCN;LRRC7;DCX;IRF5;MAPT;MAPRE1;STX1A;RHOQ;DGKH;GATA6;GSKIP;FOXO3;MACROH2A1;ZBTB4;RHOBTB1;ADD2;PRDX3;CDC42;BAG5;IRAK1;RNF138;PDCD10;CAMK2N1;RPS3;UBQLN1;PIP5K1A;MAP2K6;MPP2;TCF7L2;FZD5;GADD45A;CAV2;NEK6;STAT3;ESR1;SNAP91;USF1;WARS1;PER1;MAVS;TBL2;SNAI1;SPRY2;CALM3;GRB2;FERMT2;LIMS1;MAP3K12;CDK5R1
 
 
 
 
Protein Homodimerization Activity ( GO:0042803 )
 
 
114/680
 
 
0.0000677
 
 
0.0039755
 
 
0
 
 
0
 
 
1.527288
 
 
14.663155
 
 
SCARB2;DGKD;TFRC;PANK1;FMR1;CPOX;ENO1;AHR;YARS2;TXN2;TMEM266;ENPP1;PIP4K2B;MAP3K9;PITX2;SEPHS1;IL6R;MAP3K5;SH3GLB1;TPD52;ACVR1;PDGFRA;CEP135;SERPINF2;TPM1;ACSL6;ADAM10;RUNX1;THAP1;TPST1;CHMP4B;SNRPC;ZNF397;MTMR1;CSF1R;ABCB7;LPL;ZBTB4;RTN4;FXR1;BLOC1S6;SNX2;IRAK1;FLRT3;PDCD10;FUT9;PDGFC;NDP;SRGAP2;MORC2;PRNP;SMAD4;TGFB2;FZD4;GADD45A;CAV2;INHBB;NR2F2;GGCT;PTPRA;STK26;BHLHE40;COL9A1;MAP3K10;MAP3K11;MAP3K12;ACHE;ABCD3;MTCL1;HSPB8;BHLHE41;DPY30;ECE1;MIA3;SPPL3;GLIPR2;RBPMS2;MUC13;HIF1AN;CR2;TRPC6;CHUK;HMGCS1;STARD3NL;GRID1;NOG;CACYBP;ADRA2A;GREM1;COL2A1;MYH9;PAFAH1B2;TIMM9;VPS4B;RCHY1;ADD2;SLC25A27;PLN;DROSHA;BAK1;NTRK2;GCA;GCH1;STAT3;USF1;WARS1;VEGFA;ABCB10;NR6A1;GSTA4;XPNPEP1;VAPB;PRKRA;MGAT4A
 
 
 
 
Ubiquitin Conjugating Enzyme Activity ( GO:0061631 )
 
 
13/34
 
 
0.0000713
 
 
0.0039755
 
 
0
 
 
0
 
 
4.636014
 
 
44.266928
 
 
UBE2H;UBE2B;UBE2D2;UBE2E3;UBE2D3;UBE2D1;UBE2G1;UBE2J1;UBE2W;UBE2R2;UBE2NL;UBE2N;UBE2K
 
 
 
 
GDP Binding ( GO:0019003 )
 
 
19/66
 
 
0.0001581
 
 
0.0082255
 
 
0
 
 
0
 
 
3.030709
 
 
26.526004
 
 
RAB5B;RAB4A;DIRAS1;RRAS2;RAB22A;RAB43;RAP1B;RAB10;NRAS;MRAS;RAP2B;RAP1A;RRAGC;RRAS;RAB14;RAB12;RAB8B;TRIM23;ARL8B
 
 
 
 
Eukaryotic Initiation Factor 4E Binding ( GO:0008190 )
 
 
6/9
 
 
0.0001660
 
 
0.0082255
 
 
0
 
 
0
 
 
14.948707
 
 
130.107778
 
 
DDX3X;EIF4EBP1;EIF4EBP2;EIF4EBP3;OTX2;C8ORF88
 
 
 
 
Protein Heterodimerization Activity ( GO:0046982 )
 
 
44/216
 
 
0.0002062
 
 
0.0096826
 
 
0
 
 
0
 
 
1.924722
 
 
16.334049
 
 
ZNF397;ITGB1;PHB1;DGKD;FMR1;BHLHE41;MIA3;ATP1A2;AHR;NPAS3;ADD2;FXR1;PPP2CA;NEUROD1;SNX2;IRAK1;ERBB3;IL12B;BAK1;SEPHS1;MCL1;MEF2A;TCF7L2;GCA;CHUK;FZD4;GADD45A;TPM1;ZHX1;ADRA2A;USF1;RUNX1;NR4A2;RRAGC;VAPB;BHLHE40;BCL2;MICU3;SIM1;TP53;KCNK2;PAFAH1B2;NFE2L1;PAFAH1B1
 
 
 
 
Protein Kinase Binding ( GO:0019901 )
 
 
87/527
 
 
0.0007709
 
 
0.0337699
 
 
0
 
 
0
 
 
1.492502
 
 
10.698117
 
 
ITGB1;ERRFI1;DAZAP2;CCNK;USP37;CCNT2;TFRC;DIRAS1;RORA;PTPRK;RND2;DUSP16;PPP1CB;CDH5;PPP1CC;ZFYVE26;GRM5;SPRED1;CCND2;RB1CC1;ACVR1;PDGFRB;PRKAB2;PPP1R12A;BORA;PRKCD;ADAM10;ANK2;RHOC;SIRT1;YWHAZ;ADRA2A;CDC25B;CNKSR2;ACTA2;CCNA2;SIKE1;FRMD5;LRRC7;TRAF6;RARA;DCX;IRF5;MAPT;MAPRE1;TRIB2;RHOQ;GATA6;GSKIP;FOXO3;PPM1D;MACROH2A1;ZBTB4;RHOBTB1;ADD2;PRDX3;CDC42;BAG5;IRAK1;RNF138;PDCD10;CAMK2N1;RPS3;VHL;MAP2K6;MPP2;TCF7L2;FZD5;GCH1;CAV2;NEK6;STAT3;ESR1;SNAP91;USF1;WARS1;MAVS;TBL2;PKIA;SPRY2;CALM3;GRB2;FERMT2;MAP3K11;LIMS1;MAP3K12;CDK5R1
 
 
 
 
Phosphatidylinositol-3-Phosphate Phosphatase Activity ( GO:0004438 )
 
 
7/15
 
 
0.0008688
 
 
0.0337699
 
 
0
 
 
0
 
 
6.540978
 
 
46.103602
 
 
MTMR1;MTMR10;MTMR2;MTMR12;MTMR14;PTEN;MTMR9
 
 
 
 
Double-Stranded Methylated DNA Binding ( GO:0010385 )
 
 
4/5
 
 
0.0008833
 
 
0.0337699
 
 
0
 
 
0
 
 
29.875477
 
 
210.080302
 
 
MECP2;WT1;MBD1;MACROH2A1
 
 
 
 
Transition Metal Ion Binding ( GO:0046914 )
 
 
77/458
 
 
0.0008885
 
 
0.0337699
 
 
0
 
 
0
 
 
1.524051
 
 
10.707861
 
 
ISCA2;SUV39H2;DYRK2;GTF2B;HHIP;ECE1;CTCF;ADO;ZFYVE1;IKZF4;PHF8;IKZF5;LOXL2;ENDOU;NPEPPS;SHH;CA2;ANPEP;ENPP2;ENPP1;HIF1AN;LARGE1;ATP7A;NANOS1;ESCO1;ZNF282;TET2;TET1;BAZ1B;POMGNT1;THAP1;PLA2G15;MMP16;PLSCR3;FDX1;TET3;MPPED2;WDFY1;CDIP1;COMMD3-BMI1;PPARG;SNRPC;ZMYND11;TP53;KPNB1;KDM7A;FTL;HDAC4;ENPEP;KDM3A;TIMM9;MYCBP2;ADCY2;DNAJC24;PPM1K;RNF7;RCHY1;SAP30L;MORC2;TIMM8B;PRNP;GCH1;SLC31A1;MBD1;FBXL19;GALNT1;KLF4;XPNPEP1;WT1;SCD;ACER3;NAPEPLD;MSRB3;MDM4;POC1B-GALNT4;ADA;LIMS1
 
 
 
 
Protein Phosphatase Regulator Activity ( GO:0019888 )
 
 
19/75
 
 
0.0009240
 
 
0.0337699
 
 
0
 
 
0
 
 
2.542329
 
 
17.762875
 
 
PPP1R15B;PPP1R12A;PPP1R11;DMPK;YWHAB;LMTK2;PPP4R3A;RNF7;PPP2R3A;ARPP19;HSP90B1;RCAN1;PPP4R2;PPP6R1;PTPRA;PPP1R7;RCAN2;ENSA;CALM3
 
 
 
 
pre-miRNA Binding ( GO:0070883 )
 
 
5/8
 
 
0.0009465
 
 
0.0337699
 
 
0
 
 
0
 
 
12.451977
 
 
86.700321
 
 
TRUB1;LIN28A;PRKRA;XPO5;DICER1
 
 
 
 
Phosphatidylinositol Kinase Activity ( GO:0052742 )
 
 
9/24
 
 
0.0011019
 
 
0.0378030
 
 
0
 
 
0
 
 
4.487267
 
 
30.561588
 
 
PI4K2B;PIK3CA;PI4KA;PIK3R3;PIP5K1A;PIP4K2B;PIP5K1B;PIK3R1;PIK3C2A
 
 
 
 
Cyclin-Dependent Protein Serine/Threonine Kinase Regulator Activity ( GO:0016538 )
 
 
13/44
 
 
0.0012824
 
 
0.0423673
 
 
0
 
 
0
 
 
3.138743
 
 
20.900914
 
 
CCNK;CCNJ;CCNT2;CCNH;CCNC;CCNA2;CCND2;CCNE2;CCNY;CCNG1;CCNL2;CCNJL;CDK5R1
 
 
 
 
Phosphatidylinositol Monophosphate Phosphatase Activity ( GO:0052744 )
 
 
7/16
 
 
0.0013875
 
 
0.0442031
 
 
0
 
 
0
 
 
5.813872
 
 
38.256567
 
 
MTMR1;MTMR10;MTMR2;MTMR12;MTMR14;PTEN;MTMR9
 
 
 
 
Protein Tyrosine Kinase Activator Activity ( GO:0030296 )
 
 
10/30
 
 
0.0016790
 
 
0.0481481
 
 
0
 
 
0
 
 
3.739915
 
 
23.896397
 
 
GREM1;GRM5;ERBB3;IGF1;ERCC6;EFNA5;IL6ST;AREG;VEGFA;HBEGF
 
 
 
 
Transition Metal Ion Transmembrane Transporter Activity ( GO:0046915 )
 
 
11/35
 
 
0.0017169
 
 
0.0481481
 
 
0
 
 
0
 
 
3.428933
 
 
21.832746
 
 
SLC30A7;SLC30A6;SLC25A37;SLC30A9;SLC30A3;SLC39A9;SLC31A1;SLC30A5;SLC39A11;SLC30A10;TMEM165
 
 
 
 
RNA Polymerase II-specific DNA-binding Transcription Factor Binding ( GO:0061629 )
 
 
43/232
 
 
0.0018201
 
 
0.0481481
 
 
0
 
 
0
 
 
1.709387
 
 
10.784278
 
 
HDAC4;TCERG1;ASAH1;UHRF2;HDAC1;GATA6;BHLHE41;MIA3;AHR;AREG;DLL1;MTDH;NEUROD1;HEY2;EP300;HIF1AN;PITX2;MTA2;MEF2A;NCOA1;EOMES;TCF7L2;MYOCD;RBM15;STAT3;DNMT3A;FOS;PROX1;KLF4;MAPK14;TBX5;ISL1;SIRT1;REST;CREB1;MDFIC;BHLHE40;NCOA7;PPARG;TACC1;TAF4;TP53;TRIB2
 
 
 
 
Guanyl Ribonucleotide Binding ( GO:0032561 )
 
 
43/232
 
 
0.0018201
 
 
0.0481481
 
 
0
 
 
0
 
 
1.709387
 
 
10.784278
 
 
ARF4;RAB5B;DIRAS1;TUBD1;RND2;RAB22A;RHOBTB1;RAB43;RAP1B;CDC42;NRAS;ARL5B;RAP1A;RRAS;PIP4K2B;SEPHS1;TRIM23;RAB8B;RAB4A;GCH1;GRID1;VTA1;RAB39B;RRAS2;GTPBP2;RHOC;RAB10;MFHAS1;RAB30;ARL4A;MRAS;RAP2B;TUBB2A;RRAGC;RAB14;RAB34;RAB12;MPPED2;RAPGEF2;ARF5;RHOQ;ARF6;ARL8B
 
 
 
 
Transcription Coregulator Binding ( GO:0001221 )
 
 
23/103
 
 
0.0018347
 
 
0.0481481
 
 
0
 
 
0
 
 
2.155022
 
 
13.578495
 
 
SREBF1;SMAD4;CCNT2;UBE2I;HDAC1;NEK6;LEF1;GATA6;RORA;ENO1;CTCF;FOS;ESR1;AREG;RUNX1;PER1;NR5A2;SIX3;MTF2;RARA;EP300;PPARG;ZNF644
 
 
 
 
GTPase Activity ( GO:0003924 )
 
 
48/266
 
 
0.0018352
 
 
0.0481481
 
 
0
 
 
0
 
 
1.655130
 
 
10.428277
 
 
ARF4;RAB3C;RGS17;RAB5B;DDX3X;SAR1A;RGS14;DIRAS1;RAB3D;RND2;DNAJC27;RAB22A;RHOBTB1;RAB43;RAP1B;CDC42;GNA13;NRAS;RGS2;RAP1A;RRAS;NKIRAS2;RAB24;MFN1;RGS8;TRIM23;RAB8B;RAB4A;GCH1;VTA1;ENTPD7;RAB39B;RRAS2;RHOC;GNAO1;DNM3;RAB30;MRAS;RAP2B;RRAGC;RAB14;RAB34;RAB12;SRPRA;ABCE1;RHOQ;ARF6;ARL8B
 
 
 
 
Ribonucleoside Triphosphate Phosphatase Activity ( GO:0017111 )
 
 
49/274
 
 
0.0020098
 
 
0.0512203
 
 
0
 
 
0
 
 
1.637095
 
 
10.165929
 
 
ARF4;RAB3C;DDX3X;DIRAS1;RAB3D;RND2;RAB22A;RAB43;RGS2;NKIRAS2;RGS8;TRIM23;RAB8B;ENTPD1;ENTPD7;RRAS2;RHOC;DNM3;RAB30;MRAS;RAP2B;RRAGC;RAB34;RHOQ;ARF6;ARL8B;RGS17;RAB5B;SAR1A;RGS14;DNAJC27;RHOBTB1;RAP1B;CDC42;GNA13;NRAS;RAP1A;RRAS;RAB24;MFN1;RAB4A;GCH1;VTA1;RAB39B;GNAO1;RAB14;RAB12;SRPRA;ABCE1
 
 
 
 
Neuropilin Binding ( GO:0038191 )
 
 
7/17
 
 
0.0021196
 
 
0.0525198
 
 
0
 
 
0
 
 
5.232188
 
 
32.212030
 
 
SEMA7A;SEMA4D;SEMA4B;SEMA4C;PXDN;SEMA4F;VEGFA
 
 
 
 
Sodium Channel Regulator Activity ( GO:0017080 )
 
 
11/36
 
 
0.0022118
 
 
0.0526805
 
 
0
 
 
0
 
 
3.291589
 
 
20.124667
 
 
NANOS1;TMEM168;CAV3;FXYD3;TMPRSS3;FXYD2;NEDD4;ATP2B4;SGK3;SGK1;SCN2B
 
 
 
 
Cadherin Binding ( GO:0045296 )
 
 
55/317
 
 
0.0022442
 
 
0.0526805
 
 
0
 
 
0
 
 
1.578790
 
 
9.629652
 
 
ITGB1;DDX3X;YWHAB;CTNND2;ARHGAP1;PTPRM;ENO1;FNBP1L;BZW1;RAB43;GOLGA3;PCMT1;CDH5;MB21D2;SPTAN1;ACVR1;SH3GLB1;IST1;TMOD3;ANK3;GAPVD1;YWHAZ;SHTN1;CDH11;FSCN1;NUMB;CHMP4B;MYH9;PKP4;MAPRE1;EPHA2;DDX6;UBFD1;RTN4;CNN3;CNN2;DNAJB1;LDHA;SNX2;CDH20;RPL14;EPS15;SNX5;VASP;VASN;TJP1;RAB10;HNRNPK;FMNL2;VAPB;CAPZA1;SERBP1;PI4KA;EIF4G2;CDK5R1
 
 
 
 
mRNA 3’-UTR Binding ( GO:0003730 )
 
 
23/105
 
 
0.0023910
 
 
0.0535343
 
 
0
 
 
0
 
 
2.102221
 
 
12.689087
 
 
RBM38;DAZAP1;FMR1;ANGEL2;CELF2;SECISBP2L;LARP4B;LARP4;YBX3;ZFP36L2;QKI;ELAVL2;ZFP36L1;FXR1;CPEB1;BOLL;AUH;CSDC2;SERBP1;HNRNPA1;TP53;RBM24;ZNF385A
 
 
 
 
Fatty Acid Elongase Activity ( GO:0009922 )
 
 
4/6
 
 
0.0024006
 
 
0.0535343
 
 
0
 
 
0
 
 
14.936891
 
 
90.099626
 
 
ELOVL4;ELOVL2;ELOVL3;ELOVL6
 
 
 
 
Phosphatidylinositol Phosphate Binding ( GO:1901981 )
 
 
24/112
 
 
0.0026512
 
 
0.0576798
 
 
0
 
 
0
 
 
2.044231
 
 
12.127896
 
 
RUBCN;RNF34;GOLPH3L;SNX12;SNX24;ZFYVE1;PLEKHA8;SNX3;CYTH3;FUNDC2;ZFYVE26;GAP43;SNX27;SESTD1;TTPA;SAP30L;VEPH1;TOM1;CLVS2;ARFIP1;MAPT;TTPAL;FERMT2;CLVS1
 
 
 
 
RNA Polymerase Binding ( GO:0070063 )
 
 
7/18
 
 
0.0031175
 
 
0.0662107
 
 
0
 
 
0
 
 
4.756265
 
 
27.447027
 
 
TCERG1;CCNT2;PABPN1;BIN1;YTHDC2;NEDD4;ERCC6
 
 
 
 
Myosin V Binding ( GO:0031489 )
 
 
6/14
 
 
0.0035057
 
 
0.0712101
 
 
0
 
 
0
 
 
5.604176
 
 
31.682410
 
 
RAB10;RAB43;RAB3C;RAB14;RAB3D;RAB39B
 
 
 
 
GTP Binding ( GO:0005525 )
 
 
38/206
 
 
0.0035164
 
 
0.0712101
 
 
0
 
 
0
 
 
1.697838
 
 
9.593313
 
 
ARF4;DIRAS1;TUBD1;RND2;RAB22A;RHOBTB1;RAB43;RAP1B;CDC42;NRAS;ARL5B;RAP1A;RRAS;PIP4K2B;SEPHS1;TRIM23;RAB4A;GCH1;GRID1;VTA1;RAB39B;RRAS2;GTPBP2;RHOC;RAB10;MFHAS1;RAB30;ARL4A;MRAS;RAP2B;TUBB2A;RRAGC;RAB14;RAB34;ARF5;RHOQ;ARF6;ARL8B
 
 
 
 
Notch Binding ( GO:0005112 )
 
 
8/23
 
 
0.0035924
 
 
0.0712101
 
 
0
 
 
0
 
 
3.986989
 
 
22.442459
 
 
DLL4;NCOR2;ADAM17;JAG1;TCIM;CUL3;HIF1AN;DLL1
 
 
 
 
Aminopeptidase Activity ( GO:0004177 )
 
 
9/28
 
 
0.0037560
 
 
0.0728342
 
 
0
 
 
0
 
 
3.541775
 
 
19.778666
 
 
NPEPPS;ENPEP;MMP16;XPNPEP1;MMP15;ANPEP;BLMH;METAP1;METAP2
 
 
 
 
Protein Phosphatase 2A Binding ( GO:0051721 )
 
 
8/24
 
 
0.0048377
 
 
0.0884626
 
 
0
 
 
0
 
 
3.737590
 
 
19.926258
 
 
MFHAS1;PTPRA;ENSA;MAPT;ARPP19;TP53;CTTNBP2NL;STRN3
 
 
 
 
Zinc Ion Transmembrane Transporter Activity ( GO:0005385 )
 
 
8/24
 
 
0.0048377
 
 
0.0884626
 
 
0
 
 
0
 
 
3.737590
 
 
19.926258
 
 
SLC30A7;SLC30A6;SLC30A9;SLC30A3;SLC39A9;SLC30A5;SLC39A11;SLC30A10
 
 
 
 
Armadillo Repeat Domain Binding ( GO:0070016 )
 
 
4/7
 
 
0.0050770
 
 
0.0884626
 
 
0
 
 
0
 
 
9.957363
 
 
52.605079
 
 
TCF7L2;CTNNBIP1;LEF1;STRN3
 
 
 
 
Dinucleotide Phosphatase Activity ( GO:0004551 )
 
 
4/7
 
 
0.0050770
 
 
0.0884626
 
 
0
 
 
0
 
 
9.957363
 
 
52.605079
 
 
ENPP1;CILP2;NUDT17;NUDT13
 
 
 
 
Xylosyltransferase Activity ( GO:0042285 )
 
 
4/7
 
 
0.0050770
 
 
0.0884626
 
 
0
 
 
0
 
 
9.957363
 
 
52.605079
 
 
POGLUT2;POGLUT1;GXYLT2;LARGE1
 
 
 
 
Transmembrane Receptor Protein Tyrosine Kinase Activator Activity ( GO:0030297 )
 
 
6/15
 
 
0.0052637
 
 
0.0884626
 
 
0
 
 
0
 
 
4.981207
 
 
26.136000
 
 
GREM1;IGF1;EFNA5;AREG;VEGFA;HBEGF
 
 
 
 
Ubiquitin-Protein Transferase Activity ( GO:0004842 )
 
 
66/409
 
 
0.0053323
 
 
0.0884626
 
 
0
 
 
0
 
 
1.447292
 
 
7.575084
 
 
TRIM71;PPP1R11;UBE2D2;UBE2D3;UBE2D1;TNFAIP3;PRPF19;CDC23;RNF19A;RNF152;TRIM23;TRIM63;UBE2E3;MSL2;FBXO10;DDB2;RNF168;RNF144B;UBE2R2;HECW2;TRAF6;TRAF5;OBI1;RNF34;MARCHF1;CUL5;UHRF2;CUL3;PELI2;RNF38;MYCBP2;RNF7;RCHY1;UBE2J1;CDC42;ZNRF2;RMND5A;RNF138;UBE2NL;RNF217;RNF39;MARCHF8;VHL;MARCHF3;MARCHF5;UBE2H;TRIM41;UBE2B;MMAB;SIAH2;SYVN1;UBE2G1;CHFR;RNF145;UBE2W;NEURL4;NEDD4;RLIM;RNF182;UBE2N;MDM4;BCOR;RNF186;RNF141;UBE2K;ASB1
 
 
 
 
Hexosyltransferase Activity ( GO:0016758 )
 
 
21/99
 
 
0.0053554
 
 
0.0884626
 
 
0
 
 
0
 
 
2.016589
 
 
10.546069
 
 
B3GALNT2;B4GALT2;POMT2;DDX18;B4GALT3;POGLUT2;POGLUT1;CSGALNACT2;FUT11;TMTC3;POMGNT1;FUT4;B4GAT1;B3GNT7;FUT9;A4GNT;MGAT4A;GCNT3;LARGE1;B4GALT6;B4GALT5
 
 
 
 
Protein Serine/Threonine Kinase Activity ( GO:0004674 )
 
 
59/359
 
 
0.0054874
 
 
0.0889951
 
 
0
 
 
0
 
 
1.478411
 
 
7.695585
 
 
CCNK;DYRK2;TESK2;AKT3;RPS6KA1;PDK4;TLK2;MAP3K9;MAP3K7;MAP3K4;MAP3K5;ACVR1;PRKAB2;MAP2K1;CAMK1D;CHUK;PRKCD;DYRK1B;LMTK2;VRK3;TGFBR1;MELK;ULK3;SGK3;TOP1;SGK1;BRSK1;PRKAA1;STK39;TTK;PPM1D;NUAK2;IRAK1;STK38L;MAPK1;TRPM7;MARK3;MAP4K4;MAP2K6;MARK1;SRPK2;CDK19;DMPK;CSNK1A1;PLK2;NEK6;CDC42BPG;NEK7;LIMK1;MAPK14;ACVR2A;MAPK12;SNRK;CAMK4;STK26;NEK10;MAP3K10;MAP3K11;MAP3K12
 
 
 
 
L-arginine Transmembrane Transporter Activity ( GO:0061459 )
 
 
5/11
 
 
0.0057474
 
 
0.0915483
 
 
0
 
 
0
 
 
6.224929
 
 
32.114431
 
 
SLC25A15;SLC7A7;SLC38A9;SLC7A1;SLC7A2
 
 
 
 
Ubiquitin Protein Ligase Binding ( GO:0031625 )
 
 
46/268
 
 
0.0059716
 
 
0.0923526
 
 
0
 
 
0
 
 
1.555886
 
 
7.967286
 
 
RB1;DAZAP2;UBXN1;RNF34;CUL5;CUL3;DERL1;ARRB1;HAPSTR1;RTN4;UBE2J1;HSPD1;BAG4;BAG5;ERBB3;GPR37;SLF2;FZD5;UBE2B;FZD4;FBXW7;NEK6;USP2;LIMK1;UBE2G1;ERLIN2;TNFRSF1B;YWHAZ;TGFBR1;PER1;MFHAS1;UBE2W;TRAF4;TRAF5;BCL2;UBE2N;TMBIM6;RNF186;EIF4E2;TP53;TRIB2;UBE2K;BLZF1;HSPA1B;OTUB1;CUL4B
 
 
 
 
Transcription Corepressor Binding ( GO:0001222 )
 
 
12/46
 
 
0.0060050
 
 
0.0923526
 
 
0
 
 
0
 
 
2.640084
 
 
13.504465
 
 
PER1;SMAD4;HDAC1;NEK6;LEF1;SIX3;MTF2;RORA;ENO1;ZNF644;ESR1;RUNX1
 
 
 
 
Core Promoter Sequence-Specific DNA Binding ( GO:0001046 )
 
 
10/36
 
 
0.0073350
 
 
0.1108957
 
 
0
 
 
0
 
 
2.875878
 
 
14.135210
 
 
H2AZ1;HDAC1;GTF2B;AGO2;TBPL1;FOS;TAF4;TP53;H3-3B;FOXP1
 
 
 
 
Vascular Endothelial Growth Factor Receptor Binding ( GO:0005172 )
 
 
5/12
 
 
0.0089025
 
 
0.1303674
 
 
0
 
 
0
 
 
5.335351
 
 
25.190437
 
 
GREM1;CDH5;PDCL3;VEGFC;VEGFA
 
 
 
 
JUN Kinase Kinase Kinase Activity ( GO:0004706 )
 
 
4/8
 
 
0.0092076
 
 
0.1303674
 
 
0
 
 
0
 
 
7.467599
 
 
35.006086
 
 
MAP3K10;MAP3K9;MAP3K11;MAP3K5
 
 
 
 
UDP-xylosyltransferase Activity ( GO:0035252 )
 
 
4/8
 
 
0.0092076
 
 
0.1303674
 
 
0
 
 
0
 
 
7.467599
 
 
35.006086
 
 
POGLUT2;POGLUT1;GXYLT2;LARGE1
 
 
 
 
Vascular Endothelial Growth Factor Receptor 2 Binding ( GO:0043184 )
 
 
4/8
 
 
0.0092076
 
 
0.1303674
 
 
0
 
 
0
 
 
7.467599
 
 
35.006086
 
 
GREM1;CDH5;PDCL3;VEGFA
 
 
 
 
Protein Serine/Threonine/Tyrosine Kinase Activity ( GO:0004712 )
 
 
8/27
 
 
0.0105804
 
 
0.1474649
 
 
0
 
 
0
 
 
3.146908
 
 
14.314495
 
 
MAP2K4;MAP2K1;RPS6KA1;TTK;MAP3K9;MAPK14;SGK1;MAP2K6
 
 
 
 
Ion Channel Regulator Activity ( GO:0099106 )
 
 
23/119
 
 
0.0116997
 
 
0.1595618
 
 
0
 
 
0
 
 
1.794215
 
 
7.981014
 
 
NANOS1;DPP10;CAV3;NRXN1;TMPRSS3;KCNIP3;KCNIP4;ATP2B4;NRXN2;CABP1;ARPP19;CACNB3;TMEM168;FXYD3;FXYD2;KCNS3;ENSA;FKBP1B;PDE4B;MICU3;SGK3;SGK1;SCN2B
 
 
 
 
GTPase Regulator Activity ( GO:0030695 )
 
 
66/424
 
 
0.0118061
 
 
0.1595618
 
 
0
 
 
0
 
 
1.385448
 
 
6.150191
 
 
DOCK6;ARF4;ERRFI1;NRP1;DOCK3;FAM13B;ITSN1;ARHGAP1;ARRB1;IPO7;RAPGEFL1;RGS2;EVI5L;RGS8;SBF2;PDGFRB;ELMOD1;TBC1D8;GAPVD1;ADRA2A;TIAM1;TBC1D1;ADGRB3;TBC1D5;TBC1D7;RAPGEF2;PREB;DENND6A;RAPGEF5;SOS1;SOS2;CDC42SE1;RGS14;MYCBP2;RAP1GDS1;THY1;ASAP2;C9ORF72;RGPD4;RASGRP4;CYTH3;GNA13;ARHGAP21;ARHGAP20;RIC8B;FGD6;RAP1A;PDE6D;SLC38A9;TBC1D12;SRGAP2;SEC23B;RALGDS;CYTH1;REPS1;EIF2B5;ARFGEF1;STARD13;RIC1;ADAP1;ARHGAP26;TBCK;SH3BP5L;SGSM2;SMAP1;SIPA1
 
 
 
 
Histone Deacetylase Binding ( GO:0042826 )
 
 
20/100
 
 
0.0124616
 
 
0.1599250
 
 
0
 
 
0
 
 
1.871535
 
 
8.206875
 
 
MEF2A;HDAC4;PHB1;YWHAB;HDAC1;SATB2;LEF1;BHLHE41;USF1;HOXA10;NCOR2;KCTD21;TRAF6;MIER1;BCL3;HEY2;BCOR;MTA2;TP53;HSPA1B
 
 
 
 
RNA Polymerase II Core Promoter Sequence-Specific DNA Binding ( GO:0000979 )
 
 
5/13
 
 
0.0130764
 
 
0.1599250
 
 
0
 
 
0
 
 
4.668167
 
 
20.245597
 
 
H2AZ1;HDAC1;GTF2B;TBPL1;H3-3B
 
 
 
 
Cysteine-Type Peptidase Activity ( GO:0008234 )
 
 
27/147
 
 
0.0131243
 
 
0.1599250
 
 
0
 
 
0
 
 
1.685575
 
 
7.304088
 
 
OTUD4;USP37;USP38;USP53;USP32;USP34;TNFAIP3;CTSS;CASP7;CASP2;PDIA3;USP46;USP47;USP48;USP6;USP4;USP9X;USP3;USP2;USP45;JOSD1;SENP1;SFRP1;OTULIN;ATG4B;BLMH;OTUB1
 
 
 
 
Zinc Ion Binding ( GO:0008270 )
 
 
54/338
 
 
0.0131449
 
 
0.1599250
 
 
0
 
 
0
 
 
1.427579
 
 
6.183873
 
 
SUV39H2;HHIP;GTF2B;ECE1;CTCF;ZFYVE1;IKZF4;IKZF5;PHF8;NPEPPS;SHH;CA2;ANPEP;ENPP2;ENPP1;HIF1AN;ESCO1;ZNF282;TET2;TET1;BAZ1B;THAP1;PLA2G15;MMP16;TET3;WDFY1;CDIP1;COMMD3-BMI1;PPARG;SNRPC;ZMYND11;TP53;KPNB1;KDM7A;HDAC4;ENPEP;TIMM9;MYCBP2;DNAJC24;RCHY1;SAP30L;MORC2;TIMM8B;GCH1;MBD1;FBXL19;KLF4;WT1;ACER3;NAPEPLD;MSRB3;MDM4;ADA;LIMS1
 
 
 
 
N-acylsphingosine Amidohydrolase Activity ( GO:0017040 )
 
 
3/5
 
 
0.0137282
 
 
0.1599250
 
 
0
 
 
0
 
 
11.197926
 
 
48.020085
 
 
ACER2;ASAH1;ACER3
 
 
 
 
arachidonate-CoA Ligase Activity ( GO:0047676 )
 
 
3/5
 
 
0.0137282
 
 
0.1599250
 
 
0
 
 
0
 
 
11.197926
 
 
48.020085
 
 
ACSL1;ACSL6;ACSL4
 
 
 
 
Dihydropyrimidinase Activity ( GO:0004157 )
 
 
3/5
 
 
0.0137282
 
 
0.1599250
 
 
0
 
 
0
 
 
11.197926
 
 
48.020085
 
 
DPYSL4;DPYSL5;DPYSL3
 
 
 
 
lncRNA Binding ( GO:0106222 )
 
 
3/5
 
 
0.0137282
 
 
0.1599250
 
 
0
 
 
0
 
 
11.197926
 
 
48.020085
 
 
CSDE1;DNMT3A;STAT3
 
 
 
 
Sphingosine N-acyltransferase Activity ( GO:0050291 )
 
 
3/5
 
 
0.0137282
 
 
0.1599250
 
 
0
 
 
0
 
 
11.197926
 
 
48.020085
 
 
CERS4;CERS6;TLCD3B
 
 
 
 
Supercoiled DNA Binding ( GO:0097100 )
 
 
3/5
 
 
0.0137282
 
 
0.1599250
 
 
0
 
 
0
 
 
11.197926
 
 
48.020085
 
 
HMGB2;RPS3;TOP1
 
 
 
 
Calcium Channel Regulator Activity ( GO:0005246 )
 
 
11/45
 
 
0.0138052
 
 
0.1599250
 
 
0
 
 
0
 
 
2.419049
 
 
10.360088
 
 
NANOS1;CACNB3;CAV3;NRXN1;FKBP1B;PDE4B;NRXN2;MICU3;SGK3;CABP1;SGK1
 
 
 
 
Basic Amino Acid Transmembrane Transporter Activity ( GO:0015174 )
 
 
6/18
 
 
0.0143006
 
 
0.1614701
 
 
0
 
 
0
 
 
3.735269
 
 
15.865381
 
 
SLC25A15;SLC7A7;TMEM44;SLC38A9;SLC7A1;SLC7A2
 
 
 
 
Metalloaminopeptidase Activity ( GO:0070006 )
 
 
6/18
 
 
0.0143006
 
 
0.1614701
 
 
0
 
 
0
 
 
3.735269
 
 
15.865381
 
 
NPEPPS;ENPEP;MMP16;XPNPEP1;MMP15;METAP1
 
 
 
 
Histone H3K9 Methyltransferase Activity ( GO:0046974 )
 
 
4/9
 
 
0.0150360
 
 
0.1655813
 
 
0
 
 
0
 
 
5.973740
 
 
25.073638
 
 
SUV39H2;SETDB1;SUV39H1;PRDM16
 
 
 
 
Thioesterase Binding ( GO:0031996 )
 
 
4/9
 
 
0.0150360
 
 
0.1655813
 
 
0
 
 
0
 
 
5.973740
 
 
25.073638
 
 
CDC42;TRAF4;TRAF5;ARF6
 
 
 
 
Phosphatidylinositol Binding ( GO:0035091 )
 
 
21/109
 
 
0.0160432
 
 
0.1743392
 
 
0
 
 
0
 
 
1.786413
 
 
7.382299
 
 
PLEKHF2;SCARB1;RUBCN;RNF34;STK39;SNX12;SNX24;THY1;ZFYVE1;SNAP91;PITPNC1;GAP43;SNX18;SNX2;SNX27;SESTD1;SNX17;WDFY1;ESYT3;MAPT;SNX5
 
 
 
 
Cysteine-Type Deubiquitinase Activity ( GO:0004843 )
 
 
19/96
 
 
0.0162589
 
 
0.1743392
 
 
0
 
 
0
 
 
1.846758
 
 
7.607005
 
 
OTUD4;USP46;USP47;USP37;USP48;USP38;USP6;USP53;USP32;USP4;USP9X;USP3;USP34;USP2;USP45;TNFAIP3;JOSD1;OTULIN;OTUB1
 
 
 
 
Ubiquitin-Like Protein Ligase Binding ( GO:0044389 )
 
 
46/284
 
 
0.0165755
 
 
0.1743392
 
 
0
 
 
0
 
 
1.449955
 
 
5.944567
 
 
RB1;DAZAP2;UBXN1;RNF34;CUL5;CUL3;DERL1;ARRB1;HAPSTR1;RTN4;UBE2J1;HSPD1;BAG4;BAG5;ERBB3;GPR37;SLF2;FZD5;UBE2B;FZD4;FBXW7;NEK6;USP2;LIMK1;UBE2G1;ERLIN2;TNFRSF1B;YWHAZ;TGFBR1;PER1;MFHAS1;UBE2W;TRAF4;TRAF5;BCL2;UBE2N;TMBIM6;RNF186;EIF4E2;TP53;TRIB2;UBE2K;BLZF1;HSPA1B;OTUB1;CUL4B
 
 
 
 
Nuclear Receptor Binding ( GO:0016922 )
 
 
22/116
 
 
0.0166130
 
 
0.1743392
 
 
0
 
 
0
 
 
1.752173
 
 
7.179645
 
 
NCOA1;TCF7L2;ASAH1;KDM3A;WBP2;GTF2B;LEF1;TAF11;GTF2H1;PROX1;ISL1;ESR1;SIRT1;FOXP1;NR4A2;MED14;EP300;MMS19;NCOA7;TACC1;HMGN3;ZNF366
 
 
 
 
1-Phosphatidylinositol Binding ( GO:0005545 )
 
 
5/14
 
 
0.0183934
 
 
0.1866394
 
 
0
 
 
0
 
 
4.149247
 
 
16.579402
 
 
SCARB1;SESTD1;WDFY1;ZFYVE1;SNAP91
 
 
 
 
MAP Kinase Kinase Activity ( GO:0004708 )
 
 
5/14
 
 
0.0183934
 
 
0.1866394
 
 
0
 
 
0
 
 
4.149247
 
 
16.579402
 
 
MAP2K4;MAP2K1;MAP3K9;MAPK14;MAP2K6
 
 
 
 
GTPase Activator Activity ( GO:0005096 )
 
 
36/214
 
 
0.0184129
 
 
0.1866394
 
 
0
 
 
0
 
 
1.515942
 
 
6.055743
 
 
ERRFI1;NRP1;FAM13B;RGS14;ARHGAP1;ARRB1;THY1;ASAP2;C9ORF72;RGPD4;ARHGAP21;RGS2;ARHGAP20;TBC1D12;EVI5L;RGS8;SRGAP2;SEC23B;REPS1;PDGFRB;ARFGEF1;STARD13;ELMOD1;TBC1D8;ADAP1;ARHGAP26;TBCK;TBC1D1;ADGRB3;TBC1D5;SGSM2;TBC1D7;RAPGEF2;SOS1;SMAP1;SIPA1
 
 
 
 
miRNA Binding ( GO:0035198 )
 
 
8/30
 
 
0.0202525
 
 
0.2029804
 
 
0
 
 
0
 
 
2.717322
 
 
10.596130
 
 
TRIM71;LIN28A;AGO4;FMR1;TUT4;AGO2;HNRNPA1;QKI
 
 
 
 
Protein Serine/Threonine Phosphatase Activity ( GO:0004722 )
 
 
14/66
 
 
0.0205920
 
 
0.2040893
 
 
0
 
 
0
 
 
2.013562
 
 
7.818368
 
 
PPM1L;MTMR14;PTEN;CTDSPL2;PPM1K;PPM1D;CDC14A;PPM1F;PPM1G;PPP1CB;PPP2CA;PPP1CC;CTDSP2;ZNF367
 
 
 
 
Transcription Coactivator Binding ( GO:0001223 )
 
 
9/36
 
 
0.0215173
 
 
0.2109165
 
 
0
 
 
0
 
 
2.491228
 
 
9.563574
 
 
SMAD4;CCNT2;GATA6;RARA;EP300;RORA;AREG;ESR1;RUNX1
 
 
 
 
Cyclin-Dependent Protein Serine/Threonine Kinase Activator Activity ( GO:0061575 )
 
 
4/10
 
 
0.0227461
 
 
0.2149019
 
 
0
 
 
0
 
 
4.977834
 
 
18.832955
 
 
CCNK;CCND2;CCNT2;CDK5R1
 
 
 
 
Estrogen Response Element Binding ( GO:0034056 )
 
 
4/10
 
 
0.0227461
 
 
0.2149019
 
 
0
 
 
0
 
 
4.977834
 
 
18.832955
 
 
ESRRA;NR6A1;ESRRG;ESR1
 
 
 
 
Galactosyltransferase Activity ( GO:0008378 )
 
 
4/10
 
 
0.0227461
 
 
0.2149019
 
 
0
 
 
0
 
 
4.977834
 
 
18.832955
 
 
B4GALT2;B4GALT3;B4GALT6;B4GALT5
 
 
 
 
Sterol Binding ( GO:0032934 )
 
 
14/67
 
 
0.0232869
 
 
0.2149019
 
 
0
 
 
0
 
 
1.975458
 
 
7.427456
 
 
SCARB2;OSBPL9;STARD3NL;INSIG2;INSIG1;OSBPL3;RORA;ERLIN2;OSBPL11;NPC2;SIDT1;GAS1;SLC38A9;NFE2L1
 
 
 
 
Protein Phosphatase Binding ( GO:0019903 )
 
 
22/120
 
 
0.0238965
 
 
0.2149019
 
 
0
 
 
0
 
 
1.680272
 
 
6.274178
 
 
PPP1R11;STX17;STAT3;ATP2B4;MTMR9;PIK3R1;AKAP5;VRK3;MAPK14;ARPP19;HSP90B1;CTTNBP2NL;STRN3;SNX3;CDH5;MFHAS1;PTPRA;ENSA;PXDN;MAPT;TP53;MAP3K5
 
 
 
 
Lipid Kinase Activity ( GO:0001727 )
 
 
6/20
 
 
0.0243012
 
 
0.2149019
 
 
0
 
 
0
 
 
3.201296
 
 
11.899957
 
 
DGKD;PIP5K1A;PIP5K1B;PIK3R1;DGKZ;DGKH
 
 
 
 
Phosphatase Inhibitor Activity ( GO:0019212 )
 
 
8/31
 
 
0.0245460
 
 
0.2149019
 
 
0
 
 
0
 
 
2.599030
 
 
9.635139
 
 
PPP1R11;YWHAB;ENSA;STYX;LMTK2;RNF7;ARPP19;HSP90B1
 
 
 
 
Adrenergic Receptor Binding ( GO:0031690 )
 
 
5/15
 
 
0.0249583
 
 
0.2149019
 
 
0
 
 
0
 
 
3.734110
 
 
13.780916
 
 
NEDD4;ARRDC3;MAGI2;RAPGEF2;AKAP5
 
 
 
 
Phospholipase Activator Activity ( GO:0016004 )
 
 
5/15
 
 
0.0249583
 
 
0.2149019
 
 
0
 
 
0
 
 
3.734110
 
 
13.780916
 
 
PDGFRB;ARF4;PDGFRA;CCL8;STX4
 
 
 
 
ATPase Binding ( GO:0051117 )
 
 
15/74
 
 
0.0249850
 
 
0.2149019
 
 
0
 
 
0
 
 
1.901479
 
 
7.015469
 
 
UBXN1;DERL1;SYVN1;ANK2;METTL21A;AREG;ESR1;SVIP;DNAJB1;PLN;CLPP;ZNHIT6;VCPKMT;CHMP4B;ATP6V1E1
 
 
 
 
Morphogen Activity ( GO:0016015 )
 
 
3/6
 
 
0.0250558
 
 
0.2149019
 
 
0
 
 
0
 
 
7.464860
 
 
27.520319
 
 
GREM1;SHH;WNT1
 
 
 
 
Potassium Channel Inhibitor Activity ( GO:0019870 )
 
 
3/6
 
 
0.0250558
 
 
0.2149019
 
 
0
 
 
0
 
 
7.464860
 
 
27.520319
 
 
CAV3;ENSA;KCNK2
 
 
 
 
Tumor Necrosis Factor Binding ( GO:0043120 )
 
 
3/6
 
 
0.0250558
 
 
0.2149019
 
 
0
 
 
0
 
 
7.464860
 
 
27.520319
 
 
ADAM17;TNFRSF1B;TNFRSF1A
 
 
 
 
Regulatory RNA Binding ( GO:0061980 )
 
 
10/43
 
 
0.0257985
 
 
0.2191641
 
 
0
 
 
0
 
 
2.264942
 
 
8.283892
 
 
TRIM71;LIN28A;AGO4;PRKRA;FMR1;TUT4;AGO2;DICER1;HNRNPA1;QKI
 
 
 
 
Protein Tyrosine Phosphatase Activity ( GO:0004725 )
 
 
16/82
 
 
0.0293325
 
 
0.2449753
 
 
0
 
 
0
 
 
1.813176
 
 
6.398804
 
 
PTPN18;PTPRN2;EYA3;PTEN;PTPRM;PTPRK;DUSP16;DUSP9;CDC14A;PALD1;CDC25B;PTP4A1;PPP2CA;PTPRA;PTPN4;ZNF367
 
 
 
 
Transmembrane Receptor Protein Tyrosine Kinase Activity ( GO:0004714 )
 
 
11/50
 
 
0.0293861
 
 
0.2449753
 
 
0
 
 
0
 
 
2.108315
 
 
7.436524
 
 
PDGFRB;NRP1;PDGFRA;CSF1R;NTRK2;AXL;TYRO3;CRIM1;RORA;TEK;EPHA2
 
 
 
 
Iron Ion Binding ( GO:0005506 )
 
 
12/57
 
 
0.0323509
 
 
0.2608464
 
 
0
 
 
0
 
 
1.993483
 
 
6.839868
 
 
ISCA2;KDM3A;SCD;FDX1;TET2;TET1;DNAJC24;HIF1AN;ADO;KDM7A;PHF8;FTL
 
 
 
 
Dynactin Binding ( GO:0034452 )
 
 
4/11
 
 
0.0324596
 
 
0.2608464
 
 
0
 
 
0
 
 
4.266473
 
 
14.624443
 
 
HOOK3;MAPT;SNX5;PAFAH1B1
 
 
 
 
Fucosyltransferase Activity ( GO:0008417 )
 
 
4/11
 
 
0.0324596
 
 
0.2608464
 
 
0
 
 
0
 
 
4.266473
 
 
14.624443
 
 
FUT8;FUT9;FUT11;FUT4
 
 
 
 
Sequence-Specific mRNA Binding ( GO:1990825 )
 
 
4/11
 
 
0.0324596
 
 
0.2608464
 
 
0
 
 
0
 
 
4.266473
 
 
14.624443
 
 
LIN28A;NOVA1;FMR1;RBM24
 
 
 
 
E-box Binding ( GO:0070888 )
 
 
11/51
 
 
0.0335998
 
 
0.2660410
 
 
0
 
 
0
 
 
2.055491
 
 
6.974764
 
 
NEUROD1;SCRT2;TCF7L2;PER1;MAX;BHLHE40;BHLHE41;SNAI1;MITF;PPARG;ATOH1
 
 
 
 
Epidermal Growth Factor Receptor Binding ( GO:0005154 )
 
 
7/27
 
 
0.0337025
 
 
0.2660410
 
 
0
 
 
0
 
 
2.614610
 
 
8.864006
 
 
ARF4;ATXN2;SNX2;TGFA;GRB2;AREG;HBEGF
 
 
 
 
Single-Stranded RNA Binding ( GO:0003727 )
 
 
10/45
 
 
0.0345779
 
 
0.2705565
 
 
0
 
 
0
 
 
2.135274
 
 
7.184220
 
 
FXR1;DDX3X;AGO4;FMR1;STRBP;HNRNPF;AGO2;HNRNPA1;RBM7;SNRPC
 
 
 
 
pre-mRNA Binding ( GO:0036002 )
 
 
8/33
 
 
0.0349589
 
 
0.2711591
 
 
0
 
 
0
 
 
2.390836
 
 
8.017868
 
 
SLBP;CELF2;CELF3;RBM20;HNRNPA1;RBM7;SNRPC;RBM24
 
 
 
 
MAP Kinase Kinase Kinase Activity ( GO:0004709 )
 
 
6/22
 
 
0.0381243
 
 
0.2834576
 
 
0
 
 
0
 
 
2.800816
 
 
9.149999
 
 
MAP3K10;MAP3K9;MAP3K7;MAP3K11;MAP3K4;MAP3K5
 
 
 
 
bHLH Transcription Factor Binding ( GO:0043425 )
 
 
6/22
 
 
0.0381243
 
 
0.2834576
 
 
0
 
 
0
 
 
2.800816
 
 
9.149999
 
 
BHLHE40;BHLHE41;MAP3K10;ISL1;SIRT1;USF1
 
 
 
 
Semaphorin Receptor Binding ( GO:0030215 )
 
 
6/22
 
 
0.0381243
 
 
0.2834576
 
 
0
 
 
0
 
 
2.800816
 
 
9.149999
 
 
SEMA7A;SEMA6A;SEMA4D;SEMA4B;SEMA4C;SEMA4F
 
 
 
 
Phosphoric Ester Hydrolase Activity ( GO:0042578 )
 
 
11/52
 
 
0.0382228
 
 
0.2834576
 
 
0
 
 
0
 
 
2.005243
 
 
6.545763
 
 
PPP1CB;SMPD3;PPP1CC;PLCXD3;MPPED2;PTPRM;ENPP1;PXYLP1;ATP1A2;DUSP16;FRA10AC1
 
 
 
 
Syntaxin Binding ( GO:0019905 )
 
 
10/46
 
 
0.0396671
 
 
0.2834576
 
 
0
 
 
0
 
 
2.075843
 
 
6.699232
 
 
NAPB;SNAP25;UNC13C;BLOC1S6;SYT1;STX16;STX6;CACNA1A;SNAP29;VAMP2
 
 
 
 
1-Phosphatidylinositol-4-Phosphate 5-Kinase Activity ( GO:0016308 )
 
 
3/7
 
 
0.0400400
 
 
0.2834576
 
 
0
 
 
0
 
 
5.598328
 
 
18.014730
 
 
PIP5K1A;PIP4K2B;PIP5K1B
 
 
 
 
G-quadruplex RNA Binding ( GO:0002151 )
 
 
3/7
 
 
0.0400400
 
 
0.2834576
 
 
0
 
 
0
 
 
5.598328
 
 
18.014730
 
 
LIN28A;XRN1;FMR1
 
 
 
 
Alpha-(1->3)-Fucosyltransferase Activity ( GO:0046920 )
 
 
3/7
 
 
0.0400400
 
 
0.2834576
 
 
0
 
 
0
 
 
5.598328
 
 
18.014730
 
 
FUT9;FUT11;FUT4
 
 
 
 
Dehydroascorbic Acid Transmembrane Transporter Activity ( GO:0033300 )
 
 
3/7
 
 
0.0400400
 
 
0.2834576
 
 
0
 
 
0
 
 
5.598328
 
 
18.014730
 
 
SLC2A14;SLC2A1;SLC2A3
 
 
 
 
Histone H2A Ubiquitin Ligase Activity ( GO:0141053 )
 
 
3/7
 
 
0.0400400
 
 
0.2834576
 
 
0
 
 
0
 
 
5.598328
 
 
18.014730
 
 
RNF168;PCGF5;PCGF3
 
 
 
 
protein-N-terminal Amino-Acid Acetyltransferase Activity ( GO:0004596 )
 
 
3/7
 
 
0.0400400
 
 
0.2834576
 
 
0
 
 
0
 
 
5.598328
 
 
18.014730
 
 
NAA50;NAA40;NAA20
 
 
 
 
Tau Protein Binding ( GO:0048156 )
 
 
9/40
 
 
0.0407250
 
 
0.2860370
 
 
0
 
 
0
 
 
2.169286
 
 
6.943697
 
 
PPP2CA;BRSK1;PRKAA1;BIN1;EP300;MARK3;METAP1;MARK1;METAP2
 
 
 
 
Ubiquitin-Like Protein Conjugating Enzyme Binding ( GO:0044390 )
 
 
8/34
 
 
0.0411399
 
 
0.2866934
 
 
0
 
 
0
 
 
2.298750
 
 
7.334801
 
 
TCERG1;RNF144B;RNF19A;SIAH2;TRAF6;DCUN1D4;RNF217;RPS3
 
 
 
 
Phosphatidylinositol-5-Phosphate Binding ( GO:0010314 )
 
 
5/17
 
 
0.0421368
 
 
0.2913643
 
 
0
 
 
0
 
 
3.111405
 
 
9.853306
 
 
SNX3;SESTD1;SAP30L;VEPH1;TOM1
 
 
 
 
Chloride Channel Regulator Activity ( GO:0017081 )
 
 
4/12
 
 
0.0442381
 
 
0.2989426
 
 
0
 
 
0
 
 
3.732952
 
 
11.639972
 
 
SGK3;SGK1;STX1A;VTI1B
 
 
 
 
Hydrolase Activity, Acting on Carbon-Nitrogen (But Not Peptide) Bonds,
in Cyclic Amides ( GO:0016812 )
 
 
4/12
 
 
0.0442381
 
 
0.2989426
 
 
0
 
 
0
 
 
3.732952
 
 
11.639972
 
 
MBLAC2;DPYSL4;DPYSL5;DPYSL3
 
 
 
 
Platelet-Derived Growth Factor Receptor Binding ( GO:0005161 )
 
 
4/12
 
 
0.0442381
 
 
0.2989426
 
 
0
 
 
0
 
 
3.732952
 
 
11.639972
 
 
PDGFRB;PDGFRA;PDGFC;VEGFA
 
 
 
 
Acyltransferase Activity, Transferring Groups Other Than Amino-Acyl
Groups ( GO:0016747 )
 
 
14/73
 
 
0.0452231
 
 
0.3033007
 
 
0
 
 
0
 
 
1.773958
 
 
5.492437
 
 
ELOVL4;ESCO1;TAFAZZIN;GTF2B;ELOVL2;ELOVL3;ZDHHC13;ZDHHC7;ELOVL6;ZDHHC16;ZDHHC17;PLA2G15;KAT5;EP300
 
 
 
 
Lipoprotein Particle Binding ( GO:0071813 )
 
 
6/23
 
 
0.0465884
 
 
0.3055652
 
 
0
 
 
0
 
 
2.635912
 
 
8.082771
 
 
SCARB1;STAB2;LPL;MAPT;LDLR;HSPD1
 
 
 
 
Mitogen-Activated Protein Kinase Binding ( GO:0051019 )
 
 
6/23
 
 
0.0465884
 
 
0.3055652
 
 
0
 
 
0
 
 
2.635912
 
 
8.082771
 
 
GCH1;DIRAS1;MAPK14;PPM1D;DUSP16;SIRT1
 
 
 
 
Protein Kinase A Regulatory Subunit Binding ( GO:0034237 )
 
 
6/23
 
 
0.0465884
 
 
0.3055652
 
 
0
 
 
0
 
 
2.635912
 
 
8.082771
 
 
ARFGEF1;PRRC1;GSKIP;AKAP5;WASF1;AKAP1
 
 
 
 
Nuclear Estrogen Receptor Binding ( GO:0030331 )
 
 
8/35
 
 
0.0480045
 
 
0.3111000
 
 
0
 
 
0
 
 
2.213485
 
 
6.721161
 
 
NCOA1;WBP2;LEF1;MMS19;TACC1;ESR1;ISL1;ZNF366
 
 
 
 
Metal Ion Transmembrane Transporter Activity ( GO:0046873 )
 
 
7/29
 
 
0.0481298
 
 
0.3111000
 
 
0
 
 
0
 
 
2.376648
 
 
7.210402
 
 
MMGT1;UCP3;ATP2B4;ZDHHC13;TRPM7;CNNM4;TMEM165
 
 
 
 
Histone H4 Acetyltransferase Activity ( GO:0010485 )
 
 
5/18
 
 
0.0528430
 
 
0.3391075
 
 
0
 
 
0
 
 
2.871904
 
 
8.444632
 
 
NAA50;NAA40;KAT5;BRPF3;EP300
 
 
 
 
Chemorepellent Activity ( GO:0045499 )
 
 
6/24
 
 
0.0561266
 
 
0.3528188
 
 
0
 
 
0
 
 
2.489332
 
 
7.169638
 
 
SEMA7A;SEMA6A;SEMA4D;SEMA4B;SEMA4C;SEMA4F
 
 
 
 
Acylglycerol O-acyltransferase Activity ( GO:0016411 )
 
 
7/30
 
 
0.0566142
 
 
0.3528188
 
 
0
 
 
0
 
 
2.273187
 
 
6.527444
 
 
PLA2G15;TAFAZZIN;DGAT1;MBOAT2;AGPAT3;AGPAT4;PNPLA2
 
 
 
 
Phosphatidylinositol-3,5-Bisphosphate Phosphatase Activity ( GO:0106018 )
 
 
4/13
 
 
0.0580878
 
 
0.3528188
 
 
0
 
 
0
 
 
3.317991
 
 
9.442337
 
 
MTMR1;MTMR2;MTMR14;MTMR9
 
 
 
 
Adenylate Cyclase Inhibiting G Protein-Coupled Glutamate Receptor
Activity ( GO:0001640 )
 
 
3/8
 
 
0.0585394
 
 
0.3528188
 
 
0
 
 
0
 
 
4.478408
 
 
12.709967
 
 
GRM3;GRM5;GRM7
 
 
 
 
Adenylate Cyclase Regulator Activity ( GO:0010854 )
 
 
3/8
 
 
0.0585394
 
 
0.3528188
 
 
0
 
 
0
 
 
4.478408
 
 
12.709967
 
 
GNAO1;GRM7;CALM3
 
 
 
 
Protein N-acetyltransferase Activity ( GO:0034212 )
 
 
3/8
 
 
0.0585394
 
 
0.3528188
 
 
0
 
 
0
 
 
4.478408
 
 
12.709967
 
 
NAA50;NAA20;EP300
 
 
 
 
siRNA Binding ( GO:0035197 )
 
 
3/8
 
 
0.0585394
 
 
0.3528188
 
 
0
 
 
0
 
 
4.478408
 
 
12.709967
 
 
PRKRA;FMR1;DICER1
 
 
 
 
Tumor Necrosis Factor Receptor Activity ( GO:0005031 )
 
 
3/8
 
 
0.0585394
 
 
0.3528188
 
 
0
 
 
0
 
 
4.478408
 
 
12.709967
 
 
TNFRSF11A;TNFRSF1B;TNFRSF1A
 
 
 
 
Unmethylated CpG Binding ( GO:0045322 )
 
 
3/8
 
 
0.0585394
 
 
0.3528188
 
 
0
 
 
0
 
 
4.478408
 
 
12.709967
 
 
FBXL19;MBD1;DNMT3A
 
 
 
 
Carboxylic Ester Hydrolase Activity ( GO:0052689 )
 
 
19/111
 
 
0.0618836
 
 
0.3680013
 
 
0
 
 
0
 
 
1.544335
 
 
4.297113
 
 
ACHE;LYPLA1;PGAP1;ABHD4;TNFAIP6;ABHD3;LPL;ABHD6;ABHD5;PLA2G15;NCEH1;CA2;PTRH2;ESD;ENPP2;PNPLA6;LYPLAL1;PAFAH1B2;PNPLA2
 
 
 
 
Deubiquitinase Activity ( GO:0101005 )
 
 
19/111
 
 
0.0618836
 
 
0.3680013
 
 
0
 
 
0
 
 
1.544335
 
 
4.297113
 
 
OTUD4;USP46;USP47;USP37;USP48;USP6;USP38;USP53;USP4;USP32;USP9X;USP3;USP2;USP34;USP45;TNFAIP3;JOSD1;OTULIN;OTUB1
 
 
 
 
Transmembrane Receptor Protein Kinase Activity ( GO:0019199 )
 
 
8/37
 
 
0.0638576
 
 
0.3772253
 
 
0
 
 
0
 
 
2.060597
 
 
5.668907
 
 
PDGFRA;AXL;TYRO3;RORA;TEK;ACVR2A;TGFBR1;EPHA2
 
 
 
 
Insulin Receptor Binding ( GO:0005158 )
 
 
5/19
 
 
0.0649824
 
 
0.3813440
 
 
0
 
 
0
 
 
2.666616
 
 
7.289566
 
 
SNX2;GRB10;ENPP1;IGF1;PIK3R1
 
 
 
 
Protease Binding ( GO:0002020 )
 
 
20/119
 
 
0.0661067
 
 
0.3854065
 
 
0
 
 
0
 
 
1.510715
 
 
4.103834
 
 
ITGB1;PRNP;NTRK2;SERPINE1;SERPINF2;PTEN;DERL1;SYVN1;TYSND1;TNFAIP3;TNF;F3;COL1A1;COL3A1;COL1A2;BIN1;BCL2;FLOT2;TP53;LDLR
 
 
 
 
Acetyltransferase Activity ( GO:0016407 )
 
 
6/25
 
 
0.0667520
 
 
0.3866416
 
 
0
 
 
0
 
 
2.358180
 
 
6.383053
 
 
KAT5;ESCO1;GTF2B;EP300;NAT14;PAFAH1B2
 
 
 
 
Myosin Binding ( GO:0017022 )
 
 
11/57
 
 
0.0679783
 
 
0.3912040
 
 
0
 
 
0
 
 
1.786774
 
 
4.803861
 
 
RAB43;ARFGEF1;RAB10;RAB3C;RAB14;RAB3D;TOM1;LMTK2;RAB39B;MYL12B;TRAK2
 
 
 
 
Protein Kinase Regulator Activity ( GO:0019887 )
 
 
19/113
 
 
0.0715923
 
 
0.4093612
 
 
0
 
 
0
 
 
1.511305
 
 
3.984960
 
 
CDKN1C;MAP2K1;CCNJ;GMFB;CCNH;YWHAB;GSKIP;CCNC;CCNA2;MOB1A;CCND2;SH3BP5L;CCNE2;CCNY;PRKRA;CCNG1;CCNL2;CCNJL;CDK5R1
 
 
 
 
Wnt Receptor Activity ( GO:0042813 )
 
 
4/14
 
 
0.0739656
 
 
0.4149516
 
 
0
 
 
0
 
 
2.986023
 
 
7.776067
 
 
FZD5;FZD4;LRP5;RORA
 
 
 
 
Magnesium Ion Transmembrane Transporter Activity ( GO:0015095 )
 
 
4/14
 
 
0.0739656
 
 
0.4149516
 
 
0
 
 
0
 
 
2.986023
 
 
7.776067
 
 
MMGT1;ZDHHC13;TRPM7;CNNM4
 
 
 
 
Oxysterol Binding ( GO:0008142 )
 
 
4/14
 
 
0.0739656
 
 
0.4149516
 
 
0
 
 
0
 
 
2.986023
 
 
7.776067
 
 
INSIG2;INSIG1;GAS1;RORA
 
 
 
 
RNA Exonuclease Activity, Producing 5’-Phosphomonoesters ( GO:0016896 )
 
 
7/32
 
 
0.0761959
 
 
0.4212926
 
 
0
 
 
0
 
 
2.091094
 
 
5.383411
 
 
CNOT6L;CNOT7;XRN1;ERI2;EXOSC3;DCP2;DIS3L2
 
 
 
 
UDP-glycosyltransferase Activity ( GO:0008194 )
 
 
12/65
 
 
0.0770485
 
 
0.4212926
 
 
0
 
 
0
 
 
1.691811
 
 
4.336652
 
 
B3GALNT2;DDX18;POGLUT2;POGLUT1;B3GNT7;B4GAT1;MGAT4A;CSGALNACT2;GCNT3;GXYLT2;LARGE1;POMGNT1
 
 
 
 
Growth Factor Receptor Binding ( GO:0070851 )
 
 
17/100
 
 
0.0782102
 
 
0.4212926
 
 
0
 
 
0
 
 
1.531085
 
 
3.901749
 
 
PDGFRB;PDGFRA;ARF4;TSLP;TGFA;FRS3;AREG;VEGFA;ATXN2;ESM1;SNX2;PDGFC;IL12B;GRB2;IL6ST;IL6R;HBEGF
 
 
 
 
1-Phosphatidylinositol-3-Kinase Activity ( GO:0016303 )
 
 
3/9
 
 
0.0802912
 
 
0.4212926
 
 
0
 
 
0
 
 
3.731795
 
 
9.411943
 
 
PIK3CA;PIK3R3;PIK3C2A
 
 
 
 
cAMP Response Element Binding ( GO:0035497 )
 
 
3/9
 
 
0.0802912
 
 
0.4212926
 
 
0
 
 
0
 
 
3.731795
 
 
9.411943
 
 
CREB1;CREB3L1;CREB5
 
 
 
 
Dopamine Receptor Binding ( GO:0050780 )
 
 
3/9
 
 
0.0802912
 
 
0.4212926
 
 
0
 
 
0
 
 
3.731795
 
 
9.411943
 
 
GNA13;CAV2;DNAJC14
 
 
 
 
Phosphatidylinositol-3,5-Bisphosphate 3-Phosphatase Activity ( GO:0052629 )
 
 
3/9
 
 
0.0802912
 
 
0.4212926
 
 
0
 
 
0
 
 
3.731795
 
 
9.411943
 
 
MTMR1;MTMR2;MTMR14
 
 
 
 
Phosphoserine Residue Binding ( GO:0050815 )
 
 
3/9
 
 
0.0802912
 
 
0.4212926
 
 
0
 
 
0
 
 
3.731795
 
 
9.411943
 
 
YWHAB;NEDD4;YWHAZ
 
 
 
 
Potassium Ion Binding ( GO:0030955 )
 
 
3/9
 
 
0.0802912
 
 
0.4212926
 
 
0
 
 
0
 
 
3.731795
 
 
9.411943
 
 
HDAC4;VTA1;ATP1A2
 
 
 
 
pre-mRNA Intronic Binding ( GO:0097157 )
 
 
3/9
 
 
0.0802912
 
 
0.4212926
 
 
0
 
 
0
 
 
3.731795
 
 
9.411943
 
 
RBM20;RBM7;RBM24
 
 
 
 
Prostaglandin Receptor Activity ( GO:0004955 )
 
 
3/9
 
 
0.0802912
 
 
0.4212926
 
 
0
 
 
0
 
 
3.731795
 
 
9.411943
 
 
PTGER3;PPARG;PTGDR
 
 
 
 
Manganese Ion Binding ( GO:0030145 )
 
 
10/52
 
 
0.0809839
 
 
0.4224425
 
 
0
 
 
0
 
 
1.778688
 
 
4.470739
 
 
ENDOU;DYRK2;XPNPEP1;GALNT1;MPPED2;ADCY2;POC1B-GALNT4;PPM1K;LARGE1;POMGNT1
 
 
 
 
Pyrophosphatase Activity ( GO:0016462 )
 
 
11/59
 
 
0.0831696
 
 
0.4313216
 
 
0
 
 
0
 
 
1.712130
 
 
4.257850
 
 
NUDT11;ENTPD1;DDX3X;DOLPP1;ENTPD7;ENPP1;ALPL;CILP2;NUDT13;DCP2;NUDT4
 
 
 
 
Adenyl Ribonucleotide Binding ( GO:0032559 )
 
 
46/318
 
 
0.0860849
 
 
0.4371420
 
 
0
 
 
0
 
 
1.266231
 
 
3.105331
 
 
BRSK1;ABCD3;DYRK2;ABCB6;ATP10D;ATP1A2;YARS2;HSP90B1;NUAK2;TDG;AKT3;PDK4;PDE4B;TLK2;STK38L;ENPP1;PIP4K2B;ATP7A;N4BP2;MORC2;MAP4K4;MAP3K5;MARK1;ACVR1;SRPK2;PRNP;DMPK;GRID1;NEK6;CDC42BPG;ATP2B4;LMTK2;TGFBR1;TBCK;P2RX5;SNRK;PTPRA;STK26;MPPED2;RAPGEF2;MYH9;TOP1;ERCC6;ABCE1;HSPA1B;HCN1
 
 
 
 
Exopeptidase Activity ( GO:0008238 )
 
 
10/53
 
 
0.0897544
 
 
0.4371420
 
 
0
 
 
0
 
 
1.737224
 
 
4.187888
 
 
DPP10;CTSA;NPEPPS;ENPEP;XPNPEP1;ANPEP;PRCP;BLMH;METAP1;METAP2
 
 
 
 
Potassium Channel Regulator Activity ( GO:0015459 )
 
 
10/53
 
 
0.0897544
 
 
0.4371420
 
 
0
 
 
0
 
 
1.737224
 
 
4.187888
 
 
DPP10;CAV3;KCNIP3;KCNS3;ENSA;KCNIP4;SGK3;SGK1;ARPP19;KCNK2
 
 
 
 
ATP Binding ( GO:0005524 )
 
 
41/281
 
 
0.0906934
 
 
0.4371420
 
 
0
 
 
0
 
 
1.278677
 
 
3.069171
 
 
BRSK1;ABCD3;DYRK2;ABCB6;ATP10D;ATP1A2;YARS2;HSP90B1;NUAK2;TDG;AKT3;PDK4;TLK2;STK38L;ENPP1;PIP4K2B;ATP7A;N4BP2;MORC2;MAP4K4;MAP3K5;MARK1;ACVR1;SRPK2;PRNP;DMPK;NEK6;CDC42BPG;ATP2B4;LMTK2;TGFBR1;TBCK;P2RX5;SNRK;PTPRA;STK26;MYH9;TOP1;ERCC6;ABCE1;HSPA1B
 
 
 
 
Alkali Metal Ion Binding ( GO:0031420 )
 
 
4/15
 
 
0.0917864
 
 
0.4371420
 
 
0
 
 
0
 
 
2.714412
 
 
6.482805
 
 
HDAC4;VTA1;TDG;ATP1A2
 
 
 
 
Histone H3 Acetyltransferase Activity ( GO:0010484 )
 
 
4/15
 
 
0.0917864
 
 
0.4371420
 
 
0
 
 
0
 
 
2.714412
 
 
6.482805
 
 
MCM3AP;BRPF3;KAT6B;EP300
 
 
 
 
Protein Serine/Threonine Kinase Binding ( GO:0120283 )
 
 
4/15
 
 
0.0917864
 
 
0.4371420
 
 
0
 
 
0
 
 
2.714412
 
 
6.482805
 
 
DAZAP2;IRF5;VHL;FERMT2
 
 
 
 
Syntaxin-1 Binding ( GO:0017075 )
 
 
4/15
 
 
0.0917864
 
 
0.4371420
 
 
0
 
 
0
 
 
2.714412
 
 
6.482805
 
 
SNAP25;UNC13C;SYT1;VAMP2
 
 
 
 
Transmembrane Receptor Protein Phosphatase Activity ( GO:0019198 )
 
 
4/15
 
 
0.0917864
 
 
0.4371420
 
 
0
 
 
0
 
 
2.714412
 
 
6.482805
 
 
PTPRN2;PTPRA;PTPRM;PTPRK
 
 
 
 
Transmembrane Receptor Protein Tyrosine Phosphatase Activity ( GO:0005001 )
 
 
4/15
 
 
0.0917864
 
 
0.4371420
 
 
0
 
 
0
 
 
2.714412
 
 
6.482805
 
 
PTPRN2;PTPRA;PTPRM;PTPRK
 
 
 
 
Ubiquitin-Ubiquitin Ligase Activity ( GO:0034450 )
 
 
4/15
 
 
0.0917864
 
 
0.4371420
 
 
0
 
 
0
 
 
2.714412
 
 
6.482805
 
 
TRAF6;PELI2;PRPF19;UBE2K
 
 
 
 
Cysteine-Type Endopeptidase Activity ( GO:0004197 )
 
 
13/74
 
 
0.0927516
 
 
0.4371420
 
 
0
 
 
0
 
 
1.592380
 
 
3.786410
 
 
PDIA3;USP37;USP6;USP32;USP9X;USP34;USP2;CTSS;CASP7;SFRP1;CASP2;ATG4B;CAPN1
 
 
 
 
Acetylglucosaminyltransferase Activity ( GO:0008375 )
 
 
8/40
 
 
0.0930844
 
 
0.4371420
 
 
0
 
 
0
 
 
1.867098
 
 
4.432956
 
 
LFNG;B3GNT7;B4GAT1;MGAT4A;GCNT1;GCNT3;LARGE1;POMGNT1
 
 
 
 
R-SMAD Binding ( GO:0070412 )
 
 
5/21
 
 
0.0934912
 
 
0.4371420
 
 
0
 
 
0
 
 
2.333024
 
 
5.529007
 
 
SMAD4;DROSHA;PPARG;FOS;LDLRAD4
 
 
 
 
WW Domain Binding ( GO:0050699 )
 
 
5/21
 
 
0.0934912
 
 
0.4371420
 
 
0
 
 
0
 
 
2.333024
 
 
5.529007
 
 
DAZAP2;NDFIP2;TRAF4;RAPGEF2;PPARG
 
 
 
 
Lysophospholipase Activity ( GO:0004622 )
 
 
5/21
 
 
0.0934912
 
 
0.4371420
 
 
0
 
 
0
 
 
2.333024
 
 
5.529007
 
 
LYPLA1;ABHD4;ENPP2;PNPLA6;LYPLAL1
 
 
 
 
Phosphatidic Acid Binding ( GO:0070300 )
 
 
5/21
 
 
0.0934912
 
 
0.4371420
 
 
0
 
 
0
 
 
2.333024
 
 
5.529007
 
 
SMPD3;SESTD1;RAPGEF2;PITPNC1;MARK1
 
 
 
 
Guanyl-Nucleotide Exchange Factor Activity ( GO:0005085 )
 
 
31/206
 
 
0.0943743
 
 
0.4371420
 
 
0
 
 
0
 
 
1.325156
 
 
3.128013
 
 
DOCK6;ARF4;DOCK3;ITSN1;MYCBP2;RAP1GDS1;C9ORF72;RAPGEFL1;RASGRP4;CYTH3;GNA13;RIC8B;FGD6;RAP1A;SLC38A9;RALGDS;SBF2;CYTH1;EIF2B5;ARFGEF1;RIC1;GAPVD1;ADRA2A;TIAM1;SH3BP5L;RAPGEF2;PREB;DENND6A;RAPGEF5;SOS1;SOS2
 
 
 
 
Ubiquitin-Like Ligase-Substrate Adaptor Activity ( GO:1990756 )
 
 
19/118
 
 
0.1001851
 
 
0.4371420
 
 
0
 
 
0
 
 
1.434567
 
 
3.300561
 
 
KLHL29;SUV39H2;FBXW7;FEM1B;FBXL19;KLHL28;DAW1;KLHL2;FBXO45;ASB11;SOCS2;APPBP2;KLHL6;KLHL8;VHL;DCAF12;SKP1;ASB1;SPSB4
 
 
 
 
RNA Endonuclease Activity ( GO:0004521 )
 
 
8/41
 
 
0.1042787
 
 
0.4371420
 
 
0
 
 
0
 
 
1.810416
 
 
4.092787
 
 
ENDOU;ZC3H12C;KHNYN;AGO4;ZC3H12B;ELAC2;AGO2;DICER1
 
 
 
 
ATP-dependent Diacylglycerol Kinase Activity ( GO:0004143 )
 
 
3/10
 
 
0.1049534
 
 
0.4371420
 
 
0
 
 
0
 
 
3.198500
 
 
7.210183
 
 
DGKD;DGKZ;DGKH
 
 
 
 
UDP-glucosyltransferase Activity ( GO:0035251 )
 
 
3/10
 
 
0.1049534
 
 
0.4371420
 
 
0
 
 
0
 
 
3.198500
 
 
7.210183
 
 
UGCG;POGLUT2;POGLUT1
 
 
 
 
Aromatic Amino Acid Transmembrane Transporter Activity ( GO:0015173 )
 
 
3/10
 
 
0.1049534
 
 
0.4371420
 
 
0
 
 
0
 
 
3.198500
 
 
7.210183
 
 
SLC6A14;SLC16A10;SLC7A1
 
 
 
 
Clathrin Heavy Chain Binding ( GO:0032050 )
 
 
3/10
 
 
0.1049534
 
 
0.4371420
 
 
0
 
 
0
 
 
3.198500
 
 
7.210183
 
 
TOM1;LDLR;SNAP91
 
 
 
 
Filamin Binding ( GO:0031005 )
 
 
3/10
 
 
0.1049534
 
 
0.4371420
 
 
0
 
 
0
 
 
3.198500
 
 
7.210183
 
 
DPYSL4;DPYSL3;RFLNB
 
 
 
 
Nuclear Retinoid X Receptor Binding ( GO:0046965 )
 
 
3/10
 
 
0.1049534
 
 
0.4371420
 
 
0
 
 
0
 
 
3.198500
 
 
7.210183
 
 
NR4A2;PPARG;TACC1
 
 
 
 
Transferrin Receptor Binding ( GO:1990459 )
 
 
3/10
 
 
0.1049534
 
 
0.4371420
 
 
0
 
 
0
 
 
3.198500
 
 
7.210183
 
 
SNX2;CHUK;RGMA
 
 
 
 
Ubiquitin Conjugating Enzyme Binding ( GO:0031624 )
 
 
6/28
 
 
0.1050968
 
 
0.4371420
 
 
0
 
 
0
 
 
2.036263
 
 
4.587443
 
 
RNF144B;RNF19A;SIAH2;TRAF6;DCUN1D4;RNF217
 
 
 
 
 
 
 KEGG_2021_Human 
 This category is based on pathway annotations from the Kyoto
Encyclopedia of Genes and Genomes (KEGG), a manually curated resource
integrating molecular interaction networks, metabolic pathways, and
disease-associated signaling cascades. KEGG pathways represent canonical
biological systems including metabolism, signal transduction, cell
communication, and genetic information processing. Enrichment in KEGG
pathways suggests coordinated perturbation of well-characterized
biological networks. This category is particularly useful for
identifying conserved signaling routes and metabolic programs affected
by gene expression changes. 
 
 
 
 
Term
 
 
Overlap
 
 
P.value
 
 
Adjusted.P.value
 
 
Old.P.value
 
 
Old.Adjusted.P.value
 
 
Odds.Ratio
 
 
Combined.Score
 
 
Genes
 
 
 
 
 
 
MAPK signaling pathway
 
 
73/294
 
 
0.0000000
 
 
0.0000001
 
 
0
 
 
0
 
 
2.509658
 
 
54.3656465
 
 
ARRB1;IL1RAP;AREG;FGF2;TNF;DUSP16;AKT3;RPS6KA1;STMN1;MAP3K7;MAP3K4;MAP3K5;PDGFRB;MAP2K4;PDGFRA;MAP2K1;CHUK;DUSP1;CACNA2D3;RRAS2;FOS;DUSP9;TGFBR1;CDC25B;TNFRSF1A;CACNB3;MRAS;RASA1;TRAF6;RAPGEF2;MAPT;RAF1;SOS1;TP53;MET;SOS2;EPHA2;CSF1R;MAX;TGFA;CACNA1A;EFNA5;RASGRP4;RAP1B;CDC42;NRAS;RAP1A;RRAS;IRAK1;ERBB3;PDGFC;NTF3;MAPK1;CACNG2;MAP4K4;MAP2K6;NTRK2;TGFB2;GADD45A;BDNF;VEGFC;IGF1;GNG12;MAPK14;MAPK12;VEGFA;KITLG;FAS;GRB2;TEK;HSPA1B;MAP3K11;MAP3K12
 
 
 
 
Pathways in cancer
 
 
111/531
 
 
0.0000000
 
 
0.0000001
 
 
0
 
 
0
 
 
2.018491
 
 
41.6768437
 
 
RB1;HHIP;FGF2;EDNRA;FRAT2;CCND2;AKT3;IL12B;EP300;IL6R;IL13RA1;SKP1;PDGFRB;PDGFRA;MAP2K1;RALBP1;MITF;RUNX1;EML4;CCNE2;COL4A2;COL4A1;COL4A4;COL4A5;IL6ST;RAF1;TP53;NOTCH2;CSF1R;NOTCH1;MAX;LPAR2;PIK3R3;TGFA;PIK3R1;RASGRP4;HSP90B1;BCL2L11;PMAIP1;WNT1;RALGDS;WNT10B;SMAD4;TGFB2;JAG1;FZD5;FZD4;GADD45A;IGF1;GNG12;ESR1;LPAR6;BCL2;CYCS;CALM3;GRB2;ITGB1;PTEN;SLC2A1;LAMC1;SHH;CASP7;HEY2;NCOA1;CHUK;IL15;WNT9B;FOS;F2;TGFBR1;DDB2;CCNA2;PIK3CA;TRAF4;IFNG;TRAF6;TRAF5;RARA;RARB;PPARG;SOS1;MET;SOS2;LAMA2;HDAC1;LAMA4;PTGER3;LEF1;LRP5;ADCY3;ADCY2;DLL1;CDC42;DLL4;GNA13;NRAS;GNG2;MAPK1;E2F3;BAK1;VHL;TCF7L2;TXNRD1;STAT3;VEGFC;VEGFA;KITLG;APC;GSTA4;CCDC6;FAS
 
 
 
 
Signaling pathways regulating pluripotency of stem cells
 
 
44/143
 
 
0.0000000
 
 
0.0000001
 
 
0
 
 
0
 
 
3.357940
 
 
69.1432915
 
 
HOXD1;ONECUT1;PIK3R3;PIK3R1;BMI1;FGF2;NRAS;AKT3;MAPK1;HOXA1;WNT1;JARID2;ACVR1;WNT10B;MAP2K1;SMAD4;PCGF6;FZD5;SETDB1;FZD4;PCGF5;PCGF3;STAT3;LIF;WNT9B;INHBB;IGF1;KLF4;MAPK14;ISL1;DUSP9;ACVR2A;MAPK12;MEIS1;REST;PIK3CA;APC;ID1;ID4;COMMD3-BMI1;ID3;GRB2;IL6ST;RAF1
 
 
 
 
PI3K-Akt signaling pathway
 
 
80/354
 
 
0.0000000
 
 
0.0000005
 
 
0
 
 
0
 
 
2.218339
 
 
41.8178289
 
 
ITGB1;YWHAB;PTEN;LAMC1;AREG;FGF2;CCND2;YWHAQ;CREB3L1;AKT3;MYB;IL6R;PDGFRB;PDGFRA;MAP2K1;CHUK;MAGI2;TSC1;YWHAZ;COL2A1;CREB1;CCNE2;COL4A2;PIK3CA;COL4A1;COL4A4;COL6A2;DDIT4;COL4A5;COL6A3;SGK3;SGK1;RAF1;EIF4E2;SOS1;TP53;MET;SOS2;EPHA2;CREB5;CSF1R;PRKAA1;LAMA2;LAMA4;LPAR2;PIK3R3;TGFA;PIK3R1;EFNA5;FOXO3;HSP90B1;PPP2CA;NRAS;BCL2L11;GNG2;ERBB3;PDGFC;NTF3;EIF4EBP1;MAPK1;MCL1;NTRK2;BDNF;OSM;VEGFC;IGF1;PPP2R3A;GNG12;VEGFA;COL1A1;KITLG;G6PC2;COL1A2;G6PC3;LPAR6;ITGA11;COL9A1;BCL2;GRB2;TEK
 
 
 
 
Hepatitis B
 
 
44/162
 
 
0.0000001
 
 
0.0000041
 
 
0
 
 
0
 
 
2.814202
 
 
46.2076158
 
 
RB1;DDX3X;YWHAB;PIK3R3;PIK3R1;TNF;NRAS;IRAK1;YWHAQ;CREB3L1;AKT3;EP300;MAPK1;E2F3;MAP3K7;MAP2K6;MAP2K4;MAP2K1;SMAD4;TGFB2;EGR3;CHUK;STAT3;FOS;MAPK14;YWHAZ;TGFBR1;MAPK12;DDB2;CCNA2;MAVS;CREB1;CCNE2;PIK3CA;TRAF6;BCL2;CYCS;FAS;GRB2;RAF1;SOS1;TP53;SOS2;CREB5
 
 
 
 
Colorectal cancer
 
 
29/86
 
 
0.0000001
 
 
0.0000041
 
 
0
 
 
0
 
 
3.828422
 
 
62.4541600
 
 
LEF1;PIK3R3;TGFA;PIK3R1;AREG;NRAS;BCL2L11;AKT3;MAPK1;PMAIP1;BAK1;RALGDS;TCF7L2;MAP2K1;SMAD4;TGFB2;GADD45A;FOS;TGFBR1;DDB2;PIK3CA;APC;BCL2;CYCS;GRB2;RAF1;SOS1;SOS2;TP53
 
 
 
 
Prostate cancer
 
 
31/97
 
 
0.0000001
 
 
0.0000052
 
 
0
 
 
0
 
 
3.535607
 
 
56.3289371
 
 
RB1;LEF1;PTEN;PIK3R3;TGFA;PIK3R1;HSP90B1;NRAS;CREB3L1;AKT3;PDGFC;EP300;MAPK1;E2F3;PDGFRB;PDGFRA;TCF7L2;MAP2K1;CHUK;IGF1;CREB1;CCNE2;PIK3CA;BCL2;GRB2;ERG;RAF1;SOS1;TP53;SOS2;CREB5
 
 
 
 
Breast cancer
 
 
40/147
 
 
0.0000003
 
 
0.0000102
 
 
0
 
 
0
 
 
2.818290
 
 
42.6262486
 
 
RB1;NOTCH2;NOTCH1;LEF1;PTEN;LRP5;PIK3R3;PIK3R1;FGF2;DLL1;DLL4;NRAS;FRAT2;AKT3;HEY2;MAPK1;E2F3;BAK1;WNT1;NCOA1;WNT10B;TCF7L2;MAP2K1;JAG1;FZD5;FZD4;GADD45A;CSNK1A1;WNT9B;IGF1;FOS;ESR1;DDB2;PIK3CA;APC;GRB2;RAF1;SOS1;TP53;SOS2
 
 
 
 
Phospholipase D signaling pathway
 
 
40/148
 
 
0.0000003
 
 
0.0000110
 
 
0
 
 
0
 
 
2.792035
 
 
41.6838701
 
 
DGKD;LPAR2;PIK3R3;ADCY3;ADCY2;PIK3R1;AGPAT3;AGPAT4;GRM3;CYTH3;GNA13;GRM5;NRAS;RRAS;GRM7;AKT3;PDGFC;PIP5K1A;MAPK1;PIP5K1B;RALGDS;CYTH1;PDGFRB;PDGFRA;MAP2K1;RRAS2;TSC1;F2;DGKZ;DNM3;KITLG;MRAS;PIK3CA;LPAR6;GRB2;RAF1;SOS1;SOS2;DGKH;ARF6
 
 
 
 
Glioma
 
 
25/75
 
 
0.0000008
 
 
0.0000245
 
 
0
 
 
0
 
 
3.757479
 
 
52.7018196
 
 
RB1;PTEN;PIK3R3;TGFA;PIK3R1;NRAS;AKT3;MAPK1;E2F3;BAK1;PDGFRB;PDGFRA;MAP2K1;CAMK1D;GADD45A;IGF1;DDB2;PIK3CA;CAMK4;CALM3;GRB2;RAF1;SOS1;SOS2;TP53
 
 
 
 
Proteoglycans in cancer
 
 
49/205
 
 
0.0000010
 
 
0.0000265
 
 
0
 
 
0
 
 
2.370552
 
 
32.8382273
 
 
ITGB1;FGF2;TNF;PPP1CB;PPP1CC;SHH;AKT3;IL12B;MAP2K1;PPP1R12A;WNT9B;RRAS2;ANK2;ANK3;TIAM1;MRAS;PIK3CA;RAF1;SOS1;TP53;MET;SOS2;HBEGF;PIK3R3;PIK3R1;CDC42;NRAS;RRAS;ERBB3;DROSHA;MAPK1;WNT1;WNT10B;TGFB2;FZD5;CAV3;FZD4;CAV2;STAT3;IGF1;MAPK14;ESR1;MAPK12;VEGFA;COL1A1;COL1A2;SDC1;FAS;GRB2
 
 
 
 
Cellular senescence
 
 
40/156
 
 
0.0000014
 
 
0.0000341
 
 
0
 
 
0
 
 
2.598294
 
 
34.9544674
 
 
RB1;LIN54;SERPINE1;PTEN;PIK3R3;PIK3R1;FOXO3;ZFP36L2;ZFP36L1;PPP1CB;PPP1CC;NRAS;CCND2;RRAS;AKT3;EIF4EBP1;MAPK1;TRPM7;MYBL2;E2F3;CAPN1;E2F5;MAP2K6;MAP2K1;TGFB2;GADD45A;RRAS2;TSC1;MAPK14;SIRT1;TGFBR1;MAPK12;CCNA2;MRAS;CCNE2;PIK3CA;CALM3;RAF1;TP53;SLC25A4
 
 
 
 
Small cell lung cancer
 
 
28/92
 
 
0.0000015
 
 
0.0000341
 
 
0
 
 
0
 
 
3.289394
 
 
44.1808504
 
 
ITGB1;RB1;LAMA2;MAX;LAMA4;PTEN;PIK3R3;LAMC1;PIK3R1;AKT3;E2F3;BAK1;CHUK;GADD45A;DDB2;CCNE2;COL4A2;PIK3CA;TRAF4;COL4A1;COL4A4;TRAF6;TRAF5;BCL2;COL4A5;RARB;CYCS;TP53
 
 
 
 
Relaxin signaling pathway
 
 
35/129
 
 
0.0000016
 
 
0.0000341
 
 
0
 
 
0
 
 
2.803100
 
 
37.4395824
 
 
PIK3R3;ADCY3;ARRB1;ADCY2;PIK3R1;NRAS;GNG2;CREB3L1;AKT3;MAPK1;MAP2K4;MAP2K1;VEGFC;FOS;GNG12;MAPK14;TGFBR1;MAPK12;VEGFA;COL1A1;GNAO1;ACTA2;COL3A1;COL1A2;CREB1;COL4A2;PIK3CA;COL4A1;COL4A4;COL4A5;GRB2;RAF1;SOS1;SOS2;CREB5
 
 
 
 
Protein digestion and absorption
 
 
30/103
 
 
0.0000018
 
 
0.0000354
 
 
0
 
 
0
 
 
3.090904
 
 
40.9616688
 
 
COL15A1;COL16A1;ELN;COL11A1;COL12A1;ATP1A2;PRCP;COL19A1;COL25A1;COL22A1;ATP1B4;SLC16A10;COL1A1;COL3A1;COL1A2;COL2A1;SLC7A7;COL4A2;COL5A1;COL4A1;COL4A4;FXYD2;COL6A2;COL7A1;COL5A2;COL9A1;COL4A5;COL8A1;COL21A1;COL6A3
 
 
 
 
FoxO signaling pathway
 
 
35/131
 
 
0.0000023
 
 
0.0000439
 
 
0
 
 
0
 
 
2.744389
 
 
35.5963025
 
 
PRKAA1;PTEN;PIK3R3;PIK3R1;FOXO3;NRAS;CCND2;BCL2L11;AKT3;S1PR1;EP300;MAPK1;MAP2K1;PRKAB2;SMAD4;TGFB2;HOMER1;CHUK;GADD45A;PLK2;STAT3;IGF1;MAPK14;SIRT1;TGFBR1;MAPK12;G6PC2;G6PC3;PIK3CA;GRB2;SGK3;RAF1;SGK1;SOS1;SOS2
 
 
 
 
Gastric cancer
 
 
38/149
 
 
0.0000030
 
 
0.0000512
 
 
0
 
 
0
 
 
2.578086
 
 
32.7889070
 
 
RB1;LEF1;LRP5;PIK3R3;PIK3R1;FGF2;SHH;NRAS;FRAT2;AKT3;MAPK1;E2F3;BAK1;WNT1;WNT10B;TCF7L2;MAP2K1;SMAD4;TGFB2;FZD5;CDX2;FZD4;GADD45A;CSNK1A1;WNT9B;TGFBR1;DDB2;CCNE2;PIK3CA;APC;BCL2;RARB;GRB2;RAF1;SOS1;TP53;MET;SOS2
 
 
 
 
Focal adhesion
 
 
47/201
 
 
0.0000030
 
 
0.0000512
 
 
0
 
 
0
 
 
2.301601
 
 
29.2318178
 
 
ITGB1;LAMA2;LAMA4;PTEN;PIK3R3;PIK3R1;LAMC1;MYL12B;PPP1CB;RAP1B;CDC42;PPP1CC;CCND2;RAP1A;AKT3;PDGFC;PIP5K1A;MAPK1;PIP5K1B;VASP;PDGFRB;PDGFRA;MAP2K1;PPP1R12A;CAV3;CAV2;VEGFC;IGF1;VEGFA;COL1A1;COL1A2;COL2A1;COL4A2;PIK3CA;COL4A1;COL4A4;COL6A2;ITGA11;COL9A1;BCL2;COL4A5;COL6A3;GRB2;RAF1;SOS1;MET;SOS2
 
 
 
 
TNF signaling pathway
 
 
31/112
 
 
0.0000039
 
 
0.0000619
 
 
0
 
 
0
 
 
2.878405
 
 
35.8537419
 
 
PIK3R3;TNFAIP3;PIK3R1;TNF;BAG4;CASP7;CREB3L1;AKT3;CCL2;MAPK1;MAP3K7;MAP3K5;MAP2K6;MAP2K4;MAP2K1;JAG1;CHUK;IL15;LIF;VEGFC;FOS;TNFRSF1B;MAPK14;MAPK12;TNFRSF1A;CREB1;PIK3CA;BCL3;TRAF5;FAS;CREB5
 
 
 
 
Longevity regulating pathway
 
 
29/102
 
 
0.0000044
 
 
0.0000668
 
 
0
 
 
0
 
 
2.986595
 
 
36.8222680
 
 
PRKAA1;CLPB;HDAC1;PIK3R3;ADCY3;ADCY2;PIK3R1;FOXO3;ADIPOR2;NRAS;CREB3L1;SESN1;RB1CC1;AKT3;SESN2;EIF4EBP1;EIF4EBP2;PRKAB2;TSC1;IGF1;SIRT1;CREB1;PIK3CA;CAMK4;PPARG;EIF4E2;TP53;HSPA1B;CREB5
 
 
 
 
Non-small cell lung cancer
 
 
23/72
 
 
0.0000050
 
 
0.0000723
 
 
0
 
 
0
 
 
3.524617
 
 
43.0040934
 
 
RB1;MAP2K1;GADD45A;STAT3;PIK3R3;TGFA;PIK3R1;FOXO3;DDB2;EML4;NRAS;PIK3CA;AKT3;RARB;MAPK1;E2F3;GRB2;BAK1;RAF1;SOS1;MET;SOS2;TP53
 
 
 
 
Neurotrophin signaling pathway
 
 
32/119
 
 
0.0000054
 
 
0.0000732
 
 
0
 
 
0
 
 
2.766582
 
 
33.5806914
 
 
BEX3;ZNF274;PRDM4;PIK3R3;PIK3R1;FOXO3;RAP1B;CDC42;NRAS;RAP1A;IRAK1;AKT3;RPS6KA1;NTF3;MAPK1;MAP3K5;NTRK2;MAP2K1;BDNF;PRKCD;MAPK14;MAPK12;PIK3CA;TRAF6;CAMK4;BCL2;CALM3;GRB2;RAF1;SOS1;TP53;SOS2
 
 
 
 
Endometrial cancer
 
 
20/58
 
 
0.0000056
 
 
0.0000732
 
 
0
 
 
0
 
 
3.949501
 
 
47.7782265
 
 
TCF7L2;MAP2K1;GADD45A;LEF1;PTEN;PIK3R3;PIK3R1;FOXO3;DDB2;NRAS;PIK3CA;APC;AKT3;MAPK1;GRB2;BAK1;RAF1;SOS1;SOS2;TP53
 
 
 
 
AGE-RAGE signaling pathway in diabetic complications
 
 
28/100
 
 
0.0000088
 
 
0.0001113
 
 
0
 
 
0
 
 
2.922574
 
 
34.0060763
 
 
SERPINE1;PIK3R3;PIK3R1;TNF;CDC42;NRAS;AKT3;CCL2;MAPK1;SMAD4;TGFB2;PRKCD;STAT3;VEGFC;MAPK14;F3;TGFBR1;MAPK12;VEGFA;COL1A1;COL3A1;COL1A2;COL4A2;PIK3CA;COL4A1;COL4A4;BCL2;COL4A5
 
 
 
 
Rap1 signaling pathway
 
 
47/210
 
 
0.0000107
 
 
0.0001289
 
 
0
 
 
0
 
 
2.173399
 
 
24.8817546
 
 
ITGB1;CSF1R;RGS14;LPAR2;PIK3R3;ADCY3;ADCY2;PIK3R1;EFNA5;FGF2;RAP1B;CDC42;NRAS;PARD6B;RAP1A;RRAS;CNR1;AKT3;PDGFC;MAPK1;RALGDS;MAP2K6;VASP;PDGFRB;PDGFRA;MAP2K1;MAGI2;VEGFC;IGF1;MAPK14;MAPK12;VEGFA;GNAO1;TIAM1;KITLG;MRAS;PIK3CA;ADORA2B;ID1;RAPGEF2;CALM3;TEK;RAPGEF5;RAF1;MET;SIPA1;EPHA2
 
 
 
 
Human papillomavirus infection
 
 
66/331
 
 
0.0000130
 
 
0.0001483
 
 
0
 
 
0
 
 
1.881737
 
 
21.1639769
 
 
RB1;ITGB1;PTEN;LAMC1;TNF;LFNG;CCND2;CREB3L1;AKT3;HEY2;EP300;ATP6V1E1;PDGFRB;MAP2K1;CHUK;WNT9B;TSC1;TNFRSF1A;CCNA2;COL2A1;CREB1;CCNE2;COL4A2;PIK3CA;COL4A1;COL4A4;COL6A2;TBPL1;COL4A5;COL6A3;RAF1;SOS1;TP53;SOS2;CREB5;NOTCH2;NOTCH1;LAMA2;HDAC1;LAMA4;PIK3R3;PIK3R1;PPP2CA;CDC42;NRAS;PARD6B;EIF4EBP1;MAPK1;BAK1;WNT1;WNT10B;TCF7L2;JAG1;FZD5;FZD4;CSNK1A1;PPP2R3A;VEGFA;COL1A1;COL1A2;APC;ITGA11;COL9A1;FAS;GRB2;PALS1
 
 
 
 
Chronic myeloid leukemia
 
 
23/76
 
 
0.0000138
 
 
0.0001483
 
 
0
 
 
0
 
 
3.257867
 
 
36.4696770
 
 
RB1;MAP2K1;SMAD4;TGFB2;CHUK;HDAC1;GADD45A;PIK3R3;PIK3R1;TGFBR1;DDB2;RUNX1;NRAS;PIK3CA;AKT3;MAPK1;E2F3;GRB2;BAK1;RAF1;SOS1;SOS2;TP53
 
 
 
 
Pancreatic cancer
 
 
23/76
 
 
0.0000138
 
 
0.0001483
 
 
0
 
 
0
 
 
3.257867
 
 
36.4696770
 
 
RB1;MAP2K1;RALBP1;SMAD4;TGFB2;CHUK;GADD45A;STAT3;PIK3R3;TGFA;PIK3R1;TGFBR1;DDB2;VEGFA;CDC42;PIK3CA;AKT3;MAPK1;E2F3;BAK1;RAF1;TP53;RALGDS
 
 
 
 
Melanoma
 
 
22/72
 
 
0.0000178
 
 
0.0001849
 
 
0
 
 
0
 
 
3.302347
 
 
36.1237216
 
 
RB1;PDGFRB;PDGFRA;MAP2K1;GADD45A;PTEN;PIK3R3;MITF;PIK3R1;IGF1;FGF2;DDB2;NRAS;PIK3CA;AKT3;PDGFC;MAPK1;E2F3;BAK1;RAF1;MET;TP53
 
 
 
 
TGF-beta signaling pathway
 
 
26/94
 
 
0.0000231
 
 
0.0002327
 
 
0
 
 
0
 
 
2.871652
 
 
30.6546255
 
 
TNF;PPP2CA;EP300;MAPK1;PITX2;E2F5;SKP1;ACVR1;SMAD4;TGFB2;TGIF2;FST;NOG;INHBB;GDF6;GDF5;TGFBR1;ACVR2A;RGMA;GREM1;GREM2;IFNG;ID1;ID4;ID3;FBN1
 
 
 
 
Renal cell carcinoma
 
 
21/69
 
 
0.0000292
 
 
0.0002848
 
 
0
 
 
0
 
 
3.282557
 
 
34.2700212
 
 
MAP2K1;TGFB2;SLC2A1;PIK3R3;TGFA;PIK3R1;VEGFA;CDC42;RAP1B;NRAS;RAP1A;PIK3CA;AKT3;EP300;MAPK1;GRB2;VHL;RAF1;SOS1;MET;SOS2
 
 
 
 
Prolactin signaling pathway
 
 
21/70
 
 
0.0000372
 
 
0.0003507
 
 
0
 
 
0
 
 
3.215383
 
 
32.7980893
 
 
MAP2K1;STAT3;PIK3R3;FOS;PIK3R1;TNFRSF11A;FOXO3;MAPK14;ESR1;MAPK12;SOCS2;NRAS;CCND2;PIK3CA;ELF5;AKT3;MAPK1;GRB2;RAF1;SOS1;SOS2
 
 
 
 
Sphingolipid signaling pathway
 
 
30/119
 
 
0.0000401
 
 
0.0003671
 
 
0
 
 
0
 
 
2.532926
 
 
25.6428830
 
 
ASAH1;SGMS1;PTEN;PIK3R3;PIK3R1;SGMS2;TNF;GNA13;PPP2CA;SGPL1;NRAS;SPTLC2;AKT3;S1PR1;MAPK1;MAP3K5;CERS4;MAP2K1;CERS5;CERS6;PPP2R3A;MAPK14;MAPK12;SGPP1;TNFRSF1A;ACER2;PIK3CA;BCL2;RAF1;TP53
 
 
 
 
mTOR signaling pathway
 
 
36/154
 
 
0.0000419
 
 
0.0003723
 
 
0
 
 
0
 
 
2.294620
 
 
23.1294329
 
 
PRKAA1;PTEN;LRP5;PIK3R3;PIK3R1;TNF;NRAS;AKT3;RPS6KA1;SESN2;GRB10;EIF4EBP1;MAPK1;SLC38A9;ATP6V1E1;WNT1;RNF152;WNT10B;MAP2K1;FZD5;CHUK;FZD4;WNT9B;TSC1;IGF1;TNFRSF1A;RRAGC;PIK3CA;DDIT4;TBC1D7;GRB2;RAF1;EIF4E2;SGK1;SOS1;SOS2
 
 
 
 
Autophagy
 
 
33/137
 
 
0.0000457
 
 
0.0003944
 
 
0
 
 
0
 
 
2.385386
 
 
23.8376787
 
 
PRKAA1;STX17;MTMR14;PTEN;PIK3R3;PIK3R1;ZFYVE1;PPP2CA;NRAS;RRAS;RB1CC1;AKT3;MAPK1;NRBF2;MAP3K7;SNAP29;SH3GLB1;RUBCN;MAP2K1;PRKCD;RAB39B;RRAS2;TSC1;EIF2S1;VMP1;MRAS;RRAGC;PIK3CA;TRAF6;DDIT4;BCL2;ATG4B;RAF1
 
 
 
 
SNARE interactions in vesicular transport
 
 
13/33
 
 
0.0000494
 
 
0.0004141
 
 
0
 
 
0
 
 
4.868091
 
 
48.2733413
 
 
STX17;STX16;GOSR2;STX11;VAMP7;STX6;STX4;STX3;SNAP29;STX1A;VAMP2;VTI1B;BET1
 
 
 
 
Hepatocellular carcinoma
 
 
38/168
 
 
0.0000562
 
 
0.0004584
 
 
0
 
 
0
 
 
2.198902
 
 
21.5213028
 
 
RB1;LEF1;PTEN;LRP5;PIK3R3;TGFA;PIK3R1;NRAS;FRAT2;AKT3;MAPK1;E2F3;BAK1;ARID2;WNT1;WNT10B;TCF7L2;MAP2K1;SMAD4;TGFB2;FZD5;FZD4;GADD45A;CSNK1A1;TXNRD1;WNT9B;ARID1B;TGFBR1;DDB2;PIK3CA;APC;GSTA4;GRB2;RAF1;SOS1;TP53;MET;SOS2
 
 
 
 
Acute myeloid leukemia
 
 
20/67
 
 
0.0000611
 
 
0.0004856
 
 
0
 
 
0
 
 
3.191580
 
 
30.9678312
 
 
CSF1R;TCF7L2;MAP2K1;CHUK;LEF1;STAT3;PIK3R3;PIK3R1;RUNX1;CCNA2;NRAS;PIK3CA;AKT3;RARA;EIF4EBP1;MAPK1;GRB2;RAF1;SOS1;SOS2
 
 
 
 
MicroRNAs in cancer
 
 
60/310
 
 
0.0000771
 
 
0.0005973
 
 
0
 
 
0
 
 
1.810152
 
 
17.1421411
 
 
TRIM71;PTEN;BMI1;CCND2;STMN1;DNMT3B;EP300;SOX4;PDGFRB;PDGFRA;MAP2K1;TPM1;DNMT3A;DICER1;SIRT1;CDC25B;FOXP1;MMP16;CCNE2;PIK3CA;DDIT4;FSCN1;COMMD3-BMI1;RAF1;SOS1;TP53;MET;SOS2;NOTCH2;HDAC4;DNMT1;NOTCH1;HDAC1;PIK3R3;PIK3R1;EFNA5;SLC7A1;NRAS;BCL2L11;ERBB3;MAPK1;BMF;E2F3;BAK1;MCL1;ST14;TGFB2;UBE2I;STAT3;VEGFA;MARCKS;HNRNPK;CYP24A1;APC;CCNG1;BCL2;SPRY2;GRB2;MDM4;BCL2L2
 
 
 
 
Choline metabolism in cancer
 
 
25/98
 
 
0.0001391
 
 
0.0010255
 
 
0
 
 
0
 
 
2.570249
 
 
22.8238164
 
 
DGKD;PIK3R3;PIK3R1;NRAS;AKT3;PDGFC;EIF4EBP1;PIP5K1A;MAPK1;PIP5K1B;WASF1;RALGDS;PDGFRB;PDGFRA;MAP2K1;LYPLA1;TSC1;FOS;DGKZ;PIK3CA;GRB2;RAF1;SOS1;SOS2;DGKH
 
 
 
 
Mannose type O-glycan biosynthesis
 
 
10/23
 
 
0.0001392
 
 
0.0010255
 
 
0
 
 
0
 
 
5.756002
 
 
51.1098505
 
 
B3GALNT2;B4GALT2;POMT2;B4GALT3;B4GAT1;FUT9;CHST10;LARGE1;POMGNT1;FUT4
 
 
 
 
Ras signaling pathway
 
 
47/232
 
 
0.0001465
 
 
0.0010383
 
 
0
 
 
0
 
 
1.912529
 
 
16.8842707
 
 
CSF1R;RAB5B;PIK3R3;TGFA;RASAL1;PIK3R1;EFNA5;FGF2;RASGRP4;RAP1B;CDC42;NRAS;RAP1A;GNG2;RRAS;AKT3;PDGFC;NTF3;MAPK1;RALGDS;PDGFRB;PDGFRA;NTRK2;MAP2K1;RALBP1;CHUK;BDNF;RRAS2;VEGFC;IGF1;GNG12;VEGFA;TIAM1;KITLG;MRAS;PIK3CA;RASA1;CALM3;GRB2;TEK;RAPGEF5;RAF1;SOS1;MET;SOS2;EPHA2;ARF6
 
 
 
 
Fluid shear stress and atherosclerosis
 
 
32/139
 
 
0.0001478
 
 
0.0010383
 
 
0
 
 
0
 
 
2.246900
 
 
19.8162096
 
 
PRKAA1;PIK3R3;PIK3R1;TNF;TXN2;HSP90B1;CDH5;AKT3;CCL2;MAP3K7;MAP3K5;MAP2K6;ACVR1;MEF2A;MAP2K4;CAV3;CHUK;CAV2;DUSP1;FOS;MAPK14;ACVR2A;MAPK12;TNFRSF1A;VEGFA;IFNG;PIK3CA;GSTA4;BCL2;SDC1;CALM3;TP53
 
 
 
 
Ubiquitin mediated proteolysis
 
 
32/140
 
 
0.0001703
 
 
0.0011686
 
 
0
 
 
0
 
 
2.225969
 
 
19.3174098
 
 
CUL5;UBA6;UBE2D2;CUL3;UBE2D3;UBE2D1;CBLB;RNF7;PRPF19;RCHY1;RHOBTB1;UBE2J1;CDC23;UBE2NL;VHL;SKP1;UBE2F;UBE2H;UBE2I;UBE2B;FBXW7;UBE2E3;SYVN1;UBE2G1;DDB2;UBE2W;UBE2R2;TRAF6;NEDD4;UBE2N;UBE2K;CUL4B
 
 
 
 
Other types of O-glycan biosynthesis
 
 
15/47
 
 
0.0002194
 
 
0.0014727
 
 
0
 
 
0
 
 
3.511237
 
 
29.5800850
 
 
B4GALT2;GALNT12;POMT2;GALNT7;B4GALT3;COLGALT2;GALNT14;GALNT5;POGLUT1;GALNT1;C1GALT1;GXYLT2;LFNG;EOGT;POC1B-GALNT4
 
 
 
 
AMPK signaling pathway
 
 
28/120
 
 
0.0002942
 
 
0.0019316
 
 
0
 
 
0
 
 
2.284627
 
 
18.5767878
 
 
PRKAA1;PIK3R3;PIK3R1;HMGCR;FOXO3;ADIPOR2;PPP2CA;CREB3L1;AKT3;EIF4EBP1;MAP3K7;SREBF1;PRKAB2;TSC1;IGF1;PPP2R3A;SIRT1;CCNA2;RAB10;TBC1D1;G6PC2;G6PC3;CREB1;PIK3CA;RAB14;SCD;PPARG;CREB5
 
 
 
 
Osteoclast differentiation
 
 
29/127
 
 
0.0003417
 
 
0.0021956
 
 
0
 
 
0
 
 
2.221541
 
 
17.7314038
 
 
CSF1R;PIK3R3;TNFRSF11B;PIK3R1;TNFRSF11A;TNF;CALCR;AKT3;MAPK1;MAP3K7;MAP2K6;MAP2K1;TGFB2;CHUK;MITF;FOS;MAPK14;TGFBR1;MAPK12;TNFRSF1A;FOSL1;CREB1;IFNG;PIK3CA;TRAF6;CAMK4;FOSB;GRB2;PPARG
 
 
 
 
Sphingolipid metabolism
 
 
15/49
 
 
0.0003658
 
 
0.0023016
 
 
0
 
 
0
 
 
3.304318
 
 
26.1482749
 
 
CERS4;CERS5;ASAH1;CERS6;SGMS1;SGMS2;SGPP1;SMPD3;ACER2;UGCG;SGPL1;SPTLC2;NEU1;ACER3;B4GALT6
 
 
 
 
Toxoplasmosis
 
 
26/112
 
 
0.0005171
 
 
0.0031870
 
 
0
 
 
0
 
 
2.268282
 
 
17.1647040
 
 
ITGB1;LAMA2;LAMA4;LAMC1;TNF;IRAK1;AKT3;IL12B;MAPK1;MAP3K7;LDLR;MAP2K6;TGFB2;CHUK;STAT3;MAPK14;MAPK12;TNFRSF1A;GNAO1;IFNG;TRAF6;PPIF;BCL2;CYCS;HSPA1B;HLA-DQB1
 
 
 
 
Endocytosis
 
 
48/252
 
 
0.0005548
 
 
0.0033508
 
 
0
 
 
0
 
 
1.770139
 
 
13.2706676
 
 
ARF4;RAB5B;TFRC;VPS4B;SNX12;CBLB;ARRB1;ASAP2;RAB22A;SNX3;CDC42;CYTH3;PARD6B;SNX2;CHMP1B;PIP5K1A;PIP5K1B;VPS36;EPS15;LDLR;SNX5;CYTH1;SH3GLB1;ARFGEF1;PDGFRA;RAB4A;CAV3;IST1;CAV2;VTA1;VPS37C;ARPC5;VPS37B;TGFBR1;DNM3;RAB10;EHD2;BIN1;CAPZA1;NEDD4;TRAF6;RUFY2;CHMP4B;CHMP7;SMAP1;ARF5;HSPA1B;ARF6
 
 
 
 
Growth hormone synthesis, secretion and action
 
 
27/119
 
 
0.0005978
 
 
0.0035401
 
 
0
 
 
0
 
 
2.202091
 
 
16.3443700
 
 
PIK3R3;ADCY3;ADCY2;PIK3R1;SOCS2;GHRHR;NRAS;CREB3L1;AKT3;EP300;MAPK1;MAP2K6;MAP2K4;MAP2K1;STAT3;IGF1;FOS;MAPK14;SSTR3;MAPK12;CREB1;PIK3CA;GRB2;RAF1;SOS1;SOS2;CREB5
 
 
 
 
p53 signaling pathway
 
 
19/73
 
 
0.0006461
 
 
0.0037525
 
 
0
 
 
0
 
 
2.636789
 
 
19.3659328
 
 
GADD45A;SERPINE1;PTEN;IGF1;PPM1D;RCHY1;DDB2;CCND2;CCNE2;ZMAT3;SESN1;SESN2;CCNG1;BCL2;PMAIP1;CYCS;FAS;MDM4;TP53
 
 
 
 
Hepatitis C
 
 
33/157
 
 
0.0006849
 
 
0.0039024
 
 
0
 
 
0
 
 
1.998364
 
 
14.5606909
 
 
RB1;SCARB1;YWHAB;PIK3R3;PIK3R1;CLDN1;TNF;PPP2CA;NRAS;YWHAQ;AKT3;MAPK1;E2F3;BAK1;LDLR;MAP2K1;CHUK;STAT3;EIF2S1;YWHAZ;TNFRSF1A;MAVS;IFNG;PIK3CA;CLDN14;TRAF6;CYCS;FAS;GRB2;RAF1;SOS1;TP53;SOS2
 
 
 
 
HIF-1 signaling pathway
 
 
25/109
 
 
0.0007918
 
 
0.0044282
 
 
0
 
 
0
 
 
2.232270
 
 
15.9411142
 
 
TFRC;SERPINE1;SLC2A1;PIK3R3;ENO1;PIK3R1;PDHB;HK2;LDHA;AKT3;EIF4EBP1;EP300;MAPK1;VHL;IL6R;MAP2K1;STAT3;IGF1;VEGFA;IFNG;PIK3CA;BCL2;TEK;ALDOA;EIF4E2
 
 
 
 
Human cytomegalovirus infection
 
 
43/225
 
 
0.0009746
 
 
0.0053514
 
 
0
 
 
0
 
 
1.775845
 
 
12.3127960
 
 
RB1;PTGER3;PIK3R3;ADCY3;ADCY2;PIK3R1;TNF;GNA13;NRAS;GNG2;CREB3L1;AKT3;EIF4EBP1;CCL2;MAPK1;E2F3;BAK1;IL6R;MAP2K6;PDIA3;PDGFRA;MAP2K1;CHUK;STAT3;TSC1;GNG12;MAPK14;MAPK12;VEGFA;TNFRSF1A;GNAO1;CREB1;PIK3CA;TRAF5;FAS;CYCS;CALM3;GRB2;RAF1;SOS1;TP53;SOS2;CREB5
 
 
 
 
Apoptosis
 
 
30/142
 
 
0.0010739
 
 
0.0057915
 
 
0
 
 
0
 
 
2.010133
 
 
13.7421698
 
 
HRK;PIK3R3;PIK3R1;TNF;CTSS;CASP7;NRAS;BCL2L11;AIFM1;AKT3;PMAIP1;CASP2;MAPK1;CAPN1;BAK1;SPTAN1;MAP3K5;MCL1;MAP2K1;CHUK;GADD45A;FOS;EIF2S1;TNFRSF1A;PIK3CA;BCL2;CYCS;FAS;RAF1;TP53
 
 
 
 
Epstein-Barr virus infection
 
 
39/202
 
 
0.0013419
 
 
0.0071096
 
 
0
 
 
0
 
 
1.797256
 
 
11.8864903
 
 
RB1;PSMD12;HDAC1;PIK3R3;TNFAIP3;PIK3R1;TNF;CCND2;BCL2L11;IRAK1;SAP30L;AKT3;E2F3;BAK1;MAP3K7;MAP2K6;PDIA3;MAP2K4;ENTPD1;CR2;CHUK;GADD45A;STAT3;MAPK14;MAPK12;DDB2;NCOR2;CCNA2;MAVS;CCNE2;PIK3CA;NEDD4;TRAF6;TRAF5;BCL2;FAS;CYCS;TP53;HLA-DQB1
 
 
 
 
Protein processing in endoplasmic reticulum
 
 
34/171
 
 
0.0015814
 
 
0.0081936
 
 
0
 
 
0
 
 
1.862964
 
 
12.0151334
 
 
UBXN1;SAR1A;UBE2D2;UBE2D3;RPN1;DERL1;UBE2D1;HSP90B1;UBE2J1;DNAJB1;ERLEC1;LMAN1;LMAN2;UBQLN1;SSR1;CAPN1;BAK1;SEC23B;MAP3K5;SKP1;PDIA3;SYVN1;UBE2G1;EIF2S1;SVIP;CKAP4;DNAJB11;CANX;DNAJA2;BCL2;PLAA;PREB;MAN1B1;HSPA1B
 
 
 
 
Chagas disease
 
 
23/102
 
 
0.0016007
 
 
0.0081936
 
 
0
 
 
0
 
 
2.182425
 
 
14.0489137
 
 
MAP2K4;TGFB2;CHUK;SERPINE1;PIK3R3;PIK3R1;FOS;MAPK14;TNF;TGFBR1;MAPK12;TNFRSF1A;GNAO1;PPP2CA;IRAK1;IFNG;PIK3CA;TRAF6;AKT3;IL12B;CCL2;MAPK1;FAS
 
 
 
 
Phosphatidylinositol signaling system
 
 
22/97
 
 
0.0018482
 
 
0.0093028
 
 
0
 
 
0
 
 
2.198435
 
 
13.8359090
 
 
CDS1;IPPK;PI4K2B;MTMR1;MTMR2;DGKD;INPP1;MTMR14;PTEN;PIK3R3;PIK3R1;PIK3C2A;DGKZ;PIP4P2;PIK3CA;PI4KA;PIP5K1A;PIP4K2B;PIP5K1B;CALM3;DGKH;CDS2
 
 
 
 
Mucin type O-glycan biosynthesis
 
 
11/36
 
 
0.0022118
 
 
0.0109500
 
 
0
 
 
0
 
 
3.291589
 
 
20.1246665
 
 
GALNT12;GALNT7;GALNT14;GALNT5;GALNT1;GCNT1;C1GALT1;GCNT3;POC1B-GALNT4;ST6GALNAC3;B4GALT5
 
 
 
 
Salmonella infection
 
 
45/249
 
 
0.0024224
 
 
0.0117996
 
 
0
 
 
0
 
 
1.657359
 
 
9.9822416
 
 
DYNC1I2;RAB5B;ARHGEF26;CSE1L;LEF1;GCC2;PIK3C2A;TNF;MYL12B;TXN2;HSP90B1;CDC42;CYTH3;CASP7;RRAS;IRAK1;AKT3;RPS3;MAPK1;BAK1;MAP3K7;CYTH1;SKP1;MAP2K6;MAP2K4;TCF7L2;MAP2K1;CHUK;DYNLT1;FOS;ARPC5;MAPK14;DYNLL2;MAPK12;TNFRSF1A;SNX18;TUBB2A;PIK3CA;TRAF6;BCL2;CYCS;RAF1;ACBD3;ARF6;ARL8B
 
 
 
 
Estrogen signaling pathway
 
 
28/137
 
 
0.0025825
 
 
0.0123796
 
 
0
 
 
0
 
 
1.926441
 
 
11.4796545
 
 
PIK3R3;TGFA;ADCY3;ADCY2;PIK3R1;HSP90B1;NRAS;CREB3L1;AKT3;MAPK1;NCOA1;MAP2K1;PRKCD;FOS;ESR1;GNAO1;CREB1;PIK3CA;BCL2;RARA;CALM3;GRB2;RAF1;SOS1;SOS2;HSPA1B;CREB5;HBEGF
 
 
 
 
Central carbon metabolism in cancer
 
 
17/70
 
 
0.0027352
 
 
0.0129068
 
 
0
 
 
0
 
 
2.401835
 
 
14.1745463
 
 
PDGFRB;PDGFRA;MAP2K1;PTEN;SLC2A1;PIK3R3;PDHB;PIK3R1;HK2;LDHA;NRAS;PIK3CA;AKT3;MAPK1;RAF1;MET;TP53
 
 
 
 
Thyroid cancer
 
 
11/37
 
 
0.0028161
 
 
0.0130841
 
 
0
 
 
0
 
 
3.164810
 
 
18.5850241
 
 
TCF7L2;NRAS;MAP2K1;GADD45A;LEF1;CCDC6;MAPK1;PPARG;BAK1;TP53;DDB2
 
 
 
 
PD-L1 expression and PD-1 checkpoint pathway in cancer
 
 
20/89
 
 
0.0032594
 
 
0.0149144
 
 
0
 
 
0
 
 
2.171256
 
 
12.4330514
 
 
BATF3;MAP2K1;CHUK;CSNK2A1;STAT3;PTEN;PIK3R3;FOS;PIK3R1;MAPK14;MAPK12;EML4;NRAS;IFNG;PIK3CA;TRAF6;AKT3;MAPK1;RAF1;MAP2K6
 
 
 
 
Transcriptional misregulation in cancer
 
 
36/192
 
 
0.0033488
 
 
0.0150947
 
 
0
 
 
0
 
 
1.731909
 
 
9.8704060
 
 
CSF1R;CCNT2;MAX;HDAC1;SIX1;BMI1;MLLT3;HOXA11;HOXA10;FUT8;CCND2;BAK1;H3-3B;SS18;GADD45A;MITF;ETV1;IGF1;ETV4;KLF3;MLF1;RUNX1;DDB2;ETV6;CCNA2;H3-5;MEIS1;MAF;MYCN;WT1;RARA;COMMD3-BMI1;PPARG;ERG;TP53;MET
 
 
 
 
Insulin resistance
 
 
23/108
 
 
0.0034894
 
 
0.0154489
 
 
0
 
 
0
 
 
2.027679
 
 
11.4726729
 
 
SREBF1;PRKAB2;PRKAA1;GFPT2;PRKCD;GFPT1;STAT3;PTEN;SLC2A1;PIK3R3;PIK3R1;TNF;TNFRSF1A;PPP1CB;PPP1CC;G6PC2;G6PC3;CREB1;PIK3CA;CREB3L1;AKT3;RPS6KA1;CREB5
 
 
 
 
Wnt signaling pathway
 
 
32/166
 
 
0.0035297
 
 
0.0154489
 
 
0
 
 
0
 
 
1.791403
 
 
10.1152302
 
 
CTNND2;LEF1;LRP5;CCND2;FRAT2;CCN4;EP300;RSPO3;GPC4;WNT1;MAP3K7;RSPO4;SKP1;WNT10B;TCF7L2;SMAD4;CSNK2A1;FZD5;FZD4;CSNK1A1;CTNNBIP1;WNT9B;CACYBP;DKK2;FOSL1;SFRP1;DAAM1;APC;TBL1XR1;ROR1;TP53;LGR4
 
 
 
 
Thyroid hormone signaling pathway
 
 
25/121
 
 
0.0036592
 
 
0.0157867
 
 
0
 
 
0
 
 
1.951901
 
 
10.9511767
 
 
NOTCH2;NOTCH1;HDAC1;SLC2A1;PIK3R3;ATP1A2;PIK3R1;MED12L;MED14;NRAS;PLN;AKT3;EP300;MAPK1;NCOA1;MAP2K1;ATP1B4;SLC16A10;ESR1;RCAN1;PIK3CA;FXYD2;RCAN2;RAF1;TP53
 
 
 
 
Fc gamma R-mediated phagocytosis
 
 
21/97
 
 
0.0041750
 
 
0.0177584
 
 
0
 
 
0
 
 
2.069893
 
 
11.3402084
 
 
VASP;MAP2K1;MARCKSL1;PRKCD;LIMK1;PIK3R3;PIK3R1;ARPC5;ASAP2;CDC42;MARCKS;PIK3CA;BIN1;CFL2;AKT3;PIP5K1A;MAPK1;PIP5K1B;RAF1;WASF1;ARF6
 
 
 
 
T cell receptor signaling pathway
 
 
22/104
 
 
0.0045918
 
 
0.0192601
 
 
0
 
 
0
 
 
2.009962
 
 
10.8205948
 
 
MAP2K1;CHUK;PIK3R3;CBLB;PIK3R1;FOS;MAPK14;TNF;MAPK12;CDC42;NRAS;IFNG;PIK3CA;GRAP2;AKT3;MAPK1;GRB2;RAF1;ICOS;SOS1;MAP3K7;SOS2
 
 
 
 
Fc epsilon RI signaling pathway
 
 
16/68
 
 
0.0050151
 
 
0.0206524
 
 
0
 
 
0
 
 
2.303173
 
 
12.1959876
 
 
MAP2K4;MAP2K1;PIK3R3;PIK3R1;MAPK14;TNF;MAPK12;NRAS;PIK3CA;AKT3;MAPK1;GRB2;RAF1;SOS1;SOS2;MAP2K6
 
 
 
 
Cell cycle
 
 
25/124
 
 
0.0051050
 
 
0.0206524
 
 
0
 
 
0
 
 
1.892429
 
 
9.9873412
 
 
RB1;CDKN1C;CCNH;YWHAB;HDAC1;TTK;CDC14A;CCND2;CDC23;YWHAQ;ORC2;EP300;E2F3;E2F5;SKP1;SMAD4;TGFB2;GADD45A;YWHAZ;CDC25B;CCNA2;STAG2;CCNE2;TFDP2;TP53
 
 
 
 
Insulin signaling pathway
 
 
27/137
 
 
0.0051289
 
 
0.0206524
 
 
0
 
 
0
 
 
1.839859
 
 
9.7013272
 
 
PRKAA1;PIK3R3;CBLB;PIK3R1;HK2;PPP1CB;SOCS2;PPP1CC;NRAS;AKT3;EIF4EBP1;FLOT2;MAPK1;SREBF1;MAP2K1;PRKAB2;TSC1;G6PC2;G6PC3;PIK3CA;CALM3;GRB2;RAF1;EIF4E2;SOS1;SOS2;RHOQ
 
 
 
 
Parathyroid hormone synthesis, secretion and action
 
 
22/106
 
 
0.0058185
 
 
0.0228232
 
 
0
 
 
0
 
 
1.961882
 
 
10.0972601
 
 
MEF2A;MAP2K1;LRP5;ADCY3;ARRB1;ADCY2;FOS;PTHLH;NR4A2;GNA13;MAFB;CREB1;CYP24A1;MMP16;MMP15;CREB3L1;BCL2;PDE4B;MAPK1;RAF1;CREB5;HBEGF
 
 
 
 
Human T-cell leukemia virus 1 infection
 
 
39/219
 
 
0.0058192
 
 
0.0228232
 
 
0
 
 
0
 
 
1.625932
 
 
8.3680168
 
 
RB1;NRP1;CRTC1;PTEN;SLC2A1;PIK3R3;ADCY3;ADCY2;PIK3R1;TNF;NRAS;CDC23;CCND2;KAT5;CREB3L1;AKT3;EP300;MAPK1;E2F3;MAP2K4;MAP2K1;SMAD4;TGFB2;CHUK;IL15;FOS;TGFBR1;TNFRSF1A;FOSL1;CCNA2;CREB1;CCNE2;PIK3CA;CANX;TBPL1;TP53;SLC25A4;HLA-DQB1;CREB5
 
 
 
 
Notch signaling pathway
 
 
14/59
 
 
0.0077443
 
 
0.0299844
 
 
0
 
 
0
 
 
2.327709
 
 
11.3145178
 
 
NOTCH2;NUMBL;NOTCH1;JAG1;HDAC1;DLL1;LFNG;NCOR2;APH1A;DLL4;ADAM17;HEY2;NUMB;EP300
 
 
 
 
Circadian rhythm
 
 
9/31
 
 
0.0079088
 
 
0.0302336
 
 
0
 
 
0
 
 
3.058285
 
 
14.8014223
 
 
PER1;PRKAA1;PRKAB2;CREB1;BHLHE40;BHLHE41;RORA;RORB;SKP1
 
 
 
 
Regulation of actin cytoskeleton
 
 
38/218
 
 
0.0091016
 
 
0.0343586
 
 
0
 
 
0
 
 
1.583560
 
 
7.4416292
 
 
ITGB1;LPAR2;PIK3R3;PIK3R1;FGF2;MYL12B;PPP1CB;CDC42;GNA13;PPP1CC;NRAS;RRAS;TMSB4X;CFL2;PDGFC;PIP5K1A;MAPK1;PIP4K2B;PIP5K1B;WASF1;PDGFRB;PDGFRA;MAP2K1;PPP1R12A;LIMK1;RRAS2;ARPC5;GNG12;F2;TIAM1;MRAS;PIK3CA;APC;ITGA11;MYH9;RAF1;SOS1;SOS2
 
 
 
 
ErbB signaling pathway
 
 
18/85
 
 
0.0096409
 
 
0.0359452
 
 
0
 
 
0
 
 
2.010976
 
 
9.3344218
 
 
MAP2K4;MAP2K1;PIK3R3;TGFA;CBLB;PIK3R1;AREG;NRAS;PIK3CA;ERBB3;AKT3;EIF4EBP1;MAPK1;GRB2;RAF1;SOS1;SOS2;HBEGF
 
 
 
 
Adipocytokine signaling pathway
 
 
15/69
 
 
0.0136006
 
 
0.0499849
 
 
0
 
 
0
 
 
2.078132
 
 
8.9310622
 
 
PRKAB2;PRKAA1;CHUK;ACSL1;STAT3;SLC2A1;ACSL6;ACSL4;TNFRSF1B;TNF;ADIPOR2;TNFRSF1A;G6PC2;G6PC3;AKT3
 
 
 
 
Melanogenesis
 
 
20/101
 
 
0.0138600
 
 
0.0499849
 
 
0
 
 
0
 
 
1.848324
 
 
7.9085105
 
 
WNT10B;TCF7L2;MAP2K1;FZD5;FZD4;LEF1;WNT9B;ADCY3;MITF;ADCY2;GNAO1;NRAS;KITLG;CREB1;CREB3L1;EP300;MAPK1;CALM3;RAF1;WNT1
 
 
 
 
Basal cell carcinoma
 
 
14/63
 
 
0.0139031
 
 
0.0499849
 
 
0
 
 
0
 
 
2.137206
 
 
9.1379345
 
 
TCF7L2;WNT10B;FZD5;FZD4;GADD45A;HHIP;LEF1;WNT9B;DDB2;SHH;APC;BAK1;WNT1;TP53
 
 
 
 
Chemical carcinogenesis
 
 
40/239
 
 
0.0146999
 
 
0.0522280
 
 
0
 
 
0
 
 
1.507408
 
 
6.3611282
 
 
RB1;NOTCH2;PIK3R3;ADCY3;CACNA1A;ADCY2;ARRB1;PIK3R1;AHR;FGF2;DLL1;HSP90B1;DLL4;NRAS;CREB3L1;AKT3;RPS6KA1;EIF4EBP1;MAPK1;KPNA5;WNT1;MAP2K1;JAG1;PTGES3;STAT3;FOS;KLF4;ESR1;VEGFA;CREB1;KLF5;GSTA4;PIK3CA;BCL2;GRB2;RAF1;SOS1;SOS2;KPNB1;CREB5
 
 
 
 
Hippo signaling pathway
 
 
29/163
 
 
0.0159128
 
 
0.0558274
 
 
0
 
 
0
 
 
1.621374
 
 
6.7135137
 
 
CRB2;YWHAB;SERPINE1;LEF1;AREG;AMOT;PPP1CB;PPP2CA;PPP1CC;PARD6B;CCND2;YWHAQ;CCN2;WNT1;WNT10B;TCF7L2;SMAD4;TGFB2;FZD5;FZD4;WNT9B;GDF6;GDF5;YWHAZ;TGFBR1;MOB1A;APC;ID1;PALS1
 
 
 
 
Human immunodeficiency virus 1 infection
 
 
36/212
 
 
0.0160827
 
 
0.0558274
 
 
0
 
 
0
 
 
1.533344
 
 
6.3327258
 
 
CUL5;PIK3R3;PIK3R1;RNF7;TNF;NRAS;GNG2;IRAK1;CFL2;AKT3;MAPK1;AP1S3;BAK1;MAP3K7;SKP1;MAP2K6;PDIA3;MAP2K1;CHUK;LIMK1;FOS;GNG12;TNFRSF1B;MAPK14;MAPK12;TNFRSF1A;GNAO1;PIK3CA;TRAF6;TRAF5;BCL2;FAS;CYCS;CALM3;RAF1;CUL4B
 
 
 
 
Adherens junction
 
 
15/71
 
 
0.0175322
 
 
0.0598558
 
 
0
 
 
0
 
 
2.003685
 
 
8.1023378
 
 
TCF7L2;SMAD4;CSNK2A1;LEF1;PTPRM;TGFBR1;CDC42;TJP1;SNAI1;EP300;MAPK1;WASF1;MAP3K7;MET;SSX2IP
 
 
 
 
Apelin signaling pathway
 
 
25/137
 
 
0.0178378
 
 
0.0598558
 
 
0
 
 
0
 
 
1.671532
 
 
6.7303110
 
 
HDAC4;PRKAA1;SERPINE1;ADCY3;ADCY2;GNA13;NRAS;GNG2;RRAS;AKT3;MAPK1;CCN2;MEF2A;MAP2K1;PRKAB2;SMAD4;JAG1;RRAS2;GNG12;TGFBR1;ACTA2;MRAS;CAMK4;CALM3;RAF1
 
 
 
 
Yersinia infection
 
 
25/137
 
 
0.0178378
 
 
0.0598558
 
 
0
 
 
0
 
 
1.671532
 
 
6.7303110
 
 
ITGB1;PIK3R3;PIK3R1;TNF;CDC42;IRAK1;AKT3;RPS6KA1;PIP5K1A;CCL2;MAPK1;PIP5K1B;MAP3K7;MAP2K6;MAP2K4;MAP2K1;CHUK;LIMK1;ARPC5;FOS;MAPK14;MAPK12;PIK3CA;TRAF6;ARF6
 
 
 
 
Kaposi sarcoma-associated herpesvirus infection
 
 
33/193
 
 
0.0186636
 
 
0.0610772
 
 
0
 
 
0
 
 
1.545548
 
 
6.1531061
 
 
RB1;LEF1;PIK3R3;PIK3R1;FGF2;NRAS;GNG2;AKT3;EP300;MAPK1;E2F3;BAK1;MAP2K6;MAP2K4;TCF7L2;MAP2K1;CHUK;STAT3;FOS;GNG12;MAPK14;MAPK12;VEGFA;TNFRSF1A;RCAN1;CREB1;PIK3CA;FAS;CYCS;CALM3;IL6ST;RAF1;TP53
 
 
 
 
Platelet activation
 
 
23/124
 
 
0.0187129
 
 
0.0610772
 
 
0
 
 
0
 
 
1.704907
 
 
6.7830437
 
 
VASP;ITGB1;PPP1R12A;PIK3R3;ADCY3;ADCY2;PIK3R1;F2;MAPK14;MAPK12;MYL12B;PTGS1;PPP1CB;RAP1B;COL1A1;GNA13;PPP1CC;COL3A1;RAP1A;COL1A2;PIK3CA;AKT3;MAPK1
 
 
 
 
C-type lectin receptor signaling pathway
 
 
20/104
 
 
0.0188085
 
 
0.0610772
 
 
0
 
 
0
 
 
1.782008
 
 
7.0807103
 
 
EGR3;CHUK;PRKCD;PIK3R3;RRAS2;CBLB;PIK3R1;MAPK14;TNF;MAPK12;NRAS;MRAS;RRAS;PIK3CA;AKT3;BCL3;IL12B;MAPK1;CALM3;RAF1
 
 
 
 
Lipid and atherosclerosis
 
 
36/215
 
 
0.0196751
 
 
0.0632115
 
 
0
 
 
0
 
 
1.507387
 
 
5.9216196
 
 
PIK3R3;PIK3R1;TNF;HSP90B1;HSPD1;RAP1B;CDC42;NRAS;CASP7;RAP1A;IRAK1;AKT3;IL12B;CCL2;MAPK1;LDLR;MAP3K7;MAP3K5;MAP2K6;MAP2K4;CHUK;STAT3;FOS;MAPK14;EIF2S1;MAPK12;TNFRSF1A;PIK3CA;TRAF6;BCL2;FAS;CYCS;CALM3;PPARG;TP53;HSPA1B
 
 
 
 
Measles
 
 
25/139
 
 
0.0210942
 
 
0.0670572
 
 
0
 
 
0
 
 
1.642019
 
 
6.3361557
 
 
PIK3R3;TNFAIP3;CBLB;PIK3R1;RCHY1;CCND2;IRAK1;AKT3;IL12B;BAK1;MAP3K7;CSNK2A1;CHUK;STAT3;FOS;EIF2S1;MAVS;CCNE2;PIK3CA;TRAF6;BCL2;CYCS;FAS;TP53;HSPA1B
 
 
 
 
Shigellosis
 
 
40/246
 
 
0.0227650
 
 
0.0716149
 
 
0
 
 
0
 
 
1.455601
 
 
5.5058541
 
 
ITGB1;UBE2D2;UBE2D3;PIK3R3;UBE2D1;PIK3R1;FNBP1L;FOXO3;TNF;HK2;MYL12B;CDC42;CYTH3;UBE2NL;AKT3;MAPK1;CAPN1;WASF1;MAP3K7;CYTH1;SKP1;H3-3B;CAST;CHUK;PRKCD;ARPC5;MAPK14;MAPK12;TNFRSF1A;H3-5;RRAGC;PIK3CA;TRAF6;TRAF5;BCL2;UBE2N;CYCS;UBE2V1;TP53;ARF6
 
 
 
 
Axon guidance
 
 
31/182
 
 
0.0231805
 
 
0.0721703
 
 
0
 
 
0
 
 
1.537888
 
 
5.7892906
 
 
ITGB1;NRP1;SEMA7A;PIK3R3;PIK3R1;EFNA5;MYL12B;ROBO1;EFNB2;CDC42;SHH;NRAS;PARD6B;RRAS;DPYSL5;CFL2;MAPK1;SRGAP2;SEMA6A;TRPC6;SEMA4D;SEMA4B;LIMK1;SEMA4C;SEMA4F;RGMA;PIK3CA;RASA1;RAF1;MET;EPHA2
 
 
 
 
GnRH signaling pathway
 
 
18/93
 
 
0.0234675
 
 
0.0723182
 
 
0
 
 
0
 
 
1.795654
 
 
6.7375441
 
 
MAP2K4;MAP2K1;PRKCD;ADCY3;ADCY2;MAPK14;MAPK12;CDC42;NRAS;MAPK1;CALM3;GRB2;RAF1;SOS1;SOS2;MAP3K4;HBEGF;MAP2K6
 
 
 
 
Non-alcoholic fatty liver disease
 
 
27/155
 
 
0.0250224
 
 
0.0763309
 
 
0
 
 
0
 
 
1.579505
 
 
5.8251906
 
 
PRKAA1;PIK3R3;PIK3R1;TNF;ADIPOR2;CDC42;CASP7;BCL2L11;AKT3;UQCRFS1;IL6R;MAP3K5;SREBF1;PRKAB2;NDUFA3;SDHD;FOS;MAPK14;EIF2S1;MAPK12;TNFRSF1A;PIK3CA;NDUFS4;CYCS;FAS;PPARG;MAP3K11
 
 
 
 
Aldosterone-regulated sodium reabsorption
 
 
9/37
 
 
0.0255379
 
 
0.0771244
 
 
0
 
 
0
 
 
2.402119
 
 
8.8099941
 
 
PIK3CA;FXYD2;PIK3R3;MAPK1;ATP1B4;ATP1A2;PIK3R1;IGF1;SGK1
 
 
 
 
ECM-receptor interaction
 
 
17/88
 
 
0.0275945
 
 
0.0825103
 
 
0
 
 
0
 
 
1.791084
 
 
6.4302388
 
 
ITGB1;LAMA2;LAMA4;LAMC1;NPNT;COL1A1;COL2A1;COL1A2;COL4A2;COL4A1;COL4A4;COL6A2;ITGA11;COL9A1;COL4A5;COL6A3;SDC1
 
 
 
 
Influenza A
 
 
29/172
 
 
0.0308869
 
 
0.0914494
 
 
0
 
 
0
 
 
1.518548
 
 
5.2806359
 
 
PIK3R3;PIK3R1;NXT2;TNF;DNAJB1;NXF1;PABPN1;AKT3;EP300;IL12B;CCL2;MAPK1;KPNA5;BAK1;BCL2L2-PABPN1;RAE1;CPSF4;MAP2K1;CHUK;EIF2S1;TNFRSF1A;MAVS;IFNG;PIK3CA;CYCS;FAS;RAF1;SLC25A4;HLA-DQB1
 
 
 
 
Toll-like receptor signaling pathway
 
 
19/104
 
 
0.0351182
 
 
0.1029679
 
 
0
 
 
0
 
 
1.672183
 
 
5.6002012
 
 
MAP2K4;MAP2K1;CHUK;CD80;PIK3R3;PIK3R1;FOS;MAPK14;TNF;MAPK12;IRAK1;PIK3CA;TRAF6;AKT3;IL12B;MAPK1;IRF5;MAP3K7;MAP2K6
 
 
 
 
VEGF signaling pathway
 
 
12/59
 
 
0.0411334
 
 
0.1193084
 
 
0
 
 
0
 
 
1.908437
 
 
6.0896977
 
 
CDC42;MAP2K1;NRAS;PIK3CA;AKT3;PIK3R3;MAPK1;PIK3R1;RAF1;MAPK14;MAPK12;VEGFA
 
 
 
 
Cushing syndrome
 
 
26/155
 
 
0.0414814
 
 
0.1193084
 
 
0
 
 
0
 
 
1.508483
 
 
4.8007612
 
 
RB1;SCARB1;LEF1;ADCY3;KCNA4;ADCY2;AHR;RAP1B;RAP1A;CREB3L1;MAPK1;E2F3;WNT1;LDLR;WNT10B;TCF7L2;MAP2K1;FZD5;FZD4;WNT9B;NCEH1;CREB1;CCNE2;APC;KCNK2;CREB5
 
 
 
 
Cholinergic synapse
 
 
20/113
 
 
0.0420047
 
 
0.1196736
 
 
0
 
 
0
 
 
1.608731
 
 
5.0996355
 
 
ACHE;MAP2K1;CACNA1A;PIK3R3;ADCY3;ADCY2;PIK3R1;FOS;GNG12;GNAO1;NRAS;CREB1;GNG2;PIK3CA;CREB3L1;CAMK4;AKT3;BCL2;MAPK1;CREB5
 
 
 
 
Glycosaminoglycan biosynthesis
 
 
11/53
 
 
0.0432710
 
 
0.1221294
 
 
0
 
 
0
 
 
1.957388
 
 
6.1467306
 
 
B4GALT2;HS3ST3B1;B4GALT3;FUT8;CHST7;B3GNT7;CHSY1;CHST12;CSGALNACT2;CHST1;HS2ST1
 
 
 
 
Carbohydrate digestion and absorption
 
 
10/47
 
 
0.0452475
 
 
0.1254421
 
 
0
 
 
0
 
 
2.019625
 
 
6.2519672
 
 
G6PC2;G6PC3;PIK3CA;FXYD2;AKT3;PIK3R3;ATP1B4;ATP1A2;PIK3R1;HK2
 
 
 
 
Th17 cell differentiation
 
 
19/107
 
 
0.0452755
 
 
0.1254421
 
 
0
 
 
0
 
 
1.614901
 
 
4.9981017
 
 
SMAD4;CHUK;STAT3;RORA;AHR;IL1RAP;FOS;MAPK14;TGFBR1;MAPK12;RUNX1;IFNG;IRF4;TBX21;RARA;MAPK1;IL6ST;IL6R;HLA-DQB1
 
 
 
 
Renin-angiotensin system
 
 
6/23
 
 
0.0465884
 
 
0.1276233
 
 
0
 
 
0
 
 
2.635912
 
 
8.0827714
 
 
CTSA;NLN;ENPEP;ANPEP;ATP6AP2;PRCP
 
 
 
 
Bladder cancer
 
 
9/41
 
 
0.0469079
 
 
0.1276233
 
 
0
 
 
0
 
 
2.101377
 
 
6.4293071
 
 
RB1;NRAS;MAP2K1;MAPK1;E2F3;RAF1;TP53;VEGFA;HBEGF
 
 
 
 
Long-term potentiation
 
 
13/67
 
 
0.0482821
 
 
0.1291764
 
 
0
 
 
0
 
 
1.799517
 
 
5.4537843
 
 
MAP2K1;PPP1CB;RAP1B;PPP1CC;GRM5;NRAS;RAP1A;CAMK4;RPS6KA1;EP300;MAPK1;CALM3;RAF1
 
 
 
 
IL-17 signaling pathway
 
 
17/94
 
 
0.0483342
 
 
0.1291764
 
 
0
 
 
0
 
 
1.650955
 
 
5.0017576
 
 
CHUK;TNFAIP3;FOS;MAPK14;TNF;MAPK12;HSP90B1;FOSL1;IFNG;TRAF4;CCL7;TRAF6;TRAF5;CCL2;MAPK1;FOSB;MAP3K7
 
 
 
 
cAMP signaling pathway
 
 
34/216
 
 
0.0500218
 
 
0.1325139
 
 
0
 
 
0
 
 
1.398735
 
 
4.1896254
 
 
HHIP;PTGER3;PIK3R3;ADCY3;ADCY2;ATP1A2;PIK3R1;PPP1CB;RAP1B;PPP1CC;EDNRA;RAP1A;PLN;RRAS;CREB3L1;AKT3;PDE4B;EP300;MAPK1;MAP2K1;PPP1R12A;BDNF;ATP2B4;RRAS2;ATP1B4;FOS;TIAM1;CREB1;PIK3CA;FXYD2;CAMK4;CALM3;RAF1;CREB5
 
 
 
 
Gap junction
 
 
16/88
 
 
0.0520573
 
 
0.1367071
 
 
0
 
 
0
 
 
1.661511
 
 
4.9104448
 
 
PDGFRB;PDGFRA;MAP2K1;HTR2C;ADCY3;ADCY2;TJP1;GRM5;NRAS;TUBB2A;PDGFC;MAPK1;GRB2;RAF1;SOS1;SOS2
 
 
 
 
Amoebiasis
 
 
18/102
 
 
0.0529845
 
 
0.1379423
 
 
0
 
 
0
 
 
1.602441
 
 
4.7075806
 
 
RAB5B;TGFB2;LAMA2;LAMA4;PIK3R3;PIK3R1;LAMC1;TNF;COL1A1;COL3A1;COL1A2;COL4A2;IFNG;PIK3CA;COL4A1;COL4A4;IL12B;COL4A5
 
 
 
 
Viral carcinogenesis
 
 
32/203
 
 
0.0549468
 
 
0.1418285
 
 
0
 
 
0
 
 
1.400822
 
 
4.0643295
 
 
RB1;HDAC4;DDX3X;YWHAB;HDAC1;GTF2B;PIK3R3;PIK3R1;CDC42;NRAS;CCND2;YWHAQ;CREB3L1;EP300;PMAIP1;MAPK1;BAK1;EGR3;STAT3;GTF2H1;YWHAZ;CCNA2;HNRNPK;CREB1;CCNE2;PIK3CA;TRAF5;TBPL1;GRB2;IL6ST;TP53;CREB5
 
 
 
 
cGMP-PKG signaling pathway
 
 
27/167
 
 
0.0566287
 
 
0.1449310
 
 
0
 
 
0
 
 
1.443129
 
 
4.1435697
 
 
ADCY3;ADCY2;ATP1A2;PPP1CB;GNA13;PPP1CC;EDNRA;RGS2;PLN;CREB3L1;AKT3;MAPK1;MEF2A;VASP;MAP2K1;PPP1R12A;TRPC6;ATP2B4;ATP1B4;ADRA2A;CREB1;FXYD2;PPIF;CALM3;RAF1;SLC25A4;CREB5
 
 
 
 
Pathogenic Escherichia coli infection
 
 
31/197
 
 
0.0592321
 
 
0.1503201
 
 
0
 
 
0
 
 
1.397722
 
 
3.9503701
 
 
ITGB1;LPAR2;CLDN1;TNF;CDC42;CYTH3;GNA13;CASP7;IRAK1;RPS3;MAPK1;BAK1;WASF1;MAP3K7;CYTH1;CHUK;FOS;ARPC5;F2;MAPK14;MAPK12;TNFRSF1A;TJP1;TUBB2A;CLDN14;TRAF6;FAS;CYCS;MYH9;TMBIM6;ARF6
 
 
 
 
Leishmaniasis
 
 
14/77
 
 
0.0661250
 
 
0.1664146
 
 
0
 
 
0
 
 
1.660948
 
 
4.5114811
 
 
ITGB1;MARCKSL1;TGFB2;FOS;MAPK14;TNF;MAPK12;IFNG;IRAK1;TRAF6;IL12B;MAPK1;MAP3K7;HLA-DQB1
 
 
 
 
mRNA surveillance pathway
 
 
17/98
 
 
0.0671587
 
 
0.1676191
 
 
0
 
 
0
 
 
1.569068
 
 
4.2375791
 
 
CPSF4;CPSF7;DAZAP1;CPSF6;MSI1;PPP2R3A;MAGOHB;NXT2;PPP1CB;PPP2CA;PNN;PPP1CC;NXF1;PABPN1;WDR82;PAPOLG;BCL2L2-PABPN1
 
 
 
 
Vasopressin-regulated water reabsorption
 
 
9/44
 
 
0.0690619
 
 
0.1709565
 
 
0
 
 
0
 
 
1.920931
 
 
5.1341733
 
 
DYNC1I2;RAB5B;CREB1;CREB3L1;ADCY3;STX4;DYNLL2;VAMP2;CREB5
 
 
 
 
Adrenergic signaling in cardiomyocytes
 
 
24/150
 
 
0.0759681
 
 
0.1865234
 
 
0
 
 
0
 
 
1.424625
 
 
3.6718881
 
 
TPM1;CACNA2D3;ATP2B4;ATP1B4;ADCY3;ADCY2;ATP1A2;PPP2R3A;MAPK14;MAPK12;PPP1CB;PPP2CA;PPP1CC;CACNB3;CREB1;PLN;CREB3L1;FXYD2;AKT3;BCL2;MAPK1;CALM3;CACNG2;CREB5
 
 
 
 
Spinocerebellar ataxia
 
 
23/143
 
 
0.0773347
 
 
0.1883474
 
 
0
 
 
0
 
 
1.433408
 
 
3.6689695
 
 
PSMD12;NFYA;KCND3;GTF2B;CACNA1A;PIK3R3;RORA;PIK3R1;AFG3L2;ATXN2;PSMA4;KAT5;PIK3CA;PSMA1;RB1CC1;AKT3;PPIF;TBPL1;CYCS;NRBF2;ATXN10;SLC25A4;MAP3K5
 
 
 
 
Progesterone-mediated oocyte maturation
 
 
17/100
 
 
0.0782102
 
 
0.1889558
 
 
0
 
 
0
 
 
1.531085
 
 
3.9017494
 
 
MAP2K1;PIK3R3;ADCY3;ADCY2;PIK3R1;IGF1;MAPK14;MAPK12;CDC25B;CCNA2;CPEB1;CDC23;PIK3CA;AKT3;RPS6KA1;MAPK1;RAF1
 
 
 
 
Inositol phosphate metabolism
 
 
13/73
 
 
0.0852316
 
 
0.2042853
 
 
0
 
 
0
 
 
1.619012
 
 
3.9866280
 
 
IPPK;PI4K2B;MTMR1;MTMR2;INPP1;MTMR14;PTEN;PIK3C2A;PIK3CA;PI4KA;PIP5K1A;PIP4K2B;PIP5K1B
 
 
 
 
Type II diabetes mellitus
 
 
9/46
 
 
0.0869300
 
 
0.2067155
 
 
0
 
 
0
 
 
1.816891
 
 
4.4380316
 
 
SOCS2;PIK3CA;PRKCD;CACNA1A;PIK3R3;MAPK1;PIK3R1;TNF;HK2
 
 
 
 
B cell receptor signaling pathway
 
 
14/81
 
 
0.0926172
 
 
0.2185187
 
 
0
 
 
0
 
 
1.561432
 
 
3.7150836
 
 
CR2;MAP2K1;CHUK;PIK3R3;FOS;PIK3R1;NRAS;PIK3CA;AKT3;MAPK1;GRB2;RAF1;SOS1;SOS2
 
 
 
 
Tight junction
 
 
26/169
 
 
0.0965701
 
 
0.2260788
 
 
0
 
 
0
 
 
1.359711
 
 
3.1783053
 
 
ITGB1;PRKAA1;CLDN1;YBX3;AMOT;MYL12B;CDC42;PPP2CA;PARD6B;RAP1A;WHAMM;MAP3K5;RAB8B;VASP;PRKAB2;ARPC5;TJAP1;RUNX1;TJP1;TIAM1;MYL6;CLDN14;NEDD4;MYH9;RAPGEF2;PALS1
 
 
 
 
JAK-STAT signaling pathway
 
 
25/162
 
 
0.0988925
 
 
0.2297348
 
 
0
 
 
0
 
 
1.364558
 
 
3.1572082
 
 
PIK3R3;PIK3R1;SOCS2;CCND2;AKT3;EP300;IL12B;IL6R;IL13RA1;MCL1;PDGFRB;PDGFRA;TSLP;IL15;STAT3;LIF;OSM;IFNG;PIK3CA;BCL2;GRB2;IL6ST;RAF1;SOS1;SOS2
 
 
 
 
Thyroid hormone synthesis
 
 
13/75
 
 
0.1006677
 
 
0.2320737
 
 
0
 
 
0
 
 
1.566608
 
 
3.5968218
 
 
GPX8;ATP1B4;GPX7;ADCY3;ADCY2;ATP1A2;LRP2;HSP90B1;CREB1;CREB3L1;FXYD2;CANX;CREB5
 
 
 
 
Ferroptosis
 
 
8/41
 
 
0.1042787
 
 
0.2385770
 
 
0
 
 
0
 
 
1.810416
 
 
4.0927874
 
 
PRNP;TFRC;ACSL1;ACSL6;ACSL4;SLC7A11;TP53;FTL
 
 
 
 
Alcoholism
 
 
28/186
 
 
0.1070108
 
 
0.2400221
 
 
0
 
 
0
 
 
1.325284
 
 
2.9617798
 
 
HDAC4;HDAC1;MACROH2A1;PPP1CB;PPP1CC;NRAS;GNG2;CREB3L1;MAPK1;H3-3B;H2AZ1;NTRK2;MAP2K1;BDNF;GNG12;GNAO1;H3-5;CREB1;ADORA2B;PKIA;CAMK4;FOSB;CALM3;GRB2;RAF1;SOS1;SOS2;CREB5
 
 
 
 
RNA transport
 
 
28/186
 
 
0.1070108
 
 
0.2400221
 
 
0
 
 
0
 
 
1.325284
 
 
2.9617798
 
 
EIF4A2;FMR1;GEMIN2;PHAX;NXT2;FXR1;PNN;NXF1;POM121C;XPO5;EIF4EBP1;EIF4EBP2;EIF4EBP3;RPP14;RAE1;EIF2B5;UBE2I;NUP210;ELAC2;THOC1;MAGOHB;EIF2S1;NUP50;EIF3J;GEMIN6;EIF4E2;KPNB1;EIF4G2
 
 
 
 
Amino sugar and nucleotide sugar metabolism
 
 
9/48
 
 
0.1072947
 
 
0.2400221
 
 
0
 
 
0
 
 
1.723521
 
 
3.8472029
 
 
CYB5R4;GFPT2;GFPT1;PMM2;UAP1;NPL;GFUS;HK2;PGM1
 
 
 
 
Regulation of lipolysis in adipocytes
 
 
10/55
 
 
0.1089320
 
 
0.2414791
 
 
0
 
 
0
 
 
1.659825
 
 
3.6798845
 
 
PIK3CA;AKT3;PTGER3;ADCY3;PIK3R3;ADCY2;ABHD5;PIK3R1;PTGS1;PNPLA2
 
 
 
 
Amphetamine addiction
 
 
12/69
 
 
0.1095452
 
 
0.2414791
 
 
0
 
 
0
 
 
1.572729
 
 
3.4779621
 
 
PPP1CB;PPP1CC;CREB1;HDAC1;CREB3L1;CAMK4;FOSB;CALM3;FOS;SIRT1;STX1A;CREB5
 
 
 
 
Inflammatory mediator regulation of TRP channels
 
 
16/98
 
 
0.1127885
 
 
0.2468270
 
 
0
 
 
0
 
 
1.458057
 
 
3.1818314
 
 
PRKCD;PIK3R3;HTR2C;ADCY3;ADCY2;IL1RAP;PIK3R1;IGF1;MAPK14;MAPK12;PPP1CB;PPP1CC;PIK3CA;CALM3;ASIC2;MAP2K6
 
 
 
 
Hedgehog signaling pathway
 
 
10/56
 
 
0.1193297
 
 
0.2592631
 
 
0
 
 
0
 
 
1.623650
 
 
3.4516606
 
 
SHH;CCND2;CSNK1A1;HHIP;CUL3;BCL2;GAS1;ARRB1;LRP2;CDON
 
 
 
 
Th1 and Th2 cell differentiation
 
 
15/92
 
 
0.1227842
 
 
0.2648631
 
 
0
 
 
0
 
 
1.455485
 
 
3.0526273
 
 
NOTCH2;NOTCH1;JAG1;CHUK;FOS;MAPK14;DLL1;MAPK12;DLL4;MAF;IFNG;TBX21;IL12B;MAPK1;HLA-DQB1
 
 
 
 
Oocyte meiosis
 
 
20/129
 
 
0.1245289
 
 
0.2667215
 
 
0
 
 
0
 
 
1.371335
 
 
2.8567884
 
 
MAP2K1;YWHAB;ADCY3;ADCY2;IGF1;MAPK14;YWHAZ;MAPK12;PPP1CB;SGO1;PPP2CA;PPP1CC;CPEB1;CDC23;CCNE2;YWHAQ;RPS6KA1;MAPK1;CALM3;SKP1
 
 
 
 
Glucagon signaling pathway
 
 
17/107
 
 
0.1258866
 
 
0.2677306
 
 
0
 
 
0
 
 
1.411438
 
 
2.9250267
 
 
PRKAB2;PRKAA1;SLC2A1;PPP4R3A;ADCY2;PDHB;SIRT1;LDHA;G6PC2;G6PC3;CREB1;CREB3L1;AKT3;EP300;SIK1;CALM3;CREB5
 
 
 
 
Type I diabetes mellitus
 
 
8/43
 
 
0.1287823
 
 
0.2718106
 
 
0
 
 
0
 
 
1.706770
 
 
3.4982506
 
 
PTPRN2;IFNG;CD80;IL12B;FAS;TNF;HSPD1;HLA-DQB1
 
 
 
 
GnRH secretion
 
 
11/64
 
 
0.1296051
 
 
0.2718106
 
 
0
 
 
0
 
 
1.550167
 
 
3.1674006
 
 
HCN3;MAP2K1;NRAS;PIK3CA;AKT3;PIK3R3;MAPK1;ARRB1;PIK3R1;RAF1;HCN1
 
 
 
 
Nitrogen metabolism
 
 
4/17
 
 
0.1327521
 
 
0.2745968
 
 
0
 
 
0
 
 
2.296550
 
 
4.6373578
 
 
CA12;CPS1;CA2;CA7
 
 
 
 
Selenocompound metabolism
 
 
4/17
 
 
0.1327521
 
 
0.2745968
 
 
0
 
 
0
 
 
2.296550
 
 
4.6373578
 
 
TXNRD1;CTH;SCLY;SEPHS1
 
 
 
 
RNA degradation
 
 
13/79
 
 
0.1362575
 
 
0.2799303
 
 
0
 
 
0
 
 
1.471327
 
 
2.9326618
 
 
DDX6;BTG2;BTG1;CNOT6L;ENO1;TENT4B;HSPD1;PAN2;CNOT7;XRN1;EXOSC3;DCP1A;DCP2
 
 
 
 
Basal transcription factors
 
 
8/45
 
 
0.1559715
 
 
0.3182661
 
 
0
 
 
0
 
 
1.614329
 
 
2.9995557
 
 
CCNH;GTF2B;TAF11;TBPL1;GTF2H1;TAF5;TAF9B;TAF4
 
 
 
 
Alzheimer disease
 
 
50/369
 
 
0.1693436
 
 
0.3432333
 
 
0
 
 
0
 
 
1.172400
 
 
2.0819784
 
 
TNF;CASP7;GRM5;FRAT2;RB1CC1;AKT3;UQCRFS1;CAPN1;MAP3K5;MAP2K1;CSNK2A1;CHUK;WNT9B;ADAM10;SDHD;DKK2;TNFRSF1A;ADAM17;PSMA4;TUBB2A;PSMA1;PIK3CA;NDUFS4;PPIF;MAPT;RAF1;SLC25A4;PSMD12;LRP5;PIK3R3;ATP5MC3;LPL;PIK3R1;RTN4;APH1A;NRAS;MAPK1;NRBF2;WNT1;WNT10B;FZD5;FZD4;CSNK1A1;NDUFA3;EIF2S1;APC;FAS;CYCS;CALM3;CDK5R1
 
 
 
 
Mitophagy
 
 
11/68
 
 
0.1750903
 
 
0.3525152
 
 
0
 
 
0
 
 
1.441056
 
 
2.5109726
 
 
BCL2L13;NRAS;MRAS;CSNK2A1;RRAS;TFEB;MFN1;RRAS2;MITF;FOXO3;TP53
 
 
 
 
Pertussis
 
 
12/76
 
 
0.1826867
 
 
0.3653735
 
 
0
 
 
0
 
 
1.400154
 
 
2.3802373
 
 
ITGB1;CASP7;IRAK1;CFL2;TRAF6;IL12B;MAPK1;CALM3;FOS;MAPK14;TNF;MAPK12
 
 
 
 
Bacterial invasion of epithelial cells
 
 
12/77
 
 
0.1946872
 
 
0.3843225
 
 
0
 
 
0
 
 
1.378535
 
 
2.2557804
 
 
ITGB1;DNM3;CDC42;PIK3CA;ARHGEF26;CAV3;CAV2;PIK3R3;PIK3R1;ARPC5;WASF1;MET
 
 
 
 
Chemokine signaling pathway
 
 
27/192
 
 
0.1947064
 
 
0.3843225
 
 
0
 
 
0
 
 
1.222723
 
 
2.0006963
 
 
PIK3R3;ADCY3;ARRB1;ADCY2;PIK3R1;FOXO3;RAP1B;CDC42;NRAS;RAP1A;CCL8;GNG2;CCL7;AKT3;CCL2;MAPK1;MAP2K1;CHUK;PRKCD;STAT3;GNG12;TIAM1;PIK3CA;GRB2;RAF1;SOS1;SOS2
 
 
 
 
Epithelial cell signaling in Helicobacter pylori infection
 
 
11/70
 
 
0.2003422
 
 
0.3928789
 
 
0
 
 
0
 
 
1.392048
 
 
2.2380353
 
 
CDC42;TJP1;MAP2K4;ADAM17;CHUK;ADAM10;ATP6V1E1;MAPK14;MET;MAPK12;HBEGF
 
 
 
 
Oxytocin signaling pathway
 
 
22/154
 
 
0.2019436
 
 
0.3934643
 
 
0
 
 
0
 
 
1.245056
 
 
1.9917995
 
 
MAP2K1;PRKAB2;PRKAA1;CAMK1D;PPP1R12A;CACNA2D3;ADCY3;ADCY2;FOS;PPP1CB;GNAO1;PPP1CC;RCAN1;RGS2;CACNB3;MYL6;NRAS;CAMK4;MAPK1;CALM3;RAF1;CACNG2
 
 
 
 
Biosynthesis of unsaturated fatty acids
 
 
5/27
 
 
0.2084031
 
 
0.4008773
 
 
0
 
 
0
 
 
1.696167
 
 
2.6600668
 
 
ELOVL4;SCD;ELOVL2;ELOVL3;ELOVL6
 
 
 
 
Fatty acid elongation
 
 
5/27
 
 
0.2084031
 
 
0.4008773
 
 
0
 
 
0
 
 
1.696167
 
 
2.6600668
 
 
ELOVL4;ELOVL2;ELOVL3;PPT1;ELOVL6
 
 
 
 
Diabetic cardiomyopathy
 
 
28/203
 
 
0.2191130
 
 
0.4188110
 
 
0
 
 
0
 
 
1.195379
 
 
1.8147852
 
 
PTEN;SLC2A1;PIK3R3;ATP5MC3;PIK3R1;PDHB;PPP1CB;PPP1CC;PLN;AKT3;PDK4;UQCRFS1;TGFB2;GFPT2;NDUFA3;PRKCD;GFPT1;SDHD;MAPK14;TGFBR1;MAPK12;COL1A1;COL3A1;COL1A2;PIK3CA;NDUFS4;PPIF;SLC25A4
 
 
 
 
Tuberculosis
 
 
25/180
 
 
0.2240573
 
 
0.4255680
 
 
0
 
 
0
 
 
1.204853
 
 
1.8022825
 
 
RAB5B;CEBPG;TNF;CTSS;HSPD1;IRAK1;AKT3;MRC1;EP300;IL12B;MAPK1;TGFB2;NFYA;RFXANK;MAPK14;MAPK12;TNFRSF1A;CREB1;IFNG;TRAF6;BCL2;CYCS;CALM3;RAF1;HLA-DQB1
 
 
 
 
Cholesterol metabolism
 
 
8/50
 
 
0.2341062
 
 
0.4349672
 
 
0
 
 
0
 
 
1.421743
 
 
2.0643424
 
 
SCARB1;NCEH1;NPC2;VAPB;LPL;LRP2;LIPA;LDLR
 
 
 
 
Cysteine and methionine metabolism
 
 
8/50
 
 
0.2341062
 
 
0.4349672
 
 
0
 
 
0
 
 
1.421743
 
 
2.0643424
 
 
AHCYL2;DNMT1;LDHA;MAT2A;CTH;DNMT3A;DNMT3B;SMS
 
 
 
 
N-Glycan biosynthesis
 
 
8/50
 
 
0.2341062
 
 
0.4349672
 
 
0
 
 
0
 
 
1.421743
 
 
2.0643424
 
 
B4GALT2;B4GALT3;DPM2;FUT8;DOLPP1;MGAT4A;RPN1;MAN1B1
 
 
 
 
Cortisol synthesis and secretion
 
 
10/65
 
 
0.2347671
 
 
0.4349672
 
 
0
 
 
0
 
 
1.357267
 
 
1.9668990
 
 
SCARB1;CREB1;NCEH1;CREB3L1;ADCY3;KCNA4;ADCY2;LDLR;KCNK2;CREB5
 
 
 
 
Dilated cardiomyopathy
 
 
14/96
 
 
0.2412807
 
 
0.4443096
 
 
0
 
 
0
 
 
1.274714
 
 
1.8123817
 
 
ITGB1;TGFB2;LAMA2;TPM1;CACNA2D3;ADCY3;ADCY2;IGF1;TNF;CACNB3;PLN;SGCB;ITGA11;CACNG2
 
 
 
 
Thiamine metabolism
 
 
3/15
 
 
0.2579989
 
 
0.4699100
 
 
0
 
 
0
 
 
1.865263
 
 
2.5270575
 
 
TPK1;AK1;ALPL
 
 
 
 
Terpenoid backbone biosynthesis
 
 
4/22
 
 
0.2582949
 
 
0.4699100
 
 
0
 
 
0
 
 
1.658149
 
 
2.2445583
 
 
NUS1;HMGCS1;PDSS1;HMGCR
 
 
 
 
Thermogenesis
 
 
31/232
 
 
0.2603014
 
 
0.4703258
 
 
0
 
 
0
 
 
1.152025
 
 
1.5505275
 
 
KDM3A;PRKAA1;COX16;ADCY3;ATP5MC3;ADCY2;NRAS;CNR1;CREB3L1;RPS6KA1;PRDM16;UQCRFS1;MAP3K5;PRKAB2;ACSL1;NDUFA3;ACSL6;ACSL4;TSC1;SDHD;MAPK14;ARID1B;MAPK12;CREB1;NDUFS4;GRB2;PPARG;SOS1;SOS2;PNPLA2;CREB5
 
 
 
 
Hypertrophic cardiomyopathy
 
 
13/90
 
 
0.2631303
 
 
0.4703258
 
 
0
 
 
0
 
 
1.260348
 
 
1.6826975
 
 
ITGB1;PRKAB2;PRKAA1;TGFB2;LAMA2;TPM1;CACNA2D3;IGF1;TNF;CACNB3;SGCB;ITGA11;CACNG2
 
 
 
 
Pathways of neurodegeneration
 
 
61/475
 
 
0.2631956
 
 
0.4703258
 
 
0
 
 
0
 
 
1.101299
 
 
1.4700781
 
 
ZFYVE1;TNF;CASP7;GRM5;FRAT2;RB1CC1;UQCRFS1;CAPN1;MAP3K5;MAP2K1;GPR37;CSNK2A1;WNT9B;GPX8;GPX7;SDHD;TNFRSF1B;DKK2;TNFRSF1A;PSMA4;TUBB2A;PSMA1;NDUFS4;PPIF;MAPT;RAF1;SLC25A4;STX1A;PSMD12;LRP5;DERL1;ATP5MC3;UBE2J1;NRAS;ATXN2;MFN1;MAPK1;NRBF2;BAK1;WNT1;MAP2K6;WNT10B;PRNP;FZD5;FZD4;CSNK1A1;BDNF;NDUFA3;RAB39B;UBE2G1;MAPK14;EIF2S1;MAPK12;APC;VAPB;BCL2;MAP3K10;FAS;CYCS;CALM3;CDK5R1
 
 
 
 
Aldosterone synthesis and secretion
 
 
14/98
 
 
0.2660812
 
 
0.4726854
 
 
0
 
 
0
 
 
1.244222
 
 
1.6472928
 
 
SCARB1;CAMK1D;ATP2B4;ATP1B4;ADCY3;ADCY2;ATP1A2;NR4A2;CREB1;CREB3L1;CAMK4;CALM3;LDLR;CREB5
 
 
 
 
Leukocyte transendothelial migration
 
 
16/114
 
 
0.2704981
 
 
0.4777217
 
 
0
 
 
0
 
 
1.218895
 
 
1.5936930
 
 
VASP;ITGB1;PIK3R3;PIK3R1;THY1;MAPK14;CLDN1;MAPK12;MYL12B;RAP1B;CDC42;CDH5;RAP1A;PIK3CA;CLDN14;SIPA1
 
 
 
 
Long-term depression
 
 
9/60
 
 
0.2752216
 
 
0.4796616
 
 
0
 
 
0
 
 
1.317088
 
 
1.6992788
 
 
GNA13;PPP2CA;GNAO1;MAP2K1;NRAS;CACNA1A;MAPK1;IGF1;RAF1
 
 
 
 
Mineral absorption
 
 
9/60
 
 
0.2752216
 
 
0.4796616
 
 
0
 
 
0
 
 
1.317088
 
 
1.6992788
 
 
SLC31A1;FXYD2;ATP2B4;ATP1B4;ATP1A2;TRPM7;ATP7A;SLC26A6;FTL
 
 
 
 
Morphine addiction
 
 
13/91
 
 
0.2763613
 
 
0.4796616
 
 
0
 
 
0
 
 
1.244118
 
 
1.5999937
 
 
GABRB2;GABRP;GABRA3;ADCY3;CACNA1A;ARRB1;ADCY2;GNG12;GNAO1;GNG2;PDE4B;PDE7B;PDE7A
 
 
 
 
Proximal tubule bicarbonate reclamation
 
 
4/23
 
 
0.2858670
 
 
0.4933247
 
 
0
 
 
0
 
 
1.570788
 
 
1.9669864
 
 
CA2;FXYD2;ATP1B4;ATP1A2
 
 
 
 
Allograft rejection
 
 
6/38
 
 
0.2899882
 
 
0.4975934
 
 
0
 
 
0
 
 
1.399136
 
 
1.7320119
 
 
IFNG;CD80;IL12B;FAS;TNF;HLA-DQB1
 
 
 
 
Glycerolipid metabolism
 
 
9/61
 
 
0.2918807
 
 
0.4980111
 
 
0
 
 
0
 
 
1.291686
 
 
1.5905950
 
 
DGKD;DGAT1;LPL;MBOAT2;DGKZ;AGPAT3;DGKH;AGPAT4;PNPLA2
 
 
 
 
Proteasome
 
 
7/46
 
 
0.2984460
 
 
0.5063522
 
 
0
 
 
0
 
 
1.339379
 
 
1.6195323
 
 
PSMD12;PSMA4;IFNG;PSMA1;POMP;PSMB8;PSMB9
 
 
 
 
Galactose metabolism
 
 
5/31
 
 
0.3016480
 
 
0.5089257
 
 
0
 
 
0
 
 
1.434892
 
 
1.7197106
 
 
B4GALT2;G6PC2;G6PC3;HK2;PGM1
 
 
 
 
Various types of N-glycan biosynthesis
 
 
6/39
 
 
0.3115752
 
 
0.5186834
 
 
0
 
 
0
 
 
1.356661
 
 
1.5820221
 
 
B4GALT2;B4GALT3;FUT8;MGAT4A;RPN1;MAN1B1
 
 
 
 
RIG-I-like receptor signaling pathway
 
 
10/70
 
 
0.3119757
 
 
0.5186834
 
 
0
 
 
0
 
 
1.243807
 
 
1.4488244
 
 
SIKE1;MAVS;DDX3X;CHUK;TRAF6;IL12B;MAPK14;TNF;MAP3K7;MAPK12
 
 
 
 
Antigen processing and presentation
 
 
11/78
 
 
0.3143157
 
 
0.5186834
 
 
0
 
 
0
 
 
1.225275
 
 
1.4180818
 
 
PDIA3;CREB1;IFNG;NFYA;CANX;RFXANK;IFI30;TNF;CTSS;HSPA1B;HLA-DQB1
 
 
 
 
Synaptic vesicle cycle
 
 
11/78
 
 
0.3143157
 
 
0.5186834
 
 
0
 
 
0
 
 
1.225275
 
 
1.4180818
 
 
DNM3;SNAP25;UNC13C;SYT1;CACNA1A;STX3;SLC6A1;ATP6V1E1;CPLX2;STX1A;VAMP2
 
 
 
 
Insulin secretion
 
 
12/86
 
 
0.3160190
 
 
0.5186834
 
 
0
 
 
0
 
 
1.210255
 
 
1.3941566
 
 
SNAP25;CREB1;CREB3L1;FXYD2;SLC2A1;ATP1B4;ADCY3;ADCY2;ATP1A2;STX1A;VAMP2;CREB5
 
 
 
 
Nucleotide excision repair
 
 
7/47
 
 
0.3181896
 
 
0.5194231
 
 
0
 
 
0
 
 
1.305821
 
 
1.4953054
 
 
CCNH;RPA3;POLE3;GTF2H1;ERCC6;DDB2;CUL4B
 
 
 
 
Synthesis and degradation of ketone bodies
 
 
2/10
 
 
0.3349292
 
 
0.5438098
 
 
0
 
 
0
 
 
1.864896
 
 
2.0398909
 
 
BDH1;HMGCS1
 
 
 
 
Cocaine addiction
 
 
7/49
 
 
0.3582615
 
 
0.5732062
 
 
0
 
 
0
 
 
1.243497
 
 
1.2764401
 
 
GRM3;CREB1;BDNF;CREB3L1;FOSB;CREB5;CDK5R1
 
 
 
 
Legionellosis
 
 
8/57
 
 
0.3598545
 
 
0.5732062
 
 
0
 
 
0
 
 
1.218152
 
 
1.2450187
 
 
BCL2L13;CASP7;SAR1A;IL12B;CYCS;TNF;HSPA1B;HSPD1
 
 
 
 
Fatty acid biosynthesis
 
 
3/18
 
 
0.3599738
 
 
0.5732062
 
 
0
 
 
0
 
 
1.491956
 
 
1.5243671
 
 
ACSL1;ACSL6;ACSL4
 
 
 
 
Inflammatory bowel disease
 
 
9/65
 
 
0.3606264
 
 
0.5732062
 
 
0
 
 
0
 
 
1.199150
 
 
1.2230280
 
 
TGFB2;MAF;IFNG;TBX21;STAT3;IL12B;RORA;TNF;HLA-DQB1
 
 
 
 
Maturity onset diabetes of the young
 
 
4/26
 
 
0.3702412
 
 
0.5854075
 
 
0
 
 
0
 
 
1.356359
 
 
1.3476792
 
 
NEUROD1;NR5A2;ONECUT1;HNF4G
 
 
 
 
Bile secretion
 
 
12/90
 
 
0.3750851
 
 
0.5899775
 
 
0
 
 
0
 
 
1.147929
 
 
1.1256620
 
 
SCARB1;NCEH1;CA2;FXYD2;SLC2A1;ATP1B4;ADCY3;ADCY2;ATP1A2;HMGCR;LDLR;SLC22A7
 
 
 
 
Dopaminergic synapse
 
 
17/132
 
 
0.3924859
 
 
0.6141489
 
 
0
 
 
0
 
 
1.103029
 
 
1.0316135
 
 
CACNA1A;ARRB1;FOS;PPP2R3A;GNG12;MAPK14;MAPK12;PPP1CB;GNAO1;PPP2CA;PPP1CC;CREB1;GNG2;CREB3L1;AKT3;CALM3;CREB5
 
 
 
 
Vascular smooth muscle contraction
 
 
17/133
 
 
0.4049710
 
 
0.6304188
 
 
0
 
 
0
 
 
1.093458
 
 
0.9884202
 
 
MAP2K1;CALCRL;PPP1R12A;PRKCD;ADCY3;ADCY2;PPP1CB;ACTA2;GNA13;PPP1CC;EDNRA;MYL6;ADORA2B;MYH9;MAPK1;CALM3;RAF1
 
 
 
 
Rheumatoid arthritis
 
 
12/93
 
 
0.4201883
 
 
0.6507532
 
 
0
 
 
0
 
 
1.105224
 
 
0.9582872
 
 
TGFB2;IFNG;IL15;CD80;CCL2;FOS;TNFRSF11A;TEK;ATP6V1E1;TNF;HLA-DQB1;VEGFA
 
 
 
 
Starch and sucrose metabolism
 
 
5/36
 
 
0.4242554
 
 
0.6515562
 
 
0
 
 
0
 
 
1.203116
 
 
1.0315756
 
 
G6PC2;G6PC3;ENPP1;HK2;PGM1
 
 
 
 
Steroid biosynthesis
 
 
3/20
 
 
0.4270711
 
 
0.6515562
 
 
0
 
 
0
 
 
1.316282
 
 
1.1198992
 
 
CYP24A1;LIPA;LBR
 
 
 
 
Arrhythmogenic right ventricular cardiomyopathy
 
 
10/77
 
 
0.4271793
 
 
0.6515562
 
 
0
 
 
0
 
 
1.113414
 
 
0.9470158
 
 
ITGB1;TCF7L2;CACNB3;LAMA2;SGCB;ACTN2;LEF1;ITGA11;CACNA2D3;CACNG2
 
 
 
 
Glycosphingolipid biosynthesis
 
 
6/45
 
 
0.4436676
 
 
0.6733046
 
 
0
 
 
0
 
 
1.147553
 
 
0.9325928
 
 
B4GALT2;B4GALT3;FUT9;ST3GAL6;ST6GALNAC3;FUT4
 
 
 
 
African trypanosomiasis
 
 
5/37
 
 
0.4485564
 
 
0.6739504
 
 
0
 
 
0
 
 
1.165453
 
 
0.9343679
 
 
IFNG;LAMA4;IL12B;FAS;TNF
 
 
 
 
 
 
 Reactome_Pathways_2024 
 Reactome is an expert-curated, peer-reviewed pathway database that
describes biological processes as detailed molecular reaction networks.
It emphasizes mechanistic precision, causal relationships, and
hierarchical pathway structure. Enrichment in Reactome pathways
indicates that target genes participate in specific molecular events or
subprocesses such as signal transduction modules, DNA repair mechanisms,
immune cascades, or cell-cycle transitions. Due to its high curation
standards, Reactome enrichment results are considered highly reliable
and mechanistically interpretable. 
 
 
 
 
Term
 
 
Overlap
 
 
P.value
 
 
Adjusted.P.value
 
 
Old.P.value
 
 
Old.Adjusted.P.value
 
 
Odds.Ratio
 
 
Combined.Score
 
 
Genes
 
 
 
 
 
 
Signal Transduction
 
 
419/2613
 
 
0.0000000
 
 
0.0000000
 
 
0
 
 
0
 
 
1.515340
 
 
39.941456
 
 
ITSN2;TFRC;ITSN1;SERPINE1;STMN2;FNBP1L;AKT3;MYB;STMN1;PROK2;PIP4K2B;MEF2A;PRKAB2;CSNK2A1;PRKCD;FRS3;DKK2;MYL6;DAAM1;MTMR1;GHRHR;FLRT3;PDGFC;PIP5K1A;PIP5K1B;ST3GAL6;ICOS;WNT10B;STARD13;JAG1;FZD5;FZD4;FOSL1;COL1A1;COL1A2;AGO4;HNRNPF;AGO2;PNOC;SNAI1;FERMT2;GABRB2;ARHGAP1;OSBPL11;AREG;LRRC1;SMPD3;LFNG;SHH;PEA15;HEY2;RSPO3;RSPO4;LYPLA1;CNOT6L;GPR37;DUSP1;HSBP1;WNT9B;RRAS2;TACR3;FOS;F2;DUSP9;DYNLL2;LRRC41;NCOR2;COL2A1;TUBB2A;PSMA4;MRAS;MMP16;PSMA1;LRRC7;RARA;RAPGEF2;GAS1;RARB;PPARG;PAFAH1B1;ARF6;HBEGF;KANK1;COL11A1;PLPPR5;ADCY3;ADCY2;FSTL1;LMAN1;NRAS;NTF3;MFN1;CSNK1A1;USP9X;STAT3;SYVN1;COL3A1;SHKBP1;FOSB;FILIP1;LGR4;POGLUT1;CCNT2;DGKD;FAM13B;UBE2D2;UBE2D3;UBE2D1;IL1RAP;FGF2;TNF;CDC14A;PPP1CB;PPP1CC;CDH5;GRAP2;GRB10;MAF1;MAP3K7;MAP2K1;FBXW7;USP4;FST;CTNNBIP1;USP2;LEMD3;VRK3;DGKZ;ARMCX3;PKP4;ROR1;IL6ST;DGKH;NOTCH2;DNMT1;CALCRL;NOTCH1;CBFB;CUL5;CUL3;ABHD6;RASAL1;FOXO3;RHOBTB1;RAP1B;BAG4;RAP1A;BCL2L11;EIF4EBP1;SLC38A9;SRGAP2;RAB4A;BDNF;ARPC5;ADAP1;MOB1A;PTPRA;BCL2;MAP3K11;ITGB1;ZNF512B;PTEN;HTR2C;ECE1;ARRB1;DUSP16;CDC73;LBR;TRPC6;PPP1R12A;CHUK;ADRA2A;TNFRSF1A;TFDP2;PIK3CA;ADORA2B;TRAF6;NUMB;SOS1;MET;SOS2;HDAC4;PRKAA1;HDAC1;LRP5;PDHB;CDC42;DNAJB1;PARD6B;CCL7;CCL2;E2F3;WHAMM;RBBP7;E2F5;HNRNPA1;WASF1;NTRK2;PTPN18;APOC4;SEMA4F;APC;VAPB;TNRC6A;CCNK;SPARC;HHIP;CCNC;ZDHHC7;IKZF1;GOLGA3;EDNRA;FRAT2;KAT5;RPS6KA1;PDK4;ATP6V1E1;GOLGA7;SOX4;CDON;PTGDR;SKP1;PDHX;ACTN2;RUNX1;TIAM1;SFRP1;RRAGC;ULK3;TP53;EPHA2;BEX3;GARRE1;LPAR2;TGFA;PIK3R3;PIK3R1;MYL12B;APH1A;SNX3;NLN;RALGDS;NMB;EGR3;IGF1;GNG12;NET1;CCT6A;CYP26A1;STAG2;LPAR6;ID1;COL9A1;ID4;ID3;GRB2;NRP1;PTPRK;BMI1;PCSK5;MUC13;SPTAN1;TMED5;NCOA1;SREBF1;NOG;RHOC;CNKSR2;NR5A2;MAPRE1;SGK1;RHOQ;AMER1;DYNC1I2;PTGER3;TRAK2;PPP2CA;PGRMC2;EMC3;EPS15;PTGES3;VEGFC;VEGFA;TJP1;KITLG;RIT1;AXL;NEDD4;CAMK4;OTULIN;FAS;CDK5R1;FOXA1;PRDM4;CD80;PRDM1;AMOT;RGS2;EP300;RGS8;IL6R;PDGFRB;PDGFRA;RALBP1;TMOD3;ADAM10;ATP1B4;TSC1;RPGRIP1L;KCTD3;SGO1;ADAM17;MYCN;COL4A2;COL4A1;TBL1XR1;COL4A4;COL4A5;RAF1;PSMD12;ARHGEF26;RASGRP4;RTN4;NCKIPSD;ARHGAP21;ARHGAP20;CALCR;IRAK1;WNT1;MARK3;CPSF7;SMAD4;TGFB2;CBX4;CAV2;INHBB;ARHGAP26;ESR1;GNAO1;COL5A1;COL5A2;TCF4;FBN1;DOCK6;ABCD3;DOCK3;SOWAHC;YWHAB;USP34;TNFAIP3;LAMC1;RND2;CHD1;LTB4R;GRM3;GRM5;SPRED1;GRM7;YWHAQ;PDE4B;CASP2;FLOT2;TGIF2;ETV4;SSTR3;YWHAZ;CKAP4;TGFBR1;SENP1;ACTA2;DNM3;GREM2;CREB1;ALDH1A2;COL6A2;RASA1;COL6A3;MYH9;ITGB3BP;RGS17;KDM3A;LAMA2;RGS14;LAMA4;RAP1GDS1;LEF1;MOSPD2;ABHD17C;FAM169A;DLL1;UACA;PTHLH;GNA13;DLL4;ERLEC1;GNG2;TNKS2;ERBB3;CNR1;PDE6D;S1PR1;MAPK1;VHL;MTA2;TCF7L2;LIMK1;MAPK14;USF1;ACVR2A;MAPK12;SLC4A7;REST;FMNL2;SCD;SPRY2;SPRY1;TEK;PDE7B;PDE7A
 
 
 
 
Diseases of Signal Transduction by Growth Factor Receptors and Second
Messengers
 
 
102/451
 
 
0.0000000
 
 
0.0000000
 
 
0
 
 
0
 
 
2.232465
 
 
52.845521
 
 
RB1;CD80;CCNC;GCC2;FGF2;PPP1CB;PPP1CC;AKT3;EP300;SKP1;PDGFRB;PDGFRA;MAP2K1;FBXW7;ADAM10;FRS3;DKK2;EML4;ADAM17;BIN2;TBL1XR1;RAF1;TP53;DNMT1;PSMD12;NOTCH1;PIK3R3;TGFA;PIK3R1;FOXO3;RAP1B;APH1A;FXR1;BAG4;BCL2L11;RAP1A;ICOS;MARK3;MCL1;SMAD4;JAG1;CPSF6;FZD5;FZD4;BDNF;ERLIN2;ESR1;QKI;AGO4;AGO2;GRB2;MAP3K11;YWHAB;PTEN;ARRB1;AREG;DUSP16;MECP2;SHH;SPRED1;HEY2;CHUK;AGTRAP;DUSP9;TGFBR1;ETV6;NCOR2;CNKSR2;MRAS;PSMA4;CREB1;PSMA1;PIK3CA;IRF4;MYH9;SOS1;MET;KDM7A;HBEGF;HDAC4;AMER1;KANK1;HDAC1;LRP5;DLL1;PPP2CA;DLL4;ERLEC1;NRAS;ERBB3;TNKS2;NTF3;MAPK1;NTRK2;TCF7L2;CSNK1A1;STAT3;SYVN1;CLCN6;KITLG;APC;AGGF1
 
 
 
 
Collagen Chain Trimerization
 
 
23/44
 
 
0.0000000
 
 
0.0000000
 
 
0
 
 
0
 
 
8.237201
 
 
192.472101
 
 
COL15A1;COL16A1;COL25A1;COL11A1;COL22A1;COL12A1;COL19A1;COL1A1;COL3A1;COL1A2;COL2A1;COL4A2;COL5A1;COL4A1;COL4A4;COL6A2;COL7A1;COL5A2;COL9A1;COL21A1;COL4A5;COL6A3;COL8A1
 
 
 
 
Post-translational Protein Modification
 
 
251/1457
 
 
0.0000000
 
 
0.0000001
 
 
0
 
 
0
 
 
1.617456
 
 
36.240771
 
 
PGAP1;DCAF6;LGALS1;EVA1A;SKP1;DDX17;WSB1;NUP210;ANK2;ANK3;FDX1;PREB;TP53;NRN1;LMAN2L;RPN1;DCUN1D4;EIF5A2;TGFA;THY1;ADAMTS10;SNX3;POM121C;ARSJ;DPH2;ADAMTS17;NEU1;CHST10;ST8SIA4;ARSI;ST3GAL6;ARSB;B3GALNT2;CTSA;NUS1;SIAH2;PMM2;RAB39B;INO80;TADA2B;HNRNPK;STAG2;B4GAT1;CALU;RAET1L;ARF4;UBXN1;MIA2;MIA3;HIF3A;BMI1;AREG;RAB22A;PIGX;ING2;MUC13;TMED7;SPTAN1;RNF152;RAB8B;NCOA1;TOMM70;AHSG;COMMD2;F2;JOSD1;DYNLL2;LRRC41;COPZ1;DDB2;NCOR2;RNF168;PSMA4;NR5A2;TUBB2A;PSMA1;B3GNT7;PTRH2;CANX;RARA;WAC;PPARG;MFGE8;ARF5;MATN3;AMER1;DYNC1I2;NAPB;STX17;SATB1;SATB2;GCNT1;DERL1;WDR20;NPL;METTL21A;FSTL1;LMAN1;LMAN2;GCNT3;TAF9B;RAE1;MBD6;GOLM1;USP9X;MBD1;C1GALT1;SYVN1;KLHL2;UBE2G1;PSMB8;PSMB9;A4GNT;CAPZA1;OTULIN;SPSB4;PNPLA2;GALNT12;RAB3C;GALNT14;UBE2D2;RAB3D;UBE2D3;UBE2D1;RORA;RAB43;EP300;LARGE1;MAP3K7;FBXW4;PDIA3;FBXW7;USP4;UBE2E3;USP3;USP2;VASH2;ADAM10;MITF;FBXO10;TPST1;DPM2;RAB30;RAB34;INO80E;PSMD12;DNMT1;RAB5B;CUL5;UBA6;CUL3;UAP1;DNAJC24;HSP90B1;SOCS2;FUT8;UBE2F;TFAP2C;UBE2H;RAB4A;SMAD4;UBE2I;UBE2B;CBX4;GFPT2;GFPT1;ESR1;FBXO30;NR4A2;UBE2W;MAVS;CNIH1;UBE2N;ALPL;MDM4;CNTN4;UBE2K;SPRN;FBN1;POMT2;B4GALT2;B4GALT3;MCFD2;USP37;USP34;PTEN;TNFAIP3;ARRB1;LAMC1;CDC73;PPP6R1;DNMT3B;VCPKMT;GFUS;ADAMTS9;ADAMTS7;USP47;USP48;DNMT3A;SAFB;POMGNT1;CKAP4;TGFBR1;SENP1;CCNA2;RCN1;UBE2R2;ASPH;RTN4RL1;TRAPPC6B;GORASP1;NUP50;TRAF6;TOP1;MAN1B1;ST6GALNAC3;B4GALT6;SEC22A;OTUB1;B4GALT5;HDAC4;UHRF2;HDAC1;GOSR2;POMP;FOXK2;ASB11;RNF7;CAND1;TNKS2;TDG;RAB24;VHL;RBBP7;GALNT7;GALNT5;FEM1B;GALNT1;FBXL19;FBXL13;RAB10;APC;RAB14;COL7A1;DOLPP1;MGAT4A;RAB12;MDGA2;ASB1;BET1;CUL4B
 
 
 
 
Collagen Biosynthesis and Modifying Enzymes
 
 
27/67
 
 
0.0000000
 
 
0.0000010
 
 
0
 
 
0
 
 
5.079822
 
 
100.043216
 
 
COLGALT2;COL15A1;COL16A1;COL11A1;COL12A1;COL19A1;COL25A1;COL22A1;P3H1;COL1A1;COL3A1;COL2A1;COL1A2;COL4A2;COL5A1;COL4A1;P4HA2;COL4A4;COL6A2;COL7A1;COL5A2;COL9A1;COL21A1;COL4A5;COL6A3;COL8A1;TLL1
 
 
 
 
Collagen Formation
 
 
32/90
 
 
0.0000000
 
 
0.0000012
 
 
0
 
 
0
 
 
4.156732
 
 
80.315835
 
 
COLGALT2;COL15A1;COL16A1;COL11A1;COL12A1;CTSS;LOXL1;COL19A1;LOXL2;COL25A1;COL22A1;P3H1;COL1A1;COL3A1;COL2A1;COL1A2;COL4A2;LOX;COL5A1;COL4A1;P4HA2;COL4A4;COL6A2;COL7A1;PXDN;COL5A2;COL9A1;COL21A1;COL4A5;COL6A3;COL8A1;TLL1
 
 
 
 
Signaling by Receptor Tyrosine Kinases
 
 
108/536
 
 
0.0000000
 
 
0.0000038
 
 
0
 
 
0
 
 
1.923772
 
 
34.675926
 
 
SPARC;PRDM1;FGF2;CDH5;GRAP2;AKT3;STMN1;RPS6KA1;GRB10;EP300;ATP6V1E1;MEF2A;PDGFRB;PDGFRA;MAP2K1;PRKCD;ADAM10;VRK3;FRS3;TIAM1;ADAM17;MYCN;COL4A2;COL4A1;COL4A4;COL4A5;DNMT1;CUL5;PIK3R3;TGFA;PIK3R1;RAP1B;APH1A;RAP1A;FLRT3;PDGFC;RALGDS;RAB4A;EGR3;BDNF;IGF1;ADAP1;ESR1;COL1A1;FOSL1;COL1A2;COL5A1;HNRNPF;ID1;COL5A2;COL9A1;ID4;ID3;GRB2;ITGB1;GABRB2;NRP1;DOCK3;YWHAB;PTPRK;LAMC1;AREG;PCSK5;SPRED1;HSBP1;FOS;DNM3;COL2A1;CREB1;PIK3CA;COL6A2;RASA1;COL6A3;SOS1;SGK1;MET;ARF6;HBEGF;LAMA2;HDAC1;COL11A1;LAMA4;CDC42;PPP2CA;NRAS;ERBB3;NTF3;MAPK1;HNRNPA1;WASF1;EPS15;NTRK2;PTPN18;STAT3;VEGFC;MAPK14;MAPK12;VEGFA;COL3A1;KITLG;RIT1;REST;AXL;NEDD4;FOSB;SPRY2;SPRY1;CDK5R1
 
 
 
 
Assembly of Collagen Fibrils and Other Multimeric Structures
 
 
24/61
 
 
0.0000000
 
 
0.0000083
 
 
0
 
 
0
 
 
4.876087
 
 
83.475780
 
 
COL15A1;COL11A1;COL12A1;CTSS;LOXL1;LOXL2;COL1A1;COL3A1;COL2A1;COL1A2;COL4A2;LOX;COL5A1;COL4A1;COL4A4;COL6A2;COL7A1;PXDN;COL5A2;COL9A1;COL4A5;COL6A3;COL8A1;TLL1
 
 
 
 
Collagen Degradation
 
 
24/64
 
 
0.0000001
 
 
0.0000217
 
 
0
 
 
0
 
 
4.509611
 
 
72.313869
 
 
COL15A1;COL16A1;COL25A1;COL11A1;COL12A1;ADAM10;COL19A1;COL1A1;ADAM17;COL3A1;COL2A1;COL1A2;COL4A2;COL5A1;COL4A1;MMP15;COL4A4;COL6A2;COL7A1;COL5A2;COL9A1;COL4A5;COL6A3;COL8A1
 
 
 
 
Signaling by NTRKs
 
 
38/134
 
 
0.0000002
 
 
0.0000302
 
 
0
 
 
0
 
 
2.983464
 
 
46.548110
 
 
DOCK3;YWHAB;PIK3R1;PCSK5;PPP2CA;NRAS;RAP1A;RPS6KA1;NTF3;EP300;MAPK1;RALGDS;MEF2A;NTRK2;MAP2K1;EGR3;BDNF;STAT3;FOS;VRK3;MAPK14;FRS3;MAPK12;FOSL1;DNM3;TIAM1;REST;RIT1;CREB1;PIK3CA;ID1;ID4;ID3;FOSB;GRB2;SGK1;SOS1;CDK5R1
 
 
 
 
Membrane Trafficking
 
 
118/633
 
 
0.0000003
 
 
0.0000456
 
 
0
 
 
0
 
 
1.745723
 
 
26.349194
 
 
ITSN2;SCARB2;SYS1;TFRC;ITSN1;GCC2;FNBP1L;RAB43;AKT3;GJA9;AP1S3;VPS36;SBF2;AGFG1;TPD52;PRKAB2;ANK2;TSC1;ANK3;GAPVD1;RAB30;BIN1;CHMP4B;PREB;DENND6A;FTL;RAB5B;LMAN2L;TGFA;GNS;BLOC1S6;SNX2;BLOC1S1;SNX5;RAB4A;SYT1;FZD4;VTA1;RAB39B;TRAPPC8;ARPC5;SNAP91;CNIH1;GRB2;ACBD3;ARF4;MCFD2;YWHAB;MIA2;ARRB1;MIA3;PIK3C2A;AREG;YWHAQ;PPP6R1;TMED7;SNAP29;SPTAN1;SH3D19;RAB8B;VPS37C;VPS37B;DYNLL2;YWHAZ;COPZ1;DNM3;TBC1D1;TUBB2A;VAMP7;TRAPPC6B;GORASP1;TBC1D7;MYH9;SEC22A;CHMP7;ARF5;PAFAH1B2;VAMP2;RHOQ;ARF6;HBEGF;PAFAH1B1;DYNC1I2;NAPB;STX17;STX16;VPS4B;GOSR2;LRP2;AGPAT3;GGA2;CYTH3;LMAN1;LMAN2;STX6;UBQLN1;CLVS2;STX4;LDLR;EPS15;CYTH1;CLVS1;REPS1;RIC1;GALNT1;DTNBP1;RAB10;RGP1;TJP1;SNX18;NECAP2;NECAP1;RAB14;KIF26A;RAB12;CAPZA1;COL7A1;BET1
 
 
 
 
Crosslinking of Collagen Fibrils
 
 
11/18
 
 
0.0000009
 
 
0.0001332
 
 
0
 
 
0
 
 
11.767690
 
 
163.979297
 
 
COL1A1;COL1A2;COL4A2;LOX;COL4A1;COL4A4;PXDN;COL4A5;TLL1;LOXL1;LOXL2
 
 
 
 
Signaling by PDGF
 
 
21/58
 
 
0.0000013
 
 
0.0001823
 
 
0
 
 
0
 
 
4.261115
 
 
57.699137
 
 
PDGFRB;PDGFRA;STAT3;PIK3R1;NRAS;COL3A1;COL2A1;COL4A2;PIK3CA;COL5A1;COL4A1;COL4A4;COL6A2;RASA1;PDGFC;COL5A2;COL9A1;COL4A5;COL6A3;GRB2;SOS1
 
 
 
 
Developmental Biology
 
 
220/1385
 
 
0.0000019
 
 
0.0002308
 
 
0
 
 
0
 
 
1.449984
 
 
19.121250
 
 
EIF4A2;ITSN1;CCNC;ALCAM;DPYSL4;KAT5;DPYSL5;AKT3;DPYSL3;MYB;RPS6KA1;HOXA3;HOXA1;PKNOX1;ATP6V1E1;HOXA4;CDON;MEF2A;CSNK2A1;MED8;MED9;EBF2;ANK2;DICER1;ANK3;ISL1;RUNX1;SHTN1;TIAM1;MYL6;HOXB4;HOXB3;TP53;STX1A;EPHA2;HOXD1;GATA6;PIK3R3;PIK3R1;MED12L;MYL12B;APH1A;RRAS;FLRT3;PRDM16;ST8SIA4;VASP;WNT10B;CDK19;ST14;JAG1;FOXF1;SIAH2;SS18L1;NR2F2;MAGOHB;CPEB1;AGO4;AGO2;ID1;COL9A1;ID4;SNAI1;GRB2;SCN2B;NRP1;HNF4G;CTCF;HIF3A;ROBO1;LFNG;MED14;SHH;HEY2;SPTAN1;NCOA1;SS18;FOXD3;SREBF1;CNOT6L;ELOVL3;NOG;WNT9B;TET2;TFEB;RHOC;SIRT1;MED28;RGMA;NCOR2;PSMA4;NR5A2;COL2A1;TUBB2A;PSMA1;TET3;TFEC;RARA;RARB;PPARG;ASAH1;AKAP5;NRAS;RPS15A;RPL14;RPS3;PAIP2;CDX2;STAT3;COL3A1;KITLG;NR6A1;WT1;CDK5R1;FOXA1;MSI1;FGF2;TNF;HOXA11;SALL1;GRB10;EP300;CAPN1;IL6R;EOMES;MAP2K1;SEMA6A;ADAM10;MITF;NPNT;FOXP1;CACNB3;COL4A2;COL4A1;TBL1XR1;COL4A4;COL4A5;PKP4;PSMD12;SEMA7A;NOTCH1;CBFB;LPL;FOXO3;EFNA5;ZFP36L2;NEUROD1;EFNB2;OTX2;WNT1;SRGAP2;MAP2K6;PRNP;TFAP2C;SMAD4;ARPC5;CNOT7;MAFB;LIN28A;COL5A1;PTPRA;COL5A2;BCL2;TCF4;LHX9;ITGB1;CHD9;ONECUT1;DPY30;SIX1;LAMC1;ZIC1;DIS3L2;KDM6B;EMX2;MYOCD;TRPC6;ATP6AP2;ARID1B;GREM1;DNM3;GAP43;CREB1;PIK3CA;TFDP2;IRF4;COL6A2;RASA1;NUMB;DCX;COL6A3;MYH9;SOS1;MET;SOS2;LAMA2;HDAC1;LEF1;HMGCR;DLL1;CDC42;PABPN1;MAPK1;RBBP7;MTA2;CACNG2;LGI1;TCF7L2;SEMA4D;LIMK1;TBX5;MAPK14;KLF4;ACVR2A;MAPK12;USF1;MEIS1;KLF5;PMP22;TNRC6A
 
 
 
 
Gene Expression (Transcription)
 
 
251/1615
 
 
0.0000019
 
 
0.0002308
 
 
0
 
 
0
 
 
1.416344
 
 
18.642139
 
 
TDRKH;RB1;CCNK;CCNH;SERPINE1;PHAX;CCNC;CDC23;KAT5;AKT3;MYB;PDK4;EPC1;PIP4K2B;SKP1;ZNF287;PRKAB2;NUP210;CSNK2A1;ZNF282;ACSL1;MED8;DICER1;CDC40;RUNX1;ZNF839;RRAGC;WDR82;DDIT4;ZSCAN25;TP53;SLBP;ZNF274;ANAPC16;TGFA;ZNF23;POM121C;NRBF2;ZNF268;ZNF267;ZNF266;NR2C2AP;JAG1;SETDB1;GADD45A;PLK2;SS18L1;EAF1;MAGOHB;NR2F6;COL1A1;COL1A2;AGO4;AGO2;INTS7;ZNF136;BTG2;BTG1;SETD9;SUV39H1;HNF4G;BMI1;MECP2;ING5;MED14;ING2;XPO5;HEY2;RSPO3;JARID2;NCOA1;SREBF1;SS18;CNOT6L;TET2;TET1;FOS;BAZ1B;SIRT1;DDB2;NCOR2;PSMA4;NR5A2;PSMA1;MTF2;TET3;RARA;RARB;PPARG;SGK1;ZNF471;NFIX;ZNF470;SATB2;PPP2CA;ZFP14;TAF9B;ZNF225;RAE1;SRSF11;PCGF6;NFYA;PCGF5;USP9X;GTF2H1;VEGFA;ZNF33B;TJP1;NR6A1;RYBP;NFIA;PRKRA;RPA3;CAMK4;CCNG1;FAS;ZNF699;PNPLA2;CDK5R1;CCNT2;ZNF691;ZNF570;GTF2B;UBE2D3;UBE2D1;RORA;PRDM1;RORB;IPO8;CCND2;ZFP30;SESN1;SESN2;EP300;PITX2;ARID2;PHLDA1;LMO1;ZNF562;USP2;THOC1;ATP1B4;TSC1;MAF;CCNE2;TBL1XR1;SRSF7;SRSF9;NOTCH2;ZNF792;CSF1R;PSMD12;DNMT1;NOTCH1;CBFB;MAX;LPL;FOXO3;BCL2L11;IRAK1;ZKSCAN3;PMAIP1;ZNF304;SLC38A9;ZKSCAN4;RPRD1A;MORC2;ZNF420;MAP2K6;ESRRA;CPSF4;TFAP2C;CPSF7;SMAD4;UBE2I;CPSF6;CBX4;BDNF;BRPF3;ESRRG;ESR1;NR4A2;CNOT7;JMY;NABP1;CYCS;MDM4;DYRK2;YWHAB;PTEN;DPY30;SLC2A3;TRIAP1;CDC73;YWHAQ;DNMT3B;CASP2;MYBL2;RBM7;LBR;ZNF385A;TRIM63;TGIF2;ELAC2;DNMT3A;YWHAZ;ARID1B;TSN;CCNA2;ELF2;CREB1;TFDP2;IFNG;NUP50;POLR1D;MET;HDAC4;PRKAA1;RNF34;HDAC1;LEF1;MLLT3;PPM1D;DLL1;HSPD1;PABPN1;TDG;SAP30L;TP53INP1;DROSHA;MAPK1;ZNF624;RBBP7;E2F5;MTA2;E2F7;ZNF740;TCF7L2;KCNIP3;TXNRD1;TAF11;TBX5;MAPK14;KLF4;REST;SCD;ERCC6;TAF5;PTPN4;TAF4;TNRC6A
 
 
 
 
Signaling by NTRK1 (TRKA)
 
 
32/115
 
 
0.0000024
 
 
0.0002718
 
 
0
 
 
0
 
 
2.900573
 
 
37.515849
 
 
YWHAB;PIK3R1;PPP2CA;NRAS;RAP1A;RPS6KA1;EP300;MAPK1;RALGDS;MEF2A;NTRK2;MAP2K1;EGR3;STAT3;FOS;VRK3;MAPK14;MAPK12;FOSL1;DNM3;REST;RIT1;CREB1;PIK3CA;ID1;ID4;ID3;FOSB;GRB2;SGK1;SOS1;CDK5R1
 
 
 
 
Metabolism of Proteins
 
 
308/2067
 
 
0.0000052
 
 
0.0005470
 
 
0
 
 
0
 
 
1.351424
 
 
16.452100
 
 
EIF4A2;PGAP1;DCAF6;PCMT1;LGALS1;EVA1A;ANPEP;SKP1;DDX17;WSB1;NUP210;CSNK2A1;ANK2;ANK3;ISL1;AFG3L2;CLPP;FDX1;PREB;TP53;STX1A;ENPEP;NRN1;LMAN2L;DCUN1D4;RPN1;EIF5A2;GATA6;TGFA;MRPL17;THY1;ADAMTS10;APH1A;SNX3;POM121C;ARSJ;DPH2;ADAMTS17;NEU1;CHST10;ST8SIA4;ARSI;SSR1;ST3GAL6;ARSB;B3GALNT2;CTSA;NUS1;SIAH2;PMM2;RAB39B;IGF1;INO80;GNG12;CS;TADA2B;CCT6A;HNRNPK;STAG2;B4GAT1;CALU;RAET1L;ACHE;SFTA3;ARF4;UBXN1;MRPS10;MIA2;MIA3;HIF3A;BMI1;AREG;RAB22A;PIGX;ING2;MUC13;TMED7;SPTAN1;RNF152;RAB8B;NCOA1;TOMM70;MRPS25;AHSG;COMMD2;JOSD1;F2;DYNLL2;COPZ1;LRRC41;DDB2;NCOR2;MRPL52;RNF168;TUBB2A;PSMA4;NR5A2;BDH1;PSMA1;B3GNT7;PTRH2;CANX;RARA;WAC;PPARG;MFGE8;MATN3;ARF5;VAMP2;AMER1;DYNC1I2;NAPB;STX17;SATB1;TIMM9;SATB2;GCNT1;DERL1;WDR20;NPL;METTL21A;FSTL1;GGA2;LMAN1;RPS15A;LMAN2;RPL14;RPS3;GCNT3;TAF9B;RAE1;MBD6;CDX2;GOLM1;USP9X;MBD1;STAT3;C1GALT1;SYVN1;KLHL2;UBE2G1;PSMB8;PSMB9;CAPZA1;A4GNT;OTULIN;MSRB3;PNPLA2;SPSB4;GALNT12;SRP19;RAB3C;GALNT14;RAB3D;UBE2D2;UBE2D3;UBE2D1;RORA;YARS2;RAB43;EP300;LARGE1;MAP3K7;FBXW4;PDIA3;FBXW7;USP4;USP3;UBE2E3;SLC30A5;USP2;VASH2;ADAM10;MITF;FBXO10;TPST1;DPM2;RAB30;CCNE2;RAB34;INO80E;RAB5B;PSMD12;DNMT1;CUL5;UBA6;CUL3;UAP1;DNAJC24;HSP90B1;SOCS2;FUT8;EIF4EBP1;UBE2F;EIF2B5;TFAP2C;UBE2H;RAB4A;SMAD4;UBE2I;UBE2B;CBX4;GFPT2;GFPT1;INHBB;EIF2S1;ESR1;FBXO30;NR4A2;UBE2W;MAVS;CNIH1;UBE2N;SRPRA;ALPL;MDM4;CNTN4;UBE2K;SPRN;FBN1;POMT2;B4GALT2;B4GALT3;MCFD2;USP37;USP34;PTEN;TNFAIP3;ARRB1;LAMC1;TRIAP1;CDC73;PPP6R1;DNMT3B;VCPKMT;GFUS;ADAMTS9;ADAMTS7;USP47;USP48;ATP6AP2;DNMT3A;SAFB;POMGNT1;ADRA2A;CKAP4;TGFBR1;SENP1;CCNA2;RCN1;UBE2R2;ASPH;ADORA2B;TRAPPC6B;RTN4RL1;GORASP1;NUP50;TRAF6;ADGRF5;TOP1;MAN1B1;ST6GALNAC3;B4GALT6;SEC22A;PFDN6;OTUB1;B4GALT5;HDAC4;UHRF2;HDAC1;GOSR2;POMP;FOXK2;ASB11;RNF7;PDHB;HSPD1;CAND1;GNG2;TNKS2;TDG;RAB24;VHL;RBBP7;H2AZ1;GALNT7;TCF7L2;GALNT5;FEM1B;FBXL19;GALNT1;FBXL13;KLF4;WARS1;WARS2;RAB10;TSPAN14;RAB14;APC;COL7A1;DOLPP1;RAB12;MGAT4A;EIF3J;MDGA2;ASB1;BET1;CUL4B
 
 
 
 
NCAM Signaling for Neurite Out-Growth
 
 
21/63
 
 
0.0000060
 
 
0.0006049
 
 
0
 
 
0
 
 
3.752773
 
 
45.094080
 
 
PRNP;CACNB3;NRAS;COL3A1;COL2A1;CREB1;COL4A2;COL5A1;COL4A1;PTPRA;COL4A4;COL6A2;COL5A2;ST8SIA4;COL9A1;COL4A5;COL6A3;MAPK1;GRB2;SOS1;SPTAN1
 
 
 
 
MAPK Family Signaling Cascades
 
 
64/314
 
 
0.0000085
 
 
0.0007340
 
 
0
 
 
0
 
 
1.934185
 
 
22.591277
 
 
YWHAB;ARRB1;AREG;FGF2;DUSP16;CDC14A;PPP1CB;PPP1CC;SPRED1;PEA15;GOLGA7;SPTAN1;IL6R;PDGFRB;PDGFRA;LYPLA1;MAP2K1;ACTN2;DUSP1;HSBP1;FRS3;DUSP9;ETV4;CNKSR2;MRAS;PSMA4;PSMA1;PIK3CA;LRRC7;RASA1;RAPGEF2;IL6ST;RAF1;SOS1;MET;HBEGF;PSMD12;CUL3;TGFA;RASAL1;PIK3R1;ABHD17C;FOXO3;RASGRP4;PPP2CA;RAP1B;CDC42;DNAJB1;NRAS;RAP1A;ERBB3;PDE6D;MAPK1;RALGDS;MARK3;MAPK12;KITLG;AGO4;PTPRA;AGO2;GRB2;TEK;MAP3K11;TNRC6A
 
 
 
 
Signaling by TGFB Family Members
 
 
39/161
 
 
0.0000085
 
 
0.0007340
 
 
0
 
 
0
 
 
2.406887
 
 
28.106597
 
 
ITGB1;CCNK;CCNT2;HDAC1;SERPINE1;UBE2D3;UBE2D1;CCNC;ARRB1;FGF2;FSTL1;PPP1CB;APH1A;PPP1CC;EP300;MAPK1;E2F5;SMAD4;TGFB2;TGIF2;USP9X;FST;NOG;ATP1B4;INHBB;TGFBR1;ACVR2A;NCOR2;GREM2;MYCN;COL1A2;MMP16;TFDP2;AGO4;AGO2;RARA;TCF4;TNRC6A;FBN1
 
 
 
 
Signaling by NTRK2 (TRKB)
 
 
12/25
 
 
0.0000086
 
 
0.0007340
 
 
0
 
 
0
 
 
6.913073
 
 
80.665508
 
 
NTRK2;TIAM1;NRAS;DOCK3;PIK3CA;BDNF;NTF3;GRB2;PIK3R1;SOS1;FRS3;CDK5R1
 
 
 
 
Sphingolipid Metabolism
 
 
29/107
 
 
0.0000123
 
 
0.0010074
 
 
0
 
 
0
 
 
2.794350
 
 
31.591332
 
 
ASAH1;PPM1L;SGMS1;SGMS2;GLB1L3;GLB1L2;SMPD3;UGCG;SGPL1;SPTLC2;ARSJ;ORMDL2;NEU1;ORMDL3;ARSI;SPTSSB;SPTSSA;ARSB;CTSA;CERS4;CERS5;CERS6;ORMDL1;SGPP1;ACER2;VAPB;ACER3;B4GALT6;B4GALT5
 
 
 
 
Oncogenic MAPK Signaling
 
 
24/82
 
 
0.0000169
 
 
0.0012323
 
 
0
 
 
0
 
 
3.106895
 
 
34.144645
 
 
MAP2K1;YWHAB;AGTRAP;ARRB1;DUSP16;DUSP9;QKI;PPP1CB;CNKSR2;FXR1;RAP1B;PPP1CC;CLCN6;SPRED1;NRAS;MRAS;BCL2L11;RAP1A;MAPK1;AGGF1;RAF1;MARK3;KDM7A;MAP3K11
 
 
 
 
Constitutive Signaling by NOTCH1 t(7;9)(NOTCH1 M1580 K2555)
Translocation Mutant
 
 
6/7
 
 
0.0000171
 
 
0.0012323
 
 
0
 
 
0
 
 
44.851208
 
 
492.290830
 
 
DLL4;ADAM17;JAG1;NOTCH1;ADAM10;DLL1
 
 
 
 
Signaling by NOTCH1 t(7;9)(NOTCH1 M1580 K2555) Translocation Mutant
 
 
6/7
 
 
0.0000171
 
 
0.0012323
 
 
0
 
 
0
 
 
44.851208
 
 
492.290830
 
 
DLL4;ADAM17;JAG1;NOTCH1;ADAM10;DLL1
 
 
 
 
Vesicle-mediated Transport
 
 
127/752
 
 
0.0000187
 
 
0.0012943
 
 
0
 
 
0
 
 
1.544429
 
 
16.815406
 
 
ITSN2;SCARB2;SCARB1;SPARC;SYS1;TFRC;ITSN1;GCC2;FNBP1L;RAB43;AKT3;GJA9;AP1S3;VPS36;SBF2;AGFG1;TPD52;PRKAB2;ANK2;TSC1;ANK3;GAPVD1;RAB30;COL4A2;BIN1;COL4A1;CHMP4B;PREB;DENND6A;FTL;RAB5B;LMAN2L;TGFA;GNS;HSP90B1;BLOC1S6;SNX2;BLOC1S1;SNX5;RAB4A;SYT1;FZD4;VTA1;RAB39B;TRAPPC8;ARPC5;SNAP91;COL1A1;COL1A2;CNIH1;GRB2;ACBD3;ARF4;MCFD2;YWHAB;STAB2;MIA2;ARRB1;MIA3;PIK3C2A;AREG;YWHAQ;PPP6R1;TMED7;SNAP29;SPTAN1;SH3D19;RAB8B;VPS37C;VPS37B;DYNLL2;YWHAZ;COPZ1;DNM3;TBC1D1;TUBB2A;VAMP7;TRAPPC6B;GORASP1;TBC1D7;MYH9;SEC22A;CHMP7;ARF5;PAFAH1B2;VAMP2;RHOQ;ARF6;HBEGF;PAFAH1B1;DYNC1I2;NAPB;STX17;STX16;VPS4B;GOSR2;LRP2;AGPAT3;GGA2;CYTH3;LMAN1;LMAN2;STX6;UBQLN1;CLVS2;STX4;LDLR;EPS15;CYTH1;CLVS1;REPS1;RIC1;GALNT1;DTNBP1;RAB10;RGP1;TJP1;COL3A1;SNX18;NECAP2;NECAP1;RAB14;KIF26A;RAB12;CAPZA1;COL7A1;BET1
 
 
 
 
RNA Polymerase II Transcription
 
 
210/1360
 
 
0.0000214
 
 
0.0014272
 
 
0
 
 
0
 
 
1.396893
 
 
15.019779
 
 
RB1;CCNK;CCNH;SERPINE1;CCNC;PHAX;CDC23;KAT5;AKT3;MYB;EPC1;PIP4K2B;SKP1;ZNF287;PRKAB2;CSNK2A1;ZNF282;MED8;CDC40;RUNX1;ZNF839;RRAGC;DDIT4;ZSCAN25;TP53;SLBP;ZNF274;ANAPC16;TGFA;ZNF23;NRBF2;ZNF268;ZNF267;ZNF266;NR2C2AP;JAG1;GADD45A;PLK2;EAF1;MAGOHB;NR2F6;COL1A1;COL1A2;AGO4;AGO2;INTS7;ZNF136;BTG2;BTG1;SETD9;HNF4G;BMI1;MECP2;ING5;MED14;ING2;HEY2;RSPO3;SREBF1;CNOT6L;FOS;SIRT1;DDB2;NCOR2;PSMA4;NR5A2;PSMA1;RARA;RARB;PPARG;SGK1;ZNF471;ZNF470;SATB2;PPP2CA;ZFP14;ZNF225;TAF9B;SRSF11;PCGF6;NFYA;USP9X;PCGF5;GTF2H1;VEGFA;ZNF33B;TJP1;RYBP;NR6A1;RPA3;CAMK4;CCNG1;FAS;ZNF699;CDK5R1;CCNT2;ZNF691;ZNF570;GTF2B;UBE2D3;UBE2D1;RORA;RORB;PRDM1;CCND2;ZFP30;SESN1;SESN2;EP300;PITX2;ARID2;ZNF562;LMO1;USP2;THOC1;ATP1B4;TSC1;MAF;CCNE2;TBL1XR1;SRSF7;SRSF9;NOTCH2;CSF1R;ZNF792;PSMD12;NOTCH1;CBFB;MAX;FOXO3;BCL2L11;IRAK1;ZKSCAN3;PMAIP1;SLC38A9;ZNF304;ZKSCAN4;RPRD1A;ZNF420;MAP2K6;ESRRA;CPSF4;TFAP2C;CPSF7;SMAD4;UBE2I;CPSF6;CBX4;BDNF;BRPF3;ESRRG;ESR1;NR4A2;CNOT7;JMY;CYCS;NABP1;MDM4;DYRK2;YWHAB;PTEN;DPY30;SLC2A3;TRIAP1;CDC73;YWHAQ;CASP2;MYBL2;LBR;ZNF385A;TRIM63;TGIF2;YWHAZ;ARID1B;CCNA2;CREB1;ELF2;TFDP2;IFNG;MET;HDAC4;PRKAA1;RNF34;HDAC1;LEF1;MLLT3;PPM1D;DLL1;HSPD1;PABPN1;TP53INP1;ZNF624;MAPK1;RBBP7;E2F5;MTA2;E2F7;ZNF740;TCF7L2;KCNIP3;TXNRD1;TAF11;TBX5;MAPK14;KLF4;REST;TAF5;PTPN4;TAF4;TNRC6A
 
 
 
 
Transcriptional Regulation by MECP2
 
 
20/63
 
 
0.0000227
 
 
0.0014589
 
 
0
 
 
0
 
 
3.489265
 
 
37.314182
 
 
NOTCH1;HDAC1;BDNF;PTEN;SLC2A3;DLL1;MECP2;NCOR2;CREB1;IRAK1;TBL1XR1;AGO4;CAMK4;AGO2;PPARG;SGK1;PTPN4;LBR;MET;TNRC6A
 
 
 
 
Signaling by MET
 
 
23/79
 
 
0.0000276
 
 
0.0017125
 
 
0
 
 
0
 
 
3.082812
 
 
32.365190
 
 
ITGB1;RAB4A;LAMA2;COL11A1;LAMA4;STAT3;LAMC1;PIK3R1;RAP1B;COL1A1;NRAS;COL3A1;COL2A1;RAP1A;COL1A2;PIK3CA;COL5A1;COL5A2;GRB2;SOS1;MET;EPS15;ARF6
 
 
 
 
Degradation of the Extracellular Matrix
 
 
34/140
 
 
0.0000295
 
 
0.0017702
 
 
0
 
 
0
 
 
2.412059
 
 
25.161483
 
 
COL15A1;COL16A1;ELN;COL11A1;COL12A1;LAMC1;NID1;CTSS;COL19A1;CAPN1;ADAMTS9;CAST;COL25A1;ADAM10;COL1A1;ADAM17;COL3A1;COL1A2;COL2A1;COL4A2;COL5A1;MMP16;COL4A1;MMP15;COL4A4;COL6A2;COL7A1;COL5A2;COL9A1;COL4A5;COL8A1;COL6A3;TLL1;FBN1
 
 
 
 
Signaling by PDGFR in Disease
 
 
10/20
 
 
0.0000315
 
 
0.0018273
 
 
0
 
 
0
 
 
7.484076
 
 
77.587489
 
 
KANK1;PDGFRA;NRAS;BIN2;PIK3CA;STAT3;GRB2;PIK3R1;SOS1;ETV6
 
 
 
 
Transcriptional Regulation of Pluripotent Stem Cells
 
 
13/32
 
 
0.0000335
 
 
0.0018874
 
 
0
 
 
0
 
 
5.124597
 
 
52.798251
 
 
EOMES;FOXD3;SMAD4;CDX2;STAT3;GATA6;HIF3A;KLF4;FGF2;FOXP1;SALL1;LIN28A;NR6A1
 
 
 
 
Generic Transcription Pathway
 
 
191/1230
 
 
0.0000367
 
 
0.0019546
 
 
0
 
 
0
 
 
1.403336
 
 
14.330658
 
 
RB1;CCNK;CCNT2;ZNF691;ZNF570;CCNH;UBE2D3;SERPINE1;UBE2D1;RORA;CCNC;RORB;PRDM1;CDC23;CCND2;ZFP30;KAT5;SESN1;AKT3;SESN2;MYB;EPC1;EP300;PIP4K2B;PITX2;ARID2;SKP1;ZNF287;ZNF562;LMO1;PRKAB2;CSNK2A1;ZNF282;USP2;MED8;ATP1B4;TSC1;RUNX1;MAF;ZNF839;CCNE2;RRAGC;TBL1XR1;DDIT4;ZSCAN25;TP53;NOTCH2;CSF1R;ZNF792;ZNF274;PSMD12;NOTCH1;ANAPC16;CBFB;MAX;TGFA;ZNF23;FOXO3;BCL2L11;IRAK1;ZKSCAN3;PMAIP1;SLC38A9;ZNF304;ZKSCAN4;NRBF2;ZNF268;ZNF267;ZNF266;ZNF420;NR2C2AP;MAP2K6;ESRRA;TFAP2C;SMAD4;JAG1;UBE2I;CBX4;BDNF;BRPF3;GADD45A;PLK2;ESRRG;ESR1;NR2F6;COL1A1;NR4A2;COL1A2;CNOT7;AGO4;AGO2;JMY;CYCS;MDM4;ZNF136;BTG2;DYRK2;BTG1;YWHAB;SETD9;PTEN;HNF4G;DPY30;SLC2A3;TRIAP1;BMI1;MECP2;ING5;MED14;ING2;YWHAQ;HEY2;RSPO3;CASP2;MYBL2;LBR;ZNF385A;SREBF1;CNOT6L;TRIM63;TGIF2;FOS;SIRT1;YWHAZ;ARID1B;DDB2;CCNA2;NCOR2;PSMA4;NR5A2;CREB1;ELF2;PSMA1;TFDP2;IFNG;RARA;RARB;PPARG;SGK1;MET;HDAC4;PRKAA1;ZNF471;RNF34;ZNF470;HDAC1;SATB2;LEF1;PPM1D;DLL1;HSPD1;PPP2CA;ZFP14;TP53INP1;ZNF624;MAPK1;RBBP7;ZNF225;E2F5;MTA2;TAF9B;E2F7;ZNF740;TCF7L2;PCGF6;NFYA;USP9X;PCGF5;KCNIP3;TXNRD1;TAF11;GTF2H1;TBX5;MAPK14;KLF4;VEGFA;ZNF33B;TJP1;REST;RYBP;NR6A1;RPA3;CAMK4;CCNG1;FAS;ZNF699;TAF5;PTPN4;TAF4;TNRC6A;CDK5R1
 
 
 
 
MET Promotes Cell Motility
 
 
15/41
 
 
0.0000369
 
 
0.0019546
 
 
0
 
 
0
 
 
4.322995
 
 
44.126151
 
 
ITGB1;LAMA2;COL11A1;LAMA4;LAMC1;RAP1B;COL1A1;COL3A1;RAP1A;COL1A2;COL2A1;COL5A1;COL5A2;GRB2;MET
 
 
 
 
Transcriptional Regulation by TP53
 
 
69/362
 
 
0.0000388
 
 
0.0019978
 
 
0
 
 
0
 
 
1.778725
 
 
18.064642
 
 
CCNK;BTG2;CCNT2;DYRK2;CCNH;SETD9;YWHAB;PTEN;PRDM1;TRIAP1;ING5;ING2;KAT5;YWHAQ;SESN1;AKT3;SESN2;EP300;CASP2;PIP4K2B;ZNF385A;PRKAB2;CNOT6L;CSNK2A1;USP2;TSC1;FOS;YWHAZ;DDB2;CCNA2;RRAGC;CCNE2;TFDP2;DDIT4;SGK1;TP53;PRKAA1;RNF34;HDAC1;PPP2CA;TP53INP1;PMAIP1;SLC38A9;RBBP7;TAF9B;MTA2;E2F7;MAP2K6;ZNF420;GADD45A;BRPF3;PLK2;TXNRD1;TAF11;GTF2H1;MAPK14;CNOT7;AGO4;RPA3;AGO2;CCNG1;JMY;FAS;CYCS;MDM4;TAF5;TAF4;TNRC6A;CDK5R1
 
 
 
 
MAPK1 MAPK3 Signaling
 
 
55/271
 
 
0.0000399
 
 
0.0019978
 
 
0
 
 
0
 
 
1.920084
 
 
19.447180
 
 
YWHAB;ARRB1;AREG;FGF2;DUSP16;PPP1CB;PPP1CC;SPRED1;PEA15;GOLGA7;SPTAN1;IL6R;PDGFRB;PDGFRA;LYPLA1;MAP2K1;ACTN2;DUSP1;FRS3;DUSP9;CNKSR2;MRAS;PSMA4;PSMA1;PIK3CA;LRRC7;RASA1;RAPGEF2;IL6ST;RAF1;SOS1;MET;HBEGF;PSMD12;CUL3;TGFA;RASAL1;PIK3R1;ABHD17C;RASGRP4;PPP2CA;RAP1B;NRAS;RAP1A;ERBB3;PDE6D;MAPK1;RALGDS;MARK3;MAPK12;KITLG;PTPRA;GRB2;TEK;MAP3K11
 
 
 
 
Transcriptional Activity of SMAD2 SMAD3 SMAD4 Heterotrimer
 
 
17/51
 
 
0.0000457
 
 
0.0022252
 
 
0
 
 
0
 
 
3.748083
 
 
37.454879
 
 
CCNK;SMAD4;CCNT2;TGIF2;HDAC1;USP9X;SERPINE1;UBE2D3;UBE2D1;ATP1B4;CCNC;NCOR2;COL1A2;TFDP2;EP300;MAPK1;E2F5
 
 
 
 
Synthesis of PIPs at the Plasma Membrane
 
 
17/53
 
 
0.0000793
 
 
0.0035923
 
 
0
 
 
0
 
 
3.539454
 
 
33.419319
 
 
PI4K2B;MTMR1;MTMR2;RAB4A;MTMR14;PTEN;MTMR9;PIK3R3;PIK3R1;PIK3C2A;PLEKHA8;PIK3CA;RAB14;PIP5K1A;PIP4K2B;PIP5K1B;SBF2
 
 
 
 
MET Activates PTK2 Signaling
 
 
12/30
 
 
0.0000807
 
 
0.0035923
 
 
0
 
 
0
 
 
4.991358
 
 
47.045429
 
 
ITGB1;COL1A1;COL3A1;COL1A2;COL2A1;LAMA2;COL5A1;COL11A1;LAMA4;COL5A2;LAMC1;MET
 
 
 
 
Synthesis of Active Ubiquitin Roles of E1 and E2 Enzymes
 
 
12/30
 
 
0.0000807
 
 
0.0035923
 
 
0
 
 
0
 
 
4.991358
 
 
47.045429
 
 
UBE2W;UBE2H;UBE2B;UBE2R2;UBA6;USP9X;UBE2D2;UBE2E3;OTULIN;UBE2D1;UBE2G1;UBE2K
 
 
 
 
RAF MAP Kinase Cascade
 
 
53/265
 
 
0.0000818
 
 
0.0035923
 
 
0
 
 
0
 
 
1.883975
 
 
17.731005
 
 
YWHAB;ARRB1;AREG;FGF2;DUSP16;PPP1CB;PPP1CC;SPRED1;PEA15;GOLGA7;SPTAN1;PDGFRB;PDGFRA;LYPLA1;MAP2K1;ACTN2;DUSP1;FRS3;DUSP9;CNKSR2;MRAS;PSMA4;PSMA1;PIK3CA;LRRC7;RASA1;RAPGEF2;RAF1;SOS1;MET;HBEGF;PSMD12;CUL3;TGFA;RASAL1;PIK3R1;ABHD17C;RASGRP4;PPP2CA;RAP1B;NRAS;RAP1A;ERBB3;PDE6D;MAPK1;RALGDS;MARK3;MAPK12;KITLG;PTPRA;GRB2;TEK;MAP3K11
 
 
 
 
Negative Regulation of the PI3K AKT Network
 
 
29/118
 
 
0.0000877
 
 
0.0037623
 
 
0
 
 
0
 
 
2.447447
 
 
22.861959
 
 
CD80;PTEN;PIK3R3;TGFA;IL1RAP;PIK3R1;FGF2;AREG;PPP2CA;IRAK1;ERBB3;AKT3;NTF3;PIP5K1A;MAPK1;PIP4K2B;PIP5K1B;ICOS;PDGFRB;PDGFRA;NTRK2;BDNF;ESR1;KITLG;PIK3CA;TRAF6;GRB2;MET;HBEGF
 
 
 
 
Non-integrin membrane-ECM Interactions
 
 
18/59
 
 
0.0001029
 
 
0.0043094
 
 
0
 
 
0
 
 
3.291093
 
 
30.218313
 
 
ITGB1;LAMA2;COL11A1;LAMA4;NRXN1;LAMC1;FGF2;COL1A1;COL3A1;COL2A1;COL1A2;COL4A2;COL5A1;COL4A1;COL4A4;COL5A2;COL4A5;SDC1
 
 
 
 
PIP3 Activates AKT Signaling
 
 
55/281
 
 
0.0001106
 
 
0.0045052
 
 
0
 
 
0
 
 
1.834071
 
 
16.707203
 
 
CD80;PTEN;IL1RAP;BMI1;AREG;FGF2;AKT3;PIP4K2B;MAF1;PDGFRB;PDGFRA;CNOT6L;CSNK2A1;CHUK;PSMA4;CREB1;RRAGC;PSMA1;PIK3CA;TRAF6;PPARG;SGK1;TP53;MET;HBEGF;PSMD12;HDAC1;PIK3R3;TGFA;PIK3R1;FOXO3;PPP2CA;IRAK1;ERBB3;TNKS2;NTF3;PIP5K1A;MAPK1;PIP5K1B;SLC38A9;RBBP7;ICOS;MTA2;NTRK2;CBX4;BDNF;ESR1;KITLG;REST;AGO4;NEDD4;AGO2;SNAI1;GRB2;TNRC6A
 
 
 
 
Anchoring Fibril Formation
 
 
8/15
 
 
0.0001126
 
 
0.0045052
 
 
0
 
 
0
 
 
8.547427
 
 
77.712850
 
 
COL1A1;COL1A2;COL4A2;COL4A1;COL4A4;COL7A1;COL4A5;TLL1
 
 
 
 
Intrinsic Pathway for Apoptosis
 
 
17/55
 
 
0.0001330
 
 
0.0052061
 
 
0
 
 
0
 
 
3.352786
 
 
29.924915
 
 
YWHAB;STAT3;DYNLL2;YWHAZ;UACA;CASP7;BCL2L11;YWHAQ;TFDP2;AKT3;BCL2;MAPK1;PMAIP1;CYCS;BMF;BAK1;TP53
 
 
 
 
Signaling by Interleukins
 
 
80/452
 
 
0.0001386
 
 
0.0052159
 
 
0
 
 
0
 
 
1.624715
 
 
14.434105
 
 
ITGB1;SNAP25;CD80;RORA;IL1RAP;FGF2;TNF;NKIRAS2;RPS6KA1;IL12B;MAP3K7;IL6R;IL13RA1;SKP1;MEF2A;MAP2K4;MAP2K1;CHUK;IL15;FOS;VRK3;TNFRSF1B;YWHAZ;TNFRSF1A;PSMA4;VAMP7;CREB1;PSMA1;PIK3CA;IFNG;IRF4;TRAF6;IL1RAPL1;CANX;FSCN1;UBE2V1;IL6ST;SOS1;SOS2;TP53;VAMP2;STX1A;CSF1R;PSMD12;PELI2;NLRC5;PIK3R3;PIK3R1;FOXO3;HSP90B1;RAP1B;SOCS2;CDC42;PPP2CA;CNN2;IRAK1;S1PR1;NDN;CCL2;MAPK1;STX4;STX3;MAP2K6;MCL1;PTPN18;IL31;TSLP;STAT3;LIF;OSM;MAPK14;VEGFA;COL1A2;HNRNPF;CAPZA1;BCL2;UBE2N;SDC1;GRB2;PTPN4
 
 
 
 
ECM Proteoglycans
 
 
21/76
 
 
0.0001390
 
 
0.0052159
 
 
0
 
 
0
 
 
2.863636
 
 
25.431768
 
 
ITGB1;TGFB2;SPARC;LAMA2;LAMA4;SERPINE1;LAMC1;COL1A1;COL3A1;COL2A1;COL1A2;COL4A2;COL5A1;COL4A1;COL4A4;COL6A2;COL5A2;COL9A1;COL4A5;COL6A3;MATN3
 
 
 
 
Signaling by PDGFRA Extracellular Domain Mutants
 
 
7/12
 
 
0.0001477
 
 
0.0053192
 
 
0
 
 
0
 
 
10.467345
 
 
92.327299
 
 
PDGFRA;NRAS;PIK3CA;STAT3;GRB2;PIK3R1;SOS1
 
 
 
 
Signaling by PDGFRA Transmembrane, Juxtamembrane and Kinase Domain
Mutants
 
 
7/12
 
 
0.0001477
 
 
0.0053192
 
 
0
 
 
0
 
 
10.467345
 
 
92.327299
 
 
PDGFRA;NRAS;PIK3CA;STAT3;GRB2;PIK3R1;SOS1
 
 
 
 
Adipogenesis
 
 
27/110
 
 
0.0001547
 
 
0.0054626
 
 
0
 
 
0
 
 
2.442124
 
 
21.427446
 
 
CHD9;HDAC1;LPL;CCNC;TNF;MED14;PRDM16;EP300;RBBP7;WNT1;MTA2;NCOA1;WNT10B;SREBF1;CDK19;SMAD4;ELOVL3;MED8;EBF2;MED9;NR2F2;KLF4;MED28;NCOR2;KLF5;TBL1XR1;PPARG
 
 
 
 
SUMOylation
 
 
38/176
 
 
0.0001588
 
 
0.0054870
 
 
0
 
 
0
 
 
2.070483
 
 
18.112887
 
 
HDAC4;DNMT1;UHRF2;HDAC1;SATB1;SATB2;RORA;BMI1;ING2;POM121C;TDG;DNMT3B;EP300;VHL;RAE1;DDX17;NCOA1;TFAP2C;UBE2I;NUP210;CBX4;MBD1;DNMT3A;MITF;SAFB;ESR1;SENP1;NR4A2;NCOR2;RNF168;HNRNPK;STAG2;NR5A2;NUP50;RARA;PPARG;TOP1;TP53
 
 
 
 
SUMO E3 Ligases SUMOylate Target Proteins
 
 
37/170
 
 
0.0001621
 
 
0.0054870
 
 
0
 
 
0
 
 
2.091485
 
 
18.252649
 
 
HDAC4;DNMT1;UHRF2;HDAC1;SATB1;SATB2;RORA;BMI1;ING2;POM121C;TDG;DNMT3B;EP300;VHL;RAE1;DDX17;NCOA1;TFAP2C;UBE2I;NUP210;CBX4;MBD1;DNMT3A;MITF;SAFB;ESR1;NR4A2;NCOR2;RNF168;HNRNPK;STAG2;NR5A2;NUP50;RARA;PPARG;TOP1;TP53
 
 
 
 
Nuclear Events (Kinase and Transcription Factor Activation)
 
 
18/61
 
 
0.0001654
 
 
0.0054870
 
 
0
 
 
0
 
 
3.137662
 
 
27.320478
 
 
MEF2A;EGR3;FOS;VRK3;MAPK14;PPP2CA;FOSL1;REST;CREB1;RPS6KA1;ID1;ID4;EP300;MAPK1;FOSB;ID3;SGK1;CDK5R1
 
 
 
 
Kidney Development
 
 
15/46
 
 
0.0001676
 
 
0.0054870
 
 
0
 
 
0
 
 
3.624708
 
 
31.513696
 
 
ITGB1;EMX2;JAG1;WNT9B;SIX1;NPNT;DLL1;FGF2;HOXA11;LFNG;GREM1;SALL1;WT1;HOXB4;ID4
 
 
 
 
Asparagine N-linked Glycosylation
 
 
58/306
 
 
0.0001805
 
 
0.0057048
 
 
0
 
 
0
 
 
1.762598
 
 
15.192829
 
 
B4GALT2;ARF4;B4GALT3;MCFD2;UBXN1;MIA2;MIA3;AREG;PPP6R1;TMED7;GFUS;SPTAN1;PDIA3;ANK2;ANK3;DYNLL2;COPZ1;DPM2;TUBB2A;TRAPPC6B;GORASP1;CANX;PREB;MAN1B1;B4GALT6;ST6GALNAC3;SEC22A;ARF5;B4GALT5;DYNC1I2;NAPB;LMAN2L;STX17;GOSR2;RPN1;DERL1;TGFA;UAP1;NPL;LMAN1;FUT8;LMAN2;CHST10;NEU1;ST8SIA4;ST3GAL6;CTSA;NUS1;GFPT2;PMM2;GFPT1;SYVN1;CNIH1;CAPZA1;COL7A1;DOLPP1;MGAT4A;BET1
 
 
 
 
PI5P, PP2A and IER3 Regulate PI3K AKT Signaling
 
 
27/111
 
 
0.0001817
 
 
0.0057048
 
 
0
 
 
0
 
 
2.412914
 
 
20.782699
 
 
CD80;PIK3R3;TGFA;IL1RAP;PIK3R1;FGF2;AREG;PPP2CA;IRAK1;ERBB3;NTF3;PIP5K1A;MAPK1;PIP4K2B;PIP5K1B;ICOS;PDGFRB;PDGFRA;NTRK2;BDNF;ESR1;KITLG;PIK3CA;TRAF6;GRB2;MET;HBEGF
 
 
 
 
trans-Golgi Network Vesicle Budding
 
 
20/72
 
 
0.0001837
 
 
0.0057048
 
 
0
 
 
0
 
 
2.883877
 
 
24.807406
 
 
TPD52;TFRC;DTNBP1;ARRB1;PIK3C2A;GNS;BLOC1S6;VAMP7;SNX2;NECAP1;BLOC1S1;CLVS2;STX4;AP1S3;ACBD3;VAMP2;SNX5;SH3D19;FTL;CLVS1
 
 
 
 
Extracellular Matrix Organization
 
 
57/300
 
 
0.0001913
 
 
0.0058396
 
 
0
 
 
0
 
 
1.767593
 
 
15.133491
 
 
ITGB1;COLGALT2;SPARC;COL16A1;ELN;COL12A1;SERPINE1;LAMC1;FGF2;CTSS;LOXL1;LOXL2;CAPN1;ADAMTS9;CAST;COL25A1;P3H1;ADAM10;ADAM17;COL2A1;MMP16;COL4A2;LOX;MMP15;COL4A1;COL4A4;COL6A2;PXDN;COL8A1;COL21A1;COL4A5;COL6A3;MATN3;COL15A1;LAMA2;COL11A1;LAMA4;NRXN1;NID1;COL19A1;TGFB2;COL22A1;GDF5;COL1A1;COL3A1;COL1A2;COL5A1;P4HA2;COL7A1;MFAP3;COL5A2;ITGA11;MFAP2;COL9A1;SDC1;TLL1;FBN1
 
 
 
 
Sphingolipid De Novo Biosynthesis
 
 
13/37
 
 
0.0001945
 
 
0.0058396
 
 
0
 
 
0
 
 
4.055821
 
 
34.656399
 
 
CERS4;CERS5;CERS6;PPM1L;SGMS1;ORMDL1;SGMS2;SPTLC2;VAPB;ORMDL2;ORMDL3;SPTSSB;SPTSSA
 
 
 
 
NCAM1 Interactions
 
 
14/42
 
 
0.0002114
 
 
0.0062402
 
 
0
 
 
0
 
 
3.744577
 
 
31.686476
 
 
PRNP;CACNB3;COL3A1;COL2A1;COL4A2;COL5A1;COL4A1;COL4A4;COL6A2;COL5A2;ST8SIA4;COL9A1;COL4A5;COL6A3
 
 
 
 
Regulation of TP53 Activity
 
 
35/160
 
 
0.0002167
 
 
0.0062956
 
 
0
 
 
0
 
 
2.104206
 
 
17.752914
 
 
PRKAA1;DYRK2;RNF34;SETD9;HDAC1;PRDM1;PPP2CA;ING5;ING2;KAT5;AKT3;TP53INP1;EP300;PIP4K2B;RBBP7;MTA2;TAF9B;ZNF385A;MAP2K6;PRKAB2;CSNK2A1;BRPF3;USP2;TAF11;MAPK14;CCNA2;RPA3;JMY;CCNG1;MDM4;TAF5;SGK1;TAF4;TP53;CDK5R1
 
 
 
 
PI Metabolism
 
 
22/84
 
 
0.0002280
 
 
0.0065169
 
 
0
 
 
0
 
 
2.661366
 
 
22.319053
 
 
PI4K2B;MTMR1;MTMR10;TNFAIP8;MTMR2;RAB4A;MTMR12;MTMR14;PTEN;MTMR9;PIK3R3;PIK3R1;PIK3C2A;PLEKHA8;PIK3CA;RAB14;PI4KA;PIP5K1A;PIP4K2B;PIP5K1B;PNPLA6;SBF2
 
 
 
 
Cargo Concentration in the ER
 
 
12/33
 
 
0.0002356
 
 
0.0066294
 
 
0
 
 
0
 
 
4.277579
 
 
35.732576
 
 
LMAN1;MCFD2;CNIH1;LMAN2L;GOSR2;LMAN2;COL7A1;MIA2;TGFA;MIA3;PREB;AREG
 
 
 
 
Antigen Processing Ubiquitination & Proteasome Degradation
 
 
56/296
 
 
0.0002427
 
 
0.0067259
 
 
0
 
 
0
 
 
1.757832
 
 
14.631312
 
 
TRIM71;UBE2D2;UBE2D3;UBE2D1;CBLB;NPEPPS;CDC23;RNF19A;UNKL;SKP1;FBXW4;WSB1;TRIM63;FBXW7;UBE2E3;FBXO10;LRRC41;RNF144B;PSMA4;PSMA1;UBE2R2;HECW2;UBE2V1;PSMD12;RNF34;CUL5;UBA6;CUL3;ASB11;RNF7;RCHY1;UBE2J1;ZNRF2;RNF138;RNF217;VHL;LONRF1;UBE2F;UBE2H;TRIM41;UBE2B;SIAH2;FBXL19;KLHL2;UBE2G1;FBXL13;FBXO30;UBE2W;NEDD4;RLIM;RNF182;UBE2N;BLMH;UBE2K;SPSB4;ASB1
 
 
 
 
MAP Kinase Activation
 
 
18/63
 
 
0.0002588
 
 
0.0070628
 
 
0
 
 
0
 
 
2.997870
 
 
24.760493
 
 
MEF2A;MAP2K4;MAP2K1;CHUK;FOS;VRK3;MAPK14;PPP2CA;CREB1;IRAK1;TRAF6;RPS6KA1;UBE2N;MAPK1;UBE2V1;MAP3K7;SKP1;MAP2K6
 
 
 
 
Nuclear Receptor Transcription Pathway
 
 
16/53
 
 
0.0002814
 
 
0.0075654
 
 
0
 
 
0
 
 
3.239653
 
 
26.486030
 
 
ESRRA;HNF4G;RORA;ESRRG;RORB;ESR1;NR2F6;NCOR2;NR4A2;NR5A2;NR6A1;RARA;RARB;PPARG;NRBF2;NR2C2AP
 
 
 
 
Circadian Clock
 
 
19/70
 
 
0.0003647
 
 
0.0095083
 
 
0
 
 
0
 
 
2.792371
 
 
22.105748
 
 
NCOA1;SREBF1;CHD9;CRTC1;SERPINE1;ELOVL3;BHLHE41;RORA;KLF15;SIRT1;PPP1CB;PPP1CC;PER1;CREB1;TBL1XR1;BHLHE40;EP300;SIK1;SKP1
 
 
 
 
Signaling by TGFBR3
 
 
15/49
 
 
0.0003658
 
 
0.0095083
 
 
0
 
 
0
 
 
3.304318
 
 
26.148275
 
 
SMAD4;TGFB2;ARRB1;FGF2;TGFBR1;ACVR2A;APH1A;MYCN;MMP16;AGO4;AGO2;RARA;EP300;TCF4;TNRC6A
 
 
 
 
Intracellular Signaling by Second Messengers
 
 
59/321
 
 
0.0003696
 
 
0.0095083
 
 
0
 
 
0
 
 
1.696548
 
 
13.408142
 
 
CD80;PTEN;IL1RAP;BMI1;AREG;FGF2;AKT3;PIP4K2B;MAF1;PDGFRB;PDGFRA;CNOT6L;CSNK2A1;CHUK;PRKCD;PSMA4;CREB1;RRAGC;PSMA1;PIK3CA;TRAF6;PPARG;SGK1;TP53;MET;HBEGF;PSMD12;HDAC1;PIK3R3;TGFA;ADCY3;ADCY2;PIK3R1;FOXO3;PPP2CA;IRAK1;ERBB3;TNKS2;NTF3;PIP5K1A;MAPK1;PIP5K1B;SLC38A9;RBBP7;ICOS;MTA2;NTRK2;CBX4;BDNF;ESR1;KITLG;REST;AGO4;CAMK4;NEDD4;AGO2;SNAI1;GRB2;TNRC6A
 
 
 
 
Activation of BH3-only Proteins
 
 
11/30
 
 
0.0003932
 
 
0.0097050
 
 
0
 
 
0
 
 
4.332514
 
 
33.972245
 
 
BCL2L11;YWHAQ;TFDP2;YWHAB;AKT3;BCL2;PMAIP1;BMF;DYNLL2;YWHAZ;TP53
 
 
 
 
Regulation of TP53 Activity Through Acetylation
 
 
11/30
 
 
0.0003932
 
 
0.0097050
 
 
0
 
 
0
 
 
4.332514
 
 
33.972245
 
 
ING5;ING2;HDAC1;BRPF3;AKT3;EP300;PIP4K2B;RBBP7;MTA2;TP53;MAP2K6
 
 
 
 
Activated NTRK2 Signals Through PI3K
 
 
5/7
 
 
0.0003934
 
 
0.0097050
 
 
0
 
 
0
 
 
18.679025
 
 
146.457580
 
 
NTRK2;PIK3CA;BDNF;GRB2;PIK3R1
 
 
 
 
Nuclear Events Stimulated by ALK Signaling in Cancer
 
 
12/35
 
 
0.0004420
 
 
0.0107581
 
 
0
 
 
0
 
 
3.905172
 
 
30.164064
 
 
RB1;MECP2;DNMT1;NOTCH1;HDAC1;AGO4;IRF4;STAT3;AGO2;MAPK1;ICOS;SKP1
 
 
 
 
Signaling by TGF-beta Receptor Complex
 
 
23/94
 
 
0.0004837
 
 
0.0116154
 
 
0
 
 
0
 
 
2.429439
 
 
18.546409
 
 
ITGB1;CCNK;SMAD4;TGFB2;CCNT2;TGIF2;HDAC1;USP9X;SERPINE1;UBE2D3;UBE2D1;ATP1B4;CCNC;TGFBR1;PPP1CB;NCOR2;PPP1CC;COL1A2;TFDP2;EP300;MAPK1;E2F5;FBN1
 
 
 
 
MITF-M-regulated Melanocyte Development
 
 
30/136
 
 
0.0005089
 
 
0.0120601
 
 
0
 
 
0
 
 
2.124641
 
 
16.111617
 
 
ASAH1;HDAC1;LEF1;AKT3;RPS6KA1;ZIC1;MAPK1;ATP6V1E1;FOXD3;SS18;TCF7L2;SS18L1;ATP6AP2;TFEB;MITF;DICER1;MAPK14;ARID1B;SIRT1;USF1;KITLG;CREB1;AGO4;IRF4;AGO2;ID1;TFEC;BCL2;MET;TNRC6A
 
 
 
 
Golgi Associated Vesicle Biogenesis
 
 
16/56
 
 
0.0005595
 
 
0.0130864
 
 
0
 
 
0
 
 
2.996169
 
 
22.436724
 
 
TPD52;TFRC;DTNBP1;ARRB1;PIK3C2A;BLOC1S6;VAMP7;SNX2;NECAP1;BLOC1S1;AP1S3;ACBD3;VAMP2;SNX5;SH3D19;FTL
 
 
 
 
Keratan Sulfate Keratin Metabolism
 
 
12/36
 
 
0.0005923
 
 
0.0135041
 
 
0
 
 
0
 
 
3.742244
 
 
27.810164
 
 
B4GALT2;B4GALT3;B3GNT7;B4GAT1;OGN;CHST1;ST3GAL6;B4GALT6;GNS;GLB1L3;GLB1L2;B4GALT5
 
 
 
 
SMAD2 SMAD3 SMAD4 Heterotrimer Regulates Transcription
 
 
12/36
 
 
0.0005923
 
 
0.0135041
 
 
0
 
 
0
 
 
3.742244
 
 
27.810164
 
 
CCNK;SMAD4;CCNT2;TGIF2;COL1A2;TFDP2;HDAC1;SERPINE1;EP300;MAPK1;CCNC;E2F5
 
 
 
 
Clathrin-mediated Endocytosis
 
 
31/145
 
 
0.0007271
 
 
0.0163694
 
 
0
 
 
0
 
 
2.041338
 
 
14.751543
 
 
ITSN2;SCARB2;RAB5B;TFRC;ITSN1;TGFA;ARRB1;LRP2;FNBP1L;PIK3C2A;AREG;UBQLN1;EPS15;LDLR;AGFG1;REPS1;SYT1;FZD4;ARPC5;GAPVD1;SNAP91;DNM3;NECAP2;SNX18;VAMP7;NECAP1;BIN1;GRB2;VAMP2;ARF6;HBEGF
 
 
 
 
Activated NTRK2 Signals Through FRS2 and FRS3
 
 
6/11
 
 
0.0007390
 
 
0.0164311
 
 
0
 
 
0
 
 
8.968207
 
 
64.662826
 
 
NTRK2;NRAS;BDNF;GRB2;SOS1;FRS3
 
 
 
 
Signaling by Nuclear Receptors
 
 
50/270
 
 
0.0008354
 
 
0.0174794
 
 
0
 
 
0
 
 
1.709700
 
 
12.117687
 
 
FOXA1;ZDHHC7;AREG;CHD1;KAT5;AKT3;MYB;PDK4;EP300;NCOA1;PDHX;SREBF1;HSBP1;FOS;RUNX1;NCOR2;NR5A2;CREB1;PIK3CA;TBL1XR1;ALDH1A2;RARA;RARB;HBEGF;KANK1;KDM3A;CBFB;HDAC1;PIK3R3;TGFA;PIK3R1;PDHB;FOXO3;NRAS;GNG2;MAPK1;CAV2;PTGES3;APOC4;GNG12;ESR1;USF1;CYP26A1;STAG2;AGO4;SCD;AGO2;BCL2;FOSB;TNRC6A
 
 
 
 
RHO GTPase Cycle
 
 
76/450
 
 
0.0008379
 
 
0.0174794
 
 
0
 
 
0
 
 
1.532365
 
 
10.856208
 
 
ITSN2;ITGB1;ZNF512B;DOCK6;ABCD3;DOCK3;TFRC;FAM13B;SOWAHC;ITSN1;STMN2;ARHGAP1;FNBP1L;OSBPL11;RND2;LRRC1;GOLGA3;MUC13;FLOT2;LBR;SPTAN1;RALBP1;TMOD3;RRAS2;KCTD3;LEMD3;RHOC;FRS3;CKAP4;SENP1;ARMCX3;TIAM1;DAAM1;PIK3CA;PKP4;SOS1;SOS2;RHOQ;EPHA2;MTMR1;GARRE1;ARHGEF26;CUL3;MOSPD2;PIK3R3;PIK3R1;FAM169A;UACA;RHOBTB1;CDC42;GNA13;LMAN1;ARHGAP21;PARD6B;ARHGAP20;PGRMC2;EMC3;WHAMM;SRGAP2;WASF1;STARD13;CPSF7;JAG1;USP9X;SEMA4F;ARHGAP26;CCT6A;NET1;SLC4A7;FMNL2;VAPB;SHKBP1;FILIP1;GRB2;FERMT2;MAP3K11
 
 
 
 
Constitutive Signaling by NOTCH1 HD+PEST Domain Mutants
 
 
16/58
 
 
0.0008541
 
 
0.0174794
 
 
0
 
 
0
 
 
2.853169
 
 
20.159056
 
 
HDAC4;NOTCH1;JAG1;FBXW7;HDAC1;ADAM10;CCNC;DLL1;NCOR2;APH1A;DLL4;ADAM17;TBL1XR1;HEY2;EP300;SKP1
 
 
 
 
Constitutive Signaling by NOTCH1 PEST Domain Mutants
 
 
16/58
 
 
0.0008541
 
 
0.0174794
 
 
0
 
 
0
 
 
2.853169
 
 
20.159056
 
 
HDAC4;NOTCH1;JAG1;FBXW7;HDAC1;ADAM10;CCNC;DLL1;NCOR2;APH1A;DLL4;ADAM17;TBL1XR1;HEY2;EP300;SKP1
 
 
 
 
Signaling by NOTCH1 HD+PEST Domain Mutants in Cancer
 
 
16/58
 
 
0.0008541
 
 
0.0174794
 
 
0
 
 
0
 
 
2.853169
 
 
20.159056
 
 
HDAC4;NOTCH1;JAG1;FBXW7;HDAC1;ADAM10;CCNC;DLL1;NCOR2;APH1A;DLL4;ADAM17;TBL1XR1;HEY2;EP300;SKP1
 
 
 
 
Signaling by NOTCH1 PEST Domain Mutants in Cancer
 
 
16/58
 
 
0.0008541
 
 
0.0174794
 
 
0
 
 
0
 
 
2.853169
 
 
20.159056
 
 
HDAC4;NOTCH1;JAG1;FBXW7;HDAC1;ADAM10;CCNC;DLL1;NCOR2;APH1A;DLL4;ADAM17;TBL1XR1;HEY2;EP300;SKP1
 
 
 
 
Signaling by NOTCH1 in Cancer
 
 
16/58
 
 
0.0008541
 
 
0.0174794
 
 
0
 
 
0
 
 
2.853169
 
 
20.159056
 
 
HDAC4;NOTCH1;JAG1;FBXW7;HDAC1;ADAM10;CCNC;DLL1;NCOR2;APH1A;DLL4;ADAM17;TBL1XR1;HEY2;EP300;SKP1
 
 
 
 
Signaling by EGFR
 
 
15/53
 
 
0.0009187
 
 
0.0181339
 
 
0
 
 
0
 
 
2.955823
 
 
20.668614
 
 
ADAM10;TGFA;PTPRK;PIK3R1;AREG;CDC42;ADAM17;NRAS;PIK3CA;SPRY2;GRB2;SPRY1;SOS1;EPS15;HBEGF
 
 
 
 
Extra-nuclear Estrogen Signaling
 
 
19/75
 
 
0.0009240
 
 
0.0181339
 
 
0
 
 
0
 
 
2.542329
 
 
17.762875
 
 
HSBP1;CAV2;PIK3R3;TGFA;ZDHHC7;FOS;PIK3R1;FOXO3;GNG12;AREG;ESR1;NRAS;CREB1;GNG2;PIK3CA;AKT3;BCL2;MAPK1;HBEGF
 
 
 
 
Gain-of-function MRAS Complexes Activate RAF Signaling
 
 
5/8
 
 
0.0009465
 
 
0.0181339
 
 
0
 
 
0
 
 
12.451977
 
 
86.700321
 
 
PPP1CB;PPP1CC;MRAS;YWHAB;RAF1
 
 
 
 
MECP2 Regulates Transcription of Neuronal Ligands
 
 
5/8
 
 
0.0009465
 
 
0.0181339
 
 
0
 
 
0
 
 
12.451977
 
 
86.700321
 
 
MECP2;CREB1;BDNF;HDAC1;DLL1
 
 
 
 
SHOC2 M1731 Mutant Abolishes MRAS Complex Function
 
 
5/8
 
 
0.0009465
 
 
0.0181339
 
 
0
 
 
0
 
 
12.451977
 
 
86.700321
 
 
PPP1CB;PPP1CC;MRAS;YWHAB;RAF1
 
 
 
 
Signaling by MRAS-complex Mutants
 
 
5/8
 
 
0.0009465
 
 
0.0181339
 
 
0
 
 
0
 
 
12.451977
 
 
86.700321
 
 
PPP1CB;PPP1CC;MRAS;YWHAB;RAF1
 
 
 
 
Transport to the Golgi and Subsequent Modification
 
 
37/186
 
 
0.0010027
 
 
0.0190093
 
 
0
 
 
0
 
 
1.865190
 
 
12.879224
 
 
B4GALT2;ARF4;B4GALT3;DYNC1I2;NAPB;MCFD2;LMAN2L;STX17;GOSR2;TGFA;MIA2;MIA3;AREG;LMAN1;FUT8;PPP6R1;LMAN2;CHST10;TMED7;SPTAN1;ANK2;ANK3;DYNLL2;COPZ1;CNIH1;TUBB2A;TRAPPC6B;GORASP1;CAPZA1;COL7A1;MGAT4A;PREB;B4GALT6;SEC22A;ARF5;B4GALT5;BET1
 
 
 
 
Estrogen-dependent Nuclear Events Downstream of ESR-membrane Signaling
 
 
9/24
 
 
0.0011019
 
 
0.0206718
 
 
0
 
 
0
 
 
4.487267
 
 
30.561588
 
 
CREB1;AKT3;BCL2;TGFA;MAPK1;FOS;FOXO3;AREG;HBEGF
 
 
 
 
Signaling by BRAF and RAF1 Fusions
 
 
17/65
 
 
0.0011502
 
 
0.0213550
 
 
0
 
 
0
 
 
2.652781
 
 
17.953634
 
 
MAP2K1;YWHAB;AGTRAP;ARRB1;QKI;CNKSR2;FXR1;RAP1B;CLCN6;NRAS;BCL2L11;RAP1A;MAPK1;AGGF1;RAF1;MARK3;KDM7A
 
 
 
 
Cytokine Signaling in Immune System
 
 
120/776
 
 
0.0011780
 
 
0.0216483
 
 
0
 
 
0
 
 
1.383481
 
 
9.330139
 
 
EIF4A2;UBE2D2;CD80;UBE2D3;UBE2D1;RORA;IL1RAP;IFI30;FGF2;TNF;GRAP2;AKT3;RPS6KA1;GRB10;IL12B;KPNA5;MAP3K7;IL6R;IL13RA1;SKP1;MEF2A;MAP2K4;MAP2K1;NUP210;PRKCD;VRK3;ADAM17;FSCN1;UBE2V1;IL6ST;RAF1;TP53;KPNB1;STX1A;CSF1R;PSMD12;CUL5;PIK3R3;TNFRSF11B;TNFRSF11A;PIK3R1;FOXO3;HSP90B1;RAP1B;SOCS2;CNN2;BCL2L11;IRAK1;POM121C;NDN;MAP2K6;MCL1;UBE2I;TSLP;LIF;EIF2S1;MAVS;COL1A2;HNRNPF;TNFSF4;UBE2N;BCL2;SDC1;GRB2;EIF4G2;ITGB1;SNAP25;EIF4E3;NKIRAS2;CHUK;IL15;FOS;TNFRSF1B;YWHAZ;TNFRSF1A;PSMA4;VAMP7;CREB1;TUBB2A;PSMA1;PIK3CA;IFNG;IRF4;NUP50;TRAF6;IL1RAPL1;CANX;IRF2;IRF5;MAPT;EIF4E2;SOS1;SOS2;VAMP2;HLA-DQB1;PELI2;NLRC5;RNF7;CDC42;PPP2CA;NRAS;S1PR1;CCL2;MAPK1;STX4;STX3;RAE1;PTPN18;IL31;STAT3;OSM;MAPK14;PSMB8;VEGFA;PRKRA;NEDD4;CAPZA1;PTPN4;ABCE1;HSPA1B
 
 
 
 
Interleukin-4 and Interleukin-13 Signaling
 
 
25/112
 
 
0.0011990
 
 
0.0217523
 
 
0
 
 
0
 
 
2.154927
 
 
14.494571
 
 
ITGB1;RORA;PIK3R1;FOXO3;FGF2;TNF;HSP90B1;S1PR1;IL12B;NDN;CCL2;IL6R;IL13RA1;MCL1;STAT3;LIF;OSM;FOS;TNFRSF1B;VEGFA;COL1A2;IRF4;FSCN1;BCL2;TP53
 
 
 
 
Toll Like Receptor 3 (TLR3) Cascade
 
 
24/106
 
 
0.0012078
 
 
0.0217523
 
 
0
 
 
0
 
 
2.194559
 
 
14.745164
 
 
MEF2A;MAP2K4;MAP2K1;CHUK;UBE2D2;UBE2D3;NLRC5;UBE2D1;FOS;VRK3;MAPK14;PPP2CA;CREB1;IRAK1;NKIRAS2;TRAF6;RPS6KA1;UBE2N;MAPK1;UBE2V1;MAP3K7;TP53;SKP1;MAP2K6
 
 
 
 
Signalling to ERKs
 
 
11/34
 
 
0.0013163
 
 
0.0233176
 
 
0
 
 
0
 
 
3.578220
 
 
23.734217
 
 
NRAS;MAP2K1;RIT1;RAP1A;YWHAB;MAPK1;GRB2;MAPK14;SOS1;RALGDS;MAPK12
 
 
 
 
NGF-stimulated Transcription
 
 
12/39
 
 
0.0013206
 
 
0.0233176
 
 
0
 
 
0
 
 
3.325872
 
 
22.049440
 
 
FOSL1;REST;EGR3;CREB1;ID1;ID4;EP300;FOSB;ID3;FOS;SGK1;CDK5R1
 
 
 
 
Death Receptor Signaling
 
 
32/157
 
 
0.0014026
 
 
0.0245248
 
 
0
 
 
0
 
 
1.921372
 
 
12.622335
 
 
BEX3;PRDM4;ARHGEF26;HDAC1;UBE2D2;ITSN1;UBE2D3;UBE2D1;TNFAIP3;TNF;RTN4;APH1A;SMPD3;GNA13;BAG4;BCL2L11;IRAK1;CASP2;MAP3K7;CHUK;USP4;USP2;TNFRSF1A;NET1;TIAM1;ADAM17;TRAF6;OTULIN;FAS;SOS1;SOS2;ITGB3BP
 
 
 
 
Interleukin-17 Signaling
 
 
18/72
 
 
0.0014667
 
 
0.0253991
 
 
0
 
 
0
 
 
2.496946
 
 
16.291950
 
 
MEF2A;MAP2K4;MAP2K1;CHUK;FOS;VRK3;MAPK14;PPP2CA;CREB1;IRAK1;TRAF6;RPS6KA1;UBE2N;MAPK1;UBE2V1;MAP3K7;SKP1;MAP2K6
 
 
 
 
Transcriptional Regulation of White Adipocyte Differentiation
 
 
20/84
 
 
0.0015716
 
 
0.0269565
 
 
0
 
 
0
 
 
2.341551
 
 
15.116278
 
 
NCOA1;SREBF1;WNT10B;CDK19;CHD9;MED8;MED9;LPL;CCNC;NR2F2;KLF4;TNF;MED28;NCOR2;MED14;KLF5;TBL1XR1;EP300;PPARG;WNT1
 
 
 
 
Laminin Interactions
 
 
10/30
 
 
0.0016790
 
 
0.0285272
 
 
0
 
 
0
 
 
3.739915
 
 
23.896397
 
 
ITGB1;LAMA2;COL4A2;COL4A1;LAMA4;COL4A4;COL7A1;COL4A5;LAMC1;NID1
 
 
 
 
ESR-mediated Signaling
 
 
37/192
 
 
0.0018157
 
 
0.0305611
 
 
0
 
 
0
 
 
1.792373
 
 
11.312199
 
 
FOXA1;KANK1;CBFB;HDAC1;PIK3R3;TGFA;ZDHHC7;PIK3R1;FOXO3;AREG;CHD1;NRAS;GNG2;KAT5;AKT3;MYB;EP300;MAPK1;NCOA1;CAV2;HSBP1;PTGES3;FOS;GNG12;ESR1;USF1;RUNX1;STAG2;CREB1;NR5A2;PIK3CA;AGO4;AGO2;BCL2;FOSB;TNRC6A;HBEGF
 
 
 
 
Transcriptional and Post-Translational Regulation of MITF-M Expression
and Activity
 
 
14/51
 
 
0.0018567
 
 
0.0309076
 
 
0
 
 
0
 
 
2.832285
 
 
17.812047
 
 
FOXD3;HDAC1;LEF1;TFEB;MITF;SIRT1;KITLG;CREB1;AKT3;RPS6KA1;ZIC1;TFEC;ID1;MAPK1
 
 
 
 
Activated NTRK2 Signals Through RAS
 
 
5/9
 
 
0.0019221
 
 
0.0309076
 
 
0
 
 
0
 
 
9.338453
 
 
58.405964
 
 
NTRK2;NRAS;BDNF;GRB2;SOS1
 
 
 
 
BH3-only Proteins Associate With and Inactivate Anti-Apoptotic BCL-2
Members
 
 
5/9
 
 
0.0019221
 
 
0.0309076
 
 
0
 
 
0
 
 
9.338453
 
 
58.405964
 
 
BCL2L11;STAT3;BCL2;PMAIP1;BMF
 
 
 
 
PI5P Regulates TP53 Acetylation
 
 
5/9
 
 
0.0019221
 
 
0.0309076
 
 
0
 
 
0
 
 
9.338453
 
 
58.405964
 
 
ING2;EP300;PIP4K2B;TP53;MAP2K6
 
 
 
 
Small Interfering RNA (siRNA) Biogenesis
 
 
5/9
 
 
0.0019221
 
 
0.0309076
 
 
0
 
 
0
 
 
9.338453
 
 
58.405964
 
 
AGO4;PRKRA;AGO2;DICER1;TSN
 
 
 
 
Metabolism of Lipids
 
 
116/758
 
 
0.0020301
 
 
0.0315439
 
 
0
 
 
0
 
 
1.365223
 
 
8.463912
 
 
PI4K2B;RORA;CCNC;AHR;PPP1CB;PPP1CC;EP300;PIP4K2B;SBF2;MFSD2A;PRKAB2;CSNK2A1;DGAT1;ACSL1;MED8;ACSL6;MED9;ACSL4;CYP39A1;PLA2G15;TBL1XR1;FDX1;KPNB1;CDS1;MTMR1;MTMR2;ABHD4;ABHD3;INSIG2;INSIG1;MTMR9;PIK3R3;ABHD5;PIK3R1;GLB1L3;GLB1L2;UGCG;SGPL1;ARSJ;NEU1;PIP5K1A;ARSI;PIP5K1B;MORC2;ARSB;ESRRA;OSBPL9;CTSA;CDK19;RAB4A;UBE2I;OSBPL3;ACER2;ACER3;PPT1;PI4KA;CDS2;ACHE;MTMR10;TNFAIP8;MTMR12;CHD9;MTMR14;PTEN;PCTP;PIK3C2A;SMPD3;MED14;SPTLC2;LBR;NCOA1;CERS4;SREBF1;CERS5;CERS6;ELOVL4;HMGCS1;STARD3NL;ELOVL2;ELOVL3;ELOVL6;MED28;NCOR2;BDH1;PIK3CA;PPARG;B4GALT6;B4GALT5;ASAH1;SGMS1;PPM1L;TAFAZZIN;SGMS2;LRP2;HMGCR;AGPAT3;PTGS1;AGPAT4;ORMDL2;ORMDL3;SPTSSB;SPTSSA;RPP14;NFYA;TXNRD1;PTGES3;MBOAT2;ORMDL1;SGPP1;PLEKHA8;CYP24A1;RAB14;VAPB;SCD;PNPLA6;PNPLA2
 
 
 
 
Signaling by NOTCH1
 
 
18/74
 
 
0.0020441
 
 
0.0315439
 
 
0
 
 
0
 
 
2.407496
 
 
14.909167
 
 
HDAC4;NOTCH1;JAG1;FBXW7;HDAC1;ADAM10;ARRB1;CCNC;DLL1;NCOR2;APH1A;DLL4;ADAM17;TBL1XR1;HEY2;NUMB;EP300;SKP1
 
 
 
 
MyD88-independent TLR4 Cascade
 
 
24/110
 
 
0.0020604
 
 
0.0315439
 
 
0
 
 
0
 
 
2.092010
 
 
12.938771
 
 
MEF2A;MAP2K4;MAP2K1;CHUK;UBE2D2;UBE2D3;NLRC5;UBE2D1;FOS;VRK3;MAPK14;PPP2CA;CREB1;IRAK1;NKIRAS2;TRAF6;RPS6KA1;UBE2N;MAPK1;UBE2V1;MAP3K7;TP53;SKP1;MAP2K6
 
 
 
 
PI3K AKT Signaling in Cancer
 
 
24/110
 
 
0.0020604
 
 
0.0315439
 
 
0
 
 
0
 
 
2.092010
 
 
12.938771
 
 
PDGFRB;PDGFRA;NTRK2;CHUK;BDNF;CD80;PTEN;PIK3R3;TGFA;PIK3R1;FOXO3;FGF2;ESR1;AREG;KITLG;CREB1;PIK3CA;ERBB3;AKT3;NTF3;GRB2;ICOS;MET;HBEGF
 
 
 
 
TRIF (TICAM1)-mediated TLR4 Signaling
 
 
24/110
 
 
0.0020604
 
 
0.0315439
 
 
0
 
 
0
 
 
2.092010
 
 
12.938771
 
 
MEF2A;MAP2K4;MAP2K1;CHUK;UBE2D2;UBE2D3;NLRC5;UBE2D1;FOS;VRK3;MAPK14;PPP2CA;CREB1;IRAK1;NKIRAS2;TRAF6;RPS6KA1;UBE2N;MAPK1;UBE2V1;MAP3K7;TP53;SKP1;MAP2K6
 
 
 
 
MyD88 Cascade Initiated on Plasma Membrane
 
 
22/98
 
 
0.0021223
 
 
0.0315439
 
 
0
 
 
0
 
 
2.169385
 
 
13.353162
 
 
MEF2A;MAP2K4;MAP2K1;CHUK;PELI2;NLRC5;FOS;VRK3;MAPK14;PPP2CA;CREB1;IRAK1;NKIRAS2;TRAF6;RPS6KA1;UBE2N;MAPK1;UBE2V1;MAP3K7;TP53;SKP1;MAP2K6
 
 
 
 
Toll Like Receptor 10 (TLR10) Cascade
 
 
22/98
 
 
0.0021223
 
 
0.0315439
 
 
0
 
 
0
 
 
2.169385
 
 
13.353162
 
 
MEF2A;MAP2K4;MAP2K1;CHUK;PELI2;NLRC5;FOS;VRK3;MAPK14;PPP2CA;CREB1;IRAK1;NKIRAS2;TRAF6;RPS6KA1;UBE2N;MAPK1;UBE2V1;MAP3K7;TP53;SKP1;MAP2K6
 
 
 
 
Toll Like Receptor 5 (TLR5) Cascade
 
 
22/98
 
 
0.0021223
 
 
0.0315439
 
 
0
 
 
0
 
 
2.169385
 
 
13.353162
 
 
MEF2A;MAP2K4;MAP2K1;CHUK;PELI2;NLRC5;FOS;VRK3;MAPK14;PPP2CA;CREB1;IRAK1;NKIRAS2;TRAF6;RPS6KA1;UBE2N;MAPK1;UBE2V1;MAP3K7;TP53;SKP1;MAP2K6
 
 
 
 
Class I MHC Mediated Antigen Processing & Presentation
 
 
63/373
 
 
0.0022134
 
 
0.0315439
 
 
0
 
 
0
 
 
1.529491
 
 
9.350107
 
 
TRIM71;UBE2D2;UBE2D3;UBE2D1;CBLB;CTSS;NPEPPS;CDC23;RNF19A;MRC1;UNKL;SKP1;FBXW4;PDIA3;WSB1;TRIM63;CHUK;FBXW7;UBE2E3;FBXO10;LRRC41;RNF144B;PSMA4;PSMA1;UBE2R2;HECW2;CANX;UBE2V1;PSMD12;RNF34;CUL5;UBA6;CUL3;ASB11;RNF7;RCHY1;UBE2J1;ZNRF2;RNF138;RNF217;STX4;VHL;LONRF1;UBE2F;UBE2H;TRIM41;UBE2B;SIAH2;FBXL19;KLHL2;UBE2G1;FBXL13;FBXO30;UBE2W;NEDD4;CD207;RLIM;RNF182;UBE2N;BLMH;UBE2K;SPSB4;ASB1
 
 
 
 
Downregulation of SMAD2 3 SMAD4 Transcriptional Activity
 
 
10/31
 
 
0.0022191
 
 
0.0315439
 
 
0
 
 
0
 
 
3.561622
 
 
21.763850
 
 
NCOR2;SMAD4;TGIF2;HDAC1;USP9X;SERPINE1;UBE2D3;UBE2D1;MAPK1;ATP1B4
 
 
 
 
Disorders of Developmental Biology
 
 
6/13
 
 
0.0022244
 
 
0.0315439
 
 
0
 
 
0
 
 
6.405135
 
 
39.124402
 
 
MECP2;NCOR2;BDNF;HDAC1;TBL1XR1;CAMK4
 
 
 
 
Disorders of Nervous System Development
 
 
6/13
 
 
0.0022244
 
 
0.0315439
 
 
0
 
 
0
 
 
6.405135
 
 
39.124402
 
 
MECP2;NCOR2;BDNF;HDAC1;TBL1XR1;CAMK4
 
 
 
 
GRB2 Events in EGFR Signaling
 
 
6/13
 
 
0.0022244
 
 
0.0315439
 
 
0
 
 
0
 
 
6.405135
 
 
39.124402
 
 
NRAS;TGFA;GRB2;SOS1;AREG;HBEGF
 
 
 
 
Loss of Function of MECP2 in Rett Syndrome
 
 
6/13
 
 
0.0022244
 
 
0.0315439
 
 
0
 
 
0
 
 
6.405135
 
 
39.124402
 
 
MECP2;NCOR2;BDNF;HDAC1;TBL1XR1;CAMK4
 
 
 
 
Pervasive Developmental Disorders
 
 
6/13
 
 
0.0022244
 
 
0.0315439
 
 
0
 
 
0
 
 
6.405135
 
 
39.124402
 
 
MECP2;NCOR2;BDNF;HDAC1;TBL1XR1;CAMK4
 
 
 
 
COPII-mediated Vesicle Transport
 
 
17/69
 
 
0.0023220
 
 
0.0326710
 
 
0
 
 
0
 
 
2.448164
 
 
14.848928
 
 
NAPB;MCFD2;LMAN2L;STX17;GOSR2;TGFA;AREG;LMAN1;CNIH1;PPP6R1;TRAPPC6B;GORASP1;LMAN2;COL7A1;PREB;SEC22A;BET1
 
 
 
 
Activated NTRK2 Signals Through CDK5
 
 
4/6
 
 
0.0024006
 
 
0.0330042
 
 
0
 
 
0
 
 
14.936891
 
 
90.099626
 
 
NTRK2;TIAM1;BDNF;CDK5R1
 
 
 
 
MET Activates PI3K AKT Signaling
 
 
4/6
 
 
0.0024006
 
 
0.0330042
 
 
0
 
 
0
 
 
14.936891
 
 
90.099626
 
 
PIK3CA;GRB2;PIK3R1;MET
 
 
 
 
RUNX1 Regulates Transcription of Genes Involved in WNT Signaling
 
 
4/6
 
 
0.0024006
 
 
0.0330042
 
 
0
 
 
0
 
 
14.936891
 
 
90.099626
 
 
CBFB;RSPO3;ESR1;RUNX1
 
 
 
 
Signaling by ALK Fusions and Activated Point Mutants
 
 
21/93
 
 
0.0024538
 
 
0.0332280
 
 
0
 
 
0
 
 
2.185385
 
 
13.134403
 
 
RB1;DNMT1;NOTCH1;HDAC1;STAT3;GCC2;PIK3R1;FRS3;MECP2;EML4;PIK3CA;IRF4;AGO4;AGO2;MYH9;MAPK1;GRB2;ICOS;TP53;SKP1;MCL1
 
 
 
 
Signaling by ALK in Cancer
 
 
21/93
 
 
0.0024538
 
 
0.0332280
 
 
0
 
 
0
 
 
2.185385
 
 
13.134403
 
 
RB1;DNMT1;NOTCH1;HDAC1;STAT3;GCC2;PIK3R1;FRS3;MECP2;EML4;PIK3CA;IRF4;AGO4;AGO2;MYH9;MAPK1;GRB2;ICOS;TP53;SKP1;MCL1
 
 
 
 
Signaling by ERBB4
 
 
15/58
 
 
0.0024749
 
 
0.0332636
 
 
0
 
 
0
 
 
2.611381
 
 
15.672329
 
 
GABRB2;SPARC;PIK3R1;ADAP1;ESR1;APH1A;ADAM17;NRAS;PIK3CA;ERBB3;NEDD4;STMN1;GRB2;SOS1;HBEGF
 
 
 
 
Semaphorin Interactions
 
 
16/64
 
 
0.0026285
 
 
0.0350659
 
 
0
 
 
0
 
 
2.495672
 
 
14.827656
 
 
ITGB1;NRP1;SEMA7A;SEMA6A;SEMA4D;LIMK1;RHOC;MYL12B;MYL6;DPYSL4;RRAS;DPYSL5;DPYSL3;MYH9;MET;CDK5R1
 
 
 
 
MITF-M-dependent Gene Expression
 
 
21/94
 
 
0.0028147
 
 
0.0367523
 
 
0
 
 
0
 
 
2.155325
 
 
12.657974
 
 
SS18;TCF7L2;ASAH1;HDAC1;SS18L1;ATP6AP2;LEF1;MITF;DICER1;MAPK14;ARID1B;SIRT1;USF1;CREB1;IRF4;AGO4;AGO2;BCL2;ATP6V1E1;MET;TNRC6A
 
 
 
 
Fatty acyl-CoA Biosynthesis
 
 
11/37
 
 
0.0028161
 
 
0.0367523
 
 
0
 
 
0
 
 
3.164810
 
 
18.585024
 
 
ELOVL4;ACSL1;SCD;ELOVL2;ELOVL3;ACSL6;PPT1;ACSL4;ELOVL6;RPP14;MORC2
 
 
 
 
NOD1 2 Signaling Pathway
 
 
11/37
 
 
0.0028161
 
 
0.0367523
 
 
0
 
 
0
 
 
3.164810
 
 
18.585024
 
 
IRAK1;CHUK;TRAF6;UBE2N;TNFAIP3;CASP2;UBE2V1;MAPK14;MAP3K7;MAPK12;MAP2K6
 
 
 
 
Cardiogenesis
 
 
9/27
 
 
0.0028429
 
 
0.0368354
 
 
0
 
 
0
 
 
3.738752
 
 
21.920007
 
 
EOMES;MYOCD;SMAD4;KAT5;LEF1;HEY2;GATA6;TBX5;ISL1
 
 
 
 
Regulation of MECP2 Expression and Activity
 
 
10/32
 
 
0.0028904
 
 
0.0371826
 
 
0
 
 
0
 
 
3.399537
 
 
19.874944
 
 
MECP2;NCOR2;CREB1;HDAC1;AGO4;TBL1XR1;CAMK4;AGO2;LBR;TNRC6A
 
 
 
 
Toll Like Receptor 7 8 (TLR7 8) Cascade
 
 
23/107
 
 
0.0030839
 
 
0.0392636
 
 
0
 
 
0
 
 
2.051935
 
 
11.863355
 
 
MEF2A;MAP2K4;MAP2K1;CHUK;PELI2;NLRC5;FOS;VRK3;MAPK14;PPP2CA;CREB1;IRAK1;NKIRAS2;TRAF6;RPS6KA1;UBE2N;MAPK1;IRF5;UBE2V1;MAP3K7;TP53;SKP1;MAP2K6
 
 
 
 
Gastrin-CREB Signalling Pathway via PKC and MAPK
 
 
7/18
 
 
0.0031175
 
 
0.0392636
 
 
0
 
 
0
 
 
4.756265
 
 
27.447027
 
 
NRAS;CREB1;RPS6KA1;MAPK1;GRB2;SOS1;HBEGF
 
 
 
 
MECP2 Regulates Neuronal Receptors and Channels
 
 
7/18
 
 
0.0031175
 
 
0.0392636
 
 
0
 
 
0
 
 
4.756265
 
 
27.447027
 
 
MECP2;NOTCH1;CREB1;HDAC1;SLC2A3;PTPN4;MET
 
 
 
 
Protein Ubiquitination
 
 
18/77
 
 
0.0032599
 
 
0.0406766
 
 
0
 
 
0
 
 
2.284691
 
 
13.082287
 
 
UBE2H;UBE2B;UBA6;USP9X;UBE2D2;UBE2E3;UBE2D3;DERL1;UBE2D1;UBE2G1;CDC73;UBE2W;UBE2R2;OTULIN;WAC;UBE2N;RNF152;UBE2K
 
 
 
 
Constitutive Signaling by Aberrant PI3K in Cancer
 
 
19/83
 
 
0.0032749
 
 
0.0406766
 
 
0
 
 
0
 
 
2.223525
 
 
12.721825
 
 
PDGFRB;PDGFRA;NTRK2;BDNF;CD80;PIK3R3;TGFA;PIK3R1;AREG;FGF2;ESR1;KITLG;PIK3CA;ERBB3;NTF3;GRB2;ICOS;MET;HBEGF
 
 
 
 
RND2 GTPase Cycle
 
 
12/43
 
 
0.0033084
 
 
0.0408117
 
 
0
 
 
0
 
 
2.896069
 
 
16.540259
 
 
GOLGA3;TFRC;ARHGAP1;MUC13;PKP4;PIK3R1;RND2;LEMD3;FRS3;CKAP4;LRRC1;EPHA2
 
 
 
 
Cohesin Loading Onto Chromatin
 
 
5/10
 
 
0.0034709
 
 
0.0420921
 
 
0
 
 
0
 
 
7.470339
 
 
42.307169
 
 
MAU2;STAG2;PDS5B;PDS5A;WAPL
 
 
 
 
IRAK1 Recruits IKK Complex
 
 
6/14
 
 
0.0035057
 
 
0.0420921
 
 
0
 
 
0
 
 
5.604176
 
 
31.682410
 
 
CHUK;IRAK1;TRAF6;PELI2;UBE2N;UBE2V1
 
 
 
 
IRAK1 Recruits IKK Complex Upon TLR7 8 or 9 Stimulation
 
 
6/14
 
 
0.0035057
 
 
0.0420921
 
 
0
 
 
0
 
 
5.604176
 
 
31.682410
 
 
CHUK;IRAK1;TRAF6;PELI2;UBE2N;UBE2V1
 
 
 
 
SHC1 Events in EGFR Signaling
 
 
6/14
 
 
0.0035057
 
 
0.0420921
 
 
0
 
 
0
 
 
5.604176
 
 
31.682410
 
 
NRAS;TGFA;GRB2;SOS1;AREG;HBEGF
 
 
 
 
FLT3 Signaling
 
 
11/38
 
 
0.0035466
 
 
0.0422875
 
 
0
 
 
0
 
 
3.047421
 
 
17.192806
 
 
SOCS2;NRAS;BCL2L11;PIK3CA;GRAP2;AKT3;GRB10;GRB2;PIK3R1;FOXO3;SOS1
 
 
 
 
TRAF6 Mediated Induction of NFkB and MAP Kinases Upon TLR7 8 or 9
Activation
 
 
22/102
 
 
0.0035879
 
 
0.0422875
 
 
0
 
 
0
 
 
2.060446
 
 
11.600681
 
 
MEF2A;MAP2K4;MAP2K1;CHUK;PELI2;NLRC5;FOS;VRK3;MAPK14;PPP2CA;CREB1;IRAK1;NKIRAS2;TRAF6;RPS6KA1;UBE2N;MAPK1;UBE2V1;MAP3K7;TP53;SKP1;MAP2K6
 
 
 
 
RAF-independent MAPK1 3 Activation
 
 
8/23
 
 
0.0035924
 
 
0.0422875
 
 
0
 
 
0
 
 
3.986989
 
 
22.442459
 
 
MAP2K1;DUSP1;PEA15;MAPK1;IL6ST;DUSP16;DUSP9;IL6R
 
 
 
 
Keratan Sulfate Biosynthesis
 
 
9/28
 
 
0.0037560
 
 
0.0439260
 
 
0
 
 
0
 
 
3.541775
 
 
19.778666
 
 
B4GALT2;B4GALT3;B3GNT7;B4GAT1;OGN;CHST1;ST3GAL6;B4GALT6;B4GALT5
 
 
 
 
Platelet Activation, Signaling and Aggregation
 
 
46/262
 
 
0.0038831
 
 
0.0451187
 
 
0
 
 
0
 
 
1.599656
 
 
8.879900
 
 
SPARC;DGKD;SERPINE1;PIK3R3;ABHD6;ARRB1;A1BG;PIK3R1;RAP1B;CDC42;GNA13;RAP1A;GNG2;TMSB4X;CDC37L1;MAPK1;STX4;FAM3C;VTI1B;TGFB2;TRPC6;ACTN2;AHSG;BRPF3;PRKCD;SERPINF2;VEGFC;IGF1;GTPBP2;GNG12;F2;CYRIB;MAPK14;DGKZ;YWHAZ;ADRA2A;VEGFA;COL1A1;COL1A2;PIK3CA;CALU;GRB2;ALDOA;RAF1;SOS1;DGKH
 
 
 
 
MyD88 Dependent Cascade Initiated on Endosome
 
 
22/103
 
 
0.0040641
 
 
0.0469196
 
 
0
 
 
0
 
 
2.034893
 
 
11.203223
 
 
MEF2A;MAP2K4;MAP2K1;CHUK;PELI2;NLRC5;FOS;VRK3;MAPK14;PPP2CA;CREB1;IRAK1;NKIRAS2;TRAF6;RPS6KA1;UBE2N;MAPK1;UBE2V1;MAP3K7;TP53;SKP1;MAP2K6
 
 
 
 
FOXO-mediated Transcription
 
 
16/67
 
 
0.0042968
 
 
0.0484389
 
 
0
 
 
0
 
 
2.348467
 
 
12.798883
 
 
SREBF1;SMAD4;BTG1;TRIM63;NFYA;YWHAB;HDAC1;GADD45A;FOXO3;KLF4;SIRT1;YWHAZ;BCL2L11;YWHAQ;AKT3;EP300
 
 
 
 
Integrin Cell Surface Interactions
 
 
19/85
 
 
0.0043318
 
 
0.0484389
 
 
0
 
 
0
 
 
2.155900
 
 
11.731900
 
 
ITGB1;COL16A1;COL1A1;COL3A1;COL2A1;COL1A2;COL4A2;COL5A1;COL4A1;COL4A4;COL6A2;COL7A1;COL5A2;ITGA11;COL9A1;COL4A5;COL6A3;COL8A1;FBN1
 
 
 
 
ER to Golgi Anterograde Transport
 
 
30/155
 
 
0.0043535
 
 
0.0484389
 
 
0
 
 
0
 
 
1.799743
 
 
9.784796
 
 
ARF4;DYNC1I2;NAPB;MCFD2;LMAN2L;STX17;GOSR2;TGFA;MIA2;MIA3;AREG;LMAN1;PPP6R1;LMAN2;TMED7;SPTAN1;ANK2;ANK3;DYNLL2;COPZ1;CNIH1;TUBB2A;TRAPPC6B;GORASP1;CAPZA1;COL7A1;PREB;SEC22A;ARF5;BET1
 
 
 
 
Toll Like Receptor 9 (TLR9) Cascade
 
 
23/110
 
 
0.0044351
 
 
0.0484389
 
 
0
 
 
0
 
 
1.980840
 
 
10.732595
 
 
MEF2A;MAP2K4;MAP2K1;CHUK;PELI2;NLRC5;FOS;VRK3;MAPK14;PPP2CA;CREB1;IRAK1;NKIRAS2;TRAF6;RPS6KA1;UBE2N;MAPK1;IRF5;UBE2V1;MAP3K7;TP53;SKP1;MAP2K6
 
 
 
 
RHO GTPases Activate ROCKs
 
 
7/19
 
 
0.0044378
 
 
0.0484389
 
 
0
 
 
0
 
 
4.359662
 
 
23.618922
 
 
PPP1CB;MYL6;PPP1R12A;LIMK1;MYH9;RHOC;MYL12B
 
 
 
 
Regulation of MITF-M-dependent Genes Involved in Apoptosis
 
 
7/19
 
 
0.0044378
 
 
0.0484389
 
 
0
 
 
0
 
 
4.359662
 
 
23.618922
 
 
HDAC1;AGO4;AGO2;BCL2;MITF;DICER1;TNRC6A
 
 
 
 
SARS-CoV-1 Targets Host Intracellular Signalling and Regulatory Pathways
 
 
7/19
 
 
0.0044378
 
 
0.0484389
 
 
0
 
 
0
 
 
4.359662
 
 
23.618922
 
 
SMAD4;UBE2I;YWHAQ;YWHAB;SERPINE1;EP300;YWHAZ
 
 
 
 
Scavenging by Class A Receptors
 
 
7/19
 
 
0.0044378
 
 
0.0484389
 
 
0
 
 
0
 
 
4.359662
 
 
23.618922
 
 
COL1A1;COL3A1;COL1A2;COL4A2;COL4A1;HSP90B1;FTL
 
 
 
 
TICAM1, RIP1-mediated IKK Complex Recruitment
 
 
7/19
 
 
0.0044378
 
 
0.0484389
 
 
0
 
 
0
 
 
4.359662
 
 
23.618922
 
 
CHUK;UBE2D2;TRAF6;UBE2D3;UBE2N;UBE2D1;UBE2V1
 
 
 
 
RAF Activation
 
 
10/34
 
 
0.0047133
 
 
0.0508306
 
 
0
 
 
0
 
 
3.115888
 
 
16.692934
 
 
PPP1CB;PPP2CA;PPP1CC;NRAS;MAP2K1;MRAS;YWHAB;RAF1;MARK3;MAP3K11
 
 
 
 
Signaling by WNT in Cancer
 
 
10/34
 
 
0.0047133
 
 
0.0508306
 
 
0
 
 
0
 
 
3.115888
 
 
16.692934
 
 
AMER1;PPP2CA;TCF7L2;TNKS2;FZD5;APC;CSNK1A1;FZD4;LRP5;DKK2
 
 
 
 
Sema4D in Semaphorin Signaling
 
 
8/24
 
 
0.0048377
 
 
0.0517505
 
 
0
 
 
0
 
 
3.737590
 
 
19.926258
 
 
MYL6;SEMA4D;RRAS;LIMK1;MYH9;RHOC;MET;MYL12B
 
 
 
 
Downstream Signal Transduction
 
 
9/29
 
 
0.0048836
 
 
0.0517505
 
 
0
 
 
0
 
 
3.364495
 
 
17.905441
 
 
PDGFRB;PDGFRA;NRAS;PIK3CA;RASA1;STAT3;GRB2;PIK3R1;SOS1
 
 
 
 
Signaling by Rho GTPases, Miro GTPases and RHOBTB3
 
 
104/688
 
 
0.0048848
 
 
0.0517505
 
 
0
 
 
0
 
 
1.342981
 
 
7.146834
 
 
ITSN2;TFRC;FAM13B;ITSN1;STMN2;FNBP1L;PPP1CB;PPP1CC;GOLGA3;RALBP1;TMOD3;PRKCD;KCTD3;LEMD3;FRS3;ARMCX3;SGO1;TIAM1;MYL6;DAAM1;PKP4;EPHA2;MTMR1;GARRE1;ARHGEF26;CUL3;PIK3R3;PIK3R1;RHOBTB1;MYL12B;NCKIPSD;ARHGAP21;ARHGAP20;SRGAP2;STARD13;CPSF7;JAG1;ARPC5;ARHGAP26;CCT6A;NET1;GRB2;FERMT2;MAP3K11;ITGB1;ZNF512B;DOCK6;ABCD3;DOCK3;SOWAHC;YWHAB;ARHGAP1;OSBPL11;RND2;LRRC1;YWHAQ;MUC13;FLOT2;LBR;SPTAN1;PPP1R12A;RRAS2;RHOC;DYNLL2;YWHAZ;LRRC41;CKAP4;SENP1;TUBB2A;PIK3CA;MYH9;SOS1;MAPRE1;SOS2;RHOQ;ITGB3BP;PAFAH1B1;DYNC1I2;RAP1GDS1;MOSPD2;FAM169A;UACA;TRAK2;CDC42;PPP2CA;GNA13;LMAN1;PARD6B;PGRMC2;MFN1;EMC3;MAPK1;WHAMM;VHL;WASF1;USP9X;LIMK1;SEMA4F;MAPK14;SLC4A7;FMNL2;VAPB;SHKBP1;FILIP1
 
 
 
 
TP53 Regulates Metabolic Genes
 
 
19/86
 
 
0.0049575
 
 
0.0522134
 
 
0
 
 
0
 
 
2.123602
 
 
11.269636
 
 
PRKAB2;PRKAA1;YWHAB;TXNRD1;PTEN;TSC1;YWHAZ;YWHAQ;RRAGC;AGO4;SESN1;AKT3;SESN2;DDIT4;AGO2;CYCS;SLC38A9;TP53;TNRC6A
 
 
 
 
RHOG GTPase Cycle
 
 
17/74
 
 
0.0050459
 
 
0.0528354
 
 
0
 
 
0
 
 
2.232777
 
 
11.809556
 
 
ITGB1;DOCK3;TFRC;ARHGEF26;GARRE1;ITSN1;ARHGAP1;PIK3R1;LEMD3;CDC42;ARHGAP21;LMAN1;PGRMC2;VAPB;LBR;MAP3K11;EPHA2
 
 
 
 
Cargo Recognition for Clathrin-Mediated Endocytosis
 
 
22/105
 
 
0.0051752
 
 
0.0538754
 
 
0
 
 
0
 
 
1.985633
 
 
10.452146
 
 
ITSN2;SCARB2;TFRC;SYT1;FZD4;ITSN1;TGFA;ARRB1;LRP2;AREG;SNAP91;NECAP2;VAMP7;NECAP1;UBQLN1;GRB2;EPS15;LDLR;VAMP2;AGFG1;HBEGF;REPS1
 
 
 
 
Constitutive Signaling by NOTCH1 HD Domain Mutants
 
 
6/15
 
 
0.0052637
 
 
0.0541710
 
 
0
 
 
0
 
 
4.981207
 
 
26.136000
 
 
DLL4;ADAM17;JAG1;NOTCH1;ADAM10;DLL1
 
 
 
 
Signaling by NOTCH1 HD Domain Mutants in Cancer
 
 
6/15
 
 
0.0052637
 
 
0.0541710
 
 
0
 
 
0
 
 
4.981207
 
 
26.136000
 
 
DLL4;ADAM17;JAG1;NOTCH1;ADAM10;DLL1
 
 
 
 
Axon Guidance
 
 
84/541
 
 
0.0053674
 
 
0.0549244
 
 
0
 
 
0
 
 
1.384237
 
 
7.235974
 
 
ITGB1;NRP1;ITSN1;MSI1;LAMC1;ROBO1;ALCAM;DPYSL4;DPYSL5;DPYSL3;RPS6KA1;GRB10;SPTAN1;MAP2K1;SEMA6A;TRPC6;CSNK2A1;ADAM10;ANK2;ANK3;RHOC;ISL1;RGMA;DNM3;SHTN1;TIAM1;MYL6;CACNB3;GAP43;COL2A1;PSMA4;TUBB2A;CREB1;PSMA1;PIK3CA;COL4A2;COL4A1;COL4A4;COL6A2;RASA1;NUMB;DCX;COL4A5;COL6A3;MYH9;SOS1;MET;SOS2;EPHA2;SEMA7A;PSMD12;PIK3R3;PIK3R1;AKAP5;EFNA5;MYL12B;CDC42;APH1A;EFNB2;NRAS;RPS15A;RRAS;FLRT3;RPL14;RPS3;ST8SIA4;MAPK1;SRGAP2;VASP;PRNP;SEMA4D;SIAH2;LIMK1;ARPC5;MAGOHB;COL3A1;COL5A1;PTPRA;COL5A2;COL9A1;GRB2;LHX9;SCN2B;CDK5R1
 
 
 
 
Establishment of Sister Chromatid Cohesion
 
 
5/11
 
 
0.0057474
 
 
0.0575062
 
 
0
 
 
0
 
 
6.224929
 
 
32.114431
 
 
STAG2;ESCO1;PDS5B;PDS5A;WAPL
 
 
 
 
NOTCH4 Activation and Transmission of Signal to the Nucleus
 
 
5/11
 
 
0.0057474
 
 
0.0575062
 
 
0
 
 
0
 
 
6.224929
 
 
32.114431
 
 
APH1A;DLL4;JAG1;ADAM10;YWHAZ
 
 
 
 
STAT3 Nuclear Events Downstream of ALK Signaling
 
 
5/11
 
 
0.0057474
 
 
0.0575062
 
 
0
 
 
0
 
 
6.224929
 
 
32.114431
 
 
DNMT1;HDAC1;STAT3;EP300;PRDM1
 
 
 
 
Signaling by FGFR4 in Disease
 
 
5/11
 
 
0.0057474
 
 
0.0575062
 
 
0
 
 
0
 
 
6.224929
 
 
32.114431
 
 
NRAS;PIK3CA;GRB2;PIK3R1;SOS1
 
 
 
 
Transcriptional Regulation by NPAS4
 
 
10/35
 
 
0.0059119
 
 
0.0588250
 
 
0
 
 
0
 
 
2.991083
 
 
15.346614
 
 
REST;BDNF;AGO4;KCNIP3;PLK2;AGO2;MAPK1;FOS;TNRC6A;CDK5R1
 
 
 
 
Signaling by KIT in Disease
 
 
7/20
 
 
0.0061396
 
 
0.0604233
 
 
0
 
 
0
 
 
4.024075
 
 
20.494584
 
 
NRAS;PIK3CA;STAT3;PIK3R3;GRB2;PIK3R1;SOS1
 
 
 
 
Signaling by Phosphorylated Juxtamembrane, Extracellular and Kinase
Domain KIT Mutants
 
 
7/20
 
 
0.0061396
 
 
0.0604233
 
 
0
 
 
0
 
 
4.024075
 
 
20.494584
 
 
NRAS;PIK3CA;STAT3;PIK3R3;GRB2;PIK3R1;SOS1
 
 
 
 
Signaling by EGFR in Cancer
 
 
8/25
 
 
0.0063883
 
 
0.0622807
 
 
0
 
 
0
 
 
3.517532
 
 
17.775083
 
 
NRAS;PIK3CA;TGFA;GRB2;PIK3R1;SOS1;AREG;HBEGF
 
 
 
 
Nervous System Development
 
 
87/567
 
 
0.0064110
 
 
0.0622807
 
 
0
 
 
0
 
 
1.364945
 
 
6.892613
 
 
ITGB1;NRP1;ITSN1;MSI1;LAMC1;ROBO1;ALCAM;DPYSL4;DPYSL5;DPYSL3;RPS6KA1;GRB10;SPTAN1;MAP2K1;SEMA6A;TRPC6;CSNK2A1;ADAM10;ANK2;ANK3;RHOC;ISL1;RGMA;DNM3;SHTN1;TIAM1;MYL6;CACNB3;GAP43;COL2A1;PSMA4;TUBB2A;CREB1;PSMA1;PIK3CA;COL4A2;COL4A1;COL4A4;COL6A2;RASA1;NUMB;DCX;COL4A5;COL6A3;MYH9;SOS1;MET;SOS2;EPHA2;SEMA7A;PSMD12;LAMA2;PIK3R3;PIK3R1;AKAP5;HMGCR;EFNA5;MYL12B;CDC42;APH1A;EFNB2;NRAS;RPS15A;RRAS;FLRT3;RPL14;RPS3;ST8SIA4;MAPK1;SRGAP2;VASP;PRNP;SEMA4D;SIAH2;LIMK1;ARPC5;MAGOHB;COL3A1;COL5A1;PTPRA;COL5A2;COL9A1;PMP22;GRB2;LHX9;SCN2B;CDK5R1
 
 
 
 
RAC2 GTPase Cycle
 
 
19/88
 
 
0.0064321
 
 
0.0622807
 
 
0
 
 
0
 
 
2.061813
 
 
10.404849
 
 
ITGB1;DOCK3;TFRC;GARRE1;ARHGAP1;PIK3R3;PIK3R1;LEMD3;ARHGAP26;ARMCX3;CDC42;TIAM1;ARHGAP21;LMAN1;PGRMC2;PIK3CA;VAPB;LBR;EPHA2
 
 
 
 
Regulation of mRNA Stability by Proteins That Bind AU-rich Elements
 
 
17/76
 
 
0.0066925
 
 
0.0644553
 
 
0
 
 
0
 
 
2.156845
 
 
10.798829
 
 
PSMD12;ANP32A;YWHAB;HSBP1;PRKCD;MAPK14;YWHAZ;ZFP36L1;PSMA4;PSMA1;XRN1;ENPP2;HNRNPD;TNPO1;EXOSC3;DCP1A;DCP2
 
 
 
 
O-linked Glycosylation
 
 
23/114
 
 
0.0069714
 
 
0.0667845
 
 
0
 
 
0
 
 
1.893338
 
 
9.402203
 
 
GALNT12;B3GALNT2;POMT2;GALNT7;GALNT14;GALNT5;GALNT1;GCNT1;C1GALT1;POMGNT1;ADAMTS10;B4GAT1;B3GNT7;A4GNT;ADAMTS17;MUC13;GCNT3;LARGE1;B4GALT6;ST6GALNAC3;ADAMTS9;ADAMTS7;B4GALT5
 
 
 
 
Signaling by High-Kinase Activity BRAF Mutants
 
 
10/36
 
 
0.0073350
 
 
0.0698962
 
 
0
 
 
0
 
 
2.875878
 
 
14.135210
 
 
RAP1B;CNKSR2;NRAS;MAP2K1;RAP1A;YWHAB;MAPK1;ARRB1;RAF1;MARK3
 
 
 
 
RHOD GTPase Cycle
 
 
13/53
 
 
0.0075574
 
 
0.0708213
 
 
0
 
 
0
 
 
2.431282
 
 
11.877362
 
 
ARHGAP1;MOSPD2;PIK3R1;LEMD3;ARHGAP26;SLC4A7;ARHGAP21;LMAN1;PGRMC2;VAPB;FILIP1;WHAMM;LBR
 
 
 
 
Rap1 Signalling
 
 
6/16
 
 
0.0075894
 
 
0.0708213
 
 
0
 
 
0
 
 
4.482832
 
 
21.880715
 
 
RAP1B;RAP1A;YWHAB;RAF1;YWHAZ;SIPA1
 
 
 
 
SUMOylation of DNA Methylation Proteins
 
 
6/16
 
 
0.0075894
 
 
0.0708213
 
 
0
 
 
0
 
 
4.482832
 
 
21.880715
 
 
DNMT1;UBE2I;CBX4;DNMT3A;DNMT3B;BMI1
 
 
 
 
Transcription of E2F Targets Under Neg Control by P107 (RBL1) and P130
(RBL2) in Complex With HDAC1
 
 
6/16
 
 
0.0075894
 
 
0.0708213
 
 
0
 
 
0
 
 
4.482832
 
 
21.880715
 
 
CCNA2;LIN54;TFDP2;HDAC1;MYBL2;E2F5
 
 
 
 
TNF Signaling
 
 
14/59
 
 
0.0077443
 
 
0.0715766
 
 
0
 
 
0
 
 
2.327709
 
 
11.314518
 
 
CHUK;USP4;UBE2D2;UBE2D3;USP2;TNFAIP3;UBE2D1;TNF;TNFRSF1A;SMPD3;ADAM17;BAG4;OTULIN;MAP3K7
 
 
 
 
Activated NOTCH1 Transmits Signal to the Nucleus
 
 
9/31
 
 
0.0079088
 
 
0.0715766
 
 
0
 
 
0
 
 
3.058285
 
 
14.801422
 
 
APH1A;DLL4;ADAM17;NOTCH1;JAG1;NUMB;ADAM10;ARRB1;DLL1
 
 
 
 
EGFR Downregulation
 
 
9/31
 
 
0.0079088
 
 
0.0715766
 
 
0
 
 
0
 
 
3.058285
 
 
14.801422
 
 
CDC42;TGFA;SPRY2;GRB2;SPRY1;PTPRK;AREG;EPS15;HBEGF
 
 
 
 
RUNX2 Regulates Bone Development
 
 
9/31
 
 
0.0079088
 
 
0.0715766
 
 
0
 
 
0
 
 
3.058285
 
 
14.801422
 
 
HDAC4;RB1;COL1A1;SMAD4;MAF;CBFB;SATB2;HEY2;MAPK1
 
 
 
 
Transcriptional Regulation of Brown and Beige Adipocyte Differentiation
 
 
9/31
 
 
0.0079088
 
 
0.0715766
 
 
0
 
 
0
 
 
3.058285
 
 
14.801422
 
 
NCOA1;SMAD4;HDAC1;PRDM16;ELOVL3;EBF2;PPARG;RBBP7;MTA2
 
 
 
 
Transcriptional Regulation of Brown and Beige Adipocyte Differentiation
by EBF2
 
 
9/31
 
 
0.0079088
 
 
0.0715766
 
 
0
 
 
0
 
 
3.058285
 
 
14.801422
 
 
NCOA1;SMAD4;HDAC1;PRDM16;ELOVL3;EBF2;PPARG;RBBP7;MTA2
 
 
 
 
RHO GTPases Activate PAKs
 
 
7/21
 
 
0.0082839
 
 
0.0745966
 
 
0
 
 
0
 
 
3.736429
 
 
17.910349
 
 
CDC42;PPP1CB;MYL6;PPP1R12A;LIMK1;MYH9;MYL12B
 
 
 
 
 
 
 WikiPathways_2024_Human 
 WikiPathways is a community-curated pathway resource that complements
canonical databases by incorporating emerging biological knowledge and
specialized pathways. Pathways are contributed and maintained by domain
experts and are frequently updated to reflect current research.
Enrichment in WikiPathways highlights involvement of target genes in
less standardized or rapidly evolving biological processes, disease
mechanisms, or tissue-specific pathways. This category is particularly
useful for capturing novel or non-canonical regulatory programs not yet
represented in traditional pathway databases. 
 
 
 
 
Term
 
 
Overlap
 
 
P.value
 
 
Adjusted.P.value
 
 
Old.P.value
 
 
Old.Adjusted.P.value
 
 
Odds.Ratio
 
 
Combined.Score
 
 
Genes
 
 
 
 
 
 
EGFR Tyrosine Kinase Inhibitor Resistance WP4806
 
 
32/84
 
 
0.0000000
 
 
0.0000002
 
 
0
 
 
0
 
 
4.637937
 
 
98.963018
 
 
PTEN;PIK3R3;TGFA;PIK3R1;FOXO3;FGF2;NRAS;BCL2L11;RRAS;ERBB3;AKT3;PDGFC;EIF4EBP1;MAPK1;IL6R;PDGFRB;PDGFRA;MAP2K1;STAT3;RRAS2;IGF1;VEGFA;MRAS;PIK3CA;AXL;BCL2;GRB2;SOS1;RAF1;EIF4E2;MET;SOS2
 
 
 
 
Cancer Pathways WP5434
 
 
108/507
 
 
0.0000000
 
 
0.0000002
 
 
0
 
 
0
 
 
2.067073
 
 
43.917678
 
 
RB1;HHIP;EDNRA;FRAT2;CCND2;AKT3;IL12B;EP300;IL6R;IL13RA1;SKP1;PDGFRB;PDGFRA;MAP2K1;RALBP1;MITF;RUNX1;EML4;CCNE2;COL4A2;COL4A1;COL4A4;COL4A5;IL6ST;RAF1;TP53;NOTCH2;CSF1R;NOTCH1;MAX;PIK3R3;TGFA;LPAR2;PIK3R1;RASGRP4;HSP90B1;BCL2L11;PMAIP1;WNT1;RALGDS;WNT10B;SMAD4;TGFB2;JAG1;FZD5;FZD4;GADD45A;IGF1;GNG12;ESR1;LPAR6;BCL2;CYCS;GRB2;ITGB1;PTEN;SLC2A1;LAMC1;SHH;CASP7;HEY2;NCOA1;CHUK;IL15;WNT9B;FOS;F2;TGFBR1;DDB2;CCNA2;TRAF4;PIK3CA;IFNG;TRAF6;TRAF5;RARA;RARB;PPARG;SOS1;SOS2;MET;LAMA2;HDAC1;LAMA4;PTGER3;LEF1;LRP5;ADCY3;ADCY2;DLL1;GNA13;DLL4;NRAS;GNG2;MAPK1;E2F3;BAK1;VHL;TCF7L2;TXNRD1;STAT3;VEGFC;VEGFA;KITLG;APC;GSTA4;CCDC6;FAS
 
 
 
 
Photodynamic Therapy Induced AP 1 Survival Signaling WP3611
 
 
23/50
 
 
0.0000000
 
 
0.0000005
 
 
0
 
 
0
 
 
6.404529
 
 
128.696231
 
 
RB1;PDGFRA;MAP2K4;FOS;MAPK14;TNF;MAPK12;TNFRSF1A;CCNA2;BCL2L11;IFNG;TRAF6;TRAF5;BCL3;BCL2;FAS;BMF;BAK1;TP53;MAP3K5;HBEGF;MAP2K6;MCL1
 
 
 
 
Epithelial To Mesenchymal Transition In Colorectal Cancer WP4239
 
 
46/160
 
 
0.0000000
 
 
0.0000010
 
 
0
 
 
0
 
 
3.048675
 
 
58.000000
 
 
NOTCH2;SPARC;NOTCH1;EIF5A2;LRP5;PIK3R3;PIK3R1;CLDN1;DLL1;DLL4;AKT3;MAPK1;WNT1;MAP2K6;MAP2K4;WNT10B;MAP2K1;SMAD4;TGFB2;JAG1;FZD5;FZD4;WNT9B;PROX1;MAPK14;TGFBR1;MAPK12;TJP1;FMNL2;COL4A2;PIK3CA;COL4A1;MMP15;CLDN14;COL4A4;TRAF6;ID1;CLDN12;SNAI1;COL4A5;GRB2;RAF1;SOS1;TP53;SOS2;PALS1
 
 
 
 
Insulin Signaling WP481
 
 
45/160
 
 
0.0000000
 
 
0.0000025
 
 
0
 
 
0
 
 
2.955023
 
 
52.909352
 
 
SNAP25;PRKAA1;PTEN;SLC2A1;PIK3R3;CBLB;PIK3R1;FOXO3;PIK3C2A;CYTH3;RPS6KA1;GRB10;EIF4EBP1;ENPP1;FLOT2;MAPK1;STX4;MAP3K9;MAP3K7;MAP3K4;MAP3K5;MAP2K6;MAP4K4;MAP2K4;MAP2K1;RAB4A;PRKCD;TSC1;FOS;MAPK14;MAPK12;EHD2;PIK3CA;MAP3K10;GRB2;SGK3;RAF1;SOS1;SGK1;SOS2;MAP3K11;RHOQ;VAMP2;MAP3K12;ARF6
 
 
 
 
PI3K Akt Signaling WP4172
 
 
74/338
 
 
0.0000001
 
 
0.0000119
 
 
0
 
 
0
 
 
2.125336
 
 
34.382805
 
 
ITGB1;PTEN;LAMC1;FGF2;CCND2;CREB3L1;MYB;AKT3;IL6R;PDGFRB;PDGFRA;MAP2K1;CHUK;TSC1;COL2A1;CREB1;CCNE2;COL4A2;PIK3CA;COL4A1;COL4A4;COL6A2;DDIT4;COL4A5;COL6A3;SGK3;SGK1;RAF1;EIF4E2;SOS1;TP53;SOS2;MET;EPHA2;CREB5;CSF1R;PRKAA1;LAMA2;LAMA4;PIK3R3;TGFA;LPAR2;PIK3R1;FOXO3;EFNA5;HSP90B1;PPP2CA;NRAS;BCL2L11;GNG2;PDGFC;NTF3;EIF4EBP1;MAPK1;MCL1;NTRK2;BDNF;OSM;VEGFC;IGF1;PPP2R3A;GNG12;VEGFA;COL1A1;KITLG;G6PC2;COL1A2;G6PC3;LPAR6;ITGA11;COL9A1;BCL2;GRB2;TEK
 
 
 
 
Pleural Mesothelioma WP5087
 
 
89/437
 
 
0.0000002
 
 
0.0000180
 
 
0
 
 
0
 
 
1.942487
 
 
30.318651
 
 
ITGB1;SPARC;YWHAB;PTEN;SLC2A1;LAMC1;BMI1;AREG;FGF2;CDH7;AMOT;CDH5;FRAT2;CCND2;RB1CC1;AKT3;RPS6KA1;MAP3K9;CCN2;MAP3K4;MAP3K5;PDGFRB;MAP2K4;PDGFRA;PRKAB2;MAP2K1;CSNK2A1;TSC1;DKK2;ACTA2;SFRP1;CREB1;PIK3CA;COL4A2;COL4A1;COL4A4;CDH11;COL4A5;ROR1;SOS1;RAF1;MET;TP53;HBEGF;EPHA2;CSF1R;PRKAA1;LAMA2;MAX;LAMA4;LEF1;LRP5;TGFA;EFNA5;CDH20;PDGFC;NTF3;EIF4EBP1;CCL2;STK38L;MAPK1;BAK1;WNT1;MCL1;MAP2K6;MAP4K4;WNT10B;NTRK2;TCF7L2;FZD5;SETDB1;CSNK1A1;BDNF;VEGFC;IGF1;MAPK14;VEGFA;FOSL1;MOB1A;KITLG;LIN28B;APC;BCL2;MAP3K10;CYCS;MDM4;GRB2;TEK;MAP3K11
 
 
 
 
Small Cell Lung Cancer WP4658
 
 
30/95
 
 
0.0000003
 
 
0.0000243
 
 
0
 
 
0
 
 
3.472904
 
 
52.696505
 
 
ITGB1;RB1;CDKN1C;LAMA2;MAX;LAMA4;PTEN;PIK3R3;LAMC1;PIK3R1;AKT3;E2F3;BAK1;SKP1;CHUK;GADD45A;DDB2;TRAF4;CCNE2;PIK3CA;COL4A2;COL4A1;COL4A4;TRAF6;TRAF5;BCL2;RARB;COL4A5;CYCS;TP53
 
 
 
 
MAPK Signaling WP382
 
 
57/246
 
 
0.0000004
 
 
0.0000331
 
 
0
 
 
0
 
 
2.279676
 
 
33.617600
 
 
ARRB1;FGF2;TNF;DUSP16;STMN1;AKT3;MAP3K7;MAP3K4;MAP3K5;PDGFRB;MAP2K4;MAP2K1;CHUK;DUSP1;PRKCD;CACNA2D3;RRAS2;FOS;DUSP9;TGFBR1;CDC25B;TNFRSF1A;CACNB3;MRAS;RASA1;TRAF6;RAPGEF2;MAPT;RAF1;SOS1;TP53;SOS2;MAX;CACNA1A;RASGRP4;RAP1B;CDC42;NRAS;RAP1A;RRAS;NTF3;MAPK1;CACNG2;MAP4K4;MAP2K6;NTRK2;TGFB2;GADD45A;BDNF;GNG12;MAPK14;MAPK12;FAS;GRB2;HSPA1B;MAP3K11;MAP3K12
 
 
 
 
Focal Adhesion PI3K Akt mTOR Signaling WP3932
 
 
66/303
 
 
0.0000006
 
 
0.0000420
 
 
0
 
 
0
 
 
2.107444
 
 
30.356261
 
 
FOXA1;ITGB1;PTEN;SLC2A1;SLC2A3;HIF3A;LAMC1;PIK3C2A;FGF2;CREB3L1;AKT3;IL6R;PDGFRB;SREBF1;PDGFRA;MAP2K1;TSC1;TBC1D1;COL2A1;CREB1;COL4A2;PIK3CA;COL4A1;COL4A4;COL6A2;DDIT4;RAF1;EIF4E2;SOS1;MET;EPHA2;CREB5;CSF1R;PRKAA1;LAMA2;COL11A1;LAMA4;LPAR2;PIK3R1;EFNA5;FOXO3;HSP90B1;PPP2CA;NRAS;GNG2;PDGFC;EIF4EBP1;MAPK1;OSM;VEGFC;IGF1;PPP2R3A;GNG12;VEGFA;COL1A1;RAB10;COL3A1;KITLG;COL1A2;COL5A1;RAB14;LPAR6;COL5A2;ITGA11;GRB2;TEK
 
 
 
 
Thyroid Stimulating Hormone TSH Signaling WP2032
 
 
21/58
 
 
0.0000013
 
 
0.0000738
 
 
0
 
 
0
 
 
4.261115
 
 
57.699137
 
 
RB1;MAP2K1;KCNIP3;STAT3;ADCY3;ADCY2;PIK3R1;MAPK14;MYL12B;GNA13;RAP1B;GNAO1;CREB1;GNG2;RAP1A;PIK3CA;RPS6KA1;MAPK1;RAF1;RALGDS;MAP2K6
 
 
 
 
Glioblastoma Signaling WP2261
 
 
26/82
 
 
0.0000015
 
 
0.0000738
 
 
0
 
 
0
 
 
3.489388
 
 
46.896919
 
 
RB1;ERRFI1;PTEN;PIK3R1;PIK3C2A;FOXO3;NRAS;CCND2;ERBB3;AKT3;EP300;MAPK1;MAP2K6;PDGFRB;PDGFRA;MAP2K4;MAP2K1;PRKCD;TSC1;PIK3CA;SPRY2;GRB2;MDM4;RAF1;MET;TP53
 
 
 
 
Mesodermal Commitment Pathway WP2857
 
 
38/145
 
 
0.0000015
 
 
0.0000738
 
 
0
 
 
0
 
 
2.675074
 
 
35.931666
 
 
FOXA1;TRIM71;CRTC1;LEF1;GATA6;C9ORF72;DLL1;MBTD1;PPP2CA;CSRP2;SESN1;DNMT3B;PITX2;JARID2;EOMES;ACVR1;ZNF462;SMAD4;FZD5;FZD4;ZNF281;NOG;TET1;KLF4;ACVR2A;ARL4A;MEIS1;KLF5;DDAH1;BHLHE40;MTF2;SNAI1;CCDC6;RARB;TCF4;BCORL1;AHDC1;CUL4B
 
 
 
 
Endometrial Cancer WP4155
 
 
22/63
 
 
0.0000015
 
 
0.0000738
 
 
0
 
 
0
 
 
4.029314
 
 
54.019167
 
 
TCF7L2;MAP2K1;GADD45A;LEF1;PTEN;PIK3R3;FOS;PIK3R1;FOXO3;FGF2;DDB2;NRAS;PIK3CA;APC;AKT3;MAPK1;GRB2;BAK1;RAF1;SOS1;TP53;SOS2
 
 
 
 
Hepatitis B Infection WP4666
 
 
39/151
 
 
0.0000016
 
 
0.0000738
 
 
0
 
 
0
 
 
2.623284
 
 
35.020909
 
 
DDX3X;YWHAB;PIK3R3;PIK3R1;TNF;NRAS;IRAK1;YWHAQ;CREB3L1;AKT3;EP300;MAPK1;MAP3K7;MAP2K6;MAP2K4;MAP2K1;SMAD4;TGFB2;EGR3;CHUK;STAT3;FOS;MAPK14;YWHAZ;TGFBR1;MAPK12;DDB2;MAVS;CREB1;PIK3CA;TRAF6;BCL2;CYCS;FAS;GRB2;RAF1;SOS1;SOS2;CREB5
 
 
 
 
Endoderm Differentiation WP2853
 
 
37/140
 
 
0.0000016
 
 
0.0000738
 
 
0
 
 
0
 
 
2.705285
 
 
36.083660
 
 
FOXA1;TRIM71;NOTCH1;CRTC1;ONECUT1;LEF1;GATA6;LAMC1;CDC73;PTHLH;MBTD1;CAND1;BTAF1;SESN1;TBX21;DNMT3B;HOXA1;OTX2;JARID2;EOMES;ZNF462;SMAD4;SFMBT1;ZNF281;SIAH2;NOG;TET1;SFRP1;DDAH1;APC;PAX9;MTF2;TCF4;TAF5;BCORL1;AHDC1;CUL4B
 
 
 
 
Melanoma WP4685
 
 
23/68
 
 
0.0000017
 
 
0.0000738
 
 
0
 
 
0
 
 
3.838789
 
 
51.094062
 
 
RB1;MAP2K1;GADD45A;PTEN;PIK3R3;MITF;FOS;PIK3R1;DDB2;GRM3;NRAS;CREB1;PIK3CA;AKT3;MAPK1;E2F3;CALM3;GRB2;BAK1;RAF1;SOS1;TP53;SOS2
 
 
 
 
VEGFA VEGFR2 Signaling WP3888
 
 
84/430
 
 
0.0000022
 
 
0.0000917
 
 
0
 
 
0
 
 
1.840127
 
 
23.988326
 
 
ITGB1;ARF4;NRARP;AMOT;CDH5;CSRP2;TMSB4X;GRB10;UBAP2L;CCN2;ATP6V1E1;CCN1;TMSB10;ADAMTS9;MAP3K5;TMOD1;SLC2A14;MAP2K4;MAP2K1;TMOD3;PRKCD;ADAM10;RHOC;F3;TMEM170A;RCN1;CREB1;PIK3CA;BIN1;FSCN1;NUMB;MYH9;ERG;ALDOA;RAF1;ARF6;HBEGF;EPHA2;HDAC4;KANK1;PRKAA1;HDAC1;PBXIP1;PIK3R1;FOXO3;SLC7A1;PPM1G;RAP1B;CDC42;PPP2CA;DLL4;LMAN1;LDHA;C15ORF39;RAP1A;DNAJB4;S1PR1;CCL2;MAPK1;WASF1;EPS15;MAP2K6;RAB4A;JAG1;EGR3;SIAH2;STAT3;LIMK1;NAP1L1;MAPK14;QKI;MAPK12;VEGFA;NR4A2;RCAN1;P4HA2;PRKRA;RCAN2;BCL2;CALU;FAS;CYCS;GRB2;EIF4G2
 
 
 
 
Brain Derived Neurotrophic Factor BDNF Signaling WP2380
 
 
36/137
 
 
0.0000026
 
 
0.0001018
 
 
0
 
 
0
 
 
2.683445
 
 
34.547975
 
 
PRKAA1;DOCK3;CRTC1;PIK3R1;FOXO3;CDC42;PPP2CA;GRIP1;RAP1A;CNR1;RPS6KA1;NTF3;EIF4EBP1;MAPK1;MEF2A;NTRK2;MAP2K1;CSNK2A1;CHUK;BDNF;PRKCD;STAT3;MAPK14;FRS3;EIF2S1;TIAM1;ADAM17;MARCKS;CREB1;APC;TRAF6;CAMK4;GRB2;MAPT;RAF1;CDK5R1
 
 
 
 
Breast Cancer Pathway WP4262
 
 
39/154
 
 
0.0000027
 
 
0.0001018
 
 
0
 
 
0
 
 
2.554413
 
 
32.762806
 
 
RB1;NOTCH2;NOTCH1;LEF1;PTEN;LRP5;PIK3R3;PIK3R1;FGF2;DLL1;DLL4;NRAS;FRAT2;AKT3;HEY2;MAPK1;E2F3;BAK1;WNT1;SKP1;NCOA1;WNT10B;TCF7L2;MAP2K1;CSNK2A1;FZD5;GADD45A;CSNK1A1;IGF1;FOS;ESR1;DDB2;PIK3CA;APC;GRB2;RAF1;SOS1;TP53;SOS2
 
 
 
 
Ectoderm Differentiation WP2858
 
 
36/138
 
 
0.0000031
 
 
0.0001109
 
 
0
 
 
0
 
 
2.656985
 
 
33.721222
 
 
STX16;CTNND2;GATA6;HMGB2;STC1;TNFRSF11B;ZBTB2;PHF8;SOCS2;MECP2;SHH;ST8SIA4;CCL2;WNT1;PGM1;CDON;RAB8B;CLVS1;TFAP2C;SMAD4;KCNK10;FZD5;ELOVL4;FZD4;ELOVL2;C1GALT1;NR2F2;RGMA;PAN2;PLCXD3;RIT1;MAFB;PI4KA;SPRY2;BCOR;PDE7A
 
 
 
 
Non Small Cell Lung Cancer WP4255
 
 
23/72
 
 
0.0000050
 
 
0.0001729
 
 
0
 
 
0
 
 
3.524617
 
 
43.004093
 
 
RB1;MAP2K1;GADD45A;STAT3;PIK3R3;TGFA;PIK3R1;FOXO3;DDB2;EML4;NRAS;PIK3CA;AKT3;RARB;MAPK1;CYCS;E2F3;GRB2;BAK1;RAF1;SOS1;TP53;SOS2
 
 
 
 
Angiopoietin Like Protein 8 Regulatory Pathway WP3915
 
 
34/132
 
 
0.0000077
 
 
0.0002539
 
 
0
 
 
0
 
 
2.610152
 
 
30.727778
 
 
PRKAA1;SLC2A1;PIK3R3;LPL;CBLB;PIK3R1;FOXO3;PIK3C2A;RPS6KA1;EIF4EBP1;FLOT2;MAPK1;MAP3K9;MAP3K7;MAP3K4;MAP3K5;MAP2K6;MAP4K4;MAP2K4;SREBF1;PRKAB2;MAP2K1;TSC1;MAPK14;MAPK12;PIK3CA;SCD;MAP3K10;RAF1;SOS1;SOS2;MAP3K11;RHOQ;MAP3K12
 
 
 
 
4 Hydroxytamoxifen DEX And Retinoic Acids Regulation Of P27 WP3879
 
 
12/25
 
 
0.0000086
 
 
0.0002700
 
 
0
 
 
0
 
 
6.913073
 
 
80.665508
 
 
PRKAA1;MAP2K1;PIK3CA;EIF4EBP1;PIK3R3;MAPK1;TSC1;PIK3R1;RAF1;MAP3K11;MAP2K6;MAP3K5
 
 
 
 
MAPK Cascade WP422
 
 
14/33
 
 
0.0000092
 
 
0.0002781
 
 
0
 
 
0
 
 
5.521144
 
 
64.035037
 
 
MAP2K4;MAP2K1;MAPK14;MAPK12;NRAS;RRAS;MAPK1;GRB2;RAF1;SOS1;SOS2;SIPA1;MAP2K6;MAP3K12
 
 
 
 
miRNA Targets In ECM And Membrane Receptors WP2911
 
 
11/22
 
 
0.0000125
 
 
0.0003640
 
 
0
 
 
0
 
 
7.486831
 
 
84.522652
 
 
COL3A1;COL1A2;COL4A2;COL5A1;COL4A1;LAMA4;COL6A2;COL5A2;ITGA11;COL6A3;LAMC1
 
 
 
 
Hepatocyte Growth Factor Receptor Signaling WP313
 
 
14/34
 
 
0.0000139
 
 
0.0003902
 
 
0
 
 
0
 
 
5.244790
 
 
58.649628
 
 
ITGB1;MAP2K1;STAT3;PTEN;FOS;RAP1B;RAP1A;PIK3CA;RASA1;MAPK1;GRB2;RAF1;SOS1;MET
 
 
 
 
DNA Damage Response Only ATM Dependent WP710
 
 
29/109
 
 
0.0000181
 
 
0.0004890
 
 
0
 
 
0
 
 
2.724181
 
 
29.748984
 
 
LEF1;PTEN;PIK3R3;PIK3R1;FOXO3;PIK3C2A;CDC42;NRAS;CCND2;BCL2L11;AKT3;PMAIP1;MAPK1;BAK1;WNT1;MAP3K7;LDLR;MAP3K4;WNT10B;TCF7L2;SMAD4;FOSL1;PIK3CA;APC;BCL2;GRB2;SOS1;TP53;SOS2
 
 
 
 
Pancreatic Adenocarcinoma Pathway WP4263
 
 
25/89
 
 
0.0000247
 
 
0.0006460
 
 
0
 
 
0
 
 
2.933193
 
 
31.111898
 
 
RB1;PIK3R3;TGFA;PIK3R1;CDC42;AKT3;MAPK1;E2F3;BAK1;RALGDS;MAP2K1;RALBP1;SMAD4;TGFB2;CHUK;GADD45A;PRKCD;STAT3;TGFBR1;DDB2;VEGFA;TIAM1;PIK3CA;RAF1;TP53
 
 
 
 
Focal Adhesion WP306
 
 
44/199
 
 
0.0000275
 
 
0.0006951
 
 
0
 
 
0
 
 
2.137899
 
 
22.447281
 
 
ITGB1;LAMA2;LAMA4;PTEN;TESK2;PIK3R3;PIK3R1;LAMC1;MYL12B;PPP1CB;RAP1B;CDC42;PPP1CC;CCND2;RAP1A;AKT3;PDGFC;MAPK1;VASP;PDGFRB;PDGFRA;MAP2K1;PPP1R12A;CAV3;CAV2;STYK1;VEGFC;IGF1;VEGFA;COL1A1;COL1A2;COL2A1;COL4A2;PIK3CA;COL4A1;COL4A4;COL6A2;COL5A2;ITGA11;BCL2;GRB2;RAF1;SOS1;MET
 
 
 
 
Host Pathogen Interaction Of Human Coronaviruses MAPK Signaling WP4877
 
 
14/36
 
 
0.0000302
 
 
0.0007366
 
 
0
 
 
0
 
 
4.767449
 
 
49.623344
 
 
MAP2K4;MAP2K1;FOS;MAPK14;MAPK12;RPS6KA1;BCL2;MAPK1;MAP3K10;MAP3K9;RAF1;MAP3K4;MAP3K11;MAP2K6
 
 
 
 
Hematopoietic SC Gene Regulation By GABP Alpha Beta Complex WP3657
 
 
10/20
 
 
0.0000315
 
 
0.0007441
 
 
0
 
 
0
 
 
7.484076
 
 
77.587489
 
 
DNMT1;SMAD4;DNMT3A;PTEN;DNMT3B;BCL2;EP300;FOXO3;ETV6;MCL1
 
 
 
 
Nanoparticle Mediated Activation Of Receptor Signaling WP2643
 
 
12/28
 
 
0.0000356
 
 
0.0008165
 
 
0
 
 
0
 
 
5.615916
 
 
57.525649
 
 
ITGB1;COL1A1;NRAS;MAP2K1;AKT3;MAPK1;GRB2;MAPK14;RAF1;SOS1;AREG;MAPK12
 
 
 
 
P53 Transcriptional Gene Network WP4963
 
 
25/91
 
 
0.0000373
 
 
0.0008311
 
 
0
 
 
0
 
 
2.843985
 
 
28.996614
 
 
BTG2;PRKAA1;NOTCH1;SERPINE1;PTEN;SLC2A1;SLC7A11;TNF;ZMAT3;SESN1;SESN2;TP53INP1;CCL2;PMAIP1;E2F7;PRKAB2;GADD45A;LIF;DDB2;TRAF4;ADORA2B;DDIT4;CCNG1;FAS;IRF5
 
 
 
 
Envelope Proteins And Potential Roles In EDMD Physiopathology WP4535
 
 
16/46
 
 
0.0000421
 
 
0.0009109
 
 
0
 
 
0
 
 
3.997162
 
 
40.271742
 
 
SMAD4;MAP2K1;TGFB2;TAFAZZIN;ADCY3;ADCY2;LEMD3;NRAS;TMEM43;MAPK1;GRB2;CCN2;MAP3K9;SOS1;LBR;SOS2
 
 
 
 
BDNF TrkB Signaling WP3676
 
 
13/33
 
 
0.0000494
 
 
0.0010380
 
 
0
 
 
0
 
 
4.868091
 
 
48.273341
 
 
NTRK2;MAP2K1;TRPC6;HOMER1;BDNF;TSC1;NRAS;CREB1;RPS6KA1;EIF4EBP1;MAPK1;GRB2;SOS1
 
 
 
 
Gastrin Signaling WP4659
 
 
29/115
 
 
0.0000532
 
 
0.0010888
 
 
0
 
 
0
 
 
2.533256
 
 
24.930134
 
 
ITGB1;SERPINE1;PIK3R3;ARRB1;PIK3R1;FOXO3;CLDN1;CDC42;KAT5;EIF4EBP1;MAPK1;MAP2K1;JAG1;CHUK;PRKCD;STAT3;FOS;MAPK14;KLF4;VEGFA;TJP1;CREB1;PIK3CA;GRB2;TCF4;PPARG;RAF1;SOS1;MAP3K11
 
 
 
 
IL6 Signaling WP364
 
 
15/43
 
 
0.0000700
 
 
0.0013884
 
 
0
 
 
0
 
 
4.013754
 
 
38.397938
 
 
NCOA1;MAP2K4;MAP2K1;HDAC1;STAT3;PRKCD;PIK3R1;PRDM1;NR2F6;MAPK1;GRB2;IL6ST;SOS1;MAP3K7;IL6R
 
 
 
 
Chromosomal And Microsatellite Instability In Colorectal Cancer WP4216
 
 
21/73
 
 
0.0000736
 
 
0.0013884
 
 
0
 
 
0
 
 
3.029363
 
 
28.828528
 
 
TCF7L2;MAP2K1;SMAD4;TGFB2;GADD45A;CSNK1A1;LEF1;FOS;TGFBR1;DDB2;BCL2L11;APC;AKT3;BCL2;MAPK1;PMAIP1;CYCS;BAK1;RAF1;TP53;RALGDS
 
 
 
 
MECP2 And Associated Rett Syndrome WP3584
 
 
21/73
 
 
0.0000736
 
 
0.0013884
 
 
0
 
 
0
 
 
3.029363
 
 
28.828528
 
 
PRPF38A;ARHGEF26;GRID1;HDAC1;BDNF;PTEN;TET2;TET1;CTCF;NREP;IGF1;FGF2;MYT1;MECP2;FUT8;REST;CREB1;HNRNPF;TET3;SGK1;CDON
 
 
 
 
Microtubule Cytoskeleton Regulation WP2038
 
 
16/48
 
 
0.0000760
 
 
0.0013884
 
 
0
 
 
0
 
 
3.746914
 
 
35.536898
 
 
NTRK2;STAT3;LIMK1;PTEN;TESK2;CDC42;TIAM1;SPRED1;APC;PTPRA;CFL2;CAMK4;STMN1;MAPT;MAPRE1;MARK1
 
 
 
 
Acute Myeloid Leukemia WP5293
 
 
20/68
 
 
0.0000770
 
 
0.0013884
 
 
0
 
 
0
 
 
3.124911
 
 
29.596897
 
 
CSF1R;TCF7L2;MAP2K1;CHUK;LEF1;STAT3;PIK3R3;PIK3R1;RUNX1;CCNA2;NRAS;PIK3CA;AKT3;RARA;EIF4EBP1;MAPK1;GRB2;RAF1;SOS1;SOS2
 
 
 
 
Notch Signaling WP61
 
 
17/54
 
 
0.0001031
 
 
0.0018155
 
 
0
 
 
0
 
 
3.443598
 
 
31.610641
 
 
NOTCH2;NUMBL;NOTCH1;JAG1;FBXW7;HDAC1;STAT3;PIK3R1;DLL1;NCOR2;APH1A;DLL4;ADAM17;HEY2;NUMB;EP300;SKP1
 
 
 
 
Adipogenesis WP236
 
 
31/131
 
 
0.0001090
 
 
0.0018752
 
 
0
 
 
0
 
 
2.328985
 
 
21.250183
 
 
RB1;SERPINE1;LPL;RORA;AHR;TNF;NDN;WNT1;MEF2A;NCOA1;WNT10B;SREBF1;GADD45A;STAT3;LIF;OSM;IGF1;KLF15;NCOR2;CYP26A1;BMP3;KLF7;KLF6;CREB1;KLF5;SCD;RARA;ID3;FAS;PPARG;IL6ST
 
 
 
 
PDGF Pathway WP2526
 
 
14/40
 
 
0.0001166
 
 
0.0019618
 
 
0
 
 
0
 
 
4.033079
 
 
36.525934
 
 
PDGFRB;MAP2K4;MAP2K1;CHUK;STAT3;PIK3R1;FOS;CDC42;TIAM1;RASA1;MAPK1;GRB2;RAF1;SOS1
 
 
 
 
Effect Of Omega 3 PUFA On Huntington 39 S Disease Pathways WP5470
 
 
29/120
 
 
0.0001208
 
 
0.0019879
 
 
0
 
 
0
 
 
2.393384
 
 
21.591705
 
 
GABRB2;PRKAA1;HDAC1;PTEN;PIK3R3;PIK3R1;HMGCR;PIK3C2A;NEUROD1;CASP7;BCL2L11;AKT3;EIF4EBP1;MAPK1;MAP2K4;GABRP;NTRK2;PRKAB2;MAP2K1;HMGCS1;BDNF;GABRA3;TSC1;REST;CREB1;PIK3CA;BCL2;MAP3K10;RAF1
 
 
 
 
TGF Beta Signaling Pathway WP366
 
 
31/132
 
 
0.0001266
 
 
0.0020383
 
 
0
 
 
0
 
 
2.305794
 
 
20.694180
 
 
ITGB1;HDAC1;PIK3R1;CDC42;EP300;SNIP1;MAPK1;E2F5;MAP3K7;SKP1;MAP2K6;MEF2A;MAP2K4;KLF11;MAP2K1;SMAD4;UBE2I;FOS;MAPK14;TGFBR1;KLF6;COL1A2;TRAF6;FOSB;SIK1;GRB2;RAF1;DCP1A;SOS1;TP53;MET
 
 
 
 
Circadian Rhythm Genes WP3594
 
 
42/201
 
 
0.0001594
 
 
0.0025145
 
 
0
 
 
0
 
 
1.987216
 
 
17.375917
 
 
SUV39H2;PRKAA1;CRTC1;HDAC1;SUV39H1;CIPC;SERPINE1;BHLHE41;PTEN;RORA;AHR;RORB;TNFRSF11A;PPP1CB;PPP1CC;SIX3;PROK2;EP300;SKP1;SREBF1;EGR3;HOMER1;KCNH7;USP2;GFPT1;PROX1;SIRT1;NR2F6;PER1;CREB1;PSPC1;BHLHE40;HNRNPD;ID4;ID3;FAS;SIK1;PPARG;TOP1;TP53;ADA;LGR4
 
 
 
 
Angiotensin II Receptor Type 1 Pathway WP5036
 
 
11/28
 
 
0.0001935
 
 
0.0029890
 
 
0
 
 
0
 
 
4.842771
 
 
41.407407
 
 
COL1A1;ACTA2;SMAD4;COL1A2;RRAS;MAPK1;CCN2;IL6ST;RAF1;TGFBR1;MAP2K6
 
 
 
 
Apoptosis WP254
 
 
22/84
 
 
0.0002280
 
 
0.0034514
 
 
0
 
 
0
 
 
2.661366
 
 
22.319053
 
 
HRK;MAP2K4;CHUK;PIK3R1;IGF1;TNFRSF1B;TNF;TNFRSF1A;CASP7;BCL2L11;IRF4;IRF2;BCL2;CASP2;PMAIP1;CYCS;FAS;IRF5;BAK1;TP53;BCL2L2;MCL1
 
 
 
 
Relevant Molecular Pathways And Targeted Agents In TNBC WP5215
 
 
13/38
 
 
0.0002639
 
 
0.0039177
 
 
0
 
 
0
 
 
3.893367
 
 
32.080546
 
 
MAP2K1;PTEN;TSC1;PIK3R1;NRAS;PIK3CA;AKT3;EIF4EBP1;MAPK1;GRB2;RAF1;SOS1;SOS2
 
 
 
 
PDGFR Beta Pathway WP3972
 
 
11/29
 
 
0.0002786
 
 
0.0039633
 
 
0
 
 
0
 
 
4.573468
 
 
37.437381
 
 
PDGFRB;MAP2K4;MAP2K1;PIK3CA;RASA1;STAT3;GRB2;PIK3R1;FOS;RAF1;SOS1
 
 
 
 
NRP1 Triggered Signaling In Pancreatic Cancer WP5144
 
 
16/53
 
 
0.0002814
 
 
0.0039633
 
 
0
 
 
0
 
 
3.239653
 
 
26.486030
 
 
NRP1;MAP2K1;SMAD4;TGFB2;CHD1;TGFBR1;VEGFA;COL1A1;CDH5;COL1A2;CCNE2;AKT3;SNAI1;MAPK1;CCN2;MET
 
 
 
 
Macrophage Stimulating Protein MSP Signaling WP5353
 
 
26/108
 
 
0.0002827
 
 
0.0039633
 
 
0
 
 
0
 
 
2.379472
 
 
19.442830
 
 
ITGB1;PTEN;SLC2A1;SIX1;PIK3R1;CLDN1;TNF;HK2;LDHA;RPS6KA1;CCL2;MAPK1;PDGFRB;PDGFRA;WNT10B;MAP2K1;DUSP1;FOS;MAPK14;VEGFA;ACTA2;TJP1;COL4A1;GRB2;RAF1;TP53
 
 
 
 
Sterol Regulatory Element Binding Proteins SREBP Signaling WP1982
 
 
19/69
 
 
0.0002983
 
 
0.0041064
 
 
0
 
 
0
 
 
2.848380
 
 
23.120999
 
 
SCARB1;SREBF1;PRKAB2;PRKAA1;HMGCS1;SAR1A;NFYA;INSIG2;INSIG1;LPL;HMGCR;SIRT1;CREB1;PIK3CA;SCD;PPARG;SEC23B;LDLR;KPNB1
 
 
 
 
Sphingolipid Pathway WP1422
 
 
11/30
 
 
0.0003932
 
 
0.0053150
 
 
0
 
 
0
 
 
4.332514
 
 
33.972245
 
 
PPP2CA;UGCG;CERS4;ASAH1;SGPL1;CERS5;CERS6;SGMS1;SPTLC2;SGMS2;B4GALT6
 
 
 
 
EGF EGFR Signaling WP437
 
 
34/159
 
 
0.0004127
 
 
0.0054809
 
 
0
 
 
0
 
 
2.043209
 
 
15.922307
 
 
ERRFI1;PTEN;CBLB;PIK3R1;CDC42;ATXN2;RAP1A;STMN1;RPS6KA1;GRB10;EIF4EBP1;MAPK1;RALGDS;EPS15;MAP3K4;MEF2A;MAP2K1;RALBP1;CAV2;PRKCD;STAT3;FOS;MAPK14;CREB1;RASA1;NEDD4;PXDN;SPRY2;FOSB;GRB2;RAF1;SOS1;SOS2;ARF6
 
 
 
 
P38 MAPK Signaling WP400
 
 
12/35
 
 
0.0004420
 
 
0.0056165
 
 
0
 
 
0
 
 
3.905172
 
 
30.164064
 
 
CDC42;MAP2K4;TGFB2;CREB1;MAX;GRB2;MAP3K9;MAPK14;MAP3K7;TGFBR1;MAP2K6;MAP3K5
 
 
 
 
Leptin Signaling WP2034
 
 
19/71
 
 
0.0004435
 
 
0.0056165
 
 
0
 
 
0
 
 
2.738516
 
 
21.143760
 
 
NCOA1;MAP2K1;PRKAA1;CHUK;STAT3;PTEN;PIK3R1;MAPK14;ESR1;CDC42;SOCS2;CREB1;CFL2;RPS6KA1;EIF4EBP1;MAPK1;GRB2;RAF1;SOS1
 
 
 
 
Overlap Between Signal Transduction Pathways LMNA Laminopathies WP4879
 
 
16/55
 
 
0.0004482
 
 
0.0056165
 
 
0
 
 
0
 
 
3.073168
 
 
23.694679
 
 
RB1;SREBF1;WNT10B;TCF7L2;TGFB2;NOTCH1;HDAC1;CSNK1A1;LEF1;TNFRSF11B;NAP1L1;DICER1;LEMD3;APC;AGO2;PPARG
 
 
 
 
Sphingolipid Metabolism Integrated Pathway WP4726
 
 
10/26
 
 
0.0004627
 
 
0.0056165
 
 
0
 
 
0
 
 
4.675955
 
 
35.904356
 
 
UGCG;CERS4;ASAH1;SGPL1;CERS5;CERS6;SGMS1;SPTLC2;SGMS2;SGPP1
 
 
 
 
Synaptic Signaling Associated With Autism Spectrum Disorder WP4539
 
 
15/50
 
 
0.0004661
 
 
0.0056165
 
 
0
 
 
0
 
 
3.209726
 
 
24.622180
 
 
NTRK2;PRKAB2;PRKAA1;HOMER1;BDNF;PTEN;PIK3R3;TSC1;PIK3R1;NRAS;PIK3CA;CAMK4;AKT3;EIF4EBP1;MAPK1
 
 
 
 
Oxidative Damage Response WP3941
 
 
13/40
 
 
0.0004674
 
 
0.0056165
 
 
0
 
 
0
 
 
3.604560
 
 
27.640763
 
 
CDKN1C;MAP2K4;CR2;GADD45A;TNFRSF1B;TNF;CDC42;BAG4;TRAF6;BCL2;CYCS;MAP3K9;BAK1
 
 
 
 
miRNA Regulation Of Prostate Cancer Signaling WP3981
 
 
11/31
 
 
0.0005448
 
 
0.0063689
 
 
0
 
 
0
 
 
4.115654
 
 
30.929399
 
 
PDGFRB;MAP2K1;PIK3CA;CREB3L1;AKT3;BCL2;MAPK1;GRB2;RAF1;SOS1;TP53
 
 
 
 
Apoptosis Modulation And Signaling WP1772
 
 
22/89
 
 
0.0005469
 
 
0.0063689
 
 
0
 
 
0
 
 
2.462056
 
 
18.493252
 
 
HRK;FOS;TNFRSF1B;TNFRSF1A;CASP7;BCL2L11;AIFM1;IRAK1;TRAF6;PTRH2;PEA15;BCL2;CASP2;PMAIP1;CYCS;FAS;BMF;BAK1;TP53;BCL2L2;MAP3K5;MCL1
 
 
 
 
Translation Inhibitors In Chronically Activated PDGFRA Cells WP4566
 
 
14/46
 
 
0.0006079
 
 
0.0068683
 
 
0
 
 
0
 
 
3.275760
 
 
24.258681
 
 
EIF4A2;MAP2K4;MAP2K1;CSNK2A1;PRKCD;PIK3R3;PIK3R1;MAPK14;PIK3CA;RPS6KA1;AKT3;EIF4EBP1;MAPK1;MAP2K6
 
 
 
 
Aryl Hydrocarbon Receptor Pathway WP2586
 
 
14/46
 
 
0.0006079
 
 
0.0068683
 
 
0
 
 
0
 
 
3.275760
 
 
24.258681
 
 
RB1;MAP2K1;LPL;AHR;TNF;ESR1;VEGFA;NCOR2;NRAS;KLF6;MAPK1;EP300;NCOA7;RAF1
 
 
 
 
Head And Neck Squamous Cell Carcinoma WP4674
 
 
19/73
 
 
0.0006461
 
 
0.0071929
 
 
0
 
 
0
 
 
2.636789
 
 
19.365933
 
 
RB1;NOTCH2;PRKAA1;SMAD4;NOTCH1;CUL3;PTEN;TSC1;PIK3R1;VEGFA;NRAS;PIK3CA;SESN1;AKT3;SESN2;DDIT4;NUMB;EIF4EBP1;TP53
 
 
 
 
Canonical And Non Canonical Notch Signaling WP3845
 
 
10/27
 
 
0.0006575
 
 
0.0072134
 
 
0
 
 
0
 
 
4.400649
 
 
32.243863
 
 
NOTCH2;DLL4;NOTCH1;JAG1;RRAS;CHUK;LEF1;MFAP2;ADAM10;DLL1
 
 
 
 
Thermogenesis WP4321
 
 
25/108
 
 
0.0006859
 
 
0.0073947
 
 
0
 
 
0
 
 
2.259294
 
 
16.458423
 
 
PRKAA1;KDM3A;ADCY3;ADCY2;NRAS;CNR1;CREB3L1;RPS6KA1;PRDM16;MAP3K5;PRKAB2;ACSL1;ACSL6;ACSL4;TSC1;MAPK14;ARID1B;MAPK12;CREB1;GRB2;PPARG;SOS1;SOS2;CREB5;PNPLA2
 
 
 
 
Neurogenesis Regulation In The Olfactory Epithelium WP5265
 
 
16/57
 
 
0.0006936
 
 
0.0073947
 
 
0
 
 
0
 
 
2.922925
 
 
21.260402
 
 
NTRK2;NUMBL;NOTCH1;JAG1;BDNF;STAT3;DLL1;RTN4;MECP2;NEUROD1;ID1;NUMB;TCF4;MAPT;PAFAH1B1;CDK5R1
 
 
 
 
White Fat Cell Differentiation WP4149
 
 
11/32
 
 
0.0007423
 
 
0.0078041
 
 
0
 
 
0
 
 
3.919448
 
 
28.242765
 
 
WNT10B;SREBF1;CREB1;KLF5;IRF4;RARA;RORA;PPARG;NR2F2;KLF4;KLF15
 
 
 
 
Estrogen Signaling WP712
 
 
9/23
 
 
0.0007687
 
 
0.0079716
 
 
0
 
 
0
 
 
4.808058
 
 
34.477514
 
 
MAP2K1;CREB1;CHUK;PIK3CA;BCL2;MAPK1;FOS;MAPK14;ESR1
 
 
 
 
Androgen Receptor Network In Prostate Cancer WP2263
 
 
25/109
 
 
0.0007918
 
 
0.0080998
 
 
0
 
 
0
 
 
2.232270
 
 
15.941114
 
 
FOXA1;RB1;PTEN;RAP1B;RAP1A;EIF4EBP1;MAPK1;MAP2K1;STAT3;TSC1;FOS;MAPK14;ARID1B;ETV4;CDC25B;PIK3CA;RASA1;BCL2;SPRY2;GRB2;SPRY1;ERG;RAF1;SOS1;TP53
 
 
 
 
Ras Signaling WP4223
 
 
37/184
 
 
0.0008143
 
 
0.0082191
 
 
0
 
 
0
 
 
1.890782
 
 
13.449462
 
 
CSF1R;RAB5B;PIK3R3;RASAL1;PIK3R1;RASGRP4;RAP1B;CDC42;NRAS;RAP1A;GNG2;RRAS;AKT3;MAPK1;RALGDS;PDGFRB;PDGFRA;NTRK2;MAP2K1;RALBP1;CHUK;RRAS2;GNG12;TIAM1;MRAS;PIK3CA;RASA1;CALM3;GRB2;TEK;RAF1;SOS1;RAPGEF5;MET;SOS2;EPHA2;ARF6
 
 
 
 
Neuroinflammation And Glutamatergic Signaling WP5083
 
 
30/140
 
 
0.0008437
 
 
0.0084037
 
 
0
 
 
0
 
 
2.046915
 
 
14.487467
 
 
SLC2A1;ADCY3;SLC2A3;FGF2;TNF;PPP1CB;PPP1CC;GRM5;GRM7;IL12B;MAPK1;LRRC8B;IL6R;IL13RA1;SMAD4;TGFB2;BDNF;STAT3;LIF;IGF1;FOS;TNFRSF1B;TGFBR1;TNFRSF1A;CREB1;IFNG;CAMK4;TRAF5;BCL2;IL6ST
 
 
 
 
Fragile X Syndrome WP4549
 
 
27/122
 
 
0.0008969
 
 
0.0088173
 
 
0
 
 
0
 
 
2.132187
 
 
14.960705
 
 
GABRB2;ABCD3;FMR1;PTEN;AKAP5;SLC6A1;MECP2;GRIP1;GRM5;CNR1;EIF4EBP2;MAPK1;NTRK2;MAP2K1;HOMER1;BDNF;TSC1;DICER1;CREB1;CAMK4;AGO2;TBC1D7;GRB2;RAF1;HOXB8;SOS1;HCN1
 
 
 
 
Kit Receptor Signaling WP304
 
 
15/53
 
 
0.0009187
 
 
0.0089165
 
 
0
 
 
0
 
 
2.955823
 
 
20.668614
 
 
MAP2K1;STAT3;MITF;PIK3R1;FOXO3;MAPK14;KITLG;RPS6KA1;GRB10;EP300;MAPK1;GRB2;MAPT;RAF1;SOS1
 
 
 
 
Cytosine Methylation WP3585
 
 
5/8
 
 
0.0009465
 
 
0.0090693
 
 
0
 
 
0
 
 
12.451977
 
 
86.700321
 
 
MECP2;DNMT1;TDG;TET2;TET1
 
 
 
 
Energy Metabolism WP1541
 
 
14/48
 
 
0.0009737
 
 
0.0092132
 
 
0
 
 
0
 
 
3.082718
 
 
21.376967
 
 
NCOA1;ESRRA;MEF2A;PRKAA1;PRKAB2;HDAC1;FOXO3;MAPK14;SIRT1;CREB1;UCP3;CAMK4;EP300;PPARG
 
 
 
 
Type I Collagen Synthesis Osteogenesis Imperfecta WP4786
 
 
11/33
 
 
0.0009956
 
 
0.0093047
 
 
0
 
 
0
 
 
3.741079
 
 
25.858913
 
 
COL1A1;COL1A2;LOX;P4HA2;CREB3L1;LRP5;P3H1;TNFRSF11B;MIA3;TNFRSF11A;WNT1
 
 
 
 
Embryonic Stem Cell Pluripotency Pathways WP3931
 
 
26/117
 
 
0.0010371
 
 
0.0095316
 
 
0
 
 
0
 
 
2.143040
 
 
14.725429
 
 
PTEN;LRP5;FGF2;AKT3;MAPK1;WNT1;MAP2K6;PDGFRB;ACVR1;PDGFRA;WNT10B;MAP2K1;SMAD4;FZD5;FZD4;NOG;STAT3;LIF;WNT9B;FOS;MAPK12;APC;GRB2;IL6ST;RAF1;SOS1
 
 
 
 
MET In Type 1 Papillary Renal Cell Carcinoma WP4205
 
 
16/59
 
 
0.0010451
 
 
0.0095316
 
 
0
 
 
0
 
 
2.786658
 
 
19.126696
 
 
MAP2K1;STAT3;PIK3R3;PIK3R1;CDC42;RAP1B;NRAS;RAP1A;PIK3CA;AKT3;MAPK1;GRB2;RAF1;SOS1;MET;SOS2
 
 
 
 
Hippocampal Synaptogenesis And Neurogenesis WP5231
 
 
9/24
 
 
0.0011019
 
 
0.0099301
 
 
0
 
 
0
 
 
4.487267
 
 
30.561588
 
 
CREB1;CCND2;BDNF;NRXN1;CAMK4;RPS6KA1;BCL2;MAPK1;MAPK14
 
 
 
 
Pathways Affected In Adenoid Cystic Carcinoma WP3651
 
 
17/65
 
 
0.0011502
 
 
0.0102432
 
 
0
 
 
0
 
 
2.652781
 
 
17.953634
 
 
KDM6B;NOTCH1;MYCBP;FBXW7;MAX;MAGI2;PTEN;FOXO3;MORF4L1;MYCN;PIK3CA;MYB;EP300;BCOR;BCORL1;RAF1;TP53
 
 
 
 
Development Of Ureteric Derived Collecting System WP5053
 
 
16/60
 
 
0.0012710
 
 
0.0110317
 
 
0
 
 
0
 
 
2.723170
 
 
18.157922
 
 
ITGB1;TGFB2;FST;CRIM1;SIX1;ETV4;HOXA11;ROBO1;GRIP1;SHH;SALL1;MYCN;WT1;RARA;RARB;SPRY1
 
 
 
 
BMP Signaling In Eyelid Development WP3927
 
 
8/20
 
 
0.0012772
 
 
0.0110317
 
 
0
 
 
0
 
 
4.984585
 
 
33.212734
 
 
SMAD4;SFRP1;SHH;NOTCH1;TGFA;INHBB;PITX2;DKK2
 
 
 
 
Heart Development WP1591
 
 
13/44
 
 
0.0012824
 
 
0.0110317
 
 
0
 
 
0
 
 
3.138743
 
 
20.900914
 
 
SMAD4;NOTCH1;GATA6;VEGFC;TBX5;ISL1;VEGFA;SHH;ERBB3;HEY2;BHLHE40;MAPK1;PITX2
 
 
 
 
MAPK And NFkB Signaling Inhibited By Yersinia YopJ WP3849
 
 
6/12
 
 
0.0013303
 
 
0.0112159
 
 
0
 
 
0
 
 
7.473082
 
 
49.489339
 
 
RRAS;CHUK;TRAF6;MAPK1;RAF1;MAP2K6
 
 
 
 
MicroRNAs In Cardiomyocyte Hypertrophy WP1544
 
 
20/83
 
 
0.0013442
 
 
0.0112159
 
 
0
 
 
0
 
 
2.378854
 
 
15.728932
 
 
HDAC4;EIF2B5;MAP2K4;MAP2K1;CHUK;STAT3;LRP5;LIF;PIK3R3;PIK3R1;IGF1;MAPK14;FGF2;TNF;RCAN1;PIK3CA;MAPK1;IL6ST;RAF1;MAP2K6
 
 
 
 
SREBF And miR33 In Cholesterol And Lipid Homeostasis WP2011
 
 
7/16
 
 
0.0013875
 
 
0.0112159
 
 
0
 
 
0
 
 
5.813872
 
 
38.256567
 
 
SREBF1;PRKAA1;HMGCS1;SCD;HMGCR;SIRT1;LDLR
 
 
 
 
Galanin Receptor Pathway WP4970
 
 
7/16
 
 
0.0013875
 
 
0.0112159
 
 
0
 
 
0
 
 
5.813872
 
 
38.256567
 
 
CDKN1C;CREB1;BCL2L11;PPARG;FOS;VAMP2;VEGFA
 
 
 
 
Cardiac Hypertrophic Response WP2795
 
 
15/55
 
 
0.0013925
 
 
0.0112159
 
 
0
 
 
0
 
 
2.807713
 
 
18.465348
 
 
MEF2A;HDAC4;MAP2K4;MAP2K1;CHUK;IGF1;MAPK14;FGF2;TNF;TGFBR1;TNFRSF1A;MAPK1;RAF1;MAP3K7;MAP2K6
 
 
 
 
Neural Crest Differentiation WP2064
 
 
23/101
 
 
0.0013927
 
 
0.0112159
 
 
0
 
 
0
 
 
2.210531
 
 
14.537540
 
 
NOTCH2;ITGB1;HDAC4;FOXD3;NOTCH1;HDAC1;MITF;FGF2;ISL1;DLL1;CDH7;DLL4;COL2A1;MYB;ID1;HEY2;ZIC1;SNAI1;PMP22;HOXA1;TCF4;WNT1;SOX5
 
 
 
 
RANKL RANK Signaling WP2018
 
 
14/50
 
 
0.0015087
 
 
0.0120222
 
 
0
 
 
0
 
 
2.911125
 
 
18.912097
 
 
MAP2K1;CHUK;MITF;TNFRSF11B;FOS;PIK3R1;TNFRSF11A;MAPK14;CDC42;TRAF6;TRAF5;MAPK1;MAP3K7;MAP2K6
 
 
 
 
Oncostatin M Signaling WP2374
 
 
15/56
 
 
0.0016973
 
 
0.0132457
 
 
0
[truncated: 7,470,163 more chars]
