# Supplementary material for: Comprehensive and bias-aware analysis of small RNA NGS data for biomarker detection with caRNAge
Source: Extracell Vesicles Circ Nucl Acids. 2026 Sep 24;7(3):1554–81. doi: 10.20517/evcna.2026.78 (PMC13678173; doi:10.20517/evcna.2026.78)
Supplement: Supplementary file 1 [file evcna-7-3-1554-SupplementaryMaterials.zip › evcna7078-SupplementaryMaterials/Supplementary File 7.html]

 

 

 

 
 
 


 caRNAge - comprehensive analysis of small RNA gene expression 

 
 
 
 
 
 
 
 
 
 
 
 
 
 
 
 
 
 

 

 
 


 


 

 

 


 


 

 


 


 
 
 
 
 
 

 


 


 caRNAge - comprehensive analysis of small
RNA gene expression 
 Over representation analysis of miRNAs and their
mRNA targets 
 22 August, 2026 

 


 
 
 
 Purpose of this overview (Disclaimer) 
 This report is meant to give you a complete overview of
differentially expressed genes for the group comparison you have chosen
with their respective sources of bias you have identified in the data
overview. Please refer to the guidelines for best practice of caRNAge
and the respective manuals and vignettes of the applied tools. You are
strongly advised to thoroughly check all available information in
addition to this data overview. caRNAge is built upon the work of many
excellent scientists who developed the necessary tools to process small
RNA NGS data, so please make sure to cite the corresponding original
authors in addition to caRNAge when publishing your work. 
 If you encounter any technical problems, feel free to contact the
corresponding author at  bkirchner@tum.de . 
 
 
 Data processing 
 Raw sequencing reads were processed with  Cutadapt  to trim
adapter sequences from 3’ ends if present and to remove degraded reads
(shorter than 16nt). Trimmed reads were analysed with  FastQC 
to evaluate phred scores and read lengths before aligning them against
the human tRNA ( GtRNAdb ), miRNA
( miRBase ) including isomiRs ( isomiRROR ), piRNAs ( piRNAdb ) and refseq transcriptome
sequences ( refseq )
with  bowtie .
Finally, gene read counts were processed with  R  and mainly  DESeq2 
to normalize and transform data and calculate expression changes. 
 
  
 
 
Fig 1: Simplified workflow for NGS data analysis
 
 
   
 
 
 
 
 
 
 
 
 
  
 Tools 
 Version 
 Parameters 
 
 
 
 
 Trimming 
 Cutadapt 
 4.4 
 -a AGATCGGAAGAGCAC, -e 0.1 -m 16 
 
 
 Quality Control 
 FastQC 
 0.12.1 
  
 
 
 Alignment 
 bowtie 
 1.3.1 
 Bowtie -v mismatches: tRNA=1, isomiR=3,
transcriptome=1; –best –norc 
 
 
 Normalisation 
 DESeq2 
 R version 4.6.1 (2026-06-24), DESeq2 1.52.0 
 ~donor + analysis_group 
 
 
 DGE calling 
 DESeq2 
 R version 4.6.1 (2026-06-24), DESeq2 1.52.0 
  
 
 
 miRNA ORA 
 rbioapi, miEAA 
 R version 4.6.1 (2026-06-24), rbioapi 0.8.3, miEEA 
  
 
 
 miRNA target prediction 
 multiMiR Targetscan 
 R version 4.6.1 (2026-06-24), multiMiR 1.34.0,
TargetScan 
  
 
 
 miRNA target ORA 
 rbioapi, enrichr 
 R version 4.6.1 (2026-06-24), rbioapi 0.8.3,
enrichr 
  
 
 
 
     
 
 
 Sample overview 
 The starting point for all analyses, is a well designed study design.
In addition to the classification of the main research question, it
should also incorporate all available information that could in any way
affect the outcome. This includes potential confounding factors such as
batch effects as well as statistical relevant information such as
patient cohorts or cell lines. Please make sure that the classifications
given in your  design.tsv  are correct and that all relevant
information is present. 
   
 
 Study design 
 The row names and “Sample” column should be matching and indicate
that samples in design were correctly assigned in the analysis. A column
named “replaceable” maybe present if groups are large enough (>7) to
replace single outlier values. 
 
 
 
 
 
 
 
 
 
 
 
 
 
 
 
 
  
 Sample 
 source_name 
 cell_type 
 condition 
 compartment 
 experimental_block 
 sequencing_platform 
 donor 
 analysis_group 
 sequencing_cycles 
 
 
 
 
 wtchg_834291_209_1 
 wtchg_834291_209_1 
 CD8 EV 
 cd8_t_cell 
 ev 
 extracellular_vesicle 
 wtchg_209 
 illumina_hiseq_4000 
 donor_834291 
 cd8_ev 
 75 
 
 
 wtchg_834291_210_1 
 wtchg_834291_210_1 
 CD8 EV 
 cd8_t_cell 
 ev 
 extracellular_vesicle 
 wtchg_210 
 illumina_hiseq_4000 
 donor_834291 
 cd8_ev 
 75 
 
 
 wtchg_834291_212_1 
 wtchg_834291_212_1 
 CD8 EV 
 cd8_t_cell 
 ev 
 extracellular_vesicle 
 wtchg_212 
 illumina_hiseq_4000 
 donor_834291 
 cd8_ev 
 75 
 
 
 wtchg_834291_225_1 
 wtchg_834291_225_1 
 CD8 S1 
 cd8_t_cell 
 s1 
 trans_synaptic_vesicle 
 wtchg_225 
 illumina_hiseq_4000 
 donor_834291 
 cd8_s1 
 75 
 
 
 wtchg_834291_228_1 
 wtchg_834291_228_1 
 CD8 S1 
 cd8_t_cell 
 s1 
 trans_synaptic_vesicle 
 wtchg_228 
 illumina_hiseq_4000 
 donor_834291 
 cd8_s1 
 75 
 
 
 wtchg_834291_237_1 
 wtchg_834291_237_1 
 CD8 S0 
 cd8_t_cell 
 s0 
 bslb_background_control 
 wtchg_237 
 illumina_hiseq_4000 
 donor_834291 
 cd8_s0 
 75 
 
 
 wtchg_834291_238_1 
 wtchg_834291_238_1 
 CD8 S1 
 cd8_t_cell 
 s1 
 trans_synaptic_vesicle 
 wtchg_238 
 illumina_hiseq_4000 
 donor_834291 
 cd8_s1 
 75 
 
 
 wtchg_834292_209_1 
 wtchg_834292_209_1 
 CD8 EV 
 cd8_t_cell 
 ev 
 extracellular_vesicle 
 wtchg_209 
 illumina_hiseq_4000 
 donor_834292 
 cd8_ev 
 75 
 
 
 wtchg_834292_210_1 
 wtchg_834292_210_1 
 CD8 EV 
 cd8_t_cell 
 ev 
 extracellular_vesicle 
 wtchg_210 
 illumina_hiseq_4000 
 donor_834292 
 cd8_ev 
 75 
 
 
 wtchg_834292_212_1 
 wtchg_834292_212_1 
 CD8 EV 
 cd8_t_cell 
 ev 
 extracellular_vesicle 
 wtchg_212 
 illumina_hiseq_4000 
 donor_834292 
 cd8_ev 
 75 
 
 
 wtchg_834292_225_1 
 wtchg_834292_225_1 
 CD8 S1 
 cd8_t_cell 
 s1 
 trans_synaptic_vesicle 
 wtchg_225 
 illumina_hiseq_4000 
 donor_834292 
 cd8_s1 
 75 
 
 
 wtchg_834292_228_1 
 wtchg_834292_228_1 
 CD8 S1 
 cd8_t_cell 
 s1 
 trans_synaptic_vesicle 
 wtchg_228 
 illumina_hiseq_4000 
 donor_834292 
 cd8_s1 
 75 
 
 
 wtchg_834292_237_1 
 wtchg_834292_237_1 
 CD8 S0 
 cd8_t_cell 
 s0 
 bslb_background_control 
 wtchg_237 
 illumina_hiseq_4000 
 donor_834292 
 cd8_s0 
 75 
 
 
 wtchg_834292_238_1 
 wtchg_834292_238_1 
 CD8 S1 
 cd8_t_cell 
 s1 
 trans_synaptic_vesicle 
 wtchg_238 
 illumina_hiseq_4000 
 donor_834292 
 cd8_s1 
 75 
 
 
 wtchg_834293_209_1 
 wtchg_834293_209_1 
 CD8 EV 
 cd8_t_cell 
 ev 
 extracellular_vesicle 
 wtchg_209 
 illumina_hiseq_4000 
 donor_834293 
 cd8_ev 
 75 
 
 
 wtchg_834293_210_1 
 wtchg_834293_210_1 
 CD8 EV 
 cd8_t_cell 
 ev 
 extracellular_vesicle 
 wtchg_210 
 illumina_hiseq_4000 
 donor_834293 
 cd8_ev 
 75 
 
 
 wtchg_834293_212_1 
 wtchg_834293_212_1 
 CD8 EV 
 cd8_t_cell 
 ev 
 extracellular_vesicle 
 wtchg_212 
 illumina_hiseq_4000 
 donor_834293 
 cd8_ev 
 75 
 
 
 wtchg_834293_225_1 
 wtchg_834293_225_1 
 CD8 S1 
 cd8_t_cell 
 s1 
 trans_synaptic_vesicle 
 wtchg_225 
 illumina_hiseq_4000 
 donor_834293 
 cd8_s1 
 75 
 
 
 wtchg_834293_228_1 
 wtchg_834293_228_1 
 CD8 S1 
 cd8_t_cell 
 s1 
 trans_synaptic_vesicle 
 wtchg_228 
 illumina_hiseq_4000 
 donor_834293 
 cd8_s1 
 75 
 
 
 wtchg_834293_237_1 
 wtchg_834293_237_1 
 CD8 S0 
 cd8_t_cell 
 s0 
 bslb_background_control 
 wtchg_237 
 illumina_hiseq_4000 
 donor_834293 
 cd8_s0 
 75 
 
 
 wtchg_834293_238_1 
 wtchg_834293_238_1 
 CD8 S1 
 cd8_t_cell 
 s1 
 trans_synaptic_vesicle 
 wtchg_238 
 illumina_hiseq_4000 
 donor_834293 
 cd8_s1 
 75 
 
 
 wtchg_834294_209_1 
 wtchg_834294_209_1 
 CD8 EV 
 cd8_t_cell 
 ev 
 extracellular_vesicle 
 wtchg_209 
 illumina_hiseq_4000 
 donor_834294 
 cd8_ev 
 75 
 
 
 wtchg_834294_210_1 
 wtchg_834294_210_1 
 CD8 EV 
 cd8_t_cell 
 ev 
 extracellular_vesicle 
 wtchg_210 
 illumina_hiseq_4000 
 donor_834294 
 cd8_ev 
 75 
 
 
 wtchg_834294_212_1 
 wtchg_834294_212_1 
 CD8 EV 
 cd8_t_cell 
 ev 
 extracellular_vesicle 
 wtchg_212 
 illumina_hiseq_4000 
 donor_834294 
 cd8_ev 
 75 
 
 
 wtchg_834294_225_1 
 wtchg_834294_225_1 
 CD8 S1 
 cd8_t_cell 
 s1 
 trans_synaptic_vesicle 
 wtchg_225 
 illumina_hiseq_4000 
 donor_834294 
 cd8_s1 
 75 
 
 
 wtchg_834294_228_1 
 wtchg_834294_228_1 
 CD8 S1 
 cd8_t_cell 
 s1 
 trans_synaptic_vesicle 
 wtchg_228 
 illumina_hiseq_4000 
 donor_834294 
 cd8_s1 
 75 
 
 
 wtchg_834294_237_1 
 wtchg_834294_237_1 
 CD8 S0 
 cd8_t_cell 
 s0 
 bslb_background_control 
 wtchg_237 
 illumina_hiseq_4000 
 donor_834294 
 cd8_s0 
 75 
 
 
 wtchg_834294_238_1 
 wtchg_834294_238_1 
 CD8 S1 
 cd8_t_cell 
 s1 
 trans_synaptic_vesicle 
 wtchg_238 
 illumina_hiseq_4000 
 donor_834294 
 cd8_s1 
 75 
 
 
 wtchg_836235_209_1 
 wtchg_836235_209_1 
 CD8 EV 
 cd8_t_cell 
 ev 
 extracellular_vesicle 
 wtchg_209 
 illumina_hiseq_4000 
 donor_836235 
 cd8_ev 
 75 
 
 
 wtchg_836235_210_1 
 wtchg_836235_210_1 
 CD8 EV 
 cd8_t_cell 
 ev 
 extracellular_vesicle 
 wtchg_210 
 illumina_hiseq_4000 
 donor_836235 
 cd8_ev 
 75 
 
 
 wtchg_836235_212_1 
 wtchg_836235_212_1 
 CD8 EV 
 cd8_t_cell 
 ev 
 extracellular_vesicle 
 wtchg_212 
 illumina_hiseq_4000 
 donor_836235 
 cd8_ev 
 75 
 
 
 wtchg_836235_225_1 
 wtchg_836235_225_1 
 CD8 S1 
 cd8_t_cell 
 s1 
 trans_synaptic_vesicle 
 wtchg_225 
 illumina_hiseq_4000 
 donor_836235 
 cd8_s1 
 75 
 
 
 wtchg_836235_228_1 
 wtchg_836235_228_1 
 CD8 S1 
 cd8_t_cell 
 s1 
 trans_synaptic_vesicle 
 wtchg_228 
 illumina_hiseq_4000 
 donor_836235 
 cd8_s1 
 75 
 
 
 wtchg_836235_237_1 
 wtchg_836235_237_1 
 CD8 S0 
 cd8_t_cell 
 s0 
 bslb_background_control 
 wtchg_237 
 illumina_hiseq_4000 
 donor_836235 
 cd8_s0 
 75 
 
 
 wtchg_836235_238_1 
 wtchg_836235_238_1 
 CD8 S1 
 cd8_t_cell 
 s1 
 trans_synaptic_vesicle 
 wtchg_238 
 illumina_hiseq_4000 
 donor_836235 
 cd8_s1 
 75 
 
 
 wtchg_836236_209_1 
 wtchg_836236_209_1 
 CD8 EV 
 cd8_t_cell 
 ev 
 extracellular_vesicle 
 wtchg_209 
 illumina_hiseq_4000 
 donor_836236 
 cd8_ev 
 75 
 
 
 wtchg_836236_210_1 
 wtchg_836236_210_1 
 CD8 EV 
 cd8_t_cell 
 ev 
 extracellular_vesicle 
 wtchg_210 
 illumina_hiseq_4000 
 donor_836236 
 cd8_ev 
 75 
 
 
 wtchg_836236_212_1 
 wtchg_836236_212_1 
 CD8 EV 
 cd8_t_cell 
 ev 
 extracellular_vesicle 
 wtchg_212 
 illumina_hiseq_4000 
 donor_836236 
 cd8_ev 
 75 
 
 
 wtchg_836236_225_1 
 wtchg_836236_225_1 
 CD8 S1 
 cd8_t_cell 
 s1 
 trans_synaptic_vesicle 
 wtchg_225 
 illumina_hiseq_4000 
 donor_836236 
 cd8_s1 
 75 
 
 
 wtchg_836236_228_1 
 wtchg_836236_228_1 
 CD8 S1 
 cd8_t_cell 
 s1 
 trans_synaptic_vesicle 
 wtchg_228 
 illumina_hiseq_4000 
 donor_836236 
 cd8_s1 
 75 
 
 
 wtchg_836236_237_1 
 wtchg_836236_237_1 
 CD8 S0 
 cd8_t_cell 
 s0 
 bslb_background_control 
 wtchg_237 
 illumina_hiseq_4000 
 donor_836236 
 cd8_s0 
 75 
 
 
 wtchg_836236_238_1 
 wtchg_836236_238_1 
 CD8 S1 
 cd8_t_cell 
 s1 
 trans_synaptic_vesicle 
 wtchg_238 
 illumina_hiseq_4000 
 donor_836236 
 cd8_s1 
 75 
 
 
 wtchg_836237_209_1 
 wtchg_836237_209_1 
 CD8 EV 
 cd8_t_cell 
 ev 
 extracellular_vesicle 
 wtchg_209 
 illumina_hiseq_4000 
 donor_836237 
 cd8_ev 
 75 
 
 
 wtchg_836237_210_1 
 wtchg_836237_210_1 
 CD8 EV 
 cd8_t_cell 
 ev 
 extracellular_vesicle 
 wtchg_210 
 illumina_hiseq_4000 
 donor_836237 
 cd8_ev 
 75 
 
 
 wtchg_836237_212_1 
 wtchg_836237_212_1 
 CD8 EV 
 cd8_t_cell 
 ev 
 extracellular_vesicle 
 wtchg_212 
 illumina_hiseq_4000 
 donor_836237 
 cd8_ev 
 75 
 
 
 wtchg_836237_225_1 
 wtchg_836237_225_1 
 CD8 S1 
 cd8_t_cell 
 s1 
 trans_synaptic_vesicle 
 wtchg_225 
 illumina_hiseq_4000 
 donor_836237 
 cd8_s1 
 75 
 
 
 wtchg_836237_228_1 
 wtchg_836237_228_1 
 CD8 S1 
 cd8_t_cell 
 s1 
 trans_synaptic_vesicle 
 wtchg_228 
 illumina_hiseq_4000 
 donor_836237 
 cd8_s1 
 75 
 
 
 wtchg_836237_237_1 
 wtchg_836237_237_1 
 CD8 S0 
 cd8_t_cell 
 s0 
 bslb_background_control 
 wtchg_237 
 illumina_hiseq_4000 
 donor_836237 
 cd8_s0 
 75 
 
 
 wtchg_836237_238_1 
 wtchg_836237_238_1 
 CD8 S1 
 cd8_t_cell 
 s1 
 trans_synaptic_vesicle 
 wtchg_238 
 illumina_hiseq_4000 
 donor_836237 
 cd8_s1 
 75 
 
 
 wtchg_836238_209_1 
 wtchg_836238_209_1 
 CD8 EV 
 cd8_t_cell 
 ev 
 extracellular_vesicle 
 wtchg_209 
 illumina_hiseq_4000 
 donor_836238 
 cd8_ev 
 75 
 
 
 wtchg_836238_210_1 
 wtchg_836238_210_1 
 CD8 EV 
 cd8_t_cell 
 ev 
 extracellular_vesicle 
 wtchg_210 
 illumina_hiseq_4000 
 donor_836238 
 cd8_ev 
 75 
 
 
 wtchg_836238_212_1 
 wtchg_836238_212_1 
 CD8 EV 
 cd8_t_cell 
 ev 
 extracellular_vesicle 
 wtchg_212 
 illumina_hiseq_4000 
 donor_836238 
 cd8_ev 
 75 
 
 
 wtchg_836238_225_1 
 wtchg_836238_225_1 
 CD8 S1 
 cd8_t_cell 
 s1 
 trans_synaptic_vesicle 
 wtchg_225 
 illumina_hiseq_4000 
 donor_836238 
 cd8_s1 
 75 
 
 
 wtchg_836238_228_1 
 wtchg_836238_228_1 
 CD8 S1 
 cd8_t_cell 
 s1 
 trans_synaptic_vesicle 
 wtchg_228 
 illumina_hiseq_4000 
 donor_836238 
 cd8_s1 
 75 
 
 
 wtchg_836238_237_1 
 wtchg_836238_237_1 
 CD8 S0 
 cd8_t_cell 
 s0 
 bslb_background_control 
 wtchg_237 
 illumina_hiseq_4000 
 donor_836238 
 cd8_s0 
 75 
 
 
 wtchg_836238_238_1 
 wtchg_836238_238_1 
 CD8 S1 
 cd8_t_cell 
 s1 
 trans_synaptic_vesicle 
 wtchg_238 
 illumina_hiseq_4000 
 donor_836238 
 cd8_s1 
 75 
 
 
 
   
 
 
 Consolidated sample groups 
  $source_name

CD8 EV CD8 S0 CD8 S1 
    24      8     24 

$cell_type

cd8_t_cell 
        56 

$condition

ev s0 s1 
24  8 24 

$compartment

bslb_background_control   extracellular_vesicle  trans_synaptic_vesicle 
                      8                      24                      24 

$experimental_block

wtchg_209 wtchg_210 wtchg_212 wtchg_225 wtchg_228 wtchg_237 wtchg_238 
        8         8         8         8         8         8         8 

$sequencing_platform

illumina_hiseq_4000 
                 56 

$donor

donor_834291 donor_834292 donor_834293 donor_834294 donor_836235 donor_836236 
           7            7            7            7            7            7 
donor_836237 donor_836238 
           7            7 

$analysis_group

cd4_ev cd8_ev cd4_s0 cd8_s1 cd4_s1 cd8_s0 
     0     24      0     24      0      8 

$sequencing_cycles

75 
56   
     
 
 
 
 miRNA ORA Results 
 Over-representation analysis (ORA) is a statistical framework used to
determine whether a predefined set of genes or miRNAs is enriched for
specific biological functions, pathways, cellular processes, or disease
associations more than would be expected by chance. Instead of
evaluating individual miRNAs in isolation, ORA quantifies whether entire
categories occur disproportionately within the input list. For this
analysis, ORA was performed using miEAA (microRNA Enrichment Analysis
and Annotation), a specialized platform designed for miRNA-centric
functional enrichment. miEAA integrates heterogeneous annotation sources
including miRBase, miRPathDB, miRWalk, miRTarBase, miRandola, RNALocate,
isomiRdb, MNDR, and others. 
 The study design parameter chosen for DGE testing was
 analysis_group  and the parameter(s) chosen for
correction were ****. 
 For downstream ORA and target prediction, miRNAs are separated by
direction and must satisfy  adjusted p-value ≤ 0.05 and |log2FC|
≥ 1 . Upregulated and downregulated miRNAs are analyzed
independently. 
 
 cd8_ev_vs_cd8_s1_upregulated 
 
 
 cd8_ev_vs_cd8_s1_downregulated 
 
 
 cd8_ev_vs_cd8_s0_upregulated 
 
 
 cd8_ev_vs_cd8_s0_downregulated 
 
 
 cd8_s1_vs_cd8_s0_upregulated 
 
 
 cd8_s1_vs_cd8_s0_downregulated 
 
 
 
 miRNA Target Prediction Results 
 This section summarizes predicted miRNA–mRNA regulatory interactions
identified using TargetScan, a widely used algorithm that infers miRNA
targeting based on seed sequence complementarity, evolutionary
conservation, and contextual features of 3′UTR binding sites. The
resulting interaction set represents putative post-transcriptional
regulatory relationships through which differentially expressed miRNAs
may influence downstream gene expression programs. 
 To contextualize these predicted interactions at the tissue level,
target genes were annotated with consensus tissue expression values
(nTPM) derived from the Human Protein Atlas (HPA) and the
Genotype-Tissue Expression (GTEx) project. These resources integrate
large-scale transcriptomic measurements across a broad spectrum of human
tissues, providing a robust estimate of physiological gene expression
patterns. Predicted miRNA target genes are presented grouped by tissue,
with each table containing targets that show detectable expression in
the respective tissue context. 
 Target tables are shown as paginated interactive tables.
Enrichment-result tables are capped at the top 200 rows after sorting by
adjusted p-value. 
 The threshold chosen for tissue specificity was nTPM =
 10  and tissue specific ORA was analyzed for
 lymph node, spleen . 
 
 cd8_ev_vs_cd8_s1_upregulated 
 
 Tissue expression heatmap (log10-transformed) 
   
 
 
 liver 
 
 
 
 
target_symbol
 
 
mature_mirna_id
 
 
score
 
 
nTPM
 
 
 
 
 
 
A1BG
 
 
hsa-miR-23a-3p
 
 
-0.434
 
 
565.1
 
 
 
 
A1BG
 
 
hsa-miR-23b-3p
 
 
-0.434
 
 
565.1
 
 
 
 
AAR2
 
 
hsa-miR-29a-3p
 
 
-0.327
 
 
22.0
 
 
 
 
ABCB10
 
 
hsa-miR-148b-3p
 
 
-0.291
 
 
17.7
 
 
 
 
ABCB6
 
 
hsa-miR-29a-3p
 
 
-0.48
 
 
12.7
 
 
 
 
ABCB7
 
 
hsa-miR-148b-3p
 
 
-0.515
 
 
14.1
 
 
 
 
ABCD3
 
 
hsa-miR-148b-3p
 
 
-0.388
 
 
118.4
 
 
 
 
ABCE1
 
 
hsa-miR-29a-3p
 
 
-0.404
 
 
37.1
 
 
 
 
ABHD17B
 
 
hsa-miR-486-5p
 
 
-0.348
 
 
11.3
 
 
 
 
ABHD4
 
 
hsa-miR-29a-3p
 
 
-0.312
 
 
24.2
 
 
 
 
ABHD5
 
 
hsa-miR-132-3p
 
 
-0.323
 
 
20.3
 
 
 
 
ABHD5
 
 
hsa-miR-27a-3p
 
 
-0.407
 
 
20.3
 
 
 
 
ACTA2
 
 
hsa-miR-27a-3p
 
 
-0.577
 
 
154.5
 
 
 
 
ACTR2
 
 
hsa-miR-15b-5p
 
 
-0.293
 
 
101.7
 
 
 
 
ACTR2
 
 
hsa-miR-16-5p
 
 
-0.293
 
 
101.7
 
 
 
 
ACTR3C
 
 
hsa-miR-30b-5p
 
 
-0.448
 
 
18.8
 
 
 
 
ACVR1
 
 
hsa-miR-30b-5p
 
 
-0.29
 
 
15.3
 
 
 
 
ACVR1
 
 
hsa-miR-148b-3p
 
 
-0.397
 
 
15.3
 
 
 
 
ADAM10
 
 
hsa-miR-148b-3p
 
 
-0.356
 
 
15.0
 
 
 
 
ADAM9
 
 
hsa-miR-30b-5p
 
 
-0.312
 
 
12.7
 
 
 
 
ADGRA3
 
 
hsa-miR-27a-3p
 
 
-0.296
 
 
54.5
 
 
 
 
ADGRA3
 
 
hsa-miR-30b-5p
 
 
-0.304
 
 
54.5
 
 
 
 
ADGRF5
 
 
hsa-miR-148b-3p
 
 
-0.354
 
 
12.3
 
 
 
 
ADIPOR2
 
 
hsa-miR-150-5p
 
 
-0.287
 
 
77.7
 
 
 
 
ADNP
 
 
hsa-miR-328-3p
 
 
-0.371
 
 
28.4
 
 
 
 
ADRB2
 
 
hsa-miR-15b-5p
 
 
-0.29
 
 
13.3
 
 
 
 
ADRB2
 
 
hsa-miR-16-5p
 
 
-0.323
 
 
13.3
 
 
 
 
ADRB2
 
 
hsa-let-7b-5p
 
 
-0.635
 
 
13.3
 
 
 
 
AFG3L2
 
 
hsa-miR-181b-5p
 
 
-0.491
 
 
25.9
 
 
 
 
AFG3L2
 
 
hsa-miR-181a-5p
 
 
-0.491
 
 
25.9
 
 
 
 
AGFG1
 
 
hsa-miR-148b-3p
 
 
-0.332
 
 
33.8
 
 
 
 
AGPAT3
 
 
hsa-miR-27a-3p
 
 
-0.29
 
 
50.8
 
 
 
 
AGPAT3
 
 
hsa-miR-16-5p
 
 
-0.299
 
 
50.8
 
 
 
 
AHCTF1
 
 
hsa-let-7b-5p
 
 
-0.374
 
 
10.2
 
 
 
 
AHR
 
 
hsa-miR-29a-3p
 
 
-0.316
 
 
42.0
 
 
 
 
AHSG
 
 
hsa-miR-27a-3p
 
 
-0.29
 
 
5439.8
 
 
 
 
AIFM1
 
 
hsa-let-7b-5p
 
 
-0.314
 
 
77.0
 
 
 
 
AK1
 
 
hsa-miR-29a-3p
 
 
-0.333
 
 
35.8
 
 
 
 
AK4
 
 
hsa-miR-15b-5p
 
 
-0.316
 
 
95.6
 
 
 
 
AK4
 
 
hsa-miR-16-5p
 
 
-0.348
 
 
95.6
 
 
 
 
AKAP1
 
 
hsa-miR-148b-3p
 
 
-0.381
 
 
48.4
 
 
 
 
AKIRIN1
 
 
hsa-miR-27a-3p
 
 
-0.368
 
 
49.7
 
 
 
 
ALAD
 
 
hsa-miR-16-5p
 
 
-0.391
 
 
196.1
 
 
 
 
ALAD
 
 
hsa-miR-15b-5p
 
 
-0.446
 
 
196.1
 
 
 
 
ALCAM
 
 
hsa-miR-148b-3p
 
 
-0.339
 
 
44.8
 
 
 
 
ALPL
 
 
hsa-miR-10a-5p
 
 
-0.32
 
 
52.2
 
 
 
 
AMT
 
 
hsa-let-7b-5p
 
 
-0.463
 
 
52.5
 
 
 
 
ANKHD1
 
 
hsa-miR-23a-3p
 
 
-0.368
 
 
51.5
 
 
 
 
ANKHD1
 
 
hsa-miR-23b-3p
 
 
-0.368
 
 
51.5
 
 
 
 
ANKHD1
 
 
hsa-miR-30b-5p
 
 
-0.405
 
 
51.5
 
 
 
 
ANKRA2
 
 
hsa-miR-30b-5p
 
 
-0.354
 
 
10.1
 
 
 
 
ANKRA2
 
 
hsa-miR-30b-5p
 
 
-0.472
 
 
10.1
 
 
 
 
ANKRD17
 
 
hsa-miR-30b-5p
 
 
-0.336
 
 
19.3
 
 
 
 
ANKRD40
 
 
hsa-miR-27a-3p
 
 
-0.35
 
 
16.6
 
 
 
 
ANKRD46
 
 
hsa-let-7b-5p
 
 
-0.389
 
 
38.9
 
 
 
 
ANP32A
 
 
hsa-miR-132-3p
 
 
-0.374
 
 
71.3
 
 
 
 
ANTXR2
 
 
hsa-miR-29a-3p
 
 
-0.347
 
 
32.2
 
 
 
 
ANXA11
 
 
hsa-miR-16-5p
 
 
-0.447
 
 
46.7
 
 
 
 
ANXA11
 
 
hsa-miR-15b-5p
 
 
-0.463
 
 
46.7
 
 
 
 
AP1S1
 
 
hsa-let-7b-5p
 
 
-0.374
 
 
39.1
 
 
 
 
AP2A1
 
 
hsa-miR-15b-5p
 
 
-0.333
 
 
48.9
 
 
 
 
AP2A1
 
 
hsa-miR-16-5p
 
 
-0.333
 
 
48.9
 
 
 
 
AP2B1
 
 
hsa-miR-16-5p
 
 
-0.302
 
 
32.8
 
 
 
 
AP2B1
 
 
hsa-miR-15b-5p
 
 
-0.334
 
 
32.8
 
 
 
 
AP3S1
 
 
hsa-miR-30b-5p
 
 
-0.293
 
 
69.8
 
 
 
 
AP5S1
 
 
hsa-miR-744-5p
 
 
-0.354
 
 
13.9
 
 
 
 
APOO
 
 
hsa-miR-181b-5p
 
 
-0.5
 
 
17.0
 
 
 
 
APOO
 
 
hsa-miR-181a-5p
 
 
-0.5
 
 
17.0
 
 
 
 
APP
 
 
hsa-miR-16-5p
 
 
-0.298
 
 
251.5
 
 
 
 
APP
 
 
hsa-miR-15b-5p
 
 
-0.298
 
 
251.5
 
 
 
 
APPBP2
 
 
hsa-miR-27a-3p
 
 
-0.378
 
 
10.3
 
 
 
 
AQP11
 
 
hsa-miR-27a-3p
 
 
-0.666
 
 
15.1
 
 
 
 
AQP11
 
 
hsa-miR-15b-5p
 
 
-0.769
 
 
15.1
 
 
 
 
AQP11
 
 
hsa-miR-16-5p
 
 
-0.801
 
 
15.1
 
 
 
 
ARF4
 
 
hsa-miR-30b-5p
 
 
-0.289
 
 
161.3
 
 
 
 
ARF4
 
 
hsa-miR-148b-3p
 
 
-0.487
 
 
161.3
 
 
 
 
ARF5
 
 
hsa-miR-29a-3p
 
 
-0.491
 
 
153.7
 
 
 
 
ARF6
 
 
hsa-miR-181b-5p
 
 
-0.33
 
 
45.4
 
 
 
 
ARF6
 
 
hsa-miR-181a-5p
 
 
-0.33
 
 
45.4
 
 
 
 
ARFGEF1
 
 
hsa-miR-148b-3p
 
 
-0.306
 
 
14.2
 
 
 
 
ARFIP1
 
 
hsa-miR-320b
 
 
-0.339
 
 
14.4
 
 
 
 
ARFIP1
 
 
hsa-miR-320c
 
 
-0.339
 
 
14.4
 
 
 
 
ARFIP1
 
 
hsa-miR-148b-3p
 
 
-0.474
 
 
14.4
 
 
 
 
ARHGAP21
 
 
hsa-miR-148b-3p
 
 
-0.287
 
 
11.9
 
 
 
 
ARHGAP5
 
 
hsa-miR-486-5p
 
 
-0.3
 
 
29.8
 
 
 
 
ARID4B
 
 
hsa-miR-486-5p
 
 
-0.367
 
 
11.1
 
 
 
 
ARL2
 
 
hsa-miR-16-5p
 
 
-0.374
 
 
22.7
 
 
 
 
ARL2
 
 
hsa-miR-15b-5p
 
 
-0.374
 
 
22.7
 
 
 
 
ARL2
 
 
hsa-miR-16-5p
 
 
-0.418
 
 
22.7
 
 
 
 
ARL2
 
 
hsa-miR-15b-5p
 
 
-0.442
 
 
22.7
 
 
 
 
ARL4A
 
 
hsa-miR-29a-3p
 
 
-0.386
 
 
14.6
 
 
 
 
ARL4A
 
 
hsa-miR-30b-5p
 
 
-0.448
 
 
14.6
 
 
 
 
ARL4D
 
 
hsa-let-7b-5p
 
 
-0.417
 
 
55.4
 
 
 
 
ARL5B
 
 
hsa-miR-29a-3p
 
 
-0.304
 
 
27.9
 
 
 
 
ARL6IP1
 
 
hsa-miR-328-3p
 
 
-0.494
 
 
336.2
 
 
 
 
ARL6IP1
 
 
hsa-miR-148b-3p
 
 
-0.579
 
 
336.2
 
 
 
 
ARL8B
 
 
hsa-miR-148b-3p
 
 
-0.337
 
 
41.1
 
 
 
 
ARL8B
 
 
hsa-miR-320b
 
 
-0.376
 
 
41.1
 
 
 
 
ARL8B
 
 
hsa-miR-320c
 
 
-0.376
 
 
41.1
 
 
 
 
ARMC8
 
 
hsa-miR-486-5p
 
 
-0.321
 
 
15.8
 
 
 
 
 
 
 adipose tissue 
 
 
 
 
target_symbol
 
 
mature_mirna_id
 
 
score
 
 
nTPM
 
 
 
 
 
 
AAR2
 
 
hsa-miR-29a-3p
 
 
-0.327
 
 
21.1
 
 
 
 
ABCB7
 
 
hsa-miR-148b-3p
 
 
-0.515
 
 
12.4
 
 
 
 
ABCD3
 
 
hsa-miR-148b-3p
 
 
-0.388
 
 
15.7
 
 
 
 
ABCE1
 
 
hsa-miR-29a-3p
 
 
-0.404
 
 
28.1
 
 
 
 
ABHD4
 
 
hsa-miR-29a-3p
 
 
-0.312
 
 
24.3
 
 
 
 
ABHD5
 
 
hsa-miR-132-3p
 
 
-0.323
 
 
27.9
 
 
 
 
ABHD5
 
 
hsa-miR-27a-3p
 
 
-0.407
 
 
27.9
 
 
 
 
ACTA2
 
 
hsa-miR-27a-3p
 
 
-0.577
 
 
979.1
 
 
 
 
ACTC1
 
 
hsa-miR-30b-5p
 
 
-0.543
 
 
47.8
 
 
 
 
ACTR2
 
 
hsa-miR-15b-5p
 
 
-0.293
 
 
105.6
 
 
 
 
ACTR2
 
 
hsa-miR-16-5p
 
 
-0.293
 
 
105.6
 
 
 
 
ACVR1
 
 
hsa-miR-30b-5p
 
 
-0.29
 
 
12.6
 
 
 
 
ACVR1
 
 
hsa-miR-148b-3p
 
 
-0.397
 
 
12.6
 
 
 
 
ACVR1C
 
 
hsa-let-7b-5p
 
 
-0.457
 
 
29.3
 
 
 
 
ADA
 
 
hsa-miR-29a-3p
 
 
-0.376
 
 
16.5
 
 
 
 
ADAM10
 
 
hsa-miR-148b-3p
 
 
-0.356
 
 
14.0
 
 
 
 
ADAM9
 
 
hsa-miR-30b-5p
 
 
-0.312
 
 
31.0
 
 
 
 
ADAMTS10
 
 
hsa-miR-27a-3p
 
 
-0.354
 
 
13.8
 
 
 
 
ADAMTS10
 
 
hsa-miR-29a-3p
 
 
-0.516
 
 
13.8
 
 
 
 
ADAMTS9
 
 
hsa-miR-29a-3p
 
 
-0.305
 
 
39.1
 
 
 
 
ADAMTS9
 
 
hsa-miR-29a-3p
 
 
-0.485
 
 
39.1
 
 
 
 
ADCY3
 
 
hsa-miR-132-3p
 
 
-0.303
 
 
90.0
 
 
 
 
ADGRF5
 
 
hsa-miR-148b-3p
 
 
-0.354
 
 
66.6
 
 
 
 
ADIPOR2
 
 
hsa-miR-150-5p
 
 
-0.287
 
 
53.2
 
 
 
 
ADNP
 
 
hsa-miR-328-3p
 
 
-0.371
 
 
25.5
 
 
 
 
ADRA2A
 
 
hsa-miR-23a-3p
 
 
-0.371
 
 
46.9
 
 
 
 
ADRA2A
 
 
hsa-miR-23b-3p
 
 
-0.371
 
 
46.9
 
 
 
 
ADRB2
 
 
hsa-miR-15b-5p
 
 
-0.29
 
 
26.4
 
 
 
 
ADRB2
 
 
hsa-miR-16-5p
 
 
-0.323
 
 
26.4
 
 
 
 
ADRB2
 
 
hsa-let-7b-5p
 
 
-0.635
 
 
26.4
 
 
 
 
AFG3L2
 
 
hsa-miR-181b-5p
 
 
-0.491
 
 
17.1
 
 
 
 
AFG3L2
 
 
hsa-miR-181a-5p
 
 
-0.491
 
 
17.1
 
 
 
 
AGFG1
 
 
hsa-miR-148b-3p
 
 
-0.332
 
 
64.8
 
 
 
 
AGPAT3
 
 
hsa-miR-27a-3p
 
 
-0.29
 
 
19.6
 
 
 
 
AGPAT3
 
 
hsa-miR-16-5p
 
 
-0.299
 
 
19.6
 
 
 
 
AHCTF1
 
 
hsa-let-7b-5p
 
 
-0.374
 
 
11.8
 
 
 
 
AHDC1
 
 
hsa-miR-148b-3p
 
 
-0.413
 
 
11.7
 
 
 
 
AHR
 
 
hsa-miR-29a-3p
 
 
-0.316
 
 
21.0
 
 
 
 
AIFM1
 
 
hsa-let-7b-5p
 
 
-0.314
 
 
17.4
 
 
 
 
AK1
 
 
hsa-miR-29a-3p
 
 
-0.333
 
 
30.1
 
 
 
 
AK4
 
 
hsa-miR-15b-5p
 
 
-0.316
 
 
22.8
 
 
 
 
AK4
 
 
hsa-miR-16-5p
 
 
-0.348
 
 
22.8
 
 
 
 
AKAP1
 
 
hsa-miR-148b-3p
 
 
-0.381
 
 
38.1
 
 
 
 
AKIRIN1
 
 
hsa-miR-27a-3p
 
 
-0.368
 
 
43.1
 
 
 
 
AKT3
 
 
hsa-miR-29a-3p
 
 
-0.287
 
 
16.9
 
 
 
 
AKT3
 
 
hsa-miR-15b-5p
 
 
-0.301
 
 
16.9
 
 
 
 
AKT3
 
 
hsa-miR-16-5p
 
 
-0.309
 
 
16.9
 
 
 
 
ALAD
 
 
hsa-miR-16-5p
 
 
-0.391
 
 
51.3
 
 
 
 
ALAD
 
 
hsa-miR-15b-5p
 
 
-0.446
 
 
51.3
 
 
 
 
ALDH1A2
 
 
hsa-miR-23b-3p
 
 
-0.346
 
 
20.6
 
 
 
 
ALDH1A2
 
 
hsa-miR-23a-3p
 
 
-0.354
 
 
20.6
 
 
 
 
ALPL
 
 
hsa-miR-10a-5p
 
 
-0.32
 
 
23.7
 
 
 
 
AMOTL1
 
 
hsa-miR-16-5p
 
 
-0.297
 
 
14.5
 
 
 
 
AMOTL1
 
 
hsa-miR-15b-5p
 
 
-0.319
 
 
14.5
 
 
 
 
ANKHD1
 
 
hsa-miR-23a-3p
 
 
-0.368
 
 
37.3
 
 
 
 
ANKHD1
 
 
hsa-miR-23b-3p
 
 
-0.368
 
 
37.3
 
 
 
 
ANKHD1
 
 
hsa-miR-30b-5p
 
 
-0.405
 
 
37.3
 
 
 
 
ANKRD17
 
 
hsa-miR-30b-5p
 
 
-0.336
 
 
12.8
 
 
 
 
ANKRD29
 
 
hsa-miR-132-3p
 
 
-0.47
 
 
17.0
 
 
 
 
ANKRD40
 
 
hsa-miR-27a-3p
 
 
-0.35
 
 
40.1
 
 
 
 
ANKRD46
 
 
hsa-let-7b-5p
 
 
-0.389
 
 
18.0
 
 
 
 
ANP32A
 
 
hsa-miR-132-3p
 
 
-0.374
 
 
47.7
 
 
 
 
ANTXR2
 
 
hsa-miR-29a-3p
 
 
-0.347
 
 
57.1
 
 
 
 
ANXA11
 
 
hsa-miR-16-5p
 
 
-0.447
 
 
130.8
 
 
 
 
ANXA11
 
 
hsa-miR-15b-5p
 
 
-0.463
 
 
130.8
 
 
 
 
AP1S1
 
 
hsa-let-7b-5p
 
 
-0.374
 
 
15.1
 
 
 
 
AP2A1
 
 
hsa-miR-15b-5p
 
 
-0.333
 
 
44.9
 
 
 
 
AP2A1
 
 
hsa-miR-16-5p
 
 
-0.333
 
 
44.9
 
 
 
 
AP2B1
 
 
hsa-miR-16-5p
 
 
-0.302
 
 
43.1
 
 
 
 
AP2B1
 
 
hsa-miR-15b-5p
 
 
-0.334
 
 
43.1
 
 
 
 
AP3S1
 
 
hsa-miR-30b-5p
 
 
-0.293
 
 
73.6
 
 
 
 
APP
 
 
hsa-miR-16-5p
 
 
-0.298
 
 
230.6
 
 
 
 
APP
 
 
hsa-miR-15b-5p
 
 
-0.298
 
 
230.6
 
 
 
 
APPBP2
 
 
hsa-miR-27a-3p
 
 
-0.378
 
 
13.9
 
 
 
 
ARF4
 
 
hsa-miR-30b-5p
 
 
-0.289
 
 
118.7
 
 
 
 
ARF4
 
 
hsa-miR-148b-3p
 
 
-0.487
 
 
118.7
 
 
 
 
ARF5
 
 
hsa-miR-29a-3p
 
 
-0.491
 
 
109.3
 
 
 
 
ARF6
 
 
hsa-miR-181b-5p
 
 
-0.33
 
 
30.0
 
 
 
 
ARF6
 
 
hsa-miR-181a-5p
 
 
-0.33
 
 
30.0
 
 
 
 
ARFGEF1
 
 
hsa-miR-148b-3p
 
 
-0.306
 
 
10.7
 
 
 
 
ARFIP1
 
 
hsa-miR-320b
 
 
-0.339
 
 
15.8
 
 
 
 
ARFIP1
 
 
hsa-miR-320c
 
 
-0.339
 
 
15.8
 
 
 
 
ARFIP1
 
 
hsa-miR-148b-3p
 
 
-0.474
 
 
15.8
 
 
 
 
ARHGAP21
 
 
hsa-miR-148b-3p
 
 
-0.287
 
 
26.1
 
 
 
 
ARHGAP5
 
 
hsa-miR-486-5p
 
 
-0.3
 
 
22.2
 
 
 
 
ARID1B
 
 
hsa-miR-29a-3p
 
 
-0.342
 
 
14.1
 
 
 
 
ARID4B
 
 
hsa-miR-486-5p
 
 
-0.367
 
 
10.7
 
 
 
 
ARL2
 
 
hsa-miR-16-5p
 
 
-0.374
 
 
125.8
 
 
 
 
ARL2
 
 
hsa-miR-15b-5p
 
 
-0.374
 
 
125.8
 
 
 
 
ARL2
 
 
hsa-miR-16-5p
 
 
-0.418
 
 
125.8
 
 
 
 
ARL2
 
 
hsa-miR-15b-5p
 
 
-0.442
 
 
125.8
 
 
 
 
ARL4A
 
 
hsa-miR-29a-3p
 
 
-0.386
 
 
30.8
 
 
 
 
ARL4A
 
 
hsa-miR-30b-5p
 
 
-0.448
 
 
30.8
 
 
 
 
ARL4D
 
 
hsa-let-7b-5p
 
 
-0.417
 
 
20.3
 
 
 
 
ARL5B
 
 
hsa-miR-29a-3p
 
 
-0.304
 
 
10.5
 
 
 
 
ARL6IP1
 
 
hsa-miR-328-3p
 
 
-0.494
 
 
133.2
 
 
 
 
ARL6IP1
 
 
hsa-miR-148b-3p
 
 
-0.579
 
 
133.2
 
 
 
 
ARL6IP6
 
 
hsa-let-7b-5p
 
 
-0.293
 
 
11.0
 
 
 
 
ARL8B
 
 
hsa-miR-148b-3p
 
 
-0.337
 
 
39.1
 
 
 
 
ARL8B
 
 
hsa-miR-320b
 
 
-0.376
 
 
39.1
 
 
 
 
 
 
 adrenal gland 
 
 
 
 
target_symbol
 
 
mature_mirna_id
 
 
score
 
 
nTPM
 
 
 
 
 
 
AAR2
 
 
hsa-miR-29a-3p
 
 
-0.327
 
 
24.5
 
 
 
 
ABCB7
 
 
hsa-miR-148b-3p
 
 
-0.515
 
 
12.5
 
 
 
 
ABCD3
 
 
hsa-miR-148b-3p
 
 
-0.388
 
 
17.6
 
 
 
 
ABCE1
 
 
hsa-miR-29a-3p
 
 
-0.404
 
 
20.3
 
 
 
 
ABHD4
 
 
hsa-miR-29a-3p
 
 
-0.312
 
 
29.6
 
 
 
 
ACTA2
 
 
hsa-miR-27a-3p
 
 
-0.577
 
 
227.6
 
 
 
 
ACTR2
 
 
hsa-miR-15b-5p
 
 
-0.293
 
 
66.7
 
 
 
 
ACTR2
 
 
hsa-miR-16-5p
 
 
-0.293
 
 
66.7
 
 
 
 
ACTR3C
 
 
hsa-miR-30b-5p
 
 
-0.448
 
 
10.1
 
 
 
 
ADAM9
 
 
hsa-miR-30b-5p
 
 
-0.312
 
 
19.6
 
 
 
 
ADCY3
 
 
hsa-miR-132-3p
 
 
-0.303
 
 
16.9
 
 
 
 
ADGRF5
 
 
hsa-miR-148b-3p
 
 
-0.354
 
 
22.0
 
 
 
 
ADIPOR2
 
 
hsa-miR-150-5p
 
 
-0.287
 
 
69.8
 
 
 
 
ADNP
 
 
hsa-miR-328-3p
 
 
-0.371
 
 
29.8
 
 
 
 
AFG3L2
 
 
hsa-miR-181b-5p
 
 
-0.491
 
 
12.9
 
 
 
 
AFG3L2
 
 
hsa-miR-181a-5p
 
 
-0.491
 
 
12.9
 
 
 
 
AGFG1
 
 
hsa-miR-148b-3p
 
 
-0.332
 
 
37.2
 
 
 
 
AGPAT3
 
 
hsa-miR-27a-3p
 
 
-0.29
 
 
19.9
 
 
 
 
AGPAT3
 
 
hsa-miR-16-5p
 
 
-0.299
 
 
19.9
 
 
 
 
AHDC1
 
 
hsa-miR-148b-3p
 
 
-0.413
 
 
23.0
 
 
 
 
AHR
 
 
hsa-miR-29a-3p
 
 
-0.316
 
 
14.2
 
 
 
 
AIFM1
 
 
hsa-let-7b-5p
 
 
-0.314
 
 
50.1
 
 
 
 
AK1
 
 
hsa-miR-29a-3p
 
 
-0.333
 
 
40.4
 
 
 
 
AKAP1
 
 
hsa-miR-148b-3p
 
 
-0.381
 
 
14.9
 
 
 
 
AKIRIN1
 
 
hsa-miR-27a-3p
 
 
-0.368
 
 
28.2
 
 
 
 
ALAD
 
 
hsa-miR-16-5p
 
 
-0.391
 
 
148.4
 
 
 
 
ALAD
 
 
hsa-miR-15b-5p
 
 
-0.446
 
 
148.4
 
 
 
 
ALCAM
 
 
hsa-miR-148b-3p
 
 
-0.339
 
 
30.9
 
 
 
 
ALPL
 
 
hsa-miR-10a-5p
 
 
-0.32
 
 
57.7
 
 
 
 
AMT
 
 
hsa-let-7b-5p
 
 
-0.463
 
 
15.0
 
 
 
 
ANKHD1
 
 
hsa-miR-23a-3p
 
 
-0.368
 
 
41.3
 
 
 
 
ANKHD1
 
 
hsa-miR-23b-3p
 
 
-0.368
 
 
41.3
 
 
 
 
ANKHD1
 
 
hsa-miR-30b-5p
 
 
-0.405
 
 
41.3
 
 
 
 
ANKRA2
 
 
hsa-miR-30b-5p
 
 
-0.354
 
 
10.7
 
 
 
 
ANKRA2
 
 
hsa-miR-30b-5p
 
 
-0.472
 
 
10.7
 
 
 
 
ANKRD17
 
 
hsa-miR-30b-5p
 
 
-0.336
 
 
12.7
 
 
 
 
ANKRD40
 
 
hsa-miR-27a-3p
 
 
-0.35
 
 
18.5
 
 
 
 
ANKRD46
 
 
hsa-let-7b-5p
 
 
-0.389
 
 
36.5
 
 
 
 
ANP32A
 
 
hsa-miR-132-3p
 
 
-0.374
 
 
32.7
 
 
 
 
ANTXR2
 
 
hsa-miR-29a-3p
 
 
-0.347
 
 
28.3
 
 
 
 
ANXA11
 
 
hsa-miR-16-5p
 
 
-0.447
 
 
28.3
 
 
 
 
ANXA11
 
 
hsa-miR-15b-5p
 
 
-0.463
 
 
28.3
 
 
 
 
AP1S1
 
 
hsa-let-7b-5p
 
 
-0.374
 
 
25.7
 
 
 
 
AP2A1
 
 
hsa-miR-15b-5p
 
 
-0.333
 
 
83.5
 
 
 
 
AP2A1
 
 
hsa-miR-16-5p
 
 
-0.333
 
 
83.5
 
 
 
 
AP2B1
 
 
hsa-miR-16-5p
 
 
-0.302
 
 
39.3
 
 
 
 
AP2B1
 
 
hsa-miR-15b-5p
 
 
-0.334
 
 
39.3
 
 
 
 
AP3S1
 
 
hsa-miR-30b-5p
 
 
-0.293
 
 
42.4
 
 
 
 
AP5S1
 
 
hsa-miR-744-5p
 
 
-0.354
 
 
10.8
 
 
 
 
APOO
 
 
hsa-miR-181b-5p
 
 
-0.5
 
 
22.6
 
 
 
 
APOO
 
 
hsa-miR-181a-5p
 
 
-0.5
 
 
22.6
 
 
 
 
APP
 
 
hsa-miR-16-5p
 
 
-0.298
 
 
422.0
 
 
 
 
APP
 
 
hsa-miR-15b-5p
 
 
-0.298
 
 
422.0
 
 
 
 
APPBP2
 
 
hsa-miR-27a-3p
 
 
-0.378
 
 
10.6
 
 
 
 
AQP11
 
 
hsa-miR-27a-3p
 
 
-0.666
 
 
14.1
 
 
 
 
AQP11
 
 
hsa-miR-15b-5p
 
 
-0.769
 
 
14.1
 
 
 
 
AQP11
 
 
hsa-miR-16-5p
 
 
-0.801
 
 
14.1
 
 
 
 
ARF4
 
 
hsa-miR-30b-5p
 
 
-0.289
 
 
151.0
 
 
 
 
ARF4
 
 
hsa-miR-148b-3p
 
 
-0.487
 
 
151.0
 
 
 
 
ARF5
 
 
hsa-miR-29a-3p
 
 
-0.491
 
 
102.3
 
 
 
 
ARF6
 
 
hsa-miR-181b-5p
 
 
-0.33
 
 
24.5
 
 
 
 
ARF6
 
 
hsa-miR-181a-5p
 
 
-0.33
 
 
24.5
 
 
 
 
ARFIP1
 
 
hsa-miR-320b
 
 
-0.339
 
 
13.9
 
 
 
 
ARFIP1
 
 
hsa-miR-320c
 
 
-0.339
 
 
13.9
 
 
 
 
ARFIP1
 
 
hsa-miR-148b-3p
 
 
-0.474
 
 
13.9
 
 
 
 
ARHGAP21
 
 
hsa-miR-148b-3p
 
 
-0.287
 
 
16.9
 
 
 
 
ARHGAP5
 
 
hsa-miR-486-5p
 
 
-0.3
 
 
25.8
 
 
 
 
ARID1B
 
 
hsa-miR-29a-3p
 
 
-0.342
 
 
10.7
 
 
 
 
ARID4B
 
 
hsa-miR-486-5p
 
 
-0.367
 
 
10.2
 
 
 
 
ARL2
 
 
hsa-miR-16-5p
 
 
-0.374
 
 
94.8
 
 
 
 
ARL2
 
 
hsa-miR-15b-5p
 
 
-0.374
 
 
94.8
 
 
 
 
ARL2
 
 
hsa-miR-16-5p
 
 
-0.418
 
 
94.8
 
 
 
 
ARL2
 
 
hsa-miR-15b-5p
 
 
-0.442
 
 
94.8
 
 
 
 
ARL5B
 
 
hsa-miR-29a-3p
 
 
-0.304
 
 
19.6
 
 
 
 
ARL6IP1
 
 
hsa-miR-328-3p
 
 
-0.494
 
 
161.7
 
 
 
 
ARL6IP1
 
 
hsa-miR-148b-3p
 
 
-0.579
 
 
161.7
 
 
 
 
ARL8B
 
 
hsa-miR-148b-3p
 
 
-0.337
 
 
48.3
 
 
 
 
ARL8B
 
 
hsa-miR-320b
 
 
-0.376
 
 
48.3
 
 
 
 
ARL8B
 
 
hsa-miR-320c
 
 
-0.376
 
 
48.3
 
 
 
 
ARMC8
 
 
hsa-miR-486-5p
 
 
-0.321
 
 
13.5
 
 
 
 
ARMCX2
 
 
hsa-miR-15b-5p
 
 
-0.344
 
 
42.3
 
 
 
 
ARMCX2
 
 
hsa-miR-16-5p
 
 
-0.366
 
 
42.3
 
 
 
 
ARMCX3
 
 
hsa-miR-425-5p
 
 
-0.325
 
 
55.6
 
 
 
 
ARMT1
 
 
hsa-let-7b-5p
 
 
-0.291
 
 
15.1
 
 
 
 
ARPP19
 
 
hsa-miR-320b
 
 
-0.325
 
 
48.8
 
 
 
 
ARPP19
 
 
hsa-miR-320c
 
 
-0.325
 
 
48.8
 
 
 
 
ARPP19
 
 
hsa-miR-15b-5p
 
 
-0.346
 
 
48.8
 
 
 
 
ARPP19
 
 
hsa-miR-16-5p
 
 
-0.354
 
 
48.8
 
 
 
 
ARPP19
 
 
hsa-miR-29a-3p
 
 
-0.371
 
 
48.8
 
 
 
 
ARPP19
 
 
hsa-miR-328-3p
 
 
-0.404
 
 
48.8
 
 
 
 
ARPP19
 
 
hsa-let-7b-5p
 
 
-0.476
 
 
48.8
 
 
 
 
ARRDC3
 
 
hsa-miR-148b-3p
 
 
-0.305
 
 
75.7
 
 
 
 
ARRDC3
 
 
hsa-miR-29a-3p
 
 
-0.402
 
 
75.7
 
 
 
 
ARRDC4
 
 
hsa-let-7b-5p
 
 
-0.329
 
 
11.5
 
 
 
 
ASAH1
 
 
hsa-miR-27a-3p
 
 
-0.398
 
 
67.8
 
 
 
 
ASAP1
 
 
hsa-let-7b-5p
 
 
-0.308
 
 
20.2
 
 
 
 
ASB3
 
 
hsa-miR-30b-5p
 
 
-0.418
 
 
10.8
 
 
 
 
ASB3
 
 
hsa-miR-30b-5p
 
 
-0.418
 
 
10.8
 
 
 
 
ASH1L
 
 
hsa-miR-15b-5p
 
 
-0.325
 
 
16.2
 
 
 
 
ASH1L
 
 
hsa-miR-16-5p
 
 
-0.357
 
 
16.2
 
 
 
 
 
 
 amygdala 
 
 
 
 
target_symbol
 
 
mature_mirna_id
 
 
score
 
 
nTPM
 
 
 
 
 
 
AAR2
 
 
hsa-miR-29a-3p
 
 
-0.327
 
 
10.0
 
 
 
 
ABCD3
 
 
hsa-miR-148b-3p
 
 
-0.388
 
 
16.0
 
 
 
 
ABCE1
 
 
hsa-miR-29a-3p
 
 
-0.404
 
 
11.9
 
 
 
 
ABHD17B
 
 
hsa-miR-486-5p
 
 
-0.348
 
 
12.7
 
 
 
 
ABHD17C
 
 
hsa-let-7b-5p
 
 
-0.359
 
 
17.9
 
 
 
 
ABHD17C
 
 
hsa-miR-27a-3p
 
 
-0.435
 
 
17.9
 
 
 
 
ABHD4
 
 
hsa-miR-29a-3p
 
 
-0.312
 
 
16.0
 
 
 
 
ACSL6
 
 
hsa-let-7b-5p
 
 
-0.336
 
 
20.3
 
 
 
 
ACTA2
 
 
hsa-miR-27a-3p
 
 
-0.577
 
 
36.7
 
 
 
 
ACTR2
 
 
hsa-miR-15b-5p
 
 
-0.293
 
 
53.7
 
 
 
 
ACTR2
 
 
hsa-miR-16-5p
 
 
-0.293
 
 
53.7
 
 
 
 
ADAP1
 
 
hsa-miR-27a-3p
 
 
-0.302
 
 
109.3
 
 
 
 
ADCY2
 
 
hsa-miR-148b-3p
 
 
-0.299
 
 
28.0
 
 
 
 
ADCY3
 
 
hsa-miR-132-3p
 
 
-0.303
 
 
27.0
 
 
 
 
ADGRA3
 
 
hsa-miR-27a-3p
 
 
-0.296
 
 
19.9
 
 
 
 
ADGRA3
 
 
hsa-miR-30b-5p
 
 
-0.304
 
 
19.9
 
 
 
 
ADGRB3
 
 
hsa-miR-148b-3p
 
 
-0.524
 
 
12.8
 
 
 
 
ADIPOR2
 
 
hsa-miR-150-5p
 
 
-0.287
 
 
43.7
 
 
 
 
ADNP
 
 
hsa-miR-328-3p
 
 
-0.371
 
 
23.8
 
 
 
 
AGFG1
 
 
hsa-miR-148b-3p
 
 
-0.332
 
 
25.1
 
 
 
 
AGPAT3
 
 
hsa-miR-27a-3p
 
 
-0.29
 
 
41.1
 
 
 
 
AGPAT3
 
 
hsa-miR-16-5p
 
 
-0.299
 
 
41.1
 
 
 
 
AGPAT4
 
 
hsa-miR-29a-3p
 
 
-0.376
 
 
10.0
 
 
 
 
AHDC1
 
 
hsa-miR-148b-3p
 
 
-0.413
 
 
10.1
 
 
 
 
AIFM1
 
 
hsa-let-7b-5p
 
 
-0.314
 
 
10.3
 
 
 
 
AK1
 
 
hsa-miR-29a-3p
 
 
-0.333
 
 
104.5
 
 
 
 
AK4
 
 
hsa-miR-15b-5p
 
 
-0.316
 
 
19.8
 
 
 
 
AK4
 
 
hsa-miR-16-5p
 
 
-0.348
 
 
19.8
 
 
 
 
AKAP1
 
 
hsa-miR-148b-3p
 
 
-0.381
 
 
22.8
 
 
 
 
AKAP6
 
 
hsa-let-7b-5p
 
 
-0.307
 
 
12.4
 
 
 
 
AKIRIN1
 
 
hsa-miR-27a-3p
 
 
-0.368
 
 
32.7
 
 
 
 
AKT3
 
 
hsa-miR-29a-3p
 
 
-0.287
 
 
17.4
 
 
 
 
AKT3
 
 
hsa-miR-15b-5p
 
 
-0.301
 
 
17.4
 
 
 
 
AKT3
 
 
hsa-miR-16-5p
 
 
-0.309
 
 
17.4
 
 
 
 
ALAD
 
 
hsa-miR-16-5p
 
 
-0.391
 
 
58.3
 
 
 
 
ALAD
 
 
hsa-miR-15b-5p
 
 
-0.446
 
 
58.3
 
 
 
 
ALCAM
 
 
hsa-miR-148b-3p
 
 
-0.339
 
 
31.7
 
 
 
 
ALPL
 
 
hsa-miR-10a-5p
 
 
-0.32
 
 
10.6
 
 
 
 
AMOT
 
 
hsa-miR-29a-3p
 
 
-0.304
 
 
18.6
 
 
 
 
ANGEL2
 
 
hsa-miR-132-3p
 
 
-0.357
 
 
10.6
 
 
 
 
ANKHD1
 
 
hsa-miR-23a-3p
 
 
-0.368
 
 
18.9
 
 
 
 
ANKHD1
 
 
hsa-miR-23b-3p
 
 
-0.368
 
 
18.9
 
 
 
 
ANKHD1
 
 
hsa-miR-30b-5p
 
 
-0.405
 
 
18.9
 
 
 
 
ANKRD17
 
 
hsa-miR-30b-5p
 
 
-0.336
 
 
10.0
 
 
 
 
ANKRD40
 
 
hsa-miR-27a-3p
 
 
-0.35
 
 
96.8
 
 
 
 
ANKRD46
 
 
hsa-let-7b-5p
 
 
-0.389
 
 
28.3
 
 
 
 
ANLN
 
 
hsa-miR-16-5p
 
 
-0.31
 
 
32.3
 
 
 
 
ANLN
 
 
hsa-miR-15b-5p
 
 
-0.334
 
 
32.3
 
 
 
 
ANP32A
 
 
hsa-miR-132-3p
 
 
-0.374
 
 
49.8
 
 
 
 
ANXA11
 
 
hsa-miR-16-5p
 
 
-0.447
 
 
35.8
 
 
 
 
ANXA11
 
 
hsa-miR-15b-5p
 
 
-0.463
 
 
35.8
 
 
 
 
AP1S1
 
 
hsa-let-7b-5p
 
 
-0.374
 
 
88.4
 
 
 
 
AP2A1
 
 
hsa-miR-15b-5p
 
 
-0.333
 
 
49.9
 
 
 
 
AP2A1
 
 
hsa-miR-16-5p
 
 
-0.333
 
 
49.9
 
 
 
 
AP2B1
 
 
hsa-miR-16-5p
 
 
-0.302
 
 
69.3
 
 
 
 
AP2B1
 
 
hsa-miR-15b-5p
 
 
-0.334
 
 
69.3
 
 
 
 
AP3S1
 
 
hsa-miR-30b-5p
 
 
-0.293
 
 
82.5
 
 
 
 
APC
 
 
hsa-miR-29a-3p
 
 
-0.413
 
 
38.7
 
 
 
 
APLN
 
 
hsa-miR-16-5p
 
 
-0.31
 
 
15.8
 
 
 
 
APLN
 
 
hsa-miR-15b-5p
 
 
-0.395
 
 
15.8
 
 
 
 
APLN
 
 
hsa-miR-16-5p
 
 
-0.395
 
 
15.8
 
 
 
 
APOO
 
 
hsa-miR-181b-5p
 
 
-0.5
 
 
45.1
 
 
 
 
APOO
 
 
hsa-miR-181a-5p
 
 
-0.5
 
 
45.1
 
 
 
 
APP
 
 
hsa-miR-16-5p
 
 
-0.298
 
 
396.2
 
 
 
 
APP
 
 
hsa-miR-15b-5p
 
 
-0.298
 
 
396.2
 
 
 
 
ARF4
 
 
hsa-miR-30b-5p
 
 
-0.289
 
 
60.1
 
 
 
 
ARF4
 
 
hsa-miR-148b-3p
 
 
-0.487
 
 
60.1
 
 
 
 
ARF5
 
 
hsa-miR-29a-3p
 
 
-0.491
 
 
176.5
 
 
 
 
ARHGAP21
 
 
hsa-miR-148b-3p
 
 
-0.287
 
 
40.6
 
 
 
 
ARHGAP5
 
 
hsa-miR-486-5p
 
 
-0.3
 
 
27.1
 
 
 
 
ARL2
 
 
hsa-miR-16-5p
 
 
-0.374
 
 
171.9
 
 
 
 
ARL2
 
 
hsa-miR-15b-5p
 
 
-0.374
 
 
171.9
 
 
 
 
ARL2
 
 
hsa-miR-16-5p
 
 
-0.418
 
 
171.9
 
 
 
 
ARL2
 
 
hsa-miR-15b-5p
 
 
-0.442
 
 
171.9
 
 
 
 
ARL3
 
 
hsa-miR-16-5p
 
 
-0.496
 
 
26.9
 
 
 
 
ARL3
 
 
hsa-miR-15b-5p
 
 
-0.507
 
 
26.9
 
 
 
 
ARL4A
 
 
hsa-miR-29a-3p
 
 
-0.386
 
 
14.1
 
 
 
 
ARL4A
 
 
hsa-miR-30b-5p
 
 
-0.448
 
 
14.1
 
 
 
 
ARL6IP1
 
 
hsa-miR-328-3p
 
 
-0.494
 
 
241.3
 
 
 
 
ARL6IP1
 
 
hsa-miR-148b-3p
 
 
-0.579
 
 
241.3
 
 
 
 
ARL8B
 
 
hsa-miR-148b-3p
 
 
-0.337
 
 
53.8
 
 
 
 
ARL8B
 
 
hsa-miR-320b
 
 
-0.376
 
 
53.8
 
 
 
 
ARL8B
 
 
hsa-miR-320c
 
 
-0.376
 
 
53.8
 
 
 
 
ARMC8
 
 
hsa-miR-486-5p
 
 
-0.321
 
 
13.4
 
 
 
 
ARMCX2
 
 
hsa-miR-15b-5p
 
 
-0.344
 
 
20.2
 
 
 
 
ARMCX2
 
 
hsa-miR-16-5p
 
 
-0.366
 
 
20.2
 
 
 
 
ARMCX3
 
 
hsa-miR-425-5p
 
 
-0.325
 
 
32.9
 
 
 
 
ARPP19
 
 
hsa-miR-320b
 
 
-0.325
 
 
178.5
 
 
 
 
ARPP19
 
 
hsa-miR-320c
 
 
-0.325
 
 
178.5
 
 
 
 
ARPP19
 
 
hsa-miR-15b-5p
 
 
-0.346
 
 
178.5
 
 
 
 
ARPP19
 
 
hsa-miR-16-5p
 
 
-0.354
 
 
178.5
 
 
 
 
ARPP19
 
 
hsa-miR-29a-3p
 
 
-0.371
 
 
178.5
 
 
 
 
ARPP19
 
 
hsa-miR-328-3p
 
 
-0.404
 
 
178.5
 
 
 
 
ARPP19
 
 
hsa-let-7b-5p
 
 
-0.476
 
 
178.5
 
 
 
 
ARPP21
 
 
hsa-miR-16-5p
 
 
-0.309
 
 
38.3
 
 
 
 
ARPP21
 
 
hsa-miR-15b-5p
 
 
-0.342
 
 
38.3
 
 
 
 
ARRDC4
 
 
hsa-let-7b-5p
 
 
-0.329
 
 
13.2
 
 
 
 
ASAH1
 
 
hsa-miR-27a-3p
 
 
-0.398
 
 
50.6
 
 
 
 
ASAP1
 
 
hsa-let-7b-5p
 
 
-0.308
 
 
11.2
 
 
 
 
ASB3
 
 
hsa-miR-30b-5p
 
 
-0.418
 
 
20.8
 
 
 
 
 
 
 appendix 
 
 
 
 
target_symbol
 
 
mature_mirna_id
 
 
score
 
 
nTPM
 
 
 
 
 
 
AAR2
 
 
hsa-miR-29a-3p
 
 
-0.327
 
 
13.4
 
 
 
 
ABCD3
 
 
hsa-miR-148b-3p
 
 
-0.388
 
 
15.3
 
 
 
 
ABCE1
 
 
hsa-miR-29a-3p
 
 
-0.404
 
 
31.7
 
 
 
 
ACTA2
 
 
hsa-miR-27a-3p
 
 
-0.577
 
 
687.0
 
 
 
 
ACTC1
 
 
hsa-miR-30b-5p
 
 
-0.543
 
 
23.3
 
 
 
 
ACTR2
 
 
hsa-miR-15b-5p
 
 
-0.293
 
 
202.6
 
 
 
 
ACTR2
 
 
hsa-miR-16-5p
 
 
-0.293
 
 
202.6
 
 
 
 
ADA
 
 
hsa-miR-29a-3p
 
 
-0.376
 
 
15.1
 
 
 
 
ADAM10
 
 
hsa-miR-148b-3p
 
 
-0.356
 
 
17.9
 
 
 
 
ADAM9
 
 
hsa-miR-30b-5p
 
 
-0.312
 
 
27.3
 
 
 
 
ADAMTS9
 
 
hsa-miR-29a-3p
 
 
-0.305
 
 
11.5
 
 
 
 
ADAMTS9
 
 
hsa-miR-29a-3p
 
 
-0.485
 
 
11.5
 
 
 
 
ADAP1
 
 
hsa-miR-27a-3p
 
 
-0.302
 
 
10.6
 
 
 
 
ADCY3
 
 
hsa-miR-132-3p
 
 
-0.303
 
 
16.3
 
 
 
 
ADIPOR2
 
 
hsa-miR-150-5p
 
 
-0.287
 
 
10.0
 
 
 
 
ADNP
 
 
hsa-miR-328-3p
 
 
-0.371
 
 
19.0
 
 
 
 
AFG3L2
 
 
hsa-miR-181b-5p
 
 
-0.491
 
 
12.5
 
 
 
 
AFG3L2
 
 
hsa-miR-181a-5p
 
 
-0.491
 
 
12.5
 
 
 
 
AGFG1
 
 
hsa-miR-148b-3p
 
 
-0.332
 
 
45.7
 
 
 
 
AGPAT3
 
 
hsa-miR-27a-3p
 
 
-0.29
 
 
18.1
 
 
 
 
AGPAT3
 
 
hsa-miR-16-5p
 
 
-0.299
 
 
18.1
 
 
 
 
AHR
 
 
hsa-miR-29a-3p
 
 
-0.316
 
 
24.3
 
 
 
 
AIFM1
 
 
hsa-let-7b-5p
 
 
-0.314
 
 
16.6
 
 
 
 
AK1
 
 
hsa-miR-29a-3p
 
 
-0.333
 
 
10.8
 
 
 
 
AKAP1
 
 
hsa-miR-148b-3p
 
 
-0.381
 
 
16.8
 
 
 
 
AKIRIN1
 
 
hsa-miR-27a-3p
 
 
-0.368
 
 
31.6
 
 
 
 
ALAD
 
 
hsa-miR-16-5p
 
 
-0.391
 
 
15.7
 
 
 
 
ALAD
 
 
hsa-miR-15b-5p
 
 
-0.446
 
 
15.7
 
 
 
 
ALCAM
 
 
hsa-miR-148b-3p
 
 
-0.339
 
 
11.5
 
 
 
 
ALPL
 
 
hsa-miR-10a-5p
 
 
-0.32
 
 
26.3
 
 
 
 
ANKHD1
 
 
hsa-miR-23a-3p
 
 
-0.368
 
 
34.9
 
 
 
 
ANKHD1
 
 
hsa-miR-23b-3p
 
 
-0.368
 
 
34.9
 
 
 
 
ANKHD1
 
 
hsa-miR-30b-5p
 
 
-0.405
 
 
34.9
 
 
 
 
ANKRD17
 
 
hsa-miR-30b-5p
 
 
-0.336
 
 
12.9
 
 
 
 
ANKRD40
 
 
hsa-miR-27a-3p
 
 
-0.35
 
 
14.6
 
 
 
 
ANP32A
 
 
hsa-miR-132-3p
 
 
-0.374
 
 
82.3
 
 
 
 
ANTXR2
 
 
hsa-miR-29a-3p
 
 
-0.347
 
 
33.4
 
 
 
 
ANXA11
 
 
hsa-miR-16-5p
 
 
-0.447
 
 
93.8
 
 
 
 
ANXA11
 
 
hsa-miR-15b-5p
 
 
-0.463
 
 
93.8
 
 
 
 
AP1S1
 
 
hsa-let-7b-5p
 
 
-0.374
 
 
13.1
 
 
 
 
AP2A1
 
 
hsa-miR-15b-5p
 
 
-0.333
 
 
23.7
 
 
 
 
AP2A1
 
 
hsa-miR-16-5p
 
 
-0.333
 
 
23.7
 
 
 
 
AP2B1
 
 
hsa-miR-16-5p
 
 
-0.302
 
 
36.0
 
 
 
 
AP2B1
 
 
hsa-miR-15b-5p
 
 
-0.334
 
 
36.0
 
 
 
 
AP3S1
 
 
hsa-miR-30b-5p
 
 
-0.293
 
 
36.6
 
 
 
 
APP
 
 
hsa-miR-16-5p
 
 
-0.298
 
 
146.0
 
 
 
 
APP
 
 
hsa-miR-15b-5p
 
 
-0.298
 
 
146.0
 
 
 
 
ARF4
 
 
hsa-miR-30b-5p
 
 
-0.289
 
 
101.3
 
 
 
 
ARF4
 
 
hsa-miR-148b-3p
 
 
-0.487
 
 
101.3
 
 
 
 
ARF5
 
 
hsa-miR-29a-3p
 
 
-0.491
 
 
68.5
 
 
 
 
ARF6
 
 
hsa-miR-181b-5p
 
 
-0.33
 
 
41.0
 
 
 
 
ARF6
 
 
hsa-miR-181a-5p
 
 
-0.33
 
 
41.0
 
 
 
 
ARFGEF1
 
 
hsa-miR-148b-3p
 
 
-0.306
 
 
13.8
 
 
 
 
ARFIP1
 
 
hsa-miR-320b
 
 
-0.339
 
 
14.9
 
 
 
 
ARFIP1
 
 
hsa-miR-320c
 
 
-0.339
 
 
14.9
 
 
 
 
ARFIP1
 
 
hsa-miR-148b-3p
 
 
-0.474
 
 
14.9
 
 
 
 
ARHGAP5
 
 
hsa-miR-486-5p
 
 
-0.3
 
 
18.6
 
 
 
 
ARID1B
 
 
hsa-miR-29a-3p
 
 
-0.342
 
 
13.6
 
 
 
 
ARID4B
 
 
hsa-miR-486-5p
 
 
-0.367
 
 
13.3
 
 
 
 
ARL2
 
 
hsa-miR-16-5p
 
 
-0.374
 
 
38.3
 
 
 
 
ARL2
 
 
hsa-miR-15b-5p
 
 
-0.374
 
 
38.3
 
 
 
 
ARL2
 
 
hsa-miR-16-5p
 
 
-0.418
 
 
38.3
 
 
 
 
ARL2
 
 
hsa-miR-15b-5p
 
 
-0.442
 
 
38.3
 
 
 
 
ARL4A
 
 
hsa-miR-29a-3p
 
 
-0.386
 
 
15.8
 
 
 
 
ARL4A
 
 
hsa-miR-30b-5p
 
 
-0.448
 
 
15.8
 
 
 
 
ARL6IP1
 
 
hsa-miR-328-3p
 
 
-0.494
 
 
73.3
 
 
 
 
ARL6IP1
 
 
hsa-miR-148b-3p
 
 
-0.579
 
 
73.3
 
 
 
 
ARL6IP6
 
 
hsa-let-7b-5p
 
 
-0.293
 
 
10.9
 
 
 
 
ARL8B
 
 
hsa-miR-148b-3p
 
 
-0.337
 
 
34.1
 
 
 
 
ARL8B
 
 
hsa-miR-320b
 
 
-0.376
 
 
34.1
 
 
 
 
ARL8B
 
 
hsa-miR-320c
 
 
-0.376
 
 
34.1
 
 
 
 
ARMC8
 
 
hsa-miR-486-5p
 
 
-0.321
 
 
17.0
 
 
 
 
ARMCX2
 
 
hsa-miR-15b-5p
 
 
-0.344
 
 
10.8
 
 
 
 
ARMCX2
 
 
hsa-miR-16-5p
 
 
-0.366
 
 
10.8
 
 
 
 
ARMCX3
 
 
hsa-miR-425-5p
 
 
-0.325
 
 
23.0
 
 
 
 
ARMT1
 
 
hsa-let-7b-5p
 
 
-0.291
 
 
14.4
 
 
 
 
ARPP19
 
 
hsa-miR-320b
 
 
-0.325
 
 
36.4
 
 
 
 
ARPP19
 
 
hsa-miR-320c
 
 
-0.325
 
 
36.4
 
 
 
 
ARPP19
 
 
hsa-miR-15b-5p
 
 
-0.346
 
 
36.4
 
 
 
 
ARPP19
 
 
hsa-miR-16-5p
 
 
-0.354
 
 
36.4
 
 
 
 
ARPP19
 
 
hsa-miR-29a-3p
 
 
-0.371
 
 
36.4
 
 
 
 
ARPP19
 
 
hsa-miR-328-3p
 
 
-0.404
 
 
36.4
 
 
 
 
ARPP19
 
 
hsa-let-7b-5p
 
 
-0.476
 
 
36.4
 
 
 
 
ARRDC3
 
 
hsa-miR-148b-3p
 
 
-0.305
 
 
25.3
 
 
 
 
ARRDC3
 
 
hsa-miR-29a-3p
 
 
-0.402
 
 
25.3
 
 
 
 
ARRDC4
 
 
hsa-let-7b-5p
 
 
-0.329
 
 
15.6
 
 
 
 
ASAH1
 
 
hsa-miR-27a-3p
 
 
-0.398
 
 
48.7
 
 
 
 
ASAP1
 
 
hsa-let-7b-5p
 
 
-0.308
 
 
11.4
 
 
 
 
ASH2L
 
 
hsa-miR-320b
 
 
-0.287
 
 
17.4
 
 
 
 
ASH2L
 
 
hsa-miR-320c
 
 
-0.287
 
 
17.4
 
 
 
 
ASPH
 
 
hsa-miR-148b-3p
 
 
-0.391
 
 
47.6
 
 
 
 
ATG9A
 
 
hsa-miR-16-5p
 
 
-0.311
 
 
16.3
 
 
 
 
ATG9A
 
 
hsa-miR-15b-5p
 
 
-0.333
 
 
16.3
 
 
 
 
ATOSB
 
 
hsa-miR-328-3p
 
 
-0.358
 
 
11.5
 
 
 
 
ATOSB
 
 
hsa-let-7b-5p
 
 
-0.429
 
 
11.5
 
 
 
 
ATP2A2
 
 
hsa-let-7b-5p
 
 
-0.311
 
 
54.5
 
 
 
 
ATP2B4
 
 
hsa-miR-148b-3p
 
 
-0.337
 
 
24.2
 
 
 
 
ATP5MC3
 
 
hsa-miR-425-5p
 
 
-0.328
 
 
123.3
 
 
 
 
ATP5MC3
 
 
hsa-miR-29a-3p
 
 
-0.33
 
 
123.3
 
 
 
 
ATP5MC3
 
 
hsa-miR-27a-3p
 
 
-0.416
 
 
123.3
 
 
 
 
 
 
 basal ganglia 
 
 
 
 
target_symbol
 
 
mature_mirna_id
 
 
score
 
 
nTPM
 
 
 
 
 
 
AAR2
 
 
hsa-miR-29a-3p
 
 
-0.327
 
 
11.5
 
 
 
 
ABCB9
 
 
hsa-let-7b-5p
 
 
-0.422
 
 
13.6
 
 
 
 
ABCD3
 
 
hsa-miR-148b-3p
 
 
-0.388
 
 
17.4
 
 
 
 
ABCE1
 
 
hsa-miR-29a-3p
 
 
-0.404
 
 
11.5
 
 
 
 
ABHD17B
 
 
hsa-miR-486-5p
 
 
-0.348
 
 
13.8
 
 
 
 
ABHD17C
 
 
hsa-let-7b-5p
 
 
-0.359
 
 
20.2
 
 
 
 
ABHD17C
 
 
hsa-miR-27a-3p
 
 
-0.435
 
 
20.2
 
 
 
 
ABHD4
 
 
hsa-miR-29a-3p
 
 
-0.312
 
 
15.4
 
 
 
 
ACSL6
 
 
hsa-let-7b-5p
 
 
-0.336
 
 
18.7
 
 
 
 
ACTA2
 
 
hsa-miR-27a-3p
 
 
-0.577
 
 
45.5
 
 
 
 
ACTN2
 
 
hsa-miR-23a-3p
 
 
-0.294
 
 
36.8
 
 
 
 
ACTN2
 
 
hsa-miR-29a-3p
 
 
-0.311
 
 
36.8
 
 
 
 
ACTR2
 
 
hsa-miR-15b-5p
 
 
-0.293
 
 
55.9
 
 
 
 
ACTR2
 
 
hsa-miR-16-5p
 
 
-0.293
 
 
55.9
 
 
 
 
ADAP1
 
 
hsa-miR-27a-3p
 
 
-0.302
 
 
77.3
 
 
 
 
ADCY2
 
 
hsa-miR-148b-3p
 
 
-0.299
 
 
23.7
 
 
 
 
ADCY3
 
 
hsa-miR-132-3p
 
 
-0.303
 
 
57.0
 
 
 
 
ADGRA3
 
 
hsa-miR-27a-3p
 
 
-0.296
 
 
18.8
 
 
 
 
ADGRA3
 
 
hsa-miR-30b-5p
 
 
-0.304
 
 
18.8
 
 
 
 
ADGRB3
 
 
hsa-miR-148b-3p
 
 
-0.524
 
 
21.1
 
 
 
 
ADGRF5
 
 
hsa-miR-148b-3p
 
 
-0.354
 
 
13.3
 
 
 
 
ADIPOR2
 
 
hsa-miR-150-5p
 
 
-0.287
 
 
50.5
 
 
 
 
ADNP
 
 
hsa-miR-328-3p
 
 
-0.371
 
 
24.2
 
 
 
 
ADRB1
 
 
hsa-let-7b-5p
 
 
-0.351
 
 
10.0
 
 
 
 
AGFG1
 
 
hsa-miR-148b-3p
 
 
-0.332
 
 
27.0
 
 
 
 
AGPAT3
 
 
hsa-miR-27a-3p
 
 
-0.29
 
 
44.7
 
 
 
 
AGPAT3
 
 
hsa-miR-16-5p
 
 
-0.299
 
 
44.7
 
 
 
 
AGPAT4
 
 
hsa-miR-29a-3p
 
 
-0.376
 
 
10.7
 
 
 
 
AHDC1
 
 
hsa-miR-148b-3p
 
 
-0.413
 
 
12.3
 
 
 
 
AIFM1
 
 
hsa-let-7b-5p
 
 
-0.314
 
 
12.5
 
 
 
 
AK1
 
 
hsa-miR-29a-3p
 
 
-0.333
 
 
100.7
 
 
 
 
AK4
 
 
hsa-miR-15b-5p
 
 
-0.316
 
 
19.2
 
 
 
 
AK4
 
 
hsa-miR-16-5p
 
 
-0.348
 
 
19.2
 
 
 
 
AKAP1
 
 
hsa-miR-148b-3p
 
 
-0.381
 
 
26.7
 
 
 
 
AKAP5
 
 
hsa-miR-29a-3p
 
 
-0.298
 
 
17.6
 
 
 
 
AKAP6
 
 
hsa-let-7b-5p
 
 
-0.307
 
 
10.5
 
 
 
 
AKIRIN1
 
 
hsa-miR-27a-3p
 
 
-0.368
 
 
33.9
 
 
 
 
AKT3
 
 
hsa-miR-29a-3p
 
 
-0.287
 
 
15.0
 
 
 
 
AKT3
 
 
hsa-miR-15b-5p
 
 
-0.301
 
 
15.0
 
 
 
 
AKT3
 
 
hsa-miR-16-5p
 
 
-0.309
 
 
15.0
 
 
 
 
ALAD
 
 
hsa-miR-16-5p
 
 
-0.391
 
 
57.8
 
 
 
 
ALAD
 
 
hsa-miR-15b-5p
 
 
-0.446
 
 
57.8
 
 
 
 
ALCAM
 
 
hsa-miR-148b-3p
 
 
-0.339
 
 
37.8
 
 
 
 
ALPL
 
 
hsa-miR-10a-5p
 
 
-0.32
 
 
12.3
 
 
 
 
AMOT
 
 
hsa-miR-29a-3p
 
 
-0.304
 
 
18.8
 
 
 
 
AMT
 
 
hsa-let-7b-5p
 
 
-0.463
 
 
11.4
 
 
 
 
ANGEL2
 
 
hsa-miR-132-3p
 
 
-0.357
 
 
14.7
 
 
 
 
ANKHD1
 
 
hsa-miR-23a-3p
 
 
-0.368
 
 
20.4
 
 
 
 
ANKHD1
 
 
hsa-miR-23b-3p
 
 
-0.368
 
 
20.4
 
 
 
 
ANKHD1
 
 
hsa-miR-30b-5p
 
 
-0.405
 
 
20.4
 
 
 
 
ANKRD13B
 
 
hsa-miR-29a-3p
 
 
-0.421
 
 
12.3
 
 
 
 
ANKRD17
 
 
hsa-miR-30b-5p
 
 
-0.336
 
 
11.2
 
 
 
 
ANKRD40
 
 
hsa-miR-27a-3p
 
 
-0.35
 
 
100.5
 
 
 
 
ANKRD46
 
 
hsa-let-7b-5p
 
 
-0.389
 
 
22.6
 
 
 
 
ANLN
 
 
hsa-miR-16-5p
 
 
-0.31
 
 
27.5
 
 
 
 
ANLN
 
 
hsa-miR-15b-5p
 
 
-0.334
 
 
27.5
 
 
 
 
ANO3
 
 
hsa-miR-16-5p
 
 
-0.31
 
 
58.4
 
 
 
 
ANO3
 
 
hsa-miR-16-5p
 
 
-0.346
 
 
58.4
 
 
 
 
ANO3
 
 
hsa-miR-15b-5p
 
 
-0.357
 
 
58.4
 
 
 
 
ANP32A
 
 
hsa-miR-132-3p
 
 
-0.374
 
 
53.9
 
 
 
 
ANXA11
 
 
hsa-miR-16-5p
 
 
-0.447
 
 
35.8
 
 
 
 
ANXA11
 
 
hsa-miR-15b-5p
 
 
-0.463
 
 
35.8
 
 
 
 
AP1S1
 
 
hsa-let-7b-5p
 
 
-0.374
 
 
116.3
 
 
 
 
AP2A1
 
 
hsa-miR-15b-5p
 
 
-0.333
 
 
40.7
 
 
 
 
AP2A1
 
 
hsa-miR-16-5p
 
 
-0.333
 
 
40.7
 
 
 
 
AP2B1
 
 
hsa-miR-16-5p
 
 
-0.302
 
 
72.3
 
 
 
 
AP2B1
 
 
hsa-miR-15b-5p
 
 
-0.334
 
 
72.3
 
 
 
 
AP3S1
 
 
hsa-miR-30b-5p
 
 
-0.293
 
 
79.6
 
 
 
 
AP5S1
 
 
hsa-miR-744-5p
 
 
-0.354
 
 
10.0
 
 
 
 
APC
 
 
hsa-miR-29a-3p
 
 
-0.413
 
 
15.7
 
 
 
 
APLN
 
 
hsa-miR-16-5p
 
 
-0.31
 
 
10.2
 
 
 
 
APLN
 
 
hsa-miR-15b-5p
 
 
-0.395
 
 
10.2
 
 
 
 
APLN
 
 
hsa-miR-16-5p
 
 
-0.395
 
 
10.2
 
 
 
 
APOO
 
 
hsa-miR-181b-5p
 
 
-0.5
 
 
38.6
 
 
 
 
APOO
 
 
hsa-miR-181a-5p
 
 
-0.5
 
 
38.6
 
 
 
 
APP
 
 
hsa-miR-16-5p
 
 
-0.298
 
 
458.2
 
 
 
 
APP
 
 
hsa-miR-15b-5p
 
 
-0.298
 
 
458.2
 
 
 
 
ARF4
 
 
hsa-miR-30b-5p
 
 
-0.289
 
 
71.0
 
 
 
 
ARF4
 
 
hsa-miR-148b-3p
 
 
-0.487
 
 
71.0
 
 
 
 
ARF5
 
 
hsa-miR-29a-3p
 
 
-0.491
 
 
195.0
 
 
 
 
ARF6
 
 
hsa-miR-181b-5p
 
 
-0.33
 
 
10.2
 
 
 
 
ARF6
 
 
hsa-miR-181a-5p
 
 
-0.33
 
 
10.2
 
 
 
 
ARHGAP21
 
 
hsa-miR-148b-3p
 
 
-0.287
 
 
37.1
 
 
 
 
ARHGAP5
 
 
hsa-miR-486-5p
 
 
-0.3
 
 
22.9
 
 
 
 
ARL2
 
 
hsa-miR-16-5p
 
 
-0.374
 
 
286.3
 
 
 
 
ARL2
 
 
hsa-miR-15b-5p
 
 
-0.374
 
 
286.3
 
 
 
 
ARL2
 
 
hsa-miR-16-5p
 
 
-0.418
 
 
286.3
 
 
 
 
ARL2
 
 
hsa-miR-15b-5p
 
 
-0.442
 
 
286.3
 
 
 
 
ARL3
 
 
hsa-miR-16-5p
 
 
-0.496
 
 
31.8
 
 
 
 
ARL3
 
 
hsa-miR-15b-5p
 
 
-0.507
 
 
31.8
 
 
 
 
ARL4A
 
 
hsa-miR-29a-3p
 
 
-0.386
 
 
10.5
 
 
 
 
ARL4A
 
 
hsa-miR-30b-5p
 
 
-0.448
 
 
10.5
 
 
 
 
ARL4D
 
 
hsa-let-7b-5p
 
 
-0.417
 
 
14.0
 
 
 
 
ARL6IP1
 
 
hsa-miR-328-3p
 
 
-0.494
 
 
196.4
 
 
 
 
ARL6IP1
 
 
hsa-miR-148b-3p
 
 
-0.579
 
 
196.4
 
 
 
 
ARL8B
 
 
hsa-miR-148b-3p
 
 
-0.337
 
 
53.5
 
 
 
 
ARL8B
 
 
hsa-miR-320b
 
 
-0.376
 
 
53.5
 
 
 
 
ARL8B
 
 
hsa-miR-320c
 
 
-0.376
 
 
53.5
 
 
 
 
ARMC8
 
 
hsa-miR-486-5p
 
 
-0.321
 
 
13.3
 
 
 
 
ARMCX2
 
 
hsa-miR-15b-5p
 
 
-0.344
 
 
31.3
 
 
 
 
 
 
 blood vessel 
 
 
 
 
target_symbol
 
 
mature_mirna_id
 
 
score
 
 
nTPM
 
 
 
 
 
 
AAR2
 
 
hsa-miR-29a-3p
 
 
-0.327
 
 
28.2
 
 
 
 
ABCB7
 
 
hsa-miR-148b-3p
 
 
-0.515
 
 
12.3
 
 
 
 
ABCD3
 
 
hsa-miR-148b-3p
 
 
-0.388
 
 
14.4
 
 
 
 
ABCE1
 
 
hsa-miR-29a-3p
 
 
-0.404
 
 
15.1
 
 
 
 
ABHD4
 
 
hsa-miR-29a-3p
 
 
-0.312
 
 
38.3
 
 
 
 
ACTA2
 
 
hsa-miR-27a-3p
 
 
-0.577
 
 
10509.9
 
 
 
 
ACTC1
 
 
hsa-miR-30b-5p
 
 
-0.543
 
 
388.9
 
 
 
 
ACTN2
 
 
hsa-miR-23a-3p
 
 
-0.294
 
 
12.2
 
 
 
 
ACTN2
 
 
hsa-miR-29a-3p
 
 
-0.311
 
 
12.2
 
 
 
 
ACTR2
 
 
hsa-miR-15b-5p
 
 
-0.293
 
 
77.0
 
 
 
 
ACTR2
 
 
hsa-miR-16-5p
 
 
-0.293
 
 
77.0
 
 
 
 
ADAM9
 
 
hsa-miR-30b-5p
 
 
-0.312
 
 
36.8
 
 
 
 
ADAMTS10
 
 
hsa-miR-27a-3p
 
 
-0.354
 
 
20.0
 
 
 
 
ADAMTS10
 
 
hsa-miR-29a-3p
 
 
-0.516
 
 
20.0
 
 
 
 
ADAMTS7
 
 
hsa-miR-29a-3p
 
 
-0.427
 
 
14.2
 
 
 
 
ADAMTS8
 
 
hsa-let-7b-5p
 
 
-0.571
 
 
12.7
 
 
 
 
ADAMTS9
 
 
hsa-miR-29a-3p
 
 
-0.305
 
 
18.0
 
 
 
 
ADAMTS9
 
 
hsa-miR-29a-3p
 
 
-0.485
 
 
18.0
 
 
 
 
ADCY3
 
 
hsa-miR-132-3p
 
 
-0.303
 
 
43.2
 
 
 
 
ADGRF5
 
 
hsa-miR-148b-3p
 
 
-0.354
 
 
12.6
 
 
 
 
ADIPOR2
 
 
hsa-miR-150-5p
 
 
-0.287
 
 
23.0
 
 
 
 
ADNP
 
 
hsa-miR-328-3p
 
 
-0.371
 
 
32.1
 
 
 
 
AGFG1
 
 
hsa-miR-148b-3p
 
 
-0.332
 
 
37.3
 
 
 
 
AGPAT3
 
 
hsa-miR-27a-3p
 
 
-0.29
 
 
11.3
 
 
 
 
AGPAT3
 
 
hsa-miR-16-5p
 
 
-0.299
 
 
11.3
 
 
 
 
AHCTF1
 
 
hsa-let-7b-5p
 
 
-0.374
 
 
10.0
 
 
 
 
AHDC1
 
 
hsa-miR-148b-3p
 
 
-0.413
 
 
27.7
 
 
 
 
AHR
 
 
hsa-miR-29a-3p
 
 
-0.316
 
 
15.2
 
 
 
 
AK1
 
 
hsa-miR-29a-3p
 
 
-0.333
 
 
58.4
 
 
 
 
AK4
 
 
hsa-miR-15b-5p
 
 
-0.316
 
 
25.4
 
 
 
 
AK4
 
 
hsa-miR-16-5p
 
 
-0.348
 
 
25.4
 
 
 
 
AKAP1
 
 
hsa-miR-148b-3p
 
 
-0.381
 
 
84.5
 
 
 
 
AKAP6
 
 
hsa-let-7b-5p
 
 
-0.307
 
 
15.0
 
 
 
 
AKIRIN1
 
 
hsa-miR-27a-3p
 
 
-0.368
 
 
32.7
 
 
 
 
AKT3
 
 
hsa-miR-29a-3p
 
 
-0.287
 
 
34.5
 
 
 
 
AKT3
 
 
hsa-miR-15b-5p
 
 
-0.301
 
 
34.5
 
 
 
 
AKT3
 
 
hsa-miR-16-5p
 
 
-0.309
 
 
34.5
 
 
 
 
ALAD
 
 
hsa-miR-16-5p
 
 
-0.391
 
 
22.2
 
 
 
 
ALAD
 
 
hsa-miR-15b-5p
 
 
-0.446
 
 
22.2
 
 
 
 
ALCAM
 
 
hsa-miR-148b-3p
 
 
-0.339
 
 
85.5
 
 
 
 
ALPL
 
 
hsa-miR-10a-5p
 
 
-0.32
 
 
12.2
 
 
 
 
AMOTL1
 
 
hsa-miR-16-5p
 
 
-0.297
 
 
18.9
 
 
 
 
AMOTL1
 
 
hsa-miR-15b-5p
 
 
-0.319
 
 
18.9
 
 
 
 
AMT
 
 
hsa-let-7b-5p
 
 
-0.463
 
 
11.4
 
 
 
 
ANKHD1
 
 
hsa-miR-23a-3p
 
 
-0.368
 
 
40.7
 
 
 
 
ANKHD1
 
 
hsa-miR-23b-3p
 
 
-0.368
 
 
40.7
 
 
 
 
ANKHD1
 
 
hsa-miR-30b-5p
 
 
-0.405
 
 
40.7
 
 
 
 
ANKRD17
 
 
hsa-miR-30b-5p
 
 
-0.336
 
 
13.9
 
 
 
 
ANKRD40
 
 
hsa-miR-27a-3p
 
 
-0.35
 
 
29.5
 
 
 
 
ANP32A
 
 
hsa-miR-132-3p
 
 
-0.374
 
 
51.8
 
 
 
 
ANTXR2
 
 
hsa-miR-29a-3p
 
 
-0.347
 
 
50.3
 
 
 
 
ANXA11
 
 
hsa-miR-16-5p
 
 
-0.447
 
 
150.6
 
 
 
 
ANXA11
 
 
hsa-miR-15b-5p
 
 
-0.463
 
 
150.6
 
 
 
 
AP1S1
 
 
hsa-let-7b-5p
 
 
-0.374
 
 
13.8
 
 
 
 
AP2A1
 
 
hsa-miR-15b-5p
 
 
-0.333
 
 
48.0
 
 
 
 
AP2A1
 
 
hsa-miR-16-5p
 
 
-0.333
 
 
48.0
 
 
 
 
AP2B1
 
 
hsa-miR-16-5p
 
 
-0.302
 
 
41.4
 
 
 
 
AP2B1
 
 
hsa-miR-15b-5p
 
 
-0.334
 
 
41.4
 
 
 
 
AP3S1
 
 
hsa-miR-30b-5p
 
 
-0.293
 
 
112.6
 
 
 
 
APLN
 
 
hsa-miR-16-5p
 
 
-0.31
 
 
10.7
 
 
 
 
APLN
 
 
hsa-miR-15b-5p
 
 
-0.395
 
 
10.7
 
 
 
 
APLN
 
 
hsa-miR-16-5p
 
 
-0.395
 
 
10.7
 
 
 
 
APP
 
 
hsa-miR-16-5p
 
 
-0.298
 
 
341.0
 
 
 
 
APP
 
 
hsa-miR-15b-5p
 
 
-0.298
 
 
341.0
 
 
 
 
ARF4
 
 
hsa-miR-30b-5p
 
 
-0.289
 
 
151.4
 
 
 
 
ARF4
 
 
hsa-miR-148b-3p
 
 
-0.487
 
 
151.4
 
 
 
 
ARF5
 
 
hsa-miR-29a-3p
 
 
-0.491
 
 
115.2
 
 
 
 
ARF6
 
 
hsa-miR-181b-5p
 
 
-0.33
 
 
22.6
 
 
 
 
ARF6
 
 
hsa-miR-181a-5p
 
 
-0.33
 
 
22.6
 
 
 
 
ARFIP1
 
 
hsa-miR-320b
 
 
-0.339
 
 
14.2
 
 
 
 
ARFIP1
 
 
hsa-miR-320c
 
 
-0.339
 
 
14.2
 
 
 
 
ARFIP1
 
 
hsa-miR-148b-3p
 
 
-0.474
 
 
14.2
 
 
 
 
ARHGAP21
 
 
hsa-miR-148b-3p
 
 
-0.287
 
 
14.0
 
 
 
 
ARHGAP5
 
 
hsa-miR-486-5p
 
 
-0.3
 
 
18.0
 
 
 
 
ARID1B
 
 
hsa-miR-29a-3p
 
 
-0.342
 
 
13.6
 
 
 
 
ARID4B
 
 
hsa-miR-486-5p
 
 
-0.367
 
 
12.5
 
 
 
 
ARL2
 
 
hsa-miR-16-5p
 
 
-0.374
 
 
155.0
 
 
 
 
ARL2
 
 
hsa-miR-15b-5p
 
 
-0.374
 
 
155.0
 
 
 
 
ARL2
 
 
hsa-miR-16-5p
 
 
-0.418
 
 
155.0
 
 
 
 
ARL2
 
 
hsa-miR-15b-5p
 
 
-0.442
 
 
155.0
 
 
 
 
ARL3
 
 
hsa-miR-16-5p
 
 
-0.496
 
 
12.7
 
 
 
 
ARL3
 
 
hsa-miR-15b-5p
 
 
-0.507
 
 
12.7
 
 
 
 
ARL4A
 
 
hsa-miR-29a-3p
 
 
-0.386
 
 
19.2
 
 
 
 
ARL4A
 
 
hsa-miR-30b-5p
 
 
-0.448
 
 
19.2
 
 
 
 
ARL4D
 
 
hsa-let-7b-5p
 
 
-0.417
 
 
12.9
 
 
 
 
ARL6IP1
 
 
hsa-miR-328-3p
 
 
-0.494
 
 
52.2
 
 
 
 
ARL6IP1
 
 
hsa-miR-148b-3p
 
 
-0.579
 
 
52.2
 
 
 
 
ARL8B
 
 
hsa-miR-148b-3p
 
 
-0.337
 
 
49.5
 
 
 
 
ARL8B
 
 
hsa-miR-320b
 
 
-0.376
 
 
49.5
 
 
 
 
ARL8B
 
 
hsa-miR-320c
 
 
-0.376
 
 
49.5
 
 
 
 
ARMC8
 
 
hsa-miR-486-5p
 
 
-0.321
 
 
23.5
 
 
 
 
ARMCX2
 
 
hsa-miR-15b-5p
 
 
-0.344
 
 
27.0
 
 
 
 
ARMCX2
 
 
hsa-miR-16-5p
 
 
-0.366
 
 
27.0
 
 
 
 
ARMCX3
 
 
hsa-miR-425-5p
 
 
-0.325
 
 
32.6
 
 
 
 
ARPP19
 
 
hsa-miR-320b
 
 
-0.325
 
 
47.3
 
 
 
 
ARPP19
 
 
hsa-miR-320c
 
 
-0.325
 
 
47.3
 
 
 
 
ARPP19
 
 
hsa-miR-15b-5p
 
 
-0.346
 
 
47.3
 
 
 
 
ARPP19
 
 
hsa-miR-16-5p
 
 
-0.354
 
 
47.3
 
 
 
 
ARPP19
 
 
hsa-miR-29a-3p
 
 
-0.371
 
 
47.3
 
 
 
 
ARPP19
 
 
hsa-miR-328-3p
 
 
-0.404
 
 
47.3
 
 
 
 
 
 
 bone marrow 
 
 
 
 
target_symbol
 
 
mature_mirna_id
 
 
score
 
 
nTPM
 
 
 
 
 
 
AAR2
 
 
hsa-miR-29a-3p
 
 
-0.327
 
 
21.0
 
 
 
 
ABCB10
 
 
hsa-miR-148b-3p
 
 
-0.291
 
 
56.7
 
 
 
 
ABCB7
 
 
hsa-miR-148b-3p
 
 
-0.515
 
 
10.5
 
 
 
 
ABCD3
 
 
hsa-miR-148b-3p
 
 
-0.388
 
 
23.6
 
 
 
 
ABCE1
 
 
hsa-miR-29a-3p
 
 
-0.404
 
 
25.7
 
 
 
 
ABHD17B
 
 
hsa-miR-486-5p
 
 
-0.348
 
 
18.0
 
 
 
 
ABHD4
 
 
hsa-miR-29a-3p
 
 
-0.312
 
 
12.9
 
 
 
 
ABHD5
 
 
hsa-miR-132-3p
 
 
-0.323
 
 
37.5
 
 
 
 
ABHD5
 
 
hsa-miR-27a-3p
 
 
-0.407
 
 
37.5
 
 
 
 
ACP3
 
 
hsa-let-7b-5p
 
 
-0.298
 
 
16.5
 
 
 
 
ACSL6
 
 
hsa-let-7b-5p
 
 
-0.336
 
 
21.4
 
 
 
 
ACTR2
 
 
hsa-miR-15b-5p
 
 
-0.293
 
 
138.4
 
 
 
 
ACTR2
 
 
hsa-miR-16-5p
 
 
-0.293
 
 
138.4
 
 
 
 
ADA
 
 
hsa-miR-29a-3p
 
 
-0.376
 
 
14.3
 
 
 
 
ADAM10
 
 
hsa-miR-148b-3p
 
 
-0.356
 
 
17.3
 
 
 
 
ADCY3
 
 
hsa-miR-132-3p
 
 
-0.303
 
 
16.7
 
 
 
 
ADNP
 
 
hsa-miR-328-3p
 
 
-0.371
 
 
23.9
 
 
 
 
ADRB2
 
 
hsa-miR-15b-5p
 
 
-0.29
 
 
11.8
 
 
 
 
ADRB2
 
 
hsa-miR-16-5p
 
 
-0.323
 
 
11.8
 
 
 
 
ADRB2
 
 
hsa-let-7b-5p
 
 
-0.635
 
 
11.8
 
 
 
 
AFG3L2
 
 
hsa-miR-181b-5p
 
 
-0.491
 
 
23.4
 
 
 
 
AFG3L2
 
 
hsa-miR-181a-5p
 
 
-0.491
 
 
23.4
 
 
 
 
AGFG1
 
 
hsa-miR-148b-3p
 
 
-0.332
 
 
146.9
 
 
 
 
AGO2
 
 
hsa-miR-132-3p
 
 
-0.406
 
 
10.7
 
 
 
 
AGO2
 
 
hsa-miR-27a-3p
 
 
-0.486
 
 
10.7
 
 
 
 
AGO2
 
 
hsa-miR-23a-3p
 
 
-0.576
 
 
10.7
 
 
 
 
AGO2
 
 
hsa-miR-23b-3p
 
 
-0.587
 
 
10.7
 
 
 
 
AGO2
 
 
hsa-miR-27a-3p
 
 
-0.646
 
 
10.7
 
 
 
 
AGO2
 
 
hsa-miR-30b-5p
 
 
-1.034
 
 
10.7
 
 
 
 
AGPAT3
 
 
hsa-miR-27a-3p
 
 
-0.29
 
 
10.4
 
 
 
 
AGPAT3
 
 
hsa-miR-16-5p
 
 
-0.299
 
 
10.4
 
 
 
 
AHCTF1
 
 
hsa-let-7b-5p
 
 
-0.374
 
 
16.0
 
 
 
 
AHR
 
 
hsa-miR-29a-3p
 
 
-0.316
 
 
41.5
 
 
 
 
AIFM1
 
 
hsa-let-7b-5p
 
 
-0.314
 
 
11.2
 
 
 
 
AK1
 
 
hsa-miR-29a-3p
 
 
-0.333
 
 
27.0
 
 
 
 
AKIRIN1
 
 
hsa-miR-27a-3p
 
 
-0.368
 
 
72.8
 
 
 
 
ALAD
 
 
hsa-miR-16-5p
 
 
-0.391
 
 
77.5
 
 
 
 
ALAD
 
 
hsa-miR-15b-5p
 
 
-0.446
 
 
77.5
 
 
 
 
ANKHD1
 
 
hsa-miR-23a-3p
 
 
-0.368
 
 
69.9
 
 
 
 
ANKHD1
 
 
hsa-miR-23b-3p
 
 
-0.368
 
 
69.9
 
 
 
 
ANKHD1
 
 
hsa-miR-30b-5p
 
 
-0.405
 
 
69.9
 
 
 
 
ANKRA2
 
 
hsa-miR-30b-5p
 
 
-0.354
 
 
13.2
 
 
 
 
ANKRA2
 
 
hsa-miR-30b-5p
 
 
-0.472
 
 
13.2
 
 
 
 
ANLN
 
 
hsa-miR-16-5p
 
 
-0.31
 
 
34.3
 
 
 
 
ANLN
 
 
hsa-miR-15b-5p
 
 
-0.334
 
 
34.3
 
 
 
 
ANP32A
 
 
hsa-miR-132-3p
 
 
-0.374
 
 
129.5
 
 
 
 
ANTXR2
 
 
hsa-miR-29a-3p
 
 
-0.347
 
 
17.7
 
 
 
 
ANXA11
 
 
hsa-miR-16-5p
 
 
-0.447
 
 
182.2
 
 
 
 
ANXA11
 
 
hsa-miR-15b-5p
 
 
-0.463
 
 
182.2
 
 
 
 
AP2A1
 
 
hsa-miR-15b-5p
 
 
-0.333
 
 
51.3
 
 
 
 
AP2A1
 
 
hsa-miR-16-5p
 
 
-0.333
 
 
51.3
 
 
 
 
AP2B1
 
 
hsa-miR-16-5p
 
 
-0.302
 
 
46.4
 
 
 
 
AP2B1
 
 
hsa-miR-15b-5p
 
 
-0.334
 
 
46.4
 
 
 
 
AP3S1
 
 
hsa-miR-30b-5p
 
 
-0.293
 
 
125.9
 
 
 
 
APP
 
 
hsa-miR-16-5p
 
 
-0.298
 
 
119.8
 
 
 
 
APP
 
 
hsa-miR-15b-5p
 
 
-0.298
 
 
119.8
 
 
 
 
ARF4
 
 
hsa-miR-30b-5p
 
 
-0.289
 
 
68.5
 
 
 
 
ARF4
 
 
hsa-miR-148b-3p
 
 
-0.487
 
 
68.5
 
 
 
 
ARF5
 
 
hsa-miR-29a-3p
 
 
-0.491
 
 
135.0
 
 
 
 
ARF6
 
 
hsa-miR-181b-5p
 
 
-0.33
 
 
78.0
 
 
 
 
ARF6
 
 
hsa-miR-181a-5p
 
 
-0.33
 
 
78.0
 
 
 
 
ARFGEF1
 
 
hsa-miR-148b-3p
 
 
-0.306
 
 
26.0
 
 
 
 
ARHGAP5
 
 
hsa-miR-486-5p
 
 
-0.3
 
 
10.3
 
 
 
 
ARID1B
 
 
hsa-miR-29a-3p
 
 
-0.342
 
 
27.3
 
 
 
 
ARID2
 
 
hsa-miR-132-3p
 
 
-0.304
 
 
11.7
 
 
 
 
ARID3B
 
 
hsa-let-7b-5p
 
 
-0.322
 
 
23.2
 
 
 
 
ARID3B
 
 
hsa-let-7b-5p
 
 
-0.486
 
 
23.2
 
 
 
 
ARID4B
 
 
hsa-miR-486-5p
 
 
-0.367
 
 
31.2
 
 
 
 
ARL2
 
 
hsa-miR-16-5p
 
 
-0.374
 
 
48.4
 
 
 
 
ARL2
 
 
hsa-miR-15b-5p
 
 
-0.374
 
 
48.4
 
 
 
 
ARL2
 
 
hsa-miR-16-5p
 
 
-0.418
 
 
48.4
 
 
 
 
ARL2
 
 
hsa-miR-15b-5p
 
 
-0.442
 
 
48.4
 
 
 
 
ARL4A
 
 
hsa-miR-29a-3p
 
 
-0.386
 
 
92.7
 
 
 
 
ARL4A
 
 
hsa-miR-30b-5p
 
 
-0.448
 
 
92.7
 
 
 
 
ARL5B
 
 
hsa-miR-29a-3p
 
 
-0.304
 
 
18.5
 
 
 
 
ARL6IP1
 
 
hsa-miR-328-3p
 
 
-0.494
 
 
197.7
 
 
 
 
ARL6IP1
 
 
hsa-miR-148b-3p
 
 
-0.579
 
 
197.7
 
 
 
 
ARL6IP6
 
 
hsa-let-7b-5p
 
 
-0.293
 
 
23.4
 
 
 
 
ARL8B
 
 
hsa-miR-148b-3p
 
 
-0.337
 
 
56.4
 
 
 
 
ARL8B
 
 
hsa-miR-320b
 
 
-0.376
 
 
56.4
 
 
 
 
ARL8B
 
 
hsa-miR-320c
 
 
-0.376
 
 
56.4
 
 
 
 
ARMC8
 
 
hsa-miR-486-5p
 
 
-0.321
 
 
57.2
 
 
 
 
ARMCX3
 
 
hsa-miR-425-5p
 
 
-0.325
 
 
13.9
 
 
 
 
ARPP19
 
 
hsa-miR-320b
 
 
-0.325
 
 
97.5
 
 
 
 
ARPP19
 
 
hsa-miR-320c
 
 
-0.325
 
 
97.5
 
 
 
 
ARPP19
 
 
hsa-miR-15b-5p
 
 
-0.346
 
 
97.5
 
 
 
 
ARPP19
 
 
hsa-miR-16-5p
 
 
-0.354
 
 
97.5
 
 
 
 
ARPP19
 
 
hsa-miR-29a-3p
 
 
-0.371
 
 
97.5
 
 
 
 
ARPP19
 
 
hsa-miR-328-3p
 
 
-0.404
 
 
97.5
 
 
 
 
ARPP19
 
 
hsa-let-7b-5p
 
 
-0.476
 
 
97.5
 
 
 
 
ARRDC3
 
 
hsa-miR-148b-3p
 
 
-0.305
 
 
158.3
 
 
 
 
ARRDC3
 
 
hsa-miR-29a-3p
 
 
-0.402
 
 
158.3
 
 
 
 
ARRDC4
 
 
hsa-let-7b-5p
 
 
-0.329
 
 
10.8
 
 
 
 
ASAH1
 
 
hsa-miR-27a-3p
 
 
-0.398
 
 
101.8
 
 
 
 
ASAP1
 
 
hsa-let-7b-5p
 
 
-0.308
 
 
44.7
 
 
 
 
ASB3
 
 
hsa-miR-30b-5p
 
 
-0.418
 
 
13.9
 
 
 
 
ASB3
 
 
hsa-miR-30b-5p
 
 
-0.418
 
 
13.9
 
 
 
 
ASH1L
 
 
hsa-miR-15b-5p
 
 
-0.325
 
 
11.2
 
 
 
 
ASH1L
 
 
hsa-miR-16-5p
 
 
-0.357
 
 
11.2
 
 
 
 
ASH2L
 
 
hsa-miR-320b
 
 
-0.287
 
 
30.3
 
 
 
 
 
 
 breast 
 
 
 
 
target_symbol
 
 
mature_mirna_id
 
 
score
 
 
nTPM
 
 
 
 
 
 
AAR2
 
 
hsa-miR-29a-3p
 
 
-0.327
 
 
20.3
 
 
 
 
ABCB7
 
 
hsa-miR-148b-3p
 
 
-0.515
 
 
11.6
 
 
 
 
ABCD3
 
 
hsa-miR-148b-3p
 
 
-0.388
 
 
30.8
 
 
 
 
ABCE1
 
 
hsa-miR-29a-3p
 
 
-0.404
 
 
30.9
 
 
 
 
ABHD17C
 
 
hsa-let-7b-5p
 
 
-0.359
 
 
10.9
 
 
 
 
ABHD17C
 
 
hsa-miR-27a-3p
 
 
-0.435
 
 
10.9
 
 
 
 
ABHD4
 
 
hsa-miR-29a-3p
 
 
-0.312
 
 
18.7
 
 
 
 
ABHD5
 
 
hsa-miR-132-3p
 
 
-0.323
 
 
18.8
 
 
 
 
ABHD5
 
 
hsa-miR-27a-3p
 
 
-0.407
 
 
18.8
 
 
 
 
ACTA2
 
 
hsa-miR-27a-3p
 
 
-0.577
 
 
802.4
 
 
 
 
ACTC1
 
 
hsa-miR-30b-5p
 
 
-0.543
 
 
11.2
 
 
 
 
ACTR2
 
 
hsa-miR-15b-5p
 
 
-0.293
 
 
72.1
 
 
 
 
ACTR2
 
 
hsa-miR-16-5p
 
 
-0.293
 
 
72.1
 
 
 
 
ACVR1
 
 
hsa-miR-30b-5p
 
 
-0.29
 
 
11.1
 
 
 
 
ACVR1
 
 
hsa-miR-148b-3p
 
 
-0.397
 
 
11.1
 
 
 
 
ADA
 
 
hsa-miR-29a-3p
 
 
-0.376
 
 
11.4
 
 
 
 
ADAM10
 
 
hsa-miR-148b-3p
 
 
-0.356
 
 
14.2
 
 
 
 
ADAM9
 
 
hsa-miR-30b-5p
 
 
-0.312
 
 
20.2
 
 
 
 
ADAMTS9
 
 
hsa-miR-29a-3p
 
 
-0.305
 
 
19.0
 
 
 
 
ADAMTS9
 
 
hsa-miR-29a-3p
 
 
-0.485
 
 
19.0
 
 
 
 
ADCY3
 
 
hsa-miR-132-3p
 
 
-0.303
 
 
72.4
 
 
 
 
ADGRA3
 
 
hsa-miR-27a-3p
 
 
-0.296
 
 
10.2
 
 
 
 
ADGRA3
 
 
hsa-miR-30b-5p
 
 
-0.304
 
 
10.2
 
 
 
 
ADGRF5
 
 
hsa-miR-148b-3p
 
 
-0.354
 
 
44.5
 
 
 
 
ADIPOR2
 
 
hsa-miR-150-5p
 
 
-0.287
 
 
44.0
 
 
 
 
ADNP
 
 
hsa-miR-328-3p
 
 
-0.371
 
 
30.5
 
 
 
 
ADRA2A
 
 
hsa-miR-23a-3p
 
 
-0.371
 
 
31.1
 
 
 
 
ADRA2A
 
 
hsa-miR-23b-3p
 
 
-0.371
 
 
31.1
 
 
 
 
ADRB2
 
 
hsa-miR-15b-5p
 
 
-0.29
 
 
32.4
 
 
 
 
ADRB2
 
 
hsa-miR-16-5p
 
 
-0.323
 
 
32.4
 
 
 
 
ADRB2
 
 
hsa-let-7b-5p
 
 
-0.635
 
 
32.4
 
 
 
 
AFG3L2
 
 
hsa-miR-181b-5p
 
 
-0.491
 
 
15.5
 
 
 
 
AFG3L2
 
 
hsa-miR-181a-5p
 
 
-0.491
 
 
15.5
 
 
 
 
AGFG1
 
 
hsa-miR-148b-3p
 
 
-0.332
 
 
48.8
 
 
 
 
AGPAT3
 
 
hsa-miR-27a-3p
 
 
-0.29
 
 
14.5
 
 
 
 
AGPAT3
 
 
hsa-miR-16-5p
 
 
-0.299
 
 
14.5
 
 
 
 
AHDC1
 
 
hsa-miR-148b-3p
 
 
-0.413
 
 
11.9
 
 
 
 
AHR
 
 
hsa-miR-29a-3p
 
 
-0.316
 
 
18.4
 
 
 
 
AIFM1
 
 
hsa-let-7b-5p
 
 
-0.314
 
 
11.7
 
 
 
 
AK1
 
 
hsa-miR-29a-3p
 
 
-0.333
 
 
20.7
 
 
 
 
AK4
 
 
hsa-miR-15b-5p
 
 
-0.316
 
 
24.4
 
 
 
 
AK4
 
 
hsa-miR-16-5p
 
 
-0.348
 
 
24.4
 
 
 
 
AKAP1
 
 
hsa-miR-148b-3p
 
 
-0.381
 
 
34.0
 
 
 
 
AKIRIN1
 
 
hsa-miR-27a-3p
 
 
-0.368
 
 
21.6
 
 
 
 
AKT3
 
 
hsa-miR-29a-3p
 
 
-0.287
 
 
13.8
 
 
 
 
AKT3
 
 
hsa-miR-15b-5p
 
 
-0.301
 
 
13.8
 
 
 
 
AKT3
 
 
hsa-miR-16-5p
 
 
-0.309
 
 
13.8
 
 
 
 
ALAD
 
 
hsa-miR-16-5p
 
 
-0.391
 
 
21.7
 
 
 
 
ALAD
 
 
hsa-miR-15b-5p
 
 
-0.446
 
 
21.7
 
 
 
 
ALCAM
 
 
hsa-miR-148b-3p
 
 
-0.339
 
 
76.0
 
 
 
 
ALPL
 
 
hsa-miR-10a-5p
 
 
-0.32
 
 
21.4
 
 
 
 
AMOTL1
 
 
hsa-miR-16-5p
 
 
-0.297
 
 
14.0
 
 
 
 
AMOTL1
 
 
hsa-miR-15b-5p
 
 
-0.319
 
 
14.0
 
 
 
 
ANKHD1
 
 
hsa-miR-23a-3p
 
 
-0.368
 
 
37.8
 
 
 
 
ANKHD1
 
 
hsa-miR-23b-3p
 
 
-0.368
 
 
37.8
 
 
 
 
ANKHD1
 
 
hsa-miR-30b-5p
 
 
-0.405
 
 
37.8
 
 
 
 
ANKRD17
 
 
hsa-miR-30b-5p
 
 
-0.336
 
 
18.7
 
 
 
 
ANKRD29
 
 
hsa-miR-132-3p
 
 
-0.47
 
 
15.0
 
 
 
 
ANKRD40
 
 
hsa-miR-27a-3p
 
 
-0.35
 
 
24.7
 
 
 
 
ANKRD46
 
 
hsa-let-7b-5p
 
 
-0.389
 
 
13.6
 
 
 
 
ANP32A
 
 
hsa-miR-132-3p
 
 
-0.374
 
 
66.9
 
 
 
 
ANTXR2
 
 
hsa-miR-29a-3p
 
 
-0.347
 
 
38.4
 
 
 
 
ANXA11
 
 
hsa-miR-16-5p
 
 
-0.447
 
 
101.9
 
 
 
 
ANXA11
 
 
hsa-miR-15b-5p
 
 
-0.463
 
 
101.9
 
 
 
 
AP1S1
 
 
hsa-let-7b-5p
 
 
-0.374
 
 
17.7
 
 
 
 
AP2A1
 
 
hsa-miR-15b-5p
 
 
-0.333
 
 
39.7
 
 
 
 
AP2A1
 
 
hsa-miR-16-5p
 
 
-0.333
 
 
39.7
 
 
 
 
AP2B1
 
 
hsa-miR-16-5p
 
 
-0.302
 
 
54.6
 
 
 
 
AP2B1
 
 
hsa-miR-15b-5p
 
 
-0.334
 
 
54.6
 
 
 
 
AP3S1
 
 
hsa-miR-30b-5p
 
 
-0.293
 
 
56.3
 
 
 
 
APOO
 
 
hsa-miR-181b-5p
 
 
-0.5
 
 
12.5
 
 
 
 
APOO
 
 
hsa-miR-181a-5p
 
 
-0.5
 
 
12.5
 
 
 
 
APP
 
 
hsa-miR-16-5p
 
 
-0.298
 
 
392.7
 
 
 
 
APP
 
 
hsa-miR-15b-5p
 
 
-0.298
 
 
392.7
 
 
 
 
APPBP2
 
 
hsa-miR-27a-3p
 
 
-0.378
 
 
10.6
 
 
 
 
ARF4
 
 
hsa-miR-30b-5p
 
 
-0.289
 
 
129.6
 
 
 
 
ARF4
 
 
hsa-miR-148b-3p
 
 
-0.487
 
 
129.6
 
 
 
 
ARF5
 
 
hsa-miR-29a-3p
 
 
-0.491
 
 
108.8
 
 
 
 
ARF6
 
 
hsa-miR-181b-5p
 
 
-0.33
 
 
31.6
 
 
 
 
ARF6
 
 
hsa-miR-181a-5p
 
 
-0.33
 
 
31.6
 
 
 
 
ARFGEF1
 
 
hsa-miR-148b-3p
 
 
-0.306
 
 
12.7
 
 
 
 
ARFIP1
 
 
hsa-miR-320b
 
 
-0.339
 
 
22.0
 
 
 
 
ARFIP1
 
 
hsa-miR-320c
 
 
-0.339
 
 
22.0
 
 
 
 
ARFIP1
 
 
hsa-miR-148b-3p
 
 
-0.474
 
 
22.0
 
 
 
 
ARHGAP21
 
 
hsa-miR-148b-3p
 
 
-0.287
 
 
15.1
 
 
 
 
ARHGAP5
 
 
hsa-miR-486-5p
 
 
-0.3
 
 
25.6
 
 
 
 
ARID1B
 
 
hsa-miR-29a-3p
 
 
-0.342
 
 
11.7
 
 
 
 
ARID4B
 
 
hsa-miR-486-5p
 
 
-0.367
 
 
12.7
 
 
 
 
ARL2
 
 
hsa-miR-16-5p
 
 
-0.374
 
 
113.6
 
 
 
 
ARL2
 
 
hsa-miR-15b-5p
 
 
-0.374
 
 
113.6
 
 
 
 
ARL2
 
 
hsa-miR-16-5p
 
 
-0.418
 
 
113.6
 
 
 
 
ARL2
 
 
hsa-miR-15b-5p
 
 
-0.442
 
 
113.6
 
 
 
 
ARL4A
 
 
hsa-miR-29a-3p
 
 
-0.386
 
 
51.6
 
 
 
 
ARL4A
 
 
hsa-miR-30b-5p
 
 
-0.448
 
 
51.6
 
 
 
 
ARL6IP1
 
 
hsa-miR-328-3p
 
 
-0.494
 
 
105.7
 
 
 
 
ARL6IP1
 
 
hsa-miR-148b-3p
 
 
-0.579
 
 
105.7
 
 
 
 
ARL6IP6
 
 
hsa-let-7b-5p
 
 
-0.293
 
 
12.8
 
 
 
 
ARL8B
 
 
hsa-miR-148b-3p
 
 
-0.337
 
 
39.7
 
 
 
 
ARL8B
 
 
hsa-miR-320b
 
 
-0.376
 
 
39.7
 
 
 
 
ARL8B
 
 
hsa-miR-320c
 
 
-0.376
 
 
39.7
 
 
 
 
 
 
 cerebellum 
 
 
 
 
target_symbol
 
 
mature_mirna_id
 
 
score
 
 
nTPM
 
 
 
 
 
 
AAR2
 
 
hsa-miR-29a-3p
 
 
-0.327
 
 
10.9
 
 
 
 
ABCB10
 
 
hsa-miR-148b-3p
 
 
-0.291
 
 
10.6
 
 
 
 
ABCB9
 
 
hsa-let-7b-5p
 
 
-0.422
 
 
18.0
 
 
 
 
ABCD3
 
 
hsa-miR-148b-3p
 
 
-0.388
 
 
12.8
 
 
 
 
ABCE1
 
 
hsa-miR-29a-3p
 
 
-0.404
 
 
12.8
 
 
 
 
ABHD17B
 
 
hsa-miR-486-5p
 
 
-0.348
 
 
11.2
 
 
 
 
ACSL6
 
 
hsa-let-7b-5p
 
 
-0.336
 
 
27.8
 
 
 
 
ACTA2
 
 
hsa-miR-27a-3p
 
 
-0.577
 
 
25.2
 
 
 
 
ACTR2
 
 
hsa-miR-15b-5p
 
 
-0.293
 
 
54.0
 
 
 
 
ACTR2
 
 
hsa-miR-16-5p
 
 
-0.293
 
 
54.0
 
 
 
 
ADAM10
 
 
hsa-miR-148b-3p
 
 
-0.356
 
 
15.6
 
 
 
 
ADAP1
 
 
hsa-miR-27a-3p
 
 
-0.302
 
 
43.2
 
 
 
 
ADCY3
 
 
hsa-miR-132-3p
 
 
-0.303
 
 
33.6
 
 
 
 
ADGRB3
 
 
hsa-miR-148b-3p
 
 
-0.524
 
 
20.8
 
 
 
 
ADIPOR2
 
 
hsa-miR-150-5p
 
 
-0.287
 
 
18.7
 
 
 
 
ADNP
 
 
hsa-miR-328-3p
 
 
-0.371
 
 
38.9
 
 
 
 
AFG3L2
 
 
hsa-miR-181b-5p
 
 
-0.491
 
 
11.5
 
 
 
 
AFG3L2
 
 
hsa-miR-181a-5p
 
 
-0.491
 
 
11.5
 
 
 
 
AGFG1
 
 
hsa-miR-148b-3p
 
 
-0.332
 
 
22.4
 
 
 
 
AGPAT3
 
 
hsa-miR-27a-3p
 
 
-0.29
 
 
27.6
 
 
 
 
AGPAT3
 
 
hsa-miR-16-5p
 
 
-0.299
 
 
27.6
 
 
 
 
AHDC1
 
 
hsa-miR-148b-3p
 
 
-0.413
 
 
32.0
 
 
 
 
AK1
 
 
hsa-miR-29a-3p
 
 
-0.333
 
 
47.4
 
 
 
 
AK4
 
 
hsa-miR-15b-5p
 
 
-0.316
 
 
14.5
 
 
 
 
AK4
 
 
hsa-miR-16-5p
 
 
-0.348
 
 
14.5
 
 
 
 
AKAIN1
 
 
hsa-miR-29a-3p
 
 
-0.52
 
 
40.8
 
 
 
 
AKAP1
 
 
hsa-miR-148b-3p
 
 
-0.381
 
 
30.6
 
 
 
 
AKIRIN1
 
 
hsa-miR-27a-3p
 
 
-0.368
 
 
20.5
 
 
 
 
AKT3
 
 
hsa-miR-29a-3p
 
 
-0.287
 
 
23.2
 
 
 
 
AKT3
 
 
hsa-miR-15b-5p
 
 
-0.301
 
 
23.2
 
 
 
 
AKT3
 
 
hsa-miR-16-5p
 
 
-0.309
 
 
23.2
 
 
 
 
ALAD
 
 
hsa-miR-16-5p
 
 
-0.391
 
 
17.9
 
 
 
 
ALAD
 
 
hsa-miR-15b-5p
 
 
-0.446
 
 
17.9
 
 
 
 
AMT
 
 
hsa-let-7b-5p
 
 
-0.463
 
 
20.1
 
 
 
 
ANGEL2
 
 
hsa-miR-132-3p
 
 
-0.357
 
 
12.7
 
 
 
 
ANKHD1
 
 
hsa-miR-23a-3p
 
 
-0.368
 
 
38.6
 
 
 
 
ANKHD1
 
 
hsa-miR-23b-3p
 
 
-0.368
 
 
38.6
 
 
 
 
ANKHD1
 
 
hsa-miR-30b-5p
 
 
-0.405
 
 
38.6
 
 
 
 
ANKRD13B
 
 
hsa-miR-29a-3p
 
 
-0.421
 
 
44.3
 
 
 
 
ANKRD13C
 
 
hsa-miR-29a-3p
 
 
-0.411
 
 
12.9
 
 
 
 
ANKRD17
 
 
hsa-miR-30b-5p
 
 
-0.336
 
 
16.2
 
 
 
 
ANKRD40
 
 
hsa-miR-27a-3p
 
 
-0.35
 
 
31.7
 
 
 
 
ANKRD46
 
 
hsa-let-7b-5p
 
 
-0.389
 
 
27.5
 
 
 
 
ANP32A
 
 
hsa-miR-132-3p
 
 
-0.374
 
 
71.1
 
 
 
 
ANXA11
 
 
hsa-miR-16-5p
 
 
-0.447
 
 
22.2
 
 
 
 
ANXA11
 
 
hsa-miR-15b-5p
 
 
-0.463
 
 
22.2
 
 
 
 
AP1S1
 
 
hsa-let-7b-5p
 
 
-0.374
 
 
23.9
 
 
 
 
AP2A1
 
 
hsa-miR-15b-5p
 
 
-0.333
 
 
67.6
 
 
 
 
AP2A1
 
 
hsa-miR-16-5p
 
 
-0.333
 
 
67.6
 
 
 
 
AP2B1
 
 
hsa-miR-16-5p
 
 
-0.302
 
 
63.1
 
 
 
 
AP2B1
 
 
hsa-miR-15b-5p
 
 
-0.334
 
 
63.1
 
 
 
 
AP3S1
 
 
hsa-miR-30b-5p
 
 
-0.293
 
 
52.4
 
 
 
 
APBB3
 
 
hsa-let-7b-5p
 
 
-0.587
 
 
23.6
 
 
 
 
APC
 
 
hsa-miR-29a-3p
 
 
-0.413
 
 
12.2
 
 
 
 
APOO
 
 
hsa-miR-181b-5p
 
 
-0.5
 
 
32.8
 
 
 
 
APOO
 
 
hsa-miR-181a-5p
 
 
-0.5
 
 
32.8
 
 
 
 
APP
 
 
hsa-miR-16-5p
 
 
-0.298
 
 
316.4
 
 
 
 
APP
 
 
hsa-miR-15b-5p
 
 
-0.298
 
 
316.4
 
 
 
 
APPBP2
 
 
hsa-miR-27a-3p
 
 
-0.378
 
 
14.6
 
 
 
 
ARF4
 
 
hsa-miR-30b-5p
 
 
-0.289
 
 
50.2
 
 
 
 
ARF4
 
 
hsa-miR-148b-3p
 
 
-0.487
 
 
50.2
 
 
 
 
ARF5
 
 
hsa-miR-29a-3p
 
 
-0.491
 
 
167.9
 
 
 
 
ARF6
 
 
hsa-miR-181b-5p
 
 
-0.33
 
 
11.0
 
 
 
 
ARF6
 
 
hsa-miR-181a-5p
 
 
-0.33
 
 
11.0
 
 
 
 
ARG2
 
 
hsa-let-7b-5p
 
 
-0.468
 
 
16.4
 
 
 
 
ARHGAP21
 
 
hsa-miR-148b-3p
 
 
-0.287
 
 
50.0
 
 
 
 
ARHGAP5
 
 
hsa-miR-486-5p
 
 
-0.3
 
 
20.0
 
 
 
 
ARID1B
 
 
hsa-miR-29a-3p
 
 
-0.342
 
 
12.5
 
 
 
 
ARID4B
 
 
hsa-miR-486-5p
 
 
-0.367
 
 
15.1
 
 
 
 
ARL2
 
 
hsa-miR-16-5p
 
 
-0.374
 
 
72.8
 
 
 
 
ARL2
 
 
hsa-miR-15b-5p
 
 
-0.374
 
 
72.8
 
 
 
 
ARL2
 
 
hsa-miR-16-5p
 
 
-0.418
 
 
72.8
 
 
 
 
ARL2
 
 
hsa-miR-15b-5p
 
 
-0.442
 
 
72.8
 
 
 
 
ARL3
 
 
hsa-miR-16-5p
 
 
-0.496
 
 
25.4
 
 
 
 
ARL3
 
 
hsa-miR-15b-5p
 
 
-0.507
 
 
25.4
 
 
 
 
ARL6IP1
 
 
hsa-miR-328-3p
 
 
-0.494
 
 
234.0
 
 
 
 
ARL6IP1
 
 
hsa-miR-148b-3p
 
 
-0.579
 
 
234.0
 
 
 
 
ARL8B
 
 
hsa-miR-148b-3p
 
 
-0.337
 
 
50.7
 
 
 
 
ARL8B
 
 
hsa-miR-320b
 
 
-0.376
 
 
50.7
 
 
 
 
ARL8B
 
 
hsa-miR-320c
 
 
-0.376
 
 
50.7
 
 
 
 
ARMC8
 
 
hsa-miR-486-5p
 
 
-0.321
 
 
35.7
 
 
 
 
ARMCX2
 
 
hsa-miR-15b-5p
 
 
-0.344
 
 
20.7
 
 
 
 
ARMCX2
 
 
hsa-miR-16-5p
 
 
-0.366
 
 
20.7
 
 
 
 
ARMCX3
 
 
hsa-miR-425-5p
 
 
-0.325
 
 
24.0
 
 
 
 
ARMT1
 
 
hsa-let-7b-5p
 
 
-0.291
 
 
11.5
 
 
 
 
ARPP19
 
 
hsa-miR-320b
 
 
-0.325
 
 
66.7
 
 
 
 
ARPP19
 
 
hsa-miR-320c
 
 
-0.325
 
 
66.7
 
 
 
 
ARPP19
 
 
hsa-miR-15b-5p
 
 
-0.346
 
 
66.7
 
 
 
 
ARPP19
 
 
hsa-miR-16-5p
 
 
-0.354
 
 
66.7
 
 
 
 
ARPP19
 
 
hsa-miR-29a-3p
 
 
-0.371
 
 
66.7
 
 
 
 
ARPP19
 
 
hsa-miR-328-3p
 
 
-0.404
 
 
66.7
 
 
 
 
ARPP19
 
 
hsa-let-7b-5p
 
 
-0.476
 
 
66.7
 
 
 
 
ARPP21
 
 
hsa-miR-16-5p
 
 
-0.309
 
 
42.2
 
 
 
 
ARPP21
 
 
hsa-miR-15b-5p
 
 
-0.342
 
 
42.2
 
 
 
 
ARRDC3
 
 
hsa-miR-148b-3p
 
 
-0.305
 
 
17.8
 
 
 
 
ARRDC3
 
 
hsa-miR-29a-3p
 
 
-0.402
 
 
17.8
 
 
 
 
ASAH1
 
 
hsa-miR-27a-3p
 
 
-0.398
 
 
32.2
 
 
 
 
ASAP2
 
 
hsa-miR-29a-3p
 
 
-0.348
 
 
10.8
 
 
 
 
ASF1A
 
 
hsa-miR-23a-3p
 
 
-0.506
 
 
12.6
 
 
 
 
ASF1A
 
 
hsa-miR-23b-3p
 
 
-0.506
 
 
12.6
 
 
 
 
 
 
 cerebral cortex 
 
 
 
 
target_symbol
 
 
mature_mirna_id
 
 
score
 
 
nTPM
 
 
 
 
 
 
AAR2
 
 
hsa-miR-29a-3p
 
 
-0.327
 
 
10.6
 
 
 
 
ABCB9
 
 
hsa-let-7b-5p
 
 
-0.422
 
 
19.5
 
 
 
 
ABCD3
 
 
hsa-miR-148b-3p
 
 
-0.388
 
 
17.1
 
 
 
 
ABCE1
 
 
hsa-miR-29a-3p
 
 
-0.404
 
 
21.8
 
 
 
 
ABHD17B
 
 
hsa-miR-486-5p
 
 
-0.348
 
 
17.0
 
 
 
 
ABHD17C
 
 
hsa-let-7b-5p
 
 
-0.359
 
 
16.3
 
 
 
 
ABHD17C
 
 
hsa-miR-27a-3p
 
 
-0.435
 
 
16.3
 
 
 
 
ABHD4
 
 
hsa-miR-29a-3p
 
 
-0.312
 
 
14.6
 
 
 
 
ACSL6
 
 
hsa-let-7b-5p
 
 
-0.336
 
 
29.5
 
 
 
 
ACTA2
 
 
hsa-miR-27a-3p
 
 
-0.577
 
 
56.2
 
 
 
 
ACTN2
 
 
hsa-miR-23a-3p
 
 
-0.294
 
 
14.9
 
 
 
 
ACTN2
 
 
hsa-miR-29a-3p
 
 
-0.311
 
 
14.9
 
 
 
 
ACTR2
 
 
hsa-miR-15b-5p
 
 
-0.293
 
 
115.2
 
 
 
 
ACTR2
 
 
hsa-miR-16-5p
 
 
-0.293
 
 
115.2
 
 
 
 
ADAM10
 
 
hsa-miR-148b-3p
 
 
-0.356
 
 
16.0
 
 
 
 
ADAM9
 
 
hsa-miR-30b-5p
 
 
-0.312
 
 
16.2
 
 
 
 
ADAMTS10
 
 
hsa-miR-27a-3p
 
 
-0.354
 
 
10.8
 
 
 
 
ADAMTS10
 
 
hsa-miR-29a-3p
 
 
-0.516
 
 
10.8
 
 
 
 
ADAP1
 
 
hsa-miR-27a-3p
 
 
-0.302
 
 
126.8
 
 
 
 
ADCY2
 
 
hsa-miR-148b-3p
 
 
-0.299
 
 
35.4
 
 
 
 
ADCY3
 
 
hsa-miR-132-3p
 
 
-0.303
 
 
37.4
 
 
 
 
ADGRA3
 
 
hsa-miR-27a-3p
 
 
-0.296
 
 
17.8
 
 
 
 
ADGRA3
 
 
hsa-miR-30b-5p
 
 
-0.304
 
 
17.8
 
 
 
 
ADGRB3
 
 
hsa-miR-148b-3p
 
 
-0.524
 
 
28.2
 
 
 
 
ADIPOR2
 
 
hsa-miR-150-5p
 
 
-0.287
 
 
29.8
 
 
 
 
ADNP
 
 
hsa-miR-328-3p
 
 
-0.371
 
 
26.0
 
 
 
 
ADORA2B
 
 
hsa-miR-27a-3p
 
 
-0.638
 
 
12.5
 
 
 
 
AFG3L2
 
 
hsa-miR-181b-5p
 
 
-0.491
 
 
17.1
 
 
 
 
AFG3L2
 
 
hsa-miR-181a-5p
 
 
-0.491
 
 
17.1
 
 
 
 
AGFG1
 
 
hsa-miR-148b-3p
 
 
-0.332
 
 
33.6
 
 
 
 
AGPAT3
 
 
hsa-miR-27a-3p
 
 
-0.29
 
 
38.5
 
 
 
 
AGPAT3
 
 
hsa-miR-16-5p
 
 
-0.299
 
 
38.5
 
 
 
 
AGPAT4
 
 
hsa-miR-29a-3p
 
 
-0.376
 
 
13.7
 
 
 
 
AHDC1
 
 
hsa-miR-148b-3p
 
 
-0.413
 
 
15.5
 
 
 
 
AIFM1
 
 
hsa-let-7b-5p
 
 
-0.314
 
 
10.3
 
 
 
 
AK1
 
 
hsa-miR-29a-3p
 
 
-0.333
 
 
81.2
 
 
 
 
AK4
 
 
hsa-miR-15b-5p
 
 
-0.316
 
 
19.8
 
 
 
 
AK4
 
 
hsa-miR-16-5p
 
 
-0.348
 
 
19.8
 
 
 
 
AKAP1
 
 
hsa-miR-148b-3p
 
 
-0.381
 
 
24.9
 
 
 
 
AKAP5
 
 
hsa-miR-29a-3p
 
 
-0.298
 
 
12.9
 
 
 
 
AKAP6
 
 
hsa-let-7b-5p
 
 
-0.307
 
 
27.4
 
 
 
 
AKIRIN1
 
 
hsa-miR-27a-3p
 
 
-0.368
 
 
38.1
 
 
 
 
AKT3
 
 
hsa-miR-29a-3p
 
 
-0.287
 
 
23.7
 
 
 
 
AKT3
 
 
hsa-miR-15b-5p
 
 
-0.301
 
 
23.7
 
 
 
 
AKT3
 
 
hsa-miR-16-5p
 
 
-0.309
 
 
23.7
 
 
 
 
ALAD
 
 
hsa-miR-16-5p
 
 
-0.391
 
 
40.0
 
 
 
 
ALAD
 
 
hsa-miR-15b-5p
 
 
-0.446
 
 
40.0
 
 
 
 
ALCAM
 
 
hsa-miR-148b-3p
 
 
-0.339
 
 
54.7
 
 
 
 
ALPL
 
 
hsa-miR-10a-5p
 
 
-0.32
 
 
13.5
 
 
 
 
AMOT
 
 
hsa-miR-29a-3p
 
 
-0.304
 
 
17.9
 
 
 
 
ANKHD1
 
 
hsa-miR-23a-3p
 
 
-0.368
 
 
22.1
 
 
 
 
ANKHD1
 
 
hsa-miR-23b-3p
 
 
-0.368
 
 
22.1
 
 
 
 
ANKHD1
 
 
hsa-miR-30b-5p
 
 
-0.405
 
 
22.1
 
 
 
 
ANKRD13B
 
 
hsa-miR-29a-3p
 
 
-0.421
 
 
17.2
 
 
 
 
ANKRD13C
 
 
hsa-miR-29a-3p
 
 
-0.411
 
 
10.2
 
 
 
 
ANKRD17
 
 
hsa-miR-30b-5p
 
 
-0.336
 
 
15.6
 
 
 
 
ANKRD40
 
 
hsa-miR-27a-3p
 
 
-0.35
 
 
54.5
 
 
 
 
ANKRD46
 
 
hsa-let-7b-5p
 
 
-0.389
 
 
39.5
 
 
 
 
ANLN
 
 
hsa-miR-16-5p
 
 
-0.31
 
 
47.6
 
 
 
 
ANLN
 
 
hsa-miR-15b-5p
 
 
-0.334
 
 
47.6
 
 
 
 
ANP32A
 
 
hsa-miR-132-3p
 
 
-0.374
 
 
64.2
 
 
 
 
ANXA11
 
 
hsa-miR-16-5p
 
 
-0.447
 
 
45.1
 
 
 
 
ANXA11
 
 
hsa-miR-15b-5p
 
 
-0.463
 
 
45.1
 
 
 
 
AP1S1
 
 
hsa-let-7b-5p
 
 
-0.374
 
 
104.9
 
 
 
 
AP2A1
 
 
hsa-miR-15b-5p
 
 
-0.333
 
 
78.9
 
 
 
 
AP2A1
 
 
hsa-miR-16-5p
 
 
-0.333
 
 
78.9
 
 
 
 
AP2B1
 
 
hsa-miR-16-5p
 
 
-0.302
 
 
106.7
 
 
 
 
AP2B1
 
 
hsa-miR-15b-5p
 
 
-0.334
 
 
106.7
 
 
 
 
AP3S1
 
 
hsa-miR-30b-5p
 
 
-0.293
 
 
82.8
 
 
 
 
APBB3
 
 
hsa-let-7b-5p
 
 
-0.587
 
 
15.0
 
 
 
 
APC
 
 
hsa-miR-29a-3p
 
 
-0.413
 
 
34.5
 
 
 
 
APLN
 
 
hsa-miR-16-5p
 
 
-0.31
 
 
14.5
 
 
 
 
APLN
 
 
hsa-miR-15b-5p
 
 
-0.395
 
 
14.5
 
 
 
 
APLN
 
 
hsa-miR-16-5p
 
 
-0.395
 
 
14.5
 
 
 
 
APOO
 
 
hsa-miR-181b-5p
 
 
-0.5
 
 
56.2
 
 
 
 
APOO
 
 
hsa-miR-181a-5p
 
 
-0.5
 
 
56.2
 
 
 
 
APP
 
 
hsa-miR-16-5p
 
 
-0.298
 
 
669.1
 
 
 
 
APP
 
 
hsa-miR-15b-5p
 
 
-0.298
 
 
669.1
 
 
 
 
APPBP2
 
 
hsa-miR-27a-3p
 
 
-0.378
 
 
17.6
 
 
 
 
ARF4
 
 
hsa-miR-30b-5p
 
 
-0.289
 
 
61.5
 
 
 
 
ARF4
 
 
hsa-miR-148b-3p
 
 
-0.487
 
 
61.5
 
 
 
 
ARF5
 
 
hsa-miR-29a-3p
 
 
-0.491
 
 
222.4
 
 
 
 
ARF6
 
 
hsa-miR-181b-5p
 
 
-0.33
 
 
13.0
 
 
 
 
ARF6
 
 
hsa-miR-181a-5p
 
 
-0.33
 
 
13.0
 
 
 
 
ARHGAP21
 
 
hsa-miR-148b-3p
 
 
-0.287
 
 
47.3
 
 
 
 
ARHGAP5
 
 
hsa-miR-486-5p
 
 
-0.3
 
 
48.8
 
 
 
 
ARID1B
 
 
hsa-miR-29a-3p
 
 
-0.342
 
 
12.2
 
 
 
 
ARID4B
 
 
hsa-miR-486-5p
 
 
-0.367
 
 
11.1
 
 
 
 
ARL2
 
 
hsa-miR-16-5p
 
 
-0.374
 
 
152.3
 
 
 
 
ARL2
 
 
hsa-miR-15b-5p
 
 
-0.374
 
 
152.3
 
 
 
 
ARL2
 
 
hsa-miR-16-5p
 
 
-0.418
 
 
152.3
 
 
 
 
ARL2
 
 
hsa-miR-15b-5p
 
 
-0.442
 
 
152.3
 
 
 
 
ARL3
 
 
hsa-miR-16-5p
 
 
-0.496
 
 
30.4
 
 
 
 
ARL3
 
 
hsa-miR-15b-5p
 
 
-0.507
 
 
30.4
 
 
 
 
ARL4A
 
 
hsa-miR-29a-3p
 
 
-0.386
 
 
23.3
 
 
 
 
ARL4A
 
 
hsa-miR-30b-5p
 
 
-0.448
 
 
23.3
 
 
 
 
ARL4D
 
 
hsa-let-7b-5p
 
 
-0.417
 
 
14.3
 
 
 
 
ARL6IP1
 
 
hsa-miR-328-3p
 
 
-0.494
 
 
314.1
 
 
 
 
ARL6IP1
 
 
hsa-miR-148b-3p
 
 
-0.579
 
 
314.1
 
 
 
 
ARL8B
 
 
hsa-miR-148b-3p
 
 
-0.337
 
 
75.1
 
 
 
 
 
 
 cervix 
 
 
 
 
target_symbol
 
 
mature_mirna_id
 
 
score
 
 
nTPM
 
 
 
 
 
 
AAR2
 
 
hsa-miR-29a-3p
 
 
-0.327
 
 
19.6
 
 
 
 
ABCB7
 
 
hsa-miR-148b-3p
 
 
-0.515
 
 
10.8
 
 
 
 
ABCD3
 
 
hsa-miR-148b-3p
 
 
-0.388
 
 
21.0
 
 
 
 
ABCE1
 
 
hsa-miR-29a-3p
 
 
-0.404
 
 
19.5
 
 
 
 
ABHD17C
 
 
hsa-let-7b-5p
 
 
-0.359
 
 
18.1
 
 
 
 
ABHD17C
 
 
hsa-miR-27a-3p
 
 
-0.435
 
 
18.1
 
 
 
 
ABHD4
 
 
hsa-miR-29a-3p
 
 
-0.312
 
 
27.3
 
 
 
 
ACP3
 
 
hsa-let-7b-5p
 
 
-0.298
 
 
10.9
 
 
 
 
ACTA2
 
 
hsa-miR-27a-3p
 
 
-0.577
 
 
2602.4
 
 
 
 
ACTC1
 
 
hsa-miR-30b-5p
 
 
-0.543
 
 
44.2
 
 
 
 
ACTR2
 
 
hsa-miR-15b-5p
 
 
-0.293
 
 
76.5
 
 
 
 
ACTR2
 
 
hsa-miR-16-5p
 
 
-0.293
 
 
76.5
 
 
 
 
ACVR1
 
 
hsa-miR-30b-5p
 
 
-0.29
 
 
20.6
 
 
 
 
ACVR1
 
 
hsa-miR-148b-3p
 
 
-0.397
 
 
20.6
 
 
 
 
ADAM10
 
 
hsa-miR-148b-3p
 
 
-0.356
 
 
15.1
 
 
 
 
ADAM9
 
 
hsa-miR-30b-5p
 
 
-0.312
 
 
32.7
 
 
 
 
ADAMTS10
 
 
hsa-miR-27a-3p
 
 
-0.354
 
 
11.2
 
 
 
 
ADAMTS10
 
 
hsa-miR-29a-3p
 
 
-0.516
 
 
11.2
 
 
 
 
ADAMTS9
 
 
hsa-miR-29a-3p
 
 
-0.305
 
 
11.0
 
 
 
 
ADAMTS9
 
 
hsa-miR-29a-3p
 
 
-0.485
 
 
11.0
 
 
 
 
ADCY3
 
 
hsa-miR-132-3p
 
 
-0.303
 
 
60.4
 
 
 
 
ADGRA3
 
 
hsa-miR-27a-3p
 
 
-0.296
 
 
10.2
 
 
 
 
ADGRA3
 
 
hsa-miR-30b-5p
 
 
-0.304
 
 
10.2
 
 
 
 
ADIPOR2
 
 
hsa-miR-150-5p
 
 
-0.287
 
 
25.7
 
 
 
 
ADNP
 
 
hsa-miR-328-3p
 
 
-0.371
 
 
35.1
 
 
 
 
ADRA2A
 
 
hsa-miR-23a-3p
 
 
-0.371
 
 
35.0
 
 
 
 
ADRA2A
 
 
hsa-miR-23b-3p
 
 
-0.371
 
 
35.0
 
 
 
 
ADRB2
 
 
hsa-miR-15b-5p
 
 
-0.29
 
 
12.7
 
 
 
 
ADRB2
 
 
hsa-miR-16-5p
 
 
-0.323
 
 
12.7
 
 
 
 
ADRB2
 
 
hsa-let-7b-5p
 
 
-0.635
 
 
12.7
 
 
 
 
AFG3L2
 
 
hsa-miR-181b-5p
 
 
-0.491
 
 
14.0
 
 
 
 
AFG3L2
 
 
hsa-miR-181a-5p
 
 
-0.491
 
 
14.0
 
 
 
 
AGFG1
 
 
hsa-miR-148b-3p
 
 
-0.332
 
 
27.5
 
 
 
 
AGPAT3
 
 
hsa-miR-27a-3p
 
 
-0.29
 
 
12.1
 
 
 
 
AGPAT3
 
 
hsa-miR-16-5p
 
 
-0.299
 
 
12.1
 
 
 
 
AHDC1
 
 
hsa-miR-148b-3p
 
 
-0.413
 
 
23.6
 
 
 
 
AHR
 
 
hsa-miR-29a-3p
 
 
-0.316
 
 
13.6
 
 
 
 
AIFM1
 
 
hsa-let-7b-5p
 
 
-0.314
 
 
14.8
 
 
 
 
AK1
 
 
hsa-miR-29a-3p
 
 
-0.333
 
 
35.3
 
 
 
 
AKAP1
 
 
hsa-miR-148b-3p
 
 
-0.381
 
 
22.6
 
 
 
 
AKIRIN1
 
 
hsa-miR-27a-3p
 
 
-0.368
 
 
20.5
 
 
 
 
AKT3
 
 
hsa-miR-29a-3p
 
 
-0.287
 
 
15.6
 
 
 
 
AKT3
 
 
hsa-miR-15b-5p
 
 
-0.301
 
 
15.6
 
 
 
 
AKT3
 
 
hsa-miR-16-5p
 
 
-0.309
 
 
15.6
 
 
 
 
ALAD
 
 
hsa-miR-16-5p
 
 
-0.391
 
 
18.9
 
 
 
 
ALAD
 
 
hsa-miR-15b-5p
 
 
-0.446
 
 
18.9
 
 
 
 
ALCAM
 
 
hsa-miR-148b-3p
 
 
-0.339
 
 
26.3
 
 
 
 
ALDH1A2
 
 
hsa-miR-23b-3p
 
 
-0.346
 
 
49.1
 
 
 
 
ALDH1A2
 
 
hsa-miR-23a-3p
 
 
-0.354
 
 
49.1
 
 
 
 
ALOX12
 
 
hsa-miR-15b-5p
 
 
-0.443
 
 
78.6
 
 
 
 
ALOX12
 
 
hsa-miR-16-5p
 
 
-0.451
 
 
78.6
 
 
 
 
AMOTL1
 
 
hsa-miR-16-5p
 
 
-0.297
 
 
12.0
 
 
 
 
AMOTL1
 
 
hsa-miR-15b-5p
 
 
-0.319
 
 
12.0
 
 
 
 
AMT
 
 
hsa-let-7b-5p
 
 
-0.463
 
 
14.5
 
 
 
 
ANKHD1
 
 
hsa-miR-23a-3p
 
 
-0.368
 
 
41.2
 
 
 
 
ANKHD1
 
 
hsa-miR-23b-3p
 
 
-0.368
 
 
41.2
 
 
 
 
ANKHD1
 
 
hsa-miR-30b-5p
 
 
-0.405
 
 
41.2
 
 
 
 
ANKRD17
 
 
hsa-miR-30b-5p
 
 
-0.336
 
 
15.8
 
 
 
 
ANKRD29
 
 
hsa-miR-132-3p
 
 
-0.47
 
 
12.2
 
 
 
 
ANKRD40
 
 
hsa-miR-27a-3p
 
 
-0.35
 
 
18.9
 
 
 
 
ANKRD46
 
 
hsa-let-7b-5p
 
 
-0.389
 
 
14.1
 
 
 
 
ANP32A
 
 
hsa-miR-132-3p
 
 
-0.374
 
 
50.7
 
 
 
 
ANTXR2
 
 
hsa-miR-29a-3p
 
 
-0.347
 
 
49.9
 
 
 
 
ANXA11
 
 
hsa-miR-16-5p
 
 
-0.447
 
 
89.1
 
 
 
 
ANXA11
 
 
hsa-miR-15b-5p
 
 
-0.463
 
 
89.1
 
 
 
 
AP1S1
 
 
hsa-let-7b-5p
 
 
-0.374
 
 
20.1
 
 
 
 
AP2A1
 
 
hsa-miR-15b-5p
 
 
-0.333
 
 
42.3
 
 
 
 
AP2A1
 
 
hsa-miR-16-5p
 
 
-0.333
 
 
42.3
 
 
 
 
AP2B1
 
 
hsa-miR-16-5p
 
 
-0.302
 
 
50.8
 
 
 
 
AP2B1
 
 
hsa-miR-15b-5p
 
 
-0.334
 
 
50.8
 
 
 
 
AP3S1
 
 
hsa-miR-30b-5p
 
 
-0.293
 
 
65.9
 
 
 
 
APOO
 
 
hsa-miR-181b-5p
 
 
-0.5
 
 
12.3
 
 
 
 
APOO
 
 
hsa-miR-181a-5p
 
 
-0.5
 
 
12.3
 
 
 
 
APP
 
 
hsa-miR-16-5p
 
 
-0.298
 
 
239.9
 
 
 
 
APP
 
 
hsa-miR-15b-5p
 
 
-0.298
 
 
239.9
 
 
 
 
APPBP2
 
 
hsa-miR-27a-3p
 
 
-0.378
 
 
11.0
 
 
 
 
ARF4
 
 
hsa-miR-30b-5p
 
 
-0.289
 
 
196.1
 
 
 
 
ARF4
 
 
hsa-miR-148b-3p
 
 
-0.487
 
 
196.1
 
 
 
 
ARF5
 
 
hsa-miR-29a-3p
 
 
-0.491
 
 
129.6
 
 
 
 
ARF6
 
 
hsa-miR-181b-5p
 
 
-0.33
 
 
31.8
 
 
 
 
ARF6
 
 
hsa-miR-181a-5p
 
 
-0.33
 
 
31.8
 
 
 
 
ARFGEF1
 
 
hsa-miR-148b-3p
 
 
-0.306
 
 
13.3
 
 
 
 
ARFIP1
 
 
hsa-miR-320b
 
 
-0.339
 
 
21.1
 
 
 
 
ARFIP1
 
 
hsa-miR-320c
 
 
-0.339
 
 
21.1
 
 
 
 
ARFIP1
 
 
hsa-miR-148b-3p
 
 
-0.474
 
 
21.1
 
 
 
 
ARG2
 
 
hsa-let-7b-5p
 
 
-0.468
 
 
16.0
 
 
 
 
ARHGAP21
 
 
hsa-miR-148b-3p
 
 
-0.287
 
 
18.5
 
 
 
 
ARHGAP5
 
 
hsa-miR-486-5p
 
 
-0.3
 
 
24.7
 
 
 
 
ARID1B
 
 
hsa-miR-29a-3p
 
 
-0.342
 
 
15.1
 
 
 
 
ARID4B
 
 
hsa-miR-486-5p
 
 
-0.367
 
 
14.1
 
 
 
 
ARL2
 
 
hsa-miR-16-5p
 
 
-0.374
 
 
102.1
 
 
 
 
ARL2
 
 
hsa-miR-15b-5p
 
 
-0.374
 
 
102.1
 
 
 
 
ARL2
 
 
hsa-miR-16-5p
 
 
-0.418
 
 
102.1
 
 
 
 
ARL2
 
 
hsa-miR-15b-5p
 
 
-0.442
 
 
102.1
 
 
 
 
ARL3
 
 
hsa-miR-16-5p
 
 
-0.496
 
 
12.6
 
 
 
 
ARL3
 
 
hsa-miR-15b-5p
 
 
-0.507
 
 
12.6
 
 
 
 
ARL4A
 
 
hsa-miR-29a-3p
 
 
-0.386
 
 
46.9
 
 
 
 
ARL4A
 
 
hsa-miR-30b-5p
 
 
-0.448
 
 
46.9
 
 
 
 
ARL4D
 
 
hsa-let-7b-5p
 
 
-0.417
 
 
27.6
 
 
 
 
ARL6IP1
 
 
hsa-miR-328-3p
 
 
-0.494
 
 
74.5
 
 
 
 
 
 
 choroid plexus 
 
 
 
 
target_symbol
 
 
mature_mirna_id
 
 
score
 
 
nTPM
 
 
 
 
 
 
AAR2
 
 
hsa-miR-29a-3p
 
 
-0.327
 
 
27.3
 
 
 
 
ABCB7
 
 
hsa-miR-148b-3p
 
 
-0.515
 
 
15.2
 
 
 
 
ABCD3
 
 
hsa-miR-148b-3p
 
 
-0.388
 
 
13.3
 
 
 
 
ABHD4
 
 
hsa-miR-29a-3p
 
 
-0.312
 
 
31.8
 
 
 
 
ACTA2
 
 
hsa-miR-27a-3p
 
 
-0.577
 
 
76.7
 
 
 
 
ACTN2
 
 
hsa-miR-23a-3p
 
 
-0.294
 
 
16.8
 
 
 
 
ACTN2
 
 
hsa-miR-29a-3p
 
 
-0.311
 
 
16.8
 
 
 
 
ACTR2
 
 
hsa-miR-15b-5p
 
 
-0.293
 
 
35.9
 
 
 
 
ACTR2
 
 
hsa-miR-16-5p
 
 
-0.293
 
 
35.9
 
 
 
 
ACTR3C
 
 
hsa-miR-30b-5p
 
 
-0.448
 
 
14.9
 
 
 
 
ADAM9
 
 
hsa-miR-30b-5p
 
 
-0.312
 
 
14.9
 
 
 
 
ADCY3
 
 
hsa-miR-132-3p
 
 
-0.303
 
 
17.0
 
 
 
 
ADGRA3
 
 
hsa-miR-27a-3p
 
 
-0.296
 
 
23.6
 
 
 
 
ADGRA3
 
 
hsa-miR-30b-5p
 
 
-0.304
 
 
23.6
 
 
 
 
ADIPOR2
 
 
hsa-miR-150-5p
 
 
-0.287
 
 
17.0
 
 
 
 
ADNP
 
 
hsa-miR-328-3p
 
 
-0.371
 
 
24.5
 
 
 
 
ADORA2B
 
 
hsa-miR-27a-3p
 
 
-0.638
 
 
11.1
 
 
 
 
AGFG1
 
 
hsa-miR-148b-3p
 
 
-0.332
 
 
23.3
 
 
 
 
AGPAT3
 
 
hsa-miR-27a-3p
 
 
-0.29
 
 
12.0
 
 
 
 
AGPAT3
 
 
hsa-miR-16-5p
 
 
-0.299
 
 
12.0
 
 
 
 
AIFM1
 
 
hsa-let-7b-5p
 
 
-0.314
 
 
37.9
 
 
 
 
AK1
 
 
hsa-miR-29a-3p
 
 
-0.333
 
 
522.7
 
 
 
 
AK4
 
 
hsa-miR-15b-5p
 
 
-0.316
 
 
13.8
 
 
 
 
AK4
 
 
hsa-miR-16-5p
 
 
-0.348
 
 
13.8
 
 
 
 
AKIRIN1
 
 
hsa-miR-27a-3p
 
 
-0.368
 
 
30.6
 
 
 
 
ALAD
 
 
hsa-miR-16-5p
 
 
-0.391
 
 
29.1
 
 
 
 
ALAD
 
 
hsa-miR-15b-5p
 
 
-0.446
 
 
29.1
 
 
 
 
ALPL
 
 
hsa-miR-10a-5p
 
 
-0.32
 
 
17.0
 
 
 
 
AMT
 
 
hsa-let-7b-5p
 
 
-0.463
 
 
33.4
 
 
 
 
ANKHD1
 
 
hsa-miR-23a-3p
 
 
-0.368
 
 
42.2
 
 
 
 
ANKHD1
 
 
hsa-miR-23b-3p
 
 
-0.368
 
 
42.2
 
 
 
 
ANKHD1
 
 
hsa-miR-30b-5p
 
 
-0.405
 
 
42.2
 
 
 
 
ANKRD17
 
 
hsa-miR-30b-5p
 
 
-0.336
 
 
13.8
 
 
 
 
ANKRD40
 
 
hsa-miR-27a-3p
 
 
-0.35
 
 
17.4
 
 
 
 
ANKRD46
 
 
hsa-let-7b-5p
 
 
-0.389
 
 
11.0
 
 
 
 
ANP32A
 
 
hsa-miR-132-3p
 
 
-0.374
 
 
28.9
 
 
 
 
ANXA11
 
 
hsa-miR-16-5p
 
 
-0.447
 
 
96.6
 
 
 
 
ANXA11
 
 
hsa-miR-15b-5p
 
 
-0.463
 
 
96.6
 
 
 
 
AP1S1
 
 
hsa-let-7b-5p
 
 
-0.374
 
 
10.3
 
 
 
 
AP2A1
 
 
hsa-miR-15b-5p
 
 
-0.333
 
 
20.9
 
 
 
 
AP2A1
 
 
hsa-miR-16-5p
 
 
-0.333
 
 
20.9
 
 
 
 
AP2B1
 
 
hsa-miR-16-5p
 
 
-0.302
 
 
83.9
 
 
 
 
AP2B1
 
 
hsa-miR-15b-5p
 
 
-0.334
 
 
83.9
 
 
 
 
AP3S1
 
 
hsa-miR-30b-5p
 
 
-0.293
 
 
22.5
 
 
 
 
APOO
 
 
hsa-miR-181b-5p
 
 
-0.5
 
 
102.5
 
 
 
 
APOO
 
 
hsa-miR-181a-5p
 
 
-0.5
 
 
102.5
 
 
 
 
APP
 
 
hsa-miR-16-5p
 
 
-0.298
 
 
394.3
 
 
 
 
APP
 
 
hsa-miR-15b-5p
 
 
-0.298
 
 
394.3
 
 
 
 
ARF4
 
 
hsa-miR-30b-5p
 
 
-0.289
 
 
115.4
 
 
 
 
ARF4
 
 
hsa-miR-148b-3p
 
 
-0.487
 
 
115.4
 
 
 
 
ARF5
 
 
hsa-miR-29a-3p
 
 
-0.491
 
 
140.5
 
 
 
 
ARF6
 
 
hsa-miR-181b-5p
 
 
-0.33
 
 
20.9
 
 
 
 
ARF6
 
 
hsa-miR-181a-5p
 
 
-0.33
 
 
20.9
 
 
 
 
ARG2
 
 
hsa-let-7b-5p
 
 
-0.468
 
 
49.7
 
 
 
 
ARL2
 
 
hsa-miR-16-5p
 
 
-0.374
 
 
135.4
 
 
 
 
ARL2
 
 
hsa-miR-15b-5p
 
 
-0.374
 
 
135.4
 
 
 
 
ARL2
 
 
hsa-miR-16-5p
 
 
-0.418
 
 
135.4
 
 
 
 
ARL2
 
 
hsa-miR-15b-5p
 
 
-0.442
 
 
135.4
 
 
 
 
ARL3
 
 
hsa-miR-16-5p
 
 
-0.496
 
 
27.9
 
 
 
 
ARL3
 
 
hsa-miR-15b-5p
 
 
-0.507
 
 
27.9
 
 
 
 
ARL4A
 
 
hsa-miR-29a-3p
 
 
-0.386
 
 
29.1
 
 
 
 
ARL4A
 
 
hsa-miR-30b-5p
 
 
-0.448
 
 
29.1
 
 
 
 
ARL4D
 
 
hsa-let-7b-5p
 
 
-0.417
 
 
15.4
 
 
 
 
ARL6IP1
 
 
hsa-miR-328-3p
 
 
-0.494
 
 
185.0
 
 
 
 
ARL6IP1
 
 
hsa-miR-148b-3p
 
 
-0.579
 
 
185.0
 
 
 
 
ARL8B
 
 
hsa-miR-148b-3p
 
 
-0.337
 
 
19.0
 
 
 
 
ARL8B
 
 
hsa-miR-320b
 
 
-0.376
 
 
19.0
 
 
 
 
ARL8B
 
 
hsa-miR-320c
 
 
-0.376
 
 
19.0
 
 
 
 
ARMC8
 
 
hsa-miR-486-5p
 
 
-0.321
 
 
20.8
 
 
 
 
ARMCX2
 
 
hsa-miR-15b-5p
 
 
-0.344
 
 
19.0
 
 
 
 
ARMCX2
 
 
hsa-miR-16-5p
 
 
-0.366
 
 
19.0
 
 
 
 
ARMCX3
 
 
hsa-miR-425-5p
 
 
-0.325
 
 
25.1
 
 
 
 
ARPP19
 
 
hsa-miR-320b
 
 
-0.325
 
 
32.4
 
 
 
 
ARPP19
 
 
hsa-miR-320c
 
 
-0.325
 
 
32.4
 
 
 
 
ARPP19
 
 
hsa-miR-15b-5p
 
 
-0.346
 
 
32.4
 
 
 
 
ARPP19
 
 
hsa-miR-16-5p
 
 
-0.354
 
 
32.4
 
 
 
 
ARPP19
 
 
hsa-miR-29a-3p
 
 
-0.371
 
 
32.4
 
 
 
 
ARPP19
 
 
hsa-miR-328-3p
 
 
-0.404
 
 
32.4
 
 
 
 
ARPP19
 
 
hsa-let-7b-5p
 
 
-0.476
 
 
32.4
 
 
 
 
ARRDC3
 
 
hsa-miR-148b-3p
 
 
-0.305
 
 
24.1
 
 
 
 
ARRDC3
 
 
hsa-miR-29a-3p
 
 
-0.402
 
 
24.1
 
 
 
 
ASAH1
 
 
hsa-miR-27a-3p
 
 
-0.398
 
 
113.7
 
 
 
 
ASB3
 
 
hsa-miR-30b-5p
 
 
-0.418
 
 
14.1
 
 
 
 
ASB3
 
 
hsa-miR-30b-5p
 
 
-0.418
 
 
14.1
 
 
 
 
ASPH
 
 
hsa-miR-148b-3p
 
 
-0.391
 
 
79.9
 
 
 
 
ATG9A
 
 
hsa-miR-16-5p
 
 
-0.311
 
 
10.2
 
 
 
 
ATG9A
 
 
hsa-miR-15b-5p
 
 
-0.333
 
 
10.2
 
 
 
 
ATOSB
 
 
hsa-miR-328-3p
 
 
-0.358
 
 
16.6
 
 
 
 
ATOSB
 
 
hsa-let-7b-5p
 
 
-0.429
 
 
16.6
 
 
 
 
ATP2A2
 
 
hsa-let-7b-5p
 
 
-0.311
 
 
29.6
 
 
 
 
ATP5MC3
 
 
hsa-miR-425-5p
 
 
-0.328
 
 
264.7
 
 
 
 
ATP5MC3
 
 
hsa-miR-29a-3p
 
 
-0.33
 
 
264.7
 
 
 
 
ATP5MC3
 
 
hsa-miR-27a-3p
 
 
-0.416
 
 
264.7
 
 
 
 
ATP6AP2
 
 
hsa-miR-148b-3p
 
 
-0.592
 
 
85.2
 
 
 
 
ATP6AP2
 
 
hsa-miR-148b-3p
 
 
-0.668
 
 
85.2
 
 
 
 
ATP6V1E1
 
 
hsa-miR-23a-3p
 
 
-0.294
 
 
285.9
 
 
 
 
ATP6V1E1
 
 
hsa-miR-23b-3p
 
 
-0.294
 
 
285.9
 
 
 
 
ATPAF1
 
 
hsa-let-7b-5p
 
 
-0.498
 
 
168.0
 
 
 
 
ATXN10
 
 
hsa-miR-27a-3p
 
 
-0.295
 
 
46.3
 
 
 
 
ATXN2
 
 
hsa-miR-10a-5p
 
 
-0.362
 
 
17.3
 
 
 
 
 
 
 colon 
 
 
 
 
target_symbol
 
 
mature_mirna_id
 
 
score
 
 
nTPM
 
 
 
 
 
 
AAR2
 
 
hsa-miR-29a-3p
 
 
-0.327
 
 
25.8
 
 
 
 
ABCB10
 
 
hsa-miR-148b-3p
 
 
-0.291
 
 
10.4
 
 
 
 
ABCB7
 
 
hsa-miR-148b-3p
 
 
-0.515
 
 
13.4
 
 
 
 
ABCD3
 
 
hsa-miR-148b-3p
 
 
-0.388
 
 
41.8
 
 
 
 
ABCE1
 
 
hsa-miR-29a-3p
 
 
-0.404
 
 
21.3
 
 
 
 
ABHD17C
 
 
hsa-let-7b-5p
 
 
-0.359
 
 
61.2
 
 
 
 
ABHD17C
 
 
hsa-miR-27a-3p
 
 
-0.435
 
 
61.2
 
 
 
 
ABHD4
 
 
hsa-miR-29a-3p
 
 
-0.312
 
 
20.3
 
 
 
 
ABHD5
 
 
hsa-miR-132-3p
 
 
-0.323
 
 
11.4
 
 
 
 
ABHD5
 
 
hsa-miR-27a-3p
 
 
-0.407
 
 
11.4
 
 
 
 
ACTA2
 
 
hsa-miR-27a-3p
 
 
-0.577
 
 
2601.4
 
 
 
 
ACTR2
 
 
hsa-miR-15b-5p
 
 
-0.293
 
 
132.3
 
 
 
 
ACTR2
 
 
hsa-miR-16-5p
 
 
-0.293
 
 
132.3
 
 
 
 
ADAM10
 
 
hsa-miR-148b-3p
 
 
-0.356
 
 
20.8
 
 
 
 
ADAM9
 
 
hsa-miR-30b-5p
 
 
-0.312
 
 
30.9
 
 
 
 
ADAP1
 
 
hsa-miR-27a-3p
 
 
-0.302
 
 
41.4
 
 
 
 
ADCY3
 
 
hsa-miR-132-3p
 
 
-0.303
 
 
26.7
 
 
 
 
ADGRA3
 
 
hsa-miR-27a-3p
 
 
-0.296
 
 
16.0
 
 
 
 
ADGRA3
 
 
hsa-miR-30b-5p
 
 
-0.304
 
 
16.0
 
 
 
 
ADIPOR2
 
 
hsa-miR-150-5p
 
 
-0.287
 
 
31.0
 
 
 
 
ADNP
 
 
hsa-miR-328-3p
 
 
-0.371
 
 
32.4
 
 
 
 
AFG3L2
 
 
hsa-miR-181b-5p
 
 
-0.491
 
 
38.7
 
 
 
 
AFG3L2
 
 
hsa-miR-181a-5p
 
 
-0.491
 
 
38.7
 
 
 
 
AGFG1
 
 
hsa-miR-148b-3p
 
 
-0.332
 
 
30.2
 
 
 
 
AGPAT3
 
 
hsa-miR-27a-3p
 
 
-0.29
 
 
20.6
 
 
 
 
AGPAT3
 
 
hsa-miR-16-5p
 
 
-0.299
 
 
20.6
 
 
 
 
AHDC1
 
 
hsa-miR-148b-3p
 
 
-0.413
 
 
15.6
 
 
 
 
AHR
 
 
hsa-miR-29a-3p
 
 
-0.316
 
 
12.9
 
 
 
 
AIFM1
 
 
hsa-let-7b-5p
 
 
-0.314
 
 
23.9
 
 
 
 
AK1
 
 
hsa-miR-29a-3p
 
 
-0.333
 
 
41.6
 
 
 
 
AK4
 
 
hsa-miR-15b-5p
 
 
-0.316
 
 
17.9
 
 
 
 
AK4
 
 
hsa-miR-16-5p
 
 
-0.348
 
 
17.9
 
 
 
 
AKAP1
 
 
hsa-miR-148b-3p
 
 
-0.381
 
 
72.8
 
 
 
 
AKIRIN1
 
 
hsa-miR-27a-3p
 
 
-0.368
 
 
33.0
 
 
 
 
AKT3
 
 
hsa-miR-29a-3p
 
 
-0.287
 
 
20.3
 
 
 
 
AKT3
 
 
hsa-miR-15b-5p
 
 
-0.301
 
 
20.3
 
 
 
 
AKT3
 
 
hsa-miR-16-5p
 
 
-0.309
 
 
20.3
 
 
 
 
ALAD
 
 
hsa-miR-16-5p
 
 
-0.391
 
 
24.1
 
 
 
 
ALAD
 
 
hsa-miR-15b-5p
 
 
-0.446
 
 
24.1
 
 
 
 
ALCAM
 
 
hsa-miR-148b-3p
 
 
-0.339
 
 
15.7
 
 
 
 
AMOTL1
 
 
hsa-miR-16-5p
 
 
-0.297
 
 
34.0
 
 
 
 
AMOTL1
 
 
hsa-miR-15b-5p
 
 
-0.319
 
 
34.0
 
 
 
 
AMT
 
 
hsa-let-7b-5p
 
 
-0.463
 
 
14.4
 
 
 
 
ANKHD1
 
 
hsa-miR-23a-3p
 
 
-0.368
 
 
38.3
 
 
 
 
ANKHD1
 
 
hsa-miR-23b-3p
 
 
-0.368
 
 
38.3
 
 
 
 
ANKHD1
 
 
hsa-miR-30b-5p
 
 
-0.405
 
 
38.3
 
 
 
 
ANKRD17
 
 
hsa-miR-30b-5p
 
 
-0.336
 
 
14.9
 
 
 
 
ANKRD40
 
 
hsa-miR-27a-3p
 
 
-0.35
 
 
29.8
 
 
 
 
ANP32A
 
 
hsa-miR-132-3p
 
 
-0.374
 
 
75.8
 
 
 
 
ANTXR2
 
 
hsa-miR-29a-3p
 
 
-0.347
 
 
83.9
 
 
 
 
ANXA11
 
 
hsa-miR-16-5p
 
 
-0.447
 
 
122.1
 
 
 
 
ANXA11
 
 
hsa-miR-15b-5p
 
 
-0.463
 
 
122.1
 
 
 
 
AP1S1
 
 
hsa-let-7b-5p
 
 
-0.374
 
 
31.0
 
 
 
 
AP2A1
 
 
hsa-miR-15b-5p
 
 
-0.333
 
 
40.3
 
 
 
 
AP2A1
 
 
hsa-miR-16-5p
 
 
-0.333
 
 
40.3
 
 
 
 
AP2B1
 
 
hsa-miR-16-5p
 
 
-0.302
 
 
63.4
 
 
 
 
AP2B1
 
 
hsa-miR-15b-5p
 
 
-0.334
 
 
63.4
 
 
 
 
AP3S1
 
 
hsa-miR-30b-5p
 
 
-0.293
 
 
56.5
 
 
 
 
APOO
 
 
hsa-miR-181b-5p
 
 
-0.5
 
 
16.2
 
 
 
 
APOO
 
 
hsa-miR-181a-5p
 
 
-0.5
 
 
16.2
 
 
 
 
APP
 
 
hsa-miR-16-5p
 
 
-0.298
 
 
322.5
 
 
 
 
APP
 
 
hsa-miR-15b-5p
 
 
-0.298
 
 
322.5
 
 
 
 
ARF4
 
 
hsa-miR-30b-5p
 
 
-0.289
 
 
148.9
 
 
 
 
ARF4
 
 
hsa-miR-148b-3p
 
 
-0.487
 
 
148.9
 
 
 
 
ARF5
 
 
hsa-miR-29a-3p
 
 
-0.491
 
 
119.1
 
 
 
 
ARF6
 
 
hsa-miR-181b-5p
 
 
-0.33
 
 
50.4
 
 
 
 
ARF6
 
 
hsa-miR-181a-5p
 
 
-0.33
 
 
50.4
 
 
 
 
ARFGEF1
 
 
hsa-miR-148b-3p
 
 
-0.306
 
 
18.0
 
 
 
 
ARFIP1
 
 
hsa-miR-320b
 
 
-0.339
 
 
26.4
 
 
 
 
ARFIP1
 
 
hsa-miR-320c
 
 
-0.339
 
 
26.4
 
 
 
 
ARFIP1
 
 
hsa-miR-148b-3p
 
 
-0.474
 
 
26.4
 
 
 
 
ARHGAP21
 
 
hsa-miR-148b-3p
 
 
-0.287
 
 
15.5
 
 
 
 
ARHGAP5
 
 
hsa-miR-486-5p
 
 
-0.3
 
 
27.7
 
 
 
 
ARID1B
 
 
hsa-miR-29a-3p
 
 
-0.342
 
 
11.7
 
 
 
 
ARL2
 
 
hsa-miR-16-5p
 
 
-0.374
 
 
122.8
 
 
 
 
ARL2
 
 
hsa-miR-15b-5p
 
 
-0.374
 
 
122.8
 
 
 
 
ARL2
 
 
hsa-miR-16-5p
 
 
-0.418
 
 
122.8
 
 
 
 
ARL2
 
 
hsa-miR-15b-5p
 
 
-0.442
 
 
122.8
 
 
 
 
ARL3
 
 
hsa-miR-16-5p
 
 
-0.496
 
 
10.7
 
 
 
 
ARL3
 
 
hsa-miR-15b-5p
 
 
-0.507
 
 
10.7
 
 
 
 
ARL4A
 
 
hsa-miR-29a-3p
 
 
-0.386
 
 
38.1
 
 
 
 
ARL4A
 
 
hsa-miR-30b-5p
 
 
-0.448
 
 
38.1
 
 
 
 
ARL4D
 
 
hsa-let-7b-5p
 
 
-0.417
 
 
27.1
 
 
 
 
ARL6IP1
 
 
hsa-miR-328-3p
 
 
-0.494
 
 
132.0
 
 
 
 
ARL6IP1
 
 
hsa-miR-148b-3p
 
 
-0.579
 
 
132.0
 
 
 
 
ARL6IP6
 
 
hsa-let-7b-5p
 
 
-0.293
 
 
12.7
 
 
 
 
ARL8B
 
 
hsa-miR-148b-3p
 
 
-0.337
 
 
34.2
 
 
 
 
ARL8B
 
 
hsa-miR-320b
 
 
-0.376
 
 
34.2
 
 
 
 
ARL8B
 
 
hsa-miR-320c
 
 
-0.376
 
 
34.2
 
 
 
 
ARMC8
 
 
hsa-miR-486-5p
 
 
-0.321
 
 
15.5
 
 
 
 
ARMCX2
 
 
hsa-miR-15b-5p
 
 
-0.344
 
 
22.4
 
 
 
 
ARMCX2
 
 
hsa-miR-16-5p
 
 
-0.366
 
 
22.4
 
 
 
 
ARMCX3
 
 
hsa-miR-425-5p
 
 
-0.325
 
 
27.7
 
 
 
 
ARMT1
 
 
hsa-let-7b-5p
 
 
-0.291
 
 
15.5
 
 
 
 
ARPP19
 
 
hsa-miR-320b
 
 
-0.325
 
 
35.7
 
 
 
 
ARPP19
 
 
hsa-miR-320c
 
 
-0.325
 
 
35.7
 
 
 
 
ARPP19
 
 
hsa-miR-15b-5p
 
 
-0.346
 
 
35.7
 
 
 
 
ARPP19
 
 
hsa-miR-16-5p
 
 
-0.354
 
 
35.7
 
 
 
 
ARPP19
 
 
hsa-miR-29a-3p
 
 
-0.371
 
 
35.7
 
 
 
 
ARPP19
 
 
hsa-miR-328-3p
 
 
-0.404
 
 
35.7
 
 
 
 
 
 
 duodenum 
 
 
 
 
target_symbol
 
 
mature_mirna_id
 
 
score
 
 
nTPM
 
 
 
 
 
 
AAR2
 
 
hsa-miR-29a-3p
 
 
-0.327
 
 
14.2
 
 
 
 
ABCB10
 
 
hsa-miR-148b-3p
 
 
-0.291
 
 
16.1
 
 
 
 
ABCB7
 
 
hsa-miR-148b-3p
 
 
-0.515
 
 
15.1
 
 
 
 
ABCD3
 
 
hsa-miR-148b-3p
 
 
-0.388
 
 
42.1
 
 
 
 
ABCE1
 
 
hsa-miR-29a-3p
 
 
-0.404
 
 
19.0
 
 
 
 
ABHD17B
 
 
hsa-miR-486-5p
 
 
-0.348
 
 
15.0
 
 
 
 
ABHD17C
 
 
hsa-let-7b-5p
 
 
-0.359
 
 
21.9
 
 
 
 
ABHD17C
 
 
hsa-miR-27a-3p
 
 
-0.435
 
 
21.9
 
 
 
 
ABHD4
 
 
hsa-miR-29a-3p
 
 
-0.312
 
 
15.6
 
 
 
 
ACTA2
 
 
hsa-miR-27a-3p
 
 
-0.577
 
 
234.0
 
 
 
 
ACTR2
 
 
hsa-miR-15b-5p
 
 
-0.293
 
 
98.7
 
 
 
 
ACTR2
 
 
hsa-miR-16-5p
 
 
-0.293
 
 
98.7
 
 
 
 
ADA
 
 
hsa-miR-29a-3p
 
 
-0.376
 
 
454.0
 
 
 
 
ADAM10
 
 
hsa-miR-148b-3p
 
 
-0.356
 
 
14.7
 
 
 
 
ADAM9
 
 
hsa-miR-30b-5p
 
 
-0.312
 
 
23.1
 
 
 
 
ADAP1
 
 
hsa-miR-27a-3p
 
 
-0.302
 
 
18.5
 
 
 
 
ADCY3
 
 
hsa-miR-132-3p
 
 
-0.303
 
 
10.3
 
 
 
 
ADIPOR2
 
 
hsa-miR-150-5p
 
 
-0.287
 
 
20.9
 
 
 
 
ADNP
 
 
hsa-miR-328-3p
 
 
-0.371
 
 
18.9
 
 
 
 
AFG3L2
 
 
hsa-miR-181b-5p
 
 
-0.491
 
 
48.3
 
 
 
 
AFG3L2
 
 
hsa-miR-181a-5p
 
 
-0.491
 
 
48.3
 
 
 
 
AGFG1
 
 
hsa-miR-148b-3p
 
 
-0.332
 
 
40.8
 
 
 
 
AGPAT3
 
 
hsa-miR-27a-3p
 
 
-0.29
 
 
42.0
 
 
 
 
AGPAT3
 
 
hsa-miR-16-5p
 
 
-0.299
 
 
42.0
 
 
 
 
AHR
 
 
hsa-miR-29a-3p
 
 
-0.316
 
 
15.4
 
 
 
 
AIFM1
 
 
hsa-let-7b-5p
 
 
-0.314
 
 
49.8
 
 
 
 
AK1
 
 
hsa-miR-29a-3p
 
 
-0.333
 
 
16.7
 
 
 
 
AKAP1
 
 
hsa-miR-148b-3p
 
 
-0.381
 
 
51.5
 
 
 
 
AKIRIN1
 
 
hsa-miR-27a-3p
 
 
-0.368
 
 
22.8
 
 
 
 
ALAD
 
 
hsa-miR-16-5p
 
 
-0.391
 
 
27.7
 
 
 
 
ALAD
 
 
hsa-miR-15b-5p
 
 
-0.446
 
 
27.7
 
 
 
 
ALCAM
 
 
hsa-miR-148b-3p
 
 
-0.339
 
 
16.0
 
 
 
 
AMT
 
 
hsa-let-7b-5p
 
 
-0.463
 
 
12.9
 
 
 
 
ANKHD1
 
 
hsa-miR-23a-3p
 
 
-0.368
 
 
29.8
 
 
 
 
ANKHD1
 
 
hsa-miR-23b-3p
 
 
-0.368
 
 
29.8
 
 
 
 
ANKHD1
 
 
hsa-miR-30b-5p
 
 
-0.405
 
 
29.8
 
 
 
 
ANKRD17
 
 
hsa-miR-30b-5p
 
 
-0.336
 
 
13.9
 
 
 
 
ANKRD40
 
 
hsa-miR-27a-3p
 
 
-0.35
 
 
11.1
 
 
 
 
ANP32A
 
 
hsa-miR-132-3p
 
 
-0.374
 
 
66.5
 
 
 
 
ANTXR2
 
 
hsa-miR-29a-3p
 
 
-0.347
 
 
21.6
 
 
 
 
ANXA11
 
 
hsa-miR-16-5p
 
 
-0.447
 
 
129.7
 
 
 
 
ANXA11
 
 
hsa-miR-15b-5p
 
 
-0.463
 
 
129.7
 
 
 
 
AP1S1
 
 
hsa-let-7b-5p
 
 
-0.374
 
 
36.7
 
 
 
 
AP2A1
 
 
hsa-miR-15b-5p
 
 
-0.333
 
 
23.3
 
 
 
 
AP2A1
 
 
hsa-miR-16-5p
 
 
-0.333
 
 
23.3
 
 
 
 
AP2B1
 
 
hsa-miR-16-5p
 
 
-0.302
 
 
31.5
 
 
 
 
AP2B1
 
 
hsa-miR-15b-5p
 
 
-0.334
 
 
31.5
 
 
 
 
AP3S1
 
 
hsa-miR-30b-5p
 
 
-0.293
 
 
58.2
 
 
 
 
APOO
 
 
hsa-miR-181b-5p
 
 
-0.5
 
 
13.6
 
 
 
 
APOO
 
 
hsa-miR-181a-5p
 
 
-0.5
 
 
13.6
 
 
 
 
APP
 
 
hsa-miR-16-5p
 
 
-0.298
 
 
204.9
 
 
 
 
APP
 
 
hsa-miR-15b-5p
 
 
-0.298
 
 
204.9
 
 
 
 
AQP11
 
 
hsa-miR-27a-3p
 
 
-0.666
 
 
27.0
 
 
 
 
AQP11
 
 
hsa-miR-15b-5p
 
 
-0.769
 
 
27.0
 
 
 
 
AQP11
 
 
hsa-miR-16-5p
 
 
-0.801
 
 
27.0
 
 
 
 
ARF4
 
 
hsa-miR-30b-5p
 
 
-0.289
 
 
116.2
 
 
 
 
ARF4
 
 
hsa-miR-148b-3p
 
 
-0.487
 
 
116.2
 
 
 
 
ARF5
 
 
hsa-miR-29a-3p
 
 
-0.491
 
 
86.9
 
 
 
 
ARF6
 
 
hsa-miR-181b-5p
 
 
-0.33
 
 
43.6
 
 
 
 
ARF6
 
 
hsa-miR-181a-5p
 
 
-0.33
 
 
43.6
 
 
 
 
ARFGEF1
 
 
hsa-miR-148b-3p
 
 
-0.306
 
 
15.9
 
 
 
 
ARFIP1
 
 
hsa-miR-320b
 
 
-0.339
 
 
21.8
 
 
 
 
ARFIP1
 
 
hsa-miR-320c
 
 
-0.339
 
 
21.8
 
 
 
 
ARFIP1
 
 
hsa-miR-148b-3p
 
 
-0.474
 
 
21.8
 
 
 
 
ARHGAP21
 
 
hsa-miR-148b-3p
 
 
-0.287
 
 
12.1
 
 
 
 
ARHGAP5
 
 
hsa-miR-486-5p
 
 
-0.3
 
 
33.3
 
 
 
 
ARID1B
 
 
hsa-miR-29a-3p
 
 
-0.342
 
 
13.4
 
 
 
 
ARL2
 
 
hsa-miR-16-5p
 
 
-0.374
 
 
49.6
 
 
 
 
ARL2
 
 
hsa-miR-15b-5p
 
 
-0.374
 
 
49.6
 
 
 
 
ARL2
 
 
hsa-miR-16-5p
 
 
-0.418
 
 
49.6
 
 
 
 
ARL2
 
 
hsa-miR-15b-5p
 
 
-0.442
 
 
49.6
 
 
 
 
ARL4A
 
 
hsa-miR-29a-3p
 
 
-0.386
 
 
28.1
 
 
 
 
ARL4A
 
 
hsa-miR-30b-5p
 
 
-0.448
 
 
28.1
 
 
 
 
ARL6IP1
 
 
hsa-miR-328-3p
 
 
-0.494
 
 
130.6
 
 
 
 
ARL6IP1
 
 
hsa-miR-148b-3p
 
 
-0.579
 
 
130.6
 
 
 
 
ARL8B
 
 
hsa-miR-148b-3p
 
 
-0.337
 
 
28.9
 
 
 
 
ARL8B
 
 
hsa-miR-320b
 
 
-0.376
 
 
28.9
 
 
 
 
ARL8B
 
 
hsa-miR-320c
 
 
-0.376
 
 
28.9
 
 
 
 
ARMC8
 
 
hsa-miR-486-5p
 
 
-0.321
 
 
11.3
 
 
 
 
ARMCX3
 
 
hsa-miR-425-5p
 
 
-0.325
 
 
19.0
 
 
 
 
ARMT1
 
 
hsa-let-7b-5p
 
 
-0.291
 
 
17.8
 
 
 
 
ARPP19
 
 
hsa-miR-320b
 
 
-0.325
 
 
27.2
 
 
 
 
ARPP19
 
 
hsa-miR-320c
 
 
-0.325
 
 
27.2
 
 
 
 
ARPP19
 
 
hsa-miR-15b-5p
 
 
-0.346
 
 
27.2
 
 
 
 
ARPP19
 
 
hsa-miR-16-5p
 
 
-0.354
 
 
27.2
 
 
 
 
ARPP19
 
 
hsa-miR-29a-3p
 
 
-0.371
 
 
27.2
 
 
 
 
ARPP19
 
 
hsa-miR-328-3p
 
 
-0.404
 
 
27.2
 
 
 
 
ARPP19
 
 
hsa-let-7b-5p
 
 
-0.476
 
 
27.2
 
 
 
 
ARRDC4
 
 
hsa-let-7b-5p
 
 
-0.329
 
 
14.9
 
 
 
 
ASAH1
 
 
hsa-miR-27a-3p
 
 
-0.398
 
 
51.6
 
 
 
 
ASAP2
 
 
hsa-miR-29a-3p
 
 
-0.348
 
 
13.0
 
 
 
 
ASH2L
 
 
hsa-miR-320b
 
 
-0.287
 
 
17.2
 
 
 
 
ASH2L
 
 
hsa-miR-320c
 
 
-0.287
 
 
17.2
 
 
 
 
ASPH
 
 
hsa-miR-148b-3p
 
 
-0.391
 
 
42.9
 
 
 
 
ATG5
 
 
hsa-miR-30b-5p
 
 
-0.488
 
 
12.0
 
 
 
 
ATG9A
 
 
hsa-miR-16-5p
 
 
-0.311
 
 
29.6
 
 
 
 
ATG9A
 
 
hsa-miR-15b-5p
 
 
-0.333
 
 
29.6
 
 
 
 
ATP2A2
 
 
hsa-let-7b-5p
 
 
-0.311
 
 
44.8
 
 
 
 
ATP2B4
 
 
hsa-miR-148b-3p
 
 
-0.337
 
 
11.8
 
 
 
 
ATP5MC3
 
 
hsa-miR-425-5p
 
 
-0.328
 
 
355.5
 
 
 
 
 
 
 endometrium 
 
 
 
 
target_symbol
 
 
mature_mirna_id
 
 
score
 
 
nTPM
 
 
 
 
 
 
AAR2
 
 
hsa-miR-29a-3p
 
 
-0.327
 
 
23.5
 
 
 
 
ABCB7
 
 
hsa-miR-148b-3p
 
 
-0.515
 
 
10.5
 
 
 
 
ABCD3
 
 
hsa-miR-148b-3p
 
 
-0.388
 
 
13.8
 
 
 
 
ABCE1
 
 
hsa-miR-29a-3p
 
 
-0.404
 
 
22.8
 
 
 
 
ABHD4
 
 
hsa-miR-29a-3p
 
 
-0.312
 
 
26.6
 
 
 
 
ACTA2
 
 
hsa-miR-27a-3p
 
 
-0.577
 
 
5917.5
 
 
 
 
ACTC1
 
 
hsa-miR-30b-5p
 
 
-0.543
 
 
17.7
 
 
 
 
ACTR2
 
 
hsa-miR-15b-5p
 
 
-0.293
 
 
65.5
 
 
 
 
ACTR2
 
 
hsa-miR-16-5p
 
 
-0.293
 
 
65.5
 
 
 
 
ACVR1
 
 
hsa-miR-30b-5p
 
 
-0.29
 
 
15.9
 
 
 
 
ACVR1
 
 
hsa-miR-148b-3p
 
 
-0.397
 
 
15.9
 
 
 
 
ADAM10
 
 
hsa-miR-148b-3p
 
 
-0.356
 
 
15.2
 
 
 
 
ADAM9
 
 
hsa-miR-30b-5p
 
 
-0.312
 
 
21.6
 
 
 
 
ADAMTS10
 
 
hsa-miR-27a-3p
 
 
-0.354
 
 
10.1
 
 
 
 
ADAMTS10
 
 
hsa-miR-29a-3p
 
 
-0.516
 
 
10.1
 
 
 
 
ADAMTS9
 
 
hsa-miR-29a-3p
 
 
-0.305
 
 
12.4
 
 
 
 
ADAMTS9
 
 
hsa-miR-29a-3p
 
 
-0.485
 
 
12.4
 
 
 
 
ADCY3
 
 
hsa-miR-132-3p
 
 
-0.303
 
 
52.0
 
 
 
 
ADGRA3
 
 
hsa-miR-27a-3p
 
 
-0.296
 
 
14.1
 
 
 
 
ADGRA3
 
 
hsa-miR-30b-5p
 
 
-0.304
 
 
14.1
 
 
 
 
ADGRF5
 
 
hsa-miR-148b-3p
 
 
-0.354
 
 
16.0
 
 
 
 
ADIPOR2
 
 
hsa-miR-150-5p
 
 
-0.287
 
 
24.5
 
 
 
 
ADNP
 
 
hsa-miR-328-3p
 
 
-0.371
 
 
33.6
 
 
 
 
AFG3L2
 
 
hsa-miR-181b-5p
 
 
-0.491
 
 
12.4
 
 
 
 
AFG3L2
 
 
hsa-miR-181a-5p
 
 
-0.491
 
 
12.4
 
 
 
 
AGFG1
 
 
hsa-miR-148b-3p
 
 
-0.332
 
 
23.1
 
 
 
 
AHDC1
 
 
hsa-miR-148b-3p
 
 
-0.413
 
 
26.0
 
 
 
 
AIFM1
 
 
hsa-let-7b-5p
 
 
-0.314
 
 
10.6
 
 
 
 
AK1
 
 
hsa-miR-29a-3p
 
 
-0.333
 
 
16.9
 
 
 
 
AKAP1
 
 
hsa-miR-148b-3p
 
 
-0.381
 
 
24.8
 
 
 
 
AKIRIN1
 
 
hsa-miR-27a-3p
 
 
-0.368
 
 
26.8
 
 
 
 
AKT3
 
 
hsa-miR-29a-3p
 
 
-0.287
 
 
19.5
 
 
 
 
AKT3
 
 
hsa-miR-15b-5p
 
 
-0.301
 
 
19.5
 
 
 
 
AKT3
 
 
hsa-miR-16-5p
 
 
-0.309
 
 
19.5
 
 
 
 
ALAD
 
 
hsa-miR-16-5p
 
 
-0.391
 
 
16.4
 
 
 
 
ALAD
 
 
hsa-miR-15b-5p
 
 
-0.446
 
 
16.4
 
 
 
 
ALCAM
 
 
hsa-miR-148b-3p
 
 
-0.339
 
 
14.4
 
 
 
 
ALDH1A2
 
 
hsa-miR-23b-3p
 
 
-0.346
 
 
76.9
 
 
 
 
ALDH1A2
 
 
hsa-miR-23a-3p
 
 
-0.354
 
 
76.9
 
 
 
 
AMOTL1
 
 
hsa-miR-16-5p
 
 
-0.297
 
 
13.3
 
 
 
 
AMOTL1
 
 
hsa-miR-15b-5p
 
 
-0.319
 
 
13.3
 
 
 
 
AMT
 
 
hsa-let-7b-5p
 
 
-0.463
 
 
10.0
 
 
 
 
ANKHD1
 
 
hsa-miR-23a-3p
 
 
-0.368
 
 
40.5
 
 
 
 
ANKHD1
 
 
hsa-miR-23b-3p
 
 
-0.368
 
 
40.5
 
 
 
 
ANKHD1
 
 
hsa-miR-30b-5p
 
 
-0.405
 
 
40.5
 
 
 
 
ANKRA2
 
 
hsa-miR-30b-5p
 
 
-0.354
 
 
11.6
 
 
 
 
ANKRA2
 
 
hsa-miR-30b-5p
 
 
-0.472
 
 
11.6
 
 
 
 
ANKRD17
 
 
hsa-miR-30b-5p
 
 
-0.336
 
 
13.7
 
 
 
 
ANKRD29
 
 
hsa-miR-132-3p
 
 
-0.47
 
 
14.7
 
 
 
 
ANKRD40
 
 
hsa-miR-27a-3p
 
 
-0.35
 
 
22.4
 
 
 
 
ANKRD46
 
 
hsa-let-7b-5p
 
 
-0.389
 
 
11.8
 
 
 
 
ANP32A
 
 
hsa-miR-132-3p
 
 
-0.374
 
 
55.3
 
 
 
 
ANTXR2
 
 
hsa-miR-29a-3p
 
 
-0.347
 
 
89.2
 
 
 
 
ANXA11
 
 
hsa-miR-16-5p
 
 
-0.447
 
 
67.4
 
 
 
 
ANXA11
 
 
hsa-miR-15b-5p
 
 
-0.463
 
 
67.4
 
 
 
 
AP1S1
 
 
hsa-let-7b-5p
 
 
-0.374
 
 
16.1
 
 
 
 
AP2A1
 
 
hsa-miR-15b-5p
 
 
-0.333
 
 
53.6
 
 
 
 
AP2A1
 
 
hsa-miR-16-5p
 
 
-0.333
 
 
53.6
 
 
 
 
AP2B1
 
 
hsa-miR-16-5p
 
 
-0.302
 
 
39.0
 
 
 
 
AP2B1
 
 
hsa-miR-15b-5p
 
 
-0.334
 
 
39.0
 
 
 
 
AP3S1
 
 
hsa-miR-30b-5p
 
 
-0.293
 
 
55.6
 
 
 
 
APP
 
 
hsa-miR-16-5p
 
 
-0.298
 
 
254.0
 
 
 
 
APP
 
 
hsa-miR-15b-5p
 
 
-0.298
 
 
254.0
 
 
 
 
APPBP2
 
 
hsa-miR-27a-3p
 
 
-0.378
 
 
12.0
 
 
 
 
ARF4
 
 
hsa-miR-30b-5p
 
 
-0.289
 
 
113.9
 
 
 
 
ARF4
 
 
hsa-miR-148b-3p
 
 
-0.487
 
 
113.9
 
 
 
 
ARF5
 
 
hsa-miR-29a-3p
 
 
-0.491
 
 
111.9
 
 
 
 
ARF6
 
 
hsa-miR-181b-5p
 
 
-0.33
 
 
19.5
 
 
 
 
ARF6
 
 
hsa-miR-181a-5p
 
 
-0.33
 
 
19.5
 
 
 
 
ARFIP1
 
 
hsa-miR-320b
 
 
-0.339
 
 
20.3
 
 
 
 
ARFIP1
 
 
hsa-miR-320c
 
 
-0.339
 
 
20.3
 
 
 
 
ARFIP1
 
 
hsa-miR-148b-3p
 
 
-0.474
 
 
20.3
 
 
 
 
ARHGAP21
 
 
hsa-miR-148b-3p
 
 
-0.287
 
 
18.5
 
 
 
 
ARHGAP5
 
 
hsa-miR-486-5p
 
 
-0.3
 
 
26.2
 
 
 
 
ARID1B
 
 
hsa-miR-29a-3p
 
 
-0.342
 
 
15.0
 
 
 
 
ARID4B
 
 
hsa-miR-486-5p
 
 
-0.367
 
 
14.9
 
 
 
 
ARL2
 
 
hsa-miR-16-5p
 
 
-0.374
 
 
124.7
 
 
 
 
ARL2
 
 
hsa-miR-15b-5p
 
 
-0.374
 
 
124.7
 
 
 
 
ARL2
 
 
hsa-miR-16-5p
 
 
-0.418
 
 
124.7
 
 
 
 
ARL2
 
 
hsa-miR-15b-5p
 
 
-0.442
 
 
124.7
 
 
 
 
ARL3
 
 
hsa-miR-16-5p
 
 
-0.496
 
 
11.8
 
 
 
 
ARL3
 
 
hsa-miR-15b-5p
 
 
-0.507
 
 
11.8
 
 
 
 
ARL4A
 
 
hsa-miR-29a-3p
 
 
-0.386
 
 
17.7
 
 
 
 
ARL4A
 
 
hsa-miR-30b-5p
 
 
-0.448
 
 
17.7
 
 
 
 
ARL4D
 
 
hsa-let-7b-5p
 
 
-0.417
 
 
19.2
 
 
 
 
ARL6IP1
 
 
hsa-miR-328-3p
 
 
-0.494
 
 
66.4
 
 
 
 
ARL6IP1
 
 
hsa-miR-148b-3p
 
 
-0.579
 
 
66.4
 
 
 
 
ARL6IP6
 
 
hsa-let-7b-5p
 
 
-0.293
 
 
11.0
 
 
 
 
ARL8B
 
 
hsa-miR-148b-3p
 
 
-0.337
 
 
42.3
 
 
 
 
ARL8B
 
 
hsa-miR-320b
 
 
-0.376
 
 
42.3
 
 
 
 
ARL8B
 
 
hsa-miR-320c
 
 
-0.376
 
 
42.3
 
 
 
 
ARMC8
 
 
hsa-miR-486-5p
 
 
-0.321
 
 
16.5
 
 
 
 
ARMCX2
 
 
hsa-miR-15b-5p
 
 
-0.344
 
 
28.6
 
 
 
 
ARMCX2
 
 
hsa-miR-16-5p
 
 
-0.366
 
 
28.6
 
 
 
 
ARMCX3
 
 
hsa-miR-425-5p
 
 
-0.325
 
 
38.3
 
 
 
 
ARMT1
 
 
hsa-let-7b-5p
 
 
-0.291
 
 
15.0
 
 
 
 
ARPP19
 
 
hsa-miR-320b
 
 
-0.325
 
 
44.7
 
 
 
 
ARPP19
 
 
hsa-miR-320c
 
 
-0.325
 
 
44.7
 
 
 
 
ARPP19
 
 
hsa-miR-15b-5p
 
 
-0.346
 
 
44.7
 
 
 
 
ARPP19
 
 
hsa-miR-16-5p
 
 
-0.354
 
 
44.7
 
 
 
 
 
 
 epididymis 
 
 
 
 
target_symbol
 
 
mature_mirna_id
 
 
score
 
 
nTPM
 
 
 
 
 
 
AAR2
 
 
hsa-miR-29a-3p
 
 
-0.327
 
 
23.5
 
 
 
 
ABCD3
 
 
hsa-miR-148b-3p
 
 
-0.388
 
 
20.0
 
 
 
 
ABCE1
 
 
hsa-miR-29a-3p
 
 
-0.404
 
 
15.1
 
 
 
 
ABHD4
 
 
hsa-miR-29a-3p
 
 
-0.312
 
 
28.3
 
 
 
 
ACP3
 
 
hsa-let-7b-5p
 
 
-0.298
 
 
16.3
 
 
 
 
ACTA2
 
 
hsa-miR-27a-3p
 
 
-0.577
 
 
1284.3
 
 
 
 
ACTR2
 
 
hsa-miR-15b-5p
 
 
-0.293
 
 
60.8
 
 
 
 
ACTR2
 
 
hsa-miR-16-5p
 
 
-0.293
 
 
60.8
 
 
 
 
ACVR1
 
 
hsa-miR-30b-5p
 
 
-0.29
 
 
10.8
 
 
 
 
ACVR1
 
 
hsa-miR-148b-3p
 
 
-0.397
 
 
10.8
 
 
 
 
ADAM17
 
 
hsa-miR-148b-3p
 
 
-0.301
 
 
10.4
 
 
 
 
ADAM9
 
 
hsa-miR-30b-5p
 
 
-0.312
 
 
14.3
 
 
 
 
ADCY3
 
 
hsa-miR-132-3p
 
 
-0.303
 
 
19.3
 
 
 
 
ADNP
 
 
hsa-miR-328-3p
 
 
-0.371
 
 
39.7
 
 
 
 
AGFG1
 
 
hsa-miR-148b-3p
 
 
-0.332
 
 
18.8
 
 
 
 
AGPAT3
 
 
hsa-miR-27a-3p
 
 
-0.29
 
 
11.9
 
 
 
 
AGPAT3
 
 
hsa-miR-16-5p
 
 
-0.299
 
 
11.9
 
 
 
 
AK1
 
 
hsa-miR-29a-3p
 
 
-0.333
 
 
27.0
 
 
 
 
AKAP1
 
 
hsa-miR-148b-3p
 
 
-0.381
 
 
18.6
 
 
 
 
AKIRIN1
 
 
hsa-miR-27a-3p
 
 
-0.368
 
 
39.9
 
 
 
 
ALAD
 
 
hsa-miR-16-5p
 
 
-0.391
 
 
25.1
 
 
 
 
ALAD
 
 
hsa-miR-15b-5p
 
 
-0.446
 
 
25.1
 
 
 
 
ALCAM
 
 
hsa-miR-148b-3p
 
 
-0.339
 
 
33.4
 
 
 
 
AMOT
 
 
hsa-miR-29a-3p
 
 
-0.304
 
 
137.7
 
 
 
 
ANKHD1
 
 
hsa-miR-23a-3p
 
 
-0.368
 
 
45.6
 
 
 
 
ANKHD1
 
 
hsa-miR-23b-3p
 
 
-0.368
 
 
45.6
 
 
 
 
ANKHD1
 
 
hsa-miR-30b-5p
 
 
-0.405
 
 
45.6
 
 
 
 
ANKRA2
 
 
hsa-miR-30b-5p
 
 
-0.354
 
 
12.3
 
 
 
 
ANKRA2
 
 
hsa-miR-30b-5p
 
 
-0.472
 
 
12.3
 
 
 
 
ANKRD17
 
 
hsa-miR-30b-5p
 
 
-0.336
 
 
17.5
 
 
 
 
ANKRD40
 
 
hsa-miR-27a-3p
 
 
-0.35
 
 
13.0
 
 
 
 
ANKRD46
 
 
hsa-let-7b-5p
 
 
-0.389
 
 
15.4
 
 
 
 
ANO3
 
 
hsa-miR-16-5p
 
 
-0.31
 
 
54.2
 
 
 
 
ANO3
 
 
hsa-miR-16-5p
 
 
-0.346
 
 
54.2
 
 
 
 
ANO3
 
 
hsa-miR-15b-5p
 
 
-0.357
 
 
54.2
 
 
 
 
ANP32A
 
 
hsa-miR-132-3p
 
 
-0.374
 
 
47.9
 
 
 
 
ANTXR2
 
 
hsa-miR-29a-3p
 
 
-0.347
 
 
20.7
 
 
 
 
ANXA11
 
 
hsa-miR-16-5p
 
 
-0.447
 
 
101.5
 
 
 
 
ANXA11
 
 
hsa-miR-15b-5p
 
 
-0.463
 
 
101.5
 
 
 
 
AP1S1
 
 
hsa-let-7b-5p
 
 
-0.374
 
 
16.0
 
 
 
 
AP1S3
 
 
hsa-miR-181b-5p
 
 
-0.383
 
 
45.7
 
 
 
 
AP1S3
 
 
hsa-miR-181a-5p
 
 
-0.383
 
 
45.7
 
 
 
 
AP2B1
 
 
hsa-miR-16-5p
 
 
-0.302
 
 
66.7
 
 
 
 
AP2B1
 
 
hsa-miR-15b-5p
 
 
-0.334
 
 
66.7
 
 
 
 
AP3S1
 
 
hsa-miR-30b-5p
 
 
-0.293
 
 
107.2
 
 
 
 
APOO
 
 
hsa-miR-181b-5p
 
 
-0.5
 
 
18.5
 
 
 
 
APOO
 
 
hsa-miR-181a-5p
 
 
-0.5
 
 
18.5
 
 
 
 
APP
 
 
hsa-miR-16-5p
 
 
-0.298
 
 
214.2
 
 
 
 
APP
 
 
hsa-miR-15b-5p
 
 
-0.298
 
 
214.2
 
 
 
 
ARF4
 
 
hsa-miR-30b-5p
 
 
-0.289
 
 
214.1
 
 
 
 
ARF4
 
 
hsa-miR-148b-3p
 
 
-0.487
 
 
214.1
 
 
 
 
ARF5
 
 
hsa-miR-29a-3p
 
 
-0.491
 
 
107.7
 
 
 
 
ARF6
 
 
hsa-miR-181b-5p
 
 
-0.33
 
 
27.6
 
 
 
 
ARF6
 
 
hsa-miR-181a-5p
 
 
-0.33
 
 
27.6
 
 
 
 
ARFGEF1
 
 
hsa-miR-148b-3p
 
 
-0.306
 
 
10.4
 
 
 
 
ARFIP1
 
 
hsa-miR-320b
 
 
-0.339
 
 
19.3
 
 
 
 
ARFIP1
 
 
hsa-miR-320c
 
 
-0.339
 
 
19.3
 
 
 
 
ARFIP1
 
 
hsa-miR-148b-3p
 
 
-0.474
 
 
19.3
 
 
 
 
ARHGAP5
 
 
hsa-miR-486-5p
 
 
-0.3
 
 
23.7
 
 
 
 
ARID1B
 
 
hsa-miR-29a-3p
 
 
-0.342
 
 
10.3
 
 
 
 
ARID4B
 
 
hsa-miR-486-5p
 
 
-0.367
 
 
13.6
 
 
 
 
ARL2
 
 
hsa-miR-16-5p
 
 
-0.374
 
 
26.6
 
 
 
 
ARL2
 
 
hsa-miR-15b-5p
 
 
-0.374
 
 
26.6
 
 
 
 
ARL2
 
 
hsa-miR-16-5p
 
 
-0.418
 
 
26.6
 
 
 
 
ARL2
 
 
hsa-miR-15b-5p
 
 
-0.442
 
 
26.6
 
 
 
 
ARL3
 
 
hsa-miR-16-5p
 
 
-0.496
 
 
10.0
 
 
 
 
ARL3
 
 
hsa-miR-15b-5p
 
 
-0.507
 
 
10.0
 
 
 
 
ARL4A
 
 
hsa-miR-29a-3p
 
 
-0.386
 
 
13.0
 
 
 
 
ARL4A
 
 
hsa-miR-30b-5p
 
 
-0.448
 
 
13.0
 
 
 
 
ARL6IP1
 
 
hsa-miR-328-3p
 
 
-0.494
 
 
116.0
 
 
 
 
ARL6IP1
 
 
hsa-miR-148b-3p
 
 
-0.579
 
 
116.0
 
 
 
 
ARL8B
 
 
hsa-miR-148b-3p
 
 
-0.337
 
 
37.5
 
 
 
 
ARL8B
 
 
hsa-miR-320b
 
 
-0.376
 
 
37.5
 
 
 
 
ARL8B
 
 
hsa-miR-320c
 
 
-0.376
 
 
37.5
 
 
 
 
ARMC8
 
 
hsa-miR-486-5p
 
 
-0.321
 
 
11.5
 
 
 
 
ARMCX2
 
 
hsa-miR-15b-5p
 
 
-0.344
 
 
146.2
 
 
 
 
ARMCX2
 
 
hsa-miR-16-5p
 
 
-0.366
 
 
146.2
 
 
 
 
ARMCX3
 
 
hsa-miR-425-5p
 
 
-0.325
 
 
99.4
 
 
 
 
ARMT1
 
 
hsa-let-7b-5p
 
 
-0.291
 
 
15.2
 
 
 
 
ARPP19
 
 
hsa-miR-320b
 
 
-0.325
 
 
34.2
 
 
 
 
ARPP19
 
 
hsa-miR-320c
 
 
-0.325
 
 
34.2
 
 
 
 
ARPP19
 
 
hsa-miR-15b-5p
 
 
-0.346
 
 
34.2
 
 
 
 
ARPP19
 
 
hsa-miR-16-5p
 
 
-0.354
 
 
34.2
 
 
 
 
ARPP19
 
 
hsa-miR-29a-3p
 
 
-0.371
 
 
34.2
 
 
 
 
ARPP19
 
 
hsa-miR-328-3p
 
 
-0.404
 
 
34.2
 
 
 
 
ARPP19
 
 
hsa-let-7b-5p
 
 
-0.476
 
 
34.2
 
 
 
 
ARRDC3
 
 
hsa-miR-148b-3p
 
 
-0.305
 
 
25.6
 
 
 
 
ARRDC3
 
 
hsa-miR-29a-3p
 
 
-0.402
 
 
25.6
 
 
 
 
ARRDC4
 
 
hsa-let-7b-5p
 
 
-0.329
 
 
14.7
 
 
 
 
ASAH1
 
 
hsa-miR-27a-3p
 
 
-0.398
 
 
124.2
 
 
 
 
ASAP1
 
 
hsa-let-7b-5p
 
 
-0.308
 
 
11.5
 
 
 
 
ASB3
 
 
hsa-miR-30b-5p
 
 
-0.418
 
 
13.0
 
 
 
 
ASB3
 
 
hsa-miR-30b-5p
 
 
-0.418
 
 
13.0
 
 
 
 
ASF1A
 
 
hsa-miR-23a-3p
 
 
-0.506
 
 
12.3
 
 
 
 
ASF1A
 
 
hsa-miR-23b-3p
 
 
-0.506
 
 
12.3
 
 
 
 
ASH1L
 
 
hsa-miR-15b-5p
 
 
-0.325
 
 
19.6
 
 
 
 
ASH1L
 
 
hsa-miR-16-5p
 
 
-0.357
 
 
19.6
 
 
 
 
ASH2L
 
 
hsa-miR-320b
 
 
-0.287
 
 
16.2
 
 
 
 
ASH2L
 
 
hsa-miR-320c
 
 
-0.287
 
 
16.2
 
 
 
 
ASPH
 
 
hsa-miR-148b-3p
 
 
-0.391
 
 
42.3
 
 
 
 
 
 
 esophagus 
 
 
 
 
target_symbol
 
 
mature_mirna_id
 
 
score
 
 
nTPM
 
 
 
 
 
 
AAR2
 
 
hsa-miR-29a-3p
 
 
-0.327
 
 
16.7
 
 
 
 
ABCB7
 
 
hsa-miR-148b-3p
 
 
-0.515
 
 
11.1
 
 
 
 
ABCD3
 
 
hsa-miR-148b-3p
 
 
-0.388
 
 
25.0
 
 
 
 
ABCE1
 
 
hsa-miR-29a-3p
 
 
-0.404
 
 
33.0
 
 
 
 
ABHD17B
 
 
hsa-miR-486-5p
 
 
-0.348
 
 
12.2
 
 
 
 
ABHD17C
 
 
hsa-let-7b-5p
 
 
-0.359
 
 
45.1
 
 
 
 
ABHD17C
 
 
hsa-miR-27a-3p
 
 
-0.435
 
 
45.1
 
 
 
 
ABHD4
 
 
hsa-miR-29a-3p
 
 
-0.312
 
 
40.8
 
 
 
 
ABHD5
 
 
hsa-miR-132-3p
 
 
-0.323
 
 
18.4
 
 
 
 
ABHD5
 
 
hsa-miR-27a-3p
 
 
-0.407
 
 
18.4
 
 
 
 
ACP3
 
 
hsa-let-7b-5p
 
 
-0.298
 
 
42.9
 
 
 
 
ACTA2
 
 
hsa-miR-27a-3p
 
 
-0.577
 
 
1416.9
 
 
 
 
ACTC1
 
 
hsa-miR-30b-5p
 
 
-0.543
 
 
21.5
 
 
 
 
ACTN2
 
 
hsa-miR-23a-3p
 
 
-0.294
 
 
19.6
 
 
 
 
ACTN2
 
 
hsa-miR-29a-3p
 
 
-0.311
 
 
19.6
 
 
 
 
ACTR2
 
 
hsa-miR-15b-5p
 
 
-0.293
 
 
103.7
 
 
 
 
ACTR2
 
 
hsa-miR-16-5p
 
 
-0.293
 
 
103.7
 
 
 
 
ADAM10
 
 
hsa-miR-148b-3p
 
 
-0.356
 
 
14.2
 
 
 
 
ADAM9
 
 
hsa-miR-30b-5p
 
 
-0.312
 
 
22.2
 
 
 
 
ADCY3
 
 
hsa-miR-132-3p
 
 
-0.303
 
 
29.3
 
 
 
 
ADIPOR2
 
 
hsa-miR-150-5p
 
 
-0.287
 
 
44.0
 
 
 
 
ADNP
 
 
hsa-miR-328-3p
 
 
-0.371
 
 
30.5
 
 
 
 
ADORA2B
 
 
hsa-miR-27a-3p
 
 
-0.638
 
 
10.4
 
 
 
 
ADRB2
 
 
hsa-miR-15b-5p
 
 
-0.29
 
 
14.2
 
 
 
 
ADRB2
 
 
hsa-miR-16-5p
 
 
-0.323
 
 
14.2
 
 
 
 
ADRB2
 
 
hsa-let-7b-5p
 
 
-0.635
 
 
14.2
 
 
 
 
AFG3L2
 
 
hsa-miR-181b-5p
 
 
-0.491
 
 
25.7
 
 
 
 
AFG3L2
 
 
hsa-miR-181a-5p
 
 
-0.491
 
 
25.7
 
 
 
 
AGFG1
 
 
hsa-miR-148b-3p
 
 
-0.332
 
 
44.3
 
 
 
 
AGPAT3
 
 
hsa-miR-27a-3p
 
 
-0.29
 
 
24.6
 
 
 
 
AGPAT3
 
 
hsa-miR-16-5p
 
 
-0.299
 
 
24.6
 
 
 
 
AHDC1
 
 
hsa-miR-148b-3p
 
 
-0.413
 
 
34.3
 
 
 
 
AHR
 
 
hsa-miR-29a-3p
 
 
-0.316
 
 
16.2
 
 
 
 
AIFM1
 
 
hsa-let-7b-5p
 
 
-0.314
 
 
15.9
 
 
 
 
AK1
 
 
hsa-miR-29a-3p
 
 
-0.333
 
 
30.1
 
 
 
 
AK4
 
 
hsa-miR-15b-5p
 
 
-0.316
 
 
22.0
 
 
 
 
AK4
 
 
hsa-miR-16-5p
 
 
-0.348
 
 
22.0
 
 
 
 
AKAP1
 
 
hsa-miR-148b-3p
 
 
-0.381
 
 
34.2
 
 
 
 
AKIRIN1
 
 
hsa-miR-27a-3p
 
 
-0.368
 
 
27.4
 
 
 
 
ALAD
 
 
hsa-miR-16-5p
 
 
-0.391
 
 
34.3
 
 
 
 
ALAD
 
 
hsa-miR-15b-5p
 
 
-0.446
 
 
34.3
 
 
 
 
ALOX12
 
 
hsa-miR-15b-5p
 
 
-0.443
 
 
47.5
 
 
 
 
ALOX12
 
 
hsa-miR-16-5p
 
 
-0.451
 
 
47.5
 
 
 
 
AMOTL1
 
 
hsa-miR-16-5p
 
 
-0.297
 
 
28.5
 
 
 
 
AMOTL1
 
 
hsa-miR-15b-5p
 
 
-0.319
 
 
28.5
 
 
 
 
ANKHD1
 
 
hsa-miR-23a-3p
 
 
-0.368
 
 
42.1
 
 
 
 
ANKHD1
 
 
hsa-miR-23b-3p
 
 
-0.368
 
 
42.1
 
 
 
 
ANKHD1
 
 
hsa-miR-30b-5p
 
 
-0.405
 
 
42.1
 
 
 
 
ANKRD17
 
 
hsa-miR-30b-5p
 
 
-0.336
 
 
13.6
 
 
 
 
ANKRD40
 
 
hsa-miR-27a-3p
 
 
-0.35
 
 
17.3
 
 
 
 
ANKRD46
 
 
hsa-let-7b-5p
 
 
-0.389
 
 
11.9
 
 
 
 
ANP32A
 
 
hsa-miR-132-3p
 
 
-0.374
 
 
61.9
 
 
 
 
ANTXR2
 
 
hsa-miR-29a-3p
 
 
-0.347
 
 
23.0
 
 
 
 
ANXA11
 
 
hsa-miR-16-5p
 
 
-0.447
 
 
200.5
 
 
 
 
ANXA11
 
 
hsa-miR-15b-5p
 
 
-0.463
 
 
200.5
 
 
 
 
AP1S1
 
 
hsa-let-7b-5p
 
 
-0.374
 
 
36.5
 
 
 
 
AP2A1
 
 
hsa-miR-15b-5p
 
 
-0.333
 
 
45.1
 
 
 
 
AP2A1
 
 
hsa-miR-16-5p
 
 
-0.333
 
 
45.1
 
 
 
 
AP2B1
 
 
hsa-miR-16-5p
 
 
-0.302
 
 
23.5
 
 
 
 
AP2B1
 
 
hsa-miR-15b-5p
 
 
-0.334
 
 
23.5
 
 
 
 
AP3S1
 
 
hsa-miR-30b-5p
 
 
-0.293
 
 
75.8
 
 
 
 
APOO
 
 
hsa-miR-181b-5p
 
 
-0.5
 
 
12.1
 
 
 
 
APOO
 
 
hsa-miR-181a-5p
 
 
-0.5
 
 
12.1
 
 
 
 
APP
 
 
hsa-miR-16-5p
 
 
-0.298
 
 
163.0
 
 
 
 
APP
 
 
hsa-miR-15b-5p
 
 
-0.298
 
 
163.0
 
 
 
 
ARF4
 
 
hsa-miR-30b-5p
 
 
-0.289
 
 
102.8
 
 
 
 
ARF4
 
 
hsa-miR-148b-3p
 
 
-0.487
 
 
102.8
 
 
 
 
ARF5
 
 
hsa-miR-29a-3p
 
 
-0.491
 
 
248.7
 
 
 
 
ARF6
 
 
hsa-miR-181b-5p
 
 
-0.33
 
 
81.9
 
 
 
 
ARF6
 
 
hsa-miR-181a-5p
 
 
-0.33
 
 
81.9
 
 
 
 
ARFGEF1
 
 
hsa-miR-148b-3p
 
 
-0.306
 
 
14.2
 
 
 
 
ARFIP1
 
 
hsa-miR-320b
 
 
-0.339
 
 
19.6
 
 
 
 
ARFIP1
 
 
hsa-miR-320c
 
 
-0.339
 
 
19.6
 
 
 
 
ARFIP1
 
 
hsa-miR-148b-3p
 
 
-0.474
 
 
19.6
 
 
 
 
ARG2
 
 
hsa-let-7b-5p
 
 
-0.468
 
 
17.2
 
 
 
 
ARHGAP21
 
 
hsa-miR-148b-3p
 
 
-0.287
 
 
10.2
 
 
 
 
ARHGAP5
 
 
hsa-miR-486-5p
 
 
-0.3
 
 
62.5
 
 
 
 
ARID1B
 
 
hsa-miR-29a-3p
 
 
-0.342
 
 
13.7
 
 
 
 
ARL2
 
 
hsa-miR-16-5p
 
 
-0.374
 
 
68.0
 
 
 
 
ARL2
 
 
hsa-miR-15b-5p
 
 
-0.374
 
 
68.0
 
 
 
 
ARL2
 
 
hsa-miR-16-5p
 
 
-0.418
 
 
68.0
 
 
 
 
ARL2
 
 
hsa-miR-15b-5p
 
 
-0.442
 
 
68.0
 
 
 
 
ARL4A
 
 
hsa-miR-29a-3p
 
 
-0.386
 
 
56.3
 
 
 
 
ARL4A
 
 
hsa-miR-30b-5p
 
 
-0.448
 
 
56.3
 
 
 
 
ARL4D
 
 
hsa-let-7b-5p
 
 
-0.417
 
 
80.8
 
 
 
 
ARL6IP1
 
 
hsa-miR-328-3p
 
 
-0.494
 
 
65.8
 
 
 
 
ARL6IP1
 
 
hsa-miR-148b-3p
 
 
-0.579
 
 
65.8
 
 
 
 
ARL6IP6
 
 
hsa-let-7b-5p
 
 
-0.293
 
 
11.7
 
 
 
 
ARL8B
 
 
hsa-miR-148b-3p
 
 
-0.337
 
 
71.1
 
 
 
 
ARL8B
 
 
hsa-miR-320b
 
 
-0.376
 
 
71.1
 
 
 
 
ARL8B
 
 
hsa-miR-320c
 
 
-0.376
 
 
71.1
 
 
 
 
ARMC8
 
 
hsa-miR-486-5p
 
 
-0.321
 
 
19.8
 
 
 
 
ARMCX3
 
 
hsa-miR-425-5p
 
 
-0.325
 
 
17.0
 
 
 
 
ARMT1
 
 
hsa-let-7b-5p
 
 
-0.291
 
 
11.3
 
 
 
 
ARPP19
 
 
hsa-miR-320b
 
 
-0.325
 
 
49.0
 
 
 
 
ARPP19
 
 
hsa-miR-320c
 
 
-0.325
 
 
49.0
 
 
 
 
ARPP19
 
 
hsa-miR-15b-5p
 
 
-0.346
 
 
49.0
 
 
 
 
ARPP19
 
 
hsa-miR-16-5p
 
 
-0.354
 
 
49.0
 
 
 
 
ARPP19
 
 
hsa-miR-29a-3p
 
 
-0.371
 
 
49.0
 
 
 
 
ARPP19
 
 
hsa-miR-328-3p
 
 
-0.404
 
 
49.0
 
 
 
 
 
 
 fallopian tube 
 
 
 
 
target_symbol
 
 
mature_mirna_id
 
 
score
 
 
nTPM
 
 
 
 
 
 
AAR2
 
 
hsa-miR-29a-3p
 
 
-0.327
 
 
21.8
 
 
 
 
ABCB7
 
 
hsa-miR-148b-3p
 
 
-0.515
 
 
11.3
 
 
 
 
ABCD3
 
 
hsa-miR-148b-3p
 
 
-0.388
 
 
15.8
 
 
 
 
ABCE1
 
 
hsa-miR-29a-3p
 
 
-0.404
 
 
21.1
 
 
 
 
ABHD17C
 
 
hsa-let-7b-5p
 
 
-0.359
 
 
10.3
 
 
 
 
ABHD17C
 
 
hsa-miR-27a-3p
 
 
-0.435
 
 
10.3
 
 
 
 
ABHD4
 
 
hsa-miR-29a-3p
 
 
-0.312
 
 
18.8
 
 
 
 
ACTA2
 
 
hsa-miR-27a-3p
 
 
-0.577
 
 
3776.0
 
 
 
 
ACTR2
 
 
hsa-miR-15b-5p
 
 
-0.293
 
 
50.3
 
 
 
 
ACTR2
 
 
hsa-miR-16-5p
 
 
-0.293
 
 
50.3
 
 
 
 
ACVR1
 
 
hsa-miR-30b-5p
 
 
-0.29
 
 
13.2
 
 
 
 
ACVR1
 
 
hsa-miR-148b-3p
 
 
-0.397
 
 
13.2
 
 
 
 
ADAM10
 
 
hsa-miR-148b-3p
 
 
-0.356
 
 
13.1
 
 
 
 
ADAM9
 
 
hsa-miR-30b-5p
 
 
-0.312
 
 
15.9
 
 
 
 
ADAMTS9
 
 
hsa-miR-29a-3p
 
 
-0.305
 
 
10.3
 
 
 
 
ADAMTS9
 
 
hsa-miR-29a-3p
 
 
-0.485
 
 
10.3
 
 
 
 
ADCY3
 
 
hsa-miR-132-3p
 
 
-0.303
 
 
52.6
 
 
 
 
ADGRA3
 
 
hsa-miR-27a-3p
 
 
-0.296
 
 
20.3
 
 
 
 
ADGRA3
 
 
hsa-miR-30b-5p
 
 
-0.304
 
 
20.3
 
 
 
 
ADGRF5
 
 
hsa-miR-148b-3p
 
 
-0.354
 
 
13.6
 
 
 
 
ADIPOR2
 
 
hsa-miR-150-5p
 
 
-0.287
 
 
23.2
 
 
 
 
ADNP
 
 
hsa-miR-328-3p
 
 
-0.371
 
 
31.2
 
 
 
 
AFG3L2
 
 
hsa-miR-181b-5p
 
 
-0.491
 
 
12.9
 
 
 
 
AFG3L2
 
 
hsa-miR-181a-5p
 
 
-0.491
 
 
12.9
 
 
 
 
AGFG1
 
 
hsa-miR-148b-3p
 
 
-0.332
 
 
23.3
 
 
 
 
AGPAT3
 
 
hsa-miR-27a-3p
 
 
-0.29
 
 
11.6
 
 
 
 
AGPAT3
 
 
hsa-miR-16-5p
 
 
-0.299
 
 
11.6
 
 
 
 
AHDC1
 
 
hsa-miR-148b-3p
 
 
-0.413
 
 
19.8
 
 
 
 
AIFM1
 
 
hsa-let-7b-5p
 
 
-0.314
 
 
20.8
 
 
 
 
AK1
 
 
hsa-miR-29a-3p
 
 
-0.333
 
 
321.8
 
 
 
 
AKAP1
 
 
hsa-miR-148b-3p
 
 
-0.381
 
 
33.9
 
 
 
 
AKIRIN1
 
 
hsa-miR-27a-3p
 
 
-0.368
 
 
23.3
 
 
 
 
AKT3
 
 
hsa-miR-29a-3p
 
 
-0.287
 
 
15.1
 
 
 
 
AKT3
 
 
hsa-miR-15b-5p
 
 
-0.301
 
 
15.1
 
 
 
 
AKT3
 
 
hsa-miR-16-5p
 
 
-0.309
 
 
15.1
 
 
 
 
ALAD
 
 
hsa-miR-16-5p
 
 
-0.391
 
 
14.7
 
 
 
 
ALAD
 
 
hsa-miR-15b-5p
 
 
-0.446
 
 
14.7
 
 
 
 
ALCAM
 
 
hsa-miR-148b-3p
 
 
-0.339
 
 
46.2
 
 
 
 
ALDH1A2
 
 
hsa-miR-23b-3p
 
 
-0.346
 
 
70.1
 
 
 
 
ALDH1A2
 
 
hsa-miR-23a-3p
 
 
-0.354
 
 
70.1
 
 
 
 
AMOT
 
 
hsa-miR-29a-3p
 
 
-0.304
 
 
11.3
 
 
 
 
AMOTL1
 
 
hsa-miR-16-5p
 
 
-0.297
 
 
12.5
 
 
 
 
AMOTL1
 
 
hsa-miR-15b-5p
 
 
-0.319
 
 
12.5
 
 
 
 
AMT
 
 
hsa-let-7b-5p
 
 
-0.463
 
 
13.9
 
 
 
 
ANKHD1
 
 
hsa-miR-23a-3p
 
 
-0.368
 
 
37.7
 
 
 
 
ANKHD1
 
 
hsa-miR-23b-3p
 
 
-0.368
 
 
37.7
 
 
 
 
ANKHD1
 
 
hsa-miR-30b-5p
 
 
-0.405
 
 
37.7
 
 
 
 
ANKRA2
 
 
hsa-miR-30b-5p
 
 
-0.354
 
 
10.8
 
 
 
 
ANKRA2
 
 
hsa-miR-30b-5p
 
 
-0.472
 
 
10.8
 
 
 
 
ANKRD17
 
 
hsa-miR-30b-5p
 
 
-0.336
 
 
17.1
 
 
 
 
ANKRD29
 
 
hsa-miR-132-3p
 
 
-0.47
 
 
14.4
 
 
 
 
ANKRD40
 
 
hsa-miR-27a-3p
 
 
-0.35
 
 
18.4
 
 
 
 
ANKRD46
 
 
hsa-let-7b-5p
 
 
-0.389
 
 
10.7
 
 
 
 
ANP32A
 
 
hsa-miR-132-3p
 
 
-0.374
 
 
70.6
 
 
 
 
ANTXR2
 
 
hsa-miR-29a-3p
 
 
-0.347
 
 
42.2
 
 
 
 
ANXA11
 
 
hsa-miR-16-5p
 
 
-0.447
 
 
102.6
 
 
 
 
ANXA11
 
 
hsa-miR-15b-5p
 
 
-0.463
 
 
102.6
 
 
 
 
AP1S1
 
 
hsa-let-7b-5p
 
 
-0.374
 
 
13.4
 
 
 
 
AP2A1
 
 
hsa-miR-15b-5p
 
 
-0.333
 
 
44.6
 
 
 
 
AP2A1
 
 
hsa-miR-16-5p
 
 
-0.333
 
 
44.6
 
 
 
 
AP2B1
 
 
hsa-miR-16-5p
 
 
-0.302
 
 
43.9
 
 
 
 
AP2B1
 
 
hsa-miR-15b-5p
 
 
-0.334
 
 
43.9
 
 
 
 
AP3S1
 
 
hsa-miR-30b-5p
 
 
-0.293
 
 
44.1
 
 
 
 
APOO
 
 
hsa-miR-181b-5p
 
 
-0.5
 
 
19.5
 
 
 
 
APOO
 
 
hsa-miR-181a-5p
 
 
-0.5
 
 
19.5
 
 
 
 
APP
 
 
hsa-miR-16-5p
 
 
-0.298
 
 
289.3
 
 
 
 
APP
 
 
hsa-miR-15b-5p
 
 
-0.298
 
 
289.3
 
 
 
 
ARF4
 
 
hsa-miR-30b-5p
 
 
-0.289
 
 
79.6
 
 
 
 
ARF4
 
 
hsa-miR-148b-3p
 
 
-0.487
 
 
79.6
 
 
 
 
ARF5
 
 
hsa-miR-29a-3p
 
 
-0.491
 
 
100.8
 
 
 
 
ARF6
 
 
hsa-miR-181b-5p
 
 
-0.33
 
 
26.6
 
 
 
 
ARF6
 
 
hsa-miR-181a-5p
 
 
-0.33
 
 
26.6
 
 
 
 
ARFGEF1
 
 
hsa-miR-148b-3p
 
 
-0.306
 
 
11.1
 
 
 
 
ARFIP1
 
 
hsa-miR-320b
 
 
-0.339
 
 
17.4
 
 
 
 
ARFIP1
 
 
hsa-miR-320c
 
 
-0.339
 
 
17.4
 
 
 
 
ARFIP1
 
 
hsa-miR-148b-3p
 
 
-0.474
 
 
17.4
 
 
 
 
ARHGAP21
 
 
hsa-miR-148b-3p
 
 
-0.287
 
 
16.4
 
 
 
 
ARHGAP5
 
 
hsa-miR-486-5p
 
 
-0.3
 
 
21.8
 
 
 
 
ARID1B
 
 
hsa-miR-29a-3p
 
 
-0.342
 
 
16.6
 
 
 
 
ARL2
 
 
hsa-miR-16-5p
 
 
-0.374
 
 
96.8
 
 
 
 
ARL2
 
 
hsa-miR-15b-5p
 
 
-0.374
 
 
96.8
 
 
 
 
ARL2
 
 
hsa-miR-16-5p
 
 
-0.418
 
 
96.8
 
 
 
 
ARL2
 
 
hsa-miR-15b-5p
 
 
-0.442
 
 
96.8
 
 
 
 
ARL3
 
 
hsa-miR-16-5p
 
 
-0.496
 
 
14.3
 
 
 
 
ARL3
 
 
hsa-miR-15b-5p
 
 
-0.507
 
 
14.3
 
 
 
 
ARL4A
 
 
hsa-miR-29a-3p
 
 
-0.386
 
 
26.0
 
 
 
 
ARL4A
 
 
hsa-miR-30b-5p
 
 
-0.448
 
 
26.0
 
 
 
 
ARL4D
 
 
hsa-let-7b-5p
 
 
-0.417
 
 
20.8
 
 
 
 
ARL6IP1
 
 
hsa-miR-328-3p
 
 
-0.494
 
 
81.6
 
 
 
 
ARL6IP1
 
 
hsa-miR-148b-3p
 
 
-0.579
 
 
81.6
 
 
 
 
ARL6IP6
 
 
hsa-let-7b-5p
 
 
-0.293
 
 
10.8
 
 
 
 
ARL8B
 
 
hsa-miR-148b-3p
 
 
-0.337
 
 
29.3
 
 
 
 
ARL8B
 
 
hsa-miR-320b
 
 
-0.376
 
 
29.3
 
 
 
 
ARL8B
 
 
hsa-miR-320c
 
 
-0.376
 
 
29.3
 
 
 
 
ARMC8
 
 
hsa-miR-486-5p
 
 
-0.321
 
 
14.8
 
 
 
 
ARMCX2
 
 
hsa-miR-15b-5p
 
 
-0.344
 
 
26.8
 
 
 
 
ARMCX2
 
 
hsa-miR-16-5p
 
 
-0.366
 
 
26.8
 
 
 
 
ARMCX3
 
 
hsa-miR-425-5p
 
 
-0.325
 
 
25.7
 
 
 
 
ARMT1
 
 
hsa-let-7b-5p
 
 
-0.291
 
 
11.9
 
 
 
 
ARPP19
 
 
hsa-miR-320b
 
 
-0.325
 
 
31.4
 
 
 
 
 
 
 gallbladder 
 
 
 
 
target_symbol
 
 
mature_mirna_id
 
 
score
 
 
nTPM
 
 
 
 
 
 
AAR2
 
 
hsa-miR-29a-3p
 
 
-0.327
 
 
15.5
 
 
 
 
ABCD3
 
 
hsa-miR-148b-3p
 
 
-0.388
 
 
25.2
 
 
 
 
ABCE1
 
 
hsa-miR-29a-3p
 
 
-0.404
 
 
21.0
 
 
 
 
ABHD4
 
 
hsa-miR-29a-3p
 
 
-0.312
 
 
25.4
 
 
 
 
ABHD5
 
 
hsa-miR-132-3p
 
 
-0.323
 
 
10.2
 
 
 
 
ABHD5
 
 
hsa-miR-27a-3p
 
 
-0.407
 
 
10.2
 
 
 
 
ACTA2
 
 
hsa-miR-27a-3p
 
 
-0.577
 
 
849.5
 
 
 
 
ACTC1
 
 
hsa-miR-30b-5p
 
 
-0.543
 
 
11.1
 
 
 
 
ACTR2
 
 
hsa-miR-15b-5p
 
 
-0.293
 
 
84.9
 
 
 
 
ACTR2
 
 
hsa-miR-16-5p
 
 
-0.293
 
 
84.9
 
 
 
 
ACVR1
 
 
hsa-miR-30b-5p
 
 
-0.29
 
 
14.1
 
 
 
 
ACVR1
 
 
hsa-miR-148b-3p
 
 
-0.397
 
 
14.1
 
 
 
 
ADAM10
 
 
hsa-miR-148b-3p
 
 
-0.356
 
 
17.7
 
 
 
 
ADAM9
 
 
hsa-miR-30b-5p
 
 
-0.312
 
 
44.2
 
 
 
 
ADCY3
 
 
hsa-miR-132-3p
 
 
-0.303
 
 
21.6
 
 
 
 
ADGRF5
 
 
hsa-miR-148b-3p
 
 
-0.354
 
 
16.1
 
 
 
 
ADNP
 
 
hsa-miR-328-3p
 
 
-0.371
 
 
20.9
 
 
 
 
ADRA2A
 
 
hsa-miR-23a-3p
 
 
-0.371
 
 
18.7
 
 
 
 
ADRA2A
 
 
hsa-miR-23b-3p
 
 
-0.371
 
 
18.7
 
 
 
 
AFG3L2
 
 
hsa-miR-181b-5p
 
 
-0.491
 
 
10.6
 
 
 
 
AFG3L2
 
 
hsa-miR-181a-5p
 
 
-0.491
 
 
10.6
 
 
 
 
AGFG1
 
 
hsa-miR-148b-3p
 
 
-0.332
 
 
36.5
 
 
 
 
AGPAT3
 
 
hsa-miR-27a-3p
 
 
-0.29
 
 
12.9
 
 
 
 
AGPAT3
 
 
hsa-miR-16-5p
 
 
-0.299
 
 
12.9
 
 
 
 
AHR
 
 
hsa-miR-29a-3p
 
 
-0.316
 
 
31.5
 
 
 
 
AK1
 
 
hsa-miR-29a-3p
 
 
-0.333
 
 
21.1
 
 
 
 
AK4
 
 
hsa-miR-15b-5p
 
 
-0.316
 
 
12.5
 
 
 
 
AK4
 
 
hsa-miR-16-5p
 
 
-0.348
 
 
12.5
 
 
 
 
AKAP1
 
 
hsa-miR-148b-3p
 
 
-0.381
 
 
16.8
 
 
 
 
AKIRIN1
 
 
hsa-miR-27a-3p
 
 
-0.368
 
 
39.1
 
 
 
 
ALAD
 
 
hsa-miR-16-5p
 
 
-0.391
 
 
25.7
 
 
 
 
ALAD
 
 
hsa-miR-15b-5p
 
 
-0.446
 
 
25.7
 
 
 
 
ALCAM
 
 
hsa-miR-148b-3p
 
 
-0.339
 
 
21.9
 
 
 
 
ALDH1A2
 
 
hsa-miR-23b-3p
 
 
-0.346
 
 
22.5
 
 
 
 
ALDH1A2
 
 
hsa-miR-23a-3p
 
 
-0.354
 
 
22.5
 
 
 
 
AMOTL1
 
 
hsa-miR-16-5p
 
 
-0.297
 
 
12.5
 
 
 
 
AMOTL1
 
 
hsa-miR-15b-5p
 
 
-0.319
 
 
12.5
 
 
 
 
ANKHD1
 
 
hsa-miR-23a-3p
 
 
-0.368
 
 
23.4
 
 
 
 
ANKHD1
 
 
hsa-miR-23b-3p
 
 
-0.368
 
 
23.4
 
 
 
 
ANKHD1
 
 
hsa-miR-30b-5p
 
 
-0.405
 
 
23.4
 
 
 
 
ANKRD17
 
 
hsa-miR-30b-5p
 
 
-0.336
 
 
11.6
 
 
 
 
ANKRD40
 
 
hsa-miR-27a-3p
 
 
-0.35
 
 
15.1
 
 
 
 
ANP32A
 
 
hsa-miR-132-3p
 
 
-0.374
 
 
56.0
 
 
 
 
ANTXR2
 
 
hsa-miR-29a-3p
 
 
-0.347
 
 
51.4
 
 
 
 
ANXA11
 
 
hsa-miR-16-5p
 
 
-0.447
 
 
79.6
 
 
 
 
ANXA11
 
 
hsa-miR-15b-5p
 
 
-0.463
 
 
79.6
 
 
 
 
AP1S1
 
 
hsa-let-7b-5p
 
 
-0.374
 
 
12.0
 
 
 
 
AP2A1
 
 
hsa-miR-15b-5p
 
 
-0.333
 
 
18.3
 
 
 
 
AP2A1
 
 
hsa-miR-16-5p
 
 
-0.333
 
 
18.3
 
 
 
 
AP2B1
 
 
hsa-miR-16-5p
 
 
-0.302
 
 
39.7
 
 
 
 
AP2B1
 
 
hsa-miR-15b-5p
 
 
-0.334
 
 
39.7
 
 
 
 
AP3S1
 
 
hsa-miR-30b-5p
 
 
-0.293
 
 
46.1
 
 
 
 
APP
 
 
hsa-miR-16-5p
 
 
-0.298
 
 
321.7
 
 
 
 
APP
 
 
hsa-miR-15b-5p
 
 
-0.298
 
 
321.7
 
 
 
 
APPBP2
 
 
hsa-miR-27a-3p
 
 
-0.378
 
 
10.9
 
 
 
 
ARF4
 
 
hsa-miR-30b-5p
 
 
-0.289
 
 
145.1
 
 
 
 
ARF4
 
 
hsa-miR-148b-3p
 
 
-0.487
 
 
145.1
 
 
 
 
ARF5
 
 
hsa-miR-29a-3p
 
 
-0.491
 
 
76.6
 
 
 
 
ARF6
 
 
hsa-miR-181b-5p
 
 
-0.33
 
 
30.6
 
 
 
 
ARF6
 
 
hsa-miR-181a-5p
 
 
-0.33
 
 
30.6
 
 
 
 
ARFGEF1
 
 
hsa-miR-148b-3p
 
 
-0.306
 
 
11.7
 
 
 
 
ARFIP1
 
 
hsa-miR-320b
 
 
-0.339
 
 
18.7
 
 
 
 
ARFIP1
 
 
hsa-miR-320c
 
 
-0.339
 
 
18.7
 
 
 
 
ARFIP1
 
 
hsa-miR-148b-3p
 
 
-0.474
 
 
18.7
 
 
 
 
ARHGAP21
 
 
hsa-miR-148b-3p
 
 
-0.287
 
 
16.7
 
 
 
 
ARHGAP5
 
 
hsa-miR-486-5p
 
 
-0.3
 
 
30.8
 
 
 
 
ARID1B
 
 
hsa-miR-29a-3p
 
 
-0.342
 
 
12.6
 
 
 
 
ARID4B
 
 
hsa-miR-486-5p
 
 
-0.367
 
 
12.3
 
 
 
 
ARL2
 
 
hsa-miR-16-5p
 
 
-0.374
 
 
45.0
 
 
 
 
ARL2
 
 
hsa-miR-15b-5p
 
 
-0.374
 
 
45.0
 
 
 
 
ARL2
 
 
hsa-miR-16-5p
 
 
-0.418
 
 
45.0
 
 
 
 
ARL2
 
 
hsa-miR-15b-5p
 
 
-0.442
 
 
45.0
 
 
 
 
ARL4A
 
 
hsa-miR-29a-3p
 
 
-0.386
 
 
18.0
 
 
 
 
ARL4A
 
 
hsa-miR-30b-5p
 
 
-0.448
 
 
18.0
 
 
 
 
ARL5B
 
 
hsa-miR-29a-3p
 
 
-0.304
 
 
13.2
 
 
 
 
ARL6IP1
 
 
hsa-miR-328-3p
 
 
-0.494
 
 
123.8
 
 
 
 
ARL6IP1
 
 
hsa-miR-148b-3p
 
 
-0.579
 
 
123.8
 
 
 
 
ARL8B
 
 
hsa-miR-148b-3p
 
 
-0.337
 
 
36.3
 
 
 
 
ARL8B
 
 
hsa-miR-320b
 
 
-0.376
 
 
36.3
 
 
 
 
ARL8B
 
 
hsa-miR-320c
 
 
-0.376
 
 
36.3
 
 
 
 
ARMC8
 
 
hsa-miR-486-5p
 
 
-0.321
 
 
14.3
 
 
 
 
ARMCX2
 
 
hsa-miR-15b-5p
 
 
-0.344
 
 
19.3
 
 
 
 
ARMCX2
 
 
hsa-miR-16-5p
 
 
-0.366
 
 
19.3
 
 
 
 
ARMCX3
 
 
hsa-miR-425-5p
 
 
-0.325
 
 
31.9
 
 
 
 
ARMT1
 
 
hsa-let-7b-5p
 
 
-0.291
 
 
10.6
 
 
 
 
ARPP19
 
 
hsa-miR-320b
 
 
-0.325
 
 
36.7
 
 
 
 
ARPP19
 
 
hsa-miR-320c
 
 
-0.325
 
 
36.7
 
 
 
 
ARPP19
 
 
hsa-miR-15b-5p
 
 
-0.346
 
 
36.7
 
 
 
 
ARPP19
 
 
hsa-miR-16-5p
 
 
-0.354
 
 
36.7
 
 
 
 
ARPP19
 
 
hsa-miR-29a-3p
 
 
-0.371
 
 
36.7
 
 
 
 
ARPP19
 
 
hsa-miR-328-3p
 
 
-0.404
 
 
36.7
 
 
 
 
ARPP19
 
 
hsa-let-7b-5p
 
 
-0.476
 
 
36.7
 
 
 
 
ARRDC3
 
 
hsa-miR-148b-3p
 
 
-0.305
 
 
36.6
 
 
 
 
ARRDC3
 
 
hsa-miR-29a-3p
 
 
-0.402
 
 
36.6
 
 
 
 
ARRDC4
 
 
hsa-let-7b-5p
 
 
-0.329
 
 
16.1
 
 
 
 
ASAH1
 
 
hsa-miR-27a-3p
 
 
-0.398
 
 
92.8
 
 
 
 
ASAP1
 
 
hsa-let-7b-5p
 
 
-0.308
 
 
13.5
 
 
 
 
ASH2L
 
 
hsa-miR-320b
 
 
-0.287
 
 
16.3
 
 
 
 
ASH2L
 
 
hsa-miR-320c
 
 
-0.287
 
 
16.3
 
 
 
 
ASPH
 
 
hsa-miR-148b-3p
 
 
-0.391
 
 
56.5
 
 
 
 
 
 
 heart muscle 
 
 
 
 
target_symbol
 
 
mature_mirna_id
 
 
score
 
 
nTPM
 
 
 
 
 
 
AAR2
 
 
hsa-miR-29a-3p
 
 
-0.327
 
 
17.9
 
 
 
 
ABCB7
 
 
hsa-miR-148b-3p
 
 
-0.515
 
 
20.0
 
 
 
 
ABCD3
 
 
hsa-miR-148b-3p
 
 
-0.388
 
 
24.0
 
 
 
 
ABCE1
 
 
hsa-miR-29a-3p
 
 
-0.404
 
 
23.3
 
 
 
 
ABHD18
 
 
hsa-miR-29a-3p
 
 
-0.321
 
 
12.6
 
 
 
 
ABHD18
 
 
hsa-miR-29a-3p
 
 
-0.34
 
 
12.6
 
 
 
 
ABHD4
 
 
hsa-miR-29a-3p
 
 
-0.312
 
 
21.4
 
 
 
 
ABHD5
 
 
hsa-miR-132-3p
 
 
-0.323
 
 
10.6
 
 
 
 
ABHD5
 
 
hsa-miR-27a-3p
 
 
-0.407
 
 
10.6
 
 
 
 
ACTA2
 
 
hsa-miR-27a-3p
 
 
-0.577
 
 
1015.4
 
 
 
 
ACTC1
 
 
hsa-miR-30b-5p
 
 
-0.543
 
 
10840.1
 
 
 
 
ACTN2
 
 
hsa-miR-23a-3p
 
 
-0.294
 
 
695.9
 
 
 
 
ACTN2
 
 
hsa-miR-29a-3p
 
 
-0.311
 
 
695.9
 
 
 
 
ACTR2
 
 
hsa-miR-15b-5p
 
 
-0.293
 
 
60.7
 
 
 
 
ACTR2
 
 
hsa-miR-16-5p
 
 
-0.293
 
 
60.7
 
 
 
 
ACVR1
 
 
hsa-miR-30b-5p
 
 
-0.29
 
 
14.3
 
 
 
 
ACVR1
 
 
hsa-miR-148b-3p
 
 
-0.397
 
 
14.3
 
 
 
 
ADA
 
 
hsa-miR-29a-3p
 
 
-0.376
 
 
18.4
 
 
 
 
ADAM10
 
 
hsa-miR-148b-3p
 
 
-0.356
 
 
11.3
 
 
 
 
ADAM9
 
 
hsa-miR-30b-5p
 
 
-0.312
 
 
26.6
 
 
 
 
ADAMTS7
 
 
hsa-miR-29a-3p
 
 
-0.427
 
 
28.7
 
 
 
 
ADAMTS9
 
 
hsa-miR-29a-3p
 
 
-0.305
 
 
14.2
 
 
 
 
ADAMTS9
 
 
hsa-miR-29a-3p
 
 
-0.485
 
 
14.2
 
 
 
 
ADCY3
 
 
hsa-miR-132-3p
 
 
-0.303
 
 
35.0
 
 
 
 
ADGRA3
 
 
hsa-miR-27a-3p
 
 
-0.296
 
 
10.1
 
 
 
 
ADGRA3
 
 
hsa-miR-30b-5p
 
 
-0.304
 
 
10.1
 
 
 
 
ADGRF5
 
 
hsa-miR-148b-3p
 
 
-0.354
 
 
50.1
 
 
 
 
ADIPOR2
 
 
hsa-miR-150-5p
 
 
-0.287
 
 
31.9
 
 
 
 
ADNP
 
 
hsa-miR-328-3p
 
 
-0.371
 
 
22.3
 
 
 
 
ADRB1
 
 
hsa-let-7b-5p
 
 
-0.351
 
 
20.3
 
 
 
 
AFG3L2
 
 
hsa-miR-181b-5p
 
 
-0.491
 
 
48.7
 
 
 
 
AFG3L2
 
 
hsa-miR-181a-5p
 
 
-0.491
 
 
48.7
 
 
 
 
AGFG1
 
 
hsa-miR-148b-3p
 
 
-0.332
 
 
33.8
 
 
 
 
AGPAT3
 
 
hsa-miR-27a-3p
 
 
-0.29
 
 
25.4
 
 
 
 
AGPAT3
 
 
hsa-miR-16-5p
 
 
-0.299
 
 
25.4
 
 
 
 
AHDC1
 
 
hsa-miR-148b-3p
 
 
-0.413
 
 
17.4
 
 
 
 
AIFM1
 
 
hsa-let-7b-5p
 
 
-0.314
 
 
74.9
 
 
 
 
AK1
 
 
hsa-miR-29a-3p
 
 
-0.333
 
 
313.1
 
 
 
 
AK4
 
 
hsa-miR-15b-5p
 
 
-0.316
 
 
47.4
 
 
 
 
AK4
 
 
hsa-miR-16-5p
 
 
-0.348
 
 
47.4
 
 
 
 
AKAP1
 
 
hsa-miR-148b-3p
 
 
-0.381
 
 
78.5
 
 
 
 
AKAP6
 
 
hsa-let-7b-5p
 
 
-0.307
 
 
51.1
 
 
 
 
AKIRIN1
 
 
hsa-miR-27a-3p
 
 
-0.368
 
 
36.6
 
 
 
 
AKT3
 
 
hsa-miR-29a-3p
 
 
-0.287
 
 
10.6
 
 
 
 
AKT3
 
 
hsa-miR-15b-5p
 
 
-0.301
 
 
10.6
 
 
 
 
AKT3
 
 
hsa-miR-16-5p
 
 
-0.309
 
 
10.6
 
 
 
 
ALAD
 
 
hsa-miR-16-5p
 
 
-0.391
 
 
50.8
 
 
 
 
ALAD
 
 
hsa-miR-15b-5p
 
 
-0.446
 
 
50.8
 
 
 
 
ALDH1A2
 
 
hsa-miR-23b-3p
 
 
-0.346
 
 
27.5
 
 
 
 
ALDH1A2
 
 
hsa-miR-23a-3p
 
 
-0.354
 
 
27.5
 
 
 
 
ALPL
 
 
hsa-miR-10a-5p
 
 
-0.32
 
 
15.8
 
 
 
 
AMOTL1
 
 
hsa-miR-16-5p
 
 
-0.297
 
 
17.9
 
 
 
 
AMOTL1
 
 
hsa-miR-15b-5p
 
 
-0.319
 
 
17.9
 
 
 
 
AMT
 
 
hsa-let-7b-5p
 
 
-0.463
 
 
23.5
 
 
 
 
ANKHD1
 
 
hsa-miR-23a-3p
 
 
-0.368
 
 
34.0
 
 
 
 
ANKHD1
 
 
hsa-miR-23b-3p
 
 
-0.368
 
 
34.0
 
 
 
 
ANKHD1
 
 
hsa-miR-30b-5p
 
 
-0.405
 
 
34.0
 
 
 
 
ANKRD17
 
 
hsa-miR-30b-5p
 
 
-0.336
 
 
13.6
 
 
 
 
ANKRD40
 
 
hsa-miR-27a-3p
 
 
-0.35
 
 
25.8
 
 
 
 
ANKRD46
 
 
hsa-let-7b-5p
 
 
-0.389
 
 
18.8
 
 
 
 
ANP32A
 
 
hsa-miR-132-3p
 
 
-0.374
 
 
119.7
 
 
 
 
ANTXR2
 
 
hsa-miR-29a-3p
 
 
-0.347
 
 
26.5
 
 
 
 
ANXA11
 
 
hsa-miR-16-5p
 
 
-0.447
 
 
221.3
 
 
 
 
ANXA11
 
 
hsa-miR-15b-5p
 
 
-0.463
 
 
221.3
 
 
 
 
AP1S1
 
 
hsa-let-7b-5p
 
 
-0.374
 
 
12.9
 
 
 
 
AP2A1
 
 
hsa-miR-15b-5p
 
 
-0.333
 
 
49.3
 
 
 
 
AP2A1
 
 
hsa-miR-16-5p
 
 
-0.333
 
 
49.3
 
 
 
 
AP2B1
 
 
hsa-miR-16-5p
 
 
-0.302
 
 
63.3
 
 
 
 
AP2B1
 
 
hsa-miR-15b-5p
 
 
-0.334
 
 
63.3
 
 
 
 
AP3S1
 
 
hsa-miR-30b-5p
 
 
-0.293
 
 
53.4
 
 
 
 
AP5S1
 
 
hsa-miR-744-5p
 
 
-0.354
 
 
10.7
 
 
 
 
APLN
 
 
hsa-miR-16-5p
 
 
-0.31
 
 
12.4
 
 
 
 
APLN
 
 
hsa-miR-15b-5p
 
 
-0.395
 
 
12.4
 
 
 
 
APLN
 
 
hsa-miR-16-5p
 
 
-0.395
 
 
12.4
 
 
 
 
APOO
 
 
hsa-miR-181b-5p
 
 
-0.5
 
 
61.9
 
 
 
 
APOO
 
 
hsa-miR-181a-5p
 
 
-0.5
 
 
61.9
 
 
 
 
APP
 
 
hsa-miR-16-5p
 
 
-0.298
 
 
356.3
 
 
 
 
APP
 
 
hsa-miR-15b-5p
 
 
-0.298
 
 
356.3
 
 
 
 
ARF4
 
 
hsa-miR-30b-5p
 
 
-0.289
 
 
106.8
 
 
 
 
ARF4
 
 
hsa-miR-148b-3p
 
 
-0.487
 
 
106.8
 
 
 
 
ARF5
 
 
hsa-miR-29a-3p
 
 
-0.491
 
 
127.9
 
 
 
 
ARF6
 
 
hsa-miR-181b-5p
 
 
-0.33
 
 
15.7
 
 
 
 
ARF6
 
 
hsa-miR-181a-5p
 
 
-0.33
 
 
15.7
 
 
 
 
ARHGAP21
 
 
hsa-miR-148b-3p
 
 
-0.287
 
 
19.7
 
 
 
 
ARHGAP5
 
 
hsa-miR-486-5p
 
 
-0.3
 
 
59.2
 
 
 
 
ARID1B
 
 
hsa-miR-29a-3p
 
 
-0.342
 
 
12.9
 
 
 
 
ARL2
 
 
hsa-miR-16-5p
 
 
-0.374
 
 
169.4
 
 
 
 
ARL2
 
 
hsa-miR-15b-5p
 
 
-0.374
 
 
169.4
 
 
 
 
ARL2
 
 
hsa-miR-16-5p
 
 
-0.418
 
 
169.4
 
 
 
 
ARL2
 
 
hsa-miR-15b-5p
 
 
-0.442
 
 
169.4
 
 
 
 
ARL3
 
 
hsa-miR-16-5p
 
 
-0.496
 
 
12.8
 
 
 
 
ARL3
 
 
hsa-miR-15b-5p
 
 
-0.507
 
 
12.8
 
 
 
 
ARL4A
 
 
hsa-miR-29a-3p
 
 
-0.386
 
 
14.4
 
 
 
 
ARL4A
 
 
hsa-miR-30b-5p
 
 
-0.448
 
 
14.4
 
 
 
 
ARL4D
 
 
hsa-let-7b-5p
 
 
-0.417
 
 
12.5
 
 
 
 
ARL6IP1
 
 
hsa-miR-328-3p
 
 
-0.494
 
 
30.1
 
 
 
 
ARL6IP1
 
 
hsa-miR-148b-3p
 
 
-0.579
 
 
30.1
 
 
 
 
ARL8B
 
 
hsa-miR-148b-3p
 
 
-0.337
 
 
51.7
 
 
 
 
ARL8B
 
 
hsa-miR-320b
 
 
-0.376
 
 
51.7
 
 
 
 
ARL8B
 
 
hsa-miR-320c
 
 
-0.376
 
 
51.7
 
 
 
 
 
 
 hippocampal formation 
 
 
 
 
target_symbol
 
 
mature_mirna_id
 
 
score
 
 
nTPM
 
 
 
 
 
 
AAR2
 
 
hsa-miR-29a-3p
 
 
-0.327
 
 
10.6
 
 
 
 
ABCD3
 
 
hsa-miR-148b-3p
 
 
-0.388
 
 
15.1
 
 
 
 
ABCE1
 
 
hsa-miR-29a-3p
 
 
-0.404
 
 
13.9
 
 
 
 
ABHD17B
 
 
hsa-miR-486-5p
 
 
-0.348
 
 
18.9
 
 
 
 
ABHD17C
 
 
hsa-let-7b-5p
 
 
-0.359
 
 
14.4
 
 
 
 
ABHD17C
 
 
hsa-miR-27a-3p
 
 
-0.435
 
 
14.4
 
 
 
 
ABHD4
 
 
hsa-miR-29a-3p
 
 
-0.312
 
 
14.2
 
 
 
 
ACSL6
 
 
hsa-let-7b-5p
 
 
-0.336
 
 
15.3
 
 
 
 
ACTA2
 
 
hsa-miR-27a-3p
 
 
-0.577
 
 
32.3
 
 
 
 
ACTN2
 
 
hsa-miR-23a-3p
 
 
-0.294
 
 
10.1
 
 
 
 
ACTN2
 
 
hsa-miR-29a-3p
 
 
-0.311
 
 
10.1
 
 
 
 
ACTR2
 
 
hsa-miR-15b-5p
 
 
-0.293
 
 
54.7
 
 
 
 
ACTR2
 
 
hsa-miR-16-5p
 
 
-0.293
 
 
54.7
 
 
 
 
ADAP1
 
 
hsa-miR-27a-3p
 
 
-0.302
 
 
123.8
 
 
 
 
ADCY2
 
 
hsa-miR-148b-3p
 
 
-0.299
 
 
18.5
 
 
 
 
ADCY3
 
 
hsa-miR-132-3p
 
 
-0.303
 
 
30.1
 
 
 
 
ADGRA3
 
 
hsa-miR-27a-3p
 
 
-0.296
 
 
12.8
 
 
 
 
ADGRA3
 
 
hsa-miR-30b-5p
 
 
-0.304
 
 
12.8
 
 
 
 
ADGRB3
 
 
hsa-miR-148b-3p
 
 
-0.524
 
 
12.8
 
 
 
 
ADGRF5
 
 
hsa-miR-148b-3p
 
 
-0.354
 
 
10.4
 
 
 
 
ADIPOR2
 
 
hsa-miR-150-5p
 
 
-0.287
 
 
57.6
 
 
 
 
ADNP
 
 
hsa-miR-328-3p
 
 
-0.371
 
 
23.7
 
 
 
 
AGFG1
 
 
hsa-miR-148b-3p
 
 
-0.332
 
 
29.8
 
 
 
 
AGPAT3
 
 
hsa-miR-27a-3p
 
 
-0.29
 
 
37.1
 
 
 
 
AGPAT3
 
 
hsa-miR-16-5p
 
 
-0.299
 
 
37.1
 
 
 
 
AGPAT4
 
 
hsa-miR-29a-3p
 
 
-0.376
 
 
16.8
 
 
 
 
AIFM1
 
 
hsa-let-7b-5p
 
 
-0.314
 
 
10.5
 
 
 
 
AK1
 
 
hsa-miR-29a-3p
 
 
-0.333
 
 
93.4
 
 
 
 
AK4
 
 
hsa-miR-15b-5p
 
 
-0.316
 
 
13.3
 
 
 
 
AK4
 
 
hsa-miR-16-5p
 
 
-0.348
 
 
13.3
 
 
 
 
AKAP1
 
 
hsa-miR-148b-3p
 
 
-0.381
 
 
17.0
 
 
 
 
AKAP6
 
 
hsa-let-7b-5p
 
 
-0.307
 
 
12.9
 
 
 
 
AKIRIN1
 
 
hsa-miR-27a-3p
 
 
-0.368
 
 
35.2
 
 
 
 
AKT3
 
 
hsa-miR-29a-3p
 
 
-0.287
 
 
17.7
 
 
 
 
AKT3
 
 
hsa-miR-15b-5p
 
 
-0.301
 
 
17.7
 
 
 
 
AKT3
 
 
hsa-miR-16-5p
 
 
-0.309
 
 
17.7
 
 
 
 
ALAD
 
 
hsa-miR-16-5p
 
 
-0.391
 
 
60.3
 
 
 
 
ALAD
 
 
hsa-miR-15b-5p
 
 
-0.446
 
 
60.3
 
 
 
 
ALCAM
 
 
hsa-miR-148b-3p
 
 
-0.339
 
 
33.8
 
 
 
 
ALPL
 
 
hsa-miR-10a-5p
 
 
-0.32
 
 
13.7
 
 
 
 
AMOT
 
 
hsa-miR-29a-3p
 
 
-0.304
 
 
12.0
 
 
 
 
ANGEL2
 
 
hsa-miR-132-3p
 
 
-0.357
 
 
11.0
 
 
 
 
ANKHD1
 
 
hsa-miR-23a-3p
 
 
-0.368
 
 
18.9
 
 
 
 
ANKHD1
 
 
hsa-miR-23b-3p
 
 
-0.368
 
 
18.9
 
 
 
 
ANKHD1
 
 
hsa-miR-30b-5p
 
 
-0.405
 
 
18.9
 
 
 
 
ANKRD13B
 
 
hsa-miR-29a-3p
 
 
-0.421
 
 
10.6
 
 
 
 
ANKRD17
 
 
hsa-miR-30b-5p
 
 
-0.336
 
 
10.3
 
 
 
 
ANKRD40
 
 
hsa-miR-27a-3p
 
 
-0.35
 
 
105.0
 
 
 
 
ANKRD46
 
 
hsa-let-7b-5p
 
 
-0.389
 
 
25.5
 
 
 
 
ANLN
 
 
hsa-miR-16-5p
 
 
-0.31
 
 
54.9
 
 
 
 
ANLN
 
 
hsa-miR-15b-5p
 
 
-0.334
 
 
54.9
 
 
 
 
ANP32A
 
 
hsa-miR-132-3p
 
 
-0.374
 
 
49.8
 
 
 
 
ANXA11
 
 
hsa-miR-16-5p
 
 
-0.447
 
 
30.8
 
 
 
 
ANXA11
 
 
hsa-miR-15b-5p
 
 
-0.463
 
 
30.8
 
 
 
 
AP1S1
 
 
hsa-let-7b-5p
 
 
-0.374
 
 
67.5
 
 
 
 
AP2A1
 
 
hsa-miR-15b-5p
 
 
-0.333
 
 
44.7
 
 
 
 
AP2A1
 
 
hsa-miR-16-5p
 
 
-0.333
 
 
44.7
 
 
 
 
AP2B1
 
 
hsa-miR-16-5p
 
 
-0.302
 
 
69.4
 
 
 
 
AP2B1
 
 
hsa-miR-15b-5p
 
 
-0.334
 
 
69.4
 
 
 
 
AP3S1
 
 
hsa-miR-30b-5p
 
 
-0.293
 
 
86.3
 
 
 
 
APC
 
 
hsa-miR-29a-3p
 
 
-0.413
 
 
34.3
 
 
 
 
APLN
 
 
hsa-miR-16-5p
 
 
-0.31
 
 
14.1
 
 
 
 
APLN
 
 
hsa-miR-15b-5p
 
 
-0.395
 
 
14.1
 
 
 
 
APLN
 
 
hsa-miR-16-5p
 
 
-0.395
 
 
14.1
 
 
 
 
APOO
 
 
hsa-miR-181b-5p
 
 
-0.5
 
 
45.8
 
 
 
 
APOO
 
 
hsa-miR-181a-5p
 
 
-0.5
 
 
45.8
 
 
 
 
APP
 
 
hsa-miR-16-5p
 
 
-0.298
 
 
466.6
 
 
 
 
APP
 
 
hsa-miR-15b-5p
 
 
-0.298
 
 
466.6
 
 
 
 
ARF4
 
 
hsa-miR-30b-5p
 
 
-0.289
 
 
61.3
 
 
 
 
ARF4
 
 
hsa-miR-148b-3p
 
 
-0.487
 
 
61.3
 
 
 
 
ARF5
 
 
hsa-miR-29a-3p
 
 
-0.491
 
 
168.1
 
 
 
 
ARF6
 
 
hsa-miR-181b-5p
 
 
-0.33
 
 
10.0
 
 
 
 
ARF6
 
 
hsa-miR-181a-5p
 
 
-0.33
 
 
10.0
 
 
 
 
ARHGAP21
 
 
hsa-miR-148b-3p
 
 
-0.287
 
 
50.4
 
 
 
 
ARHGAP5
 
 
hsa-miR-486-5p
 
 
-0.3
 
 
22.7
 
 
 
 
ARL2
 
 
hsa-miR-16-5p
 
 
-0.374
 
 
164.1
 
 
 
 
ARL2
 
 
hsa-miR-15b-5p
 
 
-0.374
 
 
164.1
 
 
 
 
ARL2
 
 
hsa-miR-16-5p
 
 
-0.418
 
 
164.1
 
 
 
 
ARL2
 
 
hsa-miR-15b-5p
 
 
-0.442
 
 
164.1
 
 
 
 
ARL3
 
 
hsa-miR-16-5p
 
 
-0.496
 
 
21.1
 
 
 
 
ARL3
 
 
hsa-miR-15b-5p
 
 
-0.507
 
 
21.1
 
 
 
 
ARL4A
 
 
hsa-miR-29a-3p
 
 
-0.386
 
 
12.2
 
 
 
 
ARL4A
 
 
hsa-miR-30b-5p
 
 
-0.448
 
 
12.2
 
 
 
 
ARL6IP1
 
 
hsa-miR-328-3p
 
 
-0.494
 
 
196.8
 
 
 
 
ARL6IP1
 
 
hsa-miR-148b-3p
 
 
-0.579
 
 
196.8
 
 
 
 
ARL8B
 
 
hsa-miR-148b-3p
 
 
-0.337
 
 
53.8
 
 
 
 
ARL8B
 
 
hsa-miR-320b
 
 
-0.376
 
 
53.8
 
 
 
 
ARL8B
 
 
hsa-miR-320c
 
 
-0.376
 
 
53.8
 
 
 
 
ARMC8
 
 
hsa-miR-486-5p
 
 
-0.321
 
 
13.3
 
 
 
 
ARMCX2
 
 
hsa-miR-15b-5p
 
 
-0.344
 
 
21.8
 
 
 
 
ARMCX2
 
 
hsa-miR-16-5p
 
 
-0.366
 
 
21.8
 
 
 
 
ARMCX3
 
 
hsa-miR-425-5p
 
 
-0.325
 
 
33.7
 
 
 
 
ARPP19
 
 
hsa-miR-320b
 
 
-0.325
 
 
120.9
 
 
 
 
ARPP19
 
 
hsa-miR-320c
 
 
-0.325
 
 
120.9
 
 
 
 
ARPP19
 
 
hsa-miR-15b-5p
 
 
-0.346
 
 
120.9
 
 
 
 
ARPP19
 
 
hsa-miR-16-5p
 
 
-0.354
 
 
120.9
 
 
 
 
ARPP19
 
 
hsa-miR-29a-3p
 
 
-0.371
 
 
120.9
 
 
 
 
ARPP19
 
 
hsa-miR-328-3p
 
 
-0.404
 
 
120.9
 
 
 
 
ARPP19
 
 
hsa-let-7b-5p
 
 
-0.476
 
 
120.9
 
 
 
 
ARPP21
 
 
hsa-miR-16-5p
 
 
-0.309
 
 
19.5
 
 
 
 
 
 
 kidney 
 
 
 
 
target_symbol
 
 
mature_mirna_id
 
 
score
 
 
nTPM
 
 
 
 
 
 
AAR2
 
 
hsa-miR-29a-3p
 
 
-0.327
 
 
19.2
 
 
 
 
ABCD3
 
 
hsa-miR-148b-3p
 
 
-0.388
 
 
55.5
 
 
 
 
ABCE1
 
 
hsa-miR-29a-3p
 
 
-0.404
 
 
22.2
 
 
 
 
ABHD17B
 
 
hsa-miR-486-5p
 
 
-0.348
 
 
10.9
 
 
 
 
ABHD17C
 
 
hsa-let-7b-5p
 
 
-0.359
 
 
13.6
 
 
 
 
ABHD17C
 
 
hsa-miR-27a-3p
 
 
-0.435
 
 
13.6
 
 
 
 
ABHD4
 
 
hsa-miR-29a-3p
 
 
-0.312
 
 
26.4
 
 
 
 
ABHD5
 
 
hsa-miR-132-3p
 
 
-0.323
 
 
10.4
 
 
 
 
ABHD5
 
 
hsa-miR-27a-3p
 
 
-0.407
 
 
10.4
 
 
 
 
ACP3
 
 
hsa-let-7b-5p
 
 
-0.298
 
 
26.9
 
 
 
 
ACTA2
 
 
hsa-miR-27a-3p
 
 
-0.577
 
 
281.9
 
 
 
 
ACTR2
 
 
hsa-miR-15b-5p
 
 
-0.293
 
 
79.9
 
 
 
 
ACTR2
 
 
hsa-miR-16-5p
 
 
-0.293
 
 
79.9
 
 
 
 
ACTR3C
 
 
hsa-miR-30b-5p
 
 
-0.448
 
 
11.1
 
 
 
 
ACVR1
 
 
hsa-miR-30b-5p
 
 
-0.29
 
 
15.0
 
 
 
 
ACVR1
 
 
hsa-miR-148b-3p
 
 
-0.397
 
 
15.0
 
 
 
 
ADAM10
 
 
hsa-miR-148b-3p
 
 
-0.356
 
 
16.8
 
 
 
 
ADAM9
 
 
hsa-miR-30b-5p
 
 
-0.312
 
 
32.6
 
 
 
 
ADAP1
 
 
hsa-miR-27a-3p
 
 
-0.302
 
 
21.6
 
 
 
 
ADCY3
 
 
hsa-miR-132-3p
 
 
-0.303
 
 
27.1
 
 
 
 
ADGRF5
 
 
hsa-miR-148b-3p
 
 
-0.354
 
 
55.8
 
 
 
 
ADIPOR2
 
 
hsa-miR-150-5p
 
 
-0.287
 
 
20.9
 
 
 
 
ADNP
 
 
hsa-miR-328-3p
 
 
-0.371
 
 
28.2
 
 
 
 
AFG3L2
 
 
hsa-miR-181b-5p
 
 
-0.491
 
 
36.4
 
 
 
 
AFG3L2
 
 
hsa-miR-181a-5p
 
 
-0.491
 
 
36.4
 
 
 
 
AGFG1
 
 
hsa-miR-148b-3p
 
 
-0.332
 
 
30.6
 
 
 
 
AGPAT3
 
 
hsa-miR-27a-3p
 
 
-0.29
 
 
49.9
 
 
 
 
AGPAT3
 
 
hsa-miR-16-5p
 
 
-0.299
 
 
49.9
 
 
 
 
AHDC1
 
 
hsa-miR-148b-3p
 
 
-0.413
 
 
12.2
 
 
 
 
AIFM1
 
 
hsa-let-7b-5p
 
 
-0.314
 
 
69.0
 
 
 
 
AK1
 
 
hsa-miR-29a-3p
 
 
-0.333
 
 
37.0
 
 
 
 
AK4
 
 
hsa-miR-15b-5p
 
 
-0.316
 
 
169.3
 
 
 
 
AK4
 
 
hsa-miR-16-5p
 
 
-0.348
 
 
169.3
 
 
 
 
AKAP1
 
 
hsa-miR-148b-3p
 
 
-0.381
 
 
24.5
 
 
 
 
AKIRIN1
 
 
hsa-miR-27a-3p
 
 
-0.368
 
 
40.0
 
 
 
 
ALAD
 
 
hsa-miR-16-5p
 
 
-0.391
 
 
68.5
 
 
 
 
ALAD
 
 
hsa-miR-15b-5p
 
 
-0.446
 
 
68.5
 
 
 
 
ALCAM
 
 
hsa-miR-148b-3p
 
 
-0.339
 
 
20.8
 
 
 
 
ALDH1A2
 
 
hsa-miR-23b-3p
 
 
-0.346
 
 
19.1
 
 
 
 
ALDH1A2
 
 
hsa-miR-23a-3p
 
 
-0.354
 
 
19.1
 
 
 
 
ALPL
 
 
hsa-miR-10a-5p
 
 
-0.32
 
 
42.7
 
 
 
 
AMT
 
 
hsa-let-7b-5p
 
 
-0.463
 
 
23.7
 
 
 
 
ANKHD1
 
 
hsa-miR-23a-3p
 
 
-0.368
 
 
34.2
 
 
 
 
ANKHD1
 
 
hsa-miR-23b-3p
 
 
-0.368
 
 
34.2
 
 
 
 
ANKHD1
 
 
hsa-miR-30b-5p
 
 
-0.405
 
 
34.2
 
 
 
 
ANKRD17
 
 
hsa-miR-30b-5p
 
 
-0.336
 
 
18.3
 
 
 
 
ANKRD40
 
 
hsa-miR-27a-3p
 
 
-0.35
 
 
16.6
 
 
 
 
ANKRD46
 
 
hsa-let-7b-5p
 
 
-0.389
 
 
30.5
 
 
 
 
ANP32A
 
 
hsa-miR-132-3p
 
 
-0.374
 
 
70.0
 
 
 
 
ANTXR2
 
 
hsa-miR-29a-3p
 
 
-0.347
 
 
16.0
 
 
 
 
ANXA11
 
 
hsa-miR-16-5p
 
 
-0.447
 
 
127.2
 
 
 
 
ANXA11
 
 
hsa-miR-15b-5p
 
 
-0.463
 
 
127.2
 
 
 
 
AP1S1
 
 
hsa-let-7b-5p
 
 
-0.374
 
 
48.3
 
 
 
 
AP2A1
 
 
hsa-miR-15b-5p
 
 
-0.333
 
 
31.7
 
 
 
 
AP2A1
 
 
hsa-miR-16-5p
 
 
-0.333
 
 
31.7
 
 
 
 
AP2B1
 
 
hsa-miR-16-5p
 
 
-0.302
 
 
64.4
 
 
 
 
AP2B1
 
 
hsa-miR-15b-5p
 
 
-0.334
 
 
64.4
 
 
 
 
AP3S1
 
 
hsa-miR-30b-5p
 
 
-0.293
 
 
38.1
 
 
 
 
AP5S1
 
 
hsa-miR-744-5p
 
 
-0.354
 
 
10.3
 
 
 
 
APOO
 
 
hsa-miR-181b-5p
 
 
-0.5
 
 
43.3
 
 
 
 
APOO
 
 
hsa-miR-181a-5p
 
 
-0.5
 
 
43.3
 
 
 
 
APP
 
 
hsa-miR-16-5p
 
 
-0.298
 
 
464.8
 
 
 
 
APP
 
 
hsa-miR-15b-5p
 
 
-0.298
 
 
464.8
 
 
 
 
APPBP2
 
 
hsa-miR-27a-3p
 
 
-0.378
 
 
12.2
 
 
 
 
ARF4
 
 
hsa-miR-30b-5p
 
 
-0.289
 
 
106.7
 
 
 
 
ARF4
 
 
hsa-miR-148b-3p
 
 
-0.487
 
 
106.7
 
 
 
 
ARF5
 
 
hsa-miR-29a-3p
 
 
-0.491
 
 
140.7
 
 
 
 
ARF6
 
 
hsa-miR-181b-5p
 
 
-0.33
 
 
29.7
 
 
 
 
ARF6
 
 
hsa-miR-181a-5p
 
 
-0.33
 
 
29.7
 
 
 
 
ARFGEF1
 
 
hsa-miR-148b-3p
 
 
-0.306
 
 
21.3
 
 
 
 
ARFIP1
 
 
hsa-miR-320b
 
 
-0.339
 
 
18.5
 
 
 
 
ARFIP1
 
 
hsa-miR-320c
 
 
-0.339
 
 
18.5
 
 
 
 
ARFIP1
 
 
hsa-miR-148b-3p
 
 
-0.474
 
 
18.5
 
 
 
 
ARG2
 
 
hsa-let-7b-5p
 
 
-0.468
 
 
66.0
 
 
 
 
ARHGAP21
 
 
hsa-miR-148b-3p
 
 
-0.287
 
 
10.2
 
 
 
 
ARHGAP5
 
 
hsa-miR-486-5p
 
 
-0.3
 
 
40.2
 
 
 
 
ARID1B
 
 
hsa-miR-29a-3p
 
 
-0.342
 
 
10.7
 
 
 
 
ARL2
 
 
hsa-miR-16-5p
 
 
-0.374
 
 
162.2
 
 
 
 
ARL2
 
 
hsa-miR-15b-5p
 
 
-0.374
 
 
162.2
 
 
 
 
ARL2
 
 
hsa-miR-16-5p
 
 
-0.418
 
 
162.2
 
 
 
 
ARL2
 
 
hsa-miR-15b-5p
 
 
-0.442
 
 
162.2
 
 
 
 
ARL3
 
 
hsa-miR-16-5p
 
 
-0.496
 
 
15.3
 
 
 
 
ARL3
 
 
hsa-miR-15b-5p
 
 
-0.507
 
 
15.3
 
 
 
 
ARL4D
 
 
hsa-let-7b-5p
 
 
-0.417
 
 
35.1
 
 
 
 
ARL6IP1
 
 
hsa-miR-328-3p
 
 
-0.494
 
 
140.2
 
 
 
 
ARL6IP1
 
 
hsa-miR-148b-3p
 
 
-0.579
 
 
140.2
 
 
 
 
ARL8B
 
 
hsa-miR-148b-3p
 
 
-0.337
 
 
50.2
 
 
 
 
ARL8B
 
 
hsa-miR-320b
 
 
-0.376
 
 
50.2
 
 
 
 
ARL8B
 
 
hsa-miR-320c
 
 
-0.376
 
 
50.2
 
 
 
 
ARMC8
 
 
hsa-miR-486-5p
 
 
-0.321
 
 
17.6
 
 
 
 
ARMCX2
 
 
hsa-miR-15b-5p
 
 
-0.344
 
 
26.3
 
 
 
 
ARMCX2
 
 
hsa-miR-16-5p
 
 
-0.366
 
 
26.3
 
 
 
 
ARMCX3
 
 
hsa-miR-425-5p
 
 
-0.325
 
 
34.8
 
 
 
 
ARMT1
 
 
hsa-let-7b-5p
 
 
-0.291
 
 
38.7
 
 
 
 
ARPP19
 
 
hsa-miR-320b
 
 
-0.325
 
 
52.6
 
 
 
 
ARPP19
 
 
hsa-miR-320c
 
 
-0.325
 
 
52.6
 
 
 
 
ARPP19
 
 
hsa-miR-15b-5p
 
 
-0.346
 
 
52.6
 
 
 
 
ARPP19
 
 
hsa-miR-16-5p
 
 
-0.354
 
 
52.6
 
 
 
 
ARPP19
 
 
hsa-miR-29a-3p
 
 
-0.371
 
 
52.6
 
 
 
 
ARPP19
 
 
hsa-miR-328-3p
 
 
-0.404
 
 
52.6
 
 
 
 
 
 
 lung 
 
 
 
 
target_symbol
 
 
mature_mirna_id
 
 
score
 
 
nTPM
 
 
 
 
 
 
AAR2
 
 
hsa-miR-29a-3p
 
 
-0.327
 
 
18.1
 
 
 
 
ABCD3
 
 
hsa-miR-148b-3p
 
 
-0.388
 
 
22.0
 
 
 
 
ABCE1
 
 
hsa-miR-29a-3p
 
 
-0.404
 
 
17.4
 
 
 
 
ABHD17C
 
 
hsa-let-7b-5p
 
 
-0.359
 
 
18.6
 
 
 
 
ABHD17C
 
 
hsa-miR-27a-3p
 
 
-0.435
 
 
18.6
 
 
 
 
ABHD4
 
 
hsa-miR-29a-3p
 
 
-0.312
 
 
18.5
 
 
 
 
ABHD5
 
 
hsa-miR-132-3p
 
 
-0.323
 
 
11.2
 
 
 
 
ABHD5
 
 
hsa-miR-27a-3p
 
 
-0.407
 
 
11.2
 
 
 
 
ACTA2
 
 
hsa-miR-27a-3p
 
 
-0.577
 
 
687.9
 
 
 
 
ACTR2
 
 
hsa-miR-15b-5p
 
 
-0.293
 
 
105.4
 
 
 
 
ACTR2
 
 
hsa-miR-16-5p
 
 
-0.293
 
 
105.4
 
 
 
 
ACVR1
 
 
hsa-miR-30b-5p
 
 
-0.29
 
 
12.2
 
 
 
 
ACVR1
 
 
hsa-miR-148b-3p
 
 
-0.397
 
 
12.2
 
 
 
 
ADAM10
 
 
hsa-miR-148b-3p
 
 
-0.356
 
 
19.7
 
 
 
 
ADAM17
 
 
hsa-miR-148b-3p
 
 
-0.301
 
 
12.5
 
 
 
 
ADAM9
 
 
hsa-miR-30b-5p
 
 
-0.312
 
 
42.9
 
 
 
 
ADAMTS8
 
 
hsa-let-7b-5p
 
 
-0.571
 
 
33.2
 
 
 
 
ADAMTS9
 
 
hsa-miR-29a-3p
 
 
-0.305
 
 
13.3
 
 
 
 
ADAMTS9
 
 
hsa-miR-29a-3p
 
 
-0.485
 
 
13.3
 
 
 
 
ADAP1
 
 
hsa-miR-27a-3p
 
 
-0.302
 
 
10.1
 
 
 
 
ADCY3
 
 
hsa-miR-132-3p
 
 
-0.303
 
 
39.8
 
 
 
 
ADGRF5
 
 
hsa-miR-148b-3p
 
 
-0.354
 
 
99.9
 
 
 
 
ADIPOR2
 
 
hsa-miR-150-5p
 
 
-0.287
 
 
42.5
 
 
 
 
ADNP
 
 
hsa-miR-328-3p
 
 
-0.371
 
 
25.6
 
 
 
 
ADRB1
 
 
hsa-let-7b-5p
 
 
-0.351
 
 
11.4
 
 
 
 
ADRB2
 
 
hsa-miR-15b-5p
 
 
-0.29
 
 
18.4
 
 
 
 
ADRB2
 
 
hsa-miR-16-5p
 
 
-0.323
 
 
18.4
 
 
 
 
ADRB2
 
 
hsa-let-7b-5p
 
 
-0.635
 
 
18.4
 
 
 
 
AFG3L2
 
 
hsa-miR-181b-5p
 
 
-0.491
 
 
12.4
 
 
 
 
AFG3L2
 
 
hsa-miR-181a-5p
 
 
-0.491
 
 
12.4
 
 
 
 
AGFG1
 
 
hsa-miR-148b-3p
 
 
-0.332
 
 
51.7
 
 
 
 
AGPAT3
 
 
hsa-miR-27a-3p
 
 
-0.29
 
 
18.0
 
 
 
 
AGPAT3
 
 
hsa-miR-16-5p
 
 
-0.299
 
 
18.0
 
 
 
 
AHDC1
 
 
hsa-miR-148b-3p
 
 
-0.413
 
 
11.4
 
 
 
 
AHR
 
 
hsa-miR-29a-3p
 
 
-0.316
 
 
35.5
 
 
 
 
AK1
 
 
hsa-miR-29a-3p
 
 
-0.333
 
 
70.4
 
 
 
 
AKAP1
 
 
hsa-miR-148b-3p
 
 
-0.381
 
 
18.4
 
 
 
 
AKIRIN1
 
 
hsa-miR-27a-3p
 
 
-0.368
 
 
28.3
 
 
 
 
AKT3
 
 
hsa-miR-29a-3p
 
 
-0.287
 
 
13.9
 
 
 
 
AKT3
 
 
hsa-miR-15b-5p
 
 
-0.301
 
 
13.9
 
 
 
 
AKT3
 
 
hsa-miR-16-5p
 
 
-0.309
 
 
13.9
 
 
 
 
ALAD
 
 
hsa-miR-16-5p
 
 
-0.391
 
 
19.4
 
 
 
 
ALAD
 
 
hsa-miR-15b-5p
 
 
-0.446
 
 
19.4
 
 
 
 
ALCAM
 
 
hsa-miR-148b-3p
 
 
-0.339
 
 
69.9
 
 
 
 
ALPL
 
 
hsa-miR-10a-5p
 
 
-0.32
 
 
53.5
 
 
 
 
AMOTL1
 
 
hsa-miR-16-5p
 
 
-0.297
 
 
14.9
 
 
 
 
AMOTL1
 
 
hsa-miR-15b-5p
 
 
-0.319
 
 
14.9
 
 
 
 
ANKHD1
 
 
hsa-miR-23a-3p
 
 
-0.368
 
 
38.2
 
 
 
 
ANKHD1
 
 
hsa-miR-23b-3p
 
 
-0.368
 
 
38.2
 
 
 
 
ANKHD1
 
 
hsa-miR-30b-5p
 
 
-0.405
 
 
38.2
 
 
 
 
ANKRD17
 
 
hsa-miR-30b-5p
 
 
-0.336
 
 
13.4
 
 
 
 
ANKRD29
 
 
hsa-miR-132-3p
 
 
-0.47
 
 
29.5
 
 
 
 
ANKRD40
 
 
hsa-miR-27a-3p
 
 
-0.35
 
 
21.2
 
 
 
 
ANKRD46
 
 
hsa-let-7b-5p
 
 
-0.389
 
 
10.1
 
 
 
 
ANP32A
 
 
hsa-miR-132-3p
 
 
-0.374
 
 
66.9
 
 
 
 
ANTXR2
 
 
hsa-miR-29a-3p
 
 
-0.347
 
 
23.3
 
 
 
 
ANXA11
 
 
hsa-miR-16-5p
 
 
-0.447
 
 
103.2
 
 
 
 
ANXA11
 
 
hsa-miR-15b-5p
 
 
-0.463
 
 
103.2
 
 
 
 
AP1S1
 
 
hsa-let-7b-5p
 
 
-0.374
 
 
15.2
 
 
 
 
AP2A1
 
 
hsa-miR-15b-5p
 
 
-0.333
 
 
35.7
 
 
 
 
AP2A1
 
 
hsa-miR-16-5p
 
 
-0.333
 
 
35.7
 
 
 
 
AP2B1
 
 
hsa-miR-16-5p
 
 
-0.302
 
 
49.6
 
 
 
 
AP2B1
 
 
hsa-miR-15b-5p
 
 
-0.334
 
 
49.6
 
 
 
 
AP3S1
 
 
hsa-miR-30b-5p
 
 
-0.293
 
 
40.1
 
 
 
 
APLN
 
 
hsa-miR-16-5p
 
 
-0.31
 
 
12.7
 
 
 
 
APLN
 
 
hsa-miR-15b-5p
 
 
-0.395
 
 
12.7
 
 
 
 
APLN
 
 
hsa-miR-16-5p
 
 
-0.395
 
 
12.7
 
 
 
 
APP
 
 
hsa-miR-16-5p
 
 
-0.298
 
 
255.6
 
 
 
 
APP
 
 
hsa-miR-15b-5p
 
 
-0.298
 
 
255.6
 
 
 
 
ARF4
 
 
hsa-miR-30b-5p
 
 
-0.289
 
 
116.3
 
 
 
 
ARF4
 
 
hsa-miR-148b-3p
 
 
-0.487
 
 
116.3
 
 
 
 
ARF5
 
 
hsa-miR-29a-3p
 
 
-0.491
 
 
100.1
 
 
 
 
ARF6
 
 
hsa-miR-181b-5p
 
 
-0.33
 
 
29.7
 
 
 
 
ARF6
 
 
hsa-miR-181a-5p
 
 
-0.33
 
 
29.7
 
 
 
 
ARFGEF1
 
 
hsa-miR-148b-3p
 
 
-0.306
 
 
13.0
 
 
 
 
ARFIP1
 
 
hsa-miR-320b
 
 
-0.339
 
 
15.8
 
 
 
 
ARFIP1
 
 
hsa-miR-320c
 
 
-0.339
 
 
15.8
 
 
 
 
ARFIP1
 
 
hsa-miR-148b-3p
 
 
-0.474
 
 
15.8
 
 
 
 
ARHGAP21
 
 
hsa-miR-148b-3p
 
 
-0.287
 
 
20.4
 
 
 
 
ARHGAP5
 
 
hsa-miR-486-5p
 
 
-0.3
 
 
26.5
 
 
 
 
ARID1B
 
 
hsa-miR-29a-3p
 
 
-0.342
 
 
13.4
 
 
 
 
ARID4B
 
 
hsa-miR-486-5p
 
 
-0.367
 
 
11.3
 
 
 
 
ARL2
 
 
hsa-miR-16-5p
 
 
-0.374
 
 
91.1
 
 
 
 
ARL2
 
 
hsa-miR-15b-5p
 
 
-0.374
 
 
91.1
 
 
 
 
ARL2
 
 
hsa-miR-16-5p
 
 
-0.418
 
 
91.1
 
 
 
 
ARL2
 
 
hsa-miR-15b-5p
 
 
-0.442
 
 
91.1
 
 
 
 
ARL4A
 
 
hsa-miR-29a-3p
 
 
-0.386
 
 
18.9
 
 
 
 
ARL4A
 
 
hsa-miR-30b-5p
 
 
-0.448
 
 
18.9
 
 
 
 
ARL4D
 
 
hsa-let-7b-5p
 
 
-0.417
 
 
16.0
 
 
 
 
ARL6IP1
 
 
hsa-miR-328-3p
 
 
-0.494
 
 
75.1
 
 
 
 
ARL6IP1
 
 
hsa-miR-148b-3p
 
 
-0.579
 
 
75.1
 
 
 
 
ARL6IP6
 
 
hsa-let-7b-5p
 
 
-0.293
 
 
11.9
 
 
 
 
ARL8B
 
 
hsa-miR-148b-3p
 
 
-0.337
 
 
41.1
 
 
 
 
ARL8B
 
 
hsa-miR-320b
 
 
-0.376
 
 
41.1
 
 
 
 
ARL8B
 
 
hsa-miR-320c
 
 
-0.376
 
 
41.1
 
 
 
 
ARMC8
 
 
hsa-miR-486-5p
 
 
-0.321
 
 
15.4
 
 
 
 
ARMCX2
 
 
hsa-miR-15b-5p
 
 
-0.344
 
 
14.8
 
 
 
 
ARMCX2
 
 
hsa-miR-16-5p
 
 
-0.366
 
 
14.8
 
 
 
 
ARMCX3
 
 
hsa-miR-425-5p
 
 
-0.325
 
 
26.1
 
 
 
 
ARMT1
 
 
hsa-let-7b-5p
 
 
-0.291
 
 
18.4
 
 
 
 
 
 
 lymph node 
 
 
 
 
target_symbol
 
 
mature_mirna_id
 
 
score
 
 
nTPM
 
 
 
 
 
 
AAR2
 
 
hsa-miR-29a-3p
 
 
-0.327
 
 
16.4
 
 
 
 
ABCB10
 
 
hsa-miR-148b-3p
 
 
-0.291
 
 
10.3
 
 
 
 
ABCB7
 
 
hsa-miR-148b-3p
 
 
-0.515
 
 
11.2
 
 
 
 
ABCD3
 
 
hsa-miR-148b-3p
 
 
-0.388
 
 
12.4
 
 
 
 
ABCE1
 
 
hsa-miR-29a-3p
 
 
-0.404
 
 
37.8
 
 
 
 
ABHD17B
 
 
hsa-miR-486-5p
 
 
-0.348
 
 
10.2
 
 
 
 
ACTA2
 
 
hsa-miR-27a-3p
 
 
-0.577
 
 
162.6
 
 
 
 
ACTR2
 
 
hsa-miR-15b-5p
 
 
-0.293
 
 
229.6
 
 
 
 
ACTR2
 
 
hsa-miR-16-5p
 
 
-0.293
 
 
229.6
 
 
 
 
ADA
 
 
hsa-miR-29a-3p
 
 
-0.376
 
 
27.8
 
 
 
 
ADAM10
 
 
hsa-miR-148b-3p
 
 
-0.356
 
 
20.8
 
 
 
 
ADCY3
 
 
hsa-miR-132-3p
 
 
-0.303
 
 
17.7
 
 
 
 
ADNP
 
 
hsa-miR-328-3p
 
 
-0.371
 
 
27.0
 
 
 
 
ADRA2A
 
 
hsa-miR-23a-3p
 
 
-0.371
 
 
10.5
 
 
 
 
ADRA2A
 
 
hsa-miR-23b-3p
 
 
-0.371
 
 
10.5
 
 
 
 
AFG3L2
 
 
hsa-miR-181b-5p
 
 
-0.491
 
 
12.0
 
 
 
 
AFG3L2
 
 
hsa-miR-181a-5p
 
 
-0.491
 
 
12.0
 
 
 
 
AGFG1
 
 
hsa-miR-148b-3p
 
 
-0.332
 
 
44.0
 
 
 
 
AGPAT3
 
 
hsa-miR-27a-3p
 
 
-0.29
 
 
15.3
 
 
 
 
AGPAT3
 
 
hsa-miR-16-5p
 
 
-0.299
 
 
15.3
 
 
 
 
AHR
 
 
hsa-miR-29a-3p
 
 
-0.316
 
 
14.4
 
 
 
 
AIFM1
 
 
hsa-let-7b-5p
 
 
-0.314
 
 
15.1
 
 
 
 
AKAP1
 
 
hsa-miR-148b-3p
 
 
-0.381
 
 
10.0
 
 
 
 
AKIRIN1
 
 
hsa-miR-27a-3p
 
 
-0.368
 
 
39.5
 
 
 
 
ALAD
 
 
hsa-miR-16-5p
 
 
-0.391
 
 
14.6
 
 
 
 
ALAD
 
 
hsa-miR-15b-5p
 
 
-0.446
 
 
14.6
 
 
 
 
ANKHD1
 
 
hsa-miR-23a-3p
 
 
-0.368
 
 
38.6
 
 
 
 
ANKHD1
 
 
hsa-miR-23b-3p
 
 
-0.368
 
 
38.6
 
 
 
 
ANKHD1
 
 
hsa-miR-30b-5p
 
 
-0.405
 
 
38.6
 
 
 
 
ANKRD17
 
 
hsa-miR-30b-5p
 
 
-0.336
 
 
14.1
 
 
 
 
ANKRD29
 
 
hsa-miR-132-3p
 
 
-0.47
 
 
18.8
 
 
 
 
ANKRD40
 
 
hsa-miR-27a-3p
 
 
-0.35
 
 
15.5
 
 
 
 
ANP32A
 
 
hsa-miR-132-3p
 
 
-0.374
 
 
145.8
 
 
 
 
ANTXR2
 
 
hsa-miR-29a-3p
 
 
-0.347
 
 
15.2
 
 
 
 
ANXA11
 
 
hsa-miR-16-5p
 
 
-0.447
 
 
76.0
 
 
 
 
ANXA11
 
 
hsa-miR-15b-5p
 
 
-0.463
 
 
76.0
 
 
 
 
AP1S1
 
 
hsa-let-7b-5p
 
 
-0.374
 
 
11.1
 
 
 
 
AP2A1
 
 
hsa-miR-15b-5p
 
 
-0.333
 
 
18.3
 
 
 
 
AP2A1
 
 
hsa-miR-16-5p
 
 
-0.333
 
 
18.3
 
 
 
 
AP2B1
 
 
hsa-miR-16-5p
 
 
-0.302
 
 
36.1
 
 
 
 
AP2B1
 
 
hsa-miR-15b-5p
 
 
-0.334
 
 
36.1
 
 
 
 
AP3S1
 
 
hsa-miR-30b-5p
 
 
-0.293
 
 
60.4
 
 
 
 
APOO
 
 
hsa-miR-181b-5p
 
 
-0.5
 
 
10.9
 
 
 
 
APOO
 
 
hsa-miR-181a-5p
 
 
-0.5
 
 
10.9
 
 
 
 
APP
 
 
hsa-miR-16-5p
 
 
-0.298
 
 
68.3
 
 
 
 
APP
 
 
hsa-miR-15b-5p
 
 
-0.298
 
 
68.3
 
 
 
 
ARF4
 
 
hsa-miR-30b-5p
 
 
-0.289
 
 
69.1
 
 
 
 
ARF4
 
 
hsa-miR-148b-3p
 
 
-0.487
 
 
69.1
 
 
 
 
ARF5
 
 
hsa-miR-29a-3p
 
 
-0.491
 
 
96.1
 
 
 
 
ARF6
 
 
hsa-miR-181b-5p
 
 
-0.33
 
 
57.9
 
 
 
 
ARF6
 
 
hsa-miR-181a-5p
 
 
-0.33
 
 
57.9
 
 
 
 
ARFGEF1
 
 
hsa-miR-148b-3p
 
 
-0.306
 
 
14.4
 
 
 
 
ARFIP1
 
 
hsa-miR-320b
 
 
-0.339
 
 
14.1
 
 
 
 
ARFIP1
 
 
hsa-miR-320c
 
 
-0.339
 
 
14.1
 
 
 
 
ARFIP1
 
 
hsa-miR-148b-3p
 
 
-0.474
 
 
14.1
 
 
 
 
ARHGAP5
 
 
hsa-miR-486-5p
 
 
-0.3
 
 
13.7
 
 
 
 
ARID1B
 
 
hsa-miR-29a-3p
 
 
-0.342
 
 
16.8
 
 
 
 
ARID4B
 
 
hsa-miR-486-5p
 
 
-0.367
 
 
19.6
 
 
 
 
ARL2
 
 
hsa-miR-16-5p
 
 
-0.374
 
 
35.3
 
 
 
 
ARL2
 
 
hsa-miR-15b-5p
 
 
-0.374
 
 
35.3
 
 
 
 
ARL2
 
 
hsa-miR-16-5p
 
 
-0.418
 
 
35.3
 
 
 
 
ARL2
 
 
hsa-miR-15b-5p
 
 
-0.442
 
 
35.3
 
 
 
 
ARL4A
 
 
hsa-miR-29a-3p
 
 
-0.386
 
 
17.4
 
 
 
 
ARL4A
 
 
hsa-miR-30b-5p
 
 
-0.448
 
 
17.4
 
 
 
 
ARL6IP1
 
 
hsa-miR-328-3p
 
 
-0.494
 
 
124.0
 
 
 
 
ARL6IP1
 
 
hsa-miR-148b-3p
 
 
-0.579
 
 
124.0
 
 
 
 
ARL6IP6
 
 
hsa-let-7b-5p
 
 
-0.293
 
 
17.4
 
 
 
 
ARL8B
 
 
hsa-miR-148b-3p
 
 
-0.337
 
 
36.2
 
 
 
 
ARL8B
 
 
hsa-miR-320b
 
 
-0.376
 
 
36.2
 
 
 
 
ARL8B
 
 
hsa-miR-320c
 
 
-0.376
 
 
36.2
 
 
 
 
ARMC8
 
 
hsa-miR-486-5p
 
 
-0.321
 
 
19.8
 
 
 
 
ARMCX3
 
 
hsa-miR-425-5p
 
 
-0.325
 
 
20.4
 
 
 
 
ARMT1
 
 
hsa-let-7b-5p
 
 
-0.291
 
 
19.7
 
 
 
 
ARPP19
 
 
hsa-miR-320b
 
 
-0.325
 
 
45.1
 
 
 
 
ARPP19
 
 
hsa-miR-320c
 
 
-0.325
 
 
45.1
 
 
 
 
ARPP19
 
 
hsa-miR-15b-5p
 
 
-0.346
 
 
45.1
 
 
 
 
ARPP19
 
 
hsa-miR-16-5p
 
 
-0.354
 
 
45.1
 
 
 
 
ARPP19
 
 
hsa-miR-29a-3p
 
 
-0.371
 
 
45.1
 
 
 
 
ARPP19
 
 
hsa-miR-328-3p
 
 
-0.404
 
 
45.1
 
 
 
 
ARPP19
 
 
hsa-let-7b-5p
 
 
-0.476
 
 
45.1
 
 
 
 
ARRDC3
 
 
hsa-miR-148b-3p
 
 
-0.305
 
 
27.5
 
 
 
 
ARRDC3
 
 
hsa-miR-29a-3p
 
 
-0.402
 
 
27.5
 
 
 
 
ASAH1
 
 
hsa-miR-27a-3p
 
 
-0.398
 
 
61.7
 
 
 
 
ASAP1
 
 
hsa-let-7b-5p
 
 
-0.308
 
 
16.5
 
 
 
 
ASB3
 
 
hsa-miR-30b-5p
 
 
-0.418
 
 
10.2
 
 
 
 
ASB3
 
 
hsa-miR-30b-5p
 
 
-0.418
 
 
10.2
 
 
 
 
ASF1A
 
 
hsa-miR-23a-3p
 
 
-0.506
 
 
14.9
 
 
 
 
ASF1A
 
 
hsa-miR-23b-3p
 
 
-0.506
 
 
14.9
 
 
 
 
ASH1L
 
 
hsa-miR-15b-5p
 
 
-0.325
 
 
10.2
 
 
 
 
ASH1L
 
 
hsa-miR-16-5p
 
 
-0.357
 
 
10.2
 
 
 
 
ASH2L
 
 
hsa-miR-320b
 
 
-0.287
 
 
26.6
 
 
 
 
ASH2L
 
 
hsa-miR-320c
 
 
-0.287
 
 
26.6
 
 
 
 
ASPH
 
 
hsa-miR-148b-3p
 
 
-0.391
 
 
18.8
 
 
 
 
ATG9A
 
 
hsa-miR-16-5p
 
 
-0.311
 
 
18.2
 
 
 
 
ATG9A
 
 
hsa-miR-15b-5p
 
 
-0.333
 
 
18.2
 
 
 
 
ATP2A2
 
 
hsa-let-7b-5p
 
 
-0.311
 
 
33.7
 
 
 
 
ATP2B4
 
 
hsa-miR-148b-3p
 
 
-0.337
 
 
12.0
 
 
 
 
ATP5MC3
 
 
hsa-miR-425-5p
 
 
-0.328
 
 
118.6
 
 
 
 
ATP5MC3
 
 
hsa-miR-29a-3p
 
 
-0.33
 
 
118.6
 
 
 
 
ATP5MC3
 
 
hsa-miR-27a-3p
 
 
-0.416
 
 
118.6
 
 
 
 
 
 
 midbrain 
 
 
 
 
target_symbol
 
 
mature_mirna_id
 
 
score
 
 
nTPM
 
 
 
 
 
 
AAR2
 
 
hsa-miR-29a-3p
 
 
-0.327
 
 
11.2
 
 
 
 
ABCB9
 
 
hsa-let-7b-5p
 
 
-0.422
 
 
10.6
 
 
 
 
ABCD3
 
 
hsa-miR-148b-3p
 
 
-0.388
 
 
17.1
 
 
 
 
ABCE1
 
 
hsa-miR-29a-3p
 
 
-0.404
 
 
13.7
 
 
 
 
ABHD17B
 
 
hsa-miR-486-5p
 
 
-0.348
 
 
16.6
 
 
 
 
ABHD17C
 
 
hsa-let-7b-5p
 
 
-0.359
 
 
11.2
 
 
 
 
ABHD17C
 
 
hsa-miR-27a-3p
 
 
-0.435
 
 
11.2
 
 
 
 
ABHD4
 
 
hsa-miR-29a-3p
 
 
-0.312
 
 
18.3
 
 
 
 
ACSL6
 
 
hsa-let-7b-5p
 
 
-0.336
 
 
11.1
 
 
 
 
ACTA2
 
 
hsa-miR-27a-3p
 
 
-0.577
 
 
69.3
 
 
 
 
ACTN2
 
 
hsa-miR-23a-3p
 
 
-0.294
 
 
12.2
 
 
 
 
ACTN2
 
 
hsa-miR-29a-3p
 
 
-0.311
 
 
12.2
 
 
 
 
ACTR2
 
 
hsa-miR-15b-5p
 
 
-0.293
 
 
53.1
 
 
 
 
ACTR2
 
 
hsa-miR-16-5p
 
 
-0.293
 
 
53.1
 
 
 
 
ADAM10
 
 
hsa-miR-148b-3p
 
 
-0.356
 
 
11.1
 
 
 
 
ADAP1
 
 
hsa-miR-27a-3p
 
 
-0.302
 
 
85.8
 
 
 
 
ADCY2
 
 
hsa-miR-148b-3p
 
 
-0.299
 
 
20.4
 
 
 
 
ADCY3
 
 
hsa-miR-132-3p
 
 
-0.303
 
 
29.4
 
 
 
 
ADGRA3
 
 
hsa-miR-27a-3p
 
 
-0.296
 
 
13.9
 
 
 
 
ADGRA3
 
 
hsa-miR-30b-5p
 
 
-0.304
 
 
13.9
 
 
 
 
ADGRF5
 
 
hsa-miR-148b-3p
 
 
-0.354
 
 
13.0
 
 
 
 
ADIPOR2
 
 
hsa-miR-150-5p
 
 
-0.287
 
 
76.2
 
 
 
 
ADNP
 
 
hsa-miR-328-3p
 
 
-0.371
 
 
21.5
 
 
 
 
AGFG1
 
 
hsa-miR-148b-3p
 
 
-0.332
 
 
32.4
 
 
 
 
AGPAT3
 
 
hsa-miR-27a-3p
 
 
-0.29
 
 
44.6
 
 
 
 
AGPAT3
 
 
hsa-miR-16-5p
 
 
-0.299
 
 
44.6
 
 
 
 
AGPAT4
 
 
hsa-miR-29a-3p
 
 
-0.376
 
 
22.2
 
 
 
 
AIFM1
 
 
hsa-let-7b-5p
 
 
-0.314
 
 
12.7
 
 
 
 
AK1
 
 
hsa-miR-29a-3p
 
 
-0.333
 
 
97.2
 
 
 
 
AK4
 
 
hsa-miR-15b-5p
 
 
-0.316
 
 
11.3
 
 
 
 
AK4
 
 
hsa-miR-16-5p
 
 
-0.348
 
 
11.3
 
 
 
 
AKAP1
 
 
hsa-miR-148b-3p
 
 
-0.381
 
 
19.0
 
 
 
 
AKAP6
 
 
hsa-let-7b-5p
 
 
-0.307
 
 
17.4
 
 
 
 
AKIRIN1
 
 
hsa-miR-27a-3p
 
 
-0.368
 
 
38.9
 
 
 
 
AKT3
 
 
hsa-miR-29a-3p
 
 
-0.287
 
 
16.3
 
 
 
 
AKT3
 
 
hsa-miR-15b-5p
 
 
-0.301
 
 
16.3
 
 
 
 
AKT3
 
 
hsa-miR-16-5p
 
 
-0.309
 
 
16.3
 
 
 
 
ALAD
 
 
hsa-miR-16-5p
 
 
-0.391
 
 
105.2
 
 
 
 
ALAD
 
 
hsa-miR-15b-5p
 
 
-0.446
 
 
105.2
 
 
 
 
ALCAM
 
 
hsa-miR-148b-3p
 
 
-0.339
 
 
38.7
 
 
 
 
ALPL
 
 
hsa-miR-10a-5p
 
 
-0.32
 
 
11.9
 
 
 
 
AMOT
 
 
hsa-miR-29a-3p
 
 
-0.304
 
 
16.8
 
 
 
 
ANKHD1
 
 
hsa-miR-23a-3p
 
 
-0.368
 
 
20.5
 
 
 
 
ANKHD1
 
 
hsa-miR-23b-3p
 
 
-0.368
 
 
20.5
 
 
 
 
ANKHD1
 
 
hsa-miR-30b-5p
 
 
-0.405
 
 
20.5
 
 
 
 
ANKRD29
 
 
hsa-miR-132-3p
 
 
-0.47
 
 
11.5
 
 
 
 
ANKRD40
 
 
hsa-miR-27a-3p
 
 
-0.35
 
 
160.4
 
 
 
 
ANKRD46
 
 
hsa-let-7b-5p
 
 
-0.389
 
 
24.0
 
 
 
 
ANLN
 
 
hsa-miR-16-5p
 
 
-0.31
 
 
72.6
 
 
 
 
ANLN
 
 
hsa-miR-15b-5p
 
 
-0.334
 
 
72.6
 
 
 
 
ANP32A
 
 
hsa-miR-132-3p
 
 
-0.374
 
 
54.3
 
 
 
 
ANXA11
 
 
hsa-miR-16-5p
 
 
-0.447
 
 
20.2
 
 
 
 
ANXA11
 
 
hsa-miR-15b-5p
 
 
-0.463
 
 
20.2
 
 
 
 
AP1S1
 
 
hsa-let-7b-5p
 
 
-0.374
 
 
35.4
 
 
 
 
AP2A1
 
 
hsa-miR-15b-5p
 
 
-0.333
 
 
39.4
 
 
 
 
AP2A1
 
 
hsa-miR-16-5p
 
 
-0.333
 
 
39.4
 
 
 
 
AP2B1
 
 
hsa-miR-16-5p
 
 
-0.302
 
 
69.9
 
 
 
 
AP2B1
 
 
hsa-miR-15b-5p
 
 
-0.334
 
 
69.9
 
 
 
 
AP3S1
 
 
hsa-miR-30b-5p
 
 
-0.293
 
 
69.9
 
 
 
 
APC
 
 
hsa-miR-29a-3p
 
 
-0.413
 
 
21.2
 
 
 
 
APLN
 
 
hsa-miR-16-5p
 
 
-0.31
 
 
24.6
 
 
 
 
APLN
 
 
hsa-miR-15b-5p
 
 
-0.395
 
 
24.6
 
 
 
 
APLN
 
 
hsa-miR-16-5p
 
 
-0.395
 
 
24.6
 
 
 
 
APOO
 
 
hsa-miR-181b-5p
 
 
-0.5
 
 
44.2
 
 
 
 
APOO
 
 
hsa-miR-181a-5p
 
 
-0.5
 
 
44.2
 
 
 
 
APP
 
 
hsa-miR-16-5p
 
 
-0.298
 
 
448.8
 
 
 
 
APP
 
 
hsa-miR-15b-5p
 
 
-0.298
 
 
448.8
 
 
 
 
ARF4
 
 
hsa-miR-30b-5p
 
 
-0.289
 
 
62.8
 
 
 
 
ARF4
 
 
hsa-miR-148b-3p
 
 
-0.487
 
 
62.8
 
 
 
 
ARF5
 
 
hsa-miR-29a-3p
 
 
-0.491
 
 
120.0
 
 
 
 
ARF6
 
 
hsa-miR-181b-5p
 
 
-0.33
 
 
12.0
 
 
 
 
ARF6
 
 
hsa-miR-181a-5p
 
 
-0.33
 
 
12.0
 
 
 
 
ARHGAP21
 
 
hsa-miR-148b-3p
 
 
-0.287
 
 
53.0
 
 
 
 
ARHGAP5
 
 
hsa-miR-486-5p
 
 
-0.3
 
 
21.3
 
 
 
 
ARL2
 
 
hsa-miR-16-5p
 
 
-0.374
 
 
173.0
 
 
 
 
ARL2
 
 
hsa-miR-15b-5p
 
 
-0.374
 
 
173.0
 
 
 
 
ARL2
 
 
hsa-miR-16-5p
 
 
-0.418
 
 
173.0
 
 
 
 
ARL2
 
 
hsa-miR-15b-5p
 
 
-0.442
 
 
173.0
 
 
 
 
ARL3
 
 
hsa-miR-16-5p
 
 
-0.496
 
 
22.1
 
 
 
 
ARL3
 
 
hsa-miR-15b-5p
 
 
-0.507
 
 
22.1
 
 
 
 
ARL6IP1
 
 
hsa-miR-328-3p
 
 
-0.494
 
 
198.8
 
 
 
 
ARL6IP1
 
 
hsa-miR-148b-3p
 
 
-0.579
 
 
198.8
 
 
 
 
ARL8B
 
 
hsa-miR-148b-3p
 
 
-0.337
 
 
52.4
 
 
 
 
ARL8B
 
 
hsa-miR-320b
 
 
-0.376
 
 
52.4
 
 
 
 
ARL8B
 
 
hsa-miR-320c
 
 
-0.376
 
 
52.4
 
 
 
 
ARMC8
 
 
hsa-miR-486-5p
 
 
-0.321
 
 
14.1
 
 
 
 
ARMCX2
 
 
hsa-miR-15b-5p
 
 
-0.344
 
 
18.6
 
 
 
 
ARMCX2
 
 
hsa-miR-16-5p
 
 
-0.366
 
 
18.6
 
 
 
 
ARMCX3
 
 
hsa-miR-425-5p
 
 
-0.325
 
 
35.1
 
 
 
 
ARPP19
 
 
hsa-miR-320b
 
 
-0.325
 
 
144.2
 
 
 
 
ARPP19
 
 
hsa-miR-320c
 
 
-0.325
 
 
144.2
 
 
 
 
ARPP19
 
 
hsa-miR-15b-5p
 
 
-0.346
 
 
144.2
 
 
 
 
ARPP19
 
 
hsa-miR-16-5p
 
 
-0.354
 
 
144.2
 
 
 
 
ARPP19
 
 
hsa-miR-29a-3p
 
 
-0.371
 
 
144.2
 
 
 
 
ARPP19
 
 
hsa-miR-328-3p
 
 
-0.404
 
 
144.2
 
 
 
 
ARPP19
 
 
hsa-let-7b-5p
 
 
-0.476
 
 
144.2
 
 
 
 
ARPP21
 
 
hsa-miR-16-5p
 
 
-0.309
 
 
30.5
 
 
 
 
ARPP21
 
 
hsa-miR-15b-5p
 
 
-0.342
 
 
30.5
 
 
 
 
ARRDC3
 
 
hsa-miR-148b-3p
 
 
-0.305
 
 
13.8
 
 
 
 
ARRDC3
 
 
hsa-miR-29a-3p
 
 
-0.402
 
 
13.8
 
 
 
 
 
 
 ovary 
 
 
 
 
target_symbol
 
 
mature_mirna_id
 
 
score
 
 
nTPM
 
 
 
 
 
 
AAR2
 
 
hsa-miR-29a-3p
 
 
-0.327
 
 
22.7
 
 
 
 
ABCB6
 
 
hsa-miR-29a-3p
 
 
-0.48
 
 
12.1
 
 
 
 
ABCB7
 
 
hsa-miR-148b-3p
 
 
-0.515
 
 
11.9
 
 
 
 
ABCD3
 
 
hsa-miR-148b-3p
 
 
-0.388
 
 
11.0
 
 
 
 
ABCE1
 
 
hsa-miR-29a-3p
 
 
-0.404
 
 
25.9
 
 
 
 
ABHD4
 
 
hsa-miR-29a-3p
 
 
-0.312
 
 
17.9
 
 
 
 
ACTA2
 
 
hsa-miR-27a-3p
 
 
-0.577
 
 
689.2
 
 
 
 
ACTR2
 
 
hsa-miR-15b-5p
 
 
-0.293
 
 
47.8
 
 
 
 
ACTR2
 
 
hsa-miR-16-5p
 
 
-0.293
 
 
47.8
 
 
 
 
ACVR1
 
 
hsa-miR-30b-5p
 
 
-0.29
 
 
17.7
 
 
 
 
ACVR1
 
 
hsa-miR-148b-3p
 
 
-0.397
 
 
17.7
 
 
 
 
ADAM10
 
 
hsa-miR-148b-3p
 
 
-0.356
 
 
13.1
 
 
 
 
ADAM9
 
 
hsa-miR-30b-5p
 
 
-0.312
 
 
16.9
 
 
 
 
ADCY3
 
 
hsa-miR-132-3p
 
 
-0.303
 
 
67.7
 
 
 
 
ADIPOR2
 
 
hsa-miR-150-5p
 
 
-0.287
 
 
24.2
 
 
 
 
ADNP
 
 
hsa-miR-328-3p
 
 
-0.371
 
 
36.2
 
 
 
 
ADRA2A
 
 
hsa-miR-23a-3p
 
 
-0.371
 
 
17.5
 
 
 
 
ADRA2A
 
 
hsa-miR-23b-3p
 
 
-0.371
 
 
17.5
 
 
 
 
ADRB3
 
 
hsa-let-7b-5p
 
 
-0.415
 
 
12.8
 
 
 
 
AFG3L2
 
 
hsa-miR-181b-5p
 
 
-0.491
 
 
12.5
 
 
 
 
AFG3L2
 
 
hsa-miR-181a-5p
 
 
-0.491
 
 
12.5
 
 
 
 
AGFG1
 
 
hsa-miR-148b-3p
 
 
-0.332
 
 
22.9
 
 
 
 
AHCTF1
 
 
hsa-let-7b-5p
 
 
-0.374
 
 
13.1
 
 
 
 
AHDC1
 
 
hsa-miR-148b-3p
 
 
-0.413
 
 
21.5
 
 
 
 
AK1
 
 
hsa-miR-29a-3p
 
 
-0.333
 
 
43.4
 
 
 
 
AKAP1
 
 
hsa-miR-148b-3p
 
 
-0.381
 
 
18.8
 
 
 
 
AKIRIN1
 
 
hsa-miR-27a-3p
 
 
-0.368
 
 
18.3
 
 
 
 
AKT3
 
 
hsa-miR-29a-3p
 
 
-0.287
 
 
21.9
 
 
 
 
AKT3
 
 
hsa-miR-15b-5p
 
 
-0.301
 
 
21.9
 
 
 
 
AKT3
 
 
hsa-miR-16-5p
 
 
-0.309
 
 
21.9
 
 
 
 
ALAD
 
 
hsa-miR-16-5p
 
 
-0.391
 
 
34.6
 
 
 
 
ALAD
 
 
hsa-miR-15b-5p
 
 
-0.446
 
 
34.6
 
 
 
 
ALCAM
 
 
hsa-miR-148b-3p
 
 
-0.339
 
 
33.1
 
 
 
 
ALDH1A2
 
 
hsa-miR-23b-3p
 
 
-0.346
 
 
13.5
 
 
 
 
ALDH1A2
 
 
hsa-miR-23a-3p
 
 
-0.354
 
 
13.5
 
 
 
 
AMT
 
 
hsa-let-7b-5p
 
 
-0.463
 
 
16.7
 
 
 
 
ANKHD1
 
 
hsa-miR-23a-3p
 
 
-0.368
 
 
49.9
 
 
 
 
ANKHD1
 
 
hsa-miR-23b-3p
 
 
-0.368
 
 
49.9
 
 
 
 
ANKHD1
 
 
hsa-miR-30b-5p
 
 
-0.405
 
 
49.9
 
 
 
 
ANKRA2
 
 
hsa-miR-30b-5p
 
 
-0.354
 
 
11.1
 
 
 
 
ANKRA2
 
 
hsa-miR-30b-5p
 
 
-0.472
 
 
11.1
 
 
 
 
ANKRD17
 
 
hsa-miR-30b-5p
 
 
-0.336
 
 
15.3
 
 
 
 
ANKRD29
 
 
hsa-miR-132-3p
 
 
-0.47
 
 
15.0
 
 
 
 
ANKRD40
 
 
hsa-miR-27a-3p
 
 
-0.35
 
 
18.3
 
 
 
 
ANKRD46
 
 
hsa-let-7b-5p
 
 
-0.389
 
 
19.3
 
 
 
 
ANP32A
 
 
hsa-miR-132-3p
 
 
-0.374
 
 
51.4
 
 
 
 
ANTXR2
 
 
hsa-miR-29a-3p
 
 
-0.347
 
 
66.3
 
 
 
 
ANXA11
 
 
hsa-miR-16-5p
 
 
-0.447
 
 
68.2
 
 
 
 
ANXA11
 
 
hsa-miR-15b-5p
 
 
-0.463
 
 
68.2
 
 
 
 
AP1S1
 
 
hsa-let-7b-5p
 
 
-0.374
 
 
22.2
 
 
 
 
AP2A1
 
 
hsa-miR-15b-5p
 
 
-0.333
 
 
54.6
 
 
 
 
AP2A1
 
 
hsa-miR-16-5p
 
 
-0.333
 
 
54.6
 
 
 
 
AP2B1
 
 
hsa-miR-16-5p
 
 
-0.302
 
 
50.8
 
 
 
 
AP2B1
 
 
hsa-miR-15b-5p
 
 
-0.334
 
 
50.8
 
 
 
 
AP3S1
 
 
hsa-miR-30b-5p
 
 
-0.293
 
 
82.7
 
 
 
 
APOO
 
 
hsa-miR-181b-5p
 
 
-0.5
 
 
11.0
 
 
 
 
APOO
 
 
hsa-miR-181a-5p
 
 
-0.5
 
 
11.0
 
 
 
 
APP
 
 
hsa-miR-16-5p
 
 
-0.298
 
 
401.1
 
 
 
 
APP
 
 
hsa-miR-15b-5p
 
 
-0.298
 
 
401.1
 
 
 
 
APPBP2
 
 
hsa-miR-27a-3p
 
 
-0.378
 
 
17.5
 
 
 
 
ARF4
 
 
hsa-miR-30b-5p
 
 
-0.289
 
 
123.7
 
 
 
 
ARF4
 
 
hsa-miR-148b-3p
 
 
-0.487
 
 
123.7
 
 
 
 
ARF5
 
 
hsa-miR-29a-3p
 
 
-0.491
 
 
81.8
 
 
 
 
ARF6
 
 
hsa-miR-181b-5p
 
 
-0.33
 
 
16.9
 
 
 
 
ARF6
 
 
hsa-miR-181a-5p
 
 
-0.33
 
 
16.9
 
 
 
 
ARFIP1
 
 
hsa-miR-320b
 
 
-0.339
 
 
17.2
 
 
 
 
ARFIP1
 
 
hsa-miR-320c
 
 
-0.339
 
 
17.2
 
 
 
 
ARFIP1
 
 
hsa-miR-148b-3p
 
 
-0.474
 
 
17.2
 
 
 
 
ARG2
 
 
hsa-let-7b-5p
 
 
-0.468
 
 
18.7
 
 
 
 
ARHGAP21
 
 
hsa-miR-148b-3p
 
 
-0.287
 
 
26.5
 
 
 
 
ARHGAP5
 
 
hsa-miR-486-5p
 
 
-0.3
 
 
33.0
 
 
 
 
ARID1B
 
 
hsa-miR-29a-3p
 
 
-0.342
 
 
20.5
 
 
 
 
ARID4B
 
 
hsa-miR-486-5p
 
 
-0.367
 
 
14.0
 
 
 
 
ARL2
 
 
hsa-miR-16-5p
 
 
-0.374
 
 
95.1
 
 
 
 
ARL2
 
 
hsa-miR-15b-5p
 
 
-0.374
 
 
95.1
 
 
 
 
ARL2
 
 
hsa-miR-16-5p
 
 
-0.418
 
 
95.1
 
 
 
 
ARL2
 
 
hsa-miR-15b-5p
 
 
-0.442
 
 
95.1
 
 
 
 
ARL3
 
 
hsa-miR-16-5p
 
 
-0.496
 
 
16.3
 
 
 
 
ARL3
 
 
hsa-miR-15b-5p
 
 
-0.507
 
 
16.3
 
 
 
 
ARL4A
 
 
hsa-miR-29a-3p
 
 
-0.386
 
 
14.3
 
 
 
 
ARL4A
 
 
hsa-miR-30b-5p
 
 
-0.448
 
 
14.3
 
 
 
 
ARL4D
 
 
hsa-let-7b-5p
 
 
-0.417
 
 
20.3
 
 
 
 
ARL6IP1
 
 
hsa-miR-328-3p
 
 
-0.494
 
 
97.4
 
 
 
 
ARL6IP1
 
 
hsa-miR-148b-3p
 
 
-0.579
 
 
97.4
 
 
 
 
ARL6IP6
 
 
hsa-let-7b-5p
 
 
-0.293
 
 
11.9
 
 
 
 
ARL8B
 
 
hsa-miR-148b-3p
 
 
-0.337
 
 
35.5
 
 
 
 
ARL8B
 
 
hsa-miR-320b
 
 
-0.376
 
 
35.5
 
 
 
 
ARL8B
 
 
hsa-miR-320c
 
 
-0.376
 
 
35.5
 
 
 
 
ARMC8
 
 
hsa-miR-486-5p
 
 
-0.321
 
 
14.0
 
 
 
 
ARMCX2
 
 
hsa-miR-15b-5p
 
 
-0.344
 
 
40.5
 
 
 
 
ARMCX2
 
 
hsa-miR-16-5p
 
 
-0.366
 
 
40.5
 
 
 
 
ARMCX3
 
 
hsa-miR-425-5p
 
 
-0.325
 
 
36.1
 
 
 
 
ARMT1
 
 
hsa-let-7b-5p
 
 
-0.291
 
 
15.5
 
 
 
 
ARPP19
 
 
hsa-miR-320b
 
 
-0.325
 
 
40.6
 
 
 
 
ARPP19
 
 
hsa-miR-320c
 
 
-0.325
 
 
40.6
 
 
 
 
ARPP19
 
 
hsa-miR-15b-5p
 
 
-0.346
 
 
40.6
 
 
 
 
ARPP19
 
 
hsa-miR-16-5p
 
 
-0.354
 
 
40.6
 
 
 
 
ARPP19
 
 
hsa-miR-29a-3p
 
 
-0.371
 
 
40.6
 
 
 
 
ARPP19
 
 
hsa-miR-328-3p
 
 
-0.404
 
 
40.6
 
 
 
 
ARPP19
 
 
hsa-let-7b-5p
 
 
-0.476
 
 
40.6
 
 
 
 
 
 
 pancreas 
 
 
 
 
target_symbol
 
 
mature_mirna_id
 
 
score
 
 
nTPM
 
 
 
 
 
 
AAR2
 
 
hsa-miR-29a-3p
 
 
-0.327
 
 
17.6
 
 
 
 
ABCD3
 
 
hsa-miR-148b-3p
 
 
-0.388
 
 
27.0
 
 
 
 
ABCE1
 
 
hsa-miR-29a-3p
 
 
-0.404
 
 
27.7
 
 
 
 
ABHD4
 
 
hsa-miR-29a-3p
 
 
-0.312
 
 
15.3
 
 
 
 
ABHD5
 
 
hsa-miR-132-3p
 
 
-0.323
 
 
11.4
 
 
 
 
ABHD5
 
 
hsa-miR-27a-3p
 
 
-0.407
 
 
11.4
 
 
 
 
ACTA2
 
 
hsa-miR-27a-3p
 
 
-0.577
 
 
86.3
 
 
 
 
ACTC1
 
 
hsa-miR-30b-5p
 
 
-0.543
 
 
12.1
 
 
 
 
ACTR2
 
 
hsa-miR-15b-5p
 
 
-0.293
 
 
57.4
 
 
 
 
ACTR2
 
 
hsa-miR-16-5p
 
 
-0.293
 
 
57.4
 
 
 
 
ACTR3C
 
 
hsa-miR-30b-5p
 
 
-0.448
 
 
12.4
 
 
 
 
ADAP1
 
 
hsa-miR-27a-3p
 
 
-0.302
 
 
12.7
 
 
 
 
ADCY3
 
 
hsa-miR-132-3p
 
 
-0.303
 
 
15.3
 
 
 
 
ADGRA3
 
 
hsa-miR-27a-3p
 
 
-0.296
 
 
11.8
 
 
 
 
ADGRA3
 
 
hsa-miR-30b-5p
 
 
-0.304
 
 
11.8
 
 
 
 
ADIPOR2
 
 
hsa-miR-150-5p
 
 
-0.287
 
 
30.0
 
 
 
 
ADNP
 
 
hsa-miR-328-3p
 
 
-0.371
 
 
23.5
 
 
 
 
ADRA2A
 
 
hsa-miR-23a-3p
 
 
-0.371
 
 
28.8
 
 
 
 
ADRA2A
 
 
hsa-miR-23b-3p
 
 
-0.371
 
 
28.8
 
 
 
 
AFG3L2
 
 
hsa-miR-181b-5p
 
 
-0.491
 
 
23.6
 
 
 
 
AFG3L2
 
 
hsa-miR-181a-5p
 
 
-0.491
 
 
23.6
 
 
 
 
AGFG1
 
 
hsa-miR-148b-3p
 
 
-0.332
 
 
33.0
 
 
 
 
AGPAT3
 
 
hsa-miR-27a-3p
 
 
-0.29
 
 
20.6
 
 
 
 
AGPAT3
 
 
hsa-miR-16-5p
 
 
-0.299
 
 
20.6
 
 
 
 
AIFM1
 
 
hsa-let-7b-5p
 
 
-0.314
 
 
20.4
 
 
 
 
AK1
 
 
hsa-miR-29a-3p
 
 
-0.333
 
 
14.0
 
 
 
 
AKAP1
 
 
hsa-miR-148b-3p
 
 
-0.381
 
 
21.5
 
 
 
 
AKIRIN1
 
 
hsa-miR-27a-3p
 
 
-0.368
 
 
26.1
 
 
 
 
ALAD
 
 
hsa-miR-16-5p
 
 
-0.391
 
 
45.8
 
 
 
 
ALAD
 
 
hsa-miR-15b-5p
 
 
-0.446
 
 
45.8
 
 
 
 
ALCAM
 
 
hsa-miR-148b-3p
 
 
-0.339
 
 
30.8
 
 
 
 
AMMECR1L
 
 
hsa-miR-29a-3p
 
 
-0.42
 
 
10.5
 
 
 
 
AMOTL1
 
 
hsa-miR-16-5p
 
 
-0.297
 
 
14.7
 
 
 
 
AMOTL1
 
 
hsa-miR-15b-5p
 
 
-0.319
 
 
14.7
 
 
 
 
AMT
 
 
hsa-let-7b-5p
 
 
-0.463
 
 
21.5
 
 
 
 
ANKHD1
 
 
hsa-miR-23a-3p
 
 
-0.368
 
 
57.6
 
 
 
 
ANKHD1
 
 
hsa-miR-23b-3p
 
 
-0.368
 
 
57.6
 
 
 
 
ANKHD1
 
 
hsa-miR-30b-5p
 
 
-0.405
 
 
57.6
 
 
 
 
ANKRD17
 
 
hsa-miR-30b-5p
 
 
-0.336
 
 
17.0
 
 
 
 
ANKRD40
 
 
hsa-miR-27a-3p
 
 
-0.35
 
 
18.4
 
 
 
 
ANKRD46
 
 
hsa-let-7b-5p
 
 
-0.389
 
 
17.5
 
 
 
 
ANP32A
 
 
hsa-miR-132-3p
 
 
-0.374
 
 
50.6
 
 
 
 
ANXA11
 
 
hsa-miR-16-5p
 
 
-0.447
 
 
88.5
 
 
 
 
ANXA11
 
 
hsa-miR-15b-5p
 
 
-0.463
 
 
88.5
 
 
 
 
AP1S1
 
 
hsa-let-7b-5p
 
 
-0.374
 
 
23.9
 
 
 
 
AP2A1
 
 
hsa-miR-15b-5p
 
 
-0.333
 
 
32.4
 
 
 
 
AP2A1
 
 
hsa-miR-16-5p
 
 
-0.333
 
 
32.4
 
 
 
 
AP2B1
 
 
hsa-miR-16-5p
 
 
-0.302
 
 
31.1
 
 
 
 
AP2B1
 
 
hsa-miR-15b-5p
 
 
-0.334
 
 
31.1
 
 
 
 
AP3S1
 
 
hsa-miR-30b-5p
 
 
-0.293
 
 
66.7
 
 
 
 
APOO
 
 
hsa-miR-181b-5p
 
 
-0.5
 
 
13.3
 
 
 
 
APOO
 
 
hsa-miR-181a-5p
 
 
-0.5
 
 
13.3
 
 
 
 
APP
 
 
hsa-miR-16-5p
 
 
-0.298
 
 
305.2
 
 
 
 
APP
 
 
hsa-miR-15b-5p
 
 
-0.298
 
 
305.2
 
 
 
 
APPBP2
 
 
hsa-miR-27a-3p
 
 
-0.378
 
 
10.8
 
 
 
 
ARF4
 
 
hsa-miR-30b-5p
 
 
-0.289
 
 
138.3
 
 
 
 
ARF4
 
 
hsa-miR-148b-3p
 
 
-0.487
 
 
138.3
 
 
 
 
ARF5
 
 
hsa-miR-29a-3p
 
 
-0.491
 
 
181.7
 
 
 
 
ARF6
 
 
hsa-miR-181b-5p
 
 
-0.33
 
 
23.2
 
 
 
 
ARF6
 
 
hsa-miR-181a-5p
 
 
-0.33
 
 
23.2
 
 
 
 
ARFGEF1
 
 
hsa-miR-148b-3p
 
 
-0.306
 
 
16.5
 
 
 
 
ARHGAP21
 
 
hsa-miR-148b-3p
 
 
-0.287
 
 
13.3
 
 
 
 
ARHGAP5
 
 
hsa-miR-486-5p
 
 
-0.3
 
 
28.2
 
 
 
 
ARID1B
 
 
hsa-miR-29a-3p
 
 
-0.342
 
 
15.2
 
 
 
 
ARID4B
 
 
hsa-miR-486-5p
 
 
-0.367
 
 
10.7
 
 
 
 
ARL2
 
 
hsa-miR-16-5p
 
 
-0.374
 
 
74.9
 
 
 
 
ARL2
 
 
hsa-miR-15b-5p
 
 
-0.374
 
 
74.9
 
 
 
 
ARL2
 
 
hsa-miR-16-5p
 
 
-0.418
 
 
74.9
 
 
 
 
ARL2
 
 
hsa-miR-15b-5p
 
 
-0.442
 
 
74.9
 
 
 
 
ARL6IP1
 
 
hsa-miR-328-3p
 
 
-0.494
 
 
77.1
 
 
 
 
ARL6IP1
 
 
hsa-miR-148b-3p
 
 
-0.579
 
 
77.1
 
 
 
 
ARL8B
 
 
hsa-miR-148b-3p
 
 
-0.337
 
 
26.7
 
 
 
 
ARL8B
 
 
hsa-miR-320b
 
 
-0.376
 
 
26.7
 
 
 
 
ARL8B
 
 
hsa-miR-320c
 
 
-0.376
 
 
26.7
 
 
 
 
ARMC8
 
 
hsa-miR-486-5p
 
 
-0.321
 
 
13.4
 
 
 
 
ARMCX2
 
 
hsa-miR-15b-5p
 
 
-0.344
 
 
16.6
 
 
 
 
ARMCX2
 
 
hsa-miR-16-5p
 
 
-0.366
 
 
16.6
 
 
 
 
ARMCX3
 
 
hsa-miR-425-5p
 
 
-0.325
 
 
50.5
 
 
 
 
ARPP19
 
 
hsa-miR-320b
 
 
-0.325
 
 
43.6
 
 
 
 
ARPP19
 
 
hsa-miR-320c
 
 
-0.325
 
 
43.6
 
 
 
 
ARPP19
 
 
hsa-miR-15b-5p
 
 
-0.346
 
 
43.6
 
 
 
 
ARPP19
 
 
hsa-miR-16-5p
 
 
-0.354
 
 
43.6
 
 
 
 
ARPP19
 
 
hsa-miR-29a-3p
 
 
-0.371
 
 
43.6
 
 
 
 
ARPP19
 
 
hsa-miR-328-3p
 
 
-0.404
 
 
43.6
 
 
 
 
ARPP19
 
 
hsa-let-7b-5p
 
 
-0.476
 
 
43.6
 
 
 
 
ARRDC3
 
 
hsa-miR-148b-3p
 
 
-0.305
 
 
19.3
 
 
 
 
ARRDC3
 
 
hsa-miR-29a-3p
 
 
-0.402
 
 
19.3
 
 
 
 
ARRDC4
 
 
hsa-let-7b-5p
 
 
-0.329
 
 
32.8
 
 
 
 
ASAH1
 
 
hsa-miR-27a-3p
 
 
-0.398
 
 
46.8
 
 
 
 
ASH1L
 
 
hsa-miR-15b-5p
 
 
-0.325
 
 
16.5
 
 
 
 
ASH1L
 
 
hsa-miR-16-5p
 
 
-0.357
 
 
16.5
 
 
 
 
ASH2L
 
 
hsa-miR-320b
 
 
-0.287
 
 
16.9
 
 
 
 
ASH2L
 
 
hsa-miR-320c
 
 
-0.287
 
 
16.9
 
 
 
 
ASPH
 
 
hsa-miR-148b-3p
 
 
-0.391
 
 
42.1
 
 
 
 
ATG5
 
 
hsa-miR-30b-5p
 
 
-0.488
 
 
10.4
 
 
 
 
ATG9A
 
 
hsa-miR-16-5p
 
 
-0.311
 
 
26.5
 
 
 
 
ATG9A
 
 
hsa-miR-15b-5p
 
 
-0.333
 
 
26.5
 
 
 
 
ATP2A2
 
 
hsa-let-7b-5p
 
 
-0.311
 
 
36.0
 
 
 
 
ATP5MC3
 
 
hsa-miR-425-5p
 
 
-0.328
 
 
115.2
 
 
 
 
ATP5MC3
 
 
hsa-miR-29a-3p
 
 
-0.33
 
 
115.2
 
 
 
 
 
 
 parathyroid gland 
 
 
 
 
target_symbol
 
 
mature_mirna_id
 
 
score
 
 
nTPM
 
 
 
 
 
 
AAR2
 
 
hsa-miR-29a-3p
 
 
-0.327
 
 
23.9
 
 
 
 
ABCB7
 
 
hsa-miR-148b-3p
 
 
-0.515
 
 
10.3
 
 
 
 
ABCD3
 
 
hsa-miR-148b-3p
 
 
-0.388
 
 
27.9
 
 
 
 
ABCE1
 
 
hsa-miR-29a-3p
 
 
-0.404
 
 
25.3
 
 
 
 
ABHD4
 
 
hsa-miR-29a-3p
 
 
-0.312
 
 
23.4
 
 
 
 
ABHD5
 
 
hsa-miR-132-3p
 
 
-0.323
 
 
11.5
 
 
 
 
ABHD5
 
 
hsa-miR-27a-3p
 
 
-0.407
 
 
11.5
 
 
 
 
ACSL6
 
 
hsa-let-7b-5p
 
 
-0.336
 
 
10.6
 
 
 
 
ACTA2
 
 
hsa-miR-27a-3p
 
 
-0.577
 
 
75.8
 
 
 
 
ACTR2
 
 
hsa-miR-15b-5p
 
 
-0.293
 
 
58.1
 
 
 
 
ACTR2
 
 
hsa-miR-16-5p
 
 
-0.293
 
 
58.1
 
 
 
 
ACTR3C
 
 
hsa-miR-30b-5p
 
 
-0.448
 
 
53.3
 
 
 
 
ADAM9
 
 
hsa-miR-30b-5p
 
 
-0.312
 
 
49.4
 
 
 
 
ADCY3
 
 
hsa-miR-132-3p
 
 
-0.303
 
 
16.0
 
 
 
 
ADGRA3
 
 
hsa-miR-27a-3p
 
 
-0.296
 
 
11.5
 
 
 
 
ADGRA3
 
 
hsa-miR-30b-5p
 
 
-0.304
 
 
11.5
 
 
 
 
ADGRF5
 
 
hsa-miR-148b-3p
 
 
-0.354
 
 
12.6
 
 
 
 
ADIPOR2
 
 
hsa-miR-150-5p
 
 
-0.287
 
 
15.9
 
 
 
 
ADNP
 
 
hsa-miR-328-3p
 
 
-0.371
 
 
34.0
 
 
 
 
AFG3L2
 
 
hsa-miR-181b-5p
 
 
-0.491
 
 
41.3
 
 
 
 
AFG3L2
 
 
hsa-miR-181a-5p
 
 
-0.491
 
 
41.3
 
 
 
 
AGFG1
 
 
hsa-miR-148b-3p
 
 
-0.332
 
 
39.0
 
 
 
 
AGPAT3
 
 
hsa-miR-27a-3p
 
 
-0.29
 
 
23.1
 
 
 
 
AGPAT3
 
 
hsa-miR-16-5p
 
 
-0.299
 
 
23.1
 
 
 
 
AHCTF1
 
 
hsa-let-7b-5p
 
 
-0.374
 
 
12.3
 
 
 
 
AIFM1
 
 
hsa-let-7b-5p
 
 
-0.314
 
 
27.9
 
 
 
 
AK1
 
 
hsa-miR-29a-3p
 
 
-0.333
 
 
44.1
 
 
 
 
AK4
 
 
hsa-miR-15b-5p
 
 
-0.316
 
 
16.7
 
 
 
 
AK4
 
 
hsa-miR-16-5p
 
 
-0.348
 
 
16.7
 
 
 
 
AKAP1
 
 
hsa-miR-148b-3p
 
 
-0.381
 
 
45.1
 
 
 
 
AKIRIN1
 
 
hsa-miR-27a-3p
 
 
-0.368
 
 
40.5
 
 
 
 
ALAD
 
 
hsa-miR-16-5p
 
 
-0.391
 
 
68.8
 
 
 
 
ALAD
 
 
hsa-miR-15b-5p
 
 
-0.446
 
 
68.8
 
 
 
 
ALCAM
 
 
hsa-miR-148b-3p
 
 
-0.339
 
 
412.8
 
 
 
 
ALDH1A2
 
 
hsa-miR-23b-3p
 
 
-0.346
 
 
28.0
 
 
 
 
ALDH1A2
 
 
hsa-miR-23a-3p
 
 
-0.354
 
 
28.0
 
 
 
 
AMOT
 
 
hsa-miR-29a-3p
 
 
-0.304
 
 
13.8
 
 
 
 
AMOTL1
 
 
hsa-miR-16-5p
 
 
-0.297
 
 
13.0
 
 
 
 
AMOTL1
 
 
hsa-miR-15b-5p
 
 
-0.319
 
 
13.0
 
 
 
 
AMT
 
 
hsa-let-7b-5p
 
 
-0.463
 
 
13.3
 
 
 
 
ANKHD1
 
 
hsa-miR-23a-3p
 
 
-0.368
 
 
35.6
 
 
 
 
ANKHD1
 
 
hsa-miR-23b-3p
 
 
-0.368
 
 
35.6
 
 
 
 
ANKHD1
 
 
hsa-miR-30b-5p
 
 
-0.405
 
 
35.6
 
 
 
 
ANKRD17
 
 
hsa-miR-30b-5p
 
 
-0.336
 
 
23.0
 
 
 
 
ANKRD29
 
 
hsa-miR-132-3p
 
 
-0.47
 
 
23.3
 
 
 
 
ANKRD40
 
 
hsa-miR-27a-3p
 
 
-0.35
 
 
25.3
 
 
 
 
ANKRD46
 
 
hsa-let-7b-5p
 
 
-0.389
 
 
15.1
 
 
 
 
ANP32A
 
 
hsa-miR-132-3p
 
 
-0.374
 
 
57.6
 
 
 
 
ANTXR2
 
 
hsa-miR-29a-3p
 
 
-0.347
 
 
26.2
 
 
 
 
ANXA11
 
 
hsa-miR-16-5p
 
 
-0.447
 
 
76.9
 
 
 
 
ANXA11
 
 
hsa-miR-15b-5p
 
 
-0.463
 
 
76.9
 
 
 
 
AP1S1
 
 
hsa-let-7b-5p
 
 
-0.374
 
 
21.4
 
 
 
 
AP2A1
 
 
hsa-miR-15b-5p
 
 
-0.333
 
 
12.9
 
 
 
 
AP2A1
 
 
hsa-miR-16-5p
 
 
-0.333
 
 
12.9
 
 
 
 
AP2B1
 
 
hsa-miR-16-5p
 
 
-0.302
 
 
118.9
 
 
 
 
AP2B1
 
 
hsa-miR-15b-5p
 
 
-0.334
 
 
118.9
 
 
 
 
AP3S1
 
 
hsa-miR-30b-5p
 
 
-0.293
 
 
23.4
 
 
 
 
APOO
 
 
hsa-miR-181b-5p
 
 
-0.5
 
 
58.1
 
 
 
 
APOO
 
 
hsa-miR-181a-5p
 
 
-0.5
 
 
58.1
 
 
 
 
APP
 
 
hsa-miR-16-5p
 
 
-0.298
 
 
259.7
 
 
 
 
APP
 
 
hsa-miR-15b-5p
 
 
-0.298
 
 
259.7
 
 
 
 
APPBP2
 
 
hsa-miR-27a-3p
 
 
-0.378
 
 
13.6
 
 
 
 
ARF4
 
 
hsa-miR-30b-5p
 
 
-0.289
 
 
95.4
 
 
 
 
ARF4
 
 
hsa-miR-148b-3p
 
 
-0.487
 
 
95.4
 
 
 
 
ARF5
 
 
hsa-miR-29a-3p
 
 
-0.491
 
 
97.9
 
 
 
 
ARF6
 
 
hsa-miR-181b-5p
 
 
-0.33
 
 
19.3
 
 
 
 
ARF6
 
 
hsa-miR-181a-5p
 
 
-0.33
 
 
19.3
 
 
 
 
ARFGEF1
 
 
hsa-miR-148b-3p
 
 
-0.306
 
 
26.3
 
 
 
 
ARFIP1
 
 
hsa-miR-320b
 
 
-0.339
 
 
21.0
 
 
 
 
ARFIP1
 
 
hsa-miR-320c
 
 
-0.339
 
 
21.0
 
 
 
 
ARFIP1
 
 
hsa-miR-148b-3p
 
 
-0.474
 
 
21.0
 
 
 
 
ARG2
 
 
hsa-let-7b-5p
 
 
-0.468
 
 
206.5
 
 
 
 
ARHGAP21
 
 
hsa-miR-148b-3p
 
 
-0.287
 
 
13.6
 
 
 
 
ARHGAP5
 
 
hsa-miR-486-5p
 
 
-0.3
 
 
45.3
 
 
 
 
ARID1B
 
 
hsa-miR-29a-3p
 
 
-0.342
 
 
25.3
 
 
 
 
ARID2
 
 
hsa-miR-132-3p
 
 
-0.304
 
 
11.0
 
 
 
 
ARID4B
 
 
hsa-miR-486-5p
 
 
-0.367
 
 
12.8
 
 
 
 
ARL2
 
 
hsa-miR-16-5p
 
 
-0.374
 
 
61.4
 
 
 
 
ARL2
 
 
hsa-miR-15b-5p
 
 
-0.374
 
 
61.4
 
 
 
 
ARL2
 
 
hsa-miR-16-5p
 
 
-0.418
 
 
61.4
 
 
 
 
ARL2
 
 
hsa-miR-15b-5p
 
 
-0.442
 
 
61.4
 
 
 
 
ARL3
 
 
hsa-miR-16-5p
 
 
-0.496
 
 
18.6
 
 
 
 
ARL3
 
 
hsa-miR-15b-5p
 
 
-0.507
 
 
18.6
 
 
 
 
ARL6IP1
 
 
hsa-miR-328-3p
 
 
-0.494
 
 
169.8
 
 
 
 
ARL6IP1
 
 
hsa-miR-148b-3p
 
 
-0.579
 
 
169.8
 
 
 
 
ARL8B
 
 
hsa-miR-148b-3p
 
 
-0.337
 
 
45.2
 
 
 
 
ARL8B
 
 
hsa-miR-320b
 
 
-0.376
 
 
45.2
 
 
 
 
ARL8B
 
 
hsa-miR-320c
 
 
-0.376
 
 
45.2
 
 
 
 
ARMC8
 
 
hsa-miR-486-5p
 
 
-0.321
 
 
20.0
 
 
 
 
ARMCX2
 
 
hsa-miR-15b-5p
 
 
-0.344
 
 
67.0
 
 
 
 
ARMCX2
 
 
hsa-miR-16-5p
 
 
-0.366
 
 
67.0
 
 
 
 
ARMCX3
 
 
hsa-miR-425-5p
 
 
-0.325
 
 
97.4
 
 
 
 
ARMT1
 
 
hsa-let-7b-5p
 
 
-0.291
 
 
10.9
 
 
 
 
ARPP19
 
 
hsa-miR-320b
 
 
-0.325
 
 
102.5
 
 
 
 
ARPP19
 
 
hsa-miR-320c
 
 
-0.325
 
 
102.5
 
 
 
 
ARPP19
 
 
hsa-miR-15b-5p
 
 
-0.346
 
 
102.5
 
 
 
 
ARPP19
 
 
hsa-miR-16-5p
 
 
-0.354
 
 
102.5
 
 
 
 
ARPP19
 
 
hsa-miR-29a-3p
 
 
-0.371
 
 
102.5
 
 
 
 
ARPP19
 
 
hsa-miR-328-3p
 
 
-0.404
 
 
102.5
 
 
 
 
ARPP19
 
 
hsa-let-7b-5p
 
 
-0.476
 
 
102.5
 
 
 
 
 
 
 placenta 
 
 
 
 
target_symbol
 
 
mature_mirna_id
 
 
score
 
 
nTPM
 
 
 
 
 
 
AAR2
 
 
hsa-miR-29a-3p
 
 
-0.327
 
 
16.5
 
 
 
 
ABCB10
 
 
hsa-miR-148b-3p
 
 
-0.291
 
 
10.6
 
 
 
 
ABCD3
 
 
hsa-miR-148b-3p
 
 
-0.388
 
 
13.6
 
 
 
 
ABCE1
 
 
hsa-miR-29a-3p
 
 
-0.404
 
 
26.9
 
 
 
 
ABHD4
 
 
hsa-miR-29a-3p
 
 
-0.312
 
 
18.5
 
 
 
 
ACP3
 
 
hsa-let-7b-5p
 
 
-0.298
 
 
10.1
 
 
 
 
ACTA2
 
 
hsa-miR-27a-3p
 
 
-0.577
 
 
723.7
 
 
 
 
ACTR2
 
 
hsa-miR-15b-5p
 
 
-0.293
 
 
102.8
 
 
 
 
ACTR2
 
 
hsa-miR-16-5p
 
 
-0.293
 
 
102.8
 
 
 
 
ACVR1
 
 
hsa-miR-30b-5p
 
 
-0.29
 
 
12.5
 
 
 
 
ACVR1
 
 
hsa-miR-148b-3p
 
 
-0.397
 
 
12.5
 
 
 
 
ADAM10
 
 
hsa-miR-148b-3p
 
 
-0.356
 
 
27.1
 
 
 
 
ADAM17
 
 
hsa-miR-148b-3p
 
 
-0.301
 
 
17.9
 
 
 
 
ADAM9
 
 
hsa-miR-30b-5p
 
 
-0.312
 
 
44.6
 
 
 
 
ADAMTS9
 
 
hsa-miR-29a-3p
 
 
-0.305
 
 
29.8
 
 
 
 
ADAMTS9
 
 
hsa-miR-29a-3p
 
 
-0.485
 
 
29.8
 
 
 
 
ADCY3
 
 
hsa-miR-132-3p
 
 
-0.303
 
 
78.3
 
 
 
 
ADGRF5
 
 
hsa-miR-148b-3p
 
 
-0.354
 
 
48.3
 
 
 
 
ADIPOR2
 
 
hsa-miR-150-5p
 
 
-0.287
 
 
12.2
 
 
 
 
ADNP
 
 
hsa-miR-328-3p
 
 
-0.371
 
 
23.8
 
 
 
 
ADRB1
 
 
hsa-let-7b-5p
 
 
-0.351
 
 
27.4
 
 
 
 
AFG3L2
 
 
hsa-miR-181b-5p
 
 
-0.491
 
 
12.5
 
 
 
 
AFG3L2
 
 
hsa-miR-181a-5p
 
 
-0.491
 
 
12.5
 
 
 
 
AGFG1
 
 
hsa-miR-148b-3p
 
 
-0.332
 
 
71.2
 
 
 
 
AGPAT3
 
 
hsa-miR-27a-3p
 
 
-0.29
 
 
11.5
 
 
 
 
AGPAT3
 
 
hsa-miR-16-5p
 
 
-0.299
 
 
11.5
 
 
 
 
AHR
 
 
hsa-miR-29a-3p
 
 
-0.316
 
 
16.6
 
 
 
 
AKIRIN1
 
 
hsa-miR-27a-3p
 
 
-0.368
 
 
24.9
 
 
 
 
AKT3
 
 
hsa-miR-29a-3p
 
 
-0.287
 
 
12.3
 
 
 
 
AKT3
 
 
hsa-miR-15b-5p
 
 
-0.301
 
 
12.3
 
 
 
 
AKT3
 
 
hsa-miR-16-5p
 
 
-0.309
 
 
12.3
 
 
 
 
ALAD
 
 
hsa-miR-16-5p
 
 
-0.391
 
 
23.4
 
 
 
 
ALAD
 
 
hsa-miR-15b-5p
 
 
-0.446
 
 
23.4
 
 
 
 
ALCAM
 
 
hsa-miR-148b-3p
 
 
-0.339
 
 
11.3
 
 
 
 
AMOT
 
 
hsa-miR-29a-3p
 
 
-0.304
 
 
19.6
 
 
 
 
AMOTL1
 
 
hsa-miR-16-5p
 
 
-0.297
 
 
22.6
 
 
 
 
AMOTL1
 
 
hsa-miR-15b-5p
 
 
-0.319
 
 
22.6
 
 
 
 
ANKHD1
 
 
hsa-miR-23a-3p
 
 
-0.368
 
 
26.8
 
 
 
 
ANKHD1
 
 
hsa-miR-23b-3p
 
 
-0.368
 
 
26.8
 
 
 
 
ANKHD1
 
 
hsa-miR-30b-5p
 
 
-0.405
 
 
26.8
 
 
 
 
ANKRD17
 
 
hsa-miR-30b-5p
 
 
-0.336
 
 
17.9
 
 
 
 
ANKRD40
 
 
hsa-miR-27a-3p
 
 
-0.35
 
 
21.7
 
 
 
 
ANKRD46
 
 
hsa-let-7b-5p
 
 
-0.389
 
 
19.5
 
 
 
 
ANP32A
 
 
hsa-miR-132-3p
 
 
-0.374
 
 
95.3
 
 
 
 
ANTXR2
 
 
hsa-miR-29a-3p
 
 
-0.347
 
 
84.5
 
 
 
 
ANXA11
 
 
hsa-miR-16-5p
 
 
-0.447
 
 
85.5
 
 
 
 
ANXA11
 
 
hsa-miR-15b-5p
 
 
-0.463
 
 
85.5
 
 
 
 
AP1S1
 
 
hsa-let-7b-5p
 
 
-0.374
 
 
14.9
 
 
 
 
AP2A1
 
 
hsa-miR-15b-5p
 
 
-0.333
 
 
29.6
 
 
 
 
AP2A1
 
 
hsa-miR-16-5p
 
 
-0.333
 
 
29.6
 
 
 
 
AP2B1
 
 
hsa-miR-16-5p
 
 
-0.302
 
 
62.7
 
 
 
 
AP2B1
 
 
hsa-miR-15b-5p
 
 
-0.334
 
 
62.7
 
 
 
 
AP3S1
 
 
hsa-miR-30b-5p
 
 
-0.293
 
 
56.4
 
 
 
 
APLN
 
 
hsa-miR-16-5p
 
 
-0.31
 
 
50.8
 
 
 
 
APLN
 
 
hsa-miR-15b-5p
 
 
-0.395
 
 
50.8
 
 
 
 
APLN
 
 
hsa-miR-16-5p
 
 
-0.395
 
 
50.8
 
 
 
 
APOO
 
 
hsa-miR-181b-5p
 
 
-0.5
 
 
10.3
 
 
 
 
APOO
 
 
hsa-miR-181a-5p
 
 
-0.5
 
 
10.3
 
 
 
 
APP
 
 
hsa-miR-16-5p
 
 
-0.298
 
 
417.2
 
 
 
 
APP
 
 
hsa-miR-15b-5p
 
 
-0.298
 
 
417.2
 
 
 
 
APPBP2
 
 
hsa-miR-27a-3p
 
 
-0.378
 
 
11.5
 
 
 
 
ARF4
 
 
hsa-miR-30b-5p
 
 
-0.289
 
 
170.7
 
 
 
 
ARF4
 
 
hsa-miR-148b-3p
 
 
-0.487
 
 
170.7
 
 
 
 
ARF5
 
 
hsa-miR-29a-3p
 
 
-0.491
 
 
65.2
 
 
 
 
ARF6
 
 
hsa-miR-181b-5p
 
 
-0.33
 
 
34.8
 
 
 
 
ARF6
 
 
hsa-miR-181a-5p
 
 
-0.33
 
 
34.8
 
 
 
 
ARFGEF1
 
 
hsa-miR-148b-3p
 
 
-0.306
 
 
13.6
 
 
 
 
ARFIP1
 
 
hsa-miR-320b
 
 
-0.339
 
 
16.9
 
 
 
 
ARFIP1
 
 
hsa-miR-320c
 
 
-0.339
 
 
16.9
 
 
 
 
ARFIP1
 
 
hsa-miR-148b-3p
 
 
-0.474
 
 
16.9
 
 
 
 
ARHGAP21
 
 
hsa-miR-148b-3p
 
 
-0.287
 
 
15.6
 
 
 
 
ARHGAP28
 
 
hsa-let-7b-5p
 
 
-0.454
 
 
24.3
 
 
 
 
ARHGAP5
 
 
hsa-miR-486-5p
 
 
-0.3
 
 
35.0
 
 
 
 
ARID1B
 
 
hsa-miR-29a-3p
 
 
-0.342
 
 
10.4
 
 
 
 
ARID3A
 
 
hsa-let-7b-5p
 
 
-0.332
 
 
22.0
 
 
 
 
ARID3A
 
 
hsa-let-7b-5p
 
 
-0.347
 
 
22.0
 
 
 
 
ARID3B
 
 
hsa-let-7b-5p
 
 
-0.322
 
 
14.7
 
 
 
 
ARID3B
 
 
hsa-let-7b-5p
 
 
-0.486
 
 
14.7
 
 
 
 
ARID4B
 
 
hsa-miR-486-5p
 
 
-0.367
 
 
12.0
 
 
 
 
ARL2
 
 
hsa-miR-16-5p
 
 
-0.374
 
 
52.7
 
 
 
 
ARL2
 
 
hsa-miR-15b-5p
 
 
-0.374
 
 
52.7
 
 
 
 
ARL2
 
 
hsa-miR-16-5p
 
 
-0.418
 
 
52.7
 
 
 
 
ARL2
 
 
hsa-miR-15b-5p
 
 
-0.442
 
 
52.7
 
 
 
 
ARL4A
 
 
hsa-miR-29a-3p
 
 
-0.386
 
 
22.2
 
 
 
 
ARL4A
 
 
hsa-miR-30b-5p
 
 
-0.448
 
 
22.2
 
 
 
 
ARL5B
 
 
hsa-miR-29a-3p
 
 
-0.304
 
 
12.3
 
 
 
 
ARL6IP1
 
 
hsa-miR-328-3p
 
 
-0.494
 
 
84.9
 
 
 
 
ARL6IP1
 
 
hsa-miR-148b-3p
 
 
-0.579
 
 
84.9
 
 
 
 
ARL6IP6
 
 
hsa-let-7b-5p
 
 
-0.293
 
 
26.8
 
 
 
 
ARL8B
 
 
hsa-miR-148b-3p
 
 
-0.337
 
 
46.8
 
 
 
 
ARL8B
 
 
hsa-miR-320b
 
 
-0.376
 
 
46.8
 
 
 
 
ARL8B
 
 
hsa-miR-320c
 
 
-0.376
 
 
46.8
 
 
 
 
ARMC8
 
 
hsa-miR-486-5p
 
 
-0.321
 
 
18.5
 
 
 
 
ARMCX2
 
 
hsa-miR-15b-5p
 
 
-0.344
 
 
24.7
 
 
 
 
ARMCX2
 
 
hsa-miR-16-5p
 
 
-0.366
 
 
24.7
 
 
 
 
ARMCX3
 
 
hsa-miR-425-5p
 
 
-0.325
 
 
42.7
 
 
 
 
ARMT1
 
 
hsa-let-7b-5p
 
 
-0.291
 
 
20.8
 
 
 
 
ARPP19
 
 
hsa-miR-320b
 
 
-0.325
 
 
53.5
 
 
 
 
ARPP19
 
 
hsa-miR-320c
 
 
-0.325
 
 
53.5
 
 
 
 
ARPP19
 
 
hsa-miR-15b-5p
 
 
-0.346
 
 
53.5
 
 
 
 
 
 
 prostate 
 
 
 
 
target_symbol
 
 
mature_mirna_id
 
 
score
 
 
nTPM
 
 
 
 
 
 
AAR2
 
 
hsa-miR-29a-3p
 
 
-0.327
 
 
18.7
 
 
 
 
ABCD3
 
 
hsa-miR-148b-3p
 
 
-0.388
 
 
20.2
 
 
 
 
ABCE1
 
 
hsa-miR-29a-3p
 
 
-0.404
 
 
18.2
 
 
 
 
ABHD17C
 
 
hsa-let-7b-5p
 
 
-0.359
 
 
17.0
 
 
 
 
ABHD17C
 
 
hsa-miR-27a-3p
 
 
-0.435
 
 
17.0
 
 
 
 
ABHD4
 
 
hsa-miR-29a-3p
 
 
-0.312
 
 
20.4
 
 
 
 
ACP3
 
 
hsa-let-7b-5p
 
 
-0.298
 
 
3409.5
 
 
 
 
ACTA2
 
 
hsa-miR-27a-3p
 
 
-0.577
 
 
2476.6
 
 
 
 
ACTC1
 
 
hsa-miR-30b-5p
 
 
-0.543
 
 
68.4
 
 
 
 
ACTN2
 
 
hsa-miR-23a-3p
 
 
-0.294
 
 
14.6
 
 
 
 
ACTN2
 
 
hsa-miR-29a-3p
 
 
-0.311
 
 
14.6
 
 
 
 
ACTR2
 
 
hsa-miR-15b-5p
 
 
-0.293
 
 
67.2
 
 
 
 
ACTR2
 
 
hsa-miR-16-5p
 
 
-0.293
 
 
67.2
 
 
 
 
ADAM10
 
 
hsa-miR-148b-3p
 
 
-0.356
 
 
23.6
 
 
 
 
ADAM9
 
 
hsa-miR-30b-5p
 
 
-0.312
 
 
32.8
 
 
 
 
ADCY3
 
 
hsa-miR-132-3p
 
 
-0.303
 
 
38.0
 
 
 
 
ADGRA3
 
 
hsa-miR-27a-3p
 
 
-0.296
 
 
11.3
 
 
 
 
ADGRA3
 
 
hsa-miR-30b-5p
 
 
-0.304
 
 
11.3
 
 
 
 
ADIPOR2
 
 
hsa-miR-150-5p
 
 
-0.287
 
 
19.8
 
 
 
 
ADNP
 
 
hsa-miR-328-3p
 
 
-0.371
 
 
33.0
 
 
 
 
AFG3L2
 
 
hsa-miR-181b-5p
 
 
-0.491
 
 
13.2
 
 
 
 
AFG3L2
 
 
hsa-miR-181a-5p
 
 
-0.491
 
 
13.2
 
 
 
 
AGFG1
 
 
hsa-miR-148b-3p
 
 
-0.332
 
 
19.4
 
 
 
 
AGPAT3
 
 
hsa-miR-27a-3p
 
 
-0.29
 
 
12.9
 
 
 
 
AGPAT3
 
 
hsa-miR-16-5p
 
 
-0.299
 
 
12.9
 
 
 
 
AHDC1
 
 
hsa-miR-148b-3p
 
 
-0.413
 
 
11.3
 
 
 
 
AHR
 
 
hsa-miR-29a-3p
 
 
-0.316
 
 
10.3
 
 
 
 
AIFM1
 
 
hsa-let-7b-5p
 
 
-0.314
 
 
10.1
 
 
 
 
AK1
 
 
hsa-miR-29a-3p
 
 
-0.333
 
 
16.0
 
 
 
 
AK4
 
 
hsa-miR-15b-5p
 
 
-0.316
 
 
14.9
 
 
 
 
AK4
 
 
hsa-miR-16-5p
 
 
-0.348
 
 
14.9
 
 
 
 
AKAP1
 
 
hsa-miR-148b-3p
 
 
-0.381
 
 
33.3
 
 
 
 
AKIRIN1
 
 
hsa-miR-27a-3p
 
 
-0.368
 
 
34.0
 
 
 
 
AKT3
 
 
hsa-miR-29a-3p
 
 
-0.287
 
 
13.8
 
 
 
 
AKT3
 
 
hsa-miR-15b-5p
 
 
-0.301
 
 
13.8
 
 
 
 
AKT3
 
 
hsa-miR-16-5p
 
 
-0.309
 
 
13.8
 
 
 
 
ALAD
 
 
hsa-miR-16-5p
 
 
-0.391
 
 
32.0
 
 
 
 
ALAD
 
 
hsa-miR-15b-5p
 
 
-0.446
 
 
32.0
 
 
 
 
ALCAM
 
 
hsa-miR-148b-3p
 
 
-0.339
 
 
30.7
 
 
 
 
ALDH1A2
 
 
hsa-miR-23b-3p
 
 
-0.346
 
 
19.7
 
 
 
 
ALDH1A2
 
 
hsa-miR-23a-3p
 
 
-0.354
 
 
19.7
 
 
 
 
AMOTL1
 
 
hsa-miR-16-5p
 
 
-0.297
 
 
30.4
 
 
 
 
AMOTL1
 
 
hsa-miR-15b-5p
 
 
-0.319
 
 
30.4
 
 
 
 
AMT
 
 
hsa-let-7b-5p
 
 
-0.463
 
 
15.5
 
 
 
 
ANKHD1
 
 
hsa-miR-23a-3p
 
 
-0.368
 
 
34.8
 
 
 
 
ANKHD1
 
 
hsa-miR-23b-3p
 
 
-0.368
 
 
34.8
 
 
 
 
ANKHD1
 
 
hsa-miR-30b-5p
 
 
-0.405
 
 
34.8
 
 
 
 
ANKRA2
 
 
hsa-miR-30b-5p
 
 
-0.354
 
 
11.6
 
 
 
 
ANKRA2
 
 
hsa-miR-30b-5p
 
 
-0.472
 
 
11.6
 
 
 
 
ANKRD17
 
 
hsa-miR-30b-5p
 
 
-0.336
 
 
14.2
 
 
 
 
ANKRD40
 
 
hsa-miR-27a-3p
 
 
-0.35
 
 
17.2
 
 
 
 
ANKRD46
 
 
hsa-let-7b-5p
 
 
-0.389
 
 
12.6
 
 
 
 
ANP32A
 
 
hsa-miR-132-3p
 
 
-0.374
 
 
70.7
 
 
 
 
ANTXR2
 
 
hsa-miR-29a-3p
 
 
-0.347
 
 
103.3
 
 
 
 
ANXA11
 
 
hsa-miR-16-5p
 
 
-0.447
 
 
94.1
 
 
 
 
ANXA11
 
 
hsa-miR-15b-5p
 
 
-0.463
 
 
94.1
 
 
 
 
AP1S1
 
 
hsa-let-7b-5p
 
 
-0.374
 
 
23.9
 
 
 
 
AP2A1
 
 
hsa-miR-15b-5p
 
 
-0.333
 
 
31.4
 
 
 
 
AP2A1
 
 
hsa-miR-16-5p
 
 
-0.333
 
 
31.4
 
 
 
 
AP2B1
 
 
hsa-miR-16-5p
 
 
-0.302
 
 
38.3
 
 
 
 
AP2B1
 
 
hsa-miR-15b-5p
 
 
-0.334
 
 
38.3
 
 
 
 
AP3S1
 
 
hsa-miR-30b-5p
 
 
-0.293
 
 
40.9
 
 
 
 
APOO
 
 
hsa-miR-181b-5p
 
 
-0.5
 
 
12.8
 
 
 
 
APOO
 
 
hsa-miR-181a-5p
 
 
-0.5
 
 
12.8
 
 
 
 
APP
 
 
hsa-miR-16-5p
 
 
-0.298
 
 
232.5
 
 
 
 
APP
 
 
hsa-miR-15b-5p
 
 
-0.298
 
 
232.5
 
 
 
 
APPBP2
 
 
hsa-miR-27a-3p
 
 
-0.378
 
 
12.8
 
 
 
 
ARF4
 
 
hsa-miR-30b-5p
 
 
-0.289
 
 
129.9
 
 
 
 
ARF4
 
 
hsa-miR-148b-3p
 
 
-0.487
 
 
129.9
 
 
 
 
ARF5
 
 
hsa-miR-29a-3p
 
 
-0.491
 
 
107.0
 
 
 
 
ARF6
 
 
hsa-miR-181b-5p
 
 
-0.33
 
 
30.5
 
 
 
 
ARF6
 
 
hsa-miR-181a-5p
 
 
-0.33
 
 
30.5
 
 
 
 
ARFGEF1
 
 
hsa-miR-148b-3p
 
 
-0.306
 
 
12.2
 
 
 
 
ARFIP1
 
 
hsa-miR-320b
 
 
-0.339
 
 
19.8
 
 
 
 
ARFIP1
 
 
hsa-miR-320c
 
 
-0.339
 
 
19.8
 
 
 
 
ARFIP1
 
 
hsa-miR-148b-3p
 
 
-0.474
 
 
19.8
 
 
 
 
ARG2
 
 
hsa-let-7b-5p
 
 
-0.468
 
 
101.8
 
 
 
 
ARHGAP21
 
 
hsa-miR-148b-3p
 
 
-0.287
 
 
10.2
 
 
 
 
ARHGAP5
 
 
hsa-miR-486-5p
 
 
-0.3
 
 
28.6
 
 
 
 
ARID1B
 
 
hsa-miR-29a-3p
 
 
-0.342
 
 
11.6
 
 
 
 
ARID4B
 
 
hsa-miR-486-5p
 
 
-0.367
 
 
13.4
 
 
 
 
ARL2
 
 
hsa-miR-16-5p
 
 
-0.374
 
 
84.5
 
 
 
 
ARL2
 
 
hsa-miR-15b-5p
 
 
-0.374
 
 
84.5
 
 
 
 
ARL2
 
 
hsa-miR-16-5p
 
 
-0.418
 
 
84.5
 
 
 
 
ARL2
 
 
hsa-miR-15b-5p
 
 
-0.442
 
 
84.5
 
 
 
 
ARL4A
 
 
hsa-miR-29a-3p
 
 
-0.386
 
 
19.8
 
 
 
 
ARL4A
 
 
hsa-miR-30b-5p
 
 
-0.448
 
 
19.8
 
 
 
 
ARL4D
 
 
hsa-let-7b-5p
 
 
-0.417
 
 
10.7
 
 
 
 
ARL6IP1
 
 
hsa-miR-328-3p
 
 
-0.494
 
 
71.3
 
 
 
 
ARL6IP1
 
 
hsa-miR-148b-3p
 
 
-0.579
 
 
71.3
 
 
 
 
ARL8B
 
 
hsa-miR-148b-3p
 
 
-0.337
 
 
42.2
 
 
 
 
ARL8B
 
 
hsa-miR-320b
 
 
-0.376
 
 
42.2
 
 
 
 
ARL8B
 
 
hsa-miR-320c
 
 
-0.376
 
 
42.2
 
 
 
 
ARMC8
 
 
hsa-miR-486-5p
 
 
-0.321
 
 
15.4
 
 
 
 
ARMCX2
 
 
hsa-miR-15b-5p
 
 
-0.344
 
 
26.1
 
 
 
 
ARMCX2
 
 
hsa-miR-16-5p
 
 
-0.366
 
 
26.1
 
 
 
 
ARMCX3
 
 
hsa-miR-425-5p
 
 
-0.325
 
 
41.8
 
 
 
 
ARMT1
 
 
hsa-let-7b-5p
 
 
-0.291
 
 
11.5
 
 
 
 
ARPP19
 
 
hsa-miR-320b
 
 
-0.325
 
 
37.0
 
 
 
 
ARPP19
 
 
hsa-miR-320c
 
 
-0.325
 
 
37.0
 
 
 
 
 
 
 rectum 
 
 
 
 
target_symbol
 
 
mature_mirna_id
 
 
score
 
 
nTPM
 
 
 
 
 
 
AAR2
 
 
hsa-miR-29a-3p
 
 
-0.327
 
 
16.3
 
 
 
 
ABCB10
 
 
hsa-miR-148b-3p
 
 
-0.291
 
 
10.6
 
 
 
 
ABCD3
 
 
hsa-miR-148b-3p
 
 
-0.388
 
 
37.8
 
 
 
 
ABCE1
 
 
hsa-miR-29a-3p
 
 
-0.404
 
 
26.0
 
 
 
 
ABHD17C
 
 
hsa-let-7b-5p
 
 
-0.359
 
 
52.8
 
 
 
 
ABHD17C
 
 
hsa-miR-27a-3p
 
 
-0.435
 
 
52.8
 
 
 
 
ABHD4
 
 
hsa-miR-29a-3p
 
 
-0.312
 
 
14.1
 
 
 
 
ACTA2
 
 
hsa-miR-27a-3p
 
 
-0.577
 
 
814.5
 
 
 
 
ACTR2
 
 
hsa-miR-15b-5p
 
 
-0.293
 
 
130.5
 
 
 
 
ACTR2
 
 
hsa-miR-16-5p
 
 
-0.293
 
 
130.5
 
 
 
 
ADAM10
 
 
hsa-miR-148b-3p
 
 
-0.356
 
 
16.1
 
 
 
 
ADAM9
 
 
hsa-miR-30b-5p
 
 
-0.312
 
 
39.2
 
 
 
 
ADAP1
 
 
hsa-miR-27a-3p
 
 
-0.302
 
 
21.3
 
 
 
 
ADCY3
 
 
hsa-miR-132-3p
 
 
-0.303
 
 
14.9
 
 
 
 
ADGRA3
 
 
hsa-miR-27a-3p
 
 
-0.296
 
 
16.1
 
 
 
 
ADGRA3
 
 
hsa-miR-30b-5p
 
 
-0.304
 
 
16.1
 
 
 
 
ADIPOR2
 
 
hsa-miR-150-5p
 
 
-0.287
 
 
16.7
 
 
 
 
ADNP
 
 
hsa-miR-328-3p
 
 
-0.371
 
 
22.7
 
 
 
 
AFG3L2
 
 
hsa-miR-181b-5p
 
 
-0.491
 
 
28.8
 
 
 
 
AFG3L2
 
 
hsa-miR-181a-5p
 
 
-0.491
 
 
28.8
 
 
 
 
AGFG1
 
 
hsa-miR-148b-3p
 
 
-0.332
 
 
31.0
 
 
 
 
AGPAT3
 
 
hsa-miR-27a-3p
 
 
-0.29
 
 
11.1
 
 
 
 
AGPAT3
 
 
hsa-miR-16-5p
 
 
-0.299
 
 
11.1
 
 
 
 
AHR
 
 
hsa-miR-29a-3p
 
 
-0.316
 
 
15.3
 
 
 
 
AIFM1
 
 
hsa-let-7b-5p
 
 
-0.314
 
 
14.9
 
 
 
 
AK1
 
 
hsa-miR-29a-3p
 
 
-0.333
 
 
37.1
 
 
 
 
AK4
 
 
hsa-miR-15b-5p
 
 
-0.316
 
 
12.8
 
 
 
 
AK4
 
 
hsa-miR-16-5p
 
 
-0.348
 
 
12.8
 
 
 
 
AKAP1
 
 
hsa-miR-148b-3p
 
 
-0.381
 
 
45.9
 
 
 
 
AKIRIN1
 
 
hsa-miR-27a-3p
 
 
-0.368
 
 
34.0
 
 
 
 
ALAD
 
 
hsa-miR-16-5p
 
 
-0.391
 
 
23.0
 
 
 
 
ALAD
 
 
hsa-miR-15b-5p
 
 
-0.446
 
 
23.0
 
 
 
 
ALCAM
 
 
hsa-miR-148b-3p
 
 
-0.339
 
 
13.6
 
 
 
 
AMT
 
 
hsa-let-7b-5p
 
 
-0.463
 
 
11.1
 
 
 
 
ANKHD1
 
 
hsa-miR-23a-3p
 
 
-0.368
 
 
31.1
 
 
 
 
ANKHD1
 
 
hsa-miR-23b-3p
 
 
-0.368
 
 
31.1
 
 
 
 
ANKHD1
 
 
hsa-miR-30b-5p
 
 
-0.405
 
 
31.1
 
 
 
 
ANKRD17
 
 
hsa-miR-30b-5p
 
 
-0.336
 
 
17.8
 
 
 
 
ANKRD40
 
 
hsa-miR-27a-3p
 
 
-0.35
 
 
15.3
 
 
 
 
ANP32A
 
 
hsa-miR-132-3p
 
 
-0.374
 
 
83.5
 
 
 
 
ANTXR2
 
 
hsa-miR-29a-3p
 
 
-0.347
 
 
40.1
 
 
 
 
ANXA11
 
 
hsa-miR-16-5p
 
 
-0.447
 
 
113.3
 
 
 
 
ANXA11
 
 
hsa-miR-15b-5p
 
 
-0.463
 
 
113.3
 
 
 
 
AP1S1
 
 
hsa-let-7b-5p
 
 
-0.374
 
 
21.1
 
 
 
 
AP2B1
 
 
hsa-miR-16-5p
 
 
-0.302
 
 
78.9
 
 
 
 
AP2B1
 
 
hsa-miR-15b-5p
 
 
-0.334
 
 
78.9
 
 
 
 
AP3S1
 
 
hsa-miR-30b-5p
 
 
-0.293
 
 
56.4
 
 
 
 
APOO
 
 
hsa-miR-181b-5p
 
 
-0.5
 
 
17.6
 
 
 
 
APOO
 
 
hsa-miR-181a-5p
 
 
-0.5
 
 
17.6
 
 
 
 
APP
 
 
hsa-miR-16-5p
 
 
-0.298
 
 
296.0
 
 
 
 
APP
 
 
hsa-miR-15b-5p
 
 
-0.298
 
 
296.0
 
 
 
 
ARF4
 
 
hsa-miR-30b-5p
 
 
-0.289
 
 
185.2
 
 
 
 
ARF4
 
 
hsa-miR-148b-3p
 
 
-0.487
 
 
185.2
 
 
 
 
ARF5
 
 
hsa-miR-29a-3p
 
 
-0.491
 
 
57.6
 
 
 
 
ARF6
 
 
hsa-miR-181b-5p
 
 
-0.33
 
 
50.6
 
 
 
 
ARF6
 
 
hsa-miR-181a-5p
 
 
-0.33
 
 
50.6
 
 
 
 
ARFGEF1
 
 
hsa-miR-148b-3p
 
 
-0.306
 
 
16.1
 
 
 
 
ARFIP1
 
 
hsa-miR-320b
 
 
-0.339
 
 
26.2
 
 
 
 
ARFIP1
 
 
hsa-miR-320c
 
 
-0.339
 
 
26.2
 
 
 
 
ARFIP1
 
 
hsa-miR-148b-3p
 
 
-0.474
 
 
26.2
 
 
 
 
ARHGAP21
 
 
hsa-miR-148b-3p
 
 
-0.287
 
 
17.4
 
 
 
 
ARHGAP5
 
 
hsa-miR-486-5p
 
 
-0.3
 
 
24.8
 
 
 
 
ARID1B
 
 
hsa-miR-29a-3p
 
 
-0.342
 
 
11.4
 
 
 
 
ARL2
 
 
hsa-miR-16-5p
 
 
-0.374
 
 
30.9
 
 
 
 
ARL2
 
 
hsa-miR-15b-5p
 
 
-0.374
 
 
30.9
 
 
 
 
ARL2
 
 
hsa-miR-16-5p
 
 
-0.418
 
 
30.9
 
 
 
 
ARL2
 
 
hsa-miR-15b-5p
 
 
-0.442
 
 
30.9
 
 
 
 
ARL4A
 
 
hsa-miR-29a-3p
 
 
-0.386
 
 
32.6
 
 
 
 
ARL4A
 
 
hsa-miR-30b-5p
 
 
-0.448
 
 
32.6
 
 
 
 
ARL6IP1
 
 
hsa-miR-328-3p
 
 
-0.494
 
 
116.0
 
 
 
 
ARL6IP1
 
 
hsa-miR-148b-3p
 
 
-0.579
 
 
116.0
 
 
 
 
ARL6IP6
 
 
hsa-let-7b-5p
 
 
-0.293
 
 
13.3
 
 
 
 
ARL8B
 
 
hsa-miR-148b-3p
 
 
-0.337
 
 
32.7
 
 
 
 
ARL8B
 
 
hsa-miR-320b
 
 
-0.376
 
 
32.7
 
 
 
 
ARL8B
 
 
hsa-miR-320c
 
 
-0.376
 
 
32.7
 
 
 
 
ARMC8
 
 
hsa-miR-486-5p
 
 
-0.321
 
 
17.2
 
 
 
 
ARMCX3
 
 
hsa-miR-425-5p
 
 
-0.325
 
 
20.5
 
 
 
 
ARMT1
 
 
hsa-let-7b-5p
 
 
-0.291
 
 
16.5
 
 
 
 
ARPP19
 
 
hsa-miR-320b
 
 
-0.325
 
 
33.6
 
 
 
 
ARPP19
 
 
hsa-miR-320c
 
 
-0.325
 
 
33.6
 
 
 
 
ARPP19
 
 
hsa-miR-15b-5p
 
 
-0.346
 
 
33.6
 
 
 
 
ARPP19
 
 
hsa-miR-16-5p
 
 
-0.354
 
 
33.6
 
 
 
 
ARPP19
 
 
hsa-miR-29a-3p
 
 
-0.371
 
 
33.6
 
 
 
 
ARPP19
 
 
hsa-miR-328-3p
 
 
-0.404
 
 
33.6
 
 
 
 
ARPP19
 
 
hsa-let-7b-5p
 
 
-0.476
 
 
33.6
 
 
 
 
ARRDC3
 
 
hsa-miR-148b-3p
 
 
-0.305
 
 
25.8
 
 
 
 
ARRDC3
 
 
hsa-miR-29a-3p
 
 
-0.402
 
 
25.8
 
 
 
 
ARRDC4
 
 
hsa-let-7b-5p
 
 
-0.329
 
 
21.6
 
 
 
 
ASAH1
 
 
hsa-miR-27a-3p
 
 
-0.398
 
 
81.7
 
 
 
 
ASAP2
 
 
hsa-miR-29a-3p
 
 
-0.348
 
 
15.1
 
 
 
 
ASF1A
 
 
hsa-miR-23a-3p
 
 
-0.506
 
 
10.2
 
 
 
 
ASF1A
 
 
hsa-miR-23b-3p
 
 
-0.506
 
 
10.2
 
 
 
 
ASH1L
 
 
hsa-miR-15b-5p
 
 
-0.325
 
 
10.8
 
 
 
 
ASH1L
 
 
hsa-miR-16-5p
 
 
-0.357
 
 
10.8
 
 
 
 
ASH2L
 
 
hsa-miR-320b
 
 
-0.287
 
 
21.2
 
 
 
 
ASH2L
 
 
hsa-miR-320c
 
 
-0.287
 
 
21.2
 
 
 
 
ASPH
 
 
hsa-miR-148b-3p
 
 
-0.391
 
 
77.9
 
 
 
 
ATG5
 
 
hsa-miR-30b-5p
 
 
-0.488
 
 
14.3
 
 
 
 
ATG9A
 
 
hsa-miR-16-5p
 
 
-0.311
 
 
20.5
 
 
 
 
ATG9A
 
 
hsa-miR-15b-5p
 
 
-0.333
 
 
20.5
 
 
 
 
 
 
 salivary gland 
 
 
 
 
target_symbol
 
 
mature_mirna_id
 
 
score
 
 
nTPM
 
 
 
 
 
 
AAR2
 
 
hsa-miR-29a-3p
 
 
-0.327
 
 
17.0
 
 
 
 
ABCD3
 
 
hsa-miR-148b-3p
 
 
-0.388
 
 
16.5
 
 
 
 
ABCE1
 
 
hsa-miR-29a-3p
 
 
-0.404
 
 
22.7
 
 
 
 
ABHD17C
 
 
hsa-let-7b-5p
 
 
-0.359
 
 
22.3
 
 
 
 
ABHD17C
 
 
hsa-miR-27a-3p
 
 
-0.435
 
 
22.3
 
 
 
 
ABHD4
 
 
hsa-miR-29a-3p
 
 
-0.312
 
 
17.2
 
 
 
 
ABHD5
 
 
hsa-miR-132-3p
 
 
-0.323
 
 
19.4
 
 
 
 
ABHD5
 
 
hsa-miR-27a-3p
 
 
-0.407
 
 
19.4
 
 
 
 
ACTA2
 
 
hsa-miR-27a-3p
 
 
-0.577
 
 
390.9
 
 
 
 
ACTC1
 
 
hsa-miR-30b-5p
 
 
-0.543
 
 
41.7
 
 
 
 
ACTN2
 
 
hsa-miR-23a-3p
 
 
-0.294
 
 
18.4
 
 
 
 
ACTN2
 
 
hsa-miR-29a-3p
 
 
-0.311
 
 
18.4
 
 
 
 
ACTR2
 
 
hsa-miR-15b-5p
 
 
-0.293
 
 
60.3
 
 
 
 
ACTR2
 
 
hsa-miR-16-5p
 
 
-0.293
 
 
60.3
 
 
 
 
ADAM9
 
 
hsa-miR-30b-5p
 
 
-0.312
 
 
39.8
 
 
 
 
ADCY3
 
 
hsa-miR-132-3p
 
 
-0.303
 
 
20.4
 
 
 
 
ADIPOR2
 
 
hsa-miR-150-5p
 
 
-0.287
 
 
21.2
 
 
 
 
ADNP
 
 
hsa-miR-328-3p
 
 
-0.371
 
 
27.9
 
 
 
 
AFG3L2
 
 
hsa-miR-181b-5p
 
 
-0.491
 
 
15.5
 
 
 
 
AFG3L2
 
 
hsa-miR-181a-5p
 
 
-0.491
 
 
15.5
 
 
 
 
AGFG1
 
 
hsa-miR-148b-3p
 
 
-0.332
 
 
26.3
 
 
 
 
AGPAT3
 
 
hsa-miR-27a-3p
 
 
-0.29
 
 
28.8
 
 
 
 
AGPAT3
 
 
hsa-miR-16-5p
 
 
-0.299
 
 
28.8
 
 
 
 
AHDC1
 
 
hsa-miR-148b-3p
 
 
-0.413
 
 
16.0
 
 
 
 
AIFM1
 
 
hsa-let-7b-5p
 
 
-0.314
 
 
17.0
 
 
 
 
AK1
 
 
hsa-miR-29a-3p
 
 
-0.333
 
 
38.2
 
 
 
 
AKAP1
 
 
hsa-miR-148b-3p
 
 
-0.381
 
 
31.6
 
 
 
 
AKIRIN1
 
 
hsa-miR-27a-3p
 
 
-0.368
 
 
22.9
 
 
 
 
ALAD
 
 
hsa-miR-16-5p
 
 
-0.391
 
 
92.9
 
 
 
 
ALAD
 
 
hsa-miR-15b-5p
 
 
-0.446
 
 
92.9
 
 
 
 
ALCAM
 
 
hsa-miR-148b-3p
 
 
-0.339
 
 
31.9
 
 
 
 
ALOX12
 
 
hsa-miR-15b-5p
 
 
-0.443
 
 
17.3
 
 
 
 
ALOX12
 
 
hsa-miR-16-5p
 
 
-0.451
 
 
17.3
 
 
 
 
AMOTL1
 
 
hsa-miR-16-5p
 
 
-0.297
 
 
10.4
 
 
 
 
AMOTL1
 
 
hsa-miR-15b-5p
 
 
-0.319
 
 
10.4
 
 
 
 
AMT
 
 
hsa-let-7b-5p
 
 
-0.463
 
 
10.0
 
 
 
 
ANKHD1
 
 
hsa-miR-23a-3p
 
 
-0.368
 
 
40.4
 
 
 
 
ANKHD1
 
 
hsa-miR-23b-3p
 
 
-0.368
 
 
40.4
 
 
 
 
ANKHD1
 
 
hsa-miR-30b-5p
 
 
-0.405
 
 
40.4
 
 
 
 
ANKRD17
 
 
hsa-miR-30b-5p
 
 
-0.336
 
 
15.7
 
 
 
 
ANKRD40
 
 
hsa-miR-27a-3p
 
 
-0.35
 
 
14.2
 
 
 
 
ANKRD46
 
 
hsa-let-7b-5p
 
 
-0.389
 
 
16.1
 
 
 
 
ANP32A
 
 
hsa-miR-132-3p
 
 
-0.374
 
 
50.8
 
 
 
 
ANTXR2
 
 
hsa-miR-29a-3p
 
 
-0.347
 
 
11.4
 
 
 
 
ANXA11
 
 
hsa-miR-16-5p
 
 
-0.447
 
 
87.6
 
 
 
 
ANXA11
 
 
hsa-miR-15b-5p
 
 
-0.463
 
 
87.6
 
 
 
 
AP1S1
 
 
hsa-let-7b-5p
 
 
-0.374
 
 
21.5
 
 
 
 
AP2A1
 
 
hsa-miR-15b-5p
 
 
-0.333
 
 
27.6
 
 
 
 
AP2A1
 
 
hsa-miR-16-5p
 
 
-0.333
 
 
27.6
 
 
 
 
AP2B1
 
 
hsa-miR-16-5p
 
 
-0.302
 
 
39.5
 
 
 
 
AP2B1
 
 
hsa-miR-15b-5p
 
 
-0.334
 
 
39.5
 
 
 
 
AP3S1
 
 
hsa-miR-30b-5p
 
 
-0.293
 
 
56.0
 
 
 
 
APOO
 
 
hsa-miR-181b-5p
 
 
-0.5
 
 
18.5
 
 
 
 
APOO
 
 
hsa-miR-181a-5p
 
 
-0.5
 
 
18.5
 
 
 
 
APP
 
 
hsa-miR-16-5p
 
 
-0.298
 
 
256.4
 
 
 
 
APP
 
 
hsa-miR-15b-5p
 
 
-0.298
 
 
256.4
 
 
 
 
ARF4
 
 
hsa-miR-30b-5p
 
 
-0.289
 
 
171.4
 
 
 
 
ARF4
 
 
hsa-miR-148b-3p
 
 
-0.487
 
 
171.4
 
 
 
 
ARF5
 
 
hsa-miR-29a-3p
 
 
-0.491
 
 
110.8
 
 
 
 
ARF6
 
 
hsa-miR-181b-5p
 
 
-0.33
 
 
33.7
 
 
 
 
ARF6
 
 
hsa-miR-181a-5p
 
 
-0.33
 
 
33.7
 
 
 
 
ARFGEF1
 
 
hsa-miR-148b-3p
 
 
-0.306
 
 
13.9
 
 
 
 
ARFIP1
 
 
hsa-miR-320b
 
 
-0.339
 
 
12.9
 
 
 
 
ARFIP1
 
 
hsa-miR-320c
 
 
-0.339
 
 
12.9
 
 
 
 
ARFIP1
 
 
hsa-miR-148b-3p
 
 
-0.474
 
 
12.9
 
 
 
 
ARG2
 
 
hsa-let-7b-5p
 
 
-0.468
 
 
18.4
 
 
 
 
ARHGAP21
 
 
hsa-miR-148b-3p
 
 
-0.287
 
 
23.1
 
 
 
 
ARHGAP5
 
 
hsa-miR-486-5p
 
 
-0.3
 
 
56.3
 
 
 
 
ARID1B
 
 
hsa-miR-29a-3p
 
 
-0.342
 
 
18.1
 
 
 
 
ARID4B
 
 
hsa-miR-486-5p
 
 
-0.367
 
 
10.8
 
 
 
 
ARL2
 
 
hsa-miR-16-5p
 
 
-0.374
 
 
67.7
 
 
 
 
ARL2
 
 
hsa-miR-15b-5p
 
 
-0.374
 
 
67.7
 
 
 
 
ARL2
 
 
hsa-miR-16-5p
 
 
-0.418
 
 
67.7
 
 
 
 
ARL2
 
 
hsa-miR-15b-5p
 
 
-0.442
 
 
67.7
 
 
 
 
ARL4A
 
 
hsa-miR-29a-3p
 
 
-0.386
 
 
81.5
 
 
 
 
ARL4A
 
 
hsa-miR-30b-5p
 
 
-0.448
 
 
81.5
 
 
 
 
ARL4D
 
 
hsa-let-7b-5p
 
 
-0.417
 
 
16.7
 
 
 
 
ARL6IP1
 
 
hsa-miR-328-3p
 
 
-0.494
 
 
79.4
 
 
 
 
ARL6IP1
 
 
hsa-miR-148b-3p
 
 
-0.579
 
 
79.4
 
 
 
 
ARL8B
 
 
hsa-miR-148b-3p
 
 
-0.337
 
 
35.3
 
 
 
 
ARL8B
 
 
hsa-miR-320b
 
 
-0.376
 
 
35.3
 
 
 
 
ARL8B
 
 
hsa-miR-320c
 
 
-0.376
 
 
35.3
 
 
 
 
ARMC8
 
 
hsa-miR-486-5p
 
 
-0.321
 
 
11.8
 
 
 
 
ARMCX2
 
 
hsa-miR-15b-5p
 
 
-0.344
 
 
42.0
 
 
 
 
ARMCX2
 
 
hsa-miR-16-5p
 
 
-0.366
 
 
42.0
 
 
 
 
ARMCX3
 
 
hsa-miR-425-5p
 
 
-0.325
 
 
56.1
 
 
 
 
ARPP19
 
 
hsa-miR-320b
 
 
-0.325
 
 
28.7
 
 
 
 
ARPP19
 
 
hsa-miR-320c
 
 
-0.325
 
 
28.7
 
 
 
 
ARPP19
 
 
hsa-miR-15b-5p
 
 
-0.346
 
 
28.7
 
 
 
 
ARPP19
 
 
hsa-miR-16-5p
 
 
-0.354
 
 
28.7
 
 
 
 
ARPP19
 
 
hsa-miR-29a-3p
 
 
-0.371
 
 
28.7
 
 
 
 
ARPP19
 
 
hsa-miR-328-3p
 
 
-0.404
 
 
28.7
 
 
 
 
ARPP19
 
 
hsa-let-7b-5p
 
 
-0.476
 
 
28.7
 
 
 
 
ARRDC3
 
 
hsa-miR-148b-3p
 
 
-0.305
 
 
60.1
 
 
 
 
ARRDC3
 
 
hsa-miR-29a-3p
 
 
-0.402
 
 
60.1
 
 
 
 
ASAH1
 
 
hsa-miR-27a-3p
 
 
-0.398
 
 
61.4
 
 
 
 
ASF1A
 
 
hsa-miR-23a-3p
 
 
-0.506
 
 
10.1
 
 
 
 
ASF1A
 
 
hsa-miR-23b-3p
 
 
-0.506
 
 
10.1
 
 
 
 
ASH1L
 
 
hsa-miR-15b-5p
 
 
-0.325
 
 
17.7
 
 
 
 
ASH1L
 
 
hsa-miR-16-5p
 
 
-0.357
 
 
17.7
 
 
 
 
 
 
 seminal vesicle 
 
 
 
 
target_symbol
 
 
mature_mirna_id
 
 
score
 
 
nTPM
 
 
 
 
 
 
AAR2
 
 
hsa-miR-29a-3p
 
 
-0.327
 
 
19.9
 
 
 
 
ABCD3
 
 
hsa-miR-148b-3p
 
 
-0.388
 
 
25.5
 
 
 
 
ABCE1
 
 
hsa-miR-29a-3p
 
 
-0.404
 
 
15.9
 
 
 
 
ABHD4
 
 
hsa-miR-29a-3p
 
 
-0.312
 
 
34.6
 
 
 
 
ACSL6
 
 
hsa-let-7b-5p
 
 
-0.336
 
 
17.0
 
 
 
 
ACTA2
 
 
hsa-miR-27a-3p
 
 
-0.577
 
 
5569.5
 
 
 
 
ACTC1
 
 
hsa-miR-30b-5p
 
 
-0.543
 
 
506.6
 
 
 
 
ACTR2
 
 
hsa-miR-15b-5p
 
 
-0.293
 
 
57.7
 
 
 
 
ACTR2
 
 
hsa-miR-16-5p
 
 
-0.293
 
 
57.7
 
 
 
 
ACVR1
 
 
hsa-miR-30b-5p
 
 
-0.29
 
 
11.3
 
 
 
 
ACVR1
 
 
hsa-miR-148b-3p
 
 
-0.397
 
 
11.3
 
 
 
 
ADAM10
 
 
hsa-miR-148b-3p
 
 
-0.356
 
 
11.5
 
 
 
 
ADAM9
 
 
hsa-miR-30b-5p
 
 
-0.312
 
 
32.8
 
 
 
 
ADCY3
 
 
hsa-miR-132-3p
 
 
-0.303
 
 
31.0
 
 
 
 
ADGRA3
 
 
hsa-miR-27a-3p
 
 
-0.296
 
 
10.8
 
 
 
 
ADGRA3
 
 
hsa-miR-30b-5p
 
 
-0.304
 
 
10.8
 
 
 
 
ADIPOR2
 
 
hsa-miR-150-5p
 
 
-0.287
 
 
11.2
 
 
 
 
ADNP
 
 
hsa-miR-328-3p
 
 
-0.371
 
 
28.1
 
 
 
 
AFG3L2
 
 
hsa-miR-181b-5p
 
 
-0.491
 
 
11.8
 
 
 
 
AFG3L2
 
 
hsa-miR-181a-5p
 
 
-0.491
 
 
11.8
 
 
 
 
AGFG1
 
 
hsa-miR-148b-3p
 
 
-0.332
 
 
22.0
 
 
 
 
AGPAT3
 
 
hsa-miR-27a-3p
 
 
-0.29
 
 
10.9
 
 
 
 
AGPAT3
 
 
hsa-miR-16-5p
 
 
-0.299
 
 
10.9
 
 
 
 
AHR
 
 
hsa-miR-29a-3p
 
 
-0.316
 
 
10.6
 
 
 
 
AK1
 
 
hsa-miR-29a-3p
 
 
-0.333
 
 
33.8
 
 
 
 
AK4
 
 
hsa-miR-15b-5p
 
 
-0.316
 
 
11.8
 
 
 
 
AK4
 
 
hsa-miR-16-5p
 
 
-0.348
 
 
11.8
 
 
 
 
AKAP1
 
 
hsa-miR-148b-3p
 
 
-0.381
 
 
23.3
 
 
 
 
AKIRIN1
 
 
hsa-miR-27a-3p
 
 
-0.368
 
 
33.7
 
 
 
 
AKT3
 
 
hsa-miR-29a-3p
 
 
-0.287
 
 
14.7
 
 
 
 
AKT3
 
 
hsa-miR-15b-5p
 
 
-0.301
 
 
14.7
 
 
 
 
AKT3
 
 
hsa-miR-16-5p
 
 
-0.309
 
 
14.7
 
 
 
 
ALAD
 
 
hsa-miR-16-5p
 
 
-0.391
 
 
22.8
 
 
 
 
ALAD
 
 
hsa-miR-15b-5p
 
 
-0.446
 
 
22.8
 
 
 
 
ALCAM
 
 
hsa-miR-148b-3p
 
 
-0.339
 
 
37.3
 
 
 
 
ALDH1A2
 
 
hsa-miR-23b-3p
 
 
-0.346
 
 
50.2
 
 
 
 
ALDH1A2
 
 
hsa-miR-23a-3p
 
 
-0.354
 
 
50.2
 
 
 
 
AMOT
 
 
hsa-miR-29a-3p
 
 
-0.304
 
 
24.6
 
 
 
 
AMOTL1
 
 
hsa-miR-16-5p
 
 
-0.297
 
 
21.7
 
 
 
 
AMOTL1
 
 
hsa-miR-15b-5p
 
 
-0.319
 
 
21.7
 
 
 
 
ANKHD1
 
 
hsa-miR-23a-3p
 
 
-0.368
 
 
26.1
 
 
 
 
ANKHD1
 
 
hsa-miR-23b-3p
 
 
-0.368
 
 
26.1
 
 
 
 
ANKHD1
 
 
hsa-miR-30b-5p
 
 
-0.405
 
 
26.1
 
 
 
 
ANKRD17
 
 
hsa-miR-30b-5p
 
 
-0.336
 
 
13.0
 
 
 
 
ANKRD40
 
 
hsa-miR-27a-3p
 
 
-0.35
 
 
23.3
 
 
 
 
ANKRD46
 
 
hsa-let-7b-5p
 
 
-0.389
 
 
12.4
 
 
 
 
ANP32A
 
 
hsa-miR-132-3p
 
 
-0.374
 
 
62.9
 
 
 
 
ANTXR2
 
 
hsa-miR-29a-3p
 
 
-0.347
 
 
85.0
 
 
 
 
ANXA11
 
 
hsa-miR-16-5p
 
 
-0.447
 
 
77.1
 
 
 
 
ANXA11
 
 
hsa-miR-15b-5p
 
 
-0.463
 
 
77.1
 
 
 
 
AP2A1
 
 
hsa-miR-15b-5p
 
 
-0.333
 
 
17.5
 
 
 
 
AP2A1
 
 
hsa-miR-16-5p
 
 
-0.333
 
 
17.5
 
 
 
 
AP2B1
 
 
hsa-miR-16-5p
 
 
-0.302
 
 
47.2
 
 
 
 
AP2B1
 
 
hsa-miR-15b-5p
 
 
-0.334
 
 
47.2
 
 
 
 
AP3S1
 
 
hsa-miR-30b-5p
 
 
-0.293
 
 
80.7
 
 
 
 
APOO
 
 
hsa-miR-181b-5p
 
 
-0.5
 
 
14.1
 
 
 
 
APOO
 
 
hsa-miR-181a-5p
 
 
-0.5
 
 
14.1
 
 
 
 
APP
 
 
hsa-miR-16-5p
 
 
-0.298
 
 
315.9
 
 
 
 
APP
 
 
hsa-miR-15b-5p
 
 
-0.298
 
 
315.9
 
 
 
 
ARF4
 
 
hsa-miR-30b-5p
 
 
-0.289
 
 
125.1
 
 
 
 
ARF4
 
 
hsa-miR-148b-3p
 
 
-0.487
 
 
125.1
 
 
 
 
ARF5
 
 
hsa-miR-29a-3p
 
 
-0.491
 
 
67.1
 
 
 
 
ARF6
 
 
hsa-miR-181b-5p
 
 
-0.33
 
 
18.1
 
 
 
 
ARF6
 
 
hsa-miR-181a-5p
 
 
-0.33
 
 
18.1
 
 
 
 
ARFGEF1
 
 
hsa-miR-148b-3p
 
 
-0.306
 
 
11.4
 
 
 
 
ARFIP1
 
 
hsa-miR-320b
 
 
-0.339
 
 
16.2
 
 
 
 
ARFIP1
 
 
hsa-miR-320c
 
 
-0.339
 
 
16.2
 
 
 
 
ARFIP1
 
 
hsa-miR-148b-3p
 
 
-0.474
 
 
16.2
 
 
 
 
ARHGAP21
 
 
hsa-miR-148b-3p
 
 
-0.287
 
 
10.8
 
 
 
 
ARHGAP5
 
 
hsa-miR-486-5p
 
 
-0.3
 
 
27.6
 
 
 
 
ARID1B
 
 
hsa-miR-29a-3p
 
 
-0.342
 
 
11.6
 
 
 
 
ARID4B
 
 
hsa-miR-486-5p
 
 
-0.367
 
 
10.2
 
 
 
 
ARL2
 
 
hsa-miR-16-5p
 
 
-0.374
 
 
62.7
 
 
 
 
ARL2
 
 
hsa-miR-15b-5p
 
 
-0.374
 
 
62.7
 
 
 
 
ARL2
 
 
hsa-miR-16-5p
 
 
-0.418
 
 
62.7
 
 
 
 
ARL2
 
 
hsa-miR-15b-5p
 
 
-0.442
 
 
62.7
 
 
 
 
ARL4A
 
 
hsa-miR-29a-3p
 
 
-0.386
 
 
15.5
 
 
 
 
ARL4A
 
 
hsa-miR-30b-5p
 
 
-0.448
 
 
15.5
 
 
 
 
ARL4D
 
 
hsa-let-7b-5p
 
 
-0.417
 
 
14.3
 
 
 
 
ARL6IP1
 
 
hsa-miR-328-3p
 
 
-0.494
 
 
66.4
 
 
 
 
ARL6IP1
 
 
hsa-miR-148b-3p
 
 
-0.579
 
 
66.4
 
 
 
 
ARL8B
 
 
hsa-miR-148b-3p
 
 
-0.337
 
 
39.4
 
 
 
 
ARL8B
 
 
hsa-miR-320b
 
 
-0.376
 
 
39.4
 
 
 
 
ARL8B
 
 
hsa-miR-320c
 
 
-0.376
 
 
39.4
 
 
 
 
ARMC8
 
 
hsa-miR-486-5p
 
 
-0.321
 
 
16.5
 
 
 
 
ARMCX2
 
 
hsa-miR-15b-5p
 
 
-0.344
 
 
64.0
 
 
 
 
ARMCX2
 
 
hsa-miR-16-5p
 
 
-0.366
 
 
64.0
 
 
 
 
ARMCX3
 
 
hsa-miR-425-5p
 
 
-0.325
 
 
44.1
 
 
 
 
ARMT1
 
 
hsa-let-7b-5p
 
 
-0.291
 
 
10.5
 
 
 
 
ARPP19
 
 
hsa-miR-320b
 
 
-0.325
 
 
38.5
 
 
 
 
ARPP19
 
 
hsa-miR-320c
 
 
-0.325
 
 
38.5
 
 
 
 
ARPP19
 
 
hsa-miR-15b-5p
 
 
-0.346
 
 
38.5
 
 
 
 
ARPP19
 
 
hsa-miR-16-5p
 
 
-0.354
 
 
38.5
 
 
 
 
ARPP19
 
 
hsa-miR-29a-3p
 
 
-0.371
 
 
38.5
 
 
 
 
ARPP19
 
 
hsa-miR-328-3p
 
 
-0.404
 
 
38.5
 
 
 
 
ARPP19
 
 
hsa-let-7b-5p
 
 
-0.476
 
 
38.5
 
 
 
 
ARRDC3
 
 
hsa-miR-148b-3p
 
 
-0.305
 
 
22.9
 
 
 
 
ARRDC3
 
 
hsa-miR-29a-3p
 
 
-0.402
 
 
22.9
 
 
 
 
ARRDC4
 
 
hsa-let-7b-5p
 
 
-0.329
 
 
15.4
 
 
 
 
ASAH1
 
 
hsa-miR-27a-3p
 
 
-0.398
 
 
73.4
 
 
 
 
 
 
 skeletal muscle 
 
 
 
 
target_symbol
 
 
mature_mirna_id
 
 
score
 
 
nTPM
 
 
 
 
 
 
AAR2
 
 
hsa-miR-29a-3p
 
 
-0.327
 
 
22.2
 
 
 
 
ABCB10
 
 
hsa-miR-148b-3p
 
 
-0.291
 
 
12.0
 
 
 
 
ABCB6
 
 
hsa-miR-29a-3p
 
 
-0.48
 
 
26.6
 
 
 
 
ABCB7
 
 
hsa-miR-148b-3p
 
 
-0.515
 
 
29.2
 
 
 
 
ABCD3
 
 
hsa-miR-148b-3p
 
 
-0.388
 
 
22.0
 
 
 
 
ABCE1
 
 
hsa-miR-29a-3p
 
 
-0.404
 
 
27.0
 
 
 
 
ABHD17B
 
 
hsa-miR-486-5p
 
 
-0.348
 
 
12.5
 
 
 
 
ABHD18
 
 
hsa-miR-29a-3p
 
 
-0.321
 
 
10.1
 
 
 
 
ABHD18
 
 
hsa-miR-29a-3p
 
 
-0.34
 
 
10.1
 
 
 
 
ABHD4
 
 
hsa-miR-29a-3p
 
 
-0.312
 
 
26.2
 
 
 
 
ABHD5
 
 
hsa-miR-132-3p
 
 
-0.323
 
 
12.3
 
 
 
 
ABHD5
 
 
hsa-miR-27a-3p
 
 
-0.407
 
 
12.3
 
 
 
 
ACTA2
 
 
hsa-miR-27a-3p
 
 
-0.577
 
 
194.7
 
 
 
 
ACTC1
 
 
hsa-miR-30b-5p
 
 
-0.543
 
 
519.7
 
 
 
 
ACTN2
 
 
hsa-miR-23a-3p
 
 
-0.294
 
 
1961.6
 
 
 
 
ACTN2
 
 
hsa-miR-29a-3p
 
 
-0.311
 
 
1961.6
 
 
 
 
ACTR2
 
 
hsa-miR-15b-5p
 
 
-0.293
 
 
56.5
 
 
 
 
ACTR2
 
 
hsa-miR-16-5p
 
 
-0.293
 
 
56.5
 
 
 
 
ACVR1
 
 
hsa-miR-30b-5p
 
 
-0.29
 
 
15.5
 
 
 
 
ACVR1
 
 
hsa-miR-148b-3p
 
 
-0.397
 
 
15.5
 
 
 
 
ADAM9
 
 
hsa-miR-30b-5p
 
 
-0.312
 
 
16.2
 
 
 
 
ADCY2
 
 
hsa-miR-148b-3p
 
 
-0.299
 
 
64.1
 
 
 
 
ADCY3
 
 
hsa-miR-132-3p
 
 
-0.303
 
 
14.1
 
 
 
 
ADGRA3
 
 
hsa-miR-27a-3p
 
 
-0.296
 
 
15.9
 
 
 
 
ADGRA3
 
 
hsa-miR-30b-5p
 
 
-0.304
 
 
15.9
 
 
 
 
ADGRF5
 
 
hsa-miR-148b-3p
 
 
-0.354
 
 
29.0
 
 
 
 
ADIPOR2
 
 
hsa-miR-150-5p
 
 
-0.287
 
 
41.4
 
 
 
 
ADNP
 
 
hsa-miR-328-3p
 
 
-0.371
 
 
33.8
 
 
 
 
ADRB2
 
 
hsa-miR-15b-5p
 
 
-0.29
 
 
10.9
 
 
 
 
ADRB2
 
 
hsa-miR-16-5p
 
 
-0.323
 
 
10.9
 
 
 
 
ADRB2
 
 
hsa-let-7b-5p
 
 
-0.635
 
 
10.9
 
 
 
 
AFG3L2
 
 
hsa-miR-181b-5p
 
 
-0.491
 
 
96.6
 
 
 
 
AFG3L2
 
 
hsa-miR-181a-5p
 
 
-0.491
 
 
96.6
 
 
 
 
AGFG1
 
 
hsa-miR-148b-3p
 
 
-0.332
 
 
31.6
 
 
 
 
AGO2
 
 
hsa-miR-132-3p
 
 
-0.406
 
 
16.4
 
 
 
 
AGO2
 
 
hsa-miR-27a-3p
 
 
-0.486
 
 
16.4
 
 
 
 
AGO2
 
 
hsa-miR-23a-3p
 
 
-0.576
 
 
16.4
 
 
 
 
AGO2
 
 
hsa-miR-23b-3p
 
 
-0.587
 
 
16.4
 
 
 
 
AGO2
 
 
hsa-miR-27a-3p
 
 
-0.646
 
 
16.4
 
 
 
 
AGO2
 
 
hsa-miR-30b-5p
 
 
-1.034
 
 
16.4
 
 
 
 
AGPAT3
 
 
hsa-miR-27a-3p
 
 
-0.29
 
 
56.6
 
 
 
 
AGPAT3
 
 
hsa-miR-16-5p
 
 
-0.299
 
 
56.6
 
 
 
 
AHCTF1
 
 
hsa-let-7b-5p
 
 
-0.374
 
 
10.0
 
 
 
 
AHDC1
 
 
hsa-miR-148b-3p
 
 
-0.413
 
 
29.2
 
 
 
 
AIFM1
 
 
hsa-let-7b-5p
 
 
-0.314
 
 
58.6
 
 
 
 
AK1
 
 
hsa-miR-29a-3p
 
 
-0.333
 
 
1262.8
 
 
 
 
AKAP1
 
 
hsa-miR-148b-3p
 
 
-0.381
 
 
194.7
 
 
 
 
AKAP6
 
 
hsa-let-7b-5p
 
 
-0.307
 
 
44.4
 
 
 
 
AKIRIN1
 
 
hsa-miR-27a-3p
 
 
-0.368
 
 
28.7
 
 
 
 
ALAD
 
 
hsa-miR-16-5p
 
 
-0.391
 
 
26.9
 
 
 
 
ALAD
 
 
hsa-miR-15b-5p
 
 
-0.446
 
 
26.9
 
 
 
 
ALDH1A2
 
 
hsa-miR-23b-3p
 
 
-0.346
 
 
10.0
 
 
 
 
ALDH1A2
 
 
hsa-miR-23a-3p
 
 
-0.354
 
 
10.0
 
 
 
 
AMOT
 
 
hsa-miR-29a-3p
 
 
-0.304
 
 
37.2
 
 
 
 
AMOTL1
 
 
hsa-miR-16-5p
 
 
-0.297
 
 
104.6
 
 
 
 
AMOTL1
 
 
hsa-miR-15b-5p
 
 
-0.319
 
 
104.6
 
 
 
 
ANKHD1
 
 
hsa-miR-23a-3p
 
 
-0.368
 
 
67.6
 
 
 
 
ANKHD1
 
 
hsa-miR-23b-3p
 
 
-0.368
 
 
67.6
 
 
 
 
ANKHD1
 
 
hsa-miR-30b-5p
 
 
-0.405
 
 
67.6
 
 
 
 
ANKRD17
 
 
hsa-miR-30b-5p
 
 
-0.336
 
 
21.8
 
 
 
 
ANKRD40
 
 
hsa-miR-27a-3p
 
 
-0.35
 
 
100.2
 
 
 
 
ANKRD46
 
 
hsa-let-7b-5p
 
 
-0.389
 
 
58.3
 
 
 
 
ANP32A
 
 
hsa-miR-132-3p
 
 
-0.374
 
 
57.9
 
 
 
 
ANTXR2
 
 
hsa-miR-29a-3p
 
 
-0.347
 
 
12.3
 
 
 
 
ANXA11
 
 
hsa-miR-16-5p
 
 
-0.447
 
 
177.1
 
 
 
 
ANXA11
 
 
hsa-miR-15b-5p
 
 
-0.463
 
 
177.1
 
 
 
 
AP2A1
 
 
hsa-miR-15b-5p
 
 
-0.333
 
 
85.4
 
 
 
 
AP2A1
 
 
hsa-miR-16-5p
 
 
-0.333
 
 
85.4
 
 
 
 
AP2B1
 
 
hsa-miR-16-5p
 
 
-0.302
 
 
49.8
 
 
 
 
AP2B1
 
 
hsa-miR-15b-5p
 
 
-0.334
 
 
49.8
 
 
 
 
AP3S1
 
 
hsa-miR-30b-5p
 
 
-0.293
 
 
25.5
 
 
 
 
AP5S1
 
 
hsa-miR-744-5p
 
 
-0.354
 
 
17.9
 
 
 
 
APOO
 
 
hsa-miR-181b-5p
 
 
-0.5
 
 
43.1
 
 
 
 
APOO
 
 
hsa-miR-181a-5p
 
 
-0.5
 
 
43.1
 
 
 
 
APP
 
 
hsa-miR-16-5p
 
 
-0.298
 
 
99.7
 
 
 
 
APP
 
 
hsa-miR-15b-5p
 
 
-0.298
 
 
99.7
 
 
 
 
ARF4
 
 
hsa-miR-30b-5p
 
 
-0.289
 
 
59.5
 
 
 
 
ARF4
 
 
hsa-miR-148b-3p
 
 
-0.487
 
 
59.5
 
 
 
 
ARF5
 
 
hsa-miR-29a-3p
 
 
-0.491
 
 
145.7
 
 
 
 
ARF6
 
 
hsa-miR-181b-5p
 
 
-0.33
 
 
21.4
 
 
 
 
ARF6
 
 
hsa-miR-181a-5p
 
 
-0.33
 
 
21.4
 
 
 
 
ARFGEF1
 
 
hsa-miR-148b-3p
 
 
-0.306
 
 
12.2
 
 
 
 
ARG2
 
 
hsa-let-7b-5p
 
 
-0.468
 
 
37.4
 
 
 
 
ARHGAP21
 
 
hsa-miR-148b-3p
 
 
-0.287
 
 
15.7
 
 
 
 
ARHGAP5
 
 
hsa-miR-486-5p
 
 
-0.3
 
 
11.9
 
 
 
 
ARID1B
 
 
hsa-miR-29a-3p
 
 
-0.342
 
 
30.4
 
 
 
 
ARL2
 
 
hsa-miR-16-5p
 
 
-0.374
 
 
216.4
 
 
 
 
ARL2
 
 
hsa-miR-15b-5p
 
 
-0.374
 
 
216.4
 
 
 
 
ARL2
 
 
hsa-miR-16-5p
 
 
-0.418
 
 
216.4
 
 
 
 
ARL2
 
 
hsa-miR-15b-5p
 
 
-0.442
 
 
216.4
 
 
 
 
ARL6IP1
 
 
hsa-miR-328-3p
 
 
-0.494
 
 
21.6
 
 
 
 
ARL6IP1
 
 
hsa-miR-148b-3p
 
 
-0.579
 
 
21.6
 
 
 
 
ARL6IP6
 
 
hsa-let-7b-5p
 
 
-0.293
 
 
53.9
 
 
 
 
ARL8B
 
 
hsa-miR-148b-3p
 
 
-0.337
 
 
98.5
 
 
 
 
ARL8B
 
 
hsa-miR-320b
 
 
-0.376
 
 
98.5
 
 
 
 
ARL8B
 
 
hsa-miR-320c
 
 
-0.376
 
 
98.5
 
 
 
 
ARMC8
 
 
hsa-miR-486-5p
 
 
-0.321
 
 
36.6
 
 
 
 
ARMCX3
 
 
hsa-miR-425-5p
 
 
-0.325
 
 
28.7
 
 
 
 
ARMT1
 
 
hsa-let-7b-5p
 
 
-0.291
 
 
17.8
 
 
 
 
ARPP19
 
 
hsa-miR-320b
 
 
-0.325
 
 
225.3
 
 
 
 
 
 
 skin 
 
 
 
 
target_symbol
 
 
mature_mirna_id
 
 
score
 
 
nTPM
 
 
 
 
 
 
AAR2
 
 
hsa-miR-29a-3p
 
 
-0.327
 
 
24.6
 
 
 
 
ABCB7
 
 
hsa-miR-148b-3p
 
 
-0.515
 
 
12.7
 
 
 
 
ABCD3
 
 
hsa-miR-148b-3p
 
 
-0.388
 
 
33.6
 
 
 
 
ABCE1
 
 
hsa-miR-29a-3p
 
 
-0.404
 
 
23.8
 
 
 
 
ABHD17C
 
 
hsa-let-7b-5p
 
 
-0.359
 
 
17.9
 
 
 
 
ABHD17C
 
 
hsa-miR-27a-3p
 
 
-0.435
 
 
17.9
 
 
 
 
ABHD4
 
 
hsa-miR-29a-3p
 
 
-0.312
 
 
20.2
 
 
 
 
ABHD5
 
 
hsa-miR-132-3p
 
 
-0.323
 
 
29.7
 
 
 
 
ABHD5
 
 
hsa-miR-27a-3p
 
 
-0.407
 
 
29.7
 
 
 
 
ACP3
 
 
hsa-let-7b-5p
 
 
-0.298
 
 
25.5
 
 
 
 
ACTA2
 
 
hsa-miR-27a-3p
 
 
-0.577
 
 
331.5
 
 
 
 
ACTC1
 
 
hsa-miR-30b-5p
 
 
-0.543
 
 
14.2
 
 
 
 
ACTR2
 
 
hsa-miR-15b-5p
 
 
-0.293
 
 
65.5
 
 
 
 
ACTR2
 
 
hsa-miR-16-5p
 
 
-0.293
 
 
65.5
 
 
 
 
ADAM10
 
 
hsa-miR-148b-3p
 
 
-0.356
 
 
14.8
 
 
 
 
ADCY3
 
 
hsa-miR-132-3p
 
 
-0.303
 
 
36.0
 
 
 
 
ADIPOR2
 
 
hsa-miR-150-5p
 
 
-0.287
 
 
30.8
 
 
 
 
ADNP
 
 
hsa-miR-328-3p
 
 
-0.371
 
 
33.2
 
 
 
 
ADRA2A
 
 
hsa-miR-23a-3p
 
 
-0.371
 
 
10.2
 
 
 
 
ADRA2A
 
 
hsa-miR-23b-3p
 
 
-0.371
 
 
10.2
 
 
 
 
ADRB2
 
 
hsa-miR-15b-5p
 
 
-0.29
 
 
36.9
 
 
 
 
ADRB2
 
 
hsa-miR-16-5p
 
 
-0.323
 
 
36.9
 
 
 
 
ADRB2
 
 
hsa-let-7b-5p
 
 
-0.635
 
 
36.9
 
 
 
 
AFG3L2
 
 
hsa-miR-181b-5p
 
 
-0.491
 
 
15.1
 
 
 
 
AFG3L2
 
 
hsa-miR-181a-5p
 
 
-0.491
 
 
15.1
 
 
 
 
AGFG1
 
 
hsa-miR-148b-3p
 
 
-0.332
 
 
34.3
 
 
 
 
AGPAT3
 
 
hsa-miR-27a-3p
 
 
-0.29
 
 
23.2
 
 
 
 
AGPAT3
 
 
hsa-miR-16-5p
 
 
-0.299
 
 
23.2
 
 
 
 
AHDC1
 
 
hsa-miR-148b-3p
 
 
-0.413
 
 
29.8
 
 
 
 
AHR
 
 
hsa-miR-29a-3p
 
 
-0.316
 
 
20.7
 
 
 
 
AIFM1
 
 
hsa-let-7b-5p
 
 
-0.314
 
 
10.3
 
 
 
 
AK1
 
 
hsa-miR-29a-3p
 
 
-0.333
 
 
17.4
 
 
 
 
AK4
 
 
hsa-miR-15b-5p
 
 
-0.316
 
 
25.7
 
 
 
 
AK4
 
 
hsa-miR-16-5p
 
 
-0.348
 
 
25.7
 
 
 
 
AKAP1
 
 
hsa-miR-148b-3p
 
 
-0.381
 
 
26.4
 
 
 
 
AKIRIN1
 
 
hsa-miR-27a-3p
 
 
-0.368
 
 
16.7
 
 
 
 
ALAD
 
 
hsa-miR-16-5p
 
 
-0.391
 
 
36.8
 
 
 
 
ALAD
 
 
hsa-miR-15b-5p
 
 
-0.446
 
 
36.8
 
 
 
 
ALCAM
 
 
hsa-miR-148b-3p
 
 
-0.339
 
 
10.3
 
 
 
 
ALOX12
 
 
hsa-miR-15b-5p
 
 
-0.443
 
 
20.5
 
 
 
 
ALOX12
 
 
hsa-miR-16-5p
 
 
-0.451
 
 
20.5
 
 
 
 
AMOTL1
 
 
hsa-miR-16-5p
 
 
-0.297
 
 
20.5
 
 
 
 
AMOTL1
 
 
hsa-miR-15b-5p
 
 
-0.319
 
 
20.5
 
 
 
 
ANKHD1
 
 
hsa-miR-23a-3p
 
 
-0.368
 
 
48.3
 
 
 
 
ANKHD1
 
 
hsa-miR-23b-3p
 
 
-0.368
 
 
48.3
 
 
 
 
ANKHD1
 
 
hsa-miR-30b-5p
 
 
-0.405
 
 
48.3
 
 
 
 
ANKRD17
 
 
hsa-miR-30b-5p
 
 
-0.336
 
 
16.2
 
 
 
 
ANKRD40
 
 
hsa-miR-27a-3p
 
 
-0.35
 
 
13.5
 
 
 
 
ANKRD46
 
 
hsa-let-7b-5p
 
 
-0.389
 
 
10.5
 
 
 
 
ANLN
 
 
hsa-miR-16-5p
 
 
-0.31
 
 
12.9
 
 
 
 
ANLN
 
 
hsa-miR-15b-5p
 
 
-0.334
 
 
12.9
 
 
 
 
ANP32A
 
 
hsa-miR-132-3p
 
 
-0.374
 
 
34.1
 
 
 
 
ANTXR2
 
 
hsa-miR-29a-3p
 
 
-0.347
 
 
13.8
 
 
 
 
ANXA11
 
 
hsa-miR-16-5p
 
 
-0.447
 
 
70.8
 
 
 
 
ANXA11
 
 
hsa-miR-15b-5p
 
 
-0.463
 
 
70.8
 
 
 
 
AP1S1
 
 
hsa-let-7b-5p
 
 
-0.374
 
 
40.6
 
 
 
 
AP2A1
 
 
hsa-miR-15b-5p
 
 
-0.333
 
 
52.1
 
 
 
 
AP2A1
 
 
hsa-miR-16-5p
 
 
-0.333
 
 
52.1
 
 
 
 
AP2B1
 
 
hsa-miR-16-5p
 
 
-0.302
 
 
27.9
 
 
 
 
AP2B1
 
 
hsa-miR-15b-5p
 
 
-0.334
 
 
27.9
 
 
 
 
AP3S1
 
 
hsa-miR-30b-5p
 
 
-0.293
 
 
57.3
 
 
 
 
APP
 
 
hsa-miR-16-5p
 
 
-0.298
 
 
204.7
 
 
 
 
APP
 
 
hsa-miR-15b-5p
 
 
-0.298
 
 
204.7
 
 
 
 
ARF4
 
 
hsa-miR-30b-5p
 
 
-0.289
 
 
61.3
 
 
 
 
ARF4
 
 
hsa-miR-148b-3p
 
 
-0.487
 
 
61.3
 
 
 
 
ARF5
 
 
hsa-miR-29a-3p
 
 
-0.491
 
 
215.0
 
 
 
 
ARF6
 
 
hsa-miR-181b-5p
 
 
-0.33
 
 
50.6
 
 
 
 
ARF6
 
 
hsa-miR-181a-5p
 
 
-0.33
 
 
50.6
 
 
 
 
ARFGEF1
 
 
hsa-miR-148b-3p
 
 
-0.306
 
 
12.9
 
 
 
 
ARFIP1
 
 
hsa-miR-320b
 
 
-0.339
 
 
16.4
 
 
 
 
ARFIP1
 
 
hsa-miR-320c
 
 
-0.339
 
 
16.4
 
 
 
 
ARFIP1
 
 
hsa-miR-148b-3p
 
 
-0.474
 
 
16.4
 
 
 
 
ARHGAP5
 
 
hsa-miR-486-5p
 
 
-0.3
 
 
29.1
 
 
 
 
ARID1B
 
 
hsa-miR-29a-3p
 
 
-0.342
 
 
21.3
 
 
 
 
ARL2
 
 
hsa-miR-16-5p
 
 
-0.374
 
 
96.0
 
 
 
 
ARL2
 
 
hsa-miR-15b-5p
 
 
-0.374
 
 
96.0
 
 
 
 
ARL2
 
 
hsa-miR-16-5p
 
 
-0.418
 
 
96.0
 
 
 
 
ARL2
 
 
hsa-miR-15b-5p
 
 
-0.442
 
 
96.0
 
 
 
 
ARL4A
 
 
hsa-miR-29a-3p
 
 
-0.386
 
 
85.3
 
 
 
 
ARL4A
 
 
hsa-miR-30b-5p
 
 
-0.448
 
 
85.3
 
 
 
 
ARL4D
 
 
hsa-let-7b-5p
 
 
-0.417
 
 
44.0
 
 
 
 
ARL6IP1
 
 
hsa-miR-328-3p
 
 
-0.494
 
 
62.4
 
 
 
 
ARL6IP1
 
 
hsa-miR-148b-3p
 
 
-0.579
 
 
62.4
 
 
 
 
ARL6IP6
 
 
hsa-let-7b-5p
 
 
-0.293
 
 
23.5
 
 
 
 
ARL8B
 
 
hsa-miR-148b-3p
 
 
-0.337
 
 
64.4
 
 
 
 
ARL8B
 
 
hsa-miR-320b
 
 
-0.376
 
 
64.4
 
 
 
 
ARL8B
 
 
hsa-miR-320c
 
 
-0.376
 
 
64.4
 
 
 
 
ARMC8
 
 
hsa-miR-486-5p
 
 
-0.321
 
 
12.0
 
 
 
 
ARMCX3
 
 
hsa-miR-425-5p
 
 
-0.325
 
 
10.4
 
 
 
 
ARPP19
 
 
hsa-miR-320b
 
 
-0.325
 
 
28.9
 
 
 
 
ARPP19
 
 
hsa-miR-320c
 
 
-0.325
 
 
28.9
 
 
 
 
ARPP19
 
 
hsa-miR-15b-5p
 
 
-0.346
 
 
28.9
 
 
 
 
ARPP19
 
 
hsa-miR-16-5p
 
 
-0.354
 
 
28.9
 
 
 
 
ARPP19
 
 
hsa-miR-29a-3p
 
 
-0.371
 
 
28.9
 
 
 
 
ARPP19
 
 
hsa-miR-328-3p
 
 
-0.404
 
 
28.9
 
 
 
 
ARPP19
 
 
hsa-let-7b-5p
 
 
-0.476
 
 
28.9
 
 
 
 
ARRDC3
 
 
hsa-miR-148b-3p
 
 
-0.305
 
 
38.6
 
 
 
 
ARRDC3
 
 
hsa-miR-29a-3p
 
 
-0.402
 
 
38.6
 
 
 
 
ARRDC4
 
 
hsa-let-7b-5p
 
 
-0.329
 
 
33.5
 
 
 
 
ASAH1
 
 
hsa-miR-27a-3p
 
 
-0.398
 
 
49.7
 
 
 
 
 
 
 small intestine 
 
 
 
 
target_symbol
 
 
mature_mirna_id
 
 
score
 
 
nTPM
 
 
 
 
 
 
AAR2
 
 
hsa-miR-29a-3p
 
 
-0.327
 
 
18.1
 
 
 
 
ABCB10
 
 
hsa-miR-148b-3p
 
 
-0.291
 
 
13.1
 
 
 
 
ABCB7
 
 
hsa-miR-148b-3p
 
 
-0.515
 
 
11.2
 
 
 
 
ABCD3
 
 
hsa-miR-148b-3p
 
 
-0.388
 
 
43.4
 
 
 
 
ABCE1
 
 
hsa-miR-29a-3p
 
 
-0.404
 
 
16.7
 
 
 
 
ABHD17B
 
 
hsa-miR-486-5p
 
 
-0.348
 
 
12.6
 
 
 
 
ABHD17C
 
 
hsa-let-7b-5p
 
 
-0.359
 
 
28.8
 
 
 
 
ABHD17C
 
 
hsa-miR-27a-3p
 
 
-0.435
 
 
28.8
 
 
 
 
ABHD4
 
 
hsa-miR-29a-3p
 
 
-0.312
 
 
18.9
 
 
 
 
ACTA2
 
 
hsa-miR-27a-3p
 
 
-0.577
 
 
677.1
 
 
 
 
ACTR2
 
 
hsa-miR-15b-5p
 
 
-0.293
 
 
111.3
 
 
 
 
ACTR2
 
 
hsa-miR-16-5p
 
 
-0.293
 
 
111.3
 
 
 
 
ADA
 
 
hsa-miR-29a-3p
 
 
-0.376
 
 
15.5
 
 
 
 
ADAM10
 
 
hsa-miR-148b-3p
 
 
-0.356
 
 
15.8
 
 
 
 
ADAM9
 
 
hsa-miR-30b-5p
 
 
-0.312
 
 
21.4
 
 
 
 
ADAP1
 
 
hsa-miR-27a-3p
 
 
-0.302
 
 
25.6
 
 
 
 
ADCY3
 
 
hsa-miR-132-3p
 
 
-0.303
 
 
31.8
 
 
 
 
ADIPOR2
 
 
hsa-miR-150-5p
 
 
-0.287
 
 
31.4
 
 
 
 
ADNP
 
 
hsa-miR-328-3p
 
 
-0.371
 
 
30.1
 
 
 
 
AFG3L2
 
 
hsa-miR-181b-5p
 
 
-0.491
 
 
34.0
 
 
 
 
AFG3L2
 
 
hsa-miR-181a-5p
 
 
-0.491
 
 
34.0
 
 
 
 
AGFG1
 
 
hsa-miR-148b-3p
 
 
-0.332
 
 
33.9
 
 
 
 
AGPAT3
 
 
hsa-miR-27a-3p
 
 
-0.29
 
 
49.3
 
 
 
 
AGPAT3
 
 
hsa-miR-16-5p
 
 
-0.299
 
 
49.3
 
 
 
 
AHR
 
 
hsa-miR-29a-3p
 
 
-0.316
 
 
13.6
 
 
 
 
AIFM1
 
 
hsa-let-7b-5p
 
 
-0.314
 
 
28.2
 
 
 
 
AK1
 
 
hsa-miR-29a-3p
 
 
-0.333
 
 
15.6
 
 
 
 
AKAP1
 
 
hsa-miR-148b-3p
 
 
-0.381
 
 
52.5
 
 
 
 
AKIRIN1
 
 
hsa-miR-27a-3p
 
 
-0.368
 
 
25.6
 
 
 
 
ALAD
 
 
hsa-miR-16-5p
 
 
-0.391
 
 
25.1
 
 
 
 
ALAD
 
 
hsa-miR-15b-5p
 
 
-0.446
 
 
25.1
 
 
 
 
ALCAM
 
 
hsa-miR-148b-3p
 
 
-0.339
 
 
16.4
 
 
 
 
AMT
 
 
hsa-let-7b-5p
 
 
-0.463
 
 
11.5
 
 
 
 
ANKHD1
 
 
hsa-miR-23a-3p
 
 
-0.368
 
 
44.2
 
 
 
 
ANKHD1
 
 
hsa-miR-23b-3p
 
 
-0.368
 
 
44.2
 
 
 
 
ANKHD1
 
 
hsa-miR-30b-5p
 
 
-0.405
 
 
44.2
 
 
 
 
ANKRD17
 
 
hsa-miR-30b-5p
 
 
-0.336
 
 
14.3
 
 
 
 
ANKRD40
 
 
hsa-miR-27a-3p
 
 
-0.35
 
 
15.4
 
 
 
 
ANKRD46
 
 
hsa-let-7b-5p
 
 
-0.389
 
 
10.4
 
 
 
 
ANP32A
 
 
hsa-miR-132-3p
 
 
-0.374
 
 
72.4
 
 
 
 
ANTXR2
 
 
hsa-miR-29a-3p
 
 
-0.347
 
 
35.6
 
 
 
 
ANXA11
 
 
hsa-miR-16-5p
 
 
-0.447
 
 
129.1
 
 
 
 
ANXA11
 
 
hsa-miR-15b-5p
 
 
-0.463
 
 
129.1
 
 
 
 
AP1S1
 
 
hsa-let-7b-5p
 
 
-0.374
 
 
29.8
 
 
 
 
AP2A1
 
 
hsa-miR-15b-5p
 
 
-0.333
 
 
32.5
 
 
 
 
AP2A1
 
 
hsa-miR-16-5p
 
 
-0.333
 
 
32.5
 
 
 
 
AP2B1
 
 
hsa-miR-16-5p
 
 
-0.302
 
 
36.5
 
 
 
 
AP2B1
 
 
hsa-miR-15b-5p
 
 
-0.334
 
 
36.5
 
 
 
 
AP3S1
 
 
hsa-miR-30b-5p
 
 
-0.293
 
 
76.7
 
 
 
 
APOO
 
 
hsa-miR-181b-5p
 
 
-0.5
 
 
11.3
 
 
 
 
APOO
 
 
hsa-miR-181a-5p
 
 
-0.5
 
 
11.3
 
 
 
 
APP
 
 
hsa-miR-16-5p
 
 
-0.298
 
 
184.2
 
 
 
 
APP
 
 
hsa-miR-15b-5p
 
 
-0.298
 
 
184.2
 
 
 
 
AQP11
 
 
hsa-miR-27a-3p
 
 
-0.666
 
 
23.9
 
 
 
 
AQP11
 
 
hsa-miR-15b-5p
 
 
-0.769
 
 
23.9
 
 
 
 
AQP11
 
 
hsa-miR-16-5p
 
 
-0.801
 
 
23.9
 
 
 
 
ARF4
 
 
hsa-miR-30b-5p
 
 
-0.289
 
 
102.8
 
 
 
 
ARF4
 
 
hsa-miR-148b-3p
 
 
-0.487
 
 
102.8
 
 
 
 
ARF5
 
 
hsa-miR-29a-3p
 
 
-0.491
 
 
103.1
 
 
 
 
ARF6
 
 
hsa-miR-181b-5p
 
 
-0.33
 
 
48.2
 
 
 
 
ARF6
 
 
hsa-miR-181a-5p
 
 
-0.33
 
 
48.2
 
 
 
 
ARFGEF1
 
 
hsa-miR-148b-3p
 
 
-0.306
 
 
16.5
 
 
 
 
ARFIP1
 
 
hsa-miR-320b
 
 
-0.339
 
 
20.0
 
 
 
 
ARFIP1
 
 
hsa-miR-320c
 
 
-0.339
 
 
20.0
 
 
 
 
ARFIP1
 
 
hsa-miR-148b-3p
 
 
-0.474
 
 
20.0
 
 
 
 
ARG2
 
 
hsa-let-7b-5p
 
 
-0.468
 
 
14.4
 
 
 
 
ARHGAP21
 
 
hsa-miR-148b-3p
 
 
-0.287
 
 
15.2
 
 
 
 
ARHGAP5
 
 
hsa-miR-486-5p
 
 
-0.3
 
 
36.7
 
 
 
 
ARID1B
 
 
hsa-miR-29a-3p
 
 
-0.342
 
 
14.4
 
 
 
 
ARL2
 
 
hsa-miR-16-5p
 
 
-0.374
 
 
72.6
 
 
 
 
ARL2
 
 
hsa-miR-15b-5p
 
 
-0.374
 
 
72.6
 
 
 
 
ARL2
 
 
hsa-miR-16-5p
 
 
-0.418
 
 
72.6
 
 
 
 
ARL2
 
 
hsa-miR-15b-5p
 
 
-0.442
 
 
72.6
 
 
 
 
ARL4A
 
 
hsa-miR-29a-3p
 
 
-0.386
 
 
59.0
 
 
 
 
ARL4A
 
 
hsa-miR-30b-5p
 
 
-0.448
 
 
59.0
 
 
 
 
ARL6IP1
 
 
hsa-miR-328-3p
 
 
-0.494
 
 
116.2
 
 
 
 
ARL6IP1
 
 
hsa-miR-148b-3p
 
 
-0.579
 
 
116.2
 
 
 
 
ARL8B
 
 
hsa-miR-148b-3p
 
 
-0.337
 
 
32.3
 
 
 
 
ARL8B
 
 
hsa-miR-320b
 
 
-0.376
 
 
32.3
 
 
 
 
ARL8B
 
 
hsa-miR-320c
 
 
-0.376
 
 
32.3
 
 
 
 
ARMC8
 
 
hsa-miR-486-5p
 
 
-0.321
 
 
12.8
 
 
 
 
ARMCX2
 
 
hsa-miR-15b-5p
 
 
-0.344
 
 
11.5
 
 
 
 
ARMCX2
 
 
hsa-miR-16-5p
 
 
-0.366
 
 
11.5
 
 
 
 
ARMCX3
 
 
hsa-miR-425-5p
 
 
-0.325
 
 
19.2
 
 
 
 
ARMT1
 
 
hsa-let-7b-5p
 
 
-0.291
 
 
13.7
 
 
 
 
ARPP19
 
 
hsa-miR-320b
 
 
-0.325
 
 
28.8
 
 
 
 
ARPP19
 
 
hsa-miR-320c
 
 
-0.325
 
 
28.8
 
 
 
 
ARPP19
 
 
hsa-miR-15b-5p
 
 
-0.346
 
 
28.8
 
 
 
 
ARPP19
 
 
hsa-miR-16-5p
 
 
-0.354
 
 
28.8
 
 
 
 
ARPP19
 
 
hsa-miR-29a-3p
 
 
-0.371
 
 
28.8
 
 
 
 
ARPP19
 
 
hsa-miR-328-3p
 
 
-0.404
 
 
28.8
 
 
 
 
ARPP19
 
 
hsa-let-7b-5p
 
 
-0.476
 
 
28.8
 
 
 
 
ARRDC3
 
 
hsa-miR-148b-3p
 
 
-0.305
 
 
15.8
 
 
 
 
ARRDC3
 
 
hsa-miR-29a-3p
 
 
-0.402
 
 
15.8
 
 
 
 
ARRDC4
 
 
hsa-let-7b-5p
 
 
-0.329
 
 
22.0
 
 
 
 
ASAH1
 
 
hsa-miR-27a-3p
 
 
-0.398
 
 
53.4
 
 
 
 
ASAP1
 
 
hsa-let-7b-5p
 
 
-0.308
 
 
11.5
 
 
 
 
ASAP2
 
 
hsa-miR-29a-3p
 
 
-0.348
 
 
12.8
 
 
 
 
ASH1L
 
 
hsa-miR-15b-5p
 
 
-0.325
 
 
17.1
 
 
 
 
ASH1L
 
 
hsa-miR-16-5p
 
 
-0.357
 
 
17.1
 
 
 
 
 
 
 smooth muscle 
 
 
 
 
target_symbol
 
 
mature_mirna_id
 
 
score
 
 
nTPM
 
 
 
 
 
 
AAR2
 
 
hsa-miR-29a-3p
 
 
-0.327
 
 
26.1
 
 
 
 
ABCD3
 
 
hsa-miR-148b-3p
 
 
-0.388
 
 
16.6
 
 
 
 
ABCE1
 
 
hsa-miR-29a-3p
 
 
-0.404
 
 
27.0
 
 
 
 
ABHD4
 
 
hsa-miR-29a-3p
 
 
-0.312
 
 
25.0
 
 
 
 
ACTA2
 
 
hsa-miR-27a-3p
 
 
-0.577
 
 
7564.0
 
 
 
 
ACTR2
 
 
hsa-miR-15b-5p
 
 
-0.293
 
 
96.0
 
 
 
 
ACTR2
 
 
hsa-miR-16-5p
 
 
-0.293
 
 
96.0
 
 
 
 
ACVR1
 
 
hsa-miR-30b-5p
 
 
-0.29
 
 
26.8
 
 
 
 
ACVR1
 
 
hsa-miR-148b-3p
 
 
-0.397
 
 
26.8
 
 
 
 
ADAM10
 
 
hsa-miR-148b-3p
 
 
-0.356
 
 
12.4
 
 
 
 
ADAM9
 
 
hsa-miR-30b-5p
 
 
-0.312
 
 
48.2
 
 
 
 
ADAMTS9
 
 
hsa-miR-29a-3p
 
 
-0.305
 
 
11.3
 
 
 
 
ADAMTS9
 
 
hsa-miR-29a-3p
 
 
-0.485
 
 
11.3
 
 
 
 
ADCY3
 
 
hsa-miR-132-3p
 
 
-0.303
 
 
33.0
 
 
 
 
ADGRA3
 
 
hsa-miR-27a-3p
 
 
-0.296
 
 
13.1
 
 
 
 
ADGRA3
 
 
hsa-miR-30b-5p
 
 
-0.304
 
 
13.1
 
 
 
 
ADGRF5
 
 
hsa-miR-148b-3p
 
 
-0.354
 
 
20.6
 
 
 
 
ADIPOR2
 
 
hsa-miR-150-5p
 
 
-0.287
 
 
11.2
 
 
 
 
ADNP
 
 
hsa-miR-328-3p
 
 
-0.371
 
 
27.7
 
 
 
 
AFG3L2
 
 
hsa-miR-181b-5p
 
 
-0.491
 
 
14.9
 
 
 
 
AFG3L2
 
 
hsa-miR-181a-5p
 
 
-0.491
 
 
14.9
 
 
 
 
AGFG1
 
 
hsa-miR-148b-3p
 
 
-0.332
 
 
36.7
 
 
 
 
AHR
 
 
hsa-miR-29a-3p
 
 
-0.316
 
 
14.2
 
 
 
 
AK1
 
 
hsa-miR-29a-3p
 
 
-0.333
 
 
20.4
 
 
 
 
AK4
 
 
hsa-miR-15b-5p
 
 
-0.316
 
 
19.6
 
 
 
 
AK4
 
 
hsa-miR-16-5p
 
 
-0.348
 
 
19.6
 
 
 
 
AKAP1
 
 
hsa-miR-148b-3p
 
 
-0.381
 
 
24.6
 
 
 
 
AKIRIN1
 
 
hsa-miR-27a-3p
 
 
-0.368
 
 
39.2
 
 
 
 
AKT3
 
 
hsa-miR-29a-3p
 
 
-0.287
 
 
14.7
 
 
 
 
AKT3
 
 
hsa-miR-15b-5p
 
 
-0.301
 
 
14.7
 
 
 
 
AKT3
 
 
hsa-miR-16-5p
 
 
-0.309
 
 
14.7
 
 
 
 
ALAD
 
 
hsa-miR-16-5p
 
 
-0.391
 
 
16.4
 
 
 
 
ALAD
 
 
hsa-miR-15b-5p
 
 
-0.446
 
 
16.4
 
 
 
 
ALCAM
 
 
hsa-miR-148b-3p
 
 
-0.339
 
 
15.2
 
 
 
 
ALDH1A2
 
 
hsa-miR-23b-3p
 
 
-0.346
 
 
29.8
 
 
 
 
ALDH1A2
 
 
hsa-miR-23a-3p
 
 
-0.354
 
 
29.8
 
 
 
 
AMOTL1
 
 
hsa-miR-16-5p
 
 
-0.297
 
 
19.2
 
 
 
 
AMOTL1
 
 
hsa-miR-15b-5p
 
 
-0.319
 
 
19.2
 
 
 
 
ANKHD1
 
 
hsa-miR-23a-3p
 
 
-0.368
 
 
24.3
 
 
 
 
ANKHD1
 
 
hsa-miR-23b-3p
 
 
-0.368
 
 
24.3
 
 
 
 
ANKHD1
 
 
hsa-miR-30b-5p
 
 
-0.405
 
 
24.3
 
 
 
 
ANKRD17
 
 
hsa-miR-30b-5p
 
 
-0.336
 
 
15.3
 
 
 
 
ANKRD29
 
 
hsa-miR-132-3p
 
 
-0.47
 
 
10.9
 
 
 
 
ANKRD40
 
 
hsa-miR-27a-3p
 
 
-0.35
 
 
26.1
 
 
 
 
ANKRD46
 
 
hsa-let-7b-5p
 
 
-0.389
 
 
11.6
 
 
 
 
ANLN
 
 
hsa-miR-16-5p
 
 
-0.31
 
 
13.9
 
 
 
 
ANLN
 
 
hsa-miR-15b-5p
 
 
-0.334
 
 
13.9
 
 
 
 
ANP32A
 
 
hsa-miR-132-3p
 
 
-0.374
 
 
53.2
 
 
 
 
ANTXR2
 
 
hsa-miR-29a-3p
 
 
-0.347
 
 
101.2
 
 
 
 
ANXA11
 
 
hsa-miR-16-5p
 
 
-0.447
 
 
80.2
 
 
 
 
ANXA11
 
 
hsa-miR-15b-5p
 
 
-0.463
 
 
80.2
 
 
 
 
AP1S1
 
 
hsa-let-7b-5p
 
 
-0.374
 
 
11.9
 
 
 
 
AP2A1
 
 
hsa-miR-15b-5p
 
 
-0.333
 
 
20.0
 
 
 
 
AP2A1
 
 
hsa-miR-16-5p
 
 
-0.333
 
 
20.0
 
 
 
 
AP2B1
 
 
hsa-miR-16-5p
 
 
-0.302
 
 
52.3
 
 
 
 
AP2B1
 
 
hsa-miR-15b-5p
 
 
-0.334
 
 
52.3
 
 
 
 
AP3S1
 
 
hsa-miR-30b-5p
 
 
-0.293
 
 
59.9
 
 
 
 
APOO
 
 
hsa-miR-181b-5p
 
 
-0.5
 
 
11.2
 
 
 
 
APOO
 
 
hsa-miR-181a-5p
 
 
-0.5
 
 
11.2
 
 
 
 
APP
 
 
hsa-miR-16-5p
 
 
-0.298
 
 
353.7
 
 
 
 
APP
 
 
hsa-miR-15b-5p
 
 
-0.298
 
 
353.7
 
 
 
 
APPBP2
 
 
hsa-miR-27a-3p
 
 
-0.378
 
 
11.0
 
 
 
 
ARF4
 
 
hsa-miR-30b-5p
 
 
-0.289
 
 
157.9
 
 
 
 
ARF4
 
 
hsa-miR-148b-3p
 
 
-0.487
 
 
157.9
 
 
 
 
ARF5
 
 
hsa-miR-29a-3p
 
 
-0.491
 
 
62.0
 
 
 
 
ARF6
 
 
hsa-miR-181b-5p
 
 
-0.33
 
 
22.2
 
 
 
 
ARF6
 
 
hsa-miR-181a-5p
 
 
-0.33
 
 
22.2
 
 
 
 
ARFGEF1
 
 
hsa-miR-148b-3p
 
 
-0.306
 
 
10.1
 
 
 
 
ARFIP1
 
 
hsa-miR-320b
 
 
-0.339
 
 
18.5
 
 
 
 
ARFIP1
 
 
hsa-miR-320c
 
 
-0.339
 
 
18.5
 
 
 
 
ARFIP1
 
 
hsa-miR-148b-3p
 
 
-0.474
 
 
18.5
 
 
 
 
ARHGAP21
 
 
hsa-miR-148b-3p
 
 
-0.287
 
 
16.5
 
 
 
 
ARHGAP5
 
 
hsa-miR-486-5p
 
 
-0.3
 
 
22.0
 
 
 
 
ARID1B
 
 
hsa-miR-29a-3p
 
 
-0.342
 
 
14.1
 
 
 
 
ARL2
 
 
hsa-miR-16-5p
 
 
-0.374
 
 
56.9
 
 
 
 
ARL2
 
 
hsa-miR-15b-5p
 
 
-0.374
 
 
56.9
 
 
 
 
ARL2
 
 
hsa-miR-16-5p
 
 
-0.418
 
 
56.9
 
 
 
 
ARL2
 
 
hsa-miR-15b-5p
 
 
-0.442
 
 
56.9
 
 
 
 
ARL4A
 
 
hsa-miR-29a-3p
 
 
-0.386
 
 
23.4
 
 
 
 
ARL4A
 
 
hsa-miR-30b-5p
 
 
-0.448
 
 
23.4
 
 
 
 
ARL4D
 
 
hsa-let-7b-5p
 
 
-0.417
 
 
13.8
 
 
 
 
ARL6IP1
 
 
hsa-miR-328-3p
 
 
-0.494
 
 
64.5
 
 
 
 
ARL6IP1
 
 
hsa-miR-148b-3p
 
 
-0.579
 
 
64.5
 
 
 
 
ARL6IP6
 
 
hsa-let-7b-5p
 
 
-0.293
 
 
10.8
 
 
 
 
ARL8B
 
 
hsa-miR-148b-3p
 
 
-0.337
 
 
43.9
 
 
 
 
ARL8B
 
 
hsa-miR-320b
 
 
-0.376
 
 
43.9
 
 
 
 
ARL8B
 
 
hsa-miR-320c
 
 
-0.376
 
 
43.9
 
 
 
 
ARMC8
 
 
hsa-miR-486-5p
 
 
-0.321
 
 
22.8
 
 
 
 
ARMCX2
 
 
hsa-miR-15b-5p
 
 
-0.344
 
 
30.1
 
 
 
 
ARMCX2
 
 
hsa-miR-16-5p
 
 
-0.366
 
 
30.1
 
 
 
 
ARMCX3
 
 
hsa-miR-425-5p
 
 
-0.325
 
 
33.9
 
 
 
 
ARMT1
 
 
hsa-let-7b-5p
 
 
-0.291
 
 
13.9
 
 
 
 
ARPP19
 
 
hsa-miR-320b
 
 
-0.325
 
 
54.2
 
 
 
 
ARPP19
 
 
hsa-miR-320c
 
 
-0.325
 
 
54.2
 
 
 
 
ARPP19
 
 
hsa-miR-15b-5p
 
 
-0.346
 
 
54.2
 
 
 
 
ARPP19
 
 
hsa-miR-16-5p
 
 
-0.354
 
 
54.2
 
 
 
 
ARPP19
 
 
hsa-miR-29a-3p
 
 
-0.371
 
 
54.2
 
 
 
 
ARPP19
 
 
hsa-miR-328-3p
 
 
-0.404
 
 
54.2
 
 
 
 
ARPP19
 
 
hsa-let-7b-5p
 
 
-0.476
 
 
54.2
 
 
 
 
ARRDC3
 
 
hsa-miR-148b-3p
 
 
-0.305
 
 
30.8
 
 
 
 
 
 
 spinal cord 
 
 
 
 
target_symbol
 
 
mature_mirna_id
 
 
score
 
 
nTPM
 
 
 
 
 
 
AAR2
 
 
hsa-miR-29a-3p
 
 
-0.327
 
 
14.2
 
 
 
 
ABCD3
 
 
hsa-miR-148b-3p
 
 
-0.388
 
 
20.9
 
 
 
 
ABCE1
 
 
hsa-miR-29a-3p
 
 
-0.404
 
 
16.8
 
 
 
 
ABHD17B
 
 
hsa-miR-486-5p
 
 
-0.348
 
 
26.2
 
 
 
 
ABHD4
 
 
hsa-miR-29a-3p
 
 
-0.312
 
 
17.8
 
 
 
 
ABHD5
 
 
hsa-miR-132-3p
 
 
-0.323
 
 
19.5
 
 
 
 
ABHD5
 
 
hsa-miR-27a-3p
 
 
-0.407
 
 
19.5
 
 
 
 
ACTA2
 
 
hsa-miR-27a-3p
 
 
-0.577
 
 
47.2
 
 
 
 
ACTN2
 
 
hsa-miR-23a-3p
 
 
-0.294
 
 
14.9
 
 
 
 
ACTN2
 
 
hsa-miR-29a-3p
 
 
-0.311
 
 
14.9
 
 
 
 
ACTR2
 
 
hsa-miR-15b-5p
 
 
-0.293
 
 
65.2
 
 
 
 
ACTR2
 
 
hsa-miR-16-5p
 
 
-0.293
 
 
65.2
 
 
 
 
ADAM10
 
 
hsa-miR-148b-3p
 
 
-0.356
 
 
18.7
 
 
 
 
ADAM9
 
 
hsa-miR-30b-5p
 
 
-0.312
 
 
11.3
 
 
 
 
ADAP1
 
 
hsa-miR-27a-3p
 
 
-0.302
 
 
131.1
 
 
 
 
ADCY2
 
 
hsa-miR-148b-3p
 
 
-0.299
 
 
11.4
 
 
 
 
ADCY3
 
 
hsa-miR-132-3p
 
 
-0.303
 
 
26.0
 
 
 
 
ADIPOR2
 
 
hsa-miR-150-5p
 
 
-0.287
 
 
121.3
 
 
 
 
ADNP
 
 
hsa-miR-328-3p
 
 
-0.371
 
 
22.3
 
 
 
 
AGFG1
 
 
hsa-miR-148b-3p
 
 
-0.332
 
 
53.5
 
 
 
 
AGPAT3
 
 
hsa-miR-27a-3p
 
 
-0.29
 
 
50.1
 
 
 
 
AGPAT3
 
 
hsa-miR-16-5p
 
 
-0.299
 
 
50.1
 
 
 
 
AGPAT4
 
 
hsa-miR-29a-3p
 
 
-0.376
 
 
53.1
 
 
 
 
AIFM1
 
 
hsa-let-7b-5p
 
 
-0.314
 
 
10.1
 
 
 
 
AK1
 
 
hsa-miR-29a-3p
 
 
-0.333
 
 
67.3
 
 
 
 
AK4
 
 
hsa-miR-15b-5p
 
 
-0.316
 
 
11.3
 
 
 
 
AK4
 
 
hsa-miR-16-5p
 
 
-0.348
 
 
11.3
 
 
 
 
AKAP1
 
 
hsa-miR-148b-3p
 
 
-0.381
 
 
14.0
 
 
 
 
AKAP6
 
 
hsa-let-7b-5p
 
 
-0.307
 
 
13.3
 
 
 
 
AKIRIN1
 
 
hsa-miR-27a-3p
 
 
-0.368
 
 
52.7
 
 
 
 
AKT3
 
 
hsa-miR-29a-3p
 
 
-0.287
 
 
15.8
 
 
 
 
AKT3
 
 
hsa-miR-15b-5p
 
 
-0.301
 
 
15.8
 
 
 
 
AKT3
 
 
hsa-miR-16-5p
 
 
-0.309
 
 
15.8
 
 
 
 
ALAD
 
 
hsa-miR-16-5p
 
 
-0.391
 
 
110.8
 
 
 
 
ALAD
 
 
hsa-miR-15b-5p
 
 
-0.446
 
 
110.8
 
 
 
 
ALCAM
 
 
hsa-miR-148b-3p
 
 
-0.339
 
 
56.1
 
 
 
 
ANKHD1
 
 
hsa-miR-23a-3p
 
 
-0.368
 
 
22.6
 
 
 
 
ANKHD1
 
 
hsa-miR-23b-3p
 
 
-0.368
 
 
22.6
 
 
 
 
ANKHD1
 
 
hsa-miR-30b-5p
 
 
-0.405
 
 
22.6
 
 
 
 
ANKRD13B
 
 
hsa-miR-29a-3p
 
 
-0.421
 
 
14.7
 
 
 
 
ANKRD40
 
 
hsa-miR-27a-3p
 
 
-0.35
 
 
196.0
 
 
 
 
ANKRD46
 
 
hsa-let-7b-5p
 
 
-0.389
 
 
24.6
 
 
 
 
ANLN
 
 
hsa-miR-16-5p
 
 
-0.31
 
 
163.3
 
 
 
 
ANLN
 
 
hsa-miR-15b-5p
 
 
-0.334
 
 
163.3
 
 
 
 
ANP32A
 
 
hsa-miR-132-3p
 
 
-0.374
 
 
72.8
 
 
 
 
ANXA11
 
 
hsa-miR-16-5p
 
 
-0.447
 
 
19.0
 
 
 
 
ANXA11
 
 
hsa-miR-15b-5p
 
 
-0.463
 
 
19.0
 
 
 
 
AP1S1
 
 
hsa-let-7b-5p
 
 
-0.374
 
 
28.5
 
 
 
 
AP2A1
 
 
hsa-miR-15b-5p
 
 
-0.333
 
 
32.5
 
 
 
 
AP2A1
 
 
hsa-miR-16-5p
 
 
-0.333
 
 
32.5
 
 
 
 
AP2B1
 
 
hsa-miR-16-5p
 
 
-0.302
 
 
75.2
 
 
 
 
AP2B1
 
 
hsa-miR-15b-5p
 
 
-0.334
 
 
75.2
 
 
 
 
AP3S1
 
 
hsa-miR-30b-5p
 
 
-0.293
 
 
64.2
 
 
 
 
APLN
 
 
hsa-miR-16-5p
 
 
-0.31
 
 
29.4
 
 
 
 
APLN
 
 
hsa-miR-15b-5p
 
 
-0.395
 
 
29.4
 
 
 
 
APLN
 
 
hsa-miR-16-5p
 
 
-0.395
 
 
29.4
 
 
 
 
APOO
 
 
hsa-miR-181b-5p
 
 
-0.5
 
 
36.2
 
 
 
 
APOO
 
 
hsa-miR-181a-5p
 
 
-0.5
 
 
36.2
 
 
 
 
APP
 
 
hsa-miR-16-5p
 
 
-0.298
 
 
535.7
 
 
 
 
APP
 
 
hsa-miR-15b-5p
 
 
-0.298
 
 
535.7
 
 
 
 
APPBP2
 
 
hsa-miR-27a-3p
 
 
-0.378
 
 
10.4
 
 
 
 
ARF4
 
 
hsa-miR-30b-5p
 
 
-0.289
 
 
69.7
 
 
 
 
ARF4
 
 
hsa-miR-148b-3p
 
 
-0.487
 
 
69.7
 
 
 
 
ARF5
 
 
hsa-miR-29a-3p
 
 
-0.491
 
 
100.3
 
 
 
 
ARF6
 
 
hsa-miR-181b-5p
 
 
-0.33
 
 
13.8
 
 
 
 
ARF6
 
 
hsa-miR-181a-5p
 
 
-0.33
 
 
13.8
 
 
 
 
ARFIP1
 
 
hsa-miR-320b
 
 
-0.339
 
 
10.6
 
 
 
 
ARFIP1
 
 
hsa-miR-320c
 
 
-0.339
 
 
10.6
 
 
 
 
ARFIP1
 
 
hsa-miR-148b-3p
 
 
-0.474
 
 
10.6
 
 
 
 
ARHGAP21
 
 
hsa-miR-148b-3p
 
 
-0.287
 
 
74.7
 
 
 
 
ARHGAP5
 
 
hsa-miR-486-5p
 
 
-0.3
 
 
21.0
 
 
 
 
ARID4B
 
 
hsa-miR-486-5p
 
 
-0.367
 
 
12.3
 
 
 
 
ARL2
 
 
hsa-miR-16-5p
 
 
-0.374
 
 
234.0
 
 
 
 
ARL2
 
 
hsa-miR-15b-5p
 
 
-0.374
 
 
234.0
 
 
 
 
ARL2
 
 
hsa-miR-16-5p
 
 
-0.418
 
 
234.0
 
 
 
 
ARL2
 
 
hsa-miR-15b-5p
 
 
-0.442
 
 
234.0
 
 
 
 
ARL3
 
 
hsa-miR-16-5p
 
 
-0.496
 
 
16.0
 
 
 
 
ARL3
 
 
hsa-miR-15b-5p
 
 
-0.507
 
 
16.0
 
 
 
 
ARL4A
 
 
hsa-miR-29a-3p
 
 
-0.386
 
 
12.2
 
 
 
 
ARL4A
 
 
hsa-miR-30b-5p
 
 
-0.448
 
 
12.2
 
 
 
 
ARL6IP1
 
 
hsa-miR-328-3p
 
 
-0.494
 
 
140.2
 
 
 
 
ARL6IP1
 
 
hsa-miR-148b-3p
 
 
-0.579
 
 
140.2
 
 
 
 
ARL8B
 
 
hsa-miR-148b-3p
 
 
-0.337
 
 
57.8
 
 
 
 
ARL8B
 
 
hsa-miR-320b
 
 
-0.376
 
 
57.8
 
 
 
 
ARL8B
 
 
hsa-miR-320c
 
 
-0.376
 
 
57.8
 
 
 
 
ARMC8
 
 
hsa-miR-486-5p
 
 
-0.321
 
 
15.5
 
 
 
 
ARMCX2
 
 
hsa-miR-15b-5p
 
 
-0.344
 
 
15.1
 
 
 
 
ARMCX2
 
 
hsa-miR-16-5p
 
 
-0.366
 
 
15.1
 
 
 
 
ARMCX3
 
 
hsa-miR-425-5p
 
 
-0.325
 
 
33.1
 
 
 
 
ARPP19
 
 
hsa-miR-320b
 
 
-0.325
 
 
170.8
 
 
 
 
ARPP19
 
 
hsa-miR-320c
 
 
-0.325
 
 
170.8
 
 
 
 
ARPP19
 
 
hsa-miR-15b-5p
 
 
-0.346
 
 
170.8
 
 
 
 
ARPP19
 
 
hsa-miR-16-5p
 
 
-0.354
 
 
170.8
 
 
 
 
ARPP19
 
 
hsa-miR-29a-3p
 
 
-0.371
 
 
170.8
 
 
 
 
ARPP19
 
 
hsa-miR-328-3p
 
 
-0.404
 
 
170.8
 
 
 
 
ARPP19
 
 
hsa-let-7b-5p
 
 
-0.476
 
 
170.8
 
 
 
 
ARRDC3
 
 
hsa-miR-148b-3p
 
 
-0.305
 
 
23.5
 
 
 
 
ARRDC3
 
 
hsa-miR-29a-3p
 
 
-0.402
 
 
23.5
 
 
 
 
ARRDC4
 
 
hsa-let-7b-5p
 
 
-0.329
 
 
75.7
 
 
 
 
ASAH1
 
 
hsa-miR-27a-3p
 
 
-0.398
 
 
79.8
 
 
 
 
 
 
 spleen 
 
 
 
 
target_symbol
 
 
mature_mirna_id
 
 
score
 
 
nTPM
 
 
 
 
 
 
AAR2
 
 
hsa-miR-29a-3p
 
 
-0.327
 
 
17.2
 
 
 
 
ABCB7
 
 
hsa-miR-148b-3p
 
 
-0.515
 
 
10.6
 
 
 
 
ABCD3
 
 
hsa-miR-148b-3p
 
 
-0.388
 
 
10.3
 
 
 
 
ABCE1
 
 
hsa-miR-29a-3p
 
 
-0.404
 
 
24.9
 
 
 
 
ABHD4
 
 
hsa-miR-29a-3p
 
 
-0.312
 
 
15.7
 
 
 
 
ACTA2
 
 
hsa-miR-27a-3p
 
 
-0.577
 
 
427.6
 
 
 
 
ACTR2
 
 
hsa-miR-15b-5p
 
 
-0.293
 
 
161.4
 
 
 
 
ACTR2
 
 
hsa-miR-16-5p
 
 
-0.293
 
 
161.4
 
 
 
 
ADA
 
 
hsa-miR-29a-3p
 
 
-0.376
 
 
17.0
 
 
 
 
ADAM10
 
 
hsa-miR-148b-3p
 
 
-0.356
 
 
27.3
 
 
 
 
ADAM9
 
 
hsa-miR-30b-5p
 
 
-0.312
 
 
22.6
 
 
 
 
ADAP1
 
 
hsa-miR-27a-3p
 
 
-0.302
 
 
16.5
 
 
 
 
ADCY3
 
 
hsa-miR-132-3p
 
 
-0.303
 
 
28.8
 
 
 
 
ADGRF5
 
 
hsa-miR-148b-3p
 
 
-0.354
 
 
38.8
 
 
 
 
ADIPOR2
 
 
hsa-miR-150-5p
 
 
-0.287
 
 
19.2
 
 
 
 
ADNP
 
 
hsa-miR-328-3p
 
 
-0.371
 
 
34.9
 
 
 
 
ADRA2A
 
 
hsa-miR-23a-3p
 
 
-0.371
 
 
13.1
 
 
 
 
ADRA2A
 
 
hsa-miR-23b-3p
 
 
-0.371
 
 
13.1
 
 
 
 
AFG3L2
 
 
hsa-miR-181b-5p
 
 
-0.491
 
 
10.9
 
 
 
 
AFG3L2
 
 
hsa-miR-181a-5p
 
 
-0.491
 
 
10.9
 
 
 
 
AGFG1
 
 
hsa-miR-148b-3p
 
 
-0.332
 
 
44.5
 
 
 
 
AGPAT3
 
 
hsa-miR-27a-3p
 
 
-0.29
 
 
18.3
 
 
 
 
AGPAT3
 
 
hsa-miR-16-5p
 
 
-0.299
 
 
18.3
 
 
 
 
AHDC1
 
 
hsa-miR-148b-3p
 
 
-0.413
 
 
15.7
 
 
 
 
AHR
 
 
hsa-miR-29a-3p
 
 
-0.316
 
 
17.6
 
 
 
 
AIFM1
 
 
hsa-let-7b-5p
 
 
-0.314
 
 
12.9
 
 
 
 
AK1
 
 
hsa-miR-29a-3p
 
 
-0.333
 
 
31.5
 
 
 
 
AKAP1
 
 
hsa-miR-148b-3p
 
 
-0.381
 
 
11.0
 
 
 
 
AKIRIN1
 
 
hsa-miR-27a-3p
 
 
-0.368
 
 
29.9
 
 
 
 
ALAD
 
 
hsa-miR-16-5p
 
 
-0.391
 
 
12.8
 
 
 
 
ALAD
 
 
hsa-miR-15b-5p
 
 
-0.446
 
 
12.8
 
 
 
 
ALPL
 
 
hsa-miR-10a-5p
 
 
-0.32
 
 
44.5
 
 
 
 
ANKHD1
 
 
hsa-miR-23a-3p
 
 
-0.368
 
 
46.1
 
 
 
 
ANKHD1
 
 
hsa-miR-23b-3p
 
 
-0.368
 
 
46.1
 
 
 
 
ANKHD1
 
 
hsa-miR-30b-5p
 
 
-0.405
 
 
46.1
 
 
 
 
ANKRD17
 
 
hsa-miR-30b-5p
 
 
-0.336
 
 
14.3
 
 
 
 
ANKRD40
 
 
hsa-miR-27a-3p
 
 
-0.35
 
 
19.3
 
 
 
 
ANKRD46
 
 
hsa-let-7b-5p
 
 
-0.389
 
 
10.4
 
 
 
 
ANP32A
 
 
hsa-miR-132-3p
 
 
-0.374
 
 
80.5
 
 
 
 
ANTXR2
 
 
hsa-miR-29a-3p
 
 
-0.347
 
 
17.3
 
 
 
 
ANXA11
 
 
hsa-miR-16-5p
 
 
-0.447
 
 
73.6
 
 
 
 
ANXA11
 
 
hsa-miR-15b-5p
 
 
-0.463
 
 
73.6
 
 
 
 
AP1S1
 
 
hsa-let-7b-5p
 
 
-0.374
 
 
20.9
 
 
 
 
AP2A1
 
 
hsa-miR-15b-5p
 
 
-0.333
 
 
45.6
 
 
 
 
AP2A1
 
 
hsa-miR-16-5p
 
 
-0.333
 
 
45.6
 
 
 
 
AP2B1
 
 
hsa-miR-16-5p
 
 
-0.302
 
 
39.6
 
 
 
 
AP2B1
 
 
hsa-miR-15b-5p
 
 
-0.334
 
 
39.6
 
 
 
 
AP3S1
 
 
hsa-miR-30b-5p
 
 
-0.293
 
 
36.7
 
 
 
 
APP
 
 
hsa-miR-16-5p
 
 
-0.298
 
 
140.4
 
 
 
 
APP
 
 
hsa-miR-15b-5p
 
 
-0.298
 
 
140.4
 
 
 
 
APPBP2
 
 
hsa-miR-27a-3p
 
 
-0.378
 
 
11.3
 
 
 
 
ARF4
 
 
hsa-miR-30b-5p
 
 
-0.289
 
 
60.0
 
 
 
 
ARF4
 
 
hsa-miR-148b-3p
 
 
-0.487
 
 
60.0
 
 
 
 
ARF5
 
 
hsa-miR-29a-3p
 
 
-0.491
 
 
116.2
 
 
 
 
ARF6
 
 
hsa-miR-181b-5p
 
 
-0.33
 
 
47.5
 
 
 
 
ARF6
 
 
hsa-miR-181a-5p
 
 
-0.33
 
 
47.5
 
 
 
 
ARFGEF1
 
 
hsa-miR-148b-3p
 
 
-0.306
 
 
13.0
 
 
 
 
ARFIP1
 
 
hsa-miR-320b
 
 
-0.339
 
 
17.3
 
 
 
 
ARFIP1
 
 
hsa-miR-320c
 
 
-0.339
 
 
17.3
 
 
 
 
ARFIP1
 
 
hsa-miR-148b-3p
 
 
-0.474
 
 
17.3
 
 
 
 
ARHGAP28
 
 
hsa-let-7b-5p
 
 
-0.454
 
 
11.9
 
 
 
 
ARHGAP5
 
 
hsa-miR-486-5p
 
 
-0.3
 
 
40.5
 
 
 
 
ARID1B
 
 
hsa-miR-29a-3p
 
 
-0.342
 
 
17.2
 
 
 
 
ARID4B
 
 
hsa-miR-486-5p
 
 
-0.367
 
 
17.3
 
 
 
 
ARL2
 
 
hsa-miR-16-5p
 
 
-0.374
 
 
63.8
 
 
 
 
ARL2
 
 
hsa-miR-15b-5p
 
 
-0.374
 
 
63.8
 
 
 
 
ARL2
 
 
hsa-miR-16-5p
 
 
-0.418
 
 
63.8
 
 
 
 
ARL2
 
 
hsa-miR-15b-5p
 
 
-0.442
 
 
63.8
 
 
 
 
ARL4A
 
 
hsa-miR-29a-3p
 
 
-0.386
 
 
20.6
 
 
 
 
ARL4A
 
 
hsa-miR-30b-5p
 
 
-0.448
 
 
20.6
 
 
 
 
ARL4D
 
 
hsa-let-7b-5p
 
 
-0.417
 
 
16.1
 
 
 
 
ARL6IP1
 
 
hsa-miR-328-3p
 
 
-0.494
 
 
68.6
 
 
 
 
ARL6IP1
 
 
hsa-miR-148b-3p
 
 
-0.579
 
 
68.6
 
 
 
 
ARL6IP6
 
 
hsa-let-7b-5p
 
 
-0.293
 
 
13.7
 
 
 
 
ARL8B
 
 
hsa-miR-148b-3p
 
 
-0.337
 
 
32.0
 
 
 
 
ARL8B
 
 
hsa-miR-320b
 
 
-0.376
 
 
32.0
 
 
 
 
ARL8B
 
 
hsa-miR-320c
 
 
-0.376
 
 
32.0
 
 
 
 
ARMC8
 
 
hsa-miR-486-5p
 
 
-0.321
 
 
14.9
 
 
 
 
ARMCX2
 
 
hsa-miR-15b-5p
 
 
-0.344
 
 
19.0
 
 
 
 
ARMCX2
 
 
hsa-miR-16-5p
 
 
-0.366
 
 
19.0
 
 
 
 
ARMCX3
 
 
hsa-miR-425-5p
 
 
-0.325
 
 
38.9
 
 
 
 
ARMT1
 
 
hsa-let-7b-5p
 
 
-0.291
 
 
12.0
 
 
 
 
ARPP19
 
 
hsa-miR-320b
 
 
-0.325
 
 
41.2
 
 
 
 
ARPP19
 
 
hsa-miR-320c
 
 
-0.325
 
 
41.2
 
 
 
 
ARPP19
 
 
hsa-miR-15b-5p
 
 
-0.346
 
 
41.2
 
 
 
 
ARPP19
 
 
hsa-miR-16-5p
 
 
-0.354
 
 
41.2
 
 
 
 
ARPP19
 
 
hsa-miR-29a-3p
 
 
-0.371
 
 
41.2
 
 
 
 
ARPP19
 
 
hsa-miR-328-3p
 
 
-0.404
 
 
41.2
 
 
 
 
ARPP19
 
 
hsa-let-7b-5p
 
 
-0.476
 
 
41.2
 
 
 
 
ARRDC3
 
 
hsa-miR-148b-3p
 
 
-0.305
 
 
35.0
 
 
 
 
ARRDC3
 
 
hsa-miR-29a-3p
 
 
-0.402
 
 
35.0
 
 
 
 
ASAH1
 
 
hsa-miR-27a-3p
 
 
-0.398
 
 
80.4
 
 
 
 
ASAP1
 
 
hsa-let-7b-5p
 
 
-0.308
 
 
28.1
 
 
 
 
ASB3
 
 
hsa-miR-30b-5p
 
 
-0.418
 
 
12.1
 
 
 
 
ASB3
 
 
hsa-miR-30b-5p
 
 
-0.418
 
 
12.1
 
 
 
 
ASF1A
 
 
hsa-miR-23a-3p
 
 
-0.506
 
 
10.9
 
 
 
 
ASF1A
 
 
hsa-miR-23b-3p
 
 
-0.506
 
 
10.9
 
 
 
 
ASH1L
 
 
hsa-miR-15b-5p
 
 
-0.325
 
 
13.9
 
 
 
 
ASH1L
 
 
hsa-miR-16-5p
 
 
-0.357
 
 
13.9
 
 
 
 
ASH2L
 
 
hsa-miR-320b
 
 
-0.287
 
 
18.2
 
 
 
 
 
 
 stomach 
 
 
 
 
target_symbol
 
 
mature_mirna_id
 
 
score
 
 
nTPM
 
 
 
 
 
 
AAR2
 
 
hsa-miR-29a-3p
 
 
-0.327
 
 
21.7
 
 
 
 
ABCB7
 
 
hsa-miR-148b-3p
 
 
-0.515
 
 
11.6
 
 
 
 
ABCD3
 
 
hsa-miR-148b-3p
 
 
-0.388
 
 
41.1
 
 
 
 
ABCE1
 
 
hsa-miR-29a-3p
 
 
-0.404
 
 
19.0
 
 
 
 
ABHD17C
 
 
hsa-let-7b-5p
 
 
-0.359
 
 
34.0
 
 
 
 
ABHD17C
 
 
hsa-miR-27a-3p
 
 
-0.435
 
 
34.0
 
 
 
 
ABHD4
 
 
hsa-miR-29a-3p
 
 
-0.312
 
 
26.1
 
 
 
 
ACER2
 
 
hsa-let-7b-5p
 
 
-0.333
 
 
58.3
 
 
 
 
ACTA2
 
 
hsa-miR-27a-3p
 
 
-0.577
 
 
1353.4
 
 
 
 
ACTC1
 
 
hsa-miR-30b-5p
 
 
-0.543
 
 
32.8
 
 
 
 
ACTR2
 
 
hsa-miR-15b-5p
 
 
-0.293
 
 
100.4
 
 
 
 
ACTR2
 
 
hsa-miR-16-5p
 
 
-0.293
 
 
100.4
 
 
 
 
ACVR1
 
 
hsa-miR-30b-5p
 
 
-0.29
 
 
11.0
 
 
 
 
ACVR1
 
 
hsa-miR-148b-3p
 
 
-0.397
 
 
11.0
 
 
 
 
ADA
 
 
hsa-miR-29a-3p
 
 
-0.376
 
 
44.4
 
 
 
 
ADAM10
 
 
hsa-miR-148b-3p
 
 
-0.356
 
 
19.0
 
 
 
 
ADAM9
 
 
hsa-miR-30b-5p
 
 
-0.312
 
 
41.8
 
 
 
 
ADAP1
 
 
hsa-miR-27a-3p
 
 
-0.302
 
 
18.4
 
 
 
 
ADCY3
 
 
hsa-miR-132-3p
 
 
-0.303
 
 
23.8
 
 
 
 
ADIPOR2
 
 
hsa-miR-150-5p
 
 
-0.287
 
 
42.5
 
 
 
 
ADNP
 
 
hsa-miR-328-3p
 
 
-0.371
 
 
30.6
 
 
 
 
AFG3L2
 
 
hsa-miR-181b-5p
 
 
-0.491
 
 
26.3
 
 
 
 
AFG3L2
 
 
hsa-miR-181a-5p
 
 
-0.491
 
 
26.3
 
 
 
 
AGFG1
 
 
hsa-miR-148b-3p
 
 
-0.332
 
 
31.3
 
 
 
 
AGPAT3
 
 
hsa-miR-27a-3p
 
 
-0.29
 
 
16.9
 
 
 
 
AGPAT3
 
 
hsa-miR-16-5p
 
 
-0.299
 
 
16.9
 
 
 
 
AHDC1
 
 
hsa-miR-148b-3p
 
 
-0.413
 
 
10.8
 
 
 
 
AHR
 
 
hsa-miR-29a-3p
 
 
-0.316
 
 
23.8
 
 
 
 
AIFM1
 
 
hsa-let-7b-5p
 
 
-0.314
 
 
29.8
 
 
 
 
AK1
 
 
hsa-miR-29a-3p
 
 
-0.333
 
 
28.6
 
 
 
 
AK4
 
 
hsa-miR-15b-5p
 
 
-0.316
 
 
14.0
 
 
 
 
AK4
 
 
hsa-miR-16-5p
 
 
-0.348
 
 
14.0
 
 
 
 
AKAP1
 
 
hsa-miR-148b-3p
 
 
-0.381
 
 
53.0
 
 
 
 
AKIRIN1
 
 
hsa-miR-27a-3p
 
 
-0.368
 
 
31.7
 
 
 
 
ALAD
 
 
hsa-miR-16-5p
 
 
-0.391
 
 
49.1
 
 
 
 
ALAD
 
 
hsa-miR-15b-5p
 
 
-0.446
 
 
49.1
 
 
 
 
ALCAM
 
 
hsa-miR-148b-3p
 
 
-0.339
 
 
31.6
 
 
 
 
AMOTL1
 
 
hsa-miR-16-5p
 
 
-0.297
 
 
11.8
 
 
 
 
AMOTL1
 
 
hsa-miR-15b-5p
 
 
-0.319
 
 
11.8
 
 
 
 
AMT
 
 
hsa-let-7b-5p
 
 
-0.463
 
 
13.1
 
 
 
 
ANKHD1
 
 
hsa-miR-23a-3p
 
 
-0.368
 
 
39.4
 
 
 
 
ANKHD1
 
 
hsa-miR-23b-3p
 
 
-0.368
 
 
39.4
 
 
 
 
ANKHD1
 
 
hsa-miR-30b-5p
 
 
-0.405
 
 
39.4
 
 
 
 
ANKRD17
 
 
hsa-miR-30b-5p
 
 
-0.336
 
 
14.8
 
 
 
 
ANKRD29
 
 
hsa-miR-132-3p
 
 
-0.47
 
 
14.3
 
 
 
 
ANKRD40
 
 
hsa-miR-27a-3p
 
 
-0.35
 
 
17.6
 
 
 
 
ANKRD46
 
 
hsa-let-7b-5p
 
 
-0.389
 
 
10.9
 
 
 
 
ANP32A
 
 
hsa-miR-132-3p
 
 
-0.374
 
 
74.4
 
 
 
 
ANTXR2
 
 
hsa-miR-29a-3p
 
 
-0.347
 
 
46.6
 
 
 
 
ANXA11
 
 
hsa-miR-16-5p
 
 
-0.447
 
 
131.4
 
 
 
 
ANXA11
 
 
hsa-miR-15b-5p
 
 
-0.463
 
 
131.4
 
 
 
 
AP1S1
 
 
hsa-let-7b-5p
 
 
-0.374
 
 
23.2
 
 
 
 
AP2A1
 
 
hsa-miR-15b-5p
 
 
-0.333
 
 
34.8
 
 
 
 
AP2A1
 
 
hsa-miR-16-5p
 
 
-0.333
 
 
34.8
 
 
 
 
AP2B1
 
 
hsa-miR-16-5p
 
 
-0.302
 
 
45.1
 
 
 
 
AP2B1
 
 
hsa-miR-15b-5p
 
 
-0.334
 
 
45.1
 
 
 
 
AP3S1
 
 
hsa-miR-30b-5p
 
 
-0.293
 
 
42.9
 
 
 
 
APOO
 
 
hsa-miR-181b-5p
 
 
-0.5
 
 
17.0
 
 
 
 
APOO
 
 
hsa-miR-181a-5p
 
 
-0.5
 
 
17.0
 
 
 
 
APP
 
 
hsa-miR-16-5p
 
 
-0.298
 
 
197.5
 
 
 
 
APP
 
 
hsa-miR-15b-5p
 
 
-0.298
 
 
197.5
 
 
 
 
APPBP2
 
 
hsa-miR-27a-3p
 
 
-0.378
 
 
10.1
 
 
 
 
ARF4
 
 
hsa-miR-30b-5p
 
 
-0.289
 
 
123.5
 
 
 
 
ARF4
 
 
hsa-miR-148b-3p
 
 
-0.487
 
 
123.5
 
 
 
 
ARF5
 
 
hsa-miR-29a-3p
 
 
-0.491
 
 
126.0
 
 
 
 
ARF6
 
 
hsa-miR-181b-5p
 
 
-0.33
 
 
44.4
 
 
 
 
ARF6
 
 
hsa-miR-181a-5p
 
 
-0.33
 
 
44.4
 
 
 
 
ARFGEF1
 
 
hsa-miR-148b-3p
 
 
-0.306
 
 
18.5
 
 
 
 
ARFIP1
 
 
hsa-miR-320b
 
 
-0.339
 
 
21.5
 
 
 
 
ARFIP1
 
 
hsa-miR-320c
 
 
-0.339
 
 
21.5
 
 
 
 
ARFIP1
 
 
hsa-miR-148b-3p
 
 
-0.474
 
 
21.5
 
 
 
 
ARHGAP21
 
 
hsa-miR-148b-3p
 
 
-0.287
 
 
25.5
 
 
 
 
ARHGAP5
 
 
hsa-miR-486-5p
 
 
-0.3
 
 
27.8
 
 
 
 
ARID1B
 
 
hsa-miR-29a-3p
 
 
-0.342
 
 
11.1
 
 
 
 
ARID4B
 
 
hsa-miR-486-5p
 
 
-0.367
 
 
12.5
 
 
 
 
ARL2
 
 
hsa-miR-16-5p
 
 
-0.374
 
 
85.3
 
 
 
 
ARL2
 
 
hsa-miR-15b-5p
 
 
-0.374
 
 
85.3
 
 
 
 
ARL2
 
 
hsa-miR-16-5p
 
 
-0.418
 
 
85.3
 
 
 
 
ARL2
 
 
hsa-miR-15b-5p
 
 
-0.442
 
 
85.3
 
 
 
 
ARL4A
 
 
hsa-miR-29a-3p
 
 
-0.386
 
 
24.0
 
 
 
 
ARL4A
 
 
hsa-miR-30b-5p
 
 
-0.448
 
 
24.0
 
 
 
 
ARL6IP1
 
 
hsa-miR-328-3p
 
 
-0.494
 
 
118.7
 
 
 
 
ARL6IP1
 
 
hsa-miR-148b-3p
 
 
-0.579
 
 
118.7
 
 
 
 
ARL8B
 
 
hsa-miR-148b-3p
 
 
-0.337
 
 
32.2
 
 
 
 
ARL8B
 
 
hsa-miR-320b
 
 
-0.376
 
 
32.2
 
 
 
 
ARL8B
 
 
hsa-miR-320c
 
 
-0.376
 
 
32.2
 
 
 
 
ARMC8
 
 
hsa-miR-486-5p
 
 
-0.321
 
 
14.5
 
 
 
 
ARMCX2
 
 
hsa-miR-15b-5p
 
 
-0.344
 
 
14.4
 
 
 
 
ARMCX2
 
 
hsa-miR-16-5p
 
 
-0.366
 
 
14.4
 
 
 
 
ARMCX3
 
 
hsa-miR-425-5p
 
 
-0.325
 
 
26.2
 
 
 
 
ARMT1
 
 
hsa-let-7b-5p
 
 
-0.291
 
 
13.3
 
 
 
 
ARPP19
 
 
hsa-miR-320b
 
 
-0.325
 
 
36.4
 
 
 
 
ARPP19
 
 
hsa-miR-320c
 
 
-0.325
 
 
36.4
 
 
 
 
ARPP19
 
 
hsa-miR-15b-5p
 
 
-0.346
 
 
36.4
 
 
 
 
ARPP19
 
 
hsa-miR-16-5p
 
 
-0.354
 
 
36.4
 
 
 
 
ARPP19
 
 
hsa-miR-29a-3p
 
 
-0.371
 
 
36.4
 
 
 
 
ARPP19
 
 
hsa-miR-328-3p
 
 
-0.404
 
 
36.4
 
 
 
 
ARPP19
 
 
hsa-let-7b-5p
 
 
-0.476
 
 
36.4
 
 
 
 
ARRDC3
 
 
hsa-miR-148b-3p
 
 
-0.305
 
 
24.3
 
 
 
 
ARRDC3
 
 
hsa-miR-29a-3p
 
 
-0.402
 
 
24.3
 
 
 
 
 
 
 testis 
 
 
 
 
target_symbol
 
 
mature_mirna_id
 
 
score
 
 
nTPM
 
 
 
 
 
 
AAR2
 
 
hsa-miR-29a-3p
 
 
-0.327
 
 
15.4
 
 
 
 
ABCB6
 
 
hsa-miR-29a-3p
 
 
-0.48
 
 
15.2
 
 
 
 
ABCB9
 
 
hsa-let-7b-5p
 
 
-0.422
 
 
17.1
 
 
 
 
ABCD3
 
 
hsa-miR-148b-3p
 
 
-0.388
 
 
15.3
 
 
 
 
ABCE1
 
 
hsa-miR-29a-3p
 
 
-0.404
 
 
24.7
 
 
 
 
ABHD17B
 
 
hsa-miR-486-5p
 
 
-0.348
 
 
10.4
 
 
 
 
ABHD4
 
 
hsa-miR-29a-3p
 
 
-0.312
 
 
39.1
 
 
 
 
ACSL6
 
 
hsa-let-7b-5p
 
 
-0.336
 
 
19.3
 
 
 
 
ACTA2
 
 
hsa-miR-27a-3p
 
 
-0.577
 
 
258.1
 
 
 
 
ACTR2
 
 
hsa-miR-15b-5p
 
 
-0.293
 
 
37.7
 
 
 
 
ACTR2
 
 
hsa-miR-16-5p
 
 
-0.293
 
 
37.7
 
 
 
 
ADAM9
 
 
hsa-miR-30b-5p
 
 
-0.312
 
 
10.4
 
 
 
 
ADIPOR2
 
 
hsa-miR-150-5p
 
 
-0.287
 
 
26.9
 
 
 
 
ADNP
 
 
hsa-miR-328-3p
 
 
-0.371
 
 
37.6
 
 
 
 
AFG3L2
 
 
hsa-miR-181b-5p
 
 
-0.491
 
 
10.5
 
 
 
 
AFG3L2
 
 
hsa-miR-181a-5p
 
 
-0.491
 
 
10.5
 
 
 
 
AGFG1
 
 
hsa-miR-148b-3p
 
 
-0.332
 
 
86.7
 
 
 
 
AGPAT3
 
 
hsa-miR-27a-3p
 
 
-0.29
 
 
25.0
 
 
 
 
AGPAT3
 
 
hsa-miR-16-5p
 
 
-0.299
 
 
25.0
 
 
 
 
AHCTF1
 
 
hsa-let-7b-5p
 
 
-0.374
 
 
30.4
 
 
 
 
AIFM1
 
 
hsa-let-7b-5p
 
 
-0.314
 
 
21.3
 
 
 
 
AK1
 
 
hsa-miR-29a-3p
 
 
-0.333
 
 
64.3
 
 
 
 
AK4
 
 
hsa-miR-15b-5p
 
 
-0.316
 
 
10.1
 
 
 
 
AK4
 
 
hsa-miR-16-5p
 
 
-0.348
 
 
10.1
 
 
 
 
AKAP1
 
 
hsa-miR-148b-3p
 
 
-0.381
 
 
108.8
 
 
 
 
AKIRIN1
 
 
hsa-miR-27a-3p
 
 
-0.368
 
 
78.8
 
 
 
 
ALAD
 
 
hsa-miR-16-5p
 
 
-0.391
 
 
12.6
 
 
 
 
ALAD
 
 
hsa-miR-15b-5p
 
 
-0.446
 
 
12.6
 
 
 
 
ALDH1A2
 
 
hsa-miR-23b-3p
 
 
-0.346
 
 
53.9
 
 
 
 
ALDH1A2
 
 
hsa-miR-23a-3p
 
 
-0.354
 
 
53.9
 
 
 
 
AMOTL1
 
 
hsa-miR-16-5p
 
 
-0.297
 
 
11.0
 
 
 
 
AMOTL1
 
 
hsa-miR-15b-5p
 
 
-0.319
 
 
11.0
 
 
 
 
ANKHD1
 
 
hsa-miR-23a-3p
 
 
-0.368
 
 
29.3
 
 
 
 
ANKHD1
 
 
hsa-miR-23b-3p
 
 
-0.368
 
 
29.3
 
 
 
 
ANKHD1
 
 
hsa-miR-30b-5p
 
 
-0.405
 
 
29.3
 
 
 
 
ANKRD17
 
 
hsa-miR-30b-5p
 
 
-0.336
 
 
10.4
 
 
 
 
ANLN
 
 
hsa-miR-16-5p
 
 
-0.31
 
 
11.6
 
 
 
 
ANLN
 
 
hsa-miR-15b-5p
 
 
-0.334
 
 
11.6
 
 
 
 
ANP32A
 
 
hsa-miR-132-3p
 
 
-0.374
 
 
19.4
 
 
 
 
ANXA11
 
 
hsa-miR-16-5p
 
 
-0.447
 
 
22.9
 
 
 
 
ANXA11
 
 
hsa-miR-15b-5p
 
 
-0.463
 
 
22.9
 
 
 
 
AP1S1
 
 
hsa-let-7b-5p
 
 
-0.374
 
 
17.8
 
 
 
 
AP2A1
 
 
hsa-miR-15b-5p
 
 
-0.333
 
 
85.4
 
 
 
 
AP2A1
 
 
hsa-miR-16-5p
 
 
-0.333
 
 
85.4
 
 
 
 
AP2B1
 
 
hsa-miR-16-5p
 
 
-0.302
 
 
87.4
 
 
 
 
AP2B1
 
 
hsa-miR-15b-5p
 
 
-0.334
 
 
87.4
 
 
 
 
AP3S1
 
 
hsa-miR-30b-5p
 
 
-0.293
 
 
53.5
 
 
 
 
AP5S1
 
 
hsa-miR-744-5p
 
 
-0.354
 
 
10.7
 
 
 
 
APOO
 
 
hsa-miR-181b-5p
 
 
-0.5
 
 
15.7
 
 
 
 
APOO
 
 
hsa-miR-181a-5p
 
 
-0.5
 
 
15.7
 
 
 
 
APP
 
 
hsa-miR-16-5p
 
 
-0.298
 
 
146.3
 
 
 
 
APP
 
 
hsa-miR-15b-5p
 
 
-0.298
 
 
146.3
 
 
 
 
APPBP2
 
 
hsa-miR-27a-3p
 
 
-0.378
 
 
21.9
 
 
 
 
ARF4
 
 
hsa-miR-30b-5p
 
 
-0.289
 
 
94.2
 
 
 
 
ARF4
 
 
hsa-miR-148b-3p
 
 
-0.487
 
 
94.2
 
 
 
 
ARF5
 
 
hsa-miR-29a-3p
 
 
-0.491
 
 
89.2
 
 
 
 
ARF6
 
 
hsa-miR-181b-5p
 
 
-0.33
 
 
15.0
 
 
 
 
ARF6
 
 
hsa-miR-181a-5p
 
 
-0.33
 
 
15.0
 
 
 
 
ARFGEF1
 
 
hsa-miR-148b-3p
 
 
-0.306
 
 
26.5
 
 
 
 
ARFIP1
 
 
hsa-miR-320b
 
 
-0.339
 
 
11.3
 
 
 
 
ARFIP1
 
 
hsa-miR-320c
 
 
-0.339
 
 
11.3
 
 
 
 
ARFIP1
 
 
hsa-miR-148b-3p
 
 
-0.474
 
 
11.3
 
 
 
 
ARHGAP21
 
 
hsa-miR-148b-3p
 
 
-0.287
 
 
13.5
 
 
 
 
ARHGAP28
 
 
hsa-let-7b-5p
 
 
-0.454
 
 
72.3
 
 
 
 
ARHGAP5
 
 
hsa-miR-486-5p
 
 
-0.3
 
 
43.5
 
 
 
 
ARID2
 
 
hsa-miR-132-3p
 
 
-0.304
 
 
24.3
 
 
 
 
ARID3A
 
 
hsa-let-7b-5p
 
 
-0.332
 
 
16.1
 
 
 
 
ARID3A
 
 
hsa-let-7b-5p
 
 
-0.347
 
 
16.1
 
 
 
 
ARID3B
 
 
hsa-let-7b-5p
 
 
-0.322
 
 
21.2
 
 
 
 
ARID3B
 
 
hsa-let-7b-5p
 
 
-0.486
 
 
21.2
 
 
 
 
ARID4B
 
 
hsa-miR-486-5p
 
 
-0.367
 
 
16.0
 
 
 
 
ARL2
 
 
hsa-miR-16-5p
 
 
-0.374
 
 
115.7
 
 
 
 
ARL2
 
 
hsa-miR-15b-5p
 
 
-0.374
 
 
115.7
 
 
 
 
ARL2
 
 
hsa-miR-16-5p
 
 
-0.418
 
 
115.7
 
 
 
 
ARL2
 
 
hsa-miR-15b-5p
 
 
-0.442
 
 
115.7
 
 
 
 
ARL3
 
 
hsa-miR-16-5p
 
 
-0.496
 
 
10.4
 
 
 
 
ARL3
 
 
hsa-miR-15b-5p
 
 
-0.507
 
 
10.4
 
 
 
 
ARL4A
 
 
hsa-miR-29a-3p
 
 
-0.386
 
 
181.2
 
 
 
 
ARL4A
 
 
hsa-miR-30b-5p
 
 
-0.448
 
 
181.2
 
 
 
 
ARL4D
 
 
hsa-let-7b-5p
 
 
-0.417
 
 
31.4
 
 
 
 
ARL6IP1
 
 
hsa-miR-328-3p
 
 
-0.494
 
 
121.4
 
 
 
 
ARL6IP1
 
 
hsa-miR-148b-3p
 
 
-0.579
 
 
121.4
 
 
 
 
ARL6IP6
 
 
hsa-let-7b-5p
 
 
-0.293
 
 
10.2
 
 
 
 
ARL8B
 
 
hsa-miR-148b-3p
 
 
-0.337
 
 
30.1
 
 
 
 
ARL8B
 
 
hsa-miR-320b
 
 
-0.376
 
 
30.1
 
 
 
 
ARL8B
 
 
hsa-miR-320c
 
 
-0.376
 
 
30.1
 
 
 
 
ARMC12
 
 
hsa-miR-15b-5p
 
 
-0.485
 
 
58.4
 
 
 
 
ARMC12
 
 
hsa-miR-16-5p
 
 
-0.485
 
 
58.4
 
 
 
 
ARMC8
 
 
hsa-miR-486-5p
 
 
-0.321
 
 
17.2
 
 
 
 
ARMCX3
 
 
hsa-miR-425-5p
 
 
-0.325
 
 
12.0
 
 
 
 
ARMT1
 
 
hsa-let-7b-5p
 
 
-0.291
 
 
13.1
 
 
 
 
ARPP19
 
 
hsa-miR-320b
 
 
-0.325
 
 
79.0
 
 
 
 
ARPP19
 
 
hsa-miR-320c
 
 
-0.325
 
 
79.0
 
 
 
 
ARPP19
 
 
hsa-miR-15b-5p
 
 
-0.346
 
 
79.0
 
 
 
 
ARPP19
 
 
hsa-miR-16-5p
 
 
-0.354
 
 
79.0
 
 
 
 
ARPP19
 
 
hsa-miR-29a-3p
 
 
-0.371
 
 
79.0
 
 
 
 
ARPP19
 
 
hsa-miR-328-3p
 
 
-0.404
 
 
79.0
 
 
 
 
ARPP19
 
 
hsa-let-7b-5p
 
 
-0.476
 
 
79.0
 
 
 
 
ARRDC3
 
 
hsa-miR-148b-3p
 
 
-0.305
 
 
15.5
 
 
 
 
ARRDC3
 
 
hsa-miR-29a-3p
 
 
-0.402
 
 
15.5
 
 
 
 
 
 
 thymus 
 
 
 
 
target_symbol
 
 
mature_mirna_id
 
 
score
 
 
nTPM
 
 
 
 
 
 
AAR2
 
 
hsa-miR-29a-3p
 
 
-0.327
 
 
24.4
 
 
 
 
ABCB7
 
 
hsa-miR-148b-3p
 
 
-0.515
 
 
13.0
 
 
 
 
ABCD3
 
 
hsa-miR-148b-3p
 
 
-0.388
 
 
44.2
 
 
 
 
ABCE1
 
 
hsa-miR-29a-3p
 
 
-0.404
 
 
31.9
 
 
 
 
ABHD17B
 
 
hsa-miR-486-5p
 
 
-0.348
 
 
30.8
 
 
 
 
ABHD4
 
 
hsa-miR-29a-3p
 
 
-0.312
 
 
10.3
 
 
 
 
ACTA2
 
 
hsa-miR-27a-3p
 
 
-0.577
 
 
52.7
 
 
 
 
ACTC1
 
 
hsa-miR-30b-5p
 
 
-0.543
 
 
10.5
 
 
 
 
ACTR2
 
 
hsa-miR-15b-5p
 
 
-0.293
 
 
169.2
 
 
 
 
ACTR2
 
 
hsa-miR-16-5p
 
 
-0.293
 
 
169.2
 
 
 
 
ADA
 
 
hsa-miR-29a-3p
 
 
-0.376
 
 
228.3
 
 
 
 
ADAM10
 
 
hsa-miR-148b-3p
 
 
-0.356
 
 
38.8
 
 
 
 
ADCY3
 
 
hsa-miR-132-3p
 
 
-0.303
 
 
10.7
 
 
 
 
ADIPOR2
 
 
hsa-miR-150-5p
 
 
-0.287
 
 
11.9
 
 
 
 
ADNP
 
 
hsa-miR-328-3p
 
 
-0.371
 
 
41.7
 
 
 
 
AFG3L2
 
 
hsa-miR-181b-5p
 
 
-0.491
 
 
12.1
 
 
 
 
AFG3L2
 
 
hsa-miR-181a-5p
 
 
-0.491
 
 
12.1
 
 
 
 
AGFG1
 
 
hsa-miR-148b-3p
 
 
-0.332
 
 
45.3
 
 
 
 
AGPAT3
 
 
hsa-miR-27a-3p
 
 
-0.29
 
 
14.4
 
 
 
 
AGPAT3
 
 
hsa-miR-16-5p
 
 
-0.299
 
 
14.4
 
 
 
 
AHCTF1
 
 
hsa-let-7b-5p
 
 
-0.374
 
 
11.4
 
 
 
 
AIFM1
 
 
hsa-let-7b-5p
 
 
-0.314
 
 
17.2
 
 
 
 
AKAP1
 
 
hsa-miR-148b-3p
 
 
-0.381
 
 
10.6
 
 
 
 
AKIRIN1
 
 
hsa-miR-27a-3p
 
 
-0.368
 
 
38.6
 
 
 
 
ALAD
 
 
hsa-miR-16-5p
 
 
-0.391
 
 
18.9
 
 
 
 
ALAD
 
 
hsa-miR-15b-5p
 
 
-0.446
 
 
18.9
 
 
 
 
ALCAM
 
 
hsa-miR-148b-3p
 
 
-0.339
 
 
13.3
 
 
 
 
AMOTL1
 
 
hsa-miR-16-5p
 
 
-0.297
 
 
26.0
 
 
 
 
AMOTL1
 
 
hsa-miR-15b-5p
 
 
-0.319
 
 
26.0
 
 
 
 
ANKHD1
 
 
hsa-miR-23a-3p
 
 
-0.368
 
 
45.5
 
 
 
 
ANKHD1
 
 
hsa-miR-23b-3p
 
 
-0.368
 
 
45.5
 
 
 
 
ANKHD1
 
 
hsa-miR-30b-5p
 
 
-0.405
 
 
45.5
 
 
 
 
ANKRD17
 
 
hsa-miR-30b-5p
 
 
-0.336
 
 
25.3
 
 
 
 
ANKRD40
 
 
hsa-miR-27a-3p
 
 
-0.35
 
 
15.8
 
 
 
 
ANKRD46
 
 
hsa-let-7b-5p
 
 
-0.389
 
 
14.3
 
 
 
 
ANLN
 
 
hsa-miR-16-5p
 
 
-0.31
 
 
14.9
 
 
 
 
ANLN
 
 
hsa-miR-15b-5p
 
 
-0.334
 
 
14.9
 
 
 
 
ANP32A
 
 
hsa-miR-132-3p
 
 
-0.374
 
 
189.1
 
 
 
 
ANTXR2
 
 
hsa-miR-29a-3p
 
 
-0.347
 
 
10.0
 
 
 
 
ANXA11
 
 
hsa-miR-16-5p
 
 
-0.447
 
 
93.3
 
 
 
 
ANXA11
 
 
hsa-miR-15b-5p
 
 
-0.463
 
 
93.3
 
 
 
 
AP1S1
 
 
hsa-let-7b-5p
 
 
-0.374
 
 
17.8
 
 
 
 
AP2A1
 
 
hsa-miR-15b-5p
 
 
-0.333
 
 
14.3
 
 
 
 
AP2A1
 
 
hsa-miR-16-5p
 
 
-0.333
 
 
14.3
 
 
 
 
AP2B1
 
 
hsa-miR-16-5p
 
 
-0.302
 
 
55.6
 
 
 
 
AP2B1
 
 
hsa-miR-15b-5p
 
 
-0.334
 
 
55.6
 
 
 
 
AP3S1
 
 
hsa-miR-30b-5p
 
 
-0.293
 
 
38.3
 
 
 
 
APOO
 
 
hsa-miR-181b-5p
 
 
-0.5
 
 
13.0
 
 
 
 
APOO
 
 
hsa-miR-181a-5p
 
 
-0.5
 
 
13.0
 
 
 
 
APP
 
 
hsa-miR-16-5p
 
 
-0.298
 
 
143.9
 
 
 
 
APP
 
 
hsa-miR-15b-5p
 
 
-0.298
 
 
143.9
 
 
 
 
ARF4
 
 
hsa-miR-30b-5p
 
 
-0.289
 
 
69.9
 
 
 
 
ARF4
 
 
hsa-miR-148b-3p
 
 
-0.487
 
 
69.9
 
 
 
 
ARF5
 
 
hsa-miR-29a-3p
 
 
-0.491
 
 
89.3
 
 
 
 
ARF6
 
 
hsa-miR-181b-5p
 
 
-0.33
 
 
41.8
 
 
 
 
ARF6
 
 
hsa-miR-181a-5p
 
 
-0.33
 
 
41.8
 
 
 
 
ARFGEF1
 
 
hsa-miR-148b-3p
 
 
-0.306
 
 
20.7
 
 
 
 
ARFIP1
 
 
hsa-miR-320b
 
 
-0.339
 
 
11.2
 
 
 
 
ARFIP1
 
 
hsa-miR-320c
 
 
-0.339
 
 
11.2
 
 
 
 
ARFIP1
 
 
hsa-miR-148b-3p
 
 
-0.474
 
 
11.2
 
 
 
 
ARHGAP21
 
 
hsa-miR-148b-3p
 
 
-0.287
 
 
10.5
 
 
 
 
ARHGAP5
 
 
hsa-miR-486-5p
 
 
-0.3
 
 
17.1
 
 
 
 
ARID1B
 
 
hsa-miR-29a-3p
 
 
-0.342
 
 
23.3
 
 
 
 
ARID2
 
 
hsa-miR-132-3p
 
 
-0.304
 
 
17.5
 
 
 
 
ARID4B
 
 
hsa-miR-486-5p
 
 
-0.367
 
 
19.9
 
 
 
 
ARL2
 
 
hsa-miR-16-5p
 
 
-0.374
 
 
31.2
 
 
 
 
ARL2
 
 
hsa-miR-15b-5p
 
 
-0.374
 
 
31.2
 
 
 
 
ARL2
 
 
hsa-miR-16-5p
 
 
-0.418
 
 
31.2
 
 
 
 
ARL2
 
 
hsa-miR-15b-5p
 
 
-0.442
 
 
31.2
 
 
 
 
ARL4A
 
 
hsa-miR-29a-3p
 
 
-0.386
 
 
21.1
 
 
 
 
ARL4A
 
 
hsa-miR-30b-5p
 
 
-0.448
 
 
21.1
 
 
 
 
ARL5B
 
 
hsa-miR-29a-3p
 
 
-0.304
 
 
12.6
 
 
 
 
ARL6IP1
 
 
hsa-miR-328-3p
 
 
-0.494
 
 
147.6
 
 
 
 
ARL6IP1
 
 
hsa-miR-148b-3p
 
 
-0.579
 
 
147.6
 
 
 
 
ARL6IP6
 
 
hsa-let-7b-5p
 
 
-0.293
 
 
20.0
 
 
 
 
ARL8B
 
 
hsa-miR-148b-3p
 
 
-0.337
 
 
33.3
 
 
 
 
ARL8B
 
 
hsa-miR-320b
 
 
-0.376
 
 
33.3
 
 
 
 
ARL8B
 
 
hsa-miR-320c
 
 
-0.376
 
 
33.3
 
 
 
 
ARMC8
 
 
hsa-miR-486-5p
 
 
-0.321
 
 
20.7
 
 
 
 
ARMCX3
 
 
hsa-miR-425-5p
 
 
-0.325
 
 
18.7
 
 
 
 
ARMT1
 
 
hsa-let-7b-5p
 
 
-0.291
 
 
14.5
 
 
 
 
ARPP19
 
 
hsa-miR-320b
 
 
-0.325
 
 
49.2
 
 
 
 
ARPP19
 
 
hsa-miR-320c
 
 
-0.325
 
 
49.2
 
 
 
 
ARPP19
 
 
hsa-miR-15b-5p
 
 
-0.346
 
 
49.2
 
 
 
 
ARPP19
 
 
hsa-miR-16-5p
 
 
-0.354
 
 
49.2
 
 
 
 
ARPP19
 
 
hsa-miR-29a-3p
 
 
-0.371
 
 
49.2
 
 
 
 
ARPP19
 
 
hsa-miR-328-3p
 
 
-0.404
 
 
49.2
 
 
 
 
ARPP19
 
 
hsa-let-7b-5p
 
 
-0.476
 
 
49.2
 
 
 
 
ARPP21
 
 
hsa-miR-16-5p
 
 
-0.309
 
 
141.6
 
 
 
 
ARPP21
 
 
hsa-miR-15b-5p
 
 
-0.342
 
 
141.6
 
 
 
 
ARRDC3
 
 
hsa-miR-148b-3p
 
 
-0.305
 
 
26.1
 
 
 
 
ARRDC3
 
 
hsa-miR-29a-3p
 
 
-0.402
 
 
26.1
 
 
 
 
ASAH1
 
 
hsa-miR-27a-3p
 
 
-0.398
 
 
24.2
 
 
 
 
ASAP1
 
 
hsa-let-7b-5p
 
 
-0.308
 
 
14.9
 
 
 
 
ASB3
 
 
hsa-miR-30b-5p
 
 
-0.418
 
 
16.0
 
 
 
 
ASB3
 
 
hsa-miR-30b-5p
 
 
-0.418
 
 
16.0
 
 
 
 
ASF1A
 
 
hsa-miR-23a-3p
 
 
-0.506
 
 
22.7
 
 
 
 
ASF1A
 
 
hsa-miR-23b-3p
 
 
-0.506
 
 
22.7
 
 
 
 
ASH1L
 
 
hsa-miR-15b-5p
 
 
-0.325
 
 
11.3
 
 
 
 
ASH1L
 
 
hsa-miR-16-5p
 
 
-0.357
 
 
11.3
 
 
 
 
 
 
 thyroid gland 
 
 
 
 
target_symbol
 
 
mature_mirna_id
 
 
score
 
 
nTPM
 
 
 
 
 
 
AAR2
 
 
hsa-miR-29a-3p
 
 
-0.327
 
 
16.7
 
 
 
 
ABCD3
 
 
hsa-miR-148b-3p
 
 
-0.388
 
 
25.8
 
 
 
 
ABCE1
 
 
hsa-miR-29a-3p
 
 
-0.404
 
 
25.1
 
 
 
 
ABHD4
 
 
hsa-miR-29a-3p
 
 
-0.312
 
 
22.8
 
 
 
 
ABHD5
 
 
hsa-miR-132-3p
 
 
-0.323
 
 
14.4
 
 
 
 
ABHD5
 
 
hsa-miR-27a-3p
 
 
-0.407
 
 
14.4
 
 
 
 
ACTA2
 
 
hsa-miR-27a-3p
 
 
-0.577
 
 
297.7
 
 
 
 
ACTR2
 
 
hsa-miR-15b-5p
 
 
-0.293
 
 
68.0
 
 
 
 
ACTR2
 
 
hsa-miR-16-5p
 
 
-0.293
 
 
68.0
 
 
 
 
ACVR1
 
 
hsa-miR-30b-5p
 
 
-0.29
 
 
28.6
 
 
 
 
ACVR1
 
 
hsa-miR-148b-3p
 
 
-0.397
 
 
28.6
 
 
 
 
ADAM10
 
 
hsa-miR-148b-3p
 
 
-0.356
 
 
26.7
 
 
 
 
ADAM9
 
 
hsa-miR-30b-5p
 
 
-0.312
 
 
37.4
 
 
 
 
ADCY3
 
 
hsa-miR-132-3p
 
 
-0.303
 
 
35.2
 
 
 
 
ADGRA3
 
 
hsa-miR-27a-3p
 
 
-0.296
 
 
23.6
 
 
 
 
ADGRA3
 
 
hsa-miR-30b-5p
 
 
-0.304
 
 
23.6
 
 
 
 
ADGRF5
 
 
hsa-miR-148b-3p
 
 
-0.354
 
 
30.7
 
 
 
 
ADIPOR2
 
 
hsa-miR-150-5p
 
 
-0.287
 
 
26.8
 
 
 
 
ADNP
 
 
hsa-miR-328-3p
 
 
-0.371
 
 
27.8
 
 
 
 
AFG3L2
 
 
hsa-miR-181b-5p
 
 
-0.491
 
 
24.7
 
 
 
 
AFG3L2
 
 
hsa-miR-181a-5p
 
 
-0.491
 
 
24.7
 
 
 
 
AGFG1
 
 
hsa-miR-148b-3p
 
 
-0.332
 
 
32.1
 
 
 
 
AGPAT3
 
 
hsa-miR-27a-3p
 
 
-0.29
 
 
20.1
 
 
 
 
AGPAT3
 
 
hsa-miR-16-5p
 
 
-0.299
 
 
20.1
 
 
 
 
AHCTF1
 
 
hsa-let-7b-5p
 
 
-0.374
 
 
12.9
 
 
 
 
AHDC1
 
 
hsa-miR-148b-3p
 
 
-0.413
 
 
19.9
 
 
 
 
AHR
 
 
hsa-miR-29a-3p
 
 
-0.316
 
 
11.8
 
 
 
 
AIFM1
 
 
hsa-let-7b-5p
 
 
-0.314
 
 
10.7
 
 
 
 
AK1
 
 
hsa-miR-29a-3p
 
 
-0.333
 
 
42.1
 
 
 
 
AKAP1
 
 
hsa-miR-148b-3p
 
 
-0.381
 
 
35.0
 
 
 
 
AKIRIN1
 
 
hsa-miR-27a-3p
 
 
-0.368
 
 
33.8
 
 
 
 
AKT3
 
 
hsa-miR-29a-3p
 
 
-0.287
 
 
13.0
 
 
 
 
AKT3
 
 
hsa-miR-15b-5p
 
 
-0.301
 
 
13.0
 
 
 
 
AKT3
 
 
hsa-miR-16-5p
 
 
-0.309
 
 
13.0
 
 
 
 
ALAD
 
 
hsa-miR-16-5p
 
 
-0.391
 
 
24.2
 
 
 
 
ALAD
 
 
hsa-miR-15b-5p
 
 
-0.446
 
 
24.2
 
 
 
 
ALCAM
 
 
hsa-miR-148b-3p
 
 
-0.339
 
 
58.4
 
 
 
 
ANKHD1
 
 
hsa-miR-23a-3p
 
 
-0.368
 
 
44.4
 
 
 
 
ANKHD1
 
 
hsa-miR-23b-3p
 
 
-0.368
 
 
44.4
 
 
 
 
ANKHD1
 
 
hsa-miR-30b-5p
 
 
-0.405
 
 
44.4
 
 
 
 
ANKRA2
 
 
hsa-miR-30b-5p
 
 
-0.354
 
 
13.4
 
 
 
 
ANKRA2
 
 
hsa-miR-30b-5p
 
 
-0.472
 
 
13.4
 
 
 
 
ANKRD17
 
 
hsa-miR-30b-5p
 
 
-0.336
 
 
16.8
 
 
 
 
ANKRD40
 
 
hsa-miR-27a-3p
 
 
-0.35
 
 
15.8
 
 
 
 
ANKRD46
 
 
hsa-let-7b-5p
 
 
-0.389
 
 
18.8
 
 
 
 
ANP32A
 
 
hsa-miR-132-3p
 
 
-0.374
 
 
68.9
 
 
 
 
ANXA11
 
 
hsa-miR-16-5p
 
 
-0.447
 
 
58.5
 
 
 
 
ANXA11
 
 
hsa-miR-15b-5p
 
 
-0.463
 
 
58.5
 
 
 
 
AP1S1
 
 
hsa-let-7b-5p
 
 
-0.374
 
 
15.7
 
 
 
 
AP1S3
 
 
hsa-miR-181b-5p
 
 
-0.383
 
 
12.8
 
 
 
 
AP1S3
 
 
hsa-miR-181a-5p
 
 
-0.383
 
 
12.8
 
 
 
 
AP2A1
 
 
hsa-miR-15b-5p
 
 
-0.333
 
 
29.3
 
 
 
 
AP2A1
 
 
hsa-miR-16-5p
 
 
-0.333
 
 
29.3
 
 
 
 
AP2B1
 
 
hsa-miR-16-5p
 
 
-0.302
 
 
61.0
 
 
 
 
AP2B1
 
 
hsa-miR-15b-5p
 
 
-0.334
 
 
61.0
 
 
 
 
AP3S1
 
 
hsa-miR-30b-5p
 
 
-0.293
 
 
46.1
 
 
 
 
APOO
 
 
hsa-miR-181b-5p
 
 
-0.5
 
 
19.0
 
 
 
 
APOO
 
 
hsa-miR-181a-5p
 
 
-0.5
 
 
19.0
 
 
 
 
APP
 
 
hsa-miR-16-5p
 
 
-0.298
 
 
434.2
 
 
 
 
APP
 
 
hsa-miR-15b-5p
 
 
-0.298
 
 
434.2
 
 
 
 
APPBP2
 
 
hsa-miR-27a-3p
 
 
-0.378
 
 
12.0
 
 
 
 
ARF4
 
 
hsa-miR-30b-5p
 
 
-0.289
 
 
134.8
 
 
 
 
ARF4
 
 
hsa-miR-148b-3p
 
 
-0.487
 
 
134.8
 
 
 
 
ARF5
 
 
hsa-miR-29a-3p
 
 
-0.491
 
 
88.3
 
 
 
 
ARF6
 
 
hsa-miR-181b-5p
 
 
-0.33
 
 
20.1
 
 
 
 
ARF6
 
 
hsa-miR-181a-5p
 
 
-0.33
 
 
20.1
 
 
 
 
ARFGEF1
 
 
hsa-miR-148b-3p
 
 
-0.306
 
 
20.9
 
 
 
 
ARFIP1
 
 
hsa-miR-320b
 
 
-0.339
 
 
18.8
 
 
 
 
ARFIP1
 
 
hsa-miR-320c
 
 
-0.339
 
 
18.8
 
 
 
 
ARFIP1
 
 
hsa-miR-148b-3p
 
 
-0.474
 
 
18.8
 
 
 
 
ARG2
 
 
hsa-let-7b-5p
 
 
-0.468
 
 
97.9
 
 
 
 
ARHGAP21
 
 
hsa-miR-148b-3p
 
 
-0.287
 
 
12.0
 
 
 
 
ARHGAP5
 
 
hsa-miR-486-5p
 
 
-0.3
 
 
64.3
 
 
 
 
ARID1B
 
 
hsa-miR-29a-3p
 
 
-0.342
 
 
19.6
 
 
 
 
ARID4B
 
 
hsa-miR-486-5p
 
 
-0.367
 
 
15.9
 
 
 
 
ARL2
 
 
hsa-miR-16-5p
 
 
-0.374
 
 
86.5
 
 
 
 
ARL2
 
 
hsa-miR-15b-5p
 
 
-0.374
 
 
86.5
 
 
 
 
ARL2
 
 
hsa-miR-16-5p
 
 
-0.418
 
 
86.5
 
 
 
 
ARL2
 
 
hsa-miR-15b-5p
 
 
-0.442
 
 
86.5
 
 
 
 
ARL3
 
 
hsa-miR-16-5p
 
 
-0.496
 
 
10.7
 
 
 
 
ARL3
 
 
hsa-miR-15b-5p
 
 
-0.507
 
 
10.7
 
 
 
 
ARL4A
 
 
hsa-miR-29a-3p
 
 
-0.386
 
 
18.8
 
 
 
 
ARL4A
 
 
hsa-miR-30b-5p
 
 
-0.448
 
 
18.8
 
 
 
 
ARL4D
 
 
hsa-let-7b-5p
 
 
-0.417
 
 
35.9
 
 
 
 
ARL6IP1
 
 
hsa-miR-328-3p
 
 
-0.494
 
 
105.4
 
 
 
 
ARL6IP1
 
 
hsa-miR-148b-3p
 
 
-0.579
 
 
105.4
 
 
 
 
ARL8B
 
 
hsa-miR-148b-3p
 
 
-0.337
 
 
58.1
 
 
 
 
ARL8B
 
 
hsa-miR-320b
 
 
-0.376
 
 
58.1
 
 
 
 
ARL8B
 
 
hsa-miR-320c
 
 
-0.376
 
 
58.1
 
 
 
 
ARMC8
 
 
hsa-miR-486-5p
 
 
-0.321
 
 
20.0
 
 
 
 
ARMCX2
 
 
hsa-miR-15b-5p
 
 
-0.344
 
 
55.9
 
 
 
 
ARMCX2
 
 
hsa-miR-16-5p
 
 
-0.366
 
 
55.9
 
 
 
 
ARMCX3
 
 
hsa-miR-425-5p
 
 
-0.325
 
 
46.7
 
 
 
 
ARMT1
 
 
hsa-let-7b-5p
 
 
-0.291
 
 
25.9
 
 
 
 
ARPP19
 
 
hsa-miR-320b
 
 
-0.325
 
 
53.7
 
 
 
 
ARPP19
 
 
hsa-miR-320c
 
 
-0.325
 
 
53.7
 
 
 
 
ARPP19
 
 
hsa-miR-15b-5p
 
 
-0.346
 
 
53.7
 
 
 
 
ARPP19
 
 
hsa-miR-16-5p
 
 
-0.354
 
 
53.7
 
 
 
 
ARPP19
 
 
hsa-miR-29a-3p
 
 
-0.371
 
 
53.7
 
 
 
 
ARPP19
 
 
hsa-miR-328-3p
 
 
-0.404
 
 
53.7
 
 
 
 
 
 
 tongue 
 
 
 
 
target_symbol
 
 
mature_mirna_id
 
 
score
 
 
nTPM
 
 
 
 
 
 
AAR2
 
 
hsa-miR-29a-3p
 
 
-0.327
 
 
19.3
 
 
 
 
ABCB10
 
 
hsa-miR-148b-3p
 
 
-0.291
 
 
10.2
 
 
 
 
ABCB7
 
 
hsa-miR-148b-3p
 
 
-0.515
 
 
20.8
 
 
 
 
ABCD3
 
 
hsa-miR-148b-3p
 
 
-0.388
 
 
27.9
 
 
 
 
ABCE1
 
 
hsa-miR-29a-3p
 
 
-0.404
 
 
28.5
 
 
 
 
ABHD18
 
 
hsa-miR-29a-3p
 
 
-0.321
 
 
10.9
 
 
 
 
ABHD18
 
 
hsa-miR-29a-3p
 
 
-0.34
 
 
10.9
 
 
 
 
ABHD4
 
 
hsa-miR-29a-3p
 
 
-0.312
 
 
27.0
 
 
 
 
ABHD5
 
 
hsa-miR-132-3p
 
 
-0.323
 
 
17.3
 
 
 
 
ABHD5
 
 
hsa-miR-27a-3p
 
 
-0.407
 
 
17.3
 
 
 
 
ACTA2
 
 
hsa-miR-27a-3p
 
 
-0.577
 
 
200.0
 
 
 
 
ACTC1
 
 
hsa-miR-30b-5p
 
 
-0.543
 
 
182.1
 
 
 
 
ACTN2
 
 
hsa-miR-23a-3p
 
 
-0.294
 
 
1195.2
 
 
 
 
ACTN2
 
 
hsa-miR-29a-3p
 
 
-0.311
 
 
1195.2
 
 
 
 
ACTR2
 
 
hsa-miR-15b-5p
 
 
-0.293
 
 
60.6
 
 
 
 
ACTR2
 
 
hsa-miR-16-5p
 
 
-0.293
 
 
60.6
 
 
 
 
ADAM9
 
 
hsa-miR-30b-5p
 
 
-0.312
 
 
23.3
 
 
 
 
ADCY2
 
 
hsa-miR-148b-3p
 
 
-0.299
 
 
28.8
 
 
 
 
ADCY3
 
 
hsa-miR-132-3p
 
 
-0.303
 
 
19.0
 
 
 
 
ADGRA3
 
 
hsa-miR-27a-3p
 
 
-0.296
 
 
10.7
 
 
 
 
ADGRA3
 
 
hsa-miR-30b-5p
 
 
-0.304
 
 
10.7
 
 
 
 
ADGRF5
 
 
hsa-miR-148b-3p
 
 
-0.354
 
 
66.5
 
 
 
 
ADIPOR2
 
 
hsa-miR-150-5p
 
 
-0.287
 
 
18.5
 
 
 
 
ADNP
 
 
hsa-miR-328-3p
 
 
-0.371
 
 
26.5
 
 
 
 
AFG3L2
 
 
hsa-miR-181b-5p
 
 
-0.491
 
 
92.6
 
 
 
 
AFG3L2
 
 
hsa-miR-181a-5p
 
 
-0.491
 
 
92.6
 
 
 
 
AGFG1
 
 
hsa-miR-148b-3p
 
 
-0.332
 
 
36.1
 
 
 
 
AGPAT3
 
 
hsa-miR-27a-3p
 
 
-0.29
 
 
46.9
 
 
 
 
AGPAT3
 
 
hsa-miR-16-5p
 
 
-0.299
 
 
46.9
 
 
 
 
AHCTF1
 
 
hsa-let-7b-5p
 
 
-0.374
 
 
14.7
 
 
 
 
AHDC1
 
 
hsa-miR-148b-3p
 
 
-0.413
 
 
14.5
 
 
 
 
AIFM1
 
 
hsa-let-7b-5p
 
 
-0.314
 
 
23.4
 
 
 
 
AK1
 
 
hsa-miR-29a-3p
 
 
-0.333
 
 
730.8
 
 
 
 
AK4
 
 
hsa-miR-15b-5p
 
 
-0.316
 
 
35.9
 
 
 
 
AK4
 
 
hsa-miR-16-5p
 
 
-0.348
 
 
35.9
 
 
 
 
AKAP1
 
 
hsa-miR-148b-3p
 
 
-0.381
 
 
128.4
 
 
 
 
AKAP6
 
 
hsa-let-7b-5p
 
 
-0.307
 
 
47.4
 
 
 
 
AKIRIN1
 
 
hsa-miR-27a-3p
 
 
-0.368
 
 
28.3
 
 
 
 
AKT3
 
 
hsa-miR-29a-3p
 
 
-0.287
 
 
10.7
 
 
 
 
AKT3
 
 
hsa-miR-15b-5p
 
 
-0.301
 
 
10.7
 
 
 
 
AKT3
 
 
hsa-miR-16-5p
 
 
-0.309
 
 
10.7
 
 
 
 
ALAD
 
 
hsa-miR-16-5p
 
 
-0.391
 
 
22.3
 
 
 
 
ALAD
 
 
hsa-miR-15b-5p
 
 
-0.446
 
 
22.3
 
 
 
 
AMER1
 
 
hsa-miR-29a-3p
 
 
-0.343
 
 
14.3
 
 
 
 
AMOT
 
 
hsa-miR-29a-3p
 
 
-0.304
 
 
51.2
 
 
 
 
AMOTL1
 
 
hsa-miR-16-5p
 
 
-0.297
 
 
66.9
 
 
 
 
AMOTL1
 
 
hsa-miR-15b-5p
 
 
-0.319
 
 
66.9
 
 
 
 
ANKHD1
 
 
hsa-miR-23a-3p
 
 
-0.368
 
 
26.1
 
 
 
 
ANKHD1
 
 
hsa-miR-23b-3p
 
 
-0.368
 
 
26.1
 
 
 
 
ANKHD1
 
 
hsa-miR-30b-5p
 
 
-0.405
 
 
26.1
 
 
 
 
ANKRD17
 
 
hsa-miR-30b-5p
 
 
-0.336
 
 
24.4
 
 
 
 
ANKRD40
 
 
hsa-miR-27a-3p
 
 
-0.35
 
 
78.6
 
 
 
 
ANKRD46
 
 
hsa-let-7b-5p
 
 
-0.389
 
 
33.8
 
 
 
 
ANP32A
 
 
hsa-miR-132-3p
 
 
-0.374
 
 
60.2
 
 
 
 
ANTXR2
 
 
hsa-miR-29a-3p
 
 
-0.347
 
 
20.3
 
 
 
 
ANXA11
 
 
hsa-miR-16-5p
 
 
-0.447
 
 
155.0
 
 
 
 
ANXA11
 
 
hsa-miR-15b-5p
 
 
-0.463
 
 
155.0
 
 
 
 
AP2A1
 
 
hsa-miR-15b-5p
 
 
-0.333
 
 
24.6
 
 
 
 
AP2A1
 
 
hsa-miR-16-5p
 
 
-0.333
 
 
24.6
 
 
 
 
AP2B1
 
 
hsa-miR-16-5p
 
 
-0.302
 
 
57.0
 
 
 
 
AP2B1
 
 
hsa-miR-15b-5p
 
 
-0.334
 
 
57.0
 
 
 
 
AP3S1
 
 
hsa-miR-30b-5p
 
 
-0.293
 
 
32.1
 
 
 
 
APC
 
 
hsa-miR-29a-3p
 
 
-0.413
 
 
11.0
 
 
 
 
APOO
 
 
hsa-miR-181b-5p
 
 
-0.5
 
 
68.2
 
 
 
 
APOO
 
 
hsa-miR-181a-5p
 
 
-0.5
 
 
68.2
 
 
 
 
APP
 
 
hsa-miR-16-5p
 
 
-0.298
 
 
161.6
 
 
 
 
APP
 
 
hsa-miR-15b-5p
 
 
-0.298
 
 
161.6
 
 
 
 
ARF4
 
 
hsa-miR-30b-5p
 
 
-0.289
 
 
64.9
 
 
 
 
ARF4
 
 
hsa-miR-148b-3p
 
 
-0.487
 
 
64.9
 
 
 
 
ARF5
 
 
hsa-miR-29a-3p
 
 
-0.491
 
 
94.7
 
 
 
 
ARF6
 
 
hsa-miR-181b-5p
 
 
-0.33
 
 
15.8
 
 
 
 
ARF6
 
 
hsa-miR-181a-5p
 
 
-0.33
 
 
15.8
 
 
 
 
ARFGEF1
 
 
hsa-miR-148b-3p
 
 
-0.306
 
 
13.0
 
 
 
 
ARG2
 
 
hsa-let-7b-5p
 
 
-0.468
 
 
49.2
 
 
 
 
ARHGAP21
 
 
hsa-miR-148b-3p
 
 
-0.287
 
 
21.5
 
 
 
 
ARHGAP5
 
 
hsa-miR-486-5p
 
 
-0.3
 
 
23.1
 
 
 
 
ARID1B
 
 
hsa-miR-29a-3p
 
 
-0.342
 
 
22.6
 
 
 
 
ARL2
 
 
hsa-miR-16-5p
 
 
-0.374
 
 
147.4
 
 
 
 
ARL2
 
 
hsa-miR-15b-5p
 
 
-0.374
 
 
147.4
 
 
 
 
ARL2
 
 
hsa-miR-16-5p
 
 
-0.418
 
 
147.4
 
 
 
 
ARL2
 
 
hsa-miR-15b-5p
 
 
-0.442
 
 
147.4
 
 
 
 
ARL6IP1
 
 
hsa-miR-328-3p
 
 
-0.494
 
 
48.1
 
 
 
 
ARL6IP1
 
 
hsa-miR-148b-3p
 
 
-0.579
 
 
48.1
 
 
 
 
ARL8B
 
 
hsa-miR-148b-3p
 
 
-0.337
 
 
56.0
 
 
 
 
ARL8B
 
 
hsa-miR-320b
 
 
-0.376
 
 
56.0
 
 
 
 
ARL8B
 
 
hsa-miR-320c
 
 
-0.376
 
 
56.0
 
 
 
 
ARMC8
 
 
hsa-miR-486-5p
 
 
-0.321
 
 
32.4
 
 
 
 
ARMCX3
 
 
hsa-miR-425-5p
 
 
-0.325
 
 
59.4
 
 
 
 
ARMT1
 
 
hsa-let-7b-5p
 
 
-0.291
 
 
21.1
 
 
 
 
ARPP19
 
 
hsa-miR-320b
 
 
-0.325
 
 
158.9
 
 
 
 
ARPP19
 
 
hsa-miR-320c
 
 
-0.325
 
 
158.9
 
 
 
 
ARPP19
 
 
hsa-miR-15b-5p
 
 
-0.346
 
 
158.9
 
 
 
 
ARPP19
 
 
hsa-miR-16-5p
 
 
-0.354
 
 
158.9
 
 
 
 
ARPP19
 
 
hsa-miR-29a-3p
 
 
-0.371
 
 
158.9
 
 
 
 
ARPP19
 
 
hsa-miR-328-3p
 
 
-0.404
 
 
158.9
 
 
 
 
ARPP19
 
 
hsa-let-7b-5p
 
 
-0.476
 
 
158.9
 
 
 
 
ARPP21
 
 
hsa-miR-16-5p
 
 
-0.309
 
 
41.9
 
 
 
 
ARPP21
 
 
hsa-miR-15b-5p
 
 
-0.342
 
 
41.9
 
 
 
 
ARRDC3
 
 
hsa-miR-148b-3p
 
 
-0.305
 
 
41.9
 
 
 
 
ARRDC3
 
 
hsa-miR-29a-3p
 
 
-0.402
 
 
41.9
 
 
 
 
 
 
 tonsil 
 
 
 
 
target_symbol
 
 
mature_mirna_id
 
 
score
 
 
nTPM
 
 
 
 
 
 
AAR2
 
 
hsa-miR-29a-3p
 
 
-0.327
 
 
18.7
 
 
 
 
ABCB7
 
 
hsa-miR-148b-3p
 
 
-0.515
 
 
10.4
 
 
 
 
ABCD3
 
 
hsa-miR-148b-3p
 
 
-0.388
 
 
19.9
 
 
 
 
ABCE1
 
 
hsa-miR-29a-3p
 
 
-0.404
 
 
39.4
 
 
 
 
ABHD17C
 
 
hsa-let-7b-5p
 
 
-0.359
 
 
21.6
 
 
 
 
ABHD17C
 
 
hsa-miR-27a-3p
 
 
-0.435
 
 
21.6
 
 
 
 
ABHD4
 
 
hsa-miR-29a-3p
 
 
-0.312
 
 
15.2
 
 
 
 
ACTA2
 
 
hsa-miR-27a-3p
 
 
-0.577
 
 
45.3
 
 
 
 
ACTR2
 
 
hsa-miR-15b-5p
 
 
-0.293
 
 
239.6
 
 
 
 
ACTR2
 
 
hsa-miR-16-5p
 
 
-0.293
 
 
239.6
 
 
 
 
ADA
 
 
hsa-miR-29a-3p
 
 
-0.376
 
 
23.2
 
 
 
 
ADAM10
 
 
hsa-miR-148b-3p
 
 
-0.356
 
 
15.3
 
 
 
 
ADAM17
 
 
hsa-miR-148b-3p
 
 
-0.301
 
 
11.1
 
 
 
 
ADAM9
 
 
hsa-miR-30b-5p
 
 
-0.312
 
 
12.4
 
 
 
 
ADCY3
 
 
hsa-miR-132-3p
 
 
-0.303
 
 
16.9
 
 
 
 
ADIPOR2
 
 
hsa-miR-150-5p
 
 
-0.287
 
 
11.8
 
 
 
 
ADNP
 
 
hsa-miR-328-3p
 
 
-0.371
 
 
33.4
 
 
 
 
ADRA2A
 
 
hsa-miR-23a-3p
 
 
-0.371
 
 
12.3
 
 
 
 
ADRA2A
 
 
hsa-miR-23b-3p
 
 
-0.371
 
 
12.3
 
 
 
 
AFG3L2
 
 
hsa-miR-181b-5p
 
 
-0.491
 
 
16.2
 
 
 
 
AFG3L2
 
 
hsa-miR-181a-5p
 
 
-0.491
 
 
16.2
 
 
 
 
AGFG1
 
 
hsa-miR-148b-3p
 
 
-0.332
 
 
49.9
 
 
 
 
AHR
 
 
hsa-miR-29a-3p
 
 
-0.316
 
 
18.6
 
 
 
 
AIFM1
 
 
hsa-let-7b-5p
 
 
-0.314
 
 
10.3
 
 
 
 
AK1
 
 
hsa-miR-29a-3p
 
 
-0.333
 
 
11.8
 
 
 
 
AKIRIN1
 
 
hsa-miR-27a-3p
 
 
-0.368
 
 
39.4
 
 
 
 
ALAD
 
 
hsa-miR-16-5p
 
 
-0.391
 
 
17.5
 
 
 
 
ALAD
 
 
hsa-miR-15b-5p
 
 
-0.446
 
 
17.5
 
 
 
 
ALCAM
 
 
hsa-miR-148b-3p
 
 
-0.339
 
 
13.6
 
 
 
 
ANKHD1
 
 
hsa-miR-23a-3p
 
 
-0.368
 
 
38.8
 
 
 
 
ANKHD1
 
 
hsa-miR-23b-3p
 
 
-0.368
 
 
38.8
 
 
 
 
ANKHD1
 
 
hsa-miR-30b-5p
 
 
-0.405
 
 
38.8
 
 
 
 
ANKRD17
 
 
hsa-miR-30b-5p
 
 
-0.336
 
 
16.5
 
 
 
 
ANKRD29
 
 
hsa-miR-132-3p
 
 
-0.47
 
 
13.4
 
 
 
 
ANKRD40
 
 
hsa-miR-27a-3p
 
 
-0.35
 
 
13.5
 
 
 
 
ANKRD46
 
 
hsa-let-7b-5p
 
 
-0.389
 
 
10.3
 
 
 
 
ANP32A
 
 
hsa-miR-132-3p
 
 
-0.374
 
 
132.1
 
 
 
 
ANTXR2
 
 
hsa-miR-29a-3p
 
 
-0.347
 
 
15.1
 
 
 
 
ANXA11
 
 
hsa-miR-16-5p
 
 
-0.447
 
 
95.3
 
 
 
 
ANXA11
 
 
hsa-miR-15b-5p
 
 
-0.463
 
 
95.3
 
 
 
 
AP1S1
 
 
hsa-let-7b-5p
 
 
-0.374
 
 
12.8
 
 
 
 
AP2A1
 
 
hsa-miR-15b-5p
 
 
-0.333
 
 
12.3
 
 
 
 
AP2A1
 
 
hsa-miR-16-5p
 
 
-0.333
 
 
12.3
 
 
 
 
AP2B1
 
 
hsa-miR-16-5p
 
 
-0.302
 
 
42.7
 
 
 
 
AP2B1
 
 
hsa-miR-15b-5p
 
 
-0.334
 
 
42.7
 
 
 
 
AP3S1
 
 
hsa-miR-30b-5p
 
 
-0.293
 
 
57.3
 
 
 
 
APOO
 
 
hsa-miR-181b-5p
 
 
-0.5
 
 
10.8
 
 
 
 
APOO
 
 
hsa-miR-181a-5p
 
 
-0.5
 
 
10.8
 
 
 
 
APP
 
 
hsa-miR-16-5p
 
 
-0.298
 
 
69.8
 
 
 
 
APP
 
 
hsa-miR-15b-5p
 
 
-0.298
 
 
69.8
 
 
 
 
ARF4
 
 
hsa-miR-30b-5p
 
 
-0.289
 
 
91.2
 
 
 
 
ARF4
 
 
hsa-miR-148b-3p
 
 
-0.487
 
 
91.2
 
 
 
 
ARF5
 
 
hsa-miR-29a-3p
 
 
-0.491
 
 
69.9
 
 
 
 
ARF6
 
 
hsa-miR-181b-5p
 
 
-0.33
 
 
64.2
 
 
 
 
ARF6
 
 
hsa-miR-181a-5p
 
 
-0.33
 
 
64.2
 
 
 
 
ARFGEF1
 
 
hsa-miR-148b-3p
 
 
-0.306
 
 
16.2
 
 
 
 
ARFIP1
 
 
hsa-miR-320b
 
 
-0.339
 
 
13.9
 
 
 
 
ARFIP1
 
 
hsa-miR-320c
 
 
-0.339
 
 
13.9
 
 
 
 
ARFIP1
 
 
hsa-miR-148b-3p
 
 
-0.474
 
 
13.9
 
 
 
 
ARHGAP5
 
 
hsa-miR-486-5p
 
 
-0.3
 
 
17.4
 
 
 
 
ARID1B
 
 
hsa-miR-29a-3p
 
 
-0.342
 
 
17.5
 
 
 
 
ARID4B
 
 
hsa-miR-486-5p
 
 
-0.367
 
 
15.9
 
 
 
 
ARL2
 
 
hsa-miR-16-5p
 
 
-0.374
 
 
27.2
 
 
 
 
ARL2
 
 
hsa-miR-15b-5p
 
 
-0.374
 
 
27.2
 
 
 
 
ARL2
 
 
hsa-miR-16-5p
 
 
-0.418
 
 
27.2
 
 
 
 
ARL2
 
 
hsa-miR-15b-5p
 
 
-0.442
 
 
27.2
 
 
 
 
ARL4A
 
 
hsa-miR-29a-3p
 
 
-0.386
 
 
14.9
 
 
 
 
ARL4A
 
 
hsa-miR-30b-5p
 
 
-0.448
 
 
14.9
 
 
 
 
ARL6IP1
 
 
hsa-miR-328-3p
 
 
-0.494
 
 
106.2
 
 
 
 
ARL6IP1
 
 
hsa-miR-148b-3p
 
 
-0.579
 
 
106.2
 
 
 
 
ARL6IP6
 
 
hsa-let-7b-5p
 
 
-0.293
 
 
18.0
 
 
 
 
ARL8B
 
 
hsa-miR-148b-3p
 
 
-0.337
 
 
44.5
 
 
 
 
ARL8B
 
 
hsa-miR-320b
 
 
-0.376
 
 
44.5
 
 
 
 
ARL8B
 
 
hsa-miR-320c
 
 
-0.376
 
 
44.5
 
 
 
 
ARMC8
 
 
hsa-miR-486-5p
 
 
-0.321
 
 
21.4
 
 
 
 
ARMCX3
 
 
hsa-miR-425-5p
 
 
-0.325
 
 
20.2
 
 
 
 
ARMT1
 
 
hsa-let-7b-5p
 
 
-0.291
 
 
17.3
 
 
 
 
ARPP19
 
 
hsa-miR-320b
 
 
-0.325
 
 
48.4
 
 
 
 
ARPP19
 
 
hsa-miR-320c
 
 
-0.325
 
 
48.4
 
 
 
 
ARPP19
 
 
hsa-miR-15b-5p
 
 
-0.346
 
 
48.4
 
 
 
 
ARPP19
 
 
hsa-miR-16-5p
 
 
-0.354
 
 
48.4
 
 
 
 
ARPP19
 
 
hsa-miR-29a-3p
 
 
-0.371
 
 
48.4
 
 
 
 
ARPP19
 
 
hsa-miR-328-3p
 
 
-0.404
 
 
48.4
 
 
 
 
ARPP19
 
 
hsa-let-7b-5p
 
 
-0.476
 
 
48.4
 
 
 
 
ARRDC3
 
 
hsa-miR-148b-3p
 
 
-0.305
 
 
26.4
 
 
 
 
ARRDC3
 
 
hsa-miR-29a-3p
 
 
-0.402
 
 
26.4
 
 
 
 
ASAH1
 
 
hsa-miR-27a-3p
 
 
-0.398
 
 
54.1
 
 
 
 
ASAP1
 
 
hsa-let-7b-5p
 
 
-0.308
 
 
17.8
 
 
 
 
ASB3
 
 
hsa-miR-30b-5p
 
 
-0.418
 
 
11.5
 
 
 
 
ASB3
 
 
hsa-miR-30b-5p
 
 
-0.418
 
 
11.5
 
 
 
 
ASF1A
 
 
hsa-miR-23a-3p
 
 
-0.506
 
 
14.1
 
 
 
 
ASF1A
 
 
hsa-miR-23b-3p
 
 
-0.506
 
 
14.1
 
 
 
 
ASH2L
 
 
hsa-miR-320b
 
 
-0.287
 
 
26.7
 
 
 
 
ASH2L
 
 
hsa-miR-320c
 
 
-0.287
 
 
26.7
 
 
 
 
ASPH
 
 
hsa-miR-148b-3p
 
 
-0.391
 
 
26.4
 
 
 
 
ATG9A
 
 
hsa-miR-16-5p
 
 
-0.311
 
 
19.8
 
 
 
 
ATG9A
 
 
hsa-miR-15b-5p
 
 
-0.333
 
 
19.8
 
 
 
 
ATP2A2
 
 
hsa-let-7b-5p
 
 
-0.311
 
 
45.1
 
 
 
 
ATP2B4
 
 
hsa-miR-148b-3p
 
 
-0.337
 
 
18.2
 
 
 
 
ATP5MC3
 
 
hsa-miR-425-5p
 
 
-0.328
 
 
164.6
 
 
 
 
 
 
 urinary bladder 
 
 
 
 
target_symbol
 
 
mature_mirna_id
 
 
score
 
 
nTPM
 
 
 
 
 
 
AAR2
 
 
hsa-miR-29a-3p
 
 
-0.327
 
 
25.2
 
 
 
 
ABCB6
 
 
hsa-miR-29a-3p
 
 
-0.48
 
 
13.5
 
 
 
 
ABCB7
 
 
hsa-miR-148b-3p
 
 
-0.515
 
 
11.0
 
 
 
 
ABCD3
 
 
hsa-miR-148b-3p
 
 
-0.388
 
 
21.4
 
 
 
 
ABCE1
 
 
hsa-miR-29a-3p
 
 
-0.404
 
 
37.8
 
 
 
 
ABHD17B
 
 
hsa-miR-486-5p
 
 
-0.348
 
 
12.1
 
 
 
 
ABHD17C
 
 
hsa-let-7b-5p
 
 
-0.359
 
 
26.8
 
 
 
 
ABHD17C
 
 
hsa-miR-27a-3p
 
 
-0.435
 
 
26.8
 
 
 
 
ABHD4
 
 
hsa-miR-29a-3p
 
 
-0.312
 
 
28.1
 
 
 
 
ABHD5
 
 
hsa-miR-132-3p
 
 
-0.323
 
 
15.5
 
 
 
 
ABHD5
 
 
hsa-miR-27a-3p
 
 
-0.407
 
 
15.5
 
 
 
 
ACER2
 
 
hsa-let-7b-5p
 
 
-0.333
 
 
36.7
 
 
 
 
ACTA2
 
 
hsa-miR-27a-3p
 
 
-0.577
 
 
3161.7
 
 
 
 
ACTC1
 
 
hsa-miR-30b-5p
 
 
-0.543
 
 
667.0
 
 
 
 
ACTR2
 
 
hsa-miR-15b-5p
 
 
-0.293
 
 
119.8
 
 
 
 
ACTR2
 
 
hsa-miR-16-5p
 
 
-0.293
 
 
119.8
 
 
 
 
ACVR1
 
 
hsa-miR-30b-5p
 
 
-0.29
 
 
13.3
 
 
 
 
ACVR1
 
 
hsa-miR-148b-3p
 
 
-0.397
 
 
13.3
 
 
 
 
ADAM10
 
 
hsa-miR-148b-3p
 
 
-0.356
 
 
26.2
 
 
 
 
ADAM9
 
 
hsa-miR-30b-5p
 
 
-0.312
 
 
52.5
 
 
 
 
ADAMTS9
 
 
hsa-miR-29a-3p
 
 
-0.305
 
 
20.9
 
 
 
 
ADAMTS9
 
 
hsa-miR-29a-3p
 
 
-0.485
 
 
20.9
 
 
 
 
ADCY3
 
 
hsa-miR-132-3p
 
 
-0.303
 
 
34.2
 
 
 
 
ADGRF5
 
 
hsa-miR-148b-3p
 
 
-0.354
 
 
10.6
 
 
 
 
ADIPOR2
 
 
hsa-miR-150-5p
 
 
-0.287
 
 
21.3
 
 
 
 
ADNP
 
 
hsa-miR-328-3p
 
 
-0.371
 
 
34.8
 
 
 
 
ADORA2B
 
 
hsa-miR-27a-3p
 
 
-0.638
 
 
15.6
 
 
 
 
ADRA2A
 
 
hsa-miR-23a-3p
 
 
-0.371
 
 
14.8
 
 
 
 
ADRA2A
 
 
hsa-miR-23b-3p
 
 
-0.371
 
 
14.8
 
 
 
 
AFG3L2
 
 
hsa-miR-181b-5p
 
 
-0.491
 
 
18.0
 
 
 
 
AFG3L2
 
 
hsa-miR-181a-5p
 
 
-0.491
 
 
18.0
 
 
 
 
AGFG1
 
 
hsa-miR-148b-3p
 
 
-0.332
 
 
61.2
 
 
 
 
AGPAT3
 
 
hsa-miR-27a-3p
 
 
-0.29
 
 
14.4
 
 
 
 
AGPAT3
 
 
hsa-miR-16-5p
 
 
-0.299
 
 
14.4
 
 
 
 
AHDC1
 
 
hsa-miR-148b-3p
 
 
-0.413
 
 
14.8
 
 
 
 
AHR
 
 
hsa-miR-29a-3p
 
 
-0.316
 
 
46.3
 
 
 
 
AIFM1
 
 
hsa-let-7b-5p
 
 
-0.314
 
 
13.1
 
 
 
 
AK1
 
 
hsa-miR-29a-3p
 
 
-0.333
 
 
22.9
 
 
 
 
AK4
 
 
hsa-miR-15b-5p
 
 
-0.316
 
 
16.4
 
 
 
 
AK4
 
 
hsa-miR-16-5p
 
 
-0.348
 
 
16.4
 
 
 
 
AKAP1
 
 
hsa-miR-148b-3p
 
 
-0.381
 
 
47.8
 
 
 
 
AKIRIN1
 
 
hsa-miR-27a-3p
 
 
-0.368
 
 
38.4
 
 
 
 
AKT3
 
 
hsa-miR-29a-3p
 
 
-0.287
 
 
14.4
 
 
 
 
AKT3
 
 
hsa-miR-15b-5p
 
 
-0.301
 
 
14.4
 
 
 
 
AKT3
 
 
hsa-miR-16-5p
 
 
-0.309
 
 
14.4
 
 
 
 
ALAD
 
 
hsa-miR-16-5p
 
 
-0.391
 
 
20.6
 
 
 
 
ALAD
 
 
hsa-miR-15b-5p
 
 
-0.446
 
 
20.6
 
 
 
 
ALCAM
 
 
hsa-miR-148b-3p
 
 
-0.339
 
 
23.9
 
 
 
 
AMOTL1
 
 
hsa-miR-16-5p
 
 
-0.297
 
 
26.4
 
 
 
 
AMOTL1
 
 
hsa-miR-15b-5p
 
 
-0.319
 
 
26.4
 
 
 
 
AMT
 
 
hsa-let-7b-5p
 
 
-0.463
 
 
13.1
 
 
 
 
ANKHD1
 
 
hsa-miR-23a-3p
 
 
-0.368
 
 
34.3
 
 
 
 
ANKHD1
 
 
hsa-miR-23b-3p
 
 
-0.368
 
 
34.3
 
 
 
 
ANKHD1
 
 
hsa-miR-30b-5p
 
 
-0.405
 
 
34.3
 
 
 
 
ANKRD17
 
 
hsa-miR-30b-5p
 
 
-0.336
 
 
16.5
 
 
 
 
ANKRD40
 
 
hsa-miR-27a-3p
 
 
-0.35
 
 
22.8
 
 
 
 
ANP32A
 
 
hsa-miR-132-3p
 
 
-0.374
 
 
71.6
 
 
 
 
ANTXR2
 
 
hsa-miR-29a-3p
 
 
-0.347
 
 
82.6
 
 
 
 
ANXA11
 
 
hsa-miR-16-5p
 
 
-0.447
 
 
138.5
 
 
 
 
ANXA11
 
 
hsa-miR-15b-5p
 
 
-0.463
 
 
138.5
 
 
 
 
AP1S1
 
 
hsa-let-7b-5p
 
 
-0.374
 
 
18.6
 
 
 
 
AP2A1
 
 
hsa-miR-15b-5p
 
 
-0.333
 
 
43.6
 
 
 
 
AP2A1
 
 
hsa-miR-16-5p
 
 
-0.333
 
 
43.6
 
 
 
 
AP2B1
 
 
hsa-miR-16-5p
 
 
-0.302
 
 
44.1
 
 
 
 
AP2B1
 
 
hsa-miR-15b-5p
 
 
-0.334
 
 
44.1
 
 
 
 
AP3S1
 
 
hsa-miR-30b-5p
 
 
-0.293
 
 
56.3
 
 
 
 
APOO
 
 
hsa-miR-181b-5p
 
 
-0.5
 
 
10.5
 
 
 
 
APOO
 
 
hsa-miR-181a-5p
 
 
-0.5
 
 
10.5
 
 
 
 
APP
 
 
hsa-miR-16-5p
 
 
-0.298
 
 
275.5
 
 
 
 
APP
 
 
hsa-miR-15b-5p
 
 
-0.298
 
 
275.5
 
 
 
 
ARF4
 
 
hsa-miR-30b-5p
 
 
-0.289
 
 
190.1
 
 
 
 
ARF4
 
 
hsa-miR-148b-3p
 
 
-0.487
 
 
190.1
 
 
 
 
ARF5
 
 
hsa-miR-29a-3p
 
 
-0.491
 
 
95.3
 
 
 
 
ARF6
 
 
hsa-miR-181b-5p
 
 
-0.33
 
 
43.6
 
 
 
 
ARF6
 
 
hsa-miR-181a-5p
 
 
-0.33
 
 
43.6
 
 
 
 
ARFGEF1
 
 
hsa-miR-148b-3p
 
 
-0.306
 
 
18.4
 
 
 
 
ARFIP1
 
 
hsa-miR-320b
 
 
-0.339
 
 
22.0
 
 
 
 
ARFIP1
 
 
hsa-miR-320c
 
 
-0.339
 
 
22.0
 
 
 
 
ARFIP1
 
 
hsa-miR-148b-3p
 
 
-0.474
 
 
22.0
 
 
 
 
ARG2
 
 
hsa-let-7b-5p
 
 
-0.468
 
 
16.6
 
 
 
 
ARHGAP21
 
 
hsa-miR-148b-3p
 
 
-0.287
 
 
12.9
 
 
 
 
ARHGAP5
 
 
hsa-miR-486-5p
 
 
-0.3
 
 
30.7
 
 
 
 
ARID1B
 
 
hsa-miR-29a-3p
 
 
-0.342
 
 
13.9
 
 
 
 
ARID4B
 
 
hsa-miR-486-5p
 
 
-0.367
 
 
12.0
 
 
 
 
ARL2
 
 
hsa-miR-16-5p
 
 
-0.374
 
 
110.3
 
 
 
 
ARL2
 
 
hsa-miR-15b-5p
 
 
-0.374
 
 
110.3
 
 
 
 
ARL2
 
 
hsa-miR-16-5p
 
 
-0.418
 
 
110.3
 
 
 
 
ARL2
 
 
hsa-miR-15b-5p
 
 
-0.442
 
 
110.3
 
 
 
 
ARL4A
 
 
hsa-miR-29a-3p
 
 
-0.386
 
 
20.6
 
 
 
 
ARL4A
 
 
hsa-miR-30b-5p
 
 
-0.448
 
 
20.6
 
 
 
 
ARL4D
 
 
hsa-let-7b-5p
 
 
-0.417
 
 
26.6
 
 
 
 
ARL5B
 
 
hsa-miR-29a-3p
 
 
-0.304
 
 
11.8
 
 
 
 
ARL6IP1
 
 
hsa-miR-328-3p
 
 
-0.494
 
 
124.0
 
 
 
 
ARL6IP1
 
 
hsa-miR-148b-3p
 
 
-0.579
 
 
124.0
 
 
 
 
ARL6IP6
 
 
hsa-let-7b-5p
 
 
-0.293
 
 
12.2
 
 
 
 
ARL8B
 
 
hsa-miR-148b-3p
 
 
-0.337
 
 
46.5
 
 
 
 
ARL8B
 
 
hsa-miR-320b
 
 
-0.376
 
 
46.5
 
 
 
 
ARL8B
 
 
hsa-miR-320c
 
 
-0.376
 
 
46.5
 
 
 
 
ARMC8
 
 
hsa-miR-486-5p
 
 
-0.321
 
 
20.8
 
 
 
 
ARMCX2
 
 
hsa-miR-15b-5p
 
 
-0.344
 
 
24.0
 
 
 
 
 
 
 vagina 
 
 
 
 
target_symbol
 
 
mature_mirna_id
 
 
score
 
 
nTPM
 
 
 
 
 
 
AAR2
 
 
hsa-miR-29a-3p
 
 
-0.327
 
 
15.0
 
 
 
 
ABCB7
 
 
hsa-miR-148b-3p
 
 
-0.515
 
 
10.8
 
 
 
 
ABCD3
 
 
hsa-miR-148b-3p
 
 
-0.388
 
 
14.9
 
 
 
 
ABCE1
 
 
hsa-miR-29a-3p
 
 
-0.404
 
 
15.6
 
 
 
 
ABHD17C
 
 
hsa-let-7b-5p
 
 
-0.359
 
 
21.1
 
 
 
 
ABHD17C
 
 
hsa-miR-27a-3p
 
 
-0.435
 
 
21.1
 
 
 
 
ABHD4
 
 
hsa-miR-29a-3p
 
 
-0.312
 
 
28.3
 
 
 
 
ABHD5
 
 
hsa-miR-132-3p
 
 
-0.323
 
 
11.9
 
 
 
 
ABHD5
 
 
hsa-miR-27a-3p
 
 
-0.407
 
 
11.9
 
 
 
 
ACP3
 
 
hsa-let-7b-5p
 
 
-0.298
 
 
10.8
 
 
 
 
ACTA2
 
 
hsa-miR-27a-3p
 
 
-0.577
 
 
1234.9
 
 
 
 
ACTC1
 
 
hsa-miR-30b-5p
 
 
-0.543
 
 
50.8
 
 
 
 
ACTR2
 
 
hsa-miR-15b-5p
 
 
-0.293
 
 
44.4
 
 
 
 
ACTR2
 
 
hsa-miR-16-5p
 
 
-0.293
 
 
44.4
 
 
 
 
ADAM10
 
 
hsa-miR-148b-3p
 
 
-0.356
 
 
10.3
 
 
 
 
ADAM9
 
 
hsa-miR-30b-5p
 
 
-0.312
 
 
15.5
 
 
 
 
ADCY3
 
 
hsa-miR-132-3p
 
 
-0.303
 
 
46.2
 
 
 
 
ADIPOR2
 
 
hsa-miR-150-5p
 
 
-0.287
 
 
28.4
 
 
 
 
ADNP
 
 
hsa-miR-328-3p
 
 
-0.371
 
 
30.1
 
 
 
 
ADRA2A
 
 
hsa-miR-23a-3p
 
 
-0.371
 
 
22.2
 
 
 
 
ADRA2A
 
 
hsa-miR-23b-3p
 
 
-0.371
 
 
22.2
 
 
 
 
ADRB2
 
 
hsa-miR-15b-5p
 
 
-0.29
 
 
12.2
 
 
 
 
ADRB2
 
 
hsa-miR-16-5p
 
 
-0.323
 
 
12.2
 
 
 
 
ADRB2
 
 
hsa-let-7b-5p
 
 
-0.635
 
 
12.2
 
 
 
 
AGFG1
 
 
hsa-miR-148b-3p
 
 
-0.332
 
 
31.5
 
 
 
 
AGPAT3
 
 
hsa-miR-27a-3p
 
 
-0.29
 
 
14.4
 
 
 
 
AGPAT3
 
 
hsa-miR-16-5p
 
 
-0.299
 
 
14.4
 
 
 
 
AHDC1
 
 
hsa-miR-148b-3p
 
 
-0.413
 
 
23.0
 
 
 
 
AHR
 
 
hsa-miR-29a-3p
 
 
-0.316
 
 
13.8
 
 
 
 
AK1
 
 
hsa-miR-29a-3p
 
 
-0.333
 
 
24.0
 
 
 
 
AK4
 
 
hsa-miR-15b-5p
 
 
-0.316
 
 
11.8
 
 
 
 
AK4
 
 
hsa-miR-16-5p
 
 
-0.348
 
 
11.8
 
 
 
 
AKAP1
 
 
hsa-miR-148b-3p
 
 
-0.381
 
 
24.4
 
 
 
 
AKIRIN1
 
 
hsa-miR-27a-3p
 
 
-0.368
 
 
17.7
 
 
 
 
AKT3
 
 
hsa-miR-29a-3p
 
 
-0.287
 
 
12.8
 
 
 
 
AKT3
 
 
hsa-miR-15b-5p
 
 
-0.301
 
 
12.8
 
 
 
 
AKT3
 
 
hsa-miR-16-5p
 
 
-0.309
 
 
12.8
 
 
 
 
ALAD
 
 
hsa-miR-16-5p
 
 
-0.391
 
 
19.3
 
 
 
 
ALAD
 
 
hsa-miR-15b-5p
 
 
-0.446
 
 
19.3
 
 
 
 
ALDH1A2
 
 
hsa-miR-23b-3p
 
 
-0.346
 
 
13.2
 
 
 
 
ALDH1A2
 
 
hsa-miR-23a-3p
 
 
-0.354
 
 
13.2
 
 
 
 
ALOX12
 
 
hsa-miR-15b-5p
 
 
-0.443
 
 
95.8
 
 
 
 
ALOX12
 
 
hsa-miR-16-5p
 
 
-0.451
 
 
95.8
 
 
 
 
AMOTL1
 
 
hsa-miR-16-5p
 
 
-0.297
 
 
13.1
 
 
 
 
AMOTL1
 
 
hsa-miR-15b-5p
 
 
-0.319
 
 
13.1
 
 
 
 
ANKHD1
 
 
hsa-miR-23a-3p
 
 
-0.368
 
 
41.9
 
 
 
 
ANKHD1
 
 
hsa-miR-23b-3p
 
 
-0.368
 
 
41.9
 
 
 
 
ANKHD1
 
 
hsa-miR-30b-5p
 
 
-0.405
 
 
41.9
 
 
 
 
ANKRD17
 
 
hsa-miR-30b-5p
 
 
-0.336
 
 
12.3
 
 
 
 
ANKRD29
 
 
hsa-miR-132-3p
 
 
-0.47
 
 
12.3
 
 
 
 
ANKRD40
 
 
hsa-miR-27a-3p
 
 
-0.35
 
 
15.9
 
 
 
 
ANP32A
 
 
hsa-miR-132-3p
 
 
-0.374
 
 
40.8
 
 
 
 
ANTXR2
 
 
hsa-miR-29a-3p
 
 
-0.347
 
 
27.6
 
 
 
 
ANXA11
 
 
hsa-miR-16-5p
 
 
-0.447
 
 
98.4
 
 
 
 
ANXA11
 
 
hsa-miR-15b-5p
 
 
-0.463
 
 
98.4
 
 
 
 
AP1S1
 
 
hsa-let-7b-5p
 
 
-0.374
 
 
20.1
 
 
 
 
AP2A1
 
 
hsa-miR-15b-5p
 
 
-0.333
 
 
41.4
 
 
 
 
AP2A1
 
 
hsa-miR-16-5p
 
 
-0.333
 
 
41.4
 
 
 
 
AP2B1
 
 
hsa-miR-16-5p
 
 
-0.302
 
 
32.4
 
 
 
 
AP2B1
 
 
hsa-miR-15b-5p
 
 
-0.334
 
 
32.4
 
 
 
 
AP3S1
 
 
hsa-miR-30b-5p
 
 
-0.293
 
 
63.7
 
 
 
 
APP
 
 
hsa-miR-16-5p
 
 
-0.298
 
 
142.4
 
 
 
 
APP
 
 
hsa-miR-15b-5p
 
 
-0.298
 
 
142.4
 
 
 
 
ARF4
 
 
hsa-miR-30b-5p
 
 
-0.289
 
 
85.1
 
 
 
 
ARF4
 
 
hsa-miR-148b-3p
 
 
-0.487
 
 
85.1
 
 
 
 
ARF5
 
 
hsa-miR-29a-3p
 
 
-0.491
 
 
132.3
 
 
 
 
ARF6
 
 
hsa-miR-181b-5p
 
 
-0.33
 
 
41.6
 
 
 
 
ARF6
 
 
hsa-miR-181a-5p
 
 
-0.33
 
 
41.6
 
 
 
 
ARFIP1
 
 
hsa-miR-320b
 
 
-0.339
 
 
13.1
 
 
 
 
ARFIP1
 
 
hsa-miR-320c
 
 
-0.339
 
 
13.1
 
 
 
 
ARFIP1
 
 
hsa-miR-148b-3p
 
 
-0.474
 
 
13.1
 
 
 
 
ARG2
 
 
hsa-let-7b-5p
 
 
-0.468
 
 
11.0
 
 
 
 
ARHGAP5
 
 
hsa-miR-486-5p
 
 
-0.3
 
 
13.4
 
 
 
 
ARID1B
 
 
hsa-miR-29a-3p
 
 
-0.342
 
 
13.7
 
 
 
 
ARID4B
 
 
hsa-miR-486-5p
 
 
-0.367
 
 
10.0
 
 
 
 
ARL2
 
 
hsa-miR-16-5p
 
 
-0.374
 
 
93.4
 
 
 
 
ARL2
 
 
hsa-miR-15b-5p
 
 
-0.374
 
 
93.4
 
 
 
 
ARL2
 
 
hsa-miR-16-5p
 
 
-0.418
 
 
93.4
 
 
 
 
ARL2
 
 
hsa-miR-15b-5p
 
 
-0.442
 
 
93.4
 
 
 
 
ARL4A
 
 
hsa-miR-29a-3p
 
 
-0.386
 
 
63.2
 
 
 
 
ARL4A
 
 
hsa-miR-30b-5p
 
 
-0.448
 
 
63.2
 
 
 
 
ARL4D
 
 
hsa-let-7b-5p
 
 
-0.417
 
 
38.9
 
 
 
 
ARL6IP1
 
 
hsa-miR-328-3p
 
 
-0.494
 
 
48.0
 
 
 
 
ARL6IP1
 
 
hsa-miR-148b-3p
 
 
-0.579
 
 
48.0
 
 
 
 
ARL8B
 
 
hsa-miR-148b-3p
 
 
-0.337
 
 
51.8
 
 
 
 
ARL8B
 
 
hsa-miR-320b
 
 
-0.376
 
 
51.8
 
 
 
 
ARL8B
 
 
hsa-miR-320c
 
 
-0.376
 
 
51.8
 
 
 
 
ARMC8
 
 
hsa-miR-486-5p
 
 
-0.321
 
 
12.4
 
 
 
 
ARMCX2
 
 
hsa-miR-15b-5p
 
 
-0.344
 
 
18.0
 
 
 
 
ARMCX2
 
 
hsa-miR-16-5p
 
 
-0.366
 
 
18.0
 
 
 
 
ARMCX3
 
 
hsa-miR-425-5p
 
 
-0.325
 
 
20.2
 
 
 
 
ARPP19
 
 
hsa-miR-320b
 
 
-0.325
 
 
27.0
 
 
 
 
ARPP19
 
 
hsa-miR-320c
 
 
-0.325
 
 
27.0
 
 
 
 
ARPP19
 
 
hsa-miR-15b-5p
 
 
-0.346
 
 
27.0
 
 
 
 
ARPP19
 
 
hsa-miR-16-5p
 
 
-0.354
 
 
27.0
 
 
 
 
ARPP19
 
 
hsa-miR-29a-3p
 
 
-0.371
 
 
27.0
 
 
 
 
ARPP19
 
 
hsa-miR-328-3p
 
 
-0.404
 
 
27.0
 
 
 
 
ARPP19
 
 
hsa-let-7b-5p
 
 
-0.476
 
 
27.0
 
 
 
 
ARRDC3
 
 
hsa-miR-148b-3p
 
 
-0.305
 
 
40.5
 
 
 
 
ARRDC3
 
 
hsa-miR-29a-3p
 
 
-0.402
 
 
40.5
 
 
 
 
 
 
 hypothalamus 
 
 
 
 
target_symbol
 
 
mature_mirna_id
 
 
score
 
 
nTPM
 
 
 
 
 
 
ABCB9
 
 
hsa-let-7b-5p
 
 
-0.422
 
 
11.9
 
 
 
 
ABCD3
 
 
hsa-miR-148b-3p
 
 
-0.388
 
 
13.5
 
 
 
 
ABCE1
 
 
hsa-miR-29a-3p
 
 
-0.404
 
 
12.9
 
 
 
 
ABHD17B
 
 
hsa-miR-486-5p
 
 
-0.348
 
 
11.0
 
 
 
 
ABHD17C
 
 
hsa-let-7b-5p
 
 
-0.359
 
 
11.1
 
 
 
 
ABHD17C
 
 
hsa-miR-27a-3p
 
 
-0.435
 
 
11.1
 
 
 
 
ABHD4
 
 
hsa-miR-29a-3p
 
 
-0.312
 
 
13.5
 
 
 
 
ACSL6
 
 
hsa-let-7b-5p
 
 
-0.336
 
 
11.9
 
 
 
 
ACTA2
 
 
hsa-miR-27a-3p
 
 
-0.577
 
 
34.8
 
 
 
 
ACTR2
 
 
hsa-miR-15b-5p
 
 
-0.293
 
 
50.9
 
 
 
 
ACTR2
 
 
hsa-miR-16-5p
 
 
-0.293
 
 
50.9
 
 
 
 
ADAP1
 
 
hsa-miR-27a-3p
 
 
-0.302
 
 
58.0
 
 
 
 
ADCY2
 
 
hsa-miR-148b-3p
 
 
-0.299
 
 
14.0
 
 
 
 
ADCY3
 
 
hsa-miR-132-3p
 
 
-0.303
 
 
32.1
 
 
 
 
ADGRA3
 
 
hsa-miR-27a-3p
 
 
-0.296
 
 
10.8
 
 
 
 
ADGRA3
 
 
hsa-miR-30b-5p
 
 
-0.304
 
 
10.8
 
 
 
 
ADGRB3
 
 
hsa-miR-148b-3p
 
 
-0.524
 
 
16.0
 
 
 
 
ADIPOR2
 
 
hsa-miR-150-5p
 
 
-0.287
 
 
40.5
 
 
 
 
ADNP
 
 
hsa-miR-328-3p
 
 
-0.371
 
 
23.7
 
 
 
 
AGFG1
 
 
hsa-miR-148b-3p
 
 
-0.332
 
 
26.0
 
 
 
 
AGPAT3
 
 
hsa-miR-27a-3p
 
 
-0.29
 
 
28.2
 
 
 
 
AGPAT3
 
 
hsa-miR-16-5p
 
 
-0.299
 
 
28.2
 
 
 
 
AGPAT4
 
 
hsa-miR-29a-3p
 
 
-0.376
 
 
14.0
 
 
 
 
AHDC1
 
 
hsa-miR-148b-3p
 
 
-0.413
 
 
10.9
 
 
 
 
AK1
 
 
hsa-miR-29a-3p
 
 
-0.333
 
 
100.1
 
 
 
 
AK4
 
 
hsa-miR-15b-5p
 
 
-0.316
 
 
12.0
 
 
 
 
AK4
 
 
hsa-miR-16-5p
 
 
-0.348
 
 
12.0
 
 
 
 
AKAP1
 
 
hsa-miR-148b-3p
 
 
-0.381
 
 
15.1
 
 
 
 
AKAP6
 
 
hsa-let-7b-5p
 
 
-0.307
 
 
13.6
 
 
 
 
AKIRIN1
 
 
hsa-miR-27a-3p
 
 
-0.368
 
 
39.9
 
 
 
 
AKT3
 
 
hsa-miR-29a-3p
 
 
-0.287
 
 
14.5
 
 
 
 
AKT3
 
 
hsa-miR-15b-5p
 
 
-0.301
 
 
14.5
 
 
 
 
AKT3
 
 
hsa-miR-16-5p
 
 
-0.309
 
 
14.5
 
 
 
 
ALAD
 
 
hsa-miR-16-5p
 
 
-0.391
 
 
54.5
 
 
 
 
ALAD
 
 
hsa-miR-15b-5p
 
 
-0.446
 
 
54.5
 
 
 
 
ALCAM
 
 
hsa-miR-148b-3p
 
 
-0.339
 
 
36.1
 
 
 
 
AMOT
 
 
hsa-miR-29a-3p
 
 
-0.304
 
 
11.9
 
 
 
 
ANKHD1
 
 
hsa-miR-23a-3p
 
 
-0.368
 
 
17.8
 
 
 
 
ANKHD1
 
 
hsa-miR-23b-3p
 
 
-0.368
 
 
17.8
 
 
 
 
ANKHD1
 
 
hsa-miR-30b-5p
 
 
-0.405
 
 
17.8
 
 
 
 
ANKRD17
 
 
hsa-miR-30b-5p
 
 
-0.336
 
 
11.3
 
 
 
 
ANKRD40
 
 
hsa-miR-27a-3p
 
 
-0.35
 
 
80.8
 
 
 
 
ANKRD46
 
 
hsa-let-7b-5p
 
 
-0.389
 
 
28.7
 
 
 
 
ANLN
 
 
hsa-miR-16-5p
 
 
-0.31
 
 
30.4
 
 
 
 
ANLN
 
 
hsa-miR-15b-5p
 
 
-0.334
 
 
30.4
 
 
 
 
ANP32A
 
 
hsa-miR-132-3p
 
 
-0.374
 
 
37.9
 
 
 
 
ANXA11
 
 
hsa-miR-16-5p
 
 
-0.447
 
 
13.4
 
 
 
 
ANXA11
 
 
hsa-miR-15b-5p
 
 
-0.463
 
 
13.4
 
 
 
 
AP1S1
 
 
hsa-let-7b-5p
 
 
-0.374
 
 
44.3
 
 
 
 
AP2A1
 
 
hsa-miR-15b-5p
 
 
-0.333
 
 
44.0
 
 
 
 
AP2A1
 
 
hsa-miR-16-5p
 
 
-0.333
 
 
44.0
 
 
 
 
AP2B1
 
 
hsa-miR-16-5p
 
 
-0.302
 
 
68.4
 
 
 
 
AP2B1
 
 
hsa-miR-15b-5p
 
 
-0.334
 
 
68.4
 
 
 
 
AP3S1
 
 
hsa-miR-30b-5p
 
 
-0.293
 
 
61.9
 
 
 
 
APBB3
 
 
hsa-let-7b-5p
 
 
-0.587
 
 
11.2
 
 
 
 
APC
 
 
hsa-miR-29a-3p
 
 
-0.413
 
 
11.2
 
 
 
 
APLN
 
 
hsa-miR-16-5p
 
 
-0.31
 
 
17.3
 
 
 
 
APLN
 
 
hsa-miR-15b-5p
 
 
-0.395
 
 
17.3
 
 
 
 
APLN
 
 
hsa-miR-16-5p
 
 
-0.395
 
 
17.3
 
 
 
 
APOO
 
 
hsa-miR-181b-5p
 
 
-0.5
 
 
43.8
 
 
 
 
APOO
 
 
hsa-miR-181a-5p
 
 
-0.5
 
 
43.8
 
 
 
 
APP
 
 
hsa-miR-16-5p
 
 
-0.298
 
 
437.7
 
 
 
 
APP
 
 
hsa-miR-15b-5p
 
 
-0.298
 
 
437.7
 
 
 
 
ARF4
 
 
hsa-miR-30b-5p
 
 
-0.289
 
 
75.3
 
 
 
 
ARF4
 
 
hsa-miR-148b-3p
 
 
-0.487
 
 
75.3
 
 
 
 
ARF5
 
 
hsa-miR-29a-3p
 
 
-0.491
 
 
134.0
 
 
 
 
ARF6
 
 
hsa-miR-181b-5p
 
 
-0.33
 
 
10.2
 
 
 
 
ARF6
 
 
hsa-miR-181a-5p
 
 
-0.33
 
 
10.2
 
 
 
 
ARG2
 
 
hsa-let-7b-5p
 
 
-0.468
 
 
10.7
 
 
 
 
ARHGAP21
 
 
hsa-miR-148b-3p
 
 
-0.287
 
 
30.5
 
 
 
 
ARHGAP5
 
 
hsa-miR-486-5p
 
 
-0.3
 
 
17.7
 
 
 
 
ARL2
 
 
hsa-miR-16-5p
 
 
-0.374
 
 
156.1
 
 
 
 
ARL2
 
 
hsa-miR-15b-5p
 
 
-0.374
 
 
156.1
 
 
 
 
ARL2
 
 
hsa-miR-16-5p
 
 
-0.418
 
 
156.1
 
 
 
 
ARL2
 
 
hsa-miR-15b-5p
 
 
-0.442
 
 
156.1
 
 
 
 
ARL3
 
 
hsa-miR-16-5p
 
 
-0.496
 
 
23.1
 
 
 
 
ARL3
 
 
hsa-miR-15b-5p
 
 
-0.507
 
 
23.1
 
 
 
 
ARL6IP1
 
 
hsa-miR-328-3p
 
 
-0.494
 
 
212.8
 
 
 
 
ARL6IP1
 
 
hsa-miR-148b-3p
 
 
-0.579
 
 
212.8
 
 
 
 
ARL8B
 
 
hsa-miR-148b-3p
 
 
-0.337
 
 
54.9
 
 
 
 
ARL8B
 
 
hsa-miR-320b
 
 
-0.376
 
 
54.9
 
 
 
 
ARL8B
 
 
hsa-miR-320c
 
 
-0.376
 
 
54.9
 
 
 
 
ARMC8
 
 
hsa-miR-486-5p
 
 
-0.321
 
 
16.8
 
 
 
 
ARMCX2
 
 
hsa-miR-15b-5p
 
 
-0.344
 
 
28.0
 
 
 
 
ARMCX2
 
 
hsa-miR-16-5p
 
 
-0.366
 
 
28.0
 
 
 
 
ARMCX3
 
 
hsa-miR-425-5p
 
 
-0.325
 
 
32.9
 
 
 
 
ARPP19
 
 
hsa-miR-320b
 
 
-0.325
 
 
103.9
 
 
 
 
ARPP19
 
 
hsa-miR-320c
 
 
-0.325
 
 
103.9
 
 
 
 
ARPP19
 
 
hsa-miR-15b-5p
 
 
-0.346
 
 
103.9
 
 
 
 
ARPP19
 
 
hsa-miR-16-5p
 
 
-0.354
 
 
103.9
 
 
 
 
ARPP19
 
 
hsa-miR-29a-3p
 
 
-0.371
 
 
103.9
 
 
 
 
ARPP19
 
 
hsa-miR-328-3p
 
 
-0.404
 
 
103.9
 
 
 
 
ARPP19
 
 
hsa-let-7b-5p
 
 
-0.476
 
 
103.9
 
 
 
 
ARPP21
 
 
hsa-miR-16-5p
 
 
-0.309
 
 
24.9
 
 
 
 
ARPP21
 
 
hsa-miR-15b-5p
 
 
-0.342
 
 
24.9
 
 
 
 
ARRDC3
 
 
hsa-miR-148b-3p
 
 
-0.305
 
 
10.3
 
 
 
 
ARRDC3
 
 
hsa-miR-29a-3p
 
 
-0.402
 
 
10.3
 
 
 
 
ARRDC4
 
 
hsa-let-7b-5p
 
 
-0.329
 
 
13.4
 
 
 
 
ASAH1
 
 
hsa-miR-27a-3p
 
 
-0.398
 
 
58.0
 
 
 
 
ASAP1
 
 
hsa-let-7b-5p
 
 
-0.308
 
 
11.8
 
 
 
 
 
 
 pituitary gland 
 
 
 
 
target_symbol
 
 
mature_mirna_id
 
 
score
 
 
nTPM
 
 
 
 
 
 
ABCB9
 
 
hsa-let-7b-5p
 
 
-0.422
 
 
11.0
 
 
 
 
ABCD3
 
 
hsa-miR-148b-3p
 
 
-0.388
 
 
12.4
 
 
 
 
ABHD4
 
 
hsa-miR-29a-3p
 
 
-0.312
 
 
13.7
 
 
 
 
ACTA2
 
 
hsa-miR-27a-3p
 
 
-0.577
 
 
56.3
 
 
 
 
ACTR2
 
 
hsa-miR-15b-5p
 
 
-0.293
 
 
23.5
 
 
 
 
ACTR2
 
 
hsa-miR-16-5p
 
 
-0.293
 
 
23.5
 
 
 
 
ADCY3
 
 
hsa-miR-132-3p
 
 
-0.303
 
 
41.4
 
 
 
 
ADGRB3
 
 
hsa-miR-148b-3p
 
 
-0.524
 
 
21.3
 
 
 
 
ADIPOR2
 
 
hsa-miR-150-5p
 
 
-0.287
 
 
25.4
 
 
 
 
ADNP
 
 
hsa-miR-328-3p
 
 
-0.371
 
 
26.6
 
 
 
 
AGFG1
 
 
hsa-miR-148b-3p
 
 
-0.332
 
 
16.7
 
 
 
 
AGPAT3
 
 
hsa-miR-27a-3p
 
 
-0.29
 
 
13.9
 
 
 
 
AGPAT3
 
 
hsa-miR-16-5p
 
 
-0.299
 
 
13.9
 
 
 
 
AGPAT4
 
 
hsa-miR-29a-3p
 
 
-0.376
 
 
10.5
 
 
 
 
AHDC1
 
 
hsa-miR-148b-3p
 
 
-0.413
 
 
18.1
 
 
 
 
AIFM1
 
 
hsa-let-7b-5p
 
 
-0.314
 
 
11.0
 
 
 
 
AK1
 
 
hsa-miR-29a-3p
 
 
-0.333
 
 
40.7
 
 
 
 
AKAP1
 
 
hsa-miR-148b-3p
 
 
-0.381
 
 
16.5
 
 
 
 
AKIRIN1
 
 
hsa-miR-27a-3p
 
 
-0.368
 
 
37.8
 
 
 
 
AKT3
 
 
hsa-miR-29a-3p
 
 
-0.287
 
 
11.4
 
 
 
 
AKT3
 
 
hsa-miR-15b-5p
 
 
-0.301
 
 
11.4
 
 
 
 
AKT3
 
 
hsa-miR-16-5p
 
 
-0.309
 
 
11.4
 
 
 
 
ALAD
 
 
hsa-miR-16-5p
 
 
-0.391
 
 
16.3
 
 
 
 
ALAD
 
 
hsa-miR-15b-5p
 
 
-0.446
 
 
16.3
 
 
 
 
ALCAM
 
 
hsa-miR-148b-3p
 
 
-0.339
 
 
17.9
 
 
 
 
ALDH1A2
 
 
hsa-miR-23b-3p
 
 
-0.346
 
 
14.4
 
 
 
 
ALDH1A2
 
 
hsa-miR-23a-3p
 
 
-0.354
 
 
14.4
 
 
 
 
AMOTL1
 
 
hsa-miR-16-5p
 
 
-0.297
 
 
13.5
 
 
 
 
AMOTL1
 
 
hsa-miR-15b-5p
 
 
-0.319
 
 
13.5
 
 
 
 
AMT
 
 
hsa-let-7b-5p
 
 
-0.463
 
 
16.0
 
 
 
 
ANKHD1
 
 
hsa-miR-23a-3p
 
 
-0.368
 
 
41.2
 
 
 
 
ANKHD1
 
 
hsa-miR-23b-3p
 
 
-0.368
 
 
41.2
 
 
 
 
ANKHD1
 
 
hsa-miR-30b-5p
 
 
-0.405
 
 
41.2
 
 
 
 
ANKRD17
 
 
hsa-miR-30b-5p
 
 
-0.336
 
 
13.4
 
 
 
 
ANKRD40
 
 
hsa-miR-27a-3p
 
 
-0.35
 
 
10.1
 
 
 
 
ANP32A
 
 
hsa-miR-132-3p
 
 
-0.374
 
 
26.1
 
 
 
 
ANXA11
 
 
hsa-miR-16-5p
 
 
-0.447
 
 
25.4
 
 
 
 
ANXA11
 
 
hsa-miR-15b-5p
 
 
-0.463
 
 
25.4
 
 
 
 
AP1S1
 
 
hsa-let-7b-5p
 
 
-0.374
 
 
31.4
 
 
 
 
AP2A1
 
 
hsa-miR-15b-5p
 
 
-0.333
 
 
33.0
 
 
 
 
AP2A1
 
 
hsa-miR-16-5p
 
 
-0.333
 
 
33.0
 
 
 
 
AP2B1
 
 
hsa-miR-16-5p
 
 
-0.302
 
 
51.7
 
 
 
 
AP2B1
 
 
hsa-miR-15b-5p
 
 
-0.334
 
 
51.7
 
 
 
 
AP3S1
 
 
hsa-miR-30b-5p
 
 
-0.293
 
 
47.0
 
 
 
 
APBB3
 
 
hsa-let-7b-5p
 
 
-0.587
 
 
10.1
 
 
 
 
APOO
 
 
hsa-miR-181b-5p
 
 
-0.5
 
 
28.1
 
 
 
 
APOO
 
 
hsa-miR-181a-5p
 
 
-0.5
 
 
28.1
 
 
 
 
APP
 
 
hsa-miR-16-5p
 
 
-0.298
 
 
144.3
 
 
 
 
APP
 
 
hsa-miR-15b-5p
 
 
-0.298
 
 
144.3
 
 
 
 
ARF4
 
 
hsa-miR-30b-5p
 
 
-0.289
 
 
93.4
 
 
 
 
ARF4
 
 
hsa-miR-148b-3p
 
 
-0.487
 
 
93.4
 
 
 
 
ARF5
 
 
hsa-miR-29a-3p
 
 
-0.491
 
 
111.9
 
 
 
 
ARG2
 
 
hsa-let-7b-5p
 
 
-0.468
 
 
47.4
 
 
 
 
ARHGAP21
 
 
hsa-miR-148b-3p
 
 
-0.287
 
 
11.3
 
 
 
 
ARID4B
 
 
hsa-miR-486-5p
 
 
-0.367
 
 
10.3
 
 
 
 
ARL2
 
 
hsa-miR-16-5p
 
 
-0.374
 
 
68.9
 
 
 
 
ARL2
 
 
hsa-miR-15b-5p
 
 
-0.374
 
 
68.9
 
 
 
 
ARL2
 
 
hsa-miR-16-5p
 
 
-0.418
 
 
68.9
 
 
 
 
ARL2
 
 
hsa-miR-15b-5p
 
 
-0.442
 
 
68.9
 
 
 
 
ARL3
 
 
hsa-miR-16-5p
 
 
-0.496
 
 
19.9
 
 
 
 
ARL3
 
 
hsa-miR-15b-5p
 
 
-0.507
 
 
19.9
 
 
 
 
ARL4D
 
 
hsa-let-7b-5p
 
 
-0.417
 
 
20.8
 
 
 
 
ARL6IP1
 
 
hsa-miR-328-3p
 
 
-0.494
 
 
39.7
 
 
 
 
ARL6IP1
 
 
hsa-miR-148b-3p
 
 
-0.579
 
 
39.7
 
 
 
 
ARL8B
 
 
hsa-miR-148b-3p
 
 
-0.337
 
 
25.6
 
 
 
 
ARL8B
 
 
hsa-miR-320b
 
 
-0.376
 
 
25.6
 
 
 
 
ARL8B
 
 
hsa-miR-320c
 
 
-0.376
 
 
25.6
 
 
 
 
ARMC12
 
 
hsa-miR-15b-5p
 
 
-0.485
 
 
11.0
 
 
 
 
ARMC12
 
 
hsa-miR-16-5p
 
 
-0.485
 
 
11.0
 
 
 
 
ARMC8
 
 
hsa-miR-486-5p
 
 
-0.321
 
 
11.3
 
 
 
 
ARMCX2
 
 
hsa-miR-15b-5p
 
 
-0.344
 
 
70.4
 
 
 
 
ARMCX2
 
 
hsa-miR-16-5p
 
 
-0.366
 
 
70.4
 
 
 
 
ARMCX3
 
 
hsa-miR-425-5p
 
 
-0.325
 
 
43.1
 
 
 
 
ARPP19
 
 
hsa-miR-320b
 
 
-0.325
 
 
33.0
 
 
 
 
ARPP19
 
 
hsa-miR-320c
 
 
-0.325
 
 
33.0
 
 
 
 
ARPP19
 
 
hsa-miR-15b-5p
 
 
-0.346
 
 
33.0
 
 
 
 
ARPP19
 
 
hsa-miR-16-5p
 
 
-0.354
 
 
33.0
 
 
 
 
ARPP19
 
 
hsa-miR-29a-3p
 
 
-0.371
 
 
33.0
 
 
 
 
ARPP19
 
 
hsa-miR-328-3p
 
 
-0.404
 
 
33.0
 
 
 
 
ARPP19
 
 
hsa-let-7b-5p
 
 
-0.476
 
 
33.0
 
 
 
 
ARPP21
 
 
hsa-miR-16-5p
 
 
-0.309
 
 
12.2
 
 
 
 
ARPP21
 
 
hsa-miR-15b-5p
 
 
-0.342
 
 
12.2
 
 
 
 
ARRDC3
 
 
hsa-miR-148b-3p
 
 
-0.305
 
 
20.2
 
 
 
 
ARRDC3
 
 
hsa-miR-29a-3p
 
 
-0.402
 
 
20.2
 
 
 
 
ASAH1
 
 
hsa-miR-27a-3p
 
 
-0.398
 
 
75.8
 
 
 
 
ASB3
 
 
hsa-miR-30b-5p
 
 
-0.418
 
 
13.9
 
 
 
 
ASB3
 
 
hsa-miR-30b-5p
 
 
-0.418
 
 
13.9
 
 
 
 
ASH1L
 
 
hsa-miR-15b-5p
 
 
-0.325
 
 
17.6
 
 
 
 
ASH1L
 
 
hsa-miR-16-5p
 
 
-0.357
 
 
17.6
 
 
 
 
ASH2L
 
 
hsa-miR-320b
 
 
-0.287
 
 
11.5
 
 
 
 
ASH2L
 
 
hsa-miR-320c
 
 
-0.287
 
 
11.5
 
 
 
 
ASPH
 
 
hsa-miR-148b-3p
 
 
-0.391
 
 
33.8
 
 
 
 
ATCAY
 
 
hsa-miR-10a-5p
 
 
-0.322
 
 
24.7
 
 
 
 
ATG9A
 
 
hsa-miR-16-5p
 
 
-0.311
 
 
18.7
 
 
 
 
ATG9A
 
 
hsa-miR-15b-5p
 
 
-0.333
 
 
18.7
 
 
 
 
ATOSB
 
 
hsa-miR-328-3p
 
 
-0.358
 
 
15.4
 
 
 
 
ATOSB
 
 
hsa-let-7b-5p
 
 
-0.429
 
 
15.4
 
 
 
 
ATP2A2
 
 
hsa-let-7b-5p
 
 
-0.311
 
 
13.7
 
 
 
 
ATP5MC3
 
 
hsa-miR-425-5p
 
 
-0.328
 
 
134.5
 
 
 
 
ATP5MC3
 
 
hsa-miR-29a-3p
 
 
-0.33
 
 
134.5
 
 
 
 
 
 
 retina 
 
 
 
 
target_symbol
 
 
mature_mirna_id
 
 
score
 
 
nTPM
 
 
 
 
 
 
ABCD3
 
 
hsa-miR-148b-3p
 
 
-0.388
 
 
24.4
 
 
 
 
ABCE1
 
 
hsa-miR-29a-3p
 
 
-0.404
 
 
13.3
 
 
 
 
ABHD17B
 
 
hsa-miR-486-5p
 
 
-0.348
 
 
23.6
 
 
 
 
ABHD17C
 
 
hsa-let-7b-5p
 
 
-0.359
 
 
15.6
 
 
 
 
ABHD17C
 
 
hsa-miR-27a-3p
 
 
-0.435
 
 
15.6
 
 
 
 
ABHD4
 
 
hsa-miR-29a-3p
 
 
-0.312
 
 
21.0
 
 
 
 
ACSL6
 
 
hsa-let-7b-5p
 
 
-0.336
 
 
42.4
 
 
 
 
ACTC1
 
 
hsa-miR-30b-5p
 
 
-0.543
 
 
29.3
 
 
 
 
ACTR2
 
 
hsa-miR-15b-5p
 
 
-0.293
 
 
60.2
 
 
 
 
ACTR2
 
 
hsa-miR-16-5p
 
 
-0.293
 
 
60.2
 
 
 
 
ADAM9
 
 
hsa-miR-30b-5p
 
 
-0.312
 
 
17.2
 
 
 
 
ADIPOR2
 
 
hsa-miR-150-5p
 
 
-0.287
 
 
12.1
 
 
 
 
ADNP
 
 
hsa-miR-328-3p
 
 
-0.371
 
 
40.4
 
 
 
 
AGFG1
 
 
hsa-miR-148b-3p
 
 
-0.332
 
 
44.2
 
 
 
 
AGPAT3
 
 
hsa-miR-27a-3p
 
 
-0.29
 
 
57.5
 
 
 
 
AGPAT3
 
 
hsa-miR-16-5p
 
 
-0.299
 
 
57.5
 
 
 
 
AK1
 
 
hsa-miR-29a-3p
 
 
-0.333
 
 
27.1
 
 
 
 
AK4
 
 
hsa-miR-15b-5p
 
 
-0.316
 
 
19.7
 
 
 
 
AK4
 
 
hsa-miR-16-5p
 
 
-0.348
 
 
19.7
 
 
 
 
AKAP1
 
 
hsa-miR-148b-3p
 
 
-0.381
 
 
32.2
 
 
 
 
AKAP6
 
 
hsa-let-7b-5p
 
 
-0.307
 
 
12.6
 
 
 
 
AKIRIN1
 
 
hsa-miR-27a-3p
 
 
-0.368
 
 
34.7
 
 
 
 
AKT3
 
 
hsa-miR-29a-3p
 
 
-0.287
 
 
15.2
 
 
 
 
AKT3
 
 
hsa-miR-15b-5p
 
 
-0.301
 
 
15.2
 
 
 
 
AKT3
 
 
hsa-miR-16-5p
 
 
-0.309
 
 
15.2
 
 
 
 
ALAD
 
 
hsa-miR-16-5p
 
 
-0.391
 
 
17.6
 
 
 
 
ALAD
 
 
hsa-miR-15b-5p
 
 
-0.446
 
 
17.6
 
 
 
 
ALPL
 
 
hsa-miR-10a-5p
 
 
-0.32
 
 
17.1
 
 
 
 
ANKHD1
 
 
hsa-miR-23a-3p
 
 
-0.368
 
 
37.5
 
 
 
 
ANKHD1
 
 
hsa-miR-23b-3p
 
 
-0.368
 
 
37.5
 
 
 
 
ANKHD1
 
 
hsa-miR-30b-5p
 
 
-0.405
 
 
37.5
 
 
 
 
ANKRD13C
 
 
hsa-miR-29a-3p
 
 
-0.411
 
 
12.3
 
 
 
 
ANKRD17
 
 
hsa-miR-30b-5p
 
 
-0.336
 
 
33.3
 
 
 
 
ANKRD40
 
 
hsa-miR-27a-3p
 
 
-0.35
 
 
19.2
 
 
 
 
ANKRD46
 
 
hsa-let-7b-5p
 
 
-0.389
 
 
32.6
 
 
 
 
ANP32A
 
 
hsa-miR-132-3p
 
 
-0.374
 
 
98.2
 
 
 
 
AP1S1
 
 
hsa-let-7b-5p
 
 
-0.374
 
 
13.5
 
 
 
 
AP2A1
 
 
hsa-miR-15b-5p
 
 
-0.333
 
 
12.4
 
 
 
 
AP2A1
 
 
hsa-miR-16-5p
 
 
-0.333
 
 
12.4
 
 
 
 
AP2B1
 
 
hsa-miR-16-5p
 
 
-0.302
 
 
70.5
 
 
 
 
AP2B1
 
 
hsa-miR-15b-5p
 
 
-0.334
 
 
70.5
 
 
 
 
AP3S1
 
 
hsa-miR-30b-5p
 
 
-0.293
 
 
31.5
 
 
 
 
APC
 
 
hsa-miR-29a-3p
 
 
-0.413
 
 
18.5
 
 
 
 
APOO
 
 
hsa-miR-181b-5p
 
 
-0.5
 
 
48.9
 
 
 
 
APOO
 
 
hsa-miR-181a-5p
 
 
-0.5
 
 
48.9
 
 
 
 
APP
 
 
hsa-miR-16-5p
 
 
-0.298
 
 
189.1
 
 
 
 
APP
 
 
hsa-miR-15b-5p
 
 
-0.298
 
 
189.1
 
 
 
 
APPBP2
 
 
hsa-miR-27a-3p
 
 
-0.378
 
 
16.0
 
 
 
 
ARF4
 
 
hsa-miR-30b-5p
 
 
-0.289
 
 
50.0
 
 
 
 
ARF4
 
 
hsa-miR-148b-3p
 
 
-0.487
 
 
50.0
 
 
 
 
ARF5
 
 
hsa-miR-29a-3p
 
 
-0.491
 
 
21.3
 
 
 
 
ARF6
 
 
hsa-miR-181b-5p
 
 
-0.33
 
 
13.4
 
 
 
 
ARF6
 
 
hsa-miR-181a-5p
 
 
-0.33
 
 
13.4
 
 
 
 
ARFGEF1
 
 
hsa-miR-148b-3p
 
 
-0.306
 
 
33.8
 
 
 
 
ARFIP1
 
 
hsa-miR-320b
 
 
-0.339
 
 
18.7
 
 
 
 
ARFIP1
 
 
hsa-miR-320c
 
 
-0.339
 
 
18.7
 
 
 
 
ARFIP1
 
 
hsa-miR-148b-3p
 
 
-0.474
 
 
18.7
 
 
 
 
ARG2
 
 
hsa-let-7b-5p
 
 
-0.468
 
 
20.7
 
 
 
 
ARHGAP21
 
 
hsa-miR-148b-3p
 
 
-0.287
 
 
37.0
 
 
 
 
ARHGAP5
 
 
hsa-miR-486-5p
 
 
-0.3
 
 
53.6
 
 
 
 
ARID1B
 
 
hsa-miR-29a-3p
 
 
-0.342
 
 
10.7
 
 
 
 
ARID2
 
 
hsa-miR-132-3p
 
 
-0.304
 
 
12.3
 
 
 
 
ARID3B
 
 
hsa-let-7b-5p
 
 
-0.322
 
 
17.4
 
 
 
 
ARID3B
 
 
hsa-let-7b-5p
 
 
-0.486
 
 
17.4
 
 
 
 
ARID4B
 
 
hsa-miR-486-5p
 
 
-0.367
 
 
13.5
 
 
 
 
ARL2
 
 
hsa-miR-16-5p
 
 
-0.374
 
 
40.5
 
 
 
 
ARL2
 
 
hsa-miR-15b-5p
 
 
-0.374
 
 
40.5
 
 
 
 
ARL2
 
 
hsa-miR-16-5p
 
 
-0.418
 
 
40.5
 
 
 
 
ARL2
 
 
hsa-miR-15b-5p
 
 
-0.442
 
 
40.5
 
 
 
 
ARL3
 
 
hsa-miR-16-5p
 
 
-0.496
 
 
89.3
 
 
 
 
ARL3
 
 
hsa-miR-15b-5p
 
 
-0.507
 
 
89.3
 
 
 
 
ARL4D
 
 
hsa-let-7b-5p
 
 
-0.417
 
 
78.9
 
 
 
 
ARL6IP1
 
 
hsa-miR-328-3p
 
 
-0.494
 
 
292.6
 
 
 
 
ARL6IP1
 
 
hsa-miR-148b-3p
 
 
-0.579
 
 
292.6
 
 
 
 
ARL6IP6
 
 
hsa-let-7b-5p
 
 
-0.293
 
 
10.5
 
 
 
 
ARL8B
 
 
hsa-miR-148b-3p
 
 
-0.337
 
 
31.7
 
 
 
 
ARL8B
 
 
hsa-miR-320b
 
 
-0.376
 
 
31.7
 
 
 
 
ARL8B
 
 
hsa-miR-320c
 
 
-0.376
 
 
31.7
 
 
 
 
ARMC8
 
 
hsa-miR-486-5p
 
 
-0.321
 
 
24.3
 
 
 
 
ARMCX2
 
 
hsa-miR-15b-5p
 
 
-0.344
 
 
14.2
 
 
 
 
ARMCX2
 
 
hsa-miR-16-5p
 
 
-0.366
 
 
14.2
 
 
 
 
ARMCX3
 
 
hsa-miR-425-5p
 
 
-0.325
 
 
17.6
 
 
 
 
ARMT1
 
 
hsa-let-7b-5p
 
 
-0.291
 
 
15.2
 
 
 
 
ARPP19
 
 
hsa-miR-320b
 
 
-0.325
 
 
53.1
 
 
 
 
ARPP19
 
 
hsa-miR-320c
 
 
-0.325
 
 
53.1
 
 
 
 
ARPP19
 
 
hsa-miR-15b-5p
 
 
-0.346
 
 
53.1
 
 
 
 
ARPP19
 
 
hsa-miR-16-5p
 
 
-0.354
 
 
53.1
 
 
 
 
ARPP19
 
 
hsa-miR-29a-3p
 
 
-0.371
 
 
53.1
 
 
 
 
ARPP19
 
 
hsa-miR-328-3p
 
 
-0.404
 
 
53.1
 
 
 
 
ARPP19
 
 
hsa-let-7b-5p
 
 
-0.476
 
 
53.1
 
 
 
 
ARRDC3
 
 
hsa-miR-148b-3p
 
 
-0.305
 
 
69.6
 
 
 
 
ARRDC3
 
 
hsa-miR-29a-3p
 
 
-0.402
 
 
69.6
 
 
 
 
ASAH1
 
 
hsa-miR-27a-3p
 
 
-0.398
 
 
73.6
 
 
 
 
ASB3
 
 
hsa-miR-30b-5p
 
 
-0.418
 
 
21.2
 
 
 
 
ASB3
 
 
hsa-miR-30b-5p
 
 
-0.418
 
 
21.2
 
 
 
 
ASF1A
 
 
hsa-miR-23a-3p
 
 
-0.506
 
 
10.6
 
 
 
 
ASF1A
 
 
hsa-miR-23b-3p
 
 
-0.506
 
 
10.6
 
 
 
 
ASH1L
 
 
hsa-miR-15b-5p
 
 
-0.325
 
 
24.2
 
 
 
 
ASH1L
 
 
hsa-miR-16-5p
 
 
-0.357
 
 
24.2
 
 
 
 
ASH2L
 
 
hsa-miR-320b
 
 
-0.287
 
 
20.5
 
 
 
 
 
 
 
 cd8_ev_vs_cd8_s1_downregulated 
 
 Tissue expression heatmap (log10-transformed) 
   
 
 
 bone marrow 
 
 
 
 
target_symbol
 
 
mature_mirna_id
 
 
score
 
 
nTPM
 
 
 
 
 
 
ABCB10
 
 
hsa-miR-148a-3p
 
 
-0.291
 
 
56.7
 
 
 
 
ABCB7
 
 
hsa-miR-148a-3p
 
 
-0.515
 
 
10.5
 
 
 
 
ABCD3
 
 
hsa-miR-148a-3p
 
 
-0.388
 
 
23.6
 
 
 
 
ABHD5
 
 
hsa-miR-20a-5p
 
 
-0.397
 
 
37.5
 
 
 
 
ABHD5
 
 
hsa-miR-106b-5p
 
 
-0.413
 
 
37.5
 
 
 
 
ABHD5
 
 
hsa-miR-20b-5p
 
 
-0.418
 
 
37.5
 
 
 
 
ABHD5
 
 
hsa-miR-106a-5p
 
 
-0.447
 
 
37.5
 
 
 
 
ABHD5
 
 
hsa-miR-17-5p
 
 
-0.45
 
 
37.5
 
 
 
 
ACP3
 
 
hsa-miR-98-5p
 
 
-0.298
 
 
16.5
 
 
 
 
ACP3
 
 
hsa-let-7c-5p
 
 
-0.298
 
 
16.5
 
 
 
 
ACSL6
 
 
hsa-miR-98-5p
 
 
-0.336
 
 
21.4
 
 
 
 
ACSL6
 
 
hsa-let-7c-5p
 
 
-0.336
 
 
21.4
 
 
 
 
ADAM10
 
 
hsa-miR-148a-3p
 
 
-0.356
 
 
17.3
 
 
 
 
ADRB2
 
 
hsa-let-7c-5p
 
 
-0.635
 
 
11.8
 
 
 
 
ADRB2
 
 
hsa-miR-98-5p
 
 
-0.635
 
 
11.8
 
 
 
 
AGFG1
 
 
hsa-miR-148a-3p
 
 
-0.332
 
 
146.9
 
 
 
 
AGO2
 
 
hsa-miR-21-5p
 
 
-0.632
 
 
10.7
 
 
 
 
AGO2
 
 
hsa-miR-184
 
 
-0.831
 
 
10.7
 
 
 
 
AHCTF1
 
 
hsa-miR-98-5p
 
 
-0.374
 
 
16.0
 
 
 
 
AHCTF1
 
 
hsa-let-7c-5p
 
 
-0.374
 
 
16.0
 
 
 
 
AHNAK
 
 
hsa-miR-20a-5p
 
 
-0.316
 
 
41.6
 
 
 
 
AHNAK
 
 
hsa-miR-106b-5p
 
 
-0.316
 
 
41.6
 
 
 
 
AHNAK
 
 
hsa-miR-106a-5p
 
 
-0.334
 
 
41.6
 
 
 
 
AHNAK
 
 
hsa-miR-17-5p
 
 
-0.337
 
 
41.6
 
 
 
 
AHNAK
 
 
hsa-miR-20b-5p
 
 
-0.337
 
 
41.6
 
 
 
 
AIFM1
 
 
hsa-miR-98-5p
 
 
-0.314
 
 
11.2
 
 
 
 
AIFM1
 
 
hsa-let-7c-5p
 
 
-0.314
 
 
11.2
 
 
 
 
ALDH4A1
 
 
hsa-miR-184
 
 
-0.421
 
 
11.1
 
 
 
 
ALG3
 
 
hsa-miR-106b-5p
 
 
-0.551
 
 
14.9
 
 
 
 
ALG3
 
 
hsa-miR-20a-5p
 
 
-0.559
 
 
14.9
 
 
 
 
ALG3
 
 
hsa-miR-106a-5p
 
 
-0.569
 
 
14.9
 
 
 
 
ALG3
 
 
hsa-miR-17-5p
 
 
-0.572
 
 
14.9
 
 
 
 
ALG3
 
 
hsa-miR-20b-5p
 
 
-0.58
 
 
14.9
 
 
 
 
ANKRD12
 
 
hsa-miR-7-5p
 
 
-0.37
 
 
19.4
 
 
 
 
ANKRD9
 
 
hsa-miR-20a-5p
 
 
-0.31
 
 
17.0
 
 
 
 
ANKRD9
 
 
hsa-miR-106b-5p
 
 
-0.318
 
 
17.0
 
 
 
 
ANKRD9
 
 
hsa-miR-20b-5p
 
 
-0.331
 
 
17.0
 
 
 
 
ANKRD9
 
 
hsa-miR-106a-5p
 
 
-0.344
 
 
17.0
 
 
 
 
ANKRD9
 
 
hsa-miR-17-5p
 
 
-0.347
 
 
17.0
 
 
 
 
ARF4
 
 
hsa-miR-7-5p
 
 
-0.407
 
 
68.5
 
 
 
 
ARF4
 
 
hsa-miR-148a-3p
 
 
-0.487
 
 
68.5
 
 
 
 
ARFGEF1
 
 
hsa-miR-148a-3p
 
 
-0.306
 
 
26.0
 
 
 
 
ARHGAP1
 
 
hsa-miR-17-5p
 
 
-0.325
 
 
31.5
 
 
 
 
ARHGAP1
 
 
hsa-miR-20b-5p
 
 
-0.325
 
 
31.5
 
 
 
 
ARHGAP1
 
 
hsa-miR-106a-5p
 
 
-0.329
 
 
31.5
 
 
 
 
ARHGAP1
 
 
hsa-miR-20a-5p
 
 
-0.339
 
 
31.5
 
 
 
 
ARHGAP1
 
 
hsa-miR-106b-5p
 
 
-0.339
 
 
31.5
 
 
 
 
ARHGAP24
 
 
hsa-miR-21-5p
 
 
-0.427
 
 
17.7
 
 
 
 
ARID3B
 
 
hsa-miR-98-5p
 
 
-0.31
 
 
23.2
 
 
 
 
ARID3B
 
 
hsa-let-7c-5p
 
 
-0.31
 
 
23.2
 
 
 
 
ARID3B
 
 
hsa-miR-98-5p
 
 
-0.475
 
 
23.2
 
 
 
 
ARID3B
 
 
hsa-let-7c-5p
 
 
-0.486
 
 
23.2
 
 
 
 
ARID4A
 
 
hsa-miR-7-5p
 
 
-0.341
 
 
32.8
 
 
 
 
ARID4B
 
 
hsa-miR-20a-5p
 
 
-0.29
 
 
31.2
 
 
 
 
ARID4B
 
 
hsa-miR-106b-5p
 
 
-0.29
 
 
31.2
 
 
 
 
ARL1
 
 
hsa-miR-20a-5p
 
 
-0.403
 
 
15.6
 
 
 
 
ARL1
 
 
hsa-miR-106b-5p
 
 
-0.411
 
 
15.6
 
 
 
 
ARL1
 
 
hsa-miR-20b-5p
 
 
-0.424
 
 
15.6
 
 
 
 
ARL1
 
 
hsa-miR-106a-5p
 
 
-0.429
 
 
15.6
 
 
 
 
ARL1
 
 
hsa-miR-17-5p
 
 
-0.432
 
 
15.6
 
 
 
 
ARL6IP1
 
 
hsa-miR-7-5p
 
 
-0.374
 
 
197.7
 
 
 
 
ARL6IP1
 
 
hsa-miR-148a-3p
 
 
-0.579
 
 
197.7
 
 
 
 
ARL6IP6
 
 
hsa-miR-98-5p
 
 
-0.293
 
 
23.4
 
 
 
 
ARL6IP6
 
 
hsa-let-7c-5p
 
 
-0.293
 
 
23.4
 
 
 
 
ARL8B
 
 
hsa-miR-148a-3p
 
 
-0.337
 
 
56.4
 
 
 
 
ARMC8
 
 
hsa-miR-20a-5p
 
 
-0.357
 
 
57.2
 
 
 
 
ARMC8
 
 
hsa-miR-106b-5p
 
 
-0.357
 
 
57.2
 
 
 
 
ARMC8
 
 
hsa-miR-106a-5p
 
 
-0.375
 
 
57.2
 
 
 
 
ARMC8
 
 
hsa-miR-17-5p
 
 
-0.378
 
 
57.2
 
 
 
 
ARMC8
 
 
hsa-miR-20b-5p
 
 
-0.378
 
 
57.2
 
 
 
 
ARPP19
 
 
hsa-let-7c-5p
 
 
-0.492
 
 
97.5
 
 
 
 
ARPP19
 
 
hsa-miR-98-5p
 
 
-0.5
 
 
97.5
 
 
 
 
ARRDC3
 
 
hsa-miR-148a-3p
 
 
-0.305
 
 
158.3
 
 
 
 
ARRDC4
 
 
hsa-let-7c-5p
 
 
-0.329
 
 
10.8
 
 
 
 
ARRDC4
 
 
hsa-miR-98-5p
 
 
-0.329
 
 
10.8
 
 
 
 
ASAP1
 
 
hsa-miR-98-5p
 
 
-0.308
 
 
44.7
 
 
 
 
ASAP1
 
 
hsa-let-7c-5p
 
 
-0.308
 
 
44.7
 
 
 
 
ASPH
 
 
hsa-miR-148a-3p
 
 
-0.391
 
 
15.8
 
 
 
 
ATAD2
 
 
hsa-miR-17-5p
 
 
-0.304
 
 
25.0
 
 
 
 
ATAD2
 
 
hsa-miR-20b-5p
 
 
-0.304
 
 
25.0
 
 
 
 
ATAD2
 
 
hsa-miR-106b-5p
 
 
-0.307
 
 
25.0
 
 
 
 
ATAD2
 
 
hsa-miR-106a-5p
 
 
-0.308
 
 
25.0
 
 
 
 
ATAD2
 
 
hsa-miR-20a-5p
 
 
-0.318
 
 
25.0
 
 
 
 
ATG16L1
 
 
hsa-miR-20a-5p
 
 
-0.446
 
 
13.5
 
 
 
 
ATG16L1
 
 
hsa-miR-106b-5p
 
 
-0.446
 
 
13.5
 
 
 
 
ATG16L1
 
 
hsa-miR-106a-5p
 
 
-0.464
 
 
13.5
 
 
 
 
ATG16L1
 
 
hsa-miR-17-5p
 
 
-0.467
 
 
13.5
 
 
 
 
ATG16L1
 
 
hsa-miR-20b-5p
 
 
-0.467
 
 
13.5
 
 
 
 
ATG4D
 
 
hsa-miR-148a-3p
 
 
-0.303
 
 
31.3
 
 
 
 
ATOSB
 
 
hsa-miR-98-5p
 
 
-0.429
 
 
36.6
 
 
 
 
ATOSB
 
 
hsa-let-7c-5p
 
 
-0.429
 
 
36.6
 
 
 
 
ATP2A2
 
 
hsa-miR-98-5p
 
 
-0.311
 
 
43.4
 
 
 
 
ATP2A2
 
 
hsa-let-7c-5p
 
 
-0.311
 
 
43.4
 
 
 
 
ATP2B4
 
 
hsa-miR-148a-3p
 
 
-0.337
 
 
14.9
 
 
 
 
ATP6AP2
 
 
hsa-miR-148a-3p
 
 
-0.592
 
 
117.4
 
 
 
 
ATP6AP2
 
 
hsa-miR-148a-3p
 
 
-0.668
 
 
117.4
 
 
 
 
ATP8B4
 
 
hsa-let-7c-5p
 
 
-0.325
 
 
73.7
 
 
 
 
ATP8B4
 
 
hsa-miR-98-5p
 
 
-0.325
 
 
73.7
 
 
 
 
ATPAF1
 
 
hsa-miR-98-5p
 
 
-0.498
 
 
26.0
 
 
 
 
ATPAF1
 
 
hsa-let-7c-5p
 
 
-0.498
 
 
26.0
 
 
 
 
 
 
 cerebellum 
 
 
 
 
target_symbol
 
 
mature_mirna_id
 
 
score
 
 
nTPM
 
 
 
 
 
 
ABCB10
 
 
hsa-miR-148a-3p
 
 
-0.291
 
 
10.6
 
 
 
 
ABCB9
 
 
hsa-let-7c-5p
 
 
-0.422
 
 
18.0
 
 
 
 
ABCB9
 
 
hsa-miR-98-5p
 
 
-0.444
 
 
18.0
 
 
 
 
ABCD3
 
 
hsa-miR-148a-3p
 
 
-0.388
 
 
12.8
 
 
 
 
ACSL6
 
 
hsa-miR-98-5p
 
 
-0.336
 
 
27.8
 
 
 
 
ACSL6
 
 
hsa-let-7c-5p
 
 
-0.336
 
 
27.8
 
 
 
 
ADAM10
 
 
hsa-miR-148a-3p
 
 
-0.356
 
 
15.6
 
 
 
 
ADGRB3
 
 
hsa-miR-148a-3p
 
 
-0.524
 
 
20.8
 
 
 
 
AGFG1
 
 
hsa-miR-148a-3p
 
 
-0.332
 
 
22.4
 
 
 
 
AGFG2
 
 
hsa-miR-20a-5p
 
 
-0.365
 
 
12.7
 
 
 
 
AGFG2
 
 
hsa-miR-20b-5p
 
 
-0.386
 
 
12.7
 
 
 
 
AGFG2
 
 
hsa-miR-106b-5p
 
 
-0.397
 
 
12.7
 
 
 
 
AGFG2
 
 
hsa-miR-106a-5p
 
 
-0.399
 
 
12.7
 
 
 
 
AGFG2
 
 
hsa-miR-17-5p
 
 
-0.402
 
 
12.7
 
 
 
 
AHDC1
 
 
hsa-miR-148a-3p
 
 
-0.413
 
 
32.0
 
 
 
 
AHNAK
 
 
hsa-miR-20a-5p
 
 
-0.316
 
 
15.7
 
 
 
 
AHNAK
 
 
hsa-miR-106b-5p
 
 
-0.316
 
 
15.7
 
 
 
 
AHNAK
 
 
hsa-miR-106a-5p
 
 
-0.334
 
 
15.7
 
 
 
 
AHNAK
 
 
hsa-miR-17-5p
 
 
-0.337
 
 
15.7
 
 
 
 
AHNAK
 
 
hsa-miR-20b-5p
 
 
-0.337
 
 
15.7
 
 
 
 
AKAP1
 
 
hsa-miR-148a-3p
 
 
-0.381
 
 
30.6
 
 
 
 
ALDH4A1
 
 
hsa-miR-184
 
 
-0.421
 
 
10.9
 
 
 
 
AMT
 
 
hsa-miR-98-5p
 
 
-0.463
 
 
20.1
 
 
 
 
AMT
 
 
hsa-let-7c-5p
 
 
-0.463
 
 
20.1
 
 
 
 
ANKRD12
 
 
hsa-miR-7-5p
 
 
-0.37
 
 
13.0
 
 
 
 
ANKRD13C
 
 
hsa-miR-20a-5p
 
 
-0.505
 
 
12.9
 
 
 
 
ANKRD13C
 
 
hsa-miR-106b-5p
 
 
-0.505
 
 
12.9
 
 
 
 
ANKRD13C
 
 
hsa-miR-106a-5p
 
 
-0.523
 
 
12.9
 
 
 
 
ANKRD13C
 
 
hsa-miR-17-5p
 
 
-0.526
 
 
12.9
 
 
 
 
ANKRD13C
 
 
hsa-miR-20b-5p
 
 
-0.526
 
 
12.9
 
 
 
 
ANKRD46
 
 
hsa-miR-98-5p
 
 
-0.389
 
 
27.5
 
 
 
 
ANKRD46
 
 
hsa-let-7c-5p
 
 
-0.389
 
 
27.5
 
 
 
 
ANKRD9
 
 
hsa-miR-20a-5p
 
 
-0.31
 
 
22.1
 
 
 
 
ANKRD9
 
 
hsa-miR-106b-5p
 
 
-0.318
 
 
22.1
 
 
 
 
ANKRD9
 
 
hsa-miR-20b-5p
 
 
-0.331
 
 
22.1
 
 
 
 
ANKRD9
 
 
hsa-miR-106a-5p
 
 
-0.344
 
 
22.1
 
 
 
 
ANKRD9
 
 
hsa-miR-17-5p
 
 
-0.347
 
 
22.1
 
 
 
 
AP1AR
 
 
hsa-miR-21-5p
 
 
-0.318
 
 
13.2
 
 
 
 
AP1S1
 
 
hsa-miR-98-5p
 
 
-0.374
 
 
23.9
 
 
 
 
AP1S1
 
 
hsa-let-7c-5p
 
 
-0.374
 
 
23.9
 
 
 
 
APBB3
 
 
hsa-miR-98-5p
 
 
-0.587
 
 
23.6
 
 
 
 
APBB3
 
 
hsa-let-7c-5p
 
 
-0.587
 
 
23.6
 
 
 
 
ARF4
 
 
hsa-miR-7-5p
 
 
-0.407
 
 
50.2
 
 
 
 
ARF4
 
 
hsa-miR-148a-3p
 
 
-0.487
 
 
50.2
 
 
 
 
ARG2
 
 
hsa-miR-98-5p
 
 
-0.468
 
 
16.4
 
 
 
 
ARG2
 
 
hsa-let-7c-5p
 
 
-0.468
 
 
16.4
 
 
 
 
ARHGAP1
 
 
hsa-miR-17-5p
 
 
-0.325
 
 
19.6
 
 
 
 
ARHGAP1
 
 
hsa-miR-20b-5p
 
 
-0.325
 
 
19.6
 
 
 
 
ARHGAP1
 
 
hsa-miR-106a-5p
 
 
-0.329
 
 
19.6
 
 
 
 
ARHGAP1
 
 
hsa-miR-20a-5p
 
 
-0.339
 
 
19.6
 
 
 
 
ARHGAP1
 
 
hsa-miR-106b-5p
 
 
-0.339
 
 
19.6
 
 
 
 
ARHGAP21
 
 
hsa-miR-148a-3p
 
 
-0.287
 
 
50.0
 
 
 
 
ARID4A
 
 
hsa-miR-7-5p
 
 
-0.341
 
 
10.7
 
 
 
 
ARID4B
 
 
hsa-miR-20a-5p
 
 
-0.29
 
 
15.1
 
 
 
 
ARID4B
 
 
hsa-miR-106b-5p
 
 
-0.29
 
 
15.1
 
 
 
 
ARL1
 
 
hsa-miR-20a-5p
 
 
-0.403
 
 
20.8
 
 
 
 
ARL1
 
 
hsa-miR-106b-5p
 
 
-0.411
 
 
20.8
 
 
 
 
ARL1
 
 
hsa-miR-20b-5p
 
 
-0.424
 
 
20.8
 
 
 
 
ARL1
 
 
hsa-miR-106a-5p
 
 
-0.429
 
 
20.8
 
 
 
 
ARL1
 
 
hsa-miR-17-5p
 
 
-0.432
 
 
20.8
 
 
 
 
ARL6IP1
 
 
hsa-miR-7-5p
 
 
-0.374
 
 
234.0
 
 
 
 
ARL6IP1
 
 
hsa-miR-148a-3p
 
 
-0.579
 
 
234.0
 
 
 
 
ARL8B
 
 
hsa-miR-148a-3p
 
 
-0.337
 
 
50.7
 
 
 
 
ARMC8
 
 
hsa-miR-20a-5p
 
 
-0.357
 
 
35.7
 
 
 
 
ARMC8
 
 
hsa-miR-106b-5p
 
 
-0.357
 
 
35.7
 
 
 
 
ARMC8
 
 
hsa-miR-106a-5p
 
 
-0.375
 
 
35.7
 
 
 
 
ARMC8
 
 
hsa-miR-17-5p
 
 
-0.378
 
 
35.7
 
 
 
 
ARMC8
 
 
hsa-miR-20b-5p
 
 
-0.378
 
 
35.7
 
 
 
 
ARMCX1
 
 
hsa-miR-21-5p
 
 
-0.489
 
 
17.3
 
 
 
 
ARMT1
 
 
hsa-let-7c-5p
 
 
-0.303
 
 
11.5
 
 
 
 
ARMT1
 
 
hsa-miR-98-5p
 
 
-0.303
 
 
11.5
 
 
 
 
ARPP19
 
 
hsa-let-7c-5p
 
 
-0.492
 
 
66.7
 
 
 
 
ARPP19
 
 
hsa-miR-98-5p
 
 
-0.5
 
 
66.7
 
 
 
 
ARRDC3
 
 
hsa-miR-148a-3p
 
 
-0.305
 
 
17.8
 
 
 
 
ASF1A
 
 
hsa-miR-21-5p
 
 
-0.304
 
 
12.6
 
 
 
 
ASPH
 
 
hsa-miR-148a-3p
 
 
-0.391
 
 
80.5
 
 
 
 
ATG16L1
 
 
hsa-miR-20a-5p
 
 
-0.446
 
 
24.1
 
 
 
 
ATG16L1
 
 
hsa-miR-106b-5p
 
 
-0.446
 
 
24.1
 
 
 
 
ATG16L1
 
 
hsa-miR-106a-5p
 
 
-0.464
 
 
24.1
 
 
 
 
ATG16L1
 
 
hsa-miR-17-5p
 
 
-0.467
 
 
24.1
 
 
 
 
ATG16L1
 
 
hsa-miR-20b-5p
 
 
-0.467
 
 
24.1
 
 
 
 
ATG4D
 
 
hsa-miR-148a-3p
 
 
-0.303
 
 
20.2
 
 
 
 
ATOSB
 
 
hsa-miR-98-5p
 
 
-0.429
 
 
14.6
 
 
 
 
ATOSB
 
 
hsa-let-7c-5p
 
 
-0.429
 
 
14.6
 
 
 
 
ATP2A2
 
 
hsa-miR-98-5p
 
 
-0.311
 
 
15.0
 
 
 
 
ATP2A2
 
 
hsa-let-7c-5p
 
 
-0.311
 
 
15.0
 
 
 
 
ATP2B2
 
 
hsa-miR-7-5p
 
 
-0.297
 
 
88.0
 
 
 
 
ATP6AP2
 
 
hsa-miR-148a-3p
 
 
-0.592
 
 
52.9
 
 
 
 
ATP6AP2
 
 
hsa-miR-148a-3p
 
 
-0.668
 
 
52.9
 
 
 
 
ATPAF1
 
 
hsa-miR-98-5p
 
 
-0.498
 
 
24.4
 
 
 
 
ATPAF1
 
 
hsa-let-7c-5p
 
 
-0.498
 
 
24.4
 
 
 
 
AVPI1
 
 
hsa-miR-7-5p
 
 
-0.367
 
 
16.8
 
 
 
 
B3GAT3
 
 
hsa-miR-98-5p
 
 
-0.324
 
 
70.0
 
 
 
 
B3GAT3
 
 
hsa-let-7c-5p
 
 
-0.324
 
 
70.0
 
 
 
 
B4GALT5
 
 
hsa-miR-148a-3p
 
 
-0.44
 
 
20.4
 
 
 
 
B4GALT6
 
 
hsa-miR-148a-3p
 
 
-0.528
 
 
16.6
 
 
 
 
B4GAT1
 
 
hsa-let-7c-5p
 
 
-0.47
 
 
105.2
 
 
 
 
B4GAT1
 
 
hsa-miR-98-5p
 
 
-0.47
 
 
105.2
 
 
 
 
BARHL2
 
 
hsa-miR-148a-3p
 
 
-0.544
 
 
42.1
 
 
 
 
BCL7A
 
 
hsa-miR-21-5p
 
 
-0.31
 
 
17.6
 
 
 
 
 
 
 colon 
 
 
 
 
target_symbol
 
 
mature_mirna_id
 
 
score
 
 
nTPM
 
 
 
 
 
 
ABCB10
 
 
hsa-miR-148a-3p
 
 
-0.291
 
 
10.4
 
 
 
 
ABCB7
 
 
hsa-miR-148a-3p
 
 
-0.515
 
 
13.4
 
 
 
 
ABCD3
 
 
hsa-miR-148a-3p
 
 
-0.388
 
 
41.8
 
 
 
 
ABHD17C
 
 
hsa-let-7c-5p
 
 
-0.359
 
 
61.2
 
 
 
 
ABHD17C
 
 
hsa-miR-98-5p
 
 
-0.383
 
 
61.2
 
 
 
 
ABHD5
 
 
hsa-miR-20a-5p
 
 
-0.397
 
 
11.4
 
 
 
 
ABHD5
 
 
hsa-miR-106b-5p
 
 
-0.413
 
 
11.4
 
 
 
 
ABHD5
 
 
hsa-miR-20b-5p
 
 
-0.418
 
 
11.4
 
 
 
 
ABHD5
 
 
hsa-miR-106a-5p
 
 
-0.447
 
 
11.4
 
 
 
 
ABHD5
 
 
hsa-miR-17-5p
 
 
-0.45
 
 
11.4
 
 
 
 
ADAM10
 
 
hsa-miR-148a-3p
 
 
-0.356
 
 
20.8
 
 
 
 
ADAM9
 
 
hsa-miR-20a-5p
 
 
-0.299
 
 
30.9
 
 
 
 
ADAM9
 
 
hsa-miR-106b-5p
 
 
-0.299
 
 
30.9
 
 
 
 
ADAM9
 
 
hsa-miR-106a-5p
 
 
-0.317
 
 
30.9
 
 
 
 
ADAM9
 
 
hsa-miR-17-5p
 
 
-0.32
 
 
30.9
 
 
 
 
ADAM9
 
 
hsa-miR-20b-5p
 
 
-0.328
 
 
30.9
 
 
 
 
AGFG1
 
 
hsa-miR-148a-3p
 
 
-0.332
 
 
30.2
 
 
 
 
AGFG2
 
 
hsa-miR-20a-5p
 
 
-0.365
 
 
17.3
 
 
 
 
AGFG2
 
 
hsa-miR-20b-5p
 
 
-0.386
 
 
17.3
 
 
 
 
AGFG2
 
 
hsa-miR-106b-5p
 
 
-0.397
 
 
17.3
 
 
 
 
AGFG2
 
 
hsa-miR-106a-5p
 
 
-0.399
 
 
17.3
 
 
 
 
AGFG2
 
 
hsa-miR-17-5p
 
 
-0.402
 
 
17.3
 
 
 
 
AHDC1
 
 
hsa-miR-148a-3p
 
 
-0.413
 
 
15.6
 
 
 
 
AHNAK
 
 
hsa-miR-20a-5p
 
 
-0.316
 
 
258.8
 
 
 
 
AHNAK
 
 
hsa-miR-106b-5p
 
 
-0.316
 
 
258.8
 
 
 
 
AHNAK
 
 
hsa-miR-106a-5p
 
 
-0.334
 
 
258.8
 
 
 
 
AHNAK
 
 
hsa-miR-17-5p
 
 
-0.337
 
 
258.8
 
 
 
 
AHNAK
 
 
hsa-miR-20b-5p
 
 
-0.337
 
 
258.8
 
 
 
 
AIFM1
 
 
hsa-miR-98-5p
 
 
-0.314
 
 
23.9
 
 
 
 
AIFM1
 
 
hsa-let-7c-5p
 
 
-0.314
 
 
23.9
 
 
 
 
AKAP1
 
 
hsa-miR-148a-3p
 
 
-0.381
 
 
72.8
 
 
 
 
ALCAM
 
 
hsa-miR-148a-3p
 
 
-0.339
 
 
15.7
 
 
 
 
ALG3
 
 
hsa-miR-106b-5p
 
 
-0.551
 
 
22.6
 
 
 
 
ALG3
 
 
hsa-miR-20a-5p
 
 
-0.559
 
 
22.6
 
 
 
 
ALG3
 
 
hsa-miR-106a-5p
 
 
-0.569
 
 
22.6
 
 
 
 
ALG3
 
 
hsa-miR-17-5p
 
 
-0.572
 
 
22.6
 
 
 
 
ALG3
 
 
hsa-miR-20b-5p
 
 
-0.58
 
 
22.6
 
 
 
 
ALG9
 
 
hsa-miR-7-5p
 
 
-0.302
 
 
10.0
 
 
 
 
AMT
 
 
hsa-miR-98-5p
 
 
-0.463
 
 
14.4
 
 
 
 
AMT
 
 
hsa-let-7c-5p
 
 
-0.463
 
 
14.4
 
 
 
 
ANKRD12
 
 
hsa-miR-7-5p
 
 
-0.37
 
 
12.2
 
 
 
 
ANKRD9
 
 
hsa-miR-20a-5p
 
 
-0.31
 
 
28.9
 
 
 
 
ANKRD9
 
 
hsa-miR-106b-5p
 
 
-0.318
 
 
28.9
 
 
 
 
ANKRD9
 
 
hsa-miR-20b-5p
 
 
-0.331
 
 
28.9
 
 
 
 
ANKRD9
 
 
hsa-miR-106a-5p
 
 
-0.344
 
 
28.9
 
 
 
 
ANKRD9
 
 
hsa-miR-17-5p
 
 
-0.347
 
 
28.9
 
 
 
 
AP1AR
 
 
hsa-miR-21-5p
 
 
-0.318
 
 
16.9
 
 
 
 
AP1S1
 
 
hsa-miR-98-5p
 
 
-0.374
 
 
31.0
 
 
 
 
AP1S1
 
 
hsa-let-7c-5p
 
 
-0.374
 
 
31.0
 
 
 
 
APCDD1
 
 
hsa-miR-20a-5p
 
 
-0.404
 
 
12.1
 
 
 
 
APCDD1
 
 
hsa-miR-106b-5p
 
 
-0.404
 
 
12.1
 
 
 
 
APCDD1
 
 
hsa-miR-106a-5p
 
 
-0.422
 
 
12.1
 
 
 
 
APCDD1
 
 
hsa-miR-17-5p
 
 
-0.425
 
 
12.1
 
 
 
 
APCDD1
 
 
hsa-miR-20b-5p
 
 
-0.425
 
 
12.1
 
 
 
 
ARF4
 
 
hsa-miR-7-5p
 
 
-0.407
 
 
148.9
 
 
 
 
ARF4
 
 
hsa-miR-148a-3p
 
 
-0.487
 
 
148.9
 
 
 
 
ARFGEF1
 
 
hsa-miR-148a-3p
 
 
-0.306
 
 
18.0
 
 
 
 
ARFIP1
 
 
hsa-miR-148a-3p
 
 
-0.474
 
 
26.4
 
 
 
 
ARHGAP1
 
 
hsa-miR-17-5p
 
 
-0.325
 
 
59.8
 
 
 
 
ARHGAP1
 
 
hsa-miR-20b-5p
 
 
-0.325
 
 
59.8
 
 
 
 
ARHGAP1
 
 
hsa-miR-106a-5p
 
 
-0.329
 
 
59.8
 
 
 
 
ARHGAP1
 
 
hsa-miR-20a-5p
 
 
-0.339
 
 
59.8
 
 
 
 
ARHGAP1
 
 
hsa-miR-106b-5p
 
 
-0.339
 
 
59.8
 
 
 
 
ARHGAP21
 
 
hsa-miR-148a-3p
 
 
-0.287
 
 
15.5
 
 
 
 
ARL1
 
 
hsa-miR-20a-5p
 
 
-0.403
 
 
53.0
 
 
 
 
ARL1
 
 
hsa-miR-106b-5p
 
 
-0.411
 
 
53.0
 
 
 
 
ARL1
 
 
hsa-miR-20b-5p
 
 
-0.424
 
 
53.0
 
 
 
 
ARL1
 
 
hsa-miR-106a-5p
 
 
-0.429
 
 
53.0
 
 
 
 
ARL1
 
 
hsa-miR-17-5p
 
 
-0.432
 
 
53.0
 
 
 
 
ARL4D
 
 
hsa-miR-98-5p
 
 
-0.417
 
 
27.1
 
 
 
 
ARL4D
 
 
hsa-let-7c-5p
 
 
-0.417
 
 
27.1
 
 
 
 
ARL6IP1
 
 
hsa-miR-7-5p
 
 
-0.374
 
 
132.0
 
 
 
 
ARL6IP1
 
 
hsa-miR-148a-3p
 
 
-0.579
 
 
132.0
 
 
 
 
ARL6IP6
 
 
hsa-miR-98-5p
 
 
-0.293
 
 
12.7
 
 
 
 
ARL6IP6
 
 
hsa-let-7c-5p
 
 
-0.293
 
 
12.7
 
 
 
 
ARL8B
 
 
hsa-miR-148a-3p
 
 
-0.337
 
 
34.2
 
 
 
 
ARMC8
 
 
hsa-miR-20a-5p
 
 
-0.357
 
 
15.5
 
 
 
 
ARMC8
 
 
hsa-miR-106b-5p
 
 
-0.357
 
 
15.5
 
 
 
 
ARMC8
 
 
hsa-miR-106a-5p
 
 
-0.375
 
 
15.5
 
 
 
 
ARMC8
 
 
hsa-miR-17-5p
 
 
-0.378
 
 
15.5
 
 
 
 
ARMC8
 
 
hsa-miR-20b-5p
 
 
-0.378
 
 
15.5
 
 
 
 
ARMCX1
 
 
hsa-miR-21-5p
 
 
-0.489
 
 
29.8
 
 
 
 
ARMT1
 
 
hsa-let-7c-5p
 
 
-0.303
 
 
15.5
 
 
 
 
ARMT1
 
 
hsa-miR-98-5p
 
 
-0.303
 
 
15.5
 
 
 
 
ARPP19
 
 
hsa-let-7c-5p
 
 
-0.492
 
 
35.7
 
 
 
 
ARPP19
 
 
hsa-miR-98-5p
 
 
-0.5
 
 
35.7
 
 
 
 
ARRDC3
 
 
hsa-miR-148a-3p
 
 
-0.305
 
 
18.9
 
 
 
 
ARRDC4
 
 
hsa-let-7c-5p
 
 
-0.329
 
 
40.4
 
 
 
 
ARRDC4
 
 
hsa-miR-98-5p
 
 
-0.329
 
 
40.4
 
 
 
 
ASAP1
 
 
hsa-miR-98-5p
 
 
-0.308
 
 
12.2
 
 
 
 
ASAP1
 
 
hsa-let-7c-5p
 
 
-0.308
 
 
12.2
 
 
 
 
ASPH
 
 
hsa-miR-148a-3p
 
 
-0.391
 
 
72.8
 
 
 
 
ATG16L1
 
 
hsa-miR-20a-5p
 
 
-0.446
 
 
13.4
 
 
 
 
ATG16L1
 
 
hsa-miR-106b-5p
 
 
-0.446
 
 
13.4
 
 
 
 
ATG16L1
 
 
hsa-miR-106a-5p
 
 
-0.464
 
 
13.4
 
 
 
 
ATG16L1
 
 
hsa-miR-17-5p
 
 
-0.467
 
 
13.4
 
 
 
 
ATG16L1
 
 
hsa-miR-20b-5p
 
 
-0.467
 
 
13.4
 
 
 
 
ATG4A
 
 
hsa-miR-7-5p
 
 
-0.448
 
 
11.4
 
 
 
 
ATG4D
 
 
hsa-miR-148a-3p
 
 
-0.303
 
 
21.1
 
 
 
 
ATOSB
 
 
hsa-miR-98-5p
 
 
-0.429
 
 
14.6
 
 
 
 
 
 
 duodenum 
 
 
 
 
target_symbol
 
 
mature_mirna_id
 
 
score
 
 
nTPM
 
 
 
 
 
 
ABCB10
 
 
hsa-miR-148a-3p
 
 
-0.291
 
 
16.1
 
 
 
 
ABCB7
 
 
hsa-miR-148a-3p
 
 
-0.515
 
 
15.1
 
 
 
 
ABCD3
 
 
hsa-miR-148a-3p
 
 
-0.388
 
 
42.1
 
 
 
 
ABHD17C
 
 
hsa-let-7c-5p
 
 
-0.359
 
 
21.9
 
 
 
 
ABHD17C
 
 
hsa-miR-98-5p
 
 
-0.383
 
 
21.9
 
 
 
 
ADAM10
 
 
hsa-miR-148a-3p
 
 
-0.356
 
 
14.7
 
 
 
 
ADAM9
 
 
hsa-miR-20a-5p
 
 
-0.299
 
 
23.1
 
 
 
 
ADAM9
 
 
hsa-miR-106b-5p
 
 
-0.299
 
 
23.1
 
 
 
 
ADAM9
 
 
hsa-miR-106a-5p
 
 
-0.317
 
 
23.1
 
 
 
 
ADAM9
 
 
hsa-miR-17-5p
 
 
-0.32
 
 
23.1
 
 
 
 
ADAM9
 
 
hsa-miR-20b-5p
 
 
-0.328
 
 
23.1
 
 
 
 
AGFG1
 
 
hsa-miR-148a-3p
 
 
-0.332
 
 
40.8
 
 
 
 
AHNAK
 
 
hsa-miR-20a-5p
 
 
-0.316
 
 
46.1
 
 
 
 
AHNAK
 
 
hsa-miR-106b-5p
 
 
-0.316
 
 
46.1
 
 
 
 
AHNAK
 
 
hsa-miR-106a-5p
 
 
-0.334
 
 
46.1
 
 
 
 
AHNAK
 
 
hsa-miR-17-5p
 
 
-0.337
 
 
46.1
 
 
 
 
AHNAK
 
 
hsa-miR-20b-5p
 
 
-0.337
 
 
46.1
 
 
 
 
AIFM1
 
 
hsa-miR-98-5p
 
 
-0.314
 
 
49.8
 
 
 
 
AIFM1
 
 
hsa-let-7c-5p
 
 
-0.314
 
 
49.8
 
 
 
 
AKAP1
 
 
hsa-miR-148a-3p
 
 
-0.381
 
 
51.5
 
 
 
 
ALCAM
 
 
hsa-miR-148a-3p
 
 
-0.339
 
 
16.0
 
 
 
 
ALG3
 
 
hsa-miR-106b-5p
 
 
-0.551
 
 
15.9
 
 
 
 
ALG3
 
 
hsa-miR-20a-5p
 
 
-0.559
 
 
15.9
 
 
 
 
ALG3
 
 
hsa-miR-106a-5p
 
 
-0.569
 
 
15.9
 
 
 
 
ALG3
 
 
hsa-miR-17-5p
 
 
-0.572
 
 
15.9
 
 
 
 
ALG3
 
 
hsa-miR-20b-5p
 
 
-0.58
 
 
15.9
 
 
 
 
ALG9
 
 
hsa-miR-7-5p
 
 
-0.302
 
 
11.5
 
 
 
 
AMT
 
 
hsa-miR-98-5p
 
 
-0.463
 
 
12.9
 
 
 
 
AMT
 
 
hsa-let-7c-5p
 
 
-0.463
 
 
12.9
 
 
 
 
ANKRD12
 
 
hsa-miR-7-5p
 
 
-0.37
 
 
12.1
 
 
 
 
ANKRD9
 
 
hsa-miR-20a-5p
 
 
-0.31
 
 
47.3
 
 
 
 
ANKRD9
 
 
hsa-miR-106b-5p
 
 
-0.318
 
 
47.3
 
 
 
 
ANKRD9
 
 
hsa-miR-20b-5p
 
 
-0.331
 
 
47.3
 
 
 
 
ANKRD9
 
 
hsa-miR-106a-5p
 
 
-0.344
 
 
47.3
 
 
 
 
ANKRD9
 
 
hsa-miR-17-5p
 
 
-0.347
 
 
47.3
 
 
 
 
AP1AR
 
 
hsa-miR-21-5p
 
 
-0.318
 
 
20.9
 
 
 
 
AP1S1
 
 
hsa-miR-98-5p
 
 
-0.374
 
 
36.7
 
 
 
 
AP1S1
 
 
hsa-let-7c-5p
 
 
-0.374
 
 
36.7
 
 
 
 
ARF4
 
 
hsa-miR-7-5p
 
 
-0.407
 
 
116.2
 
 
 
 
ARF4
 
 
hsa-miR-148a-3p
 
 
-0.487
 
 
116.2
 
 
 
 
ARFGEF1
 
 
hsa-miR-148a-3p
 
 
-0.306
 
 
15.9
 
 
 
 
ARFIP1
 
 
hsa-miR-148a-3p
 
 
-0.474
 
 
21.8
 
 
 
 
ARHGAP1
 
 
hsa-miR-17-5p
 
 
-0.325
 
 
24.2
 
 
 
 
ARHGAP1
 
 
hsa-miR-20b-5p
 
 
-0.325
 
 
24.2
 
 
 
 
ARHGAP1
 
 
hsa-miR-106a-5p
 
 
-0.329
 
 
24.2
 
 
 
 
ARHGAP1
 
 
hsa-miR-20a-5p
 
 
-0.339
 
 
24.2
 
 
 
 
ARHGAP1
 
 
hsa-miR-106b-5p
 
 
-0.339
 
 
24.2
 
 
 
 
ARHGAP21
 
 
hsa-miR-148a-3p
 
 
-0.287
 
 
12.1
 
 
 
 
ARL1
 
 
hsa-miR-20a-5p
 
 
-0.403
 
 
45.2
 
 
 
 
ARL1
 
 
hsa-miR-106b-5p
 
 
-0.411
 
 
45.2
 
 
 
 
ARL1
 
 
hsa-miR-20b-5p
 
 
-0.424
 
 
45.2
 
 
 
 
ARL1
 
 
hsa-miR-106a-5p
 
 
-0.429
 
 
45.2
 
 
 
 
ARL1
 
 
hsa-miR-17-5p
 
 
-0.432
 
 
45.2
 
 
 
 
ARL6IP1
 
 
hsa-miR-7-5p
 
 
-0.374
 
 
130.6
 
 
 
 
ARL6IP1
 
 
hsa-miR-148a-3p
 
 
-0.579
 
 
130.6
 
 
 
 
ARL8B
 
 
hsa-miR-148a-3p
 
 
-0.337
 
 
28.9
 
 
 
 
ARMC8
 
 
hsa-miR-20a-5p
 
 
-0.357
 
 
11.3
 
 
 
 
ARMC8
 
 
hsa-miR-106b-5p
 
 
-0.357
 
 
11.3
 
 
 
 
ARMC8
 
 
hsa-miR-106a-5p
 
 
-0.375
 
 
11.3
 
 
 
 
ARMC8
 
 
hsa-miR-17-5p
 
 
-0.378
 
 
11.3
 
 
 
 
ARMC8
 
 
hsa-miR-20b-5p
 
 
-0.378
 
 
11.3
 
 
 
 
ARMT1
 
 
hsa-let-7c-5p
 
 
-0.303
 
 
17.8
 
 
 
 
ARMT1
 
 
hsa-miR-98-5p
 
 
-0.303
 
 
17.8
 
 
 
 
ARPP19
 
 
hsa-let-7c-5p
 
 
-0.492
 
 
27.2
 
 
 
 
ARPP19
 
 
hsa-miR-98-5p
 
 
-0.5
 
 
27.2
 
 
 
 
ARRDC4
 
 
hsa-let-7c-5p
 
 
-0.329
 
 
14.9
 
 
 
 
ARRDC4
 
 
hsa-miR-98-5p
 
 
-0.329
 
 
14.9
 
 
 
 
ASPH
 
 
hsa-miR-148a-3p
 
 
-0.391
 
 
42.9
 
 
 
 
ATG4A
 
 
hsa-miR-7-5p
 
 
-0.448
 
 
12.0
 
 
 
 
ATG4D
 
 
hsa-miR-148a-3p
 
 
-0.303
 
 
20.5
 
 
 
 
ATP2A2
 
 
hsa-miR-98-5p
 
 
-0.311
 
 
44.8
 
 
 
 
ATP2A2
 
 
hsa-let-7c-5p
 
 
-0.311
 
 
44.8
 
 
 
 
ATP2B4
 
 
hsa-miR-148a-3p
 
 
-0.337
 
 
11.8
 
 
 
 
ATP6AP2
 
 
hsa-miR-148a-3p
 
 
-0.592
 
 
30.3
 
 
 
 
ATP6AP2
 
 
hsa-miR-148a-3p
 
 
-0.668
 
 
30.3
 
 
 
 
ATPAF1
 
 
hsa-miR-98-5p
 
 
-0.498
 
 
41.3
 
 
 
 
ATPAF1
 
 
hsa-let-7c-5p
 
 
-0.498
 
 
41.3
 
 
 
 
AVPI1
 
 
hsa-miR-7-5p
 
 
-0.367
 
 
13.2
 
 
 
 
B3GAT3
 
 
hsa-miR-98-5p
 
 
-0.324
 
 
16.1
 
 
 
 
B3GAT3
 
 
hsa-let-7c-5p
 
 
-0.324
 
 
16.1
 
 
 
 
B4GALT5
 
 
hsa-miR-148a-3p
 
 
-0.44
 
 
23.8
 
 
 
 
B4GAT1
 
 
hsa-let-7c-5p
 
 
-0.47
 
 
15.2
 
 
 
 
B4GAT1
 
 
hsa-miR-98-5p
 
 
-0.47
 
 
15.2
 
 
 
 
BACH1
 
 
hsa-miR-98-5p
 
 
-0.298
 
 
15.8
 
 
 
 
BACH1
 
 
hsa-miR-98-5p
 
 
-0.298
 
 
15.8
 
 
 
 
BACH1
 
 
hsa-let-7c-5p
 
 
-0.306
 
 
15.8
 
 
 
 
BACH1
 
 
hsa-let-7c-5p
 
 
-0.306
 
 
15.8
 
 
 
 
BACH1
 
 
hsa-miR-98-5p
 
 
-0.435
 
 
15.8
 
 
 
 
BACH1
 
 
hsa-miR-98-5p
 
 
-0.435
 
 
15.8
 
 
 
 
BACH1
 
 
hsa-let-7c-5p
 
 
-0.435
 
 
15.8
 
 
 
 
BACH1
 
 
hsa-let-7c-5p
 
 
-0.435
 
 
15.8
 
 
 
 
BLOC1S6
 
 
hsa-miR-98-5p
 
 
-0.321
 
 
19.0
 
 
 
 
BLOC1S6
 
 
hsa-let-7c-5p
 
 
-0.321
 
 
19.0
 
 
 
 
BNIP3L
 
 
hsa-miR-20a-5p
 
 
-0.287
 
 
20.9
 
 
 
 
BNIP3L
 
 
hsa-miR-106b-5p
 
 
-0.298
 
 
20.9
 
 
 
 
BTAF1
 
 
hsa-miR-148a-3p
 
 
-0.401
 
 
12.8
 
 
 
 
BTBD10
 
 
hsa-miR-106b-5p
 
 
-0.388
 
 
12.4
 
 
 
 
BTBD10
 
 
hsa-miR-106a-5p
 
 
-0.406
 
 
12.4
 
 
 
 
BTBD10
 
 
hsa-miR-17-5p
 
 
-0.409
 
 
12.4
 
 
 
 
BTBD10
 
 
hsa-miR-20a-5p
 
 
-0.412
 
 
12.4
 
 
 
 
 
 
 liver 
 
 
 
 
target_symbol
 
 
mature_mirna_id
 
 
score
 
 
nTPM
 
 
 
 
 
 
ABCB10
 
 
hsa-miR-148a-3p
 
 
-0.291
 
 
17.7
 
 
 
 
ABCB7
 
 
hsa-miR-148a-3p
 
 
-0.515
 
 
14.1
 
 
 
 
ABCD3
 
 
hsa-miR-148a-3p
 
 
-0.388
 
 
118.4
 
 
 
 
ABHD5
 
 
hsa-miR-20a-5p
 
 
-0.397
 
 
20.3
 
 
 
 
ABHD5
 
 
hsa-miR-106b-5p
 
 
-0.413
 
 
20.3
 
 
 
 
ABHD5
 
 
hsa-miR-20b-5p
 
 
-0.418
 
 
20.3
 
 
 
 
ABHD5
 
 
hsa-miR-106a-5p
 
 
-0.447
 
 
20.3
 
 
 
 
ABHD5
 
 
hsa-miR-17-5p
 
 
-0.45
 
 
20.3
 
 
 
 
ACVR1
 
 
hsa-miR-148a-3p
 
 
-0.397
 
 
15.3
 
 
 
 
ADAM10
 
 
hsa-miR-148a-3p
 
 
-0.356
 
 
15.0
 
 
 
 
ADAM9
 
 
hsa-miR-20a-5p
 
 
-0.299
 
 
12.7
 
 
 
 
ADAM9
 
 
hsa-miR-106b-5p
 
 
-0.299
 
 
12.7
 
 
 
 
ADAM9
 
 
hsa-miR-106a-5p
 
 
-0.317
 
 
12.7
 
 
 
 
ADAM9
 
 
hsa-miR-17-5p
 
 
-0.32
 
 
12.7
 
 
 
 
ADAM9
 
 
hsa-miR-20b-5p
 
 
-0.328
 
 
12.7
 
 
 
 
ADGRF5
 
 
hsa-miR-148a-3p
 
 
-0.354
 
 
12.3
 
 
 
 
ADRB2
 
 
hsa-let-7c-5p
 
 
-0.635
 
 
13.3
 
 
 
 
ADRB2
 
 
hsa-miR-98-5p
 
 
-0.635
 
 
13.3
 
 
 
 
AGFG1
 
 
hsa-miR-148a-3p
 
 
-0.332
 
 
33.8
 
 
 
 
AGFG2
 
 
hsa-miR-20a-5p
 
 
-0.365
 
 
51.0
 
 
 
 
AGFG2
 
 
hsa-miR-20b-5p
 
 
-0.386
 
 
51.0
 
 
 
 
AGFG2
 
 
hsa-miR-106b-5p
 
 
-0.397
 
 
51.0
 
 
 
 
AGFG2
 
 
hsa-miR-106a-5p
 
 
-0.399
 
 
51.0
 
 
 
 
AGFG2
 
 
hsa-miR-17-5p
 
 
-0.402
 
 
51.0
 
 
 
 
AHCTF1
 
 
hsa-miR-98-5p
 
 
-0.374
 
 
10.2
 
 
 
 
AHCTF1
 
 
hsa-let-7c-5p
 
 
-0.374
 
 
10.2
 
 
 
 
AHNAK
 
 
hsa-miR-20a-5p
 
 
-0.316
 
 
56.2
 
 
 
 
AHNAK
 
 
hsa-miR-106b-5p
 
 
-0.316
 
 
56.2
 
 
 
 
AHNAK
 
 
hsa-miR-106a-5p
 
 
-0.334
 
 
56.2
 
 
 
 
AHNAK
 
 
hsa-miR-17-5p
 
 
-0.337
 
 
56.2
 
 
 
 
AHNAK
 
 
hsa-miR-20b-5p
 
 
-0.337
 
 
56.2
 
 
 
 
AIFM1
 
 
hsa-miR-98-5p
 
 
-0.314
 
 
77.0
 
 
 
 
AIFM1
 
 
hsa-let-7c-5p
 
 
-0.314
 
 
77.0
 
 
 
 
AKAP1
 
 
hsa-miR-148a-3p
 
 
-0.381
 
 
48.4
 
 
 
 
ALCAM
 
 
hsa-miR-148a-3p
 
 
-0.339
 
 
44.8
 
 
 
 
ALDH4A1
 
 
hsa-miR-184
 
 
-0.421
 
 
438.6
 
 
 
 
ALG3
 
 
hsa-miR-106b-5p
 
 
-0.551
 
 
54.4
 
 
 
 
ALG3
 
 
hsa-miR-20a-5p
 
 
-0.559
 
 
54.4
 
 
 
 
ALG3
 
 
hsa-miR-106a-5p
 
 
-0.569
 
 
54.4
 
 
 
 
ALG3
 
 
hsa-miR-17-5p
 
 
-0.572
 
 
54.4
 
 
 
 
ALG3
 
 
hsa-miR-20b-5p
 
 
-0.58
 
 
54.4
 
 
 
 
ALG9
 
 
hsa-miR-7-5p
 
 
-0.302
 
 
12.5
 
 
 
 
AMT
 
 
hsa-miR-98-5p
 
 
-0.463
 
 
52.5
 
 
 
 
AMT
 
 
hsa-let-7c-5p
 
 
-0.463
 
 
52.5
 
 
 
 
ANKRD12
 
 
hsa-miR-7-5p
 
 
-0.37
 
 
12.9
 
 
 
 
ANKRD46
 
 
hsa-miR-98-5p
 
 
-0.389
 
 
38.9
 
 
 
 
ANKRD46
 
 
hsa-let-7c-5p
 
 
-0.389
 
 
38.9
 
 
 
 
ANKRD9
 
 
hsa-miR-20a-5p
 
 
-0.31
 
 
15.7
 
 
 
 
ANKRD9
 
 
hsa-miR-106b-5p
 
 
-0.318
 
 
15.7
 
 
 
 
ANKRD9
 
 
hsa-miR-20b-5p
 
 
-0.331
 
 
15.7
 
 
 
 
ANKRD9
 
 
hsa-miR-106a-5p
 
 
-0.344
 
 
15.7
 
 
 
 
ANKRD9
 
 
hsa-miR-17-5p
 
 
-0.347
 
 
15.7
 
 
 
 
AP1AR
 
 
hsa-miR-21-5p
 
 
-0.318
 
 
19.1
 
 
 
 
AP1S1
 
 
hsa-miR-98-5p
 
 
-0.374
 
 
39.1
 
 
 
 
AP1S1
 
 
hsa-let-7c-5p
 
 
-0.374
 
 
39.1
 
 
 
 
ARF4
 
 
hsa-miR-7-5p
 
 
-0.407
 
 
161.3
 
 
 
 
ARF4
 
 
hsa-miR-148a-3p
 
 
-0.487
 
 
161.3
 
 
 
 
ARFGEF1
 
 
hsa-miR-148a-3p
 
 
-0.306
 
 
14.2
 
 
 
 
ARFIP1
 
 
hsa-miR-148a-3p
 
 
-0.474
 
 
14.4
 
 
 
 
ARHGAP1
 
 
hsa-miR-17-5p
 
 
-0.325
 
 
44.2
 
 
 
 
ARHGAP1
 
 
hsa-miR-20b-5p
 
 
-0.325
 
 
44.2
 
 
 
 
ARHGAP1
 
 
hsa-miR-106a-5p
 
 
-0.329
 
 
44.2
 
 
 
 
ARHGAP1
 
 
hsa-miR-20a-5p
 
 
-0.339
 
 
44.2
 
 
 
 
ARHGAP1
 
 
hsa-miR-106b-5p
 
 
-0.339
 
 
44.2
 
 
 
 
ARHGAP21
 
 
hsa-miR-148a-3p
 
 
-0.287
 
 
11.9
 
 
 
 
ARID4A
 
 
hsa-miR-7-5p
 
 
-0.341
 
 
17.5
 
 
 
 
ARID4B
 
 
hsa-miR-20a-5p
 
 
-0.29
 
 
11.1
 
 
 
 
ARID4B
 
 
hsa-miR-106b-5p
 
 
-0.29
 
 
11.1
 
 
 
 
ARL1
 
 
hsa-miR-20a-5p
 
 
-0.403
 
 
64.0
 
 
 
 
ARL1
 
 
hsa-miR-106b-5p
 
 
-0.411
 
 
64.0
 
 
 
 
ARL1
 
 
hsa-miR-20b-5p
 
 
-0.424
 
 
64.0
 
 
 
 
ARL1
 
 
hsa-miR-106a-5p
 
 
-0.429
 
 
64.0
 
 
 
 
ARL1
 
 
hsa-miR-17-5p
 
 
-0.432
 
 
64.0
 
 
 
 
ARL4D
 
 
hsa-miR-98-5p
 
 
-0.417
 
 
55.4
 
 
 
 
ARL4D
 
 
hsa-let-7c-5p
 
 
-0.417
 
 
55.4
 
 
 
 
ARL6IP1
 
 
hsa-miR-7-5p
 
 
-0.374
 
 
336.2
 
 
 
 
ARL6IP1
 
 
hsa-miR-148a-3p
 
 
-0.579
 
 
336.2
 
 
 
 
ARL8B
 
 
hsa-miR-148a-3p
 
 
-0.337
 
 
41.1
 
 
 
 
ARMC8
 
 
hsa-miR-20a-5p
 
 
-0.357
 
 
15.8
 
 
 
 
ARMC8
 
 
hsa-miR-106b-5p
 
 
-0.357
 
 
15.8
 
 
 
 
ARMC8
 
 
hsa-miR-106a-5p
 
 
-0.375
 
 
15.8
 
 
 
 
ARMC8
 
 
hsa-miR-17-5p
 
 
-0.378
 
 
15.8
 
 
 
 
ARMC8
 
 
hsa-miR-20b-5p
 
 
-0.378
 
 
15.8
 
 
 
 
ARMT1
 
 
hsa-let-7c-5p
 
 
-0.303
 
 
35.2
 
 
 
 
ARMT1
 
 
hsa-miR-98-5p
 
 
-0.303
 
 
35.2
 
 
 
 
ARPP19
 
 
hsa-let-7c-5p
 
 
-0.492
 
 
39.5
 
 
 
 
ARPP19
 
 
hsa-miR-98-5p
 
 
-0.5
 
 
39.5
 
 
 
 
ARRDC3
 
 
hsa-miR-148a-3p
 
 
-0.305
 
 
71.0
 
 
 
 
ARRDC4
 
 
hsa-let-7c-5p
 
 
-0.329
 
 
28.3
 
 
 
 
ARRDC4
 
 
hsa-miR-98-5p
 
 
-0.329
 
 
28.3
 
 
 
 
ASF1A
 
 
hsa-miR-21-5p
 
 
-0.304
 
 
11.3
 
 
 
 
ASPH
 
 
hsa-miR-148a-3p
 
 
-0.391
 
 
69.9
 
 
 
 
ATG16L1
 
 
hsa-miR-20a-5p
 
 
-0.446
 
 
14.9
 
 
 
 
ATG16L1
 
 
hsa-miR-106b-5p
 
 
-0.446
 
 
14.9
 
 
 
 
ATG16L1
 
 
hsa-miR-106a-5p
 
 
-0.464
 
 
14.9
 
 
 
 
ATG16L1
 
 
hsa-miR-17-5p
 
 
-0.467
 
 
14.9
 
 
 
 
ATG16L1
 
 
hsa-miR-20b-5p
 
 
-0.467
 
 
14.9
 
 
 
 
ATG4A
 
 
hsa-miR-7-5p
 
 
-0.448
 
 
16.9
 
 
 
 
ATG4D
 
 
hsa-miR-148a-3p
 
 
-0.303
 
 
13.0
 
 
 
 
ATOSB
 
 
hsa-miR-98-5p
 
 
-0.429
 
 
10.9
 
 
 
 
 
 
 lymph node 
 
 
 
 
target_symbol
 
 
mature_mirna_id
 
 
score
 
 
nTPM
 
 
 
 
 
 
ABCB10
 
 
hsa-miR-148a-3p
 
 
-0.291
 
 
10.3
 
 
 
 
ABCB7
 
 
hsa-miR-148a-3p
 
 
-0.515
 
 
11.2
 
 
 
 
ABCD3
 
 
hsa-miR-148a-3p
 
 
-0.388
 
 
12.4
 
 
 
 
ADAM10
 
 
hsa-miR-148a-3p
 
 
-0.356
 
 
20.8
 
 
 
 
AGFG1
 
 
hsa-miR-148a-3p
 
 
-0.332
 
 
44.0
 
 
 
 
AHNAK
 
 
hsa-miR-20a-5p
 
 
-0.316
 
 
34.4
 
 
 
 
AHNAK
 
 
hsa-miR-106b-5p
 
 
-0.316
 
 
34.4
 
 
 
 
AHNAK
 
 
hsa-miR-106a-5p
 
 
-0.334
 
 
34.4
 
 
 
 
AHNAK
 
 
hsa-miR-17-5p
 
 
-0.337
 
 
34.4
 
 
 
 
AHNAK
 
 
hsa-miR-20b-5p
 
 
-0.337
 
 
34.4
 
 
 
 
AIFM1
 
 
hsa-miR-98-5p
 
 
-0.314
 
 
15.1
 
 
 
 
AIFM1
 
 
hsa-let-7c-5p
 
 
-0.314
 
 
15.1
 
 
 
 
AKAP1
 
 
hsa-miR-148a-3p
 
 
-0.381
 
 
10.0
 
 
 
 
ALG3
 
 
hsa-miR-106b-5p
 
 
-0.551
 
 
12.2
 
 
 
 
ALG3
 
 
hsa-miR-20a-5p
 
 
-0.559
 
 
12.2
 
 
 
 
ALG3
 
 
hsa-miR-106a-5p
 
 
-0.569
 
 
12.2
 
 
 
 
ALG3
 
 
hsa-miR-17-5p
 
 
-0.572
 
 
12.2
 
 
 
 
ALG3
 
 
hsa-miR-20b-5p
 
 
-0.58
 
 
12.2
 
 
 
 
ANKRD12
 
 
hsa-miR-7-5p
 
 
-0.37
 
 
20.9
 
 
 
 
ANKRD29
 
 
hsa-miR-20a-5p
 
 
-0.359
 
 
18.8
 
 
 
 
ANKRD29
 
 
hsa-miR-106b-5p
 
 
-0.359
 
 
18.8
 
 
 
 
ANKRD29
 
 
hsa-miR-106a-5p
 
 
-0.369
 
 
18.8
 
 
 
 
ANKRD29
 
 
hsa-miR-17-5p
 
 
-0.372
 
 
18.8
 
 
 
 
ANKRD29
 
 
hsa-miR-20b-5p
 
 
-0.38
 
 
18.8
 
 
 
 
AP1AR
 
 
hsa-miR-21-5p
 
 
-0.318
 
 
11.3
 
 
 
 
AP1S1
 
 
hsa-miR-98-5p
 
 
-0.374
 
 
11.1
 
 
 
 
AP1S1
 
 
hsa-let-7c-5p
 
 
-0.374
 
 
11.1
 
 
 
 
ARF4
 
 
hsa-miR-7-5p
 
 
-0.407
 
 
69.1
 
 
 
 
ARF4
 
 
hsa-miR-148a-3p
 
 
-0.487
 
 
69.1
 
 
 
 
ARFGEF1
 
 
hsa-miR-148a-3p
 
 
-0.306
 
 
14.4
 
 
 
 
ARFIP1
 
 
hsa-miR-148a-3p
 
 
-0.474
 
 
14.1
 
 
 
 
ARHGAP1
 
 
hsa-miR-17-5p
 
 
-0.325
 
 
28.7
 
 
 
 
ARHGAP1
 
 
hsa-miR-20b-5p
 
 
-0.325
 
 
28.7
 
 
 
 
ARHGAP1
 
 
hsa-miR-106a-5p
 
 
-0.329
 
 
28.7
 
 
 
 
ARHGAP1
 
 
hsa-miR-20a-5p
 
 
-0.339
 
 
28.7
 
 
 
 
ARHGAP1
 
 
hsa-miR-106b-5p
 
 
-0.339
 
 
28.7
 
 
 
 
ARID4A
 
 
hsa-miR-7-5p
 
 
-0.341
 
 
14.0
 
 
 
 
ARID4B
 
 
hsa-miR-20a-5p
 
 
-0.29
 
 
19.6
 
 
 
 
ARID4B
 
 
hsa-miR-106b-5p
 
 
-0.29
 
 
19.6
 
 
 
 
ARL1
 
 
hsa-miR-20a-5p
 
 
-0.403
 
 
31.0
 
 
 
 
ARL1
 
 
hsa-miR-106b-5p
 
 
-0.411
 
 
31.0
 
 
 
 
ARL1
 
 
hsa-miR-20b-5p
 
 
-0.424
 
 
31.0
 
 
 
 
ARL1
 
 
hsa-miR-106a-5p
 
 
-0.429
 
 
31.0
 
 
 
 
ARL1
 
 
hsa-miR-17-5p
 
 
-0.432
 
 
31.0
 
 
 
 
ARL6IP1
 
 
hsa-miR-7-5p
 
 
-0.374
 
 
124.0
 
 
 
 
ARL6IP1
 
 
hsa-miR-148a-3p
 
 
-0.579
 
 
124.0
 
 
 
 
ARL6IP6
 
 
hsa-miR-98-5p
 
 
-0.293
 
 
17.4
 
 
 
 
ARL6IP6
 
 
hsa-let-7c-5p
 
 
-0.293
 
 
17.4
 
 
 
 
ARL8B
 
 
hsa-miR-148a-3p
 
 
-0.337
 
 
36.2
 
 
 
 
ARMC8
 
 
hsa-miR-20a-5p
 
 
-0.357
 
 
19.8
 
 
 
 
ARMC8
 
 
hsa-miR-106b-5p
 
 
-0.357
 
 
19.8
 
 
 
 
ARMC8
 
 
hsa-miR-106a-5p
 
 
-0.375
 
 
19.8
 
 
 
 
ARMC8
 
 
hsa-miR-17-5p
 
 
-0.378
 
 
19.8
 
 
 
 
ARMC8
 
 
hsa-miR-20b-5p
 
 
-0.378
 
 
19.8
 
 
 
 
ARMT1
 
 
hsa-let-7c-5p
 
 
-0.303
 
 
19.7
 
 
 
 
ARMT1
 
 
hsa-miR-98-5p
 
 
-0.303
 
 
19.7
 
 
 
 
ARPP19
 
 
hsa-let-7c-5p
 
 
-0.492
 
 
45.1
 
 
 
 
ARPP19
 
 
hsa-miR-98-5p
 
 
-0.5
 
 
45.1
 
 
 
 
ARRDC3
 
 
hsa-miR-148a-3p
 
 
-0.305
 
 
27.5
 
 
 
 
ASAP1
 
 
hsa-miR-98-5p
 
 
-0.308
 
 
16.5
 
 
 
 
ASAP1
 
 
hsa-let-7c-5p
 
 
-0.308
 
 
16.5
 
 
 
 
ASF1A
 
 
hsa-miR-21-5p
 
 
-0.304
 
 
14.9
 
 
 
 
ASPH
 
 
hsa-miR-148a-3p
 
 
-0.391
 
 
18.8
 
 
 
 
ATAD2
 
 
hsa-miR-17-5p
 
 
-0.304
 
 
13.4
 
 
 
 
ATAD2
 
 
hsa-miR-20b-5p
 
 
-0.304
 
 
13.4
 
 
 
 
ATAD2
 
 
hsa-miR-106b-5p
 
 
-0.307
 
 
13.4
 
 
 
 
ATAD2
 
 
hsa-miR-106a-5p
 
 
-0.308
 
 
13.4
 
 
 
 
ATAD2
 
 
hsa-miR-20a-5p
 
 
-0.318
 
 
13.4
 
 
 
 
ATP2A2
 
 
hsa-miR-98-5p
 
 
-0.311
 
 
33.7
 
 
 
 
ATP2A2
 
 
hsa-let-7c-5p
 
 
-0.311
 
 
33.7
 
 
 
 
ATP2B4
 
 
hsa-miR-148a-3p
 
 
-0.337
 
 
12.0
 
 
 
 
ATP6AP2
 
 
hsa-miR-148a-3p
 
 
-0.592
 
 
63.1
 
 
 
 
ATP6AP2
 
 
hsa-miR-148a-3p
 
 
-0.668
 
 
63.1
 
 
 
 
ATPAF1
 
 
hsa-miR-98-5p
 
 
-0.498
 
 
30.3
 
 
 
 
ATPAF1
 
 
hsa-let-7c-5p
 
 
-0.498
 
 
30.3
 
 
 
 
B3GAT3
 
 
hsa-miR-98-5p
 
 
-0.324
 
 
24.9
 
 
 
 
B3GAT3
 
 
hsa-let-7c-5p
 
 
-0.324
 
 
24.9
 
 
 
 
BACH1
 
 
hsa-miR-98-5p
 
 
-0.298
 
 
15.7
 
 
 
 
BACH1
 
 
hsa-miR-98-5p
 
 
-0.298
 
 
15.7
 
 
 
 
BACH1
 
 
hsa-let-7c-5p
 
 
-0.306
 
 
15.7
 
 
 
 
BACH1
 
 
hsa-let-7c-5p
 
 
-0.306
 
 
15.7
 
 
 
 
BACH1
 
 
hsa-miR-98-5p
 
 
-0.435
 
 
15.7
 
 
 
 
BACH1
 
 
hsa-miR-98-5p
 
 
-0.435
 
 
15.7
 
 
 
 
BACH1
 
 
hsa-let-7c-5p
 
 
-0.435
 
 
15.7
 
 
 
 
BACH1
 
 
hsa-let-7c-5p
 
 
-0.435
 
 
15.7
 
 
 
 
BCL2L11
 
 
hsa-miR-148a-3p
 
 
-0.445
 
 
23.7
 
 
 
 
BCL7A
 
 
hsa-miR-21-5p
 
 
-0.31
 
 
15.1
 
 
 
 
BIN3
 
 
hsa-miR-98-5p
 
 
-0.427
 
 
12.8
 
 
 
 
BIN3
 
 
hsa-let-7c-5p
 
 
-0.427
 
 
12.8
 
 
 
 
BLOC1S4
 
 
hsa-miR-7-5p
 
 
-0.417
 
 
11.4
 
 
 
 
BLOC1S4
 
 
hsa-miR-7-5p
 
 
-0.527
 
 
11.4
 
 
 
 
BLOC1S6
 
 
hsa-miR-98-5p
 
 
-0.321
 
 
39.2
 
 
 
 
BLOC1S6
 
 
hsa-let-7c-5p
 
 
-0.321
 
 
39.2
 
 
 
 
BNIP3L
 
 
hsa-miR-20a-5p
 
 
-0.287
 
 
36.9
 
 
 
 
BNIP3L
 
 
hsa-miR-106b-5p
 
 
-0.298
 
 
36.9
 
 
 
 
BTAF1
 
 
hsa-miR-148a-3p
 
 
-0.401
 
 
25.1
 
 
 
 
BTBD10
 
 
hsa-miR-106b-5p
 
 
-0.388
 
 
14.7
 
 
 
 
BTBD10
 
 
hsa-miR-106a-5p
 
 
-0.406
 
 
14.7
 
 
 
 
BTBD10
 
 
hsa-miR-17-5p
 
 
-0.409
 
 
14.7
 
 
 
 
BTBD10
 
 
hsa-miR-20a-5p
 
 
-0.412
 
 
14.7
 
 
 
 
 
 
 placenta 
 
 
 
 
target_symbol
 
 
mature_mirna_id
 
 
score
 
 
nTPM
 
 
 
 
 
 
ABCB10
 
 
hsa-miR-148a-3p
 
 
-0.291
 
 
10.6
 
 
 
 
ABCD3
 
 
hsa-miR-148a-3p
 
 
-0.388
 
 
13.6
 
 
 
 
ACP3
 
 
hsa-miR-98-5p
 
 
-0.298
 
 
10.1
 
 
 
 
ACP3
 
 
hsa-let-7c-5p
 
 
-0.298
 
 
10.1
 
 
 
 
ACVR1
 
 
hsa-miR-148a-3p
 
 
-0.397
 
 
12.5
 
 
 
 
ADAM10
 
 
hsa-miR-148a-3p
 
 
-0.356
 
 
27.1
 
 
 
 
ADAM17
 
 
hsa-miR-148a-3p
 
 
-0.301
 
 
17.9
 
 
 
 
ADAM9
 
 
hsa-miR-20a-5p
 
 
-0.299
 
 
44.6
 
 
 
 
ADAM9
 
 
hsa-miR-106b-5p
 
 
-0.299
 
 
44.6
 
 
 
 
ADAM9
 
 
hsa-miR-106a-5p
 
 
-0.317
 
 
44.6
 
 
 
 
ADAM9
 
 
hsa-miR-17-5p
 
 
-0.32
 
 
44.6
 
 
 
 
ADAM9
 
 
hsa-miR-20b-5p
 
 
-0.328
 
 
44.6
 
 
 
 
ADGRF5
 
 
hsa-miR-148a-3p
 
 
-0.354
 
 
48.3
 
 
 
 
ADRB1
 
 
hsa-let-7c-5p
 
 
-0.351
 
 
27.4
 
 
 
 
ADRB1
 
 
hsa-miR-98-5p
 
 
-0.367
 
 
27.4
 
 
 
 
AGFG1
 
 
hsa-miR-148a-3p
 
 
-0.332
 
 
71.2
 
 
 
 
AHNAK
 
 
hsa-miR-20a-5p
 
 
-0.316
 
 
91.4
 
 
 
 
AHNAK
 
 
hsa-miR-106b-5p
 
 
-0.316
 
 
91.4
 
 
 
 
AHNAK
 
 
hsa-miR-106a-5p
 
 
-0.334
 
 
91.4
 
 
 
 
AHNAK
 
 
hsa-miR-17-5p
 
 
-0.337
 
 
91.4
 
 
 
 
AHNAK
 
 
hsa-miR-20b-5p
 
 
-0.337
 
 
91.4
 
 
 
 
ALCAM
 
 
hsa-miR-148a-3p
 
 
-0.339
 
 
11.3
 
 
 
 
ALDH4A1
 
 
hsa-miR-184
 
 
-0.421
 
 
12.4
 
 
 
 
ALG3
 
 
hsa-miR-106b-5p
 
 
-0.551
 
 
18.4
 
 
 
 
ALG3
 
 
hsa-miR-20a-5p
 
 
-0.559
 
 
18.4
 
 
 
 
ALG3
 
 
hsa-miR-106a-5p
 
 
-0.569
 
 
18.4
 
 
 
 
ALG3
 
 
hsa-miR-17-5p
 
 
-0.572
 
 
18.4
 
 
 
 
ALG3
 
 
hsa-miR-20b-5p
 
 
-0.58
 
 
18.4
 
 
 
 
ANKRD12
 
 
hsa-miR-7-5p
 
 
-0.37
 
 
13.3
 
 
 
 
ANKRD46
 
 
hsa-miR-98-5p
 
 
-0.389
 
 
19.5
 
 
 
 
ANKRD46
 
 
hsa-let-7c-5p
 
 
-0.389
 
 
19.5
 
 
 
 
ANKRD50
 
 
hsa-miR-106a-5p
 
 
-0.29
 
 
13.9
 
 
 
 
ANKRD50
 
 
hsa-miR-20a-5p
 
 
-0.3
 
 
13.9
 
 
 
 
ANKRD50
 
 
hsa-miR-106b-5p
 
 
-0.3
 
 
13.9
 
 
 
 
AP1AR
 
 
hsa-miR-21-5p
 
 
-0.318
 
 
11.2
 
 
 
 
AP1S1
 
 
hsa-miR-98-5p
 
 
-0.374
 
 
14.9
 
 
 
 
AP1S1
 
 
hsa-let-7c-5p
 
 
-0.374
 
 
14.9
 
 
 
 
APCDD1
 
 
hsa-miR-20a-5p
 
 
-0.404
 
 
25.2
 
 
 
 
APCDD1
 
 
hsa-miR-106b-5p
 
 
-0.404
 
 
25.2
 
 
 
 
APCDD1
 
 
hsa-miR-106a-5p
 
 
-0.422
 
 
25.2
 
 
 
 
APCDD1
 
 
hsa-miR-17-5p
 
 
-0.425
 
 
25.2
 
 
 
 
APCDD1
 
 
hsa-miR-20b-5p
 
 
-0.425
 
 
25.2
 
 
 
 
ARF4
 
 
hsa-miR-7-5p
 
 
-0.407
 
 
170.7
 
 
 
 
ARF4
 
 
hsa-miR-148a-3p
 
 
-0.487
 
 
170.7
 
 
 
 
ARFGEF1
 
 
hsa-miR-148a-3p
 
 
-0.306
 
 
13.6
 
 
 
 
ARFIP1
 
 
hsa-miR-148a-3p
 
 
-0.474
 
 
16.9
 
 
 
 
ARHGAP1
 
 
hsa-miR-17-5p
 
 
-0.325
 
 
36.7
 
 
 
 
ARHGAP1
 
 
hsa-miR-20b-5p
 
 
-0.325
 
 
36.7
 
 
 
 
ARHGAP1
 
 
hsa-miR-106a-5p
 
 
-0.329
 
 
36.7
 
 
 
 
ARHGAP1
 
 
hsa-miR-20a-5p
 
 
-0.339
 
 
36.7
 
 
 
 
ARHGAP1
 
 
hsa-miR-106b-5p
 
 
-0.339
 
 
36.7
 
 
 
 
ARHGAP21
 
 
hsa-miR-148a-3p
 
 
-0.287
 
 
15.6
 
 
 
 
ARHGAP24
 
 
hsa-miR-21-5p
 
 
-0.427
 
 
21.0
 
 
 
 
ARHGAP28
 
 
hsa-let-7c-5p
 
 
-0.47
 
 
24.3
 
 
 
 
ARHGAP28
 
 
hsa-miR-98-5p
 
 
-0.47
 
 
24.3
 
 
 
 
ARID3A
 
 
hsa-miR-98-5p
 
 
-0.34
 
 
22.0
 
 
 
 
ARID3A
 
 
hsa-let-7c-5p
 
 
-0.34
 
 
22.0
 
 
 
 
ARID3A
 
 
hsa-let-7c-5p
 
 
-0.347
 
 
22.0
 
 
 
 
ARID3A
 
 
hsa-miR-98-5p
 
 
-0.358
 
 
22.0
 
 
 
 
ARID3B
 
 
hsa-miR-98-5p
 
 
-0.31
 
 
14.7
 
 
 
 
ARID3B
 
 
hsa-let-7c-5p
 
 
-0.31
 
 
14.7
 
 
 
 
ARID3B
 
 
hsa-miR-98-5p
 
 
-0.475
 
 
14.7
 
 
 
 
ARID3B
 
 
hsa-let-7c-5p
 
 
-0.486
 
 
14.7
 
 
 
 
ARID4B
 
 
hsa-miR-20a-5p
 
 
-0.29
 
 
12.0
 
 
 
 
ARID4B
 
 
hsa-miR-106b-5p
 
 
-0.29
 
 
12.0
 
 
 
 
ARL1
 
 
hsa-miR-20a-5p
 
 
-0.403
 
 
48.3
 
 
 
 
ARL1
 
 
hsa-miR-106b-5p
 
 
-0.411
 
 
48.3
 
 
 
 
ARL1
 
 
hsa-miR-20b-5p
 
 
-0.424
 
 
48.3
 
 
 
 
ARL1
 
 
hsa-miR-106a-5p
 
 
-0.429
 
 
48.3
 
 
 
 
ARL1
 
 
hsa-miR-17-5p
 
 
-0.432
 
 
48.3
 
 
 
 
ARL6IP1
 
 
hsa-miR-7-5p
 
 
-0.374
 
 
84.9
 
 
 
 
ARL6IP1
 
 
hsa-miR-148a-3p
 
 
-0.579
 
 
84.9
 
 
 
 
ARL6IP6
 
 
hsa-miR-98-5p
 
 
-0.293
 
 
26.8
 
 
 
 
ARL6IP6
 
 
hsa-let-7c-5p
 
 
-0.293
 
 
26.8
 
 
 
 
ARL8B
 
 
hsa-miR-148a-3p
 
 
-0.337
 
 
46.8
 
 
 
 
ARMC8
 
 
hsa-miR-20a-5p
 
 
-0.357
 
 
18.5
 
 
 
 
ARMC8
 
 
hsa-miR-106b-5p
 
 
-0.357
 
 
18.5
 
 
 
 
ARMC8
 
 
hsa-miR-106a-5p
 
 
-0.375
 
 
18.5
 
 
 
 
ARMC8
 
 
hsa-miR-17-5p
 
 
-0.378
 
 
18.5
 
 
 
 
ARMC8
 
 
hsa-miR-20b-5p
 
 
-0.378
 
 
18.5
 
 
 
 
ARMCX1
 
 
hsa-miR-21-5p
 
 
-0.489
 
 
14.0
 
 
 
 
ARMT1
 
 
hsa-let-7c-5p
 
 
-0.303
 
 
20.8
 
 
 
 
ARMT1
 
 
hsa-miR-98-5p
 
 
-0.303
 
 
20.8
 
 
 
 
ARPP19
 
 
hsa-let-7c-5p
 
 
-0.492
 
 
53.5
 
 
 
 
ARPP19
 
 
hsa-miR-98-5p
 
 
-0.5
 
 
53.5
 
 
 
 
ARRDC3
 
 
hsa-miR-148a-3p
 
 
-0.305
 
 
72.1
 
 
 
 
ARRDC4
 
 
hsa-let-7c-5p
 
 
-0.329
 
 
17.2
 
 
 
 
ARRDC4
 
 
hsa-miR-98-5p
 
 
-0.329
 
 
17.2
 
 
 
 
ASAP1
 
 
hsa-miR-98-5p
 
 
-0.308
 
 
25.9
 
 
 
 
ASAP1
 
 
hsa-let-7c-5p
 
 
-0.308
 
 
25.9
 
 
 
 
ASPH
 
 
hsa-miR-148a-3p
 
 
-0.391
 
 
44.6
 
 
 
 
ATP2A2
 
 
hsa-miR-98-5p
 
 
-0.311
 
 
52.0
 
 
 
 
ATP2A2
 
 
hsa-let-7c-5p
 
 
-0.311
 
 
52.0
 
 
 
 
ATP2B4
 
 
hsa-miR-148a-3p
 
 
-0.337
 
 
38.2
 
 
 
 
ATP6AP2
 
 
hsa-miR-148a-3p
 
 
-0.592
 
 
100.4
 
 
 
 
ATP6AP2
 
 
hsa-miR-148a-3p
 
 
-0.668
 
 
100.4
 
 
 
 
ATPAF1
 
 
hsa-miR-98-5p
 
 
-0.498
 
 
34.1
 
 
 
 
ATPAF1
 
 
hsa-let-7c-5p
 
 
-0.498
 
 
34.1
 
 
 
 
AVPI1
 
 
hsa-miR-7-5p
 
 
-0.367
 
 
15.9
 
 
 
 
B3GAT3
 
 
hsa-miR-98-5p
 
 
-0.324
 
 
18.0
 
 
 
 
 
 
 rectum 
 
 
 
 
target_symbol
 
 
mature_mirna_id
 
 
score
 
 
nTPM
 
 
 
 
 
 
ABCB10
 
 
hsa-miR-148a-3p
 
 
-0.291
 
 
10.6
 
 
 
 
ABCD3
 
 
hsa-miR-148a-3p
 
 
-0.388
 
 
37.8
 
 
 
 
ABHD17C
 
 
hsa-let-7c-5p
 
 
-0.359
 
 
52.8
 
 
 
 
ABHD17C
 
 
hsa-miR-98-5p
 
 
-0.383
 
 
52.8
 
 
 
 
ADAM10
 
 
hsa-miR-148a-3p
 
 
-0.356
 
 
16.1
 
 
 
 
ADAM9
 
 
hsa-miR-20a-5p
 
 
-0.299
 
 
39.2
 
 
 
 
ADAM9
 
 
hsa-miR-106b-5p
 
 
-0.299
 
 
39.2
 
 
 
 
ADAM9
 
 
hsa-miR-106a-5p
 
 
-0.317
 
 
39.2
 
 
 
 
ADAM9
 
 
hsa-miR-17-5p
 
 
-0.32
 
 
39.2
 
 
 
 
ADAM9
 
 
hsa-miR-20b-5p
 
 
-0.328
 
 
39.2
 
 
 
 
AGFG1
 
 
hsa-miR-148a-3p
 
 
-0.332
 
 
31.0
 
 
 
 
AHNAK
 
 
hsa-miR-20a-5p
 
 
-0.316
 
 
90.8
 
 
 
 
AHNAK
 
 
hsa-miR-106b-5p
 
 
-0.316
 
 
90.8
 
 
 
 
AHNAK
 
 
hsa-miR-106a-5p
 
 
-0.334
 
 
90.8
 
 
 
 
AHNAK
 
 
hsa-miR-17-5p
 
 
-0.337
 
 
90.8
 
 
 
 
AHNAK
 
 
hsa-miR-20b-5p
 
 
-0.337
 
 
90.8
 
 
 
 
AIFM1
 
 
hsa-miR-98-5p
 
 
-0.314
 
 
14.9
 
 
 
 
AIFM1
 
 
hsa-let-7c-5p
 
 
-0.314
 
 
14.9
 
 
 
 
AKAP1
 
 
hsa-miR-148a-3p
 
 
-0.381
 
 
45.9
 
 
 
 
ALCAM
 
 
hsa-miR-148a-3p
 
 
-0.339
 
 
13.6
 
 
 
 
ALG3
 
 
hsa-miR-106b-5p
 
 
-0.551
 
 
17.2
 
 
 
 
ALG3
 
 
hsa-miR-20a-5p
 
 
-0.559
 
 
17.2
 
 
 
 
ALG3
 
 
hsa-miR-106a-5p
 
 
-0.569
 
 
17.2
 
 
 
 
ALG3
 
 
hsa-miR-17-5p
 
 
-0.572
 
 
17.2
 
 
 
 
ALG3
 
 
hsa-miR-20b-5p
 
 
-0.58
 
 
17.2
 
 
 
 
AMT
 
 
hsa-miR-98-5p
 
 
-0.463
 
 
11.1
 
 
 
 
AMT
 
 
hsa-let-7c-5p
 
 
-0.463
 
 
11.1
 
 
 
 
ANKRD12
 
 
hsa-miR-7-5p
 
 
-0.37
 
 
11.3
 
 
 
 
AP1AR
 
 
hsa-miR-21-5p
 
 
-0.318
 
 
15.3
 
 
 
 
AP1S1
 
 
hsa-miR-98-5p
 
 
-0.374
 
 
21.1
 
 
 
 
AP1S1
 
 
hsa-let-7c-5p
 
 
-0.374
 
 
21.1
 
 
 
 
APCDD1
 
 
hsa-miR-20a-5p
 
 
-0.404
 
 
12.4
 
 
 
 
APCDD1
 
 
hsa-miR-106b-5p
 
 
-0.404
 
 
12.4
 
 
 
 
APCDD1
 
 
hsa-miR-106a-5p
 
 
-0.422
 
 
12.4
 
 
 
 
APCDD1
 
 
hsa-miR-17-5p
 
 
-0.425
 
 
12.4
 
 
 
 
APCDD1
 
 
hsa-miR-20b-5p
 
 
-0.425
 
 
12.4
 
 
 
 
ARF4
 
 
hsa-miR-7-5p
 
 
-0.407
 
 
185.2
 
 
 
 
ARF4
 
 
hsa-miR-148a-3p
 
 
-0.487
 
 
185.2
 
 
 
 
ARFGEF1
 
 
hsa-miR-148a-3p
 
 
-0.306
 
 
16.1
 
 
 
 
ARFIP1
 
 
hsa-miR-148a-3p
 
 
-0.474
 
 
26.2
 
 
 
 
ARHGAP1
 
 
hsa-miR-17-5p
 
 
-0.325
 
 
30.6
 
 
 
 
ARHGAP1
 
 
hsa-miR-20b-5p
 
 
-0.325
 
 
30.6
 
 
 
 
ARHGAP1
 
 
hsa-miR-106a-5p
 
 
-0.329
 
 
30.6
 
 
 
 
ARHGAP1
 
 
hsa-miR-20a-5p
 
 
-0.339
 
 
30.6
 
 
 
 
ARHGAP1
 
 
hsa-miR-106b-5p
 
 
-0.339
 
 
30.6
 
 
 
 
ARHGAP21
 
 
hsa-miR-148a-3p
 
 
-0.287
 
 
17.4
 
 
 
 
ARL1
 
 
hsa-miR-20a-5p
 
 
-0.403
 
 
56.8
 
 
 
 
ARL1
 
 
hsa-miR-106b-5p
 
 
-0.411
 
 
56.8
 
 
 
 
ARL1
 
 
hsa-miR-20b-5p
 
 
-0.424
 
 
56.8
 
 
 
 
ARL1
 
 
hsa-miR-106a-5p
 
 
-0.429
 
 
56.8
 
 
 
 
ARL1
 
 
hsa-miR-17-5p
 
 
-0.432
 
 
56.8
 
 
 
 
ARL6IP1
 
 
hsa-miR-7-5p
 
 
-0.374
 
 
116.0
 
 
 
 
ARL6IP1
 
 
hsa-miR-148a-3p
 
 
-0.579
 
 
116.0
 
 
 
 
ARL6IP6
 
 
hsa-miR-98-5p
 
 
-0.293
 
 
13.3
 
 
 
 
ARL6IP6
 
 
hsa-let-7c-5p
 
 
-0.293
 
 
13.3
 
 
 
 
ARL8B
 
 
hsa-miR-148a-3p
 
 
-0.337
 
 
32.7
 
 
 
 
ARMC8
 
 
hsa-miR-20a-5p
 
 
-0.357
 
 
17.2
 
 
 
 
ARMC8
 
 
hsa-miR-106b-5p
 
 
-0.357
 
 
17.2
 
 
 
 
ARMC8
 
 
hsa-miR-106a-5p
 
 
-0.375
 
 
17.2
 
 
 
 
ARMC8
 
 
hsa-miR-17-5p
 
 
-0.378
 
 
17.2
 
 
 
 
ARMC8
 
 
hsa-miR-20b-5p
 
 
-0.378
 
 
17.2
 
 
 
 
ARMT1
 
 
hsa-let-7c-5p
 
 
-0.303
 
 
16.5
 
 
 
 
ARMT1
 
 
hsa-miR-98-5p
 
 
-0.303
 
 
16.5
 
 
 
 
ARPP19
 
 
hsa-let-7c-5p
 
 
-0.492
 
 
33.6
 
 
 
 
ARPP19
 
 
hsa-miR-98-5p
 
 
-0.5
 
 
33.6
 
 
 
 
ARRDC3
 
 
hsa-miR-148a-3p
 
 
-0.305
 
 
25.8
 
 
 
 
ARRDC4
 
 
hsa-let-7c-5p
 
 
-0.329
 
 
21.6
 
 
 
 
ARRDC4
 
 
hsa-miR-98-5p
 
 
-0.329
 
 
21.6
 
 
 
 
ASF1A
 
 
hsa-miR-21-5p
 
 
-0.304
 
 
10.2
 
 
 
 
ASPH
 
 
hsa-miR-148a-3p
 
 
-0.391
 
 
77.9
 
 
 
 
ATG4A
 
 
hsa-miR-7-5p
 
 
-0.448
 
 
12.3
 
 
 
 
ATP2A2
 
 
hsa-miR-98-5p
 
 
-0.311
 
 
64.9
 
 
 
 
ATP2A2
 
 
hsa-let-7c-5p
 
 
-0.311
 
 
64.9
 
 
 
 
ATP2B4
 
 
hsa-miR-148a-3p
 
 
-0.337
 
 
28.6
 
 
 
 
ATP6AP2
 
 
hsa-miR-148a-3p
 
 
-0.592
 
 
83.6
 
 
 
 
ATP6AP2
 
 
hsa-miR-148a-3p
 
 
-0.668
 
 
83.6
 
 
 
 
ATPAF1
 
 
hsa-miR-98-5p
 
 
-0.498
 
 
50.0
 
 
 
 
ATPAF1
 
 
hsa-let-7c-5p
 
 
-0.498
 
 
50.0
 
 
 
 
AVPI1
 
 
hsa-miR-7-5p
 
 
-0.367
 
 
17.8
 
 
 
 
B4GALT5
 
 
hsa-miR-148a-3p
 
 
-0.44
 
 
22.3
 
 
 
 
BACH1
 
 
hsa-miR-98-5p
 
 
-0.298
 
 
12.4
 
 
 
 
BACH1
 
 
hsa-miR-98-5p
 
 
-0.298
 
 
12.4
 
 
 
 
BACH1
 
 
hsa-let-7c-5p
 
 
-0.306
 
 
12.4
 
 
 
 
BACH1
 
 
hsa-let-7c-5p
 
 
-0.306
 
 
12.4
 
 
 
 
BACH1
 
 
hsa-miR-98-5p
 
 
-0.435
 
 
12.4
 
 
 
 
BACH1
 
 
hsa-miR-98-5p
 
 
-0.435
 
 
12.4
 
 
 
 
BACH1
 
 
hsa-let-7c-5p
 
 
-0.435
 
 
12.4
 
 
 
 
BACH1
 
 
hsa-let-7c-5p
 
 
-0.435
 
 
12.4
 
 
 
 
BCL2L11
 
 
hsa-miR-148a-3p
 
 
-0.445
 
 
11.2
 
 
 
 
BHLHE41
 
 
hsa-miR-148a-3p
 
 
-0.442
 
 
10.4
 
 
 
 
BLOC1S6
 
 
hsa-miR-98-5p
 
 
-0.321
 
 
20.2
 
 
 
 
BLOC1S6
 
 
hsa-let-7c-5p
 
 
-0.321
 
 
20.2
 
 
 
 
BNIP3L
 
 
hsa-miR-20a-5p
 
 
-0.287
 
 
26.5
 
 
 
 
BNIP3L
 
 
hsa-miR-106b-5p
 
 
-0.298
 
 
26.5
 
 
 
 
BTAF1
 
 
hsa-miR-148a-3p
 
 
-0.401
 
 
12.5
 
 
 
 
BTBD10
 
 
hsa-miR-106b-5p
 
 
-0.388
 
 
19.1
 
 
 
 
BTBD10
 
 
hsa-miR-106a-5p
 
 
-0.406
 
 
19.1
 
 
 
 
BTBD10
 
 
hsa-miR-17-5p
 
 
-0.409
 
 
19.1
 
 
 
 
BTBD10
 
 
hsa-miR-20a-5p
 
 
-0.412
 
 
19.1
 
 
 
 
BTBD10
 
 
hsa-miR-20b-5p
 
 
-0.425
 
 
19.1
 
 
 
 
 
 
 skeletal muscle 
 
 
 
 
target_symbol
 
 
mature_mirna_id
 
 
score
 
 
nTPM
 
 
 
 
 
 
ABCB10
 
 
hsa-miR-148a-3p
 
 
-0.291
 
 
12.0
 
 
 
 
ABCB7
 
 
hsa-miR-148a-3p
 
 
-0.515
 
 
29.2
 
 
 
 
ABCD3
 
 
hsa-miR-148a-3p
 
 
-0.388
 
 
22.0
 
 
 
 
ABHD5
 
 
hsa-miR-20a-5p
 
 
-0.397
 
 
12.3
 
 
 
 
ABHD5
 
 
hsa-miR-106b-5p
 
 
-0.413
 
 
12.3
 
 
 
 
ABHD5
 
 
hsa-miR-20b-5p
 
 
-0.418
 
 
12.3
 
 
 
 
ABHD5
 
 
hsa-miR-106a-5p
 
 
-0.447
 
 
12.3
 
 
 
 
ABHD5
 
 
hsa-miR-17-5p
 
 
-0.45
 
 
12.3
 
 
 
 
ACTC1
 
 
hsa-miR-7-5p
 
 
-0.471
 
 
519.7
 
 
 
 
ACVR1
 
 
hsa-miR-148a-3p
 
 
-0.397
 
 
15.5
 
 
 
 
ADAM9
 
 
hsa-miR-20a-5p
 
 
-0.299
 
 
16.2
 
 
 
 
ADAM9
 
 
hsa-miR-106b-5p
 
 
-0.299
 
 
16.2
 
 
 
 
ADAM9
 
 
hsa-miR-106a-5p
 
 
-0.317
 
 
16.2
 
 
 
 
ADAM9
 
 
hsa-miR-17-5p
 
 
-0.32
 
 
16.2
 
 
 
 
ADAM9
 
 
hsa-miR-20b-5p
 
 
-0.328
 
 
16.2
 
 
 
 
ADCY2
 
 
hsa-miR-148a-3p
 
 
-0.299
 
 
64.1
 
 
 
 
ADGRF5
 
 
hsa-miR-148a-3p
 
 
-0.354
 
 
29.0
 
 
 
 
ADRB2
 
 
hsa-let-7c-5p
 
 
-0.635
 
 
10.9
 
 
 
 
ADRB2
 
 
hsa-miR-98-5p
 
 
-0.635
 
 
10.9
 
 
 
 
AGFG1
 
 
hsa-miR-148a-3p
 
 
-0.332
 
 
31.6
 
 
 
 
AGO2
 
 
hsa-miR-21-5p
 
 
-0.632
 
 
16.4
 
 
 
 
AGO2
 
 
hsa-miR-184
 
 
-0.831
 
 
16.4
 
 
 
 
AHCTF1
 
 
hsa-miR-98-5p
 
 
-0.374
 
 
10.0
 
 
 
 
AHCTF1
 
 
hsa-let-7c-5p
 
 
-0.374
 
 
10.0
 
 
 
 
AHDC1
 
 
hsa-miR-148a-3p
 
 
-0.413
 
 
29.2
 
 
 
 
AHNAK
 
 
hsa-miR-20a-5p
 
 
-0.316
 
 
178.0
 
 
 
 
AHNAK
 
 
hsa-miR-106b-5p
 
 
-0.316
 
 
178.0
 
 
 
 
AHNAK
 
 
hsa-miR-106a-5p
 
 
-0.334
 
 
178.0
 
 
 
 
AHNAK
 
 
hsa-miR-17-5p
 
 
-0.337
 
 
178.0
 
 
 
 
AHNAK
 
 
hsa-miR-20b-5p
 
 
-0.337
 
 
178.0
 
 
 
 
AIFM1
 
 
hsa-miR-98-5p
 
 
-0.314
 
 
58.6
 
 
 
 
AIFM1
 
 
hsa-let-7c-5p
 
 
-0.314
 
 
58.6
 
 
 
 
AKAP1
 
 
hsa-miR-148a-3p
 
 
-0.381
 
 
194.7
 
 
 
 
AKAP6
 
 
hsa-miR-98-5p
 
 
-0.307
 
 
44.4
 
 
 
 
AKAP6
 
 
hsa-let-7c-5p
 
 
-0.307
 
 
44.4
 
 
 
 
ALDH4A1
 
 
hsa-miR-184
 
 
-0.421
 
 
69.5
 
 
 
 
ALG3
 
 
hsa-miR-106b-5p
 
 
-0.551
 
 
29.0
 
 
 
 
ALG3
 
 
hsa-miR-20a-5p
 
 
-0.559
 
 
29.0
 
 
 
 
ALG3
 
 
hsa-miR-106a-5p
 
 
-0.569
 
 
29.0
 
 
 
 
ALG3
 
 
hsa-miR-17-5p
 
 
-0.572
 
 
29.0
 
 
 
 
ALG3
 
 
hsa-miR-20b-5p
 
 
-0.58
 
 
29.0
 
 
 
 
ANKRD12
 
 
hsa-miR-7-5p
 
 
-0.37
 
 
25.1
 
 
 
 
ANKRD46
 
 
hsa-miR-98-5p
 
 
-0.389
 
 
58.3
 
 
 
 
ANKRD46
 
 
hsa-let-7c-5p
 
 
-0.389
 
 
58.3
 
 
 
 
ANKRD9
 
 
hsa-miR-20a-5p
 
 
-0.31
 
 
148.8
 
 
 
 
ANKRD9
 
 
hsa-miR-106b-5p
 
 
-0.318
 
 
148.8
 
 
 
 
ANKRD9
 
 
hsa-miR-20b-5p
 
 
-0.331
 
 
148.8
 
 
 
 
ANKRD9
 
 
hsa-miR-106a-5p
 
 
-0.344
 
 
148.8
 
 
 
 
ANKRD9
 
 
hsa-miR-17-5p
 
 
-0.347
 
 
148.8
 
 
 
 
ARF4
 
 
hsa-miR-7-5p
 
 
-0.407
 
 
59.5
 
 
 
 
ARF4
 
 
hsa-miR-148a-3p
 
 
-0.487
 
 
59.5
 
 
 
 
ARFGEF1
 
 
hsa-miR-148a-3p
 
 
-0.306
 
 
12.2
 
 
 
 
ARG2
 
 
hsa-miR-98-5p
 
 
-0.468
 
 
37.4
 
 
 
 
ARG2
 
 
hsa-let-7c-5p
 
 
-0.468
 
 
37.4
 
 
 
 
ARHGAP1
 
 
hsa-miR-17-5p
 
 
-0.325
 
 
28.5
 
 
 
 
ARHGAP1
 
 
hsa-miR-20b-5p
 
 
-0.325
 
 
28.5
 
 
 
 
ARHGAP1
 
 
hsa-miR-106a-5p
 
 
-0.329
 
 
28.5
 
 
 
 
ARHGAP1
 
 
hsa-miR-20a-5p
 
 
-0.339
 
 
28.5
 
 
 
 
ARHGAP1
 
 
hsa-miR-106b-5p
 
 
-0.339
 
 
28.5
 
 
 
 
ARHGAP21
 
 
hsa-miR-148a-3p
 
 
-0.287
 
 
15.7
 
 
 
 
ARL1
 
 
hsa-miR-20a-5p
 
 
-0.403
 
 
48.7
 
 
 
 
ARL1
 
 
hsa-miR-106b-5p
 
 
-0.411
 
 
48.7
 
 
 
 
ARL1
 
 
hsa-miR-20b-5p
 
 
-0.424
 
 
48.7
 
 
 
 
ARL1
 
 
hsa-miR-106a-5p
 
 
-0.429
 
 
48.7
 
 
 
 
ARL1
 
 
hsa-miR-17-5p
 
 
-0.432
 
 
48.7
 
 
 
 
ARL6IP1
 
 
hsa-miR-7-5p
 
 
-0.374
 
 
21.6
 
 
 
 
ARL6IP1
 
 
hsa-miR-148a-3p
 
 
-0.579
 
 
21.6
 
 
 
 
ARL6IP6
 
 
hsa-miR-98-5p
 
 
-0.293
 
 
53.9
 
 
 
 
ARL6IP6
 
 
hsa-let-7c-5p
 
 
-0.293
 
 
53.9
 
 
 
 
ARL8B
 
 
hsa-miR-148a-3p
 
 
-0.337
 
 
98.5
 
 
 
 
ARMC8
 
 
hsa-miR-20a-5p
 
 
-0.357
 
 
36.6
 
 
 
 
ARMC8
 
 
hsa-miR-106b-5p
 
 
-0.357
 
 
36.6
 
 
 
 
ARMC8
 
 
hsa-miR-106a-5p
 
 
-0.375
 
 
36.6
 
 
 
 
ARMC8
 
 
hsa-miR-17-5p
 
 
-0.378
 
 
36.6
 
 
 
 
ARMC8
 
 
hsa-miR-20b-5p
 
 
-0.378
 
 
36.6
 
 
 
 
ARMCX1
 
 
hsa-miR-21-5p
 
 
-0.489
 
 
25.9
 
 
 
 
ARMT1
 
 
hsa-let-7c-5p
 
 
-0.303
 
 
17.8
 
 
 
 
ARMT1
 
 
hsa-miR-98-5p
 
 
-0.303
 
 
17.8
 
 
 
 
ARPP19
 
 
hsa-let-7c-5p
 
 
-0.492
 
 
225.3
 
 
 
 
ARPP19
 
 
hsa-miR-98-5p
 
 
-0.5
 
 
225.3
 
 
 
 
ARRDC3
 
 
hsa-miR-148a-3p
 
 
-0.305
 
 
31.8
 
 
 
 
ARRDC4
 
 
hsa-let-7c-5p
 
 
-0.329
 
 
13.8
 
 
 
 
ARRDC4
 
 
hsa-miR-98-5p
 
 
-0.329
 
 
13.8
 
 
 
 
ASAP1
 
 
hsa-miR-98-5p
 
 
-0.308
 
 
12.4
 
 
 
 
ASAP1
 
 
hsa-let-7c-5p
 
 
-0.308
 
 
12.4
 
 
 
 
ASF1A
 
 
hsa-miR-21-5p
 
 
-0.304
 
 
15.6
 
 
 
 
ASPH
 
 
hsa-miR-148a-3p
 
 
-0.391
 
 
331.0
 
 
 
 
ATG16L1
 
 
hsa-miR-20a-5p
 
 
-0.446
 
 
20.1
 
 
 
 
ATG16L1
 
 
hsa-miR-106b-5p
 
 
-0.446
 
 
20.1
 
 
 
 
ATG16L1
 
 
hsa-miR-106a-5p
 
 
-0.464
 
 
20.1
 
 
 
 
ATG16L1
 
 
hsa-miR-17-5p
 
 
-0.467
 
 
20.1
 
 
 
 
ATG16L1
 
 
hsa-miR-20b-5p
 
 
-0.467
 
 
20.1
 
 
 
 
ATG4A
 
 
hsa-miR-7-5p
 
 
-0.448
 
 
18.0
 
 
 
 
ATG4D
 
 
hsa-miR-148a-3p
 
 
-0.303
 
 
35.5
 
 
 
 
ATOSB
 
 
hsa-miR-98-5p
 
 
-0.429
 
 
13.5
 
 
 
 
ATOSB
 
 
hsa-let-7c-5p
 
 
-0.429
 
 
13.5
 
 
 
 
ATP2A2
 
 
hsa-miR-98-5p
 
 
-0.311
 
 
2675.7
 
 
 
 
ATP2A2
 
 
hsa-let-7c-5p
 
 
-0.311
 
 
2675.7
 
 
 
 
ATP2B2
 
 
hsa-miR-7-5p
 
 
-0.297
 
 
35.1
 
 
 
 
ATP2B4
 
 
hsa-miR-148a-3p
 
 
-0.337
 
 
13.5
 
 
 
 
 
 
 small intestine 
 
 
 
 
target_symbol
 
 
mature_mirna_id
 
 
score
 
 
nTPM
 
 
 
 
 
 
ABCB10
 
 
hsa-miR-148a-3p
 
 
-0.291
 
 
13.1
 
 
 
 
ABCB7
 
 
hsa-miR-148a-3p
 
 
-0.515
 
 
11.2
 
 
 
 
ABCD3
 
 
hsa-miR-148a-3p
 
 
-0.388
 
 
43.4
 
 
 
 
ABHD17C
 
 
hsa-let-7c-5p
 
 
-0.359
 
 
28.8
 
 
 
 
ABHD17C
 
 
hsa-miR-98-5p
 
 
-0.383
 
 
28.8
 
 
 
 
ADAM10
 
 
hsa-miR-148a-3p
 
 
-0.356
 
 
15.8
 
 
 
 
ADAM9
 
 
hsa-miR-20a-5p
 
 
-0.299
 
 
21.4
 
 
 
 
ADAM9
 
 
hsa-miR-106b-5p
 
 
-0.299
 
 
21.4
 
 
 
 
ADAM9
 
 
hsa-miR-106a-5p
 
 
-0.317
 
 
21.4
 
 
 
 
ADAM9
 
 
hsa-miR-17-5p
 
 
-0.32
 
 
21.4
 
 
 
 
ADAM9
 
 
hsa-miR-20b-5p
 
 
-0.328
 
 
21.4
 
 
 
 
AGFG1
 
 
hsa-miR-148a-3p
 
 
-0.332
 
 
33.9
 
 
 
 
AGFG2
 
 
hsa-miR-20a-5p
 
 
-0.365
 
 
12.5
 
 
 
 
AGFG2
 
 
hsa-miR-20b-5p
 
 
-0.386
 
 
12.5
 
 
 
 
AGFG2
 
 
hsa-miR-106b-5p
 
 
-0.397
 
 
12.5
 
 
 
 
AGFG2
 
 
hsa-miR-106a-5p
 
 
-0.399
 
 
12.5
 
 
 
 
AGFG2
 
 
hsa-miR-17-5p
 
 
-0.402
 
 
12.5
 
 
 
 
AHNAK
 
 
hsa-miR-20a-5p
 
 
-0.316
 
 
132.1
 
 
 
 
AHNAK
 
 
hsa-miR-106b-5p
 
 
-0.316
 
 
132.1
 
 
 
 
AHNAK
 
 
hsa-miR-106a-5p
 
 
-0.334
 
 
132.1
 
 
 
 
AHNAK
 
 
hsa-miR-17-5p
 
 
-0.337
 
 
132.1
 
 
 
 
AHNAK
 
 
hsa-miR-20b-5p
 
 
-0.337
 
 
132.1
 
 
 
 
AIFM1
 
 
hsa-miR-98-5p
 
 
-0.314
 
 
28.2
 
 
 
 
AIFM1
 
 
hsa-let-7c-5p
 
 
-0.314
 
 
28.2
 
 
 
 
AKAP1
 
 
hsa-miR-148a-3p
 
 
-0.381
 
 
52.5
 
 
 
 
ALCAM
 
 
hsa-miR-148a-3p
 
 
-0.339
 
 
16.4
 
 
 
 
ALG3
 
 
hsa-miR-106b-5p
 
 
-0.551
 
 
19.2
 
 
 
 
ALG3
 
 
hsa-miR-20a-5p
 
 
-0.559
 
 
19.2
 
 
 
 
ALG3
 
 
hsa-miR-106a-5p
 
 
-0.569
 
 
19.2
 
 
 
 
ALG3
 
 
hsa-miR-17-5p
 
 
-0.572
 
 
19.2
 
 
 
 
ALG3
 
 
hsa-miR-20b-5p
 
 
-0.58
 
 
19.2
 
 
 
 
AMT
 
 
hsa-miR-98-5p
 
 
-0.463
 
 
11.5
 
 
 
 
AMT
 
 
hsa-let-7c-5p
 
 
-0.463
 
 
11.5
 
 
 
 
ANKRD12
 
 
hsa-miR-7-5p
 
 
-0.37
 
 
12.6
 
 
 
 
ANKRD46
 
 
hsa-miR-98-5p
 
 
-0.389
 
 
10.4
 
 
 
 
ANKRD46
 
 
hsa-let-7c-5p
 
 
-0.389
 
 
10.4
 
 
 
 
ANKRD9
 
 
hsa-miR-20a-5p
 
 
-0.31
 
 
31.1
 
 
 
 
ANKRD9
 
 
hsa-miR-106b-5p
 
 
-0.318
 
 
31.1
 
 
 
 
ANKRD9
 
 
hsa-miR-20b-5p
 
 
-0.331
 
 
31.1
 
 
 
 
ANKRD9
 
 
hsa-miR-106a-5p
 
 
-0.344
 
 
31.1
 
 
 
 
ANKRD9
 
 
hsa-miR-17-5p
 
 
-0.347
 
 
31.1
 
 
 
 
AP1AR
 
 
hsa-miR-21-5p
 
 
-0.318
 
 
22.4
 
 
 
 
AP1S1
 
 
hsa-miR-98-5p
 
 
-0.374
 
 
29.8
 
 
 
 
AP1S1
 
 
hsa-let-7c-5p
 
 
-0.374
 
 
29.8
 
 
 
 
APCDD1
 
 
hsa-miR-20a-5p
 
 
-0.404
 
 
13.7
 
 
 
 
APCDD1
 
 
hsa-miR-106b-5p
 
 
-0.404
 
 
13.7
 
 
 
 
APCDD1
 
 
hsa-miR-106a-5p
 
 
-0.422
 
 
13.7
 
 
 
 
APCDD1
 
 
hsa-miR-17-5p
 
 
-0.425
 
 
13.7
 
 
 
 
APCDD1
 
 
hsa-miR-20b-5p
 
 
-0.425
 
 
13.7
 
 
 
 
ARF4
 
 
hsa-miR-7-5p
 
 
-0.407
 
 
102.8
 
 
 
 
ARF4
 
 
hsa-miR-148a-3p
 
 
-0.487
 
 
102.8
 
 
 
 
ARFGEF1
 
 
hsa-miR-148a-3p
 
 
-0.306
 
 
16.5
 
 
 
 
ARFIP1
 
 
hsa-miR-148a-3p
 
 
-0.474
 
 
20.0
 
 
 
 
ARG2
 
 
hsa-miR-98-5p
 
 
-0.468
 
 
14.4
 
 
 
 
ARG2
 
 
hsa-let-7c-5p
 
 
-0.468
 
 
14.4
 
 
 
 
ARHGAP1
 
 
hsa-miR-17-5p
 
 
-0.325
 
 
44.5
 
 
 
 
ARHGAP1
 
 
hsa-miR-20b-5p
 
 
-0.325
 
 
44.5
 
 
 
 
ARHGAP1
 
 
hsa-miR-106a-5p
 
 
-0.329
 
 
44.5
 
 
 
 
ARHGAP1
 
 
hsa-miR-20a-5p
 
 
-0.339
 
 
44.5
 
 
 
 
ARHGAP1
 
 
hsa-miR-106b-5p
 
 
-0.339
 
 
44.5
 
 
 
 
ARHGAP21
 
 
hsa-miR-148a-3p
 
 
-0.287
 
 
15.2
 
 
 
 
ARL1
 
 
hsa-miR-20a-5p
 
 
-0.403
 
 
40.7
 
 
 
 
ARL1
 
 
hsa-miR-106b-5p
 
 
-0.411
 
 
40.7
 
 
 
 
ARL1
 
 
hsa-miR-20b-5p
 
 
-0.424
 
 
40.7
 
 
 
 
ARL1
 
 
hsa-miR-106a-5p
 
 
-0.429
 
 
40.7
 
 
 
 
ARL1
 
 
hsa-miR-17-5p
 
 
-0.432
 
 
40.7
 
 
 
 
ARL6IP1
 
 
hsa-miR-7-5p
 
 
-0.374
 
 
116.2
 
 
 
 
ARL6IP1
 
 
hsa-miR-148a-3p
 
 
-0.579
 
 
116.2
 
 
 
 
ARL8B
 
 
hsa-miR-148a-3p
 
 
-0.337
 
 
32.3
 
 
 
 
ARMC8
 
 
hsa-miR-20a-5p
 
 
-0.357
 
 
12.8
 
 
 
 
ARMC8
 
 
hsa-miR-106b-5p
 
 
-0.357
 
 
12.8
 
 
 
 
ARMC8
 
 
hsa-miR-106a-5p
 
 
-0.375
 
 
12.8
 
 
 
 
ARMC8
 
 
hsa-miR-17-5p
 
 
-0.378
 
 
12.8
 
 
 
 
ARMC8
 
 
hsa-miR-20b-5p
 
 
-0.378
 
 
12.8
 
 
 
 
ARMCX1
 
 
hsa-miR-21-5p
 
 
-0.489
 
 
11.5
 
 
 
 
ARMT1
 
 
hsa-let-7c-5p
 
 
-0.303
 
 
13.7
 
 
 
 
ARMT1
 
 
hsa-miR-98-5p
 
 
-0.303
 
 
13.7
 
 
 
 
ARPP19
 
 
hsa-let-7c-5p
 
 
-0.492
 
 
28.8
 
 
 
 
ARPP19
 
 
hsa-miR-98-5p
 
 
-0.5
 
 
28.8
 
 
 
 
ARRDC3
 
 
hsa-miR-148a-3p
 
 
-0.305
 
 
15.8
 
 
 
 
ARRDC4
 
 
hsa-let-7c-5p
 
 
-0.329
 
 
22.0
 
 
 
 
ARRDC4
 
 
hsa-miR-98-5p
 
 
-0.329
 
 
22.0
 
 
 
 
ASAP1
 
 
hsa-miR-98-5p
 
 
-0.308
 
 
11.5
 
 
 
 
ASAP1
 
 
hsa-let-7c-5p
 
 
-0.308
 
 
11.5
 
 
 
 
ASPH
 
 
hsa-miR-148a-3p
 
 
-0.391
 
 
42.6
 
 
 
 
ATG16L1
 
 
hsa-miR-20a-5p
 
 
-0.446
 
 
11.3
 
 
 
 
ATG16L1
 
 
hsa-miR-106b-5p
 
 
-0.446
 
 
11.3
 
 
 
 
ATG16L1
 
 
hsa-miR-106a-5p
 
 
-0.464
 
 
11.3
 
 
 
 
ATG16L1
 
 
hsa-miR-17-5p
 
 
-0.467
 
 
11.3
 
 
 
 
ATG16L1
 
 
hsa-miR-20b-5p
 
 
-0.467
 
 
11.3
 
 
 
 
ATG4A
 
 
hsa-miR-7-5p
 
 
-0.448
 
 
10.7
 
 
 
 
ATG4D
 
 
hsa-miR-148a-3p
 
 
-0.303
 
 
21.8
 
 
 
 
ATOSB
 
 
hsa-miR-98-5p
 
 
-0.429
 
 
10.7
 
 
 
 
ATOSB
 
 
hsa-let-7c-5p
 
 
-0.429
 
 
10.7
 
 
 
 
ATP2A2
 
 
hsa-miR-98-5p
 
 
-0.311
 
 
53.2
 
 
 
 
ATP2A2
 
 
hsa-let-7c-5p
 
 
-0.311
 
 
53.2
 
 
 
 
ATP2B4
 
 
hsa-miR-148a-3p
 
 
-0.337
 
 
13.8
 
 
 
 
ATP6AP2
 
 
hsa-miR-148a-3p
 
 
-0.592
 
 
45.0
 
 
 
 
ATP6AP2
 
 
hsa-miR-148a-3p
 
 
-0.668
 
 
45.0
 
 
 
 
ATPAF1
 
 
hsa-miR-98-5p
 
 
-0.498
 
 
42.6
 
 
 
 
 
 
 tongue 
 
 
 
 
target_symbol
 
 
mature_mirna_id
 
 
score
 
 
nTPM
 
 
 
 
 
 
ABCB10
 
 
hsa-miR-148a-3p
 
 
-0.291
 
 
10.2
 
 
 
 
ABCB7
 
 
hsa-miR-148a-3p
 
 
-0.515
 
 
20.8
 
 
 
 
ABCD3
 
 
hsa-miR-148a-3p
 
 
-0.388
 
 
27.9
 
 
 
 
ABHD5
 
 
hsa-miR-20a-5p
 
 
-0.397
 
 
17.3
 
 
 
 
ABHD5
 
 
hsa-miR-106b-5p
 
 
-0.413
 
 
17.3
 
 
 
 
ABHD5
 
 
hsa-miR-20b-5p
 
 
-0.418
 
 
17.3
 
 
 
 
ABHD5
 
 
hsa-miR-106a-5p
 
 
-0.447
 
 
17.3
 
 
 
 
ABHD5
 
 
hsa-miR-17-5p
 
 
-0.45
 
 
17.3
 
 
 
 
ACTC1
 
 
hsa-miR-7-5p
 
 
-0.471
 
 
182.1
 
 
 
 
ADAM9
 
 
hsa-miR-20a-5p
 
 
-0.299
 
 
23.3
 
 
 
 
ADAM9
 
 
hsa-miR-106b-5p
 
 
-0.299
 
 
23.3
 
 
 
 
ADAM9
 
 
hsa-miR-106a-5p
 
 
-0.317
 
 
23.3
 
 
 
 
ADAM9
 
 
hsa-miR-17-5p
 
 
-0.32
 
 
23.3
 
 
 
 
ADAM9
 
 
hsa-miR-20b-5p
 
 
-0.328
 
 
23.3
 
 
 
 
ADCY2
 
 
hsa-miR-148a-3p
 
 
-0.299
 
 
28.8
 
 
 
 
ADGRF5
 
 
hsa-miR-148a-3p
 
 
-0.354
 
 
66.5
 
 
 
 
AGFG1
 
 
hsa-miR-148a-3p
 
 
-0.332
 
 
36.1
 
 
 
 
AHCTF1
 
 
hsa-miR-98-5p
 
 
-0.374
 
 
14.7
 
 
 
 
AHCTF1
 
 
hsa-let-7c-5p
 
 
-0.374
 
 
14.7
 
 
 
 
AHDC1
 
 
hsa-miR-148a-3p
 
 
-0.413
 
 
14.5
 
 
 
 
AHNAK
 
 
hsa-miR-20a-5p
 
 
-0.316
 
 
160.9
 
 
 
 
AHNAK
 
 
hsa-miR-106b-5p
 
 
-0.316
 
 
160.9
 
 
 
 
AHNAK
 
 
hsa-miR-106a-5p
 
 
-0.334
 
 
160.9
 
 
 
 
AHNAK
 
 
hsa-miR-17-5p
 
 
-0.337
 
 
160.9
 
 
 
 
AHNAK
 
 
hsa-miR-20b-5p
 
 
-0.337
 
 
160.9
 
 
 
 
AIFM1
 
 
hsa-miR-98-5p
 
 
-0.314
 
 
23.4
 
 
 
 
AIFM1
 
 
hsa-let-7c-5p
 
 
-0.314
 
 
23.4
 
 
 
 
AKAP1
 
 
hsa-miR-148a-3p
 
 
-0.381
 
 
128.4
 
 
 
 
AKAP6
 
 
hsa-miR-98-5p
 
 
-0.307
 
 
47.4
 
 
 
 
AKAP6
 
 
hsa-let-7c-5p
 
 
-0.307
 
 
47.4
 
 
 
 
ALDH4A1
 
 
hsa-miR-184
 
 
-0.421
 
 
69.9
 
 
 
 
ALG3
 
 
hsa-miR-106b-5p
 
 
-0.551
 
 
13.8
 
 
 
 
ALG3
 
 
hsa-miR-20a-5p
 
 
-0.559
 
 
13.8
 
 
 
 
ALG3
 
 
hsa-miR-106a-5p
 
 
-0.569
 
 
13.8
 
 
 
 
ALG3
 
 
hsa-miR-17-5p
 
 
-0.572
 
 
13.8
 
 
 
 
ALG3
 
 
hsa-miR-20b-5p
 
 
-0.58
 
 
13.8
 
 
 
 
ANKRD12
 
 
hsa-miR-7-5p
 
 
-0.37
 
 
11.5
 
 
 
 
ANKRD46
 
 
hsa-miR-98-5p
 
 
-0.389
 
 
33.8
 
 
 
 
ANKRD46
 
 
hsa-let-7c-5p
 
 
-0.389
 
 
33.8
 
 
 
 
ANKRD9
 
 
hsa-miR-20a-5p
 
 
-0.31
 
 
24.6
 
 
 
 
ANKRD9
 
 
hsa-miR-106b-5p
 
 
-0.318
 
 
24.6
 
 
 
 
ANKRD9
 
 
hsa-miR-20b-5p
 
 
-0.331
 
 
24.6
 
 
 
 
ANKRD9
 
 
hsa-miR-106a-5p
 
 
-0.344
 
 
24.6
 
 
 
 
ANKRD9
 
 
hsa-miR-17-5p
 
 
-0.347
 
 
24.6
 
 
 
 
APCDD1
 
 
hsa-miR-20a-5p
 
 
-0.404
 
 
17.3
 
 
 
 
APCDD1
 
 
hsa-miR-106b-5p
 
 
-0.404
 
 
17.3
 
 
 
 
APCDD1
 
 
hsa-miR-106a-5p
 
 
-0.422
 
 
17.3
 
 
 
 
APCDD1
 
 
hsa-miR-17-5p
 
 
-0.425
 
 
17.3
 
 
 
 
APCDD1
 
 
hsa-miR-20b-5p
 
 
-0.425
 
 
17.3
 
 
 
 
ARF4
 
 
hsa-miR-7-5p
 
 
-0.407
 
 
64.9
 
 
 
 
ARF4
 
 
hsa-miR-148a-3p
 
 
-0.487
 
 
64.9
 
 
 
 
ARFGEF1
 
 
hsa-miR-148a-3p
 
 
-0.306
 
 
13.0
 
 
 
 
ARG2
 
 
hsa-miR-98-5p
 
 
-0.468
 
 
49.2
 
 
 
 
ARG2
 
 
hsa-let-7c-5p
 
 
-0.468
 
 
49.2
 
 
 
 
ARHGAP1
 
 
hsa-miR-17-5p
 
 
-0.325
 
 
19.1
 
 
 
 
ARHGAP1
 
 
hsa-miR-20b-5p
 
 
-0.325
 
 
19.1
 
 
 
 
ARHGAP1
 
 
hsa-miR-106a-5p
 
 
-0.329
 
 
19.1
 
 
 
 
ARHGAP1
 
 
hsa-miR-20a-5p
 
 
-0.339
 
 
19.1
 
 
 
 
ARHGAP1
 
 
hsa-miR-106b-5p
 
 
-0.339
 
 
19.1
 
 
 
 
ARHGAP21
 
 
hsa-miR-148a-3p
 
 
-0.287
 
 
21.5
 
 
 
 
ARL1
 
 
hsa-miR-20a-5p
 
 
-0.403
 
 
53.0
 
 
 
 
ARL1
 
 
hsa-miR-106b-5p
 
 
-0.411
 
 
53.0
 
 
 
 
ARL1
 
 
hsa-miR-20b-5p
 
 
-0.424
 
 
53.0
 
 
 
 
ARL1
 
 
hsa-miR-106a-5p
 
 
-0.429
 
 
53.0
 
 
 
 
ARL1
 
 
hsa-miR-17-5p
 
 
-0.432
 
 
53.0
 
 
 
 
ARL6IP1
 
 
hsa-miR-7-5p
 
 
-0.374
 
 
48.1
 
 
 
 
ARL6IP1
 
 
hsa-miR-148a-3p
 
 
-0.579
 
 
48.1
 
 
 
 
ARL8B
 
 
hsa-miR-148a-3p
 
 
-0.337
 
 
56.0
 
 
 
 
ARMC8
 
 
hsa-miR-20a-5p
 
 
-0.357
 
 
32.4
 
 
 
 
ARMC8
 
 
hsa-miR-106b-5p
 
 
-0.357
 
 
32.4
 
 
 
 
ARMC8
 
 
hsa-miR-106a-5p
 
 
-0.375
 
 
32.4
 
 
 
 
ARMC8
 
 
hsa-miR-17-5p
 
 
-0.378
 
 
32.4
 
 
 
 
ARMC8
 
 
hsa-miR-20b-5p
 
 
-0.378
 
 
32.4
 
 
 
 
ARMCX1
 
 
hsa-miR-21-5p
 
 
-0.489
 
 
23.8
 
 
 
 
ARMT1
 
 
hsa-let-7c-5p
 
 
-0.303
 
 
21.1
 
 
 
 
ARMT1
 
 
hsa-miR-98-5p
 
 
-0.303
 
 
21.1
 
 
 
 
ARPP19
 
 
hsa-let-7c-5p
 
 
-0.492
 
 
158.9
 
 
 
 
ARPP19
 
 
hsa-miR-98-5p
 
 
-0.5
 
 
158.9
 
 
 
 
ARRDC3
 
 
hsa-miR-148a-3p
 
 
-0.305
 
 
41.9
 
 
 
 
ASF1A
 
 
hsa-miR-21-5p
 
 
-0.304
 
 
15.1
 
 
 
 
ASPH
 
 
hsa-miR-148a-3p
 
 
-0.391
 
 
155.4
 
 
 
 
ATG4A
 
 
hsa-miR-7-5p
 
 
-0.448
 
 
17.2
 
 
 
 
ATG4D
 
 
hsa-miR-148a-3p
 
 
-0.303
 
 
10.1
 
 
 
 
ATOSB
 
 
hsa-miR-98-5p
 
 
-0.429
 
 
10.9
 
 
 
 
ATOSB
 
 
hsa-let-7c-5p
 
 
-0.429
 
 
10.9
 
 
 
 
ATP2A2
 
 
hsa-miR-98-5p
 
 
-0.311
 
 
2586.2
 
 
 
 
ATP2A2
 
 
hsa-let-7c-5p
 
 
-0.311
 
 
2586.2
 
 
 
 
ATP2B4
 
 
hsa-miR-148a-3p
 
 
-0.337
 
 
25.9
 
 
 
 
ATP6AP2
 
 
hsa-miR-148a-3p
 
 
-0.592
 
 
37.6
 
 
 
 
ATP6AP2
 
 
hsa-miR-148a-3p
 
 
-0.668
 
 
37.6
 
 
 
 
ATPAF1
 
 
hsa-miR-98-5p
 
 
-0.498
 
 
295.7
 
 
 
 
ATPAF1
 
 
hsa-let-7c-5p
 
 
-0.498
 
 
295.7
 
 
 
 
B4GALT5
 
 
hsa-miR-148a-3p
 
 
-0.44
 
 
22.7
 
 
 
 
B4GAT1
 
 
hsa-let-7c-5p
 
 
-0.47
 
 
17.9
 
 
 
 
B4GAT1
 
 
hsa-miR-98-5p
 
 
-0.47
 
 
17.9
 
 
 
 
BACH1
 
 
hsa-miR-98-5p
 
 
-0.298
 
 
11.9
 
 
 
 
BACH1
 
 
hsa-miR-98-5p
 
 
-0.298
 
 
11.9
 
 
 
 
BACH1
 
 
hsa-let-7c-5p
 
 
-0.306
 
 
11.9
 
 
 
 
BACH1
 
 
hsa-let-7c-5p
 
 
-0.306
 
 
11.9
 
 
 
 
BACH1
 
 
hsa-miR-98-5p
 
 
-0.435
 
 
11.9
 
 
 
 
 
 
 adipose tissue 
 
 
 
 
target_symbol
 
 
mature_mirna_id
 
 
score
 
 
nTPM
 
 
 
 
 
 
ABCB7
 
 
hsa-miR-148a-3p
 
 
-0.515
 
 
12.4
 
 
 
 
ABCD3
 
 
hsa-miR-148a-3p
 
 
-0.388
 
 
15.7
 
 
 
 
ABHD5
 
 
hsa-miR-20a-5p
 
 
-0.397
 
 
27.9
 
 
 
 
ABHD5
 
 
hsa-miR-106b-5p
 
 
-0.413
 
 
27.9
 
 
 
 
ABHD5
 
 
hsa-miR-20b-5p
 
 
-0.418
 
 
27.9
 
 
 
 
ABHD5
 
 
hsa-miR-106a-5p
 
 
-0.447
 
 
27.9
 
 
 
 
ABHD5
 
 
hsa-miR-17-5p
 
 
-0.45
 
 
27.9
 
 
 
 
ACTC1
 
 
hsa-miR-7-5p
 
 
-0.471
 
 
47.8
 
 
 
 
ACVR1
 
 
hsa-miR-148a-3p
 
 
-0.397
 
 
12.6
 
 
 
 
ACVR1C
 
 
hsa-let-7c-5p
 
 
-0.465
 
 
29.3
 
 
 
 
ACVR1C
 
 
hsa-miR-98-5p
 
 
-0.473
 
 
29.3
 
 
 
 
ADAM10
 
 
hsa-miR-148a-3p
 
 
-0.356
 
 
14.0
 
 
 
 
ADAM9
 
 
hsa-miR-20a-5p
 
 
-0.299
 
 
31.0
 
 
 
 
ADAM9
 
 
hsa-miR-106b-5p
 
 
-0.299
 
 
31.0
 
 
 
 
ADAM9
 
 
hsa-miR-106a-5p
 
 
-0.317
 
 
31.0
 
 
 
 
ADAM9
 
 
hsa-miR-17-5p
 
 
-0.32
 
 
31.0
 
 
 
 
ADAM9
 
 
hsa-miR-20b-5p
 
 
-0.328
 
 
31.0
 
 
 
 
ADGRF5
 
 
hsa-miR-148a-3p
 
 
-0.354
 
 
66.6
 
 
 
 
ADRB2
 
 
hsa-let-7c-5p
 
 
-0.635
 
 
26.4
 
 
 
 
ADRB2
 
 
hsa-miR-98-5p
 
 
-0.635
 
 
26.4
 
 
 
 
AGFG1
 
 
hsa-miR-148a-3p
 
 
-0.332
 
 
64.8
 
 
 
 
AGFG2
 
 
hsa-miR-20a-5p
 
 
-0.365
 
 
12.0
 
 
 
 
AGFG2
 
 
hsa-miR-20b-5p
 
 
-0.386
 
 
12.0
 
 
 
 
AGFG2
 
 
hsa-miR-106b-5p
 
 
-0.397
 
 
12.0
 
 
 
 
AGFG2
 
 
hsa-miR-106a-5p
 
 
-0.399
 
 
12.0
 
 
 
 
AGFG2
 
 
hsa-miR-17-5p
 
 
-0.402
 
 
12.0
 
 
 
 
AHCTF1
 
 
hsa-miR-98-5p
 
 
-0.374
 
 
11.8
 
 
 
 
AHCTF1
 
 
hsa-let-7c-5p
 
 
-0.374
 
 
11.8
 
 
 
 
AHDC1
 
 
hsa-miR-148a-3p
 
 
-0.413
 
 
11.7
 
 
 
 
AHNAK
 
 
hsa-miR-20a-5p
 
 
-0.316
 
 
230.0
 
 
 
 
AHNAK
 
 
hsa-miR-106b-5p
 
 
-0.316
 
 
230.0
 
 
 
 
AHNAK
 
 
hsa-miR-106a-5p
 
 
-0.334
 
 
230.0
 
 
 
 
AHNAK
 
 
hsa-miR-17-5p
 
 
-0.337
 
 
230.0
 
 
 
 
AHNAK
 
 
hsa-miR-20b-5p
 
 
-0.337
 
 
230.0
 
 
 
 
AIFM1
 
 
hsa-miR-98-5p
 
 
-0.314
 
 
17.4
 
 
 
 
AIFM1
 
 
hsa-let-7c-5p
 
 
-0.314
 
 
17.4
 
 
 
 
AKAP1
 
 
hsa-miR-148a-3p
 
 
-0.381
 
 
38.1
 
 
 
 
ALDH4A1
 
 
hsa-miR-184
 
 
-0.421
 
 
66.5
 
 
 
 
ALG3
 
 
hsa-miR-106b-5p
 
 
-0.551
 
 
20.9
 
 
 
 
ALG3
 
 
hsa-miR-20a-5p
 
 
-0.559
 
 
20.9
 
 
 
 
ALG3
 
 
hsa-miR-106a-5p
 
 
-0.569
 
 
20.9
 
 
 
 
ALG3
 
 
hsa-miR-17-5p
 
 
-0.572
 
 
20.9
 
 
 
 
ALG3
 
 
hsa-miR-20b-5p
 
 
-0.58
 
 
20.9
 
 
 
 
ALG9
 
 
hsa-miR-7-5p
 
 
-0.302
 
 
12.6
 
 
 
 
ANKRD12
 
 
hsa-miR-7-5p
 
 
-0.37
 
 
14.8
 
 
 
 
ANKRD29
 
 
hsa-miR-20a-5p
 
 
-0.359
 
 
17.0
 
 
 
 
ANKRD29
 
 
hsa-miR-106b-5p
 
 
-0.359
 
 
17.0
 
 
 
 
ANKRD29
 
 
hsa-miR-106a-5p
 
 
-0.369
 
 
17.0
 
 
 
 
ANKRD29
 
 
hsa-miR-17-5p
 
 
-0.372
 
 
17.0
 
 
 
 
ANKRD29
 
 
hsa-miR-20b-5p
 
 
-0.38
 
 
17.0
 
 
 
 
ANKRD46
 
 
hsa-miR-98-5p
 
 
-0.389
 
 
18.0
 
 
 
 
ANKRD46
 
 
hsa-let-7c-5p
 
 
-0.389
 
 
18.0
 
 
 
 
ANKRD9
 
 
hsa-miR-20a-5p
 
 
-0.31
 
 
11.3
 
 
 
 
ANKRD9
 
 
hsa-miR-106b-5p
 
 
-0.318
 
 
11.3
 
 
 
 
ANKRD9
 
 
hsa-miR-20b-5p
 
 
-0.331
 
 
11.3
 
 
 
 
ANKRD9
 
 
hsa-miR-106a-5p
 
 
-0.344
 
 
11.3
 
 
 
 
ANKRD9
 
 
hsa-miR-17-5p
 
 
-0.347
 
 
11.3
 
 
 
 
AP1S1
 
 
hsa-miR-98-5p
 
 
-0.374
 
 
15.1
 
 
 
 
AP1S1
 
 
hsa-let-7c-5p
 
 
-0.374
 
 
15.1
 
 
 
 
APCDD1
 
 
hsa-miR-20a-5p
 
 
-0.404
 
 
49.2
 
 
 
 
APCDD1
 
 
hsa-miR-106b-5p
 
 
-0.404
 
 
49.2
 
 
 
 
APCDD1
 
 
hsa-miR-106a-5p
 
 
-0.422
 
 
49.2
 
 
 
 
APCDD1
 
 
hsa-miR-17-5p
 
 
-0.425
 
 
49.2
 
 
 
 
APCDD1
 
 
hsa-miR-20b-5p
 
 
-0.425
 
 
49.2
 
 
 
 
ARF4
 
 
hsa-miR-7-5p
 
 
-0.407
 
 
118.7
 
 
 
 
ARF4
 
 
hsa-miR-148a-3p
 
 
-0.487
 
 
118.7
 
 
 
 
ARFGEF1
 
 
hsa-miR-148a-3p
 
 
-0.306
 
 
10.7
 
 
 
 
ARFIP1
 
 
hsa-miR-148a-3p
 
 
-0.474
 
 
15.8
 
 
 
 
ARHGAP1
 
 
hsa-miR-17-5p
 
 
-0.325
 
 
79.6
 
 
 
 
ARHGAP1
 
 
hsa-miR-20b-5p
 
 
-0.325
 
 
79.6
 
 
 
 
ARHGAP1
 
 
hsa-miR-106a-5p
 
 
-0.329
 
 
79.6
 
 
 
 
ARHGAP1
 
 
hsa-miR-20a-5p
 
 
-0.339
 
 
79.6
 
 
 
 
ARHGAP1
 
 
hsa-miR-106b-5p
 
 
-0.339
 
 
79.6
 
 
 
 
ARHGAP21
 
 
hsa-miR-148a-3p
 
 
-0.287
 
 
26.1
 
 
 
 
ARHGAP24
 
 
hsa-miR-21-5p
 
 
-0.427
 
 
13.7
 
 
 
 
ARID4B
 
 
hsa-miR-20a-5p
 
 
-0.29
 
 
10.7
 
 
 
 
ARID4B
 
 
hsa-miR-106b-5p
 
 
-0.29
 
 
10.7
 
 
 
 
ARL1
 
 
hsa-miR-20a-5p
 
 
-0.403
 
 
43.5
 
 
 
 
ARL1
 
 
hsa-miR-106b-5p
 
 
-0.411
 
 
43.5
 
 
 
 
ARL1
 
 
hsa-miR-20b-5p
 
 
-0.424
 
 
43.5
 
 
 
 
ARL1
 
 
hsa-miR-106a-5p
 
 
-0.429
 
 
43.5
 
 
 
 
ARL1
 
 
hsa-miR-17-5p
 
 
-0.432
 
 
43.5
 
 
 
 
ARL4D
 
 
hsa-miR-98-5p
 
 
-0.417
 
 
20.3
 
 
 
 
ARL4D
 
 
hsa-let-7c-5p
 
 
-0.417
 
 
20.3
 
 
 
 
ARL6IP1
 
 
hsa-miR-7-5p
 
 
-0.374
 
 
133.2
 
 
 
 
ARL6IP1
 
 
hsa-miR-148a-3p
 
 
-0.579
 
 
133.2
 
 
 
 
ARL6IP6
 
 
hsa-miR-98-5p
 
 
-0.293
 
 
11.0
 
 
 
 
ARL6IP6
 
 
hsa-let-7c-5p
 
 
-0.293
 
 
11.0
 
 
 
 
ARL8B
 
 
hsa-miR-148a-3p
 
 
-0.337
 
 
39.1
 
 
 
 
ARMC8
 
 
hsa-miR-20a-5p
 
 
-0.357
 
 
19.5
 
 
 
 
ARMC8
 
 
hsa-miR-106b-5p
 
 
-0.357
 
 
19.5
 
 
 
 
ARMC8
 
 
hsa-miR-106a-5p
 
 
-0.375
 
 
19.5
 
 
 
 
ARMC8
 
 
hsa-miR-17-5p
 
 
-0.378
 
 
19.5
 
 
 
 
ARMC8
 
 
hsa-miR-20b-5p
 
 
-0.378
 
 
19.5
 
 
 
 
ARMCX1
 
 
hsa-miR-21-5p
 
 
-0.489
 
 
23.1
 
 
 
 
ARMT1
 
 
hsa-let-7c-5p
 
 
-0.303
 
 
16.0
 
 
 
 
ARMT1
 
 
hsa-miR-98-5p
 
 
-0.303
 
 
16.0
 
 
 
 
ARPP19
 
 
hsa-let-7c-5p
 
 
-0.492
 
 
57.4
 
 
 
 
ARPP19
 
 
hsa-miR-98-5p
 
 
-0.5
 
 
57.4
 
 
 
 
ARRDC3
 
 
hsa-miR-148a-3p
 
 
-0.305
 
 
34.5
 
 
 
 
 
 
 adrenal gland 
 
 
 
 
target_symbol
 
 
mature_mirna_id
 
 
score
 
 
nTPM
 
 
 
 
 
 
ABCB7
 
 
hsa-miR-148a-3p
 
 
-0.515
 
 
12.5
 
 
 
 
ABCD3
 
 
hsa-miR-148a-3p
 
 
-0.388
 
 
17.6
 
 
 
 
ADAM9
 
 
hsa-miR-20a-5p
 
 
-0.299
 
 
19.6
 
 
 
 
ADAM9
 
 
hsa-miR-106b-5p
 
 
-0.299
 
 
19.6
 
 
 
 
ADAM9
 
 
hsa-miR-106a-5p
 
 
-0.317
 
 
19.6
 
 
 
 
ADAM9
 
 
hsa-miR-17-5p
 
 
-0.32
 
 
19.6
 
 
 
 
ADAM9
 
 
hsa-miR-20b-5p
 
 
-0.328
 
 
19.6
 
 
 
 
ADGRF5
 
 
hsa-miR-148a-3p
 
 
-0.354
 
 
22.0
 
 
 
 
AGFG1
 
 
hsa-miR-148a-3p
 
 
-0.332
 
 
37.2
 
 
 
 
AGFG2
 
 
hsa-miR-20a-5p
 
 
-0.365
 
 
33.6
 
 
 
 
AGFG2
 
 
hsa-miR-20b-5p
 
 
-0.386
 
 
33.6
 
 
 
 
AGFG2
 
 
hsa-miR-106b-5p
 
 
-0.397
 
 
33.6
 
 
 
 
AGFG2
 
 
hsa-miR-106a-5p
 
 
-0.399
 
 
33.6
 
 
 
 
AGFG2
 
 
hsa-miR-17-5p
 
 
-0.402
 
 
33.6
 
 
 
 
AHDC1
 
 
hsa-miR-148a-3p
 
 
-0.413
 
 
23.0
 
 
 
 
AHNAK
 
 
hsa-miR-20a-5p
 
 
-0.316
 
 
26.5
 
 
 
 
AHNAK
 
 
hsa-miR-106b-5p
 
 
-0.316
 
 
26.5
 
 
 
 
AHNAK
 
 
hsa-miR-106a-5p
 
 
-0.334
 
 
26.5
 
 
 
 
AHNAK
 
 
hsa-miR-17-5p
 
 
-0.337
 
 
26.5
 
 
 
 
AHNAK
 
 
hsa-miR-20b-5p
 
 
-0.337
 
 
26.5
 
 
 
 
AIFM1
 
 
hsa-miR-98-5p
 
 
-0.314
 
 
50.1
 
 
 
 
AIFM1
 
 
hsa-let-7c-5p
 
 
-0.314
 
 
50.1
 
 
 
 
AKAP1
 
 
hsa-miR-148a-3p
 
 
-0.381
 
 
14.9
 
 
 
 
ALCAM
 
 
hsa-miR-148a-3p
 
 
-0.339
 
 
30.9
 
 
 
 
ALDH4A1
 
 
hsa-miR-184
 
 
-0.421
 
 
16.2
 
 
 
 
ALG3
 
 
hsa-miR-106b-5p
 
 
-0.551
 
 
30.6
 
 
 
 
ALG3
 
 
hsa-miR-20a-5p
 
 
-0.559
 
 
30.6
 
 
 
 
ALG3
 
 
hsa-miR-106a-5p
 
 
-0.569
 
 
30.6
 
 
 
 
ALG3
 
 
hsa-miR-17-5p
 
 
-0.572
 
 
30.6
 
 
 
 
ALG3
 
 
hsa-miR-20b-5p
 
 
-0.58
 
 
30.6
 
 
 
 
AMT
 
 
hsa-miR-98-5p
 
 
-0.463
 
 
15.0
 
 
 
 
AMT
 
 
hsa-let-7c-5p
 
 
-0.463
 
 
15.0
 
 
 
 
ANKRD46
 
 
hsa-miR-98-5p
 
 
-0.389
 
 
36.5
 
 
 
 
ANKRD46
 
 
hsa-let-7c-5p
 
 
-0.389
 
 
36.5
 
 
 
 
ANKRD9
 
 
hsa-miR-20a-5p
 
 
-0.31
 
 
20.4
 
 
 
 
ANKRD9
 
 
hsa-miR-106b-5p
 
 
-0.318
 
 
20.4
 
 
 
 
ANKRD9
 
 
hsa-miR-20b-5p
 
 
-0.331
 
 
20.4
 
 
 
 
ANKRD9
 
 
hsa-miR-106a-5p
 
 
-0.344
 
 
20.4
 
 
 
 
ANKRD9
 
 
hsa-miR-17-5p
 
 
-0.347
 
 
20.4
 
 
 
 
AP1AR
 
 
hsa-miR-21-5p
 
 
-0.318
 
 
14.8
 
 
 
 
AP1S1
 
 
hsa-miR-98-5p
 
 
-0.374
 
 
25.7
 
 
 
 
AP1S1
 
 
hsa-let-7c-5p
 
 
-0.374
 
 
25.7
 
 
 
 
APCDD1
 
 
hsa-miR-20a-5p
 
 
-0.404
 
 
13.4
 
 
 
 
APCDD1
 
 
hsa-miR-106b-5p
 
 
-0.404
 
 
13.4
 
 
 
 
APCDD1
 
 
hsa-miR-106a-5p
 
 
-0.422
 
 
13.4
 
 
 
 
APCDD1
 
 
hsa-miR-17-5p
 
 
-0.425
 
 
13.4
 
 
 
 
APCDD1
 
 
hsa-miR-20b-5p
 
 
-0.425
 
 
13.4
 
 
 
 
ARF4
 
 
hsa-miR-7-5p
 
 
-0.407
 
 
151.0
 
 
 
 
ARF4
 
 
hsa-miR-148a-3p
 
 
-0.487
 
 
151.0
 
 
 
 
ARFIP1
 
 
hsa-miR-148a-3p
 
 
-0.474
 
 
13.9
 
 
 
 
ARHGAP1
 
 
hsa-miR-17-5p
 
 
-0.325
 
 
46.3
 
 
 
 
ARHGAP1
 
 
hsa-miR-20b-5p
 
 
-0.325
 
 
46.3
 
 
 
 
ARHGAP1
 
 
hsa-miR-106a-5p
 
 
-0.329
 
 
46.3
 
 
 
 
ARHGAP1
 
 
hsa-miR-20a-5p
 
 
-0.339
 
 
46.3
 
 
 
 
ARHGAP1
 
 
hsa-miR-106b-5p
 
 
-0.339
 
 
46.3
 
 
 
 
ARHGAP21
 
 
hsa-miR-148a-3p
 
 
-0.287
 
 
16.9
 
 
 
 
ARHGEF28
 
 
hsa-miR-20b-5p
 
 
-0.352
 
 
23.1
 
 
 
 
ARHGEF28
 
 
hsa-miR-17-5p
 
 
-0.363
 
 
23.1
 
 
 
 
ARHGEF28
 
 
hsa-miR-20a-5p
 
 
-0.366
 
 
23.1
 
 
 
 
ARHGEF28
 
 
hsa-miR-106a-5p
 
 
-0.367
 
 
23.1
 
 
 
 
ARHGEF28
 
 
hsa-miR-106b-5p
 
 
-0.377
 
 
23.1
 
 
 
 
ARID4B
 
 
hsa-miR-20a-5p
 
 
-0.29
 
 
10.2
 
 
 
 
ARID4B
 
 
hsa-miR-106b-5p
 
 
-0.29
 
 
10.2
 
 
 
 
ARL1
 
 
hsa-miR-20a-5p
 
 
-0.403
 
 
62.3
 
 
 
 
ARL1
 
 
hsa-miR-106b-5p
 
 
-0.411
 
 
62.3
 
 
 
 
ARL1
 
 
hsa-miR-20b-5p
 
 
-0.424
 
 
62.3
 
 
 
 
ARL1
 
 
hsa-miR-106a-5p
 
 
-0.429
 
 
62.3
 
 
 
 
ARL1
 
 
hsa-miR-17-5p
 
 
-0.432
 
 
62.3
 
 
 
 
ARL6IP1
 
 
hsa-miR-7-5p
 
 
-0.374
 
 
161.7
 
 
 
 
ARL6IP1
 
 
hsa-miR-148a-3p
 
 
-0.579
 
 
161.7
 
 
 
 
ARL8B
 
 
hsa-miR-148a-3p
 
 
-0.337
 
 
48.3
 
 
 
 
ARMC8
 
 
hsa-miR-20a-5p
 
 
-0.357
 
 
13.5
 
 
 
 
ARMC8
 
 
hsa-miR-106b-5p
 
 
-0.357
 
 
13.5
 
 
 
 
ARMC8
 
 
hsa-miR-106a-5p
 
 
-0.375
 
 
13.5
 
 
 
 
ARMC8
 
 
hsa-miR-17-5p
 
 
-0.378
 
 
13.5
 
 
 
 
ARMC8
 
 
hsa-miR-20b-5p
 
 
-0.378
 
 
13.5
 
 
 
 
ARMCX1
 
 
hsa-miR-21-5p
 
 
-0.489
 
 
32.1
 
 
 
 
ARMT1
 
 
hsa-let-7c-5p
 
 
-0.303
 
 
15.1
 
 
 
 
ARMT1
 
 
hsa-miR-98-5p
 
 
-0.303
 
 
15.1
 
 
 
 
ARPP19
 
 
hsa-let-7c-5p
 
 
-0.492
 
 
48.8
 
 
 
 
ARPP19
 
 
hsa-miR-98-5p
 
 
-0.5
 
 
48.8
 
 
 
 
ARRDC3
 
 
hsa-miR-148a-3p
 
 
-0.305
 
 
75.7
 
 
 
 
ARRDC4
 
 
hsa-let-7c-5p
 
 
-0.329
 
 
11.5
 
 
 
 
ARRDC4
 
 
hsa-miR-98-5p
 
 
-0.329
 
 
11.5
 
 
 
 
ASAP1
 
 
hsa-miR-98-5p
 
 
-0.308
 
 
20.2
 
 
 
 
ASAP1
 
 
hsa-let-7c-5p
 
 
-0.308
 
 
20.2
 
 
 
 
ASPH
 
 
hsa-miR-148a-3p
 
 
-0.391
 
 
150.8
 
 
 
 
ATG4D
 
 
hsa-miR-148a-3p
 
 
-0.303
 
 
14.9
 
 
 
 
ATOSB
 
 
hsa-miR-98-5p
 
 
-0.429
 
 
14.2
 
 
 
 
ATOSB
 
 
hsa-let-7c-5p
 
 
-0.429
 
 
14.2
 
 
 
 
ATP2A2
 
 
hsa-miR-98-5p
 
 
-0.311
 
 
46.8
 
 
 
 
ATP2A2
 
 
hsa-let-7c-5p
 
 
-0.311
 
 
46.8
 
 
 
 
ATP2B4
 
 
hsa-miR-148a-3p
 
 
-0.337
 
 
23.7
 
 
 
 
ATP6AP2
 
 
hsa-miR-148a-3p
 
 
-0.592
 
 
67.5
 
 
 
 
ATP6AP2
 
 
hsa-miR-148a-3p
 
 
-0.668
 
 
67.5
 
 
 
 
ATPAF1
 
 
hsa-miR-98-5p
 
 
-0.498
 
 
98.1
 
 
 
 
ATPAF1
 
 
hsa-let-7c-5p
 
 
-0.498
 
 
98.1
 
 
 
 
AVPI1
 
 
hsa-miR-7-5p
 
 
-0.367
 
 
46.2
 
 
 
 
B3GAT3
 
 
hsa-miR-98-5p
 
 
-0.324
 
 
33.4
 
 
 
 
B3GAT3
 
 
hsa-let-7c-5p
 
 
-0.324
 
 
33.4
 
 
 
 
 
 
 blood vessel 
 
 
 
 
target_symbol
 
 
mature_mirna_id
 
 
score
 
 
nTPM
 
 
 
 
 
 
ABCB7
 
 
hsa-miR-148a-3p
 
 
-0.515
 
 
12.3
 
 
 
 
ABCD3
 
 
hsa-miR-148a-3p
 
 
-0.388
 
 
14.4
 
 
 
 
ACTC1
 
 
hsa-miR-7-5p
 
 
-0.471
 
 
388.9
 
 
 
 
ADAM9
 
 
hsa-miR-20a-5p
 
 
-0.299
 
 
36.8
 
 
 
 
ADAM9
 
 
hsa-miR-106b-5p
 
 
-0.299
 
 
36.8
 
 
 
 
ADAM9
 
 
hsa-miR-106a-5p
 
 
-0.317
 
 
36.8
 
 
 
 
ADAM9
 
 
hsa-miR-17-5p
 
 
-0.32
 
 
36.8
 
 
 
 
ADAM9
 
 
hsa-miR-20b-5p
 
 
-0.328
 
 
36.8
 
 
 
 
ADAMTS8
 
 
hsa-miR-98-5p
 
 
-0.571
 
 
12.7
 
 
 
 
ADAMTS8
 
 
hsa-let-7c-5p
 
 
-0.571
 
 
12.7
 
 
 
 
ADGRF5
 
 
hsa-miR-148a-3p
 
 
-0.354
 
 
12.6
 
 
 
 
AGFG1
 
 
hsa-miR-148a-3p
 
 
-0.332
 
 
37.3
 
 
 
 
AGFG2
 
 
hsa-miR-20a-5p
 
 
-0.365
 
 
11.4
 
 
 
 
AGFG2
 
 
hsa-miR-20b-5p
 
 
-0.386
 
 
11.4
 
 
 
 
AGFG2
 
 
hsa-miR-106b-5p
 
 
-0.397
 
 
11.4
 
 
 
 
AGFG2
 
 
hsa-miR-106a-5p
 
 
-0.399
 
 
11.4
 
 
 
 
AGFG2
 
 
hsa-miR-17-5p
 
 
-0.402
 
 
11.4
 
 
 
 
AHCTF1
 
 
hsa-miR-98-5p
 
 
-0.374
 
 
10.0
 
 
 
 
AHCTF1
 
 
hsa-let-7c-5p
 
 
-0.374
 
 
10.0
 
 
 
 
AHDC1
 
 
hsa-miR-148a-3p
 
 
-0.413
 
 
27.7
 
 
 
 
AHNAK
 
 
hsa-miR-20a-5p
 
 
-0.316
 
 
225.6
 
 
 
 
AHNAK
 
 
hsa-miR-106b-5p
 
 
-0.316
 
 
225.6
 
 
 
 
AHNAK
 
 
hsa-miR-106a-5p
 
 
-0.334
 
 
225.6
 
 
 
 
AHNAK
 
 
hsa-miR-17-5p
 
 
-0.337
 
 
225.6
 
 
 
 
AHNAK
 
 
hsa-miR-20b-5p
 
 
-0.337
 
 
225.6
 
 
 
 
AKAP1
 
 
hsa-miR-148a-3p
 
 
-0.381
 
 
84.5
 
 
 
 
AKAP6
 
 
hsa-miR-98-5p
 
 
-0.307
 
 
15.0
 
 
 
 
AKAP6
 
 
hsa-let-7c-5p
 
 
-0.307
 
 
15.0
 
 
 
 
ALCAM
 
 
hsa-miR-148a-3p
 
 
-0.339
 
 
85.5
 
 
 
 
ALDH4A1
 
 
hsa-miR-184
 
 
-0.421
 
 
15.0
 
 
 
 
ALG3
 
 
hsa-miR-106b-5p
 
 
-0.551
 
 
20.2
 
 
 
 
ALG3
 
 
hsa-miR-20a-5p
 
 
-0.559
 
 
20.2
 
 
 
 
ALG3
 
 
hsa-miR-106a-5p
 
 
-0.569
 
 
20.2
 
 
 
 
ALG3
 
 
hsa-miR-17-5p
 
 
-0.572
 
 
20.2
 
 
 
 
ALG3
 
 
hsa-miR-20b-5p
 
 
-0.58
 
 
20.2
 
 
 
 
ALG9
 
 
hsa-miR-7-5p
 
 
-0.302
 
 
12.3
 
 
 
 
AMT
 
 
hsa-miR-98-5p
 
 
-0.463
 
 
11.4
 
 
 
 
AMT
 
 
hsa-let-7c-5p
 
 
-0.463
 
 
11.4
 
 
 
 
ANKRD12
 
 
hsa-miR-7-5p
 
 
-0.37
 
 
11.6
 
 
 
 
ANKRD9
 
 
hsa-miR-20a-5p
 
 
-0.31
 
 
10.4
 
 
 
 
ANKRD9
 
 
hsa-miR-106b-5p
 
 
-0.318
 
 
10.4
 
 
 
 
ANKRD9
 
 
hsa-miR-20b-5p
 
 
-0.331
 
 
10.4
 
 
 
 
ANKRD9
 
 
hsa-miR-106a-5p
 
 
-0.344
 
 
10.4
 
 
 
 
ANKRD9
 
 
hsa-miR-17-5p
 
 
-0.347
 
 
10.4
 
 
 
 
AP1S1
 
 
hsa-miR-98-5p
 
 
-0.374
 
 
13.8
 
 
 
 
AP1S1
 
 
hsa-let-7c-5p
 
 
-0.374
 
 
13.8
 
 
 
 
APCDD1
 
 
hsa-miR-20a-5p
 
 
-0.404
 
 
23.7
 
 
 
 
APCDD1
 
 
hsa-miR-106b-5p
 
 
-0.404
 
 
23.7
 
 
 
 
APCDD1
 
 
hsa-miR-106a-5p
 
 
-0.422
 
 
23.7
 
 
 
 
APCDD1
 
 
hsa-miR-17-5p
 
 
-0.425
 
 
23.7
 
 
 
 
APCDD1
 
 
hsa-miR-20b-5p
 
 
-0.425
 
 
23.7
 
 
 
 
ARF4
 
 
hsa-miR-7-5p
 
 
-0.407
 
 
151.4
 
 
 
 
ARF4
 
 
hsa-miR-148a-3p
 
 
-0.487
 
 
151.4
 
 
 
 
ARFIP1
 
 
hsa-miR-148a-3p
 
 
-0.474
 
 
14.2
 
 
 
 
ARHGAP1
 
 
hsa-miR-17-5p
 
 
-0.325
 
 
119.0
 
 
 
 
ARHGAP1
 
 
hsa-miR-20b-5p
 
 
-0.325
 
 
119.0
 
 
 
 
ARHGAP1
 
 
hsa-miR-106a-5p
 
 
-0.329
 
 
119.0
 
 
 
 
ARHGAP1
 
 
hsa-miR-20a-5p
 
 
-0.339
 
 
119.0
 
 
 
 
ARHGAP1
 
 
hsa-miR-106b-5p
 
 
-0.339
 
 
119.0
 
 
 
 
ARHGAP21
 
 
hsa-miR-148a-3p
 
 
-0.287
 
 
14.0
 
 
 
 
ARHGAP24
 
 
hsa-miR-21-5p
 
 
-0.427
 
 
17.3
 
 
 
 
ARHGEF28
 
 
hsa-miR-20b-5p
 
 
-0.352
 
 
10.7
 
 
 
 
ARHGEF28
 
 
hsa-miR-17-5p
 
 
-0.363
 
 
10.7
 
 
 
 
ARHGEF28
 
 
hsa-miR-20a-5p
 
 
-0.366
 
 
10.7
 
 
 
 
ARHGEF28
 
 
hsa-miR-106a-5p
 
 
-0.367
 
 
10.7
 
 
 
 
ARHGEF28
 
 
hsa-miR-106b-5p
 
 
-0.377
 
 
10.7
 
 
 
 
ARID4B
 
 
hsa-miR-20a-5p
 
 
-0.29
 
 
12.5
 
 
 
 
ARID4B
 
 
hsa-miR-106b-5p
 
 
-0.29
 
 
12.5
 
 
 
 
ARL1
 
 
hsa-miR-20a-5p
 
 
-0.403
 
 
48.7
 
 
 
 
ARL1
 
 
hsa-miR-106b-5p
 
 
-0.411
 
 
48.7
 
 
 
 
ARL1
 
 
hsa-miR-20b-5p
 
 
-0.424
 
 
48.7
 
 
 
 
ARL1
 
 
hsa-miR-106a-5p
 
 
-0.429
 
 
48.7
 
 
 
 
ARL1
 
 
hsa-miR-17-5p
 
 
-0.432
 
 
48.7
 
 
 
 
ARL4D
 
 
hsa-miR-98-5p
 
 
-0.417
 
 
12.9
 
 
 
 
ARL4D
 
 
hsa-let-7c-5p
 
 
-0.417
 
 
12.9
 
 
 
 
ARL6IP1
 
 
hsa-miR-7-5p
 
 
-0.374
 
 
52.2
 
 
 
 
ARL6IP1
 
 
hsa-miR-148a-3p
 
 
-0.579
 
 
52.2
 
 
 
 
ARL8B
 
 
hsa-miR-148a-3p
 
 
-0.337
 
 
49.5
 
 
 
 
ARMC8
 
 
hsa-miR-20a-5p
 
 
-0.357
 
 
23.5
 
 
 
 
ARMC8
 
 
hsa-miR-106b-5p
 
 
-0.357
 
 
23.5
 
 
 
 
ARMC8
 
 
hsa-miR-106a-5p
 
 
-0.375
 
 
23.5
 
 
 
 
ARMC8
 
 
hsa-miR-17-5p
 
 
-0.378
 
 
23.5
 
 
 
 
ARMC8
 
 
hsa-miR-20b-5p
 
 
-0.378
 
 
23.5
 
 
 
 
ARMCX1
 
 
hsa-miR-21-5p
 
 
-0.489
 
 
31.6
 
 
 
 
ARPP19
 
 
hsa-let-7c-5p
 
 
-0.492
 
 
47.3
 
 
 
 
ARPP19
 
 
hsa-miR-98-5p
 
 
-0.5
 
 
47.3
 
 
 
 
ARRDC3
 
 
hsa-miR-148a-3p
 
 
-0.305
 
 
25.4
 
 
 
 
ASAP1
 
 
hsa-miR-98-5p
 
 
-0.308
 
 
18.4
 
 
 
 
ASAP1
 
 
hsa-let-7c-5p
 
 
-0.308
 
 
18.4
 
 
 
 
ASPH
 
 
hsa-miR-148a-3p
 
 
-0.391
 
 
72.5
 
 
 
 
ATG16L1
 
 
hsa-miR-20a-5p
 
 
-0.446
 
 
12.7
 
 
 
 
ATG16L1
 
 
hsa-miR-106b-5p
 
 
-0.446
 
 
12.7
 
 
 
 
ATG16L1
 
 
hsa-miR-106a-5p
 
 
-0.464
 
 
12.7
 
 
 
 
ATG16L1
 
 
hsa-miR-17-5p
 
 
-0.467
 
 
12.7
 
 
 
 
ATG16L1
 
 
hsa-miR-20b-5p
 
 
-0.467
 
 
12.7
 
 
 
 
ATOSB
 
 
hsa-miR-98-5p
 
 
-0.429
 
 
26.7
 
 
 
 
ATOSB
 
 
hsa-let-7c-5p
 
 
-0.429
 
 
26.7
 
 
 
 
ATP2A2
 
 
hsa-miR-98-5p
 
 
-0.311
 
 
84.6
 
 
 
 
ATP2A2
 
 
hsa-let-7c-5p
 
 
-0.311
 
 
84.6
 
 
 
 
ATP2B4
 
 
hsa-miR-148a-3p
 
 
-0.337
 
 
24.9
 
 
 
 
 
 
 breast 
 
 
 
 
target_symbol
 
 
mature_mirna_id
 
 
score
 
 
nTPM
 
 
 
 
 
 
ABCB7
 
 
hsa-miR-148a-3p
 
 
-0.515
 
 
11.6
 
 
 
 
ABCD3
 
 
hsa-miR-148a-3p
 
 
-0.388
 
 
30.8
 
 
 
 
ABHD17C
 
 
hsa-let-7c-5p
 
 
-0.359
 
 
10.9
 
 
 
 
ABHD17C
 
 
hsa-miR-98-5p
 
 
-0.383
 
 
10.9
 
 
 
 
ABHD5
 
 
hsa-miR-20a-5p
 
 
-0.397
 
 
18.8
 
 
 
 
ABHD5
 
 
hsa-miR-106b-5p
 
 
-0.413
 
 
18.8
 
 
 
 
ABHD5
 
 
hsa-miR-20b-5p
 
 
-0.418
 
 
18.8
 
 
 
 
ABHD5
 
 
hsa-miR-106a-5p
 
 
-0.447
 
 
18.8
 
 
 
 
ABHD5
 
 
hsa-miR-17-5p
 
 
-0.45
 
 
18.8
 
 
 
 
ACTC1
 
 
hsa-miR-7-5p
 
 
-0.471
 
 
11.2
 
 
 
 
ACVR1
 
 
hsa-miR-148a-3p
 
 
-0.397
 
 
11.1
 
 
 
 
ADAM10
 
 
hsa-miR-148a-3p
 
 
-0.356
 
 
14.2
 
 
 
 
ADAM9
 
 
hsa-miR-20a-5p
 
 
-0.299
 
 
20.2
 
 
 
 
ADAM9
 
 
hsa-miR-106b-5p
 
 
-0.299
 
 
20.2
 
 
 
 
ADAM9
 
 
hsa-miR-106a-5p
 
 
-0.317
 
 
20.2
 
 
 
 
ADAM9
 
 
hsa-miR-17-5p
 
 
-0.32
 
 
20.2
 
 
 
 
ADAM9
 
 
hsa-miR-20b-5p
 
 
-0.328
 
 
20.2
 
 
 
 
ADGRF5
 
 
hsa-miR-148a-3p
 
 
-0.354
 
 
44.5
 
 
 
 
ADRB2
 
 
hsa-let-7c-5p
 
 
-0.635
 
 
32.4
 
 
 
 
ADRB2
 
 
hsa-miR-98-5p
 
 
-0.635
 
 
32.4
 
 
 
 
AGFG1
 
 
hsa-miR-148a-3p
 
 
-0.332
 
 
48.8
 
 
 
 
AGFG2
 
 
hsa-miR-20a-5p
 
 
-0.365
 
 
10.5
 
 
 
 
AGFG2
 
 
hsa-miR-20b-5p
 
 
-0.386
 
 
10.5
 
 
 
 
AGFG2
 
 
hsa-miR-106b-5p
 
 
-0.397
 
 
10.5
 
 
 
 
AGFG2
 
 
hsa-miR-106a-5p
 
 
-0.399
 
 
10.5
 
 
 
 
AGFG2
 
 
hsa-miR-17-5p
 
 
-0.402
 
 
10.5
 
 
 
 
AHDC1
 
 
hsa-miR-148a-3p
 
 
-0.413
 
 
11.9
 
 
 
 
AHNAK
 
 
hsa-miR-20a-5p
 
 
-0.316
 
 
178.3
 
 
 
 
AHNAK
 
 
hsa-miR-106b-5p
 
 
-0.316
 
 
178.3
 
 
 
 
AHNAK
 
 
hsa-miR-106a-5p
 
 
-0.334
 
 
178.3
 
 
 
 
AHNAK
 
 
hsa-miR-17-5p
 
 
-0.337
 
 
178.3
 
 
 
 
AHNAK
 
 
hsa-miR-20b-5p
 
 
-0.337
 
 
178.3
 
 
 
 
AIFM1
 
 
hsa-miR-98-5p
 
 
-0.314
 
 
11.7
 
 
 
 
AIFM1
 
 
hsa-let-7c-5p
 
 
-0.314
 
 
11.7
 
 
 
 
AKAP1
 
 
hsa-miR-148a-3p
 
 
-0.381
 
 
34.0
 
 
 
 
ALCAM
 
 
hsa-miR-148a-3p
 
 
-0.339
 
 
76.0
 
 
 
 
ALDH4A1
 
 
hsa-miR-184
 
 
-0.421
 
 
22.5
 
 
 
 
ALG3
 
 
hsa-miR-106b-5p
 
 
-0.551
 
 
18.6
 
 
 
 
ALG3
 
 
hsa-miR-20a-5p
 
 
-0.559
 
 
18.6
 
 
 
 
ALG3
 
 
hsa-miR-106a-5p
 
 
-0.569
 
 
18.6
 
 
 
 
ALG3
 
 
hsa-miR-17-5p
 
 
-0.572
 
 
18.6
 
 
 
 
ALG3
 
 
hsa-miR-20b-5p
 
 
-0.58
 
 
18.6
 
 
 
 
ALG9
 
 
hsa-miR-7-5p
 
 
-0.302
 
 
11.3
 
 
 
 
ANKRD12
 
 
hsa-miR-7-5p
 
 
-0.37
 
 
12.8
 
 
 
 
ANKRD29
 
 
hsa-miR-20a-5p
 
 
-0.359
 
 
15.0
 
 
 
 
ANKRD29
 
 
hsa-miR-106b-5p
 
 
-0.359
 
 
15.0
 
 
 
 
ANKRD29
 
 
hsa-miR-106a-5p
 
 
-0.369
 
 
15.0
 
 
 
 
ANKRD29
 
 
hsa-miR-17-5p
 
 
-0.372
 
 
15.0
 
 
 
 
ANKRD29
 
 
hsa-miR-20b-5p
 
 
-0.38
 
 
15.0
 
 
 
 
ANKRD46
 
 
hsa-miR-98-5p
 
 
-0.389
 
 
13.6
 
 
 
 
ANKRD46
 
 
hsa-let-7c-5p
 
 
-0.389
 
 
13.6
 
 
 
 
AP1AR
 
 
hsa-miR-21-5p
 
 
-0.318
 
 
15.9
 
 
 
 
AP1S1
 
 
hsa-miR-98-5p
 
 
-0.374
 
 
17.7
 
 
 
 
AP1S1
 
 
hsa-let-7c-5p
 
 
-0.374
 
 
17.7
 
 
 
 
APCDD1
 
 
hsa-miR-20a-5p
 
 
-0.404
 
 
30.9
 
 
 
 
APCDD1
 
 
hsa-miR-106b-5p
 
 
-0.404
 
 
30.9
 
 
 
 
APCDD1
 
 
hsa-miR-106a-5p
 
 
-0.422
 
 
30.9
 
 
 
 
APCDD1
 
 
hsa-miR-17-5p
 
 
-0.425
 
 
30.9
 
 
 
 
APCDD1
 
 
hsa-miR-20b-5p
 
 
-0.425
 
 
30.9
 
 
 
 
ARF4
 
 
hsa-miR-7-5p
 
 
-0.407
 
 
129.6
 
 
 
 
ARF4
 
 
hsa-miR-148a-3p
 
 
-0.487
 
 
129.6
 
 
 
 
ARFGEF1
 
 
hsa-miR-148a-3p
 
 
-0.306
 
 
12.7
 
 
 
 
ARFIP1
 
 
hsa-miR-148a-3p
 
 
-0.474
 
 
22.0
 
 
 
 
ARHGAP1
 
 
hsa-miR-17-5p
 
 
-0.325
 
 
59.0
 
 
 
 
ARHGAP1
 
 
hsa-miR-20b-5p
 
 
-0.325
 
 
59.0
 
 
 
 
ARHGAP1
 
 
hsa-miR-106a-5p
 
 
-0.329
 
 
59.0
 
 
 
 
ARHGAP1
 
 
hsa-miR-20a-5p
 
 
-0.339
 
 
59.0
 
 
 
 
ARHGAP1
 
 
hsa-miR-106b-5p
 
 
-0.339
 
 
59.0
 
 
 
 
ARHGAP21
 
 
hsa-miR-148a-3p
 
 
-0.287
 
 
15.1
 
 
 
 
ARID4B
 
 
hsa-miR-20a-5p
 
 
-0.29
 
 
12.7
 
 
 
 
ARID4B
 
 
hsa-miR-106b-5p
 
 
-0.29
 
 
12.7
 
 
 
 
ARL1
 
 
hsa-miR-20a-5p
 
 
-0.403
 
 
58.5
 
 
 
 
ARL1
 
 
hsa-miR-106b-5p
 
 
-0.411
 
 
58.5
 
 
 
 
ARL1
 
 
hsa-miR-20b-5p
 
 
-0.424
 
 
58.5
 
 
 
 
ARL1
 
 
hsa-miR-106a-5p
 
 
-0.429
 
 
58.5
 
 
 
 
ARL1
 
 
hsa-miR-17-5p
 
 
-0.432
 
 
58.5
 
 
 
 
ARL6IP1
 
 
hsa-miR-7-5p
 
 
-0.374
 
 
105.7
 
 
 
 
ARL6IP1
 
 
hsa-miR-148a-3p
 
 
-0.579
 
 
105.7
 
 
 
 
ARL6IP6
 
 
hsa-miR-98-5p
 
 
-0.293
 
 
12.8
 
 
 
 
ARL6IP6
 
 
hsa-let-7c-5p
 
 
-0.293
 
 
12.8
 
 
 
 
ARL8B
 
 
hsa-miR-148a-3p
 
 
-0.337
 
 
39.7
 
 
 
 
ARMC8
 
 
hsa-miR-20a-5p
 
 
-0.357
 
 
15.4
 
 
 
 
ARMC8
 
 
hsa-miR-106b-5p
 
 
-0.357
 
 
15.4
 
 
 
 
ARMC8
 
 
hsa-miR-106a-5p
 
 
-0.375
 
 
15.4
 
 
 
 
ARMC8
 
 
hsa-miR-17-5p
 
 
-0.378
 
 
15.4
 
 
 
 
ARMC8
 
 
hsa-miR-20b-5p
 
 
-0.378
 
 
15.4
 
 
 
 
ARMCX1
 
 
hsa-miR-21-5p
 
 
-0.489
 
 
21.0
 
 
 
 
ARMT1
 
 
hsa-let-7c-5p
 
 
-0.303
 
 
23.4
 
 
 
 
ARMT1
 
 
hsa-miR-98-5p
 
 
-0.303
 
 
23.4
 
 
 
 
ARPP19
 
 
hsa-let-7c-5p
 
 
-0.492
 
 
43.2
 
 
 
 
ARPP19
 
 
hsa-miR-98-5p
 
 
-0.5
 
 
43.2
 
 
 
 
ARRDC3
 
 
hsa-miR-148a-3p
 
 
-0.305
 
 
88.5
 
 
 
 
ARRDC4
 
 
hsa-let-7c-5p
 
 
-0.329
 
 
18.6
 
 
 
 
ARRDC4
 
 
hsa-miR-98-5p
 
 
-0.329
 
 
18.6
 
 
 
 
ASAP1
 
 
hsa-miR-98-5p
 
 
-0.308
 
 
22.2
 
 
 
 
ASAP1
 
 
hsa-let-7c-5p
 
 
-0.308
 
 
22.2
 
 
 
 
ASF1A
 
 
hsa-miR-21-5p
 
 
-0.304
 
 
11.7
 
 
 
 
ASPH
 
 
hsa-miR-148a-3p
 
 
-0.391
 
 
99.5
 
 
 
 
ATOSB
 
 
hsa-miR-98-5p
 
 
-0.429
 
 
10.1
 
 
 
 
ATOSB
 
 
hsa-let-7c-5p
 
 
-0.429
 
 
10.1
 
 
 
 
 
 
 cervix 
 
 
 
 
target_symbol
 
 
mature_mirna_id
 
 
score
 
 
nTPM
 
 
 
 
 
 
ABCB7
 
 
hsa-miR-148a-3p
 
 
-0.515
 
 
10.8
 
 
 
 
ABCD3
 
 
hsa-miR-148a-3p
 
 
-0.388
 
 
21.0
 
 
 
 
ABHD17C
 
 
hsa-let-7c-5p
 
 
-0.359
 
 
18.1
 
 
 
 
ABHD17C
 
 
hsa-miR-98-5p
 
 
-0.383
 
 
18.1
 
 
 
 
ACP3
 
 
hsa-miR-98-5p
 
 
-0.298
 
 
10.9
 
 
 
 
ACP3
 
 
hsa-let-7c-5p
 
 
-0.298
 
 
10.9
 
 
 
 
ACTC1
 
 
hsa-miR-7-5p
 
 
-0.471
 
 
44.2
 
 
 
 
ACVR1
 
 
hsa-miR-148a-3p
 
 
-0.397
 
 
20.6
 
 
 
 
ADAM10
 
 
hsa-miR-148a-3p
 
 
-0.356
 
 
15.1
 
 
 
 
ADAM9
 
 
hsa-miR-20a-5p
 
 
-0.299
 
 
32.7
 
 
 
 
ADAM9
 
 
hsa-miR-106b-5p
 
 
-0.299
 
 
32.7
 
 
 
 
ADAM9
 
 
hsa-miR-106a-5p
 
 
-0.317
 
 
32.7
 
 
 
 
ADAM9
 
 
hsa-miR-17-5p
 
 
-0.32
 
 
32.7
 
 
 
 
ADAM9
 
 
hsa-miR-20b-5p
 
 
-0.328
 
 
32.7
 
 
 
 
ADRB2
 
 
hsa-let-7c-5p
 
 
-0.635
 
 
12.7
 
 
 
 
ADRB2
 
 
hsa-miR-98-5p
 
 
-0.635
 
 
12.7
 
 
 
 
AGFG1
 
 
hsa-miR-148a-3p
 
 
-0.332
 
 
27.5
 
 
 
 
AGFG2
 
 
hsa-miR-20a-5p
 
 
-0.365
 
 
11.6
 
 
 
 
AGFG2
 
 
hsa-miR-20b-5p
 
 
-0.386
 
 
11.6
 
 
 
 
AGFG2
 
 
hsa-miR-106b-5p
 
 
-0.397
 
 
11.6
 
 
 
 
AGFG2
 
 
hsa-miR-106a-5p
 
 
-0.399
 
 
11.6
 
 
 
 
AGFG2
 
 
hsa-miR-17-5p
 
 
-0.402
 
 
11.6
 
 
 
 
AHDC1
 
 
hsa-miR-148a-3p
 
 
-0.413
 
 
23.6
 
 
 
 
AHNAK
 
 
hsa-miR-20a-5p
 
 
-0.316
 
 
181.1
 
 
 
 
AHNAK
 
 
hsa-miR-106b-5p
 
 
-0.316
 
 
181.1
 
 
 
 
AHNAK
 
 
hsa-miR-106a-5p
 
 
-0.334
 
 
181.1
 
 
 
 
AHNAK
 
 
hsa-miR-17-5p
 
 
-0.337
 
 
181.1
 
 
 
 
AHNAK
 
 
hsa-miR-20b-5p
 
 
-0.337
 
 
181.1
 
 
 
 
AIFM1
 
 
hsa-miR-98-5p
 
 
-0.314
 
 
14.8
 
 
 
 
AIFM1
 
 
hsa-let-7c-5p
 
 
-0.314
 
 
14.8
 
 
 
 
AKAP1
 
 
hsa-miR-148a-3p
 
 
-0.381
 
 
22.6
 
 
 
 
ALCAM
 
 
hsa-miR-148a-3p
 
 
-0.339
 
 
26.3
 
 
 
 
ALDH4A1
 
 
hsa-miR-184
 
 
-0.421
 
 
15.0
 
 
 
 
ALG3
 
 
hsa-miR-106b-5p
 
 
-0.551
 
 
18.1
 
 
 
 
ALG3
 
 
hsa-miR-20a-5p
 
 
-0.559
 
 
18.1
 
 
 
 
ALG3
 
 
hsa-miR-106a-5p
 
 
-0.569
 
 
18.1
 
 
 
 
ALG3
 
 
hsa-miR-17-5p
 
 
-0.572
 
 
18.1
 
 
 
 
ALG3
 
 
hsa-miR-20b-5p
 
 
-0.58
 
 
18.1
 
 
 
 
ALG9
 
 
hsa-miR-7-5p
 
 
-0.302
 
 
10.8
 
 
 
 
AMT
 
 
hsa-miR-98-5p
 
 
-0.463
 
 
14.5
 
 
 
 
AMT
 
 
hsa-let-7c-5p
 
 
-0.463
 
 
14.5
 
 
 
 
ANKRD12
 
 
hsa-miR-7-5p
 
 
-0.37
 
 
10.5
 
 
 
 
ANKRD29
 
 
hsa-miR-20a-5p
 
 
-0.359
 
 
12.2
 
 
 
 
ANKRD29
 
 
hsa-miR-106b-5p
 
 
-0.359
 
 
12.2
 
 
 
 
ANKRD29
 
 
hsa-miR-106a-5p
 
 
-0.369
 
 
12.2
 
 
 
 
ANKRD29
 
 
hsa-miR-17-5p
 
 
-0.372
 
 
12.2
 
 
 
 
ANKRD29
 
 
hsa-miR-20b-5p
 
 
-0.38
 
 
12.2
 
 
 
 
ANKRD46
 
 
hsa-miR-98-5p
 
 
-0.389
 
 
14.1
 
 
 
 
ANKRD46
 
 
hsa-let-7c-5p
 
 
-0.389
 
 
14.1
 
 
 
 
ANKRD50
 
 
hsa-miR-106a-5p
 
 
-0.29
 
 
10.7
 
 
 
 
ANKRD50
 
 
hsa-miR-20a-5p
 
 
-0.3
 
 
10.7
 
 
 
 
ANKRD50
 
 
hsa-miR-106b-5p
 
 
-0.3
 
 
10.7
 
 
 
 
ANKRD9
 
 
hsa-miR-20a-5p
 
 
-0.31
 
 
10.4
 
 
 
 
ANKRD9
 
 
hsa-miR-106b-5p
 
 
-0.318
 
 
10.4
 
 
 
 
ANKRD9
 
 
hsa-miR-20b-5p
 
 
-0.331
 
 
10.4
 
 
 
 
ANKRD9
 
 
hsa-miR-106a-5p
 
 
-0.344
 
 
10.4
 
 
 
 
ANKRD9
 
 
hsa-miR-17-5p
 
 
-0.347
 
 
10.4
 
 
 
 
AP1S1
 
 
hsa-miR-98-5p
 
 
-0.374
 
 
20.1
 
 
 
 
AP1S1
 
 
hsa-let-7c-5p
 
 
-0.374
 
 
20.1
 
 
 
 
APCDD1
 
 
hsa-miR-20a-5p
 
 
-0.404
 
 
33.1
 
 
 
 
APCDD1
 
 
hsa-miR-106b-5p
 
 
-0.404
 
 
33.1
 
 
 
 
APCDD1
 
 
hsa-miR-106a-5p
 
 
-0.422
 
 
33.1
 
 
 
 
APCDD1
 
 
hsa-miR-17-5p
 
 
-0.425
 
 
33.1
 
 
 
 
APCDD1
 
 
hsa-miR-20b-5p
 
 
-0.425
 
 
33.1
 
 
 
 
ARF4
 
 
hsa-miR-7-5p
 
 
-0.407
 
 
196.1
 
 
 
 
ARF4
 
 
hsa-miR-148a-3p
 
 
-0.487
 
 
196.1
 
 
 
 
ARFGEF1
 
 
hsa-miR-148a-3p
 
 
-0.306
 
 
13.3
 
 
 
 
ARFIP1
 
 
hsa-miR-148a-3p
 
 
-0.474
 
 
21.1
 
 
 
 
ARG2
 
 
hsa-miR-98-5p
 
 
-0.468
 
 
16.0
 
 
 
 
ARG2
 
 
hsa-let-7c-5p
 
 
-0.468
 
 
16.0
 
 
 
 
ARHGAP1
 
 
hsa-miR-17-5p
 
 
-0.325
 
 
62.9
 
 
 
 
ARHGAP1
 
 
hsa-miR-20b-5p
 
 
-0.325
 
 
62.9
 
 
 
 
ARHGAP1
 
 
hsa-miR-106a-5p
 
 
-0.329
 
 
62.9
 
 
 
 
ARHGAP1
 
 
hsa-miR-20a-5p
 
 
-0.339
 
 
62.9
 
 
 
 
ARHGAP1
 
 
hsa-miR-106b-5p
 
 
-0.339
 
 
62.9
 
 
 
 
ARHGAP21
 
 
hsa-miR-148a-3p
 
 
-0.287
 
 
18.5
 
 
 
 
ARID4B
 
 
hsa-miR-20a-5p
 
 
-0.29
 
 
14.1
 
 
 
 
ARID4B
 
 
hsa-miR-106b-5p
 
 
-0.29
 
 
14.1
 
 
 
 
ARL1
 
 
hsa-miR-20a-5p
 
 
-0.403
 
 
81.2
 
 
 
 
ARL1
 
 
hsa-miR-106b-5p
 
 
-0.411
 
 
81.2
 
 
 
 
ARL1
 
 
hsa-miR-20b-5p
 
 
-0.424
 
 
81.2
 
 
 
 
ARL1
 
 
hsa-miR-106a-5p
 
 
-0.429
 
 
81.2
 
 
 
 
ARL1
 
 
hsa-miR-17-5p
 
 
-0.432
 
 
81.2
 
 
 
 
ARL4D
 
 
hsa-miR-98-5p
 
 
-0.417
 
 
27.6
 
 
 
 
ARL4D
 
 
hsa-let-7c-5p
 
 
-0.417
 
 
27.6
 
 
 
 
ARL6IP1
 
 
hsa-miR-7-5p
 
 
-0.374
 
 
74.5
 
 
 
 
ARL6IP1
 
 
hsa-miR-148a-3p
 
 
-0.579
 
 
74.5
 
 
 
 
ARL8B
 
 
hsa-miR-148a-3p
 
 
-0.337
 
 
42.9
 
 
 
 
ARMC8
 
 
hsa-miR-20a-5p
 
 
-0.357
 
 
13.8
 
 
 
 
ARMC8
 
 
hsa-miR-106b-5p
 
 
-0.357
 
 
13.8
 
 
 
 
ARMC8
 
 
hsa-miR-106a-5p
 
 
-0.375
 
 
13.8
 
 
 
 
ARMC8
 
 
hsa-miR-17-5p
 
 
-0.378
 
 
13.8
 
 
 
 
ARMC8
 
 
hsa-miR-20b-5p
 
 
-0.378
 
 
13.8
 
 
 
 
ARMCX1
 
 
hsa-miR-21-5p
 
 
-0.489
 
 
27.8
 
 
 
 
ARMT1
 
 
hsa-let-7c-5p
 
 
-0.303
 
 
15.1
 
 
 
 
ARMT1
 
 
hsa-miR-98-5p
 
 
-0.303
 
 
15.1
 
 
 
 
ARPP19
 
 
hsa-let-7c-5p
 
 
-0.492
 
 
35.8
 
 
 
 
ARPP19
 
 
hsa-miR-98-5p
 
 
-0.5
 
 
35.8
 
 
 
 
ARRDC3
 
 
hsa-miR-148a-3p
 
 
-0.305
 
 
42.0
 
 
 
 
ARRDC4
 
 
hsa-let-7c-5p
 
 
-0.329
 
 
14.3
 
 
 
 
 
 
 choroid plexus 
 
 
 
 
target_symbol
 
 
mature_mirna_id
 
 
score
 
 
nTPM
 
 
 
 
 
 
ABCB7
 
 
hsa-miR-148a-3p
 
 
-0.515
 
 
15.2
 
 
 
 
ABCD3
 
 
hsa-miR-148a-3p
 
 
-0.388
 
 
13.3
 
 
 
 
ADAM9
 
 
hsa-miR-20a-5p
 
 
-0.299
 
 
14.9
 
 
 
 
ADAM9
 
 
hsa-miR-106b-5p
 
 
-0.299
 
 
14.9
 
 
 
 
ADAM9
 
 
hsa-miR-106a-5p
 
 
-0.317
 
 
14.9
 
 
 
 
ADAM9
 
 
hsa-miR-17-5p
 
 
-0.32
 
 
14.9
 
 
 
 
ADAM9
 
 
hsa-miR-20b-5p
 
 
-0.328
 
 
14.9
 
 
 
 
AGFG1
 
 
hsa-miR-148a-3p
 
 
-0.332
 
 
23.3
 
 
 
 
AHNAK
 
 
hsa-miR-20a-5p
 
 
-0.316
 
 
26.0
 
 
 
 
AHNAK
 
 
hsa-miR-106b-5p
 
 
-0.316
 
 
26.0
 
 
 
 
AHNAK
 
 
hsa-miR-106a-5p
 
 
-0.334
 
 
26.0
 
 
 
 
AHNAK
 
 
hsa-miR-17-5p
 
 
-0.337
 
 
26.0
 
 
 
 
AHNAK
 
 
hsa-miR-20b-5p
 
 
-0.337
 
 
26.0
 
 
 
 
AIFM1
 
 
hsa-miR-98-5p
 
 
-0.314
 
 
37.9
 
 
 
 
AIFM1
 
 
hsa-let-7c-5p
 
 
-0.314
 
 
37.9
 
 
 
 
ALDH4A1
 
 
hsa-miR-184
 
 
-0.421
 
 
121.5
 
 
 
 
ALG3
 
 
hsa-miR-106b-5p
 
 
-0.551
 
 
25.2
 
 
 
 
ALG3
 
 
hsa-miR-20a-5p
 
 
-0.559
 
 
25.2
 
 
 
 
ALG3
 
 
hsa-miR-106a-5p
 
 
-0.569
 
 
25.2
 
 
 
 
ALG3
 
 
hsa-miR-17-5p
 
 
-0.572
 
 
25.2
 
 
 
 
ALG3
 
 
hsa-miR-20b-5p
 
 
-0.58
 
 
25.2
 
 
 
 
AMT
 
 
hsa-miR-98-5p
 
 
-0.463
 
 
33.4
 
 
 
 
AMT
 
 
hsa-let-7c-5p
 
 
-0.463
 
 
33.4
 
 
 
 
ANKRD46
 
 
hsa-miR-98-5p
 
 
-0.389
 
 
11.0
 
 
 
 
ANKRD46
 
 
hsa-let-7c-5p
 
 
-0.389
 
 
11.0
 
 
 
 
ANKRD9
 
 
hsa-miR-20a-5p
 
 
-0.31
 
 
15.2
 
 
 
 
ANKRD9
 
 
hsa-miR-106b-5p
 
 
-0.318
 
 
15.2
 
 
 
 
ANKRD9
 
 
hsa-miR-20b-5p
 
 
-0.331
 
 
15.2
 
 
 
 
ANKRD9
 
 
hsa-miR-106a-5p
 
 
-0.344
 
 
15.2
 
 
 
 
ANKRD9
 
 
hsa-miR-17-5p
 
 
-0.347
 
 
15.2
 
 
 
 
AP1S1
 
 
hsa-miR-98-5p
 
 
-0.374
 
 
10.3
 
 
 
 
AP1S1
 
 
hsa-let-7c-5p
 
 
-0.374
 
 
10.3
 
 
 
 
ARF4
 
 
hsa-miR-7-5p
 
 
-0.407
 
 
115.4
 
 
 
 
ARF4
 
 
hsa-miR-148a-3p
 
 
-0.487
 
 
115.4
 
 
 
 
ARG2
 
 
hsa-miR-98-5p
 
 
-0.468
 
 
49.7
 
 
 
 
ARG2
 
 
hsa-let-7c-5p
 
 
-0.468
 
 
49.7
 
 
 
 
ARHGAP1
 
 
hsa-miR-17-5p
 
 
-0.325
 
 
26.4
 
 
 
 
ARHGAP1
 
 
hsa-miR-20b-5p
 
 
-0.325
 
 
26.4
 
 
 
 
ARHGAP1
 
 
hsa-miR-106a-5p
 
 
-0.329
 
 
26.4
 
 
 
 
ARHGAP1
 
 
hsa-miR-20a-5p
 
 
-0.339
 
 
26.4
 
 
 
 
ARHGAP1
 
 
hsa-miR-106b-5p
 
 
-0.339
 
 
26.4
 
 
 
 
ARL1
 
 
hsa-miR-20a-5p
 
 
-0.403
 
 
84.0
 
 
 
 
ARL1
 
 
hsa-miR-106b-5p
 
 
-0.411
 
 
84.0
 
 
 
 
ARL1
 
 
hsa-miR-20b-5p
 
 
-0.424
 
 
84.0
 
 
 
 
ARL1
 
 
hsa-miR-106a-5p
 
 
-0.429
 
 
84.0
 
 
 
 
ARL1
 
 
hsa-miR-17-5p
 
 
-0.432
 
 
84.0
 
 
 
 
ARL4D
 
 
hsa-miR-98-5p
 
 
-0.417
 
 
15.4
 
 
 
 
ARL4D
 
 
hsa-let-7c-5p
 
 
-0.417
 
 
15.4
 
 
 
 
ARL6IP1
 
 
hsa-miR-7-5p
 
 
-0.374
 
 
185.0
 
 
 
 
ARL6IP1
 
 
hsa-miR-148a-3p
 
 
-0.579
 
 
185.0
 
 
 
 
ARL8B
 
 
hsa-miR-148a-3p
 
 
-0.337
 
 
19.0
 
 
 
 
ARMC8
 
 
hsa-miR-20a-5p
 
 
-0.357
 
 
20.8
 
 
 
 
ARMC8
 
 
hsa-miR-106b-5p
 
 
-0.357
 
 
20.8
 
 
 
 
ARMC8
 
 
hsa-miR-106a-5p
 
 
-0.375
 
 
20.8
 
 
 
 
ARMC8
 
 
hsa-miR-17-5p
 
 
-0.378
 
 
20.8
 
 
 
 
ARMC8
 
 
hsa-miR-20b-5p
 
 
-0.378
 
 
20.8
 
 
 
 
ARMCX1
 
 
hsa-miR-21-5p
 
 
-0.489
 
 
20.0
 
 
 
 
ARPP19
 
 
hsa-let-7c-5p
 
 
-0.492
 
 
32.4
 
 
 
 
ARPP19
 
 
hsa-miR-98-5p
 
 
-0.5
 
 
32.4
 
 
 
 
ARRDC3
 
 
hsa-miR-148a-3p
 
 
-0.305
 
 
24.1
 
 
 
 
ASPH
 
 
hsa-miR-148a-3p
 
 
-0.391
 
 
79.9
 
 
 
 
ATG16L1
 
 
hsa-miR-20a-5p
 
 
-0.446
 
 
21.8
 
 
 
 
ATG16L1
 
 
hsa-miR-106b-5p
 
 
-0.446
 
 
21.8
 
 
 
 
ATG16L1
 
 
hsa-miR-106a-5p
 
 
-0.464
 
 
21.8
 
 
 
 
ATG16L1
 
 
hsa-miR-17-5p
 
 
-0.467
 
 
21.8
 
 
 
 
ATG16L1
 
 
hsa-miR-20b-5p
 
 
-0.467
 
 
21.8
 
 
 
 
ATG4A
 
 
hsa-miR-7-5p
 
 
-0.448
 
 
16.6
 
 
 
 
ATOSB
 
 
hsa-miR-98-5p
 
 
-0.429
 
 
16.6
 
 
 
 
ATOSB
 
 
hsa-let-7c-5p
 
 
-0.429
 
 
16.6
 
 
 
 
ATP2A2
 
 
hsa-miR-98-5p
 
 
-0.311
 
 
29.6
 
 
 
 
ATP2A2
 
 
hsa-let-7c-5p
 
 
-0.311
 
 
29.6
 
 
 
 
ATP2B2
 
 
hsa-miR-7-5p
 
 
-0.297
 
 
61.0
 
 
 
 
ATP6AP2
 
 
hsa-miR-148a-3p
 
 
-0.592
 
 
85.2
 
 
 
 
ATP6AP2
 
 
hsa-miR-148a-3p
 
 
-0.668
 
 
85.2
 
 
 
 
ATPAF1
 
 
hsa-miR-98-5p
 
 
-0.498
 
 
168.0
 
 
 
 
ATPAF1
 
 
hsa-let-7c-5p
 
 
-0.498
 
 
168.0
 
 
 
 
AVPI1
 
 
hsa-miR-7-5p
 
 
-0.367
 
 
20.0
 
 
 
 
B3GAT3
 
 
hsa-miR-98-5p
 
 
-0.324
 
 
87.1
 
 
 
 
B3GAT3
 
 
hsa-let-7c-5p
 
 
-0.324
 
 
87.1
 
 
 
 
B4GAT1
 
 
hsa-let-7c-5p
 
 
-0.47
 
 
93.8
 
 
 
 
B4GAT1
 
 
hsa-miR-98-5p
 
 
-0.47
 
 
93.8
 
 
 
 
BAMBI
 
 
hsa-miR-20a-5p
 
 
-0.485
 
 
37.8
 
 
 
 
BAMBI
 
 
hsa-miR-106b-5p
 
 
-0.493
 
 
37.8
 
 
 
 
BAMBI
 
 
hsa-miR-106a-5p
 
 
-0.503
 
 
37.8
 
 
 
 
BAMBI
 
 
hsa-miR-17-5p
 
 
-0.506
 
 
37.8
 
 
 
 
BAMBI
 
 
hsa-miR-20b-5p
 
 
-0.506
 
 
37.8
 
 
 
 
BIN3
 
 
hsa-miR-98-5p
 
 
-0.427
 
 
33.7
 
 
 
 
BIN3
 
 
hsa-let-7c-5p
 
 
-0.427
 
 
33.7
 
 
 
 
BLOC1S4
 
 
hsa-miR-7-5p
 
 
-0.417
 
 
10.3
 
 
 
 
BLOC1S4
 
 
hsa-miR-7-5p
 
 
-0.527
 
 
10.3
 
 
 
 
BLOC1S6
 
 
hsa-miR-98-5p
 
 
-0.321
 
 
14.9
 
 
 
 
BLOC1S6
 
 
hsa-let-7c-5p
 
 
-0.321
 
 
14.9
 
 
 
 
BNIP3L
 
 
hsa-miR-20a-5p
 
 
-0.287
 
 
46.7
 
 
 
 
BNIP3L
 
 
hsa-miR-106b-5p
 
 
-0.298
 
 
46.7
 
 
 
 
BTBD10
 
 
hsa-miR-106b-5p
 
 
-0.388
 
 
10.3
 
 
 
 
BTBD10
 
 
hsa-miR-106a-5p
 
 
-0.406
 
 
10.3
 
 
 
 
BTBD10
 
 
hsa-miR-17-5p
 
 
-0.409
 
 
10.3
 
 
 
 
BTBD10
 
 
hsa-miR-20a-5p
 
 
-0.412
 
 
10.3
 
 
 
 
BTBD10
 
 
hsa-miR-20b-5p
 
 
-0.425
 
 
10.3
 
 
 
 
BTBD3
 
 
hsa-miR-148a-3p
 
 
-0.495
 
 
18.2
 
 
 
 
 
 
 endometrium 
 
 
 
 
target_symbol
 
 
mature_mirna_id
 
 
score
 
 
nTPM
 
 
 
 
 
 
ABCB7
 
 
hsa-miR-148a-3p
 
 
-0.515
 
 
10.5
 
 
 
 
ABCD3
 
 
hsa-miR-148a-3p
 
 
-0.388
 
 
13.8
 
 
 
 
ACTC1
 
 
hsa-miR-7-5p
 
 
-0.471
 
 
17.7
 
 
 
 
ACVR1
 
 
hsa-miR-148a-3p
 
 
-0.397
 
 
15.9
 
 
 
 
ADAM10
 
 
hsa-miR-148a-3p
 
 
-0.356
 
 
15.2
 
 
 
 
ADAM9
 
 
hsa-miR-20a-5p
 
 
-0.299
 
 
21.6
 
 
 
 
ADAM9
 
 
hsa-miR-106b-5p
 
 
-0.299
 
 
21.6
 
 
 
 
ADAM9
 
 
hsa-miR-106a-5p
 
 
-0.317
 
 
21.6
 
 
 
 
ADAM9
 
 
hsa-miR-17-5p
 
 
-0.32
 
 
21.6
 
 
 
 
ADAM9
 
 
hsa-miR-20b-5p
 
 
-0.328
 
 
21.6
 
 
 
 
ADGRF5
 
 
hsa-miR-148a-3p
 
 
-0.354
 
 
16.0
 
 
 
 
AGFG1
 
 
hsa-miR-148a-3p
 
 
-0.332
 
 
23.1
 
 
 
 
AGFG2
 
 
hsa-miR-20a-5p
 
 
-0.365
 
 
20.0
 
 
 
 
AGFG2
 
 
hsa-miR-20b-5p
 
 
-0.386
 
 
20.0
 
 
 
 
AGFG2
 
 
hsa-miR-106b-5p
 
 
-0.397
 
 
20.0
 
 
 
 
AGFG2
 
 
hsa-miR-106a-5p
 
 
-0.399
 
 
20.0
 
 
 
 
AGFG2
 
 
hsa-miR-17-5p
 
 
-0.402
 
 
20.0
 
 
 
 
AHDC1
 
 
hsa-miR-148a-3p
 
 
-0.413
 
 
26.0
 
 
 
 
AHNAK
 
 
hsa-miR-20a-5p
 
 
-0.316
 
 
187.4
 
 
 
 
AHNAK
 
 
hsa-miR-106b-5p
 
 
-0.316
 
 
187.4
 
 
 
 
AHNAK
 
 
hsa-miR-106a-5p
 
 
-0.334
 
 
187.4
 
 
 
 
AHNAK
 
 
hsa-miR-17-5p
 
 
-0.337
 
 
187.4
 
 
 
 
AHNAK
 
 
hsa-miR-20b-5p
 
 
-0.337
 
 
187.4
 
 
 
 
AIFM1
 
 
hsa-miR-98-5p
 
 
-0.314
 
 
10.6
 
 
 
 
AIFM1
 
 
hsa-let-7c-5p
 
 
-0.314
 
 
10.6
 
 
 
 
AKAP1
 
 
hsa-miR-148a-3p
 
 
-0.381
 
 
24.8
 
 
 
 
ALCAM
 
 
hsa-miR-148a-3p
 
 
-0.339
 
 
14.4
 
 
 
 
ALDH4A1
 
 
hsa-miR-184
 
 
-0.421
 
 
10.1
 
 
 
 
ALG3
 
 
hsa-miR-106b-5p
 
 
-0.551
 
 
15.9
 
 
 
 
ALG3
 
 
hsa-miR-20a-5p
 
 
-0.559
 
 
15.9
 
 
 
 
ALG3
 
 
hsa-miR-106a-5p
 
 
-0.569
 
 
15.9
 
 
 
 
ALG3
 
 
hsa-miR-17-5p
 
 
-0.572
 
 
15.9
 
 
 
 
ALG3
 
 
hsa-miR-20b-5p
 
 
-0.58
 
 
15.9
 
 
 
 
ALG9
 
 
hsa-miR-7-5p
 
 
-0.302
 
 
10.7
 
 
 
 
AMT
 
 
hsa-miR-98-5p
 
 
-0.463
 
 
10.0
 
 
 
 
AMT
 
 
hsa-let-7c-5p
 
 
-0.463
 
 
10.0
 
 
 
 
ANKRD12
 
 
hsa-miR-7-5p
 
 
-0.37
 
 
11.1
 
 
 
 
ANKRD29
 
 
hsa-miR-20a-5p
 
 
-0.359
 
 
14.7
 
 
 
 
ANKRD29
 
 
hsa-miR-106b-5p
 
 
-0.359
 
 
14.7
 
 
 
 
ANKRD29
 
 
hsa-miR-106a-5p
 
 
-0.369
 
 
14.7
 
 
 
 
ANKRD29
 
 
hsa-miR-17-5p
 
 
-0.372
 
 
14.7
 
 
 
 
ANKRD29
 
 
hsa-miR-20b-5p
 
 
-0.38
 
 
14.7
 
 
 
 
ANKRD46
 
 
hsa-miR-98-5p
 
 
-0.389
 
 
11.8
 
 
 
 
ANKRD46
 
 
hsa-let-7c-5p
 
 
-0.389
 
 
11.8
 
 
 
 
AP1AR
 
 
hsa-miR-21-5p
 
 
-0.318
 
 
10.6
 
 
 
 
AP1S1
 
 
hsa-miR-98-5p
 
 
-0.374
 
 
16.1
 
 
 
 
AP1S1
 
 
hsa-let-7c-5p
 
 
-0.374
 
 
16.1
 
 
 
 
APCDD1
 
 
hsa-miR-20a-5p
 
 
-0.404
 
 
32.5
 
 
 
 
APCDD1
 
 
hsa-miR-106b-5p
 
 
-0.404
 
 
32.5
 
 
 
 
APCDD1
 
 
hsa-miR-106a-5p
 
 
-0.422
 
 
32.5
 
 
 
 
APCDD1
 
 
hsa-miR-17-5p
 
 
-0.425
 
 
32.5
 
 
 
 
APCDD1
 
 
hsa-miR-20b-5p
 
 
-0.425
 
 
32.5
 
 
 
 
ARF4
 
 
hsa-miR-7-5p
 
 
-0.407
 
 
113.9
 
 
 
 
ARF4
 
 
hsa-miR-148a-3p
 
 
-0.487
 
 
113.9
 
 
 
 
ARFIP1
 
 
hsa-miR-148a-3p
 
 
-0.474
 
 
20.3
 
 
 
 
ARHGAP1
 
 
hsa-miR-17-5p
 
 
-0.325
 
 
81.8
 
 
 
 
ARHGAP1
 
 
hsa-miR-20b-5p
 
 
-0.325
 
 
81.8
 
 
 
 
ARHGAP1
 
 
hsa-miR-106a-5p
 
 
-0.329
 
 
81.8
 
 
 
 
ARHGAP1
 
 
hsa-miR-20a-5p
 
 
-0.339
 
 
81.8
 
 
 
 
ARHGAP1
 
 
hsa-miR-106b-5p
 
 
-0.339
 
 
81.8
 
 
 
 
ARHGAP21
 
 
hsa-miR-148a-3p
 
 
-0.287
 
 
18.5
 
 
 
 
ARID4B
 
 
hsa-miR-20a-5p
 
 
-0.29
 
 
14.9
 
 
 
 
ARID4B
 
 
hsa-miR-106b-5p
 
 
-0.29
 
 
14.9
 
 
 
 
ARL1
 
 
hsa-miR-20a-5p
 
 
-0.403
 
 
47.9
 
 
 
 
ARL1
 
 
hsa-miR-106b-5p
 
 
-0.411
 
 
47.9
 
 
 
 
ARL1
 
 
hsa-miR-20b-5p
 
 
-0.424
 
 
47.9
 
 
 
 
ARL1
 
 
hsa-miR-106a-5p
 
 
-0.429
 
 
47.9
 
 
 
 
ARL1
 
 
hsa-miR-17-5p
 
 
-0.432
 
 
47.9
 
 
 
 
ARL4D
 
 
hsa-miR-98-5p
 
 
-0.417
 
 
19.2
 
 
 
 
ARL4D
 
 
hsa-let-7c-5p
 
 
-0.417
 
 
19.2
 
 
 
 
ARL6IP1
 
 
hsa-miR-7-5p
 
 
-0.374
 
 
66.4
 
 
 
 
ARL6IP1
 
 
hsa-miR-148a-3p
 
 
-0.579
 
 
66.4
 
 
 
 
ARL6IP6
 
 
hsa-miR-98-5p
 
 
-0.293
 
 
11.0
 
 
 
 
ARL6IP6
 
 
hsa-let-7c-5p
 
 
-0.293
 
 
11.0
 
 
 
 
ARL8B
 
 
hsa-miR-148a-3p
 
 
-0.337
 
 
42.3
 
 
 
 
ARMC8
 
 
hsa-miR-20a-5p
 
 
-0.357
 
 
16.5
 
 
 
 
ARMC8
 
 
hsa-miR-106b-5p
 
 
-0.357
 
 
16.5
 
 
 
 
ARMC8
 
 
hsa-miR-106a-5p
 
 
-0.375
 
 
16.5
 
 
 
 
ARMC8
 
 
hsa-miR-17-5p
 
 
-0.378
 
 
16.5
 
 
 
 
ARMC8
 
 
hsa-miR-20b-5p
 
 
-0.378
 
 
16.5
 
 
 
 
ARMCX1
 
 
hsa-miR-21-5p
 
 
-0.489
 
 
28.5
 
 
 
 
ARMT1
 
 
hsa-let-7c-5p
 
 
-0.303
 
 
15.0
 
 
 
 
ARMT1
 
 
hsa-miR-98-5p
 
 
-0.303
 
 
15.0
 
 
 
 
ARPP19
 
 
hsa-let-7c-5p
 
 
-0.492
 
 
44.7
 
 
 
 
ARPP19
 
 
hsa-miR-98-5p
 
 
-0.5
 
 
44.7
 
 
 
 
ARRDC3
 
 
hsa-miR-148a-3p
 
 
-0.305
 
 
38.0
 
 
 
 
ARRDC4
 
 
hsa-let-7c-5p
 
 
-0.329
 
 
13.7
 
 
 
 
ARRDC4
 
 
hsa-miR-98-5p
 
 
-0.329
 
 
13.7
 
 
 
 
ASAP1
 
 
hsa-miR-98-5p
 
 
-0.308
 
 
17.2
 
 
 
 
ASAP1
 
 
hsa-let-7c-5p
 
 
-0.308
 
 
17.2
 
 
 
 
ASPH
 
 
hsa-miR-148a-3p
 
 
-0.391
 
 
45.6
 
 
 
 
ATG16L1
 
 
hsa-miR-20a-5p
 
 
-0.446
 
 
11.3
 
 
 
 
ATG16L1
 
 
hsa-miR-106b-5p
 
 
-0.446
 
 
11.3
 
 
 
 
ATG16L1
 
 
hsa-miR-106a-5p
 
 
-0.464
 
 
11.3
 
 
 
 
ATG16L1
 
 
hsa-miR-17-5p
 
 
-0.467
 
 
11.3
 
 
 
 
ATG16L1
 
 
hsa-miR-20b-5p
 
 
-0.467
 
 
11.3
 
 
 
 
ATP2A2
 
 
hsa-miR-98-5p
 
 
-0.311
 
 
43.7
 
 
 
 
ATP2A2
 
 
hsa-let-7c-5p
 
 
-0.311
 
 
43.7
 
 
 
 
ATP2B4
 
 
hsa-miR-148a-3p
 
 
-0.337
 
 
98.2
 
 
 
 
ATP6AP2
 
 
hsa-miR-148a-3p
 
 
-0.592
 
 
81.0
 
 
 
 
 
 
 esophagus 
 
 
 
 
target_symbol
 
 
mature_mirna_id
 
 
score
 
 
nTPM
 
 
 
 
 
 
ABCB7
 
 
hsa-miR-148a-3p
 
 
-0.515
 
 
11.1
 
 
 
 
ABCD3
 
 
hsa-miR-148a-3p
 
 
-0.388
 
 
25.0
 
 
 
 
ABHD17C
 
 
hsa-let-7c-5p
 
 
-0.359
 
 
45.1
 
 
 
 
ABHD17C
 
 
hsa-miR-98-5p
 
 
-0.383
 
 
45.1
 
 
 
 
ABHD5
 
 
hsa-miR-20a-5p
 
 
-0.397
 
 
18.4
 
 
 
 
ABHD5
 
 
hsa-miR-106b-5p
 
 
-0.413
 
 
18.4
 
 
 
 
ABHD5
 
 
hsa-miR-20b-5p
 
 
-0.418
 
 
18.4
 
 
 
 
ABHD5
 
 
hsa-miR-106a-5p
 
 
-0.447
 
 
18.4
 
 
 
 
ABHD5
 
 
hsa-miR-17-5p
 
 
-0.45
 
 
18.4
 
 
 
 
ACP3
 
 
hsa-miR-98-5p
 
 
-0.298
 
 
42.9
 
 
 
 
ACP3
 
 
hsa-let-7c-5p
 
 
-0.298
 
 
42.9
 
 
 
 
ACTC1
 
 
hsa-miR-7-5p
 
 
-0.471
 
 
21.5
 
 
 
 
ADAM10
 
 
hsa-miR-148a-3p
 
 
-0.356
 
 
14.2
 
 
 
 
ADAM9
 
 
hsa-miR-20a-5p
 
 
-0.299
 
 
22.2
 
 
 
 
ADAM9
 
 
hsa-miR-106b-5p
 
 
-0.299
 
 
22.2
 
 
 
 
ADAM9
 
 
hsa-miR-106a-5p
 
 
-0.317
 
 
22.2
 
 
 
 
ADAM9
 
 
hsa-miR-17-5p
 
 
-0.32
 
 
22.2
 
 
 
 
ADAM9
 
 
hsa-miR-20b-5p
 
 
-0.328
 
 
22.2
 
 
 
 
ADRB2
 
 
hsa-let-7c-5p
 
 
-0.635
 
 
14.2
 
 
 
 
ADRB2
 
 
hsa-miR-98-5p
 
 
-0.635
 
 
14.2
 
 
 
 
AGFG1
 
 
hsa-miR-148a-3p
 
 
-0.332
 
 
44.3
 
 
 
 
AGFG2
 
 
hsa-miR-20a-5p
 
 
-0.365
 
 
29.3
 
 
 
 
AGFG2
 
 
hsa-miR-20b-5p
 
 
-0.386
 
 
29.3
 
 
 
 
AGFG2
 
 
hsa-miR-106b-5p
 
 
-0.397
 
 
29.3
 
 
 
 
AGFG2
 
 
hsa-miR-106a-5p
 
 
-0.399
 
 
29.3
 
 
 
 
AGFG2
 
 
hsa-miR-17-5p
 
 
-0.402
 
 
29.3
 
 
 
 
AHDC1
 
 
hsa-miR-148a-3p
 
 
-0.413
 
 
34.3
 
 
 
 
AHNAK
 
 
hsa-miR-20a-5p
 
 
-0.316
 
 
234.9
 
 
 
 
AHNAK
 
 
hsa-miR-106b-5p
 
 
-0.316
 
 
234.9
 
 
 
 
AHNAK
 
 
hsa-miR-106a-5p
 
 
-0.334
 
 
234.9
 
 
 
 
AHNAK
 
 
hsa-miR-17-5p
 
 
-0.337
 
 
234.9
 
 
 
 
AHNAK
 
 
hsa-miR-20b-5p
 
 
-0.337
 
 
234.9
 
 
 
 
AIFM1
 
 
hsa-miR-98-5p
 
 
-0.314
 
 
15.9
 
 
 
 
AIFM1
 
 
hsa-let-7c-5p
 
 
-0.314
 
 
15.9
 
 
 
 
AKAP1
 
 
hsa-miR-148a-3p
 
 
-0.381
 
 
34.2
 
 
 
 
ALDH4A1
 
 
hsa-miR-184
 
 
-0.421
 
 
36.6
 
 
 
 
ALG3
 
 
hsa-miR-106b-5p
 
 
-0.551
 
 
29.4
 
 
 
 
ALG3
 
 
hsa-miR-20a-5p
 
 
-0.559
 
 
29.4
 
 
 
 
ALG3
 
 
hsa-miR-106a-5p
 
 
-0.569
 
 
29.4
 
 
 
 
ALG3
 
 
hsa-miR-17-5p
 
 
-0.572
 
 
29.4
 
 
 
 
ALG3
 
 
hsa-miR-20b-5p
 
 
-0.58
 
 
29.4
 
 
 
 
ALG9
 
 
hsa-miR-7-5p
 
 
-0.302
 
 
10.5
 
 
 
 
ANKRD12
 
 
hsa-miR-7-5p
 
 
-0.37
 
 
13.6
 
 
 
 
ANKRD46
 
 
hsa-miR-98-5p
 
 
-0.389
 
 
11.9
 
 
 
 
ANKRD46
 
 
hsa-let-7c-5p
 
 
-0.389
 
 
11.9
 
 
 
 
ANKRD9
 
 
hsa-miR-20a-5p
 
 
-0.31
 
 
18.5
 
 
 
 
ANKRD9
 
 
hsa-miR-106b-5p
 
 
-0.318
 
 
18.5
 
 
 
 
ANKRD9
 
 
hsa-miR-20b-5p
 
 
-0.331
 
 
18.5
 
 
 
 
ANKRD9
 
 
hsa-miR-106a-5p
 
 
-0.344
 
 
18.5
 
 
 
 
ANKRD9
 
 
hsa-miR-17-5p
 
 
-0.347
 
 
18.5
 
 
 
 
AP1AR
 
 
hsa-miR-21-5p
 
 
-0.318
 
 
12.5
 
 
 
 
AP1S1
 
 
hsa-miR-98-5p
 
 
-0.374
 
 
36.5
 
 
 
 
AP1S1
 
 
hsa-let-7c-5p
 
 
-0.374
 
 
36.5
 
 
 
 
APCDD1
 
 
hsa-miR-20a-5p
 
 
-0.404
 
 
26.2
 
 
 
 
APCDD1
 
 
hsa-miR-106b-5p
 
 
-0.404
 
 
26.2
 
 
 
 
APCDD1
 
 
hsa-miR-106a-5p
 
 
-0.422
 
 
26.2
 
 
 
 
APCDD1
 
 
hsa-miR-17-5p
 
 
-0.425
 
 
26.2
 
 
 
 
APCDD1
 
 
hsa-miR-20b-5p
 
 
-0.425
 
 
26.2
 
 
 
 
ARF4
 
 
hsa-miR-7-5p
 
 
-0.407
 
 
102.8
 
 
 
 
ARF4
 
 
hsa-miR-148a-3p
 
 
-0.487
 
 
102.8
 
 
 
 
ARFGEF1
 
 
hsa-miR-148a-3p
 
 
-0.306
 
 
14.2
 
 
 
 
ARFIP1
 
 
hsa-miR-148a-3p
 
 
-0.474
 
 
19.6
 
 
 
 
ARG2
 
 
hsa-miR-98-5p
 
 
-0.468
 
 
17.2
 
 
 
 
ARG2
 
 
hsa-let-7c-5p
 
 
-0.468
 
 
17.2
 
 
 
 
ARHGAP1
 
 
hsa-miR-17-5p
 
 
-0.325
 
 
71.8
 
 
 
 
ARHGAP1
 
 
hsa-miR-20b-5p
 
 
-0.325
 
 
71.8
 
 
 
 
ARHGAP1
 
 
hsa-miR-106a-5p
 
 
-0.329
 
 
71.8
 
 
 
 
ARHGAP1
 
 
hsa-miR-20a-5p
 
 
-0.339
 
 
71.8
 
 
 
 
ARHGAP1
 
 
hsa-miR-106b-5p
 
 
-0.339
 
 
71.8
 
 
 
 
ARHGAP21
 
 
hsa-miR-148a-3p
 
 
-0.287
 
 
10.2
 
 
 
 
ARL1
 
 
hsa-miR-20a-5p
 
 
-0.403
 
 
46.2
 
 
 
 
ARL1
 
 
hsa-miR-106b-5p
 
 
-0.411
 
 
46.2
 
 
 
 
ARL1
 
 
hsa-miR-20b-5p
 
 
-0.424
 
 
46.2
 
 
 
 
ARL1
 
 
hsa-miR-106a-5p
 
 
-0.429
 
 
46.2
 
 
 
 
ARL1
 
 
hsa-miR-17-5p
 
 
-0.432
 
 
46.2
 
 
 
 
ARL4D
 
 
hsa-miR-98-5p
 
 
-0.417
 
 
80.8
 
 
 
 
ARL4D
 
 
hsa-let-7c-5p
 
 
-0.417
 
 
80.8
 
 
 
 
ARL6IP1
 
 
hsa-miR-7-5p
 
 
-0.374
 
 
65.8
 
 
 
 
ARL6IP1
 
 
hsa-miR-148a-3p
 
 
-0.579
 
 
65.8
 
 
 
 
ARL6IP6
 
 
hsa-miR-98-5p
 
 
-0.293
 
 
11.7
 
 
 
 
ARL6IP6
 
 
hsa-let-7c-5p
 
 
-0.293
 
 
11.7
 
 
 
 
ARL8B
 
 
hsa-miR-148a-3p
 
 
-0.337
 
 
71.1
 
 
 
 
ARMC8
 
 
hsa-miR-20a-5p
 
 
-0.357
 
 
19.8
 
 
 
 
ARMC8
 
 
hsa-miR-106b-5p
 
 
-0.357
 
 
19.8
 
 
 
 
ARMC8
 
 
hsa-miR-106a-5p
 
 
-0.375
 
 
19.8
 
 
 
 
ARMC8
 
 
hsa-miR-17-5p
 
 
-0.378
 
 
19.8
 
 
 
 
ARMC8
 
 
hsa-miR-20b-5p
 
 
-0.378
 
 
19.8
 
 
 
 
ARMCX1
 
 
hsa-miR-21-5p
 
 
-0.489
 
 
10.7
 
 
 
 
ARMT1
 
 
hsa-let-7c-5p
 
 
-0.303
 
 
11.3
 
 
 
 
ARMT1
 
 
hsa-miR-98-5p
 
 
-0.303
 
 
11.3
 
 
 
 
ARPP19
 
 
hsa-let-7c-5p
 
 
-0.492
 
 
49.0
 
 
 
 
ARPP19
 
 
hsa-miR-98-5p
 
 
-0.5
 
 
49.0
 
 
 
 
ARRDC3
 
 
hsa-miR-148a-3p
 
 
-0.305
 
 
36.4
 
 
 
 
ASAP1
 
 
hsa-miR-98-5p
 
 
-0.308
 
 
10.0
 
 
 
 
ASAP1
 
 
hsa-let-7c-5p
 
 
-0.308
 
 
10.0
 
 
 
 
ASPH
 
 
hsa-miR-148a-3p
 
 
-0.391
 
 
66.3
 
 
 
 
ATG16L1
 
 
hsa-miR-20a-5p
 
 
-0.446
 
 
11.5
 
 
 
 
ATG16L1
 
 
hsa-miR-106b-5p
 
 
-0.446
 
 
11.5
 
 
 
 
ATG16L1
 
 
hsa-miR-106a-5p
 
 
-0.464
 
 
11.5
 
 
 
 
ATG16L1
 
 
hsa-miR-17-5p
 
 
-0.467
 
 
11.5
 
 
 
 
 
 
 fallopian tube 
 
 
 
 
target_symbol
 
 
mature_mirna_id
 
 
score
 
 
nTPM
 
 
 
 
 
 
ABCB7
 
 
hsa-miR-148a-3p
 
 
-0.515
 
 
11.3
 
 
 
 
ABCD3
 
 
hsa-miR-148a-3p
 
 
-0.388
 
 
15.8
 
 
 
 
ABHD17C
 
 
hsa-let-7c-5p
 
 
-0.359
 
 
10.3
 
 
 
 
ABHD17C
 
 
hsa-miR-98-5p
 
 
-0.383
 
 
10.3
 
 
 
 
ACVR1
 
 
hsa-miR-148a-3p
 
 
-0.397
 
 
13.2
 
 
 
 
ADAM10
 
 
hsa-miR-148a-3p
 
 
-0.356
 
 
13.1
 
 
 
 
ADAM9
 
 
hsa-miR-20a-5p
 
 
-0.299
 
 
15.9
 
 
 
 
ADAM9
 
 
hsa-miR-106b-5p
 
 
-0.299
 
 
15.9
 
 
 
 
ADAM9
 
 
hsa-miR-106a-5p
 
 
-0.317
 
 
15.9
 
 
 
 
ADAM9
 
 
hsa-miR-17-5p
 
 
-0.32
 
 
15.9
 
 
 
 
ADAM9
 
 
hsa-miR-20b-5p
 
 
-0.328
 
 
15.9
 
 
 
 
ADGRF5
 
 
hsa-miR-148a-3p
 
 
-0.354
 
 
13.6
 
 
 
 
ADGRG2
 
 
hsa-miR-21-5p
 
 
-0.343
 
 
15.5
 
 
 
 
AGFG1
 
 
hsa-miR-148a-3p
 
 
-0.332
 
 
23.3
 
 
 
 
AGFG2
 
 
hsa-miR-20a-5p
 
 
-0.365
 
 
14.4
 
 
 
 
AGFG2
 
 
hsa-miR-20b-5p
 
 
-0.386
 
 
14.4
 
 
 
 
AGFG2
 
 
hsa-miR-106b-5p
 
 
-0.397
 
 
14.4
 
 
 
 
AGFG2
 
 
hsa-miR-106a-5p
 
 
-0.399
 
 
14.4
 
 
 
 
AGFG2
 
 
hsa-miR-17-5p
 
 
-0.402
 
 
14.4
 
 
 
 
AHDC1
 
 
hsa-miR-148a-3p
 
 
-0.413
 
 
19.8
 
 
 
 
AHNAK
 
 
hsa-miR-20a-5p
 
 
-0.316
 
 
141.9
 
 
 
 
AHNAK
 
 
hsa-miR-106b-5p
 
 
-0.316
 
 
141.9
 
 
 
 
AHNAK
 
 
hsa-miR-106a-5p
 
 
-0.334
 
 
141.9
 
 
 
 
AHNAK
 
 
hsa-miR-17-5p
 
 
-0.337
 
 
141.9
 
 
 
 
AHNAK
 
 
hsa-miR-20b-5p
 
 
-0.337
 
 
141.9
 
 
 
 
AIFM1
 
 
hsa-miR-98-5p
 
 
-0.314
 
 
20.8
 
 
 
 
AIFM1
 
 
hsa-let-7c-5p
 
 
-0.314
 
 
20.8
 
 
 
 
AKAP1
 
 
hsa-miR-148a-3p
 
 
-0.381
 
 
33.9
 
 
 
 
ALCAM
 
 
hsa-miR-148a-3p
 
 
-0.339
 
 
46.2
 
 
 
 
ALG3
 
 
hsa-miR-106b-5p
 
 
-0.551
 
 
14.3
 
 
 
 
ALG3
 
 
hsa-miR-20a-5p
 
 
-0.559
 
 
14.3
 
 
 
 
ALG3
 
 
hsa-miR-106a-5p
 
 
-0.569
 
 
14.3
 
 
 
 
ALG3
 
 
hsa-miR-17-5p
 
 
-0.572
 
 
14.3
 
 
 
 
ALG3
 
 
hsa-miR-20b-5p
 
 
-0.58
 
 
14.3
 
 
 
 
AMT
 
 
hsa-miR-98-5p
 
 
-0.463
 
 
13.9
 
 
 
 
AMT
 
 
hsa-let-7c-5p
 
 
-0.463
 
 
13.9
 
 
 
 
ANKRD29
 
 
hsa-miR-20a-5p
 
 
-0.359
 
 
14.4
 
 
 
 
ANKRD29
 
 
hsa-miR-106b-5p
 
 
-0.359
 
 
14.4
 
 
 
 
ANKRD29
 
 
hsa-miR-106a-5p
 
 
-0.369
 
 
14.4
 
 
 
 
ANKRD29
 
 
hsa-miR-17-5p
 
 
-0.372
 
 
14.4
 
 
 
 
ANKRD29
 
 
hsa-miR-20b-5p
 
 
-0.38
 
 
14.4
 
 
 
 
ANKRD46
 
 
hsa-miR-98-5p
 
 
-0.389
 
 
10.7
 
 
 
 
ANKRD46
 
 
hsa-let-7c-5p
 
 
-0.389
 
 
10.7
 
 
 
 
AP1S1
 
 
hsa-miR-98-5p
 
 
-0.374
 
 
13.4
 
 
 
 
AP1S1
 
 
hsa-let-7c-5p
 
 
-0.374
 
 
13.4
 
 
 
 
APCDD1
 
 
hsa-miR-20a-5p
 
 
-0.404
 
 
30.6
 
 
 
 
APCDD1
 
 
hsa-miR-106b-5p
 
 
-0.404
 
 
30.6
 
 
 
 
APCDD1
 
 
hsa-miR-106a-5p
 
 
-0.422
 
 
30.6
 
 
 
 
APCDD1
 
 
hsa-miR-17-5p
 
 
-0.425
 
 
30.6
 
 
 
 
APCDD1
 
 
hsa-miR-20b-5p
 
 
-0.425
 
 
30.6
 
 
 
 
ARF4
 
 
hsa-miR-7-5p
 
 
-0.407
 
 
79.6
 
 
 
 
ARF4
 
 
hsa-miR-148a-3p
 
 
-0.487
 
 
79.6
 
 
 
 
ARFGEF1
 
 
hsa-miR-148a-3p
 
 
-0.306
 
 
11.1
 
 
 
 
ARFIP1
 
 
hsa-miR-148a-3p
 
 
-0.474
 
 
17.4
 
 
 
 
ARHGAP1
 
 
hsa-miR-17-5p
 
 
-0.325
 
 
71.5
 
 
 
 
ARHGAP1
 
 
hsa-miR-20b-5p
 
 
-0.325
 
 
71.5
 
 
 
 
ARHGAP1
 
 
hsa-miR-106a-5p
 
 
-0.329
 
 
71.5
 
 
 
 
ARHGAP1
 
 
hsa-miR-20a-5p
 
 
-0.339
 
 
71.5
 
 
 
 
ARHGAP1
 
 
hsa-miR-106b-5p
 
 
-0.339
 
 
71.5
 
 
 
 
ARHGAP21
 
 
hsa-miR-148a-3p
 
 
-0.287
 
 
16.4
 
 
 
 
ARL1
 
 
hsa-miR-20a-5p
 
 
-0.403
 
 
47.1
 
 
 
 
ARL1
 
 
hsa-miR-106b-5p
 
 
-0.411
 
 
47.1
 
 
 
 
ARL1
 
 
hsa-miR-20b-5p
 
 
-0.424
 
 
47.1
 
 
 
 
ARL1
 
 
hsa-miR-106a-5p
 
 
-0.429
 
 
47.1
 
 
 
 
ARL1
 
 
hsa-miR-17-5p
 
 
-0.432
 
 
47.1
 
 
 
 
ARL4D
 
 
hsa-miR-98-5p
 
 
-0.417
 
 
20.8
 
 
 
 
ARL4D
 
 
hsa-let-7c-5p
 
 
-0.417
 
 
20.8
 
 
 
 
ARL6IP1
 
 
hsa-miR-7-5p
 
 
-0.374
 
 
81.6
 
 
 
 
ARL6IP1
 
 
hsa-miR-148a-3p
 
 
-0.579
 
 
81.6
 
 
 
 
ARL6IP6
 
 
hsa-miR-98-5p
 
 
-0.293
 
 
10.8
 
 
 
 
ARL6IP6
 
 
hsa-let-7c-5p
 
 
-0.293
 
 
10.8
 
 
 
 
ARL8B
 
 
hsa-miR-148a-3p
 
 
-0.337
 
 
29.3
 
 
 
 
ARMC8
 
 
hsa-miR-20a-5p
 
 
-0.357
 
 
14.8
 
 
 
 
ARMC8
 
 
hsa-miR-106b-5p
 
 
-0.357
 
 
14.8
 
 
 
 
ARMC8
 
 
hsa-miR-106a-5p
 
 
-0.375
 
 
14.8
 
 
 
 
ARMC8
 
 
hsa-miR-17-5p
 
 
-0.378
 
 
14.8
 
 
 
 
ARMC8
 
 
hsa-miR-20b-5p
 
 
-0.378
 
 
14.8
 
 
 
 
ARMCX1
 
 
hsa-miR-21-5p
 
 
-0.489
 
 
23.4
 
 
 
 
ARMT1
 
 
hsa-let-7c-5p
 
 
-0.303
 
 
11.9
 
 
 
 
ARMT1
 
 
hsa-miR-98-5p
 
 
-0.303
 
 
11.9
 
 
 
 
ARPP19
 
 
hsa-let-7c-5p
 
 
-0.492
 
 
31.4
 
 
 
 
ARPP19
 
 
hsa-miR-98-5p
 
 
-0.5
 
 
31.4
 
 
 
 
ARRDC3
 
 
hsa-miR-148a-3p
 
 
-0.305
 
 
23.4
 
 
 
 
ARRDC4
 
 
hsa-let-7c-5p
 
 
-0.329
 
 
13.7
 
 
 
 
ARRDC4
 
 
hsa-miR-98-5p
 
 
-0.329
 
 
13.7
 
 
 
 
ASAP1
 
 
hsa-miR-98-5p
 
 
-0.308
 
 
15.1
 
 
 
 
ASAP1
 
 
hsa-let-7c-5p
 
 
-0.308
 
 
15.1
 
 
 
 
ASPH
 
 
hsa-miR-148a-3p
 
 
-0.391
 
 
45.0
 
 
 
 
ATP2A2
 
 
hsa-miR-98-5p
 
 
-0.311
 
 
63.0
 
 
 
 
ATP2A2
 
 
hsa-let-7c-5p
 
 
-0.311
 
 
63.0
 
 
 
 
ATP2B4
 
 
hsa-miR-148a-3p
 
 
-0.337
 
 
45.7
 
 
 
 
ATP6AP2
 
 
hsa-miR-148a-3p
 
 
-0.592
 
 
71.1
 
 
 
 
ATP6AP2
 
 
hsa-miR-148a-3p
 
 
-0.668
 
 
71.1
 
 
 
 
ATPAF1
 
 
hsa-miR-98-5p
 
 
-0.498
 
 
27.2
 
 
 
 
ATPAF1
 
 
hsa-let-7c-5p
 
 
-0.498
 
 
27.2
 
 
 
 
AVPI1
 
 
hsa-miR-7-5p
 
 
-0.367
 
 
21.5
 
 
 
 
B3GAT3
 
 
hsa-miR-98-5p
 
 
-0.324
 
 
34.0
 
 
 
 
B3GAT3
 
 
hsa-let-7c-5p
 
 
-0.324
 
 
34.0
 
 
 
 
B4GALT5
 
 
hsa-miR-148a-3p
 
 
-0.44
 
 
17.2
 
 
 
 
B4GAT1
 
 
hsa-let-7c-5p
 
 
-0.47
 
 
25.4
 
 
 
 
 
 
 heart muscle 
 
 
 
 
target_symbol
 
 
mature_mirna_id
 
 
score
 
 
nTPM
 
 
 
 
 
 
ABCB7
 
 
hsa-miR-148a-3p
 
 
-0.515
 
 
20.0
 
 
 
 
ABCD3
 
 
hsa-miR-148a-3p
 
 
-0.388
 
 
24.0
 
 
 
 
ABHD5
 
 
hsa-miR-20a-5p
 
 
-0.397
 
 
10.6
 
 
 
 
ABHD5
 
 
hsa-miR-106b-5p
 
 
-0.413
 
 
10.6
 
 
 
 
ABHD5
 
 
hsa-miR-20b-5p
 
 
-0.418
 
 
10.6
 
 
 
 
ABHD5
 
 
hsa-miR-106a-5p
 
 
-0.447
 
 
10.6
 
 
 
 
ABHD5
 
 
hsa-miR-17-5p
 
 
-0.45
 
 
10.6
 
 
 
 
ACTC1
 
 
hsa-miR-7-5p
 
 
-0.471
 
 
10840.1
 
 
 
 
ACVR1
 
 
hsa-miR-148a-3p
 
 
-0.397
 
 
14.3
 
 
 
 
ADAM10
 
 
hsa-miR-148a-3p
 
 
-0.356
 
 
11.3
 
 
 
 
ADAM9
 
 
hsa-miR-20a-5p
 
 
-0.299
 
 
26.6
 
 
 
 
ADAM9
 
 
hsa-miR-106b-5p
 
 
-0.299
 
 
26.6
 
 
 
 
ADAM9
 
 
hsa-miR-106a-5p
 
 
-0.317
 
 
26.6
 
 
 
 
ADAM9
 
 
hsa-miR-17-5p
 
 
-0.32
 
 
26.6
 
 
 
 
ADAM9
 
 
hsa-miR-20b-5p
 
 
-0.328
 
 
26.6
 
 
 
 
ADGRF5
 
 
hsa-miR-148a-3p
 
 
-0.354
 
 
50.1
 
 
 
 
ADRB1
 
 
hsa-let-7c-5p
 
 
-0.351
 
 
20.3
 
 
 
 
ADRB1
 
 
hsa-miR-98-5p
 
 
-0.367
 
 
20.3
 
 
 
 
AGFG1
 
 
hsa-miR-148a-3p
 
 
-0.332
 
 
33.8
 
 
 
 
AHDC1
 
 
hsa-miR-148a-3p
 
 
-0.413
 
 
17.4
 
 
 
 
AHNAK
 
 
hsa-miR-20a-5p
 
 
-0.316
 
 
110.3
 
 
 
 
AHNAK
 
 
hsa-miR-106b-5p
 
 
-0.316
 
 
110.3
 
 
 
 
AHNAK
 
 
hsa-miR-106a-5p
 
 
-0.334
 
 
110.3
 
 
 
 
AHNAK
 
 
hsa-miR-17-5p
 
 
-0.337
 
 
110.3
 
 
 
 
AHNAK
 
 
hsa-miR-20b-5p
 
 
-0.337
 
 
110.3
 
 
 
 
AIFM1
 
 
hsa-miR-98-5p
 
 
-0.314
 
 
74.9
 
 
 
 
AIFM1
 
 
hsa-let-7c-5p
 
 
-0.314
 
 
74.9
 
 
 
 
AKAP1
 
 
hsa-miR-148a-3p
 
 
-0.381
 
 
78.5
 
 
 
 
AKAP6
 
 
hsa-miR-98-5p
 
 
-0.307
 
 
51.1
 
 
 
 
AKAP6
 
 
hsa-let-7c-5p
 
 
-0.307
 
 
51.1
 
 
 
 
ALDH4A1
 
 
hsa-miR-184
 
 
-0.421
 
 
30.9
 
 
 
 
ALG3
 
 
hsa-miR-106b-5p
 
 
-0.551
 
 
18.7
 
 
 
 
ALG3
 
 
hsa-miR-20a-5p
 
 
-0.559
 
 
18.7
 
 
 
 
ALG3
 
 
hsa-miR-106a-5p
 
 
-0.569
 
 
18.7
 
 
 
 
ALG3
 
 
hsa-miR-17-5p
 
 
-0.572
 
 
18.7
 
 
 
 
ALG3
 
 
hsa-miR-20b-5p
 
 
-0.58
 
 
18.7
 
 
 
 
AMT
 
 
hsa-miR-98-5p
 
 
-0.463
 
 
23.5
 
 
 
 
AMT
 
 
hsa-let-7c-5p
 
 
-0.463
 
 
23.5
 
 
 
 
ANKRD12
 
 
hsa-miR-7-5p
 
 
-0.37
 
 
11.1
 
 
 
 
ANKRD46
 
 
hsa-miR-98-5p
 
 
-0.389
 
 
18.8
 
 
 
 
ANKRD46
 
 
hsa-let-7c-5p
 
 
-0.389
 
 
18.8
 
 
 
 
ANKRD9
 
 
hsa-miR-20a-5p
 
 
-0.31
 
 
104.9
 
 
 
 
ANKRD9
 
 
hsa-miR-106b-5p
 
 
-0.318
 
 
104.9
 
 
 
 
ANKRD9
 
 
hsa-miR-20b-5p
 
 
-0.331
 
 
104.9
 
 
 
 
ANKRD9
 
 
hsa-miR-106a-5p
 
 
-0.344
 
 
104.9
 
 
 
 
ANKRD9
 
 
hsa-miR-17-5p
 
 
-0.347
 
 
104.9
 
 
 
 
AP1S1
 
 
hsa-miR-98-5p
 
 
-0.374
 
 
12.9
 
 
 
 
AP1S1
 
 
hsa-let-7c-5p
 
 
-0.374
 
 
12.9
 
 
 
 
APCDD1
 
 
hsa-miR-20a-5p
 
 
-0.404
 
 
30.9
 
 
 
 
APCDD1
 
 
hsa-miR-106b-5p
 
 
-0.404
 
 
30.9
 
 
 
 
APCDD1
 
 
hsa-miR-106a-5p
 
 
-0.422
 
 
30.9
 
 
 
 
APCDD1
 
 
hsa-miR-17-5p
 
 
-0.425
 
 
30.9
 
 
 
 
APCDD1
 
 
hsa-miR-20b-5p
 
 
-0.425
 
 
30.9
 
 
 
 
ARF4
 
 
hsa-miR-7-5p
 
 
-0.407
 
 
106.8
 
 
 
 
ARF4
 
 
hsa-miR-148a-3p
 
 
-0.487
 
 
106.8
 
 
 
 
ARHGAP1
 
 
hsa-miR-17-5p
 
 
-0.325
 
 
105.1
 
 
 
 
ARHGAP1
 
 
hsa-miR-20b-5p
 
 
-0.325
 
 
105.1
 
 
 
 
ARHGAP1
 
 
hsa-miR-106a-5p
 
 
-0.329
 
 
105.1
 
 
 
 
ARHGAP1
 
 
hsa-miR-20a-5p
 
 
-0.339
 
 
105.1
 
 
 
 
ARHGAP1
 
 
hsa-miR-106b-5p
 
 
-0.339
 
 
105.1
 
 
 
 
ARHGAP21
 
 
hsa-miR-148a-3p
 
 
-0.287
 
 
19.7
 
 
 
 
ARHGAP24
 
 
hsa-miR-21-5p
 
 
-0.427
 
 
25.6
 
 
 
 
ARL1
 
 
hsa-miR-20a-5p
 
 
-0.403
 
 
61.3
 
 
 
 
ARL1
 
 
hsa-miR-106b-5p
 
 
-0.411
 
 
61.3
 
 
 
 
ARL1
 
 
hsa-miR-20b-5p
 
 
-0.424
 
 
61.3
 
 
 
 
ARL1
 
 
hsa-miR-106a-5p
 
 
-0.429
 
 
61.3
 
 
 
 
ARL1
 
 
hsa-miR-17-5p
 
 
-0.432
 
 
61.3
 
 
 
 
ARL4D
 
 
hsa-miR-98-5p
 
 
-0.417
 
 
12.5
 
 
 
 
ARL4D
 
 
hsa-let-7c-5p
 
 
-0.417
 
 
12.5
 
 
 
 
ARL6IP1
 
 
hsa-miR-7-5p
 
 
-0.374
 
 
30.1
 
 
 
 
ARL6IP1
 
 
hsa-miR-148a-3p
 
 
-0.579
 
 
30.1
 
 
 
 
ARL8B
 
 
hsa-miR-148a-3p
 
 
-0.337
 
 
51.7
 
 
 
 
ARMC8
 
 
hsa-miR-20a-5p
 
 
-0.357
 
 
16.3
 
 
 
 
ARMC8
 
 
hsa-miR-106b-5p
 
 
-0.357
 
 
16.3
 
 
 
 
ARMC8
 
 
hsa-miR-106a-5p
 
 
-0.375
 
 
16.3
 
 
 
 
ARMC8
 
 
hsa-miR-17-5p
 
 
-0.378
 
 
16.3
 
 
 
 
ARMC8
 
 
hsa-miR-20b-5p
 
 
-0.378
 
 
16.3
 
 
 
 
ARMCX1
 
 
hsa-miR-21-5p
 
 
-0.489
 
 
30.7
 
 
 
 
ARMT1
 
 
hsa-let-7c-5p
 
 
-0.303
 
 
15.7
 
 
 
 
ARMT1
 
 
hsa-miR-98-5p
 
 
-0.303
 
 
15.7
 
 
 
 
ARPP19
 
 
hsa-let-7c-5p
 
 
-0.492
 
 
76.2
 
 
 
 
ARPP19
 
 
hsa-miR-98-5p
 
 
-0.5
 
 
76.2
 
 
 
 
ARRDC3
 
 
hsa-miR-148a-3p
 
 
-0.305
 
 
23.2
 
 
 
 
ARRDC4
 
 
hsa-let-7c-5p
 
 
-0.329
 
 
11.3
 
 
 
 
ARRDC4
 
 
hsa-miR-98-5p
 
 
-0.329
 
 
11.3
 
 
 
 
ASAP1
 
 
hsa-miR-98-5p
 
 
-0.308
 
 
22.3
 
 
 
 
ASAP1
 
 
hsa-let-7c-5p
 
 
-0.308
 
 
22.3
 
 
 
 
ASF1A
 
 
hsa-miR-21-5p
 
 
-0.304
 
 
12.5
 
 
 
 
ASPH
 
 
hsa-miR-148a-3p
 
 
-0.391
 
 
100.1
 
 
 
 
ATG16L1
 
 
hsa-miR-20a-5p
 
 
-0.446
 
 
10.8
 
 
 
 
ATG16L1
 
 
hsa-miR-106b-5p
 
 
-0.446
 
 
10.8
 
 
 
 
ATG16L1
 
 
hsa-miR-106a-5p
 
 
-0.464
 
 
10.8
 
 
 
 
ATG16L1
 
 
hsa-miR-17-5p
 
 
-0.467
 
 
10.8
 
 
 
 
ATG16L1
 
 
hsa-miR-20b-5p
 
 
-0.467
 
 
10.8
 
 
 
 
ATG4A
 
 
hsa-miR-7-5p
 
 
-0.448
 
 
11.7
 
 
 
 
ATG4D
 
 
hsa-miR-148a-3p
 
 
-0.303
 
 
21.7
 
 
 
 
ATOSB
 
 
hsa-miR-98-5p
 
 
-0.429
 
 
18.9
 
 
 
 
ATOSB
 
 
hsa-let-7c-5p
 
 
-0.429
 
 
18.9
 
 
 
 
ATP2A2
 
 
hsa-miR-98-5p
 
 
-0.311
 
 
941.9
 
 
 
 
ATP2A2
 
 
hsa-let-7c-5p
 
 
-0.311
 
 
941.9
 
 
 
 
 
 
 ovary 
 
 
 
 
target_symbol
 
 
mature_mirna_id
 
 
score
 
 
nTPM
 
 
 
 
 
 
ABCB7
 
 
hsa-miR-148a-3p
 
 
-0.515
 
 
11.9
 
 
 
 
ABCD3
 
 
hsa-miR-148a-3p
 
 
-0.388
 
 
11.0
 
 
 
 
ACVR1
 
 
hsa-miR-148a-3p
 
 
-0.397
 
 
17.7
 
 
 
 
ADAM10
 
 
hsa-miR-148a-3p
 
 
-0.356
 
 
13.1
 
 
 
 
ADAM9
 
 
hsa-miR-20a-5p
 
 
-0.299
 
 
16.9
 
 
 
 
ADAM9
 
 
hsa-miR-106b-5p
 
 
-0.299
 
 
16.9
 
 
 
 
ADAM9
 
 
hsa-miR-106a-5p
 
 
-0.317
 
 
16.9
 
 
 
 
ADAM9
 
 
hsa-miR-17-5p
 
 
-0.32
 
 
16.9
 
 
 
 
ADAM9
 
 
hsa-miR-20b-5p
 
 
-0.328
 
 
16.9
 
 
 
 
ADRB3
 
 
hsa-let-7c-5p
 
 
-0.415
 
 
12.8
 
 
 
 
ADRB3
 
 
hsa-miR-98-5p
 
 
-0.423
 
 
12.8
 
 
 
 
AGFG1
 
 
hsa-miR-148a-3p
 
 
-0.332
 
 
22.9
 
 
 
 
AGFG2
 
 
hsa-miR-20a-5p
 
 
-0.365
 
 
28.5
 
 
 
 
AGFG2
 
 
hsa-miR-20b-5p
 
 
-0.386
 
 
28.5
 
 
 
 
AGFG2
 
 
hsa-miR-106b-5p
 
 
-0.397
 
 
28.5
 
 
 
 
AGFG2
 
 
hsa-miR-106a-5p
 
 
-0.399
 
 
28.5
 
 
 
 
AGFG2
 
 
hsa-miR-17-5p
 
 
-0.402
 
 
28.5
 
 
 
 
AHCTF1
 
 
hsa-miR-98-5p
 
 
-0.374
 
 
13.1
 
 
 
 
AHCTF1
 
 
hsa-let-7c-5p
 
 
-0.374
 
 
13.1
 
 
 
 
AHDC1
 
 
hsa-miR-148a-3p
 
 
-0.413
 
 
21.5
 
 
 
 
AHNAK
 
 
hsa-miR-20a-5p
 
 
-0.316
 
 
105.9
 
 
 
 
AHNAK
 
 
hsa-miR-106b-5p
 
 
-0.316
 
 
105.9
 
 
 
 
AHNAK
 
 
hsa-miR-106a-5p
 
 
-0.334
 
 
105.9
 
 
 
 
AHNAK
 
 
hsa-miR-17-5p
 
 
-0.337
 
 
105.9
 
 
 
 
AHNAK
 
 
hsa-miR-20b-5p
 
 
-0.337
 
 
105.9
 
 
 
 
AKAP1
 
 
hsa-miR-148a-3p
 
 
-0.381
 
 
18.8
 
 
 
 
ALCAM
 
 
hsa-miR-148a-3p
 
 
-0.339
 
 
33.1
 
 
 
 
ALG3
 
 
hsa-miR-106b-5p
 
 
-0.551
 
 
12.1
 
 
 
 
ALG3
 
 
hsa-miR-20a-5p
 
 
-0.559
 
 
12.1
 
 
 
 
ALG3
 
 
hsa-miR-106a-5p
 
 
-0.569
 
 
12.1
 
 
 
 
ALG3
 
 
hsa-miR-17-5p
 
 
-0.572
 
 
12.1
 
 
 
 
ALG3
 
 
hsa-miR-20b-5p
 
 
-0.58
 
 
12.1
 
 
 
 
ALG9
 
 
hsa-miR-7-5p
 
 
-0.302
 
 
11.1
 
 
 
 
AMT
 
 
hsa-miR-98-5p
 
 
-0.463
 
 
16.7
 
 
 
 
AMT
 
 
hsa-let-7c-5p
 
 
-0.463
 
 
16.7
 
 
 
 
ANKRD12
 
 
hsa-miR-7-5p
 
 
-0.37
 
 
10.6
 
 
 
 
ANKRD29
 
 
hsa-miR-20a-5p
 
 
-0.359
 
 
15.0
 
 
 
 
ANKRD29
 
 
hsa-miR-106b-5p
 
 
-0.359
 
 
15.0
 
 
 
 
ANKRD29
 
 
hsa-miR-106a-5p
 
 
-0.369
 
 
15.0
 
 
 
 
ANKRD29
 
 
hsa-miR-17-5p
 
 
-0.372
 
 
15.0
 
 
 
 
ANKRD29
 
 
hsa-miR-20b-5p
 
 
-0.38
 
 
15.0
 
 
 
 
ANKRD46
 
 
hsa-miR-98-5p
 
 
-0.389
 
 
19.3
 
 
 
 
ANKRD46
 
 
hsa-let-7c-5p
 
 
-0.389
 
 
19.3
 
 
 
 
AP1AR
 
 
hsa-miR-21-5p
 
 
-0.318
 
 
10.0
 
 
 
 
AP1S1
 
 
hsa-miR-98-5p
 
 
-0.374
 
 
22.2
 
 
 
 
AP1S1
 
 
hsa-let-7c-5p
 
 
-0.374
 
 
22.2
 
 
 
 
APCDD1
 
 
hsa-miR-20a-5p
 
 
-0.404
 
 
28.1
 
 
 
 
APCDD1
 
 
hsa-miR-106b-5p
 
 
-0.404
 
 
28.1
 
 
 
 
APCDD1
 
 
hsa-miR-106a-5p
 
 
-0.422
 
 
28.1
 
 
 
 
APCDD1
 
 
hsa-miR-17-5p
 
 
-0.425
 
 
28.1
 
 
 
 
APCDD1
 
 
hsa-miR-20b-5p
 
 
-0.425
 
 
28.1
 
 
 
 
ARF4
 
 
hsa-miR-7-5p
 
 
-0.407
 
 
123.7
 
 
 
 
ARF4
 
 
hsa-miR-148a-3p
 
 
-0.487
 
 
123.7
 
 
 
 
ARFIP1
 
 
hsa-miR-148a-3p
 
 
-0.474
 
 
17.2
 
 
 
 
ARG2
 
 
hsa-miR-98-5p
 
 
-0.468
 
 
18.7
 
 
 
 
ARG2
 
 
hsa-let-7c-5p
 
 
-0.468
 
 
18.7
 
 
 
 
ARHGAP1
 
 
hsa-miR-17-5p
 
 
-0.325
 
 
43.6
 
 
 
 
ARHGAP1
 
 
hsa-miR-20b-5p
 
 
-0.325
 
 
43.6
 
 
 
 
ARHGAP1
 
 
hsa-miR-106a-5p
 
 
-0.329
 
 
43.6
 
 
 
 
ARHGAP1
 
 
hsa-miR-20a-5p
 
 
-0.339
 
 
43.6
 
 
 
 
ARHGAP1
 
 
hsa-miR-106b-5p
 
 
-0.339
 
 
43.6
 
 
 
 
ARHGAP21
 
 
hsa-miR-148a-3p
 
 
-0.287
 
 
26.5
 
 
 
 
ARHGEF28
 
 
hsa-miR-20b-5p
 
 
-0.352
 
 
18.8
 
 
 
 
ARHGEF28
 
 
hsa-miR-17-5p
 
 
-0.363
 
 
18.8
 
 
 
 
ARHGEF28
 
 
hsa-miR-20a-5p
 
 
-0.366
 
 
18.8
 
 
 
 
ARHGEF28
 
 
hsa-miR-106a-5p
 
 
-0.367
 
 
18.8
 
 
 
 
ARHGEF28
 
 
hsa-miR-106b-5p
 
 
-0.377
 
 
18.8
 
 
 
 
ARID4A
 
 
hsa-miR-7-5p
 
 
-0.341
 
 
10.1
 
 
 
 
ARID4B
 
 
hsa-miR-20a-5p
 
 
-0.29
 
 
14.0
 
 
 
 
ARID4B
 
 
hsa-miR-106b-5p
 
 
-0.29
 
 
14.0
 
 
 
 
ARL1
 
 
hsa-miR-20a-5p
 
 
-0.403
 
 
63.4
 
 
 
 
ARL1
 
 
hsa-miR-106b-5p
 
 
-0.411
 
 
63.4
 
 
 
 
ARL1
 
 
hsa-miR-20b-5p
 
 
-0.424
 
 
63.4
 
 
 
 
ARL1
 
 
hsa-miR-106a-5p
 
 
-0.429
 
 
63.4
 
 
 
 
ARL1
 
 
hsa-miR-17-5p
 
 
-0.432
 
 
63.4
 
 
 
 
ARL4D
 
 
hsa-miR-98-5p
 
 
-0.417
 
 
20.3
 
 
 
 
ARL4D
 
 
hsa-let-7c-5p
 
 
-0.417
 
 
20.3
 
 
 
 
ARL6IP1
 
 
hsa-miR-7-5p
 
 
-0.374
 
 
97.4
 
 
 
 
ARL6IP1
 
 
hsa-miR-148a-3p
 
 
-0.579
 
 
97.4
 
 
 
 
ARL6IP6
 
 
hsa-miR-98-5p
 
 
-0.293
 
 
11.9
 
 
 
 
ARL6IP6
 
 
hsa-let-7c-5p
 
 
-0.293
 
 
11.9
 
 
 
 
ARL8B
 
 
hsa-miR-148a-3p
 
 
-0.337
 
 
35.5
 
 
 
 
ARMC8
 
 
hsa-miR-20a-5p
 
 
-0.357
 
 
14.0
 
 
 
 
ARMC8
 
 
hsa-miR-106b-5p
 
 
-0.357
 
 
14.0
 
 
 
 
ARMC8
 
 
hsa-miR-106a-5p
 
 
-0.375
 
 
14.0
 
 
 
 
ARMC8
 
 
hsa-miR-17-5p
 
 
-0.378
 
 
14.0
 
 
 
 
ARMC8
 
 
hsa-miR-20b-5p
 
 
-0.378
 
 
14.0
 
 
 
 
ARMCX1
 
 
hsa-miR-21-5p
 
 
-0.489
 
 
46.7
 
 
 
 
ARMT1
 
 
hsa-let-7c-5p
 
 
-0.303
 
 
15.5
 
 
 
 
ARMT1
 
 
hsa-miR-98-5p
 
 
-0.303
 
 
15.5
 
 
 
 
ARPP19
 
 
hsa-let-7c-5p
 
 
-0.492
 
 
40.6
 
 
 
 
ARPP19
 
 
hsa-miR-98-5p
 
 
-0.5
 
 
40.6
 
 
 
 
ARRDC3
 
 
hsa-miR-148a-3p
 
 
-0.305
 
 
64.7
 
 
 
 
ASAP1
 
 
hsa-miR-98-5p
 
 
-0.308
 
 
16.8
 
 
 
 
ASAP1
 
 
hsa-let-7c-5p
 
 
-0.308
 
 
16.8
 
 
 
 
ASF1A
 
 
hsa-miR-21-5p
 
 
-0.304
 
 
18.5
 
 
 
 
ASPH
 
 
hsa-miR-148a-3p
 
 
-0.391
 
 
58.7
 
 
 
 
ATP2A2
 
 
hsa-miR-98-5p
 
 
-0.311
 
 
34.9
 
 
 
 
ATP2A2
 
 
hsa-let-7c-5p
 
 
-0.311
 
 
34.9
 
 
 
 
ATP2B4
 
 
hsa-miR-148a-3p
 
 
-0.337
 
 
30.9
 
 
 
 
 
 
 parathyroid gland 
 
 
 
 
target_symbol
 
 
mature_mirna_id
 
 
score
 
 
nTPM
 
 
 
 
 
 
ABCB7
 
 
hsa-miR-148a-3p
 
 
-0.515
 
 
10.3
 
 
 
 
ABCD3
 
 
hsa-miR-148a-3p
 
 
-0.388
 
 
27.9
 
 
 
 
ABHD5
 
 
hsa-miR-20a-5p
 
 
-0.397
 
 
11.5
 
 
 
 
ABHD5
 
 
hsa-miR-106b-5p
 
 
-0.413
 
 
11.5
 
 
 
 
ABHD5
 
 
hsa-miR-20b-5p
 
 
-0.418
 
 
11.5
 
 
 
 
ABHD5
 
 
hsa-miR-106a-5p
 
 
-0.447
 
 
11.5
 
 
 
 
ABHD5
 
 
hsa-miR-17-5p
 
 
-0.45
 
 
11.5
 
 
 
 
ACSL6
 
 
hsa-miR-98-5p
 
 
-0.336
 
 
10.6
 
 
 
 
ACSL6
 
 
hsa-let-7c-5p
 
 
-0.336
 
 
10.6
 
 
 
 
ADAM9
 
 
hsa-miR-20a-5p
 
 
-0.299
 
 
49.4
 
 
 
 
ADAM9
 
 
hsa-miR-106b-5p
 
 
-0.299
 
 
49.4
 
 
 
 
ADAM9
 
 
hsa-miR-106a-5p
 
 
-0.317
 
 
49.4
 
 
 
 
ADAM9
 
 
hsa-miR-17-5p
 
 
-0.32
 
 
49.4
 
 
 
 
ADAM9
 
 
hsa-miR-20b-5p
 
 
-0.328
 
 
49.4
 
 
 
 
ADGRF5
 
 
hsa-miR-148a-3p
 
 
-0.354
 
 
12.6
 
 
 
 
ADGRG2
 
 
hsa-miR-21-5p
 
 
-0.343
 
 
153.8
 
 
 
 
AGFG1
 
 
hsa-miR-148a-3p
 
 
-0.332
 
 
39.0
 
 
 
 
AHCTF1
 
 
hsa-miR-98-5p
 
 
-0.374
 
 
12.3
 
 
 
 
AHCTF1
 
 
hsa-let-7c-5p
 
 
-0.374
 
 
12.3
 
 
 
 
AHNAK
 
 
hsa-miR-20a-5p
 
 
-0.316
 
 
24.7
 
 
 
 
AHNAK
 
 
hsa-miR-106b-5p
 
 
-0.316
 
 
24.7
 
 
 
 
AHNAK
 
 
hsa-miR-106a-5p
 
 
-0.334
 
 
24.7
 
 
 
 
AHNAK
 
 
hsa-miR-17-5p
 
 
-0.337
 
 
24.7
 
 
 
 
AHNAK
 
 
hsa-miR-20b-5p
 
 
-0.337
 
 
24.7
 
 
 
 
AIFM1
 
 
hsa-miR-98-5p
 
 
-0.314
 
 
27.9
 
 
 
 
AIFM1
 
 
hsa-let-7c-5p
 
 
-0.314
 
 
27.9
 
 
 
 
AKAP1
 
 
hsa-miR-148a-3p
 
 
-0.381
 
 
45.1
 
 
 
 
ALCAM
 
 
hsa-miR-148a-3p
 
 
-0.339
 
 
412.8
 
 
 
 
ALDH4A1
 
 
hsa-miR-184
 
 
-0.421
 
 
21.6
 
 
 
 
ALG3
 
 
hsa-miR-106b-5p
 
 
-0.551
 
 
21.7
 
 
 
 
ALG3
 
 
hsa-miR-20a-5p
 
 
-0.559
 
 
21.7
 
 
 
 
ALG3
 
 
hsa-miR-106a-5p
 
 
-0.569
 
 
21.7
 
 
 
 
ALG3
 
 
hsa-miR-17-5p
 
 
-0.572
 
 
21.7
 
 
 
 
ALG3
 
 
hsa-miR-20b-5p
 
 
-0.58
 
 
21.7
 
 
 
 
ALG9
 
 
hsa-miR-7-5p
 
 
-0.302
 
 
12.0
 
 
 
 
AMT
 
 
hsa-miR-98-5p
 
 
-0.463
 
 
13.3
 
 
 
 
AMT
 
 
hsa-let-7c-5p
 
 
-0.463
 
 
13.3
 
 
 
 
ANKRD29
 
 
hsa-miR-20a-5p
 
 
-0.359
 
 
23.3
 
 
 
 
ANKRD29
 
 
hsa-miR-106b-5p
 
 
-0.359
 
 
23.3
 
 
 
 
ANKRD29
 
 
hsa-miR-106a-5p
 
 
-0.369
 
 
23.3
 
 
 
 
ANKRD29
 
 
hsa-miR-17-5p
 
 
-0.372
 
 
23.3
 
 
 
 
ANKRD29
 
 
hsa-miR-20b-5p
 
 
-0.38
 
 
23.3
 
 
 
 
ANKRD46
 
 
hsa-miR-98-5p
 
 
-0.389
 
 
15.1
 
 
 
 
ANKRD46
 
 
hsa-let-7c-5p
 
 
-0.389
 
 
15.1
 
 
 
 
AP1AR
 
 
hsa-miR-21-5p
 
 
-0.318
 
 
38.5
 
 
 
 
AP1S1
 
 
hsa-miR-98-5p
 
 
-0.374
 
 
21.4
 
 
 
 
AP1S1
 
 
hsa-let-7c-5p
 
 
-0.374
 
 
21.4
 
 
 
 
ARF4
 
 
hsa-miR-7-5p
 
 
-0.407
 
 
95.4
 
 
 
 
ARF4
 
 
hsa-miR-148a-3p
 
 
-0.487
 
 
95.4
 
 
 
 
ARFGEF1
 
 
hsa-miR-148a-3p
 
 
-0.306
 
 
26.3
 
 
 
 
ARFIP1
 
 
hsa-miR-148a-3p
 
 
-0.474
 
 
21.0
 
 
 
 
ARG2
 
 
hsa-miR-98-5p
 
 
-0.468
 
 
206.5
 
 
 
 
ARG2
 
 
hsa-let-7c-5p
 
 
-0.468
 
 
206.5
 
 
 
 
ARHGAP1
 
 
hsa-miR-17-5p
 
 
-0.325
 
 
23.3
 
 
 
 
ARHGAP1
 
 
hsa-miR-20b-5p
 
 
-0.325
 
 
23.3
 
 
 
 
ARHGAP1
 
 
hsa-miR-106a-5p
 
 
-0.329
 
 
23.3
 
 
 
 
ARHGAP1
 
 
hsa-miR-20a-5p
 
 
-0.339
 
 
23.3
 
 
 
 
ARHGAP1
 
 
hsa-miR-106b-5p
 
 
-0.339
 
 
23.3
 
 
 
 
ARHGAP21
 
 
hsa-miR-148a-3p
 
 
-0.287
 
 
13.6
 
 
 
 
ARID4B
 
 
hsa-miR-20a-5p
 
 
-0.29
 
 
12.8
 
 
 
 
ARID4B
 
 
hsa-miR-106b-5p
 
 
-0.29
 
 
12.8
 
 
 
 
ARL1
 
 
hsa-miR-20a-5p
 
 
-0.403
 
 
86.8
 
 
 
 
ARL1
 
 
hsa-miR-106b-5p
 
 
-0.411
 
 
86.8
 
 
 
 
ARL1
 
 
hsa-miR-20b-5p
 
 
-0.424
 
 
86.8
 
 
 
 
ARL1
 
 
hsa-miR-106a-5p
 
 
-0.429
 
 
86.8
 
 
 
 
ARL1
 
 
hsa-miR-17-5p
 
 
-0.432
 
 
86.8
 
 
 
 
ARL6IP1
 
 
hsa-miR-7-5p
 
 
-0.374
 
 
169.8
 
 
 
 
ARL6IP1
 
 
hsa-miR-148a-3p
 
 
-0.579
 
 
169.8
 
 
 
 
ARL8B
 
 
hsa-miR-148a-3p
 
 
-0.337
 
 
45.2
 
 
 
 
ARMC8
 
 
hsa-miR-20a-5p
 
 
-0.357
 
 
20.0
 
 
 
 
ARMC8
 
 
hsa-miR-106b-5p
 
 
-0.357
 
 
20.0
 
 
 
 
ARMC8
 
 
hsa-miR-106a-5p
 
 
-0.375
 
 
20.0
 
 
 
 
ARMC8
 
 
hsa-miR-17-5p
 
 
-0.378
 
 
20.0
 
 
 
 
ARMC8
 
 
hsa-miR-20b-5p
 
 
-0.378
 
 
20.0
 
 
 
 
ARMCX1
 
 
hsa-miR-21-5p
 
 
-0.489
 
 
15.9
 
 
 
 
ARMT1
 
 
hsa-let-7c-5p
 
 
-0.303
 
 
10.9
 
 
 
 
ARMT1
 
 
hsa-miR-98-5p
 
 
-0.303
 
 
10.9
 
 
 
 
ARPP19
 
 
hsa-let-7c-5p
 
 
-0.492
 
 
102.5
 
 
 
 
ARPP19
 
 
hsa-miR-98-5p
 
 
-0.5
 
 
102.5
 
 
 
 
ARRDC3
 
 
hsa-miR-148a-3p
 
 
-0.305
 
 
51.0
 
 
 
 
ASAP1
 
 
hsa-miR-98-5p
 
 
-0.308
 
 
16.8
 
 
 
 
ASAP1
 
 
hsa-let-7c-5p
 
 
-0.308
 
 
16.8
 
 
 
 
ASF1A
 
 
hsa-miR-21-5p
 
 
-0.304
 
 
14.9
 
 
 
 
ASPH
 
 
hsa-miR-148a-3p
 
 
-0.391
 
 
35.7
 
 
 
 
ATG4A
 
 
hsa-miR-7-5p
 
 
-0.448
 
 
11.7
 
 
 
 
ATG4D
 
 
hsa-miR-148a-3p
 
 
-0.303
 
 
10.1
 
 
 
 
ATOSB
 
 
hsa-miR-98-5p
 
 
-0.429
 
 
10.5
 
 
 
 
ATOSB
 
 
hsa-let-7c-5p
 
 
-0.429
 
 
10.5
 
 
 
 
ATP2A2
 
 
hsa-miR-98-5p
 
 
-0.311
 
 
52.7
 
 
 
 
ATP2A2
 
 
hsa-let-7c-5p
 
 
-0.311
 
 
52.7
 
 
 
 
ATP2B4
 
 
hsa-miR-148a-3p
 
 
-0.337
 
 
11.1
 
 
 
 
ATP6AP2
 
 
hsa-miR-148a-3p
 
 
-0.592
 
 
719.4
 
 
 
 
ATP6AP2
 
 
hsa-miR-148a-3p
 
 
-0.668
 
 
719.4
 
 
 
 
ATPAF1
 
 
hsa-miR-98-5p
 
 
-0.498
 
 
129.7
 
 
 
 
ATPAF1
 
 
hsa-let-7c-5p
 
 
-0.498
 
 
129.7
 
 
 
 
B3GAT3
 
 
hsa-miR-98-5p
 
 
-0.324
 
 
18.1
 
 
 
 
B3GAT3
 
 
hsa-let-7c-5p
 
 
-0.324
 
 
18.1
 
 
 
 
B4GALT5
 
 
hsa-miR-148a-3p
 
 
-0.44
 
 
10.6
 
 
 
 
B4GAT1
 
 
hsa-let-7c-5p
 
 
-0.47
 
 
21.3
 
 
 
 
B4GAT1
 
 
hsa-miR-98-5p
 
 
-0.47
 
 
21.3
 
 
 
 
 
 
 skin 
 
 
 
 
target_symbol
 
 
mature_mirna_id
 
 
score
 
 
nTPM
 
 
 
 
 
 
ABCB7
 
 
hsa-miR-148a-3p
 
 
-0.515
 
 
12.7
 
 
 
 
ABCD3
 
 
hsa-miR-148a-3p
 
 
-0.388
 
 
33.6
 
 
 
 
ABHD17C
 
 
hsa-let-7c-5p
 
 
-0.359
 
 
17.9
 
 
 
 
ABHD17C
 
 
hsa-miR-98-5p
 
 
-0.383
 
 
17.9
 
 
 
 
ABHD5
 
 
hsa-miR-20a-5p
 
 
-0.397
 
 
29.7
 
 
 
 
ABHD5
 
 
hsa-miR-106b-5p
 
 
-0.413
 
 
29.7
 
 
 
 
ABHD5
 
 
hsa-miR-20b-5p
 
 
-0.418
 
 
29.7
 
 
 
 
ABHD5
 
 
hsa-miR-106a-5p
 
 
-0.447
 
 
29.7
 
 
 
 
ABHD5
 
 
hsa-miR-17-5p
 
 
-0.45
 
 
29.7
 
 
 
 
ACP3
 
 
hsa-miR-98-5p
 
 
-0.298
 
 
25.5
 
 
 
 
ACP3
 
 
hsa-let-7c-5p
 
 
-0.298
 
 
25.5
 
 
 
 
ACTC1
 
 
hsa-miR-7-5p
 
 
-0.471
 
 
14.2
 
 
 
 
ADAM10
 
 
hsa-miR-148a-3p
 
 
-0.356
 
 
14.8
 
 
 
 
ADRB2
 
 
hsa-let-7c-5p
 
 
-0.635
 
 
36.9
 
 
 
 
ADRB2
 
 
hsa-miR-98-5p
 
 
-0.635
 
 
36.9
 
 
 
 
AGFG1
 
 
hsa-miR-148a-3p
 
 
-0.332
 
 
34.3
 
 
 
 
AGFG2
 
 
hsa-miR-20a-5p
 
 
-0.365
 
 
24.5
 
 
 
 
AGFG2
 
 
hsa-miR-20b-5p
 
 
-0.386
 
 
24.5
 
 
 
 
AGFG2
 
 
hsa-miR-106b-5p
 
 
-0.397
 
 
24.5
 
 
 
 
AGFG2
 
 
hsa-miR-106a-5p
 
 
-0.399
 
 
24.5
 
 
 
 
AGFG2
 
 
hsa-miR-17-5p
 
 
-0.402
 
 
24.5
 
 
 
 
AHDC1
 
 
hsa-miR-148a-3p
 
 
-0.413
 
 
29.8
 
 
 
 
AHNAK
 
 
hsa-miR-20a-5p
 
 
-0.316
 
 
316.1
 
 
 
 
AHNAK
 
 
hsa-miR-106b-5p
 
 
-0.316
 
 
316.1
 
 
 
 
AHNAK
 
 
hsa-miR-106a-5p
 
 
-0.334
 
 
316.1
 
 
 
 
AHNAK
 
 
hsa-miR-17-5p
 
 
-0.337
 
 
316.1
 
 
 
 
AHNAK
 
 
hsa-miR-20b-5p
 
 
-0.337
 
 
316.1
 
 
 
 
AIFM1
 
 
hsa-miR-98-5p
 
 
-0.314
 
 
10.3
 
 
 
 
AIFM1
 
 
hsa-let-7c-5p
 
 
-0.314
 
 
10.3
 
 
 
 
AKAP1
 
 
hsa-miR-148a-3p
 
 
-0.381
 
 
26.4
 
 
 
 
ALCAM
 
 
hsa-miR-148a-3p
 
 
-0.339
 
 
10.3
 
 
 
 
ALDH4A1
 
 
hsa-miR-184
 
 
-0.421
 
 
18.1
 
 
 
 
ALG3
 
 
hsa-miR-106b-5p
 
 
-0.551
 
 
23.7
 
 
 
 
ALG3
 
 
hsa-miR-20a-5p
 
 
-0.559
 
 
23.7
 
 
 
 
ALG3
 
 
hsa-miR-106a-5p
 
 
-0.569
 
 
23.7
 
 
 
 
ALG3
 
 
hsa-miR-17-5p
 
 
-0.572
 
 
23.7
 
 
 
 
ALG3
 
 
hsa-miR-20b-5p
 
 
-0.58
 
 
23.7
 
 
 
 
ALG9
 
 
hsa-miR-7-5p
 
 
-0.302
 
 
13.9
 
 
 
 
ANKRD12
 
 
hsa-miR-7-5p
 
 
-0.37
 
 
19.1
 
 
 
 
ANKRD46
 
 
hsa-miR-98-5p
 
 
-0.389
 
 
10.5
 
 
 
 
ANKRD46
 
 
hsa-let-7c-5p
 
 
-0.389
 
 
10.5
 
 
 
 
ANKRD50
 
 
hsa-miR-106a-5p
 
 
-0.29
 
 
10.4
 
 
 
 
ANKRD50
 
 
hsa-miR-20a-5p
 
 
-0.3
 
 
10.4
 
 
 
 
ANKRD50
 
 
hsa-miR-106b-5p
 
 
-0.3
 
 
10.4
 
 
 
 
ANKRD9
 
 
hsa-miR-20a-5p
 
 
-0.31
 
 
24.3
 
 
 
 
ANKRD9
 
 
hsa-miR-106b-5p
 
 
-0.318
 
 
24.3
 
 
 
 
ANKRD9
 
 
hsa-miR-20b-5p
 
 
-0.331
 
 
24.3
 
 
 
 
ANKRD9
 
 
hsa-miR-106a-5p
 
 
-0.344
 
 
24.3
 
 
 
 
ANKRD9
 
 
hsa-miR-17-5p
 
 
-0.347
 
 
24.3
 
 
 
 
AP1AR
 
 
hsa-miR-21-5p
 
 
-0.318
 
 
13.4
 
 
 
 
AP1S1
 
 
hsa-miR-98-5p
 
 
-0.374
 
 
40.6
 
 
 
 
AP1S1
 
 
hsa-let-7c-5p
 
 
-0.374
 
 
40.6
 
 
 
 
APCDD1
 
 
hsa-miR-20a-5p
 
 
-0.404
 
 
122.7
 
 
 
 
APCDD1
 
 
hsa-miR-106b-5p
 
 
-0.404
 
 
122.7
 
 
 
 
APCDD1
 
 
hsa-miR-106a-5p
 
 
-0.422
 
 
122.7
 
 
 
 
APCDD1
 
 
hsa-miR-17-5p
 
 
-0.425
 
 
122.7
 
 
 
 
APCDD1
 
 
hsa-miR-20b-5p
 
 
-0.425
 
 
122.7
 
 
 
 
ARF4
 
 
hsa-miR-7-5p
 
 
-0.407
 
 
61.3
 
 
 
 
ARF4
 
 
hsa-miR-148a-3p
 
 
-0.487
 
 
61.3
 
 
 
 
ARFGEF1
 
 
hsa-miR-148a-3p
 
 
-0.306
 
 
12.9
 
 
 
 
ARFIP1
 
 
hsa-miR-148a-3p
 
 
-0.474
 
 
16.4
 
 
 
 
ARHGAP1
 
 
hsa-miR-17-5p
 
 
-0.325
 
 
61.6
 
 
 
 
ARHGAP1
 
 
hsa-miR-20b-5p
 
 
-0.325
 
 
61.6
 
 
 
 
ARHGAP1
 
 
hsa-miR-106a-5p
 
 
-0.329
 
 
61.6
 
 
 
 
ARHGAP1
 
 
hsa-miR-20a-5p
 
 
-0.339
 
 
61.6
 
 
 
 
ARHGAP1
 
 
hsa-miR-106b-5p
 
 
-0.339
 
 
61.6
 
 
 
 
ARHGEF28
 
 
hsa-miR-20b-5p
 
 
-0.352
 
 
14.0
 
 
 
 
ARHGEF28
 
 
hsa-miR-17-5p
 
 
-0.363
 
 
14.0
 
 
 
 
ARHGEF28
 
 
hsa-miR-20a-5p
 
 
-0.366
 
 
14.0
 
 
 
 
ARHGEF28
 
 
hsa-miR-106a-5p
 
 
-0.367
 
 
14.0
 
 
 
 
ARHGEF28
 
 
hsa-miR-106b-5p
 
 
-0.377
 
 
14.0
 
 
 
 
ARL1
 
 
hsa-miR-20a-5p
 
 
-0.403
 
 
26.2
 
 
 
 
ARL1
 
 
hsa-miR-106b-5p
 
 
-0.411
 
 
26.2
 
 
 
 
ARL1
 
 
hsa-miR-20b-5p
 
 
-0.424
 
 
26.2
 
 
 
 
ARL1
 
 
hsa-miR-106a-5p
 
 
-0.429
 
 
26.2
 
 
 
 
ARL1
 
 
hsa-miR-17-5p
 
 
-0.432
 
 
26.2
 
 
 
 
ARL4D
 
 
hsa-miR-98-5p
 
 
-0.417
 
 
44.0
 
 
 
 
ARL4D
 
 
hsa-let-7c-5p
 
 
-0.417
 
 
44.0
 
 
 
 
ARL6IP1
 
 
hsa-miR-7-5p
 
 
-0.374
 
 
62.4
 
 
 
 
ARL6IP1
 
 
hsa-miR-148a-3p
 
 
-0.579
 
 
62.4
 
 
 
 
ARL6IP6
 
 
hsa-miR-98-5p
 
 
-0.293
 
 
23.5
 
 
 
 
ARL6IP6
 
 
hsa-let-7c-5p
 
 
-0.293
 
 
23.5
 
 
 
 
ARL8B
 
 
hsa-miR-148a-3p
 
 
-0.337
 
 
64.4
 
 
 
 
ARMC8
 
 
hsa-miR-20a-5p
 
 
-0.357
 
 
12.0
 
 
 
 
ARMC8
 
 
hsa-miR-106b-5p
 
 
-0.357
 
 
12.0
 
 
 
 
ARMC8
 
 
hsa-miR-106a-5p
 
 
-0.375
 
 
12.0
 
 
 
 
ARMC8
 
 
hsa-miR-17-5p
 
 
-0.378
 
 
12.0
 
 
 
 
ARMC8
 
 
hsa-miR-20b-5p
 
 
-0.378
 
 
12.0
 
 
 
 
ARMCX1
 
 
hsa-miR-21-5p
 
 
-0.489
 
 
10.4
 
 
 
 
ARPP19
 
 
hsa-let-7c-5p
 
 
-0.492
 
 
28.9
 
 
 
 
ARPP19
 
 
hsa-miR-98-5p
 
 
-0.5
 
 
28.9
 
 
 
 
ARRDC3
 
 
hsa-miR-148a-3p
 
 
-0.305
 
 
38.6
 
 
 
 
ARRDC4
 
 
hsa-let-7c-5p
 
 
-0.329
 
 
33.5
 
 
 
 
ARRDC4
 
 
hsa-miR-98-5p
 
 
-0.329
 
 
33.5
 
 
 
 
ASAP1
 
 
hsa-miR-98-5p
 
 
-0.308
 
 
16.2
 
 
 
 
ASAP1
 
 
hsa-let-7c-5p
 
 
-0.308
 
 
16.2
 
 
 
 
ASF1A
 
 
hsa-miR-21-5p
 
 
-0.304
 
 
11.0
 
 
 
 
ASPH
 
 
hsa-miR-148a-3p
 
 
-0.391
 
 
33.3
 
 
 
 
ATG16L1
 
 
hsa-miR-20a-5p
 
 
-0.446
 
 
17.2
 
 
 
 
ATG16L1
 
 
hsa-miR-106b-5p
 
 
-0.446
 
 
17.2
 
 
 
 
 
 
 spleen 
 
 
 
 
target_symbol
 
 
mature_mirna_id
 
 
score
 
 
nTPM
 
 
 
 
 
 
ABCB7
 
 
hsa-miR-148a-3p
 
 
-0.515
 
 
10.6
 
 
 
 
ABCD3
 
 
hsa-miR-148a-3p
 
 
-0.388
 
 
10.3
 
 
 
 
ADAM10
 
 
hsa-miR-148a-3p
 
 
-0.356
 
 
27.3
 
 
 
 
ADAM9
 
 
hsa-miR-20a-5p
 
 
-0.299
 
 
22.6
 
 
 
 
ADAM9
 
 
hsa-miR-106b-5p
 
 
-0.299
 
 
22.6
 
 
 
 
ADAM9
 
 
hsa-miR-106a-5p
 
 
-0.317
 
 
22.6
 
 
 
 
ADAM9
 
 
hsa-miR-17-5p
 
 
-0.32
 
 
22.6
 
 
 
 
ADAM9
 
 
hsa-miR-20b-5p
 
 
-0.328
 
 
22.6
 
 
 
 
ADGRF5
 
 
hsa-miR-148a-3p
 
 
-0.354
 
 
38.8
 
 
 
 
AGFG1
 
 
hsa-miR-148a-3p
 
 
-0.332
 
 
44.5
 
 
 
 
AGFG2
 
 
hsa-miR-20a-5p
 
 
-0.365
 
 
27.6
 
 
 
 
AGFG2
 
 
hsa-miR-20b-5p
 
 
-0.386
 
 
27.6
 
 
 
 
AGFG2
 
 
hsa-miR-106b-5p
 
 
-0.397
 
 
27.6
 
 
 
 
AGFG2
 
 
hsa-miR-106a-5p
 
 
-0.399
 
 
27.6
 
 
 
 
AGFG2
 
 
hsa-miR-17-5p
 
 
-0.402
 
 
27.6
 
 
 
 
AHDC1
 
 
hsa-miR-148a-3p
 
 
-0.413
 
 
15.7
 
 
 
 
AHNAK
 
 
hsa-miR-20a-5p
 
 
-0.316
 
 
56.3
 
 
 
 
AHNAK
 
 
hsa-miR-106b-5p
 
 
-0.316
 
 
56.3
 
 
 
 
AHNAK
 
 
hsa-miR-106a-5p
 
 
-0.334
 
 
56.3
 
 
 
 
AHNAK
 
 
hsa-miR-17-5p
 
 
-0.337
 
 
56.3
 
 
 
 
AHNAK
 
 
hsa-miR-20b-5p
 
 
-0.337
 
 
56.3
 
 
 
 
AIFM1
 
 
hsa-miR-98-5p
 
 
-0.314
 
 
12.9
 
 
 
 
AIFM1
 
 
hsa-let-7c-5p
 
 
-0.314
 
 
12.9
 
 
 
 
AKAP1
 
 
hsa-miR-148a-3p
 
 
-0.381
 
 
11.0
 
 
 
 
ALG3
 
 
hsa-miR-106b-5p
 
 
-0.551
 
 
19.8
 
 
 
 
ALG3
 
 
hsa-miR-20a-5p
 
 
-0.559
 
 
19.8
 
 
 
 
ALG3
 
 
hsa-miR-106a-5p
 
 
-0.569
 
 
19.8
 
 
 
 
ALG3
 
 
hsa-miR-17-5p
 
 
-0.572
 
 
19.8
 
 
 
 
ALG3
 
 
hsa-miR-20b-5p
 
 
-0.58
 
 
19.8
 
 
 
 
ANKRD12
 
 
hsa-miR-7-5p
 
 
-0.37
 
 
18.8
 
 
 
 
ANKRD46
 
 
hsa-miR-98-5p
 
 
-0.389
 
 
10.4
 
 
 
 
ANKRD46
 
 
hsa-let-7c-5p
 
 
-0.389
 
 
10.4
 
 
 
 
AP1AR
 
 
hsa-miR-21-5p
 
 
-0.318
 
 
12.4
 
 
 
 
AP1S1
 
 
hsa-miR-98-5p
 
 
-0.374
 
 
20.9
 
 
 
 
AP1S1
 
 
hsa-let-7c-5p
 
 
-0.374
 
 
20.9
 
 
 
 
ARF4
 
 
hsa-miR-7-5p
 
 
-0.407
 
 
60.0
 
 
 
 
ARF4
 
 
hsa-miR-148a-3p
 
 
-0.487
 
 
60.0
 
 
 
 
ARFGEF1
 
 
hsa-miR-148a-3p
 
 
-0.306
 
 
13.0
 
 
 
 
ARFIP1
 
 
hsa-miR-148a-3p
 
 
-0.474
 
 
17.3
 
 
 
 
ARHGAP1
 
 
hsa-miR-17-5p
 
 
-0.325
 
 
55.1
 
 
 
 
ARHGAP1
 
 
hsa-miR-20b-5p
 
 
-0.325
 
 
55.1
 
 
 
 
ARHGAP1
 
 
hsa-miR-106a-5p
 
 
-0.329
 
 
55.1
 
 
 
 
ARHGAP1
 
 
hsa-miR-20a-5p
 
 
-0.339
 
 
55.1
 
 
 
 
ARHGAP1
 
 
hsa-miR-106b-5p
 
 
-0.339
 
 
55.1
 
 
 
 
ARHGAP24
 
 
hsa-miR-21-5p
 
 
-0.427
 
 
15.5
 
 
 
 
ARHGAP28
 
 
hsa-let-7c-5p
 
 
-0.47
 
 
11.9
 
 
 
 
ARHGAP28
 
 
hsa-miR-98-5p
 
 
-0.47
 
 
11.9
 
 
 
 
ARID4A
 
 
hsa-miR-7-5p
 
 
-0.341
 
 
13.2
 
 
 
 
ARID4B
 
 
hsa-miR-20a-5p
 
 
-0.29
 
 
17.3
 
 
 
 
ARID4B
 
 
hsa-miR-106b-5p
 
 
-0.29
 
 
17.3
 
 
 
 
ARL1
 
 
hsa-miR-20a-5p
 
 
-0.403
 
 
27.2
 
 
 
 
ARL1
 
 
hsa-miR-106b-5p
 
 
-0.411
 
 
27.2
 
 
 
 
ARL1
 
 
hsa-miR-20b-5p
 
 
-0.424
 
 
27.2
 
 
 
 
ARL1
 
 
hsa-miR-106a-5p
 
 
-0.429
 
 
27.2
 
 
 
 
ARL1
 
 
hsa-miR-17-5p
 
 
-0.432
 
 
27.2
 
 
 
 
ARL4D
 
 
hsa-miR-98-5p
 
 
-0.417
 
 
16.1
 
 
 
 
ARL4D
 
 
hsa-let-7c-5p
 
 
-0.417
 
 
16.1
 
 
 
 
ARL6IP1
 
 
hsa-miR-7-5p
 
 
-0.374
 
 
68.6
 
 
 
 
ARL6IP1
 
 
hsa-miR-148a-3p
 
 
-0.579
 
 
68.6
 
 
 
 
ARL6IP6
 
 
hsa-miR-98-5p
 
 
-0.293
 
 
13.7
 
 
 
 
ARL6IP6
 
 
hsa-let-7c-5p
 
 
-0.293
 
 
13.7
 
 
 
 
ARL8B
 
 
hsa-miR-148a-3p
 
 
-0.337
 
 
32.0
 
 
 
 
ARMC8
 
 
hsa-miR-20a-5p
 
 
-0.357
 
 
14.9
 
 
 
 
ARMC8
 
 
hsa-miR-106b-5p
 
 
-0.357
 
 
14.9
 
 
 
 
ARMC8
 
 
hsa-miR-106a-5p
 
 
-0.375
 
 
14.9
 
 
 
 
ARMC8
 
 
hsa-miR-17-5p
 
 
-0.378
 
 
14.9
 
 
 
 
ARMC8
 
 
hsa-miR-20b-5p
 
 
-0.378
 
 
14.9
 
 
 
 
ARMCX1
 
 
hsa-miR-21-5p
 
 
-0.489
 
 
13.3
 
 
 
 
ARMT1
 
 
hsa-let-7c-5p
 
 
-0.303
 
 
12.0
 
 
 
 
ARMT1
 
 
hsa-miR-98-5p
 
 
-0.303
 
 
12.0
 
 
 
 
ARPP19
 
 
hsa-let-7c-5p
 
 
-0.492
 
 
41.2
 
 
 
 
ARPP19
 
 
hsa-miR-98-5p
 
 
-0.5
 
 
41.2
 
 
 
 
ARRDC3
 
 
hsa-miR-148a-3p
 
 
-0.305
 
 
35.0
 
 
 
 
ASAP1
 
 
hsa-miR-98-5p
 
 
-0.308
 
 
28.1
 
 
 
 
ASAP1
 
 
hsa-let-7c-5p
 
 
-0.308
 
 
28.1
 
 
 
 
ASF1A
 
 
hsa-miR-21-5p
 
 
-0.304
 
 
10.9
 
 
 
 
ASPH
 
 
hsa-miR-148a-3p
 
 
-0.391
 
 
26.8
 
 
 
 
ATG16L1
 
 
hsa-miR-20a-5p
 
 
-0.446
 
 
11.4
 
 
 
 
ATG16L1
 
 
hsa-miR-106b-5p
 
 
-0.446
 
 
11.4
 
 
 
 
ATG16L1
 
 
hsa-miR-106a-5p
 
 
-0.464
 
 
11.4
 
 
 
 
ATG16L1
 
 
hsa-miR-17-5p
 
 
-0.467
 
 
11.4
 
 
 
 
ATG16L1
 
 
hsa-miR-20b-5p
 
 
-0.467
 
 
11.4
 
 
 
 
ATG4D
 
 
hsa-miR-148a-3p
 
 
-0.303
 
 
16.4
 
 
 
 
ATOSB
 
 
hsa-miR-98-5p
 
 
-0.429
 
 
26.6
 
 
 
 
ATOSB
 
 
hsa-let-7c-5p
 
 
-0.429
 
 
26.6
 
 
 
 
ATP2A2
 
 
hsa-miR-98-5p
 
 
-0.311
 
 
28.8
 
 
 
 
ATP2A2
 
 
hsa-let-7c-5p
 
 
-0.311
 
 
28.8
 
 
 
 
ATP2B4
 
 
hsa-miR-148a-3p
 
 
-0.337
 
 
11.5
 
 
 
 
ATP6AP2
 
 
hsa-miR-148a-3p
 
 
-0.592
 
 
65.0
 
 
 
 
ATP6AP2
 
 
hsa-miR-148a-3p
 
 
-0.668
 
 
65.0
 
 
 
 
ATPAF1
 
 
hsa-miR-98-5p
 
 
-0.498
 
 
23.6
 
 
 
 
ATPAF1
 
 
hsa-let-7c-5p
 
 
-0.498
 
 
23.6
 
 
 
 
AVPI1
 
 
hsa-miR-7-5p
 
 
-0.367
 
 
20.8
 
 
 
 
B3GAT3
 
 
hsa-miR-98-5p
 
 
-0.324
 
 
44.2
 
 
 
 
B3GAT3
 
 
hsa-let-7c-5p
 
 
-0.324
 
 
44.2
 
 
 
 
B4GALT5
 
 
hsa-miR-148a-3p
 
 
-0.44
 
 
26.0
 
 
 
 
B4GAT1
 
 
hsa-let-7c-5p
 
 
-0.47
 
 
10.0
 
 
 
 
B4GAT1
 
 
hsa-miR-98-5p
 
 
-0.47
 
 
10.0
 
 
 
 
BACH1
 
 
hsa-miR-98-5p
 
 
-0.298
 
 
19.4
 
 
 
 
BACH1
 
 
hsa-miR-98-5p
 
 
-0.298
 
 
19.4
 
 
 
 
 
 
 stomach 
 
 
 
 
target_symbol
 
 
mature_mirna_id
 
 
score
 
 
nTPM
 
 
 
 
 
 
ABCB7
 
 
hsa-miR-148a-3p
 
 
-0.515
 
 
11.6
 
 
 
 
ABCD3
 
 
hsa-miR-148a-3p
 
 
-0.388
 
 
41.1
 
 
 
 
ABHD17C
 
 
hsa-let-7c-5p
 
 
-0.359
 
 
34.0
 
 
 
 
ABHD17C
 
 
hsa-miR-98-5p
 
 
-0.383
 
 
34.0
 
 
 
 
ACER2
 
 
hsa-miR-20a-5p
 
 
-0.298
 
 
58.3
 
 
 
 
ACER2
 
 
hsa-miR-106b-5p
 
 
-0.298
 
 
58.3
 
 
 
 
ACER2
 
 
hsa-miR-106a-5p
 
 
-0.316
 
 
58.3
 
 
 
 
ACER2
 
 
hsa-miR-17-5p
 
 
-0.319
 
 
58.3
 
 
 
 
ACER2
 
 
hsa-miR-20b-5p
 
 
-0.319
 
 
58.3
 
 
 
 
ACER2
 
 
hsa-let-7c-5p
 
 
-0.333
 
 
58.3
 
 
 
 
ACER2
 
 
hsa-miR-98-5p
 
 
-0.344
 
 
58.3
 
 
 
 
ACTC1
 
 
hsa-miR-7-5p
 
 
-0.471
 
 
32.8
 
 
 
 
ACVR1
 
 
hsa-miR-148a-3p
 
 
-0.397
 
 
11.0
 
 
 
 
ADAM10
 
 
hsa-miR-148a-3p
 
 
-0.356
 
 
19.0
 
 
 
 
ADAM9
 
 
hsa-miR-20a-5p
 
 
-0.299
 
 
41.8
 
 
 
 
ADAM9
 
 
hsa-miR-106b-5p
 
 
-0.299
 
 
41.8
 
 
 
 
ADAM9
 
 
hsa-miR-106a-5p
 
 
-0.317
 
 
41.8
 
 
 
 
ADAM9
 
 
hsa-miR-17-5p
 
 
-0.32
 
 
41.8
 
 
 
 
ADAM9
 
 
hsa-miR-20b-5p
 
 
-0.328
 
 
41.8
 
 
 
 
AGFG1
 
 
hsa-miR-148a-3p
 
 
-0.332
 
 
31.3
 
 
 
 
AGFG2
 
 
hsa-miR-20a-5p
 
 
-0.365
 
 
12.9
 
 
 
 
AGFG2
 
 
hsa-miR-20b-5p
 
 
-0.386
 
 
12.9
 
 
 
 
AGFG2
 
 
hsa-miR-106b-5p
 
 
-0.397
 
 
12.9
 
 
 
 
AGFG2
 
 
hsa-miR-106a-5p
 
 
-0.399
 
 
12.9
 
 
 
 
AGFG2
 
 
hsa-miR-17-5p
 
 
-0.402
 
 
12.9
 
 
 
 
AHDC1
 
 
hsa-miR-148a-3p
 
 
-0.413
 
 
10.8
 
 
 
 
AHNAK
 
 
hsa-miR-20a-5p
 
 
-0.316
 
 
125.8
 
 
 
 
AHNAK
 
 
hsa-miR-106b-5p
 
 
-0.316
 
 
125.8
 
 
 
 
AHNAK
 
 
hsa-miR-106a-5p
 
 
-0.334
 
 
125.8
 
 
 
 
AHNAK
 
 
hsa-miR-17-5p
 
 
-0.337
 
 
125.8
 
 
 
 
AHNAK
 
 
hsa-miR-20b-5p
 
 
-0.337
 
 
125.8
 
 
 
 
AIFM1
 
 
hsa-miR-98-5p
 
 
-0.314
 
 
29.8
 
 
 
 
AIFM1
 
 
hsa-let-7c-5p
 
 
-0.314
 
 
29.8
 
 
 
 
AKAP1
 
 
hsa-miR-148a-3p
 
 
-0.381
 
 
53.0
 
 
 
 
ALCAM
 
 
hsa-miR-148a-3p
 
 
-0.339
 
 
31.6
 
 
 
 
ALDH4A1
 
 
hsa-miR-184
 
 
-0.421
 
 
12.6
 
 
 
 
ALG3
 
 
hsa-miR-106b-5p
 
 
-0.551
 
 
22.0
 
 
 
 
ALG3
 
 
hsa-miR-20a-5p
 
 
-0.559
 
 
22.0
 
 
 
 
ALG3
 
 
hsa-miR-106a-5p
 
 
-0.569
 
 
22.0
 
 
 
 
ALG3
 
 
hsa-miR-17-5p
 
 
-0.572
 
 
22.0
 
 
 
 
ALG3
 
 
hsa-miR-20b-5p
 
 
-0.58
 
 
22.0
 
 
 
 
ALG9
 
 
hsa-miR-7-5p
 
 
-0.302
 
 
11.1
 
 
 
 
AMT
 
 
hsa-miR-98-5p
 
 
-0.463
 
 
13.1
 
 
 
 
AMT
 
 
hsa-let-7c-5p
 
 
-0.463
 
 
13.1
 
 
 
 
ANKRD12
 
 
hsa-miR-7-5p
 
 
-0.37
 
 
13.7
 
 
 
 
ANKRD29
 
 
hsa-miR-20a-5p
 
 
-0.359
 
 
14.3
 
 
 
 
ANKRD29
 
 
hsa-miR-106b-5p
 
 
-0.359
 
 
14.3
 
 
 
 
ANKRD29
 
 
hsa-miR-106a-5p
 
 
-0.369
 
 
14.3
 
 
 
 
ANKRD29
 
 
hsa-miR-17-5p
 
 
-0.372
 
 
14.3
 
 
 
 
ANKRD29
 
 
hsa-miR-20b-5p
 
 
-0.38
 
 
14.3
 
 
 
 
ANKRD46
 
 
hsa-miR-98-5p
 
 
-0.389
 
 
10.9
 
 
 
 
ANKRD46
 
 
hsa-let-7c-5p
 
 
-0.389
 
 
10.9
 
 
 
 
ANKRD9
 
 
hsa-miR-20a-5p
 
 
-0.31
 
 
21.4
 
 
 
 
ANKRD9
 
 
hsa-miR-106b-5p
 
 
-0.318
 
 
21.4
 
 
 
 
ANKRD9
 
 
hsa-miR-20b-5p
 
 
-0.331
 
 
21.4
 
 
 
 
ANKRD9
 
 
hsa-miR-106a-5p
 
 
-0.344
 
 
21.4
 
 
 
 
ANKRD9
 
 
hsa-miR-17-5p
 
 
-0.347
 
 
21.4
 
 
 
 
AP1AR
 
 
hsa-miR-21-5p
 
 
-0.318
 
 
12.3
 
 
 
 
AP1S1
 
 
hsa-miR-98-5p
 
 
-0.374
 
 
23.2
 
 
 
 
AP1S1
 
 
hsa-let-7c-5p
 
 
-0.374
 
 
23.2
 
 
 
 
APCDD1
 
 
hsa-miR-20a-5p
 
 
-0.404
 
 
22.7
 
 
 
 
APCDD1
 
 
hsa-miR-106b-5p
 
 
-0.404
 
 
22.7
 
 
 
 
APCDD1
 
 
hsa-miR-106a-5p
 
 
-0.422
 
 
22.7
 
 
 
 
APCDD1
 
 
hsa-miR-17-5p
 
 
-0.425
 
 
22.7
 
 
 
 
APCDD1
 
 
hsa-miR-20b-5p
 
 
-0.425
 
 
22.7
 
 
 
 
ARF4
 
 
hsa-miR-7-5p
 
 
-0.407
 
 
123.5
 
 
 
 
ARF4
 
 
hsa-miR-148a-3p
 
 
-0.487
 
 
123.5
 
 
 
 
ARFGEF1
 
 
hsa-miR-148a-3p
 
 
-0.306
 
 
18.5
 
 
 
 
ARFIP1
 
 
hsa-miR-148a-3p
 
 
-0.474
 
 
21.5
 
 
 
 
ARHGAP1
 
 
hsa-miR-17-5p
 
 
-0.325
 
 
46.3
 
 
 
 
ARHGAP1
 
 
hsa-miR-20b-5p
 
 
-0.325
 
 
46.3
 
 
 
 
ARHGAP1
 
 
hsa-miR-106a-5p
 
 
-0.329
 
 
46.3
 
 
 
 
ARHGAP1
 
 
hsa-miR-20a-5p
 
 
-0.339
 
 
46.3
 
 
 
 
ARHGAP1
 
 
hsa-miR-106b-5p
 
 
-0.339
 
 
46.3
 
 
 
 
ARHGAP21
 
 
hsa-miR-148a-3p
 
 
-0.287
 
 
25.5
 
 
 
 
ARHGAP24
 
 
hsa-miR-21-5p
 
 
-0.427
 
 
24.6
 
 
 
 
ARHGEF28
 
 
hsa-miR-20b-5p
 
 
-0.352
 
 
20.6
 
 
 
 
ARHGEF28
 
 
hsa-miR-17-5p
 
 
-0.363
 
 
20.6
 
 
 
 
ARHGEF28
 
 
hsa-miR-20a-5p
 
 
-0.366
 
 
20.6
 
 
 
 
ARHGEF28
 
 
hsa-miR-106a-5p
 
 
-0.367
 
 
20.6
 
 
 
 
ARHGEF28
 
 
hsa-miR-106b-5p
 
 
-0.377
 
 
20.6
 
 
 
 
ARID4B
 
 
hsa-miR-20a-5p
 
 
-0.29
 
 
12.5
 
 
 
 
ARID4B
 
 
hsa-miR-106b-5p
 
 
-0.29
 
 
12.5
 
 
 
 
ARL1
 
 
hsa-miR-20a-5p
 
 
-0.403
 
 
54.2
 
 
 
 
ARL1
 
 
hsa-miR-106b-5p
 
 
-0.411
 
 
54.2
 
 
 
 
ARL1
 
 
hsa-miR-20b-5p
 
 
-0.424
 
 
54.2
 
 
 
 
ARL1
 
 
hsa-miR-106a-5p
 
 
-0.429
 
 
54.2
 
 
 
 
ARL1
 
 
hsa-miR-17-5p
 
 
-0.432
 
 
54.2
 
 
 
 
ARL6IP1
 
 
hsa-miR-7-5p
 
 
-0.374
 
 
118.7
 
 
 
 
ARL6IP1
 
 
hsa-miR-148a-3p
 
 
-0.579
 
 
118.7
 
 
 
 
ARL8B
 
 
hsa-miR-148a-3p
 
 
-0.337
 
 
32.2
 
 
 
 
ARMC8
 
 
hsa-miR-20a-5p
 
 
-0.357
 
 
14.5
 
 
 
 
ARMC8
 
 
hsa-miR-106b-5p
 
 
-0.357
 
 
14.5
 
 
 
 
ARMC8
 
 
hsa-miR-106a-5p
 
 
-0.375
 
 
14.5
 
 
 
 
ARMC8
 
 
hsa-miR-17-5p
 
 
-0.378
 
 
14.5
 
 
 
 
ARMC8
 
 
hsa-miR-20b-5p
 
 
-0.378
 
 
14.5
 
 
 
 
ARMCX1
 
 
hsa-miR-21-5p
 
 
-0.489
 
 
14.0
 
 
 
 
ARMT1
 
 
hsa-let-7c-5p
 
 
-0.303
 
 
13.3
 
 
 
 
ARMT1
 
 
hsa-miR-98-5p
 
 
-0.303
 
 
13.3
 
 
 
 
ARPP19
 
 
hsa-let-7c-5p
 
 
-0.492
 
 
36.4
 
 
 
 
 
 
 thymus 
 
 
 
 
target_symbol
 
 
mature_mirna_id
 
 
score
 
 
nTPM
 
 
 
 
 
 
ABCB7
 
 
hsa-miR-148a-3p
 
 
-0.515
 
 
13.0
 
 
 
 
ABCD3
 
 
hsa-miR-148a-3p
 
 
-0.388
 
 
44.2
 
 
 
 
ACTC1
 
 
hsa-miR-7-5p
 
 
-0.471
 
 
10.5
 
 
 
 
ADAM10
 
 
hsa-miR-148a-3p
 
 
-0.356
 
 
38.8
 
 
 
 
AGFG1
 
 
hsa-miR-148a-3p
 
 
-0.332
 
 
45.3
 
 
 
 
AHCTF1
 
 
hsa-miR-98-5p
 
 
-0.374
 
 
11.4
 
 
 
 
AHCTF1
 
 
hsa-let-7c-5p
 
 
-0.374
 
 
11.4
 
 
 
 
AHNAK
 
 
hsa-miR-20a-5p
 
 
-0.316
 
 
26.0
 
 
 
 
AHNAK
 
 
hsa-miR-106b-5p
 
 
-0.316
 
 
26.0
 
 
 
 
AHNAK
 
 
hsa-miR-106a-5p
 
 
-0.334
 
 
26.0
 
 
 
 
AHNAK
 
 
hsa-miR-17-5p
 
 
-0.337
 
 
26.0
 
 
 
 
AHNAK
 
 
hsa-miR-20b-5p
 
 
-0.337
 
 
26.0
 
 
 
 
AIFM1
 
 
hsa-miR-98-5p
 
 
-0.314
 
 
17.2
 
 
 
 
AIFM1
 
 
hsa-let-7c-5p
 
 
-0.314
 
 
17.2
 
 
 
 
AKAP1
 
 
hsa-miR-148a-3p
 
 
-0.381
 
 
10.6
 
 
 
 
ALCAM
 
 
hsa-miR-148a-3p
 
 
-0.339
 
 
13.3
 
 
 
 
ALDH4A1
 
 
hsa-miR-184
 
 
-0.421
 
 
10.9
 
 
 
 
ALG3
 
 
hsa-miR-106b-5p
 
 
-0.551
 
 
11.4
 
 
 
 
ALG3
 
 
hsa-miR-20a-5p
 
 
-0.559
 
 
11.4
 
 
 
 
ALG3
 
 
hsa-miR-106a-5p
 
 
-0.569
 
 
11.4
 
 
 
 
ALG3
 
 
hsa-miR-17-5p
 
 
-0.572
 
 
11.4
 
 
 
 
ALG3
 
 
hsa-miR-20b-5p
 
 
-0.58
 
 
11.4
 
 
 
 
ANKRD12
 
 
hsa-miR-7-5p
 
 
-0.37
 
 
10.7
 
 
 
 
ANKRD46
 
 
hsa-miR-98-5p
 
 
-0.389
 
 
14.3
 
 
 
 
ANKRD46
 
 
hsa-let-7c-5p
 
 
-0.389
 
 
14.3
 
 
 
 
AP1AR
 
 
hsa-miR-21-5p
 
 
-0.318
 
 
10.2
 
 
 
 
AP1S1
 
 
hsa-miR-98-5p
 
 
-0.374
 
 
17.8
 
 
 
 
AP1S1
 
 
hsa-let-7c-5p
 
 
-0.374
 
 
17.8
 
 
 
 
ARF4
 
 
hsa-miR-7-5p
 
 
-0.407
 
 
69.9
 
 
 
 
ARF4
 
 
hsa-miR-148a-3p
 
 
-0.487
 
 
69.9
 
 
 
 
ARFGEF1
 
 
hsa-miR-148a-3p
 
 
-0.306
 
 
20.7
 
 
 
 
ARFIP1
 
 
hsa-miR-148a-3p
 
 
-0.474
 
 
11.2
 
 
 
 
ARHGAP1
 
 
hsa-miR-17-5p
 
 
-0.325
 
 
21.6
 
 
 
 
ARHGAP1
 
 
hsa-miR-20b-5p
 
 
-0.325
 
 
21.6
 
 
 
 
ARHGAP1
 
 
hsa-miR-106a-5p
 
 
-0.329
 
 
21.6
 
 
 
 
ARHGAP1
 
 
hsa-miR-20a-5p
 
 
-0.339
 
 
21.6
 
 
 
 
ARHGAP1
 
 
hsa-miR-106b-5p
 
 
-0.339
 
 
21.6
 
 
 
 
ARHGAP21
 
 
hsa-miR-148a-3p
 
 
-0.287
 
 
10.5
 
 
 
 
ARID4A
 
 
hsa-miR-7-5p
 
 
-0.341
 
 
13.5
 
 
 
 
ARID4B
 
 
hsa-miR-20a-5p
 
 
-0.29
 
 
19.9
 
 
 
 
ARID4B
 
 
hsa-miR-106b-5p
 
 
-0.29
 
 
19.9
 
 
 
 
ARL1
 
 
hsa-miR-20a-5p
 
 
-0.403
 
 
30.6
 
 
 
 
ARL1
 
 
hsa-miR-106b-5p
 
 
-0.411
 
 
30.6
 
 
 
 
ARL1
 
 
hsa-miR-20b-5p
 
 
-0.424
 
 
30.6
 
 
 
 
ARL1
 
 
hsa-miR-106a-5p
 
 
-0.429
 
 
30.6
 
 
 
 
ARL1
 
 
hsa-miR-17-5p
 
 
-0.432
 
 
30.6
 
 
 
 
ARL6IP1
 
 
hsa-miR-7-5p
 
 
-0.374
 
 
147.6
 
 
 
 
ARL6IP1
 
 
hsa-miR-148a-3p
 
 
-0.579
 
 
147.6
 
 
 
 
ARL6IP6
 
 
hsa-miR-98-5p
 
 
-0.293
 
 
20.0
 
 
 
 
ARL6IP6
 
 
hsa-let-7c-5p
 
 
-0.293
 
 
20.0
 
 
 
 
ARL8B
 
 
hsa-miR-148a-3p
 
 
-0.337
 
 
33.3
 
 
 
 
ARMC8
 
 
hsa-miR-20a-5p
 
 
-0.357
 
 
20.7
 
 
 
 
ARMC8
 
 
hsa-miR-106b-5p
 
 
-0.357
 
 
20.7
 
 
 
 
ARMC8
 
 
hsa-miR-106a-5p
 
 
-0.375
 
 
20.7
 
 
 
 
ARMC8
 
 
hsa-miR-17-5p
 
 
-0.378
 
 
20.7
 
 
 
 
ARMC8
 
 
hsa-miR-20b-5p
 
 
-0.378
 
 
20.7
 
 
 
 
ARMT1
 
 
hsa-let-7c-5p
 
 
-0.303
 
 
14.5
 
 
 
 
ARMT1
 
 
hsa-miR-98-5p
 
 
-0.303
 
 
14.5
 
 
 
 
ARPP19
 
 
hsa-let-7c-5p
 
 
-0.492
 
 
49.2
 
 
 
 
ARPP19
 
 
hsa-miR-98-5p
 
 
-0.5
 
 
49.2
 
 
 
 
ARRDC3
 
 
hsa-miR-148a-3p
 
 
-0.305
 
 
26.1
 
 
 
 
ASAP1
 
 
hsa-miR-98-5p
 
 
-0.308
 
 
14.9
 
 
 
 
ASAP1
 
 
hsa-let-7c-5p
 
 
-0.308
 
 
14.9
 
 
 
 
ASF1A
 
 
hsa-miR-21-5p
 
 
-0.304
 
 
22.7
 
 
 
 
ASPH
 
 
hsa-miR-148a-3p
 
 
-0.391
 
 
15.5
 
 
 
 
ATAD2
 
 
hsa-miR-17-5p
 
 
-0.304
 
 
26.3
 
 
 
 
ATAD2
 
 
hsa-miR-20b-5p
 
 
-0.304
 
 
26.3
 
 
 
 
ATAD2
 
 
hsa-miR-106b-5p
 
 
-0.307
 
 
26.3
 
 
 
 
ATAD2
 
 
hsa-miR-106a-5p
 
 
-0.308
 
 
26.3
 
 
 
 
ATAD2
 
 
hsa-miR-20a-5p
 
 
-0.318
 
 
26.3
 
 
 
 
ATG4A
 
 
hsa-miR-7-5p
 
 
-0.448
 
 
10.5
 
 
 
 
ATG4D
 
 
hsa-miR-148a-3p
 
 
-0.303
 
 
11.2
 
 
 
 
ATP2A2
 
 
hsa-miR-98-5p
 
 
-0.311
 
 
33.8
 
 
 
 
ATP2A2
 
 
hsa-let-7c-5p
 
 
-0.311
 
 
33.8
 
 
 
 
ATP2B4
 
 
hsa-miR-148a-3p
 
 
-0.337
 
 
20.2
 
 
 
 
ATP6AP2
 
 
hsa-miR-148a-3p
 
 
-0.592
 
 
67.0
 
 
 
 
ATP6AP2
 
 
hsa-miR-148a-3p
 
 
-0.668
 
 
67.0
 
 
 
 
ATPAF1
 
 
hsa-miR-98-5p
 
 
-0.498
 
 
41.4
 
 
 
 
ATPAF1
 
 
hsa-let-7c-5p
 
 
-0.498
 
 
41.4
 
 
 
 
B3GAT3
 
 
hsa-miR-98-5p
 
 
-0.324
 
 
17.5
 
 
 
 
B3GAT3
 
 
hsa-let-7c-5p
 
 
-0.324
 
 
17.5
 
 
 
 
BACH1
 
 
hsa-miR-98-5p
 
 
-0.298
 
 
13.2
 
 
 
 
BACH1
 
 
hsa-miR-98-5p
 
 
-0.298
 
 
13.2
 
 
 
 
BACH1
 
 
hsa-let-7c-5p
 
 
-0.306
 
 
13.2
 
 
 
 
BACH1
 
 
hsa-let-7c-5p
 
 
-0.306
 
 
13.2
 
 
 
 
BACH1
 
 
hsa-miR-98-5p
 
 
-0.435
 
 
13.2
 
 
 
 
BACH1
 
 
hsa-miR-98-5p
 
 
-0.435
 
 
13.2
 
 
 
 
BACH1
 
 
hsa-let-7c-5p
 
 
-0.435
 
 
13.2
 
 
 
 
BACH1
 
 
hsa-let-7c-5p
 
 
-0.435
 
 
13.2
 
 
 
 
BCL2L11
 
 
hsa-miR-148a-3p
 
 
-0.445
 
 
18.0
 
 
 
 
BCL7A
 
 
hsa-miR-21-5p
 
 
-0.31
 
 
23.0
 
 
 
 
BIN3
 
 
hsa-miR-98-5p
 
 
-0.427
 
 
12.4
 
 
 
 
BIN3
 
 
hsa-let-7c-5p
 
 
-0.427
 
 
12.4
 
 
 
 
BLOC1S6
 
 
hsa-miR-98-5p
 
 
-0.321
 
 
23.5
 
 
 
 
BLOC1S6
 
 
hsa-let-7c-5p
 
 
-0.321
 
 
23.5
 
 
 
 
BNIP3L
 
 
hsa-miR-20a-5p
 
 
-0.287
 
 
37.1
 
 
 
 
BNIP3L
 
 
hsa-miR-106b-5p
 
 
-0.298
 
 
37.1
 
 
 
 
BTAF1
 
 
hsa-miR-148a-3p
 
 
-0.401
 
 
17.2
 
 
 
 
BTBD10
 
 
hsa-miR-106b-5p
 
 
-0.388
 
 
17.3
 
 
 
 
BTBD10
 
 
hsa-miR-106a-5p
 
 
-0.406
 
 
17.3
 
 
 
 
 
 
 tonsil 
 
 
 
 
target_symbol
 
 
mature_mirna_id
 
 
score
 
 
nTPM
 
 
 
 
 
 
ABCB7
 
 
hsa-miR-148a-3p
 
 
-0.515
 
 
10.4
 
 
 
 
ABCD3
 
 
hsa-miR-148a-3p
 
 
-0.388
 
 
19.9
 
 
 
 
ABHD17C
 
 
hsa-let-7c-5p
 
 
-0.359
 
 
21.6
 
 
 
 
ABHD17C
 
 
hsa-miR-98-5p
 
 
-0.383
 
 
21.6
 
 
 
 
ADAM10
 
 
hsa-miR-148a-3p
 
 
-0.356
 
 
15.3
 
 
 
 
ADAM17
 
 
hsa-miR-148a-3p
 
 
-0.301
 
 
11.1
 
 
 
 
ADAM9
 
 
hsa-miR-20a-5p
 
 
-0.299
 
 
12.4
 
 
 
 
ADAM9
 
 
hsa-miR-106b-5p
 
 
-0.299
 
 
12.4
 
 
 
 
ADAM9
 
 
hsa-miR-106a-5p
 
 
-0.317
 
 
12.4
 
 
 
 
ADAM9
 
 
hsa-miR-17-5p
 
 
-0.32
 
 
12.4
 
 
 
 
ADAM9
 
 
hsa-miR-20b-5p
 
 
-0.328
 
 
12.4
 
 
 
 
AGFG1
 
 
hsa-miR-148a-3p
 
 
-0.332
 
 
49.9
 
 
 
 
AHNAK
 
 
hsa-miR-20a-5p
 
 
-0.316
 
 
59.8
 
 
 
 
AHNAK
 
 
hsa-miR-106b-5p
 
 
-0.316
 
 
59.8
 
 
 
 
AHNAK
 
 
hsa-miR-106a-5p
 
 
-0.334
 
 
59.8
 
 
 
 
AHNAK
 
 
hsa-miR-17-5p
 
 
-0.337
 
 
59.8
 
 
 
 
AHNAK
 
 
hsa-miR-20b-5p
 
 
-0.337
 
 
59.8
 
 
 
 
AIFM1
 
 
hsa-miR-98-5p
 
 
-0.314
 
 
10.3
 
 
 
 
AIFM1
 
 
hsa-let-7c-5p
 
 
-0.314
 
 
10.3
 
 
 
 
ALCAM
 
 
hsa-miR-148a-3p
 
 
-0.339
 
 
13.6
 
 
 
 
ALG3
 
 
hsa-miR-106b-5p
 
 
-0.551
 
 
13.2
 
 
 
 
ALG3
 
 
hsa-miR-20a-5p
 
 
-0.559
 
 
13.2
 
 
 
 
ALG3
 
 
hsa-miR-106a-5p
 
 
-0.569
 
 
13.2
 
 
 
 
ALG3
 
 
hsa-miR-17-5p
 
 
-0.572
 
 
13.2
 
 
 
 
ALG3
 
 
hsa-miR-20b-5p
 
 
-0.58
 
 
13.2
 
 
 
 
ANKRD12
 
 
hsa-miR-7-5p
 
 
-0.37
 
 
17.3
 
 
 
 
ANKRD29
 
 
hsa-miR-20a-5p
 
 
-0.359
 
 
13.4
 
 
 
 
ANKRD29
 
 
hsa-miR-106b-5p
 
 
-0.359
 
 
13.4
 
 
 
 
ANKRD29
 
 
hsa-miR-106a-5p
 
 
-0.369
 
 
13.4
 
 
 
 
ANKRD29
 
 
hsa-miR-17-5p
 
 
-0.372
 
 
13.4
 
 
 
 
ANKRD29
 
 
hsa-miR-20b-5p
 
 
-0.38
 
 
13.4
 
 
 
 
ANKRD46
 
 
hsa-miR-98-5p
 
 
-0.389
 
 
10.3
 
 
 
 
ANKRD46
 
 
hsa-let-7c-5p
 
 
-0.389
 
 
10.3
 
 
 
 
AP1AR
 
 
hsa-miR-21-5p
 
 
-0.318
 
 
11.3
 
 
 
 
AP1S1
 
 
hsa-miR-98-5p
 
 
-0.374
 
 
12.8
 
 
 
 
AP1S1
 
 
hsa-let-7c-5p
 
 
-0.374
 
 
12.8
 
 
 
 
ARF4
 
 
hsa-miR-7-5p
 
 
-0.407
 
 
91.2
 
 
 
 
ARF4
 
 
hsa-miR-148a-3p
 
 
-0.487
 
 
91.2
 
 
 
 
ARFGEF1
 
 
hsa-miR-148a-3p
 
 
-0.306
 
 
16.2
 
 
 
 
ARFIP1
 
 
hsa-miR-148a-3p
 
 
-0.474
 
 
13.9
 
 
 
 
ARHGAP1
 
 
hsa-miR-17-5p
 
 
-0.325
 
 
32.4
 
 
 
 
ARHGAP1
 
 
hsa-miR-20b-5p
 
 
-0.325
 
 
32.4
 
 
 
 
ARHGAP1
 
 
hsa-miR-106a-5p
 
 
-0.329
 
 
32.4
 
 
 
 
ARHGAP1
 
 
hsa-miR-20a-5p
 
 
-0.339
 
 
32.4
 
 
 
 
ARHGAP1
 
 
hsa-miR-106b-5p
 
 
-0.339
 
 
32.4
 
 
 
 
ARHGAP24
 
 
hsa-miR-21-5p
 
 
-0.427
 
 
14.5
 
 
 
 
ARID4A
 
 
hsa-miR-7-5p
 
 
-0.341
 
 
10.5
 
 
 
 
ARID4B
 
 
hsa-miR-20a-5p
 
 
-0.29
 
 
15.9
 
 
 
 
ARID4B
 
 
hsa-miR-106b-5p
 
 
-0.29
 
 
15.9
 
 
 
 
ARL1
 
 
hsa-miR-20a-5p
 
 
-0.403
 
 
45.6
 
 
 
 
ARL1
 
 
hsa-miR-106b-5p
 
 
-0.411
 
 
45.6
 
 
 
 
ARL1
 
 
hsa-miR-20b-5p
 
 
-0.424
 
 
45.6
 
 
 
 
ARL1
 
 
hsa-miR-106a-5p
 
 
-0.429
 
 
45.6
 
 
 
 
ARL1
 
 
hsa-miR-17-5p
 
 
-0.432
 
 
45.6
 
 
 
 
ARL6IP1
 
 
hsa-miR-7-5p
 
 
-0.374
 
 
106.2
 
 
 
 
ARL6IP1
 
 
hsa-miR-148a-3p
 
 
-0.579
 
 
106.2
 
 
 
 
ARL6IP6
 
 
hsa-miR-98-5p
 
 
-0.293
 
 
18.0
 
 
 
 
ARL6IP6
 
 
hsa-let-7c-5p
 
 
-0.293
 
 
18.0
 
 
 
 
ARL8B
 
 
hsa-miR-148a-3p
 
 
-0.337
 
 
44.5
 
 
 
 
ARMC8
 
 
hsa-miR-20a-5p
 
 
-0.357
 
 
21.4
 
 
 
 
ARMC8
 
 
hsa-miR-106b-5p
 
 
-0.357
 
 
21.4
 
 
 
 
ARMC8
 
 
hsa-miR-106a-5p
 
 
-0.375
 
 
21.4
 
 
 
 
ARMC8
 
 
hsa-miR-17-5p
 
 
-0.378
 
 
21.4
 
 
 
 
ARMC8
 
 
hsa-miR-20b-5p
 
 
-0.378
 
 
21.4
 
 
 
 
ARMT1
 
 
hsa-let-7c-5p
 
 
-0.303
 
 
17.3
 
 
 
 
ARMT1
 
 
hsa-miR-98-5p
 
 
-0.303
 
 
17.3
 
 
 
 
ARPP19
 
 
hsa-let-7c-5p
 
 
-0.492
 
 
48.4
 
 
 
 
ARPP19
 
 
hsa-miR-98-5p
 
 
-0.5
 
 
48.4
 
 
 
 
ARRDC3
 
 
hsa-miR-148a-3p
 
 
-0.305
 
 
26.4
 
 
 
 
ASAP1
 
 
hsa-miR-98-5p
 
 
-0.308
 
 
17.8
 
 
 
 
ASAP1
 
 
hsa-let-7c-5p
 
 
-0.308
 
 
17.8
 
 
 
 
ASF1A
 
 
hsa-miR-21-5p
 
 
-0.304
 
 
14.1
 
 
 
 
ASPH
 
 
hsa-miR-148a-3p
 
 
-0.391
 
 
26.4
 
 
 
 
ATAD2
 
 
hsa-miR-17-5p
 
 
-0.304
 
 
12.0
 
 
 
 
ATAD2
 
 
hsa-miR-20b-5p
 
 
-0.304
 
 
12.0
 
 
 
 
ATAD2
 
 
hsa-miR-106b-5p
 
 
-0.307
 
 
12.0
 
 
 
 
ATAD2
 
 
hsa-miR-106a-5p
 
 
-0.308
 
 
12.0
 
 
 
 
ATAD2
 
 
hsa-miR-20a-5p
 
 
-0.318
 
 
12.0
 
 
 
 
ATG4A
 
 
hsa-miR-7-5p
 
 
-0.448
 
 
10.4
 
 
 
 
ATP2A2
 
 
hsa-miR-98-5p
 
 
-0.311
 
 
45.1
 
 
 
 
ATP2A2
 
 
hsa-let-7c-5p
 
 
-0.311
 
 
45.1
 
 
 
 
ATP2B4
 
 
hsa-miR-148a-3p
 
 
-0.337
 
 
18.2
 
 
 
 
ATP6AP2
 
 
hsa-miR-148a-3p
 
 
-0.592
 
 
67.9
 
 
 
 
ATP6AP2
 
 
hsa-miR-148a-3p
 
 
-0.668
 
 
67.9
 
 
 
 
ATPAF1
 
 
hsa-miR-98-5p
 
 
-0.498
 
 
30.0
 
 
 
 
ATPAF1
 
 
hsa-let-7c-5p
 
 
-0.498
 
 
30.0
 
 
 
 
B3GAT3
 
 
hsa-miR-98-5p
 
 
-0.324
 
 
10.3
 
 
 
 
B3GAT3
 
 
hsa-let-7c-5p
 
 
-0.324
 
 
10.3
 
 
 
 
B4GALT5
 
 
hsa-miR-148a-3p
 
 
-0.44
 
 
10.8
 
 
 
 
BACH1
 
 
hsa-miR-98-5p
 
 
-0.298
 
 
13.8
 
 
 
 
BACH1
 
 
hsa-miR-98-5p
 
 
-0.298
 
 
13.8
 
 
 
 
BACH1
 
 
hsa-let-7c-5p
 
 
-0.306
 
 
13.8
 
 
 
 
BACH1
 
 
hsa-let-7c-5p
 
 
-0.306
 
 
13.8
 
 
 
 
BACH1
 
 
hsa-miR-98-5p
 
 
-0.435
 
 
13.8
 
 
 
 
BACH1
 
 
hsa-miR-98-5p
 
 
-0.435
 
 
13.8
 
 
 
 
BACH1
 
 
hsa-let-7c-5p
 
 
-0.435
 
 
13.8
 
 
 
 
BACH1
 
 
hsa-let-7c-5p
 
 
-0.435
 
 
13.8
 
 
 
 
BCL2L11
 
 
hsa-miR-148a-3p
 
 
-0.445
 
 
21.7
 
 
 
 
BCL7A
 
 
hsa-miR-21-5p
 
 
-0.31
 
 
14.4
 
 
 
 
BHLHE41
 
 
hsa-miR-148a-3p
 
 
-0.442
 
 
16.8
 
 
 
 
 
 
 urinary bladder 
 
 
 
 
target_symbol
 
 
mature_mirna_id
 
 
score
 
 
nTPM
 
 
 
 
 
 
ABCB7
 
 
hsa-miR-148a-3p
 
 
-0.515
 
 
11.0
 
 
 
 
ABCD3
 
 
hsa-miR-148a-3p
 
 
-0.388
 
 
21.4
 
 
 
 
ABHD17C
 
 
hsa-let-7c-5p
 
 
-0.359
 
 
26.8
 
 
 
 
ABHD17C
 
 
hsa-miR-98-5p
 
 
-0.383
 
 
26.8
 
 
 
 
ABHD5
 
 
hsa-miR-20a-5p
 
 
-0.397
 
 
15.5
 
 
 
 
ABHD5
 
 
hsa-miR-106b-5p
 
 
-0.413
 
 
15.5
 
 
 
 
ABHD5
 
 
hsa-miR-20b-5p
 
 
-0.418
 
 
15.5
 
 
 
 
ABHD5
 
 
hsa-miR-106a-5p
 
 
-0.447
 
 
15.5
 
 
 
 
ABHD5
 
 
hsa-miR-17-5p
 
 
-0.45
 
 
15.5
 
 
 
 
ACER2
 
 
hsa-miR-20a-5p
 
 
-0.298
 
 
36.7
 
 
 
 
ACER2
 
 
hsa-miR-106b-5p
 
 
-0.298
 
 
36.7
 
 
 
 
ACER2
 
 
hsa-miR-106a-5p
 
 
-0.316
 
 
36.7
 
 
 
 
ACER2
 
 
hsa-miR-17-5p
 
 
-0.319
 
 
36.7
 
 
 
 
ACER2
 
 
hsa-miR-20b-5p
 
 
-0.319
 
 
36.7
 
 
 
 
ACER2
 
 
hsa-let-7c-5p
 
 
-0.333
 
 
36.7
 
 
 
 
ACER2
 
 
hsa-miR-98-5p
 
 
-0.344
 
 
36.7
 
 
 
 
ACTC1
 
 
hsa-miR-7-5p
 
 
-0.471
 
 
667.0
 
 
 
 
ACVR1
 
 
hsa-miR-148a-3p
 
 
-0.397
 
 
13.3
 
 
 
 
ADAM10
 
 
hsa-miR-148a-3p
 
 
-0.356
 
 
26.2
 
 
 
 
ADAM9
 
 
hsa-miR-20a-5p
 
 
-0.299
 
 
52.5
 
 
 
 
ADAM9
 
 
hsa-miR-106b-5p
 
 
-0.299
 
 
52.5
 
 
 
 
ADAM9
 
 
hsa-miR-106a-5p
 
 
-0.317
 
 
52.5
 
 
 
 
ADAM9
 
 
hsa-miR-17-5p
 
 
-0.32
 
 
52.5
 
 
 
 
ADAM9
 
 
hsa-miR-20b-5p
 
 
-0.328
 
 
52.5
 
 
 
 
ADGRF5
 
 
hsa-miR-148a-3p
 
 
-0.354
 
 
10.6
 
 
 
 
AGFG1
 
 
hsa-miR-148a-3p
 
 
-0.332
 
 
61.2
 
 
 
 
AGFG2
 
 
hsa-miR-20a-5p
 
 
-0.365
 
 
10.3
 
 
 
 
AGFG2
 
 
hsa-miR-20b-5p
 
 
-0.386
 
 
10.3
 
 
 
 
AGFG2
 
 
hsa-miR-106b-5p
 
 
-0.397
 
 
10.3
 
 
 
 
AGFG2
 
 
hsa-miR-106a-5p
 
 
-0.399
 
 
10.3
 
 
 
 
AGFG2
 
 
hsa-miR-17-5p
 
 
-0.402
 
 
10.3
 
 
 
 
AHDC1
 
 
hsa-miR-148a-3p
 
 
-0.413
 
 
14.8
 
 
 
 
AHNAK
 
 
hsa-miR-20a-5p
 
 
-0.316
 
 
145.5
 
 
 
 
AHNAK
 
 
hsa-miR-106b-5p
 
 
-0.316
 
 
145.5
 
 
 
 
AHNAK
 
 
hsa-miR-106a-5p
 
 
-0.334
 
 
145.5
 
 
 
 
AHNAK
 
 
hsa-miR-17-5p
 
 
-0.337
 
 
145.5
 
 
 
 
AHNAK
 
 
hsa-miR-20b-5p
 
 
-0.337
 
 
145.5
 
 
 
 
AIFM1
 
 
hsa-miR-98-5p
 
 
-0.314
 
 
13.1
 
 
 
 
AIFM1
 
 
hsa-let-7c-5p
 
 
-0.314
 
 
13.1
 
 
 
 
AKAP1
 
 
hsa-miR-148a-3p
 
 
-0.381
 
 
47.8
 
 
 
 
ALCAM
 
 
hsa-miR-148a-3p
 
 
-0.339
 
 
23.9
 
 
 
 
ALDH4A1
 
 
hsa-miR-184
 
 
-0.421
 
 
21.0
 
 
 
 
ALG3
 
 
hsa-miR-106b-5p
 
 
-0.551
 
 
20.1
 
 
 
 
ALG3
 
 
hsa-miR-20a-5p
 
 
-0.559
 
 
20.1
 
 
 
 
ALG3
 
 
hsa-miR-106a-5p
 
 
-0.569
 
 
20.1
 
 
 
 
ALG3
 
 
hsa-miR-17-5p
 
 
-0.572
 
 
20.1
 
 
 
 
ALG3
 
 
hsa-miR-20b-5p
 
 
-0.58
 
 
20.1
 
 
 
 
ALG9
 
 
hsa-miR-7-5p
 
 
-0.302
 
 
10.1
 
 
 
 
AMT
 
 
hsa-miR-98-5p
 
 
-0.463
 
 
13.1
 
 
 
 
AMT
 
 
hsa-let-7c-5p
 
 
-0.463
 
 
13.1
 
 
 
 
ANKRD12
 
 
hsa-miR-7-5p
 
 
-0.37
 
 
12.7
 
 
 
 
ANKRD50
 
 
hsa-miR-106a-5p
 
 
-0.29
 
 
13.3
 
 
 
 
ANKRD50
 
 
hsa-miR-20a-5p
 
 
-0.3
 
 
13.3
 
 
 
 
ANKRD50
 
 
hsa-miR-106b-5p
 
 
-0.3
 
 
13.3
 
 
 
 
ANKRD9
 
 
hsa-miR-20a-5p
 
 
-0.31
 
 
11.5
 
 
 
 
ANKRD9
 
 
hsa-miR-106b-5p
 
 
-0.318
 
 
11.5
 
 
 
 
ANKRD9
 
 
hsa-miR-20b-5p
 
 
-0.331
 
 
11.5
 
 
 
 
ANKRD9
 
 
hsa-miR-106a-5p
 
 
-0.344
 
 
11.5
 
 
 
 
ANKRD9
 
 
hsa-miR-17-5p
 
 
-0.347
 
 
11.5
 
 
 
 
AP1AR
 
 
hsa-miR-21-5p
 
 
-0.318
 
 
12.2
 
 
 
 
AP1S1
 
 
hsa-miR-98-5p
 
 
-0.374
 
 
18.6
 
 
 
 
AP1S1
 
 
hsa-let-7c-5p
 
 
-0.374
 
 
18.6
 
 
 
 
APCDD1
 
 
hsa-miR-20a-5p
 
 
-0.404
 
 
34.0
 
 
 
 
APCDD1
 
 
hsa-miR-106b-5p
 
 
-0.404
 
 
34.0
 
 
 
 
APCDD1
 
 
hsa-miR-106a-5p
 
 
-0.422
 
 
34.0
 
 
 
 
APCDD1
 
 
hsa-miR-17-5p
 
 
-0.425
 
 
34.0
 
 
 
 
APCDD1
 
 
hsa-miR-20b-5p
 
 
-0.425
 
 
34.0
 
 
 
 
ARF4
 
 
hsa-miR-7-5p
 
 
-0.407
 
 
190.1
 
 
 
 
ARF4
 
 
hsa-miR-148a-3p
 
 
-0.487
 
 
190.1
 
 
 
 
ARFGEF1
 
 
hsa-miR-148a-3p
 
 
-0.306
 
 
18.4
 
 
 
 
ARFIP1
 
 
hsa-miR-148a-3p
 
 
-0.474
 
 
22.0
 
 
 
 
ARG2
 
 
hsa-miR-98-5p
 
 
-0.468
 
 
16.6
 
 
 
 
ARG2
 
 
hsa-let-7c-5p
 
 
-0.468
 
 
16.6
 
 
 
 
ARHGAP1
 
 
hsa-miR-17-5p
 
 
-0.325
 
 
106.2
 
 
 
 
ARHGAP1
 
 
hsa-miR-20b-5p
 
 
-0.325
 
 
106.2
 
 
 
 
ARHGAP1
 
 
hsa-miR-106a-5p
 
 
-0.329
 
 
106.2
 
 
 
 
ARHGAP1
 
 
hsa-miR-20a-5p
 
 
-0.339
 
 
106.2
 
 
 
 
ARHGAP1
 
 
hsa-miR-106b-5p
 
 
-0.339
 
 
106.2
 
 
 
 
ARHGAP21
 
 
hsa-miR-148a-3p
 
 
-0.287
 
 
12.9
 
 
 
 
ARID4B
 
 
hsa-miR-20a-5p
 
 
-0.29
 
 
12.0
 
 
 
 
ARID4B
 
 
hsa-miR-106b-5p
 
 
-0.29
 
 
12.0
 
 
 
 
ARL1
 
 
hsa-miR-20a-5p
 
 
-0.403
 
 
49.5
 
 
 
 
ARL1
 
 
hsa-miR-106b-5p
 
 
-0.411
 
 
49.5
 
 
 
 
ARL1
 
 
hsa-miR-20b-5p
 
 
-0.424
 
 
49.5
 
 
 
 
ARL1
 
 
hsa-miR-106a-5p
 
 
-0.429
 
 
49.5
 
 
 
 
ARL1
 
 
hsa-miR-17-5p
 
 
-0.432
 
 
49.5
 
 
 
 
ARL4D
 
 
hsa-miR-98-5p
 
 
-0.417
 
 
26.6
 
 
 
 
ARL4D
 
 
hsa-let-7c-5p
 
 
-0.417
 
 
26.6
 
 
 
 
ARL6IP1
 
 
hsa-miR-7-5p
 
 
-0.374
 
 
124.0
 
 
 
 
ARL6IP1
 
 
hsa-miR-148a-3p
 
 
-0.579
 
 
124.0
 
 
 
 
ARL6IP6
 
 
hsa-miR-98-5p
 
 
-0.293
 
 
12.2
 
 
 
 
ARL6IP6
 
 
hsa-let-7c-5p
 
 
-0.293
 
 
12.2
 
 
 
 
ARL8B
 
 
hsa-miR-148a-3p
 
 
-0.337
 
 
46.5
 
 
 
 
ARMC8
 
 
hsa-miR-20a-5p
 
 
-0.357
 
 
20.8
 
 
 
 
ARMC8
 
 
hsa-miR-106b-5p
 
 
-0.357
 
 
20.8
 
 
 
 
ARMC8
 
 
hsa-miR-106a-5p
 
 
-0.375
 
 
20.8
 
 
 
 
ARMC8
 
 
hsa-miR-17-5p
 
 
-0.378
 
 
20.8
 
 
 
 
ARMC8
 
 
hsa-miR-20b-5p
 
 
-0.378
 
 
20.8
 
 
 
 
ARMCX1
 
 
hsa-miR-21-5p
 
 
-0.489
 
 
22.2
 
 
 
 
ARMT1
 
 
hsa-let-7c-5p
 
 
-0.303
 
 
16.1
 
 
 
 
 
 
 vagina 
 
 
 
 
target_symbol
 
 
mature_mirna_id
 
 
score
 
 
nTPM
 
 
 
 
 
 
ABCB7
 
 
hsa-miR-148a-3p
 
 
-0.515
 
 
10.8
 
 
 
 
ABCD3
 
 
hsa-miR-148a-3p
 
 
-0.388
 
 
14.9
 
 
 
 
ABHD17C
 
 
hsa-let-7c-5p
 
 
-0.359
 
 
21.1
 
 
 
 
ABHD17C
 
 
hsa-miR-98-5p
 
 
-0.383
 
 
21.1
 
 
 
 
ABHD5
 
 
hsa-miR-20a-5p
 
 
-0.397
 
 
11.9
 
 
 
 
ABHD5
 
 
hsa-miR-106b-5p
 
 
-0.413
 
 
11.9
 
 
 
 
ABHD5
 
 
hsa-miR-20b-5p
 
 
-0.418
 
 
11.9
 
 
 
 
ABHD5
 
 
hsa-miR-106a-5p
 
 
-0.447
 
 
11.9
 
 
 
 
ABHD5
 
 
hsa-miR-17-5p
 
 
-0.45
 
 
11.9
 
 
 
 
ACP3
 
 
hsa-miR-98-5p
 
 
-0.298
 
 
10.8
 
 
 
 
ACP3
 
 
hsa-let-7c-5p
 
 
-0.298
 
 
10.8
 
 
 
 
ACTC1
 
 
hsa-miR-7-5p
 
 
-0.471
 
 
50.8
 
 
 
 
ADAM10
 
 
hsa-miR-148a-3p
 
 
-0.356
 
 
10.3
 
 
 
 
ADAM9
 
 
hsa-miR-20a-5p
 
 
-0.299
 
 
15.5
 
 
 
 
ADAM9
 
 
hsa-miR-106b-5p
 
 
-0.299
 
 
15.5
 
 
 
 
ADAM9
 
 
hsa-miR-106a-5p
 
 
-0.317
 
 
15.5
 
 
 
 
ADAM9
 
 
hsa-miR-17-5p
 
 
-0.32
 
 
15.5
 
 
 
 
ADAM9
 
 
hsa-miR-20b-5p
 
 
-0.328
 
 
15.5
 
 
 
 
ADRB2
 
 
hsa-let-7c-5p
 
 
-0.635
 
 
12.2
 
 
 
 
ADRB2
 
 
hsa-miR-98-5p
 
 
-0.635
 
 
12.2
 
 
 
 
AGFG1
 
 
hsa-miR-148a-3p
 
 
-0.332
 
 
31.5
 
 
 
 
AGFG2
 
 
hsa-miR-20a-5p
 
 
-0.365
 
 
11.5
 
 
 
 
AGFG2
 
 
hsa-miR-20b-5p
 
 
-0.386
 
 
11.5
 
 
 
 
AGFG2
 
 
hsa-miR-106b-5p
 
 
-0.397
 
 
11.5
 
 
 
 
AGFG2
 
 
hsa-miR-106a-5p
 
 
-0.399
 
 
11.5
 
 
 
 
AGFG2
 
 
hsa-miR-17-5p
 
 
-0.402
 
 
11.5
 
 
 
 
AHDC1
 
 
hsa-miR-148a-3p
 
 
-0.413
 
 
23.0
 
 
 
 
AHNAK
 
 
hsa-miR-20a-5p
 
 
-0.316
 
 
200.6
 
 
 
 
AHNAK
 
 
hsa-miR-106b-5p
 
 
-0.316
 
 
200.6
 
 
 
 
AHNAK
 
 
hsa-miR-106a-5p
 
 
-0.334
 
 
200.6
 
 
 
 
AHNAK
 
 
hsa-miR-17-5p
 
 
-0.337
 
 
200.6
 
 
 
 
AHNAK
 
 
hsa-miR-20b-5p
 
 
-0.337
 
 
200.6
 
 
 
 
AKAP1
 
 
hsa-miR-148a-3p
 
 
-0.381
 
 
24.4
 
 
 
 
ALDH4A1
 
 
hsa-miR-184
 
 
-0.421
 
 
16.8
 
 
 
 
ALG3
 
 
hsa-miR-106b-5p
 
 
-0.551
 
 
18.9
 
 
 
 
ALG3
 
 
hsa-miR-20a-5p
 
 
-0.559
 
 
18.9
 
 
 
 
ALG3
 
 
hsa-miR-106a-5p
 
 
-0.569
 
 
18.9
 
 
 
 
ALG3
 
 
hsa-miR-17-5p
 
 
-0.572
 
 
18.9
 
 
 
 
ALG3
 
 
hsa-miR-20b-5p
 
 
-0.58
 
 
18.9
 
 
 
 
ALG9
 
 
hsa-miR-7-5p
 
 
-0.302
 
 
10.2
 
 
 
 
ANKRD29
 
 
hsa-miR-20a-5p
 
 
-0.359
 
 
12.3
 
 
 
 
ANKRD29
 
 
hsa-miR-106b-5p
 
 
-0.359
 
 
12.3
 
 
 
 
ANKRD29
 
 
hsa-miR-106a-5p
 
 
-0.369
 
 
12.3
 
 
 
 
ANKRD29
 
 
hsa-miR-17-5p
 
 
-0.372
 
 
12.3
 
 
 
 
ANKRD29
 
 
hsa-miR-20b-5p
 
 
-0.38
 
 
12.3
 
 
 
 
ANKRD9
 
 
hsa-miR-20a-5p
 
 
-0.31
 
 
14.6
 
 
 
 
ANKRD9
 
 
hsa-miR-106b-5p
 
 
-0.318
 
 
14.6
 
 
 
 
ANKRD9
 
 
hsa-miR-20b-5p
 
 
-0.331
 
 
14.6
 
 
 
 
ANKRD9
 
 
hsa-miR-106a-5p
 
 
-0.344
 
 
14.6
 
 
 
 
ANKRD9
 
 
hsa-miR-17-5p
 
 
-0.347
 
 
14.6
 
 
 
 
AP1S1
 
 
hsa-miR-98-5p
 
 
-0.374
 
 
20.1
 
 
 
 
AP1S1
 
 
hsa-let-7c-5p
 
 
-0.374
 
 
20.1
 
 
 
 
APCDD1
 
 
hsa-miR-20a-5p
 
 
-0.404
 
 
33.2
 
 
 
 
APCDD1
 
 
hsa-miR-106b-5p
 
 
-0.404
 
 
33.2
 
 
 
 
APCDD1
 
 
hsa-miR-106a-5p
 
 
-0.422
 
 
33.2
 
 
 
 
APCDD1
 
 
hsa-miR-17-5p
 
 
-0.425
 
 
33.2
 
 
 
 
APCDD1
 
 
hsa-miR-20b-5p
 
 
-0.425
 
 
33.2
 
 
 
 
ARF4
 
 
hsa-miR-7-5p
 
 
-0.407
 
 
85.1
 
 
 
 
ARF4
 
 
hsa-miR-148a-3p
 
 
-0.487
 
 
85.1
 
 
 
 
ARFIP1
 
 
hsa-miR-148a-3p
 
 
-0.474
 
 
13.1
 
 
 
 
ARG2
 
 
hsa-miR-98-5p
 
 
-0.468
 
 
11.0
 
 
 
 
ARG2
 
 
hsa-let-7c-5p
 
 
-0.468
 
 
11.0
 
 
 
 
ARHGAP1
 
 
hsa-miR-17-5p
 
 
-0.325
 
 
59.0
 
 
 
 
ARHGAP1
 
 
hsa-miR-20b-5p
 
 
-0.325
 
 
59.0
 
 
 
 
ARHGAP1
 
 
hsa-miR-106a-5p
 
 
-0.329
 
 
59.0
 
 
 
 
ARHGAP1
 
 
hsa-miR-20a-5p
 
 
-0.339
 
 
59.0
 
 
 
 
ARHGAP1
 
 
hsa-miR-106b-5p
 
 
-0.339
 
 
59.0
 
 
 
 
ARID4B
 
 
hsa-miR-20a-5p
 
 
-0.29
 
 
10.0
 
 
 
 
ARID4B
 
 
hsa-miR-106b-5p
 
 
-0.29
 
 
10.0
 
 
 
 
ARL1
 
 
hsa-miR-20a-5p
 
 
-0.403
 
 
34.0
 
 
 
 
ARL1
 
 
hsa-miR-106b-5p
 
 
-0.411
 
 
34.0
 
 
 
 
ARL1
 
 
hsa-miR-20b-5p
 
 
-0.424
 
 
34.0
 
 
 
 
ARL1
 
 
hsa-miR-106a-5p
 
 
-0.429
 
 
34.0
 
 
 
 
ARL1
 
 
hsa-miR-17-5p
 
 
-0.432
 
 
34.0
 
 
 
 
ARL4D
 
 
hsa-miR-98-5p
 
 
-0.417
 
 
38.9
 
 
 
 
ARL4D
 
 
hsa-let-7c-5p
 
 
-0.417
 
 
38.9
 
 
 
 
ARL6IP1
 
 
hsa-miR-7-5p
 
 
-0.374
 
 
48.0
 
 
 
 
ARL6IP1
 
 
hsa-miR-148a-3p
 
 
-0.579
 
 
48.0
 
 
 
 
ARL8B
 
 
hsa-miR-148a-3p
 
 
-0.337
 
 
51.8
 
 
 
 
ARMC8
 
 
hsa-miR-20a-5p
 
 
-0.357
 
 
12.4
 
 
 
 
ARMC8
 
 
hsa-miR-106b-5p
 
 
-0.357
 
 
12.4
 
 
 
 
ARMC8
 
 
hsa-miR-106a-5p
 
 
-0.375
 
 
12.4
 
 
 
 
ARMC8
 
 
hsa-miR-17-5p
 
 
-0.378
 
 
12.4
 
 
 
 
ARMC8
 
 
hsa-miR-20b-5p
 
 
-0.378
 
 
12.4
 
 
 
 
ARMCX1
 
 
hsa-miR-21-5p
 
 
-0.489
 
 
20.8
 
 
 
 
ARPP19
 
 
hsa-let-7c-5p
 
 
-0.492
 
 
27.0
 
 
 
 
ARPP19
 
 
hsa-miR-98-5p
 
 
-0.5
 
 
27.0
 
 
 
 
ARRDC3
 
 
hsa-miR-148a-3p
 
 
-0.305
 
 
40.5
 
 
 
 
ARRDC4
 
 
hsa-let-7c-5p
 
 
-0.329
 
 
10.1
 
 
 
 
ARRDC4
 
 
hsa-miR-98-5p
 
 
-0.329
 
 
10.1
 
 
 
 
ASAP1
 
 
hsa-miR-98-5p
 
 
-0.308
 
 
14.4
 
 
 
 
ASAP1
 
 
hsa-let-7c-5p
 
 
-0.308
 
 
14.4
 
 
 
 
ASPH
 
 
hsa-miR-148a-3p
 
 
-0.391
 
 
40.4
 
 
 
 
ATG4D
 
 
hsa-miR-148a-3p
 
 
-0.303
 
 
14.5
 
 
 
 
ATOSB
 
 
hsa-miR-98-5p
 
 
-0.429
 
 
14.3
 
 
 
 
ATOSB
 
 
hsa-let-7c-5p
 
 
-0.429
 
 
14.3
 
 
 
 
ATP2A2
 
 
hsa-miR-98-5p
 
 
-0.311
 
 
40.2
 
 
 
 
ATP2A2
 
 
hsa-let-7c-5p
 
 
-0.311
 
 
40.2
 
 
 
 
ATP2B4
 
 
hsa-miR-148a-3p
 
 
-0.337
 
 
14.3
 
 
 
 
ATP6AP2
 
 
hsa-miR-148a-3p
 
 
-0.592
 
 
44.8
 
 
 
 
 
 
 basal ganglia 
 
 
 
 
target_symbol
 
 
mature_mirna_id
 
 
score
 
 
nTPM
 
 
 
 
 
 
ABCB9
 
 
hsa-let-7c-5p
 
 
-0.422
 
 
13.6
 
 
 
 
ABCB9
 
 
hsa-miR-98-5p
 
 
-0.444
 
 
13.6
 
 
 
 
ABCD3
 
 
hsa-miR-148a-3p
 
 
-0.388
 
 
17.4
 
 
 
 
ABHD17C
 
 
hsa-let-7c-5p
 
 
-0.359
 
 
20.2
 
 
 
 
ABHD17C
 
 
hsa-miR-98-5p
 
 
-0.383
 
 
20.2
 
 
 
 
ACSL6
 
 
hsa-miR-98-5p
 
 
-0.336
 
 
18.7
 
 
 
 
ACSL6
 
 
hsa-let-7c-5p
 
 
-0.336
 
 
18.7
 
 
 
 
ADCY2
 
 
hsa-miR-148a-3p
 
 
-0.299
 
 
23.7
 
 
 
 
ADGRB3
 
 
hsa-miR-148a-3p
 
 
-0.524
 
 
21.1
 
 
 
 
ADGRF5
 
 
hsa-miR-148a-3p
 
 
-0.354
 
 
13.3
 
 
 
 
ADRB1
 
 
hsa-let-7c-5p
 
 
-0.351
 
 
10.0
 
 
 
 
ADRB1
 
 
hsa-miR-98-5p
 
 
-0.367
 
 
10.0
 
 
 
 
AGFG1
 
 
hsa-miR-148a-3p
 
 
-0.332
 
 
27.0
 
 
 
 
AGFG2
 
 
hsa-miR-20a-5p
 
 
-0.365
 
 
19.5
 
 
 
 
AGFG2
 
 
hsa-miR-20b-5p
 
 
-0.386
 
 
19.5
 
 
 
 
AGFG2
 
 
hsa-miR-106b-5p
 
 
-0.397
 
 
19.5
 
 
 
 
AGFG2
 
 
hsa-miR-106a-5p
 
 
-0.399
 
 
19.5
 
 
 
 
AGFG2
 
 
hsa-miR-17-5p
 
 
-0.402
 
 
19.5
 
 
 
 
AHDC1
 
 
hsa-miR-148a-3p
 
 
-0.413
 
 
12.3
 
 
 
 
AHNAK
 
 
hsa-miR-20a-5p
 
 
-0.316
 
 
22.5
 
 
 
 
AHNAK
 
 
hsa-miR-106b-5p
 
 
-0.316
 
 
22.5
 
 
 
 
AHNAK
 
 
hsa-miR-106a-5p
 
 
-0.334
 
 
22.5
 
 
 
 
AHNAK
 
 
hsa-miR-17-5p
 
 
-0.337
 
 
22.5
 
 
 
 
AHNAK
 
 
hsa-miR-20b-5p
 
 
-0.337
 
 
22.5
 
 
 
 
AIFM1
 
 
hsa-miR-98-5p
 
 
-0.314
 
 
12.5
 
 
 
 
AIFM1
 
 
hsa-let-7c-5p
 
 
-0.314
 
 
12.5
 
 
 
 
AKAP1
 
 
hsa-miR-148a-3p
 
 
-0.381
 
 
26.7
 
 
 
 
AKAP6
 
 
hsa-miR-98-5p
 
 
-0.307
 
 
10.5
 
 
 
 
AKAP6
 
 
hsa-let-7c-5p
 
 
-0.307
 
 
10.5
 
 
 
 
ALCAM
 
 
hsa-miR-148a-3p
 
 
-0.339
 
 
37.8
 
 
 
 
ALDH4A1
 
 
hsa-miR-184
 
 
-0.421
 
 
86.2
 
 
 
 
ALG3
 
 
hsa-miR-106b-5p
 
 
-0.551
 
 
10.2
 
 
 
 
ALG3
 
 
hsa-miR-20a-5p
 
 
-0.559
 
 
10.2
 
 
 
 
ALG3
 
 
hsa-miR-106a-5p
 
 
-0.569
 
 
10.2
 
 
 
 
ALG3
 
 
hsa-miR-17-5p
 
 
-0.572
 
 
10.2
 
 
 
 
ALG3
 
 
hsa-miR-20b-5p
 
 
-0.58
 
 
10.2
 
 
 
 
AMT
 
 
hsa-miR-98-5p
 
 
-0.463
 
 
11.4
 
 
 
 
AMT
 
 
hsa-let-7c-5p
 
 
-0.463
 
 
11.4
 
 
 
 
ANKRD46
 
 
hsa-miR-98-5p
 
 
-0.389
 
 
22.6
 
 
 
 
ANKRD46
 
 
hsa-let-7c-5p
 
 
-0.389
 
 
22.6
 
 
 
 
ANKRD9
 
 
hsa-miR-20a-5p
 
 
-0.31
 
 
18.5
 
 
 
 
ANKRD9
 
 
hsa-miR-106b-5p
 
 
-0.318
 
 
18.5
 
 
 
 
ANKRD9
 
 
hsa-miR-20b-5p
 
 
-0.331
 
 
18.5
 
 
 
 
ANKRD9
 
 
hsa-miR-106a-5p
 
 
-0.344
 
 
18.5
 
 
 
 
ANKRD9
 
 
hsa-miR-17-5p
 
 
-0.347
 
 
18.5
 
 
 
 
AP1AR
 
 
hsa-miR-21-5p
 
 
-0.318
 
 
13.0
 
 
 
 
AP1S1
 
 
hsa-miR-98-5p
 
 
-0.374
 
 
116.3
 
 
 
 
AP1S1
 
 
hsa-let-7c-5p
 
 
-0.374
 
 
116.3
 
 
 
 
APCDD1
 
 
hsa-miR-20a-5p
 
 
-0.404
 
 
30.3
 
 
 
 
APCDD1
 
 
hsa-miR-106b-5p
 
 
-0.404
 
 
30.3
 
 
 
 
APCDD1
 
 
hsa-miR-106a-5p
 
 
-0.422
 
 
30.3
 
 
 
 
APCDD1
 
 
hsa-miR-17-5p
 
 
-0.425
 
 
30.3
 
 
 
 
APCDD1
 
 
hsa-miR-20b-5p
 
 
-0.425
 
 
30.3
 
 
 
 
ARF4
 
 
hsa-miR-7-5p
 
 
-0.407
 
 
71.0
 
 
 
 
ARF4
 
 
hsa-miR-148a-3p
 
 
-0.487
 
 
71.0
 
 
 
 
ARHGAP1
 
 
hsa-miR-17-5p
 
 
-0.325
 
 
41.0
 
 
 
 
ARHGAP1
 
 
hsa-miR-20b-5p
 
 
-0.325
 
 
41.0
 
 
 
 
ARHGAP1
 
 
hsa-miR-106a-5p
 
 
-0.329
 
 
41.0
 
 
 
 
ARHGAP1
 
 
hsa-miR-20a-5p
 
 
-0.339
 
 
41.0
 
 
 
 
ARHGAP1
 
 
hsa-miR-106b-5p
 
 
-0.339
 
 
41.0
 
 
 
 
ARHGAP21
 
 
hsa-miR-148a-3p
 
 
-0.287
 
 
37.1
 
 
 
 
ARL1
 
 
hsa-miR-20a-5p
 
 
-0.403
 
 
28.4
 
 
 
 
ARL1
 
 
hsa-miR-106b-5p
 
 
-0.411
 
 
28.4
 
 
 
 
ARL1
 
 
hsa-miR-20b-5p
 
 
-0.424
 
 
28.4
 
 
 
 
ARL1
 
 
hsa-miR-106a-5p
 
 
-0.429
 
 
28.4
 
 
 
 
ARL1
 
 
hsa-miR-17-5p
 
 
-0.432
 
 
28.4
 
 
 
 
ARL4D
 
 
hsa-miR-98-5p
 
 
-0.417
 
 
14.0
 
 
 
 
ARL4D
 
 
hsa-let-7c-5p
 
 
-0.417
 
 
14.0
 
 
 
 
ARL6IP1
 
 
hsa-miR-7-5p
 
 
-0.374
 
 
196.4
 
 
 
 
ARL6IP1
 
 
hsa-miR-148a-3p
 
 
-0.579
 
 
196.4
 
 
 
 
ARL8B
 
 
hsa-miR-148a-3p
 
 
-0.337
 
 
53.5
 
 
 
 
ARMC8
 
 
hsa-miR-20a-5p
 
 
-0.357
 
 
13.3
 
 
 
 
ARMC8
 
 
hsa-miR-106b-5p
 
 
-0.357
 
 
13.3
 
 
 
 
ARMC8
 
 
hsa-miR-106a-5p
 
 
-0.375
 
 
13.3
 
 
 
 
ARMC8
 
 
hsa-miR-17-5p
 
 
-0.378
 
 
13.3
 
 
 
 
ARMC8
 
 
hsa-miR-20b-5p
 
 
-0.378
 
 
13.3
 
 
 
 
ARMCX1
 
 
hsa-miR-21-5p
 
 
-0.489
 
 
21.1
 
 
 
 
ARMT1
 
 
hsa-let-7c-5p
 
 
-0.303
 
 
10.6
 
 
 
 
ARMT1
 
 
hsa-miR-98-5p
 
 
-0.303
 
 
10.6
 
 
 
 
ARPP19
 
 
hsa-let-7c-5p
 
 
-0.492
 
 
481.4
 
 
 
 
ARPP19
 
 
hsa-miR-98-5p
 
 
-0.5
 
 
481.4
 
 
 
 
ARRDC3
 
 
hsa-miR-148a-3p
 
 
-0.305
 
 
10.6
 
 
 
 
ARRDC4
 
 
hsa-let-7c-5p
 
 
-0.329
 
 
10.0
 
 
 
 
ARRDC4
 
 
hsa-miR-98-5p
 
 
-0.329
 
 
10.0
 
 
 
 
ASAP1
 
 
hsa-miR-98-5p
 
 
-0.308
 
 
11.6
 
 
 
 
ASAP1
 
 
hsa-let-7c-5p
 
 
-0.308
 
 
11.6
 
 
 
 
ASF1A
 
 
hsa-miR-21-5p
 
 
-0.304
 
 
15.3
 
 
 
 
ASPH
 
 
hsa-miR-148a-3p
 
 
-0.391
 
 
62.4
 
 
 
 
ATG16L1
 
 
hsa-miR-20a-5p
 
 
-0.446
 
 
11.5
 
 
 
 
ATG16L1
 
 
hsa-miR-106b-5p
 
 
-0.446
 
 
11.5
 
 
 
 
ATG16L1
 
 
hsa-miR-106a-5p
 
 
-0.464
 
 
11.5
 
 
 
 
ATG16L1
 
 
hsa-miR-17-5p
 
 
-0.467
 
 
11.5
 
 
 
 
ATG16L1
 
 
hsa-miR-20b-5p
 
 
-0.467
 
 
11.5
 
 
 
 
ATOSB
 
 
hsa-miR-98-5p
 
 
-0.429
 
 
20.2
 
 
 
 
ATOSB
 
 
hsa-let-7c-5p
 
 
-0.429
 
 
20.2
 
 
 
 
ATP2A2
 
 
hsa-miR-98-5p
 
 
-0.311
 
 
21.1
 
 
 
 
ATP2A2
 
 
hsa-let-7c-5p
 
 
-0.311
 
 
21.1
 
 
 
 
ATP2B2
 
 
hsa-miR-7-5p
 
 
-0.297
 
 
34.6
 
 
 
 
ATP6AP2
 
 
hsa-miR-148a-3p
 
 
-0.592
 
 
77.2
 
 
 
 
ATP6AP2
 
 
hsa-miR-148a-3p
 
 
-0.668
 
 
77.2
 
 
 
 
 
 
 cerebral cortex 
 
 
 
 
target_symbol
 
 
mature_mirna_id
 
 
score
 
 
nTPM
 
 
 
 
 
 
ABCB9
 
 
hsa-let-7c-5p
 
 
-0.422
 
 
19.5
 
 
 
 
ABCB9
 
 
hsa-miR-98-5p
 
 
-0.444
 
 
19.5
 
 
 
 
ABCD3
 
 
hsa-miR-148a-3p
 
 
-0.388
 
 
17.1
 
 
 
 
ABHD17C
 
 
hsa-let-7c-5p
 
 
-0.359
 
 
16.3
 
 
 
 
ABHD17C
 
 
hsa-miR-98-5p
 
 
-0.383
 
 
16.3
 
 
 
 
ACSL6
 
 
hsa-miR-98-5p
 
 
-0.336
 
 
29.5
 
 
 
 
ACSL6
 
 
hsa-let-7c-5p
 
 
-0.336
 
 
29.5
 
 
 
 
ADAM10
 
 
hsa-miR-148a-3p
 
 
-0.356
 
 
16.0
 
 
 
 
ADAM9
 
 
hsa-miR-20a-5p
 
 
-0.299
 
 
16.2
 
 
 
 
ADAM9
 
 
hsa-miR-106b-5p
 
 
-0.299
 
 
16.2
 
 
 
 
ADAM9
 
 
hsa-miR-106a-5p
 
 
-0.317
 
 
16.2
 
 
 
 
ADAM9
 
 
hsa-miR-17-5p
 
 
-0.32
 
 
16.2
 
 
 
 
ADAM9
 
 
hsa-miR-20b-5p
 
 
-0.328
 
 
16.2
 
 
 
 
ADCY2
 
 
hsa-miR-148a-3p
 
 
-0.299
 
 
35.4
 
 
 
 
ADGRB3
 
 
hsa-miR-148a-3p
 
 
-0.524
 
 
28.2
 
 
 
 
AGFG1
 
 
hsa-miR-148a-3p
 
 
-0.332
 
 
33.6
 
 
 
 
AGFG2
 
 
hsa-miR-20a-5p
 
 
-0.365
 
 
14.0
 
 
 
 
AGFG2
 
 
hsa-miR-20b-5p
 
 
-0.386
 
 
14.0
 
 
 
 
AGFG2
 
 
hsa-miR-106b-5p
 
 
-0.397
 
 
14.0
 
 
 
 
AGFG2
 
 
hsa-miR-106a-5p
 
 
-0.399
 
 
14.0
 
 
 
 
AGFG2
 
 
hsa-miR-17-5p
 
 
-0.402
 
 
14.0
 
 
 
 
AHDC1
 
 
hsa-miR-148a-3p
 
 
-0.413
 
 
15.5
 
 
 
 
AHNAK
 
 
hsa-miR-20a-5p
 
 
-0.316
 
 
26.8
 
 
 
 
AHNAK
 
 
hsa-miR-106b-5p
 
 
-0.316
 
 
26.8
 
 
 
 
AHNAK
 
 
hsa-miR-106a-5p
 
 
-0.334
 
 
26.8
 
 
 
 
AHNAK
 
 
hsa-miR-17-5p
 
 
-0.337
 
 
26.8
 
 
 
 
AHNAK
 
 
hsa-miR-20b-5p
 
 
-0.337
 
 
26.8
 
 
 
 
AIFM1
 
 
hsa-miR-98-5p
 
 
-0.314
 
 
10.3
 
 
 
 
AIFM1
 
 
hsa-let-7c-5p
 
 
-0.314
 
 
10.3
 
 
 
 
AKAP1
 
 
hsa-miR-148a-3p
 
 
-0.381
 
 
24.9
 
 
 
 
AKAP6
 
 
hsa-miR-98-5p
 
 
-0.307
 
 
27.4
 
 
 
 
AKAP6
 
 
hsa-let-7c-5p
 
 
-0.307
 
 
27.4
 
 
 
 
ALCAM
 
 
hsa-miR-148a-3p
 
 
-0.339
 
 
54.7
 
 
 
 
ALDH4A1
 
 
hsa-miR-184
 
 
-0.421
 
 
69.8
 
 
 
 
ANKRD12
 
 
hsa-miR-7-5p
 
 
-0.37
 
 
15.1
 
 
 
 
ANKRD13C
 
 
hsa-miR-20a-5p
 
 
-0.505
 
 
10.2
 
 
 
 
ANKRD13C
 
 
hsa-miR-106b-5p
 
 
-0.505
 
 
10.2
 
 
 
 
ANKRD13C
 
 
hsa-miR-106a-5p
 
 
-0.523
 
 
10.2
 
 
 
 
ANKRD13C
 
 
hsa-miR-17-5p
 
 
-0.526
 
 
10.2
 
 
 
 
ANKRD13C
 
 
hsa-miR-20b-5p
 
 
-0.526
 
 
10.2
 
 
 
 
ANKRD46
 
 
hsa-miR-98-5p
 
 
-0.389
 
 
39.5
 
 
 
 
ANKRD46
 
 
hsa-let-7c-5p
 
 
-0.389
 
 
39.5
 
 
 
 
ANKRD9
 
 
hsa-miR-20a-5p
 
 
-0.31
 
 
28.9
 
 
 
 
ANKRD9
 
 
hsa-miR-106b-5p
 
 
-0.318
 
 
28.9
 
 
 
 
ANKRD9
 
 
hsa-miR-20b-5p
 
 
-0.331
 
 
28.9
 
 
 
 
ANKRD9
 
 
hsa-miR-106a-5p
 
 
-0.344
 
 
28.9
 
 
 
 
ANKRD9
 
 
hsa-miR-17-5p
 
 
-0.347
 
 
28.9
 
 
 
 
AP1AR
 
 
hsa-miR-21-5p
 
 
-0.318
 
 
12.2
 
 
 
 
AP1S1
 
 
hsa-miR-98-5p
 
 
-0.374
 
 
104.9
 
 
 
 
AP1S1
 
 
hsa-let-7c-5p
 
 
-0.374
 
 
104.9
 
 
 
 
APBB3
 
 
hsa-miR-98-5p
 
 
-0.587
 
 
15.0
 
 
 
 
APBB3
 
 
hsa-let-7c-5p
 
 
-0.587
 
 
15.0
 
 
 
 
APCDD1
 
 
hsa-miR-20a-5p
 
 
-0.404
 
 
33.1
 
 
 
 
APCDD1
 
 
hsa-miR-106b-5p
 
 
-0.404
 
 
33.1
 
 
 
 
APCDD1
 
 
hsa-miR-106a-5p
 
 
-0.422
 
 
33.1
 
 
 
 
APCDD1
 
 
hsa-miR-17-5p
 
 
-0.425
 
 
33.1
 
 
 
 
APCDD1
 
 
hsa-miR-20b-5p
 
 
-0.425
 
 
33.1
 
 
 
 
ARF4
 
 
hsa-miR-7-5p
 
 
-0.407
 
 
61.5
 
 
 
 
ARF4
 
 
hsa-miR-148a-3p
 
 
-0.487
 
 
61.5
 
 
 
 
ARHGAP1
 
 
hsa-miR-17-5p
 
 
-0.325
 
 
40.8
 
 
 
 
ARHGAP1
 
 
hsa-miR-20b-5p
 
 
-0.325
 
 
40.8
 
 
 
 
ARHGAP1
 
 
hsa-miR-106a-5p
 
 
-0.329
 
 
40.8
 
 
 
 
ARHGAP1
 
 
hsa-miR-20a-5p
 
 
-0.339
 
 
40.8
 
 
 
 
ARHGAP1
 
 
hsa-miR-106b-5p
 
 
-0.339
 
 
40.8
 
 
 
 
ARHGAP21
 
 
hsa-miR-148a-3p
 
 
-0.287
 
 
47.3
 
 
 
 
ARID4A
 
 
hsa-miR-7-5p
 
 
-0.341
 
 
13.3
 
 
 
 
ARID4B
 
 
hsa-miR-20a-5p
 
 
-0.29
 
 
11.1
 
 
 
 
ARID4B
 
 
hsa-miR-106b-5p
 
 
-0.29
 
 
11.1
 
 
 
 
ARL1
 
 
hsa-miR-20a-5p
 
 
-0.403
 
 
34.0
 
 
 
 
ARL1
 
 
hsa-miR-106b-5p
 
 
-0.411
 
 
34.0
 
 
 
 
ARL1
 
 
hsa-miR-20b-5p
 
 
-0.424
 
 
34.0
 
 
 
 
ARL1
 
 
hsa-miR-106a-5p
 
 
-0.429
 
 
34.0
 
 
 
 
ARL1
 
 
hsa-miR-17-5p
 
 
-0.432
 
 
34.0
 
 
 
 
ARL4D
 
 
hsa-miR-98-5p
 
 
-0.417
 
 
14.3
 
 
 
 
ARL4D
 
 
hsa-let-7c-5p
 
 
-0.417
 
 
14.3
 
 
 
 
ARL6IP1
 
 
hsa-miR-7-5p
 
 
-0.374
 
 
314.1
 
 
 
 
ARL6IP1
 
 
hsa-miR-148a-3p
 
 
-0.579
 
 
314.1
 
 
 
 
ARL8B
 
 
hsa-miR-148a-3p
 
 
-0.337
 
 
75.1
 
 
 
 
ARMC8
 
 
hsa-miR-20a-5p
 
 
-0.357
 
 
21.7
 
 
 
 
ARMC8
 
 
hsa-miR-106b-5p
 
 
-0.357
 
 
21.7
 
 
 
 
ARMC8
 
 
hsa-miR-106a-5p
 
 
-0.375
 
 
21.7
 
 
 
 
ARMC8
 
 
hsa-miR-17-5p
 
 
-0.378
 
 
21.7
 
 
 
 
ARMC8
 
 
hsa-miR-20b-5p
 
 
-0.378
 
 
21.7
 
 
 
 
ARMCX1
 
 
hsa-miR-21-5p
 
 
-0.489
 
 
22.6
 
 
 
 
ARMT1
 
 
hsa-let-7c-5p
 
 
-0.303
 
 
10.1
 
 
 
 
ARMT1
 
 
hsa-miR-98-5p
 
 
-0.303
 
 
10.1
 
 
 
 
ARPP19
 
 
hsa-let-7c-5p
 
 
-0.492
 
 
292.4
 
 
 
 
ARPP19
 
 
hsa-miR-98-5p
 
 
-0.5
 
 
292.4
 
 
 
 
ARRDC3
 
 
hsa-miR-148a-3p
 
 
-0.305
 
 
15.6
 
 
 
 
ARRDC4
 
 
hsa-let-7c-5p
 
 
-0.329
 
 
12.8
 
 
 
 
ARRDC4
 
 
hsa-miR-98-5p
 
 
-0.329
 
 
12.8
 
 
 
 
ASAP1
 
 
hsa-miR-98-5p
 
 
-0.308
 
 
21.7
 
 
 
 
ASAP1
 
 
hsa-let-7c-5p
 
 
-0.308
 
 
21.7
 
 
 
 
ASF1A
 
 
hsa-miR-21-5p
 
 
-0.304
 
 
13.7
 
 
 
 
ASPH
 
 
hsa-miR-148a-3p
 
 
-0.391
 
 
76.3
 
 
 
 
ATG16L1
 
 
hsa-miR-20a-5p
 
 
-0.446
 
 
13.9
 
 
 
 
ATG16L1
 
 
hsa-miR-106b-5p
 
 
-0.446
 
 
13.9
 
 
 
 
ATG16L1
 
 
hsa-miR-106a-5p
 
 
-0.464
 
 
13.9
 
 
 
 
ATG16L1
 
 
hsa-miR-17-5p
 
 
-0.467
 
 
13.9
 
 
 
 
ATG16L1
 
 
hsa-miR-20b-5p
 
 
-0.467
 
 
13.9
 
 
 
 
 
 
 hypothalamus 
 
 
 
 
target_symbol
 
 
mature_mirna_id
 
 
score
 
 
nTPM
 
 
 
 
 
 
ABCB9
 
 
hsa-let-7c-5p
 
 
-0.422
 
 
11.9
 
 
 
 
ABCB9
 
 
hsa-miR-98-5p
 
 
-0.444
 
 
11.9
 
 
 
 
ABCD3
 
 
hsa-miR-148a-3p
 
 
-0.388
 
 
13.5
 
 
 
 
ABHD17C
 
 
hsa-let-7c-5p
 
 
-0.359
 
 
11.1
 
 
 
 
ABHD17C
 
 
hsa-miR-98-5p
 
 
-0.383
 
 
11.1
 
 
 
 
ACSL6
 
 
hsa-miR-98-5p
 
 
-0.336
 
 
11.9
 
 
 
 
ACSL6
 
 
hsa-let-7c-5p
 
 
-0.336
 
 
11.9
 
 
 
 
ADCY2
 
 
hsa-miR-148a-3p
 
 
-0.299
 
 
14.0
 
 
 
 
ADGRB3
 
 
hsa-miR-148a-3p
 
 
-0.524
 
 
16.0
 
 
 
 
AGFG1
 
 
hsa-miR-148a-3p
 
 
-0.332
 
 
26.0
 
 
 
 
AGFG2
 
 
hsa-miR-20a-5p
 
 
-0.365
 
 
10.8
 
 
 
 
AGFG2
 
 
hsa-miR-20b-5p
 
 
-0.386
 
 
10.8
 
 
 
 
AGFG2
 
 
hsa-miR-106b-5p
 
 
-0.397
 
 
10.8
 
 
 
 
AGFG2
 
 
hsa-miR-106a-5p
 
 
-0.399
 
 
10.8
 
 
 
 
AGFG2
 
 
hsa-miR-17-5p
 
 
-0.402
 
 
10.8
 
 
 
 
AHDC1
 
 
hsa-miR-148a-3p
 
 
-0.413
 
 
10.9
 
 
 
 
AHNAK
 
 
hsa-miR-20a-5p
 
 
-0.316
 
 
17.3
 
 
 
 
AHNAK
 
 
hsa-miR-106b-5p
 
 
-0.316
 
 
17.3
 
 
 
 
AHNAK
 
 
hsa-miR-106a-5p
 
 
-0.334
 
 
17.3
 
 
 
 
AHNAK
 
 
hsa-miR-17-5p
 
 
-0.337
 
 
17.3
 
 
 
 
AHNAK
 
 
hsa-miR-20b-5p
 
 
-0.337
 
 
17.3
 
 
 
 
AKAP1
 
 
hsa-miR-148a-3p
 
 
-0.381
 
 
15.1
 
 
 
 
AKAP6
 
 
hsa-miR-98-5p
 
 
-0.307
 
 
13.6
 
 
 
 
AKAP6
 
 
hsa-let-7c-5p
 
 
-0.307
 
 
13.6
 
 
 
 
ALCAM
 
 
hsa-miR-148a-3p
 
 
-0.339
 
 
36.1
 
 
 
 
ALDH4A1
 
 
hsa-miR-184
 
 
-0.421
 
 
31.5
 
 
 
 
ANKRD46
 
 
hsa-miR-98-5p
 
 
-0.389
 
 
28.7
 
 
 
 
ANKRD46
 
 
hsa-let-7c-5p
 
 
-0.389
 
 
28.7
 
 
 
 
ANKRD9
 
 
hsa-miR-20a-5p
 
 
-0.31
 
 
16.0
 
 
 
 
ANKRD9
 
 
hsa-miR-106b-5p
 
 
-0.318
 
 
16.0
 
 
 
 
ANKRD9
 
 
hsa-miR-20b-5p
 
 
-0.331
 
 
16.0
 
 
 
 
ANKRD9
 
 
hsa-miR-106a-5p
 
 
-0.344
 
 
16.0
 
 
 
 
ANKRD9
 
 
hsa-miR-17-5p
 
 
-0.347
 
 
16.0
 
 
 
 
AP1S1
 
 
hsa-miR-98-5p
 
 
-0.374
 
 
44.3
 
 
 
 
AP1S1
 
 
hsa-let-7c-5p
 
 
-0.374
 
 
44.3
 
 
 
 
APBB3
 
 
hsa-miR-98-5p
 
 
-0.587
 
 
11.2
 
 
 
 
APBB3
 
 
hsa-let-7c-5p
 
 
-0.587
 
 
11.2
 
 
 
 
APCDD1
 
 
hsa-miR-20a-5p
 
 
-0.404
 
 
19.2
 
 
 
 
APCDD1
 
 
hsa-miR-106b-5p
 
 
-0.404
 
 
19.2
 
 
 
 
APCDD1
 
 
hsa-miR-106a-5p
 
 
-0.422
 
 
19.2
 
 
 
 
APCDD1
 
 
hsa-miR-17-5p
 
 
-0.425
 
 
19.2
 
 
 
 
APCDD1
 
 
hsa-miR-20b-5p
 
 
-0.425
 
 
19.2
 
 
 
 
ARF4
 
 
hsa-miR-7-5p
 
 
-0.407
 
 
75.3
 
 
 
 
ARF4
 
 
hsa-miR-148a-3p
 
 
-0.487
 
 
75.3
 
 
 
 
ARG2
 
 
hsa-miR-98-5p
 
 
-0.468
 
 
10.7
 
 
 
 
ARG2
 
 
hsa-let-7c-5p
 
 
-0.468
 
 
10.7
 
 
 
 
ARHGAP1
 
 
hsa-miR-17-5p
 
 
-0.325
 
 
31.0
 
 
 
 
ARHGAP1
 
 
hsa-miR-20b-5p
 
 
-0.325
 
 
31.0
 
 
 
 
ARHGAP1
 
 
hsa-miR-106a-5p
 
 
-0.329
 
 
31.0
 
 
 
 
ARHGAP1
 
 
hsa-miR-20a-5p
 
 
-0.339
 
 
31.0
 
 
 
 
ARHGAP1
 
 
hsa-miR-106b-5p
 
 
-0.339
 
 
31.0
 
 
 
 
ARHGAP21
 
 
hsa-miR-148a-3p
 
 
-0.287
 
 
30.5
 
 
 
 
ARL1
 
 
hsa-miR-20a-5p
 
 
-0.403
 
 
31.3
 
 
 
 
ARL1
 
 
hsa-miR-106b-5p
 
 
-0.411
 
 
31.3
 
 
 
 
ARL1
 
 
hsa-miR-20b-5p
 
 
-0.424
 
 
31.3
 
 
 
 
ARL1
 
 
hsa-miR-106a-5p
 
 
-0.429
 
 
31.3
 
 
 
 
ARL1
 
 
hsa-miR-17-5p
 
 
-0.432
 
 
31.3
 
 
 
 
ARL6IP1
 
 
hsa-miR-7-5p
 
 
-0.374
 
 
212.8
 
 
 
 
ARL6IP1
 
 
hsa-miR-148a-3p
 
 
-0.579
 
 
212.8
 
 
 
 
ARL8B
 
 
hsa-miR-148a-3p
 
 
-0.337
 
 
54.9
 
 
 
 
ARMC8
 
 
hsa-miR-20a-5p
 
 
-0.357
 
 
16.8
 
 
 
 
ARMC8
 
 
hsa-miR-106b-5p
 
 
-0.357
 
 
16.8
 
 
 
 
ARMC8
 
 
hsa-miR-106a-5p
 
 
-0.375
 
 
16.8
 
 
 
 
ARMC8
 
 
hsa-miR-17-5p
 
 
-0.378
 
 
16.8
 
 
 
 
ARMC8
 
 
hsa-miR-20b-5p
 
 
-0.378
 
 
16.8
 
 
 
 
ARMCX1
 
 
hsa-miR-21-5p
 
 
-0.489
 
 
24.2
 
 
 
 
ARPP19
 
 
hsa-let-7c-5p
 
 
-0.492
 
 
103.9
 
 
 
 
ARPP19
 
 
hsa-miR-98-5p
 
 
-0.5
 
 
103.9
 
 
 
 
ARRDC3
 
 
hsa-miR-148a-3p
 
 
-0.305
 
 
10.3
 
 
 
 
ARRDC4
 
 
hsa-let-7c-5p
 
 
-0.329
 
 
13.4
 
 
 
 
ARRDC4
 
 
hsa-miR-98-5p
 
 
-0.329
 
 
13.4
 
 
 
 
ASAP1
 
 
hsa-miR-98-5p
 
 
-0.308
 
 
11.8
 
 
 
 
ASAP1
 
 
hsa-let-7c-5p
 
 
-0.308
 
 
11.8
 
 
 
 
ASF1A
 
 
hsa-miR-21-5p
 
 
-0.304
 
 
13.0
 
 
 
 
ASPH
 
 
hsa-miR-148a-3p
 
 
-0.391
 
 
51.0
 
 
 
 
ATOSB
 
 
hsa-miR-98-5p
 
 
-0.429
 
 
19.8
 
 
 
 
ATOSB
 
 
hsa-let-7c-5p
 
 
-0.429
 
 
19.8
 
 
 
 
ATP2A2
 
 
hsa-miR-98-5p
 
 
-0.311
 
 
11.9
 
 
 
 
ATP2A2
 
 
hsa-let-7c-5p
 
 
-0.311
 
 
11.9
 
 
 
 
ATP2B2
 
 
hsa-miR-7-5p
 
 
-0.297
 
 
12.0
 
 
 
 
ATP6AP2
 
 
hsa-miR-148a-3p
 
 
-0.592
 
 
69.0
 
 
 
 
ATP6AP2
 
 
hsa-miR-148a-3p
 
 
-0.668
 
 
69.0
 
 
 
 
ATPAF1
 
 
hsa-miR-98-5p
 
 
-0.498
 
 
52.2
 
 
 
 
ATPAF1
 
 
hsa-let-7c-5p
 
 
-0.498
 
 
52.2
 
 
 
 
AVPI1
 
 
hsa-miR-7-5p
 
 
-0.367
 
 
15.0
 
 
 
 
B3GAT3
 
 
hsa-miR-98-5p
 
 
-0.324
 
 
60.2
 
 
 
 
B3GAT3
 
 
hsa-let-7c-5p
 
 
-0.324
 
 
60.2
 
 
 
 
B4GALT5
 
 
hsa-miR-148a-3p
 
 
-0.44
 
 
13.0
 
 
 
 
B4GAT1
 
 
hsa-let-7c-5p
 
 
-0.47
 
 
207.3
 
 
 
 
B4GAT1
 
 
hsa-miR-98-5p
 
 
-0.47
 
 
207.3
 
 
 
 
BAMBI
 
 
hsa-miR-20a-5p
 
 
-0.485
 
 
12.9
 
 
 
 
BAMBI
 
 
hsa-miR-106b-5p
 
 
-0.493
 
 
12.9
 
 
 
 
BAMBI
 
 
hsa-miR-106a-5p
 
 
-0.503
 
 
12.9
 
 
 
 
BAMBI
 
 
hsa-miR-17-5p
 
 
-0.506
 
 
12.9
 
 
 
 
BAMBI
 
 
hsa-miR-20b-5p
 
 
-0.506
 
 
12.9
 
 
 
 
BEGAIN
 
 
hsa-let-7c-5p
 
 
-0.466
 
 
15.9
 
 
 
 
BEGAIN
 
 
hsa-miR-98-5p
 
 
-0.474
 
 
15.9
 
 
 
 
BHLHE41
 
 
hsa-miR-148a-3p
 
 
-0.442
 
 
27.6
 
 
 
 
BLOC1S6
 
 
hsa-miR-98-5p
 
 
-0.321
 
 
24.8
 
 
 
 
BLOC1S6
 
 
hsa-let-7c-5p
 
 
-0.321
 
 
24.8
 
 
 
 
 
 
 midbrain 
 
 
 
 
target_symbol
 
 
mature_mirna_id
 
 
score
 
 
nTPM
 
 
 
 
 
 
ABCB9
 
 
hsa-let-7c-5p
 
 
-0.422
 
 
10.6
 
 
 
 
ABCB9
 
 
hsa-miR-98-5p
 
 
-0.444
 
 
10.6
 
 
 
 
ABCD3
 
 
hsa-miR-148a-3p
 
 
-0.388
 
 
17.1
 
 
 
 
ABHD17C
 
 
hsa-let-7c-5p
 
 
-0.359
 
 
11.2
 
 
 
 
ABHD17C
 
 
hsa-miR-98-5p
 
 
-0.383
 
 
11.2
 
 
 
 
ACSL6
 
 
hsa-miR-98-5p
 
 
-0.336
 
 
11.1
 
 
 
 
ACSL6
 
 
hsa-let-7c-5p
 
 
-0.336
 
 
11.1
 
 
 
 
ADAM10
 
 
hsa-miR-148a-3p
 
 
-0.356
 
 
11.1
 
 
 
 
ADCY2
 
 
hsa-miR-148a-3p
 
 
-0.299
 
 
20.4
 
 
 
 
ADGRF5
 
 
hsa-miR-148a-3p
 
 
-0.354
 
 
13.0
 
 
 
 
AGFG1
 
 
hsa-miR-148a-3p
 
 
-0.332
 
 
32.4
 
 
 
 
AGFG2
 
 
hsa-miR-20a-5p
 
 
-0.365
 
 
12.1
 
 
 
 
AGFG2
 
 
hsa-miR-20b-5p
 
 
-0.386
 
 
12.1
 
 
 
 
AGFG2
 
 
hsa-miR-106b-5p
 
 
-0.397
 
 
12.1
 
 
 
 
AGFG2
 
 
hsa-miR-106a-5p
 
 
-0.399
 
 
12.1
 
 
 
 
AGFG2
 
 
hsa-miR-17-5p
 
 
-0.402
 
 
12.1
 
 
 
 
AHNAK
 
 
hsa-miR-20a-5p
 
 
-0.316
 
 
22.7
 
 
 
 
AHNAK
 
 
hsa-miR-106b-5p
 
 
-0.316
 
 
22.7
 
 
 
 
AHNAK
 
 
hsa-miR-106a-5p
 
 
-0.334
 
 
22.7
 
 
 
 
AHNAK
 
 
hsa-miR-17-5p
 
 
-0.337
 
 
22.7
 
 
 
 
AHNAK
 
 
hsa-miR-20b-5p
 
 
-0.337
 
 
22.7
 
 
 
 
AIFM1
 
 
hsa-miR-98-5p
 
 
-0.314
 
 
12.7
 
 
 
 
AIFM1
 
 
hsa-let-7c-5p
 
 
-0.314
 
 
12.7
 
 
 
 
AKAP1
 
 
hsa-miR-148a-3p
 
 
-0.381
 
 
19.0
 
 
 
 
AKAP6
 
 
hsa-miR-98-5p
 
 
-0.307
 
 
17.4
 
 
 
 
AKAP6
 
 
hsa-let-7c-5p
 
 
-0.307
 
 
17.4
 
 
 
 
ALCAM
 
 
hsa-miR-148a-3p
 
 
-0.339
 
 
38.7
 
 
 
 
ALDH4A1
 
 
hsa-miR-184
 
 
-0.421
 
 
73.5
 
 
 
 
ANKRD29
 
 
hsa-miR-20a-5p
 
 
-0.359
 
 
11.5
 
 
 
 
ANKRD29
 
 
hsa-miR-106b-5p
 
 
-0.359
 
 
11.5
 
 
 
 
ANKRD29
 
 
hsa-miR-106a-5p
 
 
-0.369
 
 
11.5
 
 
 
 
ANKRD29
 
 
hsa-miR-17-5p
 
 
-0.372
 
 
11.5
 
 
 
 
ANKRD29
 
 
hsa-miR-20b-5p
 
 
-0.38
 
 
11.5
 
 
 
 
ANKRD46
 
 
hsa-miR-98-5p
 
 
-0.389
 
 
24.0
 
 
 
 
ANKRD46
 
 
hsa-let-7c-5p
 
 
-0.389
 
 
24.0
 
 
 
 
ANKRD9
 
 
hsa-miR-20a-5p
 
 
-0.31
 
 
20.0
 
 
 
 
ANKRD9
 
 
hsa-miR-106b-5p
 
 
-0.318
 
 
20.0
 
 
 
 
ANKRD9
 
 
hsa-miR-20b-5p
 
 
-0.331
 
 
20.0
 
 
 
 
ANKRD9
 
 
hsa-miR-106a-5p
 
 
-0.344
 
 
20.0
 
 
 
 
ANKRD9
 
 
hsa-miR-17-5p
 
 
-0.347
 
 
20.0
 
 
 
 
AP1AR
 
 
hsa-miR-21-5p
 
 
-0.318
 
 
10.1
 
 
 
 
AP1S1
 
 
hsa-miR-98-5p
 
 
-0.374
 
 
35.4
 
 
 
 
AP1S1
 
 
hsa-let-7c-5p
 
 
-0.374
 
 
35.4
 
 
 
 
APCDD1
 
 
hsa-miR-20a-5p
 
 
-0.404
 
 
22.5
 
 
 
 
APCDD1
 
 
hsa-miR-106b-5p
 
 
-0.404
 
 
22.5
 
 
 
 
APCDD1
 
 
hsa-miR-106a-5p
 
 
-0.422
 
 
22.5
 
 
 
 
APCDD1
 
 
hsa-miR-17-5p
 
 
-0.425
 
 
22.5
 
 
 
 
APCDD1
 
 
hsa-miR-20b-5p
 
 
-0.425
 
 
22.5
 
 
 
 
ARF4
 
 
hsa-miR-7-5p
 
 
-0.407
 
 
62.8
 
 
 
 
ARF4
 
 
hsa-miR-148a-3p
 
 
-0.487
 
 
62.8
 
 
 
 
ARHGAP1
 
 
hsa-miR-17-5p
 
 
-0.325
 
 
34.8
 
 
 
 
ARHGAP1
 
 
hsa-miR-20b-5p
 
 
-0.325
 
 
34.8
 
 
 
 
ARHGAP1
 
 
hsa-miR-106a-5p
 
 
-0.329
 
 
34.8
 
 
 
 
ARHGAP1
 
 
hsa-miR-20a-5p
 
 
-0.339
 
 
34.8
 
 
 
 
ARHGAP1
 
 
hsa-miR-106b-5p
 
 
-0.339
 
 
34.8
 
 
 
 
ARHGAP21
 
 
hsa-miR-148a-3p
 
 
-0.287
 
 
53.0
 
 
 
 
ARL1
 
 
hsa-miR-20a-5p
 
 
-0.403
 
 
27.4
 
 
 
 
ARL1
 
 
hsa-miR-106b-5p
 
 
-0.411
 
 
27.4
 
 
 
 
ARL1
 
 
hsa-miR-20b-5p
 
 
-0.424
 
 
27.4
 
 
 
 
ARL1
 
 
hsa-miR-106a-5p
 
 
-0.429
 
 
27.4
 
 
 
 
ARL1
 
 
hsa-miR-17-5p
 
 
-0.432
 
 
27.4
 
 
 
 
ARL6IP1
 
 
hsa-miR-7-5p
 
 
-0.374
 
 
198.8
 
 
 
 
ARL6IP1
 
 
hsa-miR-148a-3p
 
 
-0.579
 
 
198.8
 
 
 
 
ARL8B
 
 
hsa-miR-148a-3p
 
 
-0.337
 
 
52.4
 
 
 
 
ARMC8
 
 
hsa-miR-20a-5p
 
 
-0.357
 
 
14.1
 
 
 
 
ARMC8
 
 
hsa-miR-106b-5p
 
 
-0.357
 
 
14.1
 
 
 
 
ARMC8
 
 
hsa-miR-106a-5p
 
 
-0.375
 
 
14.1
 
 
 
 
ARMC8
 
 
hsa-miR-17-5p
 
 
-0.378
 
 
14.1
 
 
 
 
ARMC8
 
 
hsa-miR-20b-5p
 
 
-0.378
 
 
14.1
 
 
 
 
ARMCX1
 
 
hsa-miR-21-5p
 
 
-0.489
 
 
17.5
 
 
 
 
ARPP19
 
 
hsa-let-7c-5p
 
 
-0.492
 
 
144.2
 
 
 
 
ARPP19
 
 
hsa-miR-98-5p
 
 
-0.5
 
 
144.2
 
 
 
 
ARRDC3
 
 
hsa-miR-148a-3p
 
 
-0.305
 
 
13.8
 
 
 
 
ARRDC4
 
 
hsa-let-7c-5p
 
 
-0.329
 
 
17.7
 
 
 
 
ARRDC4
 
 
hsa-miR-98-5p
 
 
-0.329
 
 
17.7
 
 
 
 
ASAP1
 
 
hsa-miR-98-5p
 
 
-0.308
 
 
12.8
 
 
 
 
ASAP1
 
 
hsa-let-7c-5p
 
 
-0.308
 
 
12.8
 
 
 
 
ASF1A
 
 
hsa-miR-21-5p
 
 
-0.304
 
 
14.8
 
 
 
 
ASPH
 
 
hsa-miR-148a-3p
 
 
-0.391
 
 
48.8
 
 
 
 
ATOSB
 
 
hsa-miR-98-5p
 
 
-0.429
 
 
24.1
 
 
 
 
ATOSB
 
 
hsa-let-7c-5p
 
 
-0.429
 
 
24.1
 
 
 
 
ATP2A2
 
 
hsa-miR-98-5p
 
 
-0.311
 
 
17.1
 
 
 
 
ATP2A2
 
 
hsa-let-7c-5p
 
 
-0.311
 
 
17.1
 
 
 
 
ATP2B2
 
 
hsa-miR-7-5p
 
 
-0.297
 
 
12.7
 
 
 
 
ATP6AP2
 
 
hsa-miR-148a-3p
 
 
-0.592
 
 
65.6
 
 
 
 
ATP6AP2
 
 
hsa-miR-148a-3p
 
 
-0.668
 
 
65.6
 
 
 
 
ATPAF1
 
 
hsa-miR-98-5p
 
 
-0.498
 
 
74.9
 
 
 
 
ATPAF1
 
 
hsa-let-7c-5p
 
 
-0.498
 
 
74.9
 
 
 
 
AVPI1
 
 
hsa-miR-7-5p
 
 
-0.367
 
 
18.5
 
 
 
 
B3GAT3
 
 
hsa-miR-98-5p
 
 
-0.324
 
 
58.5
 
 
 
 
B3GAT3
 
 
hsa-let-7c-5p
 
 
-0.324
 
 
58.5
 
 
 
 
B4GALT5
 
 
hsa-miR-148a-3p
 
 
-0.44
 
 
10.3
 
 
 
 
B4GAT1
 
 
hsa-let-7c-5p
 
 
-0.47
 
 
85.6
 
 
 
 
B4GAT1
 
 
hsa-miR-98-5p
 
 
-0.47
 
 
85.6
 
 
 
 
BAMBI
 
 
hsa-miR-20a-5p
 
 
-0.485
 
 
16.8
 
 
 
 
BAMBI
 
 
hsa-miR-106b-5p
 
 
-0.493
 
 
16.8
 
 
 
 
BAMBI
 
 
hsa-miR-106a-5p
 
 
-0.503
 
 
16.8
 
 
 
 
BAMBI
 
 
hsa-miR-17-5p
 
 
-0.506
 
 
16.8
 
 
 
 
BAMBI
 
 
hsa-miR-20b-5p
 
 
-0.506
 
 
16.8
 
 
 
 
BHLHE41
 
 
hsa-miR-148a-3p
 
 
-0.442
 
 
40.4
 
 
 
 
 
 
 pituitary gland 
 
 
 
 
target_symbol
 
 
mature_mirna_id
 
 
score
 
 
nTPM
 
 
 
 
 
 
ABCB9
 
 
hsa-let-7c-5p
 
 
-0.422
 
 
11.0
 
 
 
 
ABCB9
 
 
hsa-miR-98-5p
 
 
-0.444
 
 
11.0
 
 
 
 
ABCD3
 
 
hsa-miR-148a-3p
 
 
-0.388
 
 
12.4
 
 
 
 
ADGRB3
 
 
hsa-miR-148a-3p
 
 
-0.524
 
 
21.3
 
 
 
 
AGFG1
 
 
hsa-miR-148a-3p
 
 
-0.332
 
 
16.7
 
 
 
 
AGFG2
 
 
hsa-miR-20a-5p
 
 
-0.365
 
 
16.7
 
 
 
 
AGFG2
 
 
hsa-miR-20b-5p
 
 
-0.386
 
 
16.7
 
 
 
 
AGFG2
 
 
hsa-miR-106b-5p
 
 
-0.397
 
 
16.7
 
 
 
 
AGFG2
 
 
hsa-miR-106a-5p
 
 
-0.399
 
 
16.7
 
 
 
 
AGFG2
 
 
hsa-miR-17-5p
 
 
-0.402
 
 
16.7
 
 
 
 
AHDC1
 
 
hsa-miR-148a-3p
 
 
-0.413
 
 
18.1
 
 
 
 
AHNAK
 
 
hsa-miR-20a-5p
 
 
-0.316
 
 
16.0
 
 
 
 
AHNAK
 
 
hsa-miR-106b-5p
 
 
-0.316
 
 
16.0
 
 
 
 
AHNAK
 
 
hsa-miR-106a-5p
 
 
-0.334
 
 
16.0
 
 
 
 
AHNAK
 
 
hsa-miR-17-5p
 
 
-0.337
 
 
16.0
 
 
 
 
AHNAK
 
 
hsa-miR-20b-5p
 
 
-0.337
 
 
16.0
 
 
 
 
AIFM1
 
 
hsa-miR-98-5p
 
 
-0.314
 
 
11.0
 
 
 
 
AIFM1
 
 
hsa-let-7c-5p
 
 
-0.314
 
 
11.0
 
 
 
 
AKAP1
 
 
hsa-miR-148a-3p
 
 
-0.381
 
 
16.5
 
 
 
 
ALCAM
 
 
hsa-miR-148a-3p
 
 
-0.339
 
 
17.9
 
 
 
 
ALG3
 
 
hsa-miR-106b-5p
 
 
-0.551
 
 
14.3
 
 
 
 
ALG3
 
 
hsa-miR-20a-5p
 
 
-0.559
 
 
14.3
 
 
 
 
ALG3
 
 
hsa-miR-106a-5p
 
 
-0.569
 
 
14.3
 
 
 
 
ALG3
 
 
hsa-miR-17-5p
 
 
-0.572
 
 
14.3
 
 
 
 
ALG3
 
 
hsa-miR-20b-5p
 
 
-0.58
 
 
14.3
 
 
 
 
AMT
 
 
hsa-miR-98-5p
 
 
-0.463
 
 
16.0
 
 
 
 
AMT
 
 
hsa-let-7c-5p
 
 
-0.463
 
 
16.0
 
 
 
 
AP1S1
 
 
hsa-miR-98-5p
 
 
-0.374
 
 
31.4
 
 
 
 
AP1S1
 
 
hsa-let-7c-5p
 
 
-0.374
 
 
31.4
 
 
 
 
APBB3
 
 
hsa-miR-98-5p
 
 
-0.587
 
 
10.1
 
 
 
 
APBB3
 
 
hsa-let-7c-5p
 
 
-0.587
 
 
10.1
 
 
 
 
ARF4
 
 
hsa-miR-7-5p
 
 
-0.407
 
 
93.4
 
 
 
 
ARF4
 
 
hsa-miR-148a-3p
 
 
-0.487
 
 
93.4
 
 
 
 
ARG2
 
 
hsa-miR-98-5p
 
 
-0.468
 
 
47.4
 
 
 
 
ARG2
 
 
hsa-let-7c-5p
 
 
-0.468
 
 
47.4
 
 
 
 
ARHGAP1
 
 
hsa-miR-17-5p
 
 
-0.325
 
 
35.2
 
 
 
 
ARHGAP1
 
 
hsa-miR-20b-5p
 
 
-0.325
 
 
35.2
 
 
 
 
ARHGAP1
 
 
hsa-miR-106a-5p
 
 
-0.329
 
 
35.2
 
 
 
 
ARHGAP1
 
 
hsa-miR-20a-5p
 
 
-0.339
 
 
35.2
 
 
 
 
ARHGAP1
 
 
hsa-miR-106b-5p
 
 
-0.339
 
 
35.2
 
 
 
 
ARHGAP21
 
 
hsa-miR-148a-3p
 
 
-0.287
 
 
11.3
 
 
 
 
ARID4B
 
 
hsa-miR-20a-5p
 
 
-0.29
 
 
10.3
 
 
 
 
ARID4B
 
 
hsa-miR-106b-5p
 
 
-0.29
 
 
10.3
 
 
 
 
ARL1
 
 
hsa-miR-20a-5p
 
 
-0.403
 
 
52.7
 
 
 
 
ARL1
 
 
hsa-miR-106b-5p
 
 
-0.411
 
 
52.7
 
 
 
 
ARL1
 
 
hsa-miR-20b-5p
 
 
-0.424
 
 
52.7
 
 
 
 
ARL1
 
 
hsa-miR-106a-5p
 
 
-0.429
 
 
52.7
 
 
 
 
ARL1
 
 
hsa-miR-17-5p
 
 
-0.432
 
 
52.7
 
 
 
 
ARL4D
 
 
hsa-miR-98-5p
 
 
-0.417
 
 
20.8
 
 
 
 
ARL4D
 
 
hsa-let-7c-5p
 
 
-0.417
 
 
20.8
 
 
 
 
ARL6IP1
 
 
hsa-miR-7-5p
 
 
-0.374
 
 
39.7
 
 
 
 
ARL6IP1
 
 
hsa-miR-148a-3p
 
 
-0.579
 
 
39.7
 
 
 
 
ARL8B
 
 
hsa-miR-148a-3p
 
 
-0.337
 
 
25.6
 
 
 
 
ARMC8
 
 
hsa-miR-20a-5p
 
 
-0.357
 
 
11.3
 
 
 
 
ARMC8
 
 
hsa-miR-106b-5p
 
 
-0.357
 
 
11.3
 
 
 
 
ARMC8
 
 
hsa-miR-106a-5p
 
 
-0.375
 
 
11.3
 
 
 
 
ARMC8
 
 
hsa-miR-17-5p
 
 
-0.378
 
 
11.3
 
 
 
 
ARMC8
 
 
hsa-miR-20b-5p
 
 
-0.378
 
 
11.3
 
 
 
 
ARMCX1
 
 
hsa-miR-21-5p
 
 
-0.489
 
 
24.7
 
 
 
 
ARPP19
 
 
hsa-let-7c-5p
 
 
-0.492
 
 
33.0
 
 
 
 
ARPP19
 
 
hsa-miR-98-5p
 
 
-0.5
 
 
33.0
 
 
 
 
ARRDC3
 
 
hsa-miR-148a-3p
 
 
-0.305
 
 
20.2
 
 
 
 
ASPH
 
 
hsa-miR-148a-3p
 
 
-0.391
 
 
33.8
 
 
 
 
ATG4D
 
 
hsa-miR-148a-3p
 
 
-0.303
 
 
16.1
 
 
 
 
ATOSB
 
 
hsa-miR-98-5p
 
 
-0.429
 
 
15.4
 
 
 
 
ATOSB
 
 
hsa-let-7c-5p
 
 
-0.429
 
 
15.4
 
 
 
 
ATP2A2
 
 
hsa-miR-98-5p
 
 
-0.311
 
 
13.7
 
 
 
 
ATP2A2
 
 
hsa-let-7c-5p
 
 
-0.311
 
 
13.7
 
 
 
 
ATP6AP2
 
 
hsa-miR-148a-3p
 
 
-0.592
 
 
34.0
 
 
 
 
ATP6AP2
 
 
hsa-miR-148a-3p
 
 
-0.668
 
 
34.0
 
 
 
 
ATPAF1
 
 
hsa-miR-98-5p
 
 
-0.498
 
 
31.1
 
 
 
 
ATPAF1
 
 
hsa-let-7c-5p
 
 
-0.498
 
 
31.1
 
 
 
 
AVPI1
 
 
hsa-miR-7-5p
 
 
-0.367
 
 
20.2
 
 
 
 
B3GAT3
 
 
hsa-miR-98-5p
 
 
-0.324
 
 
61.5
 
 
 
 
B3GAT3
 
 
hsa-let-7c-5p
 
 
-0.324
 
 
61.5
 
 
 
 
B4GALT5
 
 
hsa-miR-148a-3p
 
 
-0.44
 
 
11.9
 
 
 
 
B4GAT1
 
 
hsa-let-7c-5p
 
 
-0.47
 
 
60.5
 
 
 
 
B4GAT1
 
 
hsa-miR-98-5p
 
 
-0.47
 
 
60.5
 
 
 
 
BEGAIN
 
 
hsa-let-7c-5p
 
 
-0.466
 
 
18.8
 
 
 
 
BEGAIN
 
 
hsa-miR-98-5p
 
 
-0.474
 
 
18.8
 
 
 
 
BHLHE41
 
 
hsa-miR-148a-3p
 
 
-0.442
 
 
15.9
 
 
 
 
BIN3
 
 
hsa-miR-98-5p
 
 
-0.427
 
 
13.2
 
 
 
 
BIN3
 
 
hsa-let-7c-5p
 
 
-0.427
 
 
13.2
 
 
 
 
BLOC1S6
 
 
hsa-miR-98-5p
 
 
-0.321
 
 
19.0
 
 
 
 
BLOC1S6
 
 
hsa-let-7c-5p
 
 
-0.321
 
 
19.0
 
 
 
 
BNIP3L
 
 
hsa-miR-20a-5p
 
 
-0.287
 
 
23.7
 
 
 
 
BNIP3L
 
 
hsa-miR-106b-5p
 
 
-0.298
 
 
23.7
 
 
 
 
BTAF1
 
 
hsa-miR-148a-3p
 
 
-0.401
 
 
11.4
 
 
 
 
BTBD10
 
 
hsa-miR-106b-5p
 
 
-0.388
 
 
13.7
 
 
 
 
BTBD10
 
 
hsa-miR-106a-5p
 
 
-0.406
 
 
13.7
 
 
 
 
BTBD10
 
 
hsa-miR-17-5p
 
 
-0.409
 
 
13.7
 
 
 
 
BTBD10
 
 
hsa-miR-20a-5p
 
 
-0.412
 
 
13.7
 
 
 
 
BTBD10
 
 
hsa-miR-20b-5p
 
 
-0.425
 
 
13.7
 
 
 
 
BTF3L4
 
 
hsa-let-7c-5p
 
 
-0.355
 
 
28.3
 
 
 
 
BTF3L4
 
 
hsa-miR-98-5p
 
 
-0.355
 
 
28.3
 
 
 
 
BTG3
 
 
hsa-miR-106b-5p
 
 
-0.647
 
 
43.7
 
 
 
 
BTG3
 
 
hsa-miR-106a-5p
 
 
-0.665
 
 
43.7
 
 
 
 
BTG3
 
 
hsa-miR-17-5p
 
 
-0.668
 
 
43.7
 
 
 
 
BTG3
 
 
hsa-miR-20a-5p
 
 
-0.671
 
 
43.7
 
 
 
 
BTG3
 
 
hsa-miR-20b-5p
 
 
-0.684
 
 
43.7
 
 
 
 
 
 
 testis 
 
 
 
 
target_symbol
 
 
mature_mirna_id
 
 
score
 
 
nTPM
 
 
 
 
 
 
ABCB9
 
 
hsa-let-7c-5p
 
 
-0.422
 
 
17.1
 
 
 
 
ABCB9
 
 
hsa-miR-98-5p
 
 
-0.444
 
 
17.1
 
 
 
 
ABCD3
 
 
hsa-miR-148a-3p
 
 
-0.388
 
 
15.3
 
 
 
 
ACRV1
 
 
hsa-miR-7-5p
 
 
-0.3
 
 
110.6
 
 
 
 
ACSL6
 
 
hsa-miR-98-5p
 
 
-0.336
 
 
19.3
 
 
 
 
ACSL6
 
 
hsa-let-7c-5p
 
 
-0.336
 
 
19.3
 
 
 
 
ADAM9
 
 
hsa-miR-20a-5p
 
 
-0.299
 
 
10.4
 
 
 
 
ADAM9
 
 
hsa-miR-106b-5p
 
 
-0.299
 
 
10.4
 
 
 
 
ADAM9
 
 
hsa-miR-106a-5p
 
 
-0.317
 
 
10.4
 
 
 
 
ADAM9
 
 
hsa-miR-17-5p
 
 
-0.32
 
 
10.4
 
 
 
 
ADAM9
 
 
hsa-miR-20b-5p
 
 
-0.328
 
 
10.4
 
 
 
 
AGFG1
 
 
hsa-miR-148a-3p
 
 
-0.332
 
 
86.7
 
 
 
 
AGFG2
 
 
hsa-miR-20a-5p
 
 
-0.365
 
 
11.5
 
 
 
 
AGFG2
 
 
hsa-miR-20b-5p
 
 
-0.386
 
 
11.5
 
 
 
 
AGFG2
 
 
hsa-miR-106b-5p
 
 
-0.397
 
 
11.5
 
 
 
 
AGFG2
 
 
hsa-miR-106a-5p
 
 
-0.399
 
 
11.5
 
 
 
 
AGFG2
 
 
hsa-miR-17-5p
 
 
-0.402
 
 
11.5
 
 
 
 
AHCTF1
 
 
hsa-miR-98-5p
 
 
-0.374
 
 
30.4
 
 
 
 
AHCTF1
 
 
hsa-let-7c-5p
 
 
-0.374
 
 
30.4
 
 
 
 
AHNAK
 
 
hsa-miR-20a-5p
 
 
-0.316
 
 
16.9
 
 
 
 
AHNAK
 
 
hsa-miR-106b-5p
 
 
-0.316
 
 
16.9
 
 
 
 
AHNAK
 
 
hsa-miR-106a-5p
 
 
-0.334
 
 
16.9
 
 
 
 
AHNAK
 
 
hsa-miR-17-5p
 
 
-0.337
 
 
16.9
 
 
 
 
AHNAK
 
 
hsa-miR-20b-5p
 
 
-0.337
 
 
16.9
 
 
 
 
AIFM1
 
 
hsa-miR-98-5p
 
 
-0.314
 
 
21.3
 
 
 
 
AIFM1
 
 
hsa-let-7c-5p
 
 
-0.314
 
 
21.3
 
 
 
 
AKAP1
 
 
hsa-miR-148a-3p
 
 
-0.381
 
 
108.8
 
 
 
 
ALG3
 
 
hsa-miR-106b-5p
 
 
-0.551
 
 
25.2
 
 
 
 
ALG3
 
 
hsa-miR-20a-5p
 
 
-0.559
 
 
25.2
 
 
 
 
ALG3
 
 
hsa-miR-106a-5p
 
 
-0.569
 
 
25.2
 
 
 
 
ALG3
 
 
hsa-miR-17-5p
 
 
-0.572
 
 
25.2
 
 
 
 
ALG3
 
 
hsa-miR-20b-5p
 
 
-0.58
 
 
25.2
 
 
 
 
ALG9
 
 
hsa-miR-7-5p
 
 
-0.302
 
 
12.8
 
 
 
 
ANKRD12
 
 
hsa-miR-7-5p
 
 
-0.37
 
 
25.9
 
 
 
 
ANKRD50
 
 
hsa-miR-106a-5p
 
 
-0.29
 
 
14.1
 
 
 
 
ANKRD50
 
 
hsa-miR-20a-5p
 
 
-0.3
 
 
14.1
 
 
 
 
ANKRD50
 
 
hsa-miR-106b-5p
 
 
-0.3
 
 
14.1
 
 
 
 
ANKRD9
 
 
hsa-miR-20a-5p
 
 
-0.31
 
 
38.5
 
 
 
 
ANKRD9
 
 
hsa-miR-106b-5p
 
 
-0.318
 
 
38.5
 
 
 
 
ANKRD9
 
 
hsa-miR-20b-5p
 
 
-0.331
 
 
38.5
 
 
 
 
ANKRD9
 
 
hsa-miR-106a-5p
 
 
-0.344
 
 
38.5
 
 
 
 
ANKRD9
 
 
hsa-miR-17-5p
 
 
-0.347
 
 
38.5
 
 
 
 
AP1AR
 
 
hsa-miR-21-5p
 
 
-0.318
 
 
13.6
 
 
 
 
AP1S1
 
 
hsa-miR-98-5p
 
 
-0.374
 
 
17.8
 
 
 
 
AP1S1
 
 
hsa-let-7c-5p
 
 
-0.374
 
 
17.8
 
 
 
 
ARF4
 
 
hsa-miR-7-5p
 
 
-0.407
 
 
94.2
 
 
 
 
ARF4
 
 
hsa-miR-148a-3p
 
 
-0.487
 
 
94.2
 
 
 
 
ARFGEF1
 
 
hsa-miR-148a-3p
 
 
-0.306
 
 
26.5
 
 
 
 
ARFIP1
 
 
hsa-miR-148a-3p
 
 
-0.474
 
 
11.3
 
 
 
 
ARHGAP1
 
 
hsa-miR-17-5p
 
 
-0.325
 
 
15.3
 
 
 
 
ARHGAP1
 
 
hsa-miR-20b-5p
 
 
-0.325
 
 
15.3
 
 
 
 
ARHGAP1
 
 
hsa-miR-106a-5p
 
 
-0.329
 
 
15.3
 
 
 
 
ARHGAP1
 
 
hsa-miR-20a-5p
 
 
-0.339
 
 
15.3
 
 
 
 
ARHGAP1
 
 
hsa-miR-106b-5p
 
 
-0.339
 
 
15.3
 
 
 
 
ARHGAP21
 
 
hsa-miR-148a-3p
 
 
-0.287
 
 
13.5
 
 
 
 
ARHGAP28
 
 
hsa-let-7c-5p
 
 
-0.47
 
 
72.3
 
 
 
 
ARHGAP28
 
 
hsa-miR-98-5p
 
 
-0.47
 
 
72.3
 
 
 
 
ARID3A
 
 
hsa-miR-98-5p
 
 
-0.34
 
 
16.1
 
 
 
 
ARID3A
 
 
hsa-let-7c-5p
 
 
-0.34
 
 
16.1
 
 
 
 
ARID3A
 
 
hsa-let-7c-5p
 
 
-0.347
 
 
16.1
 
 
 
 
ARID3A
 
 
hsa-miR-98-5p
 
 
-0.358
 
 
16.1
 
 
 
 
ARID3B
 
 
hsa-miR-98-5p
 
 
-0.31
 
 
21.2
 
 
 
 
ARID3B
 
 
hsa-let-7c-5p
 
 
-0.31
 
 
21.2
 
 
 
 
ARID3B
 
 
hsa-miR-98-5p
 
 
-0.475
 
 
21.2
 
 
 
 
ARID3B
 
 
hsa-let-7c-5p
 
 
-0.486
 
 
21.2
 
 
 
 
ARID4B
 
 
hsa-miR-20a-5p
 
 
-0.29
 
 
16.0
 
 
 
 
ARID4B
 
 
hsa-miR-106b-5p
 
 
-0.29
 
 
16.0
 
 
 
 
ARL1
 
 
hsa-miR-20a-5p
 
 
-0.403
 
 
30.6
 
 
 
 
ARL1
 
 
hsa-miR-106b-5p
 
 
-0.411
 
 
30.6
 
 
 
 
ARL1
 
 
hsa-miR-20b-5p
 
 
-0.424
 
 
30.6
 
 
 
 
ARL1
 
 
hsa-miR-106a-5p
 
 
-0.429
 
 
30.6
 
 
 
 
ARL1
 
 
hsa-miR-17-5p
 
 
-0.432
 
 
30.6
 
 
 
 
ARL4D
 
 
hsa-miR-98-5p
 
 
-0.417
 
 
31.4
 
 
 
 
ARL4D
 
 
hsa-let-7c-5p
 
 
-0.417
 
 
31.4
 
 
 
 
ARL6IP1
 
 
hsa-miR-7-5p
 
 
-0.374
 
 
121.4
 
 
 
 
ARL6IP1
 
 
hsa-miR-148a-3p
 
 
-0.579
 
 
121.4
 
 
 
 
ARL6IP6
 
 
hsa-miR-98-5p
 
 
-0.293
 
 
10.2
 
 
 
 
ARL6IP6
 
 
hsa-let-7c-5p
 
 
-0.293
 
 
10.2
 
 
 
 
ARL8B
 
 
hsa-miR-148a-3p
 
 
-0.337
 
 
30.1
 
 
 
 
ARMC8
 
 
hsa-miR-20a-5p
 
 
-0.357
 
 
17.2
 
 
 
 
ARMC8
 
 
hsa-miR-106b-5p
 
 
-0.357
 
 
17.2
 
 
 
 
ARMC8
 
 
hsa-miR-106a-5p
 
 
-0.375
 
 
17.2
 
 
 
 
ARMC8
 
 
hsa-miR-17-5p
 
 
-0.378
 
 
17.2
 
 
 
 
ARMC8
 
 
hsa-miR-20b-5p
 
 
-0.378
 
 
17.2
 
 
 
 
ARMT1
 
 
hsa-let-7c-5p
 
 
-0.303
 
 
13.1
 
 
 
 
ARMT1
 
 
hsa-miR-98-5p
 
 
-0.303
 
 
13.1
 
 
 
 
ARPP19
 
 
hsa-let-7c-5p
 
 
-0.492
 
 
79.0
 
 
 
 
ARPP19
 
 
hsa-miR-98-5p
 
 
-0.5
 
 
79.0
 
 
 
 
ARRDC3
 
 
hsa-miR-148a-3p
 
 
-0.305
 
 
15.5
 
 
 
 
ARRDC4
 
 
hsa-let-7c-5p
 
 
-0.329
 
 
13.7
 
 
 
 
ARRDC4
 
 
hsa-miR-98-5p
 
 
-0.329
 
 
13.7
 
 
 
 
ASAP1
 
 
hsa-miR-98-5p
 
 
-0.308
 
 
26.5
 
 
 
 
ASAP1
 
 
hsa-let-7c-5p
 
 
-0.308
 
 
26.5
 
 
 
 
ASPH
 
 
hsa-miR-148a-3p
 
 
-0.391
 
 
15.3
 
 
 
 
ATAD2
 
 
hsa-miR-17-5p
 
 
-0.304
 
 
17.7
 
 
 
 
ATAD2
 
 
hsa-miR-20b-5p
 
 
-0.304
 
 
17.7
 
 
 
 
ATAD2
 
 
hsa-miR-106b-5p
 
 
-0.307
 
 
17.7
 
 
 
 
ATAD2
 
 
hsa-miR-106a-5p
 
 
-0.308
 
 
17.7
 
 
 
 
ATAD2
 
 
hsa-miR-20a-5p
 
 
-0.318
 
 
17.7
 
 
 
 
ATG16L1
 
 
hsa-miR-20a-5p
 
 
-0.446
 
 
13.1
 
 
 
 
 
 
 amygdala 
 
 
 
 
target_symbol
 
 
mature_mirna_id
 
 
score
 
 
nTPM
 
 
 
 
 
 
ABCD3
 
 
hsa-miR-148a-3p
 
 
-0.388
 
 
16.0
 
 
 
 
ABHD17C
 
 
hsa-let-7c-5p
 
 
-0.359
 
 
17.9
 
 
 
 
ABHD17C
 
 
hsa-miR-98-5p
 
 
-0.383
 
 
17.9
 
 
 
 
ACSL6
 
 
hsa-miR-98-5p
 
 
-0.336
 
 
20.3
 
 
 
 
ACSL6
 
 
hsa-let-7c-5p
 
 
-0.336
 
 
20.3
 
 
 
 
ADCY2
 
 
hsa-miR-148a-3p
 
 
-0.299
 
 
28.0
 
 
 
 
ADGRB3
 
 
hsa-miR-148a-3p
 
 
-0.524
 
 
12.8
 
 
 
 
AGFG1
 
 
hsa-miR-148a-3p
 
 
-0.332
 
 
25.1
 
 
 
 
AGFG2
 
 
hsa-miR-20a-5p
 
 
-0.365
 
 
13.3
 
 
 
 
AGFG2
 
 
hsa-miR-20b-5p
 
 
-0.386
 
 
13.3
 
 
 
 
AGFG2
 
 
hsa-miR-106b-5p
 
 
-0.397
 
 
13.3
 
 
 
 
AGFG2
 
 
hsa-miR-106a-5p
 
 
-0.399
 
 
13.3
 
 
 
 
AGFG2
 
 
hsa-miR-17-5p
 
 
-0.402
 
 
13.3
 
 
 
 
AHDC1
 
 
hsa-miR-148a-3p
 
 
-0.413
 
 
10.1
 
 
 
 
AHNAK
 
 
hsa-miR-20a-5p
 
 
-0.316
 
 
31.4
 
 
 
 
AHNAK
 
 
hsa-miR-106b-5p
 
 
-0.316
 
 
31.4
 
 
 
 
AHNAK
 
 
hsa-miR-106a-5p
 
 
-0.334
 
 
31.4
 
 
 
 
AHNAK
 
 
hsa-miR-17-5p
 
 
-0.337
 
 
31.4
 
 
 
 
AHNAK
 
 
hsa-miR-20b-5p
 
 
-0.337
 
 
31.4
 
 
 
 
AIFM1
 
 
hsa-miR-98-5p
 
 
-0.314
 
 
10.3
 
 
 
 
AIFM1
 
 
hsa-let-7c-5p
 
 
-0.314
 
 
10.3
 
 
 
 
AKAP1
 
 
hsa-miR-148a-3p
 
 
-0.381
 
 
22.8
 
 
 
 
AKAP6
 
 
hsa-miR-98-5p
 
 
-0.307
 
 
12.4
 
 
 
 
AKAP6
 
 
hsa-let-7c-5p
 
 
-0.307
 
 
12.4
 
 
 
 
ALCAM
 
 
hsa-miR-148a-3p
 
 
-0.339
 
 
31.7
 
 
 
 
ALDH4A1
 
 
hsa-miR-184
 
 
-0.421
 
 
66.7
 
 
 
 
ANKRD46
 
 
hsa-miR-98-5p
 
 
-0.389
 
 
28.3
 
 
 
 
ANKRD46
 
 
hsa-let-7c-5p
 
 
-0.389
 
 
28.3
 
 
 
 
ANKRD9
 
 
hsa-miR-20a-5p
 
 
-0.31
 
 
26.5
 
 
 
 
ANKRD9
 
 
hsa-miR-106b-5p
 
 
-0.318
 
 
26.5
 
 
 
 
ANKRD9
 
 
hsa-miR-20b-5p
 
 
-0.331
 
 
26.5
 
 
 
 
ANKRD9
 
 
hsa-miR-106a-5p
 
 
-0.344
 
 
26.5
 
 
 
 
ANKRD9
 
 
hsa-miR-17-5p
 
 
-0.347
 
 
26.5
 
 
 
 
AP1S1
 
 
hsa-miR-98-5p
 
 
-0.374
 
 
88.4
 
 
 
 
AP1S1
 
 
hsa-let-7c-5p
 
 
-0.374
 
 
88.4
 
 
 
 
APCDD1
 
 
hsa-miR-20a-5p
 
 
-0.404
 
 
28.8
 
 
 
 
APCDD1
 
 
hsa-miR-106b-5p
 
 
-0.404
 
 
28.8
 
 
 
 
APCDD1
 
 
hsa-miR-106a-5p
 
 
-0.422
 
 
28.8
 
 
 
 
APCDD1
 
 
hsa-miR-17-5p
 
 
-0.425
 
 
28.8
 
 
 
 
APCDD1
 
 
hsa-miR-20b-5p
 
 
-0.425
 
 
28.8
 
 
 
 
ARF4
 
 
hsa-miR-7-5p
 
 
-0.407
 
 
60.1
 
 
 
 
ARF4
 
 
hsa-miR-148a-3p
 
 
-0.487
 
 
60.1
 
 
 
 
ARHGAP1
 
 
hsa-miR-17-5p
 
 
-0.325
 
 
37.0
 
 
 
 
ARHGAP1
 
 
hsa-miR-20b-5p
 
 
-0.325
 
 
37.0
 
 
 
 
ARHGAP1
 
 
hsa-miR-106a-5p
 
 
-0.329
 
 
37.0
 
 
 
 
ARHGAP1
 
 
hsa-miR-20a-5p
 
 
-0.339
 
 
37.0
 
 
 
 
ARHGAP1
 
 
hsa-miR-106b-5p
 
 
-0.339
 
 
37.0
 
 
 
 
ARHGAP21
 
 
hsa-miR-148a-3p
 
 
-0.287
 
 
40.6
 
 
 
 
ARL1
 
 
hsa-miR-20a-5p
 
 
-0.403
 
 
24.9
 
 
 
 
ARL1
 
 
hsa-miR-106b-5p
 
 
-0.411
 
 
24.9
 
 
 
 
ARL1
 
 
hsa-miR-20b-5p
 
 
-0.424
 
 
24.9
 
 
 
 
ARL1
 
 
hsa-miR-106a-5p
 
 
-0.429
 
 
24.9
 
 
 
 
ARL1
 
 
hsa-miR-17-5p
 
 
-0.432
 
 
24.9
 
 
 
 
ARL6IP1
 
 
hsa-miR-7-5p
 
 
-0.374
 
 
241.3
 
 
 
 
ARL6IP1
 
 
hsa-miR-148a-3p
 
 
-0.579
 
 
241.3
 
 
 
 
ARL8B
 
 
hsa-miR-148a-3p
 
 
-0.337
 
 
53.8
 
 
 
 
ARMC8
 
 
hsa-miR-20a-5p
 
 
-0.357
 
 
13.4
 
 
 
 
ARMC8
 
 
hsa-miR-106b-5p
 
 
-0.357
 
 
13.4
 
 
 
 
ARMC8
 
 
hsa-miR-106a-5p
 
 
-0.375
 
 
13.4
 
 
 
 
ARMC8
 
 
hsa-miR-17-5p
 
 
-0.378
 
 
13.4
 
 
 
 
ARMC8
 
 
hsa-miR-20b-5p
 
 
-0.378
 
 
13.4
 
 
 
 
ARMCX1
 
 
hsa-miR-21-5p
 
 
-0.489
 
 
17.8
 
 
 
 
ARPP19
 
 
hsa-let-7c-5p
 
 
-0.492
 
 
178.5
 
 
 
 
ARPP19
 
 
hsa-miR-98-5p
 
 
-0.5
 
 
178.5
 
 
 
 
ARRDC4
 
 
hsa-let-7c-5p
 
 
-0.329
 
 
13.2
 
 
 
 
ARRDC4
 
 
hsa-miR-98-5p
 
 
-0.329
 
 
13.2
 
 
 
 
ASAP1
 
 
hsa-miR-98-5p
 
 
-0.308
 
 
11.2
 
 
 
 
ASAP1
 
 
hsa-let-7c-5p
 
 
-0.308
 
 
11.2
 
 
 
 
ASF1A
 
 
hsa-miR-21-5p
 
 
-0.304
 
 
14.0
 
 
 
 
ASPH
 
 
hsa-miR-148a-3p
 
 
-0.391
 
 
62.6
 
 
 
 
ATOSB
 
 
hsa-miR-98-5p
 
 
-0.429
 
 
20.1
 
 
 
 
ATOSB
 
 
hsa-let-7c-5p
 
 
-0.429
 
 
20.1
 
 
 
 
ATP2A2
 
 
hsa-miR-98-5p
 
 
-0.311
 
 
15.8
 
 
 
 
ATP2A2
 
 
hsa-let-7c-5p
 
 
-0.311
 
 
15.8
 
 
 
 
ATP2B2
 
 
hsa-miR-7-5p
 
 
-0.297
 
 
14.3
 
 
 
 
ATP6AP2
 
 
hsa-miR-148a-3p
 
 
-0.592
 
 
69.8
 
 
 
 
ATP6AP2
 
 
hsa-miR-148a-3p
 
 
-0.668
 
 
69.8
 
 
 
 
ATPAF1
 
 
hsa-miR-98-5p
 
 
-0.498
 
 
86.8
 
 
 
 
ATPAF1
 
 
hsa-let-7c-5p
 
 
-0.498
 
 
86.8
 
 
 
 
AVPI1
 
 
hsa-miR-7-5p
 
 
-0.367
 
 
13.3
 
 
 
 
B3GAT3
 
 
hsa-miR-98-5p
 
 
-0.324
 
 
61.0
 
 
 
 
B3GAT3
 
 
hsa-let-7c-5p
 
 
-0.324
 
 
61.0
 
 
 
 
B4GALT5
 
 
hsa-miR-148a-3p
 
 
-0.44
 
 
10.8
 
 
 
 
B4GAT1
 
 
hsa-let-7c-5p
 
 
-0.47
 
 
107.6
 
 
 
 
B4GAT1
 
 
hsa-miR-98-5p
 
 
-0.47
 
 
107.6
 
 
 
 
BAMBI
 
 
hsa-miR-20a-5p
 
 
-0.485
 
 
14.6
 
 
 
 
BAMBI
 
 
hsa-miR-106b-5p
 
 
-0.493
 
 
14.6
 
 
 
 
BAMBI
 
 
hsa-miR-106a-5p
 
 
-0.503
 
 
14.6
 
 
 
 
BAMBI
 
 
hsa-miR-17-5p
 
 
-0.506
 
 
14.6
 
 
 
 
BAMBI
 
 
hsa-miR-20b-5p
 
 
-0.506
 
 
14.6
 
 
 
 
BEGAIN
 
 
hsa-let-7c-5p
 
 
-0.466
 
 
36.9
 
 
 
 
BEGAIN
 
 
hsa-miR-98-5p
 
 
-0.474
 
 
36.9
 
 
 
 
BHLHE41
 
 
hsa-miR-148a-3p
 
 
-0.442
 
 
27.2
 
 
 
 
BIN3
 
 
hsa-miR-98-5p
 
 
-0.427
 
 
10.6
 
 
 
 
BIN3
 
 
hsa-let-7c-5p
 
 
-0.427
 
 
10.6
 
 
 
 
BLOC1S6
 
 
hsa-miR-98-5p
 
 
-0.321
 
 
20.1
 
 
 
 
BLOC1S6
 
 
hsa-let-7c-5p
 
 
-0.321
 
 
20.1
 
 
 
 
BNIP3L
 
 
hsa-miR-20a-5p
 
 
-0.287
 
 
49.1
 
 
 
 
BNIP3L
 
 
hsa-miR-106b-5p
 
 
-0.298
 
 
49.1
 
 
 
 
BRMS1L
 
 
hsa-miR-20a-5p
 
 
-0.326
 
 
10.3
 
 
 
 
 
 
 appendix 
 
 
 
 
target_symbol
 
 
mature_mirna_id
 
 
score
 
 
nTPM
 
 
 
 
 
 
ABCD3
 
 
hsa-miR-148a-3p
 
 
-0.388
 
 
15.3
 
 
 
 
ACTC1
 
 
hsa-miR-7-5p
 
 
-0.471
 
 
23.3
 
 
 
 
ADAM10
 
 
hsa-miR-148a-3p
 
 
-0.356
 
 
17.9
 
 
 
 
ADAM9
 
 
hsa-miR-20a-5p
 
 
-0.299
 
 
27.3
 
 
 
 
ADAM9
 
 
hsa-miR-106b-5p
 
 
-0.299
 
 
27.3
 
 
 
 
ADAM9
 
 
hsa-miR-106a-5p
 
 
-0.317
 
 
27.3
 
 
 
 
ADAM9
 
 
hsa-miR-17-5p
 
 
-0.32
 
 
27.3
 
 
 
 
ADAM9
 
 
hsa-miR-20b-5p
 
 
-0.328
 
 
27.3
 
 
 
 
AGFG1
 
 
hsa-miR-148a-3p
 
 
-0.332
 
 
45.7
 
 
 
 
AHNAK
 
 
hsa-miR-20a-5p
 
 
-0.316
 
 
41.0
 
 
 
 
AHNAK
 
 
hsa-miR-106b-5p
 
 
-0.316
 
 
41.0
 
 
 
 
AHNAK
 
 
hsa-miR-106a-5p
 
 
-0.334
 
 
41.0
 
 
 
 
AHNAK
 
 
hsa-miR-17-5p
 
 
-0.337
 
 
41.0
 
 
 
 
AHNAK
 
 
hsa-miR-20b-5p
 
 
-0.337
 
 
41.0
 
 
 
 
AIFM1
 
 
hsa-miR-98-5p
 
 
-0.314
 
 
16.6
 
 
 
 
AIFM1
 
 
hsa-let-7c-5p
 
 
-0.314
 
 
16.6
 
 
 
 
AKAP1
 
 
hsa-miR-148a-3p
 
 
-0.381
 
 
16.8
 
 
 
 
ALCAM
 
 
hsa-miR-148a-3p
 
 
-0.339
 
 
11.5
 
 
 
 
ALG3
 
 
hsa-miR-106b-5p
 
 
-0.551
 
 
14.1
 
 
 
 
ALG3
 
 
hsa-miR-20a-5p
 
 
-0.559
 
 
14.1
 
 
 
 
ALG3
 
 
hsa-miR-106a-5p
 
 
-0.569
 
 
14.1
 
 
 
 
ALG3
 
 
hsa-miR-17-5p
 
 
-0.572
 
 
14.1
 
 
 
 
ALG3
 
 
hsa-miR-20b-5p
 
 
-0.58
 
 
14.1
 
 
 
 
ANKRD12
 
 
hsa-miR-7-5p
 
 
-0.37
 
 
19.4
 
 
 
 
AP1AR
 
 
hsa-miR-21-5p
 
 
-0.318
 
 
11.9
 
 
 
 
AP1S1
 
 
hsa-miR-98-5p
 
 
-0.374
 
 
13.1
 
 
 
 
AP1S1
 
 
hsa-let-7c-5p
 
 
-0.374
 
 
13.1
 
 
 
 
ARF4
 
 
hsa-miR-7-5p
 
 
-0.407
 
 
101.3
 
 
 
 
ARF4
 
 
hsa-miR-148a-3p
 
 
-0.487
 
 
101.3
 
 
 
 
ARFGEF1
 
 
hsa-miR-148a-3p
 
 
-0.306
 
 
13.8
 
 
 
 
ARFIP1
 
 
hsa-miR-148a-3p
 
 
-0.474
 
 
14.9
 
 
 
 
ARHGAP1
 
 
hsa-miR-17-5p
 
 
-0.325
 
 
35.8
 
 
 
 
ARHGAP1
 
 
hsa-miR-20b-5p
 
 
-0.325
 
 
35.8
 
 
 
 
ARHGAP1
 
 
hsa-miR-106a-5p
 
 
-0.329
 
 
35.8
 
 
 
 
ARHGAP1
 
 
hsa-miR-20a-5p
 
 
-0.339
 
 
35.8
 
 
 
 
ARHGAP1
 
 
hsa-miR-106b-5p
 
 
-0.339
 
 
35.8
 
 
 
 
ARID4A
 
 
hsa-miR-7-5p
 
 
-0.341
 
 
10.5
 
 
 
 
ARID4B
 
 
hsa-miR-20a-5p
 
 
-0.29
 
 
13.3
 
 
 
 
ARID4B
 
 
hsa-miR-106b-5p
 
 
-0.29
 
 
13.3
 
 
 
 
ARL1
 
 
hsa-miR-20a-5p
 
 
-0.403
 
 
37.7
 
 
 
 
ARL1
 
 
hsa-miR-106b-5p
 
 
-0.411
 
 
37.7
 
 
 
 
ARL1
 
 
hsa-miR-20b-5p
 
 
-0.424
 
 
37.7
 
 
 
 
ARL1
 
 
hsa-miR-106a-5p
 
 
-0.429
 
 
37.7
 
 
 
 
ARL1
 
 
hsa-miR-17-5p
 
 
-0.432
 
 
37.7
 
 
 
 
ARL6IP1
 
 
hsa-miR-7-5p
 
 
-0.374
 
 
73.3
 
 
 
 
ARL6IP1
 
 
hsa-miR-148a-3p
 
 
-0.579
 
 
73.3
 
 
 
 
ARL6IP6
 
 
hsa-miR-98-5p
 
 
-0.293
 
 
10.9
 
 
 
 
ARL6IP6
 
 
hsa-let-7c-5p
 
 
-0.293
 
 
10.9
 
 
 
 
ARL8B
 
 
hsa-miR-148a-3p
 
 
-0.337
 
 
34.1
 
 
 
 
ARMC8
 
 
hsa-miR-20a-5p
 
 
-0.357
 
 
17.0
 
 
 
 
ARMC8
 
 
hsa-miR-106b-5p
 
 
-0.357
 
 
17.0
 
 
 
 
ARMC8
 
 
hsa-miR-106a-5p
 
 
-0.375
 
 
17.0
 
 
 
 
ARMC8
 
 
hsa-miR-17-5p
 
 
-0.378
 
 
17.0
 
 
 
 
ARMC8
 
 
hsa-miR-20b-5p
 
 
-0.378
 
 
17.0
 
 
 
 
ARMT1
 
 
hsa-let-7c-5p
 
 
-0.303
 
 
14.4
 
 
 
 
ARMT1
 
 
hsa-miR-98-5p
 
 
-0.303
 
 
14.4
 
 
 
 
ARPP19
 
 
hsa-let-7c-5p
 
 
-0.492
 
 
36.4
 
 
 
 
ARPP19
 
 
hsa-miR-98-5p
 
 
-0.5
 
 
36.4
 
 
 
 
ARRDC3
 
 
hsa-miR-148a-3p
 
 
-0.305
 
 
25.3
 
 
 
 
ARRDC4
 
 
hsa-let-7c-5p
 
 
-0.329
 
 
15.6
 
 
 
 
ARRDC4
 
 
hsa-miR-98-5p
 
 
-0.329
 
 
15.6
 
 
 
 
ASAP1
 
 
hsa-miR-98-5p
 
 
-0.308
 
 
11.4
 
 
 
 
ASAP1
 
 
hsa-let-7c-5p
 
 
-0.308
 
 
11.4
 
 
 
 
ASPH
 
 
hsa-miR-148a-3p
 
 
-0.391
 
 
47.6
 
 
 
 
ATOSB
 
 
hsa-miR-98-5p
 
 
-0.429
 
 
11.5
 
 
 
 
ATOSB
 
 
hsa-let-7c-5p
 
 
-0.429
 
 
11.5
 
 
 
 
ATP2A2
 
 
hsa-miR-98-5p
 
 
-0.311
 
 
54.5
 
 
 
 
ATP2A2
 
 
hsa-let-7c-5p
 
 
-0.311
 
 
54.5
 
 
 
 
ATP2B4
 
 
hsa-miR-148a-3p
 
 
-0.337
 
 
24.2
 
 
 
 
ATP6AP2
 
 
hsa-miR-148a-3p
 
 
-0.592
 
 
57.3
 
 
 
 
ATP6AP2
 
 
hsa-miR-148a-3p
 
 
-0.668
 
 
57.3
 
 
 
 
ATPAF1
 
 
hsa-miR-98-5p
 
 
-0.498
 
 
26.8
 
 
 
 
ATPAF1
 
 
hsa-let-7c-5p
 
 
-0.498
 
 
26.8
 
 
 
 
B3GAT3
 
 
hsa-miR-98-5p
 
 
-0.324
 
 
16.3
 
 
 
 
B3GAT3
 
 
hsa-let-7c-5p
 
 
-0.324
 
 
16.3
 
 
 
 
B4GALT5
 
 
hsa-miR-148a-3p
 
 
-0.44
 
 
20.5
 
 
 
 
BACH1
 
 
hsa-miR-98-5p
 
 
-0.298
 
 
26.1
 
 
 
 
BACH1
 
 
hsa-miR-98-5p
 
 
-0.298
 
 
26.1
 
 
 
 
BACH1
 
 
hsa-let-7c-5p
 
 
-0.306
 
 
26.1
 
 
 
 
BACH1
 
 
hsa-let-7c-5p
 
 
-0.306
 
 
26.1
 
 
 
 
BACH1
 
 
hsa-miR-98-5p
 
 
-0.435
 
 
26.1
 
 
 
 
BACH1
 
 
hsa-miR-98-5p
 
 
-0.435
 
 
26.1
 
 
 
 
BACH1
 
 
hsa-let-7c-5p
 
 
-0.435
 
 
26.1
 
 
 
 
BACH1
 
 
hsa-let-7c-5p
 
 
-0.435
 
 
26.1
 
 
 
 
BCL2L11
 
 
hsa-miR-148a-3p
 
 
-0.445
 
 
16.0
 
 
 
 
BIN3
 
 
hsa-miR-98-5p
 
 
-0.427
 
 
10.4
 
 
 
 
BIN3
 
 
hsa-let-7c-5p
 
 
-0.427
 
 
10.4
 
 
 
 
BLOC1S6
 
 
hsa-miR-98-5p
 
 
-0.321
 
 
29.4
 
 
 
 
BLOC1S6
 
 
hsa-let-7c-5p
 
 
-0.321
 
 
29.4
 
 
 
 
BNIP3L
 
 
hsa-miR-20a-5p
 
 
-0.287
 
 
35.1
 
 
 
 
BNIP3L
 
 
hsa-miR-106b-5p
 
 
-0.298
 
 
35.1
 
 
 
 
BTAF1
 
 
hsa-miR-148a-3p
 
 
-0.401
 
 
16.0
 
 
 
 
BTBD10
 
 
hsa-miR-106b-5p
 
 
-0.388
 
 
12.9
 
 
 
 
BTBD10
 
 
hsa-miR-106a-5p
 
 
-0.406
 
 
12.9
 
 
 
 
BTBD10
 
 
hsa-miR-17-5p
 
 
-0.409
 
 
12.9
 
 
 
 
BTBD10
 
 
hsa-miR-20a-5p
 
 
-0.412
 
 
12.9
 
 
 
 
BTBD10
 
 
hsa-miR-20b-5p
 
 
-0.425
 
 
12.9
 
 
 
 
BTF3L4
 
 
hsa-let-7c-5p
 
 
-0.355
 
 
28.6
 
 
 
 
BTF3L4
 
 
hsa-miR-98-5p
 
 
-0.355
 
 
28.6
 
 
 
 
BTG3
 
 
hsa-miR-106b-5p
 
 
-0.647
 
 
13.2
 
 
 
 
 
 
 epididymis 
 
 
 
 
target_symbol
 
 
mature_mirna_id
 
 
score
 
 
nTPM
 
 
 
 
 
 
ABCD3
 
 
hsa-miR-148a-3p
 
 
-0.388
 
 
20.0
 
 
 
 
ACP3
 
 
hsa-miR-98-5p
 
 
-0.298
 
 
16.3
 
 
 
 
ACP3
 
 
hsa-let-7c-5p
 
 
-0.298
 
 
16.3
 
 
 
 
ACVR1
 
 
hsa-miR-148a-3p
 
 
-0.397
 
 
10.8
 
 
 
 
ADAM17
 
 
hsa-miR-148a-3p
 
 
-0.301
 
 
10.4
 
 
 
 
ADAM9
 
 
hsa-miR-20a-5p
 
 
-0.299
 
 
14.3
 
 
 
 
ADAM9
 
 
hsa-miR-106b-5p
 
 
-0.299
 
 
14.3
 
 
 
 
ADAM9
 
 
hsa-miR-106a-5p
 
 
-0.317
 
 
14.3
 
 
 
 
ADAM9
 
 
hsa-miR-17-5p
 
 
-0.32
 
 
14.3
 
 
 
 
ADAM9
 
 
hsa-miR-20b-5p
 
 
-0.328
 
 
14.3
 
 
 
 
ADGRG2
 
 
hsa-miR-21-5p
 
 
-0.343
 
 
1159.4
 
 
 
 
AGFG1
 
 
hsa-miR-148a-3p
 
 
-0.332
 
 
18.8
 
 
 
 
AHNAK
 
 
hsa-miR-20a-5p
 
 
-0.316
 
 
48.9
 
 
 
 
AHNAK
 
 
hsa-miR-106b-5p
 
 
-0.316
 
 
48.9
 
 
 
 
AHNAK
 
 
hsa-miR-106a-5p
 
 
-0.334
 
 
48.9
 
 
 
 
AHNAK
 
 
hsa-miR-17-5p
 
 
-0.337
 
 
48.9
 
 
 
 
AHNAK
 
 
hsa-miR-20b-5p
 
 
-0.337
 
 
48.9
 
 
 
 
AKAP1
 
 
hsa-miR-148a-3p
 
 
-0.381
 
 
18.6
 
 
 
 
ALCAM
 
 
hsa-miR-148a-3p
 
 
-0.339
 
 
33.4
 
 
 
 
ALG3
 
 
hsa-miR-106b-5p
 
 
-0.551
 
 
11.8
 
 
 
 
ALG3
 
 
hsa-miR-20a-5p
 
 
-0.559
 
 
11.8
 
 
 
 
ALG3
 
 
hsa-miR-106a-5p
 
 
-0.569
 
 
11.8
 
 
 
 
ALG3
 
 
hsa-miR-17-5p
 
 
-0.572
 
 
11.8
 
 
 
 
ALG3
 
 
hsa-miR-20b-5p
 
 
-0.58
 
 
11.8
 
 
 
 
ALG9
 
 
hsa-miR-7-5p
 
 
-0.302
 
 
10.3
 
 
 
 
ANKRD46
 
 
hsa-miR-98-5p
 
 
-0.389
 
 
15.4
 
 
 
 
ANKRD46
 
 
hsa-let-7c-5p
 
 
-0.389
 
 
15.4
 
 
 
 
AP1S1
 
 
hsa-miR-98-5p
 
 
-0.374
 
 
16.0
 
 
 
 
AP1S1
 
 
hsa-let-7c-5p
 
 
-0.374
 
 
16.0
 
 
 
 
APCDD1
 
 
hsa-miR-20a-5p
 
 
-0.404
 
 
14.6
 
 
 
 
APCDD1
 
 
hsa-miR-106b-5p
 
 
-0.404
 
 
14.6
 
 
 
 
APCDD1
 
 
hsa-miR-106a-5p
 
 
-0.422
 
 
14.6
 
 
 
 
APCDD1
 
 
hsa-miR-17-5p
 
 
-0.425
 
 
14.6
 
 
 
 
APCDD1
 
 
hsa-miR-20b-5p
 
 
-0.425
 
 
14.6
 
 
 
 
ARF4
 
 
hsa-miR-7-5p
 
 
-0.407
 
 
214.1
 
 
 
 
ARF4
 
 
hsa-miR-148a-3p
 
 
-0.487
 
 
214.1
 
 
 
 
ARFGEF1
 
 
hsa-miR-148a-3p
 
 
-0.306
 
 
10.4
 
 
 
 
ARFIP1
 
 
hsa-miR-148a-3p
 
 
-0.474
 
 
19.3
 
 
 
 
ARHGAP1
 
 
hsa-miR-17-5p
 
 
-0.325
 
 
20.3
 
 
 
 
ARHGAP1
 
 
hsa-miR-20b-5p
 
 
-0.325
 
 
20.3
 
 
 
 
ARHGAP1
 
 
hsa-miR-106a-5p
 
 
-0.329
 
 
20.3
 
 
 
 
ARHGAP1
 
 
hsa-miR-20a-5p
 
 
-0.339
 
 
20.3
 
 
 
 
ARHGAP1
 
 
hsa-miR-106b-5p
 
 
-0.339
 
 
20.3
 
 
 
 
ARID4B
 
 
hsa-miR-20a-5p
 
 
-0.29
 
 
13.6
 
 
 
 
ARID4B
 
 
hsa-miR-106b-5p
 
 
-0.29
 
 
13.6
 
 
 
 
ARL1
 
 
hsa-miR-20a-5p
 
 
-0.403
 
 
67.1
 
 
 
 
ARL1
 
 
hsa-miR-106b-5p
 
 
-0.411
 
 
67.1
 
 
 
 
ARL1
 
 
hsa-miR-20b-5p
 
 
-0.424
 
 
67.1
 
 
 
 
ARL1
 
 
hsa-miR-106a-5p
 
 
-0.429
 
 
67.1
 
 
 
 
ARL1
 
 
hsa-miR-17-5p
 
 
-0.432
 
 
67.1
 
 
 
 
ARL6IP1
 
 
hsa-miR-7-5p
 
 
-0.374
 
 
116.0
 
 
 
 
ARL6IP1
 
 
hsa-miR-148a-3p
 
 
-0.579
 
 
116.0
 
 
 
 
ARL8B
 
 
hsa-miR-148a-3p
 
 
-0.337
 
 
37.5
 
 
 
 
ARMC8
 
 
hsa-miR-20a-5p
 
 
-0.357
 
 
11.5
 
 
 
 
ARMC8
 
 
hsa-miR-106b-5p
 
 
-0.357
 
 
11.5
 
 
 
 
ARMC8
 
 
hsa-miR-106a-5p
 
 
-0.375
 
 
11.5
 
 
 
 
ARMC8
 
 
hsa-miR-17-5p
 
 
-0.378
 
 
11.5
 
 
 
 
ARMC8
 
 
hsa-miR-20b-5p
 
 
-0.378
 
 
11.5
 
 
 
 
ARMCX1
 
 
hsa-miR-21-5p
 
 
-0.489
 
 
20.1
 
 
 
 
ARMT1
 
 
hsa-let-7c-5p
 
 
-0.303
 
 
15.2
 
 
 
 
ARMT1
 
 
hsa-miR-98-5p
 
 
-0.303
 
 
15.2
 
 
 
 
ARPP19
 
 
hsa-let-7c-5p
 
 
-0.492
 
 
34.2
 
 
 
 
ARPP19
 
 
hsa-miR-98-5p
 
 
-0.5
 
 
34.2
 
 
 
 
ARRDC3
 
 
hsa-miR-148a-3p
 
 
-0.305
 
 
25.6
 
 
 
 
ARRDC4
 
 
hsa-let-7c-5p
 
 
-0.329
 
 
14.7
 
 
 
 
ARRDC4
 
 
hsa-miR-98-5p
 
 
-0.329
 
 
14.7
 
 
 
 
ASAP1
 
 
hsa-miR-98-5p
 
 
-0.308
 
 
11.5
 
 
 
 
ASAP1
 
 
hsa-let-7c-5p
 
 
-0.308
 
 
11.5
 
 
 
 
ASF1A
 
 
hsa-miR-21-5p
 
 
-0.304
 
 
12.3
 
 
 
 
ASPH
 
 
hsa-miR-148a-3p
 
 
-0.391
 
 
42.3
 
 
 
 
ATG4A
 
 
hsa-miR-7-5p
 
 
-0.448
 
 
10.9
 
 
 
 
ATP2A2
 
 
hsa-miR-98-5p
 
 
-0.311
 
 
36.9
 
 
 
 
ATP2A2
 
 
hsa-let-7c-5p
 
 
-0.311
 
 
36.9
 
 
 
 
ATP2B4
 
 
hsa-miR-148a-3p
 
 
-0.337
 
 
18.9
 
 
 
 
ATP6AP2
 
 
hsa-miR-148a-3p
 
 
-0.592
 
 
86.1
 
 
 
 
ATP6AP2
 
 
hsa-miR-148a-3p
 
 
-0.668
 
 
86.1
 
 
 
 
ATPAF1
 
 
hsa-miR-98-5p
 
 
-0.498
 
 
33.7
 
 
 
 
ATPAF1
 
 
hsa-let-7c-5p
 
 
-0.498
 
 
33.7
 
 
 
 
B3GAT3
 
 
hsa-miR-98-5p
 
 
-0.324
 
 
11.2
 
 
 
 
B3GAT3
 
 
hsa-let-7c-5p
 
 
-0.324
 
 
11.2
 
 
 
 
B4GAT1
 
 
hsa-let-7c-5p
 
 
-0.47
 
 
14.3
 
 
 
 
B4GAT1
 
 
hsa-miR-98-5p
 
 
-0.47
 
 
14.3
 
 
 
 
BHLHE41
 
 
hsa-miR-148a-3p
 
 
-0.442
 
 
18.2
 
 
 
 
BLOC1S6
 
 
hsa-miR-98-5p
 
 
-0.321
 
 
17.3
 
 
 
 
BLOC1S6
 
 
hsa-let-7c-5p
 
 
-0.321
 
 
17.3
 
 
 
 
BNIP3L
 
 
hsa-miR-20a-5p
 
 
-0.287
 
 
62.5
 
 
 
 
BNIP3L
 
 
hsa-miR-106b-5p
 
 
-0.298
 
 
62.5
 
 
 
 
BTAF1
 
 
hsa-miR-148a-3p
 
 
-0.401
 
 
16.9
 
 
 
 
BTBD10
 
 
hsa-miR-106b-5p
 
 
-0.388
 
 
11.3
 
 
 
 
BTBD10
 
 
hsa-miR-106a-5p
 
 
-0.406
 
 
11.3
 
 
 
 
BTBD10
 
 
hsa-miR-17-5p
 
 
-0.409
 
 
11.3
 
 
 
 
BTBD10
 
 
hsa-miR-20a-5p
 
 
-0.412
 
 
11.3
 
 
 
 
BTBD10
 
 
hsa-miR-20b-5p
 
 
-0.425
 
 
11.3
 
 
 
 
BTF3L4
 
 
hsa-let-7c-5p
 
 
-0.355
 
 
28.9
 
 
 
 
BTF3L4
 
 
hsa-miR-98-5p
 
 
-0.355
 
 
28.9
 
 
 
 
BTG3
 
 
hsa-miR-106b-5p
 
 
-0.647
 
 
104.1
 
 
 
 
BTG3
 
 
hsa-miR-106a-5p
 
 
-0.665
 
 
104.1
 
 
 
 
BTG3
 
 
hsa-miR-17-5p
 
 
-0.668
 
 
104.1
 
 
 
 
BTG3
 
 
hsa-miR-20a-5p
 
 
-0.671
 
 
104.1
 
 
 
 
BTG3
 
 
hsa-miR-20b-5p
 
 
-0.684
 
 
104.1
 
 
 
 
 
 
 gallbladder 
 
 
 
 
target_symbol
 
 
mature_mirna_id
 
 
score
 
 
nTPM
 
 
 
 
 
 
ABCD3
 
 
hsa-miR-148a-3p
 
 
-0.388
 
 
25.2
 
 
 
 
ABHD5
 
 
hsa-miR-20a-5p
 
 
-0.397
 
 
10.2
 
 
 
 
ABHD5
 
 
hsa-miR-106b-5p
 
 
-0.413
 
 
10.2
 
 
 
 
ABHD5
 
 
hsa-miR-20b-5p
 
 
-0.418
 
 
10.2
 
 
 
 
ABHD5
 
 
hsa-miR-106a-5p
 
 
-0.447
 
 
10.2
 
 
 
 
ABHD5
 
 
hsa-miR-17-5p
 
 
-0.45
 
 
10.2
 
 
 
 
ACTC1
 
 
hsa-miR-7-5p
 
 
-0.471
 
 
11.1
 
 
 
 
ACVR1
 
 
hsa-miR-148a-3p
 
 
-0.397
 
 
14.1
 
 
 
 
ADAM10
 
 
hsa-miR-148a-3p
 
 
-0.356
 
 
17.7
 
 
 
 
ADAM9
 
 
hsa-miR-20a-5p
 
 
-0.299
 
 
44.2
 
 
 
 
ADAM9
 
 
hsa-miR-106b-5p
 
 
-0.299
 
 
44.2
 
 
 
 
ADAM9
 
 
hsa-miR-106a-5p
 
 
-0.317
 
 
44.2
 
 
 
 
ADAM9
 
 
hsa-miR-17-5p
 
 
-0.32
 
 
44.2
 
 
 
 
ADAM9
 
 
hsa-miR-20b-5p
 
 
-0.328
 
 
44.2
 
 
 
 
ADGRF5
 
 
hsa-miR-148a-3p
 
 
-0.354
 
 
16.1
 
 
 
 
AGFG1
 
 
hsa-miR-148a-3p
 
 
-0.332
 
 
36.5
 
 
 
 
AGFG2
 
 
hsa-miR-20a-5p
 
 
-0.365
 
 
10.4
 
 
 
 
AGFG2
 
 
hsa-miR-20b-5p
 
 
-0.386
 
 
10.4
 
 
 
 
AGFG2
 
 
hsa-miR-106b-5p
 
 
-0.397
 
 
10.4
 
 
 
 
AGFG2
 
 
hsa-miR-106a-5p
 
 
-0.399
 
 
10.4
 
 
 
 
AGFG2
 
 
hsa-miR-17-5p
 
 
-0.402
 
 
10.4
 
 
 
 
AHNAK
 
 
hsa-miR-20a-5p
 
 
-0.316
 
 
70.2
 
 
 
 
AHNAK
 
 
hsa-miR-106b-5p
 
 
-0.316
 
 
70.2
 
 
 
 
AHNAK
 
 
hsa-miR-106a-5p
 
 
-0.334
 
 
70.2
 
 
 
 
AHNAK
 
 
hsa-miR-17-5p
 
 
-0.337
 
 
70.2
 
 
 
 
AHNAK
 
 
hsa-miR-20b-5p
 
 
-0.337
 
 
70.2
 
 
 
 
AKAP1
 
 
hsa-miR-148a-3p
 
 
-0.381
 
 
16.8
 
 
 
 
ALCAM
 
 
hsa-miR-148a-3p
 
 
-0.339
 
 
21.9
 
 
 
 
ALG3
 
 
hsa-miR-106b-5p
 
 
-0.551
 
 
10.8
 
 
 
 
ALG3
 
 
hsa-miR-20a-5p
 
 
-0.559
 
 
10.8
 
 
 
 
ALG3
 
 
hsa-miR-106a-5p
 
 
-0.569
 
 
10.8
 
 
 
 
ALG3
 
 
hsa-miR-17-5p
 
 
-0.572
 
 
10.8
 
 
 
 
ALG3
 
 
hsa-miR-20b-5p
 
 
-0.58
 
 
10.8
 
 
 
 
ANKRD12
 
 
hsa-miR-7-5p
 
 
-0.37
 
 
13.7
 
 
 
 
AP1AR
 
 
hsa-miR-21-5p
 
 
-0.318
 
 
12.0
 
 
 
 
AP1S1
 
 
hsa-miR-98-5p
 
 
-0.374
 
 
12.0
 
 
 
 
AP1S1
 
 
hsa-let-7c-5p
 
 
-0.374
 
 
12.0
 
 
 
 
APCDD1
 
 
hsa-miR-20a-5p
 
 
-0.404
 
 
20.5
 
 
 
 
APCDD1
 
 
hsa-miR-106b-5p
 
 
-0.404
 
 
20.5
 
 
 
 
APCDD1
 
 
hsa-miR-106a-5p
 
 
-0.422
 
 
20.5
 
 
 
 
APCDD1
 
 
hsa-miR-17-5p
 
 
-0.425
 
 
20.5
 
 
 
 
APCDD1
 
 
hsa-miR-20b-5p
 
 
-0.425
 
 
20.5
 
 
 
 
ARF4
 
 
hsa-miR-7-5p
 
 
-0.407
 
 
145.1
 
 
 
 
ARF4
 
 
hsa-miR-148a-3p
 
 
-0.487
 
 
145.1
 
 
 
 
ARFGEF1
 
 
hsa-miR-148a-3p
 
 
-0.306
 
 
11.7
 
 
 
 
ARFIP1
 
 
hsa-miR-148a-3p
 
 
-0.474
 
 
18.7
 
 
 
 
ARHGAP1
 
 
hsa-miR-17-5p
 
 
-0.325
 
 
47.5
 
 
 
 
ARHGAP1
 
 
hsa-miR-20b-5p
 
 
-0.325
 
 
47.5
 
 
 
 
ARHGAP1
 
 
hsa-miR-106a-5p
 
 
-0.329
 
 
47.5
 
 
 
 
ARHGAP1
 
 
hsa-miR-20a-5p
 
 
-0.339
 
 
47.5
 
 
 
 
ARHGAP1
 
 
hsa-miR-106b-5p
 
 
-0.339
 
 
47.5
 
 
 
 
ARHGAP21
 
 
hsa-miR-148a-3p
 
 
-0.287
 
 
16.7
 
 
 
 
ARHGAP24
 
 
hsa-miR-21-5p
 
 
-0.427
 
 
10.2
 
 
 
 
ARID4B
 
 
hsa-miR-20a-5p
 
 
-0.29
 
 
12.3
 
 
 
 
ARID4B
 
 
hsa-miR-106b-5p
 
 
-0.29
 
 
12.3
 
 
 
 
ARL1
 
 
hsa-miR-20a-5p
 
 
-0.403
 
 
40.9
 
 
 
 
ARL1
 
 
hsa-miR-106b-5p
 
 
-0.411
 
 
40.9
 
 
 
 
ARL1
 
 
hsa-miR-20b-5p
 
 
-0.424
 
 
40.9
 
 
 
 
ARL1
 
 
hsa-miR-106a-5p
 
 
-0.429
 
 
40.9
 
 
 
 
ARL1
 
 
hsa-miR-17-5p
 
 
-0.432
 
 
40.9
 
 
 
 
ARL6IP1
 
 
hsa-miR-7-5p
 
 
-0.374
 
 
123.8
 
 
 
 
ARL6IP1
 
 
hsa-miR-148a-3p
 
 
-0.579
 
 
123.8
 
 
 
 
ARL8B
 
 
hsa-miR-148a-3p
 
 
-0.337
 
 
36.3
 
 
 
 
ARMC8
 
 
hsa-miR-20a-5p
 
 
-0.357
 
 
14.3
 
 
 
 
ARMC8
 
 
hsa-miR-106b-5p
 
 
-0.357
 
 
14.3
 
 
 
 
ARMC8
 
 
hsa-miR-106a-5p
 
 
-0.375
 
 
14.3
 
 
 
 
ARMC8
 
 
hsa-miR-17-5p
 
 
-0.378
 
 
14.3
 
 
 
 
ARMC8
 
 
hsa-miR-20b-5p
 
 
-0.378
 
 
14.3
 
 
 
 
ARMCX1
 
 
hsa-miR-21-5p
 
 
-0.489
 
 
16.4
 
 
 
 
ARMT1
 
 
hsa-let-7c-5p
 
 
-0.303
 
 
10.6
 
 
 
 
ARMT1
 
 
hsa-miR-98-5p
 
 
-0.303
 
 
10.6
 
 
 
 
ARPP19
 
 
hsa-let-7c-5p
 
 
-0.492
 
 
36.7
 
 
 
 
ARPP19
 
 
hsa-miR-98-5p
 
 
-0.5
 
 
36.7
 
 
 
 
ARRDC3
 
 
hsa-miR-148a-3p
 
 
-0.305
 
 
36.6
 
 
 
 
ARRDC4
 
 
hsa-let-7c-5p
 
 
-0.329
 
 
16.1
 
 
 
 
ARRDC4
 
 
hsa-miR-98-5p
 
 
-0.329
 
 
16.1
 
 
 
 
ASAP1
 
 
hsa-miR-98-5p
 
 
-0.308
 
 
13.5
 
 
 
 
ASAP1
 
 
hsa-let-7c-5p
 
 
-0.308
 
 
13.5
 
 
 
 
ASPH
 
 
hsa-miR-148a-3p
 
 
-0.391
 
 
56.5
 
 
 
 
ATOSB
 
 
hsa-miR-98-5p
 
 
-0.429
 
 
11.0
 
 
 
 
ATOSB
 
 
hsa-let-7c-5p
 
 
-0.429
 
 
11.0
 
 
 
 
ATP2A2
 
 
hsa-miR-98-5p
 
 
-0.311
 
 
56.7
 
 
 
 
ATP2A2
 
 
hsa-let-7c-5p
 
 
-0.311
 
 
56.7
 
 
 
 
ATP2B4
 
 
hsa-miR-148a-3p
 
 
-0.337
 
 
23.7
 
 
 
 
ATP6AP2
 
 
hsa-miR-148a-3p
 
 
-0.592
 
 
74.7
 
 
 
 
ATP6AP2
 
 
hsa-miR-148a-3p
 
 
-0.668
 
 
74.7
 
 
 
 
ATPAF1
 
 
hsa-miR-98-5p
 
 
-0.498
 
 
28.6
 
 
 
 
ATPAF1
 
 
hsa-let-7c-5p
 
 
-0.498
 
 
28.6
 
 
 
 
B3GALT2
 
 
hsa-miR-17-5p
 
 
-0.35
 
 
14.1
 
 
 
 
B3GALT2
 
 
hsa-miR-20b-5p
 
 
-0.35
 
 
14.1
 
 
 
 
B3GALT2
 
 
hsa-miR-106a-5p
 
 
-0.354
 
 
14.1
 
 
 
 
B3GALT2
 
 
hsa-miR-20a-5p
 
 
-0.364
 
 
14.1
 
 
 
 
B3GALT2
 
 
hsa-miR-106b-5p
 
 
-0.364
 
 
14.1
 
 
 
 
B3GAT3
 
 
hsa-miR-98-5p
 
 
-0.324
 
 
14.5
 
 
 
 
B3GAT3
 
 
hsa-let-7c-5p
 
 
-0.324
 
 
14.5
 
 
 
 
B4GALT5
 
 
hsa-miR-148a-3p
 
 
-0.44
 
 
29.5
 
 
 
 
BACH1
 
 
hsa-miR-98-5p
 
 
-0.298
 
 
24.3
 
 
 
 
BACH1
 
 
hsa-miR-98-5p
 
 
-0.298
 
 
24.3
 
 
 
 
BACH1
 
 
hsa-let-7c-5p
 
 
-0.306
 
 
24.3
 
 
 
 
BACH1
 
 
hsa-let-7c-5p
 
 
-0.306
 
 
24.3
 
 
 
 
 
 
 hippocampal formation 
 
 
 
 
target_symbol
 
 
mature_mirna_id
 
 
score
 
 
nTPM
 
 
 
 
 
 
ABCD3
 
 
hsa-miR-148a-3p
 
 
-0.388
 
 
15.1
 
 
 
 
ABHD17C
 
 
hsa-let-7c-5p
 
 
-0.359
 
 
14.4
 
 
 
 
ABHD17C
 
 
hsa-miR-98-5p
 
 
-0.383
 
 
14.4
 
 
 
 
ACSL6
 
 
hsa-miR-98-5p
 
 
-0.336
 
 
15.3
 
 
 
 
ACSL6
 
 
hsa-let-7c-5p
 
 
-0.336
 
 
15.3
 
 
 
 
ADCY2
 
 
hsa-miR-148a-3p
 
 
-0.299
 
 
18.5
 
 
 
 
ADGRB3
 
 
hsa-miR-148a-3p
 
 
-0.524
 
 
12.8
 
 
 
 
ADGRF5
 
 
hsa-miR-148a-3p
 
 
-0.354
 
 
10.4
 
 
 
 
AGFG1
 
 
hsa-miR-148a-3p
 
 
-0.332
 
 
29.8
 
 
 
 
AGFG2
 
 
hsa-miR-20a-5p
 
 
-0.365
 
 
13.2
 
 
 
 
AGFG2
 
 
hsa-miR-20b-5p
 
 
-0.386
 
 
13.2
 
 
 
 
AGFG2
 
 
hsa-miR-106b-5p
 
 
-0.397
 
 
13.2
 
 
 
 
AGFG2
 
 
hsa-miR-106a-5p
 
 
-0.399
 
 
13.2
 
 
 
 
AGFG2
 
 
hsa-miR-17-5p
 
 
-0.402
 
 
13.2
 
 
 
 
AHNAK
 
 
hsa-miR-20a-5p
 
 
-0.316
 
 
24.2
 
 
 
 
AHNAK
 
 
hsa-miR-106b-5p
 
 
-0.316
 
 
24.2
 
 
 
 
AHNAK
 
 
hsa-miR-106a-5p
 
 
-0.334
 
 
24.2
 
 
 
 
AHNAK
 
 
hsa-miR-17-5p
 
 
-0.337
 
 
24.2
 
 
 
 
AHNAK
 
 
hsa-miR-20b-5p
 
 
-0.337
 
 
24.2
 
 
 
 
AIFM1
 
 
hsa-miR-98-5p
 
 
-0.314
 
 
10.5
 
 
 
 
AIFM1
 
 
hsa-let-7c-5p
 
 
-0.314
 
 
10.5
 
 
 
 
AKAP1
 
 
hsa-miR-148a-3p
 
 
-0.381
 
 
17.0
 
 
 
 
AKAP6
 
 
hsa-miR-98-5p
 
 
-0.307
 
 
12.9
 
 
 
 
AKAP6
 
 
hsa-let-7c-5p
 
 
-0.307
 
 
12.9
 
 
 
 
ALCAM
 
 
hsa-miR-148a-3p
 
 
-0.339
 
 
33.8
 
 
 
 
ALDH4A1
 
 
hsa-miR-184
 
 
-0.421
 
 
39.7
 
 
 
 
ANKRD46
 
 
hsa-miR-98-5p
 
 
-0.389
 
 
25.5
 
 
 
 
ANKRD46
 
 
hsa-let-7c-5p
 
 
-0.389
 
 
25.5
 
 
 
 
ANKRD9
 
 
hsa-miR-20a-5p
 
 
-0.31
 
 
23.2
 
 
 
 
ANKRD9
 
 
hsa-miR-106b-5p
 
 
-0.318
 
 
23.2
 
 
 
 
ANKRD9
 
 
hsa-miR-20b-5p
 
 
-0.331
 
 
23.2
 
 
 
 
ANKRD9
 
 
hsa-miR-106a-5p
 
 
-0.344
 
 
23.2
 
 
 
 
ANKRD9
 
 
hsa-miR-17-5p
 
 
-0.347
 
 
23.2
 
 
 
 
AP1S1
 
 
hsa-miR-98-5p
 
 
-0.374
 
 
67.5
 
 
 
 
AP1S1
 
 
hsa-let-7c-5p
 
 
-0.374
 
 
67.5
 
 
 
 
APCDD1
 
 
hsa-miR-20a-5p
 
 
-0.404
 
 
28.0
 
 
 
 
APCDD1
 
 
hsa-miR-106b-5p
 
 
-0.404
 
 
28.0
 
 
 
 
APCDD1
 
 
hsa-miR-106a-5p
 
 
-0.422
 
 
28.0
 
 
 
 
APCDD1
 
 
hsa-miR-17-5p
 
 
-0.425
 
 
28.0
 
 
 
 
APCDD1
 
 
hsa-miR-20b-5p
 
 
-0.425
 
 
28.0
 
 
 
 
ARF4
 
 
hsa-miR-7-5p
 
 
-0.407
 
 
61.3
 
 
 
 
ARF4
 
 
hsa-miR-148a-3p
 
 
-0.487
 
 
61.3
 
 
 
 
ARHGAP1
 
 
hsa-miR-17-5p
 
 
-0.325
 
 
48.9
 
 
 
 
ARHGAP1
 
 
hsa-miR-20b-5p
 
 
-0.325
 
 
48.9
 
 
 
 
ARHGAP1
 
 
hsa-miR-106a-5p
 
 
-0.329
 
 
48.9
 
 
 
 
ARHGAP1
 
 
hsa-miR-20a-5p
 
 
-0.339
 
 
48.9
 
 
 
 
ARHGAP1
 
 
hsa-miR-106b-5p
 
 
-0.339
 
 
48.9
 
 
 
 
ARHGAP21
 
 
hsa-miR-148a-3p
 
 
-0.287
 
 
50.4
 
 
 
 
ARL1
 
 
hsa-miR-20a-5p
 
 
-0.403
 
 
24.0
 
 
 
 
ARL1
 
 
hsa-miR-106b-5p
 
 
-0.411
 
 
24.0
 
 
 
 
ARL1
 
 
hsa-miR-20b-5p
 
 
-0.424
 
 
24.0
 
 
 
 
ARL1
 
 
hsa-miR-106a-5p
 
 
-0.429
 
 
24.0
 
 
 
 
ARL1
 
 
hsa-miR-17-5p
 
 
-0.432
 
 
24.0
 
 
 
 
ARL6IP1
 
 
hsa-miR-7-5p
 
 
-0.374
 
 
196.8
 
 
 
 
ARL6IP1
 
 
hsa-miR-148a-3p
 
 
-0.579
 
 
196.8
 
 
 
 
ARL8B
 
 
hsa-miR-148a-3p
 
 
-0.337
 
 
53.8
 
 
 
 
ARMC8
 
 
hsa-miR-20a-5p
 
 
-0.357
 
 
13.3
 
 
 
 
ARMC8
 
 
hsa-miR-106b-5p
 
 
-0.357
 
 
13.3
 
 
 
 
ARMC8
 
 
hsa-miR-106a-5p
 
 
-0.375
 
 
13.3
 
 
 
 
ARMC8
 
 
hsa-miR-17-5p
 
 
-0.378
 
 
13.3
 
 
 
 
ARMC8
 
 
hsa-miR-20b-5p
 
 
-0.378
 
 
13.3
 
 
 
 
ARMCX1
 
 
hsa-miR-21-5p
 
 
-0.489
 
 
17.0
 
 
 
 
ARPP19
 
 
hsa-let-7c-5p
 
 
-0.492
 
 
120.9
 
 
 
 
ARPP19
 
 
hsa-miR-98-5p
 
 
-0.5
 
 
120.9
 
 
 
 
ARRDC3
 
 
hsa-miR-148a-3p
 
 
-0.305
 
 
11.5
 
 
 
 
ARRDC4
 
 
hsa-let-7c-5p
 
 
-0.329
 
 
23.9
 
 
 
 
ARRDC4
 
 
hsa-miR-98-5p
 
 
-0.329
 
 
23.9
 
 
 
 
ASAP1
 
 
hsa-miR-98-5p
 
 
-0.308
 
 
10.8
 
 
 
 
ASAP1
 
 
hsa-let-7c-5p
 
 
-0.308
 
 
10.8
 
 
 
 
ASF1A
 
 
hsa-miR-21-5p
 
 
-0.304
 
 
11.7
 
 
 
 
ASPH
 
 
hsa-miR-148a-3p
 
 
-0.391
 
 
45.5
 
 
 
 
ATOSB
 
 
hsa-miR-98-5p
 
 
-0.429
 
 
22.4
 
 
 
 
ATOSB
 
 
hsa-let-7c-5p
 
 
-0.429
 
 
22.4
 
 
 
 
ATP2A2
 
 
hsa-miR-98-5p
 
 
-0.311
 
 
16.6
 
 
 
 
ATP2A2
 
 
hsa-let-7c-5p
 
 
-0.311
 
 
16.6
 
 
 
 
ATP2B2
 
 
hsa-miR-7-5p
 
 
-0.297
 
 
19.0
 
 
 
 
ATP6AP2
 
 
hsa-miR-148a-3p
 
 
-0.592
 
 
69.0
 
 
 
 
ATP6AP2
 
 
hsa-miR-148a-3p
 
 
-0.668
 
 
69.0
 
 
 
 
ATPAF1
 
 
hsa-miR-98-5p
 
 
-0.498
 
 
54.4
 
 
 
 
ATPAF1
 
 
hsa-let-7c-5p
 
 
-0.498
 
 
54.4
 
 
 
 
AVPI1
 
 
hsa-miR-7-5p
 
 
-0.367
 
 
14.1
 
 
 
 
B3GAT3
 
 
hsa-miR-98-5p
 
 
-0.324
 
 
64.6
 
 
 
 
B3GAT3
 
 
hsa-let-7c-5p
 
 
-0.324
 
 
64.6
 
 
 
 
B4GALT5
 
 
hsa-miR-148a-3p
 
 
-0.44
 
 
10.8
 
 
 
 
B4GAT1
 
 
hsa-let-7c-5p
 
 
-0.47
 
 
117.5
 
 
 
 
B4GAT1
 
 
hsa-miR-98-5p
 
 
-0.47
 
 
117.5
 
 
 
 
BAMBI
 
 
hsa-miR-20a-5p
 
 
-0.485
 
 
14.9
 
 
 
 
BAMBI
 
 
hsa-miR-106b-5p
 
 
-0.493
 
 
14.9
 
 
 
 
BAMBI
 
 
hsa-miR-106a-5p
 
 
-0.503
 
 
14.9
 
 
 
 
BAMBI
 
 
hsa-miR-17-5p
 
 
-0.506
 
 
14.9
 
 
 
 
BAMBI
 
 
hsa-miR-20b-5p
 
 
-0.506
 
 
14.9
 
 
 
 
BEGAIN
 
 
hsa-let-7c-5p
 
 
-0.466
 
 
36.8
 
 
 
 
BEGAIN
 
 
hsa-miR-98-5p
 
 
-0.474
 
 
36.8
 
 
 
 
BHLHE41
 
 
hsa-miR-148a-3p
 
 
-0.442
 
 
27.9
 
 
 
 
BIN3
 
 
hsa-miR-98-5p
 
 
-0.427
 
 
10.9
 
 
 
 
BIN3
 
 
hsa-let-7c-5p
 
 
-0.427
 
 
10.9
 
 
 
 
BLOC1S6
 
 
hsa-miR-98-5p
 
 
-0.321
 
 
18.3
 
 
 
 
BLOC1S6
 
 
hsa-let-7c-5p
 
 
-0.321
 
 
18.3
 
 
 
 
BNIP3L
 
 
hsa-miR-20a-5p
 
 
-0.287
 
 
54.0
 
 
 
 
BNIP3L
 
 
hsa-miR-106b-5p
 
 
-0.298
 
 
54.0
 
 
 
 
 
 
 kidney 
 
 
 
 
target_symbol
 
 
mature_mirna_id
 
 
score
 
 
nTPM
 
 
 
 
 
 
ABCD3
 
 
hsa-miR-148a-3p
 
 
-0.388
 
 
55.5
 
 
 
 
ABHD17C
 
 
hsa-let-7c-5p
 
 
-0.359
 
 
13.6
 
 
 
 
ABHD17C
 
 
hsa-miR-98-5p
 
 
-0.383
 
 
13.6
 
 
 
 
ABHD5
 
 
hsa-miR-20a-5p
 
 
-0.397
 
 
10.4
 
 
 
 
ABHD5
 
 
hsa-miR-106b-5p
 
 
-0.413
 
 
10.4
 
 
 
 
ABHD5
 
 
hsa-miR-20b-5p
 
 
-0.418
 
 
10.4
 
 
 
 
ABHD5
 
 
hsa-miR-106a-5p
 
 
-0.447
 
 
10.4
 
 
 
 
ABHD5
 
 
hsa-miR-17-5p
 
 
-0.45
 
 
10.4
 
 
 
 
ACP3
 
 
hsa-miR-98-5p
 
 
-0.298
 
 
26.9
 
 
 
 
ACP3
 
 
hsa-let-7c-5p
 
 
-0.298
 
 
26.9
 
 
 
 
ACVR1
 
 
hsa-miR-148a-3p
 
 
-0.397
 
 
15.0
 
 
 
 
ADAM10
 
 
hsa-miR-148a-3p
 
 
-0.356
 
 
16.8
 
 
 
 
ADAM9
 
 
hsa-miR-20a-5p
 
 
-0.299
 
 
32.6
 
 
 
 
ADAM9
 
 
hsa-miR-106b-5p
 
 
-0.299
 
 
32.6
 
 
 
 
ADAM9
 
 
hsa-miR-106a-5p
 
 
-0.317
 
 
32.6
 
 
 
 
ADAM9
 
 
hsa-miR-17-5p
 
 
-0.32
 
 
32.6
 
 
 
 
ADAM9
 
 
hsa-miR-20b-5p
 
 
-0.328
 
 
32.6
 
 
 
 
ADGRF5
 
 
hsa-miR-148a-3p
 
 
-0.354
 
 
55.8
 
 
 
 
AGFG1
 
 
hsa-miR-148a-3p
 
 
-0.332
 
 
30.6
 
 
 
 
AGFG2
 
 
hsa-miR-20a-5p
 
 
-0.365
 
 
15.9
 
 
 
 
AGFG2
 
 
hsa-miR-20b-5p
 
 
-0.386
 
 
15.9
 
 
 
 
AGFG2
 
 
hsa-miR-106b-5p
 
 
-0.397
 
 
15.9
 
 
 
 
AGFG2
 
 
hsa-miR-106a-5p
 
 
-0.399
 
 
15.9
 
 
 
 
AGFG2
 
 
hsa-miR-17-5p
 
 
-0.402
 
 
15.9
 
 
 
 
AHDC1
 
 
hsa-miR-148a-3p
 
 
-0.413
 
 
12.2
 
 
 
 
AHNAK
 
 
hsa-miR-20a-5p
 
 
-0.316
 
 
55.7
 
 
 
 
AHNAK
 
 
hsa-miR-106b-5p
 
 
-0.316
 
 
55.7
 
 
 
 
AHNAK
 
 
hsa-miR-106a-5p
 
 
-0.334
 
 
55.7
 
 
 
 
AHNAK
 
 
hsa-miR-17-5p
 
 
-0.337
 
 
55.7
 
 
 
 
AHNAK
 
 
hsa-miR-20b-5p
 
 
-0.337
 
 
55.7
 
 
 
 
AIFM1
 
 
hsa-miR-98-5p
 
 
-0.314
 
 
69.0
 
 
 
 
AIFM1
 
 
hsa-let-7c-5p
 
 
-0.314
 
 
69.0
 
 
 
 
AKAP1
 
 
hsa-miR-148a-3p
 
 
-0.381
 
 
24.5
 
 
 
 
ALCAM
 
 
hsa-miR-148a-3p
 
 
-0.339
 
 
20.8
 
 
 
 
ALDH4A1
 
 
hsa-miR-184
 
 
-0.421
 
 
204.2
 
 
 
 
ALG3
 
 
hsa-miR-106b-5p
 
 
-0.551
 
 
17.8
 
 
 
 
ALG3
 
 
hsa-miR-20a-5p
 
 
-0.559
 
 
17.8
 
 
 
 
ALG3
 
 
hsa-miR-106a-5p
 
 
-0.569
 
 
17.8
 
 
 
 
ALG3
 
 
hsa-miR-17-5p
 
 
-0.572
 
 
17.8
 
 
 
 
ALG3
 
 
hsa-miR-20b-5p
 
 
-0.58
 
 
17.8
 
 
 
 
AMT
 
 
hsa-miR-98-5p
 
 
-0.463
 
 
23.7
 
 
 
 
AMT
 
 
hsa-let-7c-5p
 
 
-0.463
 
 
23.7
 
 
 
 
ANKRD12
 
 
hsa-miR-7-5p
 
 
-0.37
 
 
12.1
 
 
 
 
ANKRD46
 
 
hsa-miR-98-5p
 
 
-0.389
 
 
30.5
 
 
 
 
ANKRD46
 
 
hsa-let-7c-5p
 
 
-0.389
 
 
30.5
 
 
 
 
ANKRD9
 
 
hsa-miR-20a-5p
 
 
-0.31
 
 
22.8
 
 
 
 
ANKRD9
 
 
hsa-miR-106b-5p
 
 
-0.318
 
 
22.8
 
 
 
 
ANKRD9
 
 
hsa-miR-20b-5p
 
 
-0.331
 
 
22.8
 
 
 
 
ANKRD9
 
 
hsa-miR-106a-5p
 
 
-0.344
 
 
22.8
 
 
 
 
ANKRD9
 
 
hsa-miR-17-5p
 
 
-0.347
 
 
22.8
 
 
 
 
AP1AR
 
 
hsa-miR-21-5p
 
 
-0.318
 
 
20.6
 
 
 
 
AP1S1
 
 
hsa-miR-98-5p
 
 
-0.374
 
 
48.3
 
 
 
 
AP1S1
 
 
hsa-let-7c-5p
 
 
-0.374
 
 
48.3
 
 
 
 
ARF4
 
 
hsa-miR-7-5p
 
 
-0.407
 
 
106.7
 
 
 
 
ARF4
 
 
hsa-miR-148a-3p
 
 
-0.487
 
 
106.7
 
 
 
 
ARFGEF1
 
 
hsa-miR-148a-3p
 
 
-0.306
 
 
21.3
 
 
 
 
ARFIP1
 
 
hsa-miR-148a-3p
 
 
-0.474
 
 
18.5
 
 
 
 
ARG2
 
 
hsa-miR-98-5p
 
 
-0.468
 
 
66.0
 
 
 
 
ARG2
 
 
hsa-let-7c-5p
 
 
-0.468
 
 
66.0
 
 
 
 
ARHGAP1
 
 
hsa-miR-17-5p
 
 
-0.325
 
 
38.9
 
 
 
 
ARHGAP1
 
 
hsa-miR-20b-5p
 
 
-0.325
 
 
38.9
 
 
 
 
ARHGAP1
 
 
hsa-miR-106a-5p
 
 
-0.329
 
 
38.9
 
 
 
 
ARHGAP1
 
 
hsa-miR-20a-5p
 
 
-0.339
 
 
38.9
 
 
 
 
ARHGAP1
 
 
hsa-miR-106b-5p
 
 
-0.339
 
 
38.9
 
 
 
 
ARHGAP21
 
 
hsa-miR-148a-3p
 
 
-0.287
 
 
10.2
 
 
 
 
ARHGAP24
 
 
hsa-miR-21-5p
 
 
-0.427
 
 
93.2
 
 
 
 
ARHGEF28
 
 
hsa-miR-20b-5p
 
 
-0.352
 
 
29.5
 
 
 
 
ARHGEF28
 
 
hsa-miR-17-5p
 
 
-0.363
 
 
29.5
 
 
 
 
ARHGEF28
 
 
hsa-miR-20a-5p
 
 
-0.366
 
 
29.5
 
 
 
 
ARHGEF28
 
 
hsa-miR-106a-5p
 
 
-0.367
 
 
29.5
 
 
 
 
ARHGEF28
 
 
hsa-miR-106b-5p
 
 
-0.377
 
 
29.5
 
 
 
 
ARL1
 
 
hsa-miR-20a-5p
 
 
-0.403
 
 
71.4
 
 
 
 
ARL1
 
 
hsa-miR-106b-5p
 
 
-0.411
 
 
71.4
 
 
 
 
ARL1
 
 
hsa-miR-20b-5p
 
 
-0.424
 
 
71.4
 
 
 
 
ARL1
 
 
hsa-miR-106a-5p
 
 
-0.429
 
 
71.4
 
 
 
 
ARL1
 
 
hsa-miR-17-5p
 
 
-0.432
 
 
71.4
 
 
 
 
ARL4D
 
 
hsa-miR-98-5p
 
 
-0.417
 
 
35.1
 
 
 
 
ARL4D
 
 
hsa-let-7c-5p
 
 
-0.417
 
 
35.1
 
 
 
 
ARL6IP1
 
 
hsa-miR-7-5p
 
 
-0.374
 
 
140.2
 
 
 
 
ARL6IP1
 
 
hsa-miR-148a-3p
 
 
-0.579
 
 
140.2
 
 
 
 
ARL8B
 
 
hsa-miR-148a-3p
 
 
-0.337
 
 
50.2
 
 
 
 
ARMC8
 
 
hsa-miR-20a-5p
 
 
-0.357
 
 
17.6
 
 
 
 
ARMC8
 
 
hsa-miR-106b-5p
 
 
-0.357
 
 
17.6
 
 
 
 
ARMC8
 
 
hsa-miR-106a-5p
 
 
-0.375
 
 
17.6
 
 
 
 
ARMC8
 
 
hsa-miR-17-5p
 
 
-0.378
 
 
17.6
 
 
 
 
ARMC8
 
 
hsa-miR-20b-5p
 
 
-0.378
 
 
17.6
 
 
 
 
ARMCX1
 
 
hsa-miR-21-5p
 
 
-0.489
 
 
12.8
 
 
 
 
ARMT1
 
 
hsa-let-7c-5p
 
 
-0.303
 
 
38.7
 
 
 
 
ARMT1
 
 
hsa-miR-98-5p
 
 
-0.303
 
 
38.7
 
 
 
 
ARPP19
 
 
hsa-let-7c-5p
 
 
-0.492
 
 
52.6
 
 
 
 
ARPP19
 
 
hsa-miR-98-5p
 
 
-0.5
 
 
52.6
 
 
 
 
ARRDC3
 
 
hsa-miR-148a-3p
 
 
-0.305
 
 
49.3
 
 
 
 
ARRDC4
 
 
hsa-let-7c-5p
 
 
-0.329
 
 
23.9
 
 
 
 
ARRDC4
 
 
hsa-miR-98-5p
 
 
-0.329
 
 
23.9
 
 
 
 
ASAP1
 
 
hsa-miR-98-5p
 
 
-0.308
 
 
10.1
 
 
 
 
ASAP1
 
 
hsa-let-7c-5p
 
 
-0.308
 
 
10.1
 
 
 
 
ASF1A
 
 
hsa-miR-21-5p
 
 
-0.304
 
 
11.8
 
 
 
 
ASPH
 
 
hsa-miR-148a-3p
 
 
-0.391
 
 
55.2
 
 
 
 
ATG4A
 
 
hsa-miR-7-5p
 
 
-0.448
 
 
13.1
 
 
 
 
ATG4D
 
 
hsa-miR-148a-3p
 
 
-0.303
 
 
19.9
 
 
 
 
 
 
 lung 
 
 
 
 
target_symbol
 
 
mature_mirna_id
 
 
score
 
 
nTPM
 
 
 
 
 
 
ABCD3
 
 
hsa-miR-148a-3p
 
 
-0.388
 
 
22.0
 
 
 
 
ABHD17C
 
 
hsa-let-7c-5p
 
 
-0.359
 
 
18.6
 
 
 
 
ABHD17C
 
 
hsa-miR-98-5p
 
 
-0.383
 
 
18.6
 
 
 
 
ABHD5
 
 
hsa-miR-20a-5p
 
 
-0.397
 
 
11.2
 
 
 
 
ABHD5
 
 
hsa-miR-106b-5p
 
 
-0.413
 
 
11.2
 
 
 
 
ABHD5
 
 
hsa-miR-20b-5p
 
 
-0.418
 
 
11.2
 
 
 
 
ABHD5
 
 
hsa-miR-106a-5p
 
 
-0.447
 
 
11.2
 
 
 
 
ABHD5
 
 
hsa-miR-17-5p
 
 
-0.45
 
 
11.2
 
 
 
 
ACVR1
 
 
hsa-miR-148a-3p
 
 
-0.397
 
 
12.2
 
 
 
 
ADAM10
 
 
hsa-miR-148a-3p
 
 
-0.356
 
 
19.7
 
 
 
 
ADAM17
 
 
hsa-miR-148a-3p
 
 
-0.301
 
 
12.5
 
 
 
 
ADAM9
 
 
hsa-miR-20a-5p
 
 
-0.299
 
 
42.9
 
 
 
 
ADAM9
 
 
hsa-miR-106b-5p
 
 
-0.299
 
 
42.9
 
 
 
 
ADAM9
 
 
hsa-miR-106a-5p
 
 
-0.317
 
 
42.9
 
 
 
 
ADAM9
 
 
hsa-miR-17-5p
 
 
-0.32
 
 
42.9
 
 
 
 
ADAM9
 
 
hsa-miR-20b-5p
 
 
-0.328
 
 
42.9
 
 
 
 
ADAMTS8
 
 
hsa-miR-98-5p
 
 
-0.571
 
 
33.2
 
 
 
 
ADAMTS8
 
 
hsa-let-7c-5p
 
 
-0.571
 
 
33.2
 
 
 
 
ADGRF5
 
 
hsa-miR-148a-3p
 
 
-0.354
 
 
99.9
 
 
 
 
ADRB1
 
 
hsa-let-7c-5p
 
 
-0.351
 
 
11.4
 
 
 
 
ADRB1
 
 
hsa-miR-98-5p
 
 
-0.367
 
 
11.4
 
 
 
 
ADRB2
 
 
hsa-let-7c-5p
 
 
-0.635
 
 
18.4
 
 
 
 
ADRB2
 
 
hsa-miR-98-5p
 
 
-0.635
 
 
18.4
 
 
 
 
AGFG1
 
 
hsa-miR-148a-3p
 
 
-0.332
 
 
51.7
 
 
 
 
AHDC1
 
 
hsa-miR-148a-3p
 
 
-0.413
 
 
11.4
 
 
 
 
AHNAK
 
 
hsa-miR-20a-5p
 
 
-0.316
 
 
135.8
 
 
 
 
AHNAK
 
 
hsa-miR-106b-5p
 
 
-0.316
 
 
135.8
 
 
 
 
AHNAK
 
 
hsa-miR-106a-5p
 
 
-0.334
 
 
135.8
 
 
 
 
AHNAK
 
 
hsa-miR-17-5p
 
 
-0.337
 
 
135.8
 
 
 
 
AHNAK
 
 
hsa-miR-20b-5p
 
 
-0.337
 
 
135.8
 
 
 
 
AKAP1
 
 
hsa-miR-148a-3p
 
 
-0.381
 
 
18.4
 
 
 
 
ALCAM
 
 
hsa-miR-148a-3p
 
 
-0.339
 
 
69.9
 
 
 
 
ALG3
 
 
hsa-miR-106b-5p
 
 
-0.551
 
 
17.4
 
 
 
 
ALG3
 
 
hsa-miR-20a-5p
 
 
-0.559
 
 
17.4
 
 
 
 
ALG3
 
 
hsa-miR-106a-5p
 
 
-0.569
 
 
17.4
 
 
 
 
ALG3
 
 
hsa-miR-17-5p
 
 
-0.572
 
 
17.4
 
 
 
 
ALG3
 
 
hsa-miR-20b-5p
 
 
-0.58
 
 
17.4
 
 
 
 
ANKRD12
 
 
hsa-miR-7-5p
 
 
-0.37
 
 
10.9
 
 
 
 
ANKRD29
 
 
hsa-miR-20a-5p
 
 
-0.359
 
 
29.5
 
 
 
 
ANKRD29
 
 
hsa-miR-106b-5p
 
 
-0.359
 
 
29.5
 
 
 
 
ANKRD29
 
 
hsa-miR-106a-5p
 
 
-0.369
 
 
29.5
 
 
 
 
ANKRD29
 
 
hsa-miR-17-5p
 
 
-0.372
 
 
29.5
 
 
 
 
ANKRD29
 
 
hsa-miR-20b-5p
 
 
-0.38
 
 
29.5
 
 
 
 
ANKRD46
 
 
hsa-miR-98-5p
 
 
-0.389
 
 
10.1
 
 
 
 
ANKRD46
 
 
hsa-let-7c-5p
 
 
-0.389
 
 
10.1
 
 
 
 
ANKRD9
 
 
hsa-miR-20a-5p
 
 
-0.31
 
 
15.3
 
 
 
 
ANKRD9
 
 
hsa-miR-106b-5p
 
 
-0.318
 
 
15.3
 
 
 
 
ANKRD9
 
 
hsa-miR-20b-5p
 
 
-0.331
 
 
15.3
 
 
 
 
ANKRD9
 
 
hsa-miR-106a-5p
 
 
-0.344
 
 
15.3
 
 
 
 
ANKRD9
 
 
hsa-miR-17-5p
 
 
-0.347
 
 
15.3
 
 
 
 
AP1S1
 
 
hsa-miR-98-5p
 
 
-0.374
 
 
15.2
 
 
 
 
AP1S1
 
 
hsa-let-7c-5p
 
 
-0.374
 
 
15.2
 
 
 
 
APCDD1
 
 
hsa-miR-20a-5p
 
 
-0.404
 
 
12.6
 
 
 
 
APCDD1
 
 
hsa-miR-106b-5p
 
 
-0.404
 
 
12.6
 
 
 
 
APCDD1
 
 
hsa-miR-106a-5p
 
 
-0.422
 
 
12.6
 
 
 
 
APCDD1
 
 
hsa-miR-17-5p
 
 
-0.425
 
 
12.6
 
 
 
 
APCDD1
 
 
hsa-miR-20b-5p
 
 
-0.425
 
 
12.6
 
 
 
 
ARF4
 
 
hsa-miR-7-5p
 
 
-0.407
 
 
116.3
 
 
 
 
ARF4
 
 
hsa-miR-148a-3p
 
 
-0.487
 
 
116.3
 
 
 
 
ARFGEF1
 
 
hsa-miR-148a-3p
 
 
-0.306
 
 
13.0
 
 
 
 
ARFIP1
 
 
hsa-miR-148a-3p
 
 
-0.474
 
 
15.8
 
 
 
 
ARHGAP1
 
 
hsa-miR-17-5p
 
 
-0.325
 
 
36.0
 
 
 
 
ARHGAP1
 
 
hsa-miR-20b-5p
 
 
-0.325
 
 
36.0
 
 
 
 
ARHGAP1
 
 
hsa-miR-106a-5p
 
 
-0.329
 
 
36.0
 
 
 
 
ARHGAP1
 
 
hsa-miR-20a-5p
 
 
-0.339
 
 
36.0
 
 
 
 
ARHGAP1
 
 
hsa-miR-106b-5p
 
 
-0.339
 
 
36.0
 
 
 
 
ARHGAP21
 
 
hsa-miR-148a-3p
 
 
-0.287
 
 
20.4
 
 
 
 
ARHGAP24
 
 
hsa-miR-21-5p
 
 
-0.427
 
 
14.8
 
 
 
 
ARID4B
 
 
hsa-miR-20a-5p
 
 
-0.29
 
 
11.3
 
 
 
 
ARID4B
 
 
hsa-miR-106b-5p
 
 
-0.29
 
 
11.3
 
 
 
 
ARL1
 
 
hsa-miR-20a-5p
 
 
-0.403
 
 
34.4
 
 
 
 
ARL1
 
 
hsa-miR-106b-5p
 
 
-0.411
 
 
34.4
 
 
 
 
ARL1
 
 
hsa-miR-20b-5p
 
 
-0.424
 
 
34.4
 
 
 
 
ARL1
 
 
hsa-miR-106a-5p
 
 
-0.429
 
 
34.4
 
 
 
 
ARL1
 
 
hsa-miR-17-5p
 
 
-0.432
 
 
34.4
 
 
 
 
ARL4D
 
 
hsa-miR-98-5p
 
 
-0.417
 
 
16.0
 
 
 
 
ARL4D
 
 
hsa-let-7c-5p
 
 
-0.417
 
 
16.0
 
 
 
 
ARL6IP1
 
 
hsa-miR-7-5p
 
 
-0.374
 
 
75.1
 
 
 
 
ARL6IP1
 
 
hsa-miR-148a-3p
 
 
-0.579
 
 
75.1
 
 
 
 
ARL6IP6
 
 
hsa-miR-98-5p
 
 
-0.293
 
 
11.9
 
 
 
 
ARL6IP6
 
 
hsa-let-7c-5p
 
 
-0.293
 
 
11.9
 
 
 
 
ARL8B
 
 
hsa-miR-148a-3p
 
 
-0.337
 
 
41.1
 
 
 
 
ARMC8
 
 
hsa-miR-20a-5p
 
 
-0.357
 
 
15.4
 
 
 
 
ARMC8
 
 
hsa-miR-106b-5p
 
 
-0.357
 
 
15.4
 
 
 
 
ARMC8
 
 
hsa-miR-106a-5p
 
 
-0.375
 
 
15.4
 
 
 
 
ARMC8
 
 
hsa-miR-17-5p
 
 
-0.378
 
 
15.4
 
 
 
 
ARMC8
 
 
hsa-miR-20b-5p
 
 
-0.378
 
 
15.4
 
 
 
 
ARMCX1
 
 
hsa-miR-21-5p
 
 
-0.489
 
 
15.7
 
 
 
 
ARMT1
 
 
hsa-let-7c-5p
 
 
-0.303
 
 
18.4
 
 
 
 
ARMT1
 
 
hsa-miR-98-5p
 
 
-0.303
 
 
18.4
 
 
 
 
ARPP19
 
 
hsa-let-7c-5p
 
 
-0.492
 
 
42.5
 
 
 
 
ARPP19
 
 
hsa-miR-98-5p
 
 
-0.5
 
 
42.5
 
 
 
 
ARRDC3
 
 
hsa-miR-148a-3p
 
 
-0.305
 
 
42.0
 
 
 
 
ARRDC4
 
 
hsa-let-7c-5p
 
 
-0.329
 
 
18.7
 
 
 
 
ARRDC4
 
 
hsa-miR-98-5p
 
 
-0.329
 
 
18.7
 
 
 
 
ASAP1
 
 
hsa-miR-98-5p
 
 
-0.308
 
 
27.6
 
 
 
 
ASAP1
 
 
hsa-let-7c-5p
 
 
-0.308
 
 
27.6
 
 
 
 
ASF1A
 
 
hsa-miR-21-5p
 
 
-0.304
 
 
12.6
 
 
 
 
ASPH
 
 
hsa-miR-148a-3p
 
 
-0.391
 
 
69.4
 
 
 
 
ATG4D
 
 
hsa-miR-148a-3p
 
 
-0.303
 
 
10.3
 
 
 
 
 
 
 pancreas 
 
 
 
 
target_symbol
 
 
mature_mirna_id
 
 
score
 
 
nTPM
 
 
 
 
 
 
ABCD3
 
 
hsa-miR-148a-3p
 
 
-0.388
 
 
27.0
 
 
 
 
ABHD5
 
 
hsa-miR-20a-5p
 
 
-0.397
 
 
11.4
 
 
 
 
ABHD5
 
 
hsa-miR-106b-5p
 
 
-0.413
 
 
11.4
 
 
 
 
ABHD5
 
 
hsa-miR-20b-5p
 
 
-0.418
 
 
11.4
 
 
 
 
ABHD5
 
 
hsa-miR-106a-5p
 
 
-0.447
 
 
11.4
 
 
 
 
ABHD5
 
 
hsa-miR-17-5p
 
 
-0.45
 
 
11.4
 
 
 
 
ACTC1
 
 
hsa-miR-7-5p
 
 
-0.471
 
 
12.1
 
 
 
 
AGFG1
 
 
hsa-miR-148a-3p
 
 
-0.332
 
 
33.0
 
 
 
 
AGFG2
 
 
hsa-miR-20a-5p
 
 
-0.365
 
 
17.7
 
 
 
 
AGFG2
 
 
hsa-miR-20b-5p
 
 
-0.386
 
 
17.7
 
 
 
 
AGFG2
 
 
hsa-miR-106b-5p
 
 
-0.397
 
 
17.7
 
 
 
 
AGFG2
 
 
hsa-miR-106a-5p
 
 
-0.399
 
 
17.7
 
 
 
 
AGFG2
 
 
hsa-miR-17-5p
 
 
-0.402
 
 
17.7
 
 
 
 
AHNAK
 
 
hsa-miR-20a-5p
 
 
-0.316
 
 
28.6
 
 
 
 
AHNAK
 
 
hsa-miR-106b-5p
 
 
-0.316
 
 
28.6
 
 
 
 
AHNAK
 
 
hsa-miR-106a-5p
 
 
-0.334
 
 
28.6
 
 
 
 
AHNAK
 
 
hsa-miR-17-5p
 
 
-0.337
 
 
28.6
 
 
 
 
AHNAK
 
 
hsa-miR-20b-5p
 
 
-0.337
 
 
28.6
 
 
 
 
AIFM1
 
 
hsa-miR-98-5p
 
 
-0.314
 
 
20.4
 
 
 
 
AIFM1
 
 
hsa-let-7c-5p
 
 
-0.314
 
 
20.4
 
 
 
 
AKAP1
 
 
hsa-miR-148a-3p
 
 
-0.381
 
 
21.5
 
 
 
 
ALCAM
 
 
hsa-miR-148a-3p
 
 
-0.339
 
 
30.8
 
 
 
 
ALG3
 
 
hsa-miR-106b-5p
 
 
-0.551
 
 
35.7
 
 
 
 
ALG3
 
 
hsa-miR-20a-5p
 
 
-0.559
 
 
35.7
 
 
 
 
ALG3
 
 
hsa-miR-106a-5p
 
 
-0.569
 
 
35.7
 
 
 
 
ALG3
 
 
hsa-miR-17-5p
 
 
-0.572
 
 
35.7
 
 
 
 
ALG3
 
 
hsa-miR-20b-5p
 
 
-0.58
 
 
35.7
 
 
 
 
ALG9
 
 
hsa-miR-7-5p
 
 
-0.302
 
 
18.1
 
 
 
 
AMT
 
 
hsa-miR-98-5p
 
 
-0.463
 
 
21.5
 
 
 
 
AMT
 
 
hsa-let-7c-5p
 
 
-0.463
 
 
21.5
 
 
 
 
ANKRD12
 
 
hsa-miR-7-5p
 
 
-0.37
 
 
11.2
 
 
 
 
ANKRD46
 
 
hsa-miR-98-5p
 
 
-0.389
 
 
17.5
 
 
 
 
ANKRD46
 
 
hsa-let-7c-5p
 
 
-0.389
 
 
17.5
 
 
 
 
ANKRD9
 
 
hsa-miR-20a-5p
 
 
-0.31
 
 
10.7
 
 
 
 
ANKRD9
 
 
hsa-miR-106b-5p
 
 
-0.318
 
 
10.7
 
 
 
 
ANKRD9
 
 
hsa-miR-20b-5p
 
 
-0.331
 
 
10.7
 
 
 
 
ANKRD9
 
 
hsa-miR-106a-5p
 
 
-0.344
 
 
10.7
 
 
 
 
ANKRD9
 
 
hsa-miR-17-5p
 
 
-0.347
 
 
10.7
 
 
 
 
AP1S1
 
 
hsa-miR-98-5p
 
 
-0.374
 
 
23.9
 
 
 
 
AP1S1
 
 
hsa-let-7c-5p
 
 
-0.374
 
 
23.9
 
 
 
 
ARF4
 
 
hsa-miR-7-5p
 
 
-0.407
 
 
138.3
 
 
 
 
ARF4
 
 
hsa-miR-148a-3p
 
 
-0.487
 
 
138.3
 
 
 
 
ARFGEF1
 
 
hsa-miR-148a-3p
 
 
-0.306
 
 
16.5
 
 
 
 
ARHGAP1
 
 
hsa-miR-17-5p
 
 
-0.325
 
 
34.7
 
 
 
 
ARHGAP1
 
 
hsa-miR-20b-5p
 
 
-0.325
 
 
34.7
 
 
 
 
ARHGAP1
 
 
hsa-miR-106a-5p
 
 
-0.329
 
 
34.7
 
 
 
 
ARHGAP1
 
 
hsa-miR-20a-5p
 
 
-0.339
 
 
34.7
 
 
 
 
ARHGAP1
 
 
hsa-miR-106b-5p
 
 
-0.339
 
 
34.7
 
 
 
 
ARHGAP21
 
 
hsa-miR-148a-3p
 
 
-0.287
 
 
13.3
 
 
 
 
ARHGAP24
 
 
hsa-miR-21-5p
 
 
-0.427
 
 
28.4
 
 
 
 
ARHGEF28
 
 
hsa-miR-20b-5p
 
 
-0.352
 
 
19.9
 
 
 
 
ARHGEF28
 
 
hsa-miR-17-5p
 
 
-0.363
 
 
19.9
 
 
 
 
ARHGEF28
 
 
hsa-miR-20a-5p
 
 
-0.366
 
 
19.9
 
 
 
 
ARHGEF28
 
 
hsa-miR-106a-5p
 
 
-0.367
 
 
19.9
 
 
 
 
ARHGEF28
 
 
hsa-miR-106b-5p
 
 
-0.377
 
 
19.9
 
 
 
 
ARID4B
 
 
hsa-miR-20a-5p
 
 
-0.29
 
 
10.7
 
 
 
 
ARID4B
 
 
hsa-miR-106b-5p
 
 
-0.29
 
 
10.7
 
 
 
 
ARL1
 
 
hsa-miR-20a-5p
 
 
-0.403
 
 
41.8
 
 
 
 
ARL1
 
 
hsa-miR-106b-5p
 
 
-0.411
 
 
41.8
 
 
 
 
ARL1
 
 
hsa-miR-20b-5p
 
 
-0.424
 
 
41.8
 
 
 
 
ARL1
 
 
hsa-miR-106a-5p
 
 
-0.429
 
 
41.8
 
 
 
 
ARL1
 
 
hsa-miR-17-5p
 
 
-0.432
 
 
41.8
 
 
 
 
ARL6IP1
 
 
hsa-miR-7-5p
 
 
-0.374
 
 
77.1
 
 
 
 
ARL6IP1
 
 
hsa-miR-148a-3p
 
 
-0.579
 
 
77.1
 
 
 
 
ARL8B
 
 
hsa-miR-148a-3p
 
 
-0.337
 
 
26.7
 
 
 
 
ARMC8
 
 
hsa-miR-20a-5p
 
 
-0.357
 
 
13.4
 
 
 
 
ARMC8
 
 
hsa-miR-106b-5p
 
 
-0.357
 
 
13.4
 
 
 
 
ARMC8
 
 
hsa-miR-106a-5p
 
 
-0.375
 
 
13.4
 
 
 
 
ARMC8
 
 
hsa-miR-17-5p
 
 
-0.378
 
 
13.4
 
 
 
 
ARMC8
 
 
hsa-miR-20b-5p
 
 
-0.378
 
 
13.4
 
 
 
 
ARMCX1
 
 
hsa-miR-21-5p
 
 
-0.489
 
 
12.1
 
 
 
 
ARPP19
 
 
hsa-let-7c-5p
 
 
-0.492
 
 
43.6
 
 
 
 
ARPP19
 
 
hsa-miR-98-5p
 
 
-0.5
 
 
43.6
 
 
 
 
ARRDC3
 
 
hsa-miR-148a-3p
 
 
-0.305
 
 
19.3
 
 
 
 
ARRDC4
 
 
hsa-let-7c-5p
 
 
-0.329
 
 
32.8
 
 
 
 
ARRDC4
 
 
hsa-miR-98-5p
 
 
-0.329
 
 
32.8
 
 
 
 
ASPH
 
 
hsa-miR-148a-3p
 
 
-0.391
 
 
42.1
 
 
 
 
ATG4A
 
 
hsa-miR-7-5p
 
 
-0.448
 
 
16.3
 
 
 
 
ATG4D
 
 
hsa-miR-148a-3p
 
 
-0.303
 
 
16.0
 
 
 
 
ATP2A2
 
 
hsa-miR-98-5p
 
 
-0.311
 
 
36.0
 
 
 
 
ATP2A2
 
 
hsa-let-7c-5p
 
 
-0.311
 
 
36.0
 
 
 
 
ATP6AP2
 
 
hsa-miR-148a-3p
 
 
-0.592
 
 
50.2
 
 
 
 
ATP6AP2
 
 
hsa-miR-148a-3p
 
 
-0.668
 
 
50.2
 
 
 
 
ATPAF1
 
 
hsa-miR-98-5p
 
 
-0.498
 
 
54.8
 
 
 
 
ATPAF1
 
 
hsa-let-7c-5p
 
 
-0.498
 
 
54.8
 
 
 
 
B3GAT3
 
 
hsa-miR-98-5p
 
 
-0.324
 
 
33.7
 
 
 
 
B3GAT3
 
 
hsa-let-7c-5p
 
 
-0.324
 
 
33.7
 
 
 
 
B4GALT5
 
 
hsa-miR-148a-3p
 
 
-0.44
 
 
11.8
 
 
 
 
B4GAT1
 
 
hsa-let-7c-5p
 
 
-0.47
 
 
24.7
 
 
 
 
B4GAT1
 
 
hsa-miR-98-5p
 
 
-0.47
 
 
24.7
 
 
 
 
BACH1
 
 
hsa-miR-98-5p
 
 
-0.298
 
 
16.2
 
 
 
 
BACH1
 
 
hsa-miR-98-5p
 
 
-0.298
 
 
16.2
 
 
 
 
BACH1
 
 
hsa-let-7c-5p
 
 
-0.306
 
 
16.2
 
 
 
 
BACH1
 
 
hsa-let-7c-5p
 
 
-0.306
 
 
16.2
 
 
 
 
BACH1
 
 
hsa-miR-98-5p
 
 
-0.435
 
 
16.2
 
 
 
 
BACH1
 
 
hsa-miR-98-5p
 
 
-0.435
 
 
16.2
 
 
 
 
BACH1
 
 
hsa-let-7c-5p
 
 
-0.435
 
 
16.2
 
 
 
 
BACH1
 
 
hsa-let-7c-5p
 
 
-0.435
 
 
16.2
 
 
 
 
BCAT1
 
 
hsa-miR-98-5p
 
 
-0.394
 
 
54.8
 
 
 
 
BCAT1
 
 
hsa-let-7c-5p
 
 
-0.394
 
 
54.8
 
 
 
 
 
 
 prostate 
 
 
 
 
target_symbol
 
 
mature_mirna_id
 
 
score
 
 
nTPM
 
 
 
 
 
 
ABCD3
 
 
hsa-miR-148a-3p
 
 
-0.388
 
 
20.2
 
 
 
 
ABHD17C
 
 
hsa-let-7c-5p
 
 
-0.359
 
 
17.0
 
 
 
 
ABHD17C
 
 
hsa-miR-98-5p
 
 
-0.383
 
 
17.0
 
 
 
 
ACP3
 
 
hsa-miR-98-5p
 
 
-0.298
 
 
3409.5
 
 
 
 
ACP3
 
 
hsa-let-7c-5p
 
 
-0.298
 
 
3409.5
 
 
 
 
ACTC1
 
 
hsa-miR-7-5p
 
 
-0.471
 
 
68.4
 
 
 
 
ADAM10
 
 
hsa-miR-148a-3p
 
 
-0.356
 
 
23.6
 
 
 
 
ADAM9
 
 
hsa-miR-20a-5p
 
 
-0.299
 
 
32.8
 
 
 
 
ADAM9
 
 
hsa-miR-106b-5p
 
 
-0.299
 
 
32.8
 
 
 
 
ADAM9
 
 
hsa-miR-106a-5p
 
 
-0.317
 
 
32.8
 
 
 
 
ADAM9
 
 
hsa-miR-17-5p
 
 
-0.32
 
 
32.8
 
 
 
 
ADAM9
 
 
hsa-miR-20b-5p
 
 
-0.328
 
 
32.8
 
 
 
 
AGFG1
 
 
hsa-miR-148a-3p
 
 
-0.332
 
 
19.4
 
 
 
 
AGFG2
 
 
hsa-miR-20a-5p
 
 
-0.365
 
 
12.4
 
 
 
 
AGFG2
 
 
hsa-miR-20b-5p
 
 
-0.386
 
 
12.4
 
 
 
 
AGFG2
 
 
hsa-miR-106b-5p
 
 
-0.397
 
 
12.4
 
 
 
 
AGFG2
 
 
hsa-miR-106a-5p
 
 
-0.399
 
 
12.4
 
 
 
 
AGFG2
 
 
hsa-miR-17-5p
 
 
-0.402
 
 
12.4
 
 
 
 
AHDC1
 
 
hsa-miR-148a-3p
 
 
-0.413
 
 
11.3
 
 
 
 
AHNAK
 
 
hsa-miR-20a-5p
 
 
-0.316
 
 
83.5
 
 
 
 
AHNAK
 
 
hsa-miR-106b-5p
 
 
-0.316
 
 
83.5
 
 
 
 
AHNAK
 
 
hsa-miR-106a-5p
 
 
-0.334
 
 
83.5
 
 
 
 
AHNAK
 
 
hsa-miR-17-5p
 
 
-0.337
 
 
83.5
 
 
 
 
AHNAK
 
 
hsa-miR-20b-5p
 
 
-0.337
 
 
83.5
 
 
 
 
AIFM1
 
 
hsa-miR-98-5p
 
 
-0.314
 
 
10.1
 
 
 
 
AIFM1
 
 
hsa-let-7c-5p
 
 
-0.314
 
 
10.1
 
 
 
 
AKAP1
 
 
hsa-miR-148a-3p
 
 
-0.381
 
 
33.3
 
 
 
 
ALCAM
 
 
hsa-miR-148a-3p
 
 
-0.339
 
 
30.7
 
 
 
 
ALDH4A1
 
 
hsa-miR-184
 
 
-0.421
 
 
27.7
 
 
 
 
ALG3
 
 
hsa-miR-106b-5p
 
 
-0.551
 
 
19.0
 
 
 
 
ALG3
 
 
hsa-miR-20a-5p
 
 
-0.559
 
 
19.0
 
 
 
 
ALG3
 
 
hsa-miR-106a-5p
 
 
-0.569
 
 
19.0
 
 
 
 
ALG3
 
 
hsa-miR-17-5p
 
 
-0.572
 
 
19.0
 
 
 
 
ALG3
 
 
hsa-miR-20b-5p
 
 
-0.58
 
 
19.0
 
 
 
 
AMT
 
 
hsa-miR-98-5p
 
 
-0.463
 
 
15.5
 
 
 
 
AMT
 
 
hsa-let-7c-5p
 
 
-0.463
 
 
15.5
 
 
 
 
ANKRD12
 
 
hsa-miR-7-5p
 
 
-0.37
 
 
11.4
 
 
 
 
ANKRD46
 
 
hsa-miR-98-5p
 
 
-0.389
 
 
12.6
 
 
 
 
ANKRD46
 
 
hsa-let-7c-5p
 
 
-0.389
 
 
12.6
 
 
 
 
ANKRD9
 
 
hsa-miR-20a-5p
 
 
-0.31
 
 
18.8
 
 
 
 
ANKRD9
 
 
hsa-miR-106b-5p
 
 
-0.318
 
 
18.8
 
 
 
 
ANKRD9
 
 
hsa-miR-20b-5p
 
 
-0.331
 
 
18.8
 
 
 
 
ANKRD9
 
 
hsa-miR-106a-5p
 
 
-0.344
 
 
18.8
 
 
 
 
ANKRD9
 
 
hsa-miR-17-5p
 
 
-0.347
 
 
18.8
 
 
 
 
AP1S1
 
 
hsa-miR-98-5p
 
 
-0.374
 
 
23.9
 
 
 
 
AP1S1
 
 
hsa-let-7c-5p
 
 
-0.374
 
 
23.9
 
 
 
 
APCDD1
 
 
hsa-miR-20a-5p
 
 
-0.404
 
 
31.5
 
 
 
 
APCDD1
 
 
hsa-miR-106b-5p
 
 
-0.404
 
 
31.5
 
 
 
 
APCDD1
 
 
hsa-miR-106a-5p
 
 
-0.422
 
 
31.5
 
 
 
 
APCDD1
 
 
hsa-miR-17-5p
 
 
-0.425
 
 
31.5
 
 
 
 
APCDD1
 
 
hsa-miR-20b-5p
 
 
-0.425
 
 
31.5
 
 
 
 
ARF4
 
 
hsa-miR-7-5p
 
 
-0.407
 
 
129.9
 
 
 
 
ARF4
 
 
hsa-miR-148a-3p
 
 
-0.487
 
 
129.9
 
 
 
 
ARFGEF1
 
 
hsa-miR-148a-3p
 
 
-0.306
 
 
12.2
 
 
 
 
ARFIP1
 
 
hsa-miR-148a-3p
 
 
-0.474
 
 
19.8
 
 
 
 
ARG2
 
 
hsa-miR-98-5p
 
 
-0.468
 
 
101.8
 
 
 
 
ARG2
 
 
hsa-let-7c-5p
 
 
-0.468
 
 
101.8
 
 
 
 
ARHGAP1
 
 
hsa-miR-17-5p
 
 
-0.325
 
 
60.9
 
 
 
 
ARHGAP1
 
 
hsa-miR-20b-5p
 
 
-0.325
 
 
60.9
 
 
 
 
ARHGAP1
 
 
hsa-miR-106a-5p
 
 
-0.329
 
 
60.9
 
 
 
 
ARHGAP1
 
 
hsa-miR-20a-5p
 
 
-0.339
 
 
60.9
 
 
 
 
ARHGAP1
 
 
hsa-miR-106b-5p
 
 
-0.339
 
 
60.9
 
 
 
 
ARHGAP21
 
 
hsa-miR-148a-3p
 
 
-0.287
 
 
10.2
 
 
 
 
ARID4B
 
 
hsa-miR-20a-5p
 
 
-0.29
 
 
13.4
 
 
 
 
ARID4B
 
 
hsa-miR-106b-5p
 
 
-0.29
 
 
13.4
 
 
 
 
ARL1
 
 
hsa-miR-20a-5p
 
 
-0.403
 
 
68.8
 
 
 
 
ARL1
 
 
hsa-miR-106b-5p
 
 
-0.411
 
 
68.8
 
 
 
 
ARL1
 
 
hsa-miR-20b-5p
 
 
-0.424
 
 
68.8
 
 
 
 
ARL1
 
 
hsa-miR-106a-5p
 
 
-0.429
 
 
68.8
 
 
 
 
ARL1
 
 
hsa-miR-17-5p
 
 
-0.432
 
 
68.8
 
 
 
 
ARL4D
 
 
hsa-miR-98-5p
 
 
-0.417
 
 
10.7
 
 
 
 
ARL4D
 
 
hsa-let-7c-5p
 
 
-0.417
 
 
10.7
 
 
 
 
ARL6IP1
 
 
hsa-miR-7-5p
 
 
-0.374
 
 
71.3
 
 
 
 
ARL6IP1
 
 
hsa-miR-148a-3p
 
 
-0.579
 
 
71.3
 
 
 
 
ARL8B
 
 
hsa-miR-148a-3p
 
 
-0.337
 
 
42.2
 
 
 
 
ARMC8
 
 
hsa-miR-20a-5p
 
 
-0.357
 
 
15.4
 
 
 
 
ARMC8
 
 
hsa-miR-106b-5p
 
 
-0.357
 
 
15.4
 
 
 
 
ARMC8
 
 
hsa-miR-106a-5p
 
 
-0.375
 
 
15.4
 
 
 
 
ARMC8
 
 
hsa-miR-17-5p
 
 
-0.378
 
 
15.4
 
 
 
 
ARMC8
 
 
hsa-miR-20b-5p
 
 
-0.378
 
 
15.4
 
 
 
 
ARMCX1
 
 
hsa-miR-21-5p
 
 
-0.489
 
 
22.8
 
 
 
 
ARMT1
 
 
hsa-let-7c-5p
 
 
-0.303
 
 
11.5
 
 
 
 
ARMT1
 
 
hsa-miR-98-5p
 
 
-0.303
 
 
11.5
 
 
 
 
ARPP19
 
 
hsa-let-7c-5p
 
 
-0.492
 
 
37.0
 
 
 
 
ARPP19
 
 
hsa-miR-98-5p
 
 
-0.5
 
 
37.0
 
 
 
 
ARRDC3
 
 
hsa-miR-148a-3p
 
 
-0.305
 
 
28.5
 
 
 
 
ARRDC4
 
 
hsa-let-7c-5p
 
 
-0.329
 
 
10.3
 
 
 
 
ARRDC4
 
 
hsa-miR-98-5p
 
 
-0.329
 
 
10.3
 
 
 
 
ASAP1
 
 
hsa-miR-98-5p
 
 
-0.308
 
 
11.6
 
 
 
 
ASAP1
 
 
hsa-let-7c-5p
 
 
-0.308
 
 
11.6
 
 
 
 
ASPH
 
 
hsa-miR-148a-3p
 
 
-0.391
 
 
44.4
 
 
 
 
ATG16L1
 
 
hsa-miR-20a-5p
 
 
-0.446
 
 
12.5
 
 
 
 
ATG16L1
 
 
hsa-miR-106b-5p
 
 
-0.446
 
 
12.5
 
 
 
 
ATG16L1
 
 
hsa-miR-106a-5p
 
 
-0.464
 
 
12.5
 
 
 
 
ATG16L1
 
 
hsa-miR-17-5p
 
 
-0.467
 
 
12.5
 
 
 
 
ATG16L1
 
 
hsa-miR-20b-5p
 
 
-0.467
 
 
12.5
 
 
 
 
ATG4D
 
 
hsa-miR-148a-3p
 
 
-0.303
 
 
13.5
 
 
 
 
ATOSB
 
 
hsa-miR-98-5p
 
 
-0.429
 
 
12.4
 
 
 
 
ATOSB
 
 
hsa-let-7c-5p
 
 
-0.429
 
 
12.4
 
 
 
 
ATP2A2
 
 
hsa-miR-98-5p
 
 
-0.311
 
 
87.6
 
 
 
 
 
 
 retina 
 
 
 
 
target_symbol
 
 
mature_mirna_id
 
 
score
 
 
nTPM
 
 
 
 
 
 
ABCD3
 
 
hsa-miR-148a-3p
 
 
-0.388
 
 
24.4
 
 
 
 
ABHD17C
 
 
hsa-let-7c-5p
 
 
-0.359
 
 
15.6
 
 
 
 
ABHD17C
 
 
hsa-miR-98-5p
 
 
-0.383
 
 
15.6
 
 
 
 
ACSL6
 
 
hsa-miR-98-5p
 
 
-0.336
 
 
42.4
 
 
 
 
ACSL6
 
 
hsa-let-7c-5p
 
 
-0.336
 
 
42.4
 
 
 
 
ACTC1
 
 
hsa-miR-7-5p
 
 
-0.471
 
 
29.3
 
 
 
 
ADAM9
 
 
hsa-miR-20a-5p
 
 
-0.299
 
 
17.2
 
 
 
 
ADAM9
 
 
hsa-miR-106b-5p
 
 
-0.299
 
 
17.2
 
 
 
 
ADAM9
 
 
hsa-miR-106a-5p
 
 
-0.317
 
 
17.2
 
 
 
 
ADAM9
 
 
hsa-miR-17-5p
 
 
-0.32
 
 
17.2
 
 
 
 
ADAM9
 
 
hsa-miR-20b-5p
 
 
-0.328
 
 
17.2
 
 
 
 
AGFG1
 
 
hsa-miR-148a-3p
 
 
-0.332
 
 
44.2
 
 
 
 
AKAP1
 
 
hsa-miR-148a-3p
 
 
-0.381
 
 
32.2
 
 
 
 
AKAP6
 
 
hsa-miR-98-5p
 
 
-0.307
 
 
12.6
 
 
 
 
AKAP6
 
 
hsa-let-7c-5p
 
 
-0.307
 
 
12.6
 
 
 
 
ALG9
 
 
hsa-miR-7-5p
 
 
-0.302
 
 
11.3
 
 
 
 
ANKRD12
 
 
hsa-miR-7-5p
 
 
-0.37
 
 
27.6
 
 
 
 
ANKRD13C
 
 
hsa-miR-20a-5p
 
 
-0.505
 
 
12.3
 
 
 
 
ANKRD13C
 
 
hsa-miR-106b-5p
 
 
-0.505
 
 
12.3
 
 
 
 
ANKRD13C
 
 
hsa-miR-106a-5p
 
 
-0.523
 
 
12.3
 
 
 
 
ANKRD13C
 
 
hsa-miR-17-5p
 
 
-0.526
 
 
12.3
 
 
 
 
ANKRD13C
 
 
hsa-miR-20b-5p
 
 
-0.526
 
 
12.3
 
 
 
 
ANKRD46
 
 
hsa-miR-98-5p
 
 
-0.389
 
 
32.6
 
 
 
 
ANKRD46
 
 
hsa-let-7c-5p
 
 
-0.389
 
 
32.6
 
 
 
 
AP1S1
 
 
hsa-miR-98-5p
 
 
-0.374
 
 
13.5
 
 
 
 
AP1S1
 
 
hsa-let-7c-5p
 
 
-0.374
 
 
13.5
 
 
 
 
ARF4
 
 
hsa-miR-7-5p
 
 
-0.407
 
 
50.0
 
 
 
 
ARF4
 
 
hsa-miR-148a-3p
 
 
-0.487
 
 
50.0
 
 
 
 
ARFGEF1
 
 
hsa-miR-148a-3p
 
 
-0.306
 
 
33.8
 
 
 
 
ARFIP1
 
 
hsa-miR-148a-3p
 
 
-0.474
 
 
18.7
 
 
 
 
ARG2
 
 
hsa-miR-98-5p
 
 
-0.468
 
 
20.7
 
 
 
 
ARG2
 
 
hsa-let-7c-5p
 
 
-0.468
 
 
20.7
 
 
 
 
ARHGAP21
 
 
hsa-miR-148a-3p
 
 
-0.287
 
 
37.0
 
 
 
 
ARID3B
 
 
hsa-miR-98-5p
 
 
-0.31
 
 
17.4
 
 
 
 
ARID3B
 
 
hsa-let-7c-5p
 
 
-0.31
 
 
17.4
 
 
 
 
ARID3B
 
 
hsa-miR-98-5p
 
 
-0.475
 
 
17.4
 
 
 
 
ARID3B
 
 
hsa-let-7c-5p
 
 
-0.486
 
 
17.4
 
 
 
 
ARID4A
 
 
hsa-miR-7-5p
 
 
-0.341
 
 
13.0
 
 
 
 
ARID4B
 
 
hsa-miR-20a-5p
 
 
-0.29
 
 
13.5
 
 
 
 
ARID4B
 
 
hsa-miR-106b-5p
 
 
-0.29
 
 
13.5
 
 
 
 
ARL1
 
 
hsa-miR-20a-5p
 
 
-0.403
 
 
45.1
 
 
 
 
ARL1
 
 
hsa-miR-106b-5p
 
 
-0.411
 
 
45.1
 
 
 
 
ARL1
 
 
hsa-miR-20b-5p
 
 
-0.424
 
 
45.1
 
 
 
 
ARL1
 
 
hsa-miR-106a-5p
 
 
-0.429
 
 
45.1
 
 
 
 
ARL1
 
 
hsa-miR-17-5p
 
 
-0.432
 
 
45.1
 
 
 
 
ARL4D
 
 
hsa-miR-98-5p
 
 
-0.417
 
 
78.9
 
 
 
 
ARL4D
 
 
hsa-let-7c-5p
 
 
-0.417
 
 
78.9
 
 
 
 
ARL6IP1
 
 
hsa-miR-7-5p
 
 
-0.374
 
 
292.6
 
 
 
 
ARL6IP1
 
 
hsa-miR-148a-3p
 
 
-0.579
 
 
292.6
 
 
 
 
ARL6IP6
 
 
hsa-miR-98-5p
 
 
-0.293
 
 
10.5
 
 
 
 
ARL6IP6
 
 
hsa-let-7c-5p
 
 
-0.293
 
 
10.5
 
 
 
 
ARL8B
 
 
hsa-miR-148a-3p
 
 
-0.337
 
 
31.7
 
 
 
 
ARMC8
 
 
hsa-miR-20a-5p
 
 
-0.357
 
 
24.3
 
 
 
 
ARMC8
 
 
hsa-miR-106b-5p
 
 
-0.357
 
 
24.3
 
 
 
 
ARMC8
 
 
hsa-miR-106a-5p
 
 
-0.375
 
 
24.3
 
 
 
 
ARMC8
 
 
hsa-miR-17-5p
 
 
-0.378
 
 
24.3
 
 
 
 
ARMC8
 
 
hsa-miR-20b-5p
 
 
-0.378
 
 
24.3
 
 
 
 
ARMCX1
 
 
hsa-miR-21-5p
 
 
-0.489
 
 
11.2
 
 
 
 
ARMT1
 
 
hsa-let-7c-5p
 
 
-0.303
 
 
15.2
 
 
 
 
ARMT1
 
 
hsa-miR-98-5p
 
 
-0.303
 
 
15.2
 
 
 
 
ARPP19
 
 
hsa-let-7c-5p
 
 
-0.492
 
 
53.1
 
 
 
 
ARPP19
 
 
hsa-miR-98-5p
 
 
-0.5
 
 
53.1
 
 
 
 
ARRDC3
 
 
hsa-miR-148a-3p
 
 
-0.305
 
 
69.6
 
 
 
 
ASF1A
 
 
hsa-miR-21-5p
 
 
-0.304
 
 
10.6
 
 
 
 
ASPH
 
 
hsa-miR-148a-3p
 
 
-0.391
 
 
201.9
 
 
 
 
ATP2A2
 
 
hsa-miR-98-5p
 
 
-0.311
 
 
17.7
 
 
 
 
ATP2A2
 
 
hsa-let-7c-5p
 
 
-0.311
 
 
17.7
 
 
 
 
ATP2B2
 
 
hsa-miR-7-5p
 
 
-0.297
 
 
12.5
 
 
 
 
ATP2B4
 
 
hsa-miR-148a-3p
 
 
-0.337
 
 
39.4
 
 
 
 
ATP6AP2
 
 
hsa-miR-148a-3p
 
 
-0.592
 
 
92.0
 
 
 
 
ATP6AP2
 
 
hsa-miR-148a-3p
 
 
-0.668
 
 
92.0
 
 
 
 
ATPAF1
 
 
hsa-miR-98-5p
 
 
-0.498
 
 
65.3
 
 
 
 
ATPAF1
 
 
hsa-let-7c-5p
 
 
-0.498
 
 
65.3
 
 
 
 
AVPI1
 
 
hsa-miR-7-5p
 
 
-0.367
 
 
23.7
 
 
 
 
B3GAT3
 
 
hsa-miR-98-5p
 
 
-0.324
 
 
15.0
 
 
 
 
B3GAT3
 
 
hsa-let-7c-5p
 
 
-0.324
 
 
15.0
 
 
 
 
B4GALT6
 
 
hsa-miR-148a-3p
 
 
-0.528
 
 
24.7
 
 
 
 
B4GAT1
 
 
hsa-let-7c-5p
 
 
-0.47
 
 
42.6
 
 
 
 
B4GAT1
 
 
hsa-miR-98-5p
 
 
-0.47
 
 
42.6
 
 
 
 
BACH1
 
 
hsa-miR-98-5p
 
 
-0.298
 
 
10.9
 
 
 
 
BACH1
 
 
hsa-miR-98-5p
 
 
-0.298
 
 
10.9
 
 
 
 
BACH1
 
 
hsa-let-7c-5p
 
 
-0.306
 
 
10.9
 
 
 
 
BACH1
 
 
hsa-let-7c-5p
 
 
-0.306
 
 
10.9
 
 
 
 
BACH1
 
 
hsa-miR-98-5p
 
 
-0.435
 
 
10.9
 
 
 
 
BACH1
 
 
hsa-miR-98-5p
 
 
-0.435
 
 
10.9
 
 
 
 
BACH1
 
 
hsa-let-7c-5p
 
 
-0.435
 
 
10.9
 
 
 
 
BACH1
 
 
hsa-let-7c-5p
 
 
-0.435
 
 
10.9
 
 
 
 
BAMBI
 
 
hsa-miR-20a-5p
 
 
-0.485
 
 
12.4
 
 
 
 
BAMBI
 
 
hsa-miR-106b-5p
 
 
-0.493
 
 
12.4
 
 
 
 
BAMBI
 
 
hsa-miR-106a-5p
 
 
-0.503
 
 
12.4
 
 
 
 
BAMBI
 
 
hsa-miR-17-5p
 
 
-0.506
 
 
12.4
 
 
 
 
BAMBI
 
 
hsa-miR-20b-5p
 
 
-0.506
 
 
12.4
 
 
 
 
BCAT1
 
 
hsa-miR-98-5p
 
 
-0.394
 
 
13.4
 
 
 
 
BCAT1
 
 
hsa-let-7c-5p
 
 
-0.394
 
 
13.4
 
 
 
 
BCL2L11
 
 
hsa-miR-148a-3p
 
 
-0.445
 
 
11.9
 
 
 
 
BHLHE41
 
 
hsa-miR-148a-3p
 
 
-0.442
 
 
113.5
 
 
 
 
BLOC1S6
 
 
hsa-miR-98-5p
 
 
-0.321
 
 
22.7
 
 
 
 
BLOC1S6
 
 
hsa-let-7c-5p
 
 
-0.321
 
 
22.7
 
 
 
 
BNIP3L
 
 
hsa-miR-20a-5p
 
 
-0.287
 
 
135.8
 
 
 
 
BNIP3L
 
 
hsa-miR-106b-5p
 
 
-0.298
 
 
135.8
 
 
 
 
 
 
 salivary gland 
 
 
 
 
target_symbol
 
 
mature_mirna_id
 
 
score
 
 
nTPM
 
 
 
 
 
 
ABCD3
 
 
hsa-miR-148a-3p
 
 
-0.388
 
 
16.5
 
 
 
 
ABHD17C
 
 
hsa-let-7c-5p
 
 
-0.359
 
 
22.3
 
 
 
 
ABHD17C
 
 
hsa-miR-98-5p
 
 
-0.383
 
 
22.3
 
 
 
 
ABHD5
 
 
hsa-miR-20a-5p
 
 
-0.397
 
 
19.4
 
 
 
 
ABHD5
 
 
hsa-miR-106b-5p
 
 
-0.413
 
 
19.4
 
 
 
 
ABHD5
 
 
hsa-miR-20b-5p
 
 
-0.418
 
 
19.4
 
 
 
 
ABHD5
 
 
hsa-miR-106a-5p
 
 
-0.447
 
 
19.4
 
 
 
 
ABHD5
 
 
hsa-miR-17-5p
 
 
-0.45
 
 
19.4
 
 
 
 
ACTC1
 
 
hsa-miR-7-5p
 
 
-0.471
 
 
41.7
 
 
 
 
ADAM9
 
 
hsa-miR-20a-5p
 
 
-0.299
 
 
39.8
 
 
 
 
ADAM9
 
 
hsa-miR-106b-5p
 
 
-0.299
 
 
39.8
 
 
 
 
ADAM9
 
 
hsa-miR-106a-5p
 
 
-0.317
 
 
39.8
 
 
 
 
ADAM9
 
 
hsa-miR-17-5p
 
 
-0.32
 
 
39.8
 
 
 
 
ADAM9
 
 
hsa-miR-20b-5p
 
 
-0.328
 
 
39.8
 
 
 
 
AGFG1
 
 
hsa-miR-148a-3p
 
 
-0.332
 
 
26.3
 
 
 
 
AGFG2
 
 
hsa-miR-20a-5p
 
 
-0.365
 
 
272.4
 
 
 
 
AGFG2
 
 
hsa-miR-20b-5p
 
 
-0.386
 
 
272.4
 
 
 
 
AGFG2
 
 
hsa-miR-106b-5p
 
 
-0.397
 
 
272.4
 
 
 
 
AGFG2
 
 
hsa-miR-106a-5p
 
 
-0.399
 
 
272.4
 
 
 
 
AGFG2
 
 
hsa-miR-17-5p
 
 
-0.402
 
 
272.4
 
 
 
 
AHDC1
 
 
hsa-miR-148a-3p
 
 
-0.413
 
 
16.0
 
 
 
 
AHNAK
 
 
hsa-miR-20a-5p
 
 
-0.316
 
 
118.3
 
 
 
 
AHNAK
 
 
hsa-miR-106b-5p
 
 
-0.316
 
 
118.3
 
 
 
 
AHNAK
 
 
hsa-miR-106a-5p
 
 
-0.334
 
 
118.3
 
 
 
 
AHNAK
 
 
hsa-miR-17-5p
 
 
-0.337
 
 
118.3
 
 
 
 
AHNAK
 
 
hsa-miR-20b-5p
 
 
-0.337
 
 
118.3
 
 
 
 
AIFM1
 
 
hsa-miR-98-5p
 
 
-0.314
 
 
17.0
 
 
 
 
AIFM1
 
 
hsa-let-7c-5p
 
 
-0.314
 
 
17.0
 
 
 
 
AKAP1
 
 
hsa-miR-148a-3p
 
 
-0.381
 
 
31.6
 
 
 
 
ALCAM
 
 
hsa-miR-148a-3p
 
 
-0.339
 
 
31.9
 
 
 
 
ALG3
 
 
hsa-miR-106b-5p
 
 
-0.551
 
 
25.8
 
 
 
 
ALG3
 
 
hsa-miR-20a-5p
 
 
-0.559
 
 
25.8
 
 
 
 
ALG3
 
 
hsa-miR-106a-5p
 
 
-0.569
 
 
25.8
 
 
 
 
ALG3
 
 
hsa-miR-17-5p
 
 
-0.572
 
 
25.8
 
 
 
 
ALG3
 
 
hsa-miR-20b-5p
 
 
-0.58
 
 
25.8
 
 
 
 
ALG9
 
 
hsa-miR-7-5p
 
 
-0.302
 
 
16.6
 
 
 
 
AMT
 
 
hsa-miR-98-5p
 
 
-0.463
 
 
10.0
 
 
 
 
AMT
 
 
hsa-let-7c-5p
 
 
-0.463
 
 
10.0
 
 
 
 
ANKRD12
 
 
hsa-miR-7-5p
 
 
-0.37
 
 
11.6
 
 
 
 
ANKRD46
 
 
hsa-miR-98-5p
 
 
-0.389
 
 
16.1
 
 
 
 
ANKRD46
 
 
hsa-let-7c-5p
 
 
-0.389
 
 
16.1
 
 
 
 
ANKRD9
 
 
hsa-miR-20a-5p
 
 
-0.31
 
 
25.3
 
 
 
 
ANKRD9
 
 
hsa-miR-106b-5p
 
 
-0.318
 
 
25.3
 
 
 
 
ANKRD9
 
 
hsa-miR-20b-5p
 
 
-0.331
 
 
25.3
 
 
 
 
ANKRD9
 
 
hsa-miR-106a-5p
 
 
-0.344
 
 
25.3
 
 
 
 
ANKRD9
 
 
hsa-miR-17-5p
 
 
-0.347
 
 
25.3
 
 
 
 
AP1AR
 
 
hsa-miR-21-5p
 
 
-0.318
 
 
11.4
 
 
 
 
AP1S1
 
 
hsa-miR-98-5p
 
 
-0.374
 
 
21.5
 
 
 
 
AP1S1
 
 
hsa-let-7c-5p
 
 
-0.374
 
 
21.5
 
 
 
 
APCDD1
 
 
hsa-miR-20a-5p
 
 
-0.404
 
 
10.0
 
 
 
 
APCDD1
 
 
hsa-miR-106b-5p
 
 
-0.404
 
 
10.0
 
 
 
 
APCDD1
 
 
hsa-miR-106a-5p
 
 
-0.422
 
 
10.0
 
 
 
 
APCDD1
 
 
hsa-miR-17-5p
 
 
-0.425
 
 
10.0
 
 
 
 
APCDD1
 
 
hsa-miR-20b-5p
 
 
-0.425
 
 
10.0
 
 
 
 
ARF4
 
 
hsa-miR-7-5p
 
 
-0.407
 
 
171.4
 
 
 
 
ARF4
 
 
hsa-miR-148a-3p
 
 
-0.487
 
 
171.4
 
 
 
 
ARFGEF1
 
 
hsa-miR-148a-3p
 
 
-0.306
 
 
13.9
 
 
 
 
ARFIP1
 
 
hsa-miR-148a-3p
 
 
-0.474
 
 
12.9
 
 
 
 
ARG2
 
 
hsa-miR-98-5p
 
 
-0.468
 
 
18.4
 
 
 
 
ARG2
 
 
hsa-let-7c-5p
 
 
-0.468
 
 
18.4
 
 
 
 
ARHGAP1
 
 
hsa-miR-17-5p
 
 
-0.325
 
 
43.3
 
 
 
 
ARHGAP1
 
 
hsa-miR-20b-5p
 
 
-0.325
 
 
43.3
 
 
 
 
ARHGAP1
 
 
hsa-miR-106a-5p
 
 
-0.329
 
 
43.3
 
 
 
 
ARHGAP1
 
 
hsa-miR-20a-5p
 
 
-0.339
 
 
43.3
 
 
 
 
ARHGAP1
 
 
hsa-miR-106b-5p
 
 
-0.339
 
 
43.3
 
 
 
 
ARHGAP21
 
 
hsa-miR-148a-3p
 
 
-0.287
 
 
23.1
 
 
 
 
ARHGEF28
 
 
hsa-miR-20b-5p
 
 
-0.352
 
 
18.6
 
 
 
 
ARHGEF28
 
 
hsa-miR-17-5p
 
 
-0.363
 
 
18.6
 
 
 
 
ARHGEF28
 
 
hsa-miR-20a-5p
 
 
-0.366
 
 
18.6
 
 
 
 
ARHGEF28
 
 
hsa-miR-106a-5p
 
 
-0.367
 
 
18.6
 
 
 
 
ARHGEF28
 
 
hsa-miR-106b-5p
 
 
-0.377
 
 
18.6
 
 
 
 
ARID4B
 
 
hsa-miR-20a-5p
 
 
-0.29
 
 
10.8
 
 
 
 
ARID4B
 
 
hsa-miR-106b-5p
 
 
-0.29
 
 
10.8
 
 
 
 
ARL1
 
 
hsa-miR-20a-5p
 
 
-0.403
 
 
69.5
 
 
 
 
ARL1
 
 
hsa-miR-106b-5p
 
 
-0.411
 
 
69.5
 
 
 
 
ARL1
 
 
hsa-miR-20b-5p
 
 
-0.424
 
 
69.5
 
 
 
 
ARL1
 
 
hsa-miR-106a-5p
 
 
-0.429
 
 
69.5
 
 
 
 
ARL1
 
 
hsa-miR-17-5p
 
 
-0.432
 
 
69.5
 
 
 
 
ARL4D
 
 
hsa-miR-98-5p
 
 
-0.417
 
 
16.7
 
 
 
 
ARL4D
 
 
hsa-let-7c-5p
 
 
-0.417
 
 
16.7
 
 
 
 
ARL6IP1
 
 
hsa-miR-7-5p
 
 
-0.374
 
 
79.4
 
 
 
 
ARL6IP1
 
 
hsa-miR-148a-3p
 
 
-0.579
 
 
79.4
 
 
 
 
ARL8B
 
 
hsa-miR-148a-3p
 
 
-0.337
 
 
35.3
 
 
 
 
ARMC8
 
 
hsa-miR-20a-5p
 
 
-0.357
 
 
11.8
 
 
 
 
ARMC8
 
 
hsa-miR-106b-5p
 
 
-0.357
 
 
11.8
 
 
 
 
ARMC8
 
 
hsa-miR-106a-5p
 
 
-0.375
 
 
11.8
 
 
 
 
ARMC8
 
 
hsa-miR-17-5p
 
 
-0.378
 
 
11.8
 
 
 
 
ARMC8
 
 
hsa-miR-20b-5p
 
 
-0.378
 
 
11.8
 
 
 
 
ARMCX1
 
 
hsa-miR-21-5p
 
 
-0.489
 
 
13.3
 
 
 
 
ARPP19
 
 
hsa-let-7c-5p
 
 
-0.492
 
 
28.7
 
 
 
 
ARPP19
 
 
hsa-miR-98-5p
 
 
-0.5
 
 
28.7
 
 
 
 
ARRDC3
 
 
hsa-miR-148a-3p
 
 
-0.305
 
 
60.1
 
 
 
 
ASF1A
 
 
hsa-miR-21-5p
 
 
-0.304
 
 
10.1
 
 
 
 
ASPH
 
 
hsa-miR-148a-3p
 
 
-0.391
 
 
95.6
 
 
 
 
ATG16L1
 
 
hsa-miR-20a-5p
 
 
-0.446
 
 
11.7
 
 
 
 
ATG16L1
 
 
hsa-miR-106b-5p
 
 
-0.446
 
 
11.7
 
 
 
 
ATG16L1
 
 
hsa-miR-106a-5p
 
 
-0.464
 
 
11.7
 
 
 
 
ATG16L1
 
 
hsa-miR-17-5p
 
 
-0.467
 
 
11.7
 
 
 
 
ATG16L1
 
 
hsa-miR-20b-5p
 
 
-0.467
 
 
11.7
 
 
 
 
ATG4A
 
 
hsa-miR-7-5p
 
 
-0.448
 
 
12.3
 
 
 
 
 
 
 seminal vesicle 
 
 
 
 
target_symbol
 
 
mature_mirna_id
 
 
score
 
 
nTPM
 
 
 
 
 
 
ABCD3
 
 
hsa-miR-148a-3p
 
 
-0.388
 
 
25.5
 
 
 
 
ACSL6
 
 
hsa-miR-98-5p
 
 
-0.336
 
 
17.0
 
 
 
 
ACSL6
 
 
hsa-let-7c-5p
 
 
-0.336
 
 
17.0
 
 
 
 
ACTC1
 
 
hsa-miR-7-5p
 
 
-0.471
 
 
506.6
 
 
 
 
ACVR1
 
 
hsa-miR-148a-3p
 
 
-0.397
 
 
11.3
 
 
 
 
ADAM10
 
 
hsa-miR-148a-3p
 
 
-0.356
 
 
11.5
 
 
 
 
ADAM9
 
 
hsa-miR-20a-5p
 
 
-0.299
 
 
32.8
 
 
 
 
ADAM9
 
 
hsa-miR-106b-5p
 
 
-0.299
 
 
32.8
 
 
 
 
ADAM9
 
 
hsa-miR-106a-5p
 
 
-0.317
 
 
32.8
 
 
 
 
ADAM9
 
 
hsa-miR-17-5p
 
 
-0.32
 
 
32.8
 
 
 
 
ADAM9
 
 
hsa-miR-20b-5p
 
 
-0.328
 
 
32.8
 
 
 
 
AGFG1
 
 
hsa-miR-148a-3p
 
 
-0.332
 
 
22.0
 
 
 
 
AGFG2
 
 
hsa-miR-20a-5p
 
 
-0.365
 
 
12.7
 
 
 
 
AGFG2
 
 
hsa-miR-20b-5p
 
 
-0.386
 
 
12.7
 
 
 
 
AGFG2
 
 
hsa-miR-106b-5p
 
 
-0.397
 
 
12.7
 
 
 
 
AGFG2
 
 
hsa-miR-106a-5p
 
 
-0.399
 
 
12.7
 
 
 
 
AGFG2
 
 
hsa-miR-17-5p
 
 
-0.402
 
 
12.7
 
 
 
 
AHNAK
 
 
hsa-miR-20a-5p
 
 
-0.316
 
 
99.6
 
 
 
 
AHNAK
 
 
hsa-miR-106b-5p
 
 
-0.316
 
 
99.6
 
 
 
 
AHNAK
 
 
hsa-miR-106a-5p
 
 
-0.334
 
 
99.6
 
 
 
 
AHNAK
 
 
hsa-miR-17-5p
 
 
-0.337
 
 
99.6
 
 
 
 
AHNAK
 
 
hsa-miR-20b-5p
 
 
-0.337
 
 
99.6
 
 
 
 
AKAP1
 
 
hsa-miR-148a-3p
 
 
-0.381
 
 
23.3
 
 
 
 
ALCAM
 
 
hsa-miR-148a-3p
 
 
-0.339
 
 
37.3
 
 
 
 
ALDH4A1
 
 
hsa-miR-184
 
 
-0.421
 
 
25.6
 
 
 
 
ALG3
 
 
hsa-miR-106b-5p
 
 
-0.551
 
 
12.9
 
 
 
 
ALG3
 
 
hsa-miR-20a-5p
 
 
-0.559
 
 
12.9
 
 
 
 
ALG3
 
 
hsa-miR-106a-5p
 
 
-0.569
 
 
12.9
 
 
 
 
ALG3
 
 
hsa-miR-17-5p
 
 
-0.572
 
 
12.9
 
 
 
 
ALG3
 
 
hsa-miR-20b-5p
 
 
-0.58
 
 
12.9
 
 
 
 
ALG9
 
 
hsa-miR-7-5p
 
 
-0.302
 
 
10.6
 
 
 
 
ANKRD12
 
 
hsa-miR-7-5p
 
 
-0.37
 
 
10.0
 
 
 
 
ANKRD46
 
 
hsa-miR-98-5p
 
 
-0.389
 
 
12.4
 
 
 
 
ANKRD46
 
 
hsa-let-7c-5p
 
 
-0.389
 
 
12.4
 
 
 
 
APCDD1
 
 
hsa-miR-20a-5p
 
 
-0.404
 
 
13.3
 
 
 
 
APCDD1
 
 
hsa-miR-106b-5p
 
 
-0.404
 
 
13.3
 
 
 
 
APCDD1
 
 
hsa-miR-106a-5p
 
 
-0.422
 
 
13.3
 
 
 
 
APCDD1
 
 
hsa-miR-17-5p
 
 
-0.425
 
 
13.3
 
 
 
 
APCDD1
 
 
hsa-miR-20b-5p
 
 
-0.425
 
 
13.3
 
 
 
 
ARF4
 
 
hsa-miR-7-5p
 
 
-0.407
 
 
125.1
 
 
 
 
ARF4
 
 
hsa-miR-148a-3p
 
 
-0.487
 
 
125.1
 
 
 
 
ARFGEF1
 
 
hsa-miR-148a-3p
 
 
-0.306
 
 
11.4
 
 
 
 
ARFIP1
 
 
hsa-miR-148a-3p
 
 
-0.474
 
 
16.2
 
 
 
 
ARHGAP1
 
 
hsa-miR-17-5p
 
 
-0.325
 
 
58.9
 
 
 
 
ARHGAP1
 
 
hsa-miR-20b-5p
 
 
-0.325
 
 
58.9
 
 
 
 
ARHGAP1
 
 
hsa-miR-106a-5p
 
 
-0.329
 
 
58.9
 
 
 
 
ARHGAP1
 
 
hsa-miR-20a-5p
 
 
-0.339
 
 
58.9
 
 
 
 
ARHGAP1
 
 
hsa-miR-106b-5p
 
 
-0.339
 
 
58.9
 
 
 
 
ARHGAP21
 
 
hsa-miR-148a-3p
 
 
-0.287
 
 
10.8
 
 
 
 
ARID4B
 
 
hsa-miR-20a-5p
 
 
-0.29
 
 
10.2
 
 
 
 
ARID4B
 
 
hsa-miR-106b-5p
 
 
-0.29
 
 
10.2
 
 
 
 
ARL1
 
 
hsa-miR-20a-5p
 
 
-0.403
 
 
65.0
 
 
 
 
ARL1
 
 
hsa-miR-106b-5p
 
 
-0.411
 
 
65.0
 
 
 
 
ARL1
 
 
hsa-miR-20b-5p
 
 
-0.424
 
 
65.0
 
 
 
 
ARL1
 
 
hsa-miR-106a-5p
 
 
-0.429
 
 
65.0
 
 
 
 
ARL1
 
 
hsa-miR-17-5p
 
 
-0.432
 
 
65.0
 
 
 
 
ARL4D
 
 
hsa-miR-98-5p
 
 
-0.417
 
 
14.3
 
 
 
 
ARL4D
 
 
hsa-let-7c-5p
 
 
-0.417
 
 
14.3
 
 
 
 
ARL6IP1
 
 
hsa-miR-7-5p
 
 
-0.374
 
 
66.4
 
 
 
 
ARL6IP1
 
 
hsa-miR-148a-3p
 
 
-0.579
 
 
66.4
 
 
 
 
ARL8B
 
 
hsa-miR-148a-3p
 
 
-0.337
 
 
39.4
 
 
 
 
ARMC8
 
 
hsa-miR-20a-5p
 
 
-0.357
 
 
16.5
 
 
 
 
ARMC8
 
 
hsa-miR-106b-5p
 
 
-0.357
 
 
16.5
 
 
 
 
ARMC8
 
 
hsa-miR-106a-5p
 
 
-0.375
 
 
16.5
 
 
 
 
ARMC8
 
 
hsa-miR-17-5p
 
 
-0.378
 
 
16.5
 
 
 
 
ARMC8
 
 
hsa-miR-20b-5p
 
 
-0.378
 
 
16.5
 
 
 
 
ARMCX1
 
 
hsa-miR-21-5p
 
 
-0.489
 
 
21.7
 
 
 
 
ARMT1
 
 
hsa-let-7c-5p
 
 
-0.303
 
 
10.5
 
 
 
 
ARMT1
 
 
hsa-miR-98-5p
 
 
-0.303
 
 
10.5
 
 
 
 
ARPP19
 
 
hsa-let-7c-5p
 
 
-0.492
 
 
38.5
 
 
 
 
ARPP19
 
 
hsa-miR-98-5p
 
 
-0.5
 
 
38.5
 
 
 
 
ARRDC3
 
 
hsa-miR-148a-3p
 
 
-0.305
 
 
22.9
 
 
 
 
ARRDC4
 
 
hsa-let-7c-5p
 
 
-0.329
 
 
15.4
 
 
 
 
ARRDC4
 
 
hsa-miR-98-5p
 
 
-0.329
 
 
15.4
 
 
 
 
ASAP1
 
 
hsa-miR-98-5p
 
 
-0.308
 
 
10.7
 
 
 
 
ASAP1
 
 
hsa-let-7c-5p
 
 
-0.308
 
 
10.7
 
 
 
 
ASF1A
 
 
hsa-miR-21-5p
 
 
-0.304
 
 
10.4
 
 
 
 
ASPH
 
 
hsa-miR-148a-3p
 
 
-0.391
 
 
65.8
 
 
 
 
ATP2A2
 
 
hsa-miR-98-5p
 
 
-0.311
 
 
66.0
 
 
 
 
ATP2A2
 
 
hsa-let-7c-5p
 
 
-0.311
 
 
66.0
 
 
 
 
ATP2B4
 
 
hsa-miR-148a-3p
 
 
-0.337
 
 
66.2
 
 
 
 
ATP6AP2
 
 
hsa-miR-148a-3p
 
 
-0.592
 
 
92.2
 
 
 
 
ATP6AP2
 
 
hsa-miR-148a-3p
 
 
-0.668
 
 
92.2
 
 
 
 
ATPAF1
 
 
hsa-miR-98-5p
 
 
-0.498
 
 
31.8
 
 
 
 
ATPAF1
 
 
hsa-let-7c-5p
 
 
-0.498
 
 
31.8
 
 
 
 
AVPI1
 
 
hsa-miR-7-5p
 
 
-0.367
 
 
15.6
 
 
 
 
B3GAT3
 
 
hsa-miR-98-5p
 
 
-0.324
 
 
17.2
 
 
 
 
B3GAT3
 
 
hsa-let-7c-5p
 
 
-0.324
 
 
17.2
 
 
 
 
B4GALT5
 
 
hsa-miR-148a-3p
 
 
-0.44
 
 
20.7
 
 
 
 
B4GAT1
 
 
hsa-let-7c-5p
 
 
-0.47
 
 
21.2
 
 
 
 
B4GAT1
 
 
hsa-miR-98-5p
 
 
-0.47
 
 
21.2
 
 
 
 
BAMBI
 
 
hsa-miR-20a-5p
 
 
-0.485
 
 
15.7
 
 
 
 
BAMBI
 
 
hsa-miR-106b-5p
 
 
-0.493
 
 
15.7
 
 
 
 
BAMBI
 
 
hsa-miR-106a-5p
 
 
-0.503
 
 
15.7
 
 
 
 
BAMBI
 
 
hsa-miR-17-5p
 
 
-0.506
 
 
15.7
 
 
 
 
BAMBI
 
 
hsa-miR-20b-5p
 
 
-0.506
 
 
15.7
 
 
 
 
BHLHE41
 
 
hsa-miR-148a-3p
 
 
-0.442
 
 
13.2
 
 
 
 
BLOC1S6
 
 
hsa-miR-98-5p
 
 
-0.321
 
 
19.2
 
 
 
 
BLOC1S6
 
 
hsa-let-7c-5p
 
 
-0.321
 
 
19.2
 
 
 
 
BNIP3L
 
 
hsa-miR-20a-5p
 
 
-0.287
 
 
60.7
 
 
 
 
 
 
 smooth muscle 
 
 
 
 
target_symbol
 
 
mature_mirna_id
 
 
score
 
 
nTPM
 
 
 
 
 
 
ABCD3
 
 
hsa-miR-148a-3p
 
 
-0.388
 
 
16.6
 
 
 
 
ACVR1
 
 
hsa-miR-148a-3p
 
 
-0.397
 
 
26.8
 
 
 
 
ADAM10
 
 
hsa-miR-148a-3p
 
 
-0.356
 
 
12.4
 
 
 
 
ADAM9
 
 
hsa-miR-20a-5p
 
 
-0.299
 
 
48.2
 
 
 
 
ADAM9
 
 
hsa-miR-106b-5p
 
 
-0.299
 
 
48.2
 
 
 
 
ADAM9
 
 
hsa-miR-106a-5p
 
 
-0.317
 
 
48.2
 
 
 
 
ADAM9
 
 
hsa-miR-17-5p
 
 
-0.32
 
 
48.2
 
 
 
 
ADAM9
 
 
hsa-miR-20b-5p
 
 
-0.328
 
 
48.2
 
 
 
 
ADGRF5
 
 
hsa-miR-148a-3p
 
 
-0.354
 
 
20.6
 
 
 
 
AGFG1
 
 
hsa-miR-148a-3p
 
 
-0.332
 
 
36.7
 
 
 
 
AHNAK
 
 
hsa-miR-20a-5p
 
 
-0.316
 
 
105.6
 
 
 
 
AHNAK
 
 
hsa-miR-106b-5p
 
 
-0.316
 
 
105.6
 
 
 
 
AHNAK
 
 
hsa-miR-106a-5p
 
 
-0.334
 
 
105.6
 
 
 
 
AHNAK
 
 
hsa-miR-17-5p
 
 
-0.337
 
 
105.6
 
 
 
 
AHNAK
 
 
hsa-miR-20b-5p
 
 
-0.337
 
 
105.6
 
 
 
 
AKAP1
 
 
hsa-miR-148a-3p
 
 
-0.381
 
 
24.6
 
 
 
 
ALCAM
 
 
hsa-miR-148a-3p
 
 
-0.339
 
 
15.2
 
 
 
 
ALG3
 
 
hsa-miR-106b-5p
 
 
-0.551
 
 
15.3
 
 
 
 
ALG3
 
 
hsa-miR-20a-5p
 
 
-0.559
 
 
15.3
 
 
 
 
ALG3
 
 
hsa-miR-106a-5p
 
 
-0.569
 
 
15.3
 
 
 
 
ALG3
 
 
hsa-miR-17-5p
 
 
-0.572
 
 
15.3
 
 
 
 
ALG3
 
 
hsa-miR-20b-5p
 
 
-0.58
 
 
15.3
 
 
 
 
ALG9
 
 
hsa-miR-7-5p
 
 
-0.302
 
 
10.4
 
 
 
 
ANKRD29
 
 
hsa-miR-20a-5p
 
 
-0.359
 
 
10.9
 
 
 
 
ANKRD29
 
 
hsa-miR-106b-5p
 
 
-0.359
 
 
10.9
 
 
 
 
ANKRD29
 
 
hsa-miR-106a-5p
 
 
-0.369
 
 
10.9
 
 
 
 
ANKRD29
 
 
hsa-miR-17-5p
 
 
-0.372
 
 
10.9
 
 
 
 
ANKRD29
 
 
hsa-miR-20b-5p
 
 
-0.38
 
 
10.9
 
 
 
 
ANKRD46
 
 
hsa-miR-98-5p
 
 
-0.389
 
 
11.6
 
 
 
 
ANKRD46
 
 
hsa-let-7c-5p
 
 
-0.389
 
 
11.6
 
 
 
 
AP1S1
 
 
hsa-miR-98-5p
 
 
-0.374
 
 
11.9
 
 
 
 
AP1S1
 
 
hsa-let-7c-5p
 
 
-0.374
 
 
11.9
 
 
 
 
ARF4
 
 
hsa-miR-7-5p
 
 
-0.407
 
 
157.9
 
 
 
 
ARF4
 
 
hsa-miR-148a-3p
 
 
-0.487
 
 
157.9
 
 
 
 
ARFGEF1
 
 
hsa-miR-148a-3p
 
 
-0.306
 
 
10.1
 
 
 
 
ARFIP1
 
 
hsa-miR-148a-3p
 
 
-0.474
 
 
18.5
 
 
 
 
ARHGAP1
 
 
hsa-miR-17-5p
 
 
-0.325
 
 
75.2
 
 
 
 
ARHGAP1
 
 
hsa-miR-20b-5p
 
 
-0.325
 
 
75.2
 
 
 
 
ARHGAP1
 
 
hsa-miR-106a-5p
 
 
-0.329
 
 
75.2
 
 
 
 
ARHGAP1
 
 
hsa-miR-20a-5p
 
 
-0.339
 
 
75.2
 
 
 
 
ARHGAP1
 
 
hsa-miR-106b-5p
 
 
-0.339
 
 
75.2
 
 
 
 
ARHGAP21
 
 
hsa-miR-148a-3p
 
 
-0.287
 
 
16.5
 
 
 
 
ARHGAP24
 
 
hsa-miR-21-5p
 
 
-0.427
 
 
13.1
 
 
 
 
ARL1
 
 
hsa-miR-20a-5p
 
 
-0.403
 
 
55.7
 
 
 
 
ARL1
 
 
hsa-miR-106b-5p
 
 
-0.411
 
 
55.7
 
 
 
 
ARL1
 
 
hsa-miR-20b-5p
 
 
-0.424
 
 
55.7
 
 
 
 
ARL1
 
 
hsa-miR-106a-5p
 
 
-0.429
 
 
55.7
 
 
 
 
ARL1
 
 
hsa-miR-17-5p
 
 
-0.432
 
 
55.7
 
 
 
 
ARL4D
 
 
hsa-miR-98-5p
 
 
-0.417
 
 
13.8
 
 
 
 
ARL4D
 
 
hsa-let-7c-5p
 
 
-0.417
 
 
13.8
 
 
 
 
ARL6IP1
 
 
hsa-miR-7-5p
 
 
-0.374
 
 
64.5
 
 
 
 
ARL6IP1
 
 
hsa-miR-148a-3p
 
 
-0.579
 
 
64.5
 
 
 
 
ARL6IP6
 
 
hsa-miR-98-5p
 
 
-0.293
 
 
10.8
 
 
 
 
ARL6IP6
 
 
hsa-let-7c-5p
 
 
-0.293
 
 
10.8
 
 
 
 
ARL8B
 
 
hsa-miR-148a-3p
 
 
-0.337
 
 
43.9
 
 
 
 
ARMC8
 
 
hsa-miR-20a-5p
 
 
-0.357
 
 
22.8
 
 
 
 
ARMC8
 
 
hsa-miR-106b-5p
 
 
-0.357
 
 
22.8
 
 
 
 
ARMC8
 
 
hsa-miR-106a-5p
 
 
-0.375
 
 
22.8
 
 
 
 
ARMC8
 
 
hsa-miR-17-5p
 
 
-0.378
 
 
22.8
 
 
 
 
ARMC8
 
 
hsa-miR-20b-5p
 
 
-0.378
 
 
22.8
 
 
 
 
ARMCX1
 
 
hsa-miR-21-5p
 
 
-0.489
 
 
19.8
 
 
 
 
ARMT1
 
 
hsa-let-7c-5p
 
 
-0.303
 
 
13.9
 
 
 
 
ARMT1
 
 
hsa-miR-98-5p
 
 
-0.303
 
 
13.9
 
 
 
 
ARPP19
 
 
hsa-let-7c-5p
 
 
-0.492
 
 
54.2
 
 
 
 
ARPP19
 
 
hsa-miR-98-5p
 
 
-0.5
 
 
54.2
 
 
 
 
ARRDC3
 
 
hsa-miR-148a-3p
 
 
-0.305
 
 
30.8
 
 
 
 
ARRDC4
 
 
hsa-let-7c-5p
 
 
-0.329
 
 
16.9
 
 
 
 
ARRDC4
 
 
hsa-miR-98-5p
 
 
-0.329
 
 
16.9
 
 
 
 
ASAP1
 
 
hsa-miR-98-5p
 
 
-0.308
 
 
16.7
 
 
 
 
ASAP1
 
 
hsa-let-7c-5p
 
 
-0.308
 
 
16.7
 
 
 
 
ASPH
 
 
hsa-miR-148a-3p
 
 
-0.391
 
 
62.4
 
 
 
 
ATOSB
 
 
hsa-miR-98-5p
 
 
-0.429
 
 
10.3
 
 
 
 
ATOSB
 
 
hsa-let-7c-5p
 
 
-0.429
 
 
10.3
 
 
 
 
ATP2A2
 
 
hsa-miR-98-5p
 
 
-0.311
 
 
69.4
 
 
 
 
ATP2A2
 
 
hsa-let-7c-5p
 
 
-0.311
 
 
69.4
 
 
 
 
ATP2B4
 
 
hsa-miR-148a-3p
 
 
-0.337
 
 
164.7
 
 
 
 
ATP6AP2
 
 
hsa-miR-148a-3p
 
 
-0.592
 
 
116.5
 
 
 
 
ATP6AP2
 
 
hsa-miR-148a-3p
 
 
-0.668
 
 
116.5
 
 
 
 
ATPAF1
 
 
hsa-miR-98-5p
 
 
-0.498
 
 
30.4
 
 
 
 
ATPAF1
 
 
hsa-let-7c-5p
 
 
-0.498
 
 
30.4
 
 
 
 
B3GAT3
 
 
hsa-miR-98-5p
 
 
-0.324
 
 
13.1
 
 
 
 
B3GAT3
 
 
hsa-let-7c-5p
 
 
-0.324
 
 
13.1
 
 
 
 
B4GALT5
 
 
hsa-miR-148a-3p
 
 
-0.44
 
 
35.1
 
 
 
 
B4GAT1
 
 
hsa-let-7c-5p
 
 
-0.47
 
 
13.1
 
 
 
 
B4GAT1
 
 
hsa-miR-98-5p
 
 
-0.47
 
 
13.1
 
 
 
 
BACH1
 
 
hsa-miR-98-5p
 
 
-0.298
 
 
14.9
 
 
 
 
BACH1
 
 
hsa-miR-98-5p
 
 
-0.298
 
 
14.9
 
 
 
 
BACH1
 
 
hsa-let-7c-5p
 
 
-0.306
 
 
14.9
 
 
 
 
BACH1
 
 
hsa-let-7c-5p
 
 
-0.306
 
 
14.9
 
 
 
 
BACH1
 
 
hsa-miR-98-5p
 
 
-0.435
 
 
14.9
 
 
 
 
BACH1
 
 
hsa-miR-98-5p
 
 
-0.435
 
 
14.9
 
 
 
 
BACH1
 
 
hsa-let-7c-5p
 
 
-0.435
 
 
14.9
 
 
 
 
BACH1
 
 
hsa-let-7c-5p
 
 
-0.435
 
 
14.9
 
 
 
 
BAMBI
 
 
hsa-miR-20a-5p
 
 
-0.485
 
 
11.4
 
 
 
 
BAMBI
 
 
hsa-miR-106b-5p
 
 
-0.493
 
 
11.4
 
 
 
 
BAMBI
 
 
hsa-miR-106a-5p
 
 
-0.503
 
 
11.4
 
 
 
 
BAMBI
 
 
hsa-miR-17-5p
 
 
-0.506
 
 
11.4
 
 
 
 
BAMBI
 
 
hsa-miR-20b-5p
 
 
-0.506
 
 
11.4
 
 
 
 
BLOC1S6
 
 
hsa-miR-98-5p
 
 
-0.321
 
 
22.7
 
 
 
 
BLOC1S6
 
 
hsa-let-7c-5p
 
 
-0.321
 
 
22.7
 
 
 
 
 
 
 spinal cord 
 
 
 
 
target_symbol
 
 
mature_mirna_id
 
 
score
 
 
nTPM
 
 
 
 
 
 
ABCD3
 
 
hsa-miR-148a-3p
 
 
-0.388
 
 
20.9
 
 
 
 
ABHD5
 
 
hsa-miR-20a-5p
 
 
-0.397
 
 
19.5
 
 
 
 
ABHD5
 
 
hsa-miR-106b-5p
 
 
-0.413
 
 
19.5
 
 
 
 
ABHD5
 
 
hsa-miR-20b-5p
 
 
-0.418
 
 
19.5
 
 
 
 
ABHD5
 
 
hsa-miR-106a-5p
 
 
-0.447
 
 
19.5
 
 
 
 
ABHD5
 
 
hsa-miR-17-5p
 
 
-0.45
 
 
19.5
 
 
 
 
ADAM10
 
 
hsa-miR-148a-3p
 
 
-0.356
 
 
18.7
 
 
 
 
ADAM9
 
 
hsa-miR-20a-5p
 
 
-0.299
 
 
11.3
 
 
 
 
ADAM9
 
 
hsa-miR-106b-5p
 
 
-0.299
 
 
11.3
 
 
 
 
ADAM9
 
 
hsa-miR-106a-5p
 
 
-0.317
 
 
11.3
 
 
 
 
ADAM9
 
 
hsa-miR-17-5p
 
 
-0.32
 
 
11.3
 
 
 
 
ADAM9
 
 
hsa-miR-20b-5p
 
 
-0.328
 
 
11.3
 
 
 
 
ADCY2
 
 
hsa-miR-148a-3p
 
 
-0.299
 
 
11.4
 
 
 
 
AGFG1
 
 
hsa-miR-148a-3p
 
 
-0.332
 
 
53.5
 
 
 
 
AHNAK
 
 
hsa-miR-20a-5p
 
 
-0.316
 
 
27.2
 
 
 
 
AHNAK
 
 
hsa-miR-106b-5p
 
 
-0.316
 
 
27.2
 
 
 
 
AHNAK
 
 
hsa-miR-106a-5p
 
 
-0.334
 
 
27.2
 
 
 
 
AHNAK
 
 
hsa-miR-17-5p
 
 
-0.337
 
 
27.2
 
 
 
 
AHNAK
 
 
hsa-miR-20b-5p
 
 
-0.337
 
 
27.2
 
 
 
 
AIFM1
 
 
hsa-miR-98-5p
 
 
-0.314
 
 
10.1
 
 
 
 
AIFM1
 
 
hsa-let-7c-5p
 
 
-0.314
 
 
10.1
 
 
 
 
AKAP1
 
 
hsa-miR-148a-3p
 
 
-0.381
 
 
14.0
 
 
 
 
AKAP6
 
 
hsa-miR-98-5p
 
 
-0.307
 
 
13.3
 
 
 
 
AKAP6
 
 
hsa-let-7c-5p
 
 
-0.307
 
 
13.3
 
 
 
 
ALCAM
 
 
hsa-miR-148a-3p
 
 
-0.339
 
 
56.1
 
 
 
 
ALDH4A1
 
 
hsa-miR-184
 
 
-0.421
 
 
11.4
 
 
 
 
ALG3
 
 
hsa-miR-106b-5p
 
 
-0.551
 
 
10.8
 
 
 
 
ALG3
 
 
hsa-miR-20a-5p
 
 
-0.559
 
 
10.8
 
 
 
 
ALG3
 
 
hsa-miR-106a-5p
 
 
-0.569
 
 
10.8
 
 
 
 
ALG3
 
 
hsa-miR-17-5p
 
 
-0.572
 
 
10.8
 
 
 
 
ALG3
 
 
hsa-miR-20b-5p
 
 
-0.58
 
 
10.8
 
 
 
 
ANKRD46
 
 
hsa-miR-98-5p
 
 
-0.389
 
 
24.6
 
 
 
 
ANKRD46
 
 
hsa-let-7c-5p
 
 
-0.389
 
 
24.6
 
 
 
 
AP1S1
 
 
hsa-miR-98-5p
 
 
-0.374
 
 
28.5
 
 
 
 
AP1S1
 
 
hsa-let-7c-5p
 
 
-0.374
 
 
28.5
 
 
 
 
APCDD1
 
 
hsa-miR-20a-5p
 
 
-0.404
 
 
14.7
 
 
 
 
APCDD1
 
 
hsa-miR-106b-5p
 
 
-0.404
 
 
14.7
 
 
 
 
APCDD1
 
 
hsa-miR-106a-5p
 
 
-0.422
 
 
14.7
 
 
 
 
APCDD1
 
 
hsa-miR-17-5p
 
 
-0.425
 
 
14.7
 
 
 
 
APCDD1
 
 
hsa-miR-20b-5p
 
 
-0.425
 
 
14.7
 
 
 
 
ARF4
 
 
hsa-miR-7-5p
 
 
-0.407
 
 
69.7
 
 
 
 
ARF4
 
 
hsa-miR-148a-3p
 
 
-0.487
 
 
69.7
 
 
 
 
ARFIP1
 
 
hsa-miR-148a-3p
 
 
-0.474
 
 
10.6
 
 
 
 
ARHGAP1
 
 
hsa-miR-17-5p
 
 
-0.325
 
 
38.6
 
 
 
 
ARHGAP1
 
 
hsa-miR-20b-5p
 
 
-0.325
 
 
38.6
 
 
 
 
ARHGAP1
 
 
hsa-miR-106a-5p
 
 
-0.329
 
 
38.6
 
 
 
 
ARHGAP1
 
 
hsa-miR-20a-5p
 
 
-0.339
 
 
38.6
 
 
 
 
ARHGAP1
 
 
hsa-miR-106b-5p
 
 
-0.339
 
 
38.6
 
 
 
 
ARHGAP21
 
 
hsa-miR-148a-3p
 
 
-0.287
 
 
74.7
 
 
 
 
ARID4B
 
 
hsa-miR-20a-5p
 
 
-0.29
 
 
12.3
 
 
 
 
ARID4B
 
 
hsa-miR-106b-5p
 
 
-0.29
 
 
12.3
 
 
 
 
ARL1
 
 
hsa-miR-20a-5p
 
 
-0.403
 
 
26.5
 
 
 
 
ARL1
 
 
hsa-miR-106b-5p
 
 
-0.411
 
 
26.5
 
 
 
 
ARL1
 
 
hsa-miR-20b-5p
 
 
-0.424
 
 
26.5
 
 
 
 
ARL1
 
 
hsa-miR-106a-5p
 
 
-0.429
 
 
26.5
 
 
 
 
ARL1
 
 
hsa-miR-17-5p
 
 
-0.432
 
 
26.5
 
 
 
 
ARL6IP1
 
 
hsa-miR-7-5p
 
 
-0.374
 
 
140.2
 
 
 
 
ARL6IP1
 
 
hsa-miR-148a-3p
 
 
-0.579
 
 
140.2
 
 
 
 
ARL8B
 
 
hsa-miR-148a-3p
 
 
-0.337
 
 
57.8
 
 
 
 
ARMC8
 
 
hsa-miR-20a-5p
 
 
-0.357
 
 
15.5
 
 
 
 
ARMC8
 
 
hsa-miR-106b-5p
 
 
-0.357
 
 
15.5
 
 
 
 
ARMC8
 
 
hsa-miR-106a-5p
 
 
-0.375
 
 
15.5
 
 
 
 
ARMC8
 
 
hsa-miR-17-5p
 
 
-0.378
 
 
15.5
 
 
 
 
ARMC8
 
 
hsa-miR-20b-5p
 
 
-0.378
 
 
15.5
 
 
 
 
ARMCX1
 
 
hsa-miR-21-5p
 
 
-0.489
 
 
12.2
 
 
 
 
ARPP19
 
 
hsa-let-7c-5p
 
 
-0.492
 
 
170.8
 
 
 
 
ARPP19
 
 
hsa-miR-98-5p
 
 
-0.5
 
 
170.8
 
 
 
 
ARRDC3
 
 
hsa-miR-148a-3p
 
 
-0.305
 
 
23.5
 
 
 
 
ARRDC4
 
 
hsa-let-7c-5p
 
 
-0.329
 
 
75.7
 
 
 
 
ARRDC4
 
 
hsa-miR-98-5p
 
 
-0.329
 
 
75.7
 
 
 
 
ASAP1
 
 
hsa-miR-98-5p
 
 
-0.308
 
 
11.0
 
 
 
 
ASAP1
 
 
hsa-let-7c-5p
 
 
-0.308
 
 
11.0
 
 
 
 
ASF1A
 
 
hsa-miR-21-5p
 
 
-0.304
 
 
14.8
 
 
 
 
ASPH
 
 
hsa-miR-148a-3p
 
 
-0.391
 
 
25.3
 
 
 
 
ATG16L1
 
 
hsa-miR-20a-5p
 
 
-0.446
 
 
10.3
 
 
 
 
ATG16L1
 
 
hsa-miR-106b-5p
 
 
-0.446
 
 
10.3
 
 
 
 
ATG16L1
 
 
hsa-miR-106a-5p
 
 
-0.464
 
 
10.3
 
 
 
 
ATG16L1
 
 
hsa-miR-17-5p
 
 
-0.467
 
 
10.3
 
 
 
 
ATG16L1
 
 
hsa-miR-20b-5p
 
 
-0.467
 
 
10.3
 
 
 
 
ATOSB
 
 
hsa-miR-98-5p
 
 
-0.429
 
 
30.9
 
 
 
 
ATOSB
 
 
hsa-let-7c-5p
 
 
-0.429
 
 
30.9
 
 
 
 
ATP2A2
 
 
hsa-miR-98-5p
 
 
-0.311
 
 
19.0
 
 
 
 
ATP2A2
 
 
hsa-let-7c-5p
 
 
-0.311
 
 
19.0
 
 
 
 
ATP6AP2
 
 
hsa-miR-148a-3p
 
 
-0.592
 
 
55.3
 
 
 
 
ATP6AP2
 
 
hsa-miR-148a-3p
 
 
-0.668
 
 
55.3
 
 
 
 
ATPAF1
 
 
hsa-miR-98-5p
 
 
-0.498
 
 
50.4
 
 
 
 
ATPAF1
 
 
hsa-let-7c-5p
 
 
-0.498
 
 
50.4
 
 
 
 
AVPI1
 
 
hsa-miR-7-5p
 
 
-0.367
 
 
33.9
 
 
 
 
B3GAT3
 
 
hsa-miR-98-5p
 
 
-0.324
 
 
62.6
 
 
 
 
B3GAT3
 
 
hsa-let-7c-5p
 
 
-0.324
 
 
62.6
 
 
 
 
B4GAT1
 
 
hsa-let-7c-5p
 
 
-0.47
 
 
48.7
 
 
 
 
B4GAT1
 
 
hsa-miR-98-5p
 
 
-0.47
 
 
48.7
 
 
 
 
BAMBI
 
 
hsa-miR-20a-5p
 
 
-0.485
 
 
23.0
 
 
 
 
BAMBI
 
 
hsa-miR-106b-5p
 
 
-0.493
 
 
23.0
 
 
 
 
BAMBI
 
 
hsa-miR-106a-5p
 
 
-0.503
 
 
23.0
 
 
 
 
BAMBI
 
 
hsa-miR-17-5p
 
 
-0.506
 
 
23.0
 
 
 
 
BAMBI
 
 
hsa-miR-20b-5p
 
 
-0.506
 
 
23.0
 
 
 
 
BHLHE41
 
 
hsa-miR-148a-3p
 
 
-0.442
 
 
74.7
 
 
 
 
BLOC1S6
 
 
hsa-miR-98-5p
 
 
-0.321
 
 
15.5
 
 
 
 
BLOC1S6
 
 
hsa-let-7c-5p
 
 
-0.321
 
 
15.5
 
 
 
 
 
 
 thyroid gland 
 
 
 
 
target_symbol
 
 
mature_mirna_id
 
 
score
 
 
nTPM
 
 
 
 
 
 
ABCD3
 
 
hsa-miR-148a-3p
 
 
-0.388
 
 
25.8
 
 
 
 
ABHD5
 
 
hsa-miR-20a-5p
 
 
-0.397
 
 
14.4
 
 
 
 
ABHD5
 
 
hsa-miR-106b-5p
 
 
-0.413
 
 
14.4
 
 
 
 
ABHD5
 
 
hsa-miR-20b-5p
 
 
-0.418
 
 
14.4
 
 
 
 
ABHD5
 
 
hsa-miR-106a-5p
 
 
-0.447
 
 
14.4
 
 
 
 
ABHD5
 
 
hsa-miR-17-5p
 
 
-0.45
 
 
14.4
 
 
 
 
ACVR1
 
 
hsa-miR-148a-3p
 
 
-0.397
 
 
28.6
 
 
 
 
ADAM10
 
 
hsa-miR-148a-3p
 
 
-0.356
 
 
26.7
 
 
 
 
ADAM9
 
 
hsa-miR-20a-5p
 
 
-0.299
 
 
37.4
 
 
 
 
ADAM9
 
 
hsa-miR-106b-5p
 
 
-0.299
 
 
37.4
 
 
 
 
ADAM9
 
 
hsa-miR-106a-5p
 
 
-0.317
 
 
37.4
 
 
 
 
ADAM9
 
 
hsa-miR-17-5p
 
 
-0.32
 
 
37.4
 
 
 
 
ADAM9
 
 
hsa-miR-20b-5p
 
 
-0.328
 
 
37.4
 
 
 
 
ADGRF5
 
 
hsa-miR-148a-3p
 
 
-0.354
 
 
30.7
 
 
 
 
AGFG1
 
 
hsa-miR-148a-3p
 
 
-0.332
 
 
32.1
 
 
 
 
AGFG2
 
 
hsa-miR-20a-5p
 
 
-0.365
 
 
10.2
 
 
 
 
AGFG2
 
 
hsa-miR-20b-5p
 
 
-0.386
 
 
10.2
 
 
 
 
AGFG2
 
 
hsa-miR-106b-5p
 
 
-0.397
 
 
10.2
 
 
 
 
AGFG2
 
 
hsa-miR-106a-5p
 
 
-0.399
 
 
10.2
 
 
 
 
AGFG2
 
 
hsa-miR-17-5p
 
 
-0.402
 
 
10.2
 
 
 
 
AHCTF1
 
 
hsa-miR-98-5p
 
 
-0.374
 
 
12.9
 
 
 
 
AHCTF1
 
 
hsa-let-7c-5p
 
 
-0.374
 
 
12.9
 
 
 
 
AHDC1
 
 
hsa-miR-148a-3p
 
 
-0.413
 
 
19.9
 
 
 
 
AHNAK
 
 
hsa-miR-20a-5p
 
 
-0.316
 
 
61.4
 
 
 
 
AHNAK
 
 
hsa-miR-106b-5p
 
 
-0.316
 
 
61.4
 
 
 
 
AHNAK
 
 
hsa-miR-106a-5p
 
 
-0.334
 
 
61.4
 
 
 
 
AHNAK
 
 
hsa-miR-17-5p
 
 
-0.337
 
 
61.4
 
 
 
 
AHNAK
 
 
hsa-miR-20b-5p
 
 
-0.337
 
 
61.4
 
 
 
 
AIFM1
 
 
hsa-miR-98-5p
 
 
-0.314
 
 
10.7
 
 
 
 
AIFM1
 
 
hsa-let-7c-5p
 
 
-0.314
 
 
10.7
 
 
 
 
AKAP1
 
 
hsa-miR-148a-3p
 
 
-0.381
 
 
35.0
 
 
 
 
ALCAM
 
 
hsa-miR-148a-3p
 
 
-0.339
 
 
58.4
 
 
 
 
ALG3
 
 
hsa-miR-106b-5p
 
 
-0.551
 
 
17.5
 
 
 
 
ALG3
 
 
hsa-miR-20a-5p
 
 
-0.559
 
 
17.5
 
 
 
 
ALG3
 
 
hsa-miR-106a-5p
 
 
-0.569
 
 
17.5
 
 
 
 
ALG3
 
 
hsa-miR-17-5p
 
 
-0.572
 
 
17.5
 
 
 
 
ALG3
 
 
hsa-miR-20b-5p
 
 
-0.58
 
 
17.5
 
 
 
 
ALG9
 
 
hsa-miR-7-5p
 
 
-0.302
 
 
11.5
 
 
 
 
ANKRD12
 
 
hsa-miR-7-5p
 
 
-0.37
 
 
15.3
 
 
 
 
ANKRD46
 
 
hsa-miR-98-5p
 
 
-0.389
 
 
18.8
 
 
 
 
ANKRD46
 
 
hsa-let-7c-5p
 
 
-0.389
 
 
18.8
 
 
 
 
ANKRD9
 
 
hsa-miR-20a-5p
 
 
-0.31
 
 
10.7
 
 
 
 
ANKRD9
 
 
hsa-miR-106b-5p
 
 
-0.318
 
 
10.7
 
 
 
 
ANKRD9
 
 
hsa-miR-20b-5p
 
 
-0.331
 
 
10.7
 
 
 
 
ANKRD9
 
 
hsa-miR-106a-5p
 
 
-0.344
 
 
10.7
 
 
 
 
ANKRD9
 
 
hsa-miR-17-5p
 
 
-0.347
 
 
10.7
 
 
 
 
AP1AR
 
 
hsa-miR-21-5p
 
 
-0.318
 
 
10.1
 
 
 
 
AP1S1
 
 
hsa-miR-98-5p
 
 
-0.374
 
 
15.7
 
 
 
 
AP1S1
 
 
hsa-let-7c-5p
 
 
-0.374
 
 
15.7
 
 
 
 
ARF4
 
 
hsa-miR-7-5p
 
 
-0.407
 
 
134.8
 
 
 
 
ARF4
 
 
hsa-miR-148a-3p
 
 
-0.487
 
 
134.8
 
 
 
 
ARFGEF1
 
 
hsa-miR-148a-3p
 
 
-0.306
 
 
20.9
 
 
 
 
ARFIP1
 
 
hsa-miR-148a-3p
 
 
-0.474
 
 
18.8
 
 
 
 
ARG2
 
 
hsa-miR-98-5p
 
 
-0.468
 
 
97.9
 
 
 
 
ARG2
 
 
hsa-let-7c-5p
 
 
-0.468
 
 
97.9
 
 
 
 
ARHGAP1
 
 
hsa-miR-17-5p
 
 
-0.325
 
 
42.2
 
 
 
 
ARHGAP1
 
 
hsa-miR-20b-5p
 
 
-0.325
 
 
42.2
 
 
 
 
ARHGAP1
 
 
hsa-miR-106a-5p
 
 
-0.329
 
 
42.2
 
 
 
 
ARHGAP1
 
 
hsa-miR-20a-5p
 
 
-0.339
 
 
42.2
 
 
 
 
ARHGAP1
 
 
hsa-miR-106b-5p
 
 
-0.339
 
 
42.2
 
 
 
 
ARHGAP21
 
 
hsa-miR-148a-3p
 
 
-0.287
 
 
12.0
 
 
 
 
ARHGAP24
 
 
hsa-miR-21-5p
 
 
-0.427
 
 
26.1
 
 
 
 
ARHGEF28
 
 
hsa-miR-20b-5p
 
 
-0.352
 
 
12.7
 
 
 
 
ARHGEF28
 
 
hsa-miR-17-5p
 
 
-0.363
 
 
12.7
 
 
 
 
ARHGEF28
 
 
hsa-miR-20a-5p
 
 
-0.366
 
 
12.7
 
 
 
 
ARHGEF28
 
 
hsa-miR-106a-5p
 
 
-0.367
 
 
12.7
 
 
 
 
ARHGEF28
 
 
hsa-miR-106b-5p
 
 
-0.377
 
 
12.7
 
 
 
 
ARID4B
 
 
hsa-miR-20a-5p
 
 
-0.29
 
 
15.9
 
 
 
 
ARID4B
 
 
hsa-miR-106b-5p
 
 
-0.29
 
 
15.9
 
 
 
 
ARL1
 
 
hsa-miR-20a-5p
 
 
-0.403
 
 
79.3
 
 
 
 
ARL1
 
 
hsa-miR-106b-5p
 
 
-0.411
 
 
79.3
 
 
 
 
ARL1
 
 
hsa-miR-20b-5p
 
 
-0.424
 
 
79.3
 
 
 
 
ARL1
 
 
hsa-miR-106a-5p
 
 
-0.429
 
 
79.3
 
 
 
 
ARL1
 
 
hsa-miR-17-5p
 
 
-0.432
 
 
79.3
 
 
 
 
ARL4D
 
 
hsa-miR-98-5p
 
 
-0.417
 
 
35.9
 
 
 
 
ARL4D
 
 
hsa-let-7c-5p
 
 
-0.417
 
 
35.9
 
 
 
 
ARL6IP1
 
 
hsa-miR-7-5p
 
 
-0.374
 
 
105.4
 
 
 
 
ARL6IP1
 
 
hsa-miR-148a-3p
 
 
-0.579
 
 
105.4
 
 
 
 
ARL8B
 
 
hsa-miR-148a-3p
 
 
-0.337
 
 
58.1
 
 
 
 
ARMC8
 
 
hsa-miR-20a-5p
 
 
-0.357
 
 
20.0
 
 
 
 
ARMC8
 
 
hsa-miR-106b-5p
 
 
-0.357
 
 
20.0
 
 
 
 
ARMC8
 
 
hsa-miR-106a-5p
 
 
-0.375
 
 
20.0
 
 
 
 
ARMC8
 
 
hsa-miR-17-5p
 
 
-0.378
 
 
20.0
 
 
 
 
ARMC8
 
 
hsa-miR-20b-5p
 
 
-0.378
 
 
20.0
 
 
 
 
ARMCX1
 
 
hsa-miR-21-5p
 
 
-0.489
 
 
15.6
 
 
 
 
ARMT1
 
 
hsa-let-7c-5p
 
 
-0.303
 
 
25.9
 
 
 
 
ARMT1
 
 
hsa-miR-98-5p
 
 
-0.303
 
 
25.9
 
 
 
 
ARPP19
 
 
hsa-let-7c-5p
 
 
-0.492
 
 
53.7
 
 
 
 
ARPP19
 
 
hsa-miR-98-5p
 
 
-0.5
 
 
53.7
 
 
 
 
ARRDC3
 
 
hsa-miR-148a-3p
 
 
-0.305
 
 
78.7
 
 
 
 
ARRDC4
 
 
hsa-let-7c-5p
 
 
-0.329
 
 
16.2
 
 
 
 
ARRDC4
 
 
hsa-miR-98-5p
 
 
-0.329
 
 
16.2
 
 
 
 
ASAP1
 
 
hsa-miR-98-5p
 
 
-0.308
 
 
15.1
 
 
 
 
ASAP1
 
 
hsa-let-7c-5p
 
 
-0.308
 
 
15.1
 
 
 
 
ASF1A
 
 
hsa-miR-21-5p
 
 
-0.304
 
 
14.1
 
 
 
 
ASPH
 
 
hsa-miR-148a-3p
 
 
-0.391
 
 
90.3
 
 
 
 
ATG16L1
 
 
hsa-miR-20a-5p
 
 
-0.446
 
 
11.5
 
 
 
 
ATG16L1
 
 
hsa-miR-106b-5p
 
 
-0.446
 
 
11.5
 
 
 
 
ATG16L1
 
 
hsa-miR-106a-5p
 
 
-0.464
 
 
11.5
 
 
 
 
ATG16L1
 
 
hsa-miR-17-5p
 
 
-0.467
 
 
11.5
 
 
 
 
 
 
 
 cd8_ev_vs_cd8_s0_upregulated 
 
 Tissue expression heatmap (log10-transformed) 
   
 
 
 liver 
 
 
 
 
target_symbol
 
 
mature_mirna_id
 
 
score
 
 
nTPM
 
 
 
 
 
 
A1BG
 
 
hsa-miR-23a-3p
 
 
-0.434
 
 
565.1
 
 
 
 
AAMP
 
 
hsa-miR-326
 
 
-0.722
 
 
77.2
 
 
 
 
AAR2
 
 
hsa-miR-29a-3p
 
 
-0.327
 
 
22.0
 
 
 
 
ABAT
 
 
hsa-miR-1271-5p
 
 
-0.409
 
 
323.2
 
 
 
 
ABCB10
 
 
hsa-miR-152-3p
 
 
-0.291
 
 
17.7
 
 
 
 
ABCB10
 
 
hsa-miR-148b-3p
 
 
-0.291
 
 
17.7
 
 
 
 
ABCB6
 
 
hsa-miR-29a-3p
 
 
-0.48
 
 
12.7
 
 
 
 
ABCB7
 
 
hsa-miR-148b-3p
 
 
-0.515
 
 
14.1
 
 
 
 
ABCB7
 
 
hsa-miR-152-3p
 
 
-0.515
 
 
14.1
 
 
 
 
ABCD3
 
 
hsa-miR-148b-3p
 
 
-0.388
 
 
118.4
 
 
 
 
ABCD3
 
 
hsa-miR-152-3p
 
 
-0.388
 
 
118.4
 
 
 
 
ABCE1
 
 
hsa-miR-29a-3p
 
 
-0.404
 
 
37.1
 
 
 
 
ABHD4
 
 
hsa-miR-29a-3p
 
 
-0.312
 
 
24.2
 
 
 
 
ABHD5
 
 
hsa-miR-132-3p
 
 
-0.323
 
 
20.3
 
 
 
 
ACTR2
 
 
hsa-miR-15b-5p
 
 
-0.293
 
 
101.7
 
 
 
 
ACTR3C
 
 
hsa-miR-30b-5p
 
 
-0.448
 
 
18.8
 
 
 
 
ACTR3C
 
 
hsa-miR-30d-5p
 
 
-0.456
 
 
18.8
 
 
 
 
ACVR1
 
 
hsa-miR-30b-5p
 
 
-0.29
 
 
15.3
 
 
 
 
ACVR1
 
 
hsa-miR-152-3p
 
 
-0.397
 
 
15.3
 
 
 
 
ACVR1
 
 
hsa-miR-148b-3p
 
 
-0.397
 
 
15.3
 
 
 
 
ADAM10
 
 
hsa-miR-152-3p
 
 
-0.348
 
 
15.0
 
 
 
 
ADAM10
 
 
hsa-miR-148b-3p
 
 
-0.356
 
 
15.0
 
 
 
 
ADAM9
 
 
hsa-miR-30d-5p
 
 
-0.304
 
 
12.7
 
 
 
 
ADAM9
 
 
hsa-miR-30b-5p
 
 
-0.312
 
 
12.7
 
 
 
 
ADGRA3
 
 
hsa-miR-30d-5p
 
 
-0.296
 
 
54.5
 
 
 
 
ADGRA3
 
 
hsa-miR-30b-5p
 
 
-0.304
 
 
54.5
 
 
 
 
ADGRF5
 
 
hsa-miR-148b-3p
 
 
-0.354
 
 
12.3
 
 
 
 
ADGRF5
 
 
hsa-miR-152-3p
 
 
-0.354
 
 
12.3
 
 
 
 
ADIPOR2
 
 
hsa-miR-150-5p
 
 
-0.287
 
 
77.7
 
 
 
 
ADK
 
 
hsa-miR-1271-5p
 
 
-0.329
 
 
141.2
 
 
 
 
ADNP
 
 
hsa-miR-328-3p
 
 
-0.371
 
 
28.4
 
 
 
 
ADRB2
 
 
hsa-miR-15b-5p
 
 
-0.29
 
 
13.3
 
 
 
 
ADRB2
 
 
hsa-let-7b-5p
 
 
-0.635
 
 
13.3
 
 
 
 
AFG3L2
 
 
hsa-miR-181b-5p
 
 
-0.491
 
 
25.9
 
 
 
 
AFG3L2
 
 
hsa-miR-181a-5p
 
 
-0.491
 
 
25.9
 
 
 
 
AFG3L2
 
 
hsa-miR-181d-5p
 
 
-0.499
 
 
25.9
 
 
 
 
AGFG1
 
 
hsa-miR-148b-3p
 
 
-0.332
 
 
33.8
 
 
 
 
AGFG1
 
 
hsa-miR-152-3p
 
 
-0.332
 
 
33.8
 
 
 
 
AGFG1
 
 
hsa-miR-103a-3p
 
 
-0.389
 
 
33.8
 
 
 
 
AHCTF1
 
 
hsa-let-7b-5p
 
 
-0.374
 
 
10.2
 
 
 
 
AHR
 
 
hsa-miR-1271-5p
 
 
-0.304
 
 
42.0
 
 
 
 
AHR
 
 
hsa-miR-29a-3p
 
 
-0.316
 
 
42.0
 
 
 
 
AIFM1
 
 
hsa-let-7b-5p
 
 
-0.314
 
 
77.0
 
 
 
 
AJUBA
 
 
hsa-miR-103a-3p
 
 
-0.448
 
 
40.5
 
 
 
 
AK1
 
 
hsa-miR-29a-3p
 
 
-0.333
 
 
35.8
 
 
 
 
AK3
 
 
hsa-miR-1271-5p
 
 
-0.57
 
 
186.7
 
 
 
 
AK4
 
 
hsa-miR-15b-5p
 
 
-0.316
 
 
95.6
 
 
 
 
AKAP1
 
 
hsa-miR-152-3p
 
 
-0.381
 
 
48.4
 
 
 
 
AKAP1
 
 
hsa-miR-148b-3p
 
 
-0.381
 
 
48.4
 
 
 
 
AKIRIN2
 
 
hsa-miR-103a-3p
 
 
-0.299
 
 
29.2
 
 
 
 
ALAD
 
 
hsa-miR-326
 
 
-0.352
 
 
196.1
 
 
 
 
ALAD
 
 
hsa-miR-103a-3p
 
 
-0.385
 
 
196.1
 
 
 
 
ALAD
 
 
hsa-miR-15b-5p
 
 
-0.446
 
 
196.1
 
 
 
 
ALCAM
 
 
hsa-miR-148b-3p
 
 
-0.339
 
 
44.8
 
 
 
 
ALCAM
 
 
hsa-miR-152-3p
 
 
-0.347
 
 
44.8
 
 
 
 
ALDH6A1
 
 
hsa-miR-1271-5p
 
 
-0.301
 
 
342.8
 
 
 
 
AMT
 
 
hsa-let-7b-5p
 
 
-0.463
 
 
52.5
 
 
 
 
ANKHD1
 
 
hsa-miR-23a-3p
 
 
-0.368
 
 
51.5
 
 
 
 
ANKHD1
 
 
hsa-miR-30d-5p
 
 
-0.381
 
 
51.5
 
 
 
 
ANKHD1
 
 
hsa-miR-30b-5p
 
 
-0.405
 
 
51.5
 
 
 
 
ANKRA2
 
 
hsa-miR-30d-5p
 
 
-0.343
 
 
10.1
 
 
 
 
ANKRA2
 
 
hsa-miR-30b-5p
 
 
-0.354
 
 
10.1
 
 
 
 
ANKRA2
 
 
hsa-miR-30d-5p
 
 
-0.448
 
 
10.1
 
 
 
 
ANKRA2
 
 
hsa-miR-30b-5p
 
 
-0.472
 
 
10.1
 
 
 
 
ANKRD17
 
 
hsa-miR-30d-5p
 
 
-0.312
 
 
19.3
 
 
 
 
ANKRD17
 
 
hsa-miR-30b-5p
 
 
-0.336
 
 
19.3
 
 
 
 
ANKRD46
 
 
hsa-let-7b-5p
 
 
-0.389
 
 
38.9
 
 
 
 
ANP32A
 
 
hsa-miR-132-3p
 
 
-0.374
 
 
71.3
 
 
 
 
ANTXR2
 
 
hsa-miR-29a-3p
 
 
-0.347
 
 
32.2
 
 
 
 
ANXA11
 
 
hsa-miR-15b-5p
 
 
-0.463
 
 
46.7
 
 
 
 
AP1S1
 
 
hsa-let-7b-5p
 
 
-0.374
 
 
39.1
 
 
 
 
AP2A1
 
 
hsa-miR-15b-5p
 
 
-0.333
 
 
48.9
 
 
 
 
AP2B1
 
 
hsa-miR-1271-5p
 
 
-0.299
 
 
32.8
 
 
 
 
AP2B1
 
 
hsa-miR-15b-5p
 
 
-0.334
 
 
32.8
 
 
 
 
AP3S1
 
 
hsa-miR-30b-5p
 
 
-0.293
 
 
69.8
 
 
 
 
AP5M1
 
 
hsa-miR-1271-5p
 
 
-0.343
 
 
19.7
 
 
 
 
APOO
 
 
hsa-miR-181d-5p
 
 
-0.492
 
 
17.0
 
 
 
 
APOO
 
 
hsa-miR-181b-5p
 
 
-0.5
 
 
17.0
 
 
 
 
APOO
 
 
hsa-miR-181a-5p
 
 
-0.5
 
 
17.0
 
 
 
 
APP
 
 
hsa-miR-15b-5p
 
 
-0.298
 
 
251.5
 
 
 
 
AQP11
 
 
hsa-miR-103a-3p
 
 
-0.517
 
 
15.1
 
 
 
 
AQP11
 
 
hsa-miR-15b-5p
 
 
-0.769
 
 
15.1
 
 
 
 
AQP3
 
 
hsa-miR-874-3p
 
 
-0.48
 
 
117.4
 
 
 
 
ARF4
 
 
hsa-miR-30b-5p
 
 
-0.289
 
 
161.3
 
 
 
 
ARF4
 
 
hsa-miR-194-5p
 
 
-0.293
 
 
161.3
 
 
 
 
ARF4
 
 
hsa-miR-152-3p
 
 
-0.487
 
 
161.3
 
 
 
 
ARF4
 
 
hsa-miR-148b-3p
 
 
-0.487
 
 
161.3
 
 
 
 
ARF5
 
 
hsa-miR-29a-3p
 
 
-0.491
 
 
153.7
 
 
 
 
ARF6
 
 
hsa-miR-181b-5p
 
 
-0.33
 
 
45.4
 
 
 
 
ARF6
 
 
hsa-miR-181a-5p
 
 
-0.33
 
 
45.4
 
 
 
 
ARF6
 
 
hsa-miR-181d-5p
 
 
-0.338
 
 
45.4
 
 
 
 
ARFGEF1
 
 
hsa-miR-148b-3p
 
 
-0.306
 
 
14.2
 
 
 
 
ARFGEF1
 
 
hsa-miR-152-3p
 
 
-0.306
 
 
14.2
 
 
 
 
ARFIP1
 
 
hsa-miR-320b
 
 
-0.339
 
 
14.4
 
 
 
 
ARFIP1
 
 
hsa-miR-320c
 
 
-0.339
 
 
14.4
 
 
 
 
ARFIP1
 
 
hsa-miR-152-3p
 
 
-0.474
 
 
14.4
 
 
 
 
ARFIP1
 
 
hsa-miR-148b-3p
 
 
-0.474
 
 
14.4
 
 
 
 
ARHGAP21
 
 
hsa-miR-152-3p
 
 
-0.287
 
 
11.9
 
 
 
 
ARHGAP21
 
 
hsa-miR-148b-3p
 
 
-0.287
 
 
11.9
 
 
 
 
ARIH2
 
 
hsa-miR-103a-3p
 
 
-0.307
 
 
20.4
 
 
 
 
 
 
 adipose tissue 
 
 
 
 
target_symbol
 
 
mature_mirna_id
 
 
score
 
 
nTPM
 
 
 
 
 
 
AAMP
 
 
hsa-miR-326
 
 
-0.722
 
 
66.6
 
 
 
 
AAR2
 
 
hsa-miR-29a-3p
 
 
-0.327
 
 
21.1
 
 
 
 
ABCB7
 
 
hsa-miR-148b-3p
 
 
-0.515
 
 
12.4
 
 
 
 
ABCB7
 
 
hsa-miR-152-3p
 
 
-0.515
 
 
12.4
 
 
 
 
ABCD3
 
 
hsa-miR-148b-3p
 
 
-0.388
 
 
15.7
 
 
 
 
ABCD3
 
 
hsa-miR-152-3p
 
 
-0.388
 
 
15.7
 
 
 
 
ABCE1
 
 
hsa-miR-29a-3p
 
 
-0.404
 
 
28.1
 
 
 
 
ABHD4
 
 
hsa-miR-29a-3p
 
 
-0.312
 
 
24.3
 
 
 
 
ABHD5
 
 
hsa-miR-132-3p
 
 
-0.323
 
 
27.9
 
 
 
 
ACTC1
 
 
hsa-miR-30d-5p
 
 
-0.519
 
 
47.8
 
 
 
 
ACTC1
 
 
hsa-miR-30b-5p
 
 
-0.543
 
 
47.8
 
 
 
 
ACTR2
 
 
hsa-miR-15b-5p
 
 
-0.293
 
 
105.6
 
 
 
 
ACVR1
 
 
hsa-miR-30b-5p
 
 
-0.29
 
 
12.6
 
 
 
 
ACVR1
 
 
hsa-miR-152-3p
 
 
-0.397
 
 
12.6
 
 
 
 
ACVR1
 
 
hsa-miR-148b-3p
 
 
-0.397
 
 
12.6
 
 
 
 
ACVR1C
 
 
hsa-let-7b-5p
 
 
-0.457
 
 
29.3
 
 
 
 
ADA
 
 
hsa-miR-29a-3p
 
 
-0.376
 
 
16.5
 
 
 
 
ADAM10
 
 
hsa-miR-152-3p
 
 
-0.348
 
 
14.0
 
 
 
 
ADAM10
 
 
hsa-miR-148b-3p
 
 
-0.356
 
 
14.0
 
 
 
 
ADAM9
 
 
hsa-miR-30d-5p
 
 
-0.304
 
 
31.0
 
 
 
 
ADAM9
 
 
hsa-miR-30b-5p
 
 
-0.312
 
 
31.0
 
 
 
 
ADAMTS10
 
 
hsa-miR-29a-3p
 
 
-0.516
 
 
13.8
 
 
 
 
ADAMTS9
 
 
hsa-miR-29a-3p
 
 
-0.305
 
 
39.1
 
 
 
 
ADAMTS9
 
 
hsa-miR-29a-3p
 
 
-0.485
 
 
39.1
 
 
 
 
ADCY3
 
 
hsa-miR-132-3p
 
 
-0.303
 
 
90.0
 
 
 
 
ADCY6
 
 
hsa-miR-1271-5p
 
 
-0.336
 
 
40.6
 
 
 
 
ADGRA2
 
 
hsa-miR-1271-5p
 
 
-0.427
 
 
27.3
 
 
 
 
ADGRF5
 
 
hsa-miR-148b-3p
 
 
-0.354
 
 
66.6
 
 
 
 
ADGRF5
 
 
hsa-miR-152-3p
 
 
-0.354
 
 
66.6
 
 
 
 
ADIPOR2
 
 
hsa-miR-150-5p
 
 
-0.287
 
 
53.2
 
 
 
 
ADK
 
 
hsa-miR-1271-5p
 
 
-0.329
 
 
22.4
 
 
 
 
ADNP
 
 
hsa-miR-328-3p
 
 
-0.371
 
 
25.5
 
 
 
 
ADRA2A
 
 
hsa-miR-23a-3p
 
 
-0.371
 
 
46.9
 
 
 
 
ADRB2
 
 
hsa-miR-15b-5p
 
 
-0.29
 
 
26.4
 
 
 
 
ADRB2
 
 
hsa-let-7b-5p
 
 
-0.635
 
 
26.4
 
 
 
 
AFG3L2
 
 
hsa-miR-181b-5p
 
 
-0.491
 
 
17.1
 
 
 
 
AFG3L2
 
 
hsa-miR-181a-5p
 
 
-0.491
 
 
17.1
 
 
 
 
AFG3L2
 
 
hsa-miR-181d-5p
 
 
-0.499
 
 
17.1
 
 
 
 
AGFG1
 
 
hsa-miR-148b-3p
 
 
-0.332
 
 
64.8
 
 
 
 
AGFG1
 
 
hsa-miR-152-3p
 
 
-0.332
 
 
64.8
 
 
 
 
AGFG1
 
 
hsa-miR-103a-3p
 
 
-0.389
 
 
64.8
 
 
 
 
AHCTF1
 
 
hsa-let-7b-5p
 
 
-0.374
 
 
11.8
 
 
 
 
AHDC1
 
 
hsa-miR-152-3p
 
 
-0.413
 
 
11.7
 
 
 
 
AHDC1
 
 
hsa-miR-148b-3p
 
 
-0.413
 
 
11.7
 
 
 
 
AHR
 
 
hsa-miR-1271-5p
 
 
-0.304
 
 
21.0
 
 
 
 
AHR
 
 
hsa-miR-29a-3p
 
 
-0.316
 
 
21.0
 
 
 
 
AIFM1
 
 
hsa-let-7b-5p
 
 
-0.314
 
 
17.4
 
 
 
 
AK1
 
 
hsa-miR-29a-3p
 
 
-0.333
 
 
30.1
 
 
 
 
AK3
 
 
hsa-miR-1271-5p
 
 
-0.57
 
 
70.8
 
 
 
 
AK4
 
 
hsa-miR-15b-5p
 
 
-0.316
 
 
22.8
 
 
 
 
AKAP1
 
 
hsa-miR-152-3p
 
 
-0.381
 
 
38.1
 
 
 
 
AKAP1
 
 
hsa-miR-148b-3p
 
 
-0.381
 
 
38.1
 
 
 
 
AKIRIN2
 
 
hsa-miR-103a-3p
 
 
-0.299
 
 
30.8
 
 
 
 
AKT3
 
 
hsa-miR-29a-3p
 
 
-0.287
 
 
16.9
 
 
 
 
AKT3
 
 
hsa-miR-15b-5p
 
 
-0.301
 
 
16.9
 
 
 
 
ALAD
 
 
hsa-miR-326
 
 
-0.352
 
 
51.3
 
 
 
 
ALAD
 
 
hsa-miR-103a-3p
 
 
-0.385
 
 
51.3
 
 
 
 
ALAD
 
 
hsa-miR-15b-5p
 
 
-0.446
 
 
51.3
 
 
 
 
ALDH1A2
 
 
hsa-miR-23a-3p
 
 
-0.354
 
 
20.6
 
 
 
 
ALDH6A1
 
 
hsa-miR-1271-5p
 
 
-0.301
 
 
36.3
 
 
 
 
AMOTL1
 
 
hsa-miR-15b-5p
 
 
-0.319
 
 
14.5
 
 
 
 
ANKHD1
 
 
hsa-miR-23a-3p
 
 
-0.368
 
 
37.3
 
 
 
 
ANKHD1
 
 
hsa-miR-30d-5p
 
 
-0.381
 
 
37.3
 
 
 
 
ANKHD1
 
 
hsa-miR-30b-5p
 
 
-0.405
 
 
37.3
 
 
 
 
ANKIB1
 
 
hsa-miR-1271-5p
 
 
-0.344
 
 
11.9
 
 
 
 
ANKRD17
 
 
hsa-miR-30d-5p
 
 
-0.312
 
 
12.8
 
 
 
 
ANKRD17
 
 
hsa-miR-30b-5p
 
 
-0.336
 
 
12.8
 
 
 
 
ANKRD29
 
 
hsa-miR-132-3p
 
 
-0.47
 
 
17.0
 
 
 
 
ANKRD46
 
 
hsa-let-7b-5p
 
 
-0.389
 
 
18.0
 
 
 
 
ANP32A
 
 
hsa-miR-132-3p
 
 
-0.374
 
 
47.7
 
 
 
 
ANTXR2
 
 
hsa-miR-29a-3p
 
 
-0.347
 
 
57.1
 
 
 
 
ANXA11
 
 
hsa-miR-15b-5p
 
 
-0.463
 
 
130.8
 
 
 
 
AP1S1
 
 
hsa-let-7b-5p
 
 
-0.374
 
 
15.1
 
 
 
 
AP2A1
 
 
hsa-miR-15b-5p
 
 
-0.333
 
 
44.9
 
 
 
 
AP2B1
 
 
hsa-miR-1271-5p
 
 
-0.299
 
 
43.1
 
 
 
 
AP2B1
 
 
hsa-miR-15b-5p
 
 
-0.334
 
 
43.1
 
 
 
 
AP3S1
 
 
hsa-miR-30b-5p
 
 
-0.293
 
 
73.6
 
 
 
 
AP5M1
 
 
hsa-miR-1271-5p
 
 
-0.343
 
 
10.8
 
 
 
 
APP
 
 
hsa-miR-15b-5p
 
 
-0.298
 
 
230.6
 
 
 
 
AQP3
 
 
hsa-miR-874-3p
 
 
-0.48
 
 
10.5
 
 
 
 
ARF4
 
 
hsa-miR-30b-5p
 
 
-0.289
 
 
118.7
 
 
 
 
ARF4
 
 
hsa-miR-194-5p
 
 
-0.293
 
 
118.7
 
 
 
 
ARF4
 
 
hsa-miR-152-3p
 
 
-0.487
 
 
118.7
 
 
 
 
ARF4
 
 
hsa-miR-148b-3p
 
 
-0.487
 
 
118.7
 
 
 
 
ARF5
 
 
hsa-miR-29a-3p
 
 
-0.491
 
 
109.3
 
 
 
 
ARF6
 
 
hsa-miR-181b-5p
 
 
-0.33
 
 
30.0
 
 
 
 
ARF6
 
 
hsa-miR-181a-5p
 
 
-0.33
 
 
30.0
 
 
 
 
ARF6
 
 
hsa-miR-181d-5p
 
 
-0.338
 
 
30.0
 
 
 
 
ARFGEF1
 
 
hsa-miR-148b-3p
 
 
-0.306
 
 
10.7
 
 
 
 
ARFGEF1
 
 
hsa-miR-152-3p
 
 
-0.306
 
 
10.7
 
 
 
 
ARFIP1
 
 
hsa-miR-320b
 
 
-0.339
 
 
15.8
 
 
 
 
ARFIP1
 
 
hsa-miR-320c
 
 
-0.339
 
 
15.8
 
 
 
 
ARFIP1
 
 
hsa-miR-152-3p
 
 
-0.474
 
 
15.8
 
 
 
 
ARFIP1
 
 
hsa-miR-148b-3p
 
 
-0.474
 
 
15.8
 
 
 
 
ARHGAP21
 
 
hsa-miR-152-3p
 
 
-0.287
 
 
26.1
 
 
 
 
ARHGAP21
 
 
hsa-miR-148b-3p
 
 
-0.287
 
 
26.1
 
 
 
 
ARHGAP24
 
 
hsa-miR-1271-5p
 
 
-0.342
 
 
13.7
 
 
 
 
ARHGAP6
 
 
hsa-miR-1271-5p
 
 
-0.521
 
 
10.3
 
 
 
 
ARID1B
 
 
hsa-miR-29a-3p
 
 
-0.342
 
 
14.1
 
 
 
 
ARIH2
 
 
hsa-miR-103a-3p
 
 
-0.307
 
 
19.8
 
 
 
 
 
 
 adrenal gland 
 
 
 
 
target_symbol
 
 
mature_mirna_id
 
 
score
 
 
nTPM
 
 
 
 
 
 
AAMP
 
 
hsa-miR-326
 
 
-0.722
 
 
66.4
 
 
 
 
AAR2
 
 
hsa-miR-29a-3p
 
 
-0.327
 
 
24.5
 
 
 
 
ABAT
 
 
hsa-miR-1271-5p
 
 
-0.409
 
 
26.2
 
 
 
 
ABCB7
 
 
hsa-miR-148b-3p
 
 
-0.515
 
 
12.5
 
 
 
 
ABCB7
 
 
hsa-miR-152-3p
 
 
-0.515
 
 
12.5
 
 
 
 
ABCD3
 
 
hsa-miR-148b-3p
 
 
-0.388
 
 
17.6
 
 
 
 
ABCD3
 
 
hsa-miR-152-3p
 
 
-0.388
 
 
17.6
 
 
 
 
ABCE1
 
 
hsa-miR-29a-3p
 
 
-0.404
 
 
20.3
 
 
 
 
ABHD4
 
 
hsa-miR-29a-3p
 
 
-0.312
 
 
29.6
 
 
 
 
ACTR2
 
 
hsa-miR-15b-5p
 
 
-0.293
 
 
66.7
 
 
 
 
ACTR3C
 
 
hsa-miR-30b-5p
 
 
-0.448
 
 
10.1
 
 
 
 
ACTR3C
 
 
hsa-miR-30d-5p
 
 
-0.456
 
 
10.1
 
 
 
 
ADAM9
 
 
hsa-miR-30d-5p
 
 
-0.304
 
 
19.6
 
 
 
 
ADAM9
 
 
hsa-miR-30b-5p
 
 
-0.312
 
 
19.6
 
 
 
 
ADCY3
 
 
hsa-miR-132-3p
 
 
-0.303
 
 
16.9
 
 
 
 
ADCY6
 
 
hsa-miR-1271-5p
 
 
-0.336
 
 
14.1
 
 
 
 
ADGRF5
 
 
hsa-miR-148b-3p
 
 
-0.354
 
 
22.0
 
 
 
 
ADGRF5
 
 
hsa-miR-152-3p
 
 
-0.354
 
 
22.0
 
 
 
 
ADIPOR2
 
 
hsa-miR-150-5p
 
 
-0.287
 
 
69.8
 
 
 
 
ADK
 
 
hsa-miR-1271-5p
 
 
-0.329
 
 
14.6
 
 
 
 
ADNP
 
 
hsa-miR-328-3p
 
 
-0.371
 
 
29.8
 
 
 
 
AFG3L2
 
 
hsa-miR-181b-5p
 
 
-0.491
 
 
12.9
 
 
 
 
AFG3L2
 
 
hsa-miR-181a-5p
 
 
-0.491
 
 
12.9
 
 
 
 
AFG3L2
 
 
hsa-miR-181d-5p
 
 
-0.499
 
 
12.9
 
 
 
 
AGFG1
 
 
hsa-miR-148b-3p
 
 
-0.332
 
 
37.2
 
 
 
 
AGFG1
 
 
hsa-miR-152-3p
 
 
-0.332
 
 
37.2
 
 
 
 
AGFG1
 
 
hsa-miR-103a-3p
 
 
-0.389
 
 
37.2
 
 
 
 
AHDC1
 
 
hsa-miR-152-3p
 
 
-0.413
 
 
23.0
 
 
 
 
AHDC1
 
 
hsa-miR-148b-3p
 
 
-0.413
 
 
23.0
 
 
 
 
AHR
 
 
hsa-miR-1271-5p
 
 
-0.304
 
 
14.2
 
 
 
 
AHR
 
 
hsa-miR-29a-3p
 
 
-0.316
 
 
14.2
 
 
 
 
AIFM1
 
 
hsa-let-7b-5p
 
 
-0.314
 
 
50.1
 
 
 
 
AK1
 
 
hsa-miR-29a-3p
 
 
-0.333
 
 
40.4
 
 
 
 
AK3
 
 
hsa-miR-1271-5p
 
 
-0.57
 
 
59.3
 
 
 
 
AKAP1
 
 
hsa-miR-152-3p
 
 
-0.381
 
 
14.9
 
 
 
 
AKAP1
 
 
hsa-miR-148b-3p
 
 
-0.381
 
 
14.9
 
 
 
 
AKIRIN2
 
 
hsa-miR-103a-3p
 
 
-0.299
 
 
33.1
 
 
 
 
ALAD
 
 
hsa-miR-326
 
 
-0.352
 
 
148.4
 
 
 
 
ALAD
 
 
hsa-miR-103a-3p
 
 
-0.385
 
 
148.4
 
 
 
 
ALAD
 
 
hsa-miR-15b-5p
 
 
-0.446
 
 
148.4
 
 
 
 
ALCAM
 
 
hsa-miR-148b-3p
 
 
-0.339
 
 
30.9
 
 
 
 
ALCAM
 
 
hsa-miR-152-3p
 
 
-0.347
 
 
30.9
 
 
 
 
ALDH6A1
 
 
hsa-miR-1271-5p
 
 
-0.301
 
 
13.9
 
 
 
 
AMT
 
 
hsa-let-7b-5p
 
 
-0.463
 
 
15.0
 
 
 
 
ANKHD1
 
 
hsa-miR-23a-3p
 
 
-0.368
 
 
41.3
 
 
 
 
ANKHD1
 
 
hsa-miR-30d-5p
 
 
-0.381
 
 
41.3
 
 
 
 
ANKHD1
 
 
hsa-miR-30b-5p
 
 
-0.405
 
 
41.3
 
 
 
 
ANKIB1
 
 
hsa-miR-1271-5p
 
 
-0.344
 
 
10.1
 
 
 
 
ANKRA2
 
 
hsa-miR-30d-5p
 
 
-0.343
 
 
10.7
 
 
 
 
ANKRA2
 
 
hsa-miR-30b-5p
 
 
-0.354
 
 
10.7
 
 
 
 
ANKRA2
 
 
hsa-miR-30d-5p
 
 
-0.448
 
 
10.7
 
 
 
 
ANKRA2
 
 
hsa-miR-30b-5p
 
 
-0.472
 
 
10.7
 
 
 
 
ANKRD17
 
 
hsa-miR-30d-5p
 
 
-0.312
 
 
12.7
 
 
 
 
ANKRD17
 
 
hsa-miR-30b-5p
 
 
-0.336
 
 
12.7
 
 
 
 
ANKRD46
 
 
hsa-let-7b-5p
 
 
-0.389
 
 
36.5
 
 
 
 
ANP32A
 
 
hsa-miR-132-3p
 
 
-0.374
 
 
32.7
 
 
 
 
ANTXR2
 
 
hsa-miR-29a-3p
 
 
-0.347
 
 
28.3
 
 
 
 
ANXA11
 
 
hsa-miR-15b-5p
 
 
-0.463
 
 
28.3
 
 
 
 
AP1S1
 
 
hsa-let-7b-5p
 
 
-0.374
 
 
25.7
 
 
 
 
AP2A1
 
 
hsa-miR-15b-5p
 
 
-0.333
 
 
83.5
 
 
 
 
AP2B1
 
 
hsa-miR-1271-5p
 
 
-0.299
 
 
39.3
 
 
 
 
AP2B1
 
 
hsa-miR-15b-5p
 
 
-0.334
 
 
39.3
 
 
 
 
AP3S1
 
 
hsa-miR-30b-5p
 
 
-0.293
 
 
42.4
 
 
 
 
AP5M1
 
 
hsa-miR-1271-5p
 
 
-0.343
 
 
11.5
 
 
 
 
APOO
 
 
hsa-miR-181d-5p
 
 
-0.492
 
 
22.6
 
 
 
 
APOO
 
 
hsa-miR-181b-5p
 
 
-0.5
 
 
22.6
 
 
 
 
APOO
 
 
hsa-miR-181a-5p
 
 
-0.5
 
 
22.6
 
 
 
 
APP
 
 
hsa-miR-15b-5p
 
 
-0.298
 
 
422.0
 
 
 
 
AQP11
 
 
hsa-miR-103a-3p
 
 
-0.517
 
 
14.1
 
 
 
 
AQP11
 
 
hsa-miR-15b-5p
 
 
-0.769
 
 
14.1
 
 
 
 
ARF4
 
 
hsa-miR-30b-5p
 
 
-0.289
 
 
151.0
 
 
 
 
ARF4
 
 
hsa-miR-194-5p
 
 
-0.293
 
 
151.0
 
 
 
 
ARF4
 
 
hsa-miR-152-3p
 
 
-0.487
 
 
151.0
 
 
 
 
ARF4
 
 
hsa-miR-148b-3p
 
 
-0.487
 
 
151.0
 
 
 
 
ARF5
 
 
hsa-miR-29a-3p
 
 
-0.491
 
 
102.3
 
 
 
 
ARF6
 
 
hsa-miR-181b-5p
 
 
-0.33
 
 
24.5
 
 
 
 
ARF6
 
 
hsa-miR-181a-5p
 
 
-0.33
 
 
24.5
 
 
 
 
ARF6
 
 
hsa-miR-181d-5p
 
 
-0.338
 
 
24.5
 
 
 
 
ARFIP1
 
 
hsa-miR-320b
 
 
-0.339
 
 
13.9
 
 
 
 
ARFIP1
 
 
hsa-miR-320c
 
 
-0.339
 
 
13.9
 
 
 
 
ARFIP1
 
 
hsa-miR-152-3p
 
 
-0.474
 
 
13.9
 
 
 
 
ARFIP1
 
 
hsa-miR-148b-3p
 
 
-0.474
 
 
13.9
 
 
 
 
ARHGAP21
 
 
hsa-miR-152-3p
 
 
-0.287
 
 
16.9
 
 
 
 
ARHGAP21
 
 
hsa-miR-148b-3p
 
 
-0.287
 
 
16.9
 
 
 
 
ARID1B
 
 
hsa-miR-29a-3p
 
 
-0.342
 
 
10.7
 
 
 
 
ARIH2
 
 
hsa-miR-103a-3p
 
 
-0.307
 
 
22.0
 
 
 
 
ARL2
 
 
hsa-miR-15b-5p
 
 
-0.374
 
 
94.8
 
 
 
 
ARL2
 
 
hsa-miR-15b-5p
 
 
-0.442
 
 
94.8
 
 
 
 
ARL5B
 
 
hsa-miR-29a-3p
 
 
-0.304
 
 
19.6
 
 
 
 
ARL6IP1
 
 
hsa-miR-328-3p
 
 
-0.494
 
 
161.7
 
 
 
 
ARL6IP1
 
 
hsa-miR-148b-3p
 
 
-0.579
 
 
161.7
 
 
 
 
ARL6IP1
 
 
hsa-miR-152-3p
 
 
-0.579
 
 
161.7
 
 
 
 
ARL8A
 
 
hsa-miR-103a-3p
 
 
-0.38
 
 
38.6
 
 
 
 
ARL8B
 
 
hsa-miR-152-3p
 
 
-0.337
 
 
48.3
 
 
 
 
ARL8B
 
 
hsa-miR-148b-3p
 
 
-0.337
 
 
48.3
 
 
 
 
ARL8B
 
 
hsa-miR-320b
 
 
-0.376
 
 
48.3
 
 
 
 
ARL8B
 
 
hsa-miR-320c
 
 
-0.376
 
 
48.3
 
 
 
 
ARMC1
 
 
hsa-miR-103a-3p
 
 
-0.525
 
 
14.5
 
 
 
 
ARMC8
 
 
hsa-miR-103a-3p
 
 
-0.412
 
 
13.5
 
 
 
 
ARMCX2
 
 
hsa-miR-15b-5p
 
 
-0.344
 
 
42.3
 
 
 
 
 
 
 amygdala 
 
 
 
 
target_symbol
 
 
mature_mirna_id
 
 
score
 
 
nTPM
 
 
 
 
 
 
AAMP
 
 
hsa-miR-326
 
 
-0.722
 
 
59.2
 
 
 
 
AAR2
 
 
hsa-miR-29a-3p
 
 
-0.327
 
 
10.0
 
 
 
 
ABAT
 
 
hsa-miR-1271-5p
 
 
-0.409
 
 
90.5
 
 
 
 
ABCA2
 
 
hsa-miR-1271-5p
 
 
-0.326
 
 
49.1
 
 
 
 
ABCD3
 
 
hsa-miR-148b-3p
 
 
-0.388
 
 
16.0
 
 
 
 
ABCD3
 
 
hsa-miR-152-3p
 
 
-0.388
 
 
16.0
 
 
 
 
ABCE1
 
 
hsa-miR-29a-3p
 
 
-0.404
 
 
11.9
 
 
 
 
ABHD17C
 
 
hsa-let-7b-5p
 
 
-0.359
 
 
17.9
 
 
 
 
ABHD17C
 
 
hsa-miR-330-3p
 
 
-0.399
 
 
17.9
 
 
 
 
ABHD4
 
 
hsa-miR-29a-3p
 
 
-0.312
 
 
16.0
 
 
 
 
ACHE
 
 
hsa-miR-194-5p
 
 
-0.395
 
 
14.7
 
 
 
 
ACSL6
 
 
hsa-let-7b-5p
 
 
-0.336
 
 
20.3
 
 
 
 
ACTR2
 
 
hsa-miR-15b-5p
 
 
-0.293
 
 
53.7
 
 
 
 
ADCY2
 
 
hsa-miR-152-3p
 
 
-0.299
 
 
28.0
 
 
 
 
ADCY2
 
 
hsa-miR-148b-3p
 
 
-0.299
 
 
28.0
 
 
 
 
ADCY3
 
 
hsa-miR-132-3p
 
 
-0.303
 
 
27.0
 
 
 
 
ADGRA3
 
 
hsa-miR-30d-5p
 
 
-0.296
 
 
19.9
 
 
 
 
ADGRA3
 
 
hsa-miR-30b-5p
 
 
-0.304
 
 
19.9
 
 
 
 
ADGRB3
 
 
hsa-miR-1271-5p
 
 
-0.424
 
 
12.8
 
 
 
 
ADGRB3
 
 
hsa-miR-152-3p
 
 
-0.516
 
 
12.8
 
 
 
 
ADGRB3
 
 
hsa-miR-148b-3p
 
 
-0.524
 
 
12.8
 
 
 
 
ADIPOR2
 
 
hsa-miR-150-5p
 
 
-0.287
 
 
43.7
 
 
 
 
ADK
 
 
hsa-miR-1271-5p
 
 
-0.329
 
 
16.1
 
 
 
 
ADNP
 
 
hsa-miR-328-3p
 
 
-0.371
 
 
23.8
 
 
 
 
AGFG1
 
 
hsa-miR-148b-3p
 
 
-0.332
 
 
25.1
 
 
 
 
AGFG1
 
 
hsa-miR-152-3p
 
 
-0.332
 
 
25.1
 
 
 
 
AGFG1
 
 
hsa-miR-103a-3p
 
 
-0.389
 
 
25.1
 
 
 
 
AGPAT4
 
 
hsa-miR-29a-3p
 
 
-0.376
 
 
10.0
 
 
 
 
AHDC1
 
 
hsa-miR-152-3p
 
 
-0.413
 
 
10.1
 
 
 
 
AHDC1
 
 
hsa-miR-148b-3p
 
 
-0.413
 
 
10.1
 
 
 
 
AIFM1
 
 
hsa-let-7b-5p
 
 
-0.314
 
 
10.3
 
 
 
 
AK1
 
 
hsa-miR-29a-3p
 
 
-0.333
 
 
104.5
 
 
 
 
AK3
 
 
hsa-miR-1271-5p
 
 
-0.57
 
 
32.8
 
 
 
 
AK4
 
 
hsa-miR-15b-5p
 
 
-0.316
 
 
19.8
 
 
 
 
AKAP1
 
 
hsa-miR-152-3p
 
 
-0.381
 
 
22.8
 
 
 
 
AKAP1
 
 
hsa-miR-148b-3p
 
 
-0.381
 
 
22.8
 
 
 
 
AKAP6
 
 
hsa-let-7b-5p
 
 
-0.307
 
 
12.4
 
 
 
 
AKIRIN2
 
 
hsa-miR-103a-3p
 
 
-0.299
 
 
33.1
 
 
 
 
AKT3
 
 
hsa-miR-29a-3p
 
 
-0.287
 
 
17.4
 
 
 
 
AKT3
 
 
hsa-miR-15b-5p
 
 
-0.301
 
 
17.4
 
 
 
 
ALAD
 
 
hsa-miR-326
 
 
-0.352
 
 
58.3
 
 
 
 
ALAD
 
 
hsa-miR-103a-3p
 
 
-0.385
 
 
58.3
 
 
 
 
ALAD
 
 
hsa-miR-15b-5p
 
 
-0.446
 
 
58.3
 
 
 
 
ALCAM
 
 
hsa-miR-148b-3p
 
 
-0.339
 
 
31.7
 
 
 
 
ALCAM
 
 
hsa-miR-152-3p
 
 
-0.347
 
 
31.7
 
 
 
 
ALDH6A1
 
 
hsa-miR-1271-5p
 
 
-0.301
 
 
67.5
 
 
 
 
AMOT
 
 
hsa-miR-29a-3p
 
 
-0.304
 
 
18.6
 
 
 
 
ANGEL2
 
 
hsa-miR-132-3p
 
 
-0.357
 
 
10.6
 
 
 
 
ANKHD1
 
 
hsa-miR-23a-3p
 
 
-0.368
 
 
18.9
 
 
 
 
ANKHD1
 
 
hsa-miR-30d-5p
 
 
-0.381
 
 
18.9
 
 
 
 
ANKHD1
 
 
hsa-miR-30b-5p
 
 
-0.405
 
 
18.9
 
 
 
 
ANKIB1
 
 
hsa-miR-1271-5p
 
 
-0.344
 
 
15.2
 
 
 
 
ANKRD17
 
 
hsa-miR-30d-5p
 
 
-0.312
 
 
10.0
 
 
 
 
ANKRD17
 
 
hsa-miR-30b-5p
 
 
-0.336
 
 
10.0
 
 
 
 
ANKRD46
 
 
hsa-let-7b-5p
 
 
-0.389
 
 
28.3
 
 
 
 
ANLN
 
 
hsa-miR-15b-5p
 
 
-0.334
 
 
32.3
 
 
 
 
ANP32A
 
 
hsa-miR-132-3p
 
 
-0.374
 
 
49.8
 
 
 
 
ANXA11
 
 
hsa-miR-15b-5p
 
 
-0.463
 
 
35.8
 
 
 
 
AP1S1
 
 
hsa-let-7b-5p
 
 
-0.374
 
 
88.4
 
 
 
 
AP2A1
 
 
hsa-miR-15b-5p
 
 
-0.333
 
 
49.9
 
 
 
 
AP2B1
 
 
hsa-miR-1271-5p
 
 
-0.299
 
 
69.3
 
 
 
 
AP2B1
 
 
hsa-miR-15b-5p
 
 
-0.334
 
 
69.3
 
 
 
 
AP3S1
 
 
hsa-miR-30b-5p
 
 
-0.293
 
 
82.5
 
 
 
 
APC
 
 
hsa-miR-29a-3p
 
 
-0.413
 
 
38.7
 
 
 
 
APLN
 
 
hsa-miR-15b-5p
 
 
-0.395
 
 
15.8
 
 
 
 
APOO
 
 
hsa-miR-181d-5p
 
 
-0.492
 
 
45.1
 
 
 
 
APOO
 
 
hsa-miR-181b-5p
 
 
-0.5
 
 
45.1
 
 
 
 
APOO
 
 
hsa-miR-181a-5p
 
 
-0.5
 
 
45.1
 
 
 
 
APP
 
 
hsa-miR-15b-5p
 
 
-0.298
 
 
396.2
 
 
 
 
ARF4
 
 
hsa-miR-30b-5p
 
 
-0.289
 
 
60.1
 
 
 
 
ARF4
 
 
hsa-miR-194-5p
 
 
-0.293
 
 
60.1
 
 
 
 
ARF4
 
 
hsa-miR-152-3p
 
 
-0.487
 
 
60.1
 
 
 
 
ARF4
 
 
hsa-miR-148b-3p
 
 
-0.487
 
 
60.1
 
 
 
 
ARF5
 
 
hsa-miR-29a-3p
 
 
-0.491
 
 
176.5
 
 
 
 
ARHGAP21
 
 
hsa-miR-152-3p
 
 
-0.287
 
 
40.6
 
 
 
 
ARHGAP21
 
 
hsa-miR-148b-3p
 
 
-0.287
 
 
40.6
 
 
 
 
ARIH2
 
 
hsa-miR-103a-3p
 
 
-0.307
 
 
17.1
 
 
 
 
ARL2
 
 
hsa-miR-15b-5p
 
 
-0.374
 
 
171.9
 
 
 
 
ARL2
 
 
hsa-miR-15b-5p
 
 
-0.442
 
 
171.9
 
 
 
 
ARL3
 
 
hsa-miR-15b-5p
 
 
-0.507
 
 
26.9
 
 
 
 
ARL4A
 
 
hsa-miR-29a-3p
 
 
-0.386
 
 
14.1
 
 
 
 
ARL4A
 
 
hsa-miR-30d-5p
 
 
-0.432
 
 
14.1
 
 
 
 
ARL4A
 
 
hsa-miR-30b-5p
 
 
-0.448
 
 
14.1
 
 
 
 
ARL6IP1
 
 
hsa-miR-328-3p
 
 
-0.494
 
 
241.3
 
 
 
 
ARL6IP1
 
 
hsa-miR-148b-3p
 
 
-0.579
 
 
241.3
 
 
 
 
ARL6IP1
 
 
hsa-miR-152-3p
 
 
-0.579
 
 
241.3
 
 
 
 
ARL8A
 
 
hsa-miR-103a-3p
 
 
-0.38
 
 
68.0
 
 
 
 
ARL8B
 
 
hsa-miR-152-3p
 
 
-0.337
 
 
53.8
 
 
 
 
ARL8B
 
 
hsa-miR-148b-3p
 
 
-0.337
 
 
53.8
 
 
 
 
ARL8B
 
 
hsa-miR-320b
 
 
-0.376
 
 
53.8
 
 
 
 
ARL8B
 
 
hsa-miR-320c
 
 
-0.376
 
 
53.8
 
 
 
 
ARMC1
 
 
hsa-miR-103a-3p
 
 
-0.525
 
 
17.1
 
 
 
 
ARMC8
 
 
hsa-miR-103a-3p
 
 
-0.412
 
 
13.4
 
 
 
 
ARMCX2
 
 
hsa-miR-15b-5p
 
 
-0.344
 
 
20.2
 
 
 
 
ARMCX3
 
 
hsa-miR-425-5p
 
 
-0.325
 
 
32.9
 
 
 
 
ARPC5L
 
 
hsa-miR-326
 
 
-0.439
 
 
73.0
 
 
 
 
ARPC5L
 
 
hsa-miR-1271-5p
 
 
-0.736
 
 
73.0
 
 
 
 
ARPP19
 
 
hsa-miR-1271-5p
 
 
-0.313
 
 
178.5
 
 
 
 
ARPP19
 
 
hsa-miR-320b
 
 
-0.325
 
 
178.5
 
 
 
 
ARPP19
 
 
hsa-miR-320c
 
 
-0.325
 
 
178.5
 
 
 
 
 
 
 appendix 
 
 
 
 
target_symbol
 
 
mature_mirna_id
 
 
score
 
 
nTPM
 
 
 
 
 
 
AAMP
 
 
hsa-miR-326
 
 
-0.722
 
 
43.5
 
 
 
 
AAR2
 
 
hsa-miR-29a-3p
 
 
-0.327
 
 
13.4
 
 
 
 
ABCD3
 
 
hsa-miR-148b-3p
 
 
-0.388
 
 
15.3
 
 
 
 
ABCD3
 
 
hsa-miR-152-3p
 
 
-0.388
 
 
15.3
 
 
 
 
ABCE1
 
 
hsa-miR-29a-3p
 
 
-0.404
 
 
31.7
 
 
 
 
ACTC1
 
 
hsa-miR-30d-5p
 
 
-0.519
 
 
23.3
 
 
 
 
ACTC1
 
 
hsa-miR-30b-5p
 
 
-0.543
 
 
23.3
 
 
 
 
ACTR2
 
 
hsa-miR-15b-5p
 
 
-0.293
 
 
202.6
 
 
 
 
ADA
 
 
hsa-miR-29a-3p
 
 
-0.376
 
 
15.1
 
 
 
 
ADAM10
 
 
hsa-miR-152-3p
 
 
-0.348
 
 
17.9
 
 
 
 
ADAM10
 
 
hsa-miR-148b-3p
 
 
-0.356
 
 
17.9
 
 
 
 
ADAM19
 
 
hsa-miR-874-3p
 
 
-0.374
 
 
22.7
 
 
 
 
ADAM9
 
 
hsa-miR-30d-5p
 
 
-0.304
 
 
27.3
 
 
 
 
ADAM9
 
 
hsa-miR-30b-5p
 
 
-0.312
 
 
27.3
 
 
 
 
ADAMTS9
 
 
hsa-miR-29a-3p
 
 
-0.305
 
 
11.5
 
 
 
 
ADAMTS9
 
 
hsa-miR-29a-3p
 
 
-0.485
 
 
11.5
 
 
 
 
ADCY3
 
 
hsa-miR-132-3p
 
 
-0.303
 
 
16.3
 
 
 
 
ADCYAP1
 
 
hsa-miR-103a-3p
 
 
-0.443
 
 
10.3
 
 
 
 
ADGRA2
 
 
hsa-miR-1271-5p
 
 
-0.427
 
 
11.5
 
 
 
 
ADIPOR2
 
 
hsa-miR-150-5p
 
 
-0.287
 
 
10.0
 
 
 
 
ADK
 
 
hsa-miR-1271-5p
 
 
-0.329
 
 
15.9
 
 
 
 
ADNP
 
 
hsa-miR-328-3p
 
 
-0.371
 
 
19.0
 
 
 
 
AFG3L2
 
 
hsa-miR-181b-5p
 
 
-0.491
 
 
12.5
 
 
 
 
AFG3L2
 
 
hsa-miR-181a-5p
 
 
-0.491
 
 
12.5
 
 
 
 
AFG3L2
 
 
hsa-miR-181d-5p
 
 
-0.499
 
 
12.5
 
 
 
 
AGFG1
 
 
hsa-miR-148b-3p
 
 
-0.332
 
 
45.7
 
 
 
 
AGFG1
 
 
hsa-miR-152-3p
 
 
-0.332
 
 
45.7
 
 
 
 
AGFG1
 
 
hsa-miR-103a-3p
 
 
-0.389
 
 
45.7
 
 
 
 
AHR
 
 
hsa-miR-1271-5p
 
 
-0.304
 
 
24.3
 
 
 
 
AHR
 
 
hsa-miR-29a-3p
 
 
-0.316
 
 
24.3
 
 
 
 
AIFM1
 
 
hsa-let-7b-5p
 
 
-0.314
 
 
16.6
 
 
 
 
AK1
 
 
hsa-miR-29a-3p
 
 
-0.333
 
 
10.8
 
 
 
 
AK3
 
 
hsa-miR-1271-5p
 
 
-0.57
 
 
22.4
 
 
 
 
AKAP1
 
 
hsa-miR-152-3p
 
 
-0.381
 
 
16.8
 
 
 
 
AKAP1
 
 
hsa-miR-148b-3p
 
 
-0.381
 
 
16.8
 
 
 
 
AKIRIN2
 
 
hsa-miR-103a-3p
 
 
-0.299
 
 
22.4
 
 
 
 
ALAD
 
 
hsa-miR-326
 
 
-0.352
 
 
15.7
 
 
 
 
ALAD
 
 
hsa-miR-103a-3p
 
 
-0.385
 
 
15.7
 
 
 
 
ALAD
 
 
hsa-miR-15b-5p
 
 
-0.446
 
 
15.7
 
 
 
 
ALCAM
 
 
hsa-miR-148b-3p
 
 
-0.339
 
 
11.5
 
 
 
 
ALCAM
 
 
hsa-miR-152-3p
 
 
-0.347
 
 
11.5
 
 
 
 
ANKHD1
 
 
hsa-miR-23a-3p
 
 
-0.368
 
 
34.9
 
 
 
 
ANKHD1
 
 
hsa-miR-30d-5p
 
 
-0.381
 
 
34.9
 
 
 
 
ANKHD1
 
 
hsa-miR-30b-5p
 
 
-0.405
 
 
34.9
 
 
 
 
ANKRD17
 
 
hsa-miR-30d-5p
 
 
-0.312
 
 
12.9
 
 
 
 
ANKRD17
 
 
hsa-miR-30b-5p
 
 
-0.336
 
 
12.9
 
 
 
 
ANP32A
 
 
hsa-miR-132-3p
 
 
-0.374
 
 
82.3
 
 
 
 
ANTXR2
 
 
hsa-miR-29a-3p
 
 
-0.347
 
 
33.4
 
 
 
 
ANXA11
 
 
hsa-miR-15b-5p
 
 
-0.463
 
 
93.8
 
 
 
 
AP1S1
 
 
hsa-let-7b-5p
 
 
-0.374
 
 
13.1
 
 
 
 
AP2A1
 
 
hsa-miR-15b-5p
 
 
-0.333
 
 
23.7
 
 
 
 
AP2B1
 
 
hsa-miR-1271-5p
 
 
-0.299
 
 
36.0
 
 
 
 
AP2B1
 
 
hsa-miR-15b-5p
 
 
-0.334
 
 
36.0
 
 
 
 
AP3S1
 
 
hsa-miR-30b-5p
 
 
-0.293
 
 
36.6
 
 
 
 
AP4B1
 
 
hsa-miR-378a-3p
 
 
-0.62
 
 
10.4
 
 
 
 
AP4B1
 
 
hsa-miR-378d
 
 
-0.62
 
 
10.4
 
 
 
 
AP5M1
 
 
hsa-miR-1271-5p
 
 
-0.343
 
 
12.5
 
 
 
 
APP
 
 
hsa-miR-15b-5p
 
 
-0.298
 
 
146.0
 
 
 
 
AQP3
 
 
hsa-miR-874-3p
 
 
-0.48
 
 
15.8
 
 
 
 
ARF4
 
 
hsa-miR-30b-5p
 
 
-0.289
 
 
101.3
 
 
 
 
ARF4
 
 
hsa-miR-194-5p
 
 
-0.293
 
 
101.3
 
 
 
 
ARF4
 
 
hsa-miR-152-3p
 
 
-0.487
 
 
101.3
 
 
 
 
ARF4
 
 
hsa-miR-148b-3p
 
 
-0.487
 
 
101.3
 
 
 
 
ARF5
 
 
hsa-miR-29a-3p
 
 
-0.491
 
 
68.5
 
 
 
 
ARF6
 
 
hsa-miR-181b-5p
 
 
-0.33
 
 
41.0
 
 
 
 
ARF6
 
 
hsa-miR-181a-5p
 
 
-0.33
 
 
41.0
 
 
 
 
ARF6
 
 
hsa-miR-181d-5p
 
 
-0.338
 
 
41.0
 
 
 
 
ARFGEF1
 
 
hsa-miR-148b-3p
 
 
-0.306
 
 
13.8
 
 
 
 
ARFGEF1
 
 
hsa-miR-152-3p
 
 
-0.306
 
 
13.8
 
 
 
 
ARFIP1
 
 
hsa-miR-320b
 
 
-0.339
 
 
14.9
 
 
 
 
ARFIP1
 
 
hsa-miR-320c
 
 
-0.339
 
 
14.9
 
 
 
 
ARFIP1
 
 
hsa-miR-152-3p
 
 
-0.474
 
 
14.9
 
 
 
 
ARFIP1
 
 
hsa-miR-148b-3p
 
 
-0.474
 
 
14.9
 
 
 
 
ARID1B
 
 
hsa-miR-29a-3p
 
 
-0.342
 
 
13.6
 
 
 
 
ARIH2
 
 
hsa-miR-103a-3p
 
 
-0.307
 
 
15.1
 
 
 
 
ARL2
 
 
hsa-miR-15b-5p
 
 
-0.374
 
 
38.3
 
 
 
 
ARL2
 
 
hsa-miR-15b-5p
 
 
-0.442
 
 
38.3
 
 
 
 
ARL4A
 
 
hsa-miR-29a-3p
 
 
-0.386
 
 
15.8
 
 
 
 
ARL4A
 
 
hsa-miR-30d-5p
 
 
-0.432
 
 
15.8
 
 
 
 
ARL4A
 
 
hsa-miR-30b-5p
 
 
-0.448
 
 
15.8
 
 
 
 
ARL6IP1
 
 
hsa-miR-328-3p
 
 
-0.494
 
 
73.3
 
 
 
 
ARL6IP1
 
 
hsa-miR-148b-3p
 
 
-0.579
 
 
73.3
 
 
 
 
ARL6IP1
 
 
hsa-miR-152-3p
 
 
-0.579
 
 
73.3
 
 
 
 
ARL6IP6
 
 
hsa-let-7b-5p
 
 
-0.293
 
 
10.9
 
 
 
 
ARL8A
 
 
hsa-miR-103a-3p
 
 
-0.38
 
 
23.6
 
 
 
 
ARL8B
 
 
hsa-miR-152-3p
 
 
-0.337
 
 
34.1
 
 
 
 
ARL8B
 
 
hsa-miR-148b-3p
 
 
-0.337
 
 
34.1
 
 
 
 
ARL8B
 
 
hsa-miR-320b
 
 
-0.376
 
 
34.1
 
 
 
 
ARL8B
 
 
hsa-miR-320c
 
 
-0.376
 
 
34.1
 
 
 
 
ARMC1
 
 
hsa-miR-103a-3p
 
 
-0.525
 
 
12.8
 
 
 
 
ARMC8
 
 
hsa-miR-103a-3p
 
 
-0.412
 
 
17.0
 
 
 
 
ARMCX2
 
 
hsa-miR-15b-5p
 
 
-0.344
 
 
10.8
 
 
 
 
ARMCX3
 
 
hsa-miR-425-5p
 
 
-0.325
 
 
23.0
 
 
 
 
ARMT1
 
 
hsa-let-7b-5p
 
 
-0.291
 
 
14.4
 
 
 
 
ARNT
 
 
hsa-miR-103a-3p
 
 
-0.422
 
 
17.1
 
 
 
 
ARPC5L
 
 
hsa-miR-326
 
 
-0.439
 
 
26.1
 
 
 
 
ARPC5L
 
 
hsa-miR-1271-5p
 
 
-0.736
 
 
26.1
 
 
 
 
ARPP19
 
 
hsa-miR-1271-5p
 
 
-0.313
 
 
36.4
 
 
 
 
ARPP19
 
 
hsa-miR-320b
 
 
-0.325
 
 
36.4
 
 
 
 
ARPP19
 
 
hsa-miR-320c
 
 
-0.325
 
 
36.4
 
 
 
 
 
 
 basal ganglia 
 
 
 
 
target_symbol
 
 
mature_mirna_id
 
 
score
 
 
nTPM
 
 
 
 
 
 
AAMP
 
 
hsa-miR-326
 
 
-0.722
 
 
65.2
 
 
 
 
AAR2
 
 
hsa-miR-29a-3p
 
 
-0.327
 
 
11.5
 
 
 
 
ABAT
 
 
hsa-miR-1271-5p
 
 
-0.409
 
 
128.7
 
 
 
 
ABCA2
 
 
hsa-miR-1271-5p
 
 
-0.326
 
 
55.8
 
 
 
 
ABCB9
 
 
hsa-let-7b-5p
 
 
-0.422
 
 
13.6
 
 
 
 
ABCD3
 
 
hsa-miR-148b-3p
 
 
-0.388
 
 
17.4
 
 
 
 
ABCD3
 
 
hsa-miR-152-3p
 
 
-0.388
 
 
17.4
 
 
 
 
ABCE1
 
 
hsa-miR-29a-3p
 
 
-0.404
 
 
11.5
 
 
 
 
ABHD17C
 
 
hsa-let-7b-5p
 
 
-0.359
 
 
20.2
 
 
 
 
ABHD17C
 
 
hsa-miR-330-3p
 
 
-0.399
 
 
20.2
 
 
 
 
ABHD4
 
 
hsa-miR-29a-3p
 
 
-0.312
 
 
15.4
 
 
 
 
ACHE
 
 
hsa-miR-194-5p
 
 
-0.395
 
 
38.3
 
 
 
 
ACSL6
 
 
hsa-let-7b-5p
 
 
-0.336
 
 
18.7
 
 
 
 
ACTN2
 
 
hsa-miR-23a-3p
 
 
-0.294
 
 
36.8
 
 
 
 
ACTN2
 
 
hsa-miR-29a-3p
 
 
-0.311
 
 
36.8
 
 
 
 
ACTR2
 
 
hsa-miR-15b-5p
 
 
-0.293
 
 
55.9
 
 
 
 
ADCY2
 
 
hsa-miR-152-3p
 
 
-0.299
 
 
23.7
 
 
 
 
ADCY2
 
 
hsa-miR-148b-3p
 
 
-0.299
 
 
23.7
 
 
 
 
ADCY3
 
 
hsa-miR-132-3p
 
 
-0.303
 
 
57.0
 
 
 
 
ADGRA3
 
 
hsa-miR-30d-5p
 
 
-0.296
 
 
18.8
 
 
 
 
ADGRA3
 
 
hsa-miR-30b-5p
 
 
-0.304
 
 
18.8
 
 
 
 
ADGRB3
 
 
hsa-miR-1271-5p
 
 
-0.424
 
 
21.1
 
 
 
 
ADGRB3
 
 
hsa-miR-152-3p
 
 
-0.516
 
 
21.1
 
 
 
 
ADGRB3
 
 
hsa-miR-148b-3p
 
 
-0.524
 
 
21.1
 
 
 
 
ADGRF5
 
 
hsa-miR-148b-3p
 
 
-0.354
 
 
13.3
 
 
 
 
ADGRF5
 
 
hsa-miR-152-3p
 
 
-0.354
 
 
13.3
 
 
 
 
ADIPOR2
 
 
hsa-miR-150-5p
 
 
-0.287
 
 
50.5
 
 
 
 
ADK
 
 
hsa-miR-1271-5p
 
 
-0.329
 
 
22.3
 
 
 
 
ADNP
 
 
hsa-miR-328-3p
 
 
-0.371
 
 
24.2
 
 
 
 
ADRB1
 
 
hsa-let-7b-5p
 
 
-0.351
 
 
10.0
 
 
 
 
AGFG1
 
 
hsa-miR-148b-3p
 
 
-0.332
 
 
27.0
 
 
 
 
AGFG1
 
 
hsa-miR-152-3p
 
 
-0.332
 
 
27.0
 
 
 
 
AGFG1
 
 
hsa-miR-103a-3p
 
 
-0.389
 
 
27.0
 
 
 
 
AGPAT4
 
 
hsa-miR-29a-3p
 
 
-0.376
 
 
10.7
 
 
 
 
AHDC1
 
 
hsa-miR-152-3p
 
 
-0.413
 
 
12.3
 
 
 
 
AHDC1
 
 
hsa-miR-148b-3p
 
 
-0.413
 
 
12.3
 
 
 
 
AIFM1
 
 
hsa-let-7b-5p
 
 
-0.314
 
 
12.5
 
 
 
 
AK1
 
 
hsa-miR-29a-3p
 
 
-0.333
 
 
100.7
 
 
 
 
AK3
 
 
hsa-miR-1271-5p
 
 
-0.57
 
 
37.9
 
 
 
 
AK4
 
 
hsa-miR-15b-5p
 
 
-0.316
 
 
19.2
 
 
 
 
AKAP1
 
 
hsa-miR-152-3p
 
 
-0.381
 
 
26.7
 
 
 
 
AKAP1
 
 
hsa-miR-148b-3p
 
 
-0.381
 
 
26.7
 
 
 
 
AKAP5
 
 
hsa-miR-29a-3p
 
 
-0.298
 
 
17.6
 
 
 
 
AKAP6
 
 
hsa-let-7b-5p
 
 
-0.307
 
 
10.5
 
 
 
 
AKIRIN2
 
 
hsa-miR-103a-3p
 
 
-0.299
 
 
31.9
 
 
 
 
AKT3
 
 
hsa-miR-29a-3p
 
 
-0.287
 
 
15.0
 
 
 
 
AKT3
 
 
hsa-miR-15b-5p
 
 
-0.301
 
 
15.0
 
 
 
 
ALAD
 
 
hsa-miR-326
 
 
-0.352
 
 
57.8
 
 
 
 
ALAD
 
 
hsa-miR-103a-3p
 
 
-0.385
 
 
57.8
 
 
 
 
ALAD
 
 
hsa-miR-15b-5p
 
 
-0.446
 
 
57.8
 
 
 
 
ALCAM
 
 
hsa-miR-148b-3p
 
 
-0.339
 
 
37.8
 
 
 
 
ALCAM
 
 
hsa-miR-152-3p
 
 
-0.347
 
 
37.8
 
 
 
 
ALDH6A1
 
 
hsa-miR-1271-5p
 
 
-0.301
 
 
66.9
 
 
 
 
AMOT
 
 
hsa-miR-29a-3p
 
 
-0.304
 
 
18.8
 
 
 
 
AMT
 
 
hsa-let-7b-5p
 
 
-0.463
 
 
11.4
 
 
 
 
ANGEL2
 
 
hsa-miR-132-3p
 
 
-0.357
 
 
14.7
 
 
 
 
ANKHD1
 
 
hsa-miR-23a-3p
 
 
-0.368
 
 
20.4
 
 
 
 
ANKHD1
 
 
hsa-miR-30d-5p
 
 
-0.381
 
 
20.4
 
 
 
 
ANKHD1
 
 
hsa-miR-30b-5p
 
 
-0.405
 
 
20.4
 
 
 
 
ANKIB1
 
 
hsa-miR-1271-5p
 
 
-0.344
 
 
14.3
 
 
 
 
ANKRD13B
 
 
hsa-miR-29a-3p
 
 
-0.421
 
 
12.3
 
 
 
 
ANKRD17
 
 
hsa-miR-30d-5p
 
 
-0.312
 
 
11.2
 
 
 
 
ANKRD17
 
 
hsa-miR-30b-5p
 
 
-0.336
 
 
11.2
 
 
 
 
ANKRD46
 
 
hsa-let-7b-5p
 
 
-0.389
 
 
22.6
 
 
 
 
ANLN
 
 
hsa-miR-15b-5p
 
 
-0.334
 
 
27.5
 
 
 
 
ANO3
 
 
hsa-miR-15b-5p
 
 
-0.357
 
 
58.4
 
 
 
 
ANP32A
 
 
hsa-miR-132-3p
 
 
-0.374
 
 
53.9
 
 
 
 
ANXA11
 
 
hsa-miR-15b-5p
 
 
-0.463
 
 
35.8
 
 
 
 
AP1S1
 
 
hsa-let-7b-5p
 
 
-0.374
 
 
116.3
 
 
 
 
AP2A1
 
 
hsa-miR-15b-5p
 
 
-0.333
 
 
40.7
 
 
 
 
AP2B1
 
 
hsa-miR-1271-5p
 
 
-0.299
 
 
72.3
 
 
 
 
AP2B1
 
 
hsa-miR-15b-5p
 
 
-0.334
 
 
72.3
 
 
 
 
AP3S1
 
 
hsa-miR-30b-5p
 
 
-0.293
 
 
79.6
 
 
 
 
AP4B1
 
 
hsa-miR-378a-3p
 
 
-0.62
 
 
11.6
 
 
 
 
AP4B1
 
 
hsa-miR-378d
 
 
-0.62
 
 
11.6
 
 
 
 
APC
 
 
hsa-miR-29a-3p
 
 
-0.413
 
 
15.7
 
 
 
 
APLN
 
 
hsa-miR-15b-5p
 
 
-0.395
 
 
10.2
 
 
 
 
APOO
 
 
hsa-miR-181d-5p
 
 
-0.492
 
 
38.6
 
 
 
 
APOO
 
 
hsa-miR-181b-5p
 
 
-0.5
 
 
38.6
 
 
 
 
APOO
 
 
hsa-miR-181a-5p
 
 
-0.5
 
 
38.6
 
 
 
 
APP
 
 
hsa-miR-15b-5p
 
 
-0.298
 
 
458.2
 
 
 
 
ARF4
 
 
hsa-miR-30b-5p
 
 
-0.289
 
 
71.0
 
 
 
 
ARF4
 
 
hsa-miR-194-5p
 
 
-0.293
 
 
71.0
 
 
 
 
ARF4
 
 
hsa-miR-152-3p
 
 
-0.487
 
 
71.0
 
 
 
 
ARF4
 
 
hsa-miR-148b-3p
 
 
-0.487
 
 
71.0
 
 
 
 
ARF5
 
 
hsa-miR-29a-3p
 
 
-0.491
 
 
195.0
 
 
 
 
ARF6
 
 
hsa-miR-181b-5p
 
 
-0.33
 
 
10.2
 
 
 
 
ARF6
 
 
hsa-miR-181a-5p
 
 
-0.33
 
 
10.2
 
 
 
 
ARF6
 
 
hsa-miR-181d-5p
 
 
-0.338
 
 
10.2
 
 
 
 
ARHGAP21
 
 
hsa-miR-152-3p
 
 
-0.287
 
 
37.1
 
 
 
 
ARHGAP21
 
 
hsa-miR-148b-3p
 
 
-0.287
 
 
37.1
 
 
 
 
ARIH2
 
 
hsa-miR-103a-3p
 
 
-0.307
 
 
19.4
 
 
 
 
ARL2
 
 
hsa-miR-15b-5p
 
 
-0.374
 
 
286.3
 
 
 
 
ARL2
 
 
hsa-miR-15b-5p
 
 
-0.442
 
 
286.3
 
 
 
 
ARL3
 
 
hsa-miR-15b-5p
 
 
-0.507
 
 
31.8
 
 
 
 
ARL4A
 
 
hsa-miR-29a-3p
 
 
-0.386
 
 
10.5
 
 
 
 
ARL4A
 
 
hsa-miR-30d-5p
 
 
-0.432
 
 
10.5
 
 
 
 
ARL4A
 
 
hsa-miR-30b-5p
 
 
-0.448
 
 
10.5
 
 
 
 
ARL4D
 
 
hsa-miR-1271-5p
 
 
-0.392
 
 
14.0
 
 
 
 
ARL4D
 
 
hsa-let-7b-5p
 
 
-0.417
 
 
14.0
 
 
 
 
 
 
 blood vessel 
 
 
 
 
target_symbol
 
 
mature_mirna_id
 
 
score
 
 
nTPM
 
 
 
 
 
 
AAMP
 
 
hsa-miR-326
 
 
-0.722
 
 
57.8
 
 
 
 
AAR2
 
 
hsa-miR-29a-3p
 
 
-0.327
 
 
28.2
 
 
 
 
ABCB7
 
 
hsa-miR-148b-3p
 
 
-0.515
 
 
12.3
 
 
 
 
ABCB7
 
 
hsa-miR-152-3p
 
 
-0.515
 
 
12.3
 
 
 
 
ABCD3
 
 
hsa-miR-148b-3p
 
 
-0.388
 
 
14.4
 
 
 
 
ABCD3
 
 
hsa-miR-152-3p
 
 
-0.388
 
 
14.4
 
 
 
 
ABCE1
 
 
hsa-miR-29a-3p
 
 
-0.404
 
 
15.1
 
 
 
 
ABHD4
 
 
hsa-miR-29a-3p
 
 
-0.312
 
 
38.3
 
 
 
 
ACTC1
 
 
hsa-miR-30d-5p
 
 
-0.519
 
 
388.9
 
 
 
 
ACTC1
 
 
hsa-miR-30b-5p
 
 
-0.543
 
 
388.9
 
 
 
 
ACTN2
 
 
hsa-miR-23a-3p
 
 
-0.294
 
 
12.2
 
 
 
 
ACTN2
 
 
hsa-miR-29a-3p
 
 
-0.311
 
 
12.2
 
 
 
 
ACTR2
 
 
hsa-miR-15b-5p
 
 
-0.293
 
 
77.0
 
 
 
 
ADAM9
 
 
hsa-miR-30d-5p
 
 
-0.304
 
 
36.8
 
 
 
 
ADAM9
 
 
hsa-miR-30b-5p
 
 
-0.312
 
 
36.8
 
 
 
 
ADAMTS10
 
 
hsa-miR-29a-3p
 
 
-0.516
 
 
20.0
 
 
 
 
ADAMTS7
 
 
hsa-miR-29a-3p
 
 
-0.427
 
 
14.2
 
 
 
 
ADAMTS8
 
 
hsa-let-7b-5p
 
 
-0.571
 
 
12.7
 
 
 
 
ADAMTS9
 
 
hsa-miR-29a-3p
 
 
-0.305
 
 
18.0
 
 
 
 
ADAMTS9
 
 
hsa-miR-29a-3p
 
 
-0.485
 
 
18.0
 
 
 
 
ADCY3
 
 
hsa-miR-132-3p
 
 
-0.303
 
 
43.2
 
 
 
 
ADCY6
 
 
hsa-miR-1271-5p
 
 
-0.336
 
 
29.5
 
 
 
 
ADGRA2
 
 
hsa-miR-1271-5p
 
 
-0.427
 
 
17.2
 
 
 
 
ADGRF5
 
 
hsa-miR-148b-3p
 
 
-0.354
 
 
12.6
 
 
 
 
ADGRF5
 
 
hsa-miR-152-3p
 
 
-0.354
 
 
12.6
 
 
 
 
ADIPOR2
 
 
hsa-miR-150-5p
 
 
-0.287
 
 
23.0
 
 
 
 
ADK
 
 
hsa-miR-1271-5p
 
 
-0.329
 
 
15.2
 
 
 
 
ADNP
 
 
hsa-miR-328-3p
 
 
-0.371
 
 
32.1
 
 
 
 
AGFG1
 
 
hsa-miR-148b-3p
 
 
-0.332
 
 
37.3
 
 
 
 
AGFG1
 
 
hsa-miR-152-3p
 
 
-0.332
 
 
37.3
 
 
 
 
AGFG1
 
 
hsa-miR-103a-3p
 
 
-0.389
 
 
37.3
 
 
 
 
AHCTF1
 
 
hsa-let-7b-5p
 
 
-0.374
 
 
10.0
 
 
 
 
AHDC1
 
 
hsa-miR-152-3p
 
 
-0.413
 
 
27.7
 
 
 
 
AHDC1
 
 
hsa-miR-148b-3p
 
 
-0.413
 
 
27.7
 
 
 
 
AHR
 
 
hsa-miR-1271-5p
 
 
-0.304
 
 
15.2
 
 
 
 
AHR
 
 
hsa-miR-29a-3p
 
 
-0.316
 
 
15.2
 
 
 
 
AK1
 
 
hsa-miR-29a-3p
 
 
-0.333
 
 
58.4
 
 
 
 
AK3
 
 
hsa-miR-1271-5p
 
 
-0.57
 
 
35.8
 
 
 
 
AK4
 
 
hsa-miR-15b-5p
 
 
-0.316
 
 
25.4
 
 
 
 
AKAP1
 
 
hsa-miR-152-3p
 
 
-0.381
 
 
84.5
 
 
 
 
AKAP1
 
 
hsa-miR-148b-3p
 
 
-0.381
 
 
84.5
 
 
 
 
AKAP6
 
 
hsa-let-7b-5p
 
 
-0.307
 
 
15.0
 
 
 
 
AKIRIN2
 
 
hsa-miR-103a-3p
 
 
-0.299
 
 
22.1
 
 
 
 
AKT3
 
 
hsa-miR-29a-3p
 
 
-0.287
 
 
34.5
 
 
 
 
AKT3
 
 
hsa-miR-15b-5p
 
 
-0.301
 
 
34.5
 
 
 
 
ALAD
 
 
hsa-miR-326
 
 
-0.352
 
 
22.2
 
 
 
 
ALAD
 
 
hsa-miR-103a-3p
 
 
-0.385
 
 
22.2
 
 
 
 
ALAD
 
 
hsa-miR-15b-5p
 
 
-0.446
 
 
22.2
 
 
 
 
ALCAM
 
 
hsa-miR-148b-3p
 
 
-0.339
 
 
85.5
 
 
 
 
ALCAM
 
 
hsa-miR-152-3p
 
 
-0.347
 
 
85.5
 
 
 
 
ALDH6A1
 
 
hsa-miR-1271-5p
 
 
-0.301
 
 
13.8
 
 
 
 
AMOTL1
 
 
hsa-miR-15b-5p
 
 
-0.319
 
 
18.9
 
 
 
 
AMT
 
 
hsa-let-7b-5p
 
 
-0.463
 
 
11.4
 
 
 
 
ANKHD1
 
 
hsa-miR-23a-3p
 
 
-0.368
 
 
40.7
 
 
 
 
ANKHD1
 
 
hsa-miR-30d-5p
 
 
-0.381
 
 
40.7
 
 
 
 
ANKHD1
 
 
hsa-miR-30b-5p
 
 
-0.405
 
 
40.7
 
 
 
 
ANKIB1
 
 
hsa-miR-1271-5p
 
 
-0.344
 
 
11.2
 
 
 
 
ANKRD17
 
 
hsa-miR-30d-5p
 
 
-0.312
 
 
13.9
 
 
 
 
ANKRD17
 
 
hsa-miR-30b-5p
 
 
-0.336
 
 
13.9
 
 
 
 
ANKRD27
 
 
hsa-miR-1271-5p
 
 
-0.648
 
 
12.8
 
 
 
 
ANKRD6
 
 
hsa-miR-331-3p
 
 
-0.369
 
 
14.1
 
 
 
 
ANP32A
 
 
hsa-miR-132-3p
 
 
-0.374
 
 
51.8
 
 
 
 
ANTXR2
 
 
hsa-miR-29a-3p
 
 
-0.347
 
 
50.3
 
 
 
 
ANXA11
 
 
hsa-miR-15b-5p
 
 
-0.463
 
 
150.6
 
 
 
 
AP1S1
 
 
hsa-let-7b-5p
 
 
-0.374
 
 
13.8
 
 
 
 
AP2A1
 
 
hsa-miR-15b-5p
 
 
-0.333
 
 
48.0
 
 
 
 
AP2B1
 
 
hsa-miR-1271-5p
 
 
-0.299
 
 
41.4
 
 
 
 
AP2B1
 
 
hsa-miR-15b-5p
 
 
-0.334
 
 
41.4
 
 
 
 
AP3S1
 
 
hsa-miR-30b-5p
 
 
-0.293
 
 
112.6
 
 
 
 
APLN
 
 
hsa-miR-15b-5p
 
 
-0.395
 
 
10.7
 
 
 
 
APP
 
 
hsa-miR-15b-5p
 
 
-0.298
 
 
341.0
 
 
 
 
ARF4
 
 
hsa-miR-30b-5p
 
 
-0.289
 
 
151.4
 
 
 
 
ARF4
 
 
hsa-miR-194-5p
 
 
-0.293
 
 
151.4
 
 
 
 
ARF4
 
 
hsa-miR-152-3p
 
 
-0.487
 
 
151.4
 
 
 
 
ARF4
 
 
hsa-miR-148b-3p
 
 
-0.487
 
 
151.4
 
 
 
 
ARF5
 
 
hsa-miR-29a-3p
 
 
-0.491
 
 
115.2
 
 
 
 
ARF6
 
 
hsa-miR-181b-5p
 
 
-0.33
 
 
22.6
 
 
 
 
ARF6
 
 
hsa-miR-181a-5p
 
 
-0.33
 
 
22.6
 
 
 
 
ARF6
 
 
hsa-miR-181d-5p
 
 
-0.338
 
 
22.6
 
 
 
 
ARFIP1
 
 
hsa-miR-320b
 
 
-0.339
 
 
14.2
 
 
 
 
ARFIP1
 
 
hsa-miR-320c
 
 
-0.339
 
 
14.2
 
 
 
 
ARFIP1
 
 
hsa-miR-152-3p
 
 
-0.474
 
 
14.2
 
 
 
 
ARFIP1
 
 
hsa-miR-148b-3p
 
 
-0.474
 
 
14.2
 
 
 
 
ARHGAP21
 
 
hsa-miR-152-3p
 
 
-0.287
 
 
14.0
 
 
 
 
ARHGAP21
 
 
hsa-miR-148b-3p
 
 
-0.287
 
 
14.0
 
 
 
 
ARHGAP24
 
 
hsa-miR-1271-5p
 
 
-0.342
 
 
17.3
 
 
 
 
ARID1B
 
 
hsa-miR-29a-3p
 
 
-0.342
 
 
13.6
 
 
 
 
ARIH2
 
 
hsa-miR-103a-3p
 
 
-0.307
 
 
23.5
 
 
 
 
ARL2
 
 
hsa-miR-15b-5p
 
 
-0.374
 
 
155.0
 
 
 
 
ARL2
 
 
hsa-miR-15b-5p
 
 
-0.442
 
 
155.0
 
 
 
 
ARL3
 
 
hsa-miR-15b-5p
 
 
-0.507
 
 
12.7
 
 
 
 
ARL4A
 
 
hsa-miR-29a-3p
 
 
-0.386
 
 
19.2
 
 
 
 
ARL4A
 
 
hsa-miR-30d-5p
 
 
-0.432
 
 
19.2
 
 
 
 
ARL4A
 
 
hsa-miR-30b-5p
 
 
-0.448
 
 
19.2
 
 
 
 
ARL4D
 
 
hsa-miR-1271-5p
 
 
-0.392
 
 
12.9
 
 
 
 
ARL4D
 
 
hsa-let-7b-5p
 
 
-0.417
 
 
12.9
 
 
 
 
ARL6IP1
 
 
hsa-miR-328-3p
 
 
-0.494
 
 
52.2
 
 
 
 
ARL6IP1
 
 
hsa-miR-148b-3p
 
 
-0.579
 
 
52.2
 
 
 
 
ARL6IP1
 
 
hsa-miR-152-3p
 
 
-0.579
 
 
52.2
 
 
 
 
ARL8A
 
 
hsa-miR-103a-3p
 
 
-0.38
 
 
42.8
 
 
 
 
 
 
 bone marrow 
 
 
 
 
target_symbol
 
 
mature_mirna_id
 
 
score
 
 
nTPM
 
 
 
 
 
 
AAMP
 
 
hsa-miR-326
 
 
-0.722
 
 
25.9
 
 
 
 
AAR2
 
 
hsa-miR-29a-3p
 
 
-0.327
 
 
21.0
 
 
 
 
ABCB10
 
 
hsa-miR-152-3p
 
 
-0.291
 
 
56.7
 
 
 
 
ABCB10
 
 
hsa-miR-148b-3p
 
 
-0.291
 
 
56.7
 
 
 
 
ABCB7
 
 
hsa-miR-148b-3p
 
 
-0.515
 
 
10.5
 
 
 
 
ABCB7
 
 
hsa-miR-152-3p
 
 
-0.515
 
 
10.5
 
 
 
 
ABCD3
 
 
hsa-miR-148b-3p
 
 
-0.388
 
 
23.6
 
 
 
 
ABCD3
 
 
hsa-miR-152-3p
 
 
-0.388
 
 
23.6
 
 
 
 
ABCE1
 
 
hsa-miR-29a-3p
 
 
-0.404
 
 
25.7
 
 
 
 
ABHD4
 
 
hsa-miR-29a-3p
 
 
-0.312
 
 
12.9
 
 
 
 
ABHD5
 
 
hsa-miR-132-3p
 
 
-0.323
 
 
37.5
 
 
 
 
ACP3
 
 
hsa-let-7b-5p
 
 
-0.298
 
 
16.5
 
 
 
 
ACSL6
 
 
hsa-let-7b-5p
 
 
-0.336
 
 
21.4
 
 
 
 
ACTR2
 
 
hsa-miR-15b-5p
 
 
-0.293
 
 
138.4
 
 
 
 
ADA
 
 
hsa-miR-29a-3p
 
 
-0.376
 
 
14.3
 
 
 
 
ADAM10
 
 
hsa-miR-152-3p
 
 
-0.348
 
 
17.3
 
 
 
 
ADAM10
 
 
hsa-miR-148b-3p
 
 
-0.356
 
 
17.3
 
 
 
 
ADCY3
 
 
hsa-miR-132-3p
 
 
-0.303
 
 
16.7
 
 
 
 
ADK
 
 
hsa-miR-1271-5p
 
 
-0.329
 
 
27.0
 
 
 
 
ADNP
 
 
hsa-miR-328-3p
 
 
-0.371
 
 
23.9
 
 
 
 
ADRB2
 
 
hsa-miR-15b-5p
 
 
-0.29
 
 
11.8
 
 
 
 
ADRB2
 
 
hsa-let-7b-5p
 
 
-0.635
 
 
11.8
 
 
 
 
AFG3L2
 
 
hsa-miR-181b-5p
 
 
-0.491
 
 
23.4
 
 
 
 
AFG3L2
 
 
hsa-miR-181a-5p
 
 
-0.491
 
 
23.4
 
 
 
 
AFG3L2
 
 
hsa-miR-181d-5p
 
 
-0.499
 
 
23.4
 
 
 
 
AGFG1
 
 
hsa-miR-148b-3p
 
 
-0.332
 
 
146.9
 
 
 
 
AGFG1
 
 
hsa-miR-152-3p
 
 
-0.332
 
 
146.9
 
 
 
 
AGFG1
 
 
hsa-miR-103a-3p
 
 
-0.389
 
 
146.9
 
 
 
 
AGO2
 
 
hsa-miR-132-3p
 
 
-0.406
 
 
10.7
 
 
 
 
AGO2
 
 
hsa-miR-23a-3p
 
 
-0.576
 
 
10.7
 
 
 
 
AGO2
 
 
hsa-miR-30b-5p
 
 
-1.034
 
 
10.7
 
 
 
 
AGO2
 
 
hsa-miR-30d-5p
 
 
-1.034
 
 
10.7
 
 
 
 
AGO4
 
 
hsa-miR-1271-5p
 
 
-0.289
 
 
19.2
 
 
 
 
AGO4
 
 
hsa-miR-103a-3p
 
 
-0.32
 
 
19.2
 
 
 
 
AHCTF1
 
 
hsa-let-7b-5p
 
 
-0.374
 
 
16.0
 
 
 
 
AHR
 
 
hsa-miR-1271-5p
 
 
-0.304
 
 
41.5
 
 
 
 
AHR
 
 
hsa-miR-29a-3p
 
 
-0.316
 
 
41.5
 
 
 
 
AIFM1
 
 
hsa-let-7b-5p
 
 
-0.314
 
 
11.2
 
 
 
 
AK1
 
 
hsa-miR-29a-3p
 
 
-0.333
 
 
27.0
 
 
 
 
AK3
 
 
hsa-miR-1271-5p
 
 
-0.57
 
 
19.0
 
 
 
 
AKIRIN2
 
 
hsa-miR-103a-3p
 
 
-0.299
 
 
66.5
 
 
 
 
ALAD
 
 
hsa-miR-326
 
 
-0.352
 
 
77.5
 
 
 
 
ALAD
 
 
hsa-miR-103a-3p
 
 
-0.385
 
 
77.5
 
 
 
 
ALAD
 
 
hsa-miR-15b-5p
 
 
-0.446
 
 
77.5
 
 
 
 
ANK1
 
 
hsa-miR-103a-3p
 
 
-0.321
 
 
94.9
 
 
 
 
ANKHD1
 
 
hsa-miR-23a-3p
 
 
-0.368
 
 
69.9
 
 
 
 
ANKHD1
 
 
hsa-miR-30d-5p
 
 
-0.381
 
 
69.9
 
 
 
 
ANKHD1
 
 
hsa-miR-30b-5p
 
 
-0.405
 
 
69.9
 
 
 
 
ANKRA2
 
 
hsa-miR-30d-5p
 
 
-0.343
 
 
13.2
 
 
 
 
ANKRA2
 
 
hsa-miR-30b-5p
 
 
-0.354
 
 
13.2
 
 
 
 
ANKRA2
 
 
hsa-miR-30d-5p
 
 
-0.448
 
 
13.2
 
 
 
 
ANKRA2
 
 
hsa-miR-30b-5p
 
 
-0.472
 
 
13.2
 
 
 
 
ANLN
 
 
hsa-miR-15b-5p
 
 
-0.334
 
 
34.3
 
 
 
 
ANP32A
 
 
hsa-miR-132-3p
 
 
-0.374
 
 
129.5
 
 
 
 
ANTXR2
 
 
hsa-miR-29a-3p
 
 
-0.347
 
 
17.7
 
 
 
 
ANXA11
 
 
hsa-miR-15b-5p
 
 
-0.463
 
 
182.2
 
 
 
 
AP2A1
 
 
hsa-miR-15b-5p
 
 
-0.333
 
 
51.3
 
 
 
 
AP2B1
 
 
hsa-miR-1271-5p
 
 
-0.299
 
 
46.4
 
 
 
 
AP2B1
 
 
hsa-miR-15b-5p
 
 
-0.334
 
 
46.4
 
 
 
 
AP3S1
 
 
hsa-miR-30b-5p
 
 
-0.293
 
 
125.9
 
 
 
 
AP4B1
 
 
hsa-miR-378a-3p
 
 
-0.62
 
 
19.4
 
 
 
 
AP4B1
 
 
hsa-miR-378d
 
 
-0.62
 
 
19.4
 
 
 
 
AP5M1
 
 
hsa-miR-1271-5p
 
 
-0.343
 
 
14.5
 
 
 
 
APP
 
 
hsa-miR-15b-5p
 
 
-0.298
 
 
119.8
 
 
 
 
AQP3
 
 
hsa-miR-874-3p
 
 
-0.48
 
 
18.6
 
 
 
 
ARF4
 
 
hsa-miR-30b-5p
 
 
-0.289
 
 
68.5
 
 
 
 
ARF4
 
 
hsa-miR-194-5p
 
 
-0.293
 
 
68.5
 
 
 
 
ARF4
 
 
hsa-miR-152-3p
 
 
-0.487
 
 
68.5
 
 
 
 
ARF4
 
 
hsa-miR-148b-3p
 
 
-0.487
 
 
68.5
 
 
 
 
ARF5
 
 
hsa-miR-29a-3p
 
 
-0.491
 
 
135.0
 
 
 
 
ARF6
 
 
hsa-miR-181b-5p
 
 
-0.33
 
 
78.0
 
 
 
 
ARF6
 
 
hsa-miR-181a-5p
 
 
-0.33
 
 
78.0
 
 
 
 
ARF6
 
 
hsa-miR-181d-5p
 
 
-0.338
 
 
78.0
 
 
 
 
ARFGEF1
 
 
hsa-miR-148b-3p
 
 
-0.306
 
 
26.0
 
 
 
 
ARFGEF1
 
 
hsa-miR-152-3p
 
 
-0.306
 
 
26.0
 
 
 
 
ARHGAP24
 
 
hsa-miR-1271-5p
 
 
-0.342
 
 
17.7
 
 
 
 
ARID1B
 
 
hsa-miR-29a-3p
 
 
-0.342
 
 
27.3
 
 
 
 
ARID2
 
 
hsa-miR-132-3p
 
 
-0.304
 
 
11.7
 
 
 
 
ARID2
 
 
hsa-miR-103a-3p
 
 
-0.415
 
 
11.7
 
 
 
 
ARID3B
 
 
hsa-let-7b-5p
 
 
-0.322
 
 
23.2
 
 
 
 
ARID3B
 
 
hsa-let-7b-5p
 
 
-0.486
 
 
23.2
 
 
 
 
ARIH2
 
 
hsa-miR-103a-3p
 
 
-0.307
 
 
21.7
 
 
 
 
ARL2
 
 
hsa-miR-15b-5p
 
 
-0.374
 
 
48.4
 
 
 
 
ARL2
 
 
hsa-miR-15b-5p
 
 
-0.442
 
 
48.4
 
 
 
 
ARL4A
 
 
hsa-miR-29a-3p
 
 
-0.386
 
 
92.7
 
 
 
 
ARL4A
 
 
hsa-miR-30d-5p
 
 
-0.432
 
 
92.7
 
 
 
 
ARL4A
 
 
hsa-miR-30b-5p
 
 
-0.448
 
 
92.7
 
 
 
 
ARL5B
 
 
hsa-miR-29a-3p
 
 
-0.304
 
 
18.5
 
 
 
 
ARL6IP1
 
 
hsa-miR-328-3p
 
 
-0.494
 
 
197.7
 
 
 
 
ARL6IP1
 
 
hsa-miR-148b-3p
 
 
-0.579
 
 
197.7
 
 
 
 
ARL6IP1
 
 
hsa-miR-152-3p
 
 
-0.579
 
 
197.7
 
 
 
 
ARL6IP6
 
 
hsa-let-7b-5p
 
 
-0.293
 
 
23.4
 
 
 
 
ARL8A
 
 
hsa-miR-103a-3p
 
 
-0.38
 
 
66.1
 
 
 
 
ARL8B
 
 
hsa-miR-152-3p
 
 
-0.337
 
 
56.4
 
 
 
 
ARL8B
 
 
hsa-miR-148b-3p
 
 
-0.337
 
 
56.4
 
 
 
 
ARL8B
 
 
hsa-miR-320b
 
 
-0.376
 
 
56.4
 
 
 
 
ARL8B
 
 
hsa-miR-320c
 
 
-0.376
 
 
56.4
 
 
 
 
ARMC1
 
 
hsa-miR-103a-3p
 
 
-0.525
 
 
12.4
 
 
 
 
ARMC8
 
 
hsa-miR-103a-3p
 
 
-0.412
 
 
57.2
 
 
 
 
ARMCX3
 
 
hsa-miR-425-5p
 
 
-0.325
 
 
13.9
 
 
 
 
 
 
 breast 
 
 
 
 
target_symbol
 
 
mature_mirna_id
 
 
score
 
 
nTPM
 
 
 
 
 
 
AAMP
 
 
hsa-miR-326
 
 
-0.722
 
 
65.9
 
 
 
 
AAR2
 
 
hsa-miR-29a-3p
 
 
-0.327
 
 
20.3
 
 
 
 
ABCB7
 
 
hsa-miR-148b-3p
 
 
-0.515
 
 
11.6
 
 
 
 
ABCB7
 
 
hsa-miR-152-3p
 
 
-0.515
 
 
11.6
 
 
 
 
ABCD3
 
 
hsa-miR-148b-3p
 
 
-0.388
 
 
30.8
 
 
 
 
ABCD3
 
 
hsa-miR-152-3p
 
 
-0.388
 
 
30.8
 
 
 
 
ABCE1
 
 
hsa-miR-29a-3p
 
 
-0.404
 
 
30.9
 
 
 
 
ABHD17C
 
 
hsa-let-7b-5p
 
 
-0.359
 
 
10.9
 
 
 
 
ABHD17C
 
 
hsa-miR-330-3p
 
 
-0.399
 
 
10.9
 
 
 
 
ABHD4
 
 
hsa-miR-29a-3p
 
 
-0.312
 
 
18.7
 
 
 
 
ABHD5
 
 
hsa-miR-132-3p
 
 
-0.323
 
 
18.8
 
 
 
 
ACTC1
 
 
hsa-miR-30d-5p
 
 
-0.519
 
 
11.2
 
 
 
 
ACTC1
 
 
hsa-miR-30b-5p
 
 
-0.543
 
 
11.2
 
 
 
 
ACTR2
 
 
hsa-miR-15b-5p
 
 
-0.293
 
 
72.1
 
 
 
 
ACVR1
 
 
hsa-miR-30b-5p
 
 
-0.29
 
 
11.1
 
 
 
 
ACVR1
 
 
hsa-miR-152-3p
 
 
-0.397
 
 
11.1
 
 
 
 
ACVR1
 
 
hsa-miR-148b-3p
 
 
-0.397
 
 
11.1
 
 
 
 
ADA
 
 
hsa-miR-29a-3p
 
 
-0.376
 
 
11.4
 
 
 
 
ADAM10
 
 
hsa-miR-152-3p
 
 
-0.348
 
 
14.2
 
 
 
 
ADAM10
 
 
hsa-miR-148b-3p
 
 
-0.356
 
 
14.2
 
 
 
 
ADAM9
 
 
hsa-miR-30d-5p
 
 
-0.304
 
 
20.2
 
 
 
 
ADAM9
 
 
hsa-miR-30b-5p
 
 
-0.312
 
 
20.2
 
 
 
 
ADAMTS9
 
 
hsa-miR-29a-3p
 
 
-0.305
 
 
19.0
 
 
 
 
ADAMTS9
 
 
hsa-miR-29a-3p
 
 
-0.485
 
 
19.0
 
 
 
 
ADCY3
 
 
hsa-miR-132-3p
 
 
-0.303
 
 
72.4
 
 
 
 
ADCY6
 
 
hsa-miR-1271-5p
 
 
-0.336
 
 
22.7
 
 
 
 
ADGRA2
 
 
hsa-miR-1271-5p
 
 
-0.427
 
 
21.8
 
 
 
 
ADGRA3
 
 
hsa-miR-30d-5p
 
 
-0.296
 
 
10.2
 
 
 
 
ADGRA3
 
 
hsa-miR-30b-5p
 
 
-0.304
 
 
10.2
 
 
 
 
ADGRF5
 
 
hsa-miR-148b-3p
 
 
-0.354
 
 
44.5
 
 
 
 
ADGRF5
 
 
hsa-miR-152-3p
 
 
-0.354
 
 
44.5
 
 
 
 
ADIPOR2
 
 
hsa-miR-150-5p
 
 
-0.287
 
 
44.0
 
 
 
 
ADK
 
 
hsa-miR-1271-5p
 
 
-0.329
 
 
25.7
 
 
 
 
ADNP
 
 
hsa-miR-328-3p
 
 
-0.371
 
 
30.5
 
 
 
 
ADRA2A
 
 
hsa-miR-23a-3p
 
 
-0.371
 
 
31.1
 
 
 
 
ADRB2
 
 
hsa-miR-15b-5p
 
 
-0.29
 
 
32.4
 
 
 
 
ADRB2
 
 
hsa-let-7b-5p
 
 
-0.635
 
 
32.4
 
 
 
 
AFG3L2
 
 
hsa-miR-181b-5p
 
 
-0.491
 
 
15.5
 
 
 
 
AFG3L2
 
 
hsa-miR-181a-5p
 
 
-0.491
 
 
15.5
 
 
 
 
AFG3L2
 
 
hsa-miR-181d-5p
 
 
-0.499
 
 
15.5
 
 
 
 
AGFG1
 
 
hsa-miR-148b-3p
 
 
-0.332
 
 
48.8
 
 
 
 
AGFG1
 
 
hsa-miR-152-3p
 
 
-0.332
 
 
48.8
 
 
 
 
AGFG1
 
 
hsa-miR-103a-3p
 
 
-0.389
 
 
48.8
 
 
 
 
AHDC1
 
 
hsa-miR-152-3p
 
 
-0.413
 
 
11.9
 
 
 
 
AHDC1
 
 
hsa-miR-148b-3p
 
 
-0.413
 
 
11.9
 
 
 
 
AHR
 
 
hsa-miR-1271-5p
 
 
-0.304
 
 
18.4
 
 
 
 
AHR
 
 
hsa-miR-29a-3p
 
 
-0.316
 
 
18.4
 
 
 
 
AIFM1
 
 
hsa-let-7b-5p
 
 
-0.314
 
 
11.7
 
 
 
 
AK1
 
 
hsa-miR-29a-3p
 
 
-0.333
 
 
20.7
 
 
 
 
AK3
 
 
hsa-miR-1271-5p
 
 
-0.57
 
 
36.5
 
 
 
 
AK4
 
 
hsa-miR-15b-5p
 
 
-0.316
 
 
24.4
 
 
 
 
AKAP1
 
 
hsa-miR-152-3p
 
 
-0.381
 
 
34.0
 
 
 
 
AKAP1
 
 
hsa-miR-148b-3p
 
 
-0.381
 
 
34.0
 
 
 
 
AKIRIN2
 
 
hsa-miR-103a-3p
 
 
-0.299
 
 
23.1
 
 
 
 
AKT3
 
 
hsa-miR-29a-3p
 
 
-0.287
 
 
13.8
 
 
 
 
AKT3
 
 
hsa-miR-15b-5p
 
 
-0.301
 
 
13.8
 
 
 
 
ALAD
 
 
hsa-miR-326
 
 
-0.352
 
 
21.7
 
 
 
 
ALAD
 
 
hsa-miR-103a-3p
 
 
-0.385
 
 
21.7
 
 
 
 
ALAD
 
 
hsa-miR-15b-5p
 
 
-0.446
 
 
21.7
 
 
 
 
ALCAM
 
 
hsa-miR-148b-3p
 
 
-0.339
 
 
76.0
 
 
 
 
ALCAM
 
 
hsa-miR-152-3p
 
 
-0.347
 
 
76.0
 
 
 
 
ALDH6A1
 
 
hsa-miR-1271-5p
 
 
-0.301
 
 
13.4
 
 
 
 
ALOX15B
 
 
hsa-miR-326
 
 
-0.417
 
 
105.2
 
 
 
 
AMOTL1
 
 
hsa-miR-15b-5p
 
 
-0.319
 
 
14.0
 
 
 
 
ANKHD1
 
 
hsa-miR-23a-3p
 
 
-0.368
 
 
37.8
 
 
 
 
ANKHD1
 
 
hsa-miR-30d-5p
 
 
-0.381
 
 
37.8
 
 
 
 
ANKHD1
 
 
hsa-miR-30b-5p
 
 
-0.405
 
 
37.8
 
 
 
 
ANKIB1
 
 
hsa-miR-1271-5p
 
 
-0.344
 
 
11.5
 
 
 
 
ANKRD17
 
 
hsa-miR-30d-5p
 
 
-0.312
 
 
18.7
 
 
 
 
ANKRD17
 
 
hsa-miR-30b-5p
 
 
-0.336
 
 
18.7
 
 
 
 
ANKRD29
 
 
hsa-miR-132-3p
 
 
-0.47
 
 
15.0
 
 
 
 
ANKRD46
 
 
hsa-let-7b-5p
 
 
-0.389
 
 
13.6
 
 
 
 
ANP32A
 
 
hsa-miR-132-3p
 
 
-0.374
 
 
66.9
 
 
 
 
ANTXR2
 
 
hsa-miR-29a-3p
 
 
-0.347
 
 
38.4
 
 
 
 
ANXA11
 
 
hsa-miR-15b-5p
 
 
-0.463
 
 
101.9
 
 
 
 
AP1S1
 
 
hsa-let-7b-5p
 
 
-0.374
 
 
17.7
 
 
 
 
AP2A1
 
 
hsa-miR-15b-5p
 
 
-0.333
 
 
39.7
 
 
 
 
AP2B1
 
 
hsa-miR-1271-5p
 
 
-0.299
 
 
54.6
 
 
 
 
AP2B1
 
 
hsa-miR-15b-5p
 
 
-0.334
 
 
54.6
 
 
 
 
AP3S1
 
 
hsa-miR-30b-5p
 
 
-0.293
 
 
56.3
 
 
 
 
AP5M1
 
 
hsa-miR-1271-5p
 
 
-0.343
 
 
12.9
 
 
 
 
APOO
 
 
hsa-miR-181d-5p
 
 
-0.492
 
 
12.5
 
 
 
 
APOO
 
 
hsa-miR-181b-5p
 
 
-0.5
 
 
12.5
 
 
 
 
APOO
 
 
hsa-miR-181a-5p
 
 
-0.5
 
 
12.5
 
 
 
 
APP
 
 
hsa-miR-15b-5p
 
 
-0.298
 
 
392.7
 
 
 
 
AQP3
 
 
hsa-miR-874-3p
 
 
-0.48
 
 
34.4
 
 
 
 
ARF4
 
 
hsa-miR-30b-5p
 
 
-0.289
 
 
129.6
 
 
 
 
ARF4
 
 
hsa-miR-194-5p
 
 
-0.293
 
 
129.6
 
 
 
 
ARF4
 
 
hsa-miR-152-3p
 
 
-0.487
 
 
129.6
 
 
 
 
ARF4
 
 
hsa-miR-148b-3p
 
 
-0.487
 
 
129.6
 
 
 
 
ARF5
 
 
hsa-miR-29a-3p
 
 
-0.491
 
 
108.8
 
 
 
 
ARF6
 
 
hsa-miR-181b-5p
 
 
-0.33
 
 
31.6
 
 
 
 
ARF6
 
 
hsa-miR-181a-5p
 
 
-0.33
 
 
31.6
 
 
 
 
ARF6
 
 
hsa-miR-181d-5p
 
 
-0.338
 
 
31.6
 
 
 
 
ARFGEF1
 
 
hsa-miR-148b-3p
 
 
-0.306
 
 
12.7
 
 
 
 
ARFGEF1
 
 
hsa-miR-152-3p
 
 
-0.306
 
 
12.7
 
 
 
 
ARFIP1
 
 
hsa-miR-320b
 
 
-0.339
 
 
22.0
 
 
 
 
ARFIP1
 
 
hsa-miR-320c
 
 
-0.339
 
 
22.0
 
 
 
 
ARFIP1
 
 
hsa-miR-152-3p
 
 
-0.474
 
 
22.0
 
 
 
 
ARFIP1
 
 
hsa-miR-148b-3p
 
 
-0.474
 
 
22.0
 
 
 
 
 
 
 cerebellum 
 
 
 
 
target_symbol
 
 
mature_mirna_id
 
 
score
 
 
nTPM
 
 
 
 
 
 
AAMP
 
 
hsa-miR-326
 
 
-0.722
 
 
63.9
 
 
 
 
AAR2
 
 
hsa-miR-29a-3p
 
 
-0.327
 
 
10.9
 
 
 
 
ABAT
 
 
hsa-miR-1271-5p
 
 
-0.409
 
 
63.7
 
 
 
 
ABCA2
 
 
hsa-miR-1271-5p
 
 
-0.326
 
 
43.2
 
 
 
 
ABCB10
 
 
hsa-miR-152-3p
 
 
-0.291
 
 
10.6
 
 
 
 
ABCB10
 
 
hsa-miR-148b-3p
 
 
-0.291
 
 
10.6
 
 
 
 
ABCB9
 
 
hsa-let-7b-5p
 
 
-0.422
 
 
18.0
 
 
 
 
ABCD3
 
 
hsa-miR-148b-3p
 
 
-0.388
 
 
12.8
 
 
 
 
ABCD3
 
 
hsa-miR-152-3p
 
 
-0.388
 
 
12.8
 
 
 
 
ABCE1
 
 
hsa-miR-29a-3p
 
 
-0.404
 
 
12.8
 
 
 
 
ACHE
 
 
hsa-miR-194-5p
 
 
-0.395
 
 
59.1
 
 
 
 
ACSL6
 
 
hsa-let-7b-5p
 
 
-0.336
 
 
27.8
 
 
 
 
ACTR2
 
 
hsa-miR-15b-5p
 
 
-0.293
 
 
54.0
 
 
 
 
ADAM10
 
 
hsa-miR-152-3p
 
 
-0.348
 
 
15.6
 
 
 
 
ADAM10
 
 
hsa-miR-148b-3p
 
 
-0.356
 
 
15.6
 
 
 
 
ADCY3
 
 
hsa-miR-132-3p
 
 
-0.303
 
 
33.6
 
 
 
 
ADCY6
 
 
hsa-miR-1271-5p
 
 
-0.336
 
 
21.1
 
 
 
 
ADGRB3
 
 
hsa-miR-1271-5p
 
 
-0.424
 
 
20.8
 
 
 
 
ADGRB3
 
 
hsa-miR-152-3p
 
 
-0.516
 
 
20.8
 
 
 
 
ADGRB3
 
 
hsa-miR-148b-3p
 
 
-0.524
 
 
20.8
 
 
 
 
ADIPOR2
 
 
hsa-miR-150-5p
 
 
-0.287
 
 
18.7
 
 
 
 
ADNP
 
 
hsa-miR-328-3p
 
 
-0.371
 
 
38.9
 
 
 
 
AFG3L2
 
 
hsa-miR-181b-5p
 
 
-0.491
 
 
11.5
 
 
 
 
AFG3L2
 
 
hsa-miR-181a-5p
 
 
-0.491
 
 
11.5
 
 
 
 
AFG3L2
 
 
hsa-miR-181d-5p
 
 
-0.499
 
 
11.5
 
 
 
 
AGFG1
 
 
hsa-miR-148b-3p
 
 
-0.332
 
 
22.4
 
 
 
 
AGFG1
 
 
hsa-miR-152-3p
 
 
-0.332
 
 
22.4
 
 
 
 
AGFG1
 
 
hsa-miR-103a-3p
 
 
-0.389
 
 
22.4
 
 
 
 
AHDC1
 
 
hsa-miR-152-3p
 
 
-0.413
 
 
32.0
 
 
 
 
AHDC1
 
 
hsa-miR-148b-3p
 
 
-0.413
 
 
32.0
 
 
 
 
AK1
 
 
hsa-miR-29a-3p
 
 
-0.333
 
 
47.4
 
 
 
 
AK3
 
 
hsa-miR-1271-5p
 
 
-0.57
 
 
25.8
 
 
 
 
AK4
 
 
hsa-miR-15b-5p
 
 
-0.316
 
 
14.5
 
 
 
 
AKAIN1
 
 
hsa-miR-29a-3p
 
 
-0.52
 
 
40.8
 
 
 
 
AKAP1
 
 
hsa-miR-152-3p
 
 
-0.381
 
 
30.6
 
 
 
 
AKAP1
 
 
hsa-miR-148b-3p
 
 
-0.381
 
 
30.6
 
 
 
 
AKIRIN2
 
 
hsa-miR-103a-3p
 
 
-0.299
 
 
25.9
 
 
 
 
AKT3
 
 
hsa-miR-29a-3p
 
 
-0.287
 
 
23.2
 
 
 
 
AKT3
 
 
hsa-miR-15b-5p
 
 
-0.301
 
 
23.2
 
 
 
 
ALAD
 
 
hsa-miR-326
 
 
-0.352
 
 
17.9
 
 
 
 
ALAD
 
 
hsa-miR-103a-3p
 
 
-0.385
 
 
17.9
 
 
 
 
ALAD
 
 
hsa-miR-15b-5p
 
 
-0.446
 
 
17.9
 
 
 
 
ALDH6A1
 
 
hsa-miR-1271-5p
 
 
-0.301
 
 
16.1
 
 
 
 
ALS2
 
 
hsa-miR-378a-3p
 
 
-0.335
 
 
21.3
 
 
 
 
ALS2
 
 
hsa-miR-378d
 
 
-0.335
 
 
21.3
 
 
 
 
AMT
 
 
hsa-let-7b-5p
 
 
-0.463
 
 
20.1
 
 
 
 
ANGEL2
 
 
hsa-miR-132-3p
 
 
-0.357
 
 
12.7
 
 
 
 
ANK1
 
 
hsa-miR-103a-3p
 
 
-0.321
 
 
48.2
 
 
 
 
ANKHD1
 
 
hsa-miR-23a-3p
 
 
-0.368
 
 
38.6
 
 
 
 
ANKHD1
 
 
hsa-miR-30d-5p
 
 
-0.381
 
 
38.6
 
 
 
 
ANKHD1
 
 
hsa-miR-30b-5p
 
 
-0.405
 
 
38.6
 
 
 
 
ANKRD13B
 
 
hsa-miR-29a-3p
 
 
-0.421
 
 
44.3
 
 
 
 
ANKRD13C
 
 
hsa-miR-29a-3p
 
 
-0.411
 
 
12.9
 
 
 
 
ANKRD17
 
 
hsa-miR-30d-5p
 
 
-0.312
 
 
16.2
 
 
 
 
ANKRD17
 
 
hsa-miR-30b-5p
 
 
-0.336
 
 
16.2
 
 
 
 
ANKRD27
 
 
hsa-miR-1271-5p
 
 
-0.648
 
 
10.8
 
 
 
 
ANKRD46
 
 
hsa-let-7b-5p
 
 
-0.389
 
 
27.5
 
 
 
 
ANKRD6
 
 
hsa-miR-331-3p
 
 
-0.369
 
 
25.8
 
 
 
 
ANP32A
 
 
hsa-miR-132-3p
 
 
-0.374
 
 
71.1
 
 
 
 
ANXA11
 
 
hsa-miR-15b-5p
 
 
-0.463
 
 
22.2
 
 
 
 
AP1S1
 
 
hsa-let-7b-5p
 
 
-0.374
 
 
23.9
 
 
 
 
AP2A1
 
 
hsa-miR-15b-5p
 
 
-0.333
 
 
67.6
 
 
 
 
AP2B1
 
 
hsa-miR-1271-5p
 
 
-0.299
 
 
63.1
 
 
 
 
AP2B1
 
 
hsa-miR-15b-5p
 
 
-0.334
 
 
63.1
 
 
 
 
AP3S1
 
 
hsa-miR-30b-5p
 
 
-0.293
 
 
52.4
 
 
 
 
AP4B1
 
 
hsa-miR-378a-3p
 
 
-0.62
 
 
20.4
 
 
 
 
AP4B1
 
 
hsa-miR-378d
 
 
-0.62
 
 
20.4
 
 
 
 
APBB3
 
 
hsa-let-7b-5p
 
 
-0.587
 
 
23.6
 
 
 
 
APC
 
 
hsa-miR-29a-3p
 
 
-0.413
 
 
12.2
 
 
 
 
APOO
 
 
hsa-miR-181d-5p
 
 
-0.492
 
 
32.8
 
 
 
 
APOO
 
 
hsa-miR-181b-5p
 
 
-0.5
 
 
32.8
 
 
 
 
APOO
 
 
hsa-miR-181a-5p
 
 
-0.5
 
 
32.8
 
 
 
 
APP
 
 
hsa-miR-15b-5p
 
 
-0.298
 
 
316.4
 
 
 
 
ARF4
 
 
hsa-miR-30b-5p
 
 
-0.289
 
 
50.2
 
 
 
 
ARF4
 
 
hsa-miR-194-5p
 
 
-0.293
 
 
50.2
 
 
 
 
ARF4
 
 
hsa-miR-152-3p
 
 
-0.487
 
 
50.2
 
 
 
 
ARF4
 
 
hsa-miR-148b-3p
 
 
-0.487
 
 
50.2
 
 
 
 
ARF5
 
 
hsa-miR-29a-3p
 
 
-0.491
 
 
167.9
 
 
 
 
ARF6
 
 
hsa-miR-181b-5p
 
 
-0.33
 
 
11.0
 
 
 
 
ARF6
 
 
hsa-miR-181a-5p
 
 
-0.33
 
 
11.0
 
 
 
 
ARF6
 
 
hsa-miR-181d-5p
 
 
-0.338
 
 
11.0
 
 
 
 
ARG2
 
 
hsa-let-7b-5p
 
 
-0.468
 
 
16.4
 
 
 
 
ARHGAP21
 
 
hsa-miR-152-3p
 
 
-0.287
 
 
50.0
 
 
 
 
ARHGAP21
 
 
hsa-miR-148b-3p
 
 
-0.287
 
 
50.0
 
 
 
 
ARID1B
 
 
hsa-miR-29a-3p
 
 
-0.342
 
 
12.5
 
 
 
 
ARIH2
 
 
hsa-miR-103a-3p
 
 
-0.307
 
 
27.6
 
 
 
 
ARL2
 
 
hsa-miR-15b-5p
 
 
-0.374
 
 
72.8
 
 
 
 
ARL2
 
 
hsa-miR-15b-5p
 
 
-0.442
 
 
72.8
 
 
 
 
ARL3
 
 
hsa-miR-15b-5p
 
 
-0.507
 
 
25.4
 
 
 
 
ARL6IP1
 
 
hsa-miR-328-3p
 
 
-0.494
 
 
234.0
 
 
 
 
ARL6IP1
 
 
hsa-miR-148b-3p
 
 
-0.579
 
 
234.0
 
 
 
 
ARL6IP1
 
 
hsa-miR-152-3p
 
 
-0.579
 
 
234.0
 
 
 
 
ARL8A
 
 
hsa-miR-103a-3p
 
 
-0.38
 
 
55.9
 
 
 
 
ARL8B
 
 
hsa-miR-152-3p
 
 
-0.337
 
 
50.7
 
 
 
 
ARL8B
 
 
hsa-miR-148b-3p
 
 
-0.337
 
 
50.7
 
 
 
 
ARL8B
 
 
hsa-miR-320b
 
 
-0.376
 
 
50.7
 
 
 
 
ARL8B
 
 
hsa-miR-320c
 
 
-0.376
 
 
50.7
 
 
 
 
ARMC1
 
 
hsa-miR-103a-3p
 
 
-0.525
 
 
20.5
 
 
 
 
ARMC8
 
 
hsa-miR-103a-3p
 
 
-0.412
 
 
35.7
 
 
 
 
ARMCX2
 
 
hsa-miR-15b-5p
 
 
-0.344
 
 
20.7
 
 
 
 
 
 
 cerebral cortex 
 
 
 
 
target_symbol
 
 
mature_mirna_id
 
 
score
 
 
nTPM
 
 
 
 
 
 
AAMP
 
 
hsa-miR-326
 
 
-0.722
 
 
68.3
 
 
 
 
AAR2
 
 
hsa-miR-29a-3p
 
 
-0.327
 
 
10.6
 
 
 
 
ABAT
 
 
hsa-miR-1271-5p
 
 
-0.409
 
 
124.8
 
 
 
 
ABCA2
 
 
hsa-miR-1271-5p
 
 
-0.326
 
 
54.0
 
 
 
 
ABCB9
 
 
hsa-let-7b-5p
 
 
-0.422
 
 
19.5
 
 
 
 
ABCD3
 
 
hsa-miR-148b-3p
 
 
-0.388
 
 
17.1
 
 
 
 
ABCD3
 
 
hsa-miR-152-3p
 
 
-0.388
 
 
17.1
 
 
 
 
ABCE1
 
 
hsa-miR-29a-3p
 
 
-0.404
 
 
21.8
 
 
 
 
ABHD17C
 
 
hsa-let-7b-5p
 
 
-0.359
 
 
16.3
 
 
 
 
ABHD17C
 
 
hsa-miR-330-3p
 
 
-0.399
 
 
16.3
 
 
 
 
ABHD4
 
 
hsa-miR-29a-3p
 
 
-0.312
 
 
14.6
 
 
 
 
ACHE
 
 
hsa-miR-194-5p
 
 
-0.395
 
 
23.4
 
 
 
 
ACSL6
 
 
hsa-let-7b-5p
 
 
-0.336
 
 
29.5
 
 
 
 
ACTN2
 
 
hsa-miR-23a-3p
 
 
-0.294
 
 
14.9
 
 
 
 
ACTN2
 
 
hsa-miR-29a-3p
 
 
-0.311
 
 
14.9
 
 
 
 
ACTR2
 
 
hsa-miR-15b-5p
 
 
-0.293
 
 
115.2
 
 
 
 
ADAM10
 
 
hsa-miR-152-3p
 
 
-0.348
 
 
16.0
 
 
 
 
ADAM10
 
 
hsa-miR-148b-3p
 
 
-0.356
 
 
16.0
 
 
 
 
ADAM9
 
 
hsa-miR-30d-5p
 
 
-0.304
 
 
16.2
 
 
 
 
ADAM9
 
 
hsa-miR-30b-5p
 
 
-0.312
 
 
16.2
 
 
 
 
ADAMTS10
 
 
hsa-miR-29a-3p
 
 
-0.516
 
 
10.8
 
 
 
 
ADCY2
 
 
hsa-miR-152-3p
 
 
-0.299
 
 
35.4
 
 
 
 
ADCY2
 
 
hsa-miR-148b-3p
 
 
-0.299
 
 
35.4
 
 
 
 
ADCY3
 
 
hsa-miR-132-3p
 
 
-0.303
 
 
37.4
 
 
 
 
ADCY6
 
 
hsa-miR-1271-5p
 
 
-0.336
 
 
13.2
 
 
 
 
ADGRA3
 
 
hsa-miR-30d-5p
 
 
-0.296
 
 
17.8
 
 
 
 
ADGRA3
 
 
hsa-miR-30b-5p
 
 
-0.304
 
 
17.8
 
 
 
 
ADGRB3
 
 
hsa-miR-1271-5p
 
 
-0.424
 
 
28.2
 
 
 
 
ADGRB3
 
 
hsa-miR-152-3p
 
 
-0.516
 
 
28.2
 
 
 
 
ADGRB3
 
 
hsa-miR-148b-3p
 
 
-0.524
 
 
28.2
 
 
 
 
ADIPOR2
 
 
hsa-miR-150-5p
 
 
-0.287
 
 
29.8
 
 
 
 
ADK
 
 
hsa-miR-1271-5p
 
 
-0.329
 
 
16.5
 
 
 
 
ADNP
 
 
hsa-miR-328-3p
 
 
-0.371
 
 
26.0
 
 
 
 
AFG3L2
 
 
hsa-miR-181b-5p
 
 
-0.491
 
 
17.1
 
 
 
 
AFG3L2
 
 
hsa-miR-181a-5p
 
 
-0.491
 
 
17.1
 
 
 
 
AFG3L2
 
 
hsa-miR-181d-5p
 
 
-0.499
 
 
17.1
 
 
 
 
AGFG1
 
 
hsa-miR-148b-3p
 
 
-0.332
 
 
33.6
 
 
 
 
AGFG1
 
 
hsa-miR-152-3p
 
 
-0.332
 
 
33.6
 
 
 
 
AGFG1
 
 
hsa-miR-103a-3p
 
 
-0.389
 
 
33.6
 
 
 
 
AGPAT4
 
 
hsa-miR-29a-3p
 
 
-0.376
 
 
13.7
 
 
 
 
AHDC1
 
 
hsa-miR-152-3p
 
 
-0.413
 
 
15.5
 
 
 
 
AHDC1
 
 
hsa-miR-148b-3p
 
 
-0.413
 
 
15.5
 
 
 
 
AIFM1
 
 
hsa-let-7b-5p
 
 
-0.314
 
 
10.3
 
 
 
 
AK1
 
 
hsa-miR-29a-3p
 
 
-0.333
 
 
81.2
 
 
 
 
AK3
 
 
hsa-miR-1271-5p
 
 
-0.57
 
 
33.0
 
 
 
 
AK4
 
 
hsa-miR-15b-5p
 
 
-0.316
 
 
19.8
 
 
 
 
AKAP1
 
 
hsa-miR-152-3p
 
 
-0.381
 
 
24.9
 
 
 
 
AKAP1
 
 
hsa-miR-148b-3p
 
 
-0.381
 
 
24.9
 
 
 
 
AKAP5
 
 
hsa-miR-29a-3p
 
 
-0.298
 
 
12.9
 
 
 
 
AKAP6
 
 
hsa-let-7b-5p
 
 
-0.307
 
 
27.4
 
 
 
 
AKIRIN2
 
 
hsa-miR-103a-3p
 
 
-0.299
 
 
33.4
 
 
 
 
AKT3
 
 
hsa-miR-29a-3p
 
 
-0.287
 
 
23.7
 
 
 
 
AKT3
 
 
hsa-miR-15b-5p
 
 
-0.301
 
 
23.7
 
 
 
 
ALAD
 
 
hsa-miR-326
 
 
-0.352
 
 
40.0
 
 
 
 
ALAD
 
 
hsa-miR-103a-3p
 
 
-0.385
 
 
40.0
 
 
 
 
ALAD
 
 
hsa-miR-15b-5p
 
 
-0.446
 
 
40.0
 
 
 
 
ALCAM
 
 
hsa-miR-148b-3p
 
 
-0.339
 
 
54.7
 
 
 
 
ALCAM
 
 
hsa-miR-152-3p
 
 
-0.347
 
 
54.7
 
 
 
 
ALDH6A1
 
 
hsa-miR-1271-5p
 
 
-0.301
 
 
76.2
 
 
 
 
AMOT
 
 
hsa-miR-29a-3p
 
 
-0.304
 
 
17.9
 
 
 
 
ANKHD1
 
 
hsa-miR-23a-3p
 
 
-0.368
 
 
22.1
 
 
 
 
ANKHD1
 
 
hsa-miR-30d-5p
 
 
-0.381
 
 
22.1
 
 
 
 
ANKHD1
 
 
hsa-miR-30b-5p
 
 
-0.405
 
 
22.1
 
 
 
 
ANKIB1
 
 
hsa-miR-1271-5p
 
 
-0.344
 
 
19.3
 
 
 
 
ANKRD13B
 
 
hsa-miR-29a-3p
 
 
-0.421
 
 
17.2
 
 
 
 
ANKRD13C
 
 
hsa-miR-29a-3p
 
 
-0.411
 
 
10.2
 
 
 
 
ANKRD17
 
 
hsa-miR-30d-5p
 
 
-0.312
 
 
15.6
 
 
 
 
ANKRD17
 
 
hsa-miR-30b-5p
 
 
-0.336
 
 
15.6
 
 
 
 
ANKRD46
 
 
hsa-let-7b-5p
 
 
-0.389
 
 
39.5
 
 
 
 
ANKRD6
 
 
hsa-miR-331-3p
 
 
-0.369
 
 
13.5
 
 
 
 
ANLN
 
 
hsa-miR-15b-5p
 
 
-0.334
 
 
47.6
 
 
 
 
ANP32A
 
 
hsa-miR-132-3p
 
 
-0.374
 
 
64.2
 
 
 
 
ANXA11
 
 
hsa-miR-15b-5p
 
 
-0.463
 
 
45.1
 
 
 
 
AP1S1
 
 
hsa-let-7b-5p
 
 
-0.374
 
 
104.9
 
 
 
 
AP2A1
 
 
hsa-miR-15b-5p
 
 
-0.333
 
 
78.9
 
 
 
 
AP2B1
 
 
hsa-miR-1271-5p
 
 
-0.299
 
 
106.7
 
 
 
 
AP2B1
 
 
hsa-miR-15b-5p
 
 
-0.334
 
 
106.7
 
 
 
 
AP3S1
 
 
hsa-miR-30b-5p
 
 
-0.293
 
 
82.8
 
 
 
 
AP4B1
 
 
hsa-miR-378a-3p
 
 
-0.62
 
 
10.6
 
 
 
 
AP4B1
 
 
hsa-miR-378d
 
 
-0.62
 
 
10.6
 
 
 
 
AP5M1
 
 
hsa-miR-1271-5p
 
 
-0.343
 
 
13.5
 
 
 
 
APBB3
 
 
hsa-let-7b-5p
 
 
-0.587
 
 
15.0
 
 
 
 
APC
 
 
hsa-miR-29a-3p
 
 
-0.413
 
 
34.5
 
 
 
 
APLN
 
 
hsa-miR-15b-5p
 
 
-0.395
 
 
14.5
 
 
 
 
APOO
 
 
hsa-miR-181d-5p
 
 
-0.492
 
 
56.2
 
 
 
 
APOO
 
 
hsa-miR-181b-5p
 
 
-0.5
 
 
56.2
 
 
 
 
APOO
 
 
hsa-miR-181a-5p
 
 
-0.5
 
 
56.2
 
 
 
 
APP
 
 
hsa-miR-15b-5p
 
 
-0.298
 
 
669.1
 
 
 
 
ARF4
 
 
hsa-miR-30b-5p
 
 
-0.289
 
 
61.5
 
 
 
 
ARF4
 
 
hsa-miR-194-5p
 
 
-0.293
 
 
61.5
 
 
 
 
ARF4
 
 
hsa-miR-152-3p
 
 
-0.487
 
 
61.5
 
 
 
 
ARF4
 
 
hsa-miR-148b-3p
 
 
-0.487
 
 
61.5
 
 
 
 
ARF5
 
 
hsa-miR-29a-3p
 
 
-0.491
 
 
222.4
 
 
 
 
ARF6
 
 
hsa-miR-181b-5p
 
 
-0.33
 
 
13.0
 
 
 
 
ARF6
 
 
hsa-miR-181a-5p
 
 
-0.33
 
 
13.0
 
 
 
 
ARF6
 
 
hsa-miR-181d-5p
 
 
-0.338
 
 
13.0
 
 
 
 
ARHGAP21
 
 
hsa-miR-152-3p
 
 
-0.287
 
 
47.3
 
 
 
 
ARHGAP21
 
 
hsa-miR-148b-3p
 
 
-0.287
 
 
47.3
 
 
 
 
ARID1B
 
 
hsa-miR-29a-3p
 
 
-0.342
 
 
12.2
 
 
 
 
ARIH2
 
 
hsa-miR-103a-3p
 
 
-0.307
 
 
19.9
 
 
 
 
 
 
 cervix 
 
 
 
 
target_symbol
 
 
mature_mirna_id
 
 
score
 
 
nTPM
 
 
 
 
 
 
AAMP
 
 
hsa-miR-326
 
 
-0.722
 
 
61.6
 
 
 
 
AAR2
 
 
hsa-miR-29a-3p
 
 
-0.327
 
 
19.6
 
 
 
 
ABCB7
 
 
hsa-miR-148b-3p
 
 
-0.515
 
 
10.8
 
 
 
 
ABCB7
 
 
hsa-miR-152-3p
 
 
-0.515
 
 
10.8
 
 
 
 
ABCD3
 
 
hsa-miR-148b-3p
 
 
-0.388
 
 
21.0
 
 
 
 
ABCD3
 
 
hsa-miR-152-3p
 
 
-0.388
 
 
21.0
 
 
 
 
ABCE1
 
 
hsa-miR-29a-3p
 
 
-0.404
 
 
19.5
 
 
 
 
ABHD17C
 
 
hsa-let-7b-5p
 
 
-0.359
 
 
18.1
 
 
 
 
ABHD17C
 
 
hsa-miR-330-3p
 
 
-0.399
 
 
18.1
 
 
 
 
ABHD4
 
 
hsa-miR-29a-3p
 
 
-0.312
 
 
27.3
 
 
 
 
ACP3
 
 
hsa-let-7b-5p
 
 
-0.298
 
 
10.9
 
 
 
 
ACTC1
 
 
hsa-miR-30d-5p
 
 
-0.519
 
 
44.2
 
 
 
 
ACTC1
 
 
hsa-miR-30b-5p
 
 
-0.543
 
 
44.2
 
 
 
 
ACTR2
 
 
hsa-miR-15b-5p
 
 
-0.293
 
 
76.5
 
 
 
 
ACVR1
 
 
hsa-miR-30b-5p
 
 
-0.29
 
 
20.6
 
 
 
 
ACVR1
 
 
hsa-miR-152-3p
 
 
-0.397
 
 
20.6
 
 
 
 
ACVR1
 
 
hsa-miR-148b-3p
 
 
-0.397
 
 
20.6
 
 
 
 
ADAM10
 
 
hsa-miR-152-3p
 
 
-0.348
 
 
15.1
 
 
 
 
ADAM10
 
 
hsa-miR-148b-3p
 
 
-0.356
 
 
15.1
 
 
 
 
ADAM9
 
 
hsa-miR-30d-5p
 
 
-0.304
 
 
32.7
 
 
 
 
ADAM9
 
 
hsa-miR-30b-5p
 
 
-0.312
 
 
32.7
 
 
 
 
ADAMTS10
 
 
hsa-miR-29a-3p
 
 
-0.516
 
 
11.2
 
 
 
 
ADAMTS9
 
 
hsa-miR-29a-3p
 
 
-0.305
 
 
11.0
 
 
 
 
ADAMTS9
 
 
hsa-miR-29a-3p
 
 
-0.485
 
 
11.0
 
 
 
 
ADCY3
 
 
hsa-miR-132-3p
 
 
-0.303
 
 
60.4
 
 
 
 
ADCY6
 
 
hsa-miR-1271-5p
 
 
-0.336
 
 
19.8
 
 
 
 
ADGRA2
 
 
hsa-miR-1271-5p
 
 
-0.427
 
 
49.5
 
 
 
 
ADGRA3
 
 
hsa-miR-30d-5p
 
 
-0.296
 
 
10.2
 
 
 
 
ADGRA3
 
 
hsa-miR-30b-5p
 
 
-0.304
 
 
10.2
 
 
 
 
ADIPOR2
 
 
hsa-miR-150-5p
 
 
-0.287
 
 
25.7
 
 
 
 
ADK
 
 
hsa-miR-1271-5p
 
 
-0.329
 
 
20.2
 
 
 
 
ADNP
 
 
hsa-miR-328-3p
 
 
-0.371
 
 
35.1
 
 
 
 
ADRA2A
 
 
hsa-miR-23a-3p
 
 
-0.371
 
 
35.0
 
 
 
 
ADRB2
 
 
hsa-miR-15b-5p
 
 
-0.29
 
 
12.7
 
 
 
 
ADRB2
 
 
hsa-let-7b-5p
 
 
-0.635
 
 
12.7
 
 
 
 
AFG3L2
 
 
hsa-miR-181b-5p
 
 
-0.491
 
 
14.0
 
 
 
 
AFG3L2
 
 
hsa-miR-181a-5p
 
 
-0.491
 
 
14.0
 
 
 
 
AFG3L2
 
 
hsa-miR-181d-5p
 
 
-0.499
 
 
14.0
 
 
 
 
AGFG1
 
 
hsa-miR-148b-3p
 
 
-0.332
 
 
27.5
 
 
 
 
AGFG1
 
 
hsa-miR-152-3p
 
 
-0.332
 
 
27.5
 
 
 
 
AGFG1
 
 
hsa-miR-103a-3p
 
 
-0.389
 
 
27.5
 
 
 
 
AHDC1
 
 
hsa-miR-152-3p
 
 
-0.413
 
 
23.6
 
 
 
 
AHDC1
 
 
hsa-miR-148b-3p
 
 
-0.413
 
 
23.6
 
 
 
 
AHR
 
 
hsa-miR-1271-5p
 
 
-0.304
 
 
13.6
 
 
 
 
AHR
 
 
hsa-miR-29a-3p
 
 
-0.316
 
 
13.6
 
 
 
 
AIFM1
 
 
hsa-let-7b-5p
 
 
-0.314
 
 
14.8
 
 
 
 
AJUBA
 
 
hsa-miR-103a-3p
 
 
-0.448
 
 
14.5
 
 
 
 
AK1
 
 
hsa-miR-29a-3p
 
 
-0.333
 
 
35.3
 
 
 
 
AK3
 
 
hsa-miR-1271-5p
 
 
-0.57
 
 
33.4
 
 
 
 
AKAP1
 
 
hsa-miR-152-3p
 
 
-0.381
 
 
22.6
 
 
 
 
AKAP1
 
 
hsa-miR-148b-3p
 
 
-0.381
 
 
22.6
 
 
 
 
AKIRIN2
 
 
hsa-miR-103a-3p
 
 
-0.299
 
 
19.6
 
 
 
 
AKT3
 
 
hsa-miR-29a-3p
 
 
-0.287
 
 
15.6
 
 
 
 
AKT3
 
 
hsa-miR-15b-5p
 
 
-0.301
 
 
15.6
 
 
 
 
ALAD
 
 
hsa-miR-326
 
 
-0.352
 
 
18.9
 
 
 
 
ALAD
 
 
hsa-miR-103a-3p
 
 
-0.385
 
 
18.9
 
 
 
 
ALAD
 
 
hsa-miR-15b-5p
 
 
-0.446
 
 
18.9
 
 
 
 
ALCAM
 
 
hsa-miR-148b-3p
 
 
-0.339
 
 
26.3
 
 
 
 
ALCAM
 
 
hsa-miR-152-3p
 
 
-0.347
 
 
26.3
 
 
 
 
ALDH1A2
 
 
hsa-miR-23a-3p
 
 
-0.354
 
 
49.1
 
 
 
 
ALDH6A1
 
 
hsa-miR-1271-5p
 
 
-0.301
 
 
16.3
 
 
 
 
ALOX12
 
 
hsa-miR-15b-5p
 
 
-0.443
 
 
78.6
 
 
 
 
ALOX15B
 
 
hsa-miR-326
 
 
-0.417
 
 
18.8
 
 
 
 
AMOTL1
 
 
hsa-miR-15b-5p
 
 
-0.319
 
 
12.0
 
 
 
 
AMT
 
 
hsa-let-7b-5p
 
 
-0.463
 
 
14.5
 
 
 
 
ANKHD1
 
 
hsa-miR-23a-3p
 
 
-0.368
 
 
41.2
 
 
 
 
ANKHD1
 
 
hsa-miR-30d-5p
 
 
-0.381
 
 
41.2
 
 
 
 
ANKHD1
 
 
hsa-miR-30b-5p
 
 
-0.405
 
 
41.2
 
 
 
 
ANKIB1
 
 
hsa-miR-1271-5p
 
 
-0.344
 
 
11.9
 
 
 
 
ANKRD17
 
 
hsa-miR-30d-5p
 
 
-0.312
 
 
15.8
 
 
 
 
ANKRD17
 
 
hsa-miR-30b-5p
 
 
-0.336
 
 
15.8
 
 
 
 
ANKRD29
 
 
hsa-miR-132-3p
 
 
-0.47
 
 
12.2
 
 
 
 
ANKRD46
 
 
hsa-let-7b-5p
 
 
-0.389
 
 
14.1
 
 
 
 
ANP32A
 
 
hsa-miR-132-3p
 
 
-0.374
 
 
50.7
 
 
 
 
ANTXR2
 
 
hsa-miR-29a-3p
 
 
-0.347
 
 
49.9
 
 
 
 
ANXA11
 
 
hsa-miR-15b-5p
 
 
-0.463
 
 
89.1
 
 
 
 
AP1S1
 
 
hsa-let-7b-5p
 
 
-0.374
 
 
20.1
 
 
 
 
AP2A1
 
 
hsa-miR-15b-5p
 
 
-0.333
 
 
42.3
 
 
 
 
AP2B1
 
 
hsa-miR-1271-5p
 
 
-0.299
 
 
50.8
 
 
 
 
AP2B1
 
 
hsa-miR-15b-5p
 
 
-0.334
 
 
50.8
 
 
 
 
AP3S1
 
 
hsa-miR-30b-5p
 
 
-0.293
 
 
65.9
 
 
 
 
AP4B1
 
 
hsa-miR-378a-3p
 
 
-0.62
 
 
11.8
 
 
 
 
AP4B1
 
 
hsa-miR-378d
 
 
-0.62
 
 
11.8
 
 
 
 
AP5M1
 
 
hsa-miR-1271-5p
 
 
-0.343
 
 
13.5
 
 
 
 
APOO
 
 
hsa-miR-181d-5p
 
 
-0.492
 
 
12.3
 
 
 
 
APOO
 
 
hsa-miR-181b-5p
 
 
-0.5
 
 
12.3
 
 
 
 
APOO
 
 
hsa-miR-181a-5p
 
 
-0.5
 
 
12.3
 
 
 
 
APP
 
 
hsa-miR-15b-5p
 
 
-0.298
 
 
239.9
 
 
 
 
AQP3
 
 
hsa-miR-874-3p
 
 
-0.48
 
 
331.4
 
 
 
 
ARF4
 
 
hsa-miR-30b-5p
 
 
-0.289
 
 
196.1
 
 
 
 
ARF4
 
 
hsa-miR-194-5p
 
 
-0.293
 
 
196.1
 
 
 
 
ARF4
 
 
hsa-miR-152-3p
 
 
-0.487
 
 
196.1
 
 
 
 
ARF4
 
 
hsa-miR-148b-3p
 
 
-0.487
 
 
196.1
 
 
 
 
ARF5
 
 
hsa-miR-29a-3p
 
 
-0.491
 
 
129.6
 
 
 
 
ARF6
 
 
hsa-miR-181b-5p
 
 
-0.33
 
 
31.8
 
 
 
 
ARF6
 
 
hsa-miR-181a-5p
 
 
-0.33
 
 
31.8
 
 
 
 
ARF6
 
 
hsa-miR-181d-5p
 
 
-0.338
 
 
31.8
 
 
 
 
ARFGEF1
 
 
hsa-miR-148b-3p
 
 
-0.306
 
 
13.3
 
 
 
 
ARFGEF1
 
 
hsa-miR-152-3p
 
 
-0.306
 
 
13.3
 
 
 
 
ARFIP1
 
 
hsa-miR-320b
 
 
-0.339
 
 
21.1
 
 
 
 
 
 
 choroid plexus 
 
 
 
 
target_symbol
 
 
mature_mirna_id
 
 
score
 
 
nTPM
 
 
 
 
 
 
AAMP
 
 
hsa-miR-326
 
 
-0.722
 
 
82.1
 
 
 
 
AAR2
 
 
hsa-miR-29a-3p
 
 
-0.327
 
 
27.3
 
 
 
 
ABAT
 
 
hsa-miR-1271-5p
 
 
-0.409
 
 
47.4
 
 
 
 
ABCB7
 
 
hsa-miR-148b-3p
 
 
-0.515
 
 
15.2
 
 
 
 
ABCB7
 
 
hsa-miR-152-3p
 
 
-0.515
 
 
15.2
 
 
 
 
ABCD3
 
 
hsa-miR-148b-3p
 
 
-0.388
 
 
13.3
 
 
 
 
ABCD3
 
 
hsa-miR-152-3p
 
 
-0.388
 
 
13.3
 
 
 
 
ABHD4
 
 
hsa-miR-29a-3p
 
 
-0.312
 
 
31.8
 
 
 
 
ACTN2
 
 
hsa-miR-23a-3p
 
 
-0.294
 
 
16.8
 
 
 
 
ACTN2
 
 
hsa-miR-29a-3p
 
 
-0.311
 
 
16.8
 
 
 
 
ACTR2
 
 
hsa-miR-15b-5p
 
 
-0.293
 
 
35.9
 
 
 
 
ACTR3C
 
 
hsa-miR-30b-5p
 
 
-0.448
 
 
14.9
 
 
 
 
ACTR3C
 
 
hsa-miR-30d-5p
 
 
-0.456
 
 
14.9
 
 
 
 
ADAM9
 
 
hsa-miR-30d-5p
 
 
-0.304
 
 
14.9
 
 
 
 
ADAM9
 
 
hsa-miR-30b-5p
 
 
-0.312
 
 
14.9
 
 
 
 
ADCY3
 
 
hsa-miR-132-3p
 
 
-0.303
 
 
17.0
 
 
 
 
ADGRA3
 
 
hsa-miR-30d-5p
 
 
-0.296
 
 
23.6
 
 
 
 
ADGRA3
 
 
hsa-miR-30b-5p
 
 
-0.304
 
 
23.6
 
 
 
 
ADIPOR2
 
 
hsa-miR-150-5p
 
 
-0.287
 
 
17.0
 
 
 
 
ADK
 
 
hsa-miR-1271-5p
 
 
-0.329
 
 
13.6
 
 
 
 
ADNP
 
 
hsa-miR-328-3p
 
 
-0.371
 
 
24.5
 
 
 
 
AGFG1
 
 
hsa-miR-148b-3p
 
 
-0.332
 
 
23.3
 
 
 
 
AGFG1
 
 
hsa-miR-152-3p
 
 
-0.332
 
 
23.3
 
 
 
 
AGFG1
 
 
hsa-miR-103a-3p
 
 
-0.389
 
 
23.3
 
 
 
 
AIFM1
 
 
hsa-let-7b-5p
 
 
-0.314
 
 
37.9
 
 
 
 
AK1
 
 
hsa-miR-29a-3p
 
 
-0.333
 
 
522.7
 
 
 
 
AK3
 
 
hsa-miR-1271-5p
 
 
-0.57
 
 
52.5
 
 
 
 
AK4
 
 
hsa-miR-15b-5p
 
 
-0.316
 
 
13.8
 
 
 
 
AKIRIN2
 
 
hsa-miR-103a-3p
 
 
-0.299
 
 
23.7
 
 
 
 
ALAD
 
 
hsa-miR-326
 
 
-0.352
 
 
29.1
 
 
 
 
ALAD
 
 
hsa-miR-103a-3p
 
 
-0.385
 
 
29.1
 
 
 
 
ALAD
 
 
hsa-miR-15b-5p
 
 
-0.446
 
 
29.1
 
 
 
 
ALDH6A1
 
 
hsa-miR-1271-5p
 
 
-0.301
 
 
59.5
 
 
 
 
AMT
 
 
hsa-let-7b-5p
 
 
-0.463
 
 
33.4
 
 
 
 
ANKHD1
 
 
hsa-miR-23a-3p
 
 
-0.368
 
 
42.2
 
 
 
 
ANKHD1
 
 
hsa-miR-30d-5p
 
 
-0.381
 
 
42.2
 
 
 
 
ANKHD1
 
 
hsa-miR-30b-5p
 
 
-0.405
 
 
42.2
 
 
 
 
ANKRD17
 
 
hsa-miR-30d-5p
 
 
-0.312
 
 
13.8
 
 
 
 
ANKRD17
 
 
hsa-miR-30b-5p
 
 
-0.336
 
 
13.8
 
 
 
 
ANKRD27
 
 
hsa-miR-1271-5p
 
 
-0.648
 
 
18.6
 
 
 
 
ANKRD46
 
 
hsa-let-7b-5p
 
 
-0.389
 
 
11.0
 
 
 
 
ANP32A
 
 
hsa-miR-132-3p
 
 
-0.374
 
 
28.9
 
 
 
 
ANXA11
 
 
hsa-miR-15b-5p
 
 
-0.463
 
 
96.6
 
 
 
 
AP1S1
 
 
hsa-let-7b-5p
 
 
-0.374
 
 
10.3
 
 
 
 
AP2A1
 
 
hsa-miR-15b-5p
 
 
-0.333
 
 
20.9
 
 
 
 
AP2B1
 
 
hsa-miR-1271-5p
 
 
-0.299
 
 
83.9
 
 
 
 
AP2B1
 
 
hsa-miR-15b-5p
 
 
-0.334
 
 
83.9
 
 
 
 
AP3S1
 
 
hsa-miR-30b-5p
 
 
-0.293
 
 
22.5
 
 
 
 
AP5M1
 
 
hsa-miR-1271-5p
 
 
-0.343
 
 
13.5
 
 
 
 
APOO
 
 
hsa-miR-181d-5p
 
 
-0.492
 
 
102.5
 
 
 
 
APOO
 
 
hsa-miR-181b-5p
 
 
-0.5
 
 
102.5
 
 
 
 
APOO
 
 
hsa-miR-181a-5p
 
 
-0.5
 
 
102.5
 
 
 
 
APP
 
 
hsa-miR-15b-5p
 
 
-0.298
 
 
394.3
 
 
 
 
ARF4
 
 
hsa-miR-30b-5p
 
 
-0.289
 
 
115.4
 
 
 
 
ARF4
 
 
hsa-miR-194-5p
 
 
-0.293
 
 
115.4
 
 
 
 
ARF4
 
 
hsa-miR-152-3p
 
 
-0.487
 
 
115.4
 
 
 
 
ARF4
 
 
hsa-miR-148b-3p
 
 
-0.487
 
 
115.4
 
 
 
 
ARF5
 
 
hsa-miR-29a-3p
 
 
-0.491
 
 
140.5
 
 
 
 
ARF6
 
 
hsa-miR-181b-5p
 
 
-0.33
 
 
20.9
 
 
 
 
ARF6
 
 
hsa-miR-181a-5p
 
 
-0.33
 
 
20.9
 
 
 
 
ARF6
 
 
hsa-miR-181d-5p
 
 
-0.338
 
 
20.9
 
 
 
 
ARG2
 
 
hsa-let-7b-5p
 
 
-0.468
 
 
49.7
 
 
 
 
ARIH2
 
 
hsa-miR-103a-3p
 
 
-0.307
 
 
32.2
 
 
 
 
ARL2
 
 
hsa-miR-15b-5p
 
 
-0.374
 
 
135.4
 
 
 
 
ARL2
 
 
hsa-miR-15b-5p
 
 
-0.442
 
 
135.4
 
 
 
 
ARL3
 
 
hsa-miR-15b-5p
 
 
-0.507
 
 
27.9
 
 
 
 
ARL4A
 
 
hsa-miR-29a-3p
 
 
-0.386
 
 
29.1
 
 
 
 
ARL4A
 
 
hsa-miR-30d-5p
 
 
-0.432
 
 
29.1
 
 
 
 
ARL4A
 
 
hsa-miR-30b-5p
 
 
-0.448
 
 
29.1
 
 
 
 
ARL4D
 
 
hsa-miR-1271-5p
 
 
-0.392
 
 
15.4
 
 
 
 
ARL4D
 
 
hsa-let-7b-5p
 
 
-0.417
 
 
15.4
 
 
 
 
ARL6IP1
 
 
hsa-miR-328-3p
 
 
-0.494
 
 
185.0
 
 
 
 
ARL6IP1
 
 
hsa-miR-148b-3p
 
 
-0.579
 
 
185.0
 
 
 
 
ARL6IP1
 
 
hsa-miR-152-3p
 
 
-0.579
 
 
185.0
 
 
 
 
ARL8A
 
 
hsa-miR-103a-3p
 
 
-0.38
 
 
29.6
 
 
 
 
ARL8B
 
 
hsa-miR-152-3p
 
 
-0.337
 
 
19.0
 
 
 
 
ARL8B
 
 
hsa-miR-148b-3p
 
 
-0.337
 
 
19.0
 
 
 
 
ARL8B
 
 
hsa-miR-320b
 
 
-0.376
 
 
19.0
 
 
 
 
ARL8B
 
 
hsa-miR-320c
 
 
-0.376
 
 
19.0
 
 
 
 
ARMC8
 
 
hsa-miR-103a-3p
 
 
-0.412
 
 
20.8
 
 
 
 
ARMCX2
 
 
hsa-miR-15b-5p
 
 
-0.344
 
 
19.0
 
 
 
 
ARMCX3
 
 
hsa-miR-425-5p
 
 
-0.325
 
 
25.1
 
 
 
 
ARNT
 
 
hsa-miR-103a-3p
 
 
-0.422
 
 
16.9
 
 
 
 
ARPC5L
 
 
hsa-miR-326
 
 
-0.439
 
 
33.4
 
 
 
 
ARPC5L
 
 
hsa-miR-1271-5p
 
 
-0.736
 
 
33.4
 
 
 
 
ARPP19
 
 
hsa-miR-1271-5p
 
 
-0.313
 
 
32.4
 
 
 
 
ARPP19
 
 
hsa-miR-320b
 
 
-0.325
 
 
32.4
 
 
 
 
ARPP19
 
 
hsa-miR-320c
 
 
-0.325
 
 
32.4
 
 
 
 
ARPP19
 
 
hsa-miR-15b-5p
 
 
-0.346
 
 
32.4
 
 
 
 
ARPP19
 
 
hsa-miR-29a-3p
 
 
-0.371
 
 
32.4
 
 
 
 
ARPP19
 
 
hsa-miR-328-3p
 
 
-0.404
 
 
32.4
 
 
 
 
ARPP19
 
 
hsa-let-7b-5p
 
 
-0.476
 
 
32.4
 
 
 
 
ARRDC3
 
 
hsa-miR-152-3p
 
 
-0.305
 
 
24.1
 
 
 
 
ARRDC3
 
 
hsa-miR-148b-3p
 
 
-0.305
 
 
24.1
 
 
 
 
ARRDC3
 
 
hsa-miR-29a-3p
 
 
-0.402
 
 
24.1
 
 
 
 
ASB3
 
 
hsa-miR-30d-5p
 
 
-0.394
 
 
14.1
 
 
 
 
ASB3
 
 
hsa-miR-30d-5p
 
 
-0.394
 
 
14.1
 
 
 
 
ASB3
 
 
hsa-miR-30b-5p
 
 
-0.418
 
 
14.1
 
 
 
 
ASB3
 
 
hsa-miR-30b-5p
 
 
-0.418
 
 
14.1
 
 
 
 
ASB6
 
 
hsa-miR-874-3p
 
 
-0.368
 
 
15.8
 
 
 
 
 
 
 colon 
 
 
 
 
target_symbol
 
 
mature_mirna_id
 
 
score
 
 
nTPM
 
 
 
 
 
 
AAMP
 
 
hsa-miR-326
 
 
-0.722
 
 
71.4
 
 
 
 
AAR2
 
 
hsa-miR-29a-3p
 
 
-0.327
 
 
25.8
 
 
 
 
ABCB10
 
 
hsa-miR-152-3p
 
 
-0.291
 
 
10.4
 
 
 
 
ABCB10
 
 
hsa-miR-148b-3p
 
 
-0.291
 
 
10.4
 
 
 
 
ABCB7
 
 
hsa-miR-148b-3p
 
 
-0.515
 
 
13.4
 
 
 
 
ABCB7
 
 
hsa-miR-152-3p
 
 
-0.515
 
 
13.4
 
 
 
 
ABCD3
 
 
hsa-miR-148b-3p
 
 
-0.388
 
 
41.8
 
 
 
 
ABCD3
 
 
hsa-miR-152-3p
 
 
-0.388
 
 
41.8
 
 
 
 
ABCE1
 
 
hsa-miR-29a-3p
 
 
-0.404
 
 
21.3
 
 
 
 
ABHD17C
 
 
hsa-let-7b-5p
 
 
-0.359
 
 
61.2
 
 
 
 
ABHD17C
 
 
hsa-miR-330-3p
 
 
-0.399
 
 
61.2
 
 
 
 
ABHD4
 
 
hsa-miR-29a-3p
 
 
-0.312
 
 
20.3
 
 
 
 
ABHD5
 
 
hsa-miR-132-3p
 
 
-0.323
 
 
11.4
 
 
 
 
ACTR2
 
 
hsa-miR-15b-5p
 
 
-0.293
 
 
132.3
 
 
 
 
ADAM10
 
 
hsa-miR-152-3p
 
 
-0.348
 
 
20.8
 
 
 
 
ADAM10
 
 
hsa-miR-148b-3p
 
 
-0.356
 
 
20.8
 
 
 
 
ADAM19
 
 
hsa-miR-874-3p
 
 
-0.374
 
 
22.3
 
 
 
 
ADAM9
 
 
hsa-miR-30d-5p
 
 
-0.304
 
 
30.9
 
 
 
 
ADAM9
 
 
hsa-miR-30b-5p
 
 
-0.312
 
 
30.9
 
 
 
 
ADCY3
 
 
hsa-miR-132-3p
 
 
-0.303
 
 
26.7
 
 
 
 
ADCY6
 
 
hsa-miR-1271-5p
 
 
-0.336
 
 
24.1
 
 
 
 
ADGRA2
 
 
hsa-miR-1271-5p
 
 
-0.427
 
 
20.3
 
 
 
 
ADGRA3
 
 
hsa-miR-30d-5p
 
 
-0.296
 
 
16.0
 
 
 
 
ADGRA3
 
 
hsa-miR-30b-5p
 
 
-0.304
 
 
16.0
 
 
 
 
ADIPOR2
 
 
hsa-miR-150-5p
 
 
-0.287
 
 
31.0
 
 
 
 
ADK
 
 
hsa-miR-1271-5p
 
 
-0.329
 
 
16.7
 
 
 
 
ADNP
 
 
hsa-miR-328-3p
 
 
-0.371
 
 
32.4
 
 
 
 
AFG3L2
 
 
hsa-miR-181b-5p
 
 
-0.491
 
 
38.7
 
 
 
 
AFG3L2
 
 
hsa-miR-181a-5p
 
 
-0.491
 
 
38.7
 
 
 
 
AFG3L2
 
 
hsa-miR-181d-5p
 
 
-0.499
 
 
38.7
 
 
 
 
AGFG1
 
 
hsa-miR-148b-3p
 
 
-0.332
 
 
30.2
 
 
 
 
AGFG1
 
 
hsa-miR-152-3p
 
 
-0.332
 
 
30.2
 
 
 
 
AGFG1
 
 
hsa-miR-103a-3p
 
 
-0.389
 
 
30.2
 
 
 
 
AHDC1
 
 
hsa-miR-152-3p
 
 
-0.413
 
 
15.6
 
 
 
 
AHDC1
 
 
hsa-miR-148b-3p
 
 
-0.413
 
 
15.6
 
 
 
 
AHR
 
 
hsa-miR-1271-5p
 
 
-0.304
 
 
12.9
 
 
 
 
AHR
 
 
hsa-miR-29a-3p
 
 
-0.316
 
 
12.9
 
 
 
 
AIFM1
 
 
hsa-let-7b-5p
 
 
-0.314
 
 
23.9
 
 
 
 
AK1
 
 
hsa-miR-29a-3p
 
 
-0.333
 
 
41.6
 
 
 
 
AK3
 
 
hsa-miR-1271-5p
 
 
-0.57
 
 
63.2
 
 
 
 
AK4
 
 
hsa-miR-15b-5p
 
 
-0.316
 
 
17.9
 
 
 
 
AKAP1
 
 
hsa-miR-152-3p
 
 
-0.381
 
 
72.8
 
 
 
 
AKAP1
 
 
hsa-miR-148b-3p
 
 
-0.381
 
 
72.8
 
 
 
 
AKIRIN2
 
 
hsa-miR-103a-3p
 
 
-0.299
 
 
17.5
 
 
 
 
AKT3
 
 
hsa-miR-29a-3p
 
 
-0.287
 
 
20.3
 
 
 
 
AKT3
 
 
hsa-miR-15b-5p
 
 
-0.301
 
 
20.3
 
 
 
 
ALAD
 
 
hsa-miR-326
 
 
-0.352
 
 
24.1
 
 
 
 
ALAD
 
 
hsa-miR-103a-3p
 
 
-0.385
 
 
24.1
 
 
 
 
ALAD
 
 
hsa-miR-15b-5p
 
 
-0.446
 
 
24.1
 
 
 
 
ALCAM
 
 
hsa-miR-148b-3p
 
 
-0.339
 
 
15.7
 
 
 
 
ALCAM
 
 
hsa-miR-152-3p
 
 
-0.347
 
 
15.7
 
 
 
 
ALDH6A1
 
 
hsa-miR-1271-5p
 
 
-0.301
 
 
14.2
 
 
 
 
AMOTL1
 
 
hsa-miR-15b-5p
 
 
-0.319
 
 
34.0
 
 
 
 
AMT
 
 
hsa-let-7b-5p
 
 
-0.463
 
 
14.4
 
 
 
 
ANKHD1
 
 
hsa-miR-23a-3p
 
 
-0.368
 
 
38.3
 
 
 
 
ANKHD1
 
 
hsa-miR-30d-5p
 
 
-0.381
 
 
38.3
 
 
 
 
ANKHD1
 
 
hsa-miR-30b-5p
 
 
-0.405
 
 
38.3
 
 
 
 
ANKIB1
 
 
hsa-miR-1271-5p
 
 
-0.344
 
 
12.1
 
 
 
 
ANKRD17
 
 
hsa-miR-30d-5p
 
 
-0.312
 
 
14.9
 
 
 
 
ANKRD17
 
 
hsa-miR-30b-5p
 
 
-0.336
 
 
14.9
 
 
 
 
ANKRD27
 
 
hsa-miR-1271-5p
 
 
-0.648
 
 
16.3
 
 
 
 
ANP32A
 
 
hsa-miR-132-3p
 
 
-0.374
 
 
75.8
 
 
 
 
ANTXR2
 
 
hsa-miR-29a-3p
 
 
-0.347
 
 
83.9
 
 
 
 
ANXA11
 
 
hsa-miR-15b-5p
 
 
-0.463
 
 
122.1
 
 
 
 
AP1S1
 
 
hsa-let-7b-5p
 
 
-0.374
 
 
31.0
 
 
 
 
AP2A1
 
 
hsa-miR-15b-5p
 
 
-0.333
 
 
40.3
 
 
 
 
AP2B1
 
 
hsa-miR-1271-5p
 
 
-0.299
 
 
63.4
 
 
 
 
AP2B1
 
 
hsa-miR-15b-5p
 
 
-0.334
 
 
63.4
 
 
 
 
AP3S1
 
 
hsa-miR-30b-5p
 
 
-0.293
 
 
56.5
 
 
 
 
AP4B1
 
 
hsa-miR-378a-3p
 
 
-0.62
 
 
11.3
 
 
 
 
AP4B1
 
 
hsa-miR-378d
 
 
-0.62
 
 
11.3
 
 
 
 
AP5M1
 
 
hsa-miR-1271-5p
 
 
-0.343
 
 
24.1
 
 
 
 
APOO
 
 
hsa-miR-181d-5p
 
 
-0.492
 
 
16.2
 
 
 
 
APOO
 
 
hsa-miR-181b-5p
 
 
-0.5
 
 
16.2
 
 
 
 
APOO
 
 
hsa-miR-181a-5p
 
 
-0.5
 
 
16.2
 
 
 
 
APP
 
 
hsa-miR-15b-5p
 
 
-0.298
 
 
322.5
 
 
 
 
ARF4
 
 
hsa-miR-30b-5p
 
 
-0.289
 
 
148.9
 
 
 
 
ARF4
 
 
hsa-miR-194-5p
 
 
-0.293
 
 
148.9
 
 
 
 
ARF4
 
 
hsa-miR-152-3p
 
 
-0.487
 
 
148.9
 
 
 
 
ARF4
 
 
hsa-miR-148b-3p
 
 
-0.487
 
 
148.9
 
 
 
 
ARF5
 
 
hsa-miR-29a-3p
 
 
-0.491
 
 
119.1
 
 
 
 
ARF6
 
 
hsa-miR-181b-5p
 
 
-0.33
 
 
50.4
 
 
 
 
ARF6
 
 
hsa-miR-181a-5p
 
 
-0.33
 
 
50.4
 
 
 
 
ARF6
 
 
hsa-miR-181d-5p
 
 
-0.338
 
 
50.4
 
 
 
 
ARFGEF1
 
 
hsa-miR-148b-3p
 
 
-0.306
 
 
18.0
 
 
 
 
ARFGEF1
 
 
hsa-miR-152-3p
 
 
-0.306
 
 
18.0
 
 
 
 
ARFIP1
 
 
hsa-miR-320b
 
 
-0.339
 
 
26.4
 
 
 
 
ARFIP1
 
 
hsa-miR-320c
 
 
-0.339
 
 
26.4
 
 
 
 
ARFIP1
 
 
hsa-miR-152-3p
 
 
-0.474
 
 
26.4
 
 
 
 
ARFIP1
 
 
hsa-miR-148b-3p
 
 
-0.474
 
 
26.4
 
 
 
 
ARHGAP21
 
 
hsa-miR-152-3p
 
 
-0.287
 
 
15.5
 
 
 
 
ARHGAP21
 
 
hsa-miR-148b-3p
 
 
-0.287
 
 
15.5
 
 
 
 
ARHGAP6
 
 
hsa-miR-1271-5p
 
 
-0.521
 
 
13.0
 
 
 
 
ARID1B
 
 
hsa-miR-29a-3p
 
 
-0.342
 
 
11.7
 
 
 
 
ARIH2
 
 
hsa-miR-103a-3p
 
 
-0.307
 
 
28.0
 
 
 
 
ARL2
 
 
hsa-miR-15b-5p
 
 
-0.374
 
 
122.8
 
 
 
 
ARL2
 
 
hsa-miR-15b-5p
 
 
-0.442
 
 
122.8
 
 
 
 
ARL3
 
 
hsa-miR-15b-5p
 
 
-0.507
 
 
10.7
 
 
 
 
ARL4A
 
 
hsa-miR-29a-3p
 
 
-0.386
 
 
38.1
 
 
 
 
ARL4A
 
 
hsa-miR-30d-5p
 
 
-0.432
 
 
38.1
 
 
 
 
 
 
 duodenum 
 
 
 
 
target_symbol
 
 
mature_mirna_id
 
 
score
 
 
nTPM
 
 
 
 
 
 
AAMP
 
 
hsa-miR-326
 
 
-0.722
 
 
59.1
 
 
 
 
AAR2
 
 
hsa-miR-29a-3p
 
 
-0.327
 
 
14.2
 
 
 
 
ABAT
 
 
hsa-miR-1271-5p
 
 
-0.409
 
 
41.1
 
 
 
 
ABCB10
 
 
hsa-miR-152-3p
 
 
-0.291
 
 
16.1
 
 
 
 
ABCB10
 
 
hsa-miR-148b-3p
 
 
-0.291
 
 
16.1
 
 
 
 
ABCB7
 
 
hsa-miR-148b-3p
 
 
-0.515
 
 
15.1
 
 
 
 
ABCB7
 
 
hsa-miR-152-3p
 
 
-0.515
 
 
15.1
 
 
 
 
ABCD3
 
 
hsa-miR-148b-3p
 
 
-0.388
 
 
42.1
 
 
 
 
ABCD3
 
 
hsa-miR-152-3p
 
 
-0.388
 
 
42.1
 
 
 
 
ABCE1
 
 
hsa-miR-29a-3p
 
 
-0.404
 
 
19.0
 
 
 
 
ABHD17C
 
 
hsa-let-7b-5p
 
 
-0.359
 
 
21.9
 
 
 
 
ABHD17C
 
 
hsa-miR-330-3p
 
 
-0.399
 
 
21.9
 
 
 
 
ABHD4
 
 
hsa-miR-29a-3p
 
 
-0.312
 
 
15.6
 
 
 
 
ACTR2
 
 
hsa-miR-15b-5p
 
 
-0.293
 
 
98.7
 
 
 
 
ADA
 
 
hsa-miR-29a-3p
 
 
-0.376
 
 
454.0
 
 
 
 
ADAM10
 
 
hsa-miR-152-3p
 
 
-0.348
 
 
14.7
 
 
 
 
ADAM10
 
 
hsa-miR-148b-3p
 
 
-0.356
 
 
14.7
 
 
 
 
ADAM9
 
 
hsa-miR-30d-5p
 
 
-0.304
 
 
23.1
 
 
 
 
ADAM9
 
 
hsa-miR-30b-5p
 
 
-0.312
 
 
23.1
 
 
 
 
ADCY3
 
 
hsa-miR-132-3p
 
 
-0.303
 
 
10.3
 
 
 
 
ADCY6
 
 
hsa-miR-1271-5p
 
 
-0.336
 
 
17.6
 
 
 
 
ADIPOR2
 
 
hsa-miR-150-5p
 
 
-0.287
 
 
20.9
 
 
 
 
ADK
 
 
hsa-miR-1271-5p
 
 
-0.329
 
 
15.9
 
 
 
 
ADNP
 
 
hsa-miR-328-3p
 
 
-0.371
 
 
18.9
 
 
 
 
AFG3L2
 
 
hsa-miR-181b-5p
 
 
-0.491
 
 
48.3
 
 
 
 
AFG3L2
 
 
hsa-miR-181a-5p
 
 
-0.491
 
 
48.3
 
 
 
 
AFG3L2
 
 
hsa-miR-181d-5p
 
 
-0.499
 
 
48.3
 
 
 
 
AGFG1
 
 
hsa-miR-148b-3p
 
 
-0.332
 
 
40.8
 
 
 
 
AGFG1
 
 
hsa-miR-152-3p
 
 
-0.332
 
 
40.8
 
 
 
 
AGFG1
 
 
hsa-miR-103a-3p
 
 
-0.389
 
 
40.8
 
 
 
 
AHR
 
 
hsa-miR-1271-5p
 
 
-0.304
 
 
15.4
 
 
 
 
AHR
 
 
hsa-miR-29a-3p
 
 
-0.316
 
 
15.4
 
 
 
 
AIFM1
 
 
hsa-let-7b-5p
 
 
-0.314
 
 
49.8
 
 
 
 
AK1
 
 
hsa-miR-29a-3p
 
 
-0.333
 
 
16.7
 
 
 
 
AK3
 
 
hsa-miR-1271-5p
 
 
-0.57
 
 
64.3
 
 
 
 
AKAP1
 
 
hsa-miR-152-3p
 
 
-0.381
 
 
51.5
 
 
 
 
AKAP1
 
 
hsa-miR-148b-3p
 
 
-0.381
 
 
51.5
 
 
 
 
AKIRIN2
 
 
hsa-miR-103a-3p
 
 
-0.299
 
 
12.0
 
 
 
 
ALAD
 
 
hsa-miR-326
 
 
-0.352
 
 
27.7
 
 
 
 
ALAD
 
 
hsa-miR-103a-3p
 
 
-0.385
 
 
27.7
 
 
 
 
ALAD
 
 
hsa-miR-15b-5p
 
 
-0.446
 
 
27.7
 
 
 
 
ALCAM
 
 
hsa-miR-148b-3p
 
 
-0.339
 
 
16.0
 
 
 
 
ALCAM
 
 
hsa-miR-152-3p
 
 
-0.347
 
 
16.0
 
 
 
 
ALDH6A1
 
 
hsa-miR-1271-5p
 
 
-0.301
 
 
24.9
 
 
 
 
AMT
 
 
hsa-let-7b-5p
 
 
-0.463
 
 
12.9
 
 
 
 
ANKHD1
 
 
hsa-miR-23a-3p
 
 
-0.368
 
 
29.8
 
 
 
 
ANKHD1
 
 
hsa-miR-30d-5p
 
 
-0.381
 
 
29.8
 
 
 
 
ANKHD1
 
 
hsa-miR-30b-5p
 
 
-0.405
 
 
29.8
 
 
 
 
ANKIB1
 
 
hsa-miR-1271-5p
 
 
-0.344
 
 
10.5
 
 
 
 
ANKRD17
 
 
hsa-miR-30d-5p
 
 
-0.312
 
 
13.9
 
 
 
 
ANKRD17
 
 
hsa-miR-30b-5p
 
 
-0.336
 
 
13.9
 
 
 
 
ANP32A
 
 
hsa-miR-132-3p
 
 
-0.374
 
 
66.5
 
 
 
 
ANTXR2
 
 
hsa-miR-29a-3p
 
 
-0.347
 
 
21.6
 
 
 
 
ANXA11
 
 
hsa-miR-15b-5p
 
 
-0.463
 
 
129.7
 
 
 
 
AP1S1
 
 
hsa-let-7b-5p
 
 
-0.374
 
 
36.7
 
 
 
 
AP2A1
 
 
hsa-miR-15b-5p
 
 
-0.333
 
 
23.3
 
 
 
 
AP2B1
 
 
hsa-miR-1271-5p
 
 
-0.299
 
 
31.5
 
 
 
 
AP2B1
 
 
hsa-miR-15b-5p
 
 
-0.334
 
 
31.5
 
 
 
 
AP3S1
 
 
hsa-miR-30b-5p
 
 
-0.293
 
 
58.2
 
 
 
 
AP5M1
 
 
hsa-miR-1271-5p
 
 
-0.343
 
 
27.1
 
 
 
 
APOO
 
 
hsa-miR-181d-5p
 
 
-0.492
 
 
13.6
 
 
 
 
APOO
 
 
hsa-miR-181b-5p
 
 
-0.5
 
 
13.6
 
 
 
 
APOO
 
 
hsa-miR-181a-5p
 
 
-0.5
 
 
13.6
 
 
 
 
APP
 
 
hsa-miR-15b-5p
 
 
-0.298
 
 
204.9
 
 
 
 
AQP11
 
 
hsa-miR-103a-3p
 
 
-0.517
 
 
27.0
 
 
 
 
AQP11
 
 
hsa-miR-15b-5p
 
 
-0.769
 
 
27.0
 
 
 
 
AQP3
 
 
hsa-miR-874-3p
 
 
-0.48
 
 
69.1
 
 
 
 
ARF4
 
 
hsa-miR-30b-5p
 
 
-0.289
 
 
116.2
 
 
 
 
ARF4
 
 
hsa-miR-194-5p
 
 
-0.293
 
 
116.2
 
 
 
 
ARF4
 
 
hsa-miR-152-3p
 
 
-0.487
 
 
116.2
 
 
 
 
ARF4
 
 
hsa-miR-148b-3p
 
 
-0.487
 
 
116.2
 
 
 
 
ARF5
 
 
hsa-miR-29a-3p
 
 
-0.491
 
 
86.9
 
 
 
 
ARF6
 
 
hsa-miR-181b-5p
 
 
-0.33
 
 
43.6
 
 
 
 
ARF6
 
 
hsa-miR-181a-5p
 
 
-0.33
 
 
43.6
 
 
 
 
ARF6
 
 
hsa-miR-181d-5p
 
 
-0.338
 
 
43.6
 
 
 
 
ARFGEF1
 
 
hsa-miR-148b-3p
 
 
-0.306
 
 
15.9
 
 
 
 
ARFGEF1
 
 
hsa-miR-152-3p
 
 
-0.306
 
 
15.9
 
 
 
 
ARFIP1
 
 
hsa-miR-320b
 
 
-0.339
 
 
21.8
 
 
 
 
ARFIP1
 
 
hsa-miR-320c
 
 
-0.339
 
 
21.8
 
 
 
 
ARFIP1
 
 
hsa-miR-152-3p
 
 
-0.474
 
 
21.8
 
 
 
 
ARFIP1
 
 
hsa-miR-148b-3p
 
 
-0.474
 
 
21.8
 
 
 
 
ARHGAP21
 
 
hsa-miR-152-3p
 
 
-0.287
 
 
12.1
 
 
 
 
ARHGAP21
 
 
hsa-miR-148b-3p
 
 
-0.287
 
 
12.1
 
 
 
 
ARID1B
 
 
hsa-miR-29a-3p
 
 
-0.342
 
 
13.4
 
 
 
 
ARIH2
 
 
hsa-miR-103a-3p
 
 
-0.307
 
 
11.0
 
 
 
 
ARL2
 
 
hsa-miR-15b-5p
 
 
-0.374
 
 
49.6
 
 
 
 
ARL2
 
 
hsa-miR-15b-5p
 
 
-0.442
 
 
49.6
 
 
 
 
ARL4A
 
 
hsa-miR-29a-3p
 
 
-0.386
 
 
28.1
 
 
 
 
ARL4A
 
 
hsa-miR-30d-5p
 
 
-0.432
 
 
28.1
 
 
 
 
ARL4A
 
 
hsa-miR-30b-5p
 
 
-0.448
 
 
28.1
 
 
 
 
ARL6IP1
 
 
hsa-miR-328-3p
 
 
-0.494
 
 
130.6
 
 
 
 
ARL6IP1
 
 
hsa-miR-148b-3p
 
 
-0.579
 
 
130.6
 
 
 
 
ARL6IP1
 
 
hsa-miR-152-3p
 
 
-0.579
 
 
130.6
 
 
 
 
ARL8A
 
 
hsa-miR-103a-3p
 
 
-0.38
 
 
20.3
 
 
 
 
ARL8B
 
 
hsa-miR-152-3p
 
 
-0.337
 
 
28.9
 
 
 
 
ARL8B
 
 
hsa-miR-148b-3p
 
 
-0.337
 
 
28.9
 
 
 
 
ARL8B
 
 
hsa-miR-320b
 
 
-0.376
 
 
28.9
 
 
 
 
ARL8B
 
 
hsa-miR-320c
 
 
-0.376
 
 
28.9
 
 
 
 
ARMC1
 
 
hsa-miR-103a-3p
 
 
-0.525
 
 
14.6
 
 
 
 
ARMC8
 
 
hsa-miR-103a-3p
 
 
-0.412
 
 
11.3
 
 
 
 
 
 
 endometrium 
 
 
 
 
target_symbol
 
 
mature_mirna_id
 
 
score
 
 
nTPM
 
 
 
 
 
 
AAMP
 
 
hsa-miR-326
 
 
-0.722
 
 
58.6
 
 
 
 
AAR2
 
 
hsa-miR-29a-3p
 
 
-0.327
 
 
23.5
 
 
 
 
ABCB7
 
 
hsa-miR-148b-3p
 
 
-0.515
 
 
10.5
 
 
 
 
ABCB7
 
 
hsa-miR-152-3p
 
 
-0.515
 
 
10.5
 
 
 
 
ABCD3
 
 
hsa-miR-148b-3p
 
 
-0.388
 
 
13.8
 
 
 
 
ABCD3
 
 
hsa-miR-152-3p
 
 
-0.388
 
 
13.8
 
 
 
 
ABCE1
 
 
hsa-miR-29a-3p
 
 
-0.404
 
 
22.8
 
 
 
 
ABHD4
 
 
hsa-miR-29a-3p
 
 
-0.312
 
 
26.6
 
 
 
 
ACTC1
 
 
hsa-miR-30d-5p
 
 
-0.519
 
 
17.7
 
 
 
 
ACTC1
 
 
hsa-miR-30b-5p
 
 
-0.543
 
 
17.7
 
 
 
 
ACTR2
 
 
hsa-miR-15b-5p
 
 
-0.293
 
 
65.5
 
 
 
 
ACVR1
 
 
hsa-miR-30b-5p
 
 
-0.29
 
 
15.9
 
 
 
 
ACVR1
 
 
hsa-miR-152-3p
 
 
-0.397
 
 
15.9
 
 
 
 
ACVR1
 
 
hsa-miR-148b-3p
 
 
-0.397
 
 
15.9
 
 
 
 
ADAM10
 
 
hsa-miR-152-3p
 
 
-0.348
 
 
15.2
 
 
 
 
ADAM10
 
 
hsa-miR-148b-3p
 
 
-0.356
 
 
15.2
 
 
 
 
ADAM9
 
 
hsa-miR-30d-5p
 
 
-0.304
 
 
21.6
 
 
 
 
ADAM9
 
 
hsa-miR-30b-5p
 
 
-0.312
 
 
21.6
 
 
 
 
ADAMTS10
 
 
hsa-miR-29a-3p
 
 
-0.516
 
 
10.1
 
 
 
 
ADAMTS9
 
 
hsa-miR-29a-3p
 
 
-0.305
 
 
12.4
 
 
 
 
ADAMTS9
 
 
hsa-miR-29a-3p
 
 
-0.485
 
 
12.4
 
 
 
 
ADCY3
 
 
hsa-miR-132-3p
 
 
-0.303
 
 
52.0
 
 
 
 
ADCY6
 
 
hsa-miR-1271-5p
 
 
-0.336
 
 
19.5
 
 
 
 
ADGRA2
 
 
hsa-miR-1271-5p
 
 
-0.427
 
 
39.0
 
 
 
 
ADGRA3
 
 
hsa-miR-30d-5p
 
 
-0.296
 
 
14.1
 
 
 
 
ADGRA3
 
 
hsa-miR-30b-5p
 
 
-0.304
 
 
14.1
 
 
 
 
ADGRF5
 
 
hsa-miR-148b-3p
 
 
-0.354
 
 
16.0
 
 
 
 
ADGRF5
 
 
hsa-miR-152-3p
 
 
-0.354
 
 
16.0
 
 
 
 
ADIPOR2
 
 
hsa-miR-150-5p
 
 
-0.287
 
 
24.5
 
 
 
 
ADK
 
 
hsa-miR-1271-5p
 
 
-0.329
 
 
10.1
 
 
 
 
ADNP
 
 
hsa-miR-328-3p
 
 
-0.371
 
 
33.6
 
 
 
 
AFG3L2
 
 
hsa-miR-181b-5p
 
 
-0.491
 
 
12.4
 
 
 
 
AFG3L2
 
 
hsa-miR-181a-5p
 
 
-0.491
 
 
12.4
 
 
 
 
AFG3L2
 
 
hsa-miR-181d-5p
 
 
-0.499
 
 
12.4
 
 
 
 
AGFG1
 
 
hsa-miR-148b-3p
 
 
-0.332
 
 
23.1
 
 
 
 
AGFG1
 
 
hsa-miR-152-3p
 
 
-0.332
 
 
23.1
 
 
 
 
AGFG1
 
 
hsa-miR-103a-3p
 
 
-0.389
 
 
23.1
 
 
 
 
AHDC1
 
 
hsa-miR-152-3p
 
 
-0.413
 
 
26.0
 
 
 
 
AHDC1
 
 
hsa-miR-148b-3p
 
 
-0.413
 
 
26.0
 
 
 
 
AIFM1
 
 
hsa-let-7b-5p
 
 
-0.314
 
 
10.6
 
 
 
 
AJUBA
 
 
hsa-miR-103a-3p
 
 
-0.448
 
 
11.2
 
 
 
 
AK1
 
 
hsa-miR-29a-3p
 
 
-0.333
 
 
16.9
 
 
 
 
AK3
 
 
hsa-miR-1271-5p
 
 
-0.57
 
 
39.2
 
 
 
 
AKAP1
 
 
hsa-miR-152-3p
 
 
-0.381
 
 
24.8
 
 
 
 
AKAP1
 
 
hsa-miR-148b-3p
 
 
-0.381
 
 
24.8
 
 
 
 
AKIRIN2
 
 
hsa-miR-103a-3p
 
 
-0.299
 
 
20.8
 
 
 
 
AKT3
 
 
hsa-miR-29a-3p
 
 
-0.287
 
 
19.5
 
 
 
 
AKT3
 
 
hsa-miR-15b-5p
 
 
-0.301
 
 
19.5
 
 
 
 
ALAD
 
 
hsa-miR-326
 
 
-0.352
 
 
16.4
 
 
 
 
ALAD
 
 
hsa-miR-103a-3p
 
 
-0.385
 
 
16.4
 
 
 
 
ALAD
 
 
hsa-miR-15b-5p
 
 
-0.446
 
 
16.4
 
 
 
 
ALCAM
 
 
hsa-miR-148b-3p
 
 
-0.339
 
 
14.4
 
 
 
 
ALCAM
 
 
hsa-miR-152-3p
 
 
-0.347
 
 
14.4
 
 
 
 
ALDH1A2
 
 
hsa-miR-23a-3p
 
 
-0.354
 
 
76.9
 
 
 
 
ALDH6A1
 
 
hsa-miR-1271-5p
 
 
-0.301
 
 
10.6
 
 
 
 
AMOTL1
 
 
hsa-miR-15b-5p
 
 
-0.319
 
 
13.3
 
 
 
 
AMT
 
 
hsa-let-7b-5p
 
 
-0.463
 
 
10.0
 
 
 
 
ANKHD1
 
 
hsa-miR-23a-3p
 
 
-0.368
 
 
40.5
 
 
 
 
ANKHD1
 
 
hsa-miR-30d-5p
 
 
-0.381
 
 
40.5
 
 
 
 
ANKHD1
 
 
hsa-miR-30b-5p
 
 
-0.405
 
 
40.5
 
 
 
 
ANKIB1
 
 
hsa-miR-1271-5p
 
 
-0.344
 
 
11.2
 
 
 
 
ANKRA2
 
 
hsa-miR-30d-5p
 
 
-0.343
 
 
11.6
 
 
 
 
ANKRA2
 
 
hsa-miR-30b-5p
 
 
-0.354
 
 
11.6
 
 
 
 
ANKRA2
 
 
hsa-miR-30d-5p
 
 
-0.448
 
 
11.6
 
 
 
 
ANKRA2
 
 
hsa-miR-30b-5p
 
 
-0.472
 
 
11.6
 
 
 
 
ANKRD17
 
 
hsa-miR-30d-5p
 
 
-0.312
 
 
13.7
 
 
 
 
ANKRD17
 
 
hsa-miR-30b-5p
 
 
-0.336
 
 
13.7
 
 
 
 
ANKRD29
 
 
hsa-miR-132-3p
 
 
-0.47
 
 
14.7
 
 
 
 
ANKRD46
 
 
hsa-let-7b-5p
 
 
-0.389
 
 
11.8
 
 
 
 
ANP32A
 
 
hsa-miR-132-3p
 
 
-0.374
 
 
55.3
 
 
 
 
ANTXR2
 
 
hsa-miR-29a-3p
 
 
-0.347
 
 
89.2
 
 
 
 
ANXA11
 
 
hsa-miR-15b-5p
 
 
-0.463
 
 
67.4
 
 
 
 
AP1S1
 
 
hsa-let-7b-5p
 
 
-0.374
 
 
16.1
 
 
 
 
AP2A1
 
 
hsa-miR-15b-5p
 
 
-0.333
 
 
53.6
 
 
 
 
AP2B1
 
 
hsa-miR-1271-5p
 
 
-0.299
 
 
39.0
 
 
 
 
AP2B1
 
 
hsa-miR-15b-5p
 
 
-0.334
 
 
39.0
 
 
 
 
AP3S1
 
 
hsa-miR-30b-5p
 
 
-0.293
 
 
55.6
 
 
 
 
AP4B1
 
 
hsa-miR-378a-3p
 
 
-0.62
 
 
10.1
 
 
 
 
AP4B1
 
 
hsa-miR-378d
 
 
-0.62
 
 
10.1
 
 
 
 
AP5M1
 
 
hsa-miR-1271-5p
 
 
-0.343
 
 
11.0
 
 
 
 
APP
 
 
hsa-miR-15b-5p
 
 
-0.298
 
 
254.0
 
 
 
 
AQP3
 
 
hsa-miR-874-3p
 
 
-0.48
 
 
10.3
 
 
 
 
ARF4
 
 
hsa-miR-30b-5p
 
 
-0.289
 
 
113.9
 
 
 
 
ARF4
 
 
hsa-miR-194-5p
 
 
-0.293
 
 
113.9
 
 
 
 
ARF4
 
 
hsa-miR-152-3p
 
 
-0.487
 
 
113.9
 
 
 
 
ARF4
 
 
hsa-miR-148b-3p
 
 
-0.487
 
 
113.9
 
 
 
 
ARF5
 
 
hsa-miR-29a-3p
 
 
-0.491
 
 
111.9
 
 
 
 
ARF6
 
 
hsa-miR-181b-5p
 
 
-0.33
 
 
19.5
 
 
 
 
ARF6
 
 
hsa-miR-181a-5p
 
 
-0.33
 
 
19.5
 
 
 
 
ARF6
 
 
hsa-miR-181d-5p
 
 
-0.338
 
 
19.5
 
 
 
 
ARFIP1
 
 
hsa-miR-320b
 
 
-0.339
 
 
20.3
 
 
 
 
ARFIP1
 
 
hsa-miR-320c
 
 
-0.339
 
 
20.3
 
 
 
 
ARFIP1
 
 
hsa-miR-152-3p
 
 
-0.474
 
 
20.3
 
 
 
 
ARFIP1
 
 
hsa-miR-148b-3p
 
 
-0.474
 
 
20.3
 
 
 
 
ARHGAP21
 
 
hsa-miR-152-3p
 
 
-0.287
 
 
18.5
 
 
 
 
ARHGAP21
 
 
hsa-miR-148b-3p
 
 
-0.287
 
 
18.5
 
 
 
 
ARID1B
 
 
hsa-miR-29a-3p
 
 
-0.342
 
 
15.0
 
 
 
 
ARIH2
 
 
hsa-miR-103a-3p
 
 
-0.307
 
 
22.7
 
 
 
 
ARL2
 
 
hsa-miR-15b-5p
 
 
-0.374
 
 
124.7
 
 
 
 
ARL2
 
 
hsa-miR-15b-5p
 
 
-0.442
 
 
124.7
 
 
 
 
 
 
 epididymis 
 
 
 
 
target_symbol
 
 
mature_mirna_id
 
 
score
 
 
nTPM
 
 
 
 
 
 
AAMP
 
 
hsa-miR-326
 
 
-0.722
 
 
41.4
 
 
 
 
AAR2
 
 
hsa-miR-29a-3p
 
 
-0.327
 
 
23.5
 
 
 
 
ABCD3
 
 
hsa-miR-148b-3p
 
 
-0.388
 
 
20.0
 
 
 
 
ABCD3
 
 
hsa-miR-152-3p
 
 
-0.388
 
 
20.0
 
 
 
 
ABCE1
 
 
hsa-miR-29a-3p
 
 
-0.404
 
 
15.1
 
 
 
 
ABHD4
 
 
hsa-miR-29a-3p
 
 
-0.312
 
 
28.3
 
 
 
 
ACP3
 
 
hsa-let-7b-5p
 
 
-0.298
 
 
16.3
 
 
 
 
ACTR2
 
 
hsa-miR-15b-5p
 
 
-0.293
 
 
60.8
 
 
 
 
ACVR1
 
 
hsa-miR-30b-5p
 
 
-0.29
 
 
10.8
 
 
 
 
ACVR1
 
 
hsa-miR-152-3p
 
 
-0.397
 
 
10.8
 
 
 
 
ACVR1
 
 
hsa-miR-148b-3p
 
 
-0.397
 
 
10.8
 
 
 
 
ADAM17
 
 
hsa-miR-152-3p
 
 
-0.301
 
 
10.4
 
 
 
 
ADAM17
 
 
hsa-miR-148b-3p
 
 
-0.301
 
 
10.4
 
 
 
 
ADAM9
 
 
hsa-miR-30d-5p
 
 
-0.304
 
 
14.3
 
 
 
 
ADAM9
 
 
hsa-miR-30b-5p
 
 
-0.312
 
 
14.3
 
 
 
 
ADCY3
 
 
hsa-miR-132-3p
 
 
-0.303
 
 
19.3
 
 
 
 
ADCY6
 
 
hsa-miR-1271-5p
 
 
-0.336
 
 
17.9
 
 
 
 
ADGRA2
 
 
hsa-miR-1271-5p
 
 
-0.427
 
 
17.2
 
 
 
 
ADK
 
 
hsa-miR-1271-5p
 
 
-0.329
 
 
15.6
 
 
 
 
ADNP
 
 
hsa-miR-328-3p
 
 
-0.371
 
 
39.7
 
 
 
 
AGFG1
 
 
hsa-miR-148b-3p
 
 
-0.332
 
 
18.8
 
 
 
 
AGFG1
 
 
hsa-miR-152-3p
 
 
-0.332
 
 
18.8
 
 
 
 
AGFG1
 
 
hsa-miR-103a-3p
 
 
-0.389
 
 
18.8
 
 
 
 
AK1
 
 
hsa-miR-29a-3p
 
 
-0.333
 
 
27.0
 
 
 
 
AK3
 
 
hsa-miR-1271-5p
 
 
-0.57
 
 
29.5
 
 
 
 
AKAP1
 
 
hsa-miR-152-3p
 
 
-0.381
 
 
18.6
 
 
 
 
AKAP1
 
 
hsa-miR-148b-3p
 
 
-0.381
 
 
18.6
 
 
 
 
AKIRIN2
 
 
hsa-miR-103a-3p
 
 
-0.299
 
 
22.5
 
 
 
 
ALAD
 
 
hsa-miR-326
 
 
-0.352
 
 
25.1
 
 
 
 
ALAD
 
 
hsa-miR-103a-3p
 
 
-0.385
 
 
25.1
 
 
 
 
ALAD
 
 
hsa-miR-15b-5p
 
 
-0.446
 
 
25.1
 
 
 
 
ALCAM
 
 
hsa-miR-148b-3p
 
 
-0.339
 
 
33.4
 
 
 
 
ALCAM
 
 
hsa-miR-152-3p
 
 
-0.347
 
 
33.4
 
 
 
 
AMOT
 
 
hsa-miR-29a-3p
 
 
-0.304
 
 
137.7
 
 
 
 
ANKHD1
 
 
hsa-miR-23a-3p
 
 
-0.368
 
 
45.6
 
 
 
 
ANKHD1
 
 
hsa-miR-30d-5p
 
 
-0.381
 
 
45.6
 
 
 
 
ANKHD1
 
 
hsa-miR-30b-5p
 
 
-0.405
 
 
45.6
 
 
 
 
ANKIB1
 
 
hsa-miR-1271-5p
 
 
-0.344
 
 
15.0
 
 
 
 
ANKRA2
 
 
hsa-miR-30d-5p
 
 
-0.343
 
 
12.3
 
 
 
 
ANKRA2
 
 
hsa-miR-30b-5p
 
 
-0.354
 
 
12.3
 
 
 
 
ANKRA2
 
 
hsa-miR-30d-5p
 
 
-0.448
 
 
12.3
 
 
 
 
ANKRA2
 
 
hsa-miR-30b-5p
 
 
-0.472
 
 
12.3
 
 
 
 
ANKRD17
 
 
hsa-miR-30d-5p
 
 
-0.312
 
 
17.5
 
 
 
 
ANKRD17
 
 
hsa-miR-30b-5p
 
 
-0.336
 
 
17.5
 
 
 
 
ANKRD46
 
 
hsa-let-7b-5p
 
 
-0.389
 
 
15.4
 
 
 
 
ANO3
 
 
hsa-miR-15b-5p
 
 
-0.357
 
 
54.2
 
 
 
 
ANP32A
 
 
hsa-miR-132-3p
 
 
-0.374
 
 
47.9
 
 
 
 
ANTXR2
 
 
hsa-miR-29a-3p
 
 
-0.347
 
 
20.7
 
 
 
 
ANXA11
 
 
hsa-miR-15b-5p
 
 
-0.463
 
 
101.5
 
 
 
 
AP1S1
 
 
hsa-let-7b-5p
 
 
-0.374
 
 
16.0
 
 
 
 
AP1S3
 
 
hsa-miR-181d-5p
 
 
-0.383
 
 
45.7
 
 
 
 
AP1S3
 
 
hsa-miR-181b-5p
 
 
-0.383
 
 
45.7
 
 
 
 
AP1S3
 
 
hsa-miR-181a-5p
 
 
-0.383
 
 
45.7
 
 
 
 
AP2B1
 
 
hsa-miR-1271-5p
 
 
-0.299
 
 
66.7
 
 
 
 
AP2B1
 
 
hsa-miR-15b-5p
 
 
-0.334
 
 
66.7
 
 
 
 
AP3S1
 
 
hsa-miR-30b-5p
 
 
-0.293
 
 
107.2
 
 
 
 
AP4B1
 
 
hsa-miR-378a-3p
 
 
-0.62
 
 
13.4
 
 
 
 
AP4B1
 
 
hsa-miR-378d
 
 
-0.62
 
 
13.4
 
 
 
 
AP5M1
 
 
hsa-miR-1271-5p
 
 
-0.343
 
 
14.5
 
 
 
 
APOO
 
 
hsa-miR-181d-5p
 
 
-0.492
 
 
18.5
 
 
 
 
APOO
 
 
hsa-miR-181b-5p
 
 
-0.5
 
 
18.5
 
 
 
 
APOO
 
 
hsa-miR-181a-5p
 
 
-0.5
 
 
18.5
 
 
 
 
APP
 
 
hsa-miR-15b-5p
 
 
-0.298
 
 
214.2
 
 
 
 
ARF4
 
 
hsa-miR-30b-5p
 
 
-0.289
 
 
214.1
 
 
 
 
ARF4
 
 
hsa-miR-194-5p
 
 
-0.293
 
 
214.1
 
 
 
 
ARF4
 
 
hsa-miR-152-3p
 
 
-0.487
 
 
214.1
 
 
 
 
ARF4
 
 
hsa-miR-148b-3p
 
 
-0.487
 
 
214.1
 
 
 
 
ARF5
 
 
hsa-miR-29a-3p
 
 
-0.491
 
 
107.7
 
 
 
 
ARF6
 
 
hsa-miR-181b-5p
 
 
-0.33
 
 
27.6
 
 
 
 
ARF6
 
 
hsa-miR-181a-5p
 
 
-0.33
 
 
27.6
 
 
 
 
ARF6
 
 
hsa-miR-181d-5p
 
 
-0.338
 
 
27.6
 
 
 
 
ARFGEF1
 
 
hsa-miR-148b-3p
 
 
-0.306
 
 
10.4
 
 
 
 
ARFGEF1
 
 
hsa-miR-152-3p
 
 
-0.306
 
 
10.4
 
 
 
 
ARFIP1
 
 
hsa-miR-320b
 
 
-0.339
 
 
19.3
 
 
 
 
ARFIP1
 
 
hsa-miR-320c
 
 
-0.339
 
 
19.3
 
 
 
 
ARFIP1
 
 
hsa-miR-152-3p
 
 
-0.474
 
 
19.3
 
 
 
 
ARFIP1
 
 
hsa-miR-148b-3p
 
 
-0.474
 
 
19.3
 
 
 
 
ARID1B
 
 
hsa-miR-29a-3p
 
 
-0.342
 
 
10.3
 
 
 
 
ARIH2
 
 
hsa-miR-103a-3p
 
 
-0.307
 
 
31.9
 
 
 
 
ARL2
 
 
hsa-miR-15b-5p
 
 
-0.374
 
 
26.6
 
 
 
 
ARL2
 
 
hsa-miR-15b-5p
 
 
-0.442
 
 
26.6
 
 
 
 
ARL3
 
 
hsa-miR-15b-5p
 
 
-0.507
 
 
10.0
 
 
 
 
ARL4A
 
 
hsa-miR-29a-3p
 
 
-0.386
 
 
13.0
 
 
 
 
ARL4A
 
 
hsa-miR-30d-5p
 
 
-0.432
 
 
13.0
 
 
 
 
ARL4A
 
 
hsa-miR-30b-5p
 
 
-0.448
 
 
13.0
 
 
 
 
ARL6IP1
 
 
hsa-miR-328-3p
 
 
-0.494
 
 
116.0
 
 
 
 
ARL6IP1
 
 
hsa-miR-148b-3p
 
 
-0.579
 
 
116.0
 
 
 
 
ARL6IP1
 
 
hsa-miR-152-3p
 
 
-0.579
 
 
116.0
 
 
 
 
ARL8A
 
 
hsa-miR-103a-3p
 
 
-0.38
 
 
15.9
 
 
 
 
ARL8B
 
 
hsa-miR-152-3p
 
 
-0.337
 
 
37.5
 
 
 
 
ARL8B
 
 
hsa-miR-148b-3p
 
 
-0.337
 
 
37.5
 
 
 
 
ARL8B
 
 
hsa-miR-320b
 
 
-0.376
 
 
37.5
 
 
 
 
ARL8B
 
 
hsa-miR-320c
 
 
-0.376
 
 
37.5
 
 
 
 
ARMC1
 
 
hsa-miR-103a-3p
 
 
-0.525
 
 
15.5
 
 
 
 
ARMC8
 
 
hsa-miR-103a-3p
 
 
-0.412
 
 
11.5
 
 
 
 
ARMCX2
 
 
hsa-miR-15b-5p
 
 
-0.344
 
 
146.2
 
 
 
 
ARMCX3
 
 
hsa-miR-425-5p
 
 
-0.325
 
 
99.4
 
 
 
 
ARMT1
 
 
hsa-let-7b-5p
 
 
-0.291
 
 
15.2
 
 
 
 
ARNT
 
 
hsa-miR-103a-3p
 
 
-0.422
 
 
14.0
 
 
 
 
ARPC5L
 
 
hsa-miR-326
 
 
-0.439
 
 
21.5
 
 
 
 
 
 
 esophagus 
 
 
 
 
target_symbol
 
 
mature_mirna_id
 
 
score
 
 
nTPM
 
 
 
 
 
 
AAMP
 
 
hsa-miR-326
 
 
-0.722
 
 
75.7
 
 
 
 
AAR2
 
 
hsa-miR-29a-3p
 
 
-0.327
 
 
16.7
 
 
 
 
ABCB7
 
 
hsa-miR-148b-3p
 
 
-0.515
 
 
11.1
 
 
 
 
ABCB7
 
 
hsa-miR-152-3p
 
 
-0.515
 
 
11.1
 
 
 
 
ABCD3
 
 
hsa-miR-148b-3p
 
 
-0.388
 
 
25.0
 
 
 
 
ABCD3
 
 
hsa-miR-152-3p
 
 
-0.388
 
 
25.0
 
 
 
 
ABCE1
 
 
hsa-miR-29a-3p
 
 
-0.404
 
 
33.0
 
 
 
 
ABHD17C
 
 
hsa-let-7b-5p
 
 
-0.359
 
 
45.1
 
 
 
 
ABHD17C
 
 
hsa-miR-330-3p
 
 
-0.399
 
 
45.1
 
 
 
 
ABHD4
 
 
hsa-miR-29a-3p
 
 
-0.312
 
 
40.8
 
 
 
 
ABHD5
 
 
hsa-miR-132-3p
 
 
-0.323
 
 
18.4
 
 
 
 
ACP3
 
 
hsa-let-7b-5p
 
 
-0.298
 
 
42.9
 
 
 
 
ACTC1
 
 
hsa-miR-30d-5p
 
 
-0.519
 
 
21.5
 
 
 
 
ACTC1
 
 
hsa-miR-30b-5p
 
 
-0.543
 
 
21.5
 
 
 
 
ACTN2
 
 
hsa-miR-23a-3p
 
 
-0.294
 
 
19.6
 
 
 
 
ACTN2
 
 
hsa-miR-29a-3p
 
 
-0.311
 
 
19.6
 
 
 
 
ACTR2
 
 
hsa-miR-15b-5p
 
 
-0.293
 
 
103.7
 
 
 
 
ADAM10
 
 
hsa-miR-152-3p
 
 
-0.348
 
 
14.2
 
 
 
 
ADAM10
 
 
hsa-miR-148b-3p
 
 
-0.356
 
 
14.2
 
 
 
 
ADAM9
 
 
hsa-miR-30d-5p
 
 
-0.304
 
 
22.2
 
 
 
 
ADAM9
 
 
hsa-miR-30b-5p
 
 
-0.312
 
 
22.2
 
 
 
 
ADCY3
 
 
hsa-miR-132-3p
 
 
-0.303
 
 
29.3
 
 
 
 
ADGRA2
 
 
hsa-miR-1271-5p
 
 
-0.427
 
 
23.3
 
 
 
 
ADIPOR2
 
 
hsa-miR-150-5p
 
 
-0.287
 
 
44.0
 
 
 
 
ADK
 
 
hsa-miR-1271-5p
 
 
-0.329
 
 
46.4
 
 
 
 
ADNP
 
 
hsa-miR-328-3p
 
 
-0.371
 
 
30.5
 
 
 
 
ADRB2
 
 
hsa-miR-15b-5p
 
 
-0.29
 
 
14.2
 
 
 
 
ADRB2
 
 
hsa-let-7b-5p
 
 
-0.635
 
 
14.2
 
 
 
 
AFG3L2
 
 
hsa-miR-181b-5p
 
 
-0.491
 
 
25.7
 
 
 
 
AFG3L2
 
 
hsa-miR-181a-5p
 
 
-0.491
 
 
25.7
 
 
 
 
AFG3L2
 
 
hsa-miR-181d-5p
 
 
-0.499
 
 
25.7
 
 
 
 
AGFG1
 
 
hsa-miR-148b-3p
 
 
-0.332
 
 
44.3
 
 
 
 
AGFG1
 
 
hsa-miR-152-3p
 
 
-0.332
 
 
44.3
 
 
 
 
AGFG1
 
 
hsa-miR-103a-3p
 
 
-0.389
 
 
44.3
 
 
 
 
AHDC1
 
 
hsa-miR-152-3p
 
 
-0.413
 
 
34.3
 
 
 
 
AHDC1
 
 
hsa-miR-148b-3p
 
 
-0.413
 
 
34.3
 
 
 
 
AHR
 
 
hsa-miR-1271-5p
 
 
-0.304
 
 
16.2
 
 
 
 
AHR
 
 
hsa-miR-29a-3p
 
 
-0.316
 
 
16.2
 
 
 
 
AIFM1
 
 
hsa-let-7b-5p
 
 
-0.314
 
 
15.9
 
 
 
 
AJUBA
 
 
hsa-miR-103a-3p
 
 
-0.448
 
 
17.4
 
 
 
 
AK1
 
 
hsa-miR-29a-3p
 
 
-0.333
 
 
30.1
 
 
 
 
AK3
 
 
hsa-miR-1271-5p
 
 
-0.57
 
 
57.5
 
 
 
 
AK4
 
 
hsa-miR-15b-5p
 
 
-0.316
 
 
22.0
 
 
 
 
AKAP1
 
 
hsa-miR-152-3p
 
 
-0.381
 
 
34.2
 
 
 
 
AKAP1
 
 
hsa-miR-148b-3p
 
 
-0.381
 
 
34.2
 
 
 
 
AKIRIN2
 
 
hsa-miR-103a-3p
 
 
-0.299
 
 
29.6
 
 
 
 
ALAD
 
 
hsa-miR-326
 
 
-0.352
 
 
34.3
 
 
 
 
ALAD
 
 
hsa-miR-103a-3p
 
 
-0.385
 
 
34.3
 
 
 
 
ALAD
 
 
hsa-miR-15b-5p
 
 
-0.446
 
 
34.3
 
 
 
 
ALOX12
 
 
hsa-miR-15b-5p
 
 
-0.443
 
 
47.5
 
 
 
 
ALOX15B
 
 
hsa-miR-326
 
 
-0.417
 
 
38.1
 
 
 
 
AMOTL1
 
 
hsa-miR-15b-5p
 
 
-0.319
 
 
28.5
 
 
 
 
ANKHD1
 
 
hsa-miR-23a-3p
 
 
-0.368
 
 
42.1
 
 
 
 
ANKHD1
 
 
hsa-miR-30d-5p
 
 
-0.381
 
 
42.1
 
 
 
 
ANKHD1
 
 
hsa-miR-30b-5p
 
 
-0.405
 
 
42.1
 
 
 
 
ANKIB1
 
 
hsa-miR-1271-5p
 
 
-0.344
 
 
11.1
 
 
 
 
ANKRD17
 
 
hsa-miR-30d-5p
 
 
-0.312
 
 
13.6
 
 
 
 
ANKRD17
 
 
hsa-miR-30b-5p
 
 
-0.336
 
 
13.6
 
 
 
 
ANKRD27
 
 
hsa-miR-1271-5p
 
 
-0.648
 
 
13.5
 
 
 
 
ANKRD46
 
 
hsa-let-7b-5p
 
 
-0.389
 
 
11.9
 
 
 
 
ANP32A
 
 
hsa-miR-132-3p
 
 
-0.374
 
 
61.9
 
 
 
 
ANTXR2
 
 
hsa-miR-29a-3p
 
 
-0.347
 
 
23.0
 
 
 
 
ANXA11
 
 
hsa-miR-15b-5p
 
 
-0.463
 
 
200.5
 
 
 
 
AP1S1
 
 
hsa-let-7b-5p
 
 
-0.374
 
 
36.5
 
 
 
 
AP2A1
 
 
hsa-miR-15b-5p
 
 
-0.333
 
 
45.1
 
 
 
 
AP2B1
 
 
hsa-miR-1271-5p
 
 
-0.299
 
 
23.5
 
 
 
 
AP2B1
 
 
hsa-miR-15b-5p
 
 
-0.334
 
 
23.5
 
 
 
 
AP3S1
 
 
hsa-miR-30b-5p
 
 
-0.293
 
 
75.8
 
 
 
 
AP4B1
 
 
hsa-miR-378a-3p
 
 
-0.62
 
 
11.6
 
 
 
 
AP4B1
 
 
hsa-miR-378d
 
 
-0.62
 
 
11.6
 
 
 
 
AP5M1
 
 
hsa-miR-1271-5p
 
 
-0.343
 
 
15.4
 
 
 
 
APOO
 
 
hsa-miR-181d-5p
 
 
-0.492
 
 
12.1
 
 
 
 
APOO
 
 
hsa-miR-181b-5p
 
 
-0.5
 
 
12.1
 
 
 
 
APOO
 
 
hsa-miR-181a-5p
 
 
-0.5
 
 
12.1
 
 
 
 
APP
 
 
hsa-miR-15b-5p
 
 
-0.298
 
 
163.0
 
 
 
 
AQP3
 
 
hsa-miR-874-3p
 
 
-0.48
 
 
1457.5
 
 
 
 
ARF4
 
 
hsa-miR-30b-5p
 
 
-0.289
 
 
102.8
 
 
 
 
ARF4
 
 
hsa-miR-194-5p
 
 
-0.293
 
 
102.8
 
 
 
 
ARF4
 
 
hsa-miR-152-3p
 
 
-0.487
 
 
102.8
 
 
 
 
ARF4
 
 
hsa-miR-148b-3p
 
 
-0.487
 
 
102.8
 
 
 
 
ARF5
 
 
hsa-miR-29a-3p
 
 
-0.491
 
 
248.7
 
 
 
 
ARF6
 
 
hsa-miR-181b-5p
 
 
-0.33
 
 
81.9
 
 
 
 
ARF6
 
 
hsa-miR-181a-5p
 
 
-0.33
 
 
81.9
 
 
 
 
ARF6
 
 
hsa-miR-181d-5p
 
 
-0.338
 
 
81.9
 
 
 
 
ARFGEF1
 
 
hsa-miR-148b-3p
 
 
-0.306
 
 
14.2
 
 
 
 
ARFGEF1
 
 
hsa-miR-152-3p
 
 
-0.306
 
 
14.2
 
 
 
 
ARFIP1
 
 
hsa-miR-320b
 
 
-0.339
 
 
19.6
 
 
 
 
ARFIP1
 
 
hsa-miR-320c
 
 
-0.339
 
 
19.6
 
 
 
 
ARFIP1
 
 
hsa-miR-152-3p
 
 
-0.474
 
 
19.6
 
 
 
 
ARFIP1
 
 
hsa-miR-148b-3p
 
 
-0.474
 
 
19.6
 
 
 
 
ARG2
 
 
hsa-let-7b-5p
 
 
-0.468
 
 
17.2
 
 
 
 
ARHGAP21
 
 
hsa-miR-152-3p
 
 
-0.287
 
 
10.2
 
 
 
 
ARHGAP21
 
 
hsa-miR-148b-3p
 
 
-0.287
 
 
10.2
 
 
 
 
ARID1B
 
 
hsa-miR-29a-3p
 
 
-0.342
 
 
13.7
 
 
 
 
ARIH2
 
 
hsa-miR-103a-3p
 
 
-0.307
 
 
18.8
 
 
 
 
ARL2
 
 
hsa-miR-15b-5p
 
 
-0.374
 
 
68.0
 
 
 
 
ARL2
 
 
hsa-miR-15b-5p
 
 
-0.442
 
 
68.0
 
 
 
 
ARL4A
 
 
hsa-miR-29a-3p
 
 
-0.386
 
 
56.3
 
 
 
 
ARL4A
 
 
hsa-miR-30d-5p
 
 
-0.432
 
 
56.3
 
 
 
 
ARL4A
 
 
hsa-miR-30b-5p
 
 
-0.448
 
 
56.3
 
 
 
 
 
 
 fallopian tube 
 
 
 
 
target_symbol
 
 
mature_mirna_id
 
 
score
 
 
nTPM
 
 
 
 
 
 
AAMP
 
 
hsa-miR-326
 
 
-0.722
 
 
61.9
 
 
 
 
AAR2
 
 
hsa-miR-29a-3p
 
 
-0.327
 
 
21.8
 
 
 
 
ABCB7
 
 
hsa-miR-148b-3p
 
 
-0.515
 
 
11.3
 
 
 
 
ABCB7
 
 
hsa-miR-152-3p
 
 
-0.515
 
 
11.3
 
 
 
 
ABCD3
 
 
hsa-miR-148b-3p
 
 
-0.388
 
 
15.8
 
 
 
 
ABCD3
 
 
hsa-miR-152-3p
 
 
-0.388
 
 
15.8
 
 
 
 
ABCE1
 
 
hsa-miR-29a-3p
 
 
-0.404
 
 
21.1
 
 
 
 
ABHD17C
 
 
hsa-let-7b-5p
 
 
-0.359
 
 
10.3
 
 
 
 
ABHD17C
 
 
hsa-miR-330-3p
 
 
-0.399
 
 
10.3
 
 
 
 
ABHD4
 
 
hsa-miR-29a-3p
 
 
-0.312
 
 
18.8
 
 
 
 
ACTR2
 
 
hsa-miR-15b-5p
 
 
-0.293
 
 
50.3
 
 
 
 
ACVR1
 
 
hsa-miR-30b-5p
 
 
-0.29
 
 
13.2
 
 
 
 
ACVR1
 
 
hsa-miR-152-3p
 
 
-0.397
 
 
13.2
 
 
 
 
ACVR1
 
 
hsa-miR-148b-3p
 
 
-0.397
 
 
13.2
 
 
 
 
ADAM10
 
 
hsa-miR-152-3p
 
 
-0.348
 
 
13.1
 
 
 
 
ADAM10
 
 
hsa-miR-148b-3p
 
 
-0.356
 
 
13.1
 
 
 
 
ADAM9
 
 
hsa-miR-30d-5p
 
 
-0.304
 
 
15.9
 
 
 
 
ADAM9
 
 
hsa-miR-30b-5p
 
 
-0.312
 
 
15.9
 
 
 
 
ADAMTS9
 
 
hsa-miR-29a-3p
 
 
-0.305
 
 
10.3
 
 
 
 
ADAMTS9
 
 
hsa-miR-29a-3p
 
 
-0.485
 
 
10.3
 
 
 
 
ADCY3
 
 
hsa-miR-132-3p
 
 
-0.303
 
 
52.6
 
 
 
 
ADCY6
 
 
hsa-miR-1271-5p
 
 
-0.336
 
 
18.4
 
 
 
 
ADGRA2
 
 
hsa-miR-1271-5p
 
 
-0.427
 
 
37.9
 
 
 
 
ADGRA3
 
 
hsa-miR-30d-5p
 
 
-0.296
 
 
20.3
 
 
 
 
ADGRA3
 
 
hsa-miR-30b-5p
 
 
-0.304
 
 
20.3
 
 
 
 
ADGRF5
 
 
hsa-miR-148b-3p
 
 
-0.354
 
 
13.6
 
 
 
 
ADGRF5
 
 
hsa-miR-152-3p
 
 
-0.354
 
 
13.6
 
 
 
 
ADIPOR2
 
 
hsa-miR-150-5p
 
 
-0.287
 
 
23.2
 
 
 
 
ADK
 
 
hsa-miR-1271-5p
 
 
-0.329
 
 
15.5
 
 
 
 
ADNP
 
 
hsa-miR-328-3p
 
 
-0.371
 
 
31.2
 
 
 
 
AFG3L2
 
 
hsa-miR-181b-5p
 
 
-0.491
 
 
12.9
 
 
 
 
AFG3L2
 
 
hsa-miR-181a-5p
 
 
-0.491
 
 
12.9
 
 
 
 
AFG3L2
 
 
hsa-miR-181d-5p
 
 
-0.499
 
 
12.9
 
 
 
 
AGFG1
 
 
hsa-miR-148b-3p
 
 
-0.332
 
 
23.3
 
 
 
 
AGFG1
 
 
hsa-miR-152-3p
 
 
-0.332
 
 
23.3
 
 
 
 
AGFG1
 
 
hsa-miR-103a-3p
 
 
-0.389
 
 
23.3
 
 
 
 
AHDC1
 
 
hsa-miR-152-3p
 
 
-0.413
 
 
19.8
 
 
 
 
AHDC1
 
 
hsa-miR-148b-3p
 
 
-0.413
 
 
19.8
 
 
 
 
AIFM1
 
 
hsa-let-7b-5p
 
 
-0.314
 
 
20.8
 
 
 
 
AK1
 
 
hsa-miR-29a-3p
 
 
-0.333
 
 
321.8
 
 
 
 
AK3
 
 
hsa-miR-1271-5p
 
 
-0.57
 
 
31.0
 
 
 
 
AKAP1
 
 
hsa-miR-152-3p
 
 
-0.381
 
 
33.9
 
 
 
 
AKAP1
 
 
hsa-miR-148b-3p
 
 
-0.381
 
 
33.9
 
 
 
 
AKIRIN2
 
 
hsa-miR-103a-3p
 
 
-0.299
 
 
18.4
 
 
 
 
AKT3
 
 
hsa-miR-29a-3p
 
 
-0.287
 
 
15.1
 
 
 
 
AKT3
 
 
hsa-miR-15b-5p
 
 
-0.301
 
 
15.1
 
 
 
 
ALAD
 
 
hsa-miR-326
 
 
-0.352
 
 
14.7
 
 
 
 
ALAD
 
 
hsa-miR-103a-3p
 
 
-0.385
 
 
14.7
 
 
 
 
ALAD
 
 
hsa-miR-15b-5p
 
 
-0.446
 
 
14.7
 
 
 
 
ALCAM
 
 
hsa-miR-148b-3p
 
 
-0.339
 
 
46.2
 
 
 
 
ALCAM
 
 
hsa-miR-152-3p
 
 
-0.347
 
 
46.2
 
 
 
 
ALDH1A2
 
 
hsa-miR-23a-3p
 
 
-0.354
 
 
70.1
 
 
 
 
ALDH6A1
 
 
hsa-miR-1271-5p
 
 
-0.301
 
 
12.0
 
 
 
 
AMOT
 
 
hsa-miR-29a-3p
 
 
-0.304
 
 
11.3
 
 
 
 
AMOTL1
 
 
hsa-miR-15b-5p
 
 
-0.319
 
 
12.5
 
 
 
 
AMT
 
 
hsa-let-7b-5p
 
 
-0.463
 
 
13.9
 
 
 
 
ANKHD1
 
 
hsa-miR-23a-3p
 
 
-0.368
 
 
37.7
 
 
 
 
ANKHD1
 
 
hsa-miR-30d-5p
 
 
-0.381
 
 
37.7
 
 
 
 
ANKHD1
 
 
hsa-miR-30b-5p
 
 
-0.405
 
 
37.7
 
 
 
 
ANKRA2
 
 
hsa-miR-30d-5p
 
 
-0.343
 
 
10.8
 
 
 
 
ANKRA2
 
 
hsa-miR-30b-5p
 
 
-0.354
 
 
10.8
 
 
 
 
ANKRA2
 
 
hsa-miR-30d-5p
 
 
-0.448
 
 
10.8
 
 
 
 
ANKRA2
 
 
hsa-miR-30b-5p
 
 
-0.472
 
 
10.8
 
 
 
 
ANKRD17
 
 
hsa-miR-30d-5p
 
 
-0.312
 
 
17.1
 
 
 
 
ANKRD17
 
 
hsa-miR-30b-5p
 
 
-0.336
 
 
17.1
 
 
 
 
ANKRD29
 
 
hsa-miR-132-3p
 
 
-0.47
 
 
14.4
 
 
 
 
ANKRD46
 
 
hsa-let-7b-5p
 
 
-0.389
 
 
10.7
 
 
 
 
ANP32A
 
 
hsa-miR-132-3p
 
 
-0.374
 
 
70.6
 
 
 
 
ANTXR2
 
 
hsa-miR-29a-3p
 
 
-0.347
 
 
42.2
 
 
 
 
ANXA11
 
 
hsa-miR-15b-5p
 
 
-0.463
 
 
102.6
 
 
 
 
AP1S1
 
 
hsa-let-7b-5p
 
 
-0.374
 
 
13.4
 
 
 
 
AP2A1
 
 
hsa-miR-15b-5p
 
 
-0.333
 
 
44.6
 
 
 
 
AP2B1
 
 
hsa-miR-1271-5p
 
 
-0.299
 
 
43.9
 
 
 
 
AP2B1
 
 
hsa-miR-15b-5p
 
 
-0.334
 
 
43.9
 
 
 
 
AP3S1
 
 
hsa-miR-30b-5p
 
 
-0.293
 
 
44.1
 
 
 
 
AP4B1
 
 
hsa-miR-378a-3p
 
 
-0.62
 
 
11.8
 
 
 
 
AP4B1
 
 
hsa-miR-378d
 
 
-0.62
 
 
11.8
 
 
 
 
APOO
 
 
hsa-miR-181d-5p
 
 
-0.492
 
 
19.5
 
 
 
 
APOO
 
 
hsa-miR-181b-5p
 
 
-0.5
 
 
19.5
 
 
 
 
APOO
 
 
hsa-miR-181a-5p
 
 
-0.5
 
 
19.5
 
 
 
 
APP
 
 
hsa-miR-15b-5p
 
 
-0.298
 
 
289.3
 
 
 
 
AQP3
 
 
hsa-miR-874-3p
 
 
-0.48
 
 
23.9
 
 
 
 
ARF4
 
 
hsa-miR-30b-5p
 
 
-0.289
 
 
79.6
 
 
 
 
ARF4
 
 
hsa-miR-194-5p
 
 
-0.293
 
 
79.6
 
 
 
 
ARF4
 
 
hsa-miR-152-3p
 
 
-0.487
 
 
79.6
 
 
 
 
ARF4
 
 
hsa-miR-148b-3p
 
 
-0.487
 
 
79.6
 
 
 
 
ARF5
 
 
hsa-miR-29a-3p
 
 
-0.491
 
 
100.8
 
 
 
 
ARF6
 
 
hsa-miR-181b-5p
 
 
-0.33
 
 
26.6
 
 
 
 
ARF6
 
 
hsa-miR-181a-5p
 
 
-0.33
 
 
26.6
 
 
 
 
ARF6
 
 
hsa-miR-181d-5p
 
 
-0.338
 
 
26.6
 
 
 
 
ARFGEF1
 
 
hsa-miR-148b-3p
 
 
-0.306
 
 
11.1
 
 
 
 
ARFGEF1
 
 
hsa-miR-152-3p
 
 
-0.306
 
 
11.1
 
 
 
 
ARFIP1
 
 
hsa-miR-320b
 
 
-0.339
 
 
17.4
 
 
 
 
ARFIP1
 
 
hsa-miR-320c
 
 
-0.339
 
 
17.4
 
 
 
 
ARFIP1
 
 
hsa-miR-152-3p
 
 
-0.474
 
 
17.4
 
 
 
 
ARFIP1
 
 
hsa-miR-148b-3p
 
 
-0.474
 
 
17.4
 
 
 
 
ARHGAP21
 
 
hsa-miR-152-3p
 
 
-0.287
 
 
16.4
 
 
 
 
ARHGAP21
 
 
hsa-miR-148b-3p
 
 
-0.287
 
 
16.4
 
 
 
 
ARID1B
 
 
hsa-miR-29a-3p
 
 
-0.342
 
 
16.6
 
 
 
 
ARIH2
 
 
hsa-miR-103a-3p
 
 
-0.307
 
 
20.9
 
 
 
 
 
 
 gallbladder 
 
 
 
 
target_symbol
 
 
mature_mirna_id
 
 
score
 
 
nTPM
 
 
 
 
 
 
AAMP
 
 
hsa-miR-326
 
 
-0.722
 
 
52.6
 
 
 
 
AAR2
 
 
hsa-miR-29a-3p
 
 
-0.327
 
 
15.5
 
 
 
 
ABCD3
 
 
hsa-miR-148b-3p
 
 
-0.388
 
 
25.2
 
 
 
 
ABCD3
 
 
hsa-miR-152-3p
 
 
-0.388
 
 
25.2
 
 
 
 
ABCE1
 
 
hsa-miR-29a-3p
 
 
-0.404
 
 
21.0
 
 
 
 
ABHD4
 
 
hsa-miR-29a-3p
 
 
-0.312
 
 
25.4
 
 
 
 
ABHD5
 
 
hsa-miR-132-3p
 
 
-0.323
 
 
10.2
 
 
 
 
ACTC1
 
 
hsa-miR-30d-5p
 
 
-0.519
 
 
11.1
 
 
 
 
ACTC1
 
 
hsa-miR-30b-5p
 
 
-0.543
 
 
11.1
 
 
 
 
ACTR2
 
 
hsa-miR-15b-5p
 
 
-0.293
 
 
84.9
 
 
 
 
ACVR1
 
 
hsa-miR-30b-5p
 
 
-0.29
 
 
14.1
 
 
 
 
ACVR1
 
 
hsa-miR-152-3p
 
 
-0.397
 
 
14.1
 
 
 
 
ACVR1
 
 
hsa-miR-148b-3p
 
 
-0.397
 
 
14.1
 
 
 
 
ADAM10
 
 
hsa-miR-152-3p
 
 
-0.348
 
 
17.7
 
 
 
 
ADAM10
 
 
hsa-miR-148b-3p
 
 
-0.356
 
 
17.7
 
 
 
 
ADAM9
 
 
hsa-miR-30d-5p
 
 
-0.304
 
 
44.2
 
 
 
 
ADAM9
 
 
hsa-miR-30b-5p
 
 
-0.312
 
 
44.2
 
 
 
 
ADCY3
 
 
hsa-miR-132-3p
 
 
-0.303
 
 
21.6
 
 
 
 
ADCY6
 
 
hsa-miR-1271-5p
 
 
-0.336
 
 
13.0
 
 
 
 
ADGRA2
 
 
hsa-miR-1271-5p
 
 
-0.427
 
 
24.5
 
 
 
 
ADGRF5
 
 
hsa-miR-148b-3p
 
 
-0.354
 
 
16.1
 
 
 
 
ADGRF5
 
 
hsa-miR-152-3p
 
 
-0.354
 
 
16.1
 
 
 
 
ADK
 
 
hsa-miR-1271-5p
 
 
-0.329
 
 
10.7
 
 
 
 
ADNP
 
 
hsa-miR-328-3p
 
 
-0.371
 
 
20.9
 
 
 
 
ADRA2A
 
 
hsa-miR-23a-3p
 
 
-0.371
 
 
18.7
 
 
 
 
AFG3L2
 
 
hsa-miR-181b-5p
 
 
-0.491
 
 
10.6
 
 
 
 
AFG3L2
 
 
hsa-miR-181a-5p
 
 
-0.491
 
 
10.6
 
 
 
 
AFG3L2
 
 
hsa-miR-181d-5p
 
 
-0.499
 
 
10.6
 
 
 
 
AGFG1
 
 
hsa-miR-148b-3p
 
 
-0.332
 
 
36.5
 
 
 
 
AGFG1
 
 
hsa-miR-152-3p
 
 
-0.332
 
 
36.5
 
 
 
 
AGFG1
 
 
hsa-miR-103a-3p
 
 
-0.389
 
 
36.5
 
 
 
 
AHR
 
 
hsa-miR-1271-5p
 
 
-0.304
 
 
31.5
 
 
 
 
AHR
 
 
hsa-miR-29a-3p
 
 
-0.316
 
 
31.5
 
 
 
 
AK1
 
 
hsa-miR-29a-3p
 
 
-0.333
 
 
21.1
 
 
 
 
AK3
 
 
hsa-miR-1271-5p
 
 
-0.57
 
 
33.7
 
 
 
 
AK4
 
 
hsa-miR-15b-5p
 
 
-0.316
 
 
12.5
 
 
 
 
AKAP1
 
 
hsa-miR-152-3p
 
 
-0.381
 
 
16.8
 
 
 
 
AKAP1
 
 
hsa-miR-148b-3p
 
 
-0.381
 
 
16.8
 
 
 
 
AKIRIN2
 
 
hsa-miR-103a-3p
 
 
-0.299
 
 
14.3
 
 
 
 
ALAD
 
 
hsa-miR-326
 
 
-0.352
 
 
25.7
 
 
 
 
ALAD
 
 
hsa-miR-103a-3p
 
 
-0.385
 
 
25.7
 
 
 
 
ALAD
 
 
hsa-miR-15b-5p
 
 
-0.446
 
 
25.7
 
 
 
 
ALCAM
 
 
hsa-miR-148b-3p
 
 
-0.339
 
 
21.9
 
 
 
 
ALCAM
 
 
hsa-miR-152-3p
 
 
-0.347
 
 
21.9
 
 
 
 
ALDH1A2
 
 
hsa-miR-23a-3p
 
 
-0.354
 
 
22.5
 
 
 
 
AMOTL1
 
 
hsa-miR-15b-5p
 
 
-0.319
 
 
12.5
 
 
 
 
ANKHD1
 
 
hsa-miR-23a-3p
 
 
-0.368
 
 
23.4
 
 
 
 
ANKHD1
 
 
hsa-miR-30d-5p
 
 
-0.381
 
 
23.4
 
 
 
 
ANKHD1
 
 
hsa-miR-30b-5p
 
 
-0.405
 
 
23.4
 
 
 
 
ANKIB1
 
 
hsa-miR-1271-5p
 
 
-0.344
 
 
13.9
 
 
 
 
ANKRD17
 
 
hsa-miR-30d-5p
 
 
-0.312
 
 
11.6
 
 
 
 
ANKRD17
 
 
hsa-miR-30b-5p
 
 
-0.336
 
 
11.6
 
 
 
 
ANP32A
 
 
hsa-miR-132-3p
 
 
-0.374
 
 
56.0
 
 
 
 
ANTXR2
 
 
hsa-miR-29a-3p
 
 
-0.347
 
 
51.4
 
 
 
 
ANXA11
 
 
hsa-miR-15b-5p
 
 
-0.463
 
 
79.6
 
 
 
 
AP1S1
 
 
hsa-let-7b-5p
 
 
-0.374
 
 
12.0
 
 
 
 
AP2A1
 
 
hsa-miR-15b-5p
 
 
-0.333
 
 
18.3
 
 
 
 
AP2B1
 
 
hsa-miR-1271-5p
 
 
-0.299
 
 
39.7
 
 
 
 
AP2B1
 
 
hsa-miR-15b-5p
 
 
-0.334
 
 
39.7
 
 
 
 
AP3S1
 
 
hsa-miR-30b-5p
 
 
-0.293
 
 
46.1
 
 
 
 
AP5M1
 
 
hsa-miR-1271-5p
 
 
-0.343
 
 
11.3
 
 
 
 
APP
 
 
hsa-miR-15b-5p
 
 
-0.298
 
 
321.7
 
 
 
 
ARF4
 
 
hsa-miR-30b-5p
 
 
-0.289
 
 
145.1
 
 
 
 
ARF4
 
 
hsa-miR-194-5p
 
 
-0.293
 
 
145.1
 
 
 
 
ARF4
 
 
hsa-miR-152-3p
 
 
-0.487
 
 
145.1
 
 
 
 
ARF4
 
 
hsa-miR-148b-3p
 
 
-0.487
 
 
145.1
 
 
 
 
ARF5
 
 
hsa-miR-29a-3p
 
 
-0.491
 
 
76.6
 
 
 
 
ARF6
 
 
hsa-miR-181b-5p
 
 
-0.33
 
 
30.6
 
 
 
 
ARF6
 
 
hsa-miR-181a-5p
 
 
-0.33
 
 
30.6
 
 
 
 
ARF6
 
 
hsa-miR-181d-5p
 
 
-0.338
 
 
30.6
 
 
 
 
ARFGEF1
 
 
hsa-miR-148b-3p
 
 
-0.306
 
 
11.7
 
 
 
 
ARFGEF1
 
 
hsa-miR-152-3p
 
 
-0.306
 
 
11.7
 
 
 
 
ARFIP1
 
 
hsa-miR-320b
 
 
-0.339
 
 
18.7
 
 
 
 
ARFIP1
 
 
hsa-miR-320c
 
 
-0.339
 
 
18.7
 
 
 
 
ARFIP1
 
 
hsa-miR-152-3p
 
 
-0.474
 
 
18.7
 
 
 
 
ARFIP1
 
 
hsa-miR-148b-3p
 
 
-0.474
 
 
18.7
 
 
 
 
ARHGAP21
 
 
hsa-miR-152-3p
 
 
-0.287
 
 
16.7
 
 
 
 
ARHGAP21
 
 
hsa-miR-148b-3p
 
 
-0.287
 
 
16.7
 
 
 
 
ARHGAP24
 
 
hsa-miR-1271-5p
 
 
-0.342
 
 
10.2
 
 
 
 
ARID1B
 
 
hsa-miR-29a-3p
 
 
-0.342
 
 
12.6
 
 
 
 
ARIH2
 
 
hsa-miR-103a-3p
 
 
-0.307
 
 
14.0
 
 
 
 
ARL2
 
 
hsa-miR-15b-5p
 
 
-0.374
 
 
45.0
 
 
 
 
ARL2
 
 
hsa-miR-15b-5p
 
 
-0.442
 
 
45.0
 
 
 
 
ARL4A
 
 
hsa-miR-29a-3p
 
 
-0.386
 
 
18.0
 
 
 
 
ARL4A
 
 
hsa-miR-30d-5p
 
 
-0.432
 
 
18.0
 
 
 
 
ARL4A
 
 
hsa-miR-30b-5p
 
 
-0.448
 
 
18.0
 
 
 
 
ARL5B
 
 
hsa-miR-29a-3p
 
 
-0.304
 
 
13.2
 
 
 
 
ARL6IP1
 
 
hsa-miR-328-3p
 
 
-0.494
 
 
123.8
 
 
 
 
ARL6IP1
 
 
hsa-miR-148b-3p
 
 
-0.579
 
 
123.8
 
 
 
 
ARL6IP1
 
 
hsa-miR-152-3p
 
 
-0.579
 
 
123.8
 
 
 
 
ARL8A
 
 
hsa-miR-103a-3p
 
 
-0.38
 
 
20.1
 
 
 
 
ARL8B
 
 
hsa-miR-152-3p
 
 
-0.337
 
 
36.3
 
 
 
 
ARL8B
 
 
hsa-miR-148b-3p
 
 
-0.337
 
 
36.3
 
 
 
 
ARL8B
 
 
hsa-miR-320b
 
 
-0.376
 
 
36.3
 
 
 
 
ARL8B
 
 
hsa-miR-320c
 
 
-0.376
 
 
36.3
 
 
 
 
ARMC1
 
 
hsa-miR-103a-3p
 
 
-0.525
 
 
11.1
 
 
 
 
ARMC8
 
 
hsa-miR-103a-3p
 
 
-0.412
 
 
14.3
 
 
 
 
ARMCX2
 
 
hsa-miR-15b-5p
 
 
-0.344
 
 
19.3
 
 
 
 
ARMCX3
 
 
hsa-miR-425-5p
 
 
-0.325
 
 
31.9
 
 
 
 
ARMT1
 
 
hsa-let-7b-5p
 
 
-0.291
 
 
10.6
 
 
 
 
 
 
 heart muscle 
 
 
 
 
target_symbol
 
 
mature_mirna_id
 
 
score
 
 
nTPM
 
 
 
 
 
 
AAMP
 
 
hsa-miR-326
 
 
-0.722
 
 
66.0
 
 
 
 
AAR2
 
 
hsa-miR-29a-3p
 
 
-0.327
 
 
17.9
 
 
 
 
ABAT
 
 
hsa-miR-1271-5p
 
 
-0.409
 
 
13.2
 
 
 
 
ABCB7
 
 
hsa-miR-148b-3p
 
 
-0.515
 
 
20.0
 
 
 
 
ABCB7
 
 
hsa-miR-152-3p
 
 
-0.515
 
 
20.0
 
 
 
 
ABCD3
 
 
hsa-miR-148b-3p
 
 
-0.388
 
 
24.0
 
 
 
 
ABCD3
 
 
hsa-miR-152-3p
 
 
-0.388
 
 
24.0
 
 
 
 
ABCE1
 
 
hsa-miR-29a-3p
 
 
-0.404
 
 
23.3
 
 
 
 
ABHD18
 
 
hsa-miR-29a-3p
 
 
-0.321
 
 
12.6
 
 
 
 
ABHD18
 
 
hsa-miR-29a-3p
 
 
-0.34
 
 
12.6
 
 
 
 
ABHD4
 
 
hsa-miR-29a-3p
 
 
-0.312
 
 
21.4
 
 
 
 
ABHD5
 
 
hsa-miR-132-3p
 
 
-0.323
 
 
10.6
 
 
 
 
ACHE
 
 
hsa-miR-194-5p
 
 
-0.395
 
 
11.4
 
 
 
 
ACTC1
 
 
hsa-miR-30d-5p
 
 
-0.519
 
 
10840.1
 
 
 
 
ACTC1
 
 
hsa-miR-30b-5p
 
 
-0.543
 
 
10840.1
 
 
 
 
ACTN2
 
 
hsa-miR-23a-3p
 
 
-0.294
 
 
695.9
 
 
 
 
ACTN2
 
 
hsa-miR-29a-3p
 
 
-0.311
 
 
695.9
 
 
 
 
ACTR2
 
 
hsa-miR-15b-5p
 
 
-0.293
 
 
60.7
 
 
 
 
ACVR1
 
 
hsa-miR-30b-5p
 
 
-0.29
 
 
14.3
 
 
 
 
ACVR1
 
 
hsa-miR-152-3p
 
 
-0.397
 
 
14.3
 
 
 
 
ACVR1
 
 
hsa-miR-148b-3p
 
 
-0.397
 
 
14.3
 
 
 
 
ADA
 
 
hsa-miR-29a-3p
 
 
-0.376
 
 
18.4
 
 
 
 
ADAM10
 
 
hsa-miR-152-3p
 
 
-0.348
 
 
11.3
 
 
 
 
ADAM10
 
 
hsa-miR-148b-3p
 
 
-0.356
 
 
11.3
 
 
 
 
ADAM19
 
 
hsa-miR-874-3p
 
 
-0.374
 
 
23.8
 
 
 
 
ADAM9
 
 
hsa-miR-30d-5p
 
 
-0.304
 
 
26.6
 
 
 
 
ADAM9
 
 
hsa-miR-30b-5p
 
 
-0.312
 
 
26.6
 
 
 
 
ADAMTS7
 
 
hsa-miR-29a-3p
 
 
-0.427
 
 
28.7
 
 
 
 
ADAMTS9
 
 
hsa-miR-29a-3p
 
 
-0.305
 
 
14.2
 
 
 
 
ADAMTS9
 
 
hsa-miR-29a-3p
 
 
-0.485
 
 
14.2
 
 
 
 
ADCY3
 
 
hsa-miR-132-3p
 
 
-0.303
 
 
35.0
 
 
 
 
ADCY6
 
 
hsa-miR-1271-5p
 
 
-0.336
 
 
53.6
 
 
 
 
ADGRA2
 
 
hsa-miR-1271-5p
 
 
-0.427
 
 
15.0
 
 
 
 
ADGRA3
 
 
hsa-miR-30d-5p
 
 
-0.296
 
 
10.1
 
 
 
 
ADGRA3
 
 
hsa-miR-30b-5p
 
 
-0.304
 
 
10.1
 
 
 
 
ADGRF5
 
 
hsa-miR-148b-3p
 
 
-0.354
 
 
50.1
 
 
 
 
ADGRF5
 
 
hsa-miR-152-3p
 
 
-0.354
 
 
50.1
 
 
 
 
ADIPOR2
 
 
hsa-miR-150-5p
 
 
-0.287
 
 
31.9
 
 
 
 
ADK
 
 
hsa-miR-1271-5p
 
 
-0.329
 
 
25.4
 
 
 
 
ADNP
 
 
hsa-miR-328-3p
 
 
-0.371
 
 
22.3
 
 
 
 
ADRB1
 
 
hsa-let-7b-5p
 
 
-0.351
 
 
20.3
 
 
 
 
AFG3L2
 
 
hsa-miR-181b-5p
 
 
-0.491
 
 
48.7
 
 
 
 
AFG3L2
 
 
hsa-miR-181a-5p
 
 
-0.491
 
 
48.7
 
 
 
 
AFG3L2
 
 
hsa-miR-181d-5p
 
 
-0.499
 
 
48.7
 
 
 
 
AGFG1
 
 
hsa-miR-148b-3p
 
 
-0.332
 
 
33.8
 
 
 
 
AGFG1
 
 
hsa-miR-152-3p
 
 
-0.332
 
 
33.8
 
 
 
 
AGFG1
 
 
hsa-miR-103a-3p
 
 
-0.389
 
 
33.8
 
 
 
 
AHDC1
 
 
hsa-miR-152-3p
 
 
-0.413
 
 
17.4
 
 
 
 
AHDC1
 
 
hsa-miR-148b-3p
 
 
-0.413
 
 
17.4
 
 
 
 
AIFM1
 
 
hsa-let-7b-5p
 
 
-0.314
 
 
74.9
 
 
 
 
AK1
 
 
hsa-miR-29a-3p
 
 
-0.333
 
 
313.1
 
 
 
 
AK3
 
 
hsa-miR-1271-5p
 
 
-0.57
 
 
94.2
 
 
 
 
AK4
 
 
hsa-miR-15b-5p
 
 
-0.316
 
 
47.4
 
 
 
 
AKAP1
 
 
hsa-miR-152-3p
 
 
-0.381
 
 
78.5
 
 
 
 
AKAP1
 
 
hsa-miR-148b-3p
 
 
-0.381
 
 
78.5
 
 
 
 
AKAP6
 
 
hsa-let-7b-5p
 
 
-0.307
 
 
51.1
 
 
 
 
AKIRIN2
 
 
hsa-miR-103a-3p
 
 
-0.299
 
 
27.5
 
 
 
 
AKT3
 
 
hsa-miR-29a-3p
 
 
-0.287
 
 
10.6
 
 
 
 
AKT3
 
 
hsa-miR-15b-5p
 
 
-0.301
 
 
10.6
 
 
 
 
ALAD
 
 
hsa-miR-326
 
 
-0.352
 
 
50.8
 
 
 
 
ALAD
 
 
hsa-miR-103a-3p
 
 
-0.385
 
 
50.8
 
 
 
 
ALAD
 
 
hsa-miR-15b-5p
 
 
-0.446
 
 
50.8
 
 
 
 
ALDH1A2
 
 
hsa-miR-23a-3p
 
 
-0.354
 
 
27.5
 
 
 
 
ALDH6A1
 
 
hsa-miR-1271-5p
 
 
-0.301
 
 
37.7
 
 
 
 
AMOTL1
 
 
hsa-miR-15b-5p
 
 
-0.319
 
 
17.9
 
 
 
 
AMT
 
 
hsa-let-7b-5p
 
 
-0.463
 
 
23.5
 
 
 
 
ANK1
 
 
hsa-miR-103a-3p
 
 
-0.321
 
 
93.7
 
 
 
 
ANKHD1
 
 
hsa-miR-23a-3p
 
 
-0.368
 
 
34.0
 
 
 
 
ANKHD1
 
 
hsa-miR-30d-5p
 
 
-0.381
 
 
34.0
 
 
 
 
ANKHD1
 
 
hsa-miR-30b-5p
 
 
-0.405
 
 
34.0
 
 
 
 
ANKRD17
 
 
hsa-miR-30d-5p
 
 
-0.312
 
 
13.6
 
 
 
 
ANKRD17
 
 
hsa-miR-30b-5p
 
 
-0.336
 
 
13.6
 
 
 
 
ANKRD46
 
 
hsa-let-7b-5p
 
 
-0.389
 
 
18.8
 
 
 
 
ANP32A
 
 
hsa-miR-132-3p
 
 
-0.374
 
 
119.7
 
 
 
 
ANTXR2
 
 
hsa-miR-29a-3p
 
 
-0.347
 
 
26.5
 
 
 
 
ANXA11
 
 
hsa-miR-15b-5p
 
 
-0.463
 
 
221.3
 
 
 
 
AP1S1
 
 
hsa-let-7b-5p
 
 
-0.374
 
 
12.9
 
 
 
 
AP2A1
 
 
hsa-miR-15b-5p
 
 
-0.333
 
 
49.3
 
 
 
 
AP2B1
 
 
hsa-miR-1271-5p
 
 
-0.299
 
 
63.3
 
 
 
 
AP2B1
 
 
hsa-miR-15b-5p
 
 
-0.334
 
 
63.3
 
 
 
 
AP3S1
 
 
hsa-miR-30b-5p
 
 
-0.293
 
 
53.4
 
 
 
 
AP4B1
 
 
hsa-miR-378a-3p
 
 
-0.62
 
 
10.7
 
 
 
 
AP4B1
 
 
hsa-miR-378d
 
 
-0.62
 
 
10.7
 
 
 
 
AP5M1
 
 
hsa-miR-1271-5p
 
 
-0.343
 
 
15.4
 
 
 
 
APLN
 
 
hsa-miR-15b-5p
 
 
-0.395
 
 
12.4
 
 
 
 
APOO
 
 
hsa-miR-181d-5p
 
 
-0.492
 
 
61.9
 
 
 
 
APOO
 
 
hsa-miR-181b-5p
 
 
-0.5
 
 
61.9
 
 
 
 
APOO
 
 
hsa-miR-181a-5p
 
 
-0.5
 
 
61.9
 
 
 
 
APP
 
 
hsa-miR-15b-5p
 
 
-0.298
 
 
356.3
 
 
 
 
AQP3
 
 
hsa-miR-874-3p
 
 
-0.48
 
 
33.0
 
 
 
 
ARF4
 
 
hsa-miR-30b-5p
 
 
-0.289
 
 
106.8
 
 
 
 
ARF4
 
 
hsa-miR-194-5p
 
 
-0.293
 
 
106.8
 
 
 
 
ARF4
 
 
hsa-miR-152-3p
 
 
-0.487
 
 
106.8
 
 
 
 
ARF4
 
 
hsa-miR-148b-3p
 
 
-0.487
 
 
106.8
 
 
 
 
ARF5
 
 
hsa-miR-29a-3p
 
 
-0.491
 
 
127.9
 
 
 
 
ARF6
 
 
hsa-miR-181b-5p
 
 
-0.33
 
 
15.7
 
 
 
 
ARF6
 
 
hsa-miR-181a-5p
 
 
-0.33
 
 
15.7
 
 
 
 
ARF6
 
 
hsa-miR-181d-5p
 
 
-0.338
 
 
15.7
 
 
 
 
ARHGAP21
 
 
hsa-miR-152-3p
 
 
-0.287
 
 
19.7
 
 
 
 
ARHGAP21
 
 
hsa-miR-148b-3p
 
 
-0.287
 
 
19.7
 
 
 
 
 
 
 hippocampal formation 
 
 
 
 
target_symbol
 
 
mature_mirna_id
 
 
score
 
 
nTPM
 
 
 
 
 
 
AAMP
 
 
hsa-miR-326
 
 
-0.722
 
 
57.1
 
 
 
 
AAR2
 
 
hsa-miR-29a-3p
 
 
-0.327
 
 
10.6
 
 
 
 
ABAT
 
 
hsa-miR-1271-5p
 
 
-0.409
 
 
68.5
 
 
 
 
ABCA2
 
 
hsa-miR-1271-5p
 
 
-0.326
 
 
80.9
 
 
 
 
ABCD3
 
 
hsa-miR-148b-3p
 
 
-0.388
 
 
15.1
 
 
 
 
ABCD3
 
 
hsa-miR-152-3p
 
 
-0.388
 
 
15.1
 
 
 
 
ABCE1
 
 
hsa-miR-29a-3p
 
 
-0.404
 
 
13.9
 
 
 
 
ABHD17C
 
 
hsa-let-7b-5p
 
 
-0.359
 
 
14.4
 
 
 
 
ABHD17C
 
 
hsa-miR-330-3p
 
 
-0.399
 
 
14.4
 
 
 
 
ABHD4
 
 
hsa-miR-29a-3p
 
 
-0.312
 
 
14.2
 
 
 
 
ACHE
 
 
hsa-miR-194-5p
 
 
-0.395
 
 
14.5
 
 
 
 
ACSL6
 
 
hsa-let-7b-5p
 
 
-0.336
 
 
15.3
 
 
 
 
ACTN2
 
 
hsa-miR-23a-3p
 
 
-0.294
 
 
10.1
 
 
 
 
ACTN2
 
 
hsa-miR-29a-3p
 
 
-0.311
 
 
10.1
 
 
 
 
ACTR2
 
 
hsa-miR-15b-5p
 
 
-0.293
 
 
54.7
 
 
 
 
ADCY2
 
 
hsa-miR-152-3p
 
 
-0.299
 
 
18.5
 
 
 
 
ADCY2
 
 
hsa-miR-148b-3p
 
 
-0.299
 
 
18.5
 
 
 
 
ADCY3
 
 
hsa-miR-132-3p
 
 
-0.303
 
 
30.1
 
 
 
 
ADGRA3
 
 
hsa-miR-30d-5p
 
 
-0.296
 
 
12.8
 
 
 
 
ADGRA3
 
 
hsa-miR-30b-5p
 
 
-0.304
 
 
12.8
 
 
 
 
ADGRB3
 
 
hsa-miR-1271-5p
 
 
-0.424
 
 
12.8
 
 
 
 
ADGRB3
 
 
hsa-miR-152-3p
 
 
-0.516
 
 
12.8
 
 
 
 
ADGRB3
 
 
hsa-miR-148b-3p
 
 
-0.524
 
 
12.8
 
 
 
 
ADGRF5
 
 
hsa-miR-148b-3p
 
 
-0.354
 
 
10.4
 
 
 
 
ADGRF5
 
 
hsa-miR-152-3p
 
 
-0.354
 
 
10.4
 
 
 
 
ADIPOR2
 
 
hsa-miR-150-5p
 
 
-0.287
 
 
57.6
 
 
 
 
ADK
 
 
hsa-miR-1271-5p
 
 
-0.329
 
 
13.3
 
 
 
 
ADNP
 
 
hsa-miR-328-3p
 
 
-0.371
 
 
23.7
 
 
 
 
AGFG1
 
 
hsa-miR-148b-3p
 
 
-0.332
 
 
29.8
 
 
 
 
AGFG1
 
 
hsa-miR-152-3p
 
 
-0.332
 
 
29.8
 
 
 
 
AGFG1
 
 
hsa-miR-103a-3p
 
 
-0.389
 
 
29.8
 
 
 
 
AGPAT4
 
 
hsa-miR-29a-3p
 
 
-0.376
 
 
16.8
 
 
 
 
AIFM1
 
 
hsa-let-7b-5p
 
 
-0.314
 
 
10.5
 
 
 
 
AK1
 
 
hsa-miR-29a-3p
 
 
-0.333
 
 
93.4
 
 
 
 
AK3
 
 
hsa-miR-1271-5p
 
 
-0.57
 
 
26.9
 
 
 
 
AK4
 
 
hsa-miR-15b-5p
 
 
-0.316
 
 
13.3
 
 
 
 
AKAP1
 
 
hsa-miR-152-3p
 
 
-0.381
 
 
17.0
 
 
 
 
AKAP1
 
 
hsa-miR-148b-3p
 
 
-0.381
 
 
17.0
 
 
 
 
AKAP6
 
 
hsa-let-7b-5p
 
 
-0.307
 
 
12.9
 
 
 
 
AKIRIN2
 
 
hsa-miR-103a-3p
 
 
-0.299
 
 
31.7
 
 
 
 
AKT3
 
 
hsa-miR-29a-3p
 
 
-0.287
 
 
17.7
 
 
 
 
AKT3
 
 
hsa-miR-15b-5p
 
 
-0.301
 
 
17.7
 
 
 
 
ALAD
 
 
hsa-miR-326
 
 
-0.352
 
 
60.3
 
 
 
 
ALAD
 
 
hsa-miR-103a-3p
 
 
-0.385
 
 
60.3
 
 
 
 
ALAD
 
 
hsa-miR-15b-5p
 
 
-0.446
 
 
60.3
 
 
 
 
ALCAM
 
 
hsa-miR-148b-3p
 
 
-0.339
 
 
33.8
 
 
 
 
ALCAM
 
 
hsa-miR-152-3p
 
 
-0.347
 
 
33.8
 
 
 
 
ALDH6A1
 
 
hsa-miR-1271-5p
 
 
-0.301
 
 
38.0
 
 
 
 
AMOT
 
 
hsa-miR-29a-3p
 
 
-0.304
 
 
12.0
 
 
 
 
ANGEL2
 
 
hsa-miR-132-3p
 
 
-0.357
 
 
11.0
 
 
 
 
ANKHD1
 
 
hsa-miR-23a-3p
 
 
-0.368
 
 
18.9
 
 
 
 
ANKHD1
 
 
hsa-miR-30d-5p
 
 
-0.381
 
 
18.9
 
 
 
 
ANKHD1
 
 
hsa-miR-30b-5p
 
 
-0.405
 
 
18.9
 
 
 
 
ANKIB1
 
 
hsa-miR-1271-5p
 
 
-0.344
 
 
16.1
 
 
 
 
ANKRD13B
 
 
hsa-miR-29a-3p
 
 
-0.421
 
 
10.6
 
 
 
 
ANKRD17
 
 
hsa-miR-30d-5p
 
 
-0.312
 
 
10.3
 
 
 
 
ANKRD17
 
 
hsa-miR-30b-5p
 
 
-0.336
 
 
10.3
 
 
 
 
ANKRD46
 
 
hsa-let-7b-5p
 
 
-0.389
 
 
25.5
 
 
 
 
ANLN
 
 
hsa-miR-15b-5p
 
 
-0.334
 
 
54.9
 
 
 
 
ANP32A
 
 
hsa-miR-132-3p
 
 
-0.374
 
 
49.8
 
 
 
 
ANXA11
 
 
hsa-miR-15b-5p
 
 
-0.463
 
 
30.8
 
 
 
 
AP1S1
 
 
hsa-let-7b-5p
 
 
-0.374
 
 
67.5
 
 
 
 
AP2A1
 
 
hsa-miR-15b-5p
 
 
-0.333
 
 
44.7
 
 
 
 
AP2B1
 
 
hsa-miR-1271-5p
 
 
-0.299
 
 
69.4
 
 
 
 
AP2B1
 
 
hsa-miR-15b-5p
 
 
-0.334
 
 
69.4
 
 
 
 
AP3S1
 
 
hsa-miR-30b-5p
 
 
-0.293
 
 
86.3
 
 
 
 
AP4B1
 
 
hsa-miR-378a-3p
 
 
-0.62
 
 
10.1
 
 
 
 
AP4B1
 
 
hsa-miR-378d
 
 
-0.62
 
 
10.1
 
 
 
 
APC
 
 
hsa-miR-29a-3p
 
 
-0.413
 
 
34.3
 
 
 
 
APLN
 
 
hsa-miR-15b-5p
 
 
-0.395
 
 
14.1
 
 
 
 
APOO
 
 
hsa-miR-181d-5p
 
 
-0.492
 
 
45.8
 
 
 
 
APOO
 
 
hsa-miR-181b-5p
 
 
-0.5
 
 
45.8
 
 
 
 
APOO
 
 
hsa-miR-181a-5p
 
 
-0.5
 
 
45.8
 
 
 
 
APP
 
 
hsa-miR-15b-5p
 
 
-0.298
 
 
466.6
 
 
 
 
ARF4
 
 
hsa-miR-30b-5p
 
 
-0.289
 
 
61.3
 
 
 
 
ARF4
 
 
hsa-miR-194-5p
 
 
-0.293
 
 
61.3
 
 
 
 
ARF4
 
 
hsa-miR-152-3p
 
 
-0.487
 
 
61.3
 
 
 
 
ARF4
 
 
hsa-miR-148b-3p
 
 
-0.487
 
 
61.3
 
 
 
 
ARF5
 
 
hsa-miR-29a-3p
 
 
-0.491
 
 
168.1
 
 
 
 
ARF6
 
 
hsa-miR-181b-5p
 
 
-0.33
 
 
10.0
 
 
 
 
ARF6
 
 
hsa-miR-181a-5p
 
 
-0.33
 
 
10.0
 
 
 
 
ARF6
 
 
hsa-miR-181d-5p
 
 
-0.338
 
 
10.0
 
 
 
 
ARHGAP21
 
 
hsa-miR-152-3p
 
 
-0.287
 
 
50.4
 
 
 
 
ARHGAP21
 
 
hsa-miR-148b-3p
 
 
-0.287
 
 
50.4
 
 
 
 
ARIH2
 
 
hsa-miR-103a-3p
 
 
-0.307
 
 
19.7
 
 
 
 
ARL2
 
 
hsa-miR-15b-5p
 
 
-0.374
 
 
164.1
 
 
 
 
ARL2
 
 
hsa-miR-15b-5p
 
 
-0.442
 
 
164.1
 
 
 
 
ARL3
 
 
hsa-miR-15b-5p
 
 
-0.507
 
 
21.1
 
 
 
 
ARL4A
 
 
hsa-miR-29a-3p
 
 
-0.386
 
 
12.2
 
 
 
 
ARL4A
 
 
hsa-miR-30d-5p
 
 
-0.432
 
 
12.2
 
 
 
 
ARL4A
 
 
hsa-miR-30b-5p
 
 
-0.448
 
 
12.2
 
 
 
 
ARL6IP1
 
 
hsa-miR-328-3p
 
 
-0.494
 
 
196.8
 
 
 
 
ARL6IP1
 
 
hsa-miR-148b-3p
 
 
-0.579
 
 
196.8
 
 
 
 
ARL6IP1
 
 
hsa-miR-152-3p
 
 
-0.579
 
 
196.8
 
 
 
 
ARL8A
 
 
hsa-miR-103a-3p
 
 
-0.38
 
 
89.3
 
 
 
 
ARL8B
 
 
hsa-miR-152-3p
 
 
-0.337
 
 
53.8
 
 
 
 
ARL8B
 
 
hsa-miR-148b-3p
 
 
-0.337
 
 
53.8
 
 
 
 
ARL8B
 
 
hsa-miR-320b
 
 
-0.376
 
 
53.8
 
 
 
 
ARL8B
 
 
hsa-miR-320c
 
 
-0.376
 
 
53.8
 
 
 
 
ARMC1
 
 
hsa-miR-103a-3p
 
 
-0.525
 
 
18.1
 
 
 
 
 
 
 hypothalamus 
 
 
 
 
target_symbol
 
 
mature_mirna_id
 
 
score
 
 
nTPM
 
 
 
 
 
 
AAMP
 
 
hsa-miR-326
 
 
-0.722
 
 
57.7
 
 
 
 
ABAT
 
 
hsa-miR-1271-5p
 
 
-0.409
 
 
109.8
 
 
 
 
ABCA2
 
 
hsa-miR-1271-5p
 
 
-0.326
 
 
41.4
 
 
 
 
ABCB9
 
 
hsa-let-7b-5p
 
 
-0.422
 
 
11.9
 
 
 
 
ABCD3
 
 
hsa-miR-148b-3p
 
 
-0.388
 
 
13.5
 
 
 
 
ABCD3
 
 
hsa-miR-152-3p
 
 
-0.388
 
 
13.5
 
 
 
 
ABCE1
 
 
hsa-miR-29a-3p
 
 
-0.404
 
 
12.9
 
 
 
 
ABHD17C
 
 
hsa-let-7b-5p
 
 
-0.359
 
 
11.1
 
 
 
 
ABHD17C
 
 
hsa-miR-330-3p
 
 
-0.399
 
 
11.1
 
 
 
 
ABHD4
 
 
hsa-miR-29a-3p
 
 
-0.312
 
 
13.5
 
 
 
 
ACHE
 
 
hsa-miR-194-5p
 
 
-0.395
 
 
103.9
 
 
 
 
ACSL6
 
 
hsa-let-7b-5p
 
 
-0.336
 
 
11.9
 
 
 
 
ACTR2
 
 
hsa-miR-15b-5p
 
 
-0.293
 
 
50.9
 
 
 
 
ADCY2
 
 
hsa-miR-152-3p
 
 
-0.299
 
 
14.0
 
 
 
 
ADCY2
 
 
hsa-miR-148b-3p
 
 
-0.299
 
 
14.0
 
 
 
 
ADCY3
 
 
hsa-miR-132-3p
 
 
-0.303
 
 
32.1
 
 
 
 
ADCY6
 
 
hsa-miR-1271-5p
 
 
-0.336
 
 
10.2
 
 
 
 
ADCYAP1
 
 
hsa-miR-103a-3p
 
 
-0.443
 
 
11.5
 
 
 
 
ADGRA3
 
 
hsa-miR-30d-5p
 
 
-0.296
 
 
10.8
 
 
 
 
ADGRA3
 
 
hsa-miR-30b-5p
 
 
-0.304
 
 
10.8
 
 
 
 
ADGRB3
 
 
hsa-miR-1271-5p
 
 
-0.424
 
 
16.0
 
 
 
 
ADGRB3
 
 
hsa-miR-152-3p
 
 
-0.516
 
 
16.0
 
 
 
 
ADGRB3
 
 
hsa-miR-148b-3p
 
 
-0.524
 
 
16.0
 
 
 
 
ADIPOR2
 
 
hsa-miR-150-5p
 
 
-0.287
 
 
40.5
 
 
 
 
ADK
 
 
hsa-miR-1271-5p
 
 
-0.329
 
 
15.8
 
 
 
 
ADNP
 
 
hsa-miR-328-3p
 
 
-0.371
 
 
23.7
 
 
 
 
AGFG1
 
 
hsa-miR-148b-3p
 
 
-0.332
 
 
26.0
 
 
 
 
AGFG1
 
 
hsa-miR-152-3p
 
 
-0.332
 
 
26.0
 
 
 
 
AGFG1
 
 
hsa-miR-103a-3p
 
 
-0.389
 
 
26.0
 
 
 
 
AGPAT4
 
 
hsa-miR-29a-3p
 
 
-0.376
 
 
14.0
 
 
 
 
AHDC1
 
 
hsa-miR-152-3p
 
 
-0.413
 
 
10.9
 
 
 
 
AHDC1
 
 
hsa-miR-148b-3p
 
 
-0.413
 
 
10.9
 
 
 
 
AK1
 
 
hsa-miR-29a-3p
 
 
-0.333
 
 
100.1
 
 
 
 
AK3
 
 
hsa-miR-1271-5p
 
 
-0.57
 
 
27.7
 
 
 
 
AK4
 
 
hsa-miR-15b-5p
 
 
-0.316
 
 
12.0
 
 
 
 
AKAP1
 
 
hsa-miR-152-3p
 
 
-0.381
 
 
15.1
 
 
 
 
AKAP1
 
 
hsa-miR-148b-3p
 
 
-0.381
 
 
15.1
 
 
 
 
AKAP6
 
 
hsa-let-7b-5p
 
 
-0.307
 
 
13.6
 
 
 
 
AKIRIN2
 
 
hsa-miR-103a-3p
 
 
-0.299
 
 
29.8
 
 
 
 
AKT3
 
 
hsa-miR-29a-3p
 
 
-0.287
 
 
14.5
 
 
 
 
AKT3
 
 
hsa-miR-15b-5p
 
 
-0.301
 
 
14.5
 
 
 
 
ALAD
 
 
hsa-miR-326
 
 
-0.352
 
 
54.5
 
 
 
 
ALAD
 
 
hsa-miR-103a-3p
 
 
-0.385
 
 
54.5
 
 
 
 
ALAD
 
 
hsa-miR-15b-5p
 
 
-0.446
 
 
54.5
 
 
 
 
ALCAM
 
 
hsa-miR-148b-3p
 
 
-0.339
 
 
36.1
 
 
 
 
ALCAM
 
 
hsa-miR-152-3p
 
 
-0.347
 
 
36.1
 
 
 
 
ALDH6A1
 
 
hsa-miR-1271-5p
 
 
-0.301
 
 
32.7
 
 
 
 
AMOT
 
 
hsa-miR-29a-3p
 
 
-0.304
 
 
11.9
 
 
 
 
ANKHD1
 
 
hsa-miR-23a-3p
 
 
-0.368
 
 
17.8
 
 
 
 
ANKHD1
 
 
hsa-miR-30d-5p
 
 
-0.381
 
 
17.8
 
 
 
 
ANKHD1
 
 
hsa-miR-30b-5p
 
 
-0.405
 
 
17.8
 
 
 
 
ANKIB1
 
 
hsa-miR-1271-5p
 
 
-0.344
 
 
14.5
 
 
 
 
ANKRD17
 
 
hsa-miR-30d-5p
 
 
-0.312
 
 
11.3
 
 
 
 
ANKRD17
 
 
hsa-miR-30b-5p
 
 
-0.336
 
 
11.3
 
 
 
 
ANKRD46
 
 
hsa-let-7b-5p
 
 
-0.389
 
 
28.7
 
 
 
 
ANLN
 
 
hsa-miR-15b-5p
 
 
-0.334
 
 
30.4
 
 
 
 
ANP32A
 
 
hsa-miR-132-3p
 
 
-0.374
 
 
37.9
 
 
 
 
ANXA11
 
 
hsa-miR-15b-5p
 
 
-0.463
 
 
13.4
 
 
 
 
AP1S1
 
 
hsa-let-7b-5p
 
 
-0.374
 
 
44.3
 
 
 
 
AP2A1
 
 
hsa-miR-15b-5p
 
 
-0.333
 
 
44.0
 
 
 
 
AP2B1
 
 
hsa-miR-1271-5p
 
 
-0.299
 
 
68.4
 
 
 
 
AP2B1
 
 
hsa-miR-15b-5p
 
 
-0.334
 
 
68.4
 
 
 
 
AP3S1
 
 
hsa-miR-30b-5p
 
 
-0.293
 
 
61.9
 
 
 
 
AP4B1
 
 
hsa-miR-378a-3p
 
 
-0.62
 
 
10.0
 
 
 
 
AP4B1
 
 
hsa-miR-378d
 
 
-0.62
 
 
10.0
 
 
 
 
APBB3
 
 
hsa-let-7b-5p
 
 
-0.587
 
 
11.2
 
 
 
 
APC
 
 
hsa-miR-29a-3p
 
 
-0.413
 
 
11.2
 
 
 
 
APLN
 
 
hsa-miR-15b-5p
 
 
-0.395
 
 
17.3
 
 
 
 
APOO
 
 
hsa-miR-181d-5p
 
 
-0.492
 
 
43.8
 
 
 
 
APOO
 
 
hsa-miR-181b-5p
 
 
-0.5
 
 
43.8
 
 
 
 
APOO
 
 
hsa-miR-181a-5p
 
 
-0.5
 
 
43.8
 
 
 
 
APP
 
 
hsa-miR-15b-5p
 
 
-0.298
 
 
437.7
 
 
 
 
ARF4
 
 
hsa-miR-30b-5p
 
 
-0.289
 
 
75.3
 
 
 
 
ARF4
 
 
hsa-miR-194-5p
 
 
-0.293
 
 
75.3
 
 
 
 
ARF4
 
 
hsa-miR-152-3p
 
 
-0.487
 
 
75.3
 
 
 
 
ARF4
 
 
hsa-miR-148b-3p
 
 
-0.487
 
 
75.3
 
 
 
 
ARF5
 
 
hsa-miR-29a-3p
 
 
-0.491
 
 
134.0
 
 
 
 
ARF6
 
 
hsa-miR-181b-5p
 
 
-0.33
 
 
10.2
 
 
 
 
ARF6
 
 
hsa-miR-181a-5p
 
 
-0.33
 
 
10.2
 
 
 
 
ARF6
 
 
hsa-miR-181d-5p
 
 
-0.338
 
 
10.2
 
 
 
 
ARG2
 
 
hsa-let-7b-5p
 
 
-0.468
 
 
10.7
 
 
 
 
ARHGAP21
 
 
hsa-miR-152-3p
 
 
-0.287
 
 
30.5
 
 
 
 
ARHGAP21
 
 
hsa-miR-148b-3p
 
 
-0.287
 
 
30.5
 
 
 
 
ARIH2
 
 
hsa-miR-103a-3p
 
 
-0.307
 
 
18.5
 
 
 
 
ARL2
 
 
hsa-miR-15b-5p
 
 
-0.374
 
 
156.1
 
 
 
 
ARL2
 
 
hsa-miR-15b-5p
 
 
-0.442
 
 
156.1
 
 
 
 
ARL3
 
 
hsa-miR-15b-5p
 
 
-0.507
 
 
23.1
 
 
 
 
ARL6IP1
 
 
hsa-miR-328-3p
 
 
-0.494
 
 
212.8
 
 
 
 
ARL6IP1
 
 
hsa-miR-148b-3p
 
 
-0.579
 
 
212.8
 
 
 
 
ARL6IP1
 
 
hsa-miR-152-3p
 
 
-0.579
 
 
212.8
 
 
 
 
ARL8A
 
 
hsa-miR-103a-3p
 
 
-0.38
 
 
79.8
 
 
 
 
ARL8B
 
 
hsa-miR-152-3p
 
 
-0.337
 
 
54.9
 
 
 
 
ARL8B
 
 
hsa-miR-148b-3p
 
 
-0.337
 
 
54.9
 
 
 
 
ARL8B
 
 
hsa-miR-320b
 
 
-0.376
 
 
54.9
 
 
 
 
ARL8B
 
 
hsa-miR-320c
 
 
-0.376
 
 
54.9
 
 
 
 
ARMC1
 
 
hsa-miR-103a-3p
 
 
-0.525
 
 
16.6
 
 
 
 
ARMC8
 
 
hsa-miR-103a-3p
 
 
-0.412
 
 
16.8
 
 
 
 
ARMCX2
 
 
hsa-miR-15b-5p
 
 
-0.344
 
 
28.0
 
 
 
 
ARMCX3
 
 
hsa-miR-425-5p
 
 
-0.325
 
 
32.9
 
 
 
 
ARPC5L
 
 
hsa-miR-326
 
 
-0.439
 
 
50.5
 
 
 
 
 
 
 kidney 
 
 
 
 
target_symbol
 
 
mature_mirna_id
 
 
score
 
 
nTPM
 
 
 
 
 
 
AAMP
 
 
hsa-miR-326
 
 
-0.722
 
 
67.9
 
 
 
 
AAR2
 
 
hsa-miR-29a-3p
 
 
-0.327
 
 
19.2
 
 
 
 
ABAT
 
 
hsa-miR-1271-5p
 
 
-0.409
 
 
124.5
 
 
 
 
ABCD3
 
 
hsa-miR-148b-3p
 
 
-0.388
 
 
55.5
 
 
 
 
ABCD3
 
 
hsa-miR-152-3p
 
 
-0.388
 
 
55.5
 
 
 
 
ABCE1
 
 
hsa-miR-29a-3p
 
 
-0.404
 
 
22.2
 
 
 
 
ABHD17C
 
 
hsa-let-7b-5p
 
 
-0.359
 
 
13.6
 
 
 
 
ABHD17C
 
 
hsa-miR-330-3p
 
 
-0.399
 
 
13.6
 
 
 
 
ABHD4
 
 
hsa-miR-29a-3p
 
 
-0.312
 
 
26.4
 
 
 
 
ABHD5
 
 
hsa-miR-132-3p
 
 
-0.323
 
 
10.4
 
 
 
 
ACP3
 
 
hsa-let-7b-5p
 
 
-0.298
 
 
26.9
 
 
 
 
ACTR2
 
 
hsa-miR-15b-5p
 
 
-0.293
 
 
79.9
 
 
 
 
ACTR3C
 
 
hsa-miR-30b-5p
 
 
-0.448
 
 
11.1
 
 
 
 
ACTR3C
 
 
hsa-miR-30d-5p
 
 
-0.456
 
 
11.1
 
 
 
 
ACVR1
 
 
hsa-miR-30b-5p
 
 
-0.29
 
 
15.0
 
 
 
 
ACVR1
 
 
hsa-miR-152-3p
 
 
-0.397
 
 
15.0
 
 
 
 
ACVR1
 
 
hsa-miR-148b-3p
 
 
-0.397
 
 
15.0
 
 
 
 
ADAM10
 
 
hsa-miR-152-3p
 
 
-0.348
 
 
16.8
 
 
 
 
ADAM10
 
 
hsa-miR-148b-3p
 
 
-0.356
 
 
16.8
 
 
 
 
ADAM9
 
 
hsa-miR-30d-5p
 
 
-0.304
 
 
32.6
 
 
 
 
ADAM9
 
 
hsa-miR-30b-5p
 
 
-0.312
 
 
32.6
 
 
 
 
ADCY3
 
 
hsa-miR-132-3p
 
 
-0.303
 
 
27.1
 
 
 
 
ADCY6
 
 
hsa-miR-1271-5p
 
 
-0.336
 
 
22.6
 
 
 
 
ADGRA2
 
 
hsa-miR-1271-5p
 
 
-0.427
 
 
11.1
 
 
 
 
ADGRF5
 
 
hsa-miR-148b-3p
 
 
-0.354
 
 
55.8
 
 
 
 
ADGRF5
 
 
hsa-miR-152-3p
 
 
-0.354
 
 
55.8
 
 
 
 
ADIPOR2
 
 
hsa-miR-150-5p
 
 
-0.287
 
 
20.9
 
 
 
 
ADK
 
 
hsa-miR-1271-5p
 
 
-0.329
 
 
33.4
 
 
 
 
ADNP
 
 
hsa-miR-328-3p
 
 
-0.371
 
 
28.2
 
 
 
 
AFG3L2
 
 
hsa-miR-181b-5p
 
 
-0.491
 
 
36.4
 
 
 
 
AFG3L2
 
 
hsa-miR-181a-5p
 
 
-0.491
 
 
36.4
 
 
 
 
AFG3L2
 
 
hsa-miR-181d-5p
 
 
-0.499
 
 
36.4
 
 
 
 
AGFG1
 
 
hsa-miR-148b-3p
 
 
-0.332
 
 
30.6
 
 
 
 
AGFG1
 
 
hsa-miR-152-3p
 
 
-0.332
 
 
30.6
 
 
 
 
AGFG1
 
 
hsa-miR-103a-3p
 
 
-0.389
 
 
30.6
 
 
 
 
AHDC1
 
 
hsa-miR-152-3p
 
 
-0.413
 
 
12.2
 
 
 
 
AHDC1
 
 
hsa-miR-148b-3p
 
 
-0.413
 
 
12.2
 
 
 
 
AIFM1
 
 
hsa-let-7b-5p
 
 
-0.314
 
 
69.0
 
 
 
 
AJUBA
 
 
hsa-miR-103a-3p
 
 
-0.448
 
 
16.5
 
 
 
 
AK1
 
 
hsa-miR-29a-3p
 
 
-0.333
 
 
37.0
 
 
 
 
AK3
 
 
hsa-miR-1271-5p
 
 
-0.57
 
 
94.6
 
 
 
 
AK4
 
 
hsa-miR-15b-5p
 
 
-0.316
 
 
169.3
 
 
 
 
AKAP1
 
 
hsa-miR-152-3p
 
 
-0.381
 
 
24.5
 
 
 
 
AKAP1
 
 
hsa-miR-148b-3p
 
 
-0.381
 
 
24.5
 
 
 
 
AKIRIN2
 
 
hsa-miR-103a-3p
 
 
-0.299
 
 
17.3
 
 
 
 
ALAD
 
 
hsa-miR-326
 
 
-0.352
 
 
68.5
 
 
 
 
ALAD
 
 
hsa-miR-103a-3p
 
 
-0.385
 
 
68.5
 
 
 
 
ALAD
 
 
hsa-miR-15b-5p
 
 
-0.446
 
 
68.5
 
 
 
 
ALCAM
 
 
hsa-miR-148b-3p
 
 
-0.339
 
 
20.8
 
 
 
 
ALCAM
 
 
hsa-miR-152-3p
 
 
-0.347
 
 
20.8
 
 
 
 
ALDH1A2
 
 
hsa-miR-23a-3p
 
 
-0.354
 
 
19.1
 
 
 
 
ALDH6A1
 
 
hsa-miR-1271-5p
 
 
-0.301
 
 
353.5
 
 
 
 
AMT
 
 
hsa-let-7b-5p
 
 
-0.463
 
 
23.7
 
 
 
 
ANKHD1
 
 
hsa-miR-23a-3p
 
 
-0.368
 
 
34.2
 
 
 
 
ANKHD1
 
 
hsa-miR-30d-5p
 
 
-0.381
 
 
34.2
 
 
 
 
ANKHD1
 
 
hsa-miR-30b-5p
 
 
-0.405
 
 
34.2
 
 
 
 
ANKIB1
 
 
hsa-miR-1271-5p
 
 
-0.344
 
 
23.9
 
 
 
 
ANKRD17
 
 
hsa-miR-30d-5p
 
 
-0.312
 
 
18.3
 
 
 
 
ANKRD17
 
 
hsa-miR-30b-5p
 
 
-0.336
 
 
18.3
 
 
 
 
ANKRD46
 
 
hsa-let-7b-5p
 
 
-0.389
 
 
30.5
 
 
 
 
ANP32A
 
 
hsa-miR-132-3p
 
 
-0.374
 
 
70.0
 
 
 
 
ANTXR2
 
 
hsa-miR-29a-3p
 
 
-0.347
 
 
16.0
 
 
 
 
ANXA11
 
 
hsa-miR-15b-5p
 
 
-0.463
 
 
127.2
 
 
 
 
AP1S1
 
 
hsa-let-7b-5p
 
 
-0.374
 
 
48.3
 
 
 
 
AP2A1
 
 
hsa-miR-15b-5p
 
 
-0.333
 
 
31.7
 
 
 
 
AP2B1
 
 
hsa-miR-1271-5p
 
 
-0.299
 
 
64.4
 
 
 
 
AP2B1
 
 
hsa-miR-15b-5p
 
 
-0.334
 
 
64.4
 
 
 
 
AP3S1
 
 
hsa-miR-30b-5p
 
 
-0.293
 
 
38.1
 
 
 
 
AP5M1
 
 
hsa-miR-1271-5p
 
 
-0.343
 
 
26.6
 
 
 
 
APOO
 
 
hsa-miR-181d-5p
 
 
-0.492
 
 
43.3
 
 
 
 
APOO
 
 
hsa-miR-181b-5p
 
 
-0.5
 
 
43.3
 
 
 
 
APOO
 
 
hsa-miR-181a-5p
 
 
-0.5
 
 
43.3
 
 
 
 
APP
 
 
hsa-miR-15b-5p
 
 
-0.298
 
 
464.8
 
 
 
 
AQP3
 
 
hsa-miR-874-3p
 
 
-0.48
 
 
151.2
 
 
 
 
ARF4
 
 
hsa-miR-30b-5p
 
 
-0.289
 
 
106.7
 
 
 
 
ARF4
 
 
hsa-miR-194-5p
 
 
-0.293
 
 
106.7
 
 
 
 
ARF4
 
 
hsa-miR-152-3p
 
 
-0.487
 
 
106.7
 
 
 
 
ARF4
 
 
hsa-miR-148b-3p
 
 
-0.487
 
 
106.7
 
 
 
 
ARF5
 
 
hsa-miR-29a-3p
 
 
-0.491
 
 
140.7
 
 
 
 
ARF6
 
 
hsa-miR-181b-5p
 
 
-0.33
 
 
29.7
 
 
 
 
ARF6
 
 
hsa-miR-181a-5p
 
 
-0.33
 
 
29.7
 
 
 
 
ARF6
 
 
hsa-miR-181d-5p
 
 
-0.338
 
 
29.7
 
 
 
 
ARFGEF1
 
 
hsa-miR-148b-3p
 
 
-0.306
 
 
21.3
 
 
 
 
ARFGEF1
 
 
hsa-miR-152-3p
 
 
-0.306
 
 
21.3
 
 
 
 
ARFIP1
 
 
hsa-miR-320b
 
 
-0.339
 
 
18.5
 
 
 
 
ARFIP1
 
 
hsa-miR-320c
 
 
-0.339
 
 
18.5
 
 
 
 
ARFIP1
 
 
hsa-miR-152-3p
 
 
-0.474
 
 
18.5
 
 
 
 
ARFIP1
 
 
hsa-miR-148b-3p
 
 
-0.474
 
 
18.5
 
 
 
 
ARG2
 
 
hsa-let-7b-5p
 
 
-0.468
 
 
66.0
 
 
 
 
ARHGAP21
 
 
hsa-miR-152-3p
 
 
-0.287
 
 
10.2
 
 
 
 
ARHGAP21
 
 
hsa-miR-148b-3p
 
 
-0.287
 
 
10.2
 
 
 
 
ARHGAP24
 
 
hsa-miR-1271-5p
 
 
-0.342
 
 
93.2
 
 
 
 
ARID1B
 
 
hsa-miR-29a-3p
 
 
-0.342
 
 
10.7
 
 
 
 
ARIH2
 
 
hsa-miR-103a-3p
 
 
-0.307
 
 
21.0
 
 
 
 
ARL2
 
 
hsa-miR-15b-5p
 
 
-0.374
 
 
162.2
 
 
 
 
ARL2
 
 
hsa-miR-15b-5p
 
 
-0.442
 
 
162.2
 
 
 
 
ARL3
 
 
hsa-miR-15b-5p
 
 
-0.507
 
 
15.3
 
 
 
 
ARL4D
 
 
hsa-miR-1271-5p
 
 
-0.392
 
 
35.1
 
 
 
 
ARL4D
 
 
hsa-let-7b-5p
 
 
-0.417
 
 
35.1
 
 
 
 
ARL6IP1
 
 
hsa-miR-328-3p
 
 
-0.494
 
 
140.2
 
 
 
 
 
 
 lung 
 
 
 
 
target_symbol
 
 
mature_mirna_id
 
 
score
 
 
nTPM
 
 
 
 
 
 
AAMP
 
 
hsa-miR-326
 
 
-0.722
 
 
46.6
 
 
 
 
AAR2
 
 
hsa-miR-29a-3p
 
 
-0.327
 
 
18.1
 
 
 
 
ABCD3
 
 
hsa-miR-148b-3p
 
 
-0.388
 
 
22.0
 
 
 
 
ABCD3
 
 
hsa-miR-152-3p
 
 
-0.388
 
 
22.0
 
 
 
 
ABCE1
 
 
hsa-miR-29a-3p
 
 
-0.404
 
 
17.4
 
 
 
 
ABHD17C
 
 
hsa-let-7b-5p
 
 
-0.359
 
 
18.6
 
 
 
 
ABHD17C
 
 
hsa-miR-330-3p
 
 
-0.399
 
 
18.6
 
 
 
 
ABHD4
 
 
hsa-miR-29a-3p
 
 
-0.312
 
 
18.5
 
 
 
 
ABHD5
 
 
hsa-miR-132-3p
 
 
-0.323
 
 
11.2
 
 
 
 
ACTR2
 
 
hsa-miR-15b-5p
 
 
-0.293
 
 
105.4
 
 
 
 
ACVR1
 
 
hsa-miR-30b-5p
 
 
-0.29
 
 
12.2
 
 
 
 
ACVR1
 
 
hsa-miR-152-3p
 
 
-0.397
 
 
12.2
 
 
 
 
ACVR1
 
 
hsa-miR-148b-3p
 
 
-0.397
 
 
12.2
 
 
 
 
ADAM10
 
 
hsa-miR-152-3p
 
 
-0.348
 
 
19.7
 
 
 
 
ADAM10
 
 
hsa-miR-148b-3p
 
 
-0.356
 
 
19.7
 
 
 
 
ADAM17
 
 
hsa-miR-152-3p
 
 
-0.301
 
 
12.5
 
 
 
 
ADAM17
 
 
hsa-miR-148b-3p
 
 
-0.301
 
 
12.5
 
 
 
 
ADAM19
 
 
hsa-miR-874-3p
 
 
-0.374
 
 
12.8
 
 
 
 
ADAM9
 
 
hsa-miR-30d-5p
 
 
-0.304
 
 
42.9
 
 
 
 
ADAM9
 
 
hsa-miR-30b-5p
 
 
-0.312
 
 
42.9
 
 
 
 
ADAMTS8
 
 
hsa-let-7b-5p
 
 
-0.571
 
 
33.2
 
 
 
 
ADAMTS9
 
 
hsa-miR-29a-3p
 
 
-0.305
 
 
13.3
 
 
 
 
ADAMTS9
 
 
hsa-miR-29a-3p
 
 
-0.485
 
 
13.3
 
 
 
 
ADCY3
 
 
hsa-miR-132-3p
 
 
-0.303
 
 
39.8
 
 
 
 
ADCY6
 
 
hsa-miR-1271-5p
 
 
-0.336
 
 
12.5
 
 
 
 
ADGRA2
 
 
hsa-miR-1271-5p
 
 
-0.427
 
 
13.3
 
 
 
 
ADGRF5
 
 
hsa-miR-148b-3p
 
 
-0.354
 
 
99.9
 
 
 
 
ADGRF5
 
 
hsa-miR-152-3p
 
 
-0.354
 
 
99.9
 
 
 
 
ADIPOR2
 
 
hsa-miR-150-5p
 
 
-0.287
 
 
42.5
 
 
 
 
ADK
 
 
hsa-miR-1271-5p
 
 
-0.329
 
 
23.0
 
 
 
 
ADNP
 
 
hsa-miR-328-3p
 
 
-0.371
 
 
25.6
 
 
 
 
ADRB1
 
 
hsa-let-7b-5p
 
 
-0.351
 
 
11.4
 
 
 
 
ADRB2
 
 
hsa-miR-15b-5p
 
 
-0.29
 
 
18.4
 
 
 
 
ADRB2
 
 
hsa-let-7b-5p
 
 
-0.635
 
 
18.4
 
 
 
 
AFG3L2
 
 
hsa-miR-181b-5p
 
 
-0.491
 
 
12.4
 
 
 
 
AFG3L2
 
 
hsa-miR-181a-5p
 
 
-0.491
 
 
12.4
 
 
 
 
AFG3L2
 
 
hsa-miR-181d-5p
 
 
-0.499
 
 
12.4
 
 
 
 
AGFG1
 
 
hsa-miR-148b-3p
 
 
-0.332
 
 
51.7
 
 
 
 
AGFG1
 
 
hsa-miR-152-3p
 
 
-0.332
 
 
51.7
 
 
 
 
AGFG1
 
 
hsa-miR-103a-3p
 
 
-0.389
 
 
51.7
 
 
 
 
AGO4
 
 
hsa-miR-1271-5p
 
 
-0.289
 
 
10.5
 
 
 
 
AGO4
 
 
hsa-miR-103a-3p
 
 
-0.32
 
 
10.5
 
 
 
 
AHDC1
 
 
hsa-miR-152-3p
 
 
-0.413
 
 
11.4
 
 
 
 
AHDC1
 
 
hsa-miR-148b-3p
 
 
-0.413
 
 
11.4
 
 
 
 
AHR
 
 
hsa-miR-1271-5p
 
 
-0.304
 
 
35.5
 
 
 
 
AHR
 
 
hsa-miR-29a-3p
 
 
-0.316
 
 
35.5
 
 
 
 
AJUBA
 
 
hsa-miR-103a-3p
 
 
-0.448
 
 
14.4
 
 
 
 
AK1
 
 
hsa-miR-29a-3p
 
 
-0.333
 
 
70.4
 
 
 
 
AK3
 
 
hsa-miR-1271-5p
 
 
-0.57
 
 
32.9
 
 
 
 
AKAP1
 
 
hsa-miR-152-3p
 
 
-0.381
 
 
18.4
 
 
 
 
AKAP1
 
 
hsa-miR-148b-3p
 
 
-0.381
 
 
18.4
 
 
 
 
AKIRIN2
 
 
hsa-miR-103a-3p
 
 
-0.299
 
 
29.6
 
 
 
 
AKT3
 
 
hsa-miR-29a-3p
 
 
-0.287
 
 
13.9
 
 
 
 
AKT3
 
 
hsa-miR-15b-5p
 
 
-0.301
 
 
13.9
 
 
 
 
ALAD
 
 
hsa-miR-326
 
 
-0.352
 
 
19.4
 
 
 
 
ALAD
 
 
hsa-miR-103a-3p
 
 
-0.385
 
 
19.4
 
 
 
 
ALAD
 
 
hsa-miR-15b-5p
 
 
-0.446
 
 
19.4
 
 
 
 
ALCAM
 
 
hsa-miR-148b-3p
 
 
-0.339
 
 
69.9
 
 
 
 
ALCAM
 
 
hsa-miR-152-3p
 
 
-0.347
 
 
69.9
 
 
 
 
ALOX15B
 
 
hsa-miR-326
 
 
-0.417
 
 
23.7
 
 
 
 
AMOTL1
 
 
hsa-miR-15b-5p
 
 
-0.319
 
 
14.9
 
 
 
 
ANKHD1
 
 
hsa-miR-23a-3p
 
 
-0.368
 
 
38.2
 
 
 
 
ANKHD1
 
 
hsa-miR-30d-5p
 
 
-0.381
 
 
38.2
 
 
 
 
ANKHD1
 
 
hsa-miR-30b-5p
 
 
-0.405
 
 
38.2
 
 
 
 
ANKIB1
 
 
hsa-miR-1271-5p
 
 
-0.344
 
 
10.4
 
 
 
 
ANKRD17
 
 
hsa-miR-30d-5p
 
 
-0.312
 
 
13.4
 
 
 
 
ANKRD17
 
 
hsa-miR-30b-5p
 
 
-0.336
 
 
13.4
 
 
 
 
ANKRD29
 
 
hsa-miR-132-3p
 
 
-0.47
 
 
29.5
 
 
 
 
ANKRD46
 
 
hsa-let-7b-5p
 
 
-0.389
 
 
10.1
 
 
 
 
ANP32A
 
 
hsa-miR-132-3p
 
 
-0.374
 
 
66.9
 
 
 
 
ANTXR2
 
 
hsa-miR-29a-3p
 
 
-0.347
 
 
23.3
 
 
 
 
ANXA11
 
 
hsa-miR-15b-5p
 
 
-0.463
 
 
103.2
 
 
 
 
AP1S1
 
 
hsa-let-7b-5p
 
 
-0.374
 
 
15.2
 
 
 
 
AP2A1
 
 
hsa-miR-15b-5p
 
 
-0.333
 
 
35.7
 
 
 
 
AP2B1
 
 
hsa-miR-1271-5p
 
 
-0.299
 
 
49.6
 
 
 
 
AP2B1
 
 
hsa-miR-15b-5p
 
 
-0.334
 
 
49.6
 
 
 
 
AP3S1
 
 
hsa-miR-30b-5p
 
 
-0.293
 
 
40.1
 
 
 
 
AP5M1
 
 
hsa-miR-1271-5p
 
 
-0.343
 
 
11.1
 
 
 
 
APLN
 
 
hsa-miR-15b-5p
 
 
-0.395
 
 
12.7
 
 
 
 
APP
 
 
hsa-miR-15b-5p
 
 
-0.298
 
 
255.6
 
 
 
 
AQP3
 
 
hsa-miR-874-3p
 
 
-0.48
 
 
107.5
 
 
 
 
ARF4
 
 
hsa-miR-30b-5p
 
 
-0.289
 
 
116.3
 
 
 
 
ARF4
 
 
hsa-miR-194-5p
 
 
-0.293
 
 
116.3
 
 
 
 
ARF4
 
 
hsa-miR-152-3p
 
 
-0.487
 
 
116.3
 
 
 
 
ARF4
 
 
hsa-miR-148b-3p
 
 
-0.487
 
 
116.3
 
 
 
 
ARF5
 
 
hsa-miR-29a-3p
 
 
-0.491
 
 
100.1
 
 
 
 
ARF6
 
 
hsa-miR-181b-5p
 
 
-0.33
 
 
29.7
 
 
 
 
ARF6
 
 
hsa-miR-181a-5p
 
 
-0.33
 
 
29.7
 
 
 
 
ARF6
 
 
hsa-miR-181d-5p
 
 
-0.338
 
 
29.7
 
 
 
 
ARFGEF1
 
 
hsa-miR-148b-3p
 
 
-0.306
 
 
13.0
 
 
 
 
ARFGEF1
 
 
hsa-miR-152-3p
 
 
-0.306
 
 
13.0
 
 
 
 
ARFIP1
 
 
hsa-miR-320b
 
 
-0.339
 
 
15.8
 
 
 
 
ARFIP1
 
 
hsa-miR-320c
 
 
-0.339
 
 
15.8
 
 
 
 
ARFIP1
 
 
hsa-miR-152-3p
 
 
-0.474
 
 
15.8
 
 
 
 
ARFIP1
 
 
hsa-miR-148b-3p
 
 
-0.474
 
 
15.8
 
 
 
 
ARHGAP21
 
 
hsa-miR-152-3p
 
 
-0.287
 
 
20.4
 
 
 
 
ARHGAP21
 
 
hsa-miR-148b-3p
 
 
-0.287
 
 
20.4
 
 
 
 
ARHGAP24
 
 
hsa-miR-1271-5p
 
 
-0.342
 
 
14.8
 
 
 
 
ARID1B
 
 
hsa-miR-29a-3p
 
 
-0.342
 
 
13.4
 
 
 
 
ARIH2
 
 
hsa-miR-103a-3p
 
 
-0.307
 
 
19.0
 
 
 
 
 
 
 lymph node 
 
 
 
 
target_symbol
 
 
mature_mirna_id
 
 
score
 
 
nTPM
 
 
 
 
 
 
AAMP
 
 
hsa-miR-326
 
 
-0.722
 
 
44.7
 
 
 
 
AAR2
 
 
hsa-miR-29a-3p
 
 
-0.327
 
 
16.4
 
 
 
 
ABCB10
 
 
hsa-miR-152-3p
 
 
-0.291
 
 
10.3
 
 
 
 
ABCB10
 
 
hsa-miR-148b-3p
 
 
-0.291
 
 
10.3
 
 
 
 
ABCB7
 
 
hsa-miR-148b-3p
 
 
-0.515
 
 
11.2
 
 
 
 
ABCB7
 
 
hsa-miR-152-3p
 
 
-0.515
 
 
11.2
 
 
 
 
ABCD3
 
 
hsa-miR-148b-3p
 
 
-0.388
 
 
12.4
 
 
 
 
ABCD3
 
 
hsa-miR-152-3p
 
 
-0.388
 
 
12.4
 
 
 
 
ABCE1
 
 
hsa-miR-29a-3p
 
 
-0.404
 
 
37.8
 
 
 
 
ACTR2
 
 
hsa-miR-15b-5p
 
 
-0.293
 
 
229.6
 
 
 
 
ADA
 
 
hsa-miR-29a-3p
 
 
-0.376
 
 
27.8
 
 
 
 
ADAM10
 
 
hsa-miR-152-3p
 
 
-0.348
 
 
20.8
 
 
 
 
ADAM10
 
 
hsa-miR-148b-3p
 
 
-0.356
 
 
20.8
 
 
 
 
ADAM19
 
 
hsa-miR-874-3p
 
 
-0.374
 
 
27.1
 
 
 
 
ADCY3
 
 
hsa-miR-132-3p
 
 
-0.303
 
 
17.7
 
 
 
 
ADK
 
 
hsa-miR-1271-5p
 
 
-0.329
 
 
21.3
 
 
 
 
ADNP
 
 
hsa-miR-328-3p
 
 
-0.371
 
 
27.0
 
 
 
 
ADRA2A
 
 
hsa-miR-23a-3p
 
 
-0.371
 
 
10.5
 
 
 
 
AFG3L2
 
 
hsa-miR-181b-5p
 
 
-0.491
 
 
12.0
 
 
 
 
AFG3L2
 
 
hsa-miR-181a-5p
 
 
-0.491
 
 
12.0
 
 
 
 
AFG3L2
 
 
hsa-miR-181d-5p
 
 
-0.499
 
 
12.0
 
 
 
 
AGFG1
 
 
hsa-miR-148b-3p
 
 
-0.332
 
 
44.0
 
 
 
 
AGFG1
 
 
hsa-miR-152-3p
 
 
-0.332
 
 
44.0
 
 
 
 
AGFG1
 
 
hsa-miR-103a-3p
 
 
-0.389
 
 
44.0
 
 
 
 
AHR
 
 
hsa-miR-1271-5p
 
 
-0.304
 
 
14.4
 
 
 
 
AHR
 
 
hsa-miR-29a-3p
 
 
-0.316
 
 
14.4
 
 
 
 
AIFM1
 
 
hsa-let-7b-5p
 
 
-0.314
 
 
15.1
 
 
 
 
AK3
 
 
hsa-miR-1271-5p
 
 
-0.57
 
 
27.7
 
 
 
 
AKAP1
 
 
hsa-miR-152-3p
 
 
-0.381
 
 
10.0
 
 
 
 
AKAP1
 
 
hsa-miR-148b-3p
 
 
-0.381
 
 
10.0
 
 
 
 
AKIRIN2
 
 
hsa-miR-103a-3p
 
 
-0.299
 
 
26.6
 
 
 
 
ALAD
 
 
hsa-miR-326
 
 
-0.352
 
 
14.6
 
 
 
 
ALAD
 
 
hsa-miR-103a-3p
 
 
-0.385
 
 
14.6
 
 
 
 
ALAD
 
 
hsa-miR-15b-5p
 
 
-0.446
 
 
14.6
 
 
 
 
ANKHD1
 
 
hsa-miR-23a-3p
 
 
-0.368
 
 
38.6
 
 
 
 
ANKHD1
 
 
hsa-miR-30d-5p
 
 
-0.381
 
 
38.6
 
 
 
 
ANKHD1
 
 
hsa-miR-30b-5p
 
 
-0.405
 
 
38.6
 
 
 
 
ANKIB1
 
 
hsa-miR-1271-5p
 
 
-0.344
 
 
12.4
 
 
 
 
ANKRD17
 
 
hsa-miR-30d-5p
 
 
-0.312
 
 
14.1
 
 
 
 
ANKRD17
 
 
hsa-miR-30b-5p
 
 
-0.336
 
 
14.1
 
 
 
 
ANKRD27
 
 
hsa-miR-1271-5p
 
 
-0.648
 
 
10.4
 
 
 
 
ANKRD29
 
 
hsa-miR-132-3p
 
 
-0.47
 
 
18.8
 
 
 
 
ANP32A
 
 
hsa-miR-132-3p
 
 
-0.374
 
 
145.8
 
 
 
 
ANTXR2
 
 
hsa-miR-29a-3p
 
 
-0.347
 
 
15.2
 
 
 
 
ANXA11
 
 
hsa-miR-15b-5p
 
 
-0.463
 
 
76.0
 
 
 
 
AP1S1
 
 
hsa-let-7b-5p
 
 
-0.374
 
 
11.1
 
 
 
 
AP2A1
 
 
hsa-miR-15b-5p
 
 
-0.333
 
 
18.3
 
 
 
 
AP2B1
 
 
hsa-miR-1271-5p
 
 
-0.299
 
 
36.1
 
 
 
 
AP2B1
 
 
hsa-miR-15b-5p
 
 
-0.334
 
 
36.1
 
 
 
 
AP3S1
 
 
hsa-miR-30b-5p
 
 
-0.293
 
 
60.4
 
 
 
 
AP4B1
 
 
hsa-miR-378a-3p
 
 
-0.62
 
 
13.4
 
 
 
 
AP4B1
 
 
hsa-miR-378d
 
 
-0.62
 
 
13.4
 
 
 
 
AP5M1
 
 
hsa-miR-1271-5p
 
 
-0.343
 
 
15.9
 
 
 
 
APOO
 
 
hsa-miR-181d-5p
 
 
-0.492
 
 
10.9
 
 
 
 
APOO
 
 
hsa-miR-181b-5p
 
 
-0.5
 
 
10.9
 
 
 
 
APOO
 
 
hsa-miR-181a-5p
 
 
-0.5
 
 
10.9
 
 
 
 
APP
 
 
hsa-miR-15b-5p
 
 
-0.298
 
 
68.3
 
 
 
 
AQP3
 
 
hsa-miR-874-3p
 
 
-0.48
 
 
15.1
 
 
 
 
ARF4
 
 
hsa-miR-30b-5p
 
 
-0.289
 
 
69.1
 
 
 
 
ARF4
 
 
hsa-miR-194-5p
 
 
-0.293
 
 
69.1
 
 
 
 
ARF4
 
 
hsa-miR-152-3p
 
 
-0.487
 
 
69.1
 
 
 
 
ARF4
 
 
hsa-miR-148b-3p
 
 
-0.487
 
 
69.1
 
 
 
 
ARF5
 
 
hsa-miR-29a-3p
 
 
-0.491
 
 
96.1
 
 
 
 
ARF6
 
 
hsa-miR-181b-5p
 
 
-0.33
 
 
57.9
 
 
 
 
ARF6
 
 
hsa-miR-181a-5p
 
 
-0.33
 
 
57.9
 
 
 
 
ARF6
 
 
hsa-miR-181d-5p
 
 
-0.338
 
 
57.9
 
 
 
 
ARFGEF1
 
 
hsa-miR-148b-3p
 
 
-0.306
 
 
14.4
 
 
 
 
ARFGEF1
 
 
hsa-miR-152-3p
 
 
-0.306
 
 
14.4
 
 
 
 
ARFIP1
 
 
hsa-miR-320b
 
 
-0.339
 
 
14.1
 
 
 
 
ARFIP1
 
 
hsa-miR-320c
 
 
-0.339
 
 
14.1
 
 
 
 
ARFIP1
 
 
hsa-miR-152-3p
 
 
-0.474
 
 
14.1
 
 
 
 
ARFIP1
 
 
hsa-miR-148b-3p
 
 
-0.474
 
 
14.1
 
 
 
 
ARID1B
 
 
hsa-miR-29a-3p
 
 
-0.342
 
 
16.8
 
 
 
 
ARIH2
 
 
hsa-miR-103a-3p
 
 
-0.307
 
 
19.3
 
 
 
 
ARL2
 
 
hsa-miR-15b-5p
 
 
-0.374
 
 
35.3
 
 
 
 
ARL2
 
 
hsa-miR-15b-5p
 
 
-0.442
 
 
35.3
 
 
 
 
ARL4A
 
 
hsa-miR-29a-3p
 
 
-0.386
 
 
17.4
 
 
 
 
ARL4A
 
 
hsa-miR-30d-5p
 
 
-0.432
 
 
17.4
 
 
 
 
ARL4A
 
 
hsa-miR-30b-5p
 
 
-0.448
 
 
17.4
 
 
 
 
ARL6IP1
 
 
hsa-miR-328-3p
 
 
-0.494
 
 
124.0
 
 
 
 
ARL6IP1
 
 
hsa-miR-148b-3p
 
 
-0.579
 
 
124.0
 
 
 
 
ARL6IP1
 
 
hsa-miR-152-3p
 
 
-0.579
 
 
124.0
 
 
 
 
ARL6IP6
 
 
hsa-let-7b-5p
 
 
-0.293
 
 
17.4
 
 
 
 
ARL8A
 
 
hsa-miR-103a-3p
 
 
-0.38
 
 
19.9
 
 
 
 
ARL8B
 
 
hsa-miR-152-3p
 
 
-0.337
 
 
36.2
 
 
 
 
ARL8B
 
 
hsa-miR-148b-3p
 
 
-0.337
 
 
36.2
 
 
 
 
ARL8B
 
 
hsa-miR-320b
 
 
-0.376
 
 
36.2
 
 
 
 
ARL8B
 
 
hsa-miR-320c
 
 
-0.376
 
 
36.2
 
 
 
 
ARMC1
 
 
hsa-miR-103a-3p
 
 
-0.525
 
 
18.0
 
 
 
 
ARMC8
 
 
hsa-miR-103a-3p
 
 
-0.412
 
 
19.8
 
 
 
 
ARMCX3
 
 
hsa-miR-425-5p
 
 
-0.325
 
 
20.4
 
 
 
 
ARMT1
 
 
hsa-let-7b-5p
 
 
-0.291
 
 
19.7
 
 
 
 
ARNT
 
 
hsa-miR-103a-3p
 
 
-0.422
 
 
16.3
 
 
 
 
ARPC5L
 
 
hsa-miR-326
 
 
-0.439
 
 
41.7
 
 
 
 
ARPC5L
 
 
hsa-miR-1271-5p
 
 
-0.736
 
 
41.7
 
 
 
 
ARPP19
 
 
hsa-miR-1271-5p
 
 
-0.313
 
 
45.1
 
 
 
 
ARPP19
 
 
hsa-miR-320b
 
 
-0.325
 
 
45.1
 
 
 
 
ARPP19
 
 
hsa-miR-320c
 
 
-0.325
 
 
45.1
 
 
 
 
ARPP19
 
 
hsa-miR-15b-5p
 
 
-0.346
 
 
45.1
 
 
 
 
ARPP19
 
 
hsa-miR-29a-3p
 
 
-0.371
 
 
45.1
 
 
 
 
 
 
 midbrain 
 
 
 
 
target_symbol
 
 
mature_mirna_id
 
 
score
 
 
nTPM
 
 
 
 
 
 
AAMP
 
 
hsa-miR-326
 
 
-0.722
 
 
57.7
 
 
 
 
AAR2
 
 
hsa-miR-29a-3p
 
 
-0.327
 
 
11.2
 
 
 
 
ABAT
 
 
hsa-miR-1271-5p
 
 
-0.409
 
 
90.5
 
 
 
 
ABCA2
 
 
hsa-miR-1271-5p
 
 
-0.326
 
 
82.4
 
 
 
 
ABCB9
 
 
hsa-let-7b-5p
 
 
-0.422
 
 
10.6
 
 
 
 
ABCD3
 
 
hsa-miR-148b-3p
 
 
-0.388
 
 
17.1
 
 
 
 
ABCD3
 
 
hsa-miR-152-3p
 
 
-0.388
 
 
17.1
 
 
 
 
ABCE1
 
 
hsa-miR-29a-3p
 
 
-0.404
 
 
13.7
 
 
 
 
ABHD17C
 
 
hsa-let-7b-5p
 
 
-0.359
 
 
11.2
 
 
 
 
ABHD17C
 
 
hsa-miR-330-3p
 
 
-0.399
 
 
11.2
 
 
 
 
ABHD4
 
 
hsa-miR-29a-3p
 
 
-0.312
 
 
18.3
 
 
 
 
ACHE
 
 
hsa-miR-194-5p
 
 
-0.395
 
 
47.7
 
 
 
 
ACSL6
 
 
hsa-let-7b-5p
 
 
-0.336
 
 
11.1
 
 
 
 
ACTN2
 
 
hsa-miR-23a-3p
 
 
-0.294
 
 
12.2
 
 
 
 
ACTN2
 
 
hsa-miR-29a-3p
 
 
-0.311
 
 
12.2
 
 
 
 
ACTR2
 
 
hsa-miR-15b-5p
 
 
-0.293
 
 
53.1
 
 
 
 
ADAM10
 
 
hsa-miR-152-3p
 
 
-0.348
 
 
11.1
 
 
 
 
ADAM10
 
 
hsa-miR-148b-3p
 
 
-0.356
 
 
11.1
 
 
 
 
ADCY2
 
 
hsa-miR-152-3p
 
 
-0.299
 
 
20.4
 
 
 
 
ADCY2
 
 
hsa-miR-148b-3p
 
 
-0.299
 
 
20.4
 
 
 
 
ADCY3
 
 
hsa-miR-132-3p
 
 
-0.303
 
 
29.4
 
 
 
 
ADGRA3
 
 
hsa-miR-30d-5p
 
 
-0.296
 
 
13.9
 
 
 
 
ADGRA3
 
 
hsa-miR-30b-5p
 
 
-0.304
 
 
13.9
 
 
 
 
ADGRF5
 
 
hsa-miR-148b-3p
 
 
-0.354
 
 
13.0
 
 
 
 
ADGRF5
 
 
hsa-miR-152-3p
 
 
-0.354
 
 
13.0
 
 
 
 
ADIPOR2
 
 
hsa-miR-150-5p
 
 
-0.287
 
 
76.2
 
 
 
 
ADK
 
 
hsa-miR-1271-5p
 
 
-0.329
 
 
17.6
 
 
 
 
ADNP
 
 
hsa-miR-328-3p
 
 
-0.371
 
 
21.5
 
 
 
 
AGFG1
 
 
hsa-miR-148b-3p
 
 
-0.332
 
 
32.4
 
 
 
 
AGFG1
 
 
hsa-miR-152-3p
 
 
-0.332
 
 
32.4
 
 
 
 
AGFG1
 
 
hsa-miR-103a-3p
 
 
-0.389
 
 
32.4
 
 
 
 
AGPAT4
 
 
hsa-miR-29a-3p
 
 
-0.376
 
 
22.2
 
 
 
 
AIFM1
 
 
hsa-let-7b-5p
 
 
-0.314
 
 
12.7
 
 
 
 
AK1
 
 
hsa-miR-29a-3p
 
 
-0.333
 
 
97.2
 
 
 
 
AK3
 
 
hsa-miR-1271-5p
 
 
-0.57
 
 
40.8
 
 
 
 
AK4
 
 
hsa-miR-15b-5p
 
 
-0.316
 
 
11.3
 
 
 
 
AKAP1
 
 
hsa-miR-152-3p
 
 
-0.381
 
 
19.0
 
 
 
 
AKAP1
 
 
hsa-miR-148b-3p
 
 
-0.381
 
 
19.0
 
 
 
 
AKAP6
 
 
hsa-let-7b-5p
 
 
-0.307
 
 
17.4
 
 
 
 
AKIRIN2
 
 
hsa-miR-103a-3p
 
 
-0.299
 
 
28.8
 
 
 
 
AKT3
 
 
hsa-miR-29a-3p
 
 
-0.287
 
 
16.3
 
 
 
 
AKT3
 
 
hsa-miR-15b-5p
 
 
-0.301
 
 
16.3
 
 
 
 
ALAD
 
 
hsa-miR-326
 
 
-0.352
 
 
105.2
 
 
 
 
ALAD
 
 
hsa-miR-103a-3p
 
 
-0.385
 
 
105.2
 
 
 
 
ALAD
 
 
hsa-miR-15b-5p
 
 
-0.446
 
 
105.2
 
 
 
 
ALCAM
 
 
hsa-miR-148b-3p
 
 
-0.339
 
 
38.7
 
 
 
 
ALCAM
 
 
hsa-miR-152-3p
 
 
-0.347
 
 
38.7
 
 
 
 
ALDH6A1
 
 
hsa-miR-1271-5p
 
 
-0.301
 
 
45.2
 
 
 
 
AMOT
 
 
hsa-miR-29a-3p
 
 
-0.304
 
 
16.8
 
 
 
 
ANK1
 
 
hsa-miR-103a-3p
 
 
-0.321
 
 
14.7
 
 
 
 
ANKHD1
 
 
hsa-miR-23a-3p
 
 
-0.368
 
 
20.5
 
 
 
 
ANKHD1
 
 
hsa-miR-30d-5p
 
 
-0.381
 
 
20.5
 
 
 
 
ANKHD1
 
 
hsa-miR-30b-5p
 
 
-0.405
 
 
20.5
 
 
 
 
ANKIB1
 
 
hsa-miR-1271-5p
 
 
-0.344
 
 
19.4
 
 
 
 
ANKRD29
 
 
hsa-miR-132-3p
 
 
-0.47
 
 
11.5
 
 
 
 
ANKRD46
 
 
hsa-let-7b-5p
 
 
-0.389
 
 
24.0
 
 
 
 
ANLN
 
 
hsa-miR-15b-5p
 
 
-0.334
 
 
72.6
 
 
 
 
ANP32A
 
 
hsa-miR-132-3p
 
 
-0.374
 
 
54.3
 
 
 
 
ANXA11
 
 
hsa-miR-15b-5p
 
 
-0.463
 
 
20.2
 
 
 
 
AP1S1
 
 
hsa-let-7b-5p
 
 
-0.374
 
 
35.4
 
 
 
 
AP2A1
 
 
hsa-miR-15b-5p
 
 
-0.333
 
 
39.4
 
 
 
 
AP2B1
 
 
hsa-miR-1271-5p
 
 
-0.299
 
 
69.9
 
 
 
 
AP2B1
 
 
hsa-miR-15b-5p
 
 
-0.334
 
 
69.9
 
 
 
 
AP3S1
 
 
hsa-miR-30b-5p
 
 
-0.293
 
 
69.9
 
 
 
 
APC
 
 
hsa-miR-29a-3p
 
 
-0.413
 
 
21.2
 
 
 
 
APLN
 
 
hsa-miR-15b-5p
 
 
-0.395
 
 
24.6
 
 
 
 
APOO
 
 
hsa-miR-181d-5p
 
 
-0.492
 
 
44.2
 
 
 
 
APOO
 
 
hsa-miR-181b-5p
 
 
-0.5
 
 
44.2
 
 
 
 
APOO
 
 
hsa-miR-181a-5p
 
 
-0.5
 
 
44.2
 
 
 
 
APP
 
 
hsa-miR-15b-5p
 
 
-0.298
 
 
448.8
 
 
 
 
ARF4
 
 
hsa-miR-30b-5p
 
 
-0.289
 
 
62.8
 
 
 
 
ARF4
 
 
hsa-miR-194-5p
 
 
-0.293
 
 
62.8
 
 
 
 
ARF4
 
 
hsa-miR-152-3p
 
 
-0.487
 
 
62.8
 
 
 
 
ARF4
 
 
hsa-miR-148b-3p
 
 
-0.487
 
 
62.8
 
 
 
 
ARF5
 
 
hsa-miR-29a-3p
 
 
-0.491
 
 
120.0
 
 
 
 
ARF6
 
 
hsa-miR-181b-5p
 
 
-0.33
 
 
12.0
 
 
 
 
ARF6
 
 
hsa-miR-181a-5p
 
 
-0.33
 
 
12.0
 
 
 
 
ARF6
 
 
hsa-miR-181d-5p
 
 
-0.338
 
 
12.0
 
 
 
 
ARHGAP21
 
 
hsa-miR-152-3p
 
 
-0.287
 
 
53.0
 
 
 
 
ARHGAP21
 
 
hsa-miR-148b-3p
 
 
-0.287
 
 
53.0
 
 
 
 
ARIH2
 
 
hsa-miR-103a-3p
 
 
-0.307
 
 
21.8
 
 
 
 
ARL2
 
 
hsa-miR-15b-5p
 
 
-0.374
 
 
173.0
 
 
 
 
ARL2
 
 
hsa-miR-15b-5p
 
 
-0.442
 
 
173.0
 
 
 
 
ARL3
 
 
hsa-miR-15b-5p
 
 
-0.507
 
 
22.1
 
 
 
 
ARL6IP1
 
 
hsa-miR-328-3p
 
 
-0.494
 
 
198.8
 
 
 
 
ARL6IP1
 
 
hsa-miR-148b-3p
 
 
-0.579
 
 
198.8
 
 
 
 
ARL6IP1
 
 
hsa-miR-152-3p
 
 
-0.579
 
 
198.8
 
 
 
 
ARL8A
 
 
hsa-miR-103a-3p
 
 
-0.38
 
 
98.8
 
 
 
 
ARL8B
 
 
hsa-miR-152-3p
 
 
-0.337
 
 
52.4
 
 
 
 
ARL8B
 
 
hsa-miR-148b-3p
 
 
-0.337
 
 
52.4
 
 
 
 
ARL8B
 
 
hsa-miR-320b
 
 
-0.376
 
 
52.4
 
 
 
 
ARL8B
 
 
hsa-miR-320c
 
 
-0.376
 
 
52.4
 
 
 
 
ARMC1
 
 
hsa-miR-103a-3p
 
 
-0.525
 
 
17.2
 
 
 
 
ARMC8
 
 
hsa-miR-103a-3p
 
 
-0.412
 
 
14.1
 
 
 
 
ARMCX2
 
 
hsa-miR-15b-5p
 
 
-0.344
 
 
18.6
 
 
 
 
ARMCX3
 
 
hsa-miR-425-5p
 
 
-0.325
 
 
35.1
 
 
 
 
ARPC5L
 
 
hsa-miR-326
 
 
-0.439
 
 
49.7
 
 
 
 
ARPC5L
 
 
hsa-miR-1271-5p
 
 
-0.736
 
 
49.7
 
 
 
 
ARPP19
 
 
hsa-miR-1271-5p
 
 
-0.313
 
 
144.2
 
 
 
 
ARPP19
 
 
hsa-miR-320b
 
 
-0.325
 
 
144.2
 
 
 
 
 
 
 ovary 
 
 
 
 
target_symbol
 
 
mature_mirna_id
 
 
score
 
 
nTPM
 
 
 
 
 
 
AAMP
 
 
hsa-miR-326
 
 
-0.722
 
 
79.7
 
 
 
 
AAR2
 
 
hsa-miR-29a-3p
 
 
-0.327
 
 
22.7
 
 
 
 
ABCB6
 
 
hsa-miR-29a-3p
 
 
-0.48
 
 
12.1
 
 
 
 
ABCB7
 
 
hsa-miR-148b-3p
 
 
-0.515
 
 
11.9
 
 
 
 
ABCB7
 
 
hsa-miR-152-3p
 
 
-0.515
 
 
11.9
 
 
 
 
ABCD3
 
 
hsa-miR-148b-3p
 
 
-0.388
 
 
11.0
 
 
 
 
ABCD3
 
 
hsa-miR-152-3p
 
 
-0.388
 
 
11.0
 
 
 
 
ABCE1
 
 
hsa-miR-29a-3p
 
 
-0.404
 
 
25.9
 
 
 
 
ABHD4
 
 
hsa-miR-29a-3p
 
 
-0.312
 
 
17.9
 
 
 
 
ACTR2
 
 
hsa-miR-15b-5p
 
 
-0.293
 
 
47.8
 
 
 
 
ACVR1
 
 
hsa-miR-30b-5p
 
 
-0.29
 
 
17.7
 
 
 
 
ACVR1
 
 
hsa-miR-152-3p
 
 
-0.397
 
 
17.7
 
 
 
 
ACVR1
 
 
hsa-miR-148b-3p
 
 
-0.397
 
 
17.7
 
 
 
 
ADAM10
 
 
hsa-miR-152-3p
 
 
-0.348
 
 
13.1
 
 
 
 
ADAM10
 
 
hsa-miR-148b-3p
 
 
-0.356
 
 
13.1
 
 
 
 
ADAM9
 
 
hsa-miR-30d-5p
 
 
-0.304
 
 
16.9
 
 
 
 
ADAM9
 
 
hsa-miR-30b-5p
 
 
-0.312
 
 
16.9
 
 
 
 
ADCY3
 
 
hsa-miR-132-3p
 
 
-0.303
 
 
67.7
 
 
 
 
ADCY6
 
 
hsa-miR-1271-5p
 
 
-0.336
 
 
20.8
 
 
 
 
ADGRA2
 
 
hsa-miR-1271-5p
 
 
-0.427
 
 
29.3
 
 
 
 
ADIPOR2
 
 
hsa-miR-150-5p
 
 
-0.287
 
 
24.2
 
 
 
 
ADK
 
 
hsa-miR-1271-5p
 
 
-0.329
 
 
10.8
 
 
 
 
ADNP
 
 
hsa-miR-328-3p
 
 
-0.371
 
 
36.2
 
 
 
 
ADRA2A
 
 
hsa-miR-23a-3p
 
 
-0.371
 
 
17.5
 
 
 
 
ADRB3
 
 
hsa-let-7b-5p
 
 
-0.415
 
 
12.8
 
 
 
 
AFG3L2
 
 
hsa-miR-181b-5p
 
 
-0.491
 
 
12.5
 
 
 
 
AFG3L2
 
 
hsa-miR-181a-5p
 
 
-0.491
 
 
12.5
 
 
 
 
AFG3L2
 
 
hsa-miR-181d-5p
 
 
-0.499
 
 
12.5
 
 
 
 
AGFG1
 
 
hsa-miR-148b-3p
 
 
-0.332
 
 
22.9
 
 
 
 
AGFG1
 
 
hsa-miR-152-3p
 
 
-0.332
 
 
22.9
 
 
 
 
AGFG1
 
 
hsa-miR-103a-3p
 
 
-0.389
 
 
22.9
 
 
 
 
AHCTF1
 
 
hsa-let-7b-5p
 
 
-0.374
 
 
13.1
 
 
 
 
AHDC1
 
 
hsa-miR-152-3p
 
 
-0.413
 
 
21.5
 
 
 
 
AHDC1
 
 
hsa-miR-148b-3p
 
 
-0.413
 
 
21.5
 
 
 
 
AJUBA
 
 
hsa-miR-103a-3p
 
 
-0.448
 
 
11.2
 
 
 
 
AK1
 
 
hsa-miR-29a-3p
 
 
-0.333
 
 
43.4
 
 
 
 
AK3
 
 
hsa-miR-1271-5p
 
 
-0.57
 
 
49.7
 
 
 
 
AKAP1
 
 
hsa-miR-152-3p
 
 
-0.381
 
 
18.8
 
 
 
 
AKAP1
 
 
hsa-miR-148b-3p
 
 
-0.381
 
 
18.8
 
 
 
 
AKIRIN2
 
 
hsa-miR-103a-3p
 
 
-0.299
 
 
22.8
 
 
 
 
AKT3
 
 
hsa-miR-29a-3p
 
 
-0.287
 
 
21.9
 
 
 
 
AKT3
 
 
hsa-miR-15b-5p
 
 
-0.301
 
 
21.9
 
 
 
 
ALAD
 
 
hsa-miR-326
 
 
-0.352
 
 
34.6
 
 
 
 
ALAD
 
 
hsa-miR-103a-3p
 
 
-0.385
 
 
34.6
 
 
 
 
ALAD
 
 
hsa-miR-15b-5p
 
 
-0.446
 
 
34.6
 
 
 
 
ALCAM
 
 
hsa-miR-148b-3p
 
 
-0.339
 
 
33.1
 
 
 
 
ALCAM
 
 
hsa-miR-152-3p
 
 
-0.347
 
 
33.1
 
 
 
 
ALDH1A2
 
 
hsa-miR-23a-3p
 
 
-0.354
 
 
13.5
 
 
 
 
ALDH6A1
 
 
hsa-miR-1271-5p
 
 
-0.301
 
 
20.8
 
 
 
 
AMT
 
 
hsa-let-7b-5p
 
 
-0.463
 
 
16.7
 
 
 
 
ANKHD1
 
 
hsa-miR-23a-3p
 
 
-0.368
 
 
49.9
 
 
 
 
ANKHD1
 
 
hsa-miR-30d-5p
 
 
-0.381
 
 
49.9
 
 
 
 
ANKHD1
 
 
hsa-miR-30b-5p
 
 
-0.405
 
 
49.9
 
 
 
 
ANKIB1
 
 
hsa-miR-1271-5p
 
 
-0.344
 
 
12.5
 
 
 
 
ANKRA2
 
 
hsa-miR-30d-5p
 
 
-0.343
 
 
11.1
 
 
 
 
ANKRA2
 
 
hsa-miR-30b-5p
 
 
-0.354
 
 
11.1
 
 
 
 
ANKRA2
 
 
hsa-miR-30d-5p
 
 
-0.448
 
 
11.1
 
 
 
 
ANKRA2
 
 
hsa-miR-30b-5p
 
 
-0.472
 
 
11.1
 
 
 
 
ANKRD17
 
 
hsa-miR-30d-5p
 
 
-0.312
 
 
15.3
 
 
 
 
ANKRD17
 
 
hsa-miR-30b-5p
 
 
-0.336
 
 
15.3
 
 
 
 
ANKRD29
 
 
hsa-miR-132-3p
 
 
-0.47
 
 
15.0
 
 
 
 
ANKRD46
 
 
hsa-let-7b-5p
 
 
-0.389
 
 
19.3
 
 
 
 
ANKRD6
 
 
hsa-miR-331-3p
 
 
-0.369
 
 
13.2
 
 
 
 
ANP32A
 
 
hsa-miR-132-3p
 
 
-0.374
 
 
51.4
 
 
 
 
ANTXR2
 
 
hsa-miR-29a-3p
 
 
-0.347
 
 
66.3
 
 
 
 
ANXA11
 
 
hsa-miR-15b-5p
 
 
-0.463
 
 
68.2
 
 
 
 
AP1S1
 
 
hsa-let-7b-5p
 
 
-0.374
 
 
22.2
 
 
 
 
AP2A1
 
 
hsa-miR-15b-5p
 
 
-0.333
 
 
54.6
 
 
 
 
AP2B1
 
 
hsa-miR-1271-5p
 
 
-0.299
 
 
50.8
 
 
 
 
AP2B1
 
 
hsa-miR-15b-5p
 
 
-0.334
 
 
50.8
 
 
 
 
AP3S1
 
 
hsa-miR-30b-5p
 
 
-0.293
 
 
82.7
 
 
 
 
AP4B1
 
 
hsa-miR-378a-3p
 
 
-0.62
 
 
13.3
 
 
 
 
AP4B1
 
 
hsa-miR-378d
 
 
-0.62
 
 
13.3
 
 
 
 
APOO
 
 
hsa-miR-181d-5p
 
 
-0.492
 
 
11.0
 
 
 
 
APOO
 
 
hsa-miR-181b-5p
 
 
-0.5
 
 
11.0
 
 
 
 
APOO
 
 
hsa-miR-181a-5p
 
 
-0.5
 
 
11.0
 
 
 
 
APP
 
 
hsa-miR-15b-5p
 
 
-0.298
 
 
401.1
 
 
 
 
AQP3
 
 
hsa-miR-874-3p
 
 
-0.48
 
 
11.4
 
 
 
 
ARF4
 
 
hsa-miR-30b-5p
 
 
-0.289
 
 
123.7
 
 
 
 
ARF4
 
 
hsa-miR-194-5p
 
 
-0.293
 
 
123.7
 
 
 
 
ARF4
 
 
hsa-miR-152-3p
 
 
-0.487
 
 
123.7
 
 
 
 
ARF4
 
 
hsa-miR-148b-3p
 
 
-0.487
 
 
123.7
 
 
 
 
ARF5
 
 
hsa-miR-29a-3p
 
 
-0.491
 
 
81.8
 
 
 
 
ARF6
 
 
hsa-miR-181b-5p
 
 
-0.33
 
 
16.9
 
 
 
 
ARF6
 
 
hsa-miR-181a-5p
 
 
-0.33
 
 
16.9
 
 
 
 
ARF6
 
 
hsa-miR-181d-5p
 
 
-0.338
 
 
16.9
 
 
 
 
ARFIP1
 
 
hsa-miR-320b
 
 
-0.339
 
 
17.2
 
 
 
 
ARFIP1
 
 
hsa-miR-320c
 
 
-0.339
 
 
17.2
 
 
 
 
ARFIP1
 
 
hsa-miR-152-3p
 
 
-0.474
 
 
17.2
 
 
 
 
ARFIP1
 
 
hsa-miR-148b-3p
 
 
-0.474
 
 
17.2
 
 
 
 
ARG2
 
 
hsa-let-7b-5p
 
 
-0.468
 
 
18.7
 
 
 
 
ARHGAP21
 
 
hsa-miR-152-3p
 
 
-0.287
 
 
26.5
 
 
 
 
ARHGAP21
 
 
hsa-miR-148b-3p
 
 
-0.287
 
 
26.5
 
 
 
 
ARHGAP6
 
 
hsa-miR-1271-5p
 
 
-0.521
 
 
17.5
 
 
 
 
ARID1B
 
 
hsa-miR-29a-3p
 
 
-0.342
 
 
20.5
 
 
 
 
ARIH2
 
 
hsa-miR-103a-3p
 
 
-0.307
 
 
19.4
 
 
 
 
ARL2
 
 
hsa-miR-15b-5p
 
 
-0.374
 
 
95.1
 
 
 
 
ARL2
 
 
hsa-miR-15b-5p
 
 
-0.442
 
 
95.1
 
 
 
 
ARL3
 
 
hsa-miR-15b-5p
 
 
-0.507
 
 
16.3
 
 
 
 
ARL4A
 
 
hsa-miR-29a-3p
 
 
-0.386
 
 
14.3
 
 
 
 
 
 
 pancreas 
 
 
 
 
target_symbol
 
 
mature_mirna_id
 
 
score
 
 
nTPM
 
 
 
 
 
 
AAMP
 
 
hsa-miR-326
 
 
-0.722
 
 
69.7
 
 
 
 
AAR2
 
 
hsa-miR-29a-3p
 
 
-0.327
 
 
17.6
 
 
 
 
ABAT
 
 
hsa-miR-1271-5p
 
 
-0.409
 
 
130.4
 
 
 
 
ABCD3
 
 
hsa-miR-148b-3p
 
 
-0.388
 
 
27.0
 
 
 
 
ABCD3
 
 
hsa-miR-152-3p
 
 
-0.388
 
 
27.0
 
 
 
 
ABCE1
 
 
hsa-miR-29a-3p
 
 
-0.404
 
 
27.7
 
 
 
 
ABHD4
 
 
hsa-miR-29a-3p
 
 
-0.312
 
 
15.3
 
 
 
 
ABHD5
 
 
hsa-miR-132-3p
 
 
-0.323
 
 
11.4
 
 
 
 
ACTC1
 
 
hsa-miR-30d-5p
 
 
-0.519
 
 
12.1
 
 
 
 
ACTC1
 
 
hsa-miR-30b-5p
 
 
-0.543
 
 
12.1
 
 
 
 
ACTR2
 
 
hsa-miR-15b-5p
 
 
-0.293
 
 
57.4
 
 
 
 
ACTR3C
 
 
hsa-miR-30b-5p
 
 
-0.448
 
 
12.4
 
 
 
 
ACTR3C
 
 
hsa-miR-30d-5p
 
 
-0.456
 
 
12.4
 
 
 
 
ADCY3
 
 
hsa-miR-132-3p
 
 
-0.303
 
 
15.3
 
 
 
 
ADCY6
 
 
hsa-miR-1271-5p
 
 
-0.336
 
 
21.0
 
 
 
 
ADGRA3
 
 
hsa-miR-30d-5p
 
 
-0.296
 
 
11.8
 
 
 
 
ADGRA3
 
 
hsa-miR-30b-5p
 
 
-0.304
 
 
11.8
 
 
 
 
ADIPOR2
 
 
hsa-miR-150-5p
 
 
-0.287
 
 
30.0
 
 
 
 
ADK
 
 
hsa-miR-1271-5p
 
 
-0.329
 
 
17.7
 
 
 
 
ADNP
 
 
hsa-miR-328-3p
 
 
-0.371
 
 
23.5
 
 
 
 
ADRA2A
 
 
hsa-miR-23a-3p
 
 
-0.371
 
 
28.8
 
 
 
 
AFG3L2
 
 
hsa-miR-181b-5p
 
 
-0.491
 
 
23.6
 
 
 
 
AFG3L2
 
 
hsa-miR-181a-5p
 
 
-0.491
 
 
23.6
 
 
 
 
AFG3L2
 
 
hsa-miR-181d-5p
 
 
-0.499
 
 
23.6
 
 
 
 
AGFG1
 
 
hsa-miR-148b-3p
 
 
-0.332
 
 
33.0
 
 
 
 
AGFG1
 
 
hsa-miR-152-3p
 
 
-0.332
 
 
33.0
 
 
 
 
AGFG1
 
 
hsa-miR-103a-3p
 
 
-0.389
 
 
33.0
 
 
 
 
AIFM1
 
 
hsa-let-7b-5p
 
 
-0.314
 
 
20.4
 
 
 
 
AJUBA
 
 
hsa-miR-103a-3p
 
 
-0.448
 
 
10.9
 
 
 
 
AK1
 
 
hsa-miR-29a-3p
 
 
-0.333
 
 
14.0
 
 
 
 
AK3
 
 
hsa-miR-1271-5p
 
 
-0.57
 
 
36.7
 
 
 
 
AKAP1
 
 
hsa-miR-152-3p
 
 
-0.381
 
 
21.5
 
 
 
 
AKAP1
 
 
hsa-miR-148b-3p
 
 
-0.381
 
 
21.5
 
 
 
 
AKIRIN2
 
 
hsa-miR-103a-3p
 
 
-0.299
 
 
11.7
 
 
 
 
ALAD
 
 
hsa-miR-326
 
 
-0.352
 
 
45.8
 
 
 
 
ALAD
 
 
hsa-miR-103a-3p
 
 
-0.385
 
 
45.8
 
 
 
 
ALAD
 
 
hsa-miR-15b-5p
 
 
-0.446
 
 
45.8
 
 
 
 
ALCAM
 
 
hsa-miR-148b-3p
 
 
-0.339
 
 
30.8
 
 
 
 
ALCAM
 
 
hsa-miR-152-3p
 
 
-0.347
 
 
30.8
 
 
 
 
ALDH6A1
 
 
hsa-miR-1271-5p
 
 
-0.301
 
 
22.0
 
 
 
 
AMMECR1L
 
 
hsa-miR-29a-3p
 
 
-0.42
 
 
10.5
 
 
 
 
AMOTL1
 
 
hsa-miR-15b-5p
 
 
-0.319
 
 
14.7
 
 
 
 
AMT
 
 
hsa-let-7b-5p
 
 
-0.463
 
 
21.5
 
 
 
 
ANKHD1
 
 
hsa-miR-23a-3p
 
 
-0.368
 
 
57.6
 
 
 
 
ANKHD1
 
 
hsa-miR-30d-5p
 
 
-0.381
 
 
57.6
 
 
 
 
ANKHD1
 
 
hsa-miR-30b-5p
 
 
-0.405
 
 
57.6
 
 
 
 
ANKIB1
 
 
hsa-miR-1271-5p
 
 
-0.344
 
 
11.5
 
 
 
 
ANKRD17
 
 
hsa-miR-30d-5p
 
 
-0.312
 
 
17.0
 
 
 
 
ANKRD17
 
 
hsa-miR-30b-5p
 
 
-0.336
 
 
17.0
 
 
 
 
ANKRD46
 
 
hsa-let-7b-5p
 
 
-0.389
 
 
17.5
 
 
 
 
ANP32A
 
 
hsa-miR-132-3p
 
 
-0.374
 
 
50.6
 
 
 
 
ANXA11
 
 
hsa-miR-15b-5p
 
 
-0.463
 
 
88.5
 
 
 
 
AP1S1
 
 
hsa-let-7b-5p
 
 
-0.374
 
 
23.9
 
 
 
 
AP2A1
 
 
hsa-miR-15b-5p
 
 
-0.333
 
 
32.4
 
 
 
 
AP2B1
 
 
hsa-miR-1271-5p
 
 
-0.299
 
 
31.1
 
 
 
 
AP2B1
 
 
hsa-miR-15b-5p
 
 
-0.334
 
 
31.1
 
 
 
 
AP3S1
 
 
hsa-miR-30b-5p
 
 
-0.293
 
 
66.7
 
 
 
 
AP4B1
 
 
hsa-miR-378a-3p
 
 
-0.62
 
 
13.3
 
 
 
 
AP4B1
 
 
hsa-miR-378d
 
 
-0.62
 
 
13.3
 
 
 
 
APOO
 
 
hsa-miR-181d-5p
 
 
-0.492
 
 
13.3
 
 
 
 
APOO
 
 
hsa-miR-181b-5p
 
 
-0.5
 
 
13.3
 
 
 
 
APOO
 
 
hsa-miR-181a-5p
 
 
-0.5
 
 
13.3
 
 
 
 
APP
 
 
hsa-miR-15b-5p
 
 
-0.298
 
 
305.2
 
 
 
 
AQP3
 
 
hsa-miR-874-3p
 
 
-0.48
 
 
41.8
 
 
 
 
ARF4
 
 
hsa-miR-30b-5p
 
 
-0.289
 
 
138.3
 
 
 
 
ARF4
 
 
hsa-miR-194-5p
 
 
-0.293
 
 
138.3
 
 
 
 
ARF4
 
 
hsa-miR-152-3p
 
 
-0.487
 
 
138.3
 
 
 
 
ARF4
 
 
hsa-miR-148b-3p
 
 
-0.487
 
 
138.3
 
 
 
 
ARF5
 
 
hsa-miR-29a-3p
 
 
-0.491
 
 
181.7
 
 
 
 
ARF6
 
 
hsa-miR-181b-5p
 
 
-0.33
 
 
23.2
 
 
 
 
ARF6
 
 
hsa-miR-181a-5p
 
 
-0.33
 
 
23.2
 
 
 
 
ARF6
 
 
hsa-miR-181d-5p
 
 
-0.338
 
 
23.2
 
 
 
 
ARFGEF1
 
 
hsa-miR-148b-3p
 
 
-0.306
 
 
16.5
 
 
 
 
ARFGEF1
 
 
hsa-miR-152-3p
 
 
-0.306
 
 
16.5
 
 
 
 
ARHGAP21
 
 
hsa-miR-152-3p
 
 
-0.287
 
 
13.3
 
 
 
 
ARHGAP21
 
 
hsa-miR-148b-3p
 
 
-0.287
 
 
13.3
 
 
 
 
ARHGAP24
 
 
hsa-miR-1271-5p
 
 
-0.342
 
 
28.4
 
 
 
 
ARID1B
 
 
hsa-miR-29a-3p
 
 
-0.342
 
 
15.2
 
 
 
 
ARIH2
 
 
hsa-miR-103a-3p
 
 
-0.307
 
 
23.5
 
 
 
 
ARL2
 
 
hsa-miR-15b-5p
 
 
-0.374
 
 
74.9
 
 
 
 
ARL2
 
 
hsa-miR-15b-5p
 
 
-0.442
 
 
74.9
 
 
 
 
ARL6IP1
 
 
hsa-miR-328-3p
 
 
-0.494
 
 
77.1
 
 
 
 
ARL6IP1
 
 
hsa-miR-148b-3p
 
 
-0.579
 
 
77.1
 
 
 
 
ARL6IP1
 
 
hsa-miR-152-3p
 
 
-0.579
 
 
77.1
 
 
 
 
ARL8A
 
 
hsa-miR-103a-3p
 
 
-0.38
 
 
26.7
 
 
 
 
ARL8B
 
 
hsa-miR-152-3p
 
 
-0.337
 
 
26.7
 
 
 
 
ARL8B
 
 
hsa-miR-148b-3p
 
 
-0.337
 
 
26.7
 
 
 
 
ARL8B
 
 
hsa-miR-320b
 
 
-0.376
 
 
26.7
 
 
 
 
ARL8B
 
 
hsa-miR-320c
 
 
-0.376
 
 
26.7
 
 
 
 
ARMC1
 
 
hsa-miR-103a-3p
 
 
-0.525
 
 
14.1
 
 
 
 
ARMC8
 
 
hsa-miR-103a-3p
 
 
-0.412
 
 
13.4
 
 
 
 
ARMCX2
 
 
hsa-miR-15b-5p
 
 
-0.344
 
 
16.6
 
 
 
 
ARMCX3
 
 
hsa-miR-425-5p
 
 
-0.325
 
 
50.5
 
 
 
 
ARNT
 
 
hsa-miR-103a-3p
 
 
-0.422
 
 
27.0
 
 
 
 
ARPC5L
 
 
hsa-miR-326
 
 
-0.439
 
 
27.6
 
 
 
 
ARPC5L
 
 
hsa-miR-1271-5p
 
 
-0.736
 
 
27.6
 
 
 
 
ARPP19
 
 
hsa-miR-1271-5p
 
 
-0.313
 
 
43.6
 
 
 
 
ARPP19
 
 
hsa-miR-320b
 
 
-0.325
 
 
43.6
 
 
 
 
ARPP19
 
 
hsa-miR-320c
 
 
-0.325
 
 
43.6
 
 
 
 
ARPP19
 
 
hsa-miR-15b-5p
 
 
-0.346
 
 
43.6
 
 
 
 
 
 
 parathyroid gland 
 
 
 
 
target_symbol
 
 
mature_mirna_id
 
 
score
 
 
nTPM
 
 
 
 
 
 
AAMP
 
 
hsa-miR-326
 
 
-0.722
 
 
68.3
 
 
 
 
AAR2
 
 
hsa-miR-29a-3p
 
 
-0.327
 
 
23.9
 
 
 
 
ABCA2
 
 
hsa-miR-1271-5p
 
 
-0.326
 
 
12.5
 
 
 
 
ABCB7
 
 
hsa-miR-148b-3p
 
 
-0.515
 
 
10.3
 
 
 
 
ABCB7
 
 
hsa-miR-152-3p
 
 
-0.515
 
 
10.3
 
 
 
 
ABCD3
 
 
hsa-miR-148b-3p
 
 
-0.388
 
 
27.9
 
 
 
 
ABCD3
 
 
hsa-miR-152-3p
 
 
-0.388
 
 
27.9
 
 
 
 
ABCE1
 
 
hsa-miR-29a-3p
 
 
-0.404
 
 
25.3
 
 
 
 
ABHD4
 
 
hsa-miR-29a-3p
 
 
-0.312
 
 
23.4
 
 
 
 
ABHD5
 
 
hsa-miR-132-3p
 
 
-0.323
 
 
11.5
 
 
 
 
ACSL6
 
 
hsa-let-7b-5p
 
 
-0.336
 
 
10.6
 
 
 
 
ACTR2
 
 
hsa-miR-15b-5p
 
 
-0.293
 
 
58.1
 
 
 
 
ACTR3C
 
 
hsa-miR-30b-5p
 
 
-0.448
 
 
53.3
 
 
 
 
ACTR3C
 
 
hsa-miR-30d-5p
 
 
-0.456
 
 
53.3
 
 
 
 
ADAM9
 
 
hsa-miR-30d-5p
 
 
-0.304
 
 
49.4
 
 
 
 
ADAM9
 
 
hsa-miR-30b-5p
 
 
-0.312
 
 
49.4
 
 
 
 
ADCY3
 
 
hsa-miR-132-3p
 
 
-0.303
 
 
16.0
 
 
 
 
ADCY6
 
 
hsa-miR-1271-5p
 
 
-0.336
 
 
19.5
 
 
 
 
ADGRA3
 
 
hsa-miR-30d-5p
 
 
-0.296
 
 
11.5
 
 
 
 
ADGRA3
 
 
hsa-miR-30b-5p
 
 
-0.304
 
 
11.5
 
 
 
 
ADGRF5
 
 
hsa-miR-148b-3p
 
 
-0.354
 
 
12.6
 
 
 
 
ADGRF5
 
 
hsa-miR-152-3p
 
 
-0.354
 
 
12.6
 
 
 
 
ADIPOR2
 
 
hsa-miR-150-5p
 
 
-0.287
 
 
15.9
 
 
 
 
ADK
 
 
hsa-miR-1271-5p
 
 
-0.329
 
 
41.5
 
 
 
 
ADNP
 
 
hsa-miR-328-3p
 
 
-0.371
 
 
34.0
 
 
 
 
AFG3L2
 
 
hsa-miR-181b-5p
 
 
-0.491
 
 
41.3
 
 
 
 
AFG3L2
 
 
hsa-miR-181a-5p
 
 
-0.491
 
 
41.3
 
 
 
 
AFG3L2
 
 
hsa-miR-181d-5p
 
 
-0.499
 
 
41.3
 
 
 
 
AGFG1
 
 
hsa-miR-148b-3p
 
 
-0.332
 
 
39.0
 
 
 
 
AGFG1
 
 
hsa-miR-152-3p
 
 
-0.332
 
 
39.0
 
 
 
 
AGFG1
 
 
hsa-miR-103a-3p
 
 
-0.389
 
 
39.0
 
 
 
 
AHCTF1
 
 
hsa-let-7b-5p
 
 
-0.374
 
 
12.3
 
 
 
 
AIFM1
 
 
hsa-let-7b-5p
 
 
-0.314
 
 
27.9
 
 
 
 
AK1
 
 
hsa-miR-29a-3p
 
 
-0.333
 
 
44.1
 
 
 
 
AK3
 
 
hsa-miR-1271-5p
 
 
-0.57
 
 
62.8
 
 
 
 
AK4
 
 
hsa-miR-15b-5p
 
 
-0.316
 
 
16.7
 
 
 
 
AKAP1
 
 
hsa-miR-152-3p
 
 
-0.381
 
 
45.1
 
 
 
 
AKAP1
 
 
hsa-miR-148b-3p
 
 
-0.381
 
 
45.1
 
 
 
 
AKIRIN2
 
 
hsa-miR-103a-3p
 
 
-0.299
 
 
16.7
 
 
 
 
ALAD
 
 
hsa-miR-326
 
 
-0.352
 
 
68.8
 
 
 
 
ALAD
 
 
hsa-miR-103a-3p
 
 
-0.385
 
 
68.8
 
 
 
 
ALAD
 
 
hsa-miR-15b-5p
 
 
-0.446
 
 
68.8
 
 
 
 
ALCAM
 
 
hsa-miR-148b-3p
 
 
-0.339
 
 
412.8
 
 
 
 
ALCAM
 
 
hsa-miR-152-3p
 
 
-0.347
 
 
412.8
 
 
 
 
ALDH1A2
 
 
hsa-miR-23a-3p
 
 
-0.354
 
 
28.0
 
 
 
 
ALDH6A1
 
 
hsa-miR-1271-5p
 
 
-0.301
 
 
34.8
 
 
 
 
AMOT
 
 
hsa-miR-29a-3p
 
 
-0.304
 
 
13.8
 
 
 
 
AMOTL1
 
 
hsa-miR-15b-5p
 
 
-0.319
 
 
13.0
 
 
 
 
AMT
 
 
hsa-let-7b-5p
 
 
-0.463
 
 
13.3
 
 
 
 
ANK1
 
 
hsa-miR-103a-3p
 
 
-0.321
 
 
46.9
 
 
 
 
ANKHD1
 
 
hsa-miR-23a-3p
 
 
-0.368
 
 
35.6
 
 
 
 
ANKHD1
 
 
hsa-miR-30d-5p
 
 
-0.381
 
 
35.6
 
 
 
 
ANKHD1
 
 
hsa-miR-30b-5p
 
 
-0.405
 
 
35.6
 
 
 
 
ANKIB1
 
 
hsa-miR-1271-5p
 
 
-0.344
 
 
22.5
 
 
 
 
ANKRD17
 
 
hsa-miR-30d-5p
 
 
-0.312
 
 
23.0
 
 
 
 
ANKRD17
 
 
hsa-miR-30b-5p
 
 
-0.336
 
 
23.0
 
 
 
 
ANKRD29
 
 
hsa-miR-132-3p
 
 
-0.47
 
 
23.3
 
 
 
 
ANKRD46
 
 
hsa-let-7b-5p
 
 
-0.389
 
 
15.1
 
 
 
 
ANP32A
 
 
hsa-miR-132-3p
 
 
-0.374
 
 
57.6
 
 
 
 
ANTXR2
 
 
hsa-miR-29a-3p
 
 
-0.347
 
 
26.2
 
 
 
 
ANXA11
 
 
hsa-miR-15b-5p
 
 
-0.463
 
 
76.9
 
 
 
 
AP1S1
 
 
hsa-let-7b-5p
 
 
-0.374
 
 
21.4
 
 
 
 
AP2A1
 
 
hsa-miR-15b-5p
 
 
-0.333
 
 
12.9
 
 
 
 
AP2B1
 
 
hsa-miR-1271-5p
 
 
-0.299
 
 
118.9
 
 
 
 
AP2B1
 
 
hsa-miR-15b-5p
 
 
-0.334
 
 
118.9
 
 
 
 
AP3S1
 
 
hsa-miR-30b-5p
 
 
-0.293
 
 
23.4
 
 
 
 
AP5M1
 
 
hsa-miR-1271-5p
 
 
-0.343
 
 
29.6
 
 
 
 
APOO
 
 
hsa-miR-181d-5p
 
 
-0.492
 
 
58.1
 
 
 
 
APOO
 
 
hsa-miR-181b-5p
 
 
-0.5
 
 
58.1
 
 
 
 
APOO
 
 
hsa-miR-181a-5p
 
 
-0.5
 
 
58.1
 
 
 
 
APP
 
 
hsa-miR-15b-5p
 
 
-0.298
 
 
259.7
 
 
 
 
ARF4
 
 
hsa-miR-30b-5p
 
 
-0.289
 
 
95.4
 
 
 
 
ARF4
 
 
hsa-miR-194-5p
 
 
-0.293
 
 
95.4
 
 
 
 
ARF4
 
 
hsa-miR-152-3p
 
 
-0.487
 
 
95.4
 
 
 
 
ARF4
 
 
hsa-miR-148b-3p
 
 
-0.487
 
 
95.4
 
 
 
 
ARF5
 
 
hsa-miR-29a-3p
 
 
-0.491
 
 
97.9
 
 
 
 
ARF6
 
 
hsa-miR-181b-5p
 
 
-0.33
 
 
19.3
 
 
 
 
ARF6
 
 
hsa-miR-181a-5p
 
 
-0.33
 
 
19.3
 
 
 
 
ARF6
 
 
hsa-miR-181d-5p
 
 
-0.338
 
 
19.3
 
 
 
 
ARFGEF1
 
 
hsa-miR-148b-3p
 
 
-0.306
 
 
26.3
 
 
 
 
ARFGEF1
 
 
hsa-miR-152-3p
 
 
-0.306
 
 
26.3
 
 
 
 
ARFIP1
 
 
hsa-miR-320b
 
 
-0.339
 
 
21.0
 
 
 
 
ARFIP1
 
 
hsa-miR-320c
 
 
-0.339
 
 
21.0
 
 
 
 
ARFIP1
 
 
hsa-miR-152-3p
 
 
-0.474
 
 
21.0
 
 
 
 
ARFIP1
 
 
hsa-miR-148b-3p
 
 
-0.474
 
 
21.0
 
 
 
 
ARG2
 
 
hsa-let-7b-5p
 
 
-0.468
 
 
206.5
 
 
 
 
ARHGAP21
 
 
hsa-miR-152-3p
 
 
-0.287
 
 
13.6
 
 
 
 
ARHGAP21
 
 
hsa-miR-148b-3p
 
 
-0.287
 
 
13.6
 
 
 
 
ARID1B
 
 
hsa-miR-29a-3p
 
 
-0.342
 
 
25.3
 
 
 
 
ARID2
 
 
hsa-miR-132-3p
 
 
-0.304
 
 
11.0
 
 
 
 
ARID2
 
 
hsa-miR-103a-3p
 
 
-0.415
 
 
11.0
 
 
 
 
ARIH2
 
 
hsa-miR-103a-3p
 
 
-0.307
 
 
23.0
 
 
 
 
ARL2
 
 
hsa-miR-15b-5p
 
 
-0.374
 
 
61.4
 
 
 
 
ARL2
 
 
hsa-miR-15b-5p
 
 
-0.442
 
 
61.4
 
 
 
 
ARL3
 
 
hsa-miR-15b-5p
 
 
-0.507
 
 
18.6
 
 
 
 
ARL6IP1
 
 
hsa-miR-328-3p
 
 
-0.494
 
 
169.8
 
 
 
 
ARL6IP1
 
 
hsa-miR-148b-3p
 
 
-0.579
 
 
169.8
 
 
 
 
ARL6IP1
 
 
hsa-miR-152-3p
 
 
-0.579
 
 
169.8
 
 
 
 
ARL8A
 
 
hsa-miR-103a-3p
 
 
-0.38
 
 
24.7
 
 
 
 
ARL8B
 
 
hsa-miR-152-3p
 
 
-0.337
 
 
45.2
 
 
 
 
 
 
 pituitary gland 
 
 
 
 
target_symbol
 
 
mature_mirna_id
 
 
score
 
 
nTPM
 
 
 
 
 
 
AAMP
 
 
hsa-miR-326
 
 
-0.722
 
 
60.7
 
 
 
 
ABAT
 
 
hsa-miR-1271-5p
 
 
-0.409
 
 
53.0
 
 
 
 
ABCA2
 
 
hsa-miR-1271-5p
 
 
-0.326
 
 
19.9
 
 
 
 
ABCB9
 
 
hsa-let-7b-5p
 
 
-0.422
 
 
11.0
 
 
 
 
ABCD3
 
 
hsa-miR-148b-3p
 
 
-0.388
 
 
12.4
 
 
 
 
ABCD3
 
 
hsa-miR-152-3p
 
 
-0.388
 
 
12.4
 
 
 
 
ABHD4
 
 
hsa-miR-29a-3p
 
 
-0.312
 
 
13.7
 
 
 
 
ACTR2
 
 
hsa-miR-15b-5p
 
 
-0.293
 
 
23.5
 
 
 
 
ADCY3
 
 
hsa-miR-132-3p
 
 
-0.303
 
 
41.4
 
 
 
 
ADCY6
 
 
hsa-miR-1271-5p
 
 
-0.336
 
 
13.5
 
 
 
 
ADGRB3
 
 
hsa-miR-1271-5p
 
 
-0.424
 
 
21.3
 
 
 
 
ADGRB3
 
 
hsa-miR-152-3p
 
 
-0.516
 
 
21.3
 
 
 
 
ADGRB3
 
 
hsa-miR-148b-3p
 
 
-0.524
 
 
21.3
 
 
 
 
ADIPOR2
 
 
hsa-miR-150-5p
 
 
-0.287
 
 
25.4
 
 
 
 
ADK
 
 
hsa-miR-1271-5p
 
 
-0.329
 
 
14.1
 
 
 
 
ADNP
 
 
hsa-miR-328-3p
 
 
-0.371
 
 
26.6
 
 
 
 
AGFG1
 
 
hsa-miR-148b-3p
 
 
-0.332
 
 
16.7
 
 
 
 
AGFG1
 
 
hsa-miR-152-3p
 
 
-0.332
 
 
16.7
 
 
 
 
AGFG1
 
 
hsa-miR-103a-3p
 
 
-0.389
 
 
16.7
 
 
 
 
AGPAT4
 
 
hsa-miR-29a-3p
 
 
-0.376
 
 
10.5
 
 
 
 
AHDC1
 
 
hsa-miR-152-3p
 
 
-0.413
 
 
18.1
 
 
 
 
AHDC1
 
 
hsa-miR-148b-3p
 
 
-0.413
 
 
18.1
 
 
 
 
AIFM1
 
 
hsa-let-7b-5p
 
 
-0.314
 
 
11.0
 
 
 
 
AK1
 
 
hsa-miR-29a-3p
 
 
-0.333
 
 
40.7
 
 
 
 
AK3
 
 
hsa-miR-1271-5p
 
 
-0.57
 
 
18.8
 
 
 
 
AKAP1
 
 
hsa-miR-152-3p
 
 
-0.381
 
 
16.5
 
 
 
 
AKAP1
 
 
hsa-miR-148b-3p
 
 
-0.381
 
 
16.5
 
 
 
 
AKIRIN2
 
 
hsa-miR-103a-3p
 
 
-0.299
 
 
21.2
 
 
 
 
AKT3
 
 
hsa-miR-29a-3p
 
 
-0.287
 
 
11.4
 
 
 
 
AKT3
 
 
hsa-miR-15b-5p
 
 
-0.301
 
 
11.4
 
 
 
 
ALAD
 
 
hsa-miR-326
 
 
-0.352
 
 
16.3
 
 
 
 
ALAD
 
 
hsa-miR-103a-3p
 
 
-0.385
 
 
16.3
 
 
 
 
ALAD
 
 
hsa-miR-15b-5p
 
 
-0.446
 
 
16.3
 
 
 
 
ALCAM
 
 
hsa-miR-148b-3p
 
 
-0.339
 
 
17.9
 
 
 
 
ALCAM
 
 
hsa-miR-152-3p
 
 
-0.347
 
 
17.9
 
 
 
 
ALDH1A2
 
 
hsa-miR-23a-3p
 
 
-0.354
 
 
14.4
 
 
 
 
AMOTL1
 
 
hsa-miR-15b-5p
 
 
-0.319
 
 
13.5
 
 
 
 
AMT
 
 
hsa-let-7b-5p
 
 
-0.463
 
 
16.0
 
 
 
 
ANKHD1
 
 
hsa-miR-23a-3p
 
 
-0.368
 
 
41.2
 
 
 
 
ANKHD1
 
 
hsa-miR-30d-5p
 
 
-0.381
 
 
41.2
 
 
 
 
ANKHD1
 
 
hsa-miR-30b-5p
 
 
-0.405
 
 
41.2
 
 
 
 
ANKRD17
 
 
hsa-miR-30d-5p
 
 
-0.312
 
 
13.4
 
 
 
 
ANKRD17
 
 
hsa-miR-30b-5p
 
 
-0.336
 
 
13.4
 
 
 
 
ANP32A
 
 
hsa-miR-132-3p
 
 
-0.374
 
 
26.1
 
 
 
 
ANXA11
 
 
hsa-miR-15b-5p
 
 
-0.463
 
 
25.4
 
 
 
 
AP1S1
 
 
hsa-let-7b-5p
 
 
-0.374
 
 
31.4
 
 
 
 
AP2A1
 
 
hsa-miR-15b-5p
 
 
-0.333
 
 
33.0
 
 
 
 
AP2B1
 
 
hsa-miR-1271-5p
 
 
-0.299
 
 
51.7
 
 
 
 
AP2B1
 
 
hsa-miR-15b-5p
 
 
-0.334
 
 
51.7
 
 
 
 
AP3S1
 
 
hsa-miR-30b-5p
 
 
-0.293
 
 
47.0
 
 
 
 
AP4B1
 
 
hsa-miR-378a-3p
 
 
-0.62
 
 
13.4
 
 
 
 
AP4B1
 
 
hsa-miR-378d
 
 
-0.62
 
 
13.4
 
 
 
 
APBB3
 
 
hsa-let-7b-5p
 
 
-0.587
 
 
10.1
 
 
 
 
APOO
 
 
hsa-miR-181d-5p
 
 
-0.492
 
 
28.1
 
 
 
 
APOO
 
 
hsa-miR-181b-5p
 
 
-0.5
 
 
28.1
 
 
 
 
APOO
 
 
hsa-miR-181a-5p
 
 
-0.5
 
 
28.1
 
 
 
 
APP
 
 
hsa-miR-15b-5p
 
 
-0.298
 
 
144.3
 
 
 
 
ARF4
 
 
hsa-miR-30b-5p
 
 
-0.289
 
 
93.4
 
 
 
 
ARF4
 
 
hsa-miR-194-5p
 
 
-0.293
 
 
93.4
 
 
 
 
ARF4
 
 
hsa-miR-152-3p
 
 
-0.487
 
 
93.4
 
 
 
 
ARF4
 
 
hsa-miR-148b-3p
 
 
-0.487
 
 
93.4
 
 
 
 
ARF5
 
 
hsa-miR-29a-3p
 
 
-0.491
 
 
111.9
 
 
 
 
ARG2
 
 
hsa-let-7b-5p
 
 
-0.468
 
 
47.4
 
 
 
 
ARHGAP21
 
 
hsa-miR-152-3p
 
 
-0.287
 
 
11.3
 
 
 
 
ARHGAP21
 
 
hsa-miR-148b-3p
 
 
-0.287
 
 
11.3
 
 
 
 
ARIH2
 
 
hsa-miR-103a-3p
 
 
-0.307
 
 
25.4
 
 
 
 
ARL2
 
 
hsa-miR-15b-5p
 
 
-0.374
 
 
68.9
 
 
 
 
ARL2
 
 
hsa-miR-15b-5p
 
 
-0.442
 
 
68.9
 
 
 
 
ARL3
 
 
hsa-miR-15b-5p
 
 
-0.507
 
 
19.9
 
 
 
 
ARL4D
 
 
hsa-miR-1271-5p
 
 
-0.392
 
 
20.8
 
 
 
 
ARL4D
 
 
hsa-let-7b-5p
 
 
-0.417
 
 
20.8
 
 
 
 
ARL6IP1
 
 
hsa-miR-328-3p
 
 
-0.494
 
 
39.7
 
 
 
 
ARL6IP1
 
 
hsa-miR-148b-3p
 
 
-0.579
 
 
39.7
 
 
 
 
ARL6IP1
 
 
hsa-miR-152-3p
 
 
-0.579
 
 
39.7
 
 
 
 
ARL8A
 
 
hsa-miR-103a-3p
 
 
-0.38
 
 
30.4
 
 
 
 
ARL8B
 
 
hsa-miR-152-3p
 
 
-0.337
 
 
25.6
 
 
 
 
ARL8B
 
 
hsa-miR-148b-3p
 
 
-0.337
 
 
25.6
 
 
 
 
ARL8B
 
 
hsa-miR-320b
 
 
-0.376
 
 
25.6
 
 
 
 
ARL8B
 
 
hsa-miR-320c
 
 
-0.376
 
 
25.6
 
 
 
 
ARMC12
 
 
hsa-miR-15b-5p
 
 
-0.485
 
 
11.0
 
 
 
 
ARMC8
 
 
hsa-miR-103a-3p
 
 
-0.412
 
 
11.3
 
 
 
 
ARMCX2
 
 
hsa-miR-15b-5p
 
 
-0.344
 
 
70.4
 
 
 
 
ARMCX3
 
 
hsa-miR-425-5p
 
 
-0.325
 
 
43.1
 
 
 
 
ARNT
 
 
hsa-miR-103a-3p
 
 
-0.422
 
 
12.2
 
 
 
 
ARPC5L
 
 
hsa-miR-326
 
 
-0.439
 
 
43.0
 
 
 
 
ARPC5L
 
 
hsa-miR-1271-5p
 
 
-0.736
 
 
43.0
 
 
 
 
ARPP19
 
 
hsa-miR-1271-5p
 
 
-0.313
 
 
33.0
 
 
 
 
ARPP19
 
 
hsa-miR-320b
 
 
-0.325
 
 
33.0
 
 
 
 
ARPP19
 
 
hsa-miR-320c
 
 
-0.325
 
 
33.0
 
 
 
 
ARPP19
 
 
hsa-miR-15b-5p
 
 
-0.346
 
 
33.0
 
 
 
 
ARPP19
 
 
hsa-miR-29a-3p
 
 
-0.371
 
 
33.0
 
 
 
 
ARPP19
 
 
hsa-miR-328-3p
 
 
-0.404
 
 
33.0
 
 
 
 
ARPP19
 
 
hsa-let-7b-5p
 
 
-0.476
 
 
33.0
 
 
 
 
ARPP21
 
 
hsa-miR-15b-5p
 
 
-0.342
 
 
12.2
 
 
 
 
ARRDC3
 
 
hsa-miR-152-3p
 
 
-0.305
 
 
20.2
 
 
 
 
ARRDC3
 
 
hsa-miR-148b-3p
 
 
-0.305
 
 
20.2
 
 
 
 
ARRDC3
 
 
hsa-miR-29a-3p
 
 
-0.402
 
 
20.2
 
 
 
 
ASB3
 
 
hsa-miR-30d-5p
 
 
-0.394
 
 
13.9
 
 
 
 
ASB3
 
 
hsa-miR-30d-5p
 
 
-0.394
 
 
13.9
 
 
 
 
ASB3
 
 
hsa-miR-30b-5p
 
 
-0.418
 
 
13.9
 
 
 
 
 
 
 placenta 
 
 
 
 
target_symbol
 
 
mature_mirna_id
 
 
score
 
 
nTPM
 
 
 
 
 
 
AAMP
 
 
hsa-miR-326
 
 
-0.722
 
 
51.5
 
 
 
 
AAR2
 
 
hsa-miR-29a-3p
 
 
-0.327
 
 
16.5
 
 
 
 
ABCB10
 
 
hsa-miR-152-3p
 
 
-0.291
 
 
10.6
 
 
 
 
ABCB10
 
 
hsa-miR-148b-3p
 
 
-0.291
 
 
10.6
 
 
 
 
ABCD3
 
 
hsa-miR-148b-3p
 
 
-0.388
 
 
13.6
 
 
 
 
ABCD3
 
 
hsa-miR-152-3p
 
 
-0.388
 
 
13.6
 
 
 
 
ABCE1
 
 
hsa-miR-29a-3p
 
 
-0.404
 
 
26.9
 
 
 
 
ABHD4
 
 
hsa-miR-29a-3p
 
 
-0.312
 
 
18.5
 
 
 
 
ACP3
 
 
hsa-let-7b-5p
 
 
-0.298
 
 
10.1
 
 
 
 
ACTR2
 
 
hsa-miR-15b-5p
 
 
-0.293
 
 
102.8
 
 
 
 
ACVR1
 
 
hsa-miR-30b-5p
 
 
-0.29
 
 
12.5
 
 
 
 
ACVR1
 
 
hsa-miR-152-3p
 
 
-0.397
 
 
12.5
 
 
 
 
ACVR1
 
 
hsa-miR-148b-3p
 
 
-0.397
 
 
12.5
 
 
 
 
ADAM10
 
 
hsa-miR-152-3p
 
 
-0.348
 
 
27.1
 
 
 
 
ADAM10
 
 
hsa-miR-148b-3p
 
 
-0.356
 
 
27.1
 
 
 
 
ADAM17
 
 
hsa-miR-152-3p
 
 
-0.301
 
 
17.9
 
 
 
 
ADAM17
 
 
hsa-miR-148b-3p
 
 
-0.301
 
 
17.9
 
 
 
 
ADAM19
 
 
hsa-miR-874-3p
 
 
-0.374
 
 
12.3
 
 
 
 
ADAM9
 
 
hsa-miR-30d-5p
 
 
-0.304
 
 
44.6
 
 
 
 
ADAM9
 
 
hsa-miR-30b-5p
 
 
-0.312
 
 
44.6
 
 
 
 
ADAMTS9
 
 
hsa-miR-29a-3p
 
 
-0.305
 
 
29.8
 
 
 
 
ADAMTS9
 
 
hsa-miR-29a-3p
 
 
-0.485
 
 
29.8
 
 
 
 
ADCY3
 
 
hsa-miR-132-3p
 
 
-0.303
 
 
78.3
 
 
 
 
ADCY6
 
 
hsa-miR-1271-5p
 
 
-0.336
 
 
13.6
 
 
 
 
ADGRA2
 
 
hsa-miR-1271-5p
 
 
-0.427
 
 
45.7
 
 
 
 
ADGRF5
 
 
hsa-miR-148b-3p
 
 
-0.354
 
 
48.3
 
 
 
 
ADGRF5
 
 
hsa-miR-152-3p
 
 
-0.354
 
 
48.3
 
 
 
 
ADIPOR2
 
 
hsa-miR-150-5p
 
 
-0.287
 
 
12.2
 
 
 
 
ADK
 
 
hsa-miR-1271-5p
 
 
-0.329
 
 
25.0
 
 
 
 
ADNP
 
 
hsa-miR-328-3p
 
 
-0.371
 
 
23.8
 
 
 
 
ADRB1
 
 
hsa-let-7b-5p
 
 
-0.351
 
 
27.4
 
 
 
 
AFG3L2
 
 
hsa-miR-181b-5p
 
 
-0.491
 
 
12.5
 
 
 
 
AFG3L2
 
 
hsa-miR-181a-5p
 
 
-0.491
 
 
12.5
 
 
 
 
AFG3L2
 
 
hsa-miR-181d-5p
 
 
-0.499
 
 
12.5
 
 
 
 
AGFG1
 
 
hsa-miR-148b-3p
 
 
-0.332
 
 
71.2
 
 
 
 
AGFG1
 
 
hsa-miR-152-3p
 
 
-0.332
 
 
71.2
 
 
 
 
AGFG1
 
 
hsa-miR-103a-3p
 
 
-0.389
 
 
71.2
 
 
 
 
AHR
 
 
hsa-miR-1271-5p
 
 
-0.304
 
 
16.6
 
 
 
 
AHR
 
 
hsa-miR-29a-3p
 
 
-0.316
 
 
16.6
 
 
 
 
AJUBA
 
 
hsa-miR-103a-3p
 
 
-0.448
 
 
18.1
 
 
 
 
AK3
 
 
hsa-miR-1271-5p
 
 
-0.57
 
 
33.0
 
 
 
 
AKIRIN2
 
 
hsa-miR-103a-3p
 
 
-0.299
 
 
17.4
 
 
 
 
AKT3
 
 
hsa-miR-29a-3p
 
 
-0.287
 
 
12.3
 
 
 
 
AKT3
 
 
hsa-miR-15b-5p
 
 
-0.301
 
 
12.3
 
 
 
 
ALAD
 
 
hsa-miR-326
 
 
-0.352
 
 
23.4
 
 
 
 
ALAD
 
 
hsa-miR-103a-3p
 
 
-0.385
 
 
23.4
 
 
 
 
ALAD
 
 
hsa-miR-15b-5p
 
 
-0.446
 
 
23.4
 
 
 
 
ALCAM
 
 
hsa-miR-148b-3p
 
 
-0.339
 
 
11.3
 
 
 
 
ALCAM
 
 
hsa-miR-152-3p
 
 
-0.347
 
 
11.3
 
 
 
 
AMOT
 
 
hsa-miR-29a-3p
 
 
-0.304
 
 
19.6
 
 
 
 
AMOTL1
 
 
hsa-miR-15b-5p
 
 
-0.319
 
 
22.6
 
 
 
 
ANKHD1
 
 
hsa-miR-23a-3p
 
 
-0.368
 
 
26.8
 
 
 
 
ANKHD1
 
 
hsa-miR-30d-5p
 
 
-0.381
 
 
26.8
 
 
 
 
ANKHD1
 
 
hsa-miR-30b-5p
 
 
-0.405
 
 
26.8
 
 
 
 
ANKIB1
 
 
hsa-miR-1271-5p
 
 
-0.344
 
 
11.9
 
 
 
 
ANKRD17
 
 
hsa-miR-30d-5p
 
 
-0.312
 
 
17.9
 
 
 
 
ANKRD17
 
 
hsa-miR-30b-5p
 
 
-0.336
 
 
17.9
 
 
 
 
ANKRD27
 
 
hsa-miR-1271-5p
 
 
-0.648
 
 
12.7
 
 
 
 
ANKRD46
 
 
hsa-let-7b-5p
 
 
-0.389
 
 
19.5
 
 
 
 
ANP32A
 
 
hsa-miR-132-3p
 
 
-0.374
 
 
95.3
 
 
 
 
ANTXR2
 
 
hsa-miR-29a-3p
 
 
-0.347
 
 
84.5
 
 
 
 
ANXA11
 
 
hsa-miR-15b-5p
 
 
-0.463
 
 
85.5
 
 
 
 
AP1S1
 
 
hsa-let-7b-5p
 
 
-0.374
 
 
14.9
 
 
 
 
AP2A1
 
 
hsa-miR-15b-5p
 
 
-0.333
 
 
29.6
 
 
 
 
AP2B1
 
 
hsa-miR-1271-5p
 
 
-0.299
 
 
62.7
 
 
 
 
AP2B1
 
 
hsa-miR-15b-5p
 
 
-0.334
 
 
62.7
 
 
 
 
AP3S1
 
 
hsa-miR-30b-5p
 
 
-0.293
 
 
56.4
 
 
 
 
AP5M1
 
 
hsa-miR-1271-5p
 
 
-0.343
 
 
10.3
 
 
 
 
APLN
 
 
hsa-miR-15b-5p
 
 
-0.395
 
 
50.8
 
 
 
 
APOO
 
 
hsa-miR-181d-5p
 
 
-0.492
 
 
10.3
 
 
 
 
APOO
 
 
hsa-miR-181b-5p
 
 
-0.5
 
 
10.3
 
 
 
 
APOO
 
 
hsa-miR-181a-5p
 
 
-0.5
 
 
10.3
 
 
 
 
APP
 
 
hsa-miR-15b-5p
 
 
-0.298
 
 
417.2
 
 
 
 
ARF4
 
 
hsa-miR-30b-5p
 
 
-0.289
 
 
170.7
 
 
 
 
ARF4
 
 
hsa-miR-194-5p
 
 
-0.293
 
 
170.7
 
 
 
 
ARF4
 
 
hsa-miR-152-3p
 
 
-0.487
 
 
170.7
 
 
 
 
ARF4
 
 
hsa-miR-148b-3p
 
 
-0.487
 
 
170.7
 
 
 
 
ARF5
 
 
hsa-miR-29a-3p
 
 
-0.491
 
 
65.2
 
 
 
 
ARF6
 
 
hsa-miR-181b-5p
 
 
-0.33
 
 
34.8
 
 
 
 
ARF6
 
 
hsa-miR-181a-5p
 
 
-0.33
 
 
34.8
 
 
 
 
ARF6
 
 
hsa-miR-181d-5p
 
 
-0.338
 
 
34.8
 
 
 
 
ARFGEF1
 
 
hsa-miR-148b-3p
 
 
-0.306
 
 
13.6
 
 
 
 
ARFGEF1
 
 
hsa-miR-152-3p
 
 
-0.306
 
 
13.6
 
 
 
 
ARFIP1
 
 
hsa-miR-320b
 
 
-0.339
 
 
16.9
 
 
 
 
ARFIP1
 
 
hsa-miR-320c
 
 
-0.339
 
 
16.9
 
 
 
 
ARFIP1
 
 
hsa-miR-152-3p
 
 
-0.474
 
 
16.9
 
 
 
 
ARFIP1
 
 
hsa-miR-148b-3p
 
 
-0.474
 
 
16.9
 
 
 
 
ARHGAP21
 
 
hsa-miR-152-3p
 
 
-0.287
 
 
15.6
 
 
 
 
ARHGAP21
 
 
hsa-miR-148b-3p
 
 
-0.287
 
 
15.6
 
 
 
 
ARHGAP24
 
 
hsa-miR-1271-5p
 
 
-0.342
 
 
21.0
 
 
 
 
ARHGAP28
 
 
hsa-let-7b-5p
 
 
-0.454
 
 
24.3
 
 
 
 
ARID1B
 
 
hsa-miR-29a-3p
 
 
-0.342
 
 
10.4
 
 
 
 
ARID3A
 
 
hsa-let-7b-5p
 
 
-0.332
 
 
22.0
 
 
 
 
ARID3A
 
 
hsa-let-7b-5p
 
 
-0.347
 
 
22.0
 
 
 
 
ARID3B
 
 
hsa-let-7b-5p
 
 
-0.322
 
 
14.7
 
 
 
 
ARID3B
 
 
hsa-let-7b-5p
 
 
-0.486
 
 
14.7
 
 
 
 
ARIH2
 
 
hsa-miR-103a-3p
 
 
-0.307
 
 
17.3
 
 
 
 
ARL2
 
 
hsa-miR-15b-5p
 
 
-0.374
 
 
52.7
 
 
 
 
ARL2
 
 
hsa-miR-15b-5p
 
 
-0.442
 
 
52.7
 
 
 
 
ARL4A
 
 
hsa-miR-29a-3p
 
 
-0.386
 
 
22.2
 
 
 
 
 
 
 prostate 
 
 
 
 
target_symbol
 
 
mature_mirna_id
 
 
score
 
 
nTPM
 
 
 
 
 
 
AAMP
 
 
hsa-miR-326
 
 
-0.722
 
 
56.1
 
 
 
 
AAR2
 
 
hsa-miR-29a-3p
 
 
-0.327
 
 
18.7
 
 
 
 
ABAT
 
 
hsa-miR-1271-5p
 
 
-0.409
 
 
20.4
 
 
 
 
ABCD3
 
 
hsa-miR-148b-3p
 
 
-0.388
 
 
20.2
 
 
 
 
ABCD3
 
 
hsa-miR-152-3p
 
 
-0.388
 
 
20.2
 
 
 
 
ABCE1
 
 
hsa-miR-29a-3p
 
 
-0.404
 
 
18.2
 
 
 
 
ABHD17C
 
 
hsa-let-7b-5p
 
 
-0.359
 
 
17.0
 
 
 
 
ABHD17C
 
 
hsa-miR-330-3p
 
 
-0.399
 
 
17.0
 
 
 
 
ABHD4
 
 
hsa-miR-29a-3p
 
 
-0.312
 
 
20.4
 
 
 
 
ACP3
 
 
hsa-let-7b-5p
 
 
-0.298
 
 
3409.5
 
 
 
 
ACTC1
 
 
hsa-miR-30d-5p
 
 
-0.519
 
 
68.4
 
 
 
 
ACTC1
 
 
hsa-miR-30b-5p
 
 
-0.543
 
 
68.4
 
 
 
 
ACTN2
 
 
hsa-miR-23a-3p
 
 
-0.294
 
 
14.6
 
 
 
 
ACTN2
 
 
hsa-miR-29a-3p
 
 
-0.311
 
 
14.6
 
 
 
 
ACTR2
 
 
hsa-miR-15b-5p
 
 
-0.293
 
 
67.2
 
 
 
 
ADAM10
 
 
hsa-miR-152-3p
 
 
-0.348
 
 
23.6
 
 
 
 
ADAM10
 
 
hsa-miR-148b-3p
 
 
-0.356
 
 
23.6
 
 
 
 
ADAM9
 
 
hsa-miR-30d-5p
 
 
-0.304
 
 
32.8
 
 
 
 
ADAM9
 
 
hsa-miR-30b-5p
 
 
-0.312
 
 
32.8
 
 
 
 
ADCY3
 
 
hsa-miR-132-3p
 
 
-0.303
 
 
38.0
 
 
 
 
ADCY6
 
 
hsa-miR-1271-5p
 
 
-0.336
 
 
15.1
 
 
 
 
ADGRA2
 
 
hsa-miR-1271-5p
 
 
-0.427
 
 
36.7
 
 
 
 
ADGRA3
 
 
hsa-miR-30d-5p
 
 
-0.296
 
 
11.3
 
 
 
 
ADGRA3
 
 
hsa-miR-30b-5p
 
 
-0.304
 
 
11.3
 
 
 
 
ADIPOR2
 
 
hsa-miR-150-5p
 
 
-0.287
 
 
19.8
 
 
 
 
ADK
 
 
hsa-miR-1271-5p
 
 
-0.329
 
 
14.0
 
 
 
 
ADNP
 
 
hsa-miR-328-3p
 
 
-0.371
 
 
33.0
 
 
 
 
AFG3L2
 
 
hsa-miR-181b-5p
 
 
-0.491
 
 
13.2
 
 
 
 
AFG3L2
 
 
hsa-miR-181a-5p
 
 
-0.491
 
 
13.2
 
 
 
 
AFG3L2
 
 
hsa-miR-181d-5p
 
 
-0.499
 
 
13.2
 
 
 
 
AGFG1
 
 
hsa-miR-148b-3p
 
 
-0.332
 
 
19.4
 
 
 
 
AGFG1
 
 
hsa-miR-152-3p
 
 
-0.332
 
 
19.4
 
 
 
 
AGFG1
 
 
hsa-miR-103a-3p
 
 
-0.389
 
 
19.4
 
 
 
 
AHDC1
 
 
hsa-miR-152-3p
 
 
-0.413
 
 
11.3
 
 
 
 
AHDC1
 
 
hsa-miR-148b-3p
 
 
-0.413
 
 
11.3
 
 
 
 
AHR
 
 
hsa-miR-1271-5p
 
 
-0.304
 
 
10.3
 
 
 
 
AHR
 
 
hsa-miR-29a-3p
 
 
-0.316
 
 
10.3
 
 
 
 
AIFM1
 
 
hsa-let-7b-5p
 
 
-0.314
 
 
10.1
 
 
 
 
AK1
 
 
hsa-miR-29a-3p
 
 
-0.333
 
 
16.0
 
 
 
 
AK3
 
 
hsa-miR-1271-5p
 
 
-0.57
 
 
46.2
 
 
 
 
AK4
 
 
hsa-miR-15b-5p
 
 
-0.316
 
 
14.9
 
 
 
 
AKAP1
 
 
hsa-miR-152-3p
 
 
-0.381
 
 
33.3
 
 
 
 
AKAP1
 
 
hsa-miR-148b-3p
 
 
-0.381
 
 
33.3
 
 
 
 
AKIRIN2
 
 
hsa-miR-103a-3p
 
 
-0.299
 
 
15.2
 
 
 
 
AKT3
 
 
hsa-miR-29a-3p
 
 
-0.287
 
 
13.8
 
 
 
 
AKT3
 
 
hsa-miR-15b-5p
 
 
-0.301
 
 
13.8
 
 
 
 
ALAD
 
 
hsa-miR-326
 
 
-0.352
 
 
32.0
 
 
 
 
ALAD
 
 
hsa-miR-103a-3p
 
 
-0.385
 
 
32.0
 
 
 
 
ALAD
 
 
hsa-miR-15b-5p
 
 
-0.446
 
 
32.0
 
 
 
 
ALCAM
 
 
hsa-miR-148b-3p
 
 
-0.339
 
 
30.7
 
 
 
 
ALCAM
 
 
hsa-miR-152-3p
 
 
-0.347
 
 
30.7
 
 
 
 
ALDH1A2
 
 
hsa-miR-23a-3p
 
 
-0.354
 
 
19.7
 
 
 
 
ALDH6A1
 
 
hsa-miR-1271-5p
 
 
-0.301
 
 
23.8
 
 
 
 
ALOX15B
 
 
hsa-miR-326
 
 
-0.417
 
 
68.6
 
 
 
 
AMOTL1
 
 
hsa-miR-15b-5p
 
 
-0.319
 
 
30.4
 
 
 
 
AMT
 
 
hsa-let-7b-5p
 
 
-0.463
 
 
15.5
 
 
 
 
ANKHD1
 
 
hsa-miR-23a-3p
 
 
-0.368
 
 
34.8
 
 
 
 
ANKHD1
 
 
hsa-miR-30d-5p
 
 
-0.381
 
 
34.8
 
 
 
 
ANKHD1
 
 
hsa-miR-30b-5p
 
 
-0.405
 
 
34.8
 
 
 
 
ANKRA2
 
 
hsa-miR-30d-5p
 
 
-0.343
 
 
11.6
 
 
 
 
ANKRA2
 
 
hsa-miR-30b-5p
 
 
-0.354
 
 
11.6
 
 
 
 
ANKRA2
 
 
hsa-miR-30d-5p
 
 
-0.448
 
 
11.6
 
 
 
 
ANKRA2
 
 
hsa-miR-30b-5p
 
 
-0.472
 
 
11.6
 
 
 
 
ANKRD17
 
 
hsa-miR-30d-5p
 
 
-0.312
 
 
14.2
 
 
 
 
ANKRD17
 
 
hsa-miR-30b-5p
 
 
-0.336
 
 
14.2
 
 
 
 
ANKRD27
 
 
hsa-miR-1271-5p
 
 
-0.648
 
 
11.5
 
 
 
 
ANKRD46
 
 
hsa-let-7b-5p
 
 
-0.389
 
 
12.6
 
 
 
 
ANP32A
 
 
hsa-miR-132-3p
 
 
-0.374
 
 
70.7
 
 
 
 
ANTXR2
 
 
hsa-miR-29a-3p
 
 
-0.347
 
 
103.3
 
 
 
 
ANXA11
 
 
hsa-miR-15b-5p
 
 
-0.463
 
 
94.1
 
 
 
 
AP1S1
 
 
hsa-let-7b-5p
 
 
-0.374
 
 
23.9
 
 
 
 
AP2A1
 
 
hsa-miR-15b-5p
 
 
-0.333
 
 
31.4
 
 
 
 
AP2B1
 
 
hsa-miR-1271-5p
 
 
-0.299
 
 
38.3
 
 
 
 
AP2B1
 
 
hsa-miR-15b-5p
 
 
-0.334
 
 
38.3
 
 
 
 
AP3S1
 
 
hsa-miR-30b-5p
 
 
-0.293
 
 
40.9
 
 
 
 
AP5M1
 
 
hsa-miR-1271-5p
 
 
-0.343
 
 
11.5
 
 
 
 
APOO
 
 
hsa-miR-181d-5p
 
 
-0.492
 
 
12.8
 
 
 
 
APOO
 
 
hsa-miR-181b-5p
 
 
-0.5
 
 
12.8
 
 
 
 
APOO
 
 
hsa-miR-181a-5p
 
 
-0.5
 
 
12.8
 
 
 
 
APP
 
 
hsa-miR-15b-5p
 
 
-0.298
 
 
232.5
 
 
 
 
AQP3
 
 
hsa-miR-874-3p
 
 
-0.48
 
 
81.8
 
 
 
 
ARF4
 
 
hsa-miR-30b-5p
 
 
-0.289
 
 
129.9
 
 
 
 
ARF4
 
 
hsa-miR-194-5p
 
 
-0.293
 
 
129.9
 
 
 
 
ARF4
 
 
hsa-miR-152-3p
 
 
-0.487
 
 
129.9
 
 
 
 
ARF4
 
 
hsa-miR-148b-3p
 
 
-0.487
 
 
129.9
 
 
 
 
ARF5
 
 
hsa-miR-29a-3p
 
 
-0.491
 
 
107.0
 
 
 
 
ARF6
 
 
hsa-miR-181b-5p
 
 
-0.33
 
 
30.5
 
 
 
 
ARF6
 
 
hsa-miR-181a-5p
 
 
-0.33
 
 
30.5
 
 
 
 
ARF6
 
 
hsa-miR-181d-5p
 
 
-0.338
 
 
30.5
 
 
 
 
ARFGEF1
 
 
hsa-miR-148b-3p
 
 
-0.306
 
 
12.2
 
 
 
 
ARFGEF1
 
 
hsa-miR-152-3p
 
 
-0.306
 
 
12.2
 
 
 
 
ARFIP1
 
 
hsa-miR-320b
 
 
-0.339
 
 
19.8
 
 
 
 
ARFIP1
 
 
hsa-miR-320c
 
 
-0.339
 
 
19.8
 
 
 
 
ARFIP1
 
 
hsa-miR-152-3p
 
 
-0.474
 
 
19.8
 
 
 
 
ARFIP1
 
 
hsa-miR-148b-3p
 
 
-0.474
 
 
19.8
 
 
 
 
ARG2
 
 
hsa-let-7b-5p
 
 
-0.468
 
 
101.8
 
 
 
 
ARHGAP21
 
 
hsa-miR-152-3p
 
 
-0.287
 
 
10.2
 
 
 
 
ARHGAP21
 
 
hsa-miR-148b-3p
 
 
-0.287
 
 
10.2
 
 
 
 
ARHGAP6
 
 
hsa-miR-1271-5p
 
 
-0.521
 
 
25.9
 
 
 
 
ARID1B
 
 
hsa-miR-29a-3p
 
 
-0.342
 
 
11.6
 
 
 
 
 
 
 rectum 
 
 
 
 
target_symbol
 
 
mature_mirna_id
 
 
score
 
 
nTPM
 
 
 
 
 
 
AAMP
 
 
hsa-miR-326
 
 
-0.722
 
 
54.4
 
 
 
 
AAR2
 
 
hsa-miR-29a-3p
 
 
-0.327
 
 
16.3
 
 
 
 
ABCB10
 
 
hsa-miR-152-3p
 
 
-0.291
 
 
10.6
 
 
 
 
ABCB10
 
 
hsa-miR-148b-3p
 
 
-0.291
 
 
10.6
 
 
 
 
ABCD3
 
 
hsa-miR-148b-3p
 
 
-0.388
 
 
37.8
 
 
 
 
ABCD3
 
 
hsa-miR-152-3p
 
 
-0.388
 
 
37.8
 
 
 
 
ABCE1
 
 
hsa-miR-29a-3p
 
 
-0.404
 
 
26.0
 
 
 
 
ABHD17C
 
 
hsa-let-7b-5p
 
 
-0.359
 
 
52.8
 
 
 
 
ABHD17C
 
 
hsa-miR-330-3p
 
 
-0.399
 
 
52.8
 
 
 
 
ABHD4
 
 
hsa-miR-29a-3p
 
 
-0.312
 
 
14.1
 
 
 
 
ACTR2
 
 
hsa-miR-15b-5p
 
 
-0.293
 
 
130.5
 
 
 
 
ADAM10
 
 
hsa-miR-152-3p
 
 
-0.348
 
 
16.1
 
 
 
 
ADAM10
 
 
hsa-miR-148b-3p
 
 
-0.356
 
 
16.1
 
 
 
 
ADAM9
 
 
hsa-miR-30d-5p
 
 
-0.304
 
 
39.2
 
 
 
 
ADAM9
 
 
hsa-miR-30b-5p
 
 
-0.312
 
 
39.2
 
 
 
 
ADCY3
 
 
hsa-miR-132-3p
 
 
-0.303
 
 
14.9
 
 
 
 
ADCY6
 
 
hsa-miR-1271-5p
 
 
-0.336
 
 
19.1
 
 
 
 
ADGRA3
 
 
hsa-miR-30d-5p
 
 
-0.296
 
 
16.1
 
 
 
 
ADGRA3
 
 
hsa-miR-30b-5p
 
 
-0.304
 
 
16.1
 
 
 
 
ADIPOR2
 
 
hsa-miR-150-5p
 
 
-0.287
 
 
16.7
 
 
 
 
ADK
 
 
hsa-miR-1271-5p
 
 
-0.329
 
 
18.3
 
 
 
 
ADNP
 
 
hsa-miR-328-3p
 
 
-0.371
 
 
22.7
 
 
 
 
AFG3L2
 
 
hsa-miR-181b-5p
 
 
-0.491
 
 
28.8
 
 
 
 
AFG3L2
 
 
hsa-miR-181a-5p
 
 
-0.491
 
 
28.8
 
 
 
 
AFG3L2
 
 
hsa-miR-181d-5p
 
 
-0.499
 
 
28.8
 
 
 
 
AGFG1
 
 
hsa-miR-148b-3p
 
 
-0.332
 
 
31.0
 
 
 
 
AGFG1
 
 
hsa-miR-152-3p
 
 
-0.332
 
 
31.0
 
 
 
 
AGFG1
 
 
hsa-miR-103a-3p
 
 
-0.389
 
 
31.0
 
 
 
 
AHR
 
 
hsa-miR-1271-5p
 
 
-0.304
 
 
15.3
 
 
 
 
AHR
 
 
hsa-miR-29a-3p
 
 
-0.316
 
 
15.3
 
 
 
 
AIFM1
 
 
hsa-let-7b-5p
 
 
-0.314
 
 
14.9
 
 
 
 
AK1
 
 
hsa-miR-29a-3p
 
 
-0.333
 
 
37.1
 
 
 
 
AK3
 
 
hsa-miR-1271-5p
 
 
-0.57
 
 
55.9
 
 
 
 
AK4
 
 
hsa-miR-15b-5p
 
 
-0.316
 
 
12.8
 
 
 
 
AKAP1
 
 
hsa-miR-152-3p
 
 
-0.381
 
 
45.9
 
 
 
 
AKAP1
 
 
hsa-miR-148b-3p
 
 
-0.381
 
 
45.9
 
 
 
 
AKIRIN2
 
 
hsa-miR-103a-3p
 
 
-0.299
 
 
11.7
 
 
 
 
ALAD
 
 
hsa-miR-326
 
 
-0.352
 
 
23.0
 
 
 
 
ALAD
 
 
hsa-miR-103a-3p
 
 
-0.385
 
 
23.0
 
 
 
 
ALAD
 
 
hsa-miR-15b-5p
 
 
-0.446
 
 
23.0
 
 
 
 
ALCAM
 
 
hsa-miR-148b-3p
 
 
-0.339
 
 
13.6
 
 
 
 
ALCAM
 
 
hsa-miR-152-3p
 
 
-0.347
 
 
13.6
 
 
 
 
ALDH6A1
 
 
hsa-miR-1271-5p
 
 
-0.301
 
 
14.8
 
 
 
 
AMT
 
 
hsa-let-7b-5p
 
 
-0.463
 
 
11.1
 
 
 
 
ANKHD1
 
 
hsa-miR-23a-3p
 
 
-0.368
 
 
31.1
 
 
 
 
ANKHD1
 
 
hsa-miR-30d-5p
 
 
-0.381
 
 
31.1
 
 
 
 
ANKHD1
 
 
hsa-miR-30b-5p
 
 
-0.405
 
 
31.1
 
 
 
 
ANKIB1
 
 
hsa-miR-1271-5p
 
 
-0.344
 
 
12.0
 
 
 
 
ANKRD17
 
 
hsa-miR-30d-5p
 
 
-0.312
 
 
17.8
 
 
 
 
ANKRD17
 
 
hsa-miR-30b-5p
 
 
-0.336
 
 
17.8
 
 
 
 
ANKRD27
 
 
hsa-miR-1271-5p
 
 
-0.648
 
 
11.1
 
 
 
 
ANP32A
 
 
hsa-miR-132-3p
 
 
-0.374
 
 
83.5
 
 
 
 
ANTXR2
 
 
hsa-miR-29a-3p
 
 
-0.347
 
 
40.1
 
 
 
 
ANXA11
 
 
hsa-miR-15b-5p
 
 
-0.463
 
 
113.3
 
 
 
 
AP1S1
 
 
hsa-let-7b-5p
 
 
-0.374
 
 
21.1
 
 
 
 
AP2B1
 
 
hsa-miR-1271-5p
 
 
-0.299
 
 
78.9
 
 
 
 
AP2B1
 
 
hsa-miR-15b-5p
 
 
-0.334
 
 
78.9
 
 
 
 
AP3S1
 
 
hsa-miR-30b-5p
 
 
-0.293
 
 
56.4
 
 
 
 
AP4B1
 
 
hsa-miR-378a-3p
 
 
-0.62
 
 
12.0
 
 
 
 
AP4B1
 
 
hsa-miR-378d
 
 
-0.62
 
 
12.0
 
 
 
 
AP5M1
 
 
hsa-miR-1271-5p
 
 
-0.343
 
 
27.7
 
 
 
 
APOO
 
 
hsa-miR-181d-5p
 
 
-0.492
 
 
17.6
 
 
 
 
APOO
 
 
hsa-miR-181b-5p
 
 
-0.5
 
 
17.6
 
 
 
 
APOO
 
 
hsa-miR-181a-5p
 
 
-0.5
 
 
17.6
 
 
 
 
APP
 
 
hsa-miR-15b-5p
 
 
-0.298
 
 
296.0
 
 
 
 
ARF4
 
 
hsa-miR-30b-5p
 
 
-0.289
 
 
185.2
 
 
 
 
ARF4
 
 
hsa-miR-194-5p
 
 
-0.293
 
 
185.2
 
 
 
 
ARF4
 
 
hsa-miR-152-3p
 
 
-0.487
 
 
185.2
 
 
 
 
ARF4
 
 
hsa-miR-148b-3p
 
 
-0.487
 
 
185.2
 
 
 
 
ARF5
 
 
hsa-miR-29a-3p
 
 
-0.491
 
 
57.6
 
 
 
 
ARF6
 
 
hsa-miR-181b-5p
 
 
-0.33
 
 
50.6
 
 
 
 
ARF6
 
 
hsa-miR-181a-5p
 
 
-0.33
 
 
50.6
 
 
 
 
ARF6
 
 
hsa-miR-181d-5p
 
 
-0.338
 
 
50.6
 
 
 
 
ARFGEF1
 
 
hsa-miR-148b-3p
 
 
-0.306
 
 
16.1
 
 
 
 
ARFGEF1
 
 
hsa-miR-152-3p
 
 
-0.306
 
 
16.1
 
 
 
 
ARFIP1
 
 
hsa-miR-320b
 
 
-0.339
 
 
26.2
 
 
 
 
ARFIP1
 
 
hsa-miR-320c
 
 
-0.339
 
 
26.2
 
 
 
 
ARFIP1
 
 
hsa-miR-152-3p
 
 
-0.474
 
 
26.2
 
 
 
 
ARFIP1
 
 
hsa-miR-148b-3p
 
 
-0.474
 
 
26.2
 
 
 
 
ARHGAP21
 
 
hsa-miR-152-3p
 
 
-0.287
 
 
17.4
 
 
 
 
ARHGAP21
 
 
hsa-miR-148b-3p
 
 
-0.287
 
 
17.4
 
 
 
 
ARID1B
 
 
hsa-miR-29a-3p
 
 
-0.342
 
 
11.4
 
 
 
 
ARIH2
 
 
hsa-miR-103a-3p
 
 
-0.307
 
 
16.6
 
 
 
 
ARL2
 
 
hsa-miR-15b-5p
 
 
-0.374
 
 
30.9
 
 
 
 
ARL2
 
 
hsa-miR-15b-5p
 
 
-0.442
 
 
30.9
 
 
 
 
ARL4A
 
 
hsa-miR-29a-3p
 
 
-0.386
 
 
32.6
 
 
 
 
ARL4A
 
 
hsa-miR-30d-5p
 
 
-0.432
 
 
32.6
 
 
 
 
ARL4A
 
 
hsa-miR-30b-5p
 
 
-0.448
 
 
32.6
 
 
 
 
ARL6IP1
 
 
hsa-miR-328-3p
 
 
-0.494
 
 
116.0
 
 
 
 
ARL6IP1
 
 
hsa-miR-148b-3p
 
 
-0.579
 
 
116.0
 
 
 
 
ARL6IP1
 
 
hsa-miR-152-3p
 
 
-0.579
 
 
116.0
 
 
 
 
ARL6IP6
 
 
hsa-let-7b-5p
 
 
-0.293
 
 
13.3
 
 
 
 
ARL8A
 
 
hsa-miR-103a-3p
 
 
-0.38
 
 
10.2
 
 
 
 
ARL8B
 
 
hsa-miR-152-3p
 
 
-0.337
 
 
32.7
 
 
 
 
ARL8B
 
 
hsa-miR-148b-3p
 
 
-0.337
 
 
32.7
 
 
 
 
ARL8B
 
 
hsa-miR-320b
 
 
-0.376
 
 
32.7
 
 
 
 
ARL8B
 
 
hsa-miR-320c
 
 
-0.376
 
 
32.7
 
 
 
 
ARMC1
 
 
hsa-miR-103a-3p
 
 
-0.525
 
 
15.6
 
 
 
 
ARMC8
 
 
hsa-miR-103a-3p
 
 
-0.412
 
 
17.2
 
 
 
 
ARMCX3
 
 
hsa-miR-425-5p
 
 
-0.325
 
 
20.5
 
 
 
 
 
 
 retina 
 
 
 
 
target_symbol
 
 
mature_mirna_id
 
 
score
 
 
nTPM
 
 
 
 
 
 
AAMP
 
 
hsa-miR-326
 
 
-0.722
 
 
35.6
 
 
 
 
ABAT
 
 
hsa-miR-1271-5p
 
 
-0.409
 
 
27.5
 
 
 
 
ABCD3
 
 
hsa-miR-148b-3p
 
 
-0.388
 
 
24.4
 
 
 
 
ABCD3
 
 
hsa-miR-152-3p
 
 
-0.388
 
 
24.4
 
 
 
 
ABCE1
 
 
hsa-miR-29a-3p
 
 
-0.404
 
 
13.3
 
 
 
 
ABHD17C
 
 
hsa-let-7b-5p
 
 
-0.359
 
 
15.6
 
 
 
 
ABHD17C
 
 
hsa-miR-330-3p
 
 
-0.399
 
 
15.6
 
 
 
 
ABHD4
 
 
hsa-miR-29a-3p
 
 
-0.312
 
 
21.0
 
 
 
 
ACSL6
 
 
hsa-let-7b-5p
 
 
-0.336
 
 
42.4
 
 
 
 
ACTC1
 
 
hsa-miR-30d-5p
 
 
-0.519
 
 
29.3
 
 
 
 
ACTC1
 
 
hsa-miR-30b-5p
 
 
-0.543
 
 
29.3
 
 
 
 
ACTR2
 
 
hsa-miR-15b-5p
 
 
-0.293
 
 
60.2
 
 
 
 
ADAM19
 
 
hsa-miR-874-3p
 
 
-0.374
 
 
21.0
 
 
 
 
ADAM9
 
 
hsa-miR-30d-5p
 
 
-0.304
 
 
17.2
 
 
 
 
ADAM9
 
 
hsa-miR-30b-5p
 
 
-0.312
 
 
17.2
 
 
 
 
ADIPOR2
 
 
hsa-miR-150-5p
 
 
-0.287
 
 
12.1
 
 
 
 
ADK
 
 
hsa-miR-1271-5p
 
 
-0.329
 
 
14.9
 
 
 
 
ADNP
 
 
hsa-miR-328-3p
 
 
-0.371
 
 
40.4
 
 
 
 
AGFG1
 
 
hsa-miR-148b-3p
 
 
-0.332
 
 
44.2
 
 
 
 
AGFG1
 
 
hsa-miR-152-3p
 
 
-0.332
 
 
44.2
 
 
 
 
AGFG1
 
 
hsa-miR-103a-3p
 
 
-0.389
 
 
44.2
 
 
 
 
AGO4
 
 
hsa-miR-1271-5p
 
 
-0.289
 
 
16.7
 
 
 
 
AGO4
 
 
hsa-miR-103a-3p
 
 
-0.32
 
 
16.7
 
 
 
 
AK1
 
 
hsa-miR-29a-3p
 
 
-0.333
 
 
27.1
 
 
 
 
AK3
 
 
hsa-miR-1271-5p
 
 
-0.57
 
 
15.5
 
 
 
 
AK4
 
 
hsa-miR-15b-5p
 
 
-0.316
 
 
19.7
 
 
 
 
AKAP1
 
 
hsa-miR-152-3p
 
 
-0.381
 
 
32.2
 
 
 
 
AKAP1
 
 
hsa-miR-148b-3p
 
 
-0.381
 
 
32.2
 
 
 
 
AKAP6
 
 
hsa-let-7b-5p
 
 
-0.307
 
 
12.6
 
 
 
 
AKIRIN2
 
 
hsa-miR-103a-3p
 
 
-0.299
 
 
27.5
 
 
 
 
AKT3
 
 
hsa-miR-29a-3p
 
 
-0.287
 
 
15.2
 
 
 
 
AKT3
 
 
hsa-miR-15b-5p
 
 
-0.301
 
 
15.2
 
 
 
 
ALAD
 
 
hsa-miR-326
 
 
-0.352
 
 
17.6
 
 
 
 
ALAD
 
 
hsa-miR-103a-3p
 
 
-0.385
 
 
17.6
 
 
 
 
ALAD
 
 
hsa-miR-15b-5p
 
 
-0.446
 
 
17.6
 
 
 
 
ALDH6A1
 
 
hsa-miR-1271-5p
 
 
-0.301
 
 
14.1
 
 
 
 
ANKHD1
 
 
hsa-miR-23a-3p
 
 
-0.368
 
 
37.5
 
 
 
 
ANKHD1
 
 
hsa-miR-30d-5p
 
 
-0.381
 
 
37.5
 
 
 
 
ANKHD1
 
 
hsa-miR-30b-5p
 
 
-0.405
 
 
37.5
 
 
 
 
ANKRD13C
 
 
hsa-miR-29a-3p
 
 
-0.411
 
 
12.3
 
 
 
 
ANKRD17
 
 
hsa-miR-30d-5p
 
 
-0.312
 
 
33.3
 
 
 
 
ANKRD17
 
 
hsa-miR-30b-5p
 
 
-0.336
 
 
33.3
 
 
 
 
ANKRD27
 
 
hsa-miR-1271-5p
 
 
-0.648
 
 
10.8
 
 
 
 
ANKRD46
 
 
hsa-let-7b-5p
 
 
-0.389
 
 
32.6
 
 
 
 
ANP32A
 
 
hsa-miR-132-3p
 
 
-0.374
 
 
98.2
 
 
 
 
AP1S1
 
 
hsa-let-7b-5p
 
 
-0.374
 
 
13.5
 
 
 
 
AP2A1
 
 
hsa-miR-15b-5p
 
 
-0.333
 
 
12.4
 
 
 
 
AP2B1
 
 
hsa-miR-1271-5p
 
 
-0.299
 
 
70.5
 
 
 
 
AP2B1
 
 
hsa-miR-15b-5p
 
 
-0.334
 
 
70.5
 
 
 
 
AP3S1
 
 
hsa-miR-30b-5p
 
 
-0.293
 
 
31.5
 
 
 
 
AP4B1
 
 
hsa-miR-378a-3p
 
 
-0.62
 
 
18.5
 
 
 
 
AP4B1
 
 
hsa-miR-378d
 
 
-0.62
 
 
18.5
 
 
 
 
AP5M1
 
 
hsa-miR-1271-5p
 
 
-0.343
 
 
20.8
 
 
 
 
APC
 
 
hsa-miR-29a-3p
 
 
-0.413
 
 
18.5
 
 
 
 
APOO
 
 
hsa-miR-181d-5p
 
 
-0.492
 
 
48.9
 
 
 
 
APOO
 
 
hsa-miR-181b-5p
 
 
-0.5
 
 
48.9
 
 
 
 
APOO
 
 
hsa-miR-181a-5p
 
 
-0.5
 
 
48.9
 
 
 
 
APP
 
 
hsa-miR-15b-5p
 
 
-0.298
 
 
189.1
 
 
 
 
ARF4
 
 
hsa-miR-30b-5p
 
 
-0.289
 
 
50.0
 
 
 
 
ARF4
 
 
hsa-miR-194-5p
 
 
-0.293
 
 
50.0
 
 
 
 
ARF4
 
 
hsa-miR-152-3p
 
 
-0.487
 
 
50.0
 
 
 
 
ARF4
 
 
hsa-miR-148b-3p
 
 
-0.487
 
 
50.0
 
 
 
 
ARF5
 
 
hsa-miR-29a-3p
 
 
-0.491
 
 
21.3
 
 
 
 
ARF6
 
 
hsa-miR-181b-5p
 
 
-0.33
 
 
13.4
 
 
 
 
ARF6
 
 
hsa-miR-181a-5p
 
 
-0.33
 
 
13.4
 
 
 
 
ARF6
 
 
hsa-miR-181d-5p
 
 
-0.338
 
 
13.4
 
 
 
 
ARFGEF1
 
 
hsa-miR-148b-3p
 
 
-0.306
 
 
33.8
 
 
 
 
ARFGEF1
 
 
hsa-miR-152-3p
 
 
-0.306
 
 
33.8
 
 
 
 
ARFIP1
 
 
hsa-miR-320b
 
 
-0.339
 
 
18.7
 
 
 
 
ARFIP1
 
 
hsa-miR-320c
 
 
-0.339
 
 
18.7
 
 
 
 
ARFIP1
 
 
hsa-miR-152-3p
 
 
-0.474
 
 
18.7
 
 
 
 
ARFIP1
 
 
hsa-miR-148b-3p
 
 
-0.474
 
 
18.7
 
 
 
 
ARG2
 
 
hsa-let-7b-5p
 
 
-0.468
 
 
20.7
 
 
 
 
ARHGAP21
 
 
hsa-miR-152-3p
 
 
-0.287
 
 
37.0
 
 
 
 
ARHGAP21
 
 
hsa-miR-148b-3p
 
 
-0.287
 
 
37.0
 
 
 
 
ARID1B
 
 
hsa-miR-29a-3p
 
 
-0.342
 
 
10.7
 
 
 
 
ARID2
 
 
hsa-miR-132-3p
 
 
-0.304
 
 
12.3
 
 
 
 
ARID2
 
 
hsa-miR-103a-3p
 
 
-0.415
 
 
12.3
 
 
 
 
ARID3B
 
 
hsa-let-7b-5p
 
 
-0.322
 
 
17.4
 
 
 
 
ARID3B
 
 
hsa-let-7b-5p
 
 
-0.486
 
 
17.4
 
 
 
 
ARIH2
 
 
hsa-miR-103a-3p
 
 
-0.307
 
 
30.1
 
 
 
 
ARL2
 
 
hsa-miR-15b-5p
 
 
-0.374
 
 
40.5
 
 
 
 
ARL2
 
 
hsa-miR-15b-5p
 
 
-0.442
 
 
40.5
 
 
 
 
ARL3
 
 
hsa-miR-15b-5p
 
 
-0.507
 
 
89.3
 
 
 
 
ARL4D
 
 
hsa-miR-1271-5p
 
 
-0.392
 
 
78.9
 
 
 
 
ARL4D
 
 
hsa-let-7b-5p
 
 
-0.417
 
 
78.9
 
 
 
 
ARL6IP1
 
 
hsa-miR-328-3p
 
 
-0.494
 
 
292.6
 
 
 
 
ARL6IP1
 
 
hsa-miR-148b-3p
 
 
-0.579
 
 
292.6
 
 
 
 
ARL6IP1
 
 
hsa-miR-152-3p
 
 
-0.579
 
 
292.6
 
 
 
 
ARL6IP6
 
 
hsa-let-7b-5p
 
 
-0.293
 
 
10.5
 
 
 
 
ARL8A
 
 
hsa-miR-103a-3p
 
 
-0.38
 
 
17.4
 
 
 
 
ARL8B
 
 
hsa-miR-152-3p
 
 
-0.337
 
 
31.7
 
 
 
 
ARL8B
 
 
hsa-miR-148b-3p
 
 
-0.337
 
 
31.7
 
 
 
 
ARL8B
 
 
hsa-miR-320b
 
 
-0.376
 
 
31.7
 
 
 
 
ARL8B
 
 
hsa-miR-320c
 
 
-0.376
 
 
31.7
 
 
 
 
ARMC1
 
 
hsa-miR-103a-3p
 
 
-0.525
 
 
11.6
 
 
 
 
ARMC8
 
 
hsa-miR-103a-3p
 
 
-0.412
 
 
24.3
 
 
 
 
ARMCX2
 
 
hsa-miR-15b-5p
 
 
-0.344
 
 
14.2
 
 
 
 
ARMCX3
 
 
hsa-miR-425-5p
 
 
-0.325
 
 
17.6
 
 
 
 
ARMT1
 
 
hsa-let-7b-5p
 
 
-0.291
 
 
15.2
 
 
 
 
 
 
 salivary gland 
 
 
 
 
target_symbol
 
 
mature_mirna_id
 
 
score
 
 
nTPM
 
 
 
 
 
 
AAMP
 
 
hsa-miR-326
 
 
-0.722
 
 
63.0
 
 
 
 
AAR2
 
 
hsa-miR-29a-3p
 
 
-0.327
 
 
17.0
 
 
 
 
ABCD3
 
 
hsa-miR-148b-3p
 
 
-0.388
 
 
16.5
 
 
 
 
ABCD3
 
 
hsa-miR-152-3p
 
 
-0.388
 
 
16.5
 
 
 
 
ABCE1
 
 
hsa-miR-29a-3p
 
 
-0.404
 
 
22.7
 
 
 
 
ABHD17C
 
 
hsa-let-7b-5p
 
 
-0.359
 
 
22.3
 
 
 
 
ABHD17C
 
 
hsa-miR-330-3p
 
 
-0.399
 
 
22.3
 
 
 
 
ABHD4
 
 
hsa-miR-29a-3p
 
 
-0.312
 
 
17.2
 
 
 
 
ABHD5
 
 
hsa-miR-132-3p
 
 
-0.323
 
 
19.4
 
 
 
 
ACTC1
 
 
hsa-miR-30d-5p
 
 
-0.519
 
 
41.7
 
 
 
 
ACTC1
 
 
hsa-miR-30b-5p
 
 
-0.543
 
 
41.7
 
 
 
 
ACTN2
 
 
hsa-miR-23a-3p
 
 
-0.294
 
 
18.4
 
 
 
 
ACTN2
 
 
hsa-miR-29a-3p
 
 
-0.311
 
 
18.4
 
 
 
 
ACTR2
 
 
hsa-miR-15b-5p
 
 
-0.293
 
 
60.3
 
 
 
 
ADAM9
 
 
hsa-miR-30d-5p
 
 
-0.304
 
 
39.8
 
 
 
 
ADAM9
 
 
hsa-miR-30b-5p
 
 
-0.312
 
 
39.8
 
 
 
 
ADCY3
 
 
hsa-miR-132-3p
 
 
-0.303
 
 
20.4
 
 
 
 
ADCY6
 
 
hsa-miR-1271-5p
 
 
-0.336
 
 
31.6
 
 
 
 
ADIPOR2
 
 
hsa-miR-150-5p
 
 
-0.287
 
 
21.2
 
 
 
 
ADK
 
 
hsa-miR-1271-5p
 
 
-0.329
 
 
18.2
 
 
 
 
ADNP
 
 
hsa-miR-328-3p
 
 
-0.371
 
 
27.9
 
 
 
 
AFG3L2
 
 
hsa-miR-181b-5p
 
 
-0.491
 
 
15.5
 
 
 
 
AFG3L2
 
 
hsa-miR-181a-5p
 
 
-0.491
 
 
15.5
 
 
 
 
AFG3L2
 
 
hsa-miR-181d-5p
 
 
-0.499
 
 
15.5
 
 
 
 
AGFG1
 
 
hsa-miR-148b-3p
 
 
-0.332
 
 
26.3
 
 
 
 
AGFG1
 
 
hsa-miR-152-3p
 
 
-0.332
 
 
26.3
 
 
 
 
AGFG1
 
 
hsa-miR-103a-3p
 
 
-0.389
 
 
26.3
 
 
 
 
AHDC1
 
 
hsa-miR-152-3p
 
 
-0.413
 
 
16.0
 
 
 
 
AHDC1
 
 
hsa-miR-148b-3p
 
 
-0.413
 
 
16.0
 
 
 
 
AIFM1
 
 
hsa-let-7b-5p
 
 
-0.314
 
 
17.0
 
 
 
 
AK1
 
 
hsa-miR-29a-3p
 
 
-0.333
 
 
38.2
 
 
 
 
AK3
 
 
hsa-miR-1271-5p
 
 
-0.57
 
 
36.4
 
 
 
 
AKAP1
 
 
hsa-miR-152-3p
 
 
-0.381
 
 
31.6
 
 
 
 
AKAP1
 
 
hsa-miR-148b-3p
 
 
-0.381
 
 
31.6
 
 
 
 
AKIRIN2
 
 
hsa-miR-103a-3p
 
 
-0.299
 
 
21.8
 
 
 
 
ALAD
 
 
hsa-miR-326
 
 
-0.352
 
 
92.9
 
 
 
 
ALAD
 
 
hsa-miR-103a-3p
 
 
-0.385
 
 
92.9
 
 
 
 
ALAD
 
 
hsa-miR-15b-5p
 
 
-0.446
 
 
92.9
 
 
 
 
ALCAM
 
 
hsa-miR-148b-3p
 
 
-0.339
 
 
31.9
 
 
 
 
ALCAM
 
 
hsa-miR-152-3p
 
 
-0.347
 
 
31.9
 
 
 
 
ALDH6A1
 
 
hsa-miR-1271-5p
 
 
-0.301
 
 
18.6
 
 
 
 
ALOX12
 
 
hsa-miR-15b-5p
 
 
-0.443
 
 
17.3
 
 
 
 
AMOTL1
 
 
hsa-miR-15b-5p
 
 
-0.319
 
 
10.4
 
 
 
 
AMT
 
 
hsa-let-7b-5p
 
 
-0.463
 
 
10.0
 
 
 
 
ANKHD1
 
 
hsa-miR-23a-3p
 
 
-0.368
 
 
40.4
 
 
 
 
ANKHD1
 
 
hsa-miR-30d-5p
 
 
-0.381
 
 
40.4
 
 
 
 
ANKHD1
 
 
hsa-miR-30b-5p
 
 
-0.405
 
 
40.4
 
 
 
 
ANKIB1
 
 
hsa-miR-1271-5p
 
 
-0.344
 
 
13.1
 
 
 
 
ANKRD17
 
 
hsa-miR-30d-5p
 
 
-0.312
 
 
15.7
 
 
 
 
ANKRD17
 
 
hsa-miR-30b-5p
 
 
-0.336
 
 
15.7
 
 
 
 
ANKRD46
 
 
hsa-let-7b-5p
 
 
-0.389
 
 
16.1
 
 
 
 
ANP32A
 
 
hsa-miR-132-3p
 
 
-0.374
 
 
50.8
 
 
 
 
ANTXR2
 
 
hsa-miR-29a-3p
 
 
-0.347
 
 
11.4
 
 
 
 
ANXA11
 
 
hsa-miR-15b-5p
 
 
-0.463
 
 
87.6
 
 
 
 
AP1S1
 
 
hsa-let-7b-5p
 
 
-0.374
 
 
21.5
 
 
 
 
AP2A1
 
 
hsa-miR-15b-5p
 
 
-0.333
 
 
27.6
 
 
 
 
AP2B1
 
 
hsa-miR-1271-5p
 
 
-0.299
 
 
39.5
 
 
 
 
AP2B1
 
 
hsa-miR-15b-5p
 
 
-0.334
 
 
39.5
 
 
 
 
AP3S1
 
 
hsa-miR-30b-5p
 
 
-0.293
 
 
56.0
 
 
 
 
AP5M1
 
 
hsa-miR-1271-5p
 
 
-0.343
 
 
15.7
 
 
 
 
APOO
 
 
hsa-miR-181d-5p
 
 
-0.492
 
 
18.5
 
 
 
 
APOO
 
 
hsa-miR-181b-5p
 
 
-0.5
 
 
18.5
 
 
 
 
APOO
 
 
hsa-miR-181a-5p
 
 
-0.5
 
 
18.5
 
 
 
 
APP
 
 
hsa-miR-15b-5p
 
 
-0.298
 
 
256.4
 
 
 
 
AQP3
 
 
hsa-miR-874-3p
 
 
-0.48
 
 
376.5
 
 
 
 
ARF4
 
 
hsa-miR-30b-5p
 
 
-0.289
 
 
171.4
 
 
 
 
ARF4
 
 
hsa-miR-194-5p
 
 
-0.293
 
 
171.4
 
 
 
 
ARF4
 
 
hsa-miR-152-3p
 
 
-0.487
 
 
171.4
 
 
 
 
ARF4
 
 
hsa-miR-148b-3p
 
 
-0.487
 
 
171.4
 
 
 
 
ARF5
 
 
hsa-miR-29a-3p
 
 
-0.491
 
 
110.8
 
 
 
 
ARF6
 
 
hsa-miR-181b-5p
 
 
-0.33
 
 
33.7
 
 
 
 
ARF6
 
 
hsa-miR-181a-5p
 
 
-0.33
 
 
33.7
 
 
 
 
ARF6
 
 
hsa-miR-181d-5p
 
 
-0.338
 
 
33.7
 
 
 
 
ARFGEF1
 
 
hsa-miR-148b-3p
 
 
-0.306
 
 
13.9
 
 
 
 
ARFGEF1
 
 
hsa-miR-152-3p
 
 
-0.306
 
 
13.9
 
 
 
 
ARFIP1
 
 
hsa-miR-320b
 
 
-0.339
 
 
12.9
 
 
 
 
ARFIP1
 
 
hsa-miR-320c
 
 
-0.339
 
 
12.9
 
 
 
 
ARFIP1
 
 
hsa-miR-152-3p
 
 
-0.474
 
 
12.9
 
 
 
 
ARFIP1
 
 
hsa-miR-148b-3p
 
 
-0.474
 
 
12.9
 
 
 
 
ARG2
 
 
hsa-let-7b-5p
 
 
-0.468
 
 
18.4
 
 
 
 
ARHGAP21
 
 
hsa-miR-152-3p
 
 
-0.287
 
 
23.1
 
 
 
 
ARHGAP21
 
 
hsa-miR-148b-3p
 
 
-0.287
 
 
23.1
 
 
 
 
ARID1B
 
 
hsa-miR-29a-3p
 
 
-0.342
 
 
18.1
 
 
 
 
ARIH2
 
 
hsa-miR-103a-3p
 
 
-0.307
 
 
18.1
 
 
 
 
ARL2
 
 
hsa-miR-15b-5p
 
 
-0.374
 
 
67.7
 
 
 
 
ARL2
 
 
hsa-miR-15b-5p
 
 
-0.442
 
 
67.7
 
 
 
 
ARL4A
 
 
hsa-miR-29a-3p
 
 
-0.386
 
 
81.5
 
 
 
 
ARL4A
 
 
hsa-miR-30d-5p
 
 
-0.432
 
 
81.5
 
 
 
 
ARL4A
 
 
hsa-miR-30b-5p
 
 
-0.448
 
 
81.5
 
 
 
 
ARL4D
 
 
hsa-miR-1271-5p
 
 
-0.392
 
 
16.7
 
 
 
 
ARL4D
 
 
hsa-let-7b-5p
 
 
-0.417
 
 
16.7
 
 
 
 
ARL6IP1
 
 
hsa-miR-328-3p
 
 
-0.494
 
 
79.4
 
 
 
 
ARL6IP1
 
 
hsa-miR-148b-3p
 
 
-0.579
 
 
79.4
 
 
 
 
ARL6IP1
 
 
hsa-miR-152-3p
 
 
-0.579
 
 
79.4
 
 
 
 
ARL8A
 
 
hsa-miR-103a-3p
 
 
-0.38
 
 
29.6
 
 
 
 
ARL8B
 
 
hsa-miR-152-3p
 
 
-0.337
 
 
35.3
 
 
 
 
ARL8B
 
 
hsa-miR-148b-3p
 
 
-0.337
 
 
35.3
 
 
 
 
ARL8B
 
 
hsa-miR-320b
 
 
-0.376
 
 
35.3
 
 
 
 
ARL8B
 
 
hsa-miR-320c
 
 
-0.376
 
 
35.3
 
 
 
 
ARMC1
 
 
hsa-miR-103a-3p
 
 
-0.525
 
 
14.8
 
 
 
 
 
 
 seminal vesicle 
 
 
 
 
target_symbol
 
 
mature_mirna_id
 
 
score
 
 
nTPM
 
 
 
 
 
 
AAMP
 
 
hsa-miR-326
 
 
-0.722
 
 
55.3
 
 
 
 
AAR2
 
 
hsa-miR-29a-3p
 
 
-0.327
 
 
19.9
 
 
 
 
ABCD3
 
 
hsa-miR-148b-3p
 
 
-0.388
 
 
25.5
 
 
 
 
ABCD3
 
 
hsa-miR-152-3p
 
 
-0.388
 
 
25.5
 
 
 
 
ABCE1
 
 
hsa-miR-29a-3p
 
 
-0.404
 
 
15.9
 
 
 
 
ABHD4
 
 
hsa-miR-29a-3p
 
 
-0.312
 
 
34.6
 
 
 
 
ACSL6
 
 
hsa-let-7b-5p
 
 
-0.336
 
 
17.0
 
 
 
 
ACTC1
 
 
hsa-miR-30d-5p
 
 
-0.519
 
 
506.6
 
 
 
 
ACTC1
 
 
hsa-miR-30b-5p
 
 
-0.543
 
 
506.6
 
 
 
 
ACTR2
 
 
hsa-miR-15b-5p
 
 
-0.293
 
 
57.7
 
 
 
 
ACVR1
 
 
hsa-miR-30b-5p
 
 
-0.29
 
 
11.3
 
 
 
 
ACVR1
 
 
hsa-miR-152-3p
 
 
-0.397
 
 
11.3
 
 
 
 
ACVR1
 
 
hsa-miR-148b-3p
 
 
-0.397
 
 
11.3
 
 
 
 
ADAM10
 
 
hsa-miR-152-3p
 
 
-0.348
 
 
11.5
 
 
 
 
ADAM10
 
 
hsa-miR-148b-3p
 
 
-0.356
 
 
11.5
 
 
 
 
ADAM9
 
 
hsa-miR-30d-5p
 
 
-0.304
 
 
32.8
 
 
 
 
ADAM9
 
 
hsa-miR-30b-5p
 
 
-0.312
 
 
32.8
 
 
 
 
ADCY3
 
 
hsa-miR-132-3p
 
 
-0.303
 
 
31.0
 
 
 
 
ADCY6
 
 
hsa-miR-1271-5p
 
 
-0.336
 
 
15.5
 
 
 
 
ADGRA2
 
 
hsa-miR-1271-5p
 
 
-0.427
 
 
51.7
 
 
 
 
ADGRA3
 
 
hsa-miR-30d-5p
 
 
-0.296
 
 
10.8
 
 
 
 
ADGRA3
 
 
hsa-miR-30b-5p
 
 
-0.304
 
 
10.8
 
 
 
 
ADIPOR2
 
 
hsa-miR-150-5p
 
 
-0.287
 
 
11.2
 
 
 
 
ADK
 
 
hsa-miR-1271-5p
 
 
-0.329
 
 
15.1
 
 
 
 
ADNP
 
 
hsa-miR-328-3p
 
 
-0.371
 
 
28.1
 
 
 
 
AFG3L2
 
 
hsa-miR-181b-5p
 
 
-0.491
 
 
11.8
 
 
 
 
AFG3L2
 
 
hsa-miR-181a-5p
 
 
-0.491
 
 
11.8
 
 
 
 
AFG3L2
 
 
hsa-miR-181d-5p
 
 
-0.499
 
 
11.8
 
 
 
 
AGFG1
 
 
hsa-miR-148b-3p
 
 
-0.332
 
 
22.0
 
 
 
 
AGFG1
 
 
hsa-miR-152-3p
 
 
-0.332
 
 
22.0
 
 
 
 
AGFG1
 
 
hsa-miR-103a-3p
 
 
-0.389
 
 
22.0
 
 
 
 
AHR
 
 
hsa-miR-1271-5p
 
 
-0.304
 
 
10.6
 
 
 
 
AHR
 
 
hsa-miR-29a-3p
 
 
-0.316
 
 
10.6
 
 
 
 
AJUBA
 
 
hsa-miR-103a-3p
 
 
-0.448
 
 
14.9
 
 
 
 
AK1
 
 
hsa-miR-29a-3p
 
 
-0.333
 
 
33.8
 
 
 
 
AK3
 
 
hsa-miR-1271-5p
 
 
-0.57
 
 
40.1
 
 
 
 
AK4
 
 
hsa-miR-15b-5p
 
 
-0.316
 
 
11.8
 
 
 
 
AKAP1
 
 
hsa-miR-152-3p
 
 
-0.381
 
 
23.3
 
 
 
 
AKAP1
 
 
hsa-miR-148b-3p
 
 
-0.381
 
 
23.3
 
 
 
 
AKIRIN2
 
 
hsa-miR-103a-3p
 
 
-0.299
 
 
11.0
 
 
 
 
AKT3
 
 
hsa-miR-29a-3p
 
 
-0.287
 
 
14.7
 
 
 
 
AKT3
 
 
hsa-miR-15b-5p
 
 
-0.301
 
 
14.7
 
 
 
 
ALAD
 
 
hsa-miR-326
 
 
-0.352
 
 
22.8
 
 
 
 
ALAD
 
 
hsa-miR-103a-3p
 
 
-0.385
 
 
22.8
 
 
 
 
ALAD
 
 
hsa-miR-15b-5p
 
 
-0.446
 
 
22.8
 
 
 
 
ALCAM
 
 
hsa-miR-148b-3p
 
 
-0.339
 
 
37.3
 
 
 
 
ALCAM
 
 
hsa-miR-152-3p
 
 
-0.347
 
 
37.3
 
 
 
 
ALDH1A2
 
 
hsa-miR-23a-3p
 
 
-0.354
 
 
50.2
 
 
 
 
ALDH6A1
 
 
hsa-miR-1271-5p
 
 
-0.301
 
 
15.1
 
 
 
 
AMOT
 
 
hsa-miR-29a-3p
 
 
-0.304
 
 
24.6
 
 
 
 
AMOTL1
 
 
hsa-miR-15b-5p
 
 
-0.319
 
 
21.7
 
 
 
 
ANKHD1
 
 
hsa-miR-23a-3p
 
 
-0.368
 
 
26.1
 
 
 
 
ANKHD1
 
 
hsa-miR-30d-5p
 
 
-0.381
 
 
26.1
 
 
 
 
ANKHD1
 
 
hsa-miR-30b-5p
 
 
-0.405
 
 
26.1
 
 
 
 
ANKIB1
 
 
hsa-miR-1271-5p
 
 
-0.344
 
 
10.5
 
 
 
 
ANKRD17
 
 
hsa-miR-30d-5p
 
 
-0.312
 
 
13.0
 
 
 
 
ANKRD17
 
 
hsa-miR-30b-5p
 
 
-0.336
 
 
13.0
 
 
 
 
ANKRD27
 
 
hsa-miR-1271-5p
 
 
-0.648
 
 
12.7
 
 
 
 
ANKRD46
 
 
hsa-let-7b-5p
 
 
-0.389
 
 
12.4
 
 
 
 
ANP32A
 
 
hsa-miR-132-3p
 
 
-0.374
 
 
62.9
 
 
 
 
ANTXR2
 
 
hsa-miR-29a-3p
 
 
-0.347
 
 
85.0
 
 
 
 
ANXA11
 
 
hsa-miR-15b-5p
 
 
-0.463
 
 
77.1
 
 
 
 
AP2A1
 
 
hsa-miR-15b-5p
 
 
-0.333
 
 
17.5
 
 
 
 
AP2B1
 
 
hsa-miR-1271-5p
 
 
-0.299
 
 
47.2
 
 
 
 
AP2B1
 
 
hsa-miR-15b-5p
 
 
-0.334
 
 
47.2
 
 
 
 
AP3S1
 
 
hsa-miR-30b-5p
 
 
-0.293
 
 
80.7
 
 
 
 
AP5M1
 
 
hsa-miR-1271-5p
 
 
-0.343
 
 
11.8
 
 
 
 
APOO
 
 
hsa-miR-181d-5p
 
 
-0.492
 
 
14.1
 
 
 
 
APOO
 
 
hsa-miR-181b-5p
 
 
-0.5
 
 
14.1
 
 
 
 
APOO
 
 
hsa-miR-181a-5p
 
 
-0.5
 
 
14.1
 
 
 
 
APP
 
 
hsa-miR-15b-5p
 
 
-0.298
 
 
315.9
 
 
 
 
AQP3
 
 
hsa-miR-874-3p
 
 
-0.48
 
 
34.7
 
 
 
 
ARF4
 
 
hsa-miR-30b-5p
 
 
-0.289
 
 
125.1
 
 
 
 
ARF4
 
 
hsa-miR-194-5p
 
 
-0.293
 
 
125.1
 
 
 
 
ARF4
 
 
hsa-miR-152-3p
 
 
-0.487
 
 
125.1
 
 
 
 
ARF4
 
 
hsa-miR-148b-3p
 
 
-0.487
 
 
125.1
 
 
 
 
ARF5
 
 
hsa-miR-29a-3p
 
 
-0.491
 
 
67.1
 
 
 
 
ARF6
 
 
hsa-miR-181b-5p
 
 
-0.33
 
 
18.1
 
 
 
 
ARF6
 
 
hsa-miR-181a-5p
 
 
-0.33
 
 
18.1
 
 
 
 
ARF6
 
 
hsa-miR-181d-5p
 
 
-0.338
 
 
18.1
 
 
 
 
ARFGEF1
 
 
hsa-miR-148b-3p
 
 
-0.306
 
 
11.4
 
 
 
 
ARFGEF1
 
 
hsa-miR-152-3p
 
 
-0.306
 
 
11.4
 
 
 
 
ARFIP1
 
 
hsa-miR-320b
 
 
-0.339
 
 
16.2
 
 
 
 
ARFIP1
 
 
hsa-miR-320c
 
 
-0.339
 
 
16.2
 
 
 
 
ARFIP1
 
 
hsa-miR-152-3p
 
 
-0.474
 
 
16.2
 
 
 
 
ARFIP1
 
 
hsa-miR-148b-3p
 
 
-0.474
 
 
16.2
 
 
 
 
ARHGAP21
 
 
hsa-miR-152-3p
 
 
-0.287
 
 
10.8
 
 
 
 
ARHGAP21
 
 
hsa-miR-148b-3p
 
 
-0.287
 
 
10.8
 
 
 
 
ARHGAP6
 
 
hsa-miR-1271-5p
 
 
-0.521
 
 
29.1
 
 
 
 
ARID1B
 
 
hsa-miR-29a-3p
 
 
-0.342
 
 
11.6
 
 
 
 
ARIH2
 
 
hsa-miR-103a-3p
 
 
-0.307
 
 
20.6
 
 
 
 
ARL2
 
 
hsa-miR-15b-5p
 
 
-0.374
 
 
62.7
 
 
 
 
ARL2
 
 
hsa-miR-15b-5p
 
 
-0.442
 
 
62.7
 
 
 
 
ARL4A
 
 
hsa-miR-29a-3p
 
 
-0.386
 
 
15.5
 
 
 
 
ARL4A
 
 
hsa-miR-30d-5p
 
 
-0.432
 
 
15.5
 
 
 
 
ARL4A
 
 
hsa-miR-30b-5p
 
 
-0.448
 
 
15.5
 
 
 
 
ARL4D
 
 
hsa-miR-1271-5p
 
 
-0.392
 
 
14.3
 
 
 
 
ARL4D
 
 
hsa-let-7b-5p
 
 
-0.417
 
 
14.3
 
 
 
 
ARL6IP1
 
 
hsa-miR-328-3p
 
 
-0.494
 
 
66.4
 
 
 
 
ARL6IP1
 
 
hsa-miR-148b-3p
 
 
-0.579
 
 
66.4
 
 
 
 
 
 
 skeletal muscle 
 
 
 
 
target_symbol
 
 
mature_mirna_id
 
 
score
 
 
nTPM
 
 
 
 
 
 
AAMP
 
 
hsa-miR-326
 
 
-0.722
 
 
88.8
 
 
 
 
AAR2
 
 
hsa-miR-29a-3p
 
 
-0.327
 
 
22.2
 
 
 
 
ABCB10
 
 
hsa-miR-152-3p
 
 
-0.291
 
 
12.0
 
 
 
 
ABCB10
 
 
hsa-miR-148b-3p
 
 
-0.291
 
 
12.0
 
 
 
 
ABCB6
 
 
hsa-miR-29a-3p
 
 
-0.48
 
 
26.6
 
 
 
 
ABCB7
 
 
hsa-miR-148b-3p
 
 
-0.515
 
 
29.2
 
 
 
 
ABCB7
 
 
hsa-miR-152-3p
 
 
-0.515
 
 
29.2
 
 
 
 
ABCD3
 
 
hsa-miR-148b-3p
 
 
-0.388
 
 
22.0
 
 
 
 
ABCD3
 
 
hsa-miR-152-3p
 
 
-0.388
 
 
22.0
 
 
 
 
ABCE1
 
 
hsa-miR-29a-3p
 
 
-0.404
 
 
27.0
 
 
 
 
ABHD18
 
 
hsa-miR-29a-3p
 
 
-0.321
 
 
10.1
 
 
 
 
ABHD18
 
 
hsa-miR-29a-3p
 
 
-0.34
 
 
10.1
 
 
 
 
ABHD4
 
 
hsa-miR-29a-3p
 
 
-0.312
 
 
26.2
 
 
 
 
ABHD5
 
 
hsa-miR-132-3p
 
 
-0.323
 
 
12.3
 
 
 
 
ACHE
 
 
hsa-miR-194-5p
 
 
-0.395
 
 
152.9
 
 
 
 
ACTC1
 
 
hsa-miR-30d-5p
 
 
-0.519
 
 
519.7
 
 
 
 
ACTC1
 
 
hsa-miR-30b-5p
 
 
-0.543
 
 
519.7
 
 
 
 
ACTN2
 
 
hsa-miR-23a-3p
 
 
-0.294
 
 
1961.6
 
 
 
 
ACTN2
 
 
hsa-miR-29a-3p
 
 
-0.311
 
 
1961.6
 
 
 
 
ACTR2
 
 
hsa-miR-15b-5p
 
 
-0.293
 
 
56.5
 
 
 
 
ACVR1
 
 
hsa-miR-30b-5p
 
 
-0.29
 
 
15.5
 
 
 
 
ACVR1
 
 
hsa-miR-152-3p
 
 
-0.397
 
 
15.5
 
 
 
 
ACVR1
 
 
hsa-miR-148b-3p
 
 
-0.397
 
 
15.5
 
 
 
 
ADAM9
 
 
hsa-miR-30d-5p
 
 
-0.304
 
 
16.2
 
 
 
 
ADAM9
 
 
hsa-miR-30b-5p
 
 
-0.312
 
 
16.2
 
 
 
 
ADCY2
 
 
hsa-miR-152-3p
 
 
-0.299
 
 
64.1
 
 
 
 
ADCY2
 
 
hsa-miR-148b-3p
 
 
-0.299
 
 
64.1
 
 
 
 
ADCY3
 
 
hsa-miR-132-3p
 
 
-0.303
 
 
14.1
 
 
 
 
ADCY6
 
 
hsa-miR-1271-5p
 
 
-0.336
 
 
11.4
 
 
 
 
ADGRA3
 
 
hsa-miR-30d-5p
 
 
-0.296
 
 
15.9
 
 
 
 
ADGRA3
 
 
hsa-miR-30b-5p
 
 
-0.304
 
 
15.9
 
 
 
 
ADGRF5
 
 
hsa-miR-148b-3p
 
 
-0.354
 
 
29.0
 
 
 
 
ADGRF5
 
 
hsa-miR-152-3p
 
 
-0.354
 
 
29.0
 
 
 
 
ADIPOR2
 
 
hsa-miR-150-5p
 
 
-0.287
 
 
41.4
 
 
 
 
ADK
 
 
hsa-miR-1271-5p
 
 
-0.329
 
 
38.8
 
 
 
 
ADNP
 
 
hsa-miR-328-3p
 
 
-0.371
 
 
33.8
 
 
 
 
ADRB2
 
 
hsa-miR-15b-5p
 
 
-0.29
 
 
10.9
 
 
 
 
ADRB2
 
 
hsa-let-7b-5p
 
 
-0.635
 
 
10.9
 
 
 
 
AFG3L2
 
 
hsa-miR-181b-5p
 
 
-0.491
 
 
96.6
 
 
 
 
AFG3L2
 
 
hsa-miR-181a-5p
 
 
-0.491
 
 
96.6
 
 
 
 
AFG3L2
 
 
hsa-miR-181d-5p
 
 
-0.499
 
 
96.6
 
 
 
 
AGFG1
 
 
hsa-miR-148b-3p
 
 
-0.332
 
 
31.6
 
 
 
 
AGFG1
 
 
hsa-miR-152-3p
 
 
-0.332
 
 
31.6
 
 
 
 
AGFG1
 
 
hsa-miR-103a-3p
 
 
-0.389
 
 
31.6
 
 
 
 
AGO2
 
 
hsa-miR-132-3p
 
 
-0.406
 
 
16.4
 
 
 
 
AGO2
 
 
hsa-miR-23a-3p
 
 
-0.576
 
 
16.4
 
 
 
 
AGO2
 
 
hsa-miR-30b-5p
 
 
-1.034
 
 
16.4
 
 
 
 
AGO2
 
 
hsa-miR-30d-5p
 
 
-1.034
 
 
16.4
 
 
 
 
AGO4
 
 
hsa-miR-1271-5p
 
 
-0.289
 
 
12.4
 
 
 
 
AGO4
 
 
hsa-miR-103a-3p
 
 
-0.32
 
 
12.4
 
 
 
 
AHCTF1
 
 
hsa-let-7b-5p
 
 
-0.374
 
 
10.0
 
 
 
 
AHDC1
 
 
hsa-miR-152-3p
 
 
-0.413
 
 
29.2
 
 
 
 
AHDC1
 
 
hsa-miR-148b-3p
 
 
-0.413
 
 
29.2
 
 
 
 
AIFM1
 
 
hsa-let-7b-5p
 
 
-0.314
 
 
58.6
 
 
 
 
AK1
 
 
hsa-miR-29a-3p
 
 
-0.333
 
 
1262.8
 
 
 
 
AK3
 
 
hsa-miR-1271-5p
 
 
-0.57
 
 
227.6
 
 
 
 
AKAP1
 
 
hsa-miR-152-3p
 
 
-0.381
 
 
194.7
 
 
 
 
AKAP1
 
 
hsa-miR-148b-3p
 
 
-0.381
 
 
194.7
 
 
 
 
AKAP6
 
 
hsa-let-7b-5p
 
 
-0.307
 
 
44.4
 
 
 
 
AKIRIN2
 
 
hsa-miR-103a-3p
 
 
-0.299
 
 
85.6
 
 
 
 
ALAD
 
 
hsa-miR-326
 
 
-0.352
 
 
26.9
 
 
 
 
ALAD
 
 
hsa-miR-103a-3p
 
 
-0.385
 
 
26.9
 
 
 
 
ALAD
 
 
hsa-miR-15b-5p
 
 
-0.446
 
 
26.9
 
 
 
 
ALDH1A2
 
 
hsa-miR-23a-3p
 
 
-0.354
 
 
10.0
 
 
 
 
ALDH6A1
 
 
hsa-miR-1271-5p
 
 
-0.301
 
 
25.7
 
 
 
 
AMOT
 
 
hsa-miR-29a-3p
 
 
-0.304
 
 
37.2
 
 
 
 
AMOTL1
 
 
hsa-miR-15b-5p
 
 
-0.319
 
 
104.6
 
 
 
 
ANK1
 
 
hsa-miR-103a-3p
 
 
-0.321
 
 
922.8
 
 
 
 
ANKHD1
 
 
hsa-miR-23a-3p
 
 
-0.368
 
 
67.6
 
 
 
 
ANKHD1
 
 
hsa-miR-30d-5p
 
 
-0.381
 
 
67.6
 
 
 
 
ANKHD1
 
 
hsa-miR-30b-5p
 
 
-0.405
 
 
67.6
 
 
 
 
ANKRD17
 
 
hsa-miR-30d-5p
 
 
-0.312
 
 
21.8
 
 
 
 
ANKRD17
 
 
hsa-miR-30b-5p
 
 
-0.336
 
 
21.8
 
 
 
 
ANKRD27
 
 
hsa-miR-1271-5p
 
 
-0.648
 
 
19.9
 
 
 
 
ANKRD46
 
 
hsa-let-7b-5p
 
 
-0.389
 
 
58.3
 
 
 
 
ANP32A
 
 
hsa-miR-132-3p
 
 
-0.374
 
 
57.9
 
 
 
 
ANTXR2
 
 
hsa-miR-29a-3p
 
 
-0.347
 
 
12.3
 
 
 
 
ANXA11
 
 
hsa-miR-15b-5p
 
 
-0.463
 
 
177.1
 
 
 
 
AP2A1
 
 
hsa-miR-15b-5p
 
 
-0.333
 
 
85.4
 
 
 
 
AP2B1
 
 
hsa-miR-1271-5p
 
 
-0.299
 
 
49.8
 
 
 
 
AP2B1
 
 
hsa-miR-15b-5p
 
 
-0.334
 
 
49.8
 
 
 
 
AP3S1
 
 
hsa-miR-30b-5p
 
 
-0.293
 
 
25.5
 
 
 
 
AP4B1
 
 
hsa-miR-378a-3p
 
 
-0.62
 
 
11.0
 
 
 
 
AP4B1
 
 
hsa-miR-378d
 
 
-0.62
 
 
11.0
 
 
 
 
AP5M1
 
 
hsa-miR-1271-5p
 
 
-0.343
 
 
14.5
 
 
 
 
APOO
 
 
hsa-miR-181d-5p
 
 
-0.492
 
 
43.1
 
 
 
 
APOO
 
 
hsa-miR-181b-5p
 
 
-0.5
 
 
43.1
 
 
 
 
APOO
 
 
hsa-miR-181a-5p
 
 
-0.5
 
 
43.1
 
 
 
 
APP
 
 
hsa-miR-15b-5p
 
 
-0.298
 
 
99.7
 
 
 
 
ARF4
 
 
hsa-miR-30b-5p
 
 
-0.289
 
 
59.5
 
 
 
 
ARF4
 
 
hsa-miR-194-5p
 
 
-0.293
 
 
59.5
 
 
 
 
ARF4
 
 
hsa-miR-152-3p
 
 
-0.487
 
 
59.5
 
 
 
 
ARF4
 
 
hsa-miR-148b-3p
 
 
-0.487
 
 
59.5
 
 
 
 
ARF5
 
 
hsa-miR-29a-3p
 
 
-0.491
 
 
145.7
 
 
 
 
ARF6
 
 
hsa-miR-181b-5p
 
 
-0.33
 
 
21.4
 
 
 
 
ARF6
 
 
hsa-miR-181a-5p
 
 
-0.33
 
 
21.4
 
 
 
 
ARF6
 
 
hsa-miR-181d-5p
 
 
-0.338
 
 
21.4
 
 
 
 
ARFGEF1
 
 
hsa-miR-148b-3p
 
 
-0.306
 
 
12.2
 
 
 
 
ARFGEF1
 
 
hsa-miR-152-3p
 
 
-0.306
 
 
12.2
 
 
 
 
ARG2
 
 
hsa-let-7b-5p
 
 
-0.468
 
 
37.4
 
 
 
 
 
 
 skin 
 
 
 
 
target_symbol
 
 
mature_mirna_id
 
 
score
 
 
nTPM
 
 
 
 
 
 
AAMP
 
 
hsa-miR-326
 
 
-0.722
 
 
75.8
 
 
 
 
AAR2
 
 
hsa-miR-29a-3p
 
 
-0.327
 
 
24.6
 
 
 
 
ABCB7
 
 
hsa-miR-148b-3p
 
 
-0.515
 
 
12.7
 
 
 
 
ABCB7
 
 
hsa-miR-152-3p
 
 
-0.515
 
 
12.7
 
 
 
 
ABCD3
 
 
hsa-miR-148b-3p
 
 
-0.388
 
 
33.6
 
 
 
 
ABCD3
 
 
hsa-miR-152-3p
 
 
-0.388
 
 
33.6
 
 
 
 
ABCE1
 
 
hsa-miR-29a-3p
 
 
-0.404
 
 
23.8
 
 
 
 
ABHD17C
 
 
hsa-let-7b-5p
 
 
-0.359
 
 
17.9
 
 
 
 
ABHD17C
 
 
hsa-miR-330-3p
 
 
-0.399
 
 
17.9
 
 
 
 
ABHD4
 
 
hsa-miR-29a-3p
 
 
-0.312
 
 
20.2
 
 
 
 
ABHD5
 
 
hsa-miR-132-3p
 
 
-0.323
 
 
29.7
 
 
 
 
ACP3
 
 
hsa-let-7b-5p
 
 
-0.298
 
 
25.5
 
 
 
 
ACTC1
 
 
hsa-miR-30d-5p
 
 
-0.519
 
 
14.2
 
 
 
 
ACTC1
 
 
hsa-miR-30b-5p
 
 
-0.543
 
 
14.2
 
 
 
 
ACTR2
 
 
hsa-miR-15b-5p
 
 
-0.293
 
 
65.5
 
 
 
 
ADAM10
 
 
hsa-miR-152-3p
 
 
-0.348
 
 
14.8
 
 
 
 
ADAM10
 
 
hsa-miR-148b-3p
 
 
-0.356
 
 
14.8
 
 
 
 
ADCY3
 
 
hsa-miR-132-3p
 
 
-0.303
 
 
36.0
 
 
 
 
ADCY6
 
 
hsa-miR-1271-5p
 
 
-0.336
 
 
14.5
 
 
 
 
ADGRA2
 
 
hsa-miR-1271-5p
 
 
-0.427
 
 
12.4
 
 
 
 
ADIPOR2
 
 
hsa-miR-150-5p
 
 
-0.287
 
 
30.8
 
 
 
 
ADK
 
 
hsa-miR-1271-5p
 
 
-0.329
 
 
32.7
 
 
 
 
ADNP
 
 
hsa-miR-328-3p
 
 
-0.371
 
 
33.2
 
 
 
 
ADRA2A
 
 
hsa-miR-23a-3p
 
 
-0.371
 
 
10.2
 
 
 
 
ADRB2
 
 
hsa-miR-15b-5p
 
 
-0.29
 
 
36.9
 
 
 
 
ADRB2
 
 
hsa-let-7b-5p
 
 
-0.635
 
 
36.9
 
 
 
 
AFG3L2
 
 
hsa-miR-181b-5p
 
 
-0.491
 
 
15.1
 
 
 
 
AFG3L2
 
 
hsa-miR-181a-5p
 
 
-0.491
 
 
15.1
 
 
 
 
AFG3L2
 
 
hsa-miR-181d-5p
 
 
-0.499
 
 
15.1
 
 
 
 
AGFG1
 
 
hsa-miR-148b-3p
 
 
-0.332
 
 
34.3
 
 
 
 
AGFG1
 
 
hsa-miR-152-3p
 
 
-0.332
 
 
34.3
 
 
 
 
AGFG1
 
 
hsa-miR-103a-3p
 
 
-0.389
 
 
34.3
 
 
 
 
AGO4
 
 
hsa-miR-1271-5p
 
 
-0.289
 
 
18.9
 
 
 
 
AGO4
 
 
hsa-miR-103a-3p
 
 
-0.32
 
 
18.9
 
 
 
 
AHDC1
 
 
hsa-miR-152-3p
 
 
-0.413
 
 
29.8
 
 
 
 
AHDC1
 
 
hsa-miR-148b-3p
 
 
-0.413
 
 
29.8
 
 
 
 
AHR
 
 
hsa-miR-1271-5p
 
 
-0.304
 
 
20.7
 
 
 
 
AHR
 
 
hsa-miR-29a-3p
 
 
-0.316
 
 
20.7
 
 
 
 
AIFM1
 
 
hsa-let-7b-5p
 
 
-0.314
 
 
10.3
 
 
 
 
AJUBA
 
 
hsa-miR-103a-3p
 
 
-0.448
 
 
58.2
 
 
 
 
AK1
 
 
hsa-miR-29a-3p
 
 
-0.333
 
 
17.4
 
 
 
 
AK3
 
 
hsa-miR-1271-5p
 
 
-0.57
 
 
44.8
 
 
 
 
AK4
 
 
hsa-miR-15b-5p
 
 
-0.316
 
 
25.7
 
 
 
 
AKAP1
 
 
hsa-miR-152-3p
 
 
-0.381
 
 
26.4
 
 
 
 
AKAP1
 
 
hsa-miR-148b-3p
 
 
-0.381
 
 
26.4
 
 
 
 
AKIRIN2
 
 
hsa-miR-103a-3p
 
 
-0.299
 
 
20.2
 
 
 
 
ALAD
 
 
hsa-miR-326
 
 
-0.352
 
 
36.8
 
 
 
 
ALAD
 
 
hsa-miR-103a-3p
 
 
-0.385
 
 
36.8
 
 
 
 
ALAD
 
 
hsa-miR-15b-5p
 
 
-0.446
 
 
36.8
 
 
 
 
ALCAM
 
 
hsa-miR-148b-3p
 
 
-0.339
 
 
10.3
 
 
 
 
ALCAM
 
 
hsa-miR-152-3p
 
 
-0.347
 
 
10.3
 
 
 
 
ALOX12
 
 
hsa-miR-15b-5p
 
 
-0.443
 
 
20.5
 
 
 
 
ALOX15B
 
 
hsa-miR-326
 
 
-0.417
 
 
35.6
 
 
 
 
AMOTL1
 
 
hsa-miR-15b-5p
 
 
-0.319
 
 
20.5
 
 
 
 
ANKHD1
 
 
hsa-miR-23a-3p
 
 
-0.368
 
 
48.3
 
 
 
 
ANKHD1
 
 
hsa-miR-30d-5p
 
 
-0.381
 
 
48.3
 
 
 
 
ANKHD1
 
 
hsa-miR-30b-5p
 
 
-0.405
 
 
48.3
 
 
 
 
ANKIB1
 
 
hsa-miR-1271-5p
 
 
-0.344
 
 
13.6
 
 
 
 
ANKRD17
 
 
hsa-miR-30d-5p
 
 
-0.312
 
 
16.2
 
 
 
 
ANKRD17
 
 
hsa-miR-30b-5p
 
 
-0.336
 
 
16.2
 
 
 
 
ANKRD27
 
 
hsa-miR-1271-5p
 
 
-0.648
 
 
11.7
 
 
 
 
ANKRD46
 
 
hsa-let-7b-5p
 
 
-0.389
 
 
10.5
 
 
 
 
ANLN
 
 
hsa-miR-15b-5p
 
 
-0.334
 
 
12.9
 
 
 
 
ANP32A
 
 
hsa-miR-132-3p
 
 
-0.374
 
 
34.1
 
 
 
 
ANTXR2
 
 
hsa-miR-29a-3p
 
 
-0.347
 
 
13.8
 
 
 
 
ANXA11
 
 
hsa-miR-15b-5p
 
 
-0.463
 
 
70.8
 
 
 
 
AP1S1
 
 
hsa-let-7b-5p
 
 
-0.374
 
 
40.6
 
 
 
 
AP2A1
 
 
hsa-miR-15b-5p
 
 
-0.333
 
 
52.1
 
 
 
 
AP2B1
 
 
hsa-miR-1271-5p
 
 
-0.299
 
 
27.9
 
 
 
 
AP2B1
 
 
hsa-miR-15b-5p
 
 
-0.334
 
 
27.9
 
 
 
 
AP3S1
 
 
hsa-miR-30b-5p
 
 
-0.293
 
 
57.3
 
 
 
 
AP4B1
 
 
hsa-miR-378a-3p
 
 
-0.62
 
 
10.9
 
 
 
 
AP4B1
 
 
hsa-miR-378d
 
 
-0.62
 
 
10.9
 
 
 
 
AP5M1
 
 
hsa-miR-1271-5p
 
 
-0.343
 
 
10.6
 
 
 
 
APP
 
 
hsa-miR-15b-5p
 
 
-0.298
 
 
204.7
 
 
 
 
AQP3
 
 
hsa-miR-874-3p
 
 
-0.48
 
 
554.4
 
 
 
 
ARF4
 
 
hsa-miR-30b-5p
 
 
-0.289
 
 
61.3
 
 
 
 
ARF4
 
 
hsa-miR-194-5p
 
 
-0.293
 
 
61.3
 
 
 
 
ARF4
 
 
hsa-miR-152-3p
 
 
-0.487
 
 
61.3
 
 
 
 
ARF4
 
 
hsa-miR-148b-3p
 
 
-0.487
 
 
61.3
 
 
 
 
ARF5
 
 
hsa-miR-29a-3p
 
 
-0.491
 
 
215.0
 
 
 
 
ARF6
 
 
hsa-miR-181b-5p
 
 
-0.33
 
 
50.6
 
 
 
 
ARF6
 
 
hsa-miR-181a-5p
 
 
-0.33
 
 
50.6
 
 
 
 
ARF6
 
 
hsa-miR-181d-5p
 
 
-0.338
 
 
50.6
 
 
 
 
ARFGEF1
 
 
hsa-miR-148b-3p
 
 
-0.306
 
 
12.9
 
 
 
 
ARFGEF1
 
 
hsa-miR-152-3p
 
 
-0.306
 
 
12.9
 
 
 
 
ARFIP1
 
 
hsa-miR-320b
 
 
-0.339
 
 
16.4
 
 
 
 
ARFIP1
 
 
hsa-miR-320c
 
 
-0.339
 
 
16.4
 
 
 
 
ARFIP1
 
 
hsa-miR-152-3p
 
 
-0.474
 
 
16.4
 
 
 
 
ARFIP1
 
 
hsa-miR-148b-3p
 
 
-0.474
 
 
16.4
 
 
 
 
ARID1B
 
 
hsa-miR-29a-3p
 
 
-0.342
 
 
21.3
 
 
 
 
ARIH2
 
 
hsa-miR-103a-3p
 
 
-0.307
 
 
15.8
 
 
 
 
ARL2
 
 
hsa-miR-15b-5p
 
 
-0.374
 
 
96.0
 
 
 
 
ARL2
 
 
hsa-miR-15b-5p
 
 
-0.442
 
 
96.0
 
 
 
 
ARL4A
 
 
hsa-miR-29a-3p
 
 
-0.386
 
 
85.3
 
 
 
 
ARL4A
 
 
hsa-miR-30d-5p
 
 
-0.432
 
 
85.3
 
 
 
 
ARL4A
 
 
hsa-miR-30b-5p
 
 
-0.448
 
 
85.3
 
 
 
 
ARL4D
 
 
hsa-miR-1271-5p
 
 
-0.392
 
 
44.0
 
 
 
 
ARL4D
 
 
hsa-let-7b-5p
 
 
-0.417
 
 
44.0
 
 
 
 
ARL6IP1
 
 
hsa-miR-328-3p
 
 
-0.494
 
 
62.4
 
 
 
 
 
 
 small intestine 
 
 
 
 
target_symbol
 
 
mature_mirna_id
 
 
score
 
 
nTPM
 
 
 
 
 
 
AAMP
 
 
hsa-miR-326
 
 
-0.722
 
 
58.5
 
 
 
 
AAR2
 
 
hsa-miR-29a-3p
 
 
-0.327
 
 
18.1
 
 
 
 
ABAT
 
 
hsa-miR-1271-5p
 
 
-0.409
 
 
18.9
 
 
 
 
ABCB10
 
 
hsa-miR-152-3p
 
 
-0.291
 
 
13.1
 
 
 
 
ABCB10
 
 
hsa-miR-148b-3p
 
 
-0.291
 
 
13.1
 
 
 
 
ABCB7
 
 
hsa-miR-148b-3p
 
 
-0.515
 
 
11.2
 
 
 
 
ABCB7
 
 
hsa-miR-152-3p
 
 
-0.515
 
 
11.2
 
 
 
 
ABCD3
 
 
hsa-miR-148b-3p
 
 
-0.388
 
 
43.4
 
 
 
 
ABCD3
 
 
hsa-miR-152-3p
 
 
-0.388
 
 
43.4
 
 
 
 
ABCE1
 
 
hsa-miR-29a-3p
 
 
-0.404
 
 
16.7
 
 
 
 
ABHD17C
 
 
hsa-let-7b-5p
 
 
-0.359
 
 
28.8
 
 
 
 
ABHD17C
 
 
hsa-miR-330-3p
 
 
-0.399
 
 
28.8
 
 
 
 
ABHD4
 
 
hsa-miR-29a-3p
 
 
-0.312
 
 
18.9
 
 
 
 
ACTR2
 
 
hsa-miR-15b-5p
 
 
-0.293
 
 
111.3
 
 
 
 
ADA
 
 
hsa-miR-29a-3p
 
 
-0.376
 
 
15.5
 
 
 
 
ADAM10
 
 
hsa-miR-152-3p
 
 
-0.348
 
 
15.8
 
 
 
 
ADAM10
 
 
hsa-miR-148b-3p
 
 
-0.356
 
 
15.8
 
 
 
 
ADAM19
 
 
hsa-miR-874-3p
 
 
-0.374
 
 
17.9
 
 
 
 
ADAM9
 
 
hsa-miR-30d-5p
 
 
-0.304
 
 
21.4
 
 
 
 
ADAM9
 
 
hsa-miR-30b-5p
 
 
-0.312
 
 
21.4
 
 
 
 
ADCY3
 
 
hsa-miR-132-3p
 
 
-0.303
 
 
31.8
 
 
 
 
ADCY6
 
 
hsa-miR-1271-5p
 
 
-0.336
 
 
20.2
 
 
 
 
ADGRA2
 
 
hsa-miR-1271-5p
 
 
-0.427
 
 
11.0
 
 
 
 
ADIPOR2
 
 
hsa-miR-150-5p
 
 
-0.287
 
 
31.4
 
 
 
 
ADK
 
 
hsa-miR-1271-5p
 
 
-0.329
 
 
11.7
 
 
 
 
ADNP
 
 
hsa-miR-328-3p
 
 
-0.371
 
 
30.1
 
 
 
 
AFG3L2
 
 
hsa-miR-181b-5p
 
 
-0.491
 
 
34.0
 
 
 
 
AFG3L2
 
 
hsa-miR-181a-5p
 
 
-0.491
 
 
34.0
 
 
 
 
AFG3L2
 
 
hsa-miR-181d-5p
 
 
-0.499
 
 
34.0
 
 
 
 
AGFG1
 
 
hsa-miR-148b-3p
 
 
-0.332
 
 
33.9
 
 
 
 
AGFG1
 
 
hsa-miR-152-3p
 
 
-0.332
 
 
33.9
 
 
 
 
AGFG1
 
 
hsa-miR-103a-3p
 
 
-0.389
 
 
33.9
 
 
 
 
AHR
 
 
hsa-miR-1271-5p
 
 
-0.304
 
 
13.6
 
 
 
 
AHR
 
 
hsa-miR-29a-3p
 
 
-0.316
 
 
13.6
 
 
 
 
AIFM1
 
 
hsa-let-7b-5p
 
 
-0.314
 
 
28.2
 
 
 
 
AK1
 
 
hsa-miR-29a-3p
 
 
-0.333
 
 
15.6
 
 
 
 
AK3
 
 
hsa-miR-1271-5p
 
 
-0.57
 
 
58.9
 
 
 
 
AKAP1
 
 
hsa-miR-152-3p
 
 
-0.381
 
 
52.5
 
 
 
 
AKAP1
 
 
hsa-miR-148b-3p
 
 
-0.381
 
 
52.5
 
 
 
 
AKIRIN2
 
 
hsa-miR-103a-3p
 
 
-0.299
 
 
20.2
 
 
 
 
ALAD
 
 
hsa-miR-326
 
 
-0.352
 
 
25.1
 
 
 
 
ALAD
 
 
hsa-miR-103a-3p
 
 
-0.385
 
 
25.1
 
 
 
 
ALAD
 
 
hsa-miR-15b-5p
 
 
-0.446
 
 
25.1
 
 
 
 
ALCAM
 
 
hsa-miR-148b-3p
 
 
-0.339
 
 
16.4
 
 
 
 
ALCAM
 
 
hsa-miR-152-3p
 
 
-0.347
 
 
16.4
 
 
 
 
ALDH6A1
 
 
hsa-miR-1271-5p
 
 
-0.301
 
 
22.0
 
 
 
 
AMT
 
 
hsa-let-7b-5p
 
 
-0.463
 
 
11.5
 
 
 
 
ANKHD1
 
 
hsa-miR-23a-3p
 
 
-0.368
 
 
44.2
 
 
 
 
ANKHD1
 
 
hsa-miR-30d-5p
 
 
-0.381
 
 
44.2
 
 
 
 
ANKHD1
 
 
hsa-miR-30b-5p
 
 
-0.405
 
 
44.2
 
 
 
 
ANKIB1
 
 
hsa-miR-1271-5p
 
 
-0.344
 
 
11.3
 
 
 
 
ANKRD17
 
 
hsa-miR-30d-5p
 
 
-0.312
 
 
14.3
 
 
 
 
ANKRD17
 
 
hsa-miR-30b-5p
 
 
-0.336
 
 
14.3
 
 
 
 
ANKRD27
 
 
hsa-miR-1271-5p
 
 
-0.648
 
 
10.1
 
 
 
 
ANKRD46
 
 
hsa-let-7b-5p
 
 
-0.389
 
 
10.4
 
 
 
 
ANP32A
 
 
hsa-miR-132-3p
 
 
-0.374
 
 
72.4
 
 
 
 
ANTXR2
 
 
hsa-miR-29a-3p
 
 
-0.347
 
 
35.6
 
 
 
 
ANXA11
 
 
hsa-miR-15b-5p
 
 
-0.463
 
 
129.1
 
 
 
 
AP1S1
 
 
hsa-let-7b-5p
 
 
-0.374
 
 
29.8
 
 
 
 
AP2A1
 
 
hsa-miR-15b-5p
 
 
-0.333
 
 
32.5
 
 
 
 
AP2B1
 
 
hsa-miR-1271-5p
 
 
-0.299
 
 
36.5
 
 
 
 
AP2B1
 
 
hsa-miR-15b-5p
 
 
-0.334
 
 
36.5
 
 
 
 
AP3S1
 
 
hsa-miR-30b-5p
 
 
-0.293
 
 
76.7
 
 
 
 
AP4B1
 
 
hsa-miR-378a-3p
 
 
-0.62
 
 
12.3
 
 
 
 
AP4B1
 
 
hsa-miR-378d
 
 
-0.62
 
 
12.3
 
 
 
 
AP5M1
 
 
hsa-miR-1271-5p
 
 
-0.343
 
 
23.7
 
 
 
 
APOO
 
 
hsa-miR-181d-5p
 
 
-0.492
 
 
11.3
 
 
 
 
APOO
 
 
hsa-miR-181b-5p
 
 
-0.5
 
 
11.3
 
 
 
 
APOO
 
 
hsa-miR-181a-5p
 
 
-0.5
 
 
11.3
 
 
 
 
APP
 
 
hsa-miR-15b-5p
 
 
-0.298
 
 
184.2
 
 
 
 
AQP11
 
 
hsa-miR-103a-3p
 
 
-0.517
 
 
23.9
 
 
 
 
AQP11
 
 
hsa-miR-15b-5p
 
 
-0.769
 
 
23.9
 
 
 
 
AQP3
 
 
hsa-miR-874-3p
 
 
-0.48
 
 
94.7
 
 
 
 
ARF4
 
 
hsa-miR-30b-5p
 
 
-0.289
 
 
102.8
 
 
 
 
ARF4
 
 
hsa-miR-194-5p
 
 
-0.293
 
 
102.8
 
 
 
 
ARF4
 
 
hsa-miR-152-3p
 
 
-0.487
 
 
102.8
 
 
 
 
ARF4
 
 
hsa-miR-148b-3p
 
 
-0.487
 
 
102.8
 
 
 
 
ARF5
 
 
hsa-miR-29a-3p
 
 
-0.491
 
 
103.1
 
 
 
 
ARF6
 
 
hsa-miR-181b-5p
 
 
-0.33
 
 
48.2
 
 
 
 
ARF6
 
 
hsa-miR-181a-5p
 
 
-0.33
 
 
48.2
 
 
 
 
ARF6
 
 
hsa-miR-181d-5p
 
 
-0.338
 
 
48.2
 
 
 
 
ARFGEF1
 
 
hsa-miR-148b-3p
 
 
-0.306
 
 
16.5
 
 
 
 
ARFGEF1
 
 
hsa-miR-152-3p
 
 
-0.306
 
 
16.5
 
 
 
 
ARFIP1
 
 
hsa-miR-320b
 
 
-0.339
 
 
20.0
 
 
 
 
ARFIP1
 
 
hsa-miR-320c
 
 
-0.339
 
 
20.0
 
 
 
 
ARFIP1
 
 
hsa-miR-152-3p
 
 
-0.474
 
 
20.0
 
 
 
 
ARFIP1
 
 
hsa-miR-148b-3p
 
 
-0.474
 
 
20.0
 
 
 
 
ARG2
 
 
hsa-let-7b-5p
 
 
-0.468
 
 
14.4
 
 
 
 
ARHGAP21
 
 
hsa-miR-152-3p
 
 
-0.287
 
 
15.2
 
 
 
 
ARHGAP21
 
 
hsa-miR-148b-3p
 
 
-0.287
 
 
15.2
 
 
 
 
ARID1B
 
 
hsa-miR-29a-3p
 
 
-0.342
 
 
14.4
 
 
 
 
ARIH2
 
 
hsa-miR-103a-3p
 
 
-0.307
 
 
20.5
 
 
 
 
ARL2
 
 
hsa-miR-15b-5p
 
 
-0.374
 
 
72.6
 
 
 
 
ARL2
 
 
hsa-miR-15b-5p
 
 
-0.442
 
 
72.6
 
 
 
 
ARL4A
 
 
hsa-miR-29a-3p
 
 
-0.386
 
 
59.0
 
 
 
 
ARL4A
 
 
hsa-miR-30d-5p
 
 
-0.432
 
 
59.0
 
 
 
 
ARL4A
 
 
hsa-miR-30b-5p
 
 
-0.448
 
 
59.0
 
 
 
 
ARL6IP1
 
 
hsa-miR-328-3p
 
 
-0.494
 
 
116.2
 
 
 
 
ARL6IP1
 
 
hsa-miR-148b-3p
 
 
-0.579
 
 
116.2
 
 
 
 
ARL6IP1
 
 
hsa-miR-152-3p
 
 
-0.579
 
 
116.2
 
 
 
 
 
 
 smooth muscle 
 
 
 
 
target_symbol
 
 
mature_mirna_id
 
 
score
 
 
nTPM
 
 
 
 
 
 
AAMP
 
 
hsa-miR-326
 
 
-0.722
 
 
47.3
 
 
 
 
AAR2
 
 
hsa-miR-29a-3p
 
 
-0.327
 
 
26.1
 
 
 
 
ABCD3
 
 
hsa-miR-148b-3p
 
 
-0.388
 
 
16.6
 
 
 
 
ABCD3
 
 
hsa-miR-152-3p
 
 
-0.388
 
 
16.6
 
 
 
 
ABCE1
 
 
hsa-miR-29a-3p
 
 
-0.404
 
 
27.0
 
 
 
 
ABHD4
 
 
hsa-miR-29a-3p
 
 
-0.312
 
 
25.0
 
 
 
 
ACTR2
 
 
hsa-miR-15b-5p
 
 
-0.293
 
 
96.0
 
 
 
 
ACVR1
 
 
hsa-miR-30b-5p
 
 
-0.29
 
 
26.8
 
 
 
 
ACVR1
 
 
hsa-miR-152-3p
 
 
-0.397
 
 
26.8
 
 
 
 
ACVR1
 
 
hsa-miR-148b-3p
 
 
-0.397
 
 
26.8
 
 
 
 
ADAM10
 
 
hsa-miR-152-3p
 
 
-0.348
 
 
12.4
 
 
 
 
ADAM10
 
 
hsa-miR-148b-3p
 
 
-0.356
 
 
12.4
 
 
 
 
ADAM19
 
 
hsa-miR-874-3p
 
 
-0.374
 
 
15.3
 
 
 
 
ADAM9
 
 
hsa-miR-30d-5p
 
 
-0.304
 
 
48.2
 
 
 
 
ADAM9
 
 
hsa-miR-30b-5p
 
 
-0.312
 
 
48.2
 
 
 
 
ADAMTS9
 
 
hsa-miR-29a-3p
 
 
-0.305
 
 
11.3
 
 
 
 
ADAMTS9
 
 
hsa-miR-29a-3p
 
 
-0.485
 
 
11.3
 
 
 
 
ADCY3
 
 
hsa-miR-132-3p
 
 
-0.303
 
 
33.0
 
 
 
 
ADCY6
 
 
hsa-miR-1271-5p
 
 
-0.336
 
 
12.7
 
 
 
 
ADGRA2
 
 
hsa-miR-1271-5p
 
 
-0.427
 
 
25.9
 
 
 
 
ADGRA3
 
 
hsa-miR-30d-5p
 
 
-0.296
 
 
13.1
 
 
 
 
ADGRA3
 
 
hsa-miR-30b-5p
 
 
-0.304
 
 
13.1
 
 
 
 
ADGRF5
 
 
hsa-miR-148b-3p
 
 
-0.354
 
 
20.6
 
 
 
 
ADGRF5
 
 
hsa-miR-152-3p
 
 
-0.354
 
 
20.6
 
 
 
 
ADIPOR2
 
 
hsa-miR-150-5p
 
 
-0.287
 
 
11.2
 
 
 
 
ADK
 
 
hsa-miR-1271-5p
 
 
-0.329
 
 
13.8
 
 
 
 
ADNP
 
 
hsa-miR-328-3p
 
 
-0.371
 
 
27.7
 
 
 
 
AFG3L2
 
 
hsa-miR-181b-5p
 
 
-0.491
 
 
14.9
 
 
 
 
AFG3L2
 
 
hsa-miR-181a-5p
 
 
-0.491
 
 
14.9
 
 
 
 
AFG3L2
 
 
hsa-miR-181d-5p
 
 
-0.499
 
 
14.9
 
 
 
 
AGFG1
 
 
hsa-miR-148b-3p
 
 
-0.332
 
 
36.7
 
 
 
 
AGFG1
 
 
hsa-miR-152-3p
 
 
-0.332
 
 
36.7
 
 
 
 
AGFG1
 
 
hsa-miR-103a-3p
 
 
-0.389
 
 
36.7
 
 
 
 
AHR
 
 
hsa-miR-1271-5p
 
 
-0.304
 
 
14.2
 
 
 
 
AHR
 
 
hsa-miR-29a-3p
 
 
-0.316
 
 
14.2
 
 
 
 
AK1
 
 
hsa-miR-29a-3p
 
 
-0.333
 
 
20.4
 
 
 
 
AK3
 
 
hsa-miR-1271-5p
 
 
-0.57
 
 
38.9
 
 
 
 
AK4
 
 
hsa-miR-15b-5p
 
 
-0.316
 
 
19.6
 
 
 
 
AKAP1
 
 
hsa-miR-152-3p
 
 
-0.381
 
 
24.6
 
 
 
 
AKAP1
 
 
hsa-miR-148b-3p
 
 
-0.381
 
 
24.6
 
 
 
 
AKIRIN2
 
 
hsa-miR-103a-3p
 
 
-0.299
 
 
15.3
 
 
 
 
AKT3
 
 
hsa-miR-29a-3p
 
 
-0.287
 
 
14.7
 
 
 
 
AKT3
 
 
hsa-miR-15b-5p
 
 
-0.301
 
 
14.7
 
 
 
 
ALAD
 
 
hsa-miR-326
 
 
-0.352
 
 
16.4
 
 
 
 
ALAD
 
 
hsa-miR-103a-3p
 
 
-0.385
 
 
16.4
 
 
 
 
ALAD
 
 
hsa-miR-15b-5p
 
 
-0.446
 
 
16.4
 
 
 
 
ALCAM
 
 
hsa-miR-148b-3p
 
 
-0.339
 
 
15.2
 
 
 
 
ALCAM
 
 
hsa-miR-152-3p
 
 
-0.347
 
 
15.2
 
 
 
 
ALDH1A2
 
 
hsa-miR-23a-3p
 
 
-0.354
 
 
29.8
 
 
 
 
AMOTL1
 
 
hsa-miR-15b-5p
 
 
-0.319
 
 
19.2
 
 
 
 
ANKHD1
 
 
hsa-miR-23a-3p
 
 
-0.368
 
 
24.3
 
 
 
 
ANKHD1
 
 
hsa-miR-30d-5p
 
 
-0.381
 
 
24.3
 
 
 
 
ANKHD1
 
 
hsa-miR-30b-5p
 
 
-0.405
 
 
24.3
 
 
 
 
ANKIB1
 
 
hsa-miR-1271-5p
 
 
-0.344
 
 
12.7
 
 
 
 
ANKRD17
 
 
hsa-miR-30d-5p
 
 
-0.312
 
 
15.3
 
 
 
 
ANKRD17
 
 
hsa-miR-30b-5p
 
 
-0.336
 
 
15.3
 
 
 
 
ANKRD27
 
 
hsa-miR-1271-5p
 
 
-0.648
 
 
10.8
 
 
 
 
ANKRD29
 
 
hsa-miR-132-3p
 
 
-0.47
 
 
10.9
 
 
 
 
ANKRD46
 
 
hsa-let-7b-5p
 
 
-0.389
 
 
11.6
 
 
 
 
ANLN
 
 
hsa-miR-15b-5p
 
 
-0.334
 
 
13.9
 
 
 
 
ANP32A
 
 
hsa-miR-132-3p
 
 
-0.374
 
 
53.2
 
 
 
 
ANTXR2
 
 
hsa-miR-29a-3p
 
 
-0.347
 
 
101.2
 
 
 
 
ANXA11
 
 
hsa-miR-15b-5p
 
 
-0.463
 
 
80.2
 
 
 
 
AP1S1
 
 
hsa-let-7b-5p
 
 
-0.374
 
 
11.9
 
 
 
 
AP2A1
 
 
hsa-miR-15b-5p
 
 
-0.333
 
 
20.0
 
 
 
 
AP2B1
 
 
hsa-miR-1271-5p
 
 
-0.299
 
 
52.3
 
 
 
 
AP2B1
 
 
hsa-miR-15b-5p
 
 
-0.334
 
 
52.3
 
 
 
 
AP3S1
 
 
hsa-miR-30b-5p
 
 
-0.293
 
 
59.9
 
 
 
 
AP5M1
 
 
hsa-miR-1271-5p
 
 
-0.343
 
 
13.1
 
 
 
 
APOO
 
 
hsa-miR-181d-5p
 
 
-0.492
 
 
11.2
 
 
 
 
APOO
 
 
hsa-miR-181b-5p
 
 
-0.5
 
 
11.2
 
 
 
 
APOO
 
 
hsa-miR-181a-5p
 
 
-0.5
 
 
11.2
 
 
 
 
APP
 
 
hsa-miR-15b-5p
 
 
-0.298
 
 
353.7
 
 
 
 
ARF4
 
 
hsa-miR-30b-5p
 
 
-0.289
 
 
157.9
 
 
 
 
ARF4
 
 
hsa-miR-194-5p
 
 
-0.293
 
 
157.9
 
 
 
 
ARF4
 
 
hsa-miR-152-3p
 
 
-0.487
 
 
157.9
 
 
 
 
ARF4
 
 
hsa-miR-148b-3p
 
 
-0.487
 
 
157.9
 
 
 
 
ARF5
 
 
hsa-miR-29a-3p
 
 
-0.491
 
 
62.0
 
 
 
 
ARF6
 
 
hsa-miR-181b-5p
 
 
-0.33
 
 
22.2
 
 
 
 
ARF6
 
 
hsa-miR-181a-5p
 
 
-0.33
 
 
22.2
 
 
 
 
ARF6
 
 
hsa-miR-181d-5p
 
 
-0.338
 
 
22.2
 
 
 
 
ARFGEF1
 
 
hsa-miR-148b-3p
 
 
-0.306
 
 
10.1
 
 
 
 
ARFGEF1
 
 
hsa-miR-152-3p
 
 
-0.306
 
 
10.1
 
 
 
 
ARFIP1
 
 
hsa-miR-320b
 
 
-0.339
 
 
18.5
 
 
 
 
ARFIP1
 
 
hsa-miR-320c
 
 
-0.339
 
 
18.5
 
 
 
 
ARFIP1
 
 
hsa-miR-152-3p
 
 
-0.474
 
 
18.5
 
 
 
 
ARFIP1
 
 
hsa-miR-148b-3p
 
 
-0.474
 
 
18.5
 
 
 
 
ARHGAP21
 
 
hsa-miR-152-3p
 
 
-0.287
 
 
16.5
 
 
 
 
ARHGAP21
 
 
hsa-miR-148b-3p
 
 
-0.287
 
 
16.5
 
 
 
 
ARHGAP24
 
 
hsa-miR-1271-5p
 
 
-0.342
 
 
13.1
 
 
 
 
ARHGAP6
 
 
hsa-miR-1271-5p
 
 
-0.521
 
 
12.1
 
 
 
 
ARID1B
 
 
hsa-miR-29a-3p
 
 
-0.342
 
 
14.1
 
 
 
 
ARIH2
 
 
hsa-miR-103a-3p
 
 
-0.307
 
 
22.7
 
 
 
 
ARL2
 
 
hsa-miR-15b-5p
 
 
-0.374
 
 
56.9
 
 
 
 
ARL2
 
 
hsa-miR-15b-5p
 
 
-0.442
 
 
56.9
 
 
 
 
ARL4A
 
 
hsa-miR-29a-3p
 
 
-0.386
 
 
23.4
 
 
 
 
ARL4A
 
 
hsa-miR-30d-5p
 
 
-0.432
 
 
23.4
 
 
 
 
ARL4A
 
 
hsa-miR-30b-5p
 
 
-0.448
 
 
23.4
 
 
 
 
ARL4D
 
 
hsa-miR-1271-5p
 
 
-0.392
 
 
13.8
 
 
 
 
ARL4D
 
 
hsa-let-7b-5p
 
 
-0.417
 
 
13.8
 
 
 
 
 
 
 spinal cord 
 
 
 
 
target_symbol
 
 
mature_mirna_id
 
 
score
 
 
nTPM
 
 
 
 
 
 
AAMP
 
 
hsa-miR-326
 
 
-0.722
 
 
50.4
 
 
 
 
AAR2
 
 
hsa-miR-29a-3p
 
 
-0.327
 
 
14.2
 
 
 
 
ABAT
 
 
hsa-miR-1271-5p
 
 
-0.409
 
 
50.6
 
 
 
 
ABCA2
 
 
hsa-miR-1271-5p
 
 
-0.326
 
 
178.1
 
 
 
 
ABCD3
 
 
hsa-miR-148b-3p
 
 
-0.388
 
 
20.9
 
 
 
 
ABCD3
 
 
hsa-miR-152-3p
 
 
-0.388
 
 
20.9
 
 
 
 
ABCE1
 
 
hsa-miR-29a-3p
 
 
-0.404
 
 
16.8
 
 
 
 
ABHD4
 
 
hsa-miR-29a-3p
 
 
-0.312
 
 
17.8
 
 
 
 
ABHD5
 
 
hsa-miR-132-3p
 
 
-0.323
 
 
19.5
 
 
 
 
ACHE
 
 
hsa-miR-194-5p
 
 
-0.395
 
 
14.4
 
 
 
 
ACTN2
 
 
hsa-miR-23a-3p
 
 
-0.294
 
 
14.9
 
 
 
 
ACTN2
 
 
hsa-miR-29a-3p
 
 
-0.311
 
 
14.9
 
 
 
 
ACTR2
 
 
hsa-miR-15b-5p
 
 
-0.293
 
 
65.2
 
 
 
 
ADAM10
 
 
hsa-miR-152-3p
 
 
-0.348
 
 
18.7
 
 
 
 
ADAM10
 
 
hsa-miR-148b-3p
 
 
-0.356
 
 
18.7
 
 
 
 
ADAM9
 
 
hsa-miR-30d-5p
 
 
-0.304
 
 
11.3
 
 
 
 
ADAM9
 
 
hsa-miR-30b-5p
 
 
-0.312
 
 
11.3
 
 
 
 
ADCY2
 
 
hsa-miR-152-3p
 
 
-0.299
 
 
11.4
 
 
 
 
ADCY2
 
 
hsa-miR-148b-3p
 
 
-0.299
 
 
11.4
 
 
 
 
ADCY3
 
 
hsa-miR-132-3p
 
 
-0.303
 
 
26.0
 
 
 
 
ADIPOR2
 
 
hsa-miR-150-5p
 
 
-0.287
 
 
121.3
 
 
 
 
ADK
 
 
hsa-miR-1271-5p
 
 
-0.329
 
 
18.9
 
 
 
 
ADNP
 
 
hsa-miR-328-3p
 
 
-0.371
 
 
22.3
 
 
 
 
AGFG1
 
 
hsa-miR-148b-3p
 
 
-0.332
 
 
53.5
 
 
 
 
AGFG1
 
 
hsa-miR-152-3p
 
 
-0.332
 
 
53.5
 
 
 
 
AGFG1
 
 
hsa-miR-103a-3p
 
 
-0.389
 
 
53.5
 
 
 
 
AGPAT4
 
 
hsa-miR-29a-3p
 
 
-0.376
 
 
53.1
 
 
 
 
AIFM1
 
 
hsa-let-7b-5p
 
 
-0.314
 
 
10.1
 
 
 
 
AK1
 
 
hsa-miR-29a-3p
 
 
-0.333
 
 
67.3
 
 
 
 
AK3
 
 
hsa-miR-1271-5p
 
 
-0.57
 
 
31.0
 
 
 
 
AK4
 
 
hsa-miR-15b-5p
 
 
-0.316
 
 
11.3
 
 
 
 
AKAP1
 
 
hsa-miR-152-3p
 
 
-0.381
 
 
14.0
 
 
 
 
AKAP1
 
 
hsa-miR-148b-3p
 
 
-0.381
 
 
14.0
 
 
 
 
AKAP6
 
 
hsa-let-7b-5p
 
 
-0.307
 
 
13.3
 
 
 
 
AKIRIN2
 
 
hsa-miR-103a-3p
 
 
-0.299
 
 
29.7
 
 
 
 
AKT3
 
 
hsa-miR-29a-3p
 
 
-0.287
 
 
15.8
 
 
 
 
AKT3
 
 
hsa-miR-15b-5p
 
 
-0.301
 
 
15.8
 
 
 
 
ALAD
 
 
hsa-miR-326
 
 
-0.352
 
 
110.8
 
 
 
 
ALAD
 
 
hsa-miR-103a-3p
 
 
-0.385
 
 
110.8
 
 
 
 
ALAD
 
 
hsa-miR-15b-5p
 
 
-0.446
 
 
110.8
 
 
 
 
ALCAM
 
 
hsa-miR-148b-3p
 
 
-0.339
 
 
56.1
 
 
 
 
ALCAM
 
 
hsa-miR-152-3p
 
 
-0.347
 
 
56.1
 
 
 
 
ALDH6A1
 
 
hsa-miR-1271-5p
 
 
-0.301
 
 
27.5
 
 
 
 
ANKHD1
 
 
hsa-miR-23a-3p
 
 
-0.368
 
 
22.6
 
 
 
 
ANKHD1
 
 
hsa-miR-30d-5p
 
 
-0.381
 
 
22.6
 
 
 
 
ANKHD1
 
 
hsa-miR-30b-5p
 
 
-0.405
 
 
22.6
 
 
 
 
ANKIB1
 
 
hsa-miR-1271-5p
 
 
-0.344
 
 
17.8
 
 
 
 
ANKRD13B
 
 
hsa-miR-29a-3p
 
 
-0.421
 
 
14.7
 
 
 
 
ANKRD27
 
 
hsa-miR-1271-5p
 
 
-0.648
 
 
10.7
 
 
 
 
ANKRD46
 
 
hsa-let-7b-5p
 
 
-0.389
 
 
24.6
 
 
 
 
ANLN
 
 
hsa-miR-15b-5p
 
 
-0.334
 
 
163.3
 
 
 
 
ANP32A
 
 
hsa-miR-132-3p
 
 
-0.374
 
 
72.8
 
 
 
 
ANXA11
 
 
hsa-miR-15b-5p
 
 
-0.463
 
 
19.0
 
 
 
 
AP1S1
 
 
hsa-let-7b-5p
 
 
-0.374
 
 
28.5
 
 
 
 
AP2A1
 
 
hsa-miR-15b-5p
 
 
-0.333
 
 
32.5
 
 
 
 
AP2B1
 
 
hsa-miR-1271-5p
 
 
-0.299
 
 
75.2
 
 
 
 
AP2B1
 
 
hsa-miR-15b-5p
 
 
-0.334
 
 
75.2
 
 
 
 
AP3S1
 
 
hsa-miR-30b-5p
 
 
-0.293
 
 
64.2
 
 
 
 
APLN
 
 
hsa-miR-15b-5p
 
 
-0.395
 
 
29.4
 
 
 
 
APOO
 
 
hsa-miR-181d-5p
 
 
-0.492
 
 
36.2
 
 
 
 
APOO
 
 
hsa-miR-181b-5p
 
 
-0.5
 
 
36.2
 
 
 
 
APOO
 
 
hsa-miR-181a-5p
 
 
-0.5
 
 
36.2
 
 
 
 
APP
 
 
hsa-miR-15b-5p
 
 
-0.298
 
 
535.7
 
 
 
 
ARF4
 
 
hsa-miR-30b-5p
 
 
-0.289
 
 
69.7
 
 
 
 
ARF4
 
 
hsa-miR-194-5p
 
 
-0.293
 
 
69.7
 
 
 
 
ARF4
 
 
hsa-miR-152-3p
 
 
-0.487
 
 
69.7
 
 
 
 
ARF4
 
 
hsa-miR-148b-3p
 
 
-0.487
 
 
69.7
 
 
 
 
ARF5
 
 
hsa-miR-29a-3p
 
 
-0.491
 
 
100.3
 
 
 
 
ARF6
 
 
hsa-miR-181b-5p
 
 
-0.33
 
 
13.8
 
 
 
 
ARF6
 
 
hsa-miR-181a-5p
 
 
-0.33
 
 
13.8
 
 
 
 
ARF6
 
 
hsa-miR-181d-5p
 
 
-0.338
 
 
13.8
 
 
 
 
ARFIP1
 
 
hsa-miR-320b
 
 
-0.339
 
 
10.6
 
 
 
 
ARFIP1
 
 
hsa-miR-320c
 
 
-0.339
 
 
10.6
 
 
 
 
ARFIP1
 
 
hsa-miR-152-3p
 
 
-0.474
 
 
10.6
 
 
 
 
ARFIP1
 
 
hsa-miR-148b-3p
 
 
-0.474
 
 
10.6
 
 
 
 
ARHGAP21
 
 
hsa-miR-152-3p
 
 
-0.287
 
 
74.7
 
 
 
 
ARHGAP21
 
 
hsa-miR-148b-3p
 
 
-0.287
 
 
74.7
 
 
 
 
ARIH2
 
 
hsa-miR-103a-3p
 
 
-0.307
 
 
22.5
 
 
 
 
ARL2
 
 
hsa-miR-15b-5p
 
 
-0.374
 
 
234.0
 
 
 
 
ARL2
 
 
hsa-miR-15b-5p
 
 
-0.442
 
 
234.0
 
 
 
 
ARL3
 
 
hsa-miR-15b-5p
 
 
-0.507
 
 
16.0
 
 
 
 
ARL4A
 
 
hsa-miR-29a-3p
 
 
-0.386
 
 
12.2
 
 
 
 
ARL4A
 
 
hsa-miR-30d-5p
 
 
-0.432
 
 
12.2
 
 
 
 
ARL4A
 
 
hsa-miR-30b-5p
 
 
-0.448
 
 
12.2
 
 
 
 
ARL6IP1
 
 
hsa-miR-328-3p
 
 
-0.494
 
 
140.2
 
 
 
 
ARL6IP1
 
 
hsa-miR-148b-3p
 
 
-0.579
 
 
140.2
 
 
 
 
ARL6IP1
 
 
hsa-miR-152-3p
 
 
-0.579
 
 
140.2
 
 
 
 
ARL8A
 
 
hsa-miR-103a-3p
 
 
-0.38
 
 
157.0
 
 
 
 
ARL8B
 
 
hsa-miR-152-3p
 
 
-0.337
 
 
57.8
 
 
 
 
ARL8B
 
 
hsa-miR-148b-3p
 
 
-0.337
 
 
57.8
 
 
 
 
ARL8B
 
 
hsa-miR-320b
 
 
-0.376
 
 
57.8
 
 
 
 
ARL8B
 
 
hsa-miR-320c
 
 
-0.376
 
 
57.8
 
 
 
 
ARMC1
 
 
hsa-miR-103a-3p
 
 
-0.525
 
 
20.3
 
 
 
 
ARMC8
 
 
hsa-miR-103a-3p
 
 
-0.412
 
 
15.5
 
 
 
 
ARMCX2
 
 
hsa-miR-15b-5p
 
 
-0.344
 
 
15.1
 
 
 
 
ARMCX3
 
 
hsa-miR-425-5p
 
 
-0.325
 
 
33.1
 
 
 
 
ARPC5L
 
 
hsa-miR-326
 
 
-0.439
 
 
32.8
 
 
 
 
ARPC5L
 
 
hsa-miR-1271-5p
 
 
-0.736
 
 
32.8
 
 
 
 
ARPP19
 
 
hsa-miR-1271-5p
 
 
-0.313
 
 
170.8
 
 
 
 
ARPP19
 
 
hsa-miR-320b
 
 
-0.325
 
 
170.8
 
 
 
 
 
 
 spleen 
 
 
 
 
target_symbol
 
 
mature_mirna_id
 
 
score
 
 
nTPM
 
 
 
 
 
 
AAMP
 
 
hsa-miR-326
 
 
-0.722
 
 
52.7
 
 
 
 
AAR2
 
 
hsa-miR-29a-3p
 
 
-0.327
 
 
17.2
 
 
 
 
ABCB7
 
 
hsa-miR-148b-3p
 
 
-0.515
 
 
10.6
 
 
 
 
ABCB7
 
 
hsa-miR-152-3p
 
 
-0.515
 
 
10.6
 
 
 
 
ABCD3
 
 
hsa-miR-148b-3p
 
 
-0.388
 
 
10.3
 
 
 
 
ABCD3
 
 
hsa-miR-152-3p
 
 
-0.388
 
 
10.3
 
 
 
 
ABCE1
 
 
hsa-miR-29a-3p
 
 
-0.404
 
 
24.9
 
 
 
 
ABHD4
 
 
hsa-miR-29a-3p
 
 
-0.312
 
 
15.7
 
 
 
 
ACTR2
 
 
hsa-miR-15b-5p
 
 
-0.293
 
 
161.4
 
 
 
 
ADA
 
 
hsa-miR-29a-3p
 
 
-0.376
 
 
17.0
 
 
 
 
ADAM10
 
 
hsa-miR-152-3p
 
 
-0.348
 
 
27.3
 
 
 
 
ADAM10
 
 
hsa-miR-148b-3p
 
 
-0.356
 
 
27.3
 
 
 
 
ADAM19
 
 
hsa-miR-874-3p
 
 
-0.374
 
 
21.6
 
 
 
 
ADAM9
 
 
hsa-miR-30d-5p
 
 
-0.304
 
 
22.6
 
 
 
 
ADAM9
 
 
hsa-miR-30b-5p
 
 
-0.312
 
 
22.6
 
 
 
 
ADCY3
 
 
hsa-miR-132-3p
 
 
-0.303
 
 
28.8
 
 
 
 
ADGRA2
 
 
hsa-miR-1271-5p
 
 
-0.427
 
 
11.2
 
 
 
 
ADGRF5
 
 
hsa-miR-148b-3p
 
 
-0.354
 
 
38.8
 
 
 
 
ADGRF5
 
 
hsa-miR-152-3p
 
 
-0.354
 
 
38.8
 
 
 
 
ADIPOR2
 
 
hsa-miR-150-5p
 
 
-0.287
 
 
19.2
 
 
 
 
ADK
 
 
hsa-miR-1271-5p
 
 
-0.329
 
 
14.2
 
 
 
 
ADNP
 
 
hsa-miR-328-3p
 
 
-0.371
 
 
34.9
 
 
 
 
ADRA2A
 
 
hsa-miR-23a-3p
 
 
-0.371
 
 
13.1
 
 
 
 
AFG3L2
 
 
hsa-miR-181b-5p
 
 
-0.491
 
 
10.9
 
 
 
 
AFG3L2
 
 
hsa-miR-181a-5p
 
 
-0.491
 
 
10.9
 
 
 
 
AFG3L2
 
 
hsa-miR-181d-5p
 
 
-0.499
 
 
10.9
 
 
 
 
AGFG1
 
 
hsa-miR-148b-3p
 
 
-0.332
 
 
44.5
 
 
 
 
AGFG1
 
 
hsa-miR-152-3p
 
 
-0.332
 
 
44.5
 
 
 
 
AGFG1
 
 
hsa-miR-103a-3p
 
 
-0.389
 
 
44.5
 
 
 
 
AGO4
 
 
hsa-miR-1271-5p
 
 
-0.289
 
 
13.3
 
 
 
 
AGO4
 
 
hsa-miR-103a-3p
 
 
-0.32
 
 
13.3
 
 
 
 
AHDC1
 
 
hsa-miR-152-3p
 
 
-0.413
 
 
15.7
 
 
 
 
AHDC1
 
 
hsa-miR-148b-3p
 
 
-0.413
 
 
15.7
 
 
 
 
AHR
 
 
hsa-miR-1271-5p
 
 
-0.304
 
 
17.6
 
 
 
 
AHR
 
 
hsa-miR-29a-3p
 
 
-0.316
 
 
17.6
 
 
 
 
AIFM1
 
 
hsa-let-7b-5p
 
 
-0.314
 
 
12.9
 
 
 
 
AK1
 
 
hsa-miR-29a-3p
 
 
-0.333
 
 
31.5
 
 
 
 
AK3
 
 
hsa-miR-1271-5p
 
 
-0.57
 
 
31.1
 
 
 
 
AKAP1
 
 
hsa-miR-152-3p
 
 
-0.381
 
 
11.0
 
 
 
 
AKAP1
 
 
hsa-miR-148b-3p
 
 
-0.381
 
 
11.0
 
 
 
 
AKIRIN2
 
 
hsa-miR-103a-3p
 
 
-0.299
 
 
43.9
 
 
 
 
ALAD
 
 
hsa-miR-326
 
 
-0.352
 
 
12.8
 
 
 
 
ALAD
 
 
hsa-miR-103a-3p
 
 
-0.385
 
 
12.8
 
 
 
 
ALAD
 
 
hsa-miR-15b-5p
 
 
-0.446
 
 
12.8
 
 
 
 
ANKHD1
 
 
hsa-miR-23a-3p
 
 
-0.368
 
 
46.1
 
 
 
 
ANKHD1
 
 
hsa-miR-30d-5p
 
 
-0.381
 
 
46.1
 
 
 
 
ANKHD1
 
 
hsa-miR-30b-5p
 
 
-0.405
 
 
46.1
 
 
 
 
ANKIB1
 
 
hsa-miR-1271-5p
 
 
-0.344
 
 
11.7
 
 
 
 
ANKRD17
 
 
hsa-miR-30d-5p
 
 
-0.312
 
 
14.3
 
 
 
 
ANKRD17
 
 
hsa-miR-30b-5p
 
 
-0.336
 
 
14.3
 
 
 
 
ANKRD46
 
 
hsa-let-7b-5p
 
 
-0.389
 
 
10.4
 
 
 
 
ANP32A
 
 
hsa-miR-132-3p
 
 
-0.374
 
 
80.5
 
 
 
 
ANTXR2
 
 
hsa-miR-29a-3p
 
 
-0.347
 
 
17.3
 
 
 
 
ANXA11
 
 
hsa-miR-15b-5p
 
 
-0.463
 
 
73.6
 
 
 
 
AP1S1
 
 
hsa-let-7b-5p
 
 
-0.374
 
 
20.9
 
 
 
 
AP2A1
 
 
hsa-miR-15b-5p
 
 
-0.333
 
 
45.6
 
 
 
 
AP2B1
 
 
hsa-miR-1271-5p
 
 
-0.299
 
 
39.6
 
 
 
 
AP2B1
 
 
hsa-miR-15b-5p
 
 
-0.334
 
 
39.6
 
 
 
 
AP3S1
 
 
hsa-miR-30b-5p
 
 
-0.293
 
 
36.7
 
 
 
 
AP4B1
 
 
hsa-miR-378a-3p
 
 
-0.62
 
 
19.7
 
 
 
 
AP4B1
 
 
hsa-miR-378d
 
 
-0.62
 
 
19.7
 
 
 
 
AP5M1
 
 
hsa-miR-1271-5p
 
 
-0.343
 
 
13.6
 
 
 
 
APP
 
 
hsa-miR-15b-5p
 
 
-0.298
 
 
140.4
 
 
 
 
AQP3
 
 
hsa-miR-874-3p
 
 
-0.48
 
 
103.8
 
 
 
 
ARF4
 
 
hsa-miR-30b-5p
 
 
-0.289
 
 
60.0
 
 
 
 
ARF4
 
 
hsa-miR-194-5p
 
 
-0.293
 
 
60.0
 
 
 
 
ARF4
 
 
hsa-miR-152-3p
 
 
-0.487
 
 
60.0
 
 
 
 
ARF4
 
 
hsa-miR-148b-3p
 
 
-0.487
 
 
60.0
 
 
 
 
ARF5
 
 
hsa-miR-29a-3p
 
 
-0.491
 
 
116.2
 
 
 
 
ARF6
 
 
hsa-miR-181b-5p
 
 
-0.33
 
 
47.5
 
 
 
 
ARF6
 
 
hsa-miR-181a-5p
 
 
-0.33
 
 
47.5
 
 
 
 
ARF6
 
 
hsa-miR-181d-5p
 
 
-0.338
 
 
47.5
 
 
 
 
ARFGEF1
 
 
hsa-miR-148b-3p
 
 
-0.306
 
 
13.0
 
 
 
 
ARFGEF1
 
 
hsa-miR-152-3p
 
 
-0.306
 
 
13.0
 
 
 
 
ARFIP1
 
 
hsa-miR-320b
 
 
-0.339
 
 
17.3
 
 
 
 
ARFIP1
 
 
hsa-miR-320c
 
 
-0.339
 
 
17.3
 
 
 
 
ARFIP1
 
 
hsa-miR-152-3p
 
 
-0.474
 
 
17.3
 
 
 
 
ARFIP1
 
 
hsa-miR-148b-3p
 
 
-0.474
 
 
17.3
 
 
 
 
ARHGAP24
 
 
hsa-miR-1271-5p
 
 
-0.342
 
 
15.5
 
 
 
 
ARHGAP28
 
 
hsa-let-7b-5p
 
 
-0.454
 
 
11.9
 
 
 
 
ARID1B
 
 
hsa-miR-29a-3p
 
 
-0.342
 
 
17.2
 
 
 
 
ARIH2
 
 
hsa-miR-103a-3p
 
 
-0.307
 
 
23.5
 
 
 
 
ARL2
 
 
hsa-miR-15b-5p
 
 
-0.374
 
 
63.8
 
 
 
 
ARL2
 
 
hsa-miR-15b-5p
 
 
-0.442
 
 
63.8
 
 
 
 
ARL4A
 
 
hsa-miR-29a-3p
 
 
-0.386
 
 
20.6
 
 
 
 
ARL4A
 
 
hsa-miR-30d-5p
 
 
-0.432
 
 
20.6
 
 
 
 
ARL4A
 
 
hsa-miR-30b-5p
 
 
-0.448
 
 
20.6
 
 
 
 
ARL4D
 
 
hsa-miR-1271-5p
 
 
-0.392
 
 
16.1
 
 
 
 
ARL4D
 
 
hsa-let-7b-5p
 
 
-0.417
 
 
16.1
 
 
 
 
ARL6IP1
 
 
hsa-miR-328-3p
 
 
-0.494
 
 
68.6
 
 
 
 
ARL6IP1
 
 
hsa-miR-148b-3p
 
 
-0.579
 
 
68.6
 
 
 
 
ARL6IP1
 
 
hsa-miR-152-3p
 
 
-0.579
 
 
68.6
 
 
 
 
ARL6IP6
 
 
hsa-let-7b-5p
 
 
-0.293
 
 
13.7
 
 
 
 
ARL8A
 
 
hsa-miR-103a-3p
 
 
-0.38
 
 
36.8
 
 
 
 
ARL8B
 
 
hsa-miR-152-3p
 
 
-0.337
 
 
32.0
 
 
 
 
ARL8B
 
 
hsa-miR-148b-3p
 
 
-0.337
 
 
32.0
 
 
 
 
ARL8B
 
 
hsa-miR-320b
 
 
-0.376
 
 
32.0
 
 
 
 
ARL8B
 
 
hsa-miR-320c
 
 
-0.376
 
 
32.0
 
 
 
 
ARMC1
 
 
hsa-miR-103a-3p
 
 
-0.525
 
 
13.1
 
 
 
 
ARMC8
 
 
hsa-miR-103a-3p
 
 
-0.412
 
 
14.9
 
 
 
 
 
 
 stomach 
 
 
 
 
target_symbol
 
 
mature_mirna_id
 
 
score
 
 
nTPM
 
 
 
 
 
 
AAMP
 
 
hsa-miR-326
 
 
-0.722
 
 
87.9
 
 
 
 
AAR2
 
 
hsa-miR-29a-3p
 
 
-0.327
 
 
21.7
 
 
 
 
ABCA2
 
 
hsa-miR-1271-5p
 
 
-0.326
 
 
10.2
 
 
 
 
ABCB7
 
 
hsa-miR-148b-3p
 
 
-0.515
 
 
11.6
 
 
 
 
ABCB7
 
 
hsa-miR-152-3p
 
 
-0.515
 
 
11.6
 
 
 
 
ABCD3
 
 
hsa-miR-148b-3p
 
 
-0.388
 
 
41.1
 
 
 
 
ABCD3
 
 
hsa-miR-152-3p
 
 
-0.388
 
 
41.1
 
 
 
 
ABCE1
 
 
hsa-miR-29a-3p
 
 
-0.404
 
 
19.0
 
 
 
 
ABHD17C
 
 
hsa-let-7b-5p
 
 
-0.359
 
 
34.0
 
 
 
 
ABHD17C
 
 
hsa-miR-330-3p
 
 
-0.399
 
 
34.0
 
 
 
 
ABHD4
 
 
hsa-miR-29a-3p
 
 
-0.312
 
 
26.1
 
 
 
 
ACER2
 
 
hsa-let-7b-5p
 
 
-0.333
 
 
58.3
 
 
 
 
ACTC1
 
 
hsa-miR-30d-5p
 
 
-0.519
 
 
32.8
 
 
 
 
ACTC1
 
 
hsa-miR-30b-5p
 
 
-0.543
 
 
32.8
 
 
 
 
ACTR2
 
 
hsa-miR-15b-5p
 
 
-0.293
 
 
100.4
 
 
 
 
ACVR1
 
 
hsa-miR-30b-5p
 
 
-0.29
 
 
11.0
 
 
 
 
ACVR1
 
 
hsa-miR-152-3p
 
 
-0.397
 
 
11.0
 
 
 
 
ACVR1
 
 
hsa-miR-148b-3p
 
 
-0.397
 
 
11.0
 
 
 
 
ADA
 
 
hsa-miR-29a-3p
 
 
-0.376
 
 
44.4
 
 
 
 
ADAM10
 
 
hsa-miR-152-3p
 
 
-0.348
 
 
19.0
 
 
 
 
ADAM10
 
 
hsa-miR-148b-3p
 
 
-0.356
 
 
19.0
 
 
 
 
ADAM9
 
 
hsa-miR-30d-5p
 
 
-0.304
 
 
41.8
 
 
 
 
ADAM9
 
 
hsa-miR-30b-5p
 
 
-0.312
 
 
41.8
 
 
 
 
ADCY3
 
 
hsa-miR-132-3p
 
 
-0.303
 
 
23.8
 
 
 
 
ADCY6
 
 
hsa-miR-1271-5p
 
 
-0.336
 
 
23.9
 
 
 
 
ADGRA2
 
 
hsa-miR-1271-5p
 
 
-0.427
 
 
10.3
 
 
 
 
ADIPOR2
 
 
hsa-miR-150-5p
 
 
-0.287
 
 
42.5
 
 
 
 
ADK
 
 
hsa-miR-1271-5p
 
 
-0.329
 
 
11.3
 
 
 
 
ADNP
 
 
hsa-miR-328-3p
 
 
-0.371
 
 
30.6
 
 
 
 
AFG3L2
 
 
hsa-miR-181b-5p
 
 
-0.491
 
 
26.3
 
 
 
 
AFG3L2
 
 
hsa-miR-181a-5p
 
 
-0.491
 
 
26.3
 
 
 
 
AFG3L2
 
 
hsa-miR-181d-5p
 
 
-0.499
 
 
26.3
 
 
 
 
AGFG1
 
 
hsa-miR-148b-3p
 
 
-0.332
 
 
31.3
 
 
 
 
AGFG1
 
 
hsa-miR-152-3p
 
 
-0.332
 
 
31.3
 
 
 
 
AGFG1
 
 
hsa-miR-103a-3p
 
 
-0.389
 
 
31.3
 
 
 
 
AHDC1
 
 
hsa-miR-152-3p
 
 
-0.413
 
 
10.8
 
 
 
 
AHDC1
 
 
hsa-miR-148b-3p
 
 
-0.413
 
 
10.8
 
 
 
 
AHR
 
 
hsa-miR-1271-5p
 
 
-0.304
 
 
23.8
 
 
 
 
AHR
 
 
hsa-miR-29a-3p
 
 
-0.316
 
 
23.8
 
 
 
 
AIFM1
 
 
hsa-let-7b-5p
 
 
-0.314
 
 
29.8
 
 
 
 
AK1
 
 
hsa-miR-29a-3p
 
 
-0.333
 
 
28.6
 
 
 
 
AK3
 
 
hsa-miR-1271-5p
 
 
-0.57
 
 
65.7
 
 
 
 
AK4
 
 
hsa-miR-15b-5p
 
 
-0.316
 
 
14.0
 
 
 
 
AKAP1
 
 
hsa-miR-152-3p
 
 
-0.381
 
 
53.0
 
 
 
 
AKAP1
 
 
hsa-miR-148b-3p
 
 
-0.381
 
 
53.0
 
 
 
 
AKIRIN2
 
 
hsa-miR-103a-3p
 
 
-0.299
 
 
14.8
 
 
 
 
ALAD
 
 
hsa-miR-326
 
 
-0.352
 
 
49.1
 
 
 
 
ALAD
 
 
hsa-miR-103a-3p
 
 
-0.385
 
 
49.1
 
 
 
 
ALAD
 
 
hsa-miR-15b-5p
 
 
-0.446
 
 
49.1
 
 
 
 
ALCAM
 
 
hsa-miR-148b-3p
 
 
-0.339
 
 
31.6
 
 
 
 
ALCAM
 
 
hsa-miR-152-3p
 
 
-0.347
 
 
31.6
 
 
 
 
ALDH6A1
 
 
hsa-miR-1271-5p
 
 
-0.301
 
 
22.7
 
 
 
 
AMOTL1
 
 
hsa-miR-15b-5p
 
 
-0.319
 
 
11.8
 
 
 
 
AMT
 
 
hsa-let-7b-5p
 
 
-0.463
 
 
13.1
 
 
 
 
ANKHD1
 
 
hsa-miR-23a-3p
 
 
-0.368
 
 
39.4
 
 
 
 
ANKHD1
 
 
hsa-miR-30d-5p
 
 
-0.381
 
 
39.4
 
 
 
 
ANKHD1
 
 
hsa-miR-30b-5p
 
 
-0.405
 
 
39.4
 
 
 
 
ANKIB1
 
 
hsa-miR-1271-5p
 
 
-0.344
 
 
11.0
 
 
 
 
ANKRD17
 
 
hsa-miR-30d-5p
 
 
-0.312
 
 
14.8
 
 
 
 
ANKRD17
 
 
hsa-miR-30b-5p
 
 
-0.336
 
 
14.8
 
 
 
 
ANKRD27
 
 
hsa-miR-1271-5p
 
 
-0.648
 
 
10.5
 
 
 
 
ANKRD29
 
 
hsa-miR-132-3p
 
 
-0.47
 
 
14.3
 
 
 
 
ANKRD46
 
 
hsa-let-7b-5p
 
 
-0.389
 
 
10.9
 
 
 
 
ANP32A
 
 
hsa-miR-132-3p
 
 
-0.374
 
 
74.4
 
 
 
 
ANTXR2
 
 
hsa-miR-29a-3p
 
 
-0.347
 
 
46.6
 
 
 
 
ANXA11
 
 
hsa-miR-15b-5p
 
 
-0.463
 
 
131.4
 
 
 
 
AP1S1
 
 
hsa-let-7b-5p
 
 
-0.374
 
 
23.2
 
 
 
 
AP2A1
 
 
hsa-miR-15b-5p
 
 
-0.333
 
 
34.8
 
 
 
 
AP2B1
 
 
hsa-miR-1271-5p
 
 
-0.299
 
 
45.1
 
 
 
 
AP2B1
 
 
hsa-miR-15b-5p
 
 
-0.334
 
 
45.1
 
 
 
 
AP3S1
 
 
hsa-miR-30b-5p
 
 
-0.293
 
 
42.9
 
 
 
 
AP4B1
 
 
hsa-miR-378a-3p
 
 
-0.62
 
 
10.0
 
 
 
 
AP4B1
 
 
hsa-miR-378d
 
 
-0.62
 
 
10.0
 
 
 
 
AP5M1
 
 
hsa-miR-1271-5p
 
 
-0.343
 
 
14.1
 
 
 
 
APOO
 
 
hsa-miR-181d-5p
 
 
-0.492
 
 
17.0
 
 
 
 
APOO
 
 
hsa-miR-181b-5p
 
 
-0.5
 
 
17.0
 
 
 
 
APOO
 
 
hsa-miR-181a-5p
 
 
-0.5
 
 
17.0
 
 
 
 
APP
 
 
hsa-miR-15b-5p
 
 
-0.298
 
 
197.5
 
 
 
 
AQP3
 
 
hsa-miR-874-3p
 
 
-0.48
 
 
33.6
 
 
 
 
ARF4
 
 
hsa-miR-30b-5p
 
 
-0.289
 
 
123.5
 
 
 
 
ARF4
 
 
hsa-miR-194-5p
 
 
-0.293
 
 
123.5
 
 
 
 
ARF4
 
 
hsa-miR-152-3p
 
 
-0.487
 
 
123.5
 
 
 
 
ARF4
 
 
hsa-miR-148b-3p
 
 
-0.487
 
 
123.5
 
 
 
 
ARF5
 
 
hsa-miR-29a-3p
 
 
-0.491
 
 
126.0
 
 
 
 
ARF6
 
 
hsa-miR-181b-5p
 
 
-0.33
 
 
44.4
 
 
 
 
ARF6
 
 
hsa-miR-181a-5p
 
 
-0.33
 
 
44.4
 
 
 
 
ARF6
 
 
hsa-miR-181d-5p
 
 
-0.338
 
 
44.4
 
 
 
 
ARFGEF1
 
 
hsa-miR-148b-3p
 
 
-0.306
 
 
18.5
 
 
 
 
ARFGEF1
 
 
hsa-miR-152-3p
 
 
-0.306
 
 
18.5
 
 
 
 
ARFIP1
 
 
hsa-miR-320b
 
 
-0.339
 
 
21.5
 
 
 
 
ARFIP1
 
 
hsa-miR-320c
 
 
-0.339
 
 
21.5
 
 
 
 
ARFIP1
 
 
hsa-miR-152-3p
 
 
-0.474
 
 
21.5
 
 
 
 
ARFIP1
 
 
hsa-miR-148b-3p
 
 
-0.474
 
 
21.5
 
 
 
 
ARHGAP21
 
 
hsa-miR-152-3p
 
 
-0.287
 
 
25.5
 
 
 
 
ARHGAP21
 
 
hsa-miR-148b-3p
 
 
-0.287
 
 
25.5
 
 
 
 
ARHGAP24
 
 
hsa-miR-1271-5p
 
 
-0.342
 
 
24.6
 
 
 
 
ARID1B
 
 
hsa-miR-29a-3p
 
 
-0.342
 
 
11.1
 
 
 
 
ARIH2
 
 
hsa-miR-103a-3p
 
 
-0.307
 
 
21.8
 
 
 
 
ARL2
 
 
hsa-miR-15b-5p
 
 
-0.374
 
 
85.3
 
 
 
 
ARL2
 
 
hsa-miR-15b-5p
 
 
-0.442
 
 
85.3
 
 
 
 
 
 
 testis 
 
 
 
 
target_symbol
 
 
mature_mirna_id
 
 
score
 
 
nTPM
 
 
 
 
 
 
AAMP
 
 
hsa-miR-326
 
 
-0.722
 
 
53.9
 
 
 
 
AAR2
 
 
hsa-miR-29a-3p
 
 
-0.327
 
 
15.4
 
 
 
 
ABAT
 
 
hsa-miR-1271-5p
 
 
-0.409
 
 
12.5
 
 
 
 
ABCB6
 
 
hsa-miR-29a-3p
 
 
-0.48
 
 
15.2
 
 
 
 
ABCB9
 
 
hsa-let-7b-5p
 
 
-0.422
 
 
17.1
 
 
 
 
ABCD3
 
 
hsa-miR-148b-3p
 
 
-0.388
 
 
15.3
 
 
 
 
ABCD3
 
 
hsa-miR-152-3p
 
 
-0.388
 
 
15.3
 
 
 
 
ABCE1
 
 
hsa-miR-29a-3p
 
 
-0.404
 
 
24.7
 
 
 
 
ABHD4
 
 
hsa-miR-29a-3p
 
 
-0.312
 
 
39.1
 
 
 
 
ACSL6
 
 
hsa-let-7b-5p
 
 
-0.336
 
 
19.3
 
 
 
 
ACTR2
 
 
hsa-miR-15b-5p
 
 
-0.293
 
 
37.7
 
 
 
 
ACTRT3
 
 
hsa-miR-1271-5p
 
 
-0.499
 
 
54.1
 
 
 
 
ADAM9
 
 
hsa-miR-30d-5p
 
 
-0.304
 
 
10.4
 
 
 
 
ADAM9
 
 
hsa-miR-30b-5p
 
 
-0.312
 
 
10.4
 
 
 
 
ADIPOR2
 
 
hsa-miR-150-5p
 
 
-0.287
 
 
26.9
 
 
 
 
ADK
 
 
hsa-miR-1271-5p
 
 
-0.329
 
 
14.4
 
 
 
 
ADNP
 
 
hsa-miR-328-3p
 
 
-0.371
 
 
37.6
 
 
 
 
AFG3L2
 
 
hsa-miR-181b-5p
 
 
-0.491
 
 
10.5
 
 
 
 
AFG3L2
 
 
hsa-miR-181a-5p
 
 
-0.491
 
 
10.5
 
 
 
 
AFG3L2
 
 
hsa-miR-181d-5p
 
 
-0.499
 
 
10.5
 
 
 
 
AGFG1
 
 
hsa-miR-148b-3p
 
 
-0.332
 
 
86.7
 
 
 
 
AGFG1
 
 
hsa-miR-152-3p
 
 
-0.332
 
 
86.7
 
 
 
 
AGFG1
 
 
hsa-miR-103a-3p
 
 
-0.389
 
 
86.7
 
 
 
 
AHCTF1
 
 
hsa-let-7b-5p
 
 
-0.374
 
 
30.4
 
 
 
 
AIFM1
 
 
hsa-let-7b-5p
 
 
-0.314
 
 
21.3
 
 
 
 
AK1
 
 
hsa-miR-29a-3p
 
 
-0.333
 
 
64.3
 
 
 
 
AK3
 
 
hsa-miR-1271-5p
 
 
-0.57
 
 
10.4
 
 
 
 
AK4
 
 
hsa-miR-15b-5p
 
 
-0.316
 
 
10.1
 
 
 
 
AKAP1
 
 
hsa-miR-152-3p
 
 
-0.381
 
 
108.8
 
 
 
 
AKAP1
 
 
hsa-miR-148b-3p
 
 
-0.381
 
 
108.8
 
 
 
 
AKIRIN2
 
 
hsa-miR-103a-3p
 
 
-0.299
 
 
21.4
 
 
 
 
ALAD
 
 
hsa-miR-326
 
 
-0.352
 
 
12.6
 
 
 
 
ALAD
 
 
hsa-miR-103a-3p
 
 
-0.385
 
 
12.6
 
 
 
 
ALAD
 
 
hsa-miR-15b-5p
 
 
-0.446
 
 
12.6
 
 
 
 
ALDH1A2
 
 
hsa-miR-23a-3p
 
 
-0.354
 
 
53.9
 
 
 
 
ALDH6A1
 
 
hsa-miR-1271-5p
 
 
-0.301
 
 
16.0
 
 
 
 
AMOTL1
 
 
hsa-miR-15b-5p
 
 
-0.319
 
 
11.0
 
 
 
 
ANKHD1
 
 
hsa-miR-23a-3p
 
 
-0.368
 
 
29.3
 
 
 
 
ANKHD1
 
 
hsa-miR-30d-5p
 
 
-0.381
 
 
29.3
 
 
 
 
ANKHD1
 
 
hsa-miR-30b-5p
 
 
-0.405
 
 
29.3
 
 
 
 
ANKIB1
 
 
hsa-miR-1271-5p
 
 
-0.344
 
 
19.4
 
 
 
 
ANKRD17
 
 
hsa-miR-30d-5p
 
 
-0.312
 
 
10.4
 
 
 
 
ANKRD17
 
 
hsa-miR-30b-5p
 
 
-0.336
 
 
10.4
 
 
 
 
ANKRD27
 
 
hsa-miR-1271-5p
 
 
-0.648
 
 
16.2
 
 
 
 
ANLN
 
 
hsa-miR-15b-5p
 
 
-0.334
 
 
11.6
 
 
 
 
ANP32A
 
 
hsa-miR-132-3p
 
 
-0.374
 
 
19.4
 
 
 
 
ANXA11
 
 
hsa-miR-15b-5p
 
 
-0.463
 
 
22.9
 
 
 
 
AP1S1
 
 
hsa-let-7b-5p
 
 
-0.374
 
 
17.8
 
 
 
 
AP2A1
 
 
hsa-miR-15b-5p
 
 
-0.333
 
 
85.4
 
 
 
 
AP2B1
 
 
hsa-miR-1271-5p
 
 
-0.299
 
 
87.4
 
 
 
 
AP2B1
 
 
hsa-miR-15b-5p
 
 
-0.334
 
 
87.4
 
 
 
 
AP3S1
 
 
hsa-miR-30b-5p
 
 
-0.293
 
 
53.5
 
 
 
 
AP4B1
 
 
hsa-miR-378a-3p
 
 
-0.62
 
 
10.7
 
 
 
 
AP4B1
 
 
hsa-miR-378d
 
 
-0.62
 
 
10.7
 
 
 
 
APOO
 
 
hsa-miR-181d-5p
 
 
-0.492
 
 
15.7
 
 
 
 
APOO
 
 
hsa-miR-181b-5p
 
 
-0.5
 
 
15.7
 
 
 
 
APOO
 
 
hsa-miR-181a-5p
 
 
-0.5
 
 
15.7
 
 
 
 
APP
 
 
hsa-miR-15b-5p
 
 
-0.298
 
 
146.3
 
 
 
 
ARF4
 
 
hsa-miR-30b-5p
 
 
-0.289
 
 
94.2
 
 
 
 
ARF4
 
 
hsa-miR-194-5p
 
 
-0.293
 
 
94.2
 
 
 
 
ARF4
 
 
hsa-miR-152-3p
 
 
-0.487
 
 
94.2
 
 
 
 
ARF4
 
 
hsa-miR-148b-3p
 
 
-0.487
 
 
94.2
 
 
 
 
ARF5
 
 
hsa-miR-29a-3p
 
 
-0.491
 
 
89.2
 
 
 
 
ARF6
 
 
hsa-miR-181b-5p
 
 
-0.33
 
 
15.0
 
 
 
 
ARF6
 
 
hsa-miR-181a-5p
 
 
-0.33
 
 
15.0
 
 
 
 
ARF6
 
 
hsa-miR-181d-5p
 
 
-0.338
 
 
15.0
 
 
 
 
ARFGEF1
 
 
hsa-miR-148b-3p
 
 
-0.306
 
 
26.5
 
 
 
 
ARFGEF1
 
 
hsa-miR-152-3p
 
 
-0.306
 
 
26.5
 
 
 
 
ARFIP1
 
 
hsa-miR-320b
 
 
-0.339
 
 
11.3
 
 
 
 
ARFIP1
 
 
hsa-miR-320c
 
 
-0.339
 
 
11.3
 
 
 
 
ARFIP1
 
 
hsa-miR-152-3p
 
 
-0.474
 
 
11.3
 
 
 
 
ARFIP1
 
 
hsa-miR-148b-3p
 
 
-0.474
 
 
11.3
 
 
 
 
ARHGAP21
 
 
hsa-miR-152-3p
 
 
-0.287
 
 
13.5
 
 
 
 
ARHGAP21
 
 
hsa-miR-148b-3p
 
 
-0.287
 
 
13.5
 
 
 
 
ARHGAP28
 
 
hsa-let-7b-5p
 
 
-0.454
 
 
72.3
 
 
 
 
ARID2
 
 
hsa-miR-132-3p
 
 
-0.304
 
 
24.3
 
 
 
 
ARID2
 
 
hsa-miR-103a-3p
 
 
-0.415
 
 
24.3
 
 
 
 
ARID3A
 
 
hsa-let-7b-5p
 
 
-0.332
 
 
16.1
 
 
 
 
ARID3A
 
 
hsa-let-7b-5p
 
 
-0.347
 
 
16.1
 
 
 
 
ARID3B
 
 
hsa-let-7b-5p
 
 
-0.322
 
 
21.2
 
 
 
 
ARID3B
 
 
hsa-let-7b-5p
 
 
-0.486
 
 
21.2
 
 
 
 
ARIH2
 
 
hsa-miR-103a-3p
 
 
-0.307
 
 
25.2
 
 
 
 
ARL2
 
 
hsa-miR-15b-5p
 
 
-0.374
 
 
115.7
 
 
 
 
ARL2
 
 
hsa-miR-15b-5p
 
 
-0.442
 
 
115.7
 
 
 
 
ARL3
 
 
hsa-miR-15b-5p
 
 
-0.507
 
 
10.4
 
 
 
 
ARL4A
 
 
hsa-miR-29a-3p
 
 
-0.386
 
 
181.2
 
 
 
 
ARL4A
 
 
hsa-miR-30d-5p
 
 
-0.432
 
 
181.2
 
 
 
 
ARL4A
 
 
hsa-miR-30b-5p
 
 
-0.448
 
 
181.2
 
 
 
 
ARL4D
 
 
hsa-miR-1271-5p
 
 
-0.392
 
 
31.4
 
 
 
 
ARL4D
 
 
hsa-let-7b-5p
 
 
-0.417
 
 
31.4
 
 
 
 
ARL6IP1
 
 
hsa-miR-328-3p
 
 
-0.494
 
 
121.4
 
 
 
 
ARL6IP1
 
 
hsa-miR-148b-3p
 
 
-0.579
 
 
121.4
 
 
 
 
ARL6IP1
 
 
hsa-miR-152-3p
 
 
-0.579
 
 
121.4
 
 
 
 
ARL6IP6
 
 
hsa-let-7b-5p
 
 
-0.293
 
 
10.2
 
 
 
 
ARL8A
 
 
hsa-miR-103a-3p
 
 
-0.38
 
 
18.8
 
 
 
 
ARL8B
 
 
hsa-miR-152-3p
 
 
-0.337
 
 
30.1
 
 
 
 
ARL8B
 
 
hsa-miR-148b-3p
 
 
-0.337
 
 
30.1
 
 
 
 
ARL8B
 
 
hsa-miR-320b
 
 
-0.376
 
 
30.1
 
 
 
 
ARL8B
 
 
hsa-miR-320c
 
 
-0.376
 
 
30.1
 
 
 
 
ARMC12
 
 
hsa-miR-15b-5p
 
 
-0.485
 
 
58.4
 
 
 
 
 
 
 thymus 
 
 
 
 
target_symbol
 
 
mature_mirna_id
 
 
score
 
 
nTPM
 
 
 
 
 
 
AAMP
 
 
hsa-miR-326
 
 
-0.722
 
 
50.3
 
 
 
 
AAR2
 
 
hsa-miR-29a-3p
 
 
-0.327
 
 
24.4
 
 
 
 
ABCB7
 
 
hsa-miR-148b-3p
 
 
-0.515
 
 
13.0
 
 
 
 
ABCB7
 
 
hsa-miR-152-3p
 
 
-0.515
 
 
13.0
 
 
 
 
ABCD3
 
 
hsa-miR-148b-3p
 
 
-0.388
 
 
44.2
 
 
 
 
ABCD3
 
 
hsa-miR-152-3p
 
 
-0.388
 
 
44.2
 
 
 
 
ABCE1
 
 
hsa-miR-29a-3p
 
 
-0.404
 
 
31.9
 
 
 
 
ABHD4
 
 
hsa-miR-29a-3p
 
 
-0.312
 
 
10.3
 
 
 
 
ACTC1
 
 
hsa-miR-30d-5p
 
 
-0.519
 
 
10.5
 
 
 
 
ACTC1
 
 
hsa-miR-30b-5p
 
 
-0.543
 
 
10.5
 
 
 
 
ACTR2
 
 
hsa-miR-15b-5p
 
 
-0.293
 
 
169.2
 
 
 
 
ADA
 
 
hsa-miR-29a-3p
 
 
-0.376
 
 
228.3
 
 
 
 
ADAM10
 
 
hsa-miR-152-3p
 
 
-0.348
 
 
38.8
 
 
 
 
ADAM10
 
 
hsa-miR-148b-3p
 
 
-0.356
 
 
38.8
 
 
 
 
ADCY3
 
 
hsa-miR-132-3p
 
 
-0.303
 
 
10.7
 
 
 
 
ADIPOR2
 
 
hsa-miR-150-5p
 
 
-0.287
 
 
11.9
 
 
 
 
ADK
 
 
hsa-miR-1271-5p
 
 
-0.329
 
 
25.2
 
 
 
 
ADNP
 
 
hsa-miR-328-3p
 
 
-0.371
 
 
41.7
 
 
 
 
AFG3L2
 
 
hsa-miR-181b-5p
 
 
-0.491
 
 
12.1
 
 
 
 
AFG3L2
 
 
hsa-miR-181a-5p
 
 
-0.491
 
 
12.1
 
 
 
 
AFG3L2
 
 
hsa-miR-181d-5p
 
 
-0.499
 
 
12.1
 
 
 
 
AGFG1
 
 
hsa-miR-148b-3p
 
 
-0.332
 
 
45.3
 
 
 
 
AGFG1
 
 
hsa-miR-152-3p
 
 
-0.332
 
 
45.3
 
 
 
 
AGFG1
 
 
hsa-miR-103a-3p
 
 
-0.389
 
 
45.3
 
 
 
 
AHCTF1
 
 
hsa-let-7b-5p
 
 
-0.374
 
 
11.4
 
 
 
 
AIFM1
 
 
hsa-let-7b-5p
 
 
-0.314
 
 
17.2
 
 
 
 
AK3
 
 
hsa-miR-1271-5p
 
 
-0.57
 
 
32.7
 
 
 
 
AKAP1
 
 
hsa-miR-152-3p
 
 
-0.381
 
 
10.6
 
 
 
 
AKAP1
 
 
hsa-miR-148b-3p
 
 
-0.381
 
 
10.6
 
 
 
 
AKIRIN2
 
 
hsa-miR-103a-3p
 
 
-0.299
 
 
29.1
 
 
 
 
ALAD
 
 
hsa-miR-326
 
 
-0.352
 
 
18.9
 
 
 
 
ALAD
 
 
hsa-miR-103a-3p
 
 
-0.385
 
 
18.9
 
 
 
 
ALAD
 
 
hsa-miR-15b-5p
 
 
-0.446
 
 
18.9
 
 
 
 
ALCAM
 
 
hsa-miR-148b-3p
 
 
-0.339
 
 
13.3
 
 
 
 
ALCAM
 
 
hsa-miR-152-3p
 
 
-0.347
 
 
13.3
 
 
 
 
AMOTL1
 
 
hsa-miR-15b-5p
 
 
-0.319
 
 
26.0
 
 
 
 
ANKHD1
 
 
hsa-miR-23a-3p
 
 
-0.368
 
 
45.5
 
 
 
 
ANKHD1
 
 
hsa-miR-30d-5p
 
 
-0.381
 
 
45.5
 
 
 
 
ANKHD1
 
 
hsa-miR-30b-5p
 
 
-0.405
 
 
45.5
 
 
 
 
ANKIB1
 
 
hsa-miR-1271-5p
 
 
-0.344
 
 
13.5
 
 
 
 
ANKRD17
 
 
hsa-miR-30d-5p
 
 
-0.312
 
 
25.3
 
 
 
 
ANKRD17
 
 
hsa-miR-30b-5p
 
 
-0.336
 
 
25.3
 
 
 
 
ANKRD27
 
 
hsa-miR-1271-5p
 
 
-0.648
 
 
15.5
 
 
 
 
ANKRD46
 
 
hsa-let-7b-5p
 
 
-0.389
 
 
14.3
 
 
 
 
ANLN
 
 
hsa-miR-15b-5p
 
 
-0.334
 
 
14.9
 
 
 
 
ANP32A
 
 
hsa-miR-132-3p
 
 
-0.374
 
 
189.1
 
 
 
 
ANTXR2
 
 
hsa-miR-29a-3p
 
 
-0.347
 
 
10.0
 
 
 
 
ANXA11
 
 
hsa-miR-15b-5p
 
 
-0.463
 
 
93.3
 
 
 
 
AP1S1
 
 
hsa-let-7b-5p
 
 
-0.374
 
 
17.8
 
 
 
 
AP2A1
 
 
hsa-miR-15b-5p
 
 
-0.333
 
 
14.3
 
 
 
 
AP2B1
 
 
hsa-miR-1271-5p
 
 
-0.299
 
 
55.6
 
 
 
 
AP2B1
 
 
hsa-miR-15b-5p
 
 
-0.334
 
 
55.6
 
 
 
 
AP3S1
 
 
hsa-miR-30b-5p
 
 
-0.293
 
 
38.3
 
 
 
 
AP4B1
 
 
hsa-miR-378a-3p
 
 
-0.62
 
 
15.0
 
 
 
 
AP4B1
 
 
hsa-miR-378d
 
 
-0.62
 
 
15.0
 
 
 
 
AP5M1
 
 
hsa-miR-1271-5p
 
 
-0.343
 
 
20.7
 
 
 
 
APOO
 
 
hsa-miR-181d-5p
 
 
-0.492
 
 
13.0
 
 
 
 
APOO
 
 
hsa-miR-181b-5p
 
 
-0.5
 
 
13.0
 
 
 
 
APOO
 
 
hsa-miR-181a-5p
 
 
-0.5
 
 
13.0
 
 
 
 
APP
 
 
hsa-miR-15b-5p
 
 
-0.298
 
 
143.9
 
 
 
 
AQP3
 
 
hsa-miR-874-3p
 
 
-0.48
 
 
217.7
 
 
 
 
ARF4
 
 
hsa-miR-30b-5p
 
 
-0.289
 
 
69.9
 
 
 
 
ARF4
 
 
hsa-miR-194-5p
 
 
-0.293
 
 
69.9
 
 
 
 
ARF4
 
 
hsa-miR-152-3p
 
 
-0.487
 
 
69.9
 
 
 
 
ARF4
 
 
hsa-miR-148b-3p
 
 
-0.487
 
 
69.9
 
 
 
 
ARF5
 
 
hsa-miR-29a-3p
 
 
-0.491
 
 
89.3
 
 
 
 
ARF6
 
 
hsa-miR-181b-5p
 
 
-0.33
 
 
41.8
 
 
 
 
ARF6
 
 
hsa-miR-181a-5p
 
 
-0.33
 
 
41.8
 
 
 
 
ARF6
 
 
hsa-miR-181d-5p
 
 
-0.338
 
 
41.8
 
 
 
 
ARFGEF1
 
 
hsa-miR-148b-3p
 
 
-0.306
 
 
20.7
 
 
 
 
ARFGEF1
 
 
hsa-miR-152-3p
 
 
-0.306
 
 
20.7
 
 
 
 
ARFIP1
 
 
hsa-miR-320b
 
 
-0.339
 
 
11.2
 
 
 
 
ARFIP1
 
 
hsa-miR-320c
 
 
-0.339
 
 
11.2
 
 
 
 
ARFIP1
 
 
hsa-miR-152-3p
 
 
-0.474
 
 
11.2
 
 
 
 
ARFIP1
 
 
hsa-miR-148b-3p
 
 
-0.474
 
 
11.2
 
 
 
 
ARHGAP21
 
 
hsa-miR-152-3p
 
 
-0.287
 
 
10.5
 
 
 
 
ARHGAP21
 
 
hsa-miR-148b-3p
 
 
-0.287
 
 
10.5
 
 
 
 
ARID1B
 
 
hsa-miR-29a-3p
 
 
-0.342
 
 
23.3
 
 
 
 
ARID2
 
 
hsa-miR-132-3p
 
 
-0.304
 
 
17.5
 
 
 
 
ARID2
 
 
hsa-miR-103a-3p
 
 
-0.415
 
 
17.5
 
 
 
 
ARIH2
 
 
hsa-miR-103a-3p
 
 
-0.307
 
 
24.3
 
 
 
 
ARL2
 
 
hsa-miR-15b-5p
 
 
-0.374
 
 
31.2
 
 
 
 
ARL2
 
 
hsa-miR-15b-5p
 
 
-0.442
 
 
31.2
 
 
 
 
ARL4A
 
 
hsa-miR-29a-3p
 
 
-0.386
 
 
21.1
 
 
 
 
ARL4A
 
 
hsa-miR-30d-5p
 
 
-0.432
 
 
21.1
 
 
 
 
ARL4A
 
 
hsa-miR-30b-5p
 
 
-0.448
 
 
21.1
 
 
 
 
ARL5B
 
 
hsa-miR-29a-3p
 
 
-0.304
 
 
12.6
 
 
 
 
ARL6IP1
 
 
hsa-miR-328-3p
 
 
-0.494
 
 
147.6
 
 
 
 
ARL6IP1
 
 
hsa-miR-148b-3p
 
 
-0.579
 
 
147.6
 
 
 
 
ARL6IP1
 
 
hsa-miR-152-3p
 
 
-0.579
 
 
147.6
 
 
 
 
ARL6IP6
 
 
hsa-let-7b-5p
 
 
-0.293
 
 
20.0
 
 
 
 
ARL8A
 
 
hsa-miR-103a-3p
 
 
-0.38
 
 
16.8
 
 
 
 
ARL8B
 
 
hsa-miR-152-3p
 
 
-0.337
 
 
33.3
 
 
 
 
ARL8B
 
 
hsa-miR-148b-3p
 
 
-0.337
 
 
33.3
 
 
 
 
ARL8B
 
 
hsa-miR-320b
 
 
-0.376
 
 
33.3
 
 
 
 
ARL8B
 
 
hsa-miR-320c
 
 
-0.376
 
 
33.3
 
 
 
 
ARMC1
 
 
hsa-miR-103a-3p
 
 
-0.525
 
 
20.8
 
 
 
 
ARMC8
 
 
hsa-miR-103a-3p
 
 
-0.412
 
 
20.7
 
 
 
 
ARMCX3
 
 
hsa-miR-425-5p
 
 
-0.325
 
 
18.7
 
 
 
 
ARMT1
 
 
hsa-let-7b-5p
 
 
-0.291
 
 
14.5
 
 
 
 
 
 
 thyroid gland 
 
 
 
 
target_symbol
 
 
mature_mirna_id
 
 
score
 
 
nTPM
 
 
 
 
 
 
AAMP
 
 
hsa-miR-326
 
 
-0.722
 
 
62.3
 
 
 
 
AAR2
 
 
hsa-miR-29a-3p
 
 
-0.327
 
 
16.7
 
 
 
 
ABAT
 
 
hsa-miR-1271-5p
 
 
-0.409
 
 
27.5
 
 
 
 
ABCA2
 
 
hsa-miR-1271-5p
 
 
-0.326
 
 
13.5
 
 
 
 
ABCD3
 
 
hsa-miR-148b-3p
 
 
-0.388
 
 
25.8
 
 
 
 
ABCD3
 
 
hsa-miR-152-3p
 
 
-0.388
 
 
25.8
 
 
 
 
ABCE1
 
 
hsa-miR-29a-3p
 
 
-0.404
 
 
25.1
 
 
 
 
ABHD4
 
 
hsa-miR-29a-3p
 
 
-0.312
 
 
22.8
 
 
 
 
ABHD5
 
 
hsa-miR-132-3p
 
 
-0.323
 
 
14.4
 
 
 
 
ACTR2
 
 
hsa-miR-15b-5p
 
 
-0.293
 
 
68.0
 
 
 
 
ACVR1
 
 
hsa-miR-30b-5p
 
 
-0.29
 
 
28.6
 
 
 
 
ACVR1
 
 
hsa-miR-152-3p
 
 
-0.397
 
 
28.6
 
 
 
 
ACVR1
 
 
hsa-miR-148b-3p
 
 
-0.397
 
 
28.6
 
 
 
 
ADAM10
 
 
hsa-miR-152-3p
 
 
-0.348
 
 
26.7
 
 
 
 
ADAM10
 
 
hsa-miR-148b-3p
 
 
-0.356
 
 
26.7
 
 
 
 
ADAM9
 
 
hsa-miR-30d-5p
 
 
-0.304
 
 
37.4
 
 
 
 
ADAM9
 
 
hsa-miR-30b-5p
 
 
-0.312
 
 
37.4
 
 
 
 
ADCY3
 
 
hsa-miR-132-3p
 
 
-0.303
 
 
35.2
 
 
 
 
ADCY6
 
 
hsa-miR-1271-5p
 
 
-0.336
 
 
17.0
 
 
 
 
ADGRA3
 
 
hsa-miR-30d-5p
 
 
-0.296
 
 
23.6
 
 
 
 
ADGRA3
 
 
hsa-miR-30b-5p
 
 
-0.304
 
 
23.6
 
 
 
 
ADGRF5
 
 
hsa-miR-148b-3p
 
 
-0.354
 
 
30.7
 
 
 
 
ADGRF5
 
 
hsa-miR-152-3p
 
 
-0.354
 
 
30.7
 
 
 
 
ADIPOR2
 
 
hsa-miR-150-5p
 
 
-0.287
 
 
26.8
 
 
 
 
ADK
 
 
hsa-miR-1271-5p
 
 
-0.329
 
 
35.7
 
 
 
 
ADNP
 
 
hsa-miR-328-3p
 
 
-0.371
 
 
27.8
 
 
 
 
AFG3L2
 
 
hsa-miR-181b-5p
 
 
-0.491
 
 
24.7
 
 
 
 
AFG3L2
 
 
hsa-miR-181a-5p
 
 
-0.491
 
 
24.7
 
 
 
 
AFG3L2
 
 
hsa-miR-181d-5p
 
 
-0.499
 
 
24.7
 
 
 
 
AGFG1
 
 
hsa-miR-148b-3p
 
 
-0.332
 
 
32.1
 
 
 
 
AGFG1
 
 
hsa-miR-152-3p
 
 
-0.332
 
 
32.1
 
 
 
 
AGFG1
 
 
hsa-miR-103a-3p
 
 
-0.389
 
 
32.1
 
 
 
 
AHCTF1
 
 
hsa-let-7b-5p
 
 
-0.374
 
 
12.9
 
 
 
 
AHDC1
 
 
hsa-miR-152-3p
 
 
-0.413
 
 
19.9
 
 
 
 
AHDC1
 
 
hsa-miR-148b-3p
 
 
-0.413
 
 
19.9
 
 
 
 
AHR
 
 
hsa-miR-1271-5p
 
 
-0.304
 
 
11.8
 
 
 
 
AHR
 
 
hsa-miR-29a-3p
 
 
-0.316
 
 
11.8
 
 
 
 
AIFM1
 
 
hsa-let-7b-5p
 
 
-0.314
 
 
10.7
 
 
 
 
AJUBA
 
 
hsa-miR-103a-3p
 
 
-0.448
 
 
10.2
 
 
 
 
AK1
 
 
hsa-miR-29a-3p
 
 
-0.333
 
 
42.1
 
 
 
 
AK3
 
 
hsa-miR-1271-5p
 
 
-0.57
 
 
46.8
 
 
 
 
AKAP1
 
 
hsa-miR-152-3p
 
 
-0.381
 
 
35.0
 
 
 
 
AKAP1
 
 
hsa-miR-148b-3p
 
 
-0.381
 
 
35.0
 
 
 
 
AKIRIN2
 
 
hsa-miR-103a-3p
 
 
-0.299
 
 
15.5
 
 
 
 
AKT3
 
 
hsa-miR-29a-3p
 
 
-0.287
 
 
13.0
 
 
 
 
AKT3
 
 
hsa-miR-15b-5p
 
 
-0.301
 
 
13.0
 
 
 
 
ALAD
 
 
hsa-miR-326
 
 
-0.352
 
 
24.2
 
 
 
 
ALAD
 
 
hsa-miR-103a-3p
 
 
-0.385
 
 
24.2
 
 
 
 
ALAD
 
 
hsa-miR-15b-5p
 
 
-0.446
 
 
24.2
 
 
 
 
ALCAM
 
 
hsa-miR-148b-3p
 
 
-0.339
 
 
58.4
 
 
 
 
ALCAM
 
 
hsa-miR-152-3p
 
 
-0.347
 
 
58.4
 
 
 
 
ALDH6A1
 
 
hsa-miR-1271-5p
 
 
-0.301
 
 
26.5
 
 
 
 
ANKHD1
 
 
hsa-miR-23a-3p
 
 
-0.368
 
 
44.4
 
 
 
 
ANKHD1
 
 
hsa-miR-30d-5p
 
 
-0.381
 
 
44.4
 
 
 
 
ANKHD1
 
 
hsa-miR-30b-5p
 
 
-0.405
 
 
44.4
 
 
 
 
ANKIB1
 
 
hsa-miR-1271-5p
 
 
-0.344
 
 
16.1
 
 
 
 
ANKRA2
 
 
hsa-miR-30d-5p
 
 
-0.343
 
 
13.4
 
 
 
 
ANKRA2
 
 
hsa-miR-30b-5p
 
 
-0.354
 
 
13.4
 
 
 
 
ANKRA2
 
 
hsa-miR-30d-5p
 
 
-0.448
 
 
13.4
 
 
 
 
ANKRA2
 
 
hsa-miR-30b-5p
 
 
-0.472
 
 
13.4
 
 
 
 
ANKRD17
 
 
hsa-miR-30d-5p
 
 
-0.312
 
 
16.8
 
 
 
 
ANKRD17
 
 
hsa-miR-30b-5p
 
 
-0.336
 
 
16.8
 
 
 
 
ANKRD27
 
 
hsa-miR-1271-5p
 
 
-0.648
 
 
12.4
 
 
 
 
ANKRD46
 
 
hsa-let-7b-5p
 
 
-0.389
 
 
18.8
 
 
 
 
ANP32A
 
 
hsa-miR-132-3p
 
 
-0.374
 
 
68.9
 
 
 
 
ANXA11
 
 
hsa-miR-15b-5p
 
 
-0.463
 
 
58.5
 
 
 
 
AP1S1
 
 
hsa-let-7b-5p
 
 
-0.374
 
 
15.7
 
 
 
 
AP1S3
 
 
hsa-miR-181d-5p
 
 
-0.383
 
 
12.8
 
 
 
 
AP1S3
 
 
hsa-miR-181b-5p
 
 
-0.383
 
 
12.8
 
 
 
 
AP1S3
 
 
hsa-miR-181a-5p
 
 
-0.383
 
 
12.8
 
 
 
 
AP2A1
 
 
hsa-miR-15b-5p
 
 
-0.333
 
 
29.3
 
 
 
 
AP2B1
 
 
hsa-miR-1271-5p
 
 
-0.299
 
 
61.0
 
 
 
 
AP2B1
 
 
hsa-miR-15b-5p
 
 
-0.334
 
 
61.0
 
 
 
 
AP3S1
 
 
hsa-miR-30b-5p
 
 
-0.293
 
 
46.1
 
 
 
 
AP4B1
 
 
hsa-miR-378a-3p
 
 
-0.62
 
 
10.7
 
 
 
 
AP4B1
 
 
hsa-miR-378d
 
 
-0.62
 
 
10.7
 
 
 
 
AP5M1
 
 
hsa-miR-1271-5p
 
 
-0.343
 
 
17.2
 
 
 
 
APOO
 
 
hsa-miR-181d-5p
 
 
-0.492
 
 
19.0
 
 
 
 
APOO
 
 
hsa-miR-181b-5p
 
 
-0.5
 
 
19.0
 
 
 
 
APOO
 
 
hsa-miR-181a-5p
 
 
-0.5
 
 
19.0
 
 
 
 
APP
 
 
hsa-miR-15b-5p
 
 
-0.298
 
 
434.2
 
 
 
 
ARF4
 
 
hsa-miR-30b-5p
 
 
-0.289
 
 
134.8
 
 
 
 
ARF4
 
 
hsa-miR-194-5p
 
 
-0.293
 
 
134.8
 
 
 
 
ARF4
 
 
hsa-miR-152-3p
 
 
-0.487
 
 
134.8
 
 
 
 
ARF4
 
 
hsa-miR-148b-3p
 
 
-0.487
 
 
134.8
 
 
 
 
ARF5
 
 
hsa-miR-29a-3p
 
 
-0.491
 
 
88.3
 
 
 
 
ARF6
 
 
hsa-miR-181b-5p
 
 
-0.33
 
 
20.1
 
 
 
 
ARF6
 
 
hsa-miR-181a-5p
 
 
-0.33
 
 
20.1
 
 
 
 
ARF6
 
 
hsa-miR-181d-5p
 
 
-0.338
 
 
20.1
 
 
 
 
ARFGEF1
 
 
hsa-miR-148b-3p
 
 
-0.306
 
 
20.9
 
 
 
 
ARFGEF1
 
 
hsa-miR-152-3p
 
 
-0.306
 
 
20.9
 
 
 
 
ARFIP1
 
 
hsa-miR-320b
 
 
-0.339
 
 
18.8
 
 
 
 
ARFIP1
 
 
hsa-miR-320c
 
 
-0.339
 
 
18.8
 
 
 
 
ARFIP1
 
 
hsa-miR-152-3p
 
 
-0.474
 
 
18.8
 
 
 
 
ARFIP1
 
 
hsa-miR-148b-3p
 
 
-0.474
 
 
18.8
 
 
 
 
ARG2
 
 
hsa-let-7b-5p
 
 
-0.468
 
 
97.9
 
 
 
 
ARHGAP21
 
 
hsa-miR-152-3p
 
 
-0.287
 
 
12.0
 
 
 
 
ARHGAP21
 
 
hsa-miR-148b-3p
 
 
-0.287
 
 
12.0
 
 
 
 
ARHGAP24
 
 
hsa-miR-1271-5p
 
 
-0.342
 
 
26.1
 
 
 
 
ARHGAP6
 
 
hsa-miR-1271-5p
 
 
-0.521
 
 
23.8
 
 
 
 
 
 
 tongue 
 
 
 
 
target_symbol
 
 
mature_mirna_id
 
 
score
 
 
nTPM
 
 
 
 
 
 
AAMP
 
 
hsa-miR-326
 
 
-0.722
 
 
58.1
 
 
 
 
AAR2
 
 
hsa-miR-29a-3p
 
 
-0.327
 
 
19.3
 
 
 
 
ABCB10
 
 
hsa-miR-152-3p
 
 
-0.291
 
 
10.2
 
 
 
 
ABCB10
 
 
hsa-miR-148b-3p
 
 
-0.291
 
 
10.2
 
 
 
 
ABCB7
 
 
hsa-miR-148b-3p
 
 
-0.515
 
 
20.8
 
 
 
 
ABCB7
 
 
hsa-miR-152-3p
 
 
-0.515
 
 
20.8
 
 
 
 
ABCD3
 
 
hsa-miR-148b-3p
 
 
-0.388
 
 
27.9
 
 
 
 
ABCD3
 
 
hsa-miR-152-3p
 
 
-0.388
 
 
27.9
 
 
 
 
ABCE1
 
 
hsa-miR-29a-3p
 
 
-0.404
 
 
28.5
 
 
 
 
ABHD18
 
 
hsa-miR-29a-3p
 
 
-0.321
 
 
10.9
 
 
 
 
ABHD18
 
 
hsa-miR-29a-3p
 
 
-0.34
 
 
10.9
 
 
 
 
ABHD4
 
 
hsa-miR-29a-3p
 
 
-0.312
 
 
27.0
 
 
 
 
ABHD5
 
 
hsa-miR-132-3p
 
 
-0.323
 
 
17.3
 
 
 
 
ACHE
 
 
hsa-miR-194-5p
 
 
-0.395
 
 
44.9
 
 
 
 
ACTC1
 
 
hsa-miR-30d-5p
 
 
-0.519
 
 
182.1
 
 
 
 
ACTC1
 
 
hsa-miR-30b-5p
 
 
-0.543
 
 
182.1
 
 
 
 
ACTN2
 
 
hsa-miR-23a-3p
 
 
-0.294
 
 
1195.2
 
 
 
 
ACTN2
 
 
hsa-miR-29a-3p
 
 
-0.311
 
 
1195.2
 
 
 
 
ACTR2
 
 
hsa-miR-15b-5p
 
 
-0.293
 
 
60.6
 
 
 
 
ADAM9
 
 
hsa-miR-30d-5p
 
 
-0.304
 
 
23.3
 
 
 
 
ADAM9
 
 
hsa-miR-30b-5p
 
 
-0.312
 
 
23.3
 
 
 
 
ADCY2
 
 
hsa-miR-152-3p
 
 
-0.299
 
 
28.8
 
 
 
 
ADCY2
 
 
hsa-miR-148b-3p
 
 
-0.299
 
 
28.8
 
 
 
 
ADCY3
 
 
hsa-miR-132-3p
 
 
-0.303
 
 
19.0
 
 
 
 
ADCY6
 
 
hsa-miR-1271-5p
 
 
-0.336
 
 
11.8
 
 
 
 
ADGRA3
 
 
hsa-miR-30d-5p
 
 
-0.296
 
 
10.7
 
 
 
 
ADGRA3
 
 
hsa-miR-30b-5p
 
 
-0.304
 
 
10.7
 
 
 
 
ADGRF5
 
 
hsa-miR-148b-3p
 
 
-0.354
 
 
66.5
 
 
 
 
ADGRF5
 
 
hsa-miR-152-3p
 
 
-0.354
 
 
66.5
 
 
 
 
ADIPOR2
 
 
hsa-miR-150-5p
 
 
-0.287
 
 
18.5
 
 
 
 
ADK
 
 
hsa-miR-1271-5p
 
 
-0.329
 
 
44.0
 
 
 
 
ADNP
 
 
hsa-miR-328-3p
 
 
-0.371
 
 
26.5
 
 
 
 
AFG3L2
 
 
hsa-miR-181b-5p
 
 
-0.491
 
 
92.6
 
 
 
 
AFG3L2
 
 
hsa-miR-181a-5p
 
 
-0.491
 
 
92.6
 
 
 
 
AFG3L2
 
 
hsa-miR-181d-5p
 
 
-0.499
 
 
92.6
 
 
 
 
AGFG1
 
 
hsa-miR-148b-3p
 
 
-0.332
 
 
36.1
 
 
 
 
AGFG1
 
 
hsa-miR-152-3p
 
 
-0.332
 
 
36.1
 
 
 
 
AGFG1
 
 
hsa-miR-103a-3p
 
 
-0.389
 
 
36.1
 
 
 
 
AGO4
 
 
hsa-miR-1271-5p
 
 
-0.289
 
 
11.1
 
 
 
 
AGO4
 
 
hsa-miR-103a-3p
 
 
-0.32
 
 
11.1
 
 
 
 
AHCTF1
 
 
hsa-let-7b-5p
 
 
-0.374
 
 
14.7
 
 
 
 
AHDC1
 
 
hsa-miR-152-3p
 
 
-0.413
 
 
14.5
 
 
 
 
AHDC1
 
 
hsa-miR-148b-3p
 
 
-0.413
 
 
14.5
 
 
 
 
AIFM1
 
 
hsa-let-7b-5p
 
 
-0.314
 
 
23.4
 
 
 
 
AK1
 
 
hsa-miR-29a-3p
 
 
-0.333
 
 
730.8
 
 
 
 
AK3
 
 
hsa-miR-1271-5p
 
 
-0.57
 
 
149.2
 
 
 
 
AK4
 
 
hsa-miR-15b-5p
 
 
-0.316
 
 
35.9
 
 
 
 
AKAP1
 
 
hsa-miR-152-3p
 
 
-0.381
 
 
128.4
 
 
 
 
AKAP1
 
 
hsa-miR-148b-3p
 
 
-0.381
 
 
128.4
 
 
 
 
AKAP6
 
 
hsa-let-7b-5p
 
 
-0.307
 
 
47.4
 
 
 
 
AKIRIN2
 
 
hsa-miR-103a-3p
 
 
-0.299
 
 
39.8
 
 
 
 
AKT3
 
 
hsa-miR-29a-3p
 
 
-0.287
 
 
10.7
 
 
 
 
AKT3
 
 
hsa-miR-15b-5p
 
 
-0.301
 
 
10.7
 
 
 
 
ALAD
 
 
hsa-miR-326
 
 
-0.352
 
 
22.3
 
 
 
 
ALAD
 
 
hsa-miR-103a-3p
 
 
-0.385
 
 
22.3
 
 
 
 
ALAD
 
 
hsa-miR-15b-5p
 
 
-0.446
 
 
22.3
 
 
 
 
ALDH6A1
 
 
hsa-miR-1271-5p
 
 
-0.301
 
 
32.4
 
 
 
 
AMER1
 
 
hsa-miR-29a-3p
 
 
-0.343
 
 
14.3
 
 
 
 
AMOT
 
 
hsa-miR-29a-3p
 
 
-0.304
 
 
51.2
 
 
 
 
AMOTL1
 
 
hsa-miR-15b-5p
 
 
-0.319
 
 
66.9
 
 
 
 
ANK1
 
 
hsa-miR-103a-3p
 
 
-0.321
 
 
592.2
 
 
 
 
ANKHD1
 
 
hsa-miR-23a-3p
 
 
-0.368
 
 
26.1
 
 
 
 
ANKHD1
 
 
hsa-miR-30d-5p
 
 
-0.381
 
 
26.1
 
 
 
 
ANKHD1
 
 
hsa-miR-30b-5p
 
 
-0.405
 
 
26.1
 
 
 
 
ANKRD17
 
 
hsa-miR-30d-5p
 
 
-0.312
 
 
24.4
 
 
 
 
ANKRD17
 
 
hsa-miR-30b-5p
 
 
-0.336
 
 
24.4
 
 
 
 
ANKRD27
 
 
hsa-miR-1271-5p
 
 
-0.648
 
 
10.0
 
 
 
 
ANKRD46
 
 
hsa-let-7b-5p
 
 
-0.389
 
 
33.8
 
 
 
 
ANP32A
 
 
hsa-miR-132-3p
 
 
-0.374
 
 
60.2
 
 
 
 
ANTXR2
 
 
hsa-miR-29a-3p
 
 
-0.347
 
 
20.3
 
 
 
 
ANXA11
 
 
hsa-miR-15b-5p
 
 
-0.463
 
 
155.0
 
 
 
 
AP2A1
 
 
hsa-miR-15b-5p
 
 
-0.333
 
 
24.6
 
 
 
 
AP2B1
 
 
hsa-miR-1271-5p
 
 
-0.299
 
 
57.0
 
 
 
 
AP2B1
 
 
hsa-miR-15b-5p
 
 
-0.334
 
 
57.0
 
 
 
 
AP3S1
 
 
hsa-miR-30b-5p
 
 
-0.293
 
 
32.1
 
 
 
 
AP5M1
 
 
hsa-miR-1271-5p
 
 
-0.343
 
 
26.4
 
 
 
 
APC
 
 
hsa-miR-29a-3p
 
 
-0.413
 
 
11.0
 
 
 
 
APOO
 
 
hsa-miR-181d-5p
 
 
-0.492
 
 
68.2
 
 
 
 
APOO
 
 
hsa-miR-181b-5p
 
 
-0.5
 
 
68.2
 
 
 
 
APOO
 
 
hsa-miR-181a-5p
 
 
-0.5
 
 
68.2
 
 
 
 
APP
 
 
hsa-miR-15b-5p
 
 
-0.298
 
 
161.6
 
 
 
 
AQP3
 
 
hsa-miR-874-3p
 
 
-0.48
 
 
25.0
 
 
 
 
ARF4
 
 
hsa-miR-30b-5p
 
 
-0.289
 
 
64.9
 
 
 
 
ARF4
 
 
hsa-miR-194-5p
 
 
-0.293
 
 
64.9
 
 
 
 
ARF4
 
 
hsa-miR-152-3p
 
 
-0.487
 
 
64.9
 
 
 
 
ARF4
 
 
hsa-miR-148b-3p
 
 
-0.487
 
 
64.9
 
 
 
 
ARF5
 
 
hsa-miR-29a-3p
 
 
-0.491
 
 
94.7
 
 
 
 
ARF6
 
 
hsa-miR-181b-5p
 
 
-0.33
 
 
15.8
 
 
 
 
ARF6
 
 
hsa-miR-181a-5p
 
 
-0.33
 
 
15.8
 
 
 
 
ARF6
 
 
hsa-miR-181d-5p
 
 
-0.338
 
 
15.8
 
 
 
 
ARFGEF1
 
 
hsa-miR-148b-3p
 
 
-0.306
 
 
13.0
 
 
 
 
ARFGEF1
 
 
hsa-miR-152-3p
 
 
-0.306
 
 
13.0
 
 
 
 
ARG2
 
 
hsa-let-7b-5p
 
 
-0.468
 
 
49.2
 
 
 
 
ARHGAP21
 
 
hsa-miR-152-3p
 
 
-0.287
 
 
21.5
 
 
 
 
ARHGAP21
 
 
hsa-miR-148b-3p
 
 
-0.287
 
 
21.5
 
 
 
 
ARID1B
 
 
hsa-miR-29a-3p
 
 
-0.342
 
 
22.6
 
 
 
 
ARIH2
 
 
hsa-miR-103a-3p
 
 
-0.307
 
 
74.3
 
 
 
 
ARL2
 
 
hsa-miR-15b-5p
 
 
-0.374
 
 
147.4
 
 
 
 
ARL2
 
 
hsa-miR-15b-5p
 
 
-0.442
 
 
147.4
 
 
 
 
ARL6IP1
 
 
hsa-miR-328-3p
 
 
-0.494
 
 
48.1
 
 
 
 
 
 
 tonsil 
 
 
 
 
target_symbol
 
 
mature_mirna_id
 
 
score
 
 
nTPM
 
 
 
 
 
 
AAMP
 
 
hsa-miR-326
 
 
-0.722
 
 
49.3
 
 
 
 
AAR2
 
 
hsa-miR-29a-3p
 
 
-0.327
 
 
18.7
 
 
 
 
ABCB7
 
 
hsa-miR-148b-3p
 
 
-0.515
 
 
10.4
 
 
 
 
ABCB7
 
 
hsa-miR-152-3p
 
 
-0.515
 
 
10.4
 
 
 
 
ABCD3
 
 
hsa-miR-148b-3p
 
 
-0.388
 
 
19.9
 
 
 
 
ABCD3
 
 
hsa-miR-152-3p
 
 
-0.388
 
 
19.9
 
 
 
 
ABCE1
 
 
hsa-miR-29a-3p
 
 
-0.404
 
 
39.4
 
 
 
 
ABHD17C
 
 
hsa-let-7b-5p
 
 
-0.359
 
 
21.6
 
 
 
 
ABHD17C
 
 
hsa-miR-330-3p
 
 
-0.399
 
 
21.6
 
 
 
 
ABHD4
 
 
hsa-miR-29a-3p
 
 
-0.312
 
 
15.2
 
 
 
 
ACTR2
 
 
hsa-miR-15b-5p
 
 
-0.293
 
 
239.6
 
 
 
 
ADA
 
 
hsa-miR-29a-3p
 
 
-0.376
 
 
23.2
 
 
 
 
ADAM10
 
 
hsa-miR-152-3p
 
 
-0.348
 
 
15.3
 
 
 
 
ADAM10
 
 
hsa-miR-148b-3p
 
 
-0.356
 
 
15.3
 
 
 
 
ADAM17
 
 
hsa-miR-152-3p
 
 
-0.301
 
 
11.1
 
 
 
 
ADAM17
 
 
hsa-miR-148b-3p
 
 
-0.301
 
 
11.1
 
 
 
 
ADAM19
 
 
hsa-miR-874-3p
 
 
-0.374
 
 
28.3
 
 
 
 
ADAM9
 
 
hsa-miR-30d-5p
 
 
-0.304
 
 
12.4
 
 
 
 
ADAM9
 
 
hsa-miR-30b-5p
 
 
-0.312
 
 
12.4
 
 
 
 
ADCY3
 
 
hsa-miR-132-3p
 
 
-0.303
 
 
16.9
 
 
 
 
ADIPOR2
 
 
hsa-miR-150-5p
 
 
-0.287
 
 
11.8
 
 
 
 
ADK
 
 
hsa-miR-1271-5p
 
 
-0.329
 
 
32.3
 
 
 
 
ADNP
 
 
hsa-miR-328-3p
 
 
-0.371
 
 
33.4
 
 
 
 
ADRA2A
 
 
hsa-miR-23a-3p
 
 
-0.371
 
 
12.3
 
 
 
 
AFG3L2
 
 
hsa-miR-181b-5p
 
 
-0.491
 
 
16.2
 
 
 
 
AFG3L2
 
 
hsa-miR-181a-5p
 
 
-0.491
 
 
16.2
 
 
 
 
AFG3L2
 
 
hsa-miR-181d-5p
 
 
-0.499
 
 
16.2
 
 
 
 
AGFG1
 
 
hsa-miR-148b-3p
 
 
-0.332
 
 
49.9
 
 
 
 
AGFG1
 
 
hsa-miR-152-3p
 
 
-0.332
 
 
49.9
 
 
 
 
AGFG1
 
 
hsa-miR-103a-3p
 
 
-0.389
 
 
49.9
 
 
 
 
AHR
 
 
hsa-miR-1271-5p
 
 
-0.304
 
 
18.6
 
 
 
 
AHR
 
 
hsa-miR-29a-3p
 
 
-0.316
 
 
18.6
 
 
 
 
AIFM1
 
 
hsa-let-7b-5p
 
 
-0.314
 
 
10.3
 
 
 
 
AK1
 
 
hsa-miR-29a-3p
 
 
-0.333
 
 
11.8
 
 
 
 
AK3
 
 
hsa-miR-1271-5p
 
 
-0.57
 
 
32.2
 
 
 
 
AKIRIN2
 
 
hsa-miR-103a-3p
 
 
-0.299
 
 
28.4
 
 
 
 
ALAD
 
 
hsa-miR-326
 
 
-0.352
 
 
17.5
 
 
 
 
ALAD
 
 
hsa-miR-103a-3p
 
 
-0.385
 
 
17.5
 
 
 
 
ALAD
 
 
hsa-miR-15b-5p
 
 
-0.446
 
 
17.5
 
 
 
 
ALCAM
 
 
hsa-miR-148b-3p
 
 
-0.339
 
 
13.6
 
 
 
 
ALCAM
 
 
hsa-miR-152-3p
 
 
-0.347
 
 
13.6
 
 
 
 
ANKHD1
 
 
hsa-miR-23a-3p
 
 
-0.368
 
 
38.8
 
 
 
 
ANKHD1
 
 
hsa-miR-30d-5p
 
 
-0.381
 
 
38.8
 
 
 
 
ANKHD1
 
 
hsa-miR-30b-5p
 
 
-0.405
 
 
38.8
 
 
 
 
ANKIB1
 
 
hsa-miR-1271-5p
 
 
-0.344
 
 
10.9
 
 
 
 
ANKRD17
 
 
hsa-miR-30d-5p
 
 
-0.312
 
 
16.5
 
 
 
 
ANKRD17
 
 
hsa-miR-30b-5p
 
 
-0.336
 
 
16.5
 
 
 
 
ANKRD27
 
 
hsa-miR-1271-5p
 
 
-0.648
 
 
10.7
 
 
 
 
ANKRD29
 
 
hsa-miR-132-3p
 
 
-0.47
 
 
13.4
 
 
 
 
ANKRD46
 
 
hsa-let-7b-5p
 
 
-0.389
 
 
10.3
 
 
 
 
ANP32A
 
 
hsa-miR-132-3p
 
 
-0.374
 
 
132.1
 
 
 
 
ANTXR2
 
 
hsa-miR-29a-3p
 
 
-0.347
 
 
15.1
 
 
 
 
ANXA11
 
 
hsa-miR-15b-5p
 
 
-0.463
 
 
95.3
 
 
 
 
AP1S1
 
 
hsa-let-7b-5p
 
 
-0.374
 
 
12.8
 
 
 
 
AP2A1
 
 
hsa-miR-15b-5p
 
 
-0.333
 
 
12.3
 
 
 
 
AP2B1
 
 
hsa-miR-1271-5p
 
 
-0.299
 
 
42.7
 
 
 
 
AP2B1
 
 
hsa-miR-15b-5p
 
 
-0.334
 
 
42.7
 
 
 
 
AP3S1
 
 
hsa-miR-30b-5p
 
 
-0.293
 
 
57.3
 
 
 
 
AP4B1
 
 
hsa-miR-378a-3p
 
 
-0.62
 
 
13.0
 
 
 
 
AP4B1
 
 
hsa-miR-378d
 
 
-0.62
 
 
13.0
 
 
 
 
AP5M1
 
 
hsa-miR-1271-5p
 
 
-0.343
 
 
21.3
 
 
 
 
APOO
 
 
hsa-miR-181d-5p
 
 
-0.492
 
 
10.8
 
 
 
 
APOO
 
 
hsa-miR-181b-5p
 
 
-0.5
 
 
10.8
 
 
 
 
APOO
 
 
hsa-miR-181a-5p
 
 
-0.5
 
 
10.8
 
 
 
 
APP
 
 
hsa-miR-15b-5p
 
 
-0.298
 
 
69.8
 
 
 
 
AQP3
 
 
hsa-miR-874-3p
 
 
-0.48
 
 
161.9
 
 
 
 
ARF4
 
 
hsa-miR-30b-5p
 
 
-0.289
 
 
91.2
 
 
 
 
ARF4
 
 
hsa-miR-194-5p
 
 
-0.293
 
 
91.2
 
 
 
 
ARF4
 
 
hsa-miR-152-3p
 
 
-0.487
 
 
91.2
 
 
 
 
ARF4
 
 
hsa-miR-148b-3p
 
 
-0.487
 
 
91.2
 
 
 
 
ARF5
 
 
hsa-miR-29a-3p
 
 
-0.491
 
 
69.9
 
 
 
 
ARF6
 
 
hsa-miR-181b-5p
 
 
-0.33
 
 
64.2
 
 
 
 
ARF6
 
 
hsa-miR-181a-5p
 
 
-0.33
 
 
64.2
 
 
 
 
ARF6
 
 
hsa-miR-181d-5p
 
 
-0.338
 
 
64.2
 
 
 
 
ARFGEF1
 
 
hsa-miR-148b-3p
 
 
-0.306
 
 
16.2
 
 
 
 
ARFGEF1
 
 
hsa-miR-152-3p
 
 
-0.306
 
 
16.2
 
 
 
 
ARFIP1
 
 
hsa-miR-320b
 
 
-0.339
 
 
13.9
 
 
 
 
ARFIP1
 
 
hsa-miR-320c
 
 
-0.339
 
 
13.9
 
 
 
 
ARFIP1
 
 
hsa-miR-152-3p
 
 
-0.474
 
 
13.9
 
 
 
 
ARFIP1
 
 
hsa-miR-148b-3p
 
 
-0.474
 
 
13.9
 
 
 
 
ARHGAP24
 
 
hsa-miR-1271-5p
 
 
-0.342
 
 
14.5
 
 
 
 
ARID1B
 
 
hsa-miR-29a-3p
 
 
-0.342
 
 
17.5
 
 
 
 
ARIH2
 
 
hsa-miR-103a-3p
 
 
-0.307
 
 
20.1
 
 
 
 
ARL2
 
 
hsa-miR-15b-5p
 
 
-0.374
 
 
27.2
 
 
 
 
ARL2
 
 
hsa-miR-15b-5p
 
 
-0.442
 
 
27.2
 
 
 
 
ARL4A
 
 
hsa-miR-29a-3p
 
 
-0.386
 
 
14.9
 
 
 
 
ARL4A
 
 
hsa-miR-30d-5p
 
 
-0.432
 
 
14.9
 
 
 
 
ARL4A
 
 
hsa-miR-30b-5p
 
 
-0.448
 
 
14.9
 
 
 
 
ARL6IP1
 
 
hsa-miR-328-3p
 
 
-0.494
 
 
106.2
 
 
 
 
ARL6IP1
 
 
hsa-miR-148b-3p
 
 
-0.579
 
 
106.2
 
 
 
 
ARL6IP1
 
 
hsa-miR-152-3p
 
 
-0.579
 
 
106.2
 
 
 
 
ARL6IP6
 
 
hsa-let-7b-5p
 
 
-0.293
 
 
18.0
 
 
 
 
ARL8A
 
 
hsa-miR-103a-3p
 
 
-0.38
 
 
12.4
 
 
 
 
ARL8B
 
 
hsa-miR-152-3p
 
 
-0.337
 
 
44.5
 
 
 
 
ARL8B
 
 
hsa-miR-148b-3p
 
 
-0.337
 
 
44.5
 
 
 
 
ARL8B
 
 
hsa-miR-320b
 
 
-0.376
 
 
44.5
 
 
 
 
ARL8B
 
 
hsa-miR-320c
 
 
-0.376
 
 
44.5
 
 
 
 
ARMC1
 
 
hsa-miR-103a-3p
 
 
-0.525
 
 
19.0
 
 
 
 
ARMC8
 
 
hsa-miR-103a-3p
 
 
-0.412
 
 
21.4
 
 
 
 
ARMCX3
 
 
hsa-miR-425-5p
 
 
-0.325
 
 
20.2
 
 
 
 
 
 
 urinary bladder 
 
 
 
 
target_symbol
 
 
mature_mirna_id
 
 
score
 
 
nTPM
 
 
 
 
 
 
AAMP
 
 
hsa-miR-326
 
 
-0.722
 
 
73.9
 
 
 
 
AAR2
 
 
hsa-miR-29a-3p
 
 
-0.327
 
 
25.2
 
 
 
 
ABCB6
 
 
hsa-miR-29a-3p
 
 
-0.48
 
 
13.5
 
 
 
 
ABCB7
 
 
hsa-miR-148b-3p
 
 
-0.515
 
 
11.0
 
 
 
 
ABCB7
 
 
hsa-miR-152-3p
 
 
-0.515
 
 
11.0
 
 
 
 
ABCD3
 
 
hsa-miR-148b-3p
 
 
-0.388
 
 
21.4
 
 
 
 
ABCD3
 
 
hsa-miR-152-3p
 
 
-0.388
 
 
21.4
 
 
 
 
ABCE1
 
 
hsa-miR-29a-3p
 
 
-0.404
 
 
37.8
 
 
 
 
ABHD17C
 
 
hsa-let-7b-5p
 
 
-0.359
 
 
26.8
 
 
 
 
ABHD17C
 
 
hsa-miR-330-3p
 
 
-0.399
 
 
26.8
 
 
 
 
ABHD4
 
 
hsa-miR-29a-3p
 
 
-0.312
 
 
28.1
 
 
 
 
ABHD5
 
 
hsa-miR-132-3p
 
 
-0.323
 
 
15.5
 
 
 
 
ACER2
 
 
hsa-let-7b-5p
 
 
-0.333
 
 
36.7
 
 
 
 
ACTC1
 
 
hsa-miR-30d-5p
 
 
-0.519
 
 
667.0
 
 
 
 
ACTC1
 
 
hsa-miR-30b-5p
 
 
-0.543
 
 
667.0
 
 
 
 
ACTR2
 
 
hsa-miR-15b-5p
 
 
-0.293
 
 
119.8
 
 
 
 
ACVR1
 
 
hsa-miR-30b-5p
 
 
-0.29
 
 
13.3
 
 
 
 
ACVR1
 
 
hsa-miR-152-3p
 
 
-0.397
 
 
13.3
 
 
 
 
ACVR1
 
 
hsa-miR-148b-3p
 
 
-0.397
 
 
13.3
 
 
 
 
ADAM10
 
 
hsa-miR-152-3p
 
 
-0.348
 
 
26.2
 
 
 
 
ADAM10
 
 
hsa-miR-148b-3p
 
 
-0.356
 
 
26.2
 
 
 
 
ADAM19
 
 
hsa-miR-874-3p
 
 
-0.374
 
 
15.9
 
 
 
 
ADAM9
 
 
hsa-miR-30d-5p
 
 
-0.304
 
 
52.5
 
 
 
 
ADAM9
 
 
hsa-miR-30b-5p
 
 
-0.312
 
 
52.5
 
 
 
 
ADAMTS9
 
 
hsa-miR-29a-3p
 
 
-0.305
 
 
20.9
 
 
 
 
ADAMTS9
 
 
hsa-miR-29a-3p
 
 
-0.485
 
 
20.9
 
 
 
 
ADCY3
 
 
hsa-miR-132-3p
 
 
-0.303
 
 
34.2
 
 
 
 
ADCY6
 
 
hsa-miR-1271-5p
 
 
-0.336
 
 
12.5
 
 
 
 
ADGRA2
 
 
hsa-miR-1271-5p
 
 
-0.427
 
 
35.1
 
 
 
 
ADGRF5
 
 
hsa-miR-148b-3p
 
 
-0.354
 
 
10.6
 
 
 
 
ADGRF5
 
 
hsa-miR-152-3p
 
 
-0.354
 
 
10.6
 
 
 
 
ADIPOR2
 
 
hsa-miR-150-5p
 
 
-0.287
 
 
21.3
 
 
 
 
ADK
 
 
hsa-miR-1271-5p
 
 
-0.329
 
 
22.9
 
 
 
 
ADNP
 
 
hsa-miR-328-3p
 
 
-0.371
 
 
34.8
 
 
 
 
ADRA2A
 
 
hsa-miR-23a-3p
 
 
-0.371
 
 
14.8
 
 
 
 
AFG3L2
 
 
hsa-miR-181b-5p
 
 
-0.491
 
 
18.0
 
 
 
 
AFG3L2
 
 
hsa-miR-181a-5p
 
 
-0.491
 
 
18.0
 
 
 
 
AFG3L2
 
 
hsa-miR-181d-5p
 
 
-0.499
 
 
18.0
 
 
 
 
AGFG1
 
 
hsa-miR-148b-3p
 
 
-0.332
 
 
61.2
 
 
 
 
AGFG1
 
 
hsa-miR-152-3p
 
 
-0.332
 
 
61.2
 
 
 
 
AGFG1
 
 
hsa-miR-103a-3p
 
 
-0.389
 
 
61.2
 
 
 
 
AHDC1
 
 
hsa-miR-152-3p
 
 
-0.413
 
 
14.8
 
 
 
 
AHDC1
 
 
hsa-miR-148b-3p
 
 
-0.413
 
 
14.8
 
 
 
 
AHR
 
 
hsa-miR-1271-5p
 
 
-0.304
 
 
46.3
 
 
 
 
AHR
 
 
hsa-miR-29a-3p
 
 
-0.316
 
 
46.3
 
 
 
 
AIFM1
 
 
hsa-let-7b-5p
 
 
-0.314
 
 
13.1
 
 
 
 
AK1
 
 
hsa-miR-29a-3p
 
 
-0.333
 
 
22.9
 
 
 
 
AK3
 
 
hsa-miR-1271-5p
 
 
-0.57
 
 
45.5
 
 
 
 
AK4
 
 
hsa-miR-15b-5p
 
 
-0.316
 
 
16.4
 
 
 
 
AKAP1
 
 
hsa-miR-152-3p
 
 
-0.381
 
 
47.8
 
 
 
 
AKAP1
 
 
hsa-miR-148b-3p
 
 
-0.381
 
 
47.8
 
 
 
 
AKIRIN2
 
 
hsa-miR-103a-3p
 
 
-0.299
 
 
19.4
 
 
 
 
AKT3
 
 
hsa-miR-29a-3p
 
 
-0.287
 
 
14.4
 
 
 
 
AKT3
 
 
hsa-miR-15b-5p
 
 
-0.301
 
 
14.4
 
 
 
 
ALAD
 
 
hsa-miR-326
 
 
-0.352
 
 
20.6
 
 
 
 
ALAD
 
 
hsa-miR-103a-3p
 
 
-0.385
 
 
20.6
 
 
 
 
ALAD
 
 
hsa-miR-15b-5p
 
 
-0.446
 
 
20.6
 
 
 
 
ALCAM
 
 
hsa-miR-148b-3p
 
 
-0.339
 
 
23.9
 
 
 
 
ALCAM
 
 
hsa-miR-152-3p
 
 
-0.347
 
 
23.9
 
 
 
 
ALDH6A1
 
 
hsa-miR-1271-5p
 
 
-0.301
 
 
11.4
 
 
 
 
AMOTL1
 
 
hsa-miR-15b-5p
 
 
-0.319
 
 
26.4
 
 
 
 
AMT
 
 
hsa-let-7b-5p
 
 
-0.463
 
 
13.1
 
 
 
 
ANKHD1
 
 
hsa-miR-23a-3p
 
 
-0.368
 
 
34.3
 
 
 
 
ANKHD1
 
 
hsa-miR-30d-5p
 
 
-0.381
 
 
34.3
 
 
 
 
ANKHD1
 
 
hsa-miR-30b-5p
 
 
-0.405
 
 
34.3
 
 
 
 
ANKIB1
 
 
hsa-miR-1271-5p
 
 
-0.344
 
 
12.3
 
 
 
 
ANKRD17
 
 
hsa-miR-30d-5p
 
 
-0.312
 
 
16.5
 
 
 
 
ANKRD17
 
 
hsa-miR-30b-5p
 
 
-0.336
 
 
16.5
 
 
 
 
ANKRD27
 
 
hsa-miR-1271-5p
 
 
-0.648
 
 
17.3
 
 
 
 
ANP32A
 
 
hsa-miR-132-3p
 
 
-0.374
 
 
71.6
 
 
 
 
ANTXR2
 
 
hsa-miR-29a-3p
 
 
-0.347
 
 
82.6
 
 
 
 
ANXA11
 
 
hsa-miR-15b-5p
 
 
-0.463
 
 
138.5
 
 
 
 
AP1S1
 
 
hsa-let-7b-5p
 
 
-0.374
 
 
18.6
 
 
 
 
AP2A1
 
 
hsa-miR-15b-5p
 
 
-0.333
 
 
43.6
 
 
 
 
AP2B1
 
 
hsa-miR-1271-5p
 
 
-0.299
 
 
44.1
 
 
 
 
AP2B1
 
 
hsa-miR-15b-5p
 
 
-0.334
 
 
44.1
 
 
 
 
AP3S1
 
 
hsa-miR-30b-5p
 
 
-0.293
 
 
56.3
 
 
 
 
AP5M1
 
 
hsa-miR-1271-5p
 
 
-0.343
 
 
12.4
 
 
 
 
APOO
 
 
hsa-miR-181d-5p
 
 
-0.492
 
 
10.5
 
 
 
 
APOO
 
 
hsa-miR-181b-5p
 
 
-0.5
 
 
10.5
 
 
 
 
APOO
 
 
hsa-miR-181a-5p
 
 
-0.5
 
 
10.5
 
 
 
 
APP
 
 
hsa-miR-15b-5p
 
 
-0.298
 
 
275.5
 
 
 
 
AQP3
 
 
hsa-miR-874-3p
 
 
-0.48
 
 
642.4
 
 
 
 
ARF4
 
 
hsa-miR-30b-5p
 
 
-0.289
 
 
190.1
 
 
 
 
ARF4
 
 
hsa-miR-194-5p
 
 
-0.293
 
 
190.1
 
 
 
 
ARF4
 
 
hsa-miR-152-3p
 
 
-0.487
 
 
190.1
 
 
 
 
ARF4
 
 
hsa-miR-148b-3p
 
 
-0.487
 
 
190.1
 
 
 
 
ARF5
 
 
hsa-miR-29a-3p
 
 
-0.491
 
 
95.3
 
 
 
 
ARF6
 
 
hsa-miR-181b-5p
 
 
-0.33
 
 
43.6
 
 
 
 
ARF6
 
 
hsa-miR-181a-5p
 
 
-0.33
 
 
43.6
 
 
 
 
ARF6
 
 
hsa-miR-181d-5p
 
 
-0.338
 
 
43.6
 
 
 
 
ARFGEF1
 
 
hsa-miR-148b-3p
 
 
-0.306
 
 
18.4
 
 
 
 
ARFGEF1
 
 
hsa-miR-152-3p
 
 
-0.306
 
 
18.4
 
 
 
 
ARFIP1
 
 
hsa-miR-320b
 
 
-0.339
 
 
22.0
 
 
 
 
ARFIP1
 
 
hsa-miR-320c
 
 
-0.339
 
 
22.0
 
 
 
 
ARFIP1
 
 
hsa-miR-152-3p
 
 
-0.474
 
 
22.0
 
 
 
 
ARFIP1
 
 
hsa-miR-148b-3p
 
 
-0.474
 
 
22.0
 
 
 
 
ARG2
 
 
hsa-let-7b-5p
 
 
-0.468
 
 
16.6
 
 
 
 
ARHGAP21
 
 
hsa-miR-152-3p
 
 
-0.287
 
 
12.9
 
 
 
 
ARHGAP21
 
 
hsa-miR-148b-3p
 
 
-0.287
 
 
12.9
 
 
 
 
 
 
 vagina 
 
 
 
 
target_symbol
 
 
mature_mirna_id
 
 
score
 
 
nTPM
 
 
 
 
 
 
AAMP
 
 
hsa-miR-326
 
 
-0.722
 
 
57.2
 
 
 
 
AAR2
 
 
hsa-miR-29a-3p
 
 
-0.327
 
 
15.0
 
 
 
 
ABCB7
 
 
hsa-miR-148b-3p
 
 
-0.515
 
 
10.8
 
 
 
 
ABCB7
 
 
hsa-miR-152-3p
 
 
-0.515
 
 
10.8
 
 
 
 
ABCD3
 
 
hsa-miR-148b-3p
 
 
-0.388
 
 
14.9
 
 
 
 
ABCD3
 
 
hsa-miR-152-3p
 
 
-0.388
 
 
14.9
 
 
 
 
ABCE1
 
 
hsa-miR-29a-3p
 
 
-0.404
 
 
15.6
 
 
 
 
ABHD17C
 
 
hsa-let-7b-5p
 
 
-0.359
 
 
21.1
 
 
 
 
ABHD17C
 
 
hsa-miR-330-3p
 
 
-0.399
 
 
21.1
 
 
 
 
ABHD4
 
 
hsa-miR-29a-3p
 
 
-0.312
 
 
28.3
 
 
 
 
ABHD5
 
 
hsa-miR-132-3p
 
 
-0.323
 
 
11.9
 
 
 
 
ACP3
 
 
hsa-let-7b-5p
 
 
-0.298
 
 
10.8
 
 
 
 
ACTC1
 
 
hsa-miR-30d-5p
 
 
-0.519
 
 
50.8
 
 
 
 
ACTC1
 
 
hsa-miR-30b-5p
 
 
-0.543
 
 
50.8
 
 
 
 
ACTR2
 
 
hsa-miR-15b-5p
 
 
-0.293
 
 
44.4
 
 
 
 
ADAM10
 
 
hsa-miR-152-3p
 
 
-0.348
 
 
10.3
 
 
 
 
ADAM10
 
 
hsa-miR-148b-3p
 
 
-0.356
 
 
10.3
 
 
 
 
ADAM9
 
 
hsa-miR-30d-5p
 
 
-0.304
 
 
15.5
 
 
 
 
ADAM9
 
 
hsa-miR-30b-5p
 
 
-0.312
 
 
15.5
 
 
 
 
ADCY3
 
 
hsa-miR-132-3p
 
 
-0.303
 
 
46.2
 
 
 
 
ADCY6
 
 
hsa-miR-1271-5p
 
 
-0.336
 
 
11.8
 
 
 
 
ADGRA2
 
 
hsa-miR-1271-5p
 
 
-0.427
 
 
26.1
 
 
 
 
ADIPOR2
 
 
hsa-miR-150-5p
 
 
-0.287
 
 
28.4
 
 
 
 
ADK
 
 
hsa-miR-1271-5p
 
 
-0.329
 
 
23.3
 
 
 
 
ADNP
 
 
hsa-miR-328-3p
 
 
-0.371
 
 
30.1
 
 
 
 
ADRA2A
 
 
hsa-miR-23a-3p
 
 
-0.371
 
 
22.2
 
 
 
 
ADRB2
 
 
hsa-miR-15b-5p
 
 
-0.29
 
 
12.2
 
 
 
 
ADRB2
 
 
hsa-let-7b-5p
 
 
-0.635
 
 
12.2
 
 
 
 
AGFG1
 
 
hsa-miR-148b-3p
 
 
-0.332
 
 
31.5
 
 
 
 
AGFG1
 
 
hsa-miR-152-3p
 
 
-0.332
 
 
31.5
 
 
 
 
AGFG1
 
 
hsa-miR-103a-3p
 
 
-0.389
 
 
31.5
 
 
 
 
AHDC1
 
 
hsa-miR-152-3p
 
 
-0.413
 
 
23.0
 
 
 
 
AHDC1
 
 
hsa-miR-148b-3p
 
 
-0.413
 
 
23.0
 
 
 
 
AHR
 
 
hsa-miR-1271-5p
 
 
-0.304
 
 
13.8
 
 
 
 
AHR
 
 
hsa-miR-29a-3p
 
 
-0.316
 
 
13.8
 
 
 
 
AJUBA
 
 
hsa-miR-103a-3p
 
 
-0.448
 
 
15.1
 
 
 
 
AK1
 
 
hsa-miR-29a-3p
 
 
-0.333
 
 
24.0
 
 
 
 
AK3
 
 
hsa-miR-1271-5p
 
 
-0.57
 
 
36.6
 
 
 
 
AK4
 
 
hsa-miR-15b-5p
 
 
-0.316
 
 
11.8
 
 
 
 
AKAP1
 
 
hsa-miR-152-3p
 
 
-0.381
 
 
24.4
 
 
 
 
AKAP1
 
 
hsa-miR-148b-3p
 
 
-0.381
 
 
24.4
 
 
 
 
AKIRIN2
 
 
hsa-miR-103a-3p
 
 
-0.299
 
 
22.7
 
 
 
 
AKT3
 
 
hsa-miR-29a-3p
 
 
-0.287
 
 
12.8
 
 
 
 
AKT3
 
 
hsa-miR-15b-5p
 
 
-0.301
 
 
12.8
 
 
 
 
ALAD
 
 
hsa-miR-326
 
 
-0.352
 
 
19.3
 
 
 
 
ALAD
 
 
hsa-miR-103a-3p
 
 
-0.385
 
 
19.3
 
 
 
 
ALAD
 
 
hsa-miR-15b-5p
 
 
-0.446
 
 
19.3
 
 
 
 
ALDH1A2
 
 
hsa-miR-23a-3p
 
 
-0.354
 
 
13.2
 
 
 
 
ALOX12
 
 
hsa-miR-15b-5p
 
 
-0.443
 
 
95.8
 
 
 
 
ALOX15B
 
 
hsa-miR-326
 
 
-0.417
 
 
33.0
 
 
 
 
AMOTL1
 
 
hsa-miR-15b-5p
 
 
-0.319
 
 
13.1
 
 
 
 
ANKHD1
 
 
hsa-miR-23a-3p
 
 
-0.368
 
 
41.9
 
 
 
 
ANKHD1
 
 
hsa-miR-30d-5p
 
 
-0.381
 
 
41.9
 
 
 
 
ANKHD1
 
 
hsa-miR-30b-5p
 
 
-0.405
 
 
41.9
 
 
 
 
ANKRD17
 
 
hsa-miR-30d-5p
 
 
-0.312
 
 
12.3
 
 
 
 
ANKRD17
 
 
hsa-miR-30b-5p
 
 
-0.336
 
 
12.3
 
 
 
 
ANKRD27
 
 
hsa-miR-1271-5p
 
 
-0.648
 
 
10.7
 
 
 
 
ANKRD29
 
 
hsa-miR-132-3p
 
 
-0.47
 
 
12.3
 
 
 
 
ANP32A
 
 
hsa-miR-132-3p
 
 
-0.374
 
 
40.8
 
 
 
 
ANTXR2
 
 
hsa-miR-29a-3p
 
 
-0.347
 
 
27.6
 
 
 
 
ANXA11
 
 
hsa-miR-15b-5p
 
 
-0.463
 
 
98.4
 
 
 
 
AP1S1
 
 
hsa-let-7b-5p
 
 
-0.374
 
 
20.1
 
 
 
 
AP2A1
 
 
hsa-miR-15b-5p
 
 
-0.333
 
 
41.4
 
 
 
 
AP2B1
 
 
hsa-miR-1271-5p
 
 
-0.299
 
 
32.4
 
 
 
 
AP2B1
 
 
hsa-miR-15b-5p
 
 
-0.334
 
 
32.4
 
 
 
 
AP3S1
 
 
hsa-miR-30b-5p
 
 
-0.293
 
 
63.7
 
 
 
 
APP
 
 
hsa-miR-15b-5p
 
 
-0.298
 
 
142.4
 
 
 
 
AQP3
 
 
hsa-miR-874-3p
 
 
-0.48
 
 
505.0
 
 
 
 
ARF4
 
 
hsa-miR-30b-5p
 
 
-0.289
 
 
85.1
 
 
 
 
ARF4
 
 
hsa-miR-194-5p
 
 
-0.293
 
 
85.1
 
 
 
 
ARF4
 
 
hsa-miR-152-3p
 
 
-0.487
 
 
85.1
 
 
 
 
ARF4
 
 
hsa-miR-148b-3p
 
 
-0.487
 
 
85.1
 
 
 
 
ARF5
 
 
hsa-miR-29a-3p
 
 
-0.491
 
 
132.3
 
 
 
 
ARF6
 
 
hsa-miR-181b-5p
 
 
-0.33
 
 
41.6
 
 
 
 
ARF6
 
 
hsa-miR-181a-5p
 
 
-0.33
 
 
41.6
 
 
 
 
ARF6
 
 
hsa-miR-181d-5p
 
 
-0.338
 
 
41.6
 
 
 
 
ARFIP1
 
 
hsa-miR-320b
 
 
-0.339
 
 
13.1
 
 
 
 
ARFIP1
 
 
hsa-miR-320c
 
 
-0.339
 
 
13.1
 
 
 
 
ARFIP1
 
 
hsa-miR-152-3p
 
 
-0.474
 
 
13.1
 
 
 
 
ARFIP1
 
 
hsa-miR-148b-3p
 
 
-0.474
 
 
13.1
 
 
 
 
ARG2
 
 
hsa-let-7b-5p
 
 
-0.468
 
 
11.0
 
 
 
 
ARID1B
 
 
hsa-miR-29a-3p
 
 
-0.342
 
 
13.7
 
 
 
 
ARIH2
 
 
hsa-miR-103a-3p
 
 
-0.307
 
 
16.6
 
 
 
 
ARL2
 
 
hsa-miR-15b-5p
 
 
-0.374
 
 
93.4
 
 
 
 
ARL2
 
 
hsa-miR-15b-5p
 
 
-0.442
 
 
93.4
 
 
 
 
ARL4A
 
 
hsa-miR-29a-3p
 
 
-0.386
 
 
63.2
 
 
 
 
ARL4A
 
 
hsa-miR-30d-5p
 
 
-0.432
 
 
63.2
 
 
 
 
ARL4A
 
 
hsa-miR-30b-5p
 
 
-0.448
 
 
63.2
 
 
 
 
ARL4D
 
 
hsa-miR-1271-5p
 
 
-0.392
 
 
38.9
 
 
 
 
ARL4D
 
 
hsa-let-7b-5p
 
 
-0.417
 
 
38.9
 
 
 
 
ARL6IP1
 
 
hsa-miR-328-3p
 
 
-0.494
 
 
48.0
 
 
 
 
ARL6IP1
 
 
hsa-miR-148b-3p
 
 
-0.579
 
 
48.0
 
 
 
 
ARL6IP1
 
 
hsa-miR-152-3p
 
 
-0.579
 
 
48.0
 
 
 
 
ARL8A
 
 
hsa-miR-103a-3p
 
 
-0.38
 
 
32.8
 
 
 
 
ARL8B
 
 
hsa-miR-152-3p
 
 
-0.337
 
 
51.8
 
 
 
 
ARL8B
 
 
hsa-miR-148b-3p
 
 
-0.337
 
 
51.8
 
 
 
 
ARL8B
 
 
hsa-miR-320b
 
 
-0.376
 
 
51.8
 
 
 
 
ARL8B
 
 
hsa-miR-320c
 
 
-0.376
 
 
51.8
 
 
 
 
ARMC1
 
 
hsa-miR-103a-3p
 
 
-0.525
 
 
10.3
 
 
 
 
ARMC8
 
 
hsa-miR-103a-3p
 
 
-0.412
 
 
12.4
 
 
 
 
 
 
 
 cd8_ev_vs_cd8_s0_downregulated 
 
 Tissue expression heatmap (log10-transformed) 
   
 
 
 bone marrow 
 
 
 
 
target_symbol
 
 
mature_mirna_id
 
 
score
 
 
nTPM
 
 
 
 
 
 
ABCB10
 
 
hsa-miR-148a-3p
 
 
-0.291
 
 
56.7
 
 
 
 
ABCB7
 
 
hsa-miR-148a-3p
 
 
-0.515
 
 
10.5
 
 
 
 
ABCC5
 
 
hsa-miR-125a-5p
 
 
-0.288
 
 
21.2
 
 
 
 
ABCD3
 
 
hsa-miR-148a-3p
 
 
-0.388
 
 
23.6
 
 
 
 
ABHD3
 
 
hsa-miR-125a-5p
 
 
-0.453
 
 
16.6
 
 
 
 
ABHD5
 
 
hsa-miR-20b-5p
 
 
-0.418
 
 
37.5
 
 
 
 
ABHD5
 
 
hsa-miR-106a-5p
 
 
-0.447
 
 
37.5
 
 
 
 
ABTB1
 
 
hsa-miR-125a-5p
 
 
-0.519
 
 
48.3
 
 
 
 
ACP3
 
 
hsa-let-7e-5p
 
 
-0.298
 
 
16.5
 
 
 
 
ACP3
 
 
hsa-miR-98-5p
 
 
-0.298
 
 
16.5
 
 
 
 
ACP3
 
 
hsa-let-7c-5p
 
 
-0.298
 
 
16.5
 
 
 
 
ACSL6
 
 
hsa-miR-125a-5p
 
 
-0.307
 
 
21.4
 
 
 
 
ACSL6
 
 
hsa-let-7e-5p
 
 
-0.336
 
 
21.4
 
 
 
 
ACSL6
 
 
hsa-miR-98-5p
 
 
-0.336
 
 
21.4
 
 
 
 
ACSL6
 
 
hsa-let-7c-5p
 
 
-0.336
 
 
21.4
 
 
 
 
ADAM10
 
 
hsa-miR-148a-3p
 
 
-0.356
 
 
17.3
 
 
 
 
ADRB2
 
 
hsa-let-7c-5p
 
 
-0.635
 
 
11.8
 
 
 
 
ADRB2
 
 
hsa-let-7e-5p
 
 
-0.635
 
 
11.8
 
 
 
 
ADRB2
 
 
hsa-miR-98-5p
 
 
-0.635
 
 
11.8
 
 
 
 
AGFG1
 
 
hsa-miR-148a-3p
 
 
-0.332
 
 
146.9
 
 
 
 
AGO2
 
 
hsa-miR-21-5p
 
 
-0.632
 
 
10.7
 
 
 
 
AGO2
 
 
hsa-miR-125a-5p
 
 
-0.811
 
 
10.7
 
 
 
 
AGO2
 
 
hsa-miR-184
 
 
-0.831
 
 
10.7
 
 
 
 
AGO2
 
 
hsa-miR-99b-5p
 
 
-0.836
 
 
10.7
 
 
 
 
AHCTF1
 
 
hsa-let-7e-5p
 
 
-0.374
 
 
16.0
 
 
 
 
AHCTF1
 
 
hsa-miR-98-5p
 
 
-0.374
 
 
16.0
 
 
 
 
AHCTF1
 
 
hsa-let-7c-5p
 
 
-0.374
 
 
16.0
 
 
 
 
AHNAK
 
 
hsa-miR-106a-5p
 
 
-0.334
 
 
41.6
 
 
 
 
AHNAK
 
 
hsa-miR-20b-5p
 
 
-0.337
 
 
41.6
 
 
 
 
AIFM1
 
 
hsa-miR-125a-5p
 
 
-0.3
 
 
11.2
 
 
 
 
AIFM1
 
 
hsa-let-7e-5p
 
 
-0.314
 
 
11.2
 
 
 
 
AIFM1
 
 
hsa-miR-98-5p
 
 
-0.314
 
 
11.2
 
 
 
 
AIFM1
 
 
hsa-let-7c-5p
 
 
-0.314
 
 
11.2
 
 
 
 
ALDH4A1
 
 
hsa-miR-184
 
 
-0.421
 
 
11.1
 
 
 
 
ALG3
 
 
hsa-miR-106a-5p
 
 
-0.569
 
 
14.9
 
 
 
 
ALG3
 
 
hsa-miR-20b-5p
 
 
-0.58
 
 
14.9
 
 
 
 
ANAPC16
 
 
hsa-miR-125a-5p
 
 
-0.578
 
 
13.0
 
 
 
 
ANKRD12
 
 
hsa-miR-7-5p
 
 
-0.37
 
 
19.4
 
 
 
 
ANKRD9
 
 
hsa-miR-20b-5p
 
 
-0.331
 
 
17.0
 
 
 
 
ANKRD9
 
 
hsa-miR-106a-5p
 
 
-0.344
 
 
17.0
 
 
 
 
ANPEP
 
 
hsa-miR-125a-5p
 
 
-0.372
 
 
97.7
 
 
 
 
ARF4
 
 
hsa-miR-7-5p
 
 
-0.407
 
 
68.5
 
 
 
 
ARF4
 
 
hsa-miR-148a-3p
 
 
-0.487
 
 
68.5
 
 
 
 
ARFGEF1
 
 
hsa-miR-148a-3p
 
 
-0.306
 
 
26.0
 
 
 
 
ARHGAP1
 
 
hsa-miR-20b-5p
 
 
-0.325
 
 
31.5
 
 
 
 
ARHGAP1
 
 
hsa-miR-106a-5p
 
 
-0.329
 
 
31.5
 
 
 
 
ARHGAP24
 
 
hsa-miR-21-5p
 
 
-0.427
 
 
17.7
 
 
 
 
ARID3B
 
 
hsa-let-7e-5p
 
 
-0.31
 
 
23.2
 
 
 
 
ARID3B
 
 
hsa-miR-98-5p
 
 
-0.31
 
 
23.2
 
 
 
 
ARID3B
 
 
hsa-let-7c-5p
 
 
-0.31
 
 
23.2
 
 
 
 
ARID3B
 
 
hsa-miR-98-5p
 
 
-0.475
 
 
23.2
 
 
 
 
ARID3B
 
 
hsa-let-7e-5p
 
 
-0.486
 
 
23.2
 
 
 
 
ARID3B
 
 
hsa-let-7c-5p
 
 
-0.486
 
 
23.2
 
 
 
 
ARID4A
 
 
hsa-miR-7-5p
 
 
-0.341
 
 
32.8
 
 
 
 
ARL1
 
 
hsa-miR-20b-5p
 
 
-0.424
 
 
15.6
 
 
 
 
ARL1
 
 
hsa-miR-106a-5p
 
 
-0.429
 
 
15.6
 
 
 
 
ARL6IP1
 
 
hsa-miR-7-5p
 
 
-0.374
 
 
197.7
 
 
 
 
ARL6IP1
 
 
hsa-miR-148a-3p
 
 
-0.579
 
 
197.7
 
 
 
 
ARL6IP6
 
 
hsa-miR-98-5p
 
 
-0.293
 
 
23.4
 
 
 
 
ARL6IP6
 
 
hsa-let-7c-5p
 
 
-0.293
 
 
23.4
 
 
 
 
ARL6IP6
 
 
hsa-let-7e-5p
 
 
-0.305
 
 
23.4
 
 
 
 
ARL8B
 
 
hsa-miR-148a-3p
 
 
-0.337
 
 
56.4
 
 
 
 
ARMC8
 
 
hsa-miR-106a-5p
 
 
-0.375
 
 
57.2
 
 
 
 
ARMC8
 
 
hsa-miR-20b-5p
 
 
-0.378
 
 
57.2
 
 
 
 
ARPP19
 
 
hsa-let-7e-5p
 
 
-0.492
 
 
97.5
 
 
 
 
ARPP19
 
 
hsa-let-7c-5p
 
 
-0.492
 
 
97.5
 
 
 
 
ARPP19
 
 
hsa-miR-98-5p
 
 
-0.5
 
 
97.5
 
 
 
 
ARRDC3
 
 
hsa-miR-148a-3p
 
 
-0.305
 
 
158.3
 
 
 
 
ARRDC4
 
 
hsa-let-7c-5p
 
 
-0.329
 
 
10.8
 
 
 
 
ARRDC4
 
 
hsa-let-7e-5p
 
 
-0.329
 
 
10.8
 
 
 
 
ARRDC4
 
 
hsa-miR-98-5p
 
 
-0.329
 
 
10.8
 
 
 
 
ASAP1
 
 
hsa-let-7e-5p
 
 
-0.296
 
 
44.7
 
 
 
 
ASAP1
 
 
hsa-miR-98-5p
 
 
-0.308
 
 
44.7
 
 
 
 
ASAP1
 
 
hsa-let-7c-5p
 
 
-0.308
 
 
44.7
 
 
 
 
ASPH
 
 
hsa-miR-148a-3p
 
 
-0.391
 
 
15.8
 
 
 
 
ATAD2
 
 
hsa-miR-20b-5p
 
 
-0.304
 
 
25.0
 
 
 
 
ATAD2
 
 
hsa-miR-106a-5p
 
 
-0.308
 
 
25.0
 
 
 
 
ATG16L1
 
 
hsa-miR-106a-5p
 
 
-0.464
 
 
13.5
 
 
 
 
ATG16L1
 
 
hsa-miR-20b-5p
 
 
-0.467
 
 
13.5
 
 
 
 
ATG4D
 
 
hsa-miR-148a-3p
 
 
-0.303
 
 
31.3
 
 
 
 
ATOSB
 
 
hsa-let-7e-5p
 
 
-0.429
 
 
36.6
 
 
 
 
ATOSB
 
 
hsa-miR-98-5p
 
 
-0.429
 
 
36.6
 
 
 
 
ATOSB
 
 
hsa-let-7c-5p
 
 
-0.429
 
 
36.6
 
 
 
 
ATP2A2
 
 
hsa-let-7e-5p
 
 
-0.311
 
 
43.4
 
 
 
 
ATP2A2
 
 
hsa-miR-98-5p
 
 
-0.311
 
 
43.4
 
 
 
 
ATP2A2
 
 
hsa-let-7c-5p
 
 
-0.311
 
 
43.4
 
 
 
 
ATP2B4
 
 
hsa-miR-148a-3p
 
 
-0.337
 
 
14.9
 
 
 
 
ATP6AP2
 
 
hsa-miR-148a-3p
 
 
-0.592
 
 
117.4
 
 
 
 
ATP6AP2
 
 
hsa-miR-148a-3p
 
 
-0.668
 
 
117.4
 
 
 
 
ATP8B4
 
 
hsa-let-7c-5p
 
 
-0.325
 
 
73.7
 
 
 
 
ATP8B4
 
 
hsa-let-7e-5p
 
 
-0.325
 
 
73.7
 
 
 
 
ATP8B4
 
 
hsa-miR-98-5p
 
 
-0.325
 
 
73.7
 
 
 
 
ATPAF1
 
 
hsa-let-7e-5p
 
 
-0.498
 
 
26.0
 
 
 
 
ATPAF1
 
 
hsa-miR-98-5p
 
 
-0.498
 
 
26.0
 
 
 
 
ATPAF1
 
 
hsa-let-7c-5p
 
 
-0.498
 
 
26.0
 
 
 
 
B3GAT3
 
 
hsa-let-7e-5p
 
 
-0.324
 
 
17.5
 
 
 
 
B3GAT3
 
 
hsa-miR-98-5p
 
 
-0.324
 
 
17.5
 
 
 
 
B3GAT3
 
 
hsa-let-7c-5p
 
 
-0.324
 
 
17.5
 
 
 
 
B4GALT5
 
 
hsa-miR-148a-3p
 
 
-0.44
 
 
88.5
 
 
 
 
BACH1
 
 
hsa-let-7e-5p
 
 
-0.298
 
 
84.3
 
 
 
 
 
 
 cerebellum 
 
 
 
 
target_symbol
 
 
mature_mirna_id
 
 
score
 
 
nTPM
 
 
 
 
 
 
ABCB10
 
 
hsa-miR-148a-3p
 
 
-0.291
 
 
10.6
 
 
 
 
ABCB9
 
 
hsa-let-7e-5p
 
 
-0.422
 
 
18.0
 
 
 
 
ABCB9
 
 
hsa-let-7c-5p
 
 
-0.422
 
 
18.0
 
 
 
 
ABCB9
 
 
hsa-miR-98-5p
 
 
-0.444
 
 
18.0
 
 
 
 
ABCC5
 
 
hsa-miR-125a-5p
 
 
-0.288
 
 
101.0
 
 
 
 
ABCD3
 
 
hsa-miR-148a-3p
 
 
-0.388
 
 
12.8
 
 
 
 
ABHD3
 
 
hsa-miR-125a-5p
 
 
-0.453
 
 
13.0
 
 
 
 
ABHD6
 
 
hsa-miR-125a-5p
 
 
-0.501
 
 
24.9
 
 
 
 
ABTB1
 
 
hsa-miR-125a-5p
 
 
-0.519
 
 
100.8
 
 
 
 
ACHE
 
 
hsa-miR-125a-5p
 
 
-0.308
 
 
59.1
 
 
 
 
ACHE
 
 
hsa-miR-125a-5p
 
 
-0.361
 
 
59.1
 
 
 
 
ACSL6
 
 
hsa-miR-125a-5p
 
 
-0.307
 
 
27.8
 
 
 
 
ACSL6
 
 
hsa-let-7e-5p
 
 
-0.336
 
 
27.8
 
 
 
 
ACSL6
 
 
hsa-miR-98-5p
 
 
-0.336
 
 
27.8
 
 
 
 
ACSL6
 
 
hsa-let-7c-5p
 
 
-0.336
 
 
27.8
 
 
 
 
ADAM10
 
 
hsa-miR-148a-3p
 
 
-0.356
 
 
15.6
 
 
 
 
ADGRB3
 
 
hsa-miR-148a-3p
 
 
-0.524
 
 
20.8
 
 
 
 
AGFG1
 
 
hsa-miR-148a-3p
 
 
-0.332
 
 
22.4
 
 
 
 
AGFG2
 
 
hsa-miR-20b-5p
 
 
-0.386
 
 
12.7
 
 
 
 
AGFG2
 
 
hsa-miR-106a-5p
 
 
-0.399
 
 
12.7
 
 
 
 
AHDC1
 
 
hsa-miR-148a-3p
 
 
-0.413
 
 
32.0
 
 
 
 
AHNAK
 
 
hsa-miR-106a-5p
 
 
-0.334
 
 
15.7
 
 
 
 
AHNAK
 
 
hsa-miR-20b-5p
 
 
-0.337
 
 
15.7
 
 
 
 
AKAP1
 
 
hsa-miR-148a-3p
 
 
-0.381
 
 
30.6
 
 
 
 
ALDH4A1
 
 
hsa-miR-184
 
 
-0.421
 
 
10.9
 
 
 
 
AMT
 
 
hsa-let-7e-5p
 
 
-0.463
 
 
20.1
 
 
 
 
AMT
 
 
hsa-miR-98-5p
 
 
-0.463
 
 
20.1
 
 
 
 
AMT
 
 
hsa-let-7c-5p
 
 
-0.463
 
 
20.1
 
 
 
 
ANAPC16
 
 
hsa-miR-125a-5p
 
 
-0.578
 
 
13.9
 
 
 
 
ANKRD12
 
 
hsa-miR-7-5p
 
 
-0.37
 
 
13.0
 
 
 
 
ANKRD13C
 
 
hsa-miR-106a-5p
 
 
-0.523
 
 
12.9
 
 
 
 
ANKRD13C
 
 
hsa-miR-20b-5p
 
 
-0.526
 
 
12.9
 
 
 
 
ANKRD46
 
 
hsa-let-7e-5p
 
 
-0.389
 
 
27.5
 
 
 
 
ANKRD46
 
 
hsa-miR-98-5p
 
 
-0.389
 
 
27.5
 
 
 
 
ANKRD46
 
 
hsa-let-7c-5p
 
 
-0.389
 
 
27.5
 
 
 
 
ANKRD9
 
 
hsa-miR-20b-5p
 
 
-0.331
 
 
22.1
 
 
 
 
ANKRD9
 
 
hsa-miR-106a-5p
 
 
-0.344
 
 
22.1
 
 
 
 
AP1AR
 
 
hsa-miR-21-5p
 
 
-0.318
 
 
13.2
 
 
 
 
AP1AR
 
 
hsa-miR-99b-5p
 
 
-0.823
 
 
13.2
 
 
 
 
AP1S1
 
 
hsa-let-7e-5p
 
 
-0.374
 
 
23.9
 
 
 
 
AP1S1
 
 
hsa-miR-98-5p
 
 
-0.374
 
 
23.9
 
 
 
 
AP1S1
 
 
hsa-let-7c-5p
 
 
-0.374
 
 
23.9
 
 
 
 
APBB3
 
 
hsa-let-7e-5p
 
 
-0.587
 
 
23.6
 
 
 
 
APBB3
 
 
hsa-miR-98-5p
 
 
-0.587
 
 
23.6
 
 
 
 
APBB3
 
 
hsa-let-7c-5p
 
 
-0.587
 
 
23.6
 
 
 
 
ARF4
 
 
hsa-miR-7-5p
 
 
-0.407
 
 
50.2
 
 
 
 
ARF4
 
 
hsa-miR-148a-3p
 
 
-0.487
 
 
50.2
 
 
 
 
ARG2
 
 
hsa-let-7e-5p
 
 
-0.468
 
 
16.4
 
 
 
 
ARG2
 
 
hsa-miR-98-5p
 
 
-0.468
 
 
16.4
 
 
 
 
ARG2
 
 
hsa-let-7c-5p
 
 
-0.468
 
 
16.4
 
 
 
 
ARHGAP1
 
 
hsa-miR-20b-5p
 
 
-0.325
 
 
19.6
 
 
 
 
ARHGAP1
 
 
hsa-miR-106a-5p
 
 
-0.329
 
 
19.6
 
 
 
 
ARHGAP21
 
 
hsa-miR-148a-3p
 
 
-0.287
 
 
50.0
 
 
 
 
ARID4A
 
 
hsa-miR-7-5p
 
 
-0.341
 
 
10.7
 
 
 
 
ARL1
 
 
hsa-miR-20b-5p
 
 
-0.424
 
 
20.8
 
 
 
 
ARL1
 
 
hsa-miR-106a-5p
 
 
-0.429
 
 
20.8
 
 
 
 
ARL6IP1
 
 
hsa-miR-7-5p
 
 
-0.374
 
 
234.0
 
 
 
 
ARL6IP1
 
 
hsa-miR-148a-3p
 
 
-0.579
 
 
234.0
 
 
 
 
ARL8B
 
 
hsa-miR-148a-3p
 
 
-0.337
 
 
50.7
 
 
 
 
ARMC8
 
 
hsa-miR-106a-5p
 
 
-0.375
 
 
35.7
 
 
 
 
ARMC8
 
 
hsa-miR-20b-5p
 
 
-0.378
 
 
35.7
 
 
 
 
ARMCX1
 
 
hsa-miR-21-5p
 
 
-0.489
 
 
17.3
 
 
 
 
ARMT1
 
 
hsa-let-7c-5p
 
 
-0.303
 
 
11.5
 
 
 
 
ARMT1
 
 
hsa-let-7e-5p
 
 
-0.303
 
 
11.5
 
 
 
 
ARMT1
 
 
hsa-miR-98-5p
 
 
-0.303
 
 
11.5
 
 
 
 
ARPP19
 
 
hsa-let-7e-5p
 
 
-0.492
 
 
66.7
 
 
 
 
ARPP19
 
 
hsa-let-7c-5p
 
 
-0.492
 
 
66.7
 
 
 
 
ARPP19
 
 
hsa-miR-98-5p
 
 
-0.5
 
 
66.7
 
 
 
 
ARRDC3
 
 
hsa-miR-148a-3p
 
 
-0.305
 
 
17.8
 
 
 
 
ASF1A
 
 
hsa-miR-21-5p
 
 
-0.304
 
 
12.6
 
 
 
 
ASPH
 
 
hsa-miR-148a-3p
 
 
-0.391
 
 
80.5
 
 
 
 
ATG16L1
 
 
hsa-miR-106a-5p
 
 
-0.464
 
 
24.1
 
 
 
 
ATG16L1
 
 
hsa-miR-20b-5p
 
 
-0.467
 
 
24.1
 
 
 
 
ATG4D
 
 
hsa-miR-148a-3p
 
 
-0.303
 
 
20.2
 
 
 
 
ATOSB
 
 
hsa-let-7e-5p
 
 
-0.429
 
 
14.6
 
 
 
 
ATOSB
 
 
hsa-miR-98-5p
 
 
-0.429
 
 
14.6
 
 
 
 
ATOSB
 
 
hsa-let-7c-5p
 
 
-0.429
 
 
14.6
 
 
 
 
ATP2A2
 
 
hsa-let-7e-5p
 
 
-0.311
 
 
15.0
 
 
 
 
ATP2A2
 
 
hsa-miR-98-5p
 
 
-0.311
 
 
15.0
 
 
 
 
ATP2A2
 
 
hsa-let-7c-5p
 
 
-0.311
 
 
15.0
 
 
 
 
ATP2B2
 
 
hsa-miR-7-5p
 
 
-0.297
 
 
88.0
 
 
 
 
ATP6AP2
 
 
hsa-miR-148a-3p
 
 
-0.592
 
 
52.9
 
 
 
 
ATP6AP2
 
 
hsa-miR-148a-3p
 
 
-0.668
 
 
52.9
 
 
 
 
ATPAF1
 
 
hsa-let-7e-5p
 
 
-0.498
 
 
24.4
 
 
 
 
ATPAF1
 
 
hsa-miR-98-5p
 
 
-0.498
 
 
24.4
 
 
 
 
ATPAF1
 
 
hsa-let-7c-5p
 
 
-0.498
 
 
24.4
 
 
 
 
AVPI1
 
 
hsa-miR-7-5p
 
 
-0.367
 
 
16.8
 
 
 
 
B3GAT3
 
 
hsa-let-7e-5p
 
 
-0.324
 
 
70.0
 
 
 
 
B3GAT3
 
 
hsa-miR-98-5p
 
 
-0.324
 
 
70.0
 
 
 
 
B3GAT3
 
 
hsa-let-7c-5p
 
 
-0.324
 
 
70.0
 
 
 
 
B4GALT5
 
 
hsa-miR-148a-3p
 
 
-0.44
 
 
20.4
 
 
 
 
B4GALT6
 
 
hsa-miR-148a-3p
 
 
-0.528
 
 
16.6
 
 
 
 
B4GAT1
 
 
hsa-let-7c-5p
 
 
-0.47
 
 
105.2
 
 
 
 
B4GAT1
 
 
hsa-let-7e-5p
 
 
-0.47
 
 
105.2
 
 
 
 
B4GAT1
 
 
hsa-miR-98-5p
 
 
-0.47
 
 
105.2
 
 
 
 
BARHL2
 
 
hsa-miR-148a-3p
 
 
-0.544
 
 
42.1
 
 
 
 
BAZ2A
 
 
hsa-miR-99b-5p
 
 
-0.408
 
 
26.6
 
 
 
 
BCL7A
 
 
hsa-miR-21-5p
 
 
-0.31
 
 
17.6
 
 
 
 
BDH1
 
 
hsa-miR-125a-5p
 
 
-0.389
 
 
48.5
 
 
 
 
BEGAIN
 
 
hsa-let-7c-5p
 
 
-0.466
 
 
53.7
 
 
 
 
 
 
 colon 
 
 
 
 
target_symbol
 
 
mature_mirna_id
 
 
score
 
 
nTPM
 
 
 
 
 
 
ABCB10
 
 
hsa-miR-148a-3p
 
 
-0.291
 
 
10.4
 
 
 
 
ABCB7
 
 
hsa-miR-148a-3p
 
 
-0.515
 
 
13.4
 
 
 
 
ABCC5
 
 
hsa-miR-125a-5p
 
 
-0.288
 
 
24.4
 
 
 
 
ABCD3
 
 
hsa-miR-148a-3p
 
 
-0.388
 
 
41.8
 
 
 
 
ABHD17C
 
 
hsa-let-7c-5p
 
 
-0.359
 
 
61.2
 
 
 
 
ABHD17C
 
 
hsa-let-7e-5p
 
 
-0.359
 
 
61.2
 
 
 
 
ABHD17C
 
 
hsa-miR-98-5p
 
 
-0.383
 
 
61.2
 
 
 
 
ABHD3
 
 
hsa-miR-125a-5p
 
 
-0.453
 
 
59.5
 
 
 
 
ABHD5
 
 
hsa-miR-20b-5p
 
 
-0.418
 
 
11.4
 
 
 
 
ABHD5
 
 
hsa-miR-106a-5p
 
 
-0.447
 
 
11.4
 
 
 
 
ABHD6
 
 
hsa-miR-125a-5p
 
 
-0.501
 
 
13.2
 
 
 
 
ABTB1
 
 
hsa-miR-125a-5p
 
 
-0.519
 
 
39.8
 
 
 
 
ADAM10
 
 
hsa-miR-148a-3p
 
 
-0.356
 
 
20.8
 
 
 
 
ADAM9
 
 
hsa-miR-106a-5p
 
 
-0.317
 
 
30.9
 
 
 
 
ADAM9
 
 
hsa-miR-20b-5p
 
 
-0.328
 
 
30.9
 
 
 
 
AGFG1
 
 
hsa-miR-148a-3p
 
 
-0.332
 
 
30.2
 
 
 
 
AGFG2
 
 
hsa-miR-20b-5p
 
 
-0.386
 
 
17.3
 
 
 
 
AGFG2
 
 
hsa-miR-106a-5p
 
 
-0.399
 
 
17.3
 
 
 
 
AHDC1
 
 
hsa-miR-148a-3p
 
 
-0.413
 
 
15.6
 
 
 
 
AHNAK
 
 
hsa-miR-106a-5p
 
 
-0.334
 
 
258.8
 
 
 
 
AHNAK
 
 
hsa-miR-20b-5p
 
 
-0.337
 
 
258.8
 
 
 
 
AIFM1
 
 
hsa-miR-125a-5p
 
 
-0.3
 
 
23.9
 
 
 
 
AIFM1
 
 
hsa-let-7e-5p
 
 
-0.314
 
 
23.9
 
 
 
 
AIFM1
 
 
hsa-miR-98-5p
 
 
-0.314
 
 
23.9
 
 
 
 
AIFM1
 
 
hsa-let-7c-5p
 
 
-0.314
 
 
23.9
 
 
 
 
AKAP1
 
 
hsa-miR-148a-3p
 
 
-0.381
 
 
72.8
 
 
 
 
ALCAM
 
 
hsa-miR-148a-3p
 
 
-0.339
 
 
15.7
 
 
 
 
ALG3
 
 
hsa-miR-106a-5p
 
 
-0.569
 
 
22.6
 
 
 
 
ALG3
 
 
hsa-miR-20b-5p
 
 
-0.58
 
 
22.6
 
 
 
 
ALG9
 
 
hsa-miR-7-5p
 
 
-0.302
 
 
10.0
 
 
 
 
AMT
 
 
hsa-let-7e-5p
 
 
-0.463
 
 
14.4
 
 
 
 
AMT
 
 
hsa-miR-98-5p
 
 
-0.463
 
 
14.4
 
 
 
 
AMT
 
 
hsa-let-7c-5p
 
 
-0.463
 
 
14.4
 
 
 
 
ANAPC16
 
 
hsa-miR-125a-5p
 
 
-0.578
 
 
22.7
 
 
 
 
ANKRD12
 
 
hsa-miR-7-5p
 
 
-0.37
 
 
12.2
 
 
 
 
ANKRD9
 
 
hsa-miR-20b-5p
 
 
-0.331
 
 
28.9
 
 
 
 
ANKRD9
 
 
hsa-miR-106a-5p
 
 
-0.344
 
 
28.9
 
 
 
 
ANPEP
 
 
hsa-miR-125a-5p
 
 
-0.372
 
 
250.5
 
 
 
 
AP1AR
 
 
hsa-miR-21-5p
 
 
-0.318
 
 
16.9
 
 
 
 
AP1AR
 
 
hsa-miR-99b-5p
 
 
-0.823
 
 
16.9
 
 
 
 
AP1S1
 
 
hsa-let-7e-5p
 
 
-0.374
 
 
31.0
 
 
 
 
AP1S1
 
 
hsa-miR-98-5p
 
 
-0.374
 
 
31.0
 
 
 
 
AP1S1
 
 
hsa-let-7c-5p
 
 
-0.374
 
 
31.0
 
 
 
 
APCDD1
 
 
hsa-miR-106a-5p
 
 
-0.422
 
 
12.1
 
 
 
 
APCDD1
 
 
hsa-miR-20b-5p
 
 
-0.425
 
 
12.1
 
 
 
 
ARF4
 
 
hsa-miR-7-5p
 
 
-0.407
 
 
148.9
 
 
 
 
ARF4
 
 
hsa-miR-148a-3p
 
 
-0.487
 
 
148.9
 
 
 
 
ARFGEF1
 
 
hsa-miR-148a-3p
 
 
-0.306
 
 
18.0
 
 
 
 
ARFIP1
 
 
hsa-miR-148a-3p
 
 
-0.474
 
 
26.4
 
 
 
 
ARHGAP1
 
 
hsa-miR-20b-5p
 
 
-0.325
 
 
59.8
 
 
 
 
ARHGAP1
 
 
hsa-miR-106a-5p
 
 
-0.329
 
 
59.8
 
 
 
 
ARHGAP21
 
 
hsa-miR-148a-3p
 
 
-0.287
 
 
15.5
 
 
 
 
ARL1
 
 
hsa-miR-20b-5p
 
 
-0.424
 
 
53.0
 
 
 
 
ARL1
 
 
hsa-miR-106a-5p
 
 
-0.429
 
 
53.0
 
 
 
 
ARL4D
 
 
hsa-let-7e-5p
 
 
-0.417
 
 
27.1
 
 
 
 
ARL4D
 
 
hsa-miR-98-5p
 
 
-0.417
 
 
27.1
 
 
 
 
ARL4D
 
 
hsa-let-7c-5p
 
 
-0.417
 
 
27.1
 
 
 
 
ARL6IP1
 
 
hsa-miR-7-5p
 
 
-0.374
 
 
132.0
 
 
 
 
ARL6IP1
 
 
hsa-miR-148a-3p
 
 
-0.579
 
 
132.0
 
 
 
 
ARL6IP6
 
 
hsa-miR-98-5p
 
 
-0.293
 
 
12.7
 
 
 
 
ARL6IP6
 
 
hsa-let-7c-5p
 
 
-0.293
 
 
12.7
 
 
 
 
ARL6IP6
 
 
hsa-let-7e-5p
 
 
-0.305
 
 
12.7
 
 
 
 
ARL8B
 
 
hsa-miR-148a-3p
 
 
-0.337
 
 
34.2
 
 
 
 
ARMC8
 
 
hsa-miR-106a-5p
 
 
-0.375
 
 
15.5
 
 
 
 
ARMC8
 
 
hsa-miR-20b-5p
 
 
-0.378
 
 
15.5
 
 
 
 
ARMCX1
 
 
hsa-miR-21-5p
 
 
-0.489
 
 
29.8
 
 
 
 
ARMT1
 
 
hsa-let-7c-5p
 
 
-0.303
 
 
15.5
 
 
 
 
ARMT1
 
 
hsa-let-7e-5p
 
 
-0.303
 
 
15.5
 
 
 
 
ARMT1
 
 
hsa-miR-98-5p
 
 
-0.303
 
 
15.5
 
 
 
 
ARPP19
 
 
hsa-let-7e-5p
 
 
-0.492
 
 
35.7
 
 
 
 
ARPP19
 
 
hsa-let-7c-5p
 
 
-0.492
 
 
35.7
 
 
 
 
ARPP19
 
 
hsa-miR-98-5p
 
 
-0.5
 
 
35.7
 
 
 
 
ARRDC3
 
 
hsa-miR-148a-3p
 
 
-0.305
 
 
18.9
 
 
 
 
ARRDC4
 
 
hsa-let-7c-5p
 
 
-0.329
 
 
40.4
 
 
 
 
ARRDC4
 
 
hsa-let-7e-5p
 
 
-0.329
 
 
40.4
 
 
 
 
ARRDC4
 
 
hsa-miR-98-5p
 
 
-0.329
 
 
40.4
 
 
 
 
ASAP1
 
 
hsa-let-7e-5p
 
 
-0.296
 
 
12.2
 
 
 
 
ASAP1
 
 
hsa-miR-98-5p
 
 
-0.308
 
 
12.2
 
 
 
 
ASAP1
 
 
hsa-let-7c-5p
 
 
-0.308
 
 
12.2
 
 
 
 
ASPH
 
 
hsa-miR-148a-3p
 
 
-0.391
 
 
72.8
 
 
 
 
ATG16L1
 
 
hsa-miR-106a-5p
 
 
-0.464
 
 
13.4
 
 
 
 
ATG16L1
 
 
hsa-miR-20b-5p
 
 
-0.467
 
 
13.4
 
 
 
 
ATG4A
 
 
hsa-miR-7-5p
 
 
-0.448
 
 
11.4
 
 
 
 
ATG4D
 
 
hsa-miR-148a-3p
 
 
-0.303
 
 
21.1
 
 
 
 
ATOSB
 
 
hsa-let-7e-5p
 
 
-0.429
 
 
14.6
 
 
 
 
ATOSB
 
 
hsa-miR-98-5p
 
 
-0.429
 
 
14.6
 
 
 
 
ATOSB
 
 
hsa-let-7c-5p
 
 
-0.429
 
 
14.6
 
 
 
 
ATP2A2
 
 
hsa-let-7e-5p
 
 
-0.311
 
 
60.2
 
 
 
 
ATP2A2
 
 
hsa-miR-98-5p
 
 
-0.311
 
 
60.2
 
 
 
 
ATP2A2
 
 
hsa-let-7c-5p
 
 
-0.311
 
 
60.2
 
 
 
 
ATP2B4
 
 
hsa-miR-148a-3p
 
 
-0.337
 
 
46.5
 
 
 
 
ATP6AP2
 
 
hsa-miR-148a-3p
 
 
-0.592
 
 
77.7
 
 
 
 
ATP6AP2
 
 
hsa-miR-148a-3p
 
 
-0.668
 
 
77.7
 
 
 
 
ATPAF1
 
 
hsa-let-7e-5p
 
 
-0.498
 
 
49.0
 
 
 
 
ATPAF1
 
 
hsa-miR-98-5p
 
 
-0.498
 
 
49.0
 
 
 
 
ATPAF1
 
 
hsa-let-7c-5p
 
 
-0.498
 
 
49.0
 
 
 
 
AVPI1
 
 
hsa-miR-7-5p
 
 
-0.367
 
 
23.0
 
 
 
 
B3GALNT2
 
 
hsa-miR-125a-5p
 
 
-0.311
 
 
14.0
 
 
 
 
B3GAT3
 
 
hsa-let-7e-5p
 
 
-0.324
 
 
30.8
 
 
 
 
B3GAT3
 
 
hsa-miR-98-5p
 
 
-0.324
 
 
30.8
 
 
 
 
 
 
 duodenum 
 
 
 
 
target_symbol
 
 
mature_mirna_id
 
 
score
 
 
nTPM
 
 
 
 
 
 
ABCB10
 
 
hsa-miR-148a-3p
 
 
-0.291
 
 
16.1
 
 
 
 
ABCB7
 
 
hsa-miR-148a-3p
 
 
-0.515
 
 
15.1
 
 
 
 
ABCC5
 
 
hsa-miR-125a-5p
 
 
-0.288
 
 
10.1
 
 
 
 
ABCD3
 
 
hsa-miR-148a-3p
 
 
-0.388
 
 
42.1
 
 
 
 
ABHD17C
 
 
hsa-let-7c-5p
 
 
-0.359
 
 
21.9
 
 
 
 
ABHD17C
 
 
hsa-let-7e-5p
 
 
-0.359
 
 
21.9
 
 
 
 
ABHD17C
 
 
hsa-miR-98-5p
 
 
-0.383
 
 
21.9
 
 
 
 
ABHD3
 
 
hsa-miR-125a-5p
 
 
-0.453
 
 
41.5
 
 
 
 
ABHD6
 
 
hsa-miR-125a-5p
 
 
-0.501
 
 
37.1
 
 
 
 
ABTB1
 
 
hsa-miR-125a-5p
 
 
-0.519
 
 
11.6
 
 
 
 
ADAM10
 
 
hsa-miR-148a-3p
 
 
-0.356
 
 
14.7
 
 
 
 
ADAM9
 
 
hsa-miR-106a-5p
 
 
-0.317
 
 
23.1
 
 
 
 
ADAM9
 
 
hsa-miR-20b-5p
 
 
-0.328
 
 
23.1
 
 
 
 
AGFG1
 
 
hsa-miR-148a-3p
 
 
-0.332
 
 
40.8
 
 
 
 
AGGF1
 
 
hsa-miR-125a-5p
 
 
-0.331
 
 
13.2
 
 
 
 
AHNAK
 
 
hsa-miR-106a-5p
 
 
-0.334
 
 
46.1
 
 
 
 
AHNAK
 
 
hsa-miR-20b-5p
 
 
-0.337
 
 
46.1
 
 
 
 
AIFM1
 
 
hsa-miR-125a-5p
 
 
-0.3
 
 
49.8
 
 
 
 
AIFM1
 
 
hsa-let-7e-5p
 
 
-0.314
 
 
49.8
 
 
 
 
AIFM1
 
 
hsa-miR-98-5p
 
 
-0.314
 
 
49.8
 
 
 
 
AIFM1
 
 
hsa-let-7c-5p
 
 
-0.314
 
 
49.8
 
 
 
 
AKAP1
 
 
hsa-miR-148a-3p
 
 
-0.381
 
 
51.5
 
 
 
 
ALCAM
 
 
hsa-miR-148a-3p
 
 
-0.339
 
 
16.0
 
 
 
 
ALG3
 
 
hsa-miR-106a-5p
 
 
-0.569
 
 
15.9
 
 
 
 
ALG3
 
 
hsa-miR-20b-5p
 
 
-0.58
 
 
15.9
 
 
 
 
ALG9
 
 
hsa-miR-7-5p
 
 
-0.302
 
 
11.5
 
 
 
 
AMT
 
 
hsa-let-7e-5p
 
 
-0.463
 
 
12.9
 
 
 
 
AMT
 
 
hsa-miR-98-5p
 
 
-0.463
 
 
12.9
 
 
 
 
AMT
 
 
hsa-let-7c-5p
 
 
-0.463
 
 
12.9
 
 
 
 
ANAPC16
 
 
hsa-miR-125a-5p
 
 
-0.578
 
 
14.5
 
 
 
 
ANKRD12
 
 
hsa-miR-7-5p
 
 
-0.37
 
 
12.1
 
 
 
 
ANKRD9
 
 
hsa-miR-20b-5p
 
 
-0.331
 
 
47.3
 
 
 
 
ANKRD9
 
 
hsa-miR-106a-5p
 
 
-0.344
 
 
47.3
 
 
 
 
ANPEP
 
 
hsa-miR-125a-5p
 
 
-0.372
 
 
2245.0
 
 
 
 
AP1AR
 
 
hsa-miR-21-5p
 
 
-0.318
 
 
20.9
 
 
 
 
AP1AR
 
 
hsa-miR-99b-5p
 
 
-0.823
 
 
20.9
 
 
 
 
AP1S1
 
 
hsa-let-7e-5p
 
 
-0.374
 
 
36.7
 
 
 
 
AP1S1
 
 
hsa-miR-98-5p
 
 
-0.374
 
 
36.7
 
 
 
 
AP1S1
 
 
hsa-let-7c-5p
 
 
-0.374
 
 
36.7
 
 
 
 
ARF4
 
 
hsa-miR-7-5p
 
 
-0.407
 
 
116.2
 
 
 
 
ARF4
 
 
hsa-miR-148a-3p
 
 
-0.487
 
 
116.2
 
 
 
 
ARFGEF1
 
 
hsa-miR-148a-3p
 
 
-0.306
 
 
15.9
 
 
 
 
ARFIP1
 
 
hsa-miR-148a-3p
 
 
-0.474
 
 
21.8
 
 
 
 
ARHGAP1
 
 
hsa-miR-20b-5p
 
 
-0.325
 
 
24.2
 
 
 
 
ARHGAP1
 
 
hsa-miR-106a-5p
 
 
-0.329
 
 
24.2
 
 
 
 
ARHGAP21
 
 
hsa-miR-148a-3p
 
 
-0.287
 
 
12.1
 
 
 
 
ARL1
 
 
hsa-miR-20b-5p
 
 
-0.424
 
 
45.2
 
 
 
 
ARL1
 
 
hsa-miR-106a-5p
 
 
-0.429
 
 
45.2
 
 
 
 
ARL6IP1
 
 
hsa-miR-7-5p
 
 
-0.374
 
 
130.6
 
 
 
 
ARL6IP1
 
 
hsa-miR-148a-3p
 
 
-0.579
 
 
130.6
 
 
 
 
ARL8B
 
 
hsa-miR-148a-3p
 
 
-0.337
 
 
28.9
 
 
 
 
ARMC8
 
 
hsa-miR-106a-5p
 
 
-0.375
 
 
11.3
 
 
 
 
ARMC8
 
 
hsa-miR-20b-5p
 
 
-0.378
 
 
11.3
 
 
 
 
ARMT1
 
 
hsa-let-7c-5p
 
 
-0.303
 
 
17.8
 
 
 
 
ARMT1
 
 
hsa-let-7e-5p
 
 
-0.303
 
 
17.8
 
 
 
 
ARMT1
 
 
hsa-miR-98-5p
 
 
-0.303
 
 
17.8
 
 
 
 
ARPP19
 
 
hsa-let-7e-5p
 
 
-0.492
 
 
27.2
 
 
 
 
ARPP19
 
 
hsa-let-7c-5p
 
 
-0.492
 
 
27.2
 
 
 
 
ARPP19
 
 
hsa-miR-98-5p
 
 
-0.5
 
 
27.2
 
 
 
 
ARRDC4
 
 
hsa-let-7c-5p
 
 
-0.329
 
 
14.9
 
 
 
 
ARRDC4
 
 
hsa-let-7e-5p
 
 
-0.329
 
 
14.9
 
 
 
 
ARRDC4
 
 
hsa-miR-98-5p
 
 
-0.329
 
 
14.9
 
 
 
 
ASPH
 
 
hsa-miR-148a-3p
 
 
-0.391
 
 
42.9
 
 
 
 
ATG4A
 
 
hsa-miR-7-5p
 
 
-0.448
 
 
12.0
 
 
 
 
ATG4D
 
 
hsa-miR-148a-3p
 
 
-0.303
 
 
20.5
 
 
 
 
ATP2A2
 
 
hsa-let-7e-5p
 
 
-0.311
 
 
44.8
 
 
 
 
ATP2A2
 
 
hsa-miR-98-5p
 
 
-0.311
 
 
44.8
 
 
 
 
ATP2A2
 
 
hsa-let-7c-5p
 
 
-0.311
 
 
44.8
 
 
 
 
ATP2B4
 
 
hsa-miR-148a-3p
 
 
-0.337
 
 
11.8
 
 
 
 
ATP6AP2
 
 
hsa-miR-148a-3p
 
 
-0.592
 
 
30.3
 
 
 
 
ATP6AP2
 
 
hsa-miR-148a-3p
 
 
-0.668
 
 
30.3
 
 
 
 
ATPAF1
 
 
hsa-let-7e-5p
 
 
-0.498
 
 
41.3
 
 
 
 
ATPAF1
 
 
hsa-miR-98-5p
 
 
-0.498
 
 
41.3
 
 
 
 
ATPAF1
 
 
hsa-let-7c-5p
 
 
-0.498
 
 
41.3
 
 
 
 
AVPI1
 
 
hsa-miR-7-5p
 
 
-0.367
 
 
13.2
 
 
 
 
B3GAT3
 
 
hsa-let-7e-5p
 
 
-0.324
 
 
16.1
 
 
 
 
B3GAT3
 
 
hsa-miR-98-5p
 
 
-0.324
 
 
16.1
 
 
 
 
B3GAT3
 
 
hsa-let-7c-5p
 
 
-0.324
 
 
16.1
 
 
 
 
B4GALT5
 
 
hsa-miR-148a-3p
 
 
-0.44
 
 
23.8
 
 
 
 
B4GAT1
 
 
hsa-let-7c-5p
 
 
-0.47
 
 
15.2
 
 
 
 
B4GAT1
 
 
hsa-let-7e-5p
 
 
-0.47
 
 
15.2
 
 
 
 
B4GAT1
 
 
hsa-miR-98-5p
 
 
-0.47
 
 
15.2
 
 
 
 
BACH1
 
 
hsa-let-7e-5p
 
 
-0.298
 
 
15.8
 
 
 
 
BACH1
 
 
hsa-let-7e-5p
 
 
-0.298
 
 
15.8
 
 
 
 
BACH1
 
 
hsa-miR-98-5p
 
 
-0.298
 
 
15.8
 
 
 
 
BACH1
 
 
hsa-miR-98-5p
 
 
-0.298
 
 
15.8
 
 
 
 
BACH1
 
 
hsa-let-7c-5p
 
 
-0.306
 
 
15.8
 
 
 
 
BACH1
 
 
hsa-let-7c-5p
 
 
-0.306
 
 
15.8
 
 
 
 
BACH1
 
 
hsa-let-7e-5p
 
 
-0.435
 
 
15.8
 
 
 
 
BACH1
 
 
hsa-let-7e-5p
 
 
-0.435
 
 
15.8
 
 
 
 
BACH1
 
 
hsa-miR-98-5p
 
 
-0.435
 
 
15.8
 
 
 
 
BACH1
 
 
hsa-miR-98-5p
 
 
-0.435
 
 
15.8
 
 
 
 
BACH1
 
 
hsa-let-7c-5p
 
 
-0.435
 
 
15.8
 
 
 
 
BACH1
 
 
hsa-let-7c-5p
 
 
-0.435
 
 
15.8
 
 
 
 
BAK1
 
 
hsa-miR-125a-5p
 
 
-0.402
 
 
44.2
 
 
 
 
BAZ2A
 
 
hsa-miR-99b-5p
 
 
-0.408
 
 
23.9
 
 
 
 
BCAP29
 
 
hsa-let-7e-5p
 
 
-0.289
 
 
15.5
 
 
 
 
BDH1
 
 
hsa-miR-125a-5p
 
 
-0.389
 
 
16.3
 
 
 
 
BLOC1S6
 
 
hsa-let-7e-5p
 
 
-0.321
 
 
19.0
 
 
 
 
BLOC1S6
 
 
hsa-miR-98-5p
 
 
-0.321
 
 
19.0
 
 
 
 
 
 
 liver 
 
 
 
 
target_symbol
 
 
mature_mirna_id
 
 
score
 
 
nTPM
 
 
 
 
 
 
ABCB10
 
 
hsa-miR-148a-3p
 
 
-0.291
 
 
17.7
 
 
 
 
ABCB7
 
 
hsa-miR-148a-3p
 
 
-0.515
 
 
14.1
 
 
 
 
ABCD3
 
 
hsa-miR-148a-3p
 
 
-0.388
 
 
118.4
 
 
 
 
ABHD3
 
 
hsa-miR-125a-5p
 
 
-0.453
 
 
36.7
 
 
 
 
ABHD5
 
 
hsa-miR-20b-5p
 
 
-0.418
 
 
20.3
 
 
 
 
ABHD5
 
 
hsa-miR-106a-5p
 
 
-0.447
 
 
20.3
 
 
 
 
ABHD6
 
 
hsa-miR-125a-5p
 
 
-0.501
 
 
60.7
 
 
 
 
ABTB1
 
 
hsa-miR-125a-5p
 
 
-0.519
 
 
18.5
 
 
 
 
ACVR1
 
 
hsa-miR-148a-3p
 
 
-0.397
 
 
15.3
 
 
 
 
ADAM10
 
 
hsa-miR-148a-3p
 
 
-0.356
 
 
15.0
 
 
 
 
ADAM9
 
 
hsa-miR-106a-5p
 
 
-0.317
 
 
12.7
 
 
 
 
ADAM9
 
 
hsa-miR-20b-5p
 
 
-0.328
 
 
12.7
 
 
 
 
ADGRF5
 
 
hsa-miR-148a-3p
 
 
-0.354
 
 
12.3
 
 
 
 
ADRB2
 
 
hsa-let-7c-5p
 
 
-0.635
 
 
13.3
 
 
 
 
ADRB2
 
 
hsa-let-7e-5p
 
 
-0.635
 
 
13.3
 
 
 
 
ADRB2
 
 
hsa-miR-98-5p
 
 
-0.635
 
 
13.3
 
 
 
 
AGFG1
 
 
hsa-miR-148a-3p
 
 
-0.332
 
 
33.8
 
 
 
 
AGFG2
 
 
hsa-miR-20b-5p
 
 
-0.386
 
 
51.0
 
 
 
 
AGFG2
 
 
hsa-miR-106a-5p
 
 
-0.399
 
 
51.0
 
 
 
 
AGGF1
 
 
hsa-miR-125a-5p
 
 
-0.331
 
 
12.9
 
 
 
 
AHCTF1
 
 
hsa-let-7e-5p
 
 
-0.374
 
 
10.2
 
 
 
 
AHCTF1
 
 
hsa-miR-98-5p
 
 
-0.374
 
 
10.2
 
 
 
 
AHCTF1
 
 
hsa-let-7c-5p
 
 
-0.374
 
 
10.2
 
 
 
 
AHNAK
 
 
hsa-miR-106a-5p
 
 
-0.334
 
 
56.2
 
 
 
 
AHNAK
 
 
hsa-miR-20b-5p
 
 
-0.337
 
 
56.2
 
 
 
 
AIFM1
 
 
hsa-miR-125a-5p
 
 
-0.3
 
 
77.0
 
 
 
 
AIFM1
 
 
hsa-let-7e-5p
 
 
-0.314
 
 
77.0
 
 
 
 
AIFM1
 
 
hsa-miR-98-5p
 
 
-0.314
 
 
77.0
 
 
 
 
AIFM1
 
 
hsa-let-7c-5p
 
 
-0.314
 
 
77.0
 
 
 
 
AKAP1
 
 
hsa-miR-148a-3p
 
 
-0.381
 
 
48.4
 
 
 
 
ALCAM
 
 
hsa-miR-148a-3p
 
 
-0.339
 
 
44.8
 
 
 
 
ALDH4A1
 
 
hsa-miR-184
 
 
-0.421
 
 
438.6
 
 
 
 
ALG3
 
 
hsa-miR-106a-5p
 
 
-0.569
 
 
54.4
 
 
 
 
ALG3
 
 
hsa-miR-20b-5p
 
 
-0.58
 
 
54.4
 
 
 
 
ALG9
 
 
hsa-miR-7-5p
 
 
-0.302
 
 
12.5
 
 
 
 
AMT
 
 
hsa-let-7e-5p
 
 
-0.463
 
 
52.5
 
 
 
 
AMT
 
 
hsa-miR-98-5p
 
 
-0.463
 
 
52.5
 
 
 
 
AMT
 
 
hsa-let-7c-5p
 
 
-0.463
 
 
52.5
 
 
 
 
ANAPC16
 
 
hsa-miR-125a-5p
 
 
-0.578
 
 
25.2
 
 
 
 
ANKRD12
 
 
hsa-miR-7-5p
 
 
-0.37
 
 
12.9
 
 
 
 
ANKRD46
 
 
hsa-let-7e-5p
 
 
-0.389
 
 
38.9
 
 
 
 
ANKRD46
 
 
hsa-miR-98-5p
 
 
-0.389
 
 
38.9
 
 
 
 
ANKRD46
 
 
hsa-let-7c-5p
 
 
-0.389
 
 
38.9
 
 
 
 
ANKRD9
 
 
hsa-miR-20b-5p
 
 
-0.331
 
 
15.7
 
 
 
 
ANKRD9
 
 
hsa-miR-106a-5p
 
 
-0.344
 
 
15.7
 
 
 
 
ANPEP
 
 
hsa-miR-125a-5p
 
 
-0.372
 
 
256.4
 
 
 
 
AP1AR
 
 
hsa-miR-21-5p
 
 
-0.318
 
 
19.1
 
 
 
 
AP1AR
 
 
hsa-miR-99b-5p
 
 
-0.823
 
 
19.1
 
 
 
 
AP1S1
 
 
hsa-let-7e-5p
 
 
-0.374
 
 
39.1
 
 
 
 
AP1S1
 
 
hsa-miR-98-5p
 
 
-0.374
 
 
39.1
 
 
 
 
AP1S1
 
 
hsa-let-7c-5p
 
 
-0.374
 
 
39.1
 
 
 
 
APOC4
 
 
hsa-miR-125a-5p
 
 
-0.515
 
 
585.9
 
 
 
 
ARF4
 
 
hsa-miR-7-5p
 
 
-0.407
 
 
161.3
 
 
 
 
ARF4
 
 
hsa-miR-148a-3p
 
 
-0.487
 
 
161.3
 
 
 
 
ARFGEF1
 
 
hsa-miR-148a-3p
 
 
-0.306
 
 
14.2
 
 
 
 
ARFIP1
 
 
hsa-miR-148a-3p
 
 
-0.474
 
 
14.4
 
 
 
 
ARHGAP1
 
 
hsa-miR-20b-5p
 
 
-0.325
 
 
44.2
 
 
 
 
ARHGAP1
 
 
hsa-miR-106a-5p
 
 
-0.329
 
 
44.2
 
 
 
 
ARHGAP21
 
 
hsa-miR-148a-3p
 
 
-0.287
 
 
11.9
 
 
 
 
ARID4A
 
 
hsa-miR-7-5p
 
 
-0.341
 
 
17.5
 
 
 
 
ARL1
 
 
hsa-miR-20b-5p
 
 
-0.424
 
 
64.0
 
 
 
 
ARL1
 
 
hsa-miR-106a-5p
 
 
-0.429
 
 
64.0
 
 
 
 
ARL4D
 
 
hsa-let-7e-5p
 
 
-0.417
 
 
55.4
 
 
 
 
ARL4D
 
 
hsa-miR-98-5p
 
 
-0.417
 
 
55.4
 
 
 
 
ARL4D
 
 
hsa-let-7c-5p
 
 
-0.417
 
 
55.4
 
 
 
 
ARL6IP1
 
 
hsa-miR-7-5p
 
 
-0.374
 
 
336.2
 
 
 
 
ARL6IP1
 
 
hsa-miR-148a-3p
 
 
-0.579
 
 
336.2
 
 
 
 
ARL8B
 
 
hsa-miR-148a-3p
 
 
-0.337
 
 
41.1
 
 
 
 
ARMC8
 
 
hsa-miR-106a-5p
 
 
-0.375
 
 
15.8
 
 
 
 
ARMC8
 
 
hsa-miR-20b-5p
 
 
-0.378
 
 
15.8
 
 
 
 
ARMT1
 
 
hsa-let-7c-5p
 
 
-0.303
 
 
35.2
 
 
 
 
ARMT1
 
 
hsa-let-7e-5p
 
 
-0.303
 
 
35.2
 
 
 
 
ARMT1
 
 
hsa-miR-98-5p
 
 
-0.303
 
 
35.2
 
 
 
 
ARPP19
 
 
hsa-let-7e-5p
 
 
-0.492
 
 
39.5
 
 
 
 
ARPP19
 
 
hsa-let-7c-5p
 
 
-0.492
 
 
39.5
 
 
 
 
ARPP19
 
 
hsa-miR-98-5p
 
 
-0.5
 
 
39.5
 
 
 
 
ARRDC3
 
 
hsa-miR-148a-3p
 
 
-0.305
 
 
71.0
 
 
 
 
ARRDC4
 
 
hsa-let-7c-5p
 
 
-0.329
 
 
28.3
 
 
 
 
ARRDC4
 
 
hsa-let-7e-5p
 
 
-0.329
 
 
28.3
 
 
 
 
ARRDC4
 
 
hsa-miR-98-5p
 
 
-0.329
 
 
28.3
 
 
 
 
ASF1A
 
 
hsa-miR-21-5p
 
 
-0.304
 
 
11.3
 
 
 
 
ASPH
 
 
hsa-miR-148a-3p
 
 
-0.391
 
 
69.9
 
 
 
 
ATG16L1
 
 
hsa-miR-106a-5p
 
 
-0.464
 
 
14.9
 
 
 
 
ATG16L1
 
 
hsa-miR-20b-5p
 
 
-0.467
 
 
14.9
 
 
 
 
ATG4A
 
 
hsa-miR-7-5p
 
 
-0.448
 
 
16.9
 
 
 
 
ATG4D
 
 
hsa-miR-148a-3p
 
 
-0.303
 
 
13.0
 
 
 
 
ATOSB
 
 
hsa-let-7e-5p
 
 
-0.429
 
 
10.9
 
 
 
 
ATOSB
 
 
hsa-miR-98-5p
 
 
-0.429
 
 
10.9
 
 
 
 
ATOSB
 
 
hsa-let-7c-5p
 
 
-0.429
 
 
10.9
 
 
 
 
ATP2A2
 
 
hsa-let-7e-5p
 
 
-0.311
 
 
49.3
 
 
 
 
ATP2A2
 
 
hsa-miR-98-5p
 
 
-0.311
 
 
49.3
 
 
 
 
ATP2A2
 
 
hsa-let-7c-5p
 
 
-0.311
 
 
49.3
 
 
 
 
ATP2B2
 
 
hsa-miR-7-5p
 
 
-0.297
 
 
30.4
 
 
 
 
ATP6AP2
 
 
hsa-miR-148a-3p
 
 
-0.592
 
 
90.5
 
 
 
 
ATP6AP2
 
 
hsa-miR-148a-3p
 
 
-0.668
 
 
90.5
 
 
 
 
ATPAF1
 
 
hsa-let-7e-5p
 
 
-0.498
 
 
101.4
 
 
 
 
ATPAF1
 
 
hsa-miR-98-5p
 
 
-0.498
 
 
101.4
 
 
 
 
ATPAF1
 
 
hsa-let-7c-5p
 
 
-0.498
 
 
101.4
 
 
 
 
AVPI1
 
 
hsa-miR-7-5p
 
 
-0.367
 
 
47.4
 
 
 
 
B3GAT3
 
 
hsa-let-7e-5p
 
 
-0.324
 
 
53.3
 
 
 
 
 
 
 lymph node 
 
 
 
 
target_symbol
 
 
mature_mirna_id
 
 
score
 
 
nTPM
 
 
 
 
 
 
ABCB10
 
 
hsa-miR-148a-3p
 
 
-0.291
 
 
10.3
 
 
 
 
ABCB7
 
 
hsa-miR-148a-3p
 
 
-0.515
 
 
11.2
 
 
 
 
ABCC5
 
 
hsa-miR-125a-5p
 
 
-0.288
 
 
12.3
 
 
 
 
ABCD3
 
 
hsa-miR-148a-3p
 
 
-0.388
 
 
12.4
 
 
 
 
ABHD3
 
 
hsa-miR-125a-5p
 
 
-0.453
 
 
27.9
 
 
 
 
ABTB1
 
 
hsa-miR-125a-5p
 
 
-0.519
 
 
20.7
 
 
 
 
ADAM10
 
 
hsa-miR-148a-3p
 
 
-0.356
 
 
20.8
 
 
 
 
AGFG1
 
 
hsa-miR-148a-3p
 
 
-0.332
 
 
44.0
 
 
 
 
AGGF1
 
 
hsa-miR-125a-5p
 
 
-0.331
 
 
12.7
 
 
 
 
AHNAK
 
 
hsa-miR-106a-5p
 
 
-0.334
 
 
34.4
 
 
 
 
AHNAK
 
 
hsa-miR-20b-5p
 
 
-0.337
 
 
34.4
 
 
 
 
AIFM1
 
 
hsa-miR-125a-5p
 
 
-0.3
 
 
15.1
 
 
 
 
AIFM1
 
 
hsa-let-7e-5p
 
 
-0.314
 
 
15.1
 
 
 
 
AIFM1
 
 
hsa-miR-98-5p
 
 
-0.314
 
 
15.1
 
 
 
 
AIFM1
 
 
hsa-let-7c-5p
 
 
-0.314
 
 
15.1
 
 
 
 
AKAP1
 
 
hsa-miR-148a-3p
 
 
-0.381
 
 
10.0
 
 
 
 
ALG3
 
 
hsa-miR-106a-5p
 
 
-0.569
 
 
12.2
 
 
 
 
ALG3
 
 
hsa-miR-20b-5p
 
 
-0.58
 
 
12.2
 
 
 
 
ANAPC16
 
 
hsa-miR-125a-5p
 
 
-0.578
 
 
23.5
 
 
 
 
ANKRD12
 
 
hsa-miR-7-5p
 
 
-0.37
 
 
20.9
 
 
 
 
ANKRD29
 
 
hsa-miR-106a-5p
 
 
-0.369
 
 
18.8
 
 
 
 
ANKRD29
 
 
hsa-miR-20b-5p
 
 
-0.38
 
 
18.8
 
 
 
 
ANPEP
 
 
hsa-miR-125a-5p
 
 
-0.372
 
 
12.0
 
 
 
 
AP1AR
 
 
hsa-miR-21-5p
 
 
-0.318
 
 
11.3
 
 
 
 
AP1AR
 
 
hsa-miR-99b-5p
 
 
-0.823
 
 
11.3
 
 
 
 
AP1S1
 
 
hsa-let-7e-5p
 
 
-0.374
 
 
11.1
 
 
 
 
AP1S1
 
 
hsa-miR-98-5p
 
 
-0.374
 
 
11.1
 
 
 
 
AP1S1
 
 
hsa-let-7c-5p
 
 
-0.374
 
 
11.1
 
 
 
 
ARF4
 
 
hsa-miR-7-5p
 
 
-0.407
 
 
69.1
 
 
 
 
ARF4
 
 
hsa-miR-148a-3p
 
 
-0.487
 
 
69.1
 
 
 
 
ARFGEF1
 
 
hsa-miR-148a-3p
 
 
-0.306
 
 
14.4
 
 
 
 
ARFIP1
 
 
hsa-miR-148a-3p
 
 
-0.474
 
 
14.1
 
 
 
 
ARHGAP1
 
 
hsa-miR-20b-5p
 
 
-0.325
 
 
28.7
 
 
 
 
ARHGAP1
 
 
hsa-miR-106a-5p
 
 
-0.329
 
 
28.7
 
 
 
 
ARID4A
 
 
hsa-miR-7-5p
 
 
-0.341
 
 
14.0
 
 
 
 
ARL1
 
 
hsa-miR-20b-5p
 
 
-0.424
 
 
31.0
 
 
 
 
ARL1
 
 
hsa-miR-106a-5p
 
 
-0.429
 
 
31.0
 
 
 
 
ARL6IP1
 
 
hsa-miR-7-5p
 
 
-0.374
 
 
124.0
 
 
 
 
ARL6IP1
 
 
hsa-miR-148a-3p
 
 
-0.579
 
 
124.0
 
 
 
 
ARL6IP6
 
 
hsa-miR-98-5p
 
 
-0.293
 
 
17.4
 
 
 
 
ARL6IP6
 
 
hsa-let-7c-5p
 
 
-0.293
 
 
17.4
 
 
 
 
ARL6IP6
 
 
hsa-let-7e-5p
 
 
-0.305
 
 
17.4
 
 
 
 
ARL8B
 
 
hsa-miR-148a-3p
 
 
-0.337
 
 
36.2
 
 
 
 
ARMC8
 
 
hsa-miR-106a-5p
 
 
-0.375
 
 
19.8
 
 
 
 
ARMC8
 
 
hsa-miR-20b-5p
 
 
-0.378
 
 
19.8
 
 
 
 
ARMT1
 
 
hsa-let-7c-5p
 
 
-0.303
 
 
19.7
 
 
 
 
ARMT1
 
 
hsa-let-7e-5p
 
 
-0.303
 
 
19.7
 
 
 
 
ARMT1
 
 
hsa-miR-98-5p
 
 
-0.303
 
 
19.7
 
 
 
 
ARPP19
 
 
hsa-let-7e-5p
 
 
-0.492
 
 
45.1
 
 
 
 
ARPP19
 
 
hsa-let-7c-5p
 
 
-0.492
 
 
45.1
 
 
 
 
ARPP19
 
 
hsa-miR-98-5p
 
 
-0.5
 
 
45.1
 
 
 
 
ARRDC3
 
 
hsa-miR-148a-3p
 
 
-0.305
 
 
27.5
 
 
 
 
ASAP1
 
 
hsa-let-7e-5p
 
 
-0.296
 
 
16.5
 
 
 
 
ASAP1
 
 
hsa-miR-98-5p
 
 
-0.308
 
 
16.5
 
 
 
 
ASAP1
 
 
hsa-let-7c-5p
 
 
-0.308
 
 
16.5
 
 
 
 
ASF1A
 
 
hsa-miR-21-5p
 
 
-0.304
 
 
14.9
 
 
 
 
ASPH
 
 
hsa-miR-148a-3p
 
 
-0.391
 
 
18.8
 
 
 
 
ATAD2
 
 
hsa-miR-20b-5p
 
 
-0.304
 
 
13.4
 
 
 
 
ATAD2
 
 
hsa-miR-106a-5p
 
 
-0.308
 
 
13.4
 
 
 
 
ATP2A2
 
 
hsa-let-7e-5p
 
 
-0.311
 
 
33.7
 
 
 
 
ATP2A2
 
 
hsa-miR-98-5p
 
 
-0.311
 
 
33.7
 
 
 
 
ATP2A2
 
 
hsa-let-7c-5p
 
 
-0.311
 
 
33.7
 
 
 
 
ATP2B4
 
 
hsa-miR-148a-3p
 
 
-0.337
 
 
12.0
 
 
 
 
ATP6AP2
 
 
hsa-miR-148a-3p
 
 
-0.592
 
 
63.1
 
 
 
 
ATP6AP2
 
 
hsa-miR-148a-3p
 
 
-0.668
 
 
63.1
 
 
 
 
ATPAF1
 
 
hsa-let-7e-5p
 
 
-0.498
 
 
30.3
 
 
 
 
ATPAF1
 
 
hsa-miR-98-5p
 
 
-0.498
 
 
30.3
 
 
 
 
ATPAF1
 
 
hsa-let-7c-5p
 
 
-0.498
 
 
30.3
 
 
 
 
B3GAT3
 
 
hsa-let-7e-5p
 
 
-0.324
 
 
24.9
 
 
 
 
B3GAT3
 
 
hsa-miR-98-5p
 
 
-0.324
 
 
24.9
 
 
 
 
B3GAT3
 
 
hsa-let-7c-5p
 
 
-0.324
 
 
24.9
 
 
 
 
BACH1
 
 
hsa-let-7e-5p
 
 
-0.298
 
 
15.7
 
 
 
 
BACH1
 
 
hsa-let-7e-5p
 
 
-0.298
 
 
15.7
 
 
 
 
BACH1
 
 
hsa-miR-98-5p
 
 
-0.298
 
 
15.7
 
 
 
 
BACH1
 
 
hsa-miR-98-5p
 
 
-0.298
 
 
15.7
 
 
 
 
BACH1
 
 
hsa-let-7c-5p
 
 
-0.306
 
 
15.7
 
 
 
 
BACH1
 
 
hsa-let-7c-5p
 
 
-0.306
 
 
15.7
 
 
 
 
BACH1
 
 
hsa-let-7e-5p
 
 
-0.435
 
 
15.7
 
 
 
 
BACH1
 
 
hsa-let-7e-5p
 
 
-0.435
 
 
15.7
 
 
 
 
BACH1
 
 
hsa-miR-98-5p
 
 
-0.435
 
 
15.7
 
 
 
 
BACH1
 
 
hsa-miR-98-5p
 
 
-0.435
 
 
15.7
 
 
 
 
BACH1
 
 
hsa-let-7c-5p
 
 
-0.435
 
 
15.7
 
 
 
 
BACH1
 
 
hsa-let-7c-5p
 
 
-0.435
 
 
15.7
 
 
 
 
BAK1
 
 
hsa-miR-125a-5p
 
 
-0.402
 
 
20.6
 
 
 
 
BAZ2A
 
 
hsa-miR-99b-5p
 
 
-0.408
 
 
31.2
 
 
 
 
BCAP29
 
 
hsa-let-7e-5p
 
 
-0.289
 
 
15.9
 
 
 
 
BCL2L11
 
 
hsa-miR-148a-3p
 
 
-0.445
 
 
23.7
 
 
 
 
BCL2L12
 
 
hsa-miR-125a-5p
 
 
-0.288
 
 
19.2
 
 
 
 
BCL7A
 
 
hsa-miR-21-5p
 
 
-0.31
 
 
15.1
 
 
 
 
BIN2
 
 
hsa-miR-125a-5p
 
 
-0.679
 
 
39.3
 
 
 
 
BIN3
 
 
hsa-let-7e-5p
 
 
-0.427
 
 
12.8
 
 
 
 
BIN3
 
 
hsa-miR-98-5p
 
 
-0.427
 
 
12.8
 
 
 
 
BIN3
 
 
hsa-let-7c-5p
 
 
-0.427
 
 
12.8
 
 
 
 
BLOC1S4
 
 
hsa-miR-7-5p
 
 
-0.417
 
 
11.4
 
 
 
 
BLOC1S4
 
 
hsa-miR-7-5p
 
 
-0.527
 
 
11.4
 
 
 
 
BLOC1S6
 
 
hsa-let-7e-5p
 
 
-0.321
 
 
39.2
 
 
 
 
BLOC1S6
 
 
hsa-miR-98-5p
 
 
-0.321
 
 
39.2
 
 
 
 
BLOC1S6
 
 
hsa-let-7c-5p
 
 
-0.321
 
 
39.2
 
 
 
 
BLZF1
 
 
hsa-miR-125a-5p
 
 
-0.461
 
 
11.1
 
 
 
 
BMF
 
 
hsa-miR-125a-5p
 
 
-0.34
 
 
12.8
 
 
 
 
 
 
 placenta 
 
 
 
 
target_symbol
 
 
mature_mirna_id
 
 
score
 
 
nTPM
 
 
 
 
 
 
ABCB10
 
 
hsa-miR-148a-3p
 
 
-0.291
 
 
10.6
 
 
 
 
ABCC5
 
 
hsa-miR-125a-5p
 
 
-0.288
 
 
16.1
 
 
 
 
ABCD3
 
 
hsa-miR-148a-3p
 
 
-0.388
 
 
13.6
 
 
 
 
ABHD3
 
 
hsa-miR-125a-5p
 
 
-0.453
 
 
18.6
 
 
 
 
ABTB1
 
 
hsa-miR-125a-5p
 
 
-0.519
 
 
11.5
 
 
 
 
ACP3
 
 
hsa-let-7e-5p
 
 
-0.298
 
 
10.1
 
 
 
 
ACP3
 
 
hsa-miR-98-5p
 
 
-0.298
 
 
10.1
 
 
 
 
ACP3
 
 
hsa-let-7c-5p
 
 
-0.298
 
 
10.1
 
 
 
 
ACVR1
 
 
hsa-miR-148a-3p
 
 
-0.397
 
 
12.5
 
 
 
 
ADAM10
 
 
hsa-miR-148a-3p
 
 
-0.356
 
 
27.1
 
 
 
 
ADAM17
 
 
hsa-miR-125a-5p
 
 
-0.292
 
 
17.9
 
 
 
 
ADAM17
 
 
hsa-miR-148a-3p
 
 
-0.301
 
 
17.9
 
 
 
 
ADAM9
 
 
hsa-miR-106a-5p
 
 
-0.317
 
 
44.6
 
 
 
 
ADAM9
 
 
hsa-miR-20b-5p
 
 
-0.328
 
 
44.6
 
 
 
 
ADGRF5
 
 
hsa-miR-148a-3p
 
 
-0.354
 
 
48.3
 
 
 
 
ADRB1
 
 
hsa-let-7c-5p
 
 
-0.351
 
 
27.4
 
 
 
 
ADRB1
 
 
hsa-let-7e-5p
 
 
-0.367
 
 
27.4
 
 
 
 
ADRB1
 
 
hsa-miR-98-5p
 
 
-0.367
 
 
27.4
 
 
 
 
AGFG1
 
 
hsa-miR-148a-3p
 
 
-0.332
 
 
71.2
 
 
 
 
AGGF1
 
 
hsa-miR-125a-5p
 
 
-0.331
 
 
10.3
 
 
 
 
AHNAK
 
 
hsa-miR-106a-5p
 
 
-0.334
 
 
91.4
 
 
 
 
AHNAK
 
 
hsa-miR-20b-5p
 
 
-0.337
 
 
91.4
 
 
 
 
ALCAM
 
 
hsa-miR-148a-3p
 
 
-0.339
 
 
11.3
 
 
 
 
ALDH4A1
 
 
hsa-miR-184
 
 
-0.421
 
 
12.4
 
 
 
 
ALG3
 
 
hsa-miR-106a-5p
 
 
-0.569
 
 
18.4
 
 
 
 
ALG3
 
 
hsa-miR-20b-5p
 
 
-0.58
 
 
18.4
 
 
 
 
ANAPC16
 
 
hsa-miR-125a-5p
 
 
-0.578
 
 
11.0
 
 
 
 
ANKRD12
 
 
hsa-miR-7-5p
 
 
-0.37
 
 
13.3
 
 
 
 
ANKRD46
 
 
hsa-let-7e-5p
 
 
-0.389
 
 
19.5
 
 
 
 
ANKRD46
 
 
hsa-miR-98-5p
 
 
-0.389
 
 
19.5
 
 
 
 
ANKRD46
 
 
hsa-let-7c-5p
 
 
-0.389
 
 
19.5
 
 
 
 
ANKRD50
 
 
hsa-miR-106a-5p
 
 
-0.29
 
 
13.9
 
 
 
 
ANPEP
 
 
hsa-miR-125a-5p
 
 
-0.372
 
 
31.1
 
 
 
 
AP1AR
 
 
hsa-miR-21-5p
 
 
-0.318
 
 
11.2
 
 
 
 
AP1AR
 
 
hsa-miR-99b-5p
 
 
-0.823
 
 
11.2
 
 
 
 
AP1S1
 
 
hsa-let-7e-5p
 
 
-0.374
 
 
14.9
 
 
 
 
AP1S1
 
 
hsa-miR-98-5p
 
 
-0.374
 
 
14.9
 
 
 
 
AP1S1
 
 
hsa-let-7c-5p
 
 
-0.374
 
 
14.9
 
 
 
 
APCDD1
 
 
hsa-miR-106a-5p
 
 
-0.422
 
 
25.2
 
 
 
 
APCDD1
 
 
hsa-miR-20b-5p
 
 
-0.425
 
 
25.2
 
 
 
 
ARF4
 
 
hsa-miR-7-5p
 
 
-0.407
 
 
170.7
 
 
 
 
ARF4
 
 
hsa-miR-148a-3p
 
 
-0.487
 
 
170.7
 
 
 
 
ARFGEF1
 
 
hsa-miR-148a-3p
 
 
-0.306
 
 
13.6
 
 
 
 
ARFIP1
 
 
hsa-miR-148a-3p
 
 
-0.474
 
 
16.9
 
 
 
 
ARHGAP1
 
 
hsa-miR-20b-5p
 
 
-0.325
 
 
36.7
 
 
 
 
ARHGAP1
 
 
hsa-miR-106a-5p
 
 
-0.329
 
 
36.7
 
 
 
 
ARHGAP21
 
 
hsa-miR-148a-3p
 
 
-0.287
 
 
15.6
 
 
 
 
ARHGAP24
 
 
hsa-miR-21-5p
 
 
-0.427
 
 
21.0
 
 
 
 
ARHGAP28
 
 
hsa-let-7c-5p
 
 
-0.47
 
 
24.3
 
 
 
 
ARHGAP28
 
 
hsa-miR-98-5p
 
 
-0.47
 
 
24.3
 
 
 
 
ARHGAP28
 
 
hsa-let-7e-5p
 
 
-0.486
 
 
24.3
 
 
 
 
ARID3A
 
 
hsa-miR-98-5p
 
 
-0.34
 
 
22.0
 
 
 
 
ARID3A
 
 
hsa-let-7c-5p
 
 
-0.34
 
 
22.0
 
 
 
 
ARID3A
 
 
hsa-let-7e-5p
 
 
-0.347
 
 
22.0
 
 
 
 
ARID3A
 
 
hsa-let-7c-5p
 
 
-0.347
 
 
22.0
 
 
 
 
ARID3A
 
 
hsa-let-7e-5p
 
 
-0.348
 
 
22.0
 
 
 
 
ARID3A
 
 
hsa-miR-98-5p
 
 
-0.358
 
 
22.0
 
 
 
 
ARID3B
 
 
hsa-let-7e-5p
 
 
-0.31
 
 
14.7
 
 
 
 
ARID3B
 
 
hsa-miR-98-5p
 
 
-0.31
 
 
14.7
 
 
 
 
ARID3B
 
 
hsa-let-7c-5p
 
 
-0.31
 
 
14.7
 
 
 
 
ARID3B
 
 
hsa-miR-98-5p
 
 
-0.475
 
 
14.7
 
 
 
 
ARID3B
 
 
hsa-let-7e-5p
 
 
-0.486
 
 
14.7
 
 
 
 
ARID3B
 
 
hsa-let-7c-5p
 
 
-0.486
 
 
14.7
 
 
 
 
ARL1
 
 
hsa-miR-20b-5p
 
 
-0.424
 
 
48.3
 
 
 
 
ARL1
 
 
hsa-miR-106a-5p
 
 
-0.429
 
 
48.3
 
 
 
 
ARL6IP1
 
 
hsa-miR-7-5p
 
 
-0.374
 
 
84.9
 
 
 
 
ARL6IP1
 
 
hsa-miR-148a-3p
 
 
-0.579
 
 
84.9
 
 
 
 
ARL6IP6
 
 
hsa-miR-98-5p
 
 
-0.293
 
 
26.8
 
 
 
 
ARL6IP6
 
 
hsa-let-7c-5p
 
 
-0.293
 
 
26.8
 
 
 
 
ARL6IP6
 
 
hsa-let-7e-5p
 
 
-0.305
 
 
26.8
 
 
 
 
ARL8B
 
 
hsa-miR-148a-3p
 
 
-0.337
 
 
46.8
 
 
 
 
ARMC8
 
 
hsa-miR-106a-5p
 
 
-0.375
 
 
18.5
 
 
 
 
ARMC8
 
 
hsa-miR-20b-5p
 
 
-0.378
 
 
18.5
 
 
 
 
ARMCX1
 
 
hsa-miR-21-5p
 
 
-0.489
 
 
14.0
 
 
 
 
ARMT1
 
 
hsa-let-7c-5p
 
 
-0.303
 
 
20.8
 
 
 
 
ARMT1
 
 
hsa-let-7e-5p
 
 
-0.303
 
 
20.8
 
 
 
 
ARMT1
 
 
hsa-miR-98-5p
 
 
-0.303
 
 
20.8
 
 
 
 
ARPP19
 
 
hsa-let-7e-5p
 
 
-0.492
 
 
53.5
 
 
 
 
ARPP19
 
 
hsa-let-7c-5p
 
 
-0.492
 
 
53.5
 
 
 
 
ARPP19
 
 
hsa-miR-98-5p
 
 
-0.5
 
 
53.5
 
 
 
 
ARRDC3
 
 
hsa-miR-148a-3p
 
 
-0.305
 
 
72.1
 
 
 
 
ARRDC4
 
 
hsa-let-7c-5p
 
 
-0.329
 
 
17.2
 
 
 
 
ARRDC4
 
 
hsa-let-7e-5p
 
 
-0.329
 
 
17.2
 
 
 
 
ARRDC4
 
 
hsa-miR-98-5p
 
 
-0.329
 
 
17.2
 
 
 
 
ASAP1
 
 
hsa-let-7e-5p
 
 
-0.296
 
 
25.9
 
 
 
 
ASAP1
 
 
hsa-miR-98-5p
 
 
-0.308
 
 
25.9
 
 
 
 
ASAP1
 
 
hsa-let-7c-5p
 
 
-0.308
 
 
25.9
 
 
 
 
ASPH
 
 
hsa-miR-148a-3p
 
 
-0.391
 
 
44.6
 
 
 
 
ATP10D
 
 
hsa-miR-125a-5p
 
 
-0.375
 
 
14.0
 
 
 
 
ATP2A2
 
 
hsa-let-7e-5p
 
 
-0.311
 
 
52.0
 
 
 
 
ATP2A2
 
 
hsa-miR-98-5p
 
 
-0.311
 
 
52.0
 
 
 
 
ATP2A2
 
 
hsa-let-7c-5p
 
 
-0.311
 
 
52.0
 
 
 
 
ATP2B4
 
 
hsa-miR-148a-3p
 
 
-0.337
 
 
38.2
 
 
 
 
ATP6AP2
 
 
hsa-miR-148a-3p
 
 
-0.592
 
 
100.4
 
 
 
 
ATP6AP2
 
 
hsa-miR-148a-3p
 
 
-0.668
 
 
100.4
 
 
 
 
ATPAF1
 
 
hsa-let-7e-5p
 
 
-0.498
 
 
34.1
 
 
 
 
ATPAF1
 
 
hsa-miR-98-5p
 
 
-0.498
 
 
34.1
 
 
 
 
ATPAF1
 
 
hsa-let-7c-5p
 
 
-0.498
 
 
34.1
 
 
 
 
AVPI1
 
 
hsa-miR-7-5p
 
 
-0.367
 
 
15.9
 
 
 
 
B3GAT3
 
 
hsa-let-7e-5p
 
 
-0.324
 
 
18.0
 
 
 
 
 
 
 rectum 
 
 
 
 
target_symbol
 
 
mature_mirna_id
 
 
score
 
 
nTPM
 
 
 
 
 
 
ABCB10
 
 
hsa-miR-148a-3p
 
 
-0.291
 
 
10.6
 
 
 
 
ABCC5
 
 
hsa-miR-125a-5p
 
 
-0.288
 
 
20.8
 
 
 
 
ABCD3
 
 
hsa-miR-148a-3p
 
 
-0.388
 
 
37.8
 
 
 
 
ABHD17C
 
 
hsa-let-7c-5p
 
 
-0.359
 
 
52.8
 
 
 
 
ABHD17C
 
 
hsa-let-7e-5p
 
 
-0.359
 
 
52.8
 
 
 
 
ABHD17C
 
 
hsa-miR-98-5p
 
 
-0.383
 
 
52.8
 
 
 
 
ABHD3
 
 
hsa-miR-125a-5p
 
 
-0.453
 
 
61.7
 
 
 
 
ABHD6
 
 
hsa-miR-125a-5p
 
 
-0.501
 
 
10.4
 
 
 
 
ADAM10
 
 
hsa-miR-148a-3p
 
 
-0.356
 
 
16.1
 
 
 
 
ADAM9
 
 
hsa-miR-106a-5p
 
 
-0.317
 
 
39.2
 
 
 
 
ADAM9
 
 
hsa-miR-20b-5p
 
 
-0.328
 
 
39.2
 
 
 
 
AGFG1
 
 
hsa-miR-148a-3p
 
 
-0.332
 
 
31.0
 
 
 
 
AGGF1
 
 
hsa-miR-125a-5p
 
 
-0.331
 
 
10.9
 
 
 
 
AHNAK
 
 
hsa-miR-106a-5p
 
 
-0.334
 
 
90.8
 
 
 
 
AHNAK
 
 
hsa-miR-20b-5p
 
 
-0.337
 
 
90.8
 
 
 
 
AIFM1
 
 
hsa-miR-125a-5p
 
 
-0.3
 
 
14.9
 
 
 
 
AIFM1
 
 
hsa-let-7e-5p
 
 
-0.314
 
 
14.9
 
 
 
 
AIFM1
 
 
hsa-miR-98-5p
 
 
-0.314
 
 
14.9
 
 
 
 
AIFM1
 
 
hsa-let-7c-5p
 
 
-0.314
 
 
14.9
 
 
 
 
AKAP1
 
 
hsa-miR-148a-3p
 
 
-0.381
 
 
45.9
 
 
 
 
ALCAM
 
 
hsa-miR-148a-3p
 
 
-0.339
 
 
13.6
 
 
 
 
ALG3
 
 
hsa-miR-106a-5p
 
 
-0.569
 
 
17.2
 
 
 
 
ALG3
 
 
hsa-miR-20b-5p
 
 
-0.58
 
 
17.2
 
 
 
 
AMT
 
 
hsa-let-7e-5p
 
 
-0.463
 
 
11.1
 
 
 
 
AMT
 
 
hsa-miR-98-5p
 
 
-0.463
 
 
11.1
 
 
 
 
AMT
 
 
hsa-let-7c-5p
 
 
-0.463
 
 
11.1
 
 
 
 
ANAPC16
 
 
hsa-miR-125a-5p
 
 
-0.578
 
 
16.4
 
 
 
 
ANKRD12
 
 
hsa-miR-7-5p
 
 
-0.37
 
 
11.3
 
 
 
 
ANPEP
 
 
hsa-miR-125a-5p
 
 
-0.372
 
 
56.3
 
 
 
 
AP1AR
 
 
hsa-miR-21-5p
 
 
-0.318
 
 
15.3
 
 
 
 
AP1AR
 
 
hsa-miR-99b-5p
 
 
-0.823
 
 
15.3
 
 
 
 
AP1S1
 
 
hsa-let-7e-5p
 
 
-0.374
 
 
21.1
 
 
 
 
AP1S1
 
 
hsa-miR-98-5p
 
 
-0.374
 
 
21.1
 
 
 
 
AP1S1
 
 
hsa-let-7c-5p
 
 
-0.374
 
 
21.1
 
 
 
 
APCDD1
 
 
hsa-miR-106a-5p
 
 
-0.422
 
 
12.4
 
 
 
 
APCDD1
 
 
hsa-miR-20b-5p
 
 
-0.425
 
 
12.4
 
 
 
 
ARF4
 
 
hsa-miR-7-5p
 
 
-0.407
 
 
185.2
 
 
 
 
ARF4
 
 
hsa-miR-148a-3p
 
 
-0.487
 
 
185.2
 
 
 
 
ARFGEF1
 
 
hsa-miR-148a-3p
 
 
-0.306
 
 
16.1
 
 
 
 
ARFIP1
 
 
hsa-miR-148a-3p
 
 
-0.474
 
 
26.2
 
 
 
 
ARHGAP1
 
 
hsa-miR-20b-5p
 
 
-0.325
 
 
30.6
 
 
 
 
ARHGAP1
 
 
hsa-miR-106a-5p
 
 
-0.329
 
 
30.6
 
 
 
 
ARHGAP21
 
 
hsa-miR-148a-3p
 
 
-0.287
 
 
17.4
 
 
 
 
ARL1
 
 
hsa-miR-20b-5p
 
 
-0.424
 
 
56.8
 
 
 
 
ARL1
 
 
hsa-miR-106a-5p
 
 
-0.429
 
 
56.8
 
 
 
 
ARL6IP1
 
 
hsa-miR-7-5p
 
 
-0.374
 
 
116.0
 
 
 
 
ARL6IP1
 
 
hsa-miR-148a-3p
 
 
-0.579
 
 
116.0
 
 
 
 
ARL6IP6
 
 
hsa-miR-98-5p
 
 
-0.293
 
 
13.3
 
 
 
 
ARL6IP6
 
 
hsa-let-7c-5p
 
 
-0.293
 
 
13.3
 
 
 
 
ARL6IP6
 
 
hsa-let-7e-5p
 
 
-0.305
 
 
13.3
 
 
 
 
ARL8B
 
 
hsa-miR-148a-3p
 
 
-0.337
 
 
32.7
 
 
 
 
ARMC8
 
 
hsa-miR-106a-5p
 
 
-0.375
 
 
17.2
 
 
 
 
ARMC8
 
 
hsa-miR-20b-5p
 
 
-0.378
 
 
17.2
 
 
 
 
ARMT1
 
 
hsa-let-7c-5p
 
 
-0.303
 
 
16.5
 
 
 
 
ARMT1
 
 
hsa-let-7e-5p
 
 
-0.303
 
 
16.5
 
 
 
 
ARMT1
 
 
hsa-miR-98-5p
 
 
-0.303
 
 
16.5
 
 
 
 
ARPP19
 
 
hsa-let-7e-5p
 
 
-0.492
 
 
33.6
 
 
 
 
ARPP19
 
 
hsa-let-7c-5p
 
 
-0.492
 
 
33.6
 
 
 
 
ARPP19
 
 
hsa-miR-98-5p
 
 
-0.5
 
 
33.6
 
 
 
 
ARRDC3
 
 
hsa-miR-148a-3p
 
 
-0.305
 
 
25.8
 
 
 
 
ARRDC4
 
 
hsa-let-7c-5p
 
 
-0.329
 
 
21.6
 
 
 
 
ARRDC4
 
 
hsa-let-7e-5p
 
 
-0.329
 
 
21.6
 
 
 
 
ARRDC4
 
 
hsa-miR-98-5p
 
 
-0.329
 
 
21.6
 
 
 
 
ASF1A
 
 
hsa-miR-21-5p
 
 
-0.304
 
 
10.2
 
 
 
 
ASPH
 
 
hsa-miR-148a-3p
 
 
-0.391
 
 
77.9
 
 
 
 
ATG4A
 
 
hsa-miR-7-5p
 
 
-0.448
 
 
12.3
 
 
 
 
ATP2A2
 
 
hsa-let-7e-5p
 
 
-0.311
 
 
64.9
 
 
 
 
ATP2A2
 
 
hsa-miR-98-5p
 
 
-0.311
 
 
64.9
 
 
 
 
ATP2A2
 
 
hsa-let-7c-5p
 
 
-0.311
 
 
64.9
 
 
 
 
ATP2B4
 
 
hsa-miR-148a-3p
 
 
-0.337
 
 
28.6
 
 
 
 
ATP6AP2
 
 
hsa-miR-148a-3p
 
 
-0.592
 
 
83.6
 
 
 
 
ATP6AP2
 
 
hsa-miR-148a-3p
 
 
-0.668
 
 
83.6
 
 
 
 
ATPAF1
 
 
hsa-let-7e-5p
 
 
-0.498
 
 
50.0
 
 
 
 
ATPAF1
 
 
hsa-miR-98-5p
 
 
-0.498
 
 
50.0
 
 
 
 
ATPAF1
 
 
hsa-let-7c-5p
 
 
-0.498
 
 
50.0
 
 
 
 
AVPI1
 
 
hsa-miR-7-5p
 
 
-0.367
 
 
17.8
 
 
 
 
B4GALT5
 
 
hsa-miR-148a-3p
 
 
-0.44
 
 
22.3
 
 
 
 
BACH1
 
 
hsa-let-7e-5p
 
 
-0.298
 
 
12.4
 
 
 
 
BACH1
 
 
hsa-let-7e-5p
 
 
-0.298
 
 
12.4
 
 
 
 
BACH1
 
 
hsa-miR-98-5p
 
 
-0.298
 
 
12.4
 
 
 
 
BACH1
 
 
hsa-miR-98-5p
 
 
-0.298
 
 
12.4
 
 
 
 
BACH1
 
 
hsa-let-7c-5p
 
 
-0.306
 
 
12.4
 
 
 
 
BACH1
 
 
hsa-let-7c-5p
 
 
-0.306
 
 
12.4
 
 
 
 
BACH1
 
 
hsa-let-7e-5p
 
 
-0.435
 
 
12.4
 
 
 
 
BACH1
 
 
hsa-let-7e-5p
 
 
-0.435
 
 
12.4
 
 
 
 
BACH1
 
 
hsa-miR-98-5p
 
 
-0.435
 
 
12.4
 
 
 
 
BACH1
 
 
hsa-miR-98-5p
 
 
-0.435
 
 
12.4
 
 
 
 
BACH1
 
 
hsa-let-7c-5p
 
 
-0.435
 
 
12.4
 
 
 
 
BACH1
 
 
hsa-let-7c-5p
 
 
-0.435
 
 
12.4
 
 
 
 
BAK1
 
 
hsa-miR-125a-5p
 
 
-0.402
 
 
23.4
 
 
 
 
BAZ2A
 
 
hsa-miR-99b-5p
 
 
-0.408
 
 
24.9
 
 
 
 
BCAP29
 
 
hsa-let-7e-5p
 
 
-0.289
 
 
30.4
 
 
 
 
BCL2L11
 
 
hsa-miR-148a-3p
 
 
-0.445
 
 
11.2
 
 
 
 
BDH1
 
 
hsa-miR-125a-5p
 
 
-0.389
 
 
18.6
 
 
 
 
BHLHE41
 
 
hsa-miR-148a-3p
 
 
-0.442
 
 
10.4
 
 
 
 
BLOC1S6
 
 
hsa-let-7e-5p
 
 
-0.321
 
 
20.2
 
 
 
 
BLOC1S6
 
 
hsa-miR-98-5p
 
 
-0.321
 
 
20.2
 
 
 
 
BLOC1S6
 
 
hsa-let-7c-5p
 
 
-0.321
 
 
20.2
 
 
 
 
BLZF1
 
 
hsa-miR-125a-5p
 
 
-0.461
 
 
14.6
 
 
 
 
BTAF1
 
 
hsa-miR-148a-3p
 
 
-0.401
 
 
12.5
 
 
 
 
 
 
 skeletal muscle 
 
 
 
 
target_symbol
 
 
mature_mirna_id
 
 
score
 
 
nTPM
 
 
 
 
 
 
ABCB10
 
 
hsa-miR-148a-3p
 
 
-0.291
 
 
12.0
 
 
 
 
ABCB7
 
 
hsa-miR-148a-3p
 
 
-0.515
 
 
29.2
 
 
 
 
ABCC5
 
 
hsa-miR-125a-5p
 
 
-0.288
 
 
118.1
 
 
 
 
ABCD3
 
 
hsa-miR-148a-3p
 
 
-0.388
 
 
22.0
 
 
 
 
ABHD3
 
 
hsa-miR-125a-5p
 
 
-0.453
 
 
13.6
 
 
 
 
ABHD5
 
 
hsa-miR-20b-5p
 
 
-0.418
 
 
12.3
 
 
 
 
ABHD5
 
 
hsa-miR-106a-5p
 
 
-0.447
 
 
12.3
 
 
 
 
ABTB1
 
 
hsa-miR-125a-5p
 
 
-0.519
 
 
44.0
 
 
 
 
ACHE
 
 
hsa-miR-125a-5p
 
 
-0.308
 
 
152.9
 
 
 
 
ACHE
 
 
hsa-miR-125a-5p
 
 
-0.361
 
 
152.9
 
 
 
 
ACTC1
 
 
hsa-miR-7-5p
 
 
-0.471
 
 
519.7
 
 
 
 
ACVR1
 
 
hsa-miR-148a-3p
 
 
-0.397
 
 
15.5
 
 
 
 
ACVR2A
 
 
hsa-miR-125a-5p
 
 
-0.301
 
 
16.4
 
 
 
 
ADAM9
 
 
hsa-miR-106a-5p
 
 
-0.317
 
 
16.2
 
 
 
 
ADAM9
 
 
hsa-miR-20b-5p
 
 
-0.328
 
 
16.2
 
 
 
 
ADCY2
 
 
hsa-miR-148a-3p
 
 
-0.299
 
 
64.1
 
 
 
 
ADGRF5
 
 
hsa-miR-148a-3p
 
 
-0.354
 
 
29.0
 
 
 
 
ADRB2
 
 
hsa-let-7c-5p
 
 
-0.635
 
 
10.9
 
 
 
 
ADRB2
 
 
hsa-let-7e-5p
 
 
-0.635
 
 
10.9
 
 
 
 
ADRB2
 
 
hsa-miR-98-5p
 
 
-0.635
 
 
10.9
 
 
 
 
AGFG1
 
 
hsa-miR-148a-3p
 
 
-0.332
 
 
31.6
 
 
 
 
AGGF1
 
 
hsa-miR-125a-5p
 
 
-0.331
 
 
12.2
 
 
 
 
AGO2
 
 
hsa-miR-21-5p
 
 
-0.632
 
 
16.4
 
 
 
 
AGO2
 
 
hsa-miR-125a-5p
 
 
-0.811
 
 
16.4
 
 
 
 
AGO2
 
 
hsa-miR-184
 
 
-0.831
 
 
16.4
 
 
 
 
AGO2
 
 
hsa-miR-99b-5p
 
 
-0.836
 
 
16.4
 
 
 
 
AHCTF1
 
 
hsa-let-7e-5p
 
 
-0.374
 
 
10.0
 
 
 
 
AHCTF1
 
 
hsa-miR-98-5p
 
 
-0.374
 
 
10.0
 
 
 
 
AHCTF1
 
 
hsa-let-7c-5p
 
 
-0.374
 
 
10.0
 
 
 
 
AHDC1
 
 
hsa-miR-148a-3p
 
 
-0.413
 
 
29.2
 
 
 
 
AHNAK
 
 
hsa-miR-106a-5p
 
 
-0.334
 
 
178.0
 
 
 
 
AHNAK
 
 
hsa-miR-20b-5p
 
 
-0.337
 
 
178.0
 
 
 
 
AIFM1
 
 
hsa-miR-125a-5p
 
 
-0.3
 
 
58.6
 
 
 
 
AIFM1
 
 
hsa-let-7e-5p
 
 
-0.314
 
 
58.6
 
 
 
 
AIFM1
 
 
hsa-miR-98-5p
 
 
-0.314
 
 
58.6
 
 
 
 
AIFM1
 
 
hsa-let-7c-5p
 
 
-0.314
 
 
58.6
 
 
 
 
AKAP1
 
 
hsa-miR-148a-3p
 
 
-0.381
 
 
194.7
 
 
 
 
AKAP6
 
 
hsa-let-7e-5p
 
 
-0.307
 
 
44.4
 
 
 
 
AKAP6
 
 
hsa-miR-98-5p
 
 
-0.307
 
 
44.4
 
 
 
 
AKAP6
 
 
hsa-let-7c-5p
 
 
-0.307
 
 
44.4
 
 
 
 
ALDH4A1
 
 
hsa-miR-184
 
 
-0.421
 
 
69.5
 
 
 
 
ALG3
 
 
hsa-miR-106a-5p
 
 
-0.569
 
 
29.0
 
 
 
 
ALG3
 
 
hsa-miR-20b-5p
 
 
-0.58
 
 
29.0
 
 
 
 
ANAPC16
 
 
hsa-miR-125a-5p
 
 
-0.578
 
 
57.2
 
 
 
 
ANKRD12
 
 
hsa-miR-7-5p
 
 
-0.37
 
 
25.1
 
 
 
 
ANKRD46
 
 
hsa-let-7e-5p
 
 
-0.389
 
 
58.3
 
 
 
 
ANKRD46
 
 
hsa-miR-98-5p
 
 
-0.389
 
 
58.3
 
 
 
 
ANKRD46
 
 
hsa-let-7c-5p
 
 
-0.389
 
 
58.3
 
 
 
 
ANKRD9
 
 
hsa-miR-20b-5p
 
 
-0.331
 
 
148.8
 
 
 
 
ANKRD9
 
 
hsa-miR-106a-5p
 
 
-0.344
 
 
148.8
 
 
 
 
ARF4
 
 
hsa-miR-7-5p
 
 
-0.407
 
 
59.5
 
 
 
 
ARF4
 
 
hsa-miR-148a-3p
 
 
-0.487
 
 
59.5
 
 
 
 
ARFGEF1
 
 
hsa-miR-148a-3p
 
 
-0.306
 
 
12.2
 
 
 
 
ARG2
 
 
hsa-let-7e-5p
 
 
-0.468
 
 
37.4
 
 
 
 
ARG2
 
 
hsa-miR-98-5p
 
 
-0.468
 
 
37.4
 
 
 
 
ARG2
 
 
hsa-let-7c-5p
 
 
-0.468
 
 
37.4
 
 
 
 
ARHGAP1
 
 
hsa-miR-20b-5p
 
 
-0.325
 
 
28.5
 
 
 
 
ARHGAP1
 
 
hsa-miR-106a-5p
 
 
-0.329
 
 
28.5
 
 
 
 
ARHGAP21
 
 
hsa-miR-148a-3p
 
 
-0.287
 
 
15.7
 
 
 
 
ARL1
 
 
hsa-miR-20b-5p
 
 
-0.424
 
 
48.7
 
 
 
 
ARL1
 
 
hsa-miR-106a-5p
 
 
-0.429
 
 
48.7
 
 
 
 
ARL6IP1
 
 
hsa-miR-7-5p
 
 
-0.374
 
 
21.6
 
 
 
 
ARL6IP1
 
 
hsa-miR-148a-3p
 
 
-0.579
 
 
21.6
 
 
 
 
ARL6IP6
 
 
hsa-miR-98-5p
 
 
-0.293
 
 
53.9
 
 
 
 
ARL6IP6
 
 
hsa-let-7c-5p
 
 
-0.293
 
 
53.9
 
 
 
 
ARL6IP6
 
 
hsa-let-7e-5p
 
 
-0.305
 
 
53.9
 
 
 
 
ARL8B
 
 
hsa-miR-148a-3p
 
 
-0.337
 
 
98.5
 
 
 
 
ARMC8
 
 
hsa-miR-106a-5p
 
 
-0.375
 
 
36.6
 
 
 
 
ARMC8
 
 
hsa-miR-20b-5p
 
 
-0.378
 
 
36.6
 
 
 
 
ARMCX1
 
 
hsa-miR-21-5p
 
 
-0.489
 
 
25.9
 
 
 
 
ARMT1
 
 
hsa-let-7c-5p
 
 
-0.303
 
 
17.8
 
 
 
 
ARMT1
 
 
hsa-let-7e-5p
 
 
-0.303
 
 
17.8
 
 
 
 
ARMT1
 
 
hsa-miR-98-5p
 
 
-0.303
 
 
17.8
 
 
 
 
ARPP19
 
 
hsa-let-7e-5p
 
 
-0.492
 
 
225.3
 
 
 
 
ARPP19
 
 
hsa-let-7c-5p
 
 
-0.492
 
 
225.3
 
 
 
 
ARPP19
 
 
hsa-miR-98-5p
 
 
-0.5
 
 
225.3
 
 
 
 
ARRDC3
 
 
hsa-miR-148a-3p
 
 
-0.305
 
 
31.8
 
 
 
 
ARRDC4
 
 
hsa-let-7c-5p
 
 
-0.329
 
 
13.8
 
 
 
 
ARRDC4
 
 
hsa-let-7e-5p
 
 
-0.329
 
 
13.8
 
 
 
 
ARRDC4
 
 
hsa-miR-98-5p
 
 
-0.329
 
 
13.8
 
 
 
 
ASAP1
 
 
hsa-let-7e-5p
 
 
-0.296
 
 
12.4
 
 
 
 
ASAP1
 
 
hsa-miR-98-5p
 
 
-0.308
 
 
12.4
 
 
 
 
ASAP1
 
 
hsa-let-7c-5p
 
 
-0.308
 
 
12.4
 
 
 
 
ASF1A
 
 
hsa-miR-21-5p
 
 
-0.304
 
 
15.6
 
 
 
 
ASPH
 
 
hsa-miR-148a-3p
 
 
-0.391
 
 
331.0
 
 
 
 
ATG16L1
 
 
hsa-miR-106a-5p
 
 
-0.464
 
 
20.1
 
 
 
 
ATG16L1
 
 
hsa-miR-20b-5p
 
 
-0.467
 
 
20.1
 
 
 
 
ATG4A
 
 
hsa-miR-7-5p
 
 
-0.448
 
 
18.0
 
 
 
 
ATG4D
 
 
hsa-miR-148a-3p
 
 
-0.303
 
 
35.5
 
 
 
 
ATOSB
 
 
hsa-let-7e-5p
 
 
-0.429
 
 
13.5
 
 
 
 
ATOSB
 
 
hsa-miR-98-5p
 
 
-0.429
 
 
13.5
 
 
 
 
ATOSB
 
 
hsa-let-7c-5p
 
 
-0.429
 
 
13.5
 
 
 
 
ATP2A2
 
 
hsa-let-7e-5p
 
 
-0.311
 
 
2675.7
 
 
 
 
ATP2A2
 
 
hsa-miR-98-5p
 
 
-0.311
 
 
2675.7
 
 
 
 
ATP2A2
 
 
hsa-let-7c-5p
 
 
-0.311
 
 
2675.7
 
 
 
 
ATP2B2
 
 
hsa-miR-7-5p
 
 
-0.297
 
 
35.1
 
 
 
 
ATP2B4
 
 
hsa-miR-148a-3p
 
 
-0.337
 
 
13.5
 
 
 
 
ATP6AP2
 
 
hsa-miR-148a-3p
 
 
-0.592
 
 
20.5
 
 
 
 
ATP6AP2
 
 
hsa-miR-148a-3p
 
 
-0.668
 
 
20.5
 
 
 
 
ATPAF1
 
 
hsa-let-7e-5p
 
 
-0.498
 
 
260.2
 
 
 
 
 
 
 small intestine 
 
 
 
 
target_symbol
 
 
mature_mirna_id
 
 
score
 
 
nTPM
 
 
 
 
 
 
ABCB10
 
 
hsa-miR-148a-3p
 
 
-0.291
 
 
13.1
 
 
 
 
ABCB7
 
 
hsa-miR-148a-3p
 
 
-0.515
 
 
11.2
 
 
 
 
ABCC5
 
 
hsa-miR-125a-5p
 
 
-0.288
 
 
22.9
 
 
 
 
ABCD3
 
 
hsa-miR-148a-3p
 
 
-0.388
 
 
43.4
 
 
 
 
ABHD17C
 
 
hsa-let-7c-5p
 
 
-0.359
 
 
28.8
 
 
 
 
ABHD17C
 
 
hsa-let-7e-5p
 
 
-0.359
 
 
28.8
 
 
 
 
ABHD17C
 
 
hsa-miR-98-5p
 
 
-0.383
 
 
28.8
 
 
 
 
ABHD3
 
 
hsa-miR-125a-5p
 
 
-0.453
 
 
53.0
 
 
 
 
ABHD6
 
 
hsa-miR-125a-5p
 
 
-0.501
 
 
49.2
 
 
 
 
ABTB1
 
 
hsa-miR-125a-5p
 
 
-0.519
 
 
31.9
 
 
 
 
ADAM10
 
 
hsa-miR-148a-3p
 
 
-0.356
 
 
15.8
 
 
 
 
ADAM9
 
 
hsa-miR-106a-5p
 
 
-0.317
 
 
21.4
 
 
 
 
ADAM9
 
 
hsa-miR-20b-5p
 
 
-0.328
 
 
21.4
 
 
 
 
AGFG1
 
 
hsa-miR-148a-3p
 
 
-0.332
 
 
33.9
 
 
 
 
AGFG2
 
 
hsa-miR-20b-5p
 
 
-0.386
 
 
12.5
 
 
 
 
AGFG2
 
 
hsa-miR-106a-5p
 
 
-0.399
 
 
12.5
 
 
 
 
AGGF1
 
 
hsa-miR-125a-5p
 
 
-0.331
 
 
12.5
 
 
 
 
AHNAK
 
 
hsa-miR-106a-5p
 
 
-0.334
 
 
132.1
 
 
 
 
AHNAK
 
 
hsa-miR-20b-5p
 
 
-0.337
 
 
132.1
 
 
 
 
AIFM1
 
 
hsa-miR-125a-5p
 
 
-0.3
 
 
28.2
 
 
 
 
AIFM1
 
 
hsa-let-7e-5p
 
 
-0.314
 
 
28.2
 
 
 
 
AIFM1
 
 
hsa-miR-98-5p
 
 
-0.314
 
 
28.2
 
 
 
 
AIFM1
 
 
hsa-let-7c-5p
 
 
-0.314
 
 
28.2
 
 
 
 
AKAP1
 
 
hsa-miR-148a-3p
 
 
-0.381
 
 
52.5
 
 
 
 
ALCAM
 
 
hsa-miR-148a-3p
 
 
-0.339
 
 
16.4
 
 
 
 
ALG3
 
 
hsa-miR-106a-5p
 
 
-0.569
 
 
19.2
 
 
 
 
ALG3
 
 
hsa-miR-20b-5p
 
 
-0.58
 
 
19.2
 
 
 
 
AMT
 
 
hsa-let-7e-5p
 
 
-0.463
 
 
11.5
 
 
 
 
AMT
 
 
hsa-miR-98-5p
 
 
-0.463
 
 
11.5
 
 
 
 
AMT
 
 
hsa-let-7c-5p
 
 
-0.463
 
 
11.5
 
 
 
 
ANAPC16
 
 
hsa-miR-125a-5p
 
 
-0.578
 
 
19.0
 
 
 
 
ANKRD12
 
 
hsa-miR-7-5p
 
 
-0.37
 
 
12.6
 
 
 
 
ANKRD46
 
 
hsa-let-7e-5p
 
 
-0.389
 
 
10.4
 
 
 
 
ANKRD46
 
 
hsa-miR-98-5p
 
 
-0.389
 
 
10.4
 
 
 
 
ANKRD46
 
 
hsa-let-7c-5p
 
 
-0.389
 
 
10.4
 
 
 
 
ANKRD9
 
 
hsa-miR-20b-5p
 
 
-0.331
 
 
31.1
 
 
 
 
ANKRD9
 
 
hsa-miR-106a-5p
 
 
-0.344
 
 
31.1
 
 
 
 
ANPEP
 
 
hsa-miR-125a-5p
 
 
-0.372
 
 
1857.1
 
 
 
 
AP1AR
 
 
hsa-miR-21-5p
 
 
-0.318
 
 
22.4
 
 
 
 
AP1AR
 
 
hsa-miR-99b-5p
 
 
-0.823
 
 
22.4
 
 
 
 
AP1S1
 
 
hsa-let-7e-5p
 
 
-0.374
 
 
29.8
 
 
 
 
AP1S1
 
 
hsa-miR-98-5p
 
 
-0.374
 
 
29.8
 
 
 
 
AP1S1
 
 
hsa-let-7c-5p
 
 
-0.374
 
 
29.8
 
 
 
 
APCDD1
 
 
hsa-miR-106a-5p
 
 
-0.422
 
 
13.7
 
 
 
 
APCDD1
 
 
hsa-miR-20b-5p
 
 
-0.425
 
 
13.7
 
 
 
 
ARF4
 
 
hsa-miR-7-5p
 
 
-0.407
 
 
102.8
 
 
 
 
ARF4
 
 
hsa-miR-148a-3p
 
 
-0.487
 
 
102.8
 
 
 
 
ARFGEF1
 
 
hsa-miR-148a-3p
 
 
-0.306
 
 
16.5
 
 
 
 
ARFIP1
 
 
hsa-miR-148a-3p
 
 
-0.474
 
 
20.0
 
 
 
 
ARG2
 
 
hsa-let-7e-5p
 
 
-0.468
 
 
14.4
 
 
 
 
ARG2
 
 
hsa-miR-98-5p
 
 
-0.468
 
 
14.4
 
 
 
 
ARG2
 
 
hsa-let-7c-5p
 
 
-0.468
 
 
14.4
 
 
 
 
ARHGAP1
 
 
hsa-miR-20b-5p
 
 
-0.325
 
 
44.5
 
 
 
 
ARHGAP1
 
 
hsa-miR-106a-5p
 
 
-0.329
 
 
44.5
 
 
 
 
ARHGAP21
 
 
hsa-miR-148a-3p
 
 
-0.287
 
 
15.2
 
 
 
 
ARL1
 
 
hsa-miR-20b-5p
 
 
-0.424
 
 
40.7
 
 
 
 
ARL1
 
 
hsa-miR-106a-5p
 
 
-0.429
 
 
40.7
 
 
 
 
ARL6IP1
 
 
hsa-miR-7-5p
 
 
-0.374
 
 
116.2
 
 
 
 
ARL6IP1
 
 
hsa-miR-148a-3p
 
 
-0.579
 
 
116.2
 
 
 
 
ARL8B
 
 
hsa-miR-148a-3p
 
 
-0.337
 
 
32.3
 
 
 
 
ARMC8
 
 
hsa-miR-106a-5p
 
 
-0.375
 
 
12.8
 
 
 
 
ARMC8
 
 
hsa-miR-20b-5p
 
 
-0.378
 
 
12.8
 
 
 
 
ARMCX1
 
 
hsa-miR-21-5p
 
 
-0.489
 
 
11.5
 
 
 
 
ARMT1
 
 
hsa-let-7c-5p
 
 
-0.303
 
 
13.7
 
 
 
 
ARMT1
 
 
hsa-let-7e-5p
 
 
-0.303
 
 
13.7
 
 
 
 
ARMT1
 
 
hsa-miR-98-5p
 
 
-0.303
 
 
13.7
 
 
 
 
ARPP19
 
 
hsa-let-7e-5p
 
 
-0.492
 
 
28.8
 
 
 
 
ARPP19
 
 
hsa-let-7c-5p
 
 
-0.492
 
 
28.8
 
 
 
 
ARPP19
 
 
hsa-miR-98-5p
 
 
-0.5
 
 
28.8
 
 
 
 
ARRDC3
 
 
hsa-miR-148a-3p
 
 
-0.305
 
 
15.8
 
 
 
 
ARRDC4
 
 
hsa-let-7c-5p
 
 
-0.329
 
 
22.0
 
 
 
 
ARRDC4
 
 
hsa-let-7e-5p
 
 
-0.329
 
 
22.0
 
 
 
 
ARRDC4
 
 
hsa-miR-98-5p
 
 
-0.329
 
 
22.0
 
 
 
 
ASAP1
 
 
hsa-let-7e-5p
 
 
-0.296
 
 
11.5
 
 
 
 
ASAP1
 
 
hsa-miR-98-5p
 
 
-0.308
 
 
11.5
 
 
 
 
ASAP1
 
 
hsa-let-7c-5p
 
 
-0.308
 
 
11.5
 
 
 
 
ASPH
 
 
hsa-miR-148a-3p
 
 
-0.391
 
 
42.6
 
 
 
 
ATG16L1
 
 
hsa-miR-106a-5p
 
 
-0.464
 
 
11.3
 
 
 
 
ATG16L1
 
 
hsa-miR-20b-5p
 
 
-0.467
 
 
11.3
 
 
 
 
ATG4A
 
 
hsa-miR-7-5p
 
 
-0.448
 
 
10.7
 
 
 
 
ATG4D
 
 
hsa-miR-148a-3p
 
 
-0.303
 
 
21.8
 
 
 
 
ATOSB
 
 
hsa-let-7e-5p
 
 
-0.429
 
 
10.7
 
 
 
 
ATOSB
 
 
hsa-miR-98-5p
 
 
-0.429
 
 
10.7
 
 
 
 
ATOSB
 
 
hsa-let-7c-5p
 
 
-0.429
 
 
10.7
 
 
 
 
ATP2A2
 
 
hsa-let-7e-5p
 
 
-0.311
 
 
53.2
 
 
 
 
ATP2A2
 
 
hsa-miR-98-5p
 
 
-0.311
 
 
53.2
 
 
 
 
ATP2A2
 
 
hsa-let-7c-5p
 
 
-0.311
 
 
53.2
 
 
 
 
ATP2B4
 
 
hsa-miR-148a-3p
 
 
-0.337
 
 
13.8
 
 
 
 
ATP6AP2
 
 
hsa-miR-148a-3p
 
 
-0.592
 
 
45.0
 
 
 
 
ATP6AP2
 
 
hsa-miR-148a-3p
 
 
-0.668
 
 
45.0
 
 
 
 
ATPAF1
 
 
hsa-let-7e-5p
 
 
-0.498
 
 
42.6
 
 
 
 
ATPAF1
 
 
hsa-miR-98-5p
 
 
-0.498
 
 
42.6
 
 
 
 
ATPAF1
 
 
hsa-let-7c-5p
 
 
-0.498
 
 
42.6
 
 
 
 
AVPI1
 
 
hsa-miR-7-5p
 
 
-0.367
 
 
15.7
 
 
 
 
B3GAT3
 
 
hsa-let-7e-5p
 
 
-0.324
 
 
30.1
 
 
 
 
B3GAT3
 
 
hsa-miR-98-5p
 
 
-0.324
 
 
30.1
 
 
 
 
B3GAT3
 
 
hsa-let-7c-5p
 
 
-0.324
 
 
30.1
 
 
 
 
B4GALT5
 
 
hsa-miR-148a-3p
 
 
-0.44
 
 
20.1
 
 
 
 
B4GAT1
 
 
hsa-let-7c-5p
 
 
-0.47
 
 
14.2
 
 
 
 
B4GAT1
 
 
hsa-let-7e-5p
 
 
-0.47
 
 
14.2
 
 
 
 
 
 
 tongue 
 
 
 
 
target_symbol
 
 
mature_mirna_id
 
 
score
 
 
nTPM
 
 
 
 
 
 
ABCB10
 
 
hsa-miR-148a-3p
 
 
-0.291
 
 
10.2
 
 
 
 
ABCB7
 
 
hsa-miR-148a-3p
 
 
-0.515
 
 
20.8
 
 
 
 
ABCC5
 
 
hsa-miR-125a-5p
 
 
-0.288
 
 
37.6
 
 
 
 
ABCD3
 
 
hsa-miR-148a-3p
 
 
-0.388
 
 
27.9
 
 
 
 
ABHD5
 
 
hsa-miR-20b-5p
 
 
-0.418
 
 
17.3
 
 
 
 
ABHD5
 
 
hsa-miR-106a-5p
 
 
-0.447
 
 
17.3
 
 
 
 
ABTB1
 
 
hsa-miR-125a-5p
 
 
-0.519
 
 
24.5
 
 
 
 
ACHE
 
 
hsa-miR-125a-5p
 
 
-0.308
 
 
44.9
 
 
 
 
ACHE
 
 
hsa-miR-125a-5p
 
 
-0.361
 
 
44.9
 
 
 
 
ACTC1
 
 
hsa-miR-7-5p
 
 
-0.471
 
 
182.1
 
 
 
 
ADAM9
 
 
hsa-miR-106a-5p
 
 
-0.317
 
 
23.3
 
 
 
 
ADAM9
 
 
hsa-miR-20b-5p
 
 
-0.328
 
 
23.3
 
 
 
 
ADCY2
 
 
hsa-miR-148a-3p
 
 
-0.299
 
 
28.8
 
 
 
 
ADGRF5
 
 
hsa-miR-148a-3p
 
 
-0.354
 
 
66.5
 
 
 
 
AGFG1
 
 
hsa-miR-148a-3p
 
 
-0.332
 
 
36.1
 
 
 
 
AGGF1
 
 
hsa-miR-125a-5p
 
 
-0.331
 
 
10.4
 
 
 
 
AHCTF1
 
 
hsa-let-7e-5p
 
 
-0.374
 
 
14.7
 
 
 
 
AHCTF1
 
 
hsa-miR-98-5p
 
 
-0.374
 
 
14.7
 
 
 
 
AHCTF1
 
 
hsa-let-7c-5p
 
 
-0.374
 
 
14.7
 
 
 
 
AHDC1
 
 
hsa-miR-148a-3p
 
 
-0.413
 
 
14.5
 
 
 
 
AHNAK
 
 
hsa-miR-106a-5p
 
 
-0.334
 
 
160.9
 
 
 
 
AHNAK
 
 
hsa-miR-20b-5p
 
 
-0.337
 
 
160.9
 
 
 
 
AIFM1
 
 
hsa-miR-125a-5p
 
 
-0.3
 
 
23.4
 
 
 
 
AIFM1
 
 
hsa-let-7e-5p
 
 
-0.314
 
 
23.4
 
 
 
 
AIFM1
 
 
hsa-miR-98-5p
 
 
-0.314
 
 
23.4
 
 
 
 
AIFM1
 
 
hsa-let-7c-5p
 
 
-0.314
 
 
23.4
 
 
 
 
AKAP1
 
 
hsa-miR-148a-3p
 
 
-0.381
 
 
128.4
 
 
 
 
AKAP6
 
 
hsa-let-7e-5p
 
 
-0.307
 
 
47.4
 
 
 
 
AKAP6
 
 
hsa-miR-98-5p
 
 
-0.307
 
 
47.4
 
 
 
 
AKAP6
 
 
hsa-let-7c-5p
 
 
-0.307
 
 
47.4
 
 
 
 
ALDH4A1
 
 
hsa-miR-184
 
 
-0.421
 
 
69.9
 
 
 
 
ALG3
 
 
hsa-miR-106a-5p
 
 
-0.569
 
 
13.8
 
 
 
 
ALG3
 
 
hsa-miR-20b-5p
 
 
-0.58
 
 
13.8
 
 
 
 
ANAPC16
 
 
hsa-miR-125a-5p
 
 
-0.578
 
 
38.9
 
 
 
 
ANKRD12
 
 
hsa-miR-7-5p
 
 
-0.37
 
 
11.5
 
 
 
 
ANKRD46
 
 
hsa-let-7e-5p
 
 
-0.389
 
 
33.8
 
 
 
 
ANKRD46
 
 
hsa-miR-98-5p
 
 
-0.389
 
 
33.8
 
 
 
 
ANKRD46
 
 
hsa-let-7c-5p
 
 
-0.389
 
 
33.8
 
 
 
 
ANKRD9
 
 
hsa-miR-20b-5p
 
 
-0.331
 
 
24.6
 
 
 
 
ANKRD9
 
 
hsa-miR-106a-5p
 
 
-0.344
 
 
24.6
 
 
 
 
APCDD1
 
 
hsa-miR-106a-5p
 
 
-0.422
 
 
17.3
 
 
 
 
APCDD1
 
 
hsa-miR-20b-5p
 
 
-0.425
 
 
17.3
 
 
 
 
ARF4
 
 
hsa-miR-7-5p
 
 
-0.407
 
 
64.9
 
 
 
 
ARF4
 
 
hsa-miR-148a-3p
 
 
-0.487
 
 
64.9
 
 
 
 
ARFGEF1
 
 
hsa-miR-148a-3p
 
 
-0.306
 
 
13.0
 
 
 
 
ARG2
 
 
hsa-let-7e-5p
 
 
-0.468
 
 
49.2
 
 
 
 
ARG2
 
 
hsa-miR-98-5p
 
 
-0.468
 
 
49.2
 
 
 
 
ARG2
 
 
hsa-let-7c-5p
 
 
-0.468
 
 
49.2
 
 
 
 
ARHGAP1
 
 
hsa-miR-20b-5p
 
 
-0.325
 
 
19.1
 
 
 
 
ARHGAP1
 
 
hsa-miR-106a-5p
 
 
-0.329
 
 
19.1
 
 
 
 
ARHGAP21
 
 
hsa-miR-148a-3p
 
 
-0.287
 
 
21.5
 
 
 
 
ARL1
 
 
hsa-miR-20b-5p
 
 
-0.424
 
 
53.0
 
 
 
 
ARL1
 
 
hsa-miR-106a-5p
 
 
-0.429
 
 
53.0
 
 
 
 
ARL6IP1
 
 
hsa-miR-7-5p
 
 
-0.374
 
 
48.1
 
 
 
 
ARL6IP1
 
 
hsa-miR-148a-3p
 
 
-0.579
 
 
48.1
 
 
 
 
ARL8B
 
 
hsa-miR-148a-3p
 
 
-0.337
 
 
56.0
 
 
 
 
ARMC8
 
 
hsa-miR-106a-5p
 
 
-0.375
 
 
32.4
 
 
 
 
ARMC8
 
 
hsa-miR-20b-5p
 
 
-0.378
 
 
32.4
 
 
 
 
ARMCX1
 
 
hsa-miR-21-5p
 
 
-0.489
 
 
23.8
 
 
 
 
ARMT1
 
 
hsa-let-7c-5p
 
 
-0.303
 
 
21.1
 
 
 
 
ARMT1
 
 
hsa-let-7e-5p
 
 
-0.303
 
 
21.1
 
 
 
 
ARMT1
 
 
hsa-miR-98-5p
 
 
-0.303
 
 
21.1
 
 
 
 
ARPP19
 
 
hsa-let-7e-5p
 
 
-0.492
 
 
158.9
 
 
 
 
ARPP19
 
 
hsa-let-7c-5p
 
 
-0.492
 
 
158.9
 
 
 
 
ARPP19
 
 
hsa-miR-98-5p
 
 
-0.5
 
 
158.9
 
 
 
 
ARRDC3
 
 
hsa-miR-148a-3p
 
 
-0.305
 
 
41.9
 
 
 
 
ASF1A
 
 
hsa-miR-21-5p
 
 
-0.304
 
 
15.1
 
 
 
 
ASPH
 
 
hsa-miR-148a-3p
 
 
-0.391
 
 
155.4
 
 
 
 
ATG4A
 
 
hsa-miR-7-5p
 
 
-0.448
 
 
17.2
 
 
 
 
ATG4D
 
 
hsa-miR-148a-3p
 
 
-0.303
 
 
10.1
 
 
 
 
ATOSB
 
 
hsa-let-7e-5p
 
 
-0.429
 
 
10.9
 
 
 
 
ATOSB
 
 
hsa-miR-98-5p
 
 
-0.429
 
 
10.9
 
 
 
 
ATOSB
 
 
hsa-let-7c-5p
 
 
-0.429
 
 
10.9
 
 
 
 
ATP2A2
 
 
hsa-let-7e-5p
 
 
-0.311
 
 
2586.2
 
 
 
 
ATP2A2
 
 
hsa-miR-98-5p
 
 
-0.311
 
 
2586.2
 
 
 
 
ATP2A2
 
 
hsa-let-7c-5p
 
 
-0.311
 
 
2586.2
 
 
 
 
ATP2B4
 
 
hsa-miR-148a-3p
 
 
-0.337
 
 
25.9
 
 
 
 
ATP6AP2
 
 
hsa-miR-148a-3p
 
 
-0.592
 
 
37.6
 
 
 
 
ATP6AP2
 
 
hsa-miR-148a-3p
 
 
-0.668
 
 
37.6
 
 
 
 
ATPAF1
 
 
hsa-let-7e-5p
 
 
-0.498
 
 
295.7
 
 
 
 
ATPAF1
 
 
hsa-miR-98-5p
 
 
-0.498
 
 
295.7
 
 
 
 
ATPAF1
 
 
hsa-let-7c-5p
 
 
-0.498
 
 
295.7
 
 
 
 
B3GALNT2
 
 
hsa-miR-125a-5p
 
 
-0.311
 
 
15.5
 
 
 
 
B4GALT5
 
 
hsa-miR-148a-3p
 
 
-0.44
 
 
22.7
 
 
 
 
B4GAT1
 
 
hsa-let-7c-5p
 
 
-0.47
 
 
17.9
 
 
 
 
B4GAT1
 
 
hsa-let-7e-5p
 
 
-0.47
 
 
17.9
 
 
 
 
B4GAT1
 
 
hsa-miR-98-5p
 
 
-0.47
 
 
17.9
 
 
 
 
BACH1
 
 
hsa-let-7e-5p
 
 
-0.298
 
 
11.9
 
 
 
 
BACH1
 
 
hsa-let-7e-5p
 
 
-0.298
 
 
11.9
 
 
 
 
BACH1
 
 
hsa-miR-98-5p
 
 
-0.298
 
 
11.9
 
 
 
 
BACH1
 
 
hsa-miR-98-5p
 
 
-0.298
 
 
11.9
 
 
 
 
BACH1
 
 
hsa-let-7c-5p
 
 
-0.306
 
 
11.9
 
 
 
 
BACH1
 
 
hsa-let-7c-5p
 
 
-0.306
 
 
11.9
 
 
 
 
BACH1
 
 
hsa-let-7e-5p
 
 
-0.435
 
 
11.9
 
 
 
 
BACH1
 
 
hsa-let-7e-5p
 
 
-0.435
 
 
11.9
 
 
 
 
BACH1
 
 
hsa-miR-98-5p
 
 
-0.435
 
 
11.9
 
 
 
 
BACH1
 
 
hsa-miR-98-5p
 
 
-0.435
 
 
11.9
 
 
 
 
BACH1
 
 
hsa-let-7c-5p
 
 
-0.435
 
 
11.9
 
 
 
 
BACH1
 
 
hsa-let-7c-5p
 
 
-0.435
 
 
11.9
 
 
 
 
BAK1
 
 
hsa-miR-125a-5p
 
 
-0.402
 
 
16.9
 
 
 
 
 
 
 adipose tissue 
 
 
 
 
target_symbol
 
 
mature_mirna_id
 
 
score
 
 
nTPM
 
 
 
 
 
 
ABCB7
 
 
hsa-miR-148a-3p
 
 
-0.515
 
 
12.4
 
 
 
 
ABCC5
 
 
hsa-miR-125a-5p
 
 
-0.288
 
 
15.3
 
 
 
 
ABCD3
 
 
hsa-miR-148a-3p
 
 
-0.388
 
 
15.7
 
 
 
 
ABHD3
 
 
hsa-miR-125a-5p
 
 
-0.453
 
 
16.0
 
 
 
 
ABHD5
 
 
hsa-miR-20b-5p
 
 
-0.418
 
 
27.9
 
 
 
 
ABHD5
 
 
hsa-miR-106a-5p
 
 
-0.447
 
 
27.9
 
 
 
 
ABHD6
 
 
hsa-miR-125a-5p
 
 
-0.501
 
 
11.4
 
 
 
 
ABTB1
 
 
hsa-miR-125a-5p
 
 
-0.519
 
 
38.9
 
 
 
 
ACTC1
 
 
hsa-miR-7-5p
 
 
-0.471
 
 
47.8
 
 
 
 
ACVR1
 
 
hsa-miR-148a-3p
 
 
-0.397
 
 
12.6
 
 
 
 
ACVR1C
 
 
hsa-let-7e-5p
 
 
-0.457
 
 
29.3
 
 
 
 
ACVR1C
 
 
hsa-let-7c-5p
 
 
-0.465
 
 
29.3
 
 
 
 
ACVR1C
 
 
hsa-miR-98-5p
 
 
-0.473
 
 
29.3
 
 
 
 
ADAM10
 
 
hsa-miR-148a-3p
 
 
-0.356
 
 
14.0
 
 
 
 
ADAM9
 
 
hsa-miR-106a-5p
 
 
-0.317
 
 
31.0
 
 
 
 
ADAM9
 
 
hsa-miR-20b-5p
 
 
-0.328
 
 
31.0
 
 
 
 
ADGRF5
 
 
hsa-miR-148a-3p
 
 
-0.354
 
 
66.6
 
 
 
 
ADRB2
 
 
hsa-let-7c-5p
 
 
-0.635
 
 
26.4
 
 
 
 
ADRB2
 
 
hsa-let-7e-5p
 
 
-0.635
 
 
26.4
 
 
 
 
ADRB2
 
 
hsa-miR-98-5p
 
 
-0.635
 
 
26.4
 
 
 
 
AGFG1
 
 
hsa-miR-148a-3p
 
 
-0.332
 
 
64.8
 
 
 
 
AGFG2
 
 
hsa-miR-20b-5p
 
 
-0.386
 
 
12.0
 
 
 
 
AGFG2
 
 
hsa-miR-106a-5p
 
 
-0.399
 
 
12.0
 
 
 
 
AHCTF1
 
 
hsa-let-7e-5p
 
 
-0.374
 
 
11.8
 
 
 
 
AHCTF1
 
 
hsa-miR-98-5p
 
 
-0.374
 
 
11.8
 
 
 
 
AHCTF1
 
 
hsa-let-7c-5p
 
 
-0.374
 
 
11.8
 
 
 
 
AHDC1
 
 
hsa-miR-148a-3p
 
 
-0.413
 
 
11.7
 
 
 
 
AHNAK
 
 
hsa-miR-106a-5p
 
 
-0.334
 
 
230.0
 
 
 
 
AHNAK
 
 
hsa-miR-20b-5p
 
 
-0.337
 
 
230.0
 
 
 
 
AIFM1
 
 
hsa-miR-125a-5p
 
 
-0.3
 
 
17.4
 
 
 
 
AIFM1
 
 
hsa-let-7e-5p
 
 
-0.314
 
 
17.4
 
 
 
 
AIFM1
 
 
hsa-miR-98-5p
 
 
-0.314
 
 
17.4
 
 
 
 
AIFM1
 
 
hsa-let-7c-5p
 
 
-0.314
 
 
17.4
 
 
 
 
AKAP1
 
 
hsa-miR-148a-3p
 
 
-0.381
 
 
38.1
 
 
 
 
ALDH4A1
 
 
hsa-miR-184
 
 
-0.421
 
 
66.5
 
 
 
 
ALG3
 
 
hsa-miR-106a-5p
 
 
-0.569
 
 
20.9
 
 
 
 
ALG3
 
 
hsa-miR-20b-5p
 
 
-0.58
 
 
20.9
 
 
 
 
ALG9
 
 
hsa-miR-7-5p
 
 
-0.302
 
 
12.6
 
 
 
 
ANAPC16
 
 
hsa-miR-125a-5p
 
 
-0.578
 
 
18.9
 
 
 
 
ANKRD12
 
 
hsa-miR-7-5p
 
 
-0.37
 
 
14.8
 
 
 
 
ANKRD29
 
 
hsa-miR-106a-5p
 
 
-0.369
 
 
17.0
 
 
 
 
ANKRD29
 
 
hsa-miR-20b-5p
 
 
-0.38
 
 
17.0
 
 
 
 
ANKRD46
 
 
hsa-let-7e-5p
 
 
-0.389
 
 
18.0
 
 
 
 
ANKRD46
 
 
hsa-miR-98-5p
 
 
-0.389
 
 
18.0
 
 
 
 
ANKRD46
 
 
hsa-let-7c-5p
 
 
-0.389
 
 
18.0
 
 
 
 
ANKRD9
 
 
hsa-miR-20b-5p
 
 
-0.331
 
 
11.3
 
 
 
 
ANKRD9
 
 
hsa-miR-106a-5p
 
 
-0.344
 
 
11.3
 
 
 
 
ANPEP
 
 
hsa-miR-125a-5p
 
 
-0.372
 
 
50.1
 
 
 
 
AP1S1
 
 
hsa-let-7e-5p
 
 
-0.374
 
 
15.1
 
 
 
 
AP1S1
 
 
hsa-miR-98-5p
 
 
-0.374
 
 
15.1
 
 
 
 
AP1S1
 
 
hsa-let-7c-5p
 
 
-0.374
 
 
15.1
 
 
 
 
APCDD1
 
 
hsa-miR-106a-5p
 
 
-0.422
 
 
49.2
 
 
 
 
APCDD1
 
 
hsa-miR-20b-5p
 
 
-0.425
 
 
49.2
 
 
 
 
ARF4
 
 
hsa-miR-7-5p
 
 
-0.407
 
 
118.7
 
 
 
 
ARF4
 
 
hsa-miR-148a-3p
 
 
-0.487
 
 
118.7
 
 
 
 
ARFGEF1
 
 
hsa-miR-148a-3p
 
 
-0.306
 
 
10.7
 
 
 
 
ARFIP1
 
 
hsa-miR-148a-3p
 
 
-0.474
 
 
15.8
 
 
 
 
ARHGAP1
 
 
hsa-miR-20b-5p
 
 
-0.325
 
 
79.6
 
 
 
 
ARHGAP1
 
 
hsa-miR-106a-5p
 
 
-0.329
 
 
79.6
 
 
 
 
ARHGAP21
 
 
hsa-miR-148a-3p
 
 
-0.287
 
 
26.1
 
 
 
 
ARHGAP24
 
 
hsa-miR-21-5p
 
 
-0.427
 
 
13.7
 
 
 
 
ARL1
 
 
hsa-miR-20b-5p
 
 
-0.424
 
 
43.5
 
 
 
 
ARL1
 
 
hsa-miR-106a-5p
 
 
-0.429
 
 
43.5
 
 
 
 
ARL4D
 
 
hsa-let-7e-5p
 
 
-0.417
 
 
20.3
 
 
 
 
ARL4D
 
 
hsa-miR-98-5p
 
 
-0.417
 
 
20.3
 
 
 
 
ARL4D
 
 
hsa-let-7c-5p
 
 
-0.417
 
 
20.3
 
 
 
 
ARL6IP1
 
 
hsa-miR-7-5p
 
 
-0.374
 
 
133.2
 
 
 
 
ARL6IP1
 
 
hsa-miR-148a-3p
 
 
-0.579
 
 
133.2
 
 
 
 
ARL6IP6
 
 
hsa-miR-98-5p
 
 
-0.293
 
 
11.0
 
 
 
 
ARL6IP6
 
 
hsa-let-7c-5p
 
 
-0.293
 
 
11.0
 
 
 
 
ARL6IP6
 
 
hsa-let-7e-5p
 
 
-0.305
 
 
11.0
 
 
 
 
ARL8B
 
 
hsa-miR-148a-3p
 
 
-0.337
 
 
39.1
 
 
 
 
ARMC8
 
 
hsa-miR-106a-5p
 
 
-0.375
 
 
19.5
 
 
 
 
ARMC8
 
 
hsa-miR-20b-5p
 
 
-0.378
 
 
19.5
 
 
 
 
ARMCX1
 
 
hsa-miR-21-5p
 
 
-0.489
 
 
23.1
 
 
 
 
ARMT1
 
 
hsa-let-7c-5p
 
 
-0.303
 
 
16.0
 
 
 
 
ARMT1
 
 
hsa-let-7e-5p
 
 
-0.303
 
 
16.0
 
 
 
 
ARMT1
 
 
hsa-miR-98-5p
 
 
-0.303
 
 
16.0
 
 
 
 
ARPP19
 
 
hsa-let-7e-5p
 
 
-0.492
 
 
57.4
 
 
 
 
ARPP19
 
 
hsa-let-7c-5p
 
 
-0.492
 
 
57.4
 
 
 
 
ARPP19
 
 
hsa-miR-98-5p
 
 
-0.5
 
 
57.4
 
 
 
 
ARRDC3
 
 
hsa-miR-148a-3p
 
 
-0.305
 
 
34.5
 
 
 
 
ARRDC4
 
 
hsa-let-7c-5p
 
 
-0.329
 
 
11.8
 
 
 
 
ARRDC4
 
 
hsa-let-7e-5p
 
 
-0.329
 
 
11.8
 
 
 
 
ARRDC4
 
 
hsa-miR-98-5p
 
 
-0.329
 
 
11.8
 
 
 
 
ASAP1
 
 
hsa-let-7e-5p
 
 
-0.296
 
 
32.3
 
 
 
 
ASAP1
 
 
hsa-miR-98-5p
 
 
-0.308
 
 
32.3
 
 
 
 
ASAP1
 
 
hsa-let-7c-5p
 
 
-0.308
 
 
32.3
 
 
 
 
ASPH
 
 
hsa-miR-148a-3p
 
 
-0.391
 
 
362.0
 
 
 
 
ATOSB
 
 
hsa-let-7e-5p
 
 
-0.429
 
 
13.2
 
 
 
 
ATOSB
 
 
hsa-miR-98-5p
 
 
-0.429
 
 
13.2
 
 
 
 
ATOSB
 
 
hsa-let-7c-5p
 
 
-0.429
 
 
13.2
 
 
 
 
ATP2A2
 
 
hsa-let-7e-5p
 
 
-0.311
 
 
56.6
 
 
 
 
ATP2A2
 
 
hsa-miR-98-5p
 
 
-0.311
 
 
56.6
 
 
 
 
ATP2A2
 
 
hsa-let-7c-5p
 
 
-0.311
 
 
56.6
 
 
 
 
ATP2B4
 
 
hsa-miR-148a-3p
 
 
-0.337
 
 
55.3
 
 
 
 
ATP6AP2
 
 
hsa-miR-148a-3p
 
 
-0.592
 
 
76.1
 
 
 
 
ATP6AP2
 
 
hsa-miR-148a-3p
 
 
-0.668
 
 
76.1
 
 
 
 
ATPAF1
 
 
hsa-let-7e-5p
 
 
-0.498
 
 
81.3
 
 
 
 
ATPAF1
 
 
hsa-miR-98-5p
 
 
-0.498
 
 
81.3
 
 
 
 
 
 
 adrenal gland 
 
 
 
 
target_symbol
 
 
mature_mirna_id
 
 
score
 
 
nTPM
 
 
 
 
 
 
ABCB7
 
 
hsa-miR-148a-3p
 
 
-0.515
 
 
12.5
 
 
 
 
ABCC5
 
 
hsa-miR-125a-5p
 
 
-0.288
 
 
23.1
 
 
 
 
ABCD3
 
 
hsa-miR-148a-3p
 
 
-0.388
 
 
17.6
 
 
 
 
ABHD6
 
 
hsa-miR-125a-5p
 
 
-0.501
 
 
13.2
 
 
 
 
ABTB1
 
 
hsa-miR-125a-5p
 
 
-0.519
 
 
19.3
 
 
 
 
ADAM9
 
 
hsa-miR-106a-5p
 
 
-0.317
 
 
19.6
 
 
 
 
ADAM9
 
 
hsa-miR-20b-5p
 
 
-0.328
 
 
19.6
 
 
 
 
ADGRF5
 
 
hsa-miR-148a-3p
 
 
-0.354
 
 
22.0
 
 
 
 
AGFG1
 
 
hsa-miR-148a-3p
 
 
-0.332
 
 
37.2
 
 
 
 
AGFG2
 
 
hsa-miR-20b-5p
 
 
-0.386
 
 
33.6
 
 
 
 
AGFG2
 
 
hsa-miR-106a-5p
 
 
-0.399
 
 
33.6
 
 
 
 
AGGF1
 
 
hsa-miR-125a-5p
 
 
-0.331
 
 
10.0
 
 
 
 
AHDC1
 
 
hsa-miR-148a-3p
 
 
-0.413
 
 
23.0
 
 
 
 
AHNAK
 
 
hsa-miR-106a-5p
 
 
-0.334
 
 
26.5
 
 
 
 
AHNAK
 
 
hsa-miR-20b-5p
 
 
-0.337
 
 
26.5
 
 
 
 
AIFM1
 
 
hsa-miR-125a-5p
 
 
-0.3
 
 
50.1
 
 
 
 
AIFM1
 
 
hsa-let-7e-5p
 
 
-0.314
 
 
50.1
 
 
 
 
AIFM1
 
 
hsa-miR-98-5p
 
 
-0.314
 
 
50.1
 
 
 
 
AIFM1
 
 
hsa-let-7c-5p
 
 
-0.314
 
 
50.1
 
 
 
 
AKAP1
 
 
hsa-miR-148a-3p
 
 
-0.381
 
 
14.9
 
 
 
 
ALCAM
 
 
hsa-miR-148a-3p
 
 
-0.339
 
 
30.9
 
 
 
 
ALDH4A1
 
 
hsa-miR-184
 
 
-0.421
 
 
16.2
 
 
 
 
ALG3
 
 
hsa-miR-106a-5p
 
 
-0.569
 
 
30.6
 
 
 
 
ALG3
 
 
hsa-miR-20b-5p
 
 
-0.58
 
 
30.6
 
 
 
 
AMT
 
 
hsa-let-7e-5p
 
 
-0.463
 
 
15.0
 
 
 
 
AMT
 
 
hsa-miR-98-5p
 
 
-0.463
 
 
15.0
 
 
 
 
AMT
 
 
hsa-let-7c-5p
 
 
-0.463
 
 
15.0
 
 
 
 
ANAPC16
 
 
hsa-miR-125a-5p
 
 
-0.578
 
 
24.7
 
 
 
 
ANKRD46
 
 
hsa-let-7e-5p
 
 
-0.389
 
 
36.5
 
 
 
 
ANKRD46
 
 
hsa-miR-98-5p
 
 
-0.389
 
 
36.5
 
 
 
 
ANKRD46
 
 
hsa-let-7c-5p
 
 
-0.389
 
 
36.5
 
 
 
 
ANKRD9
 
 
hsa-miR-20b-5p
 
 
-0.331
 
 
20.4
 
 
 
 
ANKRD9
 
 
hsa-miR-106a-5p
 
 
-0.344
 
 
20.4
 
 
 
 
AP1AR
 
 
hsa-miR-21-5p
 
 
-0.318
 
 
14.8
 
 
 
 
AP1AR
 
 
hsa-miR-99b-5p
 
 
-0.823
 
 
14.8
 
 
 
 
AP1S1
 
 
hsa-let-7e-5p
 
 
-0.374
 
 
25.7
 
 
 
 
AP1S1
 
 
hsa-miR-98-5p
 
 
-0.374
 
 
25.7
 
 
 
 
AP1S1
 
 
hsa-let-7c-5p
 
 
-0.374
 
 
25.7
 
 
 
 
APCDD1
 
 
hsa-miR-106a-5p
 
 
-0.422
 
 
13.4
 
 
 
 
APCDD1
 
 
hsa-miR-20b-5p
 
 
-0.425
 
 
13.4
 
 
 
 
ARF4
 
 
hsa-miR-7-5p
 
 
-0.407
 
 
151.0
 
 
 
 
ARF4
 
 
hsa-miR-148a-3p
 
 
-0.487
 
 
151.0
 
 
 
 
ARFIP1
 
 
hsa-miR-148a-3p
 
 
-0.474
 
 
13.9
 
 
 
 
ARHGAP1
 
 
hsa-miR-20b-5p
 
 
-0.325
 
 
46.3
 
 
 
 
ARHGAP1
 
 
hsa-miR-106a-5p
 
 
-0.329
 
 
46.3
 
 
 
 
ARHGAP21
 
 
hsa-miR-148a-3p
 
 
-0.287
 
 
16.9
 
 
 
 
ARHGEF28
 
 
hsa-miR-20b-5p
 
 
-0.352
 
 
23.1
 
 
 
 
ARHGEF28
 
 
hsa-miR-106a-5p
 
 
-0.367
 
 
23.1
 
 
 
 
ARL1
 
 
hsa-miR-20b-5p
 
 
-0.424
 
 
62.3
 
 
 
 
ARL1
 
 
hsa-miR-106a-5p
 
 
-0.429
 
 
62.3
 
 
 
 
ARL6IP1
 
 
hsa-miR-7-5p
 
 
-0.374
 
 
161.7
 
 
 
 
ARL6IP1
 
 
hsa-miR-148a-3p
 
 
-0.579
 
 
161.7
 
 
 
 
ARL8B
 
 
hsa-miR-148a-3p
 
 
-0.337
 
 
48.3
 
 
 
 
ARMC8
 
 
hsa-miR-106a-5p
 
 
-0.375
 
 
13.5
 
 
 
 
ARMC8
 
 
hsa-miR-20b-5p
 
 
-0.378
 
 
13.5
 
 
 
 
ARMCX1
 
 
hsa-miR-21-5p
 
 
-0.489
 
 
32.1
 
 
 
 
ARMT1
 
 
hsa-let-7c-5p
 
 
-0.303
 
 
15.1
 
 
 
 
ARMT1
 
 
hsa-let-7e-5p
 
 
-0.303
 
 
15.1
 
 
 
 
ARMT1
 
 
hsa-miR-98-5p
 
 
-0.303
 
 
15.1
 
 
 
 
ARPP19
 
 
hsa-let-7e-5p
 
 
-0.492
 
 
48.8
 
 
 
 
ARPP19
 
 
hsa-let-7c-5p
 
 
-0.492
 
 
48.8
 
 
 
 
ARPP19
 
 
hsa-miR-98-5p
 
 
-0.5
 
 
48.8
 
 
 
 
ARRDC3
 
 
hsa-miR-148a-3p
 
 
-0.305
 
 
75.7
 
 
 
 
ARRDC4
 
 
hsa-let-7c-5p
 
 
-0.329
 
 
11.5
 
 
 
 
ARRDC4
 
 
hsa-let-7e-5p
 
 
-0.329
 
 
11.5
 
 
 
 
ARRDC4
 
 
hsa-miR-98-5p
 
 
-0.329
 
 
11.5
 
 
 
 
ASAP1
 
 
hsa-let-7e-5p
 
 
-0.296
 
 
20.2
 
 
 
 
ASAP1
 
 
hsa-miR-98-5p
 
 
-0.308
 
 
20.2
 
 
 
 
ASAP1
 
 
hsa-let-7c-5p
 
 
-0.308
 
 
20.2
 
 
 
 
ASPH
 
 
hsa-miR-148a-3p
 
 
-0.391
 
 
150.8
 
 
 
 
ATG4D
 
 
hsa-miR-148a-3p
 
 
-0.303
 
 
14.9
 
 
 
 
ATOSB
 
 
hsa-let-7e-5p
 
 
-0.429
 
 
14.2
 
 
 
 
ATOSB
 
 
hsa-miR-98-5p
 
 
-0.429
 
 
14.2
 
 
 
 
ATOSB
 
 
hsa-let-7c-5p
 
 
-0.429
 
 
14.2
 
 
 
 
ATP2A2
 
 
hsa-let-7e-5p
 
 
-0.311
 
 
46.8
 
 
 
 
ATP2A2
 
 
hsa-miR-98-5p
 
 
-0.311
 
 
46.8
 
 
 
 
ATP2A2
 
 
hsa-let-7c-5p
 
 
-0.311
 
 
46.8
 
 
 
 
ATP2B4
 
 
hsa-miR-148a-3p
 
 
-0.337
 
 
23.7
 
 
 
 
ATP6AP2
 
 
hsa-miR-148a-3p
 
 
-0.592
 
 
67.5
 
 
 
 
ATP6AP2
 
 
hsa-miR-148a-3p
 
 
-0.668
 
 
67.5
 
 
 
 
ATPAF1
 
 
hsa-let-7e-5p
 
 
-0.498
 
 
98.1
 
 
 
 
ATPAF1
 
 
hsa-miR-98-5p
 
 
-0.498
 
 
98.1
 
 
 
 
ATPAF1
 
 
hsa-let-7c-5p
 
 
-0.498
 
 
98.1
 
 
 
 
AVPI1
 
 
hsa-miR-7-5p
 
 
-0.367
 
 
46.2
 
 
 
 
B3GAT3
 
 
hsa-let-7e-5p
 
 
-0.324
 
 
33.4
 
 
 
 
B3GAT3
 
 
hsa-miR-98-5p
 
 
-0.324
 
 
33.4
 
 
 
 
B3GAT3
 
 
hsa-let-7c-5p
 
 
-0.324
 
 
33.4
 
 
 
 
B4GALT5
 
 
hsa-miR-148a-3p
 
 
-0.44
 
 
44.9
 
 
 
 
B4GALT6
 
 
hsa-miR-148a-3p
 
 
-0.528
 
 
17.1
 
 
 
 
B4GAT1
 
 
hsa-let-7c-5p
 
 
-0.47
 
 
39.1
 
 
 
 
B4GAT1
 
 
hsa-let-7e-5p
 
 
-0.47
 
 
39.1
 
 
 
 
B4GAT1
 
 
hsa-miR-98-5p
 
 
-0.47
 
 
39.1
 
 
 
 
BACH1
 
 
hsa-let-7e-5p
 
 
-0.298
 
 
11.4
 
 
 
 
BACH1
 
 
hsa-let-7e-5p
 
 
-0.298
 
 
11.4
 
 
 
 
BACH1
 
 
hsa-miR-98-5p
 
 
-0.298
 
 
11.4
 
 
 
 
BACH1
 
 
hsa-miR-98-5p
 
 
-0.298
 
 
11.4
 
 
 
 
BACH1
 
 
hsa-let-7c-5p
 
 
-0.306
 
 
11.4
 
 
 
 
BACH1
 
 
hsa-let-7c-5p
 
 
-0.306
 
 
11.4
 
 
 
 
BACH1
 
 
hsa-let-7e-5p
 
 
-0.435
 
 
11.4
 
 
 
 
BACH1
 
 
hsa-let-7e-5p
 
 
-0.435
 
 
11.4
 
 
 
 
 
 
 blood vessel 
 
 
 
 
target_symbol
 
 
mature_mirna_id
 
 
score
 
 
nTPM
 
 
 
 
 
 
ABCB7
 
 
hsa-miR-148a-3p
 
 
-0.515
 
 
12.3
 
 
 
 
ABCC5
 
 
hsa-miR-125a-5p
 
 
-0.288
 
 
17.4
 
 
 
 
ABCD3
 
 
hsa-miR-148a-3p
 
 
-0.388
 
 
14.4
 
 
 
 
ABHD3
 
 
hsa-miR-125a-5p
 
 
-0.453
 
 
10.2
 
 
 
 
ABTB1
 
 
hsa-miR-125a-5p
 
 
-0.519
 
 
47.2
 
 
 
 
ACTC1
 
 
hsa-miR-7-5p
 
 
-0.471
 
 
388.9
 
 
 
 
ADAM9
 
 
hsa-miR-106a-5p
 
 
-0.317
 
 
36.8
 
 
 
 
ADAM9
 
 
hsa-miR-20b-5p
 
 
-0.328
 
 
36.8
 
 
 
 
ADAMTS8
 
 
hsa-let-7e-5p
 
 
-0.571
 
 
12.7
 
 
 
 
ADAMTS8
 
 
hsa-miR-98-5p
 
 
-0.571
 
 
12.7
 
 
 
 
ADAMTS8
 
 
hsa-let-7c-5p
 
 
-0.571
 
 
12.7
 
 
 
 
ADGRF5
 
 
hsa-miR-148a-3p
 
 
-0.354
 
 
12.6
 
 
 
 
AGFG1
 
 
hsa-miR-148a-3p
 
 
-0.332
 
 
37.3
 
 
 
 
AGFG2
 
 
hsa-miR-20b-5p
 
 
-0.386
 
 
11.4
 
 
 
 
AGFG2
 
 
hsa-miR-106a-5p
 
 
-0.399
 
 
11.4
 
 
 
 
AHCTF1
 
 
hsa-let-7e-5p
 
 
-0.374
 
 
10.0
 
 
 
 
AHCTF1
 
 
hsa-miR-98-5p
 
 
-0.374
 
 
10.0
 
 
 
 
AHCTF1
 
 
hsa-let-7c-5p
 
 
-0.374
 
 
10.0
 
 
 
 
AHDC1
 
 
hsa-miR-148a-3p
 
 
-0.413
 
 
27.7
 
 
 
 
AHNAK
 
 
hsa-miR-106a-5p
 
 
-0.334
 
 
225.6
 
 
 
 
AHNAK
 
 
hsa-miR-20b-5p
 
 
-0.337
 
 
225.6
 
 
 
 
AKAP1
 
 
hsa-miR-148a-3p
 
 
-0.381
 
 
84.5
 
 
 
 
AKAP6
 
 
hsa-let-7e-5p
 
 
-0.307
 
 
15.0
 
 
 
 
AKAP6
 
 
hsa-miR-98-5p
 
 
-0.307
 
 
15.0
 
 
 
 
AKAP6
 
 
hsa-let-7c-5p
 
 
-0.307
 
 
15.0
 
 
 
 
ALCAM
 
 
hsa-miR-148a-3p
 
 
-0.339
 
 
85.5
 
 
 
 
ALDH4A1
 
 
hsa-miR-184
 
 
-0.421
 
 
15.0
 
 
 
 
ALG3
 
 
hsa-miR-106a-5p
 
 
-0.569
 
 
20.2
 
 
 
 
ALG3
 
 
hsa-miR-20b-5p
 
 
-0.58
 
 
20.2
 
 
 
 
ALG9
 
 
hsa-miR-7-5p
 
 
-0.302
 
 
12.3
 
 
 
 
AMT
 
 
hsa-let-7e-5p
 
 
-0.463
 
 
11.4
 
 
 
 
AMT
 
 
hsa-miR-98-5p
 
 
-0.463
 
 
11.4
 
 
 
 
AMT
 
 
hsa-let-7c-5p
 
 
-0.463
 
 
11.4
 
 
 
 
ANAPC16
 
 
hsa-miR-125a-5p
 
 
-0.578
 
 
31.1
 
 
 
 
ANKRD12
 
 
hsa-miR-7-5p
 
 
-0.37
 
 
11.6
 
 
 
 
ANKRD9
 
 
hsa-miR-20b-5p
 
 
-0.331
 
 
10.4
 
 
 
 
ANKRD9
 
 
hsa-miR-106a-5p
 
 
-0.344
 
 
10.4
 
 
 
 
ANPEP
 
 
hsa-miR-125a-5p
 
 
-0.372
 
 
14.7
 
 
 
 
AP1S1
 
 
hsa-let-7e-5p
 
 
-0.374
 
 
13.8
 
 
 
 
AP1S1
 
 
hsa-miR-98-5p
 
 
-0.374
 
 
13.8
 
 
 
 
AP1S1
 
 
hsa-let-7c-5p
 
 
-0.374
 
 
13.8
 
 
 
 
APCDD1
 
 
hsa-miR-106a-5p
 
 
-0.422
 
 
23.7
 
 
 
 
APCDD1
 
 
hsa-miR-20b-5p
 
 
-0.425
 
 
23.7
 
 
 
 
ARF4
 
 
hsa-miR-7-5p
 
 
-0.407
 
 
151.4
 
 
 
 
ARF4
 
 
hsa-miR-148a-3p
 
 
-0.487
 
 
151.4
 
 
 
 
ARFIP1
 
 
hsa-miR-148a-3p
 
 
-0.474
 
 
14.2
 
 
 
 
ARHGAP1
 
 
hsa-miR-20b-5p
 
 
-0.325
 
 
119.0
 
 
 
 
ARHGAP1
 
 
hsa-miR-106a-5p
 
 
-0.329
 
 
119.0
 
 
 
 
ARHGAP21
 
 
hsa-miR-148a-3p
 
 
-0.287
 
 
14.0
 
 
 
 
ARHGAP24
 
 
hsa-miR-21-5p
 
 
-0.427
 
 
17.3
 
 
 
 
ARHGEF28
 
 
hsa-miR-20b-5p
 
 
-0.352
 
 
10.7
 
 
 
 
ARHGEF28
 
 
hsa-miR-106a-5p
 
 
-0.367
 
 
10.7
 
 
 
 
ARL1
 
 
hsa-miR-20b-5p
 
 
-0.424
 
 
48.7
 
 
 
 
ARL1
 
 
hsa-miR-106a-5p
 
 
-0.429
 
 
48.7
 
 
 
 
ARL4D
 
 
hsa-let-7e-5p
 
 
-0.417
 
 
12.9
 
 
 
 
ARL4D
 
 
hsa-miR-98-5p
 
 
-0.417
 
 
12.9
 
 
 
 
ARL4D
 
 
hsa-let-7c-5p
 
 
-0.417
 
 
12.9
 
 
 
 
ARL6IP1
 
 
hsa-miR-7-5p
 
 
-0.374
 
 
52.2
 
 
 
 
ARL6IP1
 
 
hsa-miR-148a-3p
 
 
-0.579
 
 
52.2
 
 
 
 
ARL8B
 
 
hsa-miR-148a-3p
 
 
-0.337
 
 
49.5
 
 
 
 
ARMC8
 
 
hsa-miR-106a-5p
 
 
-0.375
 
 
23.5
 
 
 
 
ARMC8
 
 
hsa-miR-20b-5p
 
 
-0.378
 
 
23.5
 
 
 
 
ARMCX1
 
 
hsa-miR-21-5p
 
 
-0.489
 
 
31.6
 
 
 
 
ARPP19
 
 
hsa-let-7e-5p
 
 
-0.492
 
 
47.3
 
 
 
 
ARPP19
 
 
hsa-let-7c-5p
 
 
-0.492
 
 
47.3
 
 
 
 
ARPP19
 
 
hsa-miR-98-5p
 
 
-0.5
 
 
47.3
 
 
 
 
ARRDC3
 
 
hsa-miR-148a-3p
 
 
-0.305
 
 
25.4
 
 
 
 
ASAP1
 
 
hsa-let-7e-5p
 
 
-0.296
 
 
18.4
 
 
 
 
ASAP1
 
 
hsa-miR-98-5p
 
 
-0.308
 
 
18.4
 
 
 
 
ASAP1
 
 
hsa-let-7c-5p
 
 
-0.308
 
 
18.4
 
 
 
 
ASPH
 
 
hsa-miR-148a-3p
 
 
-0.391
 
 
72.5
 
 
 
 
ATG16L1
 
 
hsa-miR-106a-5p
 
 
-0.464
 
 
12.7
 
 
 
 
ATG16L1
 
 
hsa-miR-20b-5p
 
 
-0.467
 
 
12.7
 
 
 
 
ATOSB
 
 
hsa-let-7e-5p
 
 
-0.429
 
 
26.7
 
 
 
 
ATOSB
 
 
hsa-miR-98-5p
 
 
-0.429
 
 
26.7
 
 
 
 
ATOSB
 
 
hsa-let-7c-5p
 
 
-0.429
 
 
26.7
 
 
 
 
ATP10D
 
 
hsa-miR-125a-5p
 
 
-0.375
 
 
11.2
 
 
 
 
ATP2A2
 
 
hsa-let-7e-5p
 
 
-0.311
 
 
84.6
 
 
 
 
ATP2A2
 
 
hsa-miR-98-5p
 
 
-0.311
 
 
84.6
 
 
 
 
ATP2A2
 
 
hsa-let-7c-5p
 
 
-0.311
 
 
84.6
 
 
 
 
ATP2B4
 
 
hsa-miR-148a-3p
 
 
-0.337
 
 
24.9
 
 
 
 
ATP6AP2
 
 
hsa-miR-148a-3p
 
 
-0.592
 
 
93.4
 
 
 
 
ATP6AP2
 
 
hsa-miR-148a-3p
 
 
-0.668
 
 
93.4
 
 
 
 
ATPAF1
 
 
hsa-let-7e-5p
 
 
-0.498
 
 
30.6
 
 
 
 
ATPAF1
 
 
hsa-miR-98-5p
 
 
-0.498
 
 
30.6
 
 
 
 
ATPAF1
 
 
hsa-let-7c-5p
 
 
-0.498
 
 
30.6
 
 
 
 
AVPI1
 
 
hsa-miR-7-5p
 
 
-0.367
 
 
57.0
 
 
 
 
B3GALNT2
 
 
hsa-miR-125a-5p
 
 
-0.311
 
 
21.4
 
 
 
 
B3GAT3
 
 
hsa-let-7e-5p
 
 
-0.324
 
 
38.8
 
 
 
 
B3GAT3
 
 
hsa-miR-98-5p
 
 
-0.324
 
 
38.8
 
 
 
 
B3GAT3
 
 
hsa-let-7c-5p
 
 
-0.324
 
 
38.8
 
 
 
 
B4GALT5
 
 
hsa-miR-148a-3p
 
 
-0.44
 
 
15.5
 
 
 
 
B4GAT1
 
 
hsa-let-7c-5p
 
 
-0.47
 
 
18.5
 
 
 
 
B4GAT1
 
 
hsa-let-7e-5p
 
 
-0.47
 
 
18.5
 
 
 
 
B4GAT1
 
 
hsa-miR-98-5p
 
 
-0.47
 
 
18.5
 
 
 
 
BACE2
 
 
hsa-let-7e-5p
 
 
-0.364
 
 
11.5
 
 
 
 
BACE2
 
 
hsa-miR-98-5p
 
 
-0.364
 
 
11.5
 
 
 
 
BACE2
 
 
hsa-let-7c-5p
 
 
-0.364
 
 
11.5
 
 
 
 
BACH1
 
 
hsa-let-7e-5p
 
 
-0.298
 
 
10.9
 
 
 
 
BACH1
 
 
hsa-let-7e-5p
 
 
-0.298
 
 
10.9
 
 
 
 
 
 
 breast 
 
 
 
 
target_symbol
 
 
mature_mirna_id
 
 
score
 
 
nTPM
 
 
 
 
 
 
ABCB7
 
 
hsa-miR-148a-3p
 
 
-0.515
 
 
11.6
 
 
 
 
ABCC5
 
 
hsa-miR-125a-5p
 
 
-0.288
 
 
12.8
 
 
 
 
ABCD3
 
 
hsa-miR-148a-3p
 
 
-0.388
 
 
30.8
 
 
 
 
ABHD17C
 
 
hsa-let-7c-5p
 
 
-0.359
 
 
10.9
 
 
 
 
ABHD17C
 
 
hsa-let-7e-5p
 
 
-0.359
 
 
10.9
 
 
 
 
ABHD17C
 
 
hsa-miR-98-5p
 
 
-0.383
 
 
10.9
 
 
 
 
ABHD3
 
 
hsa-miR-125a-5p
 
 
-0.453
 
 
14.7
 
 
 
 
ABHD5
 
 
hsa-miR-20b-5p
 
 
-0.418
 
 
18.8
 
 
 
 
ABHD5
 
 
hsa-miR-106a-5p
 
 
-0.447
 
 
18.8
 
 
 
 
ABTB1
 
 
hsa-miR-125a-5p
 
 
-0.519
 
 
33.9
 
 
 
 
ACTC1
 
 
hsa-miR-7-5p
 
 
-0.471
 
 
11.2
 
 
 
 
ACVR1
 
 
hsa-miR-148a-3p
 
 
-0.397
 
 
11.1
 
 
 
 
ADAM10
 
 
hsa-miR-148a-3p
 
 
-0.356
 
 
14.2
 
 
 
 
ADAM9
 
 
hsa-miR-106a-5p
 
 
-0.317
 
 
20.2
 
 
 
 
ADAM9
 
 
hsa-miR-20b-5p
 
 
-0.328
 
 
20.2
 
 
 
 
ADGRF5
 
 
hsa-miR-148a-3p
 
 
-0.354
 
 
44.5
 
 
 
 
ADRB2
 
 
hsa-let-7c-5p
 
 
-0.635
 
 
32.4
 
 
 
 
ADRB2
 
 
hsa-let-7e-5p
 
 
-0.635
 
 
32.4
 
 
 
 
ADRB2
 
 
hsa-miR-98-5p
 
 
-0.635
 
 
32.4
 
 
 
 
AGFG1
 
 
hsa-miR-148a-3p
 
 
-0.332
 
 
48.8
 
 
 
 
AGFG2
 
 
hsa-miR-20b-5p
 
 
-0.386
 
 
10.5
 
 
 
 
AGFG2
 
 
hsa-miR-106a-5p
 
 
-0.399
 
 
10.5
 
 
 
 
AHDC1
 
 
hsa-miR-148a-3p
 
 
-0.413
 
 
11.9
 
 
 
 
AHNAK
 
 
hsa-miR-106a-5p
 
 
-0.334
 
 
178.3
 
 
 
 
AHNAK
 
 
hsa-miR-20b-5p
 
 
-0.337
 
 
178.3
 
 
 
 
AIFM1
 
 
hsa-miR-125a-5p
 
 
-0.3
 
 
11.7
 
 
 
 
AIFM1
 
 
hsa-let-7e-5p
 
 
-0.314
 
 
11.7
 
 
 
 
AIFM1
 
 
hsa-miR-98-5p
 
 
-0.314
 
 
11.7
 
 
 
 
AIFM1
 
 
hsa-let-7c-5p
 
 
-0.314
 
 
11.7
 
 
 
 
AKAP1
 
 
hsa-miR-148a-3p
 
 
-0.381
 
 
34.0
 
 
 
 
ALCAM
 
 
hsa-miR-148a-3p
 
 
-0.339
 
 
76.0
 
 
 
 
ALDH4A1
 
 
hsa-miR-184
 
 
-0.421
 
 
22.5
 
 
 
 
ALG3
 
 
hsa-miR-106a-5p
 
 
-0.569
 
 
18.6
 
 
 
 
ALG3
 
 
hsa-miR-20b-5p
 
 
-0.58
 
 
18.6
 
 
 
 
ALG9
 
 
hsa-miR-7-5p
 
 
-0.302
 
 
11.3
 
 
 
 
ANAPC16
 
 
hsa-miR-125a-5p
 
 
-0.578
 
 
20.2
 
 
 
 
ANKRD12
 
 
hsa-miR-7-5p
 
 
-0.37
 
 
12.8
 
 
 
 
ANKRD29
 
 
hsa-miR-106a-5p
 
 
-0.369
 
 
15.0
 
 
 
 
ANKRD29
 
 
hsa-miR-20b-5p
 
 
-0.38
 
 
15.0
 
 
 
 
ANKRD46
 
 
hsa-let-7e-5p
 
 
-0.389
 
 
13.6
 
 
 
 
ANKRD46
 
 
hsa-miR-98-5p
 
 
-0.389
 
 
13.6
 
 
 
 
ANKRD46
 
 
hsa-let-7c-5p
 
 
-0.389
 
 
13.6
 
 
 
 
ANPEP
 
 
hsa-miR-125a-5p
 
 
-0.372
 
 
49.7
 
 
 
 
AP1AR
 
 
hsa-miR-21-5p
 
 
-0.318
 
 
15.9
 
 
 
 
AP1AR
 
 
hsa-miR-99b-5p
 
 
-0.823
 
 
15.9
 
 
 
 
AP1S1
 
 
hsa-let-7e-5p
 
 
-0.374
 
 
17.7
 
 
 
 
AP1S1
 
 
hsa-miR-98-5p
 
 
-0.374
 
 
17.7
 
 
 
 
AP1S1
 
 
hsa-let-7c-5p
 
 
-0.374
 
 
17.7
 
 
 
 
APCDD1
 
 
hsa-miR-106a-5p
 
 
-0.422
 
 
30.9
 
 
 
 
APCDD1
 
 
hsa-miR-20b-5p
 
 
-0.425
 
 
30.9
 
 
 
 
ARF4
 
 
hsa-miR-7-5p
 
 
-0.407
 
 
129.6
 
 
 
 
ARF4
 
 
hsa-miR-148a-3p
 
 
-0.487
 
 
129.6
 
 
 
 
ARFGEF1
 
 
hsa-miR-148a-3p
 
 
-0.306
 
 
12.7
 
 
 
 
ARFIP1
 
 
hsa-miR-148a-3p
 
 
-0.474
 
 
22.0
 
 
 
 
ARHGAP1
 
 
hsa-miR-20b-5p
 
 
-0.325
 
 
59.0
 
 
 
 
ARHGAP1
 
 
hsa-miR-106a-5p
 
 
-0.329
 
 
59.0
 
 
 
 
ARHGAP21
 
 
hsa-miR-148a-3p
 
 
-0.287
 
 
15.1
 
 
 
 
ARL1
 
 
hsa-miR-20b-5p
 
 
-0.424
 
 
58.5
 
 
 
 
ARL1
 
 
hsa-miR-106a-5p
 
 
-0.429
 
 
58.5
 
 
 
 
ARL6IP1
 
 
hsa-miR-7-5p
 
 
-0.374
 
 
105.7
 
 
 
 
ARL6IP1
 
 
hsa-miR-148a-3p
 
 
-0.579
 
 
105.7
 
 
 
 
ARL6IP6
 
 
hsa-miR-98-5p
 
 
-0.293
 
 
12.8
 
 
 
 
ARL6IP6
 
 
hsa-let-7c-5p
 
 
-0.293
 
 
12.8
 
 
 
 
ARL6IP6
 
 
hsa-let-7e-5p
 
 
-0.305
 
 
12.8
 
 
 
 
ARL8B
 
 
hsa-miR-148a-3p
 
 
-0.337
 
 
39.7
 
 
 
 
ARMC8
 
 
hsa-miR-106a-5p
 
 
-0.375
 
 
15.4
 
 
 
 
ARMC8
 
 
hsa-miR-20b-5p
 
 
-0.378
 
 
15.4
 
 
 
 
ARMCX1
 
 
hsa-miR-21-5p
 
 
-0.489
 
 
21.0
 
 
 
 
ARMT1
 
 
hsa-let-7c-5p
 
 
-0.303
 
 
23.4
 
 
 
 
ARMT1
 
 
hsa-let-7e-5p
 
 
-0.303
 
 
23.4
 
 
 
 
ARMT1
 
 
hsa-miR-98-5p
 
 
-0.303
 
 
23.4
 
 
 
 
ARPP19
 
 
hsa-let-7e-5p
 
 
-0.492
 
 
43.2
 
 
 
 
ARPP19
 
 
hsa-let-7c-5p
 
 
-0.492
 
 
43.2
 
 
 
 
ARPP19
 
 
hsa-miR-98-5p
 
 
-0.5
 
 
43.2
 
 
 
 
ARRDC3
 
 
hsa-miR-148a-3p
 
 
-0.305
 
 
88.5
 
 
 
 
ARRDC4
 
 
hsa-let-7c-5p
 
 
-0.329
 
 
18.6
 
 
 
 
ARRDC4
 
 
hsa-let-7e-5p
 
 
-0.329
 
 
18.6
 
 
 
 
ARRDC4
 
 
hsa-miR-98-5p
 
 
-0.329
 
 
18.6
 
 
 
 
ASAP1
 
 
hsa-let-7e-5p
 
 
-0.296
 
 
22.2
 
 
 
 
ASAP1
 
 
hsa-miR-98-5p
 
 
-0.308
 
 
22.2
 
 
 
 
ASAP1
 
 
hsa-let-7c-5p
 
 
-0.308
 
 
22.2
 
 
 
 
ASF1A
 
 
hsa-miR-21-5p
 
 
-0.304
 
 
11.7
 
 
 
 
ASPH
 
 
hsa-miR-148a-3p
 
 
-0.391
 
 
99.5
 
 
 
 
ATOSB
 
 
hsa-let-7e-5p
 
 
-0.429
 
 
10.1
 
 
 
 
ATOSB
 
 
hsa-miR-98-5p
 
 
-0.429
 
 
10.1
 
 
 
 
ATOSB
 
 
hsa-let-7c-5p
 
 
-0.429
 
 
10.1
 
 
 
 
ATP2A2
 
 
hsa-let-7e-5p
 
 
-0.311
 
 
28.6
 
 
 
 
ATP2A2
 
 
hsa-miR-98-5p
 
 
-0.311
 
 
28.6
 
 
 
 
ATP2A2
 
 
hsa-let-7c-5p
 
 
-0.311
 
 
28.6
 
 
 
 
ATP2B4
 
 
hsa-miR-148a-3p
 
 
-0.337
 
 
15.5
 
 
 
 
ATP6AP2
 
 
hsa-miR-148a-3p
 
 
-0.592
 
 
89.1
 
 
 
 
ATP6AP2
 
 
hsa-miR-148a-3p
 
 
-0.668
 
 
89.1
 
 
 
 
ATPAF1
 
 
hsa-let-7e-5p
 
 
-0.498
 
 
33.1
 
 
 
 
ATPAF1
 
 
hsa-miR-98-5p
 
 
-0.498
 
 
33.1
 
 
 
 
ATPAF1
 
 
hsa-let-7c-5p
 
 
-0.498
 
 
33.1
 
 
 
 
AVPI1
 
 
hsa-miR-7-5p
 
 
-0.367
 
 
22.2
 
 
 
 
B3GAT3
 
 
hsa-let-7e-5p
 
 
-0.324
 
 
29.8
 
 
 
 
B3GAT3
 
 
hsa-miR-98-5p
 
 
-0.324
 
 
29.8
 
 
 
 
B3GAT3
 
 
hsa-let-7c-5p
 
 
-0.324
 
 
29.8
 
 
 
 
B4GALT5
 
 
hsa-miR-148a-3p
 
 
-0.44
 
 
17.9
 
 
 
 
 
 
 cervix 
 
 
 
 
target_symbol
 
 
mature_mirna_id
 
 
score
 
 
nTPM
 
 
 
 
 
 
ABCB7
 
 
hsa-miR-148a-3p
 
 
-0.515
 
 
10.8
 
 
 
 
ABCC5
 
 
hsa-miR-125a-5p
 
 
-0.288
 
 
31.4
 
 
 
 
ABCD3
 
 
hsa-miR-148a-3p
 
 
-0.388
 
 
21.0
 
 
 
 
ABHD17C
 
 
hsa-let-7c-5p
 
 
-0.359
 
 
18.1
 
 
 
 
ABHD17C
 
 
hsa-let-7e-5p
 
 
-0.359
 
 
18.1
 
 
 
 
ABHD17C
 
 
hsa-miR-98-5p
 
 
-0.383
 
 
18.1
 
 
 
 
ABHD3
 
 
hsa-miR-125a-5p
 
 
-0.453
 
 
10.5
 
 
 
 
ABTB1
 
 
hsa-miR-125a-5p
 
 
-0.519
 
 
42.5
 
 
 
 
ACP3
 
 
hsa-let-7e-5p
 
 
-0.298
 
 
10.9
 
 
 
 
ACP3
 
 
hsa-miR-98-5p
 
 
-0.298
 
 
10.9
 
 
 
 
ACP3
 
 
hsa-let-7c-5p
 
 
-0.298
 
 
10.9
 
 
 
 
ACTC1
 
 
hsa-miR-7-5p
 
 
-0.471
 
 
44.2
 
 
 
 
ACVR1
 
 
hsa-miR-148a-3p
 
 
-0.397
 
 
20.6
 
 
 
 
ADAM10
 
 
hsa-miR-148a-3p
 
 
-0.356
 
 
15.1
 
 
 
 
ADAM9
 
 
hsa-miR-106a-5p
 
 
-0.317
 
 
32.7
 
 
 
 
ADAM9
 
 
hsa-miR-20b-5p
 
 
-0.328
 
 
32.7
 
 
 
 
ADRB2
 
 
hsa-let-7c-5p
 
 
-0.635
 
 
12.7
 
 
 
 
ADRB2
 
 
hsa-let-7e-5p
 
 
-0.635
 
 
12.7
 
 
 
 
ADRB2
 
 
hsa-miR-98-5p
 
 
-0.635
 
 
12.7
 
 
 
 
AGFG1
 
 
hsa-miR-148a-3p
 
 
-0.332
 
 
27.5
 
 
 
 
AGFG2
 
 
hsa-miR-20b-5p
 
 
-0.386
 
 
11.6
 
 
 
 
AGFG2
 
 
hsa-miR-106a-5p
 
 
-0.399
 
 
11.6
 
 
 
 
AHDC1
 
 
hsa-miR-148a-3p
 
 
-0.413
 
 
23.6
 
 
 
 
AHNAK
 
 
hsa-miR-106a-5p
 
 
-0.334
 
 
181.1
 
 
 
 
AHNAK
 
 
hsa-miR-20b-5p
 
 
-0.337
 
 
181.1
 
 
 
 
AIFM1
 
 
hsa-miR-125a-5p
 
 
-0.3
 
 
14.8
 
 
 
 
AIFM1
 
 
hsa-let-7e-5p
 
 
-0.314
 
 
14.8
 
 
 
 
AIFM1
 
 
hsa-miR-98-5p
 
 
-0.314
 
 
14.8
 
 
 
 
AIFM1
 
 
hsa-let-7c-5p
 
 
-0.314
 
 
14.8
 
 
 
 
AKAP1
 
 
hsa-miR-148a-3p
 
 
-0.381
 
 
22.6
 
 
 
 
ALCAM
 
 
hsa-miR-148a-3p
 
 
-0.339
 
 
26.3
 
 
 
 
ALDH4A1
 
 
hsa-miR-184
 
 
-0.421
 
 
15.0
 
 
 
 
ALG3
 
 
hsa-miR-106a-5p
 
 
-0.569
 
 
18.1
 
 
 
 
ALG3
 
 
hsa-miR-20b-5p
 
 
-0.58
 
 
18.1
 
 
 
 
ALG9
 
 
hsa-miR-7-5p
 
 
-0.302
 
 
10.8
 
 
 
 
AMT
 
 
hsa-let-7e-5p
 
 
-0.463
 
 
14.5
 
 
 
 
AMT
 
 
hsa-miR-98-5p
 
 
-0.463
 
 
14.5
 
 
 
 
AMT
 
 
hsa-let-7c-5p
 
 
-0.463
 
 
14.5
 
 
 
 
ANAPC16
 
 
hsa-miR-125a-5p
 
 
-0.578
 
 
18.3
 
 
 
 
ANKRD12
 
 
hsa-miR-7-5p
 
 
-0.37
 
 
10.5
 
 
 
 
ANKRD29
 
 
hsa-miR-106a-5p
 
 
-0.369
 
 
12.2
 
 
 
 
ANKRD29
 
 
hsa-miR-20b-5p
 
 
-0.38
 
 
12.2
 
 
 
 
ANKRD46
 
 
hsa-let-7e-5p
 
 
-0.389
 
 
14.1
 
 
 
 
ANKRD46
 
 
hsa-miR-98-5p
 
 
-0.389
 
 
14.1
 
 
 
 
ANKRD46
 
 
hsa-let-7c-5p
 
 
-0.389
 
 
14.1
 
 
 
 
ANKRD50
 
 
hsa-miR-106a-5p
 
 
-0.29
 
 
10.7
 
 
 
 
ANKRD9
 
 
hsa-miR-20b-5p
 
 
-0.331
 
 
10.4
 
 
 
 
ANKRD9
 
 
hsa-miR-106a-5p
 
 
-0.344
 
 
10.4
 
 
 
 
ANPEP
 
 
hsa-miR-125a-5p
 
 
-0.372
 
 
38.9
 
 
 
 
AP1S1
 
 
hsa-let-7e-5p
 
 
-0.374
 
 
20.1
 
 
 
 
AP1S1
 
 
hsa-miR-98-5p
 
 
-0.374
 
 
20.1
 
 
 
 
AP1S1
 
 
hsa-let-7c-5p
 
 
-0.374
 
 
20.1
 
 
 
 
APCDD1
 
 
hsa-miR-106a-5p
 
 
-0.422
 
 
33.1
 
 
 
 
APCDD1
 
 
hsa-miR-20b-5p
 
 
-0.425
 
 
33.1
 
 
 
 
ARF4
 
 
hsa-miR-7-5p
 
 
-0.407
 
 
196.1
 
 
 
 
ARF4
 
 
hsa-miR-148a-3p
 
 
-0.487
 
 
196.1
 
 
 
 
ARFGEF1
 
 
hsa-miR-148a-3p
 
 
-0.306
 
 
13.3
 
 
 
 
ARFIP1
 
 
hsa-miR-148a-3p
 
 
-0.474
 
 
21.1
 
 
 
 
ARG2
 
 
hsa-let-7e-5p
 
 
-0.468
 
 
16.0
 
 
 
 
ARG2
 
 
hsa-miR-98-5p
 
 
-0.468
 
 
16.0
 
 
 
 
ARG2
 
 
hsa-let-7c-5p
 
 
-0.468
 
 
16.0
 
 
 
 
ARHGAP1
 
 
hsa-miR-20b-5p
 
 
-0.325
 
 
62.9
 
 
 
 
ARHGAP1
 
 
hsa-miR-106a-5p
 
 
-0.329
 
 
62.9
 
 
 
 
ARHGAP21
 
 
hsa-miR-148a-3p
 
 
-0.287
 
 
18.5
 
 
 
 
ARL1
 
 
hsa-miR-20b-5p
 
 
-0.424
 
 
81.2
 
 
 
 
ARL1
 
 
hsa-miR-106a-5p
 
 
-0.429
 
 
81.2
 
 
 
 
ARL4D
 
 
hsa-let-7e-5p
 
 
-0.417
 
 
27.6
 
 
 
 
ARL4D
 
 
hsa-miR-98-5p
 
 
-0.417
 
 
27.6
 
 
 
 
ARL4D
 
 
hsa-let-7c-5p
 
 
-0.417
 
 
27.6
 
 
 
 
ARL6IP1
 
 
hsa-miR-7-5p
 
 
-0.374
 
 
74.5
 
 
 
 
ARL6IP1
 
 
hsa-miR-148a-3p
 
 
-0.579
 
 
74.5
 
 
 
 
ARL8B
 
 
hsa-miR-148a-3p
 
 
-0.337
 
 
42.9
 
 
 
 
ARMC8
 
 
hsa-miR-106a-5p
 
 
-0.375
 
 
13.8
 
 
 
 
ARMC8
 
 
hsa-miR-20b-5p
 
 
-0.378
 
 
13.8
 
 
 
 
ARMCX1
 
 
hsa-miR-21-5p
 
 
-0.489
 
 
27.8
 
 
 
 
ARMT1
 
 
hsa-let-7c-5p
 
 
-0.303
 
 
15.1
 
 
 
 
ARMT1
 
 
hsa-let-7e-5p
 
 
-0.303
 
 
15.1
 
 
 
 
ARMT1
 
 
hsa-miR-98-5p
 
 
-0.303
 
 
15.1
 
 
 
 
ARPP19
 
 
hsa-let-7e-5p
 
 
-0.492
 
 
35.8
 
 
 
 
ARPP19
 
 
hsa-let-7c-5p
 
 
-0.492
 
 
35.8
 
 
 
 
ARPP19
 
 
hsa-miR-98-5p
 
 
-0.5
 
 
35.8
 
 
 
 
ARRDC3
 
 
hsa-miR-148a-3p
 
 
-0.305
 
 
42.0
 
 
 
 
ARRDC4
 
 
hsa-let-7c-5p
 
 
-0.329
 
 
14.3
 
 
 
 
ARRDC4
 
 
hsa-let-7e-5p
 
 
-0.329
 
 
14.3
 
 
 
 
ARRDC4
 
 
hsa-miR-98-5p
 
 
-0.329
 
 
14.3
 
 
 
 
ASAP1
 
 
hsa-let-7e-5p
 
 
-0.296
 
 
15.1
 
 
 
 
ASAP1
 
 
hsa-miR-98-5p
 
 
-0.308
 
 
15.1
 
 
 
 
ASAP1
 
 
hsa-let-7c-5p
 
 
-0.308
 
 
15.1
 
 
 
 
ASPH
 
 
hsa-miR-148a-3p
 
 
-0.391
 
 
60.9
 
 
 
 
ATAD2
 
 
hsa-miR-20b-5p
 
 
-0.304
 
 
12.1
 
 
 
 
ATAD2
 
 
hsa-miR-106a-5p
 
 
-0.308
 
 
12.1
 
 
 
 
ATG4D
 
 
hsa-miR-148a-3p
 
 
-0.303
 
 
11.7
 
 
 
 
ATOSB
 
 
hsa-let-7e-5p
 
 
-0.429
 
 
12.3
 
 
 
 
ATOSB
 
 
hsa-miR-98-5p
 
 
-0.429
 
 
12.3
 
 
 
 
ATOSB
 
 
hsa-let-7c-5p
 
 
-0.429
 
 
12.3
 
 
 
 
ATP2A2
 
 
hsa-let-7e-5p
 
 
-0.311
 
 
40.8
 
 
 
 
ATP2A2
 
 
hsa-miR-98-5p
 
 
-0.311
 
 
40.8
 
 
 
 
ATP2A2
 
 
hsa-let-7c-5p
 
 
-0.311
 
 
40.8
 
 
 
 
ATP2B4
 
 
hsa-miR-148a-3p
 
 
-0.337
 
 
44.8
 
 
 
 
ATP6AP2
 
 
hsa-miR-148a-3p
 
 
-0.592
 
 
99.7
 
 
 
 
 
 
 choroid plexus 
 
 
 
 
target_symbol
 
 
mature_mirna_id
 
 
score
 
 
nTPM
 
 
 
 
 
 
ABCB7
 
 
hsa-miR-148a-3p
 
 
-0.515
 
 
15.2
 
 
 
 
ABCC5
 
 
hsa-miR-125a-5p
 
 
-0.288
 
 
137.7
 
 
 
 
ABCD3
 
 
hsa-miR-148a-3p
 
 
-0.388
 
 
13.3
 
 
 
 
ABHD3
 
 
hsa-miR-125a-5p
 
 
-0.453
 
 
24.1
 
 
 
 
ABHD6
 
 
hsa-miR-125a-5p
 
 
-0.501
 
 
15.1
 
 
 
 
ABTB1
 
 
hsa-miR-125a-5p
 
 
-0.519
 
 
29.8
 
 
 
 
ADAM9
 
 
hsa-miR-106a-5p
 
 
-0.317
 
 
14.9
 
 
 
 
ADAM9
 
 
hsa-miR-20b-5p
 
 
-0.328
 
 
14.9
 
 
 
 
AGFG1
 
 
hsa-miR-148a-3p
 
 
-0.332
 
 
23.3
 
 
 
 
AHNAK
 
 
hsa-miR-106a-5p
 
 
-0.334
 
 
26.0
 
 
 
 
AHNAK
 
 
hsa-miR-20b-5p
 
 
-0.337
 
 
26.0
 
 
 
 
AIFM1
 
 
hsa-miR-125a-5p
 
 
-0.3
 
 
37.9
 
 
 
 
AIFM1
 
 
hsa-let-7e-5p
 
 
-0.314
 
 
37.9
 
 
 
 
AIFM1
 
 
hsa-miR-98-5p
 
 
-0.314
 
 
37.9
 
 
 
 
AIFM1
 
 
hsa-let-7c-5p
 
 
-0.314
 
 
37.9
 
 
 
 
ALDH4A1
 
 
hsa-miR-184
 
 
-0.421
 
 
121.5
 
 
 
 
ALG3
 
 
hsa-miR-106a-5p
 
 
-0.569
 
 
25.2
 
 
 
 
ALG3
 
 
hsa-miR-20b-5p
 
 
-0.58
 
 
25.2
 
 
 
 
AMT
 
 
hsa-let-7e-5p
 
 
-0.463
 
 
33.4
 
 
 
 
AMT
 
 
hsa-miR-98-5p
 
 
-0.463
 
 
33.4
 
 
 
 
AMT
 
 
hsa-let-7c-5p
 
 
-0.463
 
 
33.4
 
 
 
 
ANAPC16
 
 
hsa-miR-125a-5p
 
 
-0.578
 
 
16.8
 
 
 
 
ANKRD46
 
 
hsa-let-7e-5p
 
 
-0.389
 
 
11.0
 
 
 
 
ANKRD46
 
 
hsa-miR-98-5p
 
 
-0.389
 
 
11.0
 
 
 
 
ANKRD46
 
 
hsa-let-7c-5p
 
 
-0.389
 
 
11.0
 
 
 
 
ANKRD9
 
 
hsa-miR-20b-5p
 
 
-0.331
 
 
15.2
 
 
 
 
ANKRD9
 
 
hsa-miR-106a-5p
 
 
-0.344
 
 
15.2
 
 
 
 
AP1S1
 
 
hsa-let-7e-5p
 
 
-0.374
 
 
10.3
 
 
 
 
AP1S1
 
 
hsa-miR-98-5p
 
 
-0.374
 
 
10.3
 
 
 
 
AP1S1
 
 
hsa-let-7c-5p
 
 
-0.374
 
 
10.3
 
 
 
 
ARF4
 
 
hsa-miR-7-5p
 
 
-0.407
 
 
115.4
 
 
 
 
ARF4
 
 
hsa-miR-148a-3p
 
 
-0.487
 
 
115.4
 
 
 
 
ARG2
 
 
hsa-let-7e-5p
 
 
-0.468
 
 
49.7
 
 
 
 
ARG2
 
 
hsa-miR-98-5p
 
 
-0.468
 
 
49.7
 
 
 
 
ARG2
 
 
hsa-let-7c-5p
 
 
-0.468
 
 
49.7
 
 
 
 
ARHGAP1
 
 
hsa-miR-20b-5p
 
 
-0.325
 
 
26.4
 
 
 
 
ARHGAP1
 
 
hsa-miR-106a-5p
 
 
-0.329
 
 
26.4
 
 
 
 
ARL1
 
 
hsa-miR-20b-5p
 
 
-0.424
 
 
84.0
 
 
 
 
ARL1
 
 
hsa-miR-106a-5p
 
 
-0.429
 
 
84.0
 
 
 
 
ARL4D
 
 
hsa-let-7e-5p
 
 
-0.417
 
 
15.4
 
 
 
 
ARL4D
 
 
hsa-miR-98-5p
 
 
-0.417
 
 
15.4
 
 
 
 
ARL4D
 
 
hsa-let-7c-5p
 
 
-0.417
 
 
15.4
 
 
 
 
ARL6IP1
 
 
hsa-miR-7-5p
 
 
-0.374
 
 
185.0
 
 
 
 
ARL6IP1
 
 
hsa-miR-148a-3p
 
 
-0.579
 
 
185.0
 
 
 
 
ARL8B
 
 
hsa-miR-148a-3p
 
 
-0.337
 
 
19.0
 
 
 
 
ARMC8
 
 
hsa-miR-106a-5p
 
 
-0.375
 
 
20.8
 
 
 
 
ARMC8
 
 
hsa-miR-20b-5p
 
 
-0.378
 
 
20.8
 
 
 
 
ARMCX1
 
 
hsa-miR-21-5p
 
 
-0.489
 
 
20.0
 
 
 
 
ARPP19
 
 
hsa-let-7e-5p
 
 
-0.492
 
 
32.4
 
 
 
 
ARPP19
 
 
hsa-let-7c-5p
 
 
-0.492
 
 
32.4
 
 
 
 
ARPP19
 
 
hsa-miR-98-5p
 
 
-0.5
 
 
32.4
 
 
 
 
ARRDC3
 
 
hsa-miR-148a-3p
 
 
-0.305
 
 
24.1
 
 
 
 
ASPH
 
 
hsa-miR-148a-3p
 
 
-0.391
 
 
79.9
 
 
 
 
ATG16L1
 
 
hsa-miR-106a-5p
 
 
-0.464
 
 
21.8
 
 
 
 
ATG16L1
 
 
hsa-miR-20b-5p
 
 
-0.467
 
 
21.8
 
 
 
 
ATG4A
 
 
hsa-miR-7-5p
 
 
-0.448
 
 
16.6
 
 
 
 
ATOSB
 
 
hsa-let-7e-5p
 
 
-0.429
 
 
16.6
 
 
 
 
ATOSB
 
 
hsa-miR-98-5p
 
 
-0.429
 
 
16.6
 
 
 
 
ATOSB
 
 
hsa-let-7c-5p
 
 
-0.429
 
 
16.6
 
 
 
 
ATP2A2
 
 
hsa-let-7e-5p
 
 
-0.311
 
 
29.6
 
 
 
 
ATP2A2
 
 
hsa-miR-98-5p
 
 
-0.311
 
 
29.6
 
 
 
 
ATP2A2
 
 
hsa-let-7c-5p
 
 
-0.311
 
 
29.6
 
 
 
 
ATP2B2
 
 
hsa-miR-7-5p
 
 
-0.297
 
 
61.0
 
 
 
 
ATP6AP2
 
 
hsa-miR-148a-3p
 
 
-0.592
 
 
85.2
 
 
 
 
ATP6AP2
 
 
hsa-miR-148a-3p
 
 
-0.668
 
 
85.2
 
 
 
 
ATPAF1
 
 
hsa-let-7e-5p
 
 
-0.498
 
 
168.0
 
 
 
 
ATPAF1
 
 
hsa-miR-98-5p
 
 
-0.498
 
 
168.0
 
 
 
 
ATPAF1
 
 
hsa-let-7c-5p
 
 
-0.498
 
 
168.0
 
 
 
 
AVPI1
 
 
hsa-miR-7-5p
 
 
-0.367
 
 
20.0
 
 
 
 
B3GAT3
 
 
hsa-let-7e-5p
 
 
-0.324
 
 
87.1
 
 
 
 
B3GAT3
 
 
hsa-miR-98-5p
 
 
-0.324
 
 
87.1
 
 
 
 
B3GAT3
 
 
hsa-let-7c-5p
 
 
-0.324
 
 
87.1
 
 
 
 
B4GAT1
 
 
hsa-let-7c-5p
 
 
-0.47
 
 
93.8
 
 
 
 
B4GAT1
 
 
hsa-let-7e-5p
 
 
-0.47
 
 
93.8
 
 
 
 
B4GAT1
 
 
hsa-miR-98-5p
 
 
-0.47
 
 
93.8
 
 
 
 
BAK1
 
 
hsa-miR-125a-5p
 
 
-0.402
 
 
16.1
 
 
 
 
BAMBI
 
 
hsa-miR-106a-5p
 
 
-0.503
 
 
37.8
 
 
 
 
BAMBI
 
 
hsa-miR-20b-5p
 
 
-0.506
 
 
37.8
 
 
 
 
BCAP29
 
 
hsa-let-7e-5p
 
 
-0.289
 
 
12.5
 
 
 
 
BDH1
 
 
hsa-miR-125a-5p
 
 
-0.389
 
 
39.2
 
 
 
 
BIN3
 
 
hsa-let-7e-5p
 
 
-0.427
 
 
33.7
 
 
 
 
BIN3
 
 
hsa-miR-98-5p
 
 
-0.427
 
 
33.7
 
 
 
 
BIN3
 
 
hsa-let-7c-5p
 
 
-0.427
 
 
33.7
 
 
 
 
BLOC1S4
 
 
hsa-miR-7-5p
 
 
-0.417
 
 
10.3
 
 
 
 
BLOC1S4
 
 
hsa-miR-7-5p
 
 
-0.527
 
 
10.3
 
 
 
 
BLOC1S6
 
 
hsa-let-7e-5p
 
 
-0.321
 
 
14.9
 
 
 
 
BLOC1S6
 
 
hsa-miR-98-5p
 
 
-0.321
 
 
14.9
 
 
 
 
BLOC1S6
 
 
hsa-let-7c-5p
 
 
-0.321
 
 
14.9
 
 
 
 
BTBD10
 
 
hsa-miR-106a-5p
 
 
-0.406
 
 
10.3
 
 
 
 
BTBD10
 
 
hsa-miR-20b-5p
 
 
-0.425
 
 
10.3
 
 
 
 
BTBD3
 
 
hsa-miR-148a-3p
 
 
-0.495
 
 
18.2
 
 
 
 
BTF3L4
 
 
hsa-let-7c-5p
 
 
-0.355
 
 
26.9
 
 
 
 
BTF3L4
 
 
hsa-let-7e-5p
 
 
-0.355
 
 
26.9
 
 
 
 
BTF3L4
 
 
hsa-miR-98-5p
 
 
-0.355
 
 
26.9
 
 
 
 
BTG3
 
 
hsa-miR-106a-5p
 
 
-0.665
 
 
25.9
 
 
 
 
BTG3
 
 
hsa-miR-20b-5p
 
 
-0.684
 
 
25.9
 
 
 
 
BZW1
 
 
hsa-let-7e-5p
 
 
-0.354
 
 
50.7
 
 
 
 
BZW1
 
 
hsa-miR-98-5p
 
 
-0.354
 
 
50.7
 
 
 
 
BZW1
 
 
hsa-let-7c-5p
 
 
-0.354
 
 
50.7
 
 
 
 
BZW1
 
 
hsa-let-7e-5p
 
 
-0.375
 
 
50.7
 
 
 
 
 
 
 endometrium 
 
 
 
 
target_symbol
 
 
mature_mirna_id
 
 
score
 
 
nTPM
 
 
 
 
 
 
ABCB7
 
 
hsa-miR-148a-3p
 
 
-0.515
 
 
10.5
 
 
 
 
ABCC5
 
 
hsa-miR-125a-5p
 
 
-0.288
 
 
22.5
 
 
 
 
ABCD3
 
 
hsa-miR-148a-3p
 
 
-0.388
 
 
13.8
 
 
 
 
ABTB1
 
 
hsa-miR-125a-5p
 
 
-0.519
 
 
33.5
 
 
 
 
ACTC1
 
 
hsa-miR-7-5p
 
 
-0.471
 
 
17.7
 
 
 
 
ACVR1
 
 
hsa-miR-148a-3p
 
 
-0.397
 
 
15.9
 
 
 
 
ADAM10
 
 
hsa-miR-148a-3p
 
 
-0.356
 
 
15.2
 
 
 
 
ADAM9
 
 
hsa-miR-106a-5p
 
 
-0.317
 
 
21.6
 
 
 
 
ADAM9
 
 
hsa-miR-20b-5p
 
 
-0.328
 
 
21.6
 
 
 
 
ADGRF5
 
 
hsa-miR-148a-3p
 
 
-0.354
 
 
16.0
 
 
 
 
AGFG1
 
 
hsa-miR-148a-3p
 
 
-0.332
 
 
23.1
 
 
 
 
AGFG2
 
 
hsa-miR-20b-5p
 
 
-0.386
 
 
20.0
 
 
 
 
AGFG2
 
 
hsa-miR-106a-5p
 
 
-0.399
 
 
20.0
 
 
 
 
AHDC1
 
 
hsa-miR-148a-3p
 
 
-0.413
 
 
26.0
 
 
 
 
AHNAK
 
 
hsa-miR-106a-5p
 
 
-0.334
 
 
187.4
 
 
 
 
AHNAK
 
 
hsa-miR-20b-5p
 
 
-0.337
 
 
187.4
 
 
 
 
AIFM1
 
 
hsa-miR-125a-5p
 
 
-0.3
 
 
10.6
 
 
 
 
AIFM1
 
 
hsa-let-7e-5p
 
 
-0.314
 
 
10.6
 
 
 
 
AIFM1
 
 
hsa-miR-98-5p
 
 
-0.314
 
 
10.6
 
 
 
 
AIFM1
 
 
hsa-let-7c-5p
 
 
-0.314
 
 
10.6
 
 
 
 
AKAP1
 
 
hsa-miR-148a-3p
 
 
-0.381
 
 
24.8
 
 
 
 
ALCAM
 
 
hsa-miR-148a-3p
 
 
-0.339
 
 
14.4
 
 
 
 
ALDH4A1
 
 
hsa-miR-184
 
 
-0.421
 
 
10.1
 
 
 
 
ALG3
 
 
hsa-miR-106a-5p
 
 
-0.569
 
 
15.9
 
 
 
 
ALG3
 
 
hsa-miR-20b-5p
 
 
-0.58
 
 
15.9
 
 
 
 
ALG9
 
 
hsa-miR-7-5p
 
 
-0.302
 
 
10.7
 
 
 
 
AMT
 
 
hsa-let-7e-5p
 
 
-0.463
 
 
10.0
 
 
 
 
AMT
 
 
hsa-miR-98-5p
 
 
-0.463
 
 
10.0
 
 
 
 
AMT
 
 
hsa-let-7c-5p
 
 
-0.463
 
 
10.0
 
 
 
 
ANAPC16
 
 
hsa-miR-125a-5p
 
 
-0.578
 
 
18.6
 
 
 
 
ANKRD12
 
 
hsa-miR-7-5p
 
 
-0.37
 
 
11.1
 
 
 
 
ANKRD29
 
 
hsa-miR-106a-5p
 
 
-0.369
 
 
14.7
 
 
 
 
ANKRD29
 
 
hsa-miR-20b-5p
 
 
-0.38
 
 
14.7
 
 
 
 
ANKRD46
 
 
hsa-let-7e-5p
 
 
-0.389
 
 
11.8
 
 
 
 
ANKRD46
 
 
hsa-miR-98-5p
 
 
-0.389
 
 
11.8
 
 
 
 
ANKRD46
 
 
hsa-let-7c-5p
 
 
-0.389
 
 
11.8
 
 
 
 
AP1AR
 
 
hsa-miR-21-5p
 
 
-0.318
 
 
10.6
 
 
 
 
AP1AR
 
 
hsa-miR-99b-5p
 
 
-0.823
 
 
10.6
 
 
 
 
AP1S1
 
 
hsa-let-7e-5p
 
 
-0.374
 
 
16.1
 
 
 
 
AP1S1
 
 
hsa-miR-98-5p
 
 
-0.374
 
 
16.1
 
 
 
 
AP1S1
 
 
hsa-let-7c-5p
 
 
-0.374
 
 
16.1
 
 
 
 
APCDD1
 
 
hsa-miR-106a-5p
 
 
-0.422
 
 
32.5
 
 
 
 
APCDD1
 
 
hsa-miR-20b-5p
 
 
-0.425
 
 
32.5
 
 
 
 
ARF4
 
 
hsa-miR-7-5p
 
 
-0.407
 
 
113.9
 
 
 
 
ARF4
 
 
hsa-miR-148a-3p
 
 
-0.487
 
 
113.9
 
 
 
 
ARFIP1
 
 
hsa-miR-148a-3p
 
 
-0.474
 
 
20.3
 
 
 
 
ARHGAP1
 
 
hsa-miR-20b-5p
 
 
-0.325
 
 
81.8
 
 
 
 
ARHGAP1
 
 
hsa-miR-106a-5p
 
 
-0.329
 
 
81.8
 
 
 
 
ARHGAP21
 
 
hsa-miR-148a-3p
 
 
-0.287
 
 
18.5
 
 
 
 
ARL1
 
 
hsa-miR-20b-5p
 
 
-0.424
 
 
47.9
 
 
 
 
ARL1
 
 
hsa-miR-106a-5p
 
 
-0.429
 
 
47.9
 
 
 
 
ARL4D
 
 
hsa-let-7e-5p
 
 
-0.417
 
 
19.2
 
 
 
 
ARL4D
 
 
hsa-miR-98-5p
 
 
-0.417
 
 
19.2
 
 
 
 
ARL4D
 
 
hsa-let-7c-5p
 
 
-0.417
 
 
19.2
 
 
 
 
ARL6IP1
 
 
hsa-miR-7-5p
 
 
-0.374
 
 
66.4
 
 
 
 
ARL6IP1
 
 
hsa-miR-148a-3p
 
 
-0.579
 
 
66.4
 
 
 
 
ARL6IP6
 
 
hsa-miR-98-5p
 
 
-0.293
 
 
11.0
 
 
 
 
ARL6IP6
 
 
hsa-let-7c-5p
 
 
-0.293
 
 
11.0
 
 
 
 
ARL6IP6
 
 
hsa-let-7e-5p
 
 
-0.305
 
 
11.0
 
 
 
 
ARL8B
 
 
hsa-miR-148a-3p
 
 
-0.337
 
 
42.3
 
 
 
 
ARMC8
 
 
hsa-miR-106a-5p
 
 
-0.375
 
 
16.5
 
 
 
 
ARMC8
 
 
hsa-miR-20b-5p
 
 
-0.378
 
 
16.5
 
 
 
 
ARMCX1
 
 
hsa-miR-21-5p
 
 
-0.489
 
 
28.5
 
 
 
 
ARMT1
 
 
hsa-let-7c-5p
 
 
-0.303
 
 
15.0
 
 
 
 
ARMT1
 
 
hsa-let-7e-5p
 
 
-0.303
 
 
15.0
 
 
 
 
ARMT1
 
 
hsa-miR-98-5p
 
 
-0.303
 
 
15.0
 
 
 
 
ARPP19
 
 
hsa-let-7e-5p
 
 
-0.492
 
 
44.7
 
 
 
 
ARPP19
 
 
hsa-let-7c-5p
 
 
-0.492
 
 
44.7
 
 
 
 
ARPP19
 
 
hsa-miR-98-5p
 
 
-0.5
 
 
44.7
 
 
 
 
ARRDC3
 
 
hsa-miR-148a-3p
 
 
-0.305
 
 
38.0
 
 
 
 
ARRDC4
 
 
hsa-let-7c-5p
 
 
-0.329
 
 
13.7
 
 
 
 
ARRDC4
 
 
hsa-let-7e-5p
 
 
-0.329
 
 
13.7
 
 
 
 
ARRDC4
 
 
hsa-miR-98-5p
 
 
-0.329
 
 
13.7
 
 
 
 
ASAP1
 
 
hsa-let-7e-5p
 
 
-0.296
 
 
17.2
 
 
 
 
ASAP1
 
 
hsa-miR-98-5p
 
 
-0.308
 
 
17.2
 
 
 
 
ASAP1
 
 
hsa-let-7c-5p
 
 
-0.308
 
 
17.2
 
 
 
 
ASPH
 
 
hsa-miR-148a-3p
 
 
-0.391
 
 
45.6
 
 
 
 
ATG16L1
 
 
hsa-miR-106a-5p
 
 
-0.464
 
 
11.3
 
 
 
 
ATG16L1
 
 
hsa-miR-20b-5p
 
 
-0.467
 
 
11.3
 
 
 
 
ATP2A2
 
 
hsa-let-7e-5p
 
 
-0.311
 
 
43.7
 
 
 
 
ATP2A2
 
 
hsa-miR-98-5p
 
 
-0.311
 
 
43.7
 
 
 
 
ATP2A2
 
 
hsa-let-7c-5p
 
 
-0.311
 
 
43.7
 
 
 
 
ATP2B4
 
 
hsa-miR-148a-3p
 
 
-0.337
 
 
98.2
 
 
 
 
ATP6AP2
 
 
hsa-miR-148a-3p
 
 
-0.592
 
 
81.0
 
 
 
 
ATP6AP2
 
 
hsa-miR-148a-3p
 
 
-0.668
 
 
81.0
 
 
 
 
ATPAF1
 
 
hsa-let-7e-5p
 
 
-0.498
 
 
23.6
 
 
 
 
ATPAF1
 
 
hsa-miR-98-5p
 
 
-0.498
 
 
23.6
 
 
 
 
ATPAF1
 
 
hsa-let-7c-5p
 
 
-0.498
 
 
23.6
 
 
 
 
AVPI1
 
 
hsa-miR-7-5p
 
 
-0.367
 
 
12.2
 
 
 
 
B3GALNT2
 
 
hsa-miR-125a-5p
 
 
-0.311
 
 
10.2
 
 
 
 
B3GAT3
 
 
hsa-let-7e-5p
 
 
-0.324
 
 
33.5
 
 
 
 
B3GAT3
 
 
hsa-miR-98-5p
 
 
-0.324
 
 
33.5
 
 
 
 
B3GAT3
 
 
hsa-let-7c-5p
 
 
-0.324
 
 
33.5
 
 
 
 
B4GALT5
 
 
hsa-miR-148a-3p
 
 
-0.44
 
 
22.0
 
 
 
 
B4GAT1
 
 
hsa-let-7c-5p
 
 
-0.47
 
 
28.7
 
 
 
 
B4GAT1
 
 
hsa-let-7e-5p
 
 
-0.47
 
 
28.7
 
 
 
 
B4GAT1
 
 
hsa-miR-98-5p
 
 
-0.47
 
 
28.7
 
 
 
 
BACH1
 
 
hsa-let-7e-5p
 
 
-0.298
 
 
18.4
 
 
 
 
BACH1
 
 
hsa-let-7e-5p
 
 
-0.298
 
 
18.4
 
 
 
 
BACH1
 
 
hsa-miR-98-5p
 
 
-0.298
 
 
18.4
 
 
 
 
 
 
 esophagus 
 
 
 
 
target_symbol
 
 
mature_mirna_id
 
 
score
 
 
nTPM
 
 
 
 
 
 
ABCB7
 
 
hsa-miR-148a-3p
 
 
-0.515
 
 
11.1
 
 
 
 
ABCC5
 
 
hsa-miR-125a-5p
 
 
-0.288
 
 
44.1
 
 
 
 
ABCD3
 
 
hsa-miR-148a-3p
 
 
-0.388
 
 
25.0
 
 
 
 
ABHD17C
 
 
hsa-let-7c-5p
 
 
-0.359
 
 
45.1
 
 
 
 
ABHD17C
 
 
hsa-let-7e-5p
 
 
-0.359
 
 
45.1
 
 
 
 
ABHD17C
 
 
hsa-miR-98-5p
 
 
-0.383
 
 
45.1
 
 
 
 
ABHD3
 
 
hsa-miR-125a-5p
 
 
-0.453
 
 
17.3
 
 
 
 
ABHD5
 
 
hsa-miR-20b-5p
 
 
-0.418
 
 
18.4
 
 
 
 
ABHD5
 
 
hsa-miR-106a-5p
 
 
-0.447
 
 
18.4
 
 
 
 
ABTB1
 
 
hsa-miR-125a-5p
 
 
-0.519
 
 
13.8
 
 
 
 
ACP3
 
 
hsa-let-7e-5p
 
 
-0.298
 
 
42.9
 
 
 
 
ACP3
 
 
hsa-miR-98-5p
 
 
-0.298
 
 
42.9
 
 
 
 
ACP3
 
 
hsa-let-7c-5p
 
 
-0.298
 
 
42.9
 
 
 
 
ACTC1
 
 
hsa-miR-7-5p
 
 
-0.471
 
 
21.5
 
 
 
 
ADAM10
 
 
hsa-miR-148a-3p
 
 
-0.356
 
 
14.2
 
 
 
 
ADAM9
 
 
hsa-miR-106a-5p
 
 
-0.317
 
 
22.2
 
 
 
 
ADAM9
 
 
hsa-miR-20b-5p
 
 
-0.328
 
 
22.2
 
 
 
 
ADRB2
 
 
hsa-let-7c-5p
 
 
-0.635
 
 
14.2
 
 
 
 
ADRB2
 
 
hsa-let-7e-5p
 
 
-0.635
 
 
14.2
 
 
 
 
ADRB2
 
 
hsa-miR-98-5p
 
 
-0.635
 
 
14.2
 
 
 
 
AGFG1
 
 
hsa-miR-148a-3p
 
 
-0.332
 
 
44.3
 
 
 
 
AGFG2
 
 
hsa-miR-20b-5p
 
 
-0.386
 
 
29.3
 
 
 
 
AGFG2
 
 
hsa-miR-106a-5p
 
 
-0.399
 
 
29.3
 
 
 
 
AHDC1
 
 
hsa-miR-148a-3p
 
 
-0.413
 
 
34.3
 
 
 
 
AHNAK
 
 
hsa-miR-106a-5p
 
 
-0.334
 
 
234.9
 
 
 
 
AHNAK
 
 
hsa-miR-20b-5p
 
 
-0.337
 
 
234.9
 
 
 
 
AIFM1
 
 
hsa-miR-125a-5p
 
 
-0.3
 
 
15.9
 
 
 
 
AIFM1
 
 
hsa-let-7e-5p
 
 
-0.314
 
 
15.9
 
 
 
 
AIFM1
 
 
hsa-miR-98-5p
 
 
-0.314
 
 
15.9
 
 
 
 
AIFM1
 
 
hsa-let-7c-5p
 
 
-0.314
 
 
15.9
 
 
 
 
AKAP1
 
 
hsa-miR-148a-3p
 
 
-0.381
 
 
34.2
 
 
 
 
ALDH4A1
 
 
hsa-miR-184
 
 
-0.421
 
 
36.6
 
 
 
 
ALG3
 
 
hsa-miR-106a-5p
 
 
-0.569
 
 
29.4
 
 
 
 
ALG3
 
 
hsa-miR-20b-5p
 
 
-0.58
 
 
29.4
 
 
 
 
ALG9
 
 
hsa-miR-7-5p
 
 
-0.302
 
 
10.5
 
 
 
 
ANAPC16
 
 
hsa-miR-125a-5p
 
 
-0.578
 
 
13.6
 
 
 
 
ANKRD12
 
 
hsa-miR-7-5p
 
 
-0.37
 
 
13.6
 
 
 
 
ANKRD46
 
 
hsa-let-7e-5p
 
 
-0.389
 
 
11.9
 
 
 
 
ANKRD46
 
 
hsa-miR-98-5p
 
 
-0.389
 
 
11.9
 
 
 
 
ANKRD46
 
 
hsa-let-7c-5p
 
 
-0.389
 
 
11.9
 
 
 
 
ANKRD9
 
 
hsa-miR-20b-5p
 
 
-0.331
 
 
18.5
 
 
 
 
ANKRD9
 
 
hsa-miR-106a-5p
 
 
-0.344
 
 
18.5
 
 
 
 
AP1AR
 
 
hsa-miR-21-5p
 
 
-0.318
 
 
12.5
 
 
 
 
AP1AR
 
 
hsa-miR-99b-5p
 
 
-0.823
 
 
12.5
 
 
 
 
AP1S1
 
 
hsa-let-7e-5p
 
 
-0.374
 
 
36.5
 
 
 
 
AP1S1
 
 
hsa-miR-98-5p
 
 
-0.374
 
 
36.5
 
 
 
 
AP1S1
 
 
hsa-let-7c-5p
 
 
-0.374
 
 
36.5
 
 
 
 
APCDD1
 
 
hsa-miR-106a-5p
 
 
-0.422
 
 
26.2
 
 
 
 
APCDD1
 
 
hsa-miR-20b-5p
 
 
-0.425
 
 
26.2
 
 
 
 
ARF4
 
 
hsa-miR-7-5p
 
 
-0.407
 
 
102.8
 
 
 
 
ARF4
 
 
hsa-miR-148a-3p
 
 
-0.487
 
 
102.8
 
 
 
 
ARFGEF1
 
 
hsa-miR-148a-3p
 
 
-0.306
 
 
14.2
 
 
 
 
ARFIP1
 
 
hsa-miR-148a-3p
 
 
-0.474
 
 
19.6
 
 
 
 
ARG2
 
 
hsa-let-7e-5p
 
 
-0.468
 
 
17.2
 
 
 
 
ARG2
 
 
hsa-miR-98-5p
 
 
-0.468
 
 
17.2
 
 
 
 
ARG2
 
 
hsa-let-7c-5p
 
 
-0.468
 
 
17.2
 
 
 
 
ARHGAP1
 
 
hsa-miR-20b-5p
 
 
-0.325
 
 
71.8
 
 
 
 
ARHGAP1
 
 
hsa-miR-106a-5p
 
 
-0.329
 
 
71.8
 
 
 
 
ARHGAP21
 
 
hsa-miR-148a-3p
 
 
-0.287
 
 
10.2
 
 
 
 
ARL1
 
 
hsa-miR-20b-5p
 
 
-0.424
 
 
46.2
 
 
 
 
ARL1
 
 
hsa-miR-106a-5p
 
 
-0.429
 
 
46.2
 
 
 
 
ARL4D
 
 
hsa-let-7e-5p
 
 
-0.417
 
 
80.8
 
 
 
 
ARL4D
 
 
hsa-miR-98-5p
 
 
-0.417
 
 
80.8
 
 
 
 
ARL4D
 
 
hsa-let-7c-5p
 
 
-0.417
 
 
80.8
 
 
 
 
ARL6IP1
 
 
hsa-miR-7-5p
 
 
-0.374
 
 
65.8
 
 
 
 
ARL6IP1
 
 
hsa-miR-148a-3p
 
 
-0.579
 
 
65.8
 
 
 
 
ARL6IP6
 
 
hsa-miR-98-5p
 
 
-0.293
 
 
11.7
 
 
 
 
ARL6IP6
 
 
hsa-let-7c-5p
 
 
-0.293
 
 
11.7
 
 
 
 
ARL6IP6
 
 
hsa-let-7e-5p
 
 
-0.305
 
 
11.7
 
 
 
 
ARL8B
 
 
hsa-miR-148a-3p
 
 
-0.337
 
 
71.1
 
 
 
 
ARMC8
 
 
hsa-miR-106a-5p
 
 
-0.375
 
 
19.8
 
 
 
 
ARMC8
 
 
hsa-miR-20b-5p
 
 
-0.378
 
 
19.8
 
 
 
 
ARMCX1
 
 
hsa-miR-21-5p
 
 
-0.489
 
 
10.7
 
 
 
 
ARMT1
 
 
hsa-let-7c-5p
 
 
-0.303
 
 
11.3
 
 
 
 
ARMT1
 
 
hsa-let-7e-5p
 
 
-0.303
 
 
11.3
 
 
 
 
ARMT1
 
 
hsa-miR-98-5p
 
 
-0.303
 
 
11.3
 
 
 
 
ARPP19
 
 
hsa-let-7e-5p
 
 
-0.492
 
 
49.0
 
 
 
 
ARPP19
 
 
hsa-let-7c-5p
 
 
-0.492
 
 
49.0
 
 
 
 
ARPP19
 
 
hsa-miR-98-5p
 
 
-0.5
 
 
49.0
 
 
 
 
ARRDC3
 
 
hsa-miR-148a-3p
 
 
-0.305
 
 
36.4
 
 
 
 
ASAP1
 
 
hsa-let-7e-5p
 
 
-0.296
 
 
10.0
 
 
 
 
ASAP1
 
 
hsa-miR-98-5p
 
 
-0.308
 
 
10.0
 
 
 
 
ASAP1
 
 
hsa-let-7c-5p
 
 
-0.308
 
 
10.0
 
 
 
 
ASPH
 
 
hsa-miR-148a-3p
 
 
-0.391
 
 
66.3
 
 
 
 
ATG16L1
 
 
hsa-miR-106a-5p
 
 
-0.464
 
 
11.5
 
 
 
 
ATG16L1
 
 
hsa-miR-20b-5p
 
 
-0.467
 
 
11.5
 
 
 
 
ATG4D
 
 
hsa-miR-148a-3p
 
 
-0.303
 
 
26.4
 
 
 
 
ATOSB
 
 
hsa-let-7e-5p
 
 
-0.429
 
 
28.9
 
 
 
 
ATOSB
 
 
hsa-miR-98-5p
 
 
-0.429
 
 
28.9
 
 
 
 
ATOSB
 
 
hsa-let-7c-5p
 
 
-0.429
 
 
28.9
 
 
 
 
ATP2A2
 
 
hsa-let-7e-5p
 
 
-0.311
 
 
170.5
 
 
 
 
ATP2A2
 
 
hsa-miR-98-5p
 
 
-0.311
 
 
170.5
 
 
 
 
ATP2A2
 
 
hsa-let-7c-5p
 
 
-0.311
 
 
170.5
 
 
 
 
ATP2B4
 
 
hsa-miR-148a-3p
 
 
-0.337
 
 
30.6
 
 
 
 
ATP6AP2
 
 
hsa-miR-148a-3p
 
 
-0.592
 
 
65.8
 
 
 
 
ATP6AP2
 
 
hsa-miR-148a-3p
 
 
-0.668
 
 
65.8
 
 
 
 
ATPAF1
 
 
hsa-let-7e-5p
 
 
-0.498
 
 
36.9
 
 
 
 
ATPAF1
 
 
hsa-miR-98-5p
 
 
-0.498
 
 
36.9
 
 
 
 
ATPAF1
 
 
hsa-let-7c-5p
 
 
-0.498
 
 
36.9
 
 
 
 
AVPI1
 
 
hsa-miR-7-5p
 
 
-0.367
 
 
26.7
 
 
 
 
 
 
 fallopian tube 
 
 
 
 
target_symbol
 
 
mature_mirna_id
 
 
score
 
 
nTPM
 
 
 
 
 
 
ABCB7
 
 
hsa-miR-148a-3p
 
 
-0.515
 
 
11.3
 
 
 
 
ABCC5
 
 
hsa-miR-125a-5p
 
 
-0.288
 
 
16.2
 
 
 
 
ABCD3
 
 
hsa-miR-148a-3p
 
 
-0.388
 
 
15.8
 
 
 
 
ABHD17C
 
 
hsa-let-7c-5p
 
 
-0.359
 
 
10.3
 
 
 
 
ABHD17C
 
 
hsa-let-7e-5p
 
 
-0.359
 
 
10.3
 
 
 
 
ABHD17C
 
 
hsa-miR-98-5p
 
 
-0.383
 
 
10.3
 
 
 
 
ABTB1
 
 
hsa-miR-125a-5p
 
 
-0.519
 
 
39.7
 
 
 
 
ACVR1
 
 
hsa-miR-148a-3p
 
 
-0.397
 
 
13.2
 
 
 
 
ADAM10
 
 
hsa-miR-148a-3p
 
 
-0.356
 
 
13.1
 
 
 
 
ADAM9
 
 
hsa-miR-106a-5p
 
 
-0.317
 
 
15.9
 
 
 
 
ADAM9
 
 
hsa-miR-20b-5p
 
 
-0.328
 
 
15.9
 
 
 
 
ADGRF5
 
 
hsa-miR-148a-3p
 
 
-0.354
 
 
13.6
 
 
 
 
ADGRG2
 
 
hsa-miR-21-5p
 
 
-0.343
 
 
15.5
 
 
 
 
AGFG1
 
 
hsa-miR-148a-3p
 
 
-0.332
 
 
23.3
 
 
 
 
AGFG2
 
 
hsa-miR-20b-5p
 
 
-0.386
 
 
14.4
 
 
 
 
AGFG2
 
 
hsa-miR-106a-5p
 
 
-0.399
 
 
14.4
 
 
 
 
AHDC1
 
 
hsa-miR-148a-3p
 
 
-0.413
 
 
19.8
 
 
 
 
AHNAK
 
 
hsa-miR-106a-5p
 
 
-0.334
 
 
141.9
 
 
 
 
AHNAK
 
 
hsa-miR-20b-5p
 
 
-0.337
 
 
141.9
 
 
 
 
AIFM1
 
 
hsa-miR-125a-5p
 
 
-0.3
 
 
20.8
 
 
 
 
AIFM1
 
 
hsa-let-7e-5p
 
 
-0.314
 
 
20.8
 
 
 
 
AIFM1
 
 
hsa-miR-98-5p
 
 
-0.314
 
 
20.8
 
 
 
 
AIFM1
 
 
hsa-let-7c-5p
 
 
-0.314
 
 
20.8
 
 
 
 
AKAP1
 
 
hsa-miR-148a-3p
 
 
-0.381
 
 
33.9
 
 
 
 
ALCAM
 
 
hsa-miR-148a-3p
 
 
-0.339
 
 
46.2
 
 
 
 
ALG3
 
 
hsa-miR-106a-5p
 
 
-0.569
 
 
14.3
 
 
 
 
ALG3
 
 
hsa-miR-20b-5p
 
 
-0.58
 
 
14.3
 
 
 
 
AMT
 
 
hsa-let-7e-5p
 
 
-0.463
 
 
13.9
 
 
 
 
AMT
 
 
hsa-miR-98-5p
 
 
-0.463
 
 
13.9
 
 
 
 
AMT
 
 
hsa-let-7c-5p
 
 
-0.463
 
 
13.9
 
 
 
 
ANAPC16
 
 
hsa-miR-125a-5p
 
 
-0.578
 
 
21.7
 
 
 
 
ANKRD29
 
 
hsa-miR-106a-5p
 
 
-0.369
 
 
14.4
 
 
 
 
ANKRD29
 
 
hsa-miR-20b-5p
 
 
-0.38
 
 
14.4
 
 
 
 
ANKRD46
 
 
hsa-let-7e-5p
 
 
-0.389
 
 
10.7
 
 
 
 
ANKRD46
 
 
hsa-miR-98-5p
 
 
-0.389
 
 
10.7
 
 
 
 
ANKRD46
 
 
hsa-let-7c-5p
 
 
-0.389
 
 
10.7
 
 
 
 
AP1S1
 
 
hsa-let-7e-5p
 
 
-0.374
 
 
13.4
 
 
 
 
AP1S1
 
 
hsa-miR-98-5p
 
 
-0.374
 
 
13.4
 
 
 
 
AP1S1
 
 
hsa-let-7c-5p
 
 
-0.374
 
 
13.4
 
 
 
 
APCDD1
 
 
hsa-miR-106a-5p
 
 
-0.422
 
 
30.6
 
 
 
 
APCDD1
 
 
hsa-miR-20b-5p
 
 
-0.425
 
 
30.6
 
 
 
 
ARF4
 
 
hsa-miR-7-5p
 
 
-0.407
 
 
79.6
 
 
 
 
ARF4
 
 
hsa-miR-148a-3p
 
 
-0.487
 
 
79.6
 
 
 
 
ARFGEF1
 
 
hsa-miR-148a-3p
 
 
-0.306
 
 
11.1
 
 
 
 
ARFIP1
 
 
hsa-miR-148a-3p
 
 
-0.474
 
 
17.4
 
 
 
 
ARHGAP1
 
 
hsa-miR-20b-5p
 
 
-0.325
 
 
71.5
 
 
 
 
ARHGAP1
 
 
hsa-miR-106a-5p
 
 
-0.329
 
 
71.5
 
 
 
 
ARHGAP21
 
 
hsa-miR-148a-3p
 
 
-0.287
 
 
16.4
 
 
 
 
ARL1
 
 
hsa-miR-20b-5p
 
 
-0.424
 
 
47.1
 
 
 
 
ARL1
 
 
hsa-miR-106a-5p
 
 
-0.429
 
 
47.1
 
 
 
 
ARL4D
 
 
hsa-let-7e-5p
 
 
-0.417
 
 
20.8
 
 
 
 
ARL4D
 
 
hsa-miR-98-5p
 
 
-0.417
 
 
20.8
 
 
 
 
ARL4D
 
 
hsa-let-7c-5p
 
 
-0.417
 
 
20.8
 
 
 
 
ARL6IP1
 
 
hsa-miR-7-5p
 
 
-0.374
 
 
81.6
 
 
 
 
ARL6IP1
 
 
hsa-miR-148a-3p
 
 
-0.579
 
 
81.6
 
 
 
 
ARL6IP6
 
 
hsa-miR-98-5p
 
 
-0.293
 
 
10.8
 
 
 
 
ARL6IP6
 
 
hsa-let-7c-5p
 
 
-0.293
 
 
10.8
 
 
 
 
ARL6IP6
 
 
hsa-let-7e-5p
 
 
-0.305
 
 
10.8
 
 
 
 
ARL8B
 
 
hsa-miR-148a-3p
 
 
-0.337
 
 
29.3
 
 
 
 
ARMC8
 
 
hsa-miR-106a-5p
 
 
-0.375
 
 
14.8
 
 
 
 
ARMC8
 
 
hsa-miR-20b-5p
 
 
-0.378
 
 
14.8
 
 
 
 
ARMCX1
 
 
hsa-miR-21-5p
 
 
-0.489
 
 
23.4
 
 
 
 
ARMT1
 
 
hsa-let-7c-5p
 
 
-0.303
 
 
11.9
 
 
 
 
ARMT1
 
 
hsa-let-7e-5p
 
 
-0.303
 
 
11.9
 
 
 
 
ARMT1
 
 
hsa-miR-98-5p
 
 
-0.303
 
 
11.9
 
 
 
 
ARPP19
 
 
hsa-let-7e-5p
 
 
-0.492
 
 
31.4
 
 
 
 
ARPP19
 
 
hsa-let-7c-5p
 
 
-0.492
 
 
31.4
 
 
 
 
ARPP19
 
 
hsa-miR-98-5p
 
 
-0.5
 
 
31.4
 
 
 
 
ARRDC3
 
 
hsa-miR-148a-3p
 
 
-0.305
 
 
23.4
 
 
 
 
ARRDC4
 
 
hsa-let-7c-5p
 
 
-0.329
 
 
13.7
 
 
 
 
ARRDC4
 
 
hsa-let-7e-5p
 
 
-0.329
 
 
13.7
 
 
 
 
ARRDC4
 
 
hsa-miR-98-5p
 
 
-0.329
 
 
13.7
 
 
 
 
ASAP1
 
 
hsa-let-7e-5p
 
 
-0.296
 
 
15.1
 
 
 
 
ASAP1
 
 
hsa-miR-98-5p
 
 
-0.308
 
 
15.1
 
 
 
 
ASAP1
 
 
hsa-let-7c-5p
 
 
-0.308
 
 
15.1
 
 
 
 
ASPH
 
 
hsa-miR-148a-3p
 
 
-0.391
 
 
45.0
 
 
 
 
ATP2A2
 
 
hsa-let-7e-5p
 
 
-0.311
 
 
63.0
 
 
 
 
ATP2A2
 
 
hsa-miR-98-5p
 
 
-0.311
 
 
63.0
 
 
 
 
ATP2A2
 
 
hsa-let-7c-5p
 
 
-0.311
 
 
63.0
 
 
 
 
ATP2B4
 
 
hsa-miR-148a-3p
 
 
-0.337
 
 
45.7
 
 
 
 
ATP6AP2
 
 
hsa-miR-148a-3p
 
 
-0.592
 
 
71.1
 
 
 
 
ATP6AP2
 
 
hsa-miR-148a-3p
 
 
-0.668
 
 
71.1
 
 
 
 
ATPAF1
 
 
hsa-let-7e-5p
 
 
-0.498
 
 
27.2
 
 
 
 
ATPAF1
 
 
hsa-miR-98-5p
 
 
-0.498
 
 
27.2
 
 
 
 
ATPAF1
 
 
hsa-let-7c-5p
 
 
-0.498
 
 
27.2
 
 
 
 
AVPI1
 
 
hsa-miR-7-5p
 
 
-0.367
 
 
21.5
 
 
 
 
B3GALNT2
 
 
hsa-miR-125a-5p
 
 
-0.311
 
 
10.6
 
 
 
 
B3GAT3
 
 
hsa-let-7e-5p
 
 
-0.324
 
 
34.0
 
 
 
 
B3GAT3
 
 
hsa-miR-98-5p
 
 
-0.324
 
 
34.0
 
 
 
 
B3GAT3
 
 
hsa-let-7c-5p
 
 
-0.324
 
 
34.0
 
 
 
 
B4GALT5
 
 
hsa-miR-148a-3p
 
 
-0.44
 
 
17.2
 
 
 
 
B4GAT1
 
 
hsa-let-7c-5p
 
 
-0.47
 
 
25.4
 
 
 
 
B4GAT1
 
 
hsa-let-7e-5p
 
 
-0.47
 
 
25.4
 
 
 
 
B4GAT1
 
 
hsa-miR-98-5p
 
 
-0.47
 
 
25.4
 
 
 
 
BAZ2A
 
 
hsa-miR-99b-5p
 
 
-0.408
 
 
23.4
 
 
 
 
BCAP29
 
 
hsa-let-7e-5p
 
 
-0.289
 
 
17.1
 
 
 
 
BHLHE41
 
 
hsa-miR-148a-3p
 
 
-0.442
 
 
42.8
 
 
 
 
BIN3
 
 
hsa-let-7e-5p
 
 
-0.427
 
 
14.8
 
 
 
 
BIN3
 
 
hsa-miR-98-5p
 
 
-0.427
 
 
14.8
 
 
 
 
BIN3
 
 
hsa-let-7c-5p
 
 
-0.427
 
 
14.8
 
 
 
 
 
 
 heart muscle 
 
 
 
 
target_symbol
 
 
mature_mirna_id
 
 
score
 
 
nTPM
 
 
 
 
 
 
ABCB7
 
 
hsa-miR-148a-3p
 
 
-0.515
 
 
20.0
 
 
 
 
ABCC5
 
 
hsa-miR-125a-5p
 
 
-0.288
 
 
47.0
 
 
 
 
ABCD3
 
 
hsa-miR-148a-3p
 
 
-0.388
 
 
24.0
 
 
 
 
ABHD5
 
 
hsa-miR-20b-5p
 
 
-0.418
 
 
10.6
 
 
 
 
ABHD5
 
 
hsa-miR-106a-5p
 
 
-0.447
 
 
10.6
 
 
 
 
ABTB1
 
 
hsa-miR-125a-5p
 
 
-0.519
 
 
42.4
 
 
 
 
ACHE
 
 
hsa-miR-125a-5p
 
 
-0.308
 
 
11.4
 
 
 
 
ACHE
 
 
hsa-miR-125a-5p
 
 
-0.361
 
 
11.4
 
 
 
 
ACTC1
 
 
hsa-miR-7-5p
 
 
-0.471
 
 
10840.1
 
 
 
 
ACVR1
 
 
hsa-miR-148a-3p
 
 
-0.397
 
 
14.3
 
 
 
 
ADAM10
 
 
hsa-miR-148a-3p
 
 
-0.356
 
 
11.3
 
 
 
 
ADAM9
 
 
hsa-miR-106a-5p
 
 
-0.317
 
 
26.6
 
 
 
 
ADAM9
 
 
hsa-miR-20b-5p
 
 
-0.328
 
 
26.6
 
 
 
 
ADGRF5
 
 
hsa-miR-148a-3p
 
 
-0.354
 
 
50.1
 
 
 
 
ADRB1
 
 
hsa-let-7c-5p
 
 
-0.351
 
 
20.3
 
 
 
 
ADRB1
 
 
hsa-let-7e-5p
 
 
-0.367
 
 
20.3
 
 
 
 
ADRB1
 
 
hsa-miR-98-5p
 
 
-0.367
 
 
20.3
 
 
 
 
AGFG1
 
 
hsa-miR-148a-3p
 
 
-0.332
 
 
33.8
 
 
 
 
AHDC1
 
 
hsa-miR-148a-3p
 
 
-0.413
 
 
17.4
 
 
 
 
AHNAK
 
 
hsa-miR-106a-5p
 
 
-0.334
 
 
110.3
 
 
 
 
AHNAK
 
 
hsa-miR-20b-5p
 
 
-0.337
 
 
110.3
 
 
 
 
AIFM1
 
 
hsa-miR-125a-5p
 
 
-0.3
 
 
74.9
 
 
 
 
AIFM1
 
 
hsa-let-7e-5p
 
 
-0.314
 
 
74.9
 
 
 
 
AIFM1
 
 
hsa-miR-98-5p
 
 
-0.314
 
 
74.9
 
 
 
 
AIFM1
 
 
hsa-let-7c-5p
 
 
-0.314
 
 
74.9
 
 
 
 
AKAP1
 
 
hsa-miR-148a-3p
 
 
-0.381
 
 
78.5
 
 
 
 
AKAP6
 
 
hsa-let-7e-5p
 
 
-0.307
 
 
51.1
 
 
 
 
AKAP6
 
 
hsa-miR-98-5p
 
 
-0.307
 
 
51.1
 
 
 
 
AKAP6
 
 
hsa-let-7c-5p
 
 
-0.307
 
 
51.1
 
 
 
 
ALDH4A1
 
 
hsa-miR-184
 
 
-0.421
 
 
30.9
 
 
 
 
ALG3
 
 
hsa-miR-106a-5p
 
 
-0.569
 
 
18.7
 
 
 
 
ALG3
 
 
hsa-miR-20b-5p
 
 
-0.58
 
 
18.7
 
 
 
 
AMT
 
 
hsa-let-7e-5p
 
 
-0.463
 
 
23.5
 
 
 
 
AMT
 
 
hsa-miR-98-5p
 
 
-0.463
 
 
23.5
 
 
 
 
AMT
 
 
hsa-let-7c-5p
 
 
-0.463
 
 
23.5
 
 
 
 
ANAPC16
 
 
hsa-miR-125a-5p
 
 
-0.578
 
 
42.0
 
 
 
 
ANKRD12
 
 
hsa-miR-7-5p
 
 
-0.37
 
 
11.1
 
 
 
 
ANKRD46
 
 
hsa-let-7e-5p
 
 
-0.389
 
 
18.8
 
 
 
 
ANKRD46
 
 
hsa-miR-98-5p
 
 
-0.389
 
 
18.8
 
 
 
 
ANKRD46
 
 
hsa-let-7c-5p
 
 
-0.389
 
 
18.8
 
 
 
 
ANKRD9
 
 
hsa-miR-20b-5p
 
 
-0.331
 
 
104.9
 
 
 
 
ANKRD9
 
 
hsa-miR-106a-5p
 
 
-0.344
 
 
104.9
 
 
 
 
AP1S1
 
 
hsa-let-7e-5p
 
 
-0.374
 
 
12.9
 
 
 
 
AP1S1
 
 
hsa-miR-98-5p
 
 
-0.374
 
 
12.9
 
 
 
 
AP1S1
 
 
hsa-let-7c-5p
 
 
-0.374
 
 
12.9
 
 
 
 
APCDD1
 
 
hsa-miR-106a-5p
 
 
-0.422
 
 
30.9
 
 
 
 
APCDD1
 
 
hsa-miR-20b-5p
 
 
-0.425
 
 
30.9
 
 
 
 
ARF4
 
 
hsa-miR-7-5p
 
 
-0.407
 
 
106.8
 
 
 
 
ARF4
 
 
hsa-miR-148a-3p
 
 
-0.487
 
 
106.8
 
 
 
 
ARHGAP1
 
 
hsa-miR-20b-5p
 
 
-0.325
 
 
105.1
 
 
 
 
ARHGAP1
 
 
hsa-miR-106a-5p
 
 
-0.329
 
 
105.1
 
 
 
 
ARHGAP21
 
 
hsa-miR-148a-3p
 
 
-0.287
 
 
19.7
 
 
 
 
ARHGAP24
 
 
hsa-miR-21-5p
 
 
-0.427
 
 
25.6
 
 
 
 
ARL1
 
 
hsa-miR-20b-5p
 
 
-0.424
 
 
61.3
 
 
 
 
ARL1
 
 
hsa-miR-106a-5p
 
 
-0.429
 
 
61.3
 
 
 
 
ARL4D
 
 
hsa-let-7e-5p
 
 
-0.417
 
 
12.5
 
 
 
 
ARL4D
 
 
hsa-miR-98-5p
 
 
-0.417
 
 
12.5
 
 
 
 
ARL4D
 
 
hsa-let-7c-5p
 
 
-0.417
 
 
12.5
 
 
 
 
ARL6IP1
 
 
hsa-miR-7-5p
 
 
-0.374
 
 
30.1
 
 
 
 
ARL6IP1
 
 
hsa-miR-148a-3p
 
 
-0.579
 
 
30.1
 
 
 
 
ARL8B
 
 
hsa-miR-148a-3p
 
 
-0.337
 
 
51.7
 
 
 
 
ARMC8
 
 
hsa-miR-106a-5p
 
 
-0.375
 
 
16.3
 
 
 
 
ARMC8
 
 
hsa-miR-20b-5p
 
 
-0.378
 
 
16.3
 
 
 
 
ARMCX1
 
 
hsa-miR-21-5p
 
 
-0.489
 
 
30.7
 
 
 
 
ARMT1
 
 
hsa-let-7c-5p
 
 
-0.303
 
 
15.7
 
 
 
 
ARMT1
 
 
hsa-let-7e-5p
 
 
-0.303
 
 
15.7
 
 
 
 
ARMT1
 
 
hsa-miR-98-5p
 
 
-0.303
 
 
15.7
 
 
 
 
ARPP19
 
 
hsa-let-7e-5p
 
 
-0.492
 
 
76.2
 
 
 
 
ARPP19
 
 
hsa-let-7c-5p
 
 
-0.492
 
 
76.2
 
 
 
 
ARPP19
 
 
hsa-miR-98-5p
 
 
-0.5
 
 
76.2
 
 
 
 
ARRDC3
 
 
hsa-miR-148a-3p
 
 
-0.305
 
 
23.2
 
 
 
 
ARRDC4
 
 
hsa-let-7c-5p
 
 
-0.329
 
 
11.3
 
 
 
 
ARRDC4
 
 
hsa-let-7e-5p
 
 
-0.329
 
 
11.3
 
 
 
 
ARRDC4
 
 
hsa-miR-98-5p
 
 
-0.329
 
 
11.3
 
 
 
 
ASAP1
 
 
hsa-let-7e-5p
 
 
-0.296
 
 
22.3
 
 
 
 
ASAP1
 
 
hsa-miR-98-5p
 
 
-0.308
 
 
22.3
 
 
 
 
ASAP1
 
 
hsa-let-7c-5p
 
 
-0.308
 
 
22.3
 
 
 
 
ASF1A
 
 
hsa-miR-21-5p
 
 
-0.304
 
 
12.5
 
 
 
 
ASPH
 
 
hsa-miR-148a-3p
 
 
-0.391
 
 
100.1
 
 
 
 
ATG16L1
 
 
hsa-miR-106a-5p
 
 
-0.464
 
 
10.8
 
 
 
 
ATG16L1
 
 
hsa-miR-20b-5p
 
 
-0.467
 
 
10.8
 
 
 
 
ATG4A
 
 
hsa-miR-7-5p
 
 
-0.448
 
 
11.7
 
 
 
 
ATG4D
 
 
hsa-miR-148a-3p
 
 
-0.303
 
 
21.7
 
 
 
 
ATOSB
 
 
hsa-let-7e-5p
 
 
-0.429
 
 
18.9
 
 
 
 
ATOSB
 
 
hsa-miR-98-5p
 
 
-0.429
 
 
18.9
 
 
 
 
ATOSB
 
 
hsa-let-7c-5p
 
 
-0.429
 
 
18.9
 
 
 
 
ATP2A2
 
 
hsa-let-7e-5p
 
 
-0.311
 
 
941.9
 
 
 
 
ATP2A2
 
 
hsa-miR-98-5p
 
 
-0.311
 
 
941.9
 
 
 
 
ATP2A2
 
 
hsa-let-7c-5p
 
 
-0.311
 
 
941.9
 
 
 
 
ATP2B4
 
 
hsa-miR-148a-3p
 
 
-0.337
 
 
56.1
 
 
 
 
ATP6AP2
 
 
hsa-miR-148a-3p
 
 
-0.592
 
 
51.6
 
 
 
 
ATP6AP2
 
 
hsa-miR-148a-3p
 
 
-0.668
 
 
51.6
 
 
 
 
ATPAF1
 
 
hsa-let-7e-5p
 
 
-0.498
 
 
249.4
 
 
 
 
ATPAF1
 
 
hsa-miR-98-5p
 
 
-0.498
 
 
249.4
 
 
 
 
ATPAF1
 
 
hsa-let-7c-5p
 
 
-0.498
 
 
249.4
 
 
 
 
AVPI1
 
 
hsa-miR-7-5p
 
 
-0.367
 
 
22.9
 
 
 
 
B3GALT2
 
 
hsa-miR-20b-5p
 
 
-0.35
 
 
19.7
 
 
 
 
B3GALT2
 
 
hsa-miR-106a-5p
 
 
-0.354
 
 
19.7
 
 
 
 
B3GAT3
 
 
hsa-let-7e-5p
 
 
-0.324
 
 
25.1
 
 
 
 
B3GAT3
 
 
hsa-miR-98-5p
 
 
-0.324
 
 
25.1
 
 
 
 
 
 
 ovary 
 
 
 
 
target_symbol
 
 
mature_mirna_id
 
 
score
 
 
nTPM
 
 
 
 
 
 
ABCB7
 
 
hsa-miR-148a-3p
 
 
-0.515
 
 
11.9
 
 
 
 
ABCC5
 
 
hsa-miR-125a-5p
 
 
-0.288
 
 
23.4
 
 
 
 
ABCD3
 
 
hsa-miR-148a-3p
 
 
-0.388
 
 
11.0
 
 
 
 
ABHD3
 
 
hsa-miR-125a-5p
 
 
-0.453
 
 
12.0
 
 
 
 
ABTB1
 
 
hsa-miR-125a-5p
 
 
-0.519
 
 
31.5
 
 
 
 
ACVR1
 
 
hsa-miR-148a-3p
 
 
-0.397
 
 
17.7
 
 
 
 
ADAM10
 
 
hsa-miR-148a-3p
 
 
-0.356
 
 
13.1
 
 
 
 
ADAM9
 
 
hsa-miR-106a-5p
 
 
-0.317
 
 
16.9
 
 
 
 
ADAM9
 
 
hsa-miR-20b-5p
 
 
-0.328
 
 
16.9
 
 
 
 
ADRB3
 
 
hsa-let-7e-5p
 
 
-0.415
 
 
12.8
 
 
 
 
ADRB3
 
 
hsa-let-7c-5p
 
 
-0.415
 
 
12.8
 
 
 
 
ADRB3
 
 
hsa-miR-98-5p
 
 
-0.423
 
 
12.8
 
 
 
 
AGFG1
 
 
hsa-miR-148a-3p
 
 
-0.332
 
 
22.9
 
 
 
 
AGFG2
 
 
hsa-miR-20b-5p
 
 
-0.386
 
 
28.5
 
 
 
 
AGFG2
 
 
hsa-miR-106a-5p
 
 
-0.399
 
 
28.5
 
 
 
 
AGGF1
 
 
hsa-miR-125a-5p
 
 
-0.331
 
 
10.3
 
 
 
 
AHCTF1
 
 
hsa-let-7e-5p
 
 
-0.374
 
 
13.1
 
 
 
 
AHCTF1
 
 
hsa-miR-98-5p
 
 
-0.374
 
 
13.1
 
 
 
 
AHCTF1
 
 
hsa-let-7c-5p
 
 
-0.374
 
 
13.1
 
 
 
 
AHDC1
 
 
hsa-miR-148a-3p
 
 
-0.413
 
 
21.5
 
 
 
 
AHNAK
 
 
hsa-miR-106a-5p
 
 
-0.334
 
 
105.9
 
 
 
 
AHNAK
 
 
hsa-miR-20b-5p
 
 
-0.337
 
 
105.9
 
 
 
 
AKAP1
 
 
hsa-miR-148a-3p
 
 
-0.381
 
 
18.8
 
 
 
 
ALCAM
 
 
hsa-miR-148a-3p
 
 
-0.339
 
 
33.1
 
 
 
 
ALG3
 
 
hsa-miR-106a-5p
 
 
-0.569
 
 
12.1
 
 
 
 
ALG3
 
 
hsa-miR-20b-5p
 
 
-0.58
 
 
12.1
 
 
 
 
ALG9
 
 
hsa-miR-7-5p
 
 
-0.302
 
 
11.1
 
 
 
 
AMT
 
 
hsa-let-7e-5p
 
 
-0.463
 
 
16.7
 
 
 
 
AMT
 
 
hsa-miR-98-5p
 
 
-0.463
 
 
16.7
 
 
 
 
AMT
 
 
hsa-let-7c-5p
 
 
-0.463
 
 
16.7
 
 
 
 
ANAPC16
 
 
hsa-miR-125a-5p
 
 
-0.578
 
 
22.3
 
 
 
 
ANKRD12
 
 
hsa-miR-7-5p
 
 
-0.37
 
 
10.6
 
 
 
 
ANKRD29
 
 
hsa-miR-106a-5p
 
 
-0.369
 
 
15.0
 
 
 
 
ANKRD29
 
 
hsa-miR-20b-5p
 
 
-0.38
 
 
15.0
 
 
 
 
ANKRD46
 
 
hsa-let-7e-5p
 
 
-0.389
 
 
19.3
 
 
 
 
ANKRD46
 
 
hsa-miR-98-5p
 
 
-0.389
 
 
19.3
 
 
 
 
ANKRD46
 
 
hsa-let-7c-5p
 
 
-0.389
 
 
19.3
 
 
 
 
AP1AR
 
 
hsa-miR-21-5p
 
 
-0.318
 
 
10.0
 
 
 
 
AP1AR
 
 
hsa-miR-99b-5p
 
 
-0.823
 
 
10.0
 
 
 
 
AP1S1
 
 
hsa-let-7e-5p
 
 
-0.374
 
 
22.2
 
 
 
 
AP1S1
 
 
hsa-miR-98-5p
 
 
-0.374
 
 
22.2
 
 
 
 
AP1S1
 
 
hsa-let-7c-5p
 
 
-0.374
 
 
22.2
 
 
 
 
APCDD1
 
 
hsa-miR-106a-5p
 
 
-0.422
 
 
28.1
 
 
 
 
APCDD1
 
 
hsa-miR-20b-5p
 
 
-0.425
 
 
28.1
 
 
 
 
ARF4
 
 
hsa-miR-7-5p
 
 
-0.407
 
 
123.7
 
 
 
 
ARF4
 
 
hsa-miR-148a-3p
 
 
-0.487
 
 
123.7
 
 
 
 
ARFIP1
 
 
hsa-miR-148a-3p
 
 
-0.474
 
 
17.2
 
 
 
 
ARG2
 
 
hsa-let-7e-5p
 
 
-0.468
 
 
18.7
 
 
 
 
ARG2
 
 
hsa-miR-98-5p
 
 
-0.468
 
 
18.7
 
 
 
 
ARG2
 
 
hsa-let-7c-5p
 
 
-0.468
 
 
18.7
 
 
 
 
ARHGAP1
 
 
hsa-miR-20b-5p
 
 
-0.325
 
 
43.6
 
 
 
 
ARHGAP1
 
 
hsa-miR-106a-5p
 
 
-0.329
 
 
43.6
 
 
 
 
ARHGAP21
 
 
hsa-miR-148a-3p
 
 
-0.287
 
 
26.5
 
 
 
 
ARHGEF28
 
 
hsa-miR-20b-5p
 
 
-0.352
 
 
18.8
 
 
 
 
ARHGEF28
 
 
hsa-miR-106a-5p
 
 
-0.367
 
 
18.8
 
 
 
 
ARID4A
 
 
hsa-miR-7-5p
 
 
-0.341
 
 
10.1
 
 
 
 
ARL1
 
 
hsa-miR-20b-5p
 
 
-0.424
 
 
63.4
 
 
 
 
ARL1
 
 
hsa-miR-106a-5p
 
 
-0.429
 
 
63.4
 
 
 
 
ARL4D
 
 
hsa-let-7e-5p
 
 
-0.417
 
 
20.3
 
 
 
 
ARL4D
 
 
hsa-miR-98-5p
 
 
-0.417
 
 
20.3
 
 
 
 
ARL4D
 
 
hsa-let-7c-5p
 
 
-0.417
 
 
20.3
 
 
 
 
ARL6IP1
 
 
hsa-miR-7-5p
 
 
-0.374
 
 
97.4
 
 
 
 
ARL6IP1
 
 
hsa-miR-148a-3p
 
 
-0.579
 
 
97.4
 
 
 
 
ARL6IP6
 
 
hsa-miR-98-5p
 
 
-0.293
 
 
11.9
 
 
 
 
ARL6IP6
 
 
hsa-let-7c-5p
 
 
-0.293
 
 
11.9
 
 
 
 
ARL6IP6
 
 
hsa-let-7e-5p
 
 
-0.305
 
 
11.9
 
 
 
 
ARL8B
 
 
hsa-miR-148a-3p
 
 
-0.337
 
 
35.5
 
 
 
 
ARMC8
 
 
hsa-miR-106a-5p
 
 
-0.375
 
 
14.0
 
 
 
 
ARMC8
 
 
hsa-miR-20b-5p
 
 
-0.378
 
 
14.0
 
 
 
 
ARMCX1
 
 
hsa-miR-21-5p
 
 
-0.489
 
 
46.7
 
 
 
 
ARMT1
 
 
hsa-let-7c-5p
 
 
-0.303
 
 
15.5
 
 
 
 
ARMT1
 
 
hsa-let-7e-5p
 
 
-0.303
 
 
15.5
 
 
 
 
ARMT1
 
 
hsa-miR-98-5p
 
 
-0.303
 
 
15.5
 
 
 
 
ARPP19
 
 
hsa-let-7e-5p
 
 
-0.492
 
 
40.6
 
 
 
 
ARPP19
 
 
hsa-let-7c-5p
 
 
-0.492
 
 
40.6
 
 
 
 
ARPP19
 
 
hsa-miR-98-5p
 
 
-0.5
 
 
40.6
 
 
 
 
ARRDC3
 
 
hsa-miR-148a-3p
 
 
-0.305
 
 
64.7
 
 
 
 
ASAP1
 
 
hsa-let-7e-5p
 
 
-0.296
 
 
16.8
 
 
 
 
ASAP1
 
 
hsa-miR-98-5p
 
 
-0.308
 
 
16.8
 
 
 
 
ASAP1
 
 
hsa-let-7c-5p
 
 
-0.308
 
 
16.8
 
 
 
 
ASF1A
 
 
hsa-miR-21-5p
 
 
-0.304
 
 
18.5
 
 
 
 
ASPH
 
 
hsa-miR-148a-3p
 
 
-0.391
 
 
58.7
 
 
 
 
ATP2A2
 
 
hsa-let-7e-5p
 
 
-0.311
 
 
34.9
 
 
 
 
ATP2A2
 
 
hsa-miR-98-5p
 
 
-0.311
 
 
34.9
 
 
 
 
ATP2A2
 
 
hsa-let-7c-5p
 
 
-0.311
 
 
34.9
 
 
 
 
ATP2B4
 
 
hsa-miR-148a-3p
 
 
-0.337
 
 
30.9
 
 
 
 
ATP6AP2
 
 
hsa-miR-148a-3p
 
 
-0.592
 
 
86.3
 
 
 
 
ATP6AP2
 
 
hsa-miR-148a-3p
 
 
-0.668
 
 
86.3
 
 
 
 
ATPAF1
 
 
hsa-let-7e-5p
 
 
-0.498
 
 
33.9
 
 
 
 
ATPAF1
 
 
hsa-miR-98-5p
 
 
-0.498
 
 
33.9
 
 
 
 
ATPAF1
 
 
hsa-let-7c-5p
 
 
-0.498
 
 
33.9
 
 
 
 
AVPI1
 
 
hsa-miR-7-5p
 
 
-0.367
 
 
85.9
 
 
 
 
B3GAT3
 
 
hsa-let-7e-5p
 
 
-0.324
 
 
35.4
 
 
 
 
B3GAT3
 
 
hsa-miR-98-5p
 
 
-0.324
 
 
35.4
 
 
 
 
B3GAT3
 
 
hsa-let-7c-5p
 
 
-0.324
 
 
35.4
 
 
 
 
B4GALT5
 
 
hsa-miR-148a-3p
 
 
-0.44
 
 
19.0
 
 
 
 
B4GAT1
 
 
hsa-let-7c-5p
 
 
-0.47
 
 
33.5
 
 
 
 
B4GAT1
 
 
hsa-let-7e-5p
 
 
-0.47
 
 
33.5
 
 
 
 
B4GAT1
 
 
hsa-miR-98-5p
 
 
-0.47
 
 
33.5
 
 
 
 
BACH1
 
 
hsa-let-7e-5p
 
 
-0.298
 
 
12.0
 
 
 
 
 
 
 parathyroid gland 
 
 
 
 
target_symbol
 
 
mature_mirna_id
 
 
score
 
 
nTPM
 
 
 
 
 
 
ABCB7
 
 
hsa-miR-148a-3p
 
 
-0.515
 
 
10.3
 
 
 
 
ABCC5
 
 
hsa-miR-125a-5p
 
 
-0.288
 
 
22.6
 
 
 
 
ABCD3
 
 
hsa-miR-148a-3p
 
 
-0.388
 
 
27.9
 
 
 
 
ABHD3
 
 
hsa-miR-125a-5p
 
 
-0.453
 
 
15.0
 
 
 
 
ABHD5
 
 
hsa-miR-20b-5p
 
 
-0.418
 
 
11.5
 
 
 
 
ABHD5
 
 
hsa-miR-106a-5p
 
 
-0.447
 
 
11.5
 
 
 
 
ACSL6
 
 
hsa-miR-125a-5p
 
 
-0.307
 
 
10.6
 
 
 
 
ACSL6
 
 
hsa-let-7e-5p
 
 
-0.336
 
 
10.6
 
 
 
 
ACSL6
 
 
hsa-miR-98-5p
 
 
-0.336
 
 
10.6
 
 
 
 
ACSL6
 
 
hsa-let-7c-5p
 
 
-0.336
 
 
10.6
 
 
 
 
ADAM9
 
 
hsa-miR-106a-5p
 
 
-0.317
 
 
49.4
 
 
 
 
ADAM9
 
 
hsa-miR-20b-5p
 
 
-0.328
 
 
49.4
 
 
 
 
ADGRF5
 
 
hsa-miR-148a-3p
 
 
-0.354
 
 
12.6
 
 
 
 
ADGRG2
 
 
hsa-miR-21-5p
 
 
-0.343
 
 
153.8
 
 
 
 
AGFG1
 
 
hsa-miR-148a-3p
 
 
-0.332
 
 
39.0
 
 
 
 
AGGF1
 
 
hsa-miR-125a-5p
 
 
-0.331
 
 
15.0
 
 
 
 
AHCTF1
 
 
hsa-let-7e-5p
 
 
-0.374
 
 
12.3
 
 
 
 
AHCTF1
 
 
hsa-miR-98-5p
 
 
-0.374
 
 
12.3
 
 
 
 
AHCTF1
 
 
hsa-let-7c-5p
 
 
-0.374
 
 
12.3
 
 
 
 
AHNAK
 
 
hsa-miR-106a-5p
 
 
-0.334
 
 
24.7
 
 
 
 
AHNAK
 
 
hsa-miR-20b-5p
 
 
-0.337
 
 
24.7
 
 
 
 
AIFM1
 
 
hsa-miR-125a-5p
 
 
-0.3
 
 
27.9
 
 
 
 
AIFM1
 
 
hsa-let-7e-5p
 
 
-0.314
 
 
27.9
 
 
 
 
AIFM1
 
 
hsa-miR-98-5p
 
 
-0.314
 
 
27.9
 
 
 
 
AIFM1
 
 
hsa-let-7c-5p
 
 
-0.314
 
 
27.9
 
 
 
 
AKAP1
 
 
hsa-miR-148a-3p
 
 
-0.381
 
 
45.1
 
 
 
 
ALCAM
 
 
hsa-miR-148a-3p
 
 
-0.339
 
 
412.8
 
 
 
 
ALDH4A1
 
 
hsa-miR-184
 
 
-0.421
 
 
21.6
 
 
 
 
ALG3
 
 
hsa-miR-106a-5p
 
 
-0.569
 
 
21.7
 
 
 
 
ALG3
 
 
hsa-miR-20b-5p
 
 
-0.58
 
 
21.7
 
 
 
 
ALG9
 
 
hsa-miR-7-5p
 
 
-0.302
 
 
12.0
 
 
 
 
AMT
 
 
hsa-let-7e-5p
 
 
-0.463
 
 
13.3
 
 
 
 
AMT
 
 
hsa-miR-98-5p
 
 
-0.463
 
 
13.3
 
 
 
 
AMT
 
 
hsa-let-7c-5p
 
 
-0.463
 
 
13.3
 
 
 
 
ANAPC16
 
 
hsa-miR-125a-5p
 
 
-0.578
 
 
30.6
 
 
 
 
ANKRD29
 
 
hsa-miR-106a-5p
 
 
-0.369
 
 
23.3
 
 
 
 
ANKRD29
 
 
hsa-miR-20b-5p
 
 
-0.38
 
 
23.3
 
 
 
 
ANKRD46
 
 
hsa-let-7e-5p
 
 
-0.389
 
 
15.1
 
 
 
 
ANKRD46
 
 
hsa-miR-98-5p
 
 
-0.389
 
 
15.1
 
 
 
 
ANKRD46
 
 
hsa-let-7c-5p
 
 
-0.389
 
 
15.1
 
 
 
 
AP1AR
 
 
hsa-miR-21-5p
 
 
-0.318
 
 
38.5
 
 
 
 
AP1AR
 
 
hsa-miR-99b-5p
 
 
-0.823
 
 
38.5
 
 
 
 
AP1S1
 
 
hsa-let-7e-5p
 
 
-0.374
 
 
21.4
 
 
 
 
AP1S1
 
 
hsa-miR-98-5p
 
 
-0.374
 
 
21.4
 
 
 
 
AP1S1
 
 
hsa-let-7c-5p
 
 
-0.374
 
 
21.4
 
 
 
 
ARF4
 
 
hsa-miR-7-5p
 
 
-0.407
 
 
95.4
 
 
 
 
ARF4
 
 
hsa-miR-148a-3p
 
 
-0.487
 
 
95.4
 
 
 
 
ARFGEF1
 
 
hsa-miR-148a-3p
 
 
-0.306
 
 
26.3
 
 
 
 
ARFIP1
 
 
hsa-miR-148a-3p
 
 
-0.474
 
 
21.0
 
 
 
 
ARG2
 
 
hsa-let-7e-5p
 
 
-0.468
 
 
206.5
 
 
 
 
ARG2
 
 
hsa-miR-98-5p
 
 
-0.468
 
 
206.5
 
 
 
 
ARG2
 
 
hsa-let-7c-5p
 
 
-0.468
 
 
206.5
 
 
 
 
ARHGAP1
 
 
hsa-miR-20b-5p
 
 
-0.325
 
 
23.3
 
 
 
 
ARHGAP1
 
 
hsa-miR-106a-5p
 
 
-0.329
 
 
23.3
 
 
 
 
ARHGAP21
 
 
hsa-miR-148a-3p
 
 
-0.287
 
 
13.6
 
 
 
 
ARL1
 
 
hsa-miR-20b-5p
 
 
-0.424
 
 
86.8
 
 
 
 
ARL1
 
 
hsa-miR-106a-5p
 
 
-0.429
 
 
86.8
 
 
 
 
ARL6IP1
 
 
hsa-miR-7-5p
 
 
-0.374
 
 
169.8
 
 
 
 
ARL6IP1
 
 
hsa-miR-148a-3p
 
 
-0.579
 
 
169.8
 
 
 
 
ARL8B
 
 
hsa-miR-148a-3p
 
 
-0.337
 
 
45.2
 
 
 
 
ARMC8
 
 
hsa-miR-106a-5p
 
 
-0.375
 
 
20.0
 
 
 
 
ARMC8
 
 
hsa-miR-20b-5p
 
 
-0.378
 
 
20.0
 
 
 
 
ARMCX1
 
 
hsa-miR-21-5p
 
 
-0.489
 
 
15.9
 
 
 
 
ARMT1
 
 
hsa-let-7c-5p
 
 
-0.303
 
 
10.9
 
 
 
 
ARMT1
 
 
hsa-let-7e-5p
 
 
-0.303
 
 
10.9
 
 
 
 
ARMT1
 
 
hsa-miR-98-5p
 
 
-0.303
 
 
10.9
 
 
 
 
ARPP19
 
 
hsa-let-7e-5p
 
 
-0.492
 
 
102.5
 
 
 
 
ARPP19
 
 
hsa-let-7c-5p
 
 
-0.492
 
 
102.5
 
 
 
 
ARPP19
 
 
hsa-miR-98-5p
 
 
-0.5
 
 
102.5
 
 
 
 
ARRDC3
 
 
hsa-miR-148a-3p
 
 
-0.305
 
 
51.0
 
 
 
 
ASAP1
 
 
hsa-let-7e-5p
 
 
-0.296
 
 
16.8
 
 
 
 
ASAP1
 
 
hsa-miR-98-5p
 
 
-0.308
 
 
16.8
 
 
 
 
ASAP1
 
 
hsa-let-7c-5p
 
 
-0.308
 
 
16.8
 
 
 
 
ASF1A
 
 
hsa-miR-21-5p
 
 
-0.304
 
 
14.9
 
 
 
 
ASPH
 
 
hsa-miR-148a-3p
 
 
-0.391
 
 
35.7
 
 
 
 
ATG4A
 
 
hsa-miR-7-5p
 
 
-0.448
 
 
11.7
 
 
 
 
ATG4D
 
 
hsa-miR-148a-3p
 
 
-0.303
 
 
10.1
 
 
 
 
ATOSB
 
 
hsa-let-7e-5p
 
 
-0.429
 
 
10.5
 
 
 
 
ATOSB
 
 
hsa-miR-98-5p
 
 
-0.429
 
 
10.5
 
 
 
 
ATOSB
 
 
hsa-let-7c-5p
 
 
-0.429
 
 
10.5
 
 
 
 
ATP2A2
 
 
hsa-let-7e-5p
 
 
-0.311
 
 
52.7
 
 
 
 
ATP2A2
 
 
hsa-miR-98-5p
 
 
-0.311
 
 
52.7
 
 
 
 
ATP2A2
 
 
hsa-let-7c-5p
 
 
-0.311
 
 
52.7
 
 
 
 
ATP2B4
 
 
hsa-miR-148a-3p
 
 
-0.337
 
 
11.1
 
 
 
 
ATP6AP2
 
 
hsa-miR-148a-3p
 
 
-0.592
 
 
719.4
 
 
 
 
ATP6AP2
 
 
hsa-miR-148a-3p
 
 
-0.668
 
 
719.4
 
 
 
 
ATPAF1
 
 
hsa-let-7e-5p
 
 
-0.498
 
 
129.7
 
 
 
 
ATPAF1
 
 
hsa-miR-98-5p
 
 
-0.498
 
 
129.7
 
 
 
 
ATPAF1
 
 
hsa-let-7c-5p
 
 
-0.498
 
 
129.7
 
 
 
 
B3GAT3
 
 
hsa-let-7e-5p
 
 
-0.324
 
 
18.1
 
 
 
 
B3GAT3
 
 
hsa-miR-98-5p
 
 
-0.324
 
 
18.1
 
 
 
 
B3GAT3
 
 
hsa-let-7c-5p
 
 
-0.324
 
 
18.1
 
 
 
 
B4GALT5
 
 
hsa-miR-148a-3p
 
 
-0.44
 
 
10.6
 
 
 
 
B4GAT1
 
 
hsa-let-7c-5p
 
 
-0.47
 
 
21.3
 
 
 
 
B4GAT1
 
 
hsa-let-7e-5p
 
 
-0.47
 
 
21.3
 
 
 
 
B4GAT1
 
 
hsa-miR-98-5p
 
 
-0.47
 
 
21.3
 
 
 
 
BAK1
 
 
hsa-miR-125a-5p
 
 
-0.402
 
 
12.6
 
 
 
 
BAZ2A
 
 
hsa-miR-99b-5p
 
 
-0.408
 
 
31.4
 
 
 
 
BCAP29
 
 
hsa-let-7e-5p
 
 
-0.289
 
 
32.8
 
 
 
 
BCL2L11
 
 
hsa-miR-148a-3p
 
 
-0.445
 
 
17.6
 
 
 
 
 
 
 skin 
 
 
 
 
target_symbol
 
 
mature_mirna_id
 
 
score
 
 
nTPM
 
 
 
 
 
 
ABCB7
 
 
hsa-miR-148a-3p
 
 
-0.515
 
 
12.7
 
 
 
 
ABCC5
 
 
hsa-miR-125a-5p
 
 
-0.288
 
 
22.7
 
 
 
 
ABCD3
 
 
hsa-miR-148a-3p
 
 
-0.388
 
 
33.6
 
 
 
 
ABHD17C
 
 
hsa-let-7c-5p
 
 
-0.359
 
 
17.9
 
 
 
 
ABHD17C
 
 
hsa-let-7e-5p
 
 
-0.359
 
 
17.9
 
 
 
 
ABHD17C
 
 
hsa-miR-98-5p
 
 
-0.383
 
 
17.9
 
 
 
 
ABHD5
 
 
hsa-miR-20b-5p
 
 
-0.418
 
 
29.7
 
 
 
 
ABHD5
 
 
hsa-miR-106a-5p
 
 
-0.447
 
 
29.7
 
 
 
 
ABHD6
 
 
hsa-miR-125a-5p
 
 
-0.501
 
 
24.1
 
 
 
 
ABTB1
 
 
hsa-miR-125a-5p
 
 
-0.519
 
 
25.3
 
 
 
 
ACP3
 
 
hsa-let-7e-5p
 
 
-0.298
 
 
25.5
 
 
 
 
ACP3
 
 
hsa-miR-98-5p
 
 
-0.298
 
 
25.5
 
 
 
 
ACP3
 
 
hsa-let-7c-5p
 
 
-0.298
 
 
25.5
 
 
 
 
ACTC1
 
 
hsa-miR-7-5p
 
 
-0.471
 
 
14.2
 
 
 
 
ACVR2A
 
 
hsa-miR-125a-5p
 
 
-0.301
 
 
18.1
 
 
 
 
ADAM10
 
 
hsa-miR-148a-3p
 
 
-0.356
 
 
14.8
 
 
 
 
ADRB2
 
 
hsa-let-7c-5p
 
 
-0.635
 
 
36.9
 
 
 
 
ADRB2
 
 
hsa-let-7e-5p
 
 
-0.635
 
 
36.9
 
 
 
 
ADRB2
 
 
hsa-miR-98-5p
 
 
-0.635
 
 
36.9
 
 
 
 
AGFG1
 
 
hsa-miR-148a-3p
 
 
-0.332
 
 
34.3
 
 
 
 
AGFG2
 
 
hsa-miR-20b-5p
 
 
-0.386
 
 
24.5
 
 
 
 
AGFG2
 
 
hsa-miR-106a-5p
 
 
-0.399
 
 
24.5
 
 
 
 
AGGF1
 
 
hsa-miR-125a-5p
 
 
-0.331
 
 
11.8
 
 
 
 
AHDC1
 
 
hsa-miR-148a-3p
 
 
-0.413
 
 
29.8
 
 
 
 
AHNAK
 
 
hsa-miR-106a-5p
 
 
-0.334
 
 
316.1
 
 
 
 
AHNAK
 
 
hsa-miR-20b-5p
 
 
-0.337
 
 
316.1
 
 
 
 
AIFM1
 
 
hsa-miR-125a-5p
 
 
-0.3
 
 
10.3
 
 
 
 
AIFM1
 
 
hsa-let-7e-5p
 
 
-0.314
 
 
10.3
 
 
 
 
AIFM1
 
 
hsa-miR-98-5p
 
 
-0.314
 
 
10.3
 
 
 
 
AIFM1
 
 
hsa-let-7c-5p
 
 
-0.314
 
 
10.3
 
 
 
 
AKAP1
 
 
hsa-miR-148a-3p
 
 
-0.381
 
 
26.4
 
 
 
 
ALCAM
 
 
hsa-miR-148a-3p
 
 
-0.339
 
 
10.3
 
 
 
 
ALDH4A1
 
 
hsa-miR-184
 
 
-0.421
 
 
18.1
 
 
 
 
ALG3
 
 
hsa-miR-106a-5p
 
 
-0.569
 
 
23.7
 
 
 
 
ALG3
 
 
hsa-miR-20b-5p
 
 
-0.58
 
 
23.7
 
 
 
 
ALG9
 
 
hsa-miR-7-5p
 
 
-0.302
 
 
13.9
 
 
 
 
ANAPC16
 
 
hsa-miR-125a-5p
 
 
-0.578
 
 
14.6
 
 
 
 
ANKRD12
 
 
hsa-miR-7-5p
 
 
-0.37
 
 
19.1
 
 
 
 
ANKRD46
 
 
hsa-let-7e-5p
 
 
-0.389
 
 
10.5
 
 
 
 
ANKRD46
 
 
hsa-miR-98-5p
 
 
-0.389
 
 
10.5
 
 
 
 
ANKRD46
 
 
hsa-let-7c-5p
 
 
-0.389
 
 
10.5
 
 
 
 
ANKRD50
 
 
hsa-miR-106a-5p
 
 
-0.29
 
 
10.4
 
 
 
 
ANKRD9
 
 
hsa-miR-20b-5p
 
 
-0.331
 
 
24.3
 
 
 
 
ANKRD9
 
 
hsa-miR-106a-5p
 
 
-0.344
 
 
24.3
 
 
 
 
ANPEP
 
 
hsa-miR-125a-5p
 
 
-0.372
 
 
29.7
 
 
 
 
AP1AR
 
 
hsa-miR-21-5p
 
 
-0.318
 
 
13.4
 
 
 
 
AP1AR
 
 
hsa-miR-99b-5p
 
 
-0.823
 
 
13.4
 
 
 
 
AP1S1
 
 
hsa-let-7e-5p
 
 
-0.374
 
 
40.6
 
 
 
 
AP1S1
 
 
hsa-miR-98-5p
 
 
-0.374
 
 
40.6
 
 
 
 
AP1S1
 
 
hsa-let-7c-5p
 
 
-0.374
 
 
40.6
 
 
 
 
APCDD1
 
 
hsa-miR-106a-5p
 
 
-0.422
 
 
122.7
 
 
 
 
APCDD1
 
 
hsa-miR-20b-5p
 
 
-0.425
 
 
122.7
 
 
 
 
ARF4
 
 
hsa-miR-7-5p
 
 
-0.407
 
 
61.3
 
 
 
 
ARF4
 
 
hsa-miR-148a-3p
 
 
-0.487
 
 
61.3
 
 
 
 
ARFGEF1
 
 
hsa-miR-148a-3p
 
 
-0.306
 
 
12.9
 
 
 
 
ARFIP1
 
 
hsa-miR-148a-3p
 
 
-0.474
 
 
16.4
 
 
 
 
ARHGAP1
 
 
hsa-miR-20b-5p
 
 
-0.325
 
 
61.6
 
 
 
 
ARHGAP1
 
 
hsa-miR-106a-5p
 
 
-0.329
 
 
61.6
 
 
 
 
ARHGEF28
 
 
hsa-miR-20b-5p
 
 
-0.352
 
 
14.0
 
 
 
 
ARHGEF28
 
 
hsa-miR-106a-5p
 
 
-0.367
 
 
14.0
 
 
 
 
ARL1
 
 
hsa-miR-20b-5p
 
 
-0.424
 
 
26.2
 
 
 
 
ARL1
 
 
hsa-miR-106a-5p
 
 
-0.429
 
 
26.2
 
 
 
 
ARL4D
 
 
hsa-let-7e-5p
 
 
-0.417
 
 
44.0
 
 
 
 
ARL4D
 
 
hsa-miR-98-5p
 
 
-0.417
 
 
44.0
 
 
 
 
ARL4D
 
 
hsa-let-7c-5p
 
 
-0.417
 
 
44.0
 
 
 
 
ARL6IP1
 
 
hsa-miR-7-5p
 
 
-0.374
 
 
62.4
 
 
 
 
ARL6IP1
 
 
hsa-miR-148a-3p
 
 
-0.579
 
 
62.4
 
 
 
 
ARL6IP6
 
 
hsa-miR-98-5p
 
 
-0.293
 
 
23.5
 
 
 
 
ARL6IP6
 
 
hsa-let-7c-5p
 
 
-0.293
 
 
23.5
 
 
 
 
ARL6IP6
 
 
hsa-let-7e-5p
 
 
-0.305
 
 
23.5
 
 
 
 
ARL8B
 
 
hsa-miR-148a-3p
 
 
-0.337
 
 
64.4
 
 
 
 
ARMC8
 
 
hsa-miR-106a-5p
 
 
-0.375
 
 
12.0
 
 
 
 
ARMC8
 
 
hsa-miR-20b-5p
 
 
-0.378
 
 
12.0
 
 
 
 
ARMCX1
 
 
hsa-miR-21-5p
 
 
-0.489
 
 
10.4
 
 
 
 
ARPP19
 
 
hsa-let-7e-5p
 
 
-0.492
 
 
28.9
 
 
 
 
ARPP19
 
 
hsa-let-7c-5p
 
 
-0.492
 
 
28.9
 
 
 
 
ARPP19
 
 
hsa-miR-98-5p
 
 
-0.5
 
 
28.9
 
 
 
 
ARRDC3
 
 
hsa-miR-148a-3p
 
 
-0.305
 
 
38.6
 
 
 
 
ARRDC4
 
 
hsa-let-7c-5p
 
 
-0.329
 
 
33.5
 
 
 
 
ARRDC4
 
 
hsa-let-7e-5p
 
 
-0.329
 
 
33.5
 
 
 
 
ARRDC4
 
 
hsa-miR-98-5p
 
 
-0.329
 
 
33.5
 
 
 
 
ASAP1
 
 
hsa-let-7e-5p
 
 
-0.296
 
 
16.2
 
 
 
 
ASAP1
 
 
hsa-miR-98-5p
 
 
-0.308
 
 
16.2
 
 
 
 
ASAP1
 
 
hsa-let-7c-5p
 
 
-0.308
 
 
16.2
 
 
 
 
ASF1A
 
 
hsa-miR-21-5p
 
 
-0.304
 
 
11.0
 
 
 
 
ASPH
 
 
hsa-miR-148a-3p
 
 
-0.391
 
 
33.3
 
 
 
 
ATG16L1
 
 
hsa-miR-106a-5p
 
 
-0.464
 
 
17.2
 
 
 
 
ATG16L1
 
 
hsa-miR-20b-5p
 
 
-0.467
 
 
17.2
 
 
 
 
ATG4D
 
 
hsa-miR-148a-3p
 
 
-0.303
 
 
23.8
 
 
 
 
ATOSB
 
 
hsa-let-7e-5p
 
 
-0.429
 
 
14.2
 
 
 
 
ATOSB
 
 
hsa-miR-98-5p
 
 
-0.429
 
 
14.2
 
 
 
 
ATOSB
 
 
hsa-let-7c-5p
 
 
-0.429
 
 
14.2
 
 
 
 
ATP2A2
 
 
hsa-let-7e-5p
 
 
-0.311
 
 
41.6
 
 
 
 
ATP2A2
 
 
hsa-miR-98-5p
 
 
-0.311
 
 
41.6
 
 
 
 
ATP2A2
 
 
hsa-let-7c-5p
 
 
-0.311
 
 
41.6
 
 
 
 
ATP2B4
 
 
hsa-miR-148a-3p
 
 
-0.337
 
 
51.7
 
 
 
 
ATP6AP2
 
 
hsa-miR-148a-3p
 
 
-0.592
 
 
68.0
 
 
 
 
ATP6AP2
 
 
hsa-miR-148a-3p
 
 
-0.668
 
 
68.0
 
 
 
 
ATPAF1
 
 
hsa-let-7e-5p
 
 
-0.498
 
 
18.2
 
 
 
 
ATPAF1
 
 
hsa-miR-98-5p
 
 
-0.498
 
 
18.2
 
 
 
 
 
 
 spleen 
 
 
 
 
target_symbol
 
 
mature_mirna_id
 
 
score
 
 
nTPM
 
 
 
 
 
 
ABCB7
 
 
hsa-miR-148a-3p
 
 
-0.515
 
 
10.6
 
 
 
 
ABCC5
 
 
hsa-miR-125a-5p
 
 
-0.288
 
 
38.3
 
 
 
 
ABCD3
 
 
hsa-miR-148a-3p
 
 
-0.388
 
 
10.3
 
 
 
 
ABHD3
 
 
hsa-miR-125a-5p
 
 
-0.453
 
 
22.6
 
 
 
 
ABHD6
 
 
hsa-miR-125a-5p
 
 
-0.501
 
 
33.0
 
 
 
 
ABTB1
 
 
hsa-miR-125a-5p
 
 
-0.519
 
 
94.6
 
 
 
 
ADAM10
 
 
hsa-miR-148a-3p
 
 
-0.356
 
 
27.3
 
 
 
 
ADAM9
 
 
hsa-miR-106a-5p
 
 
-0.317
 
 
22.6
 
 
 
 
ADAM9
 
 
hsa-miR-20b-5p
 
 
-0.328
 
 
22.6
 
 
 
 
ADGRF5
 
 
hsa-miR-148a-3p
 
 
-0.354
 
 
38.8
 
 
 
 
AGFG1
 
 
hsa-miR-148a-3p
 
 
-0.332
 
 
44.5
 
 
 
 
AGFG2
 
 
hsa-miR-20b-5p
 
 
-0.386
 
 
27.6
 
 
 
 
AGFG2
 
 
hsa-miR-106a-5p
 
 
-0.399
 
 
27.6
 
 
 
 
AGGF1
 
 
hsa-miR-125a-5p
 
 
-0.331
 
 
11.9
 
 
 
 
AHDC1
 
 
hsa-miR-148a-3p
 
 
-0.413
 
 
15.7
 
 
 
 
AHNAK
 
 
hsa-miR-106a-5p
 
 
-0.334
 
 
56.3
 
 
 
 
AHNAK
 
 
hsa-miR-20b-5p
 
 
-0.337
 
 
56.3
 
 
 
 
AIFM1
 
 
hsa-miR-125a-5p
 
 
-0.3
 
 
12.9
 
 
 
 
AIFM1
 
 
hsa-let-7e-5p
 
 
-0.314
 
 
12.9
 
 
 
 
AIFM1
 
 
hsa-miR-98-5p
 
 
-0.314
 
 
12.9
 
 
 
 
AIFM1
 
 
hsa-let-7c-5p
 
 
-0.314
 
 
12.9
 
 
 
 
AKAP1
 
 
hsa-miR-148a-3p
 
 
-0.381
 
 
11.0
 
 
 
 
ALG3
 
 
hsa-miR-106a-5p
 
 
-0.569
 
 
19.8
 
 
 
 
ALG3
 
 
hsa-miR-20b-5p
 
 
-0.58
 
 
19.8
 
 
 
 
ANAPC16
 
 
hsa-miR-125a-5p
 
 
-0.578
 
 
21.2
 
 
 
 
ANKRD12
 
 
hsa-miR-7-5p
 
 
-0.37
 
 
18.8
 
 
 
 
ANKRD46
 
 
hsa-let-7e-5p
 
 
-0.389
 
 
10.4
 
 
 
 
ANKRD46
 
 
hsa-miR-98-5p
 
 
-0.389
 
 
10.4
 
 
 
 
ANKRD46
 
 
hsa-let-7c-5p
 
 
-0.389
 
 
10.4
 
 
 
 
ANPEP
 
 
hsa-miR-125a-5p
 
 
-0.372
 
 
24.0
 
 
 
 
AP1AR
 
 
hsa-miR-21-5p
 
 
-0.318
 
 
12.4
 
 
 
 
AP1AR
 
 
hsa-miR-99b-5p
 
 
-0.823
 
 
12.4
 
 
 
 
AP1S1
 
 
hsa-let-7e-5p
 
 
-0.374
 
 
20.9
 
 
 
 
AP1S1
 
 
hsa-miR-98-5p
 
 
-0.374
 
 
20.9
 
 
 
 
AP1S1
 
 
hsa-let-7c-5p
 
 
-0.374
 
 
20.9
 
 
 
 
ARF4
 
 
hsa-miR-7-5p
 
 
-0.407
 
 
60.0
 
 
 
 
ARF4
 
 
hsa-miR-148a-3p
 
 
-0.487
 
 
60.0
 
 
 
 
ARFGEF1
 
 
hsa-miR-148a-3p
 
 
-0.306
 
 
13.0
 
 
 
 
ARFIP1
 
 
hsa-miR-148a-3p
 
 
-0.474
 
 
17.3
 
 
 
 
ARHGAP1
 
 
hsa-miR-20b-5p
 
 
-0.325
 
 
55.1
 
 
 
 
ARHGAP1
 
 
hsa-miR-106a-5p
 
 
-0.329
 
 
55.1
 
 
 
 
ARHGAP24
 
 
hsa-miR-21-5p
 
 
-0.427
 
 
15.5
 
 
 
 
ARHGAP28
 
 
hsa-let-7c-5p
 
 
-0.47
 
 
11.9
 
 
 
 
ARHGAP28
 
 
hsa-miR-98-5p
 
 
-0.47
 
 
11.9
 
 
 
 
ARHGAP28
 
 
hsa-let-7e-5p
 
 
-0.486
 
 
11.9
 
 
 
 
ARID4A
 
 
hsa-miR-7-5p
 
 
-0.341
 
 
13.2
 
 
 
 
ARL1
 
 
hsa-miR-20b-5p
 
 
-0.424
 
 
27.2
 
 
 
 
ARL1
 
 
hsa-miR-106a-5p
 
 
-0.429
 
 
27.2
 
 
 
 
ARL4D
 
 
hsa-let-7e-5p
 
 
-0.417
 
 
16.1
 
 
 
 
ARL4D
 
 
hsa-miR-98-5p
 
 
-0.417
 
 
16.1
 
 
 
 
ARL4D
 
 
hsa-let-7c-5p
 
 
-0.417
 
 
16.1
 
 
 
 
ARL6IP1
 
 
hsa-miR-7-5p
 
 
-0.374
 
 
68.6
 
 
 
 
ARL6IP1
 
 
hsa-miR-148a-3p
 
 
-0.579
 
 
68.6
 
 
 
 
ARL6IP6
 
 
hsa-miR-98-5p
 
 
-0.293
 
 
13.7
 
 
 
 
ARL6IP6
 
 
hsa-let-7c-5p
 
 
-0.293
 
 
13.7
 
 
 
 
ARL6IP6
 
 
hsa-let-7e-5p
 
 
-0.305
 
 
13.7
 
 
 
 
ARL8B
 
 
hsa-miR-148a-3p
 
 
-0.337
 
 
32.0
 
 
 
 
ARMC8
 
 
hsa-miR-106a-5p
 
 
-0.375
 
 
14.9
 
 
 
 
ARMC8
 
 
hsa-miR-20b-5p
 
 
-0.378
 
 
14.9
 
 
 
 
ARMCX1
 
 
hsa-miR-21-5p
 
 
-0.489
 
 
13.3
 
 
 
 
ARMT1
 
 
hsa-let-7c-5p
 
 
-0.303
 
 
12.0
 
 
 
 
ARMT1
 
 
hsa-let-7e-5p
 
 
-0.303
 
 
12.0
 
 
 
 
ARMT1
 
 
hsa-miR-98-5p
 
 
-0.303
 
 
12.0
 
 
 
 
ARPP19
 
 
hsa-let-7e-5p
 
 
-0.492
 
 
41.2
 
 
 
 
ARPP19
 
 
hsa-let-7c-5p
 
 
-0.492
 
 
41.2
 
 
 
 
ARPP19
 
 
hsa-miR-98-5p
 
 
-0.5
 
 
41.2
 
 
 
 
ARRDC3
 
 
hsa-miR-148a-3p
 
 
-0.305
 
 
35.0
 
 
 
 
ASAP1
 
 
hsa-let-7e-5p
 
 
-0.296
 
 
28.1
 
 
 
 
ASAP1
 
 
hsa-miR-98-5p
 
 
-0.308
 
 
28.1
 
 
 
 
ASAP1
 
 
hsa-let-7c-5p
 
 
-0.308
 
 
28.1
 
 
 
 
ASF1A
 
 
hsa-miR-21-5p
 
 
-0.304
 
 
10.9
 
 
 
 
ASPH
 
 
hsa-miR-148a-3p
 
 
-0.391
 
 
26.8
 
 
 
 
ATG16L1
 
 
hsa-miR-106a-5p
 
 
-0.464
 
 
11.4
 
 
 
 
ATG16L1
 
 
hsa-miR-20b-5p
 
 
-0.467
 
 
11.4
 
 
 
 
ATG4D
 
 
hsa-miR-148a-3p
 
 
-0.303
 
 
16.4
 
 
 
 
ATOSB
 
 
hsa-let-7e-5p
 
 
-0.429
 
 
26.6
 
 
 
 
ATOSB
 
 
hsa-miR-98-5p
 
 
-0.429
 
 
26.6
 
 
 
 
ATOSB
 
 
hsa-let-7c-5p
 
 
-0.429
 
 
26.6
 
 
 
 
ATP2A2
 
 
hsa-let-7e-5p
 
 
-0.311
 
 
28.8
 
 
 
 
ATP2A2
 
 
hsa-miR-98-5p
 
 
-0.311
 
 
28.8
 
 
 
 
ATP2A2
 
 
hsa-let-7c-5p
 
 
-0.311
 
 
28.8
 
 
 
 
ATP2B4
 
 
hsa-miR-148a-3p
 
 
-0.337
 
 
11.5
 
 
 
 
ATP6AP2
 
 
hsa-miR-148a-3p
 
 
-0.592
 
 
65.0
 
 
 
 
ATP6AP2
 
 
hsa-miR-148a-3p
 
 
-0.668
 
 
65.0
 
 
 
 
ATPAF1
 
 
hsa-let-7e-5p
 
 
-0.498
 
 
23.6
 
 
 
 
ATPAF1
 
 
hsa-miR-98-5p
 
 
-0.498
 
 
23.6
 
 
 
 
ATPAF1
 
 
hsa-let-7c-5p
 
 
-0.498
 
 
23.6
 
 
 
 
AVPI1
 
 
hsa-miR-7-5p
 
 
-0.367
 
 
20.8
 
 
 
 
B3GAT3
 
 
hsa-let-7e-5p
 
 
-0.324
 
 
44.2
 
 
 
 
B3GAT3
 
 
hsa-miR-98-5p
 
 
-0.324
 
 
44.2
 
 
 
 
B3GAT3
 
 
hsa-let-7c-5p
 
 
-0.324
 
 
44.2
 
 
 
 
B4GALT5
 
 
hsa-miR-148a-3p
 
 
-0.44
 
 
26.0
 
 
 
 
B4GAT1
 
 
hsa-let-7c-5p
 
 
-0.47
 
 
10.0
 
 
 
 
B4GAT1
 
 
hsa-let-7e-5p
 
 
-0.47
 
 
10.0
 
 
 
 
B4GAT1
 
 
hsa-miR-98-5p
 
 
-0.47
 
 
10.0
 
 
 
 
BACH1
 
 
hsa-let-7e-5p
 
 
-0.298
 
 
19.4
 
 
 
 
BACH1
 
 
hsa-let-7e-5p
 
 
-0.298
 
 
19.4
 
 
 
 
BACH1
 
 
hsa-miR-98-5p
 
 
-0.298
 
 
19.4
 
 
 
 
BACH1
 
 
hsa-miR-98-5p
 
 
-0.298
 
 
19.4
 
 
 
 
BACH1
 
 
hsa-let-7c-5p
 
 
-0.306
 
 
19.4
 
 
 
 
 
 
 stomach 
 
 
 
 
target_symbol
 
 
mature_mirna_id
 
 
score
 
 
nTPM
 
 
 
 
 
 
ABCB7
 
 
hsa-miR-148a-3p
 
 
-0.515
 
 
11.6
 
 
 
 
ABCC5
 
 
hsa-miR-125a-5p
 
 
-0.288
 
 
77.7
 
 
 
 
ABCD3
 
 
hsa-miR-148a-3p
 
 
-0.388
 
 
41.1
 
 
 
 
ABHD17C
 
 
hsa-let-7c-5p
 
 
-0.359
 
 
34.0
 
 
 
 
ABHD17C
 
 
hsa-let-7e-5p
 
 
-0.359
 
 
34.0
 
 
 
 
ABHD17C
 
 
hsa-miR-98-5p
 
 
-0.383
 
 
34.0
 
 
 
 
ABHD3
 
 
hsa-miR-125a-5p
 
 
-0.453
 
 
33.6
 
 
 
 
ABHD6
 
 
hsa-miR-125a-5p
 
 
-0.501
 
 
12.2
 
 
 
 
ABTB1
 
 
hsa-miR-125a-5p
 
 
-0.519
 
 
28.0
 
 
 
 
ACER2
 
 
hsa-miR-106a-5p
 
 
-0.316
 
 
58.3
 
 
 
 
ACER2
 
 
hsa-miR-20b-5p
 
 
-0.319
 
 
58.3
 
 
 
 
ACER2
 
 
hsa-let-7e-5p
 
 
-0.333
 
 
58.3
 
 
 
 
ACER2
 
 
hsa-let-7c-5p
 
 
-0.333
 
 
58.3
 
 
 
 
ACER2
 
 
hsa-miR-125a-5p
 
 
-0.338
 
 
58.3
 
 
 
 
ACER2
 
 
hsa-miR-98-5p
 
 
-0.344
 
 
58.3
 
 
 
 
ACTC1
 
 
hsa-miR-7-5p
 
 
-0.471
 
 
32.8
 
 
 
 
ACVR1
 
 
hsa-miR-148a-3p
 
 
-0.397
 
 
11.0
 
 
 
 
ADAM10
 
 
hsa-miR-148a-3p
 
 
-0.356
 
 
19.0
 
 
 
 
ADAM9
 
 
hsa-miR-106a-5p
 
 
-0.317
 
 
41.8
 
 
 
 
ADAM9
 
 
hsa-miR-20b-5p
 
 
-0.328
 
 
41.8
 
 
 
 
AGFG1
 
 
hsa-miR-148a-3p
 
 
-0.332
 
 
31.3
 
 
 
 
AGFG2
 
 
hsa-miR-20b-5p
 
 
-0.386
 
 
12.9
 
 
 
 
AGFG2
 
 
hsa-miR-106a-5p
 
 
-0.399
 
 
12.9
 
 
 
 
AHDC1
 
 
hsa-miR-148a-3p
 
 
-0.413
 
 
10.8
 
 
 
 
AHNAK
 
 
hsa-miR-106a-5p
 
 
-0.334
 
 
125.8
 
 
 
 
AHNAK
 
 
hsa-miR-20b-5p
 
 
-0.337
 
 
125.8
 
 
 
 
AIFM1
 
 
hsa-miR-125a-5p
 
 
-0.3
 
 
29.8
 
 
 
 
AIFM1
 
 
hsa-let-7e-5p
 
 
-0.314
 
 
29.8
 
 
 
 
AIFM1
 
 
hsa-miR-98-5p
 
 
-0.314
 
 
29.8
 
 
 
 
AIFM1
 
 
hsa-let-7c-5p
 
 
-0.314
 
 
29.8
 
 
 
 
AKAP1
 
 
hsa-miR-148a-3p
 
 
-0.381
 
 
53.0
 
 
 
 
ALCAM
 
 
hsa-miR-148a-3p
 
 
-0.339
 
 
31.6
 
 
 
 
ALDH4A1
 
 
hsa-miR-184
 
 
-0.421
 
 
12.6
 
 
 
 
ALG3
 
 
hsa-miR-106a-5p
 
 
-0.569
 
 
22.0
 
 
 
 
ALG3
 
 
hsa-miR-20b-5p
 
 
-0.58
 
 
22.0
 
 
 
 
ALG9
 
 
hsa-miR-7-5p
 
 
-0.302
 
 
11.1
 
 
 
 
AMT
 
 
hsa-let-7e-5p
 
 
-0.463
 
 
13.1
 
 
 
 
AMT
 
 
hsa-miR-98-5p
 
 
-0.463
 
 
13.1
 
 
 
 
AMT
 
 
hsa-let-7c-5p
 
 
-0.463
 
 
13.1
 
 
 
 
ANAPC16
 
 
hsa-miR-125a-5p
 
 
-0.578
 
 
24.4
 
 
 
 
ANKRD12
 
 
hsa-miR-7-5p
 
 
-0.37
 
 
13.7
 
 
 
 
ANKRD29
 
 
hsa-miR-106a-5p
 
 
-0.369
 
 
14.3
 
 
 
 
ANKRD29
 
 
hsa-miR-20b-5p
 
 
-0.38
 
 
14.3
 
 
 
 
ANKRD46
 
 
hsa-let-7e-5p
 
 
-0.389
 
 
10.9
 
 
 
 
ANKRD46
 
 
hsa-miR-98-5p
 
 
-0.389
 
 
10.9
 
 
 
 
ANKRD46
 
 
hsa-let-7c-5p
 
 
-0.389
 
 
10.9
 
 
 
 
ANKRD9
 
 
hsa-miR-20b-5p
 
 
-0.331
 
 
21.4
 
 
 
 
ANKRD9
 
 
hsa-miR-106a-5p
 
 
-0.344
 
 
21.4
 
 
 
 
AP1AR
 
 
hsa-miR-21-5p
 
 
-0.318
 
 
12.3
 
 
 
 
AP1AR
 
 
hsa-miR-99b-5p
 
 
-0.823
 
 
12.3
 
 
 
 
AP1S1
 
 
hsa-let-7e-5p
 
 
-0.374
 
 
23.2
 
 
 
 
AP1S1
 
 
hsa-miR-98-5p
 
 
-0.374
 
 
23.2
 
 
 
 
AP1S1
 
 
hsa-let-7c-5p
 
 
-0.374
 
 
23.2
 
 
 
 
APCDD1
 
 
hsa-miR-106a-5p
 
 
-0.422
 
 
22.7
 
 
 
 
APCDD1
 
 
hsa-miR-20b-5p
 
 
-0.425
 
 
22.7
 
 
 
 
ARF4
 
 
hsa-miR-7-5p
 
 
-0.407
 
 
123.5
 
 
 
 
ARF4
 
 
hsa-miR-148a-3p
 
 
-0.487
 
 
123.5
 
 
 
 
ARFGEF1
 
 
hsa-miR-148a-3p
 
 
-0.306
 
 
18.5
 
 
 
 
ARFIP1
 
 
hsa-miR-148a-3p
 
 
-0.474
 
 
21.5
 
 
 
 
ARHGAP1
 
 
hsa-miR-20b-5p
 
 
-0.325
 
 
46.3
 
 
 
 
ARHGAP1
 
 
hsa-miR-106a-5p
 
 
-0.329
 
 
46.3
 
 
 
 
ARHGAP21
 
 
hsa-miR-148a-3p
 
 
-0.287
 
 
25.5
 
 
 
 
ARHGAP24
 
 
hsa-miR-21-5p
 
 
-0.427
 
 
24.6
 
 
 
 
ARHGEF28
 
 
hsa-miR-20b-5p
 
 
-0.352
 
 
20.6
 
 
 
 
ARHGEF28
 
 
hsa-miR-106a-5p
 
 
-0.367
 
 
20.6
 
 
 
 
ARL1
 
 
hsa-miR-20b-5p
 
 
-0.424
 
 
54.2
 
 
 
 
ARL1
 
 
hsa-miR-106a-5p
 
 
-0.429
 
 
54.2
 
 
 
 
ARL6IP1
 
 
hsa-miR-7-5p
 
 
-0.374
 
 
118.7
 
 
 
 
ARL6IP1
 
 
hsa-miR-148a-3p
 
 
-0.579
 
 
118.7
 
 
 
 
ARL8B
 
 
hsa-miR-148a-3p
 
 
-0.337
 
 
32.2
 
 
 
 
ARMC8
 
 
hsa-miR-106a-5p
 
 
-0.375
 
 
14.5
 
 
 
 
ARMC8
 
 
hsa-miR-20b-5p
 
 
-0.378
 
 
14.5
 
 
 
 
ARMCX1
 
 
hsa-miR-21-5p
 
 
-0.489
 
 
14.0
 
 
 
 
ARMT1
 
 
hsa-let-7c-5p
 
 
-0.303
 
 
13.3
 
 
 
 
ARMT1
 
 
hsa-let-7e-5p
 
 
-0.303
 
 
13.3
 
 
 
 
ARMT1
 
 
hsa-miR-98-5p
 
 
-0.303
 
 
13.3
 
 
 
 
ARPP19
 
 
hsa-let-7e-5p
 
 
-0.492
 
 
36.4
 
 
 
 
ARPP19
 
 
hsa-let-7c-5p
 
 
-0.492
 
 
36.4
 
 
 
 
ARPP19
 
 
hsa-miR-98-5p
 
 
-0.5
 
 
36.4
 
 
 
 
ARRDC3
 
 
hsa-miR-148a-3p
 
 
-0.305
 
 
24.3
 
 
 
 
ARRDC4
 
 
hsa-let-7c-5p
 
 
-0.329
 
 
25.2
 
 
 
 
ARRDC4
 
 
hsa-let-7e-5p
 
 
-0.329
 
 
25.2
 
 
 
 
ARRDC4
 
 
hsa-miR-98-5p
 
 
-0.329
 
 
25.2
 
 
 
 
ASF1A
 
 
hsa-miR-21-5p
 
 
-0.304
 
 
10.3
 
 
 
 
ASPH
 
 
hsa-miR-148a-3p
 
 
-0.391
 
 
96.9
 
 
 
 
ATG16L1
 
 
hsa-miR-106a-5p
 
 
-0.464
 
 
11.5
 
 
 
 
ATG16L1
 
 
hsa-miR-20b-5p
 
 
-0.467
 
 
11.5
 
 
 
 
ATG4A
 
 
hsa-miR-7-5p
 
 
-0.448
 
 
11.6
 
 
 
 
ATG4D
 
 
hsa-miR-148a-3p
 
 
-0.303
 
 
13.4
 
 
 
 
ATOSB
 
 
hsa-let-7e-5p
 
 
-0.429
 
 
11.2
 
 
 
 
ATOSB
 
 
hsa-miR-98-5p
 
 
-0.429
 
 
11.2
 
 
 
 
ATOSB
 
 
hsa-let-7c-5p
 
 
-0.429
 
 
11.2
 
 
 
 
ATP2A2
 
 
hsa-let-7e-5p
 
 
-0.311
 
 
44.5
 
 
 
 
ATP2A2
 
 
hsa-miR-98-5p
 
 
-0.311
 
 
44.5
 
 
 
 
ATP2A2
 
 
hsa-let-7c-5p
 
 
-0.311
 
 
44.5
 
 
 
 
ATP2B4
 
 
hsa-miR-148a-3p
 
 
-0.337
 
 
14.5
 
 
 
 
ATP6AP2
 
 
hsa-miR-148a-3p
 
 
-0.592
 
 
46.4
 
 
 
 
ATP6AP2
 
 
hsa-miR-148a-3p
 
 
-0.668
 
 
46.4
 
 
 
 
ATPAF1
 
 
hsa-let-7e-5p
 
 
-0.498
 
 
40.7
 
 
 
 
ATPAF1
 
 
hsa-miR-98-5p
 
 
-0.498
 
 
40.7
 
 
 
 
 
 
 thymus 
 
 
 
 
target_symbol
 
 
mature_mirna_id
 
 
score
 
 
nTPM
 
 
 
 
 
 
ABCB7
 
 
hsa-miR-148a-3p
 
 
-0.515
 
 
13.0
 
 
 
 
ABCC5
 
 
hsa-miR-125a-5p
 
 
-0.288
 
 
14.2
 
 
 
 
ABCD3
 
 
hsa-miR-148a-3p
 
 
-0.388
 
 
44.2
 
 
 
 
ABHD3
 
 
hsa-miR-125a-5p
 
 
-0.453
 
 
20.0
 
 
 
 
ABTB1
 
 
hsa-miR-125a-5p
 
 
-0.519
 
 
20.1
 
 
 
 
ACTC1
 
 
hsa-miR-7-5p
 
 
-0.471
 
 
10.5
 
 
 
 
ADAM10
 
 
hsa-miR-148a-3p
 
 
-0.356
 
 
38.8
 
 
 
 
AGFG1
 
 
hsa-miR-148a-3p
 
 
-0.332
 
 
45.3
 
 
 
 
AGGF1
 
 
hsa-miR-125a-5p
 
 
-0.331
 
 
13.4
 
 
 
 
AHCTF1
 
 
hsa-let-7e-5p
 
 
-0.374
 
 
11.4
 
 
 
 
AHCTF1
 
 
hsa-miR-98-5p
 
 
-0.374
 
 
11.4
 
 
 
 
AHCTF1
 
 
hsa-let-7c-5p
 
 
-0.374
 
 
11.4
 
 
 
 
AHNAK
 
 
hsa-miR-106a-5p
 
 
-0.334
 
 
26.0
 
 
 
 
AHNAK
 
 
hsa-miR-20b-5p
 
 
-0.337
 
 
26.0
 
 
 
 
AIFM1
 
 
hsa-miR-125a-5p
 
 
-0.3
 
 
17.2
 
 
 
 
AIFM1
 
 
hsa-let-7e-5p
 
 
-0.314
 
 
17.2
 
 
 
 
AIFM1
 
 
hsa-miR-98-5p
 
 
-0.314
 
 
17.2
 
 
 
 
AIFM1
 
 
hsa-let-7c-5p
 
 
-0.314
 
 
17.2
 
 
 
 
AKAP1
 
 
hsa-miR-148a-3p
 
 
-0.381
 
 
10.6
 
 
 
 
ALCAM
 
 
hsa-miR-148a-3p
 
 
-0.339
 
 
13.3
 
 
 
 
ALDH4A1
 
 
hsa-miR-184
 
 
-0.421
 
 
10.9
 
 
 
 
ALG3
 
 
hsa-miR-106a-5p
 
 
-0.569
 
 
11.4
 
 
 
 
ALG3
 
 
hsa-miR-20b-5p
 
 
-0.58
 
 
11.4
 
 
 
 
ANAPC16
 
 
hsa-miR-125a-5p
 
 
-0.578
 
 
29.7
 
 
 
 
ANKRD12
 
 
hsa-miR-7-5p
 
 
-0.37
 
 
10.7
 
 
 
 
ANKRD46
 
 
hsa-let-7e-5p
 
 
-0.389
 
 
14.3
 
 
 
 
ANKRD46
 
 
hsa-miR-98-5p
 
 
-0.389
 
 
14.3
 
 
 
 
ANKRD46
 
 
hsa-let-7c-5p
 
 
-0.389
 
 
14.3
 
 
 
 
AP1AR
 
 
hsa-miR-21-5p
 
 
-0.318
 
 
10.2
 
 
 
 
AP1AR
 
 
hsa-miR-99b-5p
 
 
-0.823
 
 
10.2
 
 
 
 
AP1S1
 
 
hsa-let-7e-5p
 
 
-0.374
 
 
17.8
 
 
 
 
AP1S1
 
 
hsa-miR-98-5p
 
 
-0.374
 
 
17.8
 
 
 
 
AP1S1
 
 
hsa-let-7c-5p
 
 
-0.374
 
 
17.8
 
 
 
 
ARF4
 
 
hsa-miR-7-5p
 
 
-0.407
 
 
69.9
 
 
 
 
ARF4
 
 
hsa-miR-148a-3p
 
 
-0.487
 
 
69.9
 
 
 
 
ARFGEF1
 
 
hsa-miR-148a-3p
 
 
-0.306
 
 
20.7
 
 
 
 
ARFIP1
 
 
hsa-miR-148a-3p
 
 
-0.474
 
 
11.2
 
 
 
 
ARHGAP1
 
 
hsa-miR-20b-5p
 
 
-0.325
 
 
21.6
 
 
 
 
ARHGAP1
 
 
hsa-miR-106a-5p
 
 
-0.329
 
 
21.6
 
 
 
 
ARHGAP21
 
 
hsa-miR-148a-3p
 
 
-0.287
 
 
10.5
 
 
 
 
ARID4A
 
 
hsa-miR-7-5p
 
 
-0.341
 
 
13.5
 
 
 
 
ARL1
 
 
hsa-miR-20b-5p
 
 
-0.424
 
 
30.6
 
 
 
 
ARL1
 
 
hsa-miR-106a-5p
 
 
-0.429
 
 
30.6
 
 
 
 
ARL6IP1
 
 
hsa-miR-7-5p
 
 
-0.374
 
 
147.6
 
 
 
 
ARL6IP1
 
 
hsa-miR-148a-3p
 
 
-0.579
 
 
147.6
 
 
 
 
ARL6IP6
 
 
hsa-miR-98-5p
 
 
-0.293
 
 
20.0
 
 
 
 
ARL6IP6
 
 
hsa-let-7c-5p
 
 
-0.293
 
 
20.0
 
 
 
 
ARL6IP6
 
 
hsa-let-7e-5p
 
 
-0.305
 
 
20.0
 
 
 
 
ARL8B
 
 
hsa-miR-148a-3p
 
 
-0.337
 
 
33.3
 
 
 
 
ARMC8
 
 
hsa-miR-106a-5p
 
 
-0.375
 
 
20.7
 
 
 
 
ARMC8
 
 
hsa-miR-20b-5p
 
 
-0.378
 
 
20.7
 
 
 
 
ARMT1
 
 
hsa-let-7c-5p
 
 
-0.303
 
 
14.5
 
 
 
 
ARMT1
 
 
hsa-let-7e-5p
 
 
-0.303
 
 
14.5
 
 
 
 
ARMT1
 
 
hsa-miR-98-5p
 
 
-0.303
 
 
14.5
 
 
 
 
ARPP19
 
 
hsa-let-7e-5p
 
 
-0.492
 
 
49.2
 
 
 
 
ARPP19
 
 
hsa-let-7c-5p
 
 
-0.492
 
 
49.2
 
 
 
 
ARPP19
 
 
hsa-miR-98-5p
 
 
-0.5
 
 
49.2
 
 
 
 
ARRDC3
 
 
hsa-miR-148a-3p
 
 
-0.305
 
 
26.1
 
 
 
 
ASAP1
 
 
hsa-let-7e-5p
 
 
-0.296
 
 
14.9
 
 
 
 
ASAP1
 
 
hsa-miR-98-5p
 
 
-0.308
 
 
14.9
 
 
 
 
ASAP1
 
 
hsa-let-7c-5p
 
 
-0.308
 
 
14.9
 
 
 
 
ASF1A
 
 
hsa-miR-21-5p
 
 
-0.304
 
 
22.7
 
 
 
 
ASPH
 
 
hsa-miR-148a-3p
 
 
-0.391
 
 
15.5
 
 
 
 
ATAD2
 
 
hsa-miR-20b-5p
 
 
-0.304
 
 
26.3
 
 
 
 
ATAD2
 
 
hsa-miR-106a-5p
 
 
-0.308
 
 
26.3
 
 
 
 
ATG4A
 
 
hsa-miR-7-5p
 
 
-0.448
 
 
10.5
 
 
 
 
ATG4D
 
 
hsa-miR-148a-3p
 
 
-0.303
 
 
11.2
 
 
 
 
ATP2A2
 
 
hsa-let-7e-5p
 
 
-0.311
 
 
33.8
 
 
 
 
ATP2A2
 
 
hsa-miR-98-5p
 
 
-0.311
 
 
33.8
 
 
 
 
ATP2A2
 
 
hsa-let-7c-5p
 
 
-0.311
 
 
33.8
 
 
 
 
ATP2B4
 
 
hsa-miR-148a-3p
 
 
-0.337
 
 
20.2
 
 
 
 
ATP6AP2
 
 
hsa-miR-148a-3p
 
 
-0.592
 
 
67.0
 
 
 
 
ATP6AP2
 
 
hsa-miR-148a-3p
 
 
-0.668
 
 
67.0
 
 
 
 
ATPAF1
 
 
hsa-let-7e-5p
 
 
-0.498
 
 
41.4
 
 
 
 
ATPAF1
 
 
hsa-miR-98-5p
 
 
-0.498
 
 
41.4
 
 
 
 
ATPAF1
 
 
hsa-let-7c-5p
 
 
-0.498
 
 
41.4
 
 
 
 
B3GAT3
 
 
hsa-let-7e-5p
 
 
-0.324
 
 
17.5
 
 
 
 
B3GAT3
 
 
hsa-miR-98-5p
 
 
-0.324
 
 
17.5
 
 
 
 
B3GAT3
 
 
hsa-let-7c-5p
 
 
-0.324
 
 
17.5
 
 
 
 
BACH1
 
 
hsa-let-7e-5p
 
 
-0.298
 
 
13.2
 
 
 
 
BACH1
 
 
hsa-let-7e-5p
 
 
-0.298
 
 
13.2
 
 
 
 
BACH1
 
 
hsa-miR-98-5p
 
 
-0.298
 
 
13.2
 
 
 
 
BACH1
 
 
hsa-miR-98-5p
 
 
-0.298
 
 
13.2
 
 
 
 
BACH1
 
 
hsa-let-7c-5p
 
 
-0.306
 
 
13.2
 
 
 
 
BACH1
 
 
hsa-let-7c-5p
 
 
-0.306
 
 
13.2
 
 
 
 
BACH1
 
 
hsa-let-7e-5p
 
 
-0.435
 
 
13.2
 
 
 
 
BACH1
 
 
hsa-let-7e-5p
 
 
-0.435
 
 
13.2
 
 
 
 
BACH1
 
 
hsa-miR-98-5p
 
 
-0.435
 
 
13.2
 
 
 
 
BACH1
 
 
hsa-miR-98-5p
 
 
-0.435
 
 
13.2
 
 
 
 
BACH1
 
 
hsa-let-7c-5p
 
 
-0.435
 
 
13.2
 
 
 
 
BACH1
 
 
hsa-let-7c-5p
 
 
-0.435
 
 
13.2
 
 
 
 
BAK1
 
 
hsa-miR-125a-5p
 
 
-0.402
 
 
17.9
 
 
 
 
BAZ2A
 
 
hsa-miR-99b-5p
 
 
-0.408
 
 
47.1
 
 
 
 
BCAP29
 
 
hsa-let-7e-5p
 
 
-0.289
 
 
12.4
 
 
 
 
BCL2L11
 
 
hsa-miR-148a-3p
 
 
-0.445
 
 
18.0
 
 
 
 
BCL2L12
 
 
hsa-miR-125a-5p
 
 
-0.288
 
 
16.2
 
 
 
 
BCL7A
 
 
hsa-miR-21-5p
 
 
-0.31
 
 
23.0
 
 
 
 
BIN2
 
 
hsa-miR-125a-5p
 
 
-0.679
 
 
45.3
 
 
 
 
BIN3
 
 
hsa-let-7e-5p
 
 
-0.427
 
 
12.4
 
 
 
 
BIN3
 
 
hsa-miR-98-5p
 
 
-0.427
 
 
12.4
 
 
 
 
 
 
 tonsil 
 
 
 
 
target_symbol
 
 
mature_mirna_id
 
 
score
 
 
nTPM
 
 
 
 
 
 
ABCB7
 
 
hsa-miR-148a-3p
 
 
-0.515
 
 
10.4
 
 
 
 
ABCC5
 
 
hsa-miR-125a-5p
 
 
-0.288
 
 
11.1
 
 
 
 
ABCD3
 
 
hsa-miR-148a-3p
 
 
-0.388
 
 
19.9
 
 
 
 
ABHD17C
 
 
hsa-let-7c-5p
 
 
-0.359
 
 
21.6
 
 
 
 
ABHD17C
 
 
hsa-let-7e-5p
 
 
-0.359
 
 
21.6
 
 
 
 
ABHD17C
 
 
hsa-miR-98-5p
 
 
-0.383
 
 
21.6
 
 
 
 
ABHD3
 
 
hsa-miR-125a-5p
 
 
-0.453
 
 
24.2
 
 
 
 
ADAM10
 
 
hsa-miR-148a-3p
 
 
-0.356
 
 
15.3
 
 
 
 
ADAM17
 
 
hsa-miR-125a-5p
 
 
-0.292
 
 
11.1
 
 
 
 
ADAM17
 
 
hsa-miR-148a-3p
 
 
-0.301
 
 
11.1
 
 
 
 
ADAM9
 
 
hsa-miR-106a-5p
 
 
-0.317
 
 
12.4
 
 
 
 
ADAM9
 
 
hsa-miR-20b-5p
 
 
-0.328
 
 
12.4
 
 
 
 
AGFG1
 
 
hsa-miR-148a-3p
 
 
-0.332
 
 
49.9
 
 
 
 
AGGF1
 
 
hsa-miR-125a-5p
 
 
-0.331
 
 
13.2
 
 
 
 
AHNAK
 
 
hsa-miR-106a-5p
 
 
-0.334
 
 
59.8
 
 
 
 
AHNAK
 
 
hsa-miR-20b-5p
 
 
-0.337
 
 
59.8
 
 
 
 
AIFM1
 
 
hsa-miR-125a-5p
 
 
-0.3
 
 
10.3
 
 
 
 
AIFM1
 
 
hsa-let-7e-5p
 
 
-0.314
 
 
10.3
 
 
 
 
AIFM1
 
 
hsa-miR-98-5p
 
 
-0.314
 
 
10.3
 
 
 
 
AIFM1
 
 
hsa-let-7c-5p
 
 
-0.314
 
 
10.3
 
 
 
 
ALCAM
 
 
hsa-miR-148a-3p
 
 
-0.339
 
 
13.6
 
 
 
 
ALG3
 
 
hsa-miR-106a-5p
 
 
-0.569
 
 
13.2
 
 
 
 
ALG3
 
 
hsa-miR-20b-5p
 
 
-0.58
 
 
13.2
 
 
 
 
ANAPC16
 
 
hsa-miR-125a-5p
 
 
-0.578
 
 
23.8
 
 
 
 
ANKRD12
 
 
hsa-miR-7-5p
 
 
-0.37
 
 
17.3
 
 
 
 
ANKRD29
 
 
hsa-miR-106a-5p
 
 
-0.369
 
 
13.4
 
 
 
 
ANKRD29
 
 
hsa-miR-20b-5p
 
 
-0.38
 
 
13.4
 
 
 
 
ANKRD46
 
 
hsa-let-7e-5p
 
 
-0.389
 
 
10.3
 
 
 
 
ANKRD46
 
 
hsa-miR-98-5p
 
 
-0.389
 
 
10.3
 
 
 
 
ANKRD46
 
 
hsa-let-7c-5p
 
 
-0.389
 
 
10.3
 
 
 
 
AP1AR
 
 
hsa-miR-21-5p
 
 
-0.318
 
 
11.3
 
 
 
 
AP1AR
 
 
hsa-miR-99b-5p
 
 
-0.823
 
 
11.3
 
 
 
 
AP1S1
 
 
hsa-let-7e-5p
 
 
-0.374
 
 
12.8
 
 
 
 
AP1S1
 
 
hsa-miR-98-5p
 
 
-0.374
 
 
12.8
 
 
 
 
AP1S1
 
 
hsa-let-7c-5p
 
 
-0.374
 
 
12.8
 
 
 
 
ARF4
 
 
hsa-miR-7-5p
 
 
-0.407
 
 
91.2
 
 
 
 
ARF4
 
 
hsa-miR-148a-3p
 
 
-0.487
 
 
91.2
 
 
 
 
ARFGEF1
 
 
hsa-miR-148a-3p
 
 
-0.306
 
 
16.2
 
 
 
 
ARFIP1
 
 
hsa-miR-148a-3p
 
 
-0.474
 
 
13.9
 
 
 
 
ARHGAP1
 
 
hsa-miR-20b-5p
 
 
-0.325
 
 
32.4
 
 
 
 
ARHGAP1
 
 
hsa-miR-106a-5p
 
 
-0.329
 
 
32.4
 
 
 
 
ARHGAP24
 
 
hsa-miR-21-5p
 
 
-0.427
 
 
14.5
 
 
 
 
ARID4A
 
 
hsa-miR-7-5p
 
 
-0.341
 
 
10.5
 
 
 
 
ARL1
 
 
hsa-miR-20b-5p
 
 
-0.424
 
 
45.6
 
 
 
 
ARL1
 
 
hsa-miR-106a-5p
 
 
-0.429
 
 
45.6
 
 
 
 
ARL6IP1
 
 
hsa-miR-7-5p
 
 
-0.374
 
 
106.2
 
 
 
 
ARL6IP1
 
 
hsa-miR-148a-3p
 
 
-0.579
 
 
106.2
 
 
 
 
ARL6IP6
 
 
hsa-miR-98-5p
 
 
-0.293
 
 
18.0
 
 
 
 
ARL6IP6
 
 
hsa-let-7c-5p
 
 
-0.293
 
 
18.0
 
 
 
 
ARL6IP6
 
 
hsa-let-7e-5p
 
 
-0.305
 
 
18.0
 
 
 
 
ARL8B
 
 
hsa-miR-148a-3p
 
 
-0.337
 
 
44.5
 
 
 
 
ARMC8
 
 
hsa-miR-106a-5p
 
 
-0.375
 
 
21.4
 
 
 
 
ARMC8
 
 
hsa-miR-20b-5p
 
 
-0.378
 
 
21.4
 
 
 
 
ARMT1
 
 
hsa-let-7c-5p
 
 
-0.303
 
 
17.3
 
 
 
 
ARMT1
 
 
hsa-let-7e-5p
 
 
-0.303
 
 
17.3
 
 
 
 
ARMT1
 
 
hsa-miR-98-5p
 
 
-0.303
 
 
17.3
 
 
 
 
ARPP19
 
 
hsa-let-7e-5p
 
 
-0.492
 
 
48.4
 
 
 
 
ARPP19
 
 
hsa-let-7c-5p
 
 
-0.492
 
 
48.4
 
 
 
 
ARPP19
 
 
hsa-miR-98-5p
 
 
-0.5
 
 
48.4
 
 
 
 
ARRDC3
 
 
hsa-miR-148a-3p
 
 
-0.305
 
 
26.4
 
 
 
 
ASAP1
 
 
hsa-let-7e-5p
 
 
-0.296
 
 
17.8
 
 
 
 
ASAP1
 
 
hsa-miR-98-5p
 
 
-0.308
 
 
17.8
 
 
 
 
ASAP1
 
 
hsa-let-7c-5p
 
 
-0.308
 
 
17.8
 
 
 
 
ASF1A
 
 
hsa-miR-21-5p
 
 
-0.304
 
 
14.1
 
 
 
 
ASPH
 
 
hsa-miR-148a-3p
 
 
-0.391
 
 
26.4
 
 
 
 
ATAD2
 
 
hsa-miR-20b-5p
 
 
-0.304
 
 
12.0
 
 
 
 
ATAD2
 
 
hsa-miR-106a-5p
 
 
-0.308
 
 
12.0
 
 
 
 
ATG4A
 
 
hsa-miR-7-5p
 
 
-0.448
 
 
10.4
 
 
 
 
ATP2A2
 
 
hsa-let-7e-5p
 
 
-0.311
 
 
45.1
 
 
 
 
ATP2A2
 
 
hsa-miR-98-5p
 
 
-0.311
 
 
45.1
 
 
 
 
ATP2A2
 
 
hsa-let-7c-5p
 
 
-0.311
 
 
45.1
 
 
 
 
ATP2B4
 
 
hsa-miR-148a-3p
 
 
-0.337
 
 
18.2
 
 
 
 
ATP6AP2
 
 
hsa-miR-148a-3p
 
 
-0.592
 
 
67.9
 
 
 
 
ATP6AP2
 
 
hsa-miR-148a-3p
 
 
-0.668
 
 
67.9
 
 
 
 
ATPAF1
 
 
hsa-let-7e-5p
 
 
-0.498
 
 
30.0
 
 
 
 
ATPAF1
 
 
hsa-miR-98-5p
 
 
-0.498
 
 
30.0
 
 
 
 
ATPAF1
 
 
hsa-let-7c-5p
 
 
-0.498
 
 
30.0
 
 
 
 
B3GAT3
 
 
hsa-let-7e-5p
 
 
-0.324
 
 
10.3
 
 
 
 
B3GAT3
 
 
hsa-miR-98-5p
 
 
-0.324
 
 
10.3
 
 
 
 
B3GAT3
 
 
hsa-let-7c-5p
 
 
-0.324
 
 
10.3
 
 
 
 
B4GALT5
 
 
hsa-miR-148a-3p
 
 
-0.44
 
 
10.8
 
 
 
 
BACH1
 
 
hsa-let-7e-5p
 
 
-0.298
 
 
13.8
 
 
 
 
BACH1
 
 
hsa-let-7e-5p
 
 
-0.298
 
 
13.8
 
 
 
 
BACH1
 
 
hsa-miR-98-5p
 
 
-0.298
 
 
13.8
 
 
 
 
BACH1
 
 
hsa-miR-98-5p
 
 
-0.298
 
 
13.8
 
 
 
 
BACH1
 
 
hsa-let-7c-5p
 
 
-0.306
 
 
13.8
 
 
 
 
BACH1
 
 
hsa-let-7c-5p
 
 
-0.306
 
 
13.8
 
 
 
 
BACH1
 
 
hsa-let-7e-5p
 
 
-0.435
 
 
13.8
 
 
 
 
BACH1
 
 
hsa-let-7e-5p
 
 
-0.435
 
 
13.8
 
 
 
 
BACH1
 
 
hsa-miR-98-5p
 
 
-0.435
 
 
13.8
 
 
 
 
BACH1
 
 
hsa-miR-98-5p
 
 
-0.435
 
 
13.8
 
 
 
 
BACH1
 
 
hsa-let-7c-5p
 
 
-0.435
 
 
13.8
 
 
 
 
BACH1
 
 
hsa-let-7c-5p
 
 
-0.435
 
 
13.8
 
 
 
 
BAK1
 
 
hsa-miR-125a-5p
 
 
-0.402
 
 
21.9
 
 
 
 
BAZ2A
 
 
hsa-miR-99b-5p
 
 
-0.408
 
 
28.7
 
 
 
 
BCAP29
 
 
hsa-let-7e-5p
 
 
-0.289
 
 
15.2
 
 
 
 
BCL2L11
 
 
hsa-miR-148a-3p
 
 
-0.445
 
 
21.7
 
 
 
 
BCL2L12
 
 
hsa-miR-125a-5p
 
 
-0.288
 
 
14.8
 
 
 
 
BCL7A
 
 
hsa-miR-21-5p
 
 
-0.31
 
 
14.4
 
 
 
 
BHLHE41
 
 
hsa-miR-148a-3p
 
 
-0.442
 
 
16.8
 
 
 
 
 
 
 urinary bladder 
 
 
 
 
target_symbol
 
 
mature_mirna_id
 
 
score
 
 
nTPM
 
 
 
 
 
 
ABCB7
 
 
hsa-miR-148a-3p
 
 
-0.515
 
 
11.0
 
 
 
 
ABCC5
 
 
hsa-miR-125a-5p
 
 
-0.288
 
 
38.3
 
 
 
 
ABCD3
 
 
hsa-miR-148a-3p
 
 
-0.388
 
 
21.4
 
 
 
 
ABHD17C
 
 
hsa-let-7c-5p
 
 
-0.359
 
 
26.8
 
 
 
 
ABHD17C
 
 
hsa-let-7e-5p
 
 
-0.359
 
 
26.8
 
 
 
 
ABHD17C
 
 
hsa-miR-98-5p
 
 
-0.383
 
 
26.8
 
 
 
 
ABHD3
 
 
hsa-miR-125a-5p
 
 
-0.453
 
 
14.3
 
 
 
 
ABHD5
 
 
hsa-miR-20b-5p
 
 
-0.418
 
 
15.5
 
 
 
 
ABHD5
 
 
hsa-miR-106a-5p
 
 
-0.447
 
 
15.5
 
 
 
 
ABTB1
 
 
hsa-miR-125a-5p
 
 
-0.519
 
 
36.6
 
 
 
 
ACER2
 
 
hsa-miR-106a-5p
 
 
-0.316
 
 
36.7
 
 
 
 
ACER2
 
 
hsa-miR-20b-5p
 
 
-0.319
 
 
36.7
 
 
 
 
ACER2
 
 
hsa-let-7e-5p
 
 
-0.333
 
 
36.7
 
 
 
 
ACER2
 
 
hsa-let-7c-5p
 
 
-0.333
 
 
36.7
 
 
 
 
ACER2
 
 
hsa-miR-125a-5p
 
 
-0.338
 
 
36.7
 
 
 
 
ACER2
 
 
hsa-miR-98-5p
 
 
-0.344
 
 
36.7
 
 
 
 
ACTC1
 
 
hsa-miR-7-5p
 
 
-0.471
 
 
667.0
 
 
 
 
ACVR1
 
 
hsa-miR-148a-3p
 
 
-0.397
 
 
13.3
 
 
 
 
ADAM10
 
 
hsa-miR-148a-3p
 
 
-0.356
 
 
26.2
 
 
 
 
ADAM9
 
 
hsa-miR-106a-5p
 
 
-0.317
 
 
52.5
 
 
 
 
ADAM9
 
 
hsa-miR-20b-5p
 
 
-0.328
 
 
52.5
 
 
 
 
ADGRF5
 
 
hsa-miR-148a-3p
 
 
-0.354
 
 
10.6
 
 
 
 
AGFG1
 
 
hsa-miR-148a-3p
 
 
-0.332
 
 
61.2
 
 
 
 
AGFG2
 
 
hsa-miR-20b-5p
 
 
-0.386
 
 
10.3
 
 
 
 
AGFG2
 
 
hsa-miR-106a-5p
 
 
-0.399
 
 
10.3
 
 
 
 
AHDC1
 
 
hsa-miR-148a-3p
 
 
-0.413
 
 
14.8
 
 
 
 
AHNAK
 
 
hsa-miR-106a-5p
 
 
-0.334
 
 
145.5
 
 
 
 
AHNAK
 
 
hsa-miR-20b-5p
 
 
-0.337
 
 
145.5
 
 
 
 
AIFM1
 
 
hsa-miR-125a-5p
 
 
-0.3
 
 
13.1
 
 
 
 
AIFM1
 
 
hsa-let-7e-5p
 
 
-0.314
 
 
13.1
 
 
 
 
AIFM1
 
 
hsa-miR-98-5p
 
 
-0.314
 
 
13.1
 
 
 
 
AIFM1
 
 
hsa-let-7c-5p
 
 
-0.314
 
 
13.1
 
 
 
 
AKAP1
 
 
hsa-miR-148a-3p
 
 
-0.381
 
 
47.8
 
 
 
 
ALCAM
 
 
hsa-miR-148a-3p
 
 
-0.339
 
 
23.9
 
 
 
 
ALDH4A1
 
 
hsa-miR-184
 
 
-0.421
 
 
21.0
 
 
 
 
ALG3
 
 
hsa-miR-106a-5p
 
 
-0.569
 
 
20.1
 
 
 
 
ALG3
 
 
hsa-miR-20b-5p
 
 
-0.58
 
 
20.1
 
 
 
 
ALG9
 
 
hsa-miR-7-5p
 
 
-0.302
 
 
10.1
 
 
 
 
AMT
 
 
hsa-let-7e-5p
 
 
-0.463
 
 
13.1
 
 
 
 
AMT
 
 
hsa-miR-98-5p
 
 
-0.463
 
 
13.1
 
 
 
 
AMT
 
 
hsa-let-7c-5p
 
 
-0.463
 
 
13.1
 
 
 
 
ANAPC16
 
 
hsa-miR-125a-5p
 
 
-0.578
 
 
20.8
 
 
 
 
ANKRD12
 
 
hsa-miR-7-5p
 
 
-0.37
 
 
12.7
 
 
 
 
ANKRD50
 
 
hsa-miR-106a-5p
 
 
-0.29
 
 
13.3
 
 
 
 
ANKRD9
 
 
hsa-miR-20b-5p
 
 
-0.331
 
 
11.5
 
 
 
 
ANKRD9
 
 
hsa-miR-106a-5p
 
 
-0.344
 
 
11.5
 
 
 
 
ANPEP
 
 
hsa-miR-125a-5p
 
 
-0.372
 
 
15.7
 
 
 
 
AP1AR
 
 
hsa-miR-21-5p
 
 
-0.318
 
 
12.2
 
 
 
 
AP1AR
 
 
hsa-miR-99b-5p
 
 
-0.823
 
 
12.2
 
 
 
 
AP1S1
 
 
hsa-let-7e-5p
 
 
-0.374
 
 
18.6
 
 
 
 
AP1S1
 
 
hsa-miR-98-5p
 
 
-0.374
 
 
18.6
 
 
 
 
AP1S1
 
 
hsa-let-7c-5p
 
 
-0.374
 
 
18.6
 
 
 
 
APCDD1
 
 
hsa-miR-106a-5p
 
 
-0.422
 
 
34.0
 
 
 
 
APCDD1
 
 
hsa-miR-20b-5p
 
 
-0.425
 
 
34.0
 
 
 
 
ARF4
 
 
hsa-miR-7-5p
 
 
-0.407
 
 
190.1
 
 
 
 
ARF4
 
 
hsa-miR-148a-3p
 
 
-0.487
 
 
190.1
 
 
 
 
ARFGEF1
 
 
hsa-miR-148a-3p
 
 
-0.306
 
 
18.4
 
 
 
 
ARFIP1
 
 
hsa-miR-148a-3p
 
 
-0.474
 
 
22.0
 
 
 
 
ARG2
 
 
hsa-let-7e-5p
 
 
-0.468
 
 
16.6
 
 
 
 
ARG2
 
 
hsa-miR-98-5p
 
 
-0.468
 
 
16.6
 
 
 
 
ARG2
 
 
hsa-let-7c-5p
 
 
-0.468
 
 
16.6
 
 
 
 
ARHGAP1
 
 
hsa-miR-20b-5p
 
 
-0.325
 
 
106.2
 
 
 
 
ARHGAP1
 
 
hsa-miR-106a-5p
 
 
-0.329
 
 
106.2
 
 
 
 
ARHGAP21
 
 
hsa-miR-148a-3p
 
 
-0.287
 
 
12.9
 
 
 
 
ARL1
 
 
hsa-miR-20b-5p
 
 
-0.424
 
 
49.5
 
 
 
 
ARL1
 
 
hsa-miR-106a-5p
 
 
-0.429
 
 
49.5
 
 
 
 
ARL4D
 
 
hsa-let-7e-5p
 
 
-0.417
 
 
26.6
 
 
 
 
ARL4D
 
 
hsa-miR-98-5p
 
 
-0.417
 
 
26.6
 
 
 
 
ARL4D
 
 
hsa-let-7c-5p
 
 
-0.417
 
 
26.6
 
 
 
 
ARL6IP1
 
 
hsa-miR-7-5p
 
 
-0.374
 
 
124.0
 
 
 
 
ARL6IP1
 
 
hsa-miR-148a-3p
 
 
-0.579
 
 
124.0
 
 
 
 
ARL6IP6
 
 
hsa-miR-98-5p
 
 
-0.293
 
 
12.2
 
 
 
 
ARL6IP6
 
 
hsa-let-7c-5p
 
 
-0.293
 
 
12.2
 
 
 
 
ARL6IP6
 
 
hsa-let-7e-5p
 
 
-0.305
 
 
12.2
 
 
 
 
ARL8B
 
 
hsa-miR-148a-3p
 
 
-0.337
 
 
46.5
 
 
 
 
ARMC8
 
 
hsa-miR-106a-5p
 
 
-0.375
 
 
20.8
 
 
 
 
ARMC8
 
 
hsa-miR-20b-5p
 
 
-0.378
 
 
20.8
 
 
 
 
ARMCX1
 
 
hsa-miR-21-5p
 
 
-0.489
 
 
22.2
 
 
 
 
ARMT1
 
 
hsa-let-7c-5p
 
 
-0.303
 
 
16.1
 
 
 
 
ARMT1
 
 
hsa-let-7e-5p
 
 
-0.303
 
 
16.1
 
 
 
 
ARMT1
 
 
hsa-miR-98-5p
 
 
-0.303
 
 
16.1
 
 
 
 
ARPP19
 
 
hsa-let-7e-5p
 
 
-0.492
 
 
51.7
 
 
 
 
ARPP19
 
 
hsa-let-7c-5p
 
 
-0.492
 
 
51.7
 
 
 
 
ARPP19
 
 
hsa-miR-98-5p
 
 
-0.5
 
 
51.7
 
 
 
 
ARRDC3
 
 
hsa-miR-148a-3p
 
 
-0.305
 
 
53.2
 
 
 
 
ARRDC4
 
 
hsa-let-7c-5p
 
 
-0.329
 
 
10.9
 
 
 
 
ARRDC4
 
 
hsa-let-7e-5p
 
 
-0.329
 
 
10.9
 
 
 
 
ARRDC4
 
 
hsa-miR-98-5p
 
 
-0.329
 
 
10.9
 
 
 
 
ASAP1
 
 
hsa-let-7e-5p
 
 
-0.296
 
 
14.4
 
 
 
 
ASAP1
 
 
hsa-miR-98-5p
 
 
-0.308
 
 
14.4
 
 
 
 
ASAP1
 
 
hsa-let-7c-5p
 
 
-0.308
 
 
14.4
 
 
 
 
ASPH
 
 
hsa-miR-148a-3p
 
 
-0.391
 
 
103.6
 
 
 
 
ATG16L1
 
 
hsa-miR-106a-5p
 
 
-0.464
 
 
13.3
 
 
 
 
ATG16L1
 
 
hsa-miR-20b-5p
 
 
-0.467
 
 
13.3
 
 
 
 
ATOSB
 
 
hsa-let-7e-5p
 
 
-0.429
 
 
10.8
 
 
 
 
ATOSB
 
 
hsa-miR-98-5p
 
 
-0.429
 
 
10.8
 
 
 
 
ATOSB
 
 
hsa-let-7c-5p
 
 
-0.429
 
 
10.8
 
 
 
 
ATP2A2
 
 
hsa-let-7e-5p
 
 
-0.311
 
 
84.9
 
 
 
 
ATP2A2
 
 
hsa-miR-98-5p
 
 
-0.311
 
 
84.9
 
 
 
 
ATP2A2
 
 
hsa-let-7c-5p
 
 
-0.311
 
 
84.9
 
 
 
 
 
 
 vagina 
 
 
 
 
target_symbol
 
 
mature_mirna_id
 
 
score
 
 
nTPM
 
 
 
 
 
 
ABCB7
 
 
hsa-miR-148a-3p
 
 
-0.515
 
 
10.8
 
 
 
 
ABCC5
 
 
hsa-miR-125a-5p
 
 
-0.288
 
 
35.3
 
 
 
 
ABCD3
 
 
hsa-miR-148a-3p
 
 
-0.388
 
 
14.9
 
 
 
 
ABHD17C
 
 
hsa-let-7c-5p
 
 
-0.359
 
 
21.1
 
 
 
 
ABHD17C
 
 
hsa-let-7e-5p
 
 
-0.359
 
 
21.1
 
 
 
 
ABHD17C
 
 
hsa-miR-98-5p
 
 
-0.383
 
 
21.1
 
 
 
 
ABHD3
 
 
hsa-miR-125a-5p
 
 
-0.453
 
 
12.7
 
 
 
 
ABHD5
 
 
hsa-miR-20b-5p
 
 
-0.418
 
 
11.9
 
 
 
 
ABHD5
 
 
hsa-miR-106a-5p
 
 
-0.447
 
 
11.9
 
 
 
 
ABTB1
 
 
hsa-miR-125a-5p
 
 
-0.519
 
 
32.4
 
 
 
 
ACP3
 
 
hsa-let-7e-5p
 
 
-0.298
 
 
10.8
 
 
 
 
ACP3
 
 
hsa-miR-98-5p
 
 
-0.298
 
 
10.8
 
 
 
 
ACP3
 
 
hsa-let-7c-5p
 
 
-0.298
 
 
10.8
 
 
 
 
ACTC1
 
 
hsa-miR-7-5p
 
 
-0.471
 
 
50.8
 
 
 
 
ADAM10
 
 
hsa-miR-148a-3p
 
 
-0.356
 
 
10.3
 
 
 
 
ADAM9
 
 
hsa-miR-106a-5p
 
 
-0.317
 
 
15.5
 
 
 
 
ADAM9
 
 
hsa-miR-20b-5p
 
 
-0.328
 
 
15.5
 
 
 
 
ADRB2
 
 
hsa-let-7c-5p
 
 
-0.635
 
 
12.2
 
 
 
 
ADRB2
 
 
hsa-let-7e-5p
 
 
-0.635
 
 
12.2
 
 
 
 
ADRB2
 
 
hsa-miR-98-5p
 
 
-0.635
 
 
12.2
 
 
 
 
AGFG1
 
 
hsa-miR-148a-3p
 
 
-0.332
 
 
31.5
 
 
 
 
AGFG2
 
 
hsa-miR-20b-5p
 
 
-0.386
 
 
11.5
 
 
 
 
AGFG2
 
 
hsa-miR-106a-5p
 
 
-0.399
 
 
11.5
 
 
 
 
AHDC1
 
 
hsa-miR-148a-3p
 
 
-0.413
 
 
23.0
 
 
 
 
AHNAK
 
 
hsa-miR-106a-5p
 
 
-0.334
 
 
200.6
 
 
 
 
AHNAK
 
 
hsa-miR-20b-5p
 
 
-0.337
 
 
200.6
 
 
 
 
AKAP1
 
 
hsa-miR-148a-3p
 
 
-0.381
 
 
24.4
 
 
 
 
ALDH4A1
 
 
hsa-miR-184
 
 
-0.421
 
 
16.8
 
 
 
 
ALG3
 
 
hsa-miR-106a-5p
 
 
-0.569
 
 
18.9
 
 
 
 
ALG3
 
 
hsa-miR-20b-5p
 
 
-0.58
 
 
18.9
 
 
 
 
ALG9
 
 
hsa-miR-7-5p
 
 
-0.302
 
 
10.2
 
 
 
 
ANAPC16
 
 
hsa-miR-125a-5p
 
 
-0.578
 
 
14.7
 
 
 
 
ANKRD29
 
 
hsa-miR-106a-5p
 
 
-0.369
 
 
12.3
 
 
 
 
ANKRD29
 
 
hsa-miR-20b-5p
 
 
-0.38
 
 
12.3
 
 
 
 
ANKRD9
 
 
hsa-miR-20b-5p
 
 
-0.331
 
 
14.6
 
 
 
 
ANKRD9
 
 
hsa-miR-106a-5p
 
 
-0.344
 
 
14.6
 
 
 
 
ANPEP
 
 
hsa-miR-125a-5p
 
 
-0.372
 
 
13.0
 
 
 
 
AP1S1
 
 
hsa-let-7e-5p
 
 
-0.374
 
 
20.1
 
 
 
 
AP1S1
 
 
hsa-miR-98-5p
 
 
-0.374
 
 
20.1
 
 
 
 
AP1S1
 
 
hsa-let-7c-5p
 
 
-0.374
 
 
20.1
 
 
 
 
APCDD1
 
 
hsa-miR-106a-5p
 
 
-0.422
 
 
33.2
 
 
 
 
APCDD1
 
 
hsa-miR-20b-5p
 
 
-0.425
 
 
33.2
 
 
 
 
ARF4
 
 
hsa-miR-7-5p
 
 
-0.407
 
 
85.1
 
 
 
 
ARF4
 
 
hsa-miR-148a-3p
 
 
-0.487
 
 
85.1
 
 
 
 
ARFIP1
 
 
hsa-miR-148a-3p
 
 
-0.474
 
 
13.1
 
 
 
 
ARG2
 
 
hsa-let-7e-5p
 
 
-0.468
 
 
11.0
 
 
 
 
ARG2
 
 
hsa-miR-98-5p
 
 
-0.468
 
 
11.0
 
 
 
 
ARG2
 
 
hsa-let-7c-5p
 
 
-0.468
 
 
11.0
 
 
 
 
ARHGAP1
 
 
hsa-miR-20b-5p
 
 
-0.325
 
 
59.0
 
 
 
 
ARHGAP1
 
 
hsa-miR-106a-5p
 
 
-0.329
 
 
59.0
 
 
 
 
ARL1
 
 
hsa-miR-20b-5p
 
 
-0.424
 
 
34.0
 
 
 
 
ARL1
 
 
hsa-miR-106a-5p
 
 
-0.429
 
 
34.0
 
 
 
 
ARL4D
 
 
hsa-let-7e-5p
 
 
-0.417
 
 
38.9
 
 
 
 
ARL4D
 
 
hsa-miR-98-5p
 
 
-0.417
 
 
38.9
 
 
 
 
ARL4D
 
 
hsa-let-7c-5p
 
 
-0.417
 
 
38.9
 
 
 
 
ARL6IP1
 
 
hsa-miR-7-5p
 
 
-0.374
 
 
48.0
 
 
 
 
ARL6IP1
 
 
hsa-miR-148a-3p
 
 
-0.579
 
 
48.0
 
 
 
 
ARL8B
 
 
hsa-miR-148a-3p
 
 
-0.337
 
 
51.8
 
 
 
 
ARMC8
 
 
hsa-miR-106a-5p
 
 
-0.375
 
 
12.4
 
 
 
 
ARMC8
 
 
hsa-miR-20b-5p
 
 
-0.378
 
 
12.4
 
 
 
 
ARMCX1
 
 
hsa-miR-21-5p
 
 
-0.489
 
 
20.8
 
 
 
 
ARPP19
 
 
hsa-let-7e-5p
 
 
-0.492
 
 
27.0
 
 
 
 
ARPP19
 
 
hsa-let-7c-5p
 
 
-0.492
 
 
27.0
 
 
 
 
ARPP19
 
 
hsa-miR-98-5p
 
 
-0.5
 
 
27.0
 
 
 
 
ARRDC3
 
 
hsa-miR-148a-3p
 
 
-0.305
 
 
40.5
 
 
 
 
ARRDC4
 
 
hsa-let-7c-5p
 
 
-0.329
 
 
10.1
 
 
 
 
ARRDC4
 
 
hsa-let-7e-5p
 
 
-0.329
 
 
10.1
 
 
 
 
ARRDC4
 
 
hsa-miR-98-5p
 
 
-0.329
 
 
10.1
 
 
 
 
ASAP1
 
 
hsa-let-7e-5p
 
 
-0.296
 
 
14.4
 
 
 
 
ASAP1
 
 
hsa-miR-98-5p
 
 
-0.308
 
 
14.4
 
 
 
 
ASAP1
 
 
hsa-let-7c-5p
 
 
-0.308
 
 
14.4
 
 
 
 
ASPH
 
 
hsa-miR-148a-3p
 
 
-0.391
 
 
40.4
 
 
 
 
ATG4D
 
 
hsa-miR-148a-3p
 
 
-0.303
 
 
14.5
 
 
 
 
ATOSB
 
 
hsa-let-7e-5p
 
 
-0.429
 
 
14.3
 
 
 
 
ATOSB
 
 
hsa-miR-98-5p
 
 
-0.429
 
 
14.3
 
 
 
 
ATOSB
 
 
hsa-let-7c-5p
 
 
-0.429
 
 
14.3
 
 
 
 
ATP2A2
 
 
hsa-let-7e-5p
 
 
-0.311
 
 
40.2
 
 
 
 
ATP2A2
 
 
hsa-miR-98-5p
 
 
-0.311
 
 
40.2
 
 
 
 
ATP2A2
 
 
hsa-let-7c-5p
 
 
-0.311
 
 
40.2
 
 
 
 
ATP2B4
 
 
hsa-miR-148a-3p
 
 
-0.337
 
 
14.3
 
 
 
 
ATP6AP2
 
 
hsa-miR-148a-3p
 
 
-0.592
 
 
44.8
 
 
 
 
ATP6AP2
 
 
hsa-miR-148a-3p
 
 
-0.668
 
 
44.8
 
 
 
 
ATPAF1
 
 
hsa-let-7e-5p
 
 
-0.498
 
 
15.7
 
 
 
 
ATPAF1
 
 
hsa-miR-98-5p
 
 
-0.498
 
 
15.7
 
 
 
 
ATPAF1
 
 
hsa-let-7c-5p
 
 
-0.498
 
 
15.7
 
 
 
 
AVPI1
 
 
hsa-miR-7-5p
 
 
-0.367
 
 
46.2
 
 
 
 
B3GAT3
 
 
hsa-let-7e-5p
 
 
-0.324
 
 
28.4
 
 
 
 
B3GAT3
 
 
hsa-miR-98-5p
 
 
-0.324
 
 
28.4
 
 
 
 
B3GAT3
 
 
hsa-let-7c-5p
 
 
-0.324
 
 
28.4
 
 
 
 
B4GALT5
 
 
hsa-miR-148a-3p
 
 
-0.44
 
 
16.5
 
 
 
 
B4GAT1
 
 
hsa-let-7c-5p
 
 
-0.47
 
 
15.8
 
 
 
 
B4GAT1
 
 
hsa-let-7e-5p
 
 
-0.47
 
 
15.8
 
 
 
 
B4GAT1
 
 
hsa-miR-98-5p
 
 
-0.47
 
 
15.8
 
 
 
 
BACH1
 
 
hsa-let-7e-5p
 
 
-0.298
 
 
12.6
 
 
 
 
BACH1
 
 
hsa-let-7e-5p
 
 
-0.298
 
 
12.6
 
 
 
 
BACH1
 
 
hsa-miR-98-5p
 
 
-0.298
 
 
12.6
 
 
 
 
BACH1
 
 
hsa-miR-98-5p
 
 
-0.298
 
 
12.6
 
 
 
 
BACH1
 
 
hsa-let-7c-5p
 
 
-0.306
 
 
12.6
 
 
 
 
BACH1
 
 
hsa-let-7c-5p
 
 
-0.306
 
 
12.6
 
 
 
 
BACH1
 
 
hsa-let-7e-5p
 
 
-0.435
 
 
12.6
 
 
 
 
 
 
 basal ganglia 
 
 
 
 
target_symbol
 
 
mature_mirna_id
 
 
score
 
 
nTPM
 
 
 
 
 
 
ABCB9
 
 
hsa-let-7e-5p
 
 
-0.422
 
 
13.6
 
 
 
 
ABCB9
 
 
hsa-let-7c-5p
 
 
-0.422
 
 
13.6
 
 
 
 
ABCB9
 
 
hsa-miR-98-5p
 
 
-0.444
 
 
13.6
 
 
 
 
ABCC5
 
 
hsa-miR-125a-5p
 
 
-0.288
 
 
35.1
 
 
 
 
ABCD3
 
 
hsa-miR-148a-3p
 
 
-0.388
 
 
17.4
 
 
 
 
ABHD17C
 
 
hsa-let-7c-5p
 
 
-0.359
 
 
20.2
 
 
 
 
ABHD17C
 
 
hsa-let-7e-5p
 
 
-0.359
 
 
20.2
 
 
 
 
ABHD17C
 
 
hsa-miR-98-5p
 
 
-0.383
 
 
20.2
 
 
 
 
ABHD3
 
 
hsa-miR-125a-5p
 
 
-0.453
 
 
11.4
 
 
 
 
ABHD6
 
 
hsa-miR-125a-5p
 
 
-0.501
 
 
18.4
 
 
 
 
ABTB1
 
 
hsa-miR-125a-5p
 
 
-0.519
 
 
41.3
 
 
 
 
ACHE
 
 
hsa-miR-125a-5p
 
 
-0.308
 
 
38.3
 
 
 
 
ACHE
 
 
hsa-miR-125a-5p
 
 
-0.361
 
 
38.3
 
 
 
 
ACSL6
 
 
hsa-miR-125a-5p
 
 
-0.307
 
 
18.7
 
 
 
 
ACSL6
 
 
hsa-let-7e-5p
 
 
-0.336
 
 
18.7
 
 
 
 
ACSL6
 
 
hsa-miR-98-5p
 
 
-0.336
 
 
18.7
 
 
 
 
ACSL6
 
 
hsa-let-7c-5p
 
 
-0.336
 
 
18.7
 
 
 
 
ADCY2
 
 
hsa-miR-148a-3p
 
 
-0.299
 
 
23.7
 
 
 
 
ADGRB3
 
 
hsa-miR-148a-3p
 
 
-0.524
 
 
21.1
 
 
 
 
ADGRF5
 
 
hsa-miR-148a-3p
 
 
-0.354
 
 
13.3
 
 
 
 
ADRB1
 
 
hsa-let-7c-5p
 
 
-0.351
 
 
10.0
 
 
 
 
ADRB1
 
 
hsa-let-7e-5p
 
 
-0.367
 
 
10.0
 
 
 
 
ADRB1
 
 
hsa-miR-98-5p
 
 
-0.367
 
 
10.0
 
 
 
 
AGFG1
 
 
hsa-miR-148a-3p
 
 
-0.332
 
 
27.0
 
 
 
 
AGFG2
 
 
hsa-miR-20b-5p
 
 
-0.386
 
 
19.5
 
 
 
 
AGFG2
 
 
hsa-miR-106a-5p
 
 
-0.399
 
 
19.5
 
 
 
 
AHDC1
 
 
hsa-miR-148a-3p
 
 
-0.413
 
 
12.3
 
 
 
 
AHNAK
 
 
hsa-miR-106a-5p
 
 
-0.334
 
 
22.5
 
 
 
 
AHNAK
 
 
hsa-miR-20b-5p
 
 
-0.337
 
 
22.5
 
 
 
 
AIFM1
 
 
hsa-miR-125a-5p
 
 
-0.3
 
 
12.5
 
 
 
 
AIFM1
 
 
hsa-let-7e-5p
 
 
-0.314
 
 
12.5
 
 
 
 
AIFM1
 
 
hsa-miR-98-5p
 
 
-0.314
 
 
12.5
 
 
 
 
AIFM1
 
 
hsa-let-7c-5p
 
 
-0.314
 
 
12.5
 
 
 
 
AKAP1
 
 
hsa-miR-148a-3p
 
 
-0.381
 
 
26.7
 
 
 
 
AKAP6
 
 
hsa-let-7e-5p
 
 
-0.307
 
 
10.5
 
 
 
 
AKAP6
 
 
hsa-miR-98-5p
 
 
-0.307
 
 
10.5
 
 
 
 
AKAP6
 
 
hsa-let-7c-5p
 
 
-0.307
 
 
10.5
 
 
 
 
ALCAM
 
 
hsa-miR-148a-3p
 
 
-0.339
 
 
37.8
 
 
 
 
ALDH4A1
 
 
hsa-miR-184
 
 
-0.421
 
 
86.2
 
 
 
 
ALG3
 
 
hsa-miR-106a-5p
 
 
-0.569
 
 
10.2
 
 
 
 
ALG3
 
 
hsa-miR-20b-5p
 
 
-0.58
 
 
10.2
 
 
 
 
AMT
 
 
hsa-let-7e-5p
 
 
-0.463
 
 
11.4
 
 
 
 
AMT
 
 
hsa-miR-98-5p
 
 
-0.463
 
 
11.4
 
 
 
 
AMT
 
 
hsa-let-7c-5p
 
 
-0.463
 
 
11.4
 
 
 
 
ANAPC16
 
 
hsa-miR-125a-5p
 
 
-0.578
 
 
11.6
 
 
 
 
ANKRD46
 
 
hsa-let-7e-5p
 
 
-0.389
 
 
22.6
 
 
 
 
ANKRD46
 
 
hsa-miR-98-5p
 
 
-0.389
 
 
22.6
 
 
 
 
ANKRD46
 
 
hsa-let-7c-5p
 
 
-0.389
 
 
22.6
 
 
 
 
ANKRD9
 
 
hsa-miR-20b-5p
 
 
-0.331
 
 
18.5
 
 
 
 
ANKRD9
 
 
hsa-miR-106a-5p
 
 
-0.344
 
 
18.5
 
 
 
 
AP1AR
 
 
hsa-miR-21-5p
 
 
-0.318
 
 
13.0
 
 
 
 
AP1AR
 
 
hsa-miR-99b-5p
 
 
-0.823
 
 
13.0
 
 
 
 
AP1S1
 
 
hsa-let-7e-5p
 
 
-0.374
 
 
116.3
 
 
 
 
AP1S1
 
 
hsa-miR-98-5p
 
 
-0.374
 
 
116.3
 
 
 
 
AP1S1
 
 
hsa-let-7c-5p
 
 
-0.374
 
 
116.3
 
 
 
 
APCDD1
 
 
hsa-miR-106a-5p
 
 
-0.422
 
 
30.3
 
 
 
 
APCDD1
 
 
hsa-miR-20b-5p
 
 
-0.425
 
 
30.3
 
 
 
 
ARF4
 
 
hsa-miR-7-5p
 
 
-0.407
 
 
71.0
 
 
 
 
ARF4
 
 
hsa-miR-148a-3p
 
 
-0.487
 
 
71.0
 
 
 
 
ARHGAP1
 
 
hsa-miR-20b-5p
 
 
-0.325
 
 
41.0
 
 
 
 
ARHGAP1
 
 
hsa-miR-106a-5p
 
 
-0.329
 
 
41.0
 
 
 
 
ARHGAP21
 
 
hsa-miR-148a-3p
 
 
-0.287
 
 
37.1
 
 
 
 
ARL1
 
 
hsa-miR-20b-5p
 
 
-0.424
 
 
28.4
 
 
 
 
ARL1
 
 
hsa-miR-106a-5p
 
 
-0.429
 
 
28.4
 
 
 
 
ARL4D
 
 
hsa-let-7e-5p
 
 
-0.417
 
 
14.0
 
 
 
 
ARL4D
 
 
hsa-miR-98-5p
 
 
-0.417
 
 
14.0
 
 
 
 
ARL4D
 
 
hsa-let-7c-5p
 
 
-0.417
 
 
14.0
 
 
 
 
ARL6IP1
 
 
hsa-miR-7-5p
 
 
-0.374
 
 
196.4
 
 
 
 
ARL6IP1
 
 
hsa-miR-148a-3p
 
 
-0.579
 
 
196.4
 
 
 
 
ARL8B
 
 
hsa-miR-148a-3p
 
 
-0.337
 
 
53.5
 
 
 
 
ARMC8
 
 
hsa-miR-106a-5p
 
 
-0.375
 
 
13.3
 
 
 
 
ARMC8
 
 
hsa-miR-20b-5p
 
 
-0.378
 
 
13.3
 
 
 
 
ARMCX1
 
 
hsa-miR-21-5p
 
 
-0.489
 
 
21.1
 
 
 
 
ARMT1
 
 
hsa-let-7c-5p
 
 
-0.303
 
 
10.6
 
 
 
 
ARMT1
 
 
hsa-let-7e-5p
 
 
-0.303
 
 
10.6
 
 
 
 
ARMT1
 
 
hsa-miR-98-5p
 
 
-0.303
 
 
10.6
 
 
 
 
ARPP19
 
 
hsa-let-7e-5p
 
 
-0.492
 
 
481.4
 
 
 
 
ARPP19
 
 
hsa-let-7c-5p
 
 
-0.492
 
 
481.4
 
 
 
 
ARPP19
 
 
hsa-miR-98-5p
 
 
-0.5
 
 
481.4
 
 
 
 
ARRDC3
 
 
hsa-miR-148a-3p
 
 
-0.305
 
 
10.6
 
 
 
 
ARRDC4
 
 
hsa-let-7c-5p
 
 
-0.329
 
 
10.0
 
 
 
 
ARRDC4
 
 
hsa-let-7e-5p
 
 
-0.329
 
 
10.0
 
 
 
 
ARRDC4
 
 
hsa-miR-98-5p
 
 
-0.329
 
 
10.0
 
 
 
 
ASAP1
 
 
hsa-let-7e-5p
 
 
-0.296
 
 
11.6
 
 
 
 
ASAP1
 
 
hsa-miR-98-5p
 
 
-0.308
 
 
11.6
 
 
 
 
ASAP1
 
 
hsa-let-7c-5p
 
 
-0.308
 
 
11.6
 
 
 
 
ASF1A
 
 
hsa-miR-21-5p
 
 
-0.304
 
 
15.3
 
 
 
 
ASPH
 
 
hsa-miR-148a-3p
 
 
-0.391
 
 
62.4
 
 
 
 
ATG16L1
 
 
hsa-miR-106a-5p
 
 
-0.464
 
 
11.5
 
 
 
 
ATG16L1
 
 
hsa-miR-20b-5p
 
 
-0.467
 
 
11.5
 
 
 
 
ATOSB
 
 
hsa-let-7e-5p
 
 
-0.429
 
 
20.2
 
 
 
 
ATOSB
 
 
hsa-miR-98-5p
 
 
-0.429
 
 
20.2
 
 
 
 
ATOSB
 
 
hsa-let-7c-5p
 
 
-0.429
 
 
20.2
 
 
 
 
ATP2A2
 
 
hsa-let-7e-5p
 
 
-0.311
 
 
21.1
 
 
 
 
ATP2A2
 
 
hsa-miR-98-5p
 
 
-0.311
 
 
21.1
 
 
 
 
ATP2A2
 
 
hsa-let-7c-5p
 
 
-0.311
 
 
21.1
 
 
 
 
ATP2B2
 
 
hsa-miR-7-5p
 
 
-0.297
 
 
34.6
 
 
 
 
ATP6AP2
 
 
hsa-miR-148a-3p
 
 
-0.592
 
 
77.2
 
 
 
 
ATP6AP2
 
 
hsa-miR-148a-3p
 
 
-0.668
 
 
77.2
 
 
 
 
ATPAF1
 
 
hsa-let-7e-5p
 
 
-0.498
 
 
88.4
 
 
 
 
 
 
 cerebral cortex 
 
 
 
 
target_symbol
 
 
mature_mirna_id
 
 
score
 
 
nTPM
 
 
 
 
 
 
ABCB9
 
 
hsa-let-7e-5p
 
 
-0.422
 
 
19.5
 
 
 
 
ABCB9
 
 
hsa-let-7c-5p
 
 
-0.422
 
 
19.5
 
 
 
 
ABCB9
 
 
hsa-miR-98-5p
 
 
-0.444
 
 
19.5
 
 
 
 
ABCC5
 
 
hsa-miR-125a-5p
 
 
-0.288
 
 
41.2
 
 
 
 
ABCD3
 
 
hsa-miR-148a-3p
 
 
-0.388
 
 
17.1
 
 
 
 
ABHD17C
 
 
hsa-let-7c-5p
 
 
-0.359
 
 
16.3
 
 
 
 
ABHD17C
 
 
hsa-let-7e-5p
 
 
-0.359
 
 
16.3
 
 
 
 
ABHD17C
 
 
hsa-miR-98-5p
 
 
-0.383
 
 
16.3
 
 
 
 
ABHD6
 
 
hsa-miR-125a-5p
 
 
-0.501
 
 
21.5
 
 
 
 
ABTB1
 
 
hsa-miR-125a-5p
 
 
-0.519
 
 
51.2
 
 
 
 
ACHE
 
 
hsa-miR-125a-5p
 
 
-0.308
 
 
23.4
 
 
 
 
ACHE
 
 
hsa-miR-125a-5p
 
 
-0.361
 
 
23.4
 
 
 
 
ACSL6
 
 
hsa-miR-125a-5p
 
 
-0.307
 
 
29.5
 
 
 
 
ACSL6
 
 
hsa-let-7e-5p
 
 
-0.336
 
 
29.5
 
 
 
 
ACSL6
 
 
hsa-miR-98-5p
 
 
-0.336
 
 
29.5
 
 
 
 
ACSL6
 
 
hsa-let-7c-5p
 
 
-0.336
 
 
29.5
 
 
 
 
ADAM10
 
 
hsa-miR-148a-3p
 
 
-0.356
 
 
16.0
 
 
 
 
ADAM9
 
 
hsa-miR-106a-5p
 
 
-0.317
 
 
16.2
 
 
 
 
ADAM9
 
 
hsa-miR-20b-5p
 
 
-0.328
 
 
16.2
 
 
 
 
ADCY2
 
 
hsa-miR-148a-3p
 
 
-0.299
 
 
35.4
 
 
 
 
ADGRB3
 
 
hsa-miR-148a-3p
 
 
-0.524
 
 
28.2
 
 
 
 
AGFG1
 
 
hsa-miR-148a-3p
 
 
-0.332
 
 
33.6
 
 
 
 
AGFG2
 
 
hsa-miR-20b-5p
 
 
-0.386
 
 
14.0
 
 
 
 
AGFG2
 
 
hsa-miR-106a-5p
 
 
-0.399
 
 
14.0
 
 
 
 
AGGF1
 
 
hsa-miR-125a-5p
 
 
-0.331
 
 
12.2
 
 
 
 
AHDC1
 
 
hsa-miR-148a-3p
 
 
-0.413
 
 
15.5
 
 
 
 
AHNAK
 
 
hsa-miR-106a-5p
 
 
-0.334
 
 
26.8
 
 
 
 
AHNAK
 
 
hsa-miR-20b-5p
 
 
-0.337
 
 
26.8
 
 
 
 
AIFM1
 
 
hsa-miR-125a-5p
 
 
-0.3
 
 
10.3
 
 
 
 
AIFM1
 
 
hsa-let-7e-5p
 
 
-0.314
 
 
10.3
 
 
 
 
AIFM1
 
 
hsa-miR-98-5p
 
 
-0.314
 
 
10.3
 
 
 
 
AIFM1
 
 
hsa-let-7c-5p
 
 
-0.314
 
 
10.3
 
 
 
 
AKAP1
 
 
hsa-miR-148a-3p
 
 
-0.381
 
 
24.9
 
 
 
 
AKAP6
 
 
hsa-let-7e-5p
 
 
-0.307
 
 
27.4
 
 
 
 
AKAP6
 
 
hsa-miR-98-5p
 
 
-0.307
 
 
27.4
 
 
 
 
AKAP6
 
 
hsa-let-7c-5p
 
 
-0.307
 
 
27.4
 
 
 
 
ALCAM
 
 
hsa-miR-148a-3p
 
 
-0.339
 
 
54.7
 
 
 
 
ALDH4A1
 
 
hsa-miR-184
 
 
-0.421
 
 
69.8
 
 
 
 
ANKRD12
 
 
hsa-miR-7-5p
 
 
-0.37
 
 
15.1
 
 
 
 
ANKRD13C
 
 
hsa-miR-106a-5p
 
 
-0.523
 
 
10.2
 
 
 
 
ANKRD13C
 
 
hsa-miR-20b-5p
 
 
-0.526
 
 
10.2
 
 
 
 
ANKRD46
 
 
hsa-let-7e-5p
 
 
-0.389
 
 
39.5
 
 
 
 
ANKRD46
 
 
hsa-miR-98-5p
 
 
-0.389
 
 
39.5
 
 
 
 
ANKRD46
 
 
hsa-let-7c-5p
 
 
-0.389
 
 
39.5
 
 
 
 
ANKRD9
 
 
hsa-miR-20b-5p
 
 
-0.331
 
 
28.9
 
 
 
 
ANKRD9
 
 
hsa-miR-106a-5p
 
 
-0.344
 
 
28.9
 
 
 
 
AP1AR
 
 
hsa-miR-21-5p
 
 
-0.318
 
 
12.2
 
 
 
 
AP1AR
 
 
hsa-miR-99b-5p
 
 
-0.823
 
 
12.2
 
 
 
 
AP1S1
 
 
hsa-let-7e-5p
 
 
-0.374
 
 
104.9
 
 
 
 
AP1S1
 
 
hsa-miR-98-5p
 
 
-0.374
 
 
104.9
 
 
 
 
AP1S1
 
 
hsa-let-7c-5p
 
 
-0.374
 
 
104.9
 
 
 
 
APBB3
 
 
hsa-let-7e-5p
 
 
-0.587
 
 
15.0
 
 
 
 
APBB3
 
 
hsa-miR-98-5p
 
 
-0.587
 
 
15.0
 
 
 
 
APBB3
 
 
hsa-let-7c-5p
 
 
-0.587
 
 
15.0
 
 
 
 
APCDD1
 
 
hsa-miR-106a-5p
 
 
-0.422
 
 
33.1
 
 
 
 
APCDD1
 
 
hsa-miR-20b-5p
 
 
-0.425
 
 
33.1
 
 
 
 
ARF4
 
 
hsa-miR-7-5p
 
 
-0.407
 
 
61.5
 
 
 
 
ARF4
 
 
hsa-miR-148a-3p
 
 
-0.487
 
 
61.5
 
 
 
 
ARHGAP1
 
 
hsa-miR-20b-5p
 
 
-0.325
 
 
40.8
 
 
 
 
ARHGAP1
 
 
hsa-miR-106a-5p
 
 
-0.329
 
 
40.8
 
 
 
 
ARHGAP21
 
 
hsa-miR-148a-3p
 
 
-0.287
 
 
47.3
 
 
 
 
ARID4A
 
 
hsa-miR-7-5p
 
 
-0.341
 
 
13.3
 
 
 
 
ARL1
 
 
hsa-miR-20b-5p
 
 
-0.424
 
 
34.0
 
 
 
 
ARL1
 
 
hsa-miR-106a-5p
 
 
-0.429
 
 
34.0
 
 
 
 
ARL4D
 
 
hsa-let-7e-5p
 
 
-0.417
 
 
14.3
 
 
 
 
ARL4D
 
 
hsa-miR-98-5p
 
 
-0.417
 
 
14.3
 
 
 
 
ARL4D
 
 
hsa-let-7c-5p
 
 
-0.417
 
 
14.3
 
 
 
 
ARL6IP1
 
 
hsa-miR-7-5p
 
 
-0.374
 
 
314.1
 
 
 
 
ARL6IP1
 
 
hsa-miR-148a-3p
 
 
-0.579
 
 
314.1
 
 
 
 
ARL8B
 
 
hsa-miR-148a-3p
 
 
-0.337
 
 
75.1
 
 
 
 
ARMC8
 
 
hsa-miR-106a-5p
 
 
-0.375
 
 
21.7
 
 
 
 
ARMC8
 
 
hsa-miR-20b-5p
 
 
-0.378
 
 
21.7
 
 
 
 
ARMCX1
 
 
hsa-miR-21-5p
 
 
-0.489
 
 
22.6
 
 
 
 
ARMT1
 
 
hsa-let-7c-5p
 
 
-0.303
 
 
10.1
 
 
 
 
ARMT1
 
 
hsa-let-7e-5p
 
 
-0.303
 
 
10.1
 
 
 
 
ARMT1
 
 
hsa-miR-98-5p
 
 
-0.303
 
 
10.1
 
 
 
 
ARPP19
 
 
hsa-let-7e-5p
 
 
-0.492
 
 
292.4
 
 
 
 
ARPP19
 
 
hsa-let-7c-5p
 
 
-0.492
 
 
292.4
 
 
 
 
ARPP19
 
 
hsa-miR-98-5p
 
 
-0.5
 
 
292.4
 
 
 
 
ARRDC3
 
 
hsa-miR-148a-3p
 
 
-0.305
 
 
15.6
 
 
 
 
ARRDC4
 
 
hsa-let-7c-5p
 
 
-0.329
 
 
12.8
 
 
 
 
ARRDC4
 
 
hsa-let-7e-5p
 
 
-0.329
 
 
12.8
 
 
 
 
ARRDC4
 
 
hsa-miR-98-5p
 
 
-0.329
 
 
12.8
 
 
 
 
ASAP1
 
 
hsa-let-7e-5p
 
 
-0.296
 
 
21.7
 
 
 
 
ASAP1
 
 
hsa-miR-98-5p
 
 
-0.308
 
 
21.7
 
 
 
 
ASAP1
 
 
hsa-let-7c-5p
 
 
-0.308
 
 
21.7
 
 
 
 
ASF1A
 
 
hsa-miR-21-5p
 
 
-0.304
 
 
13.7
 
 
 
 
ASPH
 
 
hsa-miR-148a-3p
 
 
-0.391
 
 
76.3
 
 
 
 
ATG16L1
 
 
hsa-miR-106a-5p
 
 
-0.464
 
 
13.9
 
 
 
 
ATG16L1
 
 
hsa-miR-20b-5p
 
 
-0.467
 
 
13.9
 
 
 
 
ATOSB
 
 
hsa-let-7e-5p
 
 
-0.429
 
 
21.2
 
 
 
 
ATOSB
 
 
hsa-miR-98-5p
 
 
-0.429
 
 
21.2
 
 
 
 
ATOSB
 
 
hsa-let-7c-5p
 
 
-0.429
 
 
21.2
 
 
 
 
ATP2A2
 
 
hsa-let-7e-5p
 
 
-0.311
 
 
54.4
 
 
 
 
ATP2A2
 
 
hsa-miR-98-5p
 
 
-0.311
 
 
54.4
 
 
 
 
ATP2A2
 
 
hsa-let-7c-5p
 
 
-0.311
 
 
54.4
 
 
 
 
ATP2B2
 
 
hsa-miR-7-5p
 
 
-0.297
 
 
63.7
 
 
 
 
ATP2B4
 
 
hsa-miR-148a-3p
 
 
-0.337
 
 
35.3
 
 
 
 
ATP6AP2
 
 
hsa-miR-148a-3p
 
 
-0.592
 
 
109.4
 
 
 
 
ATP6AP2
 
 
hsa-miR-148a-3p
 
 
-0.668
 
 
109.4
 
 
 
 
 
 
 hypothalamus 
 
 
 
 
target_symbol
 
 
mature_mirna_id
 
 
score
 
 
nTPM
 
 
 
 
 
 
ABCB9
 
 
hsa-let-7e-5p
 
 
-0.422
 
 
11.9
 
 
 
 
ABCB9
 
 
hsa-let-7c-5p
 
 
-0.422
 
 
11.9
 
 
 
 
ABCB9
 
 
hsa-miR-98-5p
 
 
-0.444
 
 
11.9
 
 
 
 
ABCC5
 
 
hsa-miR-125a-5p
 
 
-0.288
 
 
33.4
 
 
 
 
ABCD3
 
 
hsa-miR-148a-3p
 
 
-0.388
 
 
13.5
 
 
 
 
ABHD17C
 
 
hsa-let-7c-5p
 
 
-0.359
 
 
11.1
 
 
 
 
ABHD17C
 
 
hsa-let-7e-5p
 
 
-0.359
 
 
11.1
 
 
 
 
ABHD17C
 
 
hsa-miR-98-5p
 
 
-0.383
 
 
11.1
 
 
 
 
ABHD6
 
 
hsa-miR-125a-5p
 
 
-0.501
 
 
12.9
 
 
 
 
ABTB1
 
 
hsa-miR-125a-5p
 
 
-0.519
 
 
20.4
 
 
 
 
ACHE
 
 
hsa-miR-125a-5p
 
 
-0.308
 
 
103.9
 
 
 
 
ACHE
 
 
hsa-miR-125a-5p
 
 
-0.361
 
 
103.9
 
 
 
 
ACSL6
 
 
hsa-miR-125a-5p
 
 
-0.307
 
 
11.9
 
 
 
 
ACSL6
 
 
hsa-let-7e-5p
 
 
-0.336
 
 
11.9
 
 
 
 
ACSL6
 
 
hsa-miR-98-5p
 
 
-0.336
 
 
11.9
 
 
 
 
ACSL6
 
 
hsa-let-7c-5p
 
 
-0.336
 
 
11.9
 
 
 
 
ADCY2
 
 
hsa-miR-148a-3p
 
 
-0.299
 
 
14.0
 
 
 
 
ADGRB3
 
 
hsa-miR-148a-3p
 
 
-0.524
 
 
16.0
 
 
 
 
AGFG1
 
 
hsa-miR-148a-3p
 
 
-0.332
 
 
26.0
 
 
 
 
AGFG2
 
 
hsa-miR-20b-5p
 
 
-0.386
 
 
10.8
 
 
 
 
AGFG2
 
 
hsa-miR-106a-5p
 
 
-0.399
 
 
10.8
 
 
 
 
AHDC1
 
 
hsa-miR-148a-3p
 
 
-0.413
 
 
10.9
 
 
 
 
AHNAK
 
 
hsa-miR-106a-5p
 
 
-0.334
 
 
17.3
 
 
 
 
AHNAK
 
 
hsa-miR-20b-5p
 
 
-0.337
 
 
17.3
 
 
 
 
AKAP1
 
 
hsa-miR-148a-3p
 
 
-0.381
 
 
15.1
 
 
 
 
AKAP6
 
 
hsa-let-7e-5p
 
 
-0.307
 
 
13.6
 
 
 
 
AKAP6
 
 
hsa-miR-98-5p
 
 
-0.307
 
 
13.6
 
 
 
 
AKAP6
 
 
hsa-let-7c-5p
 
 
-0.307
 
 
13.6
 
 
 
 
ALCAM
 
 
hsa-miR-148a-3p
 
 
-0.339
 
 
36.1
 
 
 
 
ALDH4A1
 
 
hsa-miR-184
 
 
-0.421
 
 
31.5
 
 
 
 
ANKRD46
 
 
hsa-let-7e-5p
 
 
-0.389
 
 
28.7
 
 
 
 
ANKRD46
 
 
hsa-miR-98-5p
 
 
-0.389
 
 
28.7
 
 
 
 
ANKRD46
 
 
hsa-let-7c-5p
 
 
-0.389
 
 
28.7
 
 
 
 
ANKRD9
 
 
hsa-miR-20b-5p
 
 
-0.331
 
 
16.0
 
 
 
 
ANKRD9
 
 
hsa-miR-106a-5p
 
 
-0.344
 
 
16.0
 
 
 
 
AP1S1
 
 
hsa-let-7e-5p
 
 
-0.374
 
 
44.3
 
 
 
 
AP1S1
 
 
hsa-miR-98-5p
 
 
-0.374
 
 
44.3
 
 
 
 
AP1S1
 
 
hsa-let-7c-5p
 
 
-0.374
 
 
44.3
 
 
 
 
APBB3
 
 
hsa-let-7e-5p
 
 
-0.587
 
 
11.2
 
 
 
 
APBB3
 
 
hsa-miR-98-5p
 
 
-0.587
 
 
11.2
 
 
 
 
APBB3
 
 
hsa-let-7c-5p
 
 
-0.587
 
 
11.2
 
 
 
 
APCDD1
 
 
hsa-miR-106a-5p
 
 
-0.422
 
 
19.2
 
 
 
 
APCDD1
 
 
hsa-miR-20b-5p
 
 
-0.425
 
 
19.2
 
 
 
 
ARF4
 
 
hsa-miR-7-5p
 
 
-0.407
 
 
75.3
 
 
 
 
ARF4
 
 
hsa-miR-148a-3p
 
 
-0.487
 
 
75.3
 
 
 
 
ARG2
 
 
hsa-let-7e-5p
 
 
-0.468
 
 
10.7
 
 
 
 
ARG2
 
 
hsa-miR-98-5p
 
 
-0.468
 
 
10.7
 
 
 
 
ARG2
 
 
hsa-let-7c-5p
 
 
-0.468
 
 
10.7
 
 
 
 
ARHGAP1
 
 
hsa-miR-20b-5p
 
 
-0.325
 
 
31.0
 
 
 
 
ARHGAP1
 
 
hsa-miR-106a-5p
 
 
-0.329
 
 
31.0
 
 
 
 
ARHGAP21
 
 
hsa-miR-148a-3p
 
 
-0.287
 
 
30.5
 
 
 
 
ARL1
 
 
hsa-miR-20b-5p
 
 
-0.424
 
 
31.3
 
 
 
 
ARL1
 
 
hsa-miR-106a-5p
 
 
-0.429
 
 
31.3
 
 
 
 
ARL6IP1
 
 
hsa-miR-7-5p
 
 
-0.374
 
 
212.8
 
 
 
 
ARL6IP1
 
 
hsa-miR-148a-3p
 
 
-0.579
 
 
212.8
 
 
 
 
ARL8B
 
 
hsa-miR-148a-3p
 
 
-0.337
 
 
54.9
 
 
 
 
ARMC8
 
 
hsa-miR-106a-5p
 
 
-0.375
 
 
16.8
 
 
 
 
ARMC8
 
 
hsa-miR-20b-5p
 
 
-0.378
 
 
16.8
 
 
 
 
ARMCX1
 
 
hsa-miR-21-5p
 
 
-0.489
 
 
24.2
 
 
 
 
ARPP19
 
 
hsa-let-7e-5p
 
 
-0.492
 
 
103.9
 
 
 
 
ARPP19
 
 
hsa-let-7c-5p
 
 
-0.492
 
 
103.9
 
 
 
 
ARPP19
 
 
hsa-miR-98-5p
 
 
-0.5
 
 
103.9
 
 
 
 
ARRDC3
 
 
hsa-miR-148a-3p
 
 
-0.305
 
 
10.3
 
 
 
 
ARRDC4
 
 
hsa-let-7c-5p
 
 
-0.329
 
 
13.4
 
 
 
 
ARRDC4
 
 
hsa-let-7e-5p
 
 
-0.329
 
 
13.4
 
 
 
 
ARRDC4
 
 
hsa-miR-98-5p
 
 
-0.329
 
 
13.4
 
 
 
 
ASAP1
 
 
hsa-let-7e-5p
 
 
-0.296
 
 
11.8
 
 
 
 
ASAP1
 
 
hsa-miR-98-5p
 
 
-0.308
 
 
11.8
 
 
 
 
ASAP1
 
 
hsa-let-7c-5p
 
 
-0.308
 
 
11.8
 
 
 
 
ASF1A
 
 
hsa-miR-21-5p
 
 
-0.304
 
 
13.0
 
 
 
 
ASPH
 
 
hsa-miR-148a-3p
 
 
-0.391
 
 
51.0
 
 
 
 
ATOSB
 
 
hsa-let-7e-5p
 
 
-0.429
 
 
19.8
 
 
 
 
ATOSB
 
 
hsa-miR-98-5p
 
 
-0.429
 
 
19.8
 
 
 
 
ATOSB
 
 
hsa-let-7c-5p
 
 
-0.429
 
 
19.8
 
 
 
 
ATP2A2
 
 
hsa-let-7e-5p
 
 
-0.311
 
 
11.9
 
 
 
 
ATP2A2
 
 
hsa-miR-98-5p
 
 
-0.311
 
 
11.9
 
 
 
 
ATP2A2
 
 
hsa-let-7c-5p
 
 
-0.311
 
 
11.9
 
 
 
 
ATP2B2
 
 
hsa-miR-7-5p
 
 
-0.297
 
 
12.0
 
 
 
 
ATP6AP2
 
 
hsa-miR-148a-3p
 
 
-0.592
 
 
69.0
 
 
 
 
ATP6AP2
 
 
hsa-miR-148a-3p
 
 
-0.668
 
 
69.0
 
 
 
 
ATPAF1
 
 
hsa-let-7e-5p
 
 
-0.498
 
 
52.2
 
 
 
 
ATPAF1
 
 
hsa-miR-98-5p
 
 
-0.498
 
 
52.2
 
 
 
 
ATPAF1
 
 
hsa-let-7c-5p
 
 
-0.498
 
 
52.2
 
 
 
 
AVPI1
 
 
hsa-miR-7-5p
 
 
-0.367
 
 
15.0
 
 
 
 
B3GAT3
 
 
hsa-let-7e-5p
 
 
-0.324
 
 
60.2
 
 
 
 
B3GAT3
 
 
hsa-miR-98-5p
 
 
-0.324
 
 
60.2
 
 
 
 
B3GAT3
 
 
hsa-let-7c-5p
 
 
-0.324
 
 
60.2
 
 
 
 
B4GALT5
 
 
hsa-miR-148a-3p
 
 
-0.44
 
 
13.0
 
 
 
 
B4GAT1
 
 
hsa-let-7c-5p
 
 
-0.47
 
 
207.3
 
 
 
 
B4GAT1
 
 
hsa-let-7e-5p
 
 
-0.47
 
 
207.3
 
 
 
 
B4GAT1
 
 
hsa-miR-98-5p
 
 
-0.47
 
 
207.3
 
 
 
 
BAMBI
 
 
hsa-miR-106a-5p
 
 
-0.503
 
 
12.9
 
 
 
 
BAMBI
 
 
hsa-miR-20b-5p
 
 
-0.506
 
 
12.9
 
 
 
 
BCAP29
 
 
hsa-let-7e-5p
 
 
-0.289
 
 
16.4
 
 
 
 
BDH1
 
 
hsa-miR-125a-5p
 
 
-0.389
 
 
18.1
 
 
 
 
BEGAIN
 
 
hsa-let-7c-5p
 
 
-0.466
 
 
15.9
 
 
 
 
BEGAIN
 
 
hsa-let-7e-5p
 
 
-0.466
 
 
15.9
 
 
 
 
BEGAIN
 
 
hsa-miR-98-5p
 
 
-0.474
 
 
15.9
 
 
 
 
BHLHE41
 
 
hsa-miR-148a-3p
 
 
-0.442
 
 
27.6
 
 
 
 
BLOC1S6
 
 
hsa-let-7e-5p
 
 
-0.321
 
 
24.8
 
 
 
 
 
 
 midbrain 
 
 
 
 
target_symbol
 
 
mature_mirna_id
 
 
score
 
 
nTPM
 
 
 
 
 
 
ABCB9
 
 
hsa-let-7e-5p
 
 
-0.422
 
 
10.6
 
 
 
 
ABCB9
 
 
hsa-let-7c-5p
 
 
-0.422
 
 
10.6
 
 
 
 
ABCB9
 
 
hsa-miR-98-5p
 
 
-0.444
 
 
10.6
 
 
 
 
ABCC5
 
 
hsa-miR-125a-5p
 
 
-0.288
 
 
27.3
 
 
 
 
ABCD3
 
 
hsa-miR-148a-3p
 
 
-0.388
 
 
17.1
 
 
 
 
ABHD17C
 
 
hsa-let-7c-5p
 
 
-0.359
 
 
11.2
 
 
 
 
ABHD17C
 
 
hsa-let-7e-5p
 
 
-0.359
 
 
11.2
 
 
 
 
ABHD17C
 
 
hsa-miR-98-5p
 
 
-0.383
 
 
11.2
 
 
 
 
ABHD3
 
 
hsa-miR-125a-5p
 
 
-0.453
 
 
12.3
 
 
 
 
ABHD6
 
 
hsa-miR-125a-5p
 
 
-0.501
 
 
21.1
 
 
 
 
ABTB1
 
 
hsa-miR-125a-5p
 
 
-0.519
 
 
18.7
 
 
 
 
ACHE
 
 
hsa-miR-125a-5p
 
 
-0.308
 
 
47.7
 
 
 
 
ACHE
 
 
hsa-miR-125a-5p
 
 
-0.361
 
 
47.7
 
 
 
 
ACSL6
 
 
hsa-miR-125a-5p
 
 
-0.307
 
 
11.1
 
 
 
 
ACSL6
 
 
hsa-let-7e-5p
 
 
-0.336
 
 
11.1
 
 
 
 
ACSL6
 
 
hsa-miR-98-5p
 
 
-0.336
 
 
11.1
 
 
 
 
ACSL6
 
 
hsa-let-7c-5p
 
 
-0.336
 
 
11.1
 
 
 
 
ADAM10
 
 
hsa-miR-148a-3p
 
 
-0.356
 
 
11.1
 
 
 
 
ADCY2
 
 
hsa-miR-148a-3p
 
 
-0.299
 
 
20.4
 
 
 
 
ADGRF5
 
 
hsa-miR-148a-3p
 
 
-0.354
 
 
13.0
 
 
 
 
AGFG1
 
 
hsa-miR-148a-3p
 
 
-0.332
 
 
32.4
 
 
 
 
AGFG2
 
 
hsa-miR-20b-5p
 
 
-0.386
 
 
12.1
 
 
 
 
AGFG2
 
 
hsa-miR-106a-5p
 
 
-0.399
 
 
12.1
 
 
 
 
AGGF1
 
 
hsa-miR-125a-5p
 
 
-0.331
 
 
11.3
 
 
 
 
AHNAK
 
 
hsa-miR-106a-5p
 
 
-0.334
 
 
22.7
 
 
 
 
AHNAK
 
 
hsa-miR-20b-5p
 
 
-0.337
 
 
22.7
 
 
 
 
AIFM1
 
 
hsa-miR-125a-5p
 
 
-0.3
 
 
12.7
 
 
 
 
AIFM1
 
 
hsa-let-7e-5p
 
 
-0.314
 
 
12.7
 
 
 
 
AIFM1
 
 
hsa-miR-98-5p
 
 
-0.314
 
 
12.7
 
 
 
 
AIFM1
 
 
hsa-let-7c-5p
 
 
-0.314
 
 
12.7
 
 
 
 
AKAP1
 
 
hsa-miR-148a-3p
 
 
-0.381
 
 
19.0
 
 
 
 
AKAP6
 
 
hsa-let-7e-5p
 
 
-0.307
 
 
17.4
 
 
 
 
AKAP6
 
 
hsa-miR-98-5p
 
 
-0.307
 
 
17.4
 
 
 
 
AKAP6
 
 
hsa-let-7c-5p
 
 
-0.307
 
 
17.4
 
 
 
 
ALCAM
 
 
hsa-miR-148a-3p
 
 
-0.339
 
 
38.7
 
 
 
 
ALDH4A1
 
 
hsa-miR-184
 
 
-0.421
 
 
73.5
 
 
 
 
ANKRD29
 
 
hsa-miR-106a-5p
 
 
-0.369
 
 
11.5
 
 
 
 
ANKRD29
 
 
hsa-miR-20b-5p
 
 
-0.38
 
 
11.5
 
 
 
 
ANKRD46
 
 
hsa-let-7e-5p
 
 
-0.389
 
 
24.0
 
 
 
 
ANKRD46
 
 
hsa-miR-98-5p
 
 
-0.389
 
 
24.0
 
 
 
 
ANKRD46
 
 
hsa-let-7c-5p
 
 
-0.389
 
 
24.0
 
 
 
 
ANKRD9
 
 
hsa-miR-20b-5p
 
 
-0.331
 
 
20.0
 
 
 
 
ANKRD9
 
 
hsa-miR-106a-5p
 
 
-0.344
 
 
20.0
 
 
 
 
AP1AR
 
 
hsa-miR-21-5p
 
 
-0.318
 
 
10.1
 
 
 
 
AP1AR
 
 
hsa-miR-99b-5p
 
 
-0.823
 
 
10.1
 
 
 
 
AP1S1
 
 
hsa-let-7e-5p
 
 
-0.374
 
 
35.4
 
 
 
 
AP1S1
 
 
hsa-miR-98-5p
 
 
-0.374
 
 
35.4
 
 
 
 
AP1S1
 
 
hsa-let-7c-5p
 
 
-0.374
 
 
35.4
 
 
 
 
APCDD1
 
 
hsa-miR-106a-5p
 
 
-0.422
 
 
22.5
 
 
 
 
APCDD1
 
 
hsa-miR-20b-5p
 
 
-0.425
 
 
22.5
 
 
 
 
ARF4
 
 
hsa-miR-7-5p
 
 
-0.407
 
 
62.8
 
 
 
 
ARF4
 
 
hsa-miR-148a-3p
 
 
-0.487
 
 
62.8
 
 
 
 
ARHGAP1
 
 
hsa-miR-20b-5p
 
 
-0.325
 
 
34.8
 
 
 
 
ARHGAP1
 
 
hsa-miR-106a-5p
 
 
-0.329
 
 
34.8
 
 
 
 
ARHGAP21
 
 
hsa-miR-148a-3p
 
 
-0.287
 
 
53.0
 
 
 
 
ARL1
 
 
hsa-miR-20b-5p
 
 
-0.424
 
 
27.4
 
 
 
 
ARL1
 
 
hsa-miR-106a-5p
 
 
-0.429
 
 
27.4
 
 
 
 
ARL6IP1
 
 
hsa-miR-7-5p
 
 
-0.374
 
 
198.8
 
 
 
 
ARL6IP1
 
 
hsa-miR-148a-3p
 
 
-0.579
 
 
198.8
 
 
 
 
ARL8B
 
 
hsa-miR-148a-3p
 
 
-0.337
 
 
52.4
 
 
 
 
ARMC8
 
 
hsa-miR-106a-5p
 
 
-0.375
 
 
14.1
 
 
 
 
ARMC8
 
 
hsa-miR-20b-5p
 
 
-0.378
 
 
14.1
 
 
 
 
ARMCX1
 
 
hsa-miR-21-5p
 
 
-0.489
 
 
17.5
 
 
 
 
ARPP19
 
 
hsa-let-7e-5p
 
 
-0.492
 
 
144.2
 
 
 
 
ARPP19
 
 
hsa-let-7c-5p
 
 
-0.492
 
 
144.2
 
 
 
 
ARPP19
 
 
hsa-miR-98-5p
 
 
-0.5
 
 
144.2
 
 
 
 
ARRDC3
 
 
hsa-miR-148a-3p
 
 
-0.305
 
 
13.8
 
 
 
 
ARRDC4
 
 
hsa-let-7c-5p
 
 
-0.329
 
 
17.7
 
 
 
 
ARRDC4
 
 
hsa-let-7e-5p
 
 
-0.329
 
 
17.7
 
 
 
 
ARRDC4
 
 
hsa-miR-98-5p
 
 
-0.329
 
 
17.7
 
 
 
 
ASAP1
 
 
hsa-let-7e-5p
 
 
-0.296
 
 
12.8
 
 
 
 
ASAP1
 
 
hsa-miR-98-5p
 
 
-0.308
 
 
12.8
 
 
 
 
ASAP1
 
 
hsa-let-7c-5p
 
 
-0.308
 
 
12.8
 
 
 
 
ASF1A
 
 
hsa-miR-21-5p
 
 
-0.304
 
 
14.8
 
 
 
 
ASPH
 
 
hsa-miR-148a-3p
 
 
-0.391
 
 
48.8
 
 
 
 
ATOSB
 
 
hsa-let-7e-5p
 
 
-0.429
 
 
24.1
 
 
 
 
ATOSB
 
 
hsa-miR-98-5p
 
 
-0.429
 
 
24.1
 
 
 
 
ATOSB
 
 
hsa-let-7c-5p
 
 
-0.429
 
 
24.1
 
 
 
 
ATP2A2
 
 
hsa-let-7e-5p
 
 
-0.311
 
 
17.1
 
 
 
 
ATP2A2
 
 
hsa-miR-98-5p
 
 
-0.311
 
 
17.1
 
 
 
 
ATP2A2
 
 
hsa-let-7c-5p
 
 
-0.311
 
 
17.1
 
 
 
 
ATP2B2
 
 
hsa-miR-7-5p
 
 
-0.297
 
 
12.7
 
 
 
 
ATP6AP2
 
 
hsa-miR-148a-3p
 
 
-0.592
 
 
65.6
 
 
 
 
ATP6AP2
 
 
hsa-miR-148a-3p
 
 
-0.668
 
 
65.6
 
 
 
 
ATPAF1
 
 
hsa-let-7e-5p
 
 
-0.498
 
 
74.9
 
 
 
 
ATPAF1
 
 
hsa-miR-98-5p
 
 
-0.498
 
 
74.9
 
 
 
 
ATPAF1
 
 
hsa-let-7c-5p
 
 
-0.498
 
 
74.9
 
 
 
 
AVPI1
 
 
hsa-miR-7-5p
 
 
-0.367
 
 
18.5
 
 
 
 
B3GAT3
 
 
hsa-let-7e-5p
 
 
-0.324
 
 
58.5
 
 
 
 
B3GAT3
 
 
hsa-miR-98-5p
 
 
-0.324
 
 
58.5
 
 
 
 
B3GAT3
 
 
hsa-let-7c-5p
 
 
-0.324
 
 
58.5
 
 
 
 
B4GALT5
 
 
hsa-miR-148a-3p
 
 
-0.44
 
 
10.3
 
 
 
 
B4GAT1
 
 
hsa-let-7c-5p
 
 
-0.47
 
 
85.6
 
 
 
 
B4GAT1
 
 
hsa-let-7e-5p
 
 
-0.47
 
 
85.6
 
 
 
 
B4GAT1
 
 
hsa-miR-98-5p
 
 
-0.47
 
 
85.6
 
 
 
 
BAMBI
 
 
hsa-miR-106a-5p
 
 
-0.503
 
 
16.8
 
 
 
 
BAMBI
 
 
hsa-miR-20b-5p
 
 
-0.506
 
 
16.8
 
 
 
 
BCAP29
 
 
hsa-let-7e-5p
 
 
-0.289
 
 
19.1
 
 
 
 
BDH1
 
 
hsa-miR-125a-5p
 
 
-0.389
 
 
18.3
 
 
 
 
BHLHE41
 
 
hsa-miR-148a-3p
 
 
-0.442
 
 
40.4
 
 
 
 
 
 
 pituitary gland 
 
 
 
 
target_symbol
 
 
mature_mirna_id
 
 
score
 
 
nTPM
 
 
 
 
 
 
ABCB9
 
 
hsa-let-7e-5p
 
 
-0.422
 
 
11.0
 
 
 
 
ABCB9
 
 
hsa-let-7c-5p
 
 
-0.422
 
 
11.0
 
 
 
 
ABCB9
 
 
hsa-miR-98-5p
 
 
-0.444
 
 
11.0
 
 
 
 
ABCC5
 
 
hsa-miR-125a-5p
 
 
-0.288
 
 
41.4
 
 
 
 
ABCD3
 
 
hsa-miR-148a-3p
 
 
-0.388
 
 
12.4
 
 
 
 
ABHD3
 
 
hsa-miR-125a-5p
 
 
-0.453
 
 
10.7
 
 
 
 
ABHD6
 
 
hsa-miR-125a-5p
 
 
-0.501
 
 
17.2
 
 
 
 
ABTB1
 
 
hsa-miR-125a-5p
 
 
-0.519
 
 
33.0
 
 
 
 
ADGRB3
 
 
hsa-miR-148a-3p
 
 
-0.524
 
 
21.3
 
 
 
 
AGFG1
 
 
hsa-miR-148a-3p
 
 
-0.332
 
 
16.7
 
 
 
 
AGFG2
 
 
hsa-miR-20b-5p
 
 
-0.386
 
 
16.7
 
 
 
 
AGFG2
 
 
hsa-miR-106a-5p
 
 
-0.399
 
 
16.7
 
 
 
 
AHDC1
 
 
hsa-miR-148a-3p
 
 
-0.413
 
 
18.1
 
 
 
 
AHNAK
 
 
hsa-miR-106a-5p
 
 
-0.334
 
 
16.0
 
 
 
 
AHNAK
 
 
hsa-miR-20b-5p
 
 
-0.337
 
 
16.0
 
 
 
 
AIFM1
 
 
hsa-miR-125a-5p
 
 
-0.3
 
 
11.0
 
 
 
 
AIFM1
 
 
hsa-let-7e-5p
 
 
-0.314
 
 
11.0
 
 
 
 
AIFM1
 
 
hsa-miR-98-5p
 
 
-0.314
 
 
11.0
 
 
 
 
AIFM1
 
 
hsa-let-7c-5p
 
 
-0.314
 
 
11.0
 
 
 
 
AKAP1
 
 
hsa-miR-148a-3p
 
 
-0.381
 
 
16.5
 
 
 
 
ALCAM
 
 
hsa-miR-148a-3p
 
 
-0.339
 
 
17.9
 
 
 
 
ALG3
 
 
hsa-miR-106a-5p
 
 
-0.569
 
 
14.3
 
 
 
 
ALG3
 
 
hsa-miR-20b-5p
 
 
-0.58
 
 
14.3
 
 
 
 
AMT
 
 
hsa-let-7e-5p
 
 
-0.463
 
 
16.0
 
 
 
 
AMT
 
 
hsa-miR-98-5p
 
 
-0.463
 
 
16.0
 
 
 
 
AMT
 
 
hsa-let-7c-5p
 
 
-0.463
 
 
16.0
 
 
 
 
ANAPC16
 
 
hsa-miR-125a-5p
 
 
-0.578
 
 
16.0
 
 
 
 
AP1S1
 
 
hsa-let-7e-5p
 
 
-0.374
 
 
31.4
 
 
 
 
AP1S1
 
 
hsa-miR-98-5p
 
 
-0.374
 
 
31.4
 
 
 
 
AP1S1
 
 
hsa-let-7c-5p
 
 
-0.374
 
 
31.4
 
 
 
 
APBB3
 
 
hsa-let-7e-5p
 
 
-0.587
 
 
10.1
 
 
 
 
APBB3
 
 
hsa-miR-98-5p
 
 
-0.587
 
 
10.1
 
 
 
 
APBB3
 
 
hsa-let-7c-5p
 
 
-0.587
 
 
10.1
 
 
 
 
ARF4
 
 
hsa-miR-7-5p
 
 
-0.407
 
 
93.4
 
 
 
 
ARF4
 
 
hsa-miR-148a-3p
 
 
-0.487
 
 
93.4
 
 
 
 
ARG2
 
 
hsa-let-7e-5p
 
 
-0.468
 
 
47.4
 
 
 
 
ARG2
 
 
hsa-miR-98-5p
 
 
-0.468
 
 
47.4
 
 
 
 
ARG2
 
 
hsa-let-7c-5p
 
 
-0.468
 
 
47.4
 
 
 
 
ARHGAP1
 
 
hsa-miR-20b-5p
 
 
-0.325
 
 
35.2
 
 
 
 
ARHGAP1
 
 
hsa-miR-106a-5p
 
 
-0.329
 
 
35.2
 
 
 
 
ARHGAP21
 
 
hsa-miR-148a-3p
 
 
-0.287
 
 
11.3
 
 
 
 
ARL1
 
 
hsa-miR-20b-5p
 
 
-0.424
 
 
52.7
 
 
 
 
ARL1
 
 
hsa-miR-106a-5p
 
 
-0.429
 
 
52.7
 
 
 
 
ARL4D
 
 
hsa-let-7e-5p
 
 
-0.417
 
 
20.8
 
 
 
 
ARL4D
 
 
hsa-miR-98-5p
 
 
-0.417
 
 
20.8
 
 
 
 
ARL4D
 
 
hsa-let-7c-5p
 
 
-0.417
 
 
20.8
 
 
 
 
ARL6IP1
 
 
hsa-miR-7-5p
 
 
-0.374
 
 
39.7
 
 
 
 
ARL6IP1
 
 
hsa-miR-148a-3p
 
 
-0.579
 
 
39.7
 
 
 
 
ARL8B
 
 
hsa-miR-148a-3p
 
 
-0.337
 
 
25.6
 
 
 
 
ARMC8
 
 
hsa-miR-106a-5p
 
 
-0.375
 
 
11.3
 
 
 
 
ARMC8
 
 
hsa-miR-20b-5p
 
 
-0.378
 
 
11.3
 
 
 
 
ARMCX1
 
 
hsa-miR-21-5p
 
 
-0.489
 
 
24.7
 
 
 
 
ARPP19
 
 
hsa-let-7e-5p
 
 
-0.492
 
 
33.0
 
 
 
 
ARPP19
 
 
hsa-let-7c-5p
 
 
-0.492
 
 
33.0
 
 
 
 
ARPP19
 
 
hsa-miR-98-5p
 
 
-0.5
 
 
33.0
 
 
 
 
ARRDC3
 
 
hsa-miR-148a-3p
 
 
-0.305
 
 
20.2
 
 
 
 
ASPH
 
 
hsa-miR-148a-3p
 
 
-0.391
 
 
33.8
 
 
 
 
ATG4D
 
 
hsa-miR-148a-3p
 
 
-0.303
 
 
16.1
 
 
 
 
ATOSB
 
 
hsa-let-7e-5p
 
 
-0.429
 
 
15.4
 
 
 
 
ATOSB
 
 
hsa-miR-98-5p
 
 
-0.429
 
 
15.4
 
 
 
 
ATOSB
 
 
hsa-let-7c-5p
 
 
-0.429
 
 
15.4
 
 
 
 
ATP2A2
 
 
hsa-let-7e-5p
 
 
-0.311
 
 
13.7
 
 
 
 
ATP2A2
 
 
hsa-miR-98-5p
 
 
-0.311
 
 
13.7
 
 
 
 
ATP2A2
 
 
hsa-let-7c-5p
 
 
-0.311
 
 
13.7
 
 
 
 
ATP6AP2
 
 
hsa-miR-148a-3p
 
 
-0.592
 
 
34.0
 
 
 
 
ATP6AP2
 
 
hsa-miR-148a-3p
 
 
-0.668
 
 
34.0
 
 
 
 
ATPAF1
 
 
hsa-let-7e-5p
 
 
-0.498
 
 
31.1
 
 
 
 
ATPAF1
 
 
hsa-miR-98-5p
 
 
-0.498
 
 
31.1
 
 
 
 
ATPAF1
 
 
hsa-let-7c-5p
 
 
-0.498
 
 
31.1
 
 
 
 
AVPI1
 
 
hsa-miR-7-5p
 
 
-0.367
 
 
20.2
 
 
 
 
B3GAT3
 
 
hsa-let-7e-5p
 
 
-0.324
 
 
61.5
 
 
 
 
B3GAT3
 
 
hsa-miR-98-5p
 
 
-0.324
 
 
61.5
 
 
 
 
B3GAT3
 
 
hsa-let-7c-5p
 
 
-0.324
 
 
61.5
 
 
 
 
B4GALT5
 
 
hsa-miR-148a-3p
 
 
-0.44
 
 
11.9
 
 
 
 
B4GAT1
 
 
hsa-let-7c-5p
 
 
-0.47
 
 
60.5
 
 
 
 
B4GAT1
 
 
hsa-let-7e-5p
 
 
-0.47
 
 
60.5
 
 
 
 
B4GAT1
 
 
hsa-miR-98-5p
 
 
-0.47
 
 
60.5
 
 
 
 
BAK1
 
 
hsa-miR-125a-5p
 
 
-0.402
 
 
11.6
 
 
 
 
BAZ2A
 
 
hsa-miR-99b-5p
 
 
-0.408
 
 
25.8
 
 
 
 
BDH1
 
 
hsa-miR-125a-5p
 
 
-0.389
 
 
11.4
 
 
 
 
BEGAIN
 
 
hsa-let-7c-5p
 
 
-0.466
 
 
18.8
 
 
 
 
BEGAIN
 
 
hsa-let-7e-5p
 
 
-0.466
 
 
18.8
 
 
 
 
BEGAIN
 
 
hsa-miR-98-5p
 
 
-0.474
 
 
18.8
 
 
 
 
BHLHE41
 
 
hsa-miR-148a-3p
 
 
-0.442
 
 
15.9
 
 
 
 
BIN3
 
 
hsa-let-7e-5p
 
 
-0.427
 
 
13.2
 
 
 
 
BIN3
 
 
hsa-miR-98-5p
 
 
-0.427
 
 
13.2
 
 
 
 
BIN3
 
 
hsa-let-7c-5p
 
 
-0.427
 
 
13.2
 
 
 
 
BLOC1S6
 
 
hsa-let-7e-5p
 
 
-0.321
 
 
19.0
 
 
 
 
BLOC1S6
 
 
hsa-miR-98-5p
 
 
-0.321
 
 
19.0
 
 
 
 
BLOC1S6
 
 
hsa-let-7c-5p
 
 
-0.321
 
 
19.0
 
 
 
 
BTAF1
 
 
hsa-miR-148a-3p
 
 
-0.401
 
 
11.4
 
 
 
 
BTBD10
 
 
hsa-miR-106a-5p
 
 
-0.406
 
 
13.7
 
 
 
 
BTBD10
 
 
hsa-miR-20b-5p
 
 
-0.425
 
 
13.7
 
 
 
 
BTF3L4
 
 
hsa-let-7c-5p
 
 
-0.355
 
 
28.3
 
 
 
 
BTF3L4
 
 
hsa-let-7e-5p
 
 
-0.355
 
 
28.3
 
 
 
 
BTF3L4
 
 
hsa-miR-98-5p
 
 
-0.355
 
 
28.3
 
 
 
 
BTG3
 
 
hsa-miR-106a-5p
 
 
-0.665
 
 
43.7
 
 
 
 
BTG3
 
 
hsa-miR-20b-5p
 
 
-0.684
 
 
43.7
 
 
 
 
BZW1
 
 
hsa-let-7e-5p
 
 
-0.354
 
 
28.1
 
 
 
 
BZW1
 
 
hsa-miR-98-5p
 
 
-0.354
 
 
28.1
 
 
 
 
 
 
 testis 
 
 
 
 
target_symbol
 
 
mature_mirna_id
 
 
score
 
 
nTPM
 
 
 
 
 
 
ABCB9
 
 
hsa-let-7e-5p
 
 
-0.422
 
 
17.1
 
 
 
 
ABCB9
 
 
hsa-let-7c-5p
 
 
-0.422
 
 
17.1
 
 
 
 
ABCB9
 
 
hsa-miR-98-5p
 
 
-0.444
 
 
17.1
 
 
 
 
ABCC5
 
 
hsa-miR-125a-5p
 
 
-0.288
 
 
17.7
 
 
 
 
ABCD3
 
 
hsa-miR-148a-3p
 
 
-0.388
 
 
15.3
 
 
 
 
ACRV1
 
 
hsa-miR-7-5p
 
 
-0.3
 
 
110.6
 
 
 
 
ACSL6
 
 
hsa-miR-125a-5p
 
 
-0.307
 
 
19.3
 
 
 
 
ACSL6
 
 
hsa-let-7e-5p
 
 
-0.336
 
 
19.3
 
 
 
 
ACSL6
 
 
hsa-miR-98-5p
 
 
-0.336
 
 
19.3
 
 
 
 
ACSL6
 
 
hsa-let-7c-5p
 
 
-0.336
 
 
19.3
 
 
 
 
ADAM9
 
 
hsa-miR-106a-5p
 
 
-0.317
 
 
10.4
 
 
 
 
ADAM9
 
 
hsa-miR-20b-5p
 
 
-0.328
 
 
10.4
 
 
 
 
AGFG1
 
 
hsa-miR-148a-3p
 
 
-0.332
 
 
86.7
 
 
 
 
AGFG2
 
 
hsa-miR-20b-5p
 
 
-0.386
 
 
11.5
 
 
 
 
AGFG2
 
 
hsa-miR-106a-5p
 
 
-0.399
 
 
11.5
 
 
 
 
AGGF1
 
 
hsa-miR-125a-5p
 
 
-0.331
 
 
15.2
 
 
 
 
AHCTF1
 
 
hsa-let-7e-5p
 
 
-0.374
 
 
30.4
 
 
 
 
AHCTF1
 
 
hsa-miR-98-5p
 
 
-0.374
 
 
30.4
 
 
 
 
AHCTF1
 
 
hsa-let-7c-5p
 
 
-0.374
 
 
30.4
 
 
 
 
AHNAK
 
 
hsa-miR-106a-5p
 
 
-0.334
 
 
16.9
 
 
 
 
AHNAK
 
 
hsa-miR-20b-5p
 
 
-0.337
 
 
16.9
 
 
 
 
AIFM1
 
 
hsa-miR-125a-5p
 
 
-0.3
 
 
21.3
 
 
 
 
AIFM1
 
 
hsa-let-7e-5p
 
 
-0.314
 
 
21.3
 
 
 
 
AIFM1
 
 
hsa-miR-98-5p
 
 
-0.314
 
 
21.3
 
 
 
 
AIFM1
 
 
hsa-let-7c-5p
 
 
-0.314
 
 
21.3
 
 
 
 
AKAP1
 
 
hsa-miR-148a-3p
 
 
-0.381
 
 
108.8
 
 
 
 
ALG3
 
 
hsa-miR-106a-5p
 
 
-0.569
 
 
25.2
 
 
 
 
ALG3
 
 
hsa-miR-20b-5p
 
 
-0.58
 
 
25.2
 
 
 
 
ALG9
 
 
hsa-miR-7-5p
 
 
-0.302
 
 
12.8
 
 
 
 
ANKRD12
 
 
hsa-miR-7-5p
 
 
-0.37
 
 
25.9
 
 
 
 
ANKRD50
 
 
hsa-miR-106a-5p
 
 
-0.29
 
 
14.1
 
 
 
 
ANKRD9
 
 
hsa-miR-20b-5p
 
 
-0.331
 
 
38.5
 
 
 
 
ANKRD9
 
 
hsa-miR-106a-5p
 
 
-0.344
 
 
38.5
 
 
 
 
AP1AR
 
 
hsa-miR-21-5p
 
 
-0.318
 
 
13.6
 
 
 
 
AP1AR
 
 
hsa-miR-99b-5p
 
 
-0.823
 
 
13.6
 
 
 
 
AP1S1
 
 
hsa-let-7e-5p
 
 
-0.374
 
 
17.8
 
 
 
 
AP1S1
 
 
hsa-miR-98-5p
 
 
-0.374
 
 
17.8
 
 
 
 
AP1S1
 
 
hsa-let-7c-5p
 
 
-0.374
 
 
17.8
 
 
 
 
ARF4
 
 
hsa-miR-7-5p
 
 
-0.407
 
 
94.2
 
 
 
 
ARF4
 
 
hsa-miR-148a-3p
 
 
-0.487
 
 
94.2
 
 
 
 
ARFGEF1
 
 
hsa-miR-148a-3p
 
 
-0.306
 
 
26.5
 
 
 
 
ARFIP1
 
 
hsa-miR-148a-3p
 
 
-0.474
 
 
11.3
 
 
 
 
ARHGAP1
 
 
hsa-miR-20b-5p
 
 
-0.325
 
 
15.3
 
 
 
 
ARHGAP1
 
 
hsa-miR-106a-5p
 
 
-0.329
 
 
15.3
 
 
 
 
ARHGAP21
 
 
hsa-miR-148a-3p
 
 
-0.287
 
 
13.5
 
 
 
 
ARHGAP28
 
 
hsa-let-7c-5p
 
 
-0.47
 
 
72.3
 
 
 
 
ARHGAP28
 
 
hsa-miR-98-5p
 
 
-0.47
 
 
72.3
 
 
 
 
ARHGAP28
 
 
hsa-let-7e-5p
 
 
-0.486
 
 
72.3
 
 
 
 
ARID3A
 
 
hsa-miR-98-5p
 
 
-0.34
 
 
16.1
 
 
 
 
ARID3A
 
 
hsa-let-7c-5p
 
 
-0.34
 
 
16.1
 
 
 
 
ARID3A
 
 
hsa-let-7e-5p
 
 
-0.347
 
 
16.1
 
 
 
 
ARID3A
 
 
hsa-let-7c-5p
 
 
-0.347
 
 
16.1
 
 
 
 
ARID3A
 
 
hsa-let-7e-5p
 
 
-0.348
 
 
16.1
 
 
 
 
ARID3A
 
 
hsa-miR-98-5p
 
 
-0.358
 
 
16.1
 
 
 
 
ARID3B
 
 
hsa-let-7e-5p
 
 
-0.31
 
 
21.2
 
 
 
 
ARID3B
 
 
hsa-miR-98-5p
 
 
-0.31
 
 
21.2
 
 
 
 
ARID3B
 
 
hsa-let-7c-5p
 
 
-0.31
 
 
21.2
 
 
 
 
ARID3B
 
 
hsa-miR-98-5p
 
 
-0.475
 
 
21.2
 
 
 
 
ARID3B
 
 
hsa-let-7e-5p
 
 
-0.486
 
 
21.2
 
 
 
 
ARID3B
 
 
hsa-let-7c-5p
 
 
-0.486
 
 
21.2
 
 
 
 
ARL1
 
 
hsa-miR-20b-5p
 
 
-0.424
 
 
30.6
 
 
 
 
ARL1
 
 
hsa-miR-106a-5p
 
 
-0.429
 
 
30.6
 
 
 
 
ARL4D
 
 
hsa-let-7e-5p
 
 
-0.417
 
 
31.4
 
 
 
 
ARL4D
 
 
hsa-miR-98-5p
 
 
-0.417
 
 
31.4
 
 
 
 
ARL4D
 
 
hsa-let-7c-5p
 
 
-0.417
 
 
31.4
 
 
 
 
ARL6IP1
 
 
hsa-miR-7-5p
 
 
-0.374
 
 
121.4
 
 
 
 
ARL6IP1
 
 
hsa-miR-148a-3p
 
 
-0.579
 
 
121.4
 
 
 
 
ARL6IP6
 
 
hsa-miR-98-5p
 
 
-0.293
 
 
10.2
 
 
 
 
ARL6IP6
 
 
hsa-let-7c-5p
 
 
-0.293
 
 
10.2
 
 
 
 
ARL6IP6
 
 
hsa-let-7e-5p
 
 
-0.305
 
 
10.2
 
 
 
 
ARL8B
 
 
hsa-miR-148a-3p
 
 
-0.337
 
 
30.1
 
 
 
 
ARMC8
 
 
hsa-miR-106a-5p
 
 
-0.375
 
 
17.2
 
 
 
 
ARMC8
 
 
hsa-miR-20b-5p
 
 
-0.378
 
 
17.2
 
 
 
 
ARMT1
 
 
hsa-let-7c-5p
 
 
-0.303
 
 
13.1
 
 
 
 
ARMT1
 
 
hsa-let-7e-5p
 
 
-0.303
 
 
13.1
 
 
 
 
ARMT1
 
 
hsa-miR-98-5p
 
 
-0.303
 
 
13.1
 
 
 
 
ARPP19
 
 
hsa-let-7e-5p
 
 
-0.492
 
 
79.0
 
 
 
 
ARPP19
 
 
hsa-let-7c-5p
 
 
-0.492
 
 
79.0
 
 
 
 
ARPP19
 
 
hsa-miR-98-5p
 
 
-0.5
 
 
79.0
 
 
 
 
ARRDC3
 
 
hsa-miR-148a-3p
 
 
-0.305
 
 
15.5
 
 
 
 
ARRDC4
 
 
hsa-let-7c-5p
 
 
-0.329
 
 
13.7
 
 
 
 
ARRDC4
 
 
hsa-let-7e-5p
 
 
-0.329
 
 
13.7
 
 
 
 
ARRDC4
 
 
hsa-miR-98-5p
 
 
-0.329
 
 
13.7
 
 
 
 
ASAP1
 
 
hsa-let-7e-5p
 
 
-0.296
 
 
26.5
 
 
 
 
ASAP1
 
 
hsa-miR-98-5p
 
 
-0.308
 
 
26.5
 
 
 
 
ASAP1
 
 
hsa-let-7c-5p
 
 
-0.308
 
 
26.5
 
 
 
 
ASPH
 
 
hsa-miR-148a-3p
 
 
-0.391
 
 
15.3
 
 
 
 
ATAD2
 
 
hsa-miR-20b-5p
 
 
-0.304
 
 
17.7
 
 
 
 
ATAD2
 
 
hsa-miR-106a-5p
 
 
-0.308
 
 
17.7
 
 
 
 
ATG16L1
 
 
hsa-miR-106a-5p
 
 
-0.464
 
 
13.1
 
 
 
 
ATG16L1
 
 
hsa-miR-20b-5p
 
 
-0.467
 
 
13.1
 
 
 
 
ATG4D
 
 
hsa-miR-148a-3p
 
 
-0.303
 
 
23.6
 
 
 
 
ATP2A2
 
 
hsa-let-7e-5p
 
 
-0.311
 
 
69.3
 
 
 
 
ATP2A2
 
 
hsa-miR-98-5p
 
 
-0.311
 
 
69.3
 
 
 
 
ATP2A2
 
 
hsa-let-7c-5p
 
 
-0.311
 
 
69.3
 
 
 
 
ATP2B4
 
 
hsa-miR-148a-3p
 
 
-0.337
 
 
25.4
 
 
 
 
ATP6AP2
 
 
hsa-miR-148a-3p
 
 
-0.592
 
 
37.7
 
 
 
 
ATP6AP2
 
 
hsa-miR-148a-3p
 
 
-0.668
 
 
37.7
 
 
 
 
ATPAF1
 
 
hsa-let-7e-5p
 
 
-0.498
 
 
29.2
 
 
 
 
ATPAF1
 
 
hsa-miR-98-5p
 
 
-0.498
 
 
29.2
 
 
 
 
 
 
 amygdala 
 
 
 
 
target_symbol
 
 
mature_mirna_id
 
 
score
 
 
nTPM
 
 
 
 
 
 
ABCC5
 
 
hsa-miR-125a-5p
 
 
-0.288
 
 
24.0
 
 
 
 
ABCD3
 
 
hsa-miR-148a-3p
 
 
-0.388
 
 
16.0
 
 
 
 
ABHD17C
 
 
hsa-let-7c-5p
 
 
-0.359
 
 
17.9
 
 
 
 
ABHD17C
 
 
hsa-let-7e-5p
 
 
-0.359
 
 
17.9
 
 
 
 
ABHD17C
 
 
hsa-miR-98-5p
 
 
-0.383
 
 
17.9
 
 
 
 
ABHD3
 
 
hsa-miR-125a-5p
 
 
-0.453
 
 
10.3
 
 
 
 
ABHD6
 
 
hsa-miR-125a-5p
 
 
-0.501
 
 
15.3
 
 
 
 
ABTB1
 
 
hsa-miR-125a-5p
 
 
-0.519
 
 
23.8
 
 
 
 
ACHE
 
 
hsa-miR-125a-5p
 
 
-0.308
 
 
14.7
 
 
 
 
ACHE
 
 
hsa-miR-125a-5p
 
 
-0.361
 
 
14.7
 
 
 
 
ACSL6
 
 
hsa-miR-125a-5p
 
 
-0.307
 
 
20.3
 
 
 
 
ACSL6
 
 
hsa-let-7e-5p
 
 
-0.336
 
 
20.3
 
 
 
 
ACSL6
 
 
hsa-miR-98-5p
 
 
-0.336
 
 
20.3
 
 
 
 
ACSL6
 
 
hsa-let-7c-5p
 
 
-0.336
 
 
20.3
 
 
 
 
ADCY2
 
 
hsa-miR-148a-3p
 
 
-0.299
 
 
28.0
 
 
 
 
ADGRB3
 
 
hsa-miR-148a-3p
 
 
-0.524
 
 
12.8
 
 
 
 
AGFG1
 
 
hsa-miR-148a-3p
 
 
-0.332
 
 
25.1
 
 
 
 
AGFG2
 
 
hsa-miR-20b-5p
 
 
-0.386
 
 
13.3
 
 
 
 
AGFG2
 
 
hsa-miR-106a-5p
 
 
-0.399
 
 
13.3
 
 
 
 
AGGF1
 
 
hsa-miR-125a-5p
 
 
-0.331
 
 
10.3
 
 
 
 
AHDC1
 
 
hsa-miR-148a-3p
 
 
-0.413
 
 
10.1
 
 
 
 
AHNAK
 
 
hsa-miR-106a-5p
 
 
-0.334
 
 
31.4
 
 
 
 
AHNAK
 
 
hsa-miR-20b-5p
 
 
-0.337
 
 
31.4
 
 
 
 
AIFM1
 
 
hsa-miR-125a-5p
 
 
-0.3
 
 
10.3
 
 
 
 
AIFM1
 
 
hsa-let-7e-5p
 
 
-0.314
 
 
10.3
 
 
 
 
AIFM1
 
 
hsa-miR-98-5p
 
 
-0.314
 
 
10.3
 
 
 
 
AIFM1
 
 
hsa-let-7c-5p
 
 
-0.314
 
 
10.3
 
 
 
 
AKAP1
 
 
hsa-miR-148a-3p
 
 
-0.381
 
 
22.8
 
 
 
 
AKAP6
 
 
hsa-let-7e-5p
 
 
-0.307
 
 
12.4
 
 
 
 
AKAP6
 
 
hsa-miR-98-5p
 
 
-0.307
 
 
12.4
 
 
 
 
AKAP6
 
 
hsa-let-7c-5p
 
 
-0.307
 
 
12.4
 
 
 
 
ALCAM
 
 
hsa-miR-148a-3p
 
 
-0.339
 
 
31.7
 
 
 
 
ALDH4A1
 
 
hsa-miR-184
 
 
-0.421
 
 
66.7
 
 
 
 
ANKRD46
 
 
hsa-let-7e-5p
 
 
-0.389
 
 
28.3
 
 
 
 
ANKRD46
 
 
hsa-miR-98-5p
 
 
-0.389
 
 
28.3
 
 
 
 
ANKRD46
 
 
hsa-let-7c-5p
 
 
-0.389
 
 
28.3
 
 
 
 
ANKRD9
 
 
hsa-miR-20b-5p
 
 
-0.331
 
 
26.5
 
 
 
 
ANKRD9
 
 
hsa-miR-106a-5p
 
 
-0.344
 
 
26.5
 
 
 
 
AP1S1
 
 
hsa-let-7e-5p
 
 
-0.374
 
 
88.4
 
 
 
 
AP1S1
 
 
hsa-miR-98-5p
 
 
-0.374
 
 
88.4
 
 
 
 
AP1S1
 
 
hsa-let-7c-5p
 
 
-0.374
 
 
88.4
 
 
 
 
APCDD1
 
 
hsa-miR-106a-5p
 
 
-0.422
 
 
28.8
 
 
 
 
APCDD1
 
 
hsa-miR-20b-5p
 
 
-0.425
 
 
28.8
 
 
 
 
ARF4
 
 
hsa-miR-7-5p
 
 
-0.407
 
 
60.1
 
 
 
 
ARF4
 
 
hsa-miR-148a-3p
 
 
-0.487
 
 
60.1
 
 
 
 
ARHGAP1
 
 
hsa-miR-20b-5p
 
 
-0.325
 
 
37.0
 
 
 
 
ARHGAP1
 
 
hsa-miR-106a-5p
 
 
-0.329
 
 
37.0
 
 
 
 
ARHGAP21
 
 
hsa-miR-148a-3p
 
 
-0.287
 
 
40.6
 
 
 
 
ARL1
 
 
hsa-miR-20b-5p
 
 
-0.424
 
 
24.9
 
 
 
 
ARL1
 
 
hsa-miR-106a-5p
 
 
-0.429
 
 
24.9
 
 
 
 
ARL6IP1
 
 
hsa-miR-7-5p
 
 
-0.374
 
 
241.3
 
 
 
 
ARL6IP1
 
 
hsa-miR-148a-3p
 
 
-0.579
 
 
241.3
 
 
 
 
ARL8B
 
 
hsa-miR-148a-3p
 
 
-0.337
 
 
53.8
 
 
 
 
ARMC8
 
 
hsa-miR-106a-5p
 
 
-0.375
 
 
13.4
 
 
 
 
ARMC8
 
 
hsa-miR-20b-5p
 
 
-0.378
 
 
13.4
 
 
 
 
ARMCX1
 
 
hsa-miR-21-5p
 
 
-0.489
 
 
17.8
 
 
 
 
ARPP19
 
 
hsa-let-7e-5p
 
 
-0.492
 
 
178.5
 
 
 
 
ARPP19
 
 
hsa-let-7c-5p
 
 
-0.492
 
 
178.5
 
 
 
 
ARPP19
 
 
hsa-miR-98-5p
 
 
-0.5
 
 
178.5
 
 
 
 
ARRDC4
 
 
hsa-let-7c-5p
 
 
-0.329
 
 
13.2
 
 
 
 
ARRDC4
 
 
hsa-let-7e-5p
 
 
-0.329
 
 
13.2
 
 
 
 
ARRDC4
 
 
hsa-miR-98-5p
 
 
-0.329
 
 
13.2
 
 
 
 
ASAP1
 
 
hsa-let-7e-5p
 
 
-0.296
 
 
11.2
 
 
 
 
ASAP1
 
 
hsa-miR-98-5p
 
 
-0.308
 
 
11.2
 
 
 
 
ASAP1
 
 
hsa-let-7c-5p
 
 
-0.308
 
 
11.2
 
 
 
 
ASF1A
 
 
hsa-miR-21-5p
 
 
-0.304
 
 
14.0
 
 
 
 
ASPH
 
 
hsa-miR-148a-3p
 
 
-0.391
 
 
62.6
 
 
 
 
ATOSB
 
 
hsa-let-7e-5p
 
 
-0.429
 
 
20.1
 
 
 
 
ATOSB
 
 
hsa-miR-98-5p
 
 
-0.429
 
 
20.1
 
 
 
 
ATOSB
 
 
hsa-let-7c-5p
 
 
-0.429
 
 
20.1
 
 
 
 
ATP2A2
 
 
hsa-let-7e-5p
 
 
-0.311
 
 
15.8
 
 
 
 
ATP2A2
 
 
hsa-miR-98-5p
 
 
-0.311
 
 
15.8
 
 
 
 
ATP2A2
 
 
hsa-let-7c-5p
 
 
-0.311
 
 
15.8
 
 
 
 
ATP2B2
 
 
hsa-miR-7-5p
 
 
-0.297
 
 
14.3
 
 
 
 
ATP6AP2
 
 
hsa-miR-148a-3p
 
 
-0.592
 
 
69.8
 
 
 
 
ATP6AP2
 
 
hsa-miR-148a-3p
 
 
-0.668
 
 
69.8
 
 
 
 
ATPAF1
 
 
hsa-let-7e-5p
 
 
-0.498
 
 
86.8
 
 
 
 
ATPAF1
 
 
hsa-miR-98-5p
 
 
-0.498
 
 
86.8
 
 
 
 
ATPAF1
 
 
hsa-let-7c-5p
 
 
-0.498
 
 
86.8
 
 
 
 
AVPI1
 
 
hsa-miR-7-5p
 
 
-0.367
 
 
13.3
 
 
 
 
B3GAT3
 
 
hsa-let-7e-5p
 
 
-0.324
 
 
61.0
 
 
 
 
B3GAT3
 
 
hsa-miR-98-5p
 
 
-0.324
 
 
61.0
 
 
 
 
B3GAT3
 
 
hsa-let-7c-5p
 
 
-0.324
 
 
61.0
 
 
 
 
B4GALT5
 
 
hsa-miR-148a-3p
 
 
-0.44
 
 
10.8
 
 
 
 
B4GAT1
 
 
hsa-let-7c-5p
 
 
-0.47
 
 
107.6
 
 
 
 
B4GAT1
 
 
hsa-let-7e-5p
 
 
-0.47
 
 
107.6
 
 
 
 
B4GAT1
 
 
hsa-miR-98-5p
 
 
-0.47
 
 
107.6
 
 
 
 
BAMBI
 
 
hsa-miR-106a-5p
 
 
-0.503
 
 
14.6
 
 
 
 
BAMBI
 
 
hsa-miR-20b-5p
 
 
-0.506
 
 
14.6
 
 
 
 
BCAP29
 
 
hsa-let-7e-5p
 
 
-0.289
 
 
16.2
 
 
 
 
BDH1
 
 
hsa-miR-125a-5p
 
 
-0.389
 
 
20.7
 
 
 
 
BEGAIN
 
 
hsa-let-7c-5p
 
 
-0.466
 
 
36.9
 
 
 
 
BEGAIN
 
 
hsa-let-7e-5p
 
 
-0.466
 
 
36.9
 
 
 
 
BEGAIN
 
 
hsa-miR-98-5p
 
 
-0.474
 
 
36.9
 
 
 
 
BHLHE41
 
 
hsa-miR-148a-3p
 
 
-0.442
 
 
27.2
 
 
 
 
BIN3
 
 
hsa-let-7e-5p
 
 
-0.427
 
 
10.6
 
 
 
 
BIN3
 
 
hsa-miR-98-5p
 
 
-0.427
 
 
10.6
 
 
 
 
BIN3
 
 
hsa-let-7c-5p
 
 
-0.427
 
 
10.6
 
 
 
 
BLOC1S6
 
 
hsa-let-7e-5p
 
 
-0.321
 
 
20.1
 
 
 
 
BLOC1S6
 
 
hsa-miR-98-5p
 
 
-0.321
 
 
20.1
 
 
 
 
 
 
 appendix 
 
 
 
 
target_symbol
 
 
mature_mirna_id
 
 
score
 
 
nTPM
 
 
 
 
 
 
ABCC5
 
 
hsa-miR-125a-5p
 
 
-0.288
 
 
15.4
 
 
 
 
ABCD3
 
 
hsa-miR-148a-3p
 
 
-0.388
 
 
15.3
 
 
 
 
ABHD3
 
 
hsa-miR-125a-5p
 
 
-0.453
 
 
23.7
 
 
 
 
ABTB1
 
 
hsa-miR-125a-5p
 
 
-0.519
 
 
17.8
 
 
 
 
ACTC1
 
 
hsa-miR-7-5p
 
 
-0.471
 
 
23.3
 
 
 
 
ADAM10
 
 
hsa-miR-148a-3p
 
 
-0.356
 
 
17.9
 
 
 
 
ADAM9
 
 
hsa-miR-106a-5p
 
 
-0.317
 
 
27.3
 
 
 
 
ADAM9
 
 
hsa-miR-20b-5p
 
 
-0.328
 
 
27.3
 
 
 
 
AGFG1
 
 
hsa-miR-148a-3p
 
 
-0.332
 
 
45.7
 
 
 
 
AGGF1
 
 
hsa-miR-125a-5p
 
 
-0.331
 
 
10.9
 
 
 
 
AHNAK
 
 
hsa-miR-106a-5p
 
 
-0.334
 
 
41.0
 
 
 
 
AHNAK
 
 
hsa-miR-20b-5p
 
 
-0.337
 
 
41.0
 
 
 
 
AIFM1
 
 
hsa-miR-125a-5p
 
 
-0.3
 
 
16.6
 
 
 
 
AIFM1
 
 
hsa-let-7e-5p
 
 
-0.314
 
 
16.6
 
 
 
 
AIFM1
 
 
hsa-miR-98-5p
 
 
-0.314
 
 
16.6
 
 
 
 
AIFM1
 
 
hsa-let-7c-5p
 
 
-0.314
 
 
16.6
 
 
 
 
AKAP1
 
 
hsa-miR-148a-3p
 
 
-0.381
 
 
16.8
 
 
 
 
ALCAM
 
 
hsa-miR-148a-3p
 
 
-0.339
 
 
11.5
 
 
 
 
ALG3
 
 
hsa-miR-106a-5p
 
 
-0.569
 
 
14.1
 
 
 
 
ALG3
 
 
hsa-miR-20b-5p
 
 
-0.58
 
 
14.1
 
 
 
 
ANAPC16
 
 
hsa-miR-125a-5p
 
 
-0.578
 
 
14.0
 
 
 
 
ANKRD12
 
 
hsa-miR-7-5p
 
 
-0.37
 
 
19.4
 
 
 
 
ANPEP
 
 
hsa-miR-125a-5p
 
 
-0.372
 
 
34.6
 
 
 
 
AP1AR
 
 
hsa-miR-21-5p
 
 
-0.318
 
 
11.9
 
 
 
 
AP1AR
 
 
hsa-miR-99b-5p
 
 
-0.823
 
 
11.9
 
 
 
 
AP1S1
 
 
hsa-let-7e-5p
 
 
-0.374
 
 
13.1
 
 
 
 
AP1S1
 
 
hsa-miR-98-5p
 
 
-0.374
 
 
13.1
 
 
 
 
AP1S1
 
 
hsa-let-7c-5p
 
 
-0.374
 
 
13.1
 
 
 
 
ARF4
 
 
hsa-miR-7-5p
 
 
-0.407
 
 
101.3
 
 
 
 
ARF4
 
 
hsa-miR-148a-3p
 
 
-0.487
 
 
101.3
 
 
 
 
ARFGEF1
 
 
hsa-miR-148a-3p
 
 
-0.306
 
 
13.8
 
 
 
 
ARFIP1
 
 
hsa-miR-148a-3p
 
 
-0.474
 
 
14.9
 
 
 
 
ARHGAP1
 
 
hsa-miR-20b-5p
 
 
-0.325
 
 
35.8
 
 
 
 
ARHGAP1
 
 
hsa-miR-106a-5p
 
 
-0.329
 
 
35.8
 
 
 
 
ARID4A
 
 
hsa-miR-7-5p
 
 
-0.341
 
 
10.5
 
 
 
 
ARL1
 
 
hsa-miR-20b-5p
 
 
-0.424
 
 
37.7
 
 
 
 
ARL1
 
 
hsa-miR-106a-5p
 
 
-0.429
 
 
37.7
 
 
 
 
ARL6IP1
 
 
hsa-miR-7-5p
 
 
-0.374
 
 
73.3
 
 
 
 
ARL6IP1
 
 
hsa-miR-148a-3p
 
 
-0.579
 
 
73.3
 
 
 
 
ARL6IP6
 
 
hsa-miR-98-5p
 
 
-0.293
 
 
10.9
 
 
 
 
ARL6IP6
 
 
hsa-let-7c-5p
 
 
-0.293
 
 
10.9
 
 
 
 
ARL6IP6
 
 
hsa-let-7e-5p
 
 
-0.305
 
 
10.9
 
 
 
 
ARL8B
 
 
hsa-miR-148a-3p
 
 
-0.337
 
 
34.1
 
 
 
 
ARMC8
 
 
hsa-miR-106a-5p
 
 
-0.375
 
 
17.0
 
 
 
 
ARMC8
 
 
hsa-miR-20b-5p
 
 
-0.378
 
 
17.0
 
 
 
 
ARMT1
 
 
hsa-let-7c-5p
 
 
-0.303
 
 
14.4
 
 
 
 
ARMT1
 
 
hsa-let-7e-5p
 
 
-0.303
 
 
14.4
 
 
 
 
ARMT1
 
 
hsa-miR-98-5p
 
 
-0.303
 
 
14.4
 
 
 
 
ARPP19
 
 
hsa-let-7e-5p
 
 
-0.492
 
 
36.4
 
 
 
 
ARPP19
 
 
hsa-let-7c-5p
 
 
-0.492
 
 
36.4
 
 
 
 
ARPP19
 
 
hsa-miR-98-5p
 
 
-0.5
 
 
36.4
 
 
 
 
ARRDC3
 
 
hsa-miR-148a-3p
 
 
-0.305
 
 
25.3
 
 
 
 
ARRDC4
 
 
hsa-let-7c-5p
 
 
-0.329
 
 
15.6
 
 
 
 
ARRDC4
 
 
hsa-let-7e-5p
 
 
-0.329
 
 
15.6
 
 
 
 
ARRDC4
 
 
hsa-miR-98-5p
 
 
-0.329
 
 
15.6
 
 
 
 
ASAP1
 
 
hsa-let-7e-5p
 
 
-0.296
 
 
11.4
 
 
 
 
ASAP1
 
 
hsa-miR-98-5p
 
 
-0.308
 
 
11.4
 
 
 
 
ASAP1
 
 
hsa-let-7c-5p
 
 
-0.308
 
 
11.4
 
 
 
 
ASPH
 
 
hsa-miR-148a-3p
 
 
-0.391
 
 
47.6
 
 
 
 
ATOSB
 
 
hsa-let-7e-5p
 
 
-0.429
 
 
11.5
 
 
 
 
ATOSB
 
 
hsa-miR-98-5p
 
 
-0.429
 
 
11.5
 
 
 
 
ATOSB
 
 
hsa-let-7c-5p
 
 
-0.429
 
 
11.5
 
 
 
 
ATP2A2
 
 
hsa-let-7e-5p
 
 
-0.311
 
 
54.5
 
 
 
 
ATP2A2
 
 
hsa-miR-98-5p
 
 
-0.311
 
 
54.5
 
 
 
 
ATP2A2
 
 
hsa-let-7c-5p
 
 
-0.311
 
 
54.5
 
 
 
 
ATP2B4
 
 
hsa-miR-148a-3p
 
 
-0.337
 
 
24.2
 
 
 
 
ATP6AP2
 
 
hsa-miR-148a-3p
 
 
-0.592
 
 
57.3
 
 
 
 
ATP6AP2
 
 
hsa-miR-148a-3p
 
 
-0.668
 
 
57.3
 
 
 
 
ATPAF1
 
 
hsa-let-7e-5p
 
 
-0.498
 
 
26.8
 
 
 
 
ATPAF1
 
 
hsa-miR-98-5p
 
 
-0.498
 
 
26.8
 
 
 
 
ATPAF1
 
 
hsa-let-7c-5p
 
 
-0.498
 
 
26.8
 
 
 
 
B3GAT3
 
 
hsa-let-7e-5p
 
 
-0.324
 
 
16.3
 
 
 
 
B3GAT3
 
 
hsa-miR-98-5p
 
 
-0.324
 
 
16.3
 
 
 
 
B3GAT3
 
 
hsa-let-7c-5p
 
 
-0.324
 
 
16.3
 
 
 
 
B4GALT5
 
 
hsa-miR-148a-3p
 
 
-0.44
 
 
20.5
 
 
 
 
BACH1
 
 
hsa-let-7e-5p
 
 
-0.298
 
 
26.1
 
 
 
 
BACH1
 
 
hsa-let-7e-5p
 
 
-0.298
 
 
26.1
 
 
 
 
BACH1
 
 
hsa-miR-98-5p
 
 
-0.298
 
 
26.1
 
 
 
 
BACH1
 
 
hsa-miR-98-5p
 
 
-0.298
 
 
26.1
 
 
 
 
BACH1
 
 
hsa-let-7c-5p
 
 
-0.306
 
 
26.1
 
 
 
 
BACH1
 
 
hsa-let-7c-5p
 
 
-0.306
 
 
26.1
 
 
 
 
BACH1
 
 
hsa-let-7e-5p
 
 
-0.435
 
 
26.1
 
 
 
 
BACH1
 
 
hsa-let-7e-5p
 
 
-0.435
 
 
26.1
 
 
 
 
BACH1
 
 
hsa-miR-98-5p
 
 
-0.435
 
 
26.1
 
 
 
 
BACH1
 
 
hsa-miR-98-5p
 
 
-0.435
 
 
26.1
 
 
 
 
BACH1
 
 
hsa-let-7c-5p
 
 
-0.435
 
 
26.1
 
 
 
 
BACH1
 
 
hsa-let-7c-5p
 
 
-0.435
 
 
26.1
 
 
 
 
BAK1
 
 
hsa-miR-125a-5p
 
 
-0.402
 
 
19.5
 
 
 
 
BAZ2A
 
 
hsa-miR-99b-5p
 
 
-0.408
 
 
29.1
 
 
 
 
BCAP29
 
 
hsa-let-7e-5p
 
 
-0.289
 
 
21.6
 
 
 
 
BCL2L11
 
 
hsa-miR-148a-3p
 
 
-0.445
 
 
16.0
 
 
 
 
BCL2L12
 
 
hsa-miR-125a-5p
 
 
-0.288
 
 
13.8
 
 
 
 
BIN2
 
 
hsa-miR-125a-5p
 
 
-0.679
 
 
30.6
 
 
 
 
BIN3
 
 
hsa-let-7e-5p
 
 
-0.427
 
 
10.4
 
 
 
 
BIN3
 
 
hsa-miR-98-5p
 
 
-0.427
 
 
10.4
 
 
 
 
BIN3
 
 
hsa-let-7c-5p
 
 
-0.427
 
 
10.4
 
 
 
 
BLOC1S6
 
 
hsa-let-7e-5p
 
 
-0.321
 
 
29.4
 
 
 
 
BLOC1S6
 
 
hsa-miR-98-5p
 
 
-0.321
 
 
29.4
 
 
 
 
BLOC1S6
 
 
hsa-let-7c-5p
 
 
-0.321
 
 
29.4
 
 
 
 
BLZF1
 
 
hsa-miR-125a-5p
 
 
-0.461
 
 
11.7
 
 
 
 
 
 
 gallbladder 
 
 
 
 
target_symbol
 
 
mature_mirna_id
 
 
score
 
 
nTPM
 
 
 
 
 
 
ABCC5
 
 
hsa-miR-125a-5p
 
 
-0.288
 
 
16.8
 
 
 
 
ABCD3
 
 
hsa-miR-148a-3p
 
 
-0.388
 
 
25.2
 
 
 
 
ABHD3
 
 
hsa-miR-125a-5p
 
 
-0.453
 
 
19.5
 
 
 
 
ABHD5
 
 
hsa-miR-20b-5p
 
 
-0.418
 
 
10.2
 
 
 
 
ABHD5
 
 
hsa-miR-106a-5p
 
 
-0.447
 
 
10.2
 
 
 
 
ABTB1
 
 
hsa-miR-125a-5p
 
 
-0.519
 
 
17.0
 
 
 
 
ACTC1
 
 
hsa-miR-7-5p
 
 
-0.471
 
 
11.1
 
 
 
 
ACVR1
 
 
hsa-miR-148a-3p
 
 
-0.397
 
 
14.1
 
 
 
 
ADAM10
 
 
hsa-miR-148a-3p
 
 
-0.356
 
 
17.7
 
 
 
 
ADAM9
 
 
hsa-miR-106a-5p
 
 
-0.317
 
 
44.2
 
 
 
 
ADAM9
 
 
hsa-miR-20b-5p
 
 
-0.328
 
 
44.2
 
 
 
 
ADGRF5
 
 
hsa-miR-148a-3p
 
 
-0.354
 
 
16.1
 
 
 
 
AGFG1
 
 
hsa-miR-148a-3p
 
 
-0.332
 
 
36.5
 
 
 
 
AGFG2
 
 
hsa-miR-20b-5p
 
 
-0.386
 
 
10.4
 
 
 
 
AGFG2
 
 
hsa-miR-106a-5p
 
 
-0.399
 
 
10.4
 
 
 
 
AHNAK
 
 
hsa-miR-106a-5p
 
 
-0.334
 
 
70.2
 
 
 
 
AHNAK
 
 
hsa-miR-20b-5p
 
 
-0.337
 
 
70.2
 
 
 
 
AKAP1
 
 
hsa-miR-148a-3p
 
 
-0.381
 
 
16.8
 
 
 
 
ALCAM
 
 
hsa-miR-148a-3p
 
 
-0.339
 
 
21.9
 
 
 
 
ALG3
 
 
hsa-miR-106a-5p
 
 
-0.569
 
 
10.8
 
 
 
 
ALG3
 
 
hsa-miR-20b-5p
 
 
-0.58
 
 
10.8
 
 
 
 
ANAPC16
 
 
hsa-miR-125a-5p
 
 
-0.578
 
 
11.6
 
 
 
 
ANKRD12
 
 
hsa-miR-7-5p
 
 
-0.37
 
 
13.7
 
 
 
 
ANPEP
 
 
hsa-miR-125a-5p
 
 
-0.372
 
 
113.0
 
 
 
 
AP1AR
 
 
hsa-miR-21-5p
 
 
-0.318
 
 
12.0
 
 
 
 
AP1AR
 
 
hsa-miR-99b-5p
 
 
-0.823
 
 
12.0
 
 
 
 
AP1S1
 
 
hsa-let-7e-5p
 
 
-0.374
 
 
12.0
 
 
 
 
AP1S1
 
 
hsa-miR-98-5p
 
 
-0.374
 
 
12.0
 
 
 
 
AP1S1
 
 
hsa-let-7c-5p
 
 
-0.374
 
 
12.0
 
 
 
 
APCDD1
 
 
hsa-miR-106a-5p
 
 
-0.422
 
 
20.5
 
 
 
 
APCDD1
 
 
hsa-miR-20b-5p
 
 
-0.425
 
 
20.5
 
 
 
 
ARF4
 
 
hsa-miR-7-5p
 
 
-0.407
 
 
145.1
 
 
 
 
ARF4
 
 
hsa-miR-148a-3p
 
 
-0.487
 
 
145.1
 
 
 
 
ARFGEF1
 
 
hsa-miR-148a-3p
 
 
-0.306
 
 
11.7
 
 
 
 
ARFIP1
 
 
hsa-miR-148a-3p
 
 
-0.474
 
 
18.7
 
 
 
 
ARHGAP1
 
 
hsa-miR-20b-5p
 
 
-0.325
 
 
47.5
 
 
 
 
ARHGAP1
 
 
hsa-miR-106a-5p
 
 
-0.329
 
 
47.5
 
 
 
 
ARHGAP21
 
 
hsa-miR-148a-3p
 
 
-0.287
 
 
16.7
 
 
 
 
ARHGAP24
 
 
hsa-miR-21-5p
 
 
-0.427
 
 
10.2
 
 
 
 
ARL1
 
 
hsa-miR-20b-5p
 
 
-0.424
 
 
40.9
 
 
 
 
ARL1
 
 
hsa-miR-106a-5p
 
 
-0.429
 
 
40.9
 
 
 
 
ARL6IP1
 
 
hsa-miR-7-5p
 
 
-0.374
 
 
123.8
 
 
 
 
ARL6IP1
 
 
hsa-miR-148a-3p
 
 
-0.579
 
 
123.8
 
 
 
 
ARL8B
 
 
hsa-miR-148a-3p
 
 
-0.337
 
 
36.3
 
 
 
 
ARMC8
 
 
hsa-miR-106a-5p
 
 
-0.375
 
 
14.3
 
 
 
 
ARMC8
 
 
hsa-miR-20b-5p
 
 
-0.378
 
 
14.3
 
 
 
 
ARMCX1
 
 
hsa-miR-21-5p
 
 
-0.489
 
 
16.4
 
 
 
 
ARMT1
 
 
hsa-let-7c-5p
 
 
-0.303
 
 
10.6
 
 
 
 
ARMT1
 
 
hsa-let-7e-5p
 
 
-0.303
 
 
10.6
 
 
 
 
ARMT1
 
 
hsa-miR-98-5p
 
 
-0.303
 
 
10.6
 
 
 
 
ARPP19
 
 
hsa-let-7e-5p
 
 
-0.492
 
 
36.7
 
 
 
 
ARPP19
 
 
hsa-let-7c-5p
 
 
-0.492
 
 
36.7
 
 
 
 
ARPP19
 
 
hsa-miR-98-5p
 
 
-0.5
 
 
36.7
 
 
 
 
ARRDC3
 
 
hsa-miR-148a-3p
 
 
-0.305
 
 
36.6
 
 
 
 
ARRDC4
 
 
hsa-let-7c-5p
 
 
-0.329
 
 
16.1
 
 
 
 
ARRDC4
 
 
hsa-let-7e-5p
 
 
-0.329
 
 
16.1
 
 
 
 
ARRDC4
 
 
hsa-miR-98-5p
 
 
-0.329
 
 
16.1
 
 
 
 
ASAP1
 
 
hsa-let-7e-5p
 
 
-0.296
 
 
13.5
 
 
 
 
ASAP1
 
 
hsa-miR-98-5p
 
 
-0.308
 
 
13.5
 
 
 
 
ASAP1
 
 
hsa-let-7c-5p
 
 
-0.308
 
 
13.5
 
 
 
 
ASPH
 
 
hsa-miR-148a-3p
 
 
-0.391
 
 
56.5
 
 
 
 
ATOSB
 
 
hsa-let-7e-5p
 
 
-0.429
 
 
11.0
 
 
 
 
ATOSB
 
 
hsa-miR-98-5p
 
 
-0.429
 
 
11.0
 
 
 
 
ATOSB
 
 
hsa-let-7c-5p
 
 
-0.429
 
 
11.0
 
 
 
 
ATP2A2
 
 
hsa-let-7e-5p
 
 
-0.311
 
 
56.7
 
 
 
 
ATP2A2
 
 
hsa-miR-98-5p
 
 
-0.311
 
 
56.7
 
 
 
 
ATP2A2
 
 
hsa-let-7c-5p
 
 
-0.311
 
 
56.7
 
 
 
 
ATP2B4
 
 
hsa-miR-148a-3p
 
 
-0.337
 
 
23.7
 
 
 
 
ATP6AP2
 
 
hsa-miR-148a-3p
 
 
-0.592
 
 
74.7
 
 
 
 
ATP6AP2
 
 
hsa-miR-148a-3p
 
 
-0.668
 
 
74.7
 
 
 
 
ATPAF1
 
 
hsa-let-7e-5p
 
 
-0.498
 
 
28.6
 
 
 
 
ATPAF1
 
 
hsa-miR-98-5p
 
 
-0.498
 
 
28.6
 
 
 
 
ATPAF1
 
 
hsa-let-7c-5p
 
 
-0.498
 
 
28.6
 
 
 
 
B3GALT2
 
 
hsa-miR-20b-5p
 
 
-0.35
 
 
14.1
 
 
 
 
B3GALT2
 
 
hsa-miR-106a-5p
 
 
-0.354
 
 
14.1
 
 
 
 
B3GAT3
 
 
hsa-let-7e-5p
 
 
-0.324
 
 
14.5
 
 
 
 
B3GAT3
 
 
hsa-miR-98-5p
 
 
-0.324
 
 
14.5
 
 
 
 
B3GAT3
 
 
hsa-let-7c-5p
 
 
-0.324
 
 
14.5
 
 
 
 
B4GALT5
 
 
hsa-miR-148a-3p
 
 
-0.44
 
 
29.5
 
 
 
 
BACH1
 
 
hsa-let-7e-5p
 
 
-0.298
 
 
24.3
 
 
 
 
BACH1
 
 
hsa-let-7e-5p
 
 
-0.298
 
 
24.3
 
 
 
 
BACH1
 
 
hsa-miR-98-5p
 
 
-0.298
 
 
24.3
 
 
 
 
BACH1
 
 
hsa-miR-98-5p
 
 
-0.298
 
 
24.3
 
 
 
 
BACH1
 
 
hsa-let-7c-5p
 
 
-0.306
 
 
24.3
 
 
 
 
BACH1
 
 
hsa-let-7c-5p
 
 
-0.306
 
 
24.3
 
 
 
 
BACH1
 
 
hsa-let-7e-5p
 
 
-0.435
 
 
24.3
 
 
 
 
BACH1
 
 
hsa-let-7e-5p
 
 
-0.435
 
 
24.3
 
 
 
 
BACH1
 
 
hsa-miR-98-5p
 
 
-0.435
 
 
24.3
 
 
 
 
BACH1
 
 
hsa-miR-98-5p
 
 
-0.435
 
 
24.3
 
 
 
 
BACH1
 
 
hsa-let-7c-5p
 
 
-0.435
 
 
24.3
 
 
 
 
BACH1
 
 
hsa-let-7c-5p
 
 
-0.435
 
 
24.3
 
 
 
 
BAZ2A
 
 
hsa-miR-99b-5p
 
 
-0.408
 
 
19.6
 
 
 
 
BCAP29
 
 
hsa-let-7e-5p
 
 
-0.289
 
 
21.7
 
 
 
 
BCL2L11
 
 
hsa-miR-148a-3p
 
 
-0.445
 
 
11.1
 
 
 
 
BHLHE41
 
 
hsa-miR-148a-3p
 
 
-0.442
 
 
17.1
 
 
 
 
BICC1
 
 
hsa-miR-148a-3p
 
 
-0.442
 
 
10.9
 
 
 
 
BIN3
 
 
hsa-let-7e-5p
 
 
-0.427
 
 
11.2
 
 
 
 
BIN3
 
 
hsa-miR-98-5p
 
 
-0.427
 
 
11.2
 
 
 
 
BIN3
 
 
hsa-let-7c-5p
 
 
-0.427
 
 
11.2
 
 
 
 
BLOC1S6
 
 
hsa-let-7e-5p
 
 
-0.321
 
 
24.8
 
 
 
 
 
 
 hippocampal formation 
 
 
 
 
target_symbol
 
 
mature_mirna_id
 
 
score
 
 
nTPM
 
 
 
 
 
 
ABCC5
 
 
hsa-miR-125a-5p
 
 
-0.288
 
 
30.8
 
 
 
 
ABCD3
 
 
hsa-miR-148a-3p
 
 
-0.388
 
 
15.1
 
 
 
 
ABHD17C
 
 
hsa-let-7c-5p
 
 
-0.359
 
 
14.4
 
 
 
 
ABHD17C
 
 
hsa-let-7e-5p
 
 
-0.359
 
 
14.4
 
 
 
 
ABHD17C
 
 
hsa-miR-98-5p
 
 
-0.383
 
 
14.4
 
 
 
 
ABHD6
 
 
hsa-miR-125a-5p
 
 
-0.501
 
 
25.8
 
 
 
 
ABTB1
 
 
hsa-miR-125a-5p
 
 
-0.519
 
 
29.3
 
 
 
 
ACHE
 
 
hsa-miR-125a-5p
 
 
-0.308
 
 
14.5
 
 
 
 
ACHE
 
 
hsa-miR-125a-5p
 
 
-0.361
 
 
14.5
 
 
 
 
ACSL6
 
 
hsa-miR-125a-5p
 
 
-0.307
 
 
15.3
 
 
 
 
ACSL6
 
 
hsa-let-7e-5p
 
 
-0.336
 
 
15.3
 
 
 
 
ACSL6
 
 
hsa-miR-98-5p
 
 
-0.336
 
 
15.3
 
 
 
 
ACSL6
 
 
hsa-let-7c-5p
 
 
-0.336
 
 
15.3
 
 
 
 
ADCY2
 
 
hsa-miR-148a-3p
 
 
-0.299
 
 
18.5
 
 
 
 
ADGRB3
 
 
hsa-miR-148a-3p
 
 
-0.524
 
 
12.8
 
 
 
 
ADGRF5
 
 
hsa-miR-148a-3p
 
 
-0.354
 
 
10.4
 
 
 
 
AGFG1
 
 
hsa-miR-148a-3p
 
 
-0.332
 
 
29.8
 
 
 
 
AGFG2
 
 
hsa-miR-20b-5p
 
 
-0.386
 
 
13.2
 
 
 
 
AGFG2
 
 
hsa-miR-106a-5p
 
 
-0.399
 
 
13.2
 
 
 
 
AHNAK
 
 
hsa-miR-106a-5p
 
 
-0.334
 
 
24.2
 
 
 
 
AHNAK
 
 
hsa-miR-20b-5p
 
 
-0.337
 
 
24.2
 
 
 
 
AIFM1
 
 
hsa-miR-125a-5p
 
 
-0.3
 
 
10.5
 
 
 
 
AIFM1
 
 
hsa-let-7e-5p
 
 
-0.314
 
 
10.5
 
 
 
 
AIFM1
 
 
hsa-miR-98-5p
 
 
-0.314
 
 
10.5
 
 
 
 
AIFM1
 
 
hsa-let-7c-5p
 
 
-0.314
 
 
10.5
 
 
 
 
AKAP1
 
 
hsa-miR-148a-3p
 
 
-0.381
 
 
17.0
 
 
 
 
AKAP6
 
 
hsa-let-7e-5p
 
 
-0.307
 
 
12.9
 
 
 
 
AKAP6
 
 
hsa-miR-98-5p
 
 
-0.307
 
 
12.9
 
 
 
 
AKAP6
 
 
hsa-let-7c-5p
 
 
-0.307
 
 
12.9
 
 
 
 
ALCAM
 
 
hsa-miR-148a-3p
 
 
-0.339
 
 
33.8
 
 
 
 
ALDH4A1
 
 
hsa-miR-184
 
 
-0.421
 
 
39.7
 
 
 
 
ANKRD46
 
 
hsa-let-7e-5p
 
 
-0.389
 
 
25.5
 
 
 
 
ANKRD46
 
 
hsa-miR-98-5p
 
 
-0.389
 
 
25.5
 
 
 
 
ANKRD46
 
 
hsa-let-7c-5p
 
 
-0.389
 
 
25.5
 
 
 
 
ANKRD9
 
 
hsa-miR-20b-5p
 
 
-0.331
 
 
23.2
 
 
 
 
ANKRD9
 
 
hsa-miR-106a-5p
 
 
-0.344
 
 
23.2
 
 
 
 
AP1S1
 
 
hsa-let-7e-5p
 
 
-0.374
 
 
67.5
 
 
 
 
AP1S1
 
 
hsa-miR-98-5p
 
 
-0.374
 
 
67.5
 
 
 
 
AP1S1
 
 
hsa-let-7c-5p
 
 
-0.374
 
 
67.5
 
 
 
 
APCDD1
 
 
hsa-miR-106a-5p
 
 
-0.422
 
 
28.0
 
 
 
 
APCDD1
 
 
hsa-miR-20b-5p
 
 
-0.425
 
 
28.0
 
 
 
 
ARF4
 
 
hsa-miR-7-5p
 
 
-0.407
 
 
61.3
 
 
 
 
ARF4
 
 
hsa-miR-148a-3p
 
 
-0.487
 
 
61.3
 
 
 
 
ARHGAP1
 
 
hsa-miR-20b-5p
 
 
-0.325
 
 
48.9
 
 
 
 
ARHGAP1
 
 
hsa-miR-106a-5p
 
 
-0.329
 
 
48.9
 
 
 
 
ARHGAP21
 
 
hsa-miR-148a-3p
 
 
-0.287
 
 
50.4
 
 
 
 
ARL1
 
 
hsa-miR-20b-5p
 
 
-0.424
 
 
24.0
 
 
 
 
ARL1
 
 
hsa-miR-106a-5p
 
 
-0.429
 
 
24.0
 
 
 
 
ARL6IP1
 
 
hsa-miR-7-5p
 
 
-0.374
 
 
196.8
 
 
 
 
ARL6IP1
 
 
hsa-miR-148a-3p
 
 
-0.579
 
 
196.8
 
 
 
 
ARL8B
 
 
hsa-miR-148a-3p
 
 
-0.337
 
 
53.8
 
 
 
 
ARMC8
 
 
hsa-miR-106a-5p
 
 
-0.375
 
 
13.3
 
 
 
 
ARMC8
 
 
hsa-miR-20b-5p
 
 
-0.378
 
 
13.3
 
 
 
 
ARMCX1
 
 
hsa-miR-21-5p
 
 
-0.489
 
 
17.0
 
 
 
 
ARPP19
 
 
hsa-let-7e-5p
 
 
-0.492
 
 
120.9
 
 
 
 
ARPP19
 
 
hsa-let-7c-5p
 
 
-0.492
 
 
120.9
 
 
 
 
ARPP19
 
 
hsa-miR-98-5p
 
 
-0.5
 
 
120.9
 
 
 
 
ARRDC3
 
 
hsa-miR-148a-3p
 
 
-0.305
 
 
11.5
 
 
 
 
ARRDC4
 
 
hsa-let-7c-5p
 
 
-0.329
 
 
23.9
 
 
 
 
ARRDC4
 
 
hsa-let-7e-5p
 
 
-0.329
 
 
23.9
 
 
 
 
ARRDC4
 
 
hsa-miR-98-5p
 
 
-0.329
 
 
23.9
 
 
 
 
ASAP1
 
 
hsa-let-7e-5p
 
 
-0.296
 
 
10.8
 
 
 
 
ASAP1
 
 
hsa-miR-98-5p
 
 
-0.308
 
 
10.8
 
 
 
 
ASAP1
 
 
hsa-let-7c-5p
 
 
-0.308
 
 
10.8
 
 
 
 
ASF1A
 
 
hsa-miR-21-5p
 
 
-0.304
 
 
11.7
 
 
 
 
ASPH
 
 
hsa-miR-148a-3p
 
 
-0.391
 
 
45.5
 
 
 
 
ATOSB
 
 
hsa-let-7e-5p
 
 
-0.429
 
 
22.4
 
 
 
 
ATOSB
 
 
hsa-miR-98-5p
 
 
-0.429
 
 
22.4
 
 
 
 
ATOSB
 
 
hsa-let-7c-5p
 
 
-0.429
 
 
22.4
 
 
 
 
ATP2A2
 
 
hsa-let-7e-5p
 
 
-0.311
 
 
16.6
 
 
 
 
ATP2A2
 
 
hsa-miR-98-5p
 
 
-0.311
 
 
16.6
 
 
 
 
ATP2A2
 
 
hsa-let-7c-5p
 
 
-0.311
 
 
16.6
 
 
 
 
ATP2B2
 
 
hsa-miR-7-5p
 
 
-0.297
 
 
19.0
 
 
 
 
ATP6AP2
 
 
hsa-miR-148a-3p
 
 
-0.592
 
 
69.0
 
 
 
 
ATP6AP2
 
 
hsa-miR-148a-3p
 
 
-0.668
 
 
69.0
 
 
 
 
ATPAF1
 
 
hsa-let-7e-5p
 
 
-0.498
 
 
54.4
 
 
 
 
ATPAF1
 
 
hsa-miR-98-5p
 
 
-0.498
 
 
54.4
 
 
 
 
ATPAF1
 
 
hsa-let-7c-5p
 
 
-0.498
 
 
54.4
 
 
 
 
AVPI1
 
 
hsa-miR-7-5p
 
 
-0.367
 
 
14.1
 
 
 
 
B3GAT3
 
 
hsa-let-7e-5p
 
 
-0.324
 
 
64.6
 
 
 
 
B3GAT3
 
 
hsa-miR-98-5p
 
 
-0.324
 
 
64.6
 
 
 
 
B3GAT3
 
 
hsa-let-7c-5p
 
 
-0.324
 
 
64.6
 
 
 
 
B4GALT5
 
 
hsa-miR-148a-3p
 
 
-0.44
 
 
10.8
 
 
 
 
B4GAT1
 
 
hsa-let-7c-5p
 
 
-0.47
 
 
117.5
 
 
 
 
B4GAT1
 
 
hsa-let-7e-5p
 
 
-0.47
 
 
117.5
 
 
 
 
B4GAT1
 
 
hsa-miR-98-5p
 
 
-0.47
 
 
117.5
 
 
 
 
BAMBI
 
 
hsa-miR-106a-5p
 
 
-0.503
 
 
14.9
 
 
 
 
BAMBI
 
 
hsa-miR-20b-5p
 
 
-0.506
 
 
14.9
 
 
 
 
BAZ2A
 
 
hsa-miR-99b-5p
 
 
-0.408
 
 
10.1
 
 
 
 
BCAP29
 
 
hsa-let-7e-5p
 
 
-0.289
 
 
16.3
 
 
 
 
BDH1
 
 
hsa-miR-125a-5p
 
 
-0.389
 
 
19.7
 
 
 
 
BEGAIN
 
 
hsa-let-7c-5p
 
 
-0.466
 
 
36.8
 
 
 
 
BEGAIN
 
 
hsa-let-7e-5p
 
 
-0.466
 
 
36.8
 
 
 
 
BEGAIN
 
 
hsa-miR-98-5p
 
 
-0.474
 
 
36.8
 
 
 
 
BHLHE41
 
 
hsa-miR-148a-3p
 
 
-0.442
 
 
27.9
 
 
 
 
BIN3
 
 
hsa-let-7e-5p
 
 
-0.427
 
 
10.9
 
 
 
 
BIN3
 
 
hsa-miR-98-5p
 
 
-0.427
 
 
10.9
 
 
 
 
BIN3
 
 
hsa-let-7c-5p
 
 
-0.427
 
 
10.9
 
 
 
 
BLOC1S6
 
 
hsa-let-7e-5p
 
 
-0.321
 
 
18.3
 
 
 
 
BLOC1S6
 
 
hsa-miR-98-5p
 
 
-0.321
 
 
18.3
 
 
 
 
 
 
 kidney 
 
 
 
 
target_symbol
 
 
mature_mirna_id
 
 
score
 
 
nTPM
 
 
 
 
 
 
ABCC5
 
 
hsa-miR-125a-5p
 
 
-0.288
 
 
48.3
 
 
 
 
ABCD3
 
 
hsa-miR-148a-3p
 
 
-0.388
 
 
55.5
 
 
 
 
ABHD17C
 
 
hsa-let-7c-5p
 
 
-0.359
 
 
13.6
 
 
 
 
ABHD17C
 
 
hsa-let-7e-5p
 
 
-0.359
 
 
13.6
 
 
 
 
ABHD17C
 
 
hsa-miR-98-5p
 
 
-0.383
 
 
13.6
 
 
 
 
ABHD3
 
 
hsa-miR-125a-5p
 
 
-0.453
 
 
13.5
 
 
 
 
ABHD5
 
 
hsa-miR-20b-5p
 
 
-0.418
 
 
10.4
 
 
 
 
ABHD5
 
 
hsa-miR-106a-5p
 
 
-0.447
 
 
10.4
 
 
 
 
ABHD6
 
 
hsa-miR-125a-5p
 
 
-0.501
 
 
33.2
 
 
 
 
ABTB1
 
 
hsa-miR-125a-5p
 
 
-0.519
 
 
26.5
 
 
 
 
ACP3
 
 
hsa-let-7e-5p
 
 
-0.298
 
 
26.9
 
 
 
 
ACP3
 
 
hsa-miR-98-5p
 
 
-0.298
 
 
26.9
 
 
 
 
ACP3
 
 
hsa-let-7c-5p
 
 
-0.298
 
 
26.9
 
 
 
 
ACVR1
 
 
hsa-miR-148a-3p
 
 
-0.397
 
 
15.0
 
 
 
 
ADAM10
 
 
hsa-miR-148a-3p
 
 
-0.356
 
 
16.8
 
 
 
 
ADAM9
 
 
hsa-miR-106a-5p
 
 
-0.317
 
 
32.6
 
 
 
 
ADAM9
 
 
hsa-miR-20b-5p
 
 
-0.328
 
 
32.6
 
 
 
 
ADGRF5
 
 
hsa-miR-148a-3p
 
 
-0.354
 
 
55.8
 
 
 
 
AGFG1
 
 
hsa-miR-148a-3p
 
 
-0.332
 
 
30.6
 
 
 
 
AGFG2
 
 
hsa-miR-20b-5p
 
 
-0.386
 
 
15.9
 
 
 
 
AGFG2
 
 
hsa-miR-106a-5p
 
 
-0.399
 
 
15.9
 
 
 
 
AGGF1
 
 
hsa-miR-125a-5p
 
 
-0.331
 
 
12.1
 
 
 
 
AHDC1
 
 
hsa-miR-148a-3p
 
 
-0.413
 
 
12.2
 
 
 
 
AHNAK
 
 
hsa-miR-106a-5p
 
 
-0.334
 
 
55.7
 
 
 
 
AHNAK
 
 
hsa-miR-20b-5p
 
 
-0.337
 
 
55.7
 
 
 
 
AIFM1
 
 
hsa-miR-125a-5p
 
 
-0.3
 
 
69.0
 
 
 
 
AIFM1
 
 
hsa-let-7e-5p
 
 
-0.314
 
 
69.0
 
 
 
 
AIFM1
 
 
hsa-miR-98-5p
 
 
-0.314
 
 
69.0
 
 
 
 
AIFM1
 
 
hsa-let-7c-5p
 
 
-0.314
 
 
69.0
 
 
 
 
AKAP1
 
 
hsa-miR-148a-3p
 
 
-0.381
 
 
24.5
 
 
 
 
ALCAM
 
 
hsa-miR-148a-3p
 
 
-0.339
 
 
20.8
 
 
 
 
ALDH4A1
 
 
hsa-miR-184
 
 
-0.421
 
 
204.2
 
 
 
 
ALG3
 
 
hsa-miR-106a-5p
 
 
-0.569
 
 
17.8
 
 
 
 
ALG3
 
 
hsa-miR-20b-5p
 
 
-0.58
 
 
17.8
 
 
 
 
AMT
 
 
hsa-let-7e-5p
 
 
-0.463
 
 
23.7
 
 
 
 
AMT
 
 
hsa-miR-98-5p
 
 
-0.463
 
 
23.7
 
 
 
 
AMT
 
 
hsa-let-7c-5p
 
 
-0.463
 
 
23.7
 
 
 
 
ANAPC16
 
 
hsa-miR-125a-5p
 
 
-0.578
 
 
23.1
 
 
 
 
ANKRD12
 
 
hsa-miR-7-5p
 
 
-0.37
 
 
12.1
 
 
 
 
ANKRD46
 
 
hsa-let-7e-5p
 
 
-0.389
 
 
30.5
 
 
 
 
ANKRD46
 
 
hsa-miR-98-5p
 
 
-0.389
 
 
30.5
 
 
 
 
ANKRD46
 
 
hsa-let-7c-5p
 
 
-0.389
 
 
30.5
 
 
 
 
ANKRD9
 
 
hsa-miR-20b-5p
 
 
-0.331
 
 
22.8
 
 
 
 
ANKRD9
 
 
hsa-miR-106a-5p
 
 
-0.344
 
 
22.8
 
 
 
 
ANPEP
 
 
hsa-miR-125a-5p
 
 
-0.372
 
 
235.6
 
 
 
 
AP1AR
 
 
hsa-miR-21-5p
 
 
-0.318
 
 
20.6
 
 
 
 
AP1AR
 
 
hsa-miR-99b-5p
 
 
-0.823
 
 
20.6
 
 
 
 
AP1S1
 
 
hsa-let-7e-5p
 
 
-0.374
 
 
48.3
 
 
 
 
AP1S1
 
 
hsa-miR-98-5p
 
 
-0.374
 
 
48.3
 
 
 
 
AP1S1
 
 
hsa-let-7c-5p
 
 
-0.374
 
 
48.3
 
 
 
 
ARF4
 
 
hsa-miR-7-5p
 
 
-0.407
 
 
106.7
 
 
 
 
ARF4
 
 
hsa-miR-148a-3p
 
 
-0.487
 
 
106.7
 
 
 
 
ARFGEF1
 
 
hsa-miR-148a-3p
 
 
-0.306
 
 
21.3
 
 
 
 
ARFIP1
 
 
hsa-miR-148a-3p
 
 
-0.474
 
 
18.5
 
 
 
 
ARG2
 
 
hsa-let-7e-5p
 
 
-0.468
 
 
66.0
 
 
 
 
ARG2
 
 
hsa-miR-98-5p
 
 
-0.468
 
 
66.0
 
 
 
 
ARG2
 
 
hsa-let-7c-5p
 
 
-0.468
 
 
66.0
 
 
 
 
ARHGAP1
 
 
hsa-miR-20b-5p
 
 
-0.325
 
 
38.9
 
 
 
 
ARHGAP1
 
 
hsa-miR-106a-5p
 
 
-0.329
 
 
38.9
 
 
 
 
ARHGAP21
 
 
hsa-miR-148a-3p
 
 
-0.287
 
 
10.2
 
 
 
 
ARHGAP24
 
 
hsa-miR-21-5p
 
 
-0.427
 
 
93.2
 
 
 
 
ARHGEF28
 
 
hsa-miR-20b-5p
 
 
-0.352
 
 
29.5
 
 
 
 
ARHGEF28
 
 
hsa-miR-106a-5p
 
 
-0.367
 
 
29.5
 
 
 
 
ARL1
 
 
hsa-miR-20b-5p
 
 
-0.424
 
 
71.4
 
 
 
 
ARL1
 
 
hsa-miR-106a-5p
 
 
-0.429
 
 
71.4
 
 
 
 
ARL4D
 
 
hsa-let-7e-5p
 
 
-0.417
 
 
35.1
 
 
 
 
ARL4D
 
 
hsa-miR-98-5p
 
 
-0.417
 
 
35.1
 
 
 
 
ARL4D
 
 
hsa-let-7c-5p
 
 
-0.417
 
 
35.1
 
 
 
 
ARL6IP1
 
 
hsa-miR-7-5p
 
 
-0.374
 
 
140.2
 
 
 
 
ARL6IP1
 
 
hsa-miR-148a-3p
 
 
-0.579
 
 
140.2
 
 
 
 
ARL8B
 
 
hsa-miR-148a-3p
 
 
-0.337
 
 
50.2
 
 
 
 
ARMC8
 
 
hsa-miR-106a-5p
 
 
-0.375
 
 
17.6
 
 
 
 
ARMC8
 
 
hsa-miR-20b-5p
 
 
-0.378
 
 
17.6
 
 
 
 
ARMCX1
 
 
hsa-miR-21-5p
 
 
-0.489
 
 
12.8
 
 
 
 
ARMT1
 
 
hsa-let-7c-5p
 
 
-0.303
 
 
38.7
 
 
 
 
ARMT1
 
 
hsa-let-7e-5p
 
 
-0.303
 
 
38.7
 
 
 
 
ARMT1
 
 
hsa-miR-98-5p
 
 
-0.303
 
 
38.7
 
 
 
 
ARPP19
 
 
hsa-let-7e-5p
 
 
-0.492
 
 
52.6
 
 
 
 
ARPP19
 
 
hsa-let-7c-5p
 
 
-0.492
 
 
52.6
 
 
 
 
ARPP19
 
 
hsa-miR-98-5p
 
 
-0.5
 
 
52.6
 
 
 
 
ARRDC3
 
 
hsa-miR-148a-3p
 
 
-0.305
 
 
49.3
 
 
 
 
ARRDC4
 
 
hsa-let-7c-5p
 
 
-0.329
 
 
23.9
 
 
 
 
ARRDC4
 
 
hsa-let-7e-5p
 
 
-0.329
 
 
23.9
 
 
 
 
ARRDC4
 
 
hsa-miR-98-5p
 
 
-0.329
 
 
23.9
 
 
 
 
ASAP1
 
 
hsa-let-7e-5p
 
 
-0.296
 
 
10.1
 
 
 
 
ASAP1
 
 
hsa-miR-98-5p
 
 
-0.308
 
 
10.1
 
 
 
 
ASAP1
 
 
hsa-let-7c-5p
 
 
-0.308
 
 
10.1
 
 
 
 
ASF1A
 
 
hsa-miR-21-5p
 
 
-0.304
 
 
11.8
 
 
 
 
ASPH
 
 
hsa-miR-148a-3p
 
 
-0.391
 
 
55.2
 
 
 
 
ATG4A
 
 
hsa-miR-7-5p
 
 
-0.448
 
 
13.1
 
 
 
 
ATG4D
 
 
hsa-miR-148a-3p
 
 
-0.303
 
 
19.9
 
 
 
 
ATOSB
 
 
hsa-let-7e-5p
 
 
-0.429
 
 
14.3
 
 
 
 
ATOSB
 
 
hsa-miR-98-5p
 
 
-0.429
 
 
14.3
 
 
 
 
ATOSB
 
 
hsa-let-7c-5p
 
 
-0.429
 
 
14.3
 
 
 
 
ATP2A2
 
 
hsa-let-7e-5p
 
 
-0.311
 
 
32.2
 
 
 
 
ATP2A2
 
 
hsa-miR-98-5p
 
 
-0.311
 
 
32.2
 
 
 
 
ATP2A2
 
 
hsa-let-7c-5p
 
 
-0.311
 
 
32.2
 
 
 
 
ATP6AP2
 
 
hsa-miR-148a-3p
 
 
-0.592
 
 
154.5
 
 
 
 
ATP6AP2
 
 
hsa-miR-148a-3p
 
 
-0.668
 
 
154.5
 
 
 
 
ATPAF1
 
 
hsa-let-7e-5p
 
 
-0.498
 
 
107.2
 
 
 
 
 
 
 lung 
 
 
 
 
target_symbol
 
 
mature_mirna_id
 
 
score
 
 
nTPM
 
 
 
 
 
 
ABCC5
 
 
hsa-miR-125a-5p
 
 
-0.288
 
 
15.3
 
 
 
 
ABCD3
 
 
hsa-miR-148a-3p
 
 
-0.388
 
 
22.0
 
 
 
 
ABHD17C
 
 
hsa-let-7c-5p
 
 
-0.359
 
 
18.6
 
 
 
 
ABHD17C
 
 
hsa-let-7e-5p
 
 
-0.359
 
 
18.6
 
 
 
 
ABHD17C
 
 
hsa-miR-98-5p
 
 
-0.383
 
 
18.6
 
 
 
 
ABHD3
 
 
hsa-miR-125a-5p
 
 
-0.453
 
 
14.8
 
 
 
 
ABHD5
 
 
hsa-miR-20b-5p
 
 
-0.418
 
 
11.2
 
 
 
 
ABHD5
 
 
hsa-miR-106a-5p
 
 
-0.447
 
 
11.2
 
 
 
 
ABTB1
 
 
hsa-miR-125a-5p
 
 
-0.519
 
 
33.0
 
 
 
 
ACVR1
 
 
hsa-miR-148a-3p
 
 
-0.397
 
 
12.2
 
 
 
 
ADAM10
 
 
hsa-miR-148a-3p
 
 
-0.356
 
 
19.7
 
 
 
 
ADAM17
 
 
hsa-miR-125a-5p
 
 
-0.292
 
 
12.5
 
 
 
 
ADAM17
 
 
hsa-miR-148a-3p
 
 
-0.301
 
 
12.5
 
 
 
 
ADAM9
 
 
hsa-miR-106a-5p
 
 
-0.317
 
 
42.9
 
 
 
 
ADAM9
 
 
hsa-miR-20b-5p
 
 
-0.328
 
 
42.9
 
 
 
 
ADAMTS8
 
 
hsa-let-7e-5p
 
 
-0.571
 
 
33.2
 
 
 
 
ADAMTS8
 
 
hsa-miR-98-5p
 
 
-0.571
 
 
33.2
 
 
 
 
ADAMTS8
 
 
hsa-let-7c-5p
 
 
-0.571
 
 
33.2
 
 
 
 
ADGRF5
 
 
hsa-miR-148a-3p
 
 
-0.354
 
 
99.9
 
 
 
 
ADRB1
 
 
hsa-let-7c-5p
 
 
-0.351
 
 
11.4
 
 
 
 
ADRB1
 
 
hsa-let-7e-5p
 
 
-0.367
 
 
11.4
 
 
 
 
ADRB1
 
 
hsa-miR-98-5p
 
 
-0.367
 
 
11.4
 
 
 
 
ADRB2
 
 
hsa-let-7c-5p
 
 
-0.635
 
 
18.4
 
 
 
 
ADRB2
 
 
hsa-let-7e-5p
 
 
-0.635
 
 
18.4
 
 
 
 
ADRB2
 
 
hsa-miR-98-5p
 
 
-0.635
 
 
18.4
 
 
 
 
AGFG1
 
 
hsa-miR-148a-3p
 
 
-0.332
 
 
51.7
 
 
 
 
AHDC1
 
 
hsa-miR-148a-3p
 
 
-0.413
 
 
11.4
 
 
 
 
AHNAK
 
 
hsa-miR-106a-5p
 
 
-0.334
 
 
135.8
 
 
 
 
AHNAK
 
 
hsa-miR-20b-5p
 
 
-0.337
 
 
135.8
 
 
 
 
AKAP1
 
 
hsa-miR-148a-3p
 
 
-0.381
 
 
18.4
 
 
 
 
ALCAM
 
 
hsa-miR-148a-3p
 
 
-0.339
 
 
69.9
 
 
 
 
ALG3
 
 
hsa-miR-106a-5p
 
 
-0.569
 
 
17.4
 
 
 
 
ALG3
 
 
hsa-miR-20b-5p
 
 
-0.58
 
 
17.4
 
 
 
 
ANAPC16
 
 
hsa-miR-125a-5p
 
 
-0.578
 
 
17.6
 
 
 
 
ANKRD12
 
 
hsa-miR-7-5p
 
 
-0.37
 
 
10.9
 
 
 
 
ANKRD29
 
 
hsa-miR-106a-5p
 
 
-0.369
 
 
29.5
 
 
 
 
ANKRD29
 
 
hsa-miR-20b-5p
 
 
-0.38
 
 
29.5
 
 
 
 
ANKRD46
 
 
hsa-let-7e-5p
 
 
-0.389
 
 
10.1
 
 
 
 
ANKRD46
 
 
hsa-miR-98-5p
 
 
-0.389
 
 
10.1
 
 
 
 
ANKRD46
 
 
hsa-let-7c-5p
 
 
-0.389
 
 
10.1
 
 
 
 
ANKRD9
 
 
hsa-miR-20b-5p
 
 
-0.331
 
 
15.3
 
 
 
 
ANKRD9
 
 
hsa-miR-106a-5p
 
 
-0.344
 
 
15.3
 
 
 
 
ANPEP
 
 
hsa-miR-125a-5p
 
 
-0.372
 
 
31.9
 
 
 
 
AP1S1
 
 
hsa-let-7e-5p
 
 
-0.374
 
 
15.2
 
 
 
 
AP1S1
 
 
hsa-miR-98-5p
 
 
-0.374
 
 
15.2
 
 
 
 
AP1S1
 
 
hsa-let-7c-5p
 
 
-0.374
 
 
15.2
 
 
 
 
APCDD1
 
 
hsa-miR-106a-5p
 
 
-0.422
 
 
12.6
 
 
 
 
APCDD1
 
 
hsa-miR-20b-5p
 
 
-0.425
 
 
12.6
 
 
 
 
ARF4
 
 
hsa-miR-7-5p
 
 
-0.407
 
 
116.3
 
 
 
 
ARF4
 
 
hsa-miR-148a-3p
 
 
-0.487
 
 
116.3
 
 
 
 
ARFGEF1
 
 
hsa-miR-148a-3p
 
 
-0.306
 
 
13.0
 
 
 
 
ARFIP1
 
 
hsa-miR-148a-3p
 
 
-0.474
 
 
15.8
 
 
 
 
ARHGAP1
 
 
hsa-miR-20b-5p
 
 
-0.325
 
 
36.0
 
 
 
 
ARHGAP1
 
 
hsa-miR-106a-5p
 
 
-0.329
 
 
36.0
 
 
 
 
ARHGAP21
 
 
hsa-miR-148a-3p
 
 
-0.287
 
 
20.4
 
 
 
 
ARHGAP24
 
 
hsa-miR-21-5p
 
 
-0.427
 
 
14.8
 
 
 
 
ARL1
 
 
hsa-miR-20b-5p
 
 
-0.424
 
 
34.4
 
 
 
 
ARL1
 
 
hsa-miR-106a-5p
 
 
-0.429
 
 
34.4
 
 
 
 
ARL4D
 
 
hsa-let-7e-5p
 
 
-0.417
 
 
16.0
 
 
 
 
ARL4D
 
 
hsa-miR-98-5p
 
 
-0.417
 
 
16.0
 
 
 
 
ARL4D
 
 
hsa-let-7c-5p
 
 
-0.417
 
 
16.0
 
 
 
 
ARL6IP1
 
 
hsa-miR-7-5p
 
 
-0.374
 
 
75.1
 
 
 
 
ARL6IP1
 
 
hsa-miR-148a-3p
 
 
-0.579
 
 
75.1
 
 
 
 
ARL6IP6
 
 
hsa-miR-98-5p
 
 
-0.293
 
 
11.9
 
 
 
 
ARL6IP6
 
 
hsa-let-7c-5p
 
 
-0.293
 
 
11.9
 
 
 
 
ARL6IP6
 
 
hsa-let-7e-5p
 
 
-0.305
 
 
11.9
 
 
 
 
ARL8B
 
 
hsa-miR-148a-3p
 
 
-0.337
 
 
41.1
 
 
 
 
ARMC8
 
 
hsa-miR-106a-5p
 
 
-0.375
 
 
15.4
 
 
 
 
ARMC8
 
 
hsa-miR-20b-5p
 
 
-0.378
 
 
15.4
 
 
 
 
ARMCX1
 
 
hsa-miR-21-5p
 
 
-0.489
 
 
15.7
 
 
 
 
ARMT1
 
 
hsa-let-7c-5p
 
 
-0.303
 
 
18.4
 
 
 
 
ARMT1
 
 
hsa-let-7e-5p
 
 
-0.303
 
 
18.4
 
 
 
 
ARMT1
 
 
hsa-miR-98-5p
 
 
-0.303
 
 
18.4
 
 
 
 
ARPP19
 
 
hsa-let-7e-5p
 
 
-0.492
 
 
42.5
 
 
 
 
ARPP19
 
 
hsa-let-7c-5p
 
 
-0.492
 
 
42.5
 
 
 
 
ARPP19
 
 
hsa-miR-98-5p
 
 
-0.5
 
 
42.5
 
 
 
 
ARRDC3
 
 
hsa-miR-148a-3p
 
 
-0.305
 
 
42.0
 
 
 
 
ARRDC4
 
 
hsa-let-7c-5p
 
 
-0.329
 
 
18.7
 
 
 
 
ARRDC4
 
 
hsa-let-7e-5p
 
 
-0.329
 
 
18.7
 
 
 
 
ARRDC4
 
 
hsa-miR-98-5p
 
 
-0.329
 
 
18.7
 
 
 
 
ASAP1
 
 
hsa-let-7e-5p
 
 
-0.296
 
 
27.6
 
 
 
 
ASAP1
 
 
hsa-miR-98-5p
 
 
-0.308
 
 
27.6
 
 
 
 
ASAP1
 
 
hsa-let-7c-5p
 
 
-0.308
 
 
27.6
 
 
 
 
ASF1A
 
 
hsa-miR-21-5p
 
 
-0.304
 
 
12.6
 
 
 
 
ASPH
 
 
hsa-miR-148a-3p
 
 
-0.391
 
 
69.4
 
 
 
 
ATG4D
 
 
hsa-miR-148a-3p
 
 
-0.303
 
 
10.3
 
 
 
 
ATOSB
 
 
hsa-let-7e-5p
 
 
-0.429
 
 
20.8
 
 
 
 
ATOSB
 
 
hsa-miR-98-5p
 
 
-0.429
 
 
20.8
 
 
 
 
ATOSB
 
 
hsa-let-7c-5p
 
 
-0.429
 
 
20.8
 
 
 
 
ATP2A2
 
 
hsa-let-7e-5p
 
 
-0.311
 
 
46.2
 
 
 
 
ATP2A2
 
 
hsa-miR-98-5p
 
 
-0.311
 
 
46.2
 
 
 
 
ATP2A2
 
 
hsa-let-7c-5p
 
 
-0.311
 
 
46.2
 
 
 
 
ATP2B4
 
 
hsa-miR-148a-3p
 
 
-0.337
 
 
33.0
 
 
 
 
ATP6AP2
 
 
hsa-miR-148a-3p
 
 
-0.592
 
 
82.3
 
 
 
 
ATP6AP2
 
 
hsa-miR-148a-3p
 
 
-0.668
 
 
82.3
 
 
 
 
ATPAF1
 
 
hsa-let-7e-5p
 
 
-0.498
 
 
24.6
 
 
 
 
ATPAF1
 
 
hsa-miR-98-5p
 
 
-0.498
 
 
24.6
 
 
 
 
ATPAF1
 
 
hsa-let-7c-5p
 
 
-0.498
 
 
24.6
 
 
 
 
AVPI1
 
 
hsa-miR-7-5p
 
 
-0.367
 
 
23.0
 
 
 
 
B3GAT3
 
 
hsa-let-7e-5p
 
 
-0.324
 
 
32.1
 
 
 
 
 
 
 pancreas 
 
 
 
 
target_symbol
 
 
mature_mirna_id
 
 
score
 
 
nTPM
 
 
 
 
 
 
ABCC5
 
 
hsa-miR-125a-5p
 
 
-0.288
 
 
12.8
 
 
 
 
ABCD3
 
 
hsa-miR-148a-3p
 
 
-0.388
 
 
27.0
 
 
 
 
ABHD3
 
 
hsa-miR-125a-5p
 
 
-0.453
 
 
11.6
 
 
 
 
ABHD5
 
 
hsa-miR-20b-5p
 
 
-0.418
 
 
11.4
 
 
 
 
ABHD5
 
 
hsa-miR-106a-5p
 
 
-0.447
 
 
11.4
 
 
 
 
ABTB1
 
 
hsa-miR-125a-5p
 
 
-0.519
 
 
18.1
 
 
 
 
ACTC1
 
 
hsa-miR-7-5p
 
 
-0.471
 
 
12.1
 
 
 
 
AGFG1
 
 
hsa-miR-148a-3p
 
 
-0.332
 
 
33.0
 
 
 
 
AGFG2
 
 
hsa-miR-20b-5p
 
 
-0.386
 
 
17.7
 
 
 
 
AGFG2
 
 
hsa-miR-106a-5p
 
 
-0.399
 
 
17.7
 
 
 
 
AHNAK
 
 
hsa-miR-106a-5p
 
 
-0.334
 
 
28.6
 
 
 
 
AHNAK
 
 
hsa-miR-20b-5p
 
 
-0.337
 
 
28.6
 
 
 
 
AIFM1
 
 
hsa-miR-125a-5p
 
 
-0.3
 
 
20.4
 
 
 
 
AIFM1
 
 
hsa-let-7e-5p
 
 
-0.314
 
 
20.4
 
 
 
 
AIFM1
 
 
hsa-miR-98-5p
 
 
-0.314
 
 
20.4
 
 
 
 
AIFM1
 
 
hsa-let-7c-5p
 
 
-0.314
 
 
20.4
 
 
 
 
AKAP1
 
 
hsa-miR-148a-3p
 
 
-0.381
 
 
21.5
 
 
 
 
ALCAM
 
 
hsa-miR-148a-3p
 
 
-0.339
 
 
30.8
 
 
 
 
ALG3
 
 
hsa-miR-106a-5p
 
 
-0.569
 
 
35.7
 
 
 
 
ALG3
 
 
hsa-miR-20b-5p
 
 
-0.58
 
 
35.7
 
 
 
 
ALG9
 
 
hsa-miR-7-5p
 
 
-0.302
 
 
18.1
 
 
 
 
AMT
 
 
hsa-let-7e-5p
 
 
-0.463
 
 
21.5
 
 
 
 
AMT
 
 
hsa-miR-98-5p
 
 
-0.463
 
 
21.5
 
 
 
 
AMT
 
 
hsa-let-7c-5p
 
 
-0.463
 
 
21.5
 
 
 
 
ANAPC16
 
 
hsa-miR-125a-5p
 
 
-0.578
 
 
32.2
 
 
 
 
ANKRD12
 
 
hsa-miR-7-5p
 
 
-0.37
 
 
11.2
 
 
 
 
ANKRD46
 
 
hsa-let-7e-5p
 
 
-0.389
 
 
17.5
 
 
 
 
ANKRD46
 
 
hsa-miR-98-5p
 
 
-0.389
 
 
17.5
 
 
 
 
ANKRD46
 
 
hsa-let-7c-5p
 
 
-0.389
 
 
17.5
 
 
 
 
ANKRD9
 
 
hsa-miR-20b-5p
 
 
-0.331
 
 
10.7
 
 
 
 
ANKRD9
 
 
hsa-miR-106a-5p
 
 
-0.344
 
 
10.7
 
 
 
 
ANPEP
 
 
hsa-miR-125a-5p
 
 
-0.372
 
 
643.6
 
 
 
 
AP1S1
 
 
hsa-let-7e-5p
 
 
-0.374
 
 
23.9
 
 
 
 
AP1S1
 
 
hsa-miR-98-5p
 
 
-0.374
 
 
23.9
 
 
 
 
AP1S1
 
 
hsa-let-7c-5p
 
 
-0.374
 
 
23.9
 
 
 
 
ARF4
 
 
hsa-miR-7-5p
 
 
-0.407
 
 
138.3
 
 
 
 
ARF4
 
 
hsa-miR-148a-3p
 
 
-0.487
 
 
138.3
 
 
 
 
ARFGEF1
 
 
hsa-miR-148a-3p
 
 
-0.306
 
 
16.5
 
 
 
 
ARHGAP1
 
 
hsa-miR-20b-5p
 
 
-0.325
 
 
34.7
 
 
 
 
ARHGAP1
 
 
hsa-miR-106a-5p
 
 
-0.329
 
 
34.7
 
 
 
 
ARHGAP21
 
 
hsa-miR-148a-3p
 
 
-0.287
 
 
13.3
 
 
 
 
ARHGAP24
 
 
hsa-miR-21-5p
 
 
-0.427
 
 
28.4
 
 
 
 
ARHGEF28
 
 
hsa-miR-20b-5p
 
 
-0.352
 
 
19.9
 
 
 
 
ARHGEF28
 
 
hsa-miR-106a-5p
 
 
-0.367
 
 
19.9
 
 
 
 
ARL1
 
 
hsa-miR-20b-5p
 
 
-0.424
 
 
41.8
 
 
 
 
ARL1
 
 
hsa-miR-106a-5p
 
 
-0.429
 
 
41.8
 
 
 
 
ARL6IP1
 
 
hsa-miR-7-5p
 
 
-0.374
 
 
77.1
 
 
 
 
ARL6IP1
 
 
hsa-miR-148a-3p
 
 
-0.579
 
 
77.1
 
 
 
 
ARL8B
 
 
hsa-miR-148a-3p
 
 
-0.337
 
 
26.7
 
 
 
 
ARMC8
 
 
hsa-miR-106a-5p
 
 
-0.375
 
 
13.4
 
 
 
 
ARMC8
 
 
hsa-miR-20b-5p
 
 
-0.378
 
 
13.4
 
 
 
 
ARMCX1
 
 
hsa-miR-21-5p
 
 
-0.489
 
 
12.1
 
 
 
 
ARPP19
 
 
hsa-let-7e-5p
 
 
-0.492
 
 
43.6
 
 
 
 
ARPP19
 
 
hsa-let-7c-5p
 
 
-0.492
 
 
43.6
 
 
 
 
ARPP19
 
 
hsa-miR-98-5p
 
 
-0.5
 
 
43.6
 
 
 
 
ARRDC3
 
 
hsa-miR-148a-3p
 
 
-0.305
 
 
19.3
 
 
 
 
ARRDC4
 
 
hsa-let-7c-5p
 
 
-0.329
 
 
32.8
 
 
 
 
ARRDC4
 
 
hsa-let-7e-5p
 
 
-0.329
 
 
32.8
 
 
 
 
ARRDC4
 
 
hsa-miR-98-5p
 
 
-0.329
 
 
32.8
 
 
 
 
ASPH
 
 
hsa-miR-148a-3p
 
 
-0.391
 
 
42.1
 
 
 
 
ATG4A
 
 
hsa-miR-7-5p
 
 
-0.448
 
 
16.3
 
 
 
 
ATG4D
 
 
hsa-miR-148a-3p
 
 
-0.303
 
 
16.0
 
 
 
 
ATP2A2
 
 
hsa-let-7e-5p
 
 
-0.311
 
 
36.0
 
 
 
 
ATP2A2
 
 
hsa-miR-98-5p
 
 
-0.311
 
 
36.0
 
 
 
 
ATP2A2
 
 
hsa-let-7c-5p
 
 
-0.311
 
 
36.0
 
 
 
 
ATP6AP2
 
 
hsa-miR-148a-3p
 
 
-0.592
 
 
50.2
 
 
 
 
ATP6AP2
 
 
hsa-miR-148a-3p
 
 
-0.668
 
 
50.2
 
 
 
 
ATPAF1
 
 
hsa-let-7e-5p
 
 
-0.498
 
 
54.8
 
 
 
 
ATPAF1
 
 
hsa-miR-98-5p
 
 
-0.498
 
 
54.8
 
 
 
 
ATPAF1
 
 
hsa-let-7c-5p
 
 
-0.498
 
 
54.8
 
 
 
 
B3GALNT2
 
 
hsa-miR-125a-5p
 
 
-0.311
 
 
34.2
 
 
 
 
B3GAT3
 
 
hsa-let-7e-5p
 
 
-0.324
 
 
33.7
 
 
 
 
B3GAT3
 
 
hsa-miR-98-5p
 
 
-0.324
 
 
33.7
 
 
 
 
B3GAT3
 
 
hsa-let-7c-5p
 
 
-0.324
 
 
33.7
 
 
 
 
B4GALT5
 
 
hsa-miR-148a-3p
 
 
-0.44
 
 
11.8
 
 
 
 
B4GAT1
 
 
hsa-let-7c-5p
 
 
-0.47
 
 
24.7
 
 
 
 
B4GAT1
 
 
hsa-let-7e-5p
 
 
-0.47
 
 
24.7
 
 
 
 
B4GAT1
 
 
hsa-miR-98-5p
 
 
-0.47
 
 
24.7
 
 
 
 
BACH1
 
 
hsa-let-7e-5p
 
 
-0.298
 
 
16.2
 
 
 
 
BACH1
 
 
hsa-let-7e-5p
 
 
-0.298
 
 
16.2
 
 
 
 
BACH1
 
 
hsa-miR-98-5p
 
 
-0.298
 
 
16.2
 
 
 
 
BACH1
 
 
hsa-miR-98-5p
 
 
-0.298
 
 
16.2
 
 
 
 
BACH1
 
 
hsa-let-7c-5p
 
 
-0.306
 
 
16.2
 
 
 
 
BACH1
 
 
hsa-let-7c-5p
 
 
-0.306
 
 
16.2
 
 
 
 
BACH1
 
 
hsa-let-7e-5p
 
 
-0.435
 
 
16.2
 
 
 
 
BACH1
 
 
hsa-let-7e-5p
 
 
-0.435
 
 
16.2
 
 
 
 
BACH1
 
 
hsa-miR-98-5p
 
 
-0.435
 
 
16.2
 
 
 
 
BACH1
 
 
hsa-miR-98-5p
 
 
-0.435
 
 
16.2
 
 
 
 
BACH1
 
 
hsa-let-7c-5p
 
 
-0.435
 
 
16.2
 
 
 
 
BACH1
 
 
hsa-let-7c-5p
 
 
-0.435
 
 
16.2
 
 
 
 
BAK1
 
 
hsa-miR-125a-5p
 
 
-0.402
 
 
12.1
 
 
 
 
BAZ2A
 
 
hsa-miR-99b-5p
 
 
-0.408
 
 
29.1
 
 
 
 
BCAP29
 
 
hsa-let-7e-5p
 
 
-0.289
 
 
16.5
 
 
 
 
BCAT1
 
 
hsa-let-7e-5p
 
 
-0.394
 
 
54.8
 
 
 
 
BCAT1
 
 
hsa-miR-98-5p
 
 
-0.394
 
 
54.8
 
 
 
 
BCAT1
 
 
hsa-let-7c-5p
 
 
-0.394
 
 
54.8
 
 
 
 
BCL2L11
 
 
hsa-miR-148a-3p
 
 
-0.445
 
 
12.8
 
 
 
 
BCL2L12
 
 
hsa-miR-125a-5p
 
 
-0.288
 
 
13.7
 
 
 
 
BEGAIN
 
 
hsa-let-7c-5p
 
 
-0.466
 
 
22.6
 
 
 
 
BEGAIN
 
 
hsa-let-7e-5p
 
 
-0.466
 
 
22.6
 
 
 
 
 
 
 prostate 
 
 
 
 
target_symbol
 
 
mature_mirna_id
 
 
score
 
 
nTPM
 
 
 
 
 
 
ABCC5
 
 
hsa-miR-125a-5p
 
 
-0.288
 
 
27.9
 
 
 
 
ABCD3
 
 
hsa-miR-148a-3p
 
 
-0.388
 
 
20.2
 
 
 
 
ABHD17C
 
 
hsa-let-7c-5p
 
 
-0.359
 
 
17.0
 
 
 
 
ABHD17C
 
 
hsa-let-7e-5p
 
 
-0.359
 
 
17.0
 
 
 
 
ABHD17C
 
 
hsa-miR-98-5p
 
 
-0.383
 
 
17.0
 
 
 
 
ABHD3
 
 
hsa-miR-125a-5p
 
 
-0.453
 
 
10.4
 
 
 
 
ABTB1
 
 
hsa-miR-125a-5p
 
 
-0.519
 
 
33.0
 
 
 
 
ACP3
 
 
hsa-let-7e-5p
 
 
-0.298
 
 
3409.5
 
 
 
 
ACP3
 
 
hsa-miR-98-5p
 
 
-0.298
 
 
3409.5
 
 
 
 
ACP3
 
 
hsa-let-7c-5p
 
 
-0.298
 
 
3409.5
 
 
 
 
ACTC1
 
 
hsa-miR-7-5p
 
 
-0.471
 
 
68.4
 
 
 
 
ADAM10
 
 
hsa-miR-148a-3p
 
 
-0.356
 
 
23.6
 
 
 
 
ADAM9
 
 
hsa-miR-106a-5p
 
 
-0.317
 
 
32.8
 
 
 
 
ADAM9
 
 
hsa-miR-20b-5p
 
 
-0.328
 
 
32.8
 
 
 
 
AGFG1
 
 
hsa-miR-148a-3p
 
 
-0.332
 
 
19.4
 
 
 
 
AGFG2
 
 
hsa-miR-20b-5p
 
 
-0.386
 
 
12.4
 
 
 
 
AGFG2
 
 
hsa-miR-106a-5p
 
 
-0.399
 
 
12.4
 
 
 
 
AHDC1
 
 
hsa-miR-148a-3p
 
 
-0.413
 
 
11.3
 
 
 
 
AHNAK
 
 
hsa-miR-106a-5p
 
 
-0.334
 
 
83.5
 
 
 
 
AHNAK
 
 
hsa-miR-20b-5p
 
 
-0.337
 
 
83.5
 
 
 
 
AIFM1
 
 
hsa-miR-125a-5p
 
 
-0.3
 
 
10.1
 
 
 
 
AIFM1
 
 
hsa-let-7e-5p
 
 
-0.314
 
 
10.1
 
 
 
 
AIFM1
 
 
hsa-miR-98-5p
 
 
-0.314
 
 
10.1
 
 
 
 
AIFM1
 
 
hsa-let-7c-5p
 
 
-0.314
 
 
10.1
 
 
 
 
AKAP1
 
 
hsa-miR-148a-3p
 
 
-0.381
 
 
33.3
 
 
 
 
ALCAM
 
 
hsa-miR-148a-3p
 
 
-0.339
 
 
30.7
 
 
 
 
ALDH4A1
 
 
hsa-miR-184
 
 
-0.421
 
 
27.7
 
 
 
 
ALG3
 
 
hsa-miR-106a-5p
 
 
-0.569
 
 
19.0
 
 
 
 
ALG3
 
 
hsa-miR-20b-5p
 
 
-0.58
 
 
19.0
 
 
 
 
AMT
 
 
hsa-let-7e-5p
 
 
-0.463
 
 
15.5
 
 
 
 
AMT
 
 
hsa-miR-98-5p
 
 
-0.463
 
 
15.5
 
 
 
 
AMT
 
 
hsa-let-7c-5p
 
 
-0.463
 
 
15.5
 
 
 
 
ANAPC16
 
 
hsa-miR-125a-5p
 
 
-0.578
 
 
19.8
 
 
 
 
ANKRD12
 
 
hsa-miR-7-5p
 
 
-0.37
 
 
11.4
 
 
 
 
ANKRD46
 
 
hsa-let-7e-5p
 
 
-0.389
 
 
12.6
 
 
 
 
ANKRD46
 
 
hsa-miR-98-5p
 
 
-0.389
 
 
12.6
 
 
 
 
ANKRD46
 
 
hsa-let-7c-5p
 
 
-0.389
 
 
12.6
 
 
 
 
ANKRD9
 
 
hsa-miR-20b-5p
 
 
-0.331
 
 
18.8
 
 
 
 
ANKRD9
 
 
hsa-miR-106a-5p
 
 
-0.344
 
 
18.8
 
 
 
 
ANPEP
 
 
hsa-miR-125a-5p
 
 
-0.372
 
 
88.3
 
 
 
 
AP1S1
 
 
hsa-let-7e-5p
 
 
-0.374
 
 
23.9
 
 
 
 
AP1S1
 
 
hsa-miR-98-5p
 
 
-0.374
 
 
23.9
 
 
 
 
AP1S1
 
 
hsa-let-7c-5p
 
 
-0.374
 
 
23.9
 
 
 
 
APCDD1
 
 
hsa-miR-106a-5p
 
 
-0.422
 
 
31.5
 
 
 
 
APCDD1
 
 
hsa-miR-20b-5p
 
 
-0.425
 
 
31.5
 
 
 
 
ARF4
 
 
hsa-miR-7-5p
 
 
-0.407
 
 
129.9
 
 
 
 
ARF4
 
 
hsa-miR-148a-3p
 
 
-0.487
 
 
129.9
 
 
 
 
ARFGEF1
 
 
hsa-miR-148a-3p
 
 
-0.306
 
 
12.2
 
 
 
 
ARFIP1
 
 
hsa-miR-148a-3p
 
 
-0.474
 
 
19.8
 
 
 
 
ARG2
 
 
hsa-let-7e-5p
 
 
-0.468
 
 
101.8
 
 
 
 
ARG2
 
 
hsa-miR-98-5p
 
 
-0.468
 
 
101.8
 
 
 
 
ARG2
 
 
hsa-let-7c-5p
 
 
-0.468
 
 
101.8
 
 
 
 
ARHGAP1
 
 
hsa-miR-20b-5p
 
 
-0.325
 
 
60.9
 
 
 
 
ARHGAP1
 
 
hsa-miR-106a-5p
 
 
-0.329
 
 
60.9
 
 
 
 
ARHGAP21
 
 
hsa-miR-148a-3p
 
 
-0.287
 
 
10.2
 
 
 
 
ARL1
 
 
hsa-miR-20b-5p
 
 
-0.424
 
 
68.8
 
 
 
 
ARL1
 
 
hsa-miR-106a-5p
 
 
-0.429
 
 
68.8
 
 
 
 
ARL4D
 
 
hsa-let-7e-5p
 
 
-0.417
 
 
10.7
 
 
 
 
ARL4D
 
 
hsa-miR-98-5p
 
 
-0.417
 
 
10.7
 
 
 
 
ARL4D
 
 
hsa-let-7c-5p
 
 
-0.417
 
 
10.7
 
 
 
 
ARL6IP1
 
 
hsa-miR-7-5p
 
 
-0.374
 
 
71.3
 
 
 
 
ARL6IP1
 
 
hsa-miR-148a-3p
 
 
-0.579
 
 
71.3
 
 
 
 
ARL8B
 
 
hsa-miR-148a-3p
 
 
-0.337
 
 
42.2
 
 
 
 
ARMC8
 
 
hsa-miR-106a-5p
 
 
-0.375
 
 
15.4
 
 
 
 
ARMC8
 
 
hsa-miR-20b-5p
 
 
-0.378
 
 
15.4
 
 
 
 
ARMCX1
 
 
hsa-miR-21-5p
 
 
-0.489
 
 
22.8
 
 
 
 
ARMT1
 
 
hsa-let-7c-5p
 
 
-0.303
 
 
11.5
 
 
 
 
ARMT1
 
 
hsa-let-7e-5p
 
 
-0.303
 
 
11.5
 
 
 
 
ARMT1
 
 
hsa-miR-98-5p
 
 
-0.303
 
 
11.5
 
 
 
 
ARPP19
 
 
hsa-let-7e-5p
 
 
-0.492
 
 
37.0
 
 
 
 
ARPP19
 
 
hsa-let-7c-5p
 
 
-0.492
 
 
37.0
 
 
 
 
ARPP19
 
 
hsa-miR-98-5p
 
 
-0.5
 
 
37.0
 
 
 
 
ARRDC3
 
 
hsa-miR-148a-3p
 
 
-0.305
 
 
28.5
 
 
 
 
ARRDC4
 
 
hsa-let-7c-5p
 
 
-0.329
 
 
10.3
 
 
 
 
ARRDC4
 
 
hsa-let-7e-5p
 
 
-0.329
 
 
10.3
 
 
 
 
ARRDC4
 
 
hsa-miR-98-5p
 
 
-0.329
 
 
10.3
 
 
 
 
ASAP1
 
 
hsa-let-7e-5p
 
 
-0.296
 
 
11.6
 
 
 
 
ASAP1
 
 
hsa-miR-98-5p
 
 
-0.308
 
 
11.6
 
 
 
 
ASAP1
 
 
hsa-let-7c-5p
 
 
-0.308
 
 
11.6
 
 
 
 
ASPH
 
 
hsa-miR-148a-3p
 
 
-0.391
 
 
44.4
 
 
 
 
ATG16L1
 
 
hsa-miR-106a-5p
 
 
-0.464
 
 
12.5
 
 
 
 
ATG16L1
 
 
hsa-miR-20b-5p
 
 
-0.467
 
 
12.5
 
 
 
 
ATG4D
 
 
hsa-miR-148a-3p
 
 
-0.303
 
 
13.5
 
 
 
 
ATOSB
 
 
hsa-let-7e-5p
 
 
-0.429
 
 
12.4
 
 
 
 
ATOSB
 
 
hsa-miR-98-5p
 
 
-0.429
 
 
12.4
 
 
 
 
ATOSB
 
 
hsa-let-7c-5p
 
 
-0.429
 
 
12.4
 
 
 
 
ATP2A2
 
 
hsa-let-7e-5p
 
 
-0.311
 
 
87.6
 
 
 
 
ATP2A2
 
 
hsa-miR-98-5p
 
 
-0.311
 
 
87.6
 
 
 
 
ATP2A2
 
 
hsa-let-7c-5p
 
 
-0.311
 
 
87.6
 
 
 
 
ATP2B4
 
 
hsa-miR-148a-3p
 
 
-0.337
 
 
31.1
 
 
 
 
ATP6AP2
 
 
hsa-miR-148a-3p
 
 
-0.592
 
 
81.2
 
 
 
 
ATP6AP2
 
 
hsa-miR-148a-3p
 
 
-0.668
 
 
81.2
 
 
 
 
ATPAF1
 
 
hsa-let-7e-5p
 
 
-0.498
 
 
35.2
 
 
 
 
ATPAF1
 
 
hsa-miR-98-5p
 
 
-0.498
 
 
35.2
 
 
 
 
ATPAF1
 
 
hsa-let-7c-5p
 
 
-0.498
 
 
35.2
 
 
 
 
AVPI1
 
 
hsa-miR-7-5p
 
 
-0.367
 
 
27.2
 
 
 
 
B3GALNT2
 
 
hsa-miR-125a-5p
 
 
-0.311
 
 
11.7
 
 
 
 
B3GAT3
 
 
hsa-let-7e-5p
 
 
-0.324
 
 
40.8
 
 
 
 
B3GAT3
 
 
hsa-miR-98-5p
 
 
-0.324
 
 
40.8
 
 
 
 
B3GAT3
 
 
hsa-let-7c-5p
 
 
-0.324
 
 
40.8
 
 
 
 
 
 
 retina 
 
 
 
 
target_symbol
 
 
mature_mirna_id
 
 
score
 
 
nTPM
 
 
 
 
 
 
ABCC5
 
 
hsa-miR-125a-5p
 
 
-0.288
 
 
116.9
 
 
 
 
ABCD3
 
 
hsa-miR-148a-3p
 
 
-0.388
 
 
24.4
 
 
 
 
ABHD17C
 
 
hsa-let-7c-5p
 
 
-0.359
 
 
15.6
 
 
 
 
ABHD17C
 
 
hsa-let-7e-5p
 
 
-0.359
 
 
15.6
 
 
 
 
ABHD17C
 
 
hsa-miR-98-5p
 
 
-0.383
 
 
15.6
 
 
 
 
ACSL6
 
 
hsa-miR-125a-5p
 
 
-0.307
 
 
42.4
 
 
 
 
ACSL6
 
 
hsa-let-7e-5p
 
 
-0.336
 
 
42.4
 
 
 
 
ACSL6
 
 
hsa-miR-98-5p
 
 
-0.336
 
 
42.4
 
 
 
 
ACSL6
 
 
hsa-let-7c-5p
 
 
-0.336
 
 
42.4
 
 
 
 
ACTC1
 
 
hsa-miR-7-5p
 
 
-0.471
 
 
29.3
 
 
 
 
ADAM9
 
 
hsa-miR-106a-5p
 
 
-0.317
 
 
17.2
 
 
 
 
ADAM9
 
 
hsa-miR-20b-5p
 
 
-0.328
 
 
17.2
 
 
 
 
AGFG1
 
 
hsa-miR-148a-3p
 
 
-0.332
 
 
44.2
 
 
 
 
AGGF1
 
 
hsa-miR-125a-5p
 
 
-0.331
 
 
20.6
 
 
 
 
AKAP1
 
 
hsa-miR-148a-3p
 
 
-0.381
 
 
32.2
 
 
 
 
AKAP6
 
 
hsa-let-7e-5p
 
 
-0.307
 
 
12.6
 
 
 
 
AKAP6
 
 
hsa-miR-98-5p
 
 
-0.307
 
 
12.6
 
 
 
 
AKAP6
 
 
hsa-let-7c-5p
 
 
-0.307
 
 
12.6
 
 
 
 
ALG9
 
 
hsa-miR-7-5p
 
 
-0.302
 
 
11.3
 
 
 
 
ANAPC16
 
 
hsa-miR-125a-5p
 
 
-0.578
 
 
18.8
 
 
 
 
ANKRD12
 
 
hsa-miR-7-5p
 
 
-0.37
 
 
27.6
 
 
 
 
ANKRD13C
 
 
hsa-miR-106a-5p
 
 
-0.523
 
 
12.3
 
 
 
 
ANKRD13C
 
 
hsa-miR-20b-5p
 
 
-0.526
 
 
12.3
 
 
 
 
ANKRD33B
 
 
hsa-miR-125a-5p
 
 
-0.389
 
 
47.3
 
 
 
 
ANKRD46
 
 
hsa-let-7e-5p
 
 
-0.389
 
 
32.6
 
 
 
 
ANKRD46
 
 
hsa-miR-98-5p
 
 
-0.389
 
 
32.6
 
 
 
 
ANKRD46
 
 
hsa-let-7c-5p
 
 
-0.389
 
 
32.6
 
 
 
 
AP1S1
 
 
hsa-let-7e-5p
 
 
-0.374
 
 
13.5
 
 
 
 
AP1S1
 
 
hsa-miR-98-5p
 
 
-0.374
 
 
13.5
 
 
 
 
AP1S1
 
 
hsa-let-7c-5p
 
 
-0.374
 
 
13.5
 
 
 
 
ARF4
 
 
hsa-miR-7-5p
 
 
-0.407
 
 
50.0
 
 
 
 
ARF4
 
 
hsa-miR-148a-3p
 
 
-0.487
 
 
50.0
 
 
 
 
ARFGEF1
 
 
hsa-miR-148a-3p
 
 
-0.306
 
 
33.8
 
 
 
 
ARFIP1
 
 
hsa-miR-148a-3p
 
 
-0.474
 
 
18.7
 
 
 
 
ARG2
 
 
hsa-let-7e-5p
 
 
-0.468
 
 
20.7
 
 
 
 
ARG2
 
 
hsa-miR-98-5p
 
 
-0.468
 
 
20.7
 
 
 
 
ARG2
 
 
hsa-let-7c-5p
 
 
-0.468
 
 
20.7
 
 
 
 
ARHGAP21
 
 
hsa-miR-148a-3p
 
 
-0.287
 
 
37.0
 
 
 
 
ARID3B
 
 
hsa-let-7e-5p
 
 
-0.31
 
 
17.4
 
 
 
 
ARID3B
 
 
hsa-miR-98-5p
 
 
-0.31
 
 
17.4
 
 
 
 
ARID3B
 
 
hsa-let-7c-5p
 
 
-0.31
 
 
17.4
 
 
 
 
ARID3B
 
 
hsa-miR-98-5p
 
 
-0.475
 
 
17.4
 
 
 
 
ARID3B
 
 
hsa-let-7e-5p
 
 
-0.486
 
 
17.4
 
 
 
 
ARID3B
 
 
hsa-let-7c-5p
 
 
-0.486
 
 
17.4
 
 
 
 
ARID4A
 
 
hsa-miR-7-5p
 
 
-0.341
 
 
13.0
 
 
 
 
ARL1
 
 
hsa-miR-20b-5p
 
 
-0.424
 
 
45.1
 
 
 
 
ARL1
 
 
hsa-miR-106a-5p
 
 
-0.429
 
 
45.1
 
 
 
 
ARL4D
 
 
hsa-let-7e-5p
 
 
-0.417
 
 
78.9
 
 
 
 
ARL4D
 
 
hsa-miR-98-5p
 
 
-0.417
 
 
78.9
 
 
 
 
ARL4D
 
 
hsa-let-7c-5p
 
 
-0.417
 
 
78.9
 
 
 
 
ARL6IP1
 
 
hsa-miR-7-5p
 
 
-0.374
 
 
292.6
 
 
 
 
ARL6IP1
 
 
hsa-miR-148a-3p
 
 
-0.579
 
 
292.6
 
 
 
 
ARL6IP6
 
 
hsa-miR-98-5p
 
 
-0.293
 
 
10.5
 
 
 
 
ARL6IP6
 
 
hsa-let-7c-5p
 
 
-0.293
 
 
10.5
 
 
 
 
ARL6IP6
 
 
hsa-let-7e-5p
 
 
-0.305
 
 
10.5
 
 
 
 
ARL8B
 
 
hsa-miR-148a-3p
 
 
-0.337
 
 
31.7
 
 
 
 
ARMC8
 
 
hsa-miR-106a-5p
 
 
-0.375
 
 
24.3
 
 
 
 
ARMC8
 
 
hsa-miR-20b-5p
 
 
-0.378
 
 
24.3
 
 
 
 
ARMCX1
 
 
hsa-miR-21-5p
 
 
-0.489
 
 
11.2
 
 
 
 
ARMT1
 
 
hsa-let-7c-5p
 
 
-0.303
 
 
15.2
 
 
 
 
ARMT1
 
 
hsa-let-7e-5p
 
 
-0.303
 
 
15.2
 
 
 
 
ARMT1
 
 
hsa-miR-98-5p
 
 
-0.303
 
 
15.2
 
 
 
 
ARPP19
 
 
hsa-let-7e-5p
 
 
-0.492
 
 
53.1
 
 
 
 
ARPP19
 
 
hsa-let-7c-5p
 
 
-0.492
 
 
53.1
 
 
 
 
ARPP19
 
 
hsa-miR-98-5p
 
 
-0.5
 
 
53.1
 
 
 
 
ARRDC3
 
 
hsa-miR-148a-3p
 
 
-0.305
 
 
69.6
 
 
 
 
ASF1A
 
 
hsa-miR-21-5p
 
 
-0.304
 
 
10.6
 
 
 
 
ASPH
 
 
hsa-miR-148a-3p
 
 
-0.391
 
 
201.9
 
 
 
 
ATP2A2
 
 
hsa-let-7e-5p
 
 
-0.311
 
 
17.7
 
 
 
 
ATP2A2
 
 
hsa-miR-98-5p
 
 
-0.311
 
 
17.7
 
 
 
 
ATP2A2
 
 
hsa-let-7c-5p
 
 
-0.311
 
 
17.7
 
 
 
 
ATP2B2
 
 
hsa-miR-7-5p
 
 
-0.297
 
 
12.5
 
 
 
 
ATP2B4
 
 
hsa-miR-148a-3p
 
 
-0.337
 
 
39.4
 
 
 
 
ATP6AP2
 
 
hsa-miR-148a-3p
 
 
-0.592
 
 
92.0
 
 
 
 
ATP6AP2
 
 
hsa-miR-148a-3p
 
 
-0.668
 
 
92.0
 
 
 
 
ATPAF1
 
 
hsa-let-7e-5p
 
 
-0.498
 
 
65.3
 
 
 
 
ATPAF1
 
 
hsa-miR-98-5p
 
 
-0.498
 
 
65.3
 
 
 
 
ATPAF1
 
 
hsa-let-7c-5p
 
 
-0.498
 
 
65.3
 
 
 
 
AVPI1
 
 
hsa-miR-7-5p
 
 
-0.367
 
 
23.7
 
 
 
 
B3GAT3
 
 
hsa-let-7e-5p
 
 
-0.324
 
 
15.0
 
 
 
 
B3GAT3
 
 
hsa-miR-98-5p
 
 
-0.324
 
 
15.0
 
 
 
 
B3GAT3
 
 
hsa-let-7c-5p
 
 
-0.324
 
 
15.0
 
 
 
 
B4GALT6
 
 
hsa-miR-148a-3p
 
 
-0.528
 
 
24.7
 
 
 
 
B4GAT1
 
 
hsa-let-7c-5p
 
 
-0.47
 
 
42.6
 
 
 
 
B4GAT1
 
 
hsa-let-7e-5p
 
 
-0.47
 
 
42.6
 
 
 
 
B4GAT1
 
 
hsa-miR-98-5p
 
 
-0.47
 
 
42.6
 
 
 
 
BACH1
 
 
hsa-let-7e-5p
 
 
-0.298
 
 
10.9
 
 
 
 
BACH1
 
 
hsa-let-7e-5p
 
 
-0.298
 
 
10.9
 
 
 
 
BACH1
 
 
hsa-miR-98-5p
 
 
-0.298
 
 
10.9
 
 
 
 
BACH1
 
 
hsa-miR-98-5p
 
 
-0.298
 
 
10.9
 
 
 
 
BACH1
 
 
hsa-let-7c-5p
 
 
-0.306
 
 
10.9
 
 
 
 
BACH1
 
 
hsa-let-7c-5p
 
 
-0.306
 
 
10.9
 
 
 
 
BACH1
 
 
hsa-let-7e-5p
 
 
-0.435
 
 
10.9
 
 
 
 
BACH1
 
 
hsa-let-7e-5p
 
 
-0.435
 
 
10.9
 
 
 
 
BACH1
 
 
hsa-miR-98-5p
 
 
-0.435
 
 
10.9
 
 
 
 
BACH1
 
 
hsa-miR-98-5p
 
 
-0.435
 
 
10.9
 
 
 
 
BACH1
 
 
hsa-let-7c-5p
 
 
-0.435
 
 
10.9
 
 
 
 
BACH1
 
 
hsa-let-7c-5p
 
 
-0.435
 
 
10.9
 
 
 
 
BAMBI
 
 
hsa-miR-106a-5p
 
 
-0.503
 
 
12.4
 
 
 
 
BAMBI
 
 
hsa-miR-20b-5p
 
 
-0.506
 
 
12.4
 
 
 
 
 
 
 salivary gland 
 
 
 
 
target_symbol
 
 
mature_mirna_id
 
 
score
 
 
nTPM
 
 
 
 
 
 
ABCC5
 
 
hsa-miR-125a-5p
 
 
-0.288
 
 
18.2
 
 
 
 
ABCD3
 
 
hsa-miR-148a-3p
 
 
-0.388
 
 
16.5
 
 
 
 
ABHD17C
 
 
hsa-let-7c-5p
 
 
-0.359
 
 
22.3
 
 
 
 
ABHD17C
 
 
hsa-let-7e-5p
 
 
-0.359
 
 
22.3
 
 
 
 
ABHD17C
 
 
hsa-miR-98-5p
 
 
-0.383
 
 
22.3
 
 
 
 
ABHD3
 
 
hsa-miR-125a-5p
 
 
-0.453
 
 
13.7
 
 
 
 
ABHD5
 
 
hsa-miR-20b-5p
 
 
-0.418
 
 
19.4
 
 
 
 
ABHD5
 
 
hsa-miR-106a-5p
 
 
-0.447
 
 
19.4
 
 
 
 
ABTB1
 
 
hsa-miR-125a-5p
 
 
-0.519
 
 
25.2
 
 
 
 
ACTC1
 
 
hsa-miR-7-5p
 
 
-0.471
 
 
41.7
 
 
 
 
ADAM9
 
 
hsa-miR-106a-5p
 
 
-0.317
 
 
39.8
 
 
 
 
ADAM9
 
 
hsa-miR-20b-5p
 
 
-0.328
 
 
39.8
 
 
 
 
AGFG1
 
 
hsa-miR-148a-3p
 
 
-0.332
 
 
26.3
 
 
 
 
AGFG2
 
 
hsa-miR-20b-5p
 
 
-0.386
 
 
272.4
 
 
 
 
AGFG2
 
 
hsa-miR-106a-5p
 
 
-0.399
 
 
272.4
 
 
 
 
AHDC1
 
 
hsa-miR-148a-3p
 
 
-0.413
 
 
16.0
 
 
 
 
AHNAK
 
 
hsa-miR-106a-5p
 
 
-0.334
 
 
118.3
 
 
 
 
AHNAK
 
 
hsa-miR-20b-5p
 
 
-0.337
 
 
118.3
 
 
 
 
AIFM1
 
 
hsa-miR-125a-5p
 
 
-0.3
 
 
17.0
 
 
 
 
AIFM1
 
 
hsa-let-7e-5p
 
 
-0.314
 
 
17.0
 
 
 
 
AIFM1
 
 
hsa-miR-98-5p
 
 
-0.314
 
 
17.0
 
 
 
 
AIFM1
 
 
hsa-let-7c-5p
 
 
-0.314
 
 
17.0
 
 
 
 
AKAP1
 
 
hsa-miR-148a-3p
 
 
-0.381
 
 
31.6
 
 
 
 
ALCAM
 
 
hsa-miR-148a-3p
 
 
-0.339
 
 
31.9
 
 
 
 
ALG3
 
 
hsa-miR-106a-5p
 
 
-0.569
 
 
25.8
 
 
 
 
ALG3
 
 
hsa-miR-20b-5p
 
 
-0.58
 
 
25.8
 
 
 
 
ALG9
 
 
hsa-miR-7-5p
 
 
-0.302
 
 
16.6
 
 
 
 
AMT
 
 
hsa-let-7e-5p
 
 
-0.463
 
 
10.0
 
 
 
 
AMT
 
 
hsa-miR-98-5p
 
 
-0.463
 
 
10.0
 
 
 
 
AMT
 
 
hsa-let-7c-5p
 
 
-0.463
 
 
10.0
 
 
 
 
ANAPC16
 
 
hsa-miR-125a-5p
 
 
-0.578
 
 
29.9
 
 
 
 
ANKRD12
 
 
hsa-miR-7-5p
 
 
-0.37
 
 
11.6
 
 
 
 
ANKRD46
 
 
hsa-let-7e-5p
 
 
-0.389
 
 
16.1
 
 
 
 
ANKRD46
 
 
hsa-miR-98-5p
 
 
-0.389
 
 
16.1
 
 
 
 
ANKRD46
 
 
hsa-let-7c-5p
 
 
-0.389
 
 
16.1
 
 
 
 
ANKRD9
 
 
hsa-miR-20b-5p
 
 
-0.331
 
 
25.3
 
 
 
 
ANKRD9
 
 
hsa-miR-106a-5p
 
 
-0.344
 
 
25.3
 
 
 
 
ANPEP
 
 
hsa-miR-125a-5p
 
 
-0.372
 
 
38.3
 
 
 
 
AP1AR
 
 
hsa-miR-21-5p
 
 
-0.318
 
 
11.4
 
 
 
 
AP1AR
 
 
hsa-miR-99b-5p
 
 
-0.823
 
 
11.4
 
 
 
 
AP1S1
 
 
hsa-let-7e-5p
 
 
-0.374
 
 
21.5
 
 
 
 
AP1S1
 
 
hsa-miR-98-5p
 
 
-0.374
 
 
21.5
 
 
 
 
AP1S1
 
 
hsa-let-7c-5p
 
 
-0.374
 
 
21.5
 
 
 
 
APCDD1
 
 
hsa-miR-106a-5p
 
 
-0.422
 
 
10.0
 
 
 
 
APCDD1
 
 
hsa-miR-20b-5p
 
 
-0.425
 
 
10.0
 
 
 
 
ARF4
 
 
hsa-miR-7-5p
 
 
-0.407
 
 
171.4
 
 
 
 
ARF4
 
 
hsa-miR-148a-3p
 
 
-0.487
 
 
171.4
 
 
 
 
ARFGEF1
 
 
hsa-miR-148a-3p
 
 
-0.306
 
 
13.9
 
 
 
 
ARFIP1
 
 
hsa-miR-148a-3p
 
 
-0.474
 
 
12.9
 
 
 
 
ARG2
 
 
hsa-let-7e-5p
 
 
-0.468
 
 
18.4
 
 
 
 
ARG2
 
 
hsa-miR-98-5p
 
 
-0.468
 
 
18.4
 
 
 
 
ARG2
 
 
hsa-let-7c-5p
 
 
-0.468
 
 
18.4
 
 
 
 
ARHGAP1
 
 
hsa-miR-20b-5p
 
 
-0.325
 
 
43.3
 
 
 
 
ARHGAP1
 
 
hsa-miR-106a-5p
 
 
-0.329
 
 
43.3
 
 
 
 
ARHGAP21
 
 
hsa-miR-148a-3p
 
 
-0.287
 
 
23.1
 
 
 
 
ARHGEF28
 
 
hsa-miR-20b-5p
 
 
-0.352
 
 
18.6
 
 
 
 
ARHGEF28
 
 
hsa-miR-106a-5p
 
 
-0.367
 
 
18.6
 
 
 
 
ARL1
 
 
hsa-miR-20b-5p
 
 
-0.424
 
 
69.5
 
 
 
 
ARL1
 
 
hsa-miR-106a-5p
 
 
-0.429
 
 
69.5
 
 
 
 
ARL4D
 
 
hsa-let-7e-5p
 
 
-0.417
 
 
16.7
 
 
 
 
ARL4D
 
 
hsa-miR-98-5p
 
 
-0.417
 
 
16.7
 
 
 
 
ARL4D
 
 
hsa-let-7c-5p
 
 
-0.417
 
 
16.7
 
 
 
 
ARL6IP1
 
 
hsa-miR-7-5p
 
 
-0.374
 
 
79.4
 
 
 
 
ARL6IP1
 
 
hsa-miR-148a-3p
 
 
-0.579
 
 
79.4
 
 
 
 
ARL8B
 
 
hsa-miR-148a-3p
 
 
-0.337
 
 
35.3
 
 
 
 
ARMC8
 
 
hsa-miR-106a-5p
 
 
-0.375
 
 
11.8
 
 
 
 
ARMC8
 
 
hsa-miR-20b-5p
 
 
-0.378
 
 
11.8
 
 
 
 
ARMCX1
 
 
hsa-miR-21-5p
 
 
-0.489
 
 
13.3
 
 
 
 
ARPP19
 
 
hsa-let-7e-5p
 
 
-0.492
 
 
28.7
 
 
 
 
ARPP19
 
 
hsa-let-7c-5p
 
 
-0.492
 
 
28.7
 
 
 
 
ARPP19
 
 
hsa-miR-98-5p
 
 
-0.5
 
 
28.7
 
 
 
 
ARRDC3
 
 
hsa-miR-148a-3p
 
 
-0.305
 
 
60.1
 
 
 
 
ASF1A
 
 
hsa-miR-21-5p
 
 
-0.304
 
 
10.1
 
 
 
 
ASPH
 
 
hsa-miR-148a-3p
 
 
-0.391
 
 
95.6
 
 
 
 
ATG16L1
 
 
hsa-miR-106a-5p
 
 
-0.464
 
 
11.7
 
 
 
 
ATG16L1
 
 
hsa-miR-20b-5p
 
 
-0.467
 
 
11.7
 
 
 
 
ATG4A
 
 
hsa-miR-7-5p
 
 
-0.448
 
 
12.3
 
 
 
 
ATG4D
 
 
hsa-miR-148a-3p
 
 
-0.303
 
 
16.3
 
 
 
 
ATOSB
 
 
hsa-let-7e-5p
 
 
-0.429
 
 
10.9
 
 
 
 
ATOSB
 
 
hsa-miR-98-5p
 
 
-0.429
 
 
10.9
 
 
 
 
ATOSB
 
 
hsa-let-7c-5p
 
 
-0.429
 
 
10.9
 
 
 
 
ATP2A2
 
 
hsa-let-7e-5p
 
 
-0.311
 
 
74.5
 
 
 
 
ATP2A2
 
 
hsa-miR-98-5p
 
 
-0.311
 
 
74.5
 
 
 
 
ATP2A2
 
 
hsa-let-7c-5p
 
 
-0.311
 
 
74.5
 
 
 
 
ATP2B2
 
 
hsa-miR-7-5p
 
 
-0.297
 
 
31.3
 
 
 
 
ATP6AP2
 
 
hsa-miR-148a-3p
 
 
-0.592
 
 
39.6
 
 
 
 
ATP6AP2
 
 
hsa-miR-148a-3p
 
 
-0.668
 
 
39.6
 
 
 
 
ATPAF1
 
 
hsa-let-7e-5p
 
 
-0.498
 
 
54.3
 
 
 
 
ATPAF1
 
 
hsa-miR-98-5p
 
 
-0.498
 
 
54.3
 
 
 
 
ATPAF1
 
 
hsa-let-7c-5p
 
 
-0.498
 
 
54.3
 
 
 
 
AVPI1
 
 
hsa-miR-7-5p
 
 
-0.367
 
 
23.0
 
 
 
 
B3GAT3
 
 
hsa-let-7e-5p
 
 
-0.324
 
 
28.6
 
 
 
 
B3GAT3
 
 
hsa-miR-98-5p
 
 
-0.324
 
 
28.6
 
 
 
 
B3GAT3
 
 
hsa-let-7c-5p
 
 
-0.324
 
 
28.6
 
 
 
 
B4GALT5
 
 
hsa-miR-148a-3p
 
 
-0.44
 
 
18.2
 
 
 
 
B4GAT1
 
 
hsa-let-7c-5p
 
 
-0.47
 
 
14.9
 
 
 
 
B4GAT1
 
 
hsa-let-7e-5p
 
 
-0.47
 
 
14.9
 
 
 
 
B4GAT1
 
 
hsa-miR-98-5p
 
 
-0.47
 
 
14.9
 
 
 
 
BACE2
 
 
hsa-let-7e-5p
 
 
-0.364
 
 
16.3
 
 
 
 
BACE2
 
 
hsa-miR-98-5p
 
 
-0.364
 
 
16.3
 
 
 
 
 
 
 seminal vesicle 
 
 
 
 
target_symbol
 
 
mature_mirna_id
 
 
score
 
 
nTPM
 
 
 
 
 
 
ABCC5
 
 
hsa-miR-125a-5p
 
 
-0.288
 
 
12.9
 
 
 
 
ABCD3
 
 
hsa-miR-148a-3p
 
 
-0.388
 
 
25.5
 
 
 
 
ABHD6
 
 
hsa-miR-125a-5p
 
 
-0.501
 
 
13.5
 
 
 
 
ABTB1
 
 
hsa-miR-125a-5p
 
 
-0.519
 
 
12.8
 
 
 
 
ACSL6
 
 
hsa-miR-125a-5p
 
 
-0.307
 
 
17.0
 
 
 
 
ACSL6
 
 
hsa-let-7e-5p
 
 
-0.336
 
 
17.0
 
 
 
 
ACSL6
 
 
hsa-miR-98-5p
 
 
-0.336
 
 
17.0
 
 
 
 
ACSL6
 
 
hsa-let-7c-5p
 
 
-0.336
 
 
17.0
 
 
 
 
ACTC1
 
 
hsa-miR-7-5p
 
 
-0.471
 
 
506.6
 
 
 
 
ACVR1
 
 
hsa-miR-148a-3p
 
 
-0.397
 
 
11.3
 
 
 
 
ADAM10
 
 
hsa-miR-148a-3p
 
 
-0.356
 
 
11.5
 
 
 
 
ADAM9
 
 
hsa-miR-106a-5p
 
 
-0.317
 
 
32.8
 
 
 
 
ADAM9
 
 
hsa-miR-20b-5p
 
 
-0.328
 
 
32.8
 
 
 
 
AGFG1
 
 
hsa-miR-148a-3p
 
 
-0.332
 
 
22.0
 
 
 
 
AGFG2
 
 
hsa-miR-20b-5p
 
 
-0.386
 
 
12.7
 
 
 
 
AGFG2
 
 
hsa-miR-106a-5p
 
 
-0.399
 
 
12.7
 
 
 
 
AHNAK
 
 
hsa-miR-106a-5p
 
 
-0.334
 
 
99.6
 
 
 
 
AHNAK
 
 
hsa-miR-20b-5p
 
 
-0.337
 
 
99.6
 
 
 
 
AKAP1
 
 
hsa-miR-148a-3p
 
 
-0.381
 
 
23.3
 
 
 
 
ALCAM
 
 
hsa-miR-148a-3p
 
 
-0.339
 
 
37.3
 
 
 
 
ALDH4A1
 
 
hsa-miR-184
 
 
-0.421
 
 
25.6
 
 
 
 
ALG3
 
 
hsa-miR-106a-5p
 
 
-0.569
 
 
12.9
 
 
 
 
ALG3
 
 
hsa-miR-20b-5p
 
 
-0.58
 
 
12.9
 
 
 
 
ALG9
 
 
hsa-miR-7-5p
 
 
-0.302
 
 
10.6
 
 
 
 
ANAPC16
 
 
hsa-miR-125a-5p
 
 
-0.578
 
 
20.1
 
 
 
 
ANKRD12
 
 
hsa-miR-7-5p
 
 
-0.37
 
 
10.0
 
 
 
 
ANKRD46
 
 
hsa-let-7e-5p
 
 
-0.389
 
 
12.4
 
 
 
 
ANKRD46
 
 
hsa-miR-98-5p
 
 
-0.389
 
 
12.4
 
 
 
 
ANKRD46
 
 
hsa-let-7c-5p
 
 
-0.389
 
 
12.4
 
 
 
 
APCDD1
 
 
hsa-miR-106a-5p
 
 
-0.422
 
 
13.3
 
 
 
 
APCDD1
 
 
hsa-miR-20b-5p
 
 
-0.425
 
 
13.3
 
 
 
 
ARF4
 
 
hsa-miR-7-5p
 
 
-0.407
 
 
125.1
 
 
 
 
ARF4
 
 
hsa-miR-148a-3p
 
 
-0.487
 
 
125.1
 
 
 
 
ARFGEF1
 
 
hsa-miR-148a-3p
 
 
-0.306
 
 
11.4
 
 
 
 
ARFIP1
 
 
hsa-miR-148a-3p
 
 
-0.474
 
 
16.2
 
 
 
 
ARHGAP1
 
 
hsa-miR-20b-5p
 
 
-0.325
 
 
58.9
 
 
 
 
ARHGAP1
 
 
hsa-miR-106a-5p
 
 
-0.329
 
 
58.9
 
 
 
 
ARHGAP21
 
 
hsa-miR-148a-3p
 
 
-0.287
 
 
10.8
 
 
 
 
ARL1
 
 
hsa-miR-20b-5p
 
 
-0.424
 
 
65.0
 
 
 
 
ARL1
 
 
hsa-miR-106a-5p
 
 
-0.429
 
 
65.0
 
 
 
 
ARL4D
 
 
hsa-let-7e-5p
 
 
-0.417
 
 
14.3
 
 
 
 
ARL4D
 
 
hsa-miR-98-5p
 
 
-0.417
 
 
14.3
 
 
 
 
ARL4D
 
 
hsa-let-7c-5p
 
 
-0.417
 
 
14.3
 
 
 
 
ARL6IP1
 
 
hsa-miR-7-5p
 
 
-0.374
 
 
66.4
 
 
 
 
ARL6IP1
 
 
hsa-miR-148a-3p
 
 
-0.579
 
 
66.4
 
 
 
 
ARL8B
 
 
hsa-miR-148a-3p
 
 
-0.337
 
 
39.4
 
 
 
 
ARMC8
 
 
hsa-miR-106a-5p
 
 
-0.375
 
 
16.5
 
 
 
 
ARMC8
 
 
hsa-miR-20b-5p
 
 
-0.378
 
 
16.5
 
 
 
 
ARMCX1
 
 
hsa-miR-21-5p
 
 
-0.489
 
 
21.7
 
 
 
 
ARMT1
 
 
hsa-let-7c-5p
 
 
-0.303
 
 
10.5
 
 
 
 
ARMT1
 
 
hsa-let-7e-5p
 
 
-0.303
 
 
10.5
 
 
 
 
ARMT1
 
 
hsa-miR-98-5p
 
 
-0.303
 
 
10.5
 
 
 
 
ARPP19
 
 
hsa-let-7e-5p
 
 
-0.492
 
 
38.5
 
 
 
 
ARPP19
 
 
hsa-let-7c-5p
 
 
-0.492
 
 
38.5
 
 
 
 
ARPP19
 
 
hsa-miR-98-5p
 
 
-0.5
 
 
38.5
 
 
 
 
ARRDC3
 
 
hsa-miR-148a-3p
 
 
-0.305
 
 
22.9
 
 
 
 
ARRDC4
 
 
hsa-let-7c-5p
 
 
-0.329
 
 
15.4
 
 
 
 
ARRDC4
 
 
hsa-let-7e-5p
 
 
-0.329
 
 
15.4
 
 
 
 
ARRDC4
 
 
hsa-miR-98-5p
 
 
-0.329
 
 
15.4
 
 
 
 
ASAP1
 
 
hsa-let-7e-5p
 
 
-0.296
 
 
10.7
 
 
 
 
ASAP1
 
 
hsa-miR-98-5p
 
 
-0.308
 
 
10.7
 
 
 
 
ASAP1
 
 
hsa-let-7c-5p
 
 
-0.308
 
 
10.7
 
 
 
 
ASF1A
 
 
hsa-miR-21-5p
 
 
-0.304
 
 
10.4
 
 
 
 
ASPH
 
 
hsa-miR-148a-3p
 
 
-0.391
 
 
65.8
 
 
 
 
ATP2A2
 
 
hsa-let-7e-5p
 
 
-0.311
 
 
66.0
 
 
 
 
ATP2A2
 
 
hsa-miR-98-5p
 
 
-0.311
 
 
66.0
 
 
 
 
ATP2A2
 
 
hsa-let-7c-5p
 
 
-0.311
 
 
66.0
 
 
 
 
ATP2B4
 
 
hsa-miR-148a-3p
 
 
-0.337
 
 
66.2
 
 
 
 
ATP6AP2
 
 
hsa-miR-148a-3p
 
 
-0.592
 
 
92.2
 
 
 
 
ATP6AP2
 
 
hsa-miR-148a-3p
 
 
-0.668
 
 
92.2
 
 
 
 
ATPAF1
 
 
hsa-let-7e-5p
 
 
-0.498
 
 
31.8
 
 
 
 
ATPAF1
 
 
hsa-miR-98-5p
 
 
-0.498
 
 
31.8
 
 
 
 
ATPAF1
 
 
hsa-let-7c-5p
 
 
-0.498
 
 
31.8
 
 
 
 
AVPI1
 
 
hsa-miR-7-5p
 
 
-0.367
 
 
15.6
 
 
 
 
B3GAT3
 
 
hsa-let-7e-5p
 
 
-0.324
 
 
17.2
 
 
 
 
B3GAT3
 
 
hsa-miR-98-5p
 
 
-0.324
 
 
17.2
 
 
 
 
B3GAT3
 
 
hsa-let-7c-5p
 
 
-0.324
 
 
17.2
 
 
 
 
B4GALT5
 
 
hsa-miR-148a-3p
 
 
-0.44
 
 
20.7
 
 
 
 
B4GAT1
 
 
hsa-let-7c-5p
 
 
-0.47
 
 
21.2
 
 
 
 
B4GAT1
 
 
hsa-let-7e-5p
 
 
-0.47
 
 
21.2
 
 
 
 
B4GAT1
 
 
hsa-miR-98-5p
 
 
-0.47
 
 
21.2
 
 
 
 
BAMBI
 
 
hsa-miR-106a-5p
 
 
-0.503
 
 
15.7
 
 
 
 
BAMBI
 
 
hsa-miR-20b-5p
 
 
-0.506
 
 
15.7
 
 
 
 
BAZ2A
 
 
hsa-miR-99b-5p
 
 
-0.408
 
 
19.8
 
 
 
 
BCAP29
 
 
hsa-let-7e-5p
 
 
-0.289
 
 
24.0
 
 
 
 
BHLHE41
 
 
hsa-miR-148a-3p
 
 
-0.442
 
 
13.2
 
 
 
 
BLOC1S6
 
 
hsa-let-7e-5p
 
 
-0.321
 
 
19.2
 
 
 
 
BLOC1S6
 
 
hsa-miR-98-5p
 
 
-0.321
 
 
19.2
 
 
 
 
BLOC1S6
 
 
hsa-let-7c-5p
 
 
-0.321
 
 
19.2
 
 
 
 
BLZF1
 
 
hsa-miR-125a-5p
 
 
-0.461
 
 
15.1
 
 
 
 
BMPR2
 
 
hsa-miR-99b-5p
 
 
-0.327
 
 
10.5
 
 
 
 
BTAF1
 
 
hsa-miR-148a-3p
 
 
-0.401
 
 
10.1
 
 
 
 
BTBD10
 
 
hsa-miR-106a-5p
 
 
-0.406
 
 
16.4
 
 
 
 
BTBD10
 
 
hsa-miR-20b-5p
 
 
-0.425
 
 
16.4
 
 
 
 
BTBD3
 
 
hsa-miR-148a-3p
 
 
-0.495
 
 
10.0
 
 
 
 
BTF3L4
 
 
hsa-let-7c-5p
 
 
-0.355
 
 
23.0
 
 
 
 
BTF3L4
 
 
hsa-let-7e-5p
 
 
-0.355
 
 
23.0
 
 
 
 
BTF3L4
 
 
hsa-miR-98-5p
 
 
-0.355
 
 
23.0
 
 
 
 
BTG3
 
 
hsa-miR-106a-5p
 
 
-0.665
 
 
19.3
 
 
 
 
BTG3
 
 
hsa-miR-20b-5p
 
 
-0.684
 
 
19.3
 
 
 
 
 
 
 smooth muscle 
 
 
 
 
target_symbol
 
 
mature_mirna_id
 
 
score
 
 
nTPM
 
 
 
 
 
 
ABCC5
 
 
hsa-miR-125a-5p
 
 
-0.288
 
 
15.0
 
 
 
 
ABCD3
 
 
hsa-miR-148a-3p
 
 
-0.388
 
 
16.6
 
 
 
 
ACVR1
 
 
hsa-miR-148a-3p
 
 
-0.397
 
 
26.8
 
 
 
 
ADAM10
 
 
hsa-miR-148a-3p
 
 
-0.356
 
 
12.4
 
 
 
 
ADAM9
 
 
hsa-miR-106a-5p
 
 
-0.317
 
 
48.2
 
 
 
 
ADAM9
 
 
hsa-miR-20b-5p
 
 
-0.328
 
 
48.2
 
 
 
 
ADGRF5
 
 
hsa-miR-148a-3p
 
 
-0.354
 
 
20.6
 
 
 
 
AGFG1
 
 
hsa-miR-148a-3p
 
 
-0.332
 
 
36.7
 
 
 
 
AHNAK
 
 
hsa-miR-106a-5p
 
 
-0.334
 
 
105.6
 
 
 
 
AHNAK
 
 
hsa-miR-20b-5p
 
 
-0.337
 
 
105.6
 
 
 
 
AKAP1
 
 
hsa-miR-148a-3p
 
 
-0.381
 
 
24.6
 
 
 
 
ALCAM
 
 
hsa-miR-148a-3p
 
 
-0.339
 
 
15.2
 
 
 
 
ALG3
 
 
hsa-miR-106a-5p
 
 
-0.569
 
 
15.3
 
 
 
 
ALG3
 
 
hsa-miR-20b-5p
 
 
-0.58
 
 
15.3
 
 
 
 
ALG9
 
 
hsa-miR-7-5p
 
 
-0.302
 
 
10.4
 
 
 
 
ANAPC16
 
 
hsa-miR-125a-5p
 
 
-0.578
 
 
16.4
 
 
 
 
ANKRD29
 
 
hsa-miR-106a-5p
 
 
-0.369
 
 
10.9
 
 
 
 
ANKRD29
 
 
hsa-miR-20b-5p
 
 
-0.38
 
 
10.9
 
 
 
 
ANKRD46
 
 
hsa-let-7e-5p
 
 
-0.389
 
 
11.6
 
 
 
 
ANKRD46
 
 
hsa-miR-98-5p
 
 
-0.389
 
 
11.6
 
 
 
 
ANKRD46
 
 
hsa-let-7c-5p
 
 
-0.389
 
 
11.6
 
 
 
 
ANPEP
 
 
hsa-miR-125a-5p
 
 
-0.372
 
 
27.8
 
 
 
 
AP1S1
 
 
hsa-let-7e-5p
 
 
-0.374
 
 
11.9
 
 
 
 
AP1S1
 
 
hsa-miR-98-5p
 
 
-0.374
 
 
11.9
 
 
 
 
AP1S1
 
 
hsa-let-7c-5p
 
 
-0.374
 
 
11.9
 
 
 
 
ARF4
 
 
hsa-miR-7-5p
 
 
-0.407
 
 
157.9
 
 
 
 
ARF4
 
 
hsa-miR-148a-3p
 
 
-0.487
 
 
157.9
 
 
 
 
ARFGEF1
 
 
hsa-miR-148a-3p
 
 
-0.306
 
 
10.1
 
 
 
 
ARFIP1
 
 
hsa-miR-148a-3p
 
 
-0.474
 
 
18.5
 
 
 
 
ARHGAP1
 
 
hsa-miR-20b-5p
 
 
-0.325
 
 
75.2
 
 
 
 
ARHGAP1
 
 
hsa-miR-106a-5p
 
 
-0.329
 
 
75.2
 
 
 
 
ARHGAP21
 
 
hsa-miR-148a-3p
 
 
-0.287
 
 
16.5
 
 
 
 
ARHGAP24
 
 
hsa-miR-21-5p
 
 
-0.427
 
 
13.1
 
 
 
 
ARL1
 
 
hsa-miR-20b-5p
 
 
-0.424
 
 
55.7
 
 
 
 
ARL1
 
 
hsa-miR-106a-5p
 
 
-0.429
 
 
55.7
 
 
 
 
ARL4D
 
 
hsa-let-7e-5p
 
 
-0.417
 
 
13.8
 
 
 
 
ARL4D
 
 
hsa-miR-98-5p
 
 
-0.417
 
 
13.8
 
 
 
 
ARL4D
 
 
hsa-let-7c-5p
 
 
-0.417
 
 
13.8
 
 
 
 
ARL6IP1
 
 
hsa-miR-7-5p
 
 
-0.374
 
 
64.5
 
 
 
 
ARL6IP1
 
 
hsa-miR-148a-3p
 
 
-0.579
 
 
64.5
 
 
 
 
ARL6IP6
 
 
hsa-miR-98-5p
 
 
-0.293
 
 
10.8
 
 
 
 
ARL6IP6
 
 
hsa-let-7c-5p
 
 
-0.293
 
 
10.8
 
 
 
 
ARL6IP6
 
 
hsa-let-7e-5p
 
 
-0.305
 
 
10.8
 
 
 
 
ARL8B
 
 
hsa-miR-148a-3p
 
 
-0.337
 
 
43.9
 
 
 
 
ARMC8
 
 
hsa-miR-106a-5p
 
 
-0.375
 
 
22.8
 
 
 
 
ARMC8
 
 
hsa-miR-20b-5p
 
 
-0.378
 
 
22.8
 
 
 
 
ARMCX1
 
 
hsa-miR-21-5p
 
 
-0.489
 
 
19.8
 
 
 
 
ARMT1
 
 
hsa-let-7c-5p
 
 
-0.303
 
 
13.9
 
 
 
 
ARMT1
 
 
hsa-let-7e-5p
 
 
-0.303
 
 
13.9
 
 
 
 
ARMT1
 
 
hsa-miR-98-5p
 
 
-0.303
 
 
13.9
 
 
 
 
ARPP19
 
 
hsa-let-7e-5p
 
 
-0.492
 
 
54.2
 
 
 
 
ARPP19
 
 
hsa-let-7c-5p
 
 
-0.492
 
 
54.2
 
 
 
 
ARPP19
 
 
hsa-miR-98-5p
 
 
-0.5
 
 
54.2
 
 
 
 
ARRDC3
 
 
hsa-miR-148a-3p
 
 
-0.305
 
 
30.8
 
 
 
 
ARRDC4
 
 
hsa-let-7c-5p
 
 
-0.329
 
 
16.9
 
 
 
 
ARRDC4
 
 
hsa-let-7e-5p
 
 
-0.329
 
 
16.9
 
 
 
 
ARRDC4
 
 
hsa-miR-98-5p
 
 
-0.329
 
 
16.9
 
 
 
 
ASAP1
 
 
hsa-let-7e-5p
 
 
-0.296
 
 
16.7
 
 
 
 
ASAP1
 
 
hsa-miR-98-5p
 
 
-0.308
 
 
16.7
 
 
 
 
ASAP1
 
 
hsa-let-7c-5p
 
 
-0.308
 
 
16.7
 
 
 
 
ASPH
 
 
hsa-miR-148a-3p
 
 
-0.391
 
 
62.4
 
 
 
 
ATOSB
 
 
hsa-let-7e-5p
 
 
-0.429
 
 
10.3
 
 
 
 
ATOSB
 
 
hsa-miR-98-5p
 
 
-0.429
 
 
10.3
 
 
 
 
ATOSB
 
 
hsa-let-7c-5p
 
 
-0.429
 
 
10.3
 
 
 
 
ATP2A2
 
 
hsa-let-7e-5p
 
 
-0.311
 
 
69.4
 
 
 
 
ATP2A2
 
 
hsa-miR-98-5p
 
 
-0.311
 
 
69.4
 
 
 
 
ATP2A2
 
 
hsa-let-7c-5p
 
 
-0.311
 
 
69.4
 
 
 
 
ATP2B4
 
 
hsa-miR-148a-3p
 
 
-0.337
 
 
164.7
 
 
 
 
ATP6AP2
 
 
hsa-miR-148a-3p
 
 
-0.592
 
 
116.5
 
 
 
 
ATP6AP2
 
 
hsa-miR-148a-3p
 
 
-0.668
 
 
116.5
 
 
 
 
ATPAF1
 
 
hsa-let-7e-5p
 
 
-0.498
 
 
30.4
 
 
 
 
ATPAF1
 
 
hsa-miR-98-5p
 
 
-0.498
 
 
30.4
 
 
 
 
ATPAF1
 
 
hsa-let-7c-5p
 
 
-0.498
 
 
30.4
 
 
 
 
B3GAT3
 
 
hsa-let-7e-5p
 
 
-0.324
 
 
13.1
 
 
 
 
B3GAT3
 
 
hsa-miR-98-5p
 
 
-0.324
 
 
13.1
 
 
 
 
B3GAT3
 
 
hsa-let-7c-5p
 
 
-0.324
 
 
13.1
 
 
 
 
B4GALT5
 
 
hsa-miR-148a-3p
 
 
-0.44
 
 
35.1
 
 
 
 
B4GAT1
 
 
hsa-let-7c-5p
 
 
-0.47
 
 
13.1
 
 
 
 
B4GAT1
 
 
hsa-let-7e-5p
 
 
-0.47
 
 
13.1
 
 
 
 
B4GAT1
 
 
hsa-miR-98-5p
 
 
-0.47
 
 
13.1
 
 
 
 
BACH1
 
 
hsa-let-7e-5p
 
 
-0.298
 
 
14.9
 
 
 
 
BACH1
 
 
hsa-let-7e-5p
 
 
-0.298
 
 
14.9
 
 
 
 
BACH1
 
 
hsa-miR-98-5p
 
 
-0.298
 
 
14.9
 
 
 
 
BACH1
 
 
hsa-miR-98-5p
 
 
-0.298
 
 
14.9
 
 
 
 
BACH1
 
 
hsa-let-7c-5p
 
 
-0.306
 
 
14.9
 
 
 
 
BACH1
 
 
hsa-let-7c-5p
 
 
-0.306
 
 
14.9
 
 
 
 
BACH1
 
 
hsa-let-7e-5p
 
 
-0.435
 
 
14.9
 
 
 
 
BACH1
 
 
hsa-let-7e-5p
 
 
-0.435
 
 
14.9
 
 
 
 
BACH1
 
 
hsa-miR-98-5p
 
 
-0.435
 
 
14.9
 
 
 
 
BACH1
 
 
hsa-miR-98-5p
 
 
-0.435
 
 
14.9
 
 
 
 
BACH1
 
 
hsa-let-7c-5p
 
 
-0.435
 
 
14.9
 
 
 
 
BACH1
 
 
hsa-let-7c-5p
 
 
-0.435
 
 
14.9
 
 
 
 
BAK1
 
 
hsa-miR-125a-5p
 
 
-0.402
 
 
12.6
 
 
 
 
BAMBI
 
 
hsa-miR-106a-5p
 
 
-0.503
 
 
11.4
 
 
 
 
BAMBI
 
 
hsa-miR-20b-5p
 
 
-0.506
 
 
11.4
 
 
 
 
BAZ2A
 
 
hsa-miR-99b-5p
 
 
-0.408
 
 
21.6
 
 
 
 
BCAP29
 
 
hsa-let-7e-5p
 
 
-0.289
 
 
36.5
 
 
 
 
BLOC1S6
 
 
hsa-let-7e-5p
 
 
-0.321
 
 
22.7
 
 
 
 
BLOC1S6
 
 
hsa-miR-98-5p
 
 
-0.321
 
 
22.7
 
 
 
 
BLOC1S6
 
 
hsa-let-7c-5p
 
 
-0.321
 
 
22.7
 
 
 
 
 
 
 spinal cord 
 
 
 
 
target_symbol
 
 
mature_mirna_id
 
 
score
 
 
nTPM
 
 
 
 
 
 
ABCC5
 
 
hsa-miR-125a-5p
 
 
-0.288
 
 
25.1
 
 
 
 
ABCD3
 
 
hsa-miR-148a-3p
 
 
-0.388
 
 
20.9
 
 
 
 
ABHD5
 
 
hsa-miR-20b-5p
 
 
-0.418
 
 
19.5
 
 
 
 
ABHD5
 
 
hsa-miR-106a-5p
 
 
-0.447
 
 
19.5
 
 
 
 
ABHD6
 
 
hsa-miR-125a-5p
 
 
-0.501
 
 
36.2
 
 
 
 
ABTB1
 
 
hsa-miR-125a-5p
 
 
-0.519
 
 
20.4
 
 
 
 
ACHE
 
 
hsa-miR-125a-5p
 
 
-0.308
 
 
14.4
 
 
 
 
ACHE
 
 
hsa-miR-125a-5p
 
 
-0.361
 
 
14.4
 
 
 
 
ADAM10
 
 
hsa-miR-148a-3p
 
 
-0.356
 
 
18.7
 
 
 
 
ADAM9
 
 
hsa-miR-106a-5p
 
 
-0.317
 
 
11.3
 
 
 
 
ADAM9
 
 
hsa-miR-20b-5p
 
 
-0.328
 
 
11.3
 
 
 
 
ADCY2
 
 
hsa-miR-148a-3p
 
 
-0.299
 
 
11.4
 
 
 
 
AGFG1
 
 
hsa-miR-148a-3p
 
 
-0.332
 
 
53.5
 
 
 
 
AHNAK
 
 
hsa-miR-106a-5p
 
 
-0.334
 
 
27.2
 
 
 
 
AHNAK
 
 
hsa-miR-20b-5p
 
 
-0.337
 
 
27.2
 
 
 
 
AIFM1
 
 
hsa-miR-125a-5p
 
 
-0.3
 
 
10.1
 
 
 
 
AIFM1
 
 
hsa-let-7e-5p
 
 
-0.314
 
 
10.1
 
 
 
 
AIFM1
 
 
hsa-miR-98-5p
 
 
-0.314
 
 
10.1
 
 
 
 
AIFM1
 
 
hsa-let-7c-5p
 
 
-0.314
 
 
10.1
 
 
 
 
AKAP1
 
 
hsa-miR-148a-3p
 
 
-0.381
 
 
14.0
 
 
 
 
AKAP6
 
 
hsa-let-7e-5p
 
 
-0.307
 
 
13.3
 
 
 
 
AKAP6
 
 
hsa-miR-98-5p
 
 
-0.307
 
 
13.3
 
 
 
 
AKAP6
 
 
hsa-let-7c-5p
 
 
-0.307
 
 
13.3
 
 
 
 
ALCAM
 
 
hsa-miR-148a-3p
 
 
-0.339
 
 
56.1
 
 
 
 
ALDH4A1
 
 
hsa-miR-184
 
 
-0.421
 
 
11.4
 
 
 
 
ALG3
 
 
hsa-miR-106a-5p
 
 
-0.569
 
 
10.8
 
 
 
 
ALG3
 
 
hsa-miR-20b-5p
 
 
-0.58
 
 
10.8
 
 
 
 
ANKRD46
 
 
hsa-let-7e-5p
 
 
-0.389
 
 
24.6
 
 
 
 
ANKRD46
 
 
hsa-miR-98-5p
 
 
-0.389
 
 
24.6
 
 
 
 
ANKRD46
 
 
hsa-let-7c-5p
 
 
-0.389
 
 
24.6
 
 
 
 
AP1S1
 
 
hsa-let-7e-5p
 
 
-0.374
 
 
28.5
 
 
 
 
AP1S1
 
 
hsa-miR-98-5p
 
 
-0.374
 
 
28.5
 
 
 
 
AP1S1
 
 
hsa-let-7c-5p
 
 
-0.374
 
 
28.5
 
 
 
 
APCDD1
 
 
hsa-miR-106a-5p
 
 
-0.422
 
 
14.7
 
 
 
 
APCDD1
 
 
hsa-miR-20b-5p
 
 
-0.425
 
 
14.7
 
 
 
 
ARF4
 
 
hsa-miR-7-5p
 
 
-0.407
 
 
69.7
 
 
 
 
ARF4
 
 
hsa-miR-148a-3p
 
 
-0.487
 
 
69.7
 
 
 
 
ARFIP1
 
 
hsa-miR-148a-3p
 
 
-0.474
 
 
10.6
 
 
 
 
ARHGAP1
 
 
hsa-miR-20b-5p
 
 
-0.325
 
 
38.6
 
 
 
 
ARHGAP1
 
 
hsa-miR-106a-5p
 
 
-0.329
 
 
38.6
 
 
 
 
ARHGAP21
 
 
hsa-miR-148a-3p
 
 
-0.287
 
 
74.7
 
 
 
 
ARL1
 
 
hsa-miR-20b-5p
 
 
-0.424
 
 
26.5
 
 
 
 
ARL1
 
 
hsa-miR-106a-5p
 
 
-0.429
 
 
26.5
 
 
 
 
ARL6IP1
 
 
hsa-miR-7-5p
 
 
-0.374
 
 
140.2
 
 
 
 
ARL6IP1
 
 
hsa-miR-148a-3p
 
 
-0.579
 
 
140.2
 
 
 
 
ARL8B
 
 
hsa-miR-148a-3p
 
 
-0.337
 
 
57.8
 
 
 
 
ARMC8
 
 
hsa-miR-106a-5p
 
 
-0.375
 
 
15.5
 
 
 
 
ARMC8
 
 
hsa-miR-20b-5p
 
 
-0.378
 
 
15.5
 
 
 
 
ARMCX1
 
 
hsa-miR-21-5p
 
 
-0.489
 
 
12.2
 
 
 
 
ARPP19
 
 
hsa-let-7e-5p
 
 
-0.492
 
 
170.8
 
 
 
 
ARPP19
 
 
hsa-let-7c-5p
 
 
-0.492
 
 
170.8
 
 
 
 
ARPP19
 
 
hsa-miR-98-5p
 
 
-0.5
 
 
170.8
 
 
 
 
ARRDC3
 
 
hsa-miR-148a-3p
 
 
-0.305
 
 
23.5
 
 
 
 
ARRDC4
 
 
hsa-let-7c-5p
 
 
-0.329
 
 
75.7
 
 
 
 
ARRDC4
 
 
hsa-let-7e-5p
 
 
-0.329
 
 
75.7
 
 
 
 
ARRDC4
 
 
hsa-miR-98-5p
 
 
-0.329
 
 
75.7
 
 
 
 
ASAP1
 
 
hsa-let-7e-5p
 
 
-0.296
 
 
11.0
 
 
 
 
ASAP1
 
 
hsa-miR-98-5p
 
 
-0.308
 
 
11.0
 
 
 
 
ASAP1
 
 
hsa-let-7c-5p
 
 
-0.308
 
 
11.0
 
 
 
 
ASF1A
 
 
hsa-miR-21-5p
 
 
-0.304
 
 
14.8
 
 
 
 
ASPH
 
 
hsa-miR-148a-3p
 
 
-0.391
 
 
25.3
 
 
 
 
ATG16L1
 
 
hsa-miR-106a-5p
 
 
-0.464
 
 
10.3
 
 
 
 
ATG16L1
 
 
hsa-miR-20b-5p
 
 
-0.467
 
 
10.3
 
 
 
 
ATOSB
 
 
hsa-let-7e-5p
 
 
-0.429
 
 
30.9
 
 
 
 
ATOSB
 
 
hsa-miR-98-5p
 
 
-0.429
 
 
30.9
 
 
 
 
ATOSB
 
 
hsa-let-7c-5p
 
 
-0.429
 
 
30.9
 
 
 
 
ATP2A2
 
 
hsa-let-7e-5p
 
 
-0.311
 
 
19.0
 
 
 
 
ATP2A2
 
 
hsa-miR-98-5p
 
 
-0.311
 
 
19.0
 
 
 
 
ATP2A2
 
 
hsa-let-7c-5p
 
 
-0.311
 
 
19.0
 
 
 
 
ATP6AP2
 
 
hsa-miR-148a-3p
 
 
-0.592
 
 
55.3
 
 
 
 
ATP6AP2
 
 
hsa-miR-148a-3p
 
 
-0.668
 
 
55.3
 
 
 
 
ATPAF1
 
 
hsa-let-7e-5p
 
 
-0.498
 
 
50.4
 
 
 
 
ATPAF1
 
 
hsa-miR-98-5p
 
 
-0.498
 
 
50.4
 
 
 
 
ATPAF1
 
 
hsa-let-7c-5p
 
 
-0.498
 
 
50.4
 
 
 
 
AVPI1
 
 
hsa-miR-7-5p
 
 
-0.367
 
 
33.9
 
 
 
 
B3GAT3
 
 
hsa-let-7e-5p
 
 
-0.324
 
 
62.6
 
 
 
 
B3GAT3
 
 
hsa-miR-98-5p
 
 
-0.324
 
 
62.6
 
 
 
 
B3GAT3
 
 
hsa-let-7c-5p
 
 
-0.324
 
 
62.6
 
 
 
 
B4GAT1
 
 
hsa-let-7c-5p
 
 
-0.47
 
 
48.7
 
 
 
 
B4GAT1
 
 
hsa-let-7e-5p
 
 
-0.47
 
 
48.7
 
 
 
 
B4GAT1
 
 
hsa-miR-98-5p
 
 
-0.47
 
 
48.7
 
 
 
 
BAMBI
 
 
hsa-miR-106a-5p
 
 
-0.503
 
 
23.0
 
 
 
 
BAMBI
 
 
hsa-miR-20b-5p
 
 
-0.506
 
 
23.0
 
 
 
 
BAZ2A
 
 
hsa-miR-99b-5p
 
 
-0.408
 
 
10.5
 
 
 
 
BCAP29
 
 
hsa-let-7e-5p
 
 
-0.289
 
 
26.8
 
 
 
 
BDH1
 
 
hsa-miR-125a-5p
 
 
-0.389
 
 
11.2
 
 
 
 
BHLHE41
 
 
hsa-miR-148a-3p
 
 
-0.442
 
 
74.7
 
 
 
 
BLOC1S6
 
 
hsa-let-7e-5p
 
 
-0.321
 
 
15.5
 
 
 
 
BLOC1S6
 
 
hsa-miR-98-5p
 
 
-0.321
 
 
15.5
 
 
 
 
BLOC1S6
 
 
hsa-let-7c-5p
 
 
-0.321
 
 
15.5
 
 
 
 
BLZF1
 
 
hsa-miR-125a-5p
 
 
-0.461
 
 
10.1
 
 
 
 
BTBD10
 
 
hsa-miR-106a-5p
 
 
-0.406
 
 
15.3
 
 
 
 
BTBD10
 
 
hsa-miR-20b-5p
 
 
-0.425
 
 
15.3
 
 
 
 
BTBD3
 
 
hsa-miR-148a-3p
 
 
-0.495
 
 
32.8
 
 
 
 
BTF3L4
 
 
hsa-let-7c-5p
 
 
-0.355
 
 
36.6
 
 
 
 
BTF3L4
 
 
hsa-let-7e-5p
 
 
-0.355
 
 
36.6
 
 
 
 
BTF3L4
 
 
hsa-miR-98-5p
 
 
-0.355
 
 
36.6
 
 
 
 
BTG3
 
 
hsa-miR-106a-5p
 
 
-0.665
 
 
27.3
 
 
 
 
BTG3
 
 
hsa-miR-20b-5p
 
 
-0.684
 
 
27.3
 
 
 
 
BZW1
 
 
hsa-let-7e-5p
 
 
-0.354
 
 
46.3
 
 
 
 
 
 
 thyroid gland 
 
 
 
 
target_symbol
 
 
mature_mirna_id
 
 
score
 
 
nTPM
 
 
 
 
 
 
ABCC5
 
 
hsa-miR-125a-5p
 
 
-0.288
 
 
13.7
 
 
 
 
ABCD3
 
 
hsa-miR-148a-3p
 
 
-0.388
 
 
25.8
 
 
 
 
ABHD3
 
 
hsa-miR-125a-5p
 
 
-0.453
 
 
21.4
 
 
 
 
ABHD5
 
 
hsa-miR-20b-5p
 
 
-0.418
 
 
14.4
 
 
 
 
ABHD5
 
 
hsa-miR-106a-5p
 
 
-0.447
 
 
14.4
 
 
 
 
ABTB1
 
 
hsa-miR-125a-5p
 
 
-0.519
 
 
23.0
 
 
 
 
ACVR1
 
 
hsa-miR-148a-3p
 
 
-0.397
 
 
28.6
 
 
 
 
ADAM10
 
 
hsa-miR-148a-3p
 
 
-0.356
 
 
26.7
 
 
 
 
ADAM9
 
 
hsa-miR-106a-5p
 
 
-0.317
 
 
37.4
 
 
 
 
ADAM9
 
 
hsa-miR-20b-5p
 
 
-0.328
 
 
37.4
 
 
 
 
ADGRF5
 
 
hsa-miR-148a-3p
 
 
-0.354
 
 
30.7
 
 
 
 
AGFG1
 
 
hsa-miR-148a-3p
 
 
-0.332
 
 
32.1
 
 
 
 
AGFG2
 
 
hsa-miR-20b-5p
 
 
-0.386
 
 
10.2
 
 
 
 
AGFG2
 
 
hsa-miR-106a-5p
 
 
-0.399
 
 
10.2
 
 
 
 
AGGF1
 
 
hsa-miR-125a-5p
 
 
-0.331
 
 
10.3
 
 
 
 
AHCTF1
 
 
hsa-let-7e-5p
 
 
-0.374
 
 
12.9
 
 
 
 
AHCTF1
 
 
hsa-miR-98-5p
 
 
-0.374
 
 
12.9
 
 
 
 
AHCTF1
 
 
hsa-let-7c-5p
 
 
-0.374
 
 
12.9
 
 
 
 
AHDC1
 
 
hsa-miR-148a-3p
 
 
-0.413
 
 
19.9
 
 
 
 
AHNAK
 
 
hsa-miR-106a-5p
 
 
-0.334
 
 
61.4
 
 
 
 
AHNAK
 
 
hsa-miR-20b-5p
 
 
-0.337
 
 
61.4
 
 
 
 
AIFM1
 
 
hsa-miR-125a-5p
 
 
-0.3
 
 
10.7
 
 
 
 
AIFM1
 
 
hsa-let-7e-5p
 
 
-0.314
 
 
10.7
 
 
 
 
AIFM1
 
 
hsa-miR-98-5p
 
 
-0.314
 
 
10.7
 
 
 
 
AIFM1
 
 
hsa-let-7c-5p
 
 
-0.314
 
 
10.7
 
 
 
 
AKAP1
 
 
hsa-miR-148a-3p
 
 
-0.381
 
 
35.0
 
 
 
 
ALCAM
 
 
hsa-miR-148a-3p
 
 
-0.339
 
 
58.4
 
 
 
 
ALG3
 
 
hsa-miR-106a-5p
 
 
-0.569
 
 
17.5
 
 
 
 
ALG3
 
 
hsa-miR-20b-5p
 
 
-0.58
 
 
17.5
 
 
 
 
ALG9
 
 
hsa-miR-7-5p
 
 
-0.302
 
 
11.5
 
 
 
 
ANAPC16
 
 
hsa-miR-125a-5p
 
 
-0.578
 
 
21.8
 
 
 
 
ANKRD12
 
 
hsa-miR-7-5p
 
 
-0.37
 
 
15.3
 
 
 
 
ANKRD46
 
 
hsa-let-7e-5p
 
 
-0.389
 
 
18.8
 
 
 
 
ANKRD46
 
 
hsa-miR-98-5p
 
 
-0.389
 
 
18.8
 
 
 
 
ANKRD46
 
 
hsa-let-7c-5p
 
 
-0.389
 
 
18.8
 
 
 
 
ANKRD9
 
 
hsa-miR-20b-5p
 
 
-0.331
 
 
10.7
 
 
 
 
ANKRD9
 
 
hsa-miR-106a-5p
 
 
-0.344
 
 
10.7
 
 
 
 
AP1AR
 
 
hsa-miR-21-5p
 
 
-0.318
 
 
10.1
 
 
 
 
AP1AR
 
 
hsa-miR-99b-5p
 
 
-0.823
 
 
10.1
 
 
 
 
AP1S1
 
 
hsa-let-7e-5p
 
 
-0.374
 
 
15.7
 
 
 
 
AP1S1
 
 
hsa-miR-98-5p
 
 
-0.374
 
 
15.7
 
 
 
 
AP1S1
 
 
hsa-let-7c-5p
 
 
-0.374
 
 
15.7
 
 
 
 
ARF4
 
 
hsa-miR-7-5p
 
 
-0.407
 
 
134.8
 
 
 
 
ARF4
 
 
hsa-miR-148a-3p
 
 
-0.487
 
 
134.8
 
 
 
 
ARFGEF1
 
 
hsa-miR-148a-3p
 
 
-0.306
 
 
20.9
 
 
 
 
ARFIP1
 
 
hsa-miR-148a-3p
 
 
-0.474
 
 
18.8
 
 
 
 
ARG2
 
 
hsa-let-7e-5p
 
 
-0.468
 
 
97.9
 
 
 
 
ARG2
 
 
hsa-miR-98-5p
 
 
-0.468
 
 
97.9
 
 
 
 
ARG2
 
 
hsa-let-7c-5p
 
 
-0.468
 
 
97.9
 
 
 
 
ARHGAP1
 
 
hsa-miR-20b-5p
 
 
-0.325
 
 
42.2
 
 
 
 
ARHGAP1
 
 
hsa-miR-106a-5p
 
 
-0.329
 
 
42.2
 
 
 
 
ARHGAP21
 
 
hsa-miR-148a-3p
 
 
-0.287
 
 
12.0
 
 
 
 
ARHGAP24
 
 
hsa-miR-21-5p
 
 
-0.427
 
 
26.1
 
 
 
 
ARHGEF28
 
 
hsa-miR-20b-5p
 
 
-0.352
 
 
12.7
 
 
 
 
ARHGEF28
 
 
hsa-miR-106a-5p
 
 
-0.367
 
 
12.7
 
 
 
 
ARL1
 
 
hsa-miR-20b-5p
 
 
-0.424
 
 
79.3
 
 
 
 
ARL1
 
 
hsa-miR-106a-5p
 
 
-0.429
 
 
79.3
 
 
 
 
ARL4D
 
 
hsa-let-7e-5p
 
 
-0.417
 
 
35.9
 
 
 
 
ARL4D
 
 
hsa-miR-98-5p
 
 
-0.417
 
 
35.9
 
 
 
 
ARL4D
 
 
hsa-let-7c-5p
 
 
-0.417
 
 
35.9
 
 
 
 
ARL6IP1
 
 
hsa-miR-7-5p
 
 
-0.374
 
 
105.4
 
 
 
 
ARL6IP1
 
 
hsa-miR-148a-3p
 
 
-0.579
 
 
105.4
 
 
 
 
ARL8B
 
 
hsa-miR-148a-3p
 
 
-0.337
 
 
58.1
 
 
 
 
ARMC8
 
 
hsa-miR-106a-5p
 
 
-0.375
 
 
20.0
 
 
 
 
ARMC8
 
 
hsa-miR-20b-5p
 
 
-0.378
 
 
20.0
 
 
 
 
ARMCX1
 
 
hsa-miR-21-5p
 
 
-0.489
 
 
15.6
 
 
 
 
ARMT1
 
 
hsa-let-7c-5p
 
 
-0.303
 
 
25.9
 
 
 
 
ARMT1
 
 
hsa-let-7e-5p
 
 
-0.303
 
 
25.9
 
 
 
 
ARMT1
 
 
hsa-miR-98-5p
 
 
-0.303
 
 
25.9
 
 
 
 
ARPP19
 
 
hsa-let-7e-5p
 
 
-0.492
 
 
53.7
 
 
 
 
ARPP19
 
 
hsa-let-7c-5p
 
 
-0.492
 
 
53.7
 
 
 
 
ARPP19
 
 
hsa-miR-98-5p
 
 
-0.5
 
 
53.7
 
 
 
 
ARRDC3
 
 
hsa-miR-148a-3p
 
 
-0.305
 
 
78.7
 
 
 
 
ARRDC4
 
 
hsa-let-7c-5p
 
 
-0.329
 
 
16.2
 
 
 
 
ARRDC4
 
 
hsa-let-7e-5p
 
 
-0.329
 
 
16.2
 
 
 
 
ARRDC4
 
 
hsa-miR-98-5p
 
 
-0.329
 
 
16.2
 
 
 
 
ASAP1
 
 
hsa-let-7e-5p
 
 
-0.296
 
 
15.1
 
 
 
 
ASAP1
 
 
hsa-miR-98-5p
 
 
-0.308
 
 
15.1
 
 
 
 
ASAP1
 
 
hsa-let-7c-5p
 
 
-0.308
 
 
15.1
 
 
 
 
ASF1A
 
 
hsa-miR-21-5p
 
 
-0.304
 
 
14.1
 
 
 
 
ASPH
 
 
hsa-miR-148a-3p
 
 
-0.391
 
 
90.3
 
 
 
 
ATG16L1
 
 
hsa-miR-106a-5p
 
 
-0.464
 
 
11.5
 
 
 
 
ATG16L1
 
 
hsa-miR-20b-5p
 
 
-0.467
 
 
11.5
 
 
 
 
ATG4A
 
 
hsa-miR-7-5p
 
 
-0.448
 
 
10.7
 
 
 
 
ATG4D
 
 
hsa-miR-148a-3p
 
 
-0.303
 
 
13.0
 
 
 
 
ATOSB
 
 
hsa-let-7e-5p
 
 
-0.429
 
 
11.1
 
 
 
 
ATOSB
 
 
hsa-miR-98-5p
 
 
-0.429
 
 
11.1
 
 
 
 
ATOSB
 
 
hsa-let-7c-5p
 
 
-0.429
 
 
11.1
 
 
 
 
ATP2A2
 
 
hsa-let-7e-5p
 
 
-0.311
 
 
68.6
 
 
 
 
ATP2A2
 
 
hsa-miR-98-5p
 
 
-0.311
 
 
68.6
 
 
 
 
ATP2A2
 
 
hsa-let-7c-5p
 
 
-0.311
 
 
68.6
 
 
 
 
ATP2B4
 
 
hsa-miR-148a-3p
 
 
-0.337
 
 
14.2
 
 
 
 
ATP6AP2
 
 
hsa-miR-148a-3p
 
 
-0.592
 
 
166.0
 
 
 
 
ATP6AP2
 
 
hsa-miR-148a-3p
 
 
-0.668
 
 
166.0
 
 
 
 
ATPAF1
 
 
hsa-let-7e-5p
 
 
-0.498
 
 
39.3
 
 
 
 
ATPAF1
 
 
hsa-miR-98-5p
 
 
-0.498
 
 
39.3
 
 
 
 
ATPAF1
 
 
hsa-let-7c-5p
 
 
-0.498
 
 
39.3
 
 
 
 
B3GAT3
 
 
hsa-let-7e-5p
 
 
-0.324
 
 
33.1
 
 
 
 
B3GAT3
 
 
hsa-miR-98-5p
 
 
-0.324
 
 
33.1
 
 
 
 
B3GAT3
 
 
hsa-let-7c-5p
 
 
-0.324
 
 
33.1
 
 
 
 
 
 
 epididymis 
 
 
 
 
target_symbol
 
 
mature_mirna_id
 
 
score
 
 
nTPM
 
 
 
 
 
 
ABCD3
 
 
hsa-miR-148a-3p
 
 
-0.388
 
 
20.0
 
 
 
 
ABHD6
 
 
hsa-miR-125a-5p
 
 
-0.501
 
 
13.9
 
 
 
 
ACP3
 
 
hsa-let-7e-5p
 
 
-0.298
 
 
16.3
 
 
 
 
ACP3
 
 
hsa-miR-98-5p
 
 
-0.298
 
 
16.3
 
 
 
 
ACP3
 
 
hsa-let-7c-5p
 
 
-0.298
 
 
16.3
 
 
 
 
ACVR1
 
 
hsa-miR-148a-3p
 
 
-0.397
 
 
10.8
 
 
 
 
ADAM17
 
 
hsa-miR-125a-5p
 
 
-0.292
 
 
10.4
 
 
 
 
ADAM17
 
 
hsa-miR-148a-3p
 
 
-0.301
 
 
10.4
 
 
 
 
ADAM9
 
 
hsa-miR-106a-5p
 
 
-0.317
 
 
14.3
 
 
 
 
ADAM9
 
 
hsa-miR-20b-5p
 
 
-0.328
 
 
14.3
 
 
 
 
ADGRG2
 
 
hsa-miR-21-5p
 
 
-0.343
 
 
1159.4
 
 
 
 
AGFG1
 
 
hsa-miR-148a-3p
 
 
-0.332
 
 
18.8
 
 
 
 
AGGF1
 
 
hsa-miR-125a-5p
 
 
-0.331
 
 
20.2
 
 
 
 
AHNAK
 
 
hsa-miR-106a-5p
 
 
-0.334
 
 
48.9
 
 
 
 
AHNAK
 
 
hsa-miR-20b-5p
 
 
-0.337
 
 
48.9
 
 
 
 
AKAP1
 
 
hsa-miR-148a-3p
 
 
-0.381
 
 
18.6
 
 
 
 
ALCAM
 
 
hsa-miR-148a-3p
 
 
-0.339
 
 
33.4
 
 
 
 
ALG3
 
 
hsa-miR-106a-5p
 
 
-0.569
 
 
11.8
 
 
 
 
ALG3
 
 
hsa-miR-20b-5p
 
 
-0.58
 
 
11.8
 
 
 
 
ALG9
 
 
hsa-miR-7-5p
 
 
-0.302
 
 
10.3
 
 
 
 
ANAPC16
 
 
hsa-miR-125a-5p
 
 
-0.578
 
 
20.9
 
 
 
 
ANKRD46
 
 
hsa-let-7e-5p
 
 
-0.389
 
 
15.4
 
 
 
 
ANKRD46
 
 
hsa-miR-98-5p
 
 
-0.389
 
 
15.4
 
 
 
 
ANKRD46
 
 
hsa-let-7c-5p
 
 
-0.389
 
 
15.4
 
 
 
 
AP1S1
 
 
hsa-let-7e-5p
 
 
-0.374
 
 
16.0
 
 
 
 
AP1S1
 
 
hsa-miR-98-5p
 
 
-0.374
 
 
16.0
 
 
 
 
AP1S1
 
 
hsa-let-7c-5p
 
 
-0.374
 
 
16.0
 
 
 
 
APCDD1
 
 
hsa-miR-106a-5p
 
 
-0.422
 
 
14.6
 
 
 
 
APCDD1
 
 
hsa-miR-20b-5p
 
 
-0.425
 
 
14.6
 
 
 
 
ARF4
 
 
hsa-miR-7-5p
 
 
-0.407
 
 
214.1
 
 
 
 
ARF4
 
 
hsa-miR-148a-3p
 
 
-0.487
 
 
214.1
 
 
 
 
ARFGEF1
 
 
hsa-miR-148a-3p
 
 
-0.306
 
 
10.4
 
 
 
 
ARFIP1
 
 
hsa-miR-148a-3p
 
 
-0.474
 
 
19.3
 
 
 
 
ARHGAP1
 
 
hsa-miR-20b-5p
 
 
-0.325
 
 
20.3
 
 
 
 
ARHGAP1
 
 
hsa-miR-106a-5p
 
 
-0.329
 
 
20.3
 
 
 
 
ARL1
 
 
hsa-miR-20b-5p
 
 
-0.424
 
 
67.1
 
 
 
 
ARL1
 
 
hsa-miR-106a-5p
 
 
-0.429
 
 
67.1
 
 
 
 
ARL6IP1
 
 
hsa-miR-7-5p
 
 
-0.374
 
 
116.0
 
 
 
 
ARL6IP1
 
 
hsa-miR-148a-3p
 
 
-0.579
 
 
116.0
 
 
 
 
ARL8B
 
 
hsa-miR-148a-3p
 
 
-0.337
 
 
37.5
 
 
 
 
ARMC8
 
 
hsa-miR-106a-5p
 
 
-0.375
 
 
11.5
 
 
 
 
ARMC8
 
 
hsa-miR-20b-5p
 
 
-0.378
 
 
11.5
 
 
 
 
ARMCX1
 
 
hsa-miR-21-5p
 
 
-0.489
 
 
20.1
 
 
 
 
ARMT1
 
 
hsa-let-7c-5p
 
 
-0.303
 
 
15.2
 
 
 
 
ARMT1
 
 
hsa-let-7e-5p
 
 
-0.303
 
 
15.2
 
 
 
 
ARMT1
 
 
hsa-miR-98-5p
 
 
-0.303
 
 
15.2
 
 
 
 
ARPP19
 
 
hsa-let-7e-5p
 
 
-0.492
 
 
34.2
 
 
 
 
ARPP19
 
 
hsa-let-7c-5p
 
 
-0.492
 
 
34.2
 
 
 
 
ARPP19
 
 
hsa-miR-98-5p
 
 
-0.5
 
 
34.2
 
 
 
 
ARRDC3
 
 
hsa-miR-148a-3p
 
 
-0.305
 
 
25.6
 
 
 
 
ARRDC4
 
 
hsa-let-7c-5p
 
 
-0.329
 
 
14.7
 
 
 
 
ARRDC4
 
 
hsa-let-7e-5p
 
 
-0.329
 
 
14.7
 
 
 
 
ARRDC4
 
 
hsa-miR-98-5p
 
 
-0.329
 
 
14.7
 
 
 
 
ASAP1
 
 
hsa-let-7e-5p
 
 
-0.296
 
 
11.5
 
 
 
 
ASAP1
 
 
hsa-miR-98-5p
 
 
-0.308
 
 
11.5
 
 
 
 
ASAP1
 
 
hsa-let-7c-5p
 
 
-0.308
 
 
11.5
 
 
 
 
ASF1A
 
 
hsa-miR-21-5p
 
 
-0.304
 
 
12.3
 
 
 
 
ASPH
 
 
hsa-miR-148a-3p
 
 
-0.391
 
 
42.3
 
 
 
 
ATG4A
 
 
hsa-miR-7-5p
 
 
-0.448
 
 
10.9
 
 
 
 
ATP2A2
 
 
hsa-let-7e-5p
 
 
-0.311
 
 
36.9
 
 
 
 
ATP2A2
 
 
hsa-miR-98-5p
 
 
-0.311
 
 
36.9
 
 
 
 
ATP2A2
 
 
hsa-let-7c-5p
 
 
-0.311
 
 
36.9
 
 
 
 
ATP2B4
 
 
hsa-miR-148a-3p
 
 
-0.337
 
 
18.9
 
 
 
 
ATP6AP2
 
 
hsa-miR-148a-3p
 
 
-0.592
 
 
86.1
 
 
 
 
ATP6AP2
 
 
hsa-miR-148a-3p
 
 
-0.668
 
 
86.1
 
 
 
 
ATPAF1
 
 
hsa-let-7e-5p
 
 
-0.498
 
 
33.7
 
 
 
 
ATPAF1
 
 
hsa-miR-98-5p
 
 
-0.498
 
 
33.7
 
 
 
 
ATPAF1
 
 
hsa-let-7c-5p
 
 
-0.498
 
 
33.7
 
 
 
 
B3GAT3
 
 
hsa-let-7e-5p
 
 
-0.324
 
 
11.2
 
 
 
 
B3GAT3
 
 
hsa-miR-98-5p
 
 
-0.324
 
 
11.2
 
 
 
 
B3GAT3
 
 
hsa-let-7c-5p
 
 
-0.324
 
 
11.2
 
 
 
 
B4GAT1
 
 
hsa-let-7c-5p
 
 
-0.47
 
 
14.3
 
 
 
 
B4GAT1
 
 
hsa-let-7e-5p
 
 
-0.47
 
 
14.3
 
 
 
 
B4GAT1
 
 
hsa-miR-98-5p
 
 
-0.47
 
 
14.3
 
 
 
 
BAZ2A
 
 
hsa-miR-99b-5p
 
 
-0.408
 
 
21.6
 
 
 
 
BCAP29
 
 
hsa-let-7e-5p
 
 
-0.289
 
 
18.0
 
 
 
 
BHLHE41
 
 
hsa-miR-148a-3p
 
 
-0.442
 
 
18.2
 
 
 
 
BLOC1S6
 
 
hsa-let-7e-5p
 
 
-0.321
 
 
17.3
 
 
 
 
BLOC1S6
 
 
hsa-miR-98-5p
 
 
-0.321
 
 
17.3
 
 
 
 
BLOC1S6
 
 
hsa-let-7c-5p
 
 
-0.321
 
 
17.3
 
 
 
 
BLZF1
 
 
hsa-miR-125a-5p
 
 
-0.461
 
 
13.5
 
 
 
 
BTAF1
 
 
hsa-miR-148a-3p
 
 
-0.401
 
 
16.9
 
 
 
 
BTBD10
 
 
hsa-miR-106a-5p
 
 
-0.406
 
 
11.3
 
 
 
 
BTBD10
 
 
hsa-miR-20b-5p
 
 
-0.425
 
 
11.3
 
 
 
 
BTF3L4
 
 
hsa-let-7c-5p
 
 
-0.355
 
 
28.9
 
 
 
 
BTF3L4
 
 
hsa-let-7e-5p
 
 
-0.355
 
 
28.9
 
 
 
 
BTF3L4
 
 
hsa-miR-98-5p
 
 
-0.355
 
 
28.9
 
 
 
 
BTG3
 
 
hsa-miR-106a-5p
 
 
-0.665
 
 
104.1
 
 
 
 
BTG3
 
 
hsa-miR-20b-5p
 
 
-0.684
 
 
104.1
 
 
 
 
BZW1
 
 
hsa-let-7e-5p
 
 
-0.354
 
 
55.1
 
 
 
 
BZW1
 
 
hsa-miR-98-5p
 
 
-0.354
 
 
55.1
 
 
 
 
BZW1
 
 
hsa-let-7c-5p
 
 
-0.354
 
 
55.1
 
 
 
 
BZW1
 
 
hsa-let-7e-5p
 
 
-0.375
 
 
55.1
 
 
 
 
BZW1
 
 
hsa-miR-98-5p
 
 
-0.375
 
 
55.1
 
 
 
 
BZW1
 
 
hsa-let-7c-5p
 
 
-0.375
 
 
55.1
 
 
 
 
C17orf75
 
 
hsa-miR-21-5p
 
 
-0.36
 
 
17.7
 
 
 
 
C1orf210
 
 
hsa-miR-125a-5p
 
 
-0.584
 
 
12.2
 
 
 
 
CALD1
 
 
hsa-miR-20b-5p
 
 
-0.373
 
 
142.4
 
 
 
 
CALD1
 
 
hsa-miR-106a-5p
 
 
-0.386
 
 
142.4
 
 
 
 
CAMK2N1
 
 
hsa-miR-20b-5p
 
 
-0.367
 
 
36.0
 
 
 
 
 
 
 
 cd8_s1_vs_cd8_s0_upregulated 
 
 Tissue expression heatmap (log10-transformed) 
   
 
 
 bone marrow 
 
 
 
 
target_symbol
 
 
mature_mirna_id
 
 
score
 
 
nTPM
 
 
 
 
 
 
ABCB10
 
 
hsa-miR-152-3p
 
 
-0.291
 
 
56.7
 
 
 
 
ABCB7
 
 
hsa-miR-152-3p
 
 
-0.515
 
 
10.5
 
 
 
 
ABCD3
 
 
hsa-miR-152-3p
 
 
-0.388
 
 
23.6
 
 
 
 
ADAM10
 
 
hsa-miR-152-3p
 
 
-0.348
 
 
17.3
 
 
 
 
AGFG1
 
 
hsa-miR-152-3p
 
 
-0.332
 
 
146.9
 
 
 
 
AP4B1
 
 
hsa-miR-378a-3p
 
 
-0.62
 
 
19.4
 
 
 
 
ARF4
 
 
hsa-miR-152-3p
 
 
-0.487
 
 
68.5
 
 
 
 
ARFGEF1
 
 
hsa-miR-152-3p
 
 
-0.306
 
 
26.0
 
 
 
 
ARL6IP1
 
 
hsa-miR-152-3p
 
 
-0.579
 
 
197.7
 
 
 
 
ARL8B
 
 
hsa-miR-152-3p
 
 
-0.337
 
 
56.4
 
 
 
 
ARRDC3
 
 
hsa-miR-152-3p
 
 
-0.305
 
 
158.3
 
 
 
 
ASPH
 
 
hsa-miR-152-3p
 
 
-0.391
 
 
15.8
 
 
 
 
ATP2B4
 
 
hsa-miR-152-3p
 
 
-0.337
 
 
14.9
 
 
 
 
ATP6AP2
 
 
hsa-miR-152-3p
 
 
-0.592
 
 
117.4
 
 
 
 
ATP6AP2
 
 
hsa-miR-152-3p
 
 
-0.668
 
 
117.4
 
 
 
 
B4GALT5
 
 
hsa-miR-152-3p
 
 
-0.44
 
 
88.5
 
 
 
 
BCL2L11
 
 
hsa-miR-152-3p
 
 
-0.445
 
 
37.8
 
 
 
 
BTAF1
 
 
hsa-miR-152-3p
 
 
-0.401
 
 
25.4
 
 
 
 
BTBD3
 
 
hsa-miR-152-3p
 
 
-0.495
 
 
11.5
 
 
 
 
C1GALT1
 
 
hsa-miR-152-3p
 
 
-0.487
 
 
35.6
 
 
 
 
C1QBP
 
 
hsa-miR-330-3p
 
 
-0.412
 
 
114.2
 
 
 
 
C1orf35
 
 
hsa-miR-152-3p
 
 
-0.511
 
 
13.4
 
 
 
 
CAND1
 
 
hsa-miR-152-3p
 
 
-0.44
 
 
21.4
 
 
 
 
CANX
 
 
hsa-miR-152-3p
 
 
-0.434
 
 
70.9
 
 
 
 
CBLB
 
 
hsa-miR-152-3p
 
 
-0.424
 
 
12.6
 
 
 
 
CCDC6
 
 
hsa-miR-152-3p
 
 
-0.511
 
 
15.9
 
 
 
 
CCT6A
 
 
hsa-miR-152-3p
 
 
-0.665
 
 
64.5
 
 
 
 
CDC14A
 
 
hsa-miR-152-3p
 
 
-0.482
 
 
14.6
 
 
 
 
CDC25B
 
 
hsa-miR-152-3p
 
 
-0.298
 
 
27.7
 
 
 
 
CDK19
 
 
hsa-miR-152-3p
 
 
-0.369
 
 
11.0
 
 
 
 
CDK19
 
 
hsa-miR-152-3p
 
 
-0.405
 
 
11.0
 
 
 
 
CEBPG
 
 
hsa-miR-152-3p
 
 
-0.319
 
 
14.5
 
 
 
 
CEP63
 
 
hsa-miR-152-3p
 
 
-0.329
 
 
20.1
 
 
 
 
CFL2
 
 
hsa-miR-152-3p
 
 
-0.487
 
 
17.1
 
 
 
 
CHD7
 
 
hsa-miR-152-3p
 
 
-0.297
 
 
16.2
 
 
 
 
CHIT1
 
 
hsa-miR-378a-3p
 
 
-0.387
 
 
47.0
 
 
 
 
CHMP4B
 
 
hsa-miR-152-3p
 
 
-0.36
 
 
201.7
 
 
 
 
CNOT7
 
 
hsa-miR-152-3p
 
 
-0.31
 
 
48.1
 
 
 
 
CS
 
 
hsa-miR-152-3p
 
 
-0.3
 
 
104.8
 
 
 
 
CTSA
 
 
hsa-miR-152-3p
 
 
-0.541
 
 
74.6
 
 
 
 
CUL5
 
 
hsa-miR-152-3p
 
 
-0.389
 
 
27.0
 
 
 
 
CYB5R4
 
 
hsa-miR-152-3p
 
 
-0.454
 
 
16.0
 
 
 
 
CYB5R4
 
 
hsa-miR-152-3p
 
 
-0.454
 
 
16.0
 
 
 
 
DCP2
 
 
hsa-miR-152-3p
 
 
-0.29
 
 
13.2
 
 
 
 
DDX6
 
 
hsa-miR-152-3p
 
 
-0.363
 
 
65.1
 
 
 
 
DEDD
 
 
hsa-miR-152-3p
 
 
-0.401
 
 
28.1
 
 
 
 
DIPK2A
 
 
hsa-miR-152-3p
 
 
-0.393
 
 
30.6
 
 
 
 
DMXL1
 
 
hsa-miR-152-3p
 
 
-0.29
 
 
23.8
 
 
 
 
DNMT1
 
 
hsa-miR-152-3p
 
 
-0.421
 
 
22.0
 
 
 
 
DUSP1
 
 
hsa-miR-152-3p
 
 
-0.32
 
 
654.4
 
 
 
 
DYNLL2
 
 
hsa-miR-152-3p
 
 
-0.366
 
 
16.7
 
 
 
 
DYRK2
 
 
hsa-miR-152-3p
 
 
-0.293
 
 
14.1
 
 
 
 
EIF4E3
 
 
hsa-miR-152-3p
 
 
-0.464
 
 
25.2
 
 
 
 
EIF4G3
 
 
hsa-miR-378a-3p
 
 
-0.443
 
 
30.4
 
 
 
 
EPS15
 
 
hsa-miR-152-3p
 
 
-0.322
 
 
19.0
 
 
 
 
ESCO1
 
 
hsa-miR-152-3p
 
 
-0.34
 
 
15.4
 
 
 
 
FAM168B
 
 
hsa-miR-152-3p
 
 
-0.409
 
 
43.7
 
 
 
 
FEZ2
 
 
hsa-miR-152-3p
 
 
-0.536
 
 
15.5
 
 
 
 
FOSB
 
 
hsa-miR-152-3p
 
 
-0.311
 
 
409.7
 
 
 
 
FXR1
 
 
hsa-miR-152-3p
 
 
-0.478
 
 
45.4
 
 
 
 
GADD45A
 
 
hsa-miR-152-3p
 
 
-0.787
 
 
128.6
 
 
 
 
GAPVD1
 
 
hsa-miR-152-3p
 
 
-0.309
 
 
21.7
 
 
 
 
GLRX5
 
 
hsa-miR-152-3p
 
 
-0.381
 
 
168.5
 
 
 
 
GMFB
 
 
hsa-miR-152-3p
 
 
-0.429
 
 
27.2
 
 
 
 
GPATCH8
 
 
hsa-miR-152-3p
 
 
-0.374
 
 
17.3
 
 
 
 
GRSF1
 
 
hsa-miR-378a-3p
 
 
-0.358
 
 
21.9
 
 
 
 
HDDC2
 
 
hsa-miR-378a-3p
 
 
-0.322
 
 
18.6
 
 
 
 
HLA-DQB1
 
 
hsa-miR-152-3p
 
 
-0.37
 
 
38.4
 
 
 
 
HSP90B1
 
 
hsa-miR-152-3p
 
 
-0.304
 
 
414.1
 
 
 
 
HSPH1
 
 
hsa-miR-330-3p
 
 
-0.345
 
 
19.6
 
 
 
 
ID2
 
 
hsa-miR-330-3p
 
 
-0.293
 
 
368.2
 
 
 
 
ING2
 
 
hsa-miR-152-3p
 
 
-0.482
 
 
20.4
 
 
 
 
INO80
 
 
hsa-miR-152-3p
 
 
-0.397
 
 
11.6
 
 
 
 
ITSN2
 
 
hsa-miR-152-3p
 
 
-0.339
 
 
69.4
 
 
 
 
KLF4
 
 
hsa-miR-152-3p
 
 
-0.325
 
 
47.7
 
 
 
 
KLF5
 
 
hsa-miR-152-3p
 
 
-0.475
 
 
60.0
 
 
 
 
LBR
 
 
hsa-miR-152-3p
 
 
-0.39
 
 
90.3
 
 
 
 
LDLR
 
 
hsa-miR-152-3p
 
 
-0.323
 
 
25.8
 
 
 
 
LEPROTL1
 
 
hsa-miR-152-3p
 
 
-0.568
 
 
52.5
 
 
 
 
LIPA
 
 
hsa-miR-152-3p
 
 
-0.463
 
 
37.5
 
 
 
 
LMAN1
 
 
hsa-miR-152-3p
 
 
-0.382
 
 
20.5
 
 
 
 
LMTK2
 
 
hsa-miR-152-3p
 
 
-0.298
 
 
13.0
 
 
 
 
LRRC41
 
 
hsa-miR-152-3p
 
 
-0.392
 
 
18.9
 
 
 
 
LSM5
 
 
hsa-miR-330-3p
 
 
-0.351
 
 
72.7
 
 
 
 
LYRM2
 
 
hsa-miR-152-3p
 
 
-0.351
 
 
14.0
 
 
 
 
LYSMD2
 
 
hsa-miR-152-3p
 
 
-0.554
 
 
14.3
 
 
 
 
MACIR
 
 
hsa-miR-152-3p
 
 
-0.332
 
 
29.6
 
 
 
 
MACIR
 
 
hsa-miR-152-3p
 
 
-0.573
 
 
29.6
 
 
 
 
MACROH2A1
 
 
hsa-miR-152-3p
 
 
-0.336
 
 
222.2
 
 
 
 
MAF1
 
 
hsa-miR-152-3p
 
 
-0.366
 
 
154.9
 
 
 
 
MAFB
 
 
hsa-miR-152-3p
 
 
-0.45
 
 
55.8
 
 
 
 
MAFG
 
 
hsa-miR-152-3p
 
 
-0.328
 
 
38.3
 
 
 
 
MAP2K1
 
 
hsa-miR-152-3p
 
 
-0.295
 
 
37.4
 
 
 
 
MAPK1
 
 
hsa-miR-378a-3p
 
 
-0.314
 
 
38.9
 
 
 
 
MAT2A
 
 
hsa-miR-330-3p
 
 
-0.307
 
 
92.8
 
 
 
 
MCM3AP
 
 
hsa-miR-152-3p
 
 
-0.287
 
 
18.7
 
 
 
 
MDFIC
 
 
hsa-miR-152-3p
 
 
-0.395
 
 
10.3
 
 
 
 
MECP2
 
 
hsa-miR-152-3p
 
 
-0.3
 
 
23.5
 
 
 
 
MIER1
 
 
hsa-miR-152-3p
 
 
-0.352
 
 
92.4
 
 
 
 
MLLT10
 
 
hsa-miR-152-3p
 
 
-0.334
 
 
26.6
 
 
 
 
 
 
 cerebellum 
 
 
 
 
target_symbol
 
 
mature_mirna_id
 
 
score
 
 
nTPM
 
 
 
 
 
 
ABCB10
 
 
hsa-miR-152-3p
 
 
-0.291
 
 
10.6
 
 
 
 
ABCD3
 
 
hsa-miR-152-3p
 
 
-0.388
 
 
12.8
 
 
 
 
ADAM10
 
 
hsa-miR-152-3p
 
 
-0.348
 
 
15.6
 
 
 
 
ADGRB3
 
 
hsa-miR-152-3p
 
 
-0.516
 
 
20.8
 
 
 
 
AGFG1
 
 
hsa-miR-152-3p
 
 
-0.332
 
 
22.4
 
 
 
 
AHDC1
 
 
hsa-miR-152-3p
 
 
-0.413
 
 
32.0
 
 
 
 
AKAP1
 
 
hsa-miR-152-3p
 
 
-0.381
 
 
30.6
 
 
 
 
ALS2
 
 
hsa-miR-378a-3p
 
 
-0.335
 
 
21.3
 
 
 
 
AP4B1
 
 
hsa-miR-378a-3p
 
 
-0.62
 
 
20.4
 
 
 
 
ARF4
 
 
hsa-miR-152-3p
 
 
-0.487
 
 
50.2
 
 
 
 
ARHGAP21
 
 
hsa-miR-152-3p
 
 
-0.287
 
 
50.0
 
 
 
 
ARL6IP1
 
 
hsa-miR-152-3p
 
 
-0.579
 
 
234.0
 
 
 
 
ARL8B
 
 
hsa-miR-152-3p
 
 
-0.337
 
 
50.7
 
 
 
 
ARRDC3
 
 
hsa-miR-152-3p
 
 
-0.305
 
 
17.8
 
 
 
 
ASPH
 
 
hsa-miR-152-3p
 
 
-0.391
 
 
80.5
 
 
 
 
ATP6AP2
 
 
hsa-miR-152-3p
 
 
-0.592
 
 
52.9
 
 
 
 
ATP6AP2
 
 
hsa-miR-152-3p
 
 
-0.668
 
 
52.9
 
 
 
 
B4GALT5
 
 
hsa-miR-152-3p
 
 
-0.44
 
 
20.4
 
 
 
 
B4GALT6
 
 
hsa-miR-152-3p
 
 
-0.528
 
 
16.6
 
 
 
 
BARHL2
 
 
hsa-miR-152-3p
 
 
-0.544
 
 
42.1
 
 
 
 
BHLHE41
 
 
hsa-miR-152-3p
 
 
-0.442
 
 
31.9
 
 
 
 
BTAF1
 
 
hsa-miR-152-3p
 
 
-0.401
 
 
10.7
 
 
 
 
BTBD3
 
 
hsa-miR-152-3p
 
 
-0.495
 
 
65.8
 
 
 
 
C1QBP
 
 
hsa-miR-330-3p
 
 
-0.412
 
 
62.4
 
 
 
 
C1orf35
 
 
hsa-miR-152-3p
 
 
-0.511
 
 
23.9
 
 
 
 
C3orf62
 
 
hsa-miR-330-3p
 
 
-0.366
 
 
10.4
 
 
 
 
CABP7
 
 
hsa-miR-152-3p
 
 
-0.511
 
 
10.8
 
 
 
 
CAND1
 
 
hsa-miR-152-3p
 
 
-0.44
 
 
24.5
 
 
 
 
CANX
 
 
hsa-miR-152-3p
 
 
-0.434
 
 
74.7
 
 
 
 
CBLB
 
 
hsa-miR-152-3p
 
 
-0.424
 
 
10.1
 
 
 
 
CCT6A
 
 
hsa-miR-152-3p
 
 
-0.665
 
 
58.7
 
 
 
 
CDC25B
 
 
hsa-miR-152-3p
 
 
-0.298
 
 
87.0
 
 
 
 
CDK19
 
 
hsa-miR-152-3p
 
 
-0.369
 
 
12.8
 
 
 
 
CDK19
 
 
hsa-miR-152-3p
 
 
-0.405
 
 
12.8
 
 
 
 
CDK5R1
 
 
hsa-miR-152-3p
 
 
-0.547
 
 
56.6
 
 
 
 
CEBPG
 
 
hsa-miR-152-3p
 
 
-0.319
 
 
17.0
 
 
 
 
CERS5
 
 
hsa-miR-152-3p
 
 
-0.311
 
 
18.6
 
 
 
 
CFL2
 
 
hsa-miR-152-3p
 
 
-0.487
 
 
43.5
 
 
 
 
CHD7
 
 
hsa-miR-152-3p
 
 
-0.297
 
 
28.5
 
 
 
 
CHMP4B
 
 
hsa-miR-152-3p
 
 
-0.36
 
 
86.1
 
 
 
 
CLCN6
 
 
hsa-miR-152-3p
 
 
-0.406
 
 
21.9
 
 
 
 
CNOT7
 
 
hsa-miR-152-3p
 
 
-0.31
 
 
28.2
 
 
 
 
CS
 
 
hsa-miR-152-3p
 
 
-0.3
 
 
79.7
 
 
 
 
CTSA
 
 
hsa-miR-152-3p
 
 
-0.541
 
 
25.7
 
 
 
 
CYTH3
 
 
hsa-miR-152-3p
 
 
-0.331
 
 
18.5
 
 
 
 
DACT1
 
 
hsa-miR-378a-3p
 
 
-0.298
 
 
22.9
 
 
 
 
DCUN1D4
 
 
hsa-miR-152-3p
 
 
-0.314
 
 
12.7
 
 
 
 
DDAH1
 
 
hsa-miR-152-3p
 
 
-0.34
 
 
27.5
 
 
 
 
DDX6
 
 
hsa-miR-152-3p
 
 
-0.363
 
 
32.5
 
 
 
 
DEDD
 
 
hsa-miR-152-3p
 
 
-0.401
 
 
21.7
 
 
 
 
DIPK2A
 
 
hsa-miR-152-3p
 
 
-0.393
 
 
14.8
 
 
 
 
DMPK
 
 
hsa-miR-152-3p
 
 
-0.378
 
 
15.6
 
 
 
 
DUSP1
 
 
hsa-miR-152-3p
 
 
-0.32
 
 
77.2
 
 
 
 
DYNLL2
 
 
hsa-miR-152-3p
 
 
-0.366
 
 
69.4
 
 
 
 
DYRK1B
 
 
hsa-miR-152-3p
 
 
-0.305
 
 
17.6
 
 
 
 
EIF4E3
 
 
hsa-miR-152-3p
 
 
-0.464
 
 
11.5
 
 
 
 
EIF4G3
 
 
hsa-miR-378a-3p
 
 
-0.443
 
 
10.7
 
 
 
 
ELAVL2
 
 
hsa-miR-152-3p
 
 
-0.3
 
 
12.1
 
 
 
 
EPS15
 
 
hsa-miR-152-3p
 
 
-0.322
 
 
23.4
 
 
 
 
ERRFI1
 
 
hsa-miR-152-3p
 
 
-0.529
 
 
27.8
 
 
 
 
FAM168B
 
 
hsa-miR-152-3p
 
 
-0.409
 
 
55.1
 
 
 
 
FAM184A
 
 
hsa-miR-152-3p
 
 
-0.371
 
 
11.1
 
 
 
 
FBXL19
 
 
hsa-miR-152-3p
 
 
-0.362
 
 
17.6
 
 
 
 
FEZ2
 
 
hsa-miR-152-3p
 
 
-0.536
 
 
31.7
 
 
 
 
FOSB
 
 
hsa-miR-152-3p
 
 
-0.311
 
 
14.1
 
 
 
 
FOXK2
 
 
hsa-miR-152-3p
 
 
-0.305
 
 
17.6
 
 
 
 
FXR1
 
 
hsa-miR-152-3p
 
 
-0.478
 
 
23.8
 
 
 
 
GAP43
 
 
hsa-miR-152-3p
 
 
-0.409
 
 
186.6
 
 
 
 
GAPVD1
 
 
hsa-miR-152-3p
 
 
-0.309
 
 
10.6
 
 
 
 
GLRX5
 
 
hsa-miR-152-3p
 
 
-0.381
 
 
37.8
 
 
 
 
GMFB
 
 
hsa-miR-152-3p
 
 
-0.429
 
 
36.2
 
 
 
 
GOLT1A
 
 
hsa-miR-378a-3p
 
 
-0.574
 
 
13.0
 
 
 
 
GPATCH8
 
 
hsa-miR-152-3p
 
 
-0.374
 
 
48.7
 
 
 
 
GPR137C
 
 
hsa-miR-152-3p
 
 
-0.33
 
 
26.0
 
 
 
 
GRSF1
 
 
hsa-miR-378a-3p
 
 
-0.358
 
 
23.4
 
 
 
 
GTF2H1
 
 
hsa-miR-152-3p
 
 
-0.524
 
 
20.1
 
 
 
 
HDDC2
 
 
hsa-miR-378a-3p
 
 
-0.322
 
 
64.6
 
 
 
 
HSP90B1
 
 
hsa-miR-152-3p
 
 
-0.304
 
 
69.3
 
 
 
 
HSPH1
 
 
hsa-miR-330-3p
 
 
-0.345
 
 
48.7
 
 
 
 
ID2
 
 
hsa-miR-330-3p
 
 
-0.293
 
 
40.5
 
 
 
 
IL6ST
 
 
hsa-miR-152-3p
 
 
-0.291
 
 
15.5
 
 
 
 
JPH3
 
 
hsa-miR-152-3p
 
 
-0.303
 
 
52.2
 
 
 
 
LEPROTL1
 
 
hsa-miR-152-3p
 
 
-0.568
 
 
14.6
 
 
 
 
LIPA
 
 
hsa-miR-152-3p
 
 
-0.463
 
 
14.0
 
 
 
 
LMAN1
 
 
hsa-miR-152-3p
 
 
-0.382
 
 
16.7
 
 
 
 
LRCH1
 
 
hsa-miR-152-3p
 
 
-0.321
 
 
35.8
 
 
 
 
LRRC41
 
 
hsa-miR-152-3p
 
 
-0.392
 
 
31.3
 
 
 
 
LSM5
 
 
hsa-miR-330-3p
 
 
-0.351
 
 
18.4
 
 
 
 
LYSMD2
 
 
hsa-miR-152-3p
 
 
-0.554
 
 
36.8
 
 
 
 
MACROH2A1
 
 
hsa-miR-152-3p
 
 
-0.336
 
 
72.9
 
 
 
 
MAF1
 
 
hsa-miR-152-3p
 
 
-0.366
 
 
67.1
 
 
 
 
MAFG
 
 
hsa-miR-152-3p
 
 
-0.328
 
 
12.7
 
 
 
 
MAP2K1
 
 
hsa-miR-152-3p
 
 
-0.295
 
 
42.8
 
 
 
 
MAP3K9
 
 
hsa-miR-152-3p
 
 
-0.366
 
 
16.6
 
 
 
 
MAPK1
 
 
hsa-miR-378a-3p
 
 
-0.314
 
 
49.3
 
 
 
 
MAT2A
 
 
hsa-miR-330-3p
 
 
-0.307
 
 
163.1
 
 
 
 
MCM3AP
 
 
hsa-miR-152-3p
 
 
-0.287
 
 
14.7
 
 
 
 
MECP2
 
 
hsa-miR-152-3p
 
 
-0.3
 
 
22.0
 
 
 
 
MED19
 
 
hsa-miR-378a-3p
 
 
-0.434
 
 
10.6
 
 
 
 
MLLT10
 
 
hsa-miR-152-3p
 
 
-0.334
 
 
15.9
 
 
 
 
 
 
 colon 
 
 
 
 
target_symbol
 
 
mature_mirna_id
 
 
score
 
 
nTPM
 
 
 
 
 
 
ABCB10
 
 
hsa-miR-152-3p
 
 
-0.291
 
 
10.4
 
 
 
 
ABCB7
 
 
hsa-miR-152-3p
 
 
-0.515
 
 
13.4
 
 
 
 
ABCD3
 
 
hsa-miR-152-3p
 
 
-0.388
 
 
41.8
 
 
 
 
ABHD17C
 
 
hsa-miR-330-3p
 
 
-0.399
 
 
61.2
 
 
 
 
ADAM10
 
 
hsa-miR-152-3p
 
 
-0.348
 
 
20.8
 
 
 
 
AGFG1
 
 
hsa-miR-152-3p
 
 
-0.332
 
 
30.2
 
 
 
 
AHDC1
 
 
hsa-miR-152-3p
 
 
-0.413
 
 
15.6
 
 
 
 
AKAP1
 
 
hsa-miR-152-3p
 
 
-0.381
 
 
72.8
 
 
 
 
ALCAM
 
 
hsa-miR-152-3p
 
 
-0.347
 
 
15.7
 
 
 
 
AP4B1
 
 
hsa-miR-378a-3p
 
 
-0.62
 
 
11.3
 
 
 
 
ARF4
 
 
hsa-miR-152-3p
 
 
-0.487
 
 
148.9
 
 
 
 
ARFGEF1
 
 
hsa-miR-152-3p
 
 
-0.306
 
 
18.0
 
 
 
 
ARFIP1
 
 
hsa-miR-152-3p
 
 
-0.474
 
 
26.4
 
 
 
 
ARHGAP21
 
 
hsa-miR-152-3p
 
 
-0.287
 
 
15.5
 
 
 
 
ARL6IP1
 
 
hsa-miR-152-3p
 
 
-0.579
 
 
132.0
 
 
 
 
ARL8B
 
 
hsa-miR-152-3p
 
 
-0.337
 
 
34.2
 
 
 
 
ARRDC3
 
 
hsa-miR-152-3p
 
 
-0.305
 
 
18.9
 
 
 
 
ASPH
 
 
hsa-miR-152-3p
 
 
-0.391
 
 
72.8
 
 
 
 
ATP2B4
 
 
hsa-miR-152-3p
 
 
-0.337
 
 
46.5
 
 
 
 
ATP6AP2
 
 
hsa-miR-152-3p
 
 
-0.592
 
 
77.7
 
 
 
 
ATP6AP2
 
 
hsa-miR-152-3p
 
 
-0.668
 
 
77.7
 
 
 
 
B4GALT5
 
 
hsa-miR-152-3p
 
 
-0.44
 
 
23.3
 
 
 
 
BCL2L11
 
 
hsa-miR-152-3p
 
 
-0.445
 
 
10.9
 
 
 
 
BMP2
 
 
hsa-miR-378a-3p
 
 
-0.481
 
 
14.2
 
 
 
 
BTAF1
 
 
hsa-miR-152-3p
 
 
-0.401
 
 
16.8
 
 
 
 
BTBD3
 
 
hsa-miR-152-3p
 
 
-0.495
 
 
20.0
 
 
 
 
C1GALT1
 
 
hsa-miR-152-3p
 
 
-0.487
 
 
14.3
 
 
 
 
C1QBP
 
 
hsa-miR-330-3p
 
 
-0.412
 
 
131.3
 
 
 
 
C1orf210
 
 
hsa-miR-378a-3p
 
 
-0.307
 
 
30.4
 
 
 
 
C1orf35
 
 
hsa-miR-152-3p
 
 
-0.511
 
 
14.8
 
 
 
 
CAND1
 
 
hsa-miR-152-3p
 
 
-0.44
 
 
18.6
 
 
 
 
CANX
 
 
hsa-miR-152-3p
 
 
-0.434
 
 
96.1
 
 
 
 
CAV2
 
 
hsa-miR-152-3p
 
 
-0.32
 
 
40.6
 
 
 
 
CCDC6
 
 
hsa-miR-152-3p
 
 
-0.511
 
 
22.3
 
 
 
 
CCN2
 
 
hsa-miR-330-3p
 
 
-0.305
 
 
141.5
 
 
 
 
CCT6A
 
 
hsa-miR-152-3p
 
 
-0.665
 
 
50.1
 
 
 
 
CDC25B
 
 
hsa-miR-152-3p
 
 
-0.298
 
 
37.1
 
 
 
 
CEBPG
 
 
hsa-miR-152-3p
 
 
-0.319
 
 
28.3
 
 
 
 
CEP63
 
 
hsa-miR-152-3p
 
 
-0.329
 
 
10.9
 
 
 
 
CERS5
 
 
hsa-miR-152-3p
 
 
-0.311
 
 
16.7
 
 
 
 
CFL2
 
 
hsa-miR-152-3p
 
 
-0.487
 
 
89.0
 
 
 
 
CHMP4B
 
 
hsa-miR-152-3p
 
 
-0.36
 
 
151.1
 
 
 
 
CLCN6
 
 
hsa-miR-152-3p
 
 
-0.406
 
 
13.0
 
 
 
 
CNOT7
 
 
hsa-miR-152-3p
 
 
-0.31
 
 
40.2
 
 
 
 
COL4A1
 
 
hsa-miR-152-3p
 
 
-0.317
 
 
91.4
 
 
 
 
CS
 
 
hsa-miR-152-3p
 
 
-0.3
 
 
103.2
 
 
 
 
CTSA
 
 
hsa-miR-152-3p
 
 
-0.541
 
 
168.5
 
 
 
 
CTTNBP2NL
 
 
hsa-miR-152-3p
 
 
-0.329
 
 
14.5
 
 
 
 
CYTH3
 
 
hsa-miR-152-3p
 
 
-0.331
 
 
17.7
 
 
 
 
DCUN1D4
 
 
hsa-miR-152-3p
 
 
-0.314
 
 
14.7
 
 
 
 
DDAH1
 
 
hsa-miR-152-3p
 
 
-0.34
 
 
42.2
 
 
 
 
DDX6
 
 
hsa-miR-152-3p
 
 
-0.363
 
 
28.1
 
 
 
 
DEDD
 
 
hsa-miR-152-3p
 
 
-0.401
 
 
28.7
 
 
 
 
DMPK
 
 
hsa-miR-152-3p
 
 
-0.378
 
 
63.0
 
 
 
 
DNAJB4
 
 
hsa-miR-152-3p
 
 
-0.33
 
 
13.4
 
 
 
 
DOCK6
 
 
hsa-miR-152-3p
 
 
-0.396
 
 
16.2
 
 
 
 
DUSP1
 
 
hsa-miR-152-3p
 
 
-0.32
 
 
319.1
 
 
 
 
DYNLL2
 
 
hsa-miR-152-3p
 
 
-0.366
 
 
24.6
 
 
 
 
DYRK1B
 
 
hsa-miR-152-3p
 
 
-0.305
 
 
11.7
 
 
 
 
DYRK2
 
 
hsa-miR-152-3p
 
 
-0.293
 
 
24.1
 
 
 
 
EIF4E3
 
 
hsa-miR-152-3p
 
 
-0.464
 
 
16.7
 
 
 
 
EIF4G3
 
 
hsa-miR-378a-3p
 
 
-0.443
 
 
20.2
 
 
 
 
EOGT
 
 
hsa-miR-152-3p
 
 
-0.587
 
 
24.1
 
 
 
 
EPS15
 
 
hsa-miR-152-3p
 
 
-0.322
 
 
18.0
 
 
 
 
ERBB3
 
 
hsa-miR-152-3p
 
 
-0.399
 
 
51.6
 
 
 
 
ERRFI1
 
 
hsa-miR-152-3p
 
 
-0.529
 
 
57.2
 
 
 
 
F3
 
 
hsa-miR-152-3p
 
 
-0.331
 
 
50.2
 
 
 
 
FAM168B
 
 
hsa-miR-152-3p
 
 
-0.409
 
 
45.9
 
 
 
 
FAM43A
 
 
hsa-miR-152-3p
 
 
-0.337
 
 
14.1
 
 
 
 
FEZ2
 
 
hsa-miR-152-3p
 
 
-0.536
 
 
30.2
 
 
 
 
FMR1
 
 
hsa-miR-152-3p
 
 
-0.385
 
 
12.0
 
 
 
 
FOSB
 
 
hsa-miR-152-3p
 
 
-0.311
 
 
35.2
 
 
 
 
FOXF1
 
 
hsa-miR-152-3p
 
 
-0.374
 
 
57.8
 
 
 
 
FOXK2
 
 
hsa-miR-152-3p
 
 
-0.305
 
 
17.1
 
 
 
 
FXR1
 
 
hsa-miR-152-3p
 
 
-0.478
 
 
46.3
 
 
 
 
GADD45A
 
 
hsa-miR-152-3p
 
 
-0.787
 
 
33.2
 
 
 
 
GAP43
 
 
hsa-miR-152-3p
 
 
-0.409
 
 
11.3
 
 
 
 
GLRX5
 
 
hsa-miR-152-3p
 
 
-0.381
 
 
49.3
 
 
 
 
GMFB
 
 
hsa-miR-152-3p
 
 
-0.429
 
 
32.8
 
 
 
 
GPATCH8
 
 
hsa-miR-152-3p
 
 
-0.374
 
 
16.1
 
 
 
 
GRSF1
 
 
hsa-miR-378a-3p
 
 
-0.358
 
 
48.9
 
 
 
 
GTF2H1
 
 
hsa-miR-152-3p
 
 
-0.524
 
 
16.8
 
 
 
 
HDDC2
 
 
hsa-miR-378a-3p
 
 
-0.322
 
 
26.6
 
 
 
 
HLA-DQB1
 
 
hsa-miR-152-3p
 
 
-0.37
 
 
44.3
 
 
 
 
HSP90B1
 
 
hsa-miR-152-3p
 
 
-0.304
 
 
319.3
 
 
 
 
HSPH1
 
 
hsa-miR-330-3p
 
 
-0.345
 
 
28.7
 
 
 
 
ID2
 
 
hsa-miR-330-3p
 
 
-0.293
 
 
145.5
 
 
 
 
IL6ST
 
 
hsa-miR-152-3p
 
 
-0.291
 
 
29.8
 
 
 
 
ITSN2
 
 
hsa-miR-152-3p
 
 
-0.339
 
 
18.0
 
 
 
 
KDSR
 
 
hsa-miR-378a-3p
 
 
-0.373
 
 
11.2
 
 
 
 
KLF4
 
 
hsa-miR-152-3p
 
 
-0.325
 
 
81.8
 
 
 
 
KLF5
 
 
hsa-miR-152-3p
 
 
-0.475
 
 
151.1
 
 
 
 
LAMA4
 
 
hsa-miR-152-3p
 
 
-0.363
 
 
75.7
 
 
 
 
LBR
 
 
hsa-miR-152-3p
 
 
-0.39
 
 
56.1
 
 
 
 
LDLR
 
 
hsa-miR-152-3p
 
 
-0.323
 
 
19.2
 
 
 
 
LEPROTL1
 
 
hsa-miR-152-3p
 
 
-0.568
 
 
26.6
 
 
 
 
LIPA
 
 
hsa-miR-152-3p
 
 
-0.463
 
 
63.5
 
 
 
 
LMAN1
 
 
hsa-miR-152-3p
 
 
-0.382
 
 
32.0
 
 
 
 
LRCH1
 
 
hsa-miR-152-3p
 
 
-0.321
 
 
14.3
 
 
 
 
LRRC41
 
 
hsa-miR-152-3p
 
 
-0.392
 
 
39.1
 
 
 
 
 
 
 duodenum 
 
 
 
 
target_symbol
 
 
mature_mirna_id
 
 
score
 
 
nTPM
 
 
 
 
 
 
ABCB10
 
 
hsa-miR-152-3p
 
 
-0.291
 
 
16.1
 
 
 
 
ABCB7
 
 
hsa-miR-152-3p
 
 
-0.515
 
 
15.1
 
 
 
 
ABCD3
 
 
hsa-miR-152-3p
 
 
-0.388
 
 
42.1
 
 
 
 
ABHD17C
 
 
hsa-miR-330-3p
 
 
-0.399
 
 
21.9
 
 
 
 
ADAM10
 
 
hsa-miR-152-3p
 
 
-0.348
 
 
14.7
 
 
 
 
AGFG1
 
 
hsa-miR-152-3p
 
 
-0.332
 
 
40.8
 
 
 
 
AKAP1
 
 
hsa-miR-152-3p
 
 
-0.381
 
 
51.5
 
 
 
 
ALCAM
 
 
hsa-miR-152-3p
 
 
-0.347
 
 
16.0
 
 
 
 
ARF4
 
 
hsa-miR-152-3p
 
 
-0.487
 
 
116.2
 
 
 
 
ARFGEF1
 
 
hsa-miR-152-3p
 
 
-0.306
 
 
15.9
 
 
 
 
ARFIP1
 
 
hsa-miR-152-3p
 
 
-0.474
 
 
21.8
 
 
 
 
ARHGAP21
 
 
hsa-miR-152-3p
 
 
-0.287
 
 
12.1
 
 
 
 
ARL6IP1
 
 
hsa-miR-152-3p
 
 
-0.579
 
 
130.6
 
 
 
 
ARL8B
 
 
hsa-miR-152-3p
 
 
-0.337
 
 
28.9
 
 
 
 
ASPH
 
 
hsa-miR-152-3p
 
 
-0.391
 
 
42.9
 
 
 
 
ATP2B4
 
 
hsa-miR-152-3p
 
 
-0.337
 
 
11.8
 
 
 
 
ATP6AP2
 
 
hsa-miR-152-3p
 
 
-0.592
 
 
30.3
 
 
 
 
ATP6AP2
 
 
hsa-miR-152-3p
 
 
-0.668
 
 
30.3
 
 
 
 
B4GALT5
 
 
hsa-miR-152-3p
 
 
-0.44
 
 
23.8
 
 
 
 
BTAF1
 
 
hsa-miR-152-3p
 
 
-0.401
 
 
12.8
 
 
 
 
BTBD3
 
 
hsa-miR-152-3p
 
 
-0.495
 
 
10.1
 
 
 
 
C1GALT1
 
 
hsa-miR-152-3p
 
 
-0.487
 
 
27.7
 
 
 
 
C1QBP
 
 
hsa-miR-330-3p
 
 
-0.412
 
 
90.1
 
 
 
 
C1orf210
 
 
hsa-miR-378a-3p
 
 
-0.307
 
 
41.2
 
 
 
 
CAND1
 
 
hsa-miR-152-3p
 
 
-0.44
 
 
18.4
 
 
 
 
CANX
 
 
hsa-miR-152-3p
 
 
-0.434
 
 
41.6
 
 
 
 
CAV2
 
 
hsa-miR-152-3p
 
 
-0.32
 
 
10.6
 
 
 
 
CCDC6
 
 
hsa-miR-152-3p
 
 
-0.511
 
 
21.5
 
 
 
 
CCN2
 
 
hsa-miR-330-3p
 
 
-0.305
 
 
41.1
 
 
 
 
CCT6A
 
 
hsa-miR-152-3p
 
 
-0.665
 
 
50.6
 
 
 
 
CDC25B
 
 
hsa-miR-152-3p
 
 
-0.298
 
 
13.6
 
 
 
 
CEBPG
 
 
hsa-miR-152-3p
 
 
-0.319
 
 
32.1
 
 
 
 
CFL2
 
 
hsa-miR-152-3p
 
 
-0.487
 
 
13.4
 
 
 
 
CHMP4B
 
 
hsa-miR-152-3p
 
 
-0.36
 
 
96.9
 
 
 
 
CNOT7
 
 
hsa-miR-152-3p
 
 
-0.31
 
 
36.0
 
 
 
 
COL4A1
 
 
hsa-miR-152-3p
 
 
-0.317
 
 
23.4
 
 
 
 
CS
 
 
hsa-miR-152-3p
 
 
-0.3
 
 
92.0
 
 
 
 
CTSA
 
 
hsa-miR-152-3p
 
 
-0.541
 
 
95.9
 
 
 
 
CYTH3
 
 
hsa-miR-152-3p
 
 
-0.331
 
 
15.5
 
 
 
 
DDAH1
 
 
hsa-miR-152-3p
 
 
-0.34
 
 
37.8
 
 
 
 
DDX6
 
 
hsa-miR-152-3p
 
 
-0.363
 
 
22.3
 
 
 
 
DEDD
 
 
hsa-miR-152-3p
 
 
-0.401
 
 
19.6
 
 
 
 
DMPK
 
 
hsa-miR-152-3p
 
 
-0.378
 
 
12.2
 
 
 
 
DOCK6
 
 
hsa-miR-152-3p
 
 
-0.396
 
 
11.9
 
 
 
 
DUSP1
 
 
hsa-miR-152-3p
 
 
-0.32
 
 
62.0
 
 
 
 
DYNLL2
 
 
hsa-miR-152-3p
 
 
-0.366
 
 
23.5
 
 
 
 
DYRK2
 
 
hsa-miR-152-3p
 
 
-0.293
 
 
19.2
 
 
 
 
EIF4E3
 
 
hsa-miR-152-3p
 
 
-0.464
 
 
13.4
 
 
 
 
EIF4G3
 
 
hsa-miR-378a-3p
 
 
-0.443
 
 
13.7
 
 
 
 
EPS15
 
 
hsa-miR-152-3p
 
 
-0.322
 
 
12.1
 
 
 
 
ERBB3
 
 
hsa-miR-152-3p
 
 
-0.399
 
 
64.5
 
 
 
 
ERRFI1
 
 
hsa-miR-152-3p
 
 
-0.529
 
 
31.4
 
 
 
 
F3
 
 
hsa-miR-152-3p
 
 
-0.331
 
 
34.5
 
 
 
 
FAM168B
 
 
hsa-miR-152-3p
 
 
-0.409
 
 
24.8
 
 
 
 
FEZ2
 
 
hsa-miR-152-3p
 
 
-0.536
 
 
20.8
 
 
 
 
FMR1
 
 
hsa-miR-152-3p
 
 
-0.385
 
 
11.0
 
 
 
 
FOSB
 
 
hsa-miR-152-3p
 
 
-0.311
 
 
23.3
 
 
 
 
FXR1
 
 
hsa-miR-152-3p
 
 
-0.478
 
 
19.9
 
 
 
 
GADD45A
 
 
hsa-miR-152-3p
 
 
-0.787
 
 
23.3
 
 
 
 
GLRX5
 
 
hsa-miR-152-3p
 
 
-0.381
 
 
43.5
 
 
 
 
GMFB
 
 
hsa-miR-152-3p
 
 
-0.429
 
 
31.5
 
 
 
 
GOLT1A
 
 
hsa-miR-378a-3p
 
 
-0.574
 
 
44.6
 
 
 
 
GPATCH8
 
 
hsa-miR-152-3p
 
 
-0.374
 
 
10.4
 
 
 
 
GRSF1
 
 
hsa-miR-378a-3p
 
 
-0.358
 
 
39.0
 
 
 
 
HDDC2
 
 
hsa-miR-378a-3p
 
 
-0.322
 
 
19.1
 
 
 
 
HSP90B1
 
 
hsa-miR-152-3p
 
 
-0.304
 
 
246.2
 
 
 
 
HSPH1
 
 
hsa-miR-330-3p
 
 
-0.345
 
 
10.6
 
 
 
 
ID2
 
 
hsa-miR-330-3p
 
 
-0.293
 
 
93.2
 
 
 
 
IL6ST
 
 
hsa-miR-152-3p
 
 
-0.291
 
 
15.0
 
 
 
 
ITSN2
 
 
hsa-miR-152-3p
 
 
-0.339
 
 
16.5
 
 
 
 
KLF4
 
 
hsa-miR-152-3p
 
 
-0.325
 
 
26.7
 
 
 
 
KLF5
 
 
hsa-miR-152-3p
 
 
-0.475
 
 
122.8
 
 
 
 
LAMA4
 
 
hsa-miR-152-3p
 
 
-0.363
 
 
16.2
 
 
 
 
LBR
 
 
hsa-miR-152-3p
 
 
-0.39
 
 
38.3
 
 
 
 
LDLR
 
 
hsa-miR-152-3p
 
 
-0.323
 
 
22.2
 
 
 
 
LEPROTL1
 
 
hsa-miR-152-3p
 
 
-0.568
 
 
19.2
 
 
 
 
LIPA
 
 
hsa-miR-152-3p
 
 
-0.463
 
 
157.9
 
 
 
 
LMAN1
 
 
hsa-miR-152-3p
 
 
-0.382
 
 
43.0
 
 
 
 
LRRC41
 
 
hsa-miR-152-3p
 
 
-0.392
 
 
21.4
 
 
 
 
LSM5
 
 
hsa-miR-330-3p
 
 
-0.351
 
 
39.8
 
 
 
 
LYSMD2
 
 
hsa-miR-152-3p
 
 
-0.554
 
 
14.9
 
 
 
 
MACROH2A1
 
 
hsa-miR-152-3p
 
 
-0.336
 
 
98.4
 
 
 
 
MAF
 
 
hsa-miR-152-3p
 
 
-0.362
 
 
66.2
 
 
 
 
MAF1
 
 
hsa-miR-152-3p
 
 
-0.366
 
 
58.4
 
 
 
 
MAFG
 
 
hsa-miR-152-3p
 
 
-0.328
 
 
10.5
 
 
 
 
MAP2K1
 
 
hsa-miR-152-3p
 
 
-0.295
 
 
17.1
 
 
 
 
MAPK1
 
 
hsa-miR-378a-3p
 
 
-0.314
 
 
24.3
 
 
 
 
MAT2A
 
 
hsa-miR-330-3p
 
 
-0.307
 
 
133.3
 
 
 
 
MCM3AP
 
 
hsa-miR-152-3p
 
 
-0.287
 
 
13.1
 
 
 
 
MED19
 
 
hsa-miR-378a-3p
 
 
-0.434
 
 
10.0
 
 
 
 
MIER1
 
 
hsa-miR-152-3p
 
 
-0.352
 
 
14.6
 
 
 
 
MMD
 
 
hsa-miR-152-3p
 
 
-0.62
 
 
10.3
 
 
 
 
MMP15
 
 
hsa-miR-152-3p
 
 
-0.458
 
 
37.8
 
 
 
 
MRPS10
 
 
hsa-miR-152-3p
 
 
-0.335
 
 
17.9
 
 
 
 
MRPS25
 
 
hsa-miR-152-3p
 
 
-0.323
 
 
12.7
 
 
 
 
MTMR10
 
 
hsa-miR-152-3p
 
 
-0.302
 
 
12.2
 
 
 
 
MTMR14
 
 
hsa-miR-152-3p
 
 
-0.467
 
 
16.7
 
 
 
 
MXD1
 
 
hsa-miR-152-3p
 
 
-0.498
 
 
27.1
 
 
 
 
NAP1L1
 
 
hsa-miR-152-3p
 
 
-0.343
 
 
72.1
 
 
 
 
NCOA1
 
 
hsa-miR-152-3p
 
 
-0.313
 
 
19.6
 
 
 
 
 
 
 liver 
 
 
 
 
target_symbol
 
 
mature_mirna_id
 
 
score
 
 
nTPM
 
 
 
 
 
 
ABCB10
 
 
hsa-miR-152-3p
 
 
-0.291
 
 
17.7
 
 
 
 
ABCB7
 
 
hsa-miR-152-3p
 
 
-0.515
 
 
14.1
 
 
 
 
ABCD3
 
 
hsa-miR-152-3p
 
 
-0.388
 
 
118.4
 
 
 
 
ACVR1
 
 
hsa-miR-152-3p
 
 
-0.397
 
 
15.3
 
 
 
 
ADAM10
 
 
hsa-miR-152-3p
 
 
-0.348
 
 
15.0
 
 
 
 
ADGRF5
 
 
hsa-miR-152-3p
 
 
-0.354
 
 
12.3
 
 
 
 
AGFG1
 
 
hsa-miR-152-3p
 
 
-0.332
 
 
33.8
 
 
 
 
AKAP1
 
 
hsa-miR-152-3p
 
 
-0.381
 
 
48.4
 
 
 
 
ALCAM
 
 
hsa-miR-152-3p
 
 
-0.347
 
 
44.8
 
 
 
 
ARF4
 
 
hsa-miR-152-3p
 
 
-0.487
 
 
161.3
 
 
 
 
ARFGEF1
 
 
hsa-miR-152-3p
 
 
-0.306
 
 
14.2
 
 
 
 
ARFIP1
 
 
hsa-miR-152-3p
 
 
-0.474
 
 
14.4
 
 
 
 
ARHGAP21
 
 
hsa-miR-152-3p
 
 
-0.287
 
 
11.9
 
 
 
 
ARL6IP1
 
 
hsa-miR-152-3p
 
 
-0.579
 
 
336.2
 
 
 
 
ARL8B
 
 
hsa-miR-152-3p
 
 
-0.337
 
 
41.1
 
 
 
 
ARRDC3
 
 
hsa-miR-152-3p
 
 
-0.305
 
 
71.0
 
 
 
 
ASPH
 
 
hsa-miR-152-3p
 
 
-0.391
 
 
69.9
 
 
 
 
ATP6AP2
 
 
hsa-miR-152-3p
 
 
-0.592
 
 
90.5
 
 
 
 
ATP6AP2
 
 
hsa-miR-152-3p
 
 
-0.668
 
 
90.5
 
 
 
 
B4GALT5
 
 
hsa-miR-152-3p
 
 
-0.44
 
 
24.1
 
 
 
 
BCL2L11
 
 
hsa-miR-152-3p
 
 
-0.445
 
 
12.9
 
 
 
 
BTAF1
 
 
hsa-miR-152-3p
 
 
-0.401
 
 
12.8
 
 
 
 
C1GALT1
 
 
hsa-miR-152-3p
 
 
-0.487
 
 
20.5
 
 
 
 
C1QBP
 
 
hsa-miR-330-3p
 
 
-0.412
 
 
125.5
 
 
 
 
C1orf35
 
 
hsa-miR-152-3p
 
 
-0.511
 
 
13.7
 
 
 
 
CAND1
 
 
hsa-miR-152-3p
 
 
-0.44
 
 
25.6
 
 
 
 
CANX
 
 
hsa-miR-152-3p
 
 
-0.434
 
 
156.6
 
 
 
 
CAV2
 
 
hsa-miR-152-3p
 
 
-0.32
 
 
38.7
 
 
 
 
CCDC6
 
 
hsa-miR-152-3p
 
 
-0.511
 
 
11.3
 
 
 
 
CCDC71
 
 
hsa-miR-152-3p
 
 
-0.369
 
 
12.0
 
 
 
 
CCN2
 
 
hsa-miR-330-3p
 
 
-0.305
 
 
77.5
 
 
 
 
CCT6A
 
 
hsa-miR-152-3p
 
 
-0.665
 
 
50.4
 
 
 
 
CDC25B
 
 
hsa-miR-152-3p
 
 
-0.298
 
 
23.7
 
 
 
 
CEBPG
 
 
hsa-miR-152-3p
 
 
-0.319
 
 
32.3
 
 
 
 
CEP63
 
 
hsa-miR-152-3p
 
 
-0.329
 
 
18.9
 
 
 
 
CERS5
 
 
hsa-miR-152-3p
 
 
-0.311
 
 
11.6
 
 
 
 
CFL2
 
 
hsa-miR-152-3p
 
 
-0.487
 
 
117.6
 
 
 
 
CHMP4B
 
 
hsa-miR-152-3p
 
 
-0.36
 
 
66.3
 
 
 
 
CLCN6
 
 
hsa-miR-152-3p
 
 
-0.406
 
 
12.8
 
 
 
 
CNOT7
 
 
hsa-miR-152-3p
 
 
-0.31
 
 
45.4
 
 
 
 
COL4A1
 
 
hsa-miR-152-3p
 
 
-0.317
 
 
20.7
 
 
 
 
CS
 
 
hsa-miR-152-3p
 
 
-0.3
 
 
38.0
 
 
 
 
CTSA
 
 
hsa-miR-152-3p
 
 
-0.541
 
 
90.0
 
 
 
 
CUL5
 
 
hsa-miR-152-3p
 
 
-0.389
 
 
13.2
 
 
 
 
DCUN1D4
 
 
hsa-miR-152-3p
 
 
-0.314
 
 
27.0
 
 
 
 
DDAH1
 
 
hsa-miR-152-3p
 
 
-0.34
 
 
74.4
 
 
 
 
DDX6
 
 
hsa-miR-152-3p
 
 
-0.363
 
 
32.0
 
 
 
 
DEDD
 
 
hsa-miR-152-3p
 
 
-0.401
 
 
23.2
 
 
 
 
DICER1
 
 
hsa-miR-152-3p
 
 
-0.29
 
 
13.2
 
 
 
 
DIPK2A
 
 
hsa-miR-152-3p
 
 
-0.393
 
 
17.6
 
 
 
 
DNAJB4
 
 
hsa-miR-152-3p
 
 
-0.33
 
 
18.7
 
 
 
 
DOCK6
 
 
hsa-miR-152-3p
 
 
-0.396
 
 
12.6
 
 
 
 
DUSP1
 
 
hsa-miR-152-3p
 
 
-0.32
 
 
669.9
 
 
 
 
DYNLL2
 
 
hsa-miR-152-3p
 
 
-0.366
 
 
32.9
 
 
 
 
EIF4E3
 
 
hsa-miR-152-3p
 
 
-0.464
 
 
10.9
 
 
 
 
EIF4G3
 
 
hsa-miR-378a-3p
 
 
-0.443
 
 
13.2
 
 
 
 
EPS15
 
 
hsa-miR-152-3p
 
 
-0.322
 
 
21.4
 
 
 
 
ERBB3
 
 
hsa-miR-152-3p
 
 
-0.399
 
 
83.3
 
 
 
 
ERRFI1
 
 
hsa-miR-152-3p
 
 
-0.529
 
 
695.4
 
 
 
 
ESR1
 
 
hsa-miR-152-3p
 
 
-0.34
 
 
15.8
 
 
 
 
FAM168B
 
 
hsa-miR-152-3p
 
 
-0.409
 
 
27.8
 
 
 
 
FBXO28
 
 
hsa-miR-152-3p
 
 
-0.313
 
 
16.3
 
 
 
 
FEZ2
 
 
hsa-miR-152-3p
 
 
-0.536
 
 
27.5
 
 
 
 
FMR1
 
 
hsa-miR-152-3p
 
 
-0.385
 
 
27.1
 
 
 
 
FOSB
 
 
hsa-miR-152-3p
 
 
-0.311
 
 
79.9
 
 
 
 
FOXK2
 
 
hsa-miR-152-3p
 
 
-0.305
 
 
12.6
 
 
 
 
FXR1
 
 
hsa-miR-152-3p
 
 
-0.478
 
 
32.9
 
 
 
 
GADD45A
 
 
hsa-miR-152-3p
 
 
-0.787
 
 
154.1
 
 
 
 
GAPVD1
 
 
hsa-miR-152-3p
 
 
-0.309
 
 
10.5
 
 
 
 
GLRX5
 
 
hsa-miR-152-3p
 
 
-0.381
 
 
102.7
 
 
 
 
GMFB
 
 
hsa-miR-152-3p
 
 
-0.429
 
 
24.2
 
 
 
 
GOLT1A
 
 
hsa-miR-378a-3p
 
 
-0.574
 
 
217.2
 
 
 
 
GRSF1
 
 
hsa-miR-378a-3p
 
 
-0.358
 
 
49.0
 
 
 
 
GTF2H1
 
 
hsa-miR-152-3p
 
 
-0.524
 
 
16.1
 
 
 
 
HDDC2
 
 
hsa-miR-378a-3p
 
 
-0.322
 
 
17.3
 
 
 
 
HLA-DQB1
 
 
hsa-miR-152-3p
 
 
-0.37
 
 
35.5
 
 
 
 
HSP90B1
 
 
hsa-miR-152-3p
 
 
-0.304
 
 
688.5
 
 
 
 
HSPH1
 
 
hsa-miR-330-3p
 
 
-0.345
 
 
47.5
 
 
 
 
ID2
 
 
hsa-miR-330-3p
 
 
-0.293
 
 
539.3
 
 
 
 
IGF1
 
 
hsa-miR-152-3p
 
 
-0.43
 
 
78.0
 
 
 
 
IL6ST
 
 
hsa-miR-152-3p
 
 
-0.291
 
 
120.9
 
 
 
 
INHBB
 
 
hsa-miR-152-3p
 
 
-0.469
 
 
18.9
 
 
 
 
INSIG2
 
 
hsa-miR-152-3p
 
 
-0.403
 
 
21.4
 
 
 
 
ITSN2
 
 
hsa-miR-152-3p
 
 
-0.339
 
 
24.2
 
 
 
 
KDSR
 
 
hsa-miR-378a-3p
 
 
-0.373
 
 
21.3
 
 
 
 
KLF4
 
 
hsa-miR-152-3p
 
 
-0.325
 
 
11.5
 
 
 
 
LBR
 
 
hsa-miR-152-3p
 
 
-0.39
 
 
44.3
 
 
 
 
LDLR
 
 
hsa-miR-152-3p
 
 
-0.323
 
 
56.4
 
 
 
 
LEPROTL1
 
 
hsa-miR-152-3p
 
 
-0.568
 
 
34.8
 
 
 
 
LIPA
 
 
hsa-miR-152-3p
 
 
-0.463
 
 
204.6
 
 
 
 
LMAN1
 
 
hsa-miR-152-3p
 
 
-0.382
 
 
169.4
 
 
 
 
LONRF3
 
 
hsa-miR-152-3p
 
 
-0.3
 
 
11.9
 
 
 
 
LRCH1
 
 
hsa-miR-152-3p
 
 
-0.321
 
 
11.4
 
 
 
 
LRRC41
 
 
hsa-miR-152-3p
 
 
-0.392
 
 
32.6
 
 
 
 
LSM5
 
 
hsa-miR-330-3p
 
 
-0.351
 
 
70.8
 
 
 
 
LYRM2
 
 
hsa-miR-152-3p
 
 
-0.351
 
 
11.6
 
 
 
 
LYSMD2
 
 
hsa-miR-152-3p
 
 
-0.554
 
 
19.7
 
 
 
 
MACROH2A1
 
 
hsa-miR-152-3p
 
 
-0.336
 
 
42.9
 
 
 
 
MAF
 
 
hsa-miR-152-3p
 
 
-0.362
 
 
24.1
 
 
 
 
MAF1
 
 
hsa-miR-152-3p
 
 
-0.366
 
 
57.7
 
 
 
 
 
 
 lymph node 
 
 
 
 
target_symbol
 
 
mature_mirna_id
 
 
score
 
 
nTPM
 
 
 
 
 
 
ABCB10
 
 
hsa-miR-152-3p
 
 
-0.291
 
 
10.3
 
 
 
 
ABCB7
 
 
hsa-miR-152-3p
 
 
-0.515
 
 
11.2
 
 
 
 
ABCD3
 
 
hsa-miR-152-3p
 
 
-0.388
 
 
12.4
 
 
 
 
ADAM10
 
 
hsa-miR-152-3p
 
 
-0.348
 
 
20.8
 
 
 
 
AGFG1
 
 
hsa-miR-152-3p
 
 
-0.332
 
 
44.0
 
 
 
 
AKAP1
 
 
hsa-miR-152-3p
 
 
-0.381
 
 
10.0
 
 
 
 
AP4B1
 
 
hsa-miR-378a-3p
 
 
-0.62
 
 
13.4
 
 
 
 
ARF4
 
 
hsa-miR-152-3p
 
 
-0.487
 
 
69.1
 
 
 
 
ARFGEF1
 
 
hsa-miR-152-3p
 
 
-0.306
 
 
14.4
 
 
 
 
ARFIP1
 
 
hsa-miR-152-3p
 
 
-0.474
 
 
14.1
 
 
 
 
ARL6IP1
 
 
hsa-miR-152-3p
 
 
-0.579
 
 
124.0
 
 
 
 
ARL8B
 
 
hsa-miR-152-3p
 
 
-0.337
 
 
36.2
 
 
 
 
ARRDC3
 
 
hsa-miR-152-3p
 
 
-0.305
 
 
27.5
 
 
 
 
ASPH
 
 
hsa-miR-152-3p
 
 
-0.391
 
 
18.8
 
 
 
 
ATP2B4
 
 
hsa-miR-152-3p
 
 
-0.337
 
 
12.0
 
 
 
 
ATP6AP2
 
 
hsa-miR-152-3p
 
 
-0.592
 
 
63.1
 
 
 
 
ATP6AP2
 
 
hsa-miR-152-3p
 
 
-0.668
 
 
63.1
 
 
 
 
BCL2L11
 
 
hsa-miR-152-3p
 
 
-0.445
 
 
23.7
 
 
 
 
BTAF1
 
 
hsa-miR-152-3p
 
 
-0.401
 
 
25.1
 
 
 
 
C1GALT1
 
 
hsa-miR-152-3p
 
 
-0.487
 
 
17.8
 
 
 
 
C1QBP
 
 
hsa-miR-330-3p
 
 
-0.412
 
 
108.0
 
 
 
 
CAND1
 
 
hsa-miR-152-3p
 
 
-0.44
 
 
25.7
 
 
 
 
CANX
 
 
hsa-miR-152-3p
 
 
-0.434
 
 
16.7
 
 
 
 
CBLB
 
 
hsa-miR-152-3p
 
 
-0.424
 
 
13.8
 
 
 
 
CCDC6
 
 
hsa-miR-152-3p
 
 
-0.511
 
 
20.0
 
 
 
 
CCDC71
 
 
hsa-miR-152-3p
 
 
-0.369
 
 
12.6
 
 
 
 
CCN2
 
 
hsa-miR-330-3p
 
 
-0.305
 
 
69.0
 
 
 
 
CCT6A
 
 
hsa-miR-152-3p
 
 
-0.665
 
 
66.0
 
 
 
 
CDC25B
 
 
hsa-miR-152-3p
 
 
-0.298
 
 
44.6
 
 
 
 
CDK5R1
 
 
hsa-miR-152-3p
 
 
-0.547
 
 
13.0
 
 
 
 
CEBPG
 
 
hsa-miR-152-3p
 
 
-0.319
 
 
11.6
 
 
 
 
CEP55
 
 
hsa-miR-152-3p
 
 
-0.305
 
 
13.7
 
 
 
 
CEP63
 
 
hsa-miR-152-3p
 
 
-0.329
 
 
14.2
 
 
 
 
CFL2
 
 
hsa-miR-152-3p
 
 
-0.487
 
 
10.0
 
 
 
 
CHIT1
 
 
hsa-miR-378a-3p
 
 
-0.387
 
 
20.4
 
 
 
 
CHMP4B
 
 
hsa-miR-152-3p
 
 
-0.36
 
 
66.1
 
 
 
 
CNOT7
 
 
hsa-miR-152-3p
 
 
-0.31
 
 
48.6
 
 
 
 
COL4A1
 
 
hsa-miR-152-3p
 
 
-0.317
 
 
25.9
 
 
 
 
CS
 
 
hsa-miR-152-3p
 
 
-0.3
 
 
62.7
 
 
 
 
CTSA
 
 
hsa-miR-152-3p
 
 
-0.541
 
 
57.8
 
 
 
 
CUL5
 
 
hsa-miR-152-3p
 
 
-0.389
 
 
10.4
 
 
 
 
DCP2
 
 
hsa-miR-152-3p
 
 
-0.29
 
 
11.8
 
 
 
 
DDX6
 
 
hsa-miR-152-3p
 
 
-0.363
 
 
45.8
 
 
 
 
DEDD
 
 
hsa-miR-152-3p
 
 
-0.401
 
 
19.8
 
 
 
 
DICER1
 
 
hsa-miR-152-3p
 
 
-0.29
 
 
11.1
 
 
 
 
DIPK2A
 
 
hsa-miR-152-3p
 
 
-0.393
 
 
10.6
 
 
 
 
DNMT1
 
 
hsa-miR-152-3p
 
 
-0.421
 
 
24.4
 
 
 
 
DUSP1
 
 
hsa-miR-152-3p
 
 
-0.32
 
 
167.2
 
 
 
 
DYNLL2
 
 
hsa-miR-152-3p
 
 
-0.366
 
 
14.5
 
 
 
 
DYRK2
 
 
hsa-miR-152-3p
 
 
-0.293
 
 
20.2
 
 
 
 
EGR3
 
 
hsa-miR-152-3p
 
 
-0.315
 
 
10.7
 
 
 
 
EIF4E3
 
 
hsa-miR-152-3p
 
 
-0.464
 
 
11.9
 
 
 
 
EIF4G3
 
 
hsa-miR-378a-3p
 
 
-0.443
 
 
15.9
 
 
 
 
EPS15
 
 
hsa-miR-152-3p
 
 
-0.322
 
 
38.9
 
 
 
 
ERRFI1
 
 
hsa-miR-152-3p
 
 
-0.529
 
 
16.5
 
 
 
 
F3
 
 
hsa-miR-152-3p
 
 
-0.331
 
 
12.1
 
 
 
 
FAM168B
 
 
hsa-miR-152-3p
 
 
-0.409
 
 
26.0
 
 
 
 
FEZ2
 
 
hsa-miR-152-3p
 
 
-0.536
 
 
22.8
 
 
 
 
FMR1
 
 
hsa-miR-152-3p
 
 
-0.385
 
 
13.4
 
 
 
 
FOSB
 
 
hsa-miR-152-3p
 
 
-0.311
 
 
63.3
 
 
 
 
FOXK2
 
 
hsa-miR-152-3p
 
 
-0.305
 
 
11.7
 
 
 
 
FXR1
 
 
hsa-miR-152-3p
 
 
-0.478
 
 
28.1
 
 
 
 
GADD45A
 
 
hsa-miR-152-3p
 
 
-0.787
 
 
12.8
 
 
 
 
GAPVD1
 
 
hsa-miR-152-3p
 
 
-0.309
 
 
11.8
 
 
 
 
GLRX5
 
 
hsa-miR-152-3p
 
 
-0.381
 
 
21.6
 
 
 
 
GMFB
 
 
hsa-miR-152-3p
 
 
-0.429
 
 
23.6
 
 
 
 
GPATCH8
 
 
hsa-miR-152-3p
 
 
-0.374
 
 
14.7
 
 
 
 
GRSF1
 
 
hsa-miR-378a-3p
 
 
-0.358
 
 
42.6
 
 
 
 
GTF2H1
 
 
hsa-miR-152-3p
 
 
-0.524
 
 
10.2
 
 
 
 
HDDC2
 
 
hsa-miR-378a-3p
 
 
-0.322
 
 
25.3
 
 
 
 
HLA-DQB1
 
 
hsa-miR-152-3p
 
 
-0.37
 
 
290.6
 
 
 
 
HSP90B1
 
 
hsa-miR-152-3p
 
 
-0.304
 
 
230.2
 
 
 
 
HSPH1
 
 
hsa-miR-330-3p
 
 
-0.345
 
 
32.6
 
 
 
 
ID2
 
 
hsa-miR-330-3p
 
 
-0.293
 
 
86.7
 
 
 
 
IL6ST
 
 
hsa-miR-152-3p
 
 
-0.291
 
 
31.5
 
 
 
 
ITSN2
 
 
hsa-miR-152-3p
 
 
-0.339
 
 
49.8
 
 
 
 
LAMA4
 
 
hsa-miR-152-3p
 
 
-0.363
 
 
10.7
 
 
 
 
LBR
 
 
hsa-miR-152-3p
 
 
-0.39
 
 
76.2
 
 
 
 
LEPROTL1
 
 
hsa-miR-152-3p
 
 
-0.568
 
 
36.8
 
 
 
 
LIPA
 
 
hsa-miR-152-3p
 
 
-0.463
 
 
149.3
 
 
 
 
LMAN1
 
 
hsa-miR-152-3p
 
 
-0.382
 
 
26.6
 
 
 
 
LRCH1
 
 
hsa-miR-152-3p
 
 
-0.321
 
 
15.2
 
 
 
 
LRRC41
 
 
hsa-miR-152-3p
 
 
-0.392
 
 
19.1
 
 
 
 
LSM5
 
 
hsa-miR-330-3p
 
 
-0.351
 
 
57.5
 
 
 
 
LYRM2
 
 
hsa-miR-152-3p
 
 
-0.351
 
 
12.0
 
 
 
 
LYSMD2
 
 
hsa-miR-152-3p
 
 
-0.554
 
 
27.1
 
 
 
 
MACROH2A1
 
 
hsa-miR-152-3p
 
 
-0.336
 
 
106.1
 
 
 
 
MAF
 
 
hsa-miR-152-3p
 
 
-0.362
 
 
29.0
 
 
 
 
MAF1
 
 
hsa-miR-152-3p
 
 
-0.366
 
 
73.5
 
 
 
 
MAFB
 
 
hsa-miR-152-3p
 
 
-0.45
 
 
26.1
 
 
 
 
MAP2K1
 
 
hsa-miR-152-3p
 
 
-0.295
 
 
37.3
 
 
 
 
MAPK1
 
 
hsa-miR-378a-3p
 
 
-0.314
 
 
34.4
 
 
 
 
MAT2A
 
 
hsa-miR-330-3p
 
 
-0.307
 
 
113.9
 
 
 
 
MCM3AP
 
 
hsa-miR-152-3p
 
 
-0.287
 
 
19.7
 
 
 
 
MDFIC
 
 
hsa-miR-152-3p
 
 
-0.395
 
 
25.7
 
 
 
 
MECP2
 
 
hsa-miR-152-3p
 
 
-0.3
 
 
12.6
 
 
 
 
METTL4
 
 
hsa-miR-378a-3p
 
 
-0.486
 
 
10.8
 
 
 
 
MIER1
 
 
hsa-miR-152-3p
 
 
-0.352
 
 
19.4
 
 
 
 
MLLT10
 
 
hsa-miR-152-3p
 
 
-0.334
 
 
14.2
 
 
 
 
MMD
 
 
hsa-miR-152-3p
 
 
-0.62
 
 
16.6
 
 
 
 
 
 
 placenta 
 
 
 
 
target_symbol
 
 
mature_mirna_id
 
 
score
 
 
nTPM
 
 
 
 
 
 
ABCB10
 
 
hsa-miR-152-3p
 
 
-0.291
 
 
10.6
 
 
 
 
ABCD3
 
 
hsa-miR-152-3p
 
 
-0.388
 
 
13.6
 
 
 
 
ACVR1
 
 
hsa-miR-152-3p
 
 
-0.397
 
 
12.5
 
 
 
 
ADAM10
 
 
hsa-miR-152-3p
 
 
-0.348
 
 
27.1
 
 
 
 
ADAM17
 
 
hsa-miR-152-3p
 
 
-0.301
 
 
17.9
 
 
 
 
ADGRF5
 
 
hsa-miR-152-3p
 
 
-0.354
 
 
48.3
 
 
 
 
AGFG1
 
 
hsa-miR-152-3p
 
 
-0.332
 
 
71.2
 
 
 
 
ALCAM
 
 
hsa-miR-152-3p
 
 
-0.347
 
 
11.3
 
 
 
 
ARF4
 
 
hsa-miR-152-3p
 
 
-0.487
 
 
170.7
 
 
 
 
ARFGEF1
 
 
hsa-miR-152-3p
 
 
-0.306
 
 
13.6
 
 
 
 
ARFIP1
 
 
hsa-miR-152-3p
 
 
-0.474
 
 
16.9
 
 
 
 
ARHGAP21
 
 
hsa-miR-152-3p
 
 
-0.287
 
 
15.6
 
 
 
 
ARL6IP1
 
 
hsa-miR-152-3p
 
 
-0.579
 
 
84.9
 
 
 
 
ARL8B
 
 
hsa-miR-152-3p
 
 
-0.337
 
 
46.8
 
 
 
 
ARRDC3
 
 
hsa-miR-152-3p
 
 
-0.305
 
 
72.1
 
 
 
 
ASPH
 
 
hsa-miR-152-3p
 
 
-0.391
 
 
44.6
 
 
 
 
ATP2B4
 
 
hsa-miR-152-3p
 
 
-0.337
 
 
38.2
 
 
 
 
ATP6AP2
 
 
hsa-miR-152-3p
 
 
-0.592
 
 
100.4
 
 
 
 
ATP6AP2
 
 
hsa-miR-152-3p
 
 
-0.668
 
 
100.4
 
 
 
 
B4GALT5
 
 
hsa-miR-152-3p
 
 
-0.44
 
 
25.6
 
 
 
 
BMP2
 
 
hsa-miR-378a-3p
 
 
-0.481
 
 
10.8
 
 
 
 
BTAF1
 
 
hsa-miR-152-3p
 
 
-0.401
 
 
10.9
 
 
 
 
BTBD3
 
 
hsa-miR-152-3p
 
 
-0.495
 
 
39.5
 
 
 
 
C1GALT1
 
 
hsa-miR-152-3p
 
 
-0.487
 
 
25.3
 
 
 
 
C1QBP
 
 
hsa-miR-330-3p
 
 
-0.412
 
 
71.1
 
 
 
 
CAND1
 
 
hsa-miR-152-3p
 
 
-0.44
 
 
21.8
 
 
 
 
CANX
 
 
hsa-miR-152-3p
 
 
-0.434
 
 
70.1
 
 
 
 
CAV2
 
 
hsa-miR-152-3p
 
 
-0.32
 
 
87.6
 
 
 
 
CCDC6
 
 
hsa-miR-152-3p
 
 
-0.511
 
 
12.4
 
 
 
 
CCN2
 
 
hsa-miR-330-3p
 
 
-0.305
 
 
186.1
 
 
 
 
CCT6A
 
 
hsa-miR-152-3p
 
 
-0.665
 
 
54.0
 
 
 
 
CDC25B
 
 
hsa-miR-152-3p
 
 
-0.298
 
 
29.2
 
 
 
 
CDK19
 
 
hsa-miR-152-3p
 
 
-0.369
 
 
10.8
 
 
 
 
CDK19
 
 
hsa-miR-152-3p
 
 
-0.405
 
 
10.8
 
 
 
 
CEBPG
 
 
hsa-miR-152-3p
 
 
-0.319
 
 
15.8
 
 
 
 
CEP63
 
 
hsa-miR-152-3p
 
 
-0.329
 
 
11.9
 
 
 
 
CFL2
 
 
hsa-miR-152-3p
 
 
-0.487
 
 
33.1
 
 
 
 
CHMP4B
 
 
hsa-miR-152-3p
 
 
-0.36
 
 
70.2
 
 
 
 
CNOT7
 
 
hsa-miR-152-3p
 
 
-0.31
 
 
40.4
 
 
 
 
COL4A1
 
 
hsa-miR-152-3p
 
 
-0.317
 
 
846.2
 
 
 
 
CS
 
 
hsa-miR-152-3p
 
 
-0.3
 
 
42.9
 
 
 
 
CTSA
 
 
hsa-miR-152-3p
 
 
-0.541
 
 
72.3
 
 
 
 
CTTNBP2NL
 
 
hsa-miR-152-3p
 
 
-0.329
 
 
19.0
 
 
 
 
CUL5
 
 
hsa-miR-152-3p
 
 
-0.389
 
 
10.7
 
 
 
 
CYTH3
 
 
hsa-miR-152-3p
 
 
-0.331
 
 
29.8
 
 
 
 
DACT1
 
 
hsa-miR-378a-3p
 
 
-0.298
 
 
11.3
 
 
 
 
DCP2
 
 
hsa-miR-152-3p
 
 
-0.29
 
 
11.3
 
 
 
 
DDAH1
 
 
hsa-miR-152-3p
 
 
-0.34
 
 
10.9
 
 
 
 
DDX6
 
 
hsa-miR-152-3p
 
 
-0.363
 
 
29.7
 
 
 
 
DEDD
 
 
hsa-miR-152-3p
 
 
-0.401
 
 
20.8
 
 
 
 
DICER1
 
 
hsa-miR-152-3p
 
 
-0.29
 
 
16.5
 
 
 
 
DIPK2A
 
 
hsa-miR-152-3p
 
 
-0.393
 
 
35.7
 
 
 
 
DNAJB4
 
 
hsa-miR-152-3p
 
 
-0.33
 
 
24.4
 
 
 
 
DNMT1
 
 
hsa-miR-152-3p
 
 
-0.421
 
 
24.4
 
 
 
 
DOCK6
 
 
hsa-miR-152-3p
 
 
-0.396
 
 
11.9
 
 
 
 
DUSP1
 
 
hsa-miR-152-3p
 
 
-0.32
 
 
279.2
 
 
 
 
DYNLL2
 
 
hsa-miR-152-3p
 
 
-0.366
 
 
28.5
 
 
 
 
DYRK2
 
 
hsa-miR-152-3p
 
 
-0.293
 
 
13.3
 
 
 
 
EIF4G3
 
 
hsa-miR-378a-3p
 
 
-0.443
 
 
14.9
 
 
 
 
EOGT
 
 
hsa-miR-152-3p
 
 
-0.587
 
 
20.9
 
 
 
 
EPS15
 
 
hsa-miR-152-3p
 
 
-0.322
 
 
27.0
 
 
 
 
ERBB3
 
 
hsa-miR-152-3p
 
 
-0.399
 
 
10.1
 
 
 
 
ERRFI1
 
 
hsa-miR-152-3p
 
 
-0.529
 
 
118.0
 
 
 
 
ESRRG
 
 
hsa-miR-152-3p
 
 
-0.347
 
 
10.0
 
 
 
 
F3
 
 
hsa-miR-152-3p
 
 
-0.331
 
 
125.0
 
 
 
 
FAM168B
 
 
hsa-miR-152-3p
 
 
-0.409
 
 
27.6
 
 
 
 
FAM184A
 
 
hsa-miR-152-3p
 
 
-0.371
 
 
21.5
 
 
 
 
FAM43A
 
 
hsa-miR-152-3p
 
 
-0.337
 
 
10.5
 
 
 
 
FBN1
 
 
hsa-miR-152-3p
 
 
-0.349
 
 
61.7
 
 
 
 
FEZ2
 
 
hsa-miR-152-3p
 
 
-0.536
 
 
44.0
 
 
 
 
FMR1
 
 
hsa-miR-152-3p
 
 
-0.385
 
 
17.7
 
 
 
 
FOSB
 
 
hsa-miR-152-3p
 
 
-0.311
 
 
54.2
 
 
 
 
FOXF1
 
 
hsa-miR-152-3p
 
 
-0.374
 
 
21.7
 
 
 
 
FOXK2
 
 
hsa-miR-152-3p
 
 
-0.305
 
 
14.7
 
 
 
 
FXR1
 
 
hsa-miR-152-3p
 
 
-0.478
 
 
37.7
 
 
 
 
GADD45A
 
 
hsa-miR-152-3p
 
 
-0.787
 
 
20.8
 
 
 
 
GAPVD1
 
 
hsa-miR-152-3p
 
 
-0.309
 
 
17.3
 
 
 
 
GDF6
 
 
hsa-miR-152-3p
 
 
-0.302
 
 
10.2
 
 
 
 
GLRX5
 
 
hsa-miR-152-3p
 
 
-0.381
 
 
35.5
 
 
 
 
GMFB
 
 
hsa-miR-152-3p
 
 
-0.429
 
 
24.8
 
 
 
 
GPATCH8
 
 
hsa-miR-152-3p
 
 
-0.374
 
 
12.3
 
 
 
 
GRSF1
 
 
hsa-miR-378a-3p
 
 
-0.358
 
 
40.0
 
 
 
 
HDDC2
 
 
hsa-miR-378a-3p
 
 
-0.322
 
 
14.8
 
 
 
 
HSP90B1
 
 
hsa-miR-152-3p
 
 
-0.304
 
 
489.7
 
 
 
 
HSPH1
 
 
hsa-miR-330-3p
 
 
-0.345
 
 
35.5
 
 
 
 
ID2
 
 
hsa-miR-330-3p
 
 
-0.293
 
 
65.9
 
 
 
 
IGF1
 
 
hsa-miR-152-3p
 
 
-0.43
 
 
22.7
 
 
 
 
IL6ST
 
 
hsa-miR-152-3p
 
 
-0.291
 
 
182.1
 
 
 
 
ITSN2
 
 
hsa-miR-152-3p
 
 
-0.339
 
 
23.2
 
 
 
 
KLF4
 
 
hsa-miR-152-3p
 
 
-0.325
 
 
30.4
 
 
 
 
KLF5
 
 
hsa-miR-152-3p
 
 
-0.475
 
 
17.7
 
 
 
 
LAMA4
 
 
hsa-miR-152-3p
 
 
-0.363
 
 
63.9
 
 
 
 
LBR
 
 
hsa-miR-152-3p
 
 
-0.39
 
 
26.0
 
 
 
 
LDLR
 
 
hsa-miR-152-3p
 
 
-0.323
 
 
15.6
 
 
 
 
LEPROTL1
 
 
hsa-miR-152-3p
 
 
-0.568
 
 
71.3
 
 
 
 
LIPA
 
 
hsa-miR-152-3p
 
 
-0.463
 
 
74.3
 
 
 
 
LMAN1
 
 
hsa-miR-152-3p
 
 
-0.382
 
 
51.5
 
 
 
 
LRCH1
 
 
hsa-miR-152-3p
 
 
-0.321
 
 
17.1
 
 
 
 
LRRC41
 
 
hsa-miR-152-3p
 
 
-0.392
 
 
31.8
 
 
 
 
LSM5
 
 
hsa-miR-330-3p
 
 
-0.351
 
 
27.0
 
 
 
 
 
 
 rectum 
 
 
 
 
target_symbol
 
 
mature_mirna_id
 
 
score
 
 
nTPM
 
 
 
 
 
 
ABCB10
 
 
hsa-miR-152-3p
 
 
-0.291
 
 
10.6
 
 
 
 
ABCD3
 
 
hsa-miR-152-3p
 
 
-0.388
 
 
37.8
 
 
 
 
ABHD17C
 
 
hsa-miR-330-3p
 
 
-0.399
 
 
52.8
 
 
 
 
ADAM10
 
 
hsa-miR-152-3p
 
 
-0.348
 
 
16.1
 
 
 
 
AGFG1
 
 
hsa-miR-152-3p
 
 
-0.332
 
 
31.0
 
 
 
 
AKAP1
 
 
hsa-miR-152-3p
 
 
-0.381
 
 
45.9
 
 
 
 
ALCAM
 
 
hsa-miR-152-3p
 
 
-0.347
 
 
13.6
 
 
 
 
AP4B1
 
 
hsa-miR-378a-3p
 
 
-0.62
 
 
12.0
 
 
 
 
ARF4
 
 
hsa-miR-152-3p
 
 
-0.487
 
 
185.2
 
 
 
 
ARFGEF1
 
 
hsa-miR-152-3p
 
 
-0.306
 
 
16.1
 
 
 
 
ARFIP1
 
 
hsa-miR-152-3p
 
 
-0.474
 
 
26.2
 
 
 
 
ARHGAP21
 
 
hsa-miR-152-3p
 
 
-0.287
 
 
17.4
 
 
 
 
ARL6IP1
 
 
hsa-miR-152-3p
 
 
-0.579
 
 
116.0
 
 
 
 
ARL8B
 
 
hsa-miR-152-3p
 
 
-0.337
 
 
32.7
 
 
 
 
ARRDC3
 
 
hsa-miR-152-3p
 
 
-0.305
 
 
25.8
 
 
 
 
ASPH
 
 
hsa-miR-152-3p
 
 
-0.391
 
 
77.9
 
 
 
 
ATP2B4
 
 
hsa-miR-152-3p
 
 
-0.337
 
 
28.6
 
 
 
 
ATP6AP2
 
 
hsa-miR-152-3p
 
 
-0.592
 
 
83.6
 
 
 
 
ATP6AP2
 
 
hsa-miR-152-3p
 
 
-0.668
 
 
83.6
 
 
 
 
B4GALT5
 
 
hsa-miR-152-3p
 
 
-0.44
 
 
22.3
 
 
 
 
BCL2L11
 
 
hsa-miR-152-3p
 
 
-0.445
 
 
11.2
 
 
 
 
BHLHE41
 
 
hsa-miR-152-3p
 
 
-0.442
 
 
10.4
 
 
 
 
BMP2
 
 
hsa-miR-378a-3p
 
 
-0.481
 
 
12.8
 
 
 
 
BTAF1
 
 
hsa-miR-152-3p
 
 
-0.401
 
 
12.5
 
 
 
 
BTBD3
 
 
hsa-miR-152-3p
 
 
-0.495
 
 
16.5
 
 
 
 
C1GALT1
 
 
hsa-miR-152-3p
 
 
-0.487
 
 
16.9
 
 
 
 
C1QBP
 
 
hsa-miR-330-3p
 
 
-0.412
 
 
132.9
 
 
 
 
C1orf210
 
 
hsa-miR-378a-3p
 
 
-0.307
 
 
28.3
 
 
 
 
CAND1
 
 
hsa-miR-152-3p
 
 
-0.44
 
 
19.4
 
 
 
 
CANX
 
 
hsa-miR-152-3p
 
 
-0.434
 
 
58.8
 
 
 
 
CAV2
 
 
hsa-miR-152-3p
 
 
-0.32
 
 
15.5
 
 
 
 
CCDC6
 
 
hsa-miR-152-3p
 
 
-0.511
 
 
18.6
 
 
 
 
CCN2
 
 
hsa-miR-330-3p
 
 
-0.305
 
 
127.9
 
 
 
 
CCT6A
 
 
hsa-miR-152-3p
 
 
-0.665
 
 
57.4
 
 
 
 
CDC25B
 
 
hsa-miR-152-3p
 
 
-0.298
 
 
10.6
 
 
 
 
CEBPG
 
 
hsa-miR-152-3p
 
 
-0.319
 
 
27.1
 
 
 
 
CEP63
 
 
hsa-miR-152-3p
 
 
-0.329
 
 
12.5
 
 
 
 
CFL2
 
 
hsa-miR-152-3p
 
 
-0.487
 
 
26.4
 
 
 
 
CHMP4B
 
 
hsa-miR-152-3p
 
 
-0.36
 
 
92.7
 
 
 
 
CNOT7
 
 
hsa-miR-152-3p
 
 
-0.31
 
 
41.0
 
 
 
 
COL4A1
 
 
hsa-miR-152-3p
 
 
-0.317
 
 
28.6
 
 
 
 
CS
 
 
hsa-miR-152-3p
 
 
-0.3
 
 
116.6
 
 
 
 
CTSA
 
 
hsa-miR-152-3p
 
 
-0.541
 
 
161.4
 
 
 
 
CTTNBP2NL
 
 
hsa-miR-152-3p
 
 
-0.329
 
 
13.9
 
 
 
 
DDAH1
 
 
hsa-miR-152-3p
 
 
-0.34
 
 
42.6
 
 
 
 
DDX6
 
 
hsa-miR-152-3p
 
 
-0.363
 
 
26.2
 
 
 
 
DEDD
 
 
hsa-miR-152-3p
 
 
-0.401
 
 
12.9
 
 
 
 
DNAJB4
 
 
hsa-miR-152-3p
 
 
-0.33
 
 
10.5
 
 
 
 
DUSP1
 
 
hsa-miR-152-3p
 
 
-0.32
 
 
215.0
 
 
 
 
DYNLL2
 
 
hsa-miR-152-3p
 
 
-0.366
 
 
18.8
 
 
 
 
DYRK2
 
 
hsa-miR-152-3p
 
 
-0.293
 
 
20.5
 
 
 
 
EIF4E3
 
 
hsa-miR-152-3p
 
 
-0.464
 
 
15.9
 
 
 
 
EIF4G3
 
 
hsa-miR-378a-3p
 
 
-0.443
 
 
23.1
 
 
 
 
EPS15
 
 
hsa-miR-152-3p
 
 
-0.322
 
 
17.3
 
 
 
 
ERBB3
 
 
hsa-miR-152-3p
 
 
-0.399
 
 
49.3
 
 
 
 
ERRFI1
 
 
hsa-miR-152-3p
 
 
-0.529
 
 
22.0
 
 
 
 
F3
 
 
hsa-miR-152-3p
 
 
-0.331
 
 
39.5
 
 
 
 
FAM168B
 
 
hsa-miR-152-3p
 
 
-0.409
 
 
30.0
 
 
 
 
FBXO28
 
 
hsa-miR-152-3p
 
 
-0.313
 
 
10.0
 
 
 
 
FEZ2
 
 
hsa-miR-152-3p
 
 
-0.536
 
 
26.2
 
 
 
 
FMR1
 
 
hsa-miR-152-3p
 
 
-0.385
 
 
11.5
 
 
 
 
FOSB
 
 
hsa-miR-152-3p
 
 
-0.311
 
 
73.8
 
 
 
 
FOXF1
 
 
hsa-miR-152-3p
 
 
-0.374
 
 
10.1
 
 
 
 
FXR1
 
 
hsa-miR-152-3p
 
 
-0.478
 
 
19.2
 
 
 
 
GADD45A
 
 
hsa-miR-152-3p
 
 
-0.787
 
 
19.9
 
 
 
 
GLRX5
 
 
hsa-miR-152-3p
 
 
-0.381
 
 
35.0
 
 
 
 
GMFB
 
 
hsa-miR-152-3p
 
 
-0.429
 
 
33.0
 
 
 
 
GRSF1
 
 
hsa-miR-378a-3p
 
 
-0.358
 
 
57.0
 
 
 
 
HDDC2
 
 
hsa-miR-378a-3p
 
 
-0.322
 
 
20.7
 
 
 
 
HLA-DQB1
 
 
hsa-miR-152-3p
 
 
-0.37
 
 
37.7
 
 
 
 
HSP90B1
 
 
hsa-miR-152-3p
 
 
-0.304
 
 
364.0
 
 
 
 
HSPH1
 
 
hsa-miR-330-3p
 
 
-0.345
 
 
23.8
 
 
 
 
ID2
 
 
hsa-miR-330-3p
 
 
-0.293
 
 
117.2
 
 
 
 
IL6ST
 
 
hsa-miR-152-3p
 
 
-0.291
 
 
27.5
 
 
 
 
ITSN2
 
 
hsa-miR-152-3p
 
 
-0.339
 
 
18.8
 
 
 
 
KLF4
 
 
hsa-miR-152-3p
 
 
-0.325
 
 
90.2
 
 
 
 
KLF5
 
 
hsa-miR-152-3p
 
 
-0.475
 
 
136.6
 
 
 
 
LAMA4
 
 
hsa-miR-152-3p
 
 
-0.363
 
 
31.9
 
 
 
 
LBR
 
 
hsa-miR-152-3p
 
 
-0.39
 
 
43.4
 
 
 
 
LDLR
 
 
hsa-miR-152-3p
 
 
-0.323
 
 
11.5
 
 
 
 
LEPROTL1
 
 
hsa-miR-152-3p
 
 
-0.568
 
 
24.3
 
 
 
 
LIPA
 
 
hsa-miR-152-3p
 
 
-0.463
 
 
67.6
 
 
 
 
LMAN1
 
 
hsa-miR-152-3p
 
 
-0.382
 
 
36.9
 
 
 
 
LRRC41
 
 
hsa-miR-152-3p
 
 
-0.392
 
 
18.4
 
 
 
 
LSM5
 
 
hsa-miR-330-3p
 
 
-0.351
 
 
56.2
 
 
 
 
LYRM2
 
 
hsa-miR-152-3p
 
 
-0.351
 
 
15.1
 
 
 
 
MACIR
 
 
hsa-miR-152-3p
 
 
-0.332
 
 
20.3
 
 
 
 
MACIR
 
 
hsa-miR-152-3p
 
 
-0.573
 
 
20.3
 
 
 
 
MACROH2A1
 
 
hsa-miR-152-3p
 
 
-0.336
 
 
101.2
 
 
 
 
MAF
 
 
hsa-miR-152-3p
 
 
-0.362
 
 
10.1
 
 
 
 
MAF1
 
 
hsa-miR-152-3p
 
 
-0.366
 
 
54.7
 
 
 
 
MAP2K1
 
 
hsa-miR-152-3p
 
 
-0.295
 
 
18.4
 
 
 
 
MAPK1
 
 
hsa-miR-378a-3p
 
 
-0.314
 
 
34.0
 
 
 
 
MAT2A
 
 
hsa-miR-330-3p
 
 
-0.307
 
 
137.7
 
 
 
 
MCM3AP
 
 
hsa-miR-152-3p
 
 
-0.287
 
 
14.1
 
 
 
 
MIER1
 
 
hsa-miR-152-3p
 
 
-0.352
 
 
22.1
 
 
 
 
MLLT10
 
 
hsa-miR-152-3p
 
 
-0.334
 
 
11.9
 
 
 
 
MMD
 
 
hsa-miR-152-3p
 
 
-0.62
 
 
12.1
 
 
 
 
MMP15
 
 
hsa-miR-152-3p
 
 
-0.458
 
 
13.5
 
 
 
 
MNAT1
 
 
hsa-miR-378a-3p
 
 
-0.398
 
 
11.9
 
 
 
 
 
 
 skeletal muscle 
 
 
 
 
target_symbol
 
 
mature_mirna_id
 
 
score
 
 
nTPM
 
 
 
 
 
 
ABCB10
 
 
hsa-miR-152-3p
 
 
-0.291
 
 
12.0
 
 
 
 
ABCB7
 
 
hsa-miR-152-3p
 
 
-0.515
 
 
29.2
 
 
 
 
ABCD3
 
 
hsa-miR-152-3p
 
 
-0.388
 
 
22.0
 
 
 
 
ACVR1
 
 
hsa-miR-152-3p
 
 
-0.397
 
 
15.5
 
 
 
 
ADCY2
 
 
hsa-miR-152-3p
 
 
-0.299
 
 
64.1
 
 
 
 
ADGRF5
 
 
hsa-miR-152-3p
 
 
-0.354
 
 
29.0
 
 
 
 
AGFG1
 
 
hsa-miR-152-3p
 
 
-0.332
 
 
31.6
 
 
 
 
AHDC1
 
 
hsa-miR-152-3p
 
 
-0.413
 
 
29.2
 
 
 
 
AKAP1
 
 
hsa-miR-152-3p
 
 
-0.381
 
 
194.7
 
 
 
 
AP4B1
 
 
hsa-miR-378a-3p
 
 
-0.62
 
 
11.0
 
 
 
 
ARF4
 
 
hsa-miR-152-3p
 
 
-0.487
 
 
59.5
 
 
 
 
ARFGEF1
 
 
hsa-miR-152-3p
 
 
-0.306
 
 
12.2
 
 
 
 
ARHGAP21
 
 
hsa-miR-152-3p
 
 
-0.287
 
 
15.7
 
 
 
 
ARL6IP1
 
 
hsa-miR-152-3p
 
 
-0.579
 
 
21.6
 
 
 
 
ARL8B
 
 
hsa-miR-152-3p
 
 
-0.337
 
 
98.5
 
 
 
 
ARRDC3
 
 
hsa-miR-152-3p
 
 
-0.305
 
 
31.8
 
 
 
 
ASPH
 
 
hsa-miR-152-3p
 
 
-0.391
 
 
331.0
 
 
 
 
ATP2B4
 
 
hsa-miR-152-3p
 
 
-0.337
 
 
13.5
 
 
 
 
ATP6AP2
 
 
hsa-miR-152-3p
 
 
-0.592
 
 
20.5
 
 
 
 
ATP6AP2
 
 
hsa-miR-152-3p
 
 
-0.668
 
 
20.5
 
 
 
 
B4GALT5
 
 
hsa-miR-152-3p
 
 
-0.44
 
 
63.3
 
 
 
 
BCL2L11
 
 
hsa-miR-152-3p
 
 
-0.445
 
 
11.0
 
 
 
 
BHLHE41
 
 
hsa-miR-152-3p
 
 
-0.442
 
 
86.6
 
 
 
 
BTAF1
 
 
hsa-miR-152-3p
 
 
-0.401
 
 
11.9
 
 
 
 
BTBD3
 
 
hsa-miR-152-3p
 
 
-0.495
 
 
14.9
 
 
 
 
C15orf61
 
 
hsa-miR-378a-3p
 
 
-0.497
 
 
22.2
 
 
 
 
C1QBP
 
 
hsa-miR-330-3p
 
 
-0.412
 
 
163.3
 
 
 
 
C1orf35
 
 
hsa-miR-152-3p
 
 
-0.511
 
 
10.7
 
 
 
 
CANX
 
 
hsa-miR-152-3p
 
 
-0.434
 
 
96.0
 
 
 
 
CAV2
 
 
hsa-miR-152-3p
 
 
-0.32
 
 
21.6
 
 
 
 
CCDC6
 
 
hsa-miR-152-3p
 
 
-0.511
 
 
16.5
 
 
 
 
CCDC71
 
 
hsa-miR-152-3p
 
 
-0.369
 
 
14.1
 
 
 
 
CCN2
 
 
hsa-miR-330-3p
 
 
-0.305
 
 
49.6
 
 
 
 
CCT6A
 
 
hsa-miR-152-3p
 
 
-0.665
 
 
125.4
 
 
 
 
CDC25B
 
 
hsa-miR-152-3p
 
 
-0.298
 
 
10.2
 
 
 
 
CEBPG
 
 
hsa-miR-152-3p
 
 
-0.319
 
 
44.4
 
 
 
 
CEP63
 
 
hsa-miR-152-3p
 
 
-0.329
 
 
35.6
 
 
 
 
CFL2
 
 
hsa-miR-152-3p
 
 
-0.487
 
 
674.8
 
 
 
 
CHMP4B
 
 
hsa-miR-152-3p
 
 
-0.36
 
 
228.4
 
 
 
 
CLCN6
 
 
hsa-miR-152-3p
 
 
-0.406
 
 
10.8
 
 
 
 
CNOT7
 
 
hsa-miR-152-3p
 
 
-0.31
 
 
74.0
 
 
 
 
COL4A1
 
 
hsa-miR-152-3p
 
 
-0.317
 
 
37.5
 
 
 
 
CS
 
 
hsa-miR-152-3p
 
 
-0.3
 
 
515.2
 
 
 
 
CTSA
 
 
hsa-miR-152-3p
 
 
-0.541
 
 
50.0
 
 
 
 
CUL5
 
 
hsa-miR-152-3p
 
 
-0.389
 
 
43.4
 
 
 
 
CYTH3
 
 
hsa-miR-152-3p
 
 
-0.331
 
 
14.6
 
 
 
 
DCUN1D4
 
 
hsa-miR-152-3p
 
 
-0.314
 
 
11.4
 
 
 
 
DDAH1
 
 
hsa-miR-152-3p
 
 
-0.34
 
 
28.5
 
 
 
 
DDX6
 
 
hsa-miR-152-3p
 
 
-0.363
 
 
40.5
 
 
 
 
DEDD
 
 
hsa-miR-152-3p
 
 
-0.401
 
 
53.6
 
 
 
 
DMPK
 
 
hsa-miR-152-3p
 
 
-0.378
 
 
193.1
 
 
 
 
DNAJB4
 
 
hsa-miR-152-3p
 
 
-0.33
 
 
41.3
 
 
 
 
DOCK6
 
 
hsa-miR-152-3p
 
 
-0.396
 
 
13.2
 
 
 
 
DUSP1
 
 
hsa-miR-152-3p
 
 
-0.32
 
 
242.2
 
 
 
 
DYNLL2
 
 
hsa-miR-152-3p
 
 
-0.366
 
 
106.5
 
 
 
 
DYRK1B
 
 
hsa-miR-152-3p
 
 
-0.305
 
 
124.8
 
 
 
 
DYRK2
 
 
hsa-miR-152-3p
 
 
-0.293
 
 
37.0
 
 
 
 
EIF4E3
 
 
hsa-miR-152-3p
 
 
-0.464
 
 
57.9
 
 
 
 
EIF4G3
 
 
hsa-miR-378a-3p
 
 
-0.443
 
 
34.0
 
 
 
 
EOGT
 
 
hsa-miR-152-3p
 
 
-0.587
 
 
18.8
 
 
 
 
EPS15
 
 
hsa-miR-152-3p
 
 
-0.322
 
 
37.8
 
 
 
 
ERRFI1
 
 
hsa-miR-152-3p
 
 
-0.529
 
 
211.9
 
 
 
 
FAM168B
 
 
hsa-miR-152-3p
 
 
-0.409
 
 
56.9
 
 
 
 
FBXO28
 
 
hsa-miR-152-3p
 
 
-0.313
 
 
11.6
 
 
 
 
FEZ2
 
 
hsa-miR-152-3p
 
 
-0.536
 
 
100.3
 
 
 
 
FOXK2
 
 
hsa-miR-152-3p
 
 
-0.305
 
 
40.1
 
 
 
 
FXR1
 
 
hsa-miR-152-3p
 
 
-0.478
 
 
741.3
 
 
 
 
GADD45A
 
 
hsa-miR-152-3p
 
 
-0.787
 
 
255.1
 
 
 
 
GAPVD1
 
 
hsa-miR-152-3p
 
 
-0.309
 
 
16.9
 
 
 
 
GLRX5
 
 
hsa-miR-152-3p
 
 
-0.381
 
 
182.8
 
 
 
 
GPATCH8
 
 
hsa-miR-152-3p
 
 
-0.374
 
 
39.6
 
 
 
 
GRSF1
 
 
hsa-miR-378a-3p
 
 
-0.358
 
 
242.1
 
 
 
 
GTF2H1
 
 
hsa-miR-152-3p
 
 
-0.524
 
 
16.1
 
 
 
 
HDDC2
 
 
hsa-miR-378a-3p
 
 
-0.322
 
 
81.4
 
 
 
 
HIF1AN
 
 
hsa-miR-152-3p
 
 
-0.365
 
 
38.9
 
 
 
 
HOMER1
 
 
hsa-miR-152-3p
 
 
-0.452
 
 
31.9
 
 
 
 
HOXC8
 
 
hsa-miR-152-3p
 
 
-0.46
 
 
14.6
 
 
 
 
HSP90B1
 
 
hsa-miR-152-3p
 
 
-0.304
 
 
74.7
 
 
 
 
HSPH1
 
 
hsa-miR-330-3p
 
 
-0.345
 
 
35.3
 
 
 
 
ID2
 
 
hsa-miR-330-3p
 
 
-0.293
 
 
18.2
 
 
 
 
IL6ST
 
 
hsa-miR-152-3p
 
 
-0.291
 
 
26.2
 
 
 
 
ING2
 
 
hsa-miR-152-3p
 
 
-0.482
 
 
15.0
 
 
 
 
ITSN2
 
 
hsa-miR-152-3p
 
 
-0.339
 
 
21.7
 
 
 
 
KLF4
 
 
hsa-miR-152-3p
 
 
-0.325
 
 
10.0
 
 
 
 
KLF5
 
 
hsa-miR-152-3p
 
 
-0.475
 
 
18.3
 
 
 
 
LAMA4
 
 
hsa-miR-152-3p
 
 
-0.363
 
 
10.0
 
 
 
 
LEPROTL1
 
 
hsa-miR-152-3p
 
 
-0.568
 
 
12.2
 
 
 
 
LIPA
 
 
hsa-miR-152-3p
 
 
-0.463
 
 
11.0
 
 
 
 
LMAN1
 
 
hsa-miR-152-3p
 
 
-0.382
 
 
31.2
 
 
 
 
LMTK2
 
 
hsa-miR-152-3p
 
 
-0.298
 
 
10.4
 
 
 
 
LRRC41
 
 
hsa-miR-152-3p
 
 
-0.392
 
 
52.8
 
 
 
 
LSM5
 
 
hsa-miR-330-3p
 
 
-0.351
 
 
53.8
 
 
 
 
LYRM2
 
 
hsa-miR-152-3p
 
 
-0.351
 
 
19.9
 
 
 
 
LYSMD2
 
 
hsa-miR-152-3p
 
 
-0.554
 
 
17.2
 
 
 
 
MACROH2A1
 
 
hsa-miR-152-3p
 
 
-0.336
 
 
79.7
 
 
 
 
MAF
 
 
hsa-miR-152-3p
 
 
-0.362
 
 
46.4
 
 
 
 
MAF1
 
 
hsa-miR-152-3p
 
 
-0.366
 
 
387.2
 
 
 
 
MAFB
 
 
hsa-miR-152-3p
 
 
-0.45
 
 
14.8
 
 
 
 
MAFG
 
 
hsa-miR-152-3p
 
 
-0.328
 
 
42.1
 
 
 
 
MAP2K1
 
 
hsa-miR-152-3p
 
 
-0.295
 
 
60.2
 
 
 
 
 
 
 small intestine 
 
 
 
 
target_symbol
 
 
mature_mirna_id
 
 
score
 
 
nTPM
 
 
 
 
 
 
ABCB10
 
 
hsa-miR-152-3p
 
 
-0.291
 
 
13.1
 
 
 
 
ABCB7
 
 
hsa-miR-152-3p
 
 
-0.515
 
 
11.2
 
 
 
 
ABCD3
 
 
hsa-miR-152-3p
 
 
-0.388
 
 
43.4
 
 
 
 
ABHD17C
 
 
hsa-miR-330-3p
 
 
-0.399
 
 
28.8
 
 
 
 
ADAM10
 
 
hsa-miR-152-3p
 
 
-0.348
 
 
15.8
 
 
 
 
AGFG1
 
 
hsa-miR-152-3p
 
 
-0.332
 
 
33.9
 
 
 
 
AKAP1
 
 
hsa-miR-152-3p
 
 
-0.381
 
 
52.5
 
 
 
 
ALCAM
 
 
hsa-miR-152-3p
 
 
-0.347
 
 
16.4
 
 
 
 
AP4B1
 
 
hsa-miR-378a-3p
 
 
-0.62
 
 
12.3
 
 
 
 
ARF4
 
 
hsa-miR-152-3p
 
 
-0.487
 
 
102.8
 
 
 
 
ARFGEF1
 
 
hsa-miR-152-3p
 
 
-0.306
 
 
16.5
 
 
 
 
ARFIP1
 
 
hsa-miR-152-3p
 
 
-0.474
 
 
20.0
 
 
 
 
ARHGAP21
 
 
hsa-miR-152-3p
 
 
-0.287
 
 
15.2
 
 
 
 
ARL6IP1
 
 
hsa-miR-152-3p
 
 
-0.579
 
 
116.2
 
 
 
 
ARL8B
 
 
hsa-miR-152-3p
 
 
-0.337
 
 
32.3
 
 
 
 
ARRDC3
 
 
hsa-miR-152-3p
 
 
-0.305
 
 
15.8
 
 
 
 
ASPH
 
 
hsa-miR-152-3p
 
 
-0.391
 
 
42.6
 
 
 
 
ATP2B4
 
 
hsa-miR-152-3p
 
 
-0.337
 
 
13.8
 
 
 
 
ATP6AP2
 
 
hsa-miR-152-3p
 
 
-0.592
 
 
45.0
 
 
 
 
ATP6AP2
 
 
hsa-miR-152-3p
 
 
-0.668
 
 
45.0
 
 
 
 
B4GALT5
 
 
hsa-miR-152-3p
 
 
-0.44
 
 
20.1
 
 
 
 
BCL2L11
 
 
hsa-miR-152-3p
 
 
-0.445
 
 
11.3
 
 
 
 
BTAF1
 
 
hsa-miR-152-3p
 
 
-0.401
 
 
18.1
 
 
 
 
BTBD3
 
 
hsa-miR-152-3p
 
 
-0.495
 
 
10.3
 
 
 
 
C1GALT1
 
 
hsa-miR-152-3p
 
 
-0.487
 
 
18.1
 
 
 
 
C1QBP
 
 
hsa-miR-330-3p
 
 
-0.412
 
 
73.8
 
 
 
 
C1orf210
 
 
hsa-miR-378a-3p
 
 
-0.307
 
 
36.5
 
 
 
 
C1orf35
 
 
hsa-miR-152-3p
 
 
-0.511
 
 
11.9
 
 
 
 
CAND1
 
 
hsa-miR-152-3p
 
 
-0.44
 
 
17.4
 
 
 
 
CANX
 
 
hsa-miR-152-3p
 
 
-0.434
 
 
91.5
 
 
 
 
CAV2
 
 
hsa-miR-152-3p
 
 
-0.32
 
 
16.7
 
 
 
 
CCDC6
 
 
hsa-miR-152-3p
 
 
-0.511
 
 
15.3
 
 
 
 
CCDC71
 
 
hsa-miR-152-3p
 
 
-0.369
 
 
10.2
 
 
 
 
CCN2
 
 
hsa-miR-330-3p
 
 
-0.305
 
 
142.6
 
 
 
 
CCT6A
 
 
hsa-miR-152-3p
 
 
-0.665
 
 
49.9
 
 
 
 
CDC14A
 
 
hsa-miR-152-3p
 
 
-0.482
 
 
10.1
 
 
 
 
CDC25B
 
 
hsa-miR-152-3p
 
 
-0.298
 
 
39.8
 
 
 
 
CEBPG
 
 
hsa-miR-152-3p
 
 
-0.319
 
 
33.2
 
 
 
 
CERS5
 
 
hsa-miR-152-3p
 
 
-0.311
 
 
14.7
 
 
 
 
CFL2
 
 
hsa-miR-152-3p
 
 
-0.487
 
 
24.1
 
 
 
 
CHMP4B
 
 
hsa-miR-152-3p
 
 
-0.36
 
 
117.7
 
 
 
 
CLCN6
 
 
hsa-miR-152-3p
 
 
-0.406
 
 
11.9
 
 
 
 
CNOT7
 
 
hsa-miR-152-3p
 
 
-0.31
 
 
36.1
 
 
 
 
COL4A1
 
 
hsa-miR-152-3p
 
 
-0.317
 
 
37.1
 
 
 
 
CS
 
 
hsa-miR-152-3p
 
 
-0.3
 
 
76.5
 
 
 
 
CTSA
 
 
hsa-miR-152-3p
 
 
-0.541
 
 
94.8
 
 
 
 
CYTH3
 
 
hsa-miR-152-3p
 
 
-0.331
 
 
15.7
 
 
 
 
DDAH1
 
 
hsa-miR-152-3p
 
 
-0.34
 
 
37.4
 
 
 
 
DDX6
 
 
hsa-miR-152-3p
 
 
-0.363
 
 
24.7
 
 
 
 
DEDD
 
 
hsa-miR-152-3p
 
 
-0.401
 
 
22.3
 
 
 
 
DMPK
 
 
hsa-miR-152-3p
 
 
-0.378
 
 
18.9
 
 
 
 
DOCK6
 
 
hsa-miR-152-3p
 
 
-0.396
 
 
14.8
 
 
 
 
DUSP1
 
 
hsa-miR-152-3p
 
 
-0.32
 
 
163.1
 
 
 
 
DYNLL2
 
 
hsa-miR-152-3p
 
 
-0.366
 
 
22.9
 
 
 
 
DYRK2
 
 
hsa-miR-152-3p
 
 
-0.293
 
 
26.4
 
 
 
 
EIF4E3
 
 
hsa-miR-152-3p
 
 
-0.464
 
 
17.5
 
 
 
 
EIF4G3
 
 
hsa-miR-378a-3p
 
 
-0.443
 
 
12.9
 
 
 
 
EOGT
 
 
hsa-miR-152-3p
 
 
-0.587
 
 
10.9
 
 
 
 
EPS15
 
 
hsa-miR-152-3p
 
 
-0.322
 
 
16.0
 
 
 
 
ERBB3
 
 
hsa-miR-152-3p
 
 
-0.399
 
 
64.9
 
 
 
 
ERRFI1
 
 
hsa-miR-152-3p
 
 
-0.529
 
 
44.5
 
 
 
 
F3
 
 
hsa-miR-152-3p
 
 
-0.331
 
 
44.4
 
 
 
 
FAM168B
 
 
hsa-miR-152-3p
 
 
-0.409
 
 
29.7
 
 
 
 
FAM43A
 
 
hsa-miR-152-3p
 
 
-0.337
 
 
10.5
 
 
 
 
FEZ2
 
 
hsa-miR-152-3p
 
 
-0.536
 
 
26.9
 
 
 
 
FMR1
 
 
hsa-miR-152-3p
 
 
-0.385
 
 
11.6
 
 
 
 
FOSB
 
 
hsa-miR-152-3p
 
 
-0.311
 
 
59.3
 
 
 
 
FOXF1
 
 
hsa-miR-152-3p
 
 
-0.374
 
 
20.4
 
 
 
 
FOXK2
 
 
hsa-miR-152-3p
 
 
-0.305
 
 
11.6
 
 
 
 
FXR1
 
 
hsa-miR-152-3p
 
 
-0.478
 
 
21.3
 
 
 
 
GADD45A
 
 
hsa-miR-152-3p
 
 
-0.787
 
 
31.6
 
 
 
 
GLRX5
 
 
hsa-miR-152-3p
 
 
-0.381
 
 
36.3
 
 
 
 
GMFB
 
 
hsa-miR-152-3p
 
 
-0.429
 
 
27.8
 
 
 
 
GOLT1A
 
 
hsa-miR-378a-3p
 
 
-0.574
 
 
42.8
 
 
 
 
GPATCH8
 
 
hsa-miR-152-3p
 
 
-0.374
 
 
15.1
 
 
 
 
GRSF1
 
 
hsa-miR-378a-3p
 
 
-0.358
 
 
37.3
 
 
 
 
GTF2H1
 
 
hsa-miR-152-3p
 
 
-0.524
 
 
14.0
 
 
 
 
HDDC2
 
 
hsa-miR-378a-3p
 
 
-0.322
 
 
20.7
 
 
 
 
HLA-DQB1
 
 
hsa-miR-152-3p
 
 
-0.37
 
 
75.3
 
 
 
 
HSP90B1
 
 
hsa-miR-152-3p
 
 
-0.304
 
 
253.8
 
 
 
 
HSPH1
 
 
hsa-miR-330-3p
 
 
-0.345
 
 
19.9
 
 
 
 
ID2
 
 
hsa-miR-330-3p
 
 
-0.293
 
 
93.7
 
 
 
 
IL6ST
 
 
hsa-miR-152-3p
 
 
-0.291
 
 
23.1
 
 
 
 
ING2
 
 
hsa-miR-152-3p
 
 
-0.482
 
 
13.5
 
 
 
 
ITSN2
 
 
hsa-miR-152-3p
 
 
-0.339
 
 
21.0
 
 
 
 
KLF4
 
 
hsa-miR-152-3p
 
 
-0.325
 
 
38.1
 
 
 
 
KLF5
 
 
hsa-miR-152-3p
 
 
-0.475
 
 
137.8
 
 
 
 
LAMA4
 
 
hsa-miR-152-3p
 
 
-0.363
 
 
31.5
 
 
 
 
LBR
 
 
hsa-miR-152-3p
 
 
-0.39
 
 
37.6
 
 
 
 
LDLR
 
 
hsa-miR-152-3p
 
 
-0.323
 
 
17.2
 
 
 
 
LEPROTL1
 
 
hsa-miR-152-3p
 
 
-0.568
 
 
24.1
 
 
 
 
LIPA
 
 
hsa-miR-152-3p
 
 
-0.463
 
 
214.2
 
 
 
 
LMAN1
 
 
hsa-miR-152-3p
 
 
-0.382
 
 
46.2
 
 
 
 
LRCH1
 
 
hsa-miR-152-3p
 
 
-0.321
 
 
11.0
 
 
 
 
LRRC41
 
 
hsa-miR-152-3p
 
 
-0.392
 
 
28.1
 
 
 
 
LSM5
 
 
hsa-miR-330-3p
 
 
-0.351
 
 
37.0
 
 
 
 
LYSMD2
 
 
hsa-miR-152-3p
 
 
-0.554
 
 
13.7
 
 
 
 
MACIR
 
 
hsa-miR-152-3p
 
 
-0.332
 
 
11.0
 
 
 
 
MACIR
 
 
hsa-miR-152-3p
 
 
-0.573
 
 
11.0
 
 
 
 
MACROH2A1
 
 
hsa-miR-152-3p
 
 
-0.336
 
 
101.5
 
 
 
 
 
 
 tongue 
 
 
 
 
target_symbol
 
 
mature_mirna_id
 
 
score
 
 
nTPM
 
 
 
 
 
 
ABCB10
 
 
hsa-miR-152-3p
 
 
-0.291
 
 
10.2
 
 
 
 
ABCB7
 
 
hsa-miR-152-3p
 
 
-0.515
 
 
20.8
 
 
 
 
ABCD3
 
 
hsa-miR-152-3p
 
 
-0.388
 
 
27.9
 
 
 
 
ADCY2
 
 
hsa-miR-152-3p
 
 
-0.299
 
 
28.8
 
 
 
 
ADGRF5
 
 
hsa-miR-152-3p
 
 
-0.354
 
 
66.5
 
 
 
 
AGFG1
 
 
hsa-miR-152-3p
 
 
-0.332
 
 
36.1
 
 
 
 
AHDC1
 
 
hsa-miR-152-3p
 
 
-0.413
 
 
14.5
 
 
 
 
AKAP1
 
 
hsa-miR-152-3p
 
 
-0.381
 
 
128.4
 
 
 
 
ARF4
 
 
hsa-miR-152-3p
 
 
-0.487
 
 
64.9
 
 
 
 
ARFGEF1
 
 
hsa-miR-152-3p
 
 
-0.306
 
 
13.0
 
 
 
 
ARHGAP21
 
 
hsa-miR-152-3p
 
 
-0.287
 
 
21.5
 
 
 
 
ARL6IP1
 
 
hsa-miR-152-3p
 
 
-0.579
 
 
48.1
 
 
 
 
ARL8B
 
 
hsa-miR-152-3p
 
 
-0.337
 
 
56.0
 
 
 
 
ARRDC3
 
 
hsa-miR-152-3p
 
 
-0.305
 
 
41.9
 
 
 
 
ASPH
 
 
hsa-miR-152-3p
 
 
-0.391
 
 
155.4
 
 
 
 
ATP2B4
 
 
hsa-miR-152-3p
 
 
-0.337
 
 
25.9
 
 
 
 
ATP6AP2
 
 
hsa-miR-152-3p
 
 
-0.592
 
 
37.6
 
 
 
 
ATP6AP2
 
 
hsa-miR-152-3p
 
 
-0.668
 
 
37.6
 
 
 
 
B4GALT5
 
 
hsa-miR-152-3p
 
 
-0.44
 
 
22.7
 
 
 
 
BHLHE41
 
 
hsa-miR-152-3p
 
 
-0.442
 
 
12.1
 
 
 
 
BTBD3
 
 
hsa-miR-152-3p
 
 
-0.495
 
 
12.3
 
 
 
 
C1GALT1
 
 
hsa-miR-152-3p
 
 
-0.487
 
 
10.5
 
 
 
 
C1QBP
 
 
hsa-miR-330-3p
 
 
-0.412
 
 
149.3
 
 
 
 
CAND1
 
 
hsa-miR-152-3p
 
 
-0.44
 
 
10.6
 
 
 
 
CANX
 
 
hsa-miR-152-3p
 
 
-0.434
 
 
49.8
 
 
 
 
CAV2
 
 
hsa-miR-152-3p
 
 
-0.32
 
 
35.3
 
 
 
 
CCDC6
 
 
hsa-miR-152-3p
 
 
-0.511
 
 
11.4
 
 
 
 
CCN2
 
 
hsa-miR-330-3p
 
 
-0.305
 
 
57.8
 
 
 
 
CCT6A
 
 
hsa-miR-152-3p
 
 
-0.665
 
 
80.7
 
 
 
 
CDH20
 
 
hsa-miR-152-3p
 
 
-0.297
 
 
22.4
 
 
 
 
CEBPG
 
 
hsa-miR-152-3p
 
 
-0.319
 
 
41.4
 
 
 
 
CEP63
 
 
hsa-miR-152-3p
 
 
-0.329
 
 
25.4
 
 
 
 
CFL2
 
 
hsa-miR-152-3p
 
 
-0.487
 
 
640.8
 
 
 
 
CHMP4B
 
 
hsa-miR-152-3p
 
 
-0.36
 
 
123.8
 
 
 
 
CNOT7
 
 
hsa-miR-152-3p
 
 
-0.31
 
 
67.2
 
 
 
 
COL4A1
 
 
hsa-miR-152-3p
 
 
-0.317
 
 
43.9
 
 
 
 
CS
 
 
hsa-miR-152-3p
 
 
-0.3
 
 
561.0
 
 
 
 
CTSA
 
 
hsa-miR-152-3p
 
 
-0.541
 
 
57.9
 
 
 
 
CUL5
 
 
hsa-miR-152-3p
 
 
-0.389
 
 
35.1
 
 
 
 
CYTH3
 
 
hsa-miR-152-3p
 
 
-0.331
 
 
18.4
 
 
 
 
DCUN1D4
 
 
hsa-miR-152-3p
 
 
-0.314
 
 
10.6
 
 
 
 
DDAH1
 
 
hsa-miR-152-3p
 
 
-0.34
 
 
13.1
 
 
 
 
DDX6
 
 
hsa-miR-152-3p
 
 
-0.363
 
 
39.1
 
 
 
 
DEDD
 
 
hsa-miR-152-3p
 
 
-0.401
 
 
32.7
 
 
 
 
DICER1
 
 
hsa-miR-152-3p
 
 
-0.29
 
 
15.9
 
 
 
 
DMPK
 
 
hsa-miR-152-3p
 
 
-0.378
 
 
138.8
 
 
 
 
DNAJB4
 
 
hsa-miR-152-3p
 
 
-0.33
 
 
66.8
 
 
 
 
DUSP1
 
 
hsa-miR-152-3p
 
 
-0.32
 
 
421.5
 
 
 
 
DYNLL2
 
 
hsa-miR-152-3p
 
 
-0.366
 
 
54.9
 
 
 
 
DYRK1B
 
 
hsa-miR-152-3p
 
 
-0.305
 
 
46.7
 
 
 
 
EIF4E3
 
 
hsa-miR-152-3p
 
 
-0.464
 
 
50.9
 
 
 
 
EIF4G3
 
 
hsa-miR-378a-3p
 
 
-0.443
 
 
24.9
 
 
 
 
EOGT
 
 
hsa-miR-152-3p
 
 
-0.587
 
 
14.2
 
 
 
 
EPS15
 
 
hsa-miR-152-3p
 
 
-0.322
 
 
28.2
 
 
 
 
ERRFI1
 
 
hsa-miR-152-3p
 
 
-0.529
 
 
28.2
 
 
 
 
FAM168B
 
 
hsa-miR-152-3p
 
 
-0.409
 
 
49.4
 
 
 
 
FBN1
 
 
hsa-miR-152-3p
 
 
-0.349
 
 
16.5
 
 
 
 
FBXO28
 
 
hsa-miR-152-3p
 
 
-0.313
 
 
13.3
 
 
 
 
FEZ2
 
 
hsa-miR-152-3p
 
 
-0.536
 
 
73.3
 
 
 
 
FOSB
 
 
hsa-miR-152-3p
 
 
-0.311
 
 
76.0
 
 
 
 
FOXK2
 
 
hsa-miR-152-3p
 
 
-0.305
 
 
26.5
 
 
 
 
FXR1
 
 
hsa-miR-152-3p
 
 
-0.478
 
 
425.1
 
 
 
 
GADD45A
 
 
hsa-miR-152-3p
 
 
-0.787
 
 
24.6
 
 
 
 
GAPVD1
 
 
hsa-miR-152-3p
 
 
-0.309
 
 
17.4
 
 
 
 
GLRX5
 
 
hsa-miR-152-3p
 
 
-0.381
 
 
157.7
 
 
 
 
GPATCH8
 
 
hsa-miR-152-3p
 
 
-0.374
 
 
12.9
 
 
 
 
GRSF1
 
 
hsa-miR-378a-3p
 
 
-0.358
 
 
140.8
 
 
 
 
HDDC2
 
 
hsa-miR-378a-3p
 
 
-0.322
 
 
56.5
 
 
 
 
HIF1AN
 
 
hsa-miR-152-3p
 
 
-0.365
 
 
38.1
 
 
 
 
HOMER1
 
 
hsa-miR-152-3p
 
 
-0.452
 
 
12.0
 
 
 
 
HSP90B1
 
 
hsa-miR-152-3p
 
 
-0.304
 
 
85.2
 
 
 
 
HSPH1
 
 
hsa-miR-330-3p
 
 
-0.345
 
 
30.4
 
 
 
 
ID2
 
 
hsa-miR-330-3p
 
 
-0.293
 
 
38.4
 
 
 
 
IL6ST
 
 
hsa-miR-152-3p
 
 
-0.291
 
 
53.5
 
 
 
 
ING2
 
 
hsa-miR-152-3p
 
 
-0.482
 
 
13.8
 
 
 
 
ITSN2
 
 
hsa-miR-152-3p
 
 
-0.339
 
 
17.6
 
 
 
 
KLF4
 
 
hsa-miR-152-3p
 
 
-0.325
 
 
21.6
 
 
 
 
LAMA4
 
 
hsa-miR-152-3p
 
 
-0.363
 
 
17.4
 
 
 
 
LBR
 
 
hsa-miR-152-3p
 
 
-0.39
 
 
11.9
 
 
 
 
LEPROTL1
 
 
hsa-miR-152-3p
 
 
-0.568
 
 
13.9
 
 
 
 
LIPA
 
 
hsa-miR-152-3p
 
 
-0.463
 
 
21.0
 
 
 
 
LMAN1
 
 
hsa-miR-152-3p
 
 
-0.382
 
 
38.4
 
 
 
 
LRRC41
 
 
hsa-miR-152-3p
 
 
-0.392
 
 
31.0
 
 
 
 
LSM5
 
 
hsa-miR-330-3p
 
 
-0.351
 
 
47.6
 
 
 
 
LYRM2
 
 
hsa-miR-152-3p
 
 
-0.351
 
 
15.8
 
 
 
 
LYSMD2
 
 
hsa-miR-152-3p
 
 
-0.554
 
 
12.2
 
 
 
 
MACROH2A1
 
 
hsa-miR-152-3p
 
 
-0.336
 
 
62.0
 
 
 
 
MAF
 
 
hsa-miR-152-3p
 
 
-0.362
 
 
18.5
 
 
 
 
MAF1
 
 
hsa-miR-152-3p
 
 
-0.366
 
 
274.9
 
 
 
 
MAFB
 
 
hsa-miR-152-3p
 
 
-0.45
 
 
15.6
 
 
 
 
MAFG
 
 
hsa-miR-152-3p
 
 
-0.328
 
 
18.4
 
 
 
 
MAP2K1
 
 
hsa-miR-152-3p
 
 
-0.295
 
 
42.0
 
 
 
 
MAPK1
 
 
hsa-miR-378a-3p
 
 
-0.314
 
 
39.0
 
 
 
 
MAT2A
 
 
hsa-miR-330-3p
 
 
-0.307
 
 
104.5
 
 
 
 
MCM3AP
 
 
hsa-miR-152-3p
 
 
-0.287
 
 
26.8
 
 
 
 
MDFIC
 
 
hsa-miR-152-3p
 
 
-0.395
 
 
13.0
 
 
 
 
MECP2
 
 
hsa-miR-152-3p
 
 
-0.3
 
 
13.5
 
 
 
 
MEOX2
 
 
hsa-miR-152-3p
 
 
-0.472
 
 
12.4
 
 
 
 
MEOX2
 
 
hsa-miR-152-3p
 
 
-0.5
 
 
12.4
 
 
 
 
MIER1
 
 
hsa-miR-152-3p
 
 
-0.352
 
 
12.2
 
 
 
 
 
 
 adipose tissue 
 
 
 
 
target_symbol
 
 
mature_mirna_id
 
 
score
 
 
nTPM
 
 
 
 
 
 
ABCB7
 
 
hsa-miR-152-3p
 
 
-0.515
 
 
12.4
 
 
 
 
ABCD3
 
 
hsa-miR-152-3p
 
 
-0.388
 
 
15.7
 
 
 
 
ACVR1
 
 
hsa-miR-152-3p
 
 
-0.397
 
 
12.6
 
 
 
 
ADAM10
 
 
hsa-miR-152-3p
 
 
-0.348
 
 
14.0
 
 
 
 
ADGRF5
 
 
hsa-miR-152-3p
 
 
-0.354
 
 
66.6
 
 
 
 
AGFG1
 
 
hsa-miR-152-3p
 
 
-0.332
 
 
64.8
 
 
 
 
AHDC1
 
 
hsa-miR-152-3p
 
 
-0.413
 
 
11.7
 
 
 
 
AKAP1
 
 
hsa-miR-152-3p
 
 
-0.381
 
 
38.1
 
 
 
 
ARF4
 
 
hsa-miR-152-3p
 
 
-0.487
 
 
118.7
 
 
 
 
ARFGEF1
 
 
hsa-miR-152-3p
 
 
-0.306
 
 
10.7
 
 
 
 
ARFIP1
 
 
hsa-miR-152-3p
 
 
-0.474
 
 
15.8
 
 
 
 
ARHGAP21
 
 
hsa-miR-152-3p
 
 
-0.287
 
 
26.1
 
 
 
 
ARL6IP1
 
 
hsa-miR-152-3p
 
 
-0.579
 
 
133.2
 
 
 
 
ARL8B
 
 
hsa-miR-152-3p
 
 
-0.337
 
 
39.1
 
 
 
 
ARRDC3
 
 
hsa-miR-152-3p
 
 
-0.305
 
 
34.5
 
 
 
 
ASPH
 
 
hsa-miR-152-3p
 
 
-0.391
 
 
362.0
 
 
 
 
ATP2B4
 
 
hsa-miR-152-3p
 
 
-0.337
 
 
55.3
 
 
 
 
ATP6AP2
 
 
hsa-miR-152-3p
 
 
-0.592
 
 
76.1
 
 
 
 
ATP6AP2
 
 
hsa-miR-152-3p
 
 
-0.668
 
 
76.1
 
 
 
 
B4GALT5
 
 
hsa-miR-152-3p
 
 
-0.44
 
 
26.8
 
 
 
 
BHLHE41
 
 
hsa-miR-152-3p
 
 
-0.442
 
 
10.3
 
 
 
 
BMP2
 
 
hsa-miR-378a-3p
 
 
-0.481
 
 
17.4
 
 
 
 
BTAF1
 
 
hsa-miR-152-3p
 
 
-0.401
 
 
16.7
 
 
 
 
BTBD3
 
 
hsa-miR-152-3p
 
 
-0.495
 
 
10.5
 
 
 
 
C1GALT1
 
 
hsa-miR-152-3p
 
 
-0.487
 
 
12.1
 
 
 
 
C1QBP
 
 
hsa-miR-330-3p
 
 
-0.412
 
 
76.2
 
 
 
 
C1orf35
 
 
hsa-miR-152-3p
 
 
-0.511
 
 
12.8
 
 
 
 
CAND1
 
 
hsa-miR-152-3p
 
 
-0.44
 
 
17.2
 
 
 
 
CANX
 
 
hsa-miR-152-3p
 
 
-0.434
 
 
112.0
 
 
 
 
CAV2
 
 
hsa-miR-152-3p
 
 
-0.32
 
 
213.4
 
 
 
 
CBLB
 
 
hsa-miR-152-3p
 
 
-0.424
 
 
11.6
 
 
 
 
CCDC6
 
 
hsa-miR-152-3p
 
 
-0.511
 
 
11.1
 
 
 
 
CCDC71
 
 
hsa-miR-152-3p
 
 
-0.369
 
 
12.3
 
 
 
 
CCDC85A
 
 
hsa-miR-152-3p
 
 
-0.296
 
 
11.8
 
 
 
 
CCN2
 
 
hsa-miR-330-3p
 
 
-0.305
 
 
251.6
 
 
 
 
CCT6A
 
 
hsa-miR-152-3p
 
 
-0.665
 
 
56.1
 
 
 
 
CDC25B
 
 
hsa-miR-152-3p
 
 
-0.298
 
 
28.9
 
 
 
 
CEBPG
 
 
hsa-miR-152-3p
 
 
-0.319
 
 
17.9
 
 
 
 
CEP63
 
 
hsa-miR-152-3p
 
 
-0.329
 
 
19.5
 
 
 
 
CERS5
 
 
hsa-miR-152-3p
 
 
-0.311
 
 
19.5
 
 
 
 
CFL2
 
 
hsa-miR-152-3p
 
 
-0.487
 
 
45.7
 
 
 
 
CHIT1
 
 
hsa-miR-378a-3p
 
 
-0.387
 
 
15.5
 
 
 
 
CHMP4B
 
 
hsa-miR-152-3p
 
 
-0.36
 
 
104.4
 
 
 
 
CLCN6
 
 
hsa-miR-152-3p
 
 
-0.406
 
 
12.8
 
 
 
 
CNOT7
 
 
hsa-miR-152-3p
 
 
-0.31
 
 
29.9
 
 
 
 
COL4A1
 
 
hsa-miR-152-3p
 
 
-0.317
 
 
326.6
 
 
 
 
CS
 
 
hsa-miR-152-3p
 
 
-0.3
 
 
144.7
 
 
 
 
CTSA
 
 
hsa-miR-152-3p
 
 
-0.541
 
 
75.8
 
 
 
 
CTTNBP2NL
 
 
hsa-miR-152-3p
 
 
-0.329
 
 
13.4
 
 
 
 
CYTH3
 
 
hsa-miR-152-3p
 
 
-0.331
 
 
27.5
 
 
 
 
DCUN1D4
 
 
hsa-miR-152-3p
 
 
-0.314
 
 
13.1
 
 
 
 
DDX6
 
 
hsa-miR-152-3p
 
 
-0.363
 
 
29.6
 
 
 
 
DEDD
 
 
hsa-miR-152-3p
 
 
-0.401
 
 
32.4
 
 
 
 
DICER1
 
 
hsa-miR-152-3p
 
 
-0.29
 
 
10.8
 
 
 
 
DMPK
 
 
hsa-miR-152-3p
 
 
-0.378
 
 
13.4
 
 
 
 
DNAJB4
 
 
hsa-miR-152-3p
 
 
-0.33
 
 
29.6
 
 
 
 
DOCK6
 
 
hsa-miR-152-3p
 
 
-0.396
 
 
29.9
 
 
 
 
DUSP1
 
 
hsa-miR-152-3p
 
 
-0.32
 
 
874.7
 
 
 
 
DYNLL2
 
 
hsa-miR-152-3p
 
 
-0.366
 
 
23.0
 
 
 
 
EGR3
 
 
hsa-miR-152-3p
 
 
-0.315
 
 
26.8
 
 
 
 
EIF4E3
 
 
hsa-miR-152-3p
 
 
-0.464
 
 
25.9
 
 
 
 
EIF4G3
 
 
hsa-miR-378a-3p
 
 
-0.443
 
 
20.2
 
 
 
 
EMX2
 
 
hsa-miR-152-3p
 
 
-0.366
 
 
10.3
 
 
 
 
EOGT
 
 
hsa-miR-152-3p
 
 
-0.587
 
 
18.2
 
 
 
 
EPS15
 
 
hsa-miR-152-3p
 
 
-0.322
 
 
26.6
 
 
 
 
ERRFI1
 
 
hsa-miR-152-3p
 
 
-0.529
 
 
134.3
 
 
 
 
F3
 
 
hsa-miR-152-3p
 
 
-0.331
 
 
112.4
 
 
 
 
FAM168B
 
 
hsa-miR-152-3p
 
 
-0.409
 
 
38.9
 
 
 
 
FAM43A
 
 
hsa-miR-152-3p
 
 
-0.337
 
 
22.9
 
 
 
 
FBN1
 
 
hsa-miR-152-3p
 
 
-0.349
 
 
49.5
 
 
 
 
FBXO28
 
 
hsa-miR-152-3p
 
 
-0.313
 
 
10.3
 
 
 
 
FEZ2
 
 
hsa-miR-152-3p
 
 
-0.536
 
 
49.8
 
 
 
 
FMR1
 
 
hsa-miR-152-3p
 
 
-0.385
 
 
11.9
 
 
 
 
FOSB
 
 
hsa-miR-152-3p
 
 
-0.311
 
 
369.6
 
 
 
 
FOXK2
 
 
hsa-miR-152-3p
 
 
-0.305
 
 
13.1
 
 
 
 
FXR1
 
 
hsa-miR-152-3p
 
 
-0.478
 
 
34.0
 
 
 
 
GADD45A
 
 
hsa-miR-152-3p
 
 
-0.787
 
 
131.8
 
 
 
 
GAPVD1
 
 
hsa-miR-152-3p
 
 
-0.309
 
 
11.5
 
 
 
 
GLRX5
 
 
hsa-miR-152-3p
 
 
-0.381
 
 
37.9
 
 
 
 
GMFB
 
 
hsa-miR-152-3p
 
 
-0.429
 
 
23.6
 
 
 
 
GPATCH8
 
 
hsa-miR-152-3p
 
 
-0.374
 
 
15.1
 
 
 
 
GRSF1
 
 
hsa-miR-378a-3p
 
 
-0.358
 
 
34.1
 
 
 
 
GTF2H1
 
 
hsa-miR-152-3p
 
 
-0.524
 
 
18.2
 
 
 
 
HDDC2
 
 
hsa-miR-378a-3p
 
 
-0.322
 
 
30.4
 
 
 
 
HLA-DQB1
 
 
hsa-miR-152-3p
 
 
-0.37
 
 
55.5
 
 
 
 
HSP90B1
 
 
hsa-miR-152-3p
 
 
-0.304
 
 
309.6
 
 
 
 
HSPA12A
 
 
hsa-miR-378a-3p
 
 
-0.326
 
 
11.1
 
 
 
 
HSPH1
 
 
hsa-miR-330-3p
 
 
-0.345
 
 
50.3
 
 
 
 
ID2
 
 
hsa-miR-330-3p
 
 
-0.293
 
 
154.6
 
 
 
 
IGF1
 
 
hsa-miR-152-3p
 
 
-0.43
 
 
81.2
 
 
 
 
IL6ST
 
 
hsa-miR-152-3p
 
 
-0.291
 
 
86.4
 
 
 
 
INHBB
 
 
hsa-miR-152-3p
 
 
-0.469
 
 
49.6
 
 
 
 
ITSN2
 
 
hsa-miR-152-3p
 
 
-0.339
 
 
21.6
 
 
 
 
KDSR
 
 
hsa-miR-378a-3p
 
 
-0.373
 
 
13.9
 
 
 
 
KLF4
 
 
hsa-miR-152-3p
 
 
-0.325
 
 
68.8
 
 
 
 
LAMA4
 
 
hsa-miR-152-3p
 
 
-0.363
 
 
108.0
 
 
 
 
LBR
 
 
hsa-miR-152-3p
 
 
-0.39
 
 
26.8
 
 
 
 
LDLR
 
 
hsa-miR-152-3p
 
 
-0.323
 
 
40.8
 
 
 
 
LEPROTL1
 
 
hsa-miR-152-3p
 
 
-0.568
 
 
33.0
 
 
 
 
LIPA
 
 
hsa-miR-152-3p
 
 
-0.463
 
 
72.7
 
 
 
 
 
 
 adrenal gland 
 
 
 
 
target_symbol
 
 
mature_mirna_id
 
 
score
 
 
nTPM
 
 
 
 
 
 
ABCB7
 
 
hsa-miR-152-3p
 
 
-0.515
 
 
12.5
 
 
 
 
ABCD3
 
 
hsa-miR-152-3p
 
 
-0.388
 
 
17.6
 
 
 
 
ADGRF5
 
 
hsa-miR-152-3p
 
 
-0.354
 
 
22.0
 
 
 
 
AGFG1
 
 
hsa-miR-152-3p
 
 
-0.332
 
 
37.2
 
 
 
 
AHDC1
 
 
hsa-miR-152-3p
 
 
-0.413
 
 
23.0
 
 
 
 
AKAP1
 
 
hsa-miR-152-3p
 
 
-0.381
 
 
14.9
 
 
 
 
ALCAM
 
 
hsa-miR-152-3p
 
 
-0.347
 
 
30.9
 
 
 
 
ARF4
 
 
hsa-miR-152-3p
 
 
-0.487
 
 
151.0
 
 
 
 
ARFIP1
 
 
hsa-miR-152-3p
 
 
-0.474
 
 
13.9
 
 
 
 
ARHGAP21
 
 
hsa-miR-152-3p
 
 
-0.287
 
 
16.9
 
 
 
 
ARL6IP1
 
 
hsa-miR-152-3p
 
 
-0.579
 
 
161.7
 
 
 
 
ARL8B
 
 
hsa-miR-152-3p
 
 
-0.337
 
 
48.3
 
 
 
 
ARRDC3
 
 
hsa-miR-152-3p
 
 
-0.305
 
 
75.7
 
 
 
 
ASPH
 
 
hsa-miR-152-3p
 
 
-0.391
 
 
150.8
 
 
 
 
ATP2B4
 
 
hsa-miR-152-3p
 
 
-0.337
 
 
23.7
 
 
 
 
ATP6AP2
 
 
hsa-miR-152-3p
 
 
-0.592
 
 
67.5
 
 
 
 
ATP6AP2
 
 
hsa-miR-152-3p
 
 
-0.668
 
 
67.5
 
 
 
 
B4GALT5
 
 
hsa-miR-152-3p
 
 
-0.44
 
 
44.9
 
 
 
 
B4GALT6
 
 
hsa-miR-152-3p
 
 
-0.528
 
 
17.1
 
 
 
 
BHLHE41
 
 
hsa-miR-152-3p
 
 
-0.442
 
 
13.8
 
 
 
 
BTAF1
 
 
hsa-miR-152-3p
 
 
-0.401
 
 
11.3
 
 
 
 
BTBD3
 
 
hsa-miR-152-3p
 
 
-0.495
 
 
20.8
 
 
 
 
C1QBP
 
 
hsa-miR-330-3p
 
 
-0.412
 
 
128.1
 
 
 
 
C1orf35
 
 
hsa-miR-152-3p
 
 
-0.511
 
 
14.2
 
 
 
 
C3orf62
 
 
hsa-miR-330-3p
 
 
-0.366
 
 
10.8
 
 
 
 
CAND1
 
 
hsa-miR-152-3p
 
 
-0.44
 
 
21.8
 
 
 
 
CANX
 
 
hsa-miR-152-3p
 
 
-0.434
 
 
97.0
 
 
 
 
CCDC6
 
 
hsa-miR-152-3p
 
 
-0.511
 
 
11.5
 
 
 
 
CCDC71
 
 
hsa-miR-152-3p
 
 
-0.369
 
 
14.5
 
 
 
 
CCN2
 
 
hsa-miR-330-3p
 
 
-0.305
 
 
101.5
 
 
 
 
CCT6A
 
 
hsa-miR-152-3p
 
 
-0.665
 
 
59.0
 
 
 
 
CDC25B
 
 
hsa-miR-152-3p
 
 
-0.298
 
 
14.9
 
 
 
 
CEP63
 
 
hsa-miR-152-3p
 
 
-0.329
 
 
12.5
 
 
 
 
CERS5
 
 
hsa-miR-152-3p
 
 
-0.311
 
 
18.2
 
 
 
 
CFL2
 
 
hsa-miR-152-3p
 
 
-0.487
 
 
51.0
 
 
 
 
CHMP4B
 
 
hsa-miR-152-3p
 
 
-0.36
 
 
110.4
 
 
 
 
CLCN6
 
 
hsa-miR-152-3p
 
 
-0.406
 
 
13.9
 
 
 
 
CNOT7
 
 
hsa-miR-152-3p
 
 
-0.31
 
 
30.7
 
 
 
 
COL4A1
 
 
hsa-miR-152-3p
 
 
-0.317
 
 
27.8
 
 
 
 
CS
 
 
hsa-miR-152-3p
 
 
-0.3
 
 
99.7
 
 
 
 
CTSA
 
 
hsa-miR-152-3p
 
 
-0.541
 
 
312.4
 
 
 
 
CUL5
 
 
hsa-miR-152-3p
 
 
-0.389
 
 
10.8
 
 
 
 
DCUN1D4
 
 
hsa-miR-152-3p
 
 
-0.314
 
 
16.8
 
 
 
 
DDAH1
 
 
hsa-miR-152-3p
 
 
-0.34
 
 
27.0
 
 
 
 
DDX6
 
 
hsa-miR-152-3p
 
 
-0.363
 
 
26.8
 
 
 
 
DEDD
 
 
hsa-miR-152-3p
 
 
-0.401
 
 
20.3
 
 
 
 
DMPK
 
 
hsa-miR-152-3p
 
 
-0.378
 
 
59.4
 
 
 
 
DNAJB4
 
 
hsa-miR-152-3p
 
 
-0.33
 
 
46.9
 
 
 
 
DUSP1
 
 
hsa-miR-152-3p
 
 
-0.32
 
 
486.8
 
 
 
 
DYNLL2
 
 
hsa-miR-152-3p
 
 
-0.366
 
 
39.5
 
 
 
 
DYRK1B
 
 
hsa-miR-152-3p
 
 
-0.305
 
 
18.7
 
 
 
 
EGR3
 
 
hsa-miR-152-3p
 
 
-0.315
 
 
23.2
 
 
 
 
EIF4E3
 
 
hsa-miR-152-3p
 
 
-0.464
 
 
11.4
 
 
 
 
EIF4G3
 
 
hsa-miR-378a-3p
 
 
-0.443
 
 
19.1
 
 
 
 
EPS15
 
 
hsa-miR-152-3p
 
 
-0.322
 
 
30.3
 
 
 
 
ERRFI1
 
 
hsa-miR-152-3p
 
 
-0.529
 
 
39.3
 
 
 
 
F3
 
 
hsa-miR-152-3p
 
 
-0.331
 
 
20.2
 
 
 
 
FAM168B
 
 
hsa-miR-152-3p
 
 
-0.409
 
 
28.3
 
 
 
 
FEZ2
 
 
hsa-miR-152-3p
 
 
-0.536
 
 
35.9
 
 
 
 
FMR1
 
 
hsa-miR-152-3p
 
 
-0.385
 
 
11.3
 
 
 
 
FOSB
 
 
hsa-miR-152-3p
 
 
-0.311
 
 
114.8
 
 
 
 
FOXK2
 
 
hsa-miR-152-3p
 
 
-0.305
 
 
24.8
 
 
 
 
FXR1
 
 
hsa-miR-152-3p
 
 
-0.478
 
 
38.7
 
 
 
 
GADD45A
 
 
hsa-miR-152-3p
 
 
-0.787
 
 
18.1
 
 
 
 
GLRX5
 
 
hsa-miR-152-3p
 
 
-0.381
 
 
81.1
 
 
 
 
GMFB
 
 
hsa-miR-152-3p
 
 
-0.429
 
 
20.6
 
 
 
 
GPATCH8
 
 
hsa-miR-152-3p
 
 
-0.374
 
 
10.6
 
 
 
 
GRSF1
 
 
hsa-miR-378a-3p
 
 
-0.358
 
 
55.6
 
 
 
 
GTF2H1
 
 
hsa-miR-152-3p
 
 
-0.524
 
 
17.3
 
 
 
 
HDDC2
 
 
hsa-miR-378a-3p
 
 
-0.322
 
 
58.7
 
 
 
 
HLA-DQB1
 
 
hsa-miR-152-3p
 
 
-0.37
 
 
23.6
 
 
 
 
HSP90B1
 
 
hsa-miR-152-3p
 
 
-0.304
 
 
321.6
 
 
 
 
HSPH1
 
 
hsa-miR-330-3p
 
 
-0.345
 
 
42.5
 
 
 
 
ID2
 
 
hsa-miR-330-3p
 
 
-0.293
 
 
165.9
 
 
 
 
IL6ST
 
 
hsa-miR-152-3p
 
 
-0.291
 
 
51.8
 
 
 
 
ING2
 
 
hsa-miR-152-3p
 
 
-0.482
 
 
25.2
 
 
 
 
ITSN2
 
 
hsa-miR-152-3p
 
 
-0.339
 
 
20.1
 
 
 
 
KDSR
 
 
hsa-miR-378a-3p
 
 
-0.373
 
 
12.6
 
 
 
 
KLF4
 
 
hsa-miR-152-3p
 
 
-0.325
 
 
22.5
 
 
 
 
LDLR
 
 
hsa-miR-152-3p
 
 
-0.323
 
 
112.1
 
 
 
 
LEPROTL1
 
 
hsa-miR-152-3p
 
 
-0.568
 
 
44.0
 
 
 
 
LIPA
 
 
hsa-miR-152-3p
 
 
-0.463
 
 
43.7
 
 
 
 
LMAN1
 
 
hsa-miR-152-3p
 
 
-0.382
 
 
40.9
 
 
 
 
LRRC41
 
 
hsa-miR-152-3p
 
 
-0.392
 
 
40.8
 
 
 
 
LSM5
 
 
hsa-miR-330-3p
 
 
-0.351
 
 
39.5
 
 
 
 
LYRM2
 
 
hsa-miR-152-3p
 
 
-0.351
 
 
15.0
 
 
 
 
LYSMD2
 
 
hsa-miR-152-3p
 
 
-0.554
 
 
18.5
 
 
 
 
MACROH2A1
 
 
hsa-miR-152-3p
 
 
-0.336
 
 
53.5
 
 
 
 
MAF
 
 
hsa-miR-152-3p
 
 
-0.362
 
 
14.6
 
 
 
 
MAF1
 
 
hsa-miR-152-3p
 
 
-0.366
 
 
80.7
 
 
 
 
MAFB
 
 
hsa-miR-152-3p
 
 
-0.45
 
 
13.1
 
 
 
 
MAFG
 
 
hsa-miR-152-3p
 
 
-0.328
 
 
12.4
 
 
 
 
MAP2K1
 
 
hsa-miR-152-3p
 
 
-0.295
 
 
22.5
 
 
 
 
MAPK1
 
 
hsa-miR-378a-3p
 
 
-0.314
 
 
31.3
 
 
 
 
MAT2A
 
 
hsa-miR-330-3p
 
 
-0.307
 
 
262.0
 
 
 
 
MCM3AP
 
 
hsa-miR-152-3p
 
 
-0.287
 
 
11.4
 
 
 
 
MDFIC
 
 
hsa-miR-152-3p
 
 
-0.395
 
 
15.0
 
 
 
 
MECP2
 
 
hsa-miR-152-3p
 
 
-0.3
 
 
15.4
 
 
 
 
MED19
 
 
hsa-miR-378a-3p
 
 
-0.434
 
 
18.3
 
 
 
 
MIER1
 
 
hsa-miR-152-3p
 
 
-0.352
 
 
19.6
 
 
 
 
 
 
 blood vessel 
 
 
 
 
target_symbol
 
 
mature_mirna_id
 
 
score
 
 
nTPM
 
 
 
 
 
 
ABCB7
 
 
hsa-miR-152-3p
 
 
-0.515
 
 
12.3
 
 
 
 
ABCD3
 
 
hsa-miR-152-3p
 
 
-0.388
 
 
14.4
 
 
 
 
ADGRF5
 
 
hsa-miR-152-3p
 
 
-0.354
 
 
12.6
 
 
 
 
AGFG1
 
 
hsa-miR-152-3p
 
 
-0.332
 
 
37.3
 
 
 
 
AHDC1
 
 
hsa-miR-152-3p
 
 
-0.413
 
 
27.7
 
 
 
 
AKAP1
 
 
hsa-miR-152-3p
 
 
-0.381
 
 
84.5
 
 
 
 
ALCAM
 
 
hsa-miR-152-3p
 
 
-0.347
 
 
85.5
 
 
 
 
ARF4
 
 
hsa-miR-152-3p
 
 
-0.487
 
 
151.4
 
 
 
 
ARFIP1
 
 
hsa-miR-152-3p
 
 
-0.474
 
 
14.2
 
 
 
 
ARHGAP21
 
 
hsa-miR-152-3p
 
 
-0.287
 
 
14.0
 
 
 
 
ARL6IP1
 
 
hsa-miR-152-3p
 
 
-0.579
 
 
52.2
 
 
 
 
ARL8B
 
 
hsa-miR-152-3p
 
 
-0.337
 
 
49.5
 
 
 
 
ARRDC3
 
 
hsa-miR-152-3p
 
 
-0.305
 
 
25.4
 
 
 
 
ASPH
 
 
hsa-miR-152-3p
 
 
-0.391
 
 
72.5
 
 
 
 
ATP2B4
 
 
hsa-miR-152-3p
 
 
-0.337
 
 
24.9
 
 
 
 
ATP6AP2
 
 
hsa-miR-152-3p
 
 
-0.592
 
 
93.4
 
 
 
 
ATP6AP2
 
 
hsa-miR-152-3p
 
 
-0.668
 
 
93.4
 
 
 
 
B4GALT5
 
 
hsa-miR-152-3p
 
 
-0.44
 
 
15.5
 
 
 
 
BHLHE41
 
 
hsa-miR-152-3p
 
 
-0.442
 
 
18.7
 
 
 
 
BTAF1
 
 
hsa-miR-152-3p
 
 
-0.401
 
 
19.0
 
 
 
 
C1QBP
 
 
hsa-miR-330-3p
 
 
-0.412
 
 
60.2
 
 
 
 
C1orf35
 
 
hsa-miR-152-3p
 
 
-0.511
 
 
14.8
 
 
 
 
CAND1
 
 
hsa-miR-152-3p
 
 
-0.44
 
 
18.4
 
 
 
 
CANX
 
 
hsa-miR-152-3p
 
 
-0.434
 
 
142.5
 
 
 
 
CAV2
 
 
hsa-miR-152-3p
 
 
-0.32
 
 
88.6
 
 
 
 
CCDC6
 
 
hsa-miR-152-3p
 
 
-0.511
 
 
26.2
 
 
 
 
CCN2
 
 
hsa-miR-330-3p
 
 
-0.305
 
 
1230.3
 
 
 
 
CCT6A
 
 
hsa-miR-152-3p
 
 
-0.665
 
 
59.5
 
 
 
 
CDC25B
 
 
hsa-miR-152-3p
 
 
-0.298
 
 
31.5
 
 
 
 
CEBPG
 
 
hsa-miR-152-3p
 
 
-0.319
 
 
11.1
 
 
 
 
CERS5
 
 
hsa-miR-152-3p
 
 
-0.311
 
 
26.1
 
 
 
 
CFL2
 
 
hsa-miR-152-3p
 
 
-0.487
 
 
108.4
 
 
 
 
CHMP4B
 
 
hsa-miR-152-3p
 
 
-0.36
 
 
174.6
 
 
 
 
CHST1
 
 
hsa-miR-152-3p
 
 
-0.408
 
 
12.4
 
 
 
 
CLCN6
 
 
hsa-miR-152-3p
 
 
-0.406
 
 
12.4
 
 
 
 
CNOT7
 
 
hsa-miR-152-3p
 
 
-0.31
 
 
20.2
 
 
 
 
CNTN4
 
 
hsa-miR-152-3p
 
 
-0.415
 
 
14.4
 
 
 
 
COL4A1
 
 
hsa-miR-152-3p
 
 
-0.317
 
 
213.1
 
 
 
 
CS
 
 
hsa-miR-152-3p
 
 
-0.3
 
 
62.2
 
 
 
 
CTSA
 
 
hsa-miR-152-3p
 
 
-0.541
 
 
96.0
 
 
 
 
CTTNBP2NL
 
 
hsa-miR-152-3p
 
 
-0.329
 
 
10.5
 
 
 
 
CYTH3
 
 
hsa-miR-152-3p
 
 
-0.331
 
 
42.4
 
 
 
 
DACT1
 
 
hsa-miR-378a-3p
 
 
-0.298
 
 
26.2
 
 
 
 
DCUN1D4
 
 
hsa-miR-152-3p
 
 
-0.314
 
 
17.6
 
 
 
 
DDAH1
 
 
hsa-miR-152-3p
 
 
-0.34
 
 
18.4
 
 
 
 
DDX6
 
 
hsa-miR-152-3p
 
 
-0.363
 
 
22.9
 
 
 
 
DEDD
 
 
hsa-miR-152-3p
 
 
-0.401
 
 
33.1
 
 
 
 
DIPK2A
 
 
hsa-miR-152-3p
 
 
-0.393
 
 
12.5
 
 
 
 
DMPK
 
 
hsa-miR-152-3p
 
 
-0.378
 
 
95.9
 
 
 
 
DNAJB4
 
 
hsa-miR-152-3p
 
 
-0.33
 
 
33.2
 
 
 
 
DOCK6
 
 
hsa-miR-152-3p
 
 
-0.396
 
 
14.7
 
 
 
 
DUSP1
 
 
hsa-miR-152-3p
 
 
-0.32
 
 
1058.8
 
 
 
 
DYNLL2
 
 
hsa-miR-152-3p
 
 
-0.366
 
 
24.3
 
 
 
 
DYRK2
 
 
hsa-miR-152-3p
 
 
-0.293
 
 
11.2
 
 
 
 
EGR3
 
 
hsa-miR-152-3p
 
 
-0.315
 
 
19.1
 
 
 
 
EIF4E3
 
 
hsa-miR-152-3p
 
 
-0.464
 
 
11.5
 
 
 
 
EIF4G3
 
 
hsa-miR-378a-3p
 
 
-0.443
 
 
11.0
 
 
 
 
EMX2
 
 
hsa-miR-152-3p
 
 
-0.366
 
 
15.6
 
 
 
 
EOGT
 
 
hsa-miR-152-3p
 
 
-0.587
 
 
53.9
 
 
 
 
EPS15
 
 
hsa-miR-152-3p
 
 
-0.322
 
 
25.5
 
 
 
 
ERRFI1
 
 
hsa-miR-152-3p
 
 
-0.529
 
 
233.7
 
 
 
 
F3
 
 
hsa-miR-152-3p
 
 
-0.331
 
 
31.2
 
 
 
 
FAM168B
 
 
hsa-miR-152-3p
 
 
-0.409
 
 
46.1
 
 
 
 
FBN1
 
 
hsa-miR-152-3p
 
 
-0.349
 
 
18.6
 
 
 
 
FEZ2
 
 
hsa-miR-152-3p
 
 
-0.536
 
 
40.0
 
 
 
 
FOSB
 
 
hsa-miR-152-3p
 
 
-0.311
 
 
80.5
 
 
 
 
FOXK2
 
 
hsa-miR-152-3p
 
 
-0.305
 
 
19.0
 
 
 
 
FXR1
 
 
hsa-miR-152-3p
 
 
-0.478
 
 
42.3
 
 
 
 
GADD45A
 
 
hsa-miR-152-3p
 
 
-0.787
 
 
232.0
 
 
 
 
GAPVD1
 
 
hsa-miR-152-3p
 
 
-0.309
 
 
10.1
 
 
 
 
GLRX5
 
 
hsa-miR-152-3p
 
 
-0.381
 
 
45.4
 
 
 
 
GMFB
 
 
hsa-miR-152-3p
 
 
-0.429
 
 
11.7
 
 
 
 
GPATCH8
 
 
hsa-miR-152-3p
 
 
-0.374
 
 
16.4
 
 
 
 
GRSF1
 
 
hsa-miR-378a-3p
 
 
-0.358
 
 
36.1
 
 
 
 
GTF2H1
 
 
hsa-miR-152-3p
 
 
-0.524
 
 
19.6
 
 
 
 
HDDC2
 
 
hsa-miR-378a-3p
 
 
-0.322
 
 
34.8
 
 
 
 
HLA-DQB1
 
 
hsa-miR-152-3p
 
 
-0.37
 
 
42.5
 
 
 
 
HSP90B1
 
 
hsa-miR-152-3p
 
 
-0.304
 
 
383.5
 
 
 
 
HSPH1
 
 
hsa-miR-330-3p
 
 
-0.345
 
 
38.9
 
 
 
 
ID2
 
 
hsa-miR-330-3p
 
 
-0.293
 
 
790.2
 
 
 
 
IGF1
 
 
hsa-miR-152-3p
 
 
-0.43
 
 
30.4
 
 
 
 
IL6ST
 
 
hsa-miR-152-3p
 
 
-0.291
 
 
35.1
 
 
 
 
ITGA11
 
 
hsa-miR-152-3p
 
 
-0.348
 
 
74.1
 
 
 
 
ITSN2
 
 
hsa-miR-152-3p
 
 
-0.339
 
 
21.8
 
 
 
 
KDSR
 
 
hsa-miR-378a-3p
 
 
-0.373
 
 
16.4
 
 
 
 
KLF4
 
 
hsa-miR-152-3p
 
 
-0.325
 
 
31.5
 
 
 
 
LAMA4
 
 
hsa-miR-152-3p
 
 
-0.363
 
 
44.4
 
 
 
 
LBR
 
 
hsa-miR-152-3p
 
 
-0.39
 
 
27.2
 
 
 
 
LDLR
 
 
hsa-miR-152-3p
 
 
-0.323
 
 
14.2
 
 
 
 
LEPROTL1
 
 
hsa-miR-152-3p
 
 
-0.568
 
 
22.7
 
 
 
 
LIPA
 
 
hsa-miR-152-3p
 
 
-0.463
 
 
35.5
 
 
 
 
LMAN1
 
 
hsa-miR-152-3p
 
 
-0.382
 
 
25.0
 
 
 
 
LRCH1
 
 
hsa-miR-152-3p
 
 
-0.321
 
 
19.8
 
 
 
 
LRRC41
 
 
hsa-miR-152-3p
 
 
-0.392
 
 
43.3
 
 
 
 
LSM5
 
 
hsa-miR-330-3p
 
 
-0.351
 
 
40.2
 
 
 
 
LYSMD2
 
 
hsa-miR-152-3p
 
 
-0.554
 
 
14.2
 
 
 
 
MACROH2A1
 
 
hsa-miR-152-3p
 
 
-0.336
 
 
46.5
 
 
 
 
MAF
 
 
hsa-miR-152-3p
 
 
-0.362
 
 
11.3
 
 
 
 
MAF1
 
 
hsa-miR-152-3p
 
 
-0.366
 
 
155.9
 
 
 
 
MAFB
 
 
hsa-miR-152-3p
 
 
-0.45
 
 
21.7
 
 
 
 
 
 
 breast 
 
 
 
 
target_symbol
 
 
mature_mirna_id
 
 
score
 
 
nTPM
 
 
 
 
 
 
ABCB7
 
 
hsa-miR-152-3p
 
 
-0.515
 
 
11.6
 
 
 
 
ABCD3
 
 
hsa-miR-152-3p
 
 
-0.388
 
 
30.8
 
 
 
 
ABHD17C
 
 
hsa-miR-330-3p
 
 
-0.399
 
 
10.9
 
 
 
 
ACVR1
 
 
hsa-miR-152-3p
 
 
-0.397
 
 
11.1
 
 
 
 
ADAM10
 
 
hsa-miR-152-3p
 
 
-0.348
 
 
14.2
 
 
 
 
ADGRF5
 
 
hsa-miR-152-3p
 
 
-0.354
 
 
44.5
 
 
 
 
AGFG1
 
 
hsa-miR-152-3p
 
 
-0.332
 
 
48.8
 
 
 
 
AHDC1
 
 
hsa-miR-152-3p
 
 
-0.413
 
 
11.9
 
 
 
 
AKAP1
 
 
hsa-miR-152-3p
 
 
-0.381
 
 
34.0
 
 
 
 
ALCAM
 
 
hsa-miR-152-3p
 
 
-0.347
 
 
76.0
 
 
 
 
ARF4
 
 
hsa-miR-152-3p
 
 
-0.487
 
 
129.6
 
 
 
 
ARFGEF1
 
 
hsa-miR-152-3p
 
 
-0.306
 
 
12.7
 
 
 
 
ARFIP1
 
 
hsa-miR-152-3p
 
 
-0.474
 
 
22.0
 
 
 
 
ARHGAP21
 
 
hsa-miR-152-3p
 
 
-0.287
 
 
15.1
 
 
 
 
ARL6IP1
 
 
hsa-miR-152-3p
 
 
-0.579
 
 
105.7
 
 
 
 
ARL8B
 
 
hsa-miR-152-3p
 
 
-0.337
 
 
39.7
 
 
 
 
ARRDC3
 
 
hsa-miR-152-3p
 
 
-0.305
 
 
88.5
 
 
 
 
ASPH
 
 
hsa-miR-152-3p
 
 
-0.391
 
 
99.5
 
 
 
 
ASPRV1
 
 
hsa-miR-330-3p
 
 
-0.301
 
 
23.5
 
 
 
 
ATP2B4
 
 
hsa-miR-152-3p
 
 
-0.337
 
 
15.5
 
 
 
 
ATP6AP2
 
 
hsa-miR-152-3p
 
 
-0.592
 
 
89.1
 
 
 
 
ATP6AP2
 
 
hsa-miR-152-3p
 
 
-0.668
 
 
89.1
 
 
 
 
B4GALT5
 
 
hsa-miR-152-3p
 
 
-0.44
 
 
17.9
 
 
 
 
BCL2L11
 
 
hsa-miR-152-3p
 
 
-0.445
 
 
11.8
 
 
 
 
BHLHE41
 
 
hsa-miR-152-3p
 
 
-0.442
 
 
12.9
 
 
 
 
BTAF1
 
 
hsa-miR-152-3p
 
 
-0.401
 
 
15.3
 
 
 
 
BTBD3
 
 
hsa-miR-152-3p
 
 
-0.495
 
 
11.8
 
 
 
 
C1GALT1
 
 
hsa-miR-152-3p
 
 
-0.487
 
 
15.5
 
 
 
 
C1QBP
 
 
hsa-miR-330-3p
 
 
-0.412
 
 
101.6
 
 
 
 
C1orf210
 
 
hsa-miR-378a-3p
 
 
-0.307
 
 
13.5
 
 
 
 
C1orf35
 
 
hsa-miR-152-3p
 
 
-0.511
 
 
13.0
 
 
 
 
CAND1
 
 
hsa-miR-152-3p
 
 
-0.44
 
 
24.3
 
 
 
 
CANX
 
 
hsa-miR-152-3p
 
 
-0.434
 
 
95.6
 
 
 
 
CAV2
 
 
hsa-miR-152-3p
 
 
-0.32
 
 
76.2
 
 
 
 
CCDC6
 
 
hsa-miR-152-3p
 
 
-0.511
 
 
21.0
 
 
 
 
CCDC71
 
 
hsa-miR-152-3p
 
 
-0.369
 
 
13.8
 
 
 
 
CCN2
 
 
hsa-miR-330-3p
 
 
-0.305
 
 
193.4
 
 
 
 
CCT6A
 
 
hsa-miR-152-3p
 
 
-0.665
 
 
57.1
 
 
 
 
CDC14A
 
 
hsa-miR-152-3p
 
 
-0.482
 
 
10.6
 
 
 
 
CDC25B
 
 
hsa-miR-152-3p
 
 
-0.298
 
 
24.5
 
 
 
 
CEBPG
 
 
hsa-miR-152-3p
 
 
-0.319
 
 
13.1
 
 
 
 
CEP63
 
 
hsa-miR-152-3p
 
 
-0.329
 
 
12.7
 
 
 
 
CERS5
 
 
hsa-miR-152-3p
 
 
-0.311
 
 
20.1
 
 
 
 
CFL2
 
 
hsa-miR-152-3p
 
 
-0.487
 
 
24.6
 
 
 
 
CHMP4B
 
 
hsa-miR-152-3p
 
 
-0.36
 
 
100.8
 
 
 
 
CLCN6
 
 
hsa-miR-152-3p
 
 
-0.406
 
 
11.2
 
 
 
 
CNOT7
 
 
hsa-miR-152-3p
 
 
-0.31
 
 
39.3
 
 
 
 
COL4A1
 
 
hsa-miR-152-3p
 
 
-0.317
 
 
122.5
 
 
 
 
CS
 
 
hsa-miR-152-3p
 
 
-0.3
 
 
78.4
 
 
 
 
CTSA
 
 
hsa-miR-152-3p
 
 
-0.541
 
 
53.7
 
 
 
 
CTTNBP2NL
 
 
hsa-miR-152-3p
 
 
-0.329
 
 
10.5
 
 
 
 
CUL5
 
 
hsa-miR-152-3p
 
 
-0.389
 
 
12.1
 
 
 
 
CYTH3
 
 
hsa-miR-152-3p
 
 
-0.331
 
 
25.8
 
 
 
 
DDAH1
 
 
hsa-miR-152-3p
 
 
-0.34
 
 
17.6
 
 
 
 
DDX6
 
 
hsa-miR-152-3p
 
 
-0.363
 
 
32.7
 
 
 
 
DEDD
 
 
hsa-miR-152-3p
 
 
-0.401
 
 
27.9
 
 
 
 
DIPK2A
 
 
hsa-miR-152-3p
 
 
-0.393
 
 
14.7
 
 
 
 
DMPK
 
 
hsa-miR-152-3p
 
 
-0.378
 
 
11.1
 
 
 
 
DNAJB4
 
 
hsa-miR-152-3p
 
 
-0.33
 
 
14.5
 
 
 
 
DOCK6
 
 
hsa-miR-152-3p
 
 
-0.396
 
 
26.8
 
 
 
 
DUSP1
 
 
hsa-miR-152-3p
 
 
-0.32
 
 
560.9
 
 
 
 
DYNLL2
 
 
hsa-miR-152-3p
 
 
-0.366
 
 
15.8
 
 
 
 
DYRK1B
 
 
hsa-miR-152-3p
 
 
-0.305
 
 
10.2
 
 
 
 
EIF4E3
 
 
hsa-miR-152-3p
 
 
-0.464
 
 
13.1
 
 
 
 
EIF4G3
 
 
hsa-miR-378a-3p
 
 
-0.443
 
 
12.7
 
 
 
 
ELF5
 
 
hsa-miR-152-3p
 
 
-0.421
 
 
140.5
 
 
 
 
EOGT
 
 
hsa-miR-152-3p
 
 
-0.587
 
 
18.4
 
 
 
 
EPS15
 
 
hsa-miR-152-3p
 
 
-0.322
 
 
24.0
 
 
 
 
ERBB3
 
 
hsa-miR-152-3p
 
 
-0.399
 
 
49.3
 
 
 
 
ERRFI1
 
 
hsa-miR-152-3p
 
 
-0.529
 
 
124.6
 
 
 
 
ESR1
 
 
hsa-miR-152-3p
 
 
-0.34
 
 
27.4
 
 
 
 
F3
 
 
hsa-miR-152-3p
 
 
-0.331
 
 
67.2
 
 
 
 
FAM168B
 
 
hsa-miR-152-3p
 
 
-0.409
 
 
27.5
 
 
 
 
FAM43A
 
 
hsa-miR-152-3p
 
 
-0.337
 
 
14.3
 
 
 
 
FBN1
 
 
hsa-miR-152-3p
 
 
-0.349
 
 
11.7
 
 
 
 
FBXO28
 
 
hsa-miR-152-3p
 
 
-0.313
 
 
11.5
 
 
 
 
FEZ2
 
 
hsa-miR-152-3p
 
 
-0.536
 
 
32.2
 
 
 
 
FMR1
 
 
hsa-miR-152-3p
 
 
-0.385
 
 
11.0
 
 
 
 
FOSB
 
 
hsa-miR-152-3p
 
 
-0.311
 
 
86.1
 
 
 
 
FOXK2
 
 
hsa-miR-152-3p
 
 
-0.305
 
 
11.5
 
 
 
 
FXR1
 
 
hsa-miR-152-3p
 
 
-0.478
 
 
30.8
 
 
 
 
GADD45A
 
 
hsa-miR-152-3p
 
 
-0.787
 
 
92.9
 
 
 
 
GAPVD1
 
 
hsa-miR-152-3p
 
 
-0.309
 
 
11.2
 
 
 
 
GLRX5
 
 
hsa-miR-152-3p
 
 
-0.381
 
 
39.6
 
 
 
 
GMFB
 
 
hsa-miR-152-3p
 
 
-0.429
 
 
24.4
 
 
 
 
GPATCH8
 
 
hsa-miR-152-3p
 
 
-0.374
 
 
14.5
 
 
 
 
GRSF1
 
 
hsa-miR-378a-3p
 
 
-0.358
 
 
33.9
 
 
 
 
GTF2H1
 
 
hsa-miR-152-3p
 
 
-0.524
 
 
17.4
 
 
 
 
HDDC2
 
 
hsa-miR-378a-3p
 
 
-0.322
 
 
27.0
 
 
 
 
HLA-DQB1
 
 
hsa-miR-152-3p
 
 
-0.37
 
 
44.3
 
 
 
 
HSP90B1
 
 
hsa-miR-152-3p
 
 
-0.304
 
 
319.6
 
 
 
 
HSPH1
 
 
hsa-miR-330-3p
 
 
-0.345
 
 
26.1
 
 
 
 
ID2
 
 
hsa-miR-330-3p
 
 
-0.293
 
 
81.8
 
 
 
 
IGF1
 
 
hsa-miR-152-3p
 
 
-0.43
 
 
41.1
 
 
 
 
IL6ST
 
 
hsa-miR-152-3p
 
 
-0.291
 
 
58.7
 
 
 
 
INHBB
 
 
hsa-miR-152-3p
 
 
-0.469
 
 
42.2
 
 
 
 
ITSN2
 
 
hsa-miR-152-3p
 
 
-0.339
 
 
14.3
 
 
 
 
KDSR
 
 
hsa-miR-378a-3p
 
 
-0.373
 
 
11.8
 
 
 
 
KLF4
 
 
hsa-miR-152-3p
 
 
-0.325
 
 
59.8
 
 
 
 
KLF5
 
 
hsa-miR-152-3p
 
 
-0.475
 
 
33.9
 
 
 
 
 
 
 cervix 
 
 
 
 
target_symbol
 
 
mature_mirna_id
 
 
score
 
 
nTPM
 
 
 
 
 
 
ABCB7
 
 
hsa-miR-152-3p
 
 
-0.515
 
 
10.8
 
 
 
 
ABCD3
 
 
hsa-miR-152-3p
 
 
-0.388
 
 
21.0
 
 
 
 
ABHD17C
 
 
hsa-miR-330-3p
 
 
-0.399
 
 
18.1
 
 
 
 
ACVR1
 
 
hsa-miR-152-3p
 
 
-0.397
 
 
20.6
 
 
 
 
ADAM10
 
 
hsa-miR-152-3p
 
 
-0.348
 
 
15.1
 
 
 
 
AGFG1
 
 
hsa-miR-152-3p
 
 
-0.332
 
 
27.5
 
 
 
 
AHDC1
 
 
hsa-miR-152-3p
 
 
-0.413
 
 
23.6
 
 
 
 
AKAP1
 
 
hsa-miR-152-3p
 
 
-0.381
 
 
22.6
 
 
 
 
ALCAM
 
 
hsa-miR-152-3p
 
 
-0.347
 
 
26.3
 
 
 
 
AP4B1
 
 
hsa-miR-378a-3p
 
 
-0.62
 
 
11.8
 
 
 
 
ARF4
 
 
hsa-miR-152-3p
 
 
-0.487
 
 
196.1
 
 
 
 
ARFGEF1
 
 
hsa-miR-152-3p
 
 
-0.306
 
 
13.3
 
 
 
 
ARFIP1
 
 
hsa-miR-152-3p
 
 
-0.474
 
 
21.1
 
 
 
 
ARHGAP21
 
 
hsa-miR-152-3p
 
 
-0.287
 
 
18.5
 
 
 
 
ARL6IP1
 
 
hsa-miR-152-3p
 
 
-0.579
 
 
74.5
 
 
 
 
ARL8B
 
 
hsa-miR-152-3p
 
 
-0.337
 
 
42.9
 
 
 
 
ARRDC3
 
 
hsa-miR-152-3p
 
 
-0.305
 
 
42.0
 
 
 
 
ASPH
 
 
hsa-miR-152-3p
 
 
-0.391
 
 
60.9
 
 
 
 
ASPRV1
 
 
hsa-miR-330-3p
 
 
-0.301
 
 
24.4
 
 
 
 
ATP2B4
 
 
hsa-miR-152-3p
 
 
-0.337
 
 
44.8
 
 
 
 
ATP6AP2
 
 
hsa-miR-152-3p
 
 
-0.592
 
 
99.7
 
 
 
 
ATP6AP2
 
 
hsa-miR-152-3p
 
 
-0.668
 
 
99.7
 
 
 
 
B4GALT5
 
 
hsa-miR-152-3p
 
 
-0.44
 
 
19.9
 
 
 
 
BHLHE41
 
 
hsa-miR-152-3p
 
 
-0.442
 
 
36.5
 
 
 
 
BTAF1
 
 
hsa-miR-152-3p
 
 
-0.401
 
 
16.0
 
 
 
 
BTBD3
 
 
hsa-miR-152-3p
 
 
-0.495
 
 
14.2
 
 
 
 
C1GALT1
 
 
hsa-miR-152-3p
 
 
-0.487
 
 
17.4
 
 
 
 
C1QBP
 
 
hsa-miR-330-3p
 
 
-0.412
 
 
64.7
 
 
 
 
C1orf35
 
 
hsa-miR-152-3p
 
 
-0.511
 
 
17.8
 
 
 
 
CAND1
 
 
hsa-miR-152-3p
 
 
-0.44
 
 
20.1
 
 
 
 
CANX
 
 
hsa-miR-152-3p
 
 
-0.434
 
 
114.0
 
 
 
 
CAV2
 
 
hsa-miR-152-3p
 
 
-0.32
 
 
60.3
 
 
 
 
CCDC6
 
 
hsa-miR-152-3p
 
 
-0.511
 
 
22.0
 
 
 
 
CCDC71
 
 
hsa-miR-152-3p
 
 
-0.369
 
 
13.4
 
 
 
 
CCN2
 
 
hsa-miR-330-3p
 
 
-0.305
 
 
498.2
 
 
 
 
CCT6A
 
 
hsa-miR-152-3p
 
 
-0.665
 
 
46.2
 
 
 
 
CDC25B
 
 
hsa-miR-152-3p
 
 
-0.298
 
 
37.7
 
 
 
 
CEBPG
 
 
hsa-miR-152-3p
 
 
-0.319
 
 
10.3
 
 
 
 
CEP63
 
 
hsa-miR-152-3p
 
 
-0.329
 
 
11.1
 
 
 
 
CERS5
 
 
hsa-miR-152-3p
 
 
-0.311
 
 
25.8
 
 
 
 
CFL2
 
 
hsa-miR-152-3p
 
 
-0.487
 
 
37.4
 
 
 
 
CHMP4B
 
 
hsa-miR-152-3p
 
 
-0.36
 
 
104.8
 
 
 
 
CLCN6
 
 
hsa-miR-152-3p
 
 
-0.406
 
 
13.9
 
 
 
 
CNOT7
 
 
hsa-miR-152-3p
 
 
-0.31
 
 
26.3
 
 
 
 
COL4A1
 
 
hsa-miR-152-3p
 
 
-0.317
 
 
61.7
 
 
 
 
CS
 
 
hsa-miR-152-3p
 
 
-0.3
 
 
59.9
 
 
 
 
CTSA
 
 
hsa-miR-152-3p
 
 
-0.541
 
 
41.9
 
 
 
 
CTTNBP2NL
 
 
hsa-miR-152-3p
 
 
-0.329
 
 
11.1
 
 
 
 
CUL5
 
 
hsa-miR-152-3p
 
 
-0.389
 
 
11.1
 
 
 
 
CYTH3
 
 
hsa-miR-152-3p
 
 
-0.331
 
 
17.5
 
 
 
 
DCUN1D4
 
 
hsa-miR-152-3p
 
 
-0.314
 
 
12.8
 
 
 
 
DDAH1
 
 
hsa-miR-152-3p
 
 
-0.34
 
 
21.1
 
 
 
 
DDX6
 
 
hsa-miR-152-3p
 
 
-0.363
 
 
31.2
 
 
 
 
DEDD
 
 
hsa-miR-152-3p
 
 
-0.401
 
 
23.3
 
 
 
 
DIPK2A
 
 
hsa-miR-152-3p
 
 
-0.393
 
 
12.8
 
 
 
 
DMPK
 
 
hsa-miR-152-3p
 
 
-0.378
 
 
20.1
 
 
 
 
DNAJB4
 
 
hsa-miR-152-3p
 
 
-0.33
 
 
22.5
 
 
 
 
DOCK6
 
 
hsa-miR-152-3p
 
 
-0.396
 
 
15.1
 
 
 
 
DUSP1
 
 
hsa-miR-152-3p
 
 
-0.32
 
 
470.6
 
 
 
 
DYNLL2
 
 
hsa-miR-152-3p
 
 
-0.366
 
 
17.6
 
 
 
 
DYRK1B
 
 
hsa-miR-152-3p
 
 
-0.305
 
 
11.8
 
 
 
 
EIF4E3
 
 
hsa-miR-152-3p
 
 
-0.464
 
 
10.8
 
 
 
 
EIF4G3
 
 
hsa-miR-378a-3p
 
 
-0.443
 
 
17.9
 
 
 
 
EMX2
 
 
hsa-miR-152-3p
 
 
-0.366
 
 
64.0
 
 
 
 
EOGT
 
 
hsa-miR-152-3p
 
 
-0.587
 
 
13.3
 
 
 
 
EPS15
 
 
hsa-miR-152-3p
 
 
-0.322
 
 
21.7
 
 
 
 
ERBB3
 
 
hsa-miR-152-3p
 
 
-0.399
 
 
24.9
 
 
 
 
ERRFI1
 
 
hsa-miR-152-3p
 
 
-0.529
 
 
73.4
 
 
 
 
ESR1
 
 
hsa-miR-152-3p
 
 
-0.34
 
 
80.7
 
 
 
 
F3
 
 
hsa-miR-152-3p
 
 
-0.331
 
 
54.3
 
 
 
 
FAM168B
 
 
hsa-miR-152-3p
 
 
-0.409
 
 
31.7
 
 
 
 
FAM43A
 
 
hsa-miR-152-3p
 
 
-0.337
 
 
19.3
 
 
 
 
FBN1
 
 
hsa-miR-152-3p
 
 
-0.349
 
 
29.3
 
 
 
 
FBXL19
 
 
hsa-miR-152-3p
 
 
-0.362
 
 
10.2
 
 
 
 
FBXO28
 
 
hsa-miR-152-3p
 
 
-0.313
 
 
10.3
 
 
 
 
FEZ2
 
 
hsa-miR-152-3p
 
 
-0.536
 
 
24.3
 
 
 
 
FMR1
 
 
hsa-miR-152-3p
 
 
-0.385
 
 
14.5
 
 
 
 
FOSB
 
 
hsa-miR-152-3p
 
 
-0.311
 
 
140.8
 
 
 
 
FOXK2
 
 
hsa-miR-152-3p
 
 
-0.305
 
 
14.1
 
 
 
 
FXR1
 
 
hsa-miR-152-3p
 
 
-0.478
 
 
34.9
 
 
 
 
GADD45A
 
 
hsa-miR-152-3p
 
 
-0.787
 
 
56.8
 
 
 
 
GAPVD1
 
 
hsa-miR-152-3p
 
 
-0.309
 
 
11.5
 
 
 
 
GLRX5
 
 
hsa-miR-152-3p
 
 
-0.381
 
 
28.7
 
 
 
 
GMFB
 
 
hsa-miR-152-3p
 
 
-0.429
 
 
17.4
 
 
 
 
GOLT1A
 
 
hsa-miR-378a-3p
 
 
-0.574
 
 
10.3
 
 
 
 
GPATCH8
 
 
hsa-miR-152-3p
 
 
-0.374
 
 
18.2
 
 
 
 
GRSF1
 
 
hsa-miR-378a-3p
 
 
-0.358
 
 
33.1
 
 
 
 
GTF2H1
 
 
hsa-miR-152-3p
 
 
-0.524
 
 
16.2
 
 
 
 
HDDC2
 
 
hsa-miR-378a-3p
 
 
-0.322
 
 
29.8
 
 
 
 
HLA-DQB1
 
 
hsa-miR-152-3p
 
 
-0.37
 
 
18.6
 
 
 
 
HSP90B1
 
 
hsa-miR-152-3p
 
 
-0.304
 
 
341.4
 
 
 
 
HSPH1
 
 
hsa-miR-330-3p
 
 
-0.345
 
 
26.3
 
 
 
 
ID2
 
 
hsa-miR-330-3p
 
 
-0.293
 
 
76.7
 
 
 
 
IGF1
 
 
hsa-miR-152-3p
 
 
-0.43
 
 
83.3
 
 
 
 
IL6ST
 
 
hsa-miR-152-3p
 
 
-0.291
 
 
72.0
 
 
 
 
ITGA11
 
 
hsa-miR-152-3p
 
 
-0.348
 
 
15.8
 
 
 
 
ITSN2
 
 
hsa-miR-152-3p
 
 
-0.339
 
 
21.7
 
 
 
 
KDSR
 
 
hsa-miR-378a-3p
 
 
-0.373
 
 
11.4
 
 
 
 
KLF4
 
 
hsa-miR-152-3p
 
 
-0.325
 
 
49.9
 
 
 
 
KLF5
 
 
hsa-miR-152-3p
 
 
-0.475
 
 
61.3
 
 
 
 
 
 
 choroid plexus 
 
 
 
 
target_symbol
 
 
mature_mirna_id
 
 
score
 
 
nTPM
 
 
 
 
 
 
ABCB7
 
 
hsa-miR-152-3p
 
 
-0.515
 
 
15.2
 
 
 
 
ABCD3
 
 
hsa-miR-152-3p
 
 
-0.388
 
 
13.3
 
 
 
 
AGFG1
 
 
hsa-miR-152-3p
 
 
-0.332
 
 
23.3
 
 
 
 
ARF4
 
 
hsa-miR-152-3p
 
 
-0.487
 
 
115.4
 
 
 
 
ARL6IP1
 
 
hsa-miR-152-3p
 
 
-0.579
 
 
185.0
 
 
 
 
ARL8B
 
 
hsa-miR-152-3p
 
 
-0.337
 
 
19.0
 
 
 
 
ARRDC3
 
 
hsa-miR-152-3p
 
 
-0.305
 
 
24.1
 
 
 
 
ASPH
 
 
hsa-miR-152-3p
 
 
-0.391
 
 
79.9
 
 
 
 
ATP6AP2
 
 
hsa-miR-152-3p
 
 
-0.592
 
 
85.2
 
 
 
 
ATP6AP2
 
 
hsa-miR-152-3p
 
 
-0.668
 
 
85.2
 
 
 
 
BTBD3
 
 
hsa-miR-152-3p
 
 
-0.495
 
 
18.2
 
 
 
 
C1GALT1
 
 
hsa-miR-152-3p
 
 
-0.487
 
 
22.9
 
 
 
 
C1QBP
 
 
hsa-miR-330-3p
 
 
-0.412
 
 
247.2
 
 
 
 
C1orf35
 
 
hsa-miR-152-3p
 
 
-0.511
 
 
11.0
 
 
 
 
CAND1
 
 
hsa-miR-152-3p
 
 
-0.44
 
 
15.9
 
 
 
 
CANX
 
 
hsa-miR-152-3p
 
 
-0.434
 
 
111.7
 
 
 
 
CCN2
 
 
hsa-miR-330-3p
 
 
-0.305
 
 
58.7
 
 
 
 
CCT6A
 
 
hsa-miR-152-3p
 
 
-0.665
 
 
46.5
 
 
 
 
CDC25B
 
 
hsa-miR-152-3p
 
 
-0.298
 
 
13.6
 
 
 
 
CEP63
 
 
hsa-miR-152-3p
 
 
-0.329
 
 
13.4
 
 
 
 
CERS5
 
 
hsa-miR-152-3p
 
 
-0.311
 
 
27.4
 
 
 
 
CFL2
 
 
hsa-miR-152-3p
 
 
-0.487
 
 
25.6
 
 
 
 
CHMP4B
 
 
hsa-miR-152-3p
 
 
-0.36
 
 
101.4
 
 
 
 
CNOT7
 
 
hsa-miR-152-3p
 
 
-0.31
 
 
27.0
 
 
 
 
COL4A1
 
 
hsa-miR-152-3p
 
 
-0.317
 
 
12.1
 
 
 
 
CS
 
 
hsa-miR-152-3p
 
 
-0.3
 
 
58.5
 
 
 
 
CTSA
 
 
hsa-miR-152-3p
 
 
-0.541
 
 
164.1
 
 
 
 
DDAH1
 
 
hsa-miR-152-3p
 
 
-0.34
 
 
48.2
 
 
 
 
DDX6
 
 
hsa-miR-152-3p
 
 
-0.363
 
 
18.7
 
 
 
 
DEDD
 
 
hsa-miR-152-3p
 
 
-0.401
 
 
13.8
 
 
 
 
DUSP1
 
 
hsa-miR-152-3p
 
 
-0.32
 
 
159.8
 
 
 
 
DYNLL2
 
 
hsa-miR-152-3p
 
 
-0.366
 
 
27.9
 
 
 
 
EIF4G3
 
 
hsa-miR-378a-3p
 
 
-0.443
 
 
34.8
 
 
 
 
EOGT
 
 
hsa-miR-152-3p
 
 
-0.587
 
 
17.6
 
 
 
 
EPS15
 
 
hsa-miR-152-3p
 
 
-0.322
 
 
14.5
 
 
 
 
ERRFI1
 
 
hsa-miR-152-3p
 
 
-0.529
 
 
104.0
 
 
 
 
F3
 
 
hsa-miR-152-3p
 
 
-0.331
 
 
35.1
 
 
 
 
FAM168B
 
 
hsa-miR-152-3p
 
 
-0.409
 
 
20.4
 
 
 
 
FAM184A
 
 
hsa-miR-152-3p
 
 
-0.371
 
 
17.1
 
 
 
 
FEZ2
 
 
hsa-miR-152-3p
 
 
-0.536
 
 
28.8
 
 
 
 
FOSB
 
 
hsa-miR-152-3p
 
 
-0.311
 
 
21.2
 
 
 
 
FXR1
 
 
hsa-miR-152-3p
 
 
-0.478
 
 
49.9
 
 
 
 
GADD45A
 
 
hsa-miR-152-3p
 
 
-0.787
 
 
64.2
 
 
 
 
GAP43
 
 
hsa-miR-152-3p
 
 
-0.409
 
 
13.2
 
 
 
 
GLRX5
 
 
hsa-miR-152-3p
 
 
-0.381
 
 
57.2
 
 
 
 
GMFB
 
 
hsa-miR-152-3p
 
 
-0.429
 
 
12.3
 
 
 
 
GRSF1
 
 
hsa-miR-378a-3p
 
 
-0.358
 
 
79.9
 
 
 
 
GTF2H1
 
 
hsa-miR-152-3p
 
 
-0.524
 
 
11.0
 
 
 
 
HDDC2
 
 
hsa-miR-378a-3p
 
 
-0.322
 
 
156.6
 
 
 
 
HLA-DQB1
 
 
hsa-miR-152-3p
 
 
-0.37
 
 
10.9
 
 
 
 
HSP90B1
 
 
hsa-miR-152-3p
 
 
-0.304
 
 
319.2
 
 
 
 
HSPH1
 
 
hsa-miR-330-3p
 
 
-0.345
 
 
44.5
 
 
 
 
ID2
 
 
hsa-miR-330-3p
 
 
-0.293
 
 
60.9
 
 
 
 
ING2
 
 
hsa-miR-152-3p
 
 
-0.482
 
 
10.5
 
 
 
 
LAMA4
 
 
hsa-miR-152-3p
 
 
-0.363
 
 
10.0
 
 
 
 
LDLR
 
 
hsa-miR-152-3p
 
 
-0.323
 
 
32.8
 
 
 
 
LEPROTL1
 
 
hsa-miR-152-3p
 
 
-0.568
 
 
27.4
 
 
 
 
LIPA
 
 
hsa-miR-152-3p
 
 
-0.463
 
 
24.5
 
 
 
 
LMAN1
 
 
hsa-miR-152-3p
 
 
-0.382
 
 
15.1
 
 
 
 
LRRC41
 
 
hsa-miR-152-3p
 
 
-0.392
 
 
22.8
 
 
 
 
LSM5
 
 
hsa-miR-330-3p
 
 
-0.351
 
 
22.8
 
 
 
 
MACROH2A1
 
 
hsa-miR-152-3p
 
 
-0.336
 
 
79.8
 
 
 
 
MAF1
 
 
hsa-miR-152-3p
 
 
-0.366
 
 
67.7
 
 
 
 
MAP2K1
 
 
hsa-miR-152-3p
 
 
-0.295
 
 
18.8
 
 
 
 
MAPK1
 
 
hsa-miR-378a-3p
 
 
-0.314
 
 
35.0
 
 
 
 
MAT2A
 
 
hsa-miR-330-3p
 
 
-0.307
 
 
85.0
 
 
 
 
MDFIC
 
 
hsa-miR-152-3p
 
 
-0.395
 
 
18.4
 
 
 
 
MECP2
 
 
hsa-miR-152-3p
 
 
-0.3
 
 
13.5
 
 
 
 
MED19
 
 
hsa-miR-378a-3p
 
 
-0.434
 
 
24.4
 
 
 
 
MLLT10
 
 
hsa-miR-152-3p
 
 
-0.334
 
 
10.7
 
 
 
 
MNAT1
 
 
hsa-miR-378a-3p
 
 
-0.398
 
 
23.4
 
 
 
 
MOSPD1
 
 
hsa-miR-152-3p
 
 
-0.314
 
 
20.8
 
 
 
 
MRAS
 
 
hsa-miR-152-3p
 
 
-0.583
 
 
18.0
 
 
 
 
MRPS10
 
 
hsa-miR-152-3p
 
 
-0.335
 
 
15.7
 
 
 
 
MTMR10
 
 
hsa-miR-152-3p
 
 
-0.302
 
 
12.6
 
 
 
 
MTMR14
 
 
hsa-miR-152-3p
 
 
-0.467
 
 
43.8
 
 
 
 
NAP1L1
 
 
hsa-miR-152-3p
 
 
-0.343
 
 
168.8
 
 
 
 
NAT14
 
 
hsa-miR-152-3p
 
 
-0.324
 
 
32.4
 
 
 
 
NCKIPSD
 
 
hsa-miR-152-3p
 
 
-0.382
 
 
14.1
 
 
 
 
NCOA1
 
 
hsa-miR-152-3p
 
 
-0.313
 
 
16.8
 
 
 
 
NFKBIA
 
 
hsa-miR-378a-3p
 
 
-0.427
 
 
101.6
 
 
 
 
NPEPL1
 
 
hsa-miR-152-3p
 
 
-0.749
 
 
15.9
 
 
 
 
NPEPL1
 
 
hsa-miR-152-3p
 
 
-0.749
 
 
15.9
 
 
 
 
NPTN
 
 
hsa-miR-152-3p
 
 
-0.507
 
 
169.3
 
 
 
 
NRP1
 
 
hsa-miR-152-3p
 
 
-0.432
 
 
14.1
 
 
 
 
ORMDL1
 
 
hsa-miR-152-3p
 
 
-0.356
 
 
33.9
 
 
 
 
OTX2
 
 
hsa-miR-152-3p
 
 
-0.42
 
 
151.9
 
 
 
 
PBXIP1
 
 
hsa-miR-152-3p
 
 
-0.429
 
 
672.0
 
 
 
 
PDIA3
 
 
hsa-miR-152-3p
 
 
-0.453
 
 
17.6
 
 
 
 
PDIA4
 
 
hsa-miR-378a-3p
 
 
-0.644
 
 
42.8
 
 
 
 
PHF3
 
 
hsa-miR-152-3p
 
 
-0.372
 
 
11.3
 
 
 
 
PI4KA
 
 
hsa-miR-152-3p
 
 
-0.302
 
 
47.5
 
 
 
 
PNPLA2
 
 
hsa-miR-152-3p
 
 
-0.367
 
 
19.9
 
 
 
 
PNPLA6
 
 
hsa-miR-152-3p
 
 
-0.474
 
 
16.9
 
 
 
 
PPP1CB
 
 
hsa-miR-152-3p
 
 
-0.318
 
 
37.5
 
 
 
 
PPP1R12A
 
 
hsa-miR-152-3p
 
 
-0.288
 
 
13.5
 
 
 
 
PPP1R7
 
 
hsa-miR-152-3p
 
 
-0.303
 
 
71.4
 
 
 
 
PPP6R1
 
 
hsa-miR-152-3p
 
 
-0.513
 
 
17.6
 
 
 
 
PRKAA1
 
 
hsa-miR-152-3p
 
 
-0.381
 
 
10.1
 
 
 
 
PRNP
 
 
hsa-miR-152-3p
 
 
-0.409
 
 
480.4
 
 
 
 
 
 
 endometrium 
 
 
 
 
target_symbol
 
 
mature_mirna_id
 
 
score
 
 
nTPM
 
 
 
 
 
 
ABCB7
 
 
hsa-miR-152-3p
 
 
-0.515
 
 
10.5
 
 
 
 
ABCD3
 
 
hsa-miR-152-3p
 
 
-0.388
 
 
13.8
 
 
 
 
ACVR1
 
 
hsa-miR-152-3p
 
 
-0.397
 
 
15.9
 
 
 
 
ADAM10
 
 
hsa-miR-152-3p
 
 
-0.348
 
 
15.2
 
 
 
 
ADGRF5
 
 
hsa-miR-152-3p
 
 
-0.354
 
 
16.0
 
 
 
 
AGFG1
 
 
hsa-miR-152-3p
 
 
-0.332
 
 
23.1
 
 
 
 
AHDC1
 
 
hsa-miR-152-3p
 
 
-0.413
 
 
26.0
 
 
 
 
AKAP1
 
 
hsa-miR-152-3p
 
 
-0.381
 
 
24.8
 
 
 
 
ALCAM
 
 
hsa-miR-152-3p
 
 
-0.347
 
 
14.4
 
 
 
 
AP4B1
 
 
hsa-miR-378a-3p
 
 
-0.62
 
 
10.1
 
 
 
 
ARF4
 
 
hsa-miR-152-3p
 
 
-0.487
 
 
113.9
 
 
 
 
ARFIP1
 
 
hsa-miR-152-3p
 
 
-0.474
 
 
20.3
 
 
 
 
ARHGAP21
 
 
hsa-miR-152-3p
 
 
-0.287
 
 
18.5
 
 
 
 
ARL6IP1
 
 
hsa-miR-152-3p
 
 
-0.579
 
 
66.4
 
 
 
 
ARL8B
 
 
hsa-miR-152-3p
 
 
-0.337
 
 
42.3
 
 
 
 
ARRDC3
 
 
hsa-miR-152-3p
 
 
-0.305
 
 
38.0
 
 
 
 
ASPH
 
 
hsa-miR-152-3p
 
 
-0.391
 
 
45.6
 
 
 
 
ATP2B4
 
 
hsa-miR-152-3p
 
 
-0.337
 
 
98.2
 
 
 
 
ATP6AP2
 
 
hsa-miR-152-3p
 
 
-0.592
 
 
81.0
 
 
 
 
ATP6AP2
 
 
hsa-miR-152-3p
 
 
-0.668
 
 
81.0
 
 
 
 
B4GALT5
 
 
hsa-miR-152-3p
 
 
-0.44
 
 
22.0
 
 
 
 
BTAF1
 
 
hsa-miR-152-3p
 
 
-0.401
 
 
17.3
 
 
 
 
BTBD3
 
 
hsa-miR-152-3p
 
 
-0.495
 
 
17.5
 
 
 
 
C1QBP
 
 
hsa-miR-330-3p
 
 
-0.412
 
 
63.5
 
 
 
 
C1orf35
 
 
hsa-miR-152-3p
 
 
-0.511
 
 
15.9
 
 
 
 
CAND1
 
 
hsa-miR-152-3p
 
 
-0.44
 
 
19.9
 
 
 
 
CANX
 
 
hsa-miR-152-3p
 
 
-0.434
 
 
116.8
 
 
 
 
CAV2
 
 
hsa-miR-152-3p
 
 
-0.32
 
 
33.4
 
 
 
 
CCDC6
 
 
hsa-miR-152-3p
 
 
-0.511
 
 
22.0
 
 
 
 
CCDC71
 
 
hsa-miR-152-3p
 
 
-0.369
 
 
14.1
 
 
 
 
CCN2
 
 
hsa-miR-330-3p
 
 
-0.305
 
 
390.6
 
 
 
 
CCT6A
 
 
hsa-miR-152-3p
 
 
-0.665
 
 
51.7
 
 
 
 
CDC25B
 
 
hsa-miR-152-3p
 
 
-0.298
 
 
37.1
 
 
 
 
CEBPG
 
 
hsa-miR-152-3p
 
 
-0.319
 
 
11.2
 
 
 
 
CEP63
 
 
hsa-miR-152-3p
 
 
-0.329
 
 
10.4
 
 
 
 
CERS5
 
 
hsa-miR-152-3p
 
 
-0.311
 
 
23.0
 
 
 
 
CFL2
 
 
hsa-miR-152-3p
 
 
-0.487
 
 
69.8
 
 
 
 
CHMP4B
 
 
hsa-miR-152-3p
 
 
-0.36
 
 
98.5
 
 
 
 
CLCN6
 
 
hsa-miR-152-3p
 
 
-0.406
 
 
12.9
 
 
 
 
CNOT7
 
 
hsa-miR-152-3p
 
 
-0.31
 
 
36.9
 
 
 
 
COL4A1
 
 
hsa-miR-152-3p
 
 
-0.317
 
 
82.9
 
 
 
 
CS
 
 
hsa-miR-152-3p
 
 
-0.3
 
 
57.7
 
 
 
 
CTSA
 
 
hsa-miR-152-3p
 
 
-0.541
 
 
43.7
 
 
 
 
CTTNBP2NL
 
 
hsa-miR-152-3p
 
 
-0.329
 
 
14.3
 
 
 
 
CUL5
 
 
hsa-miR-152-3p
 
 
-0.389
 
 
12.3
 
 
 
 
CYTH3
 
 
hsa-miR-152-3p
 
 
-0.331
 
 
15.9
 
 
 
 
DCUN1D4
 
 
hsa-miR-152-3p
 
 
-0.314
 
 
12.7
 
 
 
 
DDAH1
 
 
hsa-miR-152-3p
 
 
-0.34
 
 
10.4
 
 
 
 
DDX6
 
 
hsa-miR-152-3p
 
 
-0.363
 
 
28.5
 
 
 
 
DEDD
 
 
hsa-miR-152-3p
 
 
-0.401
 
 
23.8
 
 
 
 
DIPK2A
 
 
hsa-miR-152-3p
 
 
-0.393
 
 
13.2
 
 
 
 
DMPK
 
 
hsa-miR-152-3p
 
 
-0.378
 
 
53.1
 
 
 
 
DNAJB4
 
 
hsa-miR-152-3p
 
 
-0.33
 
 
19.9
 
 
 
 
DOCK6
 
 
hsa-miR-152-3p
 
 
-0.396
 
 
13.8
 
 
 
 
DUSP1
 
 
hsa-miR-152-3p
 
 
-0.32
 
 
357.6
 
 
 
 
DYNLL2
 
 
hsa-miR-152-3p
 
 
-0.366
 
 
18.2
 
 
 
 
DYRK1B
 
 
hsa-miR-152-3p
 
 
-0.305
 
 
11.8
 
 
 
 
DYRK2
 
 
hsa-miR-152-3p
 
 
-0.293
 
 
12.2
 
 
 
 
EIF4E3
 
 
hsa-miR-152-3p
 
 
-0.464
 
 
13.5
 
 
 
 
EIF4G3
 
 
hsa-miR-378a-3p
 
 
-0.443
 
 
19.0
 
 
 
 
EMX2
 
 
hsa-miR-152-3p
 
 
-0.366
 
 
79.8
 
 
 
 
EOGT
 
 
hsa-miR-152-3p
 
 
-0.587
 
 
26.1
 
 
 
 
EPS15
 
 
hsa-miR-152-3p
 
 
-0.322
 
 
19.2
 
 
 
 
ERRFI1
 
 
hsa-miR-152-3p
 
 
-0.529
 
 
73.0
 
 
 
 
ESR1
 
 
hsa-miR-152-3p
 
 
-0.34
 
 
103.7
 
 
 
 
F3
 
 
hsa-miR-152-3p
 
 
-0.331
 
 
57.1
 
 
 
 
FAM168B
 
 
hsa-miR-152-3p
 
 
-0.409
 
 
39.5
 
 
 
 
FAM43A
 
 
hsa-miR-152-3p
 
 
-0.337
 
 
11.2
 
 
 
 
FBN1
 
 
hsa-miR-152-3p
 
 
-0.349
 
 
12.3
 
 
 
 
FBXL19
 
 
hsa-miR-152-3p
 
 
-0.362
 
 
10.4
 
 
 
 
FEZ2
 
 
hsa-miR-152-3p
 
 
-0.536
 
 
28.7
 
 
 
 
FMR1
 
 
hsa-miR-152-3p
 
 
-0.385
 
 
17.4
 
 
 
 
FOSB
 
 
hsa-miR-152-3p
 
 
-0.311
 
 
43.6
 
 
 
 
FOXK2
 
 
hsa-miR-152-3p
 
 
-0.305
 
 
17.9
 
 
 
 
FXR1
 
 
hsa-miR-152-3p
 
 
-0.478
 
 
39.7
 
 
 
 
GADD45A
 
 
hsa-miR-152-3p
 
 
-0.787
 
 
58.3
 
 
 
 
GAPVD1
 
 
hsa-miR-152-3p
 
 
-0.309
 
 
10.3
 
 
 
 
GLRX5
 
 
hsa-miR-152-3p
 
 
-0.381
 
 
31.9
 
 
 
 
GMFB
 
 
hsa-miR-152-3p
 
 
-0.429
 
 
18.0
 
 
 
 
GPATCH8
 
 
hsa-miR-152-3p
 
 
-0.374
 
 
18.5
 
 
 
 
GRSF1
 
 
hsa-miR-378a-3p
 
 
-0.358
 
 
29.3
 
 
 
 
GTF2H1
 
 
hsa-miR-152-3p
 
 
-0.524
 
 
15.2
 
 
 
 
HDDC2
 
 
hsa-miR-378a-3p
 
 
-0.322
 
 
22.6
 
 
 
 
HLA-DQB1
 
 
hsa-miR-152-3p
 
 
-0.37
 
 
18.1
 
 
 
 
HSP90B1
 
 
hsa-miR-152-3p
 
 
-0.304
 
 
283.0
 
 
 
 
HSPH1
 
 
hsa-miR-330-3p
 
 
-0.345
 
 
24.0
 
 
 
 
ID2
 
 
hsa-miR-330-3p
 
 
-0.293
 
 
65.3
 
 
 
 
IGF1
 
 
hsa-miR-152-3p
 
 
-0.43
 
 
43.8
 
 
 
 
IL6ST
 
 
hsa-miR-152-3p
 
 
-0.291
 
 
43.5
 
 
 
 
ITGA11
 
 
hsa-miR-152-3p
 
 
-0.348
 
 
28.8
 
 
 
 
ITSN2
 
 
hsa-miR-152-3p
 
 
-0.339
 
 
18.2
 
 
 
 
KDSR
 
 
hsa-miR-378a-3p
 
 
-0.373
 
 
12.9
 
 
 
 
KLF4
 
 
hsa-miR-152-3p
 
 
-0.325
 
 
14.9
 
 
 
 
LAMA4
 
 
hsa-miR-152-3p
 
 
-0.363
 
 
72.5
 
 
 
 
LBR
 
 
hsa-miR-152-3p
 
 
-0.39
 
 
44.0
 
 
 
 
LDLR
 
 
hsa-miR-152-3p
 
 
-0.323
 
 
21.7
 
 
 
 
LEPROTL1
 
 
hsa-miR-152-3p
 
 
-0.568
 
 
18.6
 
 
 
 
LIPA
 
 
hsa-miR-152-3p
 
 
-0.463
 
 
36.3
 
 
 
 
LMAN1
 
 
hsa-miR-152-3p
 
 
-0.382
 
 
43.2
 
 
 
 
LRCH1
 
 
hsa-miR-152-3p
 
 
-0.321
 
 
15.2
 
 
 
 
 
 
 esophagus 
 
 
 
 
target_symbol
 
 
mature_mirna_id
 
 
score
 
 
nTPM
 
 
 
 
 
 
ABCB7
 
 
hsa-miR-152-3p
 
 
-0.515
 
 
11.1
 
 
 
 
ABCD3
 
 
hsa-miR-152-3p
 
 
-0.388
 
 
25.0
 
 
 
 
ABHD17C
 
 
hsa-miR-330-3p
 
 
-0.399
 
 
45.1
 
 
 
 
ADAM10
 
 
hsa-miR-152-3p
 
 
-0.348
 
 
14.2
 
 
 
 
AGFG1
 
 
hsa-miR-152-3p
 
 
-0.332
 
 
44.3
 
 
 
 
AHDC1
 
 
hsa-miR-152-3p
 
 
-0.413
 
 
34.3
 
 
 
 
AKAP1
 
 
hsa-miR-152-3p
 
 
-0.381
 
 
34.2
 
 
 
 
AP4B1
 
 
hsa-miR-378a-3p
 
 
-0.62
 
 
11.6
 
 
 
 
ARF4
 
 
hsa-miR-152-3p
 
 
-0.487
 
 
102.8
 
 
 
 
ARFGEF1
 
 
hsa-miR-152-3p
 
 
-0.306
 
 
14.2
 
 
 
 
ARFIP1
 
 
hsa-miR-152-3p
 
 
-0.474
 
 
19.6
 
 
 
 
ARHGAP21
 
 
hsa-miR-152-3p
 
 
-0.287
 
 
10.2
 
 
 
 
ARL6IP1
 
 
hsa-miR-152-3p
 
 
-0.579
 
 
65.8
 
 
 
 
ARL8B
 
 
hsa-miR-152-3p
 
 
-0.337
 
 
71.1
 
 
 
 
ARRDC3
 
 
hsa-miR-152-3p
 
 
-0.305
 
 
36.4
 
 
 
 
ASPH
 
 
hsa-miR-152-3p
 
 
-0.391
 
 
66.3
 
 
 
 
ATP2B4
 
 
hsa-miR-152-3p
 
 
-0.337
 
 
30.6
 
 
 
 
ATP6AP2
 
 
hsa-miR-152-3p
 
 
-0.592
 
 
65.8
 
 
 
 
ATP6AP2
 
 
hsa-miR-152-3p
 
 
-0.668
 
 
65.8
 
 
 
 
B4GALT5
 
 
hsa-miR-152-3p
 
 
-0.44
 
 
35.5
 
 
 
 
BTAF1
 
 
hsa-miR-152-3p
 
 
-0.401
 
 
14.2
 
 
 
 
C1GALT1
 
 
hsa-miR-152-3p
 
 
-0.487
 
 
22.4
 
 
 
 
C1QBP
 
 
hsa-miR-330-3p
 
 
-0.412
 
 
133.5
 
 
 
 
C1orf210
 
 
hsa-miR-378a-3p
 
 
-0.307
 
 
16.3
 
 
 
 
C1orf35
 
 
hsa-miR-152-3p
 
 
-0.511
 
 
21.2
 
 
 
 
CAND1
 
 
hsa-miR-152-3p
 
 
-0.44
 
 
21.3
 
 
 
 
CANX
 
 
hsa-miR-152-3p
 
 
-0.434
 
 
94.4
 
 
 
 
CAV2
 
 
hsa-miR-152-3p
 
 
-0.32
 
 
40.8
 
 
 
 
CCDC6
 
 
hsa-miR-152-3p
 
 
-0.511
 
 
32.0
 
 
 
 
CCDC71
 
 
hsa-miR-152-3p
 
 
-0.369
 
 
13.2
 
 
 
 
CCN2
 
 
hsa-miR-330-3p
 
 
-0.305
 
 
255.4
 
 
 
 
CCT6A
 
 
hsa-miR-152-3p
 
 
-0.665
 
 
69.7
 
 
 
 
CDC25B
 
 
hsa-miR-152-3p
 
 
-0.298
 
 
26.7
 
 
 
 
CEBPG
 
 
hsa-miR-152-3p
 
 
-0.319
 
 
18.8
 
 
 
 
CEP63
 
 
hsa-miR-152-3p
 
 
-0.329
 
 
10.6
 
 
 
 
CERS5
 
 
hsa-miR-152-3p
 
 
-0.311
 
 
18.7
 
 
 
 
CFL2
 
 
hsa-miR-152-3p
 
 
-0.487
 
 
64.8
 
 
 
 
CHMP4B
 
 
hsa-miR-152-3p
 
 
-0.36
 
 
157.3
 
 
 
 
CNOT7
 
 
hsa-miR-152-3p
 
 
-0.31
 
 
41.1
 
 
 
 
COL4A1
 
 
hsa-miR-152-3p
 
 
-0.317
 
 
38.2
 
 
 
 
CS
 
 
hsa-miR-152-3p
 
 
-0.3
 
 
99.6
 
 
 
 
CTSA
 
 
hsa-miR-152-3p
 
 
-0.541
 
 
56.9
 
 
 
 
CTTNBP2NL
 
 
hsa-miR-152-3p
 
 
-0.329
 
 
18.3
 
 
 
 
CUL5
 
 
hsa-miR-152-3p
 
 
-0.389
 
 
10.0
 
 
 
 
CYTH3
 
 
hsa-miR-152-3p
 
 
-0.331
 
 
13.2
 
 
 
 
DDAH1
 
 
hsa-miR-152-3p
 
 
-0.34
 
 
11.9
 
 
 
 
DDX6
 
 
hsa-miR-152-3p
 
 
-0.363
 
 
31.0
 
 
 
 
DEDD
 
 
hsa-miR-152-3p
 
 
-0.401
 
 
24.7
 
 
 
 
DICER1
 
 
hsa-miR-152-3p
 
 
-0.29
 
 
11.4
 
 
 
 
DIPK2A
 
 
hsa-miR-152-3p
 
 
-0.393
 
 
13.2
 
 
 
 
DMPK
 
 
hsa-miR-152-3p
 
 
-0.378
 
 
30.2
 
 
 
 
DNAJB4
 
 
hsa-miR-152-3p
 
 
-0.33
 
 
24.8
 
 
 
 
DOCK6
 
 
hsa-miR-152-3p
 
 
-0.396
 
 
16.2
 
 
 
 
DUSP1
 
 
hsa-miR-152-3p
 
 
-0.32
 
 
495.5
 
 
 
 
DYNLL2
 
 
hsa-miR-152-3p
 
 
-0.366
 
 
19.2
 
 
 
 
DYRK1B
 
 
hsa-miR-152-3p
 
 
-0.305
 
 
11.2
 
 
 
 
DYRK2
 
 
hsa-miR-152-3p
 
 
-0.293
 
 
10.0
 
 
 
 
EIF4E3
 
 
hsa-miR-152-3p
 
 
-0.464
 
 
18.4
 
 
 
 
EIF4G3
 
 
hsa-miR-378a-3p
 
 
-0.443
 
 
15.6
 
 
 
 
EOGT
 
 
hsa-miR-152-3p
 
 
-0.587
 
 
16.4
 
 
 
 
EPS15
 
 
hsa-miR-152-3p
 
 
-0.322
 
 
15.9
 
 
 
 
ERBB3
 
 
hsa-miR-152-3p
 
 
-0.399
 
 
68.7
 
 
 
 
ERRFI1
 
 
hsa-miR-152-3p
 
 
-0.529
 
 
48.1
 
 
 
 
F3
 
 
hsa-miR-152-3p
 
 
-0.331
 
 
38.8
 
 
 
 
FAM168B
 
 
hsa-miR-152-3p
 
 
-0.409
 
 
32.3
 
 
 
 
FAM43A
 
 
hsa-miR-152-3p
 
 
-0.337
 
 
14.3
 
 
 
 
FBN1
 
 
hsa-miR-152-3p
 
 
-0.349
 
 
10.2
 
 
 
 
FBXL19
 
 
hsa-miR-152-3p
 
 
-0.362
 
 
17.2
 
 
 
 
FBXO28
 
 
hsa-miR-152-3p
 
 
-0.313
 
 
11.7
 
 
 
 
FEZ2
 
 
hsa-miR-152-3p
 
 
-0.536
 
 
29.8
 
 
 
 
FMR1
 
 
hsa-miR-152-3p
 
 
-0.385
 
 
11.2
 
 
 
 
FOSB
 
 
hsa-miR-152-3p
 
 
-0.311
 
 
176.8
 
 
 
 
FOXK2
 
 
hsa-miR-152-3p
 
 
-0.305
 
 
19.0
 
 
 
 
FXR1
 
 
hsa-miR-152-3p
 
 
-0.478
 
 
38.1
 
 
 
 
GADD45A
 
 
hsa-miR-152-3p
 
 
-0.787
 
 
41.7
 
 
 
 
GAPVD1
 
 
hsa-miR-152-3p
 
 
-0.309
 
 
16.0
 
 
 
 
GLRX5
 
 
hsa-miR-152-3p
 
 
-0.381
 
 
34.7
 
 
 
 
GMFB
 
 
hsa-miR-152-3p
 
 
-0.429
 
 
14.9
 
 
 
 
GPATCH8
 
 
hsa-miR-152-3p
 
 
-0.374
 
 
13.2
 
 
 
 
GRSF1
 
 
hsa-miR-378a-3p
 
 
-0.358
 
 
38.1
 
 
 
 
GTF2H1
 
 
hsa-miR-152-3p
 
 
-0.524
 
 
18.7
 
 
 
 
HDDC2
 
 
hsa-miR-378a-3p
 
 
-0.322
 
 
33.8
 
 
 
 
HLA-DQB1
 
 
hsa-miR-152-3p
 
 
-0.37
 
 
20.5
 
 
 
 
HSP90B1
 
 
hsa-miR-152-3p
 
 
-0.304
 
 
131.5
 
 
 
 
HSPH1
 
 
hsa-miR-330-3p
 
 
-0.345
 
 
35.3
 
 
 
 
ID2
 
 
hsa-miR-330-3p
 
 
-0.293
 
 
33.2
 
 
 
 
IL6ST
 
 
hsa-miR-152-3p
 
 
-0.291
 
 
29.9
 
 
 
 
ITSN2
 
 
hsa-miR-152-3p
 
 
-0.339
 
 
34.4
 
 
 
 
KDSR
 
 
hsa-miR-378a-3p
 
 
-0.373
 
 
11.7
 
 
 
 
KLF4
 
 
hsa-miR-152-3p
 
 
-0.325
 
 
96.0
 
 
 
 
KLF5
 
 
hsa-miR-152-3p
 
 
-0.475
 
 
226.7
 
 
 
 
LAMA4
 
 
hsa-miR-152-3p
 
 
-0.363
 
 
33.8
 
 
 
 
LBR
 
 
hsa-miR-152-3p
 
 
-0.39
 
 
27.9
 
 
 
 
LDLR
 
 
hsa-miR-152-3p
 
 
-0.323
 
 
47.9
 
 
 
 
LEPROTL1
 
 
hsa-miR-152-3p
 
 
-0.568
 
 
16.6
 
 
 
 
LIPA
 
 
hsa-miR-152-3p
 
 
-0.463
 
 
26.9
 
 
 
 
LMAN1
 
 
hsa-miR-152-3p
 
 
-0.382
 
 
29.2
 
 
 
 
LRRC41
 
 
hsa-miR-152-3p
 
 
-0.392
 
 
42.3
 
 
 
 
LSM5
 
 
hsa-miR-330-3p
 
 
-0.351
 
 
44.0
 
 
 
 
LYRM2
 
 
hsa-miR-152-3p
 
 
-0.351
 
 
11.5
 
 
 
 
 
 
 fallopian tube 
 
 
 
 
target_symbol
 
 
mature_mirna_id
 
 
score
 
 
nTPM
 
 
 
 
 
 
ABCB7
 
 
hsa-miR-152-3p
 
 
-0.515
 
 
11.3
 
 
 
 
ABCD3
 
 
hsa-miR-152-3p
 
 
-0.388
 
 
15.8
 
 
 
 
ABHD17C
 
 
hsa-miR-330-3p
 
 
-0.399
 
 
10.3
 
 
 
 
ACVR1
 
 
hsa-miR-152-3p
 
 
-0.397
 
 
13.2
 
 
 
 
ADAM10
 
 
hsa-miR-152-3p
 
 
-0.348
 
 
13.1
 
 
 
 
ADGRF5
 
 
hsa-miR-152-3p
 
 
-0.354
 
 
13.6
 
 
 
 
AGFG1
 
 
hsa-miR-152-3p
 
 
-0.332
 
 
23.3
 
 
 
 
AHDC1
 
 
hsa-miR-152-3p
 
 
-0.413
 
 
19.8
 
 
 
 
AKAP1
 
 
hsa-miR-152-3p
 
 
-0.381
 
 
33.9
 
 
 
 
ALCAM
 
 
hsa-miR-152-3p
 
 
-0.347
 
 
46.2
 
 
 
 
AP4B1
 
 
hsa-miR-378a-3p
 
 
-0.62
 
 
11.8
 
 
 
 
ARF4
 
 
hsa-miR-152-3p
 
 
-0.487
 
 
79.6
 
 
 
 
ARFGEF1
 
 
hsa-miR-152-3p
 
 
-0.306
 
 
11.1
 
 
 
 
ARFIP1
 
 
hsa-miR-152-3p
 
 
-0.474
 
 
17.4
 
 
 
 
ARHGAP21
 
 
hsa-miR-152-3p
 
 
-0.287
 
 
16.4
 
 
 
 
ARL6IP1
 
 
hsa-miR-152-3p
 
 
-0.579
 
 
81.6
 
 
 
 
ARL8B
 
 
hsa-miR-152-3p
 
 
-0.337
 
 
29.3
 
 
 
 
ARRDC3
 
 
hsa-miR-152-3p
 
 
-0.305
 
 
23.4
 
 
 
 
ASPH
 
 
hsa-miR-152-3p
 
 
-0.391
 
 
45.0
 
 
 
 
ATP2B4
 
 
hsa-miR-152-3p
 
 
-0.337
 
 
45.7
 
 
 
 
ATP6AP2
 
 
hsa-miR-152-3p
 
 
-0.592
 
 
71.1
 
 
 
 
ATP6AP2
 
 
hsa-miR-152-3p
 
 
-0.668
 
 
71.1
 
 
 
 
B4GALT5
 
 
hsa-miR-152-3p
 
 
-0.44
 
 
17.2
 
 
 
 
BHLHE41
 
 
hsa-miR-152-3p
 
 
-0.442
 
 
42.8
 
 
 
 
BTAF1
 
 
hsa-miR-152-3p
 
 
-0.401
 
 
20.1
 
 
 
 
BTBD3
 
 
hsa-miR-152-3p
 
 
-0.495
 
 
14.4
 
 
 
 
C1GALT1
 
 
hsa-miR-152-3p
 
 
-0.487
 
 
15.3
 
 
 
 
C1QBP
 
 
hsa-miR-330-3p
 
 
-0.412
 
 
70.1
 
 
 
 
C1orf35
 
 
hsa-miR-152-3p
 
 
-0.511
 
 
15.3
 
 
 
 
CAND1
 
 
hsa-miR-152-3p
 
 
-0.44
 
 
18.1
 
 
 
 
CANX
 
 
hsa-miR-152-3p
 
 
-0.434
 
 
92.6
 
 
 
 
CAV2
 
 
hsa-miR-152-3p
 
 
-0.32
 
 
29.3
 
 
 
 
CCDC6
 
 
hsa-miR-152-3p
 
 
-0.511
 
 
23.8
 
 
 
 
CCDC71
 
 
hsa-miR-152-3p
 
 
-0.369
 
 
15.4
 
 
 
 
CCN2
 
 
hsa-miR-330-3p
 
 
-0.305
 
 
261.8
 
 
 
 
CCT6A
 
 
hsa-miR-152-3p
 
 
-0.665
 
 
48.4
 
 
 
 
CDC14A
 
 
hsa-miR-152-3p
 
 
-0.482
 
 
12.2
 
 
 
 
CDC25B
 
 
hsa-miR-152-3p
 
 
-0.298
 
 
34.7
 
 
 
 
CEBPG
 
 
hsa-miR-152-3p
 
 
-0.319
 
 
11.2
 
 
 
 
CEP63
 
 
hsa-miR-152-3p
 
 
-0.329
 
 
10.5
 
 
 
 
CERS5
 
 
hsa-miR-152-3p
 
 
-0.311
 
 
21.8
 
 
 
 
CFL2
 
 
hsa-miR-152-3p
 
 
-0.487
 
 
49.9
 
 
 
 
CHMP4B
 
 
hsa-miR-152-3p
 
 
-0.36
 
 
83.0
 
 
 
 
CLCN6
 
 
hsa-miR-152-3p
 
 
-0.406
 
 
11.5
 
 
 
 
CNOT7
 
 
hsa-miR-152-3p
 
 
-0.31
 
 
34.1
 
 
 
 
COL4A1
 
 
hsa-miR-152-3p
 
 
-0.317
 
 
55.2
 
 
 
 
CS
 
 
hsa-miR-152-3p
 
 
-0.3
 
 
54.9
 
 
 
 
CTSA
 
 
hsa-miR-152-3p
 
 
-0.541
 
 
33.3
 
 
 
 
CYTH3
 
 
hsa-miR-152-3p
 
 
-0.331
 
 
15.8
 
 
 
 
DCUN1D4
 
 
hsa-miR-152-3p
 
 
-0.314
 
 
12.3
 
 
 
 
DDAH1
 
 
hsa-miR-152-3p
 
 
-0.34
 
 
34.4
 
 
 
 
DDX6
 
 
hsa-miR-152-3p
 
 
-0.363
 
 
27.4
 
 
 
 
DEDD
 
 
hsa-miR-152-3p
 
 
-0.401
 
 
24.2
 
 
 
 
DIPK2A
 
 
hsa-miR-152-3p
 
 
-0.393
 
 
12.0
 
 
 
 
DMPK
 
 
hsa-miR-152-3p
 
 
-0.378
 
 
42.8
 
 
 
 
DNAJB4
 
 
hsa-miR-152-3p
 
 
-0.33
 
 
14.0
 
 
 
 
DOCK6
 
 
hsa-miR-152-3p
 
 
-0.396
 
 
12.1
 
 
 
 
DUSP1
 
 
hsa-miR-152-3p
 
 
-0.32
 
 
680.0
 
 
 
 
DYNLL2
 
 
hsa-miR-152-3p
 
 
-0.366
 
 
18.3
 
 
 
 
DYRK1B
 
 
hsa-miR-152-3p
 
 
-0.305
 
 
12.9
 
 
 
 
EIF4E3
 
 
hsa-miR-152-3p
 
 
-0.464
 
 
14.7
 
 
 
 
EIF4G3
 
 
hsa-miR-378a-3p
 
 
-0.443
 
 
18.1
 
 
 
 
EMX2
 
 
hsa-miR-152-3p
 
 
-0.366
 
 
54.1
 
 
 
 
EOGT
 
 
hsa-miR-152-3p
 
 
-0.587
 
 
17.8
 
 
 
 
EPS15
 
 
hsa-miR-152-3p
 
 
-0.322
 
 
15.3
 
 
 
 
ERRFI1
 
 
hsa-miR-152-3p
 
 
-0.529
 
 
66.9
 
 
 
 
ESR1
 
 
hsa-miR-152-3p
 
 
-0.34
 
 
61.1
 
 
 
 
F3
 
 
hsa-miR-152-3p
 
 
-0.331
 
 
52.6
 
 
 
 
FAM168B
 
 
hsa-miR-152-3p
 
 
-0.409
 
 
31.6
 
 
 
 
FAM43A
 
 
hsa-miR-152-3p
 
 
-0.337
 
 
12.2
 
 
 
 
FBN1
 
 
hsa-miR-152-3p
 
 
-0.349
 
 
10.7
 
 
 
 
FEZ2
 
 
hsa-miR-152-3p
 
 
-0.536
 
 
23.5
 
 
 
 
FMR1
 
 
hsa-miR-152-3p
 
 
-0.385
 
 
15.9
 
 
 
 
FOSB
 
 
hsa-miR-152-3p
 
 
-0.311
 
 
190.7
 
 
 
 
FOXK2
 
 
hsa-miR-152-3p
 
 
-0.305
 
 
12.6
 
 
 
 
FXR1
 
 
hsa-miR-152-3p
 
 
-0.478
 
 
32.8
 
 
 
 
GADD45A
 
 
hsa-miR-152-3p
 
 
-0.787
 
 
42.6
 
 
 
 
GAPVD1
 
 
hsa-miR-152-3p
 
 
-0.309
 
 
10.1
 
 
 
 
GLRX5
 
 
hsa-miR-152-3p
 
 
-0.381
 
 
28.2
 
 
 
 
GMFB
 
 
hsa-miR-152-3p
 
 
-0.429
 
 
13.3
 
 
 
 
GPATCH8
 
 
hsa-miR-152-3p
 
 
-0.374
 
 
16.7
 
 
 
 
GRSF1
 
 
hsa-miR-378a-3p
 
 
-0.358
 
 
38.9
 
 
 
 
GTF2H1
 
 
hsa-miR-152-3p
 
 
-0.524
 
 
13.7
 
 
 
 
HDDC2
 
 
hsa-miR-378a-3p
 
 
-0.322
 
 
24.2
 
 
 
 
HLA-DQB1
 
 
hsa-miR-152-3p
 
 
-0.37
 
 
21.4
 
 
 
 
HSP90B1
 
 
hsa-miR-152-3p
 
 
-0.304
 
 
287.5
 
 
 
 
HSPH1
 
 
hsa-miR-330-3p
 
 
-0.345
 
 
51.5
 
 
 
 
ID2
 
 
hsa-miR-330-3p
 
 
-0.293
 
 
91.3
 
 
 
 
IGF1
 
 
hsa-miR-152-3p
 
 
-0.43
 
 
17.8
 
 
 
 
IL6ST
 
 
hsa-miR-152-3p
 
 
-0.291
 
 
23.0
 
 
 
 
ITSN2
 
 
hsa-miR-152-3p
 
 
-0.339
 
 
13.5
 
 
 
 
KLF4
 
 
hsa-miR-152-3p
 
 
-0.325
 
 
25.3
 
 
 
 
KLF5
 
 
hsa-miR-152-3p
 
 
-0.475
 
 
35.3
 
 
 
 
LAMA4
 
 
hsa-miR-152-3p
 
 
-0.363
 
 
163.3
 
 
 
 
LBR
 
 
hsa-miR-152-3p
 
 
-0.39
 
 
27.6
 
 
 
 
LDLR
 
 
hsa-miR-152-3p
 
 
-0.323
 
 
20.4
 
 
 
 
LEPROTL1
 
 
hsa-miR-152-3p
 
 
-0.568
 
 
19.8
 
 
 
 
LIPA
 
 
hsa-miR-152-3p
 
 
-0.463
 
 
35.6
 
 
 
 
LMAN1
 
 
hsa-miR-152-3p
 
 
-0.382
 
 
45.9
 
 
 
 
LRCH1
 
 
hsa-miR-152-3p
 
 
-0.321
 
 
13.0
 
 
 
 
 
 
 heart muscle 
 
 
 
 
target_symbol
 
 
mature_mirna_id
 
 
score
 
 
nTPM
 
 
 
 
 
 
ABCB7
 
 
hsa-miR-152-3p
 
 
-0.515
 
 
20.0
 
 
 
 
ABCD3
 
 
hsa-miR-152-3p
 
 
-0.388
 
 
24.0
 
 
 
 
ACVR1
 
 
hsa-miR-152-3p
 
 
-0.397
 
 
14.3
 
 
 
 
ADAM10
 
 
hsa-miR-152-3p
 
 
-0.348
 
 
11.3
 
 
 
 
ADGRF5
 
 
hsa-miR-152-3p
 
 
-0.354
 
 
50.1
 
 
 
 
AGFG1
 
 
hsa-miR-152-3p
 
 
-0.332
 
 
33.8
 
 
 
 
AHDC1
 
 
hsa-miR-152-3p
 
 
-0.413
 
 
17.4
 
 
 
 
AKAP1
 
 
hsa-miR-152-3p
 
 
-0.381
 
 
78.5
 
 
 
 
AP4B1
 
 
hsa-miR-378a-3p
 
 
-0.62
 
 
10.7
 
 
 
 
ARF4
 
 
hsa-miR-152-3p
 
 
-0.487
 
 
106.8
 
 
 
 
ARHGAP21
 
 
hsa-miR-152-3p
 
 
-0.287
 
 
19.7
 
 
 
 
ARL6IP1
 
 
hsa-miR-152-3p
 
 
-0.579
 
 
30.1
 
 
 
 
ARL8B
 
 
hsa-miR-152-3p
 
 
-0.337
 
 
51.7
 
 
 
 
ARRDC3
 
 
hsa-miR-152-3p
 
 
-0.305
 
 
23.2
 
 
 
 
ASPH
 
 
hsa-miR-152-3p
 
 
-0.391
 
 
100.1
 
 
 
 
ATP2B4
 
 
hsa-miR-152-3p
 
 
-0.337
 
 
56.1
 
 
 
 
ATP6AP2
 
 
hsa-miR-152-3p
 
 
-0.592
 
 
51.6
 
 
 
 
ATP6AP2
 
 
hsa-miR-152-3p
 
 
-0.668
 
 
51.6
 
 
 
 
B4GALT5
 
 
hsa-miR-152-3p
 
 
-0.44
 
 
19.2
 
 
 
 
BHLHE41
 
 
hsa-miR-152-3p
 
 
-0.442
 
 
18.7
 
 
 
 
BMT2
 
 
hsa-miR-152-3p
 
 
-0.4
 
 
11.9
 
 
 
 
BTAF1
 
 
hsa-miR-152-3p
 
 
-0.401
 
 
11.8
 
 
 
 
BTBD3
 
 
hsa-miR-152-3p
 
 
-0.495
 
 
13.0
 
 
 
 
C1GALT1
 
 
hsa-miR-152-3p
 
 
-0.487
 
 
11.8
 
 
 
 
C1QBP
 
 
hsa-miR-330-3p
 
 
-0.412
 
 
119.1
 
 
 
 
C1orf35
 
 
hsa-miR-152-3p
 
 
-0.511
 
 
11.1
 
 
 
 
CAND1
 
 
hsa-miR-152-3p
 
 
-0.44
 
 
12.0
 
 
 
 
CANX
 
 
hsa-miR-152-3p
 
 
-0.434
 
 
116.9
 
 
 
 
CAV2
 
 
hsa-miR-152-3p
 
 
-0.32
 
 
143.1
 
 
 
 
CBLB
 
 
hsa-miR-152-3p
 
 
-0.424
 
 
10.1
 
 
 
 
CCDC71
 
 
hsa-miR-152-3p
 
 
-0.369
 
 
11.4
 
 
 
 
CCN2
 
 
hsa-miR-330-3p
 
 
-0.305
 
 
361.9
 
 
 
 
CCT6A
 
 
hsa-miR-152-3p
 
 
-0.665
 
 
56.1
 
 
 
 
CDC25B
 
 
hsa-miR-152-3p
 
 
-0.298
 
 
15.8
 
 
 
 
CEBPG
 
 
hsa-miR-152-3p
 
 
-0.319
 
 
11.6
 
 
 
 
CEP63
 
 
hsa-miR-152-3p
 
 
-0.329
 
 
23.4
 
 
 
 
CERS5
 
 
hsa-miR-152-3p
 
 
-0.311
 
 
13.3
 
 
 
 
CFL2
 
 
hsa-miR-152-3p
 
 
-0.487
 
 
348.8
 
 
 
 
CHMP4B
 
 
hsa-miR-152-3p
 
 
-0.36
 
 
143.2
 
 
 
 
CLCN6
 
 
hsa-miR-152-3p
 
 
-0.406
 
 
11.6
 
 
 
 
CNOT7
 
 
hsa-miR-152-3p
 
 
-0.31
 
 
35.7
 
 
 
 
COL4A1
 
 
hsa-miR-152-3p
 
 
-0.317
 
 
76.3
 
 
 
 
CS
 
 
hsa-miR-152-3p
 
 
-0.3
 
 
298.9
 
 
 
 
CTSA
 
 
hsa-miR-152-3p
 
 
-0.541
 
 
85.6
 
 
 
 
CUL5
 
 
hsa-miR-152-3p
 
 
-0.389
 
 
14.3
 
 
 
 
CYTH3
 
 
hsa-miR-152-3p
 
 
-0.331
 
 
28.0
 
 
 
 
DCUN1D4
 
 
hsa-miR-152-3p
 
 
-0.314
 
 
11.3
 
 
 
 
DDAH1
 
 
hsa-miR-152-3p
 
 
-0.34
 
 
16.3
 
 
 
 
DDX6
 
 
hsa-miR-152-3p
 
 
-0.363
 
 
34.8
 
 
 
 
DEDD
 
 
hsa-miR-152-3p
 
 
-0.401
 
 
23.0
 
 
 
 
DMPK
 
 
hsa-miR-152-3p
 
 
-0.378
 
 
145.9
 
 
 
 
DNAJB4
 
 
hsa-miR-152-3p
 
 
-0.33
 
 
47.2
 
 
 
 
DOCK6
 
 
hsa-miR-152-3p
 
 
-0.396
 
 
29.4
 
 
 
 
DUSP1
 
 
hsa-miR-152-3p
 
 
-0.32
 
 
384.9
 
 
 
 
DYNLL2
 
 
hsa-miR-152-3p
 
 
-0.366
 
 
58.6
 
 
 
 
DYRK1B
 
 
hsa-miR-152-3p
 
 
-0.305
 
 
19.1
 
 
 
 
DYRK2
 
 
hsa-miR-152-3p
 
 
-0.293
 
 
22.9
 
 
 
 
EGR3
 
 
hsa-miR-152-3p
 
 
-0.315
 
 
11.4
 
 
 
 
EIF4E3
 
 
hsa-miR-152-3p
 
 
-0.464
 
 
28.0
 
 
 
 
EIF4G3
 
 
hsa-miR-378a-3p
 
 
-0.443
 
 
17.3
 
 
 
 
EOGT
 
 
hsa-miR-152-3p
 
 
-0.587
 
 
26.8
 
 
 
 
EPS15
 
 
hsa-miR-152-3p
 
 
-0.322
 
 
22.2
 
 
 
 
ERRFI1
 
 
hsa-miR-152-3p
 
 
-0.529
 
 
50.2
 
 
 
 
F3
 
 
hsa-miR-152-3p
 
 
-0.331
 
 
34.4
 
 
 
 
FAM168B
 
 
hsa-miR-152-3p
 
 
-0.409
 
 
39.9
 
 
 
 
FAM43A
 
 
hsa-miR-152-3p
 
 
-0.337
 
 
25.8
 
 
 
 
FBN1
 
 
hsa-miR-152-3p
 
 
-0.349
 
 
19.5
 
 
 
 
FEZ2
 
 
hsa-miR-152-3p
 
 
-0.536
 
 
43.7
 
 
 
 
FMR1
 
 
hsa-miR-152-3p
 
 
-0.385
 
 
13.4
 
 
 
 
FOSB
 
 
hsa-miR-152-3p
 
 
-0.311
 
 
48.5
 
 
 
 
FOXK2
 
 
hsa-miR-152-3p
 
 
-0.305
 
 
15.9
 
 
 
 
FXR1
 
 
hsa-miR-152-3p
 
 
-0.478
 
 
112.3
 
 
 
 
GADD45A
 
 
hsa-miR-152-3p
 
 
-0.787
 
 
66.1
 
 
 
 
GLRX5
 
 
hsa-miR-152-3p
 
 
-0.381
 
 
112.7
 
 
 
 
GMFB
 
 
hsa-miR-152-3p
 
 
-0.429
 
 
16.1
 
 
 
 
GPATCH8
 
 
hsa-miR-152-3p
 
 
-0.374
 
 
18.1
 
 
 
 
GRSF1
 
 
hsa-miR-378a-3p
 
 
-0.358
 
 
72.5
 
 
 
 
GTF2H1
 
 
hsa-miR-152-3p
 
 
-0.524
 
 
14.1
 
 
 
 
HDDC2
 
 
hsa-miR-378a-3p
 
 
-0.322
 
 
54.8
 
 
 
 
HIF1AN
 
 
hsa-miR-152-3p
 
 
-0.365
 
 
11.5
 
 
 
 
HLA-DQB1
 
 
hsa-miR-152-3p
 
 
-0.37
 
 
20.8
 
 
 
 
HOMER1
 
 
hsa-miR-152-3p
 
 
-0.452
 
 
19.2
 
 
 
 
HSP90B1
 
 
hsa-miR-152-3p
 
 
-0.304
 
 
235.8
 
 
 
 
HSPH1
 
 
hsa-miR-330-3p
 
 
-0.345
 
 
54.1
 
 
 
 
ID2
 
 
hsa-miR-330-3p
 
 
-0.293
 
 
173.5
 
 
 
 
IGF1
 
 
hsa-miR-152-3p
 
 
-0.43
 
 
15.5
 
 
 
 
IL6ST
 
 
hsa-miR-152-3p
 
 
-0.291
 
 
37.0
 
 
 
 
ITSN2
 
 
hsa-miR-152-3p
 
 
-0.339
 
 
18.7
 
 
 
 
KDSR
 
 
hsa-miR-378a-3p
 
 
-0.373
 
 
11.1
 
 
 
 
KLF4
 
 
hsa-miR-152-3p
 
 
-0.325
 
 
22.1
 
 
 
 
LAMA4
 
 
hsa-miR-152-3p
 
 
-0.363
 
 
50.1
 
 
 
 
LDLR
 
 
hsa-miR-152-3p
 
 
-0.323
 
 
18.9
 
 
 
 
LEPROTL1
 
 
hsa-miR-152-3p
 
 
-0.568
 
 
31.8
 
 
 
 
LIPA
 
 
hsa-miR-152-3p
 
 
-0.463
 
 
22.7
 
 
 
 
LMAN1
 
 
hsa-miR-152-3p
 
 
-0.382
 
 
39.9
 
 
 
 
LMTK2
 
 
hsa-miR-152-3p
 
 
-0.298
 
 
11.6
 
 
 
 
LRRC41
 
 
hsa-miR-152-3p
 
 
-0.392
 
 
33.4
 
 
 
 
LSM5
 
 
hsa-miR-330-3p
 
 
-0.351
 
 
46.9
 
 
 
 
LYRM2
 
 
hsa-miR-152-3p
 
 
-0.351
 
 
16.3
 
 
 
 
MACROH2A1
 
 
hsa-miR-152-3p
 
 
-0.336
 
 
59.6
 
 
 
 
 
 
 ovary 
 
 
 
 
target_symbol
 
 
mature_mirna_id
 
 
score
 
 
nTPM
 
 
 
 
 
 
ABCB7
 
 
hsa-miR-152-3p
 
 
-0.515
 
 
11.9
 
 
 
 
ABCD3
 
 
hsa-miR-152-3p
 
 
-0.388
 
 
11.0
 
 
 
 
ACVR1
 
 
hsa-miR-152-3p
 
 
-0.397
 
 
17.7
 
 
 
 
ADAM10
 
 
hsa-miR-152-3p
 
 
-0.348
 
 
13.1
 
 
 
 
AGFG1
 
 
hsa-miR-152-3p
 
 
-0.332
 
 
22.9
 
 
 
 
AHDC1
 
 
hsa-miR-152-3p
 
 
-0.413
 
 
21.5
 
 
 
 
AKAP1
 
 
hsa-miR-152-3p
 
 
-0.381
 
 
18.8
 
 
 
 
ALCAM
 
 
hsa-miR-152-3p
 
 
-0.347
 
 
33.1
 
 
 
 
AP4B1
 
 
hsa-miR-378a-3p
 
 
-0.62
 
 
13.3
 
 
 
 
ARF4
 
 
hsa-miR-152-3p
 
 
-0.487
 
 
123.7
 
 
 
 
ARFIP1
 
 
hsa-miR-152-3p
 
 
-0.474
 
 
17.2
 
 
 
 
ARHGAP21
 
 
hsa-miR-152-3p
 
 
-0.287
 
 
26.5
 
 
 
 
ARL6IP1
 
 
hsa-miR-152-3p
 
 
-0.579
 
 
97.4
 
 
 
 
ARL8B
 
 
hsa-miR-152-3p
 
 
-0.337
 
 
35.5
 
 
 
 
ARRDC3
 
 
hsa-miR-152-3p
 
 
-0.305
 
 
64.7
 
 
 
 
ASPH
 
 
hsa-miR-152-3p
 
 
-0.391
 
 
58.7
 
 
 
 
ATP2B4
 
 
hsa-miR-152-3p
 
 
-0.337
 
 
30.9
 
 
 
 
ATP6AP2
 
 
hsa-miR-152-3p
 
 
-0.592
 
 
86.3
 
 
 
 
ATP6AP2
 
 
hsa-miR-152-3p
 
 
-0.668
 
 
86.3
 
 
 
 
B4GALT5
 
 
hsa-miR-152-3p
 
 
-0.44
 
 
19.0
 
 
 
 
BICC1
 
 
hsa-miR-152-3p
 
 
-0.442
 
 
17.4
 
 
 
 
BTAF1
 
 
hsa-miR-152-3p
 
 
-0.401
 
 
21.0
 
 
 
 
BTBD3
 
 
hsa-miR-152-3p
 
 
-0.495
 
 
13.2
 
 
 
 
C1QBP
 
 
hsa-miR-330-3p
 
 
-0.412
 
 
68.6
 
 
 
 
C1orf35
 
 
hsa-miR-152-3p
 
 
-0.511
 
 
18.4
 
 
 
 
C3orf62
 
 
hsa-miR-330-3p
 
 
-0.366
 
 
11.6
 
 
 
 
CAND1
 
 
hsa-miR-152-3p
 
 
-0.44
 
 
23.6
 
 
 
 
CANX
 
 
hsa-miR-152-3p
 
 
-0.434
 
 
112.9
 
 
 
 
CAV2
 
 
hsa-miR-152-3p
 
 
-0.32
 
 
35.6
 
 
 
 
CCDC6
 
 
hsa-miR-152-3p
 
 
-0.511
 
 
15.5
 
 
 
 
CCDC71
 
 
hsa-miR-152-3p
 
 
-0.369
 
 
18.6
 
 
 
 
CCN2
 
 
hsa-miR-330-3p
 
 
-0.305
 
 
149.8
 
 
 
 
CCT6A
 
 
hsa-miR-152-3p
 
 
-0.665
 
 
43.9
 
 
 
 
CDC25B
 
 
hsa-miR-152-3p
 
 
-0.298
 
 
36.9
 
 
 
 
CEP63
 
 
hsa-miR-152-3p
 
 
-0.329
 
 
12.0
 
 
 
 
CERS5
 
 
hsa-miR-152-3p
 
 
-0.311
 
 
25.5
 
 
 
 
CFL2
 
 
hsa-miR-152-3p
 
 
-0.487
 
 
38.9
 
 
 
 
CHMP4B
 
 
hsa-miR-152-3p
 
 
-0.36
 
 
92.9
 
 
 
 
CLCN6
 
 
hsa-miR-152-3p
 
 
-0.406
 
 
17.7
 
 
 
 
CNOT7
 
 
hsa-miR-152-3p
 
 
-0.31
 
 
41.6
 
 
 
 
COL4A1
 
 
hsa-miR-152-3p
 
 
-0.317
 
 
49.1
 
 
 
 
CS
 
 
hsa-miR-152-3p
 
 
-0.3
 
 
50.8
 
 
 
 
CTSA
 
 
hsa-miR-152-3p
 
 
-0.541
 
 
56.4
 
 
 
 
CUL5
 
 
hsa-miR-152-3p
 
 
-0.389
 
 
11.0
 
 
 
 
CYTH3
 
 
hsa-miR-152-3p
 
 
-0.331
 
 
13.2
 
 
 
 
DACT1
 
 
hsa-miR-378a-3p
 
 
-0.298
 
 
36.1
 
 
 
 
DCUN1D4
 
 
hsa-miR-152-3p
 
 
-0.314
 
 
15.6
 
 
 
 
DDX6
 
 
hsa-miR-152-3p
 
 
-0.363
 
 
38.7
 
 
 
 
DEDD
 
 
hsa-miR-152-3p
 
 
-0.401
 
 
26.8
 
 
 
 
DIPK2A
 
 
hsa-miR-152-3p
 
 
-0.393
 
 
24.4
 
 
 
 
DMPK
 
 
hsa-miR-152-3p
 
 
-0.378
 
 
14.5
 
 
 
 
DNAJB4
 
 
hsa-miR-152-3p
 
 
-0.33
 
 
15.6
 
 
 
 
DOCK6
 
 
hsa-miR-152-3p
 
 
-0.396
 
 
11.6
 
 
 
 
DUSP1
 
 
hsa-miR-152-3p
 
 
-0.32
 
 
484.3
 
 
 
 
DYNLL2
 
 
hsa-miR-152-3p
 
 
-0.366
 
 
16.8
 
 
 
 
DYRK1B
 
 
hsa-miR-152-3p
 
 
-0.305
 
 
11.1
 
 
 
 
EGR3
 
 
hsa-miR-152-3p
 
 
-0.315
 
 
16.2
 
 
 
 
EIF4E3
 
 
hsa-miR-152-3p
 
 
-0.464
 
 
20.8
 
 
 
 
EIF4G3
 
 
hsa-miR-378a-3p
 
 
-0.443
 
 
15.9
 
 
 
 
EMX2
 
 
hsa-miR-152-3p
 
 
-0.366
 
 
11.3
 
 
 
 
EOGT
 
 
hsa-miR-152-3p
 
 
-0.587
 
 
10.9
 
 
 
 
EPS15
 
 
hsa-miR-152-3p
 
 
-0.322
 
 
24.3
 
 
 
 
ERRFI1
 
 
hsa-miR-152-3p
 
 
-0.529
 
 
142.3
 
 
 
 
F3
 
 
hsa-miR-152-3p
 
 
-0.331
 
 
105.1
 
 
 
 
FAM168B
 
 
hsa-miR-152-3p
 
 
-0.409
 
 
42.1
 
 
 
 
FBN1
 
 
hsa-miR-152-3p
 
 
-0.349
 
 
25.4
 
 
 
 
FEZ2
 
 
hsa-miR-152-3p
 
 
-0.536
 
 
30.6
 
 
 
 
FMR1
 
 
hsa-miR-152-3p
 
 
-0.385
 
 
17.3
 
 
 
 
FOSB
 
 
hsa-miR-152-3p
 
 
-0.311
 
 
197.2
 
 
 
 
FOXK2
 
 
hsa-miR-152-3p
 
 
-0.305
 
 
13.7
 
 
 
 
FXR1
 
 
hsa-miR-152-3p
 
 
-0.478
 
 
47.2
 
 
 
 
GADD45A
 
 
hsa-miR-152-3p
 
 
-0.787
 
 
52.9
 
 
 
 
GAPVD1
 
 
hsa-miR-152-3p
 
 
-0.309
 
 
10.1
 
 
 
 
GLRX5
 
 
hsa-miR-152-3p
 
 
-0.381
 
 
43.2
 
 
 
 
GMFB
 
 
hsa-miR-152-3p
 
 
-0.429
 
 
16.4
 
 
 
 
GPATCH8
 
 
hsa-miR-152-3p
 
 
-0.374
 
 
21.1
 
 
 
 
GRSF1
 
 
hsa-miR-378a-3p
 
 
-0.358
 
 
36.2
 
 
 
 
GTF2H1
 
 
hsa-miR-152-3p
 
 
-0.524
 
 
18.4
 
 
 
 
HDDC2
 
 
hsa-miR-378a-3p
 
 
-0.322
 
 
28.2
 
 
 
 
HSP90B1
 
 
hsa-miR-152-3p
 
 
-0.304
 
 
299.2
 
 
 
 
HSPH1
 
 
hsa-miR-330-3p
 
 
-0.345
 
 
57.8
 
 
 
 
ID2
 
 
hsa-miR-330-3p
 
 
-0.293
 
 
208.9
 
 
 
 
IGF1
 
 
hsa-miR-152-3p
 
 
-0.43
 
 
29.4
 
 
 
 
IL6ST
 
 
hsa-miR-152-3p
 
 
-0.291
 
 
46.1
 
 
 
 
ING2
 
 
hsa-miR-152-3p
 
 
-0.482
 
 
32.5
 
 
 
 
ITSN2
 
 
hsa-miR-152-3p
 
 
-0.339
 
 
15.2
 
 
 
 
KDSR
 
 
hsa-miR-378a-3p
 
 
-0.373
 
 
15.0
 
 
 
 
KLF4
 
 
hsa-miR-152-3p
 
 
-0.325
 
 
21.6
 
 
 
 
LAMA4
 
 
hsa-miR-152-3p
 
 
-0.363
 
 
35.3
 
 
 
 
LBR
 
 
hsa-miR-152-3p
 
 
-0.39
 
 
17.2
 
 
 
 
LDLR
 
 
hsa-miR-152-3p
 
 
-0.323
 
 
35.4
 
 
 
 
LEPROTL1
 
 
hsa-miR-152-3p
 
 
-0.568
 
 
21.4
 
 
 
 
LIPA
 
 
hsa-miR-152-3p
 
 
-0.463
 
 
37.4
 
 
 
 
LMAN1
 
 
hsa-miR-152-3p
 
 
-0.382
 
 
42.7
 
 
 
 
LRRC41
 
 
hsa-miR-152-3p
 
 
-0.392
 
 
46.1
 
 
 
 
LSM5
 
 
hsa-miR-330-3p
 
 
-0.351
 
 
42.6
 
 
 
 
MACROH2A1
 
 
hsa-miR-152-3p
 
 
-0.336
 
 
39.8
 
 
 
 
MAF
 
 
hsa-miR-152-3p
 
 
-0.362
 
 
36.9
 
 
 
 
MAF1
 
 
hsa-miR-152-3p
 
 
-0.366
 
 
105.6
 
 
 
 
MAFG
 
 
hsa-miR-152-3p
 
 
-0.328
 
 
13.2
 
 
 
 
 
 
 parathyroid gland 
 
 
 
 
target_symbol
 
 
mature_mirna_id
 
 
score
 
 
nTPM
 
 
 
 
 
 
ABCB7
 
 
hsa-miR-152-3p
 
 
-0.515
 
 
10.3
 
 
 
 
ABCD3
 
 
hsa-miR-152-3p
 
 
-0.388
 
 
27.9
 
 
 
 
ADGRF5
 
 
hsa-miR-152-3p
 
 
-0.354
 
 
12.6
 
 
 
 
AGFG1
 
 
hsa-miR-152-3p
 
 
-0.332
 
 
39.0
 
 
 
 
AKAP1
 
 
hsa-miR-152-3p
 
 
-0.381
 
 
45.1
 
 
 
 
ALCAM
 
 
hsa-miR-152-3p
 
 
-0.347
 
 
412.8
 
 
 
 
ARF4
 
 
hsa-miR-152-3p
 
 
-0.487
 
 
95.4
 
 
 
 
ARFGEF1
 
 
hsa-miR-152-3p
 
 
-0.306
 
 
26.3
 
 
 
 
ARFIP1
 
 
hsa-miR-152-3p
 
 
-0.474
 
 
21.0
 
 
 
 
ARHGAP21
 
 
hsa-miR-152-3p
 
 
-0.287
 
 
13.6
 
 
 
 
ARL6IP1
 
 
hsa-miR-152-3p
 
 
-0.579
 
 
169.8
 
 
 
 
ARL8B
 
 
hsa-miR-152-3p
 
 
-0.337
 
 
45.2
 
 
 
 
ARRDC3
 
 
hsa-miR-152-3p
 
 
-0.305
 
 
51.0
 
 
 
 
ASPH
 
 
hsa-miR-152-3p
 
 
-0.391
 
 
35.7
 
 
 
 
ATP2B4
 
 
hsa-miR-152-3p
 
 
-0.337
 
 
11.1
 
 
 
 
ATP6AP2
 
 
hsa-miR-152-3p
 
 
-0.592
 
 
719.4
 
 
 
 
ATP6AP2
 
 
hsa-miR-152-3p
 
 
-0.668
 
 
719.4
 
 
 
 
B4GALT5
 
 
hsa-miR-152-3p
 
 
-0.44
 
 
10.6
 
 
 
 
BCL2L11
 
 
hsa-miR-152-3p
 
 
-0.445
 
 
17.6
 
 
 
 
BTAF1
 
 
hsa-miR-152-3p
 
 
-0.401
 
 
11.8
 
 
 
 
BTBD3
 
 
hsa-miR-152-3p
 
 
-0.495
 
 
10.5
 
 
 
 
C1GALT1
 
 
hsa-miR-152-3p
 
 
-0.487
 
 
43.0
 
 
 
 
C1QBP
 
 
hsa-miR-330-3p
 
 
-0.412
 
 
119.9
 
 
 
 
CAND1
 
 
hsa-miR-152-3p
 
 
-0.44
 
 
26.6
 
 
 
 
CCDC6
 
 
hsa-miR-152-3p
 
 
-0.511
 
 
16.9
 
 
 
 
CCDC71
 
 
hsa-miR-152-3p
 
 
-0.369
 
 
12.8
 
 
 
 
CCN2
 
 
hsa-miR-330-3p
 
 
-0.305
 
 
40.4
 
 
 
 
CCT6A
 
 
hsa-miR-152-3p
 
 
-0.665
 
 
65.8
 
 
 
 
CD226
 
 
hsa-miR-378a-3p
 
 
-0.481
 
 
58.7
 
 
 
 
CEBPG
 
 
hsa-miR-152-3p
 
 
-0.319
 
 
14.2
 
 
 
 
CEP63
 
 
hsa-miR-152-3p
 
 
-0.329
 
 
15.9
 
 
 
 
CFL2
 
 
hsa-miR-152-3p
 
 
-0.487
 
 
22.9
 
 
 
 
CHMP4B
 
 
hsa-miR-152-3p
 
 
-0.36
 
 
70.2
 
 
 
 
CNOT7
 
 
hsa-miR-152-3p
 
 
-0.31
 
 
58.7
 
 
 
 
CNTN4
 
 
hsa-miR-152-3p
 
 
-0.415
 
 
21.2
 
 
 
 
COL4A1
 
 
hsa-miR-152-3p
 
 
-0.317
 
 
34.1
 
 
 
 
CS
 
 
hsa-miR-152-3p
 
 
-0.3
 
 
119.8
 
 
 
 
CTSA
 
 
hsa-miR-152-3p
 
 
-0.541
 
 
98.8
 
 
 
 
CUL5
 
 
hsa-miR-152-3p
 
 
-0.389
 
 
12.6
 
 
 
 
CYTH3
 
 
hsa-miR-152-3p
 
 
-0.331
 
 
10.8
 
 
 
 
DDAH1
 
 
hsa-miR-152-3p
 
 
-0.34
 
 
19.8
 
 
 
 
DDX6
 
 
hsa-miR-152-3p
 
 
-0.363
 
 
26.6
 
 
 
 
DEDD
 
 
hsa-miR-152-3p
 
 
-0.401
 
 
17.0
 
 
 
 
DICER1
 
 
hsa-miR-152-3p
 
 
-0.29
 
 
10.3
 
 
 
 
DIPK2A
 
 
hsa-miR-152-3p
 
 
-0.393
 
 
14.1
 
 
 
 
DMXL1
 
 
hsa-miR-152-3p
 
 
-0.29
 
 
11.0
 
 
 
 
DNAJB4
 
 
hsa-miR-152-3p
 
 
-0.33
 
 
13.3
 
 
 
 
DUSP1
 
 
hsa-miR-152-3p
 
 
-0.32
 
 
109.5
 
 
 
 
DYNLL2
 
 
hsa-miR-152-3p
 
 
-0.366
 
 
44.4
 
 
 
 
EGR3
 
 
hsa-miR-152-3p
 
 
-0.315
 
 
12.9
 
 
 
 
EIF4E3
 
 
hsa-miR-152-3p
 
 
-0.464
 
 
23.0
 
 
 
 
EIF4G3
 
 
hsa-miR-378a-3p
 
 
-0.443
 
 
37.4
 
 
 
 
EPS15
 
 
hsa-miR-152-3p
 
 
-0.322
 
 
24.4
 
 
 
 
ERBB3
 
 
hsa-miR-152-3p
 
 
-0.399
 
 
20.5
 
 
 
 
ERRFI1
 
 
hsa-miR-152-3p
 
 
-0.529
 
 
82.8
 
 
 
 
ESRRG
 
 
hsa-miR-152-3p
 
 
-0.347
 
 
43.5
 
 
 
 
F3
 
 
hsa-miR-152-3p
 
 
-0.331
 
 
16.1
 
 
 
 
FAM168B
 
 
hsa-miR-152-3p
 
 
-0.409
 
 
43.9
 
 
 
 
FBXO28
 
 
hsa-miR-152-3p
 
 
-0.313
 
 
12.9
 
 
 
 
FEZ2
 
 
hsa-miR-152-3p
 
 
-0.536
 
 
19.1
 
 
 
 
FMR1
 
 
hsa-miR-152-3p
 
 
-0.385
 
 
20.9
 
 
 
 
FOSB
 
 
hsa-miR-152-3p
 
 
-0.311
 
 
43.4
 
 
 
 
FOXK2
 
 
hsa-miR-152-3p
 
 
-0.305
 
 
18.8
 
 
 
 
FXR1
 
 
hsa-miR-152-3p
 
 
-0.478
 
 
31.6
 
 
 
 
GADD45A
 
 
hsa-miR-152-3p
 
 
-0.787
 
 
18.3
 
 
 
 
GAPVD1
 
 
hsa-miR-152-3p
 
 
-0.309
 
 
17.6
 
 
 
 
GLRX5
 
 
hsa-miR-152-3p
 
 
-0.381
 
 
43.4
 
 
 
 
GMFB
 
 
hsa-miR-152-3p
 
 
-0.429
 
 
18.9
 
 
 
 
GOLT1A
 
 
hsa-miR-378a-3p
 
 
-0.574
 
 
17.3
 
 
 
 
GPATCH8
 
 
hsa-miR-152-3p
 
 
-0.374
 
 
13.6
 
 
 
 
GRSF1
 
 
hsa-miR-378a-3p
 
 
-0.358
 
 
44.5
 
 
 
 
HDDC2
 
 
hsa-miR-378a-3p
 
 
-0.322
 
 
46.4
 
 
 
 
HIF1AN
 
 
hsa-miR-152-3p
 
 
-0.365
 
 
11.3
 
 
 
 
HLA-DQB1
 
 
hsa-miR-152-3p
 
 
-0.37
 
 
10.2
 
 
 
 
HSP90B1
 
 
hsa-miR-152-3p
 
 
-0.304
 
 
316.0
 
 
 
 
HSPA12A
 
 
hsa-miR-378a-3p
 
 
-0.326
 
 
18.8
 
 
 
 
HSPH1
 
 
hsa-miR-330-3p
 
 
-0.345
 
 
28.4
 
 
 
 
ID2
 
 
hsa-miR-330-3p
 
 
-0.293
 
 
1136.9
 
 
 
 
IL6ST
 
 
hsa-miR-152-3p
 
 
-0.291
 
 
79.5
 
 
 
 
ITSN2
 
 
hsa-miR-152-3p
 
 
-0.339
 
 
17.6
 
 
 
 
KDSR
 
 
hsa-miR-378a-3p
 
 
-0.373
 
 
12.3
 
 
 
 
LEPROTL1
 
 
hsa-miR-152-3p
 
 
-0.568
 
 
20.5
 
 
 
 
LIPA
 
 
hsa-miR-152-3p
 
 
-0.463
 
 
64.7
 
 
 
 
LMAN1
 
 
hsa-miR-152-3p
 
 
-0.382
 
 
69.9
 
 
 
 
LMTK2
 
 
hsa-miR-152-3p
 
 
-0.298
 
 
10.1
 
 
 
 
LRP2
 
 
hsa-miR-152-3p
 
 
-0.465
 
 
42.1
 
 
 
 
LRRC41
 
 
hsa-miR-152-3p
 
 
-0.392
 
 
41.4
 
 
 
 
LSM5
 
 
hsa-miR-330-3p
 
 
-0.351
 
 
28.1
 
 
 
 
LYRM2
 
 
hsa-miR-152-3p
 
 
-0.351
 
 
21.1
 
 
 
 
LYSMD2
 
 
hsa-miR-152-3p
 
 
-0.554
 
 
10.1
 
 
 
 
MACIR
 
 
hsa-miR-152-3p
 
 
-0.332
 
 
10.6
 
 
 
 
MACIR
 
 
hsa-miR-152-3p
 
 
-0.573
 
 
10.6
 
 
 
 
MACROH2A1
 
 
hsa-miR-152-3p
 
 
-0.336
 
 
74.5
 
 
 
 
MAF
 
 
hsa-miR-152-3p
 
 
-0.362
 
 
47.8
 
 
 
 
MAF1
 
 
hsa-miR-152-3p
 
 
-0.366
 
 
82.1
 
 
 
 
MAFB
 
 
hsa-miR-152-3p
 
 
-0.45
 
 
256.8
 
 
 
 
MAFG
 
 
hsa-miR-152-3p
 
 
-0.328
 
 
12.8
 
 
 
 
MAP2K1
 
 
hsa-miR-152-3p
 
 
-0.295
 
 
31.7
 
 
 
 
MAPK1
 
 
hsa-miR-378a-3p
 
 
-0.314
 
 
48.6
 
 
 
 
MAT2A
 
 
hsa-miR-330-3p
 
 
-0.307
 
 
156.0
 
 
 
 
 
 
 skin 
 
 
 
 
target_symbol
 
 
mature_mirna_id
 
 
score
 
 
nTPM
 
 
 
 
 
 
ABCB7
 
 
hsa-miR-152-3p
 
 
-0.515
 
 
12.7
 
 
 
 
ABCD3
 
 
hsa-miR-152-3p
 
 
-0.388
 
 
33.6
 
 
 
 
ABHD17C
 
 
hsa-miR-330-3p
 
 
-0.399
 
 
17.9
 
 
 
 
ADAM10
 
 
hsa-miR-152-3p
 
 
-0.348
 
 
14.8
 
 
 
 
AGFG1
 
 
hsa-miR-152-3p
 
 
-0.332
 
 
34.3
 
 
 
 
AHDC1
 
 
hsa-miR-152-3p
 
 
-0.413
 
 
29.8
 
 
 
 
AKAP1
 
 
hsa-miR-152-3p
 
 
-0.381
 
 
26.4
 
 
 
 
ALCAM
 
 
hsa-miR-152-3p
 
 
-0.347
 
 
10.3
 
 
 
 
AP4B1
 
 
hsa-miR-378a-3p
 
 
-0.62
 
 
10.9
 
 
 
 
ARF4
 
 
hsa-miR-152-3p
 
 
-0.487
 
 
61.3
 
 
 
 
ARFGEF1
 
 
hsa-miR-152-3p
 
 
-0.306
 
 
12.9
 
 
 
 
ARFIP1
 
 
hsa-miR-152-3p
 
 
-0.474
 
 
16.4
 
 
 
 
ARL6IP1
 
 
hsa-miR-152-3p
 
 
-0.579
 
 
62.4
 
 
 
 
ARL8B
 
 
hsa-miR-152-3p
 
 
-0.337
 
 
64.4
 
 
 
 
ARRDC3
 
 
hsa-miR-152-3p
 
 
-0.305
 
 
38.6
 
 
 
 
ASPH
 
 
hsa-miR-152-3p
 
 
-0.391
 
 
33.3
 
 
 
 
ASPRV1
 
 
hsa-miR-330-3p
 
 
-0.301
 
 
614.8
 
 
 
 
ATP2B4
 
 
hsa-miR-152-3p
 
 
-0.337
 
 
51.7
 
 
 
 
ATP6AP2
 
 
hsa-miR-152-3p
 
 
-0.592
 
 
68.0
 
 
 
 
ATP6AP2
 
 
hsa-miR-152-3p
 
 
-0.668
 
 
68.0
 
 
 
 
ATP7A
 
 
hsa-miR-152-3p
 
 
-0.292
 
 
11.6
 
 
 
 
BMP2
 
 
hsa-miR-378a-3p
 
 
-0.481
 
 
10.2
 
 
 
 
BTAF1
 
 
hsa-miR-152-3p
 
 
-0.401
 
 
39.9
 
 
 
 
BTBD3
 
 
hsa-miR-152-3p
 
 
-0.495
 
 
19.6
 
 
 
 
C1QBP
 
 
hsa-miR-330-3p
 
 
-0.412
 
 
78.3
 
 
 
 
C1orf210
 
 
hsa-miR-378a-3p
 
 
-0.307
 
 
15.4
 
 
 
 
C1orf35
 
 
hsa-miR-152-3p
 
 
-0.511
 
 
22.4
 
 
 
 
CAND1
 
 
hsa-miR-152-3p
 
 
-0.44
 
 
27.6
 
 
 
 
CANX
 
 
hsa-miR-152-3p
 
 
-0.434
 
 
71.7
 
 
 
 
CAV2
 
 
hsa-miR-152-3p
 
 
-0.32
 
 
44.7
 
 
 
 
CCDC6
 
 
hsa-miR-152-3p
 
 
-0.511
 
 
27.0
 
 
 
 
CCDC71
 
 
hsa-miR-152-3p
 
 
-0.369
 
 
12.4
 
 
 
 
CCN2
 
 
hsa-miR-330-3p
 
 
-0.305
 
 
147.8
 
 
 
 
CCT6A
 
 
hsa-miR-152-3p
 
 
-0.665
 
 
56.0
 
 
 
 
CDC25B
 
 
hsa-miR-152-3p
 
 
-0.298
 
 
21.4
 
 
 
 
CEBPG
 
 
hsa-miR-152-3p
 
 
-0.319
 
 
26.1
 
 
 
 
CEP63
 
 
hsa-miR-152-3p
 
 
-0.329
 
 
10.0
 
 
 
 
CERS5
 
 
hsa-miR-152-3p
 
 
-0.311
 
 
25.4
 
 
 
 
CHMP4B
 
 
hsa-miR-152-3p
 
 
-0.36
 
 
118.7
 
 
 
 
CNOT7
 
 
hsa-miR-152-3p
 
 
-0.31
 
 
26.2
 
 
 
 
COL4A1
 
 
hsa-miR-152-3p
 
 
-0.317
 
 
23.0
 
 
 
 
CS
 
 
hsa-miR-152-3p
 
 
-0.3
 
 
81.5
 
 
 
 
CTSA
 
 
hsa-miR-152-3p
 
 
-0.541
 
 
83.7
 
 
 
 
CTTNBP2NL
 
 
hsa-miR-152-3p
 
 
-0.329
 
 
14.4
 
 
 
 
CUL5
 
 
hsa-miR-152-3p
 
 
-0.389
 
 
10.7
 
 
 
 
CYTH3
 
 
hsa-miR-152-3p
 
 
-0.331
 
 
20.7
 
 
 
 
DDX6
 
 
hsa-miR-152-3p
 
 
-0.363
 
 
25.7
 
 
 
 
DEDD
 
 
hsa-miR-152-3p
 
 
-0.401
 
 
27.6
 
 
 
 
DICER1
 
 
hsa-miR-152-3p
 
 
-0.29
 
 
11.3
 
 
 
 
DIPK2A
 
 
hsa-miR-152-3p
 
 
-0.393
 
 
23.0
 
 
 
 
DOCK6
 
 
hsa-miR-152-3p
 
 
-0.396
 
 
24.4
 
 
 
 
DUSP1
 
 
hsa-miR-152-3p
 
 
-0.32
 
 
1001.4
 
 
 
 
DYNLL2
 
 
hsa-miR-152-3p
 
 
-0.366
 
 
18.1
 
 
 
 
DYRK1B
 
 
hsa-miR-152-3p
 
 
-0.305
 
 
14.1
 
 
 
 
EGR3
 
 
hsa-miR-152-3p
 
 
-0.315
 
 
41.7
 
 
 
 
EIF4G3
 
 
hsa-miR-378a-3p
 
 
-0.443
 
 
18.4
 
 
 
 
EMX2
 
 
hsa-miR-152-3p
 
 
-0.366
 
 
10.2
 
 
 
 
EPS15
 
 
hsa-miR-152-3p
 
 
-0.322
 
 
23.5
 
 
 
 
ERBB3
 
 
hsa-miR-152-3p
 
 
-0.399
 
 
63.7
 
 
 
 
ERRFI1
 
 
hsa-miR-152-3p
 
 
-0.529
 
 
118.1
 
 
 
 
F3
 
 
hsa-miR-152-3p
 
 
-0.331
 
 
53.1
 
 
 
 
FAM168B
 
 
hsa-miR-152-3p
 
 
-0.409
 
 
27.3
 
 
 
 
FAM43A
 
 
hsa-miR-152-3p
 
 
-0.337
 
 
23.8
 
 
 
 
FBXL19
 
 
hsa-miR-152-3p
 
 
-0.362
 
 
13.3
 
 
 
 
FBXO28
 
 
hsa-miR-152-3p
 
 
-0.313
 
 
19.9
 
 
 
 
FEZ2
 
 
hsa-miR-152-3p
 
 
-0.536
 
 
15.0
 
 
 
 
FOSB
 
 
hsa-miR-152-3p
 
 
-0.311
 
 
258.5
 
 
 
 
FOXK2
 
 
hsa-miR-152-3p
 
 
-0.305
 
 
18.2
 
 
 
 
FXR1
 
 
hsa-miR-152-3p
 
 
-0.478
 
 
20.8
 
 
 
 
GADD45A
 
 
hsa-miR-152-3p
 
 
-0.787
 
 
61.1
 
 
 
 
GAPVD1
 
 
hsa-miR-152-3p
 
 
-0.309
 
 
13.9
 
 
 
 
GLRX5
 
 
hsa-miR-152-3p
 
 
-0.381
 
 
34.2
 
 
 
 
GMFB
 
 
hsa-miR-152-3p
 
 
-0.429
 
 
12.9
 
 
 
 
GPATCH8
 
 
hsa-miR-152-3p
 
 
-0.374
 
 
17.6
 
 
 
 
GRSF1
 
 
hsa-miR-378a-3p
 
 
-0.358
 
 
27.6
 
 
 
 
GTF2H1
 
 
hsa-miR-152-3p
 
 
-0.524
 
 
19.1
 
 
 
 
HDDC2
 
 
hsa-miR-378a-3p
 
 
-0.322
 
 
25.9
 
 
 
 
HLA-DQB1
 
 
hsa-miR-152-3p
 
 
-0.37
 
 
30.8
 
 
 
 
HSP90B1
 
 
hsa-miR-152-3p
 
 
-0.304
 
 
100.0
 
 
 
 
HSPH1
 
 
hsa-miR-330-3p
 
 
-0.345
 
 
20.5
 
 
 
 
ID2
 
 
hsa-miR-330-3p
 
 
-0.293
 
 
68.9
 
 
 
 
IL6ST
 
 
hsa-miR-152-3p
 
 
-0.291
 
 
12.8
 
 
 
 
INHBB
 
 
hsa-miR-152-3p
 
 
-0.469
 
 
16.2
 
 
 
 
ITSN2
 
 
hsa-miR-152-3p
 
 
-0.339
 
 
24.9
 
 
 
 
KDSR
 
 
hsa-miR-378a-3p
 
 
-0.373
 
 
16.7
 
 
 
 
KLF4
 
 
hsa-miR-152-3p
 
 
-0.325
 
 
185.8
 
 
 
 
KLF5
 
 
hsa-miR-152-3p
 
 
-0.475
 
 
221.8
 
 
 
 
LAMA4
 
 
hsa-miR-152-3p
 
 
-0.363
 
 
16.4
 
 
 
 
LBR
 
 
hsa-miR-152-3p
 
 
-0.39
 
 
13.0
 
 
 
 
LDLR
 
 
hsa-miR-152-3p
 
 
-0.323
 
 
25.1
 
 
 
 
LEPROTL1
 
 
hsa-miR-152-3p
 
 
-0.568
 
 
19.8
 
 
 
 
LIPA
 
 
hsa-miR-152-3p
 
 
-0.463
 
 
17.6
 
 
 
 
LMAN1
 
 
hsa-miR-152-3p
 
 
-0.382
 
 
19.2
 
 
 
 
LRCH1
 
 
hsa-miR-152-3p
 
 
-0.321
 
 
17.2
 
 
 
 
LRRC41
 
 
hsa-miR-152-3p
 
 
-0.392
 
 
41.2
 
 
 
 
LSM5
 
 
hsa-miR-330-3p
 
 
-0.351
 
 
31.8
 
 
 
 
MACROH2A1
 
 
hsa-miR-152-3p
 
 
-0.336
 
 
82.4
 
 
 
 
MAF
 
 
hsa-miR-152-3p
 
 
-0.362
 
 
54.6
 
 
 
 
MAF1
 
 
hsa-miR-152-3p
 
 
-0.366
 
 
65.4
 
 
 
 
MAFB
 
 
hsa-miR-152-3p
 
 
-0.45
 
 
80.7
 
 
 
 
 
 
 spleen 
 
 
 
 
target_symbol
 
 
mature_mirna_id
 
 
score
 
 
nTPM
 
 
 
 
 
 
ABCB7
 
 
hsa-miR-152-3p
 
 
-0.515
 
 
10.6
 
 
 
 
ABCD3
 
 
hsa-miR-152-3p
 
 
-0.388
 
 
10.3
 
 
 
 
ADAM10
 
 
hsa-miR-152-3p
 
 
-0.348
 
 
27.3
 
 
 
 
ADGRF5
 
 
hsa-miR-152-3p
 
 
-0.354
 
 
38.8
 
 
 
 
AGFG1
 
 
hsa-miR-152-3p
 
 
-0.332
 
 
44.5
 
 
 
 
AHDC1
 
 
hsa-miR-152-3p
 
 
-0.413
 
 
15.7
 
 
 
 
AKAP1
 
 
hsa-miR-152-3p
 
 
-0.381
 
 
11.0
 
 
 
 
AP4B1
 
 
hsa-miR-378a-3p
 
 
-0.62
 
 
19.7
 
 
 
 
ARF4
 
 
hsa-miR-152-3p
 
 
-0.487
 
 
60.0
 
 
 
 
ARFGEF1
 
 
hsa-miR-152-3p
 
 
-0.306
 
 
13.0
 
 
 
 
ARFIP1
 
 
hsa-miR-152-3p
 
 
-0.474
 
 
17.3
 
 
 
 
ARL6IP1
 
 
hsa-miR-152-3p
 
 
-0.579
 
 
68.6
 
 
 
 
ARL8B
 
 
hsa-miR-152-3p
 
 
-0.337
 
 
32.0
 
 
 
 
ARRDC3
 
 
hsa-miR-152-3p
 
 
-0.305
 
 
35.0
 
 
 
 
ASPH
 
 
hsa-miR-152-3p
 
 
-0.391
 
 
26.8
 
 
 
 
ATP2B4
 
 
hsa-miR-152-3p
 
 
-0.337
 
 
11.5
 
 
 
 
ATP6AP2
 
 
hsa-miR-152-3p
 
 
-0.592
 
 
65.0
 
 
 
 
ATP6AP2
 
 
hsa-miR-152-3p
 
 
-0.668
 
 
65.0
 
 
 
 
B4GALT5
 
 
hsa-miR-152-3p
 
 
-0.44
 
 
26.0
 
 
 
 
BCL2L11
 
 
hsa-miR-152-3p
 
 
-0.445
 
 
15.1
 
 
 
 
BMP2
 
 
hsa-miR-378a-3p
 
 
-0.481
 
 
11.7
 
 
 
 
BTAF1
 
 
hsa-miR-152-3p
 
 
-0.401
 
 
17.6
 
 
 
 
C1GALT1
 
 
hsa-miR-152-3p
 
 
-0.487
 
 
14.6
 
 
 
 
C1QBP
 
 
hsa-miR-330-3p
 
 
-0.412
 
 
70.8
 
 
 
 
C1orf35
 
 
hsa-miR-152-3p
 
 
-0.511
 
 
12.6
 
 
 
 
C3orf62
 
 
hsa-miR-330-3p
 
 
-0.366
 
 
12.1
 
 
 
 
CAND1
 
 
hsa-miR-152-3p
 
 
-0.44
 
 
18.0
 
 
 
 
CANX
 
 
hsa-miR-152-3p
 
 
-0.434
 
 
63.9
 
 
 
 
CAV2
 
 
hsa-miR-152-3p
 
 
-0.32
 
 
49.2
 
 
 
 
CCDC6
 
 
hsa-miR-152-3p
 
 
-0.511
 
 
13.8
 
 
 
 
CCDC71
 
 
hsa-miR-152-3p
 
 
-0.369
 
 
12.6
 
 
 
 
CCN2
 
 
hsa-miR-330-3p
 
 
-0.305
 
 
536.5
 
 
 
 
CCT6A
 
 
hsa-miR-152-3p
 
 
-0.665
 
 
44.4
 
 
 
 
CD226
 
 
hsa-miR-378a-3p
 
 
-0.481
 
 
19.0
 
 
 
 
CDC25B
 
 
hsa-miR-152-3p
 
 
-0.298
 
 
54.7
 
 
 
 
CDK5R1
 
 
hsa-miR-152-3p
 
 
-0.547
 
 
12.4
 
 
 
 
CEP63
 
 
hsa-miR-152-3p
 
 
-0.329
 
 
12.8
 
 
 
 
CERS5
 
 
hsa-miR-152-3p
 
 
-0.311
 
 
20.6
 
 
 
 
CFL2
 
 
hsa-miR-152-3p
 
 
-0.487
 
 
12.0
 
 
 
 
CHMP4B
 
 
hsa-miR-152-3p
 
 
-0.36
 
 
81.6
 
 
 
 
CLCN6
 
 
hsa-miR-152-3p
 
 
-0.406
 
 
11.1
 
 
 
 
CNOT7
 
 
hsa-miR-152-3p
 
 
-0.31
 
 
36.7
 
 
 
 
COL4A1
 
 
hsa-miR-152-3p
 
 
-0.317
 
 
64.7
 
 
 
 
CS
 
 
hsa-miR-152-3p
 
 
-0.3
 
 
46.2
 
 
 
 
CTSA
 
 
hsa-miR-152-3p
 
 
-0.541
 
 
92.5
 
 
 
 
CTTNBP2NL
 
 
hsa-miR-152-3p
 
 
-0.329
 
 
10.0
 
 
 
 
CYTH3
 
 
hsa-miR-152-3p
 
 
-0.331
 
 
10.5
 
 
 
 
DDAH1
 
 
hsa-miR-152-3p
 
 
-0.34
 
 
10.0
 
 
 
 
DDX6
 
 
hsa-miR-152-3p
 
 
-0.363
 
 
42.7
 
 
 
 
DEDD
 
 
hsa-miR-152-3p
 
 
-0.401
 
 
26.3
 
 
 
 
DICER1
 
 
hsa-miR-152-3p
 
 
-0.29
 
 
10.2
 
 
 
 
DIPK2A
 
 
hsa-miR-152-3p
 
 
-0.393
 
 
10.1
 
 
 
 
DNAJB4
 
 
hsa-miR-152-3p
 
 
-0.33
 
 
39.7
 
 
 
 
DOCK6
 
 
hsa-miR-152-3p
 
 
-0.396
 
 
13.9
 
 
 
 
DUSP1
 
 
hsa-miR-152-3p
 
 
-0.32
 
 
305.5
 
 
 
 
DYNLL2
 
 
hsa-miR-152-3p
 
 
-0.366
 
 
17.0
 
 
 
 
DYRK1B
 
 
hsa-miR-152-3p
 
 
-0.305
 
 
13.3
 
 
 
 
DYRK2
 
 
hsa-miR-152-3p
 
 
-0.293
 
 
13.9
 
 
 
 
EIF4E3
 
 
hsa-miR-152-3p
 
 
-0.464
 
 
16.7
 
 
 
 
EIF4G3
 
 
hsa-miR-378a-3p
 
 
-0.443
 
 
15.4
 
 
 
 
EPS15
 
 
hsa-miR-152-3p
 
 
-0.322
 
 
28.6
 
 
 
 
FAM168B
 
 
hsa-miR-152-3p
 
 
-0.409
 
 
22.8
 
 
 
 
FAM43A
 
 
hsa-miR-152-3p
 
 
-0.337
 
 
47.6
 
 
 
 
FBXL19
 
 
hsa-miR-152-3p
 
 
-0.362
 
 
10.5
 
 
 
 
FEZ2
 
 
hsa-miR-152-3p
 
 
-0.536
 
 
28.5
 
 
 
 
FMR1
 
 
hsa-miR-152-3p
 
 
-0.385
 
 
16.6
 
 
 
 
FOSB
 
 
hsa-miR-152-3p
 
 
-0.311
 
 
20.6
 
 
 
 
FOXK2
 
 
hsa-miR-152-3p
 
 
-0.305
 
 
11.2
 
 
 
 
FXR1
 
 
hsa-miR-152-3p
 
 
-0.478
 
 
22.0
 
 
 
 
GADD45A
 
 
hsa-miR-152-3p
 
 
-0.787
 
 
29.6
 
 
 
 
GAPVD1
 
 
hsa-miR-152-3p
 
 
-0.309
 
 
11.9
 
 
 
 
GLRX5
 
 
hsa-miR-152-3p
 
 
-0.381
 
 
35.6
 
 
 
 
GMFB
 
 
hsa-miR-152-3p
 
 
-0.429
 
 
17.4
 
 
 
 
GPATCH8
 
 
hsa-miR-152-3p
 
 
-0.374
 
 
14.1
 
 
 
 
GRSF1
 
 
hsa-miR-378a-3p
 
 
-0.358
 
 
31.2
 
 
 
 
GTF2H1
 
 
hsa-miR-152-3p
 
 
-0.524
 
 
16.2
 
 
 
 
HDDC2
 
 
hsa-miR-378a-3p
 
 
-0.322
 
 
20.2
 
 
 
 
HLA-DQB1
 
 
hsa-miR-152-3p
 
 
-0.37
 
 
138.4
 
 
 
 
HSP90B1
 
 
hsa-miR-152-3p
 
 
-0.304
 
 
264.8
 
 
 
 
HSPH1
 
 
hsa-miR-330-3p
 
 
-0.345
 
 
32.5
 
 
 
 
ID2
 
 
hsa-miR-330-3p
 
 
-0.293
 
 
101.2
 
 
 
 
IL15
 
 
hsa-miR-152-3p
 
 
-0.652
 
 
12.8
 
 
 
 
IL6ST
 
 
hsa-miR-152-3p
 
 
-0.291
 
 
57.6
 
 
 
 
ITSN2
 
 
hsa-miR-152-3p
 
 
-0.339
 
 
40.8
 
 
 
 
KLF4
 
 
hsa-miR-152-3p
 
 
-0.325
 
 
22.7
 
 
 
 
LBR
 
 
hsa-miR-152-3p
 
 
-0.39
 
 
39.1
 
 
 
 
LEPROTL1
 
 
hsa-miR-152-3p
 
 
-0.568
 
 
30.4
 
 
 
 
LIPA
 
 
hsa-miR-152-3p
 
 
-0.463
 
 
375.3
 
 
 
 
LMAN1
 
 
hsa-miR-152-3p
 
 
-0.382
 
 
25.8
 
 
 
 
LRCH1
 
 
hsa-miR-152-3p
 
 
-0.321
 
 
13.7
 
 
 
 
LRRC41
 
 
hsa-miR-152-3p
 
 
-0.392
 
 
27.1
 
 
 
 
LSM5
 
 
hsa-miR-330-3p
 
 
-0.351
 
 
39.0
 
 
 
 
LYSMD2
 
 
hsa-miR-152-3p
 
 
-0.554
 
 
20.1
 
 
 
 
MACROH2A1
 
 
hsa-miR-152-3p
 
 
-0.336
 
 
69.2
 
 
 
 
MAF
 
 
hsa-miR-152-3p
 
 
-0.362
 
 
24.5
 
 
 
 
MAF1
 
 
hsa-miR-152-3p
 
 
-0.366
 
 
90.3
 
 
 
 
MAFB
 
 
hsa-miR-152-3p
 
 
-0.45
 
 
90.8
 
 
 
 
MAFG
 
 
hsa-miR-152-3p
 
 
-0.328
 
 
14.7
 
 
 
 
MAP2K1
 
 
hsa-miR-152-3p
 
 
-0.295
 
 
23.7
 
 
 
 
MAPK1
 
 
hsa-miR-378a-3p
 
 
-0.314
 
 
38.2
 
 
 
 
 
 
 stomach 
 
 
 
 
target_symbol
 
 
mature_mirna_id
 
 
score
 
 
nTPM
 
 
 
 
 
 
ABCB7
 
 
hsa-miR-152-3p
 
 
-0.515
 
 
11.6
 
 
 
 
ABCD3
 
 
hsa-miR-152-3p
 
 
-0.388
 
 
41.1
 
 
 
 
ABHD17C
 
 
hsa-miR-330-3p
 
 
-0.399
 
 
34.0
 
 
 
 
ACVR1
 
 
hsa-miR-152-3p
 
 
-0.397
 
 
11.0
 
 
 
 
ADAM10
 
 
hsa-miR-152-3p
 
 
-0.348
 
 
19.0
 
 
 
 
AGFG1
 
 
hsa-miR-152-3p
 
 
-0.332
 
 
31.3
 
 
 
 
AHDC1
 
 
hsa-miR-152-3p
 
 
-0.413
 
 
10.8
 
 
 
 
AKAP1
 
 
hsa-miR-152-3p
 
 
-0.381
 
 
53.0
 
 
 
 
ALCAM
 
 
hsa-miR-152-3p
 
 
-0.347
 
 
31.6
 
 
 
 
AP4B1
 
 
hsa-miR-378a-3p
 
 
-0.62
 
 
10.0
 
 
 
 
ARF4
 
 
hsa-miR-152-3p
 
 
-0.487
 
 
123.5
 
 
 
 
ARFGEF1
 
 
hsa-miR-152-3p
 
 
-0.306
 
 
18.5
 
 
 
 
ARFIP1
 
 
hsa-miR-152-3p
 
 
-0.474
 
 
21.5
 
 
 
 
ARHGAP21
 
 
hsa-miR-152-3p
 
 
-0.287
 
 
25.5
 
 
 
 
ARL6IP1
 
 
hsa-miR-152-3p
 
 
-0.579
 
 
118.7
 
 
 
 
ARL8B
 
 
hsa-miR-152-3p
 
 
-0.337
 
 
32.2
 
 
 
 
ARRDC3
 
 
hsa-miR-152-3p
 
 
-0.305
 
 
24.3
 
 
 
 
ASPH
 
 
hsa-miR-152-3p
 
 
-0.391
 
 
96.9
 
 
 
 
ATP2B4
 
 
hsa-miR-152-3p
 
 
-0.337
 
 
14.5
 
 
 
 
ATP6AP2
 
 
hsa-miR-152-3p
 
 
-0.592
 
 
46.4
 
 
 
 
ATP6AP2
 
 
hsa-miR-152-3p
 
 
-0.668
 
 
46.4
 
 
 
 
B4GALT5
 
 
hsa-miR-152-3p
 
 
-0.44
 
 
24.3
 
 
 
 
BMP2
 
 
hsa-miR-378a-3p
 
 
-0.481
 
 
19.6
 
 
 
 
BTAF1
 
 
hsa-miR-152-3p
 
 
-0.401
 
 
15.2
 
 
 
 
BTBD3
 
 
hsa-miR-152-3p
 
 
-0.495
 
 
25.7
 
 
 
 
C1GALT1
 
 
hsa-miR-152-3p
 
 
-0.487
 
 
21.1
 
 
 
 
C1QBP
 
 
hsa-miR-330-3p
 
 
-0.412
 
 
71.1
 
 
 
 
C1orf210
 
 
hsa-miR-378a-3p
 
 
-0.307
 
 
27.4
 
 
 
 
C1orf35
 
 
hsa-miR-152-3p
 
 
-0.511
 
 
13.6
 
 
 
 
CAND1
 
 
hsa-miR-152-3p
 
 
-0.44
 
 
17.2
 
 
 
 
CANX
 
 
hsa-miR-152-3p
 
 
-0.434
 
 
93.5
 
 
 
 
CAV2
 
 
hsa-miR-152-3p
 
 
-0.32
 
 
19.4
 
 
 
 
CCDC6
 
 
hsa-miR-152-3p
 
 
-0.511
 
 
17.5
 
 
 
 
CCDC71
 
 
hsa-miR-152-3p
 
 
-0.369
 
 
10.0
 
 
 
 
CCN2
 
 
hsa-miR-330-3p
 
 
-0.305
 
 
176.0
 
 
 
 
CCT6A
 
 
hsa-miR-152-3p
 
 
-0.665
 
 
52.5
 
 
 
 
CDC25B
 
 
hsa-miR-152-3p
 
 
-0.298
 
 
29.5
 
 
 
 
CEBPG
 
 
hsa-miR-152-3p
 
 
-0.319
 
 
17.8
 
 
 
 
CEP63
 
 
hsa-miR-152-3p
 
 
-0.329
 
 
10.7
 
 
 
 
CERS5
 
 
hsa-miR-152-3p
 
 
-0.311
 
 
15.9
 
 
 
 
CFL2
 
 
hsa-miR-152-3p
 
 
-0.487
 
 
34.7
 
 
 
 
CHMP4B
 
 
hsa-miR-152-3p
 
 
-0.36
 
 
115.1
 
 
 
 
CNOT7
 
 
hsa-miR-152-3p
 
 
-0.31
 
 
30.1
 
 
 
 
COL2A1
 
 
hsa-miR-152-3p
 
 
-0.385
 
 
11.6
 
 
 
 
COL4A1
 
 
hsa-miR-152-3p
 
 
-0.317
 
 
44.8
 
 
 
 
CS
 
 
hsa-miR-152-3p
 
 
-0.3
 
 
75.9
 
 
 
 
CTSA
 
 
hsa-miR-152-3p
 
 
-0.541
 
 
58.7
 
 
 
 
CYTH3
 
 
hsa-miR-152-3p
 
 
-0.331
 
 
13.3
 
 
 
 
DCUN1D4
 
 
hsa-miR-152-3p
 
 
-0.314
 
 
12.8
 
 
 
 
DDAH1
 
 
hsa-miR-152-3p
 
 
-0.34
 
 
28.1
 
 
 
 
DDX6
 
 
hsa-miR-152-3p
 
 
-0.363
 
 
28.2
 
 
 
 
DEDD
 
 
hsa-miR-152-3p
 
 
-0.401
 
 
21.4
 
 
 
 
DMPK
 
 
hsa-miR-152-3p
 
 
-0.378
 
 
30.2
 
 
 
 
DNAJB4
 
 
hsa-miR-152-3p
 
 
-0.33
 
 
11.9
 
 
 
 
DOCK6
 
 
hsa-miR-152-3p
 
 
-0.396
 
 
15.4
 
 
 
 
DUSP1
 
 
hsa-miR-152-3p
 
 
-0.32
 
 
380.0
 
 
 
 
DYNLL2
 
 
hsa-miR-152-3p
 
 
-0.366
 
 
20.2
 
 
 
 
DYRK1B
 
 
hsa-miR-152-3p
 
 
-0.305
 
 
11.4
 
 
 
 
DYRK2
 
 
hsa-miR-152-3p
 
 
-0.293
 
 
19.4
 
 
 
 
EIF4E3
 
 
hsa-miR-152-3p
 
 
-0.464
 
 
11.9
 
 
 
 
EIF4G3
 
 
hsa-miR-378a-3p
 
 
-0.443
 
 
15.4
 
 
 
 
EOGT
 
 
hsa-miR-152-3p
 
 
-0.587
 
 
14.7
 
 
 
 
EPS15
 
 
hsa-miR-152-3p
 
 
-0.322
 
 
16.0
 
 
 
 
ERBB3
 
 
hsa-miR-152-3p
 
 
-0.399
 
 
55.8
 
 
 
 
ERRFI1
 
 
hsa-miR-152-3p
 
 
-0.529
 
 
118.2
 
 
 
 
ESRRG
 
 
hsa-miR-152-3p
 
 
-0.347
 
 
33.1
 
 
 
 
F3
 
 
hsa-miR-152-3p
 
 
-0.331
 
 
39.9
 
 
 
 
FAM168B
 
 
hsa-miR-152-3p
 
 
-0.409
 
 
30.7
 
 
 
 
FEZ2
 
 
hsa-miR-152-3p
 
 
-0.536
 
 
19.4
 
 
 
 
FMR1
 
 
hsa-miR-152-3p
 
 
-0.385
 
 
11.4
 
 
 
 
FOSB
 
 
hsa-miR-152-3p
 
 
-0.311
 
 
99.1
 
 
 
 
FOXF1
 
 
hsa-miR-152-3p
 
 
-0.374
 
 
24.7
 
 
 
 
FOXK2
 
 
hsa-miR-152-3p
 
 
-0.305
 
 
13.9
 
 
 
 
FXR1
 
 
hsa-miR-152-3p
 
 
-0.478
 
 
30.8
 
 
 
 
GADD45A
 
 
hsa-miR-152-3p
 
 
-0.787
 
 
57.5
 
 
 
 
GLRX5
 
 
hsa-miR-152-3p
 
 
-0.381
 
 
43.4
 
 
 
 
GMFB
 
 
hsa-miR-152-3p
 
 
-0.429
 
 
18.0
 
 
 
 
GPATCH8
 
 
hsa-miR-152-3p
 
 
-0.374
 
 
11.6
 
 
 
 
GRSF1
 
 
hsa-miR-378a-3p
 
 
-0.358
 
 
45.9
 
 
 
 
GTF2H1
 
 
hsa-miR-152-3p
 
 
-0.524
 
 
14.4
 
 
 
 
HDDC2
 
 
hsa-miR-378a-3p
 
 
-0.322
 
 
29.4
 
 
 
 
HLA-DQB1
 
 
hsa-miR-152-3p
 
 
-0.37
 
 
123.5
 
 
 
 
HSP90B1
 
 
hsa-miR-152-3p
 
 
-0.304
 
 
280.9
 
 
 
 
HSPH1
 
 
hsa-miR-330-3p
 
 
-0.345
 
 
48.0
 
 
 
 
ID2
 
 
hsa-miR-330-3p
 
 
-0.293
 
 
63.0
 
 
 
 
IL6ST
 
 
hsa-miR-152-3p
 
 
-0.291
 
 
30.8
 
 
 
 
ITSN2
 
 
hsa-miR-152-3p
 
 
-0.339
 
 
23.3
 
 
 
 
KLF4
 
 
hsa-miR-152-3p
 
 
-0.325
 
 
54.8
 
 
 
 
KLF5
 
 
hsa-miR-152-3p
 
 
-0.475
 
 
77.6
 
 
 
 
LAMA4
 
 
hsa-miR-152-3p
 
 
-0.363
 
 
33.3
 
 
 
 
LBR
 
 
hsa-miR-152-3p
 
 
-0.39
 
 
25.7
 
 
 
 
LDLR
 
 
hsa-miR-152-3p
 
 
-0.323
 
 
34.5
 
 
 
 
LEPROTL1
 
 
hsa-miR-152-3p
 
 
-0.568
 
 
23.8
 
 
 
 
LIPA
 
 
hsa-miR-152-3p
 
 
-0.463
 
 
32.3
 
 
 
 
LMAN1
 
 
hsa-miR-152-3p
 
 
-0.382
 
 
56.0
 
 
 
 
LRCH1
 
 
hsa-miR-152-3p
 
 
-0.321
 
 
12.3
 
 
 
 
LRRC41
 
 
hsa-miR-152-3p
 
 
-0.392
 
 
33.3
 
 
 
 
LSM5
 
 
hsa-miR-330-3p
 
 
-0.351
 
 
43.9
 
 
 
 
LYRM2
 
 
hsa-miR-152-3p
 
 
-0.351
 
 
12.2
 
 
 
 
LYSMD2
 
 
hsa-miR-152-3p
 
 
-0.554
 
 
11.4
 
 
 
 
 
 
 thymus 
 
 
 
 
target_symbol
 
 
mature_mirna_id
 
 
score
 
 
nTPM
 
 
 
 
 
 
ABCB7
 
 
hsa-miR-152-3p
 
 
-0.515
 
 
13.0
 
 
 
 
ABCD3
 
 
hsa-miR-152-3p
 
 
-0.388
 
 
44.2
 
 
 
 
ADAM10
 
 
hsa-miR-152-3p
 
 
-0.348
 
 
38.8
 
 
 
 
AGFG1
 
 
hsa-miR-152-3p
 
 
-0.332
 
 
45.3
 
 
 
 
AKAP1
 
 
hsa-miR-152-3p
 
 
-0.381
 
 
10.6
 
 
 
 
ALCAM
 
 
hsa-miR-152-3p
 
 
-0.347
 
 
13.3
 
 
 
 
AP4B1
 
 
hsa-miR-378a-3p
 
 
-0.62
 
 
15.0
 
 
 
 
ARF4
 
 
hsa-miR-152-3p
 
 
-0.487
 
 
69.9
 
 
 
 
ARFGEF1
 
 
hsa-miR-152-3p
 
 
-0.306
 
 
20.7
 
 
 
 
ARFIP1
 
 
hsa-miR-152-3p
 
 
-0.474
 
 
11.2
 
 
 
 
ARHGAP21
 
 
hsa-miR-152-3p
 
 
-0.287
 
 
10.5
 
 
 
 
ARL6IP1
 
 
hsa-miR-152-3p
 
 
-0.579
 
 
147.6
 
 
 
 
ARL8B
 
 
hsa-miR-152-3p
 
 
-0.337
 
 
33.3
 
 
 
 
ARRDC3
 
 
hsa-miR-152-3p
 
 
-0.305
 
 
26.1
 
 
 
 
ASPH
 
 
hsa-miR-152-3p
 
 
-0.391
 
 
15.5
 
 
 
 
ATP2B4
 
 
hsa-miR-152-3p
 
 
-0.337
 
 
20.2
 
 
 
 
ATP6AP2
 
 
hsa-miR-152-3p
 
 
-0.592
 
 
67.0
 
 
 
 
ATP6AP2
 
 
hsa-miR-152-3p
 
 
-0.668
 
 
67.0
 
 
 
 
BCL2L11
 
 
hsa-miR-152-3p
 
 
-0.445
 
 
18.0
 
 
 
 
BTAF1
 
 
hsa-miR-152-3p
 
 
-0.401
 
 
17.2
 
 
 
 
BTBD3
 
 
hsa-miR-152-3p
 
 
-0.495
 
 
10.0
 
 
 
 
C1GALT1
 
 
hsa-miR-152-3p
 
 
-0.487
 
 
13.3
 
 
 
 
C1QBP
 
 
hsa-miR-330-3p
 
 
-0.412
 
 
91.8
 
 
 
 
CAND1
 
 
hsa-miR-152-3p
 
 
-0.44
 
 
36.1
 
 
 
 
CCDC6
 
 
hsa-miR-152-3p
 
 
-0.511
 
 
11.3
 
 
 
 
CCDC71
 
 
hsa-miR-152-3p
 
 
-0.369
 
 
12.2
 
 
 
 
CCN2
 
 
hsa-miR-330-3p
 
 
-0.305
 
 
46.3
 
 
 
 
CCT6A
 
 
hsa-miR-152-3p
 
 
-0.665
 
 
58.5
 
 
 
 
CDC25B
 
 
hsa-miR-152-3p
 
 
-0.298
 
 
41.6
 
 
 
 
CDK19
 
 
hsa-miR-152-3p
 
 
-0.369
 
 
12.7
 
 
 
 
CDK19
 
 
hsa-miR-152-3p
 
 
-0.405
 
 
12.7
 
 
 
 
CEBPG
 
 
hsa-miR-152-3p
 
 
-0.319
 
 
13.1
 
 
 
 
CEP55
 
 
hsa-miR-152-3p
 
 
-0.305
 
 
25.5
 
 
 
 
CEP63
 
 
hsa-miR-152-3p
 
 
-0.329
 
 
23.8
 
 
 
 
CHMP4B
 
 
hsa-miR-152-3p
 
 
-0.36
 
 
77.1
 
 
 
 
CNOT7
 
 
hsa-miR-152-3p
 
 
-0.31
 
 
74.6
 
 
 
 
COL4A1
 
 
hsa-miR-152-3p
 
 
-0.317
 
 
10.0
 
 
 
 
CS
 
 
hsa-miR-152-3p
 
 
-0.3
 
 
64.7
 
 
 
 
CTSA
 
 
hsa-miR-152-3p
 
 
-0.541
 
 
21.4
 
 
 
 
CUL5
 
 
hsa-miR-152-3p
 
 
-0.389
 
 
11.9
 
 
 
 
DCP2
 
 
hsa-miR-152-3p
 
 
-0.29
 
 
16.7
 
 
 
 
DDX6
 
 
hsa-miR-152-3p
 
 
-0.363
 
 
51.2
 
 
 
 
DEDD
 
 
hsa-miR-152-3p
 
 
-0.401
 
 
21.0
 
 
 
 
DIPK2A
 
 
hsa-miR-152-3p
 
 
-0.393
 
 
15.5
 
 
 
 
DNMT1
 
 
hsa-miR-152-3p
 
 
-0.421
 
 
19.2
 
 
 
 
DUSP1
 
 
hsa-miR-152-3p
 
 
-0.32
 
 
122.9
 
 
 
 
DYNLL2
 
 
hsa-miR-152-3p
 
 
-0.366
 
 
21.0
 
 
 
 
DYRK2
 
 
hsa-miR-152-3p
 
 
-0.293
 
 
22.4
 
 
 
 
EGR3
 
 
hsa-miR-152-3p
 
 
-0.315
 
 
18.2
 
 
 
 
EIF4E3
 
 
hsa-miR-152-3p
 
 
-0.464
 
 
14.7
 
 
 
 
EIF4G3
 
 
hsa-miR-378a-3p
 
 
-0.443
 
 
16.0
 
 
 
 
EOGT
 
 
hsa-miR-152-3p
 
 
-0.587
 
 
10.3
 
 
 
 
EPS15
 
 
hsa-miR-152-3p
 
 
-0.322
 
 
21.0
 
 
 
 
ERRFI1
 
 
hsa-miR-152-3p
 
 
-0.529
 
 
17.3
 
 
 
 
ESCO1
 
 
hsa-miR-152-3p
 
 
-0.34
 
 
10.4
 
 
 
 
FAM168B
 
 
hsa-miR-152-3p
 
 
-0.409
 
 
35.9
 
 
 
 
FBXO28
 
 
hsa-miR-152-3p
 
 
-0.313
 
 
10.2
 
 
 
 
FEZ2
 
 
hsa-miR-152-3p
 
 
-0.536
 
 
17.0
 
 
 
 
FMR1
 
 
hsa-miR-152-3p
 
 
-0.385
 
 
17.6
 
 
 
 
FOSB
 
 
hsa-miR-152-3p
 
 
-0.311
 
 
142.7
 
 
 
 
FOXK2
 
 
hsa-miR-152-3p
 
 
-0.305
 
 
17.2
 
 
 
 
FXR1
 
 
hsa-miR-152-3p
 
 
-0.478
 
 
47.9
 
 
 
 
GADD45A
 
 
hsa-miR-152-3p
 
 
-0.787
 
 
28.6
 
 
 
 
GAPVD1
 
 
hsa-miR-152-3p
 
 
-0.309
 
 
13.1
 
 
 
 
GLRX5
 
 
hsa-miR-152-3p
 
 
-0.381
 
 
37.5
 
 
 
 
GMFB
 
 
hsa-miR-152-3p
 
 
-0.429
 
 
15.1
 
 
 
 
GPATCH8
 
 
hsa-miR-152-3p
 
 
-0.374
 
 
19.4
 
 
 
 
GRSF1
 
 
hsa-miR-378a-3p
 
 
-0.358
 
 
68.9
 
 
 
 
HDDC2
 
 
hsa-miR-378a-3p
 
 
-0.322
 
 
20.7
 
 
 
 
HLA-DQB1
 
 
hsa-miR-152-3p
 
 
-0.37
 
 
103.6
 
 
 
 
HSP90B1
 
 
hsa-miR-152-3p
 
 
-0.304
 
 
212.8
 
 
 
 
HSPH1
 
 
hsa-miR-330-3p
 
 
-0.345
 
 
34.8
 
 
 
 
ID2
 
 
hsa-miR-330-3p
 
 
-0.293
 
 
68.9
 
 
 
 
IL6ST
 
 
hsa-miR-152-3p
 
 
-0.291
 
 
42.2
 
 
 
 
ING2
 
 
hsa-miR-152-3p
 
 
-0.482
 
 
11.7
 
 
 
 
ITSN2
 
 
hsa-miR-152-3p
 
 
-0.339
 
 
27.9
 
 
 
 
KLF4
 
 
hsa-miR-152-3p
 
 
-0.325
 
 
15.9
 
 
 
 
KLF5
 
 
hsa-miR-152-3p
 
 
-0.475
 
 
11.2
 
 
 
 
LBR
 
 
hsa-miR-152-3p
 
 
-0.39
 
 
98.3
 
 
 
 
LEPROTL1
 
 
hsa-miR-152-3p
 
 
-0.568
 
 
61.7
 
 
 
 
LIPA
 
 
hsa-miR-152-3p
 
 
-0.463
 
 
41.4
 
 
 
 
LMAN1
 
 
hsa-miR-152-3p
 
 
-0.382
 
 
29.9
 
 
 
 
LRCH1
 
 
hsa-miR-152-3p
 
 
-0.321
 
 
17.2
 
 
 
 
LRRC41
 
 
hsa-miR-152-3p
 
 
-0.392
 
 
27.9
 
 
 
 
LSM5
 
 
hsa-miR-330-3p
 
 
-0.351
 
 
63.1
 
 
 
 
LYSMD2
 
 
hsa-miR-152-3p
 
 
-0.554
 
 
16.7
 
 
 
 
MACROH2A1
 
 
hsa-miR-152-3p
 
 
-0.336
 
 
187.9
 
 
 
 
MAF
 
 
hsa-miR-152-3p
 
 
-0.362
 
 
16.7
 
 
 
 
MAF1
 
 
hsa-miR-152-3p
 
 
-0.366
 
 
84.6
 
 
 
 
MAP2K1
 
 
hsa-miR-152-3p
 
 
-0.295
 
 
26.5
 
 
 
 
MAPK1
 
 
hsa-miR-378a-3p
 
 
-0.314
 
 
41.4
 
 
 
 
MAT2A
 
 
hsa-miR-330-3p
 
 
-0.307
 
 
95.1
 
 
 
 
MCM3AP
 
 
hsa-miR-152-3p
 
 
-0.287
 
 
20.3
 
 
 
 
MDFIC
 
 
hsa-miR-152-3p
 
 
-0.395
 
 
13.5
 
 
 
 
MECP2
 
 
hsa-miR-152-3p
 
 
-0.3
 
 
14.5
 
 
 
 
MIER1
 
 
hsa-miR-152-3p
 
 
-0.352
 
 
22.7
 
 
 
 
MLLT10
 
 
hsa-miR-152-3p
 
 
-0.334
 
 
21.7
 
 
 
 
MNAT1
 
 
hsa-miR-378a-3p
 
 
-0.398
 
 
24.3
 
 
 
 
MRPS10
 
 
hsa-miR-152-3p
 
 
-0.335
 
 
23.2
 
 
 
 
MTMR1
 
 
hsa-miR-152-3p
 
 
-0.345
 
 
29.2
 
 
 
 
 
 
 tonsil 
 
 
 
 
target_symbol
 
 
mature_mirna_id
 
 
score
 
 
nTPM
 
 
 
 
 
 
ABCB7
 
 
hsa-miR-152-3p
 
 
-0.515
 
 
10.4
 
 
 
 
ABCD3
 
 
hsa-miR-152-3p
 
 
-0.388
 
 
19.9
 
 
 
 
ABHD17C
 
 
hsa-miR-330-3p
 
 
-0.399
 
 
21.6
 
 
 
 
ADAM10
 
 
hsa-miR-152-3p
 
 
-0.348
 
 
15.3
 
 
 
 
ADAM17
 
 
hsa-miR-152-3p
 
 
-0.301
 
 
11.1
 
 
 
 
AGFG1
 
 
hsa-miR-152-3p
 
 
-0.332
 
 
49.9
 
 
 
 
ALCAM
 
 
hsa-miR-152-3p
 
 
-0.347
 
 
13.6
 
 
 
 
AP4B1
 
 
hsa-miR-378a-3p
 
 
-0.62
 
 
13.0
 
 
 
 
ARF4
 
 
hsa-miR-152-3p
 
 
-0.487
 
 
91.2
 
 
 
 
ARFGEF1
 
 
hsa-miR-152-3p
 
 
-0.306
 
 
16.2
 
 
 
 
ARFIP1
 
 
hsa-miR-152-3p
 
 
-0.474
 
 
13.9
 
 
 
 
ARL6IP1
 
 
hsa-miR-152-3p
 
 
-0.579
 
 
106.2
 
 
 
 
ARL8B
 
 
hsa-miR-152-3p
 
 
-0.337
 
 
44.5
 
 
 
 
ARRDC3
 
 
hsa-miR-152-3p
 
 
-0.305
 
 
26.4
 
 
 
 
ASPH
 
 
hsa-miR-152-3p
 
 
-0.391
 
 
26.4
 
 
 
 
ATP2B4
 
 
hsa-miR-152-3p
 
 
-0.337
 
 
18.2
 
 
 
 
ATP6AP2
 
 
hsa-miR-152-3p
 
 
-0.592
 
 
67.9
 
 
 
 
ATP6AP2
 
 
hsa-miR-152-3p
 
 
-0.668
 
 
67.9
 
 
 
 
B4GALT5
 
 
hsa-miR-152-3p
 
 
-0.44
 
 
10.8
 
 
 
 
BCL2L11
 
 
hsa-miR-152-3p
 
 
-0.445
 
 
21.7
 
 
 
 
BHLHE41
 
 
hsa-miR-152-3p
 
 
-0.442
 
 
16.8
 
 
 
 
BTAF1
 
 
hsa-miR-152-3p
 
 
-0.401
 
 
20.0
 
 
 
 
C1GALT1
 
 
hsa-miR-152-3p
 
 
-0.487
 
 
13.2
 
 
 
 
C1QBP
 
 
hsa-miR-330-3p
 
 
-0.412
 
 
126.9
 
 
 
 
CAND1
 
 
hsa-miR-152-3p
 
 
-0.44
 
 
28.9
 
 
 
 
CANX
 
 
hsa-miR-152-3p
 
 
-0.434
 
 
15.5
 
 
 
 
CCDC6
 
 
hsa-miR-152-3p
 
 
-0.511
 
 
20.3
 
 
 
 
CCN2
 
 
hsa-miR-330-3p
 
 
-0.305
 
 
13.9
 
 
 
 
CCT6A
 
 
hsa-miR-152-3p
 
 
-0.665
 
 
77.0
 
 
 
 
CDC25B
 
 
hsa-miR-152-3p
 
 
-0.298
 
 
30.7
 
 
 
 
CDK5R1
 
 
hsa-miR-152-3p
 
 
-0.547
 
 
13.2
 
 
 
 
CEBPG
 
 
hsa-miR-152-3p
 
 
-0.319
 
 
12.9
 
 
 
 
CEP55
 
 
hsa-miR-152-3p
 
 
-0.305
 
 
14.5
 
 
 
 
CEP63
 
 
hsa-miR-152-3p
 
 
-0.329
 
 
15.9
 
 
 
 
CHMP4B
 
 
hsa-miR-152-3p
 
 
-0.36
 
 
66.7
 
 
 
 
CNOT7
 
 
hsa-miR-152-3p
 
 
-0.31
 
 
60.8
 
 
 
 
COL4A1
 
 
hsa-miR-152-3p
 
 
-0.317
 
 
10.4
 
 
 
 
CS
 
 
hsa-miR-152-3p
 
 
-0.3
 
 
99.4
 
 
 
 
CTSA
 
 
hsa-miR-152-3p
 
 
-0.541
 
 
55.0
 
 
 
 
DCP2
 
 
hsa-miR-152-3p
 
 
-0.29
 
 
11.5
 
 
 
 
DDX6
 
 
hsa-miR-152-3p
 
 
-0.363
 
 
57.1
 
 
 
 
DEDD
 
 
hsa-miR-152-3p
 
 
-0.401
 
 
17.2
 
 
 
 
DICER1
 
 
hsa-miR-152-3p
 
 
-0.29
 
 
10.8
 
 
 
 
DIPK2A
 
 
hsa-miR-152-3p
 
 
-0.393
 
 
10.3
 
 
 
 
DNMT1
 
 
hsa-miR-152-3p
 
 
-0.421
 
 
23.3
 
 
 
 
DUSP1
 
 
hsa-miR-152-3p
 
 
-0.32
 
 
52.6
 
 
 
 
DYNLL2
 
 
hsa-miR-152-3p
 
 
-0.366
 
 
17.3
 
 
 
 
DYRK2
 
 
hsa-miR-152-3p
 
 
-0.293
 
 
13.3
 
 
 
 
EIF4E3
 
 
hsa-miR-152-3p
 
 
-0.464
 
 
10.2
 
 
 
 
EIF4G3
 
 
hsa-miR-378a-3p
 
 
-0.443
 
 
18.9
 
 
 
 
EPS15
 
 
hsa-miR-152-3p
 
 
-0.322
 
 
34.9
 
 
 
 
ERBB3
 
 
hsa-miR-152-3p
 
 
-0.399
 
 
10.2
 
 
 
 
ERRFI1
 
 
hsa-miR-152-3p
 
 
-0.529
 
 
11.7
 
 
 
 
EYA3
 
 
hsa-miR-152-3p
 
 
-0.309
 
 
13.5
 
 
 
 
F3
 
 
hsa-miR-152-3p
 
 
-0.331
 
 
14.0
 
 
 
 
FAM168B
 
 
hsa-miR-152-3p
 
 
-0.409
 
 
29.3
 
 
 
 
FEZ2
 
 
hsa-miR-152-3p
 
 
-0.536
 
 
18.3
 
 
 
 
FMR1
 
 
hsa-miR-152-3p
 
 
-0.385
 
 
13.2
 
 
 
 
FOXK2
 
 
hsa-miR-152-3p
 
 
-0.305
 
 
11.1
 
 
 
 
FXR1
 
 
hsa-miR-152-3p
 
 
-0.478
 
 
29.0
 
 
 
 
GADD45A
 
 
hsa-miR-152-3p
 
 
-0.787
 
 
15.7
 
 
 
 
GAPVD1
 
 
hsa-miR-152-3p
 
 
-0.309
 
 
14.1
 
 
 
 
GLRX5
 
 
hsa-miR-152-3p
 
 
-0.381
 
 
25.3
 
 
 
 
GMFB
 
 
hsa-miR-152-3p
 
 
-0.429
 
 
22.9
 
 
 
 
GPATCH8
 
 
hsa-miR-152-3p
 
 
-0.374
 
 
14.4
 
 
 
 
GRSF1
 
 
hsa-miR-378a-3p
 
 
-0.358
 
 
48.8
 
 
 
 
GTF2H1
 
 
hsa-miR-152-3p
 
 
-0.524
 
 
10.4
 
 
 
 
HDDC2
 
 
hsa-miR-378a-3p
 
 
-0.322
 
 
23.2
 
 
 
 
HLA-DQB1
 
 
hsa-miR-152-3p
 
 
-0.37
 
 
117.9
 
 
 
 
HSP90B1
 
 
hsa-miR-152-3p
 
 
-0.304
 
 
307.4
 
 
 
 
HSPH1
 
 
hsa-miR-330-3p
 
 
-0.345
 
 
42.3
 
 
 
 
ID2
 
 
hsa-miR-330-3p
 
 
-0.293
 
 
44.4
 
 
 
 
IL6ST
 
 
hsa-miR-152-3p
 
 
-0.291
 
 
31.9
 
 
 
 
ITSN2
 
 
hsa-miR-152-3p
 
 
-0.339
 
 
47.6
 
 
 
 
KLF4
 
 
hsa-miR-152-3p
 
 
-0.325
 
 
13.6
 
 
 
 
KLF5
 
 
hsa-miR-152-3p
 
 
-0.475
 
 
50.3
 
 
 
 
LBR
 
 
hsa-miR-152-3p
 
 
-0.39
 
 
66.2
 
 
 
 
LEPROTL1
 
 
hsa-miR-152-3p
 
 
-0.568
 
 
33.8
 
 
 
 
LIPA
 
 
hsa-miR-152-3p
 
 
-0.463
 
 
42.7
 
 
 
 
LMAN1
 
 
hsa-miR-152-3p
 
 
-0.382
 
 
32.6
 
 
 
 
LRCH1
 
 
hsa-miR-152-3p
 
 
-0.321
 
 
14.7
 
 
 
 
LRRC41
 
 
hsa-miR-152-3p
 
 
-0.392
 
 
21.2
 
 
 
 
LSM5
 
 
hsa-miR-330-3p
 
 
-0.351
 
 
67.5
 
 
 
 
LYRM2
 
 
hsa-miR-152-3p
 
 
-0.351
 
 
10.2
 
 
 
 
LYSMD2
 
 
hsa-miR-152-3p
 
 
-0.554
 
 
24.1
 
 
 
 
MACROH2A1
 
 
hsa-miR-152-3p
 
 
-0.336
 
 
112.1
 
 
 
 
MAF
 
 
hsa-miR-152-3p
 
 
-0.362
 
 
22.4
 
 
 
 
MAF1
 
 
hsa-miR-152-3p
 
 
-0.366
 
 
65.0
 
 
 
 
MAFB
 
 
hsa-miR-152-3p
 
 
-0.45
 
 
11.9
 
 
 
 
MAP2K1
 
 
hsa-miR-152-3p
 
 
-0.295
 
 
44.3
 
 
 
 
MAPK1
 
 
hsa-miR-378a-3p
 
 
-0.314
 
 
39.3
 
 
 
 
MAT2A
 
 
hsa-miR-330-3p
 
 
-0.307
 
 
119.7
 
 
 
 
MCM3AP
 
 
hsa-miR-152-3p
 
 
-0.287
 
 
20.2
 
 
 
 
MDFIC
 
 
hsa-miR-152-3p
 
 
-0.395
 
 
25.6
 
 
 
 
MED19
 
 
hsa-miR-378a-3p
 
 
-0.434
 
 
10.1
 
 
 
 
METTL4
 
 
hsa-miR-378a-3p
 
 
-0.486
 
 
10.3
 
 
 
 
MIER1
 
 
hsa-miR-152-3p
 
 
-0.352
 
 
18.4
 
 
 
 
MLLT10
 
 
hsa-miR-152-3p
 
 
-0.334
 
 
13.6
 
 
 
 
MMD
 
 
hsa-miR-152-3p
 
 
-0.62
 
 
11.3
 
 
 
 
MNAT1
 
 
hsa-miR-378a-3p
 
 
-0.398
 
 
16.2
 
 
 
 
 
 
 urinary bladder 
 
 
 
 
target_symbol
 
 
mature_mirna_id
 
 
score
 
 
nTPM
 
 
 
 
 
 
ABCB7
 
 
hsa-miR-152-3p
 
 
-0.515
 
 
11.0
 
 
 
 
ABCD3
 
 
hsa-miR-152-3p
 
 
-0.388
 
 
21.4
 
 
 
 
ABHD17C
 
 
hsa-miR-330-3p
 
 
-0.399
 
 
26.8
 
 
 
 
ACVR1
 
 
hsa-miR-152-3p
 
 
-0.397
 
 
13.3
 
 
 
 
ADAM10
 
 
hsa-miR-152-3p
 
 
-0.348
 
 
26.2
 
 
 
 
ADGRF5
 
 
hsa-miR-152-3p
 
 
-0.354
 
 
10.6
 
 
 
 
AGFG1
 
 
hsa-miR-152-3p
 
 
-0.332
 
 
61.2
 
 
 
 
AHDC1
 
 
hsa-miR-152-3p
 
 
-0.413
 
 
14.8
 
 
 
 
AKAP1
 
 
hsa-miR-152-3p
 
 
-0.381
 
 
47.8
 
 
 
 
ALCAM
 
 
hsa-miR-152-3p
 
 
-0.347
 
 
23.9
 
 
 
 
ARF4
 
 
hsa-miR-152-3p
 
 
-0.487
 
 
190.1
 
 
 
 
ARFGEF1
 
 
hsa-miR-152-3p
 
 
-0.306
 
 
18.4
 
 
 
 
ARFIP1
 
 
hsa-miR-152-3p
 
 
-0.474
 
 
22.0
 
 
 
 
ARHGAP21
 
 
hsa-miR-152-3p
 
 
-0.287
 
 
12.9
 
 
 
 
ARL6IP1
 
 
hsa-miR-152-3p
 
 
-0.579
 
 
124.0
 
 
 
 
ARL8B
 
 
hsa-miR-152-3p
 
 
-0.337
 
 
46.5
 
 
 
 
ARRDC3
 
 
hsa-miR-152-3p
 
 
-0.305
 
 
53.2
 
 
 
 
ASPH
 
 
hsa-miR-152-3p
 
 
-0.391
 
 
103.6
 
 
 
 
ATP2B4
 
 
hsa-miR-152-3p
 
 
-0.337
 
 
34.9
 
 
 
 
ATP6AP2
 
 
hsa-miR-152-3p
 
 
-0.592
 
 
91.4
 
 
 
 
ATP6AP2
 
 
hsa-miR-152-3p
 
 
-0.668
 
 
91.4
 
 
 
 
B4GALT5
 
 
hsa-miR-152-3p
 
 
-0.44
 
 
47.9
 
 
 
 
BHLHE41
 
 
hsa-miR-152-3p
 
 
-0.442
 
 
19.9
 
 
 
 
BMP2
 
 
hsa-miR-378a-3p
 
 
-0.481
 
 
16.1
 
 
 
 
BTAF1
 
 
hsa-miR-152-3p
 
 
-0.401
 
 
16.7
 
 
 
 
BTBD3
 
 
hsa-miR-152-3p
 
 
-0.495
 
 
17.2
 
 
 
 
C1GALT1
 
 
hsa-miR-152-3p
 
 
-0.487
 
 
12.1
 
 
 
 
C1QBP
 
 
hsa-miR-330-3p
 
 
-0.412
 
 
101.2
 
 
 
 
C1orf35
 
 
hsa-miR-152-3p
 
 
-0.511
 
 
16.3
 
 
 
 
CAND1
 
 
hsa-miR-152-3p
 
 
-0.44
 
 
21.8
 
 
 
 
CANX
 
 
hsa-miR-152-3p
 
 
-0.434
 
 
92.3
 
 
 
 
CAV2
 
 
hsa-miR-152-3p
 
 
-0.32
 
 
42.0
 
 
 
 
CCDC6
 
 
hsa-miR-152-3p
 
 
-0.511
 
 
16.2
 
 
 
 
CCDC71
 
 
hsa-miR-152-3p
 
 
-0.369
 
 
12.4
 
 
 
 
CCN2
 
 
hsa-miR-330-3p
 
 
-0.305
 
 
302.6
 
 
 
 
CCT6A
 
 
hsa-miR-152-3p
 
 
-0.665
 
 
68.3
 
 
 
 
CDC25B
 
 
hsa-miR-152-3p
 
 
-0.298
 
 
32.7
 
 
 
 
CEBPG
 
 
hsa-miR-152-3p
 
 
-0.319
 
 
15.2
 
 
 
 
CEP63
 
 
hsa-miR-152-3p
 
 
-0.329
 
 
11.7
 
 
 
 
CERS5
 
 
hsa-miR-152-3p
 
 
-0.311
 
 
19.7
 
 
 
 
CFL2
 
 
hsa-miR-152-3p
 
 
-0.487
 
 
56.5
 
 
 
 
CHMP4B
 
 
hsa-miR-152-3p
 
 
-0.36
 
 
135.0
 
 
 
 
CLCN6
 
 
hsa-miR-152-3p
 
 
-0.406
 
 
11.4
 
 
 
 
CNOT7
 
 
hsa-miR-152-3p
 
 
-0.31
 
 
42.1
 
 
 
 
COL4A1
 
 
hsa-miR-152-3p
 
 
-0.317
 
 
112.8
 
 
 
 
CS
 
 
hsa-miR-152-3p
 
 
-0.3
 
 
65.6
 
 
 
 
CTSA
 
 
hsa-miR-152-3p
 
 
-0.541
 
 
77.2
 
 
 
 
CTTNBP2NL
 
 
hsa-miR-152-3p
 
 
-0.329
 
 
14.5
 
 
 
 
CYTH3
 
 
hsa-miR-152-3p
 
 
-0.331
 
 
13.2
 
 
 
 
DACT1
 
 
hsa-miR-378a-3p
 
 
-0.298
 
 
13.0
 
 
 
 
DCUN1D4
 
 
hsa-miR-152-3p
 
 
-0.314
 
 
16.7
 
 
 
 
DDAH1
 
 
hsa-miR-152-3p
 
 
-0.34
 
 
18.0
 
 
 
 
DDX6
 
 
hsa-miR-152-3p
 
 
-0.363
 
 
28.0
 
 
 
 
DEDD
 
 
hsa-miR-152-3p
 
 
-0.401
 
 
26.0
 
 
 
 
DICER1
 
 
hsa-miR-152-3p
 
 
-0.29
 
 
12.2
 
 
 
 
DIPK2A
 
 
hsa-miR-152-3p
 
 
-0.393
 
 
10.8
 
 
 
 
DMPK
 
 
hsa-miR-152-3p
 
 
-0.378
 
 
41.0
 
 
 
 
DNAJB4
 
 
hsa-miR-152-3p
 
 
-0.33
 
 
22.0
 
 
 
 
DOCK6
 
 
hsa-miR-152-3p
 
 
-0.396
 
 
12.6
 
 
 
 
DUSP1
 
 
hsa-miR-152-3p
 
 
-0.32
 
 
725.0
 
 
 
 
DYNLL2
 
 
hsa-miR-152-3p
 
 
-0.366
 
 
20.6
 
 
 
 
DYRK1B
 
 
hsa-miR-152-3p
 
 
-0.305
 
 
14.0
 
 
 
 
DYRK2
 
 
hsa-miR-152-3p
 
 
-0.293
 
 
11.1
 
 
 
 
EGR3
 
 
hsa-miR-152-3p
 
 
-0.315
 
 
13.8
 
 
 
 
EIF4E3
 
 
hsa-miR-152-3p
 
 
-0.464
 
 
14.1
 
 
 
 
EIF4G3
 
 
hsa-miR-378a-3p
 
 
-0.443
 
 
21.4
 
 
 
 
EOGT
 
 
hsa-miR-152-3p
 
 
-0.587
 
 
21.4
 
 
 
 
EPS15
 
 
hsa-miR-152-3p
 
 
-0.322
 
 
19.1
 
 
 
 
ERBB3
 
 
hsa-miR-152-3p
 
 
-0.399
 
 
18.8
 
 
 
 
ERRFI1
 
 
hsa-miR-152-3p
 
 
-0.529
 
 
81.0
 
 
 
 
F3
 
 
hsa-miR-152-3p
 
 
-0.331
 
 
61.0
 
 
 
 
FAM168B
 
 
hsa-miR-152-3p
 
 
-0.409
 
 
42.5
 
 
 
 
FAM43A
 
 
hsa-miR-152-3p
 
 
-0.337
 
 
13.6
 
 
 
 
FBN1
 
 
hsa-miR-152-3p
 
 
-0.349
 
 
15.7
 
 
 
 
FBXO28
 
 
hsa-miR-152-3p
 
 
-0.313
 
 
10.7
 
 
 
 
FEZ2
 
 
hsa-miR-152-3p
 
 
-0.536
 
 
27.2
 
 
 
 
FMR1
 
 
hsa-miR-152-3p
 
 
-0.385
 
 
12.1
 
 
 
 
FOSB
 
 
hsa-miR-152-3p
 
 
-0.311
 
 
199.2
 
 
 
 
FOXF1
 
 
hsa-miR-152-3p
 
 
-0.374
 
 
48.0
 
 
 
 
FOXK2
 
 
hsa-miR-152-3p
 
 
-0.305
 
 
14.5
 
 
 
 
FXR1
 
 
hsa-miR-152-3p
 
 
-0.478
 
 
39.7
 
 
 
 
GADD45A
 
 
hsa-miR-152-3p
 
 
-0.787
 
 
53.1
 
 
 
 
GAPVD1
 
 
hsa-miR-152-3p
 
 
-0.309
 
 
11.4
 
 
 
 
GLRX5
 
 
hsa-miR-152-3p
 
 
-0.381
 
 
37.2
 
 
 
 
GMFB
 
 
hsa-miR-152-3p
 
 
-0.429
 
 
20.8
 
 
 
 
GOLT1A
 
 
hsa-miR-378a-3p
 
 
-0.574
 
 
12.3
 
 
 
 
GPATCH8
 
 
hsa-miR-152-3p
 
 
-0.374
 
 
15.5
 
 
 
 
GRSF1
 
 
hsa-miR-378a-3p
 
 
-0.358
 
 
45.5
 
 
 
 
GTF2H1
 
 
hsa-miR-152-3p
 
 
-0.524
 
 
16.2
 
 
 
 
HDDC2
 
 
hsa-miR-378a-3p
 
 
-0.322
 
 
25.1
 
 
 
 
HLA-DQB1
 
 
hsa-miR-152-3p
 
 
-0.37
 
 
86.5
 
 
 
 
HSP90B1
 
 
hsa-miR-152-3p
 
 
-0.304
 
 
333.2
 
 
 
 
HSPH1
 
 
hsa-miR-330-3p
 
 
-0.345
 
 
54.9
 
 
 
 
ID2
 
 
hsa-miR-330-3p
 
 
-0.293
 
 
121.9
 
 
 
 
IGF1
 
 
hsa-miR-152-3p
 
 
-0.43
 
 
19.9
 
 
 
 
IL6ST
 
 
hsa-miR-152-3p
 
 
-0.291
 
 
68.0
 
 
 
 
ITSN2
 
 
hsa-miR-152-3p
 
 
-0.339
 
 
20.8
 
 
 
 
KDSR
 
 
hsa-miR-378a-3p
 
 
-0.373
 
 
12.0
 
 
 
 
KLF4
 
 
hsa-miR-152-3p
 
 
-0.325
 
 
40.9
 
 
 
 
KLF5
 
 
hsa-miR-152-3p
 
 
-0.475
 
 
104.1
 
 
 
 
 
 
 vagina 
 
 
 
 
target_symbol
 
 
mature_mirna_id
 
 
score
 
 
nTPM
 
 
 
 
 
 
ABCB7
 
 
hsa-miR-152-3p
 
 
-0.515
 
 
10.8
 
 
 
 
ABCD3
 
 
hsa-miR-152-3p
 
 
-0.388
 
 
14.9
 
 
 
 
ABHD17C
 
 
hsa-miR-330-3p
 
 
-0.399
 
 
21.1
 
 
 
 
ADAM10
 
 
hsa-miR-152-3p
 
 
-0.348
 
 
10.3
 
 
 
 
AGFG1
 
 
hsa-miR-152-3p
 
 
-0.332
 
 
31.5
 
 
 
 
AHDC1
 
 
hsa-miR-152-3p
 
 
-0.413
 
 
23.0
 
 
 
 
AKAP1
 
 
hsa-miR-152-3p
 
 
-0.381
 
 
24.4
 
 
 
 
ARF4
 
 
hsa-miR-152-3p
 
 
-0.487
 
 
85.1
 
 
 
 
ARFIP1
 
 
hsa-miR-152-3p
 
 
-0.474
 
 
13.1
 
 
 
 
ARL6IP1
 
 
hsa-miR-152-3p
 
 
-0.579
 
 
48.0
 
 
 
 
ARL8B
 
 
hsa-miR-152-3p
 
 
-0.337
 
 
51.8
 
 
 
 
ARRDC3
 
 
hsa-miR-152-3p
 
 
-0.305
 
 
40.5
 
 
 
 
ASPH
 
 
hsa-miR-152-3p
 
 
-0.391
 
 
40.4
 
 
 
 
ATP2B4
 
 
hsa-miR-152-3p
 
 
-0.337
 
 
14.3
 
 
 
 
ATP6AP2
 
 
hsa-miR-152-3p
 
 
-0.592
 
 
44.8
 
 
 
 
ATP6AP2
 
 
hsa-miR-152-3p
 
 
-0.668
 
 
44.8
 
 
 
 
B4GALT5
 
 
hsa-miR-152-3p
 
 
-0.44
 
 
16.5
 
 
 
 
BTAF1
 
 
hsa-miR-152-3p
 
 
-0.401
 
 
16.3
 
 
 
 
C1QBP
 
 
hsa-miR-330-3p
 
 
-0.412
 
 
71.3
 
 
 
 
C1orf35
 
 
hsa-miR-152-3p
 
 
-0.511
 
 
16.5
 
 
 
 
CAND1
 
 
hsa-miR-152-3p
 
 
-0.44
 
 
18.5
 
 
 
 
CANX
 
 
hsa-miR-152-3p
 
 
-0.434
 
 
85.8
 
 
 
 
CAV2
 
 
hsa-miR-152-3p
 
 
-0.32
 
 
36.5
 
 
 
 
CCDC6
 
 
hsa-miR-152-3p
 
 
-0.511
 
 
22.5
 
 
 
 
CCDC71
 
 
hsa-miR-152-3p
 
 
-0.369
 
 
11.2
 
 
 
 
CCN2
 
 
hsa-miR-330-3p
 
 
-0.305
 
 
99.4
 
 
 
 
CCT6A
 
 
hsa-miR-152-3p
 
 
-0.665
 
 
48.6
 
 
 
 
CDC25B
 
 
hsa-miR-152-3p
 
 
-0.298
 
 
30.0
 
 
 
 
CEBPG
 
 
hsa-miR-152-3p
 
 
-0.319
 
 
10.6
 
 
 
 
CERS5
 
 
hsa-miR-152-3p
 
 
-0.311
 
 
21.6
 
 
 
 
CFL2
 
 
hsa-miR-152-3p
 
 
-0.487
 
 
23.5
 
 
 
 
CHMP4B
 
 
hsa-miR-152-3p
 
 
-0.36
 
 
105.4
 
 
 
 
CLCN6
 
 
hsa-miR-152-3p
 
 
-0.406
 
 
10.6
 
 
 
 
CNOT7
 
 
hsa-miR-152-3p
 
 
-0.31
 
 
19.2
 
 
 
 
COL4A1
 
 
hsa-miR-152-3p
 
 
-0.317
 
 
34.4
 
 
 
 
CS
 
 
hsa-miR-152-3p
 
 
-0.3
 
 
65.6
 
 
 
 
CTSA
 
 
hsa-miR-152-3p
 
 
-0.541
 
 
42.8
 
 
 
 
CTTNBP2NL
 
 
hsa-miR-152-3p
 
 
-0.329
 
 
11.2
 
 
 
 
CYTH3
 
 
hsa-miR-152-3p
 
 
-0.331
 
 
15.2
 
 
 
 
DCUN1D4
 
 
hsa-miR-152-3p
 
 
-0.314
 
 
10.6
 
 
 
 
DDX6
 
 
hsa-miR-152-3p
 
 
-0.363
 
 
21.1
 
 
 
 
DEDD
 
 
hsa-miR-152-3p
 
 
-0.401
 
 
24.0
 
 
 
 
DMPK
 
 
hsa-miR-152-3p
 
 
-0.378
 
 
16.3
 
 
 
 
DNAJB4
 
 
hsa-miR-152-3p
 
 
-0.33
 
 
12.9
 
 
 
 
DOCK6
 
 
hsa-miR-152-3p
 
 
-0.396
 
 
16.1
 
 
 
 
DUSP1
 
 
hsa-miR-152-3p
 
 
-0.32
 
 
560.5
 
 
 
 
DYNLL2
 
 
hsa-miR-152-3p
 
 
-0.366
 
 
16.6
 
 
 
 
EIF4G3
 
 
hsa-miR-378a-3p
 
 
-0.443
 
 
10.0
 
 
 
 
EOGT
 
 
hsa-miR-152-3p
 
 
-0.587
 
 
12.6
 
 
 
 
EPS15
 
 
hsa-miR-152-3p
 
 
-0.322
 
 
19.9
 
 
 
 
ERBB3
 
 
hsa-miR-152-3p
 
 
-0.399
 
 
35.2
 
 
 
 
ERRFI1
 
 
hsa-miR-152-3p
 
 
-0.529
 
 
71.5
 
 
 
 
ESR1
 
 
hsa-miR-152-3p
 
 
-0.34
 
 
45.7
 
 
 
 
F3
 
 
hsa-miR-152-3p
 
 
-0.331
 
 
67.0
 
 
 
 
FAM168B
 
 
hsa-miR-152-3p
 
 
-0.409
 
 
23.7
 
 
 
 
FAM43A
 
 
hsa-miR-152-3p
 
 
-0.337
 
 
23.1
 
 
 
 
FBXL19
 
 
hsa-miR-152-3p
 
 
-0.362
 
 
10.3
 
 
 
 
FEZ2
 
 
hsa-miR-152-3p
 
 
-0.536
 
 
21.5
 
 
 
 
FOXF1
 
 
hsa-miR-152-3p
 
 
-0.374
 
 
11.8
 
 
 
 
FOXK2
 
 
hsa-miR-152-3p
 
 
-0.305
 
 
12.7
 
 
 
 
FXR1
 
 
hsa-miR-152-3p
 
 
-0.478
 
 
27.9
 
 
 
 
GADD45A
 
 
hsa-miR-152-3p
 
 
-0.787
 
 
49.6
 
 
 
 
GLRX5
 
 
hsa-miR-152-3p
 
 
-0.381
 
 
27.8
 
 
 
 
GMFB
 
 
hsa-miR-152-3p
 
 
-0.429
 
 
11.0
 
 
 
 
GPATCH8
 
 
hsa-miR-152-3p
 
 
-0.374
 
 
15.6
 
 
 
 
GRSF1
 
 
hsa-miR-378a-3p
 
 
-0.358
 
 
22.4
 
 
 
 
GTF2H1
 
 
hsa-miR-152-3p
 
 
-0.524
 
 
16.3
 
 
 
 
HDDC2
 
 
hsa-miR-378a-3p
 
 
-0.322
 
 
26.7
 
 
 
 
HLA-DQB1
 
 
hsa-miR-152-3p
 
 
-0.37
 
 
16.6
 
 
 
 
HSP90B1
 
 
hsa-miR-152-3p
 
 
-0.304
 
 
137.1
 
 
 
 
HSPH1
 
 
hsa-miR-330-3p
 
 
-0.345
 
 
39.4
 
 
 
 
ID2
 
 
hsa-miR-330-3p
 
 
-0.293
 
 
76.2
 
 
 
 
IGF1
 
 
hsa-miR-152-3p
 
 
-0.43
 
 
23.4
 
 
 
 
IL6ST
 
 
hsa-miR-152-3p
 
 
-0.291
 
 
25.7
 
 
 
 
ITSN2
 
 
hsa-miR-152-3p
 
 
-0.339
 
 
23.3
 
 
 
 
KDSR
 
 
hsa-miR-378a-3p
 
 
-0.373
 
 
10.4
 
 
 
 
KLF4
 
 
hsa-miR-152-3p
 
 
-0.325
 
 
40.6
 
 
 
 
KLF5
 
 
hsa-miR-152-3p
 
 
-0.475
 
 
120.5
 
 
 
 
LAMA4
 
 
hsa-miR-152-3p
 
 
-0.363
 
 
40.4
 
 
 
 
LBR
 
 
hsa-miR-152-3p
 
 
-0.39
 
 
21.1
 
 
 
 
LDLR
 
 
hsa-miR-152-3p
 
 
-0.323
 
 
24.4
 
 
 
 
LEPROTL1
 
 
hsa-miR-152-3p
 
 
-0.568
 
 
15.5
 
 
 
 
LIPA
 
 
hsa-miR-152-3p
 
 
-0.463
 
 
19.6
 
 
 
 
LMAN1
 
 
hsa-miR-152-3p
 
 
-0.382
 
 
23.5
 
 
 
 
LRRC41
 
 
hsa-miR-152-3p
 
 
-0.392
 
 
37.5
 
 
 
 
LSM5
 
 
hsa-miR-330-3p
 
 
-0.351
 
 
32.7
 
 
 
 
MACROH2A1
 
 
hsa-miR-152-3p
 
 
-0.336
 
 
73.1
 
 
 
 
MAF
 
 
hsa-miR-152-3p
 
 
-0.362
 
 
29.1
 
 
 
 
MAF1
 
 
hsa-miR-152-3p
 
 
-0.366
 
 
79.2
 
 
 
 
MAFB
 
 
hsa-miR-152-3p
 
 
-0.45
 
 
20.6
 
 
 
 
MAFG
 
 
hsa-miR-152-3p
 
 
-0.328
 
 
10.9
 
 
 
 
MAP2K1
 
 
hsa-miR-152-3p
 
 
-0.295
 
 
17.6
 
 
 
 
MAPK1
 
 
hsa-miR-378a-3p
 
 
-0.314
 
 
25.0
 
 
 
 
MAT2A
 
 
hsa-miR-330-3p
 
 
-0.307
 
 
161.5
 
 
 
 
MECP2
 
 
hsa-miR-152-3p
 
 
-0.3
 
 
16.5
 
 
 
 
MED19
 
 
hsa-miR-378a-3p
 
 
-0.434
 
 
12.3
 
 
 
 
MIER1
 
 
hsa-miR-152-3p
 
 
-0.352
 
 
10.9
 
 
 
 
MITF
 
 
hsa-miR-152-3p
 
 
-0.313
 
 
15.9
 
 
 
 
MLLT10
 
 
hsa-miR-152-3p
 
 
-0.334
 
 
17.5
 
 
 
 
MNAT1
 
 
hsa-miR-378a-3p
 
 
-0.398
 
 
15.8
 
 
 
 
 
 
 amygdala 
 
 
 
 
target_symbol
 
 
mature_mirna_id
 
 
score
 
 
nTPM
 
 
 
 
 
 
ABCD3
 
 
hsa-miR-152-3p
 
 
-0.388
 
 
16.0
 
 
 
 
ABHD17C
 
 
hsa-miR-330-3p
 
 
-0.399
 
 
17.9
 
 
 
 
ADCY2
 
 
hsa-miR-152-3p
 
 
-0.299
 
 
28.0
 
 
 
 
ADGRB3
 
 
hsa-miR-152-3p
 
 
-0.516
 
 
12.8
 
 
 
 
AGFG1
 
 
hsa-miR-152-3p
 
 
-0.332
 
 
25.1
 
 
 
 
AHDC1
 
 
hsa-miR-152-3p
 
 
-0.413
 
 
10.1
 
 
 
 
AKAP1
 
 
hsa-miR-152-3p
 
 
-0.381
 
 
22.8
 
 
 
 
ALCAM
 
 
hsa-miR-152-3p
 
 
-0.347
 
 
31.7
 
 
 
 
ARF4
 
 
hsa-miR-152-3p
 
 
-0.487
 
 
60.1
 
 
 
 
ARHGAP21
 
 
hsa-miR-152-3p
 
 
-0.287
 
 
40.6
 
 
 
 
ARL6IP1
 
 
hsa-miR-152-3p
 
 
-0.579
 
 
241.3
 
 
 
 
ARL8B
 
 
hsa-miR-152-3p
 
 
-0.337
 
 
53.8
 
 
 
 
ASPH
 
 
hsa-miR-152-3p
 
 
-0.391
 
 
62.6
 
 
 
 
ATP6AP2
 
 
hsa-miR-152-3p
 
 
-0.592
 
 
69.8
 
 
 
 
ATP6AP2
 
 
hsa-miR-152-3p
 
 
-0.668
 
 
69.8
 
 
 
 
B4GALT5
 
 
hsa-miR-152-3p
 
 
-0.44
 
 
10.8
 
 
 
 
BHLHE41
 
 
hsa-miR-152-3p
 
 
-0.442
 
 
27.2
 
 
 
 
BTBD3
 
 
hsa-miR-152-3p
 
 
-0.495
 
 
27.3
 
 
 
 
C1QBP
 
 
hsa-miR-330-3p
 
 
-0.412
 
 
83.1
 
 
 
 
C1orf35
 
 
hsa-miR-152-3p
 
 
-0.511
 
 
14.3
 
 
 
 
CAND1
 
 
hsa-miR-152-3p
 
 
-0.44
 
 
16.4
 
 
 
 
CANX
 
 
hsa-miR-152-3p
 
 
-0.434
 
 
108.0
 
 
 
 
CCDC6
 
 
hsa-miR-152-3p
 
 
-0.511
 
 
16.2
 
 
 
 
CCN2
 
 
hsa-miR-330-3p
 
 
-0.305
 
 
13.4
 
 
 
 
CCT6A
 
 
hsa-miR-152-3p
 
 
-0.665
 
 
42.2
 
 
 
 
CDC25B
 
 
hsa-miR-152-3p
 
 
-0.298
 
 
21.0
 
 
 
 
CDH20
 
 
hsa-miR-152-3p
 
 
-0.297
 
 
17.1
 
 
 
 
CDK19
 
 
hsa-miR-152-3p
 
 
-0.369
 
 
18.6
 
 
 
 
CDK19
 
 
hsa-miR-152-3p
 
 
-0.405
 
 
18.6
 
 
 
 
CDK5R1
 
 
hsa-miR-152-3p
 
 
-0.547
 
 
40.0
 
 
 
 
CERS5
 
 
hsa-miR-152-3p
 
 
-0.311
 
 
19.7
 
 
 
 
CFL2
 
 
hsa-miR-152-3p
 
 
-0.487
 
 
59.1
 
 
 
 
CHMP4B
 
 
hsa-miR-152-3p
 
 
-0.36
 
 
139.8
 
 
 
 
CHST1
 
 
hsa-miR-152-3p
 
 
-0.408
 
 
32.2
 
 
 
 
CLCN6
 
 
hsa-miR-152-3p
 
 
-0.406
 
 
16.5
 
 
 
 
CNOT7
 
 
hsa-miR-152-3p
 
 
-0.31
 
 
19.4
 
 
 
 
CS
 
 
hsa-miR-152-3p
 
 
-0.3
 
 
35.9
 
 
 
 
CTSA
 
 
hsa-miR-152-3p
 
 
-0.541
 
 
29.0
 
 
 
 
CYTH3
 
 
hsa-miR-152-3p
 
 
-0.331
 
 
14.1
 
 
 
 
DCUN1D4
 
 
hsa-miR-152-3p
 
 
-0.314
 
 
12.5
 
 
 
 
DDAH1
 
 
hsa-miR-152-3p
 
 
-0.34
 
 
95.2
 
 
 
 
DDX6
 
 
hsa-miR-152-3p
 
 
-0.363
 
 
20.7
 
 
 
 
DEDD
 
 
hsa-miR-152-3p
 
 
-0.401
 
 
16.0
 
 
 
 
DMPK
 
 
hsa-miR-152-3p
 
 
-0.378
 
 
16.2
 
 
 
 
DNAJB4
 
 
hsa-miR-152-3p
 
 
-0.33
 
 
13.9
 
 
 
 
DUSP1
 
 
hsa-miR-152-3p
 
 
-0.32
 
 
67.2
 
 
 
 
DYNLL2
 
 
hsa-miR-152-3p
 
 
-0.366
 
 
66.8
 
 
 
 
DYRK1B
 
 
hsa-miR-152-3p
 
 
-0.305
 
 
18.4
 
 
 
 
EGR3
 
 
hsa-miR-152-3p
 
 
-0.315
 
 
10.2
 
 
 
 
EIF4E3
 
 
hsa-miR-152-3p
 
 
-0.464
 
 
15.5
 
 
 
 
EIF4G3
 
 
hsa-miR-378a-3p
 
 
-0.443
 
 
13.3
 
 
 
 
EMX2
 
 
hsa-miR-152-3p
 
 
-0.366
 
 
24.2
 
 
 
 
EPS15
 
 
hsa-miR-152-3p
 
 
-0.322
 
 
38.4
 
 
 
 
ERBB3
 
 
hsa-miR-152-3p
 
 
-0.399
 
 
22.1
 
 
 
 
ERRFI1
 
 
hsa-miR-152-3p
 
 
-0.529
 
 
12.7
 
 
 
 
F3
 
 
hsa-miR-152-3p
 
 
-0.331
 
 
80.6
 
 
 
 
FAM168B
 
 
hsa-miR-152-3p
 
 
-0.409
 
 
49.6
 
 
 
 
FAM184A
 
 
hsa-miR-152-3p
 
 
-0.371
 
 
17.6
 
 
 
 
FAM81A
 
 
hsa-miR-152-3p
 
 
-0.315
 
 
10.7
 
 
 
 
FBXL19
 
 
hsa-miR-152-3p
 
 
-0.362
 
 
25.1
 
 
 
 
FEZ2
 
 
hsa-miR-152-3p
 
 
-0.536
 
 
45.6
 
 
 
 
FOXK2
 
 
hsa-miR-152-3p
 
 
-0.305
 
 
12.3
 
 
 
 
FXR1
 
 
hsa-miR-152-3p
 
 
-0.478
 
 
33.9
 
 
 
 
GADD45A
 
 
hsa-miR-152-3p
 
 
-0.787
 
 
41.1
 
 
 
 
GAP43
 
 
hsa-miR-152-3p
 
 
-0.409
 
 
275.0
 
 
 
 
GLRX5
 
 
hsa-miR-152-3p
 
 
-0.381
 
 
67.3
 
 
 
 
GMFB
 
 
hsa-miR-152-3p
 
 
-0.429
 
 
57.6
 
 
 
 
GPATCH8
 
 
hsa-miR-152-3p
 
 
-0.374
 
 
10.5
 
 
 
 
GRSF1
 
 
hsa-miR-378a-3p
 
 
-0.358
 
 
28.0
 
 
 
 
GTF2H1
 
 
hsa-miR-152-3p
 
 
-0.524
 
 
12.4
 
 
 
 
HDDC2
 
 
hsa-miR-378a-3p
 
 
-0.322
 
 
110.6
 
 
 
 
HSP90B1
 
 
hsa-miR-152-3p
 
 
-0.304
 
 
99.4
 
 
 
 
HSPH1
 
 
hsa-miR-330-3p
 
 
-0.345
 
 
101.0
 
 
 
 
ID2
 
 
hsa-miR-330-3p
 
 
-0.293
 
 
97.8
 
 
 
 
IL6ST
 
 
hsa-miR-152-3p
 
 
-0.291
 
 
26.4
 
 
 
 
JPH3
 
 
hsa-miR-152-3p
 
 
-0.303
 
 
87.8
 
 
 
 
KDSR
 
 
hsa-miR-378a-3p
 
 
-0.373
 
 
10.4
 
 
 
 
LAMA4
 
 
hsa-miR-152-3p
 
 
-0.363
 
 
10.9
 
 
 
 
LEPROTL1
 
 
hsa-miR-152-3p
 
 
-0.568
 
 
15.3
 
 
 
 
LIPA
 
 
hsa-miR-152-3p
 
 
-0.463
 
 
31.0
 
 
 
 
LMAN1
 
 
hsa-miR-152-3p
 
 
-0.382
 
 
10.5
 
 
 
 
LRRC41
 
 
hsa-miR-152-3p
 
 
-0.392
 
 
23.1
 
 
 
 
LSM5
 
 
hsa-miR-330-3p
 
 
-0.351
 
 
32.8
 
 
 
 
LYRM2
 
 
hsa-miR-152-3p
 
 
-0.351
 
 
13.9
 
 
 
 
LYSMD2
 
 
hsa-miR-152-3p
 
 
-0.554
 
 
30.5
 
 
 
 
MACIR
 
 
hsa-miR-152-3p
 
 
-0.332
 
 
13.9
 
 
 
 
MACIR
 
 
hsa-miR-152-3p
 
 
-0.573
 
 
13.9
 
 
 
 
MACROH2A1
 
 
hsa-miR-152-3p
 
 
-0.336
 
 
46.9
 
 
 
 
MAF1
 
 
hsa-miR-152-3p
 
 
-0.366
 
 
58.1
 
 
 
 
MAFG
 
 
hsa-miR-152-3p
 
 
-0.328
 
 
18.1
 
 
 
 
MAP2K1
 
 
hsa-miR-152-3p
 
 
-0.295
 
 
20.1
 
 
 
 
MAPK1
 
 
hsa-miR-378a-3p
 
 
-0.314
 
 
61.9
 
 
 
 
MAT2A
 
 
hsa-miR-330-3p
 
 
-0.307
 
 
116.0
 
 
 
 
MECP2
 
 
hsa-miR-152-3p
 
 
-0.3
 
 
10.2
 
 
 
 
MED19
 
 
hsa-miR-378a-3p
 
 
-0.434
 
 
15.6
 
 
 
 
MLLT10
 
 
hsa-miR-152-3p
 
 
-0.334
 
 
11.1
 
 
 
 
MMD
 
 
hsa-miR-152-3p
 
 
-0.62
 
 
85.3
 
 
 
 
MNAT1
 
 
hsa-miR-378a-3p
 
 
-0.398
 
 
19.5
 
 
 
 
MRAS
 
 
hsa-miR-152-3p
 
 
-0.583
 
 
78.4
 
 
 
 
MRPS10
 
 
hsa-miR-152-3p
 
 
-0.335
 
 
17.1
 
 
 
 
 
 
 appendix 
 
 
 
 
target_symbol
 
 
mature_mirna_id
 
 
score
 
 
nTPM
 
 
 
 
 
 
ABCD3
 
 
hsa-miR-152-3p
 
 
-0.388
 
 
15.3
 
 
 
 
ADAM10
 
 
hsa-miR-152-3p
 
 
-0.348
 
 
17.9
 
 
 
 
AGFG1
 
 
hsa-miR-152-3p
 
 
-0.332
 
 
45.7
 
 
 
 
AKAP1
 
 
hsa-miR-152-3p
 
 
-0.381
 
 
16.8
 
 
 
 
ALCAM
 
 
hsa-miR-152-3p
 
 
-0.347
 
 
11.5
 
 
 
 
AP4B1
 
 
hsa-miR-378a-3p
 
 
-0.62
 
 
10.4
 
 
 
 
ARF4
 
 
hsa-miR-152-3p
 
 
-0.487
 
 
101.3
 
 
 
 
ARFGEF1
 
 
hsa-miR-152-3p
 
 
-0.306
 
 
13.8
 
 
 
 
ARFIP1
 
 
hsa-miR-152-3p
 
 
-0.474
 
 
14.9
 
 
 
 
ARL6IP1
 
 
hsa-miR-152-3p
 
 
-0.579
 
 
73.3
 
 
 
 
ARL8B
 
 
hsa-miR-152-3p
 
 
-0.337
 
 
34.1
 
 
 
 
ARRDC3
 
 
hsa-miR-152-3p
 
 
-0.305
 
 
25.3
 
 
 
 
ASPH
 
 
hsa-miR-152-3p
 
 
-0.391
 
 
47.6
 
 
 
 
ATP2B4
 
 
hsa-miR-152-3p
 
 
-0.337
 
 
24.2
 
 
 
 
ATP6AP2
 
 
hsa-miR-152-3p
 
 
-0.592
 
 
57.3
 
 
 
 
ATP6AP2
 
 
hsa-miR-152-3p
 
 
-0.668
 
 
57.3
 
 
 
 
B4GALT5
 
 
hsa-miR-152-3p
 
 
-0.44
 
 
20.5
 
 
 
 
BCL2L11
 
 
hsa-miR-152-3p
 
 
-0.445
 
 
16.0
 
 
 
 
BTAF1
 
 
hsa-miR-152-3p
 
 
-0.401
 
 
16.0
 
 
 
 
C1GALT1
 
 
hsa-miR-152-3p
 
 
-0.487
 
 
13.4
 
 
 
 
C1QBP
 
 
hsa-miR-330-3p
 
 
-0.412
 
 
79.2
 
 
 
 
CAND1
 
 
hsa-miR-152-3p
 
 
-0.44
 
 
18.7
 
 
 
 
CANX
 
 
hsa-miR-152-3p
 
 
-0.434
 
 
34.2
 
 
 
 
CAV2
 
 
hsa-miR-152-3p
 
 
-0.32
 
 
17.7
 
 
 
 
CCDC6
 
 
hsa-miR-152-3p
 
 
-0.511
 
 
13.1
 
 
 
 
CCN2
 
 
hsa-miR-330-3p
 
 
-0.305
 
 
158.6
 
 
 
 
CCT6A
 
 
hsa-miR-152-3p
 
 
-0.665
 
 
63.9
 
 
 
 
CDC25B
 
 
hsa-miR-152-3p
 
 
-0.298
 
 
27.2
 
 
 
 
CEBPG
 
 
hsa-miR-152-3p
 
 
-0.319
 
 
13.4
 
 
 
 
CEP63
 
 
hsa-miR-152-3p
 
 
-0.329
 
 
11.8
 
 
 
 
CFL2
 
 
hsa-miR-152-3p
 
 
-0.487
 
 
27.0
 
 
 
 
CHMP4B
 
 
hsa-miR-152-3p
 
 
-0.36
 
 
71.5
 
 
 
 
CNOT7
 
 
hsa-miR-152-3p
 
 
-0.31
 
 
37.5
 
 
 
 
COL4A1
 
 
hsa-miR-152-3p
 
 
-0.317
 
 
128.3
 
 
 
 
CS
 
 
hsa-miR-152-3p
 
 
-0.3
 
 
58.2
 
 
 
 
CTSA
 
 
hsa-miR-152-3p
 
 
-0.541
 
 
72.7
 
 
 
 
CYB5R4
 
 
hsa-miR-152-3p
 
 
-0.454
 
 
11.4
 
 
 
 
CYB5R4
 
 
hsa-miR-152-3p
 
 
-0.454
 
 
11.4
 
 
 
 
DDAH1
 
 
hsa-miR-152-3p
 
 
-0.34
 
 
12.0
 
 
 
 
DDX6
 
 
hsa-miR-152-3p
 
 
-0.363
 
 
33.0
 
 
 
 
DEDD
 
 
hsa-miR-152-3p
 
 
-0.401
 
 
18.3
 
 
 
 
DICER1
 
 
hsa-miR-152-3p
 
 
-0.29
 
 
12.1
 
 
 
 
DMPK
 
 
hsa-miR-152-3p
 
 
-0.378
 
 
13.5
 
 
 
 
DNAJB4
 
 
hsa-miR-152-3p
 
 
-0.33
 
 
10.5
 
 
 
 
DNMT1
 
 
hsa-miR-152-3p
 
 
-0.421
 
 
15.8
 
 
 
 
DUSP1
 
 
hsa-miR-152-3p
 
 
-0.32
 
 
344.5
 
 
 
 
DYNLL2
 
 
hsa-miR-152-3p
 
 
-0.366
 
 
16.7
 
 
 
 
DYRK2
 
 
hsa-miR-152-3p
 
 
-0.293
 
 
13.4
 
 
 
 
EGR3
 
 
hsa-miR-152-3p
 
 
-0.315
 
 
17.9
 
 
 
 
EIF4E3
 
 
hsa-miR-152-3p
 
 
-0.464
 
 
12.0
 
 
 
 
EIF4G3
 
 
hsa-miR-378a-3p
 
 
-0.443
 
 
18.7
 
 
 
 
EOGT
 
 
hsa-miR-152-3p
 
 
-0.587
 
 
12.1
 
 
 
 
EPS15
 
 
hsa-miR-152-3p
 
 
-0.322
 
 
20.9
 
 
 
 
ERRFI1
 
 
hsa-miR-152-3p
 
 
-0.529
 
 
29.3
 
 
 
 
F3
 
 
hsa-miR-152-3p
 
 
-0.331
 
 
27.1
 
 
 
 
FAM168B
 
 
hsa-miR-152-3p
 
 
-0.409
 
 
25.3
 
 
 
 
FBN1
 
 
hsa-miR-152-3p
 
 
-0.349
 
 
11.2
 
 
 
 
FEZ2
 
 
hsa-miR-152-3p
 
 
-0.536
 
 
19.8
 
 
 
 
FMR1
 
 
hsa-miR-152-3p
 
 
-0.385
 
 
11.4
 
 
 
 
FOSB
 
 
hsa-miR-152-3p
 
 
-0.311
 
 
111.8
 
 
 
 
FOXK2
 
 
hsa-miR-152-3p
 
 
-0.305
 
 
11.9
 
 
 
 
FXR1
 
 
hsa-miR-152-3p
 
 
-0.478
 
 
21.2
 
 
 
 
GADD45A
 
 
hsa-miR-152-3p
 
 
-0.787
 
 
18.8
 
 
 
 
GAPVD1
 
 
hsa-miR-152-3p
 
 
-0.309
 
 
11.5
 
 
 
 
GLRX5
 
 
hsa-miR-152-3p
 
 
-0.381
 
 
16.6
 
 
 
 
GMFB
 
 
hsa-miR-152-3p
 
 
-0.429
 
 
22.2
 
 
 
 
GPATCH8
 
 
hsa-miR-152-3p
 
 
-0.374
 
 
10.6
 
 
 
 
GRSF1
 
 
hsa-miR-378a-3p
 
 
-0.358
 
 
33.9
 
 
 
 
HDDC2
 
 
hsa-miR-378a-3p
 
 
-0.322
 
 
18.9
 
 
 
 
HLA-DQB1
 
 
hsa-miR-152-3p
 
 
-0.37
 
 
75.3
 
 
 
 
HSP90B1
 
 
hsa-miR-152-3p
 
 
-0.304
 
 
399.7
 
 
 
 
HSPH1
 
 
hsa-miR-330-3p
 
 
-0.345
 
 
22.2
 
 
 
 
ID2
 
 
hsa-miR-330-3p
 
 
-0.293
 
 
69.8
 
 
 
 
IGF1
 
 
hsa-miR-152-3p
 
 
-0.43
 
 
25.5
 
 
 
 
IL6ST
 
 
hsa-miR-152-3p
 
 
-0.291
 
 
45.7
 
 
 
 
ITSN2
 
 
hsa-miR-152-3p
 
 
-0.339
 
 
33.7
 
 
 
 
KLF4
 
 
hsa-miR-152-3p
 
 
-0.325
 
 
19.8
 
 
 
 
KLF5
 
 
hsa-miR-152-3p
 
 
-0.475
 
 
17.0
 
 
 
 
LAMA4
 
 
hsa-miR-152-3p
 
 
-0.363
 
 
32.1
 
 
 
 
LBR
 
 
hsa-miR-152-3p
 
 
-0.39
 
 
50.2
 
 
 
 
LDLR
 
 
hsa-miR-152-3p
 
 
-0.323
 
 
25.7
 
 
 
 
LEPROTL1
 
 
hsa-miR-152-3p
 
 
-0.568
 
 
30.8
 
 
 
 
LIPA
 
 
hsa-miR-152-3p
 
 
-0.463
 
 
63.0
 
 
 
 
LMAN1
 
 
hsa-miR-152-3p
 
 
-0.382
 
 
37.2
 
 
 
 
LRCH1
 
 
hsa-miR-152-3p
 
 
-0.321
 
 
10.1
 
 
 
 
LRRC41
 
 
hsa-miR-152-3p
 
 
-0.392
 
 
17.9
 
 
 
 
LSM5
 
 
hsa-miR-330-3p
 
 
-0.351
 
 
33.7
 
 
 
 
LYSMD2
 
 
hsa-miR-152-3p
 
 
-0.554
 
 
21.0
 
 
 
 
MACROH2A1
 
 
hsa-miR-152-3p
 
 
-0.336
 
 
84.6
 
 
 
 
MAF
 
 
hsa-miR-152-3p
 
 
-0.362
 
 
19.5
 
 
 
 
MAF1
 
 
hsa-miR-152-3p
 
 
-0.366
 
 
58.2
 
 
 
 
MAFB
 
 
hsa-miR-152-3p
 
 
-0.45
 
 
35.2
 
 
 
 
MAP2K1
 
 
hsa-miR-152-3p
 
 
-0.295
 
 
27.5
 
 
 
 
MAPK1
 
 
hsa-miR-378a-3p
 
 
-0.314
 
 
33.9
 
 
 
 
MAT2A
 
 
hsa-miR-330-3p
 
 
-0.307
 
 
144.7
 
 
 
 
MCM3AP
 
 
hsa-miR-152-3p
 
 
-0.287
 
 
19.2
 
 
 
 
MDFIC
 
 
hsa-miR-152-3p
 
 
-0.395
 
 
20.3
 
 
 
 
MECP2
 
 
hsa-miR-152-3p
 
 
-0.3
 
 
12.6
 
 
 
 
MIER1
 
 
hsa-miR-152-3p
 
 
-0.352
 
 
17.3
 
 
 
 
MLLT10
 
 
hsa-miR-152-3p
 
 
-0.334
 
 
11.0
 
 
 
 
 
 
 basal ganglia 
 
 
 
 
target_symbol
 
 
mature_mirna_id
 
 
score
 
 
nTPM
 
 
 
 
 
 
ABCD3
 
 
hsa-miR-152-3p
 
 
-0.388
 
 
17.4
 
 
 
 
ABHD17C
 
 
hsa-miR-330-3p
 
 
-0.399
 
 
20.2
 
 
 
 
ADCY2
 
 
hsa-miR-152-3p
 
 
-0.299
 
 
23.7
 
 
 
 
ADGRB3
 
 
hsa-miR-152-3p
 
 
-0.516
 
 
21.1
 
 
 
 
ADGRF5
 
 
hsa-miR-152-3p
 
 
-0.354
 
 
13.3
 
 
 
 
AGFG1
 
 
hsa-miR-152-3p
 
 
-0.332
 
 
27.0
 
 
 
 
AHDC1
 
 
hsa-miR-152-3p
 
 
-0.413
 
 
12.3
 
 
 
 
AKAP1
 
 
hsa-miR-152-3p
 
 
-0.381
 
 
26.7
 
 
 
 
ALCAM
 
 
hsa-miR-152-3p
 
 
-0.347
 
 
37.8
 
 
 
 
AP4B1
 
 
hsa-miR-378a-3p
 
 
-0.62
 
 
11.6
 
 
 
 
ARF4
 
 
hsa-miR-152-3p
 
 
-0.487
 
 
71.0
 
 
 
 
ARHGAP21
 
 
hsa-miR-152-3p
 
 
-0.287
 
 
37.1
 
 
 
 
ARL6IP1
 
 
hsa-miR-152-3p
 
 
-0.579
 
 
196.4
 
 
 
 
ARL8B
 
 
hsa-miR-152-3p
 
 
-0.337
 
 
53.5
 
 
 
 
ARRDC3
 
 
hsa-miR-152-3p
 
 
-0.305
 
 
10.6
 
 
 
 
ASPH
 
 
hsa-miR-152-3p
 
 
-0.391
 
 
62.4
 
 
 
 
ATP6AP2
 
 
hsa-miR-152-3p
 
 
-0.592
 
 
77.2
 
 
 
 
ATP6AP2
 
 
hsa-miR-152-3p
 
 
-0.668
 
 
77.2
 
 
 
 
B4GALT5
 
 
hsa-miR-152-3p
 
 
-0.44
 
 
12.9
 
 
 
 
BHLHE41
 
 
hsa-miR-152-3p
 
 
-0.442
 
 
27.8
 
 
 
 
BTBD3
 
 
hsa-miR-152-3p
 
 
-0.495
 
 
16.4
 
 
 
 
C1QBP
 
 
hsa-miR-330-3p
 
 
-0.412
 
 
77.3
 
 
 
 
C1orf35
 
 
hsa-miR-152-3p
 
 
-0.511
 
 
16.0
 
 
 
 
CAND1
 
 
hsa-miR-152-3p
 
 
-0.44
 
 
18.1
 
 
 
 
CANX
 
 
hsa-miR-152-3p
 
 
-0.434
 
 
113.6
 
 
 
 
CCDC6
 
 
hsa-miR-152-3p
 
 
-0.511
 
 
25.4
 
 
 
 
CCN2
 
 
hsa-miR-330-3p
 
 
-0.305
 
 
22.4
 
 
 
 
CCT6A
 
 
hsa-miR-152-3p
 
 
-0.665
 
 
44.4
 
 
 
 
CDC25B
 
 
hsa-miR-152-3p
 
 
-0.298
 
 
35.1
 
 
 
 
CDH20
 
 
hsa-miR-152-3p
 
 
-0.297
 
 
17.3
 
 
 
 
CDK19
 
 
hsa-miR-152-3p
 
 
-0.369
 
 
21.6
 
 
 
 
CDK19
 
 
hsa-miR-152-3p
 
 
-0.405
 
 
21.6
 
 
 
 
CDK5R1
 
 
hsa-miR-152-3p
 
 
-0.547
 
 
34.2
 
 
 
 
CEBPG
 
 
hsa-miR-152-3p
 
 
-0.319
 
 
10.6
 
 
 
 
CERS5
 
 
hsa-miR-152-3p
 
 
-0.311
 
 
24.8
 
 
 
 
CFL2
 
 
hsa-miR-152-3p
 
 
-0.487
 
 
68.0
 
 
 
 
CHMP4B
 
 
hsa-miR-152-3p
 
 
-0.36
 
 
131.2
 
 
 
 
CHST1
 
 
hsa-miR-152-3p
 
 
-0.408
 
 
32.3
 
 
 
 
CLCN6
 
 
hsa-miR-152-3p
 
 
-0.406
 
 
14.9
 
 
 
 
CNOT7
 
 
hsa-miR-152-3p
 
 
-0.31
 
 
20.1
 
 
 
 
CS
 
 
hsa-miR-152-3p
 
 
-0.3
 
 
43.8
 
 
 
 
CTSA
 
 
hsa-miR-152-3p
 
 
-0.541
 
 
35.6
 
 
 
 
CYTH3
 
 
hsa-miR-152-3p
 
 
-0.331
 
 
19.1
 
 
 
 
DCUN1D4
 
 
hsa-miR-152-3p
 
 
-0.314
 
 
13.0
 
 
 
 
DDAH1
 
 
hsa-miR-152-3p
 
 
-0.34
 
 
97.9
 
 
 
 
DDX6
 
 
hsa-miR-152-3p
 
 
-0.363
 
 
22.3
 
 
 
 
DEDD
 
 
hsa-miR-152-3p
 
 
-0.401
 
 
16.3
 
 
 
 
DMPK
 
 
hsa-miR-152-3p
 
 
-0.378
 
 
17.3
 
 
 
 
DNAJB4
 
 
hsa-miR-152-3p
 
 
-0.33
 
 
17.0
 
 
 
 
DUSP1
 
 
hsa-miR-152-3p
 
 
-0.32
 
 
88.5
 
 
 
 
DYNLL2
 
 
hsa-miR-152-3p
 
 
-0.366
 
 
77.9
 
 
 
 
DYRK1B
 
 
hsa-miR-152-3p
 
 
-0.305
 
 
20.4
 
 
 
 
EGR3
 
 
hsa-miR-152-3p
 
 
-0.315
 
 
14.7
 
 
 
 
EIF4E3
 
 
hsa-miR-152-3p
 
 
-0.464
 
 
19.1
 
 
 
 
EIF4G3
 
 
hsa-miR-378a-3p
 
 
-0.443
 
 
12.0
 
 
 
 
EMX2
 
 
hsa-miR-152-3p
 
 
-0.366
 
 
24.6
 
 
 
 
EPS15
 
 
hsa-miR-152-3p
 
 
-0.322
 
 
29.8
 
 
 
 
ERBB3
 
 
hsa-miR-152-3p
 
 
-0.399
 
 
28.3
 
 
 
 
ERRFI1
 
 
hsa-miR-152-3p
 
 
-0.529
 
 
14.2
 
 
 
 
F3
 
 
hsa-miR-152-3p
 
 
-0.331
 
 
108.1
 
 
 
 
FAM168B
 
 
hsa-miR-152-3p
 
 
-0.409
 
 
63.5
 
 
 
 
FAM184A
 
 
hsa-miR-152-3p
 
 
-0.371
 
 
18.1
 
 
 
 
FBXL19
 
 
hsa-miR-152-3p
 
 
-0.362
 
 
20.7
 
 
 
 
FEZ2
 
 
hsa-miR-152-3p
 
 
-0.536
 
 
42.1
 
 
 
 
FOXK2
 
 
hsa-miR-152-3p
 
 
-0.305
 
 
12.5
 
 
 
 
FXR1
 
 
hsa-miR-152-3p
 
 
-0.478
 
 
33.4
 
 
 
 
GADD45A
 
 
hsa-miR-152-3p
 
 
-0.787
 
 
49.7
 
 
 
 
GAP43
 
 
hsa-miR-152-3p
 
 
-0.409
 
 
210.9
 
 
 
 
GLRX5
 
 
hsa-miR-152-3p
 
 
-0.381
 
 
56.3
 
 
 
 
GMFB
 
 
hsa-miR-152-3p
 
 
-0.429
 
 
42.9
 
 
 
 
GOLT1A
 
 
hsa-miR-378a-3p
 
 
-0.574
 
 
30.7
 
 
 
 
GPATCH8
 
 
hsa-miR-152-3p
 
 
-0.374
 
 
11.9
 
 
 
 
GRSF1
 
 
hsa-miR-378a-3p
 
 
-0.358
 
 
25.5
 
 
 
 
GTF2H1
 
 
hsa-miR-152-3p
 
 
-0.524
 
 
14.2
 
 
 
 
HDDC2
 
 
hsa-miR-378a-3p
 
 
-0.322
 
 
85.7
 
 
 
 
HSP90B1
 
 
hsa-miR-152-3p
 
 
-0.304
 
 
121.6
 
 
 
 
HSPH1
 
 
hsa-miR-330-3p
 
 
-0.345
 
 
156.3
 
 
 
 
ID2
 
 
hsa-miR-330-3p
 
 
-0.293
 
 
84.0
 
 
 
 
IL6ST
 
 
hsa-miR-152-3p
 
 
-0.291
 
 
27.1
 
 
 
 
JPH3
 
 
hsa-miR-152-3p
 
 
-0.303
 
 
72.3
 
 
 
 
KDSR
 
 
hsa-miR-378a-3p
 
 
-0.373
 
 
11.2
 
 
 
 
KLF5
 
 
hsa-miR-152-3p
 
 
-0.475
 
 
13.2
 
 
 
 
LEPROTL1
 
 
hsa-miR-152-3p
 
 
-0.568
 
 
17.7
 
 
 
 
LIPA
 
 
hsa-miR-152-3p
 
 
-0.463
 
 
33.9
 
 
 
 
LMAN1
 
 
hsa-miR-152-3p
 
 
-0.382
 
 
12.3
 
 
 
 
LRRC41
 
 
hsa-miR-152-3p
 
 
-0.392
 
 
25.7
 
 
 
 
LSM5
 
 
hsa-miR-330-3p
 
 
-0.351
 
 
30.4
 
 
 
 
LYRM2
 
 
hsa-miR-152-3p
 
 
-0.351
 
 
11.5
 
 
 
 
LYSMD2
 
 
hsa-miR-152-3p
 
 
-0.554
 
 
33.8
 
 
 
 
MACIR
 
 
hsa-miR-152-3p
 
 
-0.332
 
 
12.2
 
 
 
 
MACIR
 
 
hsa-miR-152-3p
 
 
-0.573
 
 
12.2
 
 
 
 
MACROH2A1
 
 
hsa-miR-152-3p
 
 
-0.336
 
 
51.5
 
 
 
 
MAF1
 
 
hsa-miR-152-3p
 
 
-0.366
 
 
58.1
 
 
 
 
MAFG
 
 
hsa-miR-152-3p
 
 
-0.328
 
 
14.7
 
 
 
 
MAP2K1
 
 
hsa-miR-152-3p
 
 
-0.295
 
 
42.9
 
 
 
 
MAPK1
 
 
hsa-miR-378a-3p
 
 
-0.314
 
 
96.0
 
 
 
 
MAT2A
 
 
hsa-miR-330-3p
 
 
-0.307
 
 
150.0
 
 
 
 
MECP2
 
 
hsa-miR-152-3p
 
 
-0.3
 
 
12.1
 
 
 
 
MED19
 
 
hsa-miR-378a-3p
 
 
-0.434
 
 
18.3
 
 
 
 
MLLT10
 
 
hsa-miR-152-3p
 
 
-0.334
 
 
10.4
 
 
 
 
 
 
 cerebral cortex 
 
 
 
 
target_symbol
 
 
mature_mirna_id
 
 
score
 
 
nTPM
 
 
 
 
 
 
ABCD3
 
 
hsa-miR-152-3p
 
 
-0.388
 
 
17.1
 
 
 
 
ABHD17C
 
 
hsa-miR-330-3p
 
 
-0.399
 
 
16.3
 
 
 
 
ADAM10
 
 
hsa-miR-152-3p
 
 
-0.348
 
 
16.0
 
 
 
 
ADCY2
 
 
hsa-miR-152-3p
 
 
-0.299
 
 
35.4
 
 
 
 
ADGRB3
 
 
hsa-miR-152-3p
 
 
-0.516
 
 
28.2
 
 
 
 
AGFG1
 
 
hsa-miR-152-3p
 
 
-0.332
 
 
33.6
 
 
 
 
AHDC1
 
 
hsa-miR-152-3p
 
 
-0.413
 
 
15.5
 
 
 
 
AKAP1
 
 
hsa-miR-152-3p
 
 
-0.381
 
 
24.9
 
 
 
 
ALCAM
 
 
hsa-miR-152-3p
 
 
-0.347
 
 
54.7
 
 
 
 
AP4B1
 
 
hsa-miR-378a-3p
 
 
-0.62
 
 
10.6
 
 
 
 
ARF4
 
 
hsa-miR-152-3p
 
 
-0.487
 
 
61.5
 
 
 
 
ARHGAP21
 
 
hsa-miR-152-3p
 
 
-0.287
 
 
47.3
 
 
 
 
ARL6IP1
 
 
hsa-miR-152-3p
 
 
-0.579
 
 
314.1
 
 
 
 
ARL8B
 
 
hsa-miR-152-3p
 
 
-0.337
 
 
75.1
 
 
 
 
ARRDC3
 
 
hsa-miR-152-3p
 
 
-0.305
 
 
15.6
 
 
 
 
ASPH
 
 
hsa-miR-152-3p
 
 
-0.391
 
 
76.3
 
 
 
 
ATP2B4
 
 
hsa-miR-152-3p
 
 
-0.337
 
 
35.3
 
 
 
 
ATP6AP2
 
 
hsa-miR-152-3p
 
 
-0.592
 
 
109.4
 
 
 
 
ATP6AP2
 
 
hsa-miR-152-3p
 
 
-0.668
 
 
109.4
 
 
 
 
B4GALT5
 
 
hsa-miR-152-3p
 
 
-0.44
 
 
22.9
 
 
 
 
B4GALT6
 
 
hsa-miR-152-3p
 
 
-0.528
 
 
15.0
 
 
 
 
BHLHE41
 
 
hsa-miR-152-3p
 
 
-0.442
 
 
27.2
 
 
 
 
BTBD3
 
 
hsa-miR-152-3p
 
 
-0.495
 
 
32.2
 
 
 
 
C1GALT1
 
 
hsa-miR-152-3p
 
 
-0.487
 
 
12.5
 
 
 
 
C1QBP
 
 
hsa-miR-330-3p
 
 
-0.412
 
 
89.5
 
 
 
 
C1orf35
 
 
hsa-miR-152-3p
 
 
-0.511
 
 
14.3
 
 
 
 
CAND1
 
 
hsa-miR-152-3p
 
 
-0.44
 
 
28.2
 
 
 
 
CANX
 
 
hsa-miR-152-3p
 
 
-0.434
 
 
104.3
 
 
 
 
CCDC6
 
 
hsa-miR-152-3p
 
 
-0.511
 
 
20.7
 
 
 
 
CCDC85A
 
 
hsa-miR-152-3p
 
 
-0.296
 
 
11.9
 
 
 
 
CCN2
 
 
hsa-miR-330-3p
 
 
-0.305
 
 
14.7
 
 
 
 
CCT6A
 
 
hsa-miR-152-3p
 
 
-0.665
 
 
52.6
 
 
 
 
CDC25B
 
 
hsa-miR-152-3p
 
 
-0.298
 
 
28.7
 
 
 
 
CDH20
 
 
hsa-miR-152-3p
 
 
-0.297
 
 
14.5
 
 
 
 
CDK19
 
 
hsa-miR-152-3p
 
 
-0.369
 
 
18.3
 
 
 
 
CDK19
 
 
hsa-miR-152-3p
 
 
-0.405
 
 
18.3
 
 
 
 
CDK5R1
 
 
hsa-miR-152-3p
 
 
-0.547
 
 
71.3
 
 
 
 
CEBPG
 
 
hsa-miR-152-3p
 
 
-0.319
 
 
11.8
 
 
 
 
CEP63
 
 
hsa-miR-152-3p
 
 
-0.329
 
 
10.5
 
 
 
 
CERS5
 
 
hsa-miR-152-3p
 
 
-0.311
 
 
18.0
 
 
 
 
CFL2
 
 
hsa-miR-152-3p
 
 
-0.487
 
 
47.1
 
 
 
 
CHMP4B
 
 
hsa-miR-152-3p
 
 
-0.36
 
 
161.8
 
 
 
 
CHST1
 
 
hsa-miR-152-3p
 
 
-0.408
 
 
41.3
 
 
 
 
CLCN6
 
 
hsa-miR-152-3p
 
 
-0.406
 
 
23.1
 
 
 
 
CNOT7
 
 
hsa-miR-152-3p
 
 
-0.31
 
 
31.5
 
 
 
 
CS
 
 
hsa-miR-152-3p
 
 
-0.3
 
 
64.9
 
 
 
 
CTSA
 
 
hsa-miR-152-3p
 
 
-0.541
 
 
41.1
 
 
 
 
CUL5
 
 
hsa-miR-152-3p
 
 
-0.389
 
 
11.5
 
 
 
 
CYTH3
 
 
hsa-miR-152-3p
 
 
-0.331
 
 
22.1
 
 
 
 
DCUN1D4
 
 
hsa-miR-152-3p
 
 
-0.314
 
 
14.3
 
 
 
 
DDAH1
 
 
hsa-miR-152-3p
 
 
-0.34
 
 
105.0
 
 
 
 
DDX6
 
 
hsa-miR-152-3p
 
 
-0.363
 
 
35.4
 
 
 
 
DEDD
 
 
hsa-miR-152-3p
 
 
-0.401
 
 
17.4
 
 
 
 
DIPK2A
 
 
hsa-miR-152-3p
 
 
-0.393
 
 
12.2
 
 
 
 
DMPK
 
 
hsa-miR-152-3p
 
 
-0.378
 
 
17.6
 
 
 
 
DNAJB4
 
 
hsa-miR-152-3p
 
 
-0.33
 
 
12.4
 
 
 
 
DUSP1
 
 
hsa-miR-152-3p
 
 
-0.32
 
 
82.4
 
 
 
 
DYNLL2
 
 
hsa-miR-152-3p
 
 
-0.366
 
 
75.0
 
 
 
 
DYRK1B
 
 
hsa-miR-152-3p
 
 
-0.305
 
 
22.1
 
 
 
 
EGR3
 
 
hsa-miR-152-3p
 
 
-0.315
 
 
26.1
 
 
 
 
EIF4E3
 
 
hsa-miR-152-3p
 
 
-0.464
 
 
18.4
 
 
 
 
EIF4G3
 
 
hsa-miR-378a-3p
 
 
-0.443
 
 
26.7
 
 
 
 
ELAVL2
 
 
hsa-miR-152-3p
 
 
-0.3
 
 
22.3
 
 
 
 
EMX2
 
 
hsa-miR-152-3p
 
 
-0.366
 
 
23.4
 
 
 
 
EPS15
 
 
hsa-miR-152-3p
 
 
-0.322
 
 
48.2
 
 
 
 
ERBB3
 
 
hsa-miR-152-3p
 
 
-0.399
 
 
12.0
 
 
 
 
ERRFI1
 
 
hsa-miR-152-3p
 
 
-0.529
 
 
16.7
 
 
 
 
F3
 
 
hsa-miR-152-3p
 
 
-0.331
 
 
78.4
 
 
 
 
FAM168B
 
 
hsa-miR-152-3p
 
 
-0.409
 
 
60.5
 
 
 
 
FAM184A
 
 
hsa-miR-152-3p
 
 
-0.371
 
 
18.6
 
 
 
 
FAM81A
 
 
hsa-miR-152-3p
 
 
-0.315
 
 
26.3
 
 
 
 
FBXL19
 
 
hsa-miR-152-3p
 
 
-0.362
 
 
35.1
 
 
 
 
FEZ2
 
 
hsa-miR-152-3p
 
 
-0.536
 
 
48.0
 
 
 
 
FMR1
 
 
hsa-miR-152-3p
 
 
-0.385
 
 
19.5
 
 
 
 
FOSB
 
 
hsa-miR-152-3p
 
 
-0.311
 
 
24.3
 
 
 
 
FOXK2
 
 
hsa-miR-152-3p
 
 
-0.305
 
 
17.0
 
 
 
 
FXR1
 
 
hsa-miR-152-3p
 
 
-0.478
 
 
29.8
 
 
 
 
GADD45A
 
 
hsa-miR-152-3p
 
 
-0.787
 
 
37.1
 
 
 
 
GAP43
 
 
hsa-miR-152-3p
 
 
-0.409
 
 
473.7
 
 
 
 
GAPVD1
 
 
hsa-miR-152-3p
 
 
-0.309
 
 
12.6
 
 
 
 
GLRX5
 
 
hsa-miR-152-3p
 
 
-0.381
 
 
67.4
 
 
 
 
GMFB
 
 
hsa-miR-152-3p
 
 
-0.429
 
 
63.3
 
 
 
 
GPATCH8
 
 
hsa-miR-152-3p
 
 
-0.374
 
 
14.6
 
 
 
 
GRSF1
 
 
hsa-miR-378a-3p
 
 
-0.358
 
 
46.8
 
 
 
 
GTF2H1
 
 
hsa-miR-152-3p
 
 
-0.524
 
 
15.8
 
 
 
 
HDDC2
 
 
hsa-miR-378a-3p
 
 
-0.322
 
 
94.3
 
 
 
 
HOMER1
 
 
hsa-miR-152-3p
 
 
-0.452
 
 
27.6
 
 
 
 
HSP90B1
 
 
hsa-miR-152-3p
 
 
-0.304
 
 
126.1
 
 
 
 
HSPA12A
 
 
hsa-miR-378a-3p
 
 
-0.326
 
 
27.0
 
 
 
 
HSPH1
 
 
hsa-miR-330-3p
 
 
-0.345
 
 
127.7
 
 
 
 
ID2
 
 
hsa-miR-330-3p
 
 
-0.293
 
 
136.9
 
 
 
 
IL6ST
 
 
hsa-miR-152-3p
 
 
-0.291
 
 
33.0
 
 
 
 
ITSN2
 
 
hsa-miR-152-3p
 
 
-0.339
 
 
13.9
 
 
 
 
JPH3
 
 
hsa-miR-152-3p
 
 
-0.303
 
 
108.5
 
 
 
 
KDSR
 
 
hsa-miR-378a-3p
 
 
-0.373
 
 
10.7
 
 
 
 
LEPROTL1
 
 
hsa-miR-152-3p
 
 
-0.568
 
 
19.8
 
 
 
 
LIPA
 
 
hsa-miR-152-3p
 
 
-0.463
 
 
53.4
 
 
 
 
LMAN1
 
 
hsa-miR-152-3p
 
 
-0.382
 
 
15.3
 
 
 
 
LMTK2
 
 
hsa-miR-152-3p
 
 
-0.298
 
 
15.9
 
 
 
 
LRRC41
 
 
hsa-miR-152-3p
 
 
-0.392
 
 
32.0
 
 
 
 
 
 
 epididymis 
 
 
 
 
target_symbol
 
 
mature_mirna_id
 
 
score
 
 
nTPM
 
 
 
 
 
 
ABCD3
 
 
hsa-miR-152-3p
 
 
-0.388
 
 
20.0
 
 
 
 
ACVR1
 
 
hsa-miR-152-3p
 
 
-0.397
 
 
10.8
 
 
 
 
ADAM17
 
 
hsa-miR-152-3p
 
 
-0.301
 
 
10.4
 
 
 
 
AGFG1
 
 
hsa-miR-152-3p
 
 
-0.332
 
 
18.8
 
 
 
 
AKAP1
 
 
hsa-miR-152-3p
 
 
-0.381
 
 
18.6
 
 
 
 
ALCAM
 
 
hsa-miR-152-3p
 
 
-0.347
 
 
33.4
 
 
 
 
AP4B1
 
 
hsa-miR-378a-3p
 
 
-0.62
 
 
13.4
 
 
 
 
ARF4
 
 
hsa-miR-152-3p
 
 
-0.487
 
 
214.1
 
 
 
 
ARFGEF1
 
 
hsa-miR-152-3p
 
 
-0.306
 
 
10.4
 
 
 
 
ARFIP1
 
 
hsa-miR-152-3p
 
 
-0.474
 
 
19.3
 
 
 
 
ARL6IP1
 
 
hsa-miR-152-3p
 
 
-0.579
 
 
116.0
 
 
 
 
ARL8B
 
 
hsa-miR-152-3p
 
 
-0.337
 
 
37.5
 
 
 
 
ARRDC3
 
 
hsa-miR-152-3p
 
 
-0.305
 
 
25.6
 
 
 
 
ASPH
 
 
hsa-miR-152-3p
 
 
-0.391
 
 
42.3
 
 
 
 
ATP2B4
 
 
hsa-miR-152-3p
 
 
-0.337
 
 
18.9
 
 
 
 
ATP6AP2
 
 
hsa-miR-152-3p
 
 
-0.592
 
 
86.1
 
 
 
 
ATP6AP2
 
 
hsa-miR-152-3p
 
 
-0.668
 
 
86.1
 
 
 
 
BHLHE41
 
 
hsa-miR-152-3p
 
 
-0.442
 
 
18.2
 
 
 
 
BTAF1
 
 
hsa-miR-152-3p
 
 
-0.401
 
 
16.9
 
 
 
 
C1QBP
 
 
hsa-miR-330-3p
 
 
-0.412
 
 
64.4
 
 
 
 
C1orf210
 
 
hsa-miR-378a-3p
 
 
-0.307
 
 
12.2
 
 
 
 
CAND1
 
 
hsa-miR-152-3p
 
 
-0.44
 
 
26.2
 
 
 
 
CANX
 
 
hsa-miR-152-3p
 
 
-0.434
 
 
162.1
 
 
 
 
CAV2
 
 
hsa-miR-152-3p
 
 
-0.32
 
 
21.8
 
 
 
 
CCDC6
 
 
hsa-miR-152-3p
 
 
-0.511
 
 
15.8
 
 
 
 
CCN2
 
 
hsa-miR-330-3p
 
 
-0.305
 
 
32.5
 
 
 
 
CCT6A
 
 
hsa-miR-152-3p
 
 
-0.665
 
 
37.2
 
 
 
 
CEP63
 
 
hsa-miR-152-3p
 
 
-0.329
 
 
11.9
 
 
 
 
CERS5
 
 
hsa-miR-152-3p
 
 
-0.311
 
 
12.3
 
 
 
 
CFL2
 
 
hsa-miR-152-3p
 
 
-0.487
 
 
18.3
 
 
 
 
CHMP4B
 
 
hsa-miR-152-3p
 
 
-0.36
 
 
55.3
 
 
 
 
CNOT7
 
 
hsa-miR-152-3p
 
 
-0.31
 
 
42.5
 
 
 
 
COL2A1
 
 
hsa-miR-152-3p
 
 
-0.385
 
 
18.0
 
 
 
 
COL4A1
 
 
hsa-miR-152-3p
 
 
-0.317
 
 
14.0
 
 
 
 
CS
 
 
hsa-miR-152-3p
 
 
-0.3
 
 
30.4
 
 
 
 
CTSA
 
 
hsa-miR-152-3p
 
 
-0.541
 
 
36.9
 
 
 
 
DCUN1D4
 
 
hsa-miR-152-3p
 
 
-0.314
 
 
10.9
 
 
 
 
DDAH1
 
 
hsa-miR-152-3p
 
 
-0.34
 
 
72.3
 
 
 
 
DDX6
 
 
hsa-miR-152-3p
 
 
-0.363
 
 
28.8
 
 
 
 
DEDD
 
 
hsa-miR-152-3p
 
 
-0.401
 
 
14.1
 
 
 
 
DNAJB4
 
 
hsa-miR-152-3p
 
 
-0.33
 
 
10.2
 
 
 
 
DUSP1
 
 
hsa-miR-152-3p
 
 
-0.32
 
 
39.7
 
 
 
 
DYNLL2
 
 
hsa-miR-152-3p
 
 
-0.366
 
 
21.2
 
 
 
 
EIF4G3
 
 
hsa-miR-378a-3p
 
 
-0.443
 
 
16.4
 
 
 
 
EMX2
 
 
hsa-miR-152-3p
 
 
-0.366
 
 
98.5
 
 
 
 
EPS15
 
 
hsa-miR-152-3p
 
 
-0.322
 
 
18.2
 
 
 
 
ERBB3
 
 
hsa-miR-152-3p
 
 
-0.399
 
 
10.2
 
 
 
 
ERRFI1
 
 
hsa-miR-152-3p
 
 
-0.529
 
 
17.3
 
 
 
 
F3
 
 
hsa-miR-152-3p
 
 
-0.331
 
 
41.7
 
 
 
 
FAM168B
 
 
hsa-miR-152-3p
 
 
-0.409
 
 
25.1
 
 
 
 
FEZ2
 
 
hsa-miR-152-3p
 
 
-0.536
 
 
17.9
 
 
 
 
FMR1
 
 
hsa-miR-152-3p
 
 
-0.385
 
 
25.5
 
 
 
 
FXR1
 
 
hsa-miR-152-3p
 
 
-0.478
 
 
25.5
 
 
 
 
GADD45A
 
 
hsa-miR-152-3p
 
 
-0.787
 
 
21.8
 
 
 
 
GLRX5
 
 
hsa-miR-152-3p
 
 
-0.381
 
 
36.9
 
 
 
 
GMFB
 
 
hsa-miR-152-3p
 
 
-0.429
 
 
13.2
 
 
 
 
GOLT1A
 
 
hsa-miR-378a-3p
 
 
-0.574
 
 
10.1
 
 
 
 
GPATCH8
 
 
hsa-miR-152-3p
 
 
-0.374
 
 
11.2
 
 
 
 
GRSF1
 
 
hsa-miR-378a-3p
 
 
-0.358
 
 
30.1
 
 
 
 
HDDC2
 
 
hsa-miR-378a-3p
 
 
-0.322
 
 
28.0
 
 
 
 
HLA-DQB1
 
 
hsa-miR-152-3p
 
 
-0.37
 
 
121.8
 
 
 
 
HOXC8
 
 
hsa-miR-152-3p
 
 
-0.46
 
 
14.6
 
 
 
 
HSP90B1
 
 
hsa-miR-152-3p
 
 
-0.304
 
 
975.0
 
 
 
 
HSPA12A
 
 
hsa-miR-378a-3p
 
 
-0.326
 
 
11.9
 
 
 
 
HSPH1
 
 
hsa-miR-330-3p
 
 
-0.345
 
 
35.5
 
 
 
 
ID2
 
 
hsa-miR-330-3p
 
 
-0.293
 
 
19.8
 
 
 
 
IGF1
 
 
hsa-miR-152-3p
 
 
-0.43
 
 
10.0
 
 
 
 
IL6ST
 
 
hsa-miR-152-3p
 
 
-0.291
 
 
19.9
 
 
 
 
ITGA11
 
 
hsa-miR-152-3p
 
 
-0.348
 
 
12.7
 
 
 
 
ITSN2
 
 
hsa-miR-152-3p
 
 
-0.339
 
 
12.1
 
 
 
 
KDSR
 
 
hsa-miR-378a-3p
 
 
-0.373
 
 
19.7
 
 
 
 
LAMA4
 
 
hsa-miR-152-3p
 
 
-0.363
 
 
17.5
 
 
 
 
LBR
 
 
hsa-miR-152-3p
 
 
-0.39
 
 
41.9
 
 
 
 
LEPROTL1
 
 
hsa-miR-152-3p
 
 
-0.568
 
 
24.2
 
 
 
 
LIPA
 
 
hsa-miR-152-3p
 
 
-0.463
 
 
20.5
 
 
 
 
LMAN1
 
 
hsa-miR-152-3p
 
 
-0.382
 
 
49.9
 
 
 
 
LRRC41
 
 
hsa-miR-152-3p
 
 
-0.392
 
 
25.1
 
 
 
 
LSM5
 
 
hsa-miR-330-3p
 
 
-0.351
 
 
33.4
 
 
 
 
LYRM2
 
 
hsa-miR-152-3p
 
 
-0.351
 
 
13.5
 
 
 
 
LYSMD2
 
 
hsa-miR-152-3p
 
 
-0.554
 
 
10.5
 
 
 
 
MACROH2A1
 
 
hsa-miR-152-3p
 
 
-0.336
 
 
43.6
 
 
 
 
MAF1
 
 
hsa-miR-152-3p
 
 
-0.366
 
 
60.0
 
 
 
 
MAP2K1
 
 
hsa-miR-152-3p
 
 
-0.295
 
 
16.4
 
 
 
 
MAPK1
 
 
hsa-miR-378a-3p
 
 
-0.314
 
 
28.5
 
 
 
 
MAT2A
 
 
hsa-miR-330-3p
 
 
-0.307
 
 
222.9
 
 
 
 
MECP2
 
 
hsa-miR-152-3p
 
 
-0.3
 
 
10.2
 
 
 
 
MED19
 
 
hsa-miR-378a-3p
 
 
-0.434
 
 
14.2
 
 
 
 
MIER1
 
 
hsa-miR-152-3p
 
 
-0.352
 
 
12.7
 
 
 
 
MLLT10
 
 
hsa-miR-152-3p
 
 
-0.334
 
 
15.7
 
 
 
 
MNAT1
 
 
hsa-miR-378a-3p
 
 
-0.398
 
 
17.9
 
 
 
 
MOSPD1
 
 
hsa-miR-152-3p
 
 
-0.314
 
 
12.1
 
 
 
 
MRPS10
 
 
hsa-miR-152-3p
 
 
-0.335
 
 
17.0
 
 
 
 
MTMR14
 
 
hsa-miR-152-3p
 
 
-0.467
 
 
10.5
 
 
 
 
NAP1L1
 
 
hsa-miR-152-3p
 
 
-0.343
 
 
162.7
 
 
 
 
NCKIPSD
 
 
hsa-miR-152-3p
 
 
-0.382
 
 
10.7
 
 
 
 
NCOA1
 
 
hsa-miR-152-3p
 
 
-0.313
 
 
11.9
 
 
 
 
NDP
 
 
hsa-miR-152-3p
 
 
-0.388
 
 
20.7
 
 
 
 
NFKBIA
 
 
hsa-miR-378a-3p
 
 
-0.427
 
 
27.3
 
 
 
 
NOL4L
 
 
hsa-miR-152-3p
 
 
-0.394
 
 
11.5
 
 
 
 
NPTN
 
 
hsa-miR-152-3p
 
 
-0.507
 
 
142.4
 
 
 
 
 
 
 gallbladder 
 
 
 
 
target_symbol
 
 
mature_mirna_id
 
 
score
 
 
nTPM
 
 
 
 
 
 
ABCD3
 
 
hsa-miR-152-3p
 
 
-0.388
 
 
25.2
 
 
 
 
ACVR1
 
 
hsa-miR-152-3p
 
 
-0.397
 
 
14.1
 
 
 
 
ADAM10
 
 
hsa-miR-152-3p
 
 
-0.348
 
 
17.7
 
 
 
 
ADGRF5
 
 
hsa-miR-152-3p
 
 
-0.354
 
 
16.1
 
 
 
 
AGFG1
 
 
hsa-miR-152-3p
 
 
-0.332
 
 
36.5
 
 
 
 
AKAP1
 
 
hsa-miR-152-3p
 
 
-0.381
 
 
16.8
 
 
 
 
ALCAM
 
 
hsa-miR-152-3p
 
 
-0.347
 
 
21.9
 
 
 
 
ARF4
 
 
hsa-miR-152-3p
 
 
-0.487
 
 
145.1
 
 
 
 
ARFGEF1
 
 
hsa-miR-152-3p
 
 
-0.306
 
 
11.7
 
 
 
 
ARFIP1
 
 
hsa-miR-152-3p
 
 
-0.474
 
 
18.7
 
 
 
 
ARHGAP21
 
 
hsa-miR-152-3p
 
 
-0.287
 
 
16.7
 
 
 
 
ARL6IP1
 
 
hsa-miR-152-3p
 
 
-0.579
 
 
123.8
 
 
 
 
ARL8B
 
 
hsa-miR-152-3p
 
 
-0.337
 
 
36.3
 
 
 
 
ARRDC3
 
 
hsa-miR-152-3p
 
 
-0.305
 
 
36.6
 
 
 
 
ASPH
 
 
hsa-miR-152-3p
 
 
-0.391
 
 
56.5
 
 
 
 
ATP2B4
 
 
hsa-miR-152-3p
 
 
-0.337
 
 
23.7
 
 
 
 
ATP6AP2
 
 
hsa-miR-152-3p
 
 
-0.592
 
 
74.7
 
 
 
 
ATP6AP2
 
 
hsa-miR-152-3p
 
 
-0.668
 
 
74.7
 
 
 
 
B4GALT5
 
 
hsa-miR-152-3p
 
 
-0.44
 
 
29.5
 
 
 
 
BCL2L11
 
 
hsa-miR-152-3p
 
 
-0.445
 
 
11.1
 
 
 
 
BHLHE41
 
 
hsa-miR-152-3p
 
 
-0.442
 
 
17.1
 
 
 
 
BICC1
 
 
hsa-miR-152-3p
 
 
-0.442
 
 
10.9
 
 
 
 
BMP2
 
 
hsa-miR-378a-3p
 
 
-0.481
 
 
15.1
 
 
 
 
BTAF1
 
 
hsa-miR-152-3p
 
 
-0.401
 
 
12.0
 
 
 
 
BTBD3
 
 
hsa-miR-152-3p
 
 
-0.495
 
 
12.5
 
 
 
 
C1GALT1
 
 
hsa-miR-152-3p
 
 
-0.487
 
 
15.6
 
 
 
 
C1QBP
 
 
hsa-miR-330-3p
 
 
-0.412
 
 
45.7
 
 
 
 
CAND1
 
 
hsa-miR-152-3p
 
 
-0.44
 
 
17.2
 
 
 
 
CAV2
 
 
hsa-miR-152-3p
 
 
-0.32
 
 
67.6
 
 
 
 
CCDC6
 
 
hsa-miR-152-3p
 
 
-0.511
 
 
15.7
 
 
 
 
CCN2
 
 
hsa-miR-330-3p
 
 
-0.305
 
 
413.0
 
 
 
 
CCT6A
 
 
hsa-miR-152-3p
 
 
-0.665
 
 
38.0
 
 
 
 
CDC25B
 
 
hsa-miR-152-3p
 
 
-0.298
 
 
16.8
 
 
 
 
CEBPG
 
 
hsa-miR-152-3p
 
 
-0.319
 
 
10.7
 
 
 
 
CFL2
 
 
hsa-miR-152-3p
 
 
-0.487
 
 
22.6
 
 
 
 
CHMP4B
 
 
hsa-miR-152-3p
 
 
-0.36
 
 
75.6
 
 
 
 
CNOT7
 
 
hsa-miR-152-3p
 
 
-0.31
 
 
33.4
 
 
 
 
COL4A1
 
 
hsa-miR-152-3p
 
 
-0.317
 
 
109.3
 
 
 
 
CS
 
 
hsa-miR-152-3p
 
 
-0.3
 
 
39.8
 
 
 
 
CTSA
 
 
hsa-miR-152-3p
 
 
-0.541
 
 
83.8
 
 
 
 
CTTNBP2NL
 
 
hsa-miR-152-3p
 
 
-0.329
 
 
15.5
 
 
 
 
CYTH3
 
 
hsa-miR-152-3p
 
 
-0.331
 
 
11.0
 
 
 
 
DACT1
 
 
hsa-miR-378a-3p
 
 
-0.298
 
 
23.3
 
 
 
 
DCUN1D4
 
 
hsa-miR-152-3p
 
 
-0.314
 
 
10.7
 
 
 
 
DDAH1
 
 
hsa-miR-152-3p
 
 
-0.34
 
 
23.9
 
 
 
 
DDX6
 
 
hsa-miR-152-3p
 
 
-0.363
 
 
22.1
 
 
 
 
DEDD
 
 
hsa-miR-152-3p
 
 
-0.401
 
 
19.9
 
 
 
 
DIPK2A
 
 
hsa-miR-152-3p
 
 
-0.393
 
 
13.3
 
 
 
 
DMPK
 
 
hsa-miR-152-3p
 
 
-0.378
 
 
10.4
 
 
 
 
DNAJB4
 
 
hsa-miR-152-3p
 
 
-0.33
 
 
30.2
 
 
 
 
DUSP1
 
 
hsa-miR-152-3p
 
 
-0.32
 
 
534.6
 
 
 
 
DYNLL2
 
 
hsa-miR-152-3p
 
 
-0.366
 
 
12.5
 
 
 
 
DYRK2
 
 
hsa-miR-152-3p
 
 
-0.293
 
 
13.4
 
 
 
 
EGR3
 
 
hsa-miR-152-3p
 
 
-0.315
 
 
28.1
 
 
 
 
EIF4E3
 
 
hsa-miR-152-3p
 
 
-0.464
 
 
10.0
 
 
 
 
EIF4G3
 
 
hsa-miR-378a-3p
 
 
-0.443
 
 
18.7
 
 
 
 
EOGT
 
 
hsa-miR-152-3p
 
 
-0.587
 
 
16.6
 
 
 
 
EPS15
 
 
hsa-miR-152-3p
 
 
-0.322
 
 
24.0
 
 
 
 
ERBB3
 
 
hsa-miR-152-3p
 
 
-0.399
 
 
24.3
 
 
 
 
ERRFI1
 
 
hsa-miR-152-3p
 
 
-0.529
 
 
122.6
 
 
 
 
F3
 
 
hsa-miR-152-3p
 
 
-0.331
 
 
55.8
 
 
 
 
FAM168B
 
 
hsa-miR-152-3p
 
 
-0.409
 
 
22.2
 
 
 
 
FBN1
 
 
hsa-miR-152-3p
 
 
-0.349
 
 
22.4
 
 
 
 
FEZ2
 
 
hsa-miR-152-3p
 
 
-0.536
 
 
22.2
 
 
 
 
FMR1
 
 
hsa-miR-152-3p
 
 
-0.385
 
 
10.1
 
 
 
 
FOSB
 
 
hsa-miR-152-3p
 
 
-0.311
 
 
324.8
 
 
 
 
FOXF1
 
 
hsa-miR-152-3p
 
 
-0.374
 
 
14.0
 
 
 
 
FXR1
 
 
hsa-miR-152-3p
 
 
-0.478
 
 
24.3
 
 
 
 
GADD45A
 
 
hsa-miR-152-3p
 
 
-0.787
 
 
41.4
 
 
 
 
GLRX5
 
 
hsa-miR-152-3p
 
 
-0.381
 
 
16.4
 
 
 
 
GMFB
 
 
hsa-miR-152-3p
 
 
-0.429
 
 
15.5
 
 
 
 
GRSF1
 
 
hsa-miR-378a-3p
 
 
-0.358
 
 
31.7
 
 
 
 
HDDC2
 
 
hsa-miR-378a-3p
 
 
-0.322
 
 
14.2
 
 
 
 
HLA-DQB1
 
 
hsa-miR-152-3p
 
 
-0.37
 
 
42.2
 
 
 
 
HSP90B1
 
 
hsa-miR-152-3p
 
 
-0.304
 
 
205.3
 
 
 
 
HSPH1
 
 
hsa-miR-330-3p
 
 
-0.345
 
 
30.3
 
 
 
 
ID2
 
 
hsa-miR-330-3p
 
 
-0.293
 
 
141.4
 
 
 
 
IGF1
 
 
hsa-miR-152-3p
 
 
-0.43
 
 
20.1
 
 
 
 
IL6ST
 
 
hsa-miR-152-3p
 
 
-0.291
 
 
55.5
 
 
 
 
ITSN2
 
 
hsa-miR-152-3p
 
 
-0.339
 
 
17.7
 
 
 
 
KDSR
 
 
hsa-miR-378a-3p
 
 
-0.373
 
 
11.8
 
 
 
 
KLF4
 
 
hsa-miR-152-3p
 
 
-0.325
 
 
41.5
 
 
 
 
KLF5
 
 
hsa-miR-152-3p
 
 
-0.475
 
 
52.4
 
 
 
 
LAMA4
 
 
hsa-miR-152-3p
 
 
-0.363
 
 
35.0
 
 
 
 
LBR
 
 
hsa-miR-152-3p
 
 
-0.39
 
 
20.4
 
 
 
 
LDLR
 
 
hsa-miR-152-3p
 
 
-0.323
 
 
34.6
 
 
 
 
LEPROTL1
 
 
hsa-miR-152-3p
 
 
-0.568
 
 
22.0
 
 
 
 
LIPA
 
 
hsa-miR-152-3p
 
 
-0.463
 
 
78.2
 
 
 
 
LMAN1
 
 
hsa-miR-152-3p
 
 
-0.382
 
 
29.9
 
 
 
 
LRCH1
 
 
hsa-miR-152-3p
 
 
-0.321
 
 
11.8
 
 
 
 
LRRC41
 
 
hsa-miR-152-3p
 
 
-0.392
 
 
28.9
 
 
 
 
LSM5
 
 
hsa-miR-330-3p
 
 
-0.351
 
 
26.3
 
 
 
 
LYSMD2
 
 
hsa-miR-152-3p
 
 
-0.554
 
 
10.7
 
 
 
 
MACROH2A1
 
 
hsa-miR-152-3p
 
 
-0.336
 
 
61.0
 
 
 
 
MAF
 
 
hsa-miR-152-3p
 
 
-0.362
 
 
15.7
 
 
 
 
MAF1
 
 
hsa-miR-152-3p
 
 
-0.366
 
 
59.1
 
 
 
 
MAFB
 
 
hsa-miR-152-3p
 
 
-0.45
 
 
22.8
 
 
 
 
MAFG
 
 
hsa-miR-152-3p
 
 
-0.328
 
 
10.1
 
 
 
 
MAP2K1
 
 
hsa-miR-152-3p
 
 
-0.295
 
 
22.0
 
 
 
 
MAPK1
 
 
hsa-miR-378a-3p
 
 
-0.314
 
 
27.1
 
 
 
 
 
 
 hippocampal formation 
 
 
 
 
target_symbol
 
 
mature_mirna_id
 
 
score
 
 
nTPM
 
 
 
 
 
 
ABCD3
 
 
hsa-miR-152-3p
 
 
-0.388
 
 
15.1
 
 
 
 
ABHD17C
 
 
hsa-miR-330-3p
 
 
-0.399
 
 
14.4
 
 
 
 
ADCY2
 
 
hsa-miR-152-3p
 
 
-0.299
 
 
18.5
 
 
 
 
ADGRB3
 
 
hsa-miR-152-3p
 
 
-0.516
 
 
12.8
 
 
 
 
ADGRF5
 
 
hsa-miR-152-3p
 
 
-0.354
 
 
10.4
 
 
 
 
AGFG1
 
 
hsa-miR-152-3p
 
 
-0.332
 
 
29.8
 
 
 
 
AKAP1
 
 
hsa-miR-152-3p
 
 
-0.381
 
 
17.0
 
 
 
 
ALCAM
 
 
hsa-miR-152-3p
 
 
-0.347
 
 
33.8
 
 
 
 
AP4B1
 
 
hsa-miR-378a-3p
 
 
-0.62
 
 
10.1
 
 
 
 
ARF4
 
 
hsa-miR-152-3p
 
 
-0.487
 
 
61.3
 
 
 
 
ARHGAP21
 
 
hsa-miR-152-3p
 
 
-0.287
 
 
50.4
 
 
 
 
ARL6IP1
 
 
hsa-miR-152-3p
 
 
-0.579
 
 
196.8
 
 
 
 
ARL8B
 
 
hsa-miR-152-3p
 
 
-0.337
 
 
53.8
 
 
 
 
ARRDC3
 
 
hsa-miR-152-3p
 
 
-0.305
 
 
11.5
 
 
 
 
ASPH
 
 
hsa-miR-152-3p
 
 
-0.391
 
 
45.5
 
 
 
 
ATP6AP2
 
 
hsa-miR-152-3p
 
 
-0.592
 
 
69.0
 
 
 
 
ATP6AP2
 
 
hsa-miR-152-3p
 
 
-0.668
 
 
69.0
 
 
 
 
B4GALT5
 
 
hsa-miR-152-3p
 
 
-0.44
 
 
10.8
 
 
 
 
BHLHE41
 
 
hsa-miR-152-3p
 
 
-0.442
 
 
27.9
 
 
 
 
BTBD3
 
 
hsa-miR-152-3p
 
 
-0.495
 
 
49.8
 
 
 
 
C1GALT1
 
 
hsa-miR-152-3p
 
 
-0.487
 
 
11.6
 
 
 
 
C1QBP
 
 
hsa-miR-330-3p
 
 
-0.412
 
 
85.7
 
 
 
 
C1orf35
 
 
hsa-miR-152-3p
 
 
-0.511
 
 
14.2
 
 
 
 
CABP7
 
 
hsa-miR-152-3p
 
 
-0.511
 
 
52.8
 
 
 
 
CAND1
 
 
hsa-miR-152-3p
 
 
-0.44
 
 
16.6
 
 
 
 
CANX
 
 
hsa-miR-152-3p
 
 
-0.434
 
 
100.3
 
 
 
 
CCDC6
 
 
hsa-miR-152-3p
 
 
-0.511
 
 
13.7
 
 
 
 
CCN2
 
 
hsa-miR-330-3p
 
 
-0.305
 
 
21.3
 
 
 
 
CCT6A
 
 
hsa-miR-152-3p
 
 
-0.665
 
 
43.6
 
 
 
 
CDC25B
 
 
hsa-miR-152-3p
 
 
-0.298
 
 
21.3
 
 
 
 
CDH20
 
 
hsa-miR-152-3p
 
 
-0.297
 
 
12.3
 
 
 
 
CDK19
 
 
hsa-miR-152-3p
 
 
-0.369
 
 
21.6
 
 
 
 
CDK19
 
 
hsa-miR-152-3p
 
 
-0.405
 
 
21.6
 
 
 
 
CDK5R1
 
 
hsa-miR-152-3p
 
 
-0.547
 
 
44.7
 
 
 
 
CERS5
 
 
hsa-miR-152-3p
 
 
-0.311
 
 
20.1
 
 
 
 
CFL2
 
 
hsa-miR-152-3p
 
 
-0.487
 
 
71.6
 
 
 
 
CHMP4B
 
 
hsa-miR-152-3p
 
 
-0.36
 
 
120.6
 
 
 
 
CHST1
 
 
hsa-miR-152-3p
 
 
-0.408
 
 
27.5
 
 
 
 
CLCN6
 
 
hsa-miR-152-3p
 
 
-0.406
 
 
17.9
 
 
 
 
CNOT7
 
 
hsa-miR-152-3p
 
 
-0.31
 
 
16.5
 
 
 
 
CS
 
 
hsa-miR-152-3p
 
 
-0.3
 
 
33.0
 
 
 
 
CTSA
 
 
hsa-miR-152-3p
 
 
-0.541
 
 
28.7
 
 
 
 
CYTH3
 
 
hsa-miR-152-3p
 
 
-0.331
 
 
15.8
 
 
 
 
DCUN1D4
 
 
hsa-miR-152-3p
 
 
-0.314
 
 
12.8
 
 
 
 
DDAH1
 
 
hsa-miR-152-3p
 
 
-0.34
 
 
66.7
 
 
 
 
DDX6
 
 
hsa-miR-152-3p
 
 
-0.363
 
 
20.4
 
 
 
 
DEDD
 
 
hsa-miR-152-3p
 
 
-0.401
 
 
15.6
 
 
 
 
DMPK
 
 
hsa-miR-152-3p
 
 
-0.378
 
 
14.7
 
 
 
 
DNAJB4
 
 
hsa-miR-152-3p
 
 
-0.33
 
 
13.5
 
 
 
 
DUSP1
 
 
hsa-miR-152-3p
 
 
-0.32
 
 
94.3
 
 
 
 
DYNLL2
 
 
hsa-miR-152-3p
 
 
-0.366
 
 
57.7
 
 
 
 
DYRK1B
 
 
hsa-miR-152-3p
 
 
-0.305
 
 
17.2
 
 
 
 
EGR3
 
 
hsa-miR-152-3p
 
 
-0.315
 
 
10.0
 
 
 
 
EIF4E3
 
 
hsa-miR-152-3p
 
 
-0.464
 
 
16.6
 
 
 
 
EIF4G3
 
 
hsa-miR-378a-3p
 
 
-0.443
 
 
15.0
 
 
 
 
EMX2
 
 
hsa-miR-152-3p
 
 
-0.366
 
 
16.3
 
 
 
 
EPS15
 
 
hsa-miR-152-3p
 
 
-0.322
 
 
42.0
 
 
 
 
ERBB3
 
 
hsa-miR-152-3p
 
 
-0.399
 
 
35.4
 
 
 
 
ERRFI1
 
 
hsa-miR-152-3p
 
 
-0.529
 
 
12.9
 
 
 
 
F3
 
 
hsa-miR-152-3p
 
 
-0.331
 
 
46.1
 
 
 
 
FAM168B
 
 
hsa-miR-152-3p
 
 
-0.409
 
 
49.3
 
 
 
 
FAM184A
 
 
hsa-miR-152-3p
 
 
-0.371
 
 
13.6
 
 
 
 
FAM81A
 
 
hsa-miR-152-3p
 
 
-0.315
 
 
11.8
 
 
 
 
FBXL19
 
 
hsa-miR-152-3p
 
 
-0.362
 
 
26.4
 
 
 
 
FEZ2
 
 
hsa-miR-152-3p
 
 
-0.536
 
 
41.7
 
 
 
 
FOXK2
 
 
hsa-miR-152-3p
 
 
-0.305
 
 
12.4
 
 
 
 
FXR1
 
 
hsa-miR-152-3p
 
 
-0.478
 
 
35.2
 
 
 
 
GADD45A
 
 
hsa-miR-152-3p
 
 
-0.787
 
 
36.4
 
 
 
 
GAP43
 
 
hsa-miR-152-3p
 
 
-0.409
 
 
161.0
 
 
 
 
GLRX5
 
 
hsa-miR-152-3p
 
 
-0.381
 
 
57.2
 
 
 
 
GMFB
 
 
hsa-miR-152-3p
 
 
-0.429
 
 
55.9
 
 
 
 
GPATCH8
 
 
hsa-miR-152-3p
 
 
-0.374
 
 
11.6
 
 
 
 
GRSF1
 
 
hsa-miR-378a-3p
 
 
-0.358
 
 
29.0
 
 
 
 
GTF2H1
 
 
hsa-miR-152-3p
 
 
-0.524
 
 
12.3
 
 
 
 
HDDC2
 
 
hsa-miR-378a-3p
 
 
-0.322
 
 
92.7
 
 
 
 
HSP90B1
 
 
hsa-miR-152-3p
 
 
-0.304
 
 
108.7
 
 
 
 
HSPH1
 
 
hsa-miR-330-3p
 
 
-0.345
 
 
119.8
 
 
 
 
ID2
 
 
hsa-miR-330-3p
 
 
-0.293
 
 
78.5
 
 
 
 
IL6ST
 
 
hsa-miR-152-3p
 
 
-0.291
 
 
23.4
 
 
 
 
JPH3
 
 
hsa-miR-152-3p
 
 
-0.303
 
 
112.1
 
 
 
 
KDSR
 
 
hsa-miR-378a-3p
 
 
-0.373
 
 
10.4
 
 
 
 
LEPROTL1
 
 
hsa-miR-152-3p
 
 
-0.568
 
 
16.6
 
 
 
 
LIPA
 
 
hsa-miR-152-3p
 
 
-0.463
 
 
46.7
 
 
 
 
LMAN1
 
 
hsa-miR-152-3p
 
 
-0.382
 
 
11.2
 
 
 
 
LRRC41
 
 
hsa-miR-152-3p
 
 
-0.392
 
 
23.7
 
 
 
 
LSM5
 
 
hsa-miR-330-3p
 
 
-0.351
 
 
27.5
 
 
 
 
LYRM2
 
 
hsa-miR-152-3p
 
 
-0.351
 
 
11.7
 
 
 
 
LYSMD2
 
 
hsa-miR-152-3p
 
 
-0.554
 
 
31.3
 
 
 
 
MACIR
 
 
hsa-miR-152-3p
 
 
-0.332
 
 
14.7
 
 
 
 
MACIR
 
 
hsa-miR-152-3p
 
 
-0.573
 
 
14.7
 
 
 
 
MACROH2A1
 
 
hsa-miR-152-3p
 
 
-0.336
 
 
41.6
 
 
 
 
MAF1
 
 
hsa-miR-152-3p
 
 
-0.366
 
 
57.8
 
 
 
 
MAFG
 
 
hsa-miR-152-3p
 
 
-0.328
 
 
17.4
 
 
 
 
MAP2K1
 
 
hsa-miR-152-3p
 
 
-0.295
 
 
25.7
 
 
 
 
MAPK1
 
 
hsa-miR-378a-3p
 
 
-0.314
 
 
57.5
 
 
 
 
MAT2A
 
 
hsa-miR-330-3p
 
 
-0.307
 
 
136.5
 
 
 
 
MECP2
 
 
hsa-miR-152-3p
 
 
-0.3
 
 
10.7
 
 
 
 
MED19
 
 
hsa-miR-378a-3p
 
 
-0.434
 
 
14.3
 
 
 
 
MLLT10
 
 
hsa-miR-152-3p
 
 
-0.334
 
 
10.7
 
 
 
 
MMD
 
 
hsa-miR-152-3p
 
 
-0.62
 
 
73.2
 
 
 
 
 
 
 hypothalamus 
 
 
 
 
target_symbol
 
 
mature_mirna_id
 
 
score
 
 
nTPM
 
 
 
 
 
 
ABCD3
 
 
hsa-miR-152-3p
 
 
-0.388
 
 
13.5
 
 
 
 
ABHD17C
 
 
hsa-miR-330-3p
 
 
-0.399
 
 
11.1
 
 
 
 
ADCY2
 
 
hsa-miR-152-3p
 
 
-0.299
 
 
14.0
 
 
 
 
ADGRB3
 
 
hsa-miR-152-3p
 
 
-0.516
 
 
16.0
 
 
 
 
AGFG1
 
 
hsa-miR-152-3p
 
 
-0.332
 
 
26.0
 
 
 
 
AHDC1
 
 
hsa-miR-152-3p
 
 
-0.413
 
 
10.9
 
 
 
 
AKAP1
 
 
hsa-miR-152-3p
 
 
-0.381
 
 
15.1
 
 
 
 
ALCAM
 
 
hsa-miR-152-3p
 
 
-0.347
 
 
36.1
 
 
 
 
AP4B1
 
 
hsa-miR-378a-3p
 
 
-0.62
 
 
10.0
 
 
 
 
ARF4
 
 
hsa-miR-152-3p
 
 
-0.487
 
 
75.3
 
 
 
 
ARHGAP21
 
 
hsa-miR-152-3p
 
 
-0.287
 
 
30.5
 
 
 
 
ARL6IP1
 
 
hsa-miR-152-3p
 
 
-0.579
 
 
212.8
 
 
 
 
ARL8B
 
 
hsa-miR-152-3p
 
 
-0.337
 
 
54.9
 
 
 
 
ARRDC3
 
 
hsa-miR-152-3p
 
 
-0.305
 
 
10.3
 
 
 
 
ASPH
 
 
hsa-miR-152-3p
 
 
-0.391
 
 
51.0
 
 
 
 
ATP6AP2
 
 
hsa-miR-152-3p
 
 
-0.592
 
 
69.0
 
 
 
 
ATP6AP2
 
 
hsa-miR-152-3p
 
 
-0.668
 
 
69.0
 
 
 
 
B4GALT5
 
 
hsa-miR-152-3p
 
 
-0.44
 
 
13.0
 
 
 
 
BHLHE41
 
 
hsa-miR-152-3p
 
 
-0.442
 
 
27.6
 
 
 
 
BTBD3
 
 
hsa-miR-152-3p
 
 
-0.495
 
 
24.8
 
 
 
 
C1QBP
 
 
hsa-miR-330-3p
 
 
-0.412
 
 
89.2
 
 
 
 
C1orf35
 
 
hsa-miR-152-3p
 
 
-0.511
 
 
12.9
 
 
 
 
CAND1
 
 
hsa-miR-152-3p
 
 
-0.44
 
 
18.8
 
 
 
 
CANX
 
 
hsa-miR-152-3p
 
 
-0.434
 
 
136.0
 
 
 
 
CCDC6
 
 
hsa-miR-152-3p
 
 
-0.511
 
 
14.2
 
 
 
 
CCN2
 
 
hsa-miR-330-3p
 
 
-0.305
 
 
14.8
 
 
 
 
CCT6A
 
 
hsa-miR-152-3p
 
 
-0.665
 
 
50.6
 
 
 
 
CDC25B
 
 
hsa-miR-152-3p
 
 
-0.298
 
 
25.5
 
 
 
 
CDK19
 
 
hsa-miR-152-3p
 
 
-0.369
 
 
11.1
 
 
 
 
CDK19
 
 
hsa-miR-152-3p
 
 
-0.405
 
 
11.1
 
 
 
 
CDK5R1
 
 
hsa-miR-152-3p
 
 
-0.547
 
 
35.3
 
 
 
 
CEBPG
 
 
hsa-miR-152-3p
 
 
-0.319
 
 
11.3
 
 
 
 
CERS5
 
 
hsa-miR-152-3p
 
 
-0.311
 
 
22.6
 
 
 
 
CFL2
 
 
hsa-miR-152-3p
 
 
-0.487
 
 
54.0
 
 
 
 
CHMP4B
 
 
hsa-miR-152-3p
 
 
-0.36
 
 
120.3
 
 
 
 
CHST1
 
 
hsa-miR-152-3p
 
 
-0.408
 
 
21.9
 
 
 
 
CLCN6
 
 
hsa-miR-152-3p
 
 
-0.406
 
 
23.6
 
 
 
 
CNOT7
 
 
hsa-miR-152-3p
 
 
-0.31
 
 
23.3
 
 
 
 
CS
 
 
hsa-miR-152-3p
 
 
-0.3
 
 
41.8
 
 
 
 
CTSA
 
 
hsa-miR-152-3p
 
 
-0.541
 
 
28.6
 
 
 
 
CYTH3
 
 
hsa-miR-152-3p
 
 
-0.331
 
 
10.1
 
 
 
 
DCUN1D4
 
 
hsa-miR-152-3p
 
 
-0.314
 
 
18.3
 
 
 
 
DDAH1
 
 
hsa-miR-152-3p
 
 
-0.34
 
 
53.9
 
 
 
 
DDX6
 
 
hsa-miR-152-3p
 
 
-0.363
 
 
21.5
 
 
 
 
DEDD
 
 
hsa-miR-152-3p
 
 
-0.401
 
 
15.2
 
 
 
 
DMPK
 
 
hsa-miR-152-3p
 
 
-0.378
 
 
13.7
 
 
 
 
DNAJB4
 
 
hsa-miR-152-3p
 
 
-0.33
 
 
14.3
 
 
 
 
DUSP1
 
 
hsa-miR-152-3p
 
 
-0.32
 
 
89.7
 
 
 
 
DYNLL2
 
 
hsa-miR-152-3p
 
 
-0.366
 
 
71.3
 
 
 
 
DYRK1B
 
 
hsa-miR-152-3p
 
 
-0.305
 
 
15.0
 
 
 
 
EIF4E3
 
 
hsa-miR-152-3p
 
 
-0.464
 
 
13.4
 
 
 
 
EIF4G3
 
 
hsa-miR-378a-3p
 
 
-0.443
 
 
15.2
 
 
 
 
ELAVL2
 
 
hsa-miR-152-3p
 
 
-0.3
 
 
20.3
 
 
 
 
EPS15
 
 
hsa-miR-152-3p
 
 
-0.322
 
 
28.5
 
 
 
 
ERBB3
 
 
hsa-miR-152-3p
 
 
-0.399
 
 
18.5
 
 
 
 
ERRFI1
 
 
hsa-miR-152-3p
 
 
-0.529
 
 
13.2
 
 
 
 
F3
 
 
hsa-miR-152-3p
 
 
-0.331
 
 
40.4
 
 
 
 
FAM168B
 
 
hsa-miR-152-3p
 
 
-0.409
 
 
40.8
 
 
 
 
FAM184A
 
 
hsa-miR-152-3p
 
 
-0.371
 
 
12.7
 
 
 
 
FBXL19
 
 
hsa-miR-152-3p
 
 
-0.362
 
 
14.4
 
 
 
 
FEZ2
 
 
hsa-miR-152-3p
 
 
-0.536
 
 
36.5
 
 
 
 
FOXK2
 
 
hsa-miR-152-3p
 
 
-0.305
 
 
12.1
 
 
 
 
FXR1
 
 
hsa-miR-152-3p
 
 
-0.478
 
 
37.5
 
 
 
 
GADD45A
 
 
hsa-miR-152-3p
 
 
-0.787
 
 
40.7
 
 
 
 
GAP43
 
 
hsa-miR-152-3p
 
 
-0.409
 
 
309.8
 
 
 
 
GLRX5
 
 
hsa-miR-152-3p
 
 
-0.381
 
 
52.8
 
 
 
 
GMFB
 
 
hsa-miR-152-3p
 
 
-0.429
 
 
48.8
 
 
 
 
GOLT1A
 
 
hsa-miR-378a-3p
 
 
-0.574
 
 
13.8
 
 
 
 
GPATCH8
 
 
hsa-miR-152-3p
 
 
-0.374
 
 
11.8
 
 
 
 
GRSF1
 
 
hsa-miR-378a-3p
 
 
-0.358
 
 
29.8
 
 
 
 
GTF2H1
 
 
hsa-miR-152-3p
 
 
-0.524
 
 
12.8
 
 
 
 
HDDC2
 
 
hsa-miR-378a-3p
 
 
-0.322
 
 
85.8
 
 
 
 
HLA-DQB1
 
 
hsa-miR-152-3p
 
 
-0.37
 
 
10.9
 
 
 
 
HSP90B1
 
 
hsa-miR-152-3p
 
 
-0.304
 
 
126.7
 
 
 
 
HSPH1
 
 
hsa-miR-330-3p
 
 
-0.345
 
 
110.3
 
 
 
 
ID2
 
 
hsa-miR-330-3p
 
 
-0.293
 
 
56.2
 
 
 
 
IL6ST
 
 
hsa-miR-152-3p
 
 
-0.291
 
 
21.2
 
 
 
 
JPH3
 
 
hsa-miR-152-3p
 
 
-0.303
 
 
45.8
 
 
 
 
KDSR
 
 
hsa-miR-378a-3p
 
 
-0.373
 
 
10.5
 
 
 
 
LEPROTL1
 
 
hsa-miR-152-3p
 
 
-0.568
 
 
17.2
 
 
 
 
LIPA
 
 
hsa-miR-152-3p
 
 
-0.463
 
 
28.8
 
 
 
 
LMAN1
 
 
hsa-miR-152-3p
 
 
-0.382
 
 
12.1
 
 
 
 
LRRC41
 
 
hsa-miR-152-3p
 
 
-0.392
 
 
24.3
 
 
 
 
LSM5
 
 
hsa-miR-330-3p
 
 
-0.351
 
 
33.4
 
 
 
 
LYRM2
 
 
hsa-miR-152-3p
 
 
-0.351
 
 
13.6
 
 
 
 
LYSMD2
 
 
hsa-miR-152-3p
 
 
-0.554
 
 
34.9
 
 
 
 
MACIR
 
 
hsa-miR-152-3p
 
 
-0.332
 
 
12.4
 
 
 
 
MACIR
 
 
hsa-miR-152-3p
 
 
-0.573
 
 
12.4
 
 
 
 
MACROH2A1
 
 
hsa-miR-152-3p
 
 
-0.336
 
 
57.1
 
 
 
 
MAF1
 
 
hsa-miR-152-3p
 
 
-0.366
 
 
50.9
 
 
 
 
MAFG
 
 
hsa-miR-152-3p
 
 
-0.328
 
 
17.3
 
 
 
 
MAP2K1
 
 
hsa-miR-152-3p
 
 
-0.295
 
 
22.7
 
 
 
 
MAPK1
 
 
hsa-miR-378a-3p
 
 
-0.314
 
 
43.9
 
 
 
 
MAT2A
 
 
hsa-miR-330-3p
 
 
-0.307
 
 
124.3
 
 
 
 
MECP2
 
 
hsa-miR-152-3p
 
 
-0.3
 
 
11.3
 
 
 
 
MED19
 
 
hsa-miR-378a-3p
 
 
-0.434
 
 
14.6
 
 
 
 
MLLT10
 
 
hsa-miR-152-3p
 
 
-0.334
 
 
10.0
 
 
 
 
MMD
 
 
hsa-miR-152-3p
 
 
-0.62
 
 
19.6
 
 
 
 
MNAT1
 
 
hsa-miR-378a-3p
 
 
-0.398
 
 
19.4
 
 
 
 
MRAS
 
 
hsa-miR-152-3p
 
 
-0.583
 
 
78.6
 
 
 
 
 
 
 kidney 
 
 
 
 
target_symbol
 
 
mature_mirna_id
 
 
score
 
 
nTPM
 
 
 
 
 
 
ABCD3
 
 
hsa-miR-152-3p
 
 
-0.388
 
 
55.5
 
 
 
 
ABHD17C
 
 
hsa-miR-330-3p
 
 
-0.399
 
 
13.6
 
 
 
 
ACVR1
 
 
hsa-miR-152-3p
 
 
-0.397
 
 
15.0
 
 
 
 
ADAM10
 
 
hsa-miR-152-3p
 
 
-0.348
 
 
16.8
 
 
 
 
ADGRF5
 
 
hsa-miR-152-3p
 
 
-0.354
 
 
55.8
 
 
 
 
AGFG1
 
 
hsa-miR-152-3p
 
 
-0.332
 
 
30.6
 
 
 
 
AHDC1
 
 
hsa-miR-152-3p
 
 
-0.413
 
 
12.2
 
 
 
 
AKAP1
 
 
hsa-miR-152-3p
 
 
-0.381
 
 
24.5
 
 
 
 
ALCAM
 
 
hsa-miR-152-3p
 
 
-0.347
 
 
20.8
 
 
 
 
ARF4
 
 
hsa-miR-152-3p
 
 
-0.487
 
 
106.7
 
 
 
 
ARFGEF1
 
 
hsa-miR-152-3p
 
 
-0.306
 
 
21.3
 
 
 
 
ARFIP1
 
 
hsa-miR-152-3p
 
 
-0.474
 
 
18.5
 
 
 
 
ARHGAP21
 
 
hsa-miR-152-3p
 
 
-0.287
 
 
10.2
 
 
 
 
ARL6IP1
 
 
hsa-miR-152-3p
 
 
-0.579
 
 
140.2
 
 
 
 
ARL8B
 
 
hsa-miR-152-3p
 
 
-0.337
 
 
50.2
 
 
 
 
ARRDC3
 
 
hsa-miR-152-3p
 
 
-0.305
 
 
49.3
 
 
 
 
ASPH
 
 
hsa-miR-152-3p
 
 
-0.391
 
 
55.2
 
 
 
 
ATP6AP2
 
 
hsa-miR-152-3p
 
 
-0.592
 
 
154.5
 
 
 
 
ATP6AP2
 
 
hsa-miR-152-3p
 
 
-0.668
 
 
154.5
 
 
 
 
B4GALT5
 
 
hsa-miR-152-3p
 
 
-0.44
 
 
21.3
 
 
 
 
BCL2L11
 
 
hsa-miR-152-3p
 
 
-0.445
 
 
10.8
 
 
 
 
BICC1
 
 
hsa-miR-152-3p
 
 
-0.442
 
 
26.8
 
 
 
 
BTBD3
 
 
hsa-miR-152-3p
 
 
-0.495
 
 
10.9
 
 
 
 
C1GALT1
 
 
hsa-miR-152-3p
 
 
-0.487
 
 
28.8
 
 
 
 
C1QBP
 
 
hsa-miR-330-3p
 
 
-0.412
 
 
127.0
 
 
 
 
C1orf210
 
 
hsa-miR-378a-3p
 
 
-0.307
 
 
20.3
 
 
 
 
C1orf35
 
 
hsa-miR-152-3p
 
 
-0.511
 
 
13.6
 
 
 
 
CAND1
 
 
hsa-miR-152-3p
 
 
-0.44
 
 
22.7
 
 
 
 
CANX
 
 
hsa-miR-152-3p
 
 
-0.434
 
 
100.9
 
 
 
 
CAV2
 
 
hsa-miR-152-3p
 
 
-0.32
 
 
15.9
 
 
 
 
CCDC6
 
 
hsa-miR-152-3p
 
 
-0.511
 
 
19.8
 
 
 
 
CCDC71
 
 
hsa-miR-152-3p
 
 
-0.369
 
 
10.6
 
 
 
 
CCN2
 
 
hsa-miR-330-3p
 
 
-0.305
 
 
220.1
 
 
 
 
CCT6A
 
 
hsa-miR-152-3p
 
 
-0.665
 
 
68.3
 
 
 
 
CDC25B
 
 
hsa-miR-152-3p
 
 
-0.298
 
 
30.2
 
 
 
 
CEBPG
 
 
hsa-miR-152-3p
 
 
-0.319
 
 
12.0
 
 
 
 
CEP63
 
 
hsa-miR-152-3p
 
 
-0.329
 
 
13.3
 
 
 
 
CERS5
 
 
hsa-miR-152-3p
 
 
-0.311
 
 
27.1
 
 
 
 
CFL2
 
 
hsa-miR-152-3p
 
 
-0.487
 
 
29.3
 
 
 
 
CHMP4B
 
 
hsa-miR-152-3p
 
 
-0.36
 
 
109.2
 
 
 
 
CLCN6
 
 
hsa-miR-152-3p
 
 
-0.406
 
 
11.0
 
 
 
 
CNOT7
 
 
hsa-miR-152-3p
 
 
-0.31
 
 
47.2
 
 
 
 
COL4A1
 
 
hsa-miR-152-3p
 
 
-0.317
 
 
42.1
 
 
 
 
CS
 
 
hsa-miR-152-3p
 
 
-0.3
 
 
80.8
 
 
 
 
CTSA
 
 
hsa-miR-152-3p
 
 
-0.541
 
 
165.5
 
 
 
 
CUL5
 
 
hsa-miR-152-3p
 
 
-0.389
 
 
15.2
 
 
 
 
CYTH3
 
 
hsa-miR-152-3p
 
 
-0.331
 
 
14.9
 
 
 
 
DCUN1D4
 
 
hsa-miR-152-3p
 
 
-0.314
 
 
13.9
 
 
 
 
DDAH1
 
 
hsa-miR-152-3p
 
 
-0.34
 
 
116.6
 
 
 
 
DDX6
 
 
hsa-miR-152-3p
 
 
-0.363
 
 
28.6
 
 
 
 
DEDD
 
 
hsa-miR-152-3p
 
 
-0.401
 
 
21.0
 
 
 
 
DICER1
 
 
hsa-miR-152-3p
 
 
-0.29
 
 
10.0
 
 
 
 
DMPK
 
 
hsa-miR-152-3p
 
 
-0.378
 
 
11.2
 
 
 
 
DMXL1
 
 
hsa-miR-152-3p
 
 
-0.29
 
 
12.6
 
 
 
 
DNAJB4
 
 
hsa-miR-152-3p
 
 
-0.33
 
 
19.2
 
 
 
 
DOCK6
 
 
hsa-miR-152-3p
 
 
-0.396
 
 
26.5
 
 
 
 
DUSP1
 
 
hsa-miR-152-3p
 
 
-0.32
 
 
261.2
 
 
 
 
DYNLL2
 
 
hsa-miR-152-3p
 
 
-0.366
 
 
39.4
 
 
 
 
DYRK1B
 
 
hsa-miR-152-3p
 
 
-0.305
 
 
18.1
 
 
 
 
EIF4E3
 
 
hsa-miR-152-3p
 
 
-0.464
 
 
13.0
 
 
 
 
EIF4G3
 
 
hsa-miR-378a-3p
 
 
-0.443
 
 
15.1
 
 
 
 
ELF5
 
 
hsa-miR-152-3p
 
 
-0.421
 
 
15.9
 
 
 
 
EMX2
 
 
hsa-miR-152-3p
 
 
-0.366
 
 
36.5
 
 
 
 
EPS15
 
 
hsa-miR-152-3p
 
 
-0.322
 
 
19.9
 
 
 
 
ERBB3
 
 
hsa-miR-152-3p
 
 
-0.399
 
 
30.8
 
 
 
 
ERRFI1
 
 
hsa-miR-152-3p
 
 
-0.529
 
 
146.6
 
 
 
 
ESRRG
 
 
hsa-miR-152-3p
 
 
-0.347
 
 
27.1
 
 
 
 
EYA3
 
 
hsa-miR-152-3p
 
 
-0.309
 
 
10.9
 
 
 
 
F3
 
 
hsa-miR-152-3p
 
 
-0.331
 
 
16.3
 
 
 
 
FAM168B
 
 
hsa-miR-152-3p
 
 
-0.409
 
 
35.9
 
 
 
 
FAM43A
 
 
hsa-miR-152-3p
 
 
-0.337
 
 
29.3
 
 
 
 
FBXL19
 
 
hsa-miR-152-3p
 
 
-0.362
 
 
11.7
 
 
 
 
FBXO28
 
 
hsa-miR-152-3p
 
 
-0.313
 
 
12.7
 
 
 
 
FEZ2
 
 
hsa-miR-152-3p
 
 
-0.536
 
 
31.4
 
 
 
 
FMR1
 
 
hsa-miR-152-3p
 
 
-0.385
 
 
26.4
 
 
 
 
FOSB
 
 
hsa-miR-152-3p
 
 
-0.311
 
 
32.5
 
 
 
 
FOXK2
 
 
hsa-miR-152-3p
 
 
-0.305
 
 
11.8
 
 
 
 
FXR1
 
 
hsa-miR-152-3p
 
 
-0.478
 
 
25.7
 
 
 
 
GADD45A
 
 
hsa-miR-152-3p
 
 
-0.787
 
 
153.3
 
 
 
 
GAPVD1
 
 
hsa-miR-152-3p
 
 
-0.309
 
 
12.1
 
 
 
 
GLRX5
 
 
hsa-miR-152-3p
 
 
-0.381
 
 
83.3
 
 
 
 
GMFB
 
 
hsa-miR-152-3p
 
 
-0.429
 
 
31.3
 
 
 
 
GOLT1A
 
 
hsa-miR-378a-3p
 
 
-0.574
 
 
17.5
 
 
 
 
GPATCH8
 
 
hsa-miR-152-3p
 
 
-0.374
 
 
11.5
 
 
 
 
GRSF1
 
 
hsa-miR-378a-3p
 
 
-0.358
 
 
49.5
 
 
 
 
GTF2H1
 
 
hsa-miR-152-3p
 
 
-0.524
 
 
15.2
 
 
 
 
HDDC2
 
 
hsa-miR-378a-3p
 
 
-0.322
 
 
54.4
 
 
 
 
HLA-DQB1
 
 
hsa-miR-152-3p
 
 
-0.37
 
 
33.5
 
 
 
 
HSP90B1
 
 
hsa-miR-152-3p
 
 
-0.304
 
 
296.1
 
 
 
 
HSPH1
 
 
hsa-miR-330-3p
 
 
-0.345
 
 
50.2
 
 
 
 
ID2
 
 
hsa-miR-330-3p
 
 
-0.293
 
 
120.3
 
 
 
 
IL6ST
 
 
hsa-miR-152-3p
 
 
-0.291
 
 
49.8
 
 
 
 
ITSN2
 
 
hsa-miR-152-3p
 
 
-0.339
 
 
19.7
 
 
 
 
KDSR
 
 
hsa-miR-378a-3p
 
 
-0.373
 
 
14.2
 
 
 
 
LAMA4
 
 
hsa-miR-152-3p
 
 
-0.363
 
 
12.7
 
 
 
 
LBR
 
 
hsa-miR-152-3p
 
 
-0.39
 
 
18.1
 
 
 
 
LDLR
 
 
hsa-miR-152-3p
 
 
-0.323
 
 
10.4
 
 
 
 
LEPROTL1
 
 
hsa-miR-152-3p
 
 
-0.568
 
 
27.3
 
 
 
 
LIPA
 
 
hsa-miR-152-3p
 
 
-0.463
 
 
41.9
 
 
 
 
LMAN1
 
 
hsa-miR-152-3p
 
 
-0.382
 
 
60.3
 
 
 
 
 
 
 lung 
 
 
 
 
target_symbol
 
 
mature_mirna_id
 
 
score
 
 
nTPM
 
 
 
 
 
 
ABCD3
 
 
hsa-miR-152-3p
 
 
-0.388
 
 
22.0
 
 
 
 
ABHD17C
 
 
hsa-miR-330-3p
 
 
-0.399
 
 
18.6
 
 
 
 
ACVR1
 
 
hsa-miR-152-3p
 
 
-0.397
 
 
12.2
 
 
 
 
ADAM10
 
 
hsa-miR-152-3p
 
 
-0.348
 
 
19.7
 
 
 
 
ADAM17
 
 
hsa-miR-152-3p
 
 
-0.301
 
 
12.5
 
 
 
 
ADGRF5
 
 
hsa-miR-152-3p
 
 
-0.354
 
 
99.9
 
 
 
 
AGFG1
 
 
hsa-miR-152-3p
 
 
-0.332
 
 
51.7
 
 
 
 
AHDC1
 
 
hsa-miR-152-3p
 
 
-0.413
 
 
11.4
 
 
 
 
AKAP1
 
 
hsa-miR-152-3p
 
 
-0.381
 
 
18.4
 
 
 
 
ALCAM
 
 
hsa-miR-152-3p
 
 
-0.347
 
 
69.9
 
 
 
 
ARF4
 
 
hsa-miR-152-3p
 
 
-0.487
 
 
116.3
 
 
 
 
ARFGEF1
 
 
hsa-miR-152-3p
 
 
-0.306
 
 
13.0
 
 
 
 
ARFIP1
 
 
hsa-miR-152-3p
 
 
-0.474
 
 
15.8
 
 
 
 
ARHGAP21
 
 
hsa-miR-152-3p
 
 
-0.287
 
 
20.4
 
 
 
 
ARL6IP1
 
 
hsa-miR-152-3p
 
 
-0.579
 
 
75.1
 
 
 
 
ARL8B
 
 
hsa-miR-152-3p
 
 
-0.337
 
 
41.1
 
 
 
 
ARRDC3
 
 
hsa-miR-152-3p
 
 
-0.305
 
 
42.0
 
 
 
 
ASPH
 
 
hsa-miR-152-3p
 
 
-0.391
 
 
69.4
 
 
 
 
ATP2B4
 
 
hsa-miR-152-3p
 
 
-0.337
 
 
33.0
 
 
 
 
ATP6AP2
 
 
hsa-miR-152-3p
 
 
-0.592
 
 
82.3
 
 
 
 
ATP6AP2
 
 
hsa-miR-152-3p
 
 
-0.668
 
 
82.3
 
 
 
 
B4GALT5
 
 
hsa-miR-152-3p
 
 
-0.44
 
 
26.0
 
 
 
 
BHLHE41
 
 
hsa-miR-152-3p
 
 
-0.442
 
 
10.9
 
 
 
 
BMP2
 
 
hsa-miR-378a-3p
 
 
-0.481
 
 
17.8
 
 
 
 
BTAF1
 
 
hsa-miR-152-3p
 
 
-0.401
 
 
15.8
 
 
 
 
BTBD3
 
 
hsa-miR-152-3p
 
 
-0.495
 
 
11.5
 
 
 
 
C1GALT1
 
 
hsa-miR-152-3p
 
 
-0.487
 
 
14.2
 
 
 
 
C1QBP
 
 
hsa-miR-330-3p
 
 
-0.412
 
 
52.8
 
 
 
 
CAND1
 
 
hsa-miR-152-3p
 
 
-0.44
 
 
15.8
 
 
 
 
CANX
 
 
hsa-miR-152-3p
 
 
-0.434
 
 
97.8
 
 
 
 
CAV2
 
 
hsa-miR-152-3p
 
 
-0.32
 
 
152.0
 
 
 
 
CCDC6
 
 
hsa-miR-152-3p
 
 
-0.511
 
 
16.3
 
 
 
 
CCDC71
 
 
hsa-miR-152-3p
 
 
-0.369
 
 
10.5
 
 
 
 
CCN2
 
 
hsa-miR-330-3p
 
 
-0.305
 
 
212.8
 
 
 
 
CCT6A
 
 
hsa-miR-152-3p
 
 
-0.665
 
 
42.2
 
 
 
 
CDC25B
 
 
hsa-miR-152-3p
 
 
-0.298
 
 
76.1
 
 
 
 
CEBPG
 
 
hsa-miR-152-3p
 
 
-0.319
 
 
12.1
 
 
 
 
CEP63
 
 
hsa-miR-152-3p
 
 
-0.329
 
 
10.0
 
 
 
 
CERS5
 
 
hsa-miR-152-3p
 
 
-0.311
 
 
16.7
 
 
 
 
CFL2
 
 
hsa-miR-152-3p
 
 
-0.487
 
 
23.4
 
 
 
 
CHIT1
 
 
hsa-miR-378a-3p
 
 
-0.387
 
 
34.3
 
 
 
 
CHMP4B
 
 
hsa-miR-152-3p
 
 
-0.36
 
 
100.8
 
 
 
 
CLCN6
 
 
hsa-miR-152-3p
 
 
-0.406
 
 
12.8
 
 
 
 
CNOT7
 
 
hsa-miR-152-3p
 
 
-0.31
 
 
31.1
 
 
 
 
COL4A1
 
 
hsa-miR-152-3p
 
 
-0.317
 
 
121.2
 
 
 
 
CS
 
 
hsa-miR-152-3p
 
 
-0.3
 
 
57.5
 
 
 
 
CTSA
 
 
hsa-miR-152-3p
 
 
-0.541
 
 
79.9
 
 
 
 
CTTNBP2NL
 
 
hsa-miR-152-3p
 
 
-0.329
 
 
12.2
 
 
 
 
CYTH3
 
 
hsa-miR-152-3p
 
 
-0.331
 
 
18.9
 
 
 
 
DDAH1
 
 
hsa-miR-152-3p
 
 
-0.34
 
 
24.9
 
 
 
 
DDX6
 
 
hsa-miR-152-3p
 
 
-0.363
 
 
25.7
 
 
 
 
DEDD
 
 
hsa-miR-152-3p
 
 
-0.401
 
 
26.9
 
 
 
 
DIPK2A
 
 
hsa-miR-152-3p
 
 
-0.393
 
 
12.2
 
 
 
 
DMPK
 
 
hsa-miR-152-3p
 
 
-0.378
 
 
19.9
 
 
 
 
DNAJB4
 
 
hsa-miR-152-3p
 
 
-0.33
 
 
16.3
 
 
 
 
DOCK6
 
 
hsa-miR-152-3p
 
 
-0.396
 
 
44.1
 
 
 
 
DUSP1
 
 
hsa-miR-152-3p
 
 
-0.32
 
 
678.2
 
 
 
 
DYNLL2
 
 
hsa-miR-152-3p
 
 
-0.366
 
 
16.9
 
 
 
 
EIF4E3
 
 
hsa-miR-152-3p
 
 
-0.464
 
 
15.2
 
 
 
 
EIF4G3
 
 
hsa-miR-378a-3p
 
 
-0.443
 
 
12.4
 
 
 
 
EOGT
 
 
hsa-miR-152-3p
 
 
-0.587
 
 
27.4
 
 
 
 
EPS15
 
 
hsa-miR-152-3p
 
 
-0.322
 
 
23.4
 
 
 
 
ERBB3
 
 
hsa-miR-152-3p
 
 
-0.399
 
 
24.6
 
 
 
 
ERRFI1
 
 
hsa-miR-152-3p
 
 
-0.529
 
 
77.1
 
 
 
 
F3
 
 
hsa-miR-152-3p
 
 
-0.331
 
 
56.8
 
 
 
 
FAM168B
 
 
hsa-miR-152-3p
 
 
-0.409
 
 
26.0
 
 
 
 
FEZ2
 
 
hsa-miR-152-3p
 
 
-0.536
 
 
25.5
 
 
 
 
FMR1
 
 
hsa-miR-152-3p
 
 
-0.385
 
 
14.0
 
 
 
 
FOSB
 
 
hsa-miR-152-3p
 
 
-0.311
 
 
106.9
 
 
 
 
FOXF1
 
 
hsa-miR-152-3p
 
 
-0.374
 
 
32.1
 
 
 
 
FOXK2
 
 
hsa-miR-152-3p
 
 
-0.305
 
 
13.6
 
 
 
 
FXR1
 
 
hsa-miR-152-3p
 
 
-0.478
 
 
23.3
 
 
 
 
GADD45A
 
 
hsa-miR-152-3p
 
 
-0.787
 
 
81.1
 
 
 
 
GAPVD1
 
 
hsa-miR-152-3p
 
 
-0.309
 
 
14.8
 
 
 
 
GLRX5
 
 
hsa-miR-152-3p
 
 
-0.381
 
 
28.7
 
 
 
 
GMFB
 
 
hsa-miR-152-3p
 
 
-0.429
 
 
22.9
 
 
 
 
GPATCH8
 
 
hsa-miR-152-3p
 
 
-0.374
 
 
11.6
 
 
 
 
GRSF1
 
 
hsa-miR-378a-3p
 
 
-0.358
 
 
32.2
 
 
 
 
GTF2H1
 
 
hsa-miR-152-3p
 
 
-0.524
 
 
13.2
 
 
 
 
HDDC2
 
 
hsa-miR-378a-3p
 
 
-0.322
 
 
22.2
 
 
 
 
HLA-DQB1
 
 
hsa-miR-152-3p
 
 
-0.37
 
 
173.2
 
 
 
 
HSP90B1
 
 
hsa-miR-152-3p
 
 
-0.304
 
 
233.6
 
 
 
 
HSPH1
 
 
hsa-miR-330-3p
 
 
-0.345
 
 
62.9
 
 
 
 
ID2
 
 
hsa-miR-330-3p
 
 
-0.293
 
 
102.3
 
 
 
 
IGF1
 
 
hsa-miR-152-3p
 
 
-0.43
 
 
11.8
 
 
 
 
IL6ST
 
 
hsa-miR-152-3p
 
 
-0.291
 
 
49.4
 
 
 
 
ITSN2
 
 
hsa-miR-152-3p
 
 
-0.339
 
 
25.4
 
 
 
 
KDSR
 
 
hsa-miR-378a-3p
 
 
-0.373
 
 
10.3
 
 
 
 
KLF4
 
 
hsa-miR-152-3p
 
 
-0.325
 
 
31.5
 
 
 
 
KLF5
 
 
hsa-miR-152-3p
 
 
-0.475
 
 
27.9
 
 
 
 
LAMA4
 
 
hsa-miR-152-3p
 
 
-0.363
 
 
81.2
 
 
 
 
LBR
 
 
hsa-miR-152-3p
 
 
-0.39
 
 
18.1
 
 
 
 
LDLR
 
 
hsa-miR-152-3p
 
 
-0.323
 
 
51.7
 
 
 
 
LEPROTL1
 
 
hsa-miR-152-3p
 
 
-0.568
 
 
27.9
 
 
 
 
LIPA
 
 
hsa-miR-152-3p
 
 
-0.463
 
 
159.7
 
 
 
 
LMAN1
 
 
hsa-miR-152-3p
 
 
-0.382
 
 
31.7
 
 
 
 
LRCH1
 
 
hsa-miR-152-3p
 
 
-0.321
 
 
13.1
 
 
 
 
LRRC41
 
 
hsa-miR-152-3p
 
 
-0.392
 
 
32.3
 
 
 
 
LSM5
 
 
hsa-miR-330-3p
 
 
-0.351
 
 
26.8
 
 
 
 
LYSMD2
 
 
hsa-miR-152-3p
 
 
-0.554
 
 
13.6
 
 
 
 
 
 
 midbrain 
 
 
 
 
target_symbol
 
 
mature_mirna_id
 
 
score
 
 
nTPM
 
 
 
 
 
 
ABCD3
 
 
hsa-miR-152-3p
 
 
-0.388
 
 
17.1
 
 
 
 
ABHD17C
 
 
hsa-miR-330-3p
 
 
-0.399
 
 
11.2
 
 
 
 
ADAM10
 
 
hsa-miR-152-3p
 
 
-0.348
 
 
11.1
 
 
 
 
ADCY2
 
 
hsa-miR-152-3p
 
 
-0.299
 
 
20.4
 
 
 
 
ADGRF5
 
 
hsa-miR-152-3p
 
 
-0.354
 
 
13.0
 
 
 
 
AGFG1
 
 
hsa-miR-152-3p
 
 
-0.332
 
 
32.4
 
 
 
 
AKAP1
 
 
hsa-miR-152-3p
 
 
-0.381
 
 
19.0
 
 
 
 
ALCAM
 
 
hsa-miR-152-3p
 
 
-0.347
 
 
38.7
 
 
 
 
ARF4
 
 
hsa-miR-152-3p
 
 
-0.487
 
 
62.8
 
 
 
 
ARHGAP21
 
 
hsa-miR-152-3p
 
 
-0.287
 
 
53.0
 
 
 
 
ARL6IP1
 
 
hsa-miR-152-3p
 
 
-0.579
 
 
198.8
 
 
 
 
ARL8B
 
 
hsa-miR-152-3p
 
 
-0.337
 
 
52.4
 
 
 
 
ARRDC3
 
 
hsa-miR-152-3p
 
 
-0.305
 
 
13.8
 
 
 
 
ASPH
 
 
hsa-miR-152-3p
 
 
-0.391
 
 
48.8
 
 
 
 
ATP6AP2
 
 
hsa-miR-152-3p
 
 
-0.592
 
 
65.6
 
 
 
 
ATP6AP2
 
 
hsa-miR-152-3p
 
 
-0.668
 
 
65.6
 
 
 
 
B4GALT5
 
 
hsa-miR-152-3p
 
 
-0.44
 
 
10.3
 
 
 
 
BHLHE41
 
 
hsa-miR-152-3p
 
 
-0.442
 
 
40.4
 
 
 
 
BTBD3
 
 
hsa-miR-152-3p
 
 
-0.495
 
 
26.0
 
 
 
 
C1GALT1
 
 
hsa-miR-152-3p
 
 
-0.487
 
 
13.7
 
 
 
 
C1QBP
 
 
hsa-miR-330-3p
 
 
-0.412
 
 
91.4
 
 
 
 
C1orf35
 
 
hsa-miR-152-3p
 
 
-0.511
 
 
13.7
 
 
 
 
CAND1
 
 
hsa-miR-152-3p
 
 
-0.44
 
 
16.6
 
 
 
 
CANX
 
 
hsa-miR-152-3p
 
 
-0.434
 
 
130.1
 
 
 
 
CAV2
 
 
hsa-miR-152-3p
 
 
-0.32
 
 
12.0
 
 
 
 
CCDC6
 
 
hsa-miR-152-3p
 
 
-0.511
 
 
10.5
 
 
 
 
CCN2
 
 
hsa-miR-330-3p
 
 
-0.305
 
 
16.3
 
 
 
 
CCT6A
 
 
hsa-miR-152-3p
 
 
-0.665
 
 
47.8
 
 
 
 
CDC25B
 
 
hsa-miR-152-3p
 
 
-0.298
 
 
21.5
 
 
 
 
CDH20
 
 
hsa-miR-152-3p
 
 
-0.297
 
 
15.5
 
 
 
 
CDK19
 
 
hsa-miR-152-3p
 
 
-0.369
 
 
17.1
 
 
 
 
CDK19
 
 
hsa-miR-152-3p
 
 
-0.405
 
 
17.1
 
 
 
 
CDK5R1
 
 
hsa-miR-152-3p
 
 
-0.547
 
 
13.7
 
 
 
 
CEBPG
 
 
hsa-miR-152-3p
 
 
-0.319
 
 
10.3
 
 
 
 
CERS5
 
 
hsa-miR-152-3p
 
 
-0.311
 
 
16.1
 
 
 
 
CFL2
 
 
hsa-miR-152-3p
 
 
-0.487
 
 
92.9
 
 
 
 
CHMP4B
 
 
hsa-miR-152-3p
 
 
-0.36
 
 
130.6
 
 
 
 
CHST1
 
 
hsa-miR-152-3p
 
 
-0.408
 
 
19.4
 
 
 
 
CLCN6
 
 
hsa-miR-152-3p
 
 
-0.406
 
 
18.5
 
 
 
 
CNOT7
 
 
hsa-miR-152-3p
 
 
-0.31
 
 
18.4
 
 
 
 
CS
 
 
hsa-miR-152-3p
 
 
-0.3
 
 
38.3
 
 
 
 
CTSA
 
 
hsa-miR-152-3p
 
 
-0.541
 
 
31.1
 
 
 
 
CYTH3
 
 
hsa-miR-152-3p
 
 
-0.331
 
 
12.7
 
 
 
 
DCUN1D4
 
 
hsa-miR-152-3p
 
 
-0.314
 
 
14.7
 
 
 
 
DDAH1
 
 
hsa-miR-152-3p
 
 
-0.34
 
 
60.4
 
 
 
 
DDX6
 
 
hsa-miR-152-3p
 
 
-0.363
 
 
20.4
 
 
 
 
DEDD
 
 
hsa-miR-152-3p
 
 
-0.401
 
 
16.1
 
 
 
 
DMPK
 
 
hsa-miR-152-3p
 
 
-0.378
 
 
16.5
 
 
 
 
DNAJB4
 
 
hsa-miR-152-3p
 
 
-0.33
 
 
16.5
 
 
 
 
DUSP1
 
 
hsa-miR-152-3p
 
 
-0.32
 
 
95.7
 
 
 
 
DYNLL2
 
 
hsa-miR-152-3p
 
 
-0.366
 
 
86.0
 
 
 
 
DYRK1B
 
 
hsa-miR-152-3p
 
 
-0.305
 
 
10.3
 
 
 
 
EIF4E3
 
 
hsa-miR-152-3p
 
 
-0.464
 
 
15.3
 
 
 
 
EIF4G3
 
 
hsa-miR-378a-3p
 
 
-0.443
 
 
13.1
 
 
 
 
ELAVL2
 
 
hsa-miR-152-3p
 
 
-0.3
 
 
15.8
 
 
 
 
EPS15
 
 
hsa-miR-152-3p
 
 
-0.322
 
 
40.8
 
 
 
 
ERBB3
 
 
hsa-miR-152-3p
 
 
-0.399
 
 
37.0
 
 
 
 
ERRFI1
 
 
hsa-miR-152-3p
 
 
-0.529
 
 
14.4
 
 
 
 
F3
 
 
hsa-miR-152-3p
 
 
-0.331
 
 
40.3
 
 
 
 
FAM168B
 
 
hsa-miR-152-3p
 
 
-0.409
 
 
49.2
 
 
 
 
FAM184A
 
 
hsa-miR-152-3p
 
 
-0.371
 
 
10.2
 
 
 
 
FBXL19
 
 
hsa-miR-152-3p
 
 
-0.362
 
 
12.3
 
 
 
 
FEZ2
 
 
hsa-miR-152-3p
 
 
-0.536
 
 
46.2
 
 
 
 
FXR1
 
 
hsa-miR-152-3p
 
 
-0.478
 
 
47.8
 
 
 
 
GADD45A
 
 
hsa-miR-152-3p
 
 
-0.787
 
 
46.5
 
 
 
 
GAP43
 
 
hsa-miR-152-3p
 
 
-0.409
 
 
161.8
 
 
 
 
GLRX5
 
 
hsa-miR-152-3p
 
 
-0.381
 
 
56.1
 
 
 
 
GMFB
 
 
hsa-miR-152-3p
 
 
-0.429
 
 
64.0
 
 
 
 
GPATCH8
 
 
hsa-miR-152-3p
 
 
-0.374
 
 
11.8
 
 
 
 
GRSF1
 
 
hsa-miR-378a-3p
 
 
-0.358
 
 
34.9
 
 
 
 
GTF2H1
 
 
hsa-miR-152-3p
 
 
-0.524
 
 
12.4
 
 
 
 
HDDC2
 
 
hsa-miR-378a-3p
 
 
-0.322
 
 
96.7
 
 
 
 
HLA-DQB1
 
 
hsa-miR-152-3p
 
 
-0.37
 
 
13.7
 
 
 
 
HSP90B1
 
 
hsa-miR-152-3p
 
 
-0.304
 
 
134.7
 
 
 
 
HSPH1
 
 
hsa-miR-330-3p
 
 
-0.345
 
 
76.2
 
 
 
 
ID2
 
 
hsa-miR-330-3p
 
 
-0.293
 
 
74.9
 
 
 
 
IL6ST
 
 
hsa-miR-152-3p
 
 
-0.291
 
 
28.6
 
 
 
 
JPH3
 
 
hsa-miR-152-3p
 
 
-0.303
 
 
22.2
 
 
 
 
KDSR
 
 
hsa-miR-378a-3p
 
 
-0.373
 
 
13.3
 
 
 
 
LAMA4
 
 
hsa-miR-152-3p
 
 
-0.363
 
 
12.3
 
 
 
 
LEPROTL1
 
 
hsa-miR-152-3p
 
 
-0.568
 
 
16.8
 
 
 
 
LIPA
 
 
hsa-miR-152-3p
 
 
-0.463
 
 
56.7
 
 
 
 
LMAN1
 
 
hsa-miR-152-3p
 
 
-0.382
 
 
12.0
 
 
 
 
LRRC41
 
 
hsa-miR-152-3p
 
 
-0.392
 
 
21.8
 
 
 
 
LSM5
 
 
hsa-miR-330-3p
 
 
-0.351
 
 
34.7
 
 
 
 
LYRM2
 
 
hsa-miR-152-3p
 
 
-0.351
 
 
12.7
 
 
 
 
LYSMD2
 
 
hsa-miR-152-3p
 
 
-0.554
 
 
24.6
 
 
 
 
MACIR
 
 
hsa-miR-152-3p
 
 
-0.332
 
 
15.1
 
 
 
 
MACIR
 
 
hsa-miR-152-3p
 
 
-0.573
 
 
15.1
 
 
 
 
MACROH2A1
 
 
hsa-miR-152-3p
 
 
-0.336
 
 
46.4
 
 
 
 
MAF1
 
 
hsa-miR-152-3p
 
 
-0.366
 
 
58.7
 
 
 
 
MAFG
 
 
hsa-miR-152-3p
 
 
-0.328
 
 
17.1
 
 
 
 
MAP2K1
 
 
hsa-miR-152-3p
 
 
-0.295
 
 
13.7
 
 
 
 
MAPK1
 
 
hsa-miR-378a-3p
 
 
-0.314
 
 
35.3
 
 
 
 
MAT2A
 
 
hsa-miR-330-3p
 
 
-0.307
 
 
147.3
 
 
 
 
MECP2
 
 
hsa-miR-152-3p
 
 
-0.3
 
 
11.1
 
 
 
 
MED19
 
 
hsa-miR-378a-3p
 
 
-0.434
 
 
12.4
 
 
 
 
MLLT10
 
 
hsa-miR-152-3p
 
 
-0.334
 
 
11.0
 
 
 
 
MMD
 
 
hsa-miR-152-3p
 
 
-0.62
 
 
33.0
 
 
 
 
MNAT1
 
 
hsa-miR-378a-3p
 
 
-0.398
 
 
19.2
 
 
 
 
 
 
 pancreas 
 
 
 
 
target_symbol
 
 
mature_mirna_id
 
 
score
 
 
nTPM
 
 
 
 
 
 
ABCD3
 
 
hsa-miR-152-3p
 
 
-0.388
 
 
27.0
 
 
 
 
AGFG1
 
 
hsa-miR-152-3p
 
 
-0.332
 
 
33.0
 
 
 
 
AKAP1
 
 
hsa-miR-152-3p
 
 
-0.381
 
 
21.5
 
 
 
 
ALCAM
 
 
hsa-miR-152-3p
 
 
-0.347
 
 
30.8
 
 
 
 
AP4B1
 
 
hsa-miR-378a-3p
 
 
-0.62
 
 
13.3
 
 
 
 
ARF4
 
 
hsa-miR-152-3p
 
 
-0.487
 
 
138.3
 
 
 
 
ARFGEF1
 
 
hsa-miR-152-3p
 
 
-0.306
 
 
16.5
 
 
 
 
ARHGAP21
 
 
hsa-miR-152-3p
 
 
-0.287
 
 
13.3
 
 
 
 
ARL6IP1
 
 
hsa-miR-152-3p
 
 
-0.579
 
 
77.1
 
 
 
 
ARL8B
 
 
hsa-miR-152-3p
 
 
-0.337
 
 
26.7
 
 
 
 
ARRDC3
 
 
hsa-miR-152-3p
 
 
-0.305
 
 
19.3
 
 
 
 
ASPH
 
 
hsa-miR-152-3p
 
 
-0.391
 
 
42.1
 
 
 
 
ATP6AP2
 
 
hsa-miR-152-3p
 
 
-0.592
 
 
50.2
 
 
 
 
ATP6AP2
 
 
hsa-miR-152-3p
 
 
-0.668
 
 
50.2
 
 
 
 
B4GALT5
 
 
hsa-miR-152-3p
 
 
-0.44
 
 
11.8
 
 
 
 
BCL2L11
 
 
hsa-miR-152-3p
 
 
-0.445
 
 
12.8
 
 
 
 
BICC1
 
 
hsa-miR-152-3p
 
 
-0.442
 
 
15.1
 
 
 
 
BTAF1
 
 
hsa-miR-152-3p
 
 
-0.401
 
 
15.0
 
 
 
 
BTBD3
 
 
hsa-miR-152-3p
 
 
-0.495
 
 
12.8
 
 
 
 
C1QBP
 
 
hsa-miR-330-3p
 
 
-0.412
 
 
70.0
 
 
 
 
C1orf210
 
 
hsa-miR-378a-3p
 
 
-0.307
 
 
21.5
 
 
 
 
C1orf35
 
 
hsa-miR-152-3p
 
 
-0.511
 
 
15.1
 
 
 
 
CAND1
 
 
hsa-miR-152-3p
 
 
-0.44
 
 
17.7
 
 
 
 
CANX
 
 
hsa-miR-152-3p
 
 
-0.434
 
 
163.1
 
 
 
 
CAV2
 
 
hsa-miR-152-3p
 
 
-0.32
 
 
26.6
 
 
 
 
CCDC6
 
 
hsa-miR-152-3p
 
 
-0.511
 
 
23.7
 
 
 
 
CCN2
 
 
hsa-miR-330-3p
 
 
-0.305
 
 
182.9
 
 
 
 
CCT6A
 
 
hsa-miR-152-3p
 
 
-0.665
 
 
61.8
 
 
 
 
CDC25B
 
 
hsa-miR-152-3p
 
 
-0.298
 
 
12.4
 
 
 
 
CEBPG
 
 
hsa-miR-152-3p
 
 
-0.319
 
 
26.7
 
 
 
 
CEP63
 
 
hsa-miR-152-3p
 
 
-0.329
 
 
14.0
 
 
 
 
CERS5
 
 
hsa-miR-152-3p
 
 
-0.311
 
 
18.4
 
 
 
 
CHMP4B
 
 
hsa-miR-152-3p
 
 
-0.36
 
 
80.8
 
 
 
 
CNOT7
 
 
hsa-miR-152-3p
 
 
-0.31
 
 
33.9
 
 
 
 
COL4A1
 
 
hsa-miR-152-3p
 
 
-0.317
 
 
34.0
 
 
 
 
CS
 
 
hsa-miR-152-3p
 
 
-0.3
 
 
70.9
 
 
 
 
CTSA
 
 
hsa-miR-152-3p
 
 
-0.541
 
 
42.6
 
 
 
 
DDAH1
 
 
hsa-miR-152-3p
 
 
-0.34
 
 
25.9
 
 
 
 
DDX6
 
 
hsa-miR-152-3p
 
 
-0.363
 
 
29.0
 
 
 
 
DEDD
 
 
hsa-miR-152-3p
 
 
-0.401
 
 
20.1
 
 
 
 
DMPK
 
 
hsa-miR-152-3p
 
 
-0.378
 
 
26.8
 
 
 
 
DMXL1
 
 
hsa-miR-152-3p
 
 
-0.29
 
 
11.2
 
 
 
 
DNAJB4
 
 
hsa-miR-152-3p
 
 
-0.33
 
 
21.4
 
 
 
 
DOCK6
 
 
hsa-miR-152-3p
 
 
-0.396
 
 
14.1
 
 
 
 
DUSP1
 
 
hsa-miR-152-3p
 
 
-0.32
 
 
226.4
 
 
 
 
DYNLL2
 
 
hsa-miR-152-3p
 
 
-0.366
 
 
16.8
 
 
 
 
EIF4E3
 
 
hsa-miR-152-3p
 
 
-0.464
 
 
15.0
 
 
 
 
EIF4G3
 
 
hsa-miR-378a-3p
 
 
-0.443
 
 
11.3
 
 
 
 
EOGT
 
 
hsa-miR-152-3p
 
 
-0.587
 
 
11.8
 
 
 
 
EPS15
 
 
hsa-miR-152-3p
 
 
-0.322
 
 
11.9
 
 
 
 
ERBB3
 
 
hsa-miR-152-3p
 
 
-0.399
 
 
44.9
 
 
 
 
ERRFI1
 
 
hsa-miR-152-3p
 
 
-0.529
 
 
640.2
 
 
 
 
F3
 
 
hsa-miR-152-3p
 
 
-0.331
 
 
93.2
 
 
 
 
FAM168B
 
 
hsa-miR-152-3p
 
 
-0.409
 
 
26.8
 
 
 
 
FOSB
 
 
hsa-miR-152-3p
 
 
-0.311
 
 
119.1
 
 
 
 
FOXK2
 
 
hsa-miR-152-3p
 
 
-0.305
 
 
14.6
 
 
 
 
FXR1
 
 
hsa-miR-152-3p
 
 
-0.478
 
 
32.0
 
 
 
 
GADD45A
 
 
hsa-miR-152-3p
 
 
-0.787
 
 
19.1
 
 
 
 
GAPVD1
 
 
hsa-miR-152-3p
 
 
-0.309
 
 
10.0
 
 
 
 
GLRX5
 
 
hsa-miR-152-3p
 
 
-0.381
 
 
36.8
 
 
 
 
GMFB
 
 
hsa-miR-152-3p
 
 
-0.429
 
 
12.0
 
 
 
 
GOLT1A
 
 
hsa-miR-378a-3p
 
 
-0.574
 
 
25.1
 
 
 
 
GPATCH8
 
 
hsa-miR-152-3p
 
 
-0.374
 
 
10.7
 
 
 
 
GRSF1
 
 
hsa-miR-378a-3p
 
 
-0.358
 
 
55.3
 
 
 
 
GTF2H1
 
 
hsa-miR-152-3p
 
 
-0.524
 
 
16.9
 
 
 
 
HDDC2
 
 
hsa-miR-378a-3p
 
 
-0.322
 
 
33.7
 
 
 
 
HLA-DQB1
 
 
hsa-miR-152-3p
 
 
-0.37
 
 
13.8
 
 
 
 
HSP90B1
 
 
hsa-miR-152-3p
 
 
-0.304
 
 
273.4
 
 
 
 
HSPH1
 
 
hsa-miR-330-3p
 
 
-0.345
 
 
26.9
 
 
 
 
ID2
 
 
hsa-miR-330-3p
 
 
-0.293
 
 
56.7
 
 
 
 
IL6ST
 
 
hsa-miR-152-3p
 
 
-0.291
 
 
26.9
 
 
 
 
ING2
 
 
hsa-miR-152-3p
 
 
-0.482
 
 
14.9
 
 
 
 
ITSN2
 
 
hsa-miR-152-3p
 
 
-0.339
 
 
63.1
 
 
 
 
KLF5
 
 
hsa-miR-152-3p
 
 
-0.475
 
 
11.3
 
 
 
 
LBR
 
 
hsa-miR-152-3p
 
 
-0.39
 
 
15.4
 
 
 
 
LDLR
 
 
hsa-miR-152-3p
 
 
-0.323
 
 
47.9
 
 
 
 
LEPROTL1
 
 
hsa-miR-152-3p
 
 
-0.568
 
 
21.2
 
 
 
 
LIPA
 
 
hsa-miR-152-3p
 
 
-0.463
 
 
20.7
 
 
 
 
LMAN1
 
 
hsa-miR-152-3p
 
 
-0.382
 
 
57.2
 
 
 
 
LMTK2
 
 
hsa-miR-152-3p
 
 
-0.298
 
 
14.0
 
 
 
 
LRRC41
 
 
hsa-miR-152-3p
 
 
-0.392
 
 
37.7
 
 
 
 
LSM5
 
 
hsa-miR-330-3p
 
 
-0.351
 
 
51.0
 
 
 
 
LYSMD2
 
 
hsa-miR-152-3p
 
 
-0.554
 
 
12.3
 
 
 
 
MACROH2A1
 
 
hsa-miR-152-3p
 
 
-0.336
 
 
28.4
 
 
 
 
MAF1
 
 
hsa-miR-152-3p
 
 
-0.366
 
 
79.7
 
 
 
 
MAFB
 
 
hsa-miR-152-3p
 
 
-0.45
 
 
14.9
 
 
 
 
MAP2K1
 
 
hsa-miR-152-3p
 
 
-0.295
 
 
19.7
 
 
 
 
MAPK1
 
 
hsa-miR-378a-3p
 
 
-0.314
 
 
35.9
 
 
 
 
MAT2A
 
 
hsa-miR-330-3p
 
 
-0.307
 
 
825.0
 
 
 
 
MCM3AP
 
 
hsa-miR-152-3p
 
 
-0.287
 
 
14.5
 
 
 
 
MECP2
 
 
hsa-miR-152-3p
 
 
-0.3
 
 
14.0
 
 
 
 
MED19
 
 
hsa-miR-378a-3p
 
 
-0.434
 
 
12.1
 
 
 
 
MLLT10
 
 
hsa-miR-152-3p
 
 
-0.334
 
 
15.4
 
 
 
 
MMP15
 
 
hsa-miR-152-3p
 
 
-0.458
 
 
18.6
 
 
 
 
MNAT1
 
 
hsa-miR-378a-3p
 
 
-0.398
 
 
13.9
 
 
 
 
MRPS10
 
 
hsa-miR-152-3p
 
 
-0.335
 
 
16.2
 
 
 
 
MRPS25
 
 
hsa-miR-152-3p
 
 
-0.323
 
 
19.9
 
 
 
 
MTMR10
 
 
hsa-miR-152-3p
 
 
-0.302
 
 
11.8
 
 
 
 
MTMR12
 
 
hsa-miR-152-3p
 
 
-0.396
 
 
23.3
 
 
 
 
MTMR14
 
 
hsa-miR-152-3p
 
 
-0.467
 
 
11.2
 
 
 
 
 
 
 pituitary gland 
 
 
 
 
target_symbol
 
 
mature_mirna_id
 
 
score
 
 
nTPM
 
 
 
 
 
 
ABCD3
 
 
hsa-miR-152-3p
 
 
-0.388
 
 
12.4
 
 
 
 
ADGRB3
 
 
hsa-miR-152-3p
 
 
-0.516
 
 
21.3
 
 
 
 
AGFG1
 
 
hsa-miR-152-3p
 
 
-0.332
 
 
16.7
 
 
 
 
AHDC1
 
 
hsa-miR-152-3p
 
 
-0.413
 
 
18.1
 
 
 
 
AKAP1
 
 
hsa-miR-152-3p
 
 
-0.381
 
 
16.5
 
 
 
 
ALCAM
 
 
hsa-miR-152-3p
 
 
-0.347
 
 
17.9
 
 
 
 
AP4B1
 
 
hsa-miR-378a-3p
 
 
-0.62
 
 
13.4
 
 
 
 
ARF4
 
 
hsa-miR-152-3p
 
 
-0.487
 
 
93.4
 
 
 
 
ARHGAP21
 
 
hsa-miR-152-3p
 
 
-0.287
 
 
11.3
 
 
 
 
ARL6IP1
 
 
hsa-miR-152-3p
 
 
-0.579
 
 
39.7
 
 
 
 
ARL8B
 
 
hsa-miR-152-3p
 
 
-0.337
 
 
25.6
 
 
 
 
ARRDC3
 
 
hsa-miR-152-3p
 
 
-0.305
 
 
20.2
 
 
 
 
ASPH
 
 
hsa-miR-152-3p
 
 
-0.391
 
 
33.8
 
 
 
 
ATP6AP2
 
 
hsa-miR-152-3p
 
 
-0.592
 
 
34.0
 
 
 
 
ATP6AP2
 
 
hsa-miR-152-3p
 
 
-0.668
 
 
34.0
 
 
 
 
B4GALT5
 
 
hsa-miR-152-3p
 
 
-0.44
 
 
11.9
 
 
 
 
BHLHE41
 
 
hsa-miR-152-3p
 
 
-0.442
 
 
15.9
 
 
 
 
BTAF1
 
 
hsa-miR-152-3p
 
 
-0.401
 
 
11.4
 
 
 
 
C1QBP
 
 
hsa-miR-330-3p
 
 
-0.412
 
 
78.4
 
 
 
 
C1orf35
 
 
hsa-miR-152-3p
 
 
-0.511
 
 
11.5
 
 
 
 
CAND1
 
 
hsa-miR-152-3p
 
 
-0.44
 
 
18.1
 
 
 
 
CANX
 
 
hsa-miR-152-3p
 
 
-0.434
 
 
83.6
 
 
 
 
CCDC6
 
 
hsa-miR-152-3p
 
 
-0.511
 
 
11.1
 
 
 
 
CCN2
 
 
hsa-miR-330-3p
 
 
-0.305
 
 
42.3
 
 
 
 
CCT6A
 
 
hsa-miR-152-3p
 
 
-0.665
 
 
39.3
 
 
 
 
CDC25B
 
 
hsa-miR-152-3p
 
 
-0.298
 
 
44.9
 
 
 
 
CERS5
 
 
hsa-miR-152-3p
 
 
-0.311
 
 
32.0
 
 
 
 
CFL2
 
 
hsa-miR-152-3p
 
 
-0.487
 
 
11.4
 
 
 
 
CHMP4B
 
 
hsa-miR-152-3p
 
 
-0.36
 
 
73.1
 
 
 
 
CLCN6
 
 
hsa-miR-152-3p
 
 
-0.406
 
 
18.4
 
 
 
 
CNOT7
 
 
hsa-miR-152-3p
 
 
-0.31
 
 
23.6
 
 
 
 
COL4A1
 
 
hsa-miR-152-3p
 
 
-0.317
 
 
11.8
 
 
 
 
CS
 
 
hsa-miR-152-3p
 
 
-0.3
 
 
36.8
 
 
 
 
CTSA
 
 
hsa-miR-152-3p
 
 
-0.541
 
 
35.6
 
 
 
 
DCUN1D4
 
 
hsa-miR-152-3p
 
 
-0.314
 
 
15.3
 
 
 
 
DDX6
 
 
hsa-miR-152-3p
 
 
-0.363
 
 
16.5
 
 
 
 
DEDD
 
 
hsa-miR-152-3p
 
 
-0.401
 
 
16.8
 
 
 
 
DMPK
 
 
hsa-miR-152-3p
 
 
-0.378
 
 
23.6
 
 
 
 
DNAJB4
 
 
hsa-miR-152-3p
 
 
-0.33
 
 
11.1
 
 
 
 
DOCK6
 
 
hsa-miR-152-3p
 
 
-0.396
 
 
11.6
 
 
 
 
DUSP1
 
 
hsa-miR-152-3p
 
 
-0.32
 
 
304.8
 
 
 
 
DYNLL2
 
 
hsa-miR-152-3p
 
 
-0.366
 
 
26.9
 
 
 
 
DYRK1B
 
 
hsa-miR-152-3p
 
 
-0.305
 
 
29.4
 
 
 
 
EIF4G3
 
 
hsa-miR-378a-3p
 
 
-0.443
 
 
16.0
 
 
 
 
EPS15
 
 
hsa-miR-152-3p
 
 
-0.322
 
 
14.6
 
 
 
 
ERRFI1
 
 
hsa-miR-152-3p
 
 
-0.529
 
 
109.5
 
 
 
 
F3
 
 
hsa-miR-152-3p
 
 
-0.331
 
 
78.6
 
 
 
 
FAM168B
 
 
hsa-miR-152-3p
 
 
-0.409
 
 
18.1
 
 
 
 
FAM184A
 
 
hsa-miR-152-3p
 
 
-0.371
 
 
12.3
 
 
 
 
FBXL19
 
 
hsa-miR-152-3p
 
 
-0.362
 
 
12.4
 
 
 
 
FEZ2
 
 
hsa-miR-152-3p
 
 
-0.536
 
 
12.7
 
 
 
 
FOSB
 
 
hsa-miR-152-3p
 
 
-0.311
 
 
25.3
 
 
 
 
FOXK2
 
 
hsa-miR-152-3p
 
 
-0.305
 
 
17.0
 
 
 
 
FXR1
 
 
hsa-miR-152-3p
 
 
-0.478
 
 
23.9
 
 
 
 
GADD45A
 
 
hsa-miR-152-3p
 
 
-0.787
 
 
20.4
 
 
 
 
GLRX5
 
 
hsa-miR-152-3p
 
 
-0.381
 
 
34.1
 
 
 
 
GMFB
 
 
hsa-miR-152-3p
 
 
-0.429
 
 
11.6
 
 
 
 
GNRHR
 
 
hsa-miR-330-3p
 
 
-0.523
 
 
11.9
 
 
 
 
GOLT1A
 
 
hsa-miR-378a-3p
 
 
-0.574
 
 
32.6
 
 
 
 
GPATCH8
 
 
hsa-miR-152-3p
 
 
-0.374
 
 
19.4
 
 
 
 
GRSF1
 
 
hsa-miR-378a-3p
 
 
-0.358
 
 
16.2
 
 
 
 
GTF2H1
 
 
hsa-miR-152-3p
 
 
-0.524
 
 
12.9
 
 
 
 
HDDC2
 
 
hsa-miR-378a-3p
 
 
-0.322
 
 
36.5
 
 
 
 
HLA-DQB1
 
 
hsa-miR-152-3p
 
 
-0.37
 
 
20.5
 
 
 
 
HSP90B1
 
 
hsa-miR-152-3p
 
 
-0.304
 
 
160.0
 
 
 
 
HSPH1
 
 
hsa-miR-330-3p
 
 
-0.345
 
 
61.0
 
 
 
 
ID2
 
 
hsa-miR-330-3p
 
 
-0.293
 
 
63.4
 
 
 
 
IL6ST
 
 
hsa-miR-152-3p
 
 
-0.291
 
 
17.9
 
 
 
 
ING2
 
 
hsa-miR-152-3p
 
 
-0.482
 
 
14.2
 
 
 
 
ITSN2
 
 
hsa-miR-152-3p
 
 
-0.339
 
 
15.3
 
 
 
 
JPH3
 
 
hsa-miR-152-3p
 
 
-0.303
 
 
11.5
 
 
 
 
KLF5
 
 
hsa-miR-152-3p
 
 
-0.475
 
 
10.3
 
 
 
 
LAMA4
 
 
hsa-miR-152-3p
 
 
-0.363
 
 
13.4
 
 
 
 
LEPROTL1
 
 
hsa-miR-152-3p
 
 
-0.568
 
 
13.4
 
 
 
 
LIPA
 
 
hsa-miR-152-3p
 
 
-0.463
 
 
12.6
 
 
 
 
LMAN1
 
 
hsa-miR-152-3p
 
 
-0.382
 
 
35.5
 
 
 
 
LRRC41
 
 
hsa-miR-152-3p
 
 
-0.392
 
 
42.5
 
 
 
 
LSM5
 
 
hsa-miR-330-3p
 
 
-0.351
 
 
27.4
 
 
 
 
LYSMD2
 
 
hsa-miR-152-3p
 
 
-0.554
 
 
23.7
 
 
 
 
MACROH2A1
 
 
hsa-miR-152-3p
 
 
-0.336
 
 
53.0
 
 
 
 
MAF1
 
 
hsa-miR-152-3p
 
 
-0.366
 
 
67.3
 
 
 
 
MAFG
 
 
hsa-miR-152-3p
 
 
-0.328
 
 
12.3
 
 
 
 
MAP2K1
 
 
hsa-miR-152-3p
 
 
-0.295
 
 
10.0
 
 
 
 
MAPK1
 
 
hsa-miR-378a-3p
 
 
-0.314
 
 
18.3
 
 
 
 
MAT2A
 
 
hsa-miR-330-3p
 
 
-0.307
 
 
273.5
 
 
 
 
MECP2
 
 
hsa-miR-152-3p
 
 
-0.3
 
 
19.5
 
 
 
 
MED19
 
 
hsa-miR-378a-3p
 
 
-0.434
 
 
16.5
 
 
 
 
MLLT10
 
 
hsa-miR-152-3p
 
 
-0.334
 
 
13.7
 
 
 
 
MNAT1
 
 
hsa-miR-378a-3p
 
 
-0.398
 
 
21.2
 
 
 
 
MNT
 
 
hsa-miR-152-3p
 
 
-0.435
 
 
14.9
 
 
 
 
MRAS
 
 
hsa-miR-152-3p
 
 
-0.583
 
 
14.2
 
 
 
 
MRPS10
 
 
hsa-miR-152-3p
 
 
-0.335
 
 
12.6
 
 
 
 
MRPS25
 
 
hsa-miR-152-3p
 
 
-0.323
 
 
30.1
 
 
 
 
NAP1L1
 
 
hsa-miR-152-3p
 
 
-0.343
 
 
179.3
 
 
 
 
NAT14
 
 
hsa-miR-152-3p
 
 
-0.324
 
 
49.7
 
 
 
 
NCKIPSD
 
 
hsa-miR-152-3p
 
 
-0.382
 
 
19.5
 
 
 
 
NCOA1
 
 
hsa-miR-152-3p
 
 
-0.313
 
 
15.6
 
 
 
 
NEURL4
 
 
hsa-miR-152-3p
 
 
-0.43
 
 
11.0
 
 
 
 
NFKBIA
 
 
hsa-miR-378a-3p
 
 
-0.427
 
 
89.3
 
 
 
 
NOL4L
 
 
hsa-miR-152-3p
 
 
-0.394
 
 
22.0
 
 
 
 
 
 
 prostate 
 
 
 
 
target_symbol
 
 
mature_mirna_id
 
 
score
 
 
nTPM
 
 
 
 
 
 
ABCD3
 
 
hsa-miR-152-3p
 
 
-0.388
 
 
20.2
 
 
 
 
ABHD17C
 
 
hsa-miR-330-3p
 
 
-0.399
 
 
17.0
 
 
 
 
ADAM10
 
 
hsa-miR-152-3p
 
 
-0.348
 
 
23.6
 
 
 
 
AGFG1
 
 
hsa-miR-152-3p
 
 
-0.332
 
 
19.4
 
 
 
 
AHDC1
 
 
hsa-miR-152-3p
 
 
-0.413
 
 
11.3
 
 
 
 
AKAP1
 
 
hsa-miR-152-3p
 
 
-0.381
 
 
33.3
 
 
 
 
ALCAM
 
 
hsa-miR-152-3p
 
 
-0.347
 
 
30.7
 
 
 
 
ARF4
 
 
hsa-miR-152-3p
 
 
-0.487
 
 
129.9
 
 
 
 
ARFGEF1
 
 
hsa-miR-152-3p
 
 
-0.306
 
 
12.2
 
 
 
 
ARFIP1
 
 
hsa-miR-152-3p
 
 
-0.474
 
 
19.8
 
 
 
 
ARHGAP21
 
 
hsa-miR-152-3p
 
 
-0.287
 
 
10.2
 
 
 
 
ARL6IP1
 
 
hsa-miR-152-3p
 
 
-0.579
 
 
71.3
 
 
 
 
ARL8B
 
 
hsa-miR-152-3p
 
 
-0.337
 
 
42.2
 
 
 
 
ARRDC3
 
 
hsa-miR-152-3p
 
 
-0.305
 
 
28.5
 
 
 
 
ASPH
 
 
hsa-miR-152-3p
 
 
-0.391
 
 
44.4
 
 
 
 
ATP2B4
 
 
hsa-miR-152-3p
 
 
-0.337
 
 
31.1
 
 
 
 
ATP6AP2
 
 
hsa-miR-152-3p
 
 
-0.592
 
 
81.2
 
 
 
 
ATP6AP2
 
 
hsa-miR-152-3p
 
 
-0.668
 
 
81.2
 
 
 
 
B4GALT5
 
 
hsa-miR-152-3p
 
 
-0.44
 
 
16.0
 
 
 
 
BTAF1
 
 
hsa-miR-152-3p
 
 
-0.401
 
 
13.7
 
 
 
 
BTBD3
 
 
hsa-miR-152-3p
 
 
-0.495
 
 
14.2
 
 
 
 
C1QBP
 
 
hsa-miR-330-3p
 
 
-0.412
 
 
59.9
 
 
 
 
C1orf35
 
 
hsa-miR-152-3p
 
 
-0.511
 
 
16.0
 
 
 
 
CAND1
 
 
hsa-miR-152-3p
 
 
-0.44
 
 
21.0
 
 
 
 
CANX
 
 
hsa-miR-152-3p
 
 
-0.434
 
 
84.4
 
 
 
 
CAV2
 
 
hsa-miR-152-3p
 
 
-0.32
 
 
30.0
 
 
 
 
CCDC6
 
 
hsa-miR-152-3p
 
 
-0.511
 
 
18.2
 
 
 
 
CCDC71
 
 
hsa-miR-152-3p
 
 
-0.369
 
 
11.5
 
 
 
 
CCN2
 
 
hsa-miR-330-3p
 
 
-0.305
 
 
341.9
 
 
 
 
CCT6A
 
 
hsa-miR-152-3p
 
 
-0.665
 
 
43.1
 
 
 
 
CDC25B
 
 
hsa-miR-152-3p
 
 
-0.298
 
 
24.9
 
 
 
 
CDK19
 
 
hsa-miR-152-3p
 
 
-0.369
 
 
25.2
 
 
 
 
CDK19
 
 
hsa-miR-152-3p
 
 
-0.405
 
 
25.2
 
 
 
 
CEBPG
 
 
hsa-miR-152-3p
 
 
-0.319
 
 
11.2
 
 
 
 
CEP63
 
 
hsa-miR-152-3p
 
 
-0.329
 
 
12.1
 
 
 
 
CERS5
 
 
hsa-miR-152-3p
 
 
-0.311
 
 
23.8
 
 
 
 
CFL2
 
 
hsa-miR-152-3p
 
 
-0.487
 
 
70.6
 
 
 
 
CHMP4B
 
 
hsa-miR-152-3p
 
 
-0.36
 
 
88.6
 
 
 
 
CLCN6
 
 
hsa-miR-152-3p
 
 
-0.406
 
 
11.3
 
 
 
 
CNOT7
 
 
hsa-miR-152-3p
 
 
-0.31
 
 
30.6
 
 
 
 
COL4A1
 
 
hsa-miR-152-3p
 
 
-0.317
 
 
50.3
 
 
 
 
CS
 
 
hsa-miR-152-3p
 
 
-0.3
 
 
55.4
 
 
 
 
CTSA
 
 
hsa-miR-152-3p
 
 
-0.541
 
 
32.7
 
 
 
 
CUL5
 
 
hsa-miR-152-3p
 
 
-0.389
 
 
11.3
 
 
 
 
DACT1
 
 
hsa-miR-378a-3p
 
 
-0.298
 
 
11.1
 
 
 
 
DCUN1D4
 
 
hsa-miR-152-3p
 
 
-0.314
 
 
11.2
 
 
 
 
DDAH1
 
 
hsa-miR-152-3p
 
 
-0.34
 
 
26.8
 
 
 
 
DDX6
 
 
hsa-miR-152-3p
 
 
-0.363
 
 
30.9
 
 
 
 
DEDD
 
 
hsa-miR-152-3p
 
 
-0.401
 
 
21.5
 
 
 
 
DIPK2A
 
 
hsa-miR-152-3p
 
 
-0.393
 
 
10.8
 
 
 
 
DMPK
 
 
hsa-miR-152-3p
 
 
-0.378
 
 
30.1
 
 
 
 
DMXL1
 
 
hsa-miR-152-3p
 
 
-0.29
 
 
25.2
 
 
 
 
DNAJB4
 
 
hsa-miR-152-3p
 
 
-0.33
 
 
21.8
 
 
 
 
DOCK6
 
 
hsa-miR-152-3p
 
 
-0.396
 
 
14.1
 
 
 
 
DUSP1
 
 
hsa-miR-152-3p
 
 
-0.32
 
 
412.6
 
 
 
 
DYNLL2
 
 
hsa-miR-152-3p
 
 
-0.366
 
 
28.5
 
 
 
 
DYRK1B
 
 
hsa-miR-152-3p
 
 
-0.305
 
 
12.5
 
 
 
 
EGR3
 
 
hsa-miR-152-3p
 
 
-0.315
 
 
13.5
 
 
 
 
EIF4E3
 
 
hsa-miR-152-3p
 
 
-0.464
 
 
15.0
 
 
 
 
EIF4G3
 
 
hsa-miR-378a-3p
 
 
-0.443
 
 
16.7
 
 
 
 
EOGT
 
 
hsa-miR-152-3p
 
 
-0.587
 
 
26.0
 
 
 
 
EPS15
 
 
hsa-miR-152-3p
 
 
-0.322
 
 
24.8
 
 
 
 
ERBB3
 
 
hsa-miR-152-3p
 
 
-0.399
 
 
32.7
 
 
 
 
ERRFI1
 
 
hsa-miR-152-3p
 
 
-0.529
 
 
49.7
 
 
 
 
F3
 
 
hsa-miR-152-3p
 
 
-0.331
 
 
53.2
 
 
 
 
FAM168B
 
 
hsa-miR-152-3p
 
 
-0.409
 
 
35.2
 
 
 
 
FAM43A
 
 
hsa-miR-152-3p
 
 
-0.337
 
 
19.8
 
 
 
 
FBXL19
 
 
hsa-miR-152-3p
 
 
-0.362
 
 
10.8
 
 
 
 
FEZ2
 
 
hsa-miR-152-3p
 
 
-0.536
 
 
22.0
 
 
 
 
FMR1
 
 
hsa-miR-152-3p
 
 
-0.385
 
 
11.1
 
 
 
 
FOSB
 
 
hsa-miR-152-3p
 
 
-0.311
 
 
111.1
 
 
 
 
FOXF1
 
 
hsa-miR-152-3p
 
 
-0.374
 
 
24.7
 
 
 
 
FOXK2
 
 
hsa-miR-152-3p
 
 
-0.305
 
 
12.6
 
 
 
 
FXR1
 
 
hsa-miR-152-3p
 
 
-0.478
 
 
36.3
 
 
 
 
GADD45A
 
 
hsa-miR-152-3p
 
 
-0.787
 
 
26.2
 
 
 
 
GLRX5
 
 
hsa-miR-152-3p
 
 
-0.381
 
 
30.0
 
 
 
 
GMFB
 
 
hsa-miR-152-3p
 
 
-0.429
 
 
13.9
 
 
 
 
GPATCH8
 
 
hsa-miR-152-3p
 
 
-0.374
 
 
12.6
 
 
 
 
GRSF1
 
 
hsa-miR-378a-3p
 
 
-0.358
 
 
36.0
 
 
 
 
GTF2H1
 
 
hsa-miR-152-3p
 
 
-0.524
 
 
14.7
 
 
 
 
HDDC2
 
 
hsa-miR-378a-3p
 
 
-0.322
 
 
28.1
 
 
 
 
HLA-DQB1
 
 
hsa-miR-152-3p
 
 
-0.37
 
 
16.3
 
 
 
 
HSP90B1
 
 
hsa-miR-152-3p
 
 
-0.304
 
 
338.0
 
 
 
 
HSPH1
 
 
hsa-miR-330-3p
 
 
-0.345
 
 
27.2
 
 
 
 
ID2
 
 
hsa-miR-330-3p
 
 
-0.293
 
 
117.4
 
 
 
 
IGF1
 
 
hsa-miR-152-3p
 
 
-0.43
 
 
36.1
 
 
 
 
IL6ST
 
 
hsa-miR-152-3p
 
 
-0.291
 
 
47.0
 
 
 
 
ITSN2
 
 
hsa-miR-152-3p
 
 
-0.339
 
 
16.7
 
 
 
 
KDSR
 
 
hsa-miR-378a-3p
 
 
-0.373
 
 
10.9
 
 
 
 
KLF4
 
 
hsa-miR-152-3p
 
 
-0.325
 
 
15.7
 
 
 
 
KLF5
 
 
hsa-miR-152-3p
 
 
-0.475
 
 
21.6
 
 
 
 
LAMA4
 
 
hsa-miR-152-3p
 
 
-0.363
 
 
33.9
 
 
 
 
LBR
 
 
hsa-miR-152-3p
 
 
-0.39
 
 
22.3
 
 
 
 
LDLR
 
 
hsa-miR-152-3p
 
 
-0.323
 
 
19.0
 
 
 
 
LEPROTL1
 
 
hsa-miR-152-3p
 
 
-0.568
 
 
16.7
 
 
 
 
LIPA
 
 
hsa-miR-152-3p
 
 
-0.463
 
 
28.8
 
 
 
 
LMAN1
 
 
hsa-miR-152-3p
 
 
-0.382
 
 
54.9
 
 
 
 
LRCH1
 
 
hsa-miR-152-3p
 
 
-0.321
 
 
10.1
 
 
 
 
LRRC41
 
 
hsa-miR-152-3p
 
 
-0.392
 
 
40.7
 
 
 
 
LSM5
 
 
hsa-miR-330-3p
 
 
-0.351
 
 
32.8
 
 
 
 
 
 
 retina 
 
 
 
 
target_symbol
 
 
mature_mirna_id
 
 
score
 
 
nTPM
 
 
 
 
 
 
ABCD3
 
 
hsa-miR-152-3p
 
 
-0.388
 
 
24.4
 
 
 
 
ABHD17C
 
 
hsa-miR-330-3p
 
 
-0.399
 
 
15.6
 
 
 
 
AGFG1
 
 
hsa-miR-152-3p
 
 
-0.332
 
 
44.2
 
 
 
 
AKAP1
 
 
hsa-miR-152-3p
 
 
-0.381
 
 
32.2
 
 
 
 
AP4B1
 
 
hsa-miR-378a-3p
 
 
-0.62
 
 
18.5
 
 
 
 
ARF4
 
 
hsa-miR-152-3p
 
 
-0.487
 
 
50.0
 
 
 
 
ARFGEF1
 
 
hsa-miR-152-3p
 
 
-0.306
 
 
33.8
 
 
 
 
ARFIP1
 
 
hsa-miR-152-3p
 
 
-0.474
 
 
18.7
 
 
 
 
ARHGAP21
 
 
hsa-miR-152-3p
 
 
-0.287
 
 
37.0
 
 
 
 
ARL6IP1
 
 
hsa-miR-152-3p
 
 
-0.579
 
 
292.6
 
 
 
 
ARL8B
 
 
hsa-miR-152-3p
 
 
-0.337
 
 
31.7
 
 
 
 
ARRDC3
 
 
hsa-miR-152-3p
 
 
-0.305
 
 
69.6
 
 
 
 
ASPH
 
 
hsa-miR-152-3p
 
 
-0.391
 
 
201.9
 
 
 
 
ATP2B4
 
 
hsa-miR-152-3p
 
 
-0.337
 
 
39.4
 
 
 
 
ATP6AP2
 
 
hsa-miR-152-3p
 
 
-0.592
 
 
92.0
 
 
 
 
ATP6AP2
 
 
hsa-miR-152-3p
 
 
-0.668
 
 
92.0
 
 
 
 
B4GALT6
 
 
hsa-miR-152-3p
 
 
-0.528
 
 
24.7
 
 
 
 
BCL2L11
 
 
hsa-miR-152-3p
 
 
-0.445
 
 
11.9
 
 
 
 
BHLHE41
 
 
hsa-miR-152-3p
 
 
-0.442
 
 
113.5
 
 
 
 
BTAF1
 
 
hsa-miR-152-3p
 
 
-0.401
 
 
27.2
 
 
 
 
BTBD3
 
 
hsa-miR-152-3p
 
 
-0.495
 
 
39.4
 
 
 
 
C15orf61
 
 
hsa-miR-378a-3p
 
 
-0.497
 
 
10.0
 
 
 
 
C1GALT1
 
 
hsa-miR-152-3p
 
 
-0.487
 
 
21.8
 
 
 
 
C1QBP
 
 
hsa-miR-330-3p
 
 
-0.412
 
 
80.3
 
 
 
 
CAND1
 
 
hsa-miR-152-3p
 
 
-0.44
 
 
34.7
 
 
 
 
CANX
 
 
hsa-miR-152-3p
 
 
-0.434
 
 
61.7
 
 
 
 
CAV2
 
 
hsa-miR-152-3p
 
 
-0.32
 
 
15.5
 
 
 
 
CCDC144A
 
 
hsa-miR-152-3p
 
 
-0.292
 
 
11.9
 
 
 
 
CCDC6
 
 
hsa-miR-152-3p
 
 
-0.511
 
 
16.9
 
 
 
 
CCT6A
 
 
hsa-miR-152-3p
 
 
-0.665
 
 
44.2
 
 
 
 
CDH20
 
 
hsa-miR-152-3p
 
 
-0.297
 
 
10.2
 
 
 
 
CEBPG
 
 
hsa-miR-152-3p
 
 
-0.319
 
 
16.0
 
 
 
 
CEP63
 
 
hsa-miR-152-3p
 
 
-0.329
 
 
15.5
 
 
 
 
CFL2
 
 
hsa-miR-152-3p
 
 
-0.487
 
 
37.9
 
 
 
 
CHD7
 
 
hsa-miR-152-3p
 
 
-0.297
 
 
14.1
 
 
 
 
CHMP4B
 
 
hsa-miR-152-3p
 
 
-0.36
 
 
49.4
 
 
 
 
CLCN6
 
 
hsa-miR-152-3p
 
 
-0.406
 
 
14.1
 
 
 
 
CNOT7
 
 
hsa-miR-152-3p
 
 
-0.31
 
 
72.2
 
 
 
 
CNTN4
 
 
hsa-miR-152-3p
 
 
-0.415
 
 
18.3
 
 
 
 
CS
 
 
hsa-miR-152-3p
 
 
-0.3
 
 
119.5
 
 
 
 
CTSA
 
 
hsa-miR-152-3p
 
 
-0.541
 
 
40.2
 
 
 
 
CUL5
 
 
hsa-miR-152-3p
 
 
-0.389
 
 
11.7
 
 
 
 
DCUN1D4
 
 
hsa-miR-152-3p
 
 
-0.314
 
 
14.5
 
 
 
 
DDAH1
 
 
hsa-miR-152-3p
 
 
-0.34
 
 
32.0
 
 
 
 
DDX6
 
 
hsa-miR-152-3p
 
 
-0.363
 
 
45.2
 
 
 
 
DEDD
 
 
hsa-miR-152-3p
 
 
-0.401
 
 
22.2
 
 
 
 
DICER1
 
 
hsa-miR-152-3p
 
 
-0.29
 
 
14.3
 
 
 
 
DNAJB4
 
 
hsa-miR-152-3p
 
 
-0.33
 
 
13.9
 
 
 
 
DUSP1
 
 
hsa-miR-152-3p
 
 
-0.32
 
 
188.0
 
 
 
 
DYNLL2
 
 
hsa-miR-152-3p
 
 
-0.366
 
 
38.2
 
 
 
 
DYRK2
 
 
hsa-miR-152-3p
 
 
-0.293
 
 
13.7
 
 
 
 
EIF4G3
 
 
hsa-miR-378a-3p
 
 
-0.443
 
 
26.3
 
 
 
 
EPS15
 
 
hsa-miR-152-3p
 
 
-0.322
 
 
19.4
 
 
 
 
ERRFI1
 
 
hsa-miR-152-3p
 
 
-0.529
 
 
33.6
 
 
 
 
ESRRG
 
 
hsa-miR-152-3p
 
 
-0.347
 
 
33.5
 
 
 
 
F3
 
 
hsa-miR-152-3p
 
 
-0.331
 
 
67.1
 
 
 
 
FAM161A
 
 
hsa-miR-152-3p
 
 
-0.295
 
 
26.9
 
 
 
 
FAM168B
 
 
hsa-miR-152-3p
 
 
-0.409
 
 
45.0
 
 
 
 
FAM184A
 
 
hsa-miR-152-3p
 
 
-0.371
 
 
15.1
 
 
 
 
FEZ2
 
 
hsa-miR-152-3p
 
 
-0.536
 
 
24.5
 
 
 
 
FMR1
 
 
hsa-miR-152-3p
 
 
-0.385
 
 
11.8
 
 
 
 
FOSB
 
 
hsa-miR-152-3p
 
 
-0.311
 
 
10.0
 
 
 
 
FOXK2
 
 
hsa-miR-152-3p
 
 
-0.305
 
 
10.8
 
 
 
 
FXR1
 
 
hsa-miR-152-3p
 
 
-0.478
 
 
39.6
 
 
 
 
GADD45A
 
 
hsa-miR-152-3p
 
 
-0.787
 
 
17.6
 
 
 
 
GAPVD1
 
 
hsa-miR-152-3p
 
 
-0.309
 
 
15.3
 
 
 
 
GMFB
 
 
hsa-miR-152-3p
 
 
-0.429
 
 
17.0
 
 
 
 
GPATCH8
 
 
hsa-miR-152-3p
 
 
-0.374
 
 
27.9
 
 
 
 
GPR137C
 
 
hsa-miR-152-3p
 
 
-0.33
 
 
15.4
 
 
 
 
GRSF1
 
 
hsa-miR-378a-3p
 
 
-0.358
 
 
22.9
 
 
 
 
GTF2H1
 
 
hsa-miR-152-3p
 
 
-0.524
 
 
12.1
 
 
 
 
HDDC2
 
 
hsa-miR-378a-3p
 
 
-0.322
 
 
29.2
 
 
 
 
HSP90B1
 
 
hsa-miR-152-3p
 
 
-0.304
 
 
171.9
 
 
 
 
HSPA12A
 
 
hsa-miR-378a-3p
 
 
-0.326
 
 
10.1
 
 
 
 
HSPH1
 
 
hsa-miR-330-3p
 
 
-0.345
 
 
24.8
 
 
 
 
ID2
 
 
hsa-miR-330-3p
 
 
-0.293
 
 
64.7
 
 
 
 
IL6ST
 
 
hsa-miR-152-3p
 
 
-0.291
 
 
57.0
 
 
 
 
IMPG1
 
 
hsa-miR-378a-3p
 
 
-0.318
 
 
621.3
 
 
 
 
ITSN2
 
 
hsa-miR-152-3p
 
 
-0.339
 
 
14.7
 
 
 
 
LEPROTL1
 
 
hsa-miR-152-3p
 
 
-0.568
 
 
23.8
 
 
 
 
LIPA
 
 
hsa-miR-152-3p
 
 
-0.463
 
 
11.6
 
 
 
 
LMAN1
 
 
hsa-miR-152-3p
 
 
-0.382
 
 
37.0
 
 
 
 
LRP2
 
 
hsa-miR-152-3p
 
 
-0.465
 
 
27.1
 
 
 
 
LRRC41
 
 
hsa-miR-152-3p
 
 
-0.392
 
 
17.9
 
 
 
 
LSM5
 
 
hsa-miR-330-3p
 
 
-0.351
 
 
26.6
 
 
 
 
MACIR
 
 
hsa-miR-152-3p
 
 
-0.332
 
 
19.2
 
 
 
 
MACIR
 
 
hsa-miR-152-3p
 
 
-0.573
 
 
19.2
 
 
 
 
MACROH2A1
 
 
hsa-miR-152-3p
 
 
-0.336
 
 
95.6
 
 
 
 
MAF1
 
 
hsa-miR-152-3p
 
 
-0.366
 
 
32.9
 
 
 
 
MAP2K1
 
 
hsa-miR-152-3p
 
 
-0.295
 
 
16.4
 
 
 
 
MAPK1
 
 
hsa-miR-378a-3p
 
 
-0.314
 
 
28.1
 
 
 
 
MAT2A
 
 
hsa-miR-330-3p
 
 
-0.307
 
 
230.6
 
 
 
 
MCM3AP
 
 
hsa-miR-152-3p
 
 
-0.287
 
 
11.9
 
 
 
 
MDFIC
 
 
hsa-miR-152-3p
 
 
-0.395
 
 
18.9
 
 
 
 
MECP2
 
 
hsa-miR-152-3p
 
 
-0.3
 
 
21.4
 
 
 
 
MED19
 
 
hsa-miR-378a-3p
 
 
-0.434
 
 
12.3
 
 
 
 
MIER1
 
 
hsa-miR-152-3p
 
 
-0.352
 
 
17.5
 
 
 
 
MLLT10
 
 
hsa-miR-152-3p
 
 
-0.334
 
 
19.1
 
 
 
 
MMD
 
 
hsa-miR-152-3p
 
 
-0.62
 
 
11.9
 
 
 
 
MNAT1
 
 
hsa-miR-378a-3p
 
 
-0.398
 
 
27.0
 
 
 
 
 
 
 salivary gland 
 
 
 
 
target_symbol
 
 
mature_mirna_id
 
 
score
 
 
nTPM
 
 
 
 
 
 
ABCD3
 
 
hsa-miR-152-3p
 
 
-0.388
 
 
16.5
 
 
 
 
ABHD17C
 
 
hsa-miR-330-3p
 
 
-0.399
 
 
22.3
 
 
 
 
AGFG1
 
 
hsa-miR-152-3p
 
 
-0.332
 
 
26.3
 
 
 
 
AHDC1
 
 
hsa-miR-152-3p
 
 
-0.413
 
 
16.0
 
 
 
 
AKAP1
 
 
hsa-miR-152-3p
 
 
-0.381
 
 
31.6
 
 
 
 
ALCAM
 
 
hsa-miR-152-3p
 
 
-0.347
 
 
31.9
 
 
 
 
ARF4
 
 
hsa-miR-152-3p
 
 
-0.487
 
 
171.4
 
 
 
 
ARFGEF1
 
 
hsa-miR-152-3p
 
 
-0.306
 
 
13.9
 
 
 
 
ARFIP1
 
 
hsa-miR-152-3p
 
 
-0.474
 
 
12.9
 
 
 
 
ARHGAP21
 
 
hsa-miR-152-3p
 
 
-0.287
 
 
23.1
 
 
 
 
ARL6IP1
 
 
hsa-miR-152-3p
 
 
-0.579
 
 
79.4
 
 
 
 
ARL8B
 
 
hsa-miR-152-3p
 
 
-0.337
 
 
35.3
 
 
 
 
ARRDC3
 
 
hsa-miR-152-3p
 
 
-0.305
 
 
60.1
 
 
 
 
ASPH
 
 
hsa-miR-152-3p
 
 
-0.391
 
 
95.6
 
 
 
 
ATP6AP2
 
 
hsa-miR-152-3p
 
 
-0.592
 
 
39.6
 
 
 
 
ATP6AP2
 
 
hsa-miR-152-3p
 
 
-0.668
 
 
39.6
 
 
 
 
B4GALT5
 
 
hsa-miR-152-3p
 
 
-0.44
 
 
18.2
 
 
 
 
BHLHE41
 
 
hsa-miR-152-3p
 
 
-0.442
 
 
17.3
 
 
 
 
BTAF1
 
 
hsa-miR-152-3p
 
 
-0.401
 
 
15.9
 
 
 
 
BTBD3
 
 
hsa-miR-152-3p
 
 
-0.495
 
 
13.1
 
 
 
 
C1GALT1
 
 
hsa-miR-152-3p
 
 
-0.487
 
 
22.0
 
 
 
 
C1QBP
 
 
hsa-miR-330-3p
 
 
-0.412
 
 
62.7
 
 
 
 
C1orf210
 
 
hsa-miR-378a-3p
 
 
-0.307
 
 
18.3
 
 
 
 
C1orf35
 
 
hsa-miR-152-3p
 
 
-0.511
 
 
13.7
 
 
 
 
CAND1
 
 
hsa-miR-152-3p
 
 
-0.44
 
 
15.7
 
 
 
 
CANX
 
 
hsa-miR-152-3p
 
 
-0.434
 
 
107.0
 
 
 
 
CAV2
 
 
hsa-miR-152-3p
 
 
-0.32
 
 
23.6
 
 
 
 
CCDC6
 
 
hsa-miR-152-3p
 
 
-0.511
 
 
27.4
 
 
 
 
CCDC71
 
 
hsa-miR-152-3p
 
 
-0.369
 
 
11.9
 
 
 
 
CCN2
 
 
hsa-miR-330-3p
 
 
-0.305
 
 
129.8
 
 
 
 
CCT6A
 
 
hsa-miR-152-3p
 
 
-0.665
 
 
46.2
 
 
 
 
CDC25B
 
 
hsa-miR-152-3p
 
 
-0.298
 
 
16.3
 
 
 
 
CEBPG
 
 
hsa-miR-152-3p
 
 
-0.319
 
 
10.0
 
 
 
 
CEP63
 
 
hsa-miR-152-3p
 
 
-0.329
 
 
13.9
 
 
 
 
CERS5
 
 
hsa-miR-152-3p
 
 
-0.311
 
 
16.5
 
 
 
 
CFL2
 
 
hsa-miR-152-3p
 
 
-0.487
 
 
16.5
 
 
 
 
CHMP4B
 
 
hsa-miR-152-3p
 
 
-0.36
 
 
116.0
 
 
 
 
CNOT7
 
 
hsa-miR-152-3p
 
 
-0.31
 
 
36.2
 
 
 
 
COL4A1
 
 
hsa-miR-152-3p
 
 
-0.317
 
 
17.1
 
 
 
 
CS
 
 
hsa-miR-152-3p
 
 
-0.3
 
 
70.9
 
 
 
 
CTSA
 
 
hsa-miR-152-3p
 
 
-0.541
 
 
51.8
 
 
 
 
CYTH3
 
 
hsa-miR-152-3p
 
 
-0.331
 
 
11.3
 
 
 
 
DCUN1D4
 
 
hsa-miR-152-3p
 
 
-0.314
 
 
12.6
 
 
 
 
DDAH1
 
 
hsa-miR-152-3p
 
 
-0.34
 
 
16.7
 
 
 
 
DDX6
 
 
hsa-miR-152-3p
 
 
-0.363
 
 
30.6
 
 
 
 
DEDD
 
 
hsa-miR-152-3p
 
 
-0.401
 
 
19.9
 
 
 
 
DMPK
 
 
hsa-miR-152-3p
 
 
-0.378
 
 
15.6
 
 
 
 
DOCK6
 
 
hsa-miR-152-3p
 
 
-0.396
 
 
11.0
 
 
 
 
DUSP1
 
 
hsa-miR-152-3p
 
 
-0.32
 
 
333.4
 
 
 
 
DYNLL2
 
 
hsa-miR-152-3p
 
 
-0.366
 
 
29.1
 
 
 
 
DYRK1B
 
 
hsa-miR-152-3p
 
 
-0.305
 
 
13.7
 
 
 
 
EIF4E3
 
 
hsa-miR-152-3p
 
 
-0.464
 
 
13.5
 
 
 
 
ELF5
 
 
hsa-miR-152-3p
 
 
-0.421
 
 
138.9
 
 
 
 
EOGT
 
 
hsa-miR-152-3p
 
 
-0.587
 
 
15.6
 
 
 
 
EPS15
 
 
hsa-miR-152-3p
 
 
-0.322
 
 
13.9
 
 
 
 
ERBB3
 
 
hsa-miR-152-3p
 
 
-0.399
 
 
65.5
 
 
 
 
ERRFI1
 
 
hsa-miR-152-3p
 
 
-0.529
 
 
74.2
 
 
 
 
ESRRG
 
 
hsa-miR-152-3p
 
 
-0.347
 
 
15.0
 
 
 
 
F3
 
 
hsa-miR-152-3p
 
 
-0.331
 
 
85.9
 
 
 
 
FAM168B
 
 
hsa-miR-152-3p
 
 
-0.409
 
 
26.5
 
 
 
 
FEZ2
 
 
hsa-miR-152-3p
 
 
-0.536
 
 
15.3
 
 
 
 
FOSB
 
 
hsa-miR-152-3p
 
 
-0.311
 
 
72.5
 
 
 
 
FOXK2
 
 
hsa-miR-152-3p
 
 
-0.305
 
 
11.7
 
 
 
 
FXR1
 
 
hsa-miR-152-3p
 
 
-0.478
 
 
28.4
 
 
 
 
GADD45A
 
 
hsa-miR-152-3p
 
 
-0.787
 
 
35.2
 
 
 
 
GLRX5
 
 
hsa-miR-152-3p
 
 
-0.381
 
 
29.8
 
 
 
 
GMFB
 
 
hsa-miR-152-3p
 
 
-0.429
 
 
10.1
 
 
 
 
GPATCH8
 
 
hsa-miR-152-3p
 
 
-0.374
 
 
12.6
 
 
 
 
GRSF1
 
 
hsa-miR-378a-3p
 
 
-0.358
 
 
74.6
 
 
 
 
GTF2H1
 
 
hsa-miR-152-3p
 
 
-0.524
 
 
17.8
 
 
 
 
HDDC2
 
 
hsa-miR-378a-3p
 
 
-0.322
 
 
37.4
 
 
 
 
HLA-DQB1
 
 
hsa-miR-152-3p
 
 
-0.37
 
 
23.4
 
 
 
 
HSP90B1
 
 
hsa-miR-152-3p
 
 
-0.304
 
 
240.2
 
 
 
 
HSPH1
 
 
hsa-miR-330-3p
 
 
-0.345
 
 
19.5
 
 
 
 
ID2
 
 
hsa-miR-330-3p
 
 
-0.293
 
 
107.6
 
 
 
 
IL6ST
 
 
hsa-miR-152-3p
 
 
-0.291
 
 
40.5
 
 
 
 
INHBB
 
 
hsa-miR-152-3p
 
 
-0.469
 
 
15.8
 
 
 
 
ITSN2
 
 
hsa-miR-152-3p
 
 
-0.339
 
 
17.4
 
 
 
 
KLF4
 
 
hsa-miR-152-3p
 
 
-0.325
 
 
18.5
 
 
 
 
KLF5
 
 
hsa-miR-152-3p
 
 
-0.475
 
 
72.9
 
 
 
 
LAMA4
 
 
hsa-miR-152-3p
 
 
-0.363
 
 
36.1
 
 
 
 
LBR
 
 
hsa-miR-152-3p
 
 
-0.39
 
 
10.1
 
 
 
 
LDLR
 
 
hsa-miR-152-3p
 
 
-0.323
 
 
18.6
 
 
 
 
LEPROTL1
 
 
hsa-miR-152-3p
 
 
-0.568
 
 
18.9
 
 
 
 
LIPA
 
 
hsa-miR-152-3p
 
 
-0.463
 
 
22.4
 
 
 
 
LMAN1
 
 
hsa-miR-152-3p
 
 
-0.382
 
 
65.1
 
 
 
 
LRRC41
 
 
hsa-miR-152-3p
 
 
-0.392
 
 
35.9
 
 
 
 
LSM5
 
 
hsa-miR-330-3p
 
 
-0.351
 
 
29.5
 
 
 
 
MACROH2A1
 
 
hsa-miR-152-3p
 
 
-0.336
 
 
59.9
 
 
 
 
MAF
 
 
hsa-miR-152-3p
 
 
-0.362
 
 
11.0
 
 
 
 
MAF1
 
 
hsa-miR-152-3p
 
 
-0.366
 
 
72.9
 
 
 
 
MAFB
 
 
hsa-miR-152-3p
 
 
-0.45
 
 
16.9
 
 
 
 
MAP2K1
 
 
hsa-miR-152-3p
 
 
-0.295
 
 
20.1
 
 
 
 
MAPK1
 
 
hsa-miR-378a-3p
 
 
-0.314
 
 
27.2
 
 
 
 
MAT2A
 
 
hsa-miR-330-3p
 
 
-0.307
 
 
134.9
 
 
 
 
MCM3AP
 
 
hsa-miR-152-3p
 
 
-0.287
 
 
13.0
 
 
 
 
MDFIC
 
 
hsa-miR-152-3p
 
 
-0.395
 
 
12.6
 
 
 
 
MECP2
 
 
hsa-miR-152-3p
 
 
-0.3
 
 
12.5
 
 
 
 
MED19
 
 
hsa-miR-378a-3p
 
 
-0.434
 
 
12.5
 
 
 
 
MIER1
 
 
hsa-miR-152-3p
 
 
-0.352
 
 
10.7
 
 
 
 
 
 
 seminal vesicle 
 
 
 
 
target_symbol
 
 
mature_mirna_id
 
 
score
 
 
nTPM
 
 
 
 
 
 
ABCD3
 
 
hsa-miR-152-3p
 
 
-0.388
 
 
25.5
 
 
 
 
ACVR1
 
 
hsa-miR-152-3p
 
 
-0.397
 
 
11.3
 
 
 
 
ADAM10
 
 
hsa-miR-152-3p
 
 
-0.348
 
 
11.5
 
 
 
 
AGFG1
 
 
hsa-miR-152-3p
 
 
-0.332
 
 
22.0
 
 
 
 
AKAP1
 
 
hsa-miR-152-3p
 
 
-0.381
 
 
23.3
 
 
 
 
ALCAM
 
 
hsa-miR-152-3p
 
 
-0.347
 
 
37.3
 
 
 
 
ARF4
 
 
hsa-miR-152-3p
 
 
-0.487
 
 
125.1
 
 
 
 
ARFGEF1
 
 
hsa-miR-152-3p
 
 
-0.306
 
 
11.4
 
 
 
 
ARFIP1
 
 
hsa-miR-152-3p
 
 
-0.474
 
 
16.2
 
 
 
 
ARHGAP21
 
 
hsa-miR-152-3p
 
 
-0.287
 
 
10.8
 
 
 
 
ARL6IP1
 
 
hsa-miR-152-3p
 
 
-0.579
 
 
66.4
 
 
 
 
ARL8B
 
 
hsa-miR-152-3p
 
 
-0.337
 
 
39.4
 
 
 
 
ARRDC3
 
 
hsa-miR-152-3p
 
 
-0.305
 
 
22.9
 
 
 
 
ASPH
 
 
hsa-miR-152-3p
 
 
-0.391
 
 
65.8
 
 
 
 
ATP2B4
 
 
hsa-miR-152-3p
 
 
-0.337
 
 
66.2
 
 
 
 
ATP6AP2
 
 
hsa-miR-152-3p
 
 
-0.592
 
 
92.2
 
 
 
 
ATP6AP2
 
 
hsa-miR-152-3p
 
 
-0.668
 
 
92.2
 
 
 
 
B4GALT5
 
 
hsa-miR-152-3p
 
 
-0.44
 
 
20.7
 
 
 
 
BHLHE41
 
 
hsa-miR-152-3p
 
 
-0.442
 
 
13.2
 
 
 
 
BTAF1
 
 
hsa-miR-152-3p
 
 
-0.401
 
 
10.1
 
 
 
 
BTBD3
 
 
hsa-miR-152-3p
 
 
-0.495
 
 
10.0
 
 
 
 
C1QBP
 
 
hsa-miR-330-3p
 
 
-0.412
 
 
68.8
 
 
 
 
CAND1
 
 
hsa-miR-152-3p
 
 
-0.44
 
 
18.8
 
 
 
 
CANX
 
 
hsa-miR-152-3p
 
 
-0.434
 
 
16.6
 
 
 
 
CAV2
 
 
hsa-miR-152-3p
 
 
-0.32
 
 
30.5
 
 
 
 
CCDC6
 
 
hsa-miR-152-3p
 
 
-0.511
 
 
13.5
 
 
 
 
CCN2
 
 
hsa-miR-330-3p
 
 
-0.305
 
 
145.0
 
 
 
 
CCT6A
 
 
hsa-miR-152-3p
 
 
-0.665
 
 
47.3
 
 
 
 
CEP63
 
 
hsa-miR-152-3p
 
 
-0.329
 
 
13.8
 
 
 
 
CFL2
 
 
hsa-miR-152-3p
 
 
-0.487
 
 
101.0
 
 
 
 
CHMP4B
 
 
hsa-miR-152-3p
 
 
-0.36
 
 
77.7
 
 
 
 
CNOT7
 
 
hsa-miR-152-3p
 
 
-0.31
 
 
27.8
 
 
 
 
COL4A1
 
 
hsa-miR-152-3p
 
 
-0.317
 
 
80.6
 
 
 
 
CS
 
 
hsa-miR-152-3p
 
 
-0.3
 
 
57.0
 
 
 
 
CTSA
 
 
hsa-miR-152-3p
 
 
-0.541
 
 
46.0
 
 
 
 
DACT1
 
 
hsa-miR-378a-3p
 
 
-0.298
 
 
12.3
 
 
 
 
DCUN1D4
 
 
hsa-miR-152-3p
 
 
-0.314
 
 
15.9
 
 
 
 
DDAH1
 
 
hsa-miR-152-3p
 
 
-0.34
 
 
17.5
 
 
 
 
DDX6
 
 
hsa-miR-152-3p
 
 
-0.363
 
 
27.5
 
 
 
 
DEDD
 
 
hsa-miR-152-3p
 
 
-0.401
 
 
14.9
 
 
 
 
DIPK2A
 
 
hsa-miR-152-3p
 
 
-0.393
 
 
11.8
 
 
 
 
DMPK
 
 
hsa-miR-152-3p
 
 
-0.378
 
 
30.9
 
 
 
 
DNAJB4
 
 
hsa-miR-152-3p
 
 
-0.33
 
 
13.3
 
 
 
 
DUSP1
 
 
hsa-miR-152-3p
 
 
-0.32
 
 
336.3
 
 
 
 
DYNLL2
 
 
hsa-miR-152-3p
 
 
-0.366
 
 
24.8
 
 
 
 
EIF4E3
 
 
hsa-miR-152-3p
 
 
-0.464
 
 
17.2
 
 
 
 
EIF4G3
 
 
hsa-miR-378a-3p
 
 
-0.443
 
 
19.6
 
 
 
 
ELF5
 
 
hsa-miR-152-3p
 
 
-0.421
 
 
28.6
 
 
 
 
EMX2
 
 
hsa-miR-152-3p
 
 
-0.366
 
 
49.6
 
 
 
 
EOGT
 
 
hsa-miR-152-3p
 
 
-0.587
 
 
31.7
 
 
 
 
EPS15
 
 
hsa-miR-152-3p
 
 
-0.322
 
 
18.5
 
 
 
 
ERBB3
 
 
hsa-miR-152-3p
 
 
-0.399
 
 
14.3
 
 
 
 
ERRFI1
 
 
hsa-miR-152-3p
 
 
-0.529
 
 
24.5
 
 
 
 
F3
 
 
hsa-miR-152-3p
 
 
-0.331
 
 
56.9
 
 
 
 
FAM168B
 
 
hsa-miR-152-3p
 
 
-0.409
 
 
44.1
 
 
 
 
FBN1
 
 
hsa-miR-152-3p
 
 
-0.349
 
 
11.2
 
 
 
 
FEZ2
 
 
hsa-miR-152-3p
 
 
-0.536
 
 
30.6
 
 
 
 
FMR1
 
 
hsa-miR-152-3p
 
 
-0.385
 
 
15.4
 
 
 
 
FOSB
 
 
hsa-miR-152-3p
 
 
-0.311
 
 
27.9
 
 
 
 
FOXK2
 
 
hsa-miR-152-3p
 
 
-0.305
 
 
11.8
 
 
 
 
FXR1
 
 
hsa-miR-152-3p
 
 
-0.478
 
 
31.3
 
 
 
 
GADD45A
 
 
hsa-miR-152-3p
 
 
-0.787
 
 
34.7
 
 
 
 
GAPVD1
 
 
hsa-miR-152-3p
 
 
-0.309
 
 
11.6
 
 
 
 
GLRX5
 
 
hsa-miR-152-3p
 
 
-0.381
 
 
27.9
 
 
 
 
GMFB
 
 
hsa-miR-152-3p
 
 
-0.429
 
 
12.2
 
 
 
 
GRSF1
 
 
hsa-miR-378a-3p
 
 
-0.358
 
 
32.2
 
 
 
 
HDDC2
 
 
hsa-miR-378a-3p
 
 
-0.322
 
 
22.4
 
 
 
 
HLA-DQB1
 
 
hsa-miR-152-3p
 
 
-0.37
 
 
11.5
 
 
 
 
HSP90B1
 
 
hsa-miR-152-3p
 
 
-0.304
 
 
360.6
 
 
 
 
HSPH1
 
 
hsa-miR-330-3p
 
 
-0.345
 
 
27.6
 
 
 
 
ID2
 
 
hsa-miR-330-3p
 
 
-0.293
 
 
134.1
 
 
 
 
IGF1
 
 
hsa-miR-152-3p
 
 
-0.43
 
 
31.4
 
 
 
 
IL6ST
 
 
hsa-miR-152-3p
 
 
-0.291
 
 
59.7
 
 
 
 
ITSN2
 
 
hsa-miR-152-3p
 
 
-0.339
 
 
14.1
 
 
 
 
KDSR
 
 
hsa-miR-378a-3p
 
 
-0.373
 
 
10.9
 
 
 
 
KLF5
 
 
hsa-miR-152-3p
 
 
-0.475
 
 
13.5
 
 
 
 
LAMA4
 
 
hsa-miR-152-3p
 
 
-0.363
 
 
38.3
 
 
 
 
LBR
 
 
hsa-miR-152-3p
 
 
-0.39
 
 
34.6
 
 
 
 
LEPROTL1
 
 
hsa-miR-152-3p
 
 
-0.568
 
 
20.8
 
 
 
 
LIPA
 
 
hsa-miR-152-3p
 
 
-0.463
 
 
31.9
 
 
 
 
LMAN1
 
 
hsa-miR-152-3p
 
 
-0.382
 
 
41.2
 
 
 
 
LRCH1
 
 
hsa-miR-152-3p
 
 
-0.321
 
 
11.3
 
 
 
 
LRRC41
 
 
hsa-miR-152-3p
 
 
-0.392
 
 
39.0
 
 
 
 
LSM5
 
 
hsa-miR-330-3p
 
 
-0.351
 
 
26.1
 
 
 
 
LYSMD2
 
 
hsa-miR-152-3p
 
 
-0.554
 
 
13.0
 
 
 
 
MACROH2A1
 
 
hsa-miR-152-3p
 
 
-0.336
 
 
32.2
 
 
 
 
MAF
 
 
hsa-miR-152-3p
 
 
-0.362
 
 
14.5
 
 
 
 
MAF1
 
 
hsa-miR-152-3p
 
 
-0.366
 
 
89.1
 
 
 
 
MAP2K1
 
 
hsa-miR-152-3p
 
 
-0.295
 
 
18.8
 
 
 
 
MAPK1
 
 
hsa-miR-378a-3p
 
 
-0.314
 
 
30.8
 
 
 
 
MAT2A
 
 
hsa-miR-330-3p
 
 
-0.307
 
 
113.1
 
 
 
 
MCM3AP
 
 
hsa-miR-152-3p
 
 
-0.287
 
 
12.7
 
 
 
 
MECP2
 
 
hsa-miR-152-3p
 
 
-0.3
 
 
14.7
 
 
 
 
MIER1
 
 
hsa-miR-152-3p
 
 
-0.352
 
 
16.1
 
 
 
 
MITF
 
 
hsa-miR-152-3p
 
 
-0.313
 
 
13.4
 
 
 
 
MLLT10
 
 
hsa-miR-152-3p
 
 
-0.334
 
 
11.1
 
 
 
 
MNAT1
 
 
hsa-miR-378a-3p
 
 
-0.398
 
 
14.8
 
 
 
 
MRAS
 
 
hsa-miR-152-3p
 
 
-0.583
 
 
14.1
 
 
 
 
MRPS10
 
 
hsa-miR-152-3p
 
 
-0.335
 
 
16.0
 
 
 
 
MTMR1
 
 
hsa-miR-152-3p
 
 
-0.345
 
 
10.4
 
 
 
 
 
 
 smooth muscle 
 
 
 
 
target_symbol
 
 
mature_mirna_id
 
 
score
 
 
nTPM
 
 
 
 
 
 
ABCD3
 
 
hsa-miR-152-3p
 
 
-0.388
 
 
16.6
 
 
 
 
ACVR1
 
 
hsa-miR-152-3p
 
 
-0.397
 
 
26.8
 
 
 
 
ADAM10
 
 
hsa-miR-152-3p
 
 
-0.348
 
 
12.4
 
 
 
 
ADGRF5
 
 
hsa-miR-152-3p
 
 
-0.354
 
 
20.6
 
 
 
 
AGFG1
 
 
hsa-miR-152-3p
 
 
-0.332
 
 
36.7
 
 
 
 
AKAP1
 
 
hsa-miR-152-3p
 
 
-0.381
 
 
24.6
 
 
 
 
ALCAM
 
 
hsa-miR-152-3p
 
 
-0.347
 
 
15.2
 
 
 
 
ARF4
 
 
hsa-miR-152-3p
 
 
-0.487
 
 
157.9
 
 
 
 
ARFGEF1
 
 
hsa-miR-152-3p
 
 
-0.306
 
 
10.1
 
 
 
 
ARFIP1
 
 
hsa-miR-152-3p
 
 
-0.474
 
 
18.5
 
 
 
 
ARHGAP21
 
 
hsa-miR-152-3p
 
 
-0.287
 
 
16.5
 
 
 
 
ARL6IP1
 
 
hsa-miR-152-3p
 
 
-0.579
 
 
64.5
 
 
 
 
ARL8B
 
 
hsa-miR-152-3p
 
 
-0.337
 
 
43.9
 
 
 
 
ARRDC3
 
 
hsa-miR-152-3p
 
 
-0.305
 
 
30.8
 
 
 
 
ASPH
 
 
hsa-miR-152-3p
 
 
-0.391
 
 
62.4
 
 
 
 
ATP2B4
 
 
hsa-miR-152-3p
 
 
-0.337
 
 
164.7
 
 
 
 
ATP6AP2
 
 
hsa-miR-152-3p
 
 
-0.592
 
 
116.5
 
 
 
 
ATP6AP2
 
 
hsa-miR-152-3p
 
 
-0.668
 
 
116.5
 
 
 
 
B4GALT5
 
 
hsa-miR-152-3p
 
 
-0.44
 
 
35.1
 
 
 
 
BTAF1
 
 
hsa-miR-152-3p
 
 
-0.401
 
 
11.0
 
 
 
 
BTBD3
 
 
hsa-miR-152-3p
 
 
-0.495
 
 
15.4
 
 
 
 
C1QBP
 
 
hsa-miR-330-3p
 
 
-0.412
 
 
75.5
 
 
 
 
CAND1
 
 
hsa-miR-152-3p
 
 
-0.44
 
 
20.2
 
 
 
 
CANX
 
 
hsa-miR-152-3p
 
 
-0.434
 
 
79.3
 
 
 
 
CAV2
 
 
hsa-miR-152-3p
 
 
-0.32
 
 
39.9
 
 
 
 
CCDC6
 
 
hsa-miR-152-3p
 
 
-0.511
 
 
28.8
 
 
 
 
CCN2
 
 
hsa-miR-330-3p
 
 
-0.305
 
 
538.3
 
 
 
 
CCT6A
 
 
hsa-miR-152-3p
 
 
-0.665
 
 
58.0
 
 
 
 
CDC25B
 
 
hsa-miR-152-3p
 
 
-0.298
 
 
13.3
 
 
 
 
CEBPG
 
 
hsa-miR-152-3p
 
 
-0.319
 
 
13.6
 
 
 
 
CEP63
 
 
hsa-miR-152-3p
 
 
-0.329
 
 
11.5
 
 
 
 
CFL2
 
 
hsa-miR-152-3p
 
 
-0.487
 
 
107.3
 
 
 
 
CHMP4B
 
 
hsa-miR-152-3p
 
 
-0.36
 
 
71.3
 
 
 
 
CNOT7
 
 
hsa-miR-152-3p
 
 
-0.31
 
 
34.1
 
 
 
 
COL4A1
 
 
hsa-miR-152-3p
 
 
-0.317
 
 
291.8
 
 
 
 
CS
 
 
hsa-miR-152-3p
 
 
-0.3
 
 
72.4
 
 
 
 
CTSA
 
 
hsa-miR-152-3p
 
 
-0.541
 
 
63.4
 
 
 
 
CTTNBP2NL
 
 
hsa-miR-152-3p
 
 
-0.329
 
 
12.6
 
 
 
 
CYTH3
 
 
hsa-miR-152-3p
 
 
-0.331
 
 
11.3
 
 
 
 
DCUN1D4
 
 
hsa-miR-152-3p
 
 
-0.314
 
 
16.1
 
 
 
 
DDAH1
 
 
hsa-miR-152-3p
 
 
-0.34
 
 
15.4
 
 
 
 
DDX6
 
 
hsa-miR-152-3p
 
 
-0.363
 
 
28.6
 
 
 
 
DEDD
 
 
hsa-miR-152-3p
 
 
-0.401
 
 
19.6
 
 
 
 
DIPK2A
 
 
hsa-miR-152-3p
 
 
-0.393
 
 
14.1
 
 
 
 
DMPK
 
 
hsa-miR-152-3p
 
 
-0.378
 
 
52.3
 
 
 
 
DNAJB4
 
 
hsa-miR-152-3p
 
 
-0.33
 
 
27.9
 
 
 
 
DUSP1
 
 
hsa-miR-152-3p
 
 
-0.32
 
 
422.8
 
 
 
 
DYNLL2
 
 
hsa-miR-152-3p
 
 
-0.366
 
 
17.7
 
 
 
 
DYRK2
 
 
hsa-miR-152-3p
 
 
-0.293
 
 
12.1
 
 
 
 
EGR3
 
 
hsa-miR-152-3p
 
 
-0.315
 
 
11.7
 
 
 
 
EIF4E3
 
 
hsa-miR-152-3p
 
 
-0.464
 
 
16.1
 
 
 
 
EIF4G3
 
 
hsa-miR-378a-3p
 
 
-0.443
 
 
26.0
 
 
 
 
EMX2
 
 
hsa-miR-152-3p
 
 
-0.366
 
 
42.0
 
 
 
 
EOGT
 
 
hsa-miR-152-3p
 
 
-0.587
 
 
23.7
 
 
 
 
EPS15
 
 
hsa-miR-152-3p
 
 
-0.322
 
 
19.1
 
 
 
 
ERRFI1
 
 
hsa-miR-152-3p
 
 
-0.529
 
 
82.6
 
 
 
 
ESR1
 
 
hsa-miR-152-3p
 
 
-0.34
 
 
47.1
 
 
 
 
F3
 
 
hsa-miR-152-3p
 
 
-0.331
 
 
10.0
 
 
 
 
FAM168B
 
 
hsa-miR-152-3p
 
 
-0.409
 
 
38.1
 
 
 
 
FBN1
 
 
hsa-miR-152-3p
 
 
-0.349
 
 
27.5
 
 
 
 
FEZ2
 
 
hsa-miR-152-3p
 
 
-0.536
 
 
38.4
 
 
 
 
FMR1
 
 
hsa-miR-152-3p
 
 
-0.385
 
 
14.1
 
 
 
 
FOSB
 
 
hsa-miR-152-3p
 
 
-0.311
 
 
330.5
 
 
 
 
FOXF1
 
 
hsa-miR-152-3p
 
 
-0.374
 
 
10.9
 
 
 
 
FOXK2
 
 
hsa-miR-152-3p
 
 
-0.305
 
 
15.3
 
 
 
 
FXR1
 
 
hsa-miR-152-3p
 
 
-0.478
 
 
34.6
 
 
 
 
GADD45A
 
 
hsa-miR-152-3p
 
 
-0.787
 
 
35.8
 
 
 
 
GAPVD1
 
 
hsa-miR-152-3p
 
 
-0.309
 
 
12.4
 
 
 
 
GLRX5
 
 
hsa-miR-152-3p
 
 
-0.381
 
 
22.7
 
 
 
 
GMFB
 
 
hsa-miR-152-3p
 
 
-0.429
 
 
17.0
 
 
 
 
GPATCH8
 
 
hsa-miR-152-3p
 
 
-0.374
 
 
11.7
 
 
 
 
GRSF1
 
 
hsa-miR-378a-3p
 
 
-0.358
 
 
36.2
 
 
 
 
HDDC2
 
 
hsa-miR-378a-3p
 
 
-0.322
 
 
12.7
 
 
 
 
HLA-DQB1
 
 
hsa-miR-152-3p
 
 
-0.37
 
 
10.7
 
 
 
 
HSP90B1
 
 
hsa-miR-152-3p
 
 
-0.304
 
 
360.0
 
 
 
 
HSPH1
 
 
hsa-miR-330-3p
 
 
-0.345
 
 
26.1
 
 
 
 
ID2
 
 
hsa-miR-330-3p
 
 
-0.293
 
 
79.7
 
 
 
 
IGF1
 
 
hsa-miR-152-3p
 
 
-0.43
 
 
69.3
 
 
 
 
IL6ST
 
 
hsa-miR-152-3p
 
 
-0.291
 
 
51.2
 
 
 
 
ITGA11
 
 
hsa-miR-152-3p
 
 
-0.348
 
 
23.4
 
 
 
 
ITSN2
 
 
hsa-miR-152-3p
 
 
-0.339
 
 
16.8
 
 
 
 
KDSR
 
 
hsa-miR-378a-3p
 
 
-0.373
 
 
12.4
 
 
 
 
KLF4
 
 
hsa-miR-152-3p
 
 
-0.325
 
 
38.0
 
 
 
 
LAMA4
 
 
hsa-miR-152-3p
 
 
-0.363
 
 
100.7
 
 
 
 
LBR
 
 
hsa-miR-152-3p
 
 
-0.39
 
 
38.6
 
 
 
 
LDLR
 
 
hsa-miR-152-3p
 
 
-0.323
 
 
26.7
 
 
 
 
LEPROTL1
 
 
hsa-miR-152-3p
 
 
-0.568
 
 
22.4
 
 
 
 
LIPA
 
 
hsa-miR-152-3p
 
 
-0.463
 
 
44.5
 
 
 
 
LMAN1
 
 
hsa-miR-152-3p
 
 
-0.382
 
 
35.2
 
 
 
 
LRCH1
 
 
hsa-miR-152-3p
 
 
-0.321
 
 
16.4
 
 
 
 
LRRC41
 
 
hsa-miR-152-3p
 
 
-0.392
 
 
32.9
 
 
 
 
LSM5
 
 
hsa-miR-330-3p
 
 
-0.351
 
 
34.0
 
 
 
 
LYRM2
 
 
hsa-miR-152-3p
 
 
-0.351
 
 
11.1
 
 
 
 
LYSMD2
 
 
hsa-miR-152-3p
 
 
-0.554
 
 
10.9
 
 
 
 
MACROH2A1
 
 
hsa-miR-152-3p
 
 
-0.336
 
 
53.3
 
 
 
 
MAF
 
 
hsa-miR-152-3p
 
 
-0.362
 
 
16.7
 
 
 
 
MAF1
 
 
hsa-miR-152-3p
 
 
-0.366
 
 
75.1
 
 
 
 
MAFB
 
 
hsa-miR-152-3p
 
 
-0.45
 
 
13.2
 
 
 
 
MAFG
 
 
hsa-miR-152-3p
 
 
-0.328
 
 
10.1
 
 
 
 
MAP2K1
 
 
hsa-miR-152-3p
 
 
-0.295
 
 
27.4
 
 
 
 
 
 
 spinal cord 
 
 
 
 
target_symbol
 
 
mature_mirna_id
 
 
score
 
 
nTPM
 
 
 
 
 
 
ABCD3
 
 
hsa-miR-152-3p
 
 
-0.388
 
 
20.9
 
 
 
 
ADAM10
 
 
hsa-miR-152-3p
 
 
-0.348
 
 
18.7
 
 
 
 
ADCY2
 
 
hsa-miR-152-3p
 
 
-0.299
 
 
11.4
 
 
 
 
AGFG1
 
 
hsa-miR-152-3p
 
 
-0.332
 
 
53.5
 
 
 
 
AKAP1
 
 
hsa-miR-152-3p
 
 
-0.381
 
 
14.0
 
 
 
 
ALCAM
 
 
hsa-miR-152-3p
 
 
-0.347
 
 
56.1
 
 
 
 
ARF4
 
 
hsa-miR-152-3p
 
 
-0.487
 
 
69.7
 
 
 
 
ARFIP1
 
 
hsa-miR-152-3p
 
 
-0.474
 
 
10.6
 
 
 
 
ARHGAP21
 
 
hsa-miR-152-3p
 
 
-0.287
 
 
74.7
 
 
 
 
ARL6IP1
 
 
hsa-miR-152-3p
 
 
-0.579
 
 
140.2
 
 
 
 
ARL8B
 
 
hsa-miR-152-3p
 
 
-0.337
 
 
57.8
 
 
 
 
ARRDC3
 
 
hsa-miR-152-3p
 
 
-0.305
 
 
23.5
 
 
 
 
ASPH
 
 
hsa-miR-152-3p
 
 
-0.391
 
 
25.3
 
 
 
 
ATP6AP2
 
 
hsa-miR-152-3p
 
 
-0.592
 
 
55.3
 
 
 
 
ATP6AP2
 
 
hsa-miR-152-3p
 
 
-0.668
 
 
55.3
 
 
 
 
BHLHE41
 
 
hsa-miR-152-3p
 
 
-0.442
 
 
74.7
 
 
 
 
BTBD3
 
 
hsa-miR-152-3p
 
 
-0.495
 
 
32.8
 
 
 
 
C1GALT1
 
 
hsa-miR-152-3p
 
 
-0.487
 
 
27.9
 
 
 
 
C1QBP
 
 
hsa-miR-330-3p
 
 
-0.412
 
 
74.8
 
 
 
 
C1orf35
 
 
hsa-miR-152-3p
 
 
-0.511
 
 
12.3
 
 
 
 
CAND1
 
 
hsa-miR-152-3p
 
 
-0.44
 
 
15.1
 
 
 
 
CANX
 
 
hsa-miR-152-3p
 
 
-0.434
 
 
133.1
 
 
 
 
CAV2
 
 
hsa-miR-152-3p
 
 
-0.32
 
 
19.3
 
 
 
 
CCN2
 
 
hsa-miR-330-3p
 
 
-0.305
 
 
13.3
 
 
 
 
CCT6A
 
 
hsa-miR-152-3p
 
 
-0.665
 
 
54.3
 
 
 
 
CDC25B
 
 
hsa-miR-152-3p
 
 
-0.298
 
 
17.5
 
 
 
 
CDH20
 
 
hsa-miR-152-3p
 
 
-0.297
 
 
14.5
 
 
 
 
CDK19
 
 
hsa-miR-152-3p
 
 
-0.369
 
 
22.9
 
 
 
 
CDK19
 
 
hsa-miR-152-3p
 
 
-0.405
 
 
22.9
 
 
 
 
CERS5
 
 
hsa-miR-152-3p
 
 
-0.311
 
 
16.1
 
 
 
 
CFL2
 
 
hsa-miR-152-3p
 
 
-0.487
 
 
98.8
 
 
 
 
CHMP4B
 
 
hsa-miR-152-3p
 
 
-0.36
 
 
109.7
 
 
 
 
CLCN6
 
 
hsa-miR-152-3p
 
 
-0.406
 
 
19.6
 
 
 
 
CNOT7
 
 
hsa-miR-152-3p
 
 
-0.31
 
 
16.9
 
 
 
 
CS
 
 
hsa-miR-152-3p
 
 
-0.3
 
 
35.0
 
 
 
 
CTSA
 
 
hsa-miR-152-3p
 
 
-0.541
 
 
30.8
 
 
 
 
DCUN1D4
 
 
hsa-miR-152-3p
 
 
-0.314
 
 
13.9
 
 
 
 
DDAH1
 
 
hsa-miR-152-3p
 
 
-0.34
 
 
18.0
 
 
 
 
DDX6
 
 
hsa-miR-152-3p
 
 
-0.363
 
 
24.8
 
 
 
 
DEDD
 
 
hsa-miR-152-3p
 
 
-0.401
 
 
17.2
 
 
 
 
DIPK2A
 
 
hsa-miR-152-3p
 
 
-0.393
 
 
10.0
 
 
 
 
DMPK
 
 
hsa-miR-152-3p
 
 
-0.378
 
 
11.3
 
 
 
 
DNAJB4
 
 
hsa-miR-152-3p
 
 
-0.33
 
 
16.1
 
 
 
 
DUSP1
 
 
hsa-miR-152-3p
 
 
-0.32
 
 
157.8
 
 
 
 
DYNLL2
 
 
hsa-miR-152-3p
 
 
-0.366
 
 
63.8
 
 
 
 
EIF4E3
 
 
hsa-miR-152-3p
 
 
-0.464
 
 
20.3
 
 
 
 
EIF4G3
 
 
hsa-miR-378a-3p
 
 
-0.443
 
 
14.3
 
 
 
 
EPS15
 
 
hsa-miR-152-3p
 
 
-0.322
 
 
43.8
 
 
 
 
ERBB3
 
 
hsa-miR-152-3p
 
 
-0.399
 
 
61.1
 
 
 
 
ERRFI1
 
 
hsa-miR-152-3p
 
 
-0.529
 
 
23.5
 
 
 
 
F3
 
 
hsa-miR-152-3p
 
 
-0.331
 
 
25.8
 
 
 
 
FAM168B
 
 
hsa-miR-152-3p
 
 
-0.409
 
 
48.4
 
 
 
 
FEZ2
 
 
hsa-miR-152-3p
 
 
-0.536
 
 
57.0
 
 
 
 
FXR1
 
 
hsa-miR-152-3p
 
 
-0.478
 
 
56.4
 
 
 
 
GADD45A
 
 
hsa-miR-152-3p
 
 
-0.787
 
 
30.0
 
 
 
 
GAP43
 
 
hsa-miR-152-3p
 
 
-0.409
 
 
37.9
 
 
 
 
GLRX5
 
 
hsa-miR-152-3p
 
 
-0.381
 
 
31.8
 
 
 
 
GMFB
 
 
hsa-miR-152-3p
 
 
-0.429
 
 
98.2
 
 
 
 
GPATCH8
 
 
hsa-miR-152-3p
 
 
-0.374
 
 
13.6
 
 
 
 
GRSF1
 
 
hsa-miR-378a-3p
 
 
-0.358
 
 
33.9
 
 
 
 
GTF2H1
 
 
hsa-miR-152-3p
 
 
-0.524
 
 
13.9
 
 
 
 
HDDC2
 
 
hsa-miR-378a-3p
 
 
-0.322
 
 
61.2
 
 
 
 
HLA-DQB1
 
 
hsa-miR-152-3p
 
 
-0.37
 
 
34.3
 
 
 
 
HSP90B1
 
 
hsa-miR-152-3p
 
 
-0.304
 
 
178.6
 
 
 
 
HSPH1
 
 
hsa-miR-330-3p
 
 
-0.345
 
 
65.4
 
 
 
 
ID2
 
 
hsa-miR-330-3p
 
 
-0.293
 
 
55.4
 
 
 
 
IL6ST
 
 
hsa-miR-152-3p
 
 
-0.291
 
 
33.8
 
 
 
 
KDSR
 
 
hsa-miR-378a-3p
 
 
-0.373
 
 
18.4
 
 
 
 
LBR
 
 
hsa-miR-152-3p
 
 
-0.39
 
 
11.4
 
 
 
 
LEPROTL1
 
 
hsa-miR-152-3p
 
 
-0.568
 
 
21.2
 
 
 
 
LIPA
 
 
hsa-miR-152-3p
 
 
-0.463
 
 
124.6
 
 
 
 
LMAN1
 
 
hsa-miR-152-3p
 
 
-0.382
 
 
13.8
 
 
 
 
LRP2
 
 
hsa-miR-152-3p
 
 
-0.465
 
 
13.9
 
 
 
 
LRRC41
 
 
hsa-miR-152-3p
 
 
-0.392
 
 
22.1
 
 
 
 
LSM5
 
 
hsa-miR-330-3p
 
 
-0.351
 
 
33.3
 
 
 
 
LYRM2
 
 
hsa-miR-152-3p
 
 
-0.351
 
 
13.1
 
 
 
 
LYSMD2
 
 
hsa-miR-152-3p
 
 
-0.554
 
 
15.0
 
 
 
 
MACIR
 
 
hsa-miR-152-3p
 
 
-0.332
 
 
17.9
 
 
 
 
MACIR
 
 
hsa-miR-152-3p
 
 
-0.573
 
 
17.9
 
 
 
 
MACROH2A1
 
 
hsa-miR-152-3p
 
 
-0.336
 
 
44.6
 
 
 
 
MAF
 
 
hsa-miR-152-3p
 
 
-0.362
 
 
10.7
 
 
 
 
MAF1
 
 
hsa-miR-152-3p
 
 
-0.366
 
 
59.1
 
 
 
 
MAFG
 
 
hsa-miR-152-3p
 
 
-0.328
 
 
13.8
 
 
 
 
MAP2K1
 
 
hsa-miR-152-3p
 
 
-0.295
 
 
15.9
 
 
 
 
MAPK1
 
 
hsa-miR-378a-3p
 
 
-0.314
 
 
36.0
 
 
 
 
MAT2A
 
 
hsa-miR-330-3p
 
 
-0.307
 
 
150.7
 
 
 
 
MECP2
 
 
hsa-miR-152-3p
 
 
-0.3
 
 
13.0
 
 
 
 
MED19
 
 
hsa-miR-378a-3p
 
 
-0.434
 
 
10.0
 
 
 
 
MLLT10
 
 
hsa-miR-152-3p
 
 
-0.334
 
 
11.4
 
 
 
 
MMD
 
 
hsa-miR-152-3p
 
 
-0.62
 
 
16.6
 
 
 
 
MNAT1
 
 
hsa-miR-378a-3p
 
 
-0.398
 
 
19.4
 
 
 
 
MRAS
 
 
hsa-miR-152-3p
 
 
-0.583
 
 
47.8
 
 
 
 
MRPS10
 
 
hsa-miR-152-3p
 
 
-0.335
 
 
13.5
 
 
 
 
MRPS25
 
 
hsa-miR-152-3p
 
 
-0.323
 
 
17.8
 
 
 
 
MTMR10
 
 
hsa-miR-152-3p
 
 
-0.302
 
 
77.6
 
 
 
 
MTMR14
 
 
hsa-miR-152-3p
 
 
-0.467
 
 
10.7
 
 
 
 
NAP1L1
 
 
hsa-miR-152-3p
 
 
-0.343
 
 
446.9
 
 
 
 
NAT14
 
 
hsa-miR-152-3p
 
 
-0.324
 
 
19.3
 
 
 
 
NCKIPSD
 
 
hsa-miR-152-3p
 
 
-0.382
 
 
17.8
 
 
 
 
NCOA1
 
 
hsa-miR-152-3p
 
 
-0.313
 
 
13.7
 
 
 
 
 
 
 testis 
 
 
 
 
target_symbol
 
 
mature_mirna_id
 
 
score
 
 
nTPM
 
 
 
 
 
 
ABCD3
 
 
hsa-miR-152-3p
 
 
-0.388
 
 
15.3
 
 
 
 
AGFG1
 
 
hsa-miR-152-3p
 
 
-0.332
 
 
86.7
 
 
 
 
AKAP1
 
 
hsa-miR-152-3p
 
 
-0.381
 
 
108.8
 
 
 
 
AP4B1
 
 
hsa-miR-378a-3p
 
 
-0.62
 
 
10.7
 
 
 
 
ARF4
 
 
hsa-miR-152-3p
 
 
-0.487
 
 
94.2
 
 
 
 
ARFGEF1
 
 
hsa-miR-152-3p
 
 
-0.306
 
 
26.5
 
 
 
 
ARFIP1
 
 
hsa-miR-152-3p
 
 
-0.474
 
 
11.3
 
 
 
 
ARHGAP21
 
 
hsa-miR-152-3p
 
 
-0.287
 
 
13.5
 
 
 
 
ARL6IP1
 
 
hsa-miR-152-3p
 
 
-0.579
 
 
121.4
 
 
 
 
ARL8B
 
 
hsa-miR-152-3p
 
 
-0.337
 
 
30.1
 
 
 
 
ARRDC3
 
 
hsa-miR-152-3p
 
 
-0.305
 
 
15.5
 
 
 
 
ASPH
 
 
hsa-miR-152-3p
 
 
-0.391
 
 
15.3
 
 
 
 
ATP2B4
 
 
hsa-miR-152-3p
 
 
-0.337
 
 
25.4
 
 
 
 
ATP6AP2
 
 
hsa-miR-152-3p
 
 
-0.592
 
 
37.7
 
 
 
 
ATP6AP2
 
 
hsa-miR-152-3p
 
 
-0.668
 
 
37.7
 
 
 
 
B4GALT5
 
 
hsa-miR-152-3p
 
 
-0.44
 
 
20.8
 
 
 
 
BTAF1
 
 
hsa-miR-152-3p
 
 
-0.401
 
 
14.0
 
 
 
 
BTBD3
 
 
hsa-miR-152-3p
 
 
-0.495
 
 
13.8
 
 
 
 
C1QBP
 
 
hsa-miR-330-3p
 
 
-0.412
 
 
58.4
 
 
 
 
C3orf62
 
 
hsa-miR-330-3p
 
 
-0.366
 
 
16.8
 
 
 
 
CAND1
 
 
hsa-miR-152-3p
 
 
-0.44
 
 
26.0
 
 
 
 
CANX
 
 
hsa-miR-152-3p
 
 
-0.434
 
 
67.8
 
 
 
 
CAV2
 
 
hsa-miR-152-3p
 
 
-0.32
 
 
14.5
 
 
 
 
CCDC6
 
 
hsa-miR-152-3p
 
 
-0.511
 
 
10.6
 
 
 
 
CCDC71
 
 
hsa-miR-152-3p
 
 
-0.369
 
 
11.7
 
 
 
 
CCN2
 
 
hsa-miR-330-3p
 
 
-0.305
 
 
24.3
 
 
 
 
CCT6A
 
 
hsa-miR-152-3p
 
 
-0.665
 
 
47.1
 
 
 
 
CDC14A
 
 
hsa-miR-152-3p
 
 
-0.482
 
 
24.8
 
 
 
 
CDC25B
 
 
hsa-miR-152-3p
 
 
-0.298
 
 
12.3
 
 
 
 
CDK19
 
 
hsa-miR-152-3p
 
 
-0.369
 
 
17.3
 
 
 
 
CDK19
 
 
hsa-miR-152-3p
 
 
-0.405
 
 
17.3
 
 
 
 
CEBPG
 
 
hsa-miR-152-3p
 
 
-0.319
 
 
11.7
 
 
 
 
CEP55
 
 
hsa-miR-152-3p
 
 
-0.305
 
 
20.3
 
 
 
 
CEP63
 
 
hsa-miR-152-3p
 
 
-0.329
 
 
33.4
 
 
 
 
CERS5
 
 
hsa-miR-152-3p
 
 
-0.311
 
 
10.2
 
 
 
 
CFL2
 
 
hsa-miR-152-3p
 
 
-0.487
 
 
20.2
 
 
 
 
CHD1
 
 
hsa-miR-152-3p
 
 
-0.303
 
 
11.6
 
 
 
 
CHMP4B
 
 
hsa-miR-152-3p
 
 
-0.36
 
 
59.4
 
 
 
 
CLCN6
 
 
hsa-miR-152-3p
 
 
-0.406
 
 
13.8
 
 
 
 
CNOT7
 
 
hsa-miR-152-3p
 
 
-0.31
 
 
48.4
 
 
 
 
CNTN4
 
 
hsa-miR-152-3p
 
 
-0.415
 
 
12.6
 
 
 
 
COL4A1
 
 
hsa-miR-152-3p
 
 
-0.317
 
 
10.0
 
 
 
 
CS
 
 
hsa-miR-152-3p
 
 
-0.3
 
 
23.0
 
 
 
 
CTSA
 
 
hsa-miR-152-3p
 
 
-0.541
 
 
31.7
 
 
 
 
CYB5R4
 
 
hsa-miR-152-3p
 
 
-0.454
 
 
11.3
 
 
 
 
CYB5R4
 
 
hsa-miR-152-3p
 
 
-0.454
 
 
11.3
 
 
 
 
CYTH3
 
 
hsa-miR-152-3p
 
 
-0.331
 
 
17.8
 
 
 
 
DCUN1D4
 
 
hsa-miR-152-3p
 
 
-0.314
 
 
15.9
 
 
 
 
DDAH1
 
 
hsa-miR-152-3p
 
 
-0.34
 
 
17.9
 
 
 
 
DDX6
 
 
hsa-miR-152-3p
 
 
-0.363
 
 
27.0
 
 
 
 
DEDD
 
 
hsa-miR-152-3p
 
 
-0.401
 
 
26.4
 
 
 
 
DICER1
 
 
hsa-miR-152-3p
 
 
-0.29
 
 
10.6
 
 
 
 
DNMT1
 
 
hsa-miR-152-3p
 
 
-0.421
 
 
11.5
 
 
 
 
DUSP1
 
 
hsa-miR-152-3p
 
 
-0.32
 
 
65.8
 
 
 
 
DYNLL2
 
 
hsa-miR-152-3p
 
 
-0.366
 
 
24.9
 
 
 
 
DYRK1B
 
 
hsa-miR-152-3p
 
 
-0.305
 
 
79.9
 
 
 
 
EIF4G3
 
 
hsa-miR-378a-3p
 
 
-0.443
 
 
76.6
 
 
 
 
ELAVL2
 
 
hsa-miR-152-3p
 
 
-0.3
 
 
40.3
 
 
 
 
EPS15
 
 
hsa-miR-152-3p
 
 
-0.322
 
 
45.2
 
 
 
 
ERRFI1
 
 
hsa-miR-152-3p
 
 
-0.529
 
 
15.8
 
 
 
 
ESCO1
 
 
hsa-miR-152-3p
 
 
-0.34
 
 
18.9
 
 
 
 
EYA3
 
 
hsa-miR-152-3p
 
 
-0.309
 
 
10.6
 
 
 
 
F3
 
 
hsa-miR-152-3p
 
 
-0.331
 
 
33.4
 
 
 
 
FAM168B
 
 
hsa-miR-152-3p
 
 
-0.409
 
 
25.0
 
 
 
 
FAM184A
 
 
hsa-miR-152-3p
 
 
-0.371
 
 
16.1
 
 
 
 
FBXL19
 
 
hsa-miR-152-3p
 
 
-0.362
 
 
30.9
 
 
 
 
FEZ2
 
 
hsa-miR-152-3p
 
 
-0.536
 
 
19.4
 
 
 
 
FMR1
 
 
hsa-miR-152-3p
 
 
-0.385
 
 
14.0
 
 
 
 
FOXK2
 
 
hsa-miR-152-3p
 
 
-0.305
 
 
26.2
 
 
 
 
FXR1
 
 
hsa-miR-152-3p
 
 
-0.478
 
 
124.5
 
 
 
 
GADD45A
 
 
hsa-miR-152-3p
 
 
-0.787
 
 
13.4
 
 
 
 
GAPVD1
 
 
hsa-miR-152-3p
 
 
-0.309
 
 
25.1
 
 
 
 
GLRX5
 
 
hsa-miR-152-3p
 
 
-0.381
 
 
49.4
 
 
 
 
GMFB
 
 
hsa-miR-152-3p
 
 
-0.429
 
 
10.4
 
 
 
 
GPATCH8
 
 
hsa-miR-152-3p
 
 
-0.374
 
 
11.2
 
 
 
 
GRSF1
 
 
hsa-miR-378a-3p
 
 
-0.358
 
 
29.5
 
 
 
 
GTF2H1
 
 
hsa-miR-152-3p
 
 
-0.524
 
 
25.0
 
 
 
 
HDDC2
 
 
hsa-miR-378a-3p
 
 
-0.322
 
 
41.9
 
 
 
 
HIF1AN
 
 
hsa-miR-152-3p
 
 
-0.365
 
 
13.0
 
 
 
 
HSP90B1
 
 
hsa-miR-152-3p
 
 
-0.304
 
 
169.0
 
 
 
 
HSPH1
 
 
hsa-miR-330-3p
 
 
-0.345
 
 
63.4
 
 
 
 
ID2
 
 
hsa-miR-330-3p
 
 
-0.293
 
 
12.9
 
 
 
 
IL6ST
 
 
hsa-miR-152-3p
 
 
-0.291
 
 
15.6
 
 
 
 
ING2
 
 
hsa-miR-152-3p
 
 
-0.482
 
 
21.0
 
 
 
 
INHBB
 
 
hsa-miR-152-3p
 
 
-0.469
 
 
16.2
 
 
 
 
ITSN2
 
 
hsa-miR-152-3p
 
 
-0.339
 
 
12.0
 
 
 
 
KLF4
 
 
hsa-miR-152-3p
 
 
-0.325
 
 
14.7
 
 
 
 
KLF5
 
 
hsa-miR-152-3p
 
 
-0.475
 
 
11.5
 
 
 
 
LAMA4
 
 
hsa-miR-152-3p
 
 
-0.363
 
 
18.6
 
 
 
 
LBR
 
 
hsa-miR-152-3p
 
 
-0.39
 
 
19.2
 
 
 
 
LEPROTL1
 
 
hsa-miR-152-3p
 
 
-0.568
 
 
31.9
 
 
 
 
LIPA
 
 
hsa-miR-152-3p
 
 
-0.463
 
 
12.5
 
 
 
 
LMAN1
 
 
hsa-miR-152-3p
 
 
-0.382
 
 
19.8
 
 
 
 
LRRC41
 
 
hsa-miR-152-3p
 
 
-0.392
 
 
31.0
 
 
 
 
LSM5
 
 
hsa-miR-330-3p
 
 
-0.351
 
 
21.0
 
 
 
 
LYSMD2
 
 
hsa-miR-152-3p
 
 
-0.554
 
 
11.1
 
 
 
 
MACROH2A1
 
 
hsa-miR-152-3p
 
 
-0.336
 
 
20.7
 
 
 
 
MAF1
 
 
hsa-miR-152-3p
 
 
-0.366
 
 
72.7
 
 
 
 
MAFG
 
 
hsa-miR-152-3p
 
 
-0.328
 
 
13.1
 
 
 
 
MAP2K1
 
 
hsa-miR-152-3p
 
 
-0.295
 
 
10.0
 
 
 
 
 
 
 thyroid gland 
 
 
 
 
target_symbol
 
 
mature_mirna_id
 
 
score
 
 
nTPM
 
 
 
 
 
 
ABCD3
 
 
hsa-miR-152-3p
 
 
-0.388
 
 
25.8
 
 
 
 
ACVR1
 
 
hsa-miR-152-3p
 
 
-0.397
 
 
28.6
 
 
 
 
ADAM10
 
 
hsa-miR-152-3p
 
 
-0.348
 
 
26.7
 
 
 
 
ADGRF5
 
 
hsa-miR-152-3p
 
 
-0.354
 
 
30.7
 
 
 
 
AGFG1
 
 
hsa-miR-152-3p
 
 
-0.332
 
 
32.1
 
 
 
 
AHDC1
 
 
hsa-miR-152-3p
 
 
-0.413
 
 
19.9
 
 
 
 
AKAP1
 
 
hsa-miR-152-3p
 
 
-0.381
 
 
35.0
 
 
 
 
ALCAM
 
 
hsa-miR-152-3p
 
 
-0.347
 
 
58.4
 
 
 
 
AP4B1
 
 
hsa-miR-378a-3p
 
 
-0.62
 
 
10.7
 
 
 
 
ARF4
 
 
hsa-miR-152-3p
 
 
-0.487
 
 
134.8
 
 
 
 
ARFGEF1
 
 
hsa-miR-152-3p
 
 
-0.306
 
 
20.9
 
 
 
 
ARFIP1
 
 
hsa-miR-152-3p
 
 
-0.474
 
 
18.8
 
 
 
 
ARHGAP21
 
 
hsa-miR-152-3p
 
 
-0.287
 
 
12.0
 
 
 
 
ARL6IP1
 
 
hsa-miR-152-3p
 
 
-0.579
 
 
105.4
 
 
 
 
ARL8B
 
 
hsa-miR-152-3p
 
 
-0.337
 
 
58.1
 
 
 
 
ARRDC3
 
 
hsa-miR-152-3p
 
 
-0.305
 
 
78.7
 
 
 
 
ASPH
 
 
hsa-miR-152-3p
 
 
-0.391
 
 
90.3
 
 
 
 
ATP2B4
 
 
hsa-miR-152-3p
 
 
-0.337
 
 
14.2
 
 
 
 
ATP6AP2
 
 
hsa-miR-152-3p
 
 
-0.592
 
 
166.0
 
 
 
 
ATP6AP2
 
 
hsa-miR-152-3p
 
 
-0.668
 
 
166.0
 
 
 
 
B4GALT5
 
 
hsa-miR-152-3p
 
 
-0.44
 
 
10.8
 
 
 
 
BCL2L11
 
 
hsa-miR-152-3p
 
 
-0.445
 
 
18.5
 
 
 
 
BHLHE41
 
 
hsa-miR-152-3p
 
 
-0.442
 
 
54.2
 
 
 
 
BMP2
 
 
hsa-miR-378a-3p
 
 
-0.481
 
 
17.7
 
 
 
 
BTAF1
 
 
hsa-miR-152-3p
 
 
-0.401
 
 
14.3
 
 
 
 
BTBD3
 
 
hsa-miR-152-3p
 
 
-0.495
 
 
10.8
 
 
 
 
C1GALT1
 
 
hsa-miR-152-3p
 
 
-0.487
 
 
11.8
 
 
 
 
C1QBP
 
 
hsa-miR-330-3p
 
 
-0.412
 
 
60.9
 
 
 
 
C1orf210
 
 
hsa-miR-378a-3p
 
 
-0.307
 
 
15.8
 
 
 
 
C1orf35
 
 
hsa-miR-152-3p
 
 
-0.511
 
 
13.7
 
 
 
 
CAND1
 
 
hsa-miR-152-3p
 
 
-0.44
 
 
24.4
 
 
 
 
CANX
 
 
hsa-miR-152-3p
 
 
-0.434
 
 
141.7
 
 
 
 
CAV2
 
 
hsa-miR-152-3p
 
 
-0.32
 
 
101.6
 
 
 
 
CCDC6
 
 
hsa-miR-152-3p
 
 
-0.511
 
 
17.2
 
 
 
 
CCDC71
 
 
hsa-miR-152-3p
 
 
-0.369
 
 
15.1
 
 
 
 
CCN2
 
 
hsa-miR-330-3p
 
 
-0.305
 
 
436.1
 
 
 
 
CCT6A
 
 
hsa-miR-152-3p
 
 
-0.665
 
 
47.1
 
 
 
 
CDC25B
 
 
hsa-miR-152-3p
 
 
-0.298
 
 
41.5
 
 
 
 
CEBPG
 
 
hsa-miR-152-3p
 
 
-0.319
 
 
24.0
 
 
 
 
CEP63
 
 
hsa-miR-152-3p
 
 
-0.329
 
 
16.8
 
 
 
 
CERS5
 
 
hsa-miR-152-3p
 
 
-0.311
 
 
18.3
 
 
 
 
CFL2
 
 
hsa-miR-152-3p
 
 
-0.487
 
 
27.5
 
 
 
 
CHMP4B
 
 
hsa-miR-152-3p
 
 
-0.36
 
 
87.1
 
 
 
 
CHST1
 
 
hsa-miR-152-3p
 
 
-0.408
 
 
13.6
 
 
 
 
CLCN6
 
 
hsa-miR-152-3p
 
 
-0.406
 
 
13.4
 
 
 
 
CNOT7
 
 
hsa-miR-152-3p
 
 
-0.31
 
 
56.1
 
 
 
 
COL4A1
 
 
hsa-miR-152-3p
 
 
-0.317
 
 
51.1
 
 
 
 
CS
 
 
hsa-miR-152-3p
 
 
-0.3
 
 
49.6
 
 
 
 
CTSA
 
 
hsa-miR-152-3p
 
 
-0.541
 
 
40.6
 
 
 
 
CUL5
 
 
hsa-miR-152-3p
 
 
-0.389
 
 
13.5
 
 
 
 
CYTH3
 
 
hsa-miR-152-3p
 
 
-0.331
 
 
26.6
 
 
 
 
DCUN1D4
 
 
hsa-miR-152-3p
 
 
-0.314
 
 
12.3
 
 
 
 
DDAH1
 
 
hsa-miR-152-3p
 
 
-0.34
 
 
27.0
 
 
 
 
DDX6
 
 
hsa-miR-152-3p
 
 
-0.363
 
 
35.1
 
 
 
 
DEDD
 
 
hsa-miR-152-3p
 
 
-0.401
 
 
23.1
 
 
 
 
DICER1
 
 
hsa-miR-152-3p
 
 
-0.29
 
 
10.5
 
 
 
 
DIPK2A
 
 
hsa-miR-152-3p
 
 
-0.393
 
 
11.9
 
 
 
 
DMPK
 
 
hsa-miR-152-3p
 
 
-0.378
 
 
12.3
 
 
 
 
DMXL1
 
 
hsa-miR-152-3p
 
 
-0.29
 
 
12.3
 
 
 
 
DNAJB4
 
 
hsa-miR-152-3p
 
 
-0.33
 
 
41.7
 
 
 
 
DOCK6
 
 
hsa-miR-152-3p
 
 
-0.396
 
 
28.0
 
 
 
 
DUSP1
 
 
hsa-miR-152-3p
 
 
-0.32
 
 
575.0
 
 
 
 
DYNLL2
 
 
hsa-miR-152-3p
 
 
-0.366
 
 
30.9
 
 
 
 
DYRK1B
 
 
hsa-miR-152-3p
 
 
-0.305
 
 
15.8
 
 
 
 
EIF4E3
 
 
hsa-miR-152-3p
 
 
-0.464
 
 
16.3
 
 
 
 
EIF4G3
 
 
hsa-miR-378a-3p
 
 
-0.443
 
 
34.3
 
 
 
 
EPS15
 
 
hsa-miR-152-3p
 
 
-0.322
 
 
22.5
 
 
 
 
ERRFI1
 
 
hsa-miR-152-3p
 
 
-0.529
 
 
109.2
 
 
 
 
F3
 
 
hsa-miR-152-3p
 
 
-0.331
 
 
58.7
 
 
 
 
FAM168B
 
 
hsa-miR-152-3p
 
 
-0.409
 
 
33.9
 
 
 
 
FBN1
 
 
hsa-miR-152-3p
 
 
-0.349
 
 
14.5
 
 
 
 
FEZ2
 
 
hsa-miR-152-3p
 
 
-0.536
 
 
22.3
 
 
 
 
FMR1
 
 
hsa-miR-152-3p
 
 
-0.385
 
 
21.1
 
 
 
 
FOSB
 
 
hsa-miR-152-3p
 
 
-0.311
 
 
296.7
 
 
 
 
FOXK2
 
 
hsa-miR-152-3p
 
 
-0.305
 
 
14.4
 
 
 
 
FXR1
 
 
hsa-miR-152-3p
 
 
-0.478
 
 
30.3
 
 
 
 
GADD45A
 
 
hsa-miR-152-3p
 
 
-0.787
 
 
68.8
 
 
 
 
GAPVD1
 
 
hsa-miR-152-3p
 
 
-0.309
 
 
17.6
 
 
 
 
GLRX5
 
 
hsa-miR-152-3p
 
 
-0.381
 
 
33.5
 
 
 
 
GMFB
 
 
hsa-miR-152-3p
 
 
-0.429
 
 
20.5
 
 
 
 
GPATCH8
 
 
hsa-miR-152-3p
 
 
-0.374
 
 
15.5
 
 
 
 
GRSF1
 
 
hsa-miR-378a-3p
 
 
-0.358
 
 
41.4
 
 
 
 
GTF2H1
 
 
hsa-miR-152-3p
 
 
-0.524
 
 
15.1
 
 
 
 
HDDC2
 
 
hsa-miR-378a-3p
 
 
-0.322
 
 
28.3
 
 
 
 
HLA-DQB1
 
 
hsa-miR-152-3p
 
 
-0.37
 
 
26.1
 
 
 
 
HOMER1
 
 
hsa-miR-152-3p
 
 
-0.452
 
 
14.0
 
 
 
 
HSP90B1
 
 
hsa-miR-152-3p
 
 
-0.304
 
 
1322.6
 
 
 
 
HSPH1
 
 
hsa-miR-330-3p
 
 
-0.345
 
 
30.9
 
 
 
 
ID2
 
 
hsa-miR-330-3p
 
 
-0.293
 
 
209.4
 
 
 
 
IGF1
 
 
hsa-miR-152-3p
 
 
-0.43
 
 
27.3
 
 
 
 
IL15
 
 
hsa-miR-152-3p
 
 
-0.652
 
 
13.1
 
 
 
 
IL6ST
 
 
hsa-miR-152-3p
 
 
-0.291
 
 
48.4
 
 
 
 
INHBB
 
 
hsa-miR-152-3p
 
 
-0.469
 
 
19.9
 
 
 
 
ITSN2
 
 
hsa-miR-152-3p
 
 
-0.339
 
 
47.4
 
 
 
 
KDSR
 
 
hsa-miR-378a-3p
 
 
-0.373
 
 
12.8
 
 
 
 
KLF4
 
 
hsa-miR-152-3p
 
 
-0.325
 
 
23.6
 
 
 
 
KLF5
 
 
hsa-miR-152-3p
 
 
-0.475
 
 
24.1
 
 
 
 
LAMA4
 
 
hsa-miR-152-3p
 
 
-0.363
 
 
22.3
 
 
 
 
LBR
 
 
hsa-miR-152-3p
 
 
-0.39
 
 
10.9
 
 
 
 
LEPROTL1
 
 
hsa-miR-152-3p
 
 
-0.568
 
 
14.0
 
 
 
 
 
 
 
 cd8_s1_vs_cd8_s0_downregulated 
 
 Tissue expression heatmap (log10-transformed) 
   
 
 
 stomach 
 
 
 
 
target_symbol
 
 
mature_mirna_id
 
 
score
 
 
nTPM
 
 
 
 
 
 
A4GNT
 
 
hsa-miR-22-3p
 
 
-0.601
 
 
22.3
 
 
 
 
ABCC5
 
 
hsa-miR-125a-5p
 
 
-0.288
 
 
77.7
 
 
 
 
ABHD17C
 
 
hsa-let-7e-5p
 
 
-0.359
 
 
34.0
 
 
 
 
ABHD17C
 
 
hsa-let-7c-5p
 
 
-0.359
 
 
34.0
 
 
 
 
ABHD3
 
 
hsa-miR-125a-5p
 
 
-0.453
 
 
33.6
 
 
 
 
ABHD6
 
 
hsa-miR-125a-5p
 
 
-0.501
 
 
12.2
 
 
 
 
ABTB1
 
 
hsa-miR-125a-5p
 
 
-0.519
 
 
28.0
 
 
 
 
ACER2
 
 
hsa-let-7e-5p
 
 
-0.333
 
 
58.3
 
 
 
 
ACER2
 
 
hsa-let-7c-5p
 
 
-0.333
 
 
58.3
 
 
 
 
ACER2
 
 
hsa-miR-125a-5p
 
 
-0.338
 
 
58.3
 
 
 
 
ADIPOR2
 
 
hsa-miR-150-5p
 
 
-0.287
 
 
42.5
 
 
 
 
AIDA
 
 
hsa-miR-22-3p
 
 
-0.316
 
 
26.6
 
 
 
 
AIFM1
 
 
hsa-miR-125a-5p
 
 
-0.3
 
 
29.8
 
 
 
 
AIFM1
 
 
hsa-let-7e-5p
 
 
-0.314
 
 
29.8
 
 
 
 
AIFM1
 
 
hsa-let-7c-5p
 
 
-0.314
 
 
29.8
 
 
 
 
ALDH4A1
 
 
hsa-miR-184
 
 
-0.421
 
 
12.6
 
 
 
 
AMT
 
 
hsa-let-7e-5p
 
 
-0.463
 
 
13.1
 
 
 
 
AMT
 
 
hsa-let-7c-5p
 
 
-0.463
 
 
13.1
 
 
 
 
ANAPC16
 
 
hsa-miR-125a-5p
 
 
-0.578
 
 
24.4
 
 
 
 
ANKHD1-EIF4EBP3
 
 
hsa-miR-22-3p
 
 
-0.479
 
 
11.7
 
 
 
 
ANKRD13A
 
 
hsa-miR-22-3p
 
 
-0.478
 
 
16.1
 
 
 
 
ANKRD46
 
 
hsa-let-7e-5p
 
 
-0.389
 
 
10.9
 
 
 
 
ANKRD46
 
 
hsa-let-7c-5p
 
 
-0.389
 
 
10.9
 
 
 
 
AP1AR
 
 
hsa-miR-99b-5p
 
 
-0.823
 
 
12.3
 
 
 
 
AP1S1
 
 
hsa-let-7e-5p
 
 
-0.374
 
 
23.2
 
 
 
 
AP1S1
 
 
hsa-let-7c-5p
 
 
-0.374
 
 
23.2
 
 
 
 
ARMT1
 
 
hsa-let-7e-5p
 
 
-0.303
 
 
13.3
 
 
 
 
ARMT1
 
 
hsa-let-7c-5p
 
 
-0.303
 
 
13.3
 
 
 
 
ARPC5
 
 
hsa-miR-22-3p
 
 
-0.679
 
 
83.5
 
 
 
 
ARPP19
 
 
hsa-let-7e-5p
 
 
-0.492
 
 
36.4
 
 
 
 
ARPP19
 
 
hsa-let-7c-5p
 
 
-0.492
 
 
36.4
 
 
 
 
ARRB1
 
 
hsa-miR-22-3p
 
 
-0.616
 
 
31.7
 
 
 
 
ARRDC4
 
 
hsa-let-7e-5p
 
 
-0.329
 
 
25.2
 
 
 
 
ARRDC4
 
 
hsa-let-7c-5p
 
 
-0.329
 
 
25.2
 
 
 
 
ATOSB
 
 
hsa-let-7e-5p
 
 
-0.429
 
 
11.2
 
 
 
 
ATOSB
 
 
hsa-let-7c-5p
 
 
-0.429
 
 
11.2
 
 
 
 
ATP2A2
 
 
hsa-let-7e-5p
 
 
-0.311
 
 
44.5
 
 
 
 
ATP2A2
 
 
hsa-let-7c-5p
 
 
-0.311
 
 
44.5
 
 
 
 
ATP8A1
 
 
hsa-miR-22-3p
 
 
-0.468
 
 
11.8
 
 
 
 
ATPAF1
 
 
hsa-let-7e-5p
 
 
-0.498
 
 
40.7
 
 
 
 
ATPAF1
 
 
hsa-let-7c-5p
 
 
-0.498
 
 
40.7
 
 
 
 
ATXN2
 
 
hsa-miR-10a-5p
 
 
-0.362
 
 
16.1
 
 
 
 
B3GALNT2
 
 
hsa-miR-125a-5p
 
 
-0.311
 
 
14.3
 
 
 
 
B3GAT3
 
 
hsa-let-7e-5p
 
 
-0.324
 
 
32.0
 
 
 
 
B3GAT3
 
 
hsa-let-7c-5p
 
 
-0.324
 
 
32.0
 
 
 
 
B4GAT1
 
 
hsa-let-7e-5p
 
 
-0.47
 
 
25.1
 
 
 
 
B4GAT1
 
 
hsa-let-7c-5p
 
 
-0.47
 
 
25.1
 
 
 
 
BACE2
 
 
hsa-let-7e-5p
 
 
-0.364
 
 
21.6
 
 
 
 
BACE2
 
 
hsa-let-7c-5p
 
 
-0.364
 
 
21.6
 
 
 
 
BACH1
 
 
hsa-let-7e-5p
 
 
-0.298
 
 
12.7
 
 
 
 
BACH1
 
 
hsa-let-7e-5p
 
 
-0.298
 
 
12.7
 
 
 
 
BACH1
 
 
hsa-let-7c-5p
 
 
-0.306
 
 
12.7
 
 
 
 
BACH1
 
 
hsa-let-7c-5p
 
 
-0.306
 
 
12.7
 
 
 
 
BACH1
 
 
hsa-let-7e-5p
 
 
-0.435
 
 
12.7
 
 
 
 
BACH1
 
 
hsa-let-7e-5p
 
 
-0.435
 
 
12.7
 
 
 
 
BACH1
 
 
hsa-let-7c-5p
 
 
-0.435
 
 
12.7
 
 
 
 
BACH1
 
 
hsa-let-7c-5p
 
 
-0.435
 
 
12.7
 
 
 
 
BAG5
 
 
hsa-miR-22-3p
 
 
-0.318
 
 
14.0
 
 
 
 
BAK1
 
 
hsa-miR-125a-5p
 
 
-0.402
 
 
20.7
 
 
 
 
BAZ1B
 
 
hsa-miR-10a-5p
 
 
-0.309
 
 
17.8
 
 
 
 
BAZ2A
 
 
hsa-miR-99b-5p
 
 
-0.408
 
 
24.7
 
 
 
 
BBX
 
 
hsa-miR-10a-5p
 
 
-0.305
 
 
11.4
 
 
 
 
BCAP29
 
 
hsa-let-7e-5p
 
 
-0.289
 
 
19.2
 
 
 
 
BCL2L12
 
 
hsa-miR-125a-5p
 
 
-0.288
 
 
10.6
 
 
 
 
BDH1
 
 
hsa-miR-125a-5p
 
 
-0.389
 
 
15.2
 
 
 
 
BIN1
 
 
hsa-miR-22-3p
 
 
-0.346
 
 
42.8
 
 
 
 
BIN3
 
 
hsa-let-7e-5p
 
 
-0.427
 
 
14.3
 
 
 
 
BIN3
 
 
hsa-let-7c-5p
 
 
-0.427
 
 
14.3
 
 
 
 
BLOC1S1
 
 
hsa-miR-22-3p
 
 
-0.346
 
 
161.2
 
 
 
 
BLOC1S6
 
 
hsa-let-7e-5p
 
 
-0.321
 
 
18.9
 
 
 
 
BLOC1S6
 
 
hsa-let-7c-5p
 
 
-0.321
 
 
18.9
 
 
 
 
BLZF1
 
 
hsa-miR-125a-5p
 
 
-0.461
 
 
12.4
 
 
 
 
BORCS6
 
 
hsa-miR-125a-5p
 
 
-0.287
 
 
11.2
 
 
 
 
BTF3L4
 
 
hsa-let-7e-5p
 
 
-0.355
 
 
26.1
 
 
 
 
BTF3L4
 
 
hsa-let-7c-5p
 
 
-0.355
 
 
26.1
 
 
 
 
BTG1
 
 
hsa-miR-22-3p
 
 
-0.468
 
 
13.2
 
 
 
 
BZW1
 
 
hsa-let-7e-5p
 
 
-0.354
 
 
65.1
 
 
 
 
BZW1
 
 
hsa-let-7c-5p
 
 
-0.354
 
 
65.1
 
 
 
 
BZW1
 
 
hsa-let-7e-5p
 
 
-0.375
 
 
65.1
 
 
 
 
BZW1
 
 
hsa-let-7c-5p
 
 
-0.375
 
 
65.1
 
 
 
 
BZW2
 
 
hsa-let-7c-5p
 
 
-0.397
 
 
47.3
 
 
 
 
BZW2
 
 
hsa-let-7e-5p
 
 
-0.408
 
 
47.3
 
 
 
 
C1GALT1
 
 
hsa-let-7e-5p
 
 
-0.322
 
 
21.1
 
 
 
 
C1GALT1
 
 
hsa-let-7c-5p
 
 
-0.322
 
 
21.1
 
 
 
 
C1orf210
 
 
hsa-miR-125a-5p
 
 
-0.584
 
 
27.4
 
 
 
 
C3orf52
 
 
hsa-let-7e-5p
 
 
-0.296
 
 
12.9
 
 
 
 
C3orf52
 
 
hsa-let-7c-5p
 
 
-0.329
 
 
12.9
 
 
 
 
C5orf24
 
 
hsa-miR-22-3p
 
 
-0.493
 
 
15.2
 
 
 
 
C6orf47
 
 
hsa-miR-125a-5p
 
 
-0.564
 
 
14.8
 
 
 
 
CACFD1
 
 
hsa-let-7e-5p
 
 
-0.296
 
 
26.9
 
 
 
 
CACFD1
 
 
hsa-let-7c-5p
 
 
-0.296
 
 
26.9
 
 
 
 
CAMK2N1
 
 
hsa-miR-22-3p
 
 
-0.302
 
 
18.2
 
 
 
 
CANT1
 
 
hsa-let-7e-5p
 
 
-0.402
 
 
48.2
 
 
 
 
CANT1
 
 
hsa-let-7c-5p
 
 
-0.402
 
 
48.2
 
 
 
 
CAPN1
 
 
hsa-miR-22-3p
 
 
-0.307
 
 
64.5
 
 
 
 
CASP3
 
 
hsa-let-7e-5p
 
 
-0.495
 
 
26.1
 
 
 
 
CASP3
 
 
hsa-let-7c-5p
 
 
-0.503
 
 
26.1
 
 
 
 
CAST
 
 
hsa-miR-150-5p
 
 
-0.354
 
 
94.9
 
 
 
 
CCDC149
 
 
hsa-miR-22-3p
 
 
-0.327
 
 
12.2
 
 
 
 
CCDC47
 
 
hsa-miR-22-3p
 
 
-0.604
 
 
72.0
 
 
 
 
 
 
 basal ganglia 
 
 
 
 
target_symbol
 
 
mature_mirna_id
 
 
score
 
 
nTPM
 
 
 
 
 
 
ABCB9
 
 
hsa-let-7e-5p
 
 
-0.422
 
 
13.6
 
 
 
 
ABCB9
 
 
hsa-let-7c-5p
 
 
-0.422
 
 
13.6
 
 
 
 
ABCC5
 
 
hsa-miR-125a-5p
 
 
-0.288
 
 
35.1
 
 
 
 
ABHD17C
 
 
hsa-let-7e-5p
 
 
-0.359
 
 
20.2
 
 
 
 
ABHD17C
 
 
hsa-let-7c-5p
 
 
-0.359
 
 
20.2
 
 
 
 
ABHD3
 
 
hsa-miR-125a-5p
 
 
-0.453
 
 
11.4
 
 
 
 
ABHD6
 
 
hsa-miR-125a-5p
 
 
-0.501
 
 
18.4
 
 
 
 
ABTB1
 
 
hsa-miR-125a-5p
 
 
-0.519
 
 
41.3
 
 
 
 
ACHE
 
 
hsa-miR-125a-5p
 
 
-0.308
 
 
38.3
 
 
 
 
ACHE
 
 
hsa-miR-125a-5p
 
 
-0.361
 
 
38.3
 
 
 
 
ACSL6
 
 
hsa-miR-125a-5p
 
 
-0.307
 
 
18.7
 
 
 
 
ACSL6
 
 
hsa-let-7e-5p
 
 
-0.336
 
 
18.7
 
 
 
 
ACSL6
 
 
hsa-let-7c-5p
 
 
-0.336
 
 
18.7
 
 
 
 
ADIPOR2
 
 
hsa-miR-150-5p
 
 
-0.287
 
 
50.5
 
 
 
 
ADRB1
 
 
hsa-let-7c-5p
 
 
-0.351
 
 
10.0
 
 
 
 
ADRB1
 
 
hsa-let-7e-5p
 
 
-0.367
 
 
10.0
 
 
 
 
AIDA
 
 
hsa-miR-22-3p
 
 
-0.316
 
 
28.2
 
 
 
 
AIFM1
 
 
hsa-miR-125a-5p
 
 
-0.3
 
 
12.5
 
 
 
 
AIFM1
 
 
hsa-let-7e-5p
 
 
-0.314
 
 
12.5
 
 
 
 
AIFM1
 
 
hsa-let-7c-5p
 
 
-0.314
 
 
12.5
 
 
 
 
AKAP6
 
 
hsa-let-7e-5p
 
 
-0.307
 
 
10.5
 
 
 
 
AKAP6
 
 
hsa-let-7c-5p
 
 
-0.307
 
 
10.5
 
 
 
 
AKT3
 
 
hsa-miR-22-3p
 
 
-0.451
 
 
15.0
 
 
 
 
ALDH4A1
 
 
hsa-miR-184
 
 
-0.421
 
 
86.2
 
 
 
 
ALPL
 
 
hsa-miR-10a-5p
 
 
-0.32
 
 
12.3
 
 
 
 
AMT
 
 
hsa-let-7e-5p
 
 
-0.463
 
 
11.4
 
 
 
 
AMT
 
 
hsa-let-7c-5p
 
 
-0.463
 
 
11.4
 
 
 
 
ANAPC16
 
 
hsa-miR-125a-5p
 
 
-0.578
 
 
11.6
 
 
 
 
ANGEL2
 
 
hsa-miR-22-3p
 
 
-0.601
 
 
14.7
 
 
 
 
ANKRD46
 
 
hsa-let-7e-5p
 
 
-0.389
 
 
22.6
 
 
 
 
ANKRD46
 
 
hsa-let-7c-5p
 
 
-0.389
 
 
22.6
 
 
 
 
AP1AR
 
 
hsa-miR-99b-5p
 
 
-0.823
 
 
13.0
 
 
 
 
AP1S1
 
 
hsa-let-7e-5p
 
 
-0.374
 
 
116.3
 
 
 
 
AP1S1
 
 
hsa-let-7c-5p
 
 
-0.374
 
 
116.3
 
 
 
 
ARL4D
 
 
hsa-let-7e-5p
 
 
-0.417
 
 
14.0
 
 
 
 
ARL4D
 
 
hsa-let-7c-5p
 
 
-0.417
 
 
14.0
 
 
 
 
ARMT1
 
 
hsa-let-7e-5p
 
 
-0.303
 
 
10.6
 
 
 
 
ARMT1
 
 
hsa-let-7c-5p
 
 
-0.303
 
 
10.6
 
 
 
 
ARPC5
 
 
hsa-miR-22-3p
 
 
-0.679
 
 
21.4
 
 
 
 
ARPP19
 
 
hsa-let-7e-5p
 
 
-0.492
 
 
481.4
 
 
 
 
ARPP19
 
 
hsa-let-7c-5p
 
 
-0.492
 
 
481.4
 
 
 
 
ARRB1
 
 
hsa-miR-22-3p
 
 
-0.616
 
 
40.7
 
 
 
 
ARRDC4
 
 
hsa-let-7e-5p
 
 
-0.329
 
 
10.0
 
 
 
 
ARRDC4
 
 
hsa-let-7c-5p
 
 
-0.329
 
 
10.0
 
 
 
 
ASAP1
 
 
hsa-let-7e-5p
 
 
-0.296
 
 
11.6
 
 
 
 
ASAP1
 
 
hsa-let-7c-5p
 
 
-0.308
 
 
11.6
 
 
 
 
ATCAY
 
 
hsa-miR-10a-5p
 
 
-0.322
 
 
37.7
 
 
 
 
ATOSB
 
 
hsa-let-7e-5p
 
 
-0.429
 
 
20.2
 
 
 
 
ATOSB
 
 
hsa-let-7c-5p
 
 
-0.429
 
 
20.2
 
 
 
 
ATP2A2
 
 
hsa-let-7e-5p
 
 
-0.311
 
 
21.1
 
 
 
 
ATP2A2
 
 
hsa-let-7c-5p
 
 
-0.311
 
 
21.1
 
 
 
 
ATPAF1
 
 
hsa-let-7e-5p
 
 
-0.498
 
 
88.4
 
 
 
 
ATPAF1
 
 
hsa-let-7c-5p
 
 
-0.498
 
 
88.4
 
 
 
 
ATXN2
 
 
hsa-miR-10a-5p
 
 
-0.362
 
 
21.8
 
 
 
 
B3GAT3
 
 
hsa-let-7e-5p
 
 
-0.324
 
 
67.8
 
 
 
 
B3GAT3
 
 
hsa-let-7c-5p
 
 
-0.324
 
 
67.8
 
 
 
 
B4GAT1
 
 
hsa-let-7e-5p
 
 
-0.47
 
 
152.4
 
 
 
 
B4GAT1
 
 
hsa-let-7c-5p
 
 
-0.47
 
 
152.4
 
 
 
 
BAG5
 
 
hsa-miR-22-3p
 
 
-0.318
 
 
10.8
 
 
 
 
BATF3
 
 
hsa-miR-22-3p
 
 
-0.786
 
 
10.3
 
 
 
 
BAZ1B
 
 
hsa-miR-10a-5p
 
 
-0.309
 
 
19.2
 
 
 
 
BCAP29
 
 
hsa-let-7e-5p
 
 
-0.289
 
 
16.0
 
 
 
 
BDH1
 
 
hsa-miR-125a-5p
 
 
-0.389
 
 
28.9
 
 
 
 
BEGAIN
 
 
hsa-let-7e-5p
 
 
-0.466
 
 
23.9
 
 
 
 
BEGAIN
 
 
hsa-let-7c-5p
 
 
-0.466
 
 
23.9
 
 
 
 
BIN1
 
 
hsa-miR-22-3p
 
 
-0.346
 
 
177.7
 
 
 
 
BIN3
 
 
hsa-let-7e-5p
 
 
-0.427
 
 
10.9
 
 
 
 
BIN3
 
 
hsa-let-7c-5p
 
 
-0.427
 
 
10.9
 
 
 
 
BLOC1S1
 
 
hsa-miR-22-3p
 
 
-0.346
 
 
224.9
 
 
 
 
BLOC1S6
 
 
hsa-let-7e-5p
 
 
-0.321
 
 
22.3
 
 
 
 
BLOC1S6
 
 
hsa-let-7c-5p
 
 
-0.321
 
 
22.3
 
 
 
 
BMPR2
 
 
hsa-miR-99b-5p
 
 
-0.327
 
 
12.6
 
 
 
 
BTBD9
 
 
hsa-let-7e-5p
 
 
-0.311
 
 
12.3
 
 
 
 
BTBD9
 
 
hsa-let-7c-5p
 
 
-0.311
 
 
12.3
 
 
 
 
BTF3L4
 
 
hsa-let-7e-5p
 
 
-0.355
 
 
27.3
 
 
 
 
BTF3L4
 
 
hsa-let-7c-5p
 
 
-0.355
 
 
27.3
 
 
 
 
BZW1
 
 
hsa-let-7e-5p
 
 
-0.354
 
 
31.9
 
 
 
 
BZW1
 
 
hsa-let-7c-5p
 
 
-0.354
 
 
31.9
 
 
 
 
BZW1
 
 
hsa-let-7e-5p
 
 
-0.375
 
 
31.9
 
 
 
 
BZW1
 
 
hsa-let-7c-5p
 
 
-0.375
 
 
31.9
 
 
 
 
BZW2
 
 
hsa-let-7c-5p
 
 
-0.397
 
 
14.1
 
 
 
 
BZW2
 
 
hsa-let-7e-5p
 
 
-0.408
 
 
14.1
 
 
 
 
C19orf47
 
 
hsa-let-7e-5p
 
 
-0.311
 
 
10.9
 
 
 
 
C19orf47
 
 
hsa-let-7c-5p
 
 
-0.311
 
 
10.9
 
 
 
 
C5orf24
 
 
hsa-miR-22-3p
 
 
-0.493
 
 
21.2
 
 
 
 
CACFD1
 
 
hsa-let-7e-5p
 
 
-0.296
 
 
10.2
 
 
 
 
CACFD1
 
 
hsa-let-7c-5p
 
 
-0.296
 
 
10.2
 
 
 
 
CACNB3
 
 
hsa-miR-125a-5p
 
 
-0.393
 
 
42.4
 
 
 
 
CACNG4
 
 
hsa-let-7e-5p
 
 
-0.33
 
 
58.1
 
 
 
 
CACNG4
 
 
hsa-let-7c-5p
 
 
-0.33
 
 
58.1
 
 
 
 
CADM2
 
 
hsa-let-7e-5p
 
 
-0.294
 
 
18.3
 
 
 
 
CADM2
 
 
hsa-let-7c-5p
 
 
-0.294
 
 
18.3
 
 
 
 
CAMK2N1
 
 
hsa-miR-22-3p
 
 
-0.302
 
 
203.3
 
 
 
 
CAPN1
 
 
hsa-miR-22-3p
 
 
-0.307
 
 
28.7
 
 
 
 
CAPN3
 
 
hsa-let-7e-5p
 
 
-0.317
 
 
125.8
 
 
 
 
CAPN3
 
 
hsa-let-7c-5p
 
 
-0.317
 
 
125.8
 
 
 
 
CASP3
 
 
hsa-let-7e-5p
 
 
-0.495
 
 
10.0
 
 
 
 
CASP3
 
 
hsa-let-7c-5p
 
 
-0.503
 
 
10.0
 
 
 
 
CAST
 
 
hsa-miR-150-5p
 
 
-0.354
 
 
27.1
 
 
 
 
CBX5
 
 
hsa-let-7e-5p
 
 
-0.307
 
 
17.3
 
 
 
 
 
 
 cerebellum 
 
 
 
 
target_symbol
 
 
mature_mirna_id
 
 
score
 
 
nTPM
 
 
 
 
 
 
ABCB9
 
 
hsa-let-7e-5p
 
 
-0.422
 
 
18.0
 
 
 
 
ABCB9
 
 
hsa-let-7c-5p
 
 
-0.422
 
 
18.0
 
 
 
 
ABCC5
 
 
hsa-miR-125a-5p
 
 
-0.288
 
 
101.0
 
 
 
 
ABHD3
 
 
hsa-miR-125a-5p
 
 
-0.453
 
 
13.0
 
 
 
 
ABHD6
 
 
hsa-miR-125a-5p
 
 
-0.501
 
 
24.9
 
 
 
 
ABTB1
 
 
hsa-miR-125a-5p
 
 
-0.519
 
 
100.8
 
 
 
 
ACHE
 
 
hsa-miR-125a-5p
 
 
-0.308
 
 
59.1
 
 
 
 
ACHE
 
 
hsa-miR-125a-5p
 
 
-0.361
 
 
59.1
 
 
 
 
ACSL6
 
 
hsa-miR-125a-5p
 
 
-0.307
 
 
27.8
 
 
 
 
ACSL6
 
 
hsa-let-7e-5p
 
 
-0.336
 
 
27.8
 
 
 
 
ACSL6
 
 
hsa-let-7c-5p
 
 
-0.336
 
 
27.8
 
 
 
 
ADIPOR2
 
 
hsa-miR-150-5p
 
 
-0.287
 
 
18.7
 
 
 
 
AIDA
 
 
hsa-miR-22-3p
 
 
-0.316
 
 
24.2
 
 
 
 
AKT3
 
 
hsa-miR-22-3p
 
 
-0.451
 
 
23.2
 
 
 
 
ALDH4A1
 
 
hsa-miR-184
 
 
-0.421
 
 
10.9
 
 
 
 
AMT
 
 
hsa-let-7e-5p
 
 
-0.463
 
 
20.1
 
 
 
 
AMT
 
 
hsa-let-7c-5p
 
 
-0.463
 
 
20.1
 
 
 
 
ANAPC16
 
 
hsa-miR-125a-5p
 
 
-0.578
 
 
13.9
 
 
 
 
ANGEL2
 
 
hsa-miR-22-3p
 
 
-0.601
 
 
12.7
 
 
 
 
ANKRD46
 
 
hsa-let-7e-5p
 
 
-0.389
 
 
27.5
 
 
 
 
ANKRD46
 
 
hsa-let-7c-5p
 
 
-0.389
 
 
27.5
 
 
 
 
AP1AR
 
 
hsa-miR-99b-5p
 
 
-0.823
 
 
13.2
 
 
 
 
AP1S1
 
 
hsa-let-7e-5p
 
 
-0.374
 
 
23.9
 
 
 
 
AP1S1
 
 
hsa-let-7c-5p
 
 
-0.374
 
 
23.9
 
 
 
 
APBB3
 
 
hsa-let-7e-5p
 
 
-0.587
 
 
23.6
 
 
 
 
APBB3
 
 
hsa-let-7c-5p
 
 
-0.587
 
 
23.6
 
 
 
 
ARG2
 
 
hsa-let-7e-5p
 
 
-0.468
 
 
16.4
 
 
 
 
ARG2
 
 
hsa-let-7c-5p
 
 
-0.468
 
 
16.4
 
 
 
 
ARMT1
 
 
hsa-let-7e-5p
 
 
-0.303
 
 
11.5
 
 
 
 
ARMT1
 
 
hsa-let-7c-5p
 
 
-0.303
 
 
11.5
 
 
 
 
ARPC5
 
 
hsa-miR-22-3p
 
 
-0.679
 
 
16.1
 
 
 
 
ARPP19
 
 
hsa-let-7e-5p
 
 
-0.492
 
 
66.7
 
 
 
 
ARPP19
 
 
hsa-let-7c-5p
 
 
-0.492
 
 
66.7
 
 
 
 
ARRB1
 
 
hsa-miR-22-3p
 
 
-0.616
 
 
26.7
 
 
 
 
ATCAY
 
 
hsa-miR-10a-5p
 
 
-0.322
 
 
31.5
 
 
 
 
ATOSB
 
 
hsa-let-7e-5p
 
 
-0.429
 
 
14.6
 
 
 
 
ATOSB
 
 
hsa-let-7c-5p
 
 
-0.429
 
 
14.6
 
 
 
 
ATP2A2
 
 
hsa-let-7e-5p
 
 
-0.311
 
 
15.0
 
 
 
 
ATP2A2
 
 
hsa-let-7c-5p
 
 
-0.311
 
 
15.0
 
 
 
 
ATPAF1
 
 
hsa-let-7e-5p
 
 
-0.498
 
 
24.4
 
 
 
 
ATPAF1
 
 
hsa-let-7c-5p
 
 
-0.498
 
 
24.4
 
 
 
 
ATXN2
 
 
hsa-miR-10a-5p
 
 
-0.362
 
 
28.2
 
 
 
 
B3GAT3
 
 
hsa-let-7e-5p
 
 
-0.324
 
 
70.0
 
 
 
 
B3GAT3
 
 
hsa-let-7c-5p
 
 
-0.324
 
 
70.0
 
 
 
 
B4GAT1
 
 
hsa-let-7e-5p
 
 
-0.47
 
 
105.2
 
 
 
 
B4GAT1
 
 
hsa-let-7c-5p
 
 
-0.47
 
 
105.2
 
 
 
 
BAG5
 
 
hsa-miR-22-3p
 
 
-0.318
 
 
17.8
 
 
 
 
BAZ1B
 
 
hsa-miR-10a-5p
 
 
-0.309
 
 
24.8
 
 
 
 
BAZ2A
 
 
hsa-miR-99b-5p
 
 
-0.408
 
 
26.6
 
 
 
 
BDH1
 
 
hsa-miR-125a-5p
 
 
-0.389
 
 
48.5
 
 
 
 
BEGAIN
 
 
hsa-let-7e-5p
 
 
-0.466
 
 
53.7
 
 
 
 
BEGAIN
 
 
hsa-let-7c-5p
 
 
-0.466
 
 
53.7
 
 
 
 
BIN1
 
 
hsa-miR-22-3p
 
 
-0.346
 
 
88.3
 
 
 
 
BLOC1S1
 
 
hsa-miR-22-3p
 
 
-0.346
 
 
82.8
 
 
 
 
BLOC1S6
 
 
hsa-let-7e-5p
 
 
-0.321
 
 
18.6
 
 
 
 
BLOC1S6
 
 
hsa-let-7c-5p
 
 
-0.321
 
 
18.6
 
 
 
 
BTF3L4
 
 
hsa-let-7e-5p
 
 
-0.355
 
 
28.2
 
 
 
 
BTF3L4
 
 
hsa-let-7c-5p
 
 
-0.355
 
 
28.2
 
 
 
 
BTG1
 
 
hsa-miR-22-3p
 
 
-0.468
 
 
10.5
 
 
 
 
BZW1
 
 
hsa-let-7e-5p
 
 
-0.354
 
 
31.0
 
 
 
 
BZW1
 
 
hsa-let-7c-5p
 
 
-0.354
 
 
31.0
 
 
 
 
BZW1
 
 
hsa-let-7e-5p
 
 
-0.375
 
 
31.0
 
 
 
 
BZW1
 
 
hsa-let-7c-5p
 
 
-0.375
 
 
31.0
 
 
 
 
C5orf24
 
 
hsa-miR-22-3p
 
 
-0.493
 
 
34.2
 
 
 
 
C8orf58
 
 
hsa-let-7e-5p
 
 
-0.612
 
 
15.2
 
 
 
 
C8orf58
 
 
hsa-let-7c-5p
 
 
-0.62
 
 
15.2
 
 
 
 
CACFD1
 
 
hsa-let-7e-5p
 
 
-0.296
 
 
12.1
 
 
 
 
CACFD1
 
 
hsa-let-7c-5p
 
 
-0.296
 
 
12.1
 
 
 
 
CACNB3
 
 
hsa-miR-125a-5p
 
 
-0.393
 
 
48.5
 
 
 
 
CADM2
 
 
hsa-let-7e-5p
 
 
-0.294
 
 
16.0
 
 
 
 
CADM2
 
 
hsa-let-7c-5p
 
 
-0.294
 
 
16.0
 
 
 
 
CAMK2N1
 
 
hsa-miR-22-3p
 
 
-0.302
 
 
53.0
 
 
 
 
CAPN1
 
 
hsa-miR-22-3p
 
 
-0.307
 
 
48.1
 
 
 
 
CAPN3
 
 
hsa-let-7e-5p
 
 
-0.317
 
 
19.1
 
 
 
 
CAPN3
 
 
hsa-let-7c-5p
 
 
-0.317
 
 
19.1
 
 
 
 
CASP3
 
 
hsa-let-7e-5p
 
 
-0.495
 
 
20.1
 
 
 
 
CASP3
 
 
hsa-let-7c-5p
 
 
-0.503
 
 
20.1
 
 
 
 
CAST
 
 
hsa-miR-150-5p
 
 
-0.354
 
 
12.8
 
 
 
 
CBFA2T3
 
 
hsa-let-7c-5p
 
 
-0.294
 
 
36.7
 
 
 
 
CBFA2T3
 
 
hsa-let-7e-5p
 
 
-0.305
 
 
36.7
 
 
 
 
CBX5
 
 
hsa-let-7e-5p
 
 
-0.307
 
 
15.7
 
 
 
 
CBX5
 
 
hsa-let-7c-5p
 
 
-0.307
 
 
15.7
 
 
 
 
CBX5
 
 
hsa-let-7e-5p
 
 
-0.472
 
 
15.7
 
 
 
 
CBX5
 
 
hsa-let-7c-5p
 
 
-0.472
 
 
15.7
 
 
 
 
CCDC149
 
 
hsa-miR-22-3p
 
 
-0.327
 
 
10.5
 
 
 
 
CCDC47
 
 
hsa-miR-22-3p
 
 
-0.604
 
 
36.6
 
 
 
 
CCNJL
 
 
hsa-miR-22-3p
 
 
-0.512
 
 
16.1
 
 
 
 
CCNY
 
 
hsa-let-7c-5p
 
 
-0.323
 
 
12.0
 
 
 
 
CCNY
 
 
hsa-let-7e-5p
 
 
-0.334
 
 
12.0
 
 
 
 
CDC34
 
 
hsa-let-7c-5p
 
 
-0.309
 
 
66.6
 
 
 
 
CDC34
 
 
hsa-let-7e-5p
 
 
-0.321
 
 
66.6
 
 
 
 
CDC34
 
 
hsa-let-7e-5p
 
 
-0.555
 
 
66.6
 
 
 
 
CDC34
 
 
hsa-let-7c-5p
 
 
-0.555
 
 
66.6
 
 
 
 
CDC37L1
 
 
hsa-miR-125a-5p
 
 
-0.401
 
 
12.6
 
 
 
 
CDC42BPG
 
 
hsa-miR-125a-5p
 
 
-0.344
 
 
28.8
 
 
 
 
CDC42SE1
 
 
hsa-miR-125a-5p
 
 
-0.501
 
 
25.6
 
 
 
 
CDH22
 
 
hsa-let-7e-5p
 
 
-0.32
 
 
44.9
 
 
 
 
CDH22
 
 
hsa-let-7c-5p
 
 
-0.32
 
 
44.9
 
 
 
 
CDKN1A
 
 
hsa-let-7e-5p
 
 
-0.434
 
 
14.6
 
 
 
 
CDKN1A
 
 
hsa-let-7c-5p
 
 
-0.434
 
 
14.6
 
 
 
 
 
 
 cerebral cortex 
 
 
 
 
target_symbol
 
 
mature_mirna_id
 
 
score
 
 
nTPM
 
 
 
 
 
 
ABCB9
 
 
hsa-let-7e-5p
 
 
-0.422
 
 
19.5
 
 
 
 
ABCB9
 
 
hsa-let-7c-5p
 
 
-0.422
 
 
19.5
 
 
 
 
ABCC5
 
 
hsa-miR-125a-5p
 
 
-0.288
 
 
41.2
 
 
 
 
ABHD17C
 
 
hsa-let-7e-5p
 
 
-0.359
 
 
16.3
 
 
 
 
ABHD17C
 
 
hsa-let-7c-5p
 
 
-0.359
 
 
16.3
 
 
 
 
ABHD6
 
 
hsa-miR-125a-5p
 
 
-0.501
 
 
21.5
 
 
 
 
ABTB1
 
 
hsa-miR-125a-5p
 
 
-0.519
 
 
51.2
 
 
 
 
ACHE
 
 
hsa-miR-125a-5p
 
 
-0.308
 
 
23.4
 
 
 
 
ACHE
 
 
hsa-miR-125a-5p
 
 
-0.361
 
 
23.4
 
 
 
 
ACSL6
 
 
hsa-miR-125a-5p
 
 
-0.307
 
 
29.5
 
 
 
 
ACSL6
 
 
hsa-let-7e-5p
 
 
-0.336
 
 
29.5
 
 
 
 
ACSL6
 
 
hsa-let-7c-5p
 
 
-0.336
 
 
29.5
 
 
 
 
ADIPOR2
 
 
hsa-miR-150-5p
 
 
-0.287
 
 
29.8
 
 
 
 
AGGF1
 
 
hsa-miR-125a-5p
 
 
-0.331
 
 
12.2
 
 
 
 
AIDA
 
 
hsa-miR-22-3p
 
 
-0.316
 
 
30.3
 
 
 
 
AIFM1
 
 
hsa-miR-125a-5p
 
 
-0.3
 
 
10.3
 
 
 
 
AIFM1
 
 
hsa-let-7e-5p
 
 
-0.314
 
 
10.3
 
 
 
 
AIFM1
 
 
hsa-let-7c-5p
 
 
-0.314
 
 
10.3
 
 
 
 
AKAP6
 
 
hsa-let-7e-5p
 
 
-0.307
 
 
27.4
 
 
 
 
AKAP6
 
 
hsa-let-7c-5p
 
 
-0.307
 
 
27.4
 
 
 
 
AKT3
 
 
hsa-miR-22-3p
 
 
-0.451
 
 
23.7
 
 
 
 
ALDH4A1
 
 
hsa-miR-184
 
 
-0.421
 
 
69.8
 
 
 
 
ALPL
 
 
hsa-miR-10a-5p
 
 
-0.32
 
 
13.5
 
 
 
 
ANKRD46
 
 
hsa-let-7e-5p
 
 
-0.389
 
 
39.5
 
 
 
 
ANKRD46
 
 
hsa-let-7c-5p
 
 
-0.389
 
 
39.5
 
 
 
 
AP1AR
 
 
hsa-miR-99b-5p
 
 
-0.823
 
 
12.2
 
 
 
 
AP1S1
 
 
hsa-let-7e-5p
 
 
-0.374
 
 
104.9
 
 
 
 
AP1S1
 
 
hsa-let-7c-5p
 
 
-0.374
 
 
104.9
 
 
 
 
APBB3
 
 
hsa-let-7e-5p
 
 
-0.587
 
 
15.0
 
 
 
 
APBB3
 
 
hsa-let-7c-5p
 
 
-0.587
 
 
15.0
 
 
 
 
ARL4D
 
 
hsa-let-7e-5p
 
 
-0.417
 
 
14.3
 
 
 
 
ARL4D
 
 
hsa-let-7c-5p
 
 
-0.417
 
 
14.3
 
 
 
 
ARMT1
 
 
hsa-let-7e-5p
 
 
-0.303
 
 
10.1
 
 
 
 
ARMT1
 
 
hsa-let-7c-5p
 
 
-0.303
 
 
10.1
 
 
 
 
ARPC5
 
 
hsa-miR-22-3p
 
 
-0.679
 
 
53.1
 
 
 
 
ARPP19
 
 
hsa-let-7e-5p
 
 
-0.492
 
 
292.4
 
 
 
 
ARPP19
 
 
hsa-let-7c-5p
 
 
-0.492
 
 
292.4
 
 
 
 
ARRB1
 
 
hsa-miR-22-3p
 
 
-0.616
 
 
40.1
 
 
 
 
ARRDC4
 
 
hsa-let-7e-5p
 
 
-0.329
 
 
12.8
 
 
 
 
ARRDC4
 
 
hsa-let-7c-5p
 
 
-0.329
 
 
12.8
 
 
 
 
ASAP1
 
 
hsa-let-7e-5p
 
 
-0.296
 
 
21.7
 
 
 
 
ASAP1
 
 
hsa-let-7c-5p
 
 
-0.308
 
 
21.7
 
 
 
 
ATCAY
 
 
hsa-miR-10a-5p
 
 
-0.322
 
 
78.9
 
 
 
 
ATOSB
 
 
hsa-let-7e-5p
 
 
-0.429
 
 
21.2
 
 
 
 
ATOSB
 
 
hsa-let-7c-5p
 
 
-0.429
 
 
21.2
 
 
 
 
ATP2A2
 
 
hsa-let-7e-5p
 
 
-0.311
 
 
54.4
 
 
 
 
ATP2A2
 
 
hsa-let-7c-5p
 
 
-0.311
 
 
54.4
 
 
 
 
ATP8A1
 
 
hsa-miR-22-3p
 
 
-0.468
 
 
32.7
 
 
 
 
ATPAF1
 
 
hsa-let-7e-5p
 
 
-0.498
 
 
74.9
 
 
 
 
ATPAF1
 
 
hsa-let-7c-5p
 
 
-0.498
 
 
74.9
 
 
 
 
ATXN2
 
 
hsa-miR-10a-5p
 
 
-0.362
 
 
26.3
 
 
 
 
B3GAT3
 
 
hsa-let-7e-5p
 
 
-0.324
 
 
63.6
 
 
 
 
B3GAT3
 
 
hsa-let-7c-5p
 
 
-0.324
 
 
63.6
 
 
 
 
B4GAT1
 
 
hsa-let-7e-5p
 
 
-0.47
 
 
171.8
 
 
 
 
B4GAT1
 
 
hsa-let-7c-5p
 
 
-0.47
 
 
171.8
 
 
 
 
BAG5
 
 
hsa-miR-22-3p
 
 
-0.318
 
 
15.1
 
 
 
 
BAZ1B
 
 
hsa-miR-10a-5p
 
 
-0.309
 
 
19.0
 
 
 
 
BAZ2A
 
 
hsa-miR-99b-5p
 
 
-0.408
 
 
13.9
 
 
 
 
BBX
 
 
hsa-miR-10a-5p
 
 
-0.305
 
 
16.6
 
 
 
 
BCAP29
 
 
hsa-let-7e-5p
 
 
-0.289
 
 
33.6
 
 
 
 
BDH1
 
 
hsa-miR-125a-5p
 
 
-0.389
 
 
28.0
 
 
 
 
BEGAIN
 
 
hsa-let-7e-5p
 
 
-0.466
 
 
42.8
 
 
 
 
BEGAIN
 
 
hsa-let-7c-5p
 
 
-0.466
 
 
42.8
 
 
 
 
BIN1
 
 
hsa-miR-22-3p
 
 
-0.346
 
 
121.8
 
 
 
 
BIN3
 
 
hsa-let-7e-5p
 
 
-0.427
 
 
10.8
 
 
 
 
BIN3
 
 
hsa-let-7c-5p
 
 
-0.427
 
 
10.8
 
 
 
 
BLOC1S1
 
 
hsa-miR-22-3p
 
 
-0.346
 
 
215.7
 
 
 
 
BLOC1S6
 
 
hsa-let-7e-5p
 
 
-0.321
 
 
30.5
 
 
 
 
BLOC1S6
 
 
hsa-let-7c-5p
 
 
-0.321
 
 
30.5
 
 
 
 
BMPR2
 
 
hsa-miR-99b-5p
 
 
-0.327
 
 
20.6
 
 
 
 
BORCS6
 
 
hsa-miR-125a-5p
 
 
-0.287
 
 
10.2
 
 
 
 
BTBD9
 
 
hsa-let-7e-5p
 
 
-0.311
 
 
12.7
 
 
 
 
BTBD9
 
 
hsa-let-7c-5p
 
 
-0.311
 
 
12.7
 
 
 
 
BTF3L4
 
 
hsa-let-7e-5p
 
 
-0.355
 
 
37.5
 
 
 
 
BTF3L4
 
 
hsa-let-7c-5p
 
 
-0.355
 
 
37.5
 
 
 
 
BZW1
 
 
hsa-let-7e-5p
 
 
-0.354
 
 
64.1
 
 
 
 
BZW1
 
 
hsa-let-7c-5p
 
 
-0.354
 
 
64.1
 
 
 
 
BZW1
 
 
hsa-let-7e-5p
 
 
-0.375
 
 
64.1
 
 
 
 
BZW1
 
 
hsa-let-7c-5p
 
 
-0.375
 
 
64.1
 
 
 
 
BZW2
 
 
hsa-let-7c-5p
 
 
-0.397
 
 
16.2
 
 
 
 
BZW2
 
 
hsa-let-7e-5p
 
 
-0.408
 
 
16.2
 
 
 
 
C19orf47
 
 
hsa-let-7e-5p
 
 
-0.311
 
 
14.5
 
 
 
 
C19orf47
 
 
hsa-let-7c-5p
 
 
-0.311
 
 
14.5
 
 
 
 
C1GALT1
 
 
hsa-let-7e-5p
 
 
-0.322
 
 
12.5
 
 
 
 
C1GALT1
 
 
hsa-let-7c-5p
 
 
-0.322
 
 
12.5
 
 
 
 
C5orf24
 
 
hsa-miR-22-3p
 
 
-0.493
 
 
23.9
 
 
 
 
CACNB3
 
 
hsa-miR-125a-5p
 
 
-0.393
 
 
73.4
 
 
 
 
CACNG4
 
 
hsa-let-7e-5p
 
 
-0.33
 
 
24.4
 
 
 
 
CACNG4
 
 
hsa-let-7c-5p
 
 
-0.33
 
 
24.4
 
 
 
 
CADM2
 
 
hsa-let-7e-5p
 
 
-0.294
 
 
48.5
 
 
 
 
CADM2
 
 
hsa-let-7c-5p
 
 
-0.294
 
 
48.5
 
 
 
 
CAMK2N1
 
 
hsa-miR-22-3p
 
 
-0.302
 
 
582.0
 
 
 
 
CAPN1
 
 
hsa-miR-22-3p
 
 
-0.307
 
 
45.4
 
 
 
 
CAPN3
 
 
hsa-let-7e-5p
 
 
-0.317
 
 
48.1
 
 
 
 
CAPN3
 
 
hsa-let-7c-5p
 
 
-0.317
 
 
48.1
 
 
 
 
CASP3
 
 
hsa-let-7e-5p
 
 
-0.495
 
 
11.2
 
 
 
 
CASP3
 
 
hsa-let-7c-5p
 
 
-0.503
 
 
11.2
 
 
 
 
CAST
 
 
hsa-miR-150-5p
 
 
-0.354
 
 
28.3
 
 
 
 
CBX5
 
 
hsa-let-7e-5p
 
 
-0.307
 
 
25.0
 
 
 
 
CBX5
 
 
hsa-let-7c-5p
 
 
-0.307
 
 
25.0
 
 
 
 
 
 
 hypothalamus 
 
 
 
 
target_symbol
 
 
mature_mirna_id
 
 
score
 
 
nTPM
 
 
 
 
 
 
ABCB9
 
 
hsa-let-7e-5p
 
 
-0.422
 
 
11.9
 
 
 
 
ABCB9
 
 
hsa-let-7c-5p
 
 
-0.422
 
 
11.9
 
 
 
 
ABCC5
 
 
hsa-miR-125a-5p
 
 
-0.288
 
 
33.4
 
 
 
 
ABHD17C
 
 
hsa-let-7e-5p
 
 
-0.359
 
 
11.1
 
 
 
 
ABHD17C
 
 
hsa-let-7c-5p
 
 
-0.359
 
 
11.1
 
 
 
 
ABHD6
 
 
hsa-miR-125a-5p
 
 
-0.501
 
 
12.9
 
 
 
 
ABTB1
 
 
hsa-miR-125a-5p
 
 
-0.519
 
 
20.4
 
 
 
 
ACHE
 
 
hsa-miR-125a-5p
 
 
-0.308
 
 
103.9
 
 
 
 
ACHE
 
 
hsa-miR-125a-5p
 
 
-0.361
 
 
103.9
 
 
 
 
ACSL6
 
 
hsa-miR-125a-5p
 
 
-0.307
 
 
11.9
 
 
 
 
ACSL6
 
 
hsa-let-7e-5p
 
 
-0.336
 
 
11.9
 
 
 
 
ACSL6
 
 
hsa-let-7c-5p
 
 
-0.336
 
 
11.9
 
 
 
 
ADIPOR2
 
 
hsa-miR-150-5p
 
 
-0.287
 
 
40.5
 
 
 
 
AIDA
 
 
hsa-miR-22-3p
 
 
-0.316
 
 
30.0
 
 
 
 
AKAP6
 
 
hsa-let-7e-5p
 
 
-0.307
 
 
13.6
 
 
 
 
AKAP6
 
 
hsa-let-7c-5p
 
 
-0.307
 
 
13.6
 
 
 
 
AKT3
 
 
hsa-miR-22-3p
 
 
-0.451
 
 
14.5
 
 
 
 
ALDH4A1
 
 
hsa-miR-184
 
 
-0.421
 
 
31.5
 
 
 
 
ANKRD46
 
 
hsa-let-7e-5p
 
 
-0.389
 
 
28.7
 
 
 
 
ANKRD46
 
 
hsa-let-7c-5p
 
 
-0.389
 
 
28.7
 
 
 
 
AP1S1
 
 
hsa-let-7e-5p
 
 
-0.374
 
 
44.3
 
 
 
 
AP1S1
 
 
hsa-let-7c-5p
 
 
-0.374
 
 
44.3
 
 
 
 
APBB3
 
 
hsa-let-7e-5p
 
 
-0.587
 
 
11.2
 
 
 
 
APBB3
 
 
hsa-let-7c-5p
 
 
-0.587
 
 
11.2
 
 
 
 
ARG2
 
 
hsa-let-7e-5p
 
 
-0.468
 
 
10.7
 
 
 
 
ARG2
 
 
hsa-let-7c-5p
 
 
-0.468
 
 
10.7
 
 
 
 
ARPC5
 
 
hsa-miR-22-3p
 
 
-0.679
 
 
21.6
 
 
 
 
ARPP19
 
 
hsa-let-7e-5p
 
 
-0.492
 
 
103.9
 
 
 
 
ARPP19
 
 
hsa-let-7c-5p
 
 
-0.492
 
 
103.9
 
 
 
 
ARRB1
 
 
hsa-miR-22-3p
 
 
-0.616
 
 
21.9
 
 
 
 
ARRDC4
 
 
hsa-let-7e-5p
 
 
-0.329
 
 
13.4
 
 
 
 
ARRDC4
 
 
hsa-let-7c-5p
 
 
-0.329
 
 
13.4
 
 
 
 
ASAP1
 
 
hsa-let-7e-5p
 
 
-0.296
 
 
11.8
 
 
 
 
ASAP1
 
 
hsa-let-7c-5p
 
 
-0.308
 
 
11.8
 
 
 
 
ATCAY
 
 
hsa-miR-10a-5p
 
 
-0.322
 
 
56.2
 
 
 
 
ATOSB
 
 
hsa-let-7e-5p
 
 
-0.429
 
 
19.8
 
 
 
 
ATOSB
 
 
hsa-let-7c-5p
 
 
-0.429
 
 
19.8
 
 
 
 
ATP2A2
 
 
hsa-let-7e-5p
 
 
-0.311
 
 
11.9
 
 
 
 
ATP2A2
 
 
hsa-let-7c-5p
 
 
-0.311
 
 
11.9
 
 
 
 
ATPAF1
 
 
hsa-let-7e-5p
 
 
-0.498
 
 
52.2
 
 
 
 
ATPAF1
 
 
hsa-let-7c-5p
 
 
-0.498
 
 
52.2
 
 
 
 
ATXN2
 
 
hsa-miR-10a-5p
 
 
-0.362
 
 
24.5
 
 
 
 
B3GAT3
 
 
hsa-let-7e-5p
 
 
-0.324
 
 
60.2
 
 
 
 
B3GAT3
 
 
hsa-let-7c-5p
 
 
-0.324
 
 
60.2
 
 
 
 
B4GAT1
 
 
hsa-let-7e-5p
 
 
-0.47
 
 
207.3
 
 
 
 
B4GAT1
 
 
hsa-let-7c-5p
 
 
-0.47
 
 
207.3
 
 
 
 
BAG5
 
 
hsa-miR-22-3p
 
 
-0.318
 
 
10.3
 
 
 
 
BAZ1B
 
 
hsa-miR-10a-5p
 
 
-0.309
 
 
17.2
 
 
 
 
BCAP29
 
 
hsa-let-7e-5p
 
 
-0.289
 
 
16.4
 
 
 
 
BDH1
 
 
hsa-miR-125a-5p
 
 
-0.389
 
 
18.1
 
 
 
 
BEGAIN
 
 
hsa-let-7e-5p
 
 
-0.466
 
 
15.9
 
 
 
 
BEGAIN
 
 
hsa-let-7c-5p
 
 
-0.466
 
 
15.9
 
 
 
 
BIN1
 
 
hsa-miR-22-3p
 
 
-0.346
 
 
127.6
 
 
 
 
BLOC1S1
 
 
hsa-miR-22-3p
 
 
-0.346
 
 
179.6
 
 
 
 
BLOC1S6
 
 
hsa-let-7e-5p
 
 
-0.321
 
 
24.8
 
 
 
 
BLOC1S6
 
 
hsa-let-7c-5p
 
 
-0.321
 
 
24.8
 
 
 
 
BMPR2
 
 
hsa-miR-99b-5p
 
 
-0.327
 
 
11.9
 
 
 
 
BTF3L4
 
 
hsa-let-7e-5p
 
 
-0.355
 
 
33.4
 
 
 
 
BTF3L4
 
 
hsa-let-7c-5p
 
 
-0.355
 
 
33.4
 
 
 
 
BZW1
 
 
hsa-let-7e-5p
 
 
-0.354
 
 
32.4
 
 
 
 
BZW1
 
 
hsa-let-7c-5p
 
 
-0.354
 
 
32.4
 
 
 
 
BZW1
 
 
hsa-let-7e-5p
 
 
-0.375
 
 
32.4
 
 
 
 
BZW1
 
 
hsa-let-7c-5p
 
 
-0.375
 
 
32.4
 
 
 
 
C19orf47
 
 
hsa-let-7e-5p
 
 
-0.311
 
 
12.4
 
 
 
 
C19orf47
 
 
hsa-let-7c-5p
 
 
-0.311
 
 
12.4
 
 
 
 
C5orf24
 
 
hsa-miR-22-3p
 
 
-0.493
 
 
18.2
 
 
 
 
CACNB3
 
 
hsa-miR-125a-5p
 
 
-0.393
 
 
32.6
 
 
 
 
CACNG4
 
 
hsa-let-7e-5p
 
 
-0.33
 
 
28.6
 
 
 
 
CACNG4
 
 
hsa-let-7c-5p
 
 
-0.33
 
 
28.6
 
 
 
 
CADM2
 
 
hsa-let-7e-5p
 
 
-0.294
 
 
11.9
 
 
 
 
CADM2
 
 
hsa-let-7c-5p
 
 
-0.294
 
 
11.9
 
 
 
 
CAMK2N1
 
 
hsa-miR-22-3p
 
 
-0.302
 
 
175.0
 
 
 
 
CAPN1
 
 
hsa-miR-22-3p
 
 
-0.307
 
 
27.9
 
 
 
 
CAPN3
 
 
hsa-let-7e-5p
 
 
-0.317
 
 
74.2
 
 
 
 
CAPN3
 
 
hsa-let-7c-5p
 
 
-0.317
 
 
74.2
 
 
 
 
CAST
 
 
hsa-miR-150-5p
 
 
-0.354
 
 
29.8
 
 
 
 
CBX5
 
 
hsa-let-7e-5p
 
 
-0.307
 
 
20.5
 
 
 
 
CBX5
 
 
hsa-let-7c-5p
 
 
-0.307
 
 
20.5
 
 
 
 
CBX5
 
 
hsa-let-7e-5p
 
 
-0.472
 
 
20.5
 
 
 
 
CBX5
 
 
hsa-let-7c-5p
 
 
-0.472
 
 
20.5
 
 
 
 
CCDC47
 
 
hsa-miR-22-3p
 
 
-0.604
 
 
27.0
 
 
 
 
CCNY
 
 
hsa-let-7c-5p
 
 
-0.323
 
 
22.8
 
 
 
 
CCNY
 
 
hsa-let-7e-5p
 
 
-0.334
 
 
22.8
 
 
 
 
CDC34
 
 
hsa-let-7c-5p
 
 
-0.309
 
 
45.1
 
 
 
 
CDC34
 
 
hsa-let-7e-5p
 
 
-0.321
 
 
45.1
 
 
 
 
CDC34
 
 
hsa-let-7e-5p
 
 
-0.555
 
 
45.1
 
 
 
 
CDC34
 
 
hsa-let-7c-5p
 
 
-0.555
 
 
45.1
 
 
 
 
CDC37L1
 
 
hsa-miR-125a-5p
 
 
-0.401
 
 
13.2
 
 
 
 
CDC42SE1
 
 
hsa-miR-125a-5p
 
 
-0.501
 
 
26.3
 
 
 
 
CDKN1A
 
 
hsa-let-7e-5p
 
 
-0.434
 
 
37.8
 
 
 
 
CDKN1A
 
 
hsa-let-7c-5p
 
 
-0.434
 
 
37.8
 
 
 
 
CDV3
 
 
hsa-let-7e-5p
 
 
-0.453
 
 
32.9
 
 
 
 
CDV3
 
 
hsa-let-7c-5p
 
 
-0.461
 
 
32.9
 
 
 
 
CEBPD
 
 
hsa-let-7e-5p
 
 
-0.309
 
 
59.6
 
 
 
 
CEBPD
 
 
hsa-let-7c-5p
 
 
-0.309
 
 
59.6
 
 
 
 
CELF2
 
 
hsa-miR-10a-5p
 
 
-0.319
 
 
12.0
 
 
 
 
CENPV
 
 
hsa-miR-22-3p
 
 
-0.491
 
 
11.5
 
 
 
 
CERCAM
 
 
hsa-let-7e-5p
 
 
-0.344
 
 
182.5
 
 
 
 
CERCAM
 
 
hsa-let-7c-5p
 
 
-0.344
 
 
182.5
 
 
 
 
CFAP36
 
 
hsa-miR-22-3p
 
 
-0.549
 
 
96.7
 
 
 
 
 
 
 midbrain 
 
 
 
 
target_symbol
 
 
mature_mirna_id
 
 
score
 
 
nTPM
 
 
 
 
 
 
ABCB9
 
 
hsa-let-7e-5p
 
 
-0.422
 
 
10.6
 
 
 
 
ABCB9
 
 
hsa-let-7c-5p
 
 
-0.422
 
 
10.6
 
 
 
 
ABCC5
 
 
hsa-miR-125a-5p
 
 
-0.288
 
 
27.3
 
 
 
 
ABHD17C
 
 
hsa-let-7e-5p
 
 
-0.359
 
 
11.2
 
 
 
 
ABHD17C
 
 
hsa-let-7c-5p
 
 
-0.359
 
 
11.2
 
 
 
 
ABHD3
 
 
hsa-miR-125a-5p
 
 
-0.453
 
 
12.3
 
 
 
 
ABHD6
 
 
hsa-miR-125a-5p
 
 
-0.501
 
 
21.1
 
 
 
 
ABTB1
 
 
hsa-miR-125a-5p
 
 
-0.519
 
 
18.7
 
 
 
 
ACHE
 
 
hsa-miR-125a-5p
 
 
-0.308
 
 
47.7
 
 
 
 
ACHE
 
 
hsa-miR-125a-5p
 
 
-0.361
 
 
47.7
 
 
 
 
ACSL6
 
 
hsa-miR-125a-5p
 
 
-0.307
 
 
11.1
 
 
 
 
ACSL6
 
 
hsa-let-7e-5p
 
 
-0.336
 
 
11.1
 
 
 
 
ACSL6
 
 
hsa-let-7c-5p
 
 
-0.336
 
 
11.1
 
 
 
 
ADIPOR2
 
 
hsa-miR-150-5p
 
 
-0.287
 
 
76.2
 
 
 
 
AGGF1
 
 
hsa-miR-125a-5p
 
 
-0.331
 
 
11.3
 
 
 
 
AIDA
 
 
hsa-miR-22-3p
 
 
-0.316
 
 
32.2
 
 
 
 
AIFM1
 
 
hsa-miR-125a-5p
 
 
-0.3
 
 
12.7
 
 
 
 
AIFM1
 
 
hsa-let-7e-5p
 
 
-0.314
 
 
12.7
 
 
 
 
AIFM1
 
 
hsa-let-7c-5p
 
 
-0.314
 
 
12.7
 
 
 
 
AKAP6
 
 
hsa-let-7e-5p
 
 
-0.307
 
 
17.4
 
 
 
 
AKAP6
 
 
hsa-let-7c-5p
 
 
-0.307
 
 
17.4
 
 
 
 
AKT3
 
 
hsa-miR-22-3p
 
 
-0.451
 
 
16.3
 
 
 
 
ALDH4A1
 
 
hsa-miR-184
 
 
-0.421
 
 
73.5
 
 
 
 
ALPL
 
 
hsa-miR-10a-5p
 
 
-0.32
 
 
11.9
 
 
 
 
ANKRD46
 
 
hsa-let-7e-5p
 
 
-0.389
 
 
24.0
 
 
 
 
ANKRD46
 
 
hsa-let-7c-5p
 
 
-0.389
 
 
24.0
 
 
 
 
AP1AR
 
 
hsa-miR-99b-5p
 
 
-0.823
 
 
10.1
 
 
 
 
AP1S1
 
 
hsa-let-7e-5p
 
 
-0.374
 
 
35.4
 
 
 
 
AP1S1
 
 
hsa-let-7c-5p
 
 
-0.374
 
 
35.4
 
 
 
 
ARPC5
 
 
hsa-miR-22-3p
 
 
-0.679
 
 
27.1
 
 
 
 
ARPP19
 
 
hsa-let-7e-5p
 
 
-0.492
 
 
144.2
 
 
 
 
ARPP19
 
 
hsa-let-7c-5p
 
 
-0.492
 
 
144.2
 
 
 
 
ARRB1
 
 
hsa-miR-22-3p
 
 
-0.616
 
 
27.6
 
 
 
 
ARRDC4
 
 
hsa-let-7e-5p
 
 
-0.329
 
 
17.7
 
 
 
 
ARRDC4
 
 
hsa-let-7c-5p
 
 
-0.329
 
 
17.7
 
 
 
 
ASAP1
 
 
hsa-let-7e-5p
 
 
-0.296
 
 
12.8
 
 
 
 
ASAP1
 
 
hsa-let-7c-5p
 
 
-0.308
 
 
12.8
 
 
 
 
ATCAY
 
 
hsa-miR-10a-5p
 
 
-0.322
 
 
24.7
 
 
 
 
ATOSB
 
 
hsa-let-7e-5p
 
 
-0.429
 
 
24.1
 
 
 
 
ATOSB
 
 
hsa-let-7c-5p
 
 
-0.429
 
 
24.1
 
 
 
 
ATP2A2
 
 
hsa-let-7e-5p
 
 
-0.311
 
 
17.1
 
 
 
 
ATP2A2
 
 
hsa-let-7c-5p
 
 
-0.311
 
 
17.1
 
 
 
 
ATPAF1
 
 
hsa-let-7e-5p
 
 
-0.498
 
 
74.9
 
 
 
 
ATPAF1
 
 
hsa-let-7c-5p
 
 
-0.498
 
 
74.9
 
 
 
 
ATXN2
 
 
hsa-miR-10a-5p
 
 
-0.362
 
 
24.2
 
 
 
 
B3GAT3
 
 
hsa-let-7e-5p
 
 
-0.324
 
 
58.5
 
 
 
 
B3GAT3
 
 
hsa-let-7c-5p
 
 
-0.324
 
 
58.5
 
 
 
 
B4GAT1
 
 
hsa-let-7e-5p
 
 
-0.47
 
 
85.6
 
 
 
 
B4GAT1
 
 
hsa-let-7c-5p
 
 
-0.47
 
 
85.6
 
 
 
 
BAG5
 
 
hsa-miR-22-3p
 
 
-0.318
 
 
10.6
 
 
 
 
BAZ1B
 
 
hsa-miR-10a-5p
 
 
-0.309
 
 
19.8
 
 
 
 
BBX
 
 
hsa-miR-10a-5p
 
 
-0.305
 
 
11.6
 
 
 
 
BCAP29
 
 
hsa-let-7e-5p
 
 
-0.289
 
 
19.1
 
 
 
 
BDH1
 
 
hsa-miR-125a-5p
 
 
-0.389
 
 
18.3
 
 
 
 
BIN1
 
 
hsa-miR-22-3p
 
 
-0.346
 
 
190.1
 
 
 
 
BLOC1S1
 
 
hsa-miR-22-3p
 
 
-0.346
 
 
246.9
 
 
 
 
BLOC1S6
 
 
hsa-let-7e-5p
 
 
-0.321
 
 
18.5
 
 
 
 
BLOC1S6
 
 
hsa-let-7c-5p
 
 
-0.321
 
 
18.5
 
 
 
 
BTF3L4
 
 
hsa-let-7e-5p
 
 
-0.355
 
 
28.8
 
 
 
 
BTF3L4
 
 
hsa-let-7c-5p
 
 
-0.355
 
 
28.8
 
 
 
 
BZW1
 
 
hsa-let-7e-5p
 
 
-0.354
 
 
39.5
 
 
 
 
BZW1
 
 
hsa-let-7c-5p
 
 
-0.354
 
 
39.5
 
 
 
 
BZW1
 
 
hsa-let-7e-5p
 
 
-0.375
 
 
39.5
 
 
 
 
BZW1
 
 
hsa-let-7c-5p
 
 
-0.375
 
 
39.5
 
 
 
 
C19orf47
 
 
hsa-let-7e-5p
 
 
-0.311
 
 
13.9
 
 
 
 
C19orf47
 
 
hsa-let-7c-5p
 
 
-0.311
 
 
13.9
 
 
 
 
C1GALT1
 
 
hsa-let-7e-5p
 
 
-0.322
 
 
13.7
 
 
 
 
C1GALT1
 
 
hsa-let-7c-5p
 
 
-0.322
 
 
13.7
 
 
 
 
C2orf88
 
 
hsa-let-7e-5p
 
 
-0.429
 
 
10.0
 
 
 
 
C2orf88
 
 
hsa-let-7c-5p
 
 
-0.429
 
 
10.0
 
 
 
 
C5orf24
 
 
hsa-miR-22-3p
 
 
-0.493
 
 
17.1
 
 
 
 
CACFD1
 
 
hsa-let-7e-5p
 
 
-0.296
 
 
10.3
 
 
 
 
CACFD1
 
 
hsa-let-7c-5p
 
 
-0.296
 
 
10.3
 
 
 
 
CACNB3
 
 
hsa-miR-125a-5p
 
 
-0.393
 
 
23.1
 
 
 
 
CACNG4
 
 
hsa-let-7e-5p
 
 
-0.33
 
 
17.0
 
 
 
 
CACNG4
 
 
hsa-let-7c-5p
 
 
-0.33
 
 
17.0
 
 
 
 
CADM2
 
 
hsa-let-7e-5p
 
 
-0.294
 
 
16.8
 
 
 
 
CADM2
 
 
hsa-let-7c-5p
 
 
-0.294
 
 
16.8
 
 
 
 
CAMK2N1
 
 
hsa-miR-22-3p
 
 
-0.302
 
 
198.9
 
 
 
 
CAPN1
 
 
hsa-miR-22-3p
 
 
-0.307
 
 
27.9
 
 
 
 
CAPN3
 
 
hsa-let-7e-5p
 
 
-0.317
 
 
184.7
 
 
 
 
CAPN3
 
 
hsa-let-7c-5p
 
 
-0.317
 
 
184.7
 
 
 
 
CAST
 
 
hsa-miR-150-5p
 
 
-0.354
 
 
34.0
 
 
 
 
CBX5
 
 
hsa-let-7e-5p
 
 
-0.307
 
 
16.9
 
 
 
 
CBX5
 
 
hsa-let-7c-5p
 
 
-0.307
 
 
16.9
 
 
 
 
CBX5
 
 
hsa-let-7e-5p
 
 
-0.472
 
 
16.9
 
 
 
 
CBX5
 
 
hsa-let-7c-5p
 
 
-0.472
 
 
16.9
 
 
 
 
CCDC47
 
 
hsa-miR-22-3p
 
 
-0.604
 
 
29.8
 
 
 
 
CCNY
 
 
hsa-let-7c-5p
 
 
-0.323
 
 
28.8
 
 
 
 
CCNY
 
 
hsa-let-7e-5p
 
 
-0.334
 
 
28.8
 
 
 
 
CDC34
 
 
hsa-let-7c-5p
 
 
-0.309
 
 
46.8
 
 
 
 
CDC34
 
 
hsa-let-7e-5p
 
 
-0.321
 
 
46.8
 
 
 
 
CDC34
 
 
hsa-let-7e-5p
 
 
-0.555
 
 
46.8
 
 
 
 
CDC34
 
 
hsa-let-7c-5p
 
 
-0.555
 
 
46.8
 
 
 
 
CDC37L1
 
 
hsa-miR-125a-5p
 
 
-0.401
 
 
10.1
 
 
 
 
CDC42SE1
 
 
hsa-miR-125a-5p
 
 
-0.501
 
 
31.7
 
 
 
 
CDH5
 
 
hsa-miR-125a-5p
 
 
-0.36
 
 
14.3
 
 
 
 
CDKN1A
 
 
hsa-let-7e-5p
 
 
-0.434
 
 
54.4
 
 
 
 
CDKN1A
 
 
hsa-let-7c-5p
 
 
-0.434
 
 
54.4
 
 
 
 
CDV3
 
 
hsa-let-7e-5p
 
 
-0.453
 
 
42.0
 
 
 
 
 
 
 pituitary gland 
 
 
 
 
target_symbol
 
 
mature_mirna_id
 
 
score
 
 
nTPM
 
 
 
 
 
 
ABCB9
 
 
hsa-let-7e-5p
 
 
-0.422
 
 
11.0
 
 
 
 
ABCB9
 
 
hsa-let-7c-5p
 
 
-0.422
 
 
11.0
 
 
 
 
ABCC5
 
 
hsa-miR-125a-5p
 
 
-0.288
 
 
41.4
 
 
 
 
ABHD3
 
 
hsa-miR-125a-5p
 
 
-0.453
 
 
10.7
 
 
 
 
ABHD6
 
 
hsa-miR-125a-5p
 
 
-0.501
 
 
17.2
 
 
 
 
ABTB1
 
 
hsa-miR-125a-5p
 
 
-0.519
 
 
33.0
 
 
 
 
ADIPOR2
 
 
hsa-miR-150-5p
 
 
-0.287
 
 
25.4
 
 
 
 
AIDA
 
 
hsa-miR-22-3p
 
 
-0.316
 
 
20.6
 
 
 
 
AIFM1
 
 
hsa-miR-125a-5p
 
 
-0.3
 
 
11.0
 
 
 
 
AIFM1
 
 
hsa-let-7e-5p
 
 
-0.314
 
 
11.0
 
 
 
 
AIFM1
 
 
hsa-let-7c-5p
 
 
-0.314
 
 
11.0
 
 
 
 
AKT3
 
 
hsa-miR-22-3p
 
 
-0.451
 
 
11.4
 
 
 
 
AMT
 
 
hsa-let-7e-5p
 
 
-0.463
 
 
16.0
 
 
 
 
AMT
 
 
hsa-let-7c-5p
 
 
-0.463
 
 
16.0
 
 
 
 
ANAPC16
 
 
hsa-miR-125a-5p
 
 
-0.578
 
 
16.0
 
 
 
 
ANKHD1-EIF4EBP3
 
 
hsa-miR-22-3p
 
 
-0.479
 
 
10.0
 
 
 
 
AP1S1
 
 
hsa-let-7e-5p
 
 
-0.374
 
 
31.4
 
 
 
 
AP1S1
 
 
hsa-let-7c-5p
 
 
-0.374
 
 
31.4
 
 
 
 
APBB3
 
 
hsa-let-7e-5p
 
 
-0.587
 
 
10.1
 
 
 
 
APBB3
 
 
hsa-let-7c-5p
 
 
-0.587
 
 
10.1
 
 
 
 
ARG2
 
 
hsa-let-7e-5p
 
 
-0.468
 
 
47.4
 
 
 
 
ARG2
 
 
hsa-let-7c-5p
 
 
-0.468
 
 
47.4
 
 
 
 
ARL4D
 
 
hsa-let-7e-5p
 
 
-0.417
 
 
20.8
 
 
 
 
ARL4D
 
 
hsa-let-7c-5p
 
 
-0.417
 
 
20.8
 
 
 
 
ARPC5
 
 
hsa-miR-22-3p
 
 
-0.679
 
 
18.4
 
 
 
 
ARPP19
 
 
hsa-let-7e-5p
 
 
-0.492
 
 
33.0
 
 
 
 
ARPP19
 
 
hsa-let-7c-5p
 
 
-0.492
 
 
33.0
 
 
 
 
ATCAY
 
 
hsa-miR-10a-5p
 
 
-0.322
 
 
24.7
 
 
 
 
ATOSB
 
 
hsa-let-7e-5p
 
 
-0.429
 
 
15.4
 
 
 
 
ATOSB
 
 
hsa-let-7c-5p
 
 
-0.429
 
 
15.4
 
 
 
 
ATP2A2
 
 
hsa-let-7e-5p
 
 
-0.311
 
 
13.7
 
 
 
 
ATP2A2
 
 
hsa-let-7c-5p
 
 
-0.311
 
 
13.7
 
 
 
 
ATPAF1
 
 
hsa-let-7e-5p
 
 
-0.498
 
 
31.1
 
 
 
 
ATPAF1
 
 
hsa-let-7c-5p
 
 
-0.498
 
 
31.1
 
 
 
 
ATXN2
 
 
hsa-miR-10a-5p
 
 
-0.362
 
 
21.6
 
 
 
 
B3GAT3
 
 
hsa-let-7e-5p
 
 
-0.324
 
 
61.5
 
 
 
 
B3GAT3
 
 
hsa-let-7c-5p
 
 
-0.324
 
 
61.5
 
 
 
 
B4GAT1
 
 
hsa-let-7e-5p
 
 
-0.47
 
 
60.5
 
 
 
 
B4GAT1
 
 
hsa-let-7c-5p
 
 
-0.47
 
 
60.5
 
 
 
 
BAG5
 
 
hsa-miR-22-3p
 
 
-0.318
 
 
10.5
 
 
 
 
BAK1
 
 
hsa-miR-125a-5p
 
 
-0.402
 
 
11.6
 
 
 
 
BAZ1B
 
 
hsa-miR-10a-5p
 
 
-0.309
 
 
18.2
 
 
 
 
BAZ2A
 
 
hsa-miR-99b-5p
 
 
-0.408
 
 
25.8
 
 
 
 
BDH1
 
 
hsa-miR-125a-5p
 
 
-0.389
 
 
11.4
 
 
 
 
BEGAIN
 
 
hsa-let-7e-5p
 
 
-0.466
 
 
18.8
 
 
 
 
BEGAIN
 
 
hsa-let-7c-5p
 
 
-0.466
 
 
18.8
 
 
 
 
BIN1
 
 
hsa-miR-22-3p
 
 
-0.346
 
 
39.7
 
 
 
 
BIN3
 
 
hsa-let-7e-5p
 
 
-0.427
 
 
13.2
 
 
 
 
BIN3
 
 
hsa-let-7c-5p
 
 
-0.427
 
 
13.2
 
 
 
 
BLOC1S1
 
 
hsa-miR-22-3p
 
 
-0.346
 
 
107.6
 
 
 
 
BLOC1S6
 
 
hsa-let-7e-5p
 
 
-0.321
 
 
19.0
 
 
 
 
BLOC1S6
 
 
hsa-let-7c-5p
 
 
-0.321
 
 
19.0
 
 
 
 
BTF3L4
 
 
hsa-let-7e-5p
 
 
-0.355
 
 
28.3
 
 
 
 
BTF3L4
 
 
hsa-let-7c-5p
 
 
-0.355
 
 
28.3
 
 
 
 
BZW1
 
 
hsa-let-7e-5p
 
 
-0.354
 
 
28.1
 
 
 
 
BZW1
 
 
hsa-let-7c-5p
 
 
-0.354
 
 
28.1
 
 
 
 
BZW1
 
 
hsa-let-7e-5p
 
 
-0.375
 
 
28.1
 
 
 
 
BZW1
 
 
hsa-let-7c-5p
 
 
-0.375
 
 
28.1
 
 
 
 
BZW2
 
 
hsa-let-7c-5p
 
 
-0.397
 
 
10.9
 
 
 
 
BZW2
 
 
hsa-let-7e-5p
 
 
-0.408
 
 
10.9
 
 
 
 
C5orf24
 
 
hsa-miR-22-3p
 
 
-0.493
 
 
14.4
 
 
 
 
C6orf47
 
 
hsa-miR-125a-5p
 
 
-0.564
 
 
11.1
 
 
 
 
CACFD1
 
 
hsa-let-7e-5p
 
 
-0.296
 
 
18.3
 
 
 
 
CACFD1
 
 
hsa-let-7c-5p
 
 
-0.296
 
 
18.3
 
 
 
 
CACNB3
 
 
hsa-miR-125a-5p
 
 
-0.393
 
 
11.5
 
 
 
 
CAMK2N1
 
 
hsa-miR-22-3p
 
 
-0.302
 
 
33.4
 
 
 
 
CANT1
 
 
hsa-let-7e-5p
 
 
-0.402
 
 
16.3
 
 
 
 
CANT1
 
 
hsa-let-7c-5p
 
 
-0.402
 
 
16.3
 
 
 
 
CAPN1
 
 
hsa-miR-22-3p
 
 
-0.307
 
 
38.7
 
 
 
 
CAPN3
 
 
hsa-let-7e-5p
 
 
-0.317
 
 
14.0
 
 
 
 
CAPN3
 
 
hsa-let-7c-5p
 
 
-0.317
 
 
14.0
 
 
 
 
CAST
 
 
hsa-miR-150-5p
 
 
-0.354
 
 
44.6
 
 
 
 
CBX5
 
 
hsa-let-7e-5p
 
 
-0.307
 
 
18.3
 
 
 
 
CBX5
 
 
hsa-let-7c-5p
 
 
-0.307
 
 
18.3
 
 
 
 
CBX5
 
 
hsa-let-7e-5p
 
 
-0.472
 
 
18.3
 
 
 
 
CBX5
 
 
hsa-let-7c-5p
 
 
-0.472
 
 
18.3
 
 
 
 
CCDC47
 
 
hsa-miR-22-3p
 
 
-0.604
 
 
20.7
 
 
 
 
CCN1
 
 
hsa-miR-22-3p
 
 
-0.601
 
 
23.5
 
 
 
 
CCNY
 
 
hsa-let-7c-5p
 
 
-0.323
 
 
13.7
 
 
 
 
CCNY
 
 
hsa-let-7e-5p
 
 
-0.334
 
 
13.7
 
 
 
 
CDC34
 
 
hsa-let-7c-5p
 
 
-0.309
 
 
44.1
 
 
 
 
CDC34
 
 
hsa-let-7e-5p
 
 
-0.321
 
 
44.1
 
 
 
 
CDC34
 
 
hsa-let-7e-5p
 
 
-0.555
 
 
44.1
 
 
 
 
CDC34
 
 
hsa-let-7c-5p
 
 
-0.555
 
 
44.1
 
 
 
 
CDC42BPG
 
 
hsa-miR-125a-5p
 
 
-0.344
 
 
10.5
 
 
 
 
CDC42SE1
 
 
hsa-miR-125a-5p
 
 
-0.501
 
 
37.5
 
 
 
 
CDKN1A
 
 
hsa-let-7e-5p
 
 
-0.434
 
 
104.4
 
 
 
 
CDKN1A
 
 
hsa-let-7c-5p
 
 
-0.434
 
 
104.4
 
 
 
 
CDV3
 
 
hsa-let-7e-5p
 
 
-0.453
 
 
37.0
 
 
 
 
CDV3
 
 
hsa-let-7c-5p
 
 
-0.461
 
 
37.0
 
 
 
 
CEBPD
 
 
hsa-let-7e-5p
 
 
-0.309
 
 
212.0
 
 
 
 
CEBPD
 
 
hsa-let-7c-5p
 
 
-0.309
 
 
212.0
 
 
 
 
CERCAM
 
 
hsa-let-7e-5p
 
 
-0.344
 
 
115.4
 
 
 
 
CERCAM
 
 
hsa-let-7c-5p
 
 
-0.344
 
 
115.4
 
 
 
 
CFAP36
 
 
hsa-miR-22-3p
 
 
-0.549
 
 
113.7
 
 
 
 
CLASP2
 
 
hsa-let-7e-5p
 
 
-0.344
 
 
20.4
 
 
 
 
CLASP2
 
 
hsa-let-7c-5p
 
 
-0.344
 
 
20.4
 
 
 
 
CLDN12
 
 
hsa-let-7e-5p
 
 
-0.511
 
 
21.0
 
 
 
 
CLDN12
 
 
hsa-let-7c-5p
 
 
-0.511
 
 
21.0
 
 
 
 
CLIC4
 
 
hsa-miR-22-3p
 
 
-0.594
 
 
13.4
 
 
 
 
 
 
 testis 
 
 
 
 
target_symbol
 
 
mature_mirna_id
 
 
score
 
 
nTPM
 
 
 
 
 
 
ABCB9
 
 
hsa-let-7e-5p
 
 
-0.422
 
 
17.1
 
 
 
 
ABCB9
 
 
hsa-let-7c-5p
 
 
-0.422
 
 
17.1
 
 
 
 
ABCC5
 
 
hsa-miR-125a-5p
 
 
-0.288
 
 
17.7
 
 
 
 
ACSL6
 
 
hsa-miR-125a-5p
 
 
-0.307
 
 
19.3
 
 
 
 
ACSL6
 
 
hsa-let-7e-5p
 
 
-0.336
 
 
19.3
 
 
 
 
ACSL6
 
 
hsa-let-7c-5p
 
 
-0.336
 
 
19.3
 
 
 
 
ADIPOR2
 
 
hsa-miR-150-5p
 
 
-0.287
 
 
26.9
 
 
 
 
AGGF1
 
 
hsa-miR-125a-5p
 
 
-0.331
 
 
15.2
 
 
 
 
AHCTF1
 
 
hsa-let-7e-5p
 
 
-0.374
 
 
30.4
 
 
 
 
AHCTF1
 
 
hsa-let-7c-5p
 
 
-0.374
 
 
30.4
 
 
 
 
AIDA
 
 
hsa-miR-22-3p
 
 
-0.316
 
 
41.4
 
 
 
 
AIFM1
 
 
hsa-miR-125a-5p
 
 
-0.3
 
 
21.3
 
 
 
 
AIFM1
 
 
hsa-let-7e-5p
 
 
-0.314
 
 
21.3
 
 
 
 
AIFM1
 
 
hsa-let-7c-5p
 
 
-0.314
 
 
21.3
 
 
 
 
ANKHD1-EIF4EBP3
 
 
hsa-miR-22-3p
 
 
-0.479
 
 
11.4
 
 
 
 
ANKRD13A
 
 
hsa-miR-22-3p
 
 
-0.478
 
 
56.9
 
 
 
 
AP1AR
 
 
hsa-miR-99b-5p
 
 
-0.823
 
 
13.6
 
 
 
 
AP1S1
 
 
hsa-let-7e-5p
 
 
-0.374
 
 
17.8
 
 
 
 
AP1S1
 
 
hsa-let-7c-5p
 
 
-0.374
 
 
17.8
 
 
 
 
ARHGAP28
 
 
hsa-let-7c-5p
 
 
-0.47
 
 
72.3
 
 
 
 
ARHGAP28
 
 
hsa-let-7e-5p
 
 
-0.486
 
 
72.3
 
 
 
 
ARID3A
 
 
hsa-let-7c-5p
 
 
-0.34
 
 
16.1
 
 
 
 
ARID3A
 
 
hsa-let-7e-5p
 
 
-0.347
 
 
16.1
 
 
 
 
ARID3A
 
 
hsa-let-7c-5p
 
 
-0.347
 
 
16.1
 
 
 
 
ARID3A
 
 
hsa-let-7e-5p
 
 
-0.348
 
 
16.1
 
 
 
 
ARID3B
 
 
hsa-let-7e-5p
 
 
-0.31
 
 
21.2
 
 
 
 
ARID3B
 
 
hsa-let-7c-5p
 
 
-0.31
 
 
21.2
 
 
 
 
ARID3B
 
 
hsa-let-7e-5p
 
 
-0.486
 
 
21.2
 
 
 
 
ARID3B
 
 
hsa-let-7c-5p
 
 
-0.486
 
 
21.2
 
 
 
 
ARL4D
 
 
hsa-let-7e-5p
 
 
-0.417
 
 
31.4
 
 
 
 
ARL4D
 
 
hsa-let-7c-5p
 
 
-0.417
 
 
31.4
 
 
 
 
ARL6IP6
 
 
hsa-let-7c-5p
 
 
-0.293
 
 
10.2
 
 
 
 
ARL6IP6
 
 
hsa-let-7e-5p
 
 
-0.305
 
 
10.2
 
 
 
 
ARMT1
 
 
hsa-let-7e-5p
 
 
-0.303
 
 
13.1
 
 
 
 
ARMT1
 
 
hsa-let-7c-5p
 
 
-0.303
 
 
13.1
 
 
 
 
ARPC5
 
 
hsa-miR-22-3p
 
 
-0.679
 
 
35.9
 
 
 
 
ARPP19
 
 
hsa-let-7e-5p
 
 
-0.492
 
 
79.0
 
 
 
 
ARPP19
 
 
hsa-let-7c-5p
 
 
-0.492
 
 
79.0
 
 
 
 
ARRDC4
 
 
hsa-let-7e-5p
 
 
-0.329
 
 
13.7
 
 
 
 
ARRDC4
 
 
hsa-let-7c-5p
 
 
-0.329
 
 
13.7
 
 
 
 
ASAP1
 
 
hsa-let-7e-5p
 
 
-0.296
 
 
26.5
 
 
 
 
ASAP1
 
 
hsa-let-7c-5p
 
 
-0.308
 
 
26.5
 
 
 
 
ATP2A2
 
 
hsa-let-7e-5p
 
 
-0.311
 
 
69.3
 
 
 
 
ATP2A2
 
 
hsa-let-7c-5p
 
 
-0.311
 
 
69.3
 
 
 
 
ATPAF1
 
 
hsa-let-7e-5p
 
 
-0.498
 
 
29.2
 
 
 
 
ATPAF1
 
 
hsa-let-7c-5p
 
 
-0.498
 
 
29.2
 
 
 
 
ATXN2
 
 
hsa-miR-10a-5p
 
 
-0.362
 
 
23.4
 
 
 
 
B3GAT3
 
 
hsa-let-7e-5p
 
 
-0.324
 
 
26.6
 
 
 
 
B3GAT3
 
 
hsa-let-7c-5p
 
 
-0.324
 
 
26.6
 
 
 
 
BACH1
 
 
hsa-let-7e-5p
 
 
-0.298
 
 
25.4
 
 
 
 
BACH1
 
 
hsa-let-7e-5p
 
 
-0.298
 
 
25.4
 
 
 
 
BACH1
 
 
hsa-let-7c-5p
 
 
-0.306
 
 
25.4
 
 
 
 
BACH1
 
 
hsa-let-7c-5p
 
 
-0.306
 
 
25.4
 
 
 
 
BACH1
 
 
hsa-let-7e-5p
 
 
-0.435
 
 
25.4
 
 
 
 
BACH1
 
 
hsa-let-7e-5p
 
 
-0.435
 
 
25.4
 
 
 
 
BACH1
 
 
hsa-let-7c-5p
 
 
-0.435
 
 
25.4
 
 
 
 
BACH1
 
 
hsa-let-7c-5p
 
 
-0.435
 
 
25.4
 
 
 
 
BAG5
 
 
hsa-miR-22-3p
 
 
-0.318
 
 
119.4
 
 
 
 
BAZ1B
 
 
hsa-miR-10a-5p
 
 
-0.309
 
 
16.1
 
 
 
 
BAZ2A
 
 
hsa-miR-99b-5p
 
 
-0.408
 
 
44.2
 
 
 
 
BBX
 
 
hsa-miR-10a-5p
 
 
-0.305
 
 
54.2
 
 
 
 
BCAP29
 
 
hsa-let-7e-5p
 
 
-0.289
 
 
65.9
 
 
 
 
BIN1
 
 
hsa-miR-22-3p
 
 
-0.346
 
 
10.0
 
 
 
 
BIN3
 
 
hsa-let-7e-5p
 
 
-0.427
 
 
13.0
 
 
 
 
BIN3
 
 
hsa-let-7c-5p
 
 
-0.427
 
 
13.0
 
 
 
 
BLOC1S1
 
 
hsa-miR-22-3p
 
 
-0.346
 
 
54.5
 
 
 
 
BLOC1S6
 
 
hsa-let-7e-5p
 
 
-0.321
 
 
15.9
 
 
 
 
BLOC1S6
 
 
hsa-let-7c-5p
 
 
-0.321
 
 
15.9
 
 
 
 
BLZF1
 
 
hsa-miR-125a-5p
 
 
-0.461
 
 
32.7
 
 
 
 
BTF3L4
 
 
hsa-let-7e-5p
 
 
-0.355
 
 
31.8
 
 
 
 
BTF3L4
 
 
hsa-let-7c-5p
 
 
-0.355
 
 
31.8
 
 
 
 
BZW1
 
 
hsa-let-7e-5p
 
 
-0.354
 
 
159.6
 
 
 
 
BZW1
 
 
hsa-let-7c-5p
 
 
-0.354
 
 
159.6
 
 
 
 
BZW1
 
 
hsa-let-7e-5p
 
 
-0.375
 
 
159.6
 
 
 
 
BZW1
 
 
hsa-let-7c-5p
 
 
-0.375
 
 
159.6
 
 
 
 
BZW2
 
 
hsa-let-7c-5p
 
 
-0.397
 
 
10.9
 
 
 
 
BZW2
 
 
hsa-let-7e-5p
 
 
-0.408
 
 
10.9
 
 
 
 
C15orf39
 
 
hsa-miR-125a-5p
 
 
-0.335
 
 
69.4
 
 
 
 
C15orf39
 
 
hsa-let-7e-5p
 
 
-0.374
 
 
69.4
 
 
 
 
C15orf39
 
 
hsa-let-7c-5p
 
 
-0.374
 
 
69.4
 
 
 
 
C19orf38
 
 
hsa-miR-125a-5p
 
 
-0.676
 
 
10.8
 
 
 
 
C19orf47
 
 
hsa-let-7e-5p
 
 
-0.311
 
 
37.1
 
 
 
 
C19orf47
 
 
hsa-let-7c-5p
 
 
-0.311
 
 
37.1
 
 
 
 
C2orf88
 
 
hsa-let-7e-5p
 
 
-0.429
 
 
68.6
 
 
 
 
C2orf88
 
 
hsa-let-7c-5p
 
 
-0.429
 
 
68.6
 
 
 
 
C5orf24
 
 
hsa-miR-22-3p
 
 
-0.493
 
 
11.6
 
 
 
 
C8orf58
 
 
hsa-let-7e-5p
 
 
-0.612
 
 
15.8
 
 
 
 
C8orf58
 
 
hsa-let-7c-5p
 
 
-0.62
 
 
15.8
 
 
 
 
C9orf40
 
 
hsa-let-7e-5p
 
 
-0.809
 
 
11.0
 
 
 
 
C9orf40
 
 
hsa-let-7c-5p
 
 
-0.809
 
 
11.0
 
 
 
 
CACFD1
 
 
hsa-let-7e-5p
 
 
-0.296
 
 
23.0
 
 
 
 
CACFD1
 
 
hsa-let-7c-5p
 
 
-0.296
 
 
23.0
 
 
 
 
CANT1
 
 
hsa-let-7e-5p
 
 
-0.402
 
 
19.3
 
 
 
 
CANT1
 
 
hsa-let-7c-5p
 
 
-0.402
 
 
19.3
 
 
 
 
CAPN3
 
 
hsa-let-7e-5p
 
 
-0.317
 
 
29.0
 
 
 
 
CAPN3
 
 
hsa-let-7c-5p
 
 
-0.317
 
 
29.0
 
 
 
 
CARMIL1
 
 
hsa-miR-22-3p
 
 
-0.329
 
 
11.5
 
 
 
 
CARNMT1
 
 
hsa-let-7e-5p
 
 
-0.307
 
 
15.3
 
 
 
 
CARNMT1
 
 
hsa-let-7c-5p
 
 
-0.307
 
 
15.3
 
 
 
 
CAST
 
 
hsa-miR-150-5p
 
 
-0.354
 
 
92.4
 
 
 
 
 
 
 adipose tissue 
 
 
 
 
target_symbol
 
 
mature_mirna_id
 
 
score
 
 
nTPM
 
 
 
 
 
 
ABCC5
 
 
hsa-miR-125a-5p
 
 
-0.288
 
 
15.3
 
 
 
 
ABHD3
 
 
hsa-miR-125a-5p
 
 
-0.453
 
 
16.0
 
 
 
 
ABHD6
 
 
hsa-miR-125a-5p
 
 
-0.501
 
 
11.4
 
 
 
 
ABTB1
 
 
hsa-miR-125a-5p
 
 
-0.519
 
 
38.9
 
 
 
 
ACER3
 
 
hsa-miR-22-3p
 
 
-0.453
 
 
10.2
 
 
 
 
ACVR1C
 
 
hsa-let-7e-5p
 
 
-0.457
 
 
29.3
 
 
 
 
ACVR1C
 
 
hsa-let-7c-5p
 
 
-0.465
 
 
29.3
 
 
 
 
ADIPOR2
 
 
hsa-miR-150-5p
 
 
-0.287
 
 
53.2
 
 
 
 
ADRB2
 
 
hsa-let-7e-5p
 
 
-0.635
 
 
26.4
 
 
 
 
ADRB2
 
 
hsa-let-7c-5p
 
 
-0.635
 
 
26.4
 
 
 
 
AHCTF1
 
 
hsa-let-7e-5p
 
 
-0.374
 
 
11.8
 
 
 
 
AHCTF1
 
 
hsa-let-7c-5p
 
 
-0.374
 
 
11.8
 
 
 
 
AIDA
 
 
hsa-miR-22-3p
 
 
-0.316
 
 
67.8
 
 
 
 
AIFM1
 
 
hsa-miR-125a-5p
 
 
-0.3
 
 
17.4
 
 
 
 
AIFM1
 
 
hsa-let-7e-5p
 
 
-0.314
 
 
17.4
 
 
 
 
AIFM1
 
 
hsa-let-7c-5p
 
 
-0.314
 
 
17.4
 
 
 
 
AKT3
 
 
hsa-miR-22-3p
 
 
-0.451
 
 
16.9
 
 
 
 
ALDH4A1
 
 
hsa-miR-184
 
 
-0.421
 
 
66.5
 
 
 
 
ALPL
 
 
hsa-miR-10a-5p
 
 
-0.32
 
 
23.7
 
 
 
 
ANAPC16
 
 
hsa-miR-125a-5p
 
 
-0.578
 
 
18.9
 
 
 
 
ANKRD13A
 
 
hsa-miR-22-3p
 
 
-0.478
 
 
12.3
 
 
 
 
ANKRD46
 
 
hsa-let-7e-5p
 
 
-0.389
 
 
18.0
 
 
 
 
ANKRD46
 
 
hsa-let-7c-5p
 
 
-0.389
 
 
18.0
 
 
 
 
ANPEP
 
 
hsa-miR-125a-5p
 
 
-0.372
 
 
50.1
 
 
 
 
AP1S1
 
 
hsa-let-7e-5p
 
 
-0.374
 
 
15.1
 
 
 
 
AP1S1
 
 
hsa-let-7c-5p
 
 
-0.374
 
 
15.1
 
 
 
 
ARL4D
 
 
hsa-let-7e-5p
 
 
-0.417
 
 
20.3
 
 
 
 
ARL4D
 
 
hsa-let-7c-5p
 
 
-0.417
 
 
20.3
 
 
 
 
ARL6IP6
 
 
hsa-let-7c-5p
 
 
-0.293
 
 
11.0
 
 
 
 
ARL6IP6
 
 
hsa-let-7e-5p
 
 
-0.305
 
 
11.0
 
 
 
 
ARMT1
 
 
hsa-let-7e-5p
 
 
-0.303
 
 
16.0
 
 
 
 
ARMT1
 
 
hsa-let-7c-5p
 
 
-0.303
 
 
16.0
 
 
 
 
ARPC5
 
 
hsa-miR-22-3p
 
 
-0.679
 
 
80.7
 
 
 
 
ARPP19
 
 
hsa-let-7e-5p
 
 
-0.492
 
 
57.4
 
 
 
 
ARPP19
 
 
hsa-let-7c-5p
 
 
-0.492
 
 
57.4
 
 
 
 
ARRB1
 
 
hsa-miR-22-3p
 
 
-0.616
 
 
28.8
 
 
 
 
ARRDC4
 
 
hsa-let-7e-5p
 
 
-0.329
 
 
11.8
 
 
 
 
ARRDC4
 
 
hsa-let-7c-5p
 
 
-0.329
 
 
11.8
 
 
 
 
ASAP1
 
 
hsa-let-7e-5p
 
 
-0.296
 
 
32.3
 
 
 
 
ASAP1
 
 
hsa-let-7c-5p
 
 
-0.308
 
 
32.3
 
 
 
 
ATOSB
 
 
hsa-let-7e-5p
 
 
-0.429
 
 
13.2
 
 
 
 
ATOSB
 
 
hsa-let-7c-5p
 
 
-0.429
 
 
13.2
 
 
 
 
ATP2A2
 
 
hsa-let-7e-5p
 
 
-0.311
 
 
56.6
 
 
 
 
ATP2A2
 
 
hsa-let-7c-5p
 
 
-0.311
 
 
56.6
 
 
 
 
ATPAF1
 
 
hsa-let-7e-5p
 
 
-0.498
 
 
81.3
 
 
 
 
ATPAF1
 
 
hsa-let-7c-5p
 
 
-0.498
 
 
81.3
 
 
 
 
ATXN2
 
 
hsa-miR-10a-5p
 
 
-0.362
 
 
19.0
 
 
 
 
B3GAT3
 
 
hsa-let-7e-5p
 
 
-0.324
 
 
30.8
 
 
 
 
B3GAT3
 
 
hsa-let-7c-5p
 
 
-0.324
 
 
30.8
 
 
 
 
B4GAT1
 
 
hsa-let-7e-5p
 
 
-0.47
 
 
16.2
 
 
 
 
B4GAT1
 
 
hsa-let-7c-5p
 
 
-0.47
 
 
16.2
 
 
 
 
BACH1
 
 
hsa-let-7e-5p
 
 
-0.298
 
 
14.8
 
 
 
 
BACH1
 
 
hsa-let-7e-5p
 
 
-0.298
 
 
14.8
 
 
 
 
BACH1
 
 
hsa-let-7c-5p
 
 
-0.306
 
 
14.8
 
 
 
 
BACH1
 
 
hsa-let-7c-5p
 
 
-0.306
 
 
14.8
 
 
 
 
BACH1
 
 
hsa-let-7e-5p
 
 
-0.435
 
 
14.8
 
 
 
 
BACH1
 
 
hsa-let-7e-5p
 
 
-0.435
 
 
14.8
 
 
 
 
BACH1
 
 
hsa-let-7c-5p
 
 
-0.435
 
 
14.8
 
 
 
 
BACH1
 
 
hsa-let-7c-5p
 
 
-0.435
 
 
14.8
 
 
 
 
BAG5
 
 
hsa-miR-22-3p
 
 
-0.318
 
 
23.0
 
 
 
 
BAZ1B
 
 
hsa-miR-10a-5p
 
 
-0.309
 
 
21.2
 
 
 
 
BAZ2A
 
 
hsa-miR-99b-5p
 
 
-0.408
 
 
26.9
 
 
 
 
BBX
 
 
hsa-miR-10a-5p
 
 
-0.305
 
 
13.1
 
 
 
 
BCAP29
 
 
hsa-let-7e-5p
 
 
-0.289
 
 
45.2
 
 
 
 
BIN1
 
 
hsa-miR-22-3p
 
 
-0.346
 
 
41.5
 
 
 
 
BIN3
 
 
hsa-let-7e-5p
 
 
-0.427
 
 
19.4
 
 
 
 
BIN3
 
 
hsa-let-7c-5p
 
 
-0.427
 
 
19.4
 
 
 
 
BLOC1S1
 
 
hsa-miR-22-3p
 
 
-0.346
 
 
122.1
 
 
 
 
BLOC1S6
 
 
hsa-let-7e-5p
 
 
-0.321
 
 
28.2
 
 
 
 
BLOC1S6
 
 
hsa-let-7c-5p
 
 
-0.321
 
 
28.2
 
 
 
 
BLZF1
 
 
hsa-miR-125a-5p
 
 
-0.461
 
 
14.0
 
 
 
 
BMPR2
 
 
hsa-miR-99b-5p
 
 
-0.327
 
 
15.1
 
 
 
 
BTF3L4
 
 
hsa-let-7e-5p
 
 
-0.355
 
 
41.8
 
 
 
 
BTF3L4
 
 
hsa-let-7c-5p
 
 
-0.355
 
 
41.8
 
 
 
 
BTG1
 
 
hsa-miR-22-3p
 
 
-0.468
 
 
17.1
 
 
 
 
BZW1
 
 
hsa-let-7e-5p
 
 
-0.354
 
 
122.3
 
 
 
 
BZW1
 
 
hsa-let-7c-5p
 
 
-0.354
 
 
122.3
 
 
 
 
BZW1
 
 
hsa-let-7e-5p
 
 
-0.375
 
 
122.3
 
 
 
 
BZW1
 
 
hsa-let-7c-5p
 
 
-0.375
 
 
122.3
 
 
 
 
BZW2
 
 
hsa-let-7c-5p
 
 
-0.397
 
 
27.0
 
 
 
 
BZW2
 
 
hsa-let-7e-5p
 
 
-0.408
 
 
27.0
 
 
 
 
C15orf39
 
 
hsa-miR-125a-5p
 
 
-0.335
 
 
14.1
 
 
 
 
C15orf39
 
 
hsa-let-7e-5p
 
 
-0.374
 
 
14.1
 
 
 
 
C15orf39
 
 
hsa-let-7c-5p
 
 
-0.374
 
 
14.1
 
 
 
 
C1GALT1
 
 
hsa-let-7e-5p
 
 
-0.322
 
 
12.1
 
 
 
 
C1GALT1
 
 
hsa-let-7c-5p
 
 
-0.322
 
 
12.1
 
 
 
 
C5orf24
 
 
hsa-miR-22-3p
 
 
-0.493
 
 
22.6
 
 
 
 
C6orf47
 
 
hsa-miR-125a-5p
 
 
-0.564
 
 
10.6
 
 
 
 
C8orf58
 
 
hsa-let-7e-5p
 
 
-0.612
 
 
10.5
 
 
 
 
C8orf58
 
 
hsa-let-7c-5p
 
 
-0.62
 
 
10.5
 
 
 
 
CAMK2N1
 
 
hsa-miR-22-3p
 
 
-0.302
 
 
19.0
 
 
 
 
CANT1
 
 
hsa-let-7e-5p
 
 
-0.402
 
 
14.4
 
 
 
 
CANT1
 
 
hsa-let-7c-5p
 
 
-0.402
 
 
14.4
 
 
 
 
CAPN1
 
 
hsa-miR-22-3p
 
 
-0.307
 
 
43.2
 
 
 
 
CAPN3
 
 
hsa-let-7e-5p
 
 
-0.317
 
 
13.6
 
 
 
 
CAPN3
 
 
hsa-let-7c-5p
 
 
-0.317
 
 
13.6
 
 
 
 
CASP2
 
 
hsa-miR-125a-5p
 
 
-0.296
 
 
10.1
 
 
 
 
CASP3
 
 
hsa-let-7e-5p
 
 
-0.495
 
 
12.6
 
 
 
 
CASP3
 
 
hsa-let-7c-5p
 
 
-0.503
 
 
12.6
 
 
 
 
CAST
 
 
hsa-miR-150-5p
 
 
-0.354
 
 
131.1
 
 
 
 
 
 
 adrenal gland 
 
 
 
 
target_symbol
 
 
mature_mirna_id
 
 
score
 
 
nTPM
 
 
 
 
 
 
ABCC5
 
 
hsa-miR-125a-5p
 
 
-0.288
 
 
23.1
 
 
 
 
ABHD6
 
 
hsa-miR-125a-5p
 
 
-0.501
 
 
13.2
 
 
 
 
ABTB1
 
 
hsa-miR-125a-5p
 
 
-0.519
 
 
19.3
 
 
 
 
ADIPOR2
 
 
hsa-miR-150-5p
 
 
-0.287
 
 
69.8
 
 
 
 
AGGF1
 
 
hsa-miR-125a-5p
 
 
-0.331
 
 
10.0
 
 
 
 
AIDA
 
 
hsa-miR-22-3p
 
 
-0.316
 
 
34.1
 
 
 
 
AIFM1
 
 
hsa-miR-125a-5p
 
 
-0.3
 
 
50.1
 
 
 
 
AIFM1
 
 
hsa-let-7e-5p
 
 
-0.314
 
 
50.1
 
 
 
 
AIFM1
 
 
hsa-let-7c-5p
 
 
-0.314
 
 
50.1
 
 
 
 
ALDH4A1
 
 
hsa-miR-184
 
 
-0.421
 
 
16.2
 
 
 
 
ALPL
 
 
hsa-miR-10a-5p
 
 
-0.32
 
 
57.7
 
 
 
 
AMT
 
 
hsa-let-7e-5p
 
 
-0.463
 
 
15.0
 
 
 
 
AMT
 
 
hsa-let-7c-5p
 
 
-0.463
 
 
15.0
 
 
 
 
ANAPC16
 
 
hsa-miR-125a-5p
 
 
-0.578
 
 
24.7
 
 
 
 
ANKHD1-EIF4EBP3
 
 
hsa-miR-22-3p
 
 
-0.479
 
 
14.6
 
 
 
 
ANKRD46
 
 
hsa-let-7e-5p
 
 
-0.389
 
 
36.5
 
 
 
 
ANKRD46
 
 
hsa-let-7c-5p
 
 
-0.389
 
 
36.5
 
 
 
 
AP1AR
 
 
hsa-miR-99b-5p
 
 
-0.823
 
 
14.8
 
 
 
 
AP1S1
 
 
hsa-let-7e-5p
 
 
-0.374
 
 
25.7
 
 
 
 
AP1S1
 
 
hsa-let-7c-5p
 
 
-0.374
 
 
25.7
 
 
 
 
ARMT1
 
 
hsa-let-7e-5p
 
 
-0.303
 
 
15.1
 
 
 
 
ARMT1
 
 
hsa-let-7c-5p
 
 
-0.303
 
 
15.1
 
 
 
 
ARPC5
 
 
hsa-miR-22-3p
 
 
-0.679
 
 
46.4
 
 
 
 
ARPP19
 
 
hsa-let-7e-5p
 
 
-0.492
 
 
48.8
 
 
 
 
ARPP19
 
 
hsa-let-7c-5p
 
 
-0.492
 
 
48.8
 
 
 
 
ARRDC4
 
 
hsa-let-7e-5p
 
 
-0.329
 
 
11.5
 
 
 
 
ARRDC4
 
 
hsa-let-7c-5p
 
 
-0.329
 
 
11.5
 
 
 
 
ASAP1
 
 
hsa-let-7e-5p
 
 
-0.296
 
 
20.2
 
 
 
 
ASAP1
 
 
hsa-let-7c-5p
 
 
-0.308
 
 
20.2
 
 
 
 
ATOSB
 
 
hsa-let-7e-5p
 
 
-0.429
 
 
14.2
 
 
 
 
ATOSB
 
 
hsa-let-7c-5p
 
 
-0.429
 
 
14.2
 
 
 
 
ATP2A2
 
 
hsa-let-7e-5p
 
 
-0.311
 
 
46.8
 
 
 
 
ATP2A2
 
 
hsa-let-7c-5p
 
 
-0.311
 
 
46.8
 
 
 
 
ATPAF1
 
 
hsa-let-7e-5p
 
 
-0.498
 
 
98.1
 
 
 
 
ATPAF1
 
 
hsa-let-7c-5p
 
 
-0.498
 
 
98.1
 
 
 
 
ATXN2
 
 
hsa-miR-10a-5p
 
 
-0.362
 
 
24.4
 
 
 
 
B3GAT3
 
 
hsa-let-7e-5p
 
 
-0.324
 
 
33.4
 
 
 
 
B3GAT3
 
 
hsa-let-7c-5p
 
 
-0.324
 
 
33.4
 
 
 
 
B4GAT1
 
 
hsa-let-7e-5p
 
 
-0.47
 
 
39.1
 
 
 
 
B4GAT1
 
 
hsa-let-7c-5p
 
 
-0.47
 
 
39.1
 
 
 
 
BACH1
 
 
hsa-let-7e-5p
 
 
-0.298
 
 
11.4
 
 
 
 
BACH1
 
 
hsa-let-7e-5p
 
 
-0.298
 
 
11.4
 
 
 
 
BACH1
 
 
hsa-let-7c-5p
 
 
-0.306
 
 
11.4
 
 
 
 
BACH1
 
 
hsa-let-7c-5p
 
 
-0.306
 
 
11.4
 
 
 
 
BACH1
 
 
hsa-let-7e-5p
 
 
-0.435
 
 
11.4
 
 
 
 
BACH1
 
 
hsa-let-7e-5p
 
 
-0.435
 
 
11.4
 
 
 
 
BACH1
 
 
hsa-let-7c-5p
 
 
-0.435
 
 
11.4
 
 
 
 
BACH1
 
 
hsa-let-7c-5p
 
 
-0.435
 
 
11.4
 
 
 
 
BAG5
 
 
hsa-miR-22-3p
 
 
-0.318
 
 
10.8
 
 
 
 
BAK1
 
 
hsa-miR-125a-5p
 
 
-0.402
 
 
27.9
 
 
 
 
BAZ1B
 
 
hsa-miR-10a-5p
 
 
-0.309
 
 
18.3
 
 
 
 
BAZ2A
 
 
hsa-miR-99b-5p
 
 
-0.408
 
 
22.2
 
 
 
 
BBX
 
 
hsa-miR-10a-5p
 
 
-0.305
 
 
23.6
 
 
 
 
BCAP29
 
 
hsa-let-7e-5p
 
 
-0.289
 
 
25.1
 
 
 
 
BIN1
 
 
hsa-miR-22-3p
 
 
-0.346
 
 
88.9
 
 
 
 
BIN3
 
 
hsa-let-7e-5p
 
 
-0.427
 
 
30.6
 
 
 
 
BIN3
 
 
hsa-let-7c-5p
 
 
-0.427
 
 
30.6
 
 
 
 
BLOC1S1
 
 
hsa-miR-22-3p
 
 
-0.346
 
 
165.7
 
 
 
 
BLOC1S6
 
 
hsa-let-7e-5p
 
 
-0.321
 
 
29.4
 
 
 
 
BLOC1S6
 
 
hsa-let-7c-5p
 
 
-0.321
 
 
29.4
 
 
 
 
BLZF1
 
 
hsa-miR-125a-5p
 
 
-0.461
 
 
12.4
 
 
 
 
BMPR2
 
 
hsa-miR-99b-5p
 
 
-0.327
 
 
17.0
 
 
 
 
BORCS6
 
 
hsa-miR-125a-5p
 
 
-0.287
 
 
11.6
 
 
 
 
BTF3L4
 
 
hsa-let-7e-5p
 
 
-0.355
 
 
49.0
 
 
 
 
BTF3L4
 
 
hsa-let-7c-5p
 
 
-0.355
 
 
49.0
 
 
 
 
BZW1
 
 
hsa-let-7e-5p
 
 
-0.354
 
 
121.8
 
 
 
 
BZW1
 
 
hsa-let-7c-5p
 
 
-0.354
 
 
121.8
 
 
 
 
BZW1
 
 
hsa-let-7e-5p
 
 
-0.375
 
 
121.8
 
 
 
 
BZW1
 
 
hsa-let-7c-5p
 
 
-0.375
 
 
121.8
 
 
 
 
BZW2
 
 
hsa-let-7c-5p
 
 
-0.397
 
 
18.5
 
 
 
 
BZW2
 
 
hsa-let-7e-5p
 
 
-0.408
 
 
18.5
 
 
 
 
C15orf39
 
 
hsa-miR-125a-5p
 
 
-0.335
 
 
14.8
 
 
 
 
C15orf39
 
 
hsa-let-7e-5p
 
 
-0.374
 
 
14.8
 
 
 
 
C15orf39
 
 
hsa-let-7c-5p
 
 
-0.374
 
 
14.8
 
 
 
 
C5orf24
 
 
hsa-miR-22-3p
 
 
-0.493
 
 
24.5
 
 
 
 
C6orf47
 
 
hsa-miR-125a-5p
 
 
-0.564
 
 
12.1
 
 
 
 
CACFD1
 
 
hsa-let-7e-5p
 
 
-0.296
 
 
10.8
 
 
 
 
CACFD1
 
 
hsa-let-7c-5p
 
 
-0.296
 
 
10.8
 
 
 
 
CAMK2N1
 
 
hsa-miR-22-3p
 
 
-0.302
 
 
58.3
 
 
 
 
CANT1
 
 
hsa-let-7e-5p
 
 
-0.402
 
 
21.8
 
 
 
 
CANT1
 
 
hsa-let-7c-5p
 
 
-0.402
 
 
21.8
 
 
 
 
CAPN1
 
 
hsa-miR-22-3p
 
 
-0.307
 
 
41.1
 
 
 
 
CAPN3
 
 
hsa-let-7e-5p
 
 
-0.317
 
 
10.1
 
 
 
 
CAPN3
 
 
hsa-let-7c-5p
 
 
-0.317
 
 
10.1
 
 
 
 
CASP3
 
 
hsa-let-7e-5p
 
 
-0.495
 
 
11.3
 
 
 
 
CASP3
 
 
hsa-let-7c-5p
 
 
-0.503
 
 
11.3
 
 
 
 
CAST
 
 
hsa-miR-150-5p
 
 
-0.354
 
 
59.1
 
 
 
 
CBX5
 
 
hsa-let-7e-5p
 
 
-0.307
 
 
12.4
 
 
 
 
CBX5
 
 
hsa-let-7c-5p
 
 
-0.307
 
 
12.4
 
 
 
 
CBX5
 
 
hsa-let-7e-5p
 
 
-0.472
 
 
12.4
 
 
 
 
CBX5
 
 
hsa-let-7c-5p
 
 
-0.472
 
 
12.4
 
 
 
 
CCDC47
 
 
hsa-miR-22-3p
 
 
-0.604
 
 
59.7
 
 
 
 
CCN1
 
 
hsa-miR-22-3p
 
 
-0.601
 
 
39.4
 
 
 
 
CCNY
 
 
hsa-let-7c-5p
 
 
-0.323
 
 
19.9
 
 
 
 
CCNY
 
 
hsa-let-7e-5p
 
 
-0.334
 
 
19.9
 
 
 
 
CDC34
 
 
hsa-let-7c-5p
 
 
-0.309
 
 
38.1
 
 
 
 
CDC34
 
 
hsa-let-7e-5p
 
 
-0.321
 
 
38.1
 
 
 
 
CDC34
 
 
hsa-let-7e-5p
 
 
-0.555
 
 
38.1
 
 
 
 
CDC34
 
 
hsa-let-7c-5p
 
 
-0.555
 
 
38.1
 
 
 
 
CDC37L1
 
 
hsa-miR-125a-5p
 
 
-0.401
 
 
14.7
 
 
 
 
 
 
 amygdala 
 
 
 
 
target_symbol
 
 
mature_mirna_id
 
 
score
 
 
nTPM
 
 
 
 
 
 
ABCC5
 
 
hsa-miR-125a-5p
 
 
-0.288
 
 
24.0
 
 
 
 
ABHD17C
 
 
hsa-let-7e-5p
 
 
-0.359
 
 
17.9
 
 
 
 
ABHD17C
 
 
hsa-let-7c-5p
 
 
-0.359
 
 
17.9
 
 
 
 
ABHD3
 
 
hsa-miR-125a-5p
 
 
-0.453
 
 
10.3
 
 
 
 
ABHD6
 
 
hsa-miR-125a-5p
 
 
-0.501
 
 
15.3
 
 
 
 
ABTB1
 
 
hsa-miR-125a-5p
 
 
-0.519
 
 
23.8
 
 
 
 
ACHE
 
 
hsa-miR-125a-5p
 
 
-0.308
 
 
14.7
 
 
 
 
ACHE
 
 
hsa-miR-125a-5p
 
 
-0.361
 
 
14.7
 
 
 
 
ACSL6
 
 
hsa-miR-125a-5p
 
 
-0.307
 
 
20.3
 
 
 
 
ACSL6
 
 
hsa-let-7e-5p
 
 
-0.336
 
 
20.3
 
 
 
 
ACSL6
 
 
hsa-let-7c-5p
 
 
-0.336
 
 
20.3
 
 
 
 
ADIPOR2
 
 
hsa-miR-150-5p
 
 
-0.287
 
 
43.7
 
 
 
 
AGGF1
 
 
hsa-miR-125a-5p
 
 
-0.331
 
 
10.3
 
 
 
 
AIDA
 
 
hsa-miR-22-3p
 
 
-0.316
 
 
28.0
 
 
 
 
AIFM1
 
 
hsa-miR-125a-5p
 
 
-0.3
 
 
10.3
 
 
 
 
AIFM1
 
 
hsa-let-7e-5p
 
 
-0.314
 
 
10.3
 
 
 
 
AIFM1
 
 
hsa-let-7c-5p
 
 
-0.314
 
 
10.3
 
 
 
 
AKAP6
 
 
hsa-let-7e-5p
 
 
-0.307
 
 
12.4
 
 
 
 
AKAP6
 
 
hsa-let-7c-5p
 
 
-0.307
 
 
12.4
 
 
 
 
AKT3
 
 
hsa-miR-22-3p
 
 
-0.451
 
 
17.4
 
 
 
 
ALDH4A1
 
 
hsa-miR-184
 
 
-0.421
 
 
66.7
 
 
 
 
ALPL
 
 
hsa-miR-10a-5p
 
 
-0.32
 
 
10.6
 
 
 
 
ANGEL2
 
 
hsa-miR-22-3p
 
 
-0.601
 
 
10.6
 
 
 
 
ANKRD46
 
 
hsa-let-7e-5p
 
 
-0.389
 
 
28.3
 
 
 
 
ANKRD46
 
 
hsa-let-7c-5p
 
 
-0.389
 
 
28.3
 
 
 
 
AP1S1
 
 
hsa-let-7e-5p
 
 
-0.374
 
 
88.4
 
 
 
 
AP1S1
 
 
hsa-let-7c-5p
 
 
-0.374
 
 
88.4
 
 
 
 
ARPC5
 
 
hsa-miR-22-3p
 
 
-0.679
 
 
19.6
 
 
 
 
ARPP19
 
 
hsa-let-7e-5p
 
 
-0.492
 
 
178.5
 
 
 
 
ARPP19
 
 
hsa-let-7c-5p
 
 
-0.492
 
 
178.5
 
 
 
 
ARRB1
 
 
hsa-miR-22-3p
 
 
-0.616
 
 
32.5
 
 
 
 
ARRDC4
 
 
hsa-let-7e-5p
 
 
-0.329
 
 
13.2
 
 
 
 
ARRDC4
 
 
hsa-let-7c-5p
 
 
-0.329
 
 
13.2
 
 
 
 
ASAP1
 
 
hsa-let-7e-5p
 
 
-0.296
 
 
11.2
 
 
 
 
ASAP1
 
 
hsa-let-7c-5p
 
 
-0.308
 
 
11.2
 
 
 
 
ATCAY
 
 
hsa-miR-10a-5p
 
 
-0.322
 
 
44.9
 
 
 
 
ATOSB
 
 
hsa-let-7e-5p
 
 
-0.429
 
 
20.1
 
 
 
 
ATOSB
 
 
hsa-let-7c-5p
 
 
-0.429
 
 
20.1
 
 
 
 
ATP2A2
 
 
hsa-let-7e-5p
 
 
-0.311
 
 
15.8
 
 
 
 
ATP2A2
 
 
hsa-let-7c-5p
 
 
-0.311
 
 
15.8
 
 
 
 
ATPAF1
 
 
hsa-let-7e-5p
 
 
-0.498
 
 
86.8
 
 
 
 
ATPAF1
 
 
hsa-let-7c-5p
 
 
-0.498
 
 
86.8
 
 
 
 
ATXN2
 
 
hsa-miR-10a-5p
 
 
-0.362
 
 
21.8
 
 
 
 
B3GAT3
 
 
hsa-let-7e-5p
 
 
-0.324
 
 
61.0
 
 
 
 
B3GAT3
 
 
hsa-let-7c-5p
 
 
-0.324
 
 
61.0
 
 
 
 
B4GAT1
 
 
hsa-let-7e-5p
 
 
-0.47
 
 
107.6
 
 
 
 
B4GAT1
 
 
hsa-let-7c-5p
 
 
-0.47
 
 
107.6
 
 
 
 
BAZ1B
 
 
hsa-miR-10a-5p
 
 
-0.309
 
 
17.0
 
 
 
 
BCAP29
 
 
hsa-let-7e-5p
 
 
-0.289
 
 
16.2
 
 
 
 
BDH1
 
 
hsa-miR-125a-5p
 
 
-0.389
 
 
20.7
 
 
 
 
BEGAIN
 
 
hsa-let-7e-5p
 
 
-0.466
 
 
36.9
 
 
 
 
BEGAIN
 
 
hsa-let-7c-5p
 
 
-0.466
 
 
36.9
 
 
 
 
BIN1
 
 
hsa-miR-22-3p
 
 
-0.346
 
 
167.4
 
 
 
 
BIN3
 
 
hsa-let-7e-5p
 
 
-0.427
 
 
10.6
 
 
 
 
BIN3
 
 
hsa-let-7c-5p
 
 
-0.427
 
 
10.6
 
 
 
 
BLOC1S1
 
 
hsa-miR-22-3p
 
 
-0.346
 
 
261.3
 
 
 
 
BLOC1S6
 
 
hsa-let-7e-5p
 
 
-0.321
 
 
20.1
 
 
 
 
BLOC1S6
 
 
hsa-let-7c-5p
 
 
-0.321
 
 
20.1
 
 
 
 
BMPR2
 
 
hsa-miR-99b-5p
 
 
-0.327
 
 
10.7
 
 
 
 
BTF3L4
 
 
hsa-let-7e-5p
 
 
-0.355
 
 
29.6
 
 
 
 
BTF3L4
 
 
hsa-let-7c-5p
 
 
-0.355
 
 
29.6
 
 
 
 
BZW1
 
 
hsa-let-7e-5p
 
 
-0.354
 
 
34.3
 
 
 
 
BZW1
 
 
hsa-let-7c-5p
 
 
-0.354
 
 
34.3
 
 
 
 
BZW1
 
 
hsa-let-7e-5p
 
 
-0.375
 
 
34.3
 
 
 
 
BZW1
 
 
hsa-let-7c-5p
 
 
-0.375
 
 
34.3
 
 
 
 
C19orf47
 
 
hsa-let-7e-5p
 
 
-0.311
 
 
13.3
 
 
 
 
C19orf47
 
 
hsa-let-7c-5p
 
 
-0.311
 
 
13.3
 
 
 
 
C5orf24
 
 
hsa-miR-22-3p
 
 
-0.493
 
 
17.3
 
 
 
 
CACFD1
 
 
hsa-let-7e-5p
 
 
-0.296
 
 
10.5
 
 
 
 
CACFD1
 
 
hsa-let-7c-5p
 
 
-0.296
 
 
10.5
 
 
 
 
CACNB3
 
 
hsa-miR-125a-5p
 
 
-0.393
 
 
74.6
 
 
 
 
CACNG4
 
 
hsa-let-7e-5p
 
 
-0.33
 
 
35.0
 
 
 
 
CACNG4
 
 
hsa-let-7c-5p
 
 
-0.33
 
 
35.0
 
 
 
 
CADM2
 
 
hsa-let-7e-5p
 
 
-0.294
 
 
18.3
 
 
 
 
CADM2
 
 
hsa-let-7c-5p
 
 
-0.294
 
 
18.3
 
 
 
 
CAMK2N1
 
 
hsa-miR-22-3p
 
 
-0.302
 
 
570.4
 
 
 
 
CAPN1
 
 
hsa-miR-22-3p
 
 
-0.307
 
 
25.9
 
 
 
 
CAPN3
 
 
hsa-let-7e-5p
 
 
-0.317
 
 
74.7
 
 
 
 
CAPN3
 
 
hsa-let-7c-5p
 
 
-0.317
 
 
74.7
 
 
 
 
CASP3
 
 
hsa-let-7e-5p
 
 
-0.495
 
 
10.5
 
 
 
 
CASP3
 
 
hsa-let-7c-5p
 
 
-0.503
 
 
10.5
 
 
 
 
CAST
 
 
hsa-miR-150-5p
 
 
-0.354
 
 
23.2
 
 
 
 
CBX5
 
 
hsa-let-7e-5p
 
 
-0.307
 
 
18.6
 
 
 
 
CBX5
 
 
hsa-let-7c-5p
 
 
-0.307
 
 
18.6
 
 
 
 
CBX5
 
 
hsa-let-7e-5p
 
 
-0.472
 
 
18.6
 
 
 
 
CBX5
 
 
hsa-let-7c-5p
 
 
-0.472
 
 
18.6
 
 
 
 
CCDC149
 
 
hsa-miR-22-3p
 
 
-0.327
 
 
10.0
 
 
 
 
CCDC47
 
 
hsa-miR-22-3p
 
 
-0.604
 
 
29.4
 
 
 
 
CCNY
 
 
hsa-let-7c-5p
 
 
-0.323
 
 
36.8
 
 
 
 
CCNY
 
 
hsa-let-7e-5p
 
 
-0.334
 
 
36.8
 
 
 
 
CDC34
 
 
hsa-let-7c-5p
 
 
-0.309
 
 
53.0
 
 
 
 
CDC34
 
 
hsa-let-7e-5p
 
 
-0.321
 
 
53.0
 
 
 
 
CDC34
 
 
hsa-let-7e-5p
 
 
-0.555
 
 
53.0
 
 
 
 
CDC34
 
 
hsa-let-7c-5p
 
 
-0.555
 
 
53.0
 
 
 
 
CDC37L1
 
 
hsa-miR-125a-5p
 
 
-0.401
 
 
10.7
 
 
 
 
CDC42SE1
 
 
hsa-miR-125a-5p
 
 
-0.501
 
 
23.8
 
 
 
 
CDH22
 
 
hsa-let-7e-5p
 
 
-0.32
 
 
12.8
 
 
 
 
CDH22
 
 
hsa-let-7c-5p
 
 
-0.32
 
 
12.8
 
 
 
 
CDKN1A
 
 
hsa-let-7e-5p
 
 
-0.434
 
 
34.5
 
 
 
 
CDKN1A
 
 
hsa-let-7c-5p
 
 
-0.434
 
 
34.5
 
 
 
 
 
 
 appendix 
 
 
 
 
target_symbol
 
 
mature_mirna_id
 
 
score
 
 
nTPM
 
 
 
 
 
 
ABCC5
 
 
hsa-miR-125a-5p
 
 
-0.288
 
 
15.4
 
 
 
 
ABHD3
 
 
hsa-miR-125a-5p
 
 
-0.453
 
 
23.7
 
 
 
 
ABTB1
 
 
hsa-miR-125a-5p
 
 
-0.519
 
 
17.8
 
 
 
 
ADIPOR2
 
 
hsa-miR-150-5p
 
 
-0.287
 
 
10.0
 
 
 
 
AGGF1
 
 
hsa-miR-125a-5p
 
 
-0.331
 
 
10.9
 
 
 
 
AIDA
 
 
hsa-miR-22-3p
 
 
-0.316
 
 
36.9
 
 
 
 
AIFM1
 
 
hsa-miR-125a-5p
 
 
-0.3
 
 
16.6
 
 
 
 
AIFM1
 
 
hsa-let-7e-5p
 
 
-0.314
 
 
16.6
 
 
 
 
AIFM1
 
 
hsa-let-7c-5p
 
 
-0.314
 
 
16.6
 
 
 
 
ALPL
 
 
hsa-miR-10a-5p
 
 
-0.32
 
 
26.3
 
 
 
 
ANAPC16
 
 
hsa-miR-125a-5p
 
 
-0.578
 
 
14.0
 
 
 
 
ANKRD13A
 
 
hsa-miR-22-3p
 
 
-0.478
 
 
34.4
 
 
 
 
ANPEP
 
 
hsa-miR-125a-5p
 
 
-0.372
 
 
34.6
 
 
 
 
AP1AR
 
 
hsa-miR-99b-5p
 
 
-0.823
 
 
11.9
 
 
 
 
AP1S1
 
 
hsa-let-7e-5p
 
 
-0.374
 
 
13.1
 
 
 
 
AP1S1
 
 
hsa-let-7c-5p
 
 
-0.374
 
 
13.1
 
 
 
 
ARL6IP6
 
 
hsa-let-7c-5p
 
 
-0.293
 
 
10.9
 
 
 
 
ARL6IP6
 
 
hsa-let-7e-5p
 
 
-0.305
 
 
10.9
 
 
 
 
ARMT1
 
 
hsa-let-7e-5p
 
 
-0.303
 
 
14.4
 
 
 
 
ARMT1
 
 
hsa-let-7c-5p
 
 
-0.303
 
 
14.4
 
 
 
 
ARPC5
 
 
hsa-miR-22-3p
 
 
-0.679
 
 
139.8
 
 
 
 
ARPP19
 
 
hsa-let-7e-5p
 
 
-0.492
 
 
36.4
 
 
 
 
ARPP19
 
 
hsa-let-7c-5p
 
 
-0.492
 
 
36.4
 
 
 
 
ARRB1
 
 
hsa-miR-22-3p
 
 
-0.616
 
 
20.7
 
 
 
 
ARRDC4
 
 
hsa-let-7e-5p
 
 
-0.329
 
 
15.6
 
 
 
 
ARRDC4
 
 
hsa-let-7c-5p
 
 
-0.329
 
 
15.6
 
 
 
 
ASAP1
 
 
hsa-let-7e-5p
 
 
-0.296
 
 
11.4
 
 
 
 
ASAP1
 
 
hsa-let-7c-5p
 
 
-0.308
 
 
11.4
 
 
 
 
ATOSB
 
 
hsa-let-7e-5p
 
 
-0.429
 
 
11.5
 
 
 
 
ATOSB
 
 
hsa-let-7c-5p
 
 
-0.429
 
 
11.5
 
 
 
 
ATP2A2
 
 
hsa-let-7e-5p
 
 
-0.311
 
 
54.5
 
 
 
 
ATP2A2
 
 
hsa-let-7c-5p
 
 
-0.311
 
 
54.5
 
 
 
 
ATPAF1
 
 
hsa-let-7e-5p
 
 
-0.498
 
 
26.8
 
 
 
 
ATPAF1
 
 
hsa-let-7c-5p
 
 
-0.498
 
 
26.8
 
 
 
 
ATXN2
 
 
hsa-miR-10a-5p
 
 
-0.362
 
 
10.6
 
 
 
 
B3GAT3
 
 
hsa-let-7e-5p
 
 
-0.324
 
 
16.3
 
 
 
 
B3GAT3
 
 
hsa-let-7c-5p
 
 
-0.324
 
 
16.3
 
 
 
 
BACH1
 
 
hsa-let-7e-5p
 
 
-0.298
 
 
26.1
 
 
 
 
BACH1
 
 
hsa-let-7e-5p
 
 
-0.298
 
 
26.1
 
 
 
 
BACH1
 
 
hsa-let-7c-5p
 
 
-0.306
 
 
26.1
 
 
 
 
BACH1
 
 
hsa-let-7c-5p
 
 
-0.306
 
 
26.1
 
 
 
 
BACH1
 
 
hsa-let-7e-5p
 
 
-0.435
 
 
26.1
 
 
 
 
BACH1
 
 
hsa-let-7e-5p
 
 
-0.435
 
 
26.1
 
 
 
 
BACH1
 
 
hsa-let-7c-5p
 
 
-0.435
 
 
26.1
 
 
 
 
BACH1
 
 
hsa-let-7c-5p
 
 
-0.435
 
 
26.1
 
 
 
 
BAG5
 
 
hsa-miR-22-3p
 
 
-0.318
 
 
14.3
 
 
 
 
BAK1
 
 
hsa-miR-125a-5p
 
 
-0.402
 
 
19.5
 
 
 
 
BAZ1B
 
 
hsa-miR-10a-5p
 
 
-0.309
 
 
20.9
 
 
 
 
BAZ2A
 
 
hsa-miR-99b-5p
 
 
-0.408
 
 
29.1
 
 
 
 
BBX
 
 
hsa-miR-10a-5p
 
 
-0.305
 
 
12.0
 
 
 
 
BCAP29
 
 
hsa-let-7e-5p
 
 
-0.289
 
 
21.6
 
 
 
 
BCL2L11
 
 
hsa-miR-10a-5p
 
 
-0.483
 
 
16.0
 
 
 
 
BCL2L12
 
 
hsa-miR-125a-5p
 
 
-0.288
 
 
13.8
 
 
 
 
BIN1
 
 
hsa-miR-22-3p
 
 
-0.346
 
 
24.3
 
 
 
 
BIN2
 
 
hsa-miR-125a-5p
 
 
-0.679
 
 
30.6
 
 
 
 
BIN3
 
 
hsa-let-7e-5p
 
 
-0.427
 
 
10.4
 
 
 
 
BIN3
 
 
hsa-let-7c-5p
 
 
-0.427
 
 
10.4
 
 
 
 
BLOC1S1
 
 
hsa-miR-22-3p
 
 
-0.346
 
 
78.5
 
 
 
 
BLOC1S6
 
 
hsa-let-7e-5p
 
 
-0.321
 
 
29.4
 
 
 
 
BLOC1S6
 
 
hsa-let-7c-5p
 
 
-0.321
 
 
29.4
 
 
 
 
BLZF1
 
 
hsa-miR-125a-5p
 
 
-0.461
 
 
11.7
 
 
 
 
BMF
 
 
hsa-miR-125a-5p
 
 
-0.34
 
 
11.8
 
 
 
 
BTF3L4
 
 
hsa-let-7e-5p
 
 
-0.355
 
 
28.6
 
 
 
 
BTF3L4
 
 
hsa-let-7c-5p
 
 
-0.355
 
 
28.6
 
 
 
 
BTG1
 
 
hsa-miR-22-3p
 
 
-0.468
 
 
18.7
 
 
 
 
BZW1
 
 
hsa-let-7e-5p
 
 
-0.354
 
 
91.9
 
 
 
 
BZW1
 
 
hsa-let-7c-5p
 
 
-0.354
 
 
91.9
 
 
 
 
BZW1
 
 
hsa-let-7e-5p
 
 
-0.375
 
 
91.9
 
 
 
 
BZW1
 
 
hsa-let-7c-5p
 
 
-0.375
 
 
91.9
 
 
 
 
BZW2
 
 
hsa-let-7c-5p
 
 
-0.397
 
 
25.1
 
 
 
 
BZW2
 
 
hsa-let-7e-5p
 
 
-0.408
 
 
25.1
 
 
 
 
C15orf39
 
 
hsa-miR-125a-5p
 
 
-0.335
 
 
12.0
 
 
 
 
C15orf39
 
 
hsa-let-7e-5p
 
 
-0.374
 
 
12.0
 
 
 
 
C15orf39
 
 
hsa-let-7c-5p
 
 
-0.374
 
 
12.0
 
 
 
 
C19orf38
 
 
hsa-miR-125a-5p
 
 
-0.676
 
 
10.6
 
 
 
 
C1GALT1
 
 
hsa-let-7e-5p
 
 
-0.322
 
 
13.4
 
 
 
 
C1GALT1
 
 
hsa-let-7c-5p
 
 
-0.322
 
 
13.4
 
 
 
 
C5orf24
 
 
hsa-miR-22-3p
 
 
-0.493
 
 
16.8
 
 
 
 
CANT1
 
 
hsa-let-7e-5p
 
 
-0.402
 
 
23.3
 
 
 
 
CANT1
 
 
hsa-let-7c-5p
 
 
-0.402
 
 
23.3
 
 
 
 
CAPN1
 
 
hsa-miR-22-3p
 
 
-0.307
 
 
48.9
 
 
 
 
CASP2
 
 
hsa-miR-125a-5p
 
 
-0.296
 
 
13.1
 
 
 
 
CASP3
 
 
hsa-let-7e-5p
 
 
-0.495
 
 
22.0
 
 
 
 
CASP3
 
 
hsa-let-7c-5p
 
 
-0.503
 
 
22.0
 
 
 
 
CASP5
 
 
hsa-let-7e-5p
 
 
-0.379
 
 
13.8
 
 
 
 
CASP5
 
 
hsa-let-7c-5p
 
 
-0.379
 
 
13.8
 
 
 
 
CAST
 
 
hsa-miR-150-5p
 
 
-0.354
 
 
81.2
 
 
 
 
CBX5
 
 
hsa-let-7e-5p
 
 
-0.307
 
 
11.9
 
 
 
 
CBX5
 
 
hsa-let-7c-5p
 
 
-0.307
 
 
11.9
 
 
 
 
CBX5
 
 
hsa-let-7e-5p
 
 
-0.472
 
 
11.9
 
 
 
 
CBX5
 
 
hsa-let-7c-5p
 
 
-0.472
 
 
11.9
 
 
 
 
CCDC47
 
 
hsa-miR-22-3p
 
 
-0.604
 
 
38.2
 
 
 
 
CCN1
 
 
hsa-miR-22-3p
 
 
-0.601
 
 
26.6
 
 
 
 
CCNY
 
 
hsa-let-7c-5p
 
 
-0.323
 
 
20.0
 
 
 
 
CCNY
 
 
hsa-let-7e-5p
 
 
-0.334
 
 
20.0
 
 
 
 
CCR7
 
 
hsa-let-7e-5p
 
 
-0.482
 
 
63.4
 
 
 
 
CCR7
 
 
hsa-let-7c-5p
 
 
-0.482
 
 
63.4
 
 
 
 
CD86
 
 
hsa-let-7e-5p
 
 
-0.323
 
 
32.5
 
 
 
 
CD86
 
 
hsa-let-7c-5p
 
 
-0.323
 
 
32.5
 
 
 
 
CDC34
 
 
hsa-let-7c-5p
 
 
-0.309
 
 
12.5
 
 
 
 
 
 
 blood vessel 
 
 
 
 
target_symbol
 
 
mature_mirna_id
 
 
score
 
 
nTPM
 
 
 
 
 
 
ABCC5
 
 
hsa-miR-125a-5p
 
 
-0.288
 
 
17.4
 
 
 
 
ABHD3
 
 
hsa-miR-125a-5p
 
 
-0.453
 
 
10.2
 
 
 
 
ABTB1
 
 
hsa-miR-125a-5p
 
 
-0.519
 
 
47.2
 
 
 
 
ADAMTS8
 
 
hsa-let-7e-5p
 
 
-0.571
 
 
12.7
 
 
 
 
ADAMTS8
 
 
hsa-let-7c-5p
 
 
-0.571
 
 
12.7
 
 
 
 
ADIPOR2
 
 
hsa-miR-150-5p
 
 
-0.287
 
 
23.0
 
 
 
 
AHCTF1
 
 
hsa-let-7e-5p
 
 
-0.374
 
 
10.0
 
 
 
 
AHCTF1
 
 
hsa-let-7c-5p
 
 
-0.374
 
 
10.0
 
 
 
 
AIDA
 
 
hsa-miR-22-3p
 
 
-0.316
 
 
49.9
 
 
 
 
AKAP6
 
 
hsa-let-7e-5p
 
 
-0.307
 
 
15.0
 
 
 
 
AKAP6
 
 
hsa-let-7c-5p
 
 
-0.307
 
 
15.0
 
 
 
 
AKT3
 
 
hsa-miR-22-3p
 
 
-0.451
 
 
34.5
 
 
 
 
ALDH4A1
 
 
hsa-miR-184
 
 
-0.421
 
 
15.0
 
 
 
 
ALPL
 
 
hsa-miR-10a-5p
 
 
-0.32
 
 
12.2
 
 
 
 
AMT
 
 
hsa-let-7e-5p
 
 
-0.463
 
 
11.4
 
 
 
 
AMT
 
 
hsa-let-7c-5p
 
 
-0.463
 
 
11.4
 
 
 
 
ANAPC16
 
 
hsa-miR-125a-5p
 
 
-0.578
 
 
31.1
 
 
 
 
ANKRD13A
 
 
hsa-miR-22-3p
 
 
-0.478
 
 
16.9
 
 
 
 
ANPEP
 
 
hsa-miR-125a-5p
 
 
-0.372
 
 
14.7
 
 
 
 
AP1S1
 
 
hsa-let-7e-5p
 
 
-0.374
 
 
13.8
 
 
 
 
AP1S1
 
 
hsa-let-7c-5p
 
 
-0.374
 
 
13.8
 
 
 
 
ARHGEF26
 
 
hsa-miR-22-3p
 
 
-0.374
 
 
10.5
 
 
 
 
ARL4D
 
 
hsa-let-7e-5p
 
 
-0.417
 
 
12.9
 
 
 
 
ARL4D
 
 
hsa-let-7c-5p
 
 
-0.417
 
 
12.9
 
 
 
 
ARPC5
 
 
hsa-miR-22-3p
 
 
-0.679
 
 
68.6
 
 
 
 
ARPP19
 
 
hsa-let-7e-5p
 
 
-0.492
 
 
47.3
 
 
 
 
ARPP19
 
 
hsa-let-7c-5p
 
 
-0.492
 
 
47.3
 
 
 
 
ARRB1
 
 
hsa-miR-22-3p
 
 
-0.616
 
 
10.5
 
 
 
 
ASAP1
 
 
hsa-let-7e-5p
 
 
-0.296
 
 
18.4
 
 
 
 
ASAP1
 
 
hsa-let-7c-5p
 
 
-0.308
 
 
18.4
 
 
 
 
ATOSB
 
 
hsa-let-7e-5p
 
 
-0.429
 
 
26.7
 
 
 
 
ATOSB
 
 
hsa-let-7c-5p
 
 
-0.429
 
 
26.7
 
 
 
 
ATP10D
 
 
hsa-miR-125a-5p
 
 
-0.375
 
 
11.2
 
 
 
 
ATP2A2
 
 
hsa-let-7e-5p
 
 
-0.311
 
 
84.6
 
 
 
 
ATP2A2
 
 
hsa-let-7c-5p
 
 
-0.311
 
 
84.6
 
 
 
 
ATPAF1
 
 
hsa-let-7e-5p
 
 
-0.498
 
 
30.6
 
 
 
 
ATPAF1
 
 
hsa-let-7c-5p
 
 
-0.498
 
 
30.6
 
 
 
 
ATXN2
 
 
hsa-miR-10a-5p
 
 
-0.362
 
 
25.1
 
 
 
 
B3GALNT2
 
 
hsa-miR-125a-5p
 
 
-0.311
 
 
21.4
 
 
 
 
B3GAT3
 
 
hsa-let-7e-5p
 
 
-0.324
 
 
38.8
 
 
 
 
B3GAT3
 
 
hsa-let-7c-5p
 
 
-0.324
 
 
38.8
 
 
 
 
B4GAT1
 
 
hsa-let-7e-5p
 
 
-0.47
 
 
18.5
 
 
 
 
B4GAT1
 
 
hsa-let-7c-5p
 
 
-0.47
 
 
18.5
 
 
 
 
BACE2
 
 
hsa-let-7e-5p
 
 
-0.364
 
 
11.5
 
 
 
 
BACE2
 
 
hsa-let-7c-5p
 
 
-0.364
 
 
11.5
 
 
 
 
BACH1
 
 
hsa-let-7e-5p
 
 
-0.298
 
 
10.9
 
 
 
 
BACH1
 
 
hsa-let-7e-5p
 
 
-0.298
 
 
10.9
 
 
 
 
BACH1
 
 
hsa-let-7c-5p
 
 
-0.306
 
 
10.9
 
 
 
 
BACH1
 
 
hsa-let-7c-5p
 
 
-0.306
 
 
10.9
 
 
 
 
BACH1
 
 
hsa-let-7e-5p
 
 
-0.435
 
 
10.9
 
 
 
 
BACH1
 
 
hsa-let-7e-5p
 
 
-0.435
 
 
10.9
 
 
 
 
BACH1
 
 
hsa-let-7c-5p
 
 
-0.435
 
 
10.9
 
 
 
 
BACH1
 
 
hsa-let-7c-5p
 
 
-0.435
 
 
10.9
 
 
 
 
BAG5
 
 
hsa-miR-22-3p
 
 
-0.318
 
 
13.3
 
 
 
 
BAZ1B
 
 
hsa-miR-10a-5p
 
 
-0.309
 
 
22.6
 
 
 
 
BAZ2A
 
 
hsa-miR-99b-5p
 
 
-0.408
 
 
25.1
 
 
 
 
BBX
 
 
hsa-miR-10a-5p
 
 
-0.305
 
 
15.7
 
 
 
 
BCAP29
 
 
hsa-let-7e-5p
 
 
-0.289
 
 
48.5
 
 
 
 
BIN1
 
 
hsa-miR-22-3p
 
 
-0.346
 
 
22.6
 
 
 
 
BIN3
 
 
hsa-let-7e-5p
 
 
-0.427
 
 
15.5
 
 
 
 
BIN3
 
 
hsa-let-7c-5p
 
 
-0.427
 
 
15.5
 
 
 
 
BLOC1S1
 
 
hsa-miR-22-3p
 
 
-0.346
 
 
131.0
 
 
 
 
BLOC1S6
 
 
hsa-let-7e-5p
 
 
-0.321
 
 
21.2
 
 
 
 
BLOC1S6
 
 
hsa-let-7c-5p
 
 
-0.321
 
 
21.2
 
 
 
 
BLZF1
 
 
hsa-miR-125a-5p
 
 
-0.461
 
 
14.2
 
 
 
 
BMPR2
 
 
hsa-miR-99b-5p
 
 
-0.327
 
 
16.9
 
 
 
 
BORCS6
 
 
hsa-miR-125a-5p
 
 
-0.287
 
 
10.3
 
 
 
 
BTF3L4
 
 
hsa-let-7e-5p
 
 
-0.355
 
 
27.9
 
 
 
 
BTF3L4
 
 
hsa-let-7c-5p
 
 
-0.355
 
 
27.9
 
 
 
 
BZW1
 
 
hsa-let-7e-5p
 
 
-0.354
 
 
72.0
 
 
 
 
BZW1
 
 
hsa-let-7c-5p
 
 
-0.354
 
 
72.0
 
 
 
 
BZW1
 
 
hsa-let-7e-5p
 
 
-0.375
 
 
72.0
 
 
 
 
BZW1
 
 
hsa-let-7c-5p
 
 
-0.375
 
 
72.0
 
 
 
 
BZW2
 
 
hsa-let-7c-5p
 
 
-0.397
 
 
19.3
 
 
 
 
BZW2
 
 
hsa-let-7e-5p
 
 
-0.408
 
 
19.3
 
 
 
 
C15orf39
 
 
hsa-miR-125a-5p
 
 
-0.335
 
 
28.4
 
 
 
 
C15orf39
 
 
hsa-let-7e-5p
 
 
-0.374
 
 
28.4
 
 
 
 
C15orf39
 
 
hsa-let-7c-5p
 
 
-0.374
 
 
28.4
 
 
 
 
C19orf47
 
 
hsa-let-7e-5p
 
 
-0.311
 
 
13.6
 
 
 
 
C19orf47
 
 
hsa-let-7c-5p
 
 
-0.311
 
 
13.6
 
 
 
 
C5orf24
 
 
hsa-miR-22-3p
 
 
-0.493
 
 
29.6
 
 
 
 
C6orf47
 
 
hsa-miR-125a-5p
 
 
-0.564
 
 
13.8
 
 
 
 
C8orf58
 
 
hsa-let-7e-5p
 
 
-0.612
 
 
11.3
 
 
 
 
C8orf58
 
 
hsa-let-7c-5p
 
 
-0.62
 
 
11.3
 
 
 
 
CACFD1
 
 
hsa-let-7e-5p
 
 
-0.296
 
 
11.6
 
 
 
 
CACFD1
 
 
hsa-let-7c-5p
 
 
-0.296
 
 
11.6
 
 
 
 
CACNB3
 
 
hsa-miR-125a-5p
 
 
-0.393
 
 
16.1
 
 
 
 
CAMK2N1
 
 
hsa-miR-22-3p
 
 
-0.302
 
 
14.9
 
 
 
 
CANT1
 
 
hsa-let-7e-5p
 
 
-0.402
 
 
16.6
 
 
 
 
CANT1
 
 
hsa-let-7c-5p
 
 
-0.402
 
 
16.6
 
 
 
 
CAPN1
 
 
hsa-miR-22-3p
 
 
-0.307
 
 
54.0
 
 
 
 
CAPN3
 
 
hsa-let-7e-5p
 
 
-0.317
 
 
16.3
 
 
 
 
CAPN3
 
 
hsa-let-7c-5p
 
 
-0.317
 
 
16.3
 
 
 
 
CASP3
 
 
hsa-let-7e-5p
 
 
-0.495
 
 
10.6
 
 
 
 
CASP3
 
 
hsa-let-7c-5p
 
 
-0.503
 
 
10.6
 
 
 
 
CAST
 
 
hsa-miR-150-5p
 
 
-0.354
 
 
133.6
 
 
 
 
CBX5
 
 
hsa-let-7e-5p
 
 
-0.307
 
 
10.4
 
 
 
 
CBX5
 
 
hsa-let-7c-5p
 
 
-0.307
 
 
10.4
 
 
 
 
CBX5
 
 
hsa-let-7e-5p
 
 
-0.472
 
 
10.4
 
 
 
 
CBX5
 
 
hsa-let-7c-5p
 
 
-0.472
 
 
10.4
 
 
 
 
 
 
 bone marrow 
 
 
 
 
target_symbol
 
 
mature_mirna_id
 
 
score
 
 
nTPM
 
 
 
 
 
 
ABCC5
 
 
hsa-miR-125a-5p
 
 
-0.288
 
 
21.2
 
 
 
 
ABHD3
 
 
hsa-miR-125a-5p
 
 
-0.453
 
 
16.6
 
 
 
 
ABTB1
 
 
hsa-miR-125a-5p
 
 
-0.519
 
 
48.3
 
 
 
 
ACP3
 
 
hsa-let-7e-5p
 
 
-0.298
 
 
16.5
 
 
 
 
ACP3
 
 
hsa-let-7c-5p
 
 
-0.298
 
 
16.5
 
 
 
 
ACSL6
 
 
hsa-miR-125a-5p
 
 
-0.307
 
 
21.4
 
 
 
 
ACSL6
 
 
hsa-let-7e-5p
 
 
-0.336
 
 
21.4
 
 
 
 
ACSL6
 
 
hsa-let-7c-5p
 
 
-0.336
 
 
21.4
 
 
 
 
ADRB2
 
 
hsa-let-7e-5p
 
 
-0.635
 
 
11.8
 
 
 
 
ADRB2
 
 
hsa-let-7c-5p
 
 
-0.635
 
 
11.8
 
 
 
 
AGO2
 
 
hsa-miR-125a-5p
 
 
-0.811
 
 
10.7
 
 
 
 
AGO2
 
 
hsa-miR-184
 
 
-0.831
 
 
10.7
 
 
 
 
AGO2
 
 
hsa-miR-99b-5p
 
 
-0.836
 
 
10.7
 
 
 
 
AHCTF1
 
 
hsa-let-7e-5p
 
 
-0.374
 
 
16.0
 
 
 
 
AHCTF1
 
 
hsa-let-7c-5p
 
 
-0.374
 
 
16.0
 
 
 
 
AIDA
 
 
hsa-miR-22-3p
 
 
-0.316
 
 
20.7
 
 
 
 
AIFM1
 
 
hsa-miR-125a-5p
 
 
-0.3
 
 
11.2
 
 
 
 
AIFM1
 
 
hsa-let-7e-5p
 
 
-0.314
 
 
11.2
 
 
 
 
AIFM1
 
 
hsa-let-7c-5p
 
 
-0.314
 
 
11.2
 
 
 
 
ALDH4A1
 
 
hsa-miR-184
 
 
-0.421
 
 
11.1
 
 
 
 
ANAPC16
 
 
hsa-miR-125a-5p
 
 
-0.578
 
 
13.0
 
 
 
 
ANKRD13A
 
 
hsa-miR-22-3p
 
 
-0.478
 
 
34.0
 
 
 
 
ANPEP
 
 
hsa-miR-125a-5p
 
 
-0.372
 
 
97.7
 
 
 
 
ARID3B
 
 
hsa-let-7e-5p
 
 
-0.31
 
 
23.2
 
 
 
 
ARID3B
 
 
hsa-let-7c-5p
 
 
-0.31
 
 
23.2
 
 
 
 
ARID3B
 
 
hsa-let-7e-5p
 
 
-0.486
 
 
23.2
 
 
 
 
ARID3B
 
 
hsa-let-7c-5p
 
 
-0.486
 
 
23.2
 
 
 
 
ARL6IP6
 
 
hsa-let-7c-5p
 
 
-0.293
 
 
23.4
 
 
 
 
ARL6IP6
 
 
hsa-let-7e-5p
 
 
-0.305
 
 
23.4
 
 
 
 
ARPC5
 
 
hsa-miR-22-3p
 
 
-0.679
 
 
65.3
 
 
 
 
ARPP19
 
 
hsa-let-7e-5p
 
 
-0.492
 
 
97.5
 
 
 
 
ARPP19
 
 
hsa-let-7c-5p
 
 
-0.492
 
 
97.5
 
 
 
 
ARRB1
 
 
hsa-miR-22-3p
 
 
-0.616
 
 
14.8
 
 
 
 
ARRDC4
 
 
hsa-let-7e-5p
 
 
-0.329
 
 
10.8
 
 
 
 
ARRDC4
 
 
hsa-let-7c-5p
 
 
-0.329
 
 
10.8
 
 
 
 
ASAP1
 
 
hsa-let-7e-5p
 
 
-0.296
 
 
44.7
 
 
 
 
ASAP1
 
 
hsa-let-7c-5p
 
 
-0.308
 
 
44.7
 
 
 
 
ATOSB
 
 
hsa-let-7e-5p
 
 
-0.429
 
 
36.6
 
 
 
 
ATOSB
 
 
hsa-let-7c-5p
 
 
-0.429
 
 
36.6
 
 
 
 
ATP2A2
 
 
hsa-let-7e-5p
 
 
-0.311
 
 
43.4
 
 
 
 
ATP2A2
 
 
hsa-let-7c-5p
 
 
-0.311
 
 
43.4
 
 
 
 
ATP8A1
 
 
hsa-miR-22-3p
 
 
-0.468
 
 
22.1
 
 
 
 
ATP8B4
 
 
hsa-let-7e-5p
 
 
-0.325
 
 
73.7
 
 
 
 
ATP8B4
 
 
hsa-let-7c-5p
 
 
-0.325
 
 
73.7
 
 
 
 
ATPAF1
 
 
hsa-let-7e-5p
 
 
-0.498
 
 
26.0
 
 
 
 
ATPAF1
 
 
hsa-let-7c-5p
 
 
-0.498
 
 
26.0
 
 
 
 
B3GAT3
 
 
hsa-let-7e-5p
 
 
-0.324
 
 
17.5
 
 
 
 
B3GAT3
 
 
hsa-let-7c-5p
 
 
-0.324
 
 
17.5
 
 
 
 
BACH1
 
 
hsa-let-7e-5p
 
 
-0.298
 
 
84.3
 
 
 
 
BACH1
 
 
hsa-let-7e-5p
 
 
-0.298
 
 
84.3
 
 
 
 
BACH1
 
 
hsa-let-7c-5p
 
 
-0.306
 
 
84.3
 
 
 
 
BACH1
 
 
hsa-let-7c-5p
 
 
-0.306
 
 
84.3
 
 
 
 
BACH1
 
 
hsa-let-7e-5p
 
 
-0.435
 
 
84.3
 
 
 
 
BACH1
 
 
hsa-let-7e-5p
 
 
-0.435
 
 
84.3
 
 
 
 
BACH1
 
 
hsa-let-7c-5p
 
 
-0.435
 
 
84.3
 
 
 
 
BACH1
 
 
hsa-let-7c-5p
 
 
-0.435
 
 
84.3
 
 
 
 
BAG5
 
 
hsa-miR-22-3p
 
 
-0.318
 
 
26.2
 
 
 
 
BAK1
 
 
hsa-miR-125a-5p
 
 
-0.402
 
 
26.1
 
 
 
 
BAZ1B
 
 
hsa-miR-10a-5p
 
 
-0.309
 
 
23.3
 
 
 
 
BAZ2A
 
 
hsa-miR-99b-5p
 
 
-0.408
 
 
62.3
 
 
 
 
BBX
 
 
hsa-miR-10a-5p
 
 
-0.305
 
 
17.4
 
 
 
 
BCAP29
 
 
hsa-let-7e-5p
 
 
-0.289
 
 
16.8
 
 
 
 
BCL2L11
 
 
hsa-miR-10a-5p
 
 
-0.483
 
 
37.8
 
 
 
 
BCL2L12
 
 
hsa-miR-125a-5p
 
 
-0.288
 
 
17.2
 
 
 
 
BIN1
 
 
hsa-miR-22-3p
 
 
-0.346
 
 
11.4
 
 
 
 
BIN2
 
 
hsa-miR-125a-5p
 
 
-0.679
 
 
95.7
 
 
 
 
BIN3
 
 
hsa-let-7e-5p
 
 
-0.427
 
 
40.9
 
 
 
 
BIN3
 
 
hsa-let-7c-5p
 
 
-0.427
 
 
40.9
 
 
 
 
BLOC1S1
 
 
hsa-miR-22-3p
 
 
-0.346
 
 
216.6
 
 
 
 
BLOC1S6
 
 
hsa-let-7e-5p
 
 
-0.321
 
 
54.9
 
 
 
 
BLOC1S6
 
 
hsa-let-7c-5p
 
 
-0.321
 
 
54.9
 
 
 
 
BLZF1
 
 
hsa-miR-125a-5p
 
 
-0.461
 
 
23.9
 
 
 
 
BMF
 
 
hsa-miR-125a-5p
 
 
-0.34
 
 
31.1
 
 
 
 
BTF3L4
 
 
hsa-let-7e-5p
 
 
-0.355
 
 
57.6
 
 
 
 
BTF3L4
 
 
hsa-let-7c-5p
 
 
-0.355
 
 
57.6
 
 
 
 
BTG1
 
 
hsa-miR-22-3p
 
 
-0.468
 
 
88.7
 
 
 
 
BZW1
 
 
hsa-let-7e-5p
 
 
-0.354
 
 
207.1
 
 
 
 
BZW1
 
 
hsa-let-7c-5p
 
 
-0.354
 
 
207.1
 
 
 
 
BZW1
 
 
hsa-let-7e-5p
 
 
-0.375
 
 
207.1
 
 
 
 
BZW1
 
 
hsa-let-7c-5p
 
 
-0.375
 
 
207.1
 
 
 
 
BZW2
 
 
hsa-let-7c-5p
 
 
-0.397
 
 
43.1
 
 
 
 
BZW2
 
 
hsa-let-7e-5p
 
 
-0.408
 
 
43.1
 
 
 
 
C15orf39
 
 
hsa-miR-125a-5p
 
 
-0.335
 
 
58.4
 
 
 
 
C15orf39
 
 
hsa-let-7e-5p
 
 
-0.374
 
 
58.4
 
 
 
 
C15orf39
 
 
hsa-let-7c-5p
 
 
-0.374
 
 
58.4
 
 
 
 
C19orf38
 
 
hsa-miR-125a-5p
 
 
-0.676
 
 
92.6
 
 
 
 
C1GALT1
 
 
hsa-let-7e-5p
 
 
-0.322
 
 
35.6
 
 
 
 
C1GALT1
 
 
hsa-let-7c-5p
 
 
-0.322
 
 
35.6
 
 
 
 
C5orf24
 
 
hsa-miR-22-3p
 
 
-0.493
 
 
23.5
 
 
 
 
CANT1
 
 
hsa-let-7e-5p
 
 
-0.402
 
 
18.0
 
 
 
 
CANT1
 
 
hsa-let-7c-5p
 
 
-0.402
 
 
18.0
 
 
 
 
CAPN1
 
 
hsa-miR-22-3p
 
 
-0.307
 
 
62.8
 
 
 
 
CAPN3
 
 
hsa-let-7e-5p
 
 
-0.317
 
 
45.0
 
 
 
 
CAPN3
 
 
hsa-let-7c-5p
 
 
-0.317
 
 
45.0
 
 
 
 
CASP2
 
 
hsa-miR-125a-5p
 
 
-0.296
 
 
37.9
 
 
 
 
CASP3
 
 
hsa-let-7e-5p
 
 
-0.495
 
 
13.3
 
 
 
 
CASP3
 
 
hsa-let-7c-5p
 
 
-0.503
 
 
13.3
 
 
 
 
CAST
 
 
hsa-miR-150-5p
 
 
-0.354
 
 
112.3
 
 
 
 
CBX5
 
 
hsa-let-7e-5p
 
 
-0.307
 
 
16.2
 
 
 
 
CBX5
 
 
hsa-let-7c-5p
 
 
-0.307
 
 
16.2
 
 
 
 
 
 
 breast 
 
 
 
 
target_symbol
 
 
mature_mirna_id
 
 
score
 
 
nTPM
 
 
 
 
 
 
ABCC5
 
 
hsa-miR-125a-5p
 
 
-0.288
 
 
12.8
 
 
 
 
ABHD17C
 
 
hsa-let-7e-5p
 
 
-0.359
 
 
10.9
 
 
 
 
ABHD17C
 
 
hsa-let-7c-5p
 
 
-0.359
 
 
10.9
 
 
 
 
ABHD3
 
 
hsa-miR-125a-5p
 
 
-0.453
 
 
14.7
 
 
 
 
ABTB1
 
 
hsa-miR-125a-5p
 
 
-0.519
 
 
33.9
 
 
 
 
ADIPOR2
 
 
hsa-miR-150-5p
 
 
-0.287
 
 
44.0
 
 
 
 
ADRB2
 
 
hsa-let-7e-5p
 
 
-0.635
 
 
32.4
 
 
 
 
ADRB2
 
 
hsa-let-7c-5p
 
 
-0.635
 
 
32.4
 
 
 
 
AIDA
 
 
hsa-miR-22-3p
 
 
-0.316
 
 
39.1
 
 
 
 
AIFM1
 
 
hsa-miR-125a-5p
 
 
-0.3
 
 
11.7
 
 
 
 
AIFM1
 
 
hsa-let-7e-5p
 
 
-0.314
 
 
11.7
 
 
 
 
AIFM1
 
 
hsa-let-7c-5p
 
 
-0.314
 
 
11.7
 
 
 
 
AKT3
 
 
hsa-miR-22-3p
 
 
-0.451
 
 
13.8
 
 
 
 
ALDH4A1
 
 
hsa-miR-184
 
 
-0.421
 
 
22.5
 
 
 
 
ALPL
 
 
hsa-miR-10a-5p
 
 
-0.32
 
 
21.4
 
 
 
 
ANAPC16
 
 
hsa-miR-125a-5p
 
 
-0.578
 
 
20.2
 
 
 
 
ANKRD13A
 
 
hsa-miR-22-3p
 
 
-0.478
 
 
15.4
 
 
 
 
ANKRD46
 
 
hsa-let-7e-5p
 
 
-0.389
 
 
13.6
 
 
 
 
ANKRD46
 
 
hsa-let-7c-5p
 
 
-0.389
 
 
13.6
 
 
 
 
ANPEP
 
 
hsa-miR-125a-5p
 
 
-0.372
 
 
49.7
 
 
 
 
AP1AR
 
 
hsa-miR-99b-5p
 
 
-0.823
 
 
15.9
 
 
 
 
AP1S1
 
 
hsa-let-7e-5p
 
 
-0.374
 
 
17.7
 
 
 
 
AP1S1
 
 
hsa-let-7c-5p
 
 
-0.374
 
 
17.7
 
 
 
 
ARL6IP6
 
 
hsa-let-7c-5p
 
 
-0.293
 
 
12.8
 
 
 
 
ARL6IP6
 
 
hsa-let-7e-5p
 
 
-0.305
 
 
12.8
 
 
 
 
ARMT1
 
 
hsa-let-7e-5p
 
 
-0.303
 
 
23.4
 
 
 
 
ARMT1
 
 
hsa-let-7c-5p
 
 
-0.303
 
 
23.4
 
 
 
 
ARPC5
 
 
hsa-miR-22-3p
 
 
-0.679
 
 
45.7
 
 
 
 
ARPP19
 
 
hsa-let-7e-5p
 
 
-0.492
 
 
43.2
 
 
 
 
ARPP19
 
 
hsa-let-7c-5p
 
 
-0.492
 
 
43.2
 
 
 
 
ARRB1
 
 
hsa-miR-22-3p
 
 
-0.616
 
 
22.5
 
 
 
 
ARRDC4
 
 
hsa-let-7e-5p
 
 
-0.329
 
 
18.6
 
 
 
 
ARRDC4
 
 
hsa-let-7c-5p
 
 
-0.329
 
 
18.6
 
 
 
 
ASAP1
 
 
hsa-let-7e-5p
 
 
-0.296
 
 
22.2
 
 
 
 
ASAP1
 
 
hsa-let-7c-5p
 
 
-0.308
 
 
22.2
 
 
 
 
ATOSB
 
 
hsa-let-7e-5p
 
 
-0.429
 
 
10.1
 
 
 
 
ATOSB
 
 
hsa-let-7c-5p
 
 
-0.429
 
 
10.1
 
 
 
 
ATP2A2
 
 
hsa-let-7e-5p
 
 
-0.311
 
 
28.6
 
 
 
 
ATP2A2
 
 
hsa-let-7c-5p
 
 
-0.311
 
 
28.6
 
 
 
 
ATPAF1
 
 
hsa-let-7e-5p
 
 
-0.498
 
 
33.1
 
 
 
 
ATPAF1
 
 
hsa-let-7c-5p
 
 
-0.498
 
 
33.1
 
 
 
 
ATXN2
 
 
hsa-miR-10a-5p
 
 
-0.362
 
 
16.9
 
 
 
 
B3GAT3
 
 
hsa-let-7e-5p
 
 
-0.324
 
 
29.8
 
 
 
 
B3GAT3
 
 
hsa-let-7c-5p
 
 
-0.324
 
 
29.8
 
 
 
 
B4GAT1
 
 
hsa-let-7e-5p
 
 
-0.47
 
 
15.6
 
 
 
 
B4GAT1
 
 
hsa-let-7c-5p
 
 
-0.47
 
 
15.6
 
 
 
 
BACH1
 
 
hsa-let-7e-5p
 
 
-0.298
 
 
12.3
 
 
 
 
BACH1
 
 
hsa-let-7e-5p
 
 
-0.298
 
 
12.3
 
 
 
 
BACH1
 
 
hsa-let-7c-5p
 
 
-0.306
 
 
12.3
 
 
 
 
BACH1
 
 
hsa-let-7c-5p
 
 
-0.306
 
 
12.3
 
 
 
 
BACH1
 
 
hsa-let-7e-5p
 
 
-0.435
 
 
12.3
 
 
 
 
BACH1
 
 
hsa-let-7e-5p
 
 
-0.435
 
 
12.3
 
 
 
 
BACH1
 
 
hsa-let-7c-5p
 
 
-0.435
 
 
12.3
 
 
 
 
BACH1
 
 
hsa-let-7c-5p
 
 
-0.435
 
 
12.3
 
 
 
 
BAG5
 
 
hsa-miR-22-3p
 
 
-0.318
 
 
17.5
 
 
 
 
BAZ1B
 
 
hsa-miR-10a-5p
 
 
-0.309
 
 
15.9
 
 
 
 
BAZ2A
 
 
hsa-miR-99b-5p
 
 
-0.408
 
 
25.2
 
 
 
 
BBX
 
 
hsa-miR-10a-5p
 
 
-0.305
 
 
13.5
 
 
 
 
BCAP29
 
 
hsa-let-7e-5p
 
 
-0.289
 
 
23.7
 
 
 
 
BCL2L11
 
 
hsa-miR-10a-5p
 
 
-0.483
 
 
11.8
 
 
 
 
BIN1
 
 
hsa-miR-22-3p
 
 
-0.346
 
 
35.8
 
 
 
 
BIN3
 
 
hsa-let-7e-5p
 
 
-0.427
 
 
19.3
 
 
 
 
BIN3
 
 
hsa-let-7c-5p
 
 
-0.427
 
 
19.3
 
 
 
 
BLOC1S1
 
 
hsa-miR-22-3p
 
 
-0.346
 
 
110.5
 
 
 
 
BLOC1S6
 
 
hsa-let-7e-5p
 
 
-0.321
 
 
23.9
 
 
 
 
BLOC1S6
 
 
hsa-let-7c-5p
 
 
-0.321
 
 
23.9
 
 
 
 
BLZF1
 
 
hsa-miR-125a-5p
 
 
-0.461
 
 
18.1
 
 
 
 
BMPR2
 
 
hsa-miR-99b-5p
 
 
-0.327
 
 
10.7
 
 
 
 
BTF3L4
 
 
hsa-let-7e-5p
 
 
-0.355
 
 
40.8
 
 
 
 
BTF3L4
 
 
hsa-let-7c-5p
 
 
-0.355
 
 
40.8
 
 
 
 
BTG1
 
 
hsa-miR-22-3p
 
 
-0.468
 
 
14.4
 
 
 
 
BZW1
 
 
hsa-let-7e-5p
 
 
-0.354
 
 
83.1
 
 
 
 
BZW1
 
 
hsa-let-7c-5p
 
 
-0.354
 
 
83.1
 
 
 
 
BZW1
 
 
hsa-let-7e-5p
 
 
-0.375
 
 
83.1
 
 
 
 
BZW1
 
 
hsa-let-7c-5p
 
 
-0.375
 
 
83.1
 
 
 
 
BZW2
 
 
hsa-let-7c-5p
 
 
-0.397
 
 
46.4
 
 
 
 
BZW2
 
 
hsa-let-7e-5p
 
 
-0.408
 
 
46.4
 
 
 
 
C15orf39
 
 
hsa-miR-125a-5p
 
 
-0.335
 
 
13.1
 
 
 
 
C15orf39
 
 
hsa-let-7e-5p
 
 
-0.374
 
 
13.1
 
 
 
 
C15orf39
 
 
hsa-let-7c-5p
 
 
-0.374
 
 
13.1
 
 
 
 
C1GALT1
 
 
hsa-let-7e-5p
 
 
-0.322
 
 
15.5
 
 
 
 
C1GALT1
 
 
hsa-let-7c-5p
 
 
-0.322
 
 
15.5
 
 
 
 
C1orf210
 
 
hsa-miR-125a-5p
 
 
-0.584
 
 
13.5
 
 
 
 
C5orf24
 
 
hsa-miR-22-3p
 
 
-0.493
 
 
18.9
 
 
 
 
C6orf47
 
 
hsa-miR-125a-5p
 
 
-0.564
 
 
10.2
 
 
 
 
C8orf58
 
 
hsa-let-7e-5p
 
 
-0.612
 
 
10.3
 
 
 
 
C8orf58
 
 
hsa-let-7c-5p
 
 
-0.62
 
 
10.3
 
 
 
 
CAMK2N1
 
 
hsa-miR-22-3p
 
 
-0.302
 
 
18.6
 
 
 
 
CANT1
 
 
hsa-let-7e-5p
 
 
-0.402
 
 
23.7
 
 
 
 
CANT1
 
 
hsa-let-7c-5p
 
 
-0.402
 
 
23.7
 
 
 
 
CAPN1
 
 
hsa-miR-22-3p
 
 
-0.307
 
 
46.1
 
 
 
 
CAPN3
 
 
hsa-let-7e-5p
 
 
-0.317
 
 
14.5
 
 
 
 
CAPN3
 
 
hsa-let-7c-5p
 
 
-0.317
 
 
14.5
 
 
 
 
CASP2
 
 
hsa-miR-125a-5p
 
 
-0.296
 
 
10.1
 
 
 
 
CASP3
 
 
hsa-let-7e-5p
 
 
-0.495
 
 
12.5
 
 
 
 
CASP3
 
 
hsa-let-7c-5p
 
 
-0.503
 
 
12.5
 
 
 
 
CAST
 
 
hsa-miR-150-5p
 
 
-0.354
 
 
86.9
 
 
 
 
CBX5
 
 
hsa-let-7e-5p
 
 
-0.307
 
 
12.9
 
 
 
 
CBX5
 
 
hsa-let-7c-5p
 
 
-0.307
 
 
12.9
 
 
 
 
CBX5
 
 
hsa-let-7e-5p
 
 
-0.472
 
 
12.9
 
 
 
 
 
 
 cervix 
 
 
 
 
target_symbol
 
 
mature_mirna_id
 
 
score
 
 
nTPM
 
 
 
 
 
 
ABCC5
 
 
hsa-miR-125a-5p
 
 
-0.288
 
 
31.4
 
 
 
 
ABHD17C
 
 
hsa-let-7e-5p
 
 
-0.359
 
 
18.1
 
 
 
 
ABHD17C
 
 
hsa-let-7c-5p
 
 
-0.359
 
 
18.1
 
 
 
 
ABHD3
 
 
hsa-miR-125a-5p
 
 
-0.453
 
 
10.5
 
 
 
 
ABTB1
 
 
hsa-miR-125a-5p
 
 
-0.519
 
 
42.5
 
 
 
 
ACP3
 
 
hsa-let-7e-5p
 
 
-0.298
 
 
10.9
 
 
 
 
ACP3
 
 
hsa-let-7c-5p
 
 
-0.298
 
 
10.9
 
 
 
 
ADIPOR2
 
 
hsa-miR-150-5p
 
 
-0.287
 
 
25.7
 
 
 
 
ADRB2
 
 
hsa-let-7e-5p
 
 
-0.635
 
 
12.7
 
 
 
 
ADRB2
 
 
hsa-let-7c-5p
 
 
-0.635
 
 
12.7
 
 
 
 
AIDA
 
 
hsa-miR-22-3p
 
 
-0.316
 
 
44.9
 
 
 
 
AIFM1
 
 
hsa-miR-125a-5p
 
 
-0.3
 
 
14.8
 
 
 
 
AIFM1
 
 
hsa-let-7e-5p
 
 
-0.314
 
 
14.8
 
 
 
 
AIFM1
 
 
hsa-let-7c-5p
 
 
-0.314
 
 
14.8
 
 
 
 
AKT3
 
 
hsa-miR-22-3p
 
 
-0.451
 
 
15.6
 
 
 
 
ALDH4A1
 
 
hsa-miR-184
 
 
-0.421
 
 
15.0
 
 
 
 
AMT
 
 
hsa-let-7e-5p
 
 
-0.463
 
 
14.5
 
 
 
 
AMT
 
 
hsa-let-7c-5p
 
 
-0.463
 
 
14.5
 
 
 
 
ANAPC16
 
 
hsa-miR-125a-5p
 
 
-0.578
 
 
18.3
 
 
 
 
ANKRD13A
 
 
hsa-miR-22-3p
 
 
-0.478
 
 
13.2
 
 
 
 
ANKRD46
 
 
hsa-let-7e-5p
 
 
-0.389
 
 
14.1
 
 
 
 
ANKRD46
 
 
hsa-let-7c-5p
 
 
-0.389
 
 
14.1
 
 
 
 
ANPEP
 
 
hsa-miR-125a-5p
 
 
-0.372
 
 
38.9
 
 
 
 
AP1S1
 
 
hsa-let-7e-5p
 
 
-0.374
 
 
20.1
 
 
 
 
AP1S1
 
 
hsa-let-7c-5p
 
 
-0.374
 
 
20.1
 
 
 
 
ARG2
 
 
hsa-let-7e-5p
 
 
-0.468
 
 
16.0
 
 
 
 
ARG2
 
 
hsa-let-7c-5p
 
 
-0.468
 
 
16.0
 
 
 
 
ARL4D
 
 
hsa-let-7e-5p
 
 
-0.417
 
 
27.6
 
 
 
 
ARL4D
 
 
hsa-let-7c-5p
 
 
-0.417
 
 
27.6
 
 
 
 
ARMT1
 
 
hsa-let-7e-5p
 
 
-0.303
 
 
15.1
 
 
 
 
ARMT1
 
 
hsa-let-7c-5p
 
 
-0.303
 
 
15.1
 
 
 
 
ARPC5
 
 
hsa-miR-22-3p
 
 
-0.679
 
 
45.8
 
 
 
 
ARPP19
 
 
hsa-let-7e-5p
 
 
-0.492
 
 
35.8
 
 
 
 
ARPP19
 
 
hsa-let-7c-5p
 
 
-0.492
 
 
35.8
 
 
 
 
ARRB1
 
 
hsa-miR-22-3p
 
 
-0.616
 
 
10.1
 
 
 
 
ARRDC4
 
 
hsa-let-7e-5p
 
 
-0.329
 
 
14.3
 
 
 
 
ARRDC4
 
 
hsa-let-7c-5p
 
 
-0.329
 
 
14.3
 
 
 
 
ASAP1
 
 
hsa-let-7e-5p
 
 
-0.296
 
 
15.1
 
 
 
 
ASAP1
 
 
hsa-let-7c-5p
 
 
-0.308
 
 
15.1
 
 
 
 
ATOSB
 
 
hsa-let-7e-5p
 
 
-0.429
 
 
12.3
 
 
 
 
ATOSB
 
 
hsa-let-7c-5p
 
 
-0.429
 
 
12.3
 
 
 
 
ATP2A2
 
 
hsa-let-7e-5p
 
 
-0.311
 
 
40.8
 
 
 
 
ATP2A2
 
 
hsa-let-7c-5p
 
 
-0.311
 
 
40.8
 
 
 
 
ATPAF1
 
 
hsa-let-7e-5p
 
 
-0.498
 
 
33.4
 
 
 
 
ATPAF1
 
 
hsa-let-7c-5p
 
 
-0.498
 
 
33.4
 
 
 
 
ATXN2
 
 
hsa-miR-10a-5p
 
 
-0.362
 
 
19.5
 
 
 
 
B3GAT3
 
 
hsa-let-7e-5p
 
 
-0.324
 
 
36.2
 
 
 
 
B3GAT3
 
 
hsa-let-7c-5p
 
 
-0.324
 
 
36.2
 
 
 
 
B4GAT1
 
 
hsa-let-7e-5p
 
 
-0.47
 
 
25.3
 
 
 
 
B4GAT1
 
 
hsa-let-7c-5p
 
 
-0.47
 
 
25.3
 
 
 
 
BACH1
 
 
hsa-let-7e-5p
 
 
-0.298
 
 
17.0
 
 
 
 
BACH1
 
 
hsa-let-7e-5p
 
 
-0.298
 
 
17.0
 
 
 
 
BACH1
 
 
hsa-let-7c-5p
 
 
-0.306
 
 
17.0
 
 
 
 
BACH1
 
 
hsa-let-7c-5p
 
 
-0.306
 
 
17.0
 
 
 
 
BACH1
 
 
hsa-let-7e-5p
 
 
-0.435
 
 
17.0
 
 
 
 
BACH1
 
 
hsa-let-7e-5p
 
 
-0.435
 
 
17.0
 
 
 
 
BACH1
 
 
hsa-let-7c-5p
 
 
-0.435
 
 
17.0
 
 
 
 
BACH1
 
 
hsa-let-7c-5p
 
 
-0.435
 
 
17.0
 
 
 
 
BAG5
 
 
hsa-miR-22-3p
 
 
-0.318
 
 
13.9
 
 
 
 
BAK1
 
 
hsa-miR-125a-5p
 
 
-0.402
 
 
10.0
 
 
 
 
BAZ1B
 
 
hsa-miR-10a-5p
 
 
-0.309
 
 
21.1
 
 
 
 
BAZ2A
 
 
hsa-miR-99b-5p
 
 
-0.408
 
 
25.9
 
 
 
 
BBX
 
 
hsa-miR-10a-5p
 
 
-0.305
 
 
16.2
 
 
 
 
BCAP29
 
 
hsa-let-7e-5p
 
 
-0.289
 
 
47.0
 
 
 
 
BDH1
 
 
hsa-miR-125a-5p
 
 
-0.389
 
 
10.5
 
 
 
 
BIN1
 
 
hsa-miR-22-3p
 
 
-0.346
 
 
42.5
 
 
 
 
BIN3
 
 
hsa-let-7e-5p
 
 
-0.427
 
 
13.1
 
 
 
 
BIN3
 
 
hsa-let-7c-5p
 
 
-0.427
 
 
13.1
 
 
 
 
BLOC1S1
 
 
hsa-miR-22-3p
 
 
-0.346
 
 
100.3
 
 
 
 
BLOC1S6
 
 
hsa-let-7e-5p
 
 
-0.321
 
 
24.2
 
 
 
 
BLOC1S6
 
 
hsa-let-7c-5p
 
 
-0.321
 
 
24.2
 
 
 
 
BLZF1
 
 
hsa-miR-125a-5p
 
 
-0.461
 
 
14.6
 
 
 
 
BMF
 
 
hsa-miR-125a-5p
 
 
-0.34
 
 
12.5
 
 
 
 
BMPR2
 
 
hsa-miR-99b-5p
 
 
-0.327
 
 
15.5
 
 
 
 
BTF3L4
 
 
hsa-let-7e-5p
 
 
-0.355
 
 
29.5
 
 
 
 
BTF3L4
 
 
hsa-let-7c-5p
 
 
-0.355
 
 
29.5
 
 
 
 
BZW1
 
 
hsa-let-7e-5p
 
 
-0.354
 
 
61.6
 
 
 
 
BZW1
 
 
hsa-let-7c-5p
 
 
-0.354
 
 
61.6
 
 
 
 
BZW1
 
 
hsa-let-7e-5p
 
 
-0.375
 
 
61.6
 
 
 
 
BZW1
 
 
hsa-let-7c-5p
 
 
-0.375
 
 
61.6
 
 
 
 
BZW2
 
 
hsa-let-7c-5p
 
 
-0.397
 
 
22.5
 
 
 
 
BZW2
 
 
hsa-let-7e-5p
 
 
-0.408
 
 
22.5
 
 
 
 
C15orf39
 
 
hsa-miR-125a-5p
 
 
-0.335
 
 
12.8
 
 
 
 
C15orf39
 
 
hsa-let-7e-5p
 
 
-0.374
 
 
12.8
 
 
 
 
C15orf39
 
 
hsa-let-7c-5p
 
 
-0.374
 
 
12.8
 
 
 
 
C1GALT1
 
 
hsa-let-7e-5p
 
 
-0.322
 
 
17.4
 
 
 
 
C1GALT1
 
 
hsa-let-7c-5p
 
 
-0.322
 
 
17.4
 
 
 
 
C2orf88
 
 
hsa-let-7e-5p
 
 
-0.429
 
 
45.4
 
 
 
 
C2orf88
 
 
hsa-let-7c-5p
 
 
-0.429
 
 
45.4
 
 
 
 
C5orf24
 
 
hsa-miR-22-3p
 
 
-0.493
 
 
19.0
 
 
 
 
C6orf47
 
 
hsa-miR-125a-5p
 
 
-0.564
 
 
12.2
 
 
 
 
CACNB3
 
 
hsa-miR-125a-5p
 
 
-0.393
 
 
33.5
 
 
 
 
CAMK2N1
 
 
hsa-miR-22-3p
 
 
-0.302
 
 
26.9
 
 
 
 
CANT1
 
 
hsa-let-7e-5p
 
 
-0.402
 
 
30.3
 
 
 
 
CANT1
 
 
hsa-let-7c-5p
 
 
-0.402
 
 
30.3
 
 
 
 
CAPN1
 
 
hsa-miR-22-3p
 
 
-0.307
 
 
97.7
 
 
 
 
CAPN3
 
 
hsa-let-7e-5p
 
 
-0.317
 
 
18.4
 
 
 
 
CAPN3
 
 
hsa-let-7c-5p
 
 
-0.317
 
 
18.4
 
 
 
 
CASP2
 
 
hsa-miR-125a-5p
 
 
-0.296
 
 
11.2
 
 
 
 
CASP3
 
 
hsa-let-7e-5p
 
 
-0.495
 
 
11.5
 
 
 
 
 
 
 choroid plexus 
 
 
 
 
target_symbol
 
 
mature_mirna_id
 
 
score
 
 
nTPM
 
 
 
 
 
 
ABCC5
 
 
hsa-miR-125a-5p
 
 
-0.288
 
 
137.7
 
 
 
 
ABHD3
 
 
hsa-miR-125a-5p
 
 
-0.453
 
 
24.1
 
 
 
 
ABHD6
 
 
hsa-miR-125a-5p
 
 
-0.501
 
 
15.1
 
 
 
 
ABTB1
 
 
hsa-miR-125a-5p
 
 
-0.519
 
 
29.8
 
 
 
 
ADIPOR2
 
 
hsa-miR-150-5p
 
 
-0.287
 
 
17.0
 
 
 
 
AIDA
 
 
hsa-miR-22-3p
 
 
-0.316
 
 
24.6
 
 
 
 
AIFM1
 
 
hsa-miR-125a-5p
 
 
-0.3
 
 
37.9
 
 
 
 
AIFM1
 
 
hsa-let-7e-5p
 
 
-0.314
 
 
37.9
 
 
 
 
AIFM1
 
 
hsa-let-7c-5p
 
 
-0.314
 
 
37.9
 
 
 
 
ALDH4A1
 
 
hsa-miR-184
 
 
-0.421
 
 
121.5
 
 
 
 
ALPL
 
 
hsa-miR-10a-5p
 
 
-0.32
 
 
17.0
 
 
 
 
AMT
 
 
hsa-let-7e-5p
 
 
-0.463
 
 
33.4
 
 
 
 
AMT
 
 
hsa-let-7c-5p
 
 
-0.463
 
 
33.4
 
 
 
 
ANAPC16
 
 
hsa-miR-125a-5p
 
 
-0.578
 
 
16.8
 
 
 
 
ANKHD1-EIF4EBP3
 
 
hsa-miR-22-3p
 
 
-0.479
 
 
11.6
 
 
 
 
ANKRD46
 
 
hsa-let-7e-5p
 
 
-0.389
 
 
11.0
 
 
 
 
ANKRD46
 
 
hsa-let-7c-5p
 
 
-0.389
 
 
11.0
 
 
 
 
AP1S1
 
 
hsa-let-7e-5p
 
 
-0.374
 
 
10.3
 
 
 
 
AP1S1
 
 
hsa-let-7c-5p
 
 
-0.374
 
 
10.3
 
 
 
 
ARG2
 
 
hsa-let-7e-5p
 
 
-0.468
 
 
49.7
 
 
 
 
ARG2
 
 
hsa-let-7c-5p
 
 
-0.468
 
 
49.7
 
 
 
 
ARL4D
 
 
hsa-let-7e-5p
 
 
-0.417
 
 
15.4
 
 
 
 
ARL4D
 
 
hsa-let-7c-5p
 
 
-0.417
 
 
15.4
 
 
 
 
ARPC5
 
 
hsa-miR-22-3p
 
 
-0.679
 
 
17.2
 
 
 
 
ARPP19
 
 
hsa-let-7e-5p
 
 
-0.492
 
 
32.4
 
 
 
 
ARPP19
 
 
hsa-let-7c-5p
 
 
-0.492
 
 
32.4
 
 
 
 
ATOSB
 
 
hsa-let-7e-5p
 
 
-0.429
 
 
16.6
 
 
 
 
ATOSB
 
 
hsa-let-7c-5p
 
 
-0.429
 
 
16.6
 
 
 
 
ATP2A2
 
 
hsa-let-7e-5p
 
 
-0.311
 
 
29.6
 
 
 
 
ATP2A2
 
 
hsa-let-7c-5p
 
 
-0.311
 
 
29.6
 
 
 
 
ATPAF1
 
 
hsa-let-7e-5p
 
 
-0.498
 
 
168.0
 
 
 
 
ATPAF1
 
 
hsa-let-7c-5p
 
 
-0.498
 
 
168.0
 
 
 
 
ATXN2
 
 
hsa-miR-10a-5p
 
 
-0.362
 
 
17.3
 
 
 
 
B3GAT3
 
 
hsa-let-7e-5p
 
 
-0.324
 
 
87.1
 
 
 
 
B3GAT3
 
 
hsa-let-7c-5p
 
 
-0.324
 
 
87.1
 
 
 
 
B4GAT1
 
 
hsa-let-7e-5p
 
 
-0.47
 
 
93.8
 
 
 
 
B4GAT1
 
 
hsa-let-7c-5p
 
 
-0.47
 
 
93.8
 
 
 
 
BAG5
 
 
hsa-miR-22-3p
 
 
-0.318
 
 
18.0
 
 
 
 
BAK1
 
 
hsa-miR-125a-5p
 
 
-0.402
 
 
16.1
 
 
 
 
BATF3
 
 
hsa-miR-22-3p
 
 
-0.786
 
 
24.8
 
 
 
 
BAZ1B
 
 
hsa-miR-10a-5p
 
 
-0.309
 
 
12.0
 
 
 
 
BCAP29
 
 
hsa-let-7e-5p
 
 
-0.289
 
 
12.5
 
 
 
 
BDH1
 
 
hsa-miR-125a-5p
 
 
-0.389
 
 
39.2
 
 
 
 
BIN1
 
 
hsa-miR-22-3p
 
 
-0.346
 
 
43.9
 
 
 
 
BIN3
 
 
hsa-let-7e-5p
 
 
-0.427
 
 
33.7
 
 
 
 
BIN3
 
 
hsa-let-7c-5p
 
 
-0.427
 
 
33.7
 
 
 
 
BLOC1S1
 
 
hsa-miR-22-3p
 
 
-0.346
 
 
256.2
 
 
 
 
BLOC1S6
 
 
hsa-let-7e-5p
 
 
-0.321
 
 
14.9
 
 
 
 
BLOC1S6
 
 
hsa-let-7c-5p
 
 
-0.321
 
 
14.9
 
 
 
 
BTF3L4
 
 
hsa-let-7e-5p
 
 
-0.355
 
 
26.9
 
 
 
 
BTF3L4
 
 
hsa-let-7c-5p
 
 
-0.355
 
 
26.9
 
 
 
 
BZW1
 
 
hsa-let-7e-5p
 
 
-0.354
 
 
50.7
 
 
 
 
BZW1
 
 
hsa-let-7c-5p
 
 
-0.354
 
 
50.7
 
 
 
 
BZW1
 
 
hsa-let-7e-5p
 
 
-0.375
 
 
50.7
 
 
 
 
BZW1
 
 
hsa-let-7c-5p
 
 
-0.375
 
 
50.7
 
 
 
 
BZW2
 
 
hsa-let-7c-5p
 
 
-0.397
 
 
19.4
 
 
 
 
BZW2
 
 
hsa-let-7e-5p
 
 
-0.408
 
 
19.4
 
 
 
 
C1GALT1
 
 
hsa-let-7e-5p
 
 
-0.322
 
 
22.9
 
 
 
 
C1GALT1
 
 
hsa-let-7c-5p
 
 
-0.322
 
 
22.9
 
 
 
 
CAMK2N1
 
 
hsa-miR-22-3p
 
 
-0.302
 
 
15.0
 
 
 
 
CANT1
 
 
hsa-let-7e-5p
 
 
-0.402
 
 
11.0
 
 
 
 
CANT1
 
 
hsa-let-7c-5p
 
 
-0.402
 
 
11.0
 
 
 
 
CAPN1
 
 
hsa-miR-22-3p
 
 
-0.307
 
 
21.6
 
 
 
 
CAPN3
 
 
hsa-let-7e-5p
 
 
-0.317
 
 
17.5
 
 
 
 
CAPN3
 
 
hsa-let-7c-5p
 
 
-0.317
 
 
17.5
 
 
 
 
CAST
 
 
hsa-miR-150-5p
 
 
-0.354
 
 
52.4
 
 
 
 
CBX5
 
 
hsa-let-7e-5p
 
 
-0.307
 
 
112.0
 
 
 
 
CBX5
 
 
hsa-let-7c-5p
 
 
-0.307
 
 
112.0
 
 
 
 
CBX5
 
 
hsa-let-7e-5p
 
 
-0.472
 
 
112.0
 
 
 
 
CBX5
 
 
hsa-let-7c-5p
 
 
-0.472
 
 
112.0
 
 
 
 
CCDC47
 
 
hsa-miR-22-3p
 
 
-0.604
 
 
79.1
 
 
 
 
CCNY
 
 
hsa-let-7c-5p
 
 
-0.323
 
 
13.3
 
 
 
 
CCNY
 
 
hsa-let-7e-5p
 
 
-0.334
 
 
13.3
 
 
 
 
CDC34
 
 
hsa-let-7c-5p
 
 
-0.309
 
 
42.2
 
 
 
 
CDC34
 
 
hsa-let-7e-5p
 
 
-0.321
 
 
42.2
 
 
 
 
CDC34
 
 
hsa-let-7e-5p
 
 
-0.555
 
 
42.2
 
 
 
 
CDC34
 
 
hsa-let-7c-5p
 
 
-0.555
 
 
42.2
 
 
 
 
CDC37L1
 
 
hsa-miR-125a-5p
 
 
-0.401
 
 
12.9
 
 
 
 
CDC42SE1
 
 
hsa-miR-125a-5p
 
 
-0.501
 
 
31.3
 
 
 
 
CDKN1A
 
 
hsa-let-7e-5p
 
 
-0.434
 
 
300.9
 
 
 
 
CDKN1A
 
 
hsa-let-7c-5p
 
 
-0.434
 
 
300.9
 
 
 
 
CDV3
 
 
hsa-let-7e-5p
 
 
-0.453
 
 
57.1
 
 
 
 
CDV3
 
 
hsa-let-7c-5p
 
 
-0.461
 
 
57.1
 
 
 
 
CEBPD
 
 
hsa-let-7e-5p
 
 
-0.309
 
 
20.5
 
 
 
 
CEBPD
 
 
hsa-let-7c-5p
 
 
-0.309
 
 
20.5
 
 
 
 
CERCAM
 
 
hsa-let-7e-5p
 
 
-0.344
 
 
29.3
 
 
 
 
CERCAM
 
 
hsa-let-7c-5p
 
 
-0.344
 
 
29.3
 
 
 
 
CFAP36
 
 
hsa-miR-22-3p
 
 
-0.549
 
 
178.7
 
 
 
 
CLASP2
 
 
hsa-let-7e-5p
 
 
-0.344
 
 
17.7
 
 
 
 
CLASP2
 
 
hsa-let-7c-5p
 
 
-0.344
 
 
17.7
 
 
 
 
CLDN12
 
 
hsa-let-7e-5p
 
 
-0.511
 
 
10.5
 
 
 
 
CLDN12
 
 
hsa-let-7c-5p
 
 
-0.511
 
 
10.5
 
 
 
 
CLIC4
 
 
hsa-miR-22-3p
 
 
-0.594
 
 
19.2
 
 
 
 
CMTM6
 
 
hsa-miR-150-5p
 
 
-0.328
 
 
30.2
 
 
 
 
COL1A2
 
 
hsa-let-7e-5p
 
 
-0.499
 
 
10.7
 
 
 
 
COL1A2
 
 
hsa-let-7c-5p
 
 
-0.499
 
 
10.7
 
 
 
 
COPZ1
 
 
hsa-miR-125a-5p
 
 
-0.296
 
 
112.4
 
 
 
 
CPNE2
 
 
hsa-miR-125a-5p
 
 
-0.357
 
 
40.7
 
 
 
 
CPSF6
 
 
hsa-miR-125a-5p
 
 
-0.384
 
 
17.7
 
 
 
 
CSF1R
 
 
hsa-miR-22-3p
 
 
-0.472
 
 
21.2
 
 
 
 
 
 
 colon 
 
 
 
 
target_symbol
 
 
mature_mirna_id
 
 
score
 
 
nTPM
 
 
 
 
 
 
ABCC5
 
 
hsa-miR-125a-5p
 
 
-0.288
 
 
24.4
 
 
 
 
ABHD17C
 
 
hsa-let-7e-5p
 
 
-0.359
 
 
61.2
 
 
 
 
ABHD17C
 
 
hsa-let-7c-5p
 
 
-0.359
 
 
61.2
 
 
 
 
ABHD3
 
 
hsa-miR-125a-5p
 
 
-0.453
 
 
59.5
 
 
 
 
ABHD6
 
 
hsa-miR-125a-5p
 
 
-0.501
 
 
13.2
 
 
 
 
ABTB1
 
 
hsa-miR-125a-5p
 
 
-0.519
 
 
39.8
 
 
 
 
ADIPOR2
 
 
hsa-miR-150-5p
 
 
-0.287
 
 
31.0
 
 
 
 
AIDA
 
 
hsa-miR-22-3p
 
 
-0.316
 
 
47.7
 
 
 
 
AIFM1
 
 
hsa-miR-125a-5p
 
 
-0.3
 
 
23.9
 
 
 
 
AIFM1
 
 
hsa-let-7e-5p
 
 
-0.314
 
 
23.9
 
 
 
 
AIFM1
 
 
hsa-let-7c-5p
 
 
-0.314
 
 
23.9
 
 
 
 
AKT3
 
 
hsa-miR-22-3p
 
 
-0.451
 
 
20.3
 
 
 
 
AMT
 
 
hsa-let-7e-5p
 
 
-0.463
 
 
14.4
 
 
 
 
AMT
 
 
hsa-let-7c-5p
 
 
-0.463
 
 
14.4
 
 
 
 
ANAPC16
 
 
hsa-miR-125a-5p
 
 
-0.578
 
 
22.7
 
 
 
 
ANKHD1-EIF4EBP3
 
 
hsa-miR-22-3p
 
 
-0.479
 
 
11.6
 
 
 
 
ANKRD13A
 
 
hsa-miR-22-3p
 
 
-0.478
 
 
20.4
 
 
 
 
ANPEP
 
 
hsa-miR-125a-5p
 
 
-0.372
 
 
250.5
 
 
 
 
AP1AR
 
 
hsa-miR-99b-5p
 
 
-0.823
 
 
16.9
 
 
 
 
AP1S1
 
 
hsa-let-7e-5p
 
 
-0.374
 
 
31.0
 
 
 
 
AP1S1
 
 
hsa-let-7c-5p
 
 
-0.374
 
 
31.0
 
 
 
 
ARHGEF26
 
 
hsa-miR-22-3p
 
 
-0.374
 
 
16.6
 
 
 
 
ARL4D
 
 
hsa-let-7e-5p
 
 
-0.417
 
 
27.1
 
 
 
 
ARL4D
 
 
hsa-let-7c-5p
 
 
-0.417
 
 
27.1
 
 
 
 
ARL6IP6
 
 
hsa-let-7c-5p
 
 
-0.293
 
 
12.7
 
 
 
 
ARL6IP6
 
 
hsa-let-7e-5p
 
 
-0.305
 
 
12.7
 
 
 
 
ARMT1
 
 
hsa-let-7e-5p
 
 
-0.303
 
 
15.5
 
 
 
 
ARMT1
 
 
hsa-let-7c-5p
 
 
-0.303
 
 
15.5
 
 
 
 
ARPC5
 
 
hsa-miR-22-3p
 
 
-0.679
 
 
98.1
 
 
 
 
ARPP19
 
 
hsa-let-7e-5p
 
 
-0.492
 
 
35.7
 
 
 
 
ARPP19
 
 
hsa-let-7c-5p
 
 
-0.492
 
 
35.7
 
 
 
 
ARRB1
 
 
hsa-miR-22-3p
 
 
-0.616
 
 
24.1
 
 
 
 
ARRDC4
 
 
hsa-let-7e-5p
 
 
-0.329
 
 
40.4
 
 
 
 
ARRDC4
 
 
hsa-let-7c-5p
 
 
-0.329
 
 
40.4
 
 
 
 
ASAP1
 
 
hsa-let-7e-5p
 
 
-0.296
 
 
12.2
 
 
 
 
ASAP1
 
 
hsa-let-7c-5p
 
 
-0.308
 
 
12.2
 
 
 
 
ATOH1
 
 
hsa-miR-22-3p
 
 
-0.566
 
 
16.9
 
 
 
 
ATOSB
 
 
hsa-let-7e-5p
 
 
-0.429
 
 
14.6
 
 
 
 
ATOSB
 
 
hsa-let-7c-5p
 
 
-0.429
 
 
14.6
 
 
 
 
ATP2A2
 
 
hsa-let-7e-5p
 
 
-0.311
 
 
60.2
 
 
 
 
ATP2A2
 
 
hsa-let-7c-5p
 
 
-0.311
 
 
60.2
 
 
 
 
ATP8A1
 
 
hsa-miR-22-3p
 
 
-0.468
 
 
12.0
 
 
 
 
ATPAF1
 
 
hsa-let-7e-5p
 
 
-0.498
 
 
49.0
 
 
 
 
ATPAF1
 
 
hsa-let-7c-5p
 
 
-0.498
 
 
49.0
 
 
 
 
ATXN2
 
 
hsa-miR-10a-5p
 
 
-0.362
 
 
22.4
 
 
 
 
B3GALNT2
 
 
hsa-miR-125a-5p
 
 
-0.311
 
 
14.0
 
 
 
 
B3GAT3
 
 
hsa-let-7e-5p
 
 
-0.324
 
 
30.8
 
 
 
 
B3GAT3
 
 
hsa-let-7c-5p
 
 
-0.324
 
 
30.8
 
 
 
 
B4GAT1
 
 
hsa-let-7e-5p
 
 
-0.47
 
 
29.4
 
 
 
 
B4GAT1
 
 
hsa-let-7c-5p
 
 
-0.47
 
 
29.4
 
 
 
 
BACH1
 
 
hsa-let-7e-5p
 
 
-0.298
 
 
12.5
 
 
 
 
BACH1
 
 
hsa-let-7e-5p
 
 
-0.298
 
 
12.5
 
 
 
 
BACH1
 
 
hsa-let-7c-5p
 
 
-0.306
 
 
12.5
 
 
 
 
BACH1
 
 
hsa-let-7c-5p
 
 
-0.306
 
 
12.5
 
 
 
 
BACH1
 
 
hsa-let-7e-5p
 
 
-0.435
 
 
12.5
 
 
 
 
BACH1
 
 
hsa-let-7e-5p
 
 
-0.435
 
 
12.5
 
 
 
 
BACH1
 
 
hsa-let-7c-5p
 
 
-0.435
 
 
12.5
 
 
 
 
BACH1
 
 
hsa-let-7c-5p
 
 
-0.435
 
 
12.5
 
 
 
 
BAG5
 
 
hsa-miR-22-3p
 
 
-0.318
 
 
13.8
 
 
 
 
BAK1
 
 
hsa-miR-125a-5p
 
 
-0.402
 
 
30.4
 
 
 
 
BAZ1B
 
 
hsa-miR-10a-5p
 
 
-0.309
 
 
21.5
 
 
 
 
BAZ2A
 
 
hsa-miR-99b-5p
 
 
-0.408
 
 
25.3
 
 
 
 
BBX
 
 
hsa-miR-10a-5p
 
 
-0.305
 
 
10.4
 
 
 
 
BCAP29
 
 
hsa-let-7e-5p
 
 
-0.289
 
 
31.3
 
 
 
 
BCL2L11
 
 
hsa-miR-10a-5p
 
 
-0.483
 
 
10.9
 
 
 
 
BCL2L12
 
 
hsa-miR-125a-5p
 
 
-0.288
 
 
10.6
 
 
 
 
BDH1
 
 
hsa-miR-125a-5p
 
 
-0.389
 
 
40.4
 
 
 
 
BIN1
 
 
hsa-miR-22-3p
 
 
-0.346
 
 
33.8
 
 
 
 
BIN3
 
 
hsa-let-7e-5p
 
 
-0.427
 
 
12.9
 
 
 
 
BIN3
 
 
hsa-let-7c-5p
 
 
-0.427
 
 
12.9
 
 
 
 
BLOC1S1
 
 
hsa-miR-22-3p
 
 
-0.346
 
 
182.5
 
 
 
 
BLOC1S6
 
 
hsa-let-7e-5p
 
 
-0.321
 
 
19.9
 
 
 
 
BLOC1S6
 
 
hsa-let-7c-5p
 
 
-0.321
 
 
19.9
 
 
 
 
BLZF1
 
 
hsa-miR-125a-5p
 
 
-0.461
 
 
13.0
 
 
 
 
BMPR2
 
 
hsa-miR-99b-5p
 
 
-0.327
 
 
10.5
 
 
 
 
BORCS6
 
 
hsa-miR-125a-5p
 
 
-0.287
 
 
11.0
 
 
 
 
BTF3L4
 
 
hsa-let-7e-5p
 
 
-0.355
 
 
22.9
 
 
 
 
BTF3L4
 
 
hsa-let-7c-5p
 
 
-0.355
 
 
22.9
 
 
 
 
BZW1
 
 
hsa-let-7e-5p
 
 
-0.354
 
 
92.2
 
 
 
 
BZW1
 
 
hsa-let-7c-5p
 
 
-0.354
 
 
92.2
 
 
 
 
BZW1
 
 
hsa-let-7e-5p
 
 
-0.375
 
 
92.2
 
 
 
 
BZW1
 
 
hsa-let-7c-5p
 
 
-0.375
 
 
92.2
 
 
 
 
BZW2
 
 
hsa-let-7c-5p
 
 
-0.397
 
 
47.9
 
 
 
 
BZW2
 
 
hsa-let-7e-5p
 
 
-0.408
 
 
47.9
 
 
 
 
C15orf39
 
 
hsa-miR-125a-5p
 
 
-0.335
 
 
10.3
 
 
 
 
C15orf39
 
 
hsa-let-7e-5p
 
 
-0.374
 
 
10.3
 
 
 
 
C15orf39
 
 
hsa-let-7c-5p
 
 
-0.374
 
 
10.3
 
 
 
 
C1GALT1
 
 
hsa-let-7e-5p
 
 
-0.322
 
 
14.3
 
 
 
 
C1GALT1
 
 
hsa-let-7c-5p
 
 
-0.322
 
 
14.3
 
 
 
 
C1orf210
 
 
hsa-miR-125a-5p
 
 
-0.584
 
 
30.4
 
 
 
 
C2orf88
 
 
hsa-let-7e-5p
 
 
-0.429
 
 
33.8
 
 
 
 
C2orf88
 
 
hsa-let-7c-5p
 
 
-0.429
 
 
33.8
 
 
 
 
C5orf24
 
 
hsa-miR-22-3p
 
 
-0.493
 
 
19.7
 
 
 
 
C6orf47
 
 
hsa-miR-125a-5p
 
 
-0.564
 
 
16.9
 
 
 
 
CACFD1
 
 
hsa-let-7e-5p
 
 
-0.296
 
 
18.7
 
 
 
 
CACFD1
 
 
hsa-let-7c-5p
 
 
-0.296
 
 
18.7
 
 
 
 
CACNB3
 
 
hsa-miR-125a-5p
 
 
-0.393
 
 
17.0
 
 
 
 
CAMK2N1
 
 
hsa-miR-22-3p
 
 
-0.302
 
 
79.7
 
 
 
 
CANT1
 
 
hsa-let-7e-5p
 
 
-0.402
 
 
83.6
 
 
 
 
CANT1
 
 
hsa-let-7c-5p
 
 
-0.402
 
 
83.6
 
 
 
 
 
 
 duodenum 
 
 
 
 
target_symbol
 
 
mature_mirna_id
 
 
score
 
 
nTPM
 
 
 
 
 
 
ABCC5
 
 
hsa-miR-125a-5p
 
 
-0.288
 
 
10.1
 
 
 
 
ABHD17C
 
 
hsa-let-7e-5p
 
 
-0.359
 
 
21.9
 
 
 
 
ABHD17C
 
 
hsa-let-7c-5p
 
 
-0.359
 
 
21.9
 
 
 
 
ABHD3
 
 
hsa-miR-125a-5p
 
 
-0.453
 
 
41.5
 
 
 
 
ABHD6
 
 
hsa-miR-125a-5p
 
 
-0.501
 
 
37.1
 
 
 
 
ABTB1
 
 
hsa-miR-125a-5p
 
 
-0.519
 
 
11.6
 
 
 
 
ADIPOR2
 
 
hsa-miR-150-5p
 
 
-0.287
 
 
20.9
 
 
 
 
AGGF1
 
 
hsa-miR-125a-5p
 
 
-0.331
 
 
13.2
 
 
 
 
AGMAT
 
 
hsa-miR-22-3p
 
 
-0.294
 
 
16.8
 
 
 
 
AIDA
 
 
hsa-miR-22-3p
 
 
-0.316
 
 
23.9
 
 
 
 
AIFM1
 
 
hsa-miR-125a-5p
 
 
-0.3
 
 
49.8
 
 
 
 
AIFM1
 
 
hsa-let-7e-5p
 
 
-0.314
 
 
49.8
 
 
 
 
AIFM1
 
 
hsa-let-7c-5p
 
 
-0.314
 
 
49.8
 
 
 
 
AMT
 
 
hsa-let-7e-5p
 
 
-0.463
 
 
12.9
 
 
 
 
AMT
 
 
hsa-let-7c-5p
 
 
-0.463
 
 
12.9
 
 
 
 
ANAPC16
 
 
hsa-miR-125a-5p
 
 
-0.578
 
 
14.5
 
 
 
 
ANKRD13A
 
 
hsa-miR-22-3p
 
 
-0.478
 
 
13.4
 
 
 
 
ANPEP
 
 
hsa-miR-125a-5p
 
 
-0.372
 
 
2245.0
 
 
 
 
AP1AR
 
 
hsa-miR-99b-5p
 
 
-0.823
 
 
20.9
 
 
 
 
AP1S1
 
 
hsa-let-7e-5p
 
 
-0.374
 
 
36.7
 
 
 
 
AP1S1
 
 
hsa-let-7c-5p
 
 
-0.374
 
 
36.7
 
 
 
 
ARMT1
 
 
hsa-let-7e-5p
 
 
-0.303
 
 
17.8
 
 
 
 
ARMT1
 
 
hsa-let-7c-5p
 
 
-0.303
 
 
17.8
 
 
 
 
ARPC5
 
 
hsa-miR-22-3p
 
 
-0.679
 
 
93.6
 
 
 
 
ARPP19
 
 
hsa-let-7e-5p
 
 
-0.492
 
 
27.2
 
 
 
 
ARPP19
 
 
hsa-let-7c-5p
 
 
-0.492
 
 
27.2
 
 
 
 
ARRB1
 
 
hsa-miR-22-3p
 
 
-0.616
 
 
20.6
 
 
 
 
ARRDC4
 
 
hsa-let-7e-5p
 
 
-0.329
 
 
14.9
 
 
 
 
ARRDC4
 
 
hsa-let-7c-5p
 
 
-0.329
 
 
14.9
 
 
 
 
ATOH1
 
 
hsa-miR-22-3p
 
 
-0.566
 
 
10.4
 
 
 
 
ATP2A2
 
 
hsa-let-7e-5p
 
 
-0.311
 
 
44.8
 
 
 
 
ATP2A2
 
 
hsa-let-7c-5p
 
 
-0.311
 
 
44.8
 
 
 
 
ATPAF1
 
 
hsa-let-7e-5p
 
 
-0.498
 
 
41.3
 
 
 
 
ATPAF1
 
 
hsa-let-7c-5p
 
 
-0.498
 
 
41.3
 
 
 
 
ATXN2
 
 
hsa-miR-10a-5p
 
 
-0.362
 
 
10.5
 
 
 
 
B3GAT3
 
 
hsa-let-7e-5p
 
 
-0.324
 
 
16.1
 
 
 
 
B3GAT3
 
 
hsa-let-7c-5p
 
 
-0.324
 
 
16.1
 
 
 
 
B4GAT1
 
 
hsa-let-7e-5p
 
 
-0.47
 
 
15.2
 
 
 
 
B4GAT1
 
 
hsa-let-7c-5p
 
 
-0.47
 
 
15.2
 
 
 
 
BACH1
 
 
hsa-let-7e-5p
 
 
-0.298
 
 
15.8
 
 
 
 
BACH1
 
 
hsa-let-7e-5p
 
 
-0.298
 
 
15.8
 
 
 
 
BACH1
 
 
hsa-let-7c-5p
 
 
-0.306
 
 
15.8
 
 
 
 
BACH1
 
 
hsa-let-7c-5p
 
 
-0.306
 
 
15.8
 
 
 
 
BACH1
 
 
hsa-let-7e-5p
 
 
-0.435
 
 
15.8
 
 
 
 
BACH1
 
 
hsa-let-7e-5p
 
 
-0.435
 
 
15.8
 
 
 
 
BACH1
 
 
hsa-let-7c-5p
 
 
-0.435
 
 
15.8
 
 
 
 
BACH1
 
 
hsa-let-7c-5p
 
 
-0.435
 
 
15.8
 
 
 
 
BAG5
 
 
hsa-miR-22-3p
 
 
-0.318
 
 
11.6
 
 
 
 
BAK1
 
 
hsa-miR-125a-5p
 
 
-0.402
 
 
44.2
 
 
 
 
BAZ1B
 
 
hsa-miR-10a-5p
 
 
-0.309
 
 
15.1
 
 
 
 
BAZ2A
 
 
hsa-miR-99b-5p
 
 
-0.408
 
 
23.9
 
 
 
 
BCAP29
 
 
hsa-let-7e-5p
 
 
-0.289
 
 
15.5
 
 
 
 
BDH1
 
 
hsa-miR-125a-5p
 
 
-0.389
 
 
16.3
 
 
 
 
BIN1
 
 
hsa-miR-22-3p
 
 
-0.346
 
 
16.4
 
 
 
 
BLOC1S1
 
 
hsa-miR-22-3p
 
 
-0.346
 
 
139.3
 
 
 
 
BLOC1S6
 
 
hsa-let-7e-5p
 
 
-0.321
 
 
19.0
 
 
 
 
BLOC1S6
 
 
hsa-let-7c-5p
 
 
-0.321
 
 
19.0
 
 
 
 
BLZF1
 
 
hsa-miR-125a-5p
 
 
-0.461
 
 
11.6
 
 
 
 
BMF
 
 
hsa-miR-125a-5p
 
 
-0.34
 
 
20.0
 
 
 
 
BTF3L4
 
 
hsa-let-7e-5p
 
 
-0.355
 
 
13.6
 
 
 
 
BTF3L4
 
 
hsa-let-7c-5p
 
 
-0.355
 
 
13.6
 
 
 
 
BZW1
 
 
hsa-let-7e-5p
 
 
-0.354
 
 
60.8
 
 
 
 
BZW1
 
 
hsa-let-7c-5p
 
 
-0.354
 
 
60.8
 
 
 
 
BZW1
 
 
hsa-let-7e-5p
 
 
-0.375
 
 
60.8
 
 
 
 
BZW1
 
 
hsa-let-7c-5p
 
 
-0.375
 
 
60.8
 
 
 
 
BZW2
 
 
hsa-let-7c-5p
 
 
-0.397
 
 
27.2
 
 
 
 
BZW2
 
 
hsa-let-7e-5p
 
 
-0.408
 
 
27.2
 
 
 
 
C15orf39
 
 
hsa-miR-125a-5p
 
 
-0.335
 
 
10.8
 
 
 
 
C15orf39
 
 
hsa-let-7e-5p
 
 
-0.374
 
 
10.8
 
 
 
 
C15orf39
 
 
hsa-let-7c-5p
 
 
-0.374
 
 
10.8
 
 
 
 
C1GALT1
 
 
hsa-let-7e-5p
 
 
-0.322
 
 
27.7
 
 
 
 
C1GALT1
 
 
hsa-let-7c-5p
 
 
-0.322
 
 
27.7
 
 
 
 
C1orf210
 
 
hsa-miR-125a-5p
 
 
-0.584
 
 
41.2
 
 
 
 
C2orf88
 
 
hsa-let-7e-5p
 
 
-0.429
 
 
32.6
 
 
 
 
C2orf88
 
 
hsa-let-7c-5p
 
 
-0.429
 
 
32.6
 
 
 
 
C5orf24
 
 
hsa-miR-22-3p
 
 
-0.493
 
 
20.8
 
 
 
 
CAMK2N1
 
 
hsa-miR-22-3p
 
 
-0.302
 
 
75.8
 
 
 
 
CANT1
 
 
hsa-let-7e-5p
 
 
-0.402
 
 
48.8
 
 
 
 
CANT1
 
 
hsa-let-7c-5p
 
 
-0.402
 
 
48.8
 
 
 
 
CAPN1
 
 
hsa-miR-22-3p
 
 
-0.307
 
 
75.6
 
 
 
 
CARMIL1
 
 
hsa-miR-22-3p
 
 
-0.329
 
 
15.2
 
 
 
 
CARNMT1
 
 
hsa-let-7e-5p
 
 
-0.307
 
 
12.0
 
 
 
 
CARNMT1
 
 
hsa-let-7c-5p
 
 
-0.307
 
 
12.0
 
 
 
 
CASP2
 
 
hsa-miR-125a-5p
 
 
-0.296
 
 
10.1
 
 
 
 
CASP3
 
 
hsa-let-7e-5p
 
 
-0.495
 
 
47.5
 
 
 
 
CASP3
 
 
hsa-let-7c-5p
 
 
-0.503
 
 
47.5
 
 
 
 
CAST
 
 
hsa-miR-150-5p
 
 
-0.354
 
 
113.3
 
 
 
 
CCDC47
 
 
hsa-miR-22-3p
 
 
-0.604
 
 
79.4
 
 
 
 
CCNY
 
 
hsa-let-7c-5p
 
 
-0.323
 
 
20.2
 
 
 
 
CCNY
 
 
hsa-let-7e-5p
 
 
-0.334
 
 
20.2
 
 
 
 
CDC34
 
 
hsa-let-7c-5p
 
 
-0.309
 
 
16.8
 
 
 
 
CDC34
 
 
hsa-let-7e-5p
 
 
-0.321
 
 
16.8
 
 
 
 
CDC34
 
 
hsa-let-7e-5p
 
 
-0.555
 
 
16.8
 
 
 
 
CDC34
 
 
hsa-let-7c-5p
 
 
-0.555
 
 
16.8
 
 
 
 
CDC42BPG
 
 
hsa-miR-125a-5p
 
 
-0.344
 
 
10.0
 
 
 
 
CDC42SE1
 
 
hsa-miR-125a-5p
 
 
-0.501
 
 
18.8
 
 
 
 
CDKN1A
 
 
hsa-let-7e-5p
 
 
-0.434
 
 
108.4
 
 
 
 
CDKN1A
 
 
hsa-let-7c-5p
 
 
-0.434
 
 
108.4
 
 
 
 
CDV3
 
 
hsa-let-7e-5p
 
 
-0.453
 
 
53.7
 
 
 
 
CDV3
 
 
hsa-let-7c-5p
 
 
-0.461
 
 
53.7
 
 
 
 
 
 
 endometrium 
 
 
 
 
target_symbol
 
 
mature_mirna_id
 
 
score
 
 
nTPM
 
 
 
 
 
 
ABCC5
 
 
hsa-miR-125a-5p
 
 
-0.288
 
 
22.5
 
 
 
 
ABTB1
 
 
hsa-miR-125a-5p
 
 
-0.519
 
 
33.5
 
 
 
 
ADIPOR2
 
 
hsa-miR-150-5p
 
 
-0.287
 
 
24.5
 
 
 
 
AIDA
 
 
hsa-miR-22-3p
 
 
-0.316
 
 
33.8
 
 
 
 
AIFM1
 
 
hsa-miR-125a-5p
 
 
-0.3
 
 
10.6
 
 
 
 
AIFM1
 
 
hsa-let-7e-5p
 
 
-0.314
 
 
10.6
 
 
 
 
AIFM1
 
 
hsa-let-7c-5p
 
 
-0.314
 
 
10.6
 
 
 
 
AKT3
 
 
hsa-miR-22-3p
 
 
-0.451
 
 
19.5
 
 
 
 
ALDH4A1
 
 
hsa-miR-184
 
 
-0.421
 
 
10.1
 
 
 
 
AMT
 
 
hsa-let-7e-5p
 
 
-0.463
 
 
10.0
 
 
 
 
AMT
 
 
hsa-let-7c-5p
 
 
-0.463
 
 
10.0
 
 
 
 
ANAPC16
 
 
hsa-miR-125a-5p
 
 
-0.578
 
 
18.6
 
 
 
 
ANKRD13A
 
 
hsa-miR-22-3p
 
 
-0.478
 
 
11.2
 
 
 
 
ANKRD46
 
 
hsa-let-7e-5p
 
 
-0.389
 
 
11.8
 
 
 
 
ANKRD46
 
 
hsa-let-7c-5p
 
 
-0.389
 
 
11.8
 
 
 
 
AP1AR
 
 
hsa-miR-99b-5p
 
 
-0.823
 
 
10.6
 
 
 
 
AP1S1
 
 
hsa-let-7e-5p
 
 
-0.374
 
 
16.1
 
 
 
 
AP1S1
 
 
hsa-let-7c-5p
 
 
-0.374
 
 
16.1
 
 
 
 
ARL4D
 
 
hsa-let-7e-5p
 
 
-0.417
 
 
19.2
 
 
 
 
ARL4D
 
 
hsa-let-7c-5p
 
 
-0.417
 
 
19.2
 
 
 
 
ARL6IP6
 
 
hsa-let-7c-5p
 
 
-0.293
 
 
11.0
 
 
 
 
ARL6IP6
 
 
hsa-let-7e-5p
 
 
-0.305
 
 
11.0
 
 
 
 
ARMT1
 
 
hsa-let-7e-5p
 
 
-0.303
 
 
15.0
 
 
 
 
ARMT1
 
 
hsa-let-7c-5p
 
 
-0.303
 
 
15.0
 
 
 
 
ARPC5
 
 
hsa-miR-22-3p
 
 
-0.679
 
 
63.7
 
 
 
 
ARPP19
 
 
hsa-let-7e-5p
 
 
-0.492
 
 
44.7
 
 
 
 
ARPP19
 
 
hsa-let-7c-5p
 
 
-0.492
 
 
44.7
 
 
 
 
ARRDC4
 
 
hsa-let-7e-5p
 
 
-0.329
 
 
13.7
 
 
 
 
ARRDC4
 
 
hsa-let-7c-5p
 
 
-0.329
 
 
13.7
 
 
 
 
ASAP1
 
 
hsa-let-7e-5p
 
 
-0.296
 
 
17.2
 
 
 
 
ASAP1
 
 
hsa-let-7c-5p
 
 
-0.308
 
 
17.2
 
 
 
 
ATP2A2
 
 
hsa-let-7e-5p
 
 
-0.311
 
 
43.7
 
 
 
 
ATP2A2
 
 
hsa-let-7c-5p
 
 
-0.311
 
 
43.7
 
 
 
 
ATPAF1
 
 
hsa-let-7e-5p
 
 
-0.498
 
 
23.6
 
 
 
 
ATPAF1
 
 
hsa-let-7c-5p
 
 
-0.498
 
 
23.6
 
 
 
 
ATXN2
 
 
hsa-miR-10a-5p
 
 
-0.362
 
 
21.0
 
 
 
 
B3GALNT2
 
 
hsa-miR-125a-5p
 
 
-0.311
 
 
10.2
 
 
 
 
B3GAT3
 
 
hsa-let-7e-5p
 
 
-0.324
 
 
33.5
 
 
 
 
B3GAT3
 
 
hsa-let-7c-5p
 
 
-0.324
 
 
33.5
 
 
 
 
B4GAT1
 
 
hsa-let-7e-5p
 
 
-0.47
 
 
28.7
 
 
 
 
B4GAT1
 
 
hsa-let-7c-5p
 
 
-0.47
 
 
28.7
 
 
 
 
BACH1
 
 
hsa-let-7e-5p
 
 
-0.298
 
 
18.4
 
 
 
 
BACH1
 
 
hsa-let-7e-5p
 
 
-0.298
 
 
18.4
 
 
 
 
BACH1
 
 
hsa-let-7c-5p
 
 
-0.306
 
 
18.4
 
 
 
 
BACH1
 
 
hsa-let-7c-5p
 
 
-0.306
 
 
18.4
 
 
 
 
BACH1
 
 
hsa-let-7e-5p
 
 
-0.435
 
 
18.4
 
 
 
 
BACH1
 
 
hsa-let-7e-5p
 
 
-0.435
 
 
18.4
 
 
 
 
BACH1
 
 
hsa-let-7c-5p
 
 
-0.435
 
 
18.4
 
 
 
 
BACH1
 
 
hsa-let-7c-5p
 
 
-0.435
 
 
18.4
 
 
 
 
BAG5
 
 
hsa-miR-22-3p
 
 
-0.318
 
 
15.1
 
 
 
 
BAZ1B
 
 
hsa-miR-10a-5p
 
 
-0.309
 
 
22.9
 
 
 
 
BAZ2A
 
 
hsa-miR-99b-5p
 
 
-0.408
 
 
24.8
 
 
 
 
BBX
 
 
hsa-miR-10a-5p
 
 
-0.305
 
 
17.7
 
 
 
 
BCAP29
 
 
hsa-let-7e-5p
 
 
-0.289
 
 
41.4
 
 
 
 
BIN1
 
 
hsa-miR-22-3p
 
 
-0.346
 
 
39.4
 
 
 
 
BIN3
 
 
hsa-let-7e-5p
 
 
-0.427
 
 
11.7
 
 
 
 
BIN3
 
 
hsa-let-7c-5p
 
 
-0.427
 
 
11.7
 
 
 
 
BLOC1S1
 
 
hsa-miR-22-3p
 
 
-0.346
 
 
110.1
 
 
 
 
BLOC1S6
 
 
hsa-let-7e-5p
 
 
-0.321
 
 
28.8
 
 
 
 
BLOC1S6
 
 
hsa-let-7c-5p
 
 
-0.321
 
 
28.8
 
 
 
 
BLZF1
 
 
hsa-miR-125a-5p
 
 
-0.461
 
 
14.6
 
 
 
 
BMF
 
 
hsa-miR-125a-5p
 
 
-0.34
 
 
14.8
 
 
 
 
BMPR2
 
 
hsa-miR-99b-5p
 
 
-0.327
 
 
14.3
 
 
 
 
BTF3L4
 
 
hsa-let-7e-5p
 
 
-0.355
 
 
34.0
 
 
 
 
BTF3L4
 
 
hsa-let-7c-5p
 
 
-0.355
 
 
34.0
 
 
 
 
BZW1
 
 
hsa-let-7e-5p
 
 
-0.354
 
 
62.3
 
 
 
 
BZW1
 
 
hsa-let-7c-5p
 
 
-0.354
 
 
62.3
 
 
 
 
BZW1
 
 
hsa-let-7e-5p
 
 
-0.375
 
 
62.3
 
 
 
 
BZW1
 
 
hsa-let-7c-5p
 
 
-0.375
 
 
62.3
 
 
 
 
BZW2
 
 
hsa-let-7c-5p
 
 
-0.397
 
 
18.0
 
 
 
 
BZW2
 
 
hsa-let-7e-5p
 
 
-0.408
 
 
18.0
 
 
 
 
C15orf39
 
 
hsa-miR-125a-5p
 
 
-0.335
 
 
14.0
 
 
 
 
C15orf39
 
 
hsa-let-7e-5p
 
 
-0.374
 
 
14.0
 
 
 
 
C15orf39
 
 
hsa-let-7c-5p
 
 
-0.374
 
 
14.0
 
 
 
 
C2orf88
 
 
hsa-let-7e-5p
 
 
-0.429
 
 
14.8
 
 
 
 
C2orf88
 
 
hsa-let-7c-5p
 
 
-0.429
 
 
14.8
 
 
 
 
C5orf24
 
 
hsa-miR-22-3p
 
 
-0.493
 
 
27.1
 
 
 
 
C6orf47
 
 
hsa-miR-125a-5p
 
 
-0.564
 
 
13.7
 
 
 
 
CACNB3
 
 
hsa-miR-125a-5p
 
 
-0.393
 
 
30.6
 
 
 
 
CAMK2N1
 
 
hsa-miR-22-3p
 
 
-0.302
 
 
25.6
 
 
 
 
CANT1
 
 
hsa-let-7e-5p
 
 
-0.402
 
 
14.8
 
 
 
 
CANT1
 
 
hsa-let-7c-5p
 
 
-0.402
 
 
14.8
 
 
 
 
CAPN1
 
 
hsa-miR-22-3p
 
 
-0.307
 
 
53.8
 
 
 
 
CAPN3
 
 
hsa-let-7e-5p
 
 
-0.317
 
 
12.3
 
 
 
 
CAPN3
 
 
hsa-let-7c-5p
 
 
-0.317
 
 
12.3
 
 
 
 
CASP3
 
 
hsa-let-7e-5p
 
 
-0.495
 
 
10.7
 
 
 
 
CASP3
 
 
hsa-let-7c-5p
 
 
-0.503
 
 
10.7
 
 
 
 
CAST
 
 
hsa-miR-150-5p
 
 
-0.354
 
 
68.1
 
 
 
 
CCDC47
 
 
hsa-miR-22-3p
 
 
-0.604
 
 
36.0
 
 
 
 
CCN1
 
 
hsa-miR-22-3p
 
 
-0.601
 
 
126.8
 
 
 
 
CCNY
 
 
hsa-let-7c-5p
 
 
-0.323
 
 
24.0
 
 
 
 
CCNY
 
 
hsa-let-7e-5p
 
 
-0.334
 
 
24.0
 
 
 
 
CDC34
 
 
hsa-let-7c-5p
 
 
-0.309
 
 
35.3
 
 
 
 
CDC34
 
 
hsa-let-7e-5p
 
 
-0.321
 
 
35.3
 
 
 
 
CDC34
 
 
hsa-let-7e-5p
 
 
-0.555
 
 
35.3
 
 
 
 
CDC34
 
 
hsa-let-7c-5p
 
 
-0.555
 
 
35.3
 
 
 
 
CDC37L1
 
 
hsa-miR-125a-5p
 
 
-0.401
 
 
11.3
 
 
 
 
CDC42SE1
 
 
hsa-miR-125a-5p
 
 
-0.501
 
 
33.8
 
 
 
 
CDH5
 
 
hsa-miR-125a-5p
 
 
-0.36
 
 
19.3
 
 
 
 
CDKN1A
 
 
hsa-let-7e-5p
 
 
-0.434
 
 
61.1
 
 
 
 
 
 
 esophagus 
 
 
 
 
target_symbol
 
 
mature_mirna_id
 
 
score
 
 
nTPM
 
 
 
 
 
 
ABCC5
 
 
hsa-miR-125a-5p
 
 
-0.288
 
 
44.1
 
 
 
 
ABHD17C
 
 
hsa-let-7e-5p
 
 
-0.359
 
 
45.1
 
 
 
 
ABHD17C
 
 
hsa-let-7c-5p
 
 
-0.359
 
 
45.1
 
 
 
 
ABHD3
 
 
hsa-miR-125a-5p
 
 
-0.453
 
 
17.3
 
 
 
 
ABTB1
 
 
hsa-miR-125a-5p
 
 
-0.519
 
 
13.8
 
 
 
 
ACP3
 
 
hsa-let-7e-5p
 
 
-0.298
 
 
42.9
 
 
 
 
ACP3
 
 
hsa-let-7c-5p
 
 
-0.298
 
 
42.9
 
 
 
 
ADIPOR2
 
 
hsa-miR-150-5p
 
 
-0.287
 
 
44.0
 
 
 
 
ADRB2
 
 
hsa-let-7e-5p
 
 
-0.635
 
 
14.2
 
 
 
 
ADRB2
 
 
hsa-let-7c-5p
 
 
-0.635
 
 
14.2
 
 
 
 
AIDA
 
 
hsa-miR-22-3p
 
 
-0.316
 
 
42.4
 
 
 
 
AIFM1
 
 
hsa-miR-125a-5p
 
 
-0.3
 
 
15.9
 
 
 
 
AIFM1
 
 
hsa-let-7e-5p
 
 
-0.314
 
 
15.9
 
 
 
 
AIFM1
 
 
hsa-let-7c-5p
 
 
-0.314
 
 
15.9
 
 
 
 
ALDH4A1
 
 
hsa-miR-184
 
 
-0.421
 
 
36.6
 
 
 
 
ANAPC16
 
 
hsa-miR-125a-5p
 
 
-0.578
 
 
13.6
 
 
 
 
ANKHD1-EIF4EBP3
 
 
hsa-miR-22-3p
 
 
-0.479
 
 
14.8
 
 
 
 
ANKRD13A
 
 
hsa-miR-22-3p
 
 
-0.478
 
 
29.8
 
 
 
 
ANKRD46
 
 
hsa-let-7e-5p
 
 
-0.389
 
 
11.9
 
 
 
 
ANKRD46
 
 
hsa-let-7c-5p
 
 
-0.389
 
 
11.9
 
 
 
 
AP1AR
 
 
hsa-miR-99b-5p
 
 
-0.823
 
 
12.5
 
 
 
 
AP1S1
 
 
hsa-let-7e-5p
 
 
-0.374
 
 
36.5
 
 
 
 
AP1S1
 
 
hsa-let-7c-5p
 
 
-0.374
 
 
36.5
 
 
 
 
ARG2
 
 
hsa-let-7e-5p
 
 
-0.468
 
 
17.2
 
 
 
 
ARG2
 
 
hsa-let-7c-5p
 
 
-0.468
 
 
17.2
 
 
 
 
ARL4D
 
 
hsa-let-7e-5p
 
 
-0.417
 
 
80.8
 
 
 
 
ARL4D
 
 
hsa-let-7c-5p
 
 
-0.417
 
 
80.8
 
 
 
 
ARL6IP6
 
 
hsa-let-7c-5p
 
 
-0.293
 
 
11.7
 
 
 
 
ARL6IP6
 
 
hsa-let-7e-5p
 
 
-0.305
 
 
11.7
 
 
 
 
ARMT1
 
 
hsa-let-7e-5p
 
 
-0.303
 
 
11.3
 
 
 
 
ARMT1
 
 
hsa-let-7c-5p
 
 
-0.303
 
 
11.3
 
 
 
 
ARPC5
 
 
hsa-miR-22-3p
 
 
-0.679
 
 
73.2
 
 
 
 
ARPP19
 
 
hsa-let-7e-5p
 
 
-0.492
 
 
49.0
 
 
 
 
ARPP19
 
 
hsa-let-7c-5p
 
 
-0.492
 
 
49.0
 
 
 
 
ARRB1
 
 
hsa-miR-22-3p
 
 
-0.616
 
 
17.9
 
 
 
 
ASAP1
 
 
hsa-let-7e-5p
 
 
-0.296
 
 
10.0
 
 
 
 
ASAP1
 
 
hsa-let-7c-5p
 
 
-0.308
 
 
10.0
 
 
 
 
ATOSB
 
 
hsa-let-7e-5p
 
 
-0.429
 
 
28.9
 
 
 
 
ATOSB
 
 
hsa-let-7c-5p
 
 
-0.429
 
 
28.9
 
 
 
 
ATP2A2
 
 
hsa-let-7e-5p
 
 
-0.311
 
 
170.5
 
 
 
 
ATP2A2
 
 
hsa-let-7c-5p
 
 
-0.311
 
 
170.5
 
 
 
 
ATPAF1
 
 
hsa-let-7e-5p
 
 
-0.498
 
 
36.9
 
 
 
 
ATPAF1
 
 
hsa-let-7c-5p
 
 
-0.498
 
 
36.9
 
 
 
 
ATXN2
 
 
hsa-miR-10a-5p
 
 
-0.362
 
 
13.9
 
 
 
 
B3GALNT2
 
 
hsa-miR-125a-5p
 
 
-0.311
 
 
11.3
 
 
 
 
B3GAT3
 
 
hsa-let-7e-5p
 
 
-0.324
 
 
23.2
 
 
 
 
B3GAT3
 
 
hsa-let-7c-5p
 
 
-0.324
 
 
23.2
 
 
 
 
BACH1
 
 
hsa-let-7e-5p
 
 
-0.298
 
 
21.6
 
 
 
 
BACH1
 
 
hsa-let-7e-5p
 
 
-0.298
 
 
21.6
 
 
 
 
BACH1
 
 
hsa-let-7c-5p
 
 
-0.306
 
 
21.6
 
 
 
 
BACH1
 
 
hsa-let-7c-5p
 
 
-0.306
 
 
21.6
 
 
 
 
BACH1
 
 
hsa-let-7e-5p
 
 
-0.435
 
 
21.6
 
 
 
 
BACH1
 
 
hsa-let-7e-5p
 
 
-0.435
 
 
21.6
 
 
 
 
BACH1
 
 
hsa-let-7c-5p
 
 
-0.435
 
 
21.6
 
 
 
 
BACH1
 
 
hsa-let-7c-5p
 
 
-0.435
 
 
21.6
 
 
 
 
BAG5
 
 
hsa-miR-22-3p
 
 
-0.318
 
 
20.5
 
 
 
 
BAK1
 
 
hsa-miR-125a-5p
 
 
-0.402
 
 
32.4
 
 
 
 
BAZ1B
 
 
hsa-miR-10a-5p
 
 
-0.309
 
 
16.9
 
 
 
 
BAZ2A
 
 
hsa-miR-99b-5p
 
 
-0.408
 
 
29.0
 
 
 
 
BBX
 
 
hsa-miR-10a-5p
 
 
-0.305
 
 
12.1
 
 
 
 
BCAP29
 
 
hsa-let-7e-5p
 
 
-0.289
 
 
32.4
 
 
 
 
BCL2L12
 
 
hsa-miR-125a-5p
 
 
-0.288
 
 
12.0
 
 
 
 
BDH1
 
 
hsa-miR-125a-5p
 
 
-0.389
 
 
43.7
 
 
 
 
BIN1
 
 
hsa-miR-22-3p
 
 
-0.346
 
 
31.5
 
 
 
 
BIN3
 
 
hsa-let-7e-5p
 
 
-0.427
 
 
17.6
 
 
 
 
BIN3
 
 
hsa-let-7c-5p
 
 
-0.427
 
 
17.6
 
 
 
 
BLOC1S1
 
 
hsa-miR-22-3p
 
 
-0.346
 
 
175.9
 
 
 
 
BLOC1S6
 
 
hsa-let-7e-5p
 
 
-0.321
 
 
19.8
 
 
 
 
BLOC1S6
 
 
hsa-let-7c-5p
 
 
-0.321
 
 
19.8
 
 
 
 
BLZF1
 
 
hsa-miR-125a-5p
 
 
-0.461
 
 
30.4
 
 
 
 
BORCS6
 
 
hsa-miR-125a-5p
 
 
-0.287
 
 
14.9
 
 
 
 
BTF3L4
 
 
hsa-let-7e-5p
 
 
-0.355
 
 
33.3
 
 
 
 
BTF3L4
 
 
hsa-let-7c-5p
 
 
-0.355
 
 
33.3
 
 
 
 
BZW1
 
 
hsa-let-7e-5p
 
 
-0.354
 
 
113.4
 
 
 
 
BZW1
 
 
hsa-let-7c-5p
 
 
-0.354
 
 
113.4
 
 
 
 
BZW1
 
 
hsa-let-7e-5p
 
 
-0.375
 
 
113.4
 
 
 
 
BZW1
 
 
hsa-let-7c-5p
 
 
-0.375
 
 
113.4
 
 
 
 
BZW2
 
 
hsa-let-7c-5p
 
 
-0.397
 
 
40.0
 
 
 
 
BZW2
 
 
hsa-let-7e-5p
 
 
-0.408
 
 
40.0
 
 
 
 
C15orf39
 
 
hsa-miR-125a-5p
 
 
-0.335
 
 
19.7
 
 
 
 
C15orf39
 
 
hsa-let-7e-5p
 
 
-0.374
 
 
19.7
 
 
 
 
C15orf39
 
 
hsa-let-7c-5p
 
 
-0.374
 
 
19.7
 
 
 
 
C19orf47
 
 
hsa-let-7e-5p
 
 
-0.311
 
 
11.8
 
 
 
 
C19orf47
 
 
hsa-let-7c-5p
 
 
-0.311
 
 
11.8
 
 
 
 
C1GALT1
 
 
hsa-let-7e-5p
 
 
-0.322
 
 
22.4
 
 
 
 
C1GALT1
 
 
hsa-let-7c-5p
 
 
-0.322
 
 
22.4
 
 
 
 
C1orf210
 
 
hsa-miR-125a-5p
 
 
-0.584
 
 
16.3
 
 
 
 
C5orf24
 
 
hsa-miR-22-3p
 
 
-0.493
 
 
19.7
 
 
 
 
C6orf47
 
 
hsa-miR-125a-5p
 
 
-0.564
 
 
20.5
 
 
 
 
CACNB3
 
 
hsa-miR-125a-5p
 
 
-0.393
 
 
21.0
 
 
 
 
CAMK2N1
 
 
hsa-miR-22-3p
 
 
-0.302
 
 
27.0
 
 
 
 
CANT1
 
 
hsa-let-7e-5p
 
 
-0.402
 
 
42.0
 
 
 
 
CANT1
 
 
hsa-let-7c-5p
 
 
-0.402
 
 
42.0
 
 
 
 
CAPN1
 
 
hsa-miR-22-3p
 
 
-0.307
 
 
267.3
 
 
 
 
CAPN3
 
 
hsa-let-7e-5p
 
 
-0.317
 
 
15.1
 
 
 
 
CAPN3
 
 
hsa-let-7c-5p
 
 
-0.317
 
 
15.1
 
 
 
 
CASP2
 
 
hsa-miR-125a-5p
 
 
-0.296
 
 
14.9
 
 
 
 
CASP3
 
 
hsa-let-7e-5p
 
 
-0.495
 
 
25.0
 
 
 
 
CASP3
 
 
hsa-let-7c-5p
 
 
-0.503
 
 
25.0
 
 
 
 
CAST
 
 
hsa-miR-150-5p
 
 
-0.354
 
 
322.1
 
 
 
 
 
 
 fallopian tube 
 
 
 
 
target_symbol
 
 
mature_mirna_id
 
 
score
 
 
nTPM
 
 
 
 
 
 
ABCC5
 
 
hsa-miR-125a-5p
 
 
-0.288
 
 
16.2
 
 
 
 
ABHD17C
 
 
hsa-let-7e-5p
 
 
-0.359
 
 
10.3
 
 
 
 
ABHD17C
 
 
hsa-let-7c-5p
 
 
-0.359
 
 
10.3
 
 
 
 
ABTB1
 
 
hsa-miR-125a-5p
 
 
-0.519
 
 
39.7
 
 
 
 
ADIPOR2
 
 
hsa-miR-150-5p
 
 
-0.287
 
 
23.2
 
 
 
 
AIDA
 
 
hsa-miR-22-3p
 
 
-0.316
 
 
29.5
 
 
 
 
AIFM1
 
 
hsa-miR-125a-5p
 
 
-0.3
 
 
20.8
 
 
 
 
AIFM1
 
 
hsa-let-7e-5p
 
 
-0.314
 
 
20.8
 
 
 
 
AIFM1
 
 
hsa-let-7c-5p
 
 
-0.314
 
 
20.8
 
 
 
 
AKT3
 
 
hsa-miR-22-3p
 
 
-0.451
 
 
15.1
 
 
 
 
AMT
 
 
hsa-let-7e-5p
 
 
-0.463
 
 
13.9
 
 
 
 
AMT
 
 
hsa-let-7c-5p
 
 
-0.463
 
 
13.9
 
 
 
 
ANAPC16
 
 
hsa-miR-125a-5p
 
 
-0.578
 
 
21.7
 
 
 
 
ANKRD13A
 
 
hsa-miR-22-3p
 
 
-0.478
 
 
11.6
 
 
 
 
ANKRD46
 
 
hsa-let-7e-5p
 
 
-0.389
 
 
10.7
 
 
 
 
ANKRD46
 
 
hsa-let-7c-5p
 
 
-0.389
 
 
10.7
 
 
 
 
AP1S1
 
 
hsa-let-7e-5p
 
 
-0.374
 
 
13.4
 
 
 
 
AP1S1
 
 
hsa-let-7c-5p
 
 
-0.374
 
 
13.4
 
 
 
 
ARL4D
 
 
hsa-let-7e-5p
 
 
-0.417
 
 
20.8
 
 
 
 
ARL4D
 
 
hsa-let-7c-5p
 
 
-0.417
 
 
20.8
 
 
 
 
ARL6IP6
 
 
hsa-let-7c-5p
 
 
-0.293
 
 
10.8
 
 
 
 
ARL6IP6
 
 
hsa-let-7e-5p
 
 
-0.305
 
 
10.8
 
 
 
 
ARMT1
 
 
hsa-let-7e-5p
 
 
-0.303
 
 
11.9
 
 
 
 
ARMT1
 
 
hsa-let-7c-5p
 
 
-0.303
 
 
11.9
 
 
 
 
ARPC5
 
 
hsa-miR-22-3p
 
 
-0.679
 
 
50.4
 
 
 
 
ARPP19
 
 
hsa-let-7e-5p
 
 
-0.492
 
 
31.4
 
 
 
 
ARPP19
 
 
hsa-let-7c-5p
 
 
-0.492
 
 
31.4
 
 
 
 
ARRB1
 
 
hsa-miR-22-3p
 
 
-0.616
 
 
15.0
 
 
 
 
ARRDC4
 
 
hsa-let-7e-5p
 
 
-0.329
 
 
13.7
 
 
 
 
ARRDC4
 
 
hsa-let-7c-5p
 
 
-0.329
 
 
13.7
 
 
 
 
ASAP1
 
 
hsa-let-7e-5p
 
 
-0.296
 
 
15.1
 
 
 
 
ASAP1
 
 
hsa-let-7c-5p
 
 
-0.308
 
 
15.1
 
 
 
 
ATP2A2
 
 
hsa-let-7e-5p
 
 
-0.311
 
 
63.0
 
 
 
 
ATP2A2
 
 
hsa-let-7c-5p
 
 
-0.311
 
 
63.0
 
 
 
 
ATPAF1
 
 
hsa-let-7e-5p
 
 
-0.498
 
 
27.2
 
 
 
 
ATPAF1
 
 
hsa-let-7c-5p
 
 
-0.498
 
 
27.2
 
 
 
 
ATXN2
 
 
hsa-miR-10a-5p
 
 
-0.362
 
 
19.4
 
 
 
 
B3GALNT2
 
 
hsa-miR-125a-5p
 
 
-0.311
 
 
10.6
 
 
 
 
B3GAT3
 
 
hsa-let-7e-5p
 
 
-0.324
 
 
34.0
 
 
 
 
B3GAT3
 
 
hsa-let-7c-5p
 
 
-0.324
 
 
34.0
 
 
 
 
B4GAT1
 
 
hsa-let-7e-5p
 
 
-0.47
 
 
25.4
 
 
 
 
B4GAT1
 
 
hsa-let-7c-5p
 
 
-0.47
 
 
25.4
 
 
 
 
BAG5
 
 
hsa-miR-22-3p
 
 
-0.318
 
 
15.0
 
 
 
 
BAZ1B
 
 
hsa-miR-10a-5p
 
 
-0.309
 
 
18.3
 
 
 
 
BAZ2A
 
 
hsa-miR-99b-5p
 
 
-0.408
 
 
23.4
 
 
 
 
BBX
 
 
hsa-miR-10a-5p
 
 
-0.305
 
 
15.8
 
 
 
 
BCAP29
 
 
hsa-let-7e-5p
 
 
-0.289
 
 
17.1
 
 
 
 
BIN1
 
 
hsa-miR-22-3p
 
 
-0.346
 
 
50.2
 
 
 
 
BIN3
 
 
hsa-let-7e-5p
 
 
-0.427
 
 
14.8
 
 
 
 
BIN3
 
 
hsa-let-7c-5p
 
 
-0.427
 
 
14.8
 
 
 
 
BLOC1S1
 
 
hsa-miR-22-3p
 
 
-0.346
 
 
129.0
 
 
 
 
BLOC1S6
 
 
hsa-let-7e-5p
 
 
-0.321
 
 
17.1
 
 
 
 
BLOC1S6
 
 
hsa-let-7c-5p
 
 
-0.321
 
 
17.1
 
 
 
 
BLZF1
 
 
hsa-miR-125a-5p
 
 
-0.461
 
 
10.7
 
 
 
 
BMF
 
 
hsa-miR-125a-5p
 
 
-0.34
 
 
11.2
 
 
 
 
BTF3L4
 
 
hsa-let-7e-5p
 
 
-0.355
 
 
34.3
 
 
 
 
BTF3L4
 
 
hsa-let-7c-5p
 
 
-0.355
 
 
34.3
 
 
 
 
BZW1
 
 
hsa-let-7e-5p
 
 
-0.354
 
 
50.2
 
 
 
 
BZW1
 
 
hsa-let-7c-5p
 
 
-0.354
 
 
50.2
 
 
 
 
BZW1
 
 
hsa-let-7e-5p
 
 
-0.375
 
 
50.2
 
 
 
 
BZW1
 
 
hsa-let-7c-5p
 
 
-0.375
 
 
50.2
 
 
 
 
BZW2
 
 
hsa-let-7c-5p
 
 
-0.397
 
 
19.0
 
 
 
 
BZW2
 
 
hsa-let-7e-5p
 
 
-0.408
 
 
19.0
 
 
 
 
C15orf39
 
 
hsa-miR-125a-5p
 
 
-0.335
 
 
15.2
 
 
 
 
C15orf39
 
 
hsa-let-7e-5p
 
 
-0.374
 
 
15.2
 
 
 
 
C15orf39
 
 
hsa-let-7c-5p
 
 
-0.374
 
 
15.2
 
 
 
 
C1GALT1
 
 
hsa-let-7e-5p
 
 
-0.322
 
 
15.3
 
 
 
 
C1GALT1
 
 
hsa-let-7c-5p
 
 
-0.322
 
 
15.3
 
 
 
 
C2orf88
 
 
hsa-let-7e-5p
 
 
-0.429
 
 
19.7
 
 
 
 
C2orf88
 
 
hsa-let-7c-5p
 
 
-0.429
 
 
19.7
 
 
 
 
C5orf24
 
 
hsa-miR-22-3p
 
 
-0.493
 
 
15.5
 
 
 
 
C6orf47
 
 
hsa-miR-125a-5p
 
 
-0.564
 
 
11.8
 
 
 
 
CACNB3
 
 
hsa-miR-125a-5p
 
 
-0.393
 
 
18.4
 
 
 
 
CAMK2N1
 
 
hsa-miR-22-3p
 
 
-0.302
 
 
18.8
 
 
 
 
CANT1
 
 
hsa-let-7e-5p
 
 
-0.402
 
 
17.1
 
 
 
 
CANT1
 
 
hsa-let-7c-5p
 
 
-0.402
 
 
17.1
 
 
 
 
CAPN1
 
 
hsa-miR-22-3p
 
 
-0.307
 
 
86.8
 
 
 
 
CAPN3
 
 
hsa-let-7e-5p
 
 
-0.317
 
 
15.7
 
 
 
 
CAPN3
 
 
hsa-let-7c-5p
 
 
-0.317
 
 
15.7
 
 
 
 
CAST
 
 
hsa-miR-150-5p
 
 
-0.354
 
 
74.8
 
 
 
 
CBX5
 
 
hsa-let-7e-5p
 
 
-0.307
 
 
15.0
 
 
 
 
CBX5
 
 
hsa-let-7c-5p
 
 
-0.307
 
 
15.0
 
 
 
 
CBX5
 
 
hsa-let-7e-5p
 
 
-0.472
 
 
15.0
 
 
 
 
CBX5
 
 
hsa-let-7c-5p
 
 
-0.472
 
 
15.0
 
 
 
 
CCDC149
 
 
hsa-miR-22-3p
 
 
-0.327
 
 
10.3
 
 
 
 
CCDC47
 
 
hsa-miR-22-3p
 
 
-0.604
 
 
41.3
 
 
 
 
CCN1
 
 
hsa-miR-22-3p
 
 
-0.601
 
 
252.9
 
 
 
 
CCNY
 
 
hsa-let-7c-5p
 
 
-0.323
 
 
20.9
 
 
 
 
CCNY
 
 
hsa-let-7e-5p
 
 
-0.334
 
 
20.9
 
 
 
 
CDC34
 
 
hsa-let-7c-5p
 
 
-0.309
 
 
38.1
 
 
 
 
CDC34
 
 
hsa-let-7e-5p
 
 
-0.321
 
 
38.1
 
 
 
 
CDC34
 
 
hsa-let-7e-5p
 
 
-0.555
 
 
38.1
 
 
 
 
CDC34
 
 
hsa-let-7c-5p
 
 
-0.555
 
 
38.1
 
 
 
 
CDC37L1
 
 
hsa-miR-125a-5p
 
 
-0.401
 
 
10.2
 
 
 
 
CDC42SE1
 
 
hsa-miR-125a-5p
 
 
-0.501
 
 
43.0
 
 
 
 
CDH5
 
 
hsa-miR-125a-5p
 
 
-0.36
 
 
17.4
 
 
 
 
CDKN1A
 
 
hsa-let-7e-5p
 
 
-0.434
 
 
168.3
 
 
 
 
CDKN1A
 
 
hsa-let-7c-5p
 
 
-0.434
 
 
168.3
 
 
 
 
CDV3
 
 
hsa-let-7e-5p
 
 
-0.453
 
 
41.3
 
 
 
 
CDV3
 
 
hsa-let-7c-5p
 
 
-0.461
 
 
41.3
 
 
 
 
 
 
 gallbladder 
 
 
 
 
target_symbol
 
 
mature_mirna_id
 
 
score
 
 
nTPM
 
 
 
 
 
 
ABCC5
 
 
hsa-miR-125a-5p
 
 
-0.288
 
 
16.8
 
 
 
 
ABHD3
 
 
hsa-miR-125a-5p
 
 
-0.453
 
 
19.5
 
 
 
 
ABTB1
 
 
hsa-miR-125a-5p
 
 
-0.519
 
 
17.0
 
 
 
 
AIDA
 
 
hsa-miR-22-3p
 
 
-0.316
 
 
38.3
 
 
 
 
ANAPC16
 
 
hsa-miR-125a-5p
 
 
-0.578
 
 
11.6
 
 
 
 
ANKRD13A
 
 
hsa-miR-22-3p
 
 
-0.478
 
 
21.4
 
 
 
 
ANPEP
 
 
hsa-miR-125a-5p
 
 
-0.372
 
 
113.0
 
 
 
 
AP1AR
 
 
hsa-miR-99b-5p
 
 
-0.823
 
 
12.0
 
 
 
 
AP1S1
 
 
hsa-let-7e-5p
 
 
-0.374
 
 
12.0
 
 
 
 
AP1S1
 
 
hsa-let-7c-5p
 
 
-0.374
 
 
12.0
 
 
 
 
ARMT1
 
 
hsa-let-7e-5p
 
 
-0.303
 
 
10.6
 
 
 
 
ARMT1
 
 
hsa-let-7c-5p
 
 
-0.303
 
 
10.6
 
 
 
 
ARPC5
 
 
hsa-miR-22-3p
 
 
-0.679
 
 
49.6
 
 
 
 
ARPP19
 
 
hsa-let-7e-5p
 
 
-0.492
 
 
36.7
 
 
 
 
ARPP19
 
 
hsa-let-7c-5p
 
 
-0.492
 
 
36.7
 
 
 
 
ARRB1
 
 
hsa-miR-22-3p
 
 
-0.616
 
 
12.1
 
 
 
 
ARRDC4
 
 
hsa-let-7e-5p
 
 
-0.329
 
 
16.1
 
 
 
 
ARRDC4
 
 
hsa-let-7c-5p
 
 
-0.329
 
 
16.1
 
 
 
 
ASAP1
 
 
hsa-let-7e-5p
 
 
-0.296
 
 
13.5
 
 
 
 
ASAP1
 
 
hsa-let-7c-5p
 
 
-0.308
 
 
13.5
 
 
 
 
ATOSB
 
 
hsa-let-7e-5p
 
 
-0.429
 
 
11.0
 
 
 
 
ATOSB
 
 
hsa-let-7c-5p
 
 
-0.429
 
 
11.0
 
 
 
 
ATP2A2
 
 
hsa-let-7e-5p
 
 
-0.311
 
 
56.7
 
 
 
 
ATP2A2
 
 
hsa-let-7c-5p
 
 
-0.311
 
 
56.7
 
 
 
 
ATPAF1
 
 
hsa-let-7e-5p
 
 
-0.498
 
 
28.6
 
 
 
 
ATPAF1
 
 
hsa-let-7c-5p
 
 
-0.498
 
 
28.6
 
 
 
 
ATXN2
 
 
hsa-miR-10a-5p
 
 
-0.362
 
 
11.2
 
 
 
 
B3GAT3
 
 
hsa-let-7e-5p
 
 
-0.324
 
 
14.5
 
 
 
 
B3GAT3
 
 
hsa-let-7c-5p
 
 
-0.324
 
 
14.5
 
 
 
 
BACH1
 
 
hsa-let-7e-5p
 
 
-0.298
 
 
24.3
 
 
 
 
BACH1
 
 
hsa-let-7e-5p
 
 
-0.298
 
 
24.3
 
 
 
 
BACH1
 
 
hsa-let-7c-5p
 
 
-0.306
 
 
24.3
 
 
 
 
BACH1
 
 
hsa-let-7c-5p
 
 
-0.306
 
 
24.3
 
 
 
 
BACH1
 
 
hsa-let-7e-5p
 
 
-0.435
 
 
24.3
 
 
 
 
BACH1
 
 
hsa-let-7e-5p
 
 
-0.435
 
 
24.3
 
 
 
 
BACH1
 
 
hsa-let-7c-5p
 
 
-0.435
 
 
24.3
 
 
 
 
BACH1
 
 
hsa-let-7c-5p
 
 
-0.435
 
 
24.3
 
 
 
 
BAG5
 
 
hsa-miR-22-3p
 
 
-0.318
 
 
12.1
 
 
 
 
BAZ1B
 
 
hsa-miR-10a-5p
 
 
-0.309
 
 
15.0
 
 
 
 
BAZ2A
 
 
hsa-miR-99b-5p
 
 
-0.408
 
 
19.6
 
 
 
 
BBX
 
 
hsa-miR-10a-5p
 
 
-0.305
 
 
15.7
 
 
 
 
BCAP29
 
 
hsa-let-7e-5p
 
 
-0.289
 
 
21.7
 
 
 
 
BCL2L11
 
 
hsa-miR-10a-5p
 
 
-0.483
 
 
11.1
 
 
 
 
BIN1
 
 
hsa-miR-22-3p
 
 
-0.346
 
 
21.2
 
 
 
 
BIN3
 
 
hsa-let-7e-5p
 
 
-0.427
 
 
11.2
 
 
 
 
BIN3
 
 
hsa-let-7c-5p
 
 
-0.427
 
 
11.2
 
 
 
 
BLOC1S1
 
 
hsa-miR-22-3p
 
 
-0.346
 
 
74.6
 
 
 
 
BLOC1S6
 
 
hsa-let-7e-5p
 
 
-0.321
 
 
24.8
 
 
 
 
BLOC1S6
 
 
hsa-let-7c-5p
 
 
-0.321
 
 
24.8
 
 
 
 
BLZF1
 
 
hsa-miR-125a-5p
 
 
-0.461
 
 
15.3
 
 
 
 
BMF
 
 
hsa-miR-125a-5p
 
 
-0.34
 
 
11.4
 
 
 
 
BMPR2
 
 
hsa-miR-99b-5p
 
 
-0.327
 
 
12.7
 
 
 
 
BTF3L4
 
 
hsa-let-7e-5p
 
 
-0.355
 
 
22.4
 
 
 
 
BTF3L4
 
 
hsa-let-7c-5p
 
 
-0.355
 
 
22.4
 
 
 
 
BZW1
 
 
hsa-let-7e-5p
 
 
-0.354
 
 
87.2
 
 
 
 
BZW1
 
 
hsa-let-7c-5p
 
 
-0.354
 
 
87.2
 
 
 
 
BZW1
 
 
hsa-let-7e-5p
 
 
-0.375
 
 
87.2
 
 
 
 
BZW1
 
 
hsa-let-7c-5p
 
 
-0.375
 
 
87.2
 
 
 
 
BZW2
 
 
hsa-let-7c-5p
 
 
-0.397
 
 
16.1
 
 
 
 
BZW2
 
 
hsa-let-7e-5p
 
 
-0.408
 
 
16.1
 
 
 
 
C1GALT1
 
 
hsa-let-7e-5p
 
 
-0.322
 
 
15.6
 
 
 
 
C1GALT1
 
 
hsa-let-7c-5p
 
 
-0.322
 
 
15.6
 
 
 
 
C5orf24
 
 
hsa-miR-22-3p
 
 
-0.493
 
 
20.0
 
 
 
 
CAMK2N1
 
 
hsa-miR-22-3p
 
 
-0.302
 
 
25.7
 
 
 
 
CANT1
 
 
hsa-let-7e-5p
 
 
-0.402
 
 
23.6
 
 
 
 
CANT1
 
 
hsa-let-7c-5p
 
 
-0.402
 
 
23.6
 
 
 
 
CAPN1
 
 
hsa-miR-22-3p
 
 
-0.307
 
 
52.1
 
 
 
 
CASP3
 
 
hsa-let-7e-5p
 
 
-0.495
 
 
15.7
 
 
 
 
CASP3
 
 
hsa-let-7c-5p
 
 
-0.503
 
 
15.7
 
 
 
 
CAST
 
 
hsa-miR-150-5p
 
 
-0.354
 
 
73.8
 
 
 
 
CCDC47
 
 
hsa-miR-22-3p
 
 
-0.604
 
 
44.0
 
 
 
 
CCN1
 
 
hsa-miR-22-3p
 
 
-0.601
 
 
60.6
 
 
 
 
CCNY
 
 
hsa-let-7c-5p
 
 
-0.323
 
 
21.3
 
 
 
 
CCNY
 
 
hsa-let-7e-5p
 
 
-0.334
 
 
21.3
 
 
 
 
CD86
 
 
hsa-let-7e-5p
 
 
-0.323
 
 
13.4
 
 
 
 
CD86
 
 
hsa-let-7c-5p
 
 
-0.323
 
 
13.4
 
 
 
 
CDC37L1
 
 
hsa-miR-125a-5p
 
 
-0.401
 
 
13.7
 
 
 
 
CDC42SE1
 
 
hsa-miR-125a-5p
 
 
-0.501
 
 
34.8
 
 
 
 
CDH5
 
 
hsa-miR-125a-5p
 
 
-0.36
 
 
27.8
 
 
 
 
CDKN1A
 
 
hsa-let-7e-5p
 
 
-0.434
 
 
124.3
 
 
 
 
CDKN1A
 
 
hsa-let-7c-5p
 
 
-0.434
 
 
124.3
 
 
 
 
CDV3
 
 
hsa-let-7e-5p
 
 
-0.453
 
 
70.6
 
 
 
 
CDV3
 
 
hsa-let-7c-5p
 
 
-0.461
 
 
70.6
 
 
 
 
CEBPD
 
 
hsa-let-7e-5p
 
 
-0.309
 
 
13.8
 
 
 
 
CEBPD
 
 
hsa-let-7c-5p
 
 
-0.309
 
 
13.8
 
 
 
 
CELF2
 
 
hsa-miR-10a-5p
 
 
-0.319
 
 
16.1
 
 
 
 
CEMIP2
 
 
hsa-let-7e-5p
 
 
-0.453
 
 
38.9
 
 
 
 
CEMIP2
 
 
hsa-let-7c-5p
 
 
-0.453
 
 
38.9
 
 
 
 
CERCAM
 
 
hsa-let-7e-5p
 
 
-0.344
 
 
29.2
 
 
 
 
CERCAM
 
 
hsa-let-7c-5p
 
 
-0.344
 
 
29.2
 
 
 
 
CFAP36
 
 
hsa-miR-22-3p
 
 
-0.549
 
 
38.3
 
 
 
 
CLASP2
 
 
hsa-let-7e-5p
 
 
-0.344
 
 
16.8
 
 
 
 
CLASP2
 
 
hsa-let-7c-5p
 
 
-0.344
 
 
16.8
 
 
 
 
CLDN12
 
 
hsa-let-7e-5p
 
 
-0.511
 
 
17.8
 
 
 
 
CLDN12
 
 
hsa-let-7c-5p
 
 
-0.511
 
 
17.8
 
 
 
 
CLIC4
 
 
hsa-miR-22-3p
 
 
-0.594
 
 
99.8
 
 
 
 
CMTM6
 
 
hsa-miR-150-5p
 
 
-0.328
 
 
47.2
 
 
 
 
CNOT6L
 
 
hsa-miR-22-3p
 
 
-0.293
 
 
12.2
 
 
 
 
COL1A2
 
 
hsa-let-7e-5p
 
 
-0.499
 
 
1249.9
 
 
 
 
COL1A2
 
 
hsa-let-7c-5p
 
 
-0.499
 
 
1249.9
 
 
 
 
 
 
 heart muscle 
 
 
 
 
target_symbol
 
 
mature_mirna_id
 
 
score
 
 
nTPM
 
 
 
 
 
 
ABCC5
 
 
hsa-miR-125a-5p
 
 
-0.288
 
 
47.0
 
 
 
 
ABTB1
 
 
hsa-miR-125a-5p
 
 
-0.519
 
 
42.4
 
 
 
 
ACHE
 
 
hsa-miR-125a-5p
 
 
-0.308
 
 
11.4
 
 
 
 
ACHE
 
 
hsa-miR-125a-5p
 
 
-0.361
 
 
11.4
 
 
 
 
ADIPOR2
 
 
hsa-miR-150-5p
 
 
-0.287
 
 
31.9
 
 
 
 
ADRB1
 
 
hsa-let-7c-5p
 
 
-0.351
 
 
20.3
 
 
 
 
ADRB1
 
 
hsa-let-7e-5p
 
 
-0.367
 
 
20.3
 
 
 
 
AIDA
 
 
hsa-miR-22-3p
 
 
-0.316
 
 
42.8
 
 
 
 
AIFM1
 
 
hsa-miR-125a-5p
 
 
-0.3
 
 
74.9
 
 
 
 
AIFM1
 
 
hsa-let-7e-5p
 
 
-0.314
 
 
74.9
 
 
 
 
AIFM1
 
 
hsa-let-7c-5p
 
 
-0.314
 
 
74.9
 
 
 
 
AKAP6
 
 
hsa-let-7e-5p
 
 
-0.307
 
 
51.1
 
 
 
 
AKAP6
 
 
hsa-let-7c-5p
 
 
-0.307
 
 
51.1
 
 
 
 
AKT3
 
 
hsa-miR-22-3p
 
 
-0.451
 
 
10.6
 
 
 
 
ALDH4A1
 
 
hsa-miR-184
 
 
-0.421
 
 
30.9
 
 
 
 
ALPL
 
 
hsa-miR-10a-5p
 
 
-0.32
 
 
15.8
 
 
 
 
AMT
 
 
hsa-let-7e-5p
 
 
-0.463
 
 
23.5
 
 
 
 
AMT
 
 
hsa-let-7c-5p
 
 
-0.463
 
 
23.5
 
 
 
 
ANAPC16
 
 
hsa-miR-125a-5p
 
 
-0.578
 
 
42.0
 
 
 
 
ANKRD13A
 
 
hsa-miR-22-3p
 
 
-0.478
 
 
10.0
 
 
 
 
ANKRD46
 
 
hsa-let-7e-5p
 
 
-0.389
 
 
18.8
 
 
 
 
ANKRD46
 
 
hsa-let-7c-5p
 
 
-0.389
 
 
18.8
 
 
 
 
AP1S1
 
 
hsa-let-7e-5p
 
 
-0.374
 
 
12.9
 
 
 
 
AP1S1
 
 
hsa-let-7c-5p
 
 
-0.374
 
 
12.9
 
 
 
 
ARL4D
 
 
hsa-let-7e-5p
 
 
-0.417
 
 
12.5
 
 
 
 
ARL4D
 
 
hsa-let-7c-5p
 
 
-0.417
 
 
12.5
 
 
 
 
ARMT1
 
 
hsa-let-7e-5p
 
 
-0.303
 
 
15.7
 
 
 
 
ARMT1
 
 
hsa-let-7c-5p
 
 
-0.303
 
 
15.7
 
 
 
 
ARPC5
 
 
hsa-miR-22-3p
 
 
-0.679
 
 
33.5
 
 
 
 
ARPP19
 
 
hsa-let-7e-5p
 
 
-0.492
 
 
76.2
 
 
 
 
ARPP19
 
 
hsa-let-7c-5p
 
 
-0.492
 
 
76.2
 
 
 
 
ARRB1
 
 
hsa-miR-22-3p
 
 
-0.616
 
 
16.5
 
 
 
 
ARRDC4
 
 
hsa-let-7e-5p
 
 
-0.329
 
 
11.3
 
 
 
 
ARRDC4
 
 
hsa-let-7c-5p
 
 
-0.329
 
 
11.3
 
 
 
 
ASAP1
 
 
hsa-let-7e-5p
 
 
-0.296
 
 
22.3
 
 
 
 
ASAP1
 
 
hsa-let-7c-5p
 
 
-0.308
 
 
22.3
 
 
 
 
ATOSB
 
 
hsa-let-7e-5p
 
 
-0.429
 
 
18.9
 
 
 
 
ATOSB
 
 
hsa-let-7c-5p
 
 
-0.429
 
 
18.9
 
 
 
 
ATP2A2
 
 
hsa-let-7e-5p
 
 
-0.311
 
 
941.9
 
 
 
 
ATP2A2
 
 
hsa-let-7c-5p
 
 
-0.311
 
 
941.9
 
 
 
 
ATPAF1
 
 
hsa-let-7e-5p
 
 
-0.498
 
 
249.4
 
 
 
 
ATPAF1
 
 
hsa-let-7c-5p
 
 
-0.498
 
 
249.4
 
 
 
 
ATXN2
 
 
hsa-miR-10a-5p
 
 
-0.362
 
 
22.3
 
 
 
 
B3GAT3
 
 
hsa-let-7e-5p
 
 
-0.324
 
 
25.1
 
 
 
 
B3GAT3
 
 
hsa-let-7c-5p
 
 
-0.324
 
 
25.1
 
 
 
 
B4GAT1
 
 
hsa-let-7e-5p
 
 
-0.47
 
 
43.6
 
 
 
 
B4GAT1
 
 
hsa-let-7c-5p
 
 
-0.47
 
 
43.6
 
 
 
 
BACH1
 
 
hsa-let-7e-5p
 
 
-0.298
 
 
12.3
 
 
 
 
BACH1
 
 
hsa-let-7e-5p
 
 
-0.298
 
 
12.3
 
 
 
 
BACH1
 
 
hsa-let-7c-5p
 
 
-0.306
 
 
12.3
 
 
 
 
BACH1
 
 
hsa-let-7c-5p
 
 
-0.306
 
 
12.3
 
 
 
 
BACH1
 
 
hsa-let-7e-5p
 
 
-0.435
 
 
12.3
 
 
 
 
BACH1
 
 
hsa-let-7e-5p
 
 
-0.435
 
 
12.3
 
 
 
 
BACH1
 
 
hsa-let-7c-5p
 
 
-0.435
 
 
12.3
 
 
 
 
BACH1
 
 
hsa-let-7c-5p
 
 
-0.435
 
 
12.3
 
 
 
 
BAG5
 
 
hsa-miR-22-3p
 
 
-0.318
 
 
12.4
 
 
 
 
BAK1
 
 
hsa-miR-125a-5p
 
 
-0.402
 
 
21.7
 
 
 
 
BAZ1B
 
 
hsa-miR-10a-5p
 
 
-0.309
 
 
18.3
 
 
 
 
BAZ2A
 
 
hsa-miR-99b-5p
 
 
-0.408
 
 
19.8
 
 
 
 
BBX
 
 
hsa-miR-10a-5p
 
 
-0.305
 
 
15.4
 
 
 
 
BCAP29
 
 
hsa-let-7e-5p
 
 
-0.289
 
 
57.5
 
 
 
 
BDH1
 
 
hsa-miR-125a-5p
 
 
-0.389
 
 
48.7
 
 
 
 
BIN1
 
 
hsa-miR-22-3p
 
 
-0.346
 
 
15.7
 
 
 
 
BIN3
 
 
hsa-let-7e-5p
 
 
-0.427
 
 
13.1
 
 
 
 
BIN3
 
 
hsa-let-7c-5p
 
 
-0.427
 
 
13.1
 
 
 
 
BLOC1S1
 
 
hsa-miR-22-3p
 
 
-0.346
 
 
165.5
 
 
 
 
BLOC1S6
 
 
hsa-let-7e-5p
 
 
-0.321
 
 
23.5
 
 
 
 
BLOC1S6
 
 
hsa-let-7c-5p
 
 
-0.321
 
 
23.5
 
 
 
 
BLZF1
 
 
hsa-miR-125a-5p
 
 
-0.461
 
 
14.9
 
 
 
 
BMPR2
 
 
hsa-miR-99b-5p
 
 
-0.327
 
 
16.2
 
 
 
 
BORCS6
 
 
hsa-miR-125a-5p
 
 
-0.287
 
 
14.0
 
 
 
 
BTF3L4
 
 
hsa-let-7e-5p
 
 
-0.355
 
 
37.6
 
 
 
 
BTF3L4
 
 
hsa-let-7c-5p
 
 
-0.355
 
 
37.6
 
 
 
 
BZW1
 
 
hsa-let-7e-5p
 
 
-0.354
 
 
53.4
 
 
 
 
BZW1
 
 
hsa-let-7c-5p
 
 
-0.354
 
 
53.4
 
 
 
 
BZW1
 
 
hsa-let-7e-5p
 
 
-0.375
 
 
53.4
 
 
 
 
BZW1
 
 
hsa-let-7c-5p
 
 
-0.375
 
 
53.4
 
 
 
 
BZW2
 
 
hsa-let-7c-5p
 
 
-0.397
 
 
248.4
 
 
 
 
BZW2
 
 
hsa-let-7e-5p
 
 
-0.408
 
 
248.4
 
 
 
 
C15orf39
 
 
hsa-miR-125a-5p
 
 
-0.335
 
 
13.8
 
 
 
 
C15orf39
 
 
hsa-let-7e-5p
 
 
-0.374
 
 
13.8
 
 
 
 
C15orf39
 
 
hsa-let-7c-5p
 
 
-0.374
 
 
13.8
 
 
 
 
C19orf47
 
 
hsa-let-7e-5p
 
 
-0.311
 
 
164.2
 
 
 
 
C19orf47
 
 
hsa-let-7c-5p
 
 
-0.311
 
 
164.2
 
 
 
 
C1GALT1
 
 
hsa-let-7e-5p
 
 
-0.322
 
 
11.8
 
 
 
 
C1GALT1
 
 
hsa-let-7c-5p
 
 
-0.322
 
 
11.8
 
 
 
 
C2orf88
 
 
hsa-let-7e-5p
 
 
-0.429
 
 
11.0
 
 
 
 
C2orf88
 
 
hsa-let-7c-5p
 
 
-0.429
 
 
11.0
 
 
 
 
C5orf24
 
 
hsa-miR-22-3p
 
 
-0.493
 
 
18.3
 
 
 
 
C6orf47
 
 
hsa-miR-125a-5p
 
 
-0.564
 
 
16.3
 
 
 
 
CACFD1
 
 
hsa-let-7e-5p
 
 
-0.296
 
 
10.5
 
 
 
 
CACFD1
 
 
hsa-let-7c-5p
 
 
-0.296
 
 
10.5
 
 
 
 
CAMK2N1
 
 
hsa-miR-22-3p
 
 
-0.302
 
 
21.2
 
 
 
 
CANT1
 
 
hsa-let-7e-5p
 
 
-0.402
 
 
15.5
 
 
 
 
CANT1
 
 
hsa-let-7c-5p
 
 
-0.402
 
 
15.5
 
 
 
 
CAPN1
 
 
hsa-miR-22-3p
 
 
-0.307
 
 
49.7
 
 
 
 
CAPN3
 
 
hsa-let-7e-5p
 
 
-0.317
 
 
16.9
 
 
 
 
CAPN3
 
 
hsa-let-7c-5p
 
 
-0.317
 
 
16.9
 
 
 
 
CASP3
 
 
hsa-let-7e-5p
 
 
-0.495
 
 
12.7
 
 
 
 
CASP3
 
 
hsa-let-7c-5p
 
 
-0.503
 
 
12.7
 
 
 
 
 
 
 hippocampal formation 
 
 
 
 
target_symbol
 
 
mature_mirna_id
 
 
score
 
 
nTPM
 
 
 
 
 
 
ABCC5
 
 
hsa-miR-125a-5p
 
 
-0.288
 
 
30.8
 
 
 
 
ABHD17C
 
 
hsa-let-7e-5p
 
 
-0.359
 
 
14.4
 
 
 
 
ABHD17C
 
 
hsa-let-7c-5p
 
 
-0.359
 
 
14.4
 
 
 
 
ABHD6
 
 
hsa-miR-125a-5p
 
 
-0.501
 
 
25.8
 
 
 
 
ABTB1
 
 
hsa-miR-125a-5p
 
 
-0.519
 
 
29.3
 
 
 
 
ACHE
 
 
hsa-miR-125a-5p
 
 
-0.308
 
 
14.5
 
 
 
 
ACHE
 
 
hsa-miR-125a-5p
 
 
-0.361
 
 
14.5
 
 
 
 
ACSL6
 
 
hsa-miR-125a-5p
 
 
-0.307
 
 
15.3
 
 
 
 
ACSL6
 
 
hsa-let-7e-5p
 
 
-0.336
 
 
15.3
 
 
 
 
ACSL6
 
 
hsa-let-7c-5p
 
 
-0.336
 
 
15.3
 
 
 
 
ADIPOR2
 
 
hsa-miR-150-5p
 
 
-0.287
 
 
57.6
 
 
 
 
AIDA
 
 
hsa-miR-22-3p
 
 
-0.316
 
 
22.9
 
 
 
 
AIFM1
 
 
hsa-miR-125a-5p
 
 
-0.3
 
 
10.5
 
 
 
 
AIFM1
 
 
hsa-let-7e-5p
 
 
-0.314
 
 
10.5
 
 
 
 
AIFM1
 
 
hsa-let-7c-5p
 
 
-0.314
 
 
10.5
 
 
 
 
AKAP6
 
 
hsa-let-7e-5p
 
 
-0.307
 
 
12.9
 
 
 
 
AKAP6
 
 
hsa-let-7c-5p
 
 
-0.307
 
 
12.9
 
 
 
 
AKT3
 
 
hsa-miR-22-3p
 
 
-0.451
 
 
17.7
 
 
 
 
ALDH4A1
 
 
hsa-miR-184
 
 
-0.421
 
 
39.7
 
 
 
 
ALPL
 
 
hsa-miR-10a-5p
 
 
-0.32
 
 
13.7
 
 
 
 
ANGEL2
 
 
hsa-miR-22-3p
 
 
-0.601
 
 
11.0
 
 
 
 
ANKRD46
 
 
hsa-let-7e-5p
 
 
-0.389
 
 
25.5
 
 
 
 
ANKRD46
 
 
hsa-let-7c-5p
 
 
-0.389
 
 
25.5
 
 
 
 
AP1S1
 
 
hsa-let-7e-5p
 
 
-0.374
 
 
67.5
 
 
 
 
AP1S1
 
 
hsa-let-7c-5p
 
 
-0.374
 
 
67.5
 
 
 
 
ARPC5
 
 
hsa-miR-22-3p
 
 
-0.679
 
 
23.8
 
 
 
 
ARPP19
 
 
hsa-let-7e-5p
 
 
-0.492
 
 
120.9
 
 
 
 
ARPP19
 
 
hsa-let-7c-5p
 
 
-0.492
 
 
120.9
 
 
 
 
ARRB1
 
 
hsa-miR-22-3p
 
 
-0.616
 
 
25.3
 
 
 
 
ARRDC4
 
 
hsa-let-7e-5p
 
 
-0.329
 
 
23.9
 
 
 
 
ARRDC4
 
 
hsa-let-7c-5p
 
 
-0.329
 
 
23.9
 
 
 
 
ASAP1
 
 
hsa-let-7e-5p
 
 
-0.296
 
 
10.8
 
 
 
 
ASAP1
 
 
hsa-let-7c-5p
 
 
-0.308
 
 
10.8
 
 
 
 
ATCAY
 
 
hsa-miR-10a-5p
 
 
-0.322
 
 
38.9
 
 
 
 
ATOSB
 
 
hsa-let-7e-5p
 
 
-0.429
 
 
22.4
 
 
 
 
ATOSB
 
 
hsa-let-7c-5p
 
 
-0.429
 
 
22.4
 
 
 
 
ATP2A2
 
 
hsa-let-7e-5p
 
 
-0.311
 
 
16.6
 
 
 
 
ATP2A2
 
 
hsa-let-7c-5p
 
 
-0.311
 
 
16.6
 
 
 
 
ATPAF1
 
 
hsa-let-7e-5p
 
 
-0.498
 
 
54.4
 
 
 
 
ATPAF1
 
 
hsa-let-7c-5p
 
 
-0.498
 
 
54.4
 
 
 
 
ATXN2
 
 
hsa-miR-10a-5p
 
 
-0.362
 
 
22.7
 
 
 
 
B3GAT3
 
 
hsa-let-7e-5p
 
 
-0.324
 
 
64.6
 
 
 
 
B3GAT3
 
 
hsa-let-7c-5p
 
 
-0.324
 
 
64.6
 
 
 
 
B4GAT1
 
 
hsa-let-7e-5p
 
 
-0.47
 
 
117.5
 
 
 
 
B4GAT1
 
 
hsa-let-7c-5p
 
 
-0.47
 
 
117.5
 
 
 
 
BAG5
 
 
hsa-miR-22-3p
 
 
-0.318
 
 
10.9
 
 
 
 
BAZ1B
 
 
hsa-miR-10a-5p
 
 
-0.309
 
 
16.8
 
 
 
 
BAZ2A
 
 
hsa-miR-99b-5p
 
 
-0.408
 
 
10.1
 
 
 
 
BBX
 
 
hsa-miR-10a-5p
 
 
-0.305
 
 
10.1
 
 
 
 
BCAP29
 
 
hsa-let-7e-5p
 
 
-0.289
 
 
16.3
 
 
 
 
BDH1
 
 
hsa-miR-125a-5p
 
 
-0.389
 
 
19.7
 
 
 
 
BEGAIN
 
 
hsa-let-7e-5p
 
 
-0.466
 
 
36.8
 
 
 
 
BEGAIN
 
 
hsa-let-7c-5p
 
 
-0.466
 
 
36.8
 
 
 
 
BIN1
 
 
hsa-miR-22-3p
 
 
-0.346
 
 
207.9
 
 
 
 
BIN3
 
 
hsa-let-7e-5p
 
 
-0.427
 
 
10.9
 
 
 
 
BIN3
 
 
hsa-let-7c-5p
 
 
-0.427
 
 
10.9
 
 
 
 
BLOC1S1
 
 
hsa-miR-22-3p
 
 
-0.346
 
 
227.5
 
 
 
 
BLOC1S6
 
 
hsa-let-7e-5p
 
 
-0.321
 
 
18.3
 
 
 
 
BLOC1S6
 
 
hsa-let-7c-5p
 
 
-0.321
 
 
18.3
 
 
 
 
BTF3L4
 
 
hsa-let-7e-5p
 
 
-0.355
 
 
28.4
 
 
 
 
BTF3L4
 
 
hsa-let-7c-5p
 
 
-0.355
 
 
28.4
 
 
 
 
BZW1
 
 
hsa-let-7e-5p
 
 
-0.354
 
 
34.8
 
 
 
 
BZW1
 
 
hsa-let-7c-5p
 
 
-0.354
 
 
34.8
 
 
 
 
BZW1
 
 
hsa-let-7e-5p
 
 
-0.375
 
 
34.8
 
 
 
 
BZW1
 
 
hsa-let-7c-5p
 
 
-0.375
 
 
34.8
 
 
 
 
C19orf47
 
 
hsa-let-7e-5p
 
 
-0.311
 
 
14.0
 
 
 
 
C19orf47
 
 
hsa-let-7c-5p
 
 
-0.311
 
 
14.0
 
 
 
 
C1GALT1
 
 
hsa-let-7e-5p
 
 
-0.322
 
 
11.6
 
 
 
 
C1GALT1
 
 
hsa-let-7c-5p
 
 
-0.322
 
 
11.6
 
 
 
 
C5orf24
 
 
hsa-miR-22-3p
 
 
-0.493
 
 
18.9
 
 
 
 
CACNB3
 
 
hsa-miR-125a-5p
 
 
-0.393
 
 
50.1
 
 
 
 
CACNG4
 
 
hsa-let-7e-5p
 
 
-0.33
 
 
24.4
 
 
 
 
CACNG4
 
 
hsa-let-7c-5p
 
 
-0.33
 
 
24.4
 
 
 
 
CADM2
 
 
hsa-let-7e-5p
 
 
-0.294
 
 
17.6
 
 
 
 
CADM2
 
 
hsa-let-7c-5p
 
 
-0.294
 
 
17.6
 
 
 
 
CAMK2N1
 
 
hsa-miR-22-3p
 
 
-0.302
 
 
353.4
 
 
 
 
CAPN1
 
 
hsa-miR-22-3p
 
 
-0.307
 
 
22.1
 
 
 
 
CAPN3
 
 
hsa-let-7e-5p
 
 
-0.317
 
 
121.1
 
 
 
 
CAPN3
 
 
hsa-let-7c-5p
 
 
-0.317
 
 
121.1
 
 
 
 
CAST
 
 
hsa-miR-150-5p
 
 
-0.354
 
 
26.3
 
 
 
 
CBX5
 
 
hsa-let-7e-5p
 
 
-0.307
 
 
16.0
 
 
 
 
CBX5
 
 
hsa-let-7c-5p
 
 
-0.307
 
 
16.0
 
 
 
 
CBX5
 
 
hsa-let-7e-5p
 
 
-0.472
 
 
16.0
 
 
 
 
CBX5
 
 
hsa-let-7c-5p
 
 
-0.472
 
 
16.0
 
 
 
 
CCDC149
 
 
hsa-miR-22-3p
 
 
-0.327
 
 
10.0
 
 
 
 
CCDC47
 
 
hsa-miR-22-3p
 
 
-0.604
 
 
30.6
 
 
 
 
CCN1
 
 
hsa-miR-22-3p
 
 
-0.601
 
 
11.7
 
 
 
 
CCNY
 
 
hsa-let-7c-5p
 
 
-0.323
 
 
33.9
 
 
 
 
CCNY
 
 
hsa-let-7e-5p
 
 
-0.334
 
 
33.9
 
 
 
 
CDC34
 
 
hsa-let-7c-5p
 
 
-0.309
 
 
52.2
 
 
 
 
CDC34
 
 
hsa-let-7e-5p
 
 
-0.321
 
 
52.2
 
 
 
 
CDC34
 
 
hsa-let-7e-5p
 
 
-0.555
 
 
52.2
 
 
 
 
CDC34
 
 
hsa-let-7c-5p
 
 
-0.555
 
 
52.2
 
 
 
 
CDC37L1
 
 
hsa-miR-125a-5p
 
 
-0.401
 
 
11.3
 
 
 
 
CDC42SE1
 
 
hsa-miR-125a-5p
 
 
-0.501
 
 
26.6
 
 
 
 
CDH5
 
 
hsa-miR-125a-5p
 
 
-0.36
 
 
10.4
 
 
 
 
CDKN1A
 
 
hsa-let-7e-5p
 
 
-0.434
 
 
35.2
 
 
 
 
CDKN1A
 
 
hsa-let-7c-5p
 
 
-0.434
 
 
35.2
 
 
 
 
CDV3
 
 
hsa-let-7e-5p
 
 
-0.453
 
 
33.9
 
 
 
 
CDV3
 
 
hsa-let-7c-5p
 
 
-0.461
 
 
33.9
 
 
 
 
 
 
 kidney 
 
 
 
 
target_symbol
 
 
mature_mirna_id
 
 
score
 
 
nTPM
 
 
 
 
 
 
ABCC5
 
 
hsa-miR-125a-5p
 
 
-0.288
 
 
48.3
 
 
 
 
ABHD17C
 
 
hsa-let-7e-5p
 
 
-0.359
 
 
13.6
 
 
 
 
ABHD17C
 
 
hsa-let-7c-5p
 
 
-0.359
 
 
13.6
 
 
 
 
ABHD3
 
 
hsa-miR-125a-5p
 
 
-0.453
 
 
13.5
 
 
 
 
ABHD6
 
 
hsa-miR-125a-5p
 
 
-0.501
 
 
33.2
 
 
 
 
ABTB1
 
 
hsa-miR-125a-5p
 
 
-0.519
 
 
26.5
 
 
 
 
ACP3
 
 
hsa-let-7e-5p
 
 
-0.298
 
 
26.9
 
 
 
 
ACP3
 
 
hsa-let-7c-5p
 
 
-0.298
 
 
26.9
 
 
 
 
ADIPOR2
 
 
hsa-miR-150-5p
 
 
-0.287
 
 
20.9
 
 
 
 
AGGF1
 
 
hsa-miR-125a-5p
 
 
-0.331
 
 
12.1
 
 
 
 
AGMAT
 
 
hsa-miR-22-3p
 
 
-0.294
 
 
125.0
 
 
 
 
AIDA
 
 
hsa-miR-22-3p
 
 
-0.316
 
 
36.7
 
 
 
 
AIFM1
 
 
hsa-miR-125a-5p
 
 
-0.3
 
 
69.0
 
 
 
 
AIFM1
 
 
hsa-let-7e-5p
 
 
-0.314
 
 
69.0
 
 
 
 
AIFM1
 
 
hsa-let-7c-5p
 
 
-0.314
 
 
69.0
 
 
 
 
ALDH4A1
 
 
hsa-miR-184
 
 
-0.421
 
 
204.2
 
 
 
 
ALPL
 
 
hsa-miR-10a-5p
 
 
-0.32
 
 
42.7
 
 
 
 
AMT
 
 
hsa-let-7e-5p
 
 
-0.463
 
 
23.7
 
 
 
 
AMT
 
 
hsa-let-7c-5p
 
 
-0.463
 
 
23.7
 
 
 
 
ANAPC16
 
 
hsa-miR-125a-5p
 
 
-0.578
 
 
23.1
 
 
 
 
ANKHD1-EIF4EBP3
 
 
hsa-miR-22-3p
 
 
-0.479
 
 
11.4
 
 
 
 
ANKRD13A
 
 
hsa-miR-22-3p
 
 
-0.478
 
 
16.4
 
 
 
 
ANKRD46
 
 
hsa-let-7e-5p
 
 
-0.389
 
 
30.5
 
 
 
 
ANKRD46
 
 
hsa-let-7c-5p
 
 
-0.389
 
 
30.5
 
 
 
 
ANPEP
 
 
hsa-miR-125a-5p
 
 
-0.372
 
 
235.6
 
 
 
 
AP1AR
 
 
hsa-miR-99b-5p
 
 
-0.823
 
 
20.6
 
 
 
 
AP1S1
 
 
hsa-let-7e-5p
 
 
-0.374
 
 
48.3
 
 
 
 
AP1S1
 
 
hsa-let-7c-5p
 
 
-0.374
 
 
48.3
 
 
 
 
ARG2
 
 
hsa-let-7e-5p
 
 
-0.468
 
 
66.0
 
 
 
 
ARG2
 
 
hsa-let-7c-5p
 
 
-0.468
 
 
66.0
 
 
 
 
ARL4D
 
 
hsa-let-7e-5p
 
 
-0.417
 
 
35.1
 
 
 
 
ARL4D
 
 
hsa-let-7c-5p
 
 
-0.417
 
 
35.1
 
 
 
 
ARMT1
 
 
hsa-let-7e-5p
 
 
-0.303
 
 
38.7
 
 
 
 
ARMT1
 
 
hsa-let-7c-5p
 
 
-0.303
 
 
38.7
 
 
 
 
ARPC5
 
 
hsa-miR-22-3p
 
 
-0.679
 
 
36.4
 
 
 
 
ARPP19
 
 
hsa-let-7e-5p
 
 
-0.492
 
 
52.6
 
 
 
 
ARPP19
 
 
hsa-let-7c-5p
 
 
-0.492
 
 
52.6
 
 
 
 
ARRB1
 
 
hsa-miR-22-3p
 
 
-0.616
 
 
16.5
 
 
 
 
ARRDC4
 
 
hsa-let-7e-5p
 
 
-0.329
 
 
23.9
 
 
 
 
ARRDC4
 
 
hsa-let-7c-5p
 
 
-0.329
 
 
23.9
 
 
 
 
ASAP1
 
 
hsa-let-7e-5p
 
 
-0.296
 
 
10.1
 
 
 
 
ASAP1
 
 
hsa-let-7c-5p
 
 
-0.308
 
 
10.1
 
 
 
 
ATOSB
 
 
hsa-let-7e-5p
 
 
-0.429
 
 
14.3
 
 
 
 
ATOSB
 
 
hsa-let-7c-5p
 
 
-0.429
 
 
14.3
 
 
 
 
ATP2A2
 
 
hsa-let-7e-5p
 
 
-0.311
 
 
32.2
 
 
 
 
ATP2A2
 
 
hsa-let-7c-5p
 
 
-0.311
 
 
32.2
 
 
 
 
ATPAF1
 
 
hsa-let-7e-5p
 
 
-0.498
 
 
107.2
 
 
 
 
ATPAF1
 
 
hsa-let-7c-5p
 
 
-0.498
 
 
107.2
 
 
 
 
ATXN2
 
 
hsa-miR-10a-5p
 
 
-0.362
 
 
16.8
 
 
 
 
B3GAT3
 
 
hsa-let-7e-5p
 
 
-0.324
 
 
48.1
 
 
 
 
B3GAT3
 
 
hsa-let-7c-5p
 
 
-0.324
 
 
48.1
 
 
 
 
B4GAT1
 
 
hsa-let-7e-5p
 
 
-0.47
 
 
24.3
 
 
 
 
B4GAT1
 
 
hsa-let-7c-5p
 
 
-0.47
 
 
24.3
 
 
 
 
BACH1
 
 
hsa-let-7e-5p
 
 
-0.298
 
 
14.8
 
 
 
 
BACH1
 
 
hsa-let-7e-5p
 
 
-0.298
 
 
14.8
 
 
 
 
BACH1
 
 
hsa-let-7c-5p
 
 
-0.306
 
 
14.8
 
 
 
 
BACH1
 
 
hsa-let-7c-5p
 
 
-0.306
 
 
14.8
 
 
 
 
BACH1
 
 
hsa-let-7e-5p
 
 
-0.435
 
 
14.8
 
 
 
 
BACH1
 
 
hsa-let-7e-5p
 
 
-0.435
 
 
14.8
 
 
 
 
BACH1
 
 
hsa-let-7c-5p
 
 
-0.435
 
 
14.8
 
 
 
 
BACH1
 
 
hsa-let-7c-5p
 
 
-0.435
 
 
14.8
 
 
 
 
BAG5
 
 
hsa-miR-22-3p
 
 
-0.318
 
 
14.8
 
 
 
 
BAK1
 
 
hsa-miR-125a-5p
 
 
-0.402
 
 
20.0
 
 
 
 
BAZ1B
 
 
hsa-miR-10a-5p
 
 
-0.309
 
 
15.7
 
 
 
 
BAZ2A
 
 
hsa-miR-99b-5p
 
 
-0.408
 
 
18.9
 
 
 
 
BBX
 
 
hsa-miR-10a-5p
 
 
-0.305
 
 
18.2
 
 
 
 
BCAP29
 
 
hsa-let-7e-5p
 
 
-0.289
 
 
19.2
 
 
 
 
BCL2L11
 
 
hsa-miR-10a-5p
 
 
-0.483
 
 
10.8
 
 
 
 
BDH1
 
 
hsa-miR-125a-5p
 
 
-0.389
 
 
21.7
 
 
 
 
BIN1
 
 
hsa-miR-22-3p
 
 
-0.346
 
 
78.7
 
 
 
 
BIN3
 
 
hsa-let-7e-5p
 
 
-0.427
 
 
17.8
 
 
 
 
BIN3
 
 
hsa-let-7c-5p
 
 
-0.427
 
 
17.8
 
 
 
 
BLOC1S1
 
 
hsa-miR-22-3p
 
 
-0.346
 
 
215.5
 
 
 
 
BLOC1S6
 
 
hsa-let-7e-5p
 
 
-0.321
 
 
22.6
 
 
 
 
BLOC1S6
 
 
hsa-let-7c-5p
 
 
-0.321
 
 
22.6
 
 
 
 
BLZF1
 
 
hsa-miR-125a-5p
 
 
-0.461
 
 
15.9
 
 
 
 
BMF
 
 
hsa-miR-125a-5p
 
 
-0.34
 
 
10.2
 
 
 
 
BMPR2
 
 
hsa-miR-99b-5p
 
 
-0.327
 
 
12.2
 
 
 
 
BORCS6
 
 
hsa-miR-125a-5p
 
 
-0.287
 
 
10.8
 
 
 
 
BTF3L4
 
 
hsa-let-7e-5p
 
 
-0.355
 
 
32.7
 
 
 
 
BTF3L4
 
 
hsa-let-7c-5p
 
 
-0.355
 
 
32.7
 
 
 
 
BZW1
 
 
hsa-let-7e-5p
 
 
-0.354
 
 
82.6
 
 
 
 
BZW1
 
 
hsa-let-7c-5p
 
 
-0.354
 
 
82.6
 
 
 
 
BZW1
 
 
hsa-let-7e-5p
 
 
-0.375
 
 
82.6
 
 
 
 
BZW1
 
 
hsa-let-7c-5p
 
 
-0.375
 
 
82.6
 
 
 
 
BZW2
 
 
hsa-let-7c-5p
 
 
-0.397
 
 
25.1
 
 
 
 
BZW2
 
 
hsa-let-7e-5p
 
 
-0.408
 
 
25.1
 
 
 
 
C1GALT1
 
 
hsa-let-7e-5p
 
 
-0.322
 
 
28.8
 
 
 
 
C1GALT1
 
 
hsa-let-7c-5p
 
 
-0.322
 
 
28.8
 
 
 
 
C1orf210
 
 
hsa-miR-125a-5p
 
 
-0.584
 
 
20.3
 
 
 
 
C5orf24
 
 
hsa-miR-22-3p
 
 
-0.493
 
 
38.3
 
 
 
 
C6orf47
 
 
hsa-miR-125a-5p
 
 
-0.564
 
 
12.9
 
 
 
 
C8orf58
 
 
hsa-let-7e-5p
 
 
-0.612
 
 
12.6
 
 
 
 
C8orf58
 
 
hsa-let-7c-5p
 
 
-0.62
 
 
12.6
 
 
 
 
CACFD1
 
 
hsa-let-7e-5p
 
 
-0.296
 
 
28.4
 
 
 
 
CACFD1
 
 
hsa-let-7c-5p
 
 
-0.296
 
 
28.4
 
 
 
 
CACNB3
 
 
hsa-miR-125a-5p
 
 
-0.393
 
 
11.4
 
 
 
 
CAMK2N1
 
 
hsa-miR-22-3p
 
 
-0.302
 
 
81.6
 
 
 
 
CANT1
 
 
hsa-let-7e-5p
 
 
-0.402
 
 
14.3
 
 
 
 
CANT1
 
 
hsa-let-7c-5p
 
 
-0.402
 
 
14.3
 
 
 
 
 
 
 lung 
 
 
 
 
target_symbol
 
 
mature_mirna_id
 
 
score
 
 
nTPM
 
 
 
 
 
 
ABCC5
 
 
hsa-miR-125a-5p
 
 
-0.288
 
 
15.3
 
 
 
 
ABHD17C
 
 
hsa-let-7e-5p
 
 
-0.359
 
 
18.6
 
 
 
 
ABHD17C
 
 
hsa-let-7c-5p
 
 
-0.359
 
 
18.6
 
 
 
 
ABHD3
 
 
hsa-miR-125a-5p
 
 
-0.453
 
 
14.8
 
 
 
 
ABTB1
 
 
hsa-miR-125a-5p
 
 
-0.519
 
 
33.0
 
 
 
 
ADAM17
 
 
hsa-miR-125a-5p
 
 
-0.292
 
 
12.5
 
 
 
 
ADAMTS8
 
 
hsa-let-7e-5p
 
 
-0.571
 
 
33.2
 
 
 
 
ADAMTS8
 
 
hsa-let-7c-5p
 
 
-0.571
 
 
33.2
 
 
 
 
ADIPOR2
 
 
hsa-miR-150-5p
 
 
-0.287
 
 
42.5
 
 
 
 
ADRB1
 
 
hsa-let-7c-5p
 
 
-0.351
 
 
11.4
 
 
 
 
ADRB1
 
 
hsa-let-7e-5p
 
 
-0.367
 
 
11.4
 
 
 
 
ADRB2
 
 
hsa-let-7e-5p
 
 
-0.635
 
 
18.4
 
 
 
 
ADRB2
 
 
hsa-let-7c-5p
 
 
-0.635
 
 
18.4
 
 
 
 
AIDA
 
 
hsa-miR-22-3p
 
 
-0.316
 
 
43.6
 
 
 
 
AKT3
 
 
hsa-miR-22-3p
 
 
-0.451
 
 
13.9
 
 
 
 
ALPL
 
 
hsa-miR-10a-5p
 
 
-0.32
 
 
53.5
 
 
 
 
ANAPC16
 
 
hsa-miR-125a-5p
 
 
-0.578
 
 
17.6
 
 
 
 
ANKHD1-EIF4EBP3
 
 
hsa-miR-22-3p
 
 
-0.479
 
 
10.9
 
 
 
 
ANKRD13A
 
 
hsa-miR-22-3p
 
 
-0.478
 
 
25.4
 
 
 
 
ANKRD46
 
 
hsa-let-7e-5p
 
 
-0.389
 
 
10.1
 
 
 
 
ANKRD46
 
 
hsa-let-7c-5p
 
 
-0.389
 
 
10.1
 
 
 
 
ANPEP
 
 
hsa-miR-125a-5p
 
 
-0.372
 
 
31.9
 
 
 
 
AP1S1
 
 
hsa-let-7e-5p
 
 
-0.374
 
 
15.2
 
 
 
 
AP1S1
 
 
hsa-let-7c-5p
 
 
-0.374
 
 
15.2
 
 
 
 
ARHGEF26
 
 
hsa-miR-22-3p
 
 
-0.374
 
 
15.4
 
 
 
 
ARL4D
 
 
hsa-let-7e-5p
 
 
-0.417
 
 
16.0
 
 
 
 
ARL4D
 
 
hsa-let-7c-5p
 
 
-0.417
 
 
16.0
 
 
 
 
ARL6IP6
 
 
hsa-let-7c-5p
 
 
-0.293
 
 
11.9
 
 
 
 
ARL6IP6
 
 
hsa-let-7e-5p
 
 
-0.305
 
 
11.9
 
 
 
 
ARMT1
 
 
hsa-let-7e-5p
 
 
-0.303
 
 
18.4
 
 
 
 
ARMT1
 
 
hsa-let-7c-5p
 
 
-0.303
 
 
18.4
 
 
 
 
ARPC5
 
 
hsa-miR-22-3p
 
 
-0.679
 
 
97.1
 
 
 
 
ARPP19
 
 
hsa-let-7e-5p
 
 
-0.492
 
 
42.5
 
 
 
 
ARPP19
 
 
hsa-let-7c-5p
 
 
-0.492
 
 
42.5
 
 
 
 
ARRB1
 
 
hsa-miR-22-3p
 
 
-0.616
 
 
50.0
 
 
 
 
ARRDC4
 
 
hsa-let-7e-5p
 
 
-0.329
 
 
18.7
 
 
 
 
ARRDC4
 
 
hsa-let-7c-5p
 
 
-0.329
 
 
18.7
 
 
 
 
ASAP1
 
 
hsa-let-7e-5p
 
 
-0.296
 
 
27.6
 
 
 
 
ASAP1
 
 
hsa-let-7c-5p
 
 
-0.308
 
 
27.6
 
 
 
 
ATOSB
 
 
hsa-let-7e-5p
 
 
-0.429
 
 
20.8
 
 
 
 
ATOSB
 
 
hsa-let-7c-5p
 
 
-0.429
 
 
20.8
 
 
 
 
ATP2A2
 
 
hsa-let-7e-5p
 
 
-0.311
 
 
46.2
 
 
 
 
ATP2A2
 
 
hsa-let-7c-5p
 
 
-0.311
 
 
46.2
 
 
 
 
ATP8A1
 
 
hsa-miR-22-3p
 
 
-0.468
 
 
11.2
 
 
 
 
ATPAF1
 
 
hsa-let-7e-5p
 
 
-0.498
 
 
24.6
 
 
 
 
ATPAF1
 
 
hsa-let-7c-5p
 
 
-0.498
 
 
24.6
 
 
 
 
ATXN2
 
 
hsa-miR-10a-5p
 
 
-0.362
 
 
18.4
 
 
 
 
B3GAT3
 
 
hsa-let-7e-5p
 
 
-0.324
 
 
32.1
 
 
 
 
B3GAT3
 
 
hsa-let-7c-5p
 
 
-0.324
 
 
32.1
 
 
 
 
B4GAT1
 
 
hsa-let-7e-5p
 
 
-0.47
 
 
16.9
 
 
 
 
B4GAT1
 
 
hsa-let-7c-5p
 
 
-0.47
 
 
16.9
 
 
 
 
BACH1
 
 
hsa-let-7e-5p
 
 
-0.298
 
 
20.2
 
 
 
 
BACH1
 
 
hsa-let-7e-5p
 
 
-0.298
 
 
20.2
 
 
 
 
BACH1
 
 
hsa-let-7c-5p
 
 
-0.306
 
 
20.2
 
 
 
 
BACH1
 
 
hsa-let-7c-5p
 
 
-0.306
 
 
20.2
 
 
 
 
BACH1
 
 
hsa-let-7e-5p
 
 
-0.435
 
 
20.2
 
 
 
 
BACH1
 
 
hsa-let-7e-5p
 
 
-0.435
 
 
20.2
 
 
 
 
BACH1
 
 
hsa-let-7c-5p
 
 
-0.435
 
 
20.2
 
 
 
 
BACH1
 
 
hsa-let-7c-5p
 
 
-0.435
 
 
20.2
 
 
 
 
BAG5
 
 
hsa-miR-22-3p
 
 
-0.318
 
 
14.6
 
 
 
 
BAK1
 
 
hsa-miR-125a-5p
 
 
-0.402
 
 
12.9
 
 
 
 
BAZ1B
 
 
hsa-miR-10a-5p
 
 
-0.309
 
 
19.1
 
 
 
 
BAZ2A
 
 
hsa-miR-99b-5p
 
 
-0.408
 
 
33.3
 
 
 
 
BBX
 
 
hsa-miR-10a-5p
 
 
-0.305
 
 
12.6
 
 
 
 
BCAP29
 
 
hsa-let-7e-5p
 
 
-0.289
 
 
24.4
 
 
 
 
BIN1
 
 
hsa-miR-22-3p
 
 
-0.346
 
 
21.8
 
 
 
 
BIN2
 
 
hsa-miR-125a-5p
 
 
-0.679
 
 
16.0
 
 
 
 
BIN3
 
 
hsa-let-7e-5p
 
 
-0.427
 
 
12.4
 
 
 
 
BIN3
 
 
hsa-let-7c-5p
 
 
-0.427
 
 
12.4
 
 
 
 
BLOC1S1
 
 
hsa-miR-22-3p
 
 
-0.346
 
 
112.1
 
 
 
 
BLOC1S6
 
 
hsa-let-7e-5p
 
 
-0.321
 
 
19.2
 
 
 
 
BLOC1S6
 
 
hsa-let-7c-5p
 
 
-0.321
 
 
19.2
 
 
 
 
BLZF1
 
 
hsa-miR-125a-5p
 
 
-0.461
 
 
12.2
 
 
 
 
BMF
 
 
hsa-miR-125a-5p
 
 
-0.34
 
 
12.0
 
 
 
 
BMP5
 
 
hsa-let-7e-5p
 
 
-0.337
 
 
12.2
 
 
 
 
BMP5
 
 
hsa-let-7c-5p
 
 
-0.337
 
 
12.2
 
 
 
 
BMPR2
 
 
hsa-miR-99b-5p
 
 
-0.327
 
 
21.8
 
 
 
 
BTF3L4
 
 
hsa-let-7e-5p
 
 
-0.355
 
 
24.8
 
 
 
 
BTF3L4
 
 
hsa-let-7c-5p
 
 
-0.355
 
 
24.8
 
 
 
 
BZW1
 
 
hsa-let-7e-5p
 
 
-0.354
 
 
69.3
 
 
 
 
BZW1
 
 
hsa-let-7c-5p
 
 
-0.354
 
 
69.3
 
 
 
 
BZW1
 
 
hsa-let-7e-5p
 
 
-0.375
 
 
69.3
 
 
 
 
BZW1
 
 
hsa-let-7c-5p
 
 
-0.375
 
 
69.3
 
 
 
 
BZW2
 
 
hsa-let-7c-5p
 
 
-0.397
 
 
20.9
 
 
 
 
BZW2
 
 
hsa-let-7e-5p
 
 
-0.408
 
 
20.9
 
 
 
 
C15orf39
 
 
hsa-miR-125a-5p
 
 
-0.335
 
 
14.5
 
 
 
 
C15orf39
 
 
hsa-let-7e-5p
 
 
-0.374
 
 
14.5
 
 
 
 
C15orf39
 
 
hsa-let-7c-5p
 
 
-0.374
 
 
14.5
 
 
 
 
C1GALT1
 
 
hsa-let-7e-5p
 
 
-0.322
 
 
14.2
 
 
 
 
C1GALT1
 
 
hsa-let-7c-5p
 
 
-0.322
 
 
14.2
 
 
 
 
C5orf24
 
 
hsa-miR-22-3p
 
 
-0.493
 
 
21.6
 
 
 
 
C6orf47
 
 
hsa-miR-125a-5p
 
 
-0.564
 
 
11.4
 
 
 
 
C8orf58
 
 
hsa-let-7e-5p
 
 
-0.612
 
 
10.1
 
 
 
 
C8orf58
 
 
hsa-let-7c-5p
 
 
-0.62
 
 
10.1
 
 
 
 
CACFD1
 
 
hsa-let-7e-5p
 
 
-0.296
 
 
12.3
 
 
 
 
CACFD1
 
 
hsa-let-7c-5p
 
 
-0.296
 
 
12.3
 
 
 
 
CACNB3
 
 
hsa-miR-125a-5p
 
 
-0.393
 
 
11.1
 
 
 
 
CAMK2N1
 
 
hsa-miR-22-3p
 
 
-0.302
 
 
63.8
 
 
 
 
CANT1
 
 
hsa-let-7e-5p
 
 
-0.402
 
 
14.8
 
 
 
 
CANT1
 
 
hsa-let-7c-5p
 
 
-0.402
 
 
14.8
 
 
 
 
 
 
 lymph node 
 
 
 
 
target_symbol
 
 
mature_mirna_id
 
 
score
 
 
nTPM
 
 
 
 
 
 
ABCC5
 
 
hsa-miR-125a-5p
 
 
-0.288
 
 
12.3
 
 
 
 
ABHD3
 
 
hsa-miR-125a-5p
 
 
-0.453
 
 
27.9
 
 
 
 
ABTB1
 
 
hsa-miR-125a-5p
 
 
-0.519
 
 
20.7
 
 
 
 
AGGF1
 
 
hsa-miR-125a-5p
 
 
-0.331
 
 
12.7
 
 
 
 
AIDA
 
 
hsa-miR-22-3p
 
 
-0.316
 
 
47.9
 
 
 
 
AIFM1
 
 
hsa-miR-125a-5p
 
 
-0.3
 
 
15.1
 
 
 
 
AIFM1
 
 
hsa-let-7e-5p
 
 
-0.314
 
 
15.1
 
 
 
 
AIFM1
 
 
hsa-let-7c-5p
 
 
-0.314
 
 
15.1
 
 
 
 
ANAPC16
 
 
hsa-miR-125a-5p
 
 
-0.578
 
 
23.5
 
 
 
 
ANKRD13A
 
 
hsa-miR-22-3p
 
 
-0.478
 
 
50.3
 
 
 
 
ANPEP
 
 
hsa-miR-125a-5p
 
 
-0.372
 
 
12.0
 
 
 
 
AP1AR
 
 
hsa-miR-99b-5p
 
 
-0.823
 
 
11.3
 
 
 
 
AP1S1
 
 
hsa-let-7e-5p
 
 
-0.374
 
 
11.1
 
 
 
 
AP1S1
 
 
hsa-let-7c-5p
 
 
-0.374
 
 
11.1
 
 
 
 
ARL6IP6
 
 
hsa-let-7c-5p
 
 
-0.293
 
 
17.4
 
 
 
 
ARL6IP6
 
 
hsa-let-7e-5p
 
 
-0.305
 
 
17.4
 
 
 
 
ARMT1
 
 
hsa-let-7e-5p
 
 
-0.303
 
 
19.7
 
 
 
 
ARMT1
 
 
hsa-let-7c-5p
 
 
-0.303
 
 
19.7
 
 
 
 
ARPC5
 
 
hsa-miR-22-3p
 
 
-0.679
 
 
148.6
 
 
 
 
ARPP19
 
 
hsa-let-7e-5p
 
 
-0.492
 
 
45.1
 
 
 
 
ARPP19
 
 
hsa-let-7c-5p
 
 
-0.492
 
 
45.1
 
 
 
 
ARRB1
 
 
hsa-miR-22-3p
 
 
-0.616
 
 
14.3
 
 
 
 
ASAP1
 
 
hsa-let-7e-5p
 
 
-0.296
 
 
16.5
 
 
 
 
ASAP1
 
 
hsa-let-7c-5p
 
 
-0.308
 
 
16.5
 
 
 
 
ATP2A2
 
 
hsa-let-7e-5p
 
 
-0.311
 
 
33.7
 
 
 
 
ATP2A2
 
 
hsa-let-7c-5p
 
 
-0.311
 
 
33.7
 
 
 
 
ATP8A1
 
 
hsa-miR-22-3p
 
 
-0.468
 
 
14.0
 
 
 
 
ATPAF1
 
 
hsa-let-7e-5p
 
 
-0.498
 
 
30.3
 
 
 
 
ATPAF1
 
 
hsa-let-7c-5p
 
 
-0.498
 
 
30.3
 
 
 
 
B3GAT3
 
 
hsa-let-7e-5p
 
 
-0.324
 
 
24.9
 
 
 
 
B3GAT3
 
 
hsa-let-7c-5p
 
 
-0.324
 
 
24.9
 
 
 
 
BACH1
 
 
hsa-let-7e-5p
 
 
-0.298
 
 
15.7
 
 
 
 
BACH1
 
 
hsa-let-7e-5p
 
 
-0.298
 
 
15.7
 
 
 
 
BACH1
 
 
hsa-let-7c-5p
 
 
-0.306
 
 
15.7
 
 
 
 
BACH1
 
 
hsa-let-7c-5p
 
 
-0.306
 
 
15.7
 
 
 
 
BACH1
 
 
hsa-let-7e-5p
 
 
-0.435
 
 
15.7
 
 
 
 
BACH1
 
 
hsa-let-7e-5p
 
 
-0.435
 
 
15.7
 
 
 
 
BACH1
 
 
hsa-let-7c-5p
 
 
-0.435
 
 
15.7
 
 
 
 
BACH1
 
 
hsa-let-7c-5p
 
 
-0.435
 
 
15.7
 
 
 
 
BAG5
 
 
hsa-miR-22-3p
 
 
-0.318
 
 
16.0
 
 
 
 
BAK1
 
 
hsa-miR-125a-5p
 
 
-0.402
 
 
20.6
 
 
 
 
BAZ1B
 
 
hsa-miR-10a-5p
 
 
-0.309
 
 
26.0
 
 
 
 
BAZ2A
 
 
hsa-miR-99b-5p
 
 
-0.408
 
 
31.2
 
 
 
 
BBX
 
 
hsa-miR-10a-5p
 
 
-0.305
 
 
13.0
 
 
 
 
BCAP29
 
 
hsa-let-7e-5p
 
 
-0.289
 
 
15.9
 
 
 
 
BCL2L11
 
 
hsa-miR-10a-5p
 
 
-0.483
 
 
23.7
 
 
 
 
BCL2L12
 
 
hsa-miR-125a-5p
 
 
-0.288
 
 
19.2
 
 
 
 
BIN1
 
 
hsa-miR-22-3p
 
 
-0.346
 
 
38.4
 
 
 
 
BIN2
 
 
hsa-miR-125a-5p
 
 
-0.679
 
 
39.3
 
 
 
 
BIN3
 
 
hsa-let-7e-5p
 
 
-0.427
 
 
12.8
 
 
 
 
BIN3
 
 
hsa-let-7c-5p
 
 
-0.427
 
 
12.8
 
 
 
 
BLOC1S1
 
 
hsa-miR-22-3p
 
 
-0.346
 
 
103.4
 
 
 
 
BLOC1S6
 
 
hsa-let-7e-5p
 
 
-0.321
 
 
39.2
 
 
 
 
BLOC1S6
 
 
hsa-let-7c-5p
 
 
-0.321
 
 
39.2
 
 
 
 
BLZF1
 
 
hsa-miR-125a-5p
 
 
-0.461
 
 
11.1
 
 
 
 
BMF
 
 
hsa-miR-125a-5p
 
 
-0.34
 
 
12.8
 
 
 
 
BTF3L4
 
 
hsa-let-7e-5p
 
 
-0.355
 
 
34.2
 
 
 
 
BTF3L4
 
 
hsa-let-7c-5p
 
 
-0.355
 
 
34.2
 
 
 
 
BTG1
 
 
hsa-miR-22-3p
 
 
-0.468
 
 
22.3
 
 
 
 
BZW1
 
 
hsa-let-7e-5p
 
 
-0.354
 
 
91.4
 
 
 
 
BZW1
 
 
hsa-let-7c-5p
 
 
-0.354
 
 
91.4
 
 
 
 
BZW1
 
 
hsa-let-7e-5p
 
 
-0.375
 
 
91.4
 
 
 
 
BZW1
 
 
hsa-let-7c-5p
 
 
-0.375
 
 
91.4
 
 
 
 
BZW2
 
 
hsa-let-7c-5p
 
 
-0.397
 
 
28.0
 
 
 
 
BZW2
 
 
hsa-let-7e-5p
 
 
-0.408
 
 
28.0
 
 
 
 
C15orf39
 
 
hsa-miR-125a-5p
 
 
-0.335
 
 
10.2
 
 
 
 
C15orf39
 
 
hsa-let-7e-5p
 
 
-0.374
 
 
10.2
 
 
 
 
C15orf39
 
 
hsa-let-7c-5p
 
 
-0.374
 
 
10.2
 
 
 
 
C1GALT1
 
 
hsa-let-7e-5p
 
 
-0.322
 
 
17.8
 
 
 
 
C1GALT1
 
 
hsa-let-7c-5p
 
 
-0.322
 
 
17.8
 
 
 
 
C5orf24
 
 
hsa-miR-22-3p
 
 
-0.493
 
 
15.3
 
 
 
 
CANT1
 
 
hsa-let-7e-5p
 
 
-0.402
 
 
14.3
 
 
 
 
CANT1
 
 
hsa-let-7c-5p
 
 
-0.402
 
 
14.3
 
 
 
 
CAPN1
 
 
hsa-miR-22-3p
 
 
-0.307
 
 
53.3
 
 
 
 
CASP2
 
 
hsa-miR-125a-5p
 
 
-0.296
 
 
20.5
 
 
 
 
CASP3
 
 
hsa-let-7e-5p
 
 
-0.495
 
 
35.8
 
 
 
 
CASP3
 
 
hsa-let-7c-5p
 
 
-0.503
 
 
35.8
 
 
 
 
CAST
 
 
hsa-miR-150-5p
 
 
-0.354
 
 
49.5
 
 
 
 
CBX5
 
 
hsa-let-7e-5p
 
 
-0.307
 
 
17.6
 
 
 
 
CBX5
 
 
hsa-let-7c-5p
 
 
-0.307
 
 
17.6
 
 
 
 
CBX5
 
 
hsa-let-7e-5p
 
 
-0.472
 
 
17.6
 
 
 
 
CBX5
 
 
hsa-let-7c-5p
 
 
-0.472
 
 
17.6
 
 
 
 
CCDC47
 
 
hsa-miR-22-3p
 
 
-0.604
 
 
30.4
 
 
 
 
CCNY
 
 
hsa-let-7c-5p
 
 
-0.323
 
 
21.9
 
 
 
 
CCNY
 
 
hsa-let-7e-5p
 
 
-0.334
 
 
21.9
 
 
 
 
CCR7
 
 
hsa-let-7e-5p
 
 
-0.482
 
 
78.7
 
 
 
 
CCR7
 
 
hsa-let-7c-5p
 
 
-0.482
 
 
78.7
 
 
 
 
CD86
 
 
hsa-let-7e-5p
 
 
-0.323
 
 
27.0
 
 
 
 
CD86
 
 
hsa-let-7c-5p
 
 
-0.323
 
 
27.0
 
 
 
 
CDC34
 
 
hsa-let-7c-5p
 
 
-0.309
 
 
11.7
 
 
 
 
CDC34
 
 
hsa-let-7e-5p
 
 
-0.321
 
 
11.7
 
 
 
 
CDC34
 
 
hsa-let-7e-5p
 
 
-0.555
 
 
11.7
 
 
 
 
CDC34
 
 
hsa-let-7c-5p
 
 
-0.555
 
 
11.7
 
 
 
 
CDC37L1
 
 
hsa-miR-125a-5p
 
 
-0.401
 
 
12.2
 
 
 
 
CDC42SE1
 
 
hsa-miR-125a-5p
 
 
-0.501
 
 
79.0
 
 
 
 
CDCA8
 
 
hsa-let-7e-5p
 
 
-0.342
 
 
18.1
 
 
 
 
CDCA8
 
 
hsa-let-7c-5p
 
 
-0.342
 
 
18.1
 
 
 
 
CDH5
 
 
hsa-miR-125a-5p
 
 
-0.36
 
 
12.3
 
 
 
 
CDKN1A
 
 
hsa-let-7e-5p
 
 
-0.434
 
 
16.1
 
 
 
 
CDKN1A
 
 
hsa-let-7c-5p
 
 
-0.434
 
 
16.1
 
 
 
 
 
 
 ovary 
 
 
 
 
target_symbol
 
 
mature_mirna_id
 
 
score
 
 
nTPM
 
 
 
 
 
 
ABCC5
 
 
hsa-miR-125a-5p
 
 
-0.288
 
 
23.4
 
 
 
 
ABHD3
 
 
hsa-miR-125a-5p
 
 
-0.453
 
 
12.0
 
 
 
 
ABTB1
 
 
hsa-miR-125a-5p
 
 
-0.519
 
 
31.5
 
 
 
 
ADIPOR2
 
 
hsa-miR-150-5p
 
 
-0.287
 
 
24.2
 
 
 
 
ADRB3
 
 
hsa-let-7e-5p
 
 
-0.415
 
 
12.8
 
 
 
 
ADRB3
 
 
hsa-let-7c-5p
 
 
-0.415
 
 
12.8
 
 
 
 
AGGF1
 
 
hsa-miR-125a-5p
 
 
-0.331
 
 
10.3
 
 
 
 
AHCTF1
 
 
hsa-let-7e-5p
 
 
-0.374
 
 
13.1
 
 
 
 
AHCTF1
 
 
hsa-let-7c-5p
 
 
-0.374
 
 
13.1
 
 
 
 
AIDA
 
 
hsa-miR-22-3p
 
 
-0.316
 
 
41.9
 
 
 
 
AKT3
 
 
hsa-miR-22-3p
 
 
-0.451
 
 
21.9
 
 
 
 
AMT
 
 
hsa-let-7e-5p
 
 
-0.463
 
 
16.7
 
 
 
 
AMT
 
 
hsa-let-7c-5p
 
 
-0.463
 
 
16.7
 
 
 
 
ANAPC16
 
 
hsa-miR-125a-5p
 
 
-0.578
 
 
22.3
 
 
 
 
ANKRD13A
 
 
hsa-miR-22-3p
 
 
-0.478
 
 
16.1
 
 
 
 
ANKRD46
 
 
hsa-let-7e-5p
 
 
-0.389
 
 
19.3
 
 
 
 
ANKRD46
 
 
hsa-let-7c-5p
 
 
-0.389
 
 
19.3
 
 
 
 
AP1AR
 
 
hsa-miR-99b-5p
 
 
-0.823
 
 
10.0
 
 
 
 
AP1S1
 
 
hsa-let-7e-5p
 
 
-0.374
 
 
22.2
 
 
 
 
AP1S1
 
 
hsa-let-7c-5p
 
 
-0.374
 
 
22.2
 
 
 
 
ARG2
 
 
hsa-let-7e-5p
 
 
-0.468
 
 
18.7
 
 
 
 
ARG2
 
 
hsa-let-7c-5p
 
 
-0.468
 
 
18.7
 
 
 
 
ARL4D
 
 
hsa-let-7e-5p
 
 
-0.417
 
 
20.3
 
 
 
 
ARL4D
 
 
hsa-let-7c-5p
 
 
-0.417
 
 
20.3
 
 
 
 
ARL6IP6
 
 
hsa-let-7c-5p
 
 
-0.293
 
 
11.9
 
 
 
 
ARL6IP6
 
 
hsa-let-7e-5p
 
 
-0.305
 
 
11.9
 
 
 
 
ARMT1
 
 
hsa-let-7e-5p
 
 
-0.303
 
 
15.5
 
 
 
 
ARMT1
 
 
hsa-let-7c-5p
 
 
-0.303
 
 
15.5
 
 
 
 
ARPC5
 
 
hsa-miR-22-3p
 
 
-0.679
 
 
45.9
 
 
 
 
ARPP19
 
 
hsa-let-7e-5p
 
 
-0.492
 
 
40.6
 
 
 
 
ARPP19
 
 
hsa-let-7c-5p
 
 
-0.492
 
 
40.6
 
 
 
 
ARRB1
 
 
hsa-miR-22-3p
 
 
-0.616
 
 
17.2
 
 
 
 
ASAP1
 
 
hsa-let-7e-5p
 
 
-0.296
 
 
16.8
 
 
 
 
ASAP1
 
 
hsa-let-7c-5p
 
 
-0.308
 
 
16.8
 
 
 
 
ATP2A2
 
 
hsa-let-7e-5p
 
 
-0.311
 
 
34.9
 
 
 
 
ATP2A2
 
 
hsa-let-7c-5p
 
 
-0.311
 
 
34.9
 
 
 
 
ATPAF1
 
 
hsa-let-7e-5p
 
 
-0.498
 
 
33.9
 
 
 
 
ATPAF1
 
 
hsa-let-7c-5p
 
 
-0.498
 
 
33.9
 
 
 
 
ATXN2
 
 
hsa-miR-10a-5p
 
 
-0.362
 
 
25.4
 
 
 
 
B3GAT3
 
 
hsa-let-7e-5p
 
 
-0.324
 
 
35.4
 
 
 
 
B3GAT3
 
 
hsa-let-7c-5p
 
 
-0.324
 
 
35.4
 
 
 
 
B4GAT1
 
 
hsa-let-7e-5p
 
 
-0.47
 
 
33.5
 
 
 
 
B4GAT1
 
 
hsa-let-7c-5p
 
 
-0.47
 
 
33.5
 
 
 
 
BACH1
 
 
hsa-let-7e-5p
 
 
-0.298
 
 
12.0
 
 
 
 
BACH1
 
 
hsa-let-7e-5p
 
 
-0.298
 
 
12.0
 
 
 
 
BACH1
 
 
hsa-let-7c-5p
 
 
-0.306
 
 
12.0
 
 
 
 
BACH1
 
 
hsa-let-7c-5p
 
 
-0.306
 
 
12.0
 
 
 
 
BACH1
 
 
hsa-let-7e-5p
 
 
-0.435
 
 
12.0
 
 
 
 
BACH1
 
 
hsa-let-7e-5p
 
 
-0.435
 
 
12.0
 
 
 
 
BACH1
 
 
hsa-let-7c-5p
 
 
-0.435
 
 
12.0
 
 
 
 
BACH1
 
 
hsa-let-7c-5p
 
 
-0.435
 
 
12.0
 
 
 
 
BAG5
 
 
hsa-miR-22-3p
 
 
-0.318
 
 
10.0
 
 
 
 
BAZ1B
 
 
hsa-miR-10a-5p
 
 
-0.309
 
 
25.4
 
 
 
 
BAZ2A
 
 
hsa-miR-99b-5p
 
 
-0.408
 
 
30.7
 
 
 
 
BBX
 
 
hsa-miR-10a-5p
 
 
-0.305
 
 
20.8
 
 
 
 
BCAP29
 
 
hsa-let-7e-5p
 
 
-0.289
 
 
22.2
 
 
 
 
BIN1
 
 
hsa-miR-22-3p
 
 
-0.346
 
 
32.3
 
 
 
 
BIN3
 
 
hsa-let-7e-5p
 
 
-0.427
 
 
11.4
 
 
 
 
BIN3
 
 
hsa-let-7c-5p
 
 
-0.427
 
 
11.4
 
 
 
 
BLOC1S1
 
 
hsa-miR-22-3p
 
 
-0.346
 
 
123.1
 
 
 
 
BLOC1S6
 
 
hsa-let-7e-5p
 
 
-0.321
 
 
33.7
 
 
 
 
BLOC1S6
 
 
hsa-let-7c-5p
 
 
-0.321
 
 
33.7
 
 
 
 
BLZF1
 
 
hsa-miR-125a-5p
 
 
-0.461
 
 
12.8
 
 
 
 
BMF
 
 
hsa-miR-125a-5p
 
 
-0.34
 
 
11.0
 
 
 
 
BMPR2
 
 
hsa-miR-99b-5p
 
 
-0.327
 
 
11.2
 
 
 
 
BTF3L4
 
 
hsa-let-7e-5p
 
 
-0.355
 
 
61.0
 
 
 
 
BTF3L4
 
 
hsa-let-7c-5p
 
 
-0.355
 
 
61.0
 
 
 
 
BZW1
 
 
hsa-let-7e-5p
 
 
-0.354
 
 
60.3
 
 
 
 
BZW1
 
 
hsa-let-7c-5p
 
 
-0.354
 
 
60.3
 
 
 
 
BZW1
 
 
hsa-let-7e-5p
 
 
-0.375
 
 
60.3
 
 
 
 
BZW1
 
 
hsa-let-7c-5p
 
 
-0.375
 
 
60.3
 
 
 
 
BZW2
 
 
hsa-let-7c-5p
 
 
-0.397
 
 
18.7
 
 
 
 
BZW2
 
 
hsa-let-7e-5p
 
 
-0.408
 
 
18.7
 
 
 
 
C15orf39
 
 
hsa-miR-125a-5p
 
 
-0.335
 
 
17.4
 
 
 
 
C15orf39
 
 
hsa-let-7e-5p
 
 
-0.374
 
 
17.4
 
 
 
 
C15orf39
 
 
hsa-let-7c-5p
 
 
-0.374
 
 
17.4
 
 
 
 
C5orf24
 
 
hsa-miR-22-3p
 
 
-0.493
 
 
19.6
 
 
 
 
C6orf47
 
 
hsa-miR-125a-5p
 
 
-0.564
 
 
10.6
 
 
 
 
CACFD1
 
 
hsa-let-7e-5p
 
 
-0.296
 
 
10.2
 
 
 
 
CACFD1
 
 
hsa-let-7c-5p
 
 
-0.296
 
 
10.2
 
 
 
 
CACNB3
 
 
hsa-miR-125a-5p
 
 
-0.393
 
 
42.6
 
 
 
 
CAMK2N1
 
 
hsa-miR-22-3p
 
 
-0.302
 
 
28.4
 
 
 
 
CANT1
 
 
hsa-let-7e-5p
 
 
-0.402
 
 
13.8
 
 
 
 
CANT1
 
 
hsa-let-7c-5p
 
 
-0.402
 
 
13.8
 
 
 
 
CAPN1
 
 
hsa-miR-22-3p
 
 
-0.307
 
 
42.4
 
 
 
 
CAPN3
 
 
hsa-let-7e-5p
 
 
-0.317
 
 
17.3
 
 
 
 
CAPN3
 
 
hsa-let-7c-5p
 
 
-0.317
 
 
17.3
 
 
 
 
CAST
 
 
hsa-miR-150-5p
 
 
-0.354
 
 
67.8
 
 
 
 
CBX5
 
 
hsa-let-7e-5p
 
 
-0.307
 
 
15.1
 
 
 
 
CBX5
 
 
hsa-let-7c-5p
 
 
-0.307
 
 
15.1
 
 
 
 
CBX5
 
 
hsa-let-7e-5p
 
 
-0.472
 
 
15.1
 
 
 
 
CBX5
 
 
hsa-let-7c-5p
 
 
-0.472
 
 
15.1
 
 
 
 
CCDC47
 
 
hsa-miR-22-3p
 
 
-0.604
 
 
32.4
 
 
 
 
CCN1
 
 
hsa-miR-22-3p
 
 
-0.601
 
 
54.1
 
 
 
 
CCNY
 
 
hsa-let-7c-5p
 
 
-0.323
 
 
25.0
 
 
 
 
CCNY
 
 
hsa-let-7e-5p
 
 
-0.334
 
 
25.0
 
 
 
 
CDC34
 
 
hsa-let-7c-5p
 
 
-0.309
 
 
36.7
 
 
 
 
CDC34
 
 
hsa-let-7e-5p
 
 
-0.321
 
 
36.7
 
 
 
 
CDC34
 
 
hsa-let-7e-5p
 
 
-0.555
 
 
36.7
 
 
 
 
CDC34
 
 
hsa-let-7c-5p
 
 
-0.555
 
 
36.7
 
 
 
 
 
 
 pancreas 
 
 
 
 
target_symbol
 
 
mature_mirna_id
 
 
score
 
 
nTPM
 
 
 
 
 
 
ABCC5
 
 
hsa-miR-125a-5p
 
 
-0.288
 
 
12.8
 
 
 
 
ABHD3
 
 
hsa-miR-125a-5p
 
 
-0.453
 
 
11.6
 
 
 
 
ABTB1
 
 
hsa-miR-125a-5p
 
 
-0.519
 
 
18.1
 
 
 
 
ADIPOR2
 
 
hsa-miR-150-5p
 
 
-0.287
 
 
30.0
 
 
 
 
AIDA
 
 
hsa-miR-22-3p
 
 
-0.316
 
 
30.6
 
 
 
 
AIFM1
 
 
hsa-miR-125a-5p
 
 
-0.3
 
 
20.4
 
 
 
 
AIFM1
 
 
hsa-let-7e-5p
 
 
-0.314
 
 
20.4
 
 
 
 
AIFM1
 
 
hsa-let-7c-5p
 
 
-0.314
 
 
20.4
 
 
 
 
AMT
 
 
hsa-let-7e-5p
 
 
-0.463
 
 
21.5
 
 
 
 
AMT
 
 
hsa-let-7c-5p
 
 
-0.463
 
 
21.5
 
 
 
 
ANAPC16
 
 
hsa-miR-125a-5p
 
 
-0.578
 
 
32.2
 
 
 
 
ANKHD1-EIF4EBP3
 
 
hsa-miR-22-3p
 
 
-0.479
 
 
16.1
 
 
 
 
ANKRD13A
 
 
hsa-miR-22-3p
 
 
-0.478
 
 
11.6
 
 
 
 
ANKRD46
 
 
hsa-let-7e-5p
 
 
-0.389
 
 
17.5
 
 
 
 
ANKRD46
 
 
hsa-let-7c-5p
 
 
-0.389
 
 
17.5
 
 
 
 
ANPEP
 
 
hsa-miR-125a-5p
 
 
-0.372
 
 
643.6
 
 
 
 
AP1S1
 
 
hsa-let-7e-5p
 
 
-0.374
 
 
23.9
 
 
 
 
AP1S1
 
 
hsa-let-7c-5p
 
 
-0.374
 
 
23.9
 
 
 
 
ARPC5
 
 
hsa-miR-22-3p
 
 
-0.679
 
 
48.7
 
 
 
 
ARPP19
 
 
hsa-let-7e-5p
 
 
-0.492
 
 
43.6
 
 
 
 
ARPP19
 
 
hsa-let-7c-5p
 
 
-0.492
 
 
43.6
 
 
 
 
ARRB1
 
 
hsa-miR-22-3p
 
 
-0.616
 
 
23.2
 
 
 
 
ARRDC4
 
 
hsa-let-7e-5p
 
 
-0.329
 
 
32.8
 
 
 
 
ARRDC4
 
 
hsa-let-7c-5p
 
 
-0.329
 
 
32.8
 
 
 
 
ATP2A2
 
 
hsa-let-7e-5p
 
 
-0.311
 
 
36.0
 
 
 
 
ATP2A2
 
 
hsa-let-7c-5p
 
 
-0.311
 
 
36.0
 
 
 
 
ATPAF1
 
 
hsa-let-7e-5p
 
 
-0.498
 
 
54.8
 
 
 
 
ATPAF1
 
 
hsa-let-7c-5p
 
 
-0.498
 
 
54.8
 
 
 
 
ATXN2
 
 
hsa-miR-10a-5p
 
 
-0.362
 
 
13.3
 
 
 
 
B3GALNT2
 
 
hsa-miR-125a-5p
 
 
-0.311
 
 
34.2
 
 
 
 
B3GAT3
 
 
hsa-let-7e-5p
 
 
-0.324
 
 
33.7
 
 
 
 
B3GAT3
 
 
hsa-let-7c-5p
 
 
-0.324
 
 
33.7
 
 
 
 
B4GAT1
 
 
hsa-let-7e-5p
 
 
-0.47
 
 
24.7
 
 
 
 
B4GAT1
 
 
hsa-let-7c-5p
 
 
-0.47
 
 
24.7
 
 
 
 
BACH1
 
 
hsa-let-7e-5p
 
 
-0.298
 
 
16.2
 
 
 
 
BACH1
 
 
hsa-let-7e-5p
 
 
-0.298
 
 
16.2
 
 
 
 
BACH1
 
 
hsa-let-7c-5p
 
 
-0.306
 
 
16.2
 
 
 
 
BACH1
 
 
hsa-let-7c-5p
 
 
-0.306
 
 
16.2
 
 
 
 
BACH1
 
 
hsa-let-7e-5p
 
 
-0.435
 
 
16.2
 
 
 
 
BACH1
 
 
hsa-let-7e-5p
 
 
-0.435
 
 
16.2
 
 
 
 
BACH1
 
 
hsa-let-7c-5p
 
 
-0.435
 
 
16.2
 
 
 
 
BACH1
 
 
hsa-let-7c-5p
 
 
-0.435
 
 
16.2
 
 
 
 
BAG5
 
 
hsa-miR-22-3p
 
 
-0.318
 
 
11.5
 
 
 
 
BAK1
 
 
hsa-miR-125a-5p
 
 
-0.402
 
 
12.1
 
 
 
 
BAZ1B
 
 
hsa-miR-10a-5p
 
 
-0.309
 
 
19.8
 
 
 
 
BAZ2A
 
 
hsa-miR-99b-5p
 
 
-0.408
 
 
29.1
 
 
 
 
BBX
 
 
hsa-miR-10a-5p
 
 
-0.305
 
 
11.6
 
 
 
 
BCAP29
 
 
hsa-let-7e-5p
 
 
-0.289
 
 
16.5
 
 
 
 
BCAT1
 
 
hsa-let-7e-5p
 
 
-0.394
 
 
54.8
 
 
 
 
BCAT1
 
 
hsa-let-7c-5p
 
 
-0.394
 
 
54.8
 
 
 
 
BCL2L11
 
 
hsa-miR-10a-5p
 
 
-0.483
 
 
12.8
 
 
 
 
BCL2L12
 
 
hsa-miR-125a-5p
 
 
-0.288
 
 
13.7
 
 
 
 
BEGAIN
 
 
hsa-let-7e-5p
 
 
-0.466
 
 
22.6
 
 
 
 
BEGAIN
 
 
hsa-let-7c-5p
 
 
-0.466
 
 
22.6
 
 
 
 
BIN1
 
 
hsa-miR-22-3p
 
 
-0.346
 
 
20.4
 
 
 
 
BIN3
 
 
hsa-let-7e-5p
 
 
-0.427
 
 
11.6
 
 
 
 
BIN3
 
 
hsa-let-7c-5p
 
 
-0.427
 
 
11.6
 
 
 
 
BLOC1S1
 
 
hsa-miR-22-3p
 
 
-0.346
 
 
108.1
 
 
 
 
BLOC1S6
 
 
hsa-let-7e-5p
 
 
-0.321
 
 
19.3
 
 
 
 
BLOC1S6
 
 
hsa-let-7c-5p
 
 
-0.321
 
 
19.3
 
 
 
 
BORCS6
 
 
hsa-miR-125a-5p
 
 
-0.287
 
 
20.2
 
 
 
 
BTF3L4
 
 
hsa-let-7e-5p
 
 
-0.355
 
 
36.1
 
 
 
 
BTF3L4
 
 
hsa-let-7c-5p
 
 
-0.355
 
 
36.1
 
 
 
 
BTG1
 
 
hsa-miR-22-3p
 
 
-0.468
 
 
18.5
 
 
 
 
BZW1
 
 
hsa-let-7e-5p
 
 
-0.354
 
 
59.0
 
 
 
 
BZW1
 
 
hsa-let-7c-5p
 
 
-0.354
 
 
59.0
 
 
 
 
BZW1
 
 
hsa-let-7e-5p
 
 
-0.375
 
 
59.0
 
 
 
 
BZW1
 
 
hsa-let-7c-5p
 
 
-0.375
 
 
59.0
 
 
 
 
BZW2
 
 
hsa-let-7c-5p
 
 
-0.397
 
 
31.2
 
 
 
 
BZW2
 
 
hsa-let-7e-5p
 
 
-0.408
 
 
31.2
 
 
 
 
C15orf39
 
 
hsa-miR-125a-5p
 
 
-0.335
 
 
13.7
 
 
 
 
C15orf39
 
 
hsa-let-7e-5p
 
 
-0.374
 
 
13.7
 
 
 
 
C15orf39
 
 
hsa-let-7c-5p
 
 
-0.374
 
 
13.7
 
 
 
 
C1orf210
 
 
hsa-miR-125a-5p
 
 
-0.584
 
 
21.5
 
 
 
 
C2orf88
 
 
hsa-let-7e-5p
 
 
-0.429
 
 
12.0
 
 
 
 
C2orf88
 
 
hsa-let-7c-5p
 
 
-0.429
 
 
12.0
 
 
 
 
C3orf52
 
 
hsa-let-7e-5p
 
 
-0.296
 
 
17.1
 
 
 
 
C3orf52
 
 
hsa-let-7c-5p
 
 
-0.329
 
 
17.1
 
 
 
 
C5orf24
 
 
hsa-miR-22-3p
 
 
-0.493
 
 
12.3
 
 
 
 
C6orf47
 
 
hsa-miR-125a-5p
 
 
-0.564
 
 
12.5
 
 
 
 
CACFD1
 
 
hsa-let-7e-5p
 
 
-0.296
 
 
18.2
 
 
 
 
CACFD1
 
 
hsa-let-7c-5p
 
 
-0.296
 
 
18.2
 
 
 
 
CANT1
 
 
hsa-let-7e-5p
 
 
-0.402
 
 
18.2
 
 
 
 
CANT1
 
 
hsa-let-7c-5p
 
 
-0.402
 
 
18.2
 
 
 
 
CAPN1
 
 
hsa-miR-22-3p
 
 
-0.307
 
 
68.8
 
 
 
 
CASP3
 
 
hsa-let-7e-5p
 
 
-0.495
 
 
11.6
 
 
 
 
CASP3
 
 
hsa-let-7c-5p
 
 
-0.503
 
 
11.6
 
 
 
 
CAST
 
 
hsa-miR-150-5p
 
 
-0.354
 
 
52.9
 
 
 
 
CBFA2T3
 
 
hsa-let-7c-5p
 
 
-0.294
 
 
25.8
 
 
 
 
CBFA2T3
 
 
hsa-let-7e-5p
 
 
-0.305
 
 
25.8
 
 
 
 
CCDC149
 
 
hsa-miR-22-3p
 
 
-0.327
 
 
17.6
 
 
 
 
CCDC47
 
 
hsa-miR-22-3p
 
 
-0.604
 
 
54.5
 
 
 
 
CCN1
 
 
hsa-miR-22-3p
 
 
-0.601
 
 
38.4
 
 
 
 
CCNY
 
 
hsa-let-7c-5p
 
 
-0.323
 
 
19.4
 
 
 
 
CCNY
 
 
hsa-let-7e-5p
 
 
-0.334
 
 
19.4
 
 
 
 
CDC34
 
 
hsa-let-7c-5p
 
 
-0.309
 
 
60.2
 
 
 
 
CDC34
 
 
hsa-let-7e-5p
 
 
-0.321
 
 
60.2
 
 
 
 
CDC34
 
 
hsa-let-7e-5p
 
 
-0.555
 
 
60.2
 
 
 
 
CDC34
 
 
hsa-let-7c-5p
 
 
-0.555
 
 
60.2
 
 
 
 
CDC37L1
 
 
hsa-miR-125a-5p
 
 
-0.401
 
 
18.5
 
 
 
 
 
 
 parathyroid gland 
 
 
 
 
target_symbol
 
 
mature_mirna_id
 
 
score
 
 
nTPM
 
 
 
 
 
 
ABCC5
 
 
hsa-miR-125a-5p
 
 
-0.288
 
 
22.6
 
 
 
 
ABHD3
 
 
hsa-miR-125a-5p
 
 
-0.453
 
 
15.0
 
 
 
 
ACSL6
 
 
hsa-miR-125a-5p
 
 
-0.307
 
 
10.6
 
 
 
 
ACSL6
 
 
hsa-let-7e-5p
 
 
-0.336
 
 
10.6
 
 
 
 
ACSL6
 
 
hsa-let-7c-5p
 
 
-0.336
 
 
10.6
 
 
 
 
ADIPOR2
 
 
hsa-miR-150-5p
 
 
-0.287
 
 
15.9
 
 
 
 
AGGF1
 
 
hsa-miR-125a-5p
 
 
-0.331
 
 
15.0
 
 
 
 
AHCTF1
 
 
hsa-let-7e-5p
 
 
-0.374
 
 
12.3
 
 
 
 
AHCTF1
 
 
hsa-let-7c-5p
 
 
-0.374
 
 
12.3
 
 
 
 
AIDA
 
 
hsa-miR-22-3p
 
 
-0.316
 
 
30.5
 
 
 
 
AIFM1
 
 
hsa-miR-125a-5p
 
 
-0.3
 
 
27.9
 
 
 
 
AIFM1
 
 
hsa-let-7e-5p
 
 
-0.314
 
 
27.9
 
 
 
 
AIFM1
 
 
hsa-let-7c-5p
 
 
-0.314
 
 
27.9
 
 
 
 
ALDH4A1
 
 
hsa-miR-184
 
 
-0.421
 
 
21.6
 
 
 
 
AMT
 
 
hsa-let-7e-5p
 
 
-0.463
 
 
13.3
 
 
 
 
AMT
 
 
hsa-let-7c-5p
 
 
-0.463
 
 
13.3
 
 
 
 
ANAPC16
 
 
hsa-miR-125a-5p
 
 
-0.578
 
 
30.6
 
 
 
 
ANKHD1-EIF4EBP3
 
 
hsa-miR-22-3p
 
 
-0.479
 
 
19.9
 
 
 
 
ANKRD13A
 
 
hsa-miR-22-3p
 
 
-0.478
 
 
16.3
 
 
 
 
ANKRD46
 
 
hsa-let-7e-5p
 
 
-0.389
 
 
15.1
 
 
 
 
ANKRD46
 
 
hsa-let-7c-5p
 
 
-0.389
 
 
15.1
 
 
 
 
AP1AR
 
 
hsa-miR-99b-5p
 
 
-0.823
 
 
38.5
 
 
 
 
AP1S1
 
 
hsa-let-7e-5p
 
 
-0.374
 
 
21.4
 
 
 
 
AP1S1
 
 
hsa-let-7c-5p
 
 
-0.374
 
 
21.4
 
 
 
 
ARG2
 
 
hsa-let-7e-5p
 
 
-0.468
 
 
206.5
 
 
 
 
ARG2
 
 
hsa-let-7c-5p
 
 
-0.468
 
 
206.5
 
 
 
 
ARMT1
 
 
hsa-let-7e-5p
 
 
-0.303
 
 
10.9
 
 
 
 
ARMT1
 
 
hsa-let-7c-5p
 
 
-0.303
 
 
10.9
 
 
 
 
ARPC5
 
 
hsa-miR-22-3p
 
 
-0.679
 
 
28.1
 
 
 
 
ARPP19
 
 
hsa-let-7e-5p
 
 
-0.492
 
 
102.5
 
 
 
 
ARPP19
 
 
hsa-let-7c-5p
 
 
-0.492
 
 
102.5
 
 
 
 
ASAP1
 
 
hsa-let-7e-5p
 
 
-0.296
 
 
16.8
 
 
 
 
ASAP1
 
 
hsa-let-7c-5p
 
 
-0.308
 
 
16.8
 
 
 
 
ATOSB
 
 
hsa-let-7e-5p
 
 
-0.429
 
 
10.5
 
 
 
 
ATOSB
 
 
hsa-let-7c-5p
 
 
-0.429
 
 
10.5
 
 
 
 
ATP2A2
 
 
hsa-let-7e-5p
 
 
-0.311
 
 
52.7
 
 
 
 
ATP2A2
 
 
hsa-let-7c-5p
 
 
-0.311
 
 
52.7
 
 
 
 
ATP8A1
 
 
hsa-miR-22-3p
 
 
-0.468
 
 
33.8
 
 
 
 
ATPAF1
 
 
hsa-let-7e-5p
 
 
-0.498
 
 
129.7
 
 
 
 
ATPAF1
 
 
hsa-let-7c-5p
 
 
-0.498
 
 
129.7
 
 
 
 
ATXN2
 
 
hsa-miR-10a-5p
 
 
-0.362
 
 
17.5
 
 
 
 
B3GAT3
 
 
hsa-let-7e-5p
 
 
-0.324
 
 
18.1
 
 
 
 
B3GAT3
 
 
hsa-let-7c-5p
 
 
-0.324
 
 
18.1
 
 
 
 
B4GAT1
 
 
hsa-let-7e-5p
 
 
-0.47
 
 
21.3
 
 
 
 
B4GAT1
 
 
hsa-let-7c-5p
 
 
-0.47
 
 
21.3
 
 
 
 
BAG5
 
 
hsa-miR-22-3p
 
 
-0.318
 
 
13.2
 
 
 
 
BAK1
 
 
hsa-miR-125a-5p
 
 
-0.402
 
 
12.6
 
 
 
 
BAZ1B
 
 
hsa-miR-10a-5p
 
 
-0.309
 
 
26.0
 
 
 
 
BAZ2A
 
 
hsa-miR-99b-5p
 
 
-0.408
 
 
31.4
 
 
 
 
BBX
 
 
hsa-miR-10a-5p
 
 
-0.305
 
 
43.6
 
 
 
 
BCAP29
 
 
hsa-let-7e-5p
 
 
-0.289
 
 
32.8
 
 
 
 
BCL2L11
 
 
hsa-miR-10a-5p
 
 
-0.483
 
 
17.6
 
 
 
 
BDH1
 
 
hsa-miR-125a-5p
 
 
-0.389
 
 
14.6
 
 
 
 
BIN1
 
 
hsa-miR-22-3p
 
 
-0.346
 
 
18.4
 
 
 
 
BIN3
 
 
hsa-let-7e-5p
 
 
-0.427
 
 
11.8
 
 
 
 
BIN3
 
 
hsa-let-7c-5p
 
 
-0.427
 
 
11.8
 
 
 
 
BLOC1S1
 
 
hsa-miR-22-3p
 
 
-0.346
 
 
168.9
 
 
 
 
BLOC1S6
 
 
hsa-let-7e-5p
 
 
-0.321
 
 
15.8
 
 
 
 
BLOC1S6
 
 
hsa-let-7c-5p
 
 
-0.321
 
 
15.8
 
 
 
 
BLZF1
 
 
hsa-miR-125a-5p
 
 
-0.461
 
 
14.2
 
 
 
 
BMPR2
 
 
hsa-miR-99b-5p
 
 
-0.327
 
 
12.6
 
 
 
 
BTBD9
 
 
hsa-let-7e-5p
 
 
-0.311
 
 
12.9
 
 
 
 
BTBD9
 
 
hsa-let-7c-5p
 
 
-0.311
 
 
12.9
 
 
 
 
BTF3L4
 
 
hsa-let-7e-5p
 
 
-0.355
 
 
39.5
 
 
 
 
BTF3L4
 
 
hsa-let-7c-5p
 
 
-0.355
 
 
39.5
 
 
 
 
BZW1
 
 
hsa-let-7e-5p
 
 
-0.354
 
 
68.1
 
 
 
 
BZW1
 
 
hsa-let-7c-5p
 
 
-0.354
 
 
68.1
 
 
 
 
BZW1
 
 
hsa-let-7e-5p
 
 
-0.375
 
 
68.1
 
 
 
 
BZW1
 
 
hsa-let-7c-5p
 
 
-0.375
 
 
68.1
 
 
 
 
C15orf39
 
 
hsa-miR-125a-5p
 
 
-0.335
 
 
14.8
 
 
 
 
C15orf39
 
 
hsa-let-7e-5p
 
 
-0.374
 
 
14.8
 
 
 
 
C15orf39
 
 
hsa-let-7c-5p
 
 
-0.374
 
 
14.8
 
 
 
 
C1GALT1
 
 
hsa-let-7e-5p
 
 
-0.322
 
 
43.0
 
 
 
 
C1GALT1
 
 
hsa-let-7c-5p
 
 
-0.322
 
 
43.0
 
 
 
 
C2orf88
 
 
hsa-let-7e-5p
 
 
-0.429
 
 
33.0
 
 
 
 
C2orf88
 
 
hsa-let-7c-5p
 
 
-0.429
 
 
33.0
 
 
 
 
C5orf24
 
 
hsa-miR-22-3p
 
 
-0.493
 
 
25.2
 
 
 
 
CAMK2N1
 
 
hsa-miR-22-3p
 
 
-0.302
 
 
39.8
 
 
 
 
CANT1
 
 
hsa-let-7e-5p
 
 
-0.402
 
 
23.0
 
 
 
 
CANT1
 
 
hsa-let-7c-5p
 
 
-0.402
 
 
23.0
 
 
 
 
CAPN1
 
 
hsa-miR-22-3p
 
 
-0.307
 
 
34.4
 
 
 
 
CARNMT1
 
 
hsa-let-7e-5p
 
 
-0.307
 
 
10.6
 
 
 
 
CARNMT1
 
 
hsa-let-7c-5p
 
 
-0.307
 
 
10.6
 
 
 
 
CASP2
 
 
hsa-miR-125a-5p
 
 
-0.296
 
 
10.5
 
 
 
 
CAST
 
 
hsa-miR-150-5p
 
 
-0.354
 
 
105.0
 
 
 
 
CBX5
 
 
hsa-let-7e-5p
 
 
-0.307
 
 
24.1
 
 
 
 
CBX5
 
 
hsa-let-7c-5p
 
 
-0.307
 
 
24.1
 
 
 
 
CBX5
 
 
hsa-let-7e-5p
 
 
-0.472
 
 
24.1
 
 
 
 
CBX5
 
 
hsa-let-7c-5p
 
 
-0.472
 
 
24.1
 
 
 
 
CCDC47
 
 
hsa-miR-22-3p
 
 
-0.604
 
 
79.5
 
 
 
 
CCN1
 
 
hsa-miR-22-3p
 
 
-0.601
 
 
13.9
 
 
 
 
CCNY
 
 
hsa-let-7c-5p
 
 
-0.323
 
 
16.8
 
 
 
 
CCNY
 
 
hsa-let-7e-5p
 
 
-0.334
 
 
16.8
 
 
 
 
CDC34
 
 
hsa-let-7c-5p
 
 
-0.309
 
 
17.6
 
 
 
 
CDC34
 
 
hsa-let-7e-5p
 
 
-0.321
 
 
17.6
 
 
 
 
CDC34
 
 
hsa-let-7e-5p
 
 
-0.555
 
 
17.6
 
 
 
 
CDC34
 
 
hsa-let-7c-5p
 
 
-0.555
 
 
17.6
 
 
 
 
CDC37L1
 
 
hsa-miR-125a-5p
 
 
-0.401
 
 
10.9
 
 
 
 
CDC42SE1
 
 
hsa-miR-125a-5p
 
 
-0.501
 
 
67.2
 
 
 
 
CDKN1A
 
 
hsa-let-7e-5p
 
 
-0.434
 
 
65.8
 
 
 
 
 
 
 placenta 
 
 
 
 
target_symbol
 
 
mature_mirna_id
 
 
score
 
 
nTPM
 
 
 
 
 
 
ABCC5
 
 
hsa-miR-125a-5p
 
 
-0.288
 
 
16.1
 
 
 
 
ABHD3
 
 
hsa-miR-125a-5p
 
 
-0.453
 
 
18.6
 
 
 
 
ABTB1
 
 
hsa-miR-125a-5p
 
 
-0.519
 
 
11.5
 
 
 
 
ACP3
 
 
hsa-let-7e-5p
 
 
-0.298
 
 
10.1
 
 
 
 
ACP3
 
 
hsa-let-7c-5p
 
 
-0.298
 
 
10.1
 
 
 
 
ADAM17
 
 
hsa-miR-125a-5p
 
 
-0.292
 
 
17.9
 
 
 
 
ADIPOR2
 
 
hsa-miR-150-5p
 
 
-0.287
 
 
12.2
 
 
 
 
ADRB1
 
 
hsa-let-7c-5p
 
 
-0.351
 
 
27.4
 
 
 
 
ADRB1
 
 
hsa-let-7e-5p
 
 
-0.367
 
 
27.4
 
 
 
 
AGGF1
 
 
hsa-miR-125a-5p
 
 
-0.331
 
 
10.3
 
 
 
 
AIDA
 
 
hsa-miR-22-3p
 
 
-0.316
 
 
44.9
 
 
 
 
AKT3
 
 
hsa-miR-22-3p
 
 
-0.451
 
 
12.3
 
 
 
 
ALDH4A1
 
 
hsa-miR-184
 
 
-0.421
 
 
12.4
 
 
 
 
ANAPC16
 
 
hsa-miR-125a-5p
 
 
-0.578
 
 
11.0
 
 
 
 
ANKRD13A
 
 
hsa-miR-22-3p
 
 
-0.478
 
 
22.9
 
 
 
 
ANKRD46
 
 
hsa-let-7e-5p
 
 
-0.389
 
 
19.5
 
 
 
 
ANKRD46
 
 
hsa-let-7c-5p
 
 
-0.389
 
 
19.5
 
 
 
 
ANPEP
 
 
hsa-miR-125a-5p
 
 
-0.372
 
 
31.1
 
 
 
 
AP1AR
 
 
hsa-miR-99b-5p
 
 
-0.823
 
 
11.2
 
 
 
 
AP1S1
 
 
hsa-let-7e-5p
 
 
-0.374
 
 
14.9
 
 
 
 
AP1S1
 
 
hsa-let-7c-5p
 
 
-0.374
 
 
14.9
 
 
 
 
ARHGAP28
 
 
hsa-let-7c-5p
 
 
-0.47
 
 
24.3
 
 
 
 
ARHGAP28
 
 
hsa-let-7e-5p
 
 
-0.486
 
 
24.3
 
 
 
 
ARID3A
 
 
hsa-let-7c-5p
 
 
-0.34
 
 
22.0
 
 
 
 
ARID3A
 
 
hsa-let-7e-5p
 
 
-0.347
 
 
22.0
 
 
 
 
ARID3A
 
 
hsa-let-7c-5p
 
 
-0.347
 
 
22.0
 
 
 
 
ARID3A
 
 
hsa-let-7e-5p
 
 
-0.348
 
 
22.0
 
 
 
 
ARID3B
 
 
hsa-let-7e-5p
 
 
-0.31
 
 
14.7
 
 
 
 
ARID3B
 
 
hsa-let-7c-5p
 
 
-0.31
 
 
14.7
 
 
 
 
ARID3B
 
 
hsa-let-7e-5p
 
 
-0.486
 
 
14.7
 
 
 
 
ARID3B
 
 
hsa-let-7c-5p
 
 
-0.486
 
 
14.7
 
 
 
 
ARL6IP6
 
 
hsa-let-7c-5p
 
 
-0.293
 
 
26.8
 
 
 
 
ARL6IP6
 
 
hsa-let-7e-5p
 
 
-0.305
 
 
26.8
 
 
 
 
ARMT1
 
 
hsa-let-7e-5p
 
 
-0.303
 
 
20.8
 
 
 
 
ARMT1
 
 
hsa-let-7c-5p
 
 
-0.303
 
 
20.8
 
 
 
 
ARPC5
 
 
hsa-miR-22-3p
 
 
-0.679
 
 
101.6
 
 
 
 
ARPP19
 
 
hsa-let-7e-5p
 
 
-0.492
 
 
53.5
 
 
 
 
ARPP19
 
 
hsa-let-7c-5p
 
 
-0.492
 
 
53.5
 
 
 
 
ARRB1
 
 
hsa-miR-22-3p
 
 
-0.616
 
 
23.6
 
 
 
 
ARRDC4
 
 
hsa-let-7e-5p
 
 
-0.329
 
 
17.2
 
 
 
 
ARRDC4
 
 
hsa-let-7c-5p
 
 
-0.329
 
 
17.2
 
 
 
 
ASAP1
 
 
hsa-let-7e-5p
 
 
-0.296
 
 
25.9
 
 
 
 
ASAP1
 
 
hsa-let-7c-5p
 
 
-0.308
 
 
25.9
 
 
 
 
ATP10D
 
 
hsa-miR-125a-5p
 
 
-0.375
 
 
14.0
 
 
 
 
ATP2A2
 
 
hsa-let-7e-5p
 
 
-0.311
 
 
52.0
 
 
 
 
ATP2A2
 
 
hsa-let-7c-5p
 
 
-0.311
 
 
52.0
 
 
 
 
ATPAF1
 
 
hsa-let-7e-5p
 
 
-0.498
 
 
34.1
 
 
 
 
ATPAF1
 
 
hsa-let-7c-5p
 
 
-0.498
 
 
34.1
 
 
 
 
ATXN2
 
 
hsa-miR-10a-5p
 
 
-0.362
 
 
15.3
 
 
 
 
B3GAT3
 
 
hsa-let-7e-5p
 
 
-0.324
 
 
18.0
 
 
 
 
B3GAT3
 
 
hsa-let-7c-5p
 
 
-0.324
 
 
18.0
 
 
 
 
B4GAT1
 
 
hsa-let-7e-5p
 
 
-0.47
 
 
21.1
 
 
 
 
B4GAT1
 
 
hsa-let-7c-5p
 
 
-0.47
 
 
21.1
 
 
 
 
BACH1
 
 
hsa-let-7e-5p
 
 
-0.298
 
 
47.5
 
 
 
 
BACH1
 
 
hsa-let-7e-5p
 
 
-0.298
 
 
47.5
 
 
 
 
BACH1
 
 
hsa-let-7c-5p
 
 
-0.306
 
 
47.5
 
 
 
 
BACH1
 
 
hsa-let-7c-5p
 
 
-0.306
 
 
47.5
 
 
 
 
BACH1
 
 
hsa-let-7e-5p
 
 
-0.435
 
 
47.5
 
 
 
 
BACH1
 
 
hsa-let-7e-5p
 
 
-0.435
 
 
47.5
 
 
 
 
BACH1
 
 
hsa-let-7c-5p
 
 
-0.435
 
 
47.5
 
 
 
 
BACH1
 
 
hsa-let-7c-5p
 
 
-0.435
 
 
47.5
 
 
 
 
BAG5
 
 
hsa-miR-22-3p
 
 
-0.318
 
 
13.6
 
 
 
 
BAK1
 
 
hsa-miR-125a-5p
 
 
-0.402
 
 
10.4
 
 
 
 
BAZ1B
 
 
hsa-miR-10a-5p
 
 
-0.309
 
 
21.3
 
 
 
 
BAZ2A
 
 
hsa-miR-99b-5p
 
 
-0.408
 
 
33.2
 
 
 
 
BBX
 
 
hsa-miR-10a-5p
 
 
-0.305
 
 
22.7
 
 
 
 
BCAP29
 
 
hsa-let-7e-5p
 
 
-0.289
 
 
25.6
 
 
 
 
BCAT1
 
 
hsa-let-7e-5p
 
 
-0.394
 
 
14.3
 
 
 
 
BCAT1
 
 
hsa-let-7c-5p
 
 
-0.394
 
 
14.3
 
 
 
 
BIN1
 
 
hsa-miR-22-3p
 
 
-0.346
 
 
22.1
 
 
 
 
BIN2
 
 
hsa-miR-125a-5p
 
 
-0.679
 
 
18.3
 
 
 
 
BLOC1S1
 
 
hsa-miR-22-3p
 
 
-0.346
 
 
69.0
 
 
 
 
BLOC1S6
 
 
hsa-let-7e-5p
 
 
-0.321
 
 
24.7
 
 
 
 
BLOC1S6
 
 
hsa-let-7c-5p
 
 
-0.321
 
 
24.7
 
 
 
 
BLZF1
 
 
hsa-miR-125a-5p
 
 
-0.461
 
 
17.1
 
 
 
 
BMP5
 
 
hsa-let-7e-5p
 
 
-0.337
 
 
66.9
 
 
 
 
BMP5
 
 
hsa-let-7c-5p
 
 
-0.337
 
 
66.9
 
 
 
 
BMPR2
 
 
hsa-miR-99b-5p
 
 
-0.327
 
 
21.8
 
 
 
 
BTF3L4
 
 
hsa-let-7e-5p
 
 
-0.355
 
 
35.1
 
 
 
 
BTF3L4
 
 
hsa-let-7c-5p
 
 
-0.355
 
 
35.1
 
 
 
 
BZW1
 
 
hsa-let-7e-5p
 
 
-0.354
 
 
98.8
 
 
 
 
BZW1
 
 
hsa-let-7c-5p
 
 
-0.354
 
 
98.8
 
 
 
 
BZW1
 
 
hsa-let-7e-5p
 
 
-0.375
 
 
98.8
 
 
 
 
BZW1
 
 
hsa-let-7c-5p
 
 
-0.375
 
 
98.8
 
 
 
 
BZW2
 
 
hsa-let-7c-5p
 
 
-0.397
 
 
75.6
 
 
 
 
BZW2
 
 
hsa-let-7e-5p
 
 
-0.408
 
 
75.6
 
 
 
 
C15orf39
 
 
hsa-miR-125a-5p
 
 
-0.335
 
 
14.3
 
 
 
 
C15orf39
 
 
hsa-let-7e-5p
 
 
-0.374
 
 
14.3
 
 
 
 
C15orf39
 
 
hsa-let-7c-5p
 
 
-0.374
 
 
14.3
 
 
 
 
C1GALT1
 
 
hsa-let-7e-5p
 
 
-0.322
 
 
25.3
 
 
 
 
C1GALT1
 
 
hsa-let-7c-5p
 
 
-0.322
 
 
25.3
 
 
 
 
C5orf24
 
 
hsa-miR-22-3p
 
 
-0.493
 
 
26.9
 
 
 
 
C8orf58
 
 
hsa-let-7e-5p
 
 
-0.612
 
 
15.5
 
 
 
 
C8orf58
 
 
hsa-let-7c-5p
 
 
-0.62
 
 
15.5
 
 
 
 
CAMK2N1
 
 
hsa-miR-22-3p
 
 
-0.302
 
 
10.9
 
 
 
 
CANT1
 
 
hsa-let-7e-5p
 
 
-0.402
 
 
24.4
 
 
 
 
CANT1
 
 
hsa-let-7c-5p
 
 
-0.402
 
 
24.4
 
 
 
 
CAPN1
 
 
hsa-miR-22-3p
 
 
-0.307
 
 
52.8
 
 
 
 
CASP2
 
 
hsa-miR-125a-5p
 
 
-0.296
 
 
11.7
 
 
 
 
CASP3
 
 
hsa-let-7e-5p
 
 
-0.495
 
 
23.2
 
 
 
 
 
 
 prostate 
 
 
 
 
target_symbol
 
 
mature_mirna_id
 
 
score
 
 
nTPM
 
 
 
 
 
 
ABCC5
 
 
hsa-miR-125a-5p
 
 
-0.288
 
 
27.9
 
 
 
 
ABHD17C
 
 
hsa-let-7e-5p
 
 
-0.359
 
 
17.0
 
 
 
 
ABHD17C
 
 
hsa-let-7c-5p
 
 
-0.359
 
 
17.0
 
 
 
 
ABHD3
 
 
hsa-miR-125a-5p
 
 
-0.453
 
 
10.4
 
 
 
 
ABTB1
 
 
hsa-miR-125a-5p
 
 
-0.519
 
 
33.0
 
 
 
 
ACP3
 
 
hsa-let-7e-5p
 
 
-0.298
 
 
3409.5
 
 
 
 
ACP3
 
 
hsa-let-7c-5p
 
 
-0.298
 
 
3409.5
 
 
 
 
ADIPOR2
 
 
hsa-miR-150-5p
 
 
-0.287
 
 
19.8
 
 
 
 
AIDA
 
 
hsa-miR-22-3p
 
 
-0.316
 
 
32.9
 
 
 
 
AIFM1
 
 
hsa-miR-125a-5p
 
 
-0.3
 
 
10.1
 
 
 
 
AIFM1
 
 
hsa-let-7e-5p
 
 
-0.314
 
 
10.1
 
 
 
 
AIFM1
 
 
hsa-let-7c-5p
 
 
-0.314
 
 
10.1
 
 
 
 
AKT3
 
 
hsa-miR-22-3p
 
 
-0.451
 
 
13.8
 
 
 
 
ALDH4A1
 
 
hsa-miR-184
 
 
-0.421
 
 
27.7
 
 
 
 
AMT
 
 
hsa-let-7e-5p
 
 
-0.463
 
 
15.5
 
 
 
 
AMT
 
 
hsa-let-7c-5p
 
 
-0.463
 
 
15.5
 
 
 
 
ANAPC16
 
 
hsa-miR-125a-5p
 
 
-0.578
 
 
19.8
 
 
 
 
ANKHD1-EIF4EBP3
 
 
hsa-miR-22-3p
 
 
-0.479
 
 
11.9
 
 
 
 
ANKRD13A
 
 
hsa-miR-22-3p
 
 
-0.478
 
 
16.8
 
 
 
 
ANKRD46
 
 
hsa-let-7e-5p
 
 
-0.389
 
 
12.6
 
 
 
 
ANKRD46
 
 
hsa-let-7c-5p
 
 
-0.389
 
 
12.6
 
 
 
 
ANPEP
 
 
hsa-miR-125a-5p
 
 
-0.372
 
 
88.3
 
 
 
 
AP1S1
 
 
hsa-let-7e-5p
 
 
-0.374
 
 
23.9
 
 
 
 
AP1S1
 
 
hsa-let-7c-5p
 
 
-0.374
 
 
23.9
 
 
 
 
ARG2
 
 
hsa-let-7e-5p
 
 
-0.468
 
 
101.8
 
 
 
 
ARG2
 
 
hsa-let-7c-5p
 
 
-0.468
 
 
101.8
 
 
 
 
ARL4D
 
 
hsa-let-7e-5p
 
 
-0.417
 
 
10.7
 
 
 
 
ARL4D
 
 
hsa-let-7c-5p
 
 
-0.417
 
 
10.7
 
 
 
 
ARMT1
 
 
hsa-let-7e-5p
 
 
-0.303
 
 
11.5
 
 
 
 
ARMT1
 
 
hsa-let-7c-5p
 
 
-0.303
 
 
11.5
 
 
 
 
ARPC5
 
 
hsa-miR-22-3p
 
 
-0.679
 
 
43.1
 
 
 
 
ARPP19
 
 
hsa-let-7e-5p
 
 
-0.492
 
 
37.0
 
 
 
 
ARPP19
 
 
hsa-let-7c-5p
 
 
-0.492
 
 
37.0
 
 
 
 
ARRB1
 
 
hsa-miR-22-3p
 
 
-0.616
 
 
15.2
 
 
 
 
ARRDC4
 
 
hsa-let-7e-5p
 
 
-0.329
 
 
10.3
 
 
 
 
ARRDC4
 
 
hsa-let-7c-5p
 
 
-0.329
 
 
10.3
 
 
 
 
ASAP1
 
 
hsa-let-7e-5p
 
 
-0.296
 
 
11.6
 
 
 
 
ASAP1
 
 
hsa-let-7c-5p
 
 
-0.308
 
 
11.6
 
 
 
 
ATOSB
 
 
hsa-let-7e-5p
 
 
-0.429
 
 
12.4
 
 
 
 
ATOSB
 
 
hsa-let-7c-5p
 
 
-0.429
 
 
12.4
 
 
 
 
ATP2A2
 
 
hsa-let-7e-5p
 
 
-0.311
 
 
87.6
 
 
 
 
ATP2A2
 
 
hsa-let-7c-5p
 
 
-0.311
 
 
87.6
 
 
 
 
ATPAF1
 
 
hsa-let-7e-5p
 
 
-0.498
 
 
35.2
 
 
 
 
ATPAF1
 
 
hsa-let-7c-5p
 
 
-0.498
 
 
35.2
 
 
 
 
ATXN2
 
 
hsa-miR-10a-5p
 
 
-0.362
 
 
18.8
 
 
 
 
B3GALNT2
 
 
hsa-miR-125a-5p
 
 
-0.311
 
 
11.7
 
 
 
 
B3GAT3
 
 
hsa-let-7e-5p
 
 
-0.324
 
 
40.8
 
 
 
 
B3GAT3
 
 
hsa-let-7c-5p
 
 
-0.324
 
 
40.8
 
 
 
 
B4GAT1
 
 
hsa-let-7e-5p
 
 
-0.47
 
 
17.0
 
 
 
 
B4GAT1
 
 
hsa-let-7c-5p
 
 
-0.47
 
 
17.0
 
 
 
 
BACH1
 
 
hsa-let-7e-5p
 
 
-0.298
 
 
12.5
 
 
 
 
BACH1
 
 
hsa-let-7e-5p
 
 
-0.298
 
 
12.5
 
 
 
 
BACH1
 
 
hsa-let-7c-5p
 
 
-0.306
 
 
12.5
 
 
 
 
BACH1
 
 
hsa-let-7c-5p
 
 
-0.306
 
 
12.5
 
 
 
 
BACH1
 
 
hsa-let-7e-5p
 
 
-0.435
 
 
12.5
 
 
 
 
BACH1
 
 
hsa-let-7e-5p
 
 
-0.435
 
 
12.5
 
 
 
 
BACH1
 
 
hsa-let-7c-5p
 
 
-0.435
 
 
12.5
 
 
 
 
BACH1
 
 
hsa-let-7c-5p
 
 
-0.435
 
 
12.5
 
 
 
 
BAG5
 
 
hsa-miR-22-3p
 
 
-0.318
 
 
13.1
 
 
 
 
BAK1
 
 
hsa-miR-125a-5p
 
 
-0.402
 
 
10.5
 
 
 
 
BAZ1B
 
 
hsa-miR-10a-5p
 
 
-0.309
 
 
21.2
 
 
 
 
BAZ2A
 
 
hsa-miR-99b-5p
 
 
-0.408
 
 
21.4
 
 
 
 
BBX
 
 
hsa-miR-10a-5p
 
 
-0.305
 
 
21.3
 
 
 
 
BCAP29
 
 
hsa-let-7e-5p
 
 
-0.289
 
 
30.7
 
 
 
 
BDH1
 
 
hsa-miR-125a-5p
 
 
-0.389
 
 
14.7
 
 
 
 
BIN1
 
 
hsa-miR-22-3p
 
 
-0.346
 
 
52.2
 
 
 
 
BIN3
 
 
hsa-let-7e-5p
 
 
-0.427
 
 
16.4
 
 
 
 
BIN3
 
 
hsa-let-7c-5p
 
 
-0.427
 
 
16.4
 
 
 
 
BLOC1S1
 
 
hsa-miR-22-3p
 
 
-0.346
 
 
112.8
 
 
 
 
BLOC1S6
 
 
hsa-let-7e-5p
 
 
-0.321
 
 
34.2
 
 
 
 
BLOC1S6
 
 
hsa-let-7c-5p
 
 
-0.321
 
 
34.2
 
 
 
 
BLZF1
 
 
hsa-miR-125a-5p
 
 
-0.461
 
 
14.6
 
 
 
 
BMP5
 
 
hsa-let-7e-5p
 
 
-0.337
 
 
13.2
 
 
 
 
BMP5
 
 
hsa-let-7c-5p
 
 
-0.337
 
 
13.2
 
 
 
 
BMPR2
 
 
hsa-miR-99b-5p
 
 
-0.327
 
 
12.9
 
 
 
 
BTF3L4
 
 
hsa-let-7e-5p
 
 
-0.355
 
 
27.4
 
 
 
 
BTF3L4
 
 
hsa-let-7c-5p
 
 
-0.355
 
 
27.4
 
 
 
 
BZW1
 
 
hsa-let-7e-5p
 
 
-0.354
 
 
93.5
 
 
 
 
BZW1
 
 
hsa-let-7c-5p
 
 
-0.354
 
 
93.5
 
 
 
 
BZW1
 
 
hsa-let-7e-5p
 
 
-0.375
 
 
93.5
 
 
 
 
BZW1
 
 
hsa-let-7c-5p
 
 
-0.375
 
 
93.5
 
 
 
 
BZW2
 
 
hsa-let-7c-5p
 
 
-0.397
 
 
18.0
 
 
 
 
BZW2
 
 
hsa-let-7e-5p
 
 
-0.408
 
 
18.0
 
 
 
 
C5orf24
 
 
hsa-miR-22-3p
 
 
-0.493
 
 
20.2
 
 
 
 
C6orf47
 
 
hsa-miR-125a-5p
 
 
-0.564
 
 
11.8
 
 
 
 
CACFD1
 
 
hsa-let-7e-5p
 
 
-0.296
 
 
12.8
 
 
 
 
CACFD1
 
 
hsa-let-7c-5p
 
 
-0.296
 
 
12.8
 
 
 
 
CACNB3
 
 
hsa-miR-125a-5p
 
 
-0.393
 
 
13.0
 
 
 
 
CACNG4
 
 
hsa-let-7e-5p
 
 
-0.33
 
 
11.0
 
 
 
 
CACNG4
 
 
hsa-let-7c-5p
 
 
-0.33
 
 
11.0
 
 
 
 
CAMK2N1
 
 
hsa-miR-22-3p
 
 
-0.302
 
 
33.6
 
 
 
 
CANT1
 
 
hsa-let-7e-5p
 
 
-0.402
 
 
67.4
 
 
 
 
CANT1
 
 
hsa-let-7c-5p
 
 
-0.402
 
 
67.4
 
 
 
 
CAPN1
 
 
hsa-miR-22-3p
 
 
-0.307
 
 
60.3
 
 
 
 
CAPN3
 
 
hsa-let-7e-5p
 
 
-0.317
 
 
17.5
 
 
 
 
CAPN3
 
 
hsa-let-7c-5p
 
 
-0.317
 
 
17.5
 
 
 
 
CAST
 
 
hsa-miR-150-5p
 
 
-0.354
 
 
94.1
 
 
 
 
CBX5
 
 
hsa-let-7e-5p
 
 
-0.307
 
 
10.8
 
 
 
 
CBX5
 
 
hsa-let-7c-5p
 
 
-0.307
 
 
10.8
 
 
 
 
CBX5
 
 
hsa-let-7e-5p
 
 
-0.472
 
 
10.8
 
 
 
 
 
 
 rectum 
 
 
 
 
target_symbol
 
 
mature_mirna_id
 
 
score
 
 
nTPM
 
 
 
 
 
 
ABCC5
 
 
hsa-miR-125a-5p
 
 
-0.288
 
 
20.8
 
 
 
 
ABHD17C
 
 
hsa-let-7e-5p
 
 
-0.359
 
 
52.8
 
 
 
 
ABHD17C
 
 
hsa-let-7c-5p
 
 
-0.359
 
 
52.8
 
 
 
 
ABHD3
 
 
hsa-miR-125a-5p
 
 
-0.453
 
 
61.7
 
 
 
 
ABHD6
 
 
hsa-miR-125a-5p
 
 
-0.501
 
 
10.4
 
 
 
 
ADIPOR2
 
 
hsa-miR-150-5p
 
 
-0.287
 
 
16.7
 
 
 
 
AGGF1
 
 
hsa-miR-125a-5p
 
 
-0.331
 
 
10.9
 
 
 
 
AIDA
 
 
hsa-miR-22-3p
 
 
-0.316
 
 
34.0
 
 
 
 
AIFM1
 
 
hsa-miR-125a-5p
 
 
-0.3
 
 
14.9
 
 
 
 
AIFM1
 
 
hsa-let-7e-5p
 
 
-0.314
 
 
14.9
 
 
 
 
AIFM1
 
 
hsa-let-7c-5p
 
 
-0.314
 
 
14.9
 
 
 
 
AMT
 
 
hsa-let-7e-5p
 
 
-0.463
 
 
11.1
 
 
 
 
AMT
 
 
hsa-let-7c-5p
 
 
-0.463
 
 
11.1
 
 
 
 
ANAPC16
 
 
hsa-miR-125a-5p
 
 
-0.578
 
 
16.4
 
 
 
 
ANKHD1-EIF4EBP3
 
 
hsa-miR-22-3p
 
 
-0.479
 
 
11.2
 
 
 
 
ANKRD13A
 
 
hsa-miR-22-3p
 
 
-0.478
 
 
21.0
 
 
 
 
ANPEP
 
 
hsa-miR-125a-5p
 
 
-0.372
 
 
56.3
 
 
 
 
AP1AR
 
 
hsa-miR-99b-5p
 
 
-0.823
 
 
15.3
 
 
 
 
AP1S1
 
 
hsa-let-7e-5p
 
 
-0.374
 
 
21.1
 
 
 
 
AP1S1
 
 
hsa-let-7c-5p
 
 
-0.374
 
 
21.1
 
 
 
 
ARL6IP6
 
 
hsa-let-7c-5p
 
 
-0.293
 
 
13.3
 
 
 
 
ARL6IP6
 
 
hsa-let-7e-5p
 
 
-0.305
 
 
13.3
 
 
 
 
ARMT1
 
 
hsa-let-7e-5p
 
 
-0.303
 
 
16.5
 
 
 
 
ARMT1
 
 
hsa-let-7c-5p
 
 
-0.303
 
 
16.5
 
 
 
 
ARPC5
 
 
hsa-miR-22-3p
 
 
-0.679
 
 
64.7
 
 
 
 
ARPP19
 
 
hsa-let-7e-5p
 
 
-0.492
 
 
33.6
 
 
 
 
ARPP19
 
 
hsa-let-7c-5p
 
 
-0.492
 
 
33.6
 
 
 
 
ARRB1
 
 
hsa-miR-22-3p
 
 
-0.616
 
 
21.2
 
 
 
 
ARRDC4
 
 
hsa-let-7e-5p
 
 
-0.329
 
 
21.6
 
 
 
 
ARRDC4
 
 
hsa-let-7c-5p
 
 
-0.329
 
 
21.6
 
 
 
 
ATOH1
 
 
hsa-miR-22-3p
 
 
-0.566
 
 
17.3
 
 
 
 
ATP2A2
 
 
hsa-let-7e-5p
 
 
-0.311
 
 
64.9
 
 
 
 
ATP2A2
 
 
hsa-let-7c-5p
 
 
-0.311
 
 
64.9
 
 
 
 
ATP8A1
 
 
hsa-miR-22-3p
 
 
-0.468
 
 
11.0
 
 
 
 
ATPAF1
 
 
hsa-let-7e-5p
 
 
-0.498
 
 
50.0
 
 
 
 
ATPAF1
 
 
hsa-let-7c-5p
 
 
-0.498
 
 
50.0
 
 
 
 
ATXN2
 
 
hsa-miR-10a-5p
 
 
-0.362
 
 
10.9
 
 
 
 
BACH1
 
 
hsa-let-7e-5p
 
 
-0.298
 
 
12.4
 
 
 
 
BACH1
 
 
hsa-let-7e-5p
 
 
-0.298
 
 
12.4
 
 
 
 
BACH1
 
 
hsa-let-7c-5p
 
 
-0.306
 
 
12.4
 
 
 
 
BACH1
 
 
hsa-let-7c-5p
 
 
-0.306
 
 
12.4
 
 
 
 
BACH1
 
 
hsa-let-7e-5p
 
 
-0.435
 
 
12.4
 
 
 
 
BACH1
 
 
hsa-let-7e-5p
 
 
-0.435
 
 
12.4
 
 
 
 
BACH1
 
 
hsa-let-7c-5p
 
 
-0.435
 
 
12.4
 
 
 
 
BACH1
 
 
hsa-let-7c-5p
 
 
-0.435
 
 
12.4
 
 
 
 
BAG5
 
 
hsa-miR-22-3p
 
 
-0.318
 
 
13.7
 
 
 
 
BAK1
 
 
hsa-miR-125a-5p
 
 
-0.402
 
 
23.4
 
 
 
 
BAZ1B
 
 
hsa-miR-10a-5p
 
 
-0.309
 
 
19.3
 
 
 
 
BAZ2A
 
 
hsa-miR-99b-5p
 
 
-0.408
 
 
24.9
 
 
 
 
BCAP29
 
 
hsa-let-7e-5p
 
 
-0.289
 
 
30.4
 
 
 
 
BCL2L11
 
 
hsa-miR-10a-5p
 
 
-0.483
 
 
11.2
 
 
 
 
BDH1
 
 
hsa-miR-125a-5p
 
 
-0.389
 
 
18.6
 
 
 
 
BIN1
 
 
hsa-miR-22-3p
 
 
-0.346
 
 
12.3
 
 
 
 
BLOC1S1
 
 
hsa-miR-22-3p
 
 
-0.346
 
 
161.5
 
 
 
 
BLOC1S6
 
 
hsa-let-7e-5p
 
 
-0.321
 
 
20.2
 
 
 
 
BLOC1S6
 
 
hsa-let-7c-5p
 
 
-0.321
 
 
20.2
 
 
 
 
BLZF1
 
 
hsa-miR-125a-5p
 
 
-0.461
 
 
14.6
 
 
 
 
BTF3L4
 
 
hsa-let-7e-5p
 
 
-0.355
 
 
22.2
 
 
 
 
BTF3L4
 
 
hsa-let-7c-5p
 
 
-0.355
 
 
22.2
 
 
 
 
BZW1
 
 
hsa-let-7e-5p
 
 
-0.354
 
 
85.0
 
 
 
 
BZW1
 
 
hsa-let-7c-5p
 
 
-0.354
 
 
85.0
 
 
 
 
BZW1
 
 
hsa-let-7e-5p
 
 
-0.375
 
 
85.0
 
 
 
 
BZW1
 
 
hsa-let-7c-5p
 
 
-0.375
 
 
85.0
 
 
 
 
BZW2
 
 
hsa-let-7c-5p
 
 
-0.397
 
 
53.4
 
 
 
 
BZW2
 
 
hsa-let-7e-5p
 
 
-0.408
 
 
53.4
 
 
 
 
C1GALT1
 
 
hsa-let-7e-5p
 
 
-0.322
 
 
16.9
 
 
 
 
C1GALT1
 
 
hsa-let-7c-5p
 
 
-0.322
 
 
16.9
 
 
 
 
C1orf210
 
 
hsa-miR-125a-5p
 
 
-0.584
 
 
28.3
 
 
 
 
C2orf88
 
 
hsa-let-7e-5p
 
 
-0.429
 
 
36.2
 
 
 
 
C2orf88
 
 
hsa-let-7c-5p
 
 
-0.429
 
 
36.2
 
 
 
 
C3orf52
 
 
hsa-let-7e-5p
 
 
-0.296
 
 
12.8
 
 
 
 
C3orf52
 
 
hsa-let-7c-5p
 
 
-0.329
 
 
12.8
 
 
 
 
C5orf24
 
 
hsa-miR-22-3p
 
 
-0.493
 
 
17.3
 
 
 
 
CAMK2N1
 
 
hsa-miR-22-3p
 
 
-0.302
 
 
72.4
 
 
 
 
CANT1
 
 
hsa-let-7e-5p
 
 
-0.402
 
 
77.7
 
 
 
 
CANT1
 
 
hsa-let-7c-5p
 
 
-0.402
 
 
77.7
 
 
 
 
CAPN1
 
 
hsa-miR-22-3p
 
 
-0.307
 
 
54.1
 
 
 
 
CASP2
 
 
hsa-miR-125a-5p
 
 
-0.296
 
 
11.9
 
 
 
 
CASP3
 
 
hsa-let-7e-5p
 
 
-0.495
 
 
28.1
 
 
 
 
CASP3
 
 
hsa-let-7c-5p
 
 
-0.503
 
 
28.1
 
 
 
 
CASP5
 
 
hsa-let-7e-5p
 
 
-0.379
 
 
12.1
 
 
 
 
CASP5
 
 
hsa-let-7c-5p
 
 
-0.379
 
 
12.1
 
 
 
 
CAST
 
 
hsa-miR-150-5p
 
 
-0.354
 
 
139.3
 
 
 
 
CBX5
 
 
hsa-let-7e-5p
 
 
-0.307
 
 
10.2
 
 
 
 
CBX5
 
 
hsa-let-7c-5p
 
 
-0.307
 
 
10.2
 
 
 
 
CBX5
 
 
hsa-let-7e-5p
 
 
-0.472
 
 
10.2
 
 
 
 
CBX5
 
 
hsa-let-7c-5p
 
 
-0.472
 
 
10.2
 
 
 
 
CCDC47
 
 
hsa-miR-22-3p
 
 
-0.604
 
 
75.6
 
 
 
 
CCNY
 
 
hsa-let-7c-5p
 
 
-0.323
 
 
18.8
 
 
 
 
CCNY
 
 
hsa-let-7e-5p
 
 
-0.334
 
 
18.8
 
 
 
 
CDC34
 
 
hsa-let-7c-5p
 
 
-0.309
 
 
10.8
 
 
 
 
CDC34
 
 
hsa-let-7e-5p
 
 
-0.321
 
 
10.8
 
 
 
 
CDC34
 
 
hsa-let-7e-5p
 
 
-0.555
 
 
10.8
 
 
 
 
CDC34
 
 
hsa-let-7c-5p
 
 
-0.555
 
 
10.8
 
 
 
 
CDC42SE1
 
 
hsa-miR-125a-5p
 
 
-0.501
 
 
31.7
 
 
 
 
CDCA8
 
 
hsa-let-7e-5p
 
 
-0.342
 
 
10.6
 
 
 
 
CDCA8
 
 
hsa-let-7c-5p
 
 
-0.342
 
 
10.6
 
 
 
 
CDKN1A
 
 
hsa-let-7e-5p
 
 
-0.434
 
 
101.7
 
 
 
 
CDKN1A
 
 
hsa-let-7c-5p
 
 
-0.434
 
 
101.7
 
 
 
 
CDV3
 
 
hsa-let-7e-5p
 
 
-0.453
 
 
65.2
 
 
 
 
 
 
 retina 
 
 
 
 
target_symbol
 
 
mature_mirna_id
 
 
score
 
 
nTPM
 
 
 
 
 
 
ABCC5
 
 
hsa-miR-125a-5p
 
 
-0.288
 
 
116.9
 
 
 
 
ABHD17C
 
 
hsa-let-7e-5p
 
 
-0.359
 
 
15.6
 
 
 
 
ABHD17C
 
 
hsa-let-7c-5p
 
 
-0.359
 
 
15.6
 
 
 
 
ACSL6
 
 
hsa-miR-125a-5p
 
 
-0.307
 
 
42.4
 
 
 
 
ACSL6
 
 
hsa-let-7e-5p
 
 
-0.336
 
 
42.4
 
 
 
 
ACSL6
 
 
hsa-let-7c-5p
 
 
-0.336
 
 
42.4
 
 
 
 
ADIPOR2
 
 
hsa-miR-150-5p
 
 
-0.287
 
 
12.1
 
 
 
 
AGGF1
 
 
hsa-miR-125a-5p
 
 
-0.331
 
 
20.6
 
 
 
 
AIDA
 
 
hsa-miR-22-3p
 
 
-0.316
 
 
18.0
 
 
 
 
AKAP6
 
 
hsa-let-7e-5p
 
 
-0.307
 
 
12.6
 
 
 
 
AKAP6
 
 
hsa-let-7c-5p
 
 
-0.307
 
 
12.6
 
 
 
 
AKT3
 
 
hsa-miR-22-3p
 
 
-0.451
 
 
15.2
 
 
 
 
ALPL
 
 
hsa-miR-10a-5p
 
 
-0.32
 
 
17.1
 
 
 
 
ANAPC16
 
 
hsa-miR-125a-5p
 
 
-0.578
 
 
18.8
 
 
 
 
ANKHD1-EIF4EBP3
 
 
hsa-miR-22-3p
 
 
-0.479
 
 
11.0
 
 
 
 
ANKRD33B
 
 
hsa-miR-125a-5p
 
 
-0.389
 
 
47.3
 
 
 
 
ANKRD46
 
 
hsa-let-7e-5p
 
 
-0.389
 
 
32.6
 
 
 
 
ANKRD46
 
 
hsa-let-7c-5p
 
 
-0.389
 
 
32.6
 
 
 
 
AP1S1
 
 
hsa-let-7e-5p
 
 
-0.374
 
 
13.5
 
 
 
 
AP1S1
 
 
hsa-let-7c-5p
 
 
-0.374
 
 
13.5
 
 
 
 
ARG2
 
 
hsa-let-7e-5p
 
 
-0.468
 
 
20.7
 
 
 
 
ARG2
 
 
hsa-let-7c-5p
 
 
-0.468
 
 
20.7
 
 
 
 
ARID3B
 
 
hsa-let-7e-5p
 
 
-0.31
 
 
17.4
 
 
 
 
ARID3B
 
 
hsa-let-7c-5p
 
 
-0.31
 
 
17.4
 
 
 
 
ARID3B
 
 
hsa-let-7e-5p
 
 
-0.486
 
 
17.4
 
 
 
 
ARID3B
 
 
hsa-let-7c-5p
 
 
-0.486
 
 
17.4
 
 
 
 
ARL4D
 
 
hsa-let-7e-5p
 
 
-0.417
 
 
78.9
 
 
 
 
ARL4D
 
 
hsa-let-7c-5p
 
 
-0.417
 
 
78.9
 
 
 
 
ARL6IP6
 
 
hsa-let-7c-5p
 
 
-0.293
 
 
10.5
 
 
 
 
ARL6IP6
 
 
hsa-let-7e-5p
 
 
-0.305
 
 
10.5
 
 
 
 
ARMT1
 
 
hsa-let-7e-5p
 
 
-0.303
 
 
15.2
 
 
 
 
ARMT1
 
 
hsa-let-7c-5p
 
 
-0.303
 
 
15.2
 
 
 
 
ARPP19
 
 
hsa-let-7e-5p
 
 
-0.492
 
 
53.1
 
 
 
 
ARPP19
 
 
hsa-let-7c-5p
 
 
-0.492
 
 
53.1
 
 
 
 
ATCAY
 
 
hsa-miR-10a-5p
 
 
-0.322
 
 
16.3
 
 
 
 
ATP2A2
 
 
hsa-let-7e-5p
 
 
-0.311
 
 
17.7
 
 
 
 
ATP2A2
 
 
hsa-let-7c-5p
 
 
-0.311
 
 
17.7
 
 
 
 
ATP8A1
 
 
hsa-miR-22-3p
 
 
-0.468
 
 
17.9
 
 
 
 
ATPAF1
 
 
hsa-let-7e-5p
 
 
-0.498
 
 
65.3
 
 
 
 
ATPAF1
 
 
hsa-let-7c-5p
 
 
-0.498
 
 
65.3
 
 
 
 
ATXN2
 
 
hsa-miR-10a-5p
 
 
-0.362
 
 
14.6
 
 
 
 
B3GAT3
 
 
hsa-let-7e-5p
 
 
-0.324
 
 
15.0
 
 
 
 
B3GAT3
 
 
hsa-let-7c-5p
 
 
-0.324
 
 
15.0
 
 
 
 
B4GAT1
 
 
hsa-let-7e-5p
 
 
-0.47
 
 
42.6
 
 
 
 
B4GAT1
 
 
hsa-let-7c-5p
 
 
-0.47
 
 
42.6
 
 
 
 
BACH1
 
 
hsa-let-7e-5p
 
 
-0.298
 
 
10.9
 
 
 
 
BACH1
 
 
hsa-let-7e-5p
 
 
-0.298
 
 
10.9
 
 
 
 
BACH1
 
 
hsa-let-7c-5p
 
 
-0.306
 
 
10.9
 
 
 
 
BACH1
 
 
hsa-let-7c-5p
 
 
-0.306
 
 
10.9
 
 
 
 
BACH1
 
 
hsa-let-7e-5p
 
 
-0.435
 
 
10.9
 
 
 
 
BACH1
 
 
hsa-let-7e-5p
 
 
-0.435
 
 
10.9
 
 
 
 
BACH1
 
 
hsa-let-7c-5p
 
 
-0.435
 
 
10.9
 
 
 
 
BACH1
 
 
hsa-let-7c-5p
 
 
-0.435
 
 
10.9
 
 
 
 
BAG5
 
 
hsa-miR-22-3p
 
 
-0.318
 
 
17.0
 
 
 
 
BAZ1B
 
 
hsa-miR-10a-5p
 
 
-0.309
 
 
26.3
 
 
 
 
BAZ2A
 
 
hsa-miR-99b-5p
 
 
-0.408
 
 
45.3
 
 
 
 
BBX
 
 
hsa-miR-10a-5p
 
 
-0.305
 
 
18.6
 
 
 
 
BCAT1
 
 
hsa-let-7e-5p
 
 
-0.394
 
 
13.4
 
 
 
 
BCAT1
 
 
hsa-let-7c-5p
 
 
-0.394
 
 
13.4
 
 
 
 
BCL2L11
 
 
hsa-miR-10a-5p
 
 
-0.483
 
 
11.9
 
 
 
 
BIN1
 
 
hsa-miR-22-3p
 
 
-0.346
 
 
49.9
 
 
 
 
BLOC1S1
 
 
hsa-miR-22-3p
 
 
-0.346
 
 
77.5
 
 
 
 
BLOC1S6
 
 
hsa-let-7e-5p
 
 
-0.321
 
 
22.7
 
 
 
 
BLOC1S6
 
 
hsa-let-7c-5p
 
 
-0.321
 
 
22.7
 
 
 
 
BLZF1
 
 
hsa-miR-125a-5p
 
 
-0.461
 
 
21.1
 
 
 
 
BMPR2
 
 
hsa-miR-99b-5p
 
 
-0.327
 
 
17.0
 
 
 
 
BTF3L4
 
 
hsa-let-7e-5p
 
 
-0.355
 
 
29.1
 
 
 
 
BTF3L4
 
 
hsa-let-7c-5p
 
 
-0.355
 
 
29.1
 
 
 
 
BZW1
 
 
hsa-let-7e-5p
 
 
-0.354
 
 
42.4
 
 
 
 
BZW1
 
 
hsa-let-7c-5p
 
 
-0.354
 
 
42.4
 
 
 
 
BZW1
 
 
hsa-let-7e-5p
 
 
-0.375
 
 
42.4
 
 
 
 
BZW1
 
 
hsa-let-7c-5p
 
 
-0.375
 
 
42.4
 
 
 
 
BZW2
 
 
hsa-let-7c-5p
 
 
-0.397
 
 
80.5
 
 
 
 
BZW2
 
 
hsa-let-7e-5p
 
 
-0.408
 
 
80.5
 
 
 
 
C1GALT1
 
 
hsa-let-7e-5p
 
 
-0.322
 
 
21.8
 
 
 
 
C1GALT1
 
 
hsa-let-7c-5p
 
 
-0.322
 
 
21.8
 
 
 
 
C5orf24
 
 
hsa-miR-22-3p
 
 
-0.493
 
 
17.8
 
 
 
 
CADM2
 
 
hsa-let-7e-5p
 
 
-0.294
 
 
87.9
 
 
 
 
CADM2
 
 
hsa-let-7c-5p
 
 
-0.294
 
 
87.9
 
 
 
 
CAMK2N1
 
 
hsa-miR-22-3p
 
 
-0.302
 
 
30.3
 
 
 
 
CAST
 
 
hsa-miR-150-5p
 
 
-0.354
 
 
15.9
 
 
 
 
CBX5
 
 
hsa-let-7e-5p
 
 
-0.307
 
 
21.2
 
 
 
 
CBX5
 
 
hsa-let-7c-5p
 
 
-0.307
 
 
21.2
 
 
 
 
CBX5
 
 
hsa-let-7e-5p
 
 
-0.472
 
 
21.2
 
 
 
 
CBX5
 
 
hsa-let-7c-5p
 
 
-0.472
 
 
21.2
 
 
 
 
CCDC47
 
 
hsa-miR-22-3p
 
 
-0.604
 
 
42.0
 
 
 
 
CCNY
 
 
hsa-let-7c-5p
 
 
-0.323
 
 
17.9
 
 
 
 
CCNY
 
 
hsa-let-7e-5p
 
 
-0.334
 
 
17.9
 
 
 
 
CDC34
 
 
hsa-let-7c-5p
 
 
-0.309
 
 
26.2
 
 
 
 
CDC34
 
 
hsa-let-7e-5p
 
 
-0.321
 
 
26.2
 
 
 
 
CDC34
 
 
hsa-let-7e-5p
 
 
-0.555
 
 
26.2
 
 
 
 
CDC34
 
 
hsa-let-7c-5p
 
 
-0.555
 
 
26.2
 
 
 
 
CDC37L1
 
 
hsa-miR-125a-5p
 
 
-0.401
 
 
12.0
 
 
 
 
CDC42SE1
 
 
hsa-miR-125a-5p
 
 
-0.501
 
 
19.1
 
 
 
 
CDKN1A
 
 
hsa-let-7e-5p
 
 
-0.434
 
 
27.1
 
 
 
 
CDKN1A
 
 
hsa-let-7c-5p
 
 
-0.434
 
 
27.1
 
 
 
 
CDV3
 
 
hsa-let-7e-5p
 
 
-0.453
 
 
45.1
 
 
 
 
CDV3
 
 
hsa-let-7c-5p
 
 
-0.461
 
 
45.1
 
 
 
 
CEBPD
 
 
hsa-let-7e-5p
 
 
-0.309
 
 
15.7
 
 
 
 
CEBPD
 
 
hsa-let-7c-5p
 
 
-0.309
 
 
15.7
 
 
 
 
 
 
 salivary gland 
 
 
 
 
target_symbol
 
 
mature_mirna_id
 
 
score
 
 
nTPM
 
 
 
 
 
 
ABCC5
 
 
hsa-miR-125a-5p
 
 
-0.288
 
 
18.2
 
 
 
 
ABHD17C
 
 
hsa-let-7e-5p
 
 
-0.359
 
 
22.3
 
 
 
 
ABHD17C
 
 
hsa-let-7c-5p
 
 
-0.359
 
 
22.3
 
 
 
 
ABHD3
 
 
hsa-miR-125a-5p
 
 
-0.453
 
 
13.7
 
 
 
 
ABTB1
 
 
hsa-miR-125a-5p
 
 
-0.519
 
 
25.2
 
 
 
 
ADIPOR2
 
 
hsa-miR-150-5p
 
 
-0.287
 
 
21.2
 
 
 
 
AIDA
 
 
hsa-miR-22-3p
 
 
-0.316
 
 
26.4
 
 
 
 
AIFM1
 
 
hsa-miR-125a-5p
 
 
-0.3
 
 
17.0
 
 
 
 
AIFM1
 
 
hsa-let-7e-5p
 
 
-0.314
 
 
17.0
 
 
 
 
AIFM1
 
 
hsa-let-7c-5p
 
 
-0.314
 
 
17.0
 
 
 
 
AMT
 
 
hsa-let-7e-5p
 
 
-0.463
 
 
10.0
 
 
 
 
AMT
 
 
hsa-let-7c-5p
 
 
-0.463
 
 
10.0
 
 
 
 
ANAPC16
 
 
hsa-miR-125a-5p
 
 
-0.578
 
 
29.9
 
 
 
 
ANKHD1-EIF4EBP3
 
 
hsa-miR-22-3p
 
 
-0.479
 
 
12.2
 
 
 
 
ANKRD13A
 
 
hsa-miR-22-3p
 
 
-0.478
 
 
25.9
 
 
 
 
ANKRD46
 
 
hsa-let-7e-5p
 
 
-0.389
 
 
16.1
 
 
 
 
ANKRD46
 
 
hsa-let-7c-5p
 
 
-0.389
 
 
16.1
 
 
 
 
ANPEP
 
 
hsa-miR-125a-5p
 
 
-0.372
 
 
38.3
 
 
 
 
AP1AR
 
 
hsa-miR-99b-5p
 
 
-0.823
 
 
11.4
 
 
 
 
AP1S1
 
 
hsa-let-7e-5p
 
 
-0.374
 
 
21.5
 
 
 
 
AP1S1
 
 
hsa-let-7c-5p
 
 
-0.374
 
 
21.5
 
 
 
 
ARG2
 
 
hsa-let-7e-5p
 
 
-0.468
 
 
18.4
 
 
 
 
ARG2
 
 
hsa-let-7c-5p
 
 
-0.468
 
 
18.4
 
 
 
 
ARL4D
 
 
hsa-let-7e-5p
 
 
-0.417
 
 
16.7
 
 
 
 
ARL4D
 
 
hsa-let-7c-5p
 
 
-0.417
 
 
16.7
 
 
 
 
ARPC5
 
 
hsa-miR-22-3p
 
 
-0.679
 
 
43.2
 
 
 
 
ARPP19
 
 
hsa-let-7e-5p
 
 
-0.492
 
 
28.7
 
 
 
 
ARPP19
 
 
hsa-let-7c-5p
 
 
-0.492
 
 
28.7
 
 
 
 
ARRB1
 
 
hsa-miR-22-3p
 
 
-0.616
 
 
11.9
 
 
 
 
ATOSB
 
 
hsa-let-7e-5p
 
 
-0.429
 
 
10.9
 
 
 
 
ATOSB
 
 
hsa-let-7c-5p
 
 
-0.429
 
 
10.9
 
 
 
 
ATP2A2
 
 
hsa-let-7e-5p
 
 
-0.311
 
 
74.5
 
 
 
 
ATP2A2
 
 
hsa-let-7c-5p
 
 
-0.311
 
 
74.5
 
 
 
 
ATPAF1
 
 
hsa-let-7e-5p
 
 
-0.498
 
 
54.3
 
 
 
 
ATPAF1
 
 
hsa-let-7c-5p
 
 
-0.498
 
 
54.3
 
 
 
 
ATXN2
 
 
hsa-miR-10a-5p
 
 
-0.362
 
 
14.4
 
 
 
 
B3GAT3
 
 
hsa-let-7e-5p
 
 
-0.324
 
 
28.6
 
 
 
 
B3GAT3
 
 
hsa-let-7c-5p
 
 
-0.324
 
 
28.6
 
 
 
 
B4GAT1
 
 
hsa-let-7e-5p
 
 
-0.47
 
 
14.9
 
 
 
 
B4GAT1
 
 
hsa-let-7c-5p
 
 
-0.47
 
 
14.9
 
 
 
 
BACE2
 
 
hsa-let-7e-5p
 
 
-0.364
 
 
16.3
 
 
 
 
BACE2
 
 
hsa-let-7c-5p
 
 
-0.364
 
 
16.3
 
 
 
 
BACH1
 
 
hsa-let-7e-5p
 
 
-0.298
 
 
16.4
 
 
 
 
BACH1
 
 
hsa-let-7e-5p
 
 
-0.298
 
 
16.4
 
 
 
 
BACH1
 
 
hsa-let-7c-5p
 
 
-0.306
 
 
16.4
 
 
 
 
BACH1
 
 
hsa-let-7c-5p
 
 
-0.306
 
 
16.4
 
 
 
 
BACH1
 
 
hsa-let-7e-5p
 
 
-0.435
 
 
16.4
 
 
 
 
BACH1
 
 
hsa-let-7e-5p
 
 
-0.435
 
 
16.4
 
 
 
 
BACH1
 
 
hsa-let-7c-5p
 
 
-0.435
 
 
16.4
 
 
 
 
BACH1
 
 
hsa-let-7c-5p
 
 
-0.435
 
 
16.4
 
 
 
 
BAG5
 
 
hsa-miR-22-3p
 
 
-0.318
 
 
15.5
 
 
 
 
BAK1
 
 
hsa-miR-125a-5p
 
 
-0.402
 
 
11.3
 
 
 
 
BAZ1B
 
 
hsa-miR-10a-5p
 
 
-0.309
 
 
16.1
 
 
 
 
BAZ2A
 
 
hsa-miR-99b-5p
 
 
-0.408
 
 
23.8
 
 
 
 
BBX
 
 
hsa-miR-10a-5p
 
 
-0.305
 
 
12.6
 
 
 
 
BCAP29
 
 
hsa-let-7e-5p
 
 
-0.289
 
 
13.9
 
 
 
 
BCL2L12
 
 
hsa-miR-125a-5p
 
 
-0.288
 
 
11.1
 
 
 
 
BDH1
 
 
hsa-miR-125a-5p
 
 
-0.389
 
 
22.2
 
 
 
 
BIN1
 
 
hsa-miR-22-3p
 
 
-0.346
 
 
40.9
 
 
 
 
BIN3
 
 
hsa-let-7e-5p
 
 
-0.427
 
 
13.3
 
 
 
 
BIN3
 
 
hsa-let-7c-5p
 
 
-0.427
 
 
13.3
 
 
 
 
BLOC1S1
 
 
hsa-miR-22-3p
 
 
-0.346
 
 
104.2
 
 
 
 
BLOC1S6
 
 
hsa-let-7e-5p
 
 
-0.321
 
 
19.5
 
 
 
 
BLOC1S6
 
 
hsa-let-7c-5p
 
 
-0.321
 
 
19.5
 
 
 
 
BLZF1
 
 
hsa-miR-125a-5p
 
 
-0.461
 
 
11.9
 
 
 
 
BMF
 
 
hsa-miR-125a-5p
 
 
-0.34
 
 
10.0
 
 
 
 
BTF3L4
 
 
hsa-let-7e-5p
 
 
-0.355
 
 
28.2
 
 
 
 
BTF3L4
 
 
hsa-let-7c-5p
 
 
-0.355
 
 
28.2
 
 
 
 
BZW1
 
 
hsa-let-7e-5p
 
 
-0.354
 
 
55.2
 
 
 
 
BZW1
 
 
hsa-let-7c-5p
 
 
-0.354
 
 
55.2
 
 
 
 
BZW1
 
 
hsa-let-7e-5p
 
 
-0.375
 
 
55.2
 
 
 
 
BZW1
 
 
hsa-let-7c-5p
 
 
-0.375
 
 
55.2
 
 
 
 
BZW2
 
 
hsa-let-7c-5p
 
 
-0.397
 
 
24.9
 
 
 
 
BZW2
 
 
hsa-let-7e-5p
 
 
-0.408
 
 
24.9
 
 
 
 
C15orf39
 
 
hsa-miR-125a-5p
 
 
-0.335
 
 
13.6
 
 
 
 
C15orf39
 
 
hsa-let-7e-5p
 
 
-0.374
 
 
13.6
 
 
 
 
C15orf39
 
 
hsa-let-7c-5p
 
 
-0.374
 
 
13.6
 
 
 
 
C19orf47
 
 
hsa-let-7e-5p
 
 
-0.311
 
 
12.5
 
 
 
 
C19orf47
 
 
hsa-let-7c-5p
 
 
-0.311
 
 
12.5
 
 
 
 
C1GALT1
 
 
hsa-let-7e-5p
 
 
-0.322
 
 
22.0
 
 
 
 
C1GALT1
 
 
hsa-let-7c-5p
 
 
-0.322
 
 
22.0
 
 
 
 
C1orf210
 
 
hsa-miR-125a-5p
 
 
-0.584
 
 
18.3
 
 
 
 
C2orf88
 
 
hsa-let-7e-5p
 
 
-0.429
 
 
13.2
 
 
 
 
C2orf88
 
 
hsa-let-7c-5p
 
 
-0.429
 
 
13.2
 
 
 
 
C5orf24
 
 
hsa-miR-22-3p
 
 
-0.493
 
 
17.2
 
 
 
 
C6orf47
 
 
hsa-miR-125a-5p
 
 
-0.564
 
 
12.8
 
 
 
 
CACFD1
 
 
hsa-let-7e-5p
 
 
-0.296
 
 
15.2
 
 
 
 
CACFD1
 
 
hsa-let-7c-5p
 
 
-0.296
 
 
15.2
 
 
 
 
CACNB3
 
 
hsa-miR-125a-5p
 
 
-0.393
 
 
12.3
 
 
 
 
CAMK2N1
 
 
hsa-miR-22-3p
 
 
-0.302
 
 
28.7
 
 
 
 
CANT1
 
 
hsa-let-7e-5p
 
 
-0.402
 
 
59.0
 
 
 
 
CANT1
 
 
hsa-let-7c-5p
 
 
-0.402
 
 
59.0
 
 
 
 
CAPN1
 
 
hsa-miR-22-3p
 
 
-0.307
 
 
113.6
 
 
 
 
CAPN3
 
 
hsa-let-7e-5p
 
 
-0.317
 
 
19.7
 
 
 
 
CAPN3
 
 
hsa-let-7c-5p
 
 
-0.317
 
 
19.7
 
 
 
 
CASP2
 
 
hsa-miR-125a-5p
 
 
-0.296
 
 
11.7
 
 
 
 
CASP3
 
 
hsa-let-7e-5p
 
 
-0.495
 
 
12.4
 
 
 
 
CASP3
 
 
hsa-let-7c-5p
 
 
-0.503
 
 
12.4
 
 
 
 
CAST
 
 
hsa-miR-150-5p
 
 
-0.354
 
 
103.1
 
 
 
 
CCDC149
 
 
hsa-miR-22-3p
 
 
-0.327
 
 
17.0
 
 
 
 
 
 
 seminal vesicle 
 
 
 
 
target_symbol
 
 
mature_mirna_id
 
 
score
 
 
nTPM
 
 
 
 
 
 
ABCC5
 
 
hsa-miR-125a-5p
 
 
-0.288
 
 
12.9
 
 
 
 
ABHD6
 
 
hsa-miR-125a-5p
 
 
-0.501
 
 
13.5
 
 
 
 
ABTB1
 
 
hsa-miR-125a-5p
 
 
-0.519
 
 
12.8
 
 
 
 
ACSL6
 
 
hsa-miR-125a-5p
 
 
-0.307
 
 
17.0
 
 
 
 
ACSL6
 
 
hsa-let-7e-5p
 
 
-0.336
 
 
17.0
 
 
 
 
ACSL6
 
 
hsa-let-7c-5p
 
 
-0.336
 
 
17.0
 
 
 
 
ADIPOR2
 
 
hsa-miR-150-5p
 
 
-0.287
 
 
11.2
 
 
 
 
AIDA
 
 
hsa-miR-22-3p
 
 
-0.316
 
 
39.7
 
 
 
 
AKT3
 
 
hsa-miR-22-3p
 
 
-0.451
 
 
14.7
 
 
 
 
ALDH4A1
 
 
hsa-miR-184
 
 
-0.421
 
 
25.6
 
 
 
 
ANAPC16
 
 
hsa-miR-125a-5p
 
 
-0.578
 
 
20.1
 
 
 
 
ANKRD13A
 
 
hsa-miR-22-3p
 
 
-0.478
 
 
23.1
 
 
 
 
ANKRD46
 
 
hsa-let-7e-5p
 
 
-0.389
 
 
12.4
 
 
 
 
ANKRD46
 
 
hsa-let-7c-5p
 
 
-0.389
 
 
12.4
 
 
 
 
ARL4D
 
 
hsa-let-7e-5p
 
 
-0.417
 
 
14.3
 
 
 
 
ARL4D
 
 
hsa-let-7c-5p
 
 
-0.417
 
 
14.3
 
 
 
 
ARMT1
 
 
hsa-let-7e-5p
 
 
-0.303
 
 
10.5
 
 
 
 
ARMT1
 
 
hsa-let-7c-5p
 
 
-0.303
 
 
10.5
 
 
 
 
ARPC5
 
 
hsa-miR-22-3p
 
 
-0.679
 
 
33.8
 
 
 
 
ARPP19
 
 
hsa-let-7e-5p
 
 
-0.492
 
 
38.5
 
 
 
 
ARPP19
 
 
hsa-let-7c-5p
 
 
-0.492
 
 
38.5
 
 
 
 
ARRDC4
 
 
hsa-let-7e-5p
 
 
-0.329
 
 
15.4
 
 
 
 
ARRDC4
 
 
hsa-let-7c-5p
 
 
-0.329
 
 
15.4
 
 
 
 
ASAP1
 
 
hsa-let-7e-5p
 
 
-0.296
 
 
10.7
 
 
 
 
ASAP1
 
 
hsa-let-7c-5p
 
 
-0.308
 
 
10.7
 
 
 
 
ATP2A2
 
 
hsa-let-7e-5p
 
 
-0.311
 
 
66.0
 
 
 
 
ATP2A2
 
 
hsa-let-7c-5p
 
 
-0.311
 
 
66.0
 
 
 
 
ATPAF1
 
 
hsa-let-7e-5p
 
 
-0.498
 
 
31.8
 
 
 
 
ATPAF1
 
 
hsa-let-7c-5p
 
 
-0.498
 
 
31.8
 
 
 
 
ATXN2
 
 
hsa-miR-10a-5p
 
 
-0.362
 
 
14.7
 
 
 
 
B3GAT3
 
 
hsa-let-7e-5p
 
 
-0.324
 
 
17.2
 
 
 
 
B3GAT3
 
 
hsa-let-7c-5p
 
 
-0.324
 
 
17.2
 
 
 
 
B4GAT1
 
 
hsa-let-7e-5p
 
 
-0.47
 
 
21.2
 
 
 
 
B4GAT1
 
 
hsa-let-7c-5p
 
 
-0.47
 
 
21.2
 
 
 
 
BAG5
 
 
hsa-miR-22-3p
 
 
-0.318
 
 
13.4
 
 
 
 
BAZ1B
 
 
hsa-miR-10a-5p
 
 
-0.309
 
 
24.0
 
 
 
 
BAZ2A
 
 
hsa-miR-99b-5p
 
 
-0.408
 
 
19.8
 
 
 
 
BBX
 
 
hsa-miR-10a-5p
 
 
-0.305
 
 
29.7
 
 
 
 
BCAP29
 
 
hsa-let-7e-5p
 
 
-0.289
 
 
24.0
 
 
 
 
BIN1
 
 
hsa-miR-22-3p
 
 
-0.346
 
 
22.7
 
 
 
 
BLOC1S1
 
 
hsa-miR-22-3p
 
 
-0.346
 
 
74.4
 
 
 
 
BLOC1S6
 
 
hsa-let-7e-5p
 
 
-0.321
 
 
19.2
 
 
 
 
BLOC1S6
 
 
hsa-let-7c-5p
 
 
-0.321
 
 
19.2
 
 
 
 
BLZF1
 
 
hsa-miR-125a-5p
 
 
-0.461
 
 
15.1
 
 
 
 
BMPR2
 
 
hsa-miR-99b-5p
 
 
-0.327
 
 
10.5
 
 
 
 
BTF3L4
 
 
hsa-let-7e-5p
 
 
-0.355
 
 
23.0
 
 
 
 
BTF3L4
 
 
hsa-let-7c-5p
 
 
-0.355
 
 
23.0
 
 
 
 
BZW1
 
 
hsa-let-7e-5p
 
 
-0.354
 
 
58.1
 
 
 
 
BZW1
 
 
hsa-let-7c-5p
 
 
-0.354
 
 
58.1
 
 
 
 
BZW1
 
 
hsa-let-7e-5p
 
 
-0.375
 
 
58.1
 
 
 
 
BZW1
 
 
hsa-let-7c-5p
 
 
-0.375
 
 
58.1
 
 
 
 
BZW2
 
 
hsa-let-7c-5p
 
 
-0.397
 
 
32.3
 
 
 
 
BZW2
 
 
hsa-let-7e-5p
 
 
-0.408
 
 
32.3
 
 
 
 
C5orf24
 
 
hsa-miR-22-3p
 
 
-0.493
 
 
17.3
 
 
 
 
CAMK2N1
 
 
hsa-miR-22-3p
 
 
-0.302
 
 
38.4
 
 
 
 
CANT1
 
 
hsa-let-7e-5p
 
 
-0.402
 
 
30.5
 
 
 
 
CANT1
 
 
hsa-let-7c-5p
 
 
-0.402
 
 
30.5
 
 
 
 
CAPN1
 
 
hsa-miR-22-3p
 
 
-0.307
 
 
63.6
 
 
 
 
CAST
 
 
hsa-miR-150-5p
 
 
-0.354
 
 
74.2
 
 
 
 
CBX5
 
 
hsa-let-7e-5p
 
 
-0.307
 
 
11.1
 
 
 
 
CBX5
 
 
hsa-let-7c-5p
 
 
-0.307
 
 
11.1
 
 
 
 
CBX5
 
 
hsa-let-7e-5p
 
 
-0.472
 
 
11.1
 
 
 
 
CBX5
 
 
hsa-let-7c-5p
 
 
-0.472
 
 
11.1
 
 
 
 
CCDC47
 
 
hsa-miR-22-3p
 
 
-0.604
 
 
49.0
 
 
 
 
CCNY
 
 
hsa-let-7c-5p
 
 
-0.323
 
 
19.0
 
 
 
 
CCNY
 
 
hsa-let-7e-5p
 
 
-0.334
 
 
19.0
 
 
 
 
CDC34
 
 
hsa-let-7c-5p
 
 
-0.309
 
 
18.2
 
 
 
 
CDC34
 
 
hsa-let-7e-5p
 
 
-0.321
 
 
18.2
 
 
 
 
CDC34
 
 
hsa-let-7e-5p
 
 
-0.555
 
 
18.2
 
 
 
 
CDC34
 
 
hsa-let-7c-5p
 
 
-0.555
 
 
18.2
 
 
 
 
CDC37L1
 
 
hsa-miR-125a-5p
 
 
-0.401
 
 
11.3
 
 
 
 
CDC42SE1
 
 
hsa-miR-125a-5p
 
 
-0.501
 
 
31.3
 
 
 
 
CDKN1A
 
 
hsa-let-7e-5p
 
 
-0.434
 
 
27.7
 
 
 
 
CDKN1A
 
 
hsa-let-7c-5p
 
 
-0.434
 
 
27.7
 
 
 
 
CDV3
 
 
hsa-let-7e-5p
 
 
-0.453
 
 
44.8
 
 
 
 
CDV3
 
 
hsa-let-7c-5p
 
 
-0.461
 
 
44.8
 
 
 
 
CEBPD
 
 
hsa-let-7e-5p
 
 
-0.309
 
 
18.3
 
 
 
 
CEBPD
 
 
hsa-let-7c-5p
 
 
-0.309
 
 
18.3
 
 
 
 
CELF2
 
 
hsa-miR-10a-5p
 
 
-0.319
 
 
11.5
 
 
 
 
CERCAM
 
 
hsa-let-7e-5p
 
 
-0.344
 
 
21.4
 
 
 
 
CERCAM
 
 
hsa-let-7c-5p
 
 
-0.344
 
 
21.4
 
 
 
 
CFAP36
 
 
hsa-miR-22-3p
 
 
-0.549
 
 
42.3
 
 
 
 
CLASP2
 
 
hsa-let-7e-5p
 
 
-0.344
 
 
19.6
 
 
 
 
CLASP2
 
 
hsa-let-7c-5p
 
 
-0.344
 
 
19.6
 
 
 
 
CLDN12
 
 
hsa-let-7e-5p
 
 
-0.511
 
 
11.7
 
 
 
 
CLDN12
 
 
hsa-let-7c-5p
 
 
-0.511
 
 
11.7
 
 
 
 
CLIC4
 
 
hsa-miR-22-3p
 
 
-0.594
 
 
143.9
 
 
 
 
CMTM6
 
 
hsa-miR-150-5p
 
 
-0.328
 
 
37.6
 
 
 
 
CNOT6L
 
 
hsa-miR-22-3p
 
 
-0.293
 
 
10.5
 
 
 
 
COL1A2
 
 
hsa-let-7e-5p
 
 
-0.499
 
 
208.6
 
 
 
 
COL1A2
 
 
hsa-let-7c-5p
 
 
-0.499
 
 
208.6
 
 
 
 
COL3A1
 
 
hsa-let-7e-5p
 
 
-0.474
 
 
260.6
 
 
 
 
COL3A1
 
 
hsa-let-7c-5p
 
 
-0.474
 
 
260.6
 
 
 
 
COPZ1
 
 
hsa-miR-125a-5p
 
 
-0.296
 
 
64.7
 
 
 
 
CPNE2
 
 
hsa-miR-125a-5p
 
 
-0.357
 
 
16.1
 
 
 
 
CPSF6
 
 
hsa-miR-125a-5p
 
 
-0.384
 
 
19.0
 
 
 
 
CREB1
 
 
hsa-miR-10a-5p
 
 
-0.416
 
 
11.8
 
 
 
 
CREB1
 
 
hsa-miR-22-3p
 
 
-0.424
 
 
11.8
 
 
 
 
CRISPLD2
 
 
hsa-miR-184
 
 
-0.293
 
 
26.1
 
 
 
 
CSF1R
 
 
hsa-miR-22-3p
 
 
-0.472
 
 
19.6
 
 
 
 
 
 
 skeletal muscle 
 
 
 
 
target_symbol
 
 
mature_mirna_id
 
 
score
 
 
nTPM
 
 
 
 
 
 
ABCC5
 
 
hsa-miR-125a-5p
 
 
-0.288
 
 
118.1
 
 
 
 
ABHD3
 
 
hsa-miR-125a-5p
 
 
-0.453
 
 
13.6
 
 
 
 
ABTB1
 
 
hsa-miR-125a-5p
 
 
-0.519
 
 
44.0
 
 
 
 
ACHE
 
 
hsa-miR-125a-5p
 
 
-0.308
 
 
152.9
 
 
 
 
ACHE
 
 
hsa-miR-125a-5p
 
 
-0.361
 
 
152.9
 
 
 
 
ACVR2A
 
 
hsa-miR-125a-5p
 
 
-0.301
 
 
16.4
 
 
 
 
ADIPOR2
 
 
hsa-miR-150-5p
 
 
-0.287
 
 
41.4
 
 
 
 
ADRB2
 
 
hsa-let-7e-5p
 
 
-0.635
 
 
10.9
 
 
 
 
ADRB2
 
 
hsa-let-7c-5p
 
 
-0.635
 
 
10.9
 
 
 
 
AGGF1
 
 
hsa-miR-125a-5p
 
 
-0.331
 
 
12.2
 
 
 
 
AGMAT
 
 
hsa-miR-22-3p
 
 
-0.294
 
 
32.1
 
 
 
 
AGO2
 
 
hsa-miR-125a-5p
 
 
-0.811
 
 
16.4
 
 
 
 
AGO2
 
 
hsa-miR-184
 
 
-0.831
 
 
16.4
 
 
 
 
AGO2
 
 
hsa-miR-99b-5p
 
 
-0.836
 
 
16.4
 
 
 
 
AHCTF1
 
 
hsa-let-7e-5p
 
 
-0.374
 
 
10.0
 
 
 
 
AHCTF1
 
 
hsa-let-7c-5p
 
 
-0.374
 
 
10.0
 
 
 
 
AIDA
 
 
hsa-miR-22-3p
 
 
-0.316
 
 
26.6
 
 
 
 
AIFM1
 
 
hsa-miR-125a-5p
 
 
-0.3
 
 
58.6
 
 
 
 
AIFM1
 
 
hsa-let-7e-5p
 
 
-0.314
 
 
58.6
 
 
 
 
AIFM1
 
 
hsa-let-7c-5p
 
 
-0.314
 
 
58.6
 
 
 
 
AKAP6
 
 
hsa-let-7e-5p
 
 
-0.307
 
 
44.4
 
 
 
 
AKAP6
 
 
hsa-let-7c-5p
 
 
-0.307
 
 
44.4
 
 
 
 
ALDH4A1
 
 
hsa-miR-184
 
 
-0.421
 
 
69.5
 
 
 
 
ANAPC16
 
 
hsa-miR-125a-5p
 
 
-0.578
 
 
57.2
 
 
 
 
ANKHD1-EIF4EBP3
 
 
hsa-miR-22-3p
 
 
-0.479
 
 
10.4
 
 
 
 
ANKRD46
 
 
hsa-let-7e-5p
 
 
-0.389
 
 
58.3
 
 
 
 
ANKRD46
 
 
hsa-let-7c-5p
 
 
-0.389
 
 
58.3
 
 
 
 
ARG2
 
 
hsa-let-7e-5p
 
 
-0.468
 
 
37.4
 
 
 
 
ARG2
 
 
hsa-let-7c-5p
 
 
-0.468
 
 
37.4
 
 
 
 
ARL6IP6
 
 
hsa-let-7c-5p
 
 
-0.293
 
 
53.9
 
 
 
 
ARL6IP6
 
 
hsa-let-7e-5p
 
 
-0.305
 
 
53.9
 
 
 
 
ARMT1
 
 
hsa-let-7e-5p
 
 
-0.303
 
 
17.8
 
 
 
 
ARMT1
 
 
hsa-let-7c-5p
 
 
-0.303
 
 
17.8
 
 
 
 
ARPC5
 
 
hsa-miR-22-3p
 
 
-0.679
 
 
17.5
 
 
 
 
ARPP19
 
 
hsa-let-7e-5p
 
 
-0.492
 
 
225.3
 
 
 
 
ARPP19
 
 
hsa-let-7c-5p
 
 
-0.492
 
 
225.3
 
 
 
 
ARRB1
 
 
hsa-miR-22-3p
 
 
-0.616
 
 
10.7
 
 
 
 
ARRDC4
 
 
hsa-let-7e-5p
 
 
-0.329
 
 
13.8
 
 
 
 
ARRDC4
 
 
hsa-let-7c-5p
 
 
-0.329
 
 
13.8
 
 
 
 
ASAP1
 
 
hsa-let-7e-5p
 
 
-0.296
 
 
12.4
 
 
 
 
ASAP1
 
 
hsa-let-7c-5p
 
 
-0.308
 
 
12.4
 
 
 
 
ATOSB
 
 
hsa-let-7e-5p
 
 
-0.429
 
 
13.5
 
 
 
 
ATOSB
 
 
hsa-let-7c-5p
 
 
-0.429
 
 
13.5
 
 
 
 
ATP2A2
 
 
hsa-let-7e-5p
 
 
-0.311
 
 
2675.7
 
 
 
 
ATP2A2
 
 
hsa-let-7c-5p
 
 
-0.311
 
 
2675.7
 
 
 
 
ATPAF1
 
 
hsa-let-7e-5p
 
 
-0.498
 
 
260.2
 
 
 
 
ATPAF1
 
 
hsa-let-7c-5p
 
 
-0.498
 
 
260.2
 
 
 
 
ATXN2
 
 
hsa-miR-10a-5p
 
 
-0.362
 
 
26.5
 
 
 
 
B3GALNT2
 
 
hsa-miR-125a-5p
 
 
-0.311
 
 
27.4
 
 
 
 
B3GAT3
 
 
hsa-let-7e-5p
 
 
-0.324
 
 
15.7
 
 
 
 
B3GAT3
 
 
hsa-let-7c-5p
 
 
-0.324
 
 
15.7
 
 
 
 
B4GAT1
 
 
hsa-let-7e-5p
 
 
-0.47
 
 
27.1
 
 
 
 
B4GAT1
 
 
hsa-let-7c-5p
 
 
-0.47
 
 
27.1
 
 
 
 
BACH1
 
 
hsa-let-7e-5p
 
 
-0.298
 
 
24.7
 
 
 
 
BACH1
 
 
hsa-let-7e-5p
 
 
-0.298
 
 
24.7
 
 
 
 
BACH1
 
 
hsa-let-7c-5p
 
 
-0.306
 
 
24.7
 
 
 
 
BACH1
 
 
hsa-let-7c-5p
 
 
-0.306
 
 
24.7
 
 
 
 
BACH1
 
 
hsa-let-7e-5p
 
 
-0.435
 
 
24.7
 
 
 
 
BACH1
 
 
hsa-let-7e-5p
 
 
-0.435
 
 
24.7
 
 
 
 
BACH1
 
 
hsa-let-7c-5p
 
 
-0.435
 
 
24.7
 
 
 
 
BACH1
 
 
hsa-let-7c-5p
 
 
-0.435
 
 
24.7
 
 
 
 
BAG5
 
 
hsa-miR-22-3p
 
 
-0.318
 
 
12.8
 
 
 
 
BAK1
 
 
hsa-miR-125a-5p
 
 
-0.402
 
 
17.7
 
 
 
 
BAZ1B
 
 
hsa-miR-10a-5p
 
 
-0.309
 
 
26.3
 
 
 
 
BAZ2A
 
 
hsa-miR-99b-5p
 
 
-0.408
 
 
26.0
 
 
 
 
BCAP29
 
 
hsa-let-7e-5p
 
 
-0.289
 
 
28.3
 
 
 
 
BCL2L11
 
 
hsa-miR-10a-5p
 
 
-0.483
 
 
11.0
 
 
 
 
BCL2L12
 
 
hsa-miR-125a-5p
 
 
-0.288
 
 
12.6
 
 
 
 
BDH1
 
 
hsa-miR-125a-5p
 
 
-0.389
 
 
20.2
 
 
 
 
BIN1
 
 
hsa-miR-22-3p
 
 
-0.346
 
 
1986.0
 
 
 
 
BIN3
 
 
hsa-let-7e-5p
 
 
-0.427
 
 
12.7
 
 
 
 
BIN3
 
 
hsa-let-7c-5p
 
 
-0.427
 
 
12.7
 
 
 
 
BLOC1S1
 
 
hsa-miR-22-3p
 
 
-0.346
 
 
308.2
 
 
 
 
BLOC1S6
 
 
hsa-let-7e-5p
 
 
-0.321
 
 
22.1
 
 
 
 
BLOC1S6
 
 
hsa-let-7c-5p
 
 
-0.321
 
 
22.1
 
 
 
 
BLZF1
 
 
hsa-miR-125a-5p
 
 
-0.461
 
 
10.5
 
 
 
 
BORCS6
 
 
hsa-miR-125a-5p
 
 
-0.287
 
 
21.4
 
 
 
 
BTF3L4
 
 
hsa-let-7e-5p
 
 
-0.355
 
 
65.0
 
 
 
 
BTF3L4
 
 
hsa-let-7c-5p
 
 
-0.355
 
 
65.0
 
 
 
 
BZW1
 
 
hsa-let-7e-5p
 
 
-0.354
 
 
93.0
 
 
 
 
BZW1
 
 
hsa-let-7c-5p
 
 
-0.354
 
 
93.0
 
 
 
 
BZW1
 
 
hsa-let-7e-5p
 
 
-0.375
 
 
93.0
 
 
 
 
BZW1
 
 
hsa-let-7c-5p
 
 
-0.375
 
 
93.0
 
 
 
 
BZW2
 
 
hsa-let-7c-5p
 
 
-0.397
 
 
344.4
 
 
 
 
BZW2
 
 
hsa-let-7e-5p
 
 
-0.408
 
 
344.4
 
 
 
 
C19orf47
 
 
hsa-let-7e-5p
 
 
-0.311
 
 
335.4
 
 
 
 
C19orf47
 
 
hsa-let-7c-5p
 
 
-0.311
 
 
335.4
 
 
 
 
C2orf88
 
 
hsa-let-7e-5p
 
 
-0.429
 
 
12.6
 
 
 
 
C2orf88
 
 
hsa-let-7c-5p
 
 
-0.429
 
 
12.6
 
 
 
 
C5orf24
 
 
hsa-miR-22-3p
 
 
-0.493
 
 
20.1
 
 
 
 
C6orf47
 
 
hsa-miR-125a-5p
 
 
-0.564
 
 
17.9
 
 
 
 
CANT1
 
 
hsa-let-7e-5p
 
 
-0.402
 
 
13.4
 
 
 
 
CANT1
 
 
hsa-let-7c-5p
 
 
-0.402
 
 
13.4
 
 
 
 
CAPN1
 
 
hsa-miR-22-3p
 
 
-0.307
 
 
79.5
 
 
 
 
CAPN3
 
 
hsa-let-7e-5p
 
 
-0.317
 
 
848.7
 
 
 
 
CAPN3
 
 
hsa-let-7c-5p
 
 
-0.317
 
 
848.7
 
 
 
 
CASP3
 
 
hsa-let-7e-5p
 
 
-0.495
 
 
13.2
 
 
 
 
CASP3
 
 
hsa-let-7c-5p
 
 
-0.503
 
 
13.2
 
 
 
 
CAST
 
 
hsa-miR-150-5p
 
 
-0.354
 
 
277.7
 
 
 
 
CAV3
 
 
hsa-miR-22-3p
 
 
-0.565
 
 
171.2
 
 
 
 
 
 
 skin 
 
 
 
 
target_symbol
 
 
mature_mirna_id
 
 
score
 
 
nTPM
 
 
 
 
 
 
ABCC5
 
 
hsa-miR-125a-5p
 
 
-0.288
 
 
22.7
 
 
 
 
ABHD17C
 
 
hsa-let-7e-5p
 
 
-0.359
 
 
17.9
 
 
 
 
ABHD17C
 
 
hsa-let-7c-5p
 
 
-0.359
 
 
17.9
 
 
 
 
ABHD6
 
 
hsa-miR-125a-5p
 
 
-0.501
 
 
24.1
 
 
 
 
ABTB1
 
 
hsa-miR-125a-5p
 
 
-0.519
 
 
25.3
 
 
 
 
ACP3
 
 
hsa-let-7e-5p
 
 
-0.298
 
 
25.5
 
 
 
 
ACP3
 
 
hsa-let-7c-5p
 
 
-0.298
 
 
25.5
 
 
 
 
ACVR2A
 
 
hsa-miR-125a-5p
 
 
-0.301
 
 
18.1
 
 
 
 
ADIPOR2
 
 
hsa-miR-150-5p
 
 
-0.287
 
 
30.8
 
 
 
 
ADRB2
 
 
hsa-let-7e-5p
 
 
-0.635
 
 
36.9
 
 
 
 
ADRB2
 
 
hsa-let-7c-5p
 
 
-0.635
 
 
36.9
 
 
 
 
AGGF1
 
 
hsa-miR-125a-5p
 
 
-0.331
 
 
11.8
 
 
 
 
AIDA
 
 
hsa-miR-22-3p
 
 
-0.316
 
 
25.1
 
 
 
 
AIFM1
 
 
hsa-miR-125a-5p
 
 
-0.3
 
 
10.3
 
 
 
 
AIFM1
 
 
hsa-let-7e-5p
 
 
-0.314
 
 
10.3
 
 
 
 
AIFM1
 
 
hsa-let-7c-5p
 
 
-0.314
 
 
10.3
 
 
 
 
ALDH4A1
 
 
hsa-miR-184
 
 
-0.421
 
 
18.1
 
 
 
 
ANAPC16
 
 
hsa-miR-125a-5p
 
 
-0.578
 
 
14.6
 
 
 
 
ANKHD1-EIF4EBP3
 
 
hsa-miR-22-3p
 
 
-0.479
 
 
15.0
 
 
 
 
ANKRD13A
 
 
hsa-miR-22-3p
 
 
-0.478
 
 
20.5
 
 
 
 
ANKRD46
 
 
hsa-let-7e-5p
 
 
-0.389
 
 
10.5
 
 
 
 
ANKRD46
 
 
hsa-let-7c-5p
 
 
-0.389
 
 
10.5
 
 
 
 
ANPEP
 
 
hsa-miR-125a-5p
 
 
-0.372
 
 
29.7
 
 
 
 
AP1AR
 
 
hsa-miR-99b-5p
 
 
-0.823
 
 
13.4
 
 
 
 
AP1S1
 
 
hsa-let-7e-5p
 
 
-0.374
 
 
40.6
 
 
 
 
AP1S1
 
 
hsa-let-7c-5p
 
 
-0.374
 
 
40.6
 
 
 
 
ARL4D
 
 
hsa-let-7e-5p
 
 
-0.417
 
 
44.0
 
 
 
 
ARL4D
 
 
hsa-let-7c-5p
 
 
-0.417
 
 
44.0
 
 
 
 
ARL6IP6
 
 
hsa-let-7c-5p
 
 
-0.293
 
 
23.5
 
 
 
 
ARL6IP6
 
 
hsa-let-7e-5p
 
 
-0.305
 
 
23.5
 
 
 
 
ARPC5
 
 
hsa-miR-22-3p
 
 
-0.679
 
 
40.3
 
 
 
 
ARPP19
 
 
hsa-let-7e-5p
 
 
-0.492
 
 
28.9
 
 
 
 
ARPP19
 
 
hsa-let-7c-5p
 
 
-0.492
 
 
28.9
 
 
 
 
ARRB1
 
 
hsa-miR-22-3p
 
 
-0.616
 
 
15.9
 
 
 
 
ARRDC4
 
 
hsa-let-7e-5p
 
 
-0.329
 
 
33.5
 
 
 
 
ARRDC4
 
 
hsa-let-7c-5p
 
 
-0.329
 
 
33.5
 
 
 
 
ASAP1
 
 
hsa-let-7e-5p
 
 
-0.296
 
 
16.2
 
 
 
 
ASAP1
 
 
hsa-let-7c-5p
 
 
-0.308
 
 
16.2
 
 
 
 
ATOSB
 
 
hsa-let-7e-5p
 
 
-0.429
 
 
14.2
 
 
 
 
ATOSB
 
 
hsa-let-7c-5p
 
 
-0.429
 
 
14.2
 
 
 
 
ATP2A2
 
 
hsa-let-7e-5p
 
 
-0.311
 
 
41.6
 
 
 
 
ATP2A2
 
 
hsa-let-7c-5p
 
 
-0.311
 
 
41.6
 
 
 
 
ATPAF1
 
 
hsa-let-7e-5p
 
 
-0.498
 
 
18.2
 
 
 
 
ATPAF1
 
 
hsa-let-7c-5p
 
 
-0.498
 
 
18.2
 
 
 
 
ATXN2
 
 
hsa-miR-10a-5p
 
 
-0.362
 
 
15.1
 
 
 
 
B3GALNT2
 
 
hsa-miR-125a-5p
 
 
-0.311
 
 
10.1
 
 
 
 
B3GAT3
 
 
hsa-let-7e-5p
 
 
-0.324
 
 
37.0
 
 
 
 
B3GAT3
 
 
hsa-let-7c-5p
 
 
-0.324
 
 
37.0
 
 
 
 
B4GAT1
 
 
hsa-let-7e-5p
 
 
-0.47
 
 
11.9
 
 
 
 
B4GAT1
 
 
hsa-let-7c-5p
 
 
-0.47
 
 
11.9
 
 
 
 
BACH1
 
 
hsa-let-7e-5p
 
 
-0.298
 
 
21.9
 
 
 
 
BACH1
 
 
hsa-let-7e-5p
 
 
-0.298
 
 
21.9
 
 
 
 
BACH1
 
 
hsa-let-7c-5p
 
 
-0.306
 
 
21.9
 
 
 
 
BACH1
 
 
hsa-let-7c-5p
 
 
-0.306
 
 
21.9
 
 
 
 
BACH1
 
 
hsa-let-7e-5p
 
 
-0.435
 
 
21.9
 
 
 
 
BACH1
 
 
hsa-let-7e-5p
 
 
-0.435
 
 
21.9
 
 
 
 
BACH1
 
 
hsa-let-7c-5p
 
 
-0.435
 
 
21.9
 
 
 
 
BACH1
 
 
hsa-let-7c-5p
 
 
-0.435
 
 
21.9
 
 
 
 
BAG5
 
 
hsa-miR-22-3p
 
 
-0.318
 
 
18.9
 
 
 
 
BAK1
 
 
hsa-miR-125a-5p
 
 
-0.402
 
 
17.5
 
 
 
 
BAZ1B
 
 
hsa-miR-10a-5p
 
 
-0.309
 
 
13.4
 
 
 
 
BAZ2A
 
 
hsa-miR-99b-5p
 
 
-0.408
 
 
27.8
 
 
 
 
BCAP29
 
 
hsa-let-7e-5p
 
 
-0.289
 
 
23.3
 
 
 
 
BDH1
 
 
hsa-miR-125a-5p
 
 
-0.389
 
 
26.5
 
 
 
 
BIN1
 
 
hsa-miR-22-3p
 
 
-0.346
 
 
12.2
 
 
 
 
BIN3
 
 
hsa-let-7e-5p
 
 
-0.427
 
 
15.8
 
 
 
 
BIN3
 
 
hsa-let-7c-5p
 
 
-0.427
 
 
15.8
 
 
 
 
BLOC1S1
 
 
hsa-miR-22-3p
 
 
-0.346
 
 
112.1
 
 
 
 
BLOC1S6
 
 
hsa-let-7e-5p
 
 
-0.321
 
 
17.2
 
 
 
 
BLOC1S6
 
 
hsa-let-7c-5p
 
 
-0.321
 
 
17.2
 
 
 
 
BLZF1
 
 
hsa-miR-125a-5p
 
 
-0.461
 
 
16.9
 
 
 
 
BORCS6
 
 
hsa-miR-125a-5p
 
 
-0.287
 
 
16.2
 
 
 
 
BTF3L4
 
 
hsa-let-7e-5p
 
 
-0.355
 
 
33.3
 
 
 
 
BTF3L4
 
 
hsa-let-7c-5p
 
 
-0.355
 
 
33.3
 
 
 
 
BTG1
 
 
hsa-miR-22-3p
 
 
-0.468
 
 
21.1
 
 
 
 
BZW1
 
 
hsa-let-7e-5p
 
 
-0.354
 
 
45.2
 
 
 
 
BZW1
 
 
hsa-let-7c-5p
 
 
-0.354
 
 
45.2
 
 
 
 
BZW1
 
 
hsa-let-7e-5p
 
 
-0.375
 
 
45.2
 
 
 
 
BZW1
 
 
hsa-let-7c-5p
 
 
-0.375
 
 
45.2
 
 
 
 
BZW2
 
 
hsa-let-7c-5p
 
 
-0.397
 
 
52.4
 
 
 
 
BZW2
 
 
hsa-let-7e-5p
 
 
-0.408
 
 
52.4
 
 
 
 
C15orf39
 
 
hsa-miR-125a-5p
 
 
-0.335
 
 
14.5
 
 
 
 
C15orf39
 
 
hsa-let-7e-5p
 
 
-0.374
 
 
14.5
 
 
 
 
C15orf39
 
 
hsa-let-7c-5p
 
 
-0.374
 
 
14.5
 
 
 
 
C19orf47
 
 
hsa-let-7e-5p
 
 
-0.311
 
 
11.4
 
 
 
 
C19orf47
 
 
hsa-let-7c-5p
 
 
-0.311
 
 
11.4
 
 
 
 
C1orf210
 
 
hsa-miR-125a-5p
 
 
-0.584
 
 
15.4
 
 
 
 
C3orf52
 
 
hsa-let-7e-5p
 
 
-0.296
 
 
25.9
 
 
 
 
C3orf52
 
 
hsa-let-7c-5p
 
 
-0.329
 
 
25.9
 
 
 
 
C5orf24
 
 
hsa-miR-22-3p
 
 
-0.493
 
 
18.0
 
 
 
 
C6orf47
 
 
hsa-miR-125a-5p
 
 
-0.564
 
 
16.9
 
 
 
 
C8orf58
 
 
hsa-let-7e-5p
 
 
-0.612
 
 
12.9
 
 
 
 
C8orf58
 
 
hsa-let-7c-5p
 
 
-0.62
 
 
12.9
 
 
 
 
CACNB3
 
 
hsa-miR-125a-5p
 
 
-0.393
 
 
10.9
 
 
 
 
CAMK2N1
 
 
hsa-miR-22-3p
 
 
-0.302
 
 
11.4
 
 
 
 
CANT1
 
 
hsa-let-7e-5p
 
 
-0.402
 
 
13.6
 
 
 
 
CANT1
 
 
hsa-let-7c-5p
 
 
-0.402
 
 
13.6
 
 
 
 
CAPN1
 
 
hsa-miR-22-3p
 
 
-0.307
 
 
118.3
 
 
 
 
CAPN3
 
 
hsa-let-7e-5p
 
 
-0.317
 
 
51.8
 
 
 
 
CAPN3
 
 
hsa-let-7c-5p
 
 
-0.317
 
 
51.8
 
 
 
 
 
 
 small intestine 
 
 
 
 
target_symbol
 
 
mature_mirna_id
 
 
score
 
 
nTPM
 
 
 
 
 
 
ABCC5
 
 
hsa-miR-125a-5p
 
 
-0.288
 
 
22.9
 
 
 
 
ABHD17C
 
 
hsa-let-7e-5p
 
 
-0.359
 
 
28.8
 
 
 
 
ABHD17C
 
 
hsa-let-7c-5p
 
 
-0.359
 
 
28.8
 
 
 
 
ABHD3
 
 
hsa-miR-125a-5p
 
 
-0.453
 
 
53.0
 
 
 
 
ABHD6
 
 
hsa-miR-125a-5p
 
 
-0.501
 
 
49.2
 
 
 
 
ABTB1
 
 
hsa-miR-125a-5p
 
 
-0.519
 
 
31.9
 
 
 
 
ADIPOR2
 
 
hsa-miR-150-5p
 
 
-0.287
 
 
31.4
 
 
 
 
AGGF1
 
 
hsa-miR-125a-5p
 
 
-0.331
 
 
12.5
 
 
 
 
AGMAT
 
 
hsa-miR-22-3p
 
 
-0.294
 
 
12.6
 
 
 
 
AIDA
 
 
hsa-miR-22-3p
 
 
-0.316
 
 
27.3
 
 
 
 
AIFM1
 
 
hsa-miR-125a-5p
 
 
-0.3
 
 
28.2
 
 
 
 
AIFM1
 
 
hsa-let-7e-5p
 
 
-0.314
 
 
28.2
 
 
 
 
AIFM1
 
 
hsa-let-7c-5p
 
 
-0.314
 
 
28.2
 
 
 
 
AMT
 
 
hsa-let-7e-5p
 
 
-0.463
 
 
11.5
 
 
 
 
AMT
 
 
hsa-let-7c-5p
 
 
-0.463
 
 
11.5
 
 
 
 
ANAPC16
 
 
hsa-miR-125a-5p
 
 
-0.578
 
 
19.0
 
 
 
 
ANKHD1-EIF4EBP3
 
 
hsa-miR-22-3p
 
 
-0.479
 
 
11.5
 
 
 
 
ANKRD13A
 
 
hsa-miR-22-3p
 
 
-0.478
 
 
15.8
 
 
 
 
ANKRD46
 
 
hsa-let-7e-5p
 
 
-0.389
 
 
10.4
 
 
 
 
ANKRD46
 
 
hsa-let-7c-5p
 
 
-0.389
 
 
10.4
 
 
 
 
ANPEP
 
 
hsa-miR-125a-5p
 
 
-0.372
 
 
1857.1
 
 
 
 
AP1AR
 
 
hsa-miR-99b-5p
 
 
-0.823
 
 
22.4
 
 
 
 
AP1S1
 
 
hsa-let-7e-5p
 
 
-0.374
 
 
29.8
 
 
 
 
AP1S1
 
 
hsa-let-7c-5p
 
 
-0.374
 
 
29.8
 
 
 
 
ARG2
 
 
hsa-let-7e-5p
 
 
-0.468
 
 
14.4
 
 
 
 
ARG2
 
 
hsa-let-7c-5p
 
 
-0.468
 
 
14.4
 
 
 
 
ARMT1
 
 
hsa-let-7e-5p
 
 
-0.303
 
 
13.7
 
 
 
 
ARMT1
 
 
hsa-let-7c-5p
 
 
-0.303
 
 
13.7
 
 
 
 
ARPC5
 
 
hsa-miR-22-3p
 
 
-0.679
 
 
110.5
 
 
 
 
ARPP19
 
 
hsa-let-7e-5p
 
 
-0.492
 
 
28.8
 
 
 
 
ARPP19
 
 
hsa-let-7c-5p
 
 
-0.492
 
 
28.8
 
 
 
 
ARRB1
 
 
hsa-miR-22-3p
 
 
-0.616
 
 
25.6
 
 
 
 
ARRDC4
 
 
hsa-let-7e-5p
 
 
-0.329
 
 
22.0
 
 
 
 
ARRDC4
 
 
hsa-let-7c-5p
 
 
-0.329
 
 
22.0
 
 
 
 
ASAP1
 
 
hsa-let-7e-5p
 
 
-0.296
 
 
11.5
 
 
 
 
ASAP1
 
 
hsa-let-7c-5p
 
 
-0.308
 
 
11.5
 
 
 
 
ATOH1
 
 
hsa-miR-22-3p
 
 
-0.566
 
 
18.9
 
 
 
 
ATOSB
 
 
hsa-let-7e-5p
 
 
-0.429
 
 
10.7
 
 
 
 
ATOSB
 
 
hsa-let-7c-5p
 
 
-0.429
 
 
10.7
 
 
 
 
ATP2A2
 
 
hsa-let-7e-5p
 
 
-0.311
 
 
53.2
 
 
 
 
ATP2A2
 
 
hsa-let-7c-5p
 
 
-0.311
 
 
53.2
 
 
 
 
ATPAF1
 
 
hsa-let-7e-5p
 
 
-0.498
 
 
42.6
 
 
 
 
ATPAF1
 
 
hsa-let-7c-5p
 
 
-0.498
 
 
42.6
 
 
 
 
ATXN2
 
 
hsa-miR-10a-5p
 
 
-0.362
 
 
14.3
 
 
 
 
B3GAT3
 
 
hsa-let-7e-5p
 
 
-0.324
 
 
30.1
 
 
 
 
B3GAT3
 
 
hsa-let-7c-5p
 
 
-0.324
 
 
30.1
 
 
 
 
B4GAT1
 
 
hsa-let-7e-5p
 
 
-0.47
 
 
14.2
 
 
 
 
B4GAT1
 
 
hsa-let-7c-5p
 
 
-0.47
 
 
14.2
 
 
 
 
BACH1
 
 
hsa-let-7e-5p
 
 
-0.298
 
 
16.3
 
 
 
 
BACH1
 
 
hsa-let-7e-5p
 
 
-0.298
 
 
16.3
 
 
 
 
BACH1
 
 
hsa-let-7c-5p
 
 
-0.306
 
 
16.3
 
 
 
 
BACH1
 
 
hsa-let-7c-5p
 
 
-0.306
 
 
16.3
 
 
 
 
BACH1
 
 
hsa-let-7e-5p
 
 
-0.435
 
 
16.3
 
 
 
 
BACH1
 
 
hsa-let-7e-5p
 
 
-0.435
 
 
16.3
 
 
 
 
BACH1
 
 
hsa-let-7c-5p
 
 
-0.435
 
 
16.3
 
 
 
 
BACH1
 
 
hsa-let-7c-5p
 
 
-0.435
 
 
16.3
 
 
 
 
BAG5
 
 
hsa-miR-22-3p
 
 
-0.318
 
 
11.3
 
 
 
 
BAK1
 
 
hsa-miR-125a-5p
 
 
-0.402
 
 
34.9
 
 
 
 
BAZ1B
 
 
hsa-miR-10a-5p
 
 
-0.309
 
 
17.3
 
 
 
 
BAZ2A
 
 
hsa-miR-99b-5p
 
 
-0.408
 
 
28.7
 
 
 
 
BCAP29
 
 
hsa-let-7e-5p
 
 
-0.289
 
 
17.9
 
 
 
 
BCL2L11
 
 
hsa-miR-10a-5p
 
 
-0.483
 
 
11.3
 
 
 
 
BCL2L12
 
 
hsa-miR-125a-5p
 
 
-0.288
 
 
10.4
 
 
 
 
BDH1
 
 
hsa-miR-125a-5p
 
 
-0.389
 
 
25.0
 
 
 
 
BIN1
 
 
hsa-miR-22-3p
 
 
-0.346
 
 
29.5
 
 
 
 
BIN2
 
 
hsa-miR-125a-5p
 
 
-0.679
 
 
10.3
 
 
 
 
BIN3
 
 
hsa-let-7e-5p
 
 
-0.427
 
 
12.8
 
 
 
 
BIN3
 
 
hsa-let-7c-5p
 
 
-0.427
 
 
12.8
 
 
 
 
BLOC1S1
 
 
hsa-miR-22-3p
 
 
-0.346
 
 
151.2
 
 
 
 
BLOC1S6
 
 
hsa-let-7e-5p
 
 
-0.321
 
 
20.1
 
 
 
 
BLOC1S6
 
 
hsa-let-7c-5p
 
 
-0.321
 
 
20.1
 
 
 
 
BLZF1
 
 
hsa-miR-125a-5p
 
 
-0.461
 
 
11.9
 
 
 
 
BMF
 
 
hsa-miR-125a-5p
 
 
-0.34
 
 
22.2
 
 
 
 
BORCS6
 
 
hsa-miR-125a-5p
 
 
-0.287
 
 
11.7
 
 
 
 
BTF3L4
 
 
hsa-let-7e-5p
 
 
-0.355
 
 
17.1
 
 
 
 
BTF3L4
 
 
hsa-let-7c-5p
 
 
-0.355
 
 
17.1
 
 
 
 
BTG1
 
 
hsa-miR-22-3p
 
 
-0.468
 
 
10.1
 
 
 
 
BZW1
 
 
hsa-let-7e-5p
 
 
-0.354
 
 
67.2
 
 
 
 
BZW1
 
 
hsa-let-7c-5p
 
 
-0.354
 
 
67.2
 
 
 
 
BZW1
 
 
hsa-let-7e-5p
 
 
-0.375
 
 
67.2
 
 
 
 
BZW1
 
 
hsa-let-7c-5p
 
 
-0.375
 
 
67.2
 
 
 
 
BZW2
 
 
hsa-let-7c-5p
 
 
-0.397
 
 
28.2
 
 
 
 
BZW2
 
 
hsa-let-7e-5p
 
 
-0.408
 
 
28.2
 
 
 
 
C1GALT1
 
 
hsa-let-7e-5p
 
 
-0.322
 
 
18.1
 
 
 
 
C1GALT1
 
 
hsa-let-7c-5p
 
 
-0.322
 
 
18.1
 
 
 
 
C1orf210
 
 
hsa-miR-125a-5p
 
 
-0.584
 
 
36.5
 
 
 
 
C2orf88
 
 
hsa-let-7e-5p
 
 
-0.429
 
 
41.8
 
 
 
 
C2orf88
 
 
hsa-let-7c-5p
 
 
-0.429
 
 
41.8
 
 
 
 
C5orf24
 
 
hsa-miR-22-3p
 
 
-0.493
 
 
16.1
 
 
 
 
C6orf47
 
 
hsa-miR-125a-5p
 
 
-0.564
 
 
16.8
 
 
 
 
C9orf40
 
 
hsa-let-7e-5p
 
 
-0.809
 
 
10.7
 
 
 
 
C9orf40
 
 
hsa-let-7c-5p
 
 
-0.809
 
 
10.7
 
 
 
 
CACFD1
 
 
hsa-let-7e-5p
 
 
-0.296
 
 
14.3
 
 
 
 
CACFD1
 
 
hsa-let-7c-5p
 
 
-0.296
 
 
14.3
 
 
 
 
CACNB3
 
 
hsa-miR-125a-5p
 
 
-0.393
 
 
10.2
 
 
 
 
CAMK2N1
 
 
hsa-miR-22-3p
 
 
-0.302
 
 
81.9
 
 
 
 
CANT1
 
 
hsa-let-7e-5p
 
 
-0.402
 
 
52.3
 
 
 
 
CANT1
 
 
hsa-let-7c-5p
 
 
-0.402
 
 
52.3
 
 
 
 
CAPN1
 
 
hsa-miR-22-3p
 
 
-0.307
 
 
83.0
 
 
 
 
CAPN3
 
 
hsa-let-7e-5p
 
 
-0.317
 
 
15.6
 
 
 
 
 
 
 smooth muscle 
 
 
 
 
target_symbol
 
 
mature_mirna_id
 
 
score
 
 
nTPM
 
 
 
 
 
 
ABCC5
 
 
hsa-miR-125a-5p
 
 
-0.288
 
 
15.0
 
 
 
 
ADIPOR2
 
 
hsa-miR-150-5p
 
 
-0.287
 
 
11.2
 
 
 
 
AIDA
 
 
hsa-miR-22-3p
 
 
-0.316
 
 
50.6
 
 
 
 
AKT3
 
 
hsa-miR-22-3p
 
 
-0.451
 
 
14.7
 
 
 
 
ANAPC16
 
 
hsa-miR-125a-5p
 
 
-0.578
 
 
16.4
 
 
 
 
ANKRD13A
 
 
hsa-miR-22-3p
 
 
-0.478
 
 
22.8
 
 
 
 
ANKRD46
 
 
hsa-let-7e-5p
 
 
-0.389
 
 
11.6
 
 
 
 
ANKRD46
 
 
hsa-let-7c-5p
 
 
-0.389
 
 
11.6
 
 
 
 
ANPEP
 
 
hsa-miR-125a-5p
 
 
-0.372
 
 
27.8
 
 
 
 
AP1S1
 
 
hsa-let-7e-5p
 
 
-0.374
 
 
11.9
 
 
 
 
AP1S1
 
 
hsa-let-7c-5p
 
 
-0.374
 
 
11.9
 
 
 
 
ARL4D
 
 
hsa-let-7e-5p
 
 
-0.417
 
 
13.8
 
 
 
 
ARL4D
 
 
hsa-let-7c-5p
 
 
-0.417
 
 
13.8
 
 
 
 
ARL6IP6
 
 
hsa-let-7c-5p
 
 
-0.293
 
 
10.8
 
 
 
 
ARL6IP6
 
 
hsa-let-7e-5p
 
 
-0.305
 
 
10.8
 
 
 
 
ARMT1
 
 
hsa-let-7e-5p
 
 
-0.303
 
 
13.9
 
 
 
 
ARMT1
 
 
hsa-let-7c-5p
 
 
-0.303
 
 
13.9
 
 
 
 
ARPC5
 
 
hsa-miR-22-3p
 
 
-0.679
 
 
73.7
 
 
 
 
ARPP19
 
 
hsa-let-7e-5p
 
 
-0.492
 
 
54.2
 
 
 
 
ARPP19
 
 
hsa-let-7c-5p
 
 
-0.492
 
 
54.2
 
 
 
 
ARRB1
 
 
hsa-miR-22-3p
 
 
-0.616
 
 
20.3
 
 
 
 
ARRDC4
 
 
hsa-let-7e-5p
 
 
-0.329
 
 
16.9
 
 
 
 
ARRDC4
 
 
hsa-let-7c-5p
 
 
-0.329
 
 
16.9
 
 
 
 
ASAP1
 
 
hsa-let-7e-5p
 
 
-0.296
 
 
16.7
 
 
 
 
ASAP1
 
 
hsa-let-7c-5p
 
 
-0.308
 
 
16.7
 
 
 
 
ATOSB
 
 
hsa-let-7e-5p
 
 
-0.429
 
 
10.3
 
 
 
 
ATOSB
 
 
hsa-let-7c-5p
 
 
-0.429
 
 
10.3
 
 
 
 
ATP2A2
 
 
hsa-let-7e-5p
 
 
-0.311
 
 
69.4
 
 
 
 
ATP2A2
 
 
hsa-let-7c-5p
 
 
-0.311
 
 
69.4
 
 
 
 
ATPAF1
 
 
hsa-let-7e-5p
 
 
-0.498
 
 
30.4
 
 
 
 
ATPAF1
 
 
hsa-let-7c-5p
 
 
-0.498
 
 
30.4
 
 
 
 
ATXN2
 
 
hsa-miR-10a-5p
 
 
-0.362
 
 
17.1
 
 
 
 
B3GAT3
 
 
hsa-let-7e-5p
 
 
-0.324
 
 
13.1
 
 
 
 
B3GAT3
 
 
hsa-let-7c-5p
 
 
-0.324
 
 
13.1
 
 
 
 
B4GAT1
 
 
hsa-let-7e-5p
 
 
-0.47
 
 
13.1
 
 
 
 
B4GAT1
 
 
hsa-let-7c-5p
 
 
-0.47
 
 
13.1
 
 
 
 
BACH1
 
 
hsa-let-7e-5p
 
 
-0.298
 
 
14.9
 
 
 
 
BACH1
 
 
hsa-let-7e-5p
 
 
-0.298
 
 
14.9
 
 
 
 
BACH1
 
 
hsa-let-7c-5p
 
 
-0.306
 
 
14.9
 
 
 
 
BACH1
 
 
hsa-let-7c-5p
 
 
-0.306
 
 
14.9
 
 
 
 
BACH1
 
 
hsa-let-7e-5p
 
 
-0.435
 
 
14.9
 
 
 
 
BACH1
 
 
hsa-let-7e-5p
 
 
-0.435
 
 
14.9
 
 
 
 
BACH1
 
 
hsa-let-7c-5p
 
 
-0.435
 
 
14.9
 
 
 
 
BACH1
 
 
hsa-let-7c-5p
 
 
-0.435
 
 
14.9
 
 
 
 
BAG5
 
 
hsa-miR-22-3p
 
 
-0.318
 
 
14.1
 
 
 
 
BAK1
 
 
hsa-miR-125a-5p
 
 
-0.402
 
 
12.6
 
 
 
 
BAZ1B
 
 
hsa-miR-10a-5p
 
 
-0.309
 
 
23.0
 
 
 
 
BAZ2A
 
 
hsa-miR-99b-5p
 
 
-0.408
 
 
21.6
 
 
 
 
BBX
 
 
hsa-miR-10a-5p
 
 
-0.305
 
 
15.4
 
 
 
 
BCAP29
 
 
hsa-let-7e-5p
 
 
-0.289
 
 
36.5
 
 
 
 
BIN1
 
 
hsa-miR-22-3p
 
 
-0.346
 
 
18.3
 
 
 
 
BLOC1S1
 
 
hsa-miR-22-3p
 
 
-0.346
 
 
95.8
 
 
 
 
BLOC1S6
 
 
hsa-let-7e-5p
 
 
-0.321
 
 
22.7
 
 
 
 
BLOC1S6
 
 
hsa-let-7c-5p
 
 
-0.321
 
 
22.7
 
 
 
 
BLZF1
 
 
hsa-miR-125a-5p
 
 
-0.461
 
 
16.6
 
 
 
 
BMPR2
 
 
hsa-miR-99b-5p
 
 
-0.327
 
 
15.4
 
 
 
 
BTF3L4
 
 
hsa-let-7e-5p
 
 
-0.355
 
 
32.5
 
 
 
 
BTF3L4
 
 
hsa-let-7c-5p
 
 
-0.355
 
 
32.5
 
 
 
 
BZW1
 
 
hsa-let-7e-5p
 
 
-0.354
 
 
74.7
 
 
 
 
BZW1
 
 
hsa-let-7c-5p
 
 
-0.354
 
 
74.7
 
 
 
 
BZW1
 
 
hsa-let-7e-5p
 
 
-0.375
 
 
74.7
 
 
 
 
BZW1
 
 
hsa-let-7c-5p
 
 
-0.375
 
 
74.7
 
 
 
 
BZW2
 
 
hsa-let-7c-5p
 
 
-0.397
 
 
36.4
 
 
 
 
BZW2
 
 
hsa-let-7e-5p
 
 
-0.408
 
 
36.4
 
 
 
 
C5orf24
 
 
hsa-miR-22-3p
 
 
-0.493
 
 
24.9
 
 
 
 
CACNB3
 
 
hsa-miR-125a-5p
 
 
-0.393
 
 
21.6
 
 
 
 
CAMK2N1
 
 
hsa-miR-22-3p
 
 
-0.302
 
 
21.2
 
 
 
 
CANT1
 
 
hsa-let-7e-5p
 
 
-0.402
 
 
14.5
 
 
 
 
CANT1
 
 
hsa-let-7c-5p
 
 
-0.402
 
 
14.5
 
 
 
 
CAPN1
 
 
hsa-miR-22-3p
 
 
-0.307
 
 
42.3
 
 
 
 
CASP3
 
 
hsa-let-7e-5p
 
 
-0.495
 
 
12.5
 
 
 
 
CASP3
 
 
hsa-let-7c-5p
 
 
-0.503
 
 
12.5
 
 
 
 
CAST
 
 
hsa-miR-150-5p
 
 
-0.354
 
 
86.2
 
 
 
 
CBX5
 
 
hsa-let-7e-5p
 
 
-0.307
 
 
10.1
 
 
 
 
CBX5
 
 
hsa-let-7c-5p
 
 
-0.307
 
 
10.1
 
 
 
 
CBX5
 
 
hsa-let-7e-5p
 
 
-0.472
 
 
10.1
 
 
 
 
CBX5
 
 
hsa-let-7c-5p
 
 
-0.472
 
 
10.1
 
 
 
 
CCDC47
 
 
hsa-miR-22-3p
 
 
-0.604
 
 
39.3
 
 
 
 
CCN1
 
 
hsa-miR-22-3p
 
 
-0.601
 
 
132.7
 
 
 
 
CCNY
 
 
hsa-let-7c-5p
 
 
-0.323
 
 
24.0
 
 
 
 
CCNY
 
 
hsa-let-7e-5p
 
 
-0.334
 
 
24.0
 
 
 
 
CDC34
 
 
hsa-let-7c-5p
 
 
-0.309
 
 
17.3
 
 
 
 
CDC34
 
 
hsa-let-7e-5p
 
 
-0.321
 
 
17.3
 
 
 
 
CDC34
 
 
hsa-let-7e-5p
 
 
-0.555
 
 
17.3
 
 
 
 
CDC34
 
 
hsa-let-7c-5p
 
 
-0.555
 
 
17.3
 
 
 
 
CDC37L1
 
 
hsa-miR-125a-5p
 
 
-0.401
 
 
11.4
 
 
 
 
CDC42SE1
 
 
hsa-miR-125a-5p
 
 
-0.501
 
 
34.8
 
 
 
 
CDH5
 
 
hsa-miR-125a-5p
 
 
-0.36
 
 
25.9
 
 
 
 
CDKN1A
 
 
hsa-let-7e-5p
 
 
-0.434
 
 
106.7
 
 
 
 
CDKN1A
 
 
hsa-let-7c-5p
 
 
-0.434
 
 
106.7
 
 
 
 
CDV3
 
 
hsa-let-7e-5p
 
 
-0.453
 
 
68.4
 
 
 
 
CDV3
 
 
hsa-let-7c-5p
 
 
-0.461
 
 
68.4
 
 
 
 
CEBPD
 
 
hsa-let-7e-5p
 
 
-0.309
 
 
20.2
 
 
 
 
CEBPD
 
 
hsa-let-7c-5p
 
 
-0.309
 
 
20.2
 
 
 
 
CELF2
 
 
hsa-miR-10a-5p
 
 
-0.319
 
 
21.7
 
 
 
 
CEMIP2
 
 
hsa-let-7e-5p
 
 
-0.453
 
 
10.9
 
 
 
 
CEMIP2
 
 
hsa-let-7c-5p
 
 
-0.453
 
 
10.9
 
 
 
 
CERCAM
 
 
hsa-let-7e-5p
 
 
-0.344
 
 
44.2
 
 
 
 
CERCAM
 
 
hsa-let-7c-5p
 
 
-0.344
 
 
44.2
 
 
 
 
CFAP36
 
 
hsa-miR-22-3p
 
 
-0.549
 
 
34.9
 
 
 
 
 
 
 spinal cord 
 
 
 
 
target_symbol
 
 
mature_mirna_id
 
 
score
 
 
nTPM
 
 
 
 
 
 
ABCC5
 
 
hsa-miR-125a-5p
 
 
-0.288
 
 
25.1
 
 
 
 
ABHD6
 
 
hsa-miR-125a-5p
 
 
-0.501
 
 
36.2
 
 
 
 
ABTB1
 
 
hsa-miR-125a-5p
 
 
-0.519
 
 
20.4
 
 
 
 
ACER3
 
 
hsa-miR-22-3p
 
 
-0.453
 
 
13.0
 
 
 
 
ACHE
 
 
hsa-miR-125a-5p
 
 
-0.308
 
 
14.4
 
 
 
 
ACHE
 
 
hsa-miR-125a-5p
 
 
-0.361
 
 
14.4
 
 
 
 
ADIPOR2
 
 
hsa-miR-150-5p
 
 
-0.287
 
 
121.3
 
 
 
 
AIDA
 
 
hsa-miR-22-3p
 
 
-0.316
 
 
29.9
 
 
 
 
AIFM1
 
 
hsa-miR-125a-5p
 
 
-0.3
 
 
10.1
 
 
 
 
AIFM1
 
 
hsa-let-7e-5p
 
 
-0.314
 
 
10.1
 
 
 
 
AIFM1
 
 
hsa-let-7c-5p
 
 
-0.314
 
 
10.1
 
 
 
 
AKAP6
 
 
hsa-let-7e-5p
 
 
-0.307
 
 
13.3
 
 
 
 
AKAP6
 
 
hsa-let-7c-5p
 
 
-0.307
 
 
13.3
 
 
 
 
AKT3
 
 
hsa-miR-22-3p
 
 
-0.451
 
 
15.8
 
 
 
 
ALDH4A1
 
 
hsa-miR-184
 
 
-0.421
 
 
11.4
 
 
 
 
ANKRD46
 
 
hsa-let-7e-5p
 
 
-0.389
 
 
24.6
 
 
 
 
ANKRD46
 
 
hsa-let-7c-5p
 
 
-0.389
 
 
24.6
 
 
 
 
AP1S1
 
 
hsa-let-7e-5p
 
 
-0.374
 
 
28.5
 
 
 
 
AP1S1
 
 
hsa-let-7c-5p
 
 
-0.374
 
 
28.5
 
 
 
 
ARPC5
 
 
hsa-miR-22-3p
 
 
-0.679
 
 
56.3
 
 
 
 
ARPP19
 
 
hsa-let-7e-5p
 
 
-0.492
 
 
170.8
 
 
 
 
ARPP19
 
 
hsa-let-7c-5p
 
 
-0.492
 
 
170.8
 
 
 
 
ARRDC4
 
 
hsa-let-7e-5p
 
 
-0.329
 
 
75.7
 
 
 
 
ARRDC4
 
 
hsa-let-7c-5p
 
 
-0.329
 
 
75.7
 
 
 
 
ASAP1
 
 
hsa-let-7e-5p
 
 
-0.296
 
 
11.0
 
 
 
 
ASAP1
 
 
hsa-let-7c-5p
 
 
-0.308
 
 
11.0
 
 
 
 
ATCAY
 
 
hsa-miR-10a-5p
 
 
-0.322
 
 
10.1
 
 
 
 
ATOSB
 
 
hsa-let-7e-5p
 
 
-0.429
 
 
30.9
 
 
 
 
ATOSB
 
 
hsa-let-7c-5p
 
 
-0.429
 
 
30.9
 
 
 
 
ATP2A2
 
 
hsa-let-7e-5p
 
 
-0.311
 
 
19.0
 
 
 
 
ATP2A2
 
 
hsa-let-7c-5p
 
 
-0.311
 
 
19.0
 
 
 
 
ATPAF1
 
 
hsa-let-7e-5p
 
 
-0.498
 
 
50.4
 
 
 
 
ATPAF1
 
 
hsa-let-7c-5p
 
 
-0.498
 
 
50.4
 
 
 
 
ATXN2
 
 
hsa-miR-10a-5p
 
 
-0.362
 
 
27.6
 
 
 
 
B3GAT3
 
 
hsa-let-7e-5p
 
 
-0.324
 
 
62.6
 
 
 
 
B3GAT3
 
 
hsa-let-7c-5p
 
 
-0.324
 
 
62.6
 
 
 
 
B4GAT1
 
 
hsa-let-7e-5p
 
 
-0.47
 
 
48.7
 
 
 
 
B4GAT1
 
 
hsa-let-7c-5p
 
 
-0.47
 
 
48.7
 
 
 
 
BAG5
 
 
hsa-miR-22-3p
 
 
-0.318
 
 
12.4
 
 
 
 
BAZ1B
 
 
hsa-miR-10a-5p
 
 
-0.309
 
 
19.2
 
 
 
 
BAZ2A
 
 
hsa-miR-99b-5p
 
 
-0.408
 
 
10.5
 
 
 
 
BBX
 
 
hsa-miR-10a-5p
 
 
-0.305
 
 
17.3
 
 
 
 
BCAP29
 
 
hsa-let-7e-5p
 
 
-0.289
 
 
26.8
 
 
 
 
BDH1
 
 
hsa-miR-125a-5p
 
 
-0.389
 
 
11.2
 
 
 
 
BIN1
 
 
hsa-miR-22-3p
 
 
-0.346
 
 
197.8
 
 
 
 
BLOC1S1
 
 
hsa-miR-22-3p
 
 
-0.346
 
 
273.9
 
 
 
 
BLOC1S6
 
 
hsa-let-7e-5p
 
 
-0.321
 
 
15.5
 
 
 
 
BLOC1S6
 
 
hsa-let-7c-5p
 
 
-0.321
 
 
15.5
 
 
 
 
BLZF1
 
 
hsa-miR-125a-5p
 
 
-0.461
 
 
10.1
 
 
 
 
BTF3L4
 
 
hsa-let-7e-5p
 
 
-0.355
 
 
36.6
 
 
 
 
BTF3L4
 
 
hsa-let-7c-5p
 
 
-0.355
 
 
36.6
 
 
 
 
BZW1
 
 
hsa-let-7e-5p
 
 
-0.354
 
 
46.3
 
 
 
 
BZW1
 
 
hsa-let-7c-5p
 
 
-0.354
 
 
46.3
 
 
 
 
BZW1
 
 
hsa-let-7e-5p
 
 
-0.375
 
 
46.3
 
 
 
 
BZW1
 
 
hsa-let-7c-5p
 
 
-0.375
 
 
46.3
 
 
 
 
C19orf47
 
 
hsa-let-7e-5p
 
 
-0.311
 
 
14.3
 
 
 
 
C19orf47
 
 
hsa-let-7c-5p
 
 
-0.311
 
 
14.3
 
 
 
 
C1GALT1
 
 
hsa-let-7e-5p
 
 
-0.322
 
 
27.9
 
 
 
 
C1GALT1
 
 
hsa-let-7c-5p
 
 
-0.322
 
 
27.9
 
 
 
 
C2orf88
 
 
hsa-let-7e-5p
 
 
-0.429
 
 
14.8
 
 
 
 
C2orf88
 
 
hsa-let-7c-5p
 
 
-0.429
 
 
14.8
 
 
 
 
C5orf24
 
 
hsa-miR-22-3p
 
 
-0.493
 
 
26.2
 
 
 
 
CACFD1
 
 
hsa-let-7e-5p
 
 
-0.296
 
 
13.3
 
 
 
 
CACFD1
 
 
hsa-let-7c-5p
 
 
-0.296
 
 
13.3
 
 
 
 
CADM2
 
 
hsa-let-7e-5p
 
 
-0.294
 
 
10.6
 
 
 
 
CADM2
 
 
hsa-let-7c-5p
 
 
-0.294
 
 
10.6
 
 
 
 
CAMK2N1
 
 
hsa-miR-22-3p
 
 
-0.302
 
 
147.9
 
 
 
 
CANT1
 
 
hsa-let-7e-5p
 
 
-0.402
 
 
15.9
 
 
 
 
CANT1
 
 
hsa-let-7c-5p
 
 
-0.402
 
 
15.9
 
 
 
 
CAPN1
 
 
hsa-miR-22-3p
 
 
-0.307
 
 
15.3
 
 
 
 
CAPN3
 
 
hsa-let-7e-5p
 
 
-0.317
 
 
318.3
 
 
 
 
CAPN3
 
 
hsa-let-7c-5p
 
 
-0.317
 
 
318.3
 
 
 
 
CAST
 
 
hsa-miR-150-5p
 
 
-0.354
 
 
42.5
 
 
 
 
CBX5
 
 
hsa-let-7e-5p
 
 
-0.307
 
 
18.6
 
 
 
 
CBX5
 
 
hsa-let-7c-5p
 
 
-0.307
 
 
18.6
 
 
 
 
CBX5
 
 
hsa-let-7e-5p
 
 
-0.472
 
 
18.6
 
 
 
 
CBX5
 
 
hsa-let-7c-5p
 
 
-0.472
 
 
18.6
 
 
 
 
CCDC47
 
 
hsa-miR-22-3p
 
 
-0.604
 
 
37.5
 
 
 
 
CCNY
 
 
hsa-let-7c-5p
 
 
-0.323
 
 
32.4
 
 
 
 
CCNY
 
 
hsa-let-7e-5p
 
 
-0.334
 
 
32.4
 
 
 
 
CDC34
 
 
hsa-let-7c-5p
 
 
-0.309
 
 
39.3
 
 
 
 
CDC34
 
 
hsa-let-7e-5p
 
 
-0.321
 
 
39.3
 
 
 
 
CDC34
 
 
hsa-let-7e-5p
 
 
-0.555
 
 
39.3
 
 
 
 
CDC34
 
 
hsa-let-7c-5p
 
 
-0.555
 
 
39.3
 
 
 
 
CDC42SE1
 
 
hsa-miR-125a-5p
 
 
-0.501
 
 
39.2
 
 
 
 
CDH5
 
 
hsa-miR-125a-5p
 
 
-0.36
 
 
11.2
 
 
 
 
CDKN1A
 
 
hsa-let-7e-5p
 
 
-0.434
 
 
49.5
 
 
 
 
CDKN1A
 
 
hsa-let-7c-5p
 
 
-0.434
 
 
49.5
 
 
 
 
CDV3
 
 
hsa-let-7e-5p
 
 
-0.453
 
 
47.4
 
 
 
 
CDV3
 
 
hsa-let-7c-5p
 
 
-0.461
 
 
47.4
 
 
 
 
CEBPD
 
 
hsa-let-7e-5p
 
 
-0.309
 
 
90.6
 
 
 
 
CEBPD
 
 
hsa-let-7c-5p
 
 
-0.309
 
 
90.6
 
 
 
 
CERCAM
 
 
hsa-let-7e-5p
 
 
-0.344
 
 
436.8
 
 
 
 
CERCAM
 
 
hsa-let-7c-5p
 
 
-0.344
 
 
436.8
 
 
 
 
CFAP36
 
 
hsa-miR-22-3p
 
 
-0.549
 
 
59.2
 
 
 
 
CLASP2
 
 
hsa-let-7e-5p
 
 
-0.344
 
 
56.4
 
 
 
 
CLASP2
 
 
hsa-let-7c-5p
 
 
-0.344
 
 
56.4
 
 
 
 
CLDN11
 
 
hsa-miR-99b-5p
 
 
-0.405
 
 
119.5
 
 
 
 
CLIC4
 
 
hsa-miR-22-3p
 
 
-0.594
 
 
80.5
 
 
 
 
CMTM6
 
 
hsa-miR-150-5p
 
 
-0.328
 
 
12.0
 
 
 
 
 
 
 spleen 
 
 
 
 
target_symbol
 
 
mature_mirna_id
 
 
score
 
 
nTPM
 
 
 
 
 
 
ABCC5
 
 
hsa-miR-125a-5p
 
 
-0.288
 
 
38.3
 
 
 
 
ABHD3
 
 
hsa-miR-125a-5p
 
 
-0.453
 
 
22.6
 
 
 
 
ABHD6
 
 
hsa-miR-125a-5p
 
 
-0.501
 
 
33.0
 
 
 
 
ABTB1
 
 
hsa-miR-125a-5p
 
 
-0.519
 
 
94.6
 
 
 
 
ADIPOR2
 
 
hsa-miR-150-5p
 
 
-0.287
 
 
19.2
 
 
 
 
AGGF1
 
 
hsa-miR-125a-5p
 
 
-0.331
 
 
11.9
 
 
 
 
AIDA
 
 
hsa-miR-22-3p
 
 
-0.316
 
 
35.3
 
 
 
 
AIFM1
 
 
hsa-miR-125a-5p
 
 
-0.3
 
 
12.9
 
 
 
 
AIFM1
 
 
hsa-let-7e-5p
 
 
-0.314
 
 
12.9
 
 
 
 
AIFM1
 
 
hsa-let-7c-5p
 
 
-0.314
 
 
12.9
 
 
 
 
ALPL
 
 
hsa-miR-10a-5p
 
 
-0.32
 
 
44.5
 
 
 
 
ANAPC16
 
 
hsa-miR-125a-5p
 
 
-0.578
 
 
21.2
 
 
 
 
ANKHD1-EIF4EBP3
 
 
hsa-miR-22-3p
 
 
-0.479
 
 
15.6
 
 
 
 
ANKRD13A
 
 
hsa-miR-22-3p
 
 
-0.478
 
 
30.9
 
 
 
 
ANKRD46
 
 
hsa-let-7e-5p
 
 
-0.389
 
 
10.4
 
 
 
 
ANKRD46
 
 
hsa-let-7c-5p
 
 
-0.389
 
 
10.4
 
 
 
 
ANPEP
 
 
hsa-miR-125a-5p
 
 
-0.372
 
 
24.0
 
 
 
 
AP1AR
 
 
hsa-miR-99b-5p
 
 
-0.823
 
 
12.4
 
 
 
 
AP1S1
 
 
hsa-let-7e-5p
 
 
-0.374
 
 
20.9
 
 
 
 
AP1S1
 
 
hsa-let-7c-5p
 
 
-0.374
 
 
20.9
 
 
 
 
ARHGAP28
 
 
hsa-let-7c-5p
 
 
-0.47
 
 
11.9
 
 
 
 
ARHGAP28
 
 
hsa-let-7e-5p
 
 
-0.486
 
 
11.9
 
 
 
 
ARL4D
 
 
hsa-let-7e-5p
 
 
-0.417
 
 
16.1
 
 
 
 
ARL4D
 
 
hsa-let-7c-5p
 
 
-0.417
 
 
16.1
 
 
 
 
ARL6IP6
 
 
hsa-let-7c-5p
 
 
-0.293
 
 
13.7
 
 
 
 
ARL6IP6
 
 
hsa-let-7e-5p
 
 
-0.305
 
 
13.7
 
 
 
 
ARMT1
 
 
hsa-let-7e-5p
 
 
-0.303
 
 
12.0
 
 
 
 
ARMT1
 
 
hsa-let-7c-5p
 
 
-0.303
 
 
12.0
 
 
 
 
ARPC5
 
 
hsa-miR-22-3p
 
 
-0.679
 
 
113.8
 
 
 
 
ARPP19
 
 
hsa-let-7e-5p
 
 
-0.492
 
 
41.2
 
 
 
 
ARPP19
 
 
hsa-let-7c-5p
 
 
-0.492
 
 
41.2
 
 
 
 
ARRB1
 
 
hsa-miR-22-3p
 
 
-0.616
 
 
37.8
 
 
 
 
ASAP1
 
 
hsa-let-7e-5p
 
 
-0.296
 
 
28.1
 
 
 
 
ASAP1
 
 
hsa-let-7c-5p
 
 
-0.308
 
 
28.1
 
 
 
 
ATOSB
 
 
hsa-let-7e-5p
 
 
-0.429
 
 
26.6
 
 
 
 
ATOSB
 
 
hsa-let-7c-5p
 
 
-0.429
 
 
26.6
 
 
 
 
ATP2A2
 
 
hsa-let-7e-5p
 
 
-0.311
 
 
28.8
 
 
 
 
ATP2A2
 
 
hsa-let-7c-5p
 
 
-0.311
 
 
28.8
 
 
 
 
ATPAF1
 
 
hsa-let-7e-5p
 
 
-0.498
 
 
23.6
 
 
 
 
ATPAF1
 
 
hsa-let-7c-5p
 
 
-0.498
 
 
23.6
 
 
 
 
ATXN2
 
 
hsa-miR-10a-5p
 
 
-0.362
 
 
13.5
 
 
 
 
B3GAT3
 
 
hsa-let-7e-5p
 
 
-0.324
 
 
44.2
 
 
 
 
B3GAT3
 
 
hsa-let-7c-5p
 
 
-0.324
 
 
44.2
 
 
 
 
B4GAT1
 
 
hsa-let-7e-5p
 
 
-0.47
 
 
10.0
 
 
 
 
B4GAT1
 
 
hsa-let-7c-5p
 
 
-0.47
 
 
10.0
 
 
 
 
BACH1
 
 
hsa-let-7e-5p
 
 
-0.298
 
 
19.4
 
 
 
 
BACH1
 
 
hsa-let-7e-5p
 
 
-0.298
 
 
19.4
 
 
 
 
BACH1
 
 
hsa-let-7c-5p
 
 
-0.306
 
 
19.4
 
 
 
 
BACH1
 
 
hsa-let-7c-5p
 
 
-0.306
 
 
19.4
 
 
 
 
BACH1
 
 
hsa-let-7e-5p
 
 
-0.435
 
 
19.4
 
 
 
 
BACH1
 
 
hsa-let-7e-5p
 
 
-0.435
 
 
19.4
 
 
 
 
BACH1
 
 
hsa-let-7c-5p
 
 
-0.435
 
 
19.4
 
 
 
 
BACH1
 
 
hsa-let-7c-5p
 
 
-0.435
 
 
19.4
 
 
 
 
BAG5
 
 
hsa-miR-22-3p
 
 
-0.318
 
 
17.2
 
 
 
 
BAK1
 
 
hsa-miR-125a-5p
 
 
-0.402
 
 
28.6
 
 
 
 
BATF3
 
 
hsa-miR-22-3p
 
 
-0.786
 
 
11.3
 
 
 
 
BAZ1B
 
 
hsa-miR-10a-5p
 
 
-0.309
 
 
22.0
 
 
 
 
BAZ2A
 
 
hsa-miR-99b-5p
 
 
-0.408
 
 
33.2
 
 
 
 
BBX
 
 
hsa-miR-10a-5p
 
 
-0.305
 
 
17.8
 
 
 
 
BCAP29
 
 
hsa-let-7e-5p
 
 
-0.289
 
 
17.6
 
 
 
 
BCL2L11
 
 
hsa-miR-10a-5p
 
 
-0.483
 
 
15.1
 
 
 
 
BCL2L12
 
 
hsa-miR-125a-5p
 
 
-0.288
 
 
15.1
 
 
 
 
BIN1
 
 
hsa-miR-22-3p
 
 
-0.346
 
 
52.9
 
 
 
 
BIN2
 
 
hsa-miR-125a-5p
 
 
-0.679
 
 
48.5
 
 
 
 
BIN3
 
 
hsa-let-7e-5p
 
 
-0.427
 
 
14.4
 
 
 
 
BIN3
 
 
hsa-let-7c-5p
 
 
-0.427
 
 
14.4
 
 
 
 
BLOC1S1
 
 
hsa-miR-22-3p
 
 
-0.346
 
 
172.7
 
 
 
 
BLOC1S6
 
 
hsa-let-7e-5p
 
 
-0.321
 
 
26.3
 
 
 
 
BLOC1S6
 
 
hsa-let-7c-5p
 
 
-0.321
 
 
26.3
 
 
 
 
BLZF1
 
 
hsa-miR-125a-5p
 
 
-0.461
 
 
11.7
 
 
 
 
BMF
 
 
hsa-miR-125a-5p
 
 
-0.34
 
 
17.8
 
 
 
 
BMPR2
 
 
hsa-miR-99b-5p
 
 
-0.327
 
 
11.5
 
 
 
 
BORCS6
 
 
hsa-miR-125a-5p
 
 
-0.287
 
 
15.9
 
 
 
 
BTF3L4
 
 
hsa-let-7e-5p
 
 
-0.355
 
 
26.5
 
 
 
 
BTF3L4
 
 
hsa-let-7c-5p
 
 
-0.355
 
 
26.5
 
 
 
 
BTG1
 
 
hsa-miR-22-3p
 
 
-0.468
 
 
22.1
 
 
 
 
BZW1
 
 
hsa-let-7e-5p
 
 
-0.354
 
 
59.1
 
 
 
 
BZW1
 
 
hsa-let-7c-5p
 
 
-0.354
 
 
59.1
 
 
 
 
BZW1
 
 
hsa-let-7e-5p
 
 
-0.375
 
 
59.1
 
 
 
 
BZW1
 
 
hsa-let-7c-5p
 
 
-0.375
 
 
59.1
 
 
 
 
BZW2
 
 
hsa-let-7c-5p
 
 
-0.397
 
 
16.1
 
 
 
 
BZW2
 
 
hsa-let-7e-5p
 
 
-0.408
 
 
16.1
 
 
 
 
C15orf39
 
 
hsa-miR-125a-5p
 
 
-0.335
 
 
24.1
 
 
 
 
C15orf39
 
 
hsa-let-7e-5p
 
 
-0.374
 
 
24.1
 
 
 
 
C15orf39
 
 
hsa-let-7c-5p
 
 
-0.374
 
 
24.1
 
 
 
 
C19orf38
 
 
hsa-miR-125a-5p
 
 
-0.676
 
 
21.3
 
 
 
 
C1GALT1
 
 
hsa-let-7e-5p
 
 
-0.322
 
 
14.6
 
 
 
 
C1GALT1
 
 
hsa-let-7c-5p
 
 
-0.322
 
 
14.6
 
 
 
 
C5orf24
 
 
hsa-miR-22-3p
 
 
-0.493
 
 
15.7
 
 
 
 
C6orf47
 
 
hsa-miR-125a-5p
 
 
-0.564
 
 
17.1
 
 
 
 
C8orf58
 
 
hsa-let-7e-5p
 
 
-0.612
 
 
24.5
 
 
 
 
C8orf58
 
 
hsa-let-7c-5p
 
 
-0.62
 
 
24.5
 
 
 
 
CAMK2N1
 
 
hsa-miR-22-3p
 
 
-0.302
 
 
11.4
 
 
 
 
CANT1
 
 
hsa-let-7e-5p
 
 
-0.402
 
 
24.4
 
 
 
 
CANT1
 
 
hsa-let-7c-5p
 
 
-0.402
 
 
24.4
 
 
 
 
CAPN1
 
 
hsa-miR-22-3p
 
 
-0.307
 
 
48.5
 
 
 
 
CAPN3
 
 
hsa-let-7e-5p
 
 
-0.317
 
 
23.6
 
 
 
 
CAPN3
 
 
hsa-let-7c-5p
 
 
-0.317
 
 
23.6
 
 
 
 
CASP2
 
 
hsa-miR-125a-5p
 
 
-0.296
 
 
18.2
 
 
 
 
CASP3
 
 
hsa-let-7e-5p
 
 
-0.495
 
 
19.4
 
 
 
 
 
 
 thymus 
 
 
 
 
target_symbol
 
 
mature_mirna_id
 
 
score
 
 
nTPM
 
 
 
 
 
 
ABCC5
 
 
hsa-miR-125a-5p
 
 
-0.288
 
 
14.2
 
 
 
 
ABHD3
 
 
hsa-miR-125a-5p
 
 
-0.453
 
 
20.0
 
 
 
 
ABTB1
 
 
hsa-miR-125a-5p
 
 
-0.519
 
 
20.1
 
 
 
 
ADIPOR2
 
 
hsa-miR-150-5p
 
 
-0.287
 
 
11.9
 
 
 
 
AGGF1
 
 
hsa-miR-125a-5p
 
 
-0.331
 
 
13.4
 
 
 
 
AHCTF1
 
 
hsa-let-7e-5p
 
 
-0.374
 
 
11.4
 
 
 
 
AHCTF1
 
 
hsa-let-7c-5p
 
 
-0.374
 
 
11.4
 
 
 
 
AIDA
 
 
hsa-miR-22-3p
 
 
-0.316
 
 
31.5
 
 
 
 
AIFM1
 
 
hsa-miR-125a-5p
 
 
-0.3
 
 
17.2
 
 
 
 
AIFM1
 
 
hsa-let-7e-5p
 
 
-0.314
 
 
17.2
 
 
 
 
AIFM1
 
 
hsa-let-7c-5p
 
 
-0.314
 
 
17.2
 
 
 
 
ALDH4A1
 
 
hsa-miR-184
 
 
-0.421
 
 
10.9
 
 
 
 
ANAPC16
 
 
hsa-miR-125a-5p
 
 
-0.578
 
 
29.7
 
 
 
 
ANKHD1-EIF4EBP3
 
 
hsa-miR-22-3p
 
 
-0.479
 
 
13.4
 
 
 
 
ANKRD13A
 
 
hsa-miR-22-3p
 
 
-0.478
 
 
26.0
 
 
 
 
ANKRD46
 
 
hsa-let-7e-5p
 
 
-0.389
 
 
14.3
 
 
 
 
ANKRD46
 
 
hsa-let-7c-5p
 
 
-0.389
 
 
14.3
 
 
 
 
AP1AR
 
 
hsa-miR-99b-5p
 
 
-0.823
 
 
10.2
 
 
 
 
AP1S1
 
 
hsa-let-7e-5p
 
 
-0.374
 
 
17.8
 
 
 
 
AP1S1
 
 
hsa-let-7c-5p
 
 
-0.374
 
 
17.8
 
 
 
 
ARL6IP6
 
 
hsa-let-7c-5p
 
 
-0.293
 
 
20.0
 
 
 
 
ARL6IP6
 
 
hsa-let-7e-5p
 
 
-0.305
 
 
20.0
 
 
 
 
ARMT1
 
 
hsa-let-7e-5p
 
 
-0.303
 
 
14.5
 
 
 
 
ARMT1
 
 
hsa-let-7c-5p
 
 
-0.303
 
 
14.5
 
 
 
 
ARPC5
 
 
hsa-miR-22-3p
 
 
-0.679
 
 
121.6
 
 
 
 
ARPP19
 
 
hsa-let-7e-5p
 
 
-0.492
 
 
49.2
 
 
 
 
ARPP19
 
 
hsa-let-7c-5p
 
 
-0.492
 
 
49.2
 
 
 
 
ASAP1
 
 
hsa-let-7e-5p
 
 
-0.296
 
 
14.9
 
 
 
 
ASAP1
 
 
hsa-let-7c-5p
 
 
-0.308
 
 
14.9
 
 
 
 
ATP2A2
 
 
hsa-let-7e-5p
 
 
-0.311
 
 
33.8
 
 
 
 
ATP2A2
 
 
hsa-let-7c-5p
 
 
-0.311
 
 
33.8
 
 
 
 
ATPAF1
 
 
hsa-let-7e-5p
 
 
-0.498
 
 
41.4
 
 
 
 
ATPAF1
 
 
hsa-let-7c-5p
 
 
-0.498
 
 
41.4
 
 
 
 
ATXN2
 
 
hsa-miR-10a-5p
 
 
-0.362
 
 
11.9
 
 
 
 
B3GAT3
 
 
hsa-let-7e-5p
 
 
-0.324
 
 
17.5
 
 
 
 
B3GAT3
 
 
hsa-let-7c-5p
 
 
-0.324
 
 
17.5
 
 
 
 
BACH1
 
 
hsa-let-7e-5p
 
 
-0.298
 
 
13.2
 
 
 
 
BACH1
 
 
hsa-let-7e-5p
 
 
-0.298
 
 
13.2
 
 
 
 
BACH1
 
 
hsa-let-7c-5p
 
 
-0.306
 
 
13.2
 
 
 
 
BACH1
 
 
hsa-let-7c-5p
 
 
-0.306
 
 
13.2
 
 
 
 
BACH1
 
 
hsa-let-7e-5p
 
 
-0.435
 
 
13.2
 
 
 
 
BACH1
 
 
hsa-let-7e-5p
 
 
-0.435
 
 
13.2
 
 
 
 
BACH1
 
 
hsa-let-7c-5p
 
 
-0.435
 
 
13.2
 
 
 
 
BACH1
 
 
hsa-let-7c-5p
 
 
-0.435
 
 
13.2
 
 
 
 
BAG5
 
 
hsa-miR-22-3p
 
 
-0.318
 
 
13.9
 
 
 
 
BAK1
 
 
hsa-miR-125a-5p
 
 
-0.402
 
 
17.9
 
 
 
 
BAZ1B
 
 
hsa-miR-10a-5p
 
 
-0.309
 
 
37.8
 
 
 
 
BAZ2A
 
 
hsa-miR-99b-5p
 
 
-0.408
 
 
47.1
 
 
 
 
BBX
 
 
hsa-miR-10a-5p
 
 
-0.305
 
 
15.2
 
 
 
 
BCAP29
 
 
hsa-let-7e-5p
 
 
-0.289
 
 
12.4
 
 
 
 
BCL2L11
 
 
hsa-miR-10a-5p
 
 
-0.483
 
 
18.0
 
 
 
 
BCL2L12
 
 
hsa-miR-125a-5p
 
 
-0.288
 
 
16.2
 
 
 
 
BIN1
 
 
hsa-miR-22-3p
 
 
-0.346
 
 
17.1
 
 
 
 
BIN2
 
 
hsa-miR-125a-5p
 
 
-0.679
 
 
45.3
 
 
 
 
BIN3
 
 
hsa-let-7e-5p
 
 
-0.427
 
 
12.4
 
 
 
 
BIN3
 
 
hsa-let-7c-5p
 
 
-0.427
 
 
12.4
 
 
 
 
BLOC1S1
 
 
hsa-miR-22-3p
 
 
-0.346
 
 
140.0
 
 
 
 
BLOC1S6
 
 
hsa-let-7e-5p
 
 
-0.321
 
 
23.5
 
 
 
 
BLOC1S6
 
 
hsa-let-7c-5p
 
 
-0.321
 
 
23.5
 
 
 
 
BLZF1
 
 
hsa-miR-125a-5p
 
 
-0.461
 
 
12.8
 
 
 
 
BMF
 
 
hsa-miR-125a-5p
 
 
-0.34
 
 
70.7
 
 
 
 
BTF3L4
 
 
hsa-let-7e-5p
 
 
-0.355
 
 
45.8
 
 
 
 
BTF3L4
 
 
hsa-let-7c-5p
 
 
-0.355
 
 
45.8
 
 
 
 
BTG1
 
 
hsa-miR-22-3p
 
 
-0.468
 
 
18.7
 
 
 
 
BZW1
 
 
hsa-let-7e-5p
 
 
-0.354
 
 
114.6
 
 
 
 
BZW1
 
 
hsa-let-7c-5p
 
 
-0.354
 
 
114.6
 
 
 
 
BZW1
 
 
hsa-let-7e-5p
 
 
-0.375
 
 
114.6
 
 
 
 
BZW1
 
 
hsa-let-7c-5p
 
 
-0.375
 
 
114.6
 
 
 
 
BZW2
 
 
hsa-let-7c-5p
 
 
-0.397
 
 
30.7
 
 
 
 
BZW2
 
 
hsa-let-7e-5p
 
 
-0.408
 
 
30.7
 
 
 
 
C1GALT1
 
 
hsa-let-7e-5p
 
 
-0.322
 
 
13.3
 
 
 
 
C1GALT1
 
 
hsa-let-7c-5p
 
 
-0.322
 
 
13.3
 
 
 
 
C5orf24
 
 
hsa-miR-22-3p
 
 
-0.493
 
 
14.0
 
 
 
 
CANT1
 
 
hsa-let-7e-5p
 
 
-0.402
 
 
13.7
 
 
 
 
CANT1
 
 
hsa-let-7c-5p
 
 
-0.402
 
 
13.7
 
 
 
 
CAPN1
 
 
hsa-miR-22-3p
 
 
-0.307
 
 
46.0
 
 
 
 
CASP2
 
 
hsa-miR-125a-5p
 
 
-0.296
 
 
25.6
 
 
 
 
CASP3
 
 
hsa-let-7e-5p
 
 
-0.495
 
 
33.6
 
 
 
 
CASP3
 
 
hsa-let-7c-5p
 
 
-0.503
 
 
33.6
 
 
 
 
CAST
 
 
hsa-miR-150-5p
 
 
-0.354
 
 
40.3
 
 
 
 
CBFA2T3
 
 
hsa-let-7c-5p
 
 
-0.294
 
 
27.4
 
 
 
 
CBFA2T3
 
 
hsa-let-7e-5p
 
 
-0.305
 
 
27.4
 
 
 
 
CBX5
 
 
hsa-let-7e-5p
 
 
-0.307
 
 
28.9
 
 
 
 
CBX5
 
 
hsa-let-7c-5p
 
 
-0.307
 
 
28.9
 
 
 
 
CBX5
 
 
hsa-let-7e-5p
 
 
-0.472
 
 
28.9
 
 
 
 
CBX5
 
 
hsa-let-7c-5p
 
 
-0.472
 
 
28.9
 
 
 
 
CCDC47
 
 
hsa-miR-22-3p
 
 
-0.604
 
 
27.6
 
 
 
 
CCN1
 
 
hsa-miR-22-3p
 
 
-0.601
 
 
10.4
 
 
 
 
CCNF
 
 
hsa-let-7e-5p
 
 
-0.474
 
 
14.1
 
 
 
 
CCNF
 
 
hsa-let-7c-5p
 
 
-0.474
 
 
14.1
 
 
 
 
CCNY
 
 
hsa-let-7c-5p
 
 
-0.323
 
 
21.0
 
 
 
 
CCNY
 
 
hsa-let-7e-5p
 
 
-0.334
 
 
21.0
 
 
 
 
CCR7
 
 
hsa-let-7e-5p
 
 
-0.482
 
 
54.9
 
 
 
 
CCR7
 
 
hsa-let-7c-5p
 
 
-0.482
 
 
54.9
 
 
 
 
CDC34
 
 
hsa-let-7c-5p
 
 
-0.309
 
 
15.3
 
 
 
 
CDC34
 
 
hsa-let-7e-5p
 
 
-0.321
 
 
15.3
 
 
 
 
CDC34
 
 
hsa-let-7e-5p
 
 
-0.555
 
 
15.3
 
 
 
 
CDC34
 
 
hsa-let-7c-5p
 
 
-0.555
 
 
15.3
 
 
 
 
CDC42SE1
 
 
hsa-miR-125a-5p
 
 
-0.501
 
 
52.6
 
 
 
 
CDCA8
 
 
hsa-let-7e-5p
 
 
-0.342
 
 
36.1
 
 
 
 
 
 
 thyroid gland 
 
 
 
 
target_symbol
 
 
mature_mirna_id
 
 
score
 
 
nTPM
 
 
 
 
 
 
ABCC5
 
 
hsa-miR-125a-5p
 
 
-0.288
 
 
13.7
 
 
 
 
ABHD3
 
 
hsa-miR-125a-5p
 
 
-0.453
 
 
21.4
 
 
 
 
ABTB1
 
 
hsa-miR-125a-5p
 
 
-0.519
 
 
23.0
 
 
 
 
ADIPOR2
 
 
hsa-miR-150-5p
 
 
-0.287
 
 
26.8
 
 
 
 
AGGF1
 
 
hsa-miR-125a-5p
 
 
-0.331
 
 
10.3
 
 
 
 
AHCTF1
 
 
hsa-let-7e-5p
 
 
-0.374
 
 
12.9
 
 
 
 
AHCTF1
 
 
hsa-let-7c-5p
 
 
-0.374
 
 
12.9
 
 
 
 
AIDA
 
 
hsa-miR-22-3p
 
 
-0.316
 
 
35.1
 
 
 
 
AIFM1
 
 
hsa-miR-125a-5p
 
 
-0.3
 
 
10.7
 
 
 
 
AIFM1
 
 
hsa-let-7e-5p
 
 
-0.314
 
 
10.7
 
 
 
 
AIFM1
 
 
hsa-let-7c-5p
 
 
-0.314
 
 
10.7
 
 
 
 
AKT3
 
 
hsa-miR-22-3p
 
 
-0.451
 
 
13.0
 
 
 
 
ANAPC16
 
 
hsa-miR-125a-5p
 
 
-0.578
 
 
21.8
 
 
 
 
ANKRD13A
 
 
hsa-miR-22-3p
 
 
-0.478
 
 
26.4
 
 
 
 
ANKRD46
 
 
hsa-let-7e-5p
 
 
-0.389
 
 
18.8
 
 
 
 
ANKRD46
 
 
hsa-let-7c-5p
 
 
-0.389
 
 
18.8
 
 
 
 
AP1AR
 
 
hsa-miR-99b-5p
 
 
-0.823
 
 
10.1
 
 
 
 
AP1S1
 
 
hsa-let-7e-5p
 
 
-0.374
 
 
15.7
 
 
 
 
AP1S1
 
 
hsa-let-7c-5p
 
 
-0.374
 
 
15.7
 
 
 
 
ARG2
 
 
hsa-let-7e-5p
 
 
-0.468
 
 
97.9
 
 
 
 
ARG2
 
 
hsa-let-7c-5p
 
 
-0.468
 
 
97.9
 
 
 
 
ARL4D
 
 
hsa-let-7e-5p
 
 
-0.417
 
 
35.9
 
 
 
 
ARL4D
 
 
hsa-let-7c-5p
 
 
-0.417
 
 
35.9
 
 
 
 
ARMT1
 
 
hsa-let-7e-5p
 
 
-0.303
 
 
25.9
 
 
 
 
ARMT1
 
 
hsa-let-7c-5p
 
 
-0.303
 
 
25.9
 
 
 
 
ARPC5
 
 
hsa-miR-22-3p
 
 
-0.679
 
 
43.0
 
 
 
 
ARPP19
 
 
hsa-let-7e-5p
 
 
-0.492
 
 
53.7
 
 
 
 
ARPP19
 
 
hsa-let-7c-5p
 
 
-0.492
 
 
53.7
 
 
 
 
ARRDC4
 
 
hsa-let-7e-5p
 
 
-0.329
 
 
16.2
 
 
 
 
ARRDC4
 
 
hsa-let-7c-5p
 
 
-0.329
 
 
16.2
 
 
 
 
ASAP1
 
 
hsa-let-7e-5p
 
 
-0.296
 
 
15.1
 
 
 
 
ASAP1
 
 
hsa-let-7c-5p
 
 
-0.308
 
 
15.1
 
 
 
 
ATOSB
 
 
hsa-let-7e-5p
 
 
-0.429
 
 
11.1
 
 
 
 
ATOSB
 
 
hsa-let-7c-5p
 
 
-0.429
 
 
11.1
 
 
 
 
ATP2A2
 
 
hsa-let-7e-5p
 
 
-0.311
 
 
68.6
 
 
 
 
ATP2A2
 
 
hsa-let-7c-5p
 
 
-0.311
 
 
68.6
 
 
 
 
ATP8A1
 
 
hsa-miR-22-3p
 
 
-0.468
 
 
37.1
 
 
 
 
ATPAF1
 
 
hsa-let-7e-5p
 
 
-0.498
 
 
39.3
 
 
 
 
ATPAF1
 
 
hsa-let-7c-5p
 
 
-0.498
 
 
39.3
 
 
 
 
ATXN2
 
 
hsa-miR-10a-5p
 
 
-0.362
 
 
16.2
 
 
 
 
B3GAT3
 
 
hsa-let-7e-5p
 
 
-0.324
 
 
33.1
 
 
 
 
B3GAT3
 
 
hsa-let-7c-5p
 
 
-0.324
 
 
33.1
 
 
 
 
B4GAT1
 
 
hsa-let-7e-5p
 
 
-0.47
 
 
20.5
 
 
 
 
B4GAT1
 
 
hsa-let-7c-5p
 
 
-0.47
 
 
20.5
 
 
 
 
BACH1
 
 
hsa-let-7e-5p
 
 
-0.298
 
 
11.8
 
 
 
 
BACH1
 
 
hsa-let-7e-5p
 
 
-0.298
 
 
11.8
 
 
 
 
BACH1
 
 
hsa-let-7c-5p
 
 
-0.306
 
 
11.8
 
 
 
 
BACH1
 
 
hsa-let-7c-5p
 
 
-0.306
 
 
11.8
 
 
 
 
BACH1
 
 
hsa-let-7e-5p
 
 
-0.435
 
 
11.8
 
 
 
 
BACH1
 
 
hsa-let-7e-5p
 
 
-0.435
 
 
11.8
 
 
 
 
BACH1
 
 
hsa-let-7c-5p
 
 
-0.435
 
 
11.8
 
 
 
 
BACH1
 
 
hsa-let-7c-5p
 
 
-0.435
 
 
11.8
 
 
 
 
BAG5
 
 
hsa-miR-22-3p
 
 
-0.318
 
 
22.1
 
 
 
 
BAZ1B
 
 
hsa-miR-10a-5p
 
 
-0.309
 
 
22.3
 
 
 
 
BAZ2A
 
 
hsa-miR-99b-5p
 
 
-0.408
 
 
25.4
 
 
 
 
BBX
 
 
hsa-miR-10a-5p
 
 
-0.305
 
 
24.5
 
 
 
 
BCAP29
 
 
hsa-let-7e-5p
 
 
-0.289
 
 
81.1
 
 
 
 
BCL2L11
 
 
hsa-miR-10a-5p
 
 
-0.483
 
 
18.5
 
 
 
 
BDH1
 
 
hsa-miR-125a-5p
 
 
-0.389
 
 
15.7
 
 
 
 
BIN1
 
 
hsa-miR-22-3p
 
 
-0.346
 
 
27.3
 
 
 
 
BIN3
 
 
hsa-let-7e-5p
 
 
-0.427
 
 
14.4
 
 
 
 
BIN3
 
 
hsa-let-7c-5p
 
 
-0.427
 
 
14.4
 
 
 
 
BLOC1S1
 
 
hsa-miR-22-3p
 
 
-0.346
 
 
116.6
 
 
 
 
BLOC1S6
 
 
hsa-let-7e-5p
 
 
-0.321
 
 
41.6
 
 
 
 
BLOC1S6
 
 
hsa-let-7c-5p
 
 
-0.321
 
 
41.6
 
 
 
 
BLZF1
 
 
hsa-miR-125a-5p
 
 
-0.461
 
 
17.5
 
 
 
 
BMF
 
 
hsa-miR-125a-5p
 
 
-0.34
 
 
16.3
 
 
 
 
BMPR2
 
 
hsa-miR-99b-5p
 
 
-0.327
 
 
13.2
 
 
 
 
BTF3L4
 
 
hsa-let-7e-5p
 
 
-0.355
 
 
40.3
 
 
 
 
BTF3L4
 
 
hsa-let-7c-5p
 
 
-0.355
 
 
40.3
 
 
 
 
BZW1
 
 
hsa-let-7e-5p
 
 
-0.354
 
 
69.6
 
 
 
 
BZW1
 
 
hsa-let-7c-5p
 
 
-0.354
 
 
69.6
 
 
 
 
BZW1
 
 
hsa-let-7e-5p
 
 
-0.375
 
 
69.6
 
 
 
 
BZW1
 
 
hsa-let-7c-5p
 
 
-0.375
 
 
69.6
 
 
 
 
BZW2
 
 
hsa-let-7c-5p
 
 
-0.397
 
 
34.2
 
 
 
 
BZW2
 
 
hsa-let-7e-5p
 
 
-0.408
 
 
34.2
 
 
 
 
C15orf39
 
 
hsa-miR-125a-5p
 
 
-0.335
 
 
10.6
 
 
 
 
C15orf39
 
 
hsa-let-7e-5p
 
 
-0.374
 
 
10.6
 
 
 
 
C15orf39
 
 
hsa-let-7c-5p
 
 
-0.374
 
 
10.6
 
 
 
 
C1GALT1
 
 
hsa-let-7e-5p
 
 
-0.322
 
 
11.8
 
 
 
 
C1GALT1
 
 
hsa-let-7c-5p
 
 
-0.322
 
 
11.8
 
 
 
 
C1orf210
 
 
hsa-miR-125a-5p
 
 
-0.584
 
 
15.8
 
 
 
 
C5orf24
 
 
hsa-miR-22-3p
 
 
-0.493
 
 
27.9
 
 
 
 
C6orf47
 
 
hsa-miR-125a-5p
 
 
-0.564
 
 
10.9
 
 
 
 
CACFD1
 
 
hsa-let-7e-5p
 
 
-0.296
 
 
16.5
 
 
 
 
CACFD1
 
 
hsa-let-7c-5p
 
 
-0.296
 
 
16.5
 
 
 
 
CAMK2N1
 
 
hsa-miR-22-3p
 
 
-0.302
 
 
10.3
 
 
 
 
CANT1
 
 
hsa-let-7e-5p
 
 
-0.402
 
 
12.7
 
 
 
 
CANT1
 
 
hsa-let-7c-5p
 
 
-0.402
 
 
12.7
 
 
 
 
CAPN1
 
 
hsa-miR-22-3p
 
 
-0.307
 
 
69.1
 
 
 
 
CAPN3
 
 
hsa-let-7e-5p
 
 
-0.317
 
 
12.0
 
 
 
 
CAPN3
 
 
hsa-let-7c-5p
 
 
-0.317
 
 
12.0
 
 
 
 
CARNMT1
 
 
hsa-let-7e-5p
 
 
-0.307
 
 
12.7
 
 
 
 
CARNMT1
 
 
hsa-let-7c-5p
 
 
-0.307
 
 
12.7
 
 
 
 
CAST
 
 
hsa-miR-150-5p
 
 
-0.354
 
 
91.6
 
 
 
 
CBX5
 
 
hsa-let-7e-5p
 
 
-0.307
 
 
22.4
 
 
 
 
CBX5
 
 
hsa-let-7c-5p
 
 
-0.307
 
 
22.4
 
 
 
 
CBX5
 
 
hsa-let-7e-5p
 
 
-0.472
 
 
22.4
 
 
 
 
CBX5
 
 
hsa-let-7c-5p
 
 
-0.472
 
 
22.4
 
 
 
 
CCDC47
 
 
hsa-miR-22-3p
 
 
-0.604
 
 
57.9
 
 
 
 
 
 
 tongue 
 
 
 
 
target_symbol
 
 
mature_mirna_id
 
 
score
 
 
nTPM
 
 
 
 
 
 
ABCC5
 
 
hsa-miR-125a-5p
 
 
-0.288
 
 
37.6
 
 
 
 
ABTB1
 
 
hsa-miR-125a-5p
 
 
-0.519
 
 
24.5
 
 
 
 
ACHE
 
 
hsa-miR-125a-5p
 
 
-0.308
 
 
44.9
 
 
 
 
ACHE
 
 
hsa-miR-125a-5p
 
 
-0.361
 
 
44.9
 
 
 
 
ADIPOR2
 
 
hsa-miR-150-5p
 
 
-0.287
 
 
18.5
 
 
 
 
AGGF1
 
 
hsa-miR-125a-5p
 
 
-0.331
 
 
10.4
 
 
 
 
AHCTF1
 
 
hsa-let-7e-5p
 
 
-0.374
 
 
14.7
 
 
 
 
AHCTF1
 
 
hsa-let-7c-5p
 
 
-0.374
 
 
14.7
 
 
 
 
AIDA
 
 
hsa-miR-22-3p
 
 
-0.316
 
 
46.5
 
 
 
 
AIFM1
 
 
hsa-miR-125a-5p
 
 
-0.3
 
 
23.4
 
 
 
 
AIFM1
 
 
hsa-let-7e-5p
 
 
-0.314
 
 
23.4
 
 
 
 
AIFM1
 
 
hsa-let-7c-5p
 
 
-0.314
 
 
23.4
 
 
 
 
AKAP6
 
 
hsa-let-7e-5p
 
 
-0.307
 
 
47.4
 
 
 
 
AKAP6
 
 
hsa-let-7c-5p
 
 
-0.307
 
 
47.4
 
 
 
 
AKT3
 
 
hsa-miR-22-3p
 
 
-0.451
 
 
10.7
 
 
 
 
ALDH4A1
 
 
hsa-miR-184
 
 
-0.421
 
 
69.9
 
 
 
 
ANAPC16
 
 
hsa-miR-125a-5p
 
 
-0.578
 
 
38.9
 
 
 
 
ANKRD46
 
 
hsa-let-7e-5p
 
 
-0.389
 
 
33.8
 
 
 
 
ANKRD46
 
 
hsa-let-7c-5p
 
 
-0.389
 
 
33.8
 
 
 
 
ARG2
 
 
hsa-let-7e-5p
 
 
-0.468
 
 
49.2
 
 
 
 
ARG2
 
 
hsa-let-7c-5p
 
 
-0.468
 
 
49.2
 
 
 
 
ARMT1
 
 
hsa-let-7e-5p
 
 
-0.303
 
 
21.1
 
 
 
 
ARMT1
 
 
hsa-let-7c-5p
 
 
-0.303
 
 
21.1
 
 
 
 
ARPC5
 
 
hsa-miR-22-3p
 
 
-0.679
 
 
21.0
 
 
 
 
ARPP19
 
 
hsa-let-7e-5p
 
 
-0.492
 
 
158.9
 
 
 
 
ARPP19
 
 
hsa-let-7c-5p
 
 
-0.492
 
 
158.9
 
 
 
 
ARRB1
 
 
hsa-miR-22-3p
 
 
-0.616
 
 
10.1
 
 
 
 
ATOSB
 
 
hsa-let-7e-5p
 
 
-0.429
 
 
10.9
 
 
 
 
ATOSB
 
 
hsa-let-7c-5p
 
 
-0.429
 
 
10.9
 
 
 
 
ATP2A2
 
 
hsa-let-7e-5p
 
 
-0.311
 
 
2586.2
 
 
 
 
ATP2A2
 
 
hsa-let-7c-5p
 
 
-0.311
 
 
2586.2
 
 
 
 
ATP8A1
 
 
hsa-miR-22-3p
 
 
-0.468
 
 
14.5
 
 
 
 
ATPAF1
 
 
hsa-let-7e-5p
 
 
-0.498
 
 
295.7
 
 
 
 
ATPAF1
 
 
hsa-let-7c-5p
 
 
-0.498
 
 
295.7
 
 
 
 
ATXN2
 
 
hsa-miR-10a-5p
 
 
-0.362
 
 
19.4
 
 
 
 
B3GALNT2
 
 
hsa-miR-125a-5p
 
 
-0.311
 
 
15.5
 
 
 
 
B4GAT1
 
 
hsa-let-7e-5p
 
 
-0.47
 
 
17.9
 
 
 
 
B4GAT1
 
 
hsa-let-7c-5p
 
 
-0.47
 
 
17.9
 
 
 
 
BACH1
 
 
hsa-let-7e-5p
 
 
-0.298
 
 
11.9
 
 
 
 
BACH1
 
 
hsa-let-7e-5p
 
 
-0.298
 
 
11.9
 
 
 
 
BACH1
 
 
hsa-let-7c-5p
 
 
-0.306
 
 
11.9
 
 
 
 
BACH1
 
 
hsa-let-7c-5p
 
 
-0.306
 
 
11.9
 
 
 
 
BACH1
 
 
hsa-let-7e-5p
 
 
-0.435
 
 
11.9
 
 
 
 
BACH1
 
 
hsa-let-7e-5p
 
 
-0.435
 
 
11.9
 
 
 
 
BACH1
 
 
hsa-let-7c-5p
 
 
-0.435
 
 
11.9
 
 
 
 
BACH1
 
 
hsa-let-7c-5p
 
 
-0.435
 
 
11.9
 
 
 
 
BAG5
 
 
hsa-miR-22-3p
 
 
-0.318
 
 
12.6
 
 
 
 
BAK1
 
 
hsa-miR-125a-5p
 
 
-0.402
 
 
16.9
 
 
 
 
BAZ1B
 
 
hsa-miR-10a-5p
 
 
-0.309
 
 
28.7
 
 
 
 
BAZ2A
 
 
hsa-miR-99b-5p
 
 
-0.408
 
 
29.9
 
 
 
 
BBX
 
 
hsa-miR-10a-5p
 
 
-0.305
 
 
15.1
 
 
 
 
BCAP29
 
 
hsa-let-7e-5p
 
 
-0.289
 
 
30.7
 
 
 
 
BCL2L12
 
 
hsa-miR-125a-5p
 
 
-0.288
 
 
12.5
 
 
 
 
BDH1
 
 
hsa-miR-125a-5p
 
 
-0.389
 
 
21.3
 
 
 
 
BIN1
 
 
hsa-miR-22-3p
 
 
-0.346
 
 
751.1
 
 
 
 
BLOC1S1
 
 
hsa-miR-22-3p
 
 
-0.346
 
 
191.8
 
 
 
 
BLOC1S6
 
 
hsa-let-7e-5p
 
 
-0.321
 
 
21.7
 
 
 
 
BLOC1S6
 
 
hsa-let-7c-5p
 
 
-0.321
 
 
21.7
 
 
 
 
BLZF1
 
 
hsa-miR-125a-5p
 
 
-0.461
 
 
10.2
 
 
 
 
BMP5
 
 
hsa-let-7e-5p
 
 
-0.337
 
 
11.4
 
 
 
 
BMP5
 
 
hsa-let-7c-5p
 
 
-0.337
 
 
11.4
 
 
 
 
BMPR2
 
 
hsa-miR-99b-5p
 
 
-0.327
 
 
15.0
 
 
 
 
BTF3L4
 
 
hsa-let-7e-5p
 
 
-0.355
 
 
55.0
 
 
 
 
BTF3L4
 
 
hsa-let-7c-5p
 
 
-0.355
 
 
55.0
 
 
 
 
BZW1
 
 
hsa-let-7e-5p
 
 
-0.354
 
 
70.0
 
 
 
 
BZW1
 
 
hsa-let-7c-5p
 
 
-0.354
 
 
70.0
 
 
 
 
BZW1
 
 
hsa-let-7e-5p
 
 
-0.375
 
 
70.0
 
 
 
 
BZW1
 
 
hsa-let-7c-5p
 
 
-0.375
 
 
70.0
 
 
 
 
BZW2
 
 
hsa-let-7c-5p
 
 
-0.397
 
 
241.7
 
 
 
 
BZW2
 
 
hsa-let-7e-5p
 
 
-0.408
 
 
241.7
 
 
 
 
C19orf47
 
 
hsa-let-7e-5p
 
 
-0.311
 
 
128.4
 
 
 
 
C19orf47
 
 
hsa-let-7c-5p
 
 
-0.311
 
 
128.4
 
 
 
 
C1GALT1
 
 
hsa-let-7e-5p
 
 
-0.322
 
 
10.5
 
 
 
 
C1GALT1
 
 
hsa-let-7c-5p
 
 
-0.322
 
 
10.5
 
 
 
 
C2orf88
 
 
hsa-let-7e-5p
 
 
-0.429
 
 
27.3
 
 
 
 
C2orf88
 
 
hsa-let-7c-5p
 
 
-0.429
 
 
27.3
 
 
 
 
C5orf24
 
 
hsa-miR-22-3p
 
 
-0.493
 
 
24.9
 
 
 
 
CANT1
 
 
hsa-let-7e-5p
 
 
-0.402
 
 
11.5
 
 
 
 
CANT1
 
 
hsa-let-7c-5p
 
 
-0.402
 
 
11.5
 
 
 
 
CAPN1
 
 
hsa-miR-22-3p
 
 
-0.307
 
 
51.5
 
 
 
 
CAPN3
 
 
hsa-let-7e-5p
 
 
-0.317
 
 
352.2
 
 
 
 
CAPN3
 
 
hsa-let-7c-5p
 
 
-0.317
 
 
352.2
 
 
 
 
CASP3
 
 
hsa-let-7e-5p
 
 
-0.495
 
 
12.2
 
 
 
 
CASP3
 
 
hsa-let-7c-5p
 
 
-0.503
 
 
12.2
 
 
 
 
CAST
 
 
hsa-miR-150-5p
 
 
-0.354
 
 
155.3
 
 
 
 
CAV3
 
 
hsa-miR-22-3p
 
 
-0.565
 
 
68.1
 
 
 
 
CAVIN4
 
 
hsa-miR-22-3p
 
 
-0.391
 
 
86.3
 
 
 
 
CCDC47
 
 
hsa-miR-22-3p
 
 
-0.604
 
 
78.1
 
 
 
 
CCN1
 
 
hsa-miR-22-3p
 
 
-0.601
 
 
16.0
 
 
 
 
CCNY
 
 
hsa-let-7c-5p
 
 
-0.323
 
 
21.4
 
 
 
 
CCNY
 
 
hsa-let-7e-5p
 
 
-0.334
 
 
21.4
 
 
 
 
CDC34
 
 
hsa-let-7c-5p
 
 
-0.309
 
 
58.9
 
 
 
 
CDC34
 
 
hsa-let-7e-5p
 
 
-0.321
 
 
58.9
 
 
 
 
CDC34
 
 
hsa-let-7e-5p
 
 
-0.555
 
 
58.9
 
 
 
 
CDC34
 
 
hsa-let-7c-5p
 
 
-0.555
 
 
58.9
 
 
 
 
CDC37L1
 
 
hsa-miR-125a-5p
 
 
-0.401
 
 
49.0
 
 
 
 
CDC42SE1
 
 
hsa-miR-125a-5p
 
 
-0.501
 
 
18.0
 
 
 
 
CDH5
 
 
hsa-miR-125a-5p
 
 
-0.36
 
 
55.0
 
 
 
 
CDIN1
 
 
hsa-let-7e-5p
 
 
-0.401
 
 
32.2
 
 
 
 
CDIN1
 
 
hsa-let-7c-5p
 
 
-0.401
 
 
32.2
 
 
 
 
 
 
 tonsil 
 
 
 
 
target_symbol
 
 
mature_mirna_id
 
 
score
 
 
nTPM
 
 
 
 
 
 
ABCC5
 
 
hsa-miR-125a-5p
 
 
-0.288
 
 
11.1
 
 
 
 
ABHD17C
 
 
hsa-let-7e-5p
 
 
-0.359
 
 
21.6
 
 
 
 
ABHD17C
 
 
hsa-let-7c-5p
 
 
-0.359
 
 
21.6
 
 
 
 
ABHD3
 
 
hsa-miR-125a-5p
 
 
-0.453
 
 
24.2
 
 
 
 
ADAM17
 
 
hsa-miR-125a-5p
 
 
-0.292
 
 
11.1
 
 
 
 
ADIPOR2
 
 
hsa-miR-150-5p
 
 
-0.287
 
 
11.8
 
 
 
 
AGGF1
 
 
hsa-miR-125a-5p
 
 
-0.331
 
 
13.2
 
 
 
 
AIDA
 
 
hsa-miR-22-3p
 
 
-0.316
 
 
49.6
 
 
 
 
AIFM1
 
 
hsa-miR-125a-5p
 
 
-0.3
 
 
10.3
 
 
 
 
AIFM1
 
 
hsa-let-7e-5p
 
 
-0.314
 
 
10.3
 
 
 
 
AIFM1
 
 
hsa-let-7c-5p
 
 
-0.314
 
 
10.3
 
 
 
 
ANAPC16
 
 
hsa-miR-125a-5p
 
 
-0.578
 
 
23.8
 
 
 
 
ANKHD1-EIF4EBP3
 
 
hsa-miR-22-3p
 
 
-0.479
 
 
12.6
 
 
 
 
ANKRD13A
 
 
hsa-miR-22-3p
 
 
-0.478
 
 
62.4
 
 
 
 
ANKRD46
 
 
hsa-let-7e-5p
 
 
-0.389
 
 
10.3
 
 
 
 
ANKRD46
 
 
hsa-let-7c-5p
 
 
-0.389
 
 
10.3
 
 
 
 
AP1AR
 
 
hsa-miR-99b-5p
 
 
-0.823
 
 
11.3
 
 
 
 
AP1S1
 
 
hsa-let-7e-5p
 
 
-0.374
 
 
12.8
 
 
 
 
AP1S1
 
 
hsa-let-7c-5p
 
 
-0.374
 
 
12.8
 
 
 
 
ARL6IP6
 
 
hsa-let-7c-5p
 
 
-0.293
 
 
18.0
 
 
 
 
ARL6IP6
 
 
hsa-let-7e-5p
 
 
-0.305
 
 
18.0
 
 
 
 
ARMT1
 
 
hsa-let-7e-5p
 
 
-0.303
 
 
17.3
 
 
 
 
ARMT1
 
 
hsa-let-7c-5p
 
 
-0.303
 
 
17.3
 
 
 
 
ARPC5
 
 
hsa-miR-22-3p
 
 
-0.679
 
 
123.0
 
 
 
 
ARPP19
 
 
hsa-let-7e-5p
 
 
-0.492
 
 
48.4
 
 
 
 
ARPP19
 
 
hsa-let-7c-5p
 
 
-0.492
 
 
48.4
 
 
 
 
ASAP1
 
 
hsa-let-7e-5p
 
 
-0.296
 
 
17.8
 
 
 
 
ASAP1
 
 
hsa-let-7c-5p
 
 
-0.308
 
 
17.8
 
 
 
 
ATP2A2
 
 
hsa-let-7e-5p
 
 
-0.311
 
 
45.1
 
 
 
 
ATP2A2
 
 
hsa-let-7c-5p
 
 
-0.311
 
 
45.1
 
 
 
 
ATP8A1
 
 
hsa-miR-22-3p
 
 
-0.468
 
 
11.0
 
 
 
 
ATPAF1
 
 
hsa-let-7e-5p
 
 
-0.498
 
 
30.0
 
 
 
 
ATPAF1
 
 
hsa-let-7c-5p
 
 
-0.498
 
 
30.0
 
 
 
 
B3GAT3
 
 
hsa-let-7e-5p
 
 
-0.324
 
 
10.3
 
 
 
 
B3GAT3
 
 
hsa-let-7c-5p
 
 
-0.324
 
 
10.3
 
 
 
 
BACH1
 
 
hsa-let-7e-5p
 
 
-0.298
 
 
13.8
 
 
 
 
BACH1
 
 
hsa-let-7e-5p
 
 
-0.298
 
 
13.8
 
 
 
 
BACH1
 
 
hsa-let-7c-5p
 
 
-0.306
 
 
13.8
 
 
 
 
BACH1
 
 
hsa-let-7c-5p
 
 
-0.306
 
 
13.8
 
 
 
 
BACH1
 
 
hsa-let-7e-5p
 
 
-0.435
 
 
13.8
 
 
 
 
BACH1
 
 
hsa-let-7e-5p
 
 
-0.435
 
 
13.8
 
 
 
 
BACH1
 
 
hsa-let-7c-5p
 
 
-0.435
 
 
13.8
 
 
 
 
BACH1
 
 
hsa-let-7c-5p
 
 
-0.435
 
 
13.8
 
 
 
 
BAG5
 
 
hsa-miR-22-3p
 
 
-0.318
 
 
17.9
 
 
 
 
BAK1
 
 
hsa-miR-125a-5p
 
 
-0.402
 
 
21.9
 
 
 
 
BAZ1B
 
 
hsa-miR-10a-5p
 
 
-0.309
 
 
27.8
 
 
 
 
BAZ2A
 
 
hsa-miR-99b-5p
 
 
-0.408
 
 
28.7
 
 
 
 
BBX
 
 
hsa-miR-10a-5p
 
 
-0.305
 
 
12.8
 
 
 
 
BCAP29
 
 
hsa-let-7e-5p
 
 
-0.289
 
 
15.2
 
 
 
 
BCL2L11
 
 
hsa-miR-10a-5p
 
 
-0.483
 
 
21.7
 
 
 
 
BCL2L12
 
 
hsa-miR-125a-5p
 
 
-0.288
 
 
14.8
 
 
 
 
BIN1
 
 
hsa-miR-22-3p
 
 
-0.346
 
 
25.8
 
 
 
 
BIN2
 
 
hsa-miR-125a-5p
 
 
-0.679
 
 
34.1
 
 
 
 
BIN3
 
 
hsa-let-7e-5p
 
 
-0.427
 
 
10.2
 
 
 
 
BIN3
 
 
hsa-let-7c-5p
 
 
-0.427
 
 
10.2
 
 
 
 
BLOC1S1
 
 
hsa-miR-22-3p
 
 
-0.346
 
 
107.4
 
 
 
 
BLOC1S6
 
 
hsa-let-7e-5p
 
 
-0.321
 
 
33.2
 
 
 
 
BLOC1S6
 
 
hsa-let-7c-5p
 
 
-0.321
 
 
33.2
 
 
 
 
BLZF1
 
 
hsa-miR-125a-5p
 
 
-0.461
 
 
15.2
 
 
 
 
BMF
 
 
hsa-miR-125a-5p
 
 
-0.34
 
 
22.0
 
 
 
 
BTF3L4
 
 
hsa-let-7e-5p
 
 
-0.355
 
 
38.2
 
 
 
 
BTF3L4
 
 
hsa-let-7c-5p
 
 
-0.355
 
 
38.2
 
 
 
 
BTG1
 
 
hsa-miR-22-3p
 
 
-0.468
 
 
17.1
 
 
 
 
BZW1
 
 
hsa-let-7e-5p
 
 
-0.354
 
 
102.5
 
 
 
 
BZW1
 
 
hsa-let-7c-5p
 
 
-0.354
 
 
102.5
 
 
 
 
BZW1
 
 
hsa-let-7e-5p
 
 
-0.375
 
 
102.5
 
 
 
 
BZW1
 
 
hsa-let-7c-5p
 
 
-0.375
 
 
102.5
 
 
 
 
BZW2
 
 
hsa-let-7c-5p
 
 
-0.397
 
 
35.9
 
 
 
 
BZW2
 
 
hsa-let-7e-5p
 
 
-0.408
 
 
35.9
 
 
 
 
C1GALT1
 
 
hsa-let-7e-5p
 
 
-0.322
 
 
13.2
 
 
 
 
C1GALT1
 
 
hsa-let-7c-5p
 
 
-0.322
 
 
13.2
 
 
 
 
C5orf24
 
 
hsa-miR-22-3p
 
 
-0.493
 
 
13.7
 
 
 
 
CANT1
 
 
hsa-let-7e-5p
 
 
-0.402
 
 
14.2
 
 
 
 
CANT1
 
 
hsa-let-7c-5p
 
 
-0.402
 
 
14.2
 
 
 
 
CAPN1
 
 
hsa-miR-22-3p
 
 
-0.307
 
 
80.9
 
 
 
 
CASP2
 
 
hsa-miR-125a-5p
 
 
-0.296
 
 
25.3
 
 
 
 
CASP3
 
 
hsa-let-7e-5p
 
 
-0.495
 
 
36.6
 
 
 
 
CASP3
 
 
hsa-let-7c-5p
 
 
-0.503
 
 
36.6
 
 
 
 
CAST
 
 
hsa-miR-150-5p
 
 
-0.354
 
 
97.9
 
 
 
 
CBX5
 
 
hsa-let-7e-5p
 
 
-0.307
 
 
20.1
 
 
 
 
CBX5
 
 
hsa-let-7c-5p
 
 
-0.307
 
 
20.1
 
 
 
 
CBX5
 
 
hsa-let-7e-5p
 
 
-0.472
 
 
20.1
 
 
 
 
CBX5
 
 
hsa-let-7c-5p
 
 
-0.472
 
 
20.1
 
 
 
 
CCDC47
 
 
hsa-miR-22-3p
 
 
-0.604
 
 
37.9
 
 
 
 
CCNY
 
 
hsa-let-7c-5p
 
 
-0.323
 
 
19.7
 
 
 
 
CCNY
 
 
hsa-let-7e-5p
 
 
-0.334
 
 
19.7
 
 
 
 
CCR7
 
 
hsa-let-7e-5p
 
 
-0.482
 
 
78.1
 
 
 
 
CCR7
 
 
hsa-let-7c-5p
 
 
-0.482
 
 
78.1
 
 
 
 
CD86
 
 
hsa-let-7e-5p
 
 
-0.323
 
 
20.6
 
 
 
 
CD86
 
 
hsa-let-7c-5p
 
 
-0.323
 
 
20.6
 
 
 
 
CDC42SE1
 
 
hsa-miR-125a-5p
 
 
-0.501
 
 
79.3
 
 
 
 
CDCA8
 
 
hsa-let-7e-5p
 
 
-0.342
 
 
21.2
 
 
 
 
CDCA8
 
 
hsa-let-7c-5p
 
 
-0.342
 
 
21.2
 
 
 
 
CDKN1A
 
 
hsa-let-7e-5p
 
 
-0.434
 
 
20.4
 
 
 
 
CDKN1A
 
 
hsa-let-7c-5p
 
 
-0.434
 
 
20.4
 
 
 
 
CDV3
 
 
hsa-let-7e-5p
 
 
-0.453
 
 
151.5
 
 
 
 
CDV3
 
 
hsa-let-7c-5p
 
 
-0.461
 
 
151.5
 
 
 
 
CELF2
 
 
hsa-miR-10a-5p
 
 
-0.319
 
 
27.6
 
 
 
 
CEMIP2
 
 
hsa-let-7e-5p
 
 
-0.453
 
 
19.4
 
 
 
 
CEMIP2
 
 
hsa-let-7c-5p
 
 
-0.453
 
 
19.4
 
 
 
 
 
 
 urinary bladder 
 
 
 
 
target_symbol
 
 
mature_mirna_id
 
 
score
 
 
nTPM
 
 
 
 
 
 
ABCC5
 
 
hsa-miR-125a-5p
 
 
-0.288
 
 
38.3
 
 
 
 
ABHD17C
 
 
hsa-let-7e-5p
 
 
-0.359
 
 
26.8
 
 
 
 
ABHD17C
 
 
hsa-let-7c-5p
 
 
-0.359
 
 
26.8
 
 
 
 
ABHD3
 
 
hsa-miR-125a-5p
 
 
-0.453
 
 
14.3
 
 
 
 
ABTB1
 
 
hsa-miR-125a-5p
 
 
-0.519
 
 
36.6
 
 
 
 
ACER2
 
 
hsa-let-7e-5p
 
 
-0.333
 
 
36.7
 
 
 
 
ACER2
 
 
hsa-let-7c-5p
 
 
-0.333
 
 
36.7
 
 
 
 
ACER2
 
 
hsa-miR-125a-5p
 
 
-0.338
 
 
36.7
 
 
 
 
ADIPOR2
 
 
hsa-miR-150-5p
 
 
-0.287
 
 
21.3
 
 
 
 
AIDA
 
 
hsa-miR-22-3p
 
 
-0.316
 
 
44.1
 
 
 
 
AIFM1
 
 
hsa-miR-125a-5p
 
 
-0.3
 
 
13.1
 
 
 
 
AIFM1
 
 
hsa-let-7e-5p
 
 
-0.314
 
 
13.1
 
 
 
 
AIFM1
 
 
hsa-let-7c-5p
 
 
-0.314
 
 
13.1
 
 
 
 
AKT3
 
 
hsa-miR-22-3p
 
 
-0.451
 
 
14.4
 
 
 
 
ALDH4A1
 
 
hsa-miR-184
 
 
-0.421
 
 
21.0
 
 
 
 
AMT
 
 
hsa-let-7e-5p
 
 
-0.463
 
 
13.1
 
 
 
 
AMT
 
 
hsa-let-7c-5p
 
 
-0.463
 
 
13.1
 
 
 
 
ANAPC16
 
 
hsa-miR-125a-5p
 
 
-0.578
 
 
20.8
 
 
 
 
ANKHD1-EIF4EBP3
 
 
hsa-miR-22-3p
 
 
-0.479
 
 
11.2
 
 
 
 
ANKRD13A
 
 
hsa-miR-22-3p
 
 
-0.478
 
 
24.2
 
 
 
 
ANPEP
 
 
hsa-miR-125a-5p
 
 
-0.372
 
 
15.7
 
 
 
 
AP1AR
 
 
hsa-miR-99b-5p
 
 
-0.823
 
 
12.2
 
 
 
 
AP1S1
 
 
hsa-let-7e-5p
 
 
-0.374
 
 
18.6
 
 
 
 
AP1S1
 
 
hsa-let-7c-5p
 
 
-0.374
 
 
18.6
 
 
 
 
ARG2
 
 
hsa-let-7e-5p
 
 
-0.468
 
 
16.6
 
 
 
 
ARG2
 
 
hsa-let-7c-5p
 
 
-0.468
 
 
16.6
 
 
 
 
ARL4D
 
 
hsa-let-7e-5p
 
 
-0.417
 
 
26.6
 
 
 
 
ARL4D
 
 
hsa-let-7c-5p
 
 
-0.417
 
 
26.6
 
 
 
 
ARL6IP6
 
 
hsa-let-7c-5p
 
 
-0.293
 
 
12.2
 
 
 
 
ARL6IP6
 
 
hsa-let-7e-5p
 
 
-0.305
 
 
12.2
 
 
 
 
ARMT1
 
 
hsa-let-7e-5p
 
 
-0.303
 
 
16.1
 
 
 
 
ARMT1
 
 
hsa-let-7c-5p
 
 
-0.303
 
 
16.1
 
 
 
 
ARPC5
 
 
hsa-miR-22-3p
 
 
-0.679
 
 
71.0
 
 
 
 
ARPP19
 
 
hsa-let-7e-5p
 
 
-0.492
 
 
51.7
 
 
 
 
ARPP19
 
 
hsa-let-7c-5p
 
 
-0.492
 
 
51.7
 
 
 
 
ARRB1
 
 
hsa-miR-22-3p
 
 
-0.616
 
 
15.3
 
 
 
 
ARRDC4
 
 
hsa-let-7e-5p
 
 
-0.329
 
 
10.9
 
 
 
 
ARRDC4
 
 
hsa-let-7c-5p
 
 
-0.329
 
 
10.9
 
 
 
 
ASAP1
 
 
hsa-let-7e-5p
 
 
-0.296
 
 
14.4
 
 
 
 
ASAP1
 
 
hsa-let-7c-5p
 
 
-0.308
 
 
14.4
 
 
 
 
ATOSB
 
 
hsa-let-7e-5p
 
 
-0.429
 
 
10.8
 
 
 
 
ATOSB
 
 
hsa-let-7c-5p
 
 
-0.429
 
 
10.8
 
 
 
 
ATP2A2
 
 
hsa-let-7e-5p
 
 
-0.311
 
 
84.9
 
 
 
 
ATP2A2
 
 
hsa-let-7c-5p
 
 
-0.311
 
 
84.9
 
 
 
 
ATPAF1
 
 
hsa-let-7e-5p
 
 
-0.498
 
 
26.7
 
 
 
 
ATPAF1
 
 
hsa-let-7c-5p
 
 
-0.498
 
 
26.7
 
 
 
 
ATXN2
 
 
hsa-miR-10a-5p
 
 
-0.362
 
 
19.6
 
 
 
 
B3GALNT2
 
 
hsa-miR-125a-5p
 
 
-0.311
 
 
12.7
 
 
 
 
B3GAT3
 
 
hsa-let-7e-5p
 
 
-0.324
 
 
28.2
 
 
 
 
B3GAT3
 
 
hsa-let-7c-5p
 
 
-0.324
 
 
28.2
 
 
 
 
B4GAT1
 
 
hsa-let-7e-5p
 
 
-0.47
 
 
21.0
 
 
 
 
B4GAT1
 
 
hsa-let-7c-5p
 
 
-0.47
 
 
21.0
 
 
 
 
BACH1
 
 
hsa-let-7e-5p
 
 
-0.298
 
 
27.7
 
 
 
 
BACH1
 
 
hsa-let-7e-5p
 
 
-0.298
 
 
27.7
 
 
 
 
BACH1
 
 
hsa-let-7c-5p
 
 
-0.306
 
 
27.7
 
 
 
 
BACH1
 
 
hsa-let-7c-5p
 
 
-0.306
 
 
27.7
 
 
 
 
BACH1
 
 
hsa-let-7e-5p
 
 
-0.435
 
 
27.7
 
 
 
 
BACH1
 
 
hsa-let-7e-5p
 
 
-0.435
 
 
27.7
 
 
 
 
BACH1
 
 
hsa-let-7c-5p
 
 
-0.435
 
 
27.7
 
 
 
 
BACH1
 
 
hsa-let-7c-5p
 
 
-0.435
 
 
27.7
 
 
 
 
BAG5
 
 
hsa-miR-22-3p
 
 
-0.318
 
 
14.3
 
 
 
 
BAK1
 
 
hsa-miR-125a-5p
 
 
-0.402
 
 
20.4
 
 
 
 
BAZ1B
 
 
hsa-miR-10a-5p
 
 
-0.309
 
 
21.9
 
 
 
 
BAZ2A
 
 
hsa-miR-99b-5p
 
 
-0.408
 
 
23.9
 
 
 
 
BBX
 
 
hsa-miR-10a-5p
 
 
-0.305
 
 
13.7
 
 
 
 
BCAP29
 
 
hsa-let-7e-5p
 
 
-0.289
 
 
29.3
 
 
 
 
BIN1
 
 
hsa-miR-22-3p
 
 
-0.346
 
 
89.3
 
 
 
 
BIN3
 
 
hsa-let-7e-5p
 
 
-0.427
 
 
13.2
 
 
 
 
BIN3
 
 
hsa-let-7c-5p
 
 
-0.427
 
 
13.2
 
 
 
 
BLOC1S1
 
 
hsa-miR-22-3p
 
 
-0.346
 
 
126.3
 
 
 
 
BLOC1S6
 
 
hsa-let-7e-5p
 
 
-0.321
 
 
26.7
 
 
 
 
BLOC1S6
 
 
hsa-let-7c-5p
 
 
-0.321
 
 
26.7
 
 
 
 
BLZF1
 
 
hsa-miR-125a-5p
 
 
-0.461
 
 
15.5
 
 
 
 
BMPR2
 
 
hsa-miR-99b-5p
 
 
-0.327
 
 
14.5
 
 
 
 
BTF3L4
 
 
hsa-let-7e-5p
 
 
-0.355
 
 
39.5
 
 
 
 
BTF3L4
 
 
hsa-let-7c-5p
 
 
-0.355
 
 
39.5
 
 
 
 
BZW1
 
 
hsa-let-7e-5p
 
 
-0.354
 
 
153.5
 
 
 
 
BZW1
 
 
hsa-let-7c-5p
 
 
-0.354
 
 
153.5
 
 
 
 
BZW1
 
 
hsa-let-7e-5p
 
 
-0.375
 
 
153.5
 
 
 
 
BZW1
 
 
hsa-let-7c-5p
 
 
-0.375
 
 
153.5
 
 
 
 
BZW2
 
 
hsa-let-7c-5p
 
 
-0.397
 
 
55.5
 
 
 
 
BZW2
 
 
hsa-let-7e-5p
 
 
-0.408
 
 
55.5
 
 
 
 
C15orf39
 
 
hsa-miR-125a-5p
 
 
-0.335
 
 
14.3
 
 
 
 
C15orf39
 
 
hsa-let-7e-5p
 
 
-0.374
 
 
14.3
 
 
 
 
C15orf39
 
 
hsa-let-7c-5p
 
 
-0.374
 
 
14.3
 
 
 
 
C1GALT1
 
 
hsa-let-7e-5p
 
 
-0.322
 
 
12.1
 
 
 
 
C1GALT1
 
 
hsa-let-7c-5p
 
 
-0.322
 
 
12.1
 
 
 
 
C5orf24
 
 
hsa-miR-22-3p
 
 
-0.493
 
 
20.7
 
 
 
 
C6orf47
 
 
hsa-miR-125a-5p
 
 
-0.564
 
 
16.6
 
 
 
 
CACFD1
 
 
hsa-let-7e-5p
 
 
-0.296
 
 
11.0
 
 
 
 
CACFD1
 
 
hsa-let-7c-5p
 
 
-0.296
 
 
11.0
 
 
 
 
CACNB3
 
 
hsa-miR-125a-5p
 
 
-0.393
 
 
12.0
 
 
 
 
CAMK2N1
 
 
hsa-miR-22-3p
 
 
-0.302
 
 
23.0
 
 
 
 
CANT1
 
 
hsa-let-7e-5p
 
 
-0.402
 
 
15.6
 
 
 
 
CANT1
 
 
hsa-let-7c-5p
 
 
-0.402
 
 
15.6
 
 
 
 
CAPN1
 
 
hsa-miR-22-3p
 
 
-0.307
 
 
78.1
 
 
 
 
CAPN3
 
 
hsa-let-7e-5p
 
 
-0.317
 
 
13.4
 
 
 
 
CAPN3
 
 
hsa-let-7c-5p
 
 
-0.317
 
 
13.4
 
 
 
 
CASP3
 
 
hsa-let-7e-5p
 
 
-0.495
 
 
22.0
 
 
 
 
CASP3
 
 
hsa-let-7c-5p
 
 
-0.503
 
 
22.0
 
 
 
 
 
 
 vagina 
 
 
 
 
target_symbol
 
 
mature_mirna_id
 
 
score
 
 
nTPM
 
 
 
 
 
 
ABCC5
 
 
hsa-miR-125a-5p
 
 
-0.288
 
 
35.3
 
 
 
 
ABHD17C
 
 
hsa-let-7e-5p
 
 
-0.359
 
 
21.1
 
 
 
 
ABHD17C
 
 
hsa-let-7c-5p
 
 
-0.359
 
 
21.1
 
 
 
 
ABHD3
 
 
hsa-miR-125a-5p
 
 
-0.453
 
 
12.7
 
 
 
 
ABTB1
 
 
hsa-miR-125a-5p
 
 
-0.519
 
 
32.4
 
 
 
 
ACP3
 
 
hsa-let-7e-5p
 
 
-0.298
 
 
10.8
 
 
 
 
ACP3
 
 
hsa-let-7c-5p
 
 
-0.298
 
 
10.8
 
 
 
 
ADIPOR2
 
 
hsa-miR-150-5p
 
 
-0.287
 
 
28.4
 
 
 
 
ADRB2
 
 
hsa-let-7e-5p
 
 
-0.635
 
 
12.2
 
 
 
 
ADRB2
 
 
hsa-let-7c-5p
 
 
-0.635
 
 
12.2
 
 
 
 
AIDA
 
 
hsa-miR-22-3p
 
 
-0.316
 
 
30.5
 
 
 
 
AKT3
 
 
hsa-miR-22-3p
 
 
-0.451
 
 
12.8
 
 
 
 
ALDH4A1
 
 
hsa-miR-184
 
 
-0.421
 
 
16.8
 
 
 
 
ANAPC16
 
 
hsa-miR-125a-5p
 
 
-0.578
 
 
14.7
 
 
 
 
ANKHD1-EIF4EBP3
 
 
hsa-miR-22-3p
 
 
-0.479
 
 
10.7
 
 
 
 
ANKRD13A
 
 
hsa-miR-22-3p
 
 
-0.478
 
 
11.8
 
 
 
 
ANPEP
 
 
hsa-miR-125a-5p
 
 
-0.372
 
 
13.0
 
 
 
 
AP1S1
 
 
hsa-let-7e-5p
 
 
-0.374
 
 
20.1
 
 
 
 
AP1S1
 
 
hsa-let-7c-5p
 
 
-0.374
 
 
20.1
 
 
 
 
ARG2
 
 
hsa-let-7e-5p
 
 
-0.468
 
 
11.0
 
 
 
 
ARG2
 
 
hsa-let-7c-5p
 
 
-0.468
 
 
11.0
 
 
 
 
ARL4D
 
 
hsa-let-7e-5p
 
 
-0.417
 
 
38.9
 
 
 
 
ARL4D
 
 
hsa-let-7c-5p
 
 
-0.417
 
 
38.9
 
 
 
 
ARPC5
 
 
hsa-miR-22-3p
 
 
-0.679
 
 
49.2
 
 
 
 
ARPP19
 
 
hsa-let-7e-5p
 
 
-0.492
 
 
27.0
 
 
 
 
ARPP19
 
 
hsa-let-7c-5p
 
 
-0.492
 
 
27.0
 
 
 
 
ARRB1
 
 
hsa-miR-22-3p
 
 
-0.616
 
 
10.2
 
 
 
 
ARRDC4
 
 
hsa-let-7e-5p
 
 
-0.329
 
 
10.1
 
 
 
 
ARRDC4
 
 
hsa-let-7c-5p
 
 
-0.329
 
 
10.1
 
 
 
 
ASAP1
 
 
hsa-let-7e-5p
 
 
-0.296
 
 
14.4
 
 
 
 
ASAP1
 
 
hsa-let-7c-5p
 
 
-0.308
 
 
14.4
 
 
 
 
ATOSB
 
 
hsa-let-7e-5p
 
 
-0.429
 
 
14.3
 
 
 
 
ATOSB
 
 
hsa-let-7c-5p
 
 
-0.429
 
 
14.3
 
 
 
 
ATP2A2
 
 
hsa-let-7e-5p
 
 
-0.311
 
 
40.2
 
 
 
 
ATP2A2
 
 
hsa-let-7c-5p
 
 
-0.311
 
 
40.2
 
 
 
 
ATPAF1
 
 
hsa-let-7e-5p
 
 
-0.498
 
 
15.7
 
 
 
 
ATPAF1
 
 
hsa-let-7c-5p
 
 
-0.498
 
 
15.7
 
 
 
 
ATXN2
 
 
hsa-miR-10a-5p
 
 
-0.362
 
 
17.8
 
 
 
 
B3GAT3
 
 
hsa-let-7e-5p
 
 
-0.324
 
 
28.4
 
 
 
 
B3GAT3
 
 
hsa-let-7c-5p
 
 
-0.324
 
 
28.4
 
 
 
 
B4GAT1
 
 
hsa-let-7e-5p
 
 
-0.47
 
 
15.8
 
 
 
 
B4GAT1
 
 
hsa-let-7c-5p
 
 
-0.47
 
 
15.8
 
 
 
 
BACH1
 
 
hsa-let-7e-5p
 
 
-0.298
 
 
12.6
 
 
 
 
BACH1
 
 
hsa-let-7e-5p
 
 
-0.298
 
 
12.6
 
 
 
 
BACH1
 
 
hsa-let-7c-5p
 
 
-0.306
 
 
12.6
 
 
 
 
BACH1
 
 
hsa-let-7c-5p
 
 
-0.306
 
 
12.6
 
 
 
 
BACH1
 
 
hsa-let-7e-5p
 
 
-0.435
 
 
12.6
 
 
 
 
BACH1
 
 
hsa-let-7e-5p
 
 
-0.435
 
 
12.6
 
 
 
 
BACH1
 
 
hsa-let-7c-5p
 
 
-0.435
 
 
12.6
 
 
 
 
BACH1
 
 
hsa-let-7c-5p
 
 
-0.435
 
 
12.6
 
 
 
 
BAG5
 
 
hsa-miR-22-3p
 
 
-0.318
 
 
12.4
 
 
 
 
BAK1
 
 
hsa-miR-125a-5p
 
 
-0.402
 
 
11.6
 
 
 
 
BAZ1B
 
 
hsa-miR-10a-5p
 
 
-0.309
 
 
16.5
 
 
 
 
BAZ2A
 
 
hsa-miR-99b-5p
 
 
-0.408
 
 
29.0
 
 
 
 
BCAP29
 
 
hsa-let-7e-5p
 
 
-0.289
 
 
25.4
 
 
 
 
BDH1
 
 
hsa-miR-125a-5p
 
 
-0.389
 
 
13.3
 
 
 
 
BIN1
 
 
hsa-miR-22-3p
 
 
-0.346
 
 
34.7
 
 
 
 
BIN3
 
 
hsa-let-7e-5p
 
 
-0.427
 
 
13.8
 
 
 
 
BIN3
 
 
hsa-let-7c-5p
 
 
-0.427
 
 
13.8
 
 
 
 
BLOC1S1
 
 
hsa-miR-22-3p
 
 
-0.346
 
 
103.3
 
 
 
 
BLOC1S6
 
 
hsa-let-7e-5p
 
 
-0.321
 
 
15.7
 
 
 
 
BLOC1S6
 
 
hsa-let-7c-5p
 
 
-0.321
 
 
15.7
 
 
 
 
BLZF1
 
 
hsa-miR-125a-5p
 
 
-0.461
 
 
15.2
 
 
 
 
BTF3L4
 
 
hsa-let-7e-5p
 
 
-0.355
 
 
27.9
 
 
 
 
BTF3L4
 
 
hsa-let-7c-5p
 
 
-0.355
 
 
27.9
 
 
 
 
BTG1
 
 
hsa-miR-22-3p
 
 
-0.468
 
 
10.6
 
 
 
 
BZW1
 
 
hsa-let-7e-5p
 
 
-0.354
 
 
58.1
 
 
 
 
BZW1
 
 
hsa-let-7c-5p
 
 
-0.354
 
 
58.1
 
 
 
 
BZW1
 
 
hsa-let-7e-5p
 
 
-0.375
 
 
58.1
 
 
 
 
BZW1
 
 
hsa-let-7c-5p
 
 
-0.375
 
 
58.1
 
 
 
 
BZW2
 
 
hsa-let-7c-5p
 
 
-0.397
 
 
27.5
 
 
 
 
BZW2
 
 
hsa-let-7e-5p
 
 
-0.408
 
 
27.5
 
 
 
 
C15orf39
 
 
hsa-miR-125a-5p
 
 
-0.335
 
 
13.6
 
 
 
 
C15orf39
 
 
hsa-let-7e-5p
 
 
-0.374
 
 
13.6
 
 
 
 
C15orf39
 
 
hsa-let-7c-5p
 
 
-0.374
 
 
13.6
 
 
 
 
C2orf88
 
 
hsa-let-7e-5p
 
 
-0.429
 
 
11.1
 
 
 
 
C2orf88
 
 
hsa-let-7c-5p
 
 
-0.429
 
 
11.1
 
 
 
 
C5orf24
 
 
hsa-miR-22-3p
 
 
-0.493
 
 
15.6
 
 
 
 
C6orf47
 
 
hsa-miR-125a-5p
 
 
-0.564
 
 
12.4
 
 
 
 
CACNB3
 
 
hsa-miR-125a-5p
 
 
-0.393
 
 
19.2
 
 
 
 
CAMK2N1
 
 
hsa-miR-22-3p
 
 
-0.302
 
 
23.6
 
 
 
 
CANT1
 
 
hsa-let-7e-5p
 
 
-0.402
 
 
20.5
 
 
 
 
CANT1
 
 
hsa-let-7c-5p
 
 
-0.402
 
 
20.5
 
 
 
 
CAPN1
 
 
hsa-miR-22-3p
 
 
-0.307
 
 
112.9
 
 
 
 
CAPN3
 
 
hsa-let-7e-5p
 
 
-0.317
 
 
16.2
 
 
 
 
CAPN3
 
 
hsa-let-7c-5p
 
 
-0.317
 
 
16.2
 
 
 
 
CASP2
 
 
hsa-miR-125a-5p
 
 
-0.296
 
 
11.0
 
 
 
 
CASP3
 
 
hsa-let-7e-5p
 
 
-0.495
 
 
11.4
 
 
 
 
CASP3
 
 
hsa-let-7c-5p
 
 
-0.503
 
 
11.4
 
 
 
 
CAST
 
 
hsa-miR-150-5p
 
 
-0.354
 
 
175.3
 
 
 
 
CCDC47
 
 
hsa-miR-22-3p
 
 
-0.604
 
 
28.3
 
 
 
 
CCN1
 
 
hsa-miR-22-3p
 
 
-0.601
 
 
85.1
 
 
 
 
CCNY
 
 
hsa-let-7c-5p
 
 
-0.323
 
 
15.1
 
 
 
 
CCNY
 
 
hsa-let-7e-5p
 
 
-0.334
 
 
15.1
 
 
 
 
CDC34
 
 
hsa-let-7c-5p
 
 
-0.309
 
 
35.6
 
 
 
 
CDC34
 
 
hsa-let-7e-5p
 
 
-0.321
 
 
35.6
 
 
 
 
CDC34
 
 
hsa-let-7e-5p
 
 
-0.555
 
 
35.6
 
 
 
 
CDC34
 
 
hsa-let-7c-5p
 
 
-0.555
 
 
35.6
 
 
 
 
CDC37L1
 
 
hsa-miR-125a-5p
 
 
-0.401
 
 
10.2
 
 
 
 
CDC42BPG
 
 
hsa-miR-125a-5p
 
 
-0.344
 
 
12.1
 
 
 
 
 
 
 liver 
 
 
 
 
target_symbol
 
 
mature_mirna_id
 
 
score
 
 
nTPM
 
 
 
 
 
 
ABHD3
 
 
hsa-miR-125a-5p
 
 
-0.453
 
 
36.7
 
 
 
 
ABHD6
 
 
hsa-miR-125a-5p
 
 
-0.501
 
 
60.7
 
 
 
 
ABTB1
 
 
hsa-miR-125a-5p
 
 
-0.519
 
 
18.5
 
 
 
 
ADIPOR2
 
 
hsa-miR-150-5p
 
 
-0.287
 
 
77.7
 
 
 
 
ADRB2
 
 
hsa-let-7e-5p
 
 
-0.635
 
 
13.3
 
 
 
 
ADRB2
 
 
hsa-let-7c-5p
 
 
-0.635
 
 
13.3
 
 
 
 
AGGF1
 
 
hsa-miR-125a-5p
 
 
-0.331
 
 
12.9
 
 
 
 
AGMAT
 
 
hsa-miR-22-3p
 
 
-0.294
 
 
58.0
 
 
 
 
AHCTF1
 
 
hsa-let-7e-5p
 
 
-0.374
 
 
10.2
 
 
 
 
AHCTF1
 
 
hsa-let-7c-5p
 
 
-0.374
 
 
10.2
 
 
 
 
AIDA
 
 
hsa-miR-22-3p
 
 
-0.316
 
 
45.8
 
 
 
 
AIFM1
 
 
hsa-miR-125a-5p
 
 
-0.3
 
 
77.0
 
 
 
 
AIFM1
 
 
hsa-let-7e-5p
 
 
-0.314
 
 
77.0
 
 
 
 
AIFM1
 
 
hsa-let-7c-5p
 
 
-0.314
 
 
77.0
 
 
 
 
ALDH4A1
 
 
hsa-miR-184
 
 
-0.421
 
 
438.6
 
 
 
 
ALPL
 
 
hsa-miR-10a-5p
 
 
-0.32
 
 
52.2
 
 
 
 
AMT
 
 
hsa-let-7e-5p
 
 
-0.463
 
 
52.5
 
 
 
 
AMT
 
 
hsa-let-7c-5p
 
 
-0.463
 
 
52.5
 
 
 
 
ANAPC16
 
 
hsa-miR-125a-5p
 
 
-0.578
 
 
25.2
 
 
 
 
ANKRD13A
 
 
hsa-miR-22-3p
 
 
-0.478
 
 
14.3
 
 
 
 
ANKRD46
 
 
hsa-let-7e-5p
 
 
-0.389
 
 
38.9
 
 
 
 
ANKRD46
 
 
hsa-let-7c-5p
 
 
-0.389
 
 
38.9
 
 
 
 
ANPEP
 
 
hsa-miR-125a-5p
 
 
-0.372
 
 
256.4
 
 
 
 
AP1AR
 
 
hsa-miR-99b-5p
 
 
-0.823
 
 
19.1
 
 
 
 
AP1S1
 
 
hsa-let-7e-5p
 
 
-0.374
 
 
39.1
 
 
 
 
AP1S1
 
 
hsa-let-7c-5p
 
 
-0.374
 
 
39.1
 
 
 
 
APOC4
 
 
hsa-miR-125a-5p
 
 
-0.515
 
 
585.9
 
 
 
 
ARHGEF26
 
 
hsa-miR-22-3p
 
 
-0.374
 
 
14.5
 
 
 
 
ARL4D
 
 
hsa-let-7e-5p
 
 
-0.417
 
 
55.4
 
 
 
 
ARL4D
 
 
hsa-let-7c-5p
 
 
-0.417
 
 
55.4
 
 
 
 
ARMT1
 
 
hsa-let-7e-5p
 
 
-0.303
 
 
35.2
 
 
 
 
ARMT1
 
 
hsa-let-7c-5p
 
 
-0.303
 
 
35.2
 
 
 
 
ARPC5
 
 
hsa-miR-22-3p
 
 
-0.679
 
 
60.1
 
 
 
 
ARPP19
 
 
hsa-let-7e-5p
 
 
-0.492
 
 
39.5
 
 
 
 
ARPP19
 
 
hsa-let-7c-5p
 
 
-0.492
 
 
39.5
 
 
 
 
ARRDC4
 
 
hsa-let-7e-5p
 
 
-0.329
 
 
28.3
 
 
 
 
ARRDC4
 
 
hsa-let-7c-5p
 
 
-0.329
 
 
28.3
 
 
 
 
ATOSB
 
 
hsa-let-7e-5p
 
 
-0.429
 
 
10.9
 
 
 
 
ATOSB
 
 
hsa-let-7c-5p
 
 
-0.429
 
 
10.9
 
 
 
 
ATP2A2
 
 
hsa-let-7e-5p
 
 
-0.311
 
 
49.3
 
 
 
 
ATP2A2
 
 
hsa-let-7c-5p
 
 
-0.311
 
 
49.3
 
 
 
 
ATPAF1
 
 
hsa-let-7e-5p
 
 
-0.498
 
 
101.4
 
 
 
 
ATPAF1
 
 
hsa-let-7c-5p
 
 
-0.498
 
 
101.4
 
 
 
 
ATXN2
 
 
hsa-miR-10a-5p
 
 
-0.362
 
 
19.6
 
 
 
 
B3GAT3
 
 
hsa-let-7e-5p
 
 
-0.324
 
 
53.3
 
 
 
 
B3GAT3
 
 
hsa-let-7c-5p
 
 
-0.324
 
 
53.3
 
 
 
 
B4GAT1
 
 
hsa-let-7e-5p
 
 
-0.47
 
 
18.5
 
 
 
 
B4GAT1
 
 
hsa-let-7c-5p
 
 
-0.47
 
 
18.5
 
 
 
 
BACH1
 
 
hsa-let-7e-5p
 
 
-0.298
 
 
28.5
 
 
 
 
BACH1
 
 
hsa-let-7e-5p
 
 
-0.298
 
 
28.5
 
 
 
 
BACH1
 
 
hsa-let-7c-5p
 
 
-0.306
 
 
28.5
 
 
 
 
BACH1
 
 
hsa-let-7c-5p
 
 
-0.306
 
 
28.5
 
 
 
 
BACH1
 
 
hsa-let-7e-5p
 
 
-0.435
 
 
28.5
 
 
 
 
BACH1
 
 
hsa-let-7e-5p
 
 
-0.435
 
 
28.5
 
 
 
 
BACH1
 
 
hsa-let-7c-5p
 
 
-0.435
 
 
28.5
 
 
 
 
BACH1
 
 
hsa-let-7c-5p
 
 
-0.435
 
 
28.5
 
 
 
 
BAG5
 
 
hsa-miR-22-3p
 
 
-0.318
 
 
11.1
 
 
 
 
BAK1
 
 
hsa-miR-125a-5p
 
 
-0.402
 
 
10.9
 
 
 
 
BAZ1B
 
 
hsa-miR-10a-5p
 
 
-0.309
 
 
22.6
 
 
 
 
BAZ2A
 
 
hsa-miR-99b-5p
 
 
-0.408
 
 
20.5
 
 
 
 
BCAP29
 
 
hsa-let-7e-5p
 
 
-0.289
 
 
31.8
 
 
 
 
BCL2L11
 
 
hsa-miR-10a-5p
 
 
-0.483
 
 
12.9
 
 
 
 
BCL2L12
 
 
hsa-miR-125a-5p
 
 
-0.288
 
 
14.0
 
 
 
 
BDH1
 
 
hsa-miR-125a-5p
 
 
-0.389
 
 
302.0
 
 
 
 
BIN1
 
 
hsa-miR-22-3p
 
 
-0.346
 
 
33.9
 
 
 
 
BIN3
 
 
hsa-let-7e-5p
 
 
-0.427
 
 
14.7
 
 
 
 
BIN3
 
 
hsa-let-7c-5p
 
 
-0.427
 
 
14.7
 
 
 
 
BLOC1S1
 
 
hsa-miR-22-3p
 
 
-0.346
 
 
217.0
 
 
 
 
BLOC1S6
 
 
hsa-let-7e-5p
 
 
-0.321
 
 
25.1
 
 
 
 
BLOC1S6
 
 
hsa-let-7c-5p
 
 
-0.321
 
 
25.1
 
 
 
 
BLZF1
 
 
hsa-miR-125a-5p
 
 
-0.461
 
 
13.9
 
 
 
 
BMPR2
 
 
hsa-miR-99b-5p
 
 
-0.327
 
 
12.0
 
 
 
 
BORCS6
 
 
hsa-miR-125a-5p
 
 
-0.287
 
 
11.0
 
 
 
 
BTF3L4
 
 
hsa-let-7e-5p
 
 
-0.355
 
 
38.1
 
 
 
 
BTF3L4
 
 
hsa-let-7c-5p
 
 
-0.355
 
 
38.1
 
 
 
 
BZW1
 
 
hsa-let-7e-5p
 
 
-0.354
 
 
212.2
 
 
 
 
BZW1
 
 
hsa-let-7c-5p
 
 
-0.354
 
 
212.2
 
 
 
 
BZW1
 
 
hsa-let-7e-5p
 
 
-0.375
 
 
212.2
 
 
 
 
BZW1
 
 
hsa-let-7c-5p
 
 
-0.375
 
 
212.2
 
 
 
 
BZW2
 
 
hsa-let-7c-5p
 
 
-0.397
 
 
17.2
 
 
 
 
BZW2
 
 
hsa-let-7e-5p
 
 
-0.408
 
 
17.2
 
 
 
 
C1GALT1
 
 
hsa-let-7e-5p
 
 
-0.322
 
 
20.5
 
 
 
 
C1GALT1
 
 
hsa-let-7c-5p
 
 
-0.322
 
 
20.5
 
 
 
 
C5orf24
 
 
hsa-miR-22-3p
 
 
-0.493
 
 
40.4
 
 
 
 
C6orf47
 
 
hsa-miR-125a-5p
 
 
-0.564
 
 
10.3
 
 
 
 
CACFD1
 
 
hsa-let-7e-5p
 
 
-0.296
 
 
56.6
 
 
 
 
CACFD1
 
 
hsa-let-7c-5p
 
 
-0.296
 
 
56.6
 
 
 
 
CAMK2N1
 
 
hsa-miR-22-3p
 
 
-0.302
 
 
50.9
 
 
 
 
CANT1
 
 
hsa-let-7e-5p
 
 
-0.402
 
 
16.0
 
 
 
 
CANT1
 
 
hsa-let-7c-5p
 
 
-0.402
 
 
16.0
 
 
 
 
CAPN1
 
 
hsa-miR-22-3p
 
 
-0.307
 
 
42.0
 
 
 
 
CAPN3
 
 
hsa-let-7e-5p
 
 
-0.317
 
 
41.4
 
 
 
 
CAPN3
 
 
hsa-let-7c-5p
 
 
-0.317
 
 
41.4
 
 
 
 
CARNMT1
 
 
hsa-let-7e-5p
 
 
-0.307
 
 
16.0
 
 
 
 
CARNMT1
 
 
hsa-let-7c-5p
 
 
-0.307
 
 
16.0
 
 
 
 
CASP3
 
 
hsa-let-7e-5p
 
 
-0.495
 
 
19.8
 
 
 
 
CASP3
 
 
hsa-let-7c-5p
 
 
-0.503
 
 
19.8
 
 
 
 
CAST
 
 
hsa-miR-150-5p
 
 
-0.354
 
 
109.3
 
 
 
 
CCDC47
 
 
hsa-miR-22-3p
 
 
-0.604
 
 
92.5
 
 
 
 
CCDC71L
 
 
hsa-miR-22-3p
 
 
-0.32
 
 
14.3
 
 
 
 
 
 
 epididymis 
 
 
 
 
target_symbol
 
 
mature_mirna_id
 
 
score
 
 
nTPM
 
 
 
 
 
 
ABHD6
 
 
hsa-miR-125a-5p
 
 
-0.501
 
 
13.9
 
 
 
 
ACP3
 
 
hsa-let-7e-5p
 
 
-0.298
 
 
16.3
 
 
 
 
ACP3
 
 
hsa-let-7c-5p
 
 
-0.298
 
 
16.3
 
 
 
 
ADAM17
 
 
hsa-miR-125a-5p
 
 
-0.292
 
 
10.4
 
 
 
 
AGGF1
 
 
hsa-miR-125a-5p
 
 
-0.331
 
 
20.2
 
 
 
 
AIDA
 
 
hsa-miR-22-3p
 
 
-0.316
 
 
52.9
 
 
 
 
ANAPC16
 
 
hsa-miR-125a-5p
 
 
-0.578
 
 
20.9
 
 
 
 
ANKHD1-EIF4EBP3
 
 
hsa-miR-22-3p
 
 
-0.479
 
 
18.5
 
 
 
 
ANKRD46
 
 
hsa-let-7e-5p
 
 
-0.389
 
 
15.4
 
 
 
 
ANKRD46
 
 
hsa-let-7c-5p
 
 
-0.389
 
 
15.4
 
 
 
 
AP1S1
 
 
hsa-let-7e-5p
 
 
-0.374
 
 
16.0
 
 
 
 
AP1S1
 
 
hsa-let-7c-5p
 
 
-0.374
 
 
16.0
 
 
 
 
ARMT1
 
 
hsa-let-7e-5p
 
 
-0.303
 
 
15.2
 
 
 
 
ARMT1
 
 
hsa-let-7c-5p
 
 
-0.303
 
 
15.2
 
 
 
 
ARPC5
 
 
hsa-miR-22-3p
 
 
-0.679
 
 
14.8
 
 
 
 
ARPP19
 
 
hsa-let-7e-5p
 
 
-0.492
 
 
34.2
 
 
 
 
ARPP19
 
 
hsa-let-7c-5p
 
 
-0.492
 
 
34.2
 
 
 
 
ARRDC4
 
 
hsa-let-7e-5p
 
 
-0.329
 
 
14.7
 
 
 
 
ARRDC4
 
 
hsa-let-7c-5p
 
 
-0.329
 
 
14.7
 
 
 
 
ASAP1
 
 
hsa-let-7e-5p
 
 
-0.296
 
 
11.5
 
 
 
 
ASAP1
 
 
hsa-let-7c-5p
 
 
-0.308
 
 
11.5
 
 
 
 
ATP2A2
 
 
hsa-let-7e-5p
 
 
-0.311
 
 
36.9
 
 
 
 
ATP2A2
 
 
hsa-let-7c-5p
 
 
-0.311
 
 
36.9
 
 
 
 
ATP8A1
 
 
hsa-miR-22-3p
 
 
-0.468
 
 
11.5
 
 
 
 
ATPAF1
 
 
hsa-let-7e-5p
 
 
-0.498
 
 
33.7
 
 
 
 
ATPAF1
 
 
hsa-let-7c-5p
 
 
-0.498
 
 
33.7
 
 
 
 
ATXN2
 
 
hsa-miR-10a-5p
 
 
-0.362
 
 
13.1
 
 
 
 
B3GAT3
 
 
hsa-let-7e-5p
 
 
-0.324
 
 
11.2
 
 
 
 
B3GAT3
 
 
hsa-let-7c-5p
 
 
-0.324
 
 
11.2
 
 
 
 
B4GAT1
 
 
hsa-let-7e-5p
 
 
-0.47
 
 
14.3
 
 
 
 
B4GAT1
 
 
hsa-let-7c-5p
 
 
-0.47
 
 
14.3
 
 
 
 
BAG5
 
 
hsa-miR-22-3p
 
 
-0.318
 
 
11.8
 
 
 
 
BAZ1B
 
 
hsa-miR-10a-5p
 
 
-0.309
 
 
16.7
 
 
 
 
BAZ2A
 
 
hsa-miR-99b-5p
 
 
-0.408
 
 
21.6
 
 
 
 
BBX
 
 
hsa-miR-10a-5p
 
 
-0.305
 
 
36.4
 
 
 
 
BCAP29
 
 
hsa-let-7e-5p
 
 
-0.289
 
 
18.0
 
 
 
 
BIN1
 
 
hsa-miR-22-3p
 
 
-0.346
 
 
15.8
 
 
 
 
BLOC1S1
 
 
hsa-miR-22-3p
 
 
-0.346
 
 
147.0
 
 
 
 
BLOC1S6
 
 
hsa-let-7e-5p
 
 
-0.321
 
 
17.3
 
 
 
 
BLOC1S6
 
 
hsa-let-7c-5p
 
 
-0.321
 
 
17.3
 
 
 
 
BLZF1
 
 
hsa-miR-125a-5p
 
 
-0.461
 
 
13.5
 
 
 
 
BTF3L4
 
 
hsa-let-7e-5p
 
 
-0.355
 
 
28.9
 
 
 
 
BTF3L4
 
 
hsa-let-7c-5p
 
 
-0.355
 
 
28.9
 
 
 
 
BZW1
 
 
hsa-let-7e-5p
 
 
-0.354
 
 
55.1
 
 
 
 
BZW1
 
 
hsa-let-7c-5p
 
 
-0.354
 
 
55.1
 
 
 
 
BZW1
 
 
hsa-let-7e-5p
 
 
-0.375
 
 
55.1
 
 
 
 
BZW1
 
 
hsa-let-7c-5p
 
 
-0.375
 
 
55.1
 
 
 
 
C1orf210
 
 
hsa-miR-125a-5p
 
 
-0.584
 
 
12.2
 
 
 
 
C5orf24
 
 
hsa-miR-22-3p
 
 
-0.493
 
 
14.9
 
 
 
 
CAMK2N1
 
 
hsa-miR-22-3p
 
 
-0.302
 
 
36.0
 
 
 
 
CAPN1
 
 
hsa-miR-22-3p
 
 
-0.307
 
 
25.9
 
 
 
 
CAPN3
 
 
hsa-let-7e-5p
 
 
-0.317
 
 
154.5
 
 
 
 
CAPN3
 
 
hsa-let-7c-5p
 
 
-0.317
 
 
154.5
 
 
 
 
CAST
 
 
hsa-miR-150-5p
 
 
-0.354
 
 
46.1
 
 
 
 
CBX5
 
 
hsa-let-7e-5p
 
 
-0.307
 
 
14.1
 
 
 
 
CBX5
 
 
hsa-let-7c-5p
 
 
-0.307
 
 
14.1
 
 
 
 
CBX5
 
 
hsa-let-7e-5p
 
 
-0.472
 
 
14.1
 
 
 
 
CBX5
 
 
hsa-let-7c-5p
 
 
-0.472
 
 
14.1
 
 
 
 
CCDC47
 
 
hsa-miR-22-3p
 
 
-0.604
 
 
47.4
 
 
 
 
CCNY
 
 
hsa-let-7c-5p
 
 
-0.323
 
 
25.4
 
 
 
 
CCNY
 
 
hsa-let-7e-5p
 
 
-0.334
 
 
25.4
 
 
 
 
CDC34
 
 
hsa-let-7c-5p
 
 
-0.309
 
 
13.3
 
 
 
 
CDC34
 
 
hsa-let-7e-5p
 
 
-0.321
 
 
13.3
 
 
 
 
CDC34
 
 
hsa-let-7e-5p
 
 
-0.555
 
 
13.3
 
 
 
 
CDC34
 
 
hsa-let-7c-5p
 
 
-0.555
 
 
13.3
 
 
 
 
CDC37L1
 
 
hsa-miR-125a-5p
 
 
-0.401
 
 
13.0
 
 
 
 
CDC42SE1
 
 
hsa-miR-125a-5p
 
 
-0.501
 
 
25.3
 
 
 
 
CDKN1A
 
 
hsa-let-7e-5p
 
 
-0.434
 
 
38.8
 
 
 
 
CDKN1A
 
 
hsa-let-7c-5p
 
 
-0.434
 
 
38.8
 
 
 
 
CDV3
 
 
hsa-let-7e-5p
 
 
-0.453
 
 
50.7
 
 
 
 
CDV3
 
 
hsa-let-7c-5p
 
 
-0.461
 
 
50.7
 
 
 
 
CFAP36
 
 
hsa-miR-22-3p
 
 
-0.549
 
 
72.7
 
 
 
 
CLASP2
 
 
hsa-let-7e-5p
 
 
-0.344
 
 
10.4
 
 
 
 
CLASP2
 
 
hsa-let-7c-5p
 
 
-0.344
 
 
10.4
 
 
 
 
CLDN12
 
 
hsa-let-7e-5p
 
 
-0.511
 
 
18.7
 
 
 
 
CLDN12
 
 
hsa-let-7c-5p
 
 
-0.511
 
 
18.7
 
 
 
 
CLIC4
 
 
hsa-miR-22-3p
 
 
-0.594
 
 
79.9
 
 
 
 
CMTM6
 
 
hsa-miR-150-5p
 
 
-0.328
 
 
38.7
 
 
 
 
COIL
 
 
hsa-let-7e-5p
 
 
-0.618
 
 
10.9
 
 
 
 
COIL
 
 
hsa-let-7c-5p
 
 
-0.618
 
 
10.9
 
 
 
 
COL1A2
 
 
hsa-let-7e-5p
 
 
-0.499
 
 
58.2
 
 
 
 
COL1A2
 
 
hsa-let-7c-5p
 
 
-0.499
 
 
58.2
 
 
 
 
COL3A1
 
 
hsa-let-7e-5p
 
 
-0.474
 
 
57.6
 
 
 
 
COL3A1
 
 
hsa-let-7c-5p
 
 
-0.474
 
 
57.6
 
 
 
 
COPZ1
 
 
hsa-miR-125a-5p
 
 
-0.296
 
 
118.3
 
 
 
 
CPSF6
 
 
hsa-miR-125a-5p
 
 
-0.384
 
 
36.3
 
 
 
 
CREB1
 
 
hsa-miR-10a-5p
 
 
-0.416
 
 
10.2
 
 
 
 
CREB1
 
 
hsa-miR-22-3p
 
 
-0.424
 
 
10.2
 
 
 
 
CRISPLD2
 
 
hsa-miR-184
 
 
-0.293
 
 
19.2
 
 
 
 
CSNK1A1
 
 
hsa-miR-22-3p
 
 
-0.361
 
 
56.8
 
 
 
 
CSNK2A1
 
 
hsa-miR-125a-5p
 
 
-0.349
 
 
25.2
 
 
 
 
CTDSPL
 
 
hsa-miR-99b-5p
 
 
-0.728
 
 
13.2
 
 
 
 
CTNNBIP1
 
 
hsa-miR-10a-5p
 
 
-0.389
 
 
73.0
 
 
 
 
CYB561D1
 
 
hsa-let-7e-5p
 
 
-0.393
 
 
10.4
 
 
 
 
CYB561D1
 
 
hsa-let-7c-5p
 
 
-0.393
 
 
10.4
 
 
 
 
CYRIB
 
 
hsa-miR-22-3p
 
 
-0.628
 
 
19.9
 
 
 
 
DAZAP2
 
 
hsa-miR-125a-5p
 
 
-0.294
 
 
115.7
 
 
 
 
DCAF6
 
 
hsa-miR-150-5p
 
 
-0.437
 
 
18.4
 
 
 
 
DDB2
 
 
hsa-miR-125a-5p
 
 
-0.505
 
 
11.1
 
 
 
 
DDIT4
 
 
hsa-miR-22-3p
 
 
-0.649
 
 
11.9
 
 
 
 
 
 
 
 
 cd8_ev_vs_cd8_s1_upregulated - Tissue specific
ORA 
 
 all_genes 
 
 GO_Biological_Process_2025 
 This category comprises Gene Ontology (GO) Biological Process terms
describing coordinated and temporally ordered biological programs such
as cell cycle progression, immune activation, apoptosis,
differentiation, and metabolic regulation. GO Biological Process
annotations are curated by the Gene Ontology Consortium and integrate
experimental evidence, computational inference, and expert review.
Enrichment in this category indicates that the differentially expressed
target genes participate disproportionately in shared biological
programs rather than isolated molecular events. These results provide
high-level functional insight into systemic biological changes and are
particularly useful for interpreting phenotypic consequences of gene
expression alterations. 
 
 
 
 
Term
 
 
Overlap
 
 
P.value
 
 
Adjusted.P.value
 
 
Old.P.value
 
 
Old.Adjusted.P.value
 
 
Odds.Ratio
 
 
Combined.Score
 
 
Genes
 
 
 
 
 
 
Regulation of Transcription by RNA Polymerase II ( GO:0006357 )
 
 
448/2250
 
 
0.0000000
 
 
0.0000000
 
 
0
 
 
0
 
 
1.638844
 
 
61.32924
 
 
RB1;PHB1;MAML1;BACH2;DCAF6;ELK4;HOXA9;MYB;HOXA3;BBX;HOXA1;HOXA5;MEF2A;ZNF287;ZNF283;MED8;SOX13;ZNF10;RFX3;SOX11;DICER1;SOX12;ISL1;RFX6;BIN1;HOXB4;COMMD3-BMI1;HOXB3;HOXB2;ZSCAN25;HOXB1;HOXB8;CGGBP1;HOXB5;ZNF397;ZNF275;ZNF395;ZNF274;HOXD1;ZNF391;GATA6;ZNF23;MTDH;PPP3R1;GALR1;ZNF268;HOXC8;NKX2-4;RREB1;ZNF267;ZNF266;FZD5;FZD4;NR2F2;INO80;PBX1;ST18;NR2F6;FOSL2;TADA2B;MLLT10;HNRNPK;AGO2;SNAI1;HBP1;CPEB3;ZBTB8B;ZNF136;PHF3;ARF4;SETD3;ZNF250;TCF24;BHLHE41;TFCP2L1;CTCF;HIF3A;MECP2;IKBKB;MED14;SHH;NKRF;HEY2;ZNF367;ZNF366;SS18;ZNF362;HSBP1;EN2;TET2;TET1;FOS;BAZ1B;MED26;SIRT1;PAX2;NCOR2;MED21;PTRH2;PAX9;TET3;RARA;RARB;PPARG;SINHCAF;MDFI;JDP2;ZNF473;ZNF594;ZNF471;NFIX;ZNF470;ZNF518B;FOXS1;PLAGL2;ZNF469;TAF9B;ZNF225;ZNF462;ZNF583;PCGF5;USP9X;PCGF3;WNT7A;ZNF33B;NFIA;WT1;FOSB;ZNF699;CRLF3;ZNF697;CCNT2;UBE2D3;ZBTB26;UBE2D1;RORA;RORB;AHR;TNF;YY1;SALL1;ZFP30;SALL3;SALL4;NFATC2IP;ZNF449;CHP2;SMARCAD1;ZNF689;PITX2;ZNF566;ZNF322;TLE4;ZBTB39;FST;SKIDA1;ZBTB34;MITF;PIAS2;FOXP2;MAF;DMTF1;KAT6B;MAPKAPK5;ADNP;ERG;DLX1;CBFB;FOXO4;FOXO3;MACROH2A1;RLF;NPAS3;GLIS2;NEUROD1;TPR;ZKSCAN3;SAFB2;DVL3;NDN;ZKSCAN4;NDP;SKIL;ZKSCAN1;ZNF784;ZNF420;ZBTB18;ESRRA;AKIRIN1;ZBTB16;FOXN4;ESRRG;SCRT1;CXCL10;CDK8;MAFB;ZFP62;COPS2;LHX1;MAFG;SP5;TADA1;SP8;MDM4;MAFK;ZNF774;NFE2L1;ZNF652;LHX8;ONECUT1;PTEN;FASLG;ZFP82;CDC73;TRO;DNMT3B;ZNF644;ACTR2;EMX2;PPP1R12A;CHUK;DNMT3A;ZFP91;TNFRSF1A;DUSP22;TFDP1;TFDP2;HAND2;HAND1;ZNF879;ITGA6;SOS1;CRK;HDAC4;CEBPD;ZBTB47;PLAG1;WBP2;CEBPG;LRP5;FOXK2;PPM1D;STOX2;DNAJB4;E2F3;RBBP7;E2F5;FNIP1;E2F6;ZNF620;FNIP2;E2F7;ZNF740;DRGX;JAZF1;H2AZ1;RNF20;PRRX1;IRX5;FBXL19;FOXJ2;TBX5;KLF4;TBX4;KLF3;KLF8;SLC25A37;KLF6;KLF5;KLF9;CCM2;INSM2;BCOR;TCERG1;APP;CCNK;CCNH;CCNC;IKZF5;RPS6KA3;ZNF608;DPF2;EPC1;EPC2;SOX9;SOX5;ACVR1;PDHX;EBF2;OVOL1;THAP1;RUNX1;SUB1;ZNF710;TP53;PIK3R1;ZBTB5;ZBTB4;MED12L;NHLH2;MIER1;ZNF821;ZBED4;EGR3;FOXF1;GADD45A;SS18L1;LIF;IGF1;FUBP1;ID1;ID4;CREBRF;ID3;MKX;RCOR3;SLC2A4RG;RCOR1;CSRNP1;BARHL2;SUV39H2;TENM2;SUV39H1;HNF4G;MIA3;MEOX2;NCOA1;SREBF1;FOXD3;TSC22D4;EDN1;FOXD1;IL13;NOG;TFEB;ZHX1;RGMA;NR5A2;TFEC;NCOA7;ZNF780B;VGLL2;YAP1;SATB1;SATB2;PURA;ZFP14;MNT;HDX;VEGFA;NR6A1;NEDD4;ZNF354A;ZNF354B;FOXA1;PRDM4;PRDM5;WWC2;IHH;ARID4B;NR3C1;LOXL2;AMOT;HOXA11;HOXA10;CCND1;EP300;ARID2;MAGEB10;LMO1;NANOS1;EOMES;MLIP;LMO3;MYCN;LMX1A;TBL1XR1;PPID;OTX2;SMAD2;PRNP;TFAP2C;TFAP2D;UBE2I;CBX5;TGFB1;CBX4;SMURF2;WNT3A;CBX2;HMGA1;HMGA2;ESR1;SMAD5;BMP5;SMAD7;NR4A2;BHLHE40;JMY;MXD1;DDX3X;YWHAB;HMGB2;SIX1;YBX1;TRIAP1;BCL7A;ZIC1;MYBL2;KDM6B;KLF10;KLF11;EED;SAFB;KLF14;ETV4;ARID1B;KLF15;SENP1;GREM1;ELF2;CREB1;IFNG;ELF4;ELF5;IRF2;CREB5;KDM3A;MOSPD1;DLL4;TBX21;SAP30L;VHL;MTA3;TCF7L2;USP21;ATP2B4;PEDS1;ARID3A;ARID3B;ACVR2B;REST;MEIS1;POLE3;PMP22;ERCC6;TAF5;TAF4
 
 
 
 
Negative Regulation of DNA-templated Transcription ( GO:0045892 )
 
 
232/1006
 
 
0.0000000
 
 
0.0000000
 
 
0
 
 
0
 
 
1.929425
 
 
67.08952
 
 
RB1;APP;TCERG1;PHB1;BACH2;IKZF5;ELK4;ZNF608;MYB;DPF2;EPC1;SOX9;MEF2A;RBFOX2;ZNF282;SFMBT1;SOX13;RFX3;SOX11;DICER1;SOX12;ISL1;THAP1;DKK3;RUNX1;SFRP1;BIN1;WDR82;COMMD3-BMI1;TP53;CGGBP1;ZNF397;ZNF274;GATA6;PDGFB;ZBTB5;ZBTB4;MTDH;MBTD1;MIER1;ZNF268;RREB1;SNX6;XRCC5;GADD45A;NR2F2;NR2F6;HNRNPK;ID1;ID4;SNAI1;CREBRF;ID3;RCOR3;CPEB3;ZNF136;RCOR1;SUV39H2;TENM2;SUV39H1;BHLHE41;CTCF;PTPRK;LIMD1;MECP2;SHH;SCML2;ZMYM2;QARS1;NKRF;HEY2;ZNF366;FOXD3;TSC22D4;EDN1;FOXD1;HSBP1;NOG;TET1;SIRT1;ZHX1;CBFA2T3;PAX2;NCOR2;MDFIC;PAX9;TFEC;RARA;RARB;PPARG;SINHCAF;MDFI;JDP2;YAP1;NFIX;SATB1;CIPC;PURA;FOXS1;MNT;BTAF1;ZNF469;TAF9B;USP9X;VEGFA;NR6A1;WT1;NEDD4;RLIM;FOSB;LIMS1;CDK5R1;ZNF354B;UBE2D3;ZBTB26;PRDM5;WWC2;UBE2D1;ARID4B;RORB;AHR;NR3C1;TNF;LOXL2;AMOT;YY1;SALL1;CCND1;CAPN3;EP300;PITX2;MAF1;MAGEB10;EOMES;TLE4;ZBTB39;MLIP;FST;KCTD1;ZBTB34;MITF;FOXP2;MAF;PSPC1;KAT6B;TBL1XR1;PPID;DLX1;CBFB;FOXO3;MACROH2A1;GLIS2;TPR;ZKSCAN3;NDN;SKIL;WNT4;ZBTB18;SMAD2;PRNP;UBE2I;CBX5;TGFB1;CBX4;SMURF2;CBX3;ZBTB16;CBX2;HMGA1;HMGA2;ESR1;SCRT1;SMAD7;NR4A2;GCLC;CNOT7;MAFB;COPS2;LHX1;MAFG;BHLHE40;BCL3;MDM4;MAFK;MXD1;NFE2L1;ZNF512B;HMGB2;FASLG;CDC73;TRO;BCL7A;YWHAQ;DNMT3B;YAF2;KLF10;KLF11;EED;DNMT3A;DUSP22;CREB1;ELF2;TFDP2;IFNG;HAND1;IRF2;ZMYND11;HDAC4;FOXK2;MOSPD1;PBXIP1;PPM1F;DLL4;TBX21;DNAJB4;SAP30L;VHL;RBBP7;FNIP1;E2F6;MTA3;FNIP2;E2F7;JAZF1;TCF7L2;KLF4;ACVR2B;KLF8;REST;POLE3;INSM2;BCOR
 
 
 
 
Regulation of DNA-templated Transcription ( GO:0006355 )
 
 
417/2139
 
 
0.0000000
 
 
0.0000000
 
 
0
 
 
0
 
 
1.583599
 
 
49.37327
 
 
RB1;PHB1;BACH2;ELK4;HOXA9;MYB;HOXA3;BBX;HOXA1;HOXA5;ZNF287;RBFOX2;ZNF283;ZNF282;MED8;SOX13;ZNF10;RFX3;DKK3;RFX6;HOXB3;HOXB2;ZSCAN25;HOXB1;HOXB8;HOXB5;ZNF397;ZNF275;ZNF395;ZNF274;HOXD1;ZNF391;GATA6;PDGFB;ZNF23;MTDH;ZNF268;HOXC8;NKX2-4;RREB1;ZNF267;ZNF266;FZD4;NR2F2;INO80;FOSL2;COL1A1;MLLT11;TADA2B;MLLT10;HNRNPK;SNAI1;HBP1;ZBTB8B;ZNF136;PHF3;LIN54;SETD3;ZNF250;TCF24;BHLHE41;TFCP2L1;CTCF;HIF3A;MECP2;IKBKB;ING5;MED14;SHH;EDRF1;NKRF;ZNF280B;ZNF280C;HEY2;ZNF367;SS18;ZNF362;EN2;FOS;ASH1L;BAZ1B;MED26;SIRT1;PAX2;NCOR2;MED21;PAX9;RARA;PPARG;MDFI;JDP2;ZNF473;ZNF594;ZNF471;NFIX;ZNF470;CIPC;ZNF518B;FOXS1;BTAF1;PLAGL2;ZNF225;ZNF341;ZNF583;PCGF5;PCGF3;WNT7A;GTF2H1;ZNF33B;NFIA;WT1;RLIM;FOSB;ZNF699;CRLF3;ZNF697;LIMS1;RORA;RORB;AHR;TNF;YY1;SALL1;ZFP30;SALL3;SALL4;ZNF449;SLC39A8;ZNF689;PITX2;ZNF566;ZNF322;MAP3K5;ZNF562;MAP2K1;SKIDA1;DYRK1B;MITF;PIAS2;FOXP2;MORF4L1;MAF;DMTF1;KAT6B;ADNP;ERG;DLX1;INO80D;CBFB;FOXO4;FOXO3;RLF;NPAS3;GLIS2;NEUROD1;ZKSCAN3;SAFB2;DVL3;ZKSCAN4;NDP;ZKSCAN1;ZNF784;ZNF420;ZBTB18;ESRRA;ZBTB16;FOXN4;ESRRG;ZBTB11;SCRT1;CNOT7;MAFB;ZFP62;LHX1;MAFG;BCL3;SP5;TADA1;SP8;MDM4;MAFK;ZNF774;NFE2L1;ZNF652;LHX8;ONECUT1;ZFP82;ZNF644;YAF2;EMX2;DNMT3A;ZFP91;TFDP1;TFDP2;ASPH;HAND2;HAND1;ZNF879;SOS1;CRK;HDAC4;PRKAA1;CEBPD;ZBTB47;PLAG1;WBP2;CEBPG;LRP5;FOXK2;PBXIP1;PPM1F;STOX2;TP53INP1;E2F3;RBBP7;E2F5;E2F6;ZNF620;E2F7;ZNF740;DRGX;RNF20;PRRX1;IRX5;FBXL19;FOXJ2;TBX5;KLF4;TBX4;KLF3;KLF8;SLC25A37;KLF6;MTRES1;KLF5;KLF9;POLR3F;BCOR;APP;CCNH;IKZF5;ZNF608;DPF2;EPC1;EPC2;SOX9;BTRC;SOX5;ACVR1;PDHX;SFMBT1;EBF2;OVOL1;THAP1;RUNX1;SFRP1;SUB1;ZNF710;TP53;IGSF1;ZBTB5;ZBTB4;MBTD1;SEC14L2;NHLH2;MIER1;ZNF821;SNX5;SNX6;EGR3;FOXF1;XRCC5;SS18L1;IGF1;GDF6;FUBP1;ID1;ID4;CREBRF;ID3;MKX;RCOR3;SLC2A4RG;RCOR1;CSRNP1;BARHL2;SUV39H2;SUV39H1;HNF4G;MIA3;PTPRK;LIMD1;MEOX2;NAT14;SCML2;ZMYM2;QARS1;IL10;NCOA1;SREBF1;FOXD3;TSC22D4;FOXD1;TFEB;ZHX1;CBFA2T3;MLF1;NR5A2;MDFIC;TFEC;NCOA7;ZNF780B;VGLL2;YAP1;MYCBP;SATB1;SATB2;PURA;ZFP14;MNT;APBB3;HDX;VEGFA;NR6A1;CAMK4;ZNF354A;CDK5R1;ZNF354B;CD86;FOXA1;PRDM5;WWC2;ARID4B;NR3C1;LOXL2;HOXA11;HOXA10;CAPN3;EP300;ARID2;NANOS1;EOMES;ATP1B4;KCTD1;MYCN;LMX1A;PSPC1;TBL1XR1;UBE2V1;TASP1;OTX2;WNT1;WNT4;SMAD2;TFAP2C;UBE2I;CBX5;TGFB1;CBX4;SMURF2;CBX3;WNT3A;HMGA1;HMGA2;ESR1;SMAD5;SMAD7;NR4A2;GCLC;BHLHE40;JMY;MXD1;CHD7;HMGB2;SIX1;YBX1;ELAVL2;BCL7A;YWHAQ;ZIC1;YWHAH;KLF10;KLF11;EED;SAFB;KLF14;ETV4;ARID1B;KLF15;TGFBR1;ELF2;CREB1;IFNG;ELF4;ELF5;IRF2;ZMYND11;CREB5;KDM3A;TBX21;SAP30L;VHL;MTA3;TCF7L2;USP21;ATP2B4;PEDS1;ARID3A;ARID3B;ACVR2B;REST;PITHD1;PMP22;ERCC6;NAA15;TAF5;TAF4
 
 
 
 
Positive Regulation of DNA-templated Transcription ( GO:0045893 )
 
 
267/1274
 
 
0.0000000
 
 
0.0000000
 
 
0
 
 
0
 
 
1.705911
 
 
47.37068
 
 
APP;TCERG1;CCNK;PHB1;MAML1;CCNC;DCAF6;ELK4;RPS6KA3;MYB;EPC1;EPC2;HOXA1;SOX9;BTRC;HOXA5;MEF2A;ACVR1;PDHX;RFX3;SOX11;SOX12;ISL1;RUNX1;SFRP1;RFX6;HOXB4;HOXB3;HOXB2;HOXB1;TP53;ZNF395;GATA6;PDGFB;PIK3R1;MED12L;MBTD1;SEC14L2;NHLH2;PPP3R1;GALR1;ZNF268;RREB1;SNX5;ZBED4;FZD5;FOXF1;FZD4;SS18L1;LIF;NR2F2;IGF1;INO80;GDF6;PBX1;ST18;FOSL2;COL1A1;MLLT11;TADA2B;MLLT10;AGO2;SNAI1;CREBRF;ARF4;CSRNP1;SETD3;HNF4G;MIA3;CTCF;MEOX2;NAT14;IKBKB;EDRF1;MED14;SHH;NKRF;HEY2;IL10;NCOA1;SS18;FOXD3;SREBF1;EDN1;IL13;TET2;TFEB;TET1;FOS;BAZ1B;SIRT1;PAX2;RGMA;NR5A2;MDFIC;MED21;PTRH2;TET3;TFEC;RARA;RARB;PPARG;NCOA7;YAP1;NFIX;SATB2;ZNF462;PCGF5;WNT7A;GTF2H1;VEGFA;NFIA;WT1;CAMK4;CRLF3;CD86;FOXA1;CCNT2;PRDM4;PRDM5;IHH;RORA;RORB;AHR;NR3C1;TNF;HOXA11;YY1;HOXA10;SALL1;NFATC2IP;CHP2;CAPN3;EP300;SMARCAD1;PITX2;MAP3K5;EOMES;LMO1;NANOS1;MAP2K1;MLIP;LMO3;DYRK1B;MITF;MORF4L1;MYCN;LMX1A;KAT6B;TBL1XR1;MAPKAPK5;UBE2V1;ERG;DLX1;INO80D;FOXO4;FOXO3;RLF;GLIS2;NPAS3;NEUROD1;ZKSCAN3;DVL3;TASP1;OTX2;NDP;WNT1;WNT4;ZBTB18;ESRRA;SMAD2;TFAP2C;AKIRIN1;TGFB2;TFAP2D;TGFB1;WNT3A;ZBTB16;HMGA1;HMGA2;FOXN4;ESRRG;ESR1;SMAD5;BMP5;NR4A2;CXCL10;CDK8;MAFB;LHX1;BCL3;TADA1;NFE2L1;DDX3X;YWHAB;ONECUT1;PTEN;HMGB2;SIX1;TRIAP1;YBX1;CDC73;ZIC1;MYBL2;YAF2;YWHAH;KDM6B;KLF10;ACTR2;PPP1R12A;CHUK;KLF14;ETV4;ARID1B;KLF15;TGFBR1;TNFRSF1A;SENP1;GREM1;CREB1;ELF2;TFDP1;ASPH;TFDP2;IFNG;ELF4;HAND2;HAND1;IRF2;ITGA6;SOS1;CREB5;HDAC4;PRKAA1;KDM3A;PLAG1;WBP2;CEBPG;LRP5;FOXK2;MOSPD1;TBX21;TP53INP1;E2F3;VHL;RBBP7;MTA3;E2F7;H2AZ1;RNF20;TCF7L2;PRRX1;USP21;FOXJ2;TBX5;KLF4;APLN;REST;KLF6;MEIS1;KLF5;PITHD1;CCM2;ERCC6;NAA15;TAF5;TAF4
 
 
 
 
Negative Regulation of Transcription by RNA Polymerase II ( GO:0000122 )
 
 
172/732
 
 
0.0000000
 
 
0.0000000
 
 
0
 
 
0
 
 
1.956831
 
 
54.08506
 
 
RB1;APP;TCERG1;PHB1;UBE2D3;ZBTB26;PRDM5;WWC2;UBE2D1;ARID4B;NR3C1;TNF;BACH2;IKZF5;AMOT;LOXL2;ELK4;YY1;SALL1;ZNF608;CCND1;MYB;DPF2;EPC1;EP300;SOX9;PITX2;MAGEB10;MEF2A;TLE4;EOMES;ZBTB39;MLIP;FST;SOX13;SOX11;MITF;ZBTB34;DICER1;SOX12;ISL1;RUNX1;THAP1;MAF;BIN1;TBL1XR1;COMMD3-BMI1;TP53;CGGBP1;PPID;DLX1;ZNF274;CBFB;GATA6;FOXO3;ZBTB5;MACROH2A1;ZBTB4;MTDH;GLIS2;TPR;MIER1;ZKSCAN3;NDN;RREB1;ZNF268;SKIL;ZBTB18;PRNP;CBX5;UBE2I;CBX4;SMURF2;GADD45A;ZBTB16;CBX2;HMGA2;NR2F2;ESR1;SCRT1;NR2F6;SMAD7;NR4A2;MAFB;COPS2;MAFG;ID1;BHLHE40;SNAI1;CREBRF;ID4;ID3;MAFK;MDM4;CPEB3;MXD1;ZNF136;NFE2L1;SUV39H2;TENM2;SUV39H1;BHLHE41;HMGB2;FASLG;CTCF;CDC73;MECP2;TRO;SHH;HEY2;DNMT3B;ZNF366;KLF10;FOXD3;TSC22D4;KLF11;EDN1;HSBP1;EED;DNMT3A;NOG;TET1;SIRT1;ZHX1;NCOR2;DUSP22;IFNG;HAND1;TFEC;IRF2;RARA;RARB;PPARG;SINHCAF;JDP2;MDFI;HDAC4;YAP1;NFIX;SATB1;MOSPD1;DLL4;PURA;MNT;DNAJB4;SAP30L;TBX21;ZNF469;RBBP7;VHL;TAF9B;FNIP1;E2F6;FNIP2;MTA3;E2F7;JAZF1;TCF7L2;USP9X;KLF4;ACVR2B;VEGFA;KLF8;REST;NR6A1;WT1;NEDD4;POLE3;FOSB;INSM2;BCOR;ZNF354B
 
 
 
 
Positive Regulation of Transcription by RNA Polymerase II ( GO:0045944 )
 
 
216/983
 
 
0.0000000
 
 
0.0000000
 
 
0
 
 
0
 
 
1.802237
 
 
49.07945
 
 
APP;TCERG1;CCNK;MAML1;CCNC;DCAF6;ELK4;RPS6KA3;MYB;EPC1;HOXA1;SOX9;HOXA5;MEF2A;ACVR1;PDHX;MED8;RFX3;MED9;SOX11;SOX12;ISL1;RUNX1;RFX6;HOXB4;HOXB3;HOXB2;HOXB1;TP53;ZNF395;MEPCE;GATA6;PIK3R1;MED12L;NHLH2;PPP3R1;GALR1;ZNF268;RREB1;ZBED4;FZD5;FOXF1;FZD4;SS18L1;LIF;NR2F2;IGF1;INO80;PBX1;ST18;FOSL2;MLLT10;AGO2;CREBRF;ARF4;CSRNP1;SETD3;HNF4G;MIA3;CTCF;MEOX2;IKBKB;MED14;SHH;NKRF;HEY2;NCOA1;SS18;FOXD3;SREBF1;EDN1;IL13;TET2;TFEB;TET1;FOS;BAZ1B;MED26;SIRT1;PAX2;MED28;RGMA;NR5A2;MED21;PTRH2;TET3;TFEC;RARA;RARB;PPARG;NCOA7;YAP1;NFIX;SATB2;ZNF462;PCGF5;WNT7A;VEGFA;NFIA;WT1;CRLF3;FOXA1;CCNT2;PRDM4;PRDM5;IHH;RORA;RORB;AHR;NR3C1;TNF;YY1;HOXA10;SALL1;NFATC2IP;CHP2;EP300;SMARCAD1;PITX2;EOMES;LMO1;NANOS1;MLIP;LMO3;MITF;MYCN;LMX1A;TBL1XR1;MAPKAPK5;ERG;DLX1;FOXO4;FOXO3;RLF;GLIS2;NEUROD1;DVL3;OTX2;NDP;ZBTB18;ESRRA;SMAD2;TFAP2C;AKIRIN1;TFAP2D;TGFB1;WNT3A;HMGA1;HMGA2;FOXN4;ESRRG;ESR1;SMAD5;BMP5;NR4A2;CXCL10;CDK8;NFE2L1;DDX3X;YWHAB;ONECUT1;PTEN;HMGB2;SIX1;TRIAP1;YBX1;CDC73;MYBL2;KDM6B;KLF10;ACTR2;PPP1R12A;CHUK;KLF14;ETV4;KLF15;TNFRSF1A;SENP1;GREM1;CREB1;TFDP1;TFDP2;IFNG;ELF4;HAND2;HAND1;IRF2;ITGA6;SOS1;HDAC4;PLAG1;WBP2;CEBPG;LRP5;FOXK2;MOSPD1;TBX21;E2F3;E2F7;H2AZ1;RNF20;TCF7L2;PRRX1;USP21;TAF11;FOXJ2;TBX5;KLF4;REST;KLF6;MEIS1;KLF5;CCM2;ERCC6;TAF5;TAF4
 
 
 
 
Negative Regulation of Gene Expression ( GO:0010629 )
 
 
101/364
 
 
0.0000000
 
 
0.0000000
 
 
0
 
 
0
 
 
2.424469
 
 
64.60121
 
 
RB1;APP;FMR1;TNF;CRKL;YY1;ENDOU;CHEK1;EPC1;SH3GL2;NANOS1;FBXW7;ADAM10;SOX11;DICER1;SFRP1;MYCN;ERP29;COMMD3-BMI1;DDX6;DNMT1;PDGFB;PLA2G3;MACROH2A1;ZFP36L2;FXR1;PDCD10;STC2;EIF4EBP1;N4BP1;MARK1;WNT4;NDFIP2;TFAP2C;TGFB2;TIA1;TGFB1;SETDB1;IGF1;ESR1;SMAD5;QKI;DHFR;CNOT7;LIN28A;AGO2;CD28;CPEB3;PHF19;DDX3Y;TRIM71;SUV39H2;BTG2;DDX3X;PPP1R11;SNX12;YBX1;ROBO1;MECP2;SYNCRIP;OTUD6B;CASP3;HEY2;NPTN;ITGB8;IL10;EDN1;SIRT1;PTBP3;IFNG;PTRH2;HAND2;MTF2;PPARG;TMBIM6;EIF4E2;RBM24;DCP2;YAP1;HDAC4;PRKAA1;PRKAA2;LRP3;PPM1D;TOB1;DLL4;CCL3;IGF2BP1;IGF2BP3;IGF2BP2;VHL;EXOSC3;PAIP2;ATP2B4;KLF4;VEGFA;APLN;REST;WT1;SERBP1;LGR4
 
 
 
 
Positive Regulation of RNA Biosynthetic Process ( GO:1902680 )
 
 
132/555
 
 
0.0000000
 
 
0.0000001
 
 
0
 
 
0
 
 
1.974282
 
 
44.11092
 
 
CD86;PHB1;RORA;AHR;RORB;TNF;HOXA11;YY1;SALL1;MYB;EPC1;CAPN3;EP300;EPC2;SOX9;BTRC;MAP3K5;ACVR1;EOMES;NANOS1;MAP2K1;RFX3;DYRK1B;MITF;RUNX1;MORF4L1;SFRP1;MYCN;RFX6;KAT6B;TBL1XR1;UBE2V1;TP53;INO80D;GATA6;PDGFB;FOXO3;RLF;GLIS2;NPAS3;NEUROD1;MBTD1;SEC14L2;ZKSCAN3;DVL3;TASP1;OTX2;NDP;WNT1;RREB1;SNX5;WNT4;SMAD2;TGFB1;FZD4;FOXF1;WNT3A;ZBTB16;SS18L1;HMGA1;HMGA2;INO80;NR2F2;IGF1;GDF6;ESR1;SMAD5;COL1A1;MLLT11;TADA2B;MAFB;LHX1;BCL3;SNAI1;TADA1;SETD3;HMGB2;SIX1;CTCF;NAT14;IKBKB;EDRF1;MED14;SHH;ZIC1;YAF2;YWHAH;IL10;NCOA1;TFEB;FOS;ARID1B;PAX2;TGFBR1;NR5A2;CREB1;ELF2;ASPH;MDFIC;ELF4;RARA;PPARG;CREB5;HDAC4;YAP1;PRKAA1;KDM3A;WBP2;LRP5;FOXK2;TBX21;TP53INP1;RBBP7;VHL;MTA3;RNF20;TCF7L2;USP21;FOXJ2;WNT7A;GTF2H1;KLF4;TBX5;KLF6;REST;WT1;PITHD1;CAMK4;NAA15;TAF5;CRLF3;TAF4
 
 
 
 
Negative Regulation of RNA Biosynthetic Process ( GO:1902679 )
 
 
112/470
 
 
0.0000000
 
 
0.0000021
 
 
0
 
 
0
 
 
1.972096
 
 
38.04038
 
 
RB1;PHB1;PRDM5;AHR;RORB;NR3C1;TNF;LOXL2;SALL1;MYB;EPC1;CAPN3;SOX9;RBFOX2;ZNF282;SFMBT1;SOX13;RFX3;KCTD1;FOXP2;DKK3;SFRP1;KAT6B;PSPC1;TP53;ZNF397;GATA6;PDGFB;ZBTB4;GLIS2;MBTD1;ZKSCAN3;SNX6;WNT4;ZBTB18;SMAD2;CBX5;TGFB1;UBE2I;CBX4;SMURF2;XRCC5;CBX3;ZBTB16;HMGA1;HMGA2;NR2F2;GCLC;HNRNPK;CNOT7;LHX1;BCL3;ID1;BHLHE40;ID4;ID3;MDM4;RCOR3;RCOR1;SUV39H2;SUV39H1;BHLHE41;HMGB2;CTCF;PTPRK;LIMD1;MECP2;SCML2;ZMYM2;QARS1;BCL7A;YWHAQ;NKRF;HEY2;YAF2;KLF10;TSC22D4;FOXD1;EED;DNMT3A;SIRT1;CBFA2T3;ZHX1;PAX2;NCOR2;ELF2;TFDP2;MDFIC;IFNG;PAX9;HAND1;RARA;PPARG;ZMYND11;MDFI;CIPC;FOXK2;PBXIP1;PPM1F;FOXS1;BTAF1;TBX21;RBBP7;MTA3;TCF7L2;KLF4;REST;WT1;RLIM;BCOR;LIMS1;CDK5R1
 
 
 
 
Positive Regulation of Cellular Process ( GO:0048522 )
 
 
136/622
 
 
0.0000000
 
 
0.0000146
 
 
0
 
 
0
 
 
1.766448
 
 
30.47620
 
 
CD86;APP;PGAP1;IRS2;TNF;NCMAP;CRKL;IGF1R;RPS6KA3;FGF7;CCND2;CHEK1;CHP2;CCN4;IL12B;CCN2;SOX9;PITX2;ARID2;MFSD2A;PDGFRB;PABIR1;RPS6;TPM1;ADAM10;DICER1;ISL1;CDC25B;TIAM1;FRMD5;SFRP1;ADAM17;IL6ST;TP53;S100A8;ANKRD17;DLX1;CUL3;PDGFB;CHRD;TGFA;LPL;TNFRSF11A;NID1;ZFP36L1;PRDX3;NEUROD1;EFNB2;PDCD10;PDGFC;SSR1;WNT1;ZNF268;SRPK2;TGFB2;TGFB1;TSLP;WNT3A;LIF;INO80;IGF1;QKI;BMP5;ACER2;CNOT7;FGF18;BCL2;CHRFAM7A;ARMC12;EIF4G2;DDX3X;DDX49;FLT3;PTEN;ASH2L;LAMC1;GHR;EPB41L4B;SHH;ESM1;BCL7A;CCR7;IKBKE;H3-3B;SS18;ARG2;EDN1;CNOT6L;IL15;TBC1D8;CCDC117;TNFRSF1B;OSMR;SIRT1;ARID1B;ADRA2A;TGFBR1;GREM1;IFNG;RARA;SINHCAF;CACUL1;CRK;HBEGF;HDAC4;YAP1;PRKAA1;PRKAA2;LAMA2;LRP5;TTK;AKAP6;PTHLH;CDC42;MAPK8;RPS15A;NTF3;RICTOR;STX3;VHL;TAF9B;LGI1;VEGFC;NAP1L1;VEGFA;TJP1;KITLG;H3-5;MEIS1;REST;GID8;DNAJA2;AQP11;CCM2;YPEL3;METAP1
 
 
 
 
Protein Ubiquitination ( GO:0016567 )
 
 
90/369
 
 
0.0000000
 
 
0.0000176
 
 
0
 
 
0
 
 
2.026299
 
 
34.31846
 
 
TRIM71;UBE2D2;CCNF;FBXO28;UBE2D3;UBE2D1;PRPF19;NPEPPS;CDC23;UBE2Q1;CDC27;BTRC;RNF152;SKP1;TRIM23;ARRDC4;WSB1;NHLRC3;FBXW7;UBE2E3;UBE4A;UBE4B;FBXO10;SIRT1;KLHL42;CDC34;RNF125;UBE2R2;GAN;UBE2V2;UBE2V1;OBI1;MARCHF1;RNF34;CBFB;CUL5;UHRF2;UBA6;CUL3;ZC4H2;MYCBP2;PELI2;CUL2;RNF38;UBR3;DTX2;FBXO45;ASB11;RNF7;RCHY1;UBE2J1;RNFT1;DCAF15;CAND1;RMND5A;RNF138;UBE2NL;RBBP6;VHL;MARCHF3;MARCHF5;RNF20;UBE2H;TRIM41;UBE2B;USP9X;SMURF1;KLHL2;BFAR;UBE2G2;FBXO32;KLHL20;UBE2W;NEDD4;KLHL8;OTULIN;RLIM;BCL2;RNF182;UBE2N;MKRN3;BLMH;MDM4;RNF183;RNF186;RNF141;UBE2K;RNF220;SPSB4;CUL4B
 
 
 
 
Cellular Response to Transforming Growth Factor Beta Stimulus ( GO:0071560 )
 
 
35/98
 
 
0.0000000
 
 
0.0000176
 
 
0
 
 
0
 
 
3.463589
 
 
58.23405
 
 
ITGB1;DLX1;USP15;PTPRK;NR3C1;ZFP36L2;CRKL;ZFP36L1;FNTA;SOX9;MAP3K7;SOX5;WNT4;SMAD2;TGFB2;TGFB1;USP9X;WNT7A;FOS;GDF5;SMAD5;SIRT1;PIAS2;TGFBR1;SMAD7;DUSP22;TGFBR3;SFRP1;COL3A1;COL1A2;ID1;PXDN;ADAM9;FERMT2;LIMS1
 
 
 
 
Extracellular Structure Organization ( GO:0043062 )
 
 
38/111
 
 
0.0000001
 
 
0.0000176
 
 
0
 
 
0
 
 
3.246984
 
 
54.57784
 
 
ITGB1;APP;COL15A1;COL16A1;HPSE2;COL13A1;COL11A1;ADAMTS10;COL19A1;ADAMTS17;SLC39A8;ADAMTS8;ADAMTS9;ADAMTS7;COL24A1;COL22A1;TGFBR1;COLQ;MMP11;COL3A1;COL1A2;COL2A1;COL4A2;DMTF1;MMP16;COL4A1;MMP15;COL7A1;SMOC1;COL5A2;PXDN;COL9A1;COL4A5;COL8A1;COL9A3;COL6A6;RECK;MATN3
 
 
 
 
Regulation of Apoptotic Process ( GO:0042981 )
 
 
149/704
 
 
0.0000001
 
 
0.0000207
 
 
0
 
 
0
 
 
1.696024
 
 
28.10637
 
 
ANKRD13C;FOXA1;APP;PHB1;TFRC;ARL6IP1;ZFAND6;TNF;IGF1R;RPS6KA3;CCND2;PROK2;EPC1;CAPN3;EPC2;CCNL2;SOX9;PHLDA1;HOXA5;MAP3K5;ACTN2;RPS6;FBXO10;MORF4L1;SFRP1;MELK;BIN1;PPIF;VDAC1;IL6ST;TP53;PPID;ANP32A;DCUN1D3;GATA6;PIK3R1;FOXO3;HIGD1A;RTN4;MTDH;HSP90B1;PRDX3;NEUROD1;SOCS2;MBTD1;SOCS3;BAG4;BCL2L11;NUAK2;PDCD10;PMAIP1;BMF;WNT1;ZNF268;MCL1;TXNDC5;HSPA9;WNT10B;PRPF38B;PLK5;EGR3;TSLP;CBX4;GADD45A;PLK2;SIAH2;SIAH1;CIDEB;HMGA2;BFAR;IGF1;SMAD5;GADD45G;KLHL20;MLLT11;ACER2;CXCL10;GCLC;HNRNPK;PTPRA;FASN;BCL3;JMY;BCL2;CDK1;CD28;TNFSF9;BCL2L2;HRK;ITGB1;ARF4;RBM25;DDX3X;FLT3;FASLG;TRIAP1;ING5;SHH;SPTLC1;AIFM1;SMPD1;IL10;TOMM70;KLF11;USP42;PAWR;SIRT1;PAX2;RHOB;GREM1;RNF144B;CREB1;PLSCR3;ITGA6;TMBIM6;SOS1;SGK1;PRKAA1;PRKAA2;GLS2;LRP2;FSTL1;HSPD1;MAPK8;ACVR1C;PNMA1;ERBB3;FRZB;NTF3;RICTOR;BAK1;ZNF622;SLIT2;VHL;TAF9B;MAP4K4;EGLN1;EGLN2;PNMA2;VEGFA;TJP1;REST;UFM1;TCIM;WT1;SPRY2;NAA15;TEK;LAMTOR5
 
 
 
 
Negative Regulation of Macromolecule Biosynthetic Process ( GO:0010558 )
 
 
57/202
 
 
0.0000001
 
 
0.0000239
 
 
0
 
 
0
 
 
2.458733
 
 
40.22080
 
 
RB1;DDX3Y;APP;DDX3X;SNX12;AP2A1;TNF;CRKL;ROBO1;MECP2;YY1;HEY2;ITGB8;SH3GL2;IL10;EDN1;FBXW7;ADAM10;SOX11;DICER1;SIRT1;SFRP1;MYCN;IFNG;PTRH2;HAND2;ERP29;PPARG;YAP1;DNMT1;PRKAA1;PRKAA2;PDGFB;STK39;PLA2G3;LRP3;DLL4;PDCD10;STC2;CCL3;VHL;MARK1;WNT4;NDFIP2;TGFB2;TGFB1;SETDB1;ATP2B4;IGF1;KLF4;ESR1;SMAD5;VEGFA;APLN;REST;CNOT7;CD28
 
 
 
 
Protein Polyubiquitination ( GO:0000209 )
 
 
62/228
 
 
0.0000001
 
 
0.0000272
 
 
0
 
 
0
 
 
2.337491
 
 
37.78717
 
 
TRIM71;UBE2D2;FBXO28;UBE2D3;UBE2D1;TNFAIP3;PRPF19;NPEPPS;CDC23;UBE2Q1;CDC27;BTRC;RNF152;SKP1;ARRDC4;WSB1;NHLRC3;UBE2E3;UBE4A;ZFP91;UBE4B;KLHL42;CDC34;RNF125;UBE2R2;UBE2V2;UBE2V1;MARCHF1;RNF34;CBFB;CUL5;CUL3;MYCBP2;PELI2;FBXO45;ASB11;RNF7;UBE2J1;DCAF15;ZNRF1;ZNRF2;RMND5A;UBE2NL;RNF39;RFFL;MARCHF5;RNF20;UBE2H;UBE2B;SMURF1;BFAR;UBE2G2;KLHL20;UBE2W;NEDD4;BCL2;UBE2N;MKRN3;BLMH;RNF183;RNF186;UBE2K
 
 
 
 
Negative Regulation of Cell Differentiation ( GO:0045596 )
 
 
60/220
 
 
0.0000001
 
 
0.0000366
 
 
0
 
 
0
 
 
2.346020
 
 
37.08518
 
 
RB1;FOXA1;TNFAIP6;PTEN;RORA;LDLRAD4;RORB;LIMD1;IPO7;TNF;CDC73;HOXA9;SHH;SPRED1;BCL7A;MYB;SOX9;NEPRO;ADAMTS7;IQCB1;SS18;NOG;WNT9A;SIRT1;TGFBR1;GREM1;SFRP1;NR5A2;IFNG;PPARG;SINHCAF;HOXB8;TRIB2;YAP1;CHRD;LRP5;LRP3;RTN4;ZFP36L2;PTHLH;PPP2CA;WNT1;MARK1;SMAD2;WNT10B;TGFB1;ZBTB16;CRIM1;TBX5;GDF5;BMP5;SMAD7;MEIS1;REST;COL5A1;COL5A2;SPRY2;SPRY1;CNTN4;FERMT2
 
 
 
 
External Encapsulating Structure Organization ( GO:0045229 )
 
 
37/111
 
 
0.0000002
 
 
0.0000404
 
 
0
 
 
0
 
 
3.117497
 
 
48.72091
 
 
ITGB1;APP;COL15A1;COL16A1;HPSE2;COL13A1;COL11A1;ADAMTS10;COL19A1;ADAMTS17;SLC39A8;ADAMTS8;ADAMTS9;ADAMTS7;COL24A1;COL22A1;COLQ;MMP11;COL3A1;COL1A2;COL2A1;COL4A2;DMTF1;MMP16;COL4A1;MMP15;COL7A1;SMOC1;COL5A2;PXDN;COL9A1;COL4A5;COL8A1;COL9A3;COL6A6;RECK;MATN3
 
 
 
 
Regulation of Cell Migration ( GO:0030334 )
 
 
108/479
 
 
0.0000002
 
 
0.0000404
 
 
0
 
 
0
 
 
1.830875
 
 
28.54024
 
 
PLXND1;SERPINE1;RORA;ARID4B;LDLRAD4;CLDN1;MYLK;AMOT;IGF1R;FGF7;FAM110C;DPYSL3;ENPP2;ARID2;TMSB10;ACVR1;PDGFRB;SEMA6D;TPM1;ADAM10;MITF;TIAM1;FRMD5;SFRP1;ADAM17;ADAM9;EPHA2;IGSF8;SEMA7A;CUL5;CCL3L1;PDGFB;CHRD;PIK3R3;STC1;FOXO3;RTN4;PDCD10;PDGFC;SRGAP2;ZNF268;SOCS4;TGFB1;UBE2I;FZD4;RDX;IGF1;SMAD7;COL1A1;FGF18;SNAI1;AMOTL1;FERMT2;ITGB1;MCTP1;PTEN;MIA3;PTPRK;ROBO1;SHH;TMSB4X;EDN1;NOG;TET1;F3;ADRA2A;TGFBR1;RHOB;DUSP22;PTP4A1;RAP2C;TGFBR3;RAP2B;CDH11;ITGA6;SGK3;SINHCAF;SOS1;SGK1;HBEGF;KANK1;RNF7;PPM1F;NKD1;CDC42;PARD6B;CCL8;RELN;CCL7;SAP30L;NTF3;TP53INP1;CCL3;CCL2;RICTOR;RBBP7;SLIT2;RFFL;MAP4K4;RNF20;SEMA4C;SEMA4F;TBX5;VEGFA;TJP1;KIF26A;SPRY2;RECK
 
 
 
 
Positive Regulation of Cell Growth ( GO:0030307 )
 
 
36/107
 
 
0.0000002
 
 
0.0000404
 
 
0
 
 
0
 
 
3.160809
 
 
49.02750
 
 
ITSN2;YAP1;SEMA7A;DDX3X;DDX49;RASAL1;AKAP6;CDC42;RPS6KA3;RICTOR;TAF9B;MFSD2A;H3-3B;LGI1;PABIR1;SYT4;SYT3;TGFB2;EDN1;BDNF;SMURF1;LIMK1;ADAM10;IGF1;INO80;TGFBR1;ADAM17;SFRP1;H3-5;BCL2;PLAA;ARMC12;MAP3K13;CRK;S100A8;EIF4G2
 
 
 
 
Extracellular Matrix Organization ( GO:0030198 )
 
 
52/182
 
 
0.0000002
 
 
0.0000404
 
 
0
 
 
0
 
 
2.499488
 
 
38.73183
 
 
ITGB1;APP;COLGALT2;COL16A1;HPSE2;LAMC1;CTSS;LOXL2;SLC39A8;ADAMTS8;ADAMTS9;ADAMTS7;DDX18;ADAM10;TGFBR1;GREM1;MMP11;COL2A1;COL4A2;DMTF1;LOX;MMP16;COL4A1;MMP15;PXDN;COL4A5;COL8A1;COL6A6;MATN3;COL15A1;COL13A1;COL11A1;NID1;ADAMTS10;COL19A1;ADAMTS17;TGFB2;TGFB1;COL24A1;COL22A1;COLQ;COL1A1;COL3A1;COL1A2;COL5A1;COL7A1;SMOC1;COL5A2;COL9A1;COL9A3;TLL1;RECK
 
 
 
 
 
 Reg of Transmembrane Receptor Prot Serine/Threonine Kinase Sgnlng
Pway ( GO:0090101 )
 
 
39/122
 
 
0.0000003
 
 
0.0000553
 
 
0
 
 
0
 
 
2.930290
 
 
44.35517
 
 
DLX1;USP15;TNFAIP6;IGSF1;UBE2D3;CHRD;UBE2D1;ARID4B;LDLRAD4;LRP2;CRKL;SPRED1;SAP30L;RBPMS2;VWC2L;RBBP7;WNT1;SKIL;SNX6;SMURF2;SMURF1;FST;NOG;CRIM1;TET1;LEMD3;SIRT1;DKK3;SMAD7;GREM1;TGFBR3;FKBP1A;ADAM17;FKBP1C;VEPH1;SPRY2;PPARG;SPRY1;SINHCAF
 
 
 
 
Positive Regulation of Cell Differentiation ( GO:0045597 )
 
 
74/297
 
 
0.0000003
 
 
0.0000554
 
 
0
 
 
0
 
 
2.079008
 
 
31.37149
 
 
APP;RIPOR2;LAMC1;TNF;HOXA11;LOXL2;TMEM100;RPS6KA3;CCN4;CCN2;SOX9;HIF1AN;ARID2;HOXA5;ZNF385A;SOX5;ACVR1;MMD;LMO3;CTNNBIP1;SOX11;AXIN2;NGF;ISL1;ARID1B;PAX2;TGFBR1;TIAM1;SFRP1;NR5A2;CREB1;HOXB4;RAPGEF2;PPARG;IL6ST;SOS1;RBM24;YAP1;DLX1;PKDCC;LAMA2;ZC4H2;LRP5;PLA2G3;LPL;LRP3;NID1;MACROH2A1;ZFP36L1;NEUROD1;BLOC1S6;RAP1A;FRZB;VHL;ZNF268;WNT4;SMAD2;TCF7L2;AKIRIN1;TGFB2;TGFB1;ZBTB16;SUCO;IGF1;GDF6;GDF5;SMAD5;ACVR2B;COL1A1;XKR8;KITLG;REST;SNAI1;FERMT2
 
 
 
 
Protein Modification Process ( GO:0036211 )
 
 
117/545
 
 
0.0000008
 
 
0.0001488
 
 
0
 
 
0
 
 
1.719257
 
 
24.17074
 
 
ERO1A;GMFB;MAML1;UBE2D2;UBE2D3;TESK2;PPP2R2A;MYLK;LOXL2;PPP1CB;PPP1CC;PPP4R2;AKT3;CHEK1;MAP3K9;NEK3;LARGE1;BTRC;USP6;PRKCD;DYRK1B;P3H1;ADAM10;LMTK2;CDC25B;TPST1;PLPP6;CDC34;LOX;CCNE1;LORICRIN;ERG;TRIB2;BRSK1;MTMR3;PDGFB;STK39;FUT4;UGCG;NUAK2;IRAK2;MAP3K21;STK38L;ST3GAL6;TRPM7;MARK3;ATG5;MARK1;CDK17;SRPK2;UBE2F;ICMT;PTPRN2;UBE2I;DMPK;PLK2;NEK6;NEK7;SRPK1;SSH1;MEX3B;PPP5C;COPS2;OSTC;MAP3K13;PTPRR;SETD3;DYRK2;PTEN;PTPRK;NAGPA;IKBKB;PPP6C;SERP1;TLK2;TLK1;ZNF367;PPP1R12A;STRADB;BAZ1B;DUSP9;CTTNBP2NL;CCNA2;CREB1;MMP15;B3GNT5;RARA;SGK3;SGK1;B4GALT5;PRKAA1;PRKAA2;RNF7;PPM1D;PPM1G;PPP2CA;MAPK8;ACVR1C;MKNK1;CCL2;MAPK6;FNIP2;MAP4K3;MAP4K4;ARFGEF1;USP25;MAP3K1;CSNK1A1;LIMK1;COL3A1;MGAT4A;CAMK4;NEK10;AQP11;METAP1;METAP2;PTPN3
 
 
 
 
Regulation of miRNA Transcription ( GO:1902893 )
 
 
25/66
 
 
0.0000011
 
 
0.0002052
 
 
0
 
 
0
 
 
3.792590
 
 
51.94572
 
 
FOXA1;ZNF512B;APP;PDGFB;NR3C1;FOXO3;TNF;MYB;SOX9;IL10;SREBF1;TGFB2;TGFB1;FOS;KLF4;ESR1;APLN;VEGFA;NCOR2;REST;MYCN;WT1;RARA;PPARG;TP53
 
 
 
 
Negative Regulation of Translation ( GO:0017148 )
 
 
33/102
 
 
0.0000016
 
 
0.0002799
 
 
0
 
 
0
 
 
2.978490
 
 
39.75425
 
 
TRIM71;DDX6;BTG2;DDX3X;FMR1;YBX1;LIMD1;TOB1;SYNCRIP;OTUD6B;TPR;HABP4;EIF4EBP1;IGF2BP1;IGF2BP3;IGF2BP2;PAIP2;C8ORF88;NANOS1;TIA1;PARP16;QKI;DHFR;CPEB1;LIN28A;WT1;AGO2;SERBP1;CPEB3;EIF4E2;CPEB2;RBM24;TNRC6A
 
 
 
 
Regulation of Chondrocyte Differentiation ( GO:0032330 )
 
 
14/26
 
 
0.0000019
 
 
0.0003129
 
 
0
 
 
0
 
 
7.239899
 
 
95.55135
 
 
PKDCC;ZBTB16;WNT9A;GDF6;GDF5;PTHLH;TGFBR1;HOXA11;LOXL2;GREM1;NR5A2;SOX9;ADAMTS7;SOX5
 
 
 
 
Positive Regulation of Cell-Substrate Junction Organization ( GO:0150117 )
 
 
13/23
 
 
0.0000021
 
 
0.0003419
 
 
0
 
 
0
 
 
8.065344
 
 
105.43795
 
 
NRP1;COL16A1;TSC1;PIK3R1;PPM1F;VEGFA;SFRP1;TEK;MAPRE2;FERMT2;ARF6;LIMS1;MAP4K4
 
 
 
 
Negative Regulation of Apoptotic Process ( GO:0043066 )
 
 
103/475
 
 
0.0000022
 
 
0.0003463
 
 
0
 
 
0
 
 
1.738070
 
 
22.63864
 
 
RB1;TFRC;ARL6IP1;ZFAND6;SERPINE1;TNF;IGF1R;RPS6KA3;CCND2;PDK4;PROK2;CAPN3;SOX9;PRKCD;ISL1;SFRP1;PPIF;VDAC1;IL6ST;TP53;GATA6;PLA2G3;PIK3R3;ALOX12;PIK3R1;HIGD1A;MTDH;HSP90B1;PRDX3;SOCS2;SOCS3;BAG4;NUAK2;PDCD10;WNT1;ZNF268;MCL1;TXNDC5;SNX6;HSPA9;EGR3;TSLP;CBX4;BDNF;PLK2;SIAH2;HMGA2;BFAR;IGF1;SMAD5;KLHL20;NR4A2;GCLC;HNRNPK;FASN;BCL3;BCL2;CDK1;CD28;BCL2L2;ITGB1;ARF4;DDX3X;SIX1;TNFAIP3;TRIAP1;SHH;QARS1;IL10;IL13;NGF;SIRT1;PAX2;GREM1;RNF144B;CREB1;PIK3CA;PTRH2;TMBIM6;SOS1;YAP1;PRKAA1;PRKAA2;LRP2;FSTL1;HSPD1;MAPK8;ERBB3;NTF3;CCL2;RICTOR;VHL;TAF9B;MAP4K4;VEGFA;TJP1;UFM1;TCIM;WT1;SPRY2;NAA15;TEK;LAMTOR5
 
 
 
 
Positive Regulation of Apoptotic Process ( GO:0043065 )
 
 
74/313
 
 
0.0000024
 
 
0.0003559
 
 
0
 
 
0
 
 
1.938001
 
 
25.10969
 
 
HRK;FOXA1;ITGB1;APP;PHB1;DDX3X;FASLG;TNF;ING5;CASP7;AIFM1;CASP5;SMPD1;CASP3;PHLDA1;HOXA5;MAP3K5;NANOS1;KLF11;EPHA7;RPS6;PAWR;F3;SIRT1;TGFBR1;RHOB;SFRP1;MELK;BIN1;PTRH2;VDAC1;PPARG;ITGA6;TP53;PPID;DCUN1D3;FOXO3;HSPD1;NEUROD1;MAPK8;BCL2L11;ACVR1C;PNMA1;FRZB;CCL3;PMAIP1;CCL2;BMF;BAK1;SLIT2;ZNF622;ZNF268;MCL1;SRPK2;HSPA9;WNT10B;PRNP;PRPF38B;TGFB2;TGFB1;GADD45A;SIAH1;CIDEB;PNMA2;GADD45G;MLLT11;REST;WT1;PTPRA;FASN;JMY;BCL2;BCL2L2;CDK5R1
 
 
 
 
Mitotic Cell Cycle Phase Transition ( GO:0044772 )
 
 
35/113
 
 
0.0000024
 
 
0.0003559
 
 
0
 
 
0
 
 
2.795068
 
 
36.12619
 
 
TRIM71;BRSK1;USP37;CCNJ;CUL5;CUL3;CCNF;CUL2;ARPP19;PPP6C;CCND2;CDC23;SPAST;CCND1;CDC27;ENSA;EIF4EBP1;E2F3;CLASP2;PLK2;CDC25B;CCNA2;WEE1;CDC34;MELK;DBF4;CCNE2;CCNY;CCNE1;CCNG1;CDK1;CHMP4B;BCAT1;CACUL1;CUL4B
 
 
 
 
Protein Transport ( GO:0015031 )
 
 
73/308
 
 
0.0000025
 
 
0.0003559
 
 
0
 
 
0
 
 
1.944094
 
 
25.07726
 
 
ARF4;SYS1;TFRC;TMEM167A;GCC2;AP2A1;MIA3;RAB22A;SCAMP5;ARL5B;CHMP1B;TIMM17B;TLK1;ARFIP1;EIF2D;TMED7;GOLGA7;TMED5;TRIM23;YWHAH;ARRDC4;ARRDC3;LMTK2;SCAMP1;FAM91A1;ZDHHC17;RAB32;ARL4D;ARL4A;RAB34;CANX;ERP29;PPARG;RAB38;ARF5;ARF6;NAPB;ZNF395;CALCRL;LMAN2L;STXBP3;STX17;STX16;ASAP1;LRP2;SNX30;DNAJC27;NAPG;STX11;HSP90B1;SNX27;RAB23;GRPEL2;RAB24;SYNDIG1;STX6;STX3;STX2;SNX6;CTSA;HSPA9;RIC1;SYNRG;SURF4;CMTM6;TVP23B;VMP1;RAB10;SNX18;RAB14;SNX17;CHMP2B;RAB18
 
 
 
 
Negative Regulation of Cellular Response to Growth Factor Stimulus ( GO:0090288 )
 
 
26/73
 
 
0.0000027
 
 
0.0003724
 
 
0
 
 
0
 
 
3.440811
 
 
44.12239
 
 
DLX1;TNFAIP6;UBE2D3;CHRD;UBE2D1;LRP2;RBPMS2;VWC2L;SLIT2;WNT1;SKIL;CADM4;SMURF2;SMURF1;NOG;ATP2B4;CRIM1;CASK;LEMD3;APLN;SMAD7;GREM1;NEDD4;SPRY2;PPARG;SPRY1
 
 
 
 
Positive Regulation of Developmental Process ( GO:0051094 )
 
 
61/245
 
 
0.0000031
 
 
0.0004126
 
 
0
 
 
0
 
 
2.071859
 
 
26.29378
 
 
TFRC;FMR1;ARID4B;TNF;RPS6KA3;SHH;BCL7A;DPF2;ENPP2;CCN4;KMT5B;CCN2;SOX9;ARID2;SOX5;ACVR1;NCOA1;SS18;FOXD1;KMT5C;FST;TET1;ISL1;SIRT1;ARID1B;PAX2;TGFBR1;PPARG;SINHCAF;B4GALT5;DLX1;PRKAA1;PDGFB;LPL;RTN4;NEUROD1;RELN;SAP30L;DVL3;OTX2;RBBP7;VHL;RREB1;ZNF268;MARCHF5;CLASP2;REPS1;TGFB1;SMURF2;FZD4;ZBTB16;DDHD1;VEGFA;BMP5;REST;WT1;LHX1;LEP;AGO2;CPEB3;MAP3K13
 
 
 
 
Negative Regulation of BMP Signaling Pathway ( GO:0030514 )
 
 
19/45
 
 
0.0000033
 
 
0.0004256
 
 
0
 
 
0
 
 
4.539379
 
 
57.33660
 
 
DLX1;TNFAIP6;SMURF2;SMURF1;UBE2D3;NOG;CHRD;CRIM1;UBE2D1;LRP2;LEMD3;SMAD7;GREM1;RBPMS2;VWC2L;PPARG;WNT1;SKIL;FBN1
 
 
 
 
Positive Regulation of Chondrocyte Differentiation ( GO:0032332 )
 
 
8/10
 
 
0.0000049
 
 
0.0006041
 
 
0
 
 
0
 
 
24.783297
 
 
303.21138
 
 
PKDCC;ZBTB16;SOX9;GDF6;GDF5;HOXA11;LOXL2;SOX5
 
 
 
 
Negative Regulation of Multicellular Organismal Process ( GO:0051241 )
 
 
60/243
 
 
0.0000049
 
 
0.0006041
 
 
0
 
 
0
 
 
2.048398
 
 
25.04191
 
 
RB1;FOXA1;ITGB1;APP;PTPRR;PPP1R11;WWC2;DOCK7;PTEN;ARID4B;PCTP;LDLRAD4;TNF;ROBO1;LOXL2;RPS6KA6;SHH;SPRED1;CASP3;NPTN;SVEP1;SOX9;IL10;SFMBT1;IL15;ARRDC3;IL13;WNT9B;TET1;SFRP1;ADAM17;IFNG;SINHCAF;PFN2;DLX1;LAMA4;STC1;HMGCR;PPP2CA;ACVR1C;CHSY1;SAP30L;RBBP7;N4BP1;MAP2K6;MARK1;SMAD2;WNT10B;PLCL2;TBX5;ACVR2B;QKI;BMP5;SMAD7;BMP3;NOVA1;ID1;SPRY2;SPRY1;LGR4
 
 
 
 
Regulation of Gene Expression ( GO:0010468 )
 
 
209/1127
 
 
0.0000054
 
 
0.0006534
 
 
0
 
 
0
 
 
1.439694
 
 
17.44904
 
 
RB1;APP;PHB1;TFRC;CRKL;MYB;CCN4;IL12B;CCNL2;SOX9;HOXA5;MBNL2;ZNF282;SOX13;RFX3;SOX11;DICER1;ATG14;THAP1;SFRP1;COMMD3-BMI1;HOXB1;ANKRA2;TP53;CGGBP1;ZNF397;ZNF274;ZNF395;IGSF1;PDGFB;PLA2G3;PIK3R3;ZBTB4;PDCD10;STC2;MIER1;ZNF268;RREB1;SRPK2;NDFIP2;SETDB1;RDX;IGF1;LARP4;ST18;TADA2B;HNRNPF;FUBP1;CD28;SNAI1;SLC2A4RG;LIN54;SNX12;CTCF;LIMD1;ROBO1;MECP2;ING5;EPB41L4B;SHH;ZNF280B;HEY2;ZNF280C;JARID2;IL10;EDN1;FOXD1;IL13;NOG;TFEB;ASH1L;F3;MED26;SIRT1;MLF1;NR5A2;PTRH2;MTF2;RBM12B;PPARG;YAP1;MYCBP;NRAS;STX3;APBB3;ZNF341;MOG;VEGFA;PDCL3;NFIA;WT1;LIMS1;LAMTOR5;PRDM4;ZBTB26;WWC2;IHH;RORA;RORB;AHR;NR3C1;TNF;YY1;SLC39A8;PITX2;SH3GL2;ZNF562;NANOS1;ZBTB39;MAP2K1;FBXW7;BTN1A1;ADAM10;ATP1B4;ZBTB34;MITF;MYCN;DMTF1;PSPC1;KAT6B;ERP29;UBE2V1;ADNP;DNMT1;YTHDC1;FOXO4;MACROH2A1;ZFP36L1;CALCR;CD276;WNT4;MARK1;ESRRA;SMAD2;SEC16B;TGFB2;TGFB1;BTN2A1;WNT3A;HMGA1;HMGA2;FOXN4;ESRRG;ZBTB11;ESR1;SMAD5;SCRT1;CNOT7;MAFB;LHX1;BHLHE40;TADA1;TRIM71;DDX3Y;DDX3X;ONECUT1;CHD7;SIX1;YBX1;YBX3;ELAVL2;DNMT3B;ITGB8;YAF2;KDM6B;NGF;TGFBR1;PTBP3;ACTA2;TGFBR3;TFDP1;IFNG;HAND2;IRF2;HDAC4;PRKAA1;PRKAA2;PLAG1;FOXK2;LRP3;PBXIP1;TOB1;PPM1F;PTHLH;DLL4;MAPK8;TDG;SAP30L;CCL3;IGF2BP1;CCL2;IGF2BP3;IGF2BP2;VHL;RBBP7;RBM38;RNF20;ATP2B4;ZNF804A;DTNBP1;KLF4;ACVR2B;APLN;SLC25A37;REST;ACTC1;DNAJA4;SPRY2;ERCC6
 
 
 
 
Negative Regulation of Programmed Cell Death ( GO:0043069 )
 
 
85/382
 
 
0.0000059
 
 
0.0006847
 
 
0
 
 
0
 
 
1.792509
 
 
21.59470
 
 
ARF4;DDX3X;ARL6IP1;TFRC;ZFAND6;SLC7A11;TRIAP1;TM7SF3;IGF1R;RPS6KA3;SHH;CCND2;PROK2;CAPN3;SOX9;IL10;SIRT1;PAX2;GREM1;RNF144B;SFRP1;CREB1;PPIF;VDAC1;TMBIM6;IL6ST;SOS1;TP53;BECN1;PRKAA1;PRKAA2;GATA6;PIK3R1;LRP2;HIGD1A;FSTL1;MTDH;HSP90B1;HSPD1;PRDX3;SOCS2;SOCS3;BAG4;MAPK8;NUAK2;ERBB3;PDCD10;RICTOR;VHL;TAF9B;WNT1;ZNF268;ATG5;MAP4K4;MCL1;TXNDC5;HSPA9;EGR3;TSLP;CBX4;PLK2;SIAH2;HMGA2;BFAR;IGF1;SMAD5;VEGFA;KLHL20;TJP1;GCLC;UFM1;HNRNPK;TCIM;XPNPEP1;WT1;FASN;BCL3;CD28;BCL2;CDK1;SPRY2;NAA15;TEK;BCL2L2;LAMTOR5
 
 
 
 
Protein Deubiquitination ( GO:0016579 )
 
 
26/76
 
 
0.0000063
 
 
0.0007216
 
 
0
 
 
0
 
 
3.233797
 
 
38.70637
 
 
OTUD4;USP14;USP37;USP15;USP38;USP32;USP34;USP12;TNFAIP3;USP19;OTUD6B;USP46;USP47;USP8;USP25;USP6;USP42;USP4;USP21;USP9X;USP44;YOD1;JOSD1;OTULIN;CDK1;OTUB1
 
 
 
 
G1/S Transition of Mitotic Cell Cycle ( GO:0000082 )
 
 
23/64
 
 
0.0000084
 
 
0.0009207
 
 
0
 
 
0
 
 
3.486657
 
 
40.73767
 
 
TRIM71;USP37;CCNJ;CUL5;CUL3;PLK2;CCNF;CUL2;CCNA2;PPP6C;CCND2;CDC34;DBF4;CCND1;CCNE2;CCNE1;CCNG1;EIF4EBP1;CDK1;E2F3;BCAT1;CACUL1;CUL4B
 
 
 
 
Transforming Growth Factor Beta Receptor Superfamily Signaling Pathway
( GO:0141091 )
 
 
34/114
 
 
0.0000085
 
 
0.0009207
 
 
0
 
 
0
 
 
2.646057
 
 
30.89767
 
 
ITGB1;USP15;CHRD;PTPRK;TMEM100;ACVR1C;FNTA;MAP3K7;SMAD2;TGFB2;TGFB1;SMURF1;USP9X;INHBB;FOS;GDF6;GDF5;SMAD5;SIRT1;ACVR2B;PIAS2;TGFBR1;BMP5;SMAD7;RGMA;DUSP22;TGFBR3;COL3A1;COL1A2;ID1;PXDN;ADAM9;PPARG;FERMT2
 
 
 
 
Ubiquitin-Dependent Protein Catabolic Process ( GO:0006511 )
 
 
97/455
 
 
0.0000091
 
 
0.0009646
 
 
0
 
 
0
 
 
1.698449
 
 
19.71347
 
 
UBE2D2;UBE2D3;UBE2D1;FBXO21;PSMD7;CDC23;RNF19A;BTRC;SKP1;NHLRC3;FBXW7;UBE4B;ARMC8;FBXO10;KCTD5;KLHL42;RNF125;CDC34;TBL1XR1;HECW2;CHMP4B;PLAA;KCTD17;PSMD12;CUL5;UBA6;CUL3;UBR3;FBXO45;SOCS2;RNF217;SOCS4;KLHL29;NDFIP2;UBE2H;UBE2I;SMURF2;UBE2B;SMURF1;SIAH2;SIAH1;KLHL28;BFAR;KLHL23;KLHL20;UBE2W;APPBP2;CHMP2B;UBE2N;RNF186;UBE2K;TRIM71;UBXN1;PPP1R11;TNFAIP3;CHMP1B;USP44;VPS37C;VPS37D;AXIN2;SIRT1;RAD23B;PSMA5;RNF144B;PSMA1;UBE2R2;GAN;RNF34;VPS4A;ASB11;RNF7;RCHY1;ZNRF1;ZNRF2;RMND5A;HECTD3;KCTD21;TBX21;UBQLN1;RBBP6;VHL;RFFL;RNF20;CSNK1A1;USP9X;FEM1B;FBXL19;KLHL2;UBE2G2;CHFR;RNF145;UBQLNL;NEDD4;GID8;KLHL8;RLIM;SPSB4
 
 
 
 
Negative Regulation of Cell Motility ( GO:2000146 )
 
 
39/139
 
 
0.0000097
 
 
0.0010046
 
 
0
 
 
0
 
 
2.429727
 
 
28.04665
 
 
KANK1;MCTP1;SERPINE1;CHRD;PTEN;STC1;ARID4B;MIA3;LDLRAD4;PTPRK;FOXO3;ROBO1;SHH;SAP30L;DPYSL3;TP53INP1;RBBP7;SLIT2;ARID2;SRGAP2;RNF20;TPM1;NOG;TET1;MITF;TBX5;TGFBR1;SMAD7;RHOB;DUSP22;RAP2C;TGFBR3;FRMD5;SFRP1;RAP2B;CDH11;SINHCAF;CRK;RECK
 
 
 
 
Intracellular Protein Transport ( GO:0006886 )
 
 
74/326
 
 
0.0000111
 
 
0.0011230
 
 
0
 
 
0
 
 
1.836617
 
 
20.95452
 
 
STEEP1;ARF4;IPO11;PPP1R10;CSE1L;AP2A1;IPO7;RAB22A;IPO8;ARL5B;XPO4;TIMM17B;TLK1;ARFIP1;EIF2D;KPNA5;TMED7;TNPO1;VPS36;KPNA3;TMED5;TRIM23;KPNA1;YWHAH;TOMM70;DESI2;STRADB;VPS37C;VPS37D;FAM91A1;RAB32;ARL4D;ARL4A;NUP50;ERP29;RAB38;ARF5;KPNB1;ARF6;NAPB;STXBP3;STX17;STX16;TIMM9;DNAJC27;NAPG;STX11;LMAN1;POM121C;SNX27;RAB23;GRPEL2;TPR;RAB24;SYNDIG1;STX6;E2F3;STX3;STX2;SNX6;CTSA;HSPA9;TIMM8B;RIC1;TGFB1;NUP155;SMURF1;SYNRG;SURF4;XPO7;RAB14;SNX17;BCL3;RAB18
 
 
 
 
Wnt Signaling Pathway ( GO:0016055 )
 
 
27/83
 
 
0.0000123
 
 
0.0012177
 
 
0
 
 
0
 
 
2.998408
 
 
33.90116
 
 
DDX3X;USP34;LRP5;FRAT2;FRZB;DVL3;GPC4;WNT1;WNT4;TCF7L2;WNT10B;FZD5;CSNK1A1;FZD4;WNT3A;SIAH2;FZD6;SIAH1;WNT9B;WNT7A;AXIN2;WNT9A;NR4A2;SFRP1;SNAI1;STRN;FERMT2
 
 
 
 
Regulation of Cytoplasmic Translation ( GO:2000765 )
 
 
13/26
 
 
0.0000127
 
 
0.0012290
 
 
0
 
 
0
 
 
6.203029
 
 
69.94293
 
 
FMR1;PARP16;YBX1;YBX3;SYNCRIP;CPEB1;LIN28A;CSDE1;IGF2BP1;CPEB3;CPEB2;RBM24;ZNF385A
 
 
 
 
Positive Regulation of Growth ( GO:0045927 )
 
 
26/79
 
 
0.0000140
 
 
0.0013300
 
 
0
 
 
0
 
 
3.050219
 
 
34.08803
 
 
YAP1;DDX3X;DDX49;AKAP6;PPM1F;CDC42;RPS6KA3;RICTOR;TAF9B;H3-3B;MFSD2A;LGI1;PABIR1;TGFB2;ADAM10;INO80;TGFBR1;ADAM17;SFRP1;H3-5;LEP;BCL2;ARMC12;CRK;S100A8;EIF4G2
 
 
 
 
Cell Cycle G1/S Phase Transition ( GO:0044843 )
 
 
23/66
 
 
0.0000151
 
 
0.0014061
 
 
0
 
 
0
 
 
3.324100
 
 
36.89535
 
 
TRIM71;USP37;CCNJ;CUL5;CUL3;PLK2;CCNF;CUL2;CCNA2;PPP6C;CCND2;CDC34;DBF4;CCND1;CCNE2;CCNE1;CCNG1;EIF4EBP1;CDK1;E2F3;BCAT1;CACUL1;CUL4B
 
 
 
 
Small GTPase-mediated Signal Transduction ( GO:0007264 )
 
 
48/188
 
 
0.0000164
 
 
0.0014947
 
 
0
 
 
0
 
 
2.138036
 
 
23.55690
 
 
RB1;RALB;GARRE1;RRAD;ARL3;DOCK7;ARHGAP5;RASGRP1;RAPGEFL1;CRKL;RAP1B;GNA13;NRAS;RAP1A;RASGEF1B;GRAP2;NKIRAS2;G3BP1;WASF1;REPS1;VAV3;USP8;PLEKHG7;PLK2;SIAH2;LIMK1;RRAS2;IGF1;ADRA2A;RHOB;CCNA2;RAP2C;TIAM1;COL1A2;MRAS;RAP2B;CDC42EP2;RAB18;RAPGEF2;MAPKAPK5;RAB38;GRB2;SOS1;RHEBL1;TP53;RGL2;SOS2;RAPGEF4
 
 
 
 
Nervous System Development ( GO:0007399 )
 
 
100/480
 
 
0.0000182
 
 
0.0016272
 
 
0
 
 
0
 
 
1.649293
 
 
17.99918
 
 
APP;GMFB;PLXND1;MSI2;CYP46A1;RAPGEFL1;RPS6KA3;FGF7;RPS6KA6;DPYSL5;DPF2;EP300;CHAC1;PCDHAC2;SH3GL2;CDON;RBFOX2;SEMA6A;TMOD2;SOX11;HPCAL4;FEZ2;HOXB2;IGSF8;ZC4H2;EFNA5;GLIS2;NEUROD1;NHLH2;FLRT3;BTBD3;PDGFC;NDP;NPTX1;PRNP;TGFB1;FZD5;BDNF;ZBTB16;SIAH1;CRIM1;NR2F2;NR2F6;CYP26A1;FGF14;LHX1;FGF18;CSPG5;SP8;CNTN4;FGF11;TRIM71;NRSN1;CHD7;PTEN;SLC2A1;CXCL1;HAPLN3;NRGN;ROBO1;SHH;CYP26B1;SMPD1;ZIC1;JARID2;EMX2;NOG;GFRA1;PAX2;TGFBR1;NR5A2;IL1RAPL2;B3GNT5;ALDH1A2;HDAC4;PLPPR1;LRP5;TRAK2;RELN;ACVR1C;ERBB3;SLITRK1;NTF3;IGF2BP1;IGF2BP3;IGF2BP2;LGI1;MPP2;MARCKSL1;MOG;LIMK1;SEMA4F;VEGFA;MARCKS;P2RX5;MAB21L2;ATXN10;ERCC6;MDGA2;CBLN1
 
 
 
 
Regulation of Cell Population Proliferation ( GO:0042127 )
 
 
148/769
 
 
0.0000195
 
 
0.0016966
 
 
0
 
 
0
 
 
1.499064
 
 
16.25565
 
 
CD86;APP;PHB1;PGAP1;TFRC;WWC2;IRS2;CRKL;IGF1R;FGF7;CCND2;CHP2;SOX9;PITX2;ARID2;PDGFRB;MAP2K1;RBFOX2;RPS6;ADAM10;P3H1;TSC1;CDC25B;RUNX1;TIAM1;SFRP1;ADAM17;IL6ST;TP53;PLPP1;CCL3L1;CUL3;PDGFB;TGFA;FOXO4;TNFRSF11A;PDS5B;ZFP36L1;PRDX3;EFNB2;PDCD10;PDGFC;SSR1;WNT1;ZNF268;SRPK2;TFAP2C;TGFB2;CADM4;TFAP2D;TGFB1;TSLP;XRCC5;WNT3A;HMGA1;THAP12;LIF;IGF1;ST18;BMP5;ACER2;CXCL10;CNOT7;FGF18;BCL2;STRN;MDM4;CHRFAM7A;TRIM71;BTG2;FLT3;PTEN;HMGB2;SIX1;ASH2L;CXCL1;CTCF;PTPRK;CDC73;GHR;ING5;SHH;ESM1;BCL7A;RB1CC1;ADAMTS8;IL10;KLF10;SS18;KLF11;EDN1;CNOT6L;IL15;TBC1D8;WNT9A;CCDC117;OSMR;NGF;SIRT1;CBFA2T3;ADRA2A;TGFBR1;GREM1;DDAH1;IFNG;ALDH1A2;RARA;RAPGEF2;SGK3;SINHCAF;SOS1;SGK1;CACUL1;HBEGF;YAP1;HDAC4;LRP5;TTK;PPM1D;TOB1;PTHLH;DLL4;TBRG1;RPS15A;ERBB3;FRZB;CLMN;NTF3;TP53INP1;STX3;VHL;RBM38;TCF7L2;VEGFC;NAP1L1;TBX5;VEGFA;TJP1;KITLG;REST;MEIS1;WT1;PRKRA;GID8;DNAJA2;AQP11;CCM2;LIMS1
 
 
 
 
Negative Regulation of Cell Migration ( GO:0030336 )
 
 
47/184
 
 
0.0000197
 
 
0.0016966
 
 
0
 
 
0
 
 
2.138931
 
 
23.17112
 
 
KANK1;PTPRR;MCTP1;MACIR;SERPINE1;CHRD;PTEN;STC1;ARID4B;MIA3;LDLRAD4;PTPRK;FOXO3;ROBO1;DLL4;SHH;SAP30L;DPYSL3;TP53INP1;RBBP7;SLIT2;ARID2;SRGAP2;ADAMTS9;CLASP2;RNF20;DDX18;TPM1;NOG;TET1;MITF;NR2F2;TBX5;TGFBR1;SMAD7;RHOB;DUSP22;RAP2C;TGFBR3;SFRP1;COL3A1;RAP2B;DNAJA4;CDH11;SINHCAF;RECK;PFN2
 
 
 
 
Negative Regulation of miRNA Metabolic Process ( GO:2000629 )
 
 
13/27
 
 
0.0000213
 
 
0.0018000
 
 
0
 
 
0
 
 
5.759621
 
 
61.94594
 
 
ZNF512B;TGFB1;PDGFB;TNF;ESR1;QKI;VEGFA;NCOR2;REST;RARA;PPARG;SOX9;TENT2
 
 
 
 
Canonical Wnt Signaling Pathway ( GO:0060070 )
 
 
22/63
 
 
0.0000222
 
 
0.0018382
 
 
0
 
 
0
 
 
3.333857
 
 
35.72500
 
 
TCF7L2;WNT10B;FZD5;FZD4;WNT3A;SIAH2;FZD6;SIAH1;WNT9B;LRP5;WNT7A;AXIN2;WNT9A;SMAD7;NR4A2;SFRP1;FRAT2;FRZB;SNAI1;DVL3;WNT1;WNT4
 
 
 
 
Ras Protein Signal Transduction ( GO:0007265 )
 
 
23/68
 
 
0.0000263
 
 
0.0021137
 
 
0
 
 
0
 
 
3.175992
 
 
33.49530
 
 
RB1;USP8;RALB;PLK2;RRAS2;IGF1;RASGRP1;RAPGEFL1;CRKL;CCNA2;NRAS;MRAS;RASGEF1B;GRAP2;G3BP1;RAPGEF2;MAPKAPK5;GRB2;SOS1;SOS2;TP53;RGL2;RAPGEF4
 
 
 
 
Skeletal System Development ( GO:0001501 )
 
 
42/160
 
 
0.0000266
 
 
0.0021137
 
 
0
 
 
0
 
 
2.217572
 
 
23.35728
 
 
DLX1;PKDCC;COL13A1;HHIP;CHD7;IHH;CHRD;STC1;TNFRSF11B;HAPLN3;PCSK5;COL19A1;PTHLH;HOXA11;RPS6KA3;FRZB;ITGB8;SOX9;WNT1;SOX5;TGFB2;SLC10A7;AHSG;ZBTB16;NOG;RFLNB;P3H1;IGF1;TBX4;TGFBR1;BMP5;COL1A1;BMP3;COL1A2;COL2A1;MMP16;PTRH2;CDH11;ALPL;TLL1;MATN3;FBN1
 
 
 
 
Positive Regulation of Signal Transduction ( GO:0009967 )
 
 
60/256
 
 
0.0000269
 
 
0.0021137
 
 
0
 
 
0
 
 
1.911075
 
 
20.11040
 
 
ITGB1;APP;PHB1;POGLUT1;IHH;PTEN;TNFAIP3;MIA3;LAMC1;TXNDC15;CDC73;ROBO1;ING5;ESM1;SHH;SALL1;NETO1;RSPO2;NPTN;RSPO3;NEPRO;ACVR1;ATP6AP2;F3;SIRT1;TGFBR1;SFRP1;RAB34;HAND2;PPARG;UBE2V1;IL6ST;KANK1;LAMA2;TUBD1;NID1;HSP90B1;ENHO;DLL4;RELN;PDCD10;TSPAN5;WNT1;SNX5;PRNP;TGFB2;TGFB1;HOMER1;TSLP;BDNF;LIF;WNT7A;IGF1;VEGFA;TSPAN14;FASN;UBE2N;CHRFAM7A;TEK;LIMS1
 
 
 
 
Neuron Differentiation ( GO:0030182 )
 
 
46/181
 
 
0.0000276
 
 
0.0021314
 
 
0
 
 
0
 
 
2.123909
 
 
22.29599
 
 
FOXA1;BRSK1;TENM2;RTN4;UGCG;SHH;AIFM1;ERBB3;CASP3;OTX2;PITX2;WNT1;WNT4;WNT10B;MAP2K1;TGFB2;EMX2;FZD5;IRX5;FZD4;WNT3A;PBX3;EN2;WNT9B;ATP2B4;WNT7A;SOX11;SOX12;WNT9A;PBX1;TGFBR1;RUNX1;VEGFA;NR4A2;LMX1A;LHX1;HAND2;DDIT4;ID1;IL1RAPL1;ID4;ID3;INSM2;ERCC6;LHX8;CDK5R1
 
 
 
 
Zinc Ion Import Into Organelle ( GO:0062111 )
 
 
7/9
 
 
0.0000281
 
 
0.0021332
 
 
0
 
 
0
 
 
21.677582
 
 
227.18039
 
 
SLC30A7;SLC30A6;SLC30A8;SLC30A3;SLC30A5;SLC30A1;SLC30A10
 
 
 
 
Positive Regulation of Multicellular Organismal Process ( GO:0051240 )
 
 
86/407
 
 
0.0000402
 
 
0.0029780
 
 
0
 
 
0
 
 
1.676241
 
 
16.96773
 
 
APP;SERPINE1;SIX1;ARID4B;TNF;IGF1R;LOXL2;SCAMP5;GHR;SHH;BCL7A;DPF2;IL12B;SOX9;SOX5;ACVR1;IL10;NCOA1;KDM6B;SS18;PRKAB2;FOXD1;IL15;FST;ELOVL3;IL13;NOG;RFX3;TET1;EBF2;SOX11;AXIN2;SIRT1;ETV4;ADRA2A;TGFBR1;VAMP8;TIAM1;VAMP7;ADRB3;IFNG;PPARG;SINHCAF;B4GALT5;PRKAA1;KDM3A;PDGFB;PLA2G3;LPL;ADRB1;TNFRSF11A;ADRB2;MACROH2A1;ADIPOR2;RTN4;SAP30L;PDGFC;OTX2;RBBP7;RREB1;LDLR;LURAP1;SMAD2;TCF7L2;TGFB2;TGFB1;SMURF2;ZBTB16;G0S2;ESRRG;INHBB;GADD45G;VEGFA;COL1A1;GATM;REST;SCD;LHX1;LEP;AGO2;MFAP2;SNAI1;CD28;ALPL;MAP3K13;FERMT2
 
 
 
 
Positive Regulation of Focal Adhesion Assembly ( GO:0051894 )
 
 
10/18
 
 
0.0000405
 
 
0.0029780
 
 
0
 
 
0
 
 
7.747659
 
 
78.35636
 
 
NRP1;SFRP1;COL16A1;TSC1;TEK;PPM1F;FERMT2;VEGFA;LIMS1;MAP4K4
 
 
 
 
Negative Regulation of Chondrocyte Differentiation ( GO:0032331 )
 
 
9/15
 
 
0.0000440
 
 
0.0031818
 
 
0
 
 
0
 
 
9.294923
 
 
93.24082
 
 
GREM1;NR5A2;RFLNB;WNT9A;SOX9;GDF5;PTHLH;TGFBR1;ADAMTS7
 
 
 
 
Negative Regulation of Regulated Secretory Pathway ( GO:1903306 )
 
 
6/7
 
 
0.0000448
 
 
0.0031920
 
 
0
 
 
0
 
 
37.150360
 
 
371.96535
 
 
RAP1B;SYT4;REST;RAP1A;STXBP3;FMR1
 
 
 
 
Generation of Neurons ( GO:0048699 )
 
 
47/192
 
 
0.0000624
 
 
0.0043542
 
 
0
 
 
0
 
 
2.019975
 
 
19.55567
 
 
BRSK1;NRP1;SIX1;RTN4;SHH;RELN;AIFM1;ERBB3;CASP3;WNT1;MARK1;WNT4;WNT10B;MAP2K1;TGFB2;EMX2;FZD5;IRX5;FZD4;WNT3A;USP9X;EN2;WNT9B;ATP2B4;WNT7A;SOX11;SOX12;WNT9A;EML1;PEX13;RUNX1;NR4A2;TUBB2A;LMX1A;LHX1;DDIT4;ID1;IL1RAPL1;ID4;ID3;RAPGEF2;INSM2;ERCC6;LHX8;PALS1;PAFAH1B1;CDK5R1
 
 
 
 
Transforming Growth Factor Beta Receptor Signaling Pathway ( GO:0007179 )
 
 
24/76
 
 
0.0000631
 
 
0.0043542
 
 
0
 
 
0
 
 
2.867821
 
 
27.73518
 
 
ITGB1;SMAD2;USP15;TGFB2;TGFB1;USP9X;PTPRK;FOS;SIRT1;GDF5;SMAD5;PIAS2;TGFBR1;SMAD7;DUSP22;TGFBR3;COL3A1;COL1A2;FNTA;PXDN;ID1;ADAM9;MAP3K7;FERMT2
 
 
 
 
Negative Regulation of Intracellular Signal Transduction ( GO:1902532 )
 
 
66/297
 
 
0.0000642
 
 
0.0043673
 
 
0
 
 
0
 
 
1.783929
 
 
17.22049
 
 
DGKD;DDX3X;USP32;WWC2;PTEN;TNFAIP3;RORA;LDLRAD4;LIMD1;HAPSTR1;DUSP16;CRKL;IGF1R;CYP26B1;SPRED1;DEPTOR;NKIRAS2;SMPD1;TMSB4X;SLC39A8;SH3GL2;RUBCN;EDN1;FBXW7;PAQR3;PRKCD;NOG;TSC1;SIRT1;CD2AP;GREM1;TGFBR3;SIKE1;DDIT4;TBC1D7;PPIF;PPARG;MAPKAPK5;ZMYND11;PRKAA1;PRKAA2;RNF34;UACA;PPP2CA;STRIP1;IRAK2;PRKAR2A;RNF39;SLIT2;FNIP1;RFFL;MARK3;PLEKHA1;RIOK3;KLF4;ESR1;TARBP2;SMAD7;MOB4;CYP26A1;PIK3IP1;PKIA;VEPH1;CREBRF;BCL2;CHRFAM7A
 
 
 
 
Proteasomal Protein Catabolic Process ( GO:0010498 )
 
 
79/372
 
 
0.0000680
 
 
0.0045122
 
 
0
 
 
0
 
 
1.685378
 
 
16.17283
 
 
TRIM71;UBXN1;UBE2D3;UBE2D1;PRPF19;PSMD7;DDI2;BTRC;SKP1;NHLRC3;FBXW7;USP44;UBE4A;YOD1;UBE4B;ARMC8;AXIN2;SIRT1;KCTD5;RAD23B;KLHL42;PSMA5;CDC34;PSMA1;GAN;TBL1XR1;CANX;PLAA;KCTD17;PSMD12;RNF34;CUL5;CUL3;DERL1;FBXO45;ASB11;RNF7;USP19;UBE2J1;HSP90B1;SOCS2;ZNRF1;ZNRF2;RMND5A;HECTD3;TBX21;MAN1C1;UBQLN1;PMAIP1;VHL;SOCS6;RFFL;SOCS4;KLHL29;SMURF2;UBE2B;CSNK1A1;SMURF1;FEM1B;SIAH2;FBXL19;SIAH1;KLHL28;TMUB1;KLHL2;BFAR;KLHL23;ERLIN2;UBE2G2;KLHL20;RNF145;UBE2W;APPBP2;GID8;KLHL8;UBE2N;RNF186;SPSB4;CUL4B
 
 
 
 
Protein Modification by Small Protein Removal ( GO:0070646 )
 
 
25/81
 
 
0.0000683
 
 
0.0045122
 
 
0
 
 
0
 
 
2.774292
 
 
26.60841
 
 
USP37;USP15;USP32;USP34;USP12;TNFAIP3;COPS7B;USP19;OTUD6B;USP46;USP47;USP8;USP25;USP6;USP42;USP4;USP21;USP9X;USP44;JOSD1;SENP2;SENP1;COPS2;CDK1;OTUB1
 
 
 
 
Phosphorylation ( GO:0016310 )
 
 
66/298
 
 
0.0000715
 
 
0.0046511
 
 
0
 
 
0
 
 
1.776135
 
 
16.95560
 
 
GMFB;PANK3;DGKD;DYRK2;MAML1;TESK2;LIMD1;MYLK;IKBKB;GNPTAB;AKT3;CHEK1;TLK2;TLK1;MAP3K9;NEK3;EPHA7;STRADB;PRKCD;ADAM10;DYRK1B;LMTK2;CDC25B;CREB1;CCNE1;RARA;SGK3;ERG;SGK1;TRIB2;DGKH;BRSK1;PRKAA1;PRKAA2;PDGFB;STK39;MAPK8;NUAK2;ACVR1C;IRAK2;MKNK1;STK38L;MAP3K21;CCL2;TRPM7;MAPK6;MARK3;FNIP2;MAP4K3;MAP4K4;MARK1;CDK17;SRPK2;MAP3K1;DMPK;CSNK1A1;PLK2;NEK6;NEK7;LIMK1;SRPK1;MEX3B;CAMK4;COPS2;NEK10;MAP3K13
 
 
 
 
Positive Regulation of Filopodium Assembly ( GO:0051491 )
 
 
12/26
 
 
0.0000750
 
 
0.0047538
 
 
0
 
 
0
 
 
5.314656
 
 
50.47857
 
 
CDC42;NRP1;RIPOR2;TENM2;FMR1;PLPPR5;DPYSL3;PALM;FSCN1;CCR7;PIK3R1;FNBP1L
 
 
 
 
Positive Regulation of Programmed Cell Death ( GO:0043068 )
 
 
57/248
 
 
0.0000751
 
 
0.0047538
 
 
0
 
 
0
 
 
1.861547
 
 
17.67790
 
 
HRK;FOXA1;ITGB1;APP;DDX3X;FASLG;TNF;ING5;AIFM1;SMPD1;PHLDA1;HOXA5;MAP3K5;KLF11;RPS6;PAWR;SIRT1;RHOB;SFRP1;MELK;BIN1;VDAC1;ITGA6;TP53;PPID;ZNF395;DCUN1D3;FOXO3;NEUROD1;MAPK8;BCL2L11;ACVR1C;PNMA1;FRZB;PMAIP1;BMF;BAK1;SLIT2;ZNF622;ZNF268;MCL1;HSPA9;WNT10B;PRPF38B;GADD45A;SIAH1;CIDEB;PNMA2;GADD45G;MLLT11;REST;WT1;PTPRA;FASN;JMY;BCL2;BCL2L2
 
 
 
 
Regulation of BMP Signaling Pathway ( GO:0030510 )
 
 
24/77
 
 
0.0000797
 
 
0.0049715
 
 
0
 
 
0
 
 
2.813548
 
 
26.55361
 
 
SMAD2;DLX1;FOXD1;TNFAIP6;SMURF2;SMURF1;FST;UBE2D3;NOG;CHRD;CRIM1;UBE2D1;SOX11;LRP2;LEMD3;FSTL1;GDF5;SMAD7;GREM1;RBPMS2;VWC2L;PPARG;WNT1;SKIL
 
 
 
 
 
 Reg of Phosphatidylinositol 3-Kinase/Prot Kinase B Signal
Transduction ( GO:0051897 )
 
 
41/162
 
 
0.0000811
 
 
0.0049915
 
 
0
 
 
0
 
 
2.109961
 
 
19.87616
 
 
ITGB1;APP;BECN1;PHB1;PIGU;FLT3;PDGFB;EFNA5;TNF;RTN4;IGF1R;TXN2;MTDH;CALCR;RELN;ERBB3;FAM110C;PDGFC;NTF3;CCL3;CCR7;RICTOR;PDGFRB;VAV3;TCF7L2;TGFB2;TGFB1;IGF1;SIRT1;TGFBR1;VEGFA;LIN28A;PIK3CA;LEP;NEDD4;SPRY2;CCM2;TEK;SOS1;FERMT2;HBEGF
 
 
 
 
pre-miRNA Processing ( GO:0031054 )
 
 
8/13
 
 
0.0000938
 
 
0.0056377
 
 
0
 
 
0
 
 
9.911591
 
 
91.92353
 
 
LIN28B;LIN28A;PRKRA;TUT4;AGO2;DROSHA;DICER1;TARBP2
 
 
 
 
Regulation of Osteoblast Differentiation ( GO:0045667 )
 
 
27/92
 
 
0.0000940
 
 
0.0056377
 
 
0
 
 
0
 
 
2.581889
 
 
23.93860
 
 
YAP1;TNFAIP6;PTEN;CHRD;LRP5;LRP3;RORB;LIMD1;IPO7;CCN4;SOX9;WNT4;CTNNBIP1;NOG;CRIM1;SUCO;SOX11;IGF1;ACVR2B;SMAD5;GREM1;REST;SMOC1;PPARG;IL6ST;SOS1;FERMT2
 
 
 
 
Cellular Response to Decreased Oxygen Levels ( GO:0036294 )
 
 
24/78
 
 
0.0001000
 
 
0.0058988
 
 
0
 
 
0
 
 
2.761284
 
 
25.43168
 
 
EGLN1;SUV39H2;EGLN2;TGFB1;SUV39H1;GATA6;RORA;TSC1;AK4;HIF3A;SIRT1;RTN4;ZFP36L1;VEGFA;NPEPPS;SFRP1;CPEB1;UBQLN1;PMAIP1;PPARG;MDM4;VHL;TP53;CPEB2
 
 
 
 
Regulation of Translation ( GO:0006417 )
 
 
48/201
 
 
0.0001010
 
 
0.0058988
 
 
0
 
 
0
 
 
1.954883
 
 
17.98591
 
 
EIF4A2;TRIM71;APP;DDX6;BTG2;DDX3X;FMR1;STK39;MSI2;YBX1;BZW2;FOXO3;BZW1;TOB1;LARP1B;FXR1;RPS6KA3;SYNCRIP;ATXN2;OTUD6B;EIF4EBP1;IGF2BP1;IGF2BP3;IGF2BP2;PAIP2;ZNF385A;NANOS1;TIA1;NSUN3;SAMD4A;LARP4B;LARP4;QKI;DHFR;LIN28A;WT1;CSDE1;AGO2;SERBP1;BCL3;PRR16;CD28;MAPKAPK5;CPEB3;EIF4E2;DUS3L;EIF4G2;METAP1
 
 
 
 
Autophagosome Maturation ( GO:0097352 )
 
 
18/51
 
 
0.0001036
 
 
0.0059726
 
 
0
 
 
0
 
 
3.385641
 
 
31.06438
 
 
BECN1;GABARAPL1;STX17;LIX1L;VPS4A;ATP2A2;WIPI2;ATG14;VAMP8;VMP1;VAMP7;CHMP1B;CHMP2B;RUBCNL;UBQLN1;CHMP4B;SNAP29;VTI1B
 
 
 
 
Positive Regulation of Gene Expression ( GO:0010628 )
 
 
89/436
 
 
0.0001100
 
 
0.0062657
 
 
0
 
 
0
 
 
1.604048
 
 
14.62065
 
 
APP;PHB1;DDX3X;TFRC;FMR1;CTCF;TNF;TENT4B;LARP1B;ROBO1;SCAMP5;EPB41L4B;OTUD6B;DNMT3B;HABP4;ITGB8;SOX9;HOXA5;IL10;MAP2K1;FOXD1;IL15;IL13;NOG;SOX11;MITF;LARP4B;NGF;F3;MED26;ADRA2A;TGFBR1;ACTA2;TGFBR3;NR5A2;MYCN;IFNG;HAND2;CSDE1;PRR16;ERP29;PPARG;TP53;RBM24;YAP1;DNMT1;PDGFB;STK39;PIK3R3;LRP3;PIK3R1;PPM1F;FXR1;DLL4;NRAS;MAPK8;CALCR;PDCD10;CCL3;CCL2;LURAP1;MARK1;SRPK2;SEC16B;TGFB1;WNT3A;RDX;SAMD4A;DTNBP1;ZNF804A;HMGA2;IGF1;LARP4;KLF4;VEGFA;PDCL3;REST;ACTC1;WT1;DNAJA4;FUBP1;AGO2;BCL3;CD28;SPRY2;ERCC6;CPEB3;LAMTOR5;LIMS1
 
 
 
 
Positive Regulation of miRNA Transcription ( GO:1902895 )
 
 
17/47
 
 
0.0001131
 
 
0.0063095
 
 
0
 
 
0
 
 
3.516649
 
 
31.95808
 
 
FOXA1;IL10;SREBF1;TGFB2;TGFB1;PDGFB;FOS;FOXO3;NR3C1;KLF4;TNF;APLN;MYCN;WT1;MYB;PPARG;TP53
 
 
 
 
Positive Regulation of Cell Population Proliferation ( GO:0008284 )
 
 
97/484
 
 
0.0001136
 
 
0.0063095
 
 
0
 
 
0
 
 
1.568472
 
 
14.24672
 
 
CD86;PHB1;PGAP1;IRS2;TNF;CRKL;IGF1R;FGF7;CCND2;CHP2;CCN4;SOX9;PITX2;PDGFRB;RPS6;ADAM10;SOX11;CDC25B;TIAM1;SFRP1;ADAM17;COMMD3-BMI1;IL6ST;CUL3;PDGFB;CHRD;TGFA;TNFRSF11A;PIK3R1;PRDX3;EFNB2;PDCD10;PDGFC;SSR1;WNT1;ZNF268;SRPK2;TGFB2;TGFB1;TSLP;WNT3A;LIF;HMGA2;IGF1;BMP5;CNOT7;FGF18;BCL2;CHRFAM7A;FLT3;PTEN;ASH2L;LAMC1;GHR;SHH;ESM1;BCL7A;RBPMS2;SS18;EDN1;CNOT6L;IL15;TBC1D8;CCDC117;OSMR;SIRT1;PAX2;ADRA2A;TGFBR1;GREM1;TGFBR3;IFNG;RARA;SINHCAF;CACUL1;HBEGF;HDAC4;YAP1;LRP5;TTK;PTHLH;DLL4;RPS15A;ERBB3;NTF3;STX3;TCF7L2;VEGFC;NAP1L1;VEGFA;TJP1;KITLG;MEIS1;GID8;DNAJA2;AQP11;CCM2
 
 
 
 
Positive Regulation of Epithelial Cell Proliferation ( GO:0050679 )
 
 
32/118
 
 
0.0001196
 
 
0.0065266
 
 
0
 
 
0
 
 
2.314165
 
 
20.89998
 
 
YAP1;NRP1;PDGFB;HMGB2;TGFA;MIA3;NRARP;LAMC1;CLDN1;TNF;RTN4;FGF7;NRAS;ERBB3;AKT3;SOX9;RREB1;TCF7L2;EGR3;VASH2;WNT7A;SOX11;IGF1;F3;SIRT1;PAX2;TGFBR1;BMP5;VEGFA;PDCL3;SFRP1;TEK
 
 
 
 
Regulation of Peptidyl-Tyrosine Phosphorylation ( GO:0050730 )
 
 
16/43
 
 
0.0001213
 
 
0.0065266
 
 
0
 
 
0
 
 
3.676855
 
 
33.15420
 
 
EPHA7;PGAP1;CADM4;TGFB1;IL15;LIF;EFNA5;VEGFA;RAP2C;PDCL3;FGF7;SFRP1;CNOT7;RAP2B;ENPP2;CCM2
 
 
 
 
Positive Regulation of Macromolecule Biosynthetic Process ( GO:0010557 )
 
 
75/355
 
 
0.0001218
 
 
0.0065266
 
 
0
 
 
0
 
 
1.673144
 
 
15.08076
 
 
APP;PHB1;DDX3X;DYRK2;TFRC;IRS2;CTCF;TNF;ROBO1;EPB41L4B;DNMT3B;ITGB8;SOX9;HOXA5;PDGFRB;MAP2K1;FOXD1;IL13;NOG;SOX11;MITF;NGF;F3;MED26;SIRT1;TGFBR1;ACTA2;TGFBR3;MYCN;IFNG;PTRH2;HAND2;ERP29;PPARG;SOS1;TP53;YAP1;DNMT1;PDGFB;PIK3R3;LRP3;PPM1F;DLL4;NRAS;MAPK8;CALCR;PDCD10;CCL3;CCL2;MARK1;SRPK2;SEC16B;TGFB2;TGFB1;WNT3A;RDX;DTNBP1;ZNF804A;HMGA2;IGF1;KLF4;VEGFA;PDCL3;SLC25A37;REST;ACTC1;WT1;DNAJA4;FUBP1;CD28;SPRY2;ERCC6;CDK5R1;LAMTOR5;LIMS1
 
 
 
 
Positive Regulation of Neuron Apoptotic Process ( GO:0043525 )
 
 
15/39
 
 
0.0001274
 
 
0.0067482
 
 
0
 
 
0
 
 
3.877210
 
 
34.77211
 
 
NANOS1;SRPK2;PRNP;PHB1;EPHA7;TGFB2;FBXW7;FOXO3;TNF;CASP7;CASP5;CASP3;CCL3;MCL1;CDK5R1
 
 
 
 
Intrinsic Apoptotic Signaling Pathway in Response to DNA Damage ( GO:0008630 )
 
 
18/52
 
 
0.0001379
 
 
0.0072235
 
 
0
 
 
0
 
 
3.285872
 
 
29.20717
 
 
DYRK2;CIDEB;TNFRSF1B;SIRT1;UACA;BCL2L11;DDIT4;BCL3;BCL2;EP300;CDIP1;BAK1;IKBKE;TP53;FNIP2;BCL2L2;EPHA2;MCL1
 
 
 
 
Protein Modification by Small Protein Conjugation ( GO:0032446 )
 
 
68/316
 
 
0.0001401
 
 
0.0072559
 
 
0
 
 
0
 
 
1.711541
 
 
15.18617
 
 
UBE2D2;CCNF;UBE2D3;CDC23;CDC27;BTRC;RNF152;SKP1;TRIM23;ARRDC4;FBXW7;NSMCE2;FBXO10;SENP2;SIRT1;PIAS2;SENP1;CDC34;GAN;UBE2V2;HDAC4;RNF34;CUL5;UHRF2;UBA6;DCUN1D3;CUL3;DCUN1D4;MYCBP2;CUL2;RNF38;DCUN1D2;UBR3;DTX2;FBXO45;ASB11;RNF7;RCHY1;DCAF15;CAND1;RNF138;RBBP6;VHL;MARCHF3;UBE2F;SLF2;UBE2I;CBX4;UBE2B;EYA1;USP9X;KLHL2;FBXO32;KLHL20;UFM1;NEDD4;COPS2;KLHL8;OTULIN;RLIM;RNF182;UBE2N;MKRN3;MDM4;RNF183;RNF220;SPSB4;CUL4B
 
 
 
 
Chromatin Organization ( GO:0006325 )
 
 
69/323
 
 
0.0001581
 
 
0.0080924
 
 
0
 
 
0
 
 
1.695710
 
 
14.84152
 
 
RB1;FOXA1;SUV39H2;SUV39H1;CHD7;HMGB2;CTCF;NUDT5;CHD1;LOXL2;YY1;ING5;BCL7A;CHEK1;DPF2;EPC1;SMARCAD1;SOX9;JARID2;ARID2;HMGN2;HMGN3;SKP1;H3-3B;KDM6B;SS18;NSMCE2;TET1;BAZ1B;SAFB;SIRT1;ARID1B;CFDP1;NR5A2;KAT6B;HAT1;COMMD3-BMI1;TOP1;ASF1A;HDAC4;ZNF274;DNMT1;INO80D;SATB1;SATB2;MACROH2A1;ATAD2B;CECR2;MIER1;RBBP7;MTA3;H2AZ1;SLF2;SETDB1;CBX3;FBXL19;HMGA2;NAP1L1;INO80;NPM3;ESR1;SRPK1;TADA2B;REST;NASP;POLE3;SNAI1;BCOR;ERCC6
 
 
 
 
Cellular Response to Starvation ( GO:0009267 )
 
 
36/141
 
 
0.0001772
 
 
0.0088986
 
 
0
 
 
0
 
 
2.133070
 
 
18.42549
 
 
BECN1;PRKAA1;MTMR3;SEH1L;PRKAA2;RALB;SUV39H1;PRKAG2;FOXO3;MAPK8;NUAK2;SESN1;SESN2;PDK4;PMAIP1;FNIP1;RNF152;MAP3K5;MFSD2A;ATG5;SH3GLB1;KLF10;SREBF1;GABARAPL1;TFEB;TSC1;INHBB;WIPI2;ATG14;EIF2S1;SIRT1;SFRP1;FASN;JMY;TBC1D7;TP53
 
 
 
 
Regulation of Filopodium Assembly ( GO:0051489 )
 
 
15/40
 
 
0.0001777
 
 
0.0088986
 
 
0
 
 
0
 
 
3.721905
 
 
32.13941
 
 
NRP1;RIPOR2;TENM2;FMR1;PLPPR5;PRKCD;PIK3R1;FNBP1L;CDC42;GAP43;DPYSL3;PALM;FSCN1;CCR7;ARF6
 
 
 
 
Positive Regulation of miRNA Metabolic Process ( GO:2000630 )
 
 
18/53
 
 
0.0001819
 
 
0.0090090
 
 
0
 
 
0
 
 
3.191804
 
 
27.48762
 
 
FOXA1;IL10;SREBF1;TGFB2;TGFB1;PDGFB;FOS;NR3C1;FOXO3;KLF4;TNF;APLN;LIN28B;MYCN;WT1;MYB;PPARG;TP53
 
 
 
 
miRNA Processing ( GO:0035196 )
 
 
13/32
 
 
0.0001872
 
 
0.0091709
 
 
0
 
 
0
 
 
4.242697
 
 
36.41644
 
 
TRIM71;SMAD2;TUT4;DICER1;TARBP2;LIN28B;LIN28A;PRKRA;AGO2;HNRNPA2B1;DROSHA;SNIP1;SRSF3
 
 
 
 
Negative Regulation of Cartilage Development ( GO:0061037 )
 
 
8/14
 
 
0.0001921
 
 
0.0093096
 
 
0
 
 
0
 
 
8.259179
 
 
70.67887
 
 
GREM1;NR5A2;WNT9A;SOX9;GDF5;PTHLH;TGFBR1;ADAMTS7
 
 
 
 
Regulation of Epithelial to Mesenchymal Transition ( GO:0010717 )
 
 
26/91
 
 
0.0002032
 
 
0.0097444
 
 
0
 
 
0
 
 
2.485362
 
 
21.12902
 
 
FOXA1;PTEN;LDLRAD4;LOXL2;PPP2CA;SPRED1;CLASP2;MARK1;SMAD2;TCF7L2;TGFB2;TGFB1;AXIN2;TBX5;TGFBR1;QKI;BMP5;SMAD7;GREM1;COL1A1;TIAM1;SFRP1;SNAI1;SPRY2;SPRY1;FERMT2
 
 
 
 
Sprouting Angiogenesis ( GO:0002040 )
 
 
19/58
 
 
0.0002085
 
 
0.0098971
 
 
0
 
 
0
 
 
3.023964
 
 
25.62919
 
 
NRP1;EGR3;TGFB1;PIK3R3;VEGFC;MIA3;NRARP;LOXL2;ROBO1;VEGFA;GREM1;EFNB2;ESM1;OTULIN;RSPO3;SLIT2;TEK;RECK;E2F7
 
 
 
 
Positive Regulation of Cell Migration ( GO:0030335 )
 
 
72/344
 
 
0.0002210
 
 
0.0103778
 
 
0
 
 
0
 
 
1.652412
 
 
13.90930
 
 
ITGB1;NRP1;SPARC;MIA3;RORA;CLDN1;CRKL;MYLK;IGF1R;FAM110C;ENPP2;CCN4;SOX9;ACVR1;PDGFRB;EDN1;SEMA6A;SEMA6D;ADAM10;F3;ADRA2A;TGFBR1;RHOB;PTP4A1;TIAM1;ADAM17;IFNG;RAPGEF2;ADAM9;PLAA;ITGA6;SOS1;EPHA2;HBEGF;IGSF8;SEMA7A;CCL3L1;PDGFB;PIK3R3;RTN4;PPM1F;CDC42;CCL8;CCL7;PDCD10;PDGFC;NTF3;CCL3;CCL2;RICTOR;RREB1;ZNF268;MAP4K4;CLASP2;TGFB2;TGFB1;UBE2I;SMURF2;FZD4;RDX;SEMA4C;SEMA4F;IGF1;VEGFA;COL1A1;TJP1;GNPDA1;AGO2;SNAI1;SPRY2;TEK;FERMT2
 
 
 
 
Cellular Response to Hypoxia ( GO:0071456 )
 
 
23/77
 
 
0.0002265
 
 
0.0105306
 
 
0
 
 
0
 
 
2.645273
 
 
22.20098
 
 
EGLN1;SUV39H2;EGLN2;TGFB1;SUV39H1;GATA6;RORA;AK4;HIF3A;SIRT1;RTN4;ZFP36L1;VEGFA;NPEPPS;SFRP1;CPEB1;UBQLN1;PMAIP1;PPARG;MDM4;VHL;TP53;CPEB2
 
 
 
 
Regulation of Cold-Induced Thermogenesis ( GO:0120161 )
 
 
37/148
 
 
0.0002294
 
 
0.0105564
 
 
0
 
 
0
 
 
2.073845
 
 
17.37904
 
 
RB1;KDM3A;LAMA4;DOCK7;ADRB1;PCTP;ADRB2;ADIPOR2;IGF1R;PDGFC;LDLR;MAP2K6;KDM6B;WNT10B;PRKAB2;IL15;ARRDC3;ELOVL3;IL13;PLCL2;EBF2;G0S2;ESRRG;ACVR2B;QKI;GADD45G;VEGFA;ADAM17;GATM;ADRB3;NOVA1;SCD;LEP;ID1;MFAP2;ALPL;LGR4
 
 
 
 
Regulation of Autophagy ( GO:0010506 )
 
 
52/230
 
 
0.0002336
 
 
0.0106406
 
 
0
 
 
0
 
 
1.820340
 
 
15.22188
 
 
USP32;PIK3C2A;DEPTOR;CASP3;RB1CC1;TLK2;GPR137;EP300;PIP4K2B;ATP6V1E1;RNF152;RAB8A;TRIM23;IL10;SH3GLB1;RUBCN;TFEB;ATG14;CCNY;IFNG;ULK2;VDAC1;SOS1;BECN1;PRKAA1;PRKAA2;MTMR9;FOXK2;FOXO3;C9ORF72;MTDH;MAPK8;TP53INP1;ZKSCAN3;UBQLN1;STK38L;BMF;VHL;DCAF12;SNX5;SNX6;MCL1;CTSA;PLK2;RAB39B;SVIP;ACER2;NEDD4;LEP;BCL2;EIF4G2;CDK5R1
 
 
 
 
Regulation of TORC1 Signaling ( GO:1903432 )
 
 
27/97
 
 
0.0002481
 
 
0.0111904
 
 
0
 
 
0
 
 
2.396769
 
 
19.89745
 
 
PRKAA1;SEH1L;PRKAA2;USP32;CUL3;UBE2D1;C9ORF72;DEPTOR;PIP4P1;SESN1;SESN2;EP300;GPR137;VHL;FNIP1;RNF152;FNIP2;USP4;CSNK1A1;TSC1;GPR137C;UBE2W;TBC1D7;UBE2N;LAMTOR3;OTUB1;LAMTOR5
 
 
 
 
Negative Regulation of miRNA Transcription ( GO:1902894 )
 
 
11/25
 
 
0.0002533
 
 
0.0112040
 
 
0
 
 
0
 
 
4.870013
 
 
40.32837
 
 
ZNF512B;NCOR2;REST;TGFB1;PDGFB;RARA;PPARG;SOX9;TNF;ESR1;VEGFA
 
 
 
 
Stress Granule Assembly ( GO:0034063 )
 
 
11/25
 
 
0.0002533
 
 
0.0112040
 
 
0
 
 
0
 
 
4.870013
 
 
40.32837
 
 
DAZAP2;DDX6;ATXN2;TIA1;DDX3X;FMR1;CSDE1;G3BP1;UBAP2L;C9ORF72;EIF2S1
 
 
 
 
Positive Regulation of TOR Signaling ( GO:0032008 )
 
 
22/73
 
 
0.0002672
 
 
0.0117042
 
 
0
 
 
0
 
 
2.678599
 
 
22.03849
 
 
SEH1L;USP32;USP4;CSNK1A1;USP9X;CUL3;F3;GPR137C;PIP4P1;RELN;LIN28A;PIK3CA;LEP;SESN2;EP300;GPR137;RICTOR;FNIP1;FNIP2;LAMTOR3;OTUB1;LAMTOR5
 
 
 
 
Autophagosome Membrane Docking ( GO:0016240 )
 
 
5/6
 
 
0.0002774
 
 
0.0120349
 
 
0
 
 
0
 
 
30.947501
 
 
253.46565
 
 
VAMP8;VMP1;STX17;ATP2A2;SNAP29
 
 
 
 
Regulation of Activin Receptor Signaling Pathway ( GO:0032925 )
 
 
9/18
 
 
0.0002876
 
 
0.0123613
 
 
0
 
 
0
 
 
6.195535
 
 
50.51804
 
 
FKBP1A;SHH;SMURF1;FST;IGSF1;FKBP1C;LEMD3;ACVR2B;SMAD7
 
 
 
 
Protein Glycosylation ( GO:0006486 )
 
 
36/145
 
 
0.0003194
 
 
0.0136017
 
 
0
 
 
0
 
 
2.054312
 
 
16.53501
 
 
GALNT12;POGLUT1;B4GALT1;GALNT15;RPN1;FUT4;TMEM165;FUT8;SERP1;PGM3;MAN1C1;GCNT3;SLC39A8;ST3GAL6;GCNT4;LARGE1;LARGE2;ARFGEF1;GALNT5;PMM1;ENTPD5;GFPT2;GALNT1;GFPT1;TET2;TET1;B3GNT7;OSTC;B3GNT5;DOLPP1;MGAT4A;EOGT;TET3;AQP11;POC1B-GALNT4;B4GALT5
 
 
 
 
Regulation of Angiogenesis ( GO:0045765 )
 
 
48/211
 
 
0.0003420
 
 
0.0144291
 
 
0
 
 
0
 
 
1.833876
 
 
14.63539
 
 
ITGB1;PIGU;SPARC;PLXND1;SERPINE1;GATA6;TNFAIP3;FASLG;FOXO4;CXCL13;RTN4;MTDH;AMOT;MECP2;SPRED1;PDCD10;ITGB8;HOXA5;EGLN1;TGFB2;SEMA6A;CEMIP2;VASH2;ATP2B4;FOXJ2;HMGA2;KLF4;ISL1;F3;SIRT1;QKI;RUNX1;RHOB;VEGFA;PDCL3;CXCL10;SFRP1;COL4A2;LEP;TSPAN18;AGO2;FGF18;SPRY2;CHRFAM7A;PPARG;CCM2;TEK;EPHA2
 
 
 
 
Intrinsic Apoptotic Signaling Pathway ( GO:0097193 )
 
 
30/115
 
 
0.0003987
 
 
0.0165693
 
 
0
 
 
0
 
 
2.193588
 
 
17.17004
 
 
ERO1A;DDX3X;CUL5;CUL3;CUL2;GSKIP;SELENOK;UACA;BCL2L11;AIFM1;CASP3;PMAIP1;CHAC1;BAK1;ZNF622;IKBKE;FNIP2;MAP3K5;MCL1;PRKCD;CIDEB;TNFRSF1B;MLLT11;JMY;BCL2;CYCS;RNF186;TP53;BCL2L2;EPHA2
 
 
 
 
Peptidyl-Serine Modification ( GO:0018209 )
 
 
22/75
 
 
0.0004073
 
 
0.0165693
 
 
0
 
 
0
 
 
2.577219
 
 
20.11754
 
 
SRPK2;BRSK1;POGLUT2;POGLUT1;GALNT15;GALNT1;NEK6;PLK2;PRKCD;STK39;LMTK2;TGFBR1;IKBKB;MAPK8;MKNK1;TLK2;CDK1;POC1B-GALNT4;TOP1;MAP3K13;MARK3;CDK5R1
 
 
 
 
Positive Regulation of Protein Serine/Threonine Kinase Activity ( GO:0071902 )
 
 
22/75
 
 
0.0004073
 
 
0.0165693
 
 
0
 
 
0
 
 
2.577219
 
 
20.11754
 
 
PDGFRB;EDN1;MAP2K1;RALB;FLT3;PDGFB;HMGA2;TNF;SIRT1;RASGRP1;ADRA2A;ROBO1;GHR;PPP2CA;CCNY;IFNG;TCIM;PDCD10;FGF18;NEK10;RAPGEF2;DIPK2A
 
 
 
 
Regulation of Fat Cell Differentiation ( GO:0045598 )
 
 
22/75
 
 
0.0004073
 
 
0.0165693
 
 
0
 
 
0
 
 
2.577219
 
 
20.11754
 
 
YAP1;WNT10B;TGFB1;LMO3;ZBTB16;LRP5;LPL;RORA;LRP3;TNF;SIRT1;ZFP36L2;ZFP36L1;SFRP1;CREB1;FRZB;LEP;PPARG;WNT1;TRIB2;ZNF385A;FERMT2
 
 
 
 
Cell Differentiation in Spinal Cord ( GO:0021515 )
 
 
6/9
 
 
0.0004179
 
 
0.0167030
 
 
0
 
 
0
 
 
12.382014
 
 
96.33417
 
 
LHX1;ZC4H2;SOX13;FOXN4;MDGA2;ISL1
 
 
 
 
Negative Regulation of Cytoplasmic Translation ( GO:2000766 )
 
 
6/9
 
 
0.0004179
 
 
0.0167030
 
 
0
 
 
0
 
 
12.382014
 
 
96.33417
 
 
CPEB1;FMR1;PARP16;CPEB3;CPEB2;RBM24
 
 
 
 
Response to UV ( GO:0009411 )
 
 
27/100
 
 
0.0004234
 
 
0.0167758
 
 
0
 
 
0
 
 
2.297870
 
 
17.84775
 
 
BRSK1;DCUN1D3;PTPRK;TRIAP1;YY1;MAPK8;CCND1;SMPD1;PCLAF;EP300;IL12B;BMF;BAK1;MAP3K4;N4BP1;MAP4K3;UBE2B;FBXW7;PRKCD;REV1;UBE4B;INO80;EIF2S1;NEDD4;BCL3;TP53;CUL4B
 
 
 
 
Protein Maturation ( GO:0051604 )
 
 
38/158
 
 
0.0004367
 
 
0.0171535
 
 
0
 
 
0
 
 
1.969833
 
 
15.23894
 
 
LIPT2;ISCA2;PIGU;PGAP1;NAA20;RAP1GDS1;PCSK5;CISD3;CTSS;ZMPSTE24;HSPD1;IKBKB;CASP7;FNTA;CASP5;CASP3;DDI2;TASP1;TSPAN5;PSENEN;GLRX5;ADAM10;F3;KLK10;FKBP1A;AFG3L2;BACE2;ADAM17;TSPAN14;MMP16;BOLA3;DNAJB11;ADAM9;MMS19;TLL1;NAA15;METAP1;METAP2
 
 
 
 
Cellular Response to Glucose Starvation ( GO:0042149 )
 
 
15/43
 
 
0.0004444
 
 
0.0173049
 
 
0
 
 
0
 
 
3.322550
 
 
25.64606
 
 
SH3GLB1;BECN1;PRKAA1;MTMR3;PRKAA2;SUV39H1;PRKAG2;FOXO3;ATG14;SIRT1;NUAK2;SESN1;SESN2;PMAIP1;TP53
 
 
 
 
Embryonic Digestive Tract Development ( GO:0048566 )
 
 
9/19
 
 
0.0004791
 
 
0.0184072
 
 
0
 
 
0
 
 
5.575657
 
 
42.61854
 
 
TGFB2;PKDCC;SALL1;FOXF1;RBPMS2;RARB;SOX11;PCSK5;TNF
 
 
 
 
Regulation of G1/S Transition of Mitotic Cell Cycle ( GO:2000045 )
 
 
29/111
 
 
0.0004808
 
 
0.0184072
 
 
0
 
 
0
 
 
2.197634
 
 
16.79013
 
 
RB1;ANKRD17;DDX3X;CCNH;DCUN1D3;PTEN;CDC73;PPP2CA;CCND2;CCND1;BCL7A;DPF2;CCL2;ARID2;E2F7;ACVR1;KLF11;PLK5;RRM2;RDX;INO80;KLF4;ARID1B;ADAM17;WEE1;TFDP1;CCNE2;CCNE1;CRLF3
 
 
 
 
Negative Regulation of Signal Transduction ( GO:0009968 )
 
 
59/277
 
 
0.0004912
 
 
0.0186507
 
 
0
 
 
0
 
 
1.686775
 
 
12.85083
 
 
RB1;OTUD4;YWHAB;HHIP;TNFAIP3;CCNC;NRARP;TNF;SPRED1;QARS1;SMPD1;HEY2;IL12B;PIP4K2B;CHAC1;HIF1AN;BTRC;ACVR1;IL10;SEMA6A;AHSG;FBXW7;PRKCD;NOG;CTNNBIP1;RPGRIP1L;SFRP1;ADRB3;PPARG;TMBIM6;MDFI;KANK1;PALM3;ADRB2;NKD1;GLIS2;SOCS2;DLL4;SOCS1;ERBB3;FRZB;WIF1;KCTD21;LEPROTL1;VHL;CADM4;BDNF;TMED7-TICAM2;SMAD5;BMP5;APLN;NR4A2;TCIM;KIF26A;NEDD4;BCL2;SPRY2;SPRY1;LGR4
 
 
 
 
Positive Regulation of Intracellular Signal Transduction ( GO:1902533 )
 
 
121/652
 
 
0.0004959
 
 
0.0186709
 
 
0
 
 
0
 
 
1.426487
 
 
10.85442
 
 
CD86;APP;PHB1;TFRC;GOLT1B;RORA;TNF;IGF1R;TXN2;FGF7;FAM110C;IL12B;MAP3K7;ACVR1;PDGFRB;LMO3;ARRDC3;PAQR3;SOX11;ERP29;WDFY1;UBE2V1;TP53;S100A8;ANKRD17;PDGFB;TGFA;ADRB1;PIK3R1;ADRB2;EFNA5;RTN4;MTDH;BCL2L11;CALCR;IRAK2;PDGFC;MIER1;PMAIP1;ZNF268;SLC15A4;WNT4;PRNP;NDFIP2;TGFB2;TGFB1;CAV2;PLK2;NEK6;SIAH1;LIF;IGF1;GDF6;GDF5;BMP5;PPP5C;LIN28A;FGF18;UBE2N;CHRFAM7A;ZCCHC3;FERMT2;TMEM9B;ITGB1;PIGU;DDX3X;FLT3;PTEN;FASLG;IKBKB;DHX15;CCR7;IKBKE;VAV3;EDN1;GPR37;CHUK;ZDHHC13;F3;SIRT1;ZDHHC17;ADRA2A;TGFBR1;TNFRSF1A;TGFBR3;PIK3CA;ADRB3;TRAF5;SOS1;HBEGF;BECN1;USP15;CANT1;SLC20A1;PELI2;TTK;AKAP6;RELN;ERBB3;NTF3;CCL3;RNF39;RICTOR;ZNF622;FNIP1;LURAP1;MAP4K4;TCF7L2;TMED7-TICAM2;VEGFA;FKBP1A;P2RX5;TCIM;LEP;NEDD4;SPRY2;CCM2;TEK;LAMTOR3;LAMTOR5;LIMS1
 
 
 
 
Protein O-linked Glycosylation ( GO:0006493 )
 
 
25/91
 
 
0.0005132
 
 
0.0191658
 
 
0
 
 
0
 
 
2.352573
 
 
17.82028
 
 
GALNT12;POMT2;POGLUT2;POGLUT1;GALNT15;GXYLT1;TMTC3;GXYLT2;FUT4;PGM3;GCNT3;GCNT4;LARGE1;LARGE2;GALNT5;GALNT1;TET2;TET1;B3GNT7;B4GAT1;B3GNT5;EOGT;TET3;POC1B-GALNT4;B4GALT5
 
 
 
 
Response to Transforming Growth Factor Beta ( GO:0071559 )
 
 
13/35
 
 
0.0005300
 
 
0.0196324
 
 
0
 
 
0
 
 
3.663508
 
 
27.63234
 
 
DLX1;TGFB1;WNT7A;NR3C1;ZFP36L2;TGFBR1;CRKL;ZFP36L1;SFRP1;SOX9;SOX5;WNT4;LIMS1
 
 
 
 
Apoptotic Process ( GO:0006915 )
 
 
51/232
 
 
0.0005369
 
 
0.0197257
 
 
0
 
 
0
 
 
1.754791
 
 
13.21314
 
 
CSRNP1;FASLG;AHR;CASP7;ATCAY;AIFM1;CASP5;CASP3;CHEK1;DPF2;CAPN3;EP300;RNF152;MAP3K7;ZNF385A;MAP3K5;IL10;SEMA6A;PRKCD;PAWR;NGF;RHOB;MELK;PLSCR3;ESPL1;IFNG;PIK3CA;DDIT4;VDAC1;ZNF395;EFNA5;RTN4;AKAP1;HSPD1;FXR1;BCL2L11;ERBB3;PMAIP1;BMF;BAK1;TGFB1;DMPK;GADD45A;FEM1B;ZBTB16;SIAH1;CIDEB;NSG1;GDF6;FASN;BCL2
 
 
 
 
Transition Metal Ion Transport ( GO:0000041 )
 
 
12/31
 
 
0.0005585
 
 
0.0203557
 
 
0
 
 
0
 
 
3.914924
 
 
29.32389
 
 
SLC30A6;SLC30A9;SLC30A8;TFRC;SLC31A1;SLC41A2;SLC30A5;SLC39A8;TRPM7;SLC30A1;SLC30A10;TMEM165
 
 
 
 
Protein Phosphorylation ( GO:0006468 )
 
 
75/373
 
 
0.0005809
 
 
0.0210047
 
 
0
 
 
0
 
 
1.570410
 
 
11.70101
 
 
GMFB;DYRK2;MAML1;FLT3;TESK2;MYLK;IGF1R;IKBKB;EEF2K;AKT3;CHEK1;TLK2;TLK1;MAP3K9;NEK3;MAP3K7;PDGFRB;STRADB;PRKCD;ADAM10;DYRK1B;LMTK2;TGFBR1;CDC25B;MELK;CREB1;CCNE1;RARA;ULK2;SGK3;MAPKAPK5;ERG;TOP1;SGK1;TRIB2;BRSK1;PRKAA1;PKDCC;PRKAA2;PDGFB;STK39;STK33;MAPK8;NUAK2;RELN;ACVR1C;IRAK2;MKNK1;STK38L;MAP3K21;CCL2;TRPM7;MAPK6;MARK3;FNIP2;MAP4K3;MAP4K4;MARK1;CDK17;SRPK2;MAP3K1;DMPK;CSNK1A1;PLK2;NEK6;NEK7;LIMK1;SRPK1;MEX3B;CAMK4;COPS2;NEK10;CDK1;MAP3K13;CDK5R1
 
 
 
 
Chromatin Remodeling ( GO:0006338 )
 
 
56/262
 
 
0.0006155
 
 
0.0219411
 
 
0
 
 
0
 
 
1.693602
 
 
12.52106
 
 
RB1;FOXA1;LIN54;SUV39H2;PHB1;CHD7;CTCF;NUDT5;CHD1;YY1;BCL7A;CHEK1;DPF2;SMARCAD1;SOX9;JARID2;ARID2;SKP1;KDM6B;SS18;USP3;TET1;BAZ1B;SIRT1;ARID1B;CFDP1;NR5A2;MTF2;COMMD3-BMI1;TOP1;ASF1A;CGGBP1;HDAC4;ZNF274;USP15;INO80D;SATB1;SATB2;MACROH2A1;CECR2;TDG;MIER1;RBBP7;MTA3;SRPK2;CBX3;FBXL19;INO80;NPM3;ESR1;TADA2B;MCM3AP;REST;POLE3;BCOR;ERCC6
 
 
 
 
Cellular Response to Tumor Necrosis Factor ( GO:0071356 )
 
 
24/87
 
 
0.0006164
 
 
0.0219411
 
 
0
 
 
0
 
 
2.365574
 
 
17.48529
 
 
ASAH1;CHUK;ZFAND6;TNFRSF11A;TNFRSF1B;FOXO3;TNF;YBX3;SIRT1;ZFP36L2;ST18;TNFRSF1A;ZFP36L1;IKBKB;SFRP1;BAG4;TMSB4X;CCL3;CCL2;TP53;GBP1;ZNF268;ADAMTS7;LIMS1
 
 
 
 
Neurotrophin Signaling Pathway ( GO:0038179 )
 
 
8/16
 
 
0.0006345
 
 
0.0222462
 
 
0
 
 
0
 
 
6.193664
 
 
45.60174
 
 
DOCK3;RAP1A;BDNF;CASP3;DDIT4;NTF3;RAPGEF2;NGF
 
 
 
 
Cellular Response to Growth Factor Stimulus ( GO:0071363 )
 
 
36/150
 
 
0.0006348
 
 
0.0222462
 
 
0
 
 
0
 
 
1.963636
 
 
14.45678
 
 
DLX1;NRP1;STMN2;PDGFB;LRP2;NR3C1;ZFP36L2;CRKL;ZFP36L1;TMEM100;DLL4;RAP1A;ACVR1C;CCL2;SOX9;SOX5;WNT4;ADAMTS7;ACVR1;NANOS1;DDX18;SEMA6A;EGR3;PLK5;TGFB1;WNT7A;KLF4;ACVR2B;TGFBR1;VEGFA;DUSP22;SFRP1;RAPGEF2;SPRY2;SOS1;LIMS1
 
 
 
 
Axonogenesis ( GO:0007409 )
 
 
45/200
 
 
0.0006596
 
 
0.0229406
 
 
0
 
 
0
 
 
1.806863
 
 
13.23320
 
 
APP;SPTBN4;NRP1;BRSK1;SEMA7A;MYCBP2;DOCK7;PTEN;NRXN3;EFNA5;C9ORF72;EFNB2;SHH;PARD6B;RELN;DPYSL5;KIF5C;SLITRK1;NPTN;OTX2;SLIT2;RAB8A;LGI1;EPHA7;SEMA6A;USP9X;BDNF;SEMA6D;SIAH1;SEMA4C;WNT7A;SEMA4F;ISL1;ZDHHC17;PAX2;RAB10;AFG3L2;FEZ2;ULK2;CNTN4;B4GALT6;SOS1;METAP1;CDK5R1;B4GALT5
 
 
 
 
Negative Regulation of Epithelial to Mesenchymal Transition ( GO:0010719 )
 
 
13/36
 
 
0.0007248
 
 
0.0248275
 
 
0
 
 
0
 
 
3.504022
 
 
25.33287
 
 
FOXA1;NOG;PTEN;LDLRAD4;TBX5;BMP5;SMAD7;PPP2CA;SPRED1;SFRP1;SPRY2;SPRY1;MARK1
 
 
 
 
Positive Regulation of Epithelial Cell Migration ( GO:0010634 )
 
 
13/36
 
 
0.0007248
 
 
0.0248275
 
 
0
 
 
0
 
 
3.504022
 
 
25.33287
 
 
TGFB2;PPM1F;RTN4;EPB41L4B;FGF7;IFNG;ENPP2;SOX9;RREB1;MAPRE2;CLASP2;MAP4K4;ARF6
 
 
 
 
Cellular Response to BMP Stimulus ( GO:0071773 )
 
 
17/54
 
 
0.0007426
 
 
0.0252400
 
 
0
 
 
0
 
 
2.850175
 
 
20.53645
 
 
DLX1;USP15;SMURF1;USP9X;CHRD;GDF6;ACVR2B;GDF5;SMAD5;RGMA;BMP5;TMEM100;TGFBR3;SFRP1;PPARG;SOX9;ADAMTS7
 
 
 
 
Heart Trabecula Morphogenesis ( GO:0061384 )
 
 
9/20
 
 
0.0007638
 
 
0.0252400
 
 
0
 
 
0
 
 
5.068485
 
 
36.37749
 
 
DLL4;FKBP1A;TGFBR3;TGFB2;NOG;S1PR1;TEK;TGFBR1;BMP5
 
 
 
 
Regulation of Cellular Response to Transforming Growth Factor Beta
Stimulus ( GO:1903844 )
 
 
9/20
 
 
0.0007638
 
 
0.0252400
 
 
0
 
 
0
 
 
5.068485
 
 
36.37749
 
 
SMAD2;TGFBR3;DLX1;SMURF2;SOX11;PPARG;NREP;DKK3;PALS1
 
 
 
 
Regulation of Nuclear-Transcribed mRNA Catabolic Process,
Deadenylation-Dependent Decay ( GO:1900151 )
 
 
9/20
 
 
0.0007638
 
 
0.0252400
 
 
0
 
 
0
 
 
5.068485
 
 
36.37749
 
 
SYNCRIP;CNOT7;CSDE1;IGF2BP1;YBX1;CPEB3;TOB1;ZFP36L2;ZFP36L1
 
 
 
 
Negative Regulation of Protein Metabolic Process ( GO:0051248 )
 
 
35/146
 
 
0.0007655
 
 
0.0252400
 
 
0
 
 
0
 
 
1.960319
 
 
14.06520
 
 
TRIM71;DDX6;PHB1;BTG2;DDX3X;FMR1;SNX12;YBX1;HMGCR;TOB1;SNX3;SYNCRIP;OTUD6B;EIF4EBP1;IGF2BP1;IGF2BP3;IGF2BP2;PAIP2;LDLR;NANOS1;PRNP;TIA1;EDN1;RIC1;INHBB;AZIN1;QKI;DHFR;DNAJC1;LIN28A;WT1;SERBP1;MDM4;CPEB3;EIF4E2
 
 
 
 
Collagen Fibril Organization ( GO:0030199 )
 
 
15/45
 
 
0.0007701
 
 
0.0252400
 
 
0
 
 
0
 
 
3.100686
 
 
22.22898
 
 
COLGALT2;TGFB2;COL11A1;TGFBR1;LOXL2;COL1A1;GREM1;COL3A1;COL1A2;COL2A1;COL5A1;LOX;COL5A2;PXDN;TLL1
 
 
 
 
Neuron Projection Morphogenesis ( GO:0048812 )
 
 
37/157
 
 
0.0007760
 
 
0.0252543
 
 
0
 
 
0
 
 
1.917297
 
 
13.73037
 
 
APP;BRSK1;SPTBN4;NRN1;DOCK7;PTEN;LRP2;NYAP2;CDC42;PARD6B;ALCAM;FLRT3;SLITRK1;BTBD3;NTF3;SRGAP2;RAB8A;MAP4K4;MEF2A;TSC22D4;PPP1R12A;BDNF;DTNBP1;WNT7A;DICER1;NGF;ISL1;ZDHHC17;PAX2;RAB10;AFG3L2;TMEM106B;CNTN4;SGK1;METAP1;GAS7;METAP2
 
 
 
 
Response to Endoplasmic Reticulum Stress ( GO:0034976 )
 
 
44/196
 
 
0.0007903
 
 
0.0255353
 
 
0
 
 
0
 
 
1.801240
 
 
12.86650
 
 
ERO1A;PPP1R15B;FICD;TMTC3;DERL1;ATP2A2;ASB11;PIK3R1;RNF7;SELENOK;USP19;UBE2J1;HSP90B1;SCAMP5;SERP1;ERMP1;BCL2L11;AIFM1;MAN1C1;UBQLN1;CHAC1;BAK1;MAP3K5;PDIA3;TMUB1;UBE4A;YOD1;PARP16;UBE4B;ERLIN2;UBE2G2;PDIA6;EIF2S1;PARP8;RNF145;UFM1;TMEM33;VAPB;CANX;CREBRF;BCL2;RNF183;RNF186;TP53
 
 
 
 
Negative Regulation of Protein Polymerization ( GO:0032272 )
 
 
11/28
 
 
0.0008172
 
 
0.0260156
 
 
0
 
 
0
 
 
4.009899
 
 
28.50895
 
 
KANK1;TMSB4X;STMN1;PRKCD;STMN2;TWF1;SLIT2;MAPRE1;SSH1;METAP2;PFN2
 
 
 
 
Regulation of Extracellular Matrix Organization ( GO:1903053 )
 
 
10/24
 
 
0.0008178
 
 
0.0260156
 
 
0
 
 
0
 
 
4.425690
 
 
31.46184
 
 
RB1;RIC1;LAMA2;PRDM5;ZNF469;AXIN2;LAMC1;NID1;RECK;RUNX1
 
 
 
 
Regulation of Phosphatidylinositol 3-Kinase/Protein Kinase B Signal
Transduction ( GO:0051896 )
 
 
48/219
 
 
0.0008223
 
 
0.0260156
 
 
0
 
 
0
 
 
1.747261
 
 
12.41157
 
 
ITGB1;APP;BECN1;PHB1;PIGU;FLT3;PDGFB;PTEN;EFNA5;TNF;RTN4;MTDH;IGF1R;TXN2;PPP2CA;RELN;CALCR;ERBB3;FAM110C;PDGFC;NTF3;CCL3;PIP5K1B;CCR7;RICTOR;VAV3;PDGFRB;RUBCN;TCF7L2;TGFB2;TGFB1;PLEKHA1;IGF1;SIRT1;TGFBR1;VEGFA;LIN28A;PIK3CA;PIK3IP1;NEDD4;LEP;SPRY2;CCM2;TEK;DIPK2A;SOS1;FERMT2;HBEGF
 
 
 
 
Coronary Vasculature Morphogenesis ( GO:0060977 )
 
 
5/7
 
 
0.0008587
 
 
0.0269803
 
 
0
 
 
0
 
 
15.472852
 
 
109.24011
 
 
TGFBR3;NRP1;SPRED1;TGFBR1;VEGFA
 
 
 
 
Negative Regulation of Secretion by Cell ( GO:1903531 )
 
 
14/41
 
 
0.0008726
 
 
0.0272313
 
 
0
 
 
0
 
 
3.214927
 
 
22.64589
 
 
SYT4;LIF;SLC30A1;HMGCR;TNFRSF1B;C9ORF72;TNFRSF1A;VAMP8;GRM7;DPH3;IL1RAPL1;ERP29;IL12B;CCM2
 
 
 
 
Negative Regulation of Apoptotic Signaling Pathway ( GO:2001234 )
 
 
21/74
 
 
0.0008822
 
 
0.0273434
 
 
0
 
 
0
 
 
2.459183
 
 
17.29555
 
 
RB1;ACVR1;TCF7L2;DDX3X;BDNF;SIAH2;GHITM;TRIAP1;IGF1;TNF;TGFBR1;NR4A2;QARS1;PPIF;BCL2;ITGA6;TMBIM6;BAK1;SOS1;ZMYND11;SLC25A4
 
 
 
 
Response to Sterol ( GO:0036314 )
 
 
6/10
 
 
0.0009214
 
 
0.0283641
 
 
0
 
 
0
 
 
9.285971
 
 
64.90541
 
 
SMAD2;TGFB1;INSIG1;CCL3;RORA;TGFBR1
 
 
 
 
Positive Regulation of Cell Motility ( GO:2000147 )
 
 
53/249
 
 
0.0009362
 
 
0.0286262
 
 
0
 
 
0
 
 
1.683793
 
 
11.74224
 
 
ITGB1;RORA;CLDN1;MYLK;IGF1R;FAM110C;CCR7;ACVR1;PDGFRB;EDN1;SEMA6D;ADAM10;F3;ADRA2A;TGFBR1;PTP4A1;TIAM1;ADAM17;ADAM9;ITGA6;SOS1;EPHA2;HBEGF;IGSF8;SEMA7A;CCL3L1;PDGFB;PIK3R3;PPM1F;CDC42;CCL8;CCL7;PDCD10;PDGFC;NTF3;CCL3;CCL2;RICTOR;ZNF268;MAP4K4;TGFB1;UBE2I;FZD4;RDX;SEMA4C;SEMA4F;IGF1;VEGFA;COL1A1;TJP1;SNAI1;SPRY2;FERMT2
 
 
 
 
Regulation of Release of Cytochrome C From Mitochondria ( GO:0090199 )
 
 
13/37
 
 
0.0009763
 
 
0.0296540
 
 
0
 
 
0
 
 
3.357826
 
 
23.27552
 
 
HRK;GHITM;TRIAP1;IGF1;MLLT11;BCL2L11;PLSCR3;PPIF;PMAIP1;BMF;BAK1;SOS1;TP53
 
 
 
 
Central Nervous System Neuron Differentiation ( GO:0021953 )
 
 
15/46
 
 
0.0009972
 
 
0.0300892
 
 
0
 
 
0
 
 
3.000489
 
 
20.73491
 
 
WNT3A;ZC4H2;WNT9B;FOXN4;RORB;ISL1;NEUROD1;NR4A2;LMX1A;LHX1;WNT1;MDGA2;CBLN1;METAP2;PALS1
 
 
 
 
Positive Regulation of Cartilage Development ( GO:0061036 )
 
 
8/17
 
 
0.0010527
 
 
0.0315534
 
 
0
 
 
0
 
 
5.505160
 
 
37.74554
 
 
PKDCC;ZBTB16;SOX9;GDF6;GDF5;HOXA11;LOXL2;SOX5
 
 
 
 
Response to Tumor Necrosis Factor ( GO:0034612 )
 
 
22/80
 
 
0.0010672
 
 
0.0315557
 
 
0
 
 
0
 
 
2.354359
 
 
16.11021
 
 
ASAH1;CHUK;ZFAND6;YTHDC2;ADAM10;TNFRSF11A;YBX3;SIRT1;ZFP36L2;ZFP36L1;IKBKB;SFRP1;BAG4;CASP3;SMPD1;CCL3;ADAM9;CCL2;GBP1;ZNF268;MAP4K3;ADAMTS7
 
 
 
 
Vasculogenesis ( GO:0001570 )
 
 
12/33
 
 
0.0010736
 
 
0.0315557
 
 
0
 
 
0
 
 
3.541662
 
 
24.21355
 
 
PDGFRB;TGFBR3;NRP1;SHH;SPRED1;TGFB1;WT1;HEY2;ITGB8;CCM2;RTN4;VEGFA
 
 
 
 
Ventricular Cardiac Muscle Tissue Morphogenesis ( GO:0055010 )
 
 
12/33
 
 
0.0010736
 
 
0.0315557
 
 
0
 
 
0
 
 
3.541662
 
 
24.21355
 
 
DLL4;FKBP1A;TGFBR3;TGFB2;TGFB1;NOG;TPM1;HAND1;LRP2;ISL1;TGFBR1;SMAD7
 
 
 
 
Blood Vessel Morphogenesis ( GO:0048514 )
 
 
16/51
 
 
0.0010972
 
 
0.0320452
 
 
0
 
 
0
 
 
2.835111
 
 
19.32114
 
 
NRP1;TGFB1;LRP5;TGFBR1;SMAD7;VEGFA;EFNB2;SHH;LOX;COL4A1;WT1;HEY2;ID1;ITGB8;HOXA1;CCM2
 
 
 
 
Regulation of Macroautophagy ( GO:0016241 )
 
 
27/106
 
 
0.0011221
 
 
0.0325622
 
 
0
 
 
0
 
 
2.122605
 
 
14.41791
 
 
BECN1;SCOC;PRKAA2;GNAI3;MTMR9;C9ORF72;MAPK8;SESN1;CASP3;SESN2;UBQLN1;ATP6V1E1;MAP3K7;SNX5;ATG5;SNX6;SH3GLB1;SREBF1;FZD5;FBXW7;TSC1;ATG14;SIRT1;PIK3CA;NEDD4;PAFAH1B2;CDK5R1
 
 
 
 
Hemopoiesis ( GO:0030097 )
 
 
26/101
 
 
0.0011625
 
 
0.0331730
 
 
0
 
 
0
 
 
2.152725
 
 
14.54639
 
 
FLT3;PDGFB;ASH2L;PBXIP1;ZFP36L2;PRDX3;MYB;PGM3;IL10;WNT10B;TGFB2;TGFB1;FST;ZBTB16;GLRX5;TET2;KLF3;RUNX1;TGFBR3;SFRP1;KITLG;MEIS1;MELK;COMMD3-BMI1;TEK;TP53
 
 
 
 
Regulation of Signal Transduction ( GO:0009966 )
 
 
46/211
 
 
0.0011713
 
 
0.0331730
 
 
0
 
 
0
 
 
1.734691
 
 
11.70859
 
 
APP;NRP1;CCNK;PGAP1;FRMPD1;HHIP;RGS16;GSKIP;ZDHHC7;RORA;PPM1K;PPM1D;SNX3;SOCS2;UGCG;ERBB3;IRAK2;PPP6R1;ZIC1;OTX2;HIF1AN;VHL;ACVR1;TSPYL2;PRNP;HOMER1;DYNLT1;NOG;KCNIP4;DTNBP1;TSPEAR;SENP2;HPCAL4;ZDHHC17;MDFIC;VSNL1;KIF26A;VEPH1;PXDN;CHRFAM7A;STRN;ZMYND11;CRK;MDFI;KCTD16;CDC42SE2
 
 
 
 
Protein K48-linked Deubiquitination ( GO:0071108 )
 
 
9/21
 
 
0.0011724
 
 
0.0331730
 
 
0
 
 
0
 
 
4.645841
 
 
31.35328
 
 
OTUD4;USP14;USP25;USP8;USP37;USP34;TNFAIP3;YOD1;OTUB1
 
 
 
 
Proteasome-Mediated Ubiquitin-Dependent Protein Catabolic Process ( GO:0043161 )
 
 
68/340
 
 
0.0011786
 
 
0.0331730
 
 
0
 
 
0
 
 
1.558315
 
 
10.50845
 
 
TRIM71;UBXN1;CCNF;UBE2D3;UBE2D1;CDC23;PSMD7;CDC27;BTRC;SKP1;NHLRC3;FBXW7;USP44;UBE4B;ARMC8;AXIN2;SIRT1;KCTD5;RAD23B;KLHL42;PSMA5;CDC34;PSMA1;GAN;TBL1XR1;PLAA;KCTD17;PSMD12;RNF34;CUL5;CUL3;CUL2;UBR3;FBXO45;ASB11;RNF7;SOCS2;ZNRF1;ZNRF2;RMND5A;HECTD3;TBX21;VHL;RFFL;DCAF12;SOCS4;KLHL29;SMURF2;UBE2B;CSNK1A1;SMURF1;FEM1B;SIAH2;FBXL19;SIAH1;KLHL28;KLHL2;BFAR;KLHL23;KLHL20;RNF145;UBE2W;APPBP2;GID8;KLHL8;UBE2N;RNF186;SPSB4
 
 
 
 
Regulation of Membrane Protein Ectodomain Proteolysis ( GO:0051043 )
 
 
10/25
 
 
0.0011941
 
 
0.0331730
 
 
0
 
 
0
 
 
4.130403
 
 
27.79907
 
 
IL10;APP;IFNG;TSPAN5;ADAM9;TNFRSF1B;TNF;ADRA2A;SH3D19;PTPN3
 
 
 
 
Response to Glucocorticoid ( GO:0051384 )
 
 
10/25
 
 
0.0011941
 
 
0.0331730
 
 
0
 
 
0
 
 
4.130403
 
 
27.79907
 
 
IL10;REST;BCL2L11;CALCR;SLIT2;NR3C1;TNF;ISL1;ZFP36L2;ZFP36L1
 
 
 
 
Zinc Ion Transport ( GO:0006829 )
 
 
10/25
 
 
0.0011941
 
 
0.0331730
 
 
0
 
 
0
 
 
4.130403
 
 
27.79907
 
 
SLC30A6;SLC30A9;SLC30A8;SLC30A3;SLC30A5;SLC39A11;SLC39A8;TRPM7;SLC30A1;SLC30A10
 
 
 
 
Protein K48-linked Ubiquitination ( GO:0070936 )
 
 
24/91
 
 
0.0012315
 
 
0.0340031
 
 
0
 
 
0
 
 
2.223827
 
 
14.89866
 
 
UBE2H;RNF34;UBE2B;UBE2D2;UBE2E3;CUL3;UBE2D3;MYCBP2;TNFAIP3;UBE2D1;FBXO45;BFAR;UBE2G2;ZNRF1;ZNRF2;CDC23;CDC34;UBE2R2;CDC27;BTRC;RFFL;RNF152;UBE2K;SKP1
 
 
 
 
Negative Regulation of Cold-Induced Thermogenesis ( GO:0120163 )
 
 
15/47
 
 
0.0012785
 
 
0.0350891
 
 
0
 
 
0
 
 
2.906554
 
 
19.36369
 
 
RB1;WNT10B;IL15;LAMA4;DOCK7;ARRDC3;PLCL2;PCTP;ACVR2B;QKI;ADAM17;NOVA1;ID1;LGR4;MAP2K6
 
 
 
 
Phospholipid Dephosphorylation ( GO:0046839 )
 
 
12/34
 
 
0.0014511
 
 
0.0395459
 
 
0
 
 
0
 
 
3.380481
 
 
22.09291
 
 
MTMR1;PLPP6;MTMR10;PIP4P2;MTMR3;PLPPR1;PIP4P1;MTMR12;PLPPR5;PTEN;MTMR9;PLPP1
 
 
 
 
Negative Regulation of Response to Stimulus ( GO:0048585 )
 
 
17/57
 
 
0.0014582
 
 
0.0395459
 
 
0
 
 
0
 
 
2.635952
 
 
17.21416
 
 
USP25;MCTP1;HHIP;CASK;KLF4;SIRT1;C9ORF72;MACROH2A1;ADRA2A;SOCS2;COL3A1;DDAH1;ERBB3;KIF26A;AQP11;SLIT2;VHL
 
 
 
 
RNA Transport ( GO:0050658 )
 
 
14/43
 
 
0.0014804
 
 
0.0399103
 
 
0
 
 
0
 
 
2.992860
 
 
19.49975
 
 
SLBP;NUP155;FMR1;YBX1;QKI;ZFP36L1;FXR1;ATXN2;POM121C;KIF5C;TPR;HNRNPA2B1;KPNB1;SIDT2
 
 
 
 
Regulation of MAPK Cascade ( GO:0043408 )
 
 
54/260
 
 
0.0015151
 
 
0.0406048
 
 
0
 
 
0
 
 
1.631921
 
 
10.59487
 
 
APP;FLT3;TNF;DUSP16;IGF1R;FGF7;SPRED1;SMPD1;GRAP2;SH3GL2;EPHA7;EDN1;MAP2K1;GPR37;PRKCD;ATP6AP2;ZDHHC17;ADRA2A;GREM1;TGFBR3;ADRB3;MDFIC;ERP29;PPARG;SOS1;MDFI;STK40;EPHA2;PELI2;PDGFB;TGFA;ADRB1;ADRB2;RASGRP1;ERBB3;NTF3;SHOC2;ZNF622;MAP4K4;WNT4;TGFB1;CAV2;STYX;LIF;IGF1;VEGFA;FASN;LEP;FGF18;NEK10;CHRFAM7A;GRB2;CCM2;TEK
 
 
 
 
Modification-Dependent Protein Catabolic Process ( GO:0019941 )
 
 
45/208
 
 
0.0015475
 
 
0.0412297
 
 
0
 
 
0
 
 
1.717377
 
 
11.11338
 
 
RNF34;PPP1R11;UBA6;UBE2D2;CUL3;UBE2D3;UBE2D1;UBR3;FBXO45;RNF7;FBXO21;RCHY1;CDC23;KCTD21;RNF19A;RNF217;UBQLN1;RBBP6;BTRC;RFFL;RNF20;NDFIP2;UBE2H;UBE2I;SMURF2;UBE2B;SMURF1;SIAH2;SIAH1;UBE4B;BFAR;UBE2G2;FBXO10;CHFR;RNF144B;CDC34;RNF125;UBE2R2;UBQLNL;HECW2;NEDD4;KLHL8;RLIM;UBE2K;SPSB4
 
 
 
 
Negative Regulation of T Cell Migration ( GO:2000405 )
 
 
4/5
 
 
0.0016700
 
 
0.0437269
 
 
0
 
 
0
 
 
24.749101
 
 
158.26894
 
 
RIPOR2;CD200R1;LRCH1;CD69
 
 
 
 
Regulation of Peptidyl-Lysine Acetylation ( GO:2000756 )
 
 
4/5
 
 
0.0016700
 
 
0.0437269
 
 
0
 
 
0
 
 
24.749101
 
 
158.26894
 
 
PRKAA1;PRKAA2;KLF15;SIRT1
 
 
 
 
Ventricular Compact Myocardium Morphogenesis ( GO:0003223 )
 
 
4/5
 
 
0.0016700
 
 
0.0437269
 
 
0
 
 
0
 
 
24.749101
 
 
158.26894
 
 
TGFBR3;NOG;LRP2;TGFBR1
 
 
 
 
Phosphatidylinositol 3-Kinase/Protein Kinase B Signal Transduction ( GO:0043491 )
 
 
13/39
 
 
0.0017003
 
 
0.0440174
 
 
0
 
 
0
 
 
3.099171
 
 
19.76319
 
 
EDN1;PLEKHA1;PTEN;PIK3R3;LRP2;PIK3R1;NYAP2;PIK3C2A;ATG14;IGF1R;ZFP36L1;ERBB3;PIK3CA
 
 
 
 
Blood Vessel Endothelial Cell Migration ( GO:0043534 )
 
 
10/26
 
 
0.0017004
 
 
0.0440174
 
 
0
 
 
0
 
 
3.872028
 
 
24.69149
 
 
EFNB2;GREM1;NRP1;EGR3;ID1;PIK3R3;MIA3;SLIT2;ROBO1;VEGFA
 
 
 
 
Cell Migration Involved in Sprouting Angiogenesis ( GO:0002042 )
 
 
9/22
 
 
0.0017408
 
 
0.0443067
 
 
0
 
 
0
 
 
4.288219
 
 
27.24492
 
 
EFNB2;GREM1;NRP1;EGR3;PIK3R3;MIA3;SLIT2;ROBO1;VEGFA
 
 
 
 
Negative Regulation of Myeloid Cell Differentiation ( GO:0045638 )
 
 
9/22
 
 
0.0017408
 
 
0.0443067
 
 
0
 
 
0
 
 
4.288219
 
 
27.24492
 
 
HSPA9;HOXA9;MEIS1;MAFB;ZBTB16;MYB;HOXB8;HOXA5;ZFP36L1
 
 
 
 
Regulation of Calcium Ion Import ( GO:0090279 )
 
 
9/22
 
 
0.0017408
 
 
0.0443067
 
 
0
 
 
0
 
 
4.288219
 
 
27.24492
 
 
PDGFRB;PRNP;PLN;PDGFB;CCL3;STC1;CCL2;SLC30A1;KCNN4
 
 
 
 
Regulation of Keratinocyte Migration ( GO:0051547 )
 
 
6/11
 
 
0.0017883
 
 
0.0452634
 
 
0
 
 
0
 
 
7.428345
 
 
46.99543
 
 
EPB41L4B;FGF7;PTEN;MAPRE2;ARF6;MAP4K4
 
 
 
 
Negative Regulation of Catabolic Process ( GO:0009895 )
 
 
29/120
 
 
0.0018162
 
 
0.0454658
 
 
0
 
 
0
 
 
1.979246
 
 
12.49100
 
 
USP14;PHB1;PRKAA1;SNX12;MTMR9;FOXK2;ZDHHC7;HMGCR;TNF;SNX3;ENDOU;ZKSCAN3;TLK2;EP300;STK38L;BMF;VHL;MCL1;IL10;RUBCN;RIC1;AZIN1;ADRA2A;PDCL3;LEP;BCL2;MDM4;SOS1;EIF4G2
 
 
 
 
Regulation of Transforming Growth Factor Beta Receptor Signaling Pathway
( GO:0017015 )
 
 
29/120
 
 
0.0018162
 
 
0.0454658
 
 
0
 
 
0
 
 
1.979246
 
 
12.49100
 
 
USP15;ARID4B;LDLRAD4;NREP;SPRED1;SAP30L;EP300;RBBP7;SKIL;SNX6;SMAD2;SMURF2;SMURF1;TET1;SOX11;LEMD3;SIRT1;DKK3;SMAD7;TGFBR3;FKBP1A;GIPC1;FKBP1C;VEPH1;SPRY2;PPARG;SPRY1;SINHCAF;PALS1
 
 
 
 
Lysosome Organization ( GO:0007040 )
 
 
14/44
 
 
0.0018968
 
 
0.0463299
 
 
0
 
 
0
 
 
2.892929
 
 
18.13175
 
 
SNAPIN;TFEB;NAGPA;LYSET;TMEM165;ZFYVE26;GNPTAB;RAB14;HOOK1;FHIP1B;CLVS2;ZKSCAN3;TMEM106B;CLVS1
 
 
 
 
Positive Regulation of Autophagy ( GO:0010508 )
 
 
25/99
 
 
0.0019111
 
 
0.0463299
 
 
0
 
 
0
 
 
2.097262
 
 
13.12900
 
 
BECN1;SCOC;PRKAA1;PRKAA2;GNAI3;FOXO3;PIK3C2A;C9ORF72;MTDH;DEPTOR;SESN1;RB1CC1;SESN2;TP53INP1;MAP3K7;RNF152;TRIM23;SH3GLB1;PLK2;TFEB;SIRT1;SVIP;CCNY;IFNG;PAFAH1B2
 
 
 
 
Regulation of Protein Secretion ( GO:0050708 )
 
 
25/99
 
 
0.0019111
 
 
0.0463299
 
 
0
 
 
0
 
 
2.097262
 
 
13.12900
 
 
SNAP25;HMGCR;TNF;NEUROD1;DPH3;ENSA;IL12B;ARFIP1;APBB3;TGFB2;TGFB1;KCNJ11;P3H1;RFX3;IGF1;CD2AP;G6PC2;DNAJC1;RFX6;IFNG;ERP29;ADAM9;PPID;ARF6;RAPGEF4
 
 
 
 
Positive Regulation of Cell Projection Organization ( GO:0031346 )
 
 
28/115
 
 
0.0019158
 
 
0.0463299
 
 
0
 
 
0
 
 
1.998600
 
 
12.50649
 
 
PLXND1;PLPPR5;STMN2;PLA2G3;TWF1;ROBO1;SNX3;RAP1A;RELN;SLITRK1;DPYSL3;NPTN;EP300;DVL3;SLIT2;SKIL;PLK5;CAMK1D;BDNF;FZD4;SS18L1;ZNF804A;P3H1;TIAM1;RAPGEF2;ITGA6;MAP3K13;ARF6
 
 
 
 
Neuron Development ( GO:0048666 )
 
 
36/159
 
 
0.0019255
 
 
0.0463299
 
 
0
 
 
0
 
 
1.818998
 
 
11.37341
 
 
RB1;APP;BTG2;TENM2;ARL3;DOCK7;STMN2;BICDL1;PTPRK;FRY;UGCG;BLOC1S6;ATCAY;FLRT3;STMN1;MAP4;STX3;SH3GL2;LGI1;TGFB2;SNAPIN;PBX3;DTNBP1;PBX1;RUNX1;NEDD4;RAPGEF2;CNTN4;ATXN10;ERCC6;B4GALT6;MAPRE2;PALS1;B4GALT5;CDK5R1;METAP2
 
 
 
 
Negative Regulation of Osteoblast Differentiation ( GO:0045668 )
 
 
12/35
 
 
0.0019317
 
 
0.0463299
 
 
0
 
 
0
 
 
3.233316
 
 
20.20620
 
 
GREM1;TNFAIP6;NOG;CHRD;LRP5;PTEN;CRIM1;PPARG;RORB;SOX9;LIMD1;IPO7
 
 
 
 
Positive Regulation of Mitotic Nuclear Division ( GO:0045840 )
 
 
12/35
 
 
0.0019317
 
 
0.0463299
 
 
0
 
 
0
 
 
3.233316
 
 
20.20620
 
 
RB1;PDGFRB;EDN1;CDC23;ESPL1;CUL3;NSMCE2;LRP5;PDGFB;CD28;TGFA;IGF1
 
 
 
 
Regulation of Canonical Wnt Signaling Pathway ( GO:0060828 )
 
 
46/216
 
 
0.0019321
 
 
0.0463299
 
 
0
 
 
0
 
 
1.683177
 
 
10.51842
 
 
INVS;AMER1;APP;KANK1;DDX3X;USP34;GSKIP;NRARP;TMEM131L;LIMD1;NKD1;FRZB;RSPO2;RSPO3;SOX9;BTRC;TLE4;USP8;USP47;TCF7L2;TGFB1;SMURF2;UBE2B;CSNK1A1;WNT3A;SIAH2;FZD6;NOG;ATP6AP2;SOX13;AXIN2;TMEM170B;DKK2;BICC1;DKK3;COL1A1;GREM1;SFRP1;CCNY;TBL1XR1;GID8;OTULIN;CCNYL1;RECK;LGR4;RNF220
 
 
 
 
Regulation of Cell Growth ( GO:0001558 )
 
 
47/222
 
 
0.0019707
 
 
0.0470075
 
 
0
 
 
0
 
 
1.670752
 
 
10.40774
 
 
RB1;YAP1;PHB1;DDX3X;DDX49;TFRC;DCUN1D3;AKAP6;RTN4;SOCS2;CDC42;RPS6KA3;ING5;FRZB;SESN2;RICTOR;RBBP7;SLIT2;TAF9B;MFSD2A;H3-3B;TSPYL2;LGI1;PABIR1;PRPF38B;TGFB2;EGLN2;TGFB1;ADAM10;INO80;BLTP1;TGFBR1;SFRP1;ADAM17;H3-5;NME6;WT1;BCL2;ARMC12;SGK3;CHPT1;SGK1;CRLF3;TP53;CRK;S100A8;EIF4G2
 
 
 
 
UDP-N-acetylglucosamine Biosynthetic Process ( GO:0006048 )
 
 
5/8
 
 
0.0020264
 
 
0.0473454
 
 
0
 
 
0
 
 
10.314635
 
 
63.96608
 
 
GNPDA1;GFPT2;GFPT1;PGM3;UAP1
 
 
 
 
Monoubiquitinated Protein Deubiquitination ( GO:0035520 )
 
 
5/8
 
 
0.0020264
 
 
0.0473454
 
 
0
 
 
0
 
 
10.314635
 
 
63.96608
 
 
USP47;USP15;USP21;USP32;USP9X
 
 
 
 
Positive Regulation of Transporter Activity ( GO:0032411 )
 
 
5/8
 
 
0.0020264
 
 
0.0473454
 
 
0
 
 
0
 
 
10.314635
 
 
63.96608
 
 
TRPC6;STK39;SGK3;SGK1;SYNGR3
 
 
 
 
Skin Morphogenesis ( GO:0043589 )
 
 
5/8
 
 
0.0020264
 
 
0.0473454
 
 
0
 
 
0
 
 
10.314635
 
 
63.96608
 
 
COL1A1;ERRFI1;COL1A2;ITGA6;AHDC1
 
 
 
 
Regulation of Smooth Muscle Cell Proliferation ( GO:0048660 )
 
 
16/54
 
 
0.0021434
 
 
0.0498229
 
 
0
 
 
0
 
 
2.610830
 
 
16.04451
 
 
PDGFRB;TCF7L2;PHB1;EDN1;TGFB1;XRCC5;PDGFB;TNFAIP3;PIK3R1;IGF1;TNF;IFNG;RBPMS2;CCN4;IL12B;PPARG
 
 
 
 
Artery Morphogenesis ( GO:0048844 )
 
 
11/31
 
 
0.0021616
 
 
0.0499902
 
 
0
 
 
0
 
 
3.407820
 
 
20.91353
 
 
PDGFRB;NRP1;TGFB2;FOXF1;HEY2;NOG;HOXA1;LRP2;TGFBR1;SMAD7;VEGFA
 
 
 
 
Branching Morphogenesis of an Epithelial Tube ( GO:0048754 )
 
 
13/40
 
 
0.0022021
 
 
0.0504169
 
 
0
 
 
0
 
 
2.984213
 
 
18.25838
 
 
NRP1;SIX1;NRARP;PAX2;HOXA11;VEGFA;DLL4;SHH;SALL1;COL4A1;WT1;SLIT2;EPHA2
 
 
 
 
Regulation of Apoptotic Signaling Pathway ( GO:2001233 )
 
 
13/40
 
 
0.0022021
 
 
0.0504169
 
 
0
 
 
0
 
 
2.984213
 
 
18.25838
 
 
RB1;SGMS1;BDNF;TNF;TGFBR1;NR4A2;ING5;DEPTOR;QARS1;FASN;BCL2;PPARG;TMBIM6
 
 
 
 
Central Nervous System Neuron Development ( GO:0021954 )
 
 
7/15
 
 
0.0023261
 
 
0.0527244
 
 
0
 
 
0
 
 
5.417506
 
 
32.84944
 
 
PTEN;RAPGEF2;B4GALT6;B4GALT5;LHX8;METAP2;PALS1
 
 
 
  
  
 
 
 
 GO_Cellular_Component_2025 
 This category captures Gene Ontology Cellular Component annotations
describing the subcellular localization of gene products, including
organelles, macromolecular complexes, membranes, and specialized
cellular structures. GO Cellular Component terms are curated through
experimental localization studies and integrative annotation pipelines.
Enrichment reveals whether target genes preferentially localize to
specific cellular compartments, suggesting spatial organization of
regulatory effects. These insights are valuable for understanding
compartment-specific processes such as mitochondrial regulation, vesicle
trafficking, nuclear control mechanisms, or membrane-associated
signaling. 
 
 
 
 
Term
 
 
Overlap
 
 
P.value
 
 
Adjusted.P.value
 
 
Old.P.value
 
 
Old.Adjusted.P.value
 
 
Odds.Ratio
 
 
Combined.Score
 
 
Genes
 
 
 
 
 
 
Intracellular Membrane-Bounded Organelle ( GO:0043231 )
 
 
972/5597
 
 
0.0000000
 
 
0.0000000
 
 
0
 
 
0
 
 
1.458507
 
 
58.0480009
 
 
ITSN2;CHIC2;RAMAC;TFRC;TESK2;ZC3H12C;PSMD7;PPP4R2;BBX;MEF2A;PRKAB2;GABARAPL1;RBFOX2;ZNF283;ZNF10;RFX3;DICER1;ISL1;RFX6;CDIP1;CGGBP1;SLBP;CDCA4;MEPCE;GATA6;COPS7B;ZNF23;ABCB9;RDH10;SYNDIG1;ZNF268;ZNF267;VTI1B;ZNF266;NR2F2;MAGOHB;PBX1;NR2F6;CS;PKIB;MLLT10;PKIA;AGO2;C19ORF47;SFXN4;CDIN1;ZBTB8B;FERMT2;RNF220;LIN54;ZNF250;BHLHE41;PRKAG2;SNX10;TFCP2L1;CTCF;MECP2;MED14;TUBA1A;NKRF;HEY2;OLR1;IKBKE;C11ORF24;SS18;CNOT6L;SLC39A11;FOS;BAZ1B;DYNLL1;DUSP9;MED26;DYNLL2;SIRT1;LRRC41;MED28;PSMA5;VAMP7;PLSCR3;PSMA1;MED21;PLSCR4;PPARG;ERGIC2;ERGIC1;MDFI;SLC29A3;ZNF473;KANK1;ZNF471;ZNF470;CIPC;DLST;FOXS1;GLRA2;MYCT1;BTAF1;SNX27;TMEM65;PLAGL2;STX2;ZNF225;SRSF11;EGLN1;ZNF462;EGLN2;SLF2;MDH2;USP9X;TARBP2;SNX16;SNX17;WT1;GID8;SERBP1;ZNF699;CRLF3;YPEL5;ERO1A;CCNT2;CSE1L;UBE2D2;UBE2D3;UBE2D1;RORA;MSI2;RORB;NUDT11;YY1;SESN1;KMT5B;ZNF689;NEK3;KPNA5;PITX2;MAP3K7;KPNA3;NEPRO;KPNA1;ZNF200;KHNYN;FBXW7;IST1;KMT5C;UBE2E3;CTNNBIP1;PIAS2;FOXP2;CDC25B;MAF;DMTF1;HAT1;PKP4;MAPKAPK5;SRSF7;KPNB1;S100A8;DLX1;INO80D;ZC4H2;ARL3;ISG20L2;STK39;ARL2;FBXO45;FOXO4;FOXO3;HSP90B1;GLIS2;STK33;SOCS1;ZKSCAN3;ZNF420;ZBTB18;TIA1;NEK6;ZBTB16;NEK7;STYX;FOXN4;FBXO32;QKI;PTPRA;MDM4;NFE2L1;ZNF652;CELF2;ONECUT1;CELF3;PTEN;FASLG;NXT2;CDC73;TNFSF13B;ALAD;FUNDC2;PPP6R1;ACP3;ZNF644;YAF2;ACTR2;EMX2;VPS37C;RAD23B;ARL4A;HAND1;ZNF879;TMBIM6;TOP1;AHDC1;PRKAA1;PRKAA2;CARNMT1;UHRF2;ZBTB47;FOXK2;PBXIP1;RCHY1;PPM1D;PPM1F;PPM1G;ZNRF1;CECR2;ZNRF2;PARD6B;TP53INP1;IGF2BP1;E2F3;IGF2BP3;IGF2BP2;E2F5;E2F6;ZNF620;H2AZ1;TRIM41;PRRX1;FEM1B;FOXJ2;TBX5;TBX4;SLC25A37;MCM3AP;NOVA1;JPT1;CCNK;CCNJ;CCNH;RTCA;CCNF;RTCB;CCNC;LDLRAD4;NUDT5;IFI30;IKZF5;RASSF3;CDC27;EPC1;CCN2;CCNL2;PABIR1;KCTD19;SFMBT1;NSMCE2;PISD;THAP1;CYP39A1;CDC34;CCNY;SUB1;CCDC82;TP53;TRIB2;RRAD;MYCBP2;PEX11B;PIK3R1;ZBTB5;ZBTB4;UBE2J1;MBTD1;SEC14L2;PPCS;MIER1;KCNN4;N4BP1;RAB11FIP4;SNX5;ZBED4;CTSA;FOXF1;XRCC5;ATOSB;LPAR6;ID1;ID4;ID3;GRB2;ARMC12;CALM3;MKX;SLC2A4RG;RCOR1;CSRNP1;BARHL2;SUV39H2;PPP1R11;PPP1R10;SUV39H1;MIA3;PTPRK;NAT14;RAB22A;CTSS;LARP1B;SYNCRIP;SCML2;DHX15;REV3L;SPTAN1;NCOA1;SREBF1;GRIPAP1;FOXD1;STARD3NL;CACYBP;KIF23;ZHX1;NR5A2;PDIK1L;G2E3;FAM193B;NCOA7;ZNF780B;YAP1;MARCHF1;SATB1;STX16;ZFP14;GPC4;EXOSC3;EPS15;MARCHF3;DYNC1I1;SPRYD4;NEDD4;HNRNPA2B1;CCNG1;FOXA1;PRDM4;GEMIN2;PRDM5;NR3C1;IGF1R;LOXL2;TXN2;KLHL31;TEPSIN;AMMECR1L;CCND2;CCND1;CAPN3;EP300;SNIP1;ARID2;RGS8;EOMES;PDGFRB;SLC30A9;BORA;SLC30A8;ATG9A;ADAM10;UBE4A;UBE4B;WBP11;CCNE2;PSPC1;CCNE1;HNRNPUL2;WDFY1;UBE2V2;PLAA;DENND6A;UBE2V1;PPID;PPIC;DDX6;UBA6;YTHDC1;CACNA1A;PDS5B;IRAK2;RAI2;UBE2F;PRNP;TFAP2C;UBE2H;TFAP2D;UBE2I;UBE2B;VTA1;SLC52A3;HMGA1;HMGA2;BLTP1;UBE2W;FGF14;NASP;APPBP2;BHLHE40;UBE2N;NABP1;CYCS;MXD1;UBE2K;FGF11;PHF19;RERE;DDX3Y;DYRK2;DDX3X;DDX49;NAA40;YWHAB;CHD7;HMGB2;DPY30;TNFAIP3;TRIAP1;SOBP;CHD1;UBE2Q1;YWHAQ;ZIC1;MYBL2;HORMAD2;ZNF385D;CFAP45;ZNF385A;EED;ETV4;CCNA2;ELF2;CREB1;ELF4;TBC1D5;ELF5;GORASP1;IRF2;ZMYND11;RBM24;CREB5;PARG;NAA20;RAP1GDS1;RNF7;ATAD2B;CAND1;PABPN1;FAM241B;MKNK1;TBX21;TDG;UBE2NL;FAM241A;DROSHA;VHL;MTA3;RBM38;NIPSNAP3A;EYA1;EYA3;SWT1;TAF11;ZNF804A;ATP2B4;PEDS1;CHFR;CLCN6;REST;UFM1;CLCN4;TCIM;KATNBL1;POLE3;CHPT1;ERCC6;NAA15;TAF5;TAF4;RB1;PHB1;MAML1;GPATCH2;PHAX;FNBP1L;GPATCH8;BACH2;DCAF6;ELK4;GPR174;HOXA9;MYB;HOXA3;AP1S1;HOXA1;PIP4K2B;AP1S3;HOXA5;TSPYL2;PRKCD;MED8;SOX13;MED9;SOX11;ARMC8;FNDC3A;SOX12;NISCH;RNF125;BIN1;COMMD3-BMI1;HOXB3;HOXB2;HOXB1;HOXB8;ASF1A;HOXB5;ZNF397;OSER1;ZNF395;HOXD1;ZNF391;HSPA4L;C9ORF72;MTDH;PRDX3;MCMBP;HOXC8;NKX2-4;RREB1;CDK17;NDFIP2;CDK19;PLK5;PLK2;SLC16A10;INO80;ST18;FOSL2;TADA2B;EHD2;CPEB1;PPP5C;HNRNPK;HNRNPF;SNAI1;MMS19;HBP1;CPEB3;CPEB2;ZNF136;PHF3;BTG2;ASH2L;ING5;CSRP2;AIFM1;TMSB4X;RBPMS2;AP3S1;JARID2;ZNF367;ZNF366;SLC2A14;WDR37;HSBP1;EN2;TET2;PAWR;TET1;ASH1L;PAX2;NCOR2;TUBB2A;MTF2;TET3;RARA;RARB;SINHCAF;JDP2;ZNF594;ASAH1;NFIX;SGMS1;WDR20;MIPOL1;TMEM165;RNF138;PPP1R7;LDLR;ZNF341;ZNF583;CSNK1A1;PCGF5;SEPTIN2;PCGF3;ZNF33B;NFIA;RLIM;FOSB;ANKRD13B;DGKD;FMR1;AHR;TM7SF2;PPP1CB;PPP1CC;SALL1;ZFP30;SALL3;PAPOLG;SALL4;GRAP2;CHP2;SMARCAD1;CLCC1;HMGN2;MAF1;ZNF566;HMGN3;PHLDA1;TLE4;MAP2K1;USP4;USP3;IPMK;RPS6;THOC1;DYRK1B;MITF;VRK3;RAB30;KAT6B;ADNP;ERG;SNRPC;DGKH;IPPK;ANKRD17;BRSK1;DNMT1;PARM1;CUL5;CUL3;AK1;ABHD5;MACROH2A1;RLF;NEUROD1;BAG4;BAG5;BTBD3;TPR;SAFB2;NDN;SKIL;ZNF784;MCL1;OSBPL9;ESRRA;SEC16B;AKIRIN1;ESRRG;NSG1;ADAP1;CDK8;MOB1A;CNOT7;MAFB;P4HA2;ZFP62;COPS2;LHX1;MAFG;RCAN2;BCL3;BCL2;CDK1;TADA1;MAFK;ZNF774;LHX8;ZC3H3;ZFP82;PIK3C2A;DUSP16;TRO;TLK2;DNMT3B;TLK1;HIF1AN;TNPO1;ADAMTS9;LBR;CEMIP2;DNMT3A;ZFP91;PLRG1;SMC1A;PTBP3;PTP4A1;NPEPL1;TFDP1;ESPL1;TFDP2;TRAF4;POLR1D;CRK;HDAC4;RNF34;CEBPD;NUFIP2;WBP2;CEBPG;VPS4A;RNF38;TTK;PDHB;STOX2;RSBN1L;POLR2D;RBBP6;RBBP7;JAZF1;RNF20;IRX5;OXR1;PNRC1;PNRC2;KLF6;EVI2A;KLF5;RAB14;MAB21L2;KLF9;INSM2;BCOR;PPIL4;TNRC6A;BET1;CUL4B;STEEP1;TCERG1;APP;PANK3;SPARC;PANK1;FRAT2;ZNF608;CHEK1;PCLAF;DPF2;HABP4;UBAP2L;SOX9;AGFG1;SKP1;SOX5;ARRDC4;PDHX;ESCO1;P3H1;OVOL1;RUNX1;XRN1;RUBCNL;VDAC1;DNA2;ANKRA2;IGSF8;ANP32A;DCUN1D3;DCUN1D4;EIF5A2;DCUN1D2;GSKIP;STC1;FXR1;STRIP1;SPAST;PDCD11;DPH3;TRPM7;SRPK2;SETDB1;GADD45A;SIAH2;SS18L1;SIAH1;GADD45G;SRPK1;STAG2;FUBP1;RAET1L;CREBRF;DAZAP2;AHCTF1;TENM2;DAZAP1;UBXN1;LIMD1;MEOX2;PRPF19;SPPL3;COIL;ZMYM2;XPO4;TMED5;H3-3B;TSC22D4;STRADB;TFEB;CBFA2T3;ZDHHC17;MLF1;PALD1;RHOB;MDFIC;TFEC;RGS7BP;VGLL2;SGK1;AMER1;MYCBP;SLC66A1;PPP2CA;PURA;FAM184A;SHOC2;APBB3;GINS2;PTGES3;XPO7;NR6A1;PRKRA;CAMK4;ZNF354A;FRA10AC1;CDK5R1;ZNF354B;ARID4B;HAPSTR1;HOXA11;HOXA10;SVEP1;MAGEB10;LMO1;NANOS1;PDIA3;MLIP;LMO3;ANXA11;KCTD1;TSC1;MAU2;MYCN;LMX1A;TBL1XR1;CMPK1;CHMP4B;ZFP36L2;ZFP36L1;ZMAT2;PMAIP1;OTX2;RPRD1A;SMAD2;CPSF4;CPSF7;CBX5;TGFB1;CBX4;SMURF2;CBX3;CBX2;DNAJC13;EIF2S2;ESR1;SMAD5;AZIN1;SMAD7;NR4A2;DIAPH2;LIN28B;RALYL;LIN28A;JMY;BLMH;C2ORF42;USP37;ABCD3;USP38;USP32;HSPB8;USP34;SIX1;YBX1;YBX3;DTWD2;CASP7;CASP3;RBM6;KDM6B;KLF10;USP46;KLF11;RUBCN;USP47;USP48;CAMK1D;USP42;USP44;AXIN2;SAFB;SENP2;KLF15;TGFBR1;SENP1;DDX19A;DDX19B;TRAPPC6B;ZPBP2;RBMS1;RBMS2;KDM3A;USP15;POMP;USP12;MOSPD1;TOB1;UACA;GNA13;TBRG1;NXF1;MAPK8;RMND5A;SAP30L;LRIG1;PDE6D;S1PR1;LRIG3;MAPK6;LURAP1;REPS1;TCF7L2;USP25;WTAP;B3GAT3;USP21;LIMK1;DTNBP1;ARID3A;NAP1L1;ARID3B;WEE1;H3-5;PITHD1;PMP22;AQP11;SPRY2
 
 
 
 
Nucleus ( GO:0005634 )
 
 
849/4879
 
 
0.0000000
 
 
0.0000000
 
 
0
 
 
0
 
 
1.433904
 
 
48.6579020
 
 
RAMAC;TESK2;ZC3H12C;PSMD7;PPP4R2;BBX;MEF2A;PRKAB2;RBFOX2;ZNF283;ZNF10;RFX3;DICER1;ISL1;RFX6;CDIP1;CGGBP1;SLBP;CDCA4;MEPCE;GATA6;COPS7B;ZNF23;RDH10;ZNF268;ZNF267;ZNF266;NR2F2;MAGOHB;PBX1;NR2F6;CS;PKIB;MLLT10;PKIA;AGO2;C19ORF47;CDIN1;ZBTB8B;FERMT2;RNF220;LIN54;ZNF250;BHLHE41;PRKAG2;SNX10;TFCP2L1;CTCF;MECP2;MED14;TUBA1A;NKRF;HEY2;IKBKE;SS18;CNOT6L;SLC39A11;FOS;BAZ1B;DYNLL1;DUSP9;MED26;DYNLL2;SIRT1;LRRC41;MED28;PSMA5;PLSCR3;PSMA1;MED21;PLSCR4;PPARG;ERGIC2;MDFI;ZNF473;KANK1;ZNF471;ZNF470;CIPC;DLST;FOXS1;PLAGL2;ZNF225;SRSF11;EGLN1;ZNF462;EGLN2;SLF2;MDH2;USP9X;TARBP2;WT1;GID8;SERBP1;ZNF699;CRLF3;YPEL5;CCNT2;CSE1L;UBE2D2;UBE2D3;UBE2D1;RORA;RORB;NUDT11;YY1;SESN1;KMT5B;ZNF689;NEK3;KPNA5;PITX2;MAP3K7;KPNA3;NEPRO;KPNA1;ZNF200;KHNYN;FBXW7;KMT5C;UBE2E3;CTNNBIP1;PIAS2;FOXP2;CDC25B;MAF;DMTF1;HAT1;PKP4;MAPKAPK5;SRSF7;KPNB1;S100A8;DLX1;INO80D;ZC4H2;ARL3;ISG20L2;STK39;ARL2;FOXO4;FOXO3;HSP90B1;GLIS2;STK33;ZKSCAN3;ZNF420;ZBTB18;TIA1;NEK6;ZBTB16;NEK7;STYX;FOXN4;FBXO32;QKI;PTPRA;MDM4;NFE2L1;ZNF652;CELF2;ONECUT1;CELF3;PTEN;FASLG;NXT2;CDC73;ALAD;FUNDC2;PPP6R1;ACP3;ZNF644;YAF2;ACTR2;EMX2;RAD23B;ARL4A;HAND1;ZNF879;TMBIM6;TOP1;AHDC1;PRKAA1;PRKAA2;CARNMT1;UHRF2;ZBTB47;FOXK2;PBXIP1;RCHY1;PPM1D;PPM1F;PPM1G;CECR2;PARD6B;TP53INP1;IGF2BP1;E2F3;IGF2BP3;IGF2BP2;E2F6;ZNF620;H2AZ1;PRRX1;FEM1B;FOXJ2;TBX5;TBX4;SLC25A37;MCM3AP;NOVA1;JPT1;CCNK;CCNJ;CCNH;RTCA;CCNF;RTCB;CCNC;NUDT5;IKZF5;RASSF3;CDC27;EPC1;CCNL2;PABIR1;KCTD19;SFMBT1;NSMCE2;PISD;THAP1;CDC34;CCNY;SUB1;CCDC82;TP53;TRIB2;MYCBP2;PIK3R1;ZBTB5;ZBTB4;UBE2J1;MBTD1;SEC14L2;PPCS;MIER1;KCNN4;N4BP1;ZBED4;FOXF1;XRCC5;ATOSB;ID1;ID4;ID3;GRB2;ARMC12;CALM3;MKX;SLC2A4RG;RCOR1;CSRNP1;BARHL2;SUV39H2;PPP1R11;PPP1R10;SUV39H1;MIA3;NAT14;LARP1B;SYNCRIP;SCML2;DHX15;REV3L;NCOA1;SREBF1;FOXD1;CACYBP;KIF23;ZHX1;NR5A2;PDIK1L;G2E3;FAM193B;NCOA7;ZNF780B;YAP1;SATB1;ZFP14;GPC4;EXOSC3;DYNC1I1;SPRYD4;NEDD4;HNRNPA2B1;CCNG1;FOXA1;PRDM4;GEMIN2;PRDM5;NR3C1;LOXL2;TXN2;KLHL31;AMMECR1L;CCND2;CCND1;CAPN3;EP300;SNIP1;ARID2;RGS8;EOMES;PDGFRB;SLC30A9;BORA;ADAM10;UBE4A;UBE4B;WBP11;CCNE2;PSPC1;CCNE1;HNRNPUL2;WDFY1;UBE2V2;PLAA;UBE2V1;PPID;DDX6;UBA6;YTHDC1;CACNA1A;PDS5B;IRAK2;RAI2;UBE2F;TFAP2C;UBE2H;TFAP2D;UBE2I;UBE2B;VTA1;SLC52A3;HMGA1;HMGA2;BLTP1;UBE2W;FGF14;NASP;APPBP2;BHLHE40;UBE2N;NABP1;CYCS;MXD1;UBE2K;FGF11;PHF19;RERE;DDX3Y;DYRK2;DDX3X;DDX49;NAA40;YWHAB;CHD7;HMGB2;DPY30;TNFAIP3;TRIAP1;SOBP;CHD1;UBE2Q1;YWHAQ;ZIC1;MYBL2;HORMAD2;ZNF385D;CFAP45;ZNF385A;EED;ETV4;CCNA2;ELF2;CREB1;ELF4;ELF5;IRF2;ZMYND11;RBM24;CREB5;PARG;NAA20;RAP1GDS1;RNF7;ATAD2B;CAND1;PABPN1;MKNK1;TBX21;TDG;UBE2NL;DROSHA;VHL;MTA3;RBM38;NIPSNAP3A;EYA1;EYA3;SWT1;TAF11;ZNF804A;PEDS1;CHFR;REST;UFM1;TCIM;KATNBL1;POLE3;ERCC6;NAA15;TAF5;TAF4;RB1;PHB1;MAML1;GPATCH2;PHAX;GPATCH8;BACH2;DCAF6;ELK4;HOXA9;MYB;HOXA3;HOXA1;PIP4K2B;HOXA5;TSPYL2;PRKCD;MED8;SOX13;MED9;SOX11;ARMC8;SOX12;BIN1;COMMD3-BMI1;HOXB3;HOXB2;HOXB1;HOXB8;ASF1A;HOXB5;ZNF397;OSER1;ZNF395;HOXD1;ZNF391;HSPA4L;C9ORF72;MTDH;MCMBP;HOXC8;NKX2-4;RREB1;CDK17;CDK19;PLK5;PLK2;INO80;ST18;FOSL2;TADA2B;EHD2;CPEB1;PPP5C;HNRNPK;HNRNPF;SNAI1;MMS19;HBP1;CPEB3;CPEB2;ZNF136;PHF3;BTG2;ASH2L;ING5;CSRP2;AIFM1;TMSB4X;RBPMS2;JARID2;ZNF367;ZNF366;SLC2A14;WDR37;HSBP1;EN2;TET2;PAWR;TET1;ASH1L;PAX2;NCOR2;TUBB2A;MTF2;TET3;RARA;RARB;SINHCAF;JDP2;ZNF594;ASAH1;NFIX;SGMS1;WDR20;MIPOL1;RNF138;PPP1R7;LDLR;ZNF341;ZNF583;CSNK1A1;PCGF5;SEPTIN2;PCGF3;ZNF33B;NFIA;RLIM;FOSB;DGKD;FMR1;AHR;PPP1CB;PPP1CC;SALL1;ZFP30;SALL3;PAPOLG;SALL4;GRAP2;CHP2;SMARCAD1;HMGN2;MAF1;ZNF566;HMGN3;PHLDA1;TLE4;MAP2K1;USP4;USP3;IPMK;RPS6;THOC1;DYRK1B;MITF;VRK3;KAT6B;ADNP;ERG;SNRPC;IPPK;ANKRD17;BRSK1;DNMT1;CUL5;CUL3;AK1;MACROH2A1;RLF;NEUROD1;BAG4;BAG5;BTBD3;TPR;SAFB2;NDN;SKIL;ZNF784;MCL1;ESRRA;AKIRIN1;ESRRG;NSG1;ADAP1;CDK8;MOB1A;CNOT7;MAFB;ZFP62;COPS2;LHX1;MAFG;RCAN2;BCL3;BCL2;CDK1;TADA1;MAFK;ZNF774;LHX8;ZC3H3;ZFP82;DUSP16;TRO;TLK2;DNMT3B;TLK1;HIF1AN;TNPO1;LBR;DNMT3A;ZFP91;PLRG1;SMC1A;PTBP3;PTP4A1;NPEPL1;TFDP1;ESPL1;TFDP2;TRAF4;POLR1D;CRK;HDAC4;RNF34;CEBPD;NUFIP2;WBP2;CEBPG;VPS4A;RNF38;TTK;PDHB;STOX2;RSBN1L;POLR2D;RBBP6;RBBP7;JAZF1;RNF20;IRX5;OXR1;PNRC1;PNRC2;KLF6;MAB21L2;KLF9;INSM2;BCOR;PPIL4;CUL4B;STEEP1;TCERG1;APP;PANK3;PANK1;FRAT2;ZNF608;CHEK1;PCLAF;DPF2;HABP4;UBAP2L;SOX9;SKP1;SOX5;PDHX;ESCO1;P3H1;OVOL1;RUNX1;XRN1;VDAC1;DNA2;ANKRA2;ANP32A;DCUN1D3;DCUN1D4;DCUN1D2;GSKIP;STC1;FXR1;STRIP1;SPAST;PDCD11;DPH3;TRPM7;SRPK2;SETDB1;GADD45A;SS18L1;SIAH1;GADD45G;SRPK1;STAG2;FUBP1;CREBRF;DAZAP2;AHCTF1;TENM2;DAZAP1;UBXN1;LIMD1;MEOX2;PRPF19;COIL;ZMYM2;XPO4;H3-3B;TSC22D4;STRADB;TFEB;CBFA2T3;MLF1;PALD1;RHOB;MDFIC;TFEC;RGS7BP;VGLL2;SGK1;MYCBP;PPP2CA;PURA;SHOC2;APBB3;GINS2;PTGES3;XPO7;NR6A1;PRKRA;CAMK4;ZNF354A;FRA10AC1;CDK5R1;ZNF354B;ARID4B;HAPSTR1;HOXA11;HOXA10;SVEP1;MAGEB10;LMO1;NANOS1;PDIA3;MLIP;LMO3;ANXA11;KCTD1;TSC1;MAU2;MYCN;LMX1A;TBL1XR1;CMPK1;CHMP4B;ZFP36L2;ZFP36L1;ZMAT2;PMAIP1;OTX2;RPRD1A;SMAD2;CPSF7;CBX5;TGFB1;CBX4;SMURF2;CBX3;CBX2;EIF2S2;ESR1;SMAD5;AZIN1;SMAD7;NR4A2;LIN28B;RALYL;LIN28A;JMY;BLMH;C2ORF42;USP37;USP38;USP32;HSPB8;USP34;SIX1;YBX1;YBX3;DTWD2;CASP7;CASP3;RBM6;KDM6B;KLF10;USP46;KLF11;USP47;USP48;CAMK1D;USP42;USP44;AXIN2;SAFB;SENP2;KLF15;TGFBR1;SENP1;DDX19A;DDX19B;ZPBP2;RBMS1;RBMS2;KDM3A;USP15;POMP;USP12;MOSPD1;TOB1;UACA;GNA13;TBRG1;NXF1;MAPK8;RMND5A;SAP30L;LRIG1;LRIG3;MAPK6;TCF7L2;USP25;WTAP;USP21;LIMK1;DTNBP1;ARID3A;NAP1L1;ARID3B;WEE1;H3-5;PITHD1;PMP22;SPRY2
 
 
 
 
Bounding Membrane of Organelle ( GO:0098588 )
 
 
189/881
 
 
0.0000000
 
 
0.0000000
 
 
0
 
 
0
 
 
1.737586
 
 
37.9400472
 
 
GALNT12;PI4K2B;SYS1;PGAP2;TFRC;GALNT15;HHIP;ZDHHC7;LDLRAD4;ANTXR2;NDST2;SCAMP5;TEPSIN;GOLGA3;GNPTAB;AP1S1;ARFIP1;AP1S3;LARGE1;MAP3K7;LARGE2;GOLGA7;SH3GL2;TRIM23;SLC30A7;SLC30A8;SLC30A5;PAQR3;ATG9A;CSGALNACT2;ENTPD7;CASK;TPST1;ADAM17;RAB30;RNF125;ABCB6;LMAN2L;MYCBP2;SLC35D2;PDGFB;TGFA;ADRB2;CABP1;FUT4;PLD3;RHOBTB1;SEC14L2;SNX3;APOO;UGCG;FUT8;RAP1A;SNX2;IRAK2;MICAL1;PDGFC;CHST10;ST3GAL6;WNT1;WNT4;SYT4;CD163;RAB4B;PTPRN2;DMPK;WNT3A;SURF4;DNAJC13;TVP23B;ACER2;EHD2;CNIH1;B4GAT1;PI4KA;CSPG5;POC1B-GALNT4;RNF183;CNIH3;PALS1;ITGB1;ARF4;B4GALT3;MCFD2;B4GALT1;FLT3;STAB2;SLC2A1;ATP2A2;AP2A1;SLC2A3;PCSK5;SPPL3;WDR44;CHMP1B;MRC1;TMED7;SNAP29;RAB8A;HS3ST3B1;PSENEN;SREBF1;CHST7;ATP6AP2;VPS37C;VPS37D;ZDHHC13;ZDHHC17;TNFRSF1A;RHOB;VAMP8;VAMP7;ASPH;B3GNT7;TBC1D5;GORASP1;B3GNT5;CHST1;ST6GALNAC3;DIPK2A;B4GALT6;CHST2;ERGIC1;ST6GALNAC6;HBEGF;B4GALT5;STX17;SGMS1;STX16;GOSR2;PTAFR;VPS4A;GNAI3;ASAP1;ABHD17C;SGMS2;AGPAT3;TMEM165;CDC42;CYTH3;CYTH2;LMAN1;NRAS;PLN;CHSY1;LMAN2;RAB23;RAB24;STX6;MAN1C1;GCNT3;GCNT4;WHAMM;CACNG2;RFFL;LDLR;CYTH1;CACNG4;GPR157;ARFGEF1;GALNT5;RIC1;SEPTIN2;B3GAT3;GALNT1;TMED7-TICAM2;C1GALT1;WNT7A;LYSMD3;AP2B1;SVIP;PLEKHA8;FKBP1A;RAB10;CLCN6;CLCN5;CLCN4;SNX16;ABHD17B;SNX17;RAB15;FKBP1C;COL7A1;MGAT4A;RAB18;CHPT1;LAMTOR3;RAB9B;BET1
 
 
 
 
Endoplasmic Reticulum Lumen ( GO:0005788 )
 
 
78/289
 
 
0.0000000
 
 
0.0000003
 
 
0
 
 
0
 
 
2.321074
 
 
45.3855376
 
 
ERO1A;APP;POGLUT2;COLGALT2;COL16A1;POGLUT1;FKBP14;TXNDC12;MIA3;LAMC1;SHH;IL12B;ADAMTS7;PDIA3;AHSG;COL25A1;GPX8;P3H1;ADAM10;GPX7;CNPY3;PDIA6;PDIA5;CKAP4;MINPP1;ADAM17;RCN1;COL2A1;COL4A2;DMTF1;ASPH;COL4A1;COL4A4;COL6A2;CANX;DNAJB11;EOGT;ERP29;COL8A1;COL21A1;COL4A5;COL6A3;MATN3;COL15A1;COL13A1;COL11A1;PDGFB;FSTL1;COL19A1;HSP90B1;ARSJ;STC2;PDGFC;ESD;TSPAN5;WNT1;TXNDC5;WNT4;COL24A1;GOLM1;BDNF;WNT3A;COL22A1;WNT7A;COL1A1;COL3A1;TSPAN14;COL1A2;COL5A1;P4HA2;COL7A1;MGAT4A;COL5A2;CALU;COL9A1;COL9A3;PNPLA2;FBN1
 
 
 
 
Golgi Membrane ( GO:0000139 )
 
 
105/476
 
 
0.0000008
 
 
0.0000598
 
 
0
 
 
0
 
 
1.778026
 
 
25.0494930
 
 
GALNT12;PI4K2B;SYS1;PGAP2;GALNT15;ZDHHC7;LDLRAD4;NDST2;SCAMP5;GOLGA3;GNPTAB;AP1S1;ARFIP1;AP1S3;LARGE1;LARGE2;GOLGA7;SH3GL2;TRIM23;SLC30A8;SLC30A5;CSGALNACT2;PAQR3;ATG9A;TPST1;RAB30;ADAM17;RNF125;LMAN2L;SLC35D2;PDGFB;CABP1;FUT4;PLD3;APOO;UGCG;FUT8;CHST10;PDGFC;ST3GAL6;SYT4;SURF4;TVP23B;ACER2;B4GAT1;POC1B-GALNT4;ARF4;B4GALT3;B4GALT1;SLC2A1;PCSK5;TMED7;SNAP29;RAB8A;PSENEN;HS3ST3B1;SREBF1;CHST7;ATP6AP2;ZDHHC13;ZDHHC17;TNFRSF1A;B3GNT7;B3GNT5;GORASP1;CHST1;ST6GALNAC3;B4GALT6;DIPK2A;CHST2;ERGIC1;ST6GALNAC6;B4GALT5;SGMS1;STX16;GOSR2;GNAI3;ASAP1;SGMS2;AGPAT3;CDC42;CYTH3;LMAN1;CYTH2;NRAS;CHSY1;LMAN2;STX6;MAN1C1;GCNT3;GCNT4;WHAMM;RFFL;CYTH1;ARFGEF1;GALNT5;RIC1;B3GAT3;GALNT1;C1GALT1;LYSMD3;PLEKHA8;MGAT4A;CHPT1;BET1
 
 
 
 
Basement Membrane ( GO:0005604 )
 
 
21/50
 
 
0.0000011
 
 
0.0000729
 
 
0
 
 
0
 
 
4.500655
 
 
61.6954628
 
 
COL15A1;COL16A1;COL13A1;LAMA2;COL24A1;LAMA4;COL22A1;CASK;LAMC1;NID1;COLQ;LOXL2;COL4A2;COL4A1;COL4A4;COL7A1;SMOC1;PXDN;COL8A1;COL9A3;FBN1
 
 
 
 
Cytoplasmic Stress Granule ( GO:0010494 )
 
 
27/84
 
 
0.0000157
 
 
0.0008105
 
 
0
 
 
0
 
 
2.945632
 
 
32.5777540
 
 
DAZAP2;DDX6;DDX3X;PRKAA2;NUFIP2;FMR1;YBX1;C9ORF72;LARP1B;FXR1;ATXN2;G3BP1;HABP4;RBPMS2;IGF2BP1;IGF2BP3;IGF2BP2;TIA1;LARP4B;LARP4;EIF2S1;QKI;DDX19A;DDX19B;HNRNPK;LIN28A;KPNB1
 
 
 
 
Endosome Membrane ( GO:0010008 )
 
 
85/392
 
 
0.0000165
 
 
0.0008105
 
 
0
 
 
0
 
 
1.733096
 
 
19.0853344
 
 
ITGB1;PI4K2B;APP;TFRC;FLT3;SNX12;SNX10;AP2A1;LDLRAD4;ANTXR2;WDR44;SCAMP5;EEA1;PIP4P2;PIP4P1;CHMP1B;MRC1;VPS36;MAP3K7;RAB8A;PSENEN;PDIA3;STARD3NL;ATG9A;ATP6AP2;VPS37C;VPS37D;SCAMP1;RHOB;VAMP8;RAP2C;VAMP7;RAP2B;TBC1D5;SLC29A3;ARF6;ARL8B;MARCHF1;ABCB6;TMEM63B;GOSR2;VPS4A;ADRB2;ABHD17C;SLC30A10;RHOBTB1;PLD3;TMEM165;SNX3;RAP1A;SNX2;IRAK2;RAB23;MICAL1;SYNDIG1;RFFL;LDLR;SLC15A4;RAB11FIP4;VTI1B;SNX6;RAB4B;FZD5;SLC31A1;WNT3A;CMTM6;TMED7-TICAM2;DNAJC13;NSG1;AP2B1;RAB10;EHD2;CLCN6;SNX18;CLCN5;CLCN4;SNX16;RAB14;ABHD17B;SNX17;RAB15;RAB12;LAMTOR3;TMEM9B;LAMTOR5
 
 
 
 
Intracellular Organelle Lumen ( GO:0070013 )
 
 
170/912
 
 
0.0000310
 
 
0.0013543
 
 
0
 
 
0
 
 
1.442625
 
 
14.9759916
 
 
ERO1A;APP;ISCA2;POGLUT2;COL16A1;POGLUT1;SPARC;TXNDC12;NR3C1;YARS2;TXN2;PSMD7;PDK4;IL12B;OSTF1;GOLGA7;NUDT13;PDIA3;PDHX;MLIP;ANGEL2;ADAM10;P3H1;CNPY3;ARMC8;PDIA6;PDIA5;MINPP1;PLPP6;OXA1L;ADAM17;DMTF1;COL4A2;PDE12;COL4A1;CLPP;BOLA3;COL4A4;DNAJB11;FDX1;EOGT;ERP29;COL8A1;COL4A5;KPNB1;LIPT2;PSMD12;COL13A1;PDGFB;ARL2;AK4;FOXO3;GNS;HSP90B1;CNN2;PRDX3;ARSJ;STC2;PDGFC;ESD;TSPAN5;WNT1;TXNDC5;WNT4;HSPA9;TGFB1;COL24A1;BDNF;WNT3A;IDH2;COL1A1;CS;COL1A2;COL5A1;P4HA2;COL5A2;CALU;COL9A1;CDK1;SDC1;CSPG5;SP8;COL9A3;NIT2;FBN1;COLGALT2;DDX3X;TNFAIP6;FKBP14;MRPS10;C1ORF35;ETFA;MIA3;CXCL1;LAMC1;PCSK5;CTSS;ALAD;SHH;AUH;QARS1;PGM2;MUC13;UQCRFS1;SPTAN1;PGM1;ADAMTS7;ACTR2;ARG2;DYNLT1;AHSG;COL25A1;GLRX5;MARS2;NSUN3;SIRT4;AMT;GPX8;GPX7;NGF;CKAP4;PSMA5;MMP11;RCN1;COL2A1;MMP16;NME6;COL6A2;CANX;FASTKD5;COL21A1;COL6A3;SDHAF4;NAT8L;PAFAH1B2;MATN3;COL15A1;ASAH1;CANT1;GLS2;COL11A1;DLST;PPM1K;PDHB;FSTL1;COL19A1;HSPD1;CAND1;PDPR;RNF138;IARS2;PDSS1;GPC4;LDLR;GOLM1;MDH2;MMAB;COL22A1;WNT7A;WARS2;COL3A1;TSPAN14;MTRES1;PDP2;GNPDA1;COL7A1;MGAT4A;YPEL5;ABCE1;PNPLA2
 
 
 
 
RISC Complex ( GO:0016442 )
 
 
8/13
 
 
0.0000938
 
 
0.0036863
 
 
0
 
 
0
 
 
9.911591
 
 
91.9235344
 
 
DDX6;PRKRA;AGO2;DICER1;LIMD1;TARBP2;DCP2;TNRC6A
 
 
 
 
Endoplasmic Reticulum Membrane ( GO:0005789 )
 
 
160/872
 
 
0.0001195
 
 
0.0042682
 
 
0
 
 
0
 
 
1.412153
 
 
12.7552403
 
 
ERO1A;STEEP1;PI4K2B;VKORC1L1;PGAP1;LPGAT1;PGAP2;ARL6IP1;GHITM;CYP46A1;TM7SF2;CLCC1;MAP3K7;SLC30A7;MMGT1;SLC30A5;ATG9A;ACSL6;STIMATE;ATG14;TMEM170A;PDIA6;PDIA5;CYP39A1;PLPP6;ABCB6;FICD;ABHD4;LMAN2L;INSIG2;FAXDC2;INSIG1;RPN1;TGFA;ABCB9;DNAJC25;CYP19A1;PLD3;RTN4;HSP90B1;UBE2J1;ZMPSTE24;MTDH;SEC14L2;APOO;CYB5R4;RDH10;SSR1;KDSR;PDZD8;VTI1B;ICMT;DMPK;SRD5A3;SURF4;FZD6;CIDEB;BFAR;ERLIN2;DNAJC18;CYP26A1;G6PC2;CNIH1;OSTC;CALU;BCL2;CDK1;SRPRA;RNF183;RNF186;CNIH3;NFE2L1;CNIH4;CDS2;POMT2;PIGU;MCFD2;TRAM1;MCTP1;ATP2A2;MIA3;SPPL3;PIGX;JPH4;CYP26B1;JPH3;SPTLC1;SERP1;RB1CC1;TMED7;LBR;SH3D19;PSENEN;SREBF1;ELOVL4;STARD3NL;ELOVL5;ELOVL2;ELOVL3;ATP6AP2;SLC35G1;MBLAC2;VAMP7;ASPH;TMEM33;PIGA;CANX;TMBIM6;ERGIC1;SEC23A;MARCHF1;STX17;SGMS1;TMPRSS3;GOSR2;DERL1;GNAI3;GDPD1;MOSPD2;HMGCR;SGMS2;SELENOK;AGPAT3;USP19;AGPAT4;CDC42;LMAN1;EXTL2;NRAS;PLN;GPAT4;LMAN2;EMC4;GPAT3;SPTSSB;SPTSSA;MARCHF5;HSD3B7;GALNT1;DTNBP1;ORMDL1;PEDS1;SVIP;FKBP1A;VMP1;RAB10;DPY19L1;XK;CLCN4;DPY19L4;VAPB;SCD;FKBP1C;VMA21;DOLPP1;RAB18;CHPT1;PNPLA6;PLP2;PNPLA2
 
 
 
 
PTW/PP1 Phosphatase Complex ( GO:0072357 )
 
 
5/6
 
 
0.0002774
 
 
0.0090837
 
 
0
 
 
0
 
 
30.947501
 
 
253.4656491
 
 
PPP1CB;PPP1CC;PPP1R12A;PPP1R10;WDR82
 
 
 
 
Cyclin-Dependent Protein Kinase Holoenzyme Complex ( GO:0000307 )
 
 
16/47
 
 
0.0003973
 
 
0.0120097
 
 
0
 
 
0
 
 
3.201677
 
 
25.0720019
 
 
CCNK;CCNJ;CCNH;CCNF;CCNC;CCNA2;CDK8;CCND2;CCND1;CCNE2;CCNY;CCNE1;CCNG1;CDK1;CCNL2;CDK5R1
 
 
 
 
Autophagosome ( GO:0005776 )
 
 
23/81
 
 
0.0005086
 
 
0.0142776
 
 
0
 
 
0
 
 
2.462266
 
 
18.6733805
 
 
SH3GLB1;BECN1;GABARAPL1;STX17;ATG9A;WIPI2;ATG14;C9ORF72;VMP1;TBC1D5;CHMP1B;RAB23;RAB24;TP53INP1;CHMP2B;JMY;RUBCNL;UBQLN1;CHMP4B;ULK2;PIP4K2B;SNAP29;ATG5
 
 
 
 
Serine/Threonine Protein Kinase Complex ( GO:1902554 )
 
 
21/72
 
 
0.0005938
 
 
0.0155583
 
 
0
 
 
0
 
 
2.555920
 
 
18.9877152
 
 
CCNJ;CCNH;STRADB;CCNF;CCNC;ACVR2B;TGFBR1;CCNA2;CDK8;PARD6B;CCND2;ACVR1C;CCND1;CCNY;CCNG1;CDK1;RICTOR;TBKBP1;CCNL2;IKBKE;CDK5R1
 
 
 
 
Recycling Endosome ( GO:0055037 )
 
 
36/155
 
 
0.0011984
 
 
0.0294364
 
 
0
 
 
0
 
 
1.880581
 
 
12.6501855
 
 
APP;MCTP1;TFRC;PLA2G3;AKAP5;SLC30A10;TNF;SCAMP5;TUBA1A;STX6;RAB11FIP4;RAB8A;DYNC1I1;VTI1B;PDIA3;USP6;RAB4B;SLC31A1;ATG9A;CMTM6;LMTK2;NSG1;SCAMP1;RAP2C;VAMP8;RAB10;EHD2;SNX18;FCHSD2;RAP2B;CLCN4;RAB14;RAB12;RELCH;DENND6A;ARF6
 
 
 
 
Filopodium ( GO:0030175 )
 
 
17/57
 
 
0.0014582
 
 
0.0337110
 
 
0
 
 
0
 
 
2.635952
 
 
17.2141569
 
 
ITGB1;RIPOR2;TENM2;ACTN2;FMR1;RDX;TWF1;ACTA2;CDC42;GAP43;KITLG;ACTC1;PALM;FSCN1;UTRN;ARF6;METAP2
 
 
 
 
Coated Vesicle ( GO:0030135 )
 
 
22/84
 
 
0.0021213
 
 
0.0463144
 
 
0
 
 
0
 
 
2.201951
 
 
13.5546427
 
 
LMAN2L;STX17;SURF4;PIK3C2A;SCAMP1;SNAP91;HSPD1;TEPSIN;SNX3;LMAN1;SNX18;LMAN2;COL7A1;CLVS2;STX6;TMED7;ERGIC2;TMED5;ERGIC1;CNIH4;GAS7;CLVS1
 
 
 
 
Neuron Projection ( GO:0043005 )
 
 
102/560
 
 
0.0024304
 
 
0.0502709
 
 
0
 
 
0
 
 
1.390345
 
 
8.3694596
 
 
APP;FMR1;STMN2;RORA;HTR4;CYP46A1;IGF1R;TMEM266;ALCAM;ATCAY;SCN11A;STMN1;PALM;SCN9A;RGS8;VSTM5;KPNA1;EPHA7;SEMA6A;SLC30A3;RPS6;CD2AP;BIN1;RGS9BP;FEZ2;SPTBN4;TMEM185A;C9ORF72;RTN4;FXR1;SPAST;CALCR;MAP7;MAP4;KCNN4;MARK3;MARK1;PRNP;SYT4;TGFB2;HOMER1;HTR1E;FZD4;BDNF;PLK2;RAB39B;NSG1;CPEB1;CHRFAM7A;STRN;CNTN4;CPEB3;CPEB2;CNIH3;SNAP25;NRP1;TENM2;NRSN1;DOCK7;PTEN;HTR2A;SLC6A1;GRM3;GRM7;NPTN;NEFM;ZNF385A;TSC22D4;NGF;CCSAP;VAMP7;IL1RAPL1;RAPGEF2;RGS7BP;PAFAH1B1;PRKAA1;PLPPR1;PRKAA2;KCNA4;ADCY2;LRP2;AKAP5;TRAK2;CDC42;PGRMC1;MAPK8;RELN;NTF3;STX3;CTHRC1;MPP2;KCNB1;LIMK1;CRIPT;DTNBP1;ATXN10;VIM;KCNK2;METAP1;METAP2;CDK5R1;HCN1
 
 
 
 
Recycling Endosome Membrane ( GO:0055038 )
 
 
19/70
 
 
0.0026729
 
 
0.0525227
 
 
0
 
 
0
 
 
2.310827
 
 
13.6906958
 
 
PDIA3;SLC31A1;ATG9A;CMTM6;NSG1;SCAMP1;SLC30A10;SCAMP5;RAP2C;VAMP8;EHD2;SNX18;RAP2B;RAB14;RAB12;RAB8A;RAB11FIP4;ARF6;VTI1B
 
 
 
 
COPII-coated ER to Golgi Transport Vesicle ( GO:0030134 )
 
 
20/77
 
 
0.0036617
 
 
0.0685270
 
 
0
 
 
0
 
 
2.176428
 
 
12.2093587
 
 
SEC16B;MCFD2;SEC23A;LMAN2L;STX17;SLC30A5;GOSR2;SURF4;TGFA;LMAN1;CNIH1;LMAN2;COL7A1;TMED7;ERGIC2;CNIH3;TMED5;ERGIC1;CNIH4;VTI1B
 
 
 
 
Endoplasmic reticulum-Golgi Intermediate Compartment Membrane ( GO:0033116 )
 
 
15/52
 
 
0.0038705
 
 
0.0691406
 
 
0
 
 
0
 
 
2.513045
 
 
13.9584102
 
 
MCFD2;GOSR2;GALNT1;TGFA;SPPL3;SEC14L2;LMAN1;CNIH1;GORASP1;COL7A1;WHAMM;TMED7;CNIH3;BET1;PALS1
 
 
 
 
P-body ( GO:0000932 )
 
 
21/83
 
 
0.0041264
 
 
0.0705081
 
 
0
 
 
0
 
 
2.101103
 
 
11.5357749
 
 
TRIM71;DDX6;GARRE1;SAMD4A;LIMD1;C9ORF72;PNRC1;ZFP36L1;PNRC2;MEX3B;CNOT7;LIN28A;FAM184A;IGF2BP1;IGF2BP3;TOP1;IGF2BP2;EIF4E2;DCP1A;DCP2;TNRC6A
 
 
 
 
Cytoplasmic Vesicle Membrane ( GO:0030659 )
 
 
77/415
 
 
0.0048095
 
 
0.0787562
 
 
0
 
 
0
 
 
1.419170
 
 
7.5743304
 
 
ITGB1;B4GALT1;TFRC;FLT3;STAB2;AP2A1;SLC2A3;ANTXR2;SPPL3;WDR44;TEPSIN;CHMP1B;MRC1;AP1S1;AP1S3;MAP3K7;RAB8A;PSENEN;SLC30A8;SLC30A5;ATP6AP2;VPS37C;ENTPD7;VPS37D;ZDHHC13;SLC30A1;ZDHHC17;RHOB;VAMP8;VAMP7;TBC1D5;HBEGF;PTAFR;VPS4A;MYCBP2;ADRB2;ABHD17C;RHOBTB1;TMEM165;SNX3;ZNRF2;RAP1A;SNX2;IRAK2;RAB23;MICAL1;RAB24;CACNG2;RFFL;WNT1;LDLR;CACNG4;WNT4;CD163;RAB4B;PTPRN2;WNT3A;TMED7-TICAM2;WNT7A;DNAJC13;AP2B1;SVIP;RAB10;EHD2;CLCN6;CLCN5;CLCN4;SNX16;APPBP2;ABHD17B;SNX17;RAB15;RAB18;PI4KA;CSPG5;LAMTOR3;RAB9B
 
 
 
 
Pericentric Heterochromatin ( GO:0005721 )
 
 
8/21
 
 
0.0052598
 
 
0.0826840
 
 
0
 
 
0
 
 
3.810378
 
 
19.9955803
 
 
CBX5;UHRF2;CBX3;KMT5C;POLE3;SNAI1;BAZ1B;MACROH2A1
 
 
 
 
Early Endosome ( GO:0005769 )
 
 
61/320
 
 
0.0062266
 
 
0.0941173
 
 
0
 
 
0
 
 
1.465417
 
 
7.4427479
 
 
PLEKHF2;APP;PI4K2B;NRP1;ANKRD13B;PHB1;USP32;SNX12;LDLRAD4;RAB22A;EEA1;ZFYVE26;AP1S1;AP1S3;AP3S1;SH3GL2;USP8;RUBCN;MAP2K1;MMGT1;LMTK2;VAMP8;RAB32;WDFY1;RAB38;ARL8B;ASAH1;MARCHF1;PARM1;TMEM63B;VPS4A;ADRB1;ABCB9;SLC30A10;SNX30;PLD3;HSPD1;PRDX3;SNX3;RAP1A;SNX27;SYNDIG1;STX6;LDLR;SNX6;VTI1B;RAB4B;FZD5;SLC31A1;WNT3A;CMTM6;TMED7-TICAM2;DNAJC13;NSG1;EHD2;CLCN5;CLCN4;SNX16;RAB14;SNX17;TMEM9B
 
 
 
 
Palmitoyltransferase Complex ( GO:0002178 )
 
 
5/10
 
 
0.0071526
 
 
0.1041107
 
 
0
 
 
0
 
 
6.188062
 
 
30.5707142
 
 
SPTLC1;ORMDL2;ORMDL1;SPTSSB;SPTSSA
 
 
 
 
Dendrite ( GO:0030425 )
 
 
54/281
 
 
0.0081712
 
 
0.1143846
 
 
0
 
 
0
 
 
1.479122
 
 
7.1103371
 
 
APP;TENM2;FMR1;HTR2A;HTR4;CYP46A1;TMEM266;ATCAY;GRM7;RGS8;VSTM5;ZNF385A;KPNA1;TSC22D4;EPHA7;RPS6;NGF;CD2AP;BIN1;IL1RAPL1;TMEM185A;PRKAA1;PRKAA2;ADCY2;AKAP5;LRP2;C9ORF72;TRAK2;MAPK8;RELN;NTF3;MARK3;MARK1;MPP2;PRNP;HOMER1;HTR1E;FZD4;KCNB1;BDNF;PLK2;CRIPT;ZNF804A;NSG1;CHRFAM7A;STRN;ATXN10;CPEB3;CNIH3;KCNK2;METAP1;CDK5R1;HCN1;METAP2
 
 
 
 
trans-Golgi Network ( GO:0005802 )
 
 
49/251
 
 
0.0084406
 
 
0.1143846
 
 
0
 
 
0
 
 
1.507736
 
 
7.1989906
 
 
APP;PI4K2B;SCOC;SYS1;DPY30;GCC2;PIK3C2A;PCSK5;SCAMP5;TEPSIN;ARL5B;AP1S1;ARFIP1;AP1S3;CHAC1;RAB8A;SLC30A6;SLC30A5;ATG9A;SCAMP1;FAM91A1;RAB32;BACE2;RAB30;VAMP7;TRAPPC6B;RELCH;RAB38;CHST2;MARCHF1;STX16;CABP7;ASAP1;FUT4;TMEM165;ATXN2;STX6;CLVS2;CLASP2;CLVS1;ARFGEF1;ATP8B4;RIC1;NSG1;TJAP1;PLEKHA8;KLHL20;RAB10;RAB9A
 
 
 
 
RISC-loading Complex ( GO:0070578 )
 
 
4/7
 
 
0.0092507
 
 
0.1172753
 
 
0
 
 
0
 
 
8.248742
 
 
38.6292982
 
 
PRKRA;AGO2;DICER1;TARBP2
 
 
 
 
Serine Palmitoyltransferase Complex ( GO:0017059 )
 
 
4/7
 
 
0.0092507
 
 
0.1172753
 
 
0
 
 
0
 
 
8.248742
 
 
38.6292982
 
 
SPTLC1;ORMDL2;SPTSSB;SPTSSA
 
 
 
 
Endoplasmic Reticulum Tubular Network ( GO:0071782 )
 
 
9/29
 
 
0.0141304
 
 
0.1735386
 
 
0
 
 
0
 
 
2.786208
 
 
11.8676563
 
 
RAB10;SPAST;ASPH;RAB18;STIMATE;PARP16;RTN4;KPNB1;PARP8
 
 
 
 
Axon ( GO:0030424 )
 
 
42/217
 
 
0.0159839
 
 
0.1903534
 
 
0
 
 
0
 
 
1.490292
 
 
6.1641070
 
 
APP;NRP1;PRKAA1;PRKAA2;FMR1;DOCK7;KCNA4;RORA;LRP2;SLC6A1;IGF1R;GRM3;MAPK8;ALCAM;SPAST;ATCAY;CALCR;GRM7;SCN11A;NTF3;SCN9A;MAP7;NPTN;NEFM;MAP4;VSTM5;SYT4;TGFB2;SEMA6A;KCNB1;BDNF;DTNBP1;NGF;CCSAP;CD2AP;BIN1;FEZ2;CNTN4;VIM;METAP1;CDK5R1;HCN1
 
 
 
 
Rough Endoplasmic Reticulum ( GO:0005791 )
 
 
8/25
 
 
0.0167719
 
 
0.1938635
 
 
0
 
 
0
 
 
2.913141
 
 
11.9090672
 
 
LIN28A;STX17;RAB14;RPN1;SUCO;TMCC1;SPPL3;CKAP4
 
 
 
 
Cytoplasmic Dynein Complex ( GO:0005868 )
 
 
7/21
 
 
0.0197166
 
 
0.2172704
 
 
0
 
 
0
 
 
3.094638
 
 
12.1504550
 
 
DYNC1I2;DYNC1LI2;DYNLT1;TPR;DYNLL1;DYNLL2;DYNC1I1
 
 
 
 
Lysosomal Membrane ( GO:0005765 )
 
 
68/383
 
 
0.0199026
 
 
0.2172704
 
 
0
 
 
0
 
 
1.342177
 
 
5.2571792
 
 
B4GALT1;AP2A1;ZFYVE26;PIP4P2;PIP4P1;DEPTOR;CHMP1B;SESN2;AP1S1;GPR137;SLC39A8;AP1S3;ACP3;ATP6V1E1;RNF152;SNAP29;BORCS6;TRIM23;STARD3NL;ATP6AP2;TFEB;MITF;TSC1;CKAP4;VAMP8;VAMP7;TBC1D7;CDIP1;KXD1;TMEM106B;CHMP4B;SLC29A3;ARL8B;SIDT2;SEH1L;MARCHF1;ABCB6;STX17;SLC66A1;GNAI3;ABCB9;LRP2;GNAI1;NAPG;TMEM165;RAP1B;FNIP1;LDLR;SLC15A4;VTI1B;CTSA;SNAPIN;SURF4;CMTM6;DNAJC13;AP2B1;GDAP2;GPR137C;CLCN6;CLCN5;CLCN4;RAB14;CHMP2B;RNF183;CYB561;LAMTOR3;TMEM9B;LAMTOR5
 
 
 
 
Sex Chromosome ( GO:0000803 )
 
 
3/5
 
 
0.0216814
 
 
0.2180598
 
 
0
 
 
0
 
 
9.277039
 
 
35.5431198
 
 
PCGF5;PCGF3;MACROH2A1
 
 
 
 
Sarcoplasmic Reticulum Membrane ( GO:0033017 )
 
 
9/31
 
 
0.0219993
 
 
0.2180598
 
 
0
 
 
0
 
 
2.532622
 
 
9.6663762
 
 
JPH4;SLC30A7;FKBP1A;JPH3;PLN;DMPK;ASPH;FKBP1C;ATP2A2
 
 
 
 
Autophagosome Membrane ( GO:0000421 )
 
 
10/36
 
 
0.0220197
 
 
0.2180598
 
 
0
 
 
0
 
 
2.381401
 
 
9.0869973
 
 
SH3GLB1;VMP1;GABARAPL1;STX17;CHMP1B;ATG9A;CHMP2B;JMY;RUBCNL;CHMP4B
 
 
 
 
Tight Junction ( GO:0070160 )
 
 
21/96
 
 
0.0221944
 
 
0.2180598
 
 
0
 
 
0
 
 
1.735595
 
 
6.6089997
 
 
MAP3K1;USP53;FRMD4B;CLDN1;YBX3;TGFBR1;MPP7;AMOT;MTDH;RAP2C;CYTH3;TJP1;PARD6B;RAP2B;CLDN14;CLDN12;RAPGEF2;STRN;AMOTL1;CYTH1;EPHA2
 
 
 
 
ESC/E(Z) Complex ( GO:0035098 )
 
 
5/13
 
 
0.0254795
 
 
0.2332844
 
 
0
 
 
0
 
 
3.866864
 
 
14.1909308
 
 
EED;MTF2;RBBP7;SIRT1;PHF19
 
 
 
 
Voltage-Gated Sodium Channel Complex ( GO:0001518 )
 
 
5/13
 
 
0.0254795
 
 
0.2332844
 
 
0
 
 
0
 
 
3.866864
 
 
14.1909308
 
 
SCN11A;SCN9A;SCN3A;SCN4B;SCN1A
 
 
 
 
Protein Kinase Complex ( GO:1902911 )
 
 
7/22
 
 
0.0255248
 
 
0.2332844
 
 
0
 
 
0
 
 
2.888161
 
 
10.5940822
 
 
STRADB;NEK10;RICTOR;TBKBP1;IKBKE;IGF1R;MAP3K5
 
 
 
 
Lysosome ( GO:0005764 )
 
 
90/532
 
 
0.0276360
 
 
0.2401839
 
 
0
 
 
0
 
 
1.266733
 
 
4.5458471
 
 
APP;IFI30;CTSS;ZFYVE26;PIP4P2;PIP4P1;DEPTOR;SMPD1;CHMP1B;RB1CC1;SESN2;AP1S1;GPR137;SLC39A8;AP1S3;ACP3;ATP6V1E1;RNF152;AP5S1;TRIM23;USP6;STARD3NL;USP4;PRKCD;ATP6AP2;TFEB;MITF;TSC1;VAMP8;VAMP7;NPC2;TBC1D7;TMEM106B;CHMP4B;RAB38;SLC29A3;ARL8B;SIDT2;ZNF395;ASAH1;SEH1L;CALCRL;MARCHF1;ABCB6;STX17;SLC66A1;VPS4A;GNAI3;ABCB9;LRP2;ADRB2;C9ORF72;GNS;GNAI1;NAPG;PLD3;TMEM165;SNX2;RICTOR;GPC4;FNIP1;RFFL;LDLR;SLC15A4;MARCHF3;VTI1B;TXNDC5;SNX6;CTSA;DNAJC13;GDAP2;GPR137C;GALC;CLCN6;CLCN5;CLCN4;RAB14;VMA21;RAB12;CHMP2B;CSPG5;SDC1;RAB9A;RNF183;CYB561;LAMTOR3;ADA;TMEM9B;RAB9B;LAMTOR5
 
 
 
 
Collagen-Containing Extracellular Matrix ( GO:0062023 )
 
 
67/383
 
 
0.0276982
 
 
0.2401839
 
 
0
 
 
0
 
 
1.317692
 
 
4.7257542
 
 
SPARC;COL16A1;SERPINE1;LAMC1;CTSS;LOXL2;ESM1;SHH;PODNL1;CCN2;ADAMTS8;ADAMTS9;CDON;AHSG;COL25A1;P3H1;ANXA11;CASK;F3;GREM1;SFRP1;COL2A1;COL4A2;LOX;COL4A1;COL4A4;COL6A2;PXDN;COL8A1;COL21A1;COL4A5;COL6A3;COL6A6;MATN3;S100A8;SEMA7A;COL15A1;COL13A1;LAMA2;COL11A1;LAMA4;PDGFB;NID1;ADAMTS10;COL19A1;HSP90B1;LMAN1;NDP;CPA4;CTHRC1;TGFB2;TGFB1;COL24A1;COL22A1;COLQ;COL1A1;COL3A1;COL1A2;COL5A1;SMOC1;COL7A1;COL5A2;MFAP2;COL9A1;COL9A3;FBN1;CBLN1
 
 
 
 
Bicellular Tight Junction ( GO:0005923 )
 
 
18/81
 
 
0.0281131
 
 
0.2401839
 
 
0
 
 
0
 
 
1.770334
 
 
6.3227821
 
 
USP53;FRMD4B;CLDN1;YBX3;TGFBR1;MPP7;MTDH;RAP2C;CYTH3;TJP1;PARD6B;RAP2B;CLDN14;CLDN12;RAPGEF2;STRN;AMOTL1;CYTH1
 
 
 
 
Cell-Cell Junction ( GO:0005911 )
 
 
56/316
 
 
0.0330536
 
 
0.2763844
 
 
0
 
 
0
 
 
1.337594
 
 
4.5606926
 
 
PIGU;TENM2;B4GALT1;PTPRK;LIMD1;CLDN1;PNN;TMOD3;CASK;FRS2;FRMD4B;RPGRIP1L;RAP2C;TIAM1;ADAM17;FRMD5;RAP2B;CNKSR3;PXDN;CDH11;FSCN1;RAPGEF2;PKP4;EPHA2;YAP1;SPTBN4;COL13A1;USP53;TWF1;LIN7A;LIN7C;MTDH;CNN3;CNN2;RAP1B;CYTH3;PARD6B;CCDC85C;CDH20;CCDC85A;CDH22;STX3;MPP2;CADM4;MAP3K1;RDX;MPP7;VEGFA;SMAD7;TJP1;RAB10;TEK;FERMT2;EIF4G2;LIMS1;PALS1
 
 
 
 
BLOC-1 Complex ( GO:0031083 )
 
 
5/14
 
 
0.0351898
 
 
0.2789667
 
 
0
 
 
0
 
 
3.437013
 
 
11.5036753
 
 
SNAP25;BLOC1S6;SNAPIN;DTNBP1;KXD1
 
 
 
 
Cul5-RING Ubiquitin Ligase Complex ( GO:0031466 )
 
 
5/14
 
 
0.0351898
 
 
0.2789667
 
 
0
 
 
0
 
 
3.437013
 
 
11.5036753
 
 
SOCS2;CUL5;ASB11;RNF7;SOCS4
 
 
 
 
Platelet Alpha Granule Lumen ( GO:0031093 )
 
 
15/66
 
 
0.0354920
 
 
0.2789667
 
 
0
 
 
0
 
 
1.821704
 
 
6.0816657
 
 
APP;TGFB2;SPARC;TGFB1;ACTN2;AHSG;SERPINE1;PDGFB;VEGFC;IGF1;VEGFA;ISLR;TMSB4X;SOS1;VTI1B
 
 
 
 
X Chromosome ( GO:0000805 )
 
 
3/6
 
 
0.0389076
 
 
0.2940517
 
 
0
 
 
0
 
 
6.184334
 
 
20.0778450
 
 
PCGF5;PCGF3;MACROH2A1
 
 
 
 
Activin Receptor Complex ( GO:0048179 )
 
 
3/6
 
 
0.0389076
 
 
0.2940517
 
 
0
 
 
0
 
 
6.184334
 
 
20.0778450
 
 
ACVR1C;ACVR2B;TGFBR1
 
 
 
 
Platelet Alpha Granule ( GO:0031091 )
 
 
19/90
 
 
0.0398438
 
 
0.2954456
 
 
0
 
 
0
 
 
1.657956
 
 
5.3432401
 
 
APP;TGFB2;SPARC;TGFB1;STXBP3;ACTN2;AHSG;SERPINE1;PDGFB;VEGFC;IGF1;LHFPL2;VEGFA;ISLR;VAMP7;TMSB4X;PHACTR2;SOS1;VTI1B
 
 
 
 
trans-Golgi Network Membrane ( GO:0032588 )
 
 
22/108
 
 
0.0410403
 
 
0.2986821
 
 
0
 
 
0
 
 
1.585232
 
 
5.0619645
 
 
APP;SLC30A6;SYS1;MARCHF1;RIC1;CABP7;STX16;SLC30A5;ASAP1;NSG1;SCAMP1;TMEM165;SCAMP5;TEPSIN;CLVS2;STX6;AP1S1;RAB9A;ARFIP1;AP1S3;RAB8A;CLVS1
 
 
 
 
Clathrin Adaptor Complex ( GO:0030131 )
 
 
6/20
 
 
0.0495318
 
 
0.3539273
 
 
0
 
 
0
 
 
2.651593
 
 
7.9684086
 
 
TBC1D5;SYNRG;AP1S1;AP2A1;AP2B1;AP1S3
 
 
 
 
Filopodium Membrane ( GO:0031527 )
 
 
4/11
 
 
0.0550748
 
 
0.3814093
 
 
0
 
 
0
 
 
3.534353
 
 
10.2463149
 
 
GAP43;PALM;UTRN;ARF6
 
 
 
 
Early Endosome Membrane ( GO:0031901 )
 
 
24/124
 
 
0.0572214
 
 
0.3814093
 
 
0
 
 
0
 
 
1.487096
 
 
4.2543249
 
 
APP;PI4K2B;RAB4B;MARCHF1;TMEM63B;FZD5;WNT3A;SLC31A1;CMTM6;SNX12;DNAJC13;LDLRAD4;NSG1;SLC30A10;PLD3;SNX3;EEA1;RAB14;SYNDIG1;SNX5;TMEM9B;ARL8B;SNX6;VTI1B
 
 
 
 
Lytic Vacuole Membrane ( GO:0098852 )
 
 
54/314
 
 
0.0574300
 
 
0.3814093
 
 
0
 
 
0
 
 
1.288878
 
 
3.6825689
 
 
ZFYVE26;PIP4P2;PIP4P1;DEPTOR;CHMP1B;SESN2;AP1S1;GPR137;SLC39A8;AP1S3;ACP3;ATP6V1E1;RNF152;TRIM23;STARD3NL;ATP6AP2;TFEB;MITF;TSC1;VAMP8;VAMP7;TBC1D7;TMEM106B;CHMP4B;SLC29A3;ARL8B;SIDT2;SEH1L;MARCHF1;ABCB6;STX17;SLC66A1;GNAI3;ABCB9;LRP2;GNAI1;NAPG;TMEM165;FNIP1;SLC15A4;VTI1B;DNAJC13;GDAP2;GPR137C;CLCN6;CLCN5;CLCN4;RAB14;CHMP2B;RNF183;CYB561;LAMTOR3;TMEM9B;LAMTOR5
 
 
 
 
Mitotic Spindle ( GO:0072686 )
 
 
29/155
 
 
0.0579785
 
 
0.3814093
 
 
0
 
 
0
 
 
1.426533
 
 
4.0623152
 
 
CUL3;HNF4G;ADRB2;CDC14A;CDC42;CDC27;TPR;KMT5B;MAP4;ZNF367;CLASP2;USP44;KIF23;CCDC117;DYNLL1;EML1;CCSAP;HAUS1;EML4;TUBB2A;FAM161A;ESPL1;TFDP2;TBL1XR1;KATNBL1;HECW2;CDK1;PKP4;MAPRE1
 
 
 
 
Phagocytic Vesicle ( GO:0045335 )
 
 
20/100
 
 
0.0584373
 
 
0.3814093
 
 
0
 
 
0
 
 
1.548626
 
 
4.3977910
 
 
PDIA3;ANXA11;CTSS;RAB22A;RAB32;SEC14L2;SNX3;VAMP8;PIP4P2;PIP4P1;VAMP7;RAB14;RAB34;RAB23;RAB9A;RAB38;SRGAP2;RAB8A;RAB9B;ATG5
 
 
 
 
Endolysosome ( GO:0036019 )
 
 
7/26
 
 
0.0600115
 
 
0.3814093
 
 
0
 
 
0
 
 
2.279597
 
 
6.4130050
 
 
SMPD1;PRKCD;AP2A1;AP2B1;LDLR;SLC15A4;CTSS
 
 
 
 
Focal Adhesion ( GO:0005925 )
 
 
65/387
 
 
0.0608589
 
 
0.3814093
 
 
0
 
 
0
 
 
1.253170
 
 
3.5078692
 
 
ITGB1;NRP1;YWHAB;DOCK7;LIMD1;TNFSF13B;PPP1CB;PPP1CC;ALCAM;CSRP2;YWHAQ;CAPN5;ITGB8;PDGFRB;PDIA3;ACTR2;KLF11;MAP2K1;PPP1R12A;ACTN2;RRAS2;ADAM10;CASK;KIF23;RHOB;SENP1;ADAM17;PXDN;IRF2;ITGA8;ADAM9;ITGA6;MAPRE1;MAPRE2;ARF6;EPHA2;STX16;TWF1;HSP90B1;CNN3;CDC42;CNN2;EFNB2;GNA13;NRAS;PRKAR2A;G3BP1;MAP4K4;CLASP2;HSPA9;CAV2;RDX;HMGA1;RAB10;MARCKS;HNRNPK;ACTC1;PTPRA;ITGA11;PI4KA;TADA1;VIM;LAMTOR3;FERMT2;LIMS1
 
 
 
 
Cell-Cell Contact Zone ( GO:0044291 )
 
 
11/48
 
 
0.0624303
 
 
0.3814093
 
 
0
 
 
0
 
 
1.840243
 
 
5.1042903
 
 
RAP2C;TIAM1;CADM4;RAP2B;TMEM65;PIK3CA;ATP2A2;AKAP6;PKP4;SCN4B;YWHAH
 
 
 
 
Apical Junction Complex ( GO:0043296 )
 
 
21/107
 
 
0.0633988
 
 
0.3814093
 
 
0
 
 
0
 
 
1.512629
 
 
4.1722993
 
 
USP53;FRMD4B;CLDN1;YBX3;TGFBR1;MPP7;AMOT;MTDH;RAP2C;CYTH3;TJP1;PARD6B;RAP2B;CCDC85C;CLDN14;CLDN12;RAPGEF2;STRN;AMOTL1;CYTH1;PALS1
 
 
 
 
Nuclear Membrane ( GO:0031965 )
 
 
42/239
 
 
0.0647586
 
 
0.3814093
 
 
0
 
 
0
 
 
1.322154
 
 
3.6188527
 
 
ANKRD17;AHCTF1;GATA6;TXLNG;DTX2;C9ORF72;TM7SF2;SPAST;CCND2;POM121C;TMEM43;CLMN;RB1CC1;TPR;HABP4;EPC1;SH3BGRL2;TNRC18;LBR;ARL6IP6;SH3D19;PRNP;LYPLA1;AKIRIN1;DMPK;NUP155;WTAP;CBX3;OSBPL3;SURF4;HMGA1;ATP1B4;SLC30A1;PLRG1;LEMD3;SIRT1;SENP1;PLPP6;MCM3AP;NUP50;BCL2;KPNB1
 
 
 
 
ER to Golgi Transport Vesicle Membrane ( GO:0012507 )
 
 
12/54
 
 
0.0650240
 
 
0.3814093
 
 
0
 
 
0
 
 
1.768668
 
 
4.8337688
 
 
LMAN1;SEC16B;MCFD2;SEC23A;CNIH1;SLC30A5;GOSR2;TGFA;CIDEB;TMED7;CNIH3;VTI1B
 
 
 
 
Tertiary Granule Lumen ( GO:1904724 )
 
 
12/54
 
 
0.0650240
 
 
0.3814093
 
 
0
 
 
0
 
 
1.768668
 
 
4.8337688
 
 
CNN2;ASAH1;CANT1;TNFAIP6;CXCL1;ARMC8;YPEL5;GOLGA7;SPTAN1;NIT2;CTSS;PGM1
 
 
 
 
Tertiary Granule Membrane ( GO:0070821 )
 
 
15/72
 
 
0.0691399
 
 
0.3947131
 
 
0
 
 
0
 
 
1.629376
 
 
4.3530778
 
 
SNAP25;ATP8B4;ATP6AP2;PTAFR;SLC2A3;DYNLL1;SVIP;RAP2C;VAMP8;NRAS;TSPAN14;RAP2B;RAB14;OLR1;LAMTOR3
 
 
 
 
Glutamatergic Synapse ( GO:0098978 )
 
 
20/102
 
 
0.0693008
 
 
0.3947131
 
 
0
 
 
0
 
 
1.510678
 
 
4.0324528
 
 
GRIPAP1;USP6;WNT3A;LIMK1;ATP2B4;WNT7A;DYNLL2;EFNB2;TANC1;RAP1A;SNX27;SLITRK1;IL1RAPL2;AGO2;IL1RAPL1;DLGAP1;NPTX1;SH3GL2;CLASP2;CBLN1
 
 
 
 
Cytoplasmic Side of Lysosomal Membrane ( GO:0098574 )
 
 
4/12
 
 
0.0737770
 
 
0.4142050
 
 
0
 
 
0
 
 
3.092380
 
 
8.0609324
 
 
SNAPIN;CDIP1;KXD1;BORCS6
 
 
 
 
Vacuolar Membrane ( GO:0005774 )
 
 
12/56
 
 
0.0817536
 
 
0.4525232
 
 
0
 
 
0
 
 
1.688078
 
 
4.2270238
 
 
SH3GLB1;VMP1;GABARAPL1;STX17;CHMP1B;ATG9A;ATP6AP2;VPS4A;CHMP2B;JMY;RUBCNL;CHMP4B
 
 
 
 
Cell-Substrate Junction ( GO:0030055 )
 
 
65/395
 
 
0.0843031
 
 
0.4548223
 
 
0
 
 
0
 
 
1.222211
 
 
3.0229398
 
 
ITGB1;NRP1;YWHAB;DOCK7;LIMD1;TNFSF13B;PPP1CB;PPP1CC;ALCAM;CSRP2;YWHAQ;CAPN5;ITGB8;PDGFRB;PDIA3;ACTR2;KLF11;MAP2K1;PPP1R12A;ACTN2;RRAS2;ADAM10;CASK;KIF23;RHOB;SENP1;ADAM17;PXDN;IRF2;ITGA8;ADAM9;ITGA6;MAPRE1;MAPRE2;ARF6;EPHA2;STX16;TWF1;HSP90B1;CNN3;CDC42;CNN2;EFNB2;GNA13;NRAS;PRKAR2A;G3BP1;MAP4K4;CLASP2;HSPA9;CAV2;RDX;HMGA1;RAB10;MARCKS;HNRNPK;ACTC1;PTPRA;ITGA11;PI4KA;TADA1;VIM;LAMTOR3;FERMT2;LIMS1
 
 
 
 
cullin-RING Ubiquitin Ligase Complex ( GO:0031461 )
 
 
38/219
 
 
0.0877413
 
 
0.4548223
 
 
0
 
 
0
 
 
1.301306
 
 
3.1665492
 
 
TMEM183A;CUL5;CUL3;CCNF;DCAF12L1;CUL2;FBXO45;ASB11;RNF7;DCAF6;SOCS2;DCAF15;CDC23;CAND1;CDC27;BTRC;ZSWIM5;DCAF12;SOCS4;SKP1;KLHL29;USP47;FBXW7;FEM1B;FBXL19;KLHL28;KLHL2;KLHL23;FBXO32;KCTD5;KLHL42;KLHL20;APPBP2;GAN;KLHL8;KCTD17;SPSB4;CUL4B
 
 
 
 
Phosphatidylinositol 3-Kinase Complex, Class I ( GO:0097651 )
 
 
3/8
 
 
0.0879554
 
 
0.4548223
 
 
0
 
 
0
 
 
3.710169
 
 
9.0191418
 
 
PIK3CA;PIK3R3;PIK3R1
 
 
 
 
Phosphatidylinositol 3-Kinase Complex, Class IA ( GO:0005943 )
 
 
3/8
 
 
0.0879554
 
 
0.4548223
 
 
0
 
 
0
 
 
3.710169
 
 
9.0191418
 
 
PIK3CA;PIK3R3;PIK3R1
 
 
 
 
Transferase Complex, Transferring Phosphorus-Containing Groups ( GO:0061695 )
 
 
3/8
 
 
0.0879554
 
 
0.4548223
 
 
0
 
 
0
 
 
3.710169
 
 
9.0191418
 
 
NEK10;IGF1R;MAP3K5
 
 
 
 
Secretory Granule Membrane ( GO:0030667 )
 
 
47/279
 
 
0.0941111
 
 
0.4616511
 
 
0
 
 
0
 
 
1.256049
 
 
2.9683949
 
 
SNAP25;PGAP1;SPARC;B4GALT1;MYCBP2;PTAFR;MOSPD2;SLC2A3;RAP1B;NRAS;PGRMC1;RAP1A;RAB24;OLR1;PHACTR2;ACP3;SLC15A4;SNAP29;SYT4;ATP8B4;PTPRN2;SLC30A8;RAB4B;SLC30A5;SURF4;ATP6AP2;CMTM6;TMED7-TICAM2;ADAM10;DNAJC13;LHFPL2;SCAMP1;DYNLL1;TNFRSF1B;SVIP;CKAP4;RAP2C;VAMP8;RAB10;TSPAN14;RAP2B;VAMP7;RAB14;RAB18;CYB561;LAMTOR3;RAB9B
 
 
 
 
Endocytic Vesicle ( GO:0030139 )
 
 
35/201
 
 
0.0942352
 
 
0.4616511
 
 
0
 
 
0
 
 
1.306600
 
 
3.0861400
 
 
SPARC;STAB2;AP2A1;CTSS;RAB22A;AMOT;HSP90B1;RAB23;RAB24;SRGAP2;WNT1;CACNG2;RAB11FIP4;RAB8A;CACNG4;WNT4;PDIA3;CD163;WNT3A;ENTPD7;WNT7A;ANXA11;AP2B1;GAPVD1;RAB32;EHD2;VAMP7;RAB14;RAB34;RAPGEF2;RAB9A;RAB38;RAB9B;ARF6;HBEGF
 
 
 
 
Clathrin Coat of trans-Golgi Network Vesicle ( GO:0030130 )
 
 
4/13
 
 
0.0952425
 
 
0.4616511
 
 
0
 
 
0
 
 
2.748622
 
 
6.4629140
 
 
SYNRG;AP1S1;AP2A1;AP1S3
 
 
 
 
Tertiary Granule ( GO:0070820 )
 
 
29/163
 
 
0.0968586
 
 
0.4616511
 
 
0
 
 
0
 
 
1.340739
 
 
3.1299601
 
 
SNAP25;ASAH1;CANT1;STXBP3;TNFAIP6;PTAFR;CXCL1;SLC2A3;CTSS;CNN2;NRAS;OLR1;GOLGA7;SPTAN1;PGM1;ATP8B4;PTPRN2;ATP6AP2;ARMC8;DYNLL1;SVIP;RAP2C;VAMP8;TSPAN14;RAP2B;RAB14;YPEL5;NIT2;LAMTOR3
 
 
 
 
Exocytic Vesicle ( GO:0070382 )
 
 
7/29
 
 
0.0986909
 
 
0.4616511
 
 
0
 
 
0
 
 
1.968399
 
 
4.5583447
 
 
RAB10;APP;SYT4;SYT3;SYT10;DPYSL3;IGF1
 
 
 
 
Myofibril ( GO:0030016 )
 
 
7/29
 
 
0.0986909
 
 
0.4616511
 
 
0
 
 
0
 
 
1.968399
 
 
4.5583447
 
 
TMOD1;TMOD3;TMOD2;TWF1;CAPN3;MYH4;MYL12B
 
 
 
 
Nuclear Inner Membrane ( GO:0005637 )
 
 
7/29
 
 
0.0986909
 
 
0.4616511
 
 
0
 
 
0
 
 
1.968399
 
 
4.5583447
 
 
TMEM43;CBX3;ATP1B4;LEMD3;LBR;SIRT1;TM7SF2
 
 
 
 
Actin-Based Cell Projection ( GO:0098858 )
 
 
17/88
 
 
0.0988212
 
 
0.4616511
 
 
0
 
 
0
 
 
1.482363
 
 
3.4308446
 
 
ITGB1;RIPOR2;TENM2;TGFB1;ACTN2;RDX;TWF1;ACTA2;CDC42;KITLG;ACTC1;PALM;FSCN1;TEK;UTRN;MYO1F;CLRN1
 
 
 
 
SCF Ubiquitin Ligase Complex ( GO:0019005 )
 
 
12/58
 
 
0.1009546
 
 
0.4616511
 
 
0
 
 
0
 
 
1.614495
 
 
3.7021728
 
 
USP47;TMEM183A;CUL5;FBXW7;FBXL19;CCNF;CUL2;FBXO45;BTRC;FBXO32;SKP1;SPSB4
 
 
 
 
Adherens Junction ( GO:0005912 )
 
 
27/151
 
 
0.1012941
 
 
0.4616511
 
 
0
 
 
0
 
 
1.348753
 
 
3.0882771
 
 
SPTBN4;LIN7A;LIN7C;LIMD1;CNN3;CYTH3;CCDC85C;CDH20;CCDC85A;CDH22;MPP2;TMOD3;RDX;FRS2;FRMD4B;MPP7;SMAD7;VEGFA;RAB10;TJP1;FRMD5;CDH11;CNKSR3;PKP4;EIF4G2;FERMT2;PALS1
 
 
 
 
Late Endosome ( GO:0005770 )
 
 
38/222
 
 
0.1021976
 
 
0.4616511
 
 
0
 
 
0
 
 
1.279864
 
 
2.9191735
 
 
ANKRD13B;MARCHF1;PARM1;GOSR2;VPS4A;CTSS;PLD3;ZFYVE26;PIP4P2;PIP4P1;RAP1A;TTPA;CHMP1B;IL12B;VPS36;LDLR;AP5S1;VTI1B;RUBCN;MAP2K1;STARD3NL;SLC30A3;VTA1;ATG9A;TMED7-TICAM2;NSG1;VAMP8;VAMP7;SNX16;RAB14;CHMP2B;RAPGEF2;RAB9A;CHMP4B;LAMTOR3;SLC29A3;RAB9B;LAMTOR5
 
 
 
 
Actin Cytoskeleton ( GO:0015629 )
 
 
59/361
 
 
0.1055406
 
 
0.4713345
 
 
0
 
 
0
 
 
1.211588
 
 
2.7244491
 
 
GMFB;WIPF1;SLC2A1;MYLK;LIMD2;AMOT;CAPZB;CFL2;SPTAN1;RAB8A;TMOD1;ACTR2;PPP1R12A;ACTN2;TPM3;TPM1;PAWR;CASK;TSC1;IQCG;CD2AP;CTTNBP2NL;ACTA2;ADAM17;BIN3;BIN1;RARA;FSCN1;DGKH;SPTBN4;TMEM63B;TWF1;ASAP1;CNN3;CNN2;ARHGAP21;ARSJ;MICAL1;PCDHA4;STK38L;WASF1;APBB3;GBP1;SEPTIN2;RDX;KLHL2;NPM3;KLHL20;ANLN;DIAPH2;MARCKS;ACTC1;CAPZA1;CAPZA2;SPRY2;FILIP1;TAF5;TEK;MYO1F
 
 
 
 
Lytic Vacuole ( GO:0000323 )
 
 
40/236
 
 
0.1070874
 
 
0.4728693
 
 
0
 
 
0
 
 
1.264771
 
 
2.8256370
 
 
APP;ASAH1;MARCHF1;CALCRL;VPS4A;ABCB9;ADRB2;LRP2;IFI30;C9ORF72;CTSS;ZFYVE26;SNX2;SMPD1;RB1CC1;RICTOR;RFFL;RNF152;LDLR;MARCHF3;AP5S1;SNX6;CTSA;USP6;USP4;ATP6AP2;GALC;RAB14;NPC2;VMA21;RAB12;CHMP2B;TMEM106B;RAB9A;RAB38;ADA;RAB9B;LAMTOR5;ARL8B;SIDT2
 
 
 
 
Specific Granule ( GO:0042581 )
 
 
28/159
 
 
0.1113914
 
 
0.4864093
 
 
0
 
 
0
 
 
1.323904
 
 
2.9055793
 
 
SNAP25;PGAP1;CANT1;STXBP3;MOSPD2;CXCL1;SLC2A3;CNN2;PGRMC1;RAP1A;OLR1;STX3;SLC15A4;SPTAN1;ATP8B4;CMTM6;ADAM10;ANXA11;ARMC8;SCAMP1;TNFRSF1B;CKAP4;VAMP8;TSPAN14;RAP2B;NIT2;LAMTOR3;KPNB1
 
 
 
 
Protein Serine/Threonine Phosphatase Complex ( GO:0008287 )
 
 
7/30
 
 
0.1140282
 
 
0.4880459
 
 
0
 
 
0
 
 
1.882707
 
 
4.0879397
 
 
PPP2CA;PPP3R1;PPP4R2;PTPRA;PPP2R2A;PPP4R3A;NKD1
 
 
 
 
Specific Granule Membrane ( GO:0035579 )
 
 
17/90
 
 
0.1157335
 
 
0.4880459
 
 
0
 
 
0
 
 
1.441582
 
 
3.1087211
 
 
SNAP25;ATP8B4;PGAP1;CMTM6;MOSPD2;SLC2A3;SCAMP1;TNFRSF1B;CKAP4;VAMP8;PGRMC1;TSPAN14;RAP2B;RAP1A;OLR1;SLC15A4;LAMTOR3
 
 
 
 
FNIP-folliculin RagC/D GAP ( GO:1990877 )
 
 
3/9
 
 
0.1187204
 
 
0.4880459
 
 
0
 
 
0
 
 
3.091628
 
 
6.5882086
 
 
FNIP2;LAMTOR3;LAMTOR5
 
 
 
 
Amphisome Membrane ( GO:1904930 )
 
 
3/9
 
 
0.1187204
 
 
0.4880459
 
 
0
 
 
0
 
 
3.091628
 
 
6.5882086
 
 
CHMP1B;CHMP2B;CHMP4B
 
 
 
 
Junctional Sarcoplasmic Reticulum Membrane ( GO:0014701 )
 
 
3/9
 
 
0.1187204
 
 
0.4880459
 
 
0
 
 
0
 
 
3.091628
 
 
6.5882086
 
 
JPH4;JPH3;ASPH
 
 
 
 
ISWI-type Complex ( GO:0031010 )
 
 
4/14
 
 
0.1192636
 
 
0.4880459
 
 
0
 
 
0
 
 
2.473616
 
 
5.2599441
 
 
CECR2;POLE3;RBBP7;BAZ1B
 
 
 
 
Clathrin-Coated Endocytic Vesicle Membrane ( GO:0030669 )
 
 
13/66
 
 
0.1218115
 
 
0.4880459
 
 
0
 
 
0
 
 
1.517960
 
 
3.1957306
 
 
TFRC;FZD5;FZD4;TGFA;AP2A1;RORA;LRP2;AP2B1;VAMP8;VAMP7;LDLR;SH3GL2;HBEGF
 
 
 
 
Golgi-associated Vesicle ( GO:0005798 )
 
 
10/48
 
 
0.1225817
 
 
0.4880459
 
 
0
 
 
0
 
 
1.628242
 
 
3.4176439
 
 
APP;CHIC2;RAB14;RAB12;PI4KA;ZDHHC13;CSPG5;SPPL3;ZDHHC17;RAB8A
 
 
 
 
Coated Vesicle Membrane ( GO:0030662 )
 
 
11/54
 
 
0.1229429
 
 
0.4880459
 
 
0
 
 
0
 
 
1.582912
 
 
3.3178403
 
 
TEPSIN;LMAN1;SEC16B;MCFD2;SEC23A;CNIH1;SLC30A5;GOSR2;TGFA;CNIH3;VTI1B
 
 
 
 
Core Mediator Complex ( GO:0070847 )
 
 
6/25
 
 
0.1244114
 
 
0.4889368
 
 
0
 
 
0
 
 
1.953237
 
 
4.0708620
 
 
MED14;MED21;MED8;MED9;MED26;MED28
 
 
 
 
Golgi Stack ( GO:0005795 )
 
 
7/31
 
 
0.1305319
 
 
0.5029319
 
 
0
 
 
0
 
 
1.804156
 
 
3.6735098
 
 
RAB30;RAB14;STX16;MGAT4A;RAB34;LYSET;GOLGA7
 
 
 
 
Secretory Vesicle ( GO:0099503 )
 
 
7/31
 
 
0.1305319
 
 
0.5029319
 
 
0
 
 
0
 
 
1.804156
 
 
3.6735098
 
 
RAB10;SYT4;SYT3;DYNLT1;SYT10;DPYSL3;IGF1
 
 
 
 
H3 Histone Acetyltransferase Complex ( GO:0070775 )
 
 
2/5
 
 
0.1454021
 
 
0.5347016
 
 
0
 
 
0
 
 
4.121408
 
 
7.9471147
 
 
ING5;KAT6B
 
 
 
 
MOZ/MORF Histone Acetyltransferase Complex ( GO:0070776 )
 
 
2/5
 
 
0.1454021
 
 
0.5347016
 
 
0
 
 
0
 
 
4.121408
 
 
7.9471147
 
 
ING5;KAT6B
 
 
 
 
Neuron Spine ( GO:0044309 )
 
 
2/5
 
 
0.1454021
 
 
0.5347016
 
 
0
 
 
0
 
 
4.121408
 
 
7.9471147
 
 
HOMER1;PALM
 
 
 
 
Protein Phosphatase 4 Complex ( GO:0030289 )
 
 
2/5
 
 
0.1454021
 
 
0.5347016
 
 
0
 
 
0
 
 
4.121408
 
 
7.9471147
 
 
PPP4R2;PPP4R3A
 
 
 
 
Glial Cell Projection ( GO:0097386 )
 
 
4/15
 
 
0.1455803
 
 
0.5347016
 
 
0
 
 
0
 
 
2.248611
 
 
4.3331349
 
 
ITGB1;GRM3;FMR1;KCNK2
 
 
 
 
CD40 Receptor Complex ( GO:0035631 )
 
 
3/10
 
 
0.1527689
 
 
0.5460657
 
 
0
 
 
0
 
 
2.649813
 
 
4.9785450
 
 
IKBKB;CHUK;TRAF5
 
 
 
 
cis-Golgi Network Membrane ( GO:0033106 )
 
 
3/10
 
 
0.1527689
 
 
0.5460657
 
 
0
 
 
0
 
 
2.649813
 
 
4.9785450
 
 
RAB18;RNF183;TMEM165
 
 
 
 
Cytoplasmic Side of Plasma Membrane ( GO:0009898 )
 
 
20/113
 
 
0.1528428
 
 
0.5460657
 
 
0
 
 
0
 
 
1.331141
 
 
2.5003421
 
 
CHUK;STAC;PTEN;AP2A1;AKAP5;AP2B1;IKBKB;PTP4A1;SOCS3;NRAS;SNX18;PALM;TRAF5;CHMP4B;PKP4;RGS8;SNX5;FERMT2;CYTH1;PTPN3
 
 
 
 
SAGA-type Complex ( GO:0070461 )
 
 
8/39
 
 
0.1672084
 
 
0.5920081
 
 
0
 
 
0
 
 
1.596228
 
 
2.8548775
 
 
TADA2B;POLE3;TADA1;TAF5;TAF9B;TAF4;MAP3K7;PAAF1
 
 
 
 
Spindle ( GO:0005819 )
 
 
37/227
 
 
0.1726511
 
 
0.6047922
 
 
0
 
 
0
 
 
1.205966
 
 
2.1182578
 
 
MTCL1;ARL3;CUL3;HNF4G;TTK;ADRB2;CDC14A;CDC42;CDC27;TPR;KMT5B;MAP4;ZNF367;CLASP2;CSNK1A1;CBX3;USP44;ANXA11;KIF23;CCDC117;DYNLL1;EML1;CCSAP;MZT1;EML4;TUBB2A;FAM161A;ESPL1;TFDP2;TBL1XR1;KATNBL1;HECW2;CDK1;CALM3;MMS19;PKP4;MAPRE1
 
 
 
 
Axonal Growth Cone ( GO:0044295 )
 
 
4/16
 
 
0.1738970
 
 
0.6047922
 
 
0
 
 
0
 
 
2.061107
 
 
3.6054786
 
 
FLRT3;KIF5C;KIF21A;CLASP2
 
 
 
 
Cul3-RING Ubiquitin Ligase Complex ( GO:0031463 )
 
 
11/58
 
 
0.1766189
 
 
0.6088705
 
 
0
 
 
0
 
 
1.447859
 
 
2.5102411
 
 
KLHL29;GAN;CUL3;KLHL28;KLHL8;KLHL2;KLHL23;KCTD17;KCTD5;KLHL42;KLHL20
 
 
 
 
Sodium Channel Complex ( GO:0034706 )
 
 
5/22
 
 
0.1827724
 
 
0.6245536
 
 
0
 
 
0
 
 
1.818749
 
 
3.0909892
 
 
SCN11A;SCN9A;SCN3A;SCN4B;SCN1A
 
 
 
 
Heterotrimeric G-protein Complex ( GO:0005834 )
 
 
7/34
 
 
0.1864502
 
 
0.6245536
 
 
0
 
 
0
 
 
1.603414
 
 
2.6930808
 
 
GNA13;GNG10;GNG2;GNG5;GNAI3;GNG12;GNAI1
 
 
 
 
Ficolin-1-Rich Granule Lumen ( GO:1904813 )
 
 
21/123
 
 
0.1875305
 
 
0.6245536
 
 
0
 
 
0
 
 
1.274162
 
 
2.1327108
 
 
ACTR2;PSMD12;ASAH1;DDX3X;CANT1;TNFAIP6;DYNLT1;C1ORF35;GNS;CTSS;ALAD;PSMA5;PSMD7;CAND1;GNPDA1;PGM2;OSTF1;YPEL5;KPNB1;PGM1;PAFAH1B2
 
 
 
 
AP-1 Adaptor Complex ( GO:0030121 )
 
 
3/11
 
 
0.1894042
 
 
0.6245536
 
 
0
 
 
0
 
 
2.318451
 
 
3.8576062
 
 
SYNRG;AP1S1;AP1S3
 
 
 
 
Amphisome ( GO:0044753 )
 
 
3/11
 
 
0.1894042
 
 
0.6245536
 
 
0
 
 
0
 
 
2.318451
 
 
3.8576062
 
 
CHMP1B;CHMP2B;CHMP4B
 
 
 
 
Actin Filament ( GO:0005884 )
 
 
14/78
 
 
0.1909911
 
 
0.6245536
 
 
0
 
 
0
 
 
1.353377
 
 
2.2405550
 
 
TMOD1;SPTBN4;WIPF1;ACTN2;TPM3;TPM1;PAWR;TWF1;TSC1;AMOT;DIAPH2;ACTC1;MICAL1;TEK
 
 
 
 
Apicolateral Plasma Membrane ( GO:0016327 )
 
 
2/6
 
 
0.1986494
 
 
0.6245536
 
 
0
 
 
0
 
 
3.090876
 
 
4.9955179
 
 
MTCL1;NEDD4
 
 
 
 
Astral Microtubule ( GO:0000235 )
 
 
2/6
 
 
0.1986494
 
 
0.6245536
 
 
0
 
 
0
 
 
3.090876
 
 
4.9955179
 
 
FAM161A;PAFAH1B1
 
 
 
 
Insulin-Responsive Compartment ( GO:0032593 )
 
 
2/6
 
 
0.1986494
 
 
0.6245536
 
 
0
 
 
0
 
 
3.090876
 
 
4.9955179
 
 
RAB10;RAB4B
 
 
 
 
Phosphatidylinositol 3-Kinase Complex, Class III ( GO:0035032 )
 
 
2/6
 
 
0.1986494
 
 
0.6245536
 
 
0
 
 
0
 
 
3.090876
 
 
4.9955179
 
 
BECN1;NRBF2
 
 
 
 
Pyruvate Dehydrogenase Complex ( GO:0045254 )
 
 
2/6
 
 
0.1986494
 
 
0.6245536
 
 
0
 
 
0
 
 
3.090876
 
 
4.9955179
 
 
PDHX;PDHB
 
 
 
 
Postsynaptic Density ( GO:0014069 )
 
 
27/164
 
 
0.2014574
 
 
0.6260262
 
 
0
 
 
0
 
 
1.219841
 
 
1.9544008
 
 
FMR1;CABP1;AKAP5;RTN4;NETO1;SLITRK1;SYNDIG1;RPL14;GSG1L;SRGAP2;CACNG2;FAM81A;CACNG4;NANOS1;USP8;PRNP;MPP2;USP6;HOMER1;SEMA4C;ATP2B4;CRIPT;DTNBP1;LRFN2;GAP43;STRN;CDK5R1
 
 
 
 
PRC1 Complex ( GO:0035102 )
 
 
4/17
 
 
0.2038966
 
 
0.6260262
 
 
0
 
 
0
 
 
1.902450
 
 
3.0251663
 
 
CBX4;PCGF5;PCGF3;COMMD3-BMI1
 
 
 
 
Protein Phosphatase Type 2A Complex ( GO:0000159 )
 
 
4/17
 
 
0.2038966
 
 
0.6260262
 
 
0
 
 
0
 
 
1.902450
 
 
3.0251663
 
 
PPP2CA;PTPRA;PPP2R2A;NKD1
 
 
 
 
Golgi-associated Vesicle Membrane ( GO:0030660 )
 
 
5/23
 
 
0.2085579
 
 
0.6290959
 
 
0
 
 
0
 
 
1.717608
 
 
2.6924161
 
 
PI4KA;ZDHHC13;CSPG5;SPPL3;ZDHHC17
 
 
 
 
SWI/SNF Complex ( GO:0016514 )
 
 
6/29
 
 
0.2088712
 
 
0.6290959
 
 
0
 
 
0
 
 
1.613169
 
 
2.5262824
 
 
RB1;SS18;BCL7A;DPF2;ARID2;ARID1B
 
 
 
 
Endocytic Vesicle Membrane ( GO:0030666 )
 
 
27/165
 
 
0.2107484
 
 
0.6290959
 
 
0
 
 
0
 
 
1.210930
 
 
1.8855279
 
 
TFRC;STAB2;TGFA;RORA;AP2A1;ADRB2;LRP2;SEC14L2;PIP4P2;PIP4P1;WNT1;CACNG2;LDLR;SH3GL2;CACNG4;ATG5;WNT4;CD163;FZD5;FZD4;WNT3A;ENTPD7;WNT7A;AP2B1;VAMP8;VAMP7;HBEGF
 
 
 
 
Late Endosome Membrane ( GO:0031902 )
 
 
17/99
 
 
0.2112994
 
 
0.6290959
 
 
0
 
 
0
 
 
1.282685
 
 
1.9939080
 
 
MARCHF1;STARD3NL;GOSR2;PLD3;VAMP8;PIP4P2;PIP4P1;VAMP7;CHMP1B;CHMP2B;CDIP1;CHMP4B;VPS36;LAMTOR3;SLC29A3;VTI1B;LAMTOR5
 
 
 
 
Exocytic Vesicle Membrane ( GO:0099501 )
 
 
12/67
 
 
0.2165585
 
 
0.6399059
 
 
0
 
 
0
 
 
1.349597
 
 
2.0647411
 
 
SYT4;MCTP1;SYNGR2;PTPRN2;SYPL1;SEMA4C;STX6;SYNDIG1;DTNBP1;SLC35F1;SYPL2;SYNGR3
 
 
 
 
SUMO Ligase Complex ( GO:0106068 )
 
 
3/12
 
 
0.2279466
 
 
0.6651402
 
 
0
 
 
0
 
 
2.060726
 
 
3.0470797
 
 
SLF2;UBE2I;NSMCE2
 
 
 
 
Synaptic Vesicle Membrane ( GO:0030672 )
 
 
12/68
 
 
0.2319883
 
 
0.6651402
 
 
0
 
 
0
 
 
1.325420
 
 
1.9365285
 
 
SYT4;MCTP1;SYNGR2;PTPRN2;SYPL1;SEMA4C;STX6;SYNDIG1;DTNBP1;SLC35F1;SYPL2;SYNGR3
 
 
 
 
GABA-A Receptor Complex ( GO:1902711 )
 
 
4/18
 
 
0.2352532
 
 
0.6651402
 
 
0
 
 
0
 
 
1.766458
 
 
2.5562289
 
 
GABRB3;GABRB2;GABRP;GABRB1
 
 
 
 
Endolysosome Membrane ( GO:0036020 )
 
 
4/18
 
 
0.2352532
 
 
0.6651402
 
 
0
 
 
0
 
 
1.766458
 
 
2.5562289
 
 
AP2A1;AP2B1;LDLR;SLC15A4
 
 
 
 
Endoribonuclease Complex ( GO:1902555 )
 
 
4/18
 
 
0.2352532
 
 
0.6651402
 
 
0
 
 
0
 
 
1.766458
 
 
2.5562289
 
 
PRKRA;AGO2;DICER1;TARBP2
 
 
 
 
Kinetochore Microtubule ( GO:0005828 )
 
 
4/18
 
 
0.2352532
 
 
0.6651402
 
 
0
 
 
0
 
 
1.766458
 
 
2.5562289
 
 
CHMP1B;CHMP2B;CHMP4B;CLASP2
 
 
 
 
TORC2 Complex ( GO:0031932 )
 
 
2/7
 
 
0.2536490
 
 
0.6970913
 
 
0
 
 
0
 
 
2.472558
 
 
3.3918640
 
 
SESN2;RICTOR
 
 
 
 
Cortical Endoplasmic Reticulum ( GO:0032541 )
 
 
2/7
 
 
0.2536490
 
 
0.6970913
 
 
0
 
 
0
 
 
2.472558
 
 
3.3918640
 
 
ASPH;STIMATE
 
 
 
 
cyclin/CDK Positive Transcription Elongation Factor Complex ( GO:0008024 )
 
 
2/7
 
 
0.2536490
 
 
0.6970913
 
 
0
 
 
0
 
 
2.472558
 
 
3.3918640
 
 
RB1;CCNT2
 
 
 
 
Oligosaccharyltransferase Complex A ( GO:0160226 )
 
 
2/7
 
 
0.2536490
 
 
0.6970913
 
 
0
 
 
0
 
 
2.472558
 
 
3.3918640
 
 
OSTC;RPN1
 
 
 
 
Nuclear Lumen ( GO:0031981 )
 
 
120/814
 
 
0.2616438
 
 
0.7133099
 
 
0
 
 
0
 
 
1.071449
 
 
1.4365687
 
 
SRP19;GEMIN2;FMR1;PRDM5;RORA;CDC14A;IGF1R;TXN2;PPP1CC;GOLGA3;RPS6KA3;RPS6KA6;CCND2;CHP2;HABP4;EPC1;CAPN3;KMT5B;NEPRO;PHLDA1;TSPYL2;DDX18;MLIP;FBXW7;SLC30A5;RPS6;CASK;LARP4B;MYCN;CCNY;WDR82;SUB1;WDFY1;CMPK1;TP53;PPID;SRSF9;ZNF397;IPPK;SLBP;CDCA8;ARL2;MACROH2A1;FXR1;PDCD11;DNAJC21;KCNN4;N4BP1;SRPK2;CBX5;XRCC5;HMGA2;CDK8;DIAPH2;LIN28B;STAG2;LIN28A;RCAN2;FGF18;POMT2;TUT4;SUV39H1;CHD7;HMGB2;SIX1;CTCF;CHD1;TENT4B;COIL;NKRF;TNPO1;ZNF367;RAB8A;PPP1R12A;CEMIP2;EN2;ZFP91;BAZ1B;SIRT1;DDX50;ETV4;ARL4A;MDFIC;CNKSR3;PAX9;RARA;FAM193B;TOP1;ERGIC2;PAFAH1B2;USP14;CIPC;GNAI3;SDAD1;PPM1D;GNAI1;TMEM65;PNMA1;RNF138;SAP30L;ZNF622;E2F5;EXOSC3;RPP14;DEDD;ARFGEF1;RNF20;NPM3;VMP1;SMUG1;KLF6;WEE1;TCIM;NOVA1;KATNBL1;SCD;FILIP1;ERCC6;TAF5;ZNF354A
 
 
 
 
Endosome Lumen ( GO:0031904 )
 
 
5/25
 
 
0.2631805
 
 
0.7133099
 
 
0
 
 
0
 
 
1.545667
 
 
2.0633345
 
 
RAB32;APP;IL12B;RAB38;CTSS
 
 
 
 
Postsynaptic Specialization Membrane ( GO:0099634 )
 
 
10/57
 
 
0.2653613
 
 
0.7142945
 
 
0
 
 
0
 
 
1.315761
 
 
1.7455717
 
 
GABRB3;GABRB2;MPP2;LRFN2;SLITRK1;SYNDIG1;ATP2B4;GSG1L;CACNG2;CACNG4
 
 
 
 
Clathrin Coat of Coated Pit ( GO:0030132 )
 
 
3/13
 
 
0.2677578
 
 
0.7158423
 
 
0
 
 
0
 
 
1.854545
 
 
2.4436834
 
 
TBC1D5;AP2B1;EPS15
 
 
 
 
Membrane Raft ( GO:0045121 )
 
 
27/171
 
 
0.2705302
 
 
0.7183673
 
 
0
 
 
0
 
 
1.160067
 
 
1.5166388
 
 
ITGB1;APP;ADCY2;TNFRSF11A;AKAP5;TNF;RAP1B;PPP2CA;SHH;PRKAR2A;NANOS1;PRNP;CAV2;MMAB;ATP2B4;ARID3A;ERLIN2;TNFRSF1B;TGFBR1;TNFRSF1A;ADAM17;RAP2B;FASN;CHRFAM7A;TEK;IL6ST;PLPP1
 
 
 
 
Clathrin-Coated Vesicle ( GO:0030136 )
 
 
14/84
 
 
0.2764782
 
 
0.7271550
 
 
0
 
 
0
 
 
1.236941
 
 
1.5902400
 
 
AP2A1;AP2B1;PIK3C2A;SCAMP1;SNAP91;SNX3;SNX18;RAB14;RAB12;CLVS2;STX6;RAB8A;GAS7;CLVS1
 
 
 
 
Asymmetric Synapse ( GO:0032279 )
 
 
21/131
 
 
0.2775401
 
 
0.7271550
 
 
0
 
 
0
 
 
1.180944
 
 
1.5137217
 
 
NANOS1;USP8;PRNP;MPP2;HOMER1;FMR1;SEMA4C;CRIPT;DTNBP1;CABP1;AKAP5;RTN4;GAP43;GRM7;NETO1;SYNDIG1;RPL14;STRN;SRGAP2;FAM81A;CDK5R1
 
 
 
 
Mitochondrial Intermembrane Space ( GO:0005758 )
 
 
12/71
 
 
0.2806343
 
 
0.7291031
 
 
0
 
 
0
 
 
1.257805
 
 
1.5982971
 
 
TIMM8B;NLN;GATM;AIFM1;STMP1;TIMM9;CPOX;ARL2;ALPL;CYCS;NME4;TRIAP1
 
 
 
 
Azurophil Granule Membrane ( GO:0035577 )
 
 
10/58
 
 
0.2838493
 
 
0.7291031
 
 
0
 
 
0
 
 
1.288274
 
 
1.6223393
 
 
VAMP8;RAP1B;B4GALT1;VAMP7;CMTM6;SURF4;DNAJC13;ACP3;SNAP29;CKAP4
 
 
 
 
Transport Vesicle Membrane ( GO:0030658 )
 
 
10/58
 
 
0.2838493
 
 
0.7291031
 
 
0
 
 
0
 
 
1.288274
 
 
1.6223393
 
 
LMAN1;SEC16B;MCFD2;SEC23A;CNIH1;SLC30A5;GOSR2;TGFA;CNIH3;VTI1B
 
 
 
 
Golgi Lumen ( GO:0005796 )
 
 
16/99
 
 
0.3004191
 
 
0.7666540
 
 
0
 
 
0
 
 
1.192188
 
 
1.4336978
 
 
ERO1A;APP;TGFB1;WNT3A;PDGFB;WNT7A;NGF;PCSK5;MMP11;MMP16;CSPG5;SDC1;MUC13;GPC4;WNT1;WNT4
 
 
 
 
Intracellular Membraneless Organelle ( GO:0043232 )
 
 
189/1310
 
 
0.3074390
 
 
0.7682905
 
 
0
 
 
0
 
 
1.044771
 
 
1.2322856
 
 
SRP19;RIPOR2;GEMIN2;FMR1;PRDM5;RORA;CDC14A;IGF1R;TXN2;PPP1CC;GOLGA3;RPS6KA3;RPS6KA6;CCND2;SALL1;CDC27;CHP2;HABP4;EPC1;CAPN3;KMT5B;NEPRO;PHLDA1;TMOD1;TSPYL2;NANOS1;DDX18;SLC30A9;ESCO1;FBXW7;ACTN2;TPM3;SLC30A5;TMOD3;TMOD2;RPS6;TPM1;CASK;DYRK1B;ANXA11;TSC1;LARP4B;PISD;FRMD5;MYCN;CCNY;BIN1;WDR82;SUB1;FSCN1;WDFY1;CMPK1;PKP4;UTRN;TP53;S100A8;PPID;SRSF9;ZNF397;IPPK;SLBP;ABHD4;RRAD;CDCA8;ARL2;PDS5B;ABHD5;MACROH2A1;RHOBTB1;RAP1B;CNN2;FXR1;PDCD11;RDH10;DNAJC21;N4BP1;MARK1;SRPK2;CBX5;XRCC5;CBX3;CIDEB;CDK8;DIAPH2;LIN28B;CNOT7;STAG2;LIN28A;RCAN2;FGF18;MMS19;POMT2;SNAP25;DYRK2;MTCL1;TUT4;SUV39H1;CHD7;HMGB2;SIX1;CTCF;TENT4B;COIL;EPB41L4B;EPB41L4A;SERP1;CAPZB;NKRF;HORMAD2;TNPO1;ZNF367;RAB8A;PPP1R12A;CEMIP2;EN2;ZFP91;KIF23;BAZ1B;DYNLL1;SMC1A;CCSAP;DYNLL2;SIRT1;DDX50;ETV4;CKAP4;ARL4A;MDFIC;CNKSR3;PAX9;CDC42EP2;RARA;FAM193B;TOP1;SAXO1;MAPRE1;ERGIC2;PAFAH1B2;KANK1;USP14;NUFIP2;CIPC;GNAI3;ASAP1;TTK;SDAD1;PPM1D;GNAI1;RAB40A;TMEM65;PNMA1;RAB40C;RNF138;PPP1R7;SAP30L;IGF2BP2;ZNF622;VHL;E2F5;EXOSC3;RPP14;WASF1;DEDD;ARFGEF1;RNF20;HSD3B7;MPP2;CSNK1A1;G0S2;UBE2G2;NPM3;MZT1;VMP1;SMUG1;KLF6;WEE1;TCIM;NOVA1;KATNBL1;SCD;CAPZA1;RAB18;SPRY2;FILIP1;ERCC6;TAF5;VIM;ZNF354A;PNPLA2
 
 
 
 
Smc5-Smc6 Complex ( GO:0030915 )
 
 
2/8
 
 
0.3088802
 
 
0.7682905
 
 
0
 
 
0
 
 
2.060345
 
 
2.4204970
 
 
SLF2;NSMCE2
 
 
 
 
Death-Inducing Signaling Complex ( GO:0031264 )
 
 
2/8
 
 
0.3088802
 
 
0.7682905
 
 
0
 
 
0
 
 
2.060345
 
 
2.4204970
 
 
CASP3;FASN
 
 
 
 
Pseudopodium ( GO:0031143 )
 
 
2/8
 
 
0.3088802
 
 
0.7682905
 
 
0
 
 
0
 
 
2.060345
 
 
2.4204970
 
 
VAMP7;ACTN2
 
 
 
 
Nucleolus ( GO:0005730 )
 
 
117/805
 
 
0.3218790
 
 
0.7955877
 
 
0
 
 
0
 
 
1.052971
 
 
1.1936270
 
 
SRP19;GEMIN2;FMR1;PRDM5;RORA;CDC14A;IGF1R;TXN2;PPP1CC;GOLGA3;RPS6KA3;RPS6KA6;CCND2;CHP2;HABP4;EPC1;CAPN3;KMT5B;NEPRO;PHLDA1;TSPYL2;DDX18;FBXW7;SLC30A5;RPS6;CASK;LARP4B;MYCN;CCNY;WDR82;SUB1;WDFY1;CMPK1;TP53;PPID;SRSF9;ZNF397;IPPK;SLBP;CDCA8;ARL2;MACROH2A1;FXR1;PDCD11;DNAJC21;N4BP1;SRPK2;CBX5;XRCC5;CDK8;DIAPH2;LIN28B;STAG2;LIN28A;RCAN2;FGF18;POMT2;TUT4;SUV39H1;CHD7;HMGB2;SIX1;CTCF;TENT4B;COIL;NKRF;TNPO1;ZNF367;RAB8A;PPP1R12A;CEMIP2;EN2;ZFP91;BAZ1B;SIRT1;DDX50;ETV4;ARL4A;MDFIC;CNKSR3;PAX9;RARA;POLR1D;FAM193B;TOP1;ERGIC2;PAFAH1B2;USP14;CIPC;GNAI3;SDAD1;PPM1D;GNAI1;TMEM65;PNMA1;RNF138;SAP30L;ZNF622;E2F5;EXOSC3;RPP14;DEDD;ARFGEF1;RNF20;NPM3;VMP1;SMUG1;KLF6;WEE1;TCIM;NOVA1;KATNBL1;SCD;FILIP1;ERCC6;TAF5;ZNF354A
 
 
 
 
Postsynaptic Density Membrane ( GO:0098839 )
 
 
8/47
 
 
0.3285613
 
 
0.8070286
 
 
0
 
 
0
 
 
1.268206
 
 
1.4115540
 
 
MPP2;LRFN2;SLITRK1;SYNDIG1;ATP2B4;GSG1L;CACNG2;CACNG4
 
 
 
 
Cytoskeleton ( GO:0005856 )
 
 
101/694
 
 
0.3310424
 
 
0.8080724
 
 
0
 
 
0
 
 
1.054337
 
 
1.1655792
 
 
JPT1;RIPOR2;WIPF1;MYLK;CFL2;KMT5B;NANOS1;TMOD1;SLC30A9;TPM3;ACTN2;TMOD3;TPM1;TMOD2;CASK;EML1;CD2AP;EML4;NISCH;FRMD5;ADAM17;BIN3;BIN1;FSCN1;PKP4;UTRN;S100A8;DGKH;ZNF397;TMEM63B;ARL3;CDCA8;ARL2;TWF1;ADRB2;RHOBTB1;CNN3;CNN2;ARHGAP21;SPAST;ARSJ;MICAL1;MAP7;PCDHA4;STK38L;MAP4;HOXC8;MARK1;ESRRA;KLHL20;ANLN;SESTD1;SNAP25;DYRK2;MTCL1;USP32;SEPTIN11;LIMD2;EPB41L4B;EPB41L4A;TUBA1A;CAPZB;NEFM;SPTAN1;RAB8A;ACTR2;PPP1R12A;PAWR;DYNLL1;DYNLL2;IQCG;CTTNBP2NL;ACTA2;TUBB2A;CDC42EP2;RARA;SAXO1;MAPRE2;KANK1;PPP2CA;IGF2BP2;VHL;WASF1;APBB3;GBP1;MPP2;SEPTIN2;DTNBP1;KLHL2;KLF4;NPM3;MARCKS;ACTC1;KATNBL1;CAPZA1;CAPZA2;SPRY2;FILIP1;TAF5;VIM;MYO1F
 
 
 
 
Aggresome ( GO:0016235 )
 
 
6/34
 
 
0.3338697
 
 
0.8083091
 
 
0
 
 
0
 
 
1.324717
 
 
1.4532209
 
 
KLF8;SLC2A14;STRADB;UBQLN1;SLC2A3;EPS15
 
 
 
 
Secretory Granule Lumen ( GO:0034774 )
 
 
47/316
 
 
0.3364652
 
 
0.8083091
 
 
0
 
 
0
 
 
1.080924
 
 
1.1774077
 
 
APP;PSMD12;SPARC;DDX3X;CANT1;SERPINE1;PDGFB;C1ORF35;CXCL1;GNS;CNN2;ALAD;ISLR;PSMD7;CAND1;TMSB4X;CDC37L1;PGM2;FAM3C;OSTF1;SPTAN1;VTI1B;TXNDC5;CTSA;ACTR2;TGFB2;TGFB1;NHLRC3;GCA;DYNLT1;ACTN2;XRCC5;AHSG;IST1;PRKCD;VEGFC;ARMC8;IGF1;VEGFA;PSMA5;GNPDA1;NPC2;SOS1;NIT2;PAFAH1B2;KPNB1;S100A8
 
 
 
 
Clathrin-Coated Vesicle Membrane ( GO:0030665 )
 
 
14/88
 
 
0.3393664
 
 
0.8083091
 
 
0
 
 
0
 
 
1.169806
 
 
1.2641802
 
 
TFRC;FZD5;FZD4;TGFA;AP2A1;RORA;LRP2;AP2B1;VAMP8;NECAP2;VAMP7;LDLR;SH3GL2;HBEGF
 
 
 
 
Lipid Droplet ( GO:0005811 )
 
 
14/88
 
 
0.3393664
 
 
0.8083091
 
 
0
 
 
0
 
 
1.169806
 
 
1.2641802
 
 
HSD3B7;CIDEB;G0S2;TSC1;ABHD5;UBE2G2;CKAP4;PISD;RAP1B;RAB40A;RAB40C;RDH10;RAB18;PNPLA2
 
 
 
 
Synaptic Membrane ( GO:0097060 )
 
 
7/41
 
 
0.3434571
 
 
0.8106853
 
 
0
 
 
0
 
 
1.272781
 
 
1.3602124
 
 
GABRB3;SYDE2;APP;GABRB2;FLRT3;PALM;ATP2B4
 
 
 
 
Ino80 Complex ( GO:0031011 )
 
 
3/15
 
 
0.3489252
 
 
0.8106853
 
 
0
 
 
0
 
 
1.545275
 
 
1.6270165
 
 
YY1;INO80D;INO80
 
 
 
 
TIM23 Mitochondrial Import Inner Membrane Translocase Complex ( GO:0005744 )
 
 
3/15
 
 
0.3489252
 
 
0.8106853
 
 
0
 
 
0
 
 
1.545275
 
 
1.6270165
 
 
HSPA9;GRPEL2;TIMM17B
 
 
 
 
trans-Golgi Network Transport Vesicle ( GO:0030140 )
 
 
3/15
 
 
0.3489252
 
 
0.8106853
 
 
0
 
 
0
 
 
1.545275
 
 
1.6270165
 
 
RAB14;RAB12;RAB8A
 
 
 
 
Sarcoplasmic Reticulum ( GO:0016529 )
 
 
8/48
 
 
0.3506781
 
 
0.8106853
 
 
0
 
 
0
 
 
1.236429
 
 
1.2956373
 
 
NANOS1;SLC30A7;FKBP1A;PLN;DMPK;ASPH;FKBP1C;ATP2A2
 
 
 
 
Clathrin-Coated Endocytic Vesicle ( GO:0045334 )
 
 
13/82
 
 
0.3528408
 
 
0.8109148
 
 
0
 
 
0
 
 
1.164882
 
 
1.2135020
 
 
TFRC;FZD5;FZD4;TGFA;AP2A1;RORA;LRP2;AP2B1;VAMP8;VAMP7;LDLR;SH3GL2;HBEGF
 
 
 
 
AP-2 Adaptor Complex ( GO:0030122 )
 
 
2/9
 
 
0.3632115
 
 
0.8203570
 
 
0
 
 
0
 
 
1.765907
 
 
1.7884578
 
 
TBC1D5;AP2B1
 
 
 
 
Apical Dendrite ( GO:0097440 )
 
 
2/9
 
 
0.3632115
 
 
0.8203570
 
 
0
 
 
0
 
 
1.765907
 
 
1.7884578
 
 
CPEB3;METAP2
 
 
 
 
Clathrin Coat of Endocytic Vesicle ( GO:0030128 )
 
 
2/9
 
 
0.3632115
 
 
0.8203570
 
 
0
 
 
0
 
 
1.765907
 
 
1.7884578
 
 
TBC1D5;AP2B1
 
 
 
 
AMPA Glutamate Receptor Complex ( GO:0032281 )
 
 
4/22
 
 
0.3679427
 
 
0.8262942
 
 
0
 
 
0
 
 
1.373592
 
 
1.3733559
 
 
VWC2L;CACNG2;CNIH3;CACNG4
 
 
 
 
Nuclear Chromosome ( GO:0000228 )
 
 
14/90
 
 
0.3718915
 
 
0.8304168
 
 
0
 
 
0
 
 
1.138889
 
 
1.1265355
 
 
H2AZ1;GINS2;INO80D;SUV39H1;HMGA2;INO80;SMC1A;CHD1;MACROH2A1;YY1;CFDP1;CHEK1;TOP1;KCNN4
 
 
 
 
RNA Polymerase III Complex ( GO:0005666 )
 
 
3/16
 
 
0.3893163
 
 
0.8453111
 
 
0
 
 
0
 
 
1.426325
 
 
1.3455422
 
 
POLR3E;POLR1D;POLR3F
 
 
 
 
Chitosome ( GO:0045009 )
 
 
3/16
 
 
0.3893163
 
 
0.8453111
 
 
0
 
 
0
 
 
1.426325
 
 
1.3455422
 
 
RAB32;BACE2;RAB38
 
 
 
 
Melanosome Membrane ( GO:0033162 )
 
 
3/16
 
 
0.3893163
 
 
0.8453111
 
 
0
 
 
0
 
 
1.426325
 
 
1.3455422
 
 
RAB32;BACE2;RAB38
 
 
 
 
Pigment Granule Membrane ( GO:0090741 )
 
 
3/16
 
 
0.3893163
 
 
0.8453111
 
 
0
 
 
0
 
 
1.426325
 
 
1.3455422
 
 
RAB32;BACE2;RAB38
 
 
 
 
Platelet Alpha Granule Membrane ( GO:0031092 )
 
 
3/16
 
 
0.3893163
 
 
0.8453111
 
 
0
 
 
0
 
 
1.426325
 
 
1.3455422
 
 
SPARC;PHACTR2;LHFPL2
 
 
 
 
Spindle Microtubule ( GO:0005876 )
 
 
11/71
 
 
0.4018714
 
 
0.8677772
 
 
0
 
 
0
 
 
1.133297
 
 
1.0331401
 
 
FAM161A;MTCL1;CHMP1B;CHMP2B;CDK1;CHMP4B;CALM3;MAPRE1;HAUS1;PAFAH1B1;CLASP2
 
 
 
 
Astrocyte Projection ( GO:0097449 )
 
 
2/10
 
 
0.4158221
 
 
0.8826630
 
 
0
 
 
0
 
 
1.545079
 
 
1.3558032
 
 
GRM3;KCNK2
 
 
 
 
Neuron Projection Cytoplasm ( GO:0120111 )
 
 
2/10
 
 
0.4158221
 
 
0.8826630
 
 
0
 
 
0
 
 
1.545079
 
 
1.3558032
 
 
FMR1;METAP2
 
 
 
 
Nuclear Inclusion Body ( GO:0042405 )
 
 
2/10
 
 
0.4158221
 
 
0.8826630
 
 
0
 
 
0
 
 
1.545079
 
 
1.3558032
 
 
PABPN1;TPR
 
 
 
 
Calcium Channel Complex ( GO:0034704 )
 
 
8/51
 
 
0.4177489
 
 
0.8826630
 
 
0
 
 
0
 
 
1.149966
 
 
1.0037760
 
 
ASPH;PTPRA;SESTD1;PDE4B;CACNA2D3;CACNA1A;MICU3;CALM3
 
 
 
 
Lysosomal Lumen ( GO:0043202 )
 
 
13/86
 
 
0.4212855
 
 
0.8853754
 
 
0
 
 
0
 
 
1.100796
 
 
0.9515770
 
 
CTSA;ASAH1;IFI30;GNS;CTSS;PLD3;GALC;NPC2;SMPD1;CSPG5;SDC1;GPC4;TXNDC5
 
 
 
 
Alpha-Ketoacid Dehydrogenase Complex ( GO:0045240 )
 
 
3/17
 
 
0.4290491
 
 
0.8894383
 
 
0
 
 
0
 
 
1.324367
 
 
1.1206584
 
 
PDHX;DLST;PDHB
 
 
 
 
Transcription Factor TFTC Complex ( GO:0033276 )
 
 
3/17
 
 
0.4290491
 
 
0.8894383
 
 
0
 
 
0
 
 
1.324367
 
 
1.1206584
 
 
TAF5;TAF4;TAF9B
 
 
 
 
Mitochondrial Outer Membrane ( GO:0005741 )
 
 
25/171
 
 
0.4300083
 
 
0.8894383
 
 
0
 
 
0
 
 
1.058531
 
 
0.8933476
 
 
TRABD;BECN1;FOXO3;AGPAT4;FUNDC2;NRAS;PGRMC1;BCL2L11;STMP1;PMAIP1;BMF;BAK1;MCL1;MARCHF5;TOMM70;PRNP;DMPK;RAB32;PTRH2;BCL2;VDAC1;ARMC12;SFXN2;BCL2L2;SLC29A3
 
 
 
 
Pigment Granule ( GO:0048770 )
 
 
4/24
 
 
0.4347116
 
 
0.8940630
 
 
0
 
 
0
 
 
1.236089
 
 
1.0297519
 
 
RAB32;TMEM33;RAB9A;RAB38
 
 
 
 
Melanosome ( GO:0042470 )
 
 
5/31
 
 
0.4375679
 
 
0.8940630
 
 
0
 
 
0
 
 
1.188560
 
 
0.9823724
 
 
RAB32;BACE2;TMEM33;RAB9A;RAB38
 
 
 
 
Neuromuscular Junction ( GO:0031594 )
 
 
6/38
 
 
0.4390691
 
 
0.8940630
 
 
0
 
 
0
 
 
1.158858
 
 
0.9538542
 
 
ITGB1;MPP2;SYNGR2;FCHSD2;SYNGR3;CDK5R1
 
 
 
 
Extrinsic Component of Cytoplasmic Side of Plasma Membrane ( GO:0031234 )
 
 
7/46
 
 
0.4637079
 
 
0.9093166
 
 
0
 
 
0
 
 
1.109281
 
 
0.8524830
 
 
GNA13;GNG10;GNG2;GNG5;GNAI3;GNG12;GNAI1
 
 
 
 
MLL3/4 Complex ( GO:0044666 )
 
 
2/11
 
 
0.4661372
 
 
0.9093166
 
 
0
 
 
0
 
 
1.373324
 
 
1.0482240
 
 
KDM6B;DPY30
 
 
 
 
U2-type Catalytic Step 1 Spliceosome ( GO:0071006 )
 
 
2/11
 
 
0.4661372
 
 
0.9093166
 
 
0
 
 
0
 
 
1.373324
 
 
1.0482240
 
 
MAGOHB;PRPF19
 
 
 
 
beta-catenin-TCF Complex ( GO:1990907 )
 
 
2/11
 
 
0.4661372
 
 
0.9093166
 
 
0
 
 
0
 
 
1.373324
 
 
1.0482240
 
 
TLE4;TCF7L2
 
 
 
 
Microfibril ( GO:0001527 )
 
 
2/11
 
 
0.4661372
 
 
0.9093166
 
 
0
 
 
0
 
 
1.373324
 
 
1.0482240
 
 
MFAP2;FBN1
 
 
 
 
Primary Lysosome ( GO:0005766 )
 
 
2/11
 
 
0.4661372
 
 
0.9093166
 
 
0
 
 
0
 
 
1.373324
 
 
1.0482240
 
 
STX3;GNS
 
 
 
 
Transcription Factor TFIIH Holo Complex ( GO:0005675 )
 
 
2/11
 
 
0.4661372
 
 
0.9093166
 
 
0
 
 
0
 
 
1.373324
 
 
1.0482240
 
 
GTF2H1;MMS19
 
 
 
 
 
 
 GO_Molecular_Function_2025 
 GO Molecular Function terms describe the elemental biochemical
activities of gene products, including binding affinities, catalytic
activities, transporter functions, and receptor interactions. These
annotations are curated by the Gene Ontology Consortium based on
experimental data and validated inference. Enrichment in this category
highlights specific molecular activities that are over-represented among
the target genes, offering mechanistic insight into how regulatory
changes may alter protein function or signaling capacity. This category
is especially informative for identifying enzymatic classes, signaling
mediators, or binding modules affected by miRNA regulation. 
 
 
 
 
Term
 
 
Overlap
 
 
P.value
 
 
Adjusted.P.value
 
 
Old.P.value
 
 
Old.Adjusted.P.value
 
 
Odds.Ratio
 
 
Combined.Score
 
 
Genes
 
 
 
 
 
 
RNA Polymerase II Cis-Regulatory Region Sequence-Specific DNA Binding
( GO:0000978 )
 
 
218/1054
 
 
0.0000000
 
 
0.0000003
 
 
0
 
 
0
 
 
1.663091
 
 
35.825466
 
 
BACH2;IKZF5;ELK4;HOXA9;MYB;HOXA3;HOXA1;SOX9;HOXA5;SOX5;MEF2A;ZNF287;PDHX;ZNF283;MED8;SOX13;RFX3;EBF2;SOX11;SOX12;OVOL1;ISL1;THAP1;RUNX1;RFX6;HOXB4;HOXB3;HOXB2;ZNF710;HOXB1;ZSCAN25;TP53;HOXB5;ZNF397;ZNF275;ZNF274;ZNF395;HOXD1;GATA6;ZNF23;ZBTB4;NHLH2;ZNF821;NKX2-4;RREB1;EGR3;FOXF1;NR2F2;ST18;NR2F6;FOSL2;SNAI1;HBP1;MKX;ZNF136;SLC2A4RG;ZNF250;BHLHE41;HNF4G;MIA3;CTCF;TFCP2L1;HIF3A;MEOX2;NKRF;ZNF280B;HEY2;ZNF280C;ZNF367;ZNF366;H3-3B;FOXD3;SREBF1;ZNF362;FOXD1;EN2;TFEB;TET1;FOS;PAX2;NR5A2;PAX9;TET3;TFEC;RARA;RARB;PPARG;ZNF780B;JDP2;ZNF471;NFIX;ZNF470;SATB1;SATB2;FOXS1;MNT;ZFP14;HDX;ZNF341;ZNF583;NR6A1;NFIA;WT1;FOSB;ZNF699;ZNF354A;ZNF697;ZNF354B;FOXA1;PRDM4;ZBTB26;PRDM5;RORA;RORB;NR3C1;HOXA11;YY1;HOXA10;ZFP30;ZNF449;ZNF689;PITX2;ZNF322;EOMES;ZNF562;ZBTB39;ZBTB34;MITF;FOXP2;MAF;MYCN;DMTF1;ERG;DLX1;FOXO4;FOXO3;GLIS2;NEUROD1;ZKSCAN3;OTX2;ZKSCAN4;SKIL;ZKSCAN1;ZNF784;ZNF420;ESRRA;SMAD2;TFAP2C;ZBTB16;HMGA1;HMGA2;ESRRG;ZBTB11;ESR1;SMAD5;SCRT1;NR4A2;MAFB;MAFG;BHLHE40;SP5;SP8;MAFK;MXD1;ZNF774;NFE2L1;ONECUT1;SIX1;ZFP82;ZIC1;MYBL2;ZNF644;KDM6B;KLF10;KLF11;EMX2;DNMT3A;KLF14;SAFB;ETV4;KLF15;CREB1;ELF4;HAND2;HAND1;ZNF879;IRF2;TOP1;CREB5;HDAC4;CEBPD;PLAG1;WBP2;CEBPG;FOXK2;TBX21;E2F3;E2F5;E2F6;E2F7;ZNF740;H2AZ1;TCF7L2;PRRX1;IRX5;FOXJ2;PEDS1;TBX5;KLF4;TBX4;KLF3;KLF8;SLC25A37;REST;KLF6;KLF5;KLF9;INSM2
 
 
 
 
Sequence-Specific DNA Binding ( GO:0043565 )
 
 
159/718
 
 
0.0000000
 
 
0.0000003
 
 
0
 
 
0
 
 
1.803308
 
 
37.692590
 
 
FOXA1;PRDM4;ZBTB26;PRDM5;RORA;AHR;RORB;NR3C1;BACH2;HOXA11;ELK4;YY1;HOXA10;HOXA9;ZNF449;BBX;HOXA1;SOX9;PKNOX1;PITX2;HOXA5;MEF2A;EOMES;PDHX;MBNL2;ZNF282;SOX13;RFX3;SOX12;OVOL1;ISL1;FOXP2;THAP1;MAF;MYCN;LMX1A;THAP9;HOXB4;HOXB2;HOXB1;ERG;HOXB8;HOXB5;DLX1;ZNF274;CBFB;HOXD1;GATA6;ZNF23;FOXO4;FOXO3;ZBTB4;GLIS2;NEUROD1;NHLH2;SAFB2;ZKSCAN3;ZNF821;OTX2;HOXC8;ZNF784;ESRRA;ZBTB18;CPSF4;TFAP2C;EGR3;FOXF1;XRCC5;ESRRG;NR2F2;ESR1;SMAD5;PBX1;SCRT1;NR2F6;NR4A2;LIN28B;MAFB;LHX1;MAFG;BHLHE40;SNAI1;BCL2;MAFK;SP8;LHX8;BARHL2;CSRNP1;ZNF250;ONECUT1;BHLHE41;SIX1;MIA3;CTCF;TFCP2L1;YBX1;MEOX2;MECP2;HEY2;ZIC1;MYBL2;ZNF385D;KLF10;FOXD3;SREBF1;KLF11;EMX2;FOXD1;DNMT3A;EN2;TFEB;TET1;FOS;KLF14;SAFB;ETV4;KLF15;PAX2;NR5A2;CREB1;ELF2;ELF4;HAND2;ELF5;PAX9;TET3;TFEC;IRF2;RARA;RARB;PPARG;JDP2;CREB5;NFIX;CEBPD;SATB1;CEBPG;FOXK2;HLX;TBX21;PLAGL2;E2F3;E2F7;ZNF740;DRGX;TCF7L2;IRX5;FBXL19;FOXJ2;KLF4;TBX5;KLF3;SLC25A37;KLF6;KLF5;NR6A1;WT1;FOSB;ERCC6
 
 
 
 
Cis-Regulatory Region Sequence-Specific DNA Binding ( GO:0000987 )
 
 
213/1035
 
 
0.0000000
 
 
0.0000003
 
 
0
 
 
0
 
 
1.650821
 
 
34.036152
 
 
BACH2;IKZF5;ELK4;HOXA9;MYB;HOXA3;HOXA1;SOX9;HOXA5;SOX5;MEF2A;ZNF287;PDHX;ZNF283;MED8;SOX13;RFX3;EBF2;SOX11;SOX12;OVOL1;ISL1;THAP1;RUNX1;RFX6;HOXB4;HOXB3;HOXB2;ZNF710;HOXB1;ZSCAN25;TP53;HOXB5;ZNF397;ZNF275;ZNF274;ZNF395;HOXD1;GATA6;ZNF23;ZBTB4;NHLH2;ZNF821;NKX2-4;RREB1;EGR3;FOXF1;NR2F2;ST18;NR2F6;FOSL2;SNAI1;HBP1;MKX;ZNF136;SLC2A4RG;ZNF250;BHLHE41;HNF4G;MIA3;CTCF;TFCP2L1;HIF3A;MEOX2;NKRF;ZNF280B;HEY2;ZNF280C;ZNF367;ZNF366;H3-3B;FOXD3;SREBF1;ZNF362;FOXD1;EN2;TFEB;TET1;FOS;PAX2;NR5A2;PAX9;TET3;TFEC;RARA;RARB;PPARG;ZNF780B;JDP2;ZNF471;NFIX;ZNF470;SATB1;SATB2;FOXS1;MNT;ZFP14;HDX;ZNF341;ZNF583;NFIA;WT1;FOSB;ZNF699;ZNF354A;ZNF697;ZNF354B;FOXA1;PRDM4;ZBTB26;PRDM5;RORA;RORB;NR3C1;HOXA11;YY1;HOXA10;ZFP30;ZNF449;ZNF689;PITX2;ZNF322;EOMES;ZNF562;ZBTB39;ZBTB34;MITF;FOXP2;MAF;MYCN;DMTF1;ERG;DLX1;FOXO4;FOXO3;GLIS2;ZKSCAN3;OTX2;ZKSCAN4;SKIL;ZKSCAN1;ZNF784;ZNF420;SMAD2;TFAP2C;ZBTB16;HMGA1;FOXN4;ZBTB11;ESR1;SMAD5;SCRT1;NR4A2;MAFB;MAFG;BHLHE40;SP5;SP8;MAFK;MXD1;ZNF774;NFE2L1;ONECUT1;HMGB2;SIX1;ZFP82;ZIC1;MYBL2;ZNF644;KDM6B;KLF10;KLF11;EMX2;DNMT3A;KLF14;SAFB;ETV4;KLF15;CREB1;ELF4;HAND1;ZNF879;IRF2;TOP1;HDAC4;CEBPD;PLAG1;WBP2;CEBPG;FOXK2;TBX21;E2F3;E2F5;E2F6;E2F7;ZNF740;H2AZ1;TCF7L2;PRRX1;IRX5;FOXJ2;PEDS1;TBX5;KLF4;TBX4;KLF3;KLF8;SLC25A37;REST;KLF6;KLF5;KLF9;INSM2
 
 
 
 
Sequence-Specific Double-Stranded DNA Binding ( GO:1990837 )
 
 
155/706
 
 
0.0000000
 
 
0.0000006
 
 
0
 
 
0
 
 
1.781609
 
 
35.228766
 
 
FOXA1;PRDM4;ZBTB26;PRDM5;ARID4B;AHR;RORB;NR3C1;TNF;BACH2;HOXA11;ELK4;YY1;HOXA10;HOXA9;ZNF449;BBX;HOXA1;SOX9;PKNOX1;PITX2;HOXA5;SOX5;TSPYL2;EOMES;PDHX;MBNL2;ZNF282;SOX13;RFX3;SOX11;SOX12;OVOL1;ISL1;RUNX1;MAF;MYCN;RFX6;LMX1A;TBL1XR1;HOXB4;HOXB2;HOXB1;ERG;HOXB8;TP53;HOXB5;DLX1;ZNF274;HOXD1;GATA6;ZNF23;FOXO4;FOXO3;MACROH2A1;GLIS2;NEUROD1;NHLH2;ZNF821;OTX2;HOXC8;ZNF784;ZBED4;ESRRA;ZBTB18;CPSF4;TFAP2C;EGR3;XRCC5;HMGA2;ESRRG;ESR1;SMAD5;PBX1;SCRT1;NR2F6;NR4A2;LIN28B;LHX1;MAFG;BHLHE40;SNAI1;MAFK;SP8;LHX8;BARHL2;SUV39H2;ZNF250;SUV39H1;ONECUT1;BHLHE41;HMGB2;SIX1;ASH2L;MIA3;CTCF;TFCP2L1;YBX1;MEOX2;HEY2;ZIC1;MYBL2;ZNF385D;KLF10;FOXD3;SREBF1;KLF11;EMX2;EN2;TFEB;FOS;KLF14;ETV4;KLF15;PAX2;NR5A2;CREB1;ELF2;ELF4;HAND2;ELF5;PAX9;HAND1;TFEC;IRF2;RARA;RARB;PPARG;JDP2;CREB5;HDAC4;YAP1;NFIX;CEBPD;CEBPG;FOXK2;HSPD1;TBX21;E2F3;E2F7;ZNF740;DRGX;TCF7L2;IRX5;FOXJ2;KLF4;KLF3;SLC25A37;KLF6;REST;KLF5;NR6A1;WT1;FOSB;BCOR
 
 
 
 
Double-Stranded DNA Binding ( GO:0003690 )
 
 
143/651
 
 
0.0000000
 
 
0.0000019
 
 
0
 
 
0
 
 
1.779294
 
 
32.703206
 
 
FOXA1;PRDM4;ZBTB26;AHR;RORB;NR3C1;BACH2;HOXA11;ELK4;YY1;HOXA10;HOXA9;ZNF449;BBX;HOXA1;SOX9;PKNOX1;PITX2;HOXA5;EOMES;PDHX;MBNL2;ZNF282;RFX3;SOX12;OVOL1;ISL1;MAF;MYCN;LMX1A;HOXB4;HOXB2;HOXB1;ERG;HOXB8;CGGBP1;HOXB5;DLX1;ZNF274;HOXD1;GATA6;ZNF23;FOXO4;FOXO3;MACROH2A1;GLIS2;NEUROD1;NHLH2;ZNF821;OTX2;HOXC8;ZNF784;ESRRA;ZBTB18;SMAD2;CPSF4;TFAP2C;EGR3;XRCC5;ESRRG;ESR1;SMAD5;PBX1;SCRT1;NR2F6;NR4A2;LIN28B;LHX1;MAFG;BHLHE40;SNAI1;SP8;ZCCHC3;LHX8;BARHL2;ZNF250;ONECUT1;BHLHE41;HMGB2;SIX1;MIA3;CTCF;TFCP2L1;YBX1;MEOX2;MECP2;HEY2;ZIC1;MYBL2;ZNF385D;KLF10;FOXD3;SREBF1;KLF11;EMX2;EN2;TFEB;FOS;KLF14;SAFB;ETV4;KLF15;PAX2;NR5A2;CREB1;ELF2;ELF4;HAND2;ELF5;PAX9;TFEC;IRF2;RARA;RARB;RBMS1;PPARG;TOP1;ZMYND11;JDP2;CREB5;NFIX;CEBPD;SATB1;CEBPG;PURA;TBX21;TDG;E2F3;E2F7;ZNF740;DRGX;TCF7L2;IRX5;FOXJ2;KLF4;KLF3;SLC25A37;KLF6;KLF5;NR6A1;WT1;POLR3F;FOSB
 
 
 
 
DNA-binding Transcription Activator Activity, RNA Polymerase II-specific
( GO:0001228 )
 
 
82/318
 
 
0.0000000
 
 
0.0000019
 
 
0
 
 
0
 
 
2.181633
 
 
39.679536
 
 
FOXA1;PRDM4;ONECUT1;HNF4G;SIX1;TFCP2L1;MEOX2;NR3C1;ELK4;HOXA10;YY1;NKRF;MYB;HOXA1;MYBL2;SOX9;HOXA5;MEF2A;KLF10;SREBF1;SOX11;MITF;SOX12;FOS;ETV4;KLF15;RUNX1;MYCN;CREB1;TFDP1;RFX6;LMX1A;TFDP2;ELF4;HAND2;TFEC;HAND1;IRF2;HOXB3;HOXB2;PPARG;ZNF780B;HOXB1;ERG;TP53;ZNF395;PLAG1;SATB2;CEBPG;FOXK2;FOXO4;FOXO3;RLF;GLIS2;NEUROD1;PLAGL2;OTX2;E2F3;RREB1;ZBED4;ZNF784;SMAD2;TCF7L2;TFAP2C;TFAP2D;PRRX1;FOXF1;ZBTB16;FOXJ2;PEDS1;KLF4;TBX5;ESR1;FOSL2;NR4A2;KLF6;MEIS1;KLF5;NFIA;WT1;CREBRF;NFE2L1
 
 
 
 
RNA Polymerase II Transcription Regulatory Region Sequence-Specific DNA
Binding ( GO:0000977 )
 
 
241/1236
 
 
0.0000000
 
 
0.0000019
 
 
0
 
 
0
 
 
1.543584
 
 
27.858508
 
 
RB1;BACH2;IKZF5;ELK4;HOXA9;MYB;HOXA3;BBX;HOXA1;SOX9;HOXA5;SOX5;MEF2A;ZNF287;PDHX;ZNF283;MED8;SOX13;RFX3;ZNF10;EBF2;SOX11;SOX12;OVOL1;ISL1;THAP1;RUNX1;RFX6;HOXB4;TBPL1;HOXB3;HOXB2;ZNF710;HOXB1;ZSCAN25;HOXB8;TP53;HOXB5;ZNF397;ZNF275;ZNF274;ZNF395;HOXD1;ZNF391;GATA6;ZNF23;ZBTB5;ZBTB4;NHLH2;ZNF821;HOXC8;NKX2-4;RREB1;ZBED4;ZNF266;EGR3;FOXF1;NR2F2;PBX1;ST18;NR2F6;FOSL2;SNAI1;CREBRF;HBP1;MKX;ZNF136;SLC2A4RG;BARHL2;ZNF250;TCF24;BHLHE41;HNF4G;MIA3;CTCF;TFCP2L1;HIF3A;MEOX2;NKRF;ZNF280B;HEY2;ZNF280C;ZNF367;ZNF366;H3-3B;FOXD3;SREBF1;ZNF362;FOXD1;EN2;TFEB;TET1;FOS;PAX2;NR5A2;PAX9;TET3;TFEC;RARA;RARB;PPARG;ZNF780B;JDP2;ZNF471;NFIX;ZNF470;SATB1;SATB2;PURA;FOXS1;MNT;ZFP14;HDX;ZNF341;ZNF583;NFIA;WT1;FOSB;ZNF699;ZNF354A;ZNF697;ZNF354B;FOXA1;PRDM4;ZBTB26;PRDM5;RORA;RORB;NR3C1;HOXA11;YY1;HOXA10;ZFP30;ZNF449;ZNF689;PITX2;ZNF566;ZNF322;EOMES;ZNF562;ZBTB39;ZBTB34;MITF;FOXP2;MAF;MYCN;DMTF1;LMX1A;ADNP;ERG;DLX1;FOXO4;FOXO3;MACROH2A1;GLIS2;NPAS3;ZKSCAN3;OTX2;ZKSCAN4;SKIL;ZKSCAN1;ZNF784;ZNF420;SMAD2;TFAP2C;TFAP2D;ZBTB16;HMGA1;ZBTB11;ESR1;SMAD5;SCRT1;NR4A2;MAFB;LHX1;MAFG;BHLHE40;SP5;SP8;MAFK;MXD1;ZNF774;NFE2L1;LHX8;ZNF512B;ONECUT1;SIX1;ZFP82;ZIC1;MYBL2;ZNF644;KDM6B;KLF10;KLF11;EMX2;DNMT3A;KLF14;SAFB;ETV4;KLF15;CREB1;TFDP1;TFDP2;ELF4;HAND2;HAND1;ZNF879;IRF2;TOP1;HDAC4;CEBPD;PLAG1;WBP2;CEBPG;FOXK2;STOX2;TBX21;E2F3;E2F5;E2F6;ZNF620;E2F7;ZNF740;DRGX;H2AZ1;TCF7L2;PRRX1;IRX5;FOXJ2;PEDS1;TBX5;KLF4;TBX4;KLF3;KLF8;SLC25A37;REST;KLF6;KLF5;KLF9;INSM2
 
 
 
 
GDP Binding ( GO:0019003 )
 
 
24/66
 
 
0.0000043
 
 
0.0004955
 
 
0
 
 
0
 
 
3.552705
 
 
43.935197
 
 
RALB;ARL3;RASEF;GNAI3;RRAS2;RAB22A;GNAI1;RAP2C;RAP1B;RAB10;NRAS;MRAS;RAP2B;RAP1A;RAB14;RAB12;RAB18;RAB9A;RHEBL1;GBP1;RAB8A;RAB9B;TRIM23;ARL8B
 
 
 
 
mRNA Binding ( GO:0003729 )
 
 
72/310
 
 
0.0000063
 
 
0.0005922
 
 
0
 
 
0
 
 
1.892261
 
 
22.668538
 
 
DDX3Y;DAZAP1;RBM25;DDX3X;FMR1;CELF2;CELF3;MSI2;YBX3;ELAVL2;ZC3H12C;SYNCRIP;BOLL;AUH;RBPMS2;SNIP1;ZNF385A;NANOS1;RBFOX2;KHNYN;ANGEL2;SECISBP2L;LARP4B;DDX50;CDC40;PTBP3;DDX19A;DDX19B;CSDE1;SRSF3;RBMS1;EIF4E2;TP53;SRSF7;RBM24;RBMS2;SLBP;DDX6;YTHDC1;IREB2;ZFP36L1;FXR1;ATXN2;G3BP1;TPR;IGF2BP1;IGF2BP3;IGF2BP2;N4BP1;RBM38;RNF20;CPSF7;TIA1;SAMD4A;LARP4;EIF2S2;QKI;DHFR;CPEB1;LIN28B;HNRNPK;LIN28A;CSDC2;NOVA1;FUBP1;AGO2;HNRNPA2B1;SERBP1;CPEB3;DCP1A;CPEB2;EIF4G2
 
 
 
 
Ubiquitin-Like Protein Conjugating Enzyme Activity ( GO:0061650 )
 
 
17/39
 
 
0.0000064
 
 
0.0005922
 
 
0
 
 
0
 
 
4.797662
 
 
57.405522
 
 
UBE2F;UBE2H;UBE2I;UBE2B;UBE2D2;UBE2E3;UBE2D3;UBE2D1;UBE2G2;UBE2J1;UBE2W;CDC34;UBE2Q1;UBE2R2;UBE2NL;UBE2N;UBE2K
 
 
 
 
mRNA 3’-UTR Binding ( GO:0003730 )
 
 
32/105
 
 
0.0000094
 
 
0.0007979
 
 
0
 
 
0
 
 
2.728345
 
 
31.572150
 
 
DAZAP1;FMR1;CELF2;YBX3;ZFP36L2;ELAVL2;ZFP36L1;FXR1;BOLL;AUH;IGF2BP1;IGF2BP3;IGF2BP2;ZNF385A;RBM38;RNF20;TIA1;ANGEL2;SECISBP2L;LARP4B;LARP4;QKI;CPEB1;CSDC2;HNRNPA2B1;SERBP1;RBMS1;CPEB3;TP53;CPEB2;RBM24;RBMS2
 
 
 
 
Cysteine-Type Peptidase Activity ( GO:0008234 )
 
 
40/147
 
 
0.0000166
 
 
0.0012912
 
 
0
 
 
0
 
 
2.328893
 
 
25.626017
 
 
OTUD4;USP14;USP37;USP15;USP38;USP53;USP32;USP34;USP12;TNFAIP3;CTSS;USP19;CASP7;CASP5;CASP3;OTUD6B;CAPN3;PDIA3;USP46;USP8;USP47;USP25;USP48;USP6;USP42;USP4;DESI2;USP21;USP9X;USP3;USP44;YOD1;JOSD1;SENP2;SENP1;SFRP1;ESPL1;OTULIN;BLMH;OTUB1
 
 
 
 
Ubiquitin Conjugating Enzyme Activity ( GO:0061631 )
 
 
15/34
 
 
0.0000187
 
 
0.0013375
 
 
0
 
 
0
 
 
4.898953
 
 
53.341221
 
 
UBE2H;UBE2B;UBE2D2;UBE2E3;UBE2D3;UBE2D1;UBE2G2;UBE2J1;UBE2W;CDC34;UBE2Q1;UBE2R2;UBE2NL;UBE2N;UBE2K
 
 
 
 
Cysteine-Type Deubiquitinase Activity ( GO:0004843 )
 
 
29/96
 
 
0.0000292
 
 
0.0019390
 
 
0
 
 
0
 
 
2.691997
 
 
28.111992
 
 
OTUD4;USP14;USP37;USP15;USP38;USP53;USP32;USP34;USP12;TNFAIP3;USP19;OTUD6B;USP46;USP47;USP8;USP25;USP48;USP6;USP42;DESI2;USP4;USP21;USP9X;USP3;USP44;YOD1;JOSD1;OTULIN;OTUB1
 
 
 
 
Transcription Cis-Regulatory Region Binding ( GO:0000976 )
 
 
109/548
 
 
0.0000611
 
 
0.0037899
 
 
0
 
 
0
 
 
1.555880
 
 
15.097689
 
 
RB1;FOXA1;PRDM5;RORA;ARID4B;AHR;NR3C1;TNF;IKZF5;YY1;BBX;SOX9;PITX2;ZNF566;SOX5;MEF2A;SOX13;RFX3;ZNF10;SOX11;SOX12;ISL1;RUNX1;RFX6;LMX1A;TBL1XR1;HOXB1;ADNP;HOXB8;TP53;ZNF395;ZNF391;GATA6;FOXO3;ZBTB5;MACROH2A1;ZBTB4;GLIS2;NPAS3;HOXC8;RREB1;ZBED4;ZNF266;SMAD2;TFAP2C;TFAP2D;FOXF1;XRCC5;HMGA1;HMGA2;FOXN4;PBX1;SCRT1;FOSL2;LHX1;AGO2;SNAI1;CREBRF;MAFK;LHX8;ZNF512B;BARHL2;SUV39H2;TCF24;SUV39H1;HMGB2;SIX1;ASH2L;MIA3;CTCF;HIF3A;HEY2;KLF10;FOXD3;SREBF1;KLF11;TFEB;FOS;KLF15;PAX2;TFDP1;NR5A2;TFDP2;HAND2;HAND1;IRF2;RARA;PPARG;HDAC4;YAP1;SATB1;SATB2;FOXK2;STOX2;PURA;MNT;TBX21;E2F3;E2F6;ZNF620;E2F7;DRGX;TCF7L2;KLF4;SLC25A37;REST;WT1;BCOR;TAF4
 
 
 
 
Platelet-Derived Growth Factor Binding ( GO:0048407 )
 
 
7/10
 
 
0.0000823
 
 
0.0045955
 
 
0
 
 
0
 
 
14.450882
 
 
135.911455
 
 
PDGFRB;COL3A1;COL1A2;COL2A1;COL5A1;COL4A1;PDGFB
 
 
 
 
Protein Homodimerization Activity ( GO:0042803 )
 
 
130/680
 
 
0.0000839
 
 
0.0045955
 
 
0
 
 
0
 
 
1.482968
 
 
13.918728
 
 
APP;DGKD;PANK3;TFRC;PANK1;FMR1;CPOX;AHR;NUDT5;YARS2;TXN2;TMEM266;PSMD7;PIP4K2B;MAP3K9;PITX2;MAP3K5;SH3GLB1;ACVR1;CEP135;SLC30A8;MIGA1;TPM1;ACSL6;ADAM10;FOXP2;NRBP1;RUNX1;THAP1;TPST1;OXA1L;CHMP4B;SNRPC;CARS1;ZNF397;MTMR1;FICD;ABCB7;PEX11B;PDGFB;LPL;ABCB9;ADRB2;ZBTB4;RTN4;FXR1;SEC14L2;BLOC1S6;SNX2;FLRT3;PDCD10;PPCS;IRAK2;STC2;PDGFC;TPR;SYNDIG1;MAP3K21;NDP;SRGAP2;KCNN4;RAB11FIP4;SNX6;PRNP;TGFB2;FZD4;GADD45A;CAV2;ZBTB16;INHBB;NR2F2;GGCT;PTPRA;HOOK1;BHLHE40;COL9A1;CHRFAM7A;COL9A3;MAP3K13;TENM2;ABCD3;MTCL1;HSPB8;BHLHE41;DPY30;MIA3;SPPL3;EEA1;IKBKB;GHR;RBPMS2;MUC13;ACP3;HIF1AN;TRPC6;CHUK;HMGCS1;STARD3NL;NOG;CACYBP;ADRA2A;GREM1;COL2A1;ADRB3;HAND2;PAFAH1B2;SLC25A14;CANT1;CARNMT1;TIMM9;PON2;RCHY1;SLC25A27;PGRMC1;PLN;DROSHA;BAK1;SLIT2;GBP1;GCA;TARBP2;VEGFA;GALE;ABCB10;NR6A1;GSTA4;XPNPEP1;VAPB;PRKRA;MGAT4A
 
 
 
 
Transforming Growth Factor Beta Receptor Binding ( GO:0005160 )
 
 
10/20
 
 
0.0001310
 
 
0.0066952
 
 
0
 
 
0
 
 
6.197406
 
 
55.404899
 
 
SMAD2;FKBP1A;TGFBR3;TGFB2;USP15;TGFB1;SMURF1;TGFBR1;FERMT2;SMAD7
 
 
 
 
Ubiquitin-Protein Transferase Activity ( GO:0004842 )
 
 
84/409
 
 
0.0001366
 
 
0.0066952
 
 
0
 
 
0
 
 
1.615528
 
 
14.375256
 
 
TRIM71;PPP1R11;UBE2D2;UBE2D3;UBE2D1;TNFAIP3;FBXO21;PRPF19;CDC23;UBE2Q1;RNF19A;RNF152;TRIM23;TRIM63;NHLRC3;UBE2E3;ZFP91;FBXO10;KLHL42;RNF144B;RNF125;CDC34;UBE2R2;HECW2;TRAF5;OBI1;RNF34;MARCHF1;CUL5;UHRF2;CUL3;PELI2;CUL2;RNF38;MYCBP2;UBR3;DTX2;RNF7;RCHY1;RNFT1;UBE2J1;CDC42;ZNRF1;ZNRF2;RMND5A;RNF138;HECTD3;UBE2NL;RNF217;RNF39;RBBP6;VHL;RFFL;MARCHF3;MARCHF5;RNF20;UBE2H;TRIM41;SMURF2;UBE2B;MMAB;SMURF1;SIAH2;SIAH1;BFAR;UBE2G2;CHFR;KLHL20;RNF145;UBE2W;NEURL4;NEDD4;RLIM;RNF182;UBE2N;MKRN3;MDM4;RNF183;BCOR;RNF186;RNF141;UBE2K;RNF220;LNX2
 
 
 
 
Deubiquitinase Activity ( GO:0101005 )
 
 
30/111
 
 
0.0002051
 
 
0.0095454
 
 
0
 
 
0
 
 
2.302451
 
 
19.552927
 
 
OTUD4;USP14;USP37;USP15;USP38;USP53;USP32;USP34;USP12;TNFAIP3;USP19;PSMD7;OTUD6B;USP46;USP8;USP47;USP25;USP48;USP6;USP42;USP4;DESI2;USP21;USP9X;USP3;USP44;YOD1;JOSD1;OTULIN;OTUB1
 
 
 
 
Protein Phosphatase Regulator Activity ( GO:0019888 )
 
 
22/75
 
 
0.0004073
 
 
0.0180579
 
 
0
 
 
0
 
 
2.577219
 
 
20.117536
 
 
PABIR1;PPP1R15B;PPP1R12A;PPP1R11;DMPK;YWHAB;B3GAT3;LMTK2;PPP2R2A;PPP4R3A;RNF7;ARPP19;HSP90B1;PPP3R1;PPP4R2;PPP6R1;PTPRA;PPP1R7;RCAN2;ENSA;SHOC2;CALM3
 
 
 
 
Protein Serine/Threonine Kinase Activity ( GO:0004674 )
 
 
73/359
 
 
0.0004889
 
 
0.0206888
 
 
0
 
 
0
 
 
1.592622
 
 
12.141165
 
 
CCNK;DYRK2;TESK2;MYLK;IKBKB;RPS6KA3;RPS6KA6;AKT3;CHEK1;PDK4;TLK2;TLK1;MAP3K9;NEK3;IKBKE;MAP3K7;MAP3K4;MAP3K5;ACVR1;PRKAB2;MAP2K1;CAMK1D;CHUK;PRKCD;CASK;DYRK1B;LMTK2;VRK3;NRBP1;TGFBR1;MELK;ULK2;SGK3;MAPKAPK5;TOP1;SGK1;BRSK1;PRKAA1;PRKAA2;STK39;TTK;PPM1D;STK33;MAPK8;NUAK2;ACVR1C;MKNK1;STK38L;TRPM7;MAPK6;MARK3;MAP4K3;MAP4K4;MAP2K6;MARK1;CDK17;SRPK2;CDK19;MAP3K1;DMPK;CSNK1A1;RIOK3;PLK2;NEK6;NEK7;LIMK1;ACVR2B;SRPK1;CDK8;CAMK4;NEK10;CDK1;MAP3K13
 
 
 
 
Transition Metal Ion Transmembrane Transporter Activity ( GO:0046915 )
 
 
13/35
 
 
0.0005300
 
 
0.0214544
 
 
0
 
 
0
 
 
3.663508
 
 
27.632344
 
 
SLC30A7;SLC30A6;SLC30A9;SLC30A8;SLC30A3;SLC31A1;SLC30A5;SLC39A11;SLC30A1;SLC30A10;TMEM165;SLC25A37;SLC39A8
 
 
 
 
Cyclin-Dependent Protein Serine/Threonine Kinase Regulator Activity ( GO:0016538 )
 
 
15/44
 
 
0.0005883
 
 
0.0227207
 
 
0
 
 
0
 
 
3.207793
 
 
23.860434
 
 
CCNK;CCNJ;CCNT2;CCNH;CCNF;CCNC;CCNA2;CCND2;CCND1;CCNE2;CCNY;CCNE1;CCNG1;CCNL2;CDK5R1
 
 
 
 
Ubiquitin Protein Ligase Binding ( GO:0031625 )
 
 
57/268
 
 
0.0006286
 
 
0.0227207
 
 
0
 
 
0
 
 
1.683117
 
 
12.407851
 
 
RB1;DAZAP2;UBXN1;HAPSTR1;IKBKE;GABARAPL1;GPR37;FBXW7;YOD1;AXIN2;TNFRSF1B;TGFBR1;TRAF4;TRAF5;TMBIM6;ANKRA2;EIF4E2;TP53;TRIB2;OTUB1;BECN1;RALB;RNF34;CUL5;CUL3;CUL2;DERL1;RTN4;UBE2J1;HSPD1;BAG4;BAG5;ERBB3;PRKAR2A;RFFL;HSPA9;SMAD2;RNF20;USP25;SLF2;FZD5;UBE2B;FZD4;XRCC5;NEK6;FZD6;LIMK1;ERLIN2;SMAD5;SMAD7;UBE2W;GNPDA1;BCL2;UBE2N;RNF186;UBE2K;CUL4B
 
 
 
 
K63-linked Deubiquitinase Activity ( GO:0061578 )
 
 
8/16
 
 
0.0006345
 
 
0.0227207
 
 
0
 
 
0
 
 
6.193664
 
 
45.601744
 
 
OTUD4;USP14;USP8;USP53;DESI2;USP9X;TNFAIP3;YOD1
 
 
 
 
Sodium Channel Regulator Activity ( GO:0017080 )
 
 
13/36
 
 
0.0007248
 
 
0.0249912
 
 
0
 
 
0
 
 
3.504022
 
 
25.332871
 
 
NANOS1;TMPRSS3;ATP2B4;GLRX;FGF14;FXYD3;NEDD4;SGK3;SGK1;SCN4B;FGF11;YWHAH;PTPN3
 
 
 
 
DNA-binding Transcription Factor Binding ( GO:0140297 )
 
 
61/294
 
 
0.0008059
 
 
0.0262537
 
 
0
 
 
0
 
 
1.631438
 
 
11.621622
 
 
RB1;TCERG1;DAZAP2;PRDM5;BHLHE41;MIA3;AHR;CRKL;YY1;HEY2;EP300;PITX2;MEF2A;EOMES;LMO1;RBFOX2;LMO3;DNMT3A;FOS;ISL1;SIRT1;PIAS2;RUNX1;CREB1;TFDP1;MDFIC;HAND2;HAND1;PPARG;TP53;TRIB2;CGGBP1;YAP1;HDAC4;UHRF2;CEBPG;GATA6;TXLNG;PBXIP1;FOXO4;MTDH;NEUROD1;NHLH2;PURA;VHL;SMAD2;TCF7L2;CBX5;PRRX1;NEK6;HMGA2;KLF4;TBX5;PBX1;REST;CNOT7;NFIA;BCL3;BHLHE40;BCL2;BCOR
 
 
 
 
Zinc Ion Transmembrane Transporter Activity ( GO:0005385 )
 
 
10/24
 
 
0.0008178
 
 
0.0262537
 
 
0
 
 
0
 
 
4.425690
 
 
31.461836
 
 
SLC30A7;SLC30A6;SLC30A9;SLC30A8;SLC30A3;SLC30A5;SLC39A11;SLC30A1;SLC39A8;SLC30A10
 
 
 
 
Xylosyltransferase Activity ( GO:0042285 )
 
 
5/7
 
 
0.0008587
 
 
0.0266477
 
 
0
 
 
0
 
 
15.472852
 
 
109.240111
 
 
POGLUT2;POGLUT1;GXYLT2;LARGE1;LARGE2
 
 
 
 
GTPase Activity ( GO:0003924 )
 
 
56/266
 
 
0.0009006
 
 
0.0270460
 
 
0
 
 
0
 
 
1.660952
 
 
11.647411
 
 
ARF4;DDX3X;RASEF;ARHGAP5;RAB22A;SEPTIN11;NKIRAS2;RGS8;RAB8A;TRIM23;ENTPD7;RRAS2;RHOB;RAP2C;RAB32;RAB30;MRAS;RAP2B;RGS9BP;RAB34;RAB38;ARF6;ARL8B;RGS17;RALB;RGS16;GNAI3;ARL2;DNAJC27;GNAI1;RHOBTB1;RAP1B;CDC42;GNA13;GNG10;NRAS;RAB40A;RAP1A;GNG5;RAB40C;RAB23;RAB24;GBP1;RAB4B;SEPTIN2;VTA1;RAB39B;GPN1;RAB14;RAB12;SRPRA;RAB18;RAB9A;ABCE1;RHEBL1;RAB40AL
 
 
 
 
Ubiquitin-Like Protein Peptidase Activity ( GO:0019783 )
 
 
12/33
 
 
0.0010736
 
 
0.0307012
 
 
0
 
 
0
 
 
3.541662
 
 
24.213551
 
 
USP14;USP25;USP47;USP15;USP48;DESI2;USP9X;USP44;YOD1;SENP2;SENP1;OTUB1
 
 
 
 
Protein Heterodimerization Activity ( GO:0046982 )
 
 
47/216
 
 
0.0010882
 
 
0.0307012
 
 
0
 
 
0
 
 
1.730677
 
 
11.808766
 
 
ZNF397;ITGB1;APP;PHB1;TENM2;DGKD;FMR1;PDGFB;BHLHE41;MIA3;ADRB1;AHR;NPAS3;FXR1;IKBKB;PPP2CA;NEUROD1;SNX2;ERBB3;IRAK2;IL12B;BAK1;MCL1;YWHAH;MEF2A;TCF7L2;GCA;MIGA1;CHUK;FZD4;GADD45A;KCNB1;TPM1;SMC1A;ZHX1;ADRA2A;RUNX1;NR4A2;VAPB;BHLHE40;BCL2;MICU3;TP53;KCNK2;PAFAH1B2;NFE2L1;PAFAH1B1
 
 
 
 
Magnesium Ion Transmembrane Transporter Activity ( GO:0015095 )
 
 
7/14
 
 
0.0014092
 
 
0.0385868
 
 
0
 
 
0
 
 
6.191796
 
 
40.647555
 
 
MRS2;MMGT1;SLC41A2;NIPA1;ZDHHC13;TRPM7;CNNM4
 
 
 
 
Ion Channel Regulator Activity ( GO:0099106 )
 
 
29/119
 
 
0.0015842
 
 
0.0421400
 
 
0
 
 
0
 
 
2.001354
 
 
12.904067
 
 
RRAD;TMPRSS3;GLRX;ADRB2;CABP1;ARPP19;ENSA;PDE4B;BSND;CACNG4;YWHAH;NANOS1;KCNB1;KCNIP4;ATP2B4;STIMATE;TMC7;KCNAB3;FKBP1A;FGF14;FXYD3;KCNS3;CHRFAM7A;MICU3;SGK3;SGK1;SCN4B;FGF11;PTPN3
 
 
 
 
N6-methyladenosine-containing RNA Reader Activity ( GO:1990247 )
 
 
6/11
 
 
0.0017883
 
 
0.0462469
 
 
0
 
 
0
 
 
7.428345
 
 
46.995429
 
 
YTHDC1;YTHDC2;HNRNPA2B1;IGF2BP1;IGF2BP3;IGF2BP2
 
 
 
 
Ribonucleoside Triphosphate Phosphatase Activity ( GO:0017111 )
 
 
56/274
 
 
0.0018412
 
 
0.0463085
 
 
0
 
 
0
 
 
1.599247
 
 
10.071010
 
 
ARF4;DDX3X;RASEF;ARHGAP5;RAB22A;SEPTIN11;NKIRAS2;RGS8;RAB8A;TRIM23;ENTPD7;RRAS2;RHOB;RAP2C;RAB32;RAB30;MRAS;RAP2B;RGS9BP;RAB34;RAB38;ARF6;ARL8B;RGS17;RALB;RGS16;GNAI3;ARL2;DNAJC27;GNAI1;RHOBTB1;RAP1B;CDC42;GNA13;GNG10;NRAS;RAB40A;RAP1A;GNG5;RAB40C;RAB23;RAB24;GBP1;RAB4B;SEPTIN2;VTA1;RAB39B;GPN1;RAB14;RAB12;SRPRA;RAB18;RAB9A;ABCE1;RHEBL1;RAB40AL
 
 
 
 
DNA-binding Transcription Repressor Activity, RNA Polymerase II-specific
( GO:0001227 )
 
 
45/210
 
 
0.0018901
 
 
0.0463085
 
 
0
 
 
0
 
 
1.696362
 
 
10.638059
 
 
ZNF512B;SATB1;PRDM5;ZBTB26;BHLHE41;FOXK2;CTCF;ZBTB5;NR3C1;FOXO3;ZBTB4;IKZF5;YY1;PURA;MNT;TBX21;HEY2;ZKSCAN3;PITX2;SKIL;E2F6;ZBTB18;FOXD3;ZBTB39;TFAP2C;ZBTB16;SOX13;MITF;ZBTB34;FOXP2;SCRT1;THAP1;KLF8;REST;TFEC;BHLHE40;HAND1;SNAI1;PPARG;MAFK;INSM2;MXD1;TP53;JDP2;NFE2L1
 
 
 
 
UDP-xylosyltransferase Activity ( GO:0035252 )
 
 
5/8
 
 
0.0020264
 
 
0.0483742
 
 
0
 
 
0
 
 
10.314635
 
 
63.966076
 
 
POGLUT2;POGLUT1;GXYLT2;LARGE1;LARGE2
 
 
 
 
I-SMAD Binding ( GO:0070411 )
 
 
7/15
 
 
0.0023261
 
 
0.0523975
 
 
0
 
 
0
 
 
5.417506
 
 
32.849441
 
 
SMAD2;FKBP1A;SMURF1;AXIN2;SMAD5;TGFBR1;SMAD7
 
 
 
 
Phosphatidylinositol-3-Phosphate Phosphatase Activity ( GO:0004438 )
 
 
7/15
 
 
0.0023261
 
 
0.0523975
 
 
0
 
 
0
 
 
5.417506
 
 
32.849441
 
 
MTMR1;MTMR10;MTMR3;MTMR12;MTMR14;PTEN;MTMR9
 
 
 
 
Kinase Binding ( GO:0019900 )
 
 
88/472
 
 
0.0023638
 
 
0.0523975
 
 
0
 
 
0
 
 
1.429531
 
 
8.645069
 
 
RB1;ITGB1;ERRFI1;CCNK;PTPRR;USP37;CCNT2;DGKD;TFRC;MAML1;WWC2;PRKAG2;AP2A1;PTPRK;NR3C1;IKBKB;PPP1CB;GHR;PPP1CC;ZFYVE26;SPRED1;CCND2;QARS1;CCND1;RB1CC1;PDGFRB;PRKAB2;PPP1R12A;BORA;PRKCD;RPS6;ADAM10;AXIN2;ADRA2A;CDC25B;PARP8;CNKSR2;ACTA2;CCNA2;TIAM1;SIKE1;FRMD5;MYCN;VDAC1;UTRN;ANKRA2;MAPRE1;CACUL1;CRK;DGKH;BECN1;GATA6;GSKIP;FOXO3;MACROH2A1;ZBTB4;RHOBTB1;PRDX3;CDC42;BAG5;SOCS1;RNF138;PDCD10;CAMK2N1;UBQLN1;RICTOR;RBBP6;RFFL;MAP2K6;MPP2;TCF7L2;MAP3K1;FZD5;GADD45A;CAV2;NEK6;PARP16;ESR1;SNAP91;FASN;SNAI1;SPRY2;CALM3;GRB2;MAP3K13;FERMT2;LIMS1;CDK5R1
 
 
 
 
Ubiquitin-Like Protein Ligase Binding ( GO:0044389 )
 
 
57/284
 
 
0.0025823
 
 
0.0559100
 
 
0
 
 
0
 
 
1.563011
 
 
9.314097
 
 
RB1;DAZAP2;UBXN1;HAPSTR1;IKBKE;GABARAPL1;GPR37;FBXW7;YOD1;AXIN2;TNFRSF1B;TGFBR1;TRAF4;TRAF5;TMBIM6;ANKRA2;EIF4E2;TP53;TRIB2;OTUB1;BECN1;RALB;RNF34;CUL5;CUL3;CUL2;DERL1;RTN4;UBE2J1;HSPD1;BAG4;BAG5;ERBB3;PRKAR2A;RFFL;HSPA9;SMAD2;RNF20;USP25;SLF2;FZD5;UBE2B;FZD4;XRCC5;NEK6;FZD6;LIMK1;ERLIN2;SMAD5;SMAD7;UBE2W;GNPDA1;BCL2;UBE2N;RNF186;UBE2K;CUL4B
 
 
 
 
Guanyl Ribonucleotide Binding ( GO:0032561 )
 
 
48/232
 
 
0.0028938
 
 
0.0612298
 
 
0
 
 
0
 
 
1.622574
 
 
9.484257
 
 
ARF4;RALB;RRAD;ARL3;RASEF;GNAI3;TUBD1;ARL2;RAB22A;GNAI1;RHOBTB1;RAP1B;CDC42;NRAS;ARL5B;TUBA1A;RAP1A;PIP4K2B;GBP1;RAB8A;TRIM23;RAB4B;VTA1;RAB39B;RRAS2;RHOB;RAP2C;RAB32;RAB10;ARL4D;RAB30;ARL4A;MRAS;RAP2B;TUBB2A;RAB14;RAB34;RAB12;MPPED2;RAB18;RAPGEF2;RAB9A;RAB38;RHEBL1;ARF5;RAB9B;ARF6;ARL8B
 
 
 
 
Chloride Channel Regulator Activity ( GO:0017081 )
 
 
6/12
 
 
0.0031565
 
 
0.0639747
 
 
0
 
 
0
 
 
6.189928
 
 
35.643331
 
 
VAMP8;BSND;CHRFAM7A;SGK3;SGK1;VTI1B
 
 
 
 
Ubiquitin Conjugating Enzyme Binding ( GO:0031624 )
 
 
10/28
 
 
0.0032282
 
 
0.0639747
 
 
0
 
 
0
 
 
3.441403
 
 
19.739296
 
 
RNF144B;ZMYM2;RNF125;DCUN1D3;RNF19A;SIAH2;DCUN1D4;RNF217;SIAH1;DCUN1D2
 
 
 
 
Protein Serine/Threonine Phosphatase Activity ( GO:0004722 )
 
 
18/66
 
 
0.0032297
 
 
0.0639747
 
 
0
 
 
0
 
 
2.325596
 
 
13.338174
 
 
MTMR3;MTMR14;PTEN;CTDSPL2;PPM1K;PPM1D;CDC14A;PPM1F;PPM1G;PPP1CB;PPP2CA;PPP1CC;PPP6C;PLPP6;PPP3R1;PPP5C;PDP2;ZNF367
 
 
 
 
Phosphatidylinositol Kinase Activity ( GO:0052742 )
 
 
9/24
 
 
0.0035249
 
 
0.0683677
 
 
0
 
 
0
 
 
3.716025
 
 
20.987784
 
 
PI4K2B;PIK3CA;IPMK;PI4KA;PIK3R3;PIP4K2B;PIP5K1B;PIK3R1;PIK3C2A
 
 
 
 
Phosphatidylinositol Monophosphate Phosphatase Activity ( GO:0052744 )
 
 
7/16
 
 
0.0036417
 
 
0.0691930
 
 
0
 
 
0
 
 
4.815281
 
 
27.039222
 
 
MTMR1;MTMR10;MTMR3;MTMR12;MTMR14;PTEN;MTMR9
 
 
 
 
Phosphatidylinositol 3-Kinase Regulator Activity ( GO:0035014 )
 
 
5/9
 
 
0.0040370
 
 
0.0736953
 
 
0
 
 
0
 
 
7.735527
 
 
42.640153
 
 
RUBCN;PIK3IP1;FLT3;PIK3R1;ATG14
 
 
 
 
Type I Transforming Growth Factor Beta Receptor Binding ( GO:0034713 )
 
 
5/9
 
 
0.0040370
 
 
0.0736953
 
 
0
 
 
0
 
 
7.735527
 
 
42.640153
 
 
FKBP1A;SMAD2;TGFB1;FERMT2;SMAD7
 
 
 
 
Metal Ion Transmembrane Transporter Activity ( GO:0046873 )
 
 
10/29
 
 
0.0043208
 
 
0.0773587
 
 
0
 
 
0
 
 
3.260086
 
 
17.748945
 
 
MRS2;MMGT1;SLC41A2;UCP3;ATP2B4;NIPA1;ZDHHC13;TRPM7;CNNM4;TMEM165
 
 
 
 
Fatty Acid Elongase Activity ( GO:0009922 )
 
 
4/6
 
 
0.0044553
 
 
0.0782613
 
 
0
 
 
0
 
 
12.373832
 
 
66.987833
 
 
ELOVL4;ELOVL5;ELOVL2;ELOVL3
 
 
 
 
Protein Kinase Binding ( GO:0019901 )
 
 
95/527
 
 
0.0045394
 
 
0.0782621
 
 
0
 
 
0
 
 
1.371418
 
 
7.398756
 
 
ITGB1;APP;ERRFI1;DAZAP2;CCNK;PTPRR;USP37;CCNT2;TFRC;MAML1;PRKAG2;RORA;AP2A1;PTPRK;NR3C1;DUSP16;IKBKB;PPP1CB;GHR;PPP1CC;ZFYVE26;SPRED1;CCND2;QARS1;CCND1;RB1CC1;SOX9;ACVR1;PDGFRB;PRKAB2;PPP1R12A;BORA;PRKCD;RPS6;ADAM10;AXIN2;SIRT1;ADRA2A;CDC25B;DUSP22;CNKSR2;ACTA2;CCNA2;SIKE1;FRMD5;RARA;ADAM9;MAPKAPK5;VDAC1;UTRN;ANKRA2;MAPRE1;CACUL1;CRK;TRIB2;BECN1;GATA6;GSKIP;FOXO3;PPM1D;MACROH2A1;ZBTB4;RHOBTB1;PRDX3;CDC42;MAPK8;BAG5;SOCS1;RNF138;PDCD10;CAMK2N1;PRKAR2A;TPR;RICTOR;RBBP6;VHL;RFFL;MAP2K6;CLASP2;MPP2;TCF7L2;MAP3K1;FZD5;CAV2;NEK6;ESR1;SNAP91;PKIA;SPRY2;CALM3;GRB2;MAP3K13;FERMT2;LIMS1;CDK5R1
 
 
 
 
Ubiquitin-Like Protein Conjugating Enzyme Binding ( GO:0044390 )
 
 
11/34
 
 
0.0049087
 
 
0.0830916
 
 
0
 
 
0
 
 
2.962804
 
 
15.752454
 
 
TCERG1;RNF144B;ZMYM2;RNF125;DCUN1D3;RNF19A;SIAH2;DCUN1D4;RNF217;SIAH1;DCUN1D2
 
 
 
 
Cysteine-Type Endopeptidase Activity ( GO:0004197 )
 
 
19/74
 
 
0.0052132
 
 
0.0866698
 
 
0
 
 
0
 
 
2.142268
 
 
11.260952
 
 
PDIA3;USP8;USP14;USP37;USP15;USP6;USP32;USP21;USP9X;USP34;USP12;CTSS;CASP7;SFRP1;CASP5;ESPL1;CASP3;CAPN5;CAPN3
 
 
 
 
Phosphatidylinositol-5-Phosphate Binding ( GO:0010314 )
 
 
7/17
 
 
0.0054541
 
 
0.0890831
 
 
0
 
 
0
 
 
4.333501
 
 
22.583582
 
 
SNX3;SESTD1;SAP30L;VEPH1;PLEKHA5;RUBCNL;WIPI2
 
 
 
 
Protein Kinase Regulator Activity ( GO:0019887 )
 
 
26/113
 
 
0.0061004
 
 
0.0979217
 
 
0
 
 
0
 
 
1.854498
 
 
9.456831
 
 
CCNJ;GMFB;CCNH;YWHAB;CCNF;GSKIP;PRKAG2;CCNC;SOCS3;CCND2;DEPTOR;SOCS1;CCND1;QARS1;PRKAR2A;CCNL2;MAP2K1;CCNA2;CXCL10;MOB1A;CCNE2;CCNY;CCNE1;PRKRA;CCNG1;CDK5R1
 
 
 
 
Frizzled Binding ( GO:0005109 )
 
 
11/35
 
 
0.0062732
 
 
0.0983254
 
 
0
 
 
0
 
 
2.839189
 
 
14.398841
 
 
WNT10B;SFRP1;WNT3A;WNT9B;DVL3;RSPO3;WNT7A;NDP;WNT9A;WNT1;WNT4
 
 
 
 
Protein Serine/Threonine Kinase Activator Activity ( GO:0043539 )
 
 
18/70
 
 
0.0063368
 
 
0.0983254
 
 
0
 
 
0
 
 
2.146204
 
 
10.862770
 
 
CCNK;MAP2K1;CCNT2;DDX3X;TGFB1;STRADB;PARP16;PARP8;MOB1A;CCND2;DBF4;CCND1;SPRY2;RICTOR;CALM3;MAP3K13;FERMT2;CDK5R1
 
 
 
 
GTP Binding ( GO:0005525 )
 
 
42/206
 
 
0.0066158
 
 
0.1009716
 
 
0
 
 
0
 
 
1.591277
 
 
7.985507
 
 
ARF4;RALB;RRAD;ARL3;RASEF;TUBD1;ARL2;RAB22A;GNAI1;RHOBTB1;RAP1B;CDC42;NRAS;ARL5B;TUBA1A;RAP1A;PIP4K2B;GBP1;RAB8A;TRIM23;RAB4B;VTA1;RAB39B;RRAS2;RHOB;RAP2C;RAB32;RAB10;ARL4D;RAB30;ARL4A;MRAS;RAP2B;TUBB2A;RAB14;RAB34;RAB9A;RAB38;RHEBL1;ARF5;ARF6;ARL8B
 
 
 
 
Anion Binding ( GO:0043168 )
 
 
74/402
 
 
0.0068227
 
 
0.1024504
 
 
0
 
 
0
 
 
1.404737
 
 
7.006127
 
 
GLTP;RASEF;PRKAG2;ETFA;RAB22A;SMPD3;FUNDC2;DEPTOR;PDE4B;HIF1AN;LBR;RAB8A;CFAP45;TRIM23;NANOS1;TRPC6;ACTN2;SIRT4;RRAS2;WIPI2;SIRT1;RAP2C;MRAS;RAP2B;FCHSD2;MPPED2;RAPGEF2;ARF6;RAPGEF4;ARL8B;PFN2;AMER1;RALB;ARL3;GNAI3;TWF1;HMGCR;GNAI1;RAP1B;SEC14L2;SNX3;CYTH3;CYTH2;NRAS;RAP1A;PRKAR2A;TTPA;TDG;CLVS2;KDSR;GBP1;KCNJ2;MARK1;CLVS1;PLEKHA1;PLEKHA5;ADAP1;SNAP91;PLEKHA8;DHFR;RAB10;PPP5C;SNX18;GCLC;RAB14;PSAT1;SESTD1;RAB12;RAB18;RAB9A;RHEBL1;FERMT2;RAB9B;HCN1
 
 
 
 
Cyclin-Dependent Protein Serine/Threonine Kinase Activator Activity ( GO:0061575 )
 
 
5/10
 
 
0.0071526
 
 
0.1040486
 
 
0
 
 
0
 
 
6.188062
 
 
30.570714
 
 
CCNK;CCND2;CCNT2;CCND1;CDK5R1
 
 
 
 
Estrogen Response Element Binding ( GO:0034056 )
 
 
5/10
 
 
0.0071526
 
 
0.1040486
 
 
0
 
 
0
 
 
6.188062
 
 
30.570714
 
 
ESRRA;NR6A1;ESRRG;NR3C1;ESR1
 
 
 
 
RNA Polymerase Binding ( GO:0070063 )
 
 
7/18
 
 
0.0078654
 
 
0.1126571
 
 
0
 
 
0
 
 
3.939318
 
 
19.087090
 
 
TCERG1;CCNT2;PABPN1;BIN1;YTHDC2;NEDD4;ERCC6
 
 
 
 
Phosphatidylinositol Phosphate Binding ( GO:1901981 )
 
 
25/112
 
 
0.0104731
 
 
0.1477338
 
 
0
 
 
0
 
 
1.782525
 
 
8.126439
 
 
RNF34;SNX12;SNX10;SNX11;SNX3;CYTH3;FUNDC2;ZFYVE26;SNX27;TTPA;SAP30L;CLVS2;ARFIP1;CLVS1;RUBCN;PLEKHA5;SNX24;WIPI2;PLEKHA8;GAP43;FCHSD2;SESTD1;VEPH1;RUBCNL;FERMT2
 
 
 
 
Transcription Coregulator Binding ( GO:0001221 )
 
 
23/103
 
 
0.0136128
 
 
0.1891565
 
 
0
 
 
0
 
 
1.782854
 
 
7.660472
 
 
SREBF1;CCNT2;UBE2I;CBX3;EED;NEK6;ZBTB16;GATA6;HMGA1;RORA;CTCF;FOS;ESR1;PBX1;RUNX1;NR5A2;HAND2;HAND1;MTF2;RARA;EP300;PPARG;ZNF644
 
 
 
 
UDP-glycosyltransferase Activity ( GO:0008194 )
 
 
16/65
 
 
0.0148607
 
 
0.2034607
 
 
0
 
 
0
 
 
2.023429
 
 
8.516681
 
 
DDX18;POGLUT2;B4GALT1;POGLUT1;B3GAT3;CSGALNACT2;GXYLT1;GXYLT2;B3GNT7;B4GAT1;PIGA;MGAT4A;GCNT3;GCNT4;LARGE1;LARGE2
 
 
 
 
Cytokine Activity ( GO:0005125 )
 
 
37/186
 
 
0.0150874
 
 
0.2035704
 
 
0
 
 
0
 
 
1.541512
 
 
6.464943
 
 
CCL3L1;TNFRSF11B;FASLG;CXCL13;TNF;CCL8;CCL7;CCL3;IL12B;CCL2;FAM3C;NDP;WNT1;WNT4;IL10;WNT10B;TGFB2;EDN1;TGFB1;TSLP;WNT3A;IL15;IL13;LIF;WNT9B;WNT7A;INHBB;WNT9A;GDF6;GDF5;BMP5;VEGFA;BMP3;CXCL10;KITLG;IFNG;CCM2
 
 
 
 
Intramolecular Phosphotransferase Activity ( GO:0016868 )
 
 
4/8
 
 
0.0164751
 
 
0.2101134
 
 
0
 
 
0
 
 
6.186197
 
 
25.399954
 
 
PMM1;PGM3;PGM2;PGM1
 
 
 
 
pre-miRNA Binding ( GO:0070883 )
 
 
4/8
 
 
0.0164751
 
 
0.2101134
 
 
0
 
 
0
 
 
6.186197
 
 
25.399954
 
 
LIN28A;PRKRA;DICER1;TARBP2
 
 
 
 
siRNA Binding ( GO:0035197 )
 
 
4/8
 
 
0.0164751
 
 
0.2101134
 
 
0
 
 
0
 
 
6.186197
 
 
25.399954
 
 
PRKRA;FMR1;DICER1;TARBP2
 
 
 
 
Tumor Necrosis Factor Receptor Activity ( GO:0005031 )
 
 
4/8
 
 
0.0164751
 
 
0.2101134
 
 
0
 
 
0
 
 
6.186197
 
 
25.399954
 
 
FASN;TNFRSF11A;TNFRSF1B;TNFRSF1A
 
 
 
 
Receptor Ligand Activity ( GO:0048018 )
 
 
68/380
 
 
0.0169295
 
 
0.2114426
 
 
0
 
 
0
 
 
1.355323
 
 
5.527957
 
 
CD86;APP;HMGB2;CXCL1;FASLG;CXCL13;TNF;TNFSF13B;ENDOU;SHH;FGF7;IL12B;FAM3C;IL10;EPHA7;EDN1;SEMA6A;IL15;SEMA6D;IL13;WNT9B;WNT9A;NGF;GREM1;IFNG;PTRH2;SOS1;HBEGF;SEMA7A;PDGFB;TGFA;TNFRSF11B;PTHLH;DLL4;RELN;ITPRIPL1;NTF3;CCL3;CCL2;NDP;WNT1;WNT4;WNT10B;TGFB2;TGFB1;TSLP;BDNF;WNT3A;SEMA4C;LIF;VEGFC;WNT7A;INHBB;SEMA4F;IGF1;GDF6;GDF5;VEGFA;BMP5;APLN;BMP3;CXCL10;KITLG;LEP;FGF18;CSPG5;CCM2;FBN1
 
 
 
 
Cadherin Binding ( GO:0045296 )
 
 
58/317
 
 
0.0170335
 
 
0.2114426
 
 
0
 
 
0
 
 
1.391816
 
 
5.668270
 
 
ITGB1;DDX3X;YWHAB;FNBP1L;BZW2;BZW1;CRKL;GOLGA3;PCMT1;CAPZB;EFHD2;MB21D2;SPTAN1;ACVR1;SH3GLB1;USP8;IST1;CEMIP2;TMOD3;GAPVD1;CD2AP;CDH11;FSCN1;CHMP4B;PKP4;ITGA6;MAPRE1;EPHA2;DDX6;TWF1;UBFD1;ASAP1;RTN4;CNN3;CNN2;SNX2;CDH20;COBLL1;CDH22;RPL14;EPS15;SNX5;SEPTIN2;RDX;MPP7;TJP1;RAB10;ANLN;HNRNPK;VAPB;FASN;GIPC1;CAPZA1;SERBP1;CHMP2B;PI4KA;EIF4G2;CDK5R1
 
 
 
 
Tau Protein Binding ( GO:0048156 )
 
 
11/40
 
 
0.0180258
 
 
0.2188760
 
 
0
 
 
0
 
 
2.348990
 
 
9.433425
 
 
PPP2CA;SMAD2;BRSK1;PRKAA1;PPP5C;BIN1;EP300;MARK3;METAP1;MARK1;METAP2
 
 
 
 
Alpha-Tubulin Binding ( GO:0043014 )
 
 
10/35
 
 
0.0181025
 
 
0.2188760
 
 
0
 
 
0
 
 
2.476801
 
 
9.936192
 
 
EML4;TTLL7;TBCEL;B4GALT1;SPAST;FNTA;FAM110C;INO80;ARL8B;CDK5R1
 
 
 
 
Ubiquitin-Like Ligase-Substrate Adaptor Activity ( GO:1990756 )
 
 
25/118
 
 
0.0198149
 
 
0.2365080
 
 
0
 
 
0
 
 
1.666939
 
 
6.536608
 
 
SUV39H2;FBXO45;ASB11;SOCS2;VHL;BTRC;DCAF12;SOCS4;SKP1;ARRDC4;KLHL29;FBXW7;FEM1B;FBXL19;PAQR3;KLHL28;KLHL2;KLHL23;KLHL20;SMAD7;APPBP2;GAN;KLHL8;KCTD17;SPSB4
 
 
 
 
Double-Stranded Methylated DNA Binding ( GO:0010385 )
 
 
3/5
 
 
0.0216814
 
 
0.2469915
 
 
0
 
 
0
 
 
9.277039
 
 
35.543120
 
 
MECP2;WT1;MACROH2A1
 
 
 
 
Neurotrophin TRK Receptor Binding ( GO:0005167 )
 
 
3/5
 
 
0.0216814
 
 
0.2469915
 
 
0
 
 
0
 
 
9.277039
 
 
35.543120
 
 
FRS2;GRB2;PIK3R1
 
 
 
 
Neurotrophin TRKA Receptor Binding ( GO:0005168 )
 
 
3/5
 
 
0.0216814
 
 
0.2469915
 
 
0
 
 
0
 
 
9.277039
 
 
35.543120
 
 
FRS2;GRB2;PIK3R1
 
 
 
 
Phosphatase Inhibitor Activity ( GO:0019212 )
 
 
9/31
 
 
0.0219993
 
 
0.2469915
 
 
0
 
 
0
 
 
2.532622
 
 
9.666376
 
 
PABIR1;PPP1R11;YWHAB;ENSA;STYX;LMTK2;RNF7;ARPP19;HSP90B1
 
 
 
 
Chemoattractant Activity ( GO:0042056 )
 
 
10/36
 
 
0.0220197
 
 
0.2469915
 
 
0
 
 
0
 
 
2.381401
 
 
9.086997
 
 
CXCL10;FGF7;NTF3;HMGB2;PDGFB;CCL3;CCL2;VEGFC;SOS1;VEGFA
 
 
 
 
DNA Binding, Bending ( GO:0008301 )
 
 
6/17
 
 
0.0227873
 
 
0.2495880
 
 
0
 
 
0
 
 
3.375343
 
 
12.764035
 
 
FOXD1;SAP30L;HMGA1;HMGB2;HMGA2;TOP1
 
 
 
 
Transmembrane Receptor Protein Serine/Threonine Kinase Binding ( GO:0070696 )
 
 
6/17
 
 
0.0227873
 
 
0.2495880
 
 
0
 
 
0
 
 
3.375343
 
 
12.764035
 
 
FKBP1A;BMP3;SMURF1;RSPO2;BMP5;SMAD7
 
 
 
 
Insulin-Like Growth Factor Receptor Binding ( GO:0005159 )
 
 
5/13
 
 
0.0254795
 
 
0.2711909
 
 
0
 
 
0
 
 
3.866864
 
 
14.190931
 
 
SOCS2;SOCS1;IGF1;PIK3R1;YWHAH
 
 
 
 
MAP Kinase Kinase Kinase Activity ( GO:0004709 )
 
 
7/22
 
 
0.0255248
 
 
0.2711909
 
 
0
 
 
0
 
 
2.888161
 
 
10.594082
 
 
MAP3K1;MAP3K21;MAP3K9;MAP3K13;MAP3K7;MAP3K4;MAP3K5
 
 
 
 
Eukaryotic Initiation Factor 4E Binding ( GO:0008190 )
 
 
4/9
 
 
0.0264255
 
 
0.2711909
 
 
0
 
 
0
 
 
4.948670
 
 
17.980624
 
 
DDX3X;EIF4EBP1;OTX2;C8ORF88
 
 
 
 
Thioesterase Binding ( GO:0031996 )
 
 
4/9
 
 
0.0264255
 
 
0.2711909
 
 
0
 
 
0
 
 
4.948670
 
 
17.980624
 
 
CDC42;TRAF4;TRAF5;ARF6
 
 
 
 
Type II Transforming Growth Factor Beta Receptor Binding ( GO:0005114 )
 
 
4/9
 
 
0.0264255
 
 
0.2711909
 
 
0
 
 
0
 
 
4.948670
 
 
17.980624
 
 
TGFBR3;TGFB2;TGFB1;TGFBR1
 
 
 
 
Protein Serine/Threonine/Tyrosine Kinase Activity ( GO:0004712 )
 
 
8/27
 
 
0.0267196
 
 
0.2711909
 
 
0
 
 
0
 
 
2.606191
 
 
9.440563
 
 
MAP2K4;MAP2K1;PRKAA2;TTK;MAPKAPK5;MAP3K9;SGK1;MAP2K6
 
 
 
 
Growth Factor Activity ( GO:0008083 )
 
 
20/92
 
 
0.0267987
 
 
0.2711909
 
 
0
 
 
0
 
 
1.721499
 
 
6.230799
 
 
IL10;TGFB1;BDNF;PDGFB;LIF;TGFA;VEGFC;CXCL1;IGF1;NGF;VEGFA;ENDOU;FGF7;NTF3;FGF18;IL12B;CSPG5;CCM2;SOS1;HBEGF
 
 
 
 
RNA Polymerase II-specific DNA-binding Transcription Factor Binding ( GO:0061629 )
 
 
43/232
 
 
0.0294723
 
 
0.2950401
 
 
0
 
 
0
 
 
1.412108
 
 
4.976698
 
 
HDAC4;TCERG1;ASAH1;UHRF2;GATA6;BHLHE41;MIA3;AHR;CRKL;MTDH;NEUROD1;NHLH2;PDCD11;HEY2;EP300;HIF1AN;PITX2;MEF2A;NCOA1;EOMES;TCF7L2;GABARAPL1;PRRX1;DNMT3A;HMGA2;FOS;KLF4;TBX5;ISL1;SIRT1;PIAS2;REST;CREB1;MDFIC;HAND2;BHLHE40;HAND1;NCOA7;PPARG;TACC1;TAF4;TP53;TRIB2
 
 
 
 
Metalloaminopeptidase Activity ( GO:0070006 )
 
 
6/18
 
 
0.0302483
 
 
0.2951552
 
 
0
 
 
0
 
 
3.093885
 
 
10.823389
 
 
NPEPPS;MMP16;XPNPEP1;MMP15;TRHDE;METAP1
 
 
 
 
Regulatory RNA Binding ( GO:0061980 )
 
 
11/43
 
 
0.0303343
 
 
0.2951552
 
 
0
 
 
0
 
 
2.128401
 
 
7.439776
 
 
TRIM71;LIN28A;PRKRA;FMR1;TUT4;AGO2;HNRNPA2B1;YBX1;DICER1;QKI;TARBP2
 
 
 
 
Hexosyltransferase Activity ( GO:0016758 )
 
 
21/99
 
 
0.0304349
 
 
0.2951552
 
 
0
 
 
0
 
 
1.668549
 
 
5.826849
 
 
POMT2;DDX18;B4GALT3;POGLUT2;POGLUT1;B4GALT1;B3GAT3;CSGALNACT2;TMTC3;FUT4;DPY19L1;B4GAT1;B3GNT7;DPY19L4;MGAT4A;GCNT3;GCNT4;LARGE1;B4GALT6;LARGE2;B4GALT5
 
 
 
 
Protein Tyrosine Phosphatase Activity ( GO:0004725 )
 
 
18/82
 
 
0.0314840
 
 
0.3021819
 
 
0
 
 
0
 
 
1.742571
 
 
6.026290
 
 
PTPRR;MTMR3;PTPRN2;EYA1;EYA3;PTEN;PTPRK;DUSP16;DUSP9;CDC14A;PALD1;CDC25B;DUSP22;PTP4A1;PPP2CA;PTPRA;ZNF367;PTPN3
 
 
 
 
Notch Binding ( GO:0005112 )
 
 
7/23
 
 
0.0324000
 
 
0.3039390
 
 
0
 
 
0
 
 
2.707494
 
 
9.285609
 
 
DLL4;NCOR2;ADAM17;TCIM;CUL3;CHAC1;HIF1AN
 
 
 
 
Nuclear Localization Sequence Binding ( GO:0008139 )
 
 
7/23
 
 
0.0324000
 
 
0.3039390
 
 
0
 
 
0
 
 
2.707494
 
 
9.285609
 
 
POM121C;CABP1;KPNA5;TNPO1;KPNA3;KPNB1;KPNA1
 
 
 
 
Phosphatase Activity ( GO:0016791 )
 
 
16/71
 
 
0.0328584
 
 
0.3039390
 
 
0
 
 
0
 
 
1.802061
 
 
6.155025
 
 
ARMT1;PLPPR1;PTEN;DUSP16;PPP1CB;PPP1CC;MINPP1;PLPP6;PPP5C;PUDP;PXYLP1;ALPL;CILP2;ACP3;PLPP1;FRA10AC1
 
 
 
 
Aminopeptidase Activity ( GO:0004177 )
 
 
8/28
 
 
0.0329730
 
 
0.3039390
 
 
0
 
 
0
 
 
2.475738
 
 
8.447384
 
 
NPEPPS;MMP16;XPNPEP1;MMP15;BLMH;TRHDE;METAP1;METAP2
 
 
 
 
MAP Kinase Kinase Activity ( GO:0004708 )
 
 
5/14
 
 
0.0351898
 
 
0.3150168
 
 
0
 
 
0
 
 
3.437013
 
 
11.503675
 
 
MAP2K4;MAP2K1;MAPKAPK5;MAP3K9;MAP2K6
 
 
 
 
SUMO Binding ( GO:0032183 )
 
 
5/14
 
 
0.0351898
 
 
0.3150168
 
 
0
 
 
0
 
 
3.437013
 
 
11.503675
 
 
USP25;CBX4;TDG;SERBP1;HABP4
 
 
 
 
Wnt Receptor Activity ( GO:0042813 )
 
 
5/14
 
 
0.0351898
 
 
0.3150168
 
 
0
 
 
0
 
 
3.437013
 
 
11.503675
 
 
FZD5;FZD4;FZD6;LRP5;RORA
 
 
 
 
Adenyl Ribonucleotide Binding ( GO:0032559 )
 
 
56/318
 
 
0.0367567
 
 
0.3202338
 
 
0
 
 
0
 
 
1.327227
 
 
4.384406
 
 
PANK3;ABCD3;DYRK2;PRKAG2;YARS2;AKT3;PDK4;PDE4B;TLK2;TLK1;PIP4K2B;CFAP45;MAP3K5;ACVR1;LMTK2;TGFBR1;MPPED2;RAPGEF2;TOP1;CARS1;RAPGEF4;BRSK1;ABCB6;FICD;HSPA4L;VPS4A;TWF1;ABCB9;HSP90B1;SEC14L2;NUAK2;PRKAR2A;MKNK1;TDG;STK38L;RICTOR;N4BP2;MAP4K3;MAP4K4;MARK1;SRPK2;PRNP;DMPK;KCNJ11;NEK6;ATP2B4;SRPK1;PPP5C;GCLC;P2RX5;ACTC1;PTPRA;ERCC6;ABCE1;MYO1F;HCN1
 
 
 
 
Arachidonate Binding ( GO:0050544 )
 
 
3/6
 
 
0.0389076
 
 
0.3202338
 
 
0
 
 
0
 
 
6.184334
 
 
20.077845
 
 
PPARG;STX3;S100A8
 
 
 
 
Icosatetraenoic Acid Binding ( GO:0050543 )
 
 
3/6
 
 
0.0389076
 
 
0.3202338
 
 
0
 
 
0
 
 
6.184334
 
 
20.077845
 
 
PPARG;STX3;S100A8
 
 
 
 
Minor Groove of Adenine-Thymine-Rich DNA Binding ( GO:0003680 )
 
 
3/6
 
 
0.0389076
 
 
0.3202338
 
 
0
 
 
0
 
 
6.184334
 
 
20.077845
 
 
LIN54;HMGA1;HMGA2
 
 
 
 
Morphogen Activity ( GO:0016015 )
 
 
3/6
 
 
0.0389076
 
 
0.3202338
 
 
0
 
 
0
 
 
6.184334
 
 
20.077845
 
 
GREM1;SHH;WNT1
 
 
 
 
Tumor Necrosis Factor Binding ( GO:0043120 )
 
 
3/6
 
 
0.0389076
 
 
0.3202338
 
 
0
 
 
0
 
 
6.184334
 
 
20.077845
 
 
ADAM17;TNFRSF1B;TNFRSF1A
 
 
 
 
UDP-glucosyltransferase Activity ( GO:0035251 )
 
 
4/10
 
 
0.0392741
 
 
0.3202338
 
 
0
 
 
0
 
 
4.123652
 
 
13.349045
 
 
UGCG;POGLUT2;POGLUT1;GYG2
 
 
 
 
Galactosyltransferase Activity ( GO:0008378 )
 
 
4/10
 
 
0.0392741
 
 
0.3202338
 
 
0
 
 
0
 
 
4.123652
 
 
13.349045
 
 
B4GALT3;B4GALT1;B4GALT6;B4GALT5
 
 
 
 
Nuclear Retinoid X Receptor Binding ( GO:0046965 )
 
 
4/10
 
 
0.0392741
 
 
0.3202338
 
 
0
 
 
0
 
 
4.123652
 
 
13.349045
 
 
NR4A2;HMGA1;PPARG;TACC1
 
 
 
 
Transferrin Receptor Binding ( GO:1990459 )
 
 
4/10
 
 
0.0392741
 
 
0.3202338
 
 
0
 
 
0
 
 
4.123652
 
 
13.349045
 
 
IKBKB;SNX2;CHUK;RGMA
 
 
 
 
Ubiquitin Protein Ligase Activity ( GO:0061630 )
 
 
55/313
 
 
0.0395563
 
 
0.3202338
 
 
0
 
 
0
 
 
1.323563
 
 
4.275150
 
 
TRIM71;PPP1R11;UBE2D2;UBE2D3;UBE2D1;PRPF19;RNF19A;RNF152;TRIM23;TRIM63;NHLRC3;UBE4A;UBE4B;RNF144B;RNF125;HECW2;TRAF5;MARCHF1;RNF34;UHRF2;CUL3;MYCBP2;PELI2;RNF38;UBR3;DTX2;RNF7;RCHY1;RNFT1;CDC42;ZNRF1;ZNRF2;RNF138;RNF217;RNF39;RBBP6;RFFL;MARCHF5;RNF20;TRIM41;SMURF2;SMURF1;MMAB;SIAH2;SIAH1;BFAR;RNF145;NEURL4;NEDD4;RLIM;MKRN3;RNF183;RNF186;UBE2K;RNF220
 
 
 
 
Protein Phosphatase 2A Binding ( GO:0051721 )
 
 
7/24
 
 
0.0404079
 
 
0.3243082
 
 
0
 
 
0
 
 
2.548081
 
 
8.176104
 
 
PABIR1;PTPRA;ENSA;STRN;ARPP19;TP53;CTTNBP2NL
 
 
 
 
Protein Phosphatase Binding ( GO:0019903 )
 
 
24/120
 
 
0.0412108
 
 
0.3258523
 
 
0
 
 
0
 
 
1.549421
 
 
4.941187
 
 
PABIR1;MTMR3;PPP1R11;STX17;ATP2B4;MTMR9;PIK3R1;AKAP5;VRK3;ARPP19;HSP90B1;CTTNBP2NL;SNX3;MAPK8;PTPRA;CDC27;ENSA;PXDN;STRN;SHOC2;KCNN4;TP53;IKBKE;MAP3K5
 
 
 
 
Calcium Channel Regulator Activity ( GO:0005246 )
 
 
11/45
 
 
0.0413003
 
 
0.3258523
 
 
0
 
 
0
 
 
2.002968
 
 
6.383229
 
 
NANOS1;FKBP1A;RRAD;PDE4B;STIMATE;SLC30A1;MICU3;SGK3;CABP1;SGK1;CACNG4
 
 
 
 
Cytokine Receptor Binding ( GO:0005126 )
 
 
22/109
 
 
0.0448983
 
 
0.3492567
 
 
0
 
 
0
 
 
1.566920
 
 
4.862708
 
 
SMAD2;IL10;TGFB2;USP15;TSLP;SMURF1;IL13;LIF;OSMR;VEGFA;SOCS2;TGFBR3;FKBP1A;SOCS3;SPRED1;KITLG;SOCS1;PXDN;IL12B;TNFSF9;CCM2;IL6ST
 
 
 
 
Nuclear Estrogen Receptor Binding ( GO:0030331 )
 
 
9/35
 
 
0.0462644
 
 
0.3492567
 
 
0
 
 
0
 
 
2.142489
 
 
6.584689
 
 
NCOA1;WBP2;MMS19;STRN;TACC1;ESR1;ISL1;ZNF366;PPID
 
 
 
 
ATPase Binding ( GO:0051117 )
 
 
16/74
 
 
0.0462916
 
 
0.3492567
 
 
0
 
 
0
 
 
1.708552
 
 
5.250029
 
 
USP25;UBXN1;RALB;RDX;DERL1;SNX10;METTL21A;ESR1;SVIP;PLN;CLPP;VCPKMT;CHMP4B;TMEM106B;ATP6V1E1;PTPN3
 
 
 
 
Adrenergic Receptor Binding ( GO:0031690 )
 
 
5/15
 
 
0.0468927
 
 
0.3492567
 
 
0
 
 
0
 
 
3.093132
 
 
9.464655
 
 
NEDD4;ARRDC3;RAPGEF2;ADRB1;AKAP5
 
 
 
 
Alkali Metal Ion Binding ( GO:0031420 )
 
 
5/15
 
 
0.0468927
 
 
0.3492567
 
 
0
 
 
0
 
 
3.093132
 
 
9.464655
 
 
HDAC4;KCNJ11;VTA1;TDG;CAPN3
 
 
 
 
Transmembrane Receptor Protein Tyrosine Kinase Activator Activity ( GO:0030297 )
 
 
5/15
 
 
0.0468927
 
 
0.3492567
 
 
0
 
 
0
 
 
3.093132
 
 
9.464655
 
 
GREM1;IGF1;EFNA5;VEGFA;HBEGF
 
 
 
 
Ubiquitin-Ubiquitin Ligase Activity ( GO:0034450 )
 
 
5/15
 
 
0.0468927
 
 
0.3492567
 
 
0
 
 
0
 
 
3.093132
 
 
9.464655
 
 
PELI2;UBE4A;UBE4B;PRPF19;UBE2K
 
 
 
 
miRNA Binding ( GO:0035198 )
 
 
8/30
 
 
0.0482970
 
 
0.3512854
 
 
0
 
 
0
 
 
2.250409
 
 
6.819606
 
 
TRIM71;LIN28A;FMR1;TUT4;AGO2;HNRNPA2B1;YBX1;QKI
 
 
 
 
Poly-Purine Tract Binding ( GO:0070717 )
 
 
8/30
 
 
0.0482970
 
 
0.3512854
 
 
0
 
 
0
 
 
2.250409
 
 
6.819606
 
 
DAZAP1;TIA1;DDX3X;PABPN1;FMR1;RBMS1;LARP4;RBMS2
 
 
 
 
Protein Tyrosine Kinase Activator Activity ( GO:0030296 )
 
 
8/30
 
 
0.0482970
 
 
0.3512854
 
 
0
 
 
0
 
 
2.250409
 
 
6.819606
 
 
GREM1;ERBB3;IGF1;ERCC6;EFNA5;IL6ST;VEGFA;HBEGF
 
 
 
 
mRNA 3’-UTR AU-rich Region Binding ( GO:0035925 )
 
 
7/25
 
 
0.0495997
 
 
0.3579636
 
 
0
 
 
0
 
 
2.406381
 
 
7.228218
 
 
FXR1;CPEB1;CPEB3;CPEB2;RBM24;ZFP36L2;ZFP36L1
 
 
 
 
Manganese Ion Binding ( GO:0030145 )
 
 
12/52
 
 
0.0507106
 
 
0.3603938
 
 
0
 
 
0
 
 
1.857318
 
 
5.537816
 
 
ENDOU;ARG2;B4GALT1;DYRK2;XPNPEP1;GALNT1;MPPED2;ADCY2;POC1B-GALNT4;PPM1K;LARGE1;LARGE2
 
 
 
 
Phosphoric Ester Hydrolase Activity ( GO:0042578 )
 
 
12/52
 
 
0.0507106
 
 
0.3603938
 
 
0
 
 
0
 
 
1.857318
 
 
5.537816
 
 
PPP1CB;SMPD3;PPP1CC;PLCXD3;PPP5C;ARMT1;PUDP;MPPED2;GDPD1;PXYLP1;DUSP16;FRA10AC1
 
 
 
 
PDZ Domain Binding ( GO:0030165 )
 
 
13/58
 
 
0.0530530
 
 
0.3715552
 
 
0
 
 
0
 
 
1.788652
 
 
5.252315
 
 
PLEKHA1;FZD4;PTEN;ATP2B4;CRIM1;CRIPT;ADRB1;GNG12;TGFBR3;ADAM17;GRM7;GNG5;RAPGEF2
 
 
 
 
Core Promoter Sequence-Specific DNA Binding ( GO:0001046 )
 
 
9/36
 
 
0.0543460
 
 
0.3715552
 
 
0
 
 
0
 
 
2.063018
 
 
6.008301
 
 
H2AZ1;KLF10;AGO2;TBPL1;FOS;NR3C1;TAF4;TP53;H3-3B
 
 
 
 
Ubiquitin-Like Protein Ligase Activity ( GO:0061659 )
 
 
56/326
 
 
0.0548466
 
 
0.3715552
 
 
0
 
 
0
 
 
1.287293
 
 
3.737289
 
 
TRIM71;PPP1R11;UBE2D2;UBE2D3;UBE2D1;PRPF19;RNF19A;RNF152;TRIM23;TRIM63;NHLRC3;NSMCE2;PIAS2;RNF144B;RNF125;HECW2;TRAF5;MARCHF1;RNF34;UHRF2;CUL3;MYCBP2;PELI2;RNF38;UBR3;DTX2;RNF7;RCHY1;RNFT1;CDC42;ZNRF1;ZNRF2;RNF138;RNF217;RNF39;RBBP6;RFFL;ATG5;MARCHF5;RNF20;TRIM41;CBX4;SMURF2;SMURF1;MMAB;SIAH2;SIAH1;BFAR;RNF145;NEURL4;NEDD4;RLIM;MKRN3;RNF183;RNF186;RNF220
 
 
 
 
cAMP-dependent Protein Kinase Regulator Activity ( GO:0008603 )
 
 
4/11
 
 
0.0550748
 
 
0.3715552
 
 
0
 
 
0
 
 
3.534353
 
 
10.246315
 
 
PKIB;PKIA;PRKAR2A;PRKAG2
 
 
 
 
Death Receptor Activity ( GO:0005035 )
 
 
4/11
 
 
0.0550748
 
 
0.3715552
 
 
0
 
 
0
 
 
3.534353
 
 
10.246315
 
 
FASN;TNFRSF11A;TNFRSF1B;TNFRSF1A
 
 
 
 
Dynactin Binding ( GO:0034452 )
 
 
4/11
 
 
0.0550748
 
 
0.3715552
 
 
0
 
 
0
 
 
3.534353
 
 
10.246315
 
 
BICDL1;SNX5;PAFAH1B1;SNX6
 
 
 
 
Sequence-Specific mRNA Binding ( GO:1990825 )
 
 
4/11
 
 
0.0550748
 
 
0.3715552
 
 
0
 
 
0
 
 
3.534353
 
 
10.246315
 
 
LIN28A;NOVA1;FMR1;RBM24
 
 
 
 
G-quadruplex RNA Binding ( GO:0002151 )
 
 
3/7
 
 
0.0611503
 
 
0.3946021
 
 
0
 
 
0
 
 
4.637981
 
 
12.960469
 
 
LIN28A;XRN1;FMR1
 
 
 
 
Armadillo Repeat Domain Binding ( GO:0070016 )
 
 
3/7
 
 
0.0611503
 
 
0.3946021
 
 
0
 
 
0
 
 
4.637981
 
 
12.960469
 
 
TCF7L2;CTNNBIP1;STRN
 
 
 
 
Dehydroascorbic Acid Transmembrane Transporter Activity ( GO:0033300 )
 
 
3/7
 
 
0.0611503
 
 
0.3946021
 
 
0
 
 
0
 
 
4.637981
 
 
12.960469
 
 
SLC2A14;SLC2A1;SLC2A3
 
 
 
 
Icosanoid Binding ( GO:0050542 )
 
 
3/7
 
 
0.0611503
 
 
0.3946021
 
 
0
 
 
0
 
 
4.637981
 
 
12.960469
 
 
PPARG;STX3;S100A8
 
 
 
 
Nuclear Glucocorticoid Receptor Binding ( GO:0035259 )
 
 
3/7
 
 
0.0611503
 
 
0.3946021
 
 
0
 
 
0
 
 
4.637981
 
 
12.960469
 
 
NR4A2;TACC1;YWHAH
 
 
 
 
R-SMAD Binding ( GO:0070412 )
 
 
6/21
 
 
0.0614579
 
 
0.3946021
 
 
0
 
 
0
 
 
2.474676
 
 
6.902869
 
 
SMAD2;SMURF1;DROSHA;PPARG;FOS;LDLRAD4
 
 
 
 
WW Domain Binding ( GO:0050699 )
 
 
6/21
 
 
0.0614579
 
 
0.3946021
 
 
0
 
 
0
 
 
2.474676
 
 
6.902869
 
 
DAZAP2;NDFIP2;TRAF4;RAPGEF2;PPARG;WBP11
 
 
 
 
Protein Kinase Activator Activity ( GO:0030295 )
 
 
21/107
 
 
0.0633988
 
 
0.4042760
 
 
0
 
 
0
 
 
1.512629
 
 
4.172299
 
 
MAP2K1;TGFB1;DDX3X;STRADB;PRKAG2;PARP16;PARP8;MOB1A;CCND2;DBF4;CCND1;ERBB3;PRKRA;SPRY2;CALM3;RICTOR;ERCC6;IL6ST;MAP3K13;FERMT2;CDK5R1
 
 
 
 
Calcium Ion Binding ( GO:0005509 )
 
 
55/324
 
 
0.0676434
 
 
0.4284080
 
 
0
 
 
0
 
 
1.268616
 
 
3.417026
 
 
ITGB1;SPARC;MCTP1;TNFAIP6;IHH;ATP2A2;LOXL2;SHH;GRM7;CHP2;ENPP2;EFHD2;CAPN3;CEMIP2;EML1;HPCAL4;RUNX1;RCN1;MELK;PLSCR3;VSNL1;DMTF1;ASPH;PITPNM3;CANX;PLSCR4;RAPGEF2;MICU3;SLC25A12;S100A8;ZNF395;CANT1;MYCBP2;LPL;LRP2;RASGRP1;HSP90B1;PPP3R1;MAN1C1;PCDHA4;SLIT2;PCDHA7;SLC25A24;GCA;KCNIP4;ATP2B4;MEX3B;COPS2;RCAN2;SYT10;CALU;ALPL;CALM3;CDK5R1;FBN1
 
 
 
 
Epidermal Growth Factor Receptor Binding ( GO:0005154 )
 
 
7/27
 
 
0.0716637
 
 
0.4445431
 
 
0
 
 
0
 
 
2.165491
 
 
5.707738
 
 
ARF4;LINGO1;ATXN2;SNX2;TGFA;GRB2;HBEGF
 
 
 
 
Nucleotidyltransferase Activity ( GO:0016779 )
 
 
4/12
 
 
0.0737770
 
 
0.4445431
 
 
0
 
 
0
 
 
3.092380
 
 
8.060932
 
 
YRDC;REV1;NUDT5;TENT4B
 
 
 
 
Platelet-Derived Growth Factor Receptor Binding ( GO:0005161 )
 
 
4/12
 
 
0.0737770
 
 
0.4445431
 
 
0
 
 
0
 
 
3.092380
 
 
8.060932
 
 
PDGFRB;PDGFC;PDGFB;VEGFA
 
 
 
 
Vascular Endothelial Growth Factor Receptor Binding ( GO:0005172 )
 
 
4/12
 
 
0.0737770
 
 
0.4445431
 
 
0
 
 
0
 
 
3.092380
 
 
8.060932
 
 
GREM1;PDCL3;VEGFC;VEGFA
 
 
 
 
Phosphatidylinositol Binding ( GO:0035091 )
 
 
21/109
 
 
0.0744985
 
 
0.4445431
 
 
0
 
 
0
 
 
1.478078
 
 
3.838534
 
 
PLEKHF2;RUBCN;PIGU;RNF34;STK39;SNX12;SNX24;SNX10;SNX11;SNAP91;EEA1;GAP43;SNX18;SNX2;SNX27;SNX16;SESTD1;SNX17;RUBCNL;WDFY1;SNX5
 
 
 
 
D-glucose Transmembrane Transporter Activity ( GO:0055056 )
 
 
6/22
 
 
0.0749328
 
 
0.4445431
 
 
0
 
 
0
 
 
2.319874
 
 
6.011172
 
 
SLC2A14;SLC5A9;SLC2A12;SLC2A1;SLC5A11;SLC2A3
 
 
 
 
Lysophosphatidic Acid Acyltransferase Activity ( GO:0042171 )
 
 
6/22
 
 
0.0749328
 
 
0.4445431
 
 
0
 
 
0
 
 
2.319874
 
 
6.011172
 
 
LPGAT1;GPAT4;GPAT3;ABHD5;AGPAT3;AGPAT4
 
 
 
 
mRNA 5’-UTR Binding ( GO:0048027 )
 
 
6/22
 
 
0.0749328
 
 
0.4445431
 
 
0
 
 
0
 
 
2.319874
 
 
6.011172
 
 
SYNCRIP;DDX3X;FMR1;IGF2BP1;IGF2BP3;IGF2BP2
 
 
 
 
ATP Binding ( GO:0005524 )
 
 
48/281
 
 
0.0763095
 
 
0.4445431
 
 
0
 
 
0
 
 
1.277659
 
 
3.287364
 
 
BRSK1;PANK3;ABCD3;DYRK2;ABCB6;FICD;HSPA4L;VPS4A;TWF1;PRKAG2;ABCB9;YARS2;HSP90B1;SEC14L2;NUAK2;MKNK1;TDG;AKT3;PDK4;TLK2;STK38L;TLK1;PIP4K2B;RICTOR;N4BP2;MAP4K3;MAP4K4;MAP3K5;MARK1;ACVR1;SRPK2;PRNP;DMPK;KCNJ11;NEK6;ATP2B4;LMTK2;TGFBR1;SRPK1;PPP5C;P2RX5;ACTC1;PTPRA;TOP1;ERCC6;CARS1;ABCE1;MYO1F
 
 
 
 
K48-linked Deubiquitinase Activity ( GO:1990380 )
 
 
5/17
 
 
0.0763984
 
 
0.4445431
 
 
0
 
 
0
 
 
2.577310
 
 
6.628310
 
 
USP8;USP15;DESI2;USP9X;YOD1
 
 
 
 
Neuropilin Binding ( GO:0038191 )
 
 
5/17
 
 
0.0763984
 
 
0.4445431
 
 
0
 
 
0
 
 
2.577310
 
 
6.628310
 
 
SEMA7A;SEMA4C;PXDN;SEMA4F;VEGFA
 
 
 
 
Phospholipid Scramblase Activity ( GO:0017128 )
 
 
5/17
 
 
0.0763984
 
 
0.4445431
 
 
0
 
 
0
 
 
2.577310
 
 
6.628310
 
 
VMP1;XKR8;PLSCR3;ATG9A;PLSCR4
 
 
 
 
Receptor Antagonist Activity ( GO:0048019 )
 
 
5/17
 
 
0.0763984
 
 
0.4445431
 
 
0
 
 
0
 
 
2.577310
 
 
6.628310
 
 
FST;IGSF1;PXDN;DKK2;DKK3
 
 
 
 
Beta-Tubulin Binding ( GO:0048487 )
 
 
8/33
 
 
0.0788234
 
 
0.4558049
 
 
0
 
 
0
 
 
1.980014
 
 
5.030316
 
 
EML4;TTLL7;GABARAPL1;B4GALT1;SPAST;VAPB;ARL8B;CDK5R1
 
 
 
 
Nuclear Receptor Binding ( GO:0016922 )
 
 
22/116
 
 
0.0794746
 
 
0.4567336
 
 
0
 
 
0
 
 
1.449641
 
 
3.670953
 
 
NCOA1;TCF7L2;ASAH1;KDM3A;WBP2;TAF11;HMGA1;GTF2H1;ISL1;ESR1;SIRT1;NR4A2;MED14;EP300;MMS19;STRN;NCOA7;TACC1;HMGN3;PPID;ZNF366;YWHAH
 
 
 
 
Monoatomic Anion Channel Activity ( GO:0005253 )
 
 
17/86
 
 
0.0835465
 
 
0.4760844
 
 
0
 
 
0
 
 
1.525508
 
 
3.786847
 
 
GABRB3;GABRB2;GABRP;GABRB1;SLC1A4;ANO3;SLC5A8;GLRA2;P2RX5;CLCN4;FXYD3;BSND;LRRC8D;VDAC1;SHOC2;CLCC1;LRRC8B
 
 
 
 
Protein Phosphorylated Amino Acid Binding ( GO:0045309 )
 
 
10/45
 
 
0.0872844
 
 
0.4760844
 
 
0
 
 
0
 
 
1.768115
 
 
4.311694
 
 
YWHAB;FBXW7;GRAP2;NEDD4;PIK3R3;GRB2;PIK3R1;CRK;CRKL;PTPN3
 
 
 
 
JUN Kinase Kinase Kinase Activity ( GO:0004706 )
 
 
3/8
 
 
0.0879554
 
 
0.4760844
 
 
0
 
 
0
 
 
3.710169
 
 
9.019142
 
 
MAP3K21;MAP3K9;MAP3K5
 
 
 
 
L-aspartate Transmembrane Transporter Activity ( GO:0015183 )
 
 
3/8
 
 
0.0879554
 
 
0.4760844
 
 
0
 
 
0
 
 
3.710169
 
 
9.019142
 
 
SLC25A18;SLC1A4;SLC25A12
 
 
 
 
Activin Receptor Activity ( GO:0017002 )
 
 
3/8
 
 
0.0879554
 
 
0.4760844
 
 
0
 
 
0
 
 
3.710169
 
 
9.019142
 
 
ACVR1C;ACVR2B;TGFBR1
 
 
 
 
Adenylate Cyclase Binding ( GO:0008179 )
 
 
3/8
 
 
0.0879554
 
 
0.4760844
 
 
0
 
 
0
 
 
3.710169
 
 
9.019142
 
 
AKAP6;CALM3;AKAP5
 
 
 
 
Adenylate Cyclase Regulator Activity ( GO:0010854 )
 
 
3/8
 
 
0.0879554
 
 
0.4760844
 
 
0
 
 
0
 
 
3.710169
 
 
9.019142
 
 
GRM7;CALM3;GNAI1
 
 
 
 
Cell-Matrix Adhesion Mediator Activity ( GO:0098634 )
 
 
3/8
 
 
0.0879554
 
 
0.4760844
 
 
0
 
 
0
 
 
3.710169
 
 
9.019142
 
 
ITGB1;ITGA11;SVEP1
 
 
 
 
Sulfur Amino Acid Transmembrane Transporter Activity ( GO:0000099 )
 
 
3/8
 
 
0.0879554
 
 
0.4760844
 
 
0
 
 
0
 
 
3.710169
 
 
9.019142
 
 
SLC1A4;SLC7A11;SLC25A12
 
 
 
 
Vascular Endothelial Growth Factor Receptor 2 Binding ( GO:0043184 )
 
 
3/8
 
 
0.0879554
 
 
0.4760844
 
 
0
 
 
0
 
 
3.710169
 
 
9.019142
 
 
GREM1;PDCL3;VEGFA
 
 
 
 
SH3 Domain Binding ( GO:0017124 )
 
 
11/51
 
 
0.0895935
 
 
0.4812543
 
 
0
 
 
0
 
 
1.701928
 
 
4.105854
 
 
WIPF1;MICAL1;DPYSL3;RUFY2;ADAM9;GRB2;AKAP5;CRK;SOCS4;CD2AP;REPS1
 
 
 
 
Protein Kinase A Regulatory Subunit Binding ( GO:0034237 )
 
 
6/23
 
 
0.0899444
 
 
0.4812543
 
 
0
 
 
0
 
 
2.183284
 
 
5.258578
 
 
ARFGEF1;GSKIP;AKAP6;AKAP5;WASF1;AKAP1
 
 
 
 
Protein Phosphatase Inhibitor Activity ( GO:0004864 )
 
 
8/34
 
 
0.0910698
 
 
0.4844911
 
 
0
 
 
0
 
 
1.903749
 
 
4.561630
 
 
PABIR1;PPP1R14C;PPP1R11;YWHAB;ENSA;LMTK2;RNF7;HSP90B1
 
 
 
 
cAMP Binding ( GO:0030552 )
 
 
5/18
 
 
0.0941485
 
 
0.4881839
 
 
0
 
 
0
 
 
2.378917
 
 
5.621102
 
 
PRKAR2A;PDE4B;RAPGEF2;HCN1;RAPGEF4
 
 
 
 
Voltage-Gated Sodium Channel Activity ( GO:0005248 )
 
 
5/18
 
 
0.0941485
 
 
0.4881839
 
 
0
 
 
0
 
 
2.378917
 
 
5.621102
 
 
SCN11A;SCN9A;SCN3A;SCN4B;SCN1A
 
 
 
 
Hyaluronic Acid Binding ( GO:0005540 )
 
 
4/13
 
 
0.0952425
 
 
0.4881839
 
 
0
 
 
0
 
 
2.748622
 
 
6.462914
 
 
TNFAIP6;STAB2;LYVE1;IMPG2
 
 
 
 
Phosphatidate Phosphatase Activity ( GO:0008195 )
 
 
4/13
 
 
0.0952425
 
 
0.4881839
 
 
0
 
 
0
 
 
2.748622
 
 
6.462914
 
 
PLPP6;PLPPR1;PLPPR5;PLPP1
 
 
 
 
Phosphatidylinositol-3,5-Bisphosphate Phosphatase Activity ( GO:0106018 )
 
 
4/13
 
 
0.0952425
 
 
0.4881839
 
 
0
 
 
0
 
 
2.748622
 
 
6.462914
 
 
MTMR1;MTMR3;MTMR14;MTMR9
 
 
 
 
Tropomyosin Binding ( GO:0005523 )
 
 
4/13
 
 
0.0952425
 
 
0.4881839
 
 
0
 
 
0
 
 
2.748622
 
 
6.462914
 
 
TMOD1;NEBL;TMOD3;TMOD2
 
 
 
 
Acetylglucosaminyltransferase Activity ( GO:0008375 )
 
 
9/40
 
 
0.0954344
 
 
0.4881839
 
 
0
 
 
0
 
 
1.796404
 
 
4.220319
 
 
EXTL2;B3GNT7;B4GAT1;B3GNT5;MGAT4A;PIGA;GCNT3;GCNT4;LARGE1
 
 
 
 
Growth Factor Receptor Binding ( GO:0070851 )
 
 
19/100
 
 
0.0963550
 
 
0.4901994
 
 
0
 
 
0
 
 
1.452422
 
 
3.398255
 
 
PDGFRB;IL10;ARF4;LINGO1;TSLP;PDGFB;TGFA;FRS2;FRS3;VEGFA;ATXN2;ESM1;SNX2;FGF14;PDGFC;IL12B;GRB2;IL6ST;HBEGF
 
 
 
 
Syntaxin Binding ( GO:0019905 )
 
 
10/46
 
 
0.0982808
 
 
0.4972797
 
 
0
 
 
0
 
 
1.718900
 
 
3.987721
 
 
VAMP8;NAPB;SNAP25;BLOC1S6;STXBP3;STX16;STX6;CACNA1A;SNAP29;NAPG
 
 
 
 
Calcium/Calmodulin-Dependent Protein Kinase Activity ( GO:0004683 )
 
 
6/24
 
 
0.1064566
 
 
0.5271866
 
 
0
 
 
0
 
 
2.061870
 
 
4.618627
 
 
CAMK1D;EEF2K;CAMK4;MKNK1;PHKA1;MAPKAPK5
 
 
 
 
Chemorepellent Activity ( GO:0045499 )
 
 
6/24
 
 
0.1064566
 
 
0.5271866
 
 
0
 
 
0
 
 
2.061870
 
 
4.618627
 
 
EPHA7;SEMA7A;SEMA6A;SEMA6D;SEMA4C;SEMA4F
 
 
 
 
Phosphatidylinositol-3,5-Bisphosphate Binding ( GO:0080025 )
 
 
6/24
 
 
0.1064566
 
 
0.5271866
 
 
0
 
 
0
 
 
2.061870
 
 
4.618627
 
 
SNX3;SESTD1;PLEKHA5;CLVS2;WIPI2;CLVS1
 
 
 
 
poly(A) Binding ( GO:0008143 )
 
 
6/24
 
 
0.1064566
 
 
0.5271866
 
 
0
 
 
0
 
 
2.061870
 
 
4.618627
 
 
TIA1;DDX3X;PABPN1;RBMS1;LARP4;RBMS2
 
 
 
 
Insulin Receptor Binding ( GO:0005158 )
 
 
5/19
 
 
0.1137938
 
 
0.5338194
 
 
0
 
 
0
 
 
2.208866
 
 
4.800678
 
 
SNX2;IRS2;IGF1;PIK3R1;IGF1R
 
 
 
 
Transition Metal Ion Binding ( GO:0046914 )
 
 
73/458
 
 
0.1185697
 
 
0.5338194
 
 
0
 
 
0
 
 
1.176171
 
 
2.507897
 
 
ISCA2;SUV39H2;B4GALT1;DYRK2;HHIP;CTCF;IKZF5;LOXL2;EEA1;ALAD;ENDOU;NPEPPS;SHH;ZMYM2;CA2;SMPD1;ENPP2;HIF1AN;LARGE1;LARGE2;NANOS1;ARG2;SLC30A8;ESCO1;ZNF282;TET2;TET1;BAZ1B;THAP1;RNF125;MMP16;PLSCR3;FDX1;TET3;MPPED2;WDFY1;CDIP1;COMMD3-BMI1;PPARG;SNRPC;ZMYND11;TP53;KPNB1;S100A8;HDAC4;SEC23A;KDM3A;TIMM9;MYCBP2;ADCY2;PPM1K;RNF7;RCHY1;RASGRP1;TRHDE;DPP3;SAP30L;RICTOR;TIMM8B;PRNP;SLC31A1;USP21;FBXL19;GALNT1;SIAH1;KLF4;XPNPEP1;WT1;SCD;MDM4;POC1B-GALNT4;ADA;LIMS1
 
 
 
 
Transcription Coactivator Binding ( GO:0001223 )
 
 
8/36
 
 
0.1186310
 
 
0.5338194
 
 
0
 
 
0
 
 
1.767562
 
 
3.767977
 
 
CCNT2;HAND2;GATA6;RARA;EP300;RORA;ESR1;RUNX1
 
 
 
 
1-Phosphatidylinositol-3-Kinase Activity ( GO:0016303 )
 
 
3/9
 
 
0.1187204
 
 
0.5338194
 
 
0
 
 
0
 
 
3.091628
 
 
6.588209
 
 
PIK3CA;PIK3R3;PIK3C2A
 
 
 
 
AMP Binding ( GO:0016208 )
 
 
3/9
 
 
0.1187204
 
 
0.5338194
 
 
0
 
 
0
 
 
3.091628
 
 
6.588209
 
 
MPPED2;PRKAG2;CFAP45
 
 
 
 
GDP Phosphatase Activity ( GO:0004382 )
 
 
3/9
 
 
0.1187204
 
 
0.5338194
 
 
0
 
 
0
 
 
3.091628
 
 
6.588209
 
 
ENTPD5;ENTPD7;GBP1
 
 
 
 
Activin Receptor Binding ( GO:0070697 )
 
 
3/9
 
 
0.1187204
 
 
0.5338194
 
 
0
 
 
0
 
 
3.091628
 
 
6.588209
 
 
FKBP1A;SMURF1;SMAD7
 
 
 
 
cAMP Response Element Binding ( GO:0035497 )
 
 
3/9
 
 
0.1187204
 
 
0.5338194
 
 
0
 
 
0
 
 
3.091628
 
 
6.588209
 
 
CREB1;HMGA2;CREB5
 
 
 
 
cAMP-dependent Protein Kinase Inhibitor Activity ( GO:0004862 )
 
 
3/9
 
 
0.1187204
 
 
0.5338194
 
 
0
 
 
0
 
 
3.091628
 
 
6.588209
 
 
PKIB;PRKAR2A;PRKAG2
 
 
 
 
Dopamine Receptor Binding ( GO:0050780 )
 
 
3/9
 
 
0.1187204
 
 
0.5338194
 
 
0
 
 
0
 
 
3.091628
 
 
6.588209
 
 
GNA13;CAV2;GNAI1
 
 
 
 
Histone H3K9 Methyltransferase Activity ( GO:0046974 )
 
 
3/9
 
 
0.1187204
 
 
0.5338194
 
 
0
 
 
0
 
 
3.091628
 
 
6.588209
 
 
SUV39H2;SETDB1;SUV39H1
 
 
 
 
Phosphatidylinositol-3,5-Bisphosphate 3-Phosphatase Activity ( GO:0052629 )
 
 
3/9
 
 
0.1187204
 
 
0.5338194
 
 
0
 
 
0
 
 
3.091628
 
 
6.588209
 
 
MTMR1;MTMR3;MTMR14
 
 
 
 
 
 
 KEGG_2021_Human 
 This category is based on pathway annotations from the Kyoto
Encyclopedia of Genes and Genomes (KEGG), a manually curated resource
integrating molecular interaction networks, metabolic pathways, and
disease-associated signaling cascades. KEGG pathways represent canonical
biological systems including metabolism, signal transduction, cell
communication, and genetic information processing. Enrichment in KEGG
pathways suggests coordinated perturbation of well-characterized
biological networks. This category is particularly useful for
identifying conserved signaling routes and metabolic programs affected
by gene expression changes. 
 
 
 
 
Term
 
 
Overlap
 
 
P.value
 
 
Adjusted.P.value
 
 
Old.P.value
 
 
Old.Adjusted.P.value
 
 
Odds.Ratio
 
 
Combined.Score
 
 
Genes
 
 
 
 
 
 
Signaling pathways regulating pluripotency of stem cells
 
 
56/143
 
 
0.0000000
 
 
0.0000000
 
 
0
 
 
0
 
 
4.038196
 
 
122.6628328
 
 
ONECUT1;BMI1;FGF2;IGF1R;AKT3;SMARCAD1;HOXA1;JARID2;ACVR1;MAP2K1;WNT9B;AXIN2;WNT9A;ISL1;DUSP9;PIK3CA;HAND1;COMMD3-BMI1;HOXB1;IL6ST;RAF1;HOXD1;PIK3R3;PIK3R1;NRAS;ACVR1C;DVL3;WNT1;SKIL;WNT4;SMAD2;WNT10B;FZD3;FZD5;SETDB1;FZD4;WNT3A;PCGF5;FZD6;PCGF3;LIF;WNT7A;INHBB;IGF1;KLF4;SMAD5;ACVR2B;MEIS1;REST;APC;ID1;ID4;ID3;GRB2;KRAS;TCF3
 
 
 
 
Pathways in cancer
 
 
133/531
 
 
0.0000000
 
 
0.0000000
 
 
0
 
 
0
 
 
2.118137
 
 
55.7364068
 
 
RB1;HHIP;FGF2;CRKL;IGF1R;FGF7;FRAT2;CCND2;CCND1;RASSF5;AKT3;PIM1;IL12B;EP300;SKP1;PDGFRB;MAP2K1;RALBP1;TPM3;MITF;RUNX1;EML4;CCNE2;COL4A2;CCNE1;COL4A1;COL4A4;COL4A5;IL6ST;RAF1;TP53;CUL2;PDGFB;PIK3R3;TGFA;PIK3R1;RASGRP1;HSP90B1;GNG10;BCL2L11;TPR;DVL3;PMAIP1;WNT1;WNT4;SMAD2;WNT10B;FZD3;TGFB2;TGFB1;FZD5;FZD4;GADD45A;WNT3A;ZBTB16;FZD6;IGF1;GNG12;ESR1;GADD45G;LPAR6;FGF18;BCL2;CYCS;CALM3;GRB2;ITGB1;CDKN1A;FLT3;PTEN;SLC2A1;FASLG;LAMC1;IKBKB;SHH;CASP7;CASP3;HEY2;RAC1;NCOA1;EDN1;CHUK;IL15;IL13;WNT9B;FOS;WNT9A;AXIN2;TGFBR1;CCNA2;PIK3CA;TRAF4;IFNG;TRAF5;RARA;RARB;PPARG;ITGA6;SOS1;CRK;SOS2;RALB;LAMA2;LAMA4;PTGER3;LRP5;GNAI3;ADCY3;ADCY2;GNAI1;CDC42;DLL4;GNA13;NRAS;MAPK8;GNG2;GNG5;E2F3;BAK1;VHL;EGLN1;TCF7L2;EGLN2;TXNRD1;WNT7A;VEGFC;VEGFA;KITLG;APC;GSTA4;CCDC6;FAS;KRAS
 
 
 
 
mTOR signaling pathway
 
 
54/154
 
 
0.0000000
 
 
0.0000000
 
 
0
 
 
0
 
 
3.382709
 
 
82.0273405
 
 
PTEN;TNF;IGF1R;IKBKB;RPS6KA3;RPS6KA6;DEPTOR;AKT3;SESN2;ATP6V1E1;RNF152;MAP2K1;CHUK;STRADB;RPS6;WNT9B;TSC1;WNT9A;TNFRSF1A;PIK3CA;DDIT4;TBC1D7;ULK2;RAF1;EIF4E2;SGK1;SOS1;SOS2;PRKAA1;SEH1L;PRKAA2;LRP5;PIK3R3;PIK3R1;NRAS;EIF4EBP1;DVL3;RICTOR;WNT1;FNIP1;FNIP2;WNT4;WNT10B;FZD3;FZD5;FZD4;WNT3A;FZD6;WNT7A;IGF1;GRB2;KRAS;LAMTOR3;LAMTOR5
 
 
 
 
MAPK signaling pathway
 
 
83/294
 
 
0.0000000
 
 
0.0000000
 
 
0
 
 
0
 
 
2.474430
 
 
57.1887966
 
 
PTPRR;FLT3;FASLG;IL1RAP;FGF2;TNF;DUSP16;CRKL;IGF1R;ELK4;IKBKB;RPS6KA3;RPS6KA6;FGF7;CASP3;AKT3;STMN1;RAC1;MAP3K7;MAP3K4;MAP3K5;PDGFRB;MAP2K4;MAP2K1;CHUK;DUSP1;CACNA2D3;RRAS2;FOS;NGF;DUSP9;TGFBR1;CDC25B;TNFRSF1A;MRAS;RASA1;RAPGEF2;MAPKAPK5;RAF1;SOS1;TP53;CRK;SOS2;EPHA2;PDGFB;TGFA;CACNA1A;EFNA5;RASGRP1;RAP1B;CDC42;PPP3R1;NRAS;MAPK8;RAP1A;ERBB3;MKNK1;PDGFC;NTF3;CACNG2;MAP4K3;CACNG4;MAP4K4;MAP2K6;TGFB2;MAP3K1;TGFB1;GADD45A;BDNF;VEGFC;IGF1;GNG12;GADD45G;VEGFA;PPP5C;KITLG;FGF18;FAS;GRB2;KRAS;TEK;MAP3K13;LAMTOR3
 
 
 
 
PI3K-Akt signaling pathway
 
 
93/354
 
 
0.0000000
 
 
0.0000000
 
 
0
 
 
0
 
 
2.243121
 
 
48.1371432
 
 
ITGB1;CDKN1A;FLT3;YWHAB;PTEN;PPP2R2A;FASLG;LAMC1;FGF2;IGF1R;IKBKB;GHR;FGF7;CCND2;CCND1;YWHAQ;AKT3;MYB;ITGB8;RAC1;YWHAH;PDGFRB;MAP2K1;CHUK;RPS6;TSC1;OSMR;NGF;COL2A1;CREB1;CCNE2;COL4A2;PIK3CA;CCNE1;COL4A1;COL4A4;COL6A2;DDIT4;ITGA8;COL4A5;COL6A3;SGK3;COL6A6;ITGA6;SGK1;RAF1;EIF4E2;SOS1;TP53;SOS2;EPHA2;CREB5;PRKAA1;PRKAA2;LAMA2;LAMA4;PDGFB;PIK3R3;TGFA;PIK3R1;EFNA5;FOXO3;HSP90B1;PPP2CA;GNG10;NRAS;RELN;BCL2L11;GNG2;ERBB3;GNG5;PDGFC;NTF3;EIF4EBP1;MCL1;BDNF;VEGFC;IGF1;GNG12;VEGFA;COL1A1;KITLG;G6PC2;COL1A2;LPAR6;FGF18;ITGA11;COL9A1;BCL2;COL9A3;GRB2;KRAS;TEK
 
 
 
 
FoxO signaling pathway
 
 
46/131
 
 
0.0000000
 
 
0.0000000
 
 
0
 
 
0
 
 
3.383143
 
 
70.8842963
 
 
CDKN1A;PRKAA1;PRKAA2;PTEN;PIK3R3;PRKAG2;IRS2;FASLG;FOXO4;PIK3R1;FOXO3;IGF1R;IKBKB;MAPK8;NRAS;CCND2;BCL2L11;CCND1;AKT3;S1PR1;EP300;IL10;GABARAPL1;MAP2K1;PRKAB2;TGFB2;TGFB1;HOMER1;CHUK;GADD45A;PLK2;IGF1;FBXO32;SIRT1;TGFBR1;GADD45G;G6PC2;PIK3CA;CAT;GRB2;SGK3;KRAS;RAF1;SGK1;SOS1;SOS2
 
 
 
 
Renal cell carcinoma
 
 
30/69
 
 
0.0000000
 
 
0.0000001
 
 
0
 
 
0
 
 
4.793737
 
 
95.6066377
 
 
CDKN1A;CUL2;SLC2A1;PDGFB;PIK3R3;TGFA;PIK3R1;CRKL;CDC42;RAP1B;NRAS;RAP1A;AKT3;EP300;RAC1;VHL;PAK5;EGLN1;MAP2K1;TGFB2;EGLN2;TGFB1;VEGFA;PIK3CA;GRB2;KRAS;RAF1;SOS1;CRK;SOS2
 
 
 
 
Gastric cancer
 
 
49/149
 
 
0.0000000
 
 
0.0000001
 
 
0
 
 
0
 
 
3.063887
 
 
60.2444507
 
 
RB1;CDKN1A;FGF2;FGF7;SHH;FRAT2;CCND1;AKT3;MAP2K1;WNT9B;AXIN2;WNT9A;TGFBR1;CCNE2;PIK3CA;CCNE1;RARB;RAF1;SOS1;TP53;SOS2;LRP5;PIK3R3;PIK3R1;NRAS;DVL3;E2F3;BAK1;WNT1;WNT4;SMAD2;WNT10B;TCF7L2;FZD3;TGFB2;TGFB1;FZD5;FZD4;GADD45A;WNT3A;CSNK1A1;FZD6;WNT7A;GADD45G;APC;FGF18;BCL2;GRB2;KRAS
 
 
 
 
Proteoglycans in cancer
 
 
61/205
 
 
0.0000000
 
 
0.0000001
 
 
0
 
 
0
 
 
2.653593
 
 
51.9986944
 
 
ITGB1;CDKN1A;HPSE2;IHH;FASLG;FGF2;TNF;IGF1R;PPP1CB;PPP1CC;SHH;CCND1;CASP3;AKT3;IL12B;RAC1;VAV3;MAP2K1;PPP1R12A;RPS6;WNT9B;RRAS2;FRS2;WNT9A;TIAM1;MRAS;PIK3CA;RAF1;SOS1;TP53;SOS2;HBEGF;PIK3R3;PIK3R1;CDC42;NRAS;ERBB3;DROSHA;WNT1;WNT4;SMAD2;WNT10B;FZD3;TGFB2;TGFB1;FZD5;FZD4;CAV2;WNT3A;FZD6;RDX;WNT7A;IGF1;ESR1;VEGFA;COL1A1;COL1A2;SDC1;FAS;GRB2;KRAS
 
 
 
 
Colorectal cancer
 
 
34/86
 
 
0.0000000
 
 
0.0000001
 
 
0
 
 
0
 
 
4.077510
 
 
78.9738909
 
 
CDKN1A;RALB;PIK3R3;TGFA;PIK3R1;MAPK8;NRAS;BCL2L11;CCND1;CASP3;AKT3;PMAIP1;RAC1;BAK1;SMAD2;TCF7L2;MAP2K1;TGFB2;TGFB1;GADD45A;AXIN2;FOS;TGFBR1;GADD45G;PIK3CA;APC;BCL2;CYCS;GRB2;KRAS;RAF1;SOS1;SOS2;TP53
 
 
 
 
Breast cancer
 
 
48/147
 
 
0.0000000
 
 
0.0000002
 
 
0
 
 
0
 
 
3.030746
 
 
57.6834521
 
 
RB1;CDKN1A;PTEN;LRP5;PIK3R3;PIK3R1;FGF2;IGF1R;DLL4;FGF7;NRAS;FRAT2;CCND1;AKT3;HEY2;DVL3;E2F3;BAK1;WNT1;WNT4;NCOA1;WNT10B;TCF7L2;MAP2K1;FZD3;FZD5;FZD4;GADD45A;WNT3A;CSNK1A1;FZD6;WNT9B;WNT7A;IGF1;AXIN2;FOS;WNT9A;ESR1;GADD45G;PIK3CA;APC;FGF18;GRB2;KRAS;RAF1;SOS1;TP53;SOS2
 
 
 
 
Focal adhesion
 
 
59/201
 
 
0.0000000
 
 
0.0000002
 
 
0
 
 
0
 
 
2.601135
 
 
47.9856457
 
 
ITGB1;PTEN;LAMC1;ARHGAP5;CRKL;MYLK;IGF1R;PPP1CB;PPP1CC;CCND2;CCND1;AKT3;ITGB8;RAC1;VAV3;PDGFRB;MAP2K1;PPP1R12A;COL2A1;COL4A2;PIK3CA;COL4A1;COL4A4;COL6A2;ITGA8;COL4A5;COL6A3;COL6A6;ITGA6;RAF1;SOS1;CRK;SOS2;LAMA2;LAMA4;PDGFB;PIK3R3;PIK3R1;MYL12A;MYL12B;RAP1B;CDC42;MAPK8;RELN;RAP1A;PDGFC;PIP5K1B;PAK5;CAV2;VEGFC;IGF1;VEGFA;COL1A1;COL1A2;ITGA11;COL9A1;BCL2;COL9A3;GRB2
 
 
 
 
Cellular senescence
 
 
49/156
 
 
0.0000000
 
 
0.0000004
 
 
0
 
 
0
 
 
2.862275
 
 
51.4232722
 
 
RB1;LIN54;CDKN1A;SERPINE1;PTEN;PPP1CB;PPP1CC;CCND2;CCND1;RASSF5;AKT3;CHEK1;MYBL2;BTRC;MAP2K1;RRAS2;TSC1;SIRT1;TGFBR1;CCNA2;MRAS;CCNE2;PIK3CA;CCNE1;VDAC1;RAF1;TP53;SLC25A4;PPID;PIK3R3;PIK3R1;FOXO3;ZFP36L2;ZFP36L1;PPP3R1;NRAS;EIF4EBP1;TRPM7;E2F3;E2F5;MAP2K6;SMAD2;TGFB2;TGFB1;GADD45A;GADD45G;CDK1;CALM3;KRAS
 
 
 
 
Ubiquitin mediated proteolysis
 
 
45/140
 
 
0.0000000
 
 
0.0000006
 
 
0
 
 
0
 
 
2.958409
 
 
51.4932482
 
 
CUL5;UBA6;UBE2D2;CUL3;UBE2D3;CUL2;CBLC;UBE2D1;CBLB;RNF7;PRPF19;RCHY1;RHOBTB1;UBE2J1;SOCS3;COP1;CDC23;SOCS1;UBE2Q1;UBE2NL;CDC27;VHL;BTRC;SKP1;UBE2F;UBE2H;UBE2I;MAP3K1;SMURF2;UBE2B;FBXW7;SMURF1;UBE2E3;SIAH1;UBE4A;UBE4B;UBE2G2;PIAS2;UBE2W;CDC34;UBE2R2;NEDD4;UBE2N;UBE2K;CUL4B
 
 
 
 
Prostate cancer
 
 
35/97
 
 
0.0000000
 
 
0.0000008
 
 
0
 
 
0
 
 
3.519658
 
 
60.2293239
 
 
RB1;CDKN1A;PDGFB;PTEN;PIK3R3;TGFA;TMPRSS2;PIK3R1;IGF1R;HSP90B1;IKBKB;NRAS;CCND1;AKT3;PDGFC;EP300;E2F3;PDGFRB;TCF7L2;MAP2K1;CHUK;IGF1;CREB1;CCNE2;PIK3CA;CCNE1;BCL2;GRB2;KRAS;ERG;RAF1;SOS1;TP53;SOS2;CREB5
 
 
 
 
Human papillomavirus infection
 
 
82/331
 
 
0.0000001
 
 
0.0000016
 
 
0
 
 
0
 
 
2.066149
 
 
33.6450700
 
 
RB1;ITGB1;CDKN1A;MAML1;PTEN;PPP2R2A;FASLG;LAMC1;TNF;IKBKB;CCND2;CCND1;CASP3;AKT3;HEY2;EP300;ITGB8;ATP6V1E1;IKBKE;PDGFRB;MAP2K1;CHUK;WNT9B;TSC1;WNT9A;AXIN2;TNFRSF1A;CCNA2;COL2A1;CREB1;CCNE2;COL4A2;PIK3CA;CCNE1;COL4A1;COL4A4;COL6A2;ITGA8;TBPL1;COL4A5;COL6A3;COL6A6;ITGA6;RAF1;SOS1;TP53;SOS2;CREB5;LAMA2;LAMA4;PIK3R3;PIK3R1;PPP2CA;CDC42;NRAS;PARD6B;RELN;EIF4EBP1;DVL3;BAK1;WNT1;WNT4;WNT10B;TCF7L2;FZD3;FZD5;FZD4;CSNK1A1;WNT3A;FZD6;WNT7A;VEGFA;COL1A1;COL1A2;APC;ITGA11;COL9A1;FAS;COL9A3;GRB2;KRAS;PALS1
 
 
 
 
Autophagy
 
 
43/137
 
 
0.0000001
 
 
0.0000021
 
 
0
 
 
0
 
 
2.855082
 
 
45.5082926
 
 
BECN1;PRKAA1;MTMR3;PRKAA2;STX17;MTMR14;PTEN;PIK3R3;IRS2;PIK3R1;IGF1R;PPP2CA;MAPK8;NRAS;DEPTOR;RB1CC1;AKT3;NRBF2;MAP3K7;SNAP29;RAB8A;ATG5;SH3GLB1;RUBCN;GABARAPL1;MAP2K1;ATG9A;PRKCD;RAB39B;RRAS2;TSC1;WIPI2;ATG14;EIF2S1;VAMP8;VMP1;MRAS;PIK3CA;DDIT4;BCL2;ULK2;KRAS;RAF1
 
 
 
 
p53 signaling pathway
 
 
28/73
 
 
0.0000002
 
 
0.0000032
 
 
0
 
 
0
 
 
3.873435
 
 
59.9143691
 
 
CDKN1A;SERPINE1;PTEN;PPM1D;RCHY1;COP1;CCND2;CCND1;ZMAT3;CASP3;SESN1;CHEK1;SESN2;PMAIP1;RRM2;GADD45A;SIAH1;IGF1;GADD45G;CCNE2;CCNE1;CCNG1;BCL2;CDK1;CYCS;FAS;MDM4;TP53
 
 
 
 
Small cell lung cancer
 
 
32/92
 
 
0.0000004
 
 
0.0000056
 
 
0
 
 
0
 
 
3.322004
 
 
49.2044583
 
 
ITGB1;RB1;CDKN1A;LAMA2;LAMA4;PTEN;PIK3R3;LAMC1;PIK3R1;IKBKB;CCND1;CASP3;AKT3;E2F3;BAK1;CHUK;GADD45A;GADD45G;CCNE2;COL4A2;PIK3CA;TRAF4;CCNE1;COL4A1;COL4A4;TRAF5;BCL2;COL4A5;RARB;CYCS;ITGA6;TP53
 
 
 
 
Glioma
 
 
28/75
 
 
0.0000004
 
 
0.0000056
 
 
0
 
 
0
 
 
3.708176
 
 
54.9138783
 
 
RB1;CDKN1A;PTEN;PDGFB;PIK3R3;TGFA;PIK3R1;IGF1R;NRAS;CCND1;AKT3;E2F3;BAK1;PDGFRB;MAP2K1;CAMK1D;GADD45A;IGF1;GADD45G;PIK3CA;CAMK4;CALM3;GRB2;KRAS;RAF1;SOS1;SOS2;TP53
 
 
 
 
Neurotrophin signaling pathway
 
 
38/119
 
 
0.0000004
 
 
0.0000057
 
 
0
 
 
0
 
 
2.924929
 
 
43.1669269
 
 
BEX3;ZNF274;PRDM4;PIK3R3;FASLG;PIK3R1;FOXO3;CRKL;RAP1B;IKBKB;CDC42;RPS6KA3;RPS6KA6;MAPK8;NRAS;RAP1A;IRAK2;AKT3;NTF3;RAC1;MAP3K5;MAP2K1;MAP3K1;BDNF;PRKCD;FRS2;NGF;PIK3CA;CAMK4;BCL2;CALM3;GRB2;KRAS;RAF1;SOS1;TP53;CRK;SOS2
 
 
 
 
Pancreatic cancer
 
 
28/76
 
 
0.0000005
 
 
0.0000071
 
 
0
 
 
0
 
 
3.630711
 
 
52.6129235
 
 
RB1;CDKN1A;RALB;PIK3R3;TGFA;PIK3R1;IKBKB;CDC42;MAPK8;CCND1;AKT3;E2F3;RAC1;BAK1;SMAD2;MAP2K1;RALBP1;TGFB2;TGFB1;CHUK;GADD45A;TGFBR1;GADD45G;VEGFA;PIK3CA;KRAS;RAF1;TP53
 
 
 
 
Hepatocellular carcinoma
 
 
48/168
 
 
0.0000005
 
 
0.0000072
 
 
0
 
 
0
 
 
2.497297
 
 
36.0279156
 
 
RB1;CDKN1A;PTEN;LRP5;PIK3R3;TGFA;PIK3R1;IGF1R;NRAS;FRAT2;CCND1;AKT3;DVL3;E2F3;BAK1;ARID2;WNT1;WNT4;SMAD2;WNT10B;TCF7L2;MAP2K1;FZD3;TGFB2;TGFB1;FZD5;FZD4;GADD45A;WNT3A;CSNK1A1;TXNRD1;FZD6;WNT9B;WNT7A;AXIN2;WNT9A;ARID1B;TGFBR1;GADD45G;PIK3CA;APC;GSTA4;GRB2;KRAS;RAF1;SOS1;TP53;SOS2
 
 
 
 
TGF-beta signaling pathway
 
 
32/94
 
 
0.0000006
 
 
0.0000082
 
 
0
 
 
0
 
 
3.214468
 
 
45.8327935
 
 
CHRD;TNF;PPP2CA;ACVR1C;EP300;PITX2;E2F5;SKP1;ACVR1;SMAD2;TGFB2;TGFB1;SMURF2;SMURF1;FST;NOG;INHBB;GDF6;ACVR2B;GDF5;SMAD5;TGFBR1;RGMA;BMP5;SMAD7;GREM1;TFDP1;IFNG;ID1;ID4;ID3;FBN1
 
 
 
 
Chronic myeloid leukemia
 
 
27/76
 
 
0.0000018
 
 
0.0000225
 
 
0
 
 
0
 
 
3.428150
 
 
45.2605626
 
 
RB1;CDKN1A;PIK3R3;PIK3R1;CRKL;IKBKB;NRAS;CCND1;AKT3;E2F3;BAK1;MAP2K1;TGFB2;TGFB1;CHUK;GADD45A;TGFBR1;RUNX1;GADD45G;PIK3CA;GRB2;KRAS;RAF1;SOS1;CRK;SOS2;TP53
 
 
 
 
Melanoma
 
 
26/72
 
 
0.0000020
 
 
0.0000235
 
 
0
 
 
0
 
 
3.515816
 
 
46.1358217
 
 
RB1;CDKN1A;PTEN;PDGFB;PIK3R3;PIK3R1;FGF2;IGF1R;FGF7;NRAS;CCND1;AKT3;PDGFC;E2F3;BAK1;PDGFRB;MAP2K1;GADD45A;MITF;IGF1;GADD45G;PIK3CA;FGF18;KRAS;RAF1;TP53
 
 
 
 
Ras signaling pathway
 
 
59/232
 
 
0.0000022
 
 
0.0000243
 
 
0
 
 
0
 
 
2.131159
 
 
27.8087618
 
 
FLT3;FASLG;FGF2;IGF1R;IKBKB;FGF7;RASSF5;AKT3;RAC1;PDGFRB;MAP2K1;RALBP1;PLA2G12A;CHUK;RRAS2;NGF;TIAM1;MRAS;PIK3CA;RASA1;RAF1;SOS1;SOS2;EPHA2;ARF6;RALB;PDGFB;PIK3R3;PLA2G3;TGFA;RASAL1;PIK3R1;FOXO4;EFNA5;RASGRP1;RAP1B;CDC42;GNG10;NRAS;MAPK8;RAP1A;GNG2;GNG5;PDGFC;NTF3;SHOC2;PAK5;BDNF;VEGFC;IGF1;GNG12;VEGFA;KITLG;FGF18;CALM3;GRB2;KRAS;TEK;RGL2
 
 
 
 
AGE-RAGE signaling pathway in diabetic complications
 
 
32/100
 
 
0.0000030
 
 
0.0000325
 
 
0
 
 
0
 
 
2.929813
 
 
37.2761338
 
 
SERPINE1;PIK3R3;PIK3R1;TNF;CDC42;MAPK8;NRAS;CCND1;CASP3;AKT3;PIM1;CCL2;RAC1;SMAD2;TGFB2;EDN1;TGFB1;PRKCD;VEGFC;F3;TGFBR1;VEGFA;COL1A1;COL3A1;COL1A2;COL4A2;PIK3CA;COL4A1;COL4A4;BCL2;COL4A5;KRAS
 
 
 
 
Cell cycle
 
 
37/124
 
 
0.0000034
 
 
0.0000360
 
 
0
 
 
0
 
 
2.649653
 
 
33.3445856
 
 
RB1;CDKN1A;CCNH;YWHAB;TTK;CDC14A;CCND2;CDC23;CCND1;YWHAQ;CDC27;CHEK1;ORC2;EP300;E2F3;E2F5;SKP1;YWHAH;SMAD2;TGFB2;TGFB1;GADD45A;SMC1A;CDC25B;GADD45G;CCNA2;STAG1;WEE1;STAG2;DBF4;TFDP1;CCNE2;ESPL1;TFDP2;CCNE1;CDK1;TP53
 
 
 
 
Relaxin signaling pathway
 
 
38/129
 
 
0.0000036
 
 
0.0000364
 
 
0
 
 
0
 
 
2.601988
 
 
32.6312215
 
 
GNAI3;PIK3R3;ADCY3;ADCY2;PIK3R1;GNAI1;GNG10;MAPK8;NRAS;GNG2;GNG5;AKT3;SMAD2;MAP2K4;MAP2K1;EDN1;TGFB1;VEGFC;FOS;GNG12;TGFBR1;VEGFA;COL1A1;ACTA2;COL3A1;COL1A2;CREB1;COL4A2;PIK3CA;COL4A1;COL4A4;COL4A5;GRB2;KRAS;RAF1;SOS1;SOS2;CREB5
 
 
 
 
Endometrial cancer
 
 
22/58
 
 
0.0000048
 
 
0.0000469
 
 
0
 
 
0
 
 
3.797998
 
 
46.5399792
 
 
TCF7L2;CDKN1A;MAP2K1;GADD45A;PTEN;PIK3R3;AXIN2;PIK3R1;FOXO3;GADD45G;NRAS;CCND1;PIK3CA;APC;AKT3;GRB2;KRAS;BAK1;RAF1;SOS1;SOS2;TP53
 
 
 
 
TNF signaling pathway
 
 
34/112
 
 
0.0000055
 
 
0.0000525
 
 
0
 
 
0
 
 
2.714222
 
 
32.8550056
 
 
PIK3R3;TNFAIP3;CXCL1;PIK3R1;TNF;CXCL2;IKBKB;SOCS3;BAG4;CASP7;MAPK8;CASP3;AKT3;CCL2;MAP3K7;MAP3K5;MAP2K6;MAP2K4;MAP2K1;EDN1;CHUK;IL15;LIF;VEGFC;FOS;TNFRSF1B;TNFRSF1A;CXCL10;CREB1;PIK3CA;BCL3;TRAF5;FAS;CREB5
 
 
 
 
Wnt signaling pathway
 
 
45/166
 
 
0.0000057
 
 
0.0000525
 
 
0
 
 
0
 
 
2.319190
 
 
28.0121873
 
 
INVS;LRP5;NKD1;PPP3R1;MAPK8;CCND2;FRAT2;CCND1;WIF1;RSPO2;CCN4;EP300;DVL3;RSPO3;RAC1;GPC4;BTRC;WNT1;MAP3K7;SKP1;WNT4;TLE4;WNT10B;TCF7L2;FZD3;FZD5;FZD4;WNT3A;CSNK1A1;CTNNBIP1;FZD6;SIAH1;WNT9B;WNT7A;CACYBP;AXIN2;WNT9A;SENP2;DKK2;SFRP1;APC;TBL1XR1;ROR1;TP53;LGR4
 
 
 
 
Non-small cell lung cancer
 
 
25/72
 
 
0.0000070
 
 
0.0000631
 
 
0
 
 
0
 
 
3.307274
 
 
39.2404312
 
 
RB1;CDKN1A;PIK3R3;TGFA;PIK3R1;FOXO3;NRAS;CCND1;KIF5C;RASSF5;AKT3;E2F3;BAK1;MAP2K1;GADD45A;GADD45G;EML4;PIK3CA;RARB;GRB2;KRAS;RAF1;SOS1;SOS2;TP53
 
 
 
 
AMPK signaling pathway
 
 
35/120
 
 
0.0000108
 
 
0.0000945
 
 
0
 
 
0
 
 
2.563838
 
 
29.3085181
 
 
PRKAA1;PRKAA2;PIK3R3;PRKAG2;IRS2;PPP2R2A;PIK3R1;HMGCR;FOXO3;ADIPOR2;IGF1R;PPP2CA;EEF2K;CCND1;AKT3;EIF4EBP1;MAP3K7;RAB8A;SREBF1;PRKAB2;STRADB;TSC1;IGF1;SIRT1;CCNA2;RAB10;G6PC2;CREB1;PIK3CA;RAB14;SCD;FASN;LEP;PPARG;CREB5
 
 
 
 
Prolactin signaling pathway
 
 
24/70
 
 
0.0000136
 
 
0.0001132
 
 
0
 
 
0
 
 
3.243019
 
 
36.3316499
 
 
MAP2K1;PIK3R3;FOS;PIK3R1;TNFRSF11A;FOXO3;ESR1;SOCS2;SOCS3;MAPK8;NRAS;CCND2;SOCS1;CCND1;PIK3CA;ELF5;AKT3;GRB2;KRAS;RAF1;SOS1;SOCS6;SOS2;SOCS4
 
 
 
 
Longevity regulating pathway
 
 
31/102
 
 
0.0000137
 
 
0.0001132
 
 
0
 
 
0
 
 
2.716868
 
 
30.4169453
 
 
PRKAA1;PRKAA2;PIK3R3;PRKAG2;ADCY3;IRS2;ADCY2;PIK3R1;FOXO3;ADIPOR2;IGF1R;NRAS;SESN1;RB1CC1;AKT3;SESN2;EIF4EBP1;ATG5;PRKAB2;TSC1;IGF1;SIRT1;CREB1;PIK3CA;CAMK4;CAT;PPARG;KRAS;EIF4E2;TP53;CREB5
 
 
 
 
Hepatitis B
 
 
43/162
 
 
0.0000160
 
 
0.0001286
 
 
0
 
 
0
 
 
2.251981
 
 
24.8656382
 
 
RB1;CDKN1A;DDX3X;YWHAB;PIK3R3;FASLG;PIK3R1;TNF;IKBKB;MAPK8;NRAS;YWHAQ;CASP3;AKT3;EP300;E2F3;MAP3K7;IKBKE;MAP2K6;MAP2K4;MAP2K1;TGFB2;EGR3;TGFB1;MAP3K1;CHUK;FOS;TGFBR1;CCNA2;CREB1;CCNE2;PIK3CA;CCNE1;BCL2;CYCS;FAS;GRB2;KRAS;RAF1;SOS1;TP53;SOS2;CREB5
 
 
 
 
Insulin signaling pathway
 
 
38/137
 
 
0.0000167
 
 
0.0001307
 
 
0
 
 
0
 
 
2.390609
 
 
26.2959385
 
 
PRKAA1;PRKAA2;PIK3R3;PRKAG2;CBLB;IRS2;PIK3R1;CRKL;PPP1CB;SOCS2;IKBKB;PPP1CC;SOCS3;MAPK8;NRAS;SOCS1;PRKAR2A;MKNK1;AKT3;EIF4EBP1;SOCS4;SREBF1;MAP2K1;PRKAB2;RPS6;PHKA1;TSC1;G6PC2;PIK3CA;FASN;CALM3;GRB2;KRAS;RAF1;EIF4E2;SOS1;CRK;SOS2
 
 
 
 
Hippo signaling pathway
 
 
43/163
 
 
0.0000189
 
 
0.0001439
 
 
0
 
 
0
 
 
2.233084
 
 
24.2918067
 
 
YAP1;YWHAB;SERPINE1;PPP2R2A;LIMD1;NKD1;AMOT;PPP1CB;PPP2CA;PPP1CC;PARD6B;CCND2;CCND1;YWHAQ;DVL3;CCN2;BTRC;WNT1;WNT4;YWHAH;SMAD2;WNT10B;TCF7L2;FZD3;TGFB2;TGFB1;FZD5;FZD4;WNT3A;FZD6;WNT9B;WNT7A;AXIN2;WNT9A;GDF6;GDF5;TGFBR1;BMP5;SMAD7;MOB1A;APC;ID1;PALS1
 
 
 
 
Basal cell carcinoma
 
 
22/63
 
 
0.0000222
 
 
0.0001648
 
 
0
 
 
0
 
 
3.333857
 
 
35.7249993
 
 
TCF7L2;WNT10B;CDKN1A;FZD3;FZD5;FZD4;GADD45A;WNT3A;HHIP;FZD6;WNT9B;WNT7A;AXIN2;WNT9A;GADD45G;SHH;APC;DVL3;BAK1;WNT1;TP53;WNT4
 
 
 
 
MicroRNAs in cancer
 
 
70/310
 
 
0.0000227
 
 
0.0001648
 
 
0
 
 
0
 
 
1.822809
 
 
19.4917894
 
 
TRIM71;CDKN1A;PTEN;IRS2;BMI1;CRKL;IKBKB;CCND2;CCND1;CASP3;STMN1;PIM1;DNMT3B;EP300;PDGFRB;MAP2K1;TPM1;DNMT3A;KIF23;DICER1;SIRT1;CDC25B;MMP16;CCNE2;PIK3CA;CCNE1;DDIT4;FSCN1;COMMD3-BMI1;RAF1;SOS1;TP53;CRK;SOS2;HDAC4;DNMT1;GLS2;PDGFB;PIK3R3;PIK3R1;EFNA5;NRAS;BCL2L11;SOCS1;ERBB3;IGF2BP1;BMF;E2F3;BAK1;MCL1;FZD3;ST14;TGFB2;UBE2I;WNT3A;RDX;HMGA2;VEGFA;MARCKS;HNRNPK;APC;CCNG1;BCL2;SPRY2;GRB2;MDM4;KRAS;VIM;RECK;BCL2L2
 
 
 
 
Endocytosis
 
 
59/252
 
 
0.0000323
 
 
0.0002291
 
 
0
 
 
0
 
 
1.908071
 
 
19.7302698
 
 
ARF4;TFRC;WIPF1;SNX12;CBLC;CBLB;AP2A1;RAB22A;IGF1R;EEA1;CAPZB;KIF5C;CHMP1B;VPS36;RAB8A;SH3GL2;SH3GLB1;USP8;ACTR2;IST1;VPS37C;VPS37D;TGFBR1;BIN1;RUFY2;CHMP4B;ARF5;ARF6;VPS4A;ASAP1;ASAP2;SNX3;CDC42;CYTH3;CYTH2;ACTR3C;PARD6B;SNX2;PIP5K1B;EPS15;LDLR;RAB11FIP4;SNX5;CYTH1;SNX6;SMAD2;ARFGEF1;SMURF2;CAV2;SMURF1;VTA1;AP2B1;RAB10;EHD2;CAPZA1;NEDD4;CAPZA2;CHMP2B;SMAP1
 
 
 
 
Protein digestion and absorption
 
 
30/103
 
 
0.0000462
 
 
0.0003201
 
 
0
 
 
0
 
 
2.555968
 
 
25.5161131
 
 
COL15A1;COL16A1;COL13A1;ELN;COL11A1;COL19A1;KCNN4;COL24A1;COL25A1;COL22A1;ATP1B4;SLC16A10;COL1A1;COL3A1;COL1A2;COL2A1;COL4A2;COL5A1;COL4A1;COL4A4;COL6A2;COL7A1;COL5A2;COL9A1;COL4A5;COL8A1;COL21A1;COL6A3;COL9A3;COL6A6
 
 
 
 
Acute myeloid leukemia
 
 
22/67
 
 
0.0000653
 
 
0.0004423
 
 
0
 
 
0
 
 
3.036807
 
 
29.2661568
 
 
TCF7L2;MAP2K1;CHUK;FLT3;ZBTB16;PIK3R3;PIK3R1;RUNX1;IKBKB;CCNA2;NRAS;CCND1;PIK3CA;AKT3;PIM1;RARA;EIF4EBP1;GRB2;KRAS;RAF1;SOS1;SOS2
 
 
 
 
Human cytomegalovirus infection
 
 
53/225
 
 
0.0000668
 
 
0.0004432
 
 
0
 
 
0
 
 
1.921447
 
 
18.4712436
 
 
RB1;CDKN1A;FASLG;TNF;CRKL;IKBKB;CCND1;CASP3;AKT3;RAC1;PDIA3;MAP2K1;CHUK;TAP1;TSC1;TNFRSF1A;CREB1;PIK3CA;TRAF5;RAF1;SOS1;TP53;CRK;SOS2;CREB5;CCL3L1;CCL3L3;PTGER3;GNAI3;PIK3R3;ADCY3;ADCY2;PIK3R1;GNAI1;GNA13;GNG10;PPP3R1;NRAS;GNG2;GNG5;EIF4EBP1;CCL3;CCL2;E2F3;BAK1;MAP2K6;GNG12;VEGFA;FAS;CYCS;CALM3;GRB2;KRAS
 
 
 
 
Chagas disease
 
 
29/102
 
 
0.0000989
 
 
0.0006420
 
 
0
 
 
0
 
 
2.469873
 
 
22.7750569
 
 
CCL3L1;CCL3L3;SERPINE1;GNAI3;PIK3R3;PPP2R2A;FASLG;PIK3R1;TNF;GNAI1;IKBKB;PPP2CA;MAPK8;AKT3;CCL3;IL12B;CCL2;SMAD2;IL10;MAP2K4;TGFB2;TGFB1;CHUK;FOS;TGFBR1;TNFRSF1A;IFNG;PIK3CA;FAS
 
 
 
 
Human immunodeficiency virus 1 infection
 
 
50/212
 
 
0.0001023
 
 
0.0006503
 
 
0
 
 
0
 
 
1.923598
 
 
17.6724523
 
 
FASLG;TNF;CRKL;IKBKB;CFL2;CASP3;AKT3;CHEK1;AP1S1;AP1S3;RAC1;BTRC;MAP3K7;SKP1;PDIA3;MAP2K1;CHUK;TAP1;FOS;TNFRSF1B;TNFRSF1A;PIK3CA;TRAF5;RAF1;CRK;CUL5;GNAI3;PIK3R3;PIK3R1;RNF7;GNAI1;GNG10;PPP3R1;NRAS;MAPK8;GNG2;GNG5;BAK1;PAK5;MAP2K6;LIMK1;GNG12;WEE1;BCL2;CDK1;FAS;CYCS;CALM3;KRAS;CUL4B
 
 
 
 
Kaposi sarcoma-associated herpesvirus infection
 
 
46/193
 
 
0.0001462
 
 
0.0009098
 
 
0
 
 
0
 
 
1.949158
 
 
17.2125892
 
 
RB1;CD86;BECN1;CDKN1A;PDGFB;PIK3R3;CXCL1;PIK3R1;FGF2;CXCL2;IKBKB;GNG10;PPP3R1;NRAS;MAPK8;GNG2;CCND1;GNG5;CASP3;AKT3;EP300;E2F3;RAC1;BAK1;IKBKE;MAP2K6;MAP2K4;TCF7L2;GABARAPL1;MAP2K1;CHUK;FOS;ATG14;GNG12;VEGFA;TNFRSF1A;CD200R1;CREB1;PIK3CA;FAS;CYCS;CALM3;KRAS;IL6ST;RAF1;TP53
 
 
 
 
Apoptosis
 
 
36/142
 
 
0.0002061
 
 
0.0012295
 
 
0
 
 
0
 
 
2.112823
 
 
17.9321293
 
 
HRK;BECN1;PIK3R3;FASLG;PIK3R1;TNF;CTSS;IKBKB;CASP7;MAPK8;NRAS;BCL2L11;TUBA1A;AIFM1;CASP3;AKT3;PMAIP1;BAK1;SPTAN1;MAP3K5;MCL1;MAP2K1;CHUK;GADD45A;FOS;NGF;EIF2S1;GADD45G;TNFRSF1A;PIK3CA;BCL2;CYCS;FAS;KRAS;RAF1;TP53
 
 
 
 
Regulation of actin cytoskeleton
 
 
50/218
 
 
0.0002118
 
 
0.0012295
 
 
0
 
 
0
 
 
1.854245
 
 
15.6866340
 
 
ITGB1;FGF2;CRKL;MYLK;PPP1CB;PPP1CC;FGF7;TMSB4X;CFL2;ITGB8;PIP4K2B;RAC1;VAV3;PDGFRB;ACTR2;MAP2K1;PPP1R12A;RRAS2;TIAM1;MRAS;PIK3CA;ITGA8;ITGA6;RAF1;SOS1;CRK;SOS2;PFN2;PDGFB;PIK3R3;PIK3R1;MYL12A;MYL12B;CDC42;GNA13;ACTR3C;NRAS;PDGFC;PIP5K1B;WASF1;PAK5;RDX;LIMK1;GNG12;SSH1;DIAPH2;APC;FGF18;ITGA11;KRAS
 
 
 
 
Shigellosis
 
 
55/246
 
 
0.0002127
 
 
0.0012295
 
 
0
 
 
0
 
 
1.794914
 
 
15.1774624
 
 
ITGB1;UBE2D2;UBE2D3;UBE2D1;FNBP1L;TNF;SEPTIN11;CRKL;IKBKB;AKT3;RAC1;BTRC;MAP3K7;SKP1;H3-3B;CAST;ACTR2;GABARAPL1;CHUK;PRKCD;WIPI2;ATG14;TNFRSF1A;PIK3CA;TRAF5;UBE2V2;VDAC1;UBE2V1;TP53;CRK;PPID;PFN2;ARF6;BECN1;PIK3R3;PIK3R1;FOXO4;FOXO3;MYL12A;MYL12B;CDC42;CYTH3;CYTH2;ACTR3C;MAPK8;UBE2NL;WASF1;CYTH1;ATG5;SEPTIN2;CBX3;H3-5;BCL2;UBE2N;CYCS
 
 
 
 
Apelin signaling pathway
 
 
35/137
 
 
0.0002137
 
 
0.0012295
 
 
0
 
 
0
 
 
2.134411
 
 
18.0381927
 
 
HDAC4;BECN1;PRKAA1;PRKAA2;SERPINE1;GNAI3;PRKAG2;ADCY3;ADCY2;GNAI1;MYLK;GNA13;GNG10;NRAS;GNG2;CCND1;GNG5;AKT3;CCN2;MEF2A;SMAD2;GABARAPL1;MAP2K1;PRKAB2;RPS6;RRAS2;GNG12;TGFBR1;APLN;ACTA2;MRAS;CAMK4;CALM3;KRAS;RAF1
 
 
 
 
Thyroid cancer
 
 
14/37
 
 
0.0002604
 
 
0.0014707
 
 
0
 
 
0
 
 
3.774923
 
 
31.1558441
 
 
TCF7L2;CDKN1A;MAP2K1;GADD45A;TPM3;GADD45G;NRAS;CCND1;TPR;CCDC6;KRAS;PPARG;BAK1;TP53
 
 
 
 
Fluid shear stress and atherosclerosis
 
 
35/139
 
 
0.0002883
 
 
0.0015844
 
 
0
 
 
0
 
 
2.093120
 
 
17.0620241
 
 
PRKAA1;PRKAA2;PDGFB;PIK3R3;PIK3R1;TNF;TXN2;HSP90B1;IKBKB;MAPK8;SUMO3;AKT3;CCL2;RAC1;MAP3K7;MAP3K5;MAP2K6;ACVR1;MEF2A;MAP2K4;EDN1;CHUK;CAV2;DUSP1;FOS;ACVR2B;TNFRSF1A;VEGFA;IFNG;PIK3CA;GSTA4;BCL2;SDC1;CALM3;TP53
 
 
 
 
Cushing syndrome
 
 
38/155
 
 
0.0002909
 
 
0.0015844
 
 
0
 
 
0
 
 
2.020696
 
 
16.4534972
 
 
RB1;CDKN1A;GNAI3;ADCY3;KCNA4;ASH2L;ADCY2;AHR;GNAI1;RAP1B;RAP1A;RASD1;CCND1;DVL3;E2F3;WNT1;LDLR;WNT4;USP8;WNT10B;TCF7L2;MAP2K1;FZD3;FZD5;FZD4;WNT3A;FZD6;WNT9B;WNT7A;AXIN2;WNT9A;PBX1;CREB1;CCNE2;APC;CCNE1;KCNK2;CREB5
 
 
 
 
Rap1 signaling pathway
 
 
48/210
 
 
0.0003047
 
 
0.0016304
 
 
0
 
 
0
 
 
1.845305
 
 
14.9399201
 
 
ITGB1;RALB;PDGFB;GNAI3;PIK3R3;ADCY3;ADCY2;PIK3R1;EFNA5;FGF2;GNAI1;CRKL;IGF1R;RAP1B;CDC42;NRAS;FGF7;PARD6B;RAP1A;CNR1;RASSF5;AKT3;PDGFC;RAC1;MAP2K6;VAV3;PDGFRB;MAP2K1;VEGFC;IGF1;NGF;VEGFA;TIAM1;KITLG;MRAS;PIK3CA;ADORA2B;ID1;FGF18;RAPGEF2;CALM3;KRAS;TEK;RAF1;CRK;EPHA2;PFN2;RAPGEF4
 
 
 
 
Growth hormone synthesis, secretion and action
 
 
31/119
 
 
0.0003307
 
 
0.0017388
 
 
0
 
 
0
 
 
2.189845
 
 
17.5503484
 
 
GNAI3;PIK3R3;ADCY3;IRS2;ADCY2;PIK3R1;GNAI1;CRKL;SOCS2;GHR;SOCS3;MAPK8;NRAS;SOCS1;AKT3;EP300;MAP2K6;MAP2K4;MAP2K1;MAP3K1;IGF1;FOS;CREB1;PIK3CA;GRB2;KRAS;RAF1;SOS1;CRK;SOS2;CREB5
 
 
 
 
T cell receptor signaling pathway
 
 
28/104
 
 
0.0003520
 
 
0.0018197
 
 
0
 
 
0
 
 
2.289340
 
 
18.2045553
 
 
PIK3R3;CBLB;PIK3R1;TNF;RASGRP1;IKBKB;CDC42;PPP3R1;MAPK8;NRAS;GRAP2;AKT3;ICOS;MAP3K7;PAK5;IL10;VAV3;MAP2K1;CHUK;FOS;IFNG;PIK3CA;CD28;GRB2;KRAS;RAF1;SOS1;SOS2
 
 
 
 
Hepatitis C
 
 
38/157
 
 
0.0003822
 
 
0.0019428
 
 
0
 
 
0
 
 
1.986502
 
 
15.6330053
 
 
RB1;CDKN1A;YWHAB;PIK3R3;PPP2R2A;FASLG;PIK3R1;CLDN1;TNF;IKBKB;PPP2CA;SOCS3;NRAS;CCND1;YWHAQ;CASP3;AKT3;E2F3;BAK1;IKBKE;LDLR;YWHAH;MAP2K1;CHUK;EIF2S1;TNFRSF1A;CXCL10;IFNG;PIK3CA;CLDN14;CYCS;FAS;GRB2;KRAS;RAF1;SOS1;TP53;SOS2
 
 
 
 
Human T-cell leukemia virus 1 infection
 
 
49/219
 
 
0.0004479
 
 
0.0022393
 
 
0
 
 
0
 
 
1.794915
 
 
13.8406388
 
 
RB1;NRP1;CDKN1A;PTEN;SLC2A1;TNF;ELK4;IKBKB;CDC23;CCND2;CCND1;AKT3;CDC27;CHEK1;EP300;MAP2K4;MAP2K1;CHUK;IL15;FOS;TGFBR1;TNFRSF1A;CCNA2;CREB1;CCNE2;ESPL1;PIK3CA;CCNE1;CANX;TBPL1;VDAC1;TP53;SLC25A4;HLA-DQB1;CREB5;PIK3R3;ADCY3;ADCY2;PIK3R1;PPP3R1;NRAS;MAPK8;E2F3;SMAD2;TGFB2;MAP3K1;TGFB1;KRAS;TCF3
 
 
 
 
Phospholipase D signaling pathway
 
 
36/148
 
 
0.0004854
 
 
0.0023878
 
 
0
 
 
0
 
 
1.998935
 
 
15.2529701
 
 
DGKD;RALB;PDGFB;PIK3R3;ADCY3;ADCY2;PIK3R1;AGPAT3;AGPAT4;GRM3;CYTH3;GNA13;CYTH2;NRAS;GRM7;AKT3;PDGFC;PIP5K1B;CYTH1;PDGFRB;MAP2K1;RRAS2;TSC1;KITLG;MRAS;PIK3CA;LPAR6;GRB2;KRAS;RAF1;SOS1;SOS2;PLPP1;DGKH;ARF6;RAPGEF4
 
 
 
 
Melanogenesis
 
 
27/101
 
 
0.0005024
 
 
0.0024321
 
 
0
 
 
0
 
 
2.266685
 
 
17.2181008
 
 
GNAI3;ADCY3;ADCY2;GNAI1;NRAS;EP300;DVL3;WNT1;WNT4;WNT10B;TCF7L2;MAP2K1;FZD3;EDN1;FZD5;FZD4;WNT3A;FZD6;WNT9B;WNT7A;MITF;WNT9A;KITLG;CREB1;CALM3;KRAS;RAF1
 
 
 
 
Choline metabolism in cancer
 
 
26/98
 
 
0.0007162
 
 
0.0034130
 
 
0
 
 
0
 
 
2.242814
 
 
16.2415368
 
 
DGKD;PDGFB;PIK3R3;PIK3R1;MAPK8;NRAS;AKT3;PDGFC;EIF4EBP1;PIP5K1B;RAC1;WASF1;PDGFRB;MAP2K1;LYPLA1;TSC1;FOS;PIK3CA;GRB2;CHPT1;KRAS;RAF1;SOS1;SOS2;PLPP1;DGKH
 
 
 
 
Salmonella infection
 
 
53/249
 
 
0.0009362
 
 
0.0043929
 
 
0
 
 
0
 
 
1.683793
 
 
11.7422431
 
 
CSE1L;GCC2;PIK3C2A;TNF;TXN2;IKBKB;CASP7;TUBA1A;CASP5;KIF5C;CASP3;AKT3;RAC1;KPNA3;MAP3K7;SKP1;KPNA1;MAP2K4;ACTR2;MAP2K1;CHUK;DYNLT1;FOS;DYNLL1;DYNLL2;RHOB;TNFRSF1A;DYNC1LI2;TUBB2A;PIK3CA;RAF1;PFN2;ARF6;ARL8B;DYNC1I2;MYL12A;MYL12B;HSP90B1;CDC42;CYTH3;CYTH2;ACTR3C;MAPK8;BAK1;DYNC1I1;CYTH1;MAP2K6;TCF7L2;SNX18;BCL2;RAB9A;CYCS;RAB9B
 
 
 
 
SNARE interactions in vesicular transport
 
 
12/33
 
 
0.0010736
 
 
0.0049374
 
 
0
 
 
0
 
 
3.541662
 
 
24.2135514
 
 
VAMP8;VAMP7;STX17;STX16;GOSR2;STX6;STX3;STX2;SNAP29;STX11;VTI1B;BET1
 
 
 
 
Osteoclast differentiation
 
 
31/127
 
 
0.0010846
 
 
0.0049374
 
 
0
 
 
0
 
 
2.006420
 
 
13.6968963
 
 
PIK3R3;TNFRSF11B;PIK3R1;TNFRSF11A;TNF;IKBKB;SOCS3;PPP3R1;MAPK8;CALCR;SOCS1;AKT3;RAC1;MAP3K7;MAP2K6;MAP2K1;TGFB2;TGFB1;CHUK;MITF;FOS;TGFBR1;FOSL2;TNFRSF1A;CREB1;IFNG;PIK3CA;CAMK4;FOSB;GRB2;PPARG
 
 
 
 
Other types of O-glycan biosynthesis
 
 
15/47
 
 
0.0012785
 
 
0.0057344
 
 
0
 
 
0
 
 
2.906554
 
 
19.3636869
 
 
GALNT12;POMT2;B4GALT3;COLGALT2;GALNT5;B4GALT1;GALNT13;POGLUT1;GALNT15;GALNT1;C1GALT1;GXYLT1;GXYLT2;EOGT;POC1B-GALNT4
 
 
 
 
Fc gamma R-mediated phagocytosis
 
 
25/97
 
 
0.0014057
 
 
0.0062134
 
 
0
 
 
0
 
 
2.155771
 
 
14.1574738
 
 
PIK3R3;ASAP1;PIK3R1;ASAP2;CRKL;CDC42;ACTR3C;CFL2;AKT3;PIP5K1B;RAC1;WASF1;VAV3;ACTR2;MAP2K1;MARCKSL1;PRKCD;LIMK1;MARCKS;PIK3CA;BIN1;RAF1;CRK;PLPP1;ARF6
 
 
 
 
Insulin resistance
 
 
27/108
 
 
0.0015132
 
 
0.0065934
 
 
0
 
 
0
 
 
2.069953
 
 
13.4412307
 
 
PRKAA1;PRKAA2;PTEN;SLC2A1;PIK3R3;PRKAG2;IRS2;PIK3R1;TNF;PPP1CB;IKBKB;PPP1CC;RPS6KA3;SOCS3;RPS6KA6;MAPK8;AKT3;SREBF1;PRKAB2;GFPT2;PRKCD;GFPT1;TNFRSF1A;G6PC2;CREB1;PIK3CA;CREB5
 
 
 
 
Sphingolipid signaling pathway
 
 
29/119
 
 
0.0015842
 
 
0.0068054
 
 
0
 
 
0
 
 
2.001354
 
 
12.9040672
 
 
ASAH1;SGMS1;PTEN;GNAI3;PIK3R3;PPP2R2A;PIK3R1;SGMS2;TNF;GNAI1;GNA13;PPP2CA;MAPK8;NRAS;SPTLC1;SMPD1;AKT3;S1PR1;RAC1;MAP3K5;MAP2K1;CERS5;TNFRSF1A;ACER2;PIK3CA;BCL2;KRAS;RAF1;TP53
 
 
 
 
Tight junction
 
 
38/169
 
 
0.0016770
 
 
0.0070362
 
 
0
 
 
0
 
 
1.803266
 
 
11.5242609
 
 
ITGB1;PRKAA1;PRKAA2;PRKAG2;PPP2R2A;CLDN1;YBX3;MYL12A;AMOT;MYL12B;CDC42;PPP2CA;EPB41L4B;ACTR3C;MAPK8;PARD6B;RAP1A;TUBA1A;CCND1;RAC1;WHAMM;RAB8A;MAP3K5;ACTR2;PRKAB2;MAP3K1;RDX;TJAP1;RUNX1;RAP2C;TJP1;TIAM1;MYL6;CLDN14;NEDD4;RAPGEF2;AMOTL1;PALS1
 
 
 
 
Alzheimer disease
 
 
72/369
 
 
0.0017153
 
 
0.0070362
 
 
0
 
 
0
 
 
1.511087
 
 
9.6228718
 
 
APP;ATP2A2;IRS2;TNF;IKBKB;CASP7;FRAT2;PSMD7;TUBA1A;KIF5C;CASP3;RB1CC1;AKT3;UQCRFS1;MAP3K5;PSENEN;MAP2K1;CHUK;WNT9B;ADAM10;WNT9A;SDHD;AXIN2;WIPI2;ATG14;DKK2;TNFRSF1A;PSMA5;BACE2;ADAM17;TUBB2A;PSMA1;PIK3CA;NDUFS4;PPIF;ULK2;VDAC1;RAF1;SLC25A4;PPID;BECN1;PSMD12;CHRNA7;LRP5;PIK3R3;ATP5MC3;LPL;PIK3R1;RTN4;PPP3R1;NRAS;MAPK8;DVL3;NRBF2;WNT1;WNT4;WNT10B;FZD3;FZD5;FZD4;CSNK1A1;WNT3A;NDUFA3;FZD6;WNT7A;EIF2S1;APC;FAS;CYCS;CALM3;KRAS;CDK5R1
 
 
 
 
Chemokine signaling pathway
 
 
42/192
 
 
0.0017432
 
 
0.0070362
 
 
0
 
 
0
 
 
1.741224
 
 
11.0603169
 
 
CCL3L1;CCL3L3;GNAI3;PIK3R3;ADCY3;CXCL1;ADCY2;PIK3R1;CXCL13;FOXO3;CXCL2;GNAI1;CRKL;RAP1B;IKBKB;CDC42;GNG10;NRAS;RAP1A;CCL8;GNG2;CCL7;GNG5;AKT3;CCL3;CCL2;CCR7;RAC1;VAV3;MAP2K1;CHUK;PRKCD;GNG12;TIAM1;CXCL10;PIK3CA;GRB2;KRAS;RAF1;SOS1;CRK;SOS2
 
 
 
 
Transcriptional misregulation in cancer
 
 
42/192
 
 
0.0017432
 
 
0.0070362
 
 
0
 
 
0
 
 
1.741224
 
 
11.0603169
 
 
CD86;CDKN1A;CCNT2;FLT3;SIX1;TMPRSS2;BMI1;HOXA11;IGF1R;ELK4;HOXA10;HOXA9;FUT8;CCND2;BAK1;H3-3B;SS18;EYA1;GADD45A;ZBTB16;PBX3;HMGA2;MITF;IGF1;ETV4;KLF3;PBX1;MLF1;RUNX1;GADD45G;CCNA2;H3-5;MEIS1;MAF;MYCN;WT1;RARA;COMMD3-BMI1;PPARG;ERG;TCF3;TP53
 
 
 
 
ECM-receptor interaction
 
 
23/88
 
 
0.0017533
 
 
0.0070362
 
 
0
 
 
0
 
 
2.196203
 
 
13.9376982
 
 
ITGB1;LAMA2;LAMA4;DMP1;LAMC1;COL1A1;COL2A1;RELN;COL1A2;COL4A2;COL4A1;COL4A4;COL6A2;ITGA11;ITGA8;COL9A1;ITGB8;COL4A5;COL6A3;SDC1;COL6A6;ITGA6;COL9A3
 
 
 
 
Axon guidance
 
 
40/182
 
 
0.0020181
 
 
0.0079937
 
 
0
 
 
0
 
 
1.751280
 
 
10.8677615
 
 
ITGB1;NRP1;SEMA7A;GNAI3;PIK3R3;PIK3R1;EFNA5;MYL12A;GNAI1;MYL12B;ROBO1;EFNB2;CDC42;PPP3R1;SHH;NRAS;PARD6B;DPYSL5;CFL2;RAC1;LRIG2;SLIT2;SRGAP2;PAK5;WNT4;EPHA7;FZD3;SEMA6A;TRPC6;SEMA6D;LIMK1;SEMA4C;SEMA4F;SSH1;RGMA;PIK3CA;RASA1;KRAS;RAF1;EPHA2
 
 
 
 
Protein processing in endoplasmic reticulum
 
 
38/171
 
 
0.0020939
 
 
0.0081878
 
 
0
 
 
0
 
 
1.775941
 
 
10.9552612
 
 
ERO1A;TRAM1;SEC23A;UBXN1;UBE2D2;UBE2D3;HSPA4L;RPN1;DERL1;UBE2D1;HSP90B1;UBE2J1;LMAN1;MAPK8;LMAN2;MAN1C1;UBQLN1;SSR1;BAK1;MAP3K5;SKP1;TXNDC5;PDIA3;UBE4B;YOD1;UBE2G2;PDIA6;EIF2S1;RAD23B;SVIP;CKAP4;DNAJC1;DNAJB11;CANX;DNAJA2;ERP29;BCL2;PLAA
 
 
 
 
Circadian rhythm
 
 
11/31
 
 
0.0021616
 
 
0.0083453
 
 
0
 
 
0
 
 
3.407820
 
 
20.9135337
 
 
PRKAA1;PRKAB2;PRKAA2;CREB1;BHLHE40;BHLHE41;PRKAG2;RORA;RORB;BTRC;SKP1
 
 
 
 
Hypertrophic cardiomyopathy
 
 
23/90
 
 
0.0024070
 
 
0.0091768
 
 
0
 
 
0
 
 
2.130396
 
 
12.8449385
 
 
ITGB1;EDN1;PRKAB2;PRKAA1;TGFB2;PRKAA2;TGFB1;LAMA2;TPM3;TPM1;CACNA2D3;ATP2A2;PRKAG2;IGF1;TNF;ACTC1;SGCB;ITGA11;ITGA8;ITGB8;ITGA6;CACNG2;CACNG4
 
 
 
 
ErbB signaling pathway
 
 
22/85
 
 
0.0024924
 
 
0.0093851
 
 
0
 
 
0
 
 
2.166873
 
 
12.9893045
 
 
MAP2K4;CDKN1A;MAP2K1;PIK3R3;TGFA;CBLB;PIK3R1;CRKL;MAPK8;NRAS;PIK3CA;ERBB3;AKT3;EIF4EBP1;GRB2;KRAS;RAF1;SOS1;CRK;SOS2;PAK5;HBEGF
 
 
 
 
Toxoplasmosis
 
 
27/112
 
 
0.0026567
 
 
0.0098815
 
 
0
 
 
0
 
 
1.972083
 
 
11.6957962
 
 
ITGB1;LAMA2;LAMA4;GNAI3;LAMC1;TNF;GNAI1;IKBKB;MAPK8;SOCS1;CASP3;AKT3;IL12B;MAP3K7;LDLR;MAP2K6;IL10;TGFB2;TGFB1;CHUK;TNFRSF1A;IFNG;PPIF;BCL2;CYCS;ITGA6;HLA-DQB1
 
 
 
 
Type II diabetes mellitus
 
 
14/46
 
 
0.0030217
 
 
0.0110730
 
 
0
 
 
0
 
 
2.711806
 
 
15.7336813
 
 
KCNJ11;PRKCD;CACNA1A;PIK3R3;IRS2;PIK3R1;TNF;IKBKB;SOCS2;SOCS3;MAPK8;SOCS1;PIK3CA;SOCS4
 
 
 
 
Lipid and atherosclerosis
 
 
45/215
 
 
0.0030496
 
 
0.0110730
 
 
0
 
 
0
 
 
1.645986
 
 
9.5347695
 
 
ERO1A;CCL3L1;CCL3L3;PIK3R3;CXCL1;FASLG;PIK3R1;TNF;CXCL2;HSP90B1;HSPD1;IKBKB;RAP1B;CDC42;PPP3R1;NRAS;CASP7;MAPK8;RAP1A;CASP3;AKT3;CCL3;IL12B;OLR1;CCL2;RAC1;IKBKE;LDLR;MAP3K7;MAP3K5;MAP2K6;VAV3;MAP2K4;CHUK;FOS;EIF2S1;TNFRSF1A;PIK3CA;BCL2;FAS;CYCS;CALM3;PPARG;KRAS;TP53
 
 
 
 
HIF-1 signaling pathway
 
 
26/109
 
 
0.0036745
 
 
0.0131851
 
 
0
 
 
0
 
 
1.944325
 
 
10.9005315
 
 
CDKN1A;TFRC;SERPINE1;CUL2;SLC2A1;PIK3R3;PIK3R1;PDHB;IGF1R;MKNK1;AKT3;EIF4EBP1;EP300;VHL;EGLN1;MAP2K1;EDN1;EGLN2;RPS6;IGF1;VEGFA;IFNG;PIK3CA;BCL2;TEK;EIF4E2
 
 
 
 
Non-alcoholic fatty liver disease
 
 
34/155
 
 
0.0043167
 
 
0.0152292
 
 
0
 
 
0
 
 
1.745273
 
 
9.5034564
 
 
PRKAA1;PRKAA2;PIK3R3;PRKAG2;IRS2;FASLG;PIK3R1;TNF;ADIPOR2;IKBKB;CDC42;SOCS3;CASP7;MAPK8;BCL2L11;CASP3;AKT3;UQCRFS1;RAC1;MAP3K5;SREBF1;PRKAB2;TGFB1;NDUFA3;SDHD;FOS;EIF2S1;TNFRSF1A;PIK3CA;LEP;NDUFS4;CYCS;FAS;PPARG
 
 
 
 
IL-17 signaling pathway
 
 
23/94
 
 
0.0043441
 
 
0.0152292
 
 
0
 
 
0
 
 
2.009905
 
 
10.9317595
 
 
USP25;CHUK;IL13;TNFAIP3;CXCL1;FOS;CXCL2;TNF;HSP90B1;IKBKB;CXCL10;MAPK8;IFNG;TRAF4;CCL7;CASP3;TRAF5;CCL2;FOSB;MAPK6;IKBKE;MAP3K7;S100A8
 
 
 
 
Fc epsilon RI signaling pathway
 
 
18/68
 
 
0.0045701
 
 
0.0158396
 
 
0
 
 
0
 
 
2.232312
 
 
12.0281875
 
 
VAV3;MAP2K4;MAP2K1;IL13;PIK3R3;PIK3R1;TNF;MAPK8;NRAS;PIK3CA;AKT3;GRB2;KRAS;RAC1;RAF1;SOS1;SOS2;MAP2K6
 
 
 
 
Epstein-Barr virus infection
 
 
42/202
 
 
0.0046365
 
 
0.0158891
 
 
0
 
 
0
 
 
1.631441
 
 
8.7670347
 
 
RB1;CDKN1A;PSMD12;PIK3R3;TNFAIP3;PIK3R1;TNF;IKBKB;MAPK8;PSMD7;CCND2;BCL2L11;CCND1;CASP3;SAP30L;AKT3;E2F3;RAC1;BAK1;IKBKE;MAP3K7;MAP2K6;PDIA3;MAP2K4;CHUK;GADD45A;TAP1;GADD45G;NCOR2;CCNA2;CXCL10;CCNE2;PIK3CA;CCNE1;NEDD4;TRAF5;BCL2;FAS;CYCS;VIM;TP53;HLA-DQB1
 
 
 
 
Progesterone-mediated oocyte maturation
 
 
24/100
 
 
0.0047173
 
 
0.0159865
 
 
0
 
 
0
 
 
1.959450
 
 
10.4958196
 
 
MAP2K1;GNAI3;PIK3R3;ADCY3;ADCY2;PIK3R1;IGF1;GNAI1;IGF1R;CDC25B;CCNA2;RPS6KA3;RPS6KA6;CPEB1;MAPK8;CDC23;PIK3CA;AKT3;CDC27;CDK1;KRAS;RAF1;CPEB3;CPEB2
 
 
 
 
Adipocytokine signaling pathway
 
 
18/69
 
 
0.0053946
 
 
0.0180809
 
 
0
 
 
0
 
 
2.188414
 
 
11.4286676
 
 
PRKAB2;PRKAA1;PRKAA2;CHUK;SLC2A1;ACSL6;PRKAG2;IRS2;TNFRSF1B;TNF;ADIPOR2;TNFRSF1A;IKBKB;SOCS3;MAPK8;G6PC2;LEP;AKT3
 
 
 
 
Regulation of lipolysis in adipocytes
 
 
15/55
 
 
0.0068557
 
 
0.0227283
 
 
0
 
 
0
 
 
2.324161
 
 
11.5805214
 
 
PTGER3;GNAI3;ADCY3;PIK3R3;IRS2;ADCY2;ADRB1;ABHD5;ADRB2;PIK3R1;GNAI1;ADRB3;PIK3CA;AKT3;PNPLA2
 
 
 
 
Mucin type O-glycan biosynthesis
 
 
11/36
 
 
0.0079180
 
 
0.0259677
 
 
0
 
 
0
 
 
2.725463
 
 
13.1874639
 
 
GALNT12;GALNT5;GALNT13;GALNT15;GALNT1;C1GALT1;GCNT3;GCNT4;POC1B-GALNT4;ST6GALNAC3;B4GALT5
 
 
 
 
Chemical carcinogenesis
 
 
47/239
 
 
0.0085071
 
 
0.0276028
 
 
0
 
 
0
 
 
1.521301
 
 
7.2518194
 
 
RB1;CHRNA7;GNAI3;PIK3R3;ADCY3;CACNA1A;ADCY2;ADRB1;PIK3R1;AHR;ADRB2;FGF2;GNAI1;HSP90B1;DLL4;RPS6KA3;RPS6KA6;NRAS;FGF7;CCND1;AKT3;EIF4EBP1;KPNA5;WNT1;IKBKE;KPNA3;KPNA1;MAP2K1;PTGES3;FOS;KLF4;ESR1;VEGFA;CREB1;KLF5;ADRB3;GSTA4;PIK3CA;FGF18;BCL2;GRB2;KRAS;RAF1;SOS1;SOS2;KPNB1;CREB5
 
 
 
 
JAK-STAT signaling pathway
 
 
34/162
 
 
0.0087297
 
 
0.0280271
 
 
0
 
 
0
 
 
1.649153
 
 
7.8186649
 
 
CDKN1A;PDGFB;PIK3R3;PIK3R1;SOCS2;GHR;SOCS3;CCND2;SOCS1;CCND1;AKT3;PIM1;EP300;IL12B;SOCS6;SOCS4;MCL1;PDGFRB;IL10;TSLP;IL15;IL13;LIF;OSMR;PIAS2;IFNG;PIK3CA;LEP;BCL2;GRB2;IL6ST;RAF1;SOS1;SOS2
 
 
 
 
Mannose type O-glycan biosynthesis
 
 
8/23
 
 
0.0098099
 
 
0.0311668
 
 
0
 
 
0
 
 
3.301944
 
 
15.2693969
 
 
POMT2;B4GALT3;B4GALT1;B4GAT1;CHST10;LARGE1;LARGE2;FUT4
 
 
 
 
Dilated cardiomyopathy
 
 
22/96
 
 
0.0115648
 
 
0.0363634
 
 
0
 
 
0
 
 
1.843587
 
 
8.2220185
 
 
ITGB1;TGFB2;TGFB1;LAMA2;TPM3;TPM1;CACNA2D3;ATP2A2;ADCY3;ADCY2;ADRB1;IGF1;TNF;PLN;ACTC1;SGCB;ITGA11;ITGA8;ITGB8;ITGA6;CACNG2;CACNG4
 
 
 
 
Amoebiasis
 
 
23/102
 
 
0.0121292
 
 
0.0377489
 
 
0
 
 
0
 
 
1.805527
 
 
7.9662411
 
 
IL10;TGFB2;ARG2;TGFB1;LAMA2;LAMA4;PIK3R3;CXCL1;PIK3R1;LAMC1;TNF;CXCL2;COL1A1;COL3A1;COL1A2;COL4A2;IFNG;PIK3CA;COL4A1;COL4A4;CASP3;IL12B;COL4A5
 
 
 
 
Yersinia infection
 
 
29/137
 
 
0.0130036
 
 
0.0397762
 
 
0
 
 
0
 
 
1.666042
 
 
7.2348345
 
 
ITGB1;WIPF1;PIK3R3;PIK3R1;TNF;CRKL;IKBKB;CDC42;RPS6KA3;RPS6KA6;ACTR3C;MAPK8;AKT3;CCL2;PIP5K1B;RAC1;MAP3K7;MAP2K6;IL10;VAV3;MAP2K4;ACTR2;MAP2K1;CHUK;LIMK1;FOS;PIK3CA;CRK;ARF6
 
 
 
 
Phosphatidylinositol signaling system
 
 
22/97
 
 
0.0130414
 
 
0.0397762
 
 
0
 
 
0
 
 
1.818900
 
 
7.8933487
 
 
CDS1;IPPK;PI4K2B;MTMR1;DGKD;MTMR3;INPP1;MTMR14;IPMK;PTEN;PIK3R3;PIK3R1;PIK3C2A;PIP4P2;PIP4P1;PIK3CA;PI4KA;PIP4K2B;PIP5K1B;CALM3;DGKH;CDS2
 
 
 
 
Sphingolipid metabolism
 
 
13/49
 
 
0.0144469
 
 
0.0436268
 
 
0
 
 
0
 
 
2.236988
 
 
9.4787302
 
 
CERS5;ASAH1;SGMS1;SGMS2;SMPD3;ACER2;GALC;UGCG;SPTLC1;SMPD1;KDSR;B4GALT6;PLPP1
 
 
 
 
Measles
 
 
29/139
 
 
0.0157668
 
 
0.0467519
 
 
0
 
 
0
 
 
1.635559
 
 
6.7873184
 
 
PIK3R3;TNFAIP3;CBLB;FASLG;PIK3R1;RCHY1;IKBKB;MAPK8;CCND2;CCND1;CASP3;AKT3;IL12B;BAK1;MAP3K7;IKBKE;CHUK;FOS;EIF2S1;CCNE2;PIK3CA;CCNE1;BCL2;CD28;CYCS;FAS;RAB9A;TP53;RAB9B
 
 
 
 
Rheumatoid arthritis
 
 
21/93
 
 
0.0157883
 
 
0.0467519
 
 
0
 
 
0
 
 
1.808228
 
 
7.5014046
 
 
CD86;TGFB2;TGFB1;CCL3L1;IL15;PTH;CCL3L3;CXCL1;FOS;TNFRSF11A;CXCL2;TNF;TNFSF13B;VEGFA;IFNG;CCL3;CD28;CCL2;TEK;ATP6V1E1;HLA-DQB1
 
 
 
 
Gap junction
 
 
20/88
 
 
0.0169871
 
 
0.0498178
 
 
0
 
 
0
 
 
1.823189
 
 
7.4300489
 
 
PDGFRB;MAP2K1;GNAI3;PDGFB;ADCY3;ADCY2;ADRB1;HTR2A;GNAI1;TJP1;NRAS;TUBA1A;TUBB2A;PDGFC;CDK1;GRB2;KRAS;RAF1;SOS1;SOS2
 
 
 
 
Oocyte meiosis
 
 
27/129
 
 
0.0185594
 
 
0.0539105
 
 
0
 
 
0
 
 
1.641771
 
 
6.5453820
 
 
YWHAB;ADCY3;ADCY2;IGF1R;PPP1CB;PPP2CA;PPP1CC;RPS6KA3;RPS6KA6;PPP3R1;CDC23;YWHAQ;CDC27;BTRC;SKP1;YWHAH;MAP2K1;IGF1;SMC1A;CPEB1;CCNE2;ESPL1;CCNE1;CDK1;CALM3;CPEB3;CPEB2
 
 
 
 
Influenza A
 
 
34/172
 
 
0.0209382
 
 
0.0602468
 
 
0
 
 
0
 
 
1.528754
 
 
5.9104348
 
 
PIK3R3;TMPRSS2;FASLG;PIK3R1;NXT2;TNF;IKBKB;SOCS3;NXF1;PABPN1;CASP3;AKT3;EP300;IL12B;CCL2;KPNA5;BAK1;BCL2L2-PABPN1;IKBKE;KPNA1;CPSF4;MAP2K1;CHUK;EIF2S1;TNFRSF1A;CXCL10;IFNG;PIK3CA;CYCS;FAS;VDAC1;RAF1;SLC25A4;HLA-DQB1
 
 
 
 
Bladder cancer
 
 
11/41
 
 
0.0216191
 
 
0.0616245
 
 
0
 
 
0
 
 
2.270559
 
 
8.7057262
 
 
RB1;CDKN1A;NRAS;MAP2K1;CCND1;E2F3;KRAS;RAF1;TP53;VEGFA;HBEGF
 
 
 
 
Spinocerebellar ataxia
 
 
29/143
 
 
0.0226906
 
 
0.0637626
 
 
0
 
 
0
 
 
1.577802
 
 
5.9732495
 
 
BECN1;PSMD12;CACNA1A;PIK3R3;ATP2A2;RORA;PIK3R1;ATXN2;MAPK8;PSMD7;RELN;RB1CC1;AKT3;NRBF2;MAP3K5;WIPI2;ATG14;PSMA5;AFG3L2;FGF14;PIK3CA;PSMA1;PPIF;TBPL1;ULK2;CYCS;VDAC1;ATXN10;SLC25A4
 
 
 
 
Nitrogen metabolism
 
 
6/17
 
 
0.0227873
 
 
0.0637626
 
 
0
 
 
0
 
 
3.375343
 
 
12.7640353
 
 
CA12;GLUD1;CA3;CPS1;CA2;CA8
 
 
 
 
cAMP signaling pathway
 
 
41/216
 
 
0.0231267
 
 
0.0641242
 
 
0
 
 
0
 
 
1.454278
 
 
5.4779256
 
 
HHIP;PTGER3;GNAI3;PIK3R3;ADCY3;ATP2A2;ADCY2;ADRB1;PIK3R1;ADRB2;HTR4;GNAI1;PPP1CB;RAP1B;PPP1CC;MAPK8;RAP1A;PLN;AKT3;PDE4B;EP300;SOX9;RAC1;VAV3;EDN1;MAP2K1;PPP1R12A;HTR1E;BDNF;ATP2B4;RRAS2;ATP1B4;FOS;TIAM1;CREB1;PIK3CA;CAMK4;CALM3;RAF1;RAPGEF4;CREB5
 
 
 
 
Glycosaminoglycan biosynthesis
 
 
13/53
 
 
0.0272714
 
 
0.0749349
 
 
0
 
 
0
 
 
2.012820
 
 
7.2500115
 
 
HS3ST3B1;B4GALT3;B4GALT1;CHST7;B3GAT3;CSGALNACT2;NDST2;EXTL2;FUT8;B3GNT7;CHSY1;CHST1;CHST2
 
 
 
 
C-type lectin receptor signaling pathway
 
 
22/104
 
 
0.0280092
 
 
0.0762752
 
 
0
 
 
0
 
 
1.662949
 
 
5.9454089
 
 
IL10;EGR3;CHUK;PRKCD;PIK3R3;RRAS2;CBLB;PIK3R1;TNF;IKBKB;PPP3R1;MAPK8;NRAS;MRAS;PIK3CA;AKT3;BCL3;IL12B;CALM3;KRAS;RAF1;IKBKE
 
 
 
 
Central carbon metabolism in cancer
 
 
16/70
 
 
0.0290908
 
 
0.0785194
 
 
0
 
 
0
 
 
1.835540
 
 
6.4929158
 
 
PDGFRB;MAP2K1;FLT3;GLS2;IDH2;PTEN;SLC2A1;PIK3R3;PDHB;PIK3R1;NRAS;PIK3CA;AKT3;KRAS;RAF1;TP53
 
 
 
 
Pathways of neurodegeneration
 
 
81/475
 
 
0.0297151
 
 
0.0795009
 
 
0
 
 
0
 
 
1.278343
 
 
4.4947831
 
 
APP;ATP2A2;FASLG;TNF;CASP7;PSMD7;TUBA1A;FRAT2;KIF5C;CASP3;RB1CC1;UQCRFS1;NEFM;RAC1;RAB8A;MAP3K5;GABARAPL1;MAP2K1;GPR37;WNT9B;GPX8;GPX7;WNT9A;WIPI2;SDHD;AXIN2;ATG14;TNFRSF1B;DKK2;TNFRSF1A;PSMA5;TUBB2A;PSMA1;CAT;NDUFS4;PPIF;ULK2;VDAC1;RAF1;SLC25A4;PPID;BECN1;PSMD12;CHRNA7;LRP5;DERL1;ATP5MC3;UBE2J1;PPP3R1;NRAS;ATXN2;MAPK8;DVL3;NRBF2;BAK1;WNT1;MAP2K6;WNT4;WNT10B;PRNP;FZD3;FZD5;FZD4;CSNK1A1;BDNF;WNT3A;NDUFA3;FZD6;WNT7A;RAB39B;UBE2G2;EIF2S1;APC;VAPB;CHMP2B;BCL2;FAS;CYCS;CALM3;KRAS;CDK5R1
 
 
 
 
Parathyroid hormone synthesis, secretion and action
 
 
22/106
 
 
0.0340618
 
 
0.0903379
 
 
0
 
 
0
 
 
1.623165
 
 
5.4856134
 
 
MEF2A;MAP2K1;CDKN1A;PTH;GNAI3;LRP5;ADCY3;ADCY2;FOS;GNAI1;PTHLH;NR4A2;GNA13;MAFB;CREB1;MMP16;MMP15;BCL2;PDE4B;RAF1;CREB5;HBEGF
 
 
 
 
Cholinergic synapse
 
 
23/113
 
 
0.0376590
 
 
0.0981873
 
 
0
 
 
0
 
 
1.583834
 
 
5.1936829
 
 
MAP2K1;CHRNA7;GNAI3;CACNA1A;PIK3R3;ADCY3;ADCY2;PIK3R1;FOS;GNG12;GNAI1;GNG10;NRAS;CREB1;GNG2;PIK3CA;GNG5;CAMK4;AKT3;BCL2;KRAS;KCNJ2;CREB5
 
 
 
 
Estrogen signaling pathway
 
 
27/137
 
 
0.0376653
 
 
0.0981873
 
 
0
 
 
0
 
 
1.521658
 
 
4.9895421
 
 
GNAI3;PIK3R3;TGFA;ADCY3;ADCY2;PIK3R1;GNAI1;HSP90B1;NRAS;AKT3;NCOA1;MAP2K1;PRKCD;FOS;ESR1;CREB1;PIK3CA;BCL2;RARA;CALM3;GRB2;KRAS;RAF1;SOS1;SOS2;CREB5;HBEGF
 
 
 
 
Hedgehog signaling pathway
 
 
13/56
 
 
0.0412862
 
 
0.1067143
 
 
0
 
 
0
 
 
1.872064
 
 
5.9666932
 
 
SMURF2;CSNK1A1;SMURF1;HHIP;CUL3;IHH;LRP2;SHH;CCND2;CCND1;BCL2;BTRC;CDON
 
 
 
 
Mitophagy
 
 
15/68
 
 
0.0450062
 
 
0.1153519
 
 
0
 
 
0
 
 
1.752756
 
 
5.4352189
 
 
USP8;BECN1;GABARAPL1;USP15;ATG9A;TFEB;RRAS2;MITF;FOXO3;MAPK8;NRAS;MRAS;KRAS;TP53;ATG5
 
 
 
 
Toll-like receptor signaling pathway
 
 
21/104
 
 
0.0489848
 
 
0.1245031
 
 
0
 
 
0
 
 
1.567577
 
 
4.7281952
 
 
CD86;MAP2K4;MAP2K1;CCL3L1;CHUK;CCL3L3;PIK3R3;PIK3R1;FOS;TNF;IKBKB;CXCL10;MAPK8;PIK3CA;AKT3;CCL3;IL12B;RAC1;MAP3K7;IKBKE;MAP2K6
 
 
 
 
B cell receptor signaling pathway
 
 
17/81
 
 
0.0523620
 
 
0.1319870
 
 
0
 
 
0
 
 
1.645167
 
 
4.8525421
 
 
VAV3;MAP2K1;CHUK;PIK3R3;FOS;PIK3R1;IKBKB;NRAS;PPP3R1;PIK3CA;AKT3;GRB2;KRAS;RAC1;RAF1;SOS1;SOS2
 
 
 
 
Oxytocin signaling pathway
 
 
29/154
 
 
0.0540546
 
 
0.1351366
 
 
0
 
 
0
 
 
1.438030
 
 
4.1958252
 
 
CDKN1A;PRKAA1;PRKAA2;GNAI3;PRKAG2;ADCY3;ADCY2;GNAI1;MYLK;PPP1CB;PPP1CC;PPP3R1;NRAS;EEF2K;CCND1;CACNG2;CACNG4;KCNJ2;MAP2K1;PRKAB2;CAMK1D;PPP1R12A;CACNA2D3;FOS;MYL6;CAMK4;CALM3;KRAS;RAF1
 
 
 
 
VEGF signaling pathway
 
 
13/59
 
 
0.0597162
 
 
0.1480768
 
 
0
 
 
0
 
 
1.749667
 
 
4.9308261
 
 
MAP2K1;SH2D2A;PIK3R3;PIK3R1;VEGFA;CDC42;NRAS;PPP3R1;PIK3CA;AKT3;KRAS;RAC1;RAF1
 
 
 
 
PD-L1 expression and PD-1 checkpoint pathway in cancer
 
 
18/89
 
 
0.0638740
 
 
0.1571096
 
 
0
 
 
0
 
 
1.570127
 
 
4.3191727
 
 
MAP2K1;CHUK;PTEN;PIK3R3;FOS;PIK3R1;RASGRP1;IKBKB;EML4;NRAS;PPP3R1;IFNG;PIK3CA;AKT3;CD28;KRAS;RAF1;MAP2K6
 
 
 
 
Type I diabetes mellitus
 
 
10/43
 
 
0.0676238
 
 
0.1650020
 
 
0
 
 
0
 
 
1.875491
 
 
5.0521900
 
 
CD86;PTPRN2;IFNG;CD28;IL12B;FAS;FASLG;TNF;HSPD1;HLA-DQB1
 
 
 
 
Ether lipid metabolism
 
 
11/49
 
 
0.0707994
 
 
0.1713796
 
 
0
 
 
0
 
 
1.791712
 
 
4.7442812
 
 
PLA2G12A;ENPP2;GDPD1;PLA2G3;PEDS1;CHPT1;PAFAH1B2;PLPP1;PLD3;PAFAH1B1;PEDS1-UBE2V1
 
 
 
 
Allograft rejection
 
 
9/38
 
 
0.0731166
 
 
0.1755950
 
 
0
 
 
0
 
 
1.920517
 
 
5.0234965
 
 
CD86;IL10;IFNG;CD28;IL12B;FAS;FASLG;TNF;HLA-DQB1
 
 
 
 
Terpenoid backbone biosynthesis
 
 
6/22
 
 
0.0749328
 
 
0.1785509
 
 
0
 
 
0
 
 
2.319874
 
 
6.0111724
 
 
ICMT;HMGCS1;FNTA;PDSS1;HMGCR;ZMPSTE24
 
 
 
 
Long-term potentiation
 
 
14/67
 
 
0.0757035
 
 
0.1789888
 
 
0
 
 
0
 
 
1.635315
 
 
4.2206369
 
 
MAP2K1;PPP1CB;RAP1B;PPP1CC;RPS6KA3;RPS6KA6;NRAS;PPP3R1;RAP1A;CAMK4;EP300;CALM3;KRAS;RAF1
 
 
 
 
mRNA surveillance pathway
 
 
19/98
 
 
0.0823042
 
 
0.1930984
 
 
0
 
 
0
 
 
1.489366
 
 
3.7194428
 
 
CPSF4;CPSF7;DAZAP1;PPP2R2A;MSI2;MAGOHB;NXT2;PPP1CB;PPP2CA;PNN;PPP1CC;DDX19A;NXF1;DDX19B;PABPN1;CLP1;WDR82;PAPOLG;BCL2L2-PABPN1
 
 
 
 
cGMP-PKG signaling pathway
 
 
30/167
 
 
0.0840637
 
 
0.1957208
 
 
0
 
 
0
 
 
1.356854
 
 
3.3598153
 
 
GNAI3;ATP2A2;ADCY3;IRS2;ADRB1;ADCY2;ADRB2;GNAI1;MYLK;PPP1CB;GNA13;PPP1CC;PPP3R1;PLN;AKT3;MEF2A;MAP2K1;PPP1R12A;TRPC6;ATP2B4;ATP1B4;ADRA2A;CREB1;ADRB3;PPIF;VDAC1;CALM3;RAF1;SLC25A4;CREB5
 
 
 
 
GnRH signaling pathway
 
 
18/93
 
 
0.0901405
 
 
0.2072787
 
 
0
 
 
0
 
 
1.486041
 
 
3.5759870
 
 
MAP2K4;MAP2K1;MAP3K1;PRKCD;ADCY3;ADCY2;CDC42;MAPK8;NRAS;CALM3;GRB2;KRAS;RAF1;SOS1;SOS2;MAP3K4;HBEGF;MAP2K6
 
 
 
 
Platelet activation
 
 
23/124
 
 
0.0903871
 
 
0.2072787
 
 
0
 
 
0
 
 
1.410431
 
 
3.3901866
 
 
ITGB1;PPP1R12A;GNAI3;PIK3R3;ADCY3;ADCY2;PIK3R1;RASGRP1;MYL12A;GNAI1;MYLK;MYL12B;PPP1CB;RAP1B;COL1A1;VAMP8;GNA13;PPP1CC;COL3A1;RAP1A;COL1A2;PIK3CA;AKT3
 
 
 
 
Adrenergic signaling in cardiomyocytes
 
 
27/150
 
 
0.0952615
 
 
0.2168266
 
 
0
 
 
0
 
 
1.359798
 
 
3.1970611
 
 
GNAI3;ATP2A2;ADCY3;PPP2R2A;ADRB1;ADCY2;ADRB2;GNAI1;PPP1CB;PPP2CA;PPP1CC;PLN;AKT3;CACNG2;CACNG4;TPM3;TPM1;CACNA2D3;ATP2B4;ATP1B4;CREB1;ACTC1;BCL2;CALM3;SCN4B;CREB5;RAPGEF4
 
 
 
 
Serotonergic synapse
 
 
21/113
 
 
0.1004514
 
 
0.2251198
 
 
0
 
 
0
 
 
1.413484
 
 
3.2483010
 
 
GABRB3;APP;GABRB2;MAP2K1;GABRB1;HTR1E;DUSP1;GNAI3;CACNA1A;ALOX12;HTR2A;HTR4;GNG12;GNAI1;GNG10;NRAS;GNG2;GNG5;CASP3;KRAS;RAF1
 
 
 
 
Epithelial cell signaling in Helicobacter pylori infection
 
 
14/70
 
 
0.1011194
 
 
0.2251198
 
 
0
 
 
0
 
 
1.547439
 
 
3.5458838
 
 
MAP2K4;CHUK;ADAM10;CXCL1;CXCL2;IKBKB;CDC42;TJP1;ADAM17;MAPK8;CASP3;RAC1;ATP6V1E1;HBEGF
 
 
 
 
RIG-I-like receptor signaling pathway
 
 
14/70
 
 
0.1011194
 
 
0.2251198
 
 
0
 
 
0
 
 
1.547439
 
 
3.5458838
 
 
DDX3X;MAP3K1;CHUK;TNF;IKBKB;CXCL10;SIKE1;MAPK8;RNF125;IL12B;TBKBP1;IKBKE;MAP3K7;ATG5
 
 
 
 
Viral carcinogenesis
 
 
35/203
 
 
0.1046655
 
 
0.2313260
 
 
0
 
 
0
 
 
1.290894
 
 
2.9135295
 
 
RB1;HDAC4;CDKN1A;DDX3X;YWHAB;PIK3R3;PIK3R1;CDC42;NRAS;CCND2;CCND1;YWHAQ;CASP3;CHEK1;EP300;PMAIP1;RAC1;BAK1;YWHAH;EGR3;GTF2H1;CCNA2;HNRNPK;CREB1;CCNE2;PIK3CA;CCNE1;TRAF5;CDK1;TBPL1;GRB2;KRAS;IL6ST;TP53;CREB5
 
 
 
 
GABAergic synapse
 
 
17/89
 
 
0.1070726
 
 
0.2349434
 
 
0
 
 
0
 
 
1.461689
 
 
3.2657764
 
 
GABRB3;GABRB2;GABRP;GABRB1;GABARAPL1;GLS2;GNAI3;ADCY3;CACNA1A;ADCY2;SLC6A1;GNG12;GNAI1;TRAK2;GNG10;GNG2;GNG5
 
 
 
 
Bacterial invasion of epithelial cells
 
 
15/77
 
 
0.1096598
 
 
0.2389018
 
 
0
 
 
0
 
 
1.497538
 
 
3.3101158
 
 
ITGB1;ACTR2;CAV2;SEPTIN2;PIK3R3;PIK3R1;SEPTIN11;CRKL;CD2AP;CDC42;ACTR3C;PIK3CA;RAC1;WASF1;CRK
 
 
 
 
Thyroid hormone signaling pathway
 
 
22/121
 
 
0.1129533
 
 
0.2443317
 
 
0
 
 
0
 
 
1.376025
 
 
3.0008088
 
 
NCOA1;MAP2K1;SLC2A1;ATP1B4;PIK3R3;ATP2A2;SLC16A10;PIK3R1;ESR1;MED12L;MED14;NRAS;PLN;CCND1;PIK3CA;RCAN2;AKT3;EP300;KRAS;RAF1;TP53;WNT4
 
 
 
 
Amino sugar and nucleotide sugar metabolism
 
 
10/48
 
 
0.1225817
 
 
0.2632916
 
 
0
 
 
0
 
 
1.628242
 
 
3.4176439
 
 
CYB5R4;GALE;GNPDA1;PMM1;GFPT2;GFPT1;PGM3;PGM2;UAP1;PGM1
 
 
 
 
Inositol phosphate metabolism
 
 
14/73
 
 
0.1311515
 
 
0.2797286
 
 
0
 
 
0
 
 
1.468498
 
 
2.9831115
 
 
IPPK;PI4K2B;MTMR1;MTMR3;INPP1;MTMR14;IPMK;PTEN;PIK3C2A;MINPP1;PIK3CA;PI4KA;PIP4K2B;PIP5K1B
 
 
 
 
Alanine, aspartate and glutamate metabolism
 
 
8/37
 
 
0.1338971
 
 
0.2836015
 
 
0
 
 
0
 
 
1.706512
 
 
3.4312551
 
 
GLUD1;CPS1;GLS2;GFPT2;GFPT1;NAT8L;RIMKLB;NIT2
 
 
 
 
D-Glutamine and D-glutamate metabolism
 
 
2/5
 
 
0.1454021
 
 
0.3041233
 
 
0
 
 
0
 
 
4.121408
 
 
7.9471147
 
 
GLUD1;GLS2
 
 
 
 
Thiamine metabolism
 
 
4/15
 
 
0.1455803
 
 
0.3041233
 
 
0
 
 
0
 
 
2.248611
 
 
4.3331349
 
 
TPK1;AK1;ALPL;AK4
 
 
 
 
RNA transport
 
 
31/186
 
 
0.1638956
 
 
0.3400555
 
 
0
 
 
0
 
 
1.238403
 
 
2.2396832
 
 
EIF4A2;SEH1L;FMR1;GEMIN2;POP4;PHAX;NXT2;FXR1;PNN;NXF1;POM121C;SUMO3;TPR;EIF4EBP1;RPP14;UBE2I;NUP155;THOC1;EIF2S2;MAGOHB;SENP2;EIF2S1;DDX19A;DDX19B;NUP50;EIF3J;GEMIN6;GEMIN7;EIF4E2;KPNB1;EIF4G2
 
 
 
 
Inflammatory bowel disease
 
 
12/65
 
 
0.1870741
 
 
0.3855244
 
 
0
 
 
0
 
 
1.400688
 
 
2.3479042
 
 
IL10;SMAD2;TGFB2;MAF;TGFB1;IFNG;TBX21;IL13;IL12B;RORA;TNF;HLA-DQB1
 
 
 
 
Morphine addiction
 
 
16/91
 
 
0.1928869
 
 
0.3948356
 
 
0
 
 
0
 
 
1.319971
 
 
2.1722120
 
 
GABRB3;GABRB2;GABRP;GABRB1;GNAI3;ADCY3;CACNA1A;ADCY2;GNG12;GNAI1;GNG10;GNG2;GNG5;PDE4B;PDE7B;PDE7A
 
 
 
 
Pathogenic Escherichia coli infection
 
 
32/197
 
 
0.1985940
 
 
0.4023956
 
 
0
 
 
0
 
 
1.200607
 
 
1.9407732
 
 
ITGB1;WIPF1;FASLG;CLDN1;TNF;IKBKB;CDC42;CYTH3;GNA13;CYTH2;ACTR3C;CASP7;MAPK8;TUBA1A;CASP3;RAC1;BAK1;WASF1;MAP3K7;CYTH1;ACTR2;CHUK;FOS;TNFRSF1A;TJP1;TUBB2A;CLDN14;FAS;CYCS;TMBIM6;MYO1F;ARF6
 
 
 
 
Glycerophospholipid metabolism
 
 
17/98
 
 
0.1992188
 
 
0.4023956
 
 
0
 
 
0
 
 
1.298597
 
 
2.0950935
 
 
CDS1;LYPLA1;PLA2G12A;DGKD;LPGAT1;PLA2G3;PLD3;AGPAT3;PISD;AGPAT4;GPAT4;GPAT3;CHPT1;PNPLA6;PLPP1;DGKH;CDS2
 
 
 
 
Ferroptosis
 
 
8/41
 
 
0.2038745
 
 
0.4090903
 
 
0
 
 
0
 
 
1.499313
 
 
2.3842832
 
 
PRNP;GCLC;TFRC;ACSL6;SLC39A8;SLC7A11;TP53;ATG5
 
 
 
 
Long-term depression
 
 
11/60
 
 
0.2070178
 
 
0.4126825
 
 
0
 
 
0
 
 
1.388601
 
 
2.1869778
 
 
GNA13;PPP2CA;MAP2K1;NRAS;GNAI3;CACNA1A;KRAS;IGF1;RAF1;GNAI1;IGF1R
 
 
 
 
Proximal tubule bicarbonate reclamation
 
 
5/23
 
 
0.2085579
 
 
0.4130530
 
 
0
 
 
0
 
 
1.717608
 
 
2.6924161
 
 
GLUD1;GLS2;CA2;ATP1B4;SLC4A4
 
 
 
 
Alcoholism
 
 
30/186
 
 
0.2193345
 
 
0.4315937
 
 
0
 
 
0
 
 
1.190270
 
 
1.8058271
 
 
HDAC4;GNAI3;MACROH2A1;GNAI1;PPP1CB;PPP1CC;GNG10;NRAS;GNG2;GNG5;H3-3B;H2AZ1;MAP2K1;BDNF;GNG12;H3-5;CREB1;ADORA2B;PKIA;CAMK4;HAT1;FOSB;CALM3;GRB2;KRAS;RAF1;SOS1;SOS2;SLC29A3;CREB5
 
 
 
 
Glucagon signaling pathway
 
 
18/107
 
 
0.2289371
 
 
0.4476014
 
 
0
 
 
0
 
 
1.251258
 
 
1.8447402
 
 
PRKAB2;PRKAA1;PRKAA2;SLC2A1;PRKAG2;PHKA1;PPP4R3A;ADCY2;PDHB;SIRT1;PPP3R1;G6PC2;CREB1;AKT3;EP300;SIK1;CALM3;CREB5
 
 
 
 
Citrate cycle (TCA cycle)
 
 
6/30
 
 
0.2325820
 
 
0.4518312
 
 
0
 
 
0
 
 
1.545863
 
 
2.2546611
 
 
CS;MDH2;IDH2;DLST;PDHB;SDHD
 
 
 
 
African trypanosomiasis
 
 
7/37
 
 
0.2503287
 
 
0.4792353
 
 
0
 
 
0
 
 
1.442821
 
 
1.9982788
 
 
IL10;IFNG;LAMA4;IL12B;FAS;FASLG;TNF
 
 
 
 
Aldosterone-regulated sodium reabsorption
 
 
7/37
 
 
0.2503287
 
 
0.4792353
 
 
0
 
 
0
 
 
1.442821
 
 
1.9982788
 
 
PIK3CA;PIK3R3;ATP1B4;KRAS;PIK3R1;IGF1;SGK1
 
 
 
 
Diabetic cardiomyopathy
 
 
32/203
 
 
0.2514021
 
 
0.4792353
 
 
0
 
 
0
 
 
1.158073
 
 
1.5989533
 
 
PTEN;SLC2A1;PIK3R3;ATP5MC3;ATP2A2;PIK3R1;PDHB;PPP1CB;PPP1CC;MAPK8;PLN;AKT3;PDK4;UQCRFS1;RAC1;SMAD2;TGFB2;TGFB1;GFPT2;NDUFA3;PRKCD;GFPT1;SDHD;TGFBR1;COL1A1;COL3A1;COL1A2;PIK3CA;NDUFS4;PPIF;VDAC1;SLC25A4
 
 
 
 
Cysteine and methionine metabolism
 
 
9/50
 
 
0.2554617
 
 
0.4839491
 
 
0
 
 
0
 
 
1.357466
 
 
1.8525106
 
 
DNMT1;GCLC;MDH2;PSAT1;CTH;DNMT3A;DNMT3B;SMS;BCAT1
 
 
 
 
Vasopressin-regulated water reabsorption
 
 
8/44
 
 
0.2640262
 
 
0.4965350
 
 
0
 
 
0
 
 
1.374130
 
 
1.8299385
 
 
DYNC1I2;DYNC1LI2;CREB1;ADCY3;DYNLL1;DYNLL2;DYNC1I1;CREB5
 
 
 
 
Glutathione metabolism
 
 
10/57
 
 
0.2653613
 
 
0.4965350
 
 
0
 
 
0
 
 
1.315761
 
 
1.7455717
 
 
GGCT;GCLC;RRM2;GSTA4;IDH2;GPX8;SMS;GPX7;TXNDC12;CHAC1
 
 
 
 
Arrhythmogenic right ventricular cardiomyopathy
 
 
13/77
 
 
0.2707031
 
 
0.5034417
 
 
0
 
 
0
 
 
1.256255
 
 
1.6415889
 
 
ITGB1;TCF7L2;LAMA2;ACTN2;CACNA2D3;ATP2A2;SGCB;ITGA11;ITGA8;ITGB8;ITGA6;CACNG2;CACNG4
 
 
 
 
GnRH secretion
 
 
11/64
 
 
0.2736279
 
 
0.5057970
 
 
0
 
 
0
 
 
1.283502
 
 
1.6634003
 
 
MAP2K1;NRAS;PIK3CA;KCNJ11;AKT3;PIK3R3;KRAS;PIK3R1;RAF1;KCNN4;HCN1
 
 
 
 
Amyotrophic lateral sclerosis
 
 
55/364
 
 
0.2771232
 
 
0.5091721
 
 
0
 
 
0
 
 
1.101787
 
 
1.4139158
 
 
NXT2;TNF;PSMD7;TUBA1A;KIF5C;CASP3;RB1CC1;UQCRFS1;NEFM;RAC1;RAB8A;MAP3K5;GABARAPL1;GPX8;ANXA11;GPX7;SDHD;WIPI2;ATG14;TNFRSF1B;TNFRSF1A;PSMA5;TUBB2A;PSMA1;NUP50;CAT;NDUFS4;ULK2;SRSF3;VDAC1;TP53;SRSF7;PFN2;BECN1;PSMD12;SEH1L;DERL1;ATP5MC3;NXF1;PPP3R1;ATXN2;POM121C;TPR;UBQLN1;NRBF2;MAP2K6;NUP155;NDUFA3;RAB39B;EIF2S1;VAPB;HNRNPA2B1;CHMP2B;BCL2;CYCS
 
 
 
 
Inflammatory mediator regulation of TRP channels
 
 
16/98
 
 
0.2860159
 
 
0.5223644
 
 
0
 
 
0
 
 
1.206798
 
 
1.5105579
 
 
PRKCD;PIK3R3;ADCY3;ALOX12;ADCY2;IL1RAP;PIK3R1;IGF1;HTR2A;NGF;PPP1CB;PPP1CC;MAPK8;PIK3CA;CALM3;MAP2K6
 
 
 
 
Maturity onset diabetes of the young
 
 
5/26
 
 
0.2916207
 
 
0.5294305
 
 
0
 
 
0
 
 
1.471978
 
 
1.8139204
 
 
NEUROD1;NR5A2;RFX6;ONECUT1;HNF4G
 
 
 
 
Notch signaling pathway
 
 
10/59
 
 
0.3026868
 
 
0.5433736
 
 
0
 
 
0
 
 
1.261910
 
 
1.5080538
 
 
PSENEN;TLE4;NCOR2;DLL4;ADAM17;MAML1;HEY2;EP300;DVL3;DTX2
 
 
 
 
RNA degradation
 
 
13/79
 
 
0.3028640
 
 
0.5433736
 
 
0
 
 
0
 
 
1.218044
 
 
1.4549191
 
 
HSPA9;DDX6;BTG2;CNOT6L;TENT4B;TOB1;HSPD1;PAN2;CNOT7;XRN1;EXOSC3;DCP1A;DCP2
 
 
 
 
Viral protein interaction with cytokine and cytokine receptor
 
 
16/100
 
 
0.3150316
 
 
0.5608872
 
 
0
 
 
0
 
 
1.177927
 
 
1.3606026
 
 
IL10;CCL3L1;CCL3L3;CXCL1;CXCL13;TNFRSF1B;TNF;CXCL2;TNFRSF1A;CXCL10;CCL8;CCL7;CCL3;CCL2;CCR7;IL6ST
 
 
 
 
Th17 cell differentiation
 
 
17/107
 
 
0.3181450
 
 
0.5608872
 
 
0
 
 
0
 
 
1.168123
 
 
1.3377909
 
 
SMAD2;TGFB1;CHUK;RORA;AHR;IL1RAP;FOS;TGFBR1;RUNX1;IKBKB;PPP3R1;MAPK8;IFNG;TBX21;RARA;IL6ST;HLA-DQB1
 
 
 
 
Biosynthesis of unsaturated fatty acids
 
 
5/27
 
 
0.3205522
 
 
0.5608872
 
 
0
 
 
0
 
 
1.404988
 
 
1.5984694
 
 
ELOVL4;ELOVL5;SCD;ELOVL2;ELOVL3
 
 
 
 
Leukocyte transendothelial migration
 
 
18/114
 
 
0.3209072
 
 
0.5608872
 
 
0
 
 
0
 
 
1.159547
 
 
1.3179445
 
 
ITGB1;VAV3;GNAI3;PIK3R3;PIK3R1;ARHGAP5;CLDN1;MYL12A;GNAI1;MYL12B;RAP1B;CDC42;RAP1A;PIK3CA;CLDN14;RASSF5;RAC1;RAPGEF4
 
 
 
 
Viral myocarditis
 
 
10/60
 
 
0.3218205
 
 
0.5608872
 
 
0
 
 
0
 
 
1.236599
 
 
1.4020085
 
 
CD86;CCND1;LAMA2;SGCB;CASP3;CD28;CYCS;RAC1;EIF4G2;HLA-DQB1
 
 
 
 
Pantothenate and CoA biosynthesis
 
 
4/21
 
 
0.3342754
 
 
0.5785961
 
 
0
 
 
0
 
 
1.454476
 
 
1.5938007
 
 
PANK3;PANK1;PPCS;BCAT1
 
 
 
 
Cytokine-cytokine receptor interaction
 
 
44/295
 
 
0.3357754
 
 
0.5785961
 
 
0
 
 
0
 
 
1.084462
 
 
1.1834867
 
 
CCL3L1;CCL3L3;TNFRSF11B;CXCL1;FASLG;IL1RAP;TNFRSF11A;CXCL13;TNF;CXCL2;TNFSF13B;GHR;ACVR1C;CCL8;CCL7;CCL3;IL12B;CCL2;CCR7;ACVR1;IL10;TGFB2;TGFB1;TSLP;IL15;IL13;LIF;INHBB;GDF6;OSMR;NGF;TNFRSF1B;GDF5;ACVR2B;TGFBR1;BMP5;TNFRSF1A;BMP3;CXCL10;IFNG;LEP;TNFSF9;FAS;IL6ST
 
 
 
 
Calcium signaling pathway
 
 
36/240
 
 
0.3422223
 
 
0.5863921
 
 
0
 
 
0
 
 
1.091551
 
 
1.1704642
 
 
CHRNA7;PTAFR;PTGER3;PDGFB;ADCY3;CACNA1A;ATP2A2;ADCY2;ADRB1;ADRB2;HTR2A;HTR4;FGF2;MYLK;PPP3R1;FGF7;PLN;ERBB3;PDGFC;PDGFRB;CAMK1D;ATP2B4;VEGFC;PHKA1;NGF;VEGFA;P2RX5;ADRB3;ASPH;ADORA2B;CAMK4;FGF18;PPIF;VDAC1;CALM3;SLC25A4
 
 
 
 
Intestinal immune network for IgA production
 
 
8/48
 
 
0.3506781
 
 
0.5975242
 
 
0
 
 
0
 
 
1.236429
 
 
1.2956373
 
 
CD86;IL10;TGFB1;IL15;CD28;ICOS;TNFSF13B;HLA-DQB1
 
 
 
 
Pertussis
 
 
12/76
 
 
0.3673582
 
 
0.6166073
 
 
0
 
 
0
 
 
1.159201
 
 
1.1608453
 
 
ITGB1;IL10;CASP7;MAPK8;CASP3;CFL2;GNAI3;IL12B;CALM3;FOS;TNF;GNAI1
 
 
 
 
Graft-versus-host disease
 
 
7/42
 
 
0.3674867
 
 
0.6166073
 
 
0
 
 
0
 
 
1.236344
 
 
1.2376648
 
 
CD86;IFNG;CD28;FAS;FASLG;TNF;HLA-DQB1
 
 
 
 
Arginine biosynthesis
 
 
4/22
 
 
0.3679427
 
 
0.6166073
 
 
0
 
 
0
 
 
1.373592
 
 
1.3733559
 
 
GLUD1;ARG2;CPS1;GLS2
 
 
 
 
Cocaine addiction
 
 
8/49
 
 
0.3729658
 
 
0.6216097
 
 
0
 
 
0
 
 
1.206202
 
 
1.1896390
 
 
GRM3;CREB1;BDNF;GNAI3;FOSB;GNAI1;CREB5;CDK5R1
 
 
 
 
NF-kappa B signaling pathway
 
 
16/104
 
 
0.3750984
 
 
0.6217663
 
 
0
 
 
0
 
 
1.124122
 
 
1.1022769
 
 
EDARADD;UBE2I;CHUK;GADD45A;TNFAIP3;CXCL1;TNFRSF11A;TNF;CXCL2;GADD45G;TNFSF13B;TNFRSF1A;IKBKB;TRAF5;BCL2;MAP3K7
 
 
 
 
N-Glycan biosynthesis
 
 
8/50
 
 
0.3953476
 
 
0.6517893
 
 
0
 
 
0
 
 
1.177414
 
 
1.0926287
 
 
B4GALT3;FUT8;B4GALT1;DOLPP1;MGAT4A;RPN1;SRD5A3;MAN1C1
 
 
 
 
Adherens junction
 
 
11/71
 
 
0.4018714
 
 
0.6589826
 
 
0
 
 
0
 
 
1.133297
 
 
1.0331401
 
 
CDC42;TJP1;TCF7L2;SNAI1;EP300;RAC1;WASF1;MAP3K7;SSX2IP;TGFBR1;IGF1R
 
 
 
 
Primary bile acid biosynthesis
 
 
3/17
 
 
0.4290491
 
 
0.6960637
 
 
0
 
 
0
 
 
1.324367
 
 
1.1206584
 
 
CYP39A1;HSD3B7;CYP46A1
 
 
 
 
Selenocompound metabolism
 
 
3/17
 
 
0.4290491
 
 
0.6960637
 
 
0
 
 
0
 
 
1.324367
 
 
1.1206584
 
 
MARS2;TXNRD1;CTH
 
 
 
 
Galactose metabolism
 
 
5/31
 
 
0.4375679
 
 
0.6986636
 
 
0
 
 
0
 
 
1.188560
 
 
0.9823724
 
 
G6PC2;GALE;B4GALT1;PGM2;PGM1
 
 
 
 
RNA polymerase
 
 
5/31
 
 
0.4375679
 
 
0.6986636
 
 
0
 
 
0
 
 
1.188560
 
 
0.9823724
 
 
POLR2D;POLR1C;POLR3E;POLR1D;POLR3F
 
 
 
 
Basal transcription factors
 
 
7/45
 
 
0.4398145
 
 
0.6986636
 
 
0
 
 
0
 
 
1.138539
 
 
0.9351986
 
 
CCNH;TAF11;TBPL1;GTF2H1;TAF5;TAF9B;TAF4
 
 
 
 
Glycosphingolipid biosynthesis
 
 
7/45
 
 
0.4398145
 
 
0.6986636
 
 
0
 
 
0
 
 
1.138539
 
 
0.9351986
 
 
B4GALT3;B4GALT1;B3GNT5;ST3GAL6;ST6GALNAC3;FUT4;ST6GALNAC6
 
 
 
 
Necroptosis
 
 
23/159
 
 
0.4570466
 
 
0.7222757
 
 
0
 
 
0
 
 
1.045310
 
 
0.8184462
 
 
H2AZ1;USP21;VPS4A;TNFAIP3;FASLG;TNF;MACROH2A1;TNFRSF1A;GLUD1;MAPK8;AIFM1;IFNG;CHMP1B;SMPD1;CHMP2B;TRAF5;BCL2;CHMP4B;FAS;VDAC1;TRPM7;SLC25A4;PPID
 
 
 
 
Natural killer cell mediated cytotoxicity
 
 
19/131
 
 
0.4626498
 
 
0.7273618
 
 
0
 
 
0
 
 
1.048512
 
 
0.8081770
 
 
VAV3;MAP2K1;PIK3R3;FASLG;PIK3R1;TNF;PPP3R1;NRAS;IFNG;PIK3CA;CASP3;RAET1L;FAS;GRB2;KRAS;RAC1;RAF1;SOS1;SOS2
 
 
 
 
Various types of N-glycan biosynthesis
 
 
6/39
 
 
0.4650648
 
 
0.7274090
 
 
0
 
 
0
 
 
1.123676
 
 
0.8602620
 
 
B4GALT3;FUT8;B4GALT1;MGAT4A;RPN1;MAN1C1
 
 
 
 
Dopaminergic synapse
 
 
19/132
 
 
0.4767056
 
 
0.7418122
 
 
0
 
 
0
 
 
1.039172
 
 
0.7698772
 
 
GNAI3;CACNA1A;PPP2R2A;FOS;GNG12;GNAI1;PPP1CB;PPP2CA;PPP1CC;GNG10;MAPK8;CREB1;GNG2;GNG5;KIF5C;AKT3;CALM3;SCN1A;CREB5
 
 
 
 
Glycerolipid metabolism
 
 
9/61
 
 
0.4816787
 
 
0.7457462
 
 
0
 
 
0
 
 
1.069624
 
 
0.7813369
 
 
DGKD;GPAT4;LPL;GPAT3;PLPP1;AGPAT3;DGKH;AGPAT4;PNPLA2
 
 
 
 
Nicotine addiction
 
 
6/40
 
 
0.4907304
 
 
0.7539556
 
 
0
 
 
0
 
 
1.090563
 
 
0.7763284
 
 
GABRB3;GABRB2;GABRP;GABRB1;CHRNA7;CACNA1A
 
 
 
 
Gastric acid secretion
 
 
11/76
 
 
0.4954750
 
 
0.7539556
 
 
0
 
 
0
 
 
1.045816
 
 
0.7344119
 
 
KCNK10;CA2;GNAI3;ATP1B4;ADCY3;ADCY2;CALM3;KCNK2;GNAI1;KCNJ2;MYLK
 
 
 
 
Amphetamine addiction
 
 
10/69
 
 
0.4986757
 
 
0.7539556
 
 
0
 
 
0
 
 
1.047416
 
 
0.7287914
 
 
PPP1CB;PPP1CC;PPP3R1;CREB1;CAMK4;FOSB;CALM3;FOS;SIRT1;CREB5
 
 
 
 
 
 
 Reactome_Pathways_2024 
 Reactome is an expert-curated, peer-reviewed pathway database that
describes biological processes as detailed molecular reaction networks.
It emphasizes mechanistic precision, causal relationships, and
hierarchical pathway structure. Enrichment in Reactome pathways
indicates that target genes participate in specific molecular events or
subprocesses such as signal transduction modules, DNA repair mechanisms,
immune cascades, or cell-cycle transitions. Due to its high curation
standards, Reactome enrichment results are considered highly reliable
and mechanistically interpretable. 
 
 
 
 
Term
 
 
Overlap
 
 
P.value
 
 
Adjusted.P.value
 
 
Old.P.value
 
 
Old.Adjusted.P.value
 
 
Odds.Ratio
 
 
Combined.Score
 
 
Genes
 
 
 
 
 
 
Signal Transduction
 
 
527/2613
 
 
0.0000000
 
 
0.0000000
 
 
0
 
 
0
 
 
1.691849
 
 
80.410227
 
 
ITSN2;TFRC;WIPF1;MAML1;SERPINE1;STMN2;HTR4;FNBP1L;MYLK;PSMD7;AKT3;MYB;STMN1;PROK2;PIP4K2B;MEF2A;PRKAB2;RBFOX2;PRKCD;SOX13;FRS2;FRS3;DKK2;NISCH;MYL6;DYNC1LI2;MTMR1;SEH1L;PDGFB;CDCA8;PPP3R1;FLRT3;RDH10;PRKAR2A;GALR1;PDGFC;PIP5K1B;ST3GAL6;ICOS;WNT10B;STARD13;FZD3;ICMT;COL24A1;FZD5;FZD4;FZD6;PBX1;COL1A1;PPP5C;COL1A2;FAM135A;FASN;HNRNPF;GIPC1;AGO2;SNAI1;STRN;FERMT2;CDC42SE2;GABRB3;GABRB2;CDKN1A;GABRB1;PRKAG2;ASH2L;ARHGAP5;OSBPL11;SMPD3;IKBKB;SHH;TUBA1A;HEY2;RSPO2;RSPO3;IKBKE;LYPLA1;CNOT6L;GPR37;DUSP1;HSBP1;WNT9B;RRAS2;FOS;WNT9A;DYNLL1;DUSP9;DYNLL2;LRRC41;NCOR2;PSMA5;COL2A1;TUBB2A;MRAS;MMP16;PSMA1;RARA;CDC42EP2;RAPGEF2;RARB;PPARG;PAFAH1B1;RAPGEF4;ARF6;HBEGF;KANK1;PLPPR1;COL11A1;PTAFR;PLPPR5;ADCY3;ADCY2;FSTL1;LMAN1;NRAS;GPR150;NTF3;RICTOR;CSNK1A1;USP9X;WNT7A;COL3A1;FOSB;FILIP1;LAMTOR3;LGR4;LAMTOR5;POGLUT1;CCNT2;DGKD;PLXND1;FAM13B;UBE2D2;UBE2D3;UBE2D1;IRS2;IL1RAP;MSI2;CXCL13;FGF2;TNF;CDC14A;PPP1CB;YY1;PPP1CC;FGF7;FNTA;SALL4;GRAP2;MAF1;MAP3K7;TLE4;USP8;MAP2K1;FBXW7;TPM3;USP4;FST;CTNNBIP1;RPS6;FAM91A1;LEMD3;VRK3;ARMCX3;PKP4;MAPKAPK5;ROR1;IL6ST;S100A8;DGKH;DNMT1;CALCRL;CBFB;CUL5;CUL3;ARL2;RASAL1;FOXO4;FOXO3;RHOBTB1;RAP1B;SOCS3;BAG4;RAP1A;BCL2L11;SOCS1;EIF4EBP1;DVL3;SRGAP2;SOCS6;SKIL;TIA1;RAB4B;HTR1E;BDNF;ADAP1;CXCL10;CDK8;MOB1A;PTPRA;COPS2;BCL2;CDK1;ITGB1;ZNF512B;FLT3;PTEN;FASLG;HTR2A;DUSP16;CDC73;CAPZB;EFHD2;ITGB8;LBR;ACTR2;TRPC6;PPP1R12A;CHUK;SMC1A;NGF;ADRA2A;TNFRSF1A;TFDP1;TFDP2;PIK3CA;ADORA2B;ITGA8;SOS1;CRK;SOS2;HDAC4;PRKAA1;PRKAA2;LRP5;PDHB;CDC42;PARD6B;CCL7;POLR2D;IGF2BP1;CCL3;CCL2;E2F3;RBBP6;WHAMM;RBBP7;E2F5;WASF1;RNF20;LINGO1;SEMA4F;APLN;APC;VAPB;LEP;TNRC6A;PTPN3;CPNE8;APP;CCNK;SPARC;HHIP;CCNC;ZDHHC7;CRKL;RPS6KA3;GOLGA3;FRAT2;CHEK1;PDK4;SOX9;ATP6V1E1;BTRC;GOLGA7;CDON;PTGDR;SKP1;PDHX;ACTN2;PAQR3;OMG;RUNX1;TIAM1;SFRP1;TP53;EPHA2;PFN2;SPTBN4;TMEM87A;BEX3;GARRE1;RRAD;SH2D2A;TGFA;PIK3R3;TWF1;PIK3R1;MYL12B;SNX3;NLN;GNG10;NMB;EGR3;IGF1;GNG12;CCT6A;CYP26A1;ANLN;STAG1;STAG2;LPAR6;ID1;CD28;COL9A1;ID4;ID3;GRB2;COL9A3;RGL2;RCOR1;NRP1;AHCTF1;AP2A1;PTPRK;BMI1;PCSK5;MUC13;CCR7;RAC1;SPTAN1;TMED5;NDEL1;PSENEN;VAV3;NCOA1;SREBF1;EDN1;STRADB;NOG;GFRA1;RHOB;CNKSR2;NR5A2;ADRB3;MAPRE1;SGK1;YAP1;AMER1;DYNC1I2;PTH;PTGER3;GNAI3;GNAI1;TRAK2;PPP2CA;PGRMC2;WIF1;SHOC2;EPS15;DYNC1I1;PAK5;PTGES3;VEGFC;AP2B1;MPP7;VEGFA;FKBP1A;TJP1;KITLG;RIT1;XK;NEDD4;CAMK4;OTULIN;RAB9A;FAS;RAB9B;CDK5R1;CD86;FOXA1;PRDM4;IHH;NR3C1;IGF1R;AMOT;SYDE2;CCND1;EP300;RGS8;SH3GL2;PDGFRB;RALBP1;TMOD3;ADAM10;ATP1B4;TSC1;RPGRIP1L;KCTD3;ADAM17;MYCN;COL4A2;CCNE1;COL4A1;TBL1XR1;COL4A4;COL4A5;RAF1;PPID;PSMD12;CCL3L1;ADRB1;DTX2;ADRB2;RASGRP1;RTN4;NCKIPSD;ARHGAP21;ARHGAP20;CALCR;WNT1;MARK3;WNT4;SMAD2;CPSF7;TGFB2;TGFB1;CBX4;SMURF2;CAV2;WNT3A;SMURF1;CBX2;ARHGEF38;INHBB;ARHGAP28;ESR1;SMAD5;SMAD7;DIAPH2;COL5A1;FGF18;COL5A2;TCF4;AMOTL1;TCF3;FBN1;DOCK6;ABCD3;B4GALT1;DOCK3;SOWAHC;YWHAB;DOCK7;USP34;TNFAIP3;ATP2A2;CXCL1;YBX1;LAMC1;CHD1;CXCL2;GRM3;CYP26B1;SPRED1;EEF2K;GRM7;YWHAQ;CASP3;PDE4B;YWHAH;EED;AXIN2;ETV4;CKAP4;TGFBR1;SENP1;ACTA2;TGFBR3;CREB1;ALDH1A2;COL6A2;RASA1;COL6A3;COL6A6;RGS17;KDM3A;RALB;LAMA2;LAMA4;RGS16;RAP1GDS1;MOSPD2;ABHD17C;UACA;PTHLH;GNA13;DLL4;MAPK8;GNG2;ACVR1C;GNG5;ERBB3;CNR1;MKNK1;LRIG1;PDE6D;S1PR1;VHL;MTA3;CLASP2;TCF7L2;USP25;USP21;LIMK1;ACVR2B;REST;ACTC1;ABHD17B;SCD;SPRY2;KRAS;SPRY1;TEK;VIM;PDE7B;PDE7A
 
 
 
 
Post-translational Protein Modification
 
 
304/1457
 
 
0.0000000
 
 
0.0000000
 
 
0
 
 
0
 
 
1.706142
 
 
52.934010
 
 
APP;PGAP1;WIPF1;CCNF;DMP1;DCAF6;PSMD7;BTRC;SKP1;WSB1;NSMCE2;YOD1;DYNC1LI2;CDC34;FDX1;VDAC1;TP53;LIPT2;SPTBN4;NRN1;SEH1L;LMAN2L;DCUN1D3;RPN1;DCUN1D4;EIF5A2;CDCA8;COPS7B;TGFA;DCUN1D2;ADAMTS10;SNX3;POM121C;ARSJ;STC2;ADAMTS17;CHST10;DPH3;DPH5;ST3GAL6;ARSB;CTSA;ICMT;PMM1;SIAH2;RAB39B;INO80;TADA2B;STAG1;HNRNPK;STAG2;B4GAT1;CALU;RAET1L;ARF4;PIGU;CDKN1A;UBXN1;MIA2;MIA3;HIF3A;BMI1;RAB22A;PIGX;ING2;TUBA1A;MUC13;TMED7;IKBKE;SPTAN1;RNF152;RAB8A;NCOA1;TOMM70;AHSG;COMMD2;DYNLL1;JOSD1;DYNLL2;LRRC41;PSMA5;NCOR2;NR5A2;TUBB2A;PSMA1;B3GNT7;GAN;PIGA;PTRH2;B3GNT5;CANX;RARA;PPARG;ARF5;MATN3;AMER1;DYNC1I2;NAPB;STX17;SATB1;SATB2;DERL1;DLST;WDR20;METTL21A;FSTL1;NAPG;LMAN1;LMAN2;GCNT3;GCNT4;TAF9B;DYNC1I1;GOLM1;USP9X;C1GALT1;KLHL2;UBE2G2;PEX13;TTLL7;TTLL4;CAPZA1;OTULIN;CAPZA2;RAB9A;RAB9B;SPSB4;PNPLA2;GALNT12;GALNT13;GALNT15;UBE2D2;UBE2D3;UBE2D1;RORA;FBXO21;NR3C1;YY1;EP300;LARGE1;MAP3K7;LARGE2;PDIA3;USP8;FBXW7;USP4;UBE2E3;USP3;RPS6;VASH2;ADAM10;MITF;FBXO10;PDIA6;PIAS2;KLHL42;RAB32;TPST1;RAB30;RAB34;UBE2V2;RAB38;PSMD12;DNMT1;INO80D;CUL5;UBA6;CUL3;CUL2;UAP1;FOXO4;ADRB2;HSP90B1;DPP3;SOCS2;SOCS3;FUT8;TPR;SOCS6;UBE2F;SMAD2;SEC16B;TFAP2C;UBE2H;UBE2I;RAB4B;CBX5;TGFB1;NUP155;UBE2B;CBX4;SMURF2;GFPT2;ZBTB16;CBX2;GFPT1;SRD5A3;FBXO32;ESR1;KLHL20;SMAD7;NR4A2;UBE2W;CNIH1;COPS2;UBE2N;CDK1;ALPL;MDM4;CNTN4;UBE2K;CNIH3;FBN1;POMT2;B4GALT3;MCFD2;USP37;B4GALT1;USP34;PTEN;TNFAIP3;LAMC1;CDC73;PPP6C;CAPZB;PPP6R1;SUMO3;PGM3;DNMT3B;VCPKMT;ADAMTS8;ADAMTS9;PAAF1;ADAMTS7;USP47;USP48;USP42;USP44;DNMT3A;AXIN2;SMC1A;SAFB;SENP2;RAD23B;CKAP4;TGFBR1;SENP1;CCNA2;RCN1;UBE2R2;ASPH;TRAPPC6B;GORASP1;NUP50;TOP1;ST6GALNAC3;B4GALT6;SEC22A;SEC22C;ST6GALNAC6;OTUB1;B4GALT5;HDAC4;BECN1;USP14;SEC23A;UHRF2;GOSR2;POMP;USP12;FOXK2;ASB11;RNF7;USP19;RAB40A;CAND1;RAB40C;TDG;RAB23;RAB24;MAN1C1;VHL;RBBP7;RNF20;USP25;GALNT5;FEM1B;USP21;GALNT1;FBXL19;RAB10;APC;RAB14;RAB15;COL7A1;DOLPP1;MGAT4A;RAB12;RAB18;MDGA2;RECK;ASB3;BET1;CUL4B
 
 
 
 
Collagen Chain Trimerization
 
 
26/44
 
 
0.0000000
 
 
0.0000000
 
 
0
 
 
0
 
 
8.999517
 
 
236.293970
 
 
COL15A1;COL16A1;COL13A1;COL11A1;COL19A1;COL24A1;COL25A1;COL22A1;COL1A1;COL3A1;COL1A2;COL2A1;COL4A2;COL5A1;COL4A1;COL4A4;COL6A2;COL7A1;COL5A2;COL9A1;COL21A1;COL4A5;COL6A3;COL8A1;COL6A6;COL9A3
 
 
 
 
Signaling by Receptor Tyrosine Kinases
 
 
129/536
 
 
0.0000000
 
 
0.0000001
 
 
0
 
 
0
 
 
2.004906
 
 
45.488344
 
 
SPARC;IRS2;FGF2;CRKL;IGF1R;RPS6KA3;FGF7;GRAP2;AKT3;STMN1;CHEK1;EP300;ATP6V1E1;SH3GL2;MEF2A;PDGFRB;USP8;MAP2K1;RBFOX2;PRKCD;ADAM10;FRS2;VRK3;FRS3;TIAM1;ADAM17;MYCN;COL4A2;COL4A1;COL4A4;COL4A5;DNMT1;CUL5;RRAD;SH2D2A;PDGFB;PIK3R3;TGFA;PIK3R1;RAP1B;RAP1A;SOCS1;FLRT3;PDGFC;SOCS6;TIA1;RAB4B;EGR3;COL24A1;BDNF;IGF1;ADAP1;ESR1;COL1A1;COL1A2;COL5A1;GIPC1;HNRNPF;ID1;COL5A2;FGF18;COL9A1;ID4;ID3;COL9A3;GRB2;GABRB3;ITGB1;GABRB2;NRP1;GABRB1;DOCK3;FLT3;YWHAB;DOCK7;AP2A1;PTPRK;LAMC1;PCSK5;SPRED1;RAC1;PSENEN;VAV3;HSBP1;FOS;NGF;TGFBR3;COL2A1;CREB1;PIK3CA;COL6A2;RASA1;COL6A3;COL6A6;SOS1;SGK1;CRK;ARF6;HBEGF;YAP1;RALB;LAMA2;COL11A1;LAMA4;CDC42;PPP2CA;NRAS;ERBB3;MKNK1;NTF3;POLR2D;LRIG1;RICTOR;WASF1;EPS15;VEGFC;AP2B1;VEGFA;COL3A1;KITLG;RIT1;REST;NEDD4;FOSB;SPRY2;KRAS;SPRY1;PTPN3;CDK5R1
 
 
 
 
Collagen Biosynthesis and Modifying Enzymes
 
 
30/67
 
 
0.0000000
 
 
0.0000003
 
 
0
 
 
0
 
 
5.053446
 
 
105.169837
 
 
COLGALT2;COL15A1;COL16A1;COL13A1;COL11A1;COL19A1;COL24A1;COL25A1;COL22A1;P3H1;COL1A1;COL3A1;COL2A1;COL1A2;COL4A2;COL5A1;COL4A1;P4HA2;COL4A4;COL6A2;COL7A1;COL5A2;COL9A1;COL21A1;COL4A5;COL6A3;COL8A1;COL6A6;COL9A3;TLL1
 
 
 
 
Diseases of Signal Transduction by Growth Factor Receptors and Second
Messengers
 
 
110/451
 
 
0.0000000
 
 
0.0000004
 
 
0
 
 
0
 
 
2.033970
 
 
41.414201
 
 
RB1;CD86;MAML1;IHH;IRS2;CCNC;GCC2;FGF2;PPP1CB;PPP1CC;FGF7;PSMD7;AKT3;PIM1;EP300;SKP1;PDGFRB;MAP2K1;FBXW7;TPM3;RPS6;ADAM10;FRS2;FRS3;DKK2;EML4;ADAM17;TBL1XR1;CARS1;RAF1;TP53;DNMT1;PSMD12;PDGFB;PIK3R3;TGFA;FOXO4;PIK3R1;FOXO3;RAP1B;FXR1;BAG4;BCL2L11;RAP1A;TPR;ICOS;MARK3;MCL1;SMAD2;TGFB1;FZD5;FZD4;BDNF;WNT3A;FZD6;ERLIN2;ESR1;QKI;CDK8;AGO2;FGF18;CD28;STRN;GRB2;CDKN1A;FLT3;YWHAB;PTEN;DUSP16;MECP2;SHH;SPRED1;ZMYM2;HEY2;CNTRL;RAC1;IL10;PSENEN;CHUK;DUSP9;TGFBR1;NCOR2;PSMA5;CNKSR2;MRAS;CREB1;PSMA1;PIK3CA;SOS1;HBEGF;HDAC4;AMER1;KANK1;LRP5;PPP2CA;DLL4;NRAS;MAPK8;ERBB3;NTF3;POLR2D;RICTOR;SHOC2;TCF7L2;CSNK1A1;FKBP1A;CLCN6;KITLG;APC;KRAS
 
 
 
 
Collagen Formation
 
 
35/90
 
 
0.0000000
 
 
0.0000010
 
 
0
 
 
0
 
 
3.969234
 
 
76.903508
 
 
COLGALT2;COL15A1;COL16A1;COL13A1;COL11A1;CTSS;COL19A1;LOXL2;COL24A1;COL25A1;COL22A1;P3H1;COL1A1;COL3A1;COL2A1;COL1A2;COL4A2;LOX;COL5A1;COL4A1;P4HA2;COL4A4;COL6A2;COL7A1;PXDN;COL5A2;COL9A1;COL21A1;COL4A5;COL6A3;COL8A1;COL6A6;ITGA6;COL9A3;TLL1
 
 
 
 
Collagen Degradation
 
 
28/64
 
 
0.0000000
 
 
0.0000014
 
 
0
 
 
0
 
 
4.844332
 
 
91.492026
 
 
COL15A1;COL16A1;COL13A1;COL11A1;COL19A1;COL25A1;ADAM10;COL1A1;MMP11;ADAM17;COL3A1;COL2A1;COL1A2;COL4A2;COL5A1;COL4A1;MMP15;COL4A4;COL6A2;COL7A1;COL5A2;COL9A1;ADAM9;COL4A5;COL6A3;COL8A1;COL6A6;COL9A3
 
 
 
 
Signaling by TGFB Family Members
 
 
50/161
 
 
0.0000000
 
 
0.0000035
 
 
0
 
 
0
 
 
2.815809
 
 
50.355002
 
 
ITGB1;CCNK;CCNT2;SERPINE1;UBE2D3;UBE2D1;CCNC;YBX1;FGF2;PPP1CB;PPP1CC;EP300;ITGB8;PSENEN;FST;NOG;ATP1B4;TGFBR1;NCOR2;TGFBR3;MYCN;TFDP1;MMP16;TFDP2;ITGA8;RARA;FSTL1;ACVR1C;E2F5;SKIL;SMAD2;USP25;TGFB2;TGFB1;SMURF2;SMURF1;USP9X;INHBB;SMAD5;ACVR2B;SMAD7;FKBP1A;CDK8;COL1A2;GIPC1;AGO2;TCF4;TCF3;TNRC6A;FBN1
 
 
 
 
Antigen Processing Ubiquitination & Proteasome Degradation
 
 
77/296
 
 
0.0000000
 
 
0.0000043
 
 
0
 
 
0
 
 
2.205763
 
 
38.776796
 
 
TRIM71;UBE2D2;CCNF;UBE2D3;UBE2D1;CBLB;FBXO21;NPEPPS;CDC23;PSMD7;UBE2Q1;RNF19A;CDC27;BTRC;UNKL;SKP1;WSB1;TRIM63;FBXW7;UBE2E3;UBE4A;FBXO10;LRRC41;KLHL42;PSMA5;RNF144B;CDC34;PSMA1;UBE2R2;GAN;HECW2;UBE2V2;UBE2V1;PSMD12;RNF34;CUL5;UBA6;CUL3;CUL2;ASB11;RNF7;RCHY1;UBE2J1;SOCS3;ZNRF1;ZNRF2;SOCS1;RNF138;HECTD3;RNF217;RBBP6;VHL;LONRF1;UBE2F;UBE2H;TRIM41;SMURF2;UBE2B;SMURF1;SIAH2;ZBTB16;FBXL19;SIAH1;KLHL2;UBE2G2;FBXO32;KLHL20;UBE2W;NEDD4;RLIM;RNF182;UBE2N;BLMH;UBE2K;ASB3;RNF220;SPSB4
 
 
 
 
Assembly of Collagen Fibrils and Other Multimeric Structures
 
 
26/61
 
 
0.0000000
 
 
0.0000070
 
 
0
 
 
0
 
 
4.623747
 
 
78.553850
 
 
COL15A1;COL11A1;CTSS;LOXL2;COL24A1;COL1A1;COL3A1;COL2A1;COL1A2;COL4A2;LOX;COL5A1;COL4A1;COL4A4;COL6A2;COL7A1;PXDN;COL5A2;COL9A1;COL4A5;COL6A3;COL8A1;COL6A6;ITGA6;COL9A3;TLL1
 
 
 
 
Signaling by PDGF
 
 
25/58
 
 
0.0000001
 
 
0.0000089
 
 
0
 
 
0
 
 
4.714201
 
 
78.549683
 
 
PDGFB;PIK3R1;CRKL;NRAS;PDGFC;PDGFRB;COL3A1;COL2A1;COL4A2;PIK3CA;COL5A1;COL4A1;COL4A4;COL6A2;RASA1;COL5A2;COL9A1;COL4A5;COL6A3;COL6A6;COL9A3;GRB2;KRAS;SOS1;CRK
 
 
 
 
Signaling by MET
 
 
30/79
 
 
0.0000001
 
 
0.0000131
 
 
0
 
 
0
 
 
3.813202
 
 
61.777335
 
 
ITGB1;LAMA2;COL11A1;LAMA4;DOCK7;LAMC1;PIK3R1;CRKL;RAP1B;NRAS;RAP1A;LRIG1;RAC1;EPS15;SH3GL2;USP8;RAB4B;COL24A1;COL1A1;COL3A1;COL2A1;COL1A2;PIK3CA;COL5A1;COL5A2;GRB2;KRAS;SOS1;CRK;ARF6
 
 
 
 
Metabolism of Proteins
 
 
368/2067
 
 
0.0000001
 
 
0.0000145
 
 
0
 
 
0
 
 
1.389792
 
 
22.268137
 
 
EIF4A2;APP;PGAP1;WIPF1;CCNF;DMP1;DCAF6;PCMT1;PSMD7;BTRC;SKP1;WSB1;NSMCE2;YOD1;ISL1;AFG3L2;DYNC1LI2;CDC34;CLPP;FDX1;VDAC1;TP53;LIPT2;SPTBN4;NRN1;SEH1L;LMAN2L;DCUN1D3;MRPL18;DCUN1D4;RPN1;EIF5A2;GATA6;CDCA8;TGFA;DCUN1D2;COPS7B;ADAMTS10;SNX3;GNG10;POM121C;ARSJ;STC2;ADAMTS17;CHST10;DPH3;TSPAN5;SSR1;DPH5;ST3GAL6;ARSB;CTSA;ICMT;PMM1;SIAH2;SIAH1;RAB39B;IGF1;INO80;GNG12;CS;TADA2B;CCT6A;GLUD1;STAG1;HNRNPK;STAG2;B4GAT1;CALU;RAET1L;SFTA3;ARF4;CDKN1A;PIGU;TRAM1;UBXN1;MRPS10;MIA2;MIA3;HIF3A;BMI1;RAB22A;PIGX;ING2;TUBA1A;QARS1;MUC13;TMED7;SPTAN1;IKBKE;RNF152;RAB8A;PSENEN;NCOA1;TOMM70;ARG2;MRPS25;AHSG;COMMD2;JOSD1;DYNLL1;DYNLL2;LRRC41;NCOR2;PSMA5;TUBB2A;NR5A2;PSMA1;B3GNT7;GAN;PTRH2;PIGA;B3GNT5;CANX;RARA;PPARG;MATN3;ARF5;AMER1;DYNC1I2;NAPB;STX17;SATB1;TIMM9;SATB2;DERL1;DLST;WDR20;METTL21A;FSTL1;NAPG;LMAN1;RPS15A;LMAN2;RPL14;GCNT3;GCNT4;TAF9B;DYNC1I1;GOLM1;MDH2;USP9X;C1GALT1;KLHL2;UBE2G2;PEX13;TTLL7;TTLL4;CAPZA1;OTULIN;CAPZA2;RAB9A;RAB9B;PNPLA2;SPSB4;GALNT12;SRP19;GALNT13;GALNT15;UBE2D2;UBE2D3;UBE2D1;RORA;FBXO21;NR3C1;YARS2;YY1;KIF5C;EP300;LARGE1;MAP3K7;LARGE2;PDIA3;USP8;SLC30A8;FBXW7;USP4;USP3;UBE2E3;SLC30A5;RPS6;VASH2;ADAM10;MITF;FBXO10;PDIA6;PIAS2;KLHL42;RAB32;TPST1;OXA1L;RAB30;CCNE2;CCNE1;RAB34;UBE2V2;RAB38;CARS1;PSMD12;DNMT1;INO80D;CUL5;UBA6;CUL3;CUL2;ARL2;UAP1;FOXO4;ADRB2;HSP90B1;DPP3;SOCS2;SOCS3;FUT8;TPR;EIF4EBP1;SOCS6;UBE2F;HSPA9;SMAD2;SEC16B;TFAP2C;UBE2H;UBE2I;RAB4B;CBX5;TGFB1;NUP155;UBE2B;CBX4;SMURF2;CBX2;GFPT2;ZBTB16;IDH2;GFPT1;SRD5A3;INHBB;FBXO32;EIF2S2;EIF2S1;ESR1;KLHL20;SMAD7;NR4A2;UBE2W;CNIH1;COPS2;UBE2N;CDK1;SRPRA;ALPL;MDM4;CNTN4;UBE2K;CNIH3;FBN1;POMT2;B4GALT3;MCFD2;USP37;B4GALT1;USP34;PTEN;TNFAIP3;LAMC1;TRIAP1;CDC73;PPP6C;CAPZB;PPP6R1;SUMO3;PGM3;DNMT3B;VCPKMT;ADAMTS8;ADAMTS9;PAAF1;ADAMTS7;USP47;USP48;USP42;MARS2;USP44;ATP6AP2;DNMT3A;AXIN2;SMC1A;SAFB;SENP2;RAD23B;ADRA2A;CKAP4;TGFBR1;SENP1;CCNA2;RCN1;UBE2R2;ASPH;ADORA2B;TRAPPC6B;GORASP1;NUP50;ADGRF5;TOP1;ST6GALNAC3;B4GALT6;SEC22A;OTUB1;SEC22C;ST6GALNAC6;B4GALT5;HDAC4;BECN1;USP14;SEC23A;UHRF2;GOSR2;POMP;USP12;FOXK2;ASB11;RNF7;PDHB;USP19;HSPD1;RAB40A;CAND1;GNG2;GNG5;RAB40C;TDG;RAB23;RAB24;MAN1C1;IARS2;IARS1;VHL;RBBP7;H2AZ1;RNF20;TCF7L2;USP25;GALNT5;FEM1B;USP21;FBXL19;GALNT1;KLF4;WARS2;RAB10;EIF3M;TSPAN14;RAB14;APC;RAB15;COL7A1;DOLPP1;LEP;RAB12;MGAT4A;EIF3J;RAB18;MDGA2;RECK;ASB3;BET1;CUL4B
 
 
 
 
Class I MHC Mediated Antigen Processing & Presentation
 
 
89/373
 
 
0.0000002
 
 
0.0000186
 
 
0
 
 
0
 
 
1.967196
 
 
30.889929
 
 
TRIM71;UBE2D2;CCNF;UBE2D3;UBE2D1;CBLB;FBXO21;CTSS;IKBKB;NPEPPS;CDC23;PSMD7;UBE2Q1;RNF19A;CDC27;MRC1;BTRC;UNKL;SKP1;PDIA3;WSB1;TRIM63;CHUK;FBXW7;UBE2E3;TAP1;UBE4A;ATG14;FBXO10;LRRC41;KLHL42;PSMA5;VAMP8;RNF144B;CDC34;PSMA1;UBE2R2;GAN;HECW2;CANX;UBE2V2;UBE2V1;S100A8;BECN1;PSMD12;SEC23A;RNF34;CUL5;UBA6;CUL3;CUL2;ASB11;RNF7;RCHY1;UBE2J1;SOCS3;ZNRF1;ZNRF2;SOCS1;RNF138;HECTD3;RNF217;RBBP6;VHL;LONRF1;UBE2F;UBE2H;TRIM41;SMURF2;UBE2B;SMURF1;SIAH2;ZBTB16;FBXL19;SIAH1;KLHL2;UBE2G2;FBXO32;KLHL20;UBE2W;NEDD4;RLIM;RNF182;UBE2N;BLMH;UBE2K;ASB3;RNF220;SPSB4
 
 
 
 
Signaling by NTRKs
 
 
42/134
 
 
0.0000002
 
 
0.0000201
 
 
0
 
 
0
 
 
2.848603
 
 
44.332034
 
 
DOCK3;RALB;RRAD;YWHAB;IRS2;AP2A1;PIK3R1;PCSK5;CRKL;PPP2CA;RPS6KA3;NRAS;RAP1A;NTF3;EP300;RAC1;SH3GL2;MEF2A;MAP2K1;EGR3;BDNF;FRS2;FOS;AP2B1;VRK3;NGF;FRS3;TIAM1;REST;RIT1;CREB1;PIK3CA;ID1;ID4;ID3;FOSB;GRB2;KRAS;SGK1;SOS1;CRK;CDK5R1
 
 
 
 
Signaling by TGF-beta Receptor Complex
 
 
33/94
 
 
0.0000002
 
 
0.0000207
 
 
0
 
 
0
 
 
3.370684
 
 
52.162099
 
 
ITGB1;CCNK;CCNT2;SERPINE1;UBE2D3;UBE2D1;CCNC;YBX1;PPP1CB;PPP1CC;EP300;ITGB8;E2F5;SKIL;SMAD2;USP25;TGFB2;TGFB1;SMURF2;SMURF1;USP9X;ATP1B4;TGFBR1;SMAD7;NCOR2;TGFBR3;FKBP1A;CDK8;TFDP1;COL1A2;TFDP2;ITGA8;FBN1
 
 
 
 
Membrane Trafficking
 
 
134/633
 
 
0.0000003
 
 
0.0000297
 
 
0
 
 
0
 
 
1.692533
 
 
25.480213
 
 
ITSN2;APP;SCOC;SYS1;TFRC;GCC2;FNBP1L;KIF5C;AKT3;AP1S1;KIF21A;AP1S3;VPS36;SBF2;SH3GL2;AGFG1;PRKAB2;TSC1;GAPVD1;RAB32;DYNC1LI2;RAB30;BIN1;CHMP4B;RAB38;DENND6A;SPTBN4;LMAN2L;STXBP3;COPS7B;TGFA;ADRB2;GNS;GJC1;BLOC1S6;SNX2;SNX5;TXNDC5;SEC16B;SNAPIN;FZD4;VTA1;SURF4;RAB39B;TRAPPC8;SNAP91;CNIH1;COPS2;CHMP2B;GRB2;CNIH3;ARF4;MCFD2;YWHAB;PRKAG2;MIA2;AP2A1;MIA3;PIK3C2A;PPP6C;TUBA1A;CAPZB;YWHAQ;PPP6R1;AP3S1;RAC1;TMED7;SNAP29;SPTAN1;RAB8A;SH3D19;YWHAH;ACTR2;VPS37C;VPS37D;KIF23;DYNLL1;DYNLL2;VAMP8;TUBB2A;VAMP7;TRAPPC6B;GORASP1;TBC1D7;SEC22A;ARF5;PAFAH1B2;ARF6;HBEGF;PAFAH1B1;SEC22C;DYNC1I2;NAPB;SEC23A;PRKAA2;STX17;STX16;GOSR2;VPS4A;LRP2;NAPG;AGPAT3;CYTH3;LMAN1;CYTH2;LMAN2;STX6;UBQLN1;CLVS2;MAN1C1;LDLR;EPS15;DYNC1I1;CYTH1;CLVS1;REPS1;RIC1;GALNT1;DTNBP1;AP2B1;RAB10;TJP1;SNX18;NECAP2;RAB14;KIF26A;RAB12;CAPZA1;COL7A1;CAPZA2;RAB18;RAB9A;RAB9B;BET1
 
 
 
 
MET Promotes Cell Motility
 
 
19/41
 
 
0.0000006
 
 
0.0000557
 
 
0
 
 
0
 
 
5.365969
 
 
77.117504
 
 
ITGB1;LAMA2;COL24A1;COL11A1;LAMA4;DOCK7;LAMC1;CRKL;RAP1B;COL1A1;COL3A1;RAP1A;COL1A2;COL2A1;COL5A1;COL5A2;GRB2;RAC1;CRK
 
 
 
 
MAPK Family Signaling Cascades
 
 
76/314
 
 
0.0000007
 
 
0.0000613
 
 
0
 
 
0
 
 
2.000335
 
 
28.454598
 
 
FLT3;YWHAB;IRS2;FGF2;DUSP16;CDC14A;PPP1CB;PPP1CC;FGF7;SPRED1;PSMD7;FNTA;RAC1;GOLGA7;SPTAN1;PDGFRB;LYPLA1;MAP2K1;ACTN2;DUSP1;HSBP1;PAQR3;FRS2;GFRA1;FRS3;DUSP9;ETV4;CNKSR2;PSMA5;MRAS;PSMA1;PIK3CA;RASA1;CDC42EP2;RAPGEF2;MAPKAPK5;IL6ST;RAF1;SOS1;HBEGF;SPTBN4;PSMD12;CUL3;PDGFB;TGFA;ARL2;RASAL1;PIK3R1;ABHD17C;FOXO3;RASGRP1;PPP2CA;RAP1B;CDC42;NRAS;RAP1A;ERBB3;IGF2BP1;PDE6D;SHOC2;MARK3;ICMT;PPP5C;KITLG;ABHD17B;PTPRA;AGO2;FGF18;CDK1;GRB2;KRAS;TEK;RGL2;LAMTOR3;TNRC6A;PTPN3
 
 
 
 
Signaling by NTRK2 (TRKB)
 
 
14/25
 
 
0.0000010
 
 
0.0000863
 
 
0
 
 
0
 
 
7.898531
 
 
109.264761
 
 
DOCK3;BDNF;FRS2;PIK3R1;FRS3;TIAM1;NRAS;PIK3CA;NTF3;GRB2;KRAS;RAC1;SOS1;CDK5R1
 
 
 
 
Degradation of the Extracellular Matrix
 
 
41/140
 
 
0.0000018
 
 
0.0001481
 
 
0
 
 
0
 
 
2.582160
 
 
34.193572
 
 
COL15A1;COL16A1;COL13A1;ELN;COL11A1;LAMC1;NID1;CTSS;COL19A1;CASP3;CAPN5;CAPN3;ADAMTS8;ADAMTS9;CAST;COL25A1;ADAM10;COL1A1;MMP11;ADAM17;COL3A1;COL1A2;COL2A1;COL4A2;COL5A1;MMP16;COL4A1;MMP15;COL4A4;COL6A2;COL7A1;COL5A2;COL9A1;ADAM9;COL4A5;COL8A1;COL6A3;COL9A3;COL6A6;TLL1;FBN1
 
 
 
 
ECM Proteoglycans
 
 
27/76
 
 
0.0000018
 
 
0.0001481
 
 
0
 
 
0
 
 
3.428150
 
 
45.260563
 
 
ITGB1;APP;SPARC;LAMA2;LAMA4;SERPINE1;DMP1;LAMC1;TGFB2;TGFB1;COL1A1;COL3A1;COL2A1;COL1A2;COL4A2;COL5A1;COL4A1;COL4A4;COL6A2;COL5A2;ITGA8;COL9A1;COL4A5;COL6A3;COL6A6;COL9A3;MATN3
 
 
 
 
Developmental Biology
 
 
252/1385
 
 
0.0000026
 
 
0.0001999
 
 
0
 
 
0
 
 
1.411488
 
 
18.147457
 
 
EIF4A2;MAML1;CCNC;RPS6KA3;RPS6KA6;PSMD7;ALCAM;DPYSL5;SCN11A;AKT3;DPYSL3;MYB;HOXA3;DPF2;HOXA1;SOX9;PKNOX1;ATP6V1E1;SCN1A;CDON;MEF2A;EPHA7;MED8;MED9;EBF2;FRS2;DICER1;ISL1;RUNX1;TIAM1;MYL6;RFX6;HOXB4;LORICRIN;HOXB3;HOXB2;HOXB1;TP53;EPHA2;PFN2;SPTBN4;HOXD1;GATA6;PIK3R3;PIK3R1;MED12L;MYL12A;MYL12B;FLRT3;PRKAR2A;SCN3A;WNT10B;CDK19;ST14;FOXF1;SIAH2;SS18L1;RDX;SIAH1;NR2F2;MAGOHB;PBX1;SRPK1;CPEB1;AGO2;ID1;COL9A1;ID4;SNAI1;COL9A3;GRB2;NRP1;CDKN1A;HNF4G;AP2A1;ASH2L;CTCF;HIF3A;ROBO1;MED14;SHH;TUBA1A;QARS1;HEY2;RAC1;SPTAN1;NCOA1;PSENEN;VAV3;SS18;FOXD3;SREBF1;EDN1;CNOT6L;ELOVL3;NOG;WNT9B;TET2;TFEB;GFRA1;MED26;SIRT1;PAX2;MED28;RGMA;RHOB;PSMA5;NCOR2;NR5A2;COL2A1;TUBB2A;PSMA1;MED21;TET3;TFEC;RARA;RARB;PPARG;YAP1;ASAH1;AKAP5;NRAS;RELN;RPS15A;RPL14;PAIP2;PAK5;AP2B1;COL3A1;KITLG;NR6A1;WT1;CDK5R1;FOXA1;PLXND1;IHH;IRS2;FGF2;TNF;HOXA11;YY1;SALL1;CCND1;SALL4;SCN9A;EP300;SH3GL2;EOMES;MAP2K1;SEMA6A;SEMA6D;RPS6;ADAM10;MITF;PIAS2;COL4A2;COL4A1;TBL1XR1;COL4A4;COL4A5;PKP4;UTRN;PSMD12;SEMA7A;CBFB;CUL2;LPL;FOXO4;FOXO3;EFNA5;ZFP36L2;NEUROD1;EFNB2;KRTAP4-4;OTX2;WNT1;SRGAP2;WNT4;MAP2K6;SMAD2;PRNP;TFAP2C;TGFB1;WNT3A;CDK8;CNOT7;MAFB;LIN28A;COL5A1;PTPRA;LHX1;COL5A2;BCL2;TCF4;TCF3;ITGB1;ONECUT1;DPY30;SIX1;LAMC1;BCL7A;ZIC1;KDM6B;ACTR2;EMX2;TRPC6;EED;ATP6AP2;ARID1B;GREM1;GAP43;CREB1;TFDP1;PIK3CA;TFDP2;HAND2;COL6A2;RASA1;HAND1;ITGA8;COL6A3;COL6A6;SOS1;SCN4B;SOS2;LAMA2;CEBPD;HMGCR;CDC42;ACVR1C;PABPN1;POLR2D;IARS1;SLIT2;RBBP7;CACNG2;MTA3;CACNG4;CLASP2;LGI1;TCF7L2;EYA1;LIMK1;TBX5;KLF4;ACVR2B;MEIS1;KLF5;LEP;PMP22;KRAS;TNRC6A
 
 
 
 
Activation of BH3-only Proteins
 
 
15/30
 
 
0.0000027
 
 
0.0001999
 
 
0
 
 
0
 
 
6.206785
 
 
79.567155
 
 
YWHAB;DYNLL1;DYNLL2;MAPK8;PPP3R1;BCL2L11;TFDP1;YWHAQ;TFDP2;AKT3;BCL2;PMAIP1;BMF;TP53;YWHAH
 
 
 
 
Extracellular Matrix Organization
 
 
71/300
 
 
0.0000036
 
 
0.0002559
 
 
0
 
 
0
 
 
1.939629
 
 
24.310110
 
 
ITGB1;APP;COLGALT2;SPARC;COL16A1;ELN;SERPINE1;DMP1;LAMC1;FGF2;CTSS;LOXL2;CASP3;CAPN5;CAPN3;ITGB8;ADAMTS8;ADAMTS9;CAST;COL25A1;P3H1;ADAM10;CASK;MMP11;ADAM17;COL2A1;MMP16;COL4A2;LOX;MMP15;COL4A1;COL4A4;COL6A2;PXDN;ITGA8;COL8A1;COL21A1;ADAM9;COL4A5;COL6A3;COL6A6;ITGA6;MATN3;COL15A1;COL13A1;LAMA2;COL11A1;LAMA4;PDGFB;NID1;COL19A1;TGFB2;TGFB1;COL24A1;COL22A1;GDF5;COL1A1;COL3A1;COL1A2;COL5A1;P4HA2;COL7A1;MFAP3;COL5A2;ITGA11;MFAP2;COL9A1;SDC1;COL9A3;TLL1;FBN1
 
 
 
 
Signaling by NTRK1 (TRKA)
 
 
35/115
 
 
0.0000038
 
 
0.0002599
 
 
0
 
 
0
 
 
2.724873
 
 
34.006369
 
 
RALB;RRAD;YWHAB;IRS2;AP2A1;PIK3R1;CRKL;PPP2CA;RPS6KA3;NRAS;RAP1A;EP300;SH3GL2;MEF2A;MAP2K1;EGR3;FRS2;FOS;AP2B1;VRK3;NGF;REST;RIT1;CREB1;PIK3CA;ID1;ID4;ID3;FOSB;GRB2;KRAS;SGK1;SOS1;CRK;CDK5R1
 
 
 
 
Gene Expression (Transcription)
 
 
286/1615
 
 
0.0000053
 
 
0.0003483
 
 
0
 
 
0
 
 
1.367377
 
 
16.614840
 
 
RB1;CCNK;CCNH;MAML1;SERPINE1;PHAX;CCNC;CDC23;PSMD7;CLP1;AKT3;CDC27;MYB;CHEK1;PDK4;DPF2;EPC1;PIP4K2B;SOX9;SKP1;ZNF287;PRKAB2;ZNF282;MED8;ZNF10;DICER1;CDC40;RUNX1;ZNF839;WDR82;DDIT4;ZSCAN25;DNA2;ZNF710;TP53;SLBP;ZNF274;SEH1L;TGFA;ZNF23;POM121C;NRBF2;ZNF268;ZNF267;ZNF266;NR2C2AP;SETDB1;GADD45A;PLK2;SS18L1;MAGOHB;NR2F6;COL1A1;COL1A2;AGO2;ZNF136;BTG2;CDKN1A;ZNF250;SETD9;SUV39H1;HNF4G;PRKAG2;ASH2L;BMI1;MECP2;ING5;MED14;ING2;HEY2;RSPO3;JARID2;NCOA1;SREBF1;SS18;CNOT6L;ELOVL5;TET2;TET1;FOS;BAZ1B;MED26;SIRT1;NCOR2;PSMA5;NR5A2;PSMA1;MTF2;TET3;RARA;RARB;PPARG;SGK1;YAP1;ZNF473;ZNF471;NFIX;ZNF470;SATB2;PPP2CA;ZFP14;RICTOR;TAF9B;ZNF225;SRSF11;ZNF583;PCGF5;USP9X;MBD2;GTF2H1;TARBP2;VEGFA;ZNF33B;TJP1;NR6A1;NFIA;PRKRA;CAMK4;CCNG1;FAS;ZNF699;ZNF354A;ZNF697;LAMTOR3;PNPLA2;LAMTOR5;CDK5R1;ZNF354B;CCNT2;UBE2D3;IHH;UBE2D1;RORA;ARID4B;RORB;NR3C1;YY1;IPO8;CCND2;CCND1;ZFP30;SESN1;SESN2;EP300;ZNF689;PITX2;ARID2;ZNF566;PHLDA1;ZNF200;LMO1;ZNF562;THOC1;ATP1B4;KCTD1;TSC1;NRBP1;MAF;CCNE2;CCNE1;TBL1XR1;SRSF3;MAPKAPK5;SRSF7;SRSF9;PSMD12;DNMT1;CBFB;LPL;FOXO4;FOXO3;SOCS3;BCL2L11;TPR;ZKSCAN3;PMAIP1;TASP1;ZKSCAN4;RPRD1A;SKIL;ZKSCAN1;SOCS4;ZNF420;MAP2K6;ESRRA;SMAD2;CPSF4;TFAP2C;CPSF7;TFAP2D;UBE2I;CBX5;TGFB1;NUP155;CBX4;SMURF2;CBX3;BDNF;SMURF1;CBX2;ESRRG;FBXO32;ESR1;SMAD7;NR4A2;CDK8;CNOT7;JMY;CDK1;NABP1;CYCS;MDM4;TCF3;ZNF774;PHF19;DYRK2;YWHAB;PTEN;DPY30;FASLG;SLC2A3;YBX1;TRIAP1;CDC73;BCL7A;YWHAQ;DNMT3B;MYBL2;YAF2;LBR;ZNF385A;YWHAH;TRIM63;EED;DNMT3A;ARID1B;CCNA2;ELF2;TFDP1;CREB1;TFDP2;IFNG;HAND2;NUP50;CAT;POLR1C;POLR1D;HDAC4;PRKAA1;PRKAA2;RNF34;GLS2;PPM1D;HSPD1;PABPN1;TDG;SAP30L;POLR2D;TP53INP1;DROSHA;RBBP7;E2F5;RFFL;E2F6;MTA3;ZNF620;E2F7;ZNF740;TCF7L2;RRM2;TXNRD1;TAF11;ARID3A;TBX5;KLF4;REST;SCD;SYT10;POLR3E;POLR3F;KRAS;THRSP;ERCC6;TAF5;TAF4;TNRC6A
 
 
 
 
MAPK1 MAPK3 Signaling
 
 
65/271
 
 
0.0000057
 
 
0.0003641
 
 
0
 
 
0
 
 
1.972290
 
 
23.808806
 
 
FLT3;YWHAB;IRS2;FGF2;DUSP16;PPP1CB;PPP1CC;FGF7;SPRED1;PSMD7;FNTA;GOLGA7;SPTAN1;PDGFRB;LYPLA1;MAP2K1;ACTN2;DUSP1;PAQR3;FRS2;GFRA1;FRS3;DUSP9;CNKSR2;PSMA5;MRAS;PSMA1;PIK3CA;RASA1;RAPGEF2;IL6ST;RAF1;SOS1;HBEGF;SPTBN4;PSMD12;CUL3;PDGFB;TGFA;ARL2;RASAL1;PIK3R1;ABHD17C;RASGRP1;PPP2CA;RAP1B;NRAS;RAP1A;ERBB3;PDE6D;SHOC2;MARK3;ICMT;PPP5C;KITLG;ABHD17B;PTPRA;FGF18;CDK1;GRB2;KRAS;TEK;RGL2;LAMTOR3;PTPN3
 
 
 
 
NCAM Signaling for Neurite Out-Growth
 
 
23/63
 
 
0.0000062
 
 
0.0003823
 
 
0
 
 
0
 
 
3.574032
 
 
42.848264
 
 
SPTBN4;PRNP;GFRA1;NRAS;COL3A1;COL2A1;CREB1;COL4A2;COL5A1;COL4A1;PTPRA;COL4A4;COL6A2;COL5A2;COL9A1;COL4A5;COL6A3;COL6A6;COL9A3;GRB2;KRAS;SOS1;SPTAN1
 
 
 
 
Transcriptional Activity of SMAD2 SMAD3 SMAD4 Heterotrimer
 
 
20/51
 
 
0.0000071
 
 
0.0004216
 
 
0
 
 
0
 
 
4.007884
 
 
47.526506
 
 
SMAD2;CCNK;CCNT2;SMURF2;USP9X;SERPINE1;UBE2D3;UBE2D1;ATP1B4;CCNC;YBX1;SMAD7;NCOR2;CDK8;TFDP1;COL1A2;TFDP2;EP300;E2F5;SKIL
 
 
 
 
RAF MAP Kinase Cascade
 
 
63/265
 
 
0.0000107
 
 
0.0006159
 
 
0
 
 
0
 
 
1.948484
 
 
22.305223
 
 
FLT3;YWHAB;IRS2;FGF2;DUSP16;PPP1CB;PPP1CC;FGF7;SPRED1;PSMD7;FNTA;GOLGA7;SPTAN1;PDGFRB;LYPLA1;MAP2K1;ACTN2;DUSP1;PAQR3;FRS2;GFRA1;FRS3;DUSP9;CNKSR2;PSMA5;MRAS;PSMA1;PIK3CA;RASA1;RAPGEF2;RAF1;SOS1;HBEGF;SPTBN4;PSMD12;CUL3;PDGFB;TGFA;ARL2;RASAL1;PIK3R1;ABHD17C;RASGRP1;PPP2CA;RAP1B;NRAS;RAP1A;ERBB3;PDE6D;SHOC2;MARK3;ICMT;PPP5C;KITLG;ABHD17B;PTPRA;FGF18;GRB2;KRAS;TEK;RGL2;LAMTOR3;PTPN3
 
 
 
 
SUMOylation
 
 
46/176
 
 
0.0000128
 
 
0.0007144
 
 
0
 
 
0
 
 
2.206244
 
 
24.860824
 
 
HDAC4;DNMT1;SEH1L;UHRF2;SATB1;SATB2;CDCA8;RORA;NR3C1;BMI1;ING2;POM121C;SUMO3;TDG;TPR;DNMT3B;EP300;VHL;IKBKE;NCOA1;TFAP2C;CBX5;UBE2I;CBX4;NUP155;CBX2;NSMCE2;DNMT3A;MITF;SAFB;SENP2;SMC1A;ESR1;PIAS2;SENP1;NR4A2;NCOR2;STAG1;HNRNPK;STAG2;NR5A2;NUP50;RARA;PPARG;TOP1;TP53
 
 
 
 
Extra-nuclear Estrogen Signaling
 
 
25/75
 
 
0.0000159
 
 
0.0008624
 
 
0
 
 
0
 
 
3.108294
 
 
34.347192
 
 
GNAI3;PIK3R3;TGFA;ZDHHC7;PIK3R1;FOXO3;GNAI1;IGF1R;GNG10;NRAS;GNG2;CCND1;GNG5;AKT3;HSBP1;CAV2;FOS;GNG12;ESR1;CREB1;PIK3CA;BCL2;KRAS;STRN;HBEGF
 
 
 
 
Generic Transcription Pathway
 
 
222/1230
 
 
0.0000183
 
 
0.0009656
 
 
0
 
 
0
 
 
1.392035
 
 
15.184622
 
 
RB1;CCNK;CCNH;MAML1;SERPINE1;CCNC;PSMD7;CDC23;AKT3;CDC27;MYB;CHEK1;EPC1;PIP4K2B;SOX9;SKP1;ZNF287;PRKAB2;ZNF282;MED8;ZNF10;RUNX1;ZNF839;DDIT4;ZNF710;ZSCAN25;DNA2;TP53;ZNF274;TGFA;ZNF23;NRBF2;ZNF268;ZNF267;ZNF266;NR2C2AP;GADD45A;PLK2;NR2F6;COL1A1;COL1A2;AGO2;ZNF136;BTG2;CDKN1A;ZNF250;SETD9;HNF4G;PRKAG2;ASH2L;BMI1;MECP2;ING5;MED14;ING2;HEY2;RSPO3;SREBF1;CNOT6L;FOS;MED26;SIRT1;PSMA5;NCOR2;NR5A2;PSMA1;RARA;RARB;PPARG;SGK1;YAP1;ZNF473;ZNF471;ZNF470;SATB2;PPP2CA;ZFP14;RICTOR;ZNF225;TAF9B;ZNF583;USP9X;PCGF5;GTF2H1;VEGFA;ZNF33B;TJP1;NR6A1;CAMK4;CCNG1;FAS;ZNF699;ZNF354A;ZNF697;LAMTOR3;LAMTOR5;CDK5R1;ZNF354B;CCNT2;UBE2D3;IHH;UBE2D1;RORA;RORB;NR3C1;YY1;CCND2;CCND1;ZFP30;SESN1;SESN2;EP300;ZNF689;PITX2;ARID2;ZNF566;ZNF200;ZNF562;LMO1;ATP1B4;KCTD1;TSC1;NRBP1;MAF;CCNE2;CCNE1;TBL1XR1;MAPKAPK5;PSMD12;CBFB;FOXO4;FOXO3;SOCS3;BCL2L11;ZKSCAN3;PMAIP1;ZKSCAN4;SKIL;SOCS4;ZKSCAN1;ZNF420;MAP2K6;ESRRA;SMAD2;TFAP2C;TFAP2D;UBE2I;CBX5;TGFB1;CBX4;SMURF2;BDNF;CBX3;SMURF1;CBX2;ESRRG;FBXO32;ESR1;SMAD7;NR4A2;CDK8;CNOT7;JMY;CDK1;CYCS;MDM4;TCF3;ZNF774;DYRK2;YWHAB;PTEN;DPY30;FASLG;SLC2A3;TRIAP1;YBX1;YWHAQ;MYBL2;YAF2;LBR;ZNF385A;YWHAH;TRIM63;EED;ARID1B;CCNA2;CREB1;ELF2;TFDP1;TFDP2;IFNG;HAND2;CAT;HDAC4;PRKAA1;PRKAA2;RNF34;GLS2;PPM1D;HSPD1;TP53INP1;POLR2D;RBBP7;E2F5;RFFL;E2F6;ZNF620;E2F7;ZNF740;TCF7L2;RRM2;TXNRD1;TAF11;ARID3A;TBX5;KLF4;REST;SYT10;KRAS;TAF5;TAF4;TNRC6A
 
 
 
 
Cytokine Signaling in Immune System
 
 
149/776
 
 
0.0000207
 
 
0.0010436
 
 
0
 
 
0
 
 
1.494777
 
 
16.119210
 
 
EIF4A2;CD86;APP;UBE2D2;UBE2D3;UBE2D1;RORA;IRS2;IL1RAP;IFI30;FGF2;TNF;CRKL;RPS6KA3;PSMD7;CCND1;GRAP2;AKT3;PIM1;IL12B;KPNA5;BTRC;KPNA3;MAP3K7;KPNA1;SKP1;MEF2A;MAP2K4;MAP2K1;PRKCD;VRK3;ADAM17;PDE12;FSCN1;UBE2V1;IL6ST;RAF1;TP53;KPNB1;PSMD12;SEH1L;CCL3L1;CUL5;PIK3R3;TNFRSF11B;TNFRSF11A;PIK3R1;FOXO3;HSP90B1;RAP1B;SOCS2;CNN2;SOCS3;BCL2L11;SOCS1;POM121C;IRAK2;TPR;NDN;SOCS6;N4BP1;MAP2K6;MCL1;HSPA9;UBE2I;TGFB1;TSLP;NUP155;LIF;FANCB;EIF2S2;EIF2S1;FANCF;SMAD7;CXCL10;COL1A2;HNRNPF;UBE2N;BCL2;CDK1;SDC1;TNFSF9;GRB2;EIF4G2;ITGB1;SNAP25;CDKN1A;EIF4E3;FLT3;FASLG;CXCL1;YBX1;CXCL2;TNFSF13B;IKBKB;GHR;TUBA1A;NKIRAS2;CASP3;IL10;EDARADD;CHUK;IL15;IL13;FOS;TNFRSF1B;OSMR;TNFRSF1A;PSMA5;VAMP7;CREB1;TUBB2A;PSMA1;PIK3CA;IFNG;NUP50;IL1RAPL1;CANX;IRF2;EIF4E2;SOS1;CRK;SOS2;HLA-DQB1;BECN1;USP14;CEBPD;PELI2;PTAFR;RNF7;CDC42;PPP2CA;NRAS;MAPK8;CCL3;S1PR1;CCL2;STX3;GBP1;GBP3;TARBP2;VEGFA;PRKRA;NEDD4;CAPZA1;HNRNPA2B1;KRAS;VIM;ABCE1
 
 
 
 
Kidney Development
 
 
18/46
 
 
0.0000209
 
 
0.0010436
 
 
0
 
 
0
 
 
3.991381
 
 
43.006854
 
 
ITGB1;EMX2;EYA1;WNT9B;SIX1;GFRA1;FGF2;PAX2;HOXA11;GREM1;SALL1;WT1;LHX1;HOXB4;ID4;ITGA8;SLIT2;WNT4
 
 
 
 
Non-integrin membrane-ECM Interactions
 
 
21/59
 
 
0.0000244
 
 
0.0011830
 
 
0
 
 
0
 
 
3.432911
 
 
36.467359
 
 
ITGB1;TGFB1;LAMA2;COL11A1;LAMA4;PDGFB;CASK;LAMC1;FGF2;COL1A1;COL3A1;COL2A1;COL1A2;COL4A2;COL5A1;COL4A1;COL4A4;COL5A2;COL4A5;SDC1;ITGA6
 
 
 
 
SUMO E3 Ligases SUMOylate Target Proteins
 
 
44/170
 
 
0.0000253
 
 
0.0011971
 
 
0
 
 
0
 
 
2.176236
 
 
23.035681
 
 
HDAC4;DNMT1;SEH1L;UHRF2;SATB1;SATB2;CDCA8;RORA;NR3C1;BMI1;ING2;POM121C;SUMO3;TDG;TPR;DNMT3B;EP300;VHL;IKBKE;NCOA1;TFAP2C;CBX5;UBE2I;CBX4;NUP155;CBX2;NSMCE2;DNMT3A;MITF;SAFB;SMC1A;ESR1;PIAS2;NR4A2;NCOR2;STAG1;HNRNPK;STAG2;NR5A2;NUP50;RARA;PPARG;TOP1;TP53
 
 
 
 
Intrinsic Pathway for Apoptosis
 
 
20/55
 
 
0.0000264
 
 
0.0012114
 
 
0
 
 
0
 
 
3.549014
 
 
37.415023
 
 
YWHAB;DYNLL1;DYNLL2;UACA;CASP7;MAPK8;PPP3R1;TFDP1;BCL2L11;YWHAQ;TFDP2;CASP3;AKT3;BCL2;PMAIP1;CYCS;BMF;BAK1;TP53;YWHAH
 
 
 
 
PI3K AKT Signaling in Cancer
 
 
32/110
 
 
0.0000269
 
 
0.0012114
 
 
0
 
 
0
 
 
2.552707
 
 
26.862596
 
 
CD86;CDKN1A;FLT3;PDGFB;PTEN;PIK3R3;TGFA;IRS2;FOXO4;PIK3R1;FOXO3;FGF2;FGF7;ERBB3;AKT3;NTF3;RICTOR;RAC1;ICOS;PDGFRB;CHUK;BDNF;FRS2;ESR1;KITLG;CREB1;PIK3CA;FGF18;CD28;GRB2;STRN;HBEGF
 
 
 
 
Signaling by Interleukins
 
 
94/452
 
 
0.0000344
 
 
0.0015130
 
 
0
 
 
0
 
 
1.644085
 
 
16.895883
 
 
ITGB1;CD86;SNAP25;APP;CDKN1A;IRS2;RORA;FASLG;CXCL1;IL1RAP;FGF2;TNF;CXCL2;CRKL;IKBKB;RPS6KA3;PSMD7;CCND1;NKIRAS2;CASP3;PIM1;IL12B;BTRC;MAP3K7;SKP1;MEF2A;IL10;MAP2K4;MAP2K1;CHUK;IL15;IL13;FOS;VRK3;TNFRSF1B;OSMR;TNFRSF1A;PSMA5;VAMP7;CREB1;PSMA1;PIK3CA;IFNG;IL1RAPL1;CANX;FSCN1;UBE2V1;IL6ST;SOS1;CRK;SOS2;TP53;USP14;PSMD12;CEBPD;CCL3L1;PELI2;PTAFR;PIK3R3;PIK3R1;FOXO3;HSP90B1;RAP1B;SOCS2;CDC42;PPP2CA;CNN2;SOCS3;MAPK8;SOCS1;IRAK2;CCL3;S1PR1;NDN;CCL2;STX3;N4BP1;MAP2K6;MCL1;HSPA9;TGFB1;TSLP;LIF;VEGFA;CXCL10;COL1A2;HNRNPF;CAPZA1;HNRNPA2B1;BCL2;UBE2N;SDC1;GRB2;VIM
 
 
 
 
Crosslinking of Collagen Fibrils
 
 
10/18
 
 
0.0000405
 
 
0.0017398
 
 
0
 
 
0
 
 
7.747659
 
 
78.356355
 
 
COL1A1;COL1A2;COL4A2;LOX;COL4A1;COL4A4;PXDN;COL4A5;TLL1;LOXL2
 
 
 
 
Vesicle-mediated Transport
 
 
143/752
 
 
0.0000493
 
 
0.0020703
 
 
0
 
 
0
 
 
1.475232
 
 
14.629344
 
 
ITSN2;APP;SCOC;SPARC;SYS1;TFRC;GCC2;FNBP1L;KIF5C;AKT3;AP1S1;KIF21A;AP1S3;VPS36;SBF2;SH3GL2;AGFG1;PRKAB2;TSC1;GAPVD1;RAB32;DYNC1LI2;RAB30;COL4A2;BIN1;COL4A1;CHMP4B;RAB38;DENND6A;SPTBN4;LMAN2L;STXBP3;COPS7B;TGFA;ADRB2;GNS;HSP90B1;GJC1;BLOC1S6;SNX2;SNX5;TXNDC5;SEC16B;CD163;SNAPIN;FZD4;VTA1;SURF4;RAB39B;TRAPPC8;SNAP91;COL1A1;COL1A2;CNIH1;COPS2;CHMP2B;GRB2;CNIH3;ARF4;MCFD2;YWHAB;STAB2;PRKAG2;MIA2;AP2A1;MIA3;PIK3C2A;PPP6C;TUBA1A;CAPZB;YWHAQ;PPP6R1;AP3S1;RAC1;TMED7;SNAP29;SPTAN1;RAB8A;SH3D19;YWHAH;ACTR2;VPS37C;VPS37D;KIF23;DYNLL1;DYNLL2;VAMP8;TUBB2A;VAMP7;TRAPPC6B;GORASP1;TBC1D7;SEC22A;ARF5;PAFAH1B2;ARF6;HBEGF;PAFAH1B1;SEC22C;DYNC1I2;NAPB;SEC23A;PRKAA2;STX17;STX16;GOSR2;VPS4A;LRP2;NAPG;AGPAT3;CYTH3;LMAN1;CYTH2;LMAN2;STX6;UBQLN1;CLVS2;MAN1C1;LDLR;EPS15;DYNC1I1;CYTH1;CLVS1;REPS1;RIC1;GALNT1;DTNBP1;AP2B1;RAB10;TJP1;COL3A1;SNX18;NECAP2;RAB14;KIF26A;RAB12;CAPZA1;COL7A1;CAPZA2;RAB18;RAB9A;RAB9B;BET1
 
 
 
 
Transport to the Golgi and Subsequent Modification
 
 
46/186
 
 
0.0000570
 
 
0.0023369
 
 
0
 
 
0
 
 
2.047456
 
 
20.009890
 
 
SPTBN4;ARF4;B4GALT3;DYNC1I2;NAPB;MCFD2;SEC23A;B4GALT1;LMAN2L;STX17;GOSR2;TGFA;MIA2;MIA3;NAPG;PPP6C;LMAN1;FUT8;TUBA1A;CAPZB;PPP6R1;LMAN2;CHST10;MAN1C1;TMED7;SPTAN1;DYNC1I1;SEC16B;DYNLL1;DYNLL2;DYNC1LI2;CNIH1;TUBB2A;TRAPPC6B;GORASP1;CAPZA1;COL7A1;MGAT4A;CAPZA2;B4GALT6;CNIH3;SEC22A;ARF5;B4GALT5;SEC22C;BET1
 
 
 
 
RNA Polymerase II Transcription
 
 
238/1360
 
 
0.0000751
 
 
0.0030132
 
 
0
 
 
0
 
 
1.339660
 
 
12.722586
 
 
RB1;CCNK;CCNH;MAML1;SERPINE1;CCNC;PHAX;PSMD7;CDC23;CLP1;AKT3;CDC27;MYB;CHEK1;EPC1;PIP4K2B;SOX9;SKP1;ZNF287;PRKAB2;ZNF282;MED8;ZNF10;CDC40;RUNX1;ZNF839;DDIT4;ZNF710;ZSCAN25;DNA2;TP53;SLBP;ZNF274;TGFA;ZNF23;NRBF2;ZNF268;ZNF267;ZNF266;NR2C2AP;GADD45A;PLK2;MAGOHB;NR2F6;COL1A1;COL1A2;AGO2;ZNF136;BTG2;CDKN1A;ZNF250;SETD9;HNF4G;PRKAG2;ASH2L;BMI1;MECP2;ING5;MED14;ING2;HEY2;RSPO3;SREBF1;CNOT6L;FOS;MED26;SIRT1;PSMA5;NCOR2;NR5A2;PSMA1;RARA;RARB;PPARG;SGK1;YAP1;ZNF473;ZNF471;ZNF470;SATB2;PPP2CA;ZFP14;RICTOR;ZNF225;TAF9B;SRSF11;ZNF583;USP9X;PCGF5;GTF2H1;VEGFA;ZNF33B;TJP1;NR6A1;CAMK4;CCNG1;FAS;ZNF699;ZNF354A;ZNF697;LAMTOR3;LAMTOR5;CDK5R1;ZNF354B;CCNT2;UBE2D3;IHH;UBE2D1;RORA;RORB;NR3C1;YY1;CCND2;CCND1;ZFP30;SESN1;SESN2;EP300;ZNF689;PITX2;ARID2;ZNF566;ZNF200;ZNF562;LMO1;THOC1;ATP1B4;KCTD1;TSC1;NRBP1;MAF;CCNE2;CCNE1;TBL1XR1;SRSF3;MAPKAPK5;SRSF7;SRSF9;PSMD12;CBFB;FOXO4;FOXO3;SOCS3;BCL2L11;ZKSCAN3;PMAIP1;ZKSCAN4;RPRD1A;SKIL;SOCS4;ZKSCAN1;ZNF420;MAP2K6;ESRRA;SMAD2;CPSF4;TFAP2C;CPSF7;TFAP2D;UBE2I;CBX5;TGFB1;CBX4;SMURF2;BDNF;CBX3;SMURF1;CBX2;ESRRG;FBXO32;ESR1;SMAD7;NR4A2;CDK8;CNOT7;JMY;CDK1;CYCS;NABP1;MDM4;TCF3;ZNF774;DYRK2;YWHAB;PTEN;DPY30;FASLG;SLC2A3;TRIAP1;YBX1;CDC73;YWHAQ;MYBL2;YAF2;LBR;ZNF385A;YWHAH;TRIM63;EED;ARID1B;CCNA2;CREB1;ELF2;TFDP1;TFDP2;IFNG;HAND2;CAT;HDAC4;PRKAA1;PRKAA2;RNF34;GLS2;PPM1D;HSPD1;PABPN1;TP53INP1;POLR2D;RBBP7;E2F5;RFFL;E2F6;ZNF620;E2F7;ZNF740;TCF7L2;RRM2;TXNRD1;TAF11;ARID3A;TBX5;KLF4;REST;SYT10;KRAS;TAF5;TAF4;TNRC6A
 
 
 
 
RUNX3 Regulates p14-ARF
 
 
7/10
 
 
0.0000823
 
 
0.0032327
 
 
0
 
 
0
 
 
14.450882
 
 
135.911455
 
 
HDAC4;TGFB1;CBFB;CCND1;EP300;KRAS;RUNX1
 
 
 
 
Synthesis of Active Ubiquitin Roles of E1 and E2 Enzymes
 
 
13/30
 
 
0.0000847
 
 
0.0032564
 
 
0
 
 
0
 
 
4.742390
 
 
44.468001
 
 
UBE2H;UBE2B;UBA6;USP9X;UBE2D2;UBE2E3;UBE2D1;UBE2G2;UBE2W;CDC34;UBE2R2;OTULIN;UBE2K
 
 
 
 
Signaling by FLT3 ITD and TKD Mutants
 
 
9/16
 
 
0.0000881
 
 
0.0033192
 
 
0
 
 
0
 
 
7.966614
 
 
74.384090
 
 
CDKN1A;NRAS;PIK3CA;FLT3;PIM1;GRB2;KRAS;PIK3R1;SOS1
 
 
 
 
Interleukin-4 and Interleukin-13 Signaling
 
 
31/112
 
 
0.0001006
 
 
0.0037137
 
 
0
 
 
0
 
 
2.380063
 
 
21.907272
 
 
ITGB1;CDKN1A;CEBPD;RORA;FASLG;PIK3R1;FOXO3;FGF2;TNF;HSP90B1;SOCS3;SOCS1;CCND1;PIM1;S1PR1;IL12B;NDN;CCL2;MCL1;IL10;TGFB1;IL13;LIF;FOS;TNFRSF1B;VEGFA;COL1A2;FSCN1;BCL2;VIM;TP53
 
 
 
 
Transcriptional Regulation of White Adipocyte Differentiation
 
 
25/84
 
 
0.0001315
 
 
0.0047601
 
 
0
 
 
0
 
 
2.632766
 
 
23.527572
 
 
CEBPD;LPL;CCNC;TNF;MED14;EP300;WNT1;NCOA1;SREBF1;WNT10B;CDK19;TGFB1;MED8;MED9;NR2F2;KLF4;MED26;MED28;NCOR2;CDK8;KLF5;TBL1XR1;MED21;LEP;PPARG
 
 
 
 
Signaling by Nuclear Receptors
 
 
60/270
 
 
0.0001348
 
 
0.0047841
 
 
0
 
 
0
 
 
1.782203
 
 
15.882967
 
 
FOXA1;ZDHHC7;CHD1;IGF1R;YY1;CYP26B1;CCND1;AKT3;MYB;PDK4;EP300;NCOA1;PDHX;SREBF1;HSBP1;FOS;SMC1A;RUNX1;NCOR2;NR5A2;CREB1;PIK3CA;TBL1XR1;ALDH1A2;RARA;RARB;PPID;HBEGF;KANK1;KDM3A;CBFB;GNAI3;PIK3R3;TGFA;PIK3R1;PDHB;FOXO3;GNAI1;GNG10;NRAS;GNG2;GNG5;RDH10;POLR2D;CAV2;PTGES3;GNG12;ESR1;CYP26A1;PPP5C;STAG1;STAG2;SCD;FASN;AGO2;BCL2;FOSB;STRN;KRAS;TNRC6A
 
 
 
 
Gain-of-function MRAS Complexes Activate RAF Signaling
 
 
6/8
 
 
0.0001580
 
 
0.0052217
 
 
0
 
 
0
 
 
18.574101
 
 
162.572585
 
 
PPP1CB;PPP1CC;MRAS;YWHAB;SHOC2;RAF1
 
 
 
 
SHOC2 M1731 Mutant Abolishes MRAS Complex Function
 
 
6/8
 
 
0.0001580
 
 
0.0052217
 
 
0
 
 
0
 
 
18.574101
 
 
162.572585
 
 
PPP1CB;PPP1CC;MRAS;YWHAB;SHOC2;RAF1
 
 
 
 
Signaling by MRAS-complex Mutants
 
 
6/8
 
 
0.0001580
 
 
0.0052217
 
 
0
 
 
0
 
 
18.574101
 
 
162.572585
 
 
PPP1CB;PPP1CC;MRAS;YWHAB;SHOC2;RAF1
 
 
 
 
Integrin Cell Surface Interactions
 
 
25/85
 
 
0.0001619
 
 
0.0052217
 
 
0
 
 
0
 
 
2.588736
 
 
22.595159
 
 
ITGB1;COL16A1;COL13A1;ITGB8;COL1A1;COL3A1;COL2A1;COL1A2;COL4A2;COL5A1;COL4A1;COL4A4;COL6A2;COL7A1;COL5A2;ITGA11;ITGA8;COL9A1;COL4A5;COL6A3;COL8A1;COL6A6;ITGA6;COL9A3;FBN1
 
 
 
 
Aberrant Regulation of Mitotic G1 S Transition in Cancer Due to RB1
Defects
 
 
9/17
 
 
0.0001641
 
 
0.0052217
 
 
0
 
 
0
 
 
6.970382
 
 
60.748715
 
 
RB1;CDKN1A;CCND2;TFDP1;CCND1;CCNE2;TFDP2;CCNE1;E2F3
 
 
 
 
Defective Binding of RB1 Mutants to E2F1,(E2F2, E2F3)
 
 
9/17
 
 
0.0001641
 
 
0.0052217
 
 
0
 
 
0
 
 
6.970382
 
 
60.748715
 
 
RB1;CDKN1A;CCND2;TFDP1;CCND1;CCNE2;TFDP2;CCNE1;E2F3
 
 
 
 
Nuclear Receptor Transcription Pathway
 
 
18/53
 
 
0.0001819
 
 
0.0055971
 
 
0
 
 
0
 
 
3.191804
 
 
27.487623
 
 
ESRRA;HNF4G;RORA;ESRRG;RORB;NR3C1;ESR1;NRBP1;NR2F6;NCOR2;NR4A2;NR5A2;NR6A1;RARA;RARB;PPARG;NRBF2;NR2C2AP
 
 
 
 
Signaling by EGFR
 
 
18/53
 
 
0.0001819
 
 
0.0055971
 
 
0
 
 
0
 
 
3.191804
 
 
27.487623
 
 
ADAM10;TGFA;PTPRK;PIK3R1;CDC42;ADAM17;NRAS;PIK3CA;LRIG1;SPRY2;GRB2;KRAS;SPRY1;SOS1;EPS15;SH3GL2;HBEGF;PTPN3
 
 
 
 
TP53 Regulates Transcription of Genes Involved in G1 Cell Cycle Arrest
 
 
8/14
 
 
0.0001921
 
 
0.0057398
 
 
0
 
 
0
 
 
8.259179
 
 
70.678872
 
 
CCNA2;CDKN1A;CCNE2;CCNE1;ARID3A;TP53;E2F7;ZNF385A
 
 
 
 
Activated NTRK2 Signals Through FRS2 and FRS3
 
 
7/11
 
 
0.0001990
 
 
0.0057398
 
 
0
 
 
0
 
 
10.837532
 
 
92.359822
 
 
NRAS;BDNF;FRS2;GRB2;KRAS;SOS1;FRS3
 
 
 
 
MET Activates RAP1 and RAC1
 
 
7/11
 
 
0.0001990
 
 
0.0057398
 
 
0
 
 
0
 
 
10.837532
 
 
92.359822
 
 
RAP1B;RAP1A;DOCK7;GRB2;RAC1;CRK;CRKL
 
 
 
 
Signaling by FGFR4 in Disease
 
 
7/11
 
 
0.0001990
 
 
0.0057398
 
 
0
 
 
0
 
 
10.837532
 
 
92.359822
 
 
NRAS;PIK3CA;FRS2;GRB2;KRAS;PIK3R1;SOS1
 
 
 
 
TP53 Regulates Transcription of Cell Cycle Genes
 
 
17/49
 
 
0.0002035
 
 
0.0057788
 
 
0
 
 
0
 
 
3.296474
 
 
28.019837
 
 
BTG2;CDKN1A;CNOT6L;GADD45A;PLK2;ARID3A;CCNA2;TFDP1;CNOT7;CCNE2;TFDP2;CCNE1;CDK1;EP300;TP53;ZNF385A;E2F7
 
 
 
 
Signaling by ERBB4
 
 
19/58
 
 
0.0002085
 
 
0.0058329
 
 
0
 
 
0
 
 
3.023964
 
 
25.629185
 
 
GABRB3;PSENEN;YAP1;GABRB2;GABRB1;SPARC;PIK3R1;ADAP1;ESR1;ADAM17;NRAS;PIK3CA;ERBB3;NEDD4;STMN1;GRB2;KRAS;SOS1;HBEGF
 
 
 
 
Formation of the Ureteric Bud
 
 
10/21
 
 
0.0002190
 
 
0.0060336
 
 
0
 
 
0
 
 
5.633678
 
 
47.472185
 
 
ITGB1;GREM1;SALL1;EYA1;ITGA8;SIX1;GFRA1;SLIT2;PAX2;HOXA11
 
 
 
 
ESR-mediated Signaling
 
 
45/192
 
 
0.0002569
 
 
0.0069731
 
 
0
 
 
0
 
 
1.906090
 
 
15.757564
 
 
FOXA1;KANK1;CBFB;GNAI3;PIK3R3;TGFA;ZDHHC7;PIK3R1;FOXO3;CHD1;GNAI1;IGF1R;YY1;GNG10;NRAS;GNG2;CCND1;GNG5;AKT3;MYB;POLR2D;EP300;NCOA1;CAV2;HSBP1;PTGES3;FOS;GNG12;SMC1A;ESR1;RUNX1;PPP5C;STAG1;STAG2;CREB1;NR5A2;PIK3CA;AGO2;BCL2;FOSB;STRN;KRAS;PPID;TNRC6A;HBEGF
 
 
 
 
Constitutive Signaling by Aberrant PI3K in Cancer
 
 
24/83
 
 
0.0002883
 
 
0.0076718
 
 
0
 
 
0
 
 
2.526541
 
 
20.595369
 
 
CD86;PDGFRB;FLT3;BDNF;PDGFB;PIK3R3;TGFA;IRS2;FRS2;PIK3R1;FGF2;ESR1;FGF7;KITLG;PIK3CA;ERBB3;NTF3;FGF18;CD28;GRB2;STRN;RAC1;ICOS;HBEGF
 
 
 
 
ER to Golgi Anterograde Transport
 
 
38/155
 
 
0.0002909
 
 
0.0076718
 
 
0
 
 
0
 
 
2.020696
 
 
16.453497
 
 
SPTBN4;ARF4;DYNC1I2;NAPB;MCFD2;SEC23A;LMAN2L;STX17;GOSR2;TGFA;MIA2;MIA3;NAPG;PPP6C;LMAN1;TUBA1A;CAPZB;PPP6R1;LMAN2;TMED7;SPTAN1;DYNC1I1;SEC16B;DYNLL1;DYNLL2;DYNC1LI2;CNIH1;TUBB2A;TRAPPC6B;GORASP1;CAPZA1;COL7A1;CAPZA2;CNIH3;SEC22A;ARF5;SEC22C;BET1
 
 
 
 
COPII-mediated Vesicle Transport
 
 
21/69
 
 
0.0003144
 
 
0.0081750
 
 
0
 
 
0
 
 
2.716139
 
 
21.905033
 
 
NAPB;SEC16B;MCFD2;SEC23A;LMAN2L;STX17;GOSR2;TGFA;NAPG;PPP6C;LMAN1;CNIH1;PPP6R1;TRAPPC6B;GORASP1;LMAN2;COL7A1;SEC22A;CNIH3;SEC22C;BET1
 
 
 
 
NCAM1 Interactions
 
 
15/42
 
 
0.0003317
 
 
0.0085044
 
 
0
 
 
0
 
 
3.445808
 
 
27.605334
 
 
PRNP;GFRA1;COL3A1;COL2A1;COL4A2;COL5A1;COL4A1;COL4A4;COL6A2;COL5A2;COL9A1;COL4A5;COL6A3;COL6A6;COL9A3
 
 
 
 
RHO GTPase Cycle
 
 
89/450
 
 
0.0003374
 
 
0.0085327
 
 
0
 
 
0
 
 
1.540562
 
 
12.315500
 
 
ITSN2;ITGB1;ZNF512B;DOCK6;CPNE8;ABCD3;DOCK3;TFRC;FAM13B;PLXND1;WIPF1;SOWAHC;DOCK7;STMN2;MSI2;FNBP1L;ARHGAP5;OSBPL11;SYDE2;GOLGA3;CAPZB;EFHD2;MUC13;RAC1;LBR;SPTAN1;VAV3;RALBP1;TPM3;TMOD3;RRAS2;FRS2;KCTD3;FAM91A1;LEMD3;FRS3;CKAP4;RHOB;SENP1;NISCH;ARMCX3;TIAM1;PIK3CA;CDC42EP2;PKP4;SOS1;SOS2;EPHA2;MTMR1;TMEM87A;GARRE1;CUL3;MOSPD2;PIK3R3;TWF1;PIK3R1;UACA;RHOBTB1;CDC42;GNA13;LMAN1;ARHGAP21;PARD6B;ARHGAP20;PGRMC2;RBBP6;WHAMM;SRGAP2;WASF1;PAK5;RNF20;STARD13;CPSF7;USP9X;SEMA4F;ARHGAP28;MPP7;CCT6A;ANLN;DIAPH2;FAM135A;ACTC1;VAPB;COPS2;FILIP1;GRB2;VIM;FERMT2;CDC42SE2
 
 
 
 
Anchoring Fibril Formation
 
 
8/15
 
 
0.0003613
 
 
0.0090133
 
 
0
 
 
0
 
 
7.078885
 
 
56.105618
 
 
COL1A1;COL1A2;COL4A2;COL4A1;COL4A4;COL7A1;COL4A5;TLL1
 
 
 
 
Signalling to ERKs
 
 
13/34
 
 
0.0003814
 
 
0.0093876
 
 
0
 
 
0
 
 
3.838185
 
 
30.212850
 
 
MAP2K1;RALB;YWHAB;FRS2;NGF;CRKL;NRAS;RIT1;RAP1A;GRB2;KRAS;SOS1;CRK
 
 
 
 
MET Activates PTK2 Signaling
 
 
12/30
 
 
0.0003916
 
 
0.0095112
 
 
0
 
 
0
 
 
4.132660
 
 
32.422084
 
 
ITGB1;COL1A1;COL3A1;COL1A2;COL2A1;LAMA2;COL24A1;COL5A1;COL11A1;LAMA4;COL5A2;LAMC1
 
 
 
 
TGF-beta Receptor Signaling Activates SMADs
 
 
16/47
 
 
0.0003973
 
 
0.0095241
 
 
0
 
 
0
 
 
3.201677
 
 
25.072002
 
 
ITGB1;SMAD2;USP25;TGFB2;TGFB1;SMURF2;SMURF1;TGFBR1;SMAD7;PPP1CB;FKBP1A;TGFBR3;PPP1CC;ITGA8;ITGB8;FBN1
 
 
 
 
Adipogenesis
 
 
29/110
 
 
0.0004087
 
 
0.0096303
 
 
0
 
 
0
 
 
2.224896
 
 
17.359553
 
 
CEBPD;LPL;CCNC;TNF;MED14;EP300;RBBP7;WNT1;MTA3;NCOA1;WNT10B;SREBF1;CDK19;TGFB1;ELOVL3;MED8;EBF2;MED9;NR2F2;KLF4;MED26;MED28;NCOR2;CDK8;KLF5;TBL1XR1;MED21;LEP;PPARG
 
 
 
 
PIP3 Activates AKT Signaling
 
 
60/281
 
 
0.0004138
 
 
0.0096303
 
 
0
 
 
0
 
 
1.692401
 
 
13.183847
 
 
CD86;CDKN1A;FLT3;PTEN;IRS2;IL1RAP;BMI1;FGF2;FGF7;PSMD7;SALL4;AKT3;PIP4K2B;RAC1;MAF1;PDGFRB;CNOT6L;CHUK;EED;FRS2;PSMA5;CREB1;PSMA1;PIK3CA;PPARG;SGK1;TP53;HBEGF;PSMD12;PDGFB;PIK3R3;TGFA;PIK3R1;FOXO4;FOXO3;PPP2CA;ERBB3;NTF3;PIP5K1B;RICTOR;RBBP7;ICOS;MTA3;CBX4;BDNF;CBX2;ESR1;KITLG;REST;NEDD4;AGO2;FGF18;CD28;SNAI1;GRB2;STRN;LAMTOR3;RCOR1;TNRC6A;LAMTOR5
 
 
 
 
SHC-related Events Triggered by IGF1R
 
 
6/9
 
 
0.0004179
 
 
0.0096303
 
 
0
 
 
0
 
 
12.382014
 
 
96.334172
 
 
NRAS;GRB2;KRAS;IGF1;SOS1;IGF1R
 
 
 
 
Signaling by WNT
 
 
61/287
 
 
0.0004226
 
 
0.0096303
 
 
0
 
 
0
 
 
1.682663
 
 
13.072896
 
 
USP34;AP2A1;ASH2L;CDC73;FRAT2;PSMD7;RSPO2;RSPO3;EP300;SOX9;RAC1;BTRC;MAP3K7;TMED5;SKP1;TLE4;USP8;CTNNBIP1;SOX13;WNT9B;WNT9A;AXIN2;DKK2;PSMA5;SFRP1;PSMA1;ROR1;AMER1;PSMD12;CUL3;LRP5;SNX3;PPP2CA;GNG10;PPP3R1;GNG2;GNG5;WIF1;DVL3;PIP5K1B;WNT1;WNT4;WNT10B;TCF7L2;FZD3;FZD5;SMURF2;FZD4;CSNK1A1;SMURF1;WNT3A;FZD6;WNT7A;AP2B1;GNG12;APC;AGO2;TCF4;KRAS;LGR4;TNRC6A
 
 
 
 
Signaling by Rho GTPases, Miro GTPases and RHOBTB3
 
 
127/688
 
 
0.0004544
 
 
0.0101152
 
 
0
 
 
0
 
 
1.417800
 
 
10.912168
 
 
ITSN2;CPNE8;TFRC;FAM13B;PLXND1;WIPF1;STMN2;MSI2;FNBP1L;MYLK;PPP1CB;SYDE2;PPP1CC;GOLGA3;RALBP1;TPM3;TMOD3;PRKCD;FRS2;KCTD3;FAM91A1;LEMD3;FRS3;NISCH;ARMCX3;TIAM1;DYNC1LI2;MYL6;CCNE1;PKP4;S100A8;EPHA2;PFN2;MTMR1;TMEM87A;SEH1L;GARRE1;CUL3;CDCA8;PIK3R3;TWF1;PIK3R1;RHOBTB1;MYL12B;NCKIPSD;ARHGAP21;ARHGAP20;DVL3;SRGAP2;STARD13;CPSF7;ARHGAP28;CCT6A;ANLN;DIAPH2;FAM135A;COPS2;GRB2;FERMT2;CDC42SE2;ITGB1;ZNF512B;DOCK6;AHCTF1;ABCD3;DOCK3;SOWAHC;YWHAB;DOCK7;ARHGAP5;OSBPL11;TUBA1A;CAPZB;YWHAQ;EFHD2;MUC13;RAC1;LBR;SPTAN1;NDEL1;YWHAH;VAV3;ACTR2;PPP1R12A;RRAS2;DYNLL1;DYNLL2;LRRC41;CKAP4;RHOB;SENP1;TUBB2A;PIK3CA;CDC42EP2;SOS1;MAPRE1;SOS2;PAFAH1B1;DYNC1I2;RAP1GDS1;MOSPD2;UACA;TRAK2;CDC42;PPP2CA;GNA13;LMAN1;PARD6B;PGRMC2;RBBP6;WHAMM;VHL;WASF1;DYNC1I1;PAK5;CLASP2;RNF20;USP9X;LIMK1;SEMA4F;MPP7;ACTC1;VAPB;RAB9A;FILIP1;VIM;RAB9B
 
 
 
 
Signaling by FGFR1
 
 
17/52
 
 
0.0004548
 
 
0.0101152
 
 
0
 
 
0
 
 
3.013393
 
 
23.190031
 
 
FRS2;PIK3R1;FGF2;FRS3;TGFBR3;PPP2CA;SPRED1;NRAS;PIK3CA;FLRT3;MKNK1;GIPC1;FGF18;SPRY2;GRB2;KRAS;SOS1
 
 
 
 
Signaling by FLT3 Fusion Proteins
 
 
9/19
 
 
0.0004791
 
 
0.0105280
 
 
0
 
 
0
 
 
5.575657
 
 
42.618539
 
 
CDKN1A;NRAS;ZMYM2;PIK3CA;PIM1;GRB2;KRAS;PIK3R1;SOS1
 
 
 
 
Syndecan Interactions
 
 
11/27
 
 
0.0005670
 
 
0.0123137
 
 
0
 
 
0
 
 
4.260766
 
 
31.849934
 
 
ITGB1;COL1A1;COL3A1;TGFB1;COL1A2;COL5A1;COL5A2;CASK;SDC1;ITGA6;FGF2
 
 
 
 
Synthesis of PIPs at the Plasma Membrane
 
 
17/53
 
 
0.0005837
 
 
0.0125283
 
 
0
 
 
0
 
 
2.929517
 
 
21.813752
 
 
PI4K2B;MTMR1;MTMR3;PLEKHA1;MTMR14;PTEN;PLEKHA5;MTMR9;PIK3R3;PIK3R1;PIK3C2A;PLEKHA8;PIK3CA;RAB14;PIP4K2B;PIP5K1B;SBF2
 
 
 
 
Negative Regulation of the PI3K AKT Network
 
 
30/118
 
 
0.0006360
 
 
0.0134948
 
 
0
 
 
0
 
 
2.118436
 
 
15.592370
 
 
CD86;FLT3;PDGFB;PTEN;PIK3R3;TGFA;IRS2;IL1RAP;PIK3R1;FGF2;PPP2CA;FGF7;ERBB3;AKT3;NTF3;PIP4K2B;PIP5K1B;RAC1;ICOS;PDGFRB;BDNF;FRS2;ESR1;KITLG;PIK3CA;FGF18;CD28;GRB2;STRN;HBEGF
 
 
 
 
SMAD2 SMAD3 SMAD4 Heterotrimer Regulates Transcription
 
 
13/36
 
 
0.0007248
 
 
0.0150329
 
 
0
 
 
0
 
 
3.504022
 
 
25.332871
 
 
SMAD2;CCNK;CCNT2;SERPINE1;CCNC;YBX1;SMAD7;CDK8;COL1A2;TFDP1;TFDP2;EP300;E2F5
 
 
 
 
Transcriptional Regulation by the AP-2 (TFAP2) Family of Transcription
Factors
 
 
13/36
 
 
0.0007248
 
 
0.0150329
 
 
0
 
 
0
 
 
3.504022
 
 
25.332871
 
 
CDKN1A;TFAP2C;TFAP2D;UBE2I;TGFA;KCTD1;ESR1;HSPD1;VEGFA;YY1;EP300;MYBL2;PITX2
 
 
 
 
Transcriptional Regulation of Pluripotent Stem Cells
 
 
12/32
 
 
0.0007813
 
 
0.0160244
 
 
0
 
 
0
 
 
3.718962
 
 
26.607709
 
 
EOMES;SMAD2;FOXD3;SALL1;LIN28A;NR6A1;SALL4;GATA6;HIF3A;KLF4;FGF2;PBX1
 
 
 
 
RAS Processing
 
 
10/24
 
 
0.0008178
 
 
0.0165199
 
 
0
 
 
0
 
 
4.425690
 
 
31.461836
 
 
LYPLA1;NRAS;ICMT;ABHD17B;FNTA;PDE6D;ARL2;KRAS;ABHD17C;GOLGA7
 
 
 
 
Asparagine N-linked Glycosylation
 
 
63/306
 
 
0.0008233
 
 
0.0165199
 
 
0
 
 
0
 
 
1.615824
 
 
11.475869
 
 
ARF4;B4GALT3;MCFD2;UBXN1;B4GALT1;MIA2;MIA3;PPP6C;TUBA1A;CAPZB;PPP6R1;PGM3;TMED7;SPTAN1;PDIA3;DYNLL1;DYNLL2;RAD23B;DYNC1LI2;TUBB2A;TRAPPC6B;GORASP1;CANX;B4GALT6;ST6GALNAC3;SEC22A;ARF5;B4GALT5;SEC22C;ST6GALNAC6;SPTBN4;DYNC1I2;NAPB;SEC23A;LMAN2L;STX17;GOSR2;RPN1;DERL1;TGFA;UAP1;NAPG;LMAN1;FUT8;LMAN2;CHST10;MAN1C1;ST3GAL6;DYNC1I1;CTSA;SEC16B;PMM1;GFPT2;GFPT1;SRD5A3;CNIH1;CAPZA1;COL7A1;DOLPP1;MGAT4A;CAPZA2;CNIH3;BET1
 
 
 
 
SOS-mediated Signalling
 
 
5/7
 
 
0.0008587
 
 
0.0170443
 
 
0
 
 
0
 
 
15.472852
 
 
109.240111
 
 
NRAS;GRB2;IRS2;KRAS;SOS1
 
 
 
 
RHOBTB3 ATPase Cycle
 
 
6/10
 
 
0.0009214
 
 
0.0180947
 
 
0
 
 
0
 
 
9.285971
 
 
64.905406
 
 
CCNE1;CUL3;RAB9A;VHL;LRRC41;RAB9B
 
 
 
 
RAB Geranylgeranylation
 
 
19/65
 
 
0.0010317
 
 
0.0200472
 
 
0
 
 
0
 
 
2.562750
 
 
17.622917
 
 
RAB4B;RAB39B;RAB22A;RAB32;RAB10;RAB30;RAB40A;RAB40C;RAB14;RAB15;RAB34;RAB23;RAB12;RAB24;RAB18;RAB9A;RAB38;RAB8A;RAB9B
 
 
 
 
Cargo Concentration in the ER
 
 
12/33
 
 
0.0010736
 
 
0.0203290
 
 
0
 
 
0
 
 
3.541662
 
 
24.213551
 
 
LMAN1;MCFD2;SEC23A;CNIH1;LMAN2L;GOSR2;LMAN2;COL7A1;MIA2;TGFA;MIA3;CNIH3
 
 
 
 
Intracellular Signaling by Second Messengers
 
 
65/321
 
 
0.0010771
 
 
0.0203290
 
 
0
 
 
0
 
 
1.582412
 
 
10.813310
 
 
CD86;CDKN1A;FLT3;PTEN;IRS2;IL1RAP;BMI1;FGF2;FGF7;PSMD7;SALL4;AKT3;PIP4K2B;RAC1;MAF1;PDGFRB;CNOT6L;CHUK;EED;PRKCD;FRS2;PSMA5;CREB1;PSMA1;PIK3CA;PPARG;SGK1;TP53;HBEGF;PSMD12;PDGFB;PIK3R3;TGFA;ADCY3;ADCY2;PIK3R1;FOXO4;FOXO3;PPP2CA;ERBB3;PRKAR2A;NTF3;PIP5K1B;RICTOR;RBBP7;ICOS;MTA3;CBX4;BDNF;CBX2;ESR1;KITLG;REST;CAMK4;NEDD4;AGO2;FGF18;CD28;SNAI1;GRB2;STRN;LAMTOR3;RCOR1;TNRC6A;LAMTOR5
 
 
 
 
PI5P, PP2A and IER3 Regulate PI3K AKT Signaling
 
 
28/111
 
 
0.0010792
 
 
0.0203290
 
 
0
 
 
0
 
 
2.095407
 
 
14.314810
 
 
CD86;FLT3;PDGFB;PIK3R3;TGFA;IRS2;IL1RAP;PIK3R1;FGF2;PPP2CA;FGF7;ERBB3;NTF3;PIP4K2B;PIP5K1B;RAC1;ICOS;PDGFRB;BDNF;FRS2;ESR1;KITLG;PIK3CA;FGF18;CD28;GRB2;STRN;HBEGF
 
 
 
 
Toll Like Receptor 3 (TLR3) Cascade
 
 
27/106
 
 
0.0011221
 
 
0.0209231
 
 
0
 
 
0
 
 
2.122605
 
 
14.417912
 
 
APP;USP14;UBE2D2;UBE2D3;UBE2D1;IKBKB;PPP2CA;RPS6KA3;MAPK8;IRAK2;NKIRAS2;BTRC;MAP3K7;IKBKE;N4BP1;SKP1;MAP2K6;MEF2A;MAP2K4;MAP2K1;CHUK;FOS;VRK3;CREB1;UBE2N;UBE2V1;TP53
 
 
 
 
Downstream Signal Transduction
 
 
11/29
 
 
0.0011523
 
 
0.0212545
 
 
0
 
 
0
 
 
3.786907
 
 
25.622181
 
 
PDGFRB;NRAS;PIK3CA;RASA1;PDGFB;GRB2;KRAS;PIK3R1;SOS1;CRK;CRKL
 
 
 
 
Regulation of FZD by Ubiquitination
 
 
9/21
 
 
0.0011724
 
 
0.0212545
 
 
0
 
 
0
 
 
4.645841
 
 
31.353284
 
 
USP8;FZD5;FZD4;WNT3A;FZD6;LRP5;RSPO2;RSPO3;LGR4
 
 
 
 
Neddylation
 
 
50/234
 
 
0.0011744
 
 
0.0212545
 
 
0
 
 
0
 
 
1.691417
 
 
11.411973
 
 
CDKN1A;WIPF1;UBE2D2;CCNF;UBE2D3;UBE2D1;HIF3A;FBXO21;DCAF6;PSMD7;BTRC;SKP1;WSB1;FBXW7;COMMD2;FBXO10;LRRC41;KLHL42;PSMA5;PSMA1;GAN;AMER1;PSMD12;CUL5;DCUN1D3;CUL3;DCUN1D4;CUL2;COPS7B;DCUN1D2;ASB11;RNF7;DPP3;SOCS2;SOCS3;CAND1;RBBP7;VHL;SOCS6;UBE2F;FEM1B;ZBTB16;FBXL19;KLHL2;FBXO32;KLHL20;COPS2;ASB3;SPSB4;CUL4B
 
 
 
 
Downstream Signaling of Activated FGFR3
 
 
10/25
 
 
0.0011941
 
 
0.0214013
 
 
0
 
 
0
 
 
4.130403
 
 
27.799069
 
 
NRAS;PIK3CA;FGF18;FRS2;GRB2;KRAS;PIK3R1;SOS1;FGF2;FRS3
 
 
 
 
FLT3 Signaling
 
 
13/38
 
 
0.0012969
 
 
0.0230198
 
 
0
 
 
0
 
 
3.223325
 
 
21.427971
 
 
FLT3;PIK3R1;FOXO3;SOCS2;NRAS;BCL2L11;PIK3CA;GRAP2;AKT3;GRB2;KRAS;SOCS6;SOS1
 
 
 
 
Prolonged ERK Activation Events
 
 
7/14
 
 
0.0014092
 
 
0.0247748
 
 
0
 
 
0
 
 
6.191796
 
 
40.647555
 
 
MAP2K1;RAP1A;YWHAB;FRS2;NGF;CRK;CRKL
 
 
 
 
Transcriptional Regulation by E2F6
 
 
12/34
 
 
0.0014511
 
 
0.0252710
 
 
0
 
 
0
 
 
3.380481
 
 
22.092910
 
 
CBX5;RRM2;TFDP1;TFDP2;CBX3;EED;CHEK1;EPC1;RBBP7;BMI1;E2F6;YAF2
 
 
 
 
Protein Ubiquitination
 
 
21/77
 
 
0.0015373
 
 
0.0265228
 
 
0
 
 
0
 
 
2.327034
 
 
15.073824
 
 
RNF20;UBE2H;UBE2B;UBA6;USP9X;UBE2D2;UBE2E3;UBE2D3;DERL1;UBE2D1;UBE2G2;PEX13;CDC73;UBE2W;CDC34;UBE2R2;OTULIN;UBE2V2;UBE2N;RNF152;UBE2K
 
 
 
 
Downstream Signaling of Activated FGFR2
 
 
11/30
 
 
0.0015927
 
 
0.0265759
 
 
0
 
 
0
 
 
3.587387
 
 
23.111054
 
 
FGF7;NRAS;PIK3CA;FGF18;FRS2;GRB2;KRAS;PIK3R1;SOS1;FGF2;FRS3
 
 
 
 
Laminin Interactions
 
 
11/30
 
 
0.0015927
 
 
0.0265759
 
 
0
 
 
0
 
 
3.587387
 
 
23.111054
 
 
ITGB1;LAMA2;COL4A2;COL4A1;LAMA4;COL4A4;COL7A1;COL4A5;ITGA6;LAMC1;NID1
 
 
 
 
SUMOylation of Intracellular Receptors
 
 
11/30
 
 
0.0015927
 
 
0.0265759
 
 
0
 
 
0
 
 
3.587387
 
 
23.111054
 
 
HDAC4;NR4A2;UBE2I;NR5A2;SUMO3;RARA;RORA;PPARG;NR3C1;ESR1;PIAS2
 
 
 
 
Transcriptional Regulation by TP53
 
 
71/362
 
 
0.0015991
 
 
0.0265759
 
 
0
 
 
0
 
 
1.520803
 
 
9.791368
 
 
CCNK;BTG2;CDKN1A;CCNT2;DYRK2;CCNH;SETD9;YWHAB;PTEN;PRKAG2;TRIAP1;ING5;ING2;YWHAQ;SESN1;AKT3;CHEK1;SESN2;EP300;PIP4K2B;ZNF385A;YWHAH;PRKAB2;CNOT6L;TSC1;FOS;CCNA2;TFDP1;CCNE2;TFDP2;CCNE1;DDIT4;MAPKAPK5;DNA2;SGK1;TP53;PRKAA1;PRKAA2;RNF34;GLS2;PPP2CA;POLR2D;TP53INP1;PMAIP1;RICTOR;RBBP7;TAF9B;RFFL;E2F7;MAP2K6;ZNF420;GADD45A;PLK2;TXNRD1;TAF11;GTF2H1;ARID3A;CNOT7;AGO2;CCNG1;JMY;CDK1;FAS;CYCS;MDM4;TAF5;TAF4;LAMTOR3;TNRC6A;CDK5R1;LAMTOR5
 
 
 
 
MyD88 Cascade Initiated on Plasma Membrane
 
 
25/98
 
 
0.0016418
 
 
0.0265759
 
 
0
 
 
0
 
 
2.126116
 
 
13.632581
 
 
APP;USP14;PELI2;IKBKB;PPP2CA;RPS6KA3;MAPK8;IRAK2;NKIRAS2;BTRC;MAP3K7;N4BP1;SKP1;MAP2K6;MEF2A;MAP2K4;MAP2K1;MAP3K1;CHUK;FOS;VRK3;CREB1;UBE2N;UBE2V1;TP53
 
 
 
 
Toll Like Receptor 10 (TLR10) Cascade
 
 
25/98
 
 
0.0016418
 
 
0.0265759
 
 
0
 
 
0
 
 
2.126116
 
 
13.632581
 
 
APP;USP14;PELI2;IKBKB;PPP2CA;RPS6KA3;MAPK8;IRAK2;NKIRAS2;BTRC;MAP3K7;N4BP1;SKP1;MAP2K6;MEF2A;MAP2K4;MAP2K1;MAP3K1;CHUK;FOS;VRK3;CREB1;UBE2N;UBE2V1;TP53
 
 
 
 
Toll Like Receptor 5 (TLR5) Cascade
 
 
25/98
 
 
0.0016418
 
 
0.0265759
 
 
0
 
 
0
 
 
2.126116
 
 
13.632581
 
 
APP;USP14;PELI2;IKBKB;PPP2CA;RPS6KA3;MAPK8;IRAK2;NKIRAS2;BTRC;MAP3K7;N4BP1;SKP1;MAP2K6;MEF2A;MAP2K4;MAP2K1;MAP3K1;CHUK;FOS;VRK3;CREB1;UBE2N;UBE2V1;TP53
 
 
 
 
Activation of NOXA and Translocation to Mitochondria
 
 
4/5
 
 
0.0016700
 
 
0.0265759
 
 
0
 
 
0
 
 
24.749101
 
 
158.268940
 
 
TFDP1;TFDP2;PMAIP1;TP53
 
 
 
 
Zinc Efflux and Compartmentalization by the SLC30 Family
 
 
4/5
 
 
0.0016700
 
 
0.0265759
 
 
0
 
 
0
 
 
24.749101
 
 
158.268940
 
 
SLC30A8;SLC30A3;SLC30A5;SLC30A1
 
 
 
 
RHOD GTPase Cycle
 
 
16/53
 
 
0.0017283
 
 
0.0272325
 
 
0
 
 
0
 
 
2.681549
 
 
17.056238
 
 
CPNE8;MOSPD2;PIK3R1;ARHGAP5;LEMD3;ARHGAP21;LMAN1;DIAPH2;PGRMC2;CAPZB;VAPB;EFHD2;FILIP1;WHAMM;LBR;PAK5
 
 
 
 
Signaling by FGFR3 in Disease
 
 
9/22
 
 
0.0017408
 
 
0.0272325
 
 
0
 
 
0
 
 
4.288219
 
 
27.244919
 
 
NRAS;PIK3CA;FGF18;FRS2;GRB2;KRAS;PIK3R1;SOS1;FGF2
 
 
 
 
Deubiquitination
 
 
55/268
 
 
0.0018266
 
 
0.0283157
 
 
0
 
 
0
 
 
1.607444
 
 
10.135381
 
 
USP37;USP34;PTEN;UBE2D1;TNFAIP3;YY1;PSMD7;EP300;MAP3K7;TOMM70;USP8;USP47;USP48;USP42;USP4;USP3;USP44;YOD1;AXIN2;JOSD1;RAD23B;TGFBR1;PSMA5;CCNA2;PSMA1;PTRH2;VDAC1;TP53;OTUB1;BECN1;USP14;PSMD12;INO80D;USP12;WDR20;FOXK2;ADRB2;FOXO4;USP19;SNX3;TAF9B;SMAD2;USP25;TGFB1;SMURF2;USP9X;USP21;SIAH2;INO80;ESR1;SMAD7;TADA2B;APC;CDK1;MDM4
 
 
 
 
MAP Kinase Activation
 
 
18/63
 
 
0.0018407
 
 
0.0283157
 
 
0
 
 
0
 
 
2.481069
 
 
15.624837
 
 
MEF2A;MAP2K4;MAP2K1;CHUK;FOS;VRK3;IKBKB;PPP2CA;RPS6KA3;MAPK8;CREB1;IRAK2;UBE2N;UBE2V1;BTRC;MAP3K7;SKP1;MAP2K6
 
 
 
 
Cellular Responses to Stimuli
 
 
154/887
 
 
0.0019041
 
 
0.0290488
 
 
0
 
 
0
 
 
1.315571
 
 
8.240426
 
 
ERO1A;RB1;WIPF1;UBE2D2;UBE2D3;UBE2D1;RORA;PPP2R2A;NR3C1;BACH1;TXN2;RPS6KA3;PSMD7;CDC23;SESN1;AKT3;SESN2;CDC27;EP300;CHAC1;ATP6V1E1;BTRC;MAP3K5;SKP1;TSPYL2;MAP2K4;PRKCD;RPS6;PDIA6;PDIA5;DYNC1LI2;CCNE2;CCNE1;TBL1XR1;DNAJB11;MAPKAPK5;TP53;ASF1A;PSMD12;SEH1L;CALCRL;MRPL18;CUL3;HSPA4L;CUL2;FOXO3;HIGD1A;HSP90B1;DPP3;PRDX3;BAG4;GNG10;BAG5;POM121C;PRKAR2A;TPR;SSR1;MAP2K6;HSPA9;NUP155;CBX4;CBX2;GFPT1;HMGA1;HMGA2;GNG12;EIF2S2;EIF2S1;ESR1;GCLC;MAFG;ID1;BCL2;CREBRF;SRPRA;CYCS;MDM4;MAFK;ITGB1;CDKN1A;HSPB8;SLC7A11;HIF3A;BMI1;LIMD1;IKBKB;TUBA1A;SERP1;CAPZB;HIF1AN;IKBKE;NCOA1;KDM6B;CHUK;EED;HSBP1;GPX8;GPX7;FOS;DYNLL1;DYNLL2;SIRT1;PSMA5;CCNA2;NCOR2;CREB1;TUBB2A;TFDP1;PSMA1;PIK3CA;TFDP2;NUP50;CAT;DCP2;YAP1;AMER1;DYNC1I2;PRKAA2;GOSR2;CEBPG;ADCY3;ADCY2;HSPD1;PPP2CA;EXTL2;MAPK8;PGRMC2;GNG2;RPS15A;GNG5;RPL14;CCL2;RICTOR;E2F3;VHL;RBBP7;EXOSC3;FNIP1;FNIP2;DYNC1I1;MAP4K4;EGLN1;EGLN2;TXNRD1;PTGES3;VEGFA;DNAJA4;BMT2;CAPZA1;DNAJA2;CAPZA2;LAMTOR3;TNRC6A;LAMTOR5
 
 
 
 
MyD88-independent TLR4 Cascade
 
 
27/110
 
 
0.0020165
 
 
0.0292247
 
 
0
 
 
0
 
 
2.019839
 
 
12.535879
 
 
APP;USP14;UBE2D2;UBE2D3;UBE2D1;IKBKB;PPP2CA;RPS6KA3;MAPK8;IRAK2;NKIRAS2;BTRC;MAP3K7;IKBKE;N4BP1;SKP1;MAP2K6;MEF2A;MAP2K4;MAP2K1;CHUK;FOS;VRK3;CREB1;UBE2N;UBE2V1;TP53
 
 
 
 
TRIF (TICAM1)-mediated TLR4 Signaling
 
 
27/110
 
 
0.0020165
 
 
0.0292247
 
 
0
 
 
0
 
 
2.019839
 
 
12.535879
 
 
APP;USP14;UBE2D2;UBE2D3;UBE2D1;IKBKB;PPP2CA;RPS6KA3;MAPK8;IRAK2;NKIRAS2;BTRC;MAP3K7;IKBKE;N4BP1;SKP1;MAP2K6;MEF2A;MAP2K4;MAP2K1;CHUK;FOS;VRK3;CREB1;UBE2N;UBE2V1;TP53
 
 
 
 
Toll Like Receptor 9 (TLR9) Cascade
 
 
27/110
 
 
0.0020165
 
 
0.0292247
 
 
0
 
 
0
 
 
2.019839
 
 
12.535879
 
 
APP;USP14;PELI2;EEA1;IKBKB;PPP2CA;RPS6KA3;MAPK8;IRAK2;NKIRAS2;BTRC;MAP3K7;SLC15A4;N4BP1;SKP1;MAP2K6;MEF2A;MAP2K4;MAP2K1;MAP3K1;CHUK;FOS;VRK3;CREB1;UBE2N;UBE2V1;TP53
 
 
 
 
Activated NTRK3 Signals Through RAS
 
 
5/8
 
 
0.0020264
 
 
0.0292247
 
 
0
 
 
0
 
 
10.314635
 
 
63.966076
 
 
NRAS;NTF3;GRB2;KRAS;SOS1
 
 
 
 
Inactivation of CDC42 and RAC1
 
 
5/8
 
 
0.0020264
 
 
0.0292247
 
 
0
 
 
0
 
 
10.314635
 
 
63.966076
 
 
CDC42;RAC1;SLIT2;SRGAP2;ROBO1
 
 
 
 
Signaling by RNF43 Mutants
 
 
5/8
 
 
0.0020264
 
 
0.0292247
 
 
0
 
 
0
 
 
10.314635
 
 
63.966076
 
 
FZD5;FZD4;WNT3A;FZD6;LRP5
 
 
 
 
TGFBR3 Regulates TGF-beta Signaling
 
 
5/8
 
 
0.0020264
 
 
0.0292247
 
 
0
 
 
0
 
 
10.314635
 
 
63.966076
 
 
TGFBR3;TGFB2;TGFB1;GIPC1;TGFBR1
 
 
 
 
Signaling by TGFBR3
 
 
15/49
 
 
0.0020427
 
 
0.0292318
 
 
0
 
 
0
 
 
2.735262
 
 
16.940739
 
 
PSENEN;TGFB2;TGFB1;FGF2;TGFBR1;TGFBR3;MYCN;MMP16;GIPC1;AGO2;RARA;EP300;TCF4;TCF3;TNRC6A
 
 
 
 
PI Metabolism
 
 
22/84
 
 
0.0021213
 
 
0.0301220
 
 
0
 
 
0
 
 
2.201951
 
 
13.554643
 
 
PI4K2B;MTMR1;MTMR10;MTMR3;MTMR12;PLEKHA1;MTMR14;PTEN;GDPD1;PLEKHA5;MTMR9;PIK3R3;PIK3R1;PIK3C2A;PLEKHA8;PIK3CA;RAB14;PI4KA;PIP4K2B;PIP5K1B;PNPLA6;SBF2
 
 
 
 
Signaling by Rho GTPases
 
 
120/672
 
 
0.0021967
 
 
0.0307966
 
 
0
 
 
0
 
 
1.358655
 
 
8.316067
 
 
ITSN2;CPNE8;TFRC;FAM13B;PLXND1;WIPF1;STMN2;MSI2;FNBP1L;MYLK;PPP1CB;SYDE2;PPP1CC;GOLGA3;RALBP1;TPM3;TMOD3;PRKCD;FRS2;KCTD3;FAM91A1;LEMD3;FRS3;NISCH;ARMCX3;TIAM1;DYNC1LI2;MYL6;PKP4;S100A8;EPHA2;PFN2;MTMR1;TMEM87A;SEH1L;GARRE1;CUL3;CDCA8;PIK3R3;TWF1;PIK3R1;RHOBTB1;MYL12B;NCKIPSD;ARHGAP21;ARHGAP20;DVL3;SRGAP2;STARD13;CPSF7;ARHGAP28;CCT6A;ANLN;DIAPH2;FAM135A;COPS2;GRB2;FERMT2;CDC42SE2;ITGB1;ZNF512B;DOCK6;AHCTF1;ABCD3;DOCK3;SOWAHC;YWHAB;DOCK7;ARHGAP5;OSBPL11;TUBA1A;CAPZB;YWHAQ;EFHD2;MUC13;RAC1;LBR;SPTAN1;NDEL1;YWHAH;VAV3;ACTR2;PPP1R12A;RRAS2;DYNLL1;DYNLL2;CKAP4;RHOB;SENP1;TUBB2A;PIK3CA;CDC42EP2;SOS1;MAPRE1;SOS2;PAFAH1B1;DYNC1I2;MOSPD2;UACA;CDC42;PPP2CA;GNA13;LMAN1;PARD6B;PGRMC2;RBBP6;WHAMM;WASF1;DYNC1I1;PAK5;CLASP2;RNF20;USP9X;LIMK1;SEMA4F;MPP7;ACTC1;VAPB;FILIP1;VIM
 
 
 
 
Signaling by FGFR3
 
 
13/40
 
 
0.0022021
 
 
0.0307966
 
 
0
 
 
0
 
 
2.984213
 
 
18.258384
 
 
FRS2;PIK3R1;FGF2;FRS3;PPP2CA;NRAS;PIK3CA;MKNK1;FGF18;SPRY2;GRB2;KRAS;SOS1
 
 
 
 
Negative Regulation of TCF-dependent Signaling by WNT Ligand Antagonists
 
 
7/15
 
 
0.0023261
 
 
0.0322852
 
 
0
 
 
0
 
 
5.417506
 
 
32.849441
 
 
SFRP1;WNT3A;WIF1;LRP5;WNT9A;DKK2;WNT4
 
 
 
 
Downstream Signaling of Activated FGFR4
 
 
10/27
 
 
0.0023671
 
 
0.0326098
 
 
0
 
 
0
 
 
3.644050
 
 
22.032212
 
 
NRAS;PIK3CA;FGF18;FRS2;GRB2;KRAS;PIK3R1;SOS1;FGF2;FRS3
 
 
 
 
Cell Cycle, Mitotic
 
 
95/516
 
 
0.0024171
 
 
0.0330521
 
 
0
 
 
0
 
 
1.408173
 
 
8.484486
 
 
RB1;LIN54;CDKN1A;AHCTF1;CCNH;UBE2D1;PPP2R2A;CDC14A;PPP1CB;PPP1CC;PSMD7;TUBA1A;CDC23;CCND2;CCND1;AKT3;ENSA;CDC27;CNTRL;EP300;MYBL2;TNPO1;BTRC;PHLDA1;LBR;RAB8A;SKP1;NDEL1;CEP135;PPP1R12A;BORA;ESCO1;IST1;KIF23;DYNLL1;LEMD3;SMC1A;DYNLL2;CDC25B;HAUS1;EML4;PSMA5;CCNA2;DYNC1LI2;MAU2;TUBB2A;DBF4;TFDP1;PSMA1;ESPL1;TFDP2;CCNE2;CCNE1;GORASP1;NUP50;CHMP4B;CEP63;DNA2;MAPRE1;TP53;KPNB1;PAFAH1B1;DYNC1I2;PSMD12;SEH1L;VPS4A;CDCA8;PDS5B;ARPP19;PPP2CA;SPAST;POM121C;ORC2;TPR;E2F3;E2F5;CEP76;E2F6;DYNC1I1;CLASP2;GINS2;UBE2I;RRM2;NUP155;NEK6;NEK7;MZT1;DHFR;STAG1;WEE1;STAG2;CHMP2B;POLE3;CDK1;CEP41
 
 
 
 
Transcriptional Regulation by RUNX2
 
 
26/106
 
 
0.0024410
 
 
0.0331324
 
 
0
 
 
0
 
 
2.017591
 
 
12.136544
 
 
RB1;HDAC4;YAP1;PSMD12;CDKN1A;CBFB;SATB2;IHH;NR3C1;PPM1D;PSMD7;CCND1;AKT3;HEY2;SOX9;SKP1;ESRRA;SMURF1;ESR1;RUNX1;COL1A1;PSMA5;MAF;PSMA1;HAND2;CDK1
 
 
 
 
Aberrant Regulation of Mitotic Cell Cycle Due to RB1 Defects
 
 
12/36
 
 
0.0025351
 
 
0.0339117
 
 
0
 
 
0
 
 
3.098414
 
 
18.520824
 
 
RB1;CDKN1A;CCND2;CDC23;TFDP1;CCND1;CCNE2;TFDP2;CCNE1;CDC27;UBE2D1;E2F3
 
 
 
 
Keratan Sulfate Keratin Metabolism
 
 
12/36
 
 
0.0025351
 
 
0.0339117
 
 
0
 
 
0
 
 
3.098414
 
 
18.520824
 
 
B4GALT3;B4GALT1;B3GNT7;B4GAT1;SLC35D2;CHST1;ST3GAL6;B4GALT6;GNS;GLB1L3;CHST2;B4GALT5
 
 
 
 
Ub-specific Processing Proteases
 
 
41/190
 
 
0.0025563
 
 
0.0339498
 
 
0
 
 
0
 
 
1.710651
 
 
10.211178
 
 
BECN1;USP14;PSMD12;USP37;USP34;USP12;PTEN;WDR20;ADRB2;FOXO4;USP19;SNX3;PSMD7;TAF9B;MAP3K7;SMAD2;TOMM70;USP8;USP47;USP25;USP48;SMURF2;USP42;USP4;USP21;USP9X;USP3;SIAH2;USP44;AXIN2;TGFBR1;SMAD7;TADA2B;CCNA2;PSMA5;PSMA1;PTRH2;VDAC1;MDM4;TP53;OTUB1
 
 
 
 
Toll Like Receptor 7 8 (TLR7 8) Cascade
 
 
26/107
 
 
0.0028056
 
 
0.0366523
 
 
0
 
 
0
 
 
1.992566
 
 
11.708617
 
 
APP;USP14;PELI2;IKBKB;PPP2CA;RPS6KA3;MAPK8;IRAK2;NKIRAS2;BTRC;MAP3K7;SLC15A4;N4BP1;SKP1;MAP2K6;MEF2A;MAP2K4;MAP2K1;MAP3K1;CHUK;FOS;VRK3;CREB1;UBE2N;UBE2V1;TP53
 
 
 
 
MTOR Signalling
 
 
13/41
 
 
0.0028194
 
 
0.0366523
 
 
0
 
 
0
 
 
2.877466
 
 
16.894260
 
 
PRKAA1;PRKAB2;PRKAA2;YWHAB;STRADB;RPS6;PRKAG2;TSC1;FKBP1A;EEF2K;EIF4EBP1;LAMTOR3;LAMTOR5
 
 
 
 
Signaling by FGFR4
 
 
13/41
 
 
0.0028194
 
 
0.0366523
 
 
0
 
 
0
 
 
2.877466
 
 
16.894260
 
 
FRS2;PIK3R1;FGF2;FRS3;PPP2CA;NRAS;PIK3CA;MKNK1;FGF18;SPRY2;GRB2;KRAS;SOS1
 
 
 
 
Death Receptor Signaling
 
 
35/157
 
 
0.0028972
 
 
0.0374004
 
 
0
 
 
0
 
 
1.782422
 
 
10.416483
 
 
BEX3;PRDM4;UBE2D2;UBE2D3;UBE2D1;TNFAIP3;FASLG;TNF;RTN4;IKBKB;SMPD3;GNA13;BAG4;MAPK8;BCL2L11;CASP3;RAC1;MAP3K7;IKBKE;PSENEN;VAV3;LINGO1;CHUK;USP4;USP21;ARHGEF38;OMG;NGF;TNFRSF1A;TIAM1;ADAM17;OTULIN;FAS;SOS1;SOS2
 
 
 
 
TRAF6 Mediated Induction of NFkB and MAP Kinases Upon TLR7 8 or 9
Activation
 
 
25/102
 
 
0.0029563
 
 
0.0378979
 
 
0
 
 
0
 
 
2.015198
 
 
11.736153
 
 
APP;USP14;PELI2;IKBKB;PPP2CA;RPS6KA3;MAPK8;IRAK2;NKIRAS2;BTRC;MAP3K7;N4BP1;SKP1;MAP2K6;MEF2A;MAP2K4;MAP2K1;MAP3K1;CHUK;FOS;VRK3;CREB1;UBE2N;UBE2V1;TP53
 
 
 
 
Cellular Responses to Stress
 
 
137/787
 
 
0.0029825
 
 
0.0379708
 
 
0
 
 
0
 
 
1.317924
 
 
7.663706
 
 
ERO1A;RB1;WIPF1;UBE2D2;UBE2D3;UBE2D1;RORA;NR3C1;BACH1;TXN2;RPS6KA3;PSMD7;CDC23;SESN1;AKT3;SESN2;CDC27;EP300;CHAC1;ATP6V1E1;BTRC;MAP3K5;SKP1;TSPYL2;MAP2K4;PRKCD;RPS6;PDIA6;PDIA5;DYNC1LI2;CCNE2;CCNE1;TBL1XR1;DNAJB11;MAPKAPK5;TP53;ASF1A;PSMD12;SEH1L;MRPL18;CUL3;HSPA4L;CUL2;FOXO3;HIGD1A;HSP90B1;DPP3;PRDX3;BAG4;BAG5;POM121C;TPR;SSR1;MAP2K6;HSPA9;NUP155;CBX4;CBX2;GFPT1;HMGA1;HMGA2;EIF2S2;EIF2S1;ESR1;GCLC;MAFG;ID1;BCL2;CREBRF;SRPRA;CYCS;MDM4;MAFK;CDKN1A;HSPB8;SLC7A11;HIF3A;BMI1;LIMD1;TUBA1A;SERP1;CAPZB;HIF1AN;NCOA1;KDM6B;EED;HSBP1;GPX8;GPX7;FOS;DYNLL1;DYNLL2;SIRT1;PSMA5;CCNA2;NCOR2;CREB1;TUBB2A;TFDP1;PSMA1;TFDP2;NUP50;CAT;DCP2;AMER1;DYNC1I2;PRKAA2;GOSR2;CEBPG;HSPD1;EXTL2;MAPK8;PGRMC2;RPS15A;RPL14;CCL2;E2F3;VHL;RBBP7;EXOSC3;FNIP1;FNIP2;DYNC1I1;MAP4K4;EGLN1;EGLN2;TXNRD1;PTGES3;VEGFA;DNAJA4;BMT2;CAPZA1;DNAJA2;CAPZA2;LAMTOR3;TNRC6A;LAMTOR5
 
 
 
 
Interleukin-10 Signaling
 
 
14/46
 
 
0.0030217
 
 
0.0382064
 
 
0
 
 
0
 
 
2.711806
 
 
15.733681
 
 
CD86;IL10;CCL3L1;PTAFR;LIF;CXCL1;TNFRSF1B;CXCL2;TNF;TNFRSF1A;CXCL10;CCL3;CCL2;IL12B
 
 
 
 
Transcriptional and Post-Translational Regulation of MITF-M Expression
and Activity
 
 
15/51
 
 
0.0031529
 
 
0.0388465
 
 
0
 
 
0
 
 
2.583003
 
 
14.876660
 
 
FOXD3;EDN1;WNT3A;TFEB;MITF;SIRT1;KITLG;CREB1;QARS1;AKT3;ZIC1;TFEC;ID1;SOX9;IARS1
 
 
 
 
Frs2-mediated Activation
 
 
6/12
 
 
0.0031565
 
 
0.0388465
 
 
0
 
 
0
 
 
6.189928
 
 
35.643331
 
 
MAP2K1;RAP1A;YWHAB;FRS2;NGF;CRKL
 
 
 
 
Signaling by PDGFRA Extracellular Domain Mutants
 
 
6/12
 
 
0.0031565
 
 
0.0388465
 
 
0
 
 
0
 
 
6.189928
 
 
35.643331
 
 
NRAS;PIK3CA;GRB2;KRAS;PIK3R1;SOS1
 
 
 
 
Signaling by PDGFRA Transmembrane, Juxtamembrane and Kinase Domain
Mutants
 
 
6/12
 
 
0.0031565
 
 
0.0388465
 
 
0
 
 
0
 
 
6.189928
 
 
35.643331
 
 
NRAS;PIK3CA;GRB2;KRAS;PIK3R1;SOS1
 
 
 
 
FLT3 Signaling in Disease
 
 
10/28
 
 
0.0032282
 
 
0.0392057
 
 
0
 
 
0
 
 
3.441403
 
 
19.739296
 
 
CDKN1A;NRAS;ZMYM2;PIK3CA;FLT3;PIM1;GRB2;KRAS;PIK3R1;SOS1
 
 
 
 
Keratan Sulfate Biosynthesis
 
 
10/28
 
 
0.0032282
 
 
0.0392057
 
 
0
 
 
0
 
 
3.441403
 
 
19.739296
 
 
B4GALT3;B4GALT1;B3GNT7;B4GAT1;SLC35D2;CHST1;ST3GAL6;B4GALT6;CHST2;B4GALT5
 
 
 
 
MyD88 Dependent Cascade Initiated on Endosome
 
 
25/103
 
 
0.0033978
 
 
0.0409951
 
 
0
 
 
0
 
 
1.989246
 
 
11.308146
 
 
APP;USP14;PELI2;IKBKB;PPP2CA;RPS6KA3;MAPK8;IRAK2;NKIRAS2;BTRC;MAP3K7;N4BP1;SKP1;MAP2K6;MEF2A;MAP2K4;MAP2K1;MAP3K1;CHUK;FOS;VRK3;CREB1;UBE2N;UBE2V1;TP53
 
 
 
 
SUMOylation of DNA Methylation Proteins
 
 
7/16
 
 
0.0036417
 
 
0.0429457
 
 
0
 
 
0
 
 
4.815281
 
 
27.039222
 
 
DNMT1;UBE2I;CBX4;CBX2;DNMT3A;DNMT3B;BMI1
 
 
 
 
Transcription of E2F Targets Under Neg Control by P107 (RBL1) and P130
(RBL2) in Complex With HDAC1
 
 
7/16
 
 
0.0036417
 
 
0.0429457
 
 
0
 
 
0
 
 
4.815281
 
 
27.039222
 
 
CCNA2;LIN54;TFDP1;TFDP2;CDK1;MYBL2;E2F5
 
 
 
 
FRS-mediated FGFR3 Signaling
 
 
8/20
 
 
0.0037027
 
 
0.0429457
 
 
0
 
 
0
 
 
4.128150
 
 
23.112241
 
 
NRAS;FGF18;FRS2;GRB2;KRAS;SOS1;FGF2;FRS3
 
 
 
 
Signaling by PDGFR in Disease
 
 
8/20
 
 
0.0037027
 
 
0.0429457
 
 
0
 
 
0
 
 
4.128150
 
 
23.112241
 
 
KANK1;NRAS;PIK3CA;GRB2;KRAS;STRN;PIK3R1;SOS1
 
 
 
 
Cyclin D Associated Events in G1
 
 
14/47
 
 
0.0037613
 
 
0.0429457
 
 
0
 
 
0
 
 
2.629477
 
 
14.680337
 
 
RB1;CDKN1A;CCNH;PPP2R2A;PPP2CA;CCND2;TFDP1;CCND1;CCNE2;TFDP2;CCNE1;E2F3;E2F5;SKP1
 
 
 
 
G1 Phase
 
 
14/47
 
 
0.0037613
 
 
0.0429457
 
 
0
 
 
0
 
 
2.629477
 
 
14.680337
 
 
RB1;CDKN1A;CCNH;PPP2R2A;PPP2CA;CCND2;TFDP1;CCND1;CCNE2;TFDP2;CCNE1;E2F3;E2F5;SKP1
 
 
 
 
Signaling by ALK Fusions and Activated Point Mutants
 
 
23/93
 
 
0.0037674
 
 
0.0429457
 
 
0
 
 
0
 
 
2.038736
 
 
11.378927
 
 
RB1;IL10;CDKN1A;DNMT1;TPM3;RPS6;GCC2;FRS2;PIK3R1;FRS3;MECP2;EML4;MAPK8;PIK3CA;TPR;AGO2;GRB2;STRN;CARS1;ICOS;TP53;SKP1;MCL1
 
 
 
 
Signaling by ALK in Cancer
 
 
23/93
 
 
0.0037674
 
 
0.0429457
 
 
0
 
 
0
 
 
2.038736
 
 
11.378927
 
 
RB1;IL10;CDKN1A;DNMT1;TPM3;RPS6;GCC2;FRS2;PIK3R1;FRS3;MECP2;EML4;MAPK8;PIK3CA;TPR;AGO2;GRB2;STRN;CARS1;ICOS;TP53;SKP1;MCL1
 
 
 
 
trans-Golgi Network Vesicle Budding
 
 
19/72
 
 
0.0037688
 
 
0.0429457
 
 
0
 
 
0
 
 
2.223367
 
 
12.408609
 
 
APP;SNAPIN;TFRC;DTNBP1;PIK3C2A;GNS;VAMP8;BLOC1S6;VAMP7;SNX2;CLVS2;AP1S1;AP1S3;AP3S1;SH3GL2;SNX5;SH3D19;CLVS1;TXNDC5
 
 
 
 
FOXO-mediated Transcription
 
 
18/67
 
 
0.0038520
 
 
0.0435117
 
 
0
 
 
0
 
 
2.278002
 
 
12.663793
 
 
SMAD2;SREBF1;CDKN1A;TRIM63;YWHAB;GADD45A;FASLG;FOXO4;NR3C1;FOXO3;FBXO32;KLF4;SIRT1;BCL2L11;YWHAQ;AKT3;CAT;EP300
 
 
 
 
COPI-independent Golgi-to-ER Retrograde Traffic
 
 
15/52
 
 
0.0038705
 
 
0.0435117
 
 
0
 
 
0
 
 
2.513045
 
 
13.958410
 
 
DYNC1I2;GALNT1;DYNLL1;DYNLL2;AGPAT3;DYNC1LI2;TUBA1A;TUBB2A;CAPZB;CAPZA1;CAPZA2;RAB18;PAFAH1B2;DYNC1I1;PAFAH1B1
 
 
 
 
Cellular Response to Hypoxia
 
 
17/62
 
 
0.0039034
 
 
0.0435117
 
 
0
 
 
0
 
 
2.342386
 
 
12.990669
 
 
EGLN1;PSMD12;EGLN2;UBE2D2;UBE2D3;CUL2;UBE2D1;HIF3A;LIMD1;HIGD1A;VEGFA;PSMA5;PSMD7;PSMA1;EP300;HIF1AN;VHL
 
 
 
 
MyD88 MAL(TIRAP) Cascade Initiated on Plasma Membrane
 
 
27/115
 
 
0.0039363
 
 
0.0435117
 
 
0
 
 
0
 
 
1.904519
 
 
10.546290
 
 
APP;USP14;PELI2;IKBKB;PPP2CA;RPS6KA3;MAPK8;SOCS1;IRAK2;NKIRAS2;BTRC;MAP3K7;N4BP1;SKP1;MAP2K6;MEF2A;MAP2K4;MAP2K1;MAP3K1;CHUK;FOS;VRK3;CREB1;UBE2N;UBE2V1;TP53;S100A8
 
 
 
 
Toll Like Receptor TLR6 TLR2 Cascade
 
 
27/115
 
 
0.0039363
 
 
0.0435117
 
 
0
 
 
0
 
 
1.904519
 
 
10.546290
 
 
APP;USP14;PELI2;IKBKB;PPP2CA;RPS6KA3;MAPK8;SOCS1;IRAK2;NKIRAS2;BTRC;MAP3K7;N4BP1;SKP1;MAP2K6;MEF2A;MAP2K4;MAP2K1;MAP3K1;CHUK;FOS;VRK3;CREB1;UBE2N;UBE2V1;TP53;S100A8
 
 
 
 
Macroautophagy
 
 
32/143
 
 
0.0040138
 
 
0.0435809
 
 
0
 
 
0
 
 
1.790339
 
 
9.879128
 
 
BECN1;DYNC1I2;PRKAA1;MTMR3;PRKAA2;UBE2D2;MTMR14;UBE2D3;PRKAG2;TUBA1A;RB1CC1;DYNC1I1;ATG5;TOMM70;GABARAPL1;PRKAB2;ATG9A;TSC1;WIPI2;ATG14;DYNLL1;DYNLL2;DYNC1LI2;TUBB2A;CHMP2B;UBE2N;CHMP4B;VDAC1;UBE2V1;VIM;LAMTOR3;LAMTOR5
 
 
 
 
Activated NTRK2 Signals Through RAS
 
 
5/9
 
 
0.0040370
 
 
0.0435809
 
 
0
 
 
0
 
 
7.735527
 
 
42.640153
 
 
NRAS;BDNF;GRB2;KRAS;SOS1
 
 
 
 
EGFR Transactivation by Gastrin
 
 
5/9
 
 
0.0040370
 
 
0.0435809
 
 
0
 
 
0
 
 
7.735527
 
 
42.640153
 
 
NRAS;GRB2;KRAS;SOS1;HBEGF
 
 
 
 
PI5P Regulates TP53 Acetylation
 
 
5/9
 
 
0.0040370
 
 
0.0435809
 
 
0
 
 
0
 
 
7.735527
 
 
42.640153
 
 
ING2;EP300;PIP4K2B;TP53;MAP2K6
 
 
 
 
TCF Dependent Signaling in Response to WNT
 
 
40/189
 
 
0.0040934
 
 
0.0439330
 
 
0
 
 
0
 
 
1.668321
 
 
9.173046
 
 
AMER1;PSMD12;CUL3;USP34;LRP5;ASH2L;CDC73;PPP2CA;FRAT2;PSMD7;WIF1;RSPO2;EP300;DVL3;RSPO3;PIP5K1B;SOX9;BTRC;WNT1;WNT4;TLE4;USP8;TCF7L2;FZD5;SMURF2;FZD4;WNT3A;CSNK1A1;CTNNBIP1;FZD6;SOX13;AXIN2;WNT9A;DKK2;PSMA5;SFRP1;PSMA1;APC;TCF4;LGR4
 
 
 
 
Diseases of Mitotic Cell Cycle
 
 
12/38
 
 
0.0042002
 
 
0.0445603
 
 
0
 
 
0
 
 
2.859742
 
 
15.650318
 
 
RB1;CDKN1A;CCND2;CDC23;TFDP1;CCND1;CCNE2;TFDP2;CCNE1;CDC27;UBE2D1;E2F3
 
 
 
 
Signaling by FGFR1 in Disease
 
 
12/38
 
 
0.0042002
 
 
0.0445603
 
 
0
 
 
0
 
 
2.859742
 
 
15.650318
 
 
BAG4;NRAS;ZMYM2;PIK3CA;CNTRL;FRS2;GRB2;KRAS;PIK3R1;ERLIN2;SOS1;FGF2
 
 
 
 
Activated NTRK2 Signals Through CDK5
 
 
4/6
 
 
0.0044553
 
 
0.0461676
 
 
0
 
 
0
 
 
12.373832
 
 
66.987833
 
 
TIAM1;BDNF;RAC1;CDK5R1
 
 
 
 
RUNX1 Regulates Transcription of Genes Involved in WNT Signaling
 
 
4/6
 
 
0.0044553
 
 
0.0461676
 
 
0
 
 
0
 
 
12.373832
 
 
66.987833
 
 
CBFB;RSPO3;ESR1;RUNX1
 
 
 
 
Negative Regulation of MAPK Pathway
 
 
13/43
 
 
0.0044767
 
 
0.0461676
 
 
0
 
 
0
 
 
2.685323
 
 
14.524555
 
 
MAP2K1;YWHAB;DUSP1;PAQR3;DUSP16;DUSP9;PPP2CA;NRAS;PPP5C;KRAS;RAF1;MARK3;PTPN3
 
 
 
 
RND2 GTPase Cycle
 
 
13/43
 
 
0.0044767
 
 
0.0461676
 
 
0
 
 
0
 
 
2.685323
 
 
14.524555
 
 
PLXND1;TFRC;FRS2;PIK3R1;ARHGAP5;LEMD3;FRS3;CKAP4;NISCH;GOLGA3;MUC13;PKP4;EPHA2
 
 
 
 
Signaling by SCF-KIT
 
 
13/43
 
 
0.0044767
 
 
0.0461676
 
 
0
 
 
0
 
 
2.685323
 
 
14.524555
 
 
PIK3R3;PIK3R1;NRAS;KITLG;SOCS1;PIK3CA;GRAP2;CHEK1;GRB2;KRAS;RAC1;SOCS6;SOS1
 
 
 
 
FRS-mediated FGFR2 Signaling
 
 
9/25
 
 
0.0048359
 
 
0.0495950
 
 
0
 
 
0
 
 
3.483570
 
 
18.573299
 
 
FGF7;NRAS;FGF18;FRS2;GRB2;KRAS;SOS1;FGF2;FRS3
 
 
 
 
RAF Activation
 
 
11/34
 
 
0.0049087
 
 
0.0497887
 
 
0
 
 
0
 
 
2.962804
 
 
15.752454
 
 
PPP1CB;PPP2CA;PPP1CC;NRAS;MAP2K1;MRAS;YWHAB;SHOC2;KRAS;RAF1;MARK3
 
 
 
 
Signaling by WNT in Cancer
 
 
11/34
 
 
0.0049087
 
 
0.0497887
 
 
0
 
 
0
 
 
2.962804
 
 
15.752454
 
 
AMER1;PPP2CA;TCF7L2;FZD5;APC;CSNK1A1;FZD4;WNT3A;FZD6;LRP5;DKK2
 
 
 
 
Nervous System Development
 
 
101/567
 
 
0.0050379
 
 
0.0507213
 
 
0
 
 
0
 
 
1.351930
 
 
7.152734
 
 
PLXND1;IRS2;RPS6KA3;RPS6KA6;ALCAM;PSMD7;DPYSL5;SCN11A;DPYSL3;SCN9A;SH3GL2;SCN1A;MAP2K1;EPHA7;SEMA6A;SEMA6D;RPS6;ADAM10;FRS2;ISL1;TIAM1;MYL6;COL4A2;COL4A1;COL4A4;COL4A5;UTRN;EPHA2;PFN2;SPTBN4;SEMA7A;PSMD12;CUL2;PIK3R3;PIK3R1;EFNA5;MYL12A;MYL12B;EFNB2;FLRT3;PRKAR2A;SRGAP2;SCN3A;PRNP;SIAH2;RDX;SIAH1;MAGOHB;COL5A1;PTPRA;COL5A2;COL9A1;COL9A3;GRB2;ITGB1;NRP1;AP2A1;LAMC1;ROBO1;TUBA1A;RAC1;SPTAN1;PSENEN;VAV3;ACTR2;TRPC6;GFRA1;RGMA;RHOB;PSMA5;GAP43;COL2A1;TUBB2A;CREB1;PSMA1;PIK3CA;COL6A2;RASA1;COL6A3;COL6A6;SOS1;SOS2;SCN4B;YAP1;LAMA2;AKAP5;HMGCR;CDC42;NRAS;RPS15A;RELN;RPL14;SLIT2;PAK5;CLASP2;LIMK1;AP2B1;COL3A1;PMP22;KRAS;CDK5R1
 
 
 
 
Apoptosis
 
 
36/168
 
 
0.0050556
 
 
0.0507213
 
 
0
 
 
0
 
 
1.694083
 
 
8.957037
 
 
PSMD12;YWHAB;SATB1;HMGB2;FASLG;UACA;PPP3R1;CASP7;MAPK8;PSMD7;BCL2L11;FNTA;YWHAQ;CASP3;AKT3;PMAIP1;BMF;BAK1;SPTAN1;KPNA1;YWHAH;PRKCD;DYNLL1;DYNLL2;PSMA5;TJP1;TFDP1;PSMA1;TFDP2;APC;BCL2;CYCS;FAS;VIM;TP53;KPNB1
 
 
 
 
Activation of RAC1
 
 
6/13
 
 
0.0051759
 
 
0.0508229
 
 
0
 
 
0
 
 
5.305344
 
 
27.925964
 
 
RAC1;SLIT2;SOS1;SOS2;PAK5;ROBO1
 
 
 
 
Chk1 Chk2(Cds1) Mediated Inactivation of Cyclin B Cdk1 Complex
 
 
6/13
 
 
0.0051759
 
 
0.0508229
 
 
0
 
 
0
 
 
5.305344
 
 
27.925964
 
 
WEE1;YWHAQ;YWHAB;CHEK1;CDK1;YWHAH
 
 
 
 
GRB2 Events in EGFR Signaling
 
 
6/13
 
 
0.0051759
 
 
0.0508229
 
 
0
 
 
0
 
 
5.305344
 
 
27.925964
 
 
NRAS;TGFA;GRB2;KRAS;SOS1;HBEGF
 
 
 
 
Mitotic Telophase Cytokinesis
 
 
6/13
 
 
0.0051759
 
 
0.0508229
 
 
0
 
 
0
 
 
5.305344
 
 
27.925964
 
 
MAU2;STAG1;STAG2;KIF23;PDS5B;SMC1A
 
 
 
 
RHO GTPases Activate PAKs
 
 
8/21
 
 
0.0052598
 
 
0.0513735
 
 
0
 
 
0
 
 
3.810378
 
 
19.995580
 
 
CDC42;PPP1CB;MYL6;PPP1R12A;LIMK1;RAC1;MYL12B;MYLK
 
 
 
 
NGF-stimulated Transcription
 
 
12/39
 
 
0.0053112
 
 
0.0516024
 
 
0
 
 
0
 
 
2.753665
 
 
14.423531
 
 
REST;EGR3;CREB1;RRAD;ID1;ID4;EP300;FOSB;ID3;FOS;SGK1;CDK5R1
 
 
 
 
Signaling by NTRK3 (TRKC)
 
 
7/17
 
 
0.0054541
 
 
0.0527131
 
 
0
 
 
0
 
 
4.333501
 
 
22.583582
 
 
NRAS;PIK3CA;NTF3;GRB2;KRAS;PIK3R1;SOS1
 
 
 
 
TNF Signaling
 
 
16/59
 
 
0.0056542
 
 
0.0542303
 
 
0
 
 
0
 
 
2.306574
 
 
11.937352
 
 
CHUK;USP4;USP21;UBE2D2;UBE2D3;TNFAIP3;UBE2D1;TNF;TNFRSF1A;IKBKB;SMPD3;ADAM17;BAG4;OTULIN;IKBKE;MAP3K7
 
 
 
 
Toll Like Receptor 2 (TLR2) Cascade
 
 
27/118
 
 
0.0056992
 
 
0.0542303
 
 
0
 
 
0
 
 
1.841410
 
 
9.515366
 
 
APP;USP14;PELI2;IKBKB;PPP2CA;RPS6KA3;MAPK8;SOCS1;IRAK2;NKIRAS2;BTRC;MAP3K7;N4BP1;SKP1;MAP2K6;MEF2A;MAP2K4;MAP2K1;MAP3K1;CHUK;FOS;VRK3;CREB1;UBE2N;UBE2V1;TP53;S100A8
 
 
 
 
Toll Like Receptor TLR1 TLR2 Cascade
 
 
27/118
 
 
0.0056992
 
 
0.0542303
 
 
0
 
 
0
 
 
1.841410
 
 
9.515366
 
 
APP;USP14;PELI2;IKBKB;PPP2CA;RPS6KA3;MAPK8;SOCS1;IRAK2;NKIRAS2;BTRC;MAP3K7;N4BP1;SKP1;MAP2K6;MEF2A;MAP2K4;MAP2K1;MAP3K1;CHUK;FOS;VRK3;CREB1;UBE2N;UBE2V1;TP53;S100A8
 
 
 
 
Sphingolipid Metabolism
 
 
25/107
 
 
0.0057579
 
 
0.0545079
 
 
0
 
 
0
 
 
1.891768
 
 
9.756198
 
 
ASAH1;SGMS1;SGMS2;GLB1L3;SMPD3;UGCG;SPTLC1;ARSJ;SMPD1;ORMDL2;SPTSSB;KDSR;SPTSSA;ARSB;CTSA;CERS5;ORMDL1;ACER2;GALC;VAPB;B3GNT5;B4GALT6;PLPP1;B4GALT5;ST6GALNAC6
 
 
 
 
Adaptive Immune System
 
 
145/854
 
 
0.0058257
 
 
0.0548687
 
 
0
 
 
0
 
 
1.278112
 
 
6.576492
[truncated: 7,422,196 more chars]
